# Role of the Extracytoplasmic Function Sigma Factor SigE in the Stringent Response of Mycobacterium tuberculosis

**DOI:** 10.1128/spectrum.02944-22

**Published:** 2023-03-22

**Authors:** Giacomo Baruzzo, Agnese Serafini, Francesca Finotello, Tiziana Sanavia, Laura Cioetto-Mazzabò, Francesca Boldrin, Enrico Lavezzo, Luisa Barzon, Stefano Toppo, Roberta Provvedi, Riccardo Manganelli, Barbara Di Camillo

**Affiliations:** a Department of Information Engineering, University of Padova, Padua, Italy; b Department of Molecular Medicine, University of Padova, Padua, Italy; c Department of Biology, University of Padova, Padua, Italy; d Department of Comparative Biomedicine and Food Science, University of Padova, Padua, Italy; University Roma Tre

**Keywords:** *Mycobacterium tuberculosis*, sigma factors, stringent response, transcriptional regulation

## Abstract

Bacteria respond to nutrient starvation implementing the stringent response, a stress signaling system resulting in metabolic remodeling leading to decreased growth rate and energy requirements. A well-characterized model of stringent response in Mycobacterium tuberculosis is the one induced by growth in low phosphate. The extracytoplasmic function (ECF) sigma factor SigE was previously suggested as having a key role in the activation of stringent response. In this study, we challenge this hypothesis by analyzing the temporal dynamics of the transcriptional response of a *sigE* mutant and its wild-type parental strain to low phosphate using RNA sequencing. We found that both strains responded to low phosphate with a typical stringent response trait, including the downregulation of genes encoding ribosomal proteins and RNA polymerase. We also observed transcriptional changes that support the occurring of an energetics imbalance, compensated by a reduced activity of the electron transport chain, decreased export of protons, and a remodeling of central metabolism. The most striking difference between the two strains was the induction in the *sigE* mutant of several stress-related genes, in particular, the genes encoding the ECF sigma factor SigH and the transcriptional regulator WhiB6. Since both proteins respond to redox unbalances, their induction suggests that the *sigE* mutant is not able to maintain redox homeostasis in response to the energetics imbalance induced by low phosphate. In conclusion, our data suggest that SigE is not directly involved in initiating stringent response but in protecting the cell from stress consequent to the low phosphate exposure and activation of stringent response.

**IMPORTANCE** Mycobacterium tuberculosis can enter a dormant state enabling it to establish latent infections and to become tolerant to antibacterial drugs. Dormant bacteria’s physiology and the mechanism(s) used by bacteria to enter dormancy during infection are still unknown due to the lack of reliable animal models. However, several *in vitro* models, mimicking conditions encountered during infection, can reproduce different aspects of dormancy (growth arrest, metabolic slowdown, drug tolerance). The stringent response, a stress response program enabling bacteria to cope with nutrient starvation, is one of them. In this study, we provide evidence suggesting that the sigma factor SigE is not directly involved in the activation of stringent response as previously hypothesized, but it is important to help the bacteria to handle the metabolic stress related to the adaptation to low phosphate and activation of stringent response, thus giving an important contribution to our understanding of the mechanism behind stringent response development.

## INTRODUCTION

Tuberculosis is an airborne infectious disease caused by Mycobacterium tuberculosis. In 2020, about 10 million new cases of tuberculosis were reported, and 1.3 million people died from the disease (1).

**FIG 1:**
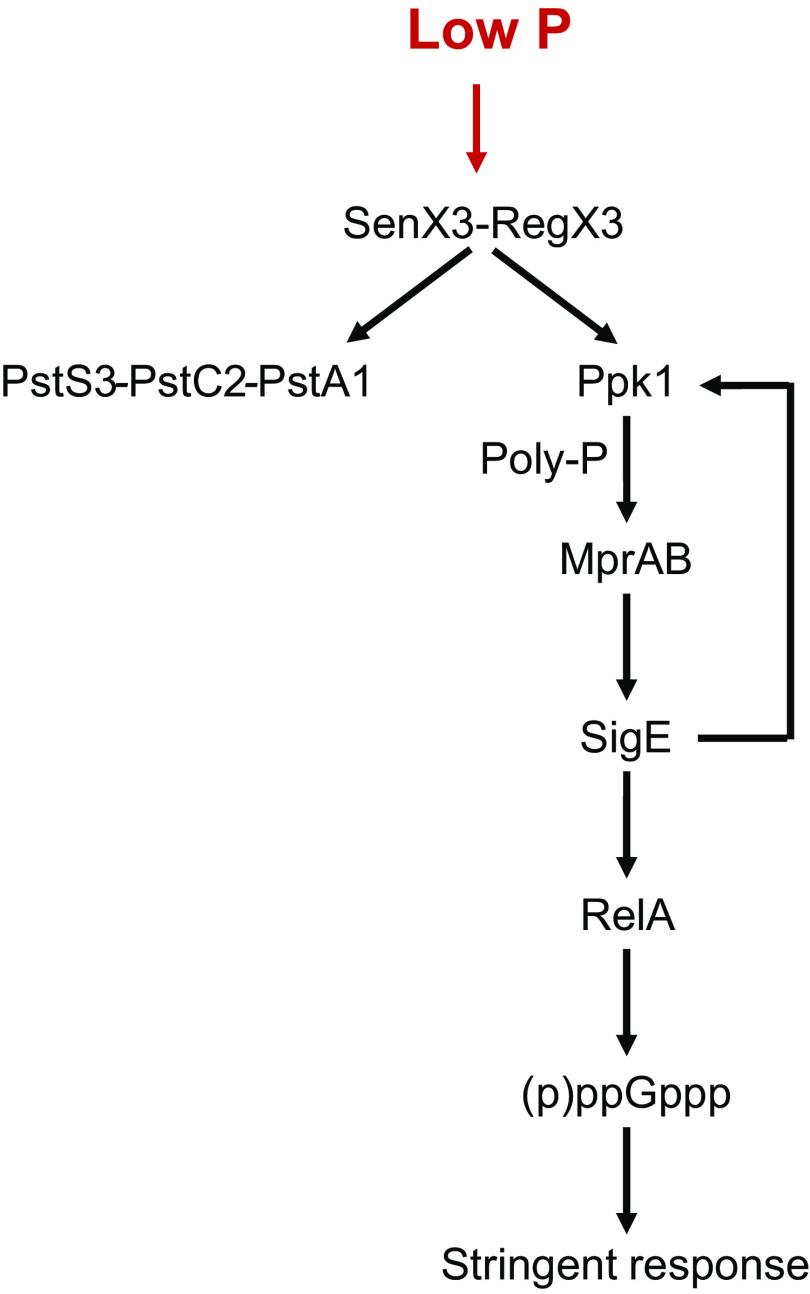
Schematic representation of the current stringent response network in M. tuberculosis. Low phosphate activates the two-component system SenX3-RegX3, which leads to the induction of the phosphate-specific transport operon *pstS3-pstC2-pstA1* and of *ppk1.* PPK1 synthesizes PolyP, resulting in a more efficient activation of the MprAB two-component system (using PolyP as a substrate for MprB phosphorylation). MprB phosphorylation allows *sigE* induction. SigE increases the expression of the genes included in its regulon, including *ppk1* and *relA*.

Tuberculosis treatment requires the administration of multiple antimicrobials for months to cope with the ability of M. tuberculosis to survive in a dormant state for prolonged periods of time, during which it develops tolerance and persistence to drugs (2). One of the key mechanisms used by M. tuberculosis to develop drug tolerance is the stringent response, a complex remodeling of metabolism with the aim to slow down growth and energy requirements to survive for long periods in conditions of starvation (3). The key molecule for the development of stringent response is the alarmone (p)ppGpp, produced by Rel, a ribosome-associated protein, in response to specific signals like the binding of a deacylated tRNA to the ribosome site A (4). One of the most characterized models of stringent response in M. tuberculosis is the one induced after exposure of bacteria to a low-phosphate environment (Fig. 1). In these conditions, the two-component regulatory system SenX3-RegX3 activates the transcription of the phosphate-specific transport operon, *pstS3-pstC2-pstA1*, and of *ppk1*, a gene encoding a polyphosphate (polyP) kinase, leading to an increase of the polyP levels in the cell, a well-known stress signal. Accumulation of polyP facilitates the phosphorylation of MprB, the response regulator of the two-component system MprAB, which positively regulates the structural gene of the extracytoplasmic sigma factor SigE (5), involved together with SenX3-RegX3 in the induction of *ppk* expression (3, 6–8). It has been proposed that in these conditions SigE is also able to drive the expression of *relA*, thus activating the stringent response (5). However, most of the experiments suggesting the direct dependence of *relA* transcription from SigE were performed in Mycobacterium smegmatis and not in M. tuberculosis. Moreover, the activation of the stringent response is known to be mostly regulated at the posttranslational level and not at the transcriptional level (9). These evidences motivated us to explore whether the real role of SigE induction in conditions of low phosphate was to initiate the stringent response. To address this question, we compared the transcriptional response dynamics to low phosphate of a *sigE* mutant and its wild-type parental strain using RNA sequencing (RNA-seq) data.

We found evidence that SigE is not directly involved in the development of the stringent response as previously hypothesized, since the main transcriptional signatures characterizing this response, like the downregulation of genes encoding ribosomal proteins and RNA polymerase (RNApol), were found conserved in the two strains. However, we found evidence that the role of SigE in these conditions is to protect the bacterium from stress induced from the metabolic and structural changes caused by the stringent response.

## RESULTS AND DISCUSSION

In this section, we first show and discuss the dynamics of the transcriptional response to low phosphate of a wild-type strain of M. tuberculosis, identifying the main genes involved in the development of the stringent response, e.g., *sigE*-regulated genes, and characterizing the main changes in the electron transport chain and the alternations in the central carbon metabolism pathways. Second, we study the stringent response in low phosphate in the *sigE* mutant strain, characterizing the main transcriptional activities and focusing on the alterations that are specific to this strain, e.g., alteration in the oxidative and acid stress. To this end, we considered the wild-type H37Rv strain (WT) and its isogenic *sigE*-null mutant ST28 (MU) in which SigE is rendered nonfunctional. Triplicate WT and MU cultures grown in phosphate-rich substrate were washed and resuspended in low-phosphate substrate. RNA extraction was performed before exposure to low phosphate (time zero) (high phosphate) and after 6, 12, and 24 h of exposure to low phosphate and subjected to paired-end Illumina RNA-seq. The resulting sequencing data are available on NCBI's Gene Expression Omnibus (GEO) (10) under accession number GSE211141. Bacteria exposed to low-phosphate media remained viable for at least 48 h (see Fig. S1 in the supplemental material). Finally, we report a validation of the robustness of the results obtained from the bioinformatics analysis.

### Analysis of H37Rv wild-type strain. (i) Differentially expressed genes and functional annotation.

Differential expression analysis was performed using both edgeR and FunPat (11), considering the entire time course, i.e., at time 6, 12, and 24 h versus time zero, to define a gene as differentially expressed (see Materials and Methods for further details).

We found 2,087 differentially expressed (DE) genes, i.e., significantly affected by phosphate starvation in the M. tuberculosis wild-type at 6, 12, and 24 h versus 0 h (referred from here on as “WT versus T0”) (see Data Set S1 in the supplemental material).

**FIG 2:**
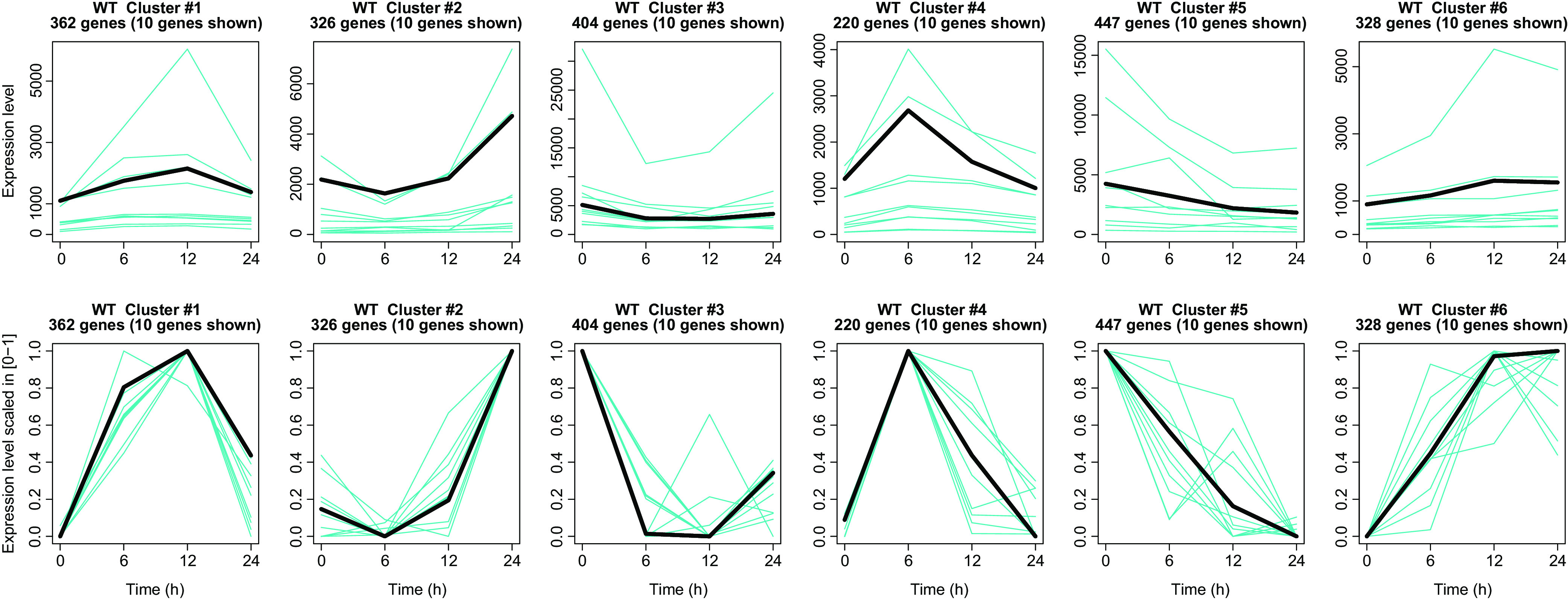
Clustering of differentially expressed genes in WT versus T0. Figure shows the 6 clusters of genes resulting from the *k*-means clustering. For each cluster, the gene expression profile of the centroid, i.e., gene expression profile obtained from the average of all the gene expression profiles in the cluster, is shown in black, together with 10 (randomly chosen) gene expression profiles from the same cluster (cyan). First row, gene expression profiles are plotted in the original scale to show the difference in gene expression level intensities. Second row, the same gene expression profiles are scaled in 0 to 1 to highlight the shape of the gene expression profiles.

Gene expression profiles were scaled to their maximum and clustered using *k*-means into 6 different clusters of 362, 326, 404, 220, 447, and 238 genes (Fig. 2; see also Data Set S1). The different clusters were characterized by specific patterns and were functionally annotated using DAVID functional annotation clustering (12) for Mycobacterium tuberculosis H37Rv. The complete list of WT versus T0 DE genes, the list of genes in each cluster, and the significantly enriched functional terms associated to each cluster are available in Data Set S1.

Cluster 1 is characterized by a peak of upregulation at 12 h with respect to time zero. Significantly enriched functional terms included lipids catabolism (enoyl-coenzyme A [enoyl-CoA] hydratase genes) and protein biosynthesis (aminoacyl-tRNA synthetases).

Cluster 2 is characterized by an increasing pattern of expression with a peak at 24 h. Significantly enriched functional terms correspond to lipid biosynthesis (i.e., triacyclglycerols and 2,3-diacyltrehaloses), response to nitric oxide and to hypoxia, and PPE family proteins.

Cluster 3, conversely, is characterized by a decreasing pattern of expression from 6 h after phosphate deprivation and is significantly enriched with functional terms related to electron transport chain (menaquinone biosynthesis, NADH dehydrogenases), redox proteins (iron-sulfur cluster assembly), aromatic amino acid, and histidine biosynthesis.

Cluster 4 is characterized by upregulation with respect to time zero with a peak at 6 h and significant association to functional terms related to phthiocerol dimycocerosate (PDIM) synthesis.

Cluster 5 shows a decreasing pattern of expression with downregulation with respect to time zero and significantly enriched annotation of the following terms: ribosome synthesis and activity (ribosomal proteins, ribonucleoproteins, RNA-binding proteins, structural constituent, cytosolic large ribosomal subunit), DNA modification enzymes (nucleases, transposases), and toxins (VapC family).

Cluster 6 shows an increasing pattern of expression, reaching a plateau at 12 h. Enriched annotation terms include the following: enzymes involved in metabolism of fatty acids and amino acids, core metabolism (tricarboxylic acids [TCA] cycle, glycolysis, glyoxylate shunt), cofactors, and nucleotide biosynthesis.

Taken together, these clusters confirm that M. tuberculosis develops a transcriptional metabolic change in response to phosphate starvation with the trait of the stringent response, characterized by deep remodeling of lipid metabolism, as well as a decrease in ribosome biogenesis and RNA transcription (9). Detailed examples are the repression of *rpoAB* (see Data Set S2 in the supplemental material), encoding the RNA polymerase, indicating a general decrease in transcription, and in the expression of several genes encoding ribosomal proteins.

### (ii) SigE network and SenX3-RegX3 regulon in H37Rv.

In agreement with the previous findings highlighting the involvement of SigE in the development of the stringent response (6, 7), exposure to a low-phosphate environment showed the induction of several genes known to be directly regulated by SigE (*sigE*, *hsp*, *htpX*, *sigB*, *rv2743-pspA-clgR*, *rv2052c-rv2053c*, and *rv1072-rv1073*) (Data Set S2) as well as genes indirectly regulated by SigE through the action of other regulators that are part of its regulon such as SigB (*hsp* and *pks2*) or ClgR (*rv1043c* and *clpP1-clpP2*) (13) (Data Set S2). These genes encode for chaperons, proteases, proteins involved in sulfolipids biosynthesis, and proteins involved in membrane stabilization and abnormal membrane protein degradation. These data suggest that in a low-phosphate environment the cell experiences surface stress, probably due to the unfolding of surface proteins, that activates the SigE response.

**FIG 3:**
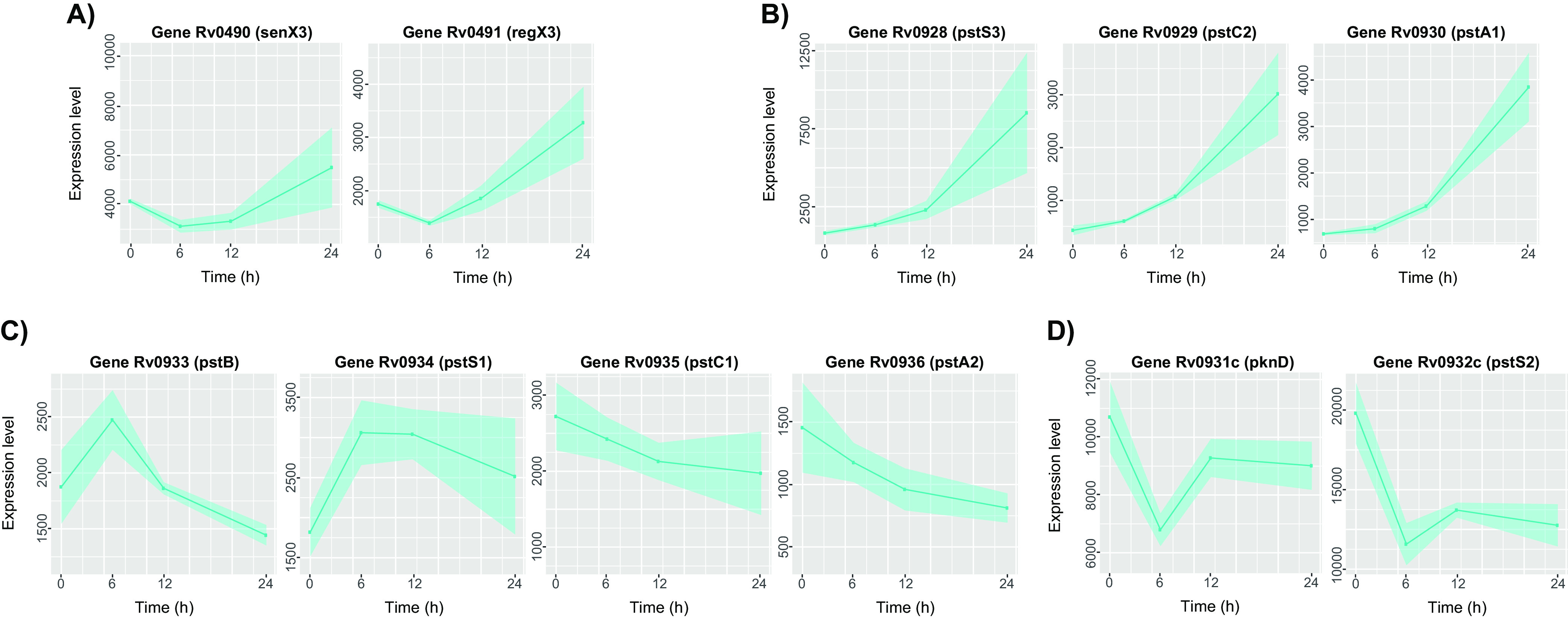
Differential expression of gene involved in phosphate homeostasis in the wild-type strain. The charts report the expression level profiles of genes encoding proteins directly involved in the sensing of phosphate availability and phosphate uptake. (A) Two-component system. *senX3*, sensor histidine kinase; *regX3*, sensory transduction protein. (B) Phosphate transport operon. *pstS*3, periplasmic phosphate-binding lipoprotein; *pstC2*-*pstA1*, phosphate transporter, ABC type. (C) Phosphate transport operon. *pstB*, phosphate-transport ATP-binding protein; *pstS1*, periplasmic phosphate-binding lipoprotein; *pstC1*-*pstA2*, phosphate-transport integral membrane ABC transporters. (D) *pstS2*, periplasmic phosphate-binding lipoprotein; *pknD*, transmembrane serine/threonine-protein kinase.

In agreement with the model of Sanyal et al. (6), in low-phosphate environments, an increased transcription of the genes encoding the two-component system SenX3-RegX3 (even if the increase of *senX3* did not reach statistical significance) was observed in the WT. The expression started to increase after 6 h from exposure to low phosphate and continued to increase until the end of the experiment (Fig. 3A; see also Data Set S2). The same pattern was observed for the phosphate transport operon *pstS3*-*pstC2*-*pstA1* (Fig. 3B; Data Set S2), known to be under transcriptional control of the two-components system SenX3-RegX3 (7, 14), but not for the other phosphate transport operons *pstB-pstS1-pstC1-pstA2* (Fig. 3C; Data Set S2) and *pknD-pstS2* (Fig. 3D; Data Set S2). In this case, the former decreased its expression from the beginning of the experiment, while the latter showed a decreased expression in the first 6 h, with a recovery after an additional 6 h of exposure to the low-phosphate environment. These data are in contrast with previous studies where the latter two operons were shown to be not differentially expressed or slightly induced in low phosphate (7, 15), possibly due to differences in strains and experimental conditions.

Another gene known to be regulated by SenX3-RegX3 is *ppk1*, encoding a polyphosphate kinase, whose SigE-dependent transcription is activated through binding of phosphorylated RegX3 to its upstream region (6). Increased Ppk1 levels cause an increase in cytoplasmic polyP, a well-known stress signal (6), which through the activation of the two-component system MprAB leads to *sigE* induction generating a feed forward loop between Ppk1 and SigE. Indeed, *ppk1* was induced after 12 h of exposure to low phosphate, when *sigE* expression reached its peak (Data Set S2).

Ppk1 was also reported to be essential for the activation of the stringent response through the SigE-dependent induction of *relA* (5, 6); indeed, in our experiment, *relA* expression showed an increasing trend starting from 6 h after exposure to the low-phosphate environment, even if this increase did not reach statistical significance (Data Set S2).

### (iii) Changes in electron-transport chain.

**FIG 4:**
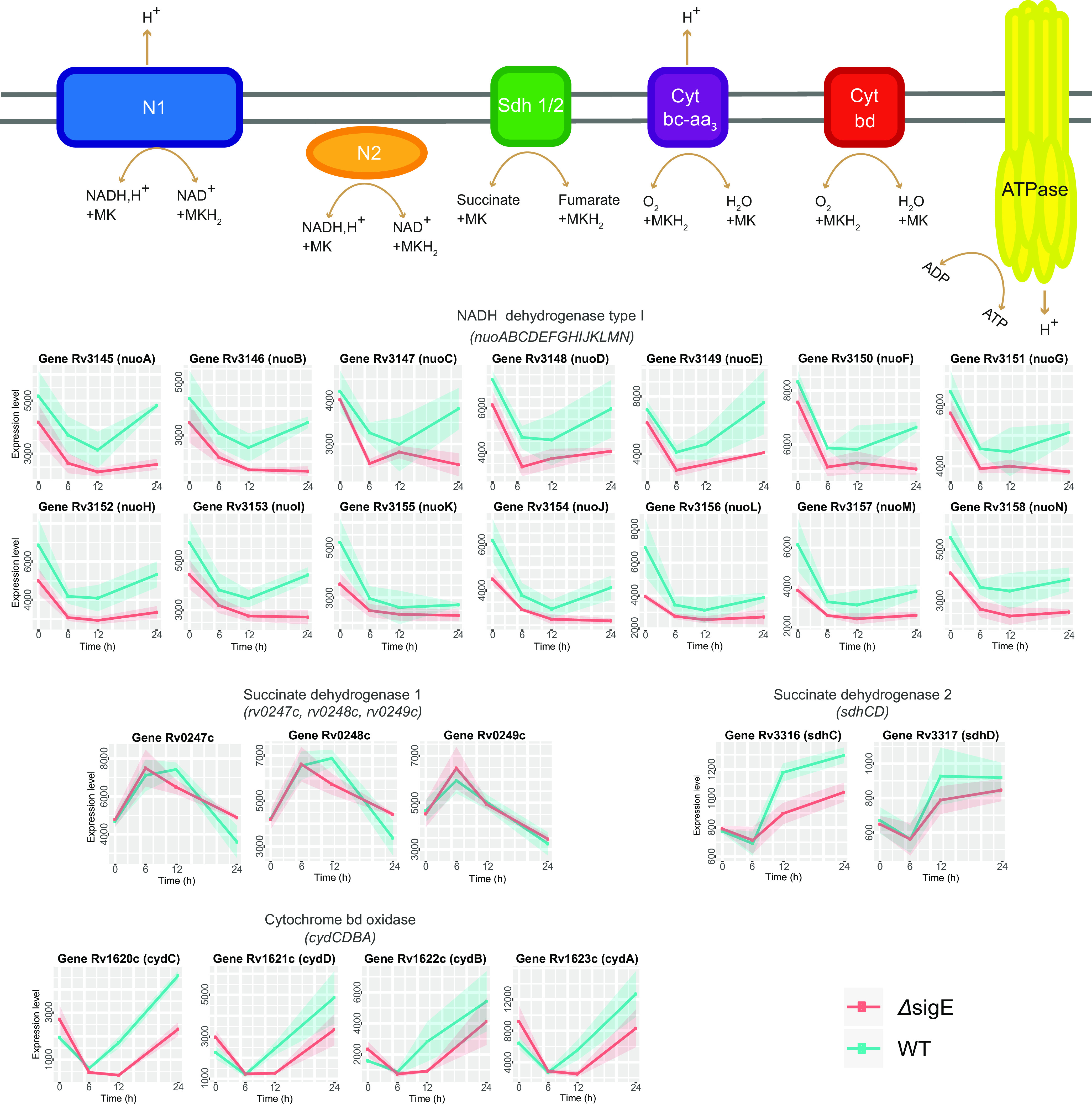
Differential expression of electron transport chain genes. At the top, schematic representation of respiratory chain enzymatic complexes. The charts with cyan/pink lines report the expression level profiles of involved genes in both wild-type (WT) (cyan) and *sigE*-null mutant (Δ*sigE*) (pink). Cyt bc-aa_3_, cytochrome *bc-aa_3_* oxidase; Cyt bd, cytochrome *bd* oxidase; N1, NADH dehydrogenase type 1; N2, NADH dehydrogenase type 2, *ndh* and *ndA*; Sdh1, succinate dehydrogenase 1; Sdh2, succinate dehydrogenase 2. MK, oxidized menaquinone; MKH_2_, reduced menaquinone.

An intuitive consequence of phosphate starvation is the reduced supply of cells with Pi for ATP-synthase, causing a drop in ATP production. A diminished activity of this enzyme implies that protons released in the periplasmic space by NADH dehydrogenase type I and cytochrome *bc-aa* cannot be reimported by ATP synthase. This results in the accumulation of protons in the periplasmic space triggering acidification and alteration of proton motive force (*pmf*). Consistent with this hypothesis, M. tuberculosis exposed to phosphate limitation induces the transcription of the gene encoding the membrane carbonic anhydrase CanA (*rv1284*), which functions as sink of protons (see Data Sets S3 and S4 in the supplemental material) (16–18). Additionally, several genes from the respiratory chain are differentially expressed (Fig. 4; Data Sets S3 and S4), which could contribute to the protection of cells from acidification and altered *pmf*. (i) The operon encoding the proton-translocating NADH dehydrogenase type I (*nuoABCDEFGHJKLMN*) is downregulated, inducing the reduction of the export of protons into the periplasmic space. NADH reoxidation can be supplied by two non-proton-translocating NADH dehydrogenases (type II) Ndh and NdhA (19), whose expressions do not change. (ii) The genes involved in the menaquinone biosynthetic pathway (*entC*-*menD*-*menE*) are downregulated, suggesting that the density of electron flux across the respiratory chain diminishes too, indirectly reducing the load of the export of protons. (iii) The cytochrome *bd* oxidase genes (*cydCDBA*), encoding a non-proton-translocating terminal oxidase with high oxygen affinity (20, 21), are strongly induced, contributing to further reduce the load of exported protons and guaranteeing the continuity of electron flux.

### (iv) Central carbon metabolism pathways alteration.

**FIG 5:**
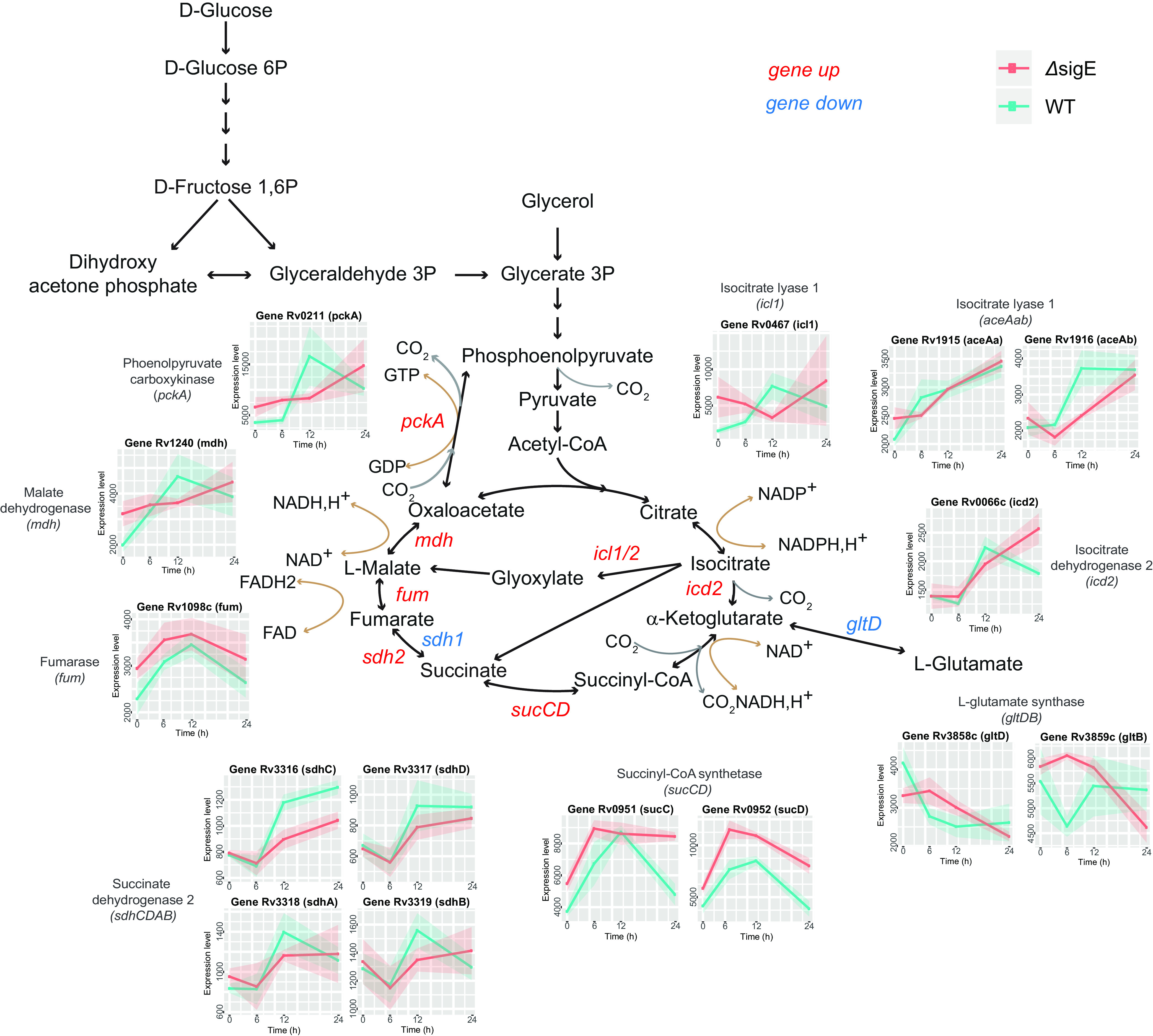
Differential expression of genes involved in the core of central carbon metabolism. The charts with cyan/pink lines report the expression level profiles of involved genes in both wild-type (WT) (cyan) and the *sigE*-null mutant (Δ*sigE*) (pink). Genes that are up-expressed at 6, 12, and 24 h compared to time zero are highlighted in red; genes that are down-expressed at 6, 12, and 24 h compared to time zero are highlighted in blue.

Another interesting transcriptional response to low phosphate regards the expression of genes encoding enzymes involved in the Krebs cycle. Genes involved in the direct production of succinate, like isocitrate lyases (*icl1*, *aceAa*, *aceAb*) and succinyl-CoA synthetase (*sucCD*), were induced (Fig. 5; see Data Set S5 in the supplemental material). It is known that when there is a significant drop in ATP levels and a rise of *pmf* as hypoxia (22, 23) or iron starvation (24), the pathways involved in succinate production are overexpressed and a fraction of succinate may be secreted to maintain the *pmf*. Our data strongly support a reduced electron transport chain (ETC) activity that likely leads to reduction of ATP production and an alteration of *pmf*. It might be possible that additionally in conditions of low phosphate, M. tuberculosis needs to secrete succinate to maintain the *pmf* and for this reason it increases its production.

Interestingly, we found a sharp and prolonged downregulation of the gene encoding the small subunit of glutamate synthase (*gltD*) (Fig. 5; Data Set S5). Comparing this pattern of expression to those of isocitrate dehydrogenase 2 (*icd2*) and *sucCD* (Fig. 5), which show only temporary induction, suggests that the cell might accumulate α-ketoglutarate. This metabolite is an important metabolic regulator (25), and a recent study in Bacteroides thetaiotaomicron revealed that during stringent response, α-ketoglutarate pool size increases, altering the metabolic processes and promoting growth arrest (26). It is possible that when phosphate is low in M. tuberculosis, the accumulated α-ketoglutarate is partially transformed in succinate and partially used as metabolic regulator.

Between 6 and 12 h, succinate dehydrogenase 1 complex (Sdh1/*rv0247c*-*rv0249c*) expression decreased, while the expression of succinate dehydrogenase 2 (Sdh2/*sdhCDAB*) increased (Fig. 5; Data Set S5). This switch is consistent with a previous demonstrated role of Sdh2 in stress conditions, causing a drop in ATP production as in hypoxia (27, 28).

Finally, the genes encoding the phosphoenolpyruvate carboxykinase PckA, the malate dehydrogenase Mdh, and the fumarase Fum were found to be induced (Fig. 5; Data Set S5). In a condition where the high-ATP yielding pathway is compromised, the induction of *pckA* may represent a substrate-level phosphorylation mechanisms to supply ATP (24, 29), which results in the production of oxaloacetate. In these conditions, the phosphoenolpyruvate-derived oxaloacetate can be used by Fum and Mdh to implement a reverse Krebs cycle toward succinate production and NADH reoxidation (Fig. 5). It is worth noting that a reverse operation of oxaloacetate-malate-succinate branch of Krebs cycle, together with glyoxylate shunt, has been demonstrated to occur in M. tuberculosis exposed to hypoxia to increase the succinate production (22, 23).

Targeted metabolomics investigations will be necessary to confirm these hypotheses. However, recent transcriptomic, proteomics, and metabolomics studies performed on M. tuberculosis exposed to bedaquiline (BDQ) (30, 31), an inhibitor of membrane-embedded F_o_ domain of ATPase (32) and hence mimicking the low phosphate effect, partially confirm our hypothesis. In response to BDQ exposure, (i) glyoxylate shunt and anaplerotic PckA are both active, (ii) succinate is secreted (31), and (iii) together with succinate, malate and fumarate are also secreted, raising the thought that the postulated malate and fumarate produced by a reductive Krebs cycle branch in low phosphate can be secreted to maintain an energized membrane. Additionally, non-proton-translocating cytochrome *bd* oxidase (*cydAB*) is upregulated following exposure to BDQ as in low phosphate (30).

In support of our hypothesis regarding the acidification of periplasmic space, a transcriptomic study shows that M. tuberculosis exposed to mild-low pH downregulates NADH dehydrogenase I and upregulates cytochrome *bd* oxidase (33) similarly to what we observed after low-phosphate exposure.

In summary, our transcriptomic data indicate that during growth in a low-phosphate environment, M. tuberculosis suffers from acidic and high *pmf* stress, and to contrast these perturbations, it (i) stimulates carbonic anhydrase expression as a sink of protons, (ii) reduces the electron flux across the respiratory chain to reduce the proton export, (iii) secretes tricarboxylic acids to maintain an energized membrane, and (iv) utilizes substrate-level phosphorylation to maintain ATP production.

### Analysis of the *sigE*-null mutant strain. (i) Differentially expressed genes and functional annotation.

When we performed the same analyses in the mutant strain, we found 1,734 genes significantly affected by phosphate starvation at 6, 12, and 24 h compared to 0 h (referred from here on as “MU versus T0”) (see Data Set S6 in the supplemental material).

**FIG 6:**
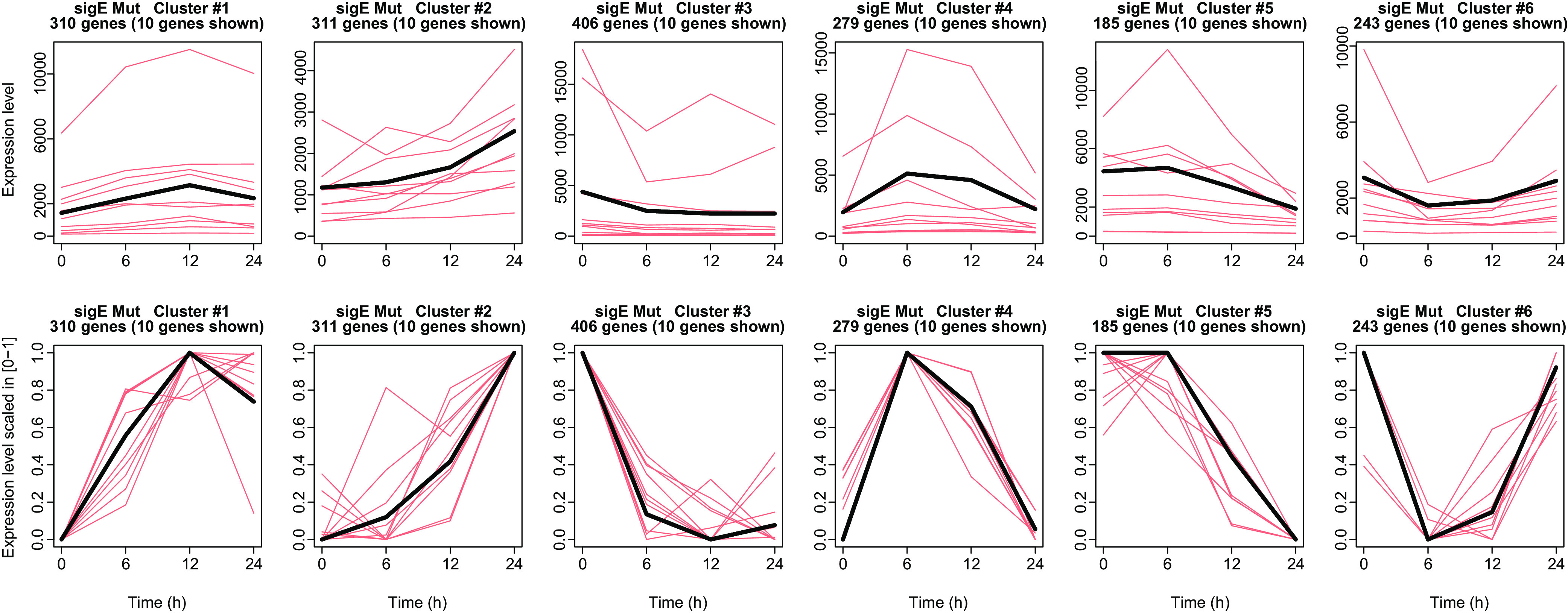
Clustering of differentially expressed genes in MU versus T0. Six clusters of genes resulting from the *k*-means clustering. For each cluster, the gene expression profile of the centroid, i.e., gene expression profile obtained from the average of all of the gene expression profiles in the cluster, is shown in black, together with 10 (randomly chosen) gene expression profiles from the same cluster (salmon/pink). First row, gene expression profiles are plotted in the original scale to show the difference in gene expression level intensities. Second row, the same gene expression profiles are scaled in 0 to 1 to highlight the shape of the gene expression profiles.

Gene expression profiles were scaled to their maximum and clustered using *k*-means in 6 different clusters of 310, 311, 406, 279, 185, and 243 genes (Fig. 6), identifying clusters of genes sharing similar expression profiles. We found that several clusters showed the same temporal patterns of the clusters found in WT. Some of them were significantly enriched with similar functional terms. The complete list of MU versus T0 DE genes, the list of genes in each cluster, and the significantly enriched functional terms associated with each cluster are available in Data Set S6.

Cluster 1 was characterized by temporal profiles similar to the ones observed in the same cluster in WT, with significant associations to functional terms related to DNA binding and transcriptional regulation, including several genes encoding toxin/antitoxin systems and histone-like proteins.

Also, clusters 2, 3, 4, and 5 showed temporal profiles similar to the correspondent profiles in WT clusters, as well as sharing the same enriched functional terms.

Cluster 6 showed a decreasing pattern of expression with respect to time zero at times 6 and 12, an increased expression between 12 and 24 h, and showed significant enrichment terms similar to cluster 2 of the WT.

Interestingly, several typical biological processes related to the stringent response, such as the downregulation of transcription of ribosomal proteins and RNA polymerase, were also present in the *sigE* mutant, suggesting that the basic stringent response does not depend on this sigma factor. This was also supported by the fact that, even if expressed at lower level in the mutant strain, *relA* was induced following a similar pattern in both WT and MU strains.

### (ii) SigE network and SenX3-RegX3 regulon in *sigE* mutant.

**FIG 7:**
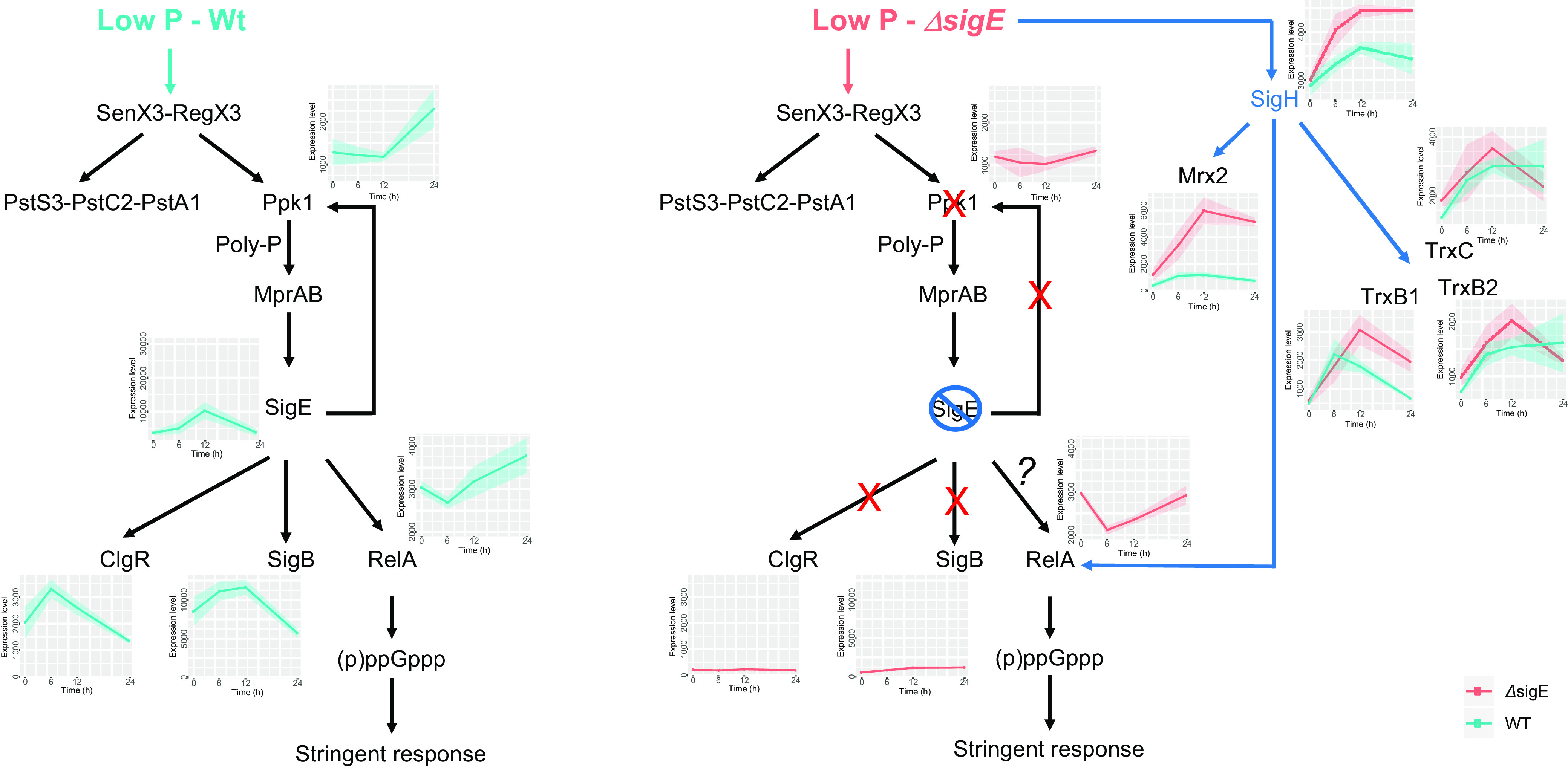
Schematic representation of the stringent response network in M. tuberculosis WT and *sigE* mutant strain showing the induction of the SigE-dependent genes in response to low pH, totally abrogated in the *sigE*-null mutant, as well as the induction of *sigH* and related genes in the *sigE* mutant. The charts with cyan/pink lines report the expression level profiles of involved genes in both wild-type (WT) (cyan) and the *sigE*-null mutant (Δ*sigE*) (pink). Explanation in text.

The genes induced in WT under the control of SigE, SigB, and ClgR were, as expected, not induced in MU, and in some cases, they showed a very low level of expression even at time zero (*sigB*, *clgR*) (Fig. 7; see also Data Set S7 in the supplemental material). An exception was the *sigE* gene, which was induced and highly expressed. This apparent incongruence was because in our *sigE*-null mutant, the *sigE* gene was disrupted by an Hyg cassette and not totally deleted, so the first part of the gene was still expressed. The overexpression of this part of the gene in the mutant was already noted using DNA microarrays (13, 34).

In the *sigE* mutant strain, the transcriptional profiles of the genes encoding the two-component system SenX3-RegX3 and the phosphate-transport systems were similar to those observed in the WT strain, with the exception of the operon *pknD-pstS2*, as in the mutant the peak of expression at 12 h was absent, suggesting a role of SigE in its regulation (Data Set S7). As expected, the induction of *ppk1* in the mutant was totally abrogated, confirming its dependence on SigE (Data Set S7). However, *relA* followed a similar temporal pattern in the two strains, but its level of expression in the mutant was significantly lower (Fig. 7), suggesting that even if SigE has some role in the conditions of phosphate starvation, some other sigma factors can, at least in part, compensate for its absence. A candidate is SigH, whose consensus sequence is very similar to that of SigE (35) and that was induced in the *sigE* mutant but not in the WT strain (Fig. 7; Data Set S7). It is not clear whether this decreased expression of *relA* in the *sigE* mutant has an effect on the activation of the stringent response, since RelA is mainly regulated at the posttranslational level (36). The expression of WT levels of *sigB* and the induction of *ppK1* were restored in a *sigE* mutant complemented strain (see Fig. S2 and S3 in the supplemental material).

### (iii) Oxidative and acid stress in *sigE* mutant.

One of the most striking differences observed between the transcriptional profiles of the two strains was the induction in the *sigE* mutant of *sigH* and its regulon (see Data Set S3 and S8 in the supplemental material), known to be important for protection from oxidative stress (37).

Genes of the SigH regulon found to be upregulated in the *sigE* mutant (Fig. 7) in response to low phosphate included those encoding the protein disaggregase ClpB (rv0384c), an important regulator of the stress response (38, 39); three genes encoding the TRX system designed to protect cells from oxidative damage *(trxB-rv1471, trxB2-rv3913, trxC-rv3914); and rv2466c-mrx2*, encoding a mycoredoxin (40) (Fig. 7; Data Set S8). Of note, genes involved in mycothiol biology as *rv0486-mshA* (41), and *rv1082-mca* (42) were induced in this mutant even if not known to be directly regulated by SigH (Data Set S8). Induction of *sigH* and *mrx2* were abrogated in a *sigE* mutant complemented strain (see Fig. S4 in the supplemental material). While SigH and mycothiol have always been associated with the oxidative stress response, several studies have also linked them to the response to low pH (33, 43, 44), suggesting that *sigE* exposed to low phosphate may experience one or both of these stress conditions. The reason why the absence of SigE may lead to acidic and/or oxidative stress is not clear, and further experiments need to be performed to clarify this matter. The main role of SigE is the protection of the cell envelope from surface stress (13, 45, 46). It is possible that the changes in the electron transport chain induced in low phosphate to reduce the electron flux and control the pH causes the membrane to lose its homeostasis, thus activating the SigE response, which, in turn, is able to maintain membrane integrity. However, in its absence, the envelope could be damaged, thus interfering with the proper functionality of the electron transport chain and of the system involved in the acid-protection, causing the generation of reactive oxygen species (ROS) and the alteration of *pmf*. The strong induction in the *sigE* mutant of the genes encoding the lipoprotein Rv1540 and the phospholipase Rv2037c (Data Set S8), both involved in the cell envelope integrity maintenance under stress (47, 48), while not part of the SigE regulon, confirms that this mutant experiences cell envelope stress under phosphate limitation.

### (iv) Other alterations specific to the *sigE* mutant.

In support of our hypothesis that the *sigE* mutant experiences a strong alteration of electron transport chain activity, we found induction of the gene encoding the well-known transcriptional factor WhiB6 (see Data Set S9 in the supplemental material), which responds to changes of redox potential (49).

Additionally, we found other genes specifically induced in the *sigE* mutant that may suggest that this strain experiences a stronger stress compared to WT, although it is not clear how these alterations are functionally connected to the absence of SigE. In particular, we found the following to be induced: (i) *whiB7*, which encodes a transcriptional regulator (Data Set S9) that modulates the expression of several genes involved in intrinsic resistance to translation-targeting antibiotics such as *eis*, *rv1258*, and the operon *rv0492c-rv0493c* (50); (ii) genes encoding multidrug-efflux pumps, such as *mmpL5-mmpS5* (51), r*v2686-rv2687*, or *mmr*, and two adjacent putative operons, *rv2640c-cadI* encoding for a regulator of the ArsR family and *rv2642-arsC* probably involved in detoxification; and (iii) several genes encoding toxin-antitoxin systems, such as *rv0299*, *vapB4-C4*, *vapB27-C27*, *vapB29-C29*, *mazF5-E5*, and *relF-G* (Data Set S9).

### Robustness and reproducibility of the RNA-seq results.

To assess the reproducibility of the main findings of this study, we first analyzed the reliability of RNA-seq data, and then evaluated the robustness of the bioinformatics pipeline.

More than 99.8% of the RNA-seq reads passed the read quality control check and were considered for read alignment (see Table S1 in the supplemental material). Read alignment step resulted in a high fraction of mapped reads (52), ranging between 97.41% and 99.66%; read alignment statistics are reported in Table S1. The read mapping statistics confirmed the absence of contaminants, a high efficiency of rRNA depletion, with a low fraction of reads mapping to rRNA genes (range, between 0.59% and 2.31%), and in stranded library preparation (see Tables S1 and S2 in the supplemental material).

The robustness of the bioinformatics pipeline was assessed through the evaluation of different strategies for both gene expression level quantification and differential gene expression analysis.

In terms of gene expression level quantification, we used two alternative strategies, namely, “totcounts” and “maxcounts” (53) (see Materials and Methods). Independently from the adopted gene expression level quantification strategy, there is a large agreement across biological replicates, i.e., average Pearson correlation of ~0.95 and ~0.96 for maxcounts and totcounts, respectively (see Fig. S5 to S8 in the supplemental material). These results suggest both the lack of batch effects in the data and the robustness of the computed gene expression levels to the choice of the quantification strategy. Remarkably, the adoption of a stranded protocol and a high sequencing depth further assisted the accuracy of the estimated gene expression levels. Considering the large agreement between the two quantification strategies and the proven robustness of the maxcounts strategy to read length bias and uneven coverage (53), maxcounts gene expression level quantifications were used in this study.

The other critical step in the bioinformatics analysis is the identification of differentially expressed genes (DEGs), since the analysis of the RNA-seq time-series can results in the selection of a high number of DEGs that is likely to include false-positives. Differential expression analysis was performed using both edgeR (54), one of the most widely used tools for RNA-seq data analysis, and FunPat (11), a tool specifically developed for the analysis of time series RNA-seq data. Compared to edgeR, FunPat selected a lower number of DEGs (see Fig. S9A in the supplemental material). Notably, 93.84% to 97.65% of all DEGs selected by FunPat were also selected by edgeR. Furthermore, we evaluated the false positives (FP) made by FunPat and edgeR by looking at external RNA controls consortium (ERCC) spike-in RNAs (*n* = 23) with constant concentration in all RNA-seq libraries detected as differentially expressed by the two methods. FunPat proved a higher capability to control the FP-rate with respect to edgeR (see Table S3 in the supplemental material). Considering the large agreement between DEGs identified by both methods and the better control of the FP-rate provided by FunPat, the DEGs identified by FunPat were used in this study. To further assess the robustness of differential expression analysis, FunPat was also tested on totcounts data. The list of DEGs obtained with the two methodologies showed a large overlap (Fig. S9B) but a slightly higher number of FPs identified from totcounts data compared to maxcounts data (Table S3).

Overall, the above tests confirmed the reliability of the produced RNA-seq data, the robustness of the adopted bioinformatics pipeline, and the reproducibility of the main finding from the bioinformatics analysis.

### Conclusions.

Our data suggest the hypothesis that SigE is not directly involved in the activation of the stringent response, even if its induction together with the genes belonging to its regulon was clearly confirmed at low phosphate. However, our data suggest that in these conditions its role is to counteract the stress that the strong modification of cellular metabolism imposes on the cells. Indeed, in the absence of SigE, another sigma factor, SigH, together with other transcriptional regulators, such as WhiB6 and WhiB7, plays as a backup system to help the cell to adapt to the new physiologic conditions.

## MATERIALS AND METHODS

To increase the reliability and reproducibility of the study, we followed the main best practices for the design of RNA-seq experiments.

First, bacteria were cultured in triplicate at each time point for each strain. Replicates are of fundamental importance in the downstream bioinformatics analyses, especially when dealing with time series data.

ERCC spike-in control mixes were added to each sample before sequencing (see Fig. S6 in the supplemental material). The predefined molar concentration ratios of the spike-in RNAs were used for assessing the quality control of samples and a fine tuning of the bioinformatics analyses.

Samples were distributed across different sequencing lanes following a strategy to minimize potential batch effects (see Fig. S10 in the supplemental material). Specifically, samples containing different strains and time points were distributed across different sequencing lanes, such that a potential batch effect in a sequencing lane would affect only one biological replicate.

Paired-end stranded protocol was used for the sequencing processes. Paired-end distance and read strandness were used during preprocessing (i.e., read alignment and gene expression level quantification) of the sequencing data to improve the accuracy of the bioinformatics pipeline.

Finally, an extremely large sequencing depth was used, resulting in almost 63 million reads per sample; the amount of reads for each sample is available in Table S1 in the supplemental material.

### Bacteria cultures.

We used three strains of M. tuberculosis as follows: the wild-type H37RV, a *sigE* mutant (ST28) in which the *sigE* gene was rendered nonfunctional, and a complemented strain (ST29) in which the WT *sigE* gene was reintroduced in an ectopic locus of the chromosome (13).

The strains were routinely growth at 37°C in 7H9 medium supplemented with ADN (5% albumin, 0.2% dextrose, 0.85% sodium chloride), 0.05% Tween 80, and 0.2% glycerol.

For all of the experiments, cells were grown in rolling bottles (225-mL volume capacity) in 30 to 35 mL modified 7H9 broth containing 20 mM MOPS (morpholinepropanesulfonic acid), pH 6.6, and 25 mM Pi (NaH2PO4) and supplemented with 0.05% Tween 80, 0.02% glycerol, and 0.2% glucose until an optical density at 600 nm (OD_600_) of 0.6 to 0.9. Then, the cells were harvested, washed three times in Pi-free broth, and resuspended in Pi-free modified Middlebrook 7H9 broth (20 mM MOPS, pH 6.6, 1.46 g sodium chloride, 0.05% Tween 80, 0.02% glycerol, and 0.2% glucose). Cells were harvested and collected for all of the experiments at time zero (in high-phosphate conditions, before the washings) and after 6, 12, 24, and 48 h of incubation.

Viability of cultures grown in Pi-free broth (see Fig. S7 in the supplemental material) was evaluated performing 1:10 serial dilutions at each time point and spotting 10 μL of each dilution on 7H10 plates supplemented with ADN, 0.05% Tween 80, and 0.2% glycerol, in duplicate. CFU were recorded after 21 days of incubation at 37°C.

### RNA sample preparation.

Total RNA was extracted from 35-mL cultures as previously describe (55). Two or three DNase treatments were performed to remove DNA contamination from the samples. The DNA contamination was verified using 5 ng of nucleic acids samples. RNA was quantified by spectrophotometer (Nanodrop), and its quality was verified by bioanalyzer (Agilent RNA 6000 Nano kit). Only samples with RNA integrity number (RIN) values of 8/9 were used for the following steps.

For RNA-seq, samples were prepared from three independent experiments (in total 24 samples). Before sequencing, the Ambion ERCC spike-in control mixes from Thermo Fisher Scientific (Waltham, MA) were added to the M. tuberculosis RNAs following the manufacturer instructions. The ERCC standards consist of two mixtures of spike-in RNAs in figure, present at defined molar concentration ratios, described by four subgroups. Each subgroup contains 23 transcripts that cover a 1e6-fold concentration range and have different lengths and GC-contents. The two mixtures of ERCC spike-ins were distributed across the 24 samples as shown in Fig. S10 in the supplemental material.

### RNA sequencing.

All samples, together with the admixed spike-in RNAs, were subjected to RNA-seq with the Illumina HiSeq sequencer (Illumina, San Diego, CA). The sequencing was run in multiplexing with 5 libraries per lane (Fig. S10). Tagged libraries were prepared with the Illumina TruSeq stranded protocol with depletion of rRNA, pooled, and subjected to 2× 100-bp paired-end sequencing.

### Read processing, mapping, and counting.

Reads were preprocessed with the FASTX toolkit 0.0.13.2 (http://hannonlab.cshl.edu/fastx_toolkit); “fastx_clipper” was used to remove adapter sequences, whereas “fastq_quality_trimmer” was used to trim read ends with Phred-scores lower than 20 and remove reads shorter than 70 bp. For each library, paired reads (i.e., read pairs passing data preprocessing) and singletons (i.e., reads whose mate was discarded by data preprocessing) were stored in separated files.

The reference for read mapping was built merging the M. tuberculosis H37Rv complete genome (GenBank accession number AL123456.3) with the FASTA sequences of the 92 ERCC RNAs. Paired reads and singletons were mapped separately to the reference using Bowtie 2 (56) version 2.2.1, which is suited for unspliced reads of 50 to 100 bp in length with the options “–end-to-end” and “–very-sensitive.” Finally, mapped paired reads and singletons were merged in a single BAM file with samtools (57).

To build the GTF file of gene coordinates, we gathered the H37Rv annotations from TubercuList (58) (http://tuberculist.epfl.ch/), selected the coding sequences (CDS), and merged them with the ERCC RNA annotations.

To further assess the reliability of the data and avoid any bias in the bioinformatics analysis, we quantified the gene expression level in two ways.

First, we used the “maxcounts” strategy described in Finotello et al. (53). By computing gene expression as the maximum coverage along each CDS (or any genomic feature of interest), maxcounts reduces the length bias and is robust to situations in which the reads are not uniformly distributed along sequences, as it happens due to sequencing errors and ambiguity in the read mapping.

In addition, the total number of reads mapped to a CDS (referred from now on as “totcounts”) was also computed. Maxcounts and totcounts were computed considering the read strandness. For our analysis, we used the –Ss option to handle stranded paired-end reads, available on Github (https://gitlab.com/sysbiobig/maxcounts).

Counts *c_gl_* for each gene *g* and library *l* were normalized as follows:

$$c{\text{′}}_{\mathrm{gl}}=\frac{c{\text{′}}_{\mathrm{gl}}\cdot \mathrm{median}\left(S_{j}\right)}{S_{l}\cdot N_{l}}$$

where *S_l_* and *N_l_* are the library sizes and normalization factors, respectively, estimated with edgeR (54) for all of the libraries (*l*).

### Differential expression analysis.

To assess the effect of phosphate starvation on M. tuberculosis gene expression, RNA-seq data from samples collected at 6, 12, and 24 h were compared to 0 h in the wild-type (“WT_vs_T0”) and in the mutant (“MU_vs_T0”).

As the analysis of RNA-seq time-series can results in the selection of a high number of differentially expressed genes (DEGs), likely to include false-positives, we analyzed RNA-seq data using FunPat, which has been shown to be robust to noise oscillations in time series experimental data (11). Funpat was used for the selection step. It implements the bounded-area method (59), which calculates for each gene the area A of the region bounded by the time series expression profile and a baseline, set at the corresponding expression level at 0 h. Specifically, in this study, two experimental conditions were analyzed as follows: WT(*t* > 0) verus WT(*t* = 0) and MU(*t* > 0) versus MU(*t* = 0). A *P* value was assigned to each gene by evaluating the significance of its bounded area against a null hypothesis distribution described by a model of the biological-plus-technical variability and its dependency on the mean gene expression level, which was derived using a negative binomial model with the tag-wise dispersion, whose parameters were estimates obtained using edgeR (54).

For the sake of comparison, differential expression analysis was also performed with edgeR using generalized linear models (“glmFit” and “glmLRT” functions) (60). For both edgeR and FunPat analyses, differentially expressed genes were selected with a significance level of 5% on *P* values adjusted for multiple testing with the Benjamini-Hochberg approach. Analyses were performed with R (https://www.R-project.org/).

### Gene clustering and functional annotation.

For each gene in WT (MU) condition, we computed the average gene expression profile, i.e., at each time point, we computed the average expression level across the three replicates of WT (MU) conditions. Considering only those genes that were identified as differentially expressed in WT versus T0 (MU versus T0), we performed a *k*-means clustering of their average gene expression profiles. A value of *k* = 6 was chosen, calculating the within-cluster-sum of squares (WSS) for different values of *k* and choosing the *k* for which WSS curve showed a clear elbow. *k*-means clustering was performed using the *kmeans* function of the “stats” R package, setting the maximum number of iterations (*max.iter*) to 10,000 and the number of restart (*nstart*) to 1,000. Average gene expression profiles were scaled and centered prior to *k*-means clustering, such that clusters contain genes sharing similar expression profiles rather than similar expression levels. The results of *k*-means clustering are available in Data Sets S1 and S6 in the supplemental material.

The different clusters of genes were functionally annotated using DAVID functional annotation clustering (https://david.ncifcrf.gov/). DAVID takes as input a list of genes and organizes them in subsets of genes with similar biological annotation based on multiple co-occurrences of the functional annotation terms found in multiple sources of biological annotation such as Gene Ontology, KEGG Pathways, BioCarta Pathways, Swiss-Prot Keywords, BBID Pathways, SMART Domains, NIH Genetic Association DB, UniProt Sequence Features, COG/KOG Ontology, NCBI OMIM, InterPro Domains, and PIR Super-Family Names. For each functional subset, we report the main annotation terms, indicating them as significant if their false discovery rate (Benjamini-Hochberg correction of functional enrichment test *P* values) is lower than 10%. The results of functional annotation analysis are available in Data Sets S1 and S6. Analyses were performed with R (https://www.R-project.org/).

### Data availability.

Sequencing reads and the gene expression count table (both raw and normalized count data) are available on NCBI's Gene Expression Omnibus (GEO) (10) under accession number GSE211141.
