# Supplementary material for: Role of the Extracytoplasmic Function Sigma Factor SigE in the Stringent Response of Mycobacterium tuberculosis
Source: Microbiol Spectr. 2023 Mar 22;11(2):e02944-22. doi: 10.1128/spectrum.02944-22 (PMC10100808; doi:10.1128/spectrum.02944-22)
Supplement: Supplemental file 10 — Tables S1 to S3 and Fig. S1 to S10. Download spectrum.02944-22-s0001.pdf, PDF file, 2.1 MB [file spectrum.02944-22-s0001.pdf]

Role of the extracytoplasmic function sigma factor SigE in the stringent response of *Mycobacterium tuberculosis*

Giacomo Baruzzo[a+], Agnese Serafini[b+], Francesca Finotello[a*], Tiziana Sanavia[a*], Laura Cioetto-Mazzabò[b], Francesca Boldrin[b], Enrico Lavezzo[b], Luisa Barzon[b], Stefano Toppo[b], Roberta Provvedi[c], Riccardo Manganelli[b,#], Barbara Di Camillo[a,d,#]

[a]Department of Information Engineering, University of Padova, Padua, Italy
[b]Department of Molecular Medicine, University of Padova, Padua, Italy
[c]Department of Biology, University of Padova, Padua, Italy
[d]Department of Comparative Biomedicine and Food Science, University of Padova, Padua, Italy

# Address correspondence to Barbara Di Camillo, barbara.dicamillo@unipd.it, and Riccardo Manganelli, riccardo.manganelli@unipd.it

*Present address:
Francesca Finotello, Institute of Molecular Biology and Digital Science Center, University of Innsbruck, Innsbruck, Austria.
Tiziana Sanavia, Department of Medical Sciences, University of Torino, Torino, Italy.

[+]Giacomo Baruzzo and Agnese Serafini contributed equally to this work. Author order was determined on the basis of alphabetical order.

Supplementary Tables and Figures

Supplementary Table S1. Read statistics: total sequenced reads, paired- (PE) and single-end (SE) reads after filtering, and mapped reads; percentage of total sequenced reads are shown in brackets.

Library	Total reads	Filtered PE + SE (% of total)	Mapped (% of total)
MU_R1_T0	60,055,782	59,925,114 + 64,468 (99.89%)	59,793,239 (99.56%)
MU_R2_T0	61,671,752	61,542,842 + 64,051 (99.89%)	61,430,496 (99.61%)
MU_R3_T0	89,894,434	89,673,970 + 92,909 (99.86%)	89,416,251 (99.47%)
MU_R1_T6	72,957,066	72,800,062 + 77,472 (99.89%)	72,468,128 (99.33%)
MU_R2_T6	62,195,838	62,066,722 + 64,132 (99.90%)	61,903,437 (99.53%)
MU_R3_T6	53,340,962	53,210,502 + 54,864 (99.86%)	52,883,988 (99.14%)
MU_R1_T12	57,888,366	57,764,606 + 61,040 (99.89%)	57,127,502 (98.69%)
MU_R2_T12	64,947,708	64,813,238 + 66,769 (99.90%)	63,985,943 (98.52%)
MU_R3_T12	52,792,224	52,663,218 + 54,525 (99.86%)	51,781,653 (98.09%)
MU_R1_T24	55,265,484	55,148,400 + 57,731 (99.89%)	54,152,830 (97.99%)
MU_R2_T24	76,637,472	76,478,328 + 79,028 (99.90%)	74,648,932 (97.41%)
MU_R3_T24	46,345,420	46,233,860 + 46,869 (99.86%)	45,553,224 (98.29%)
WT_R1_T0	69,956,752	69,785,968 + 84,596 (99.88%)	69,704,110 (99.64%)
WT_R2_T0	71,660,932	71,513,324 + 73,303 (99.90%)	71,414,087 (99.66%)
WT_R3_T0	81,559,422	81,345,084 + 106,158 (99.87%)	81,233,119 (99.60%)
WT_R1_T6	66,395,308	66,233,430 + 80,235 (99.88%)	66,018,839 (99.43%)
WT_R2_T6	58,686,434	58,566,402 + 59,576 (99.90%)	58,382,594 (99.48%)
WT_R3_T6	59,124,336	58,968,310 + 77,283 (99.87%)	58,629,359 (99.16%)
WT_R1_T12	43,472,074	43,366,706 + 52,221 (99.88%)	43,040,447 (99.01%)
WT_R2_T12	57,754,810	57,636,704 + 58,585 (99.90%)	57,444,839 (99.46%)
WT_R3_T12	70,540,880	70,355,544 + 91,804 (99.87%)	70,105,307 (99.38%)
WT_R1_T24	62,563,904	62,413,592 + 74,528 (99.88%)	61,944,386 (99.01%)
WT_R2_T24	60,777,168	60,653,828 + 61,226 (99.90%)	60,258,785 (99.15%)
WT_R3_T24	56,495,252	56,347,994 + 72,934 (99.87%)	56,103,161 (99.31%)

Supplementary Table S2. Fraction of reads mapped on the correct strand over total read mapped on each gene or ERCC RNA (median across genes/RNAs with non-null coverage).

Library	All genes [% reads]	ERCC RNAs [% reads]
MU_R1_T0	92.21	100
MU_R2_T0	91.74	100
MU_R3_T0	91.89	100
MU_R1_T6	91.83	100
MU_R2_T6	90.90	100
MU_R3_T6	91.10	100
MU_R1_T12	92.56	100
MU_R2_T12	92.15	100
MU_R3_T12	92.20	100
MU_R1_T24	92.74	100
MU_R2_T24	91.96	100
MU_R3_T24	93.25	100
WT_R1_T0	91.75	100
WT_R2_T0	93.18	100
WT_R3_T0	91.86	100
WT_R1_T6	90.57	100
WT_R2_T6	92.10	100
WT_R3_T6	91.36	100
WT_R1_T12	91.76	100
WT_R2_T12	93.42	100
WT_R3_T12	93.28	100
WT_R1_T24	91.46	100
WT_R2_T24	92.49	100
WT_R3_T24	93.79	100

Supplementary Table S3. Number of ERCC RNAs with constant concentration across all libraries (n=23) that have been wrongly selected as differentially expressed by edgeR (applied to maxcounts) and DETECTOR (applied to maxcounts or totcounts).

Comparison	edgeR maxcounts	DETECTOR maxcounts	DETECTOR totcounts
WT vs. T0	9	0	2
MU vs. T0	0	0	0
**Total**	**9**	**0**	**2**

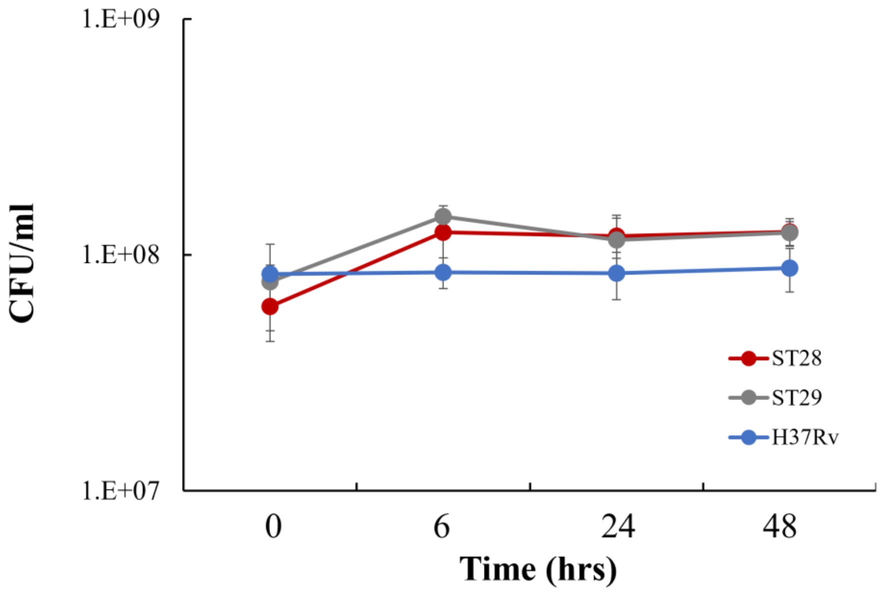

Supplementary Figure S1. Viable count of wt (H37Rv), *sigE* mutant (ST28) and *sigE* complemented strain (ST29) in low phosphate condition. Cultures were grown in modified 7H9 without phosphate supplementation. The samples were collected after 6, 24 and 48 hours and plated in 7H10 medium. T0 represents the sample collected before the transferring of cells in 7H9 without phosphate. The curves represent the average of two biological replicates and two technical replicates. The error bars represent the standard deviation.

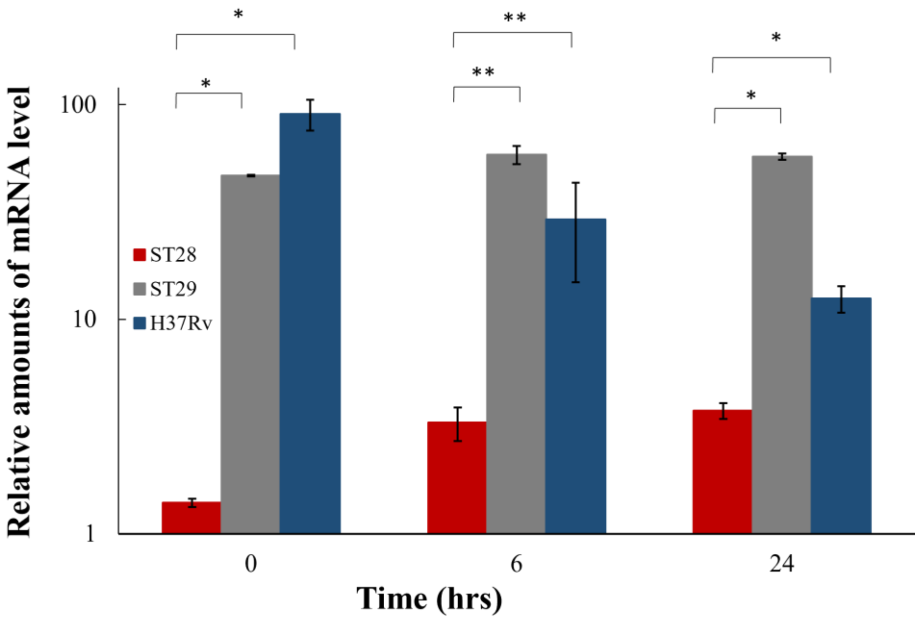

Supplementary Figure S2. Relative amounts of *sigB* mRNA level in wt (H37Rv), *sigE* mutant (ST28) and *sigE* complemented strain (ST29) grown without phosphate supplementation. Data were normalized to the level of *sigA* cDNA that represented the internal invariant control. The reported values derive from at least two independent experiments and two technical replicates. The error bars represent the standard deviation. *$P < 0.05$ **$P < 0.005$ ST28 versus ST29 (Student's *t*-test).

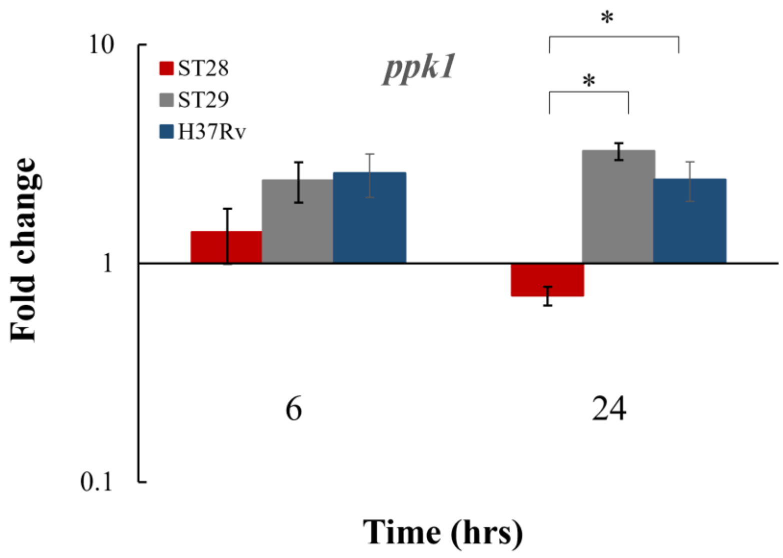

Supplementary Figure S3. Fold change of *ppk1* mRNA in wt (H37Rv), *sigE* mutant (ST28) and *sigE* complemented strain (ST29) grown without phosphate supplementation. Data were normalized to the level of *sigA* cDNA that represented the internal invariant control. Fold change values are calculated versus time '0'. The reported values derive from at least two independent experiments and two technical replicates. The error bars represent the standard. *P < 0.05 **P < 0.005 ST28 versus ST29 (Student's *t*-test).

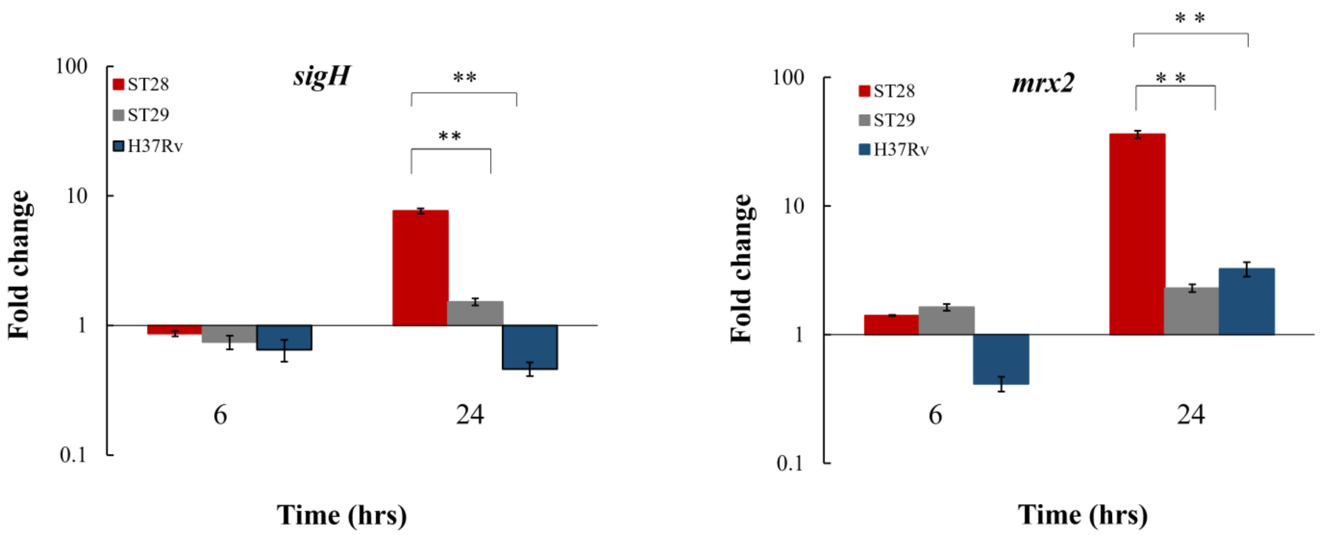

Supplementary Figure S4. Fold-changes of *sigH* and *mrx2* mRNA level in wt (H37Rv), *sigE* mutant (ST28) and *sigE* complemented strain (ST29) grown without phosphate supplementation. Fold change values are calculated versus time '0'. The reported values derive from at least two independent experiments and two technical replicates. The error bars represent the standard deviation. *P < 0.05 **P < 0.005 ST28 versus ST29 (Student's *t*-test).

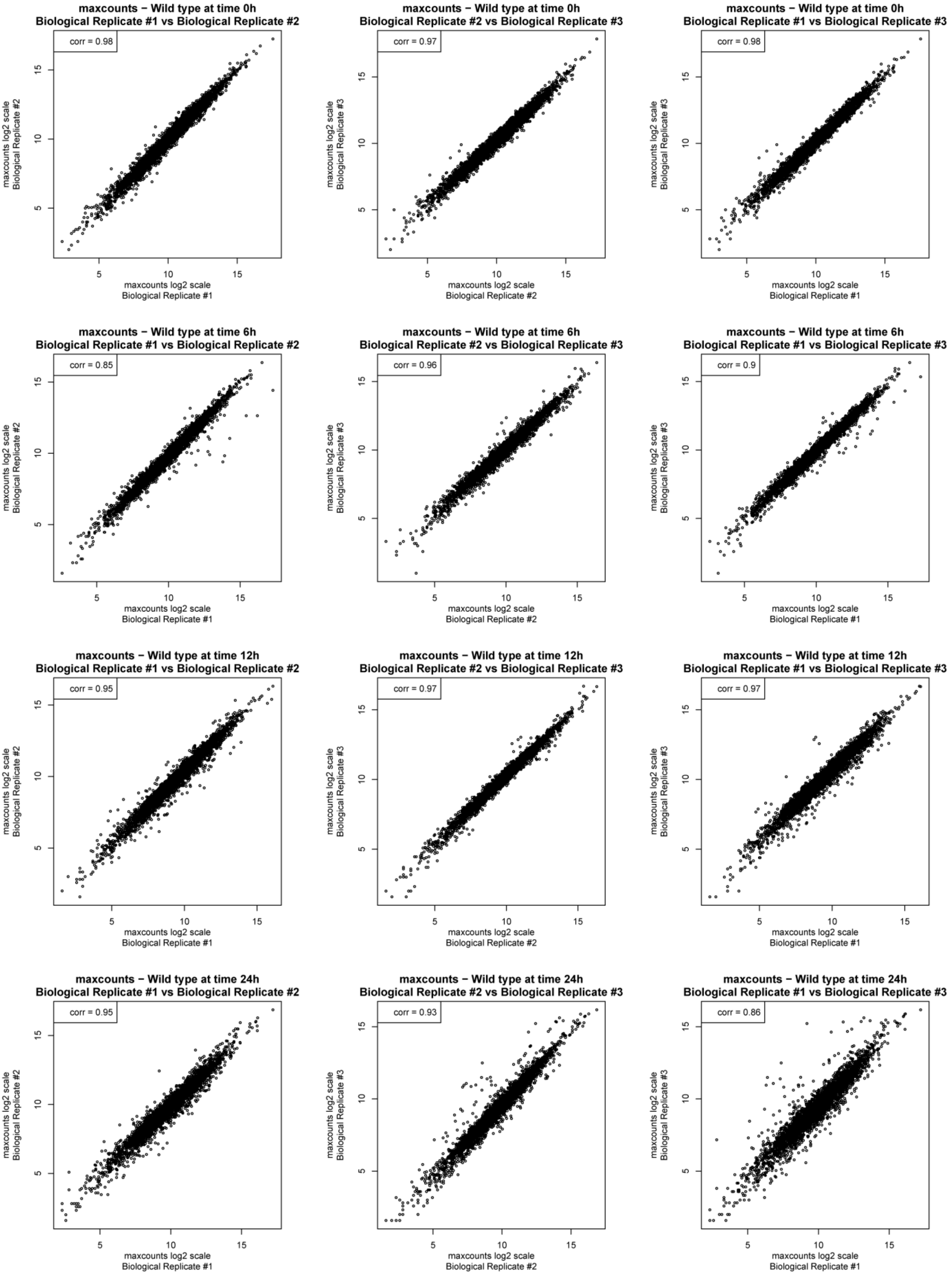

Supplementary Figure S5. Gene expression levels across biological replicates (WT) using the maxcounts quantification strategy. For each plot, the Pearson correlation between the expression levels of the two replicates is shown in the top left corner. Gene expression levels (plus a pseudo-count of 1) are shown in log2 scale.

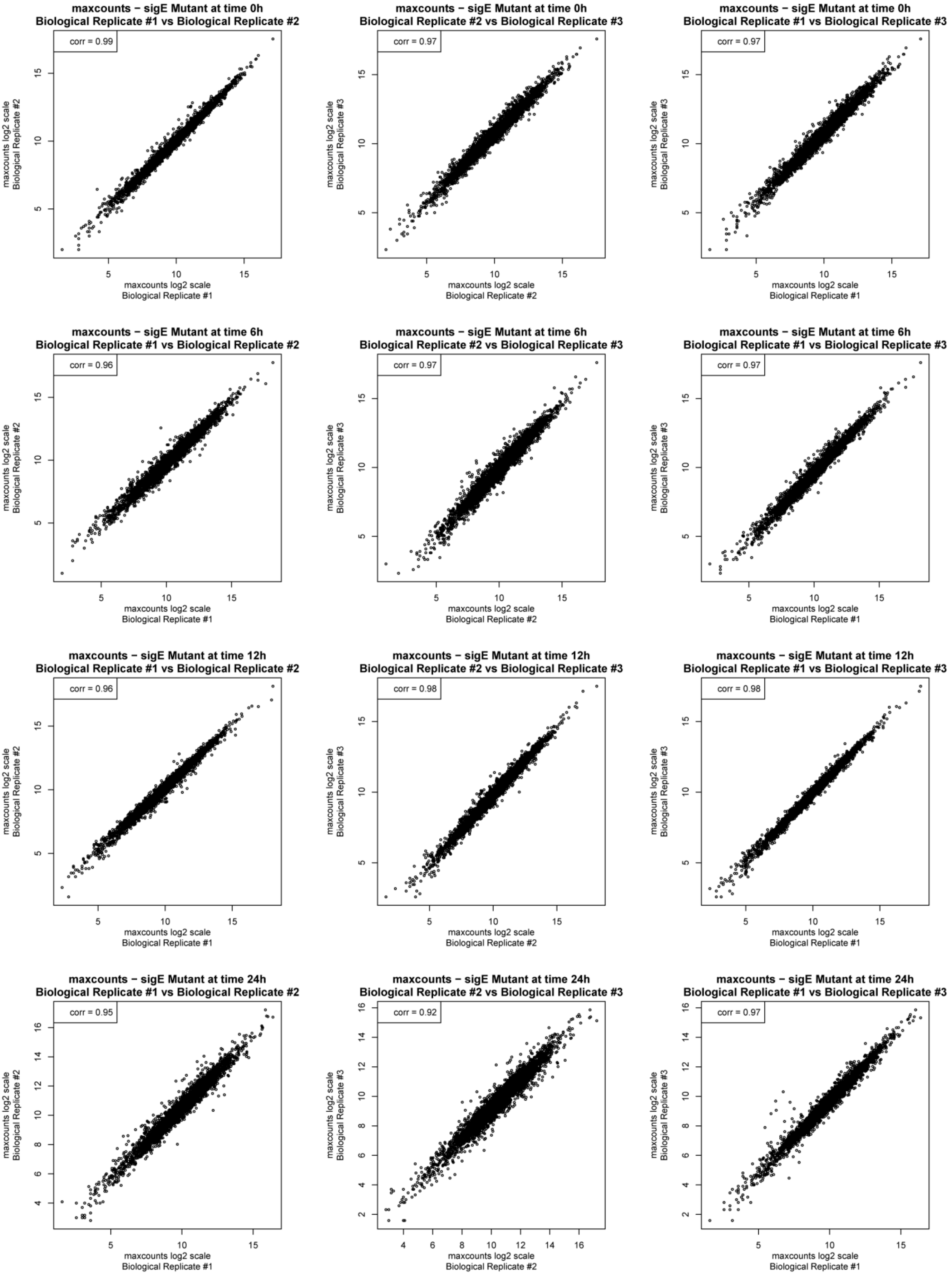

Supplementary Figure S6. Gene expression levels across biological replicates (WT) using the maxcounts quantification strategy. For each plot, the Pearson correlation between the expression levels of the two replicates is shown in the top left corner. Gene expression levels (plus a pseudo-count of 1) are shown in log2 scale.

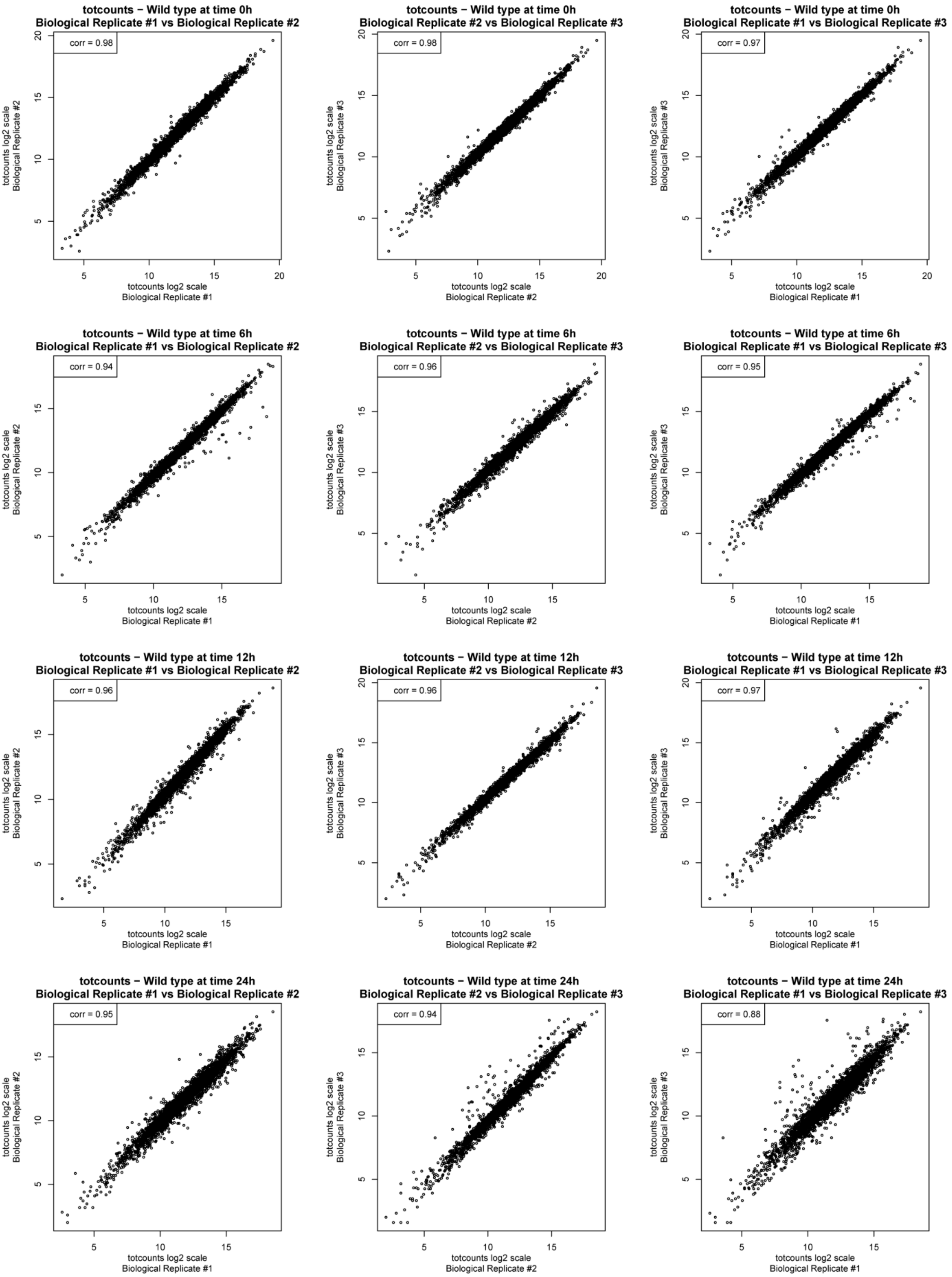

Supplementary Figure S7. Gene expression levels across biological replicates (MU) using the totcounts quantification strategy. For each plot, the Pearson correlation between the expression levels of the two replicates is shown in the top left corner. Gene expression levels (plus a pseudo-count of 1) are shown in log2 scale.

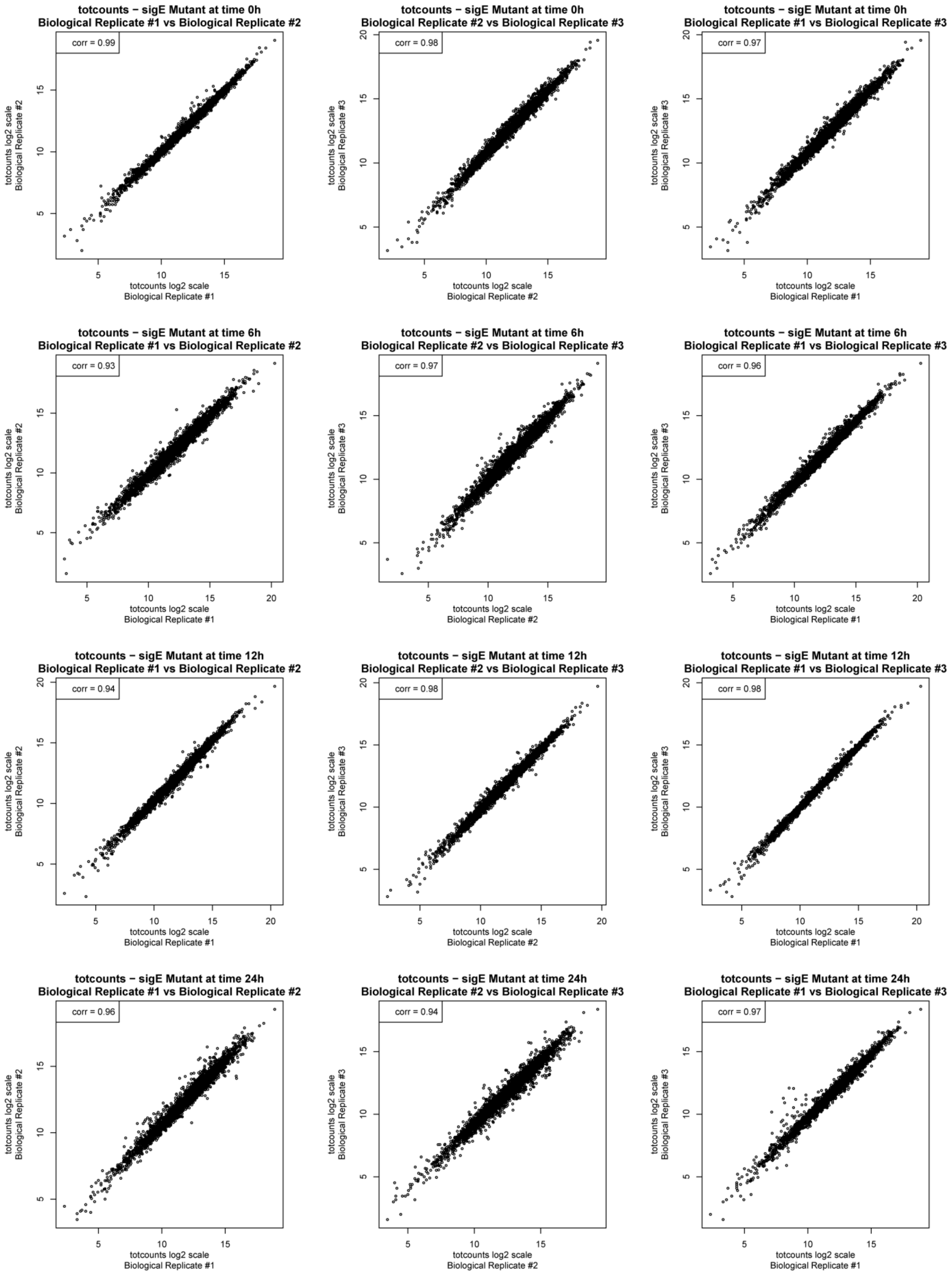

Supplementary Figure S8. Gene expression levels across biological replicates (MU) using the totcounts quantification strategy. For each plot, the Pearson correlation between the expression levels of the two replicates is shown in the top left corner. Gene expression levels (plus a pseudo-count of 1) are shown in log2 scale.

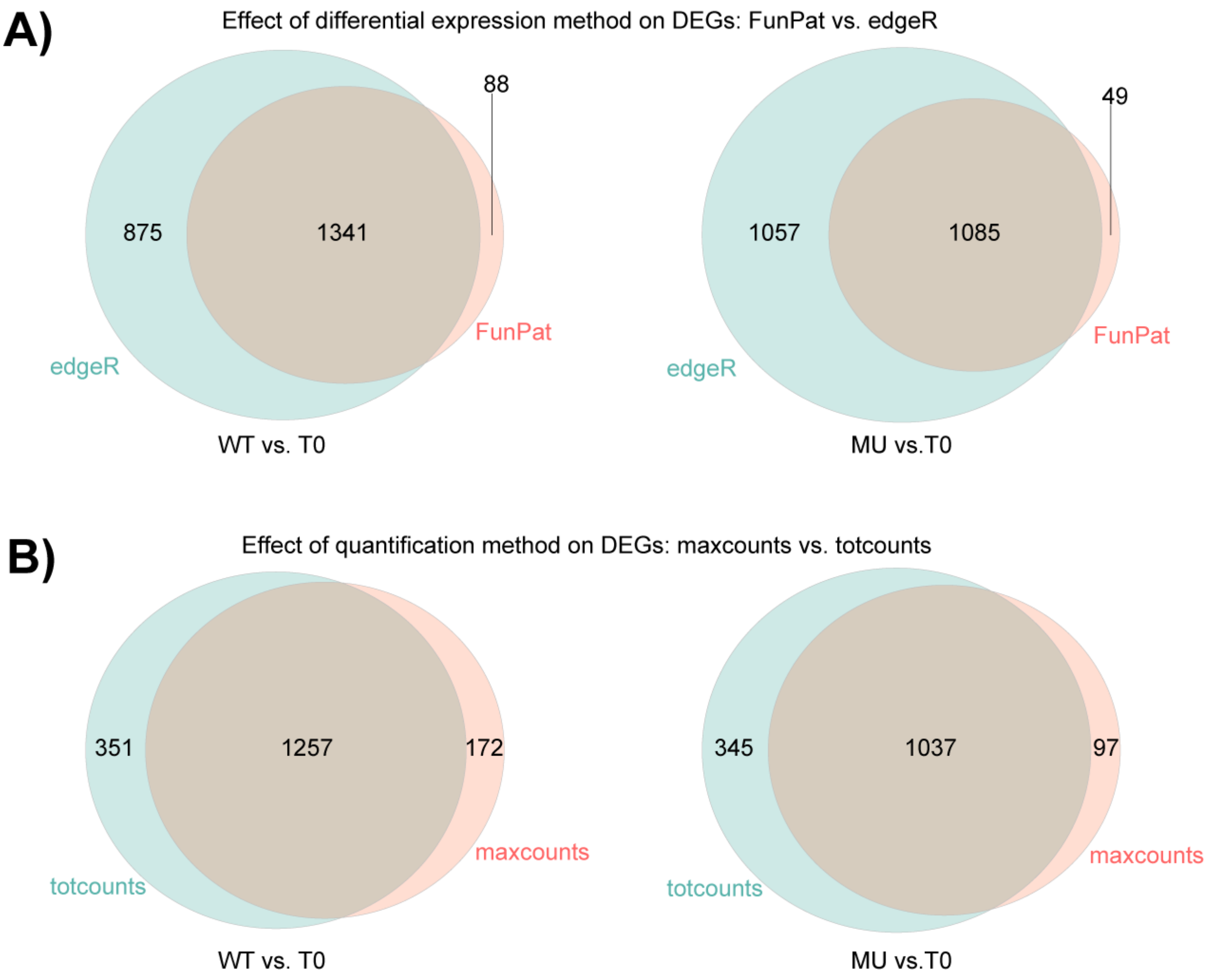

Supplementary Figure S9. Effects of differential expression methods and quantification methods on the identification of differentially expressed genes. A) Effect of different differential expression analysis tools (i.e. FunPat and edgeR) on the identification of differentially expressed genes. The Venn diagrams show the agreement between the two methods. B) Effect of different gene expression quantification tools (i.e. totcounts and maxcounts) on the identification of differentially expressed genes. The Venn diagram show the agreement between the two methods.

Lane 1	Lane 2	Lane 3	Lane 4	Lane 5	Lane 6
WT_T0_R1	WT_T0_R2	WT_T0_R3	MU_T0_R1	MU_T0_R2	MU_T0_R3
WT_T6_R1	WT_T6_R2	WT_T6_R3	MU_T6_R1	MU_T6_R2	MU_T6_R3
WT_T12_R1	WT_T12_R2	WT_T12_R3	MU_T12_R1	MU_T12_R2	MU_T12_R3
WT_T24_R1	WT_T24_R2	WT_T24_R3	MU_T24_R1	MU_T24_R2	MU_T24_R3

Spike-in Mix 1

Spike-in Mix 2

Supplementary Figure S10. Distribution of samples and ERCC Spike-In control mixes across sequencing lanes.
