# Supplementary material for: Role of the Extracytoplasmic Function Sigma Factor SigE in the Stringent Response of Mycobacterium tuberculosis
Source: Microbiol Spectr. 2023 Mar 22;11(2):e02944-22. doi: 10.1128/spectrum.02944-22 (PMC10100808; doi:10.1128/spectrum.02944-22)
Supplement: Supplemental file 2 — Data S2. Download spectrum.02944-22-s0003.pdf, PDF file, 0.5 MB [file spectrum.02944-22-s0003.pdf]

<u>Supplementary Data S2:</u> Gene expression profiles over time of genes *rpoA* and *rpoB*, and genes discussed in section "SigE network and SenX3-RegX3 regulon in H37Rv", i.e. *sigE*, *hsp*, *htpX*, *sigB*, *rv2743*, *pspA*, *clgR*, *rv2052c*, *rv2053c*, *rv1072*, *rv1073*, *pks2*, *rv1043c*, *clpP1-clpP2*, *SenX3-RegX3*, *pstS3-pstC2-pstA1*, *pstB-pstS1-pstC1-pstA2*, *pknD-pstS2*, *ppk1* and *relA*. For each gene, the plot shows the average expression level and the standard deviation (shaded area) for both the wild-type (cyan color) and *sigE*-mutant (salmon/pink color) strains.

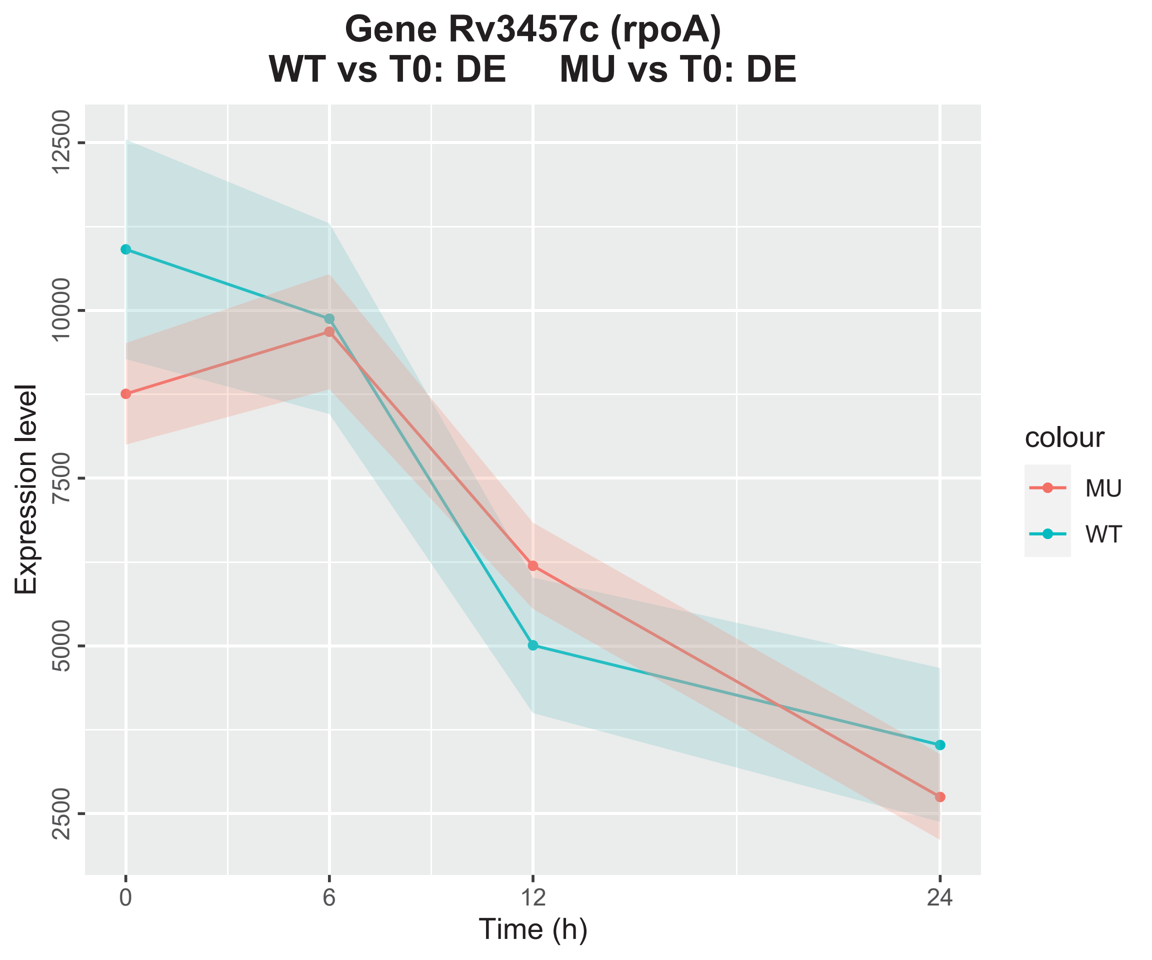

Gene Rv3457c (rpoA)
WT vs T0: DE   MU vs T0: DE
Expression level
12500
10000
7500
5000
2500
0
6
12
24
Time (h)
colour
MU
WT

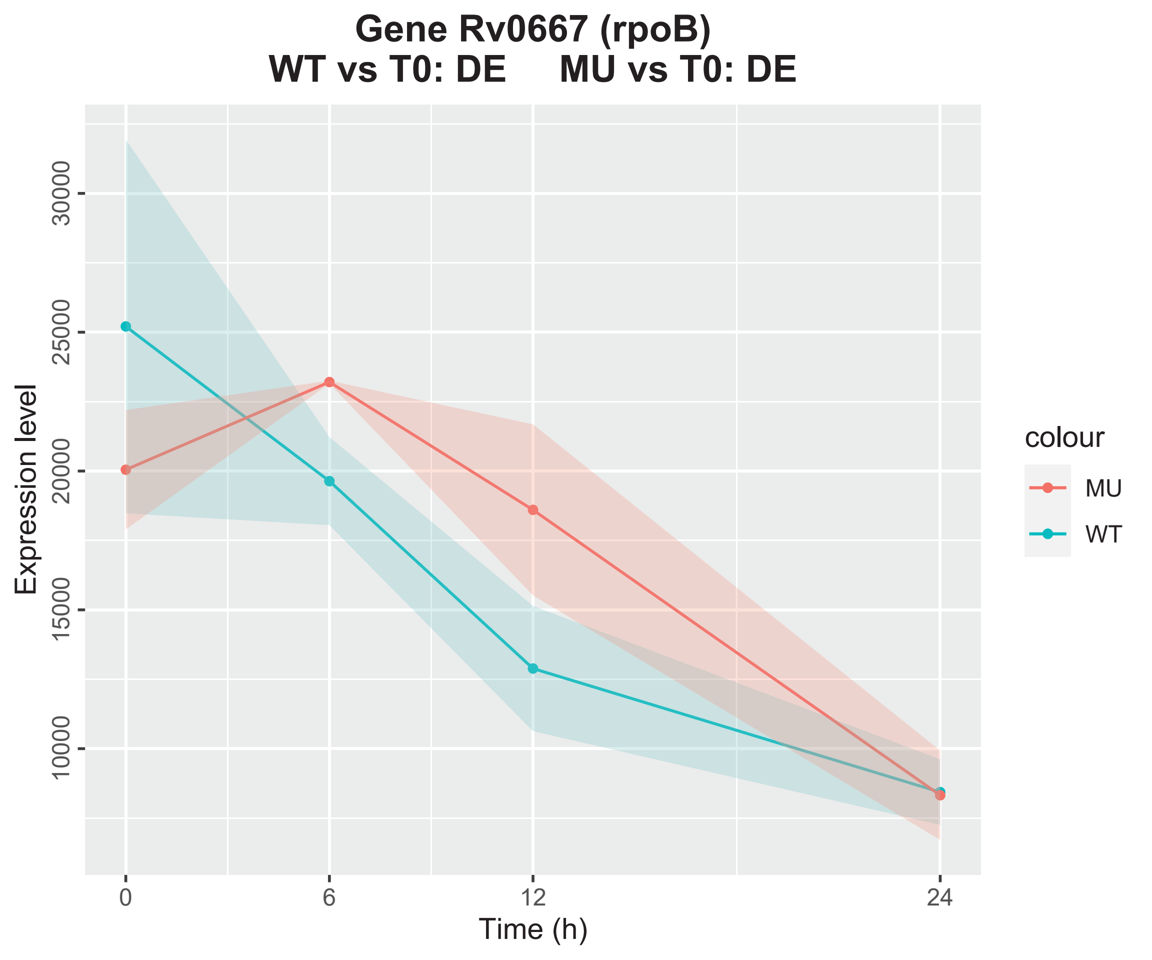
Gene Rv0667 (rpoB)
WT vs T0: DE   MU vs T0: DE
Expression level
30000
25000
20000
15000
10000
0
6
12
24
Time (h)
colour
MU
WT

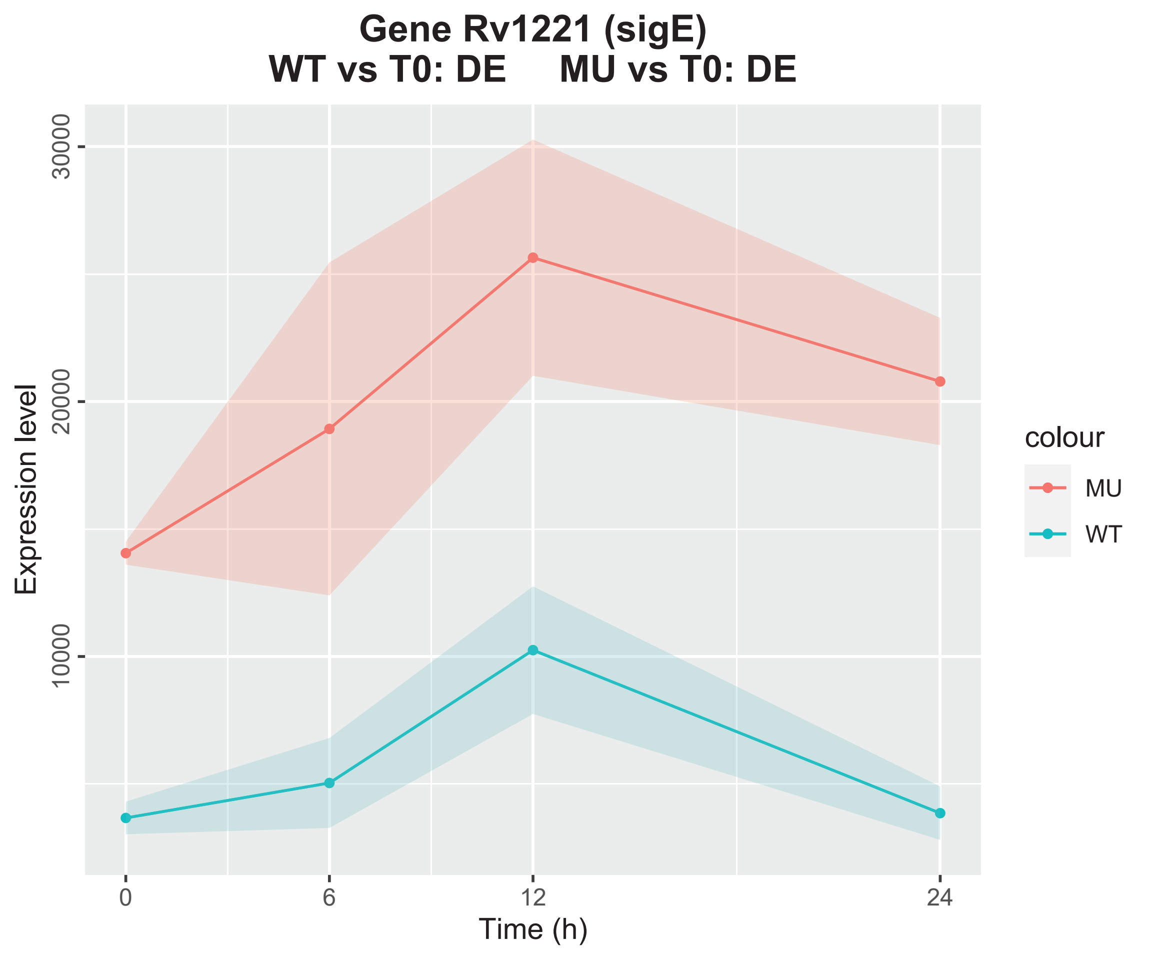
Gene Rv1221 (sigE)
WT vs T0: DE   MU vs T0: DE
Expression level
30000
20000
10000
0
6
12
24
Time (h)
colour
MU
WT

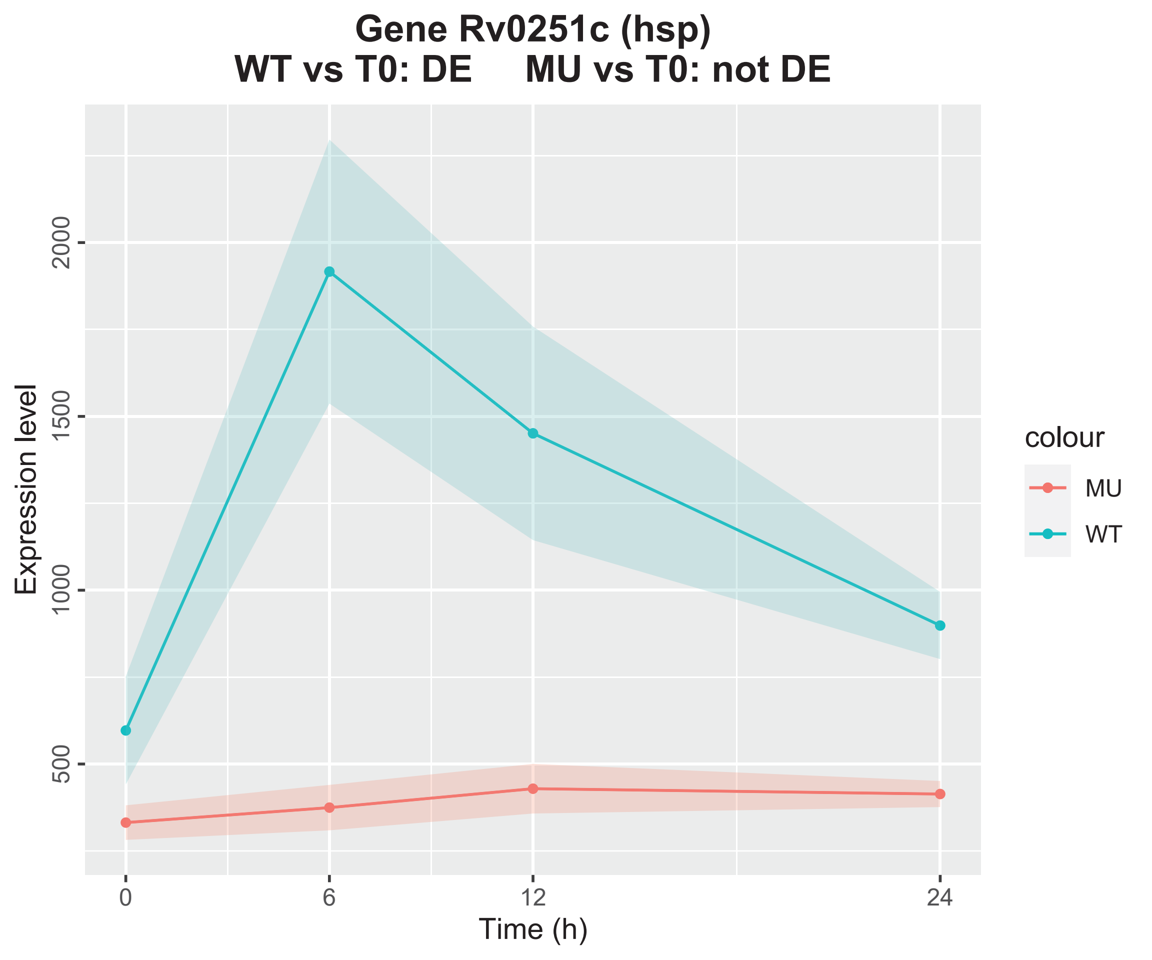

Gene Rv0251c (hsp)
WT vs T0: DE   MU vs T0: not DE
Expression level
2000
1500
1000
500
0
6
12
24
Time (h)
colour
MU
WT

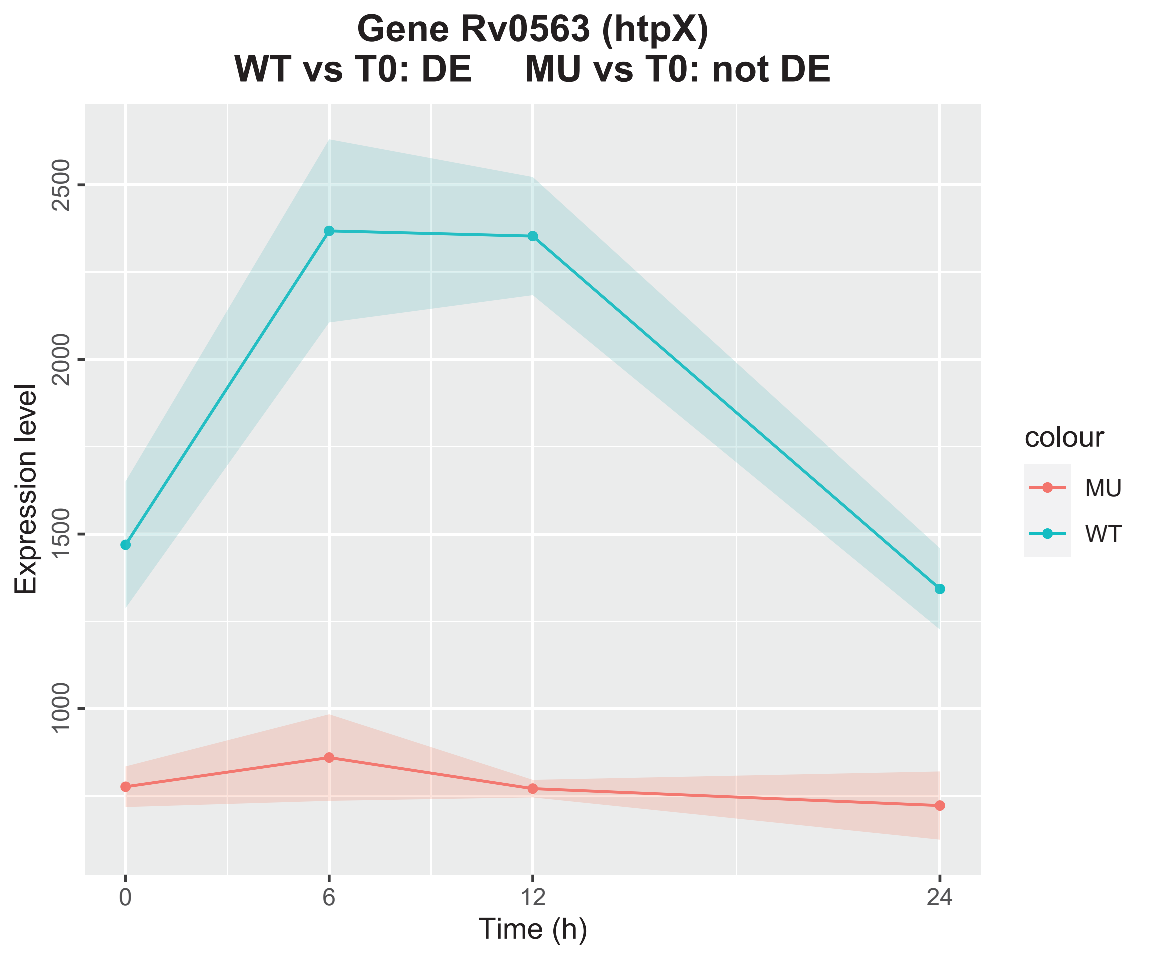
Gene Rv0563 (htpX)
WT vs T0: DE   MU vs T0: not DE
Expression level
2500
2000
1500
1000
0
6
12
24
Time (h)
colour
MU
WT

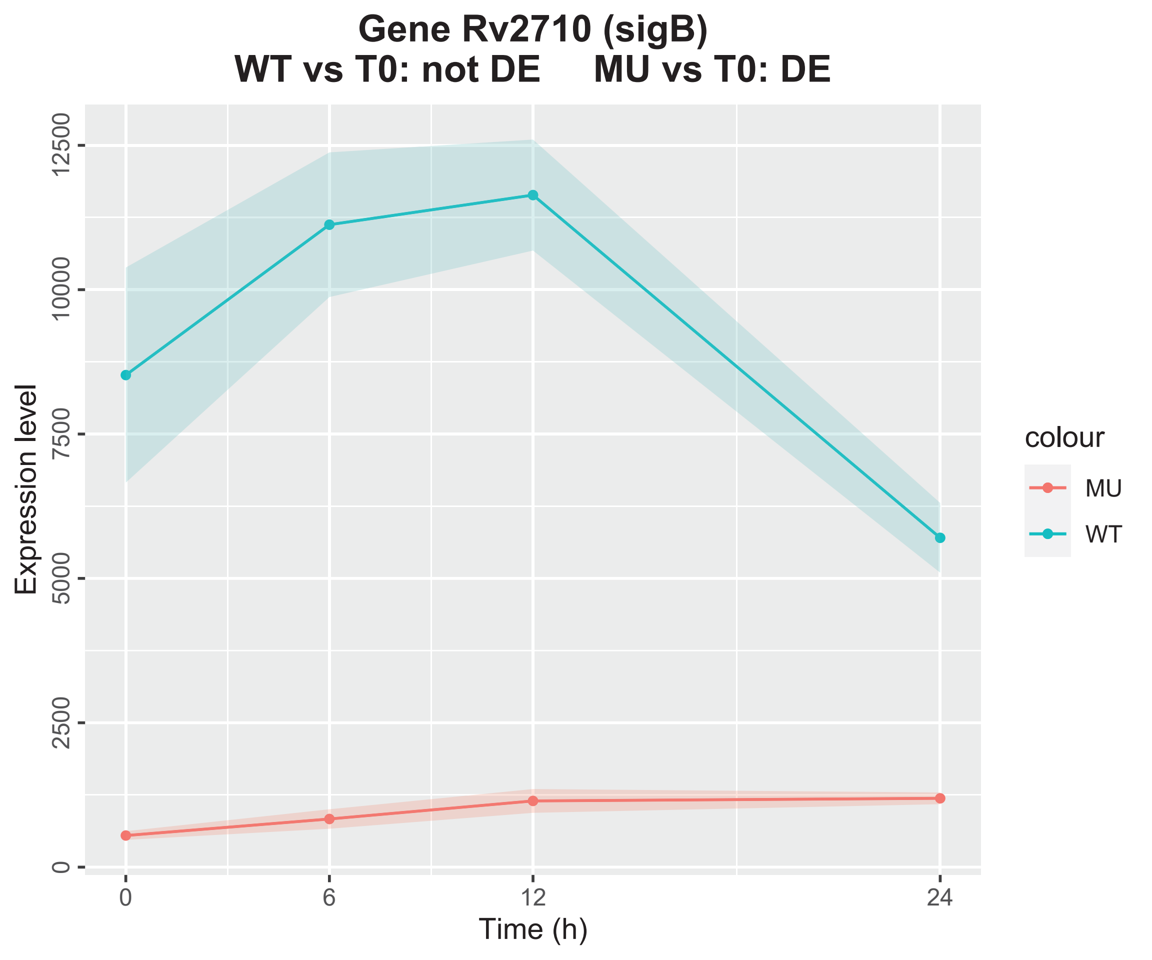
Gene Rv2710 (sigB)
WT vs T0: not DE MU vs T0: DE
Expression level
12500
10000
7500
5000
2500
0
0
6
12
24
Time (h)
colour
MU
WT

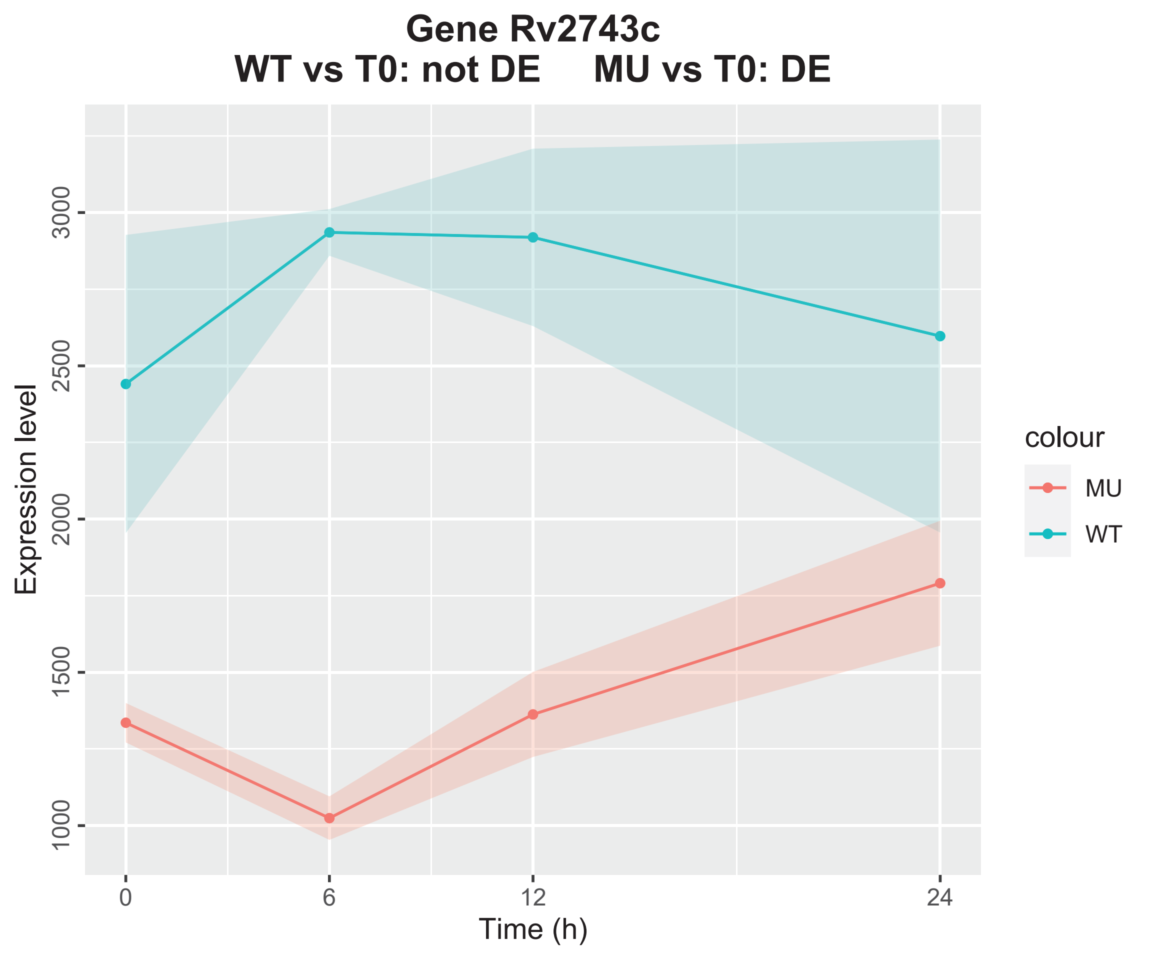

Gene Rv2743c
WT vs T0: not DE MU vs T0: DE
Expression level
3000
2500
2000
1500
1000
0
6
12
24
Time (h)
colour
MU
WT

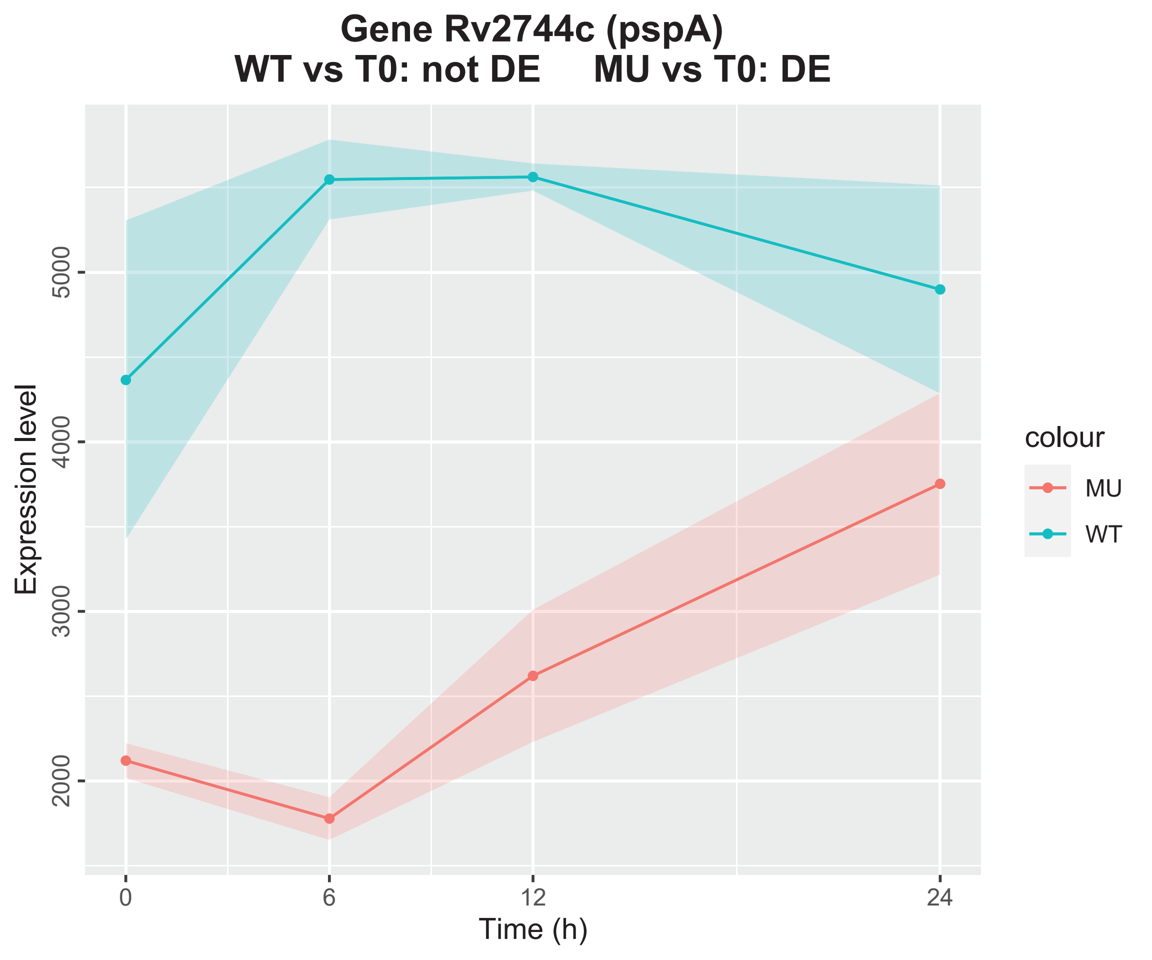

Gene Rv2744c (pspA)
WT vs T0: not DE MU vs T0: DE
Expression level
5000
4000
3000
2000
0
6
12
24
Time (h)
colour
MU
WT

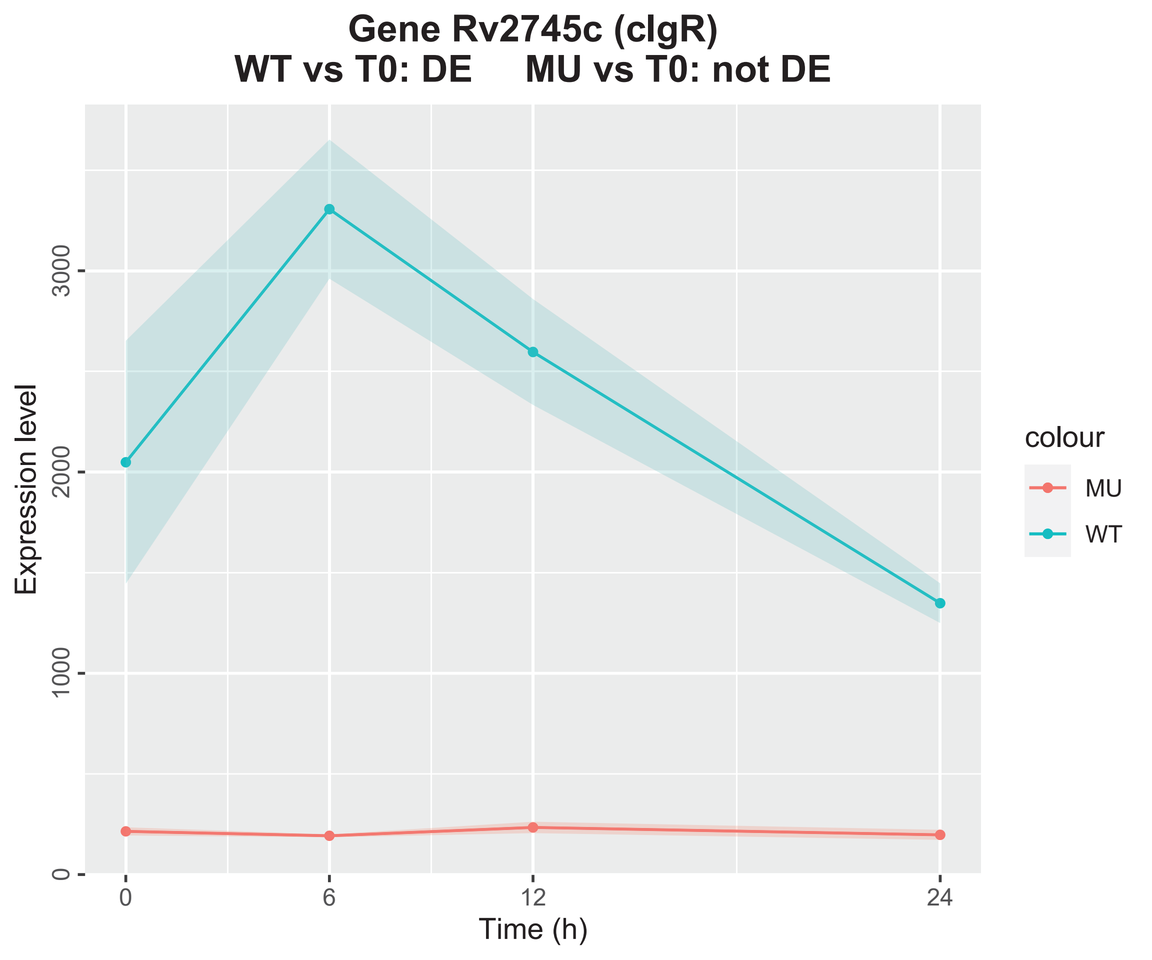
Gene Rv2745c (clgR)
WT vs T0: DE   MU vs T0: not DE
Expression level
3000
2000
1000
0
0
6
12
24
Time (h)
colour
MU
WT

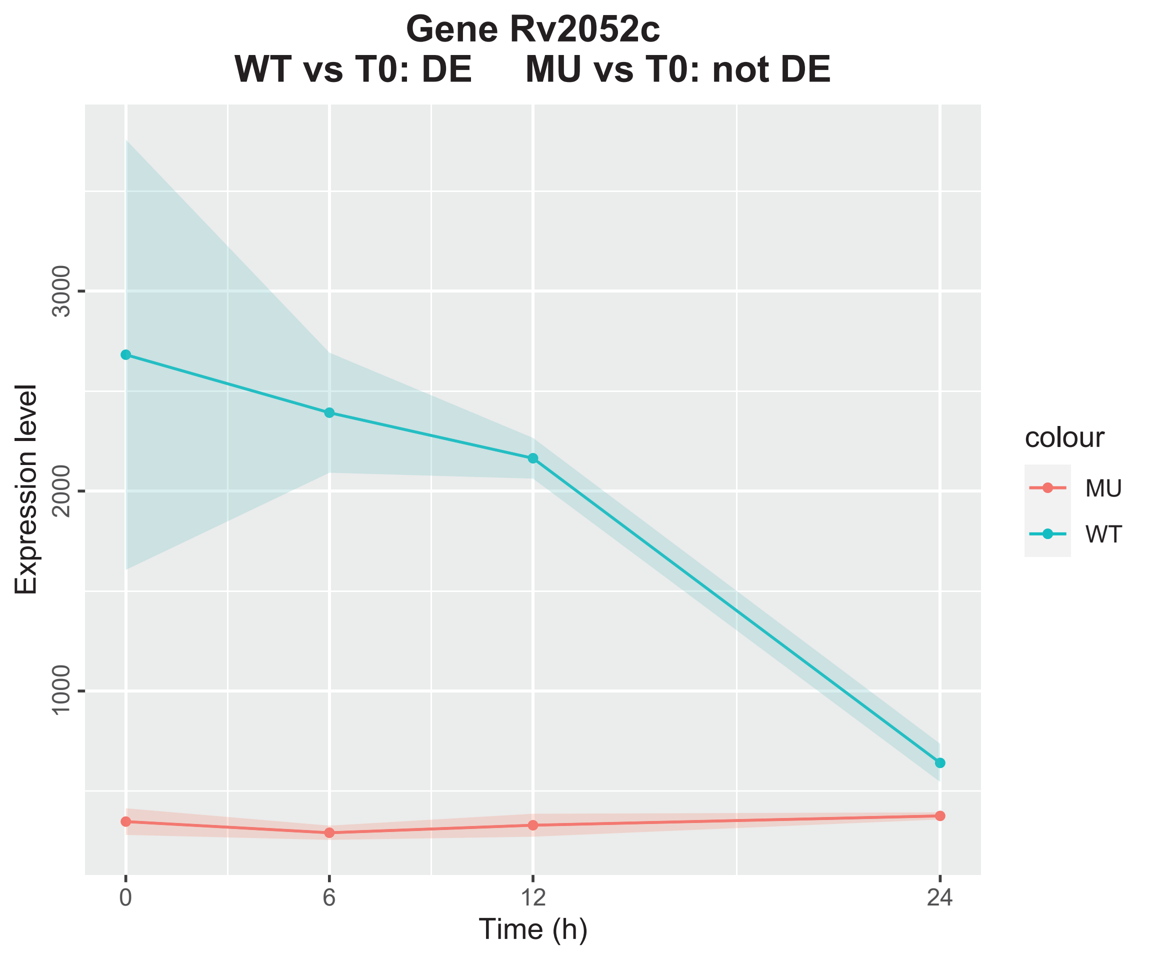
Gene Rv2052c
WT vs T0: DE   MU vs T0: not DE
Expression level
3000
2000
1000
0
6
12
24
Time (h)
colour
MU
WT

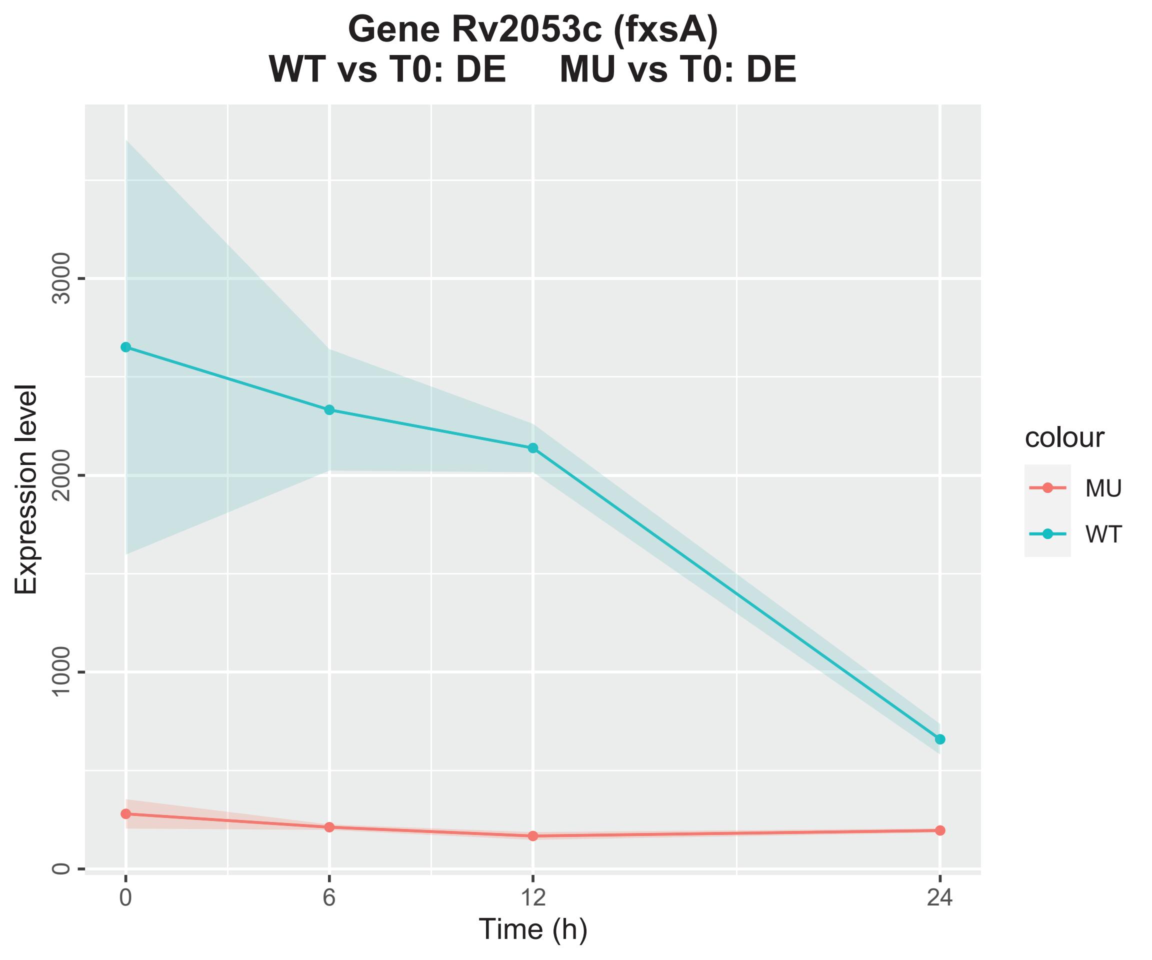
Gene Rv2053c (fxsA)
WT vs T0: DE MU vs T0: DE
Expression level
3000
2000
1000
0
0
6
12
24
Time (h)
colour
MU
WT

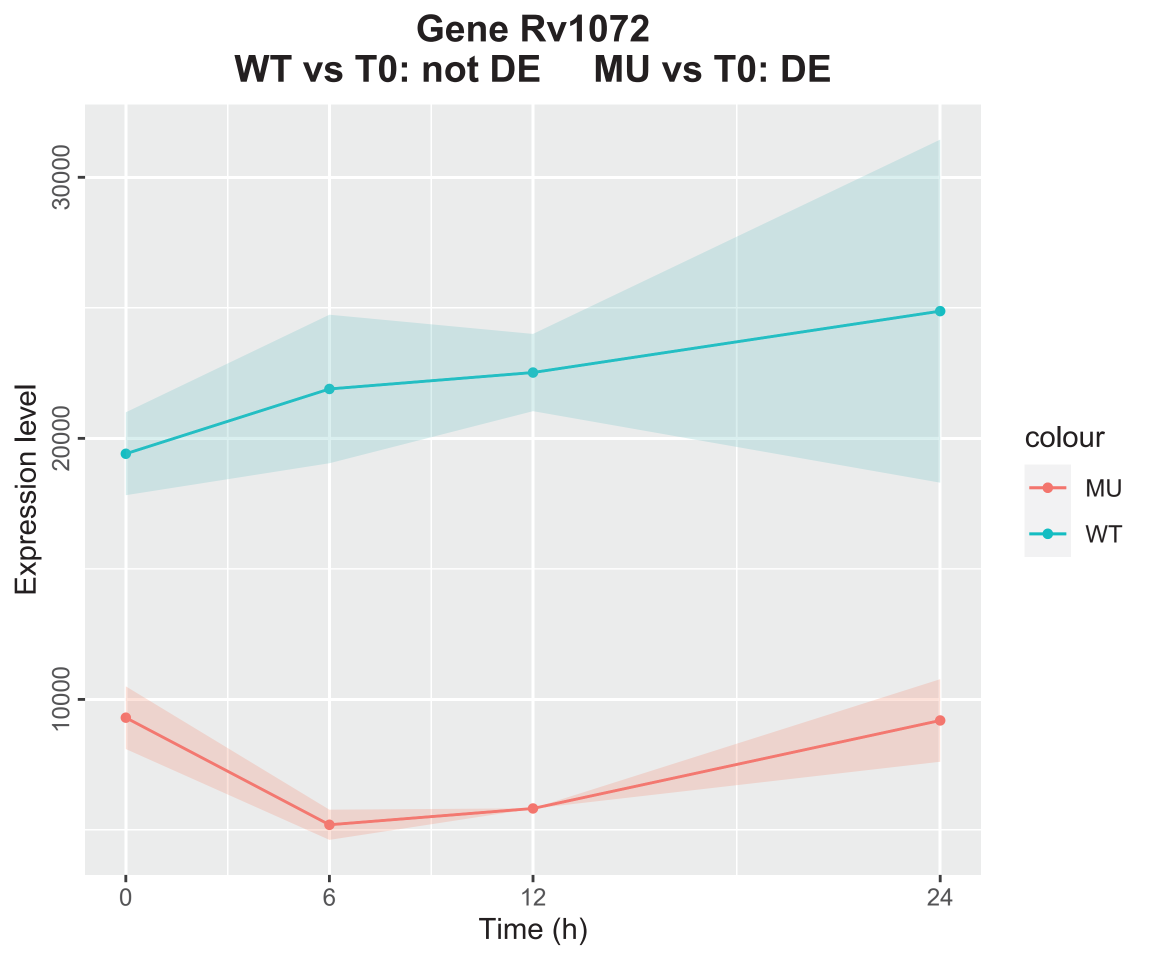
Gene Rv1072
WT vs T0: not DE MU vs T0: DE
30000
20000
10000
Expression level
0
6
12
24
Time (h)
colour
MU
WT

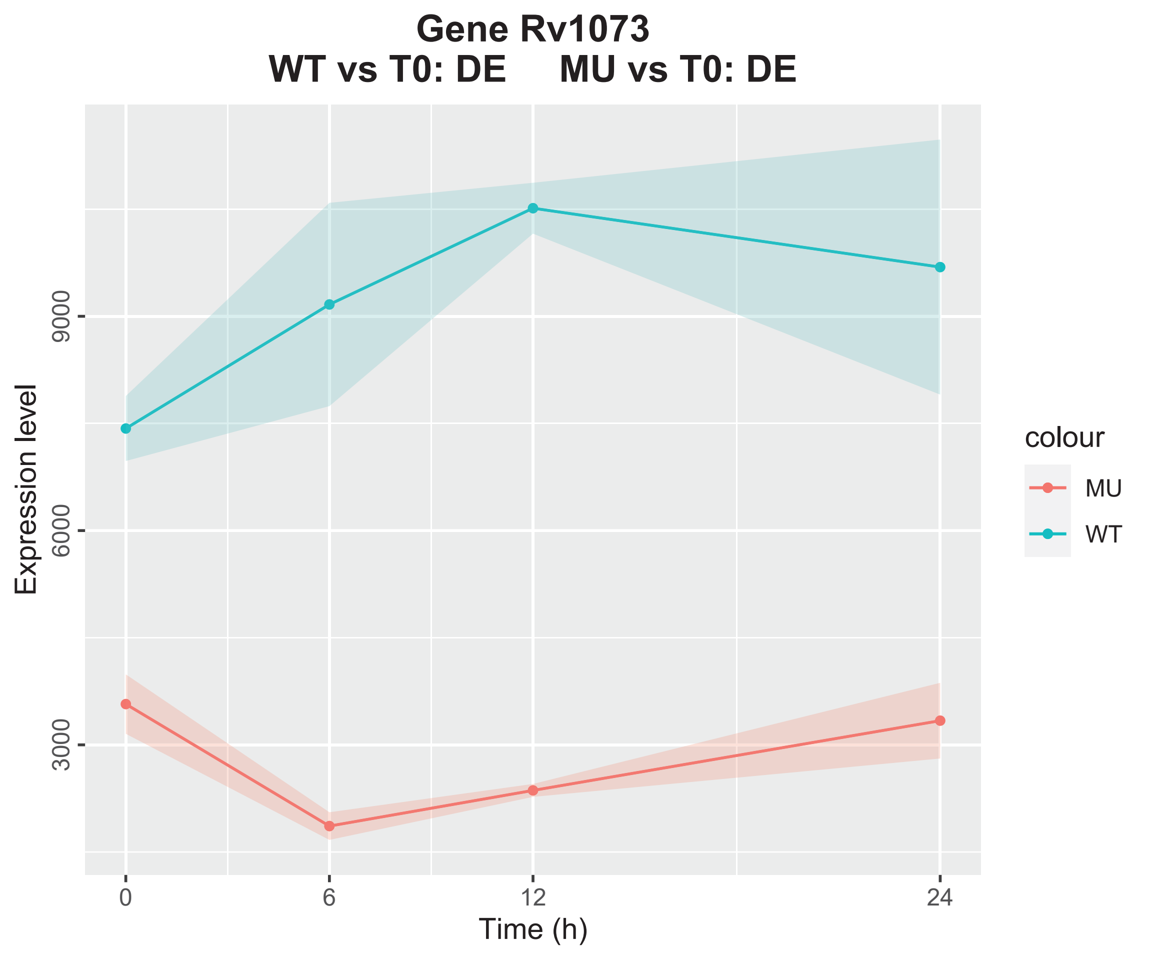

Gene Rv1073
WT vs T0: DE MU vs T0: DE
Expression level
9000
6000
3000
0
6
12
24
Time (h)
colour
MU
WT

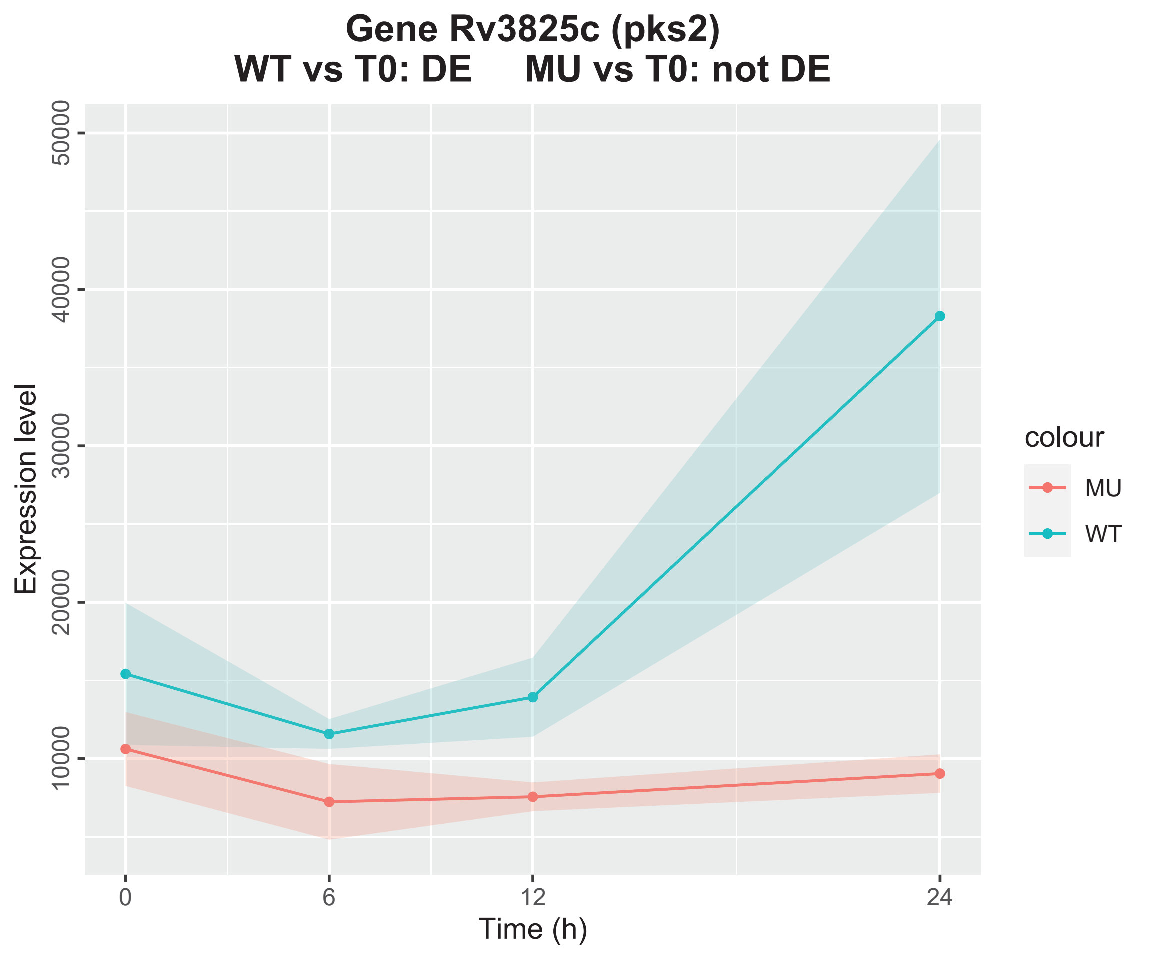
Gene Rv3825c (pks2)
WT vs T0: DE MU vs T0: not DE
Expression level
50000
40000
30000
20000
10000
0
6
12
24
Time (h)
colour
MU
WT

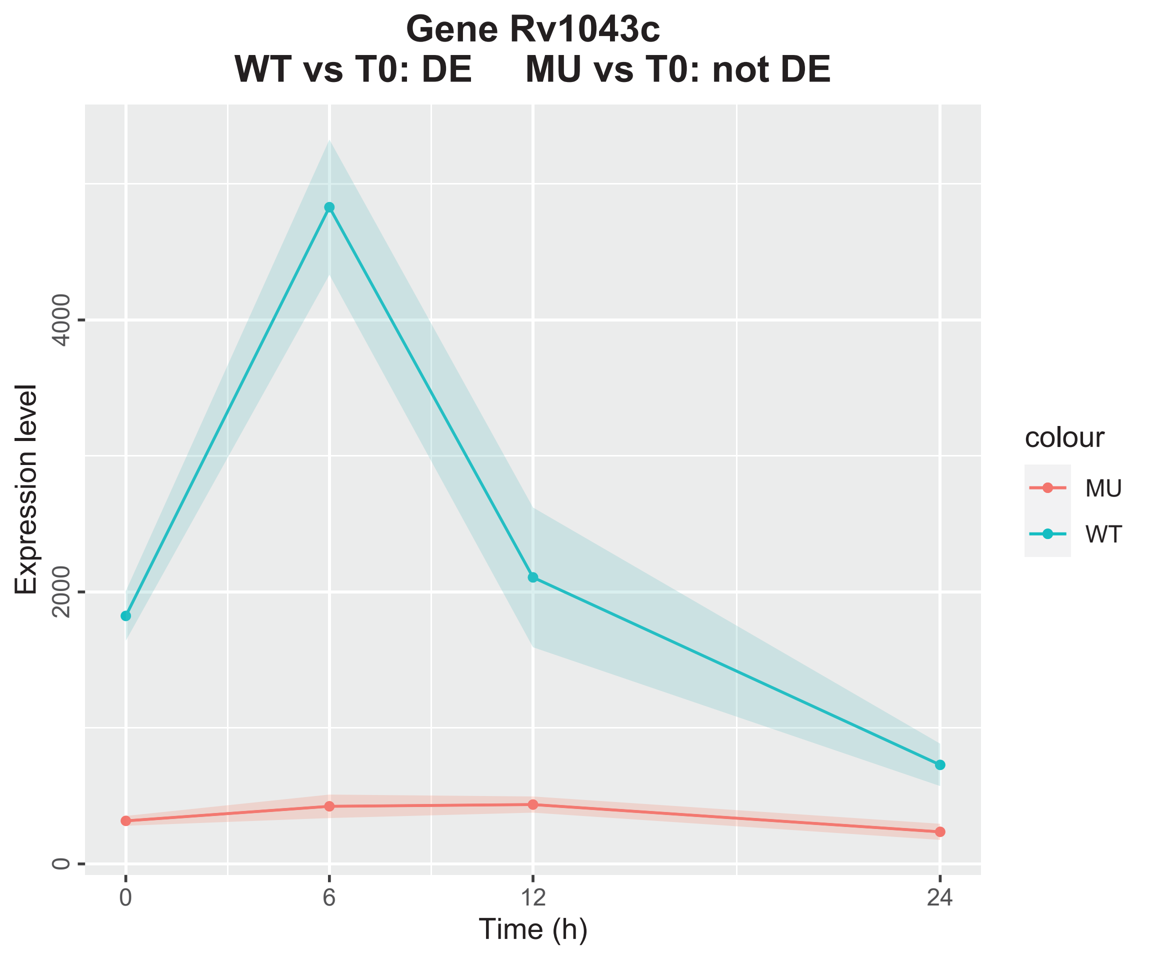
Gene Rv1043c
WT vs T0: DE MU vs T0: not DE
Expression level
0
2000
4000
Time (h)
0
6
12
24
colour
MU
WT

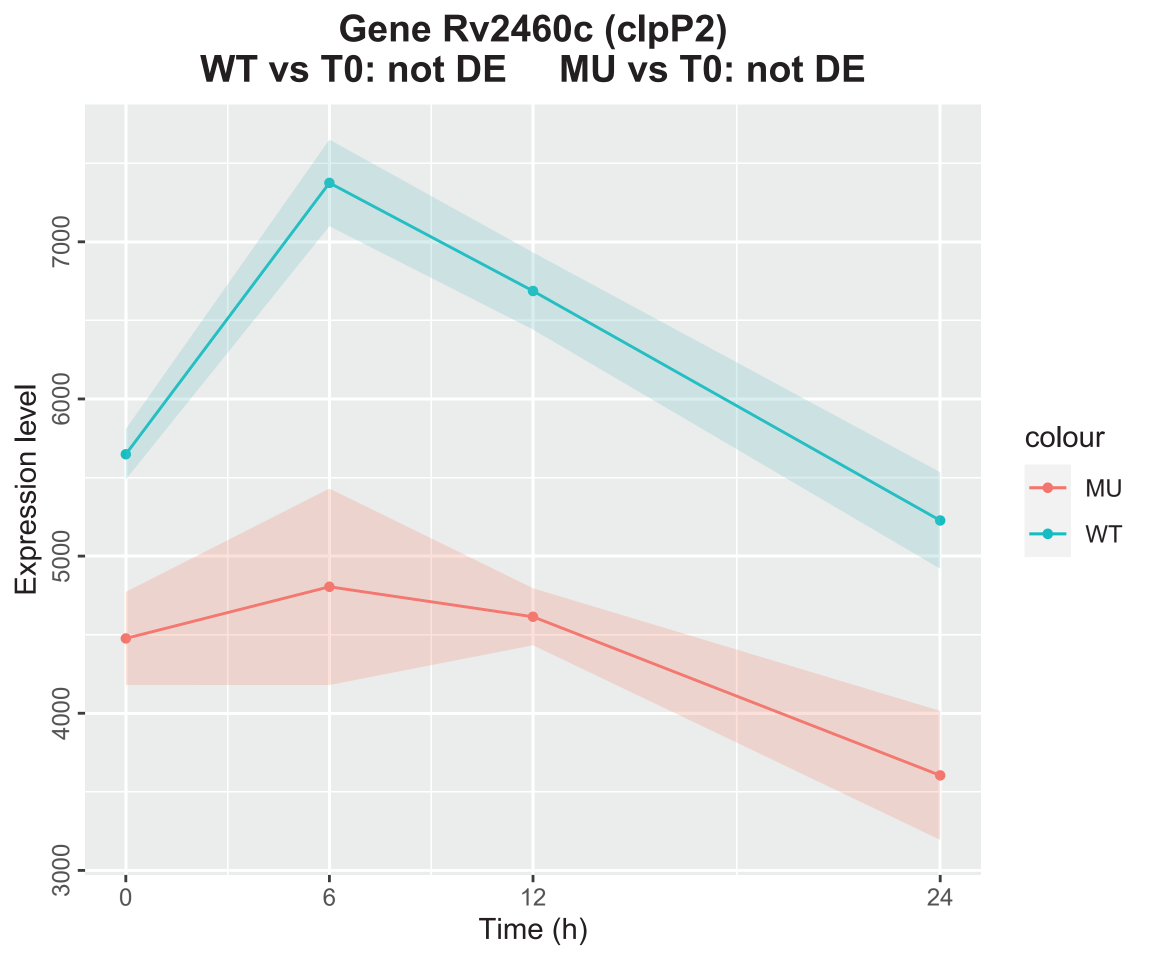

Gene Rv2460c (clpP2)
WT vs T0: not DE   MU vs T0: not DE
Expression level
7000
6000
5000
4000
3000
0
6
12
24
Time (h)
colour
MU
WT

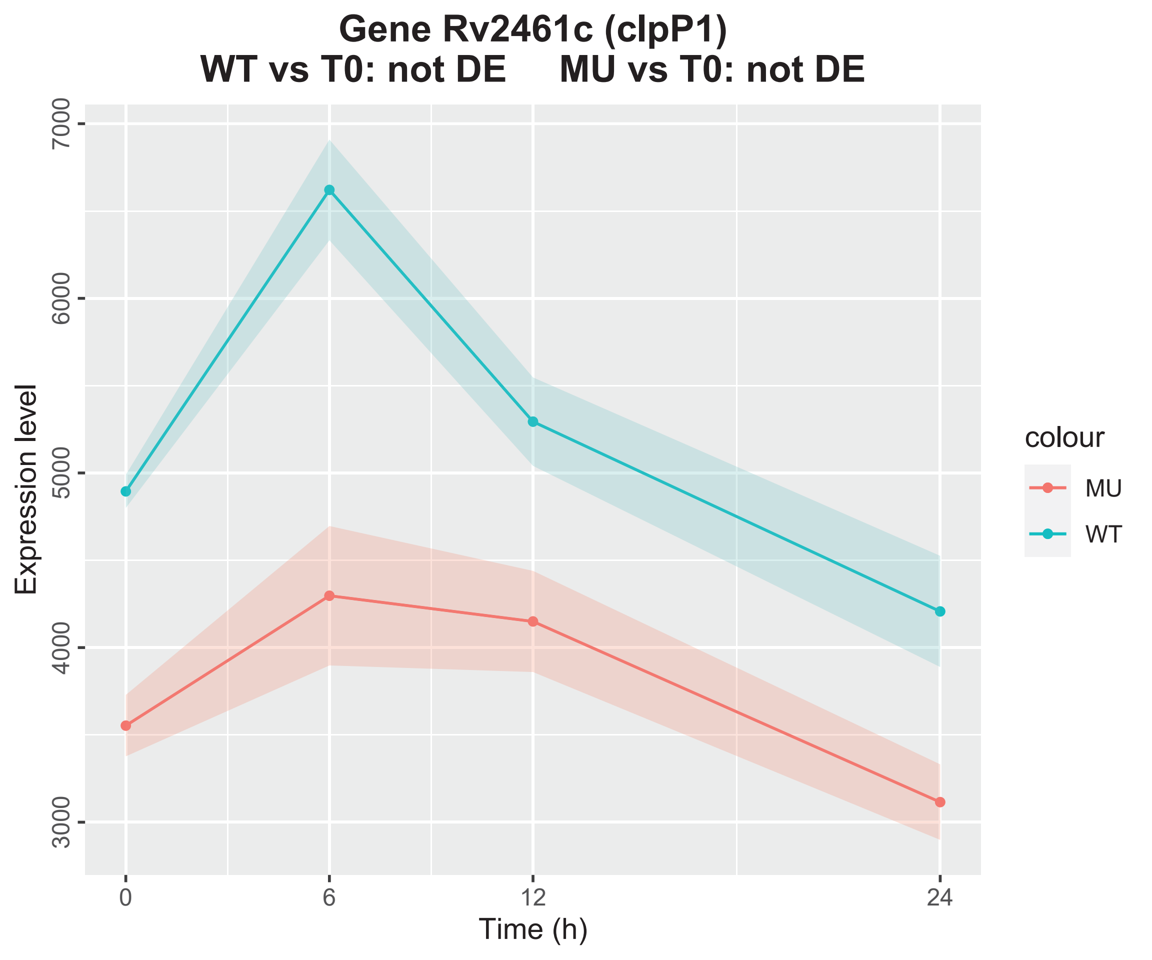
Gene Rv2461c (clpP1)
WT vs T0: not DE   MU vs T0: not DE
Expression level
7000
6000
5000
4000
3000
0
6
12
24
Time (h)
colour
MU
WT

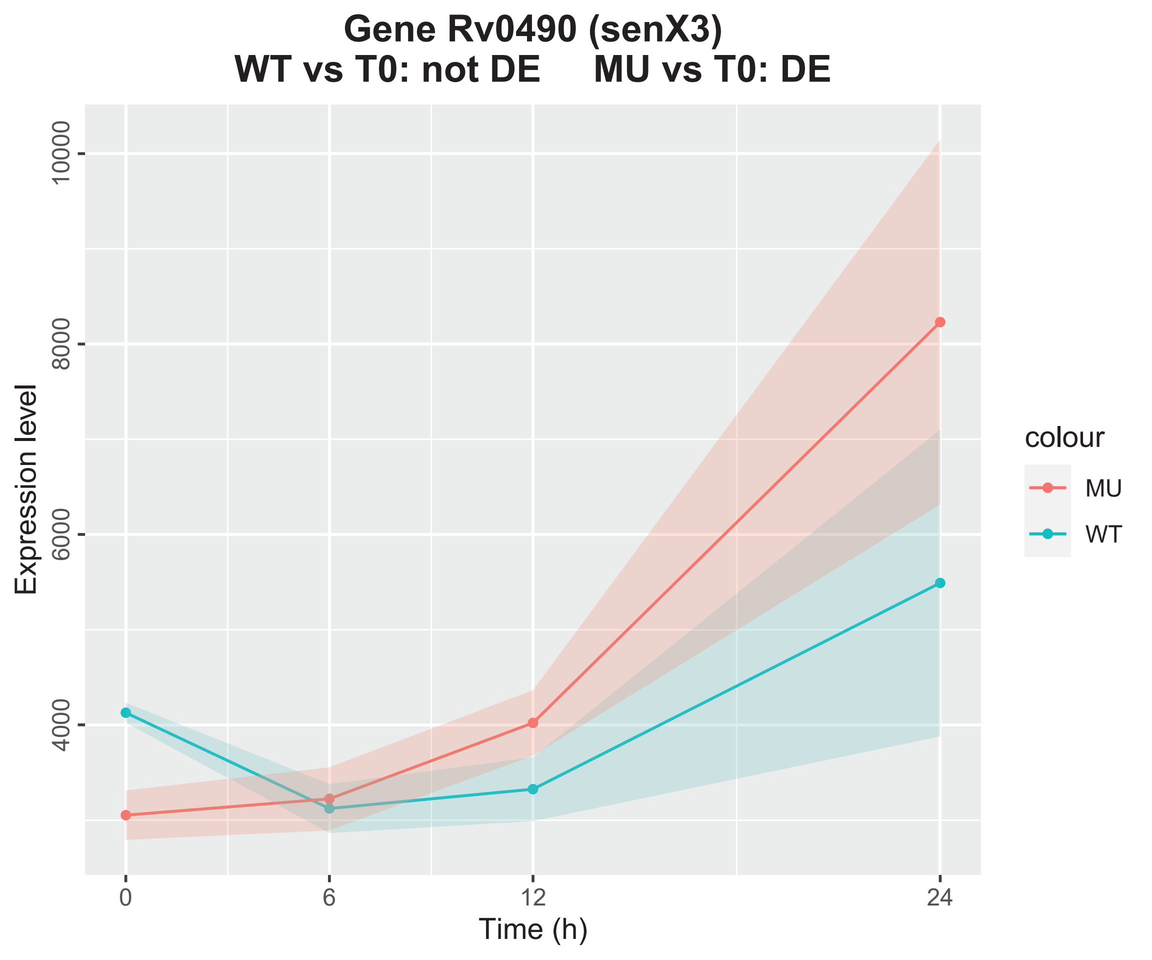
Gene Rv0490 (senX3)
WT vs T0: not DE   MU vs T0: DE
Expression level
10000
8000
6000
4000
0
6
12
24
Time (h)
colour
MU
WT

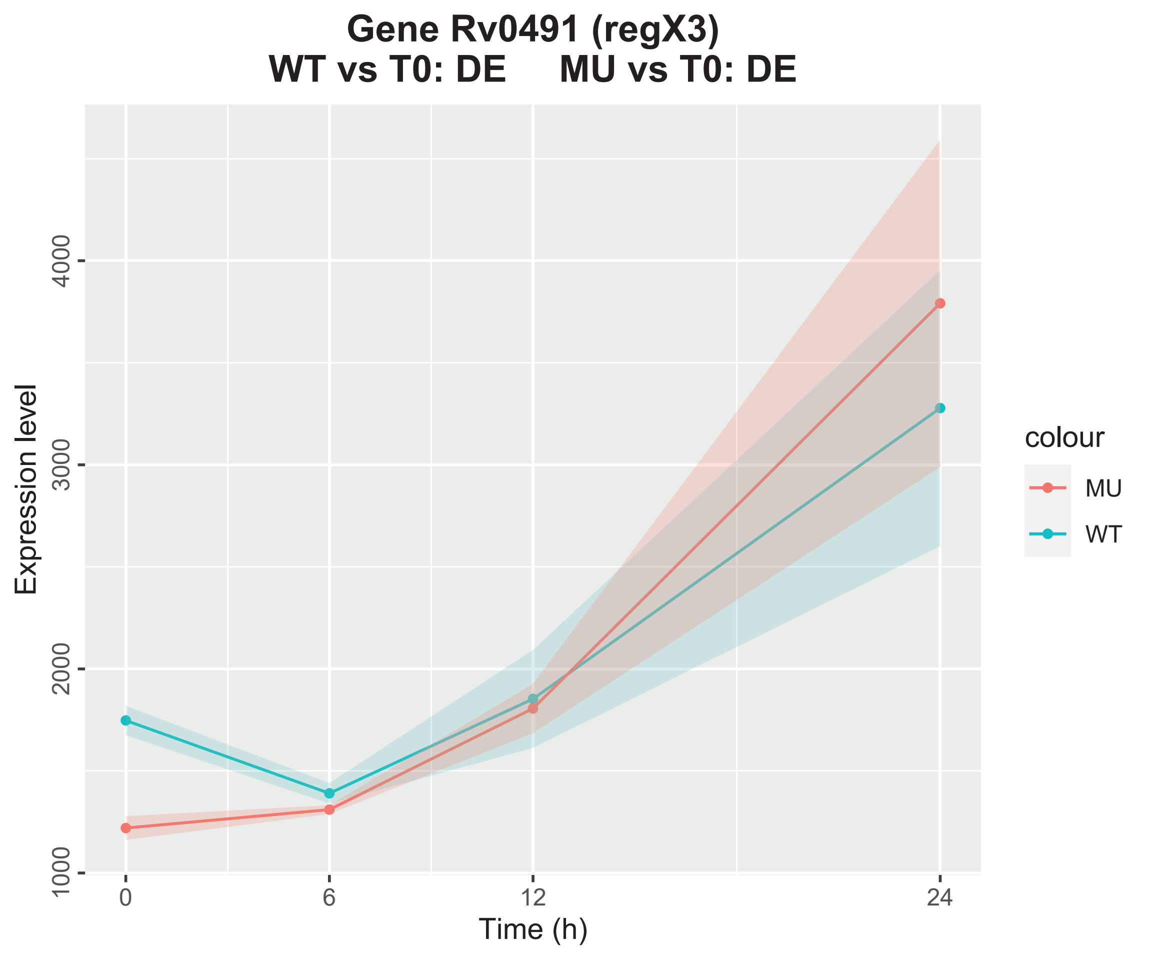

Gene Rv0491 (regX3)
WT vs T0: DE MU vs T0: DE
Expression level
1000
2000
3000
4000
0
6
12
24
Time (h)
colour
MU
WT

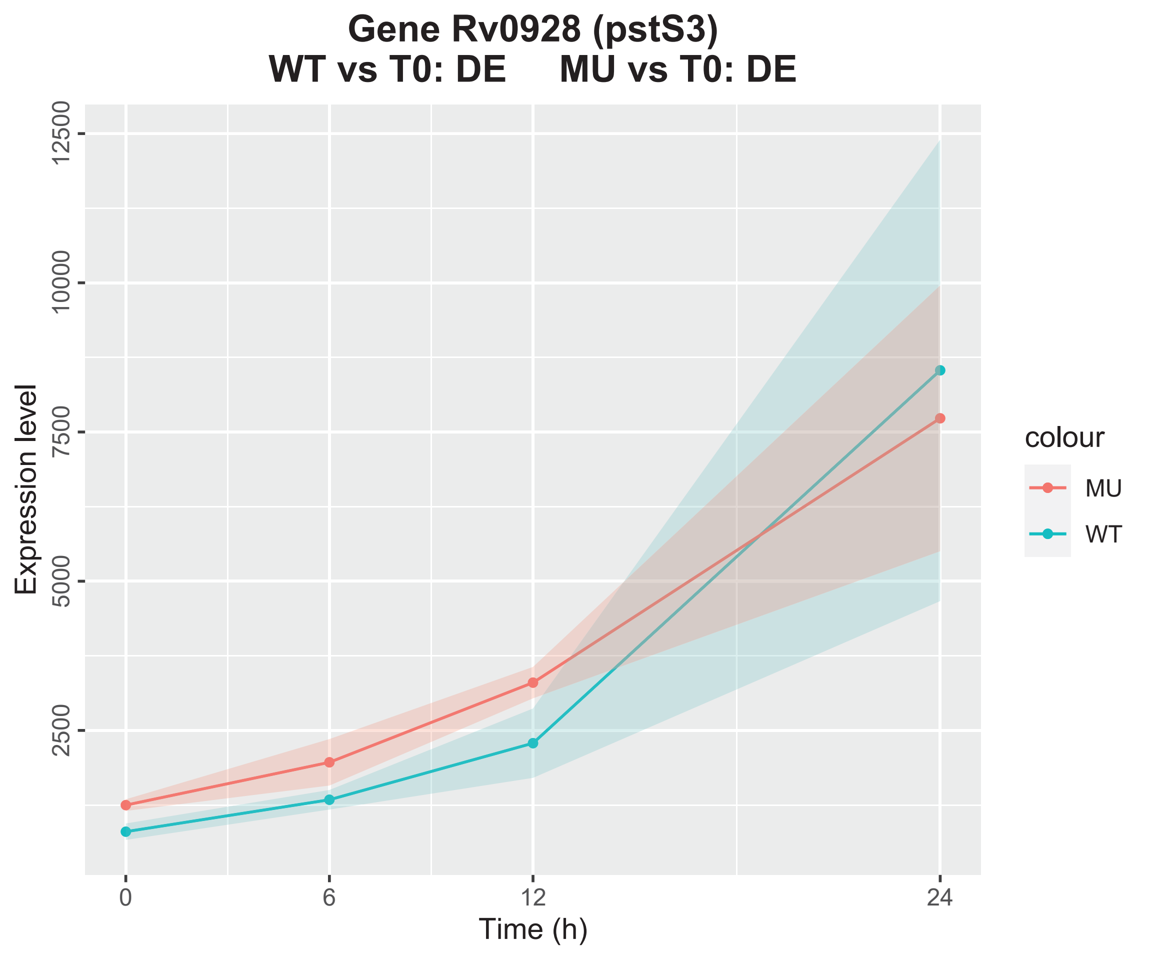

Gene Rv0928 (pstS3)
WT vs T0: DE   MU vs T0: DE
Expression level
12500
10000
7500
5000
2500
0
6
12
24
Time (h)
colour
MU
WT

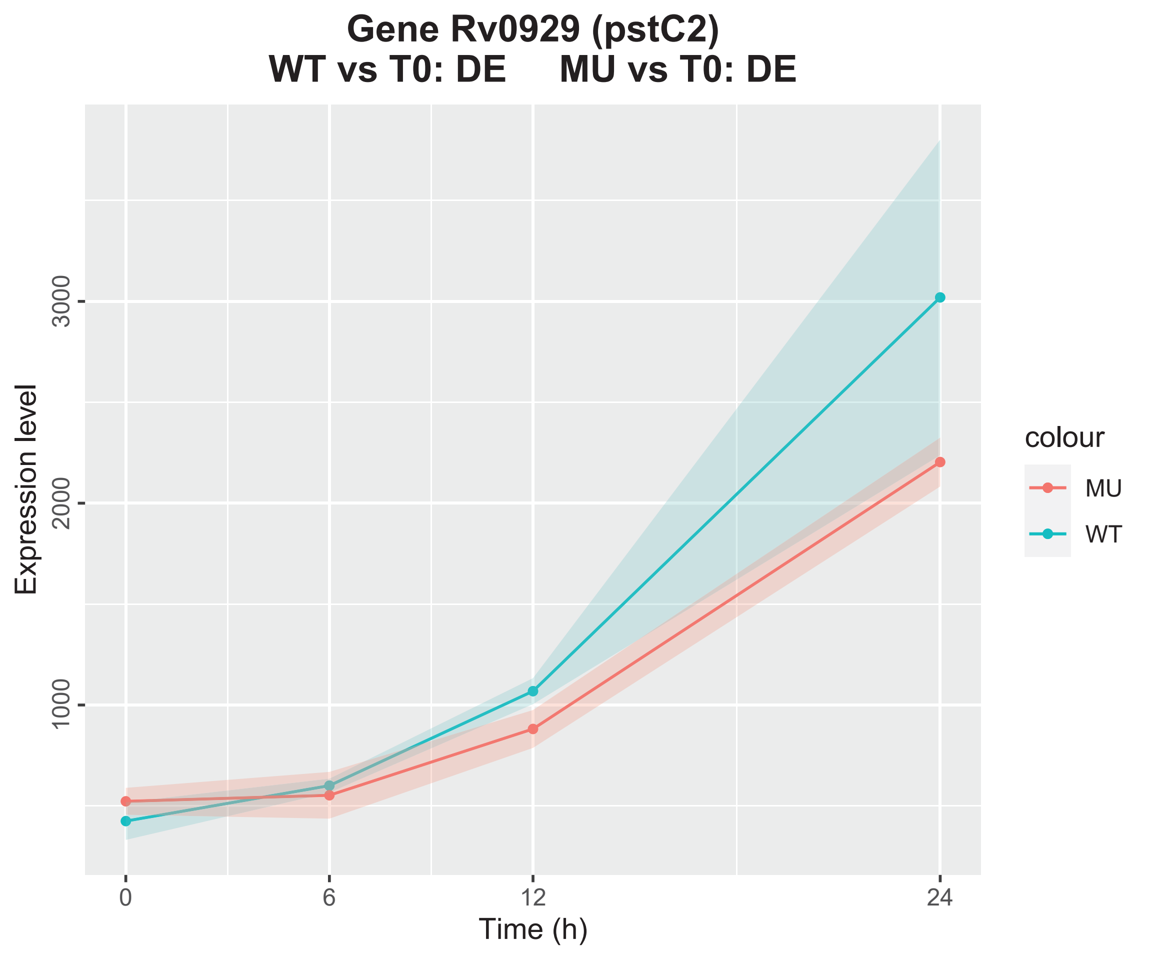

Gene Rv0929 (pstC2)
WT vs T0: DE   MU vs T0: DE
Expression level
3000
2000
1000
0
6
12
24
Time (h)
colour
MU
WT

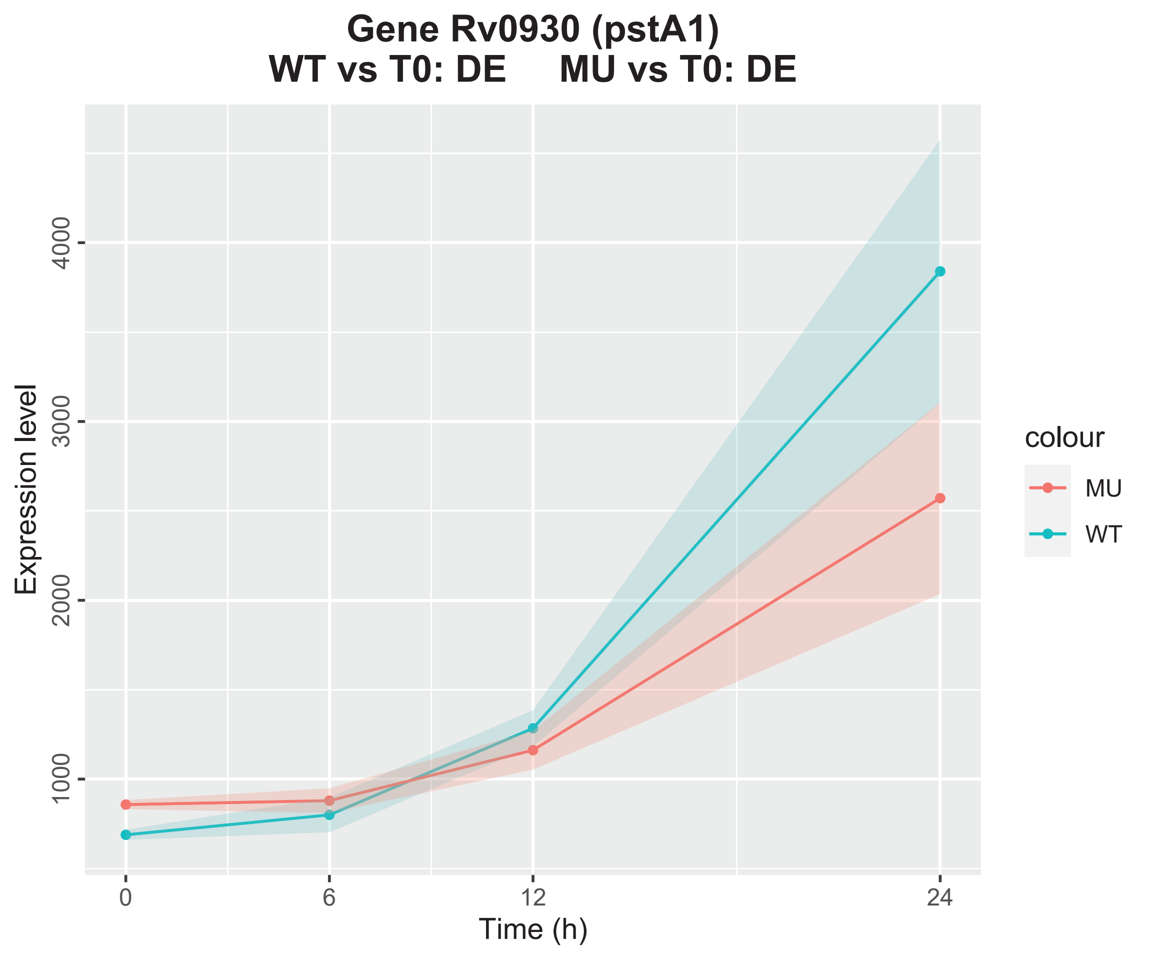

Gene Rv0930 (pstA1)
WT vs T0: DE MU vs T0: DE
Expression level
4000
3000
2000
1000
0
6
12
24
Time (h)
colour
MU
WT

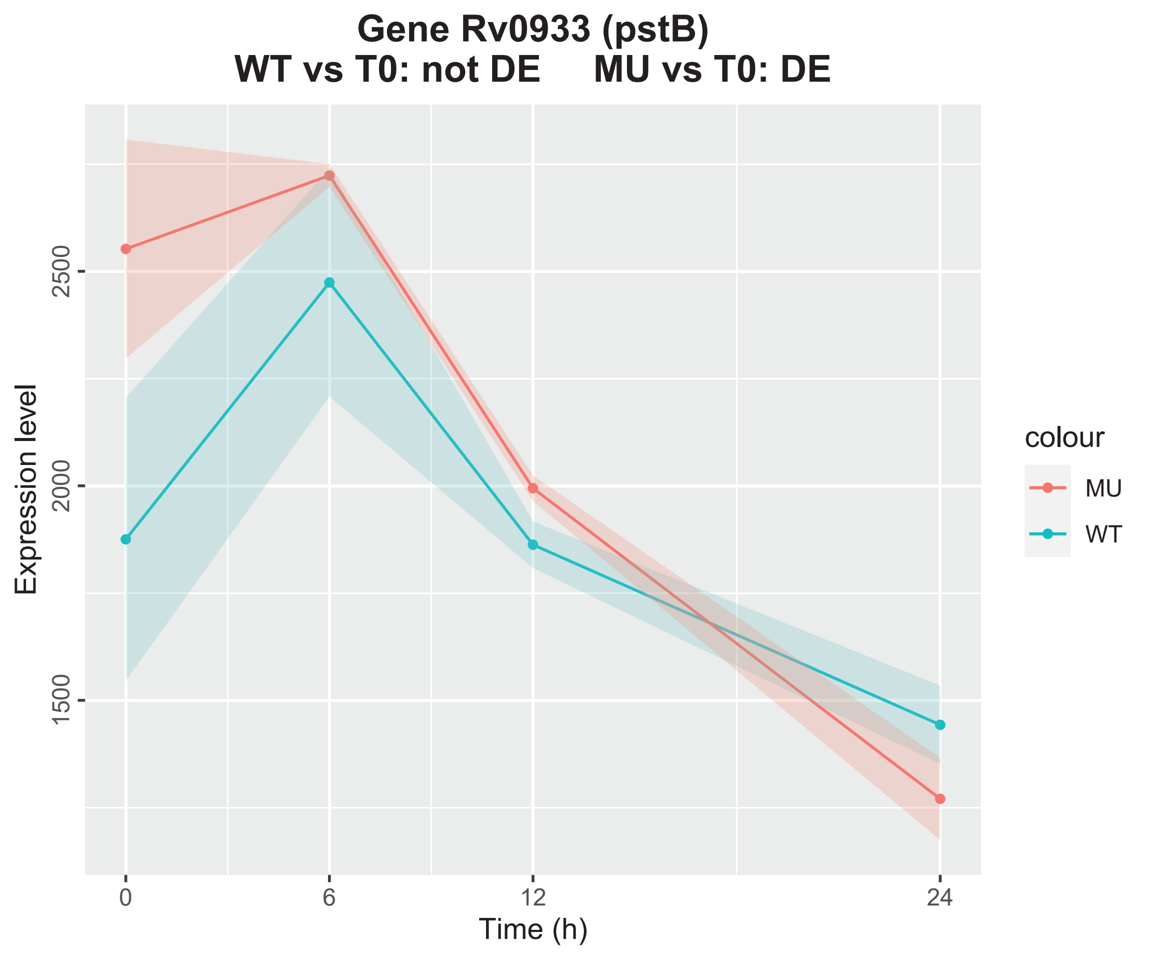
Gene Rv0933 (pstB)
WT vs T0: not DE MU vs T0: DE
Expression level
2500
2000
1500
0
6
12
24
Time (h)
colour
MU
WT

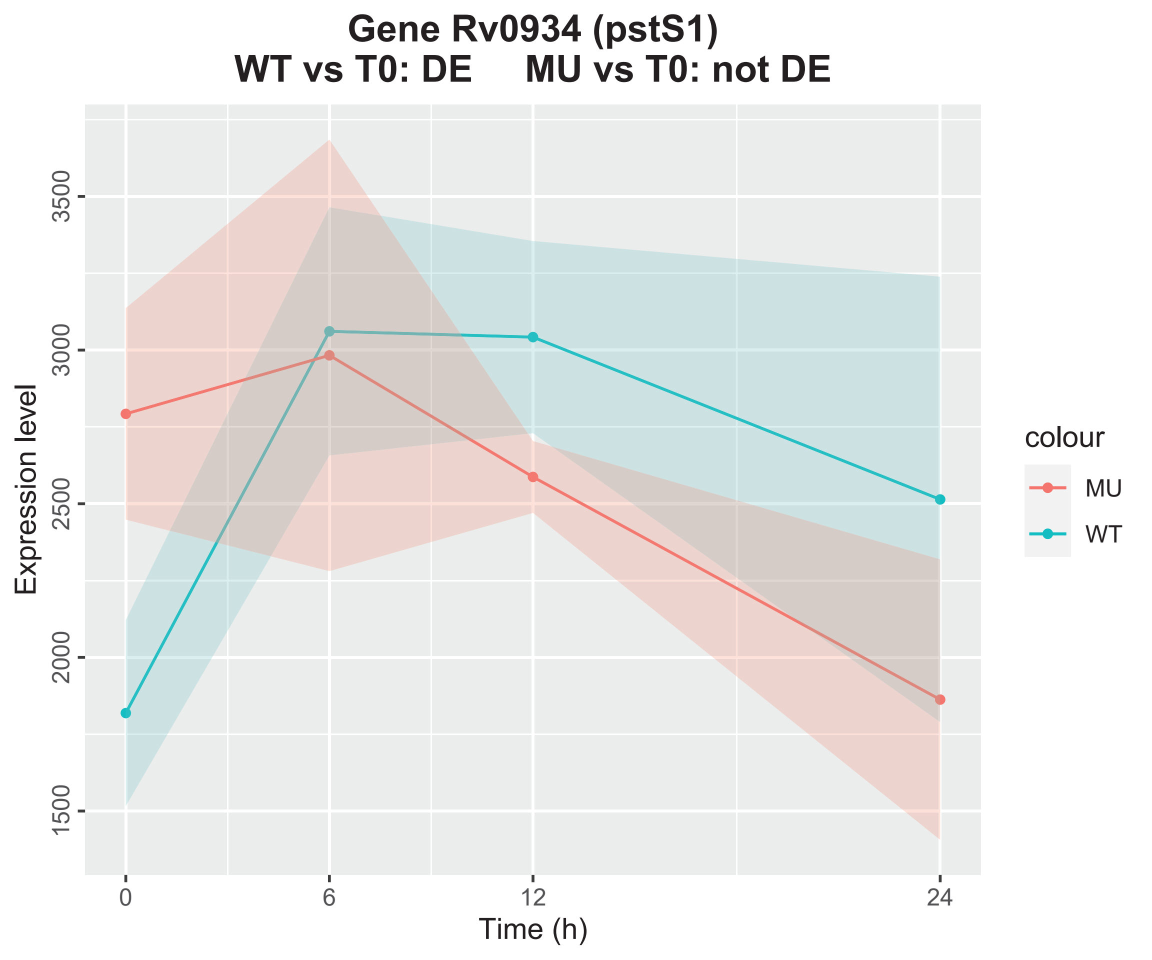

Gene Rv0934 (pstS1)
WT vs T0: DE   MU vs T0: not DE
Expression level
3500
3000
2500
2000
1500
0
6
12
24
Time (h)
colour
MU
WT

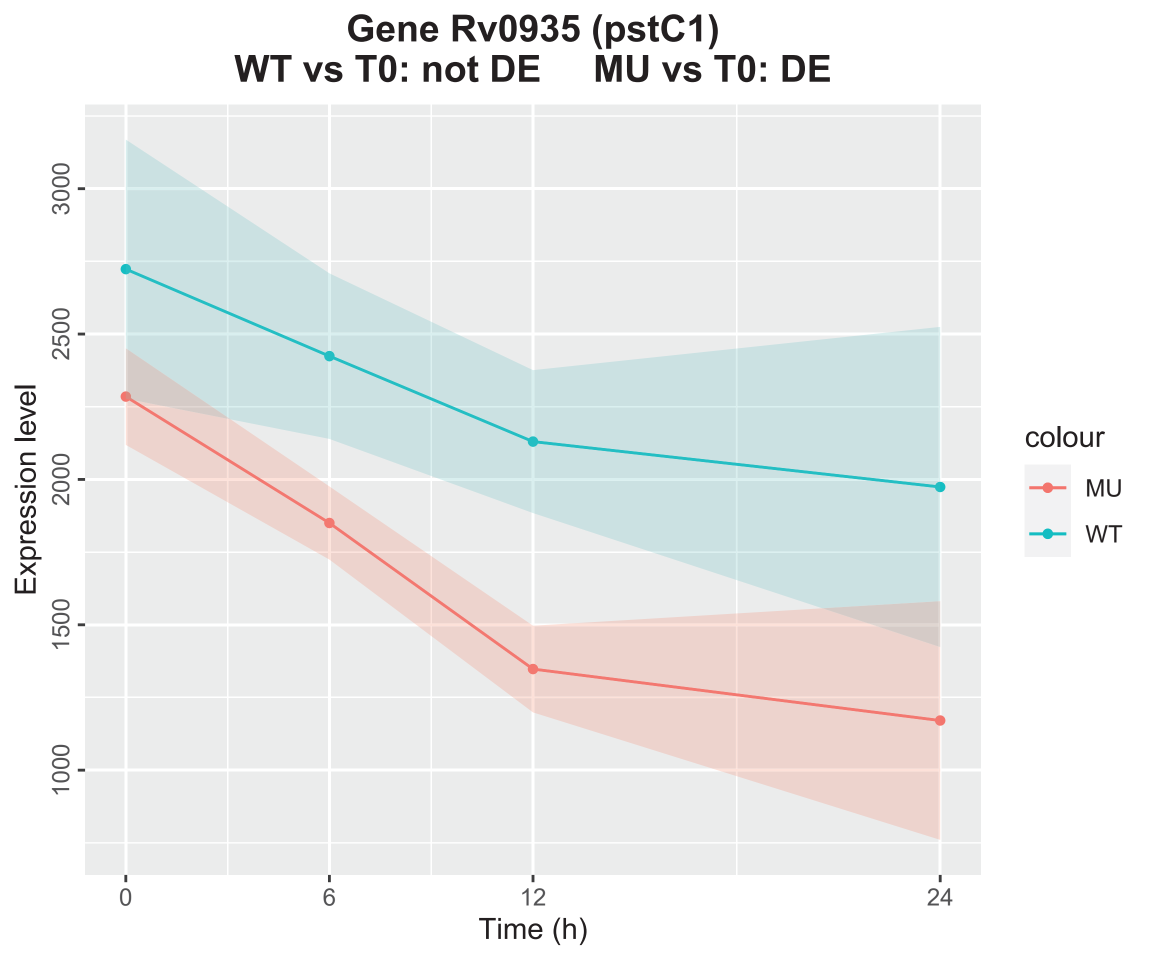
Gene Rv0935 (pstC1)
WT vs T0: not DE   MU vs T0: DE
Expression level
3000
2500
2000
1500
1000
0
6
12
24
Time (h)
colour
MU
WT

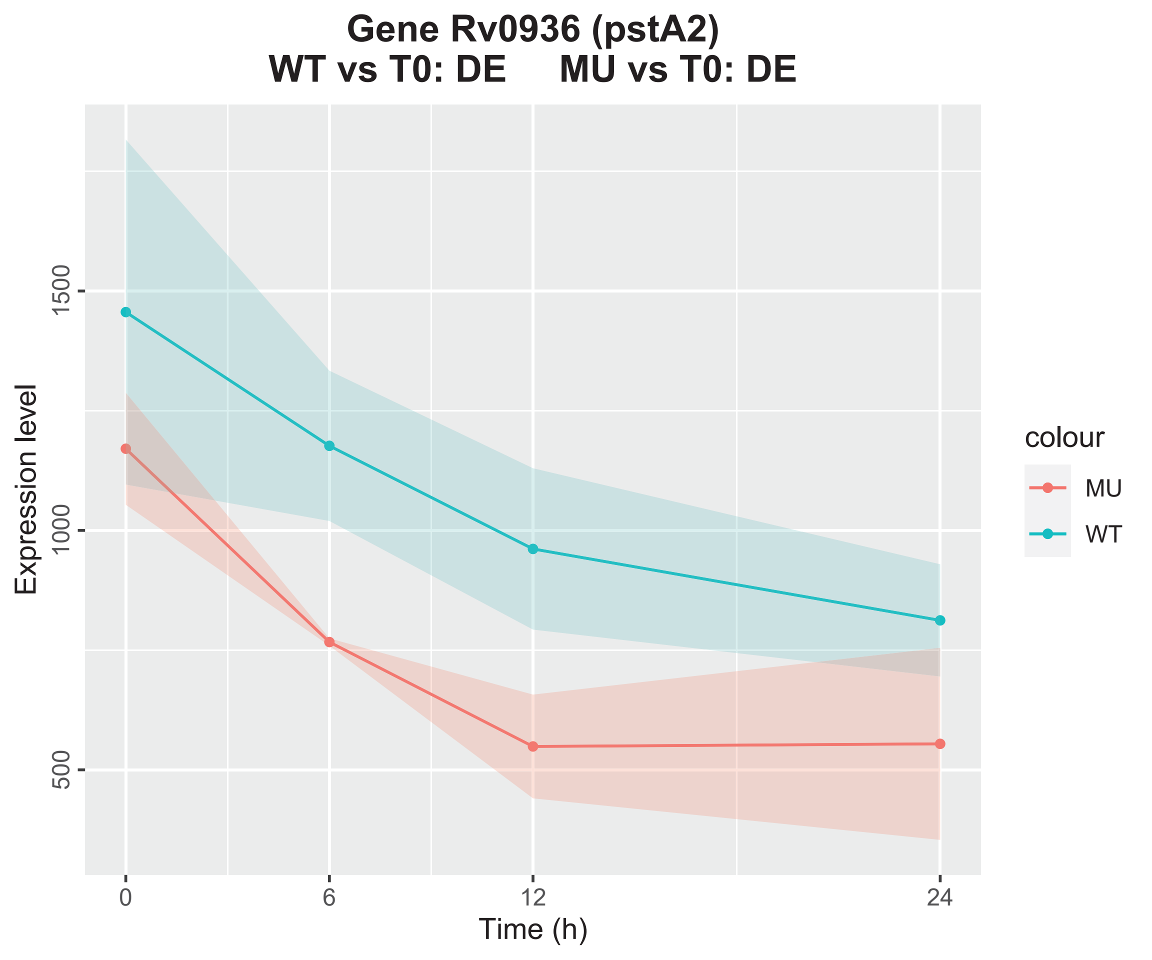
Gene Rv0936 (pstA2)
WT vs T0: DE MU vs T0: DE
Expression level
1500
1000
500
0
6
12
24
Time (h)
colour
MU
WT

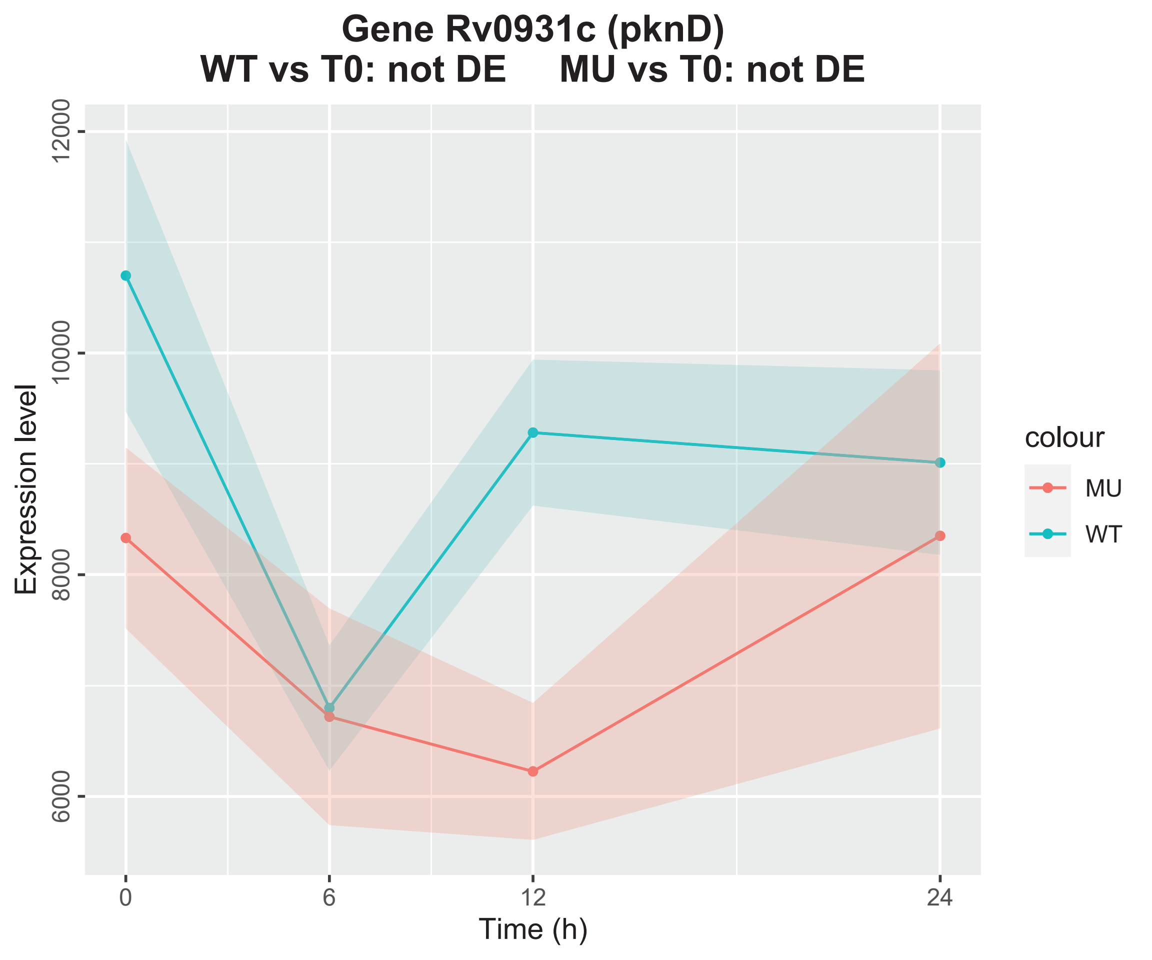
Gene Rv0931c (pknD)
WT vs T0: not DE   MU vs T0: not DE
Expression level
12000
10000
8000
6000
0
6
12
24
Time (h)
colour
MU
WT

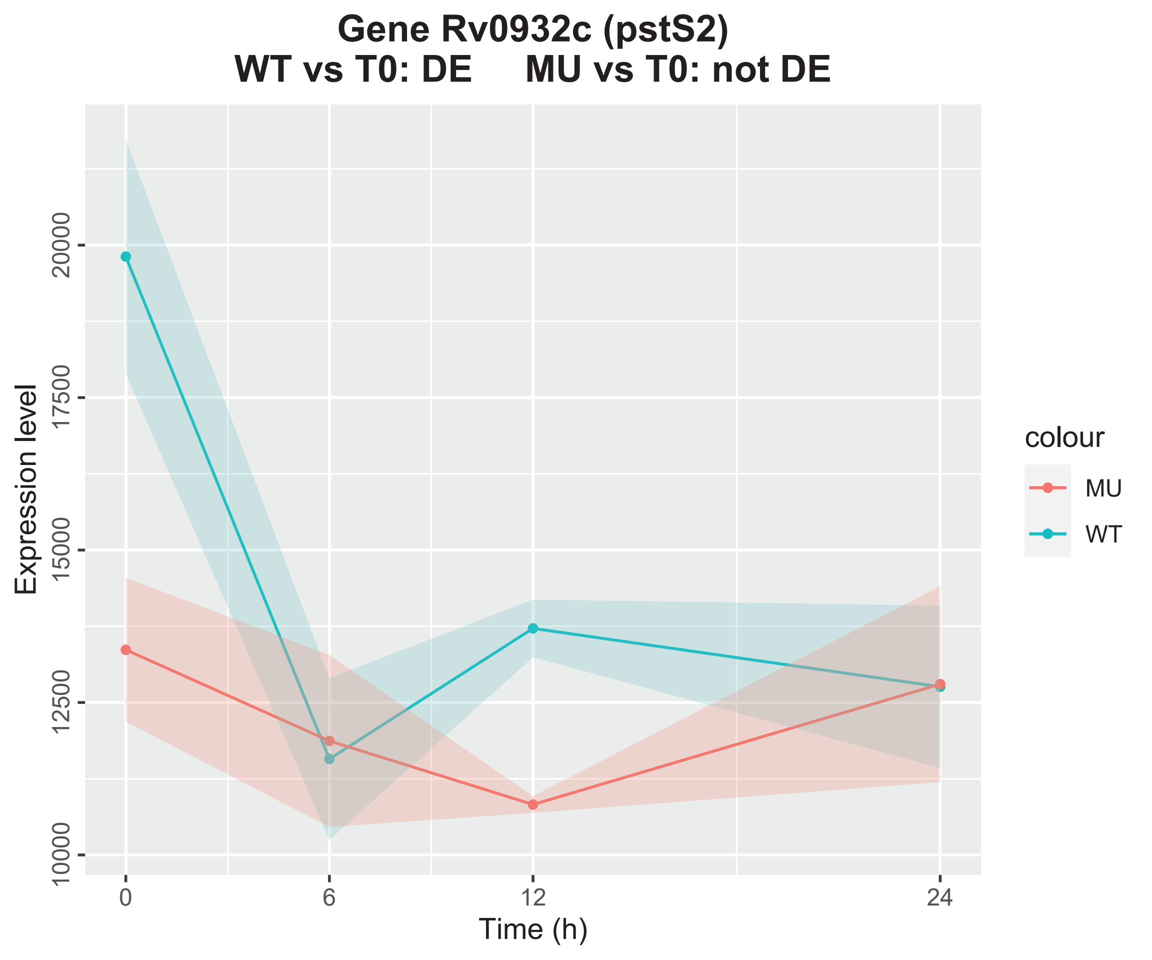
Gene Rv0932c (pstS2)
WT vs T0: DE   MU vs T0: not DE
Expression level
10000
12500
15000
17500
20000
0
6
12
24
Time (h)
colour
MU
WT

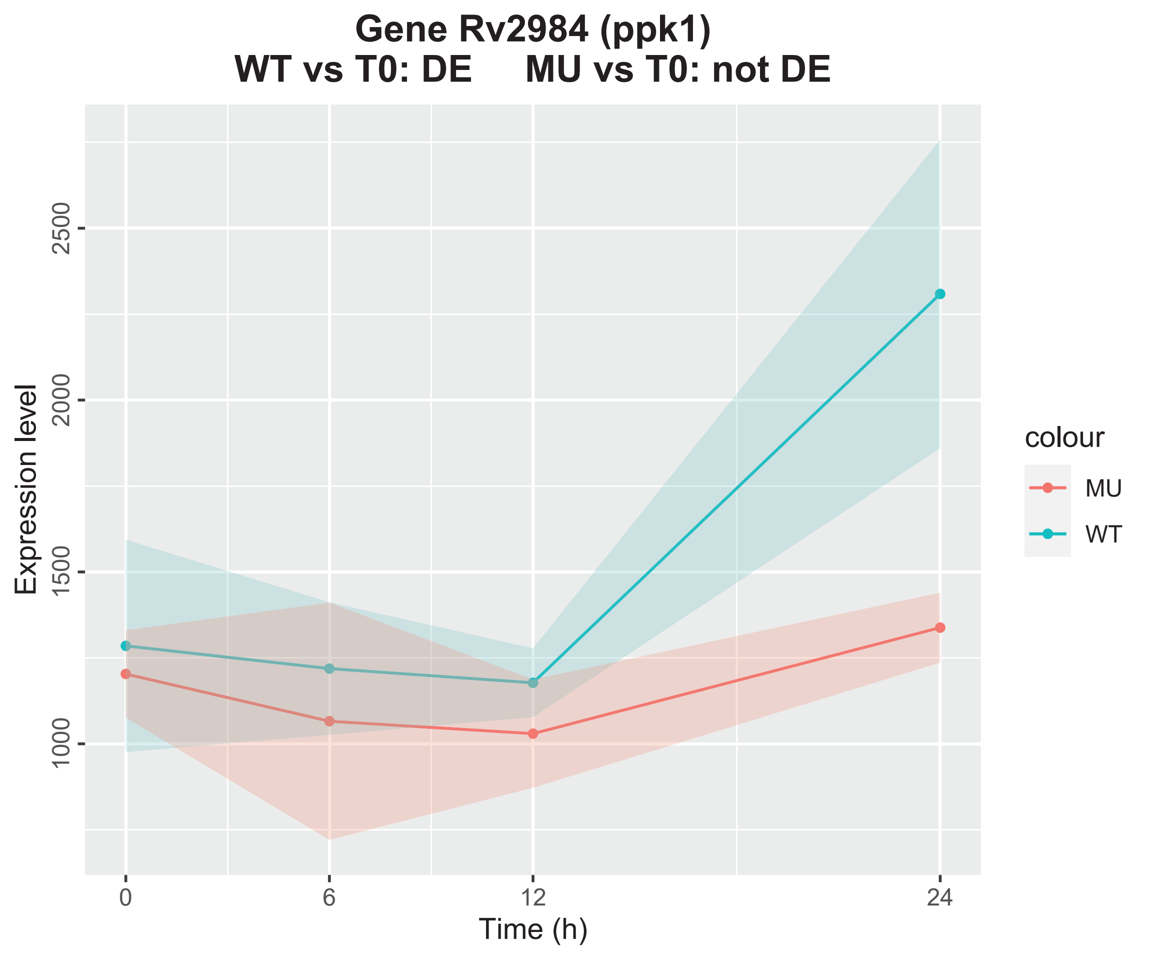
Gene Rv2984 (ppk1)
WT vs T0: DE MU vs T0: not DE
Expression level
2500
2000
1500
1000
0
6
12
24
Time (h)
colour
MU
WT

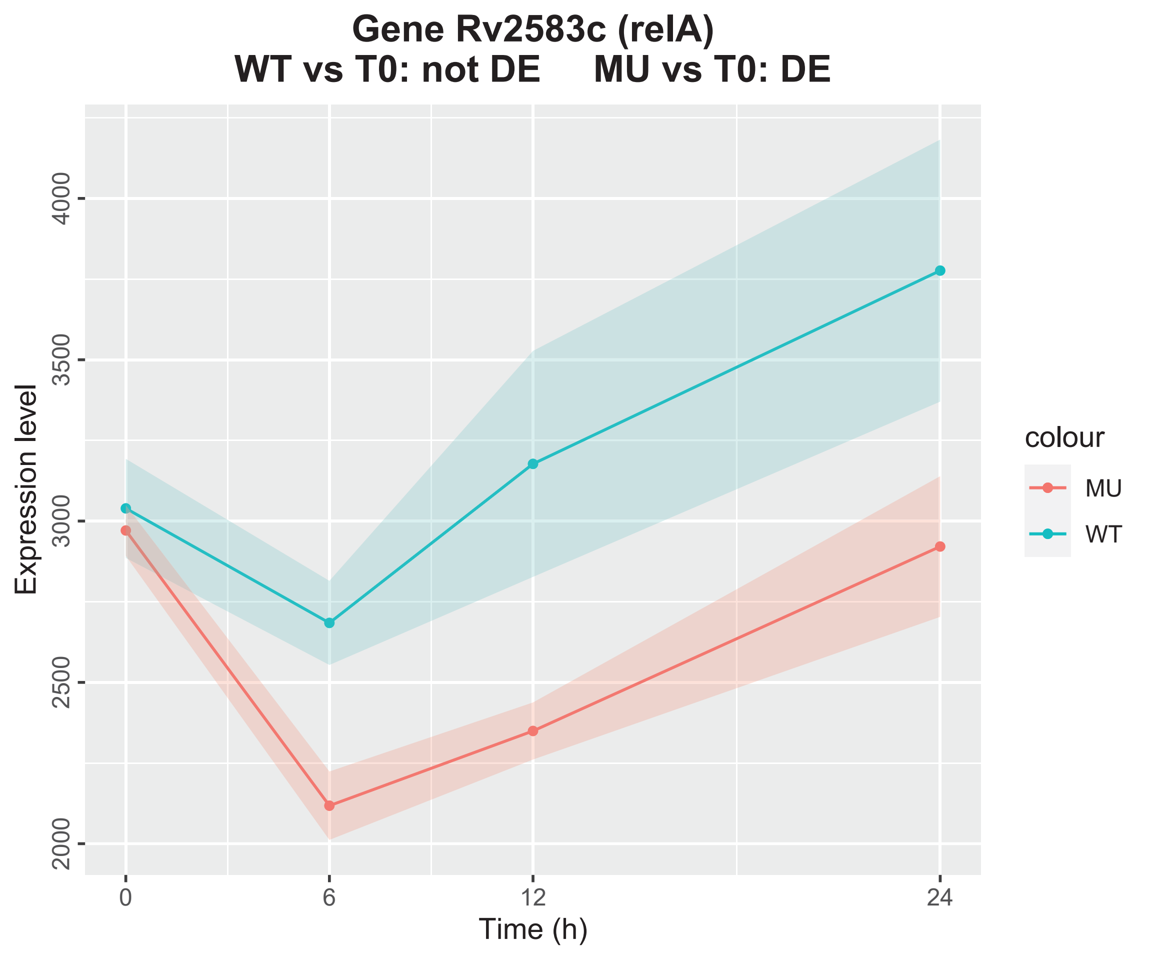

Gene Rv2583c (relA)
WT vs T0: not DE MU vs T0: DE
Expression level
4000
3500
3000
2500
2000
0
6
12
24
Time (h)
colour
MU
WT
