# Supplementary material for: Role of the Extracytoplasmic Function Sigma Factor SigE in the Stringent Response of Mycobacterium tuberculosis
Source: Microbiol Spectr. 2023 Mar 22;11(2):e02944-22. doi: 10.1128/spectrum.02944-22 (PMC10100808; doi:10.1128/spectrum.02944-22)
Supplement: Supplemental file 4 — Data S4. Download spectrum.02944-22-s0005.pdf, PDF file, 0.4 MB [file spectrum.02944-22-s0005.pdf]

<u>Supplementary Data S4:</u> Gene expression profiles over time of genes discussed in section "Changes in electron-transport chain", i.e. *canA*, *nuoABCDEFGHJKLMN*, *ndh*, *ndhA*, *entC*, *menD*, *menE*, *cydCDBA*, *rv0247c*, *rv0248c*, *rv0249c*, and *sdhCD*. For each gene, the plot shows the average expression level and the standard deviation (shaded area) for both the wild-type (cyan color) and *sigE*-mutant (salmon/pink color) strains.

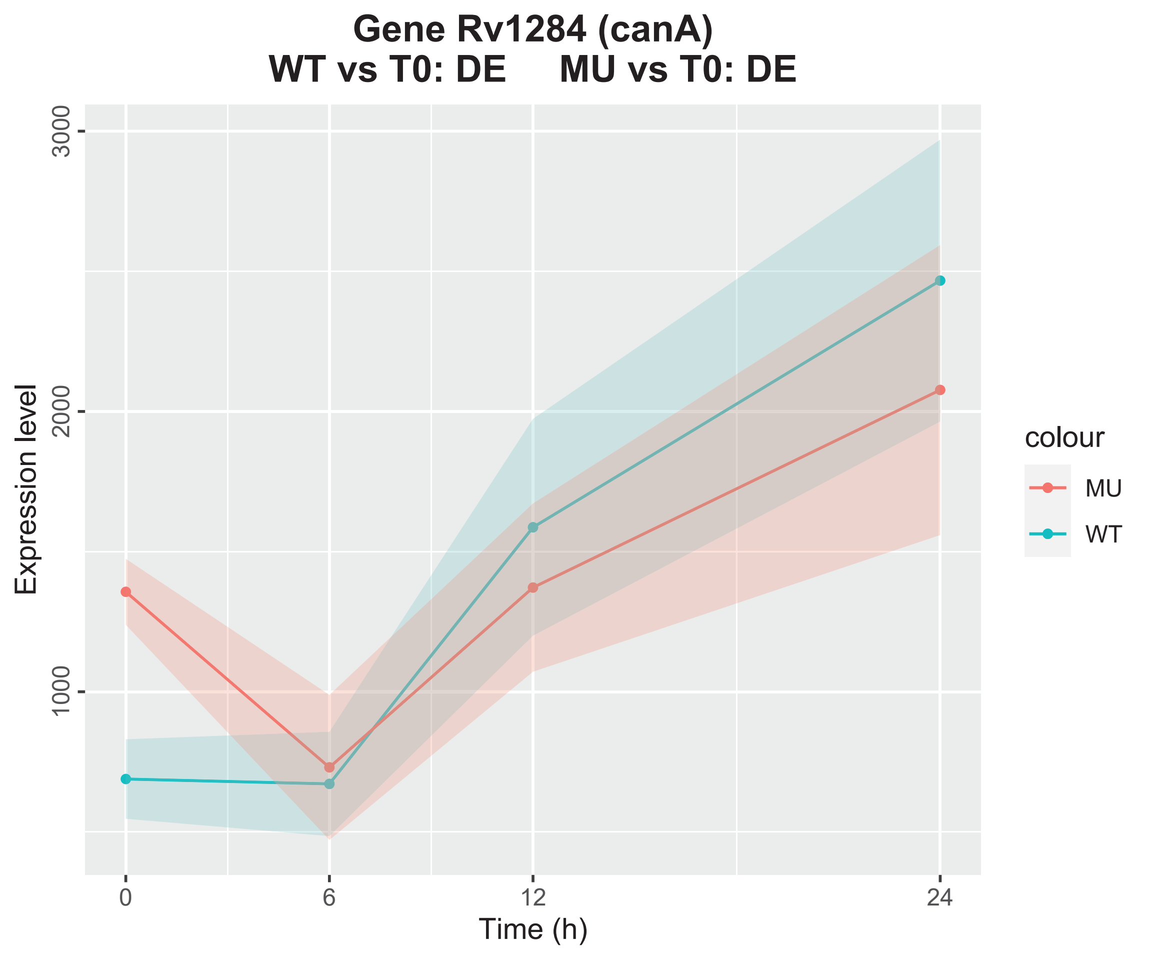
Gene Rv1284 (canA)
WT vs T0: DE MU vs T0: DE
Expression level
3000
2000
1000
0
6
12
24
Time (h)
colour
MU
WT

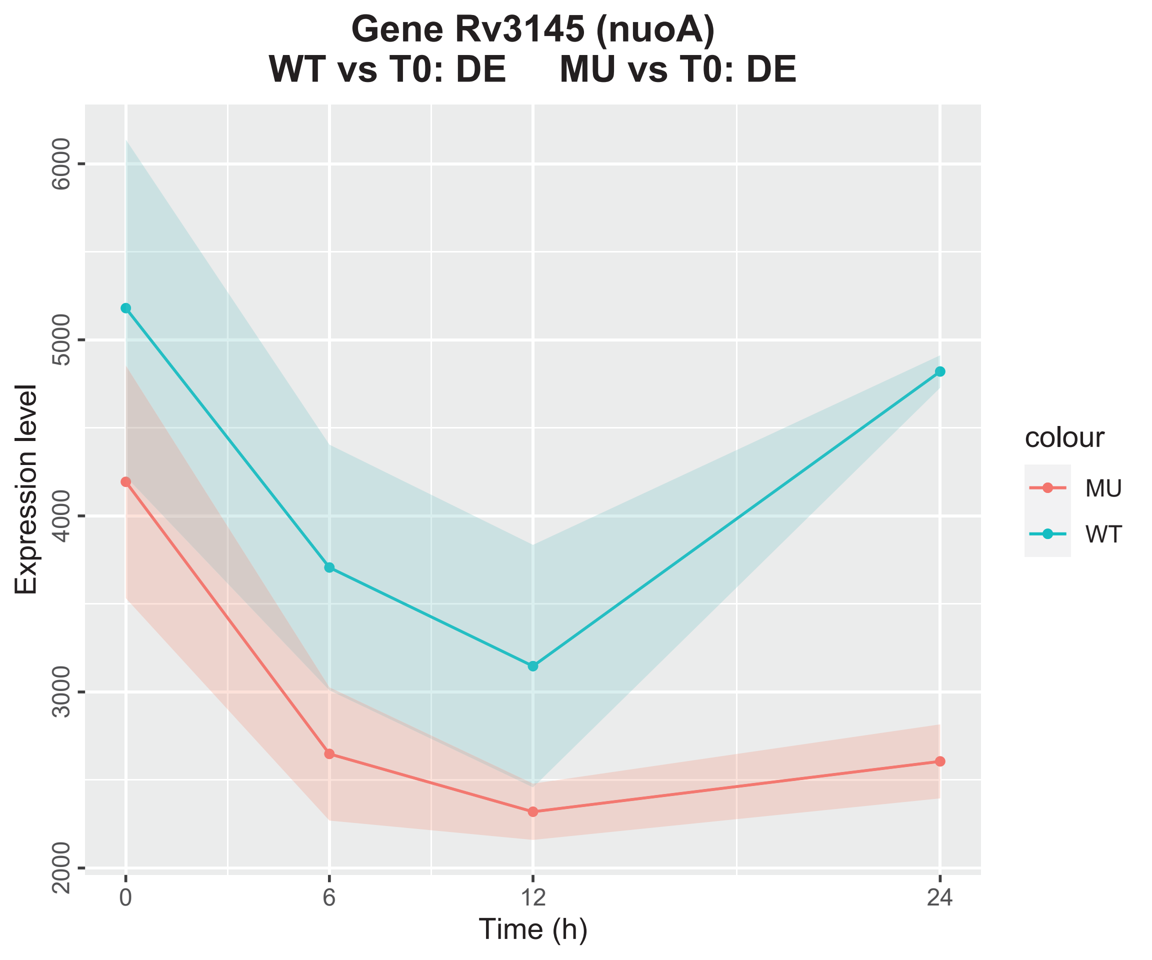
Gene Rv3145 (nuoA)
WT vs T0: DE MU vs T0: DE
Expression level
2000
3000
4000
5000
6000
0
6
12
24
Time (h)
colour
MU
WT

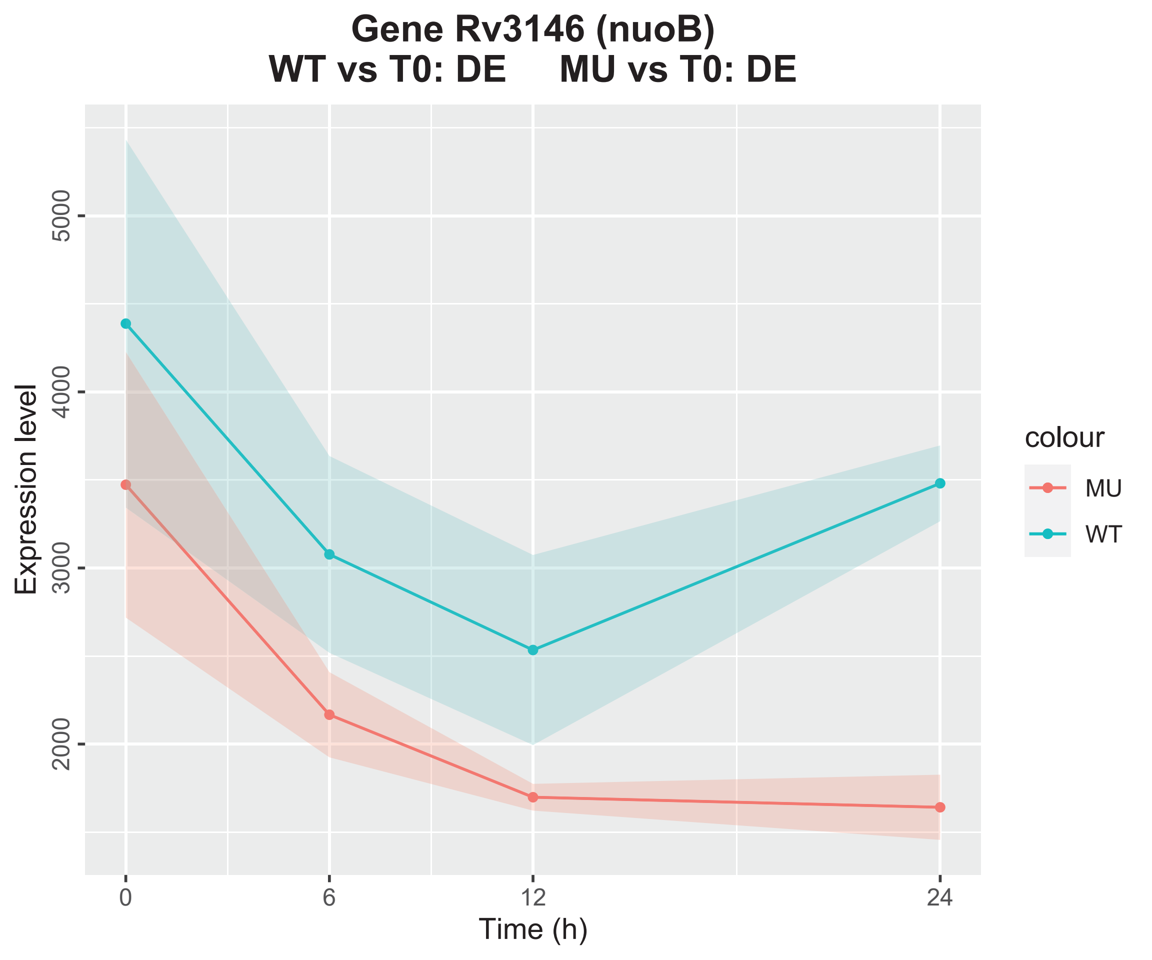

Gene Rv3146 (nuoB)
WT vs T0: DE MU vs T0: DE
Expression level
5000
4000
3000
2000
0
6
12
24
Time (h)
colour
MU
WT

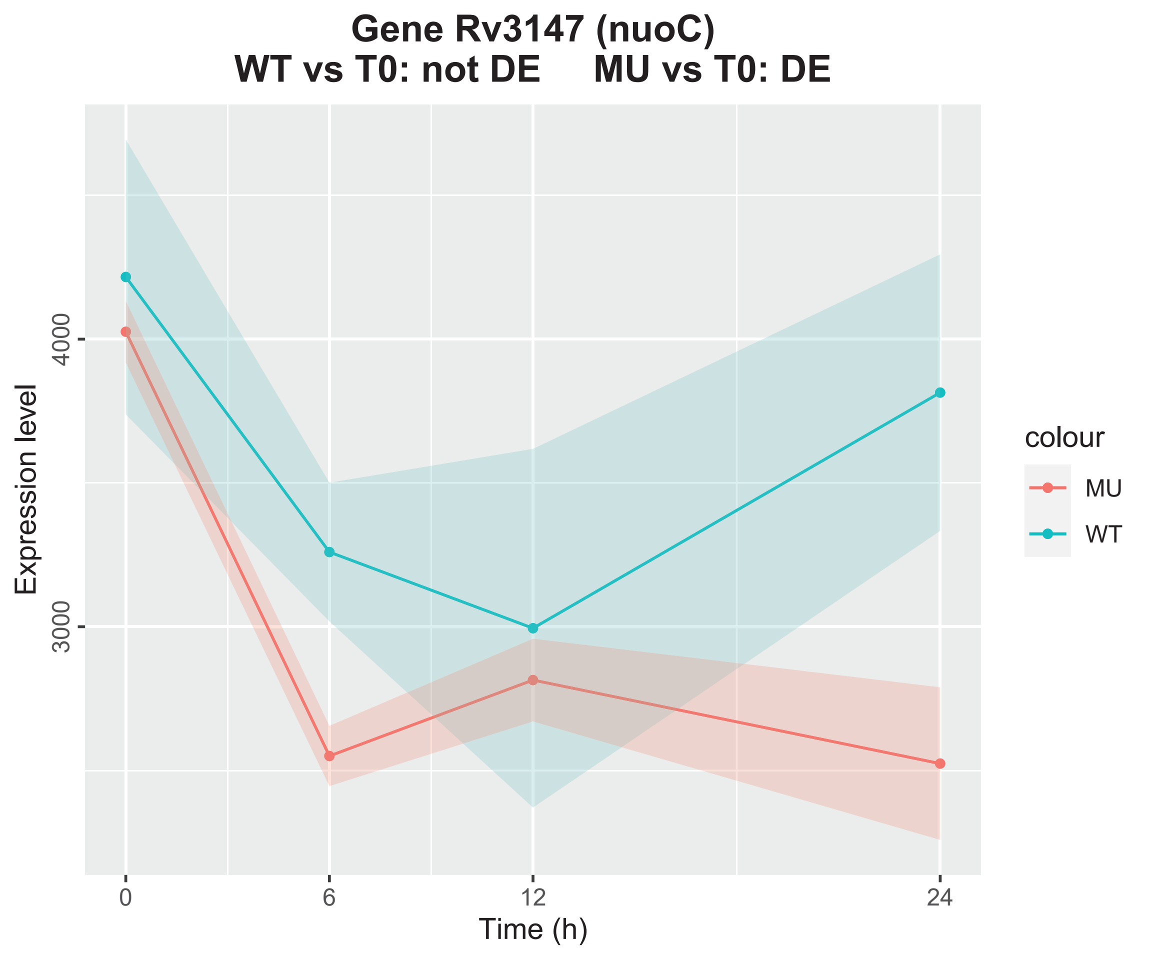

Gene Rv3147 (nuoC)
WT vs T0: not DE   MU vs T0: DE
Expression level
4000
3000
0
6
12
24
Time (h)
colour
MU
WT

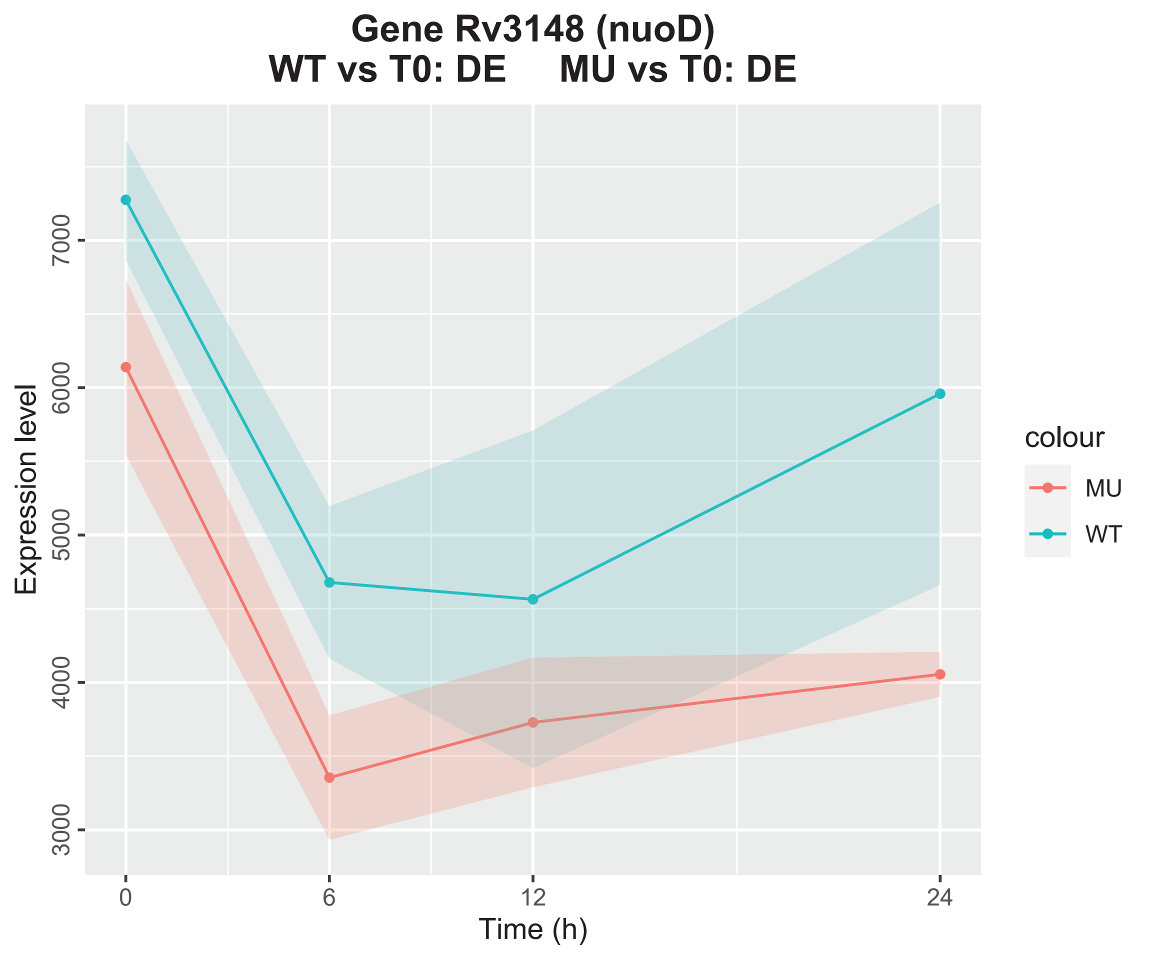
Gene Rv3148 (nuoD)
WT vs T0: DE MU vs T0: DE
Expression level
7000
6000
5000
4000
3000
0
6
12
24
Time (h)
colour
MU
WT

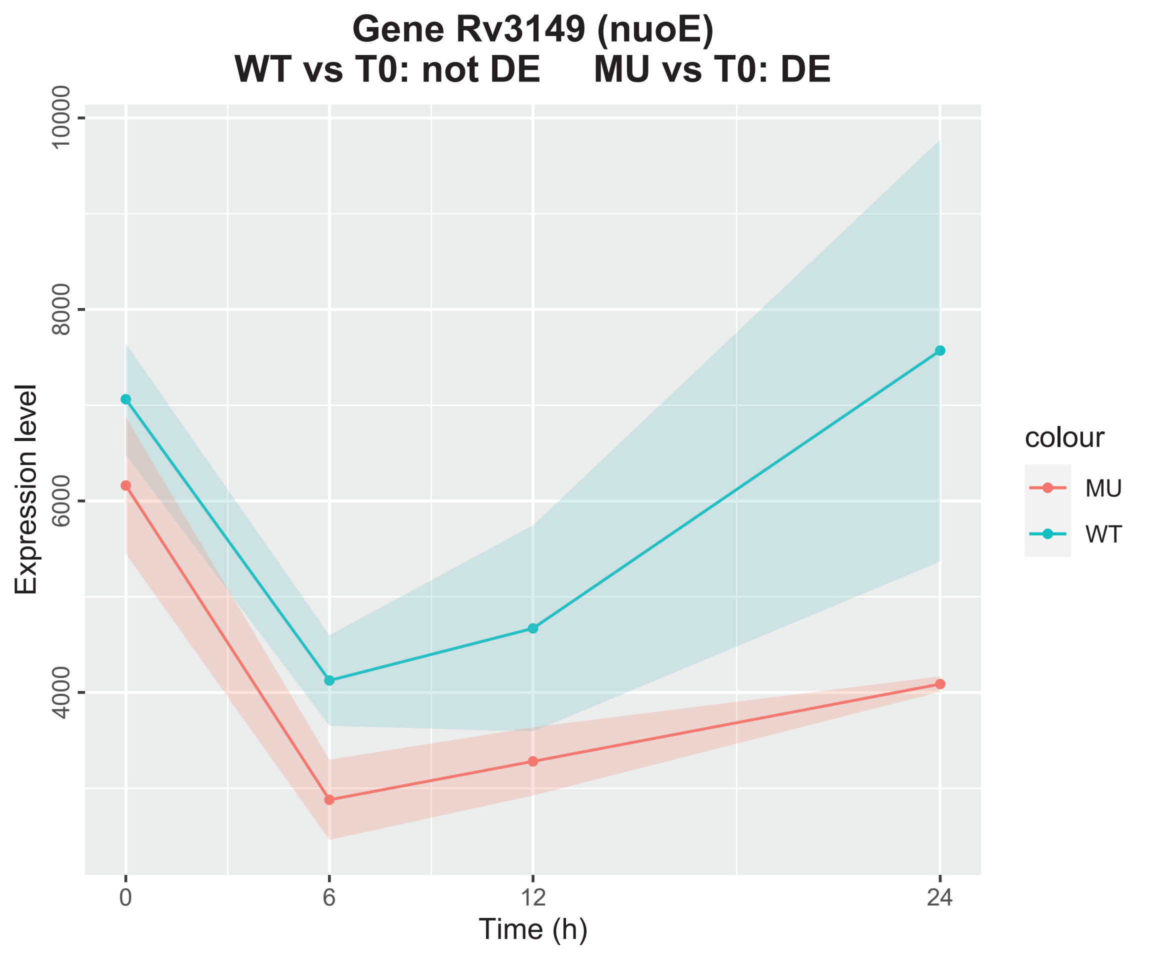
Gene Rv3149 (nuoE)
WT vs T0: not DE   MU vs T0: DE
Expression level
10000
8000
6000
4000
0
6
12
24
Time (h)
colour
MU
WT

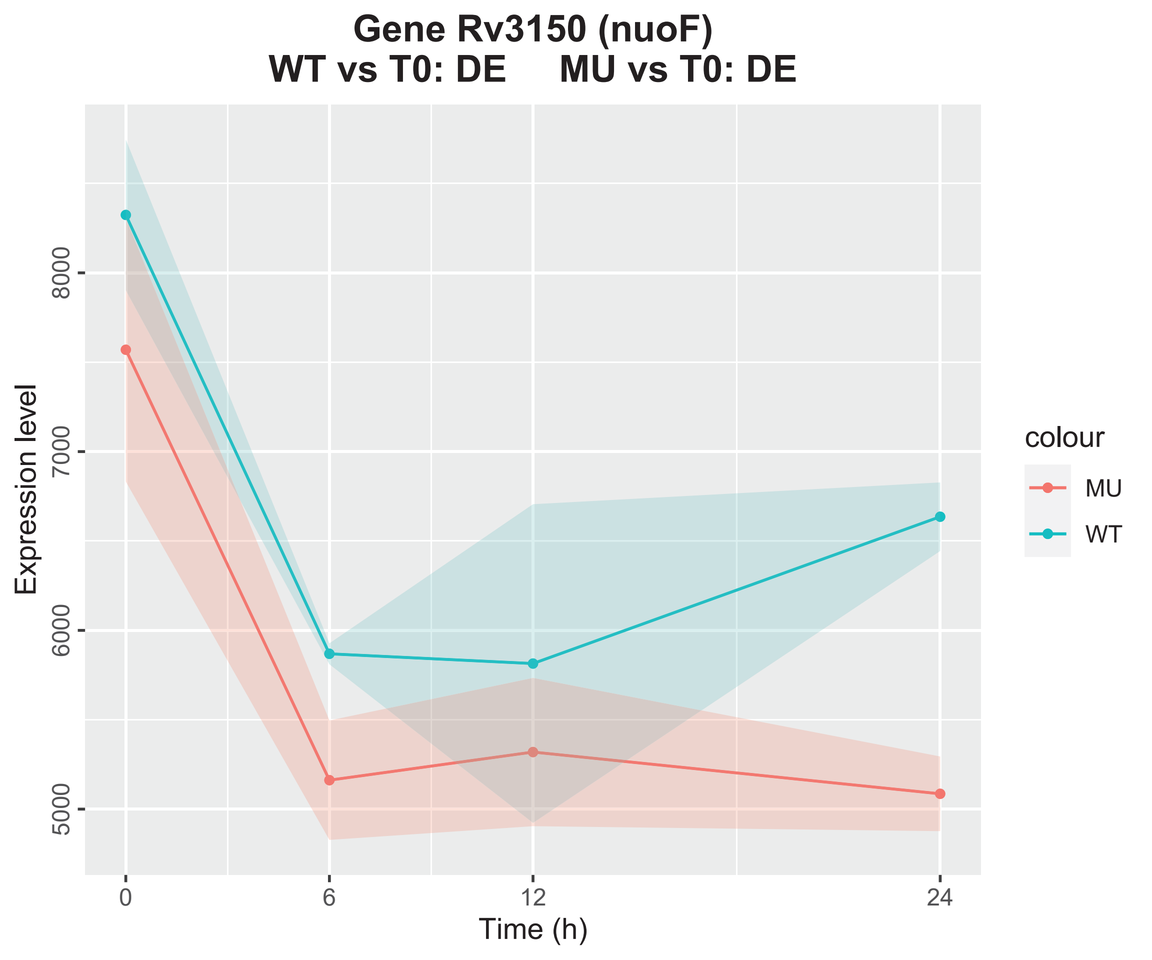

Gene Rv3150 (nuoF)
WT vs T0: DE   MU vs T0: DE
Expression level
8000
7000
6000
5000
0
6
12
24
Time (h)
colour
MU
WT

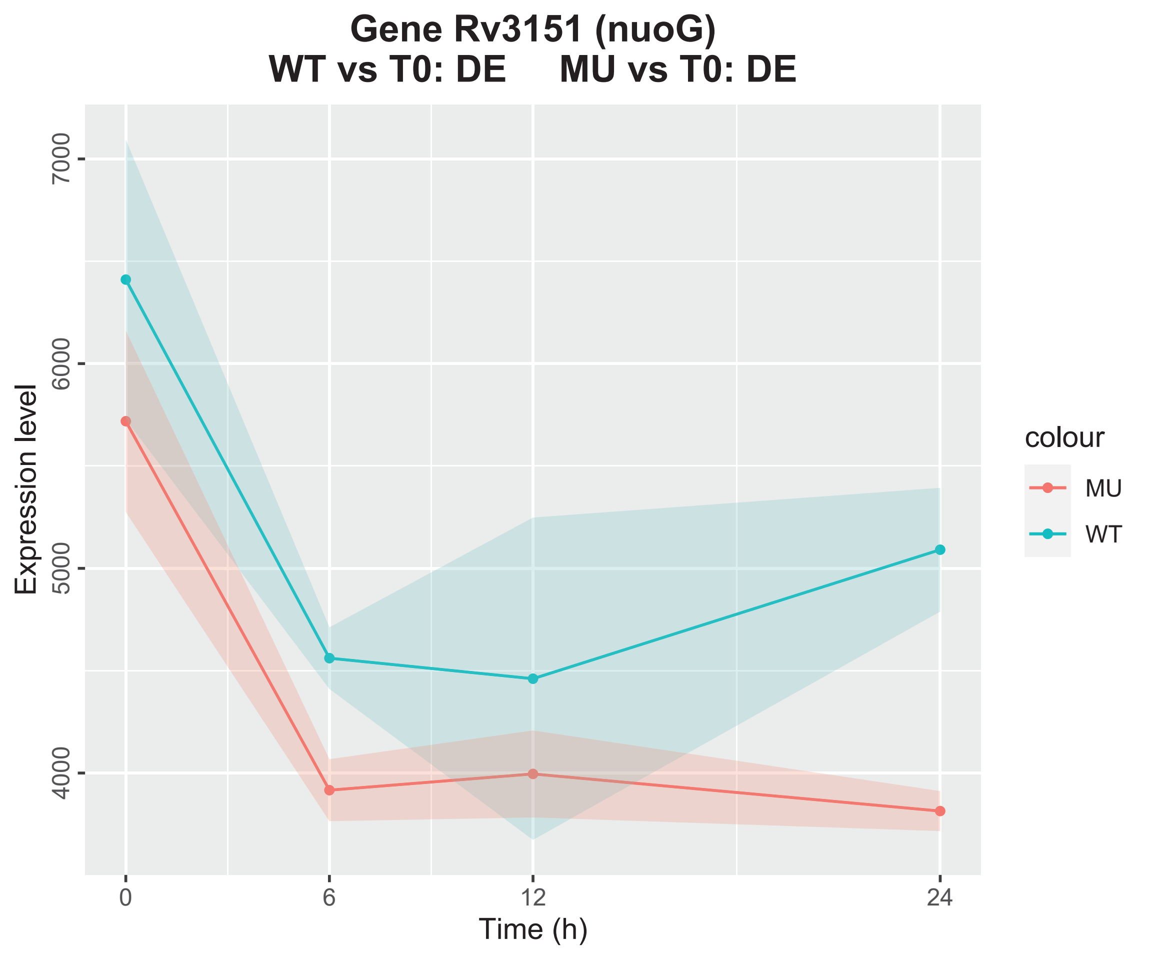
Gene Rv3151 (nuoG)
WT vs T0: DE   MU vs T0: DE
Expression level
7000
6000
5000
4000
0
6
12
24
Time (h)
colour
MU
WT

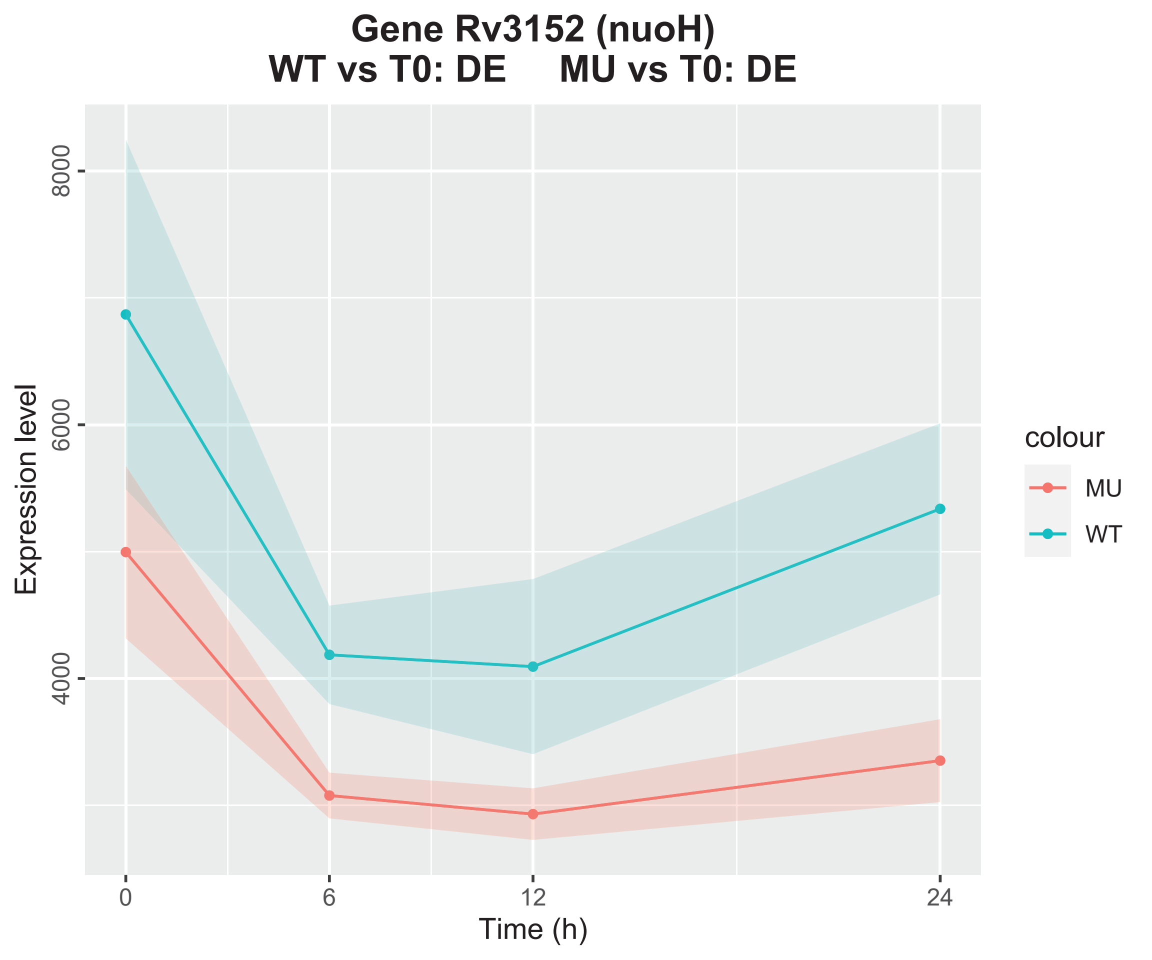
Gene Rv3152 (nuoH)
WT vs T0: DE MU vs T0: DE
Expression level
8000
6000
4000
0
6
12
24
Time (h)
colour
MU
WT

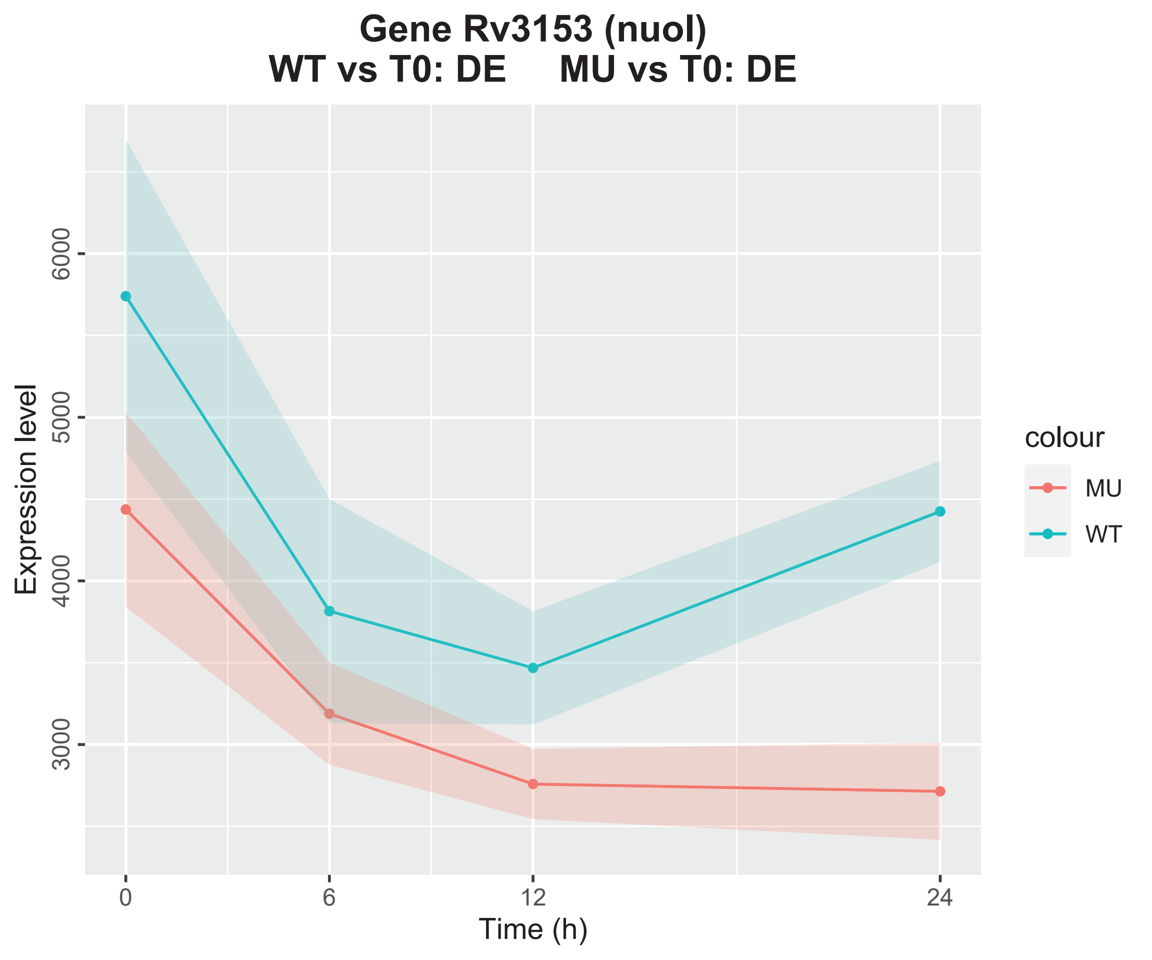

Gene Rv3153 (nuoI)
WT vs T0: DE   MU vs T0: DE
Expression level
6000
5000
4000
3000
0
6
12
24
Time (h)
colour
MU
WT

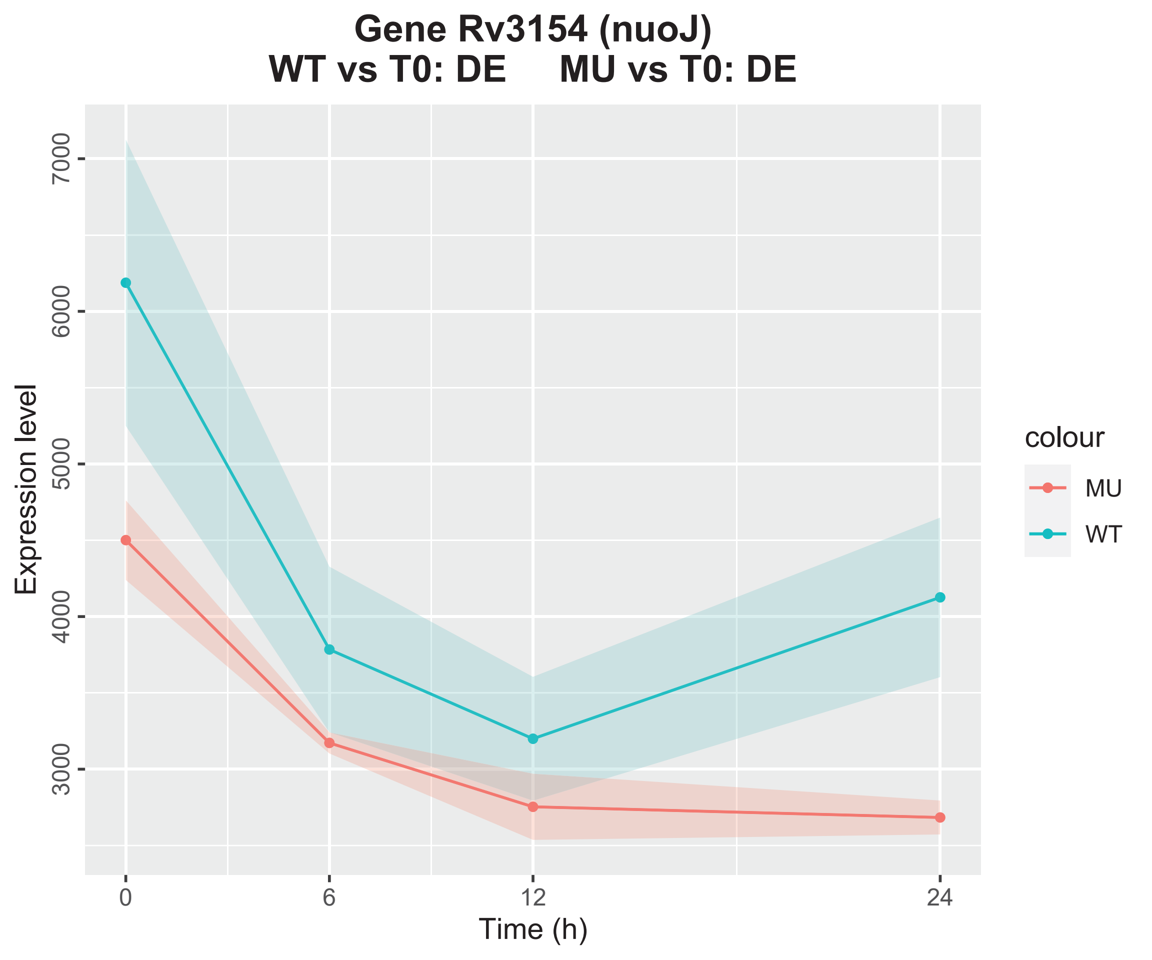
Gene Rv3154 (nuoJ)
WT vs T0: DE   MU vs T0: DE
Expression level
7000
6000
5000
4000
3000
0
6
12
24
Time (h)
colour
MU
WT

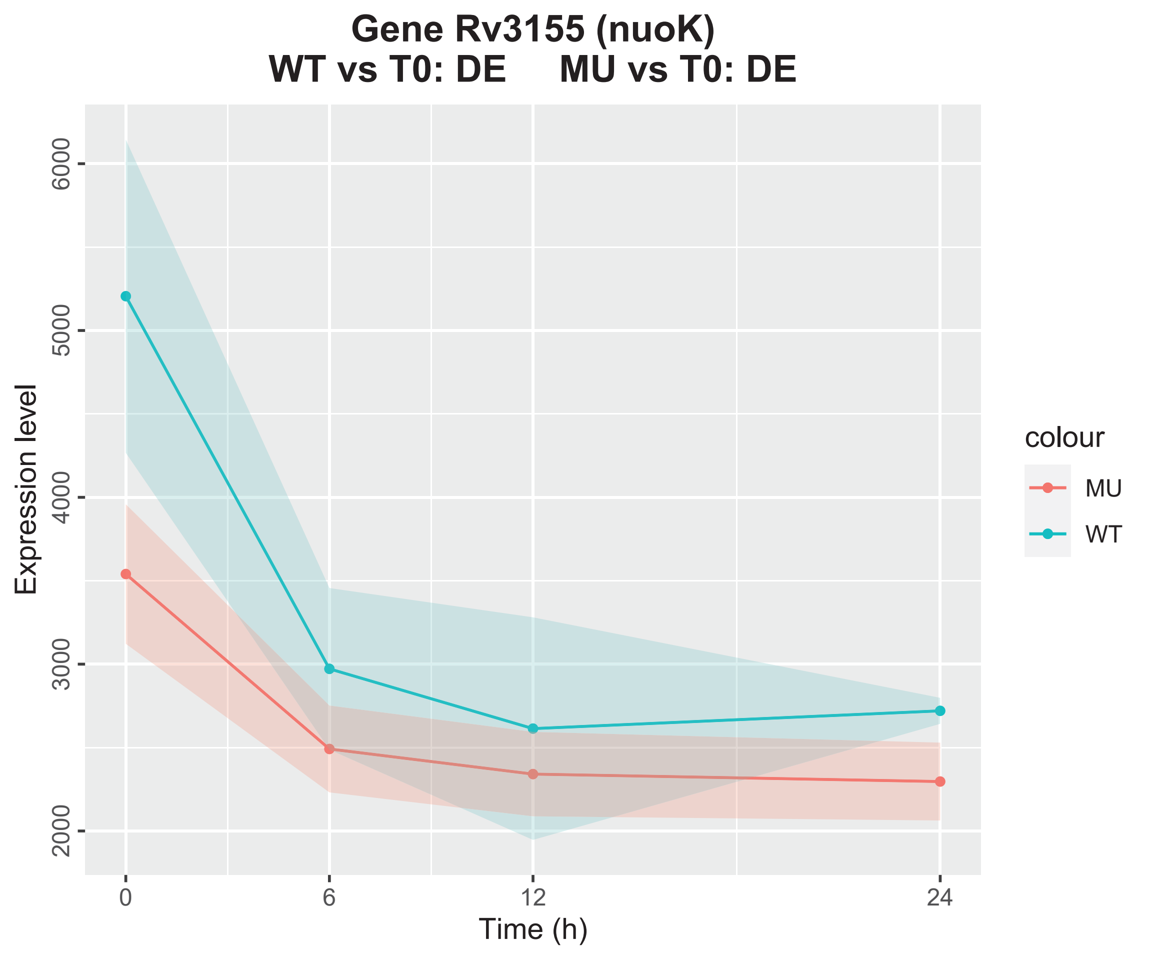
Gene Rv3155 (nuoK)
WT vs T0: DE   MU vs T0: DE
Expression level
6000
5000
4000
3000
2000
0
6
12
24
Time (h)
colour
MU
WT

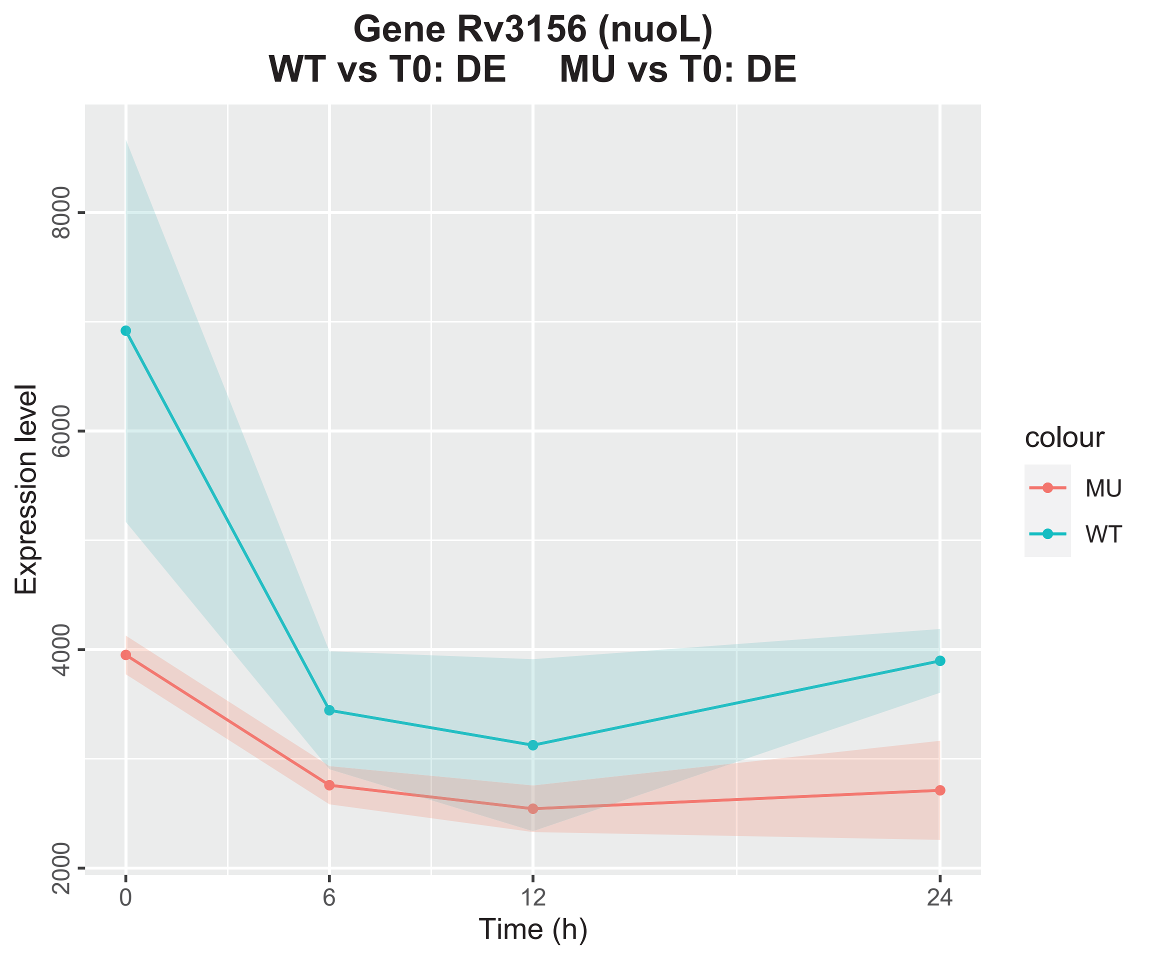
Gene Rv3156 (nuoL)
WT vs T0: DE   MU vs T0: DE
Expression level
8000
6000
4000
2000
0
6
12
24
Time (h)
colour
MU
WT

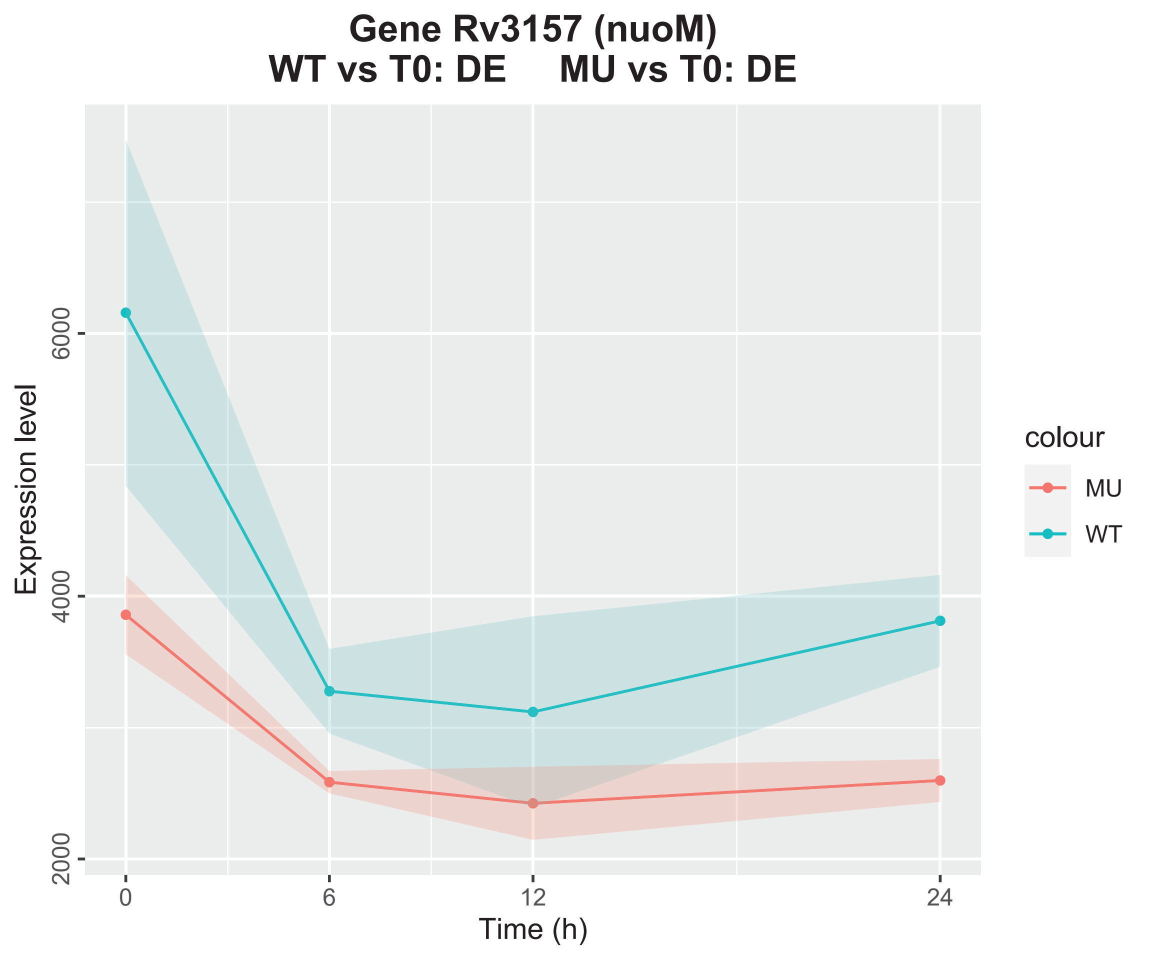

Gene Rv3157 (nuoM)
WT vs T0: DE   MU vs T0: DE
Expression level
2000
4000
6000
0
6
12
24
Time (h)
colour
MU
WT

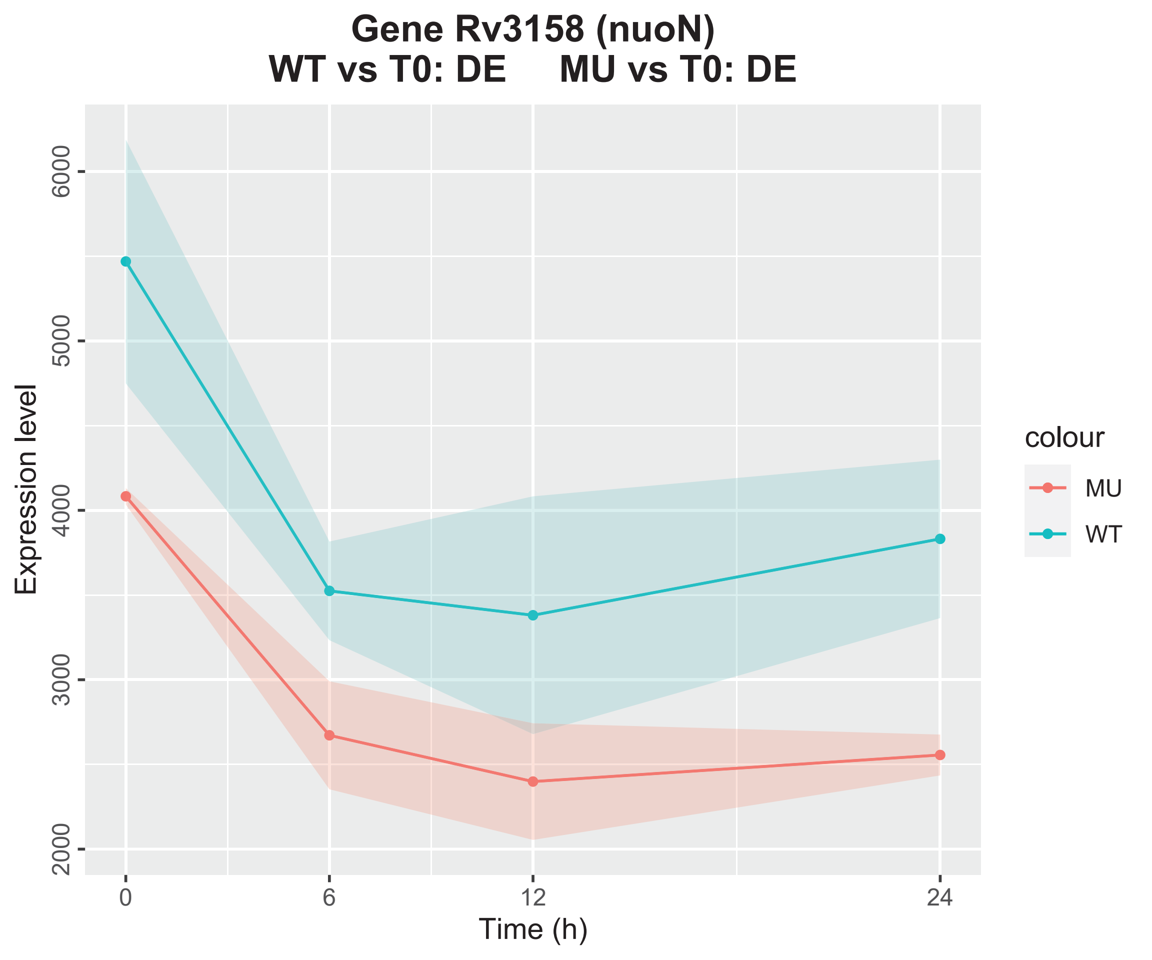
Gene Rv3158 (nuoN)
WT vs T0: DE   MU vs T0: DE
Expression level
6000
5000
4000
3000
2000
Time (h)
0
6
12
24
colour
MU
WT

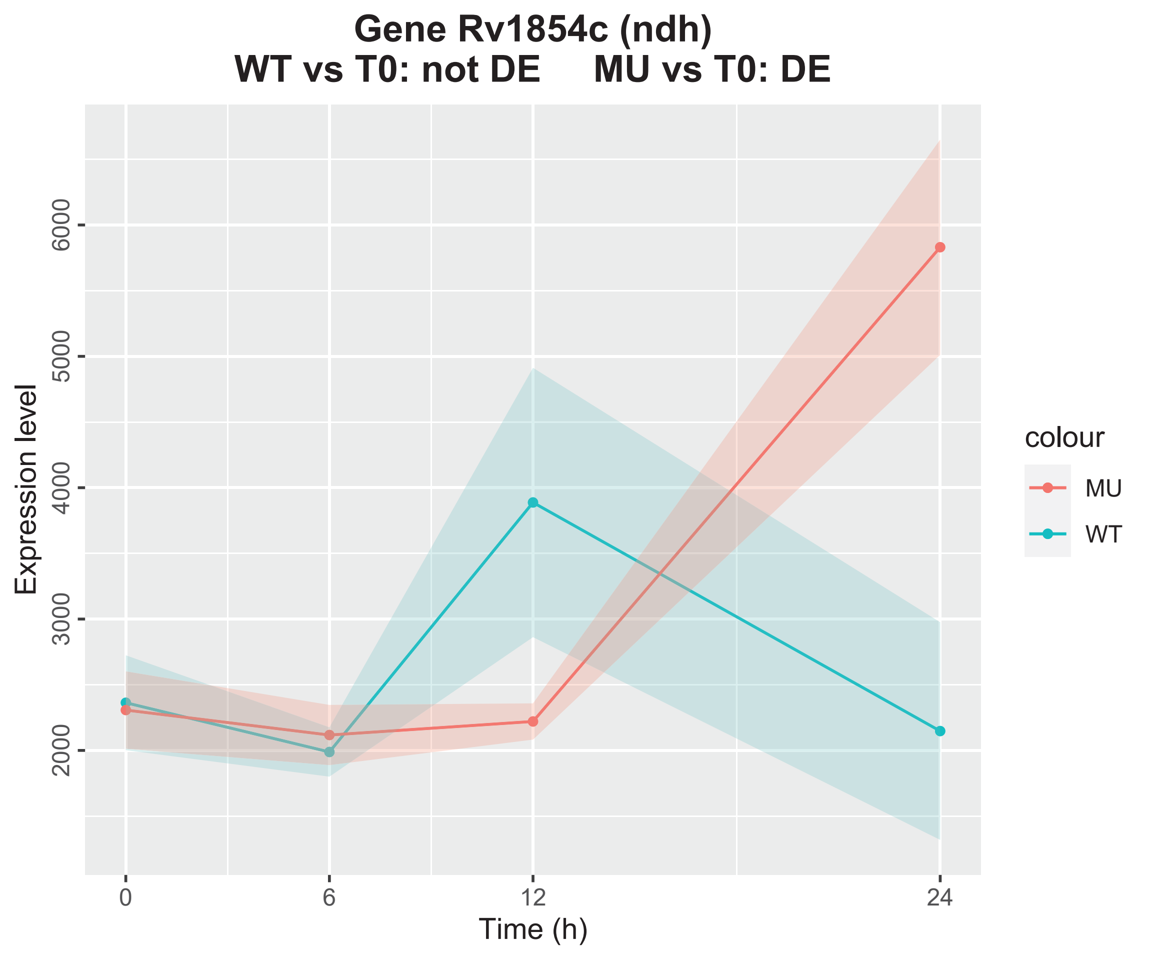
Gene Rv1854c (ndh)
WT vs T0: not DE   MU vs T0: DE
Expression level
2000
3000
4000
5000
6000
0
6
12
24
Time (h)
colour
MU
WT

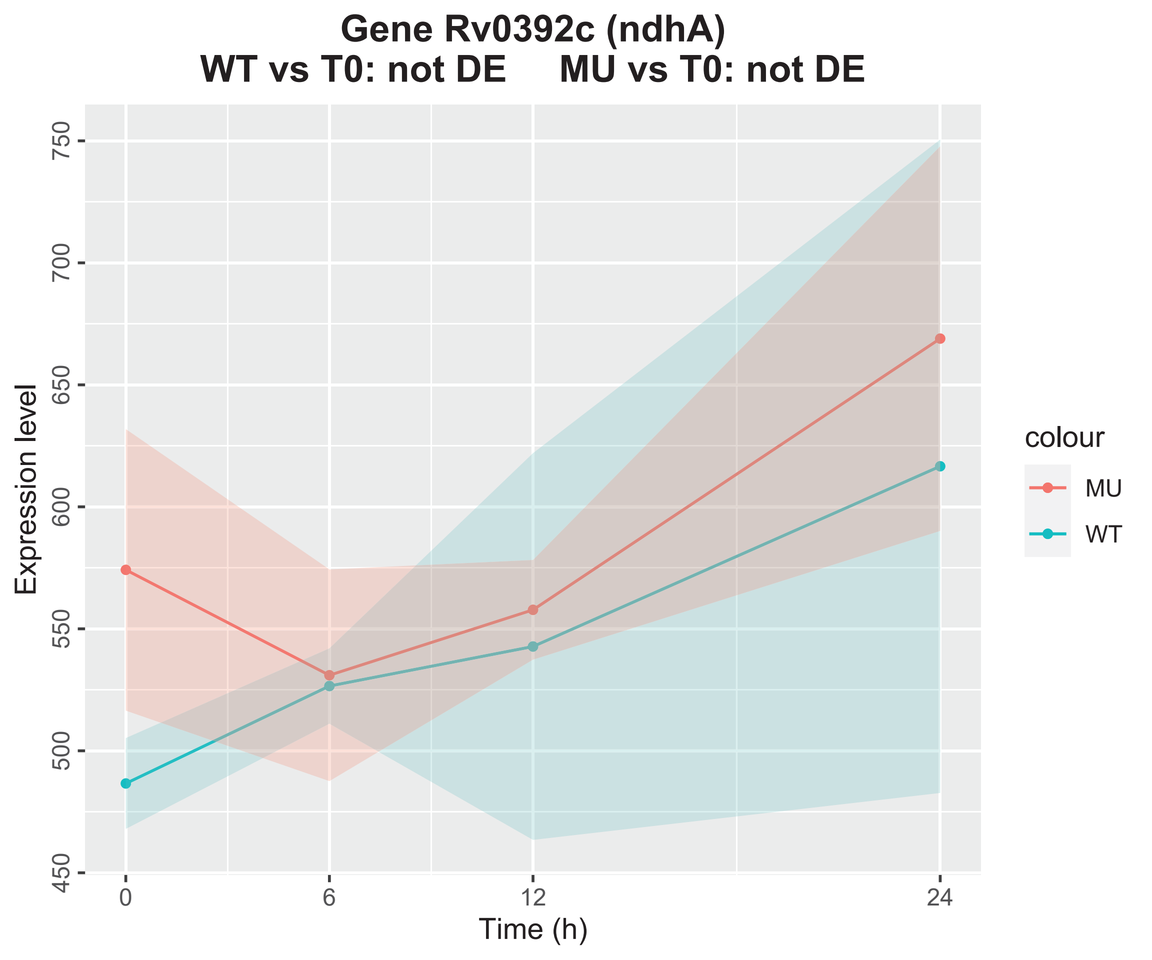
Gene Rv0392c (ndhA)
WT vs T0: not DE   MU vs T0: not DE
Expression level
750
700
650
600
550
500
450
0
6
12
24
Time (h)
colour
MU
WT

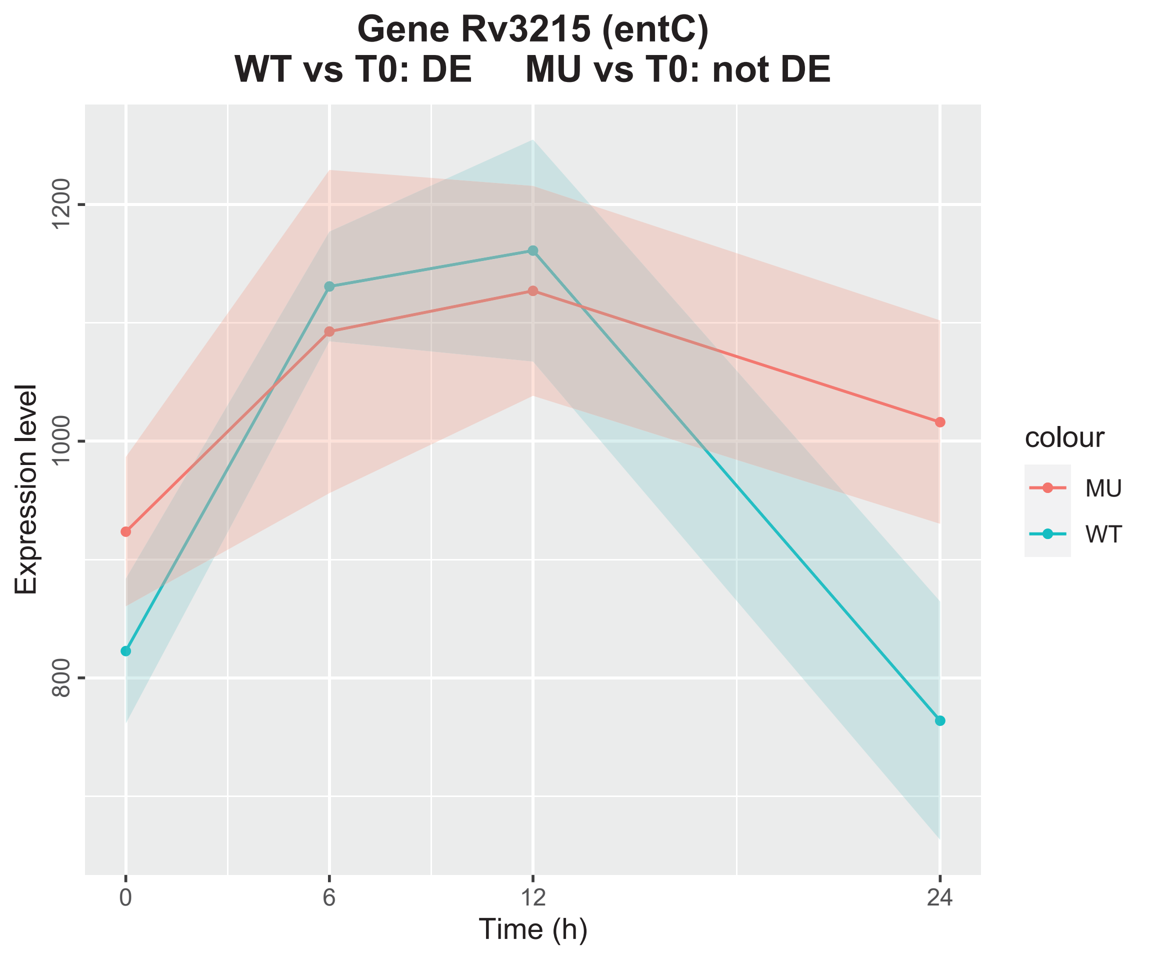
Gene Rv3215 (entC)
WT vs T0: DE   MU vs T0: not DE
Expression level
1200
1000
800
0
6
12
24
Time (h)
colour
MU
WT

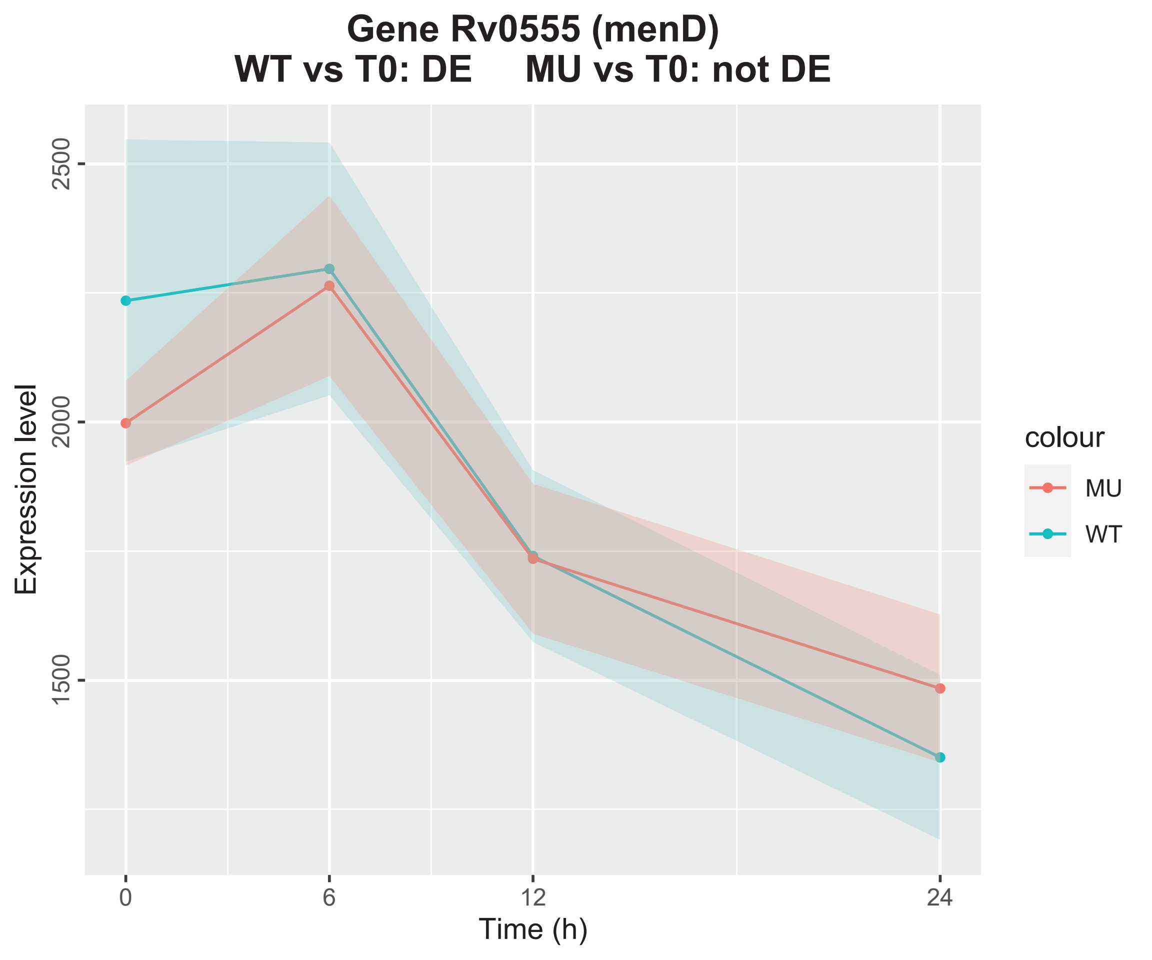
Gene Rv0555 (menD)
WT vs T0: DE MU vs T0: not DE
2500
2000
1500
Expression level
0
6
12
24
Time (h)
colour
MU
WT

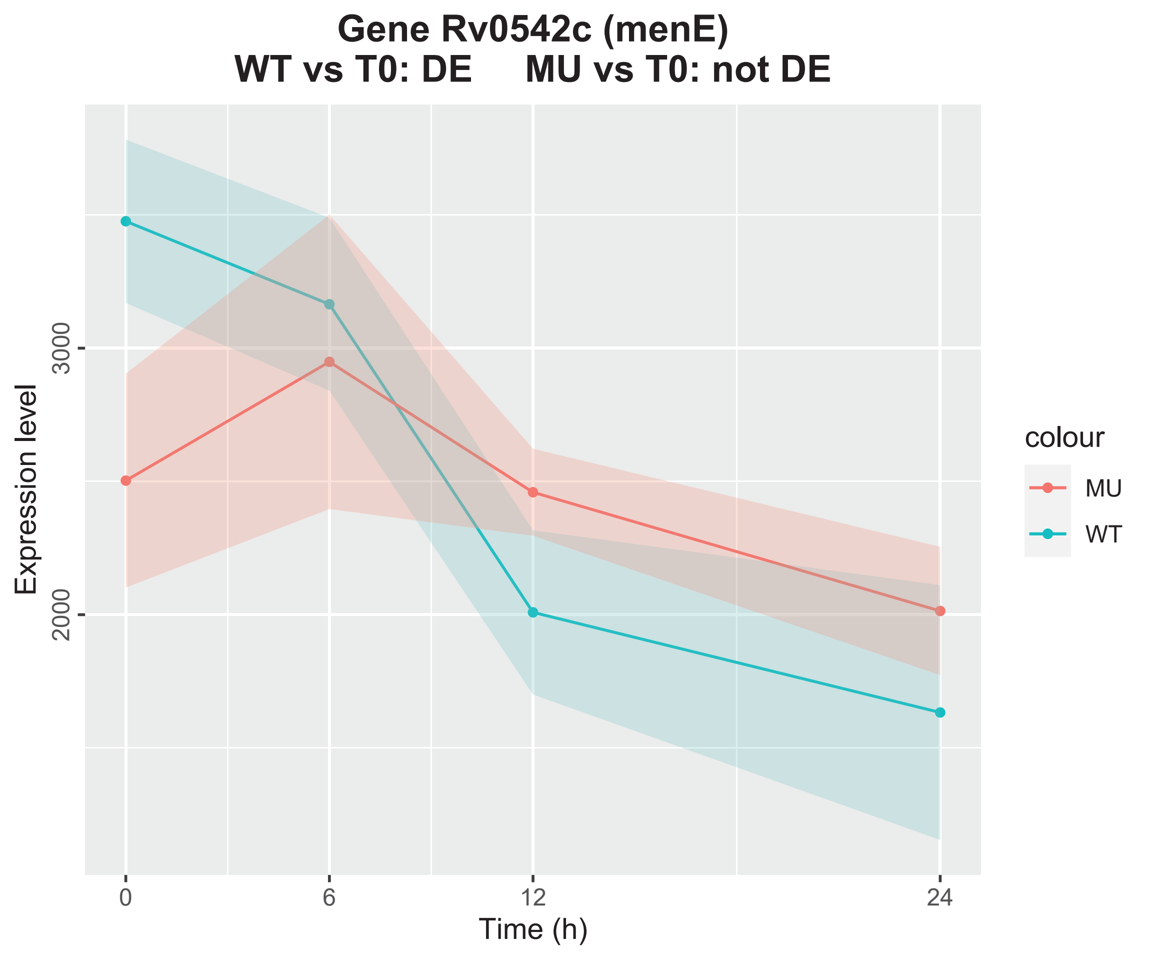

Gene Rv0542c (menE)
WT vs T0: DE MU vs T0: not DE
Expression level
3000
2000
0
6
12
24
Time (h)
colour
MU
WT

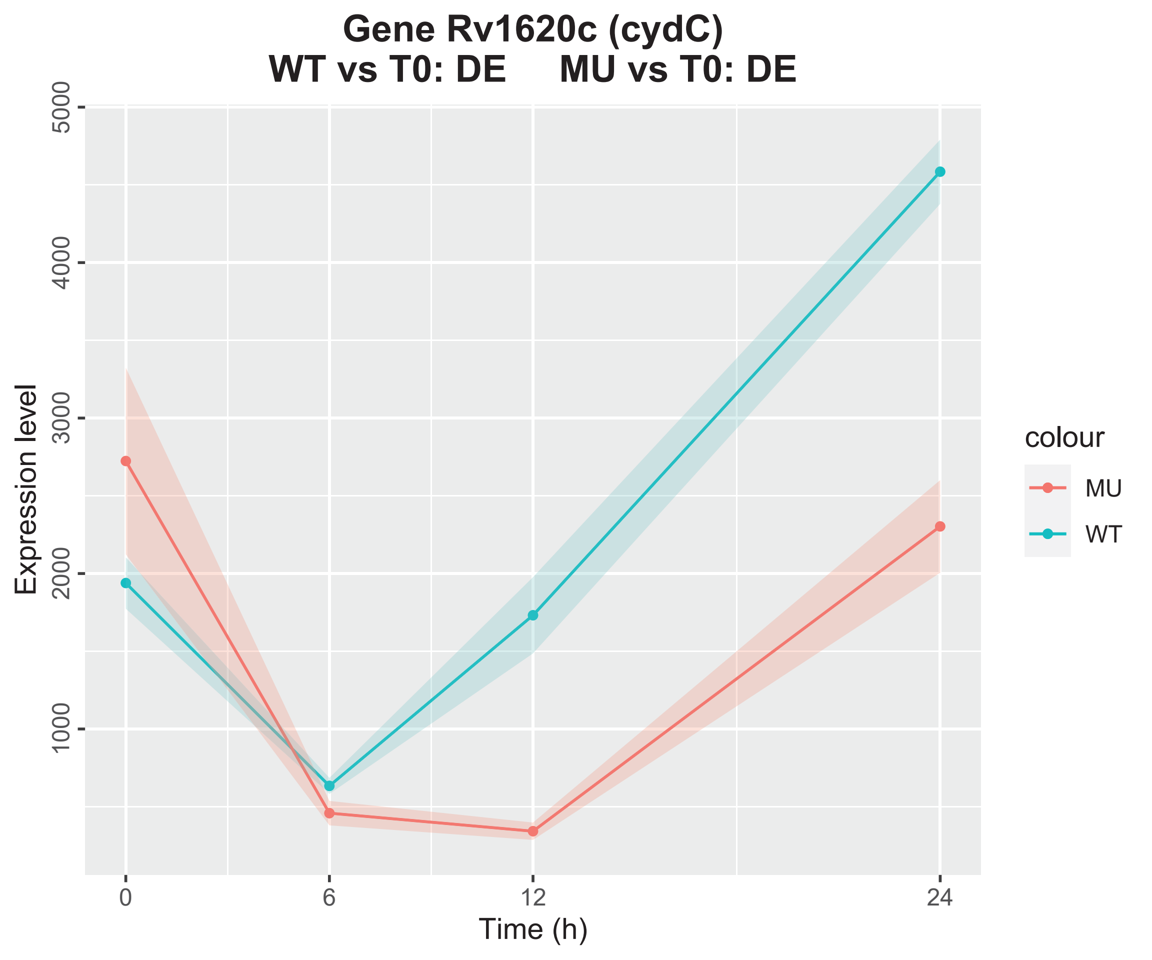

Gene Rv1620c (cydC)
WT vs T0: DE MU vs T0: DE
Expression level
5000
4000
3000
2000
1000
0
6
12
24
Time (h)
colour
MU
WT

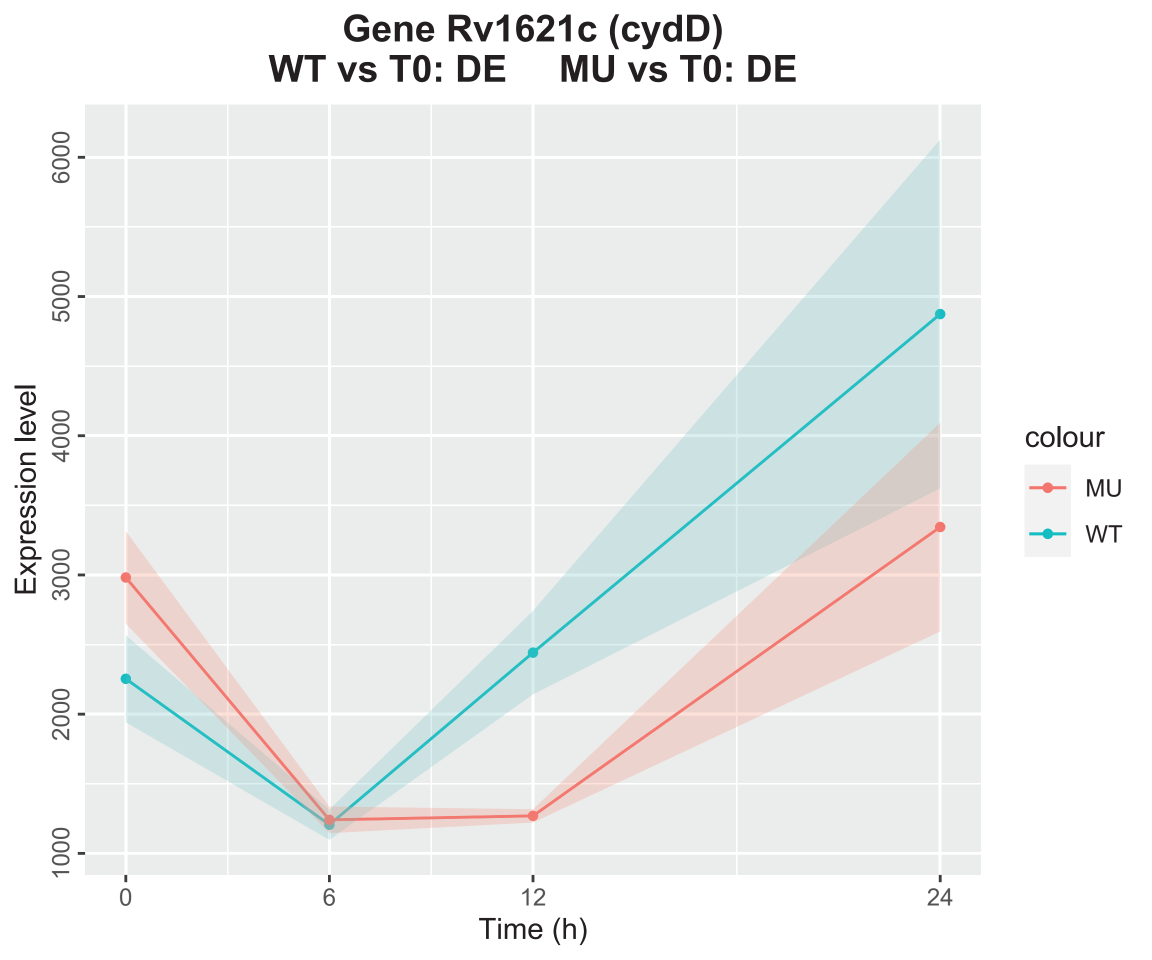
Gene Rv1621c (cydD)
WT vs T0: DE MU vs T0: DE
Expression level
1000
2000
3000
4000
5000
6000
0
6
12
24
Time (h)
colour
MU
WT

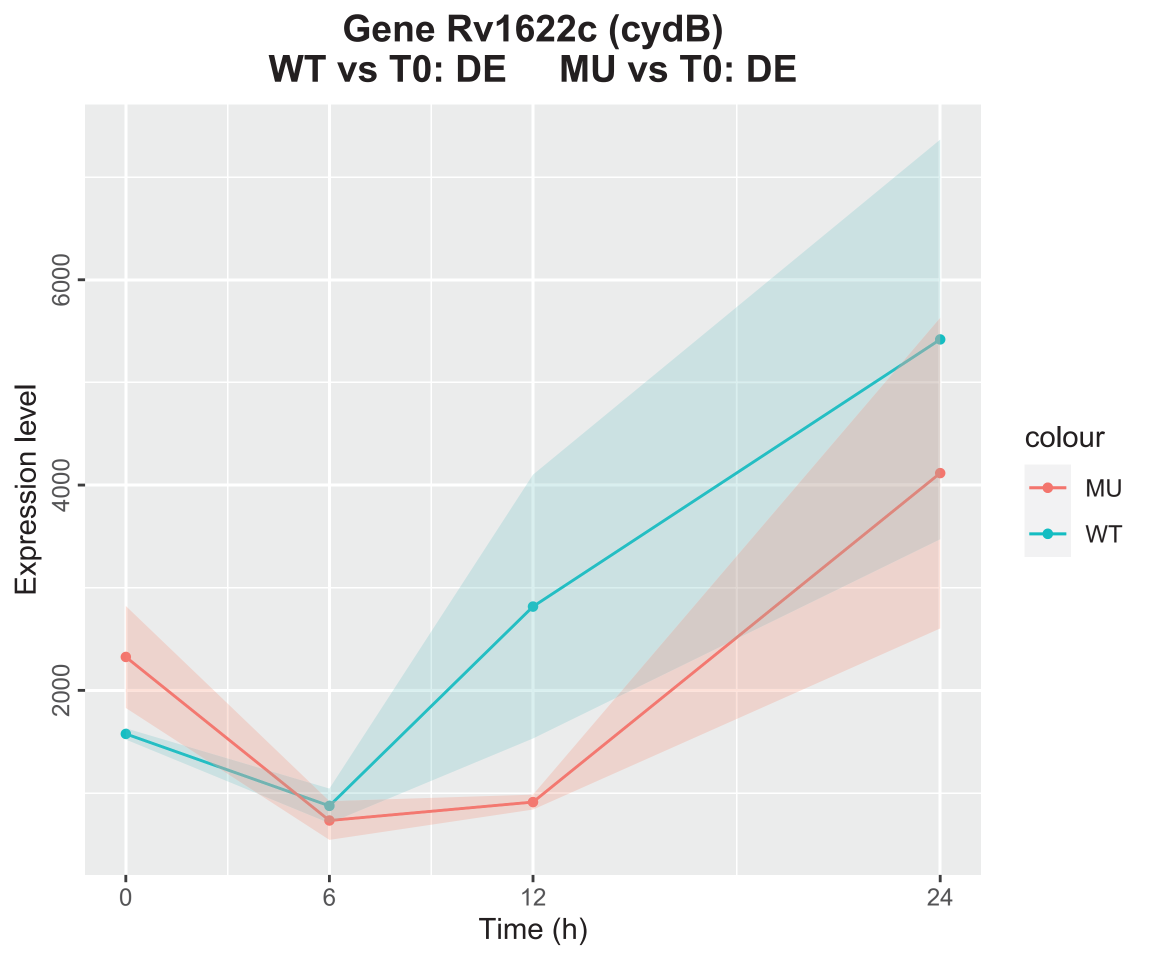
Gene Rv1622c (cydB)
WT vs T0: DE MU vs T0: DE
Expression level
6000
4000
2000
0
6
12
24
Time (h)
colour
MU
WT

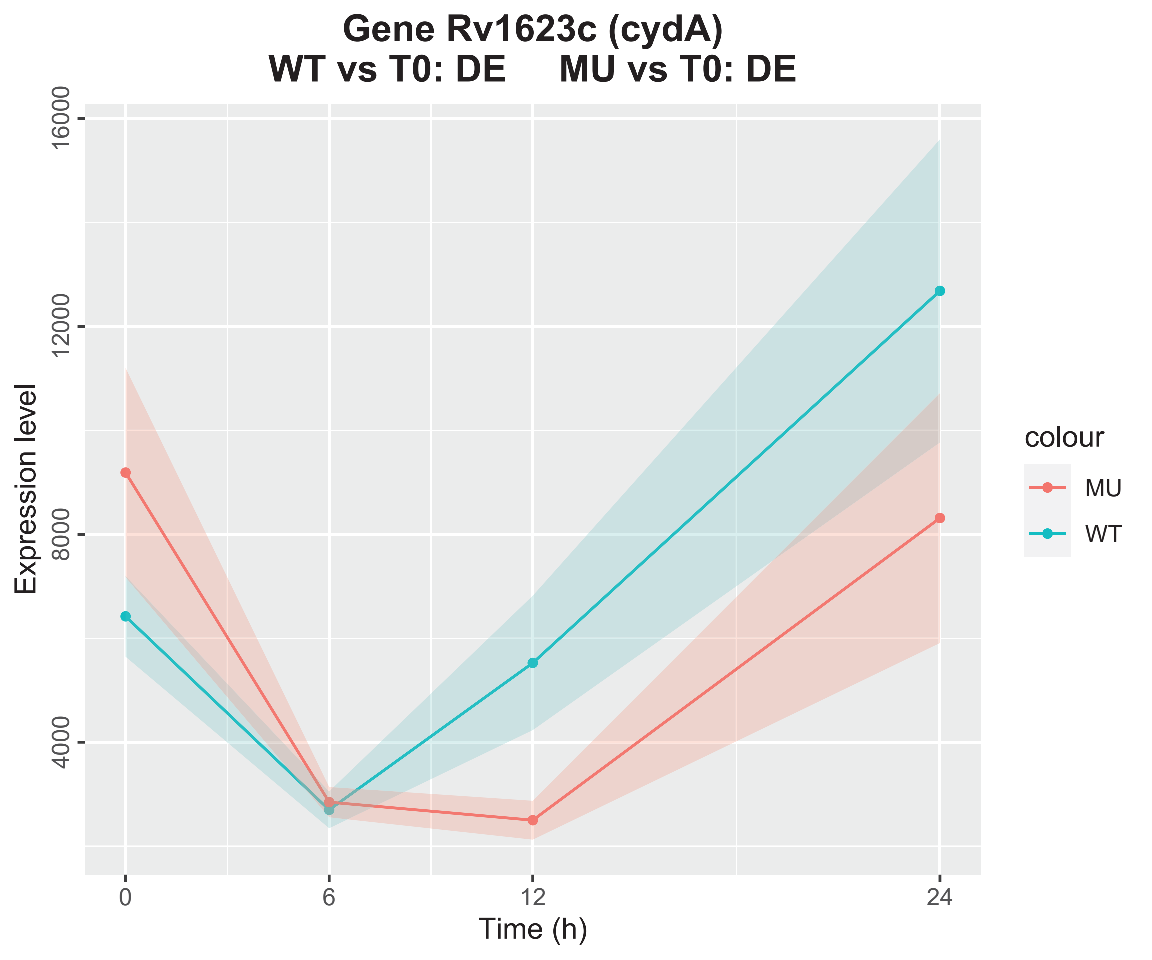

Gene Rv1623c (cydA)
WT vs T0: DE   MU vs T0: DE
Expression level
16000
12000
8000
4000
0
6
12
24
Time (h)
colour
MU
WT

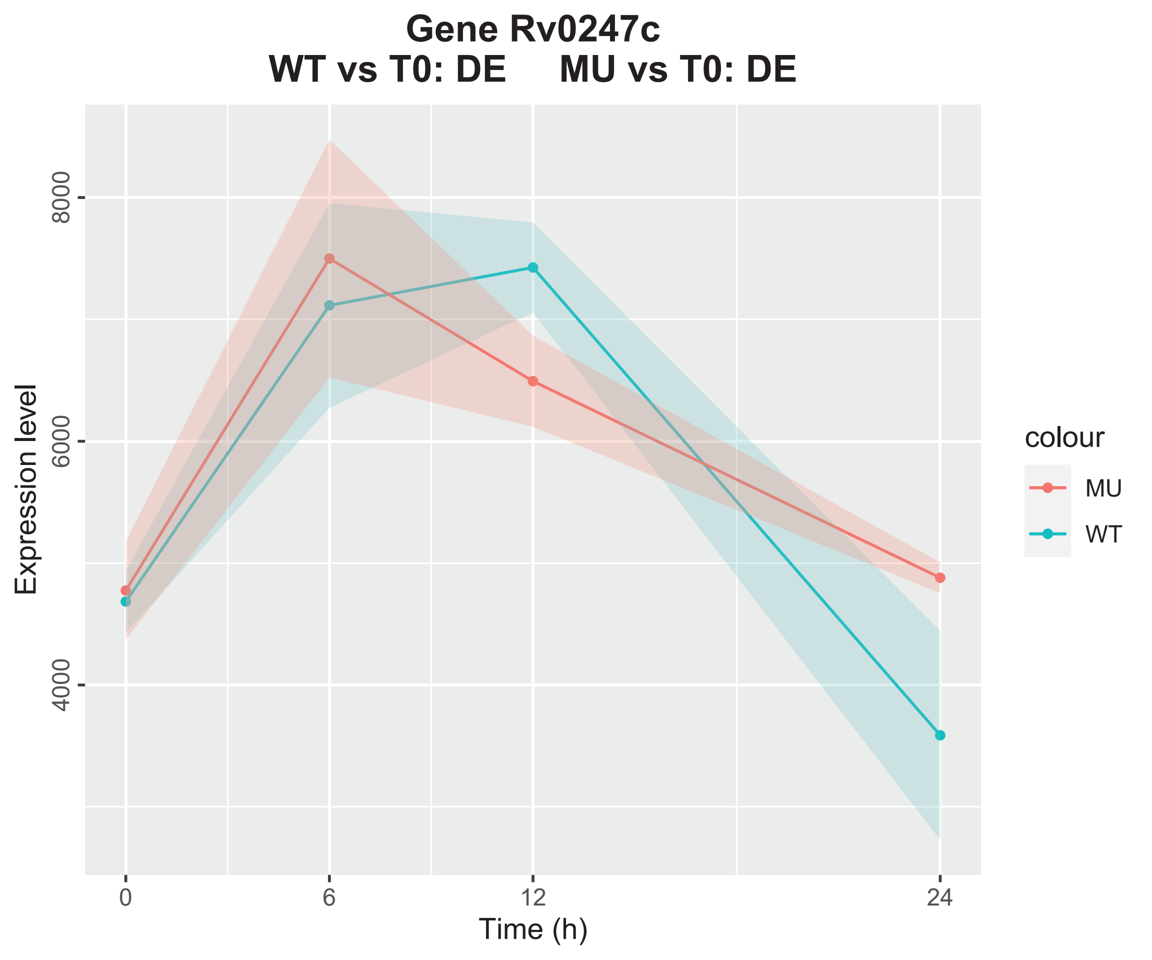
Gene Rv0247c
WT vs T0: DE   MU vs T0: DE
Expression level
8000
6000
4000
Time (h)
0
6
12
24
colour
MU
WT

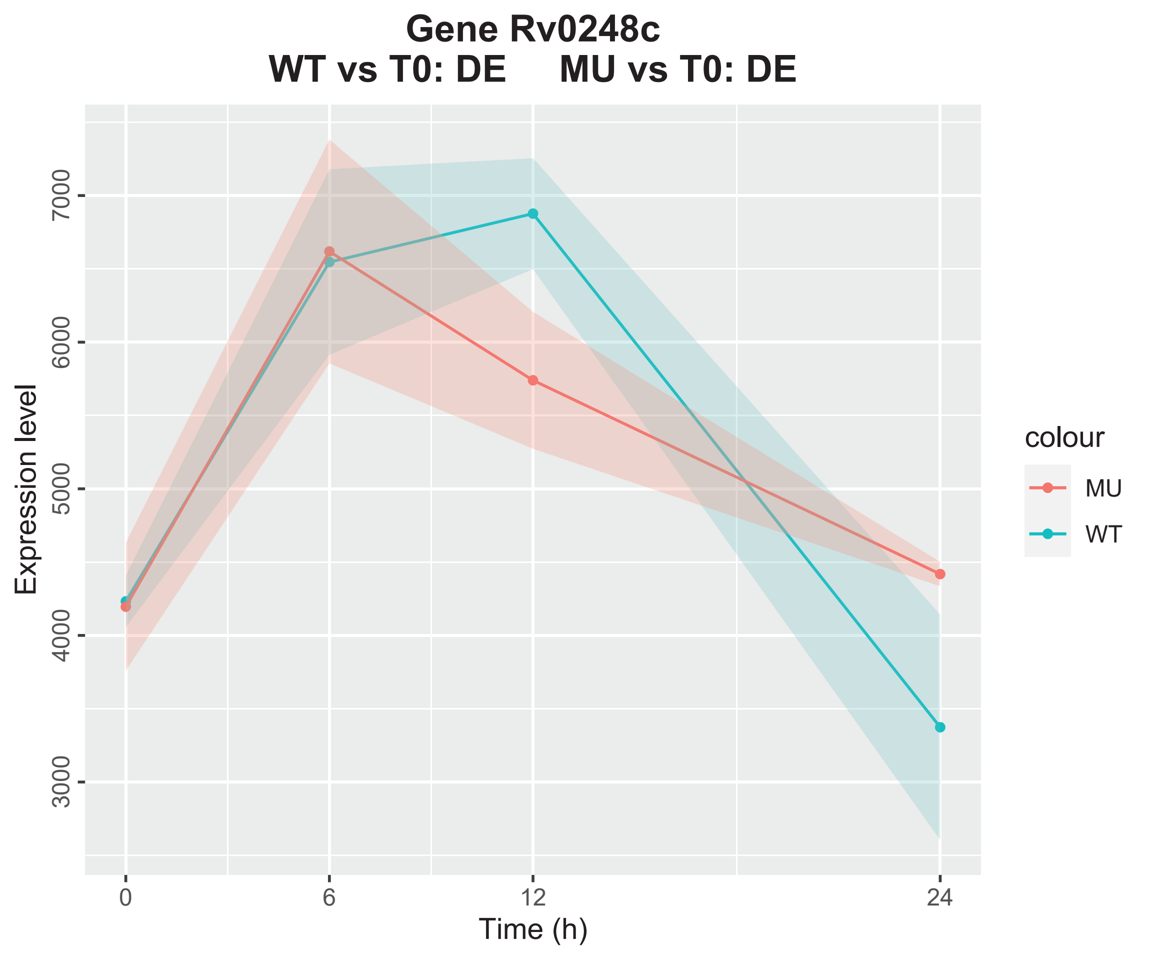
Gene Rv0248c
WT vs T0: DE   MU vs T0: DE
Expression level
Time (h)
0
6
12
24
3000
4000
5000
6000
7000
colour
MU
WT

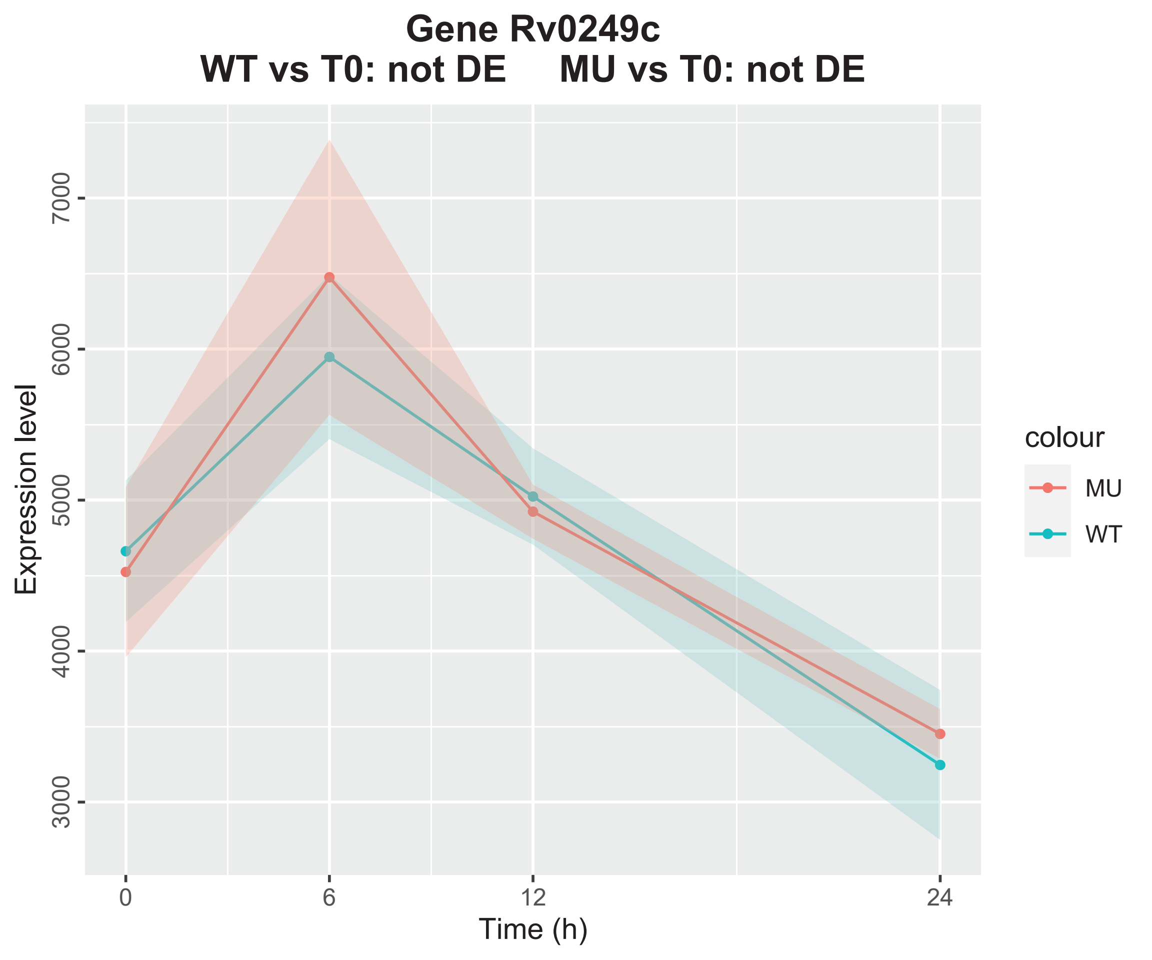
Gene Rv0249c
WT vs T0: not DE
MU vs T0: not DE
Expression level
7000
6000
5000
4000
3000
0
6
12
24
Time (h)
colour
MU
WT

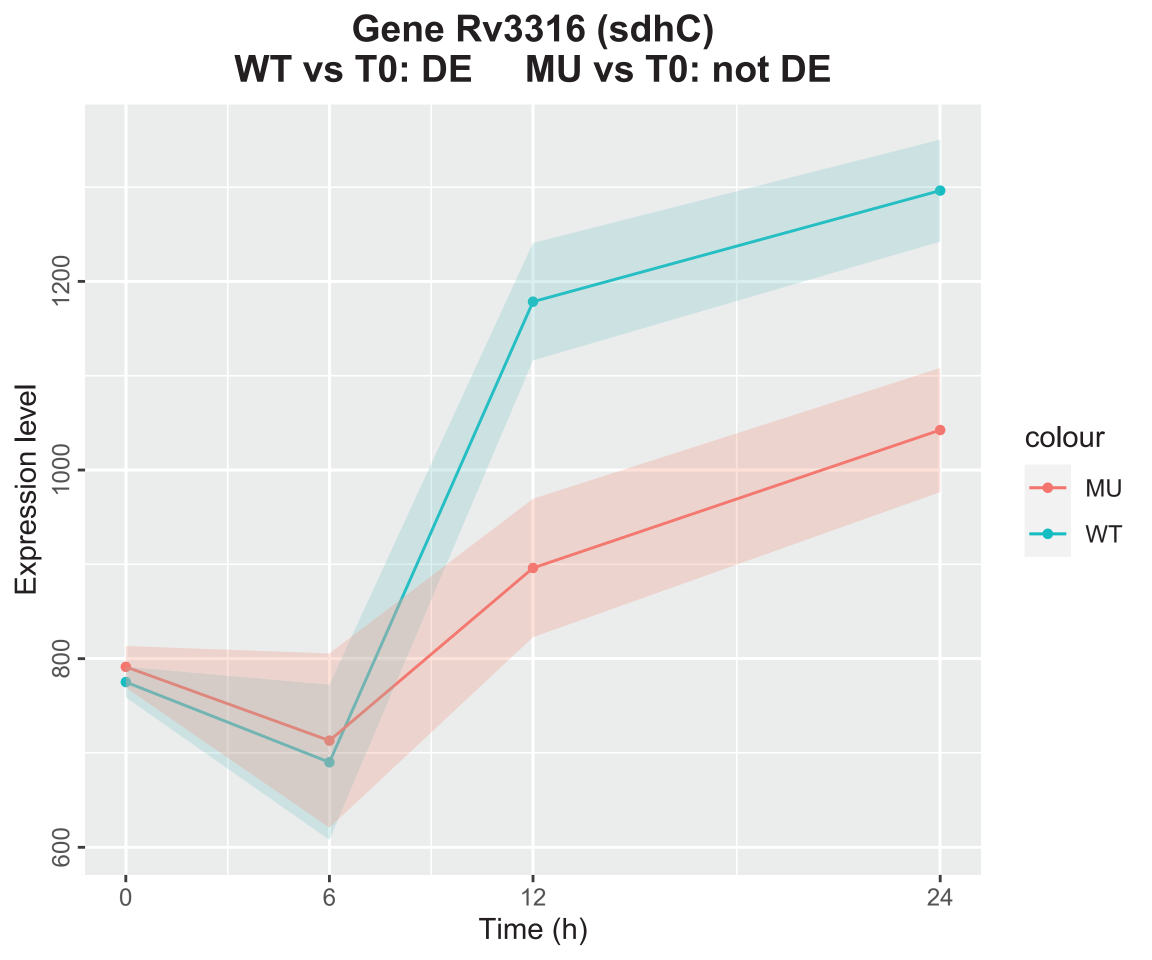
Gene Rv3316 (sdhC)
WT vs T0: DE MU vs T0: not DE
Expression level
600
800
1000
1200
0
6
12
24
Time (h)
colour
MU
WT

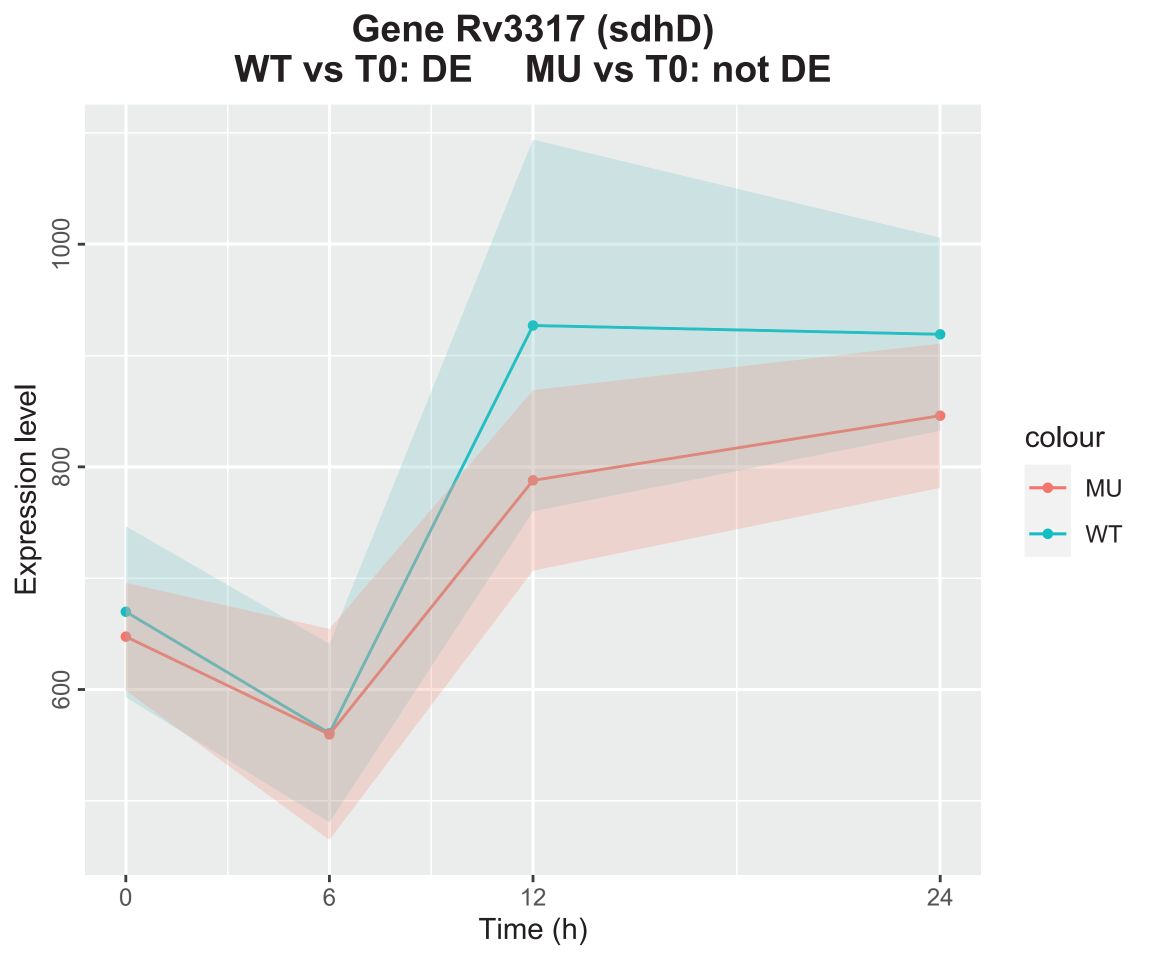

Gene Rv3317 (sdhD)
WT vs T0: DE MU vs T0: not DE
Expression level
1000
800
600
0
6
12
24
Time (h)
colour
MU
WT
