# Supplementary material for: Role of the Extracytoplasmic Function Sigma Factor SigE in the Stringent Response of Mycobacterium tuberculosis
Source: Microbiol Spectr. 2023 Mar 22;11(2):e02944-22. doi: 10.1128/spectrum.02944-22 (PMC10100808; doi:10.1128/spectrum.02944-22)
Supplement: Supplemental file 5 — Data S5. Download spectrum.02944-22-s0006.pdf, PDF file, 0.3 MB [file spectrum.02944-22-s0006.pdf]

**<u>Supplementary Data S5:</u>** Gene expression profiles over time of genes discussed in section "Central carbon metabolism pathways alteration", i.e. *sucCD*, *icl1*, *aceAa*-*aceAb*, *gltD*, *icd2*, *rv0247c*-*rv0248c*-*rv0249c*, *sdhCDAB*, *fum*, *mdh*, *PckA* and *cydBA*. For each gene, the plot shows the average expression level and the standard deviation (shaded area) for both the wild-type (cyan color) and *sigE*-mutant (salmon/pink color) strains.

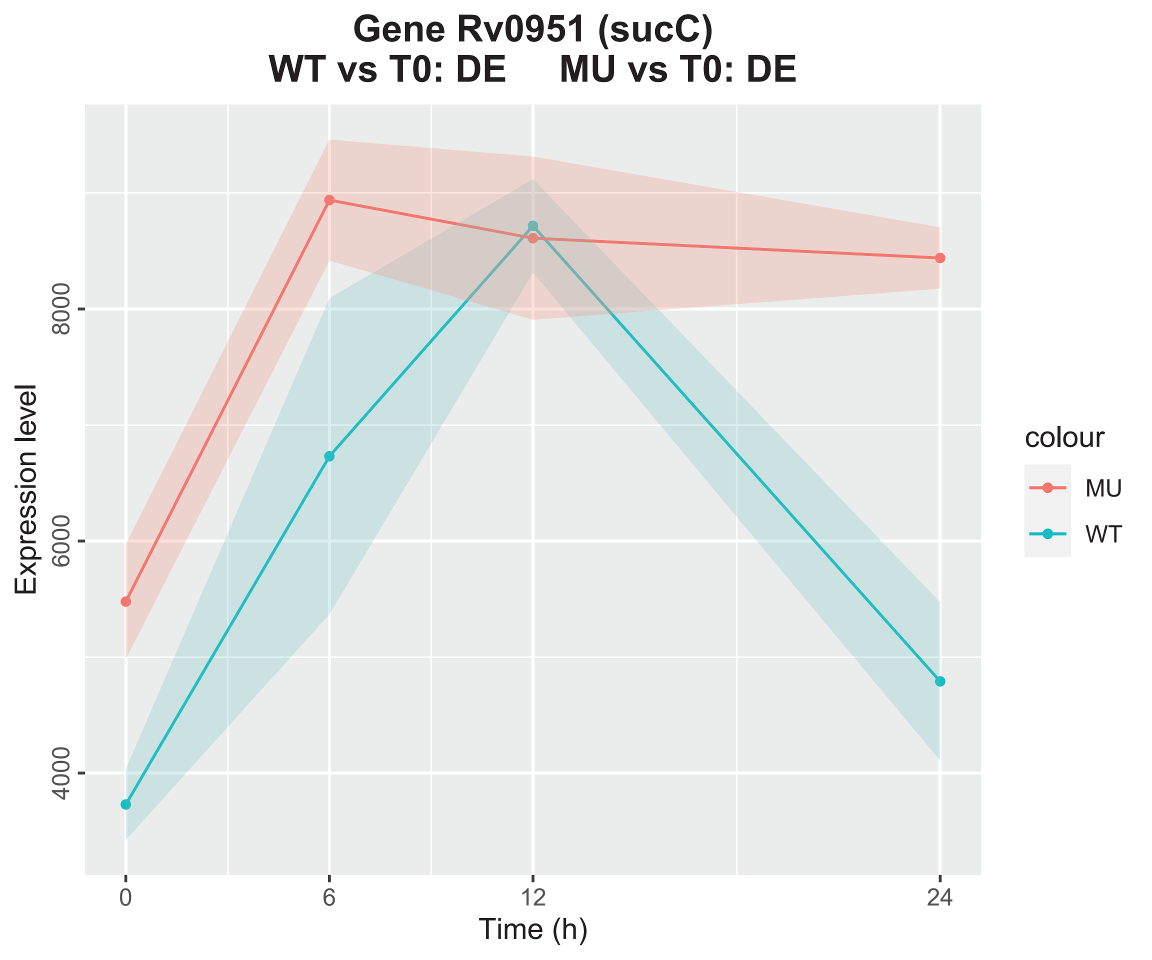
Gene Rv0951 (sucC)
WT vs T0: DE     MU vs T0: DE
Expression level
8000
6000
4000
0
6
12
24
Time (h)
colour
MU
WT

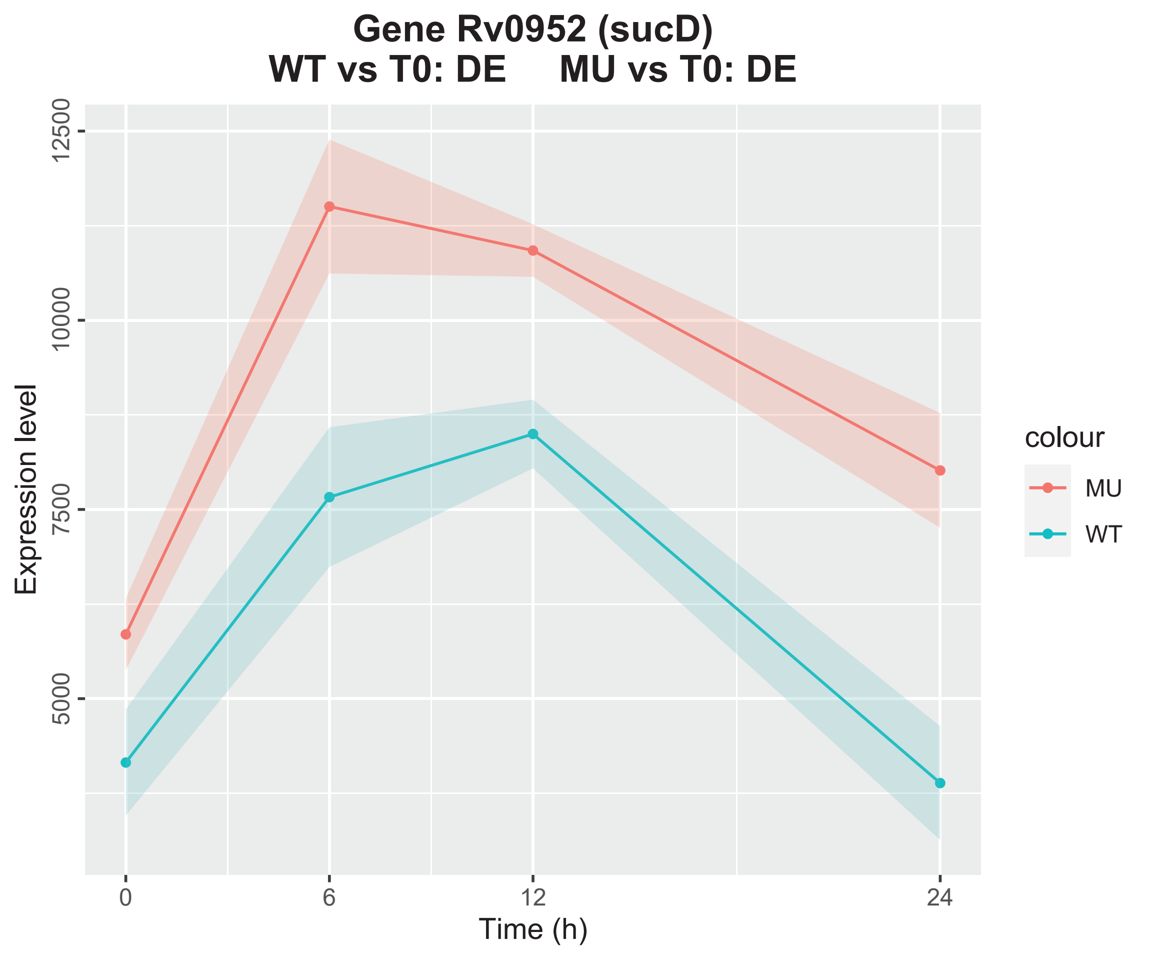
Gene Rv0952 (sucD)
WT vs T0: DE     MU vs T0: DE
Expression level
12500
10000
7500
5000
0
6
12
24
Time (h)
colour
MU
WT

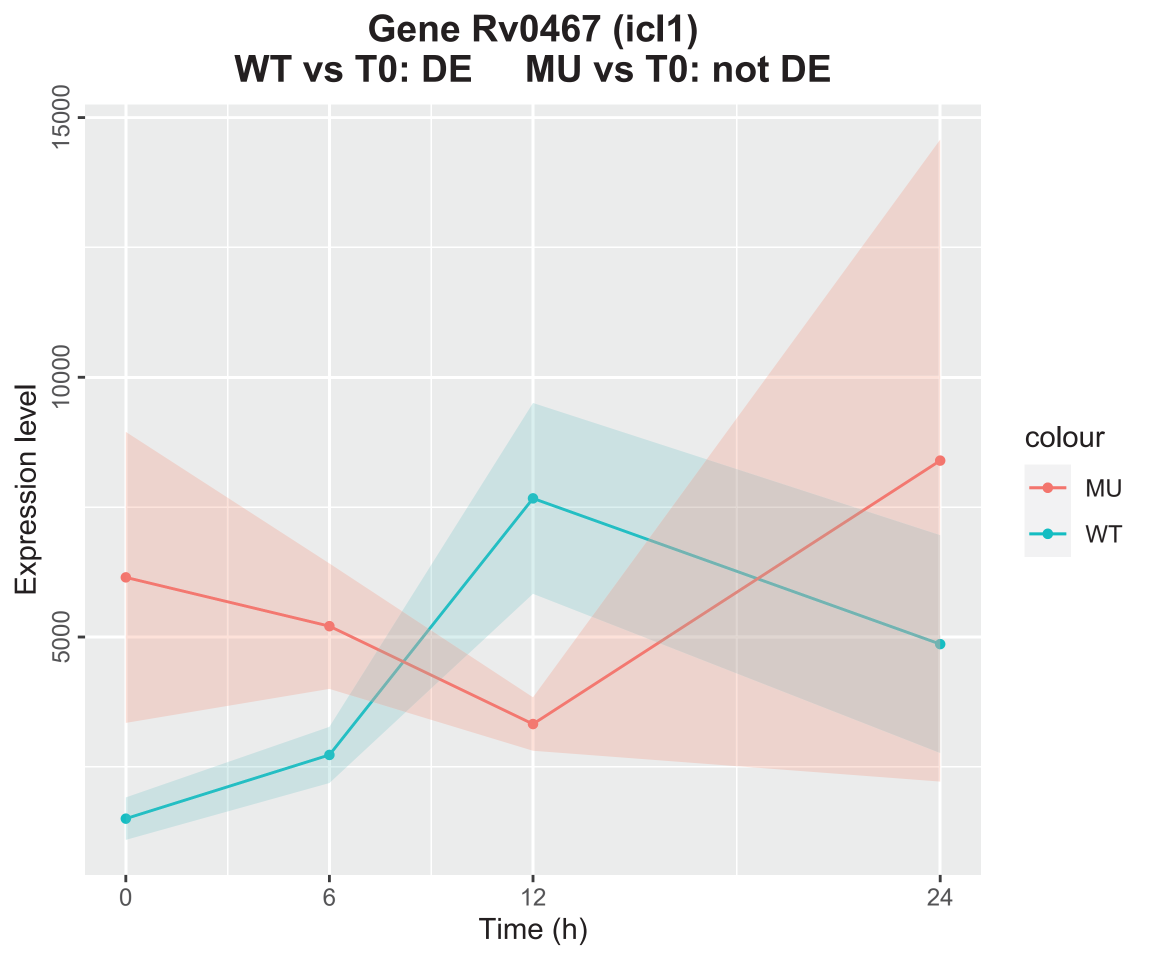
Gene Rv0467 (icl1)
WT vs T0: DE     MU vs T0: not DE
Expression level
15000
10000
5000
0
6
12
24
Time (h)
colour
MU
WT

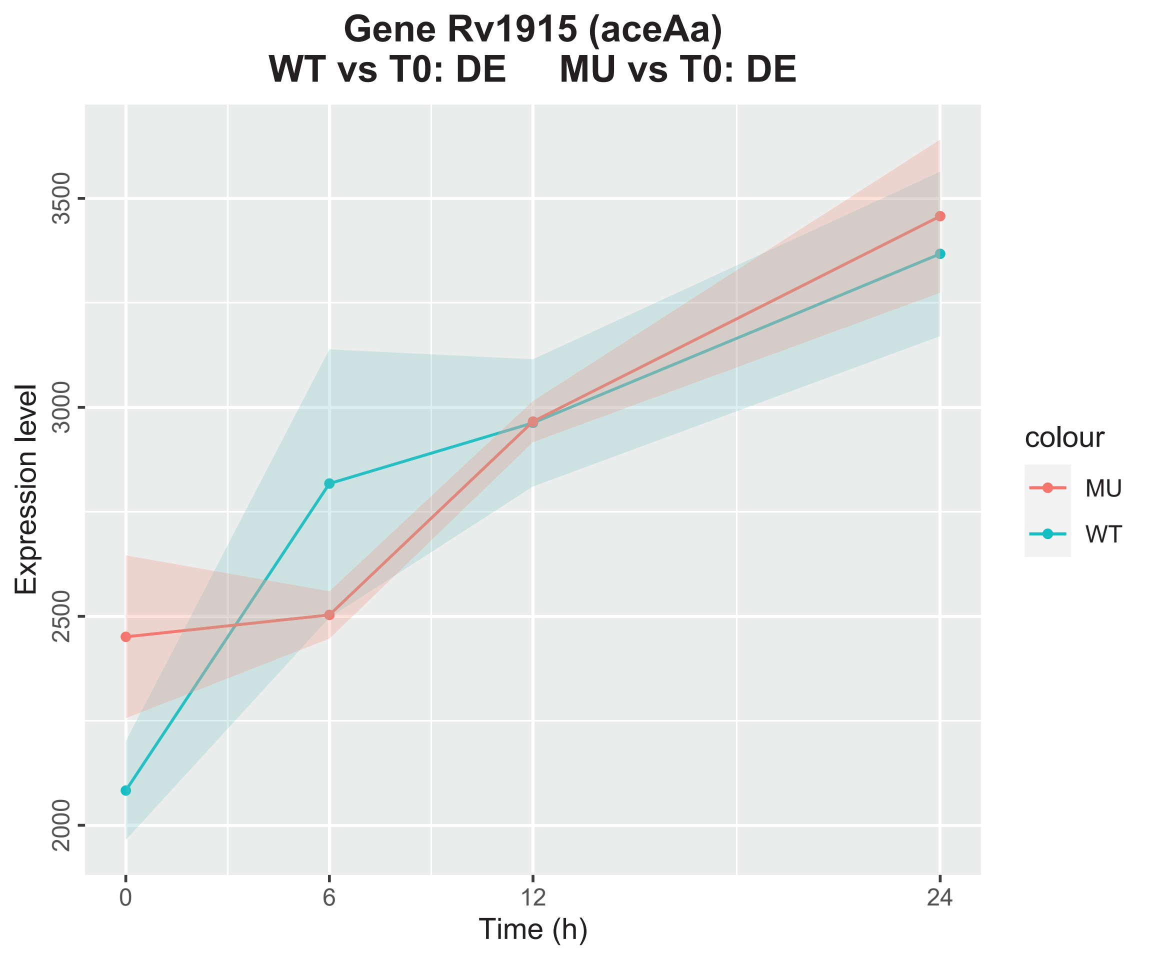
Gene Rv1915 (aceAa)
WT vs T0: DE     MU vs T0: DE
Expression level
2000
2500
3000
3500
0
6
12
24
Time (h)
colour
MU
WT

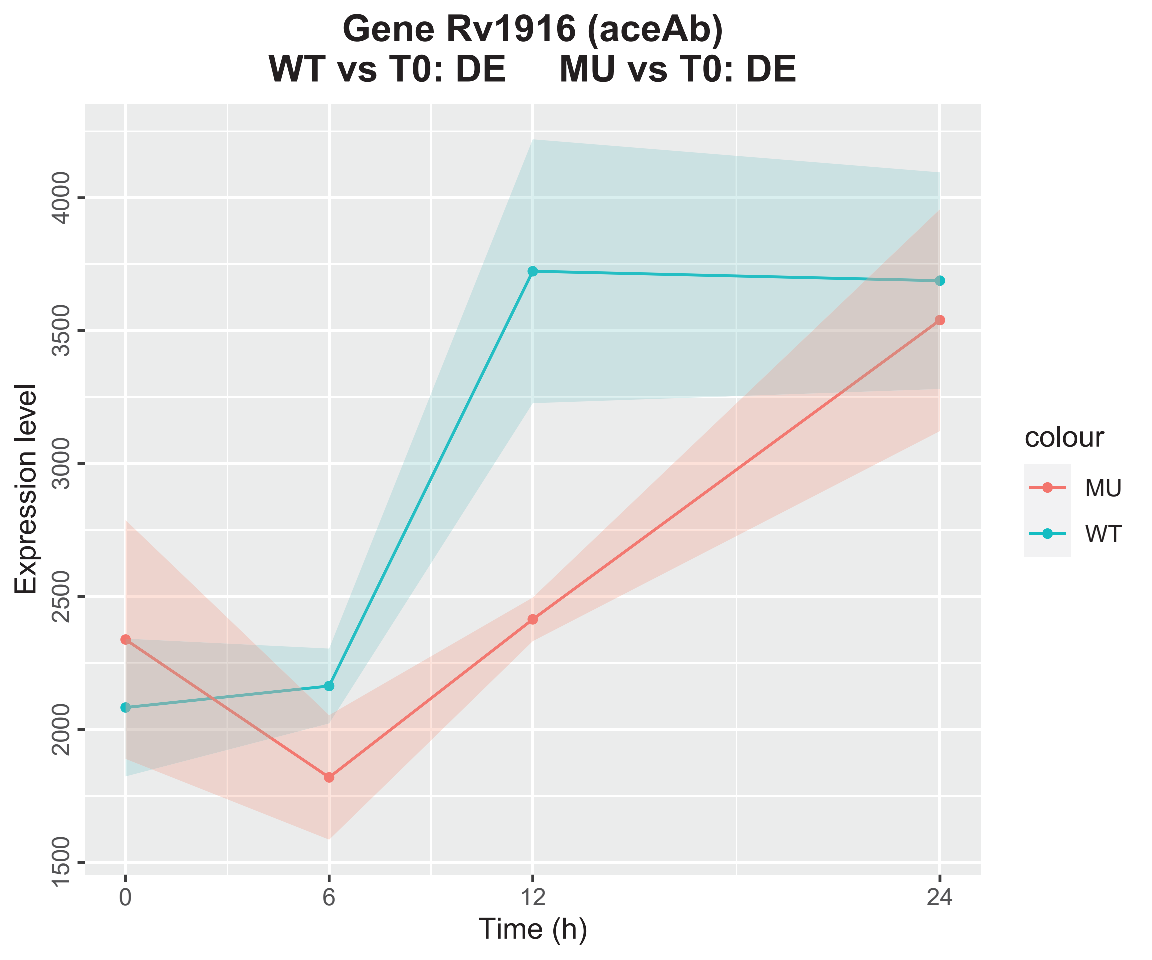
Gene Rv1916 (aceAb)
WT vs T0: DE     MU vs T0: DE
Expression level
1500
2000
2500
3000
3500
4000
0
6
12
24
Time (h)
colour
MU
WT

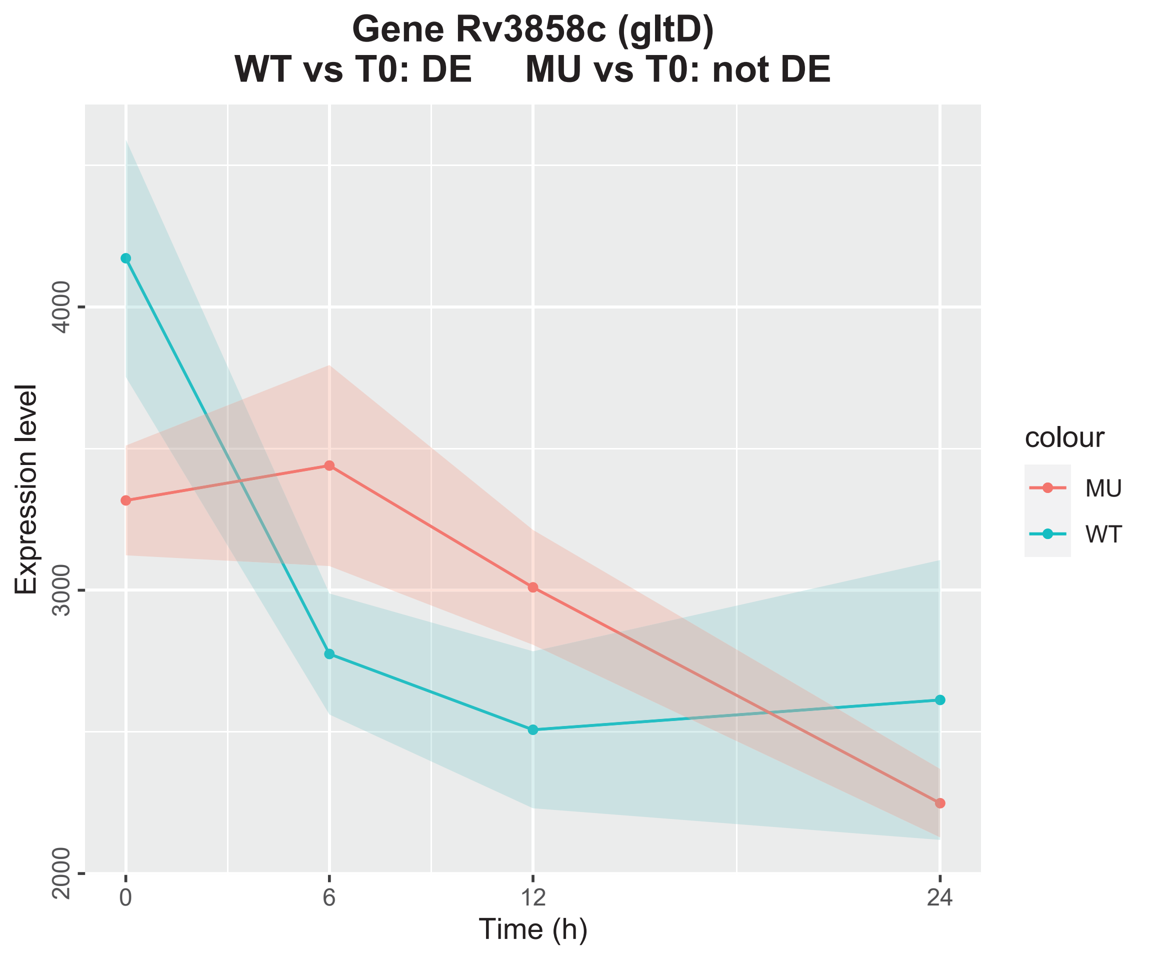
Gene Rv3858c (gltD)
WT vs T0: DE     MU vs T0: not DE
Expression level
2000
3000
4000
0
6
12
24
Time (h)
colour
MU
WT

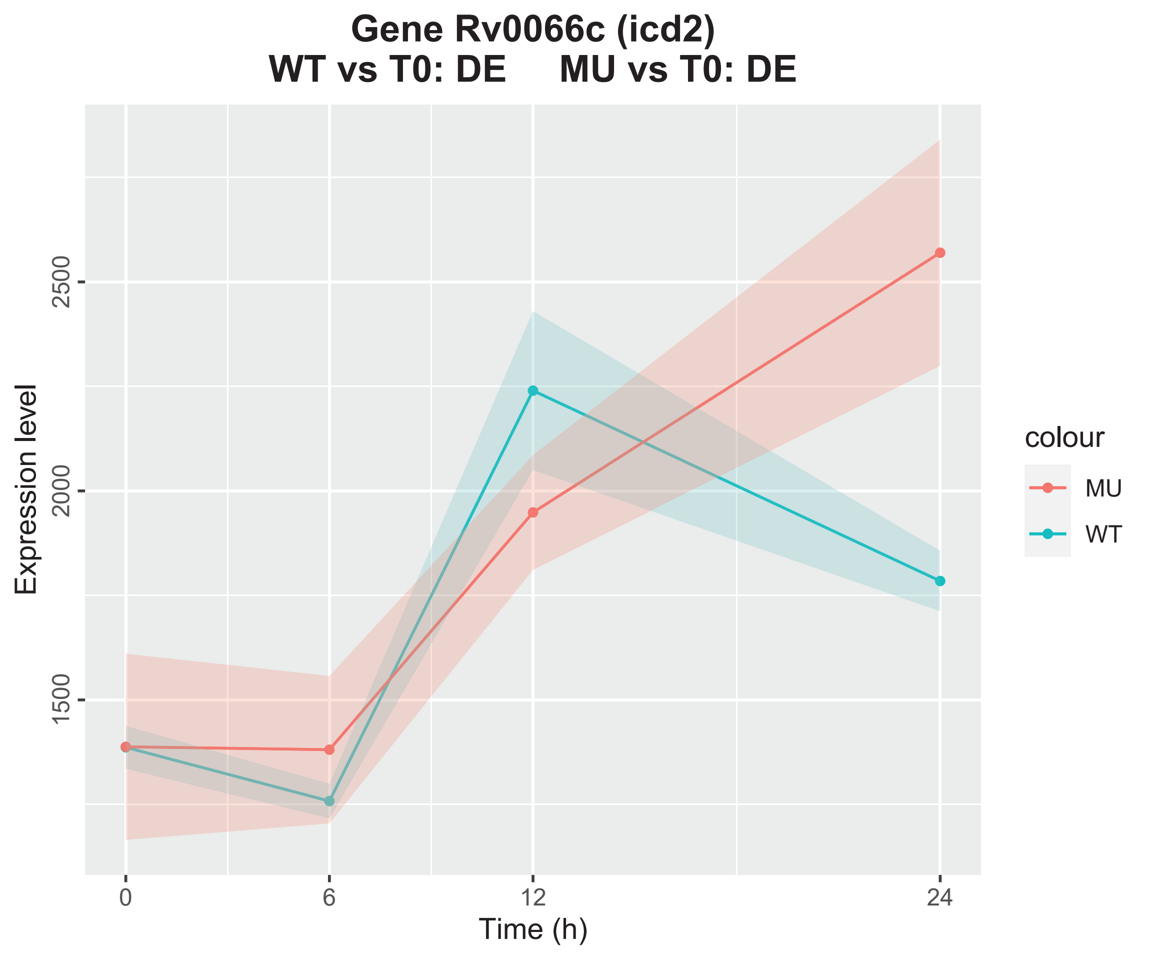
Gene Rv0066c (icd2)
WT vs T0: DE     MU vs T0: DE
Expression level
2500
2000
1500
0
6
12
24
Time (h)
colour
MU
WT

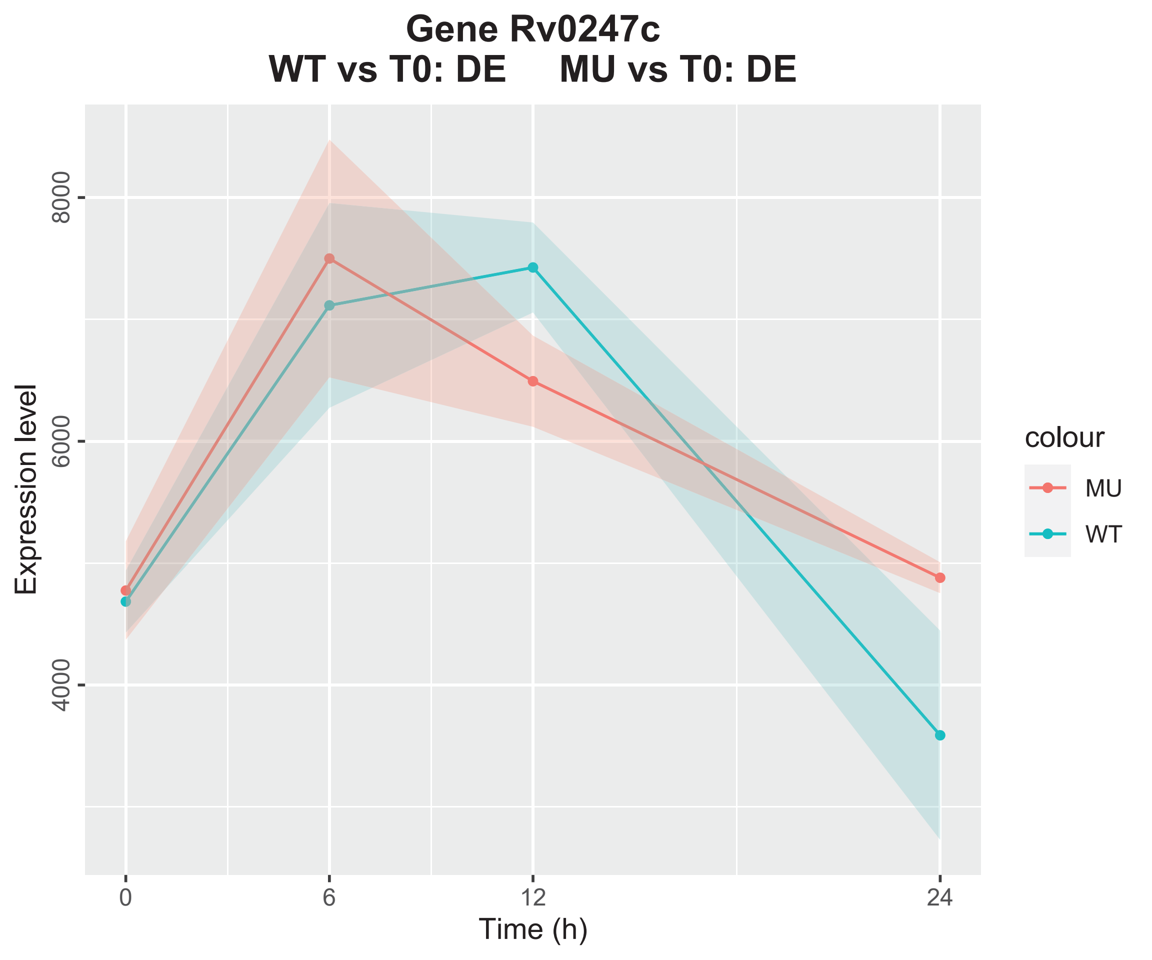
Gene Rv0247c
WT vs T0: DE     MU vs T0: DE
Expression level
8000
6000
4000
0
6
12
24
Time (h)
colour
MU
WT

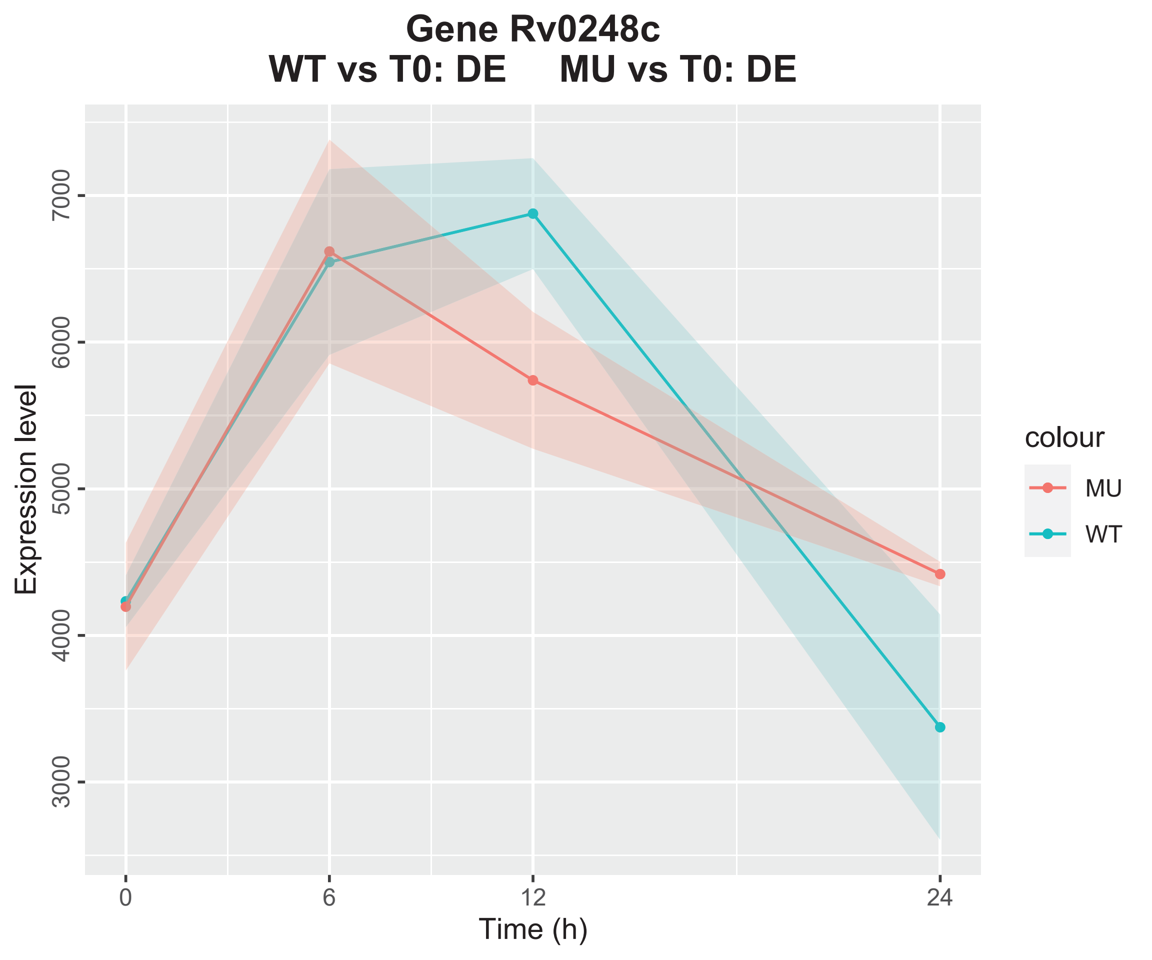
Gene Rv0248c
WT vs T0: DE     MU vs T0: DE
Expression level
7000
6000
5000
4000
3000
0
6
12
24
Time (h)
colour
MU
WT

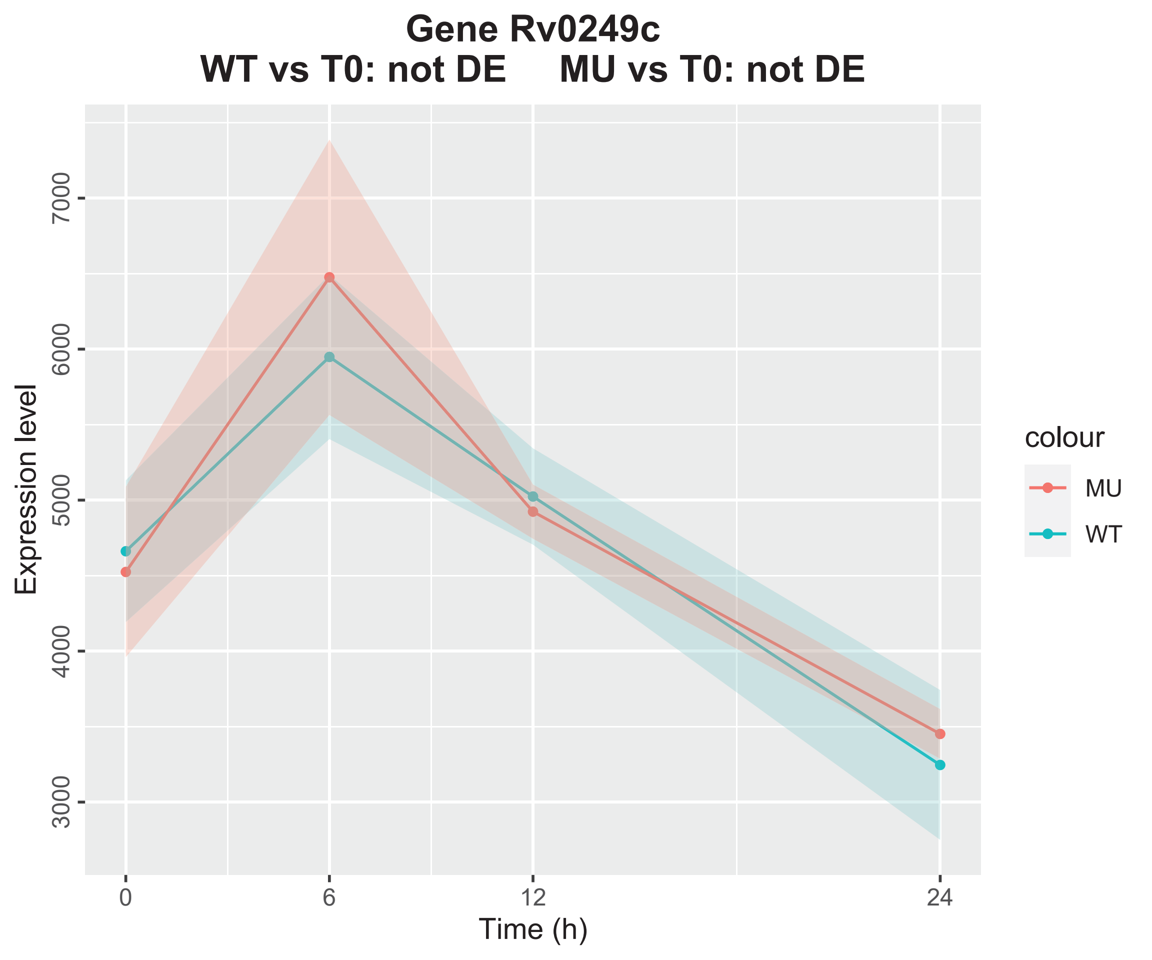
Gene Rv0249c
WT vs T0: not DE MU vs T0: not DE
Expression level
7000
6000
5000
4000
3000
0
6
12
24
Time (h)
colour
MU
WT

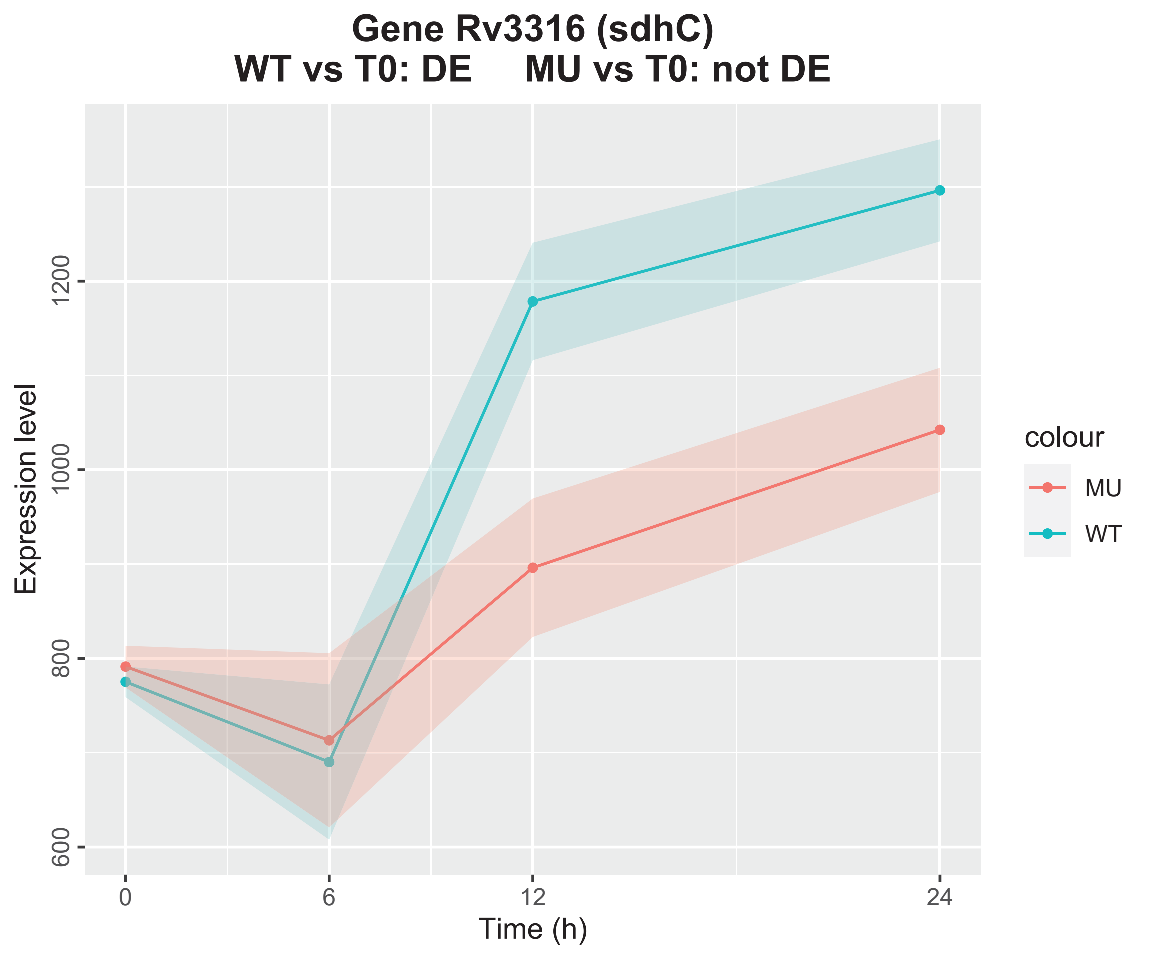

Gene Rv3316 (sdhC)
WT vs T0: DE MU vs T0: not DE
Expression level
600
800
1000
1200
0
6
12
24
Time (h)
colour
MU
WT

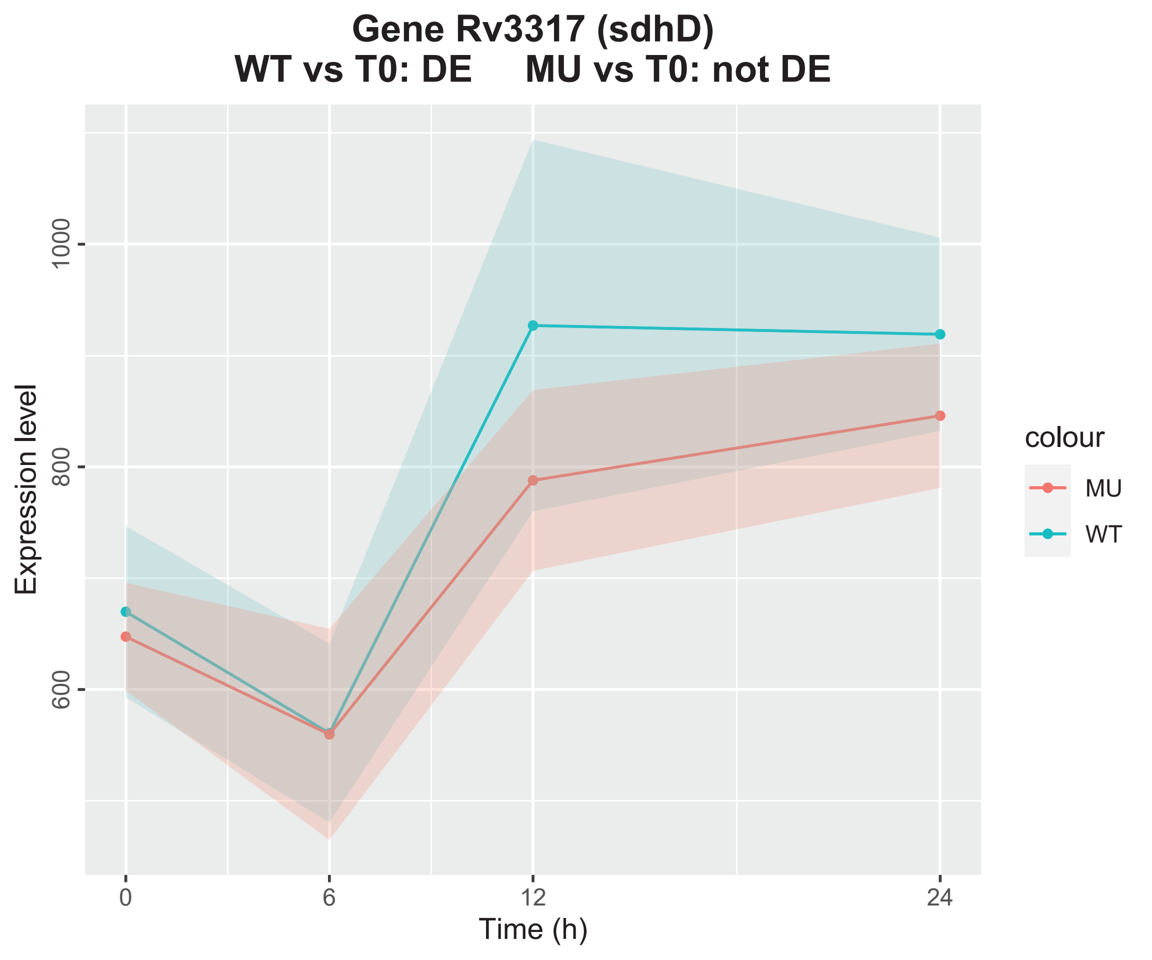

Gene Rv3317 (sdhD)
WT vs T0: DE     MU vs T0: not DE
Expression level
1000
800
600
0
6
12
24
Time (h)
colour
MU
WT

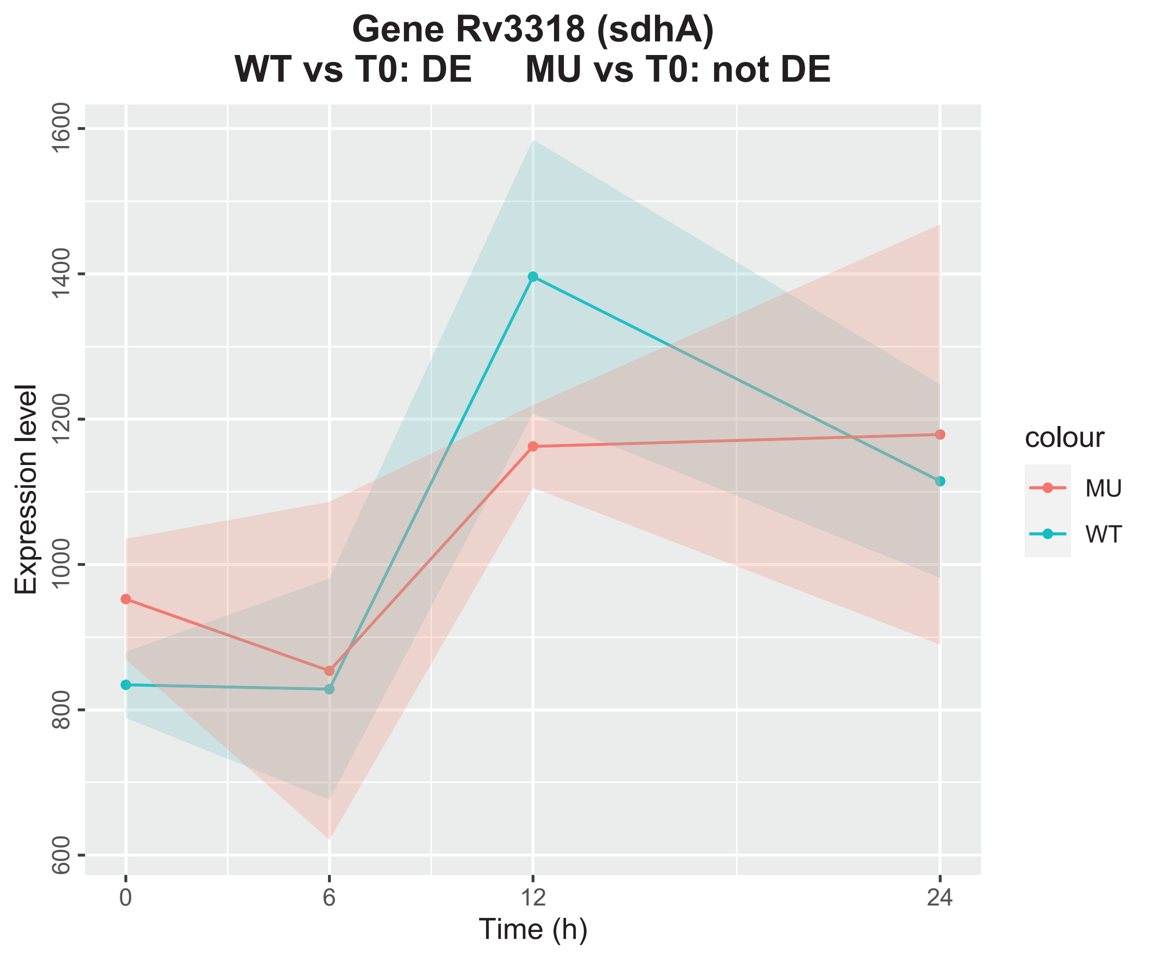

Gene Rv3318 (sdhA)
WT vs T0: DE     MU vs T0: not DE
Expression level
1600
1400
1200
1000
800
600
0
6
12
24
Time (h)
colour
MU
WT

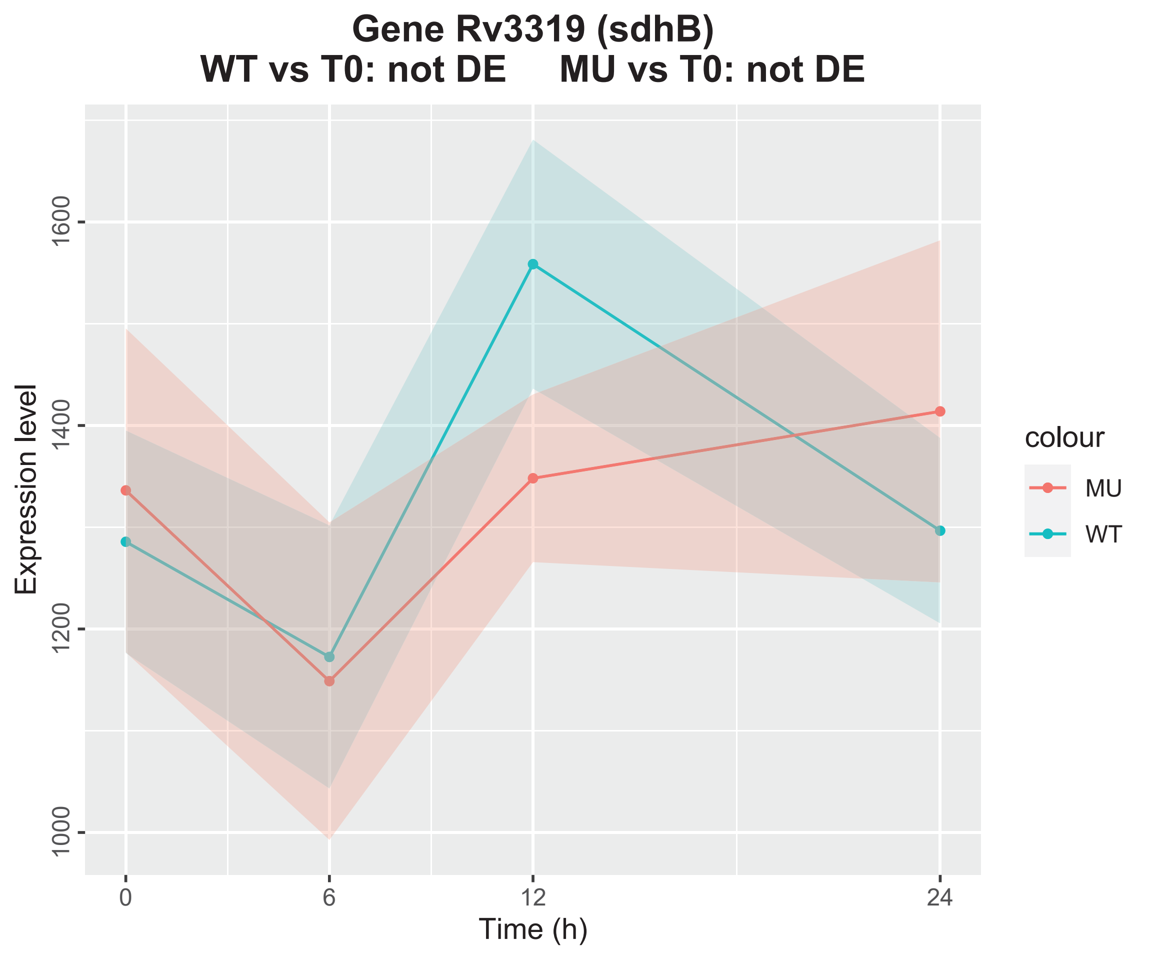

Gene Rv3319 (sdhB)
WT vs T0: not DE     MU vs T0: not DE
Expression level
1000
1200
1400
1600
0
6
12
24
Time (h)
colour
MU
WT

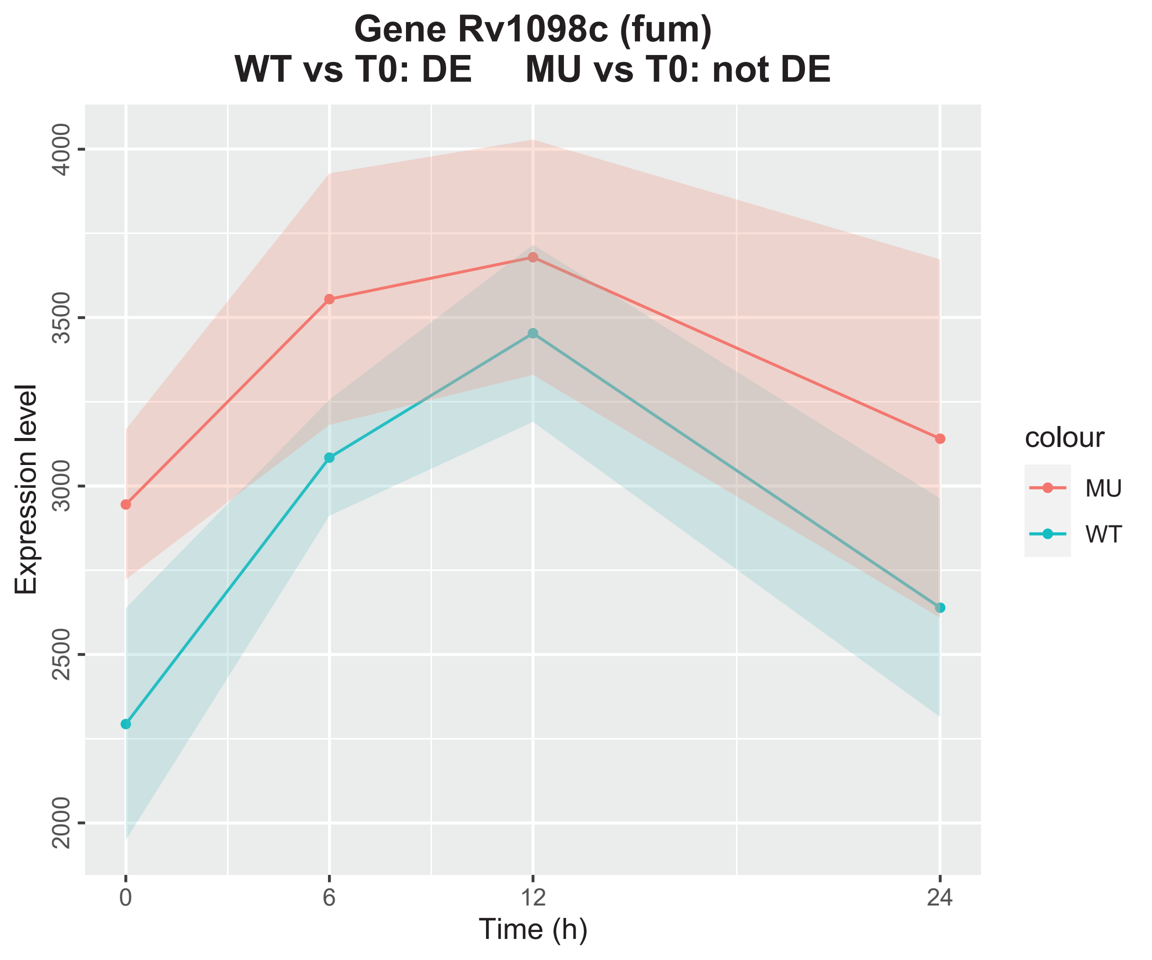
Gene Rv1098c (fum)
WT vs T0: DE     MU vs T0: not DE
Expression level
4000
3500
3000
2500
2000
0
6
12
24
Time (h)
colour
MU
WT

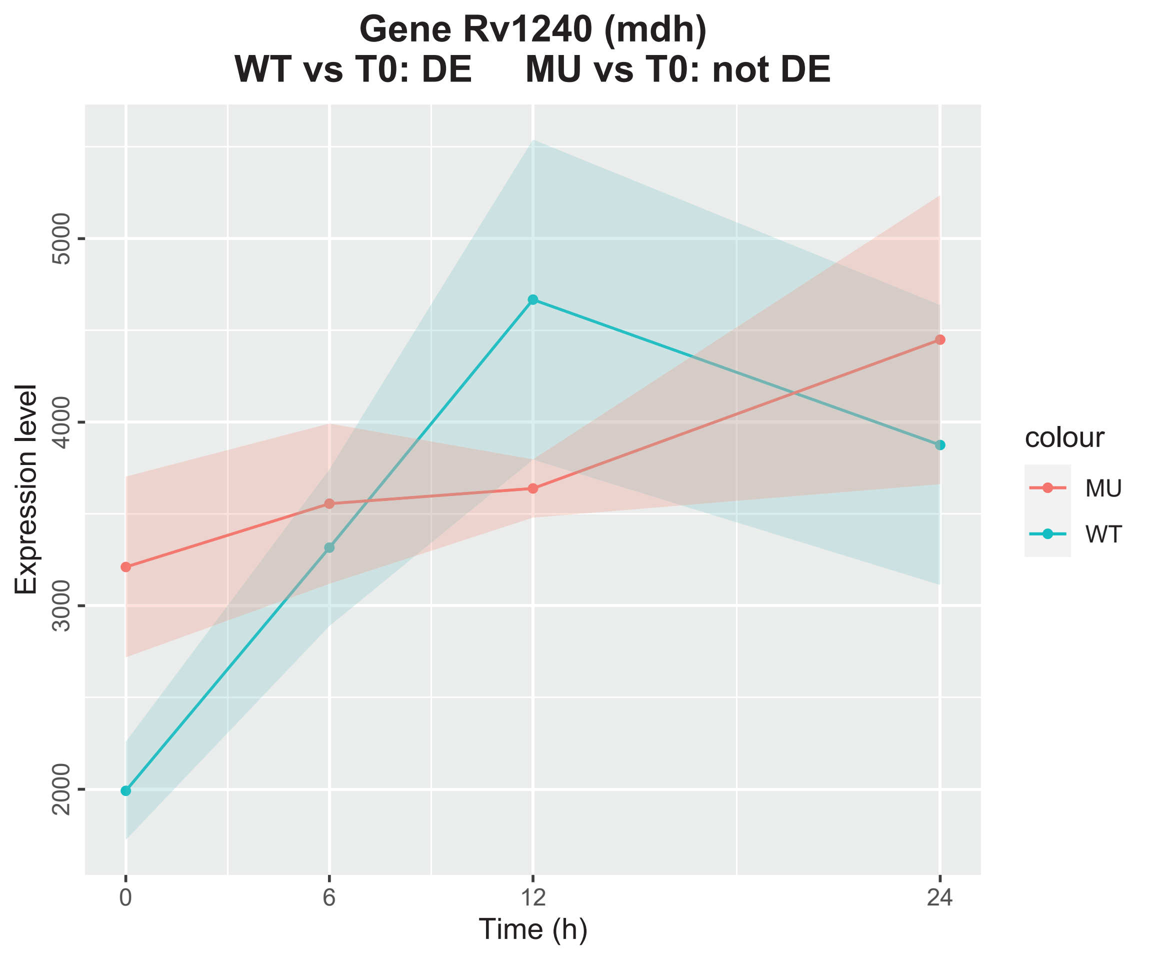
Gene Rv1240 (mdh)
WT vs T0: DE     MU vs T0: not DE
Expression level
5000
4000
3000
2000
0
6
12
24
Time (h)
colour
MU
WT

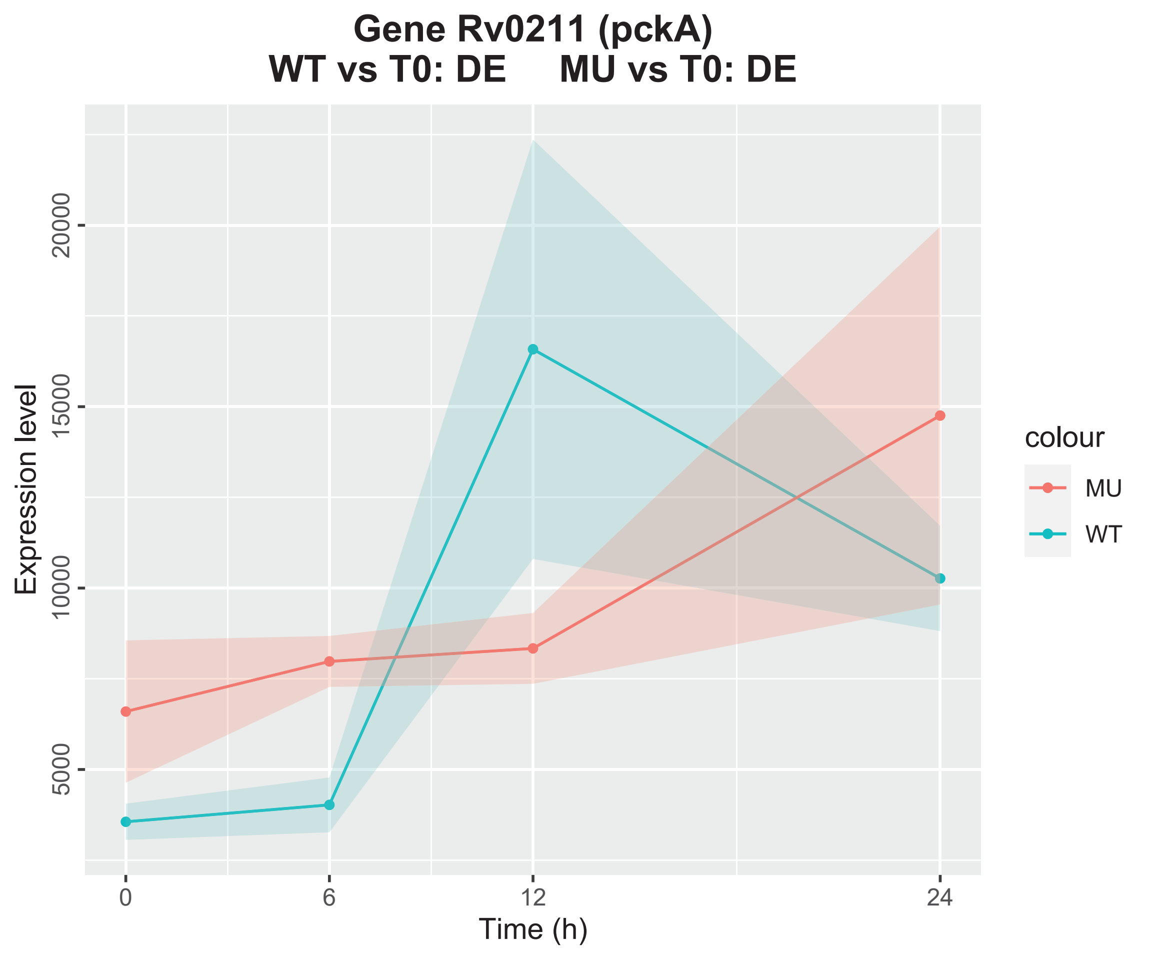
Gene Rv0211 (pckA)
WT vs T0: DE     MU vs T0: DE
Expression level
20000
15000
10000
5000
0
6
12
24
Time (h)
colour
MU
WT

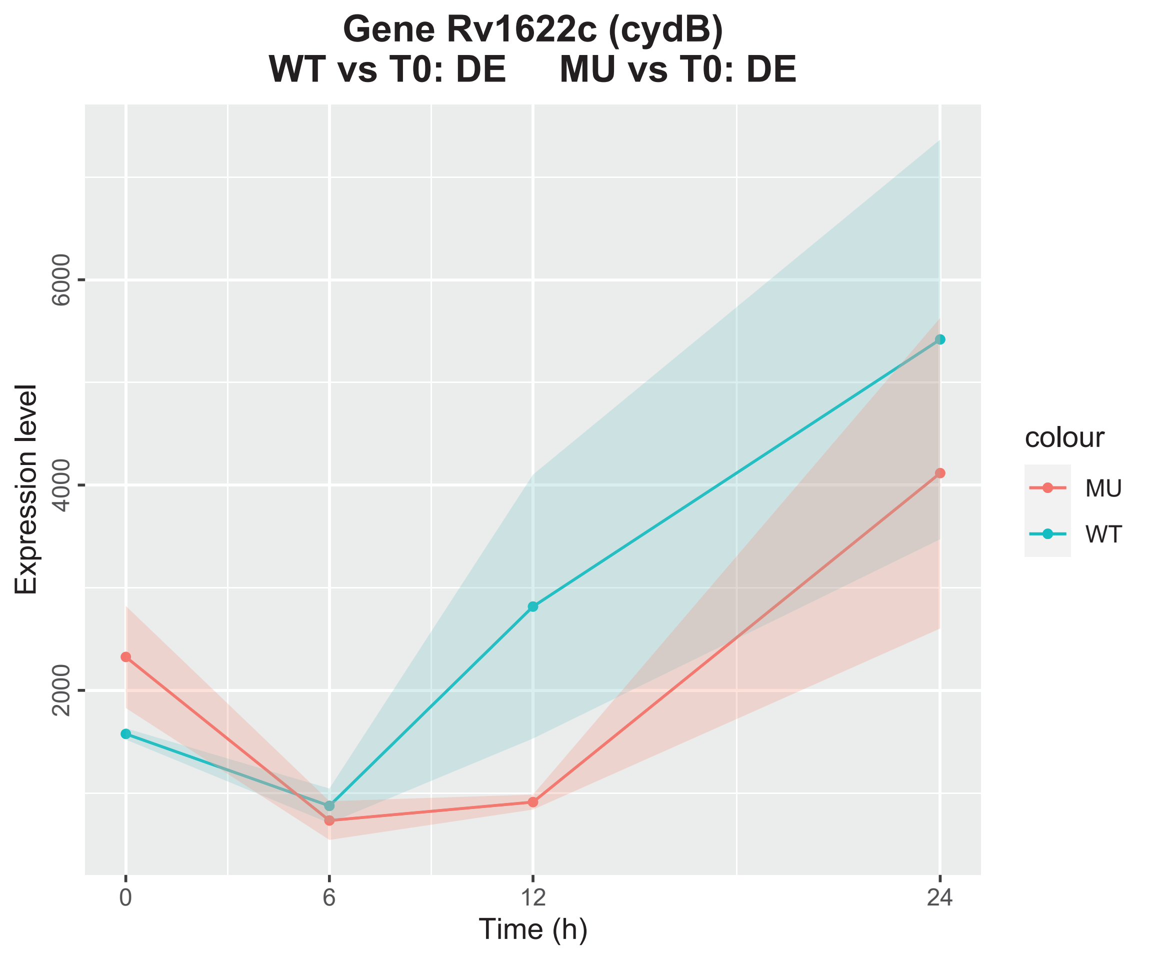

Gene Rv1622c (cydB)
WT vs T0: DE MU vs T0: DE
Expression level
6000
4000
2000
0
6
12
24
Time (h)
colour
MU
WT

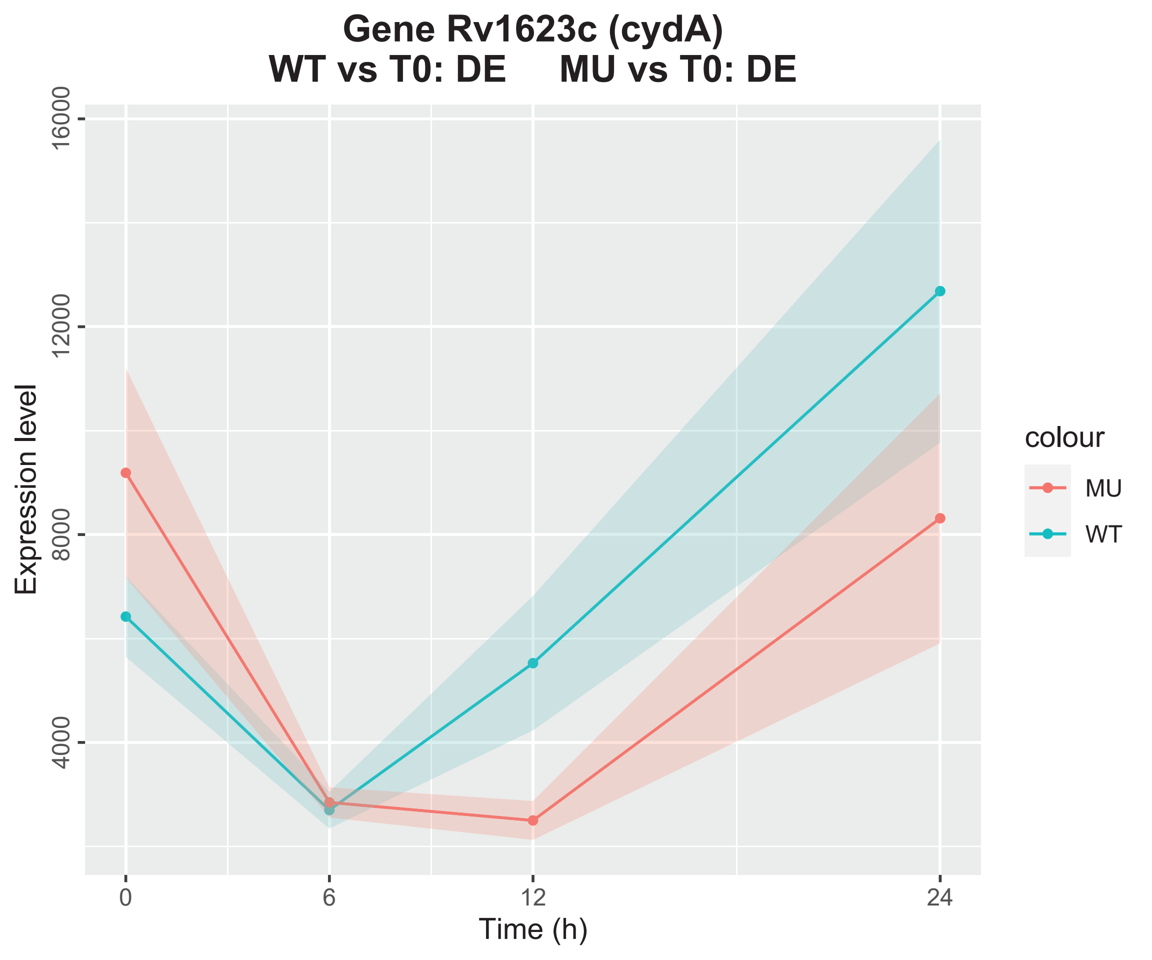
Gene Rv1623c (cydA)
WT vs T0: DE MU vs T0: DE
16000
12000
8000
4000
Expression level
0
6
12
24
Time (h)
colour
MU
WT
