# Supplementary material for: Role of the Extracytoplasmic Function Sigma Factor SigE in the Stringent Response of Mycobacterium tuberculosis
Source: Microbiol Spectr. 2023 Mar 22;11(2):e02944-22. doi: 10.1128/spectrum.02944-22 (PMC10100808; doi:10.1128/spectrum.02944-22)
Supplement: Supplemental file 7 — Data S7. Download spectrum.02944-22-s0008.pdf, PDF file, 0.2 MB [file spectrum.02944-22-s0008.pdf]

Supplementary Data S7: Gene expression profiles over time of genes discussed in section “SigE network and SenX3-RegX3 regulon in sigE mutant”, i.e. *sigE*, *sigB*, *clgR*, *SenX3-RegX3*, *pknD-pstS2*, *ppk1*, *relA* and *SigH*. For each gene, the plot shows the average expression level and the standard deviation (shaded area) for both the wild-type (cyan color) and *sigE*-mutant (salmon/pink color) strains.

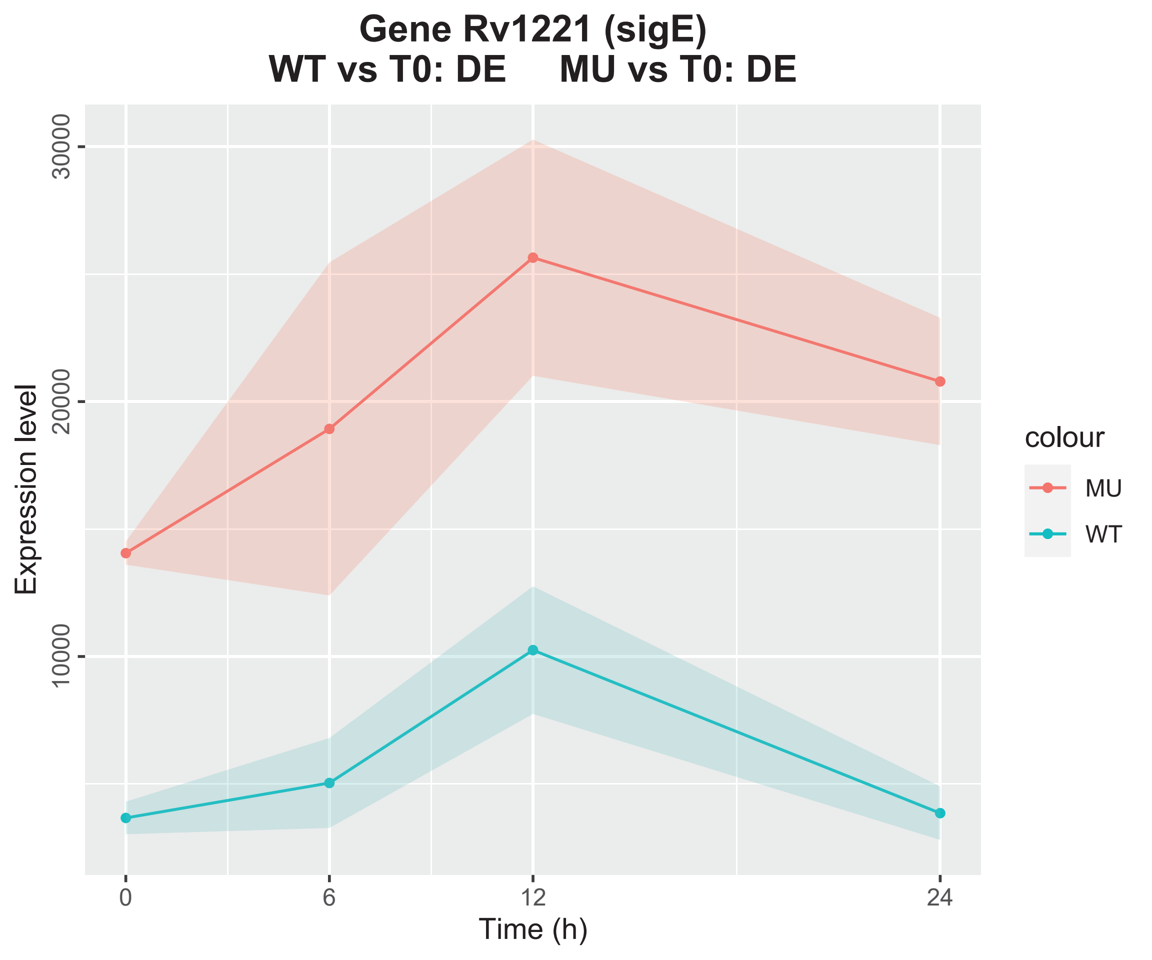
Gene Rv1221 (sigE)
WT vs T0: DE   MU vs T0: DE
Expression level
30000
20000
10000
0
6
12
24
Time (h)
colour
MU
WT

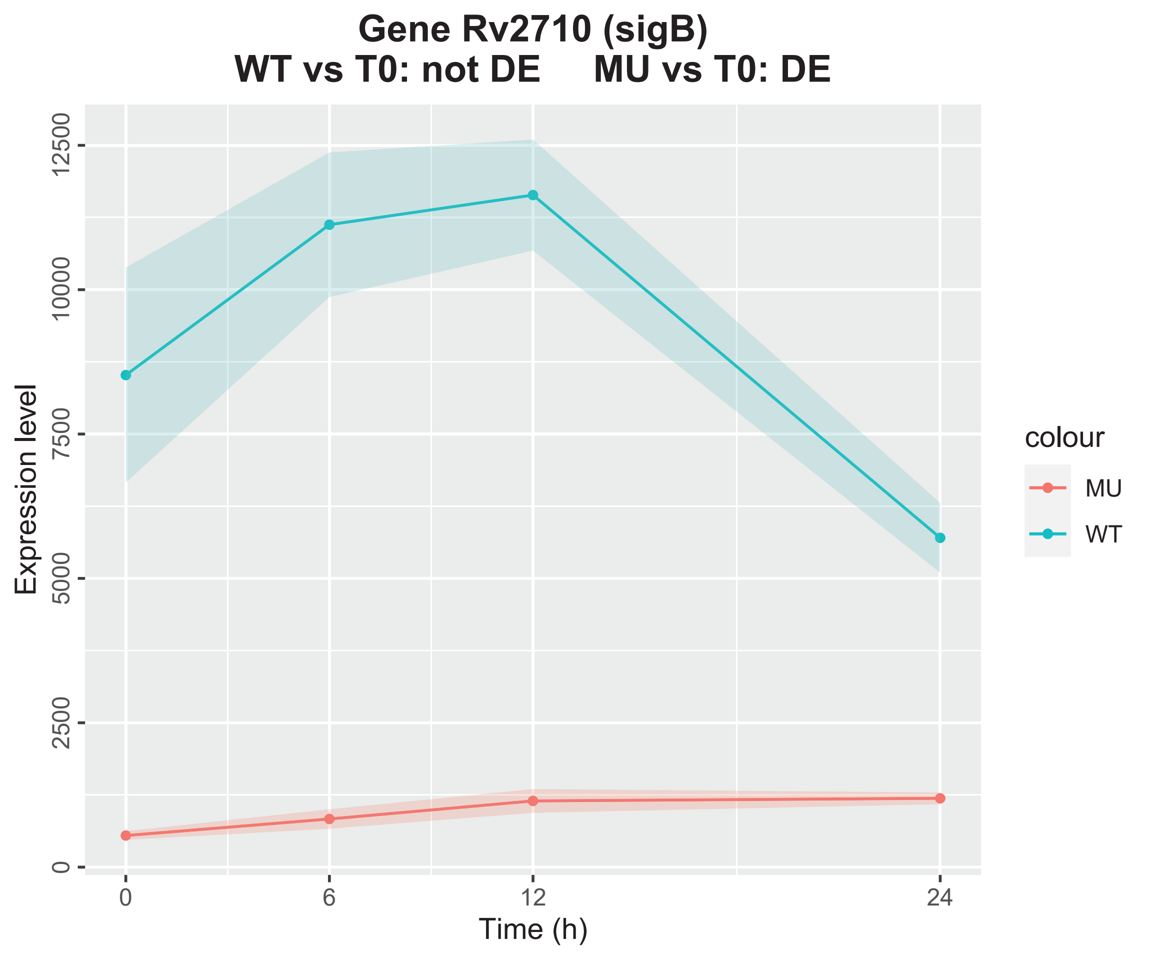
Gene Rv2710 (sigB)
WT vs T0: not DE   MU vs T0: DE
Expression level
0
2500
5000
7500
10000
12500
0
6
12
24
Time (h)
colour
MU
WT

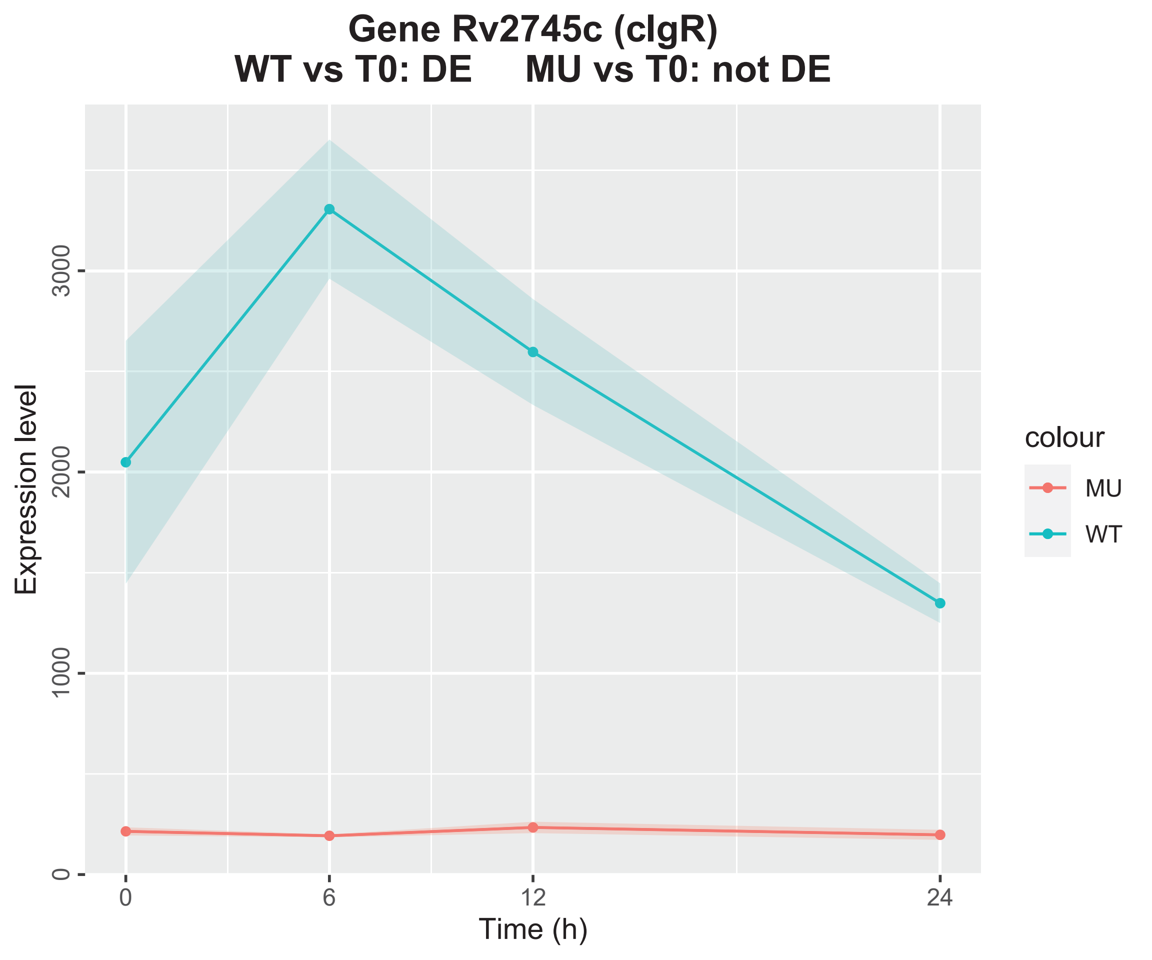
Gene Rv2745c (clgR)
WT vs T0: DE   MU vs T0: not DE
Expression level
3000
2000
1000
0
0
6
12
24
Time (h)
colour
MU
WT

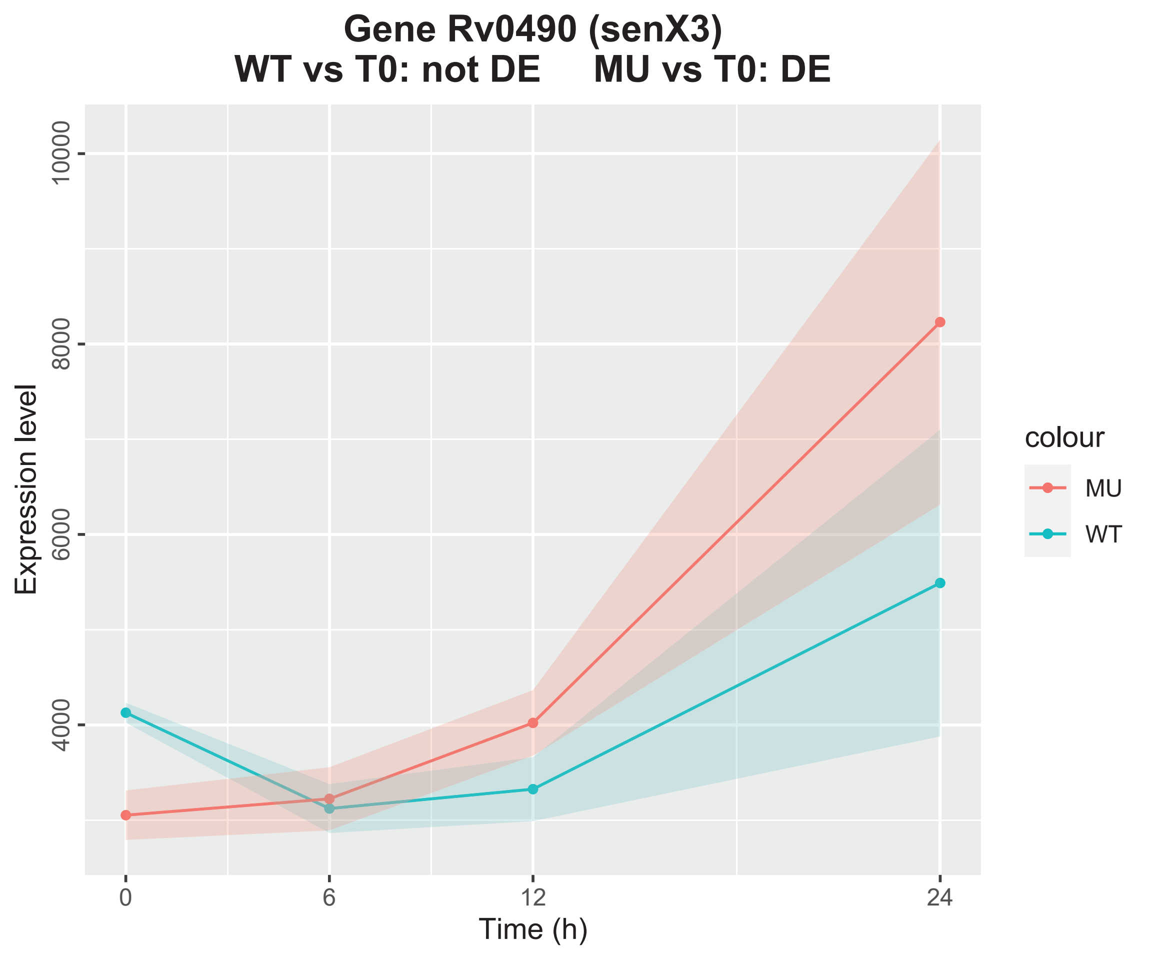
Gene Rv0490 (senX3)
WT vs T0: not DE MU vs T0: DE
Expression level
10000
8000
6000
4000
0
6
12
24
Time (h)
colour
MU
WT

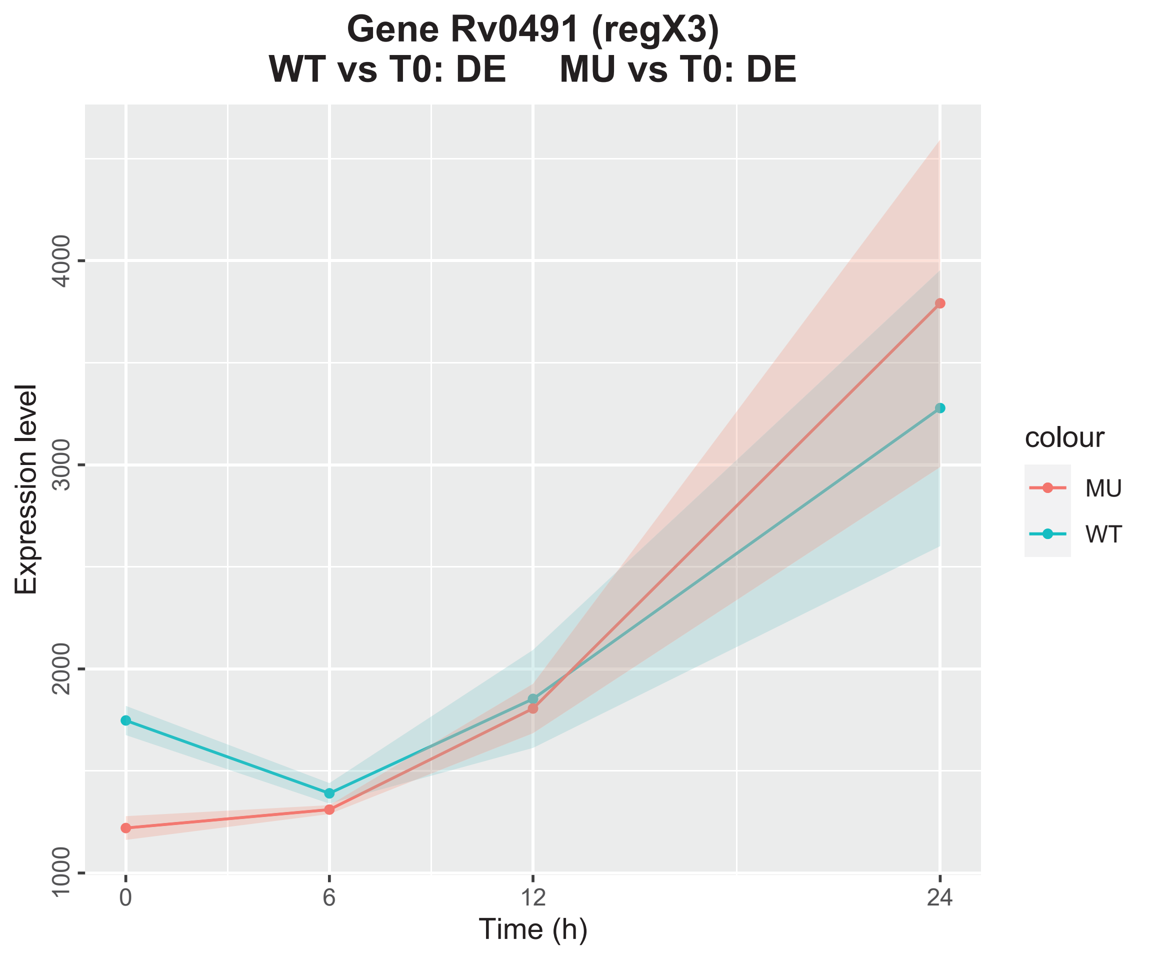

Gene Rv0491 (regX3)
WT vs T0: DE MU vs T0: DE
Expression level
4000
3000
2000
1000
0
6
12
24
Time (h)
colour
MU
WT

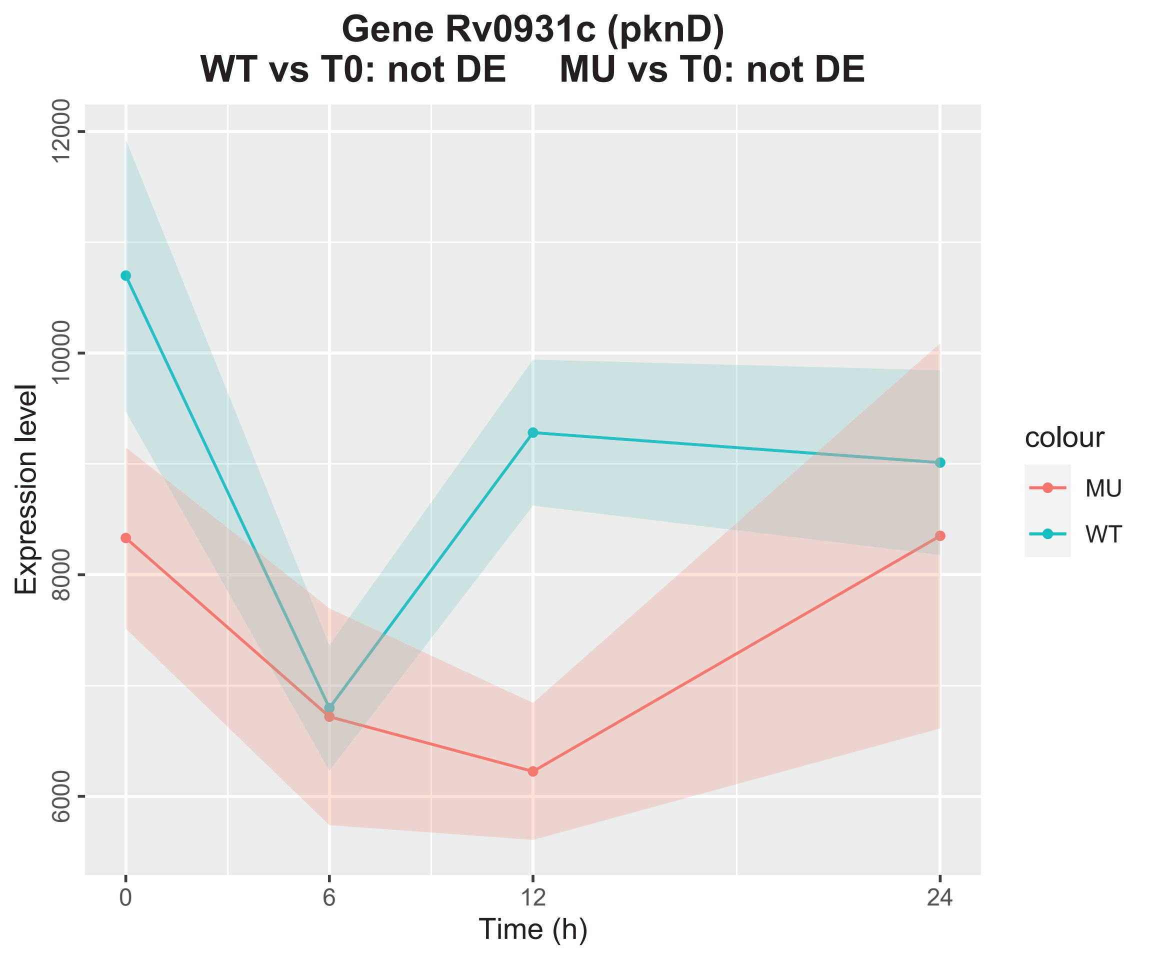

Gene Rv0931c (pknD)
WT vs T0: not DE MU vs T0: not DE
Expression level
12000
10000
8000
6000
0
6
12
24
Time (h)
colour
MU
WT

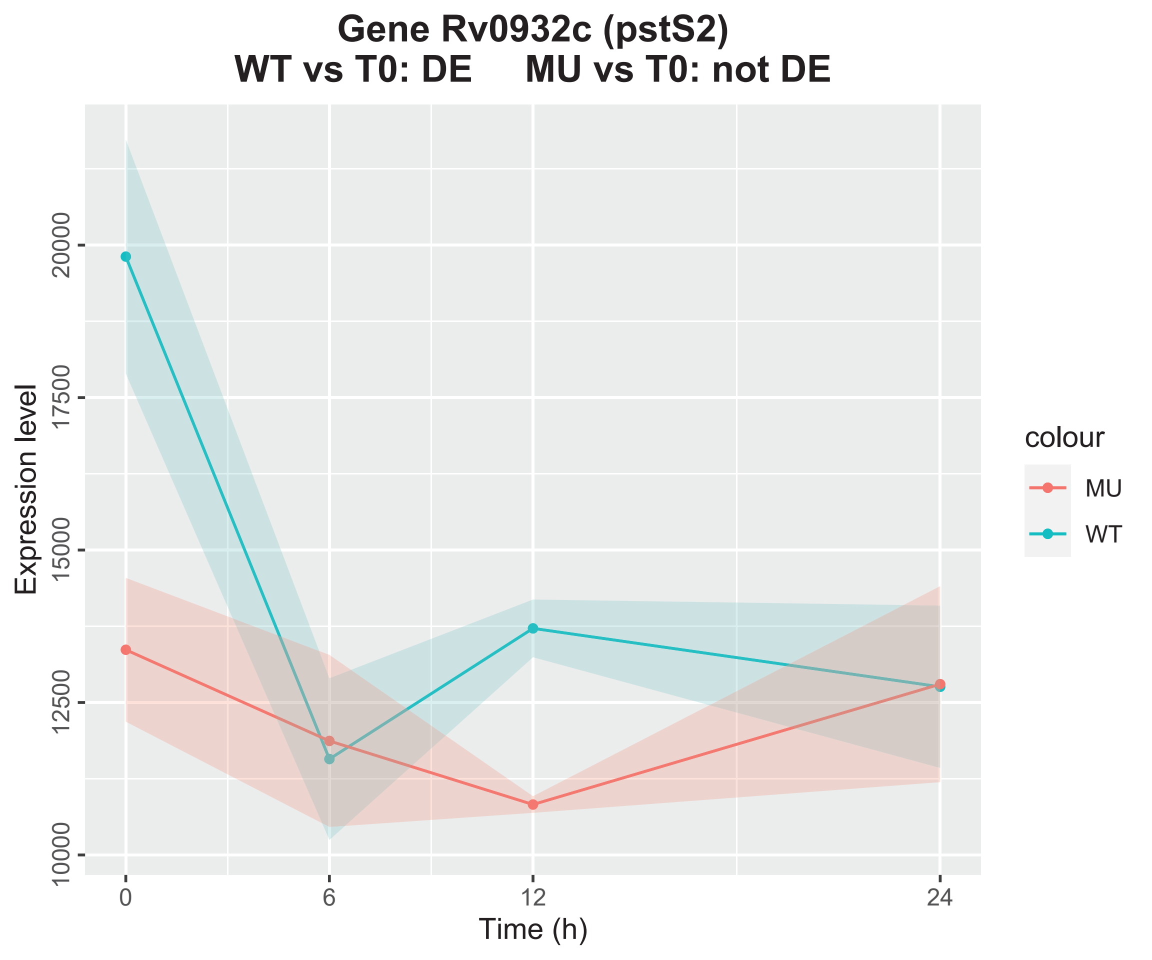

Gene Rv0932c (pstS2)
WT vs T0: DE MU vs T0: not DE
Expression level
20000
17500
15000
12500
10000
0
6
12
24
Time (h)
colour
MU
WT

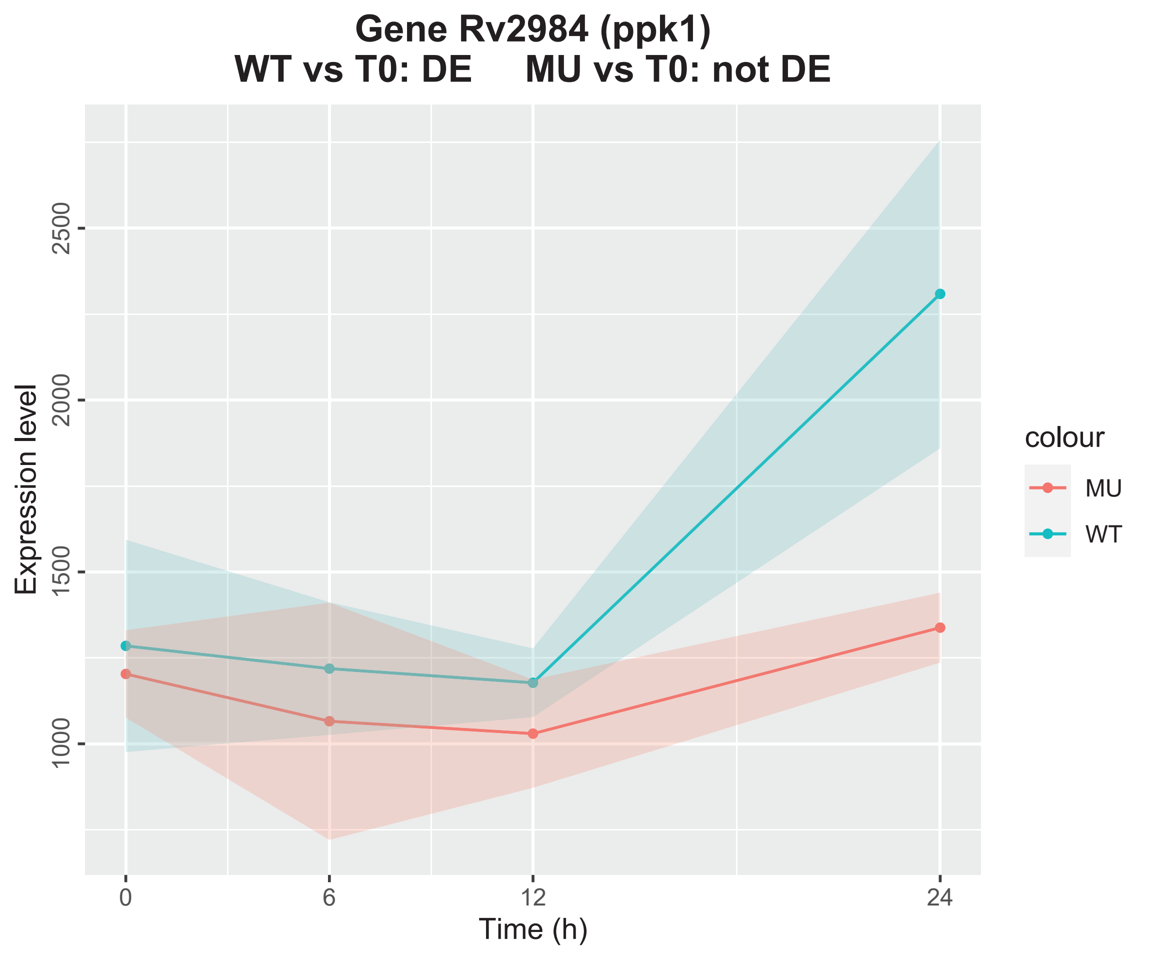

Gene Rv2984 (ppk1)
WT vs T0: DE   MU vs T0: not DE
Expression level
2500
2000
1500
1000
0
6
12
24
Time (h)
colour
MU
WT

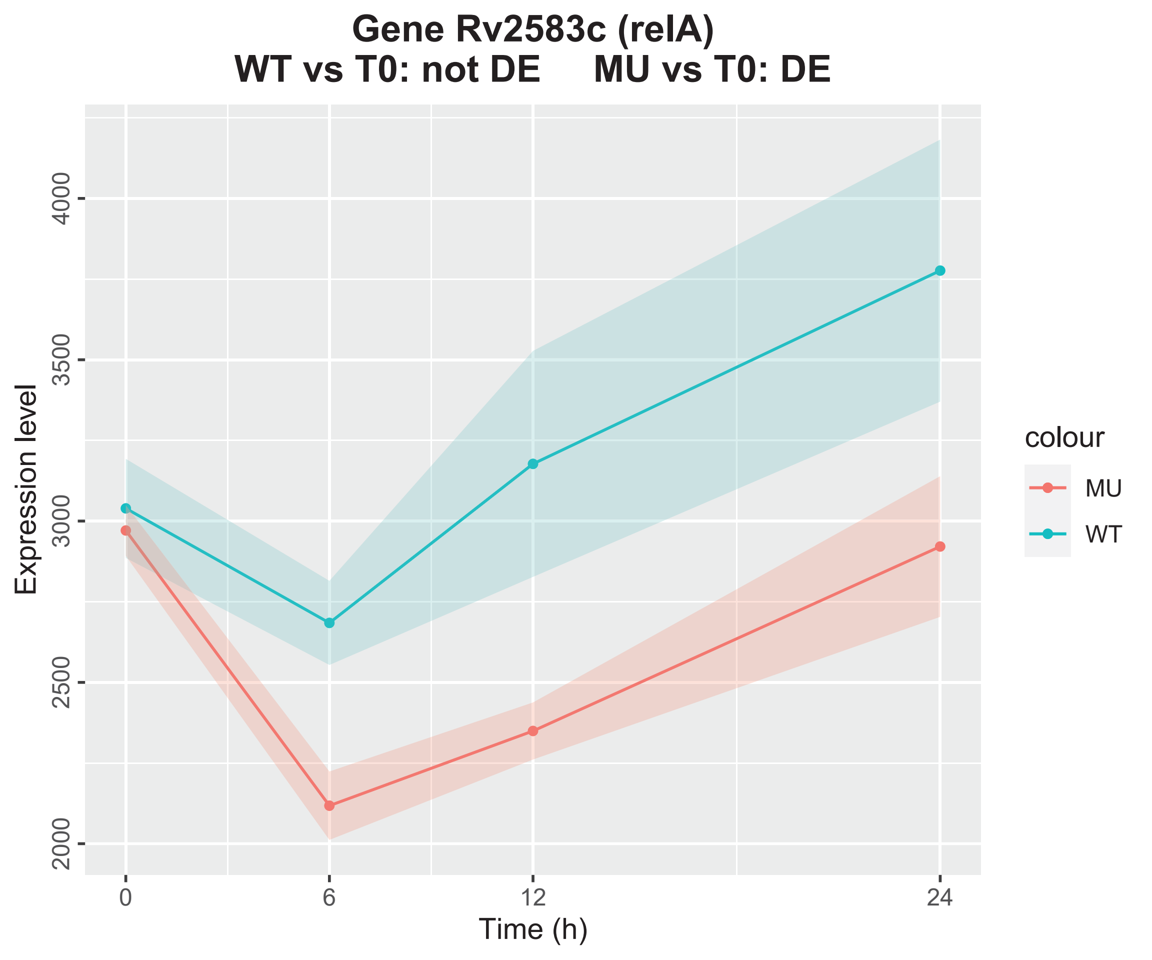
Gene Rv2583c (relA)
WT vs T0: not DE   MU vs T0: DE
Expression level
4000
3500
3000
2500
2000
0
6
12
24
Time (h)
colour
MU
WT

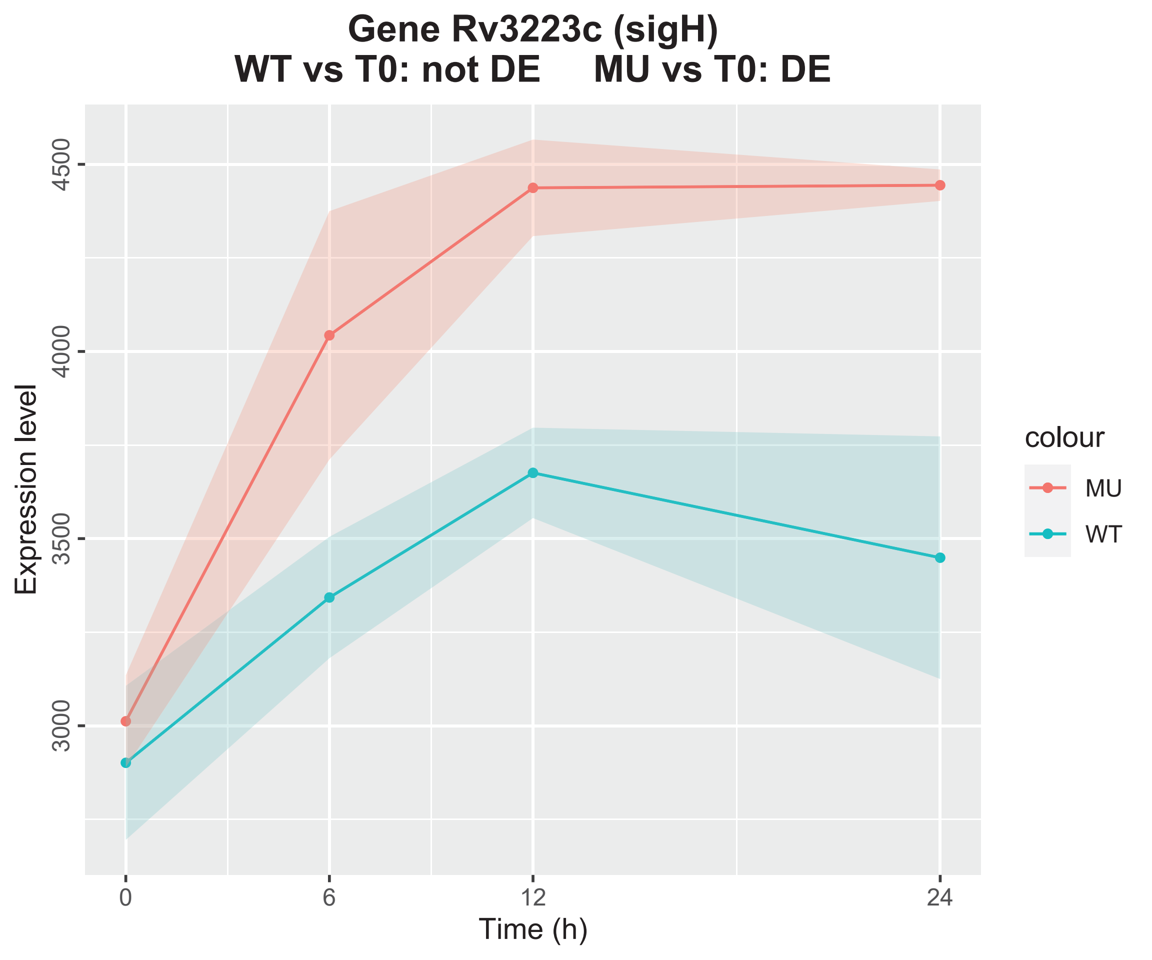
Gene Rv3223c (sigH)
WT vs T0: not DE   MU vs T0: DE
Expression level
4500
4000
3500
3000
0
6
12
24
Time (h)
colour
MU
WT
