# Supplementary material for: Role of the Extracytoplasmic Function Sigma Factor SigE in the Stringent Response of Mycobacterium tuberculosis
Source: Microbiol Spectr. 2023 Mar 22;11(2):e02944-22. doi: 10.1128/spectrum.02944-22 (PMC10100808; doi:10.1128/spectrum.02944-22)
Supplement: Supplemental file 8 — Data S8. Download spectrum.02944-22-s0009.pdf, PDF file, 0.2 MB [file spectrum.02944-22-s0009.pdf]

<u>Supplementary Data S8:</u> Gene expression profiles over time of genes discussed in section "Oxidative and acid stress in *sigE* mutant", i.e. *sigE*, *sigH*, *clpB*, *trxB1*, *trxB2*, *trxC*, *mrx2*, *mshA*, mca, Rv1540 and Rv2037c. For each gene, the plot shows the average expression level and the standard deviation (shaded area) for both the wild-type (cyan color) and *sigE*-mutant (salmon/pink color) strains.

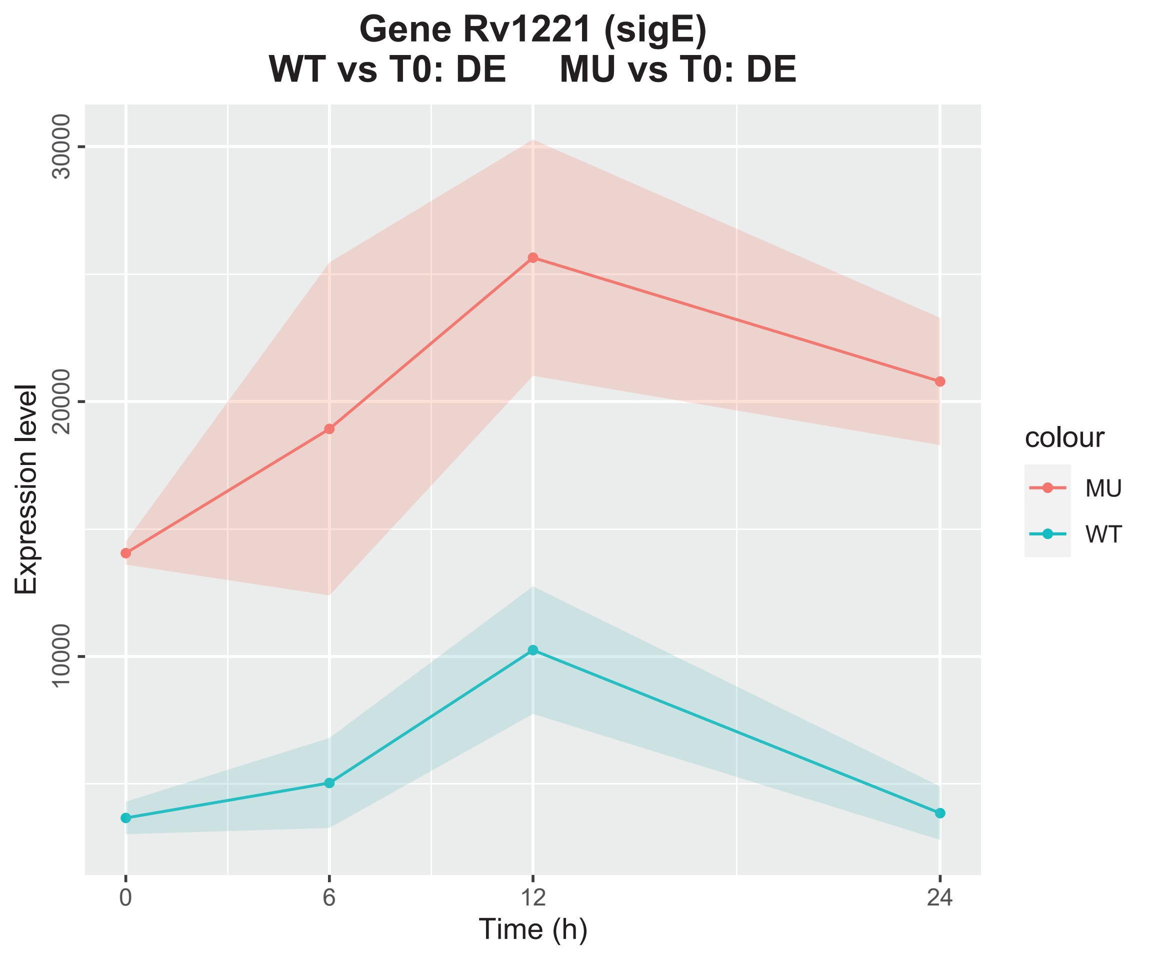

Gene Rv1221 (sigE)
WT vs T0: DE MU vs T0: DE
30000
20000
10000
0
6
12
24
Time (h)
Expression level
colour
MU
WT

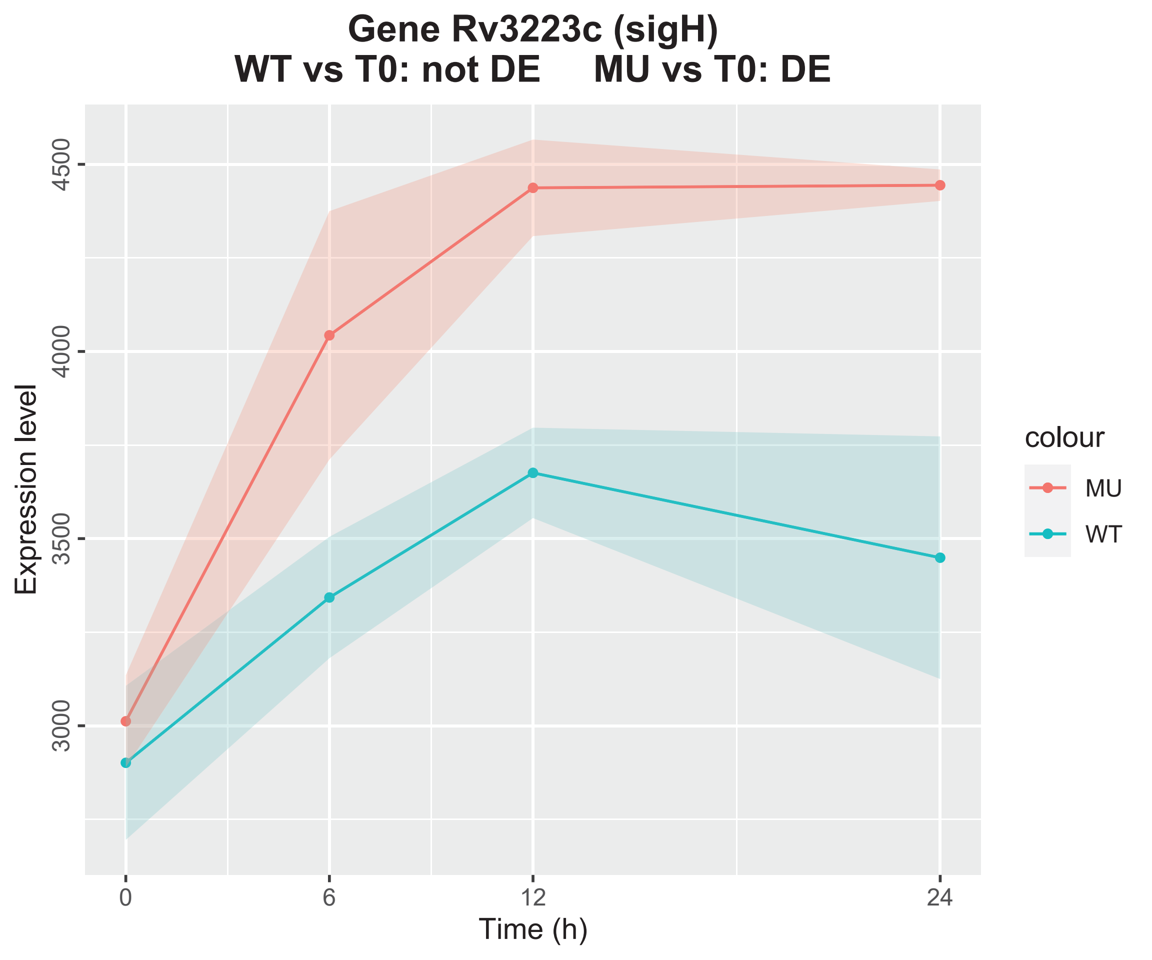
Gene Rv3223c (sigH)
WT vs T0: not DE MU vs T0: DE
Expression level
4500
4000
3500
3000
0
6
12
24
Time (h)
colour
MU
WT

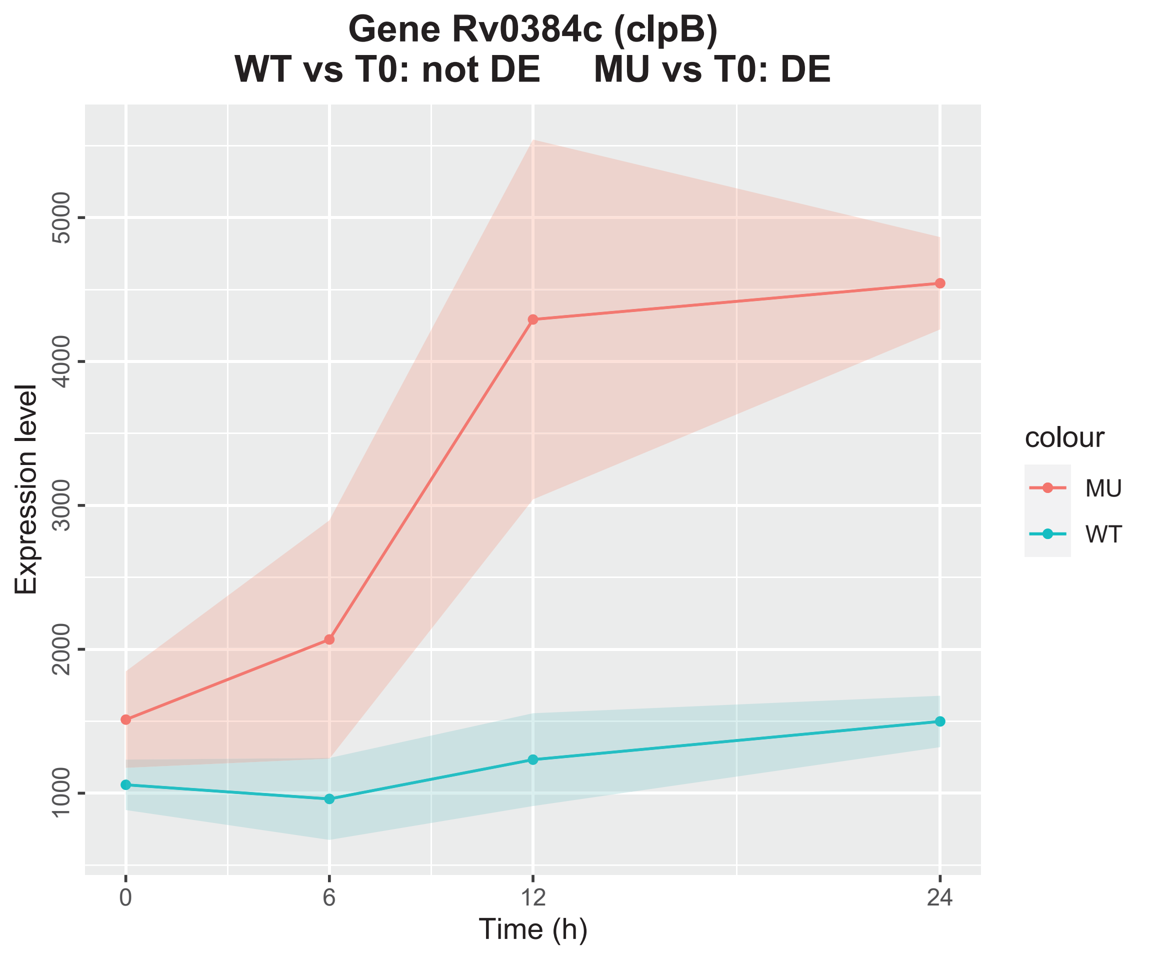

Gene Rv0384c (clpB)
WT vs T0: not DE MU vs T0: DE
Expression level
1000
2000
3000
4000
5000
0
6
12
24
Time (h)
colour
MU
WT

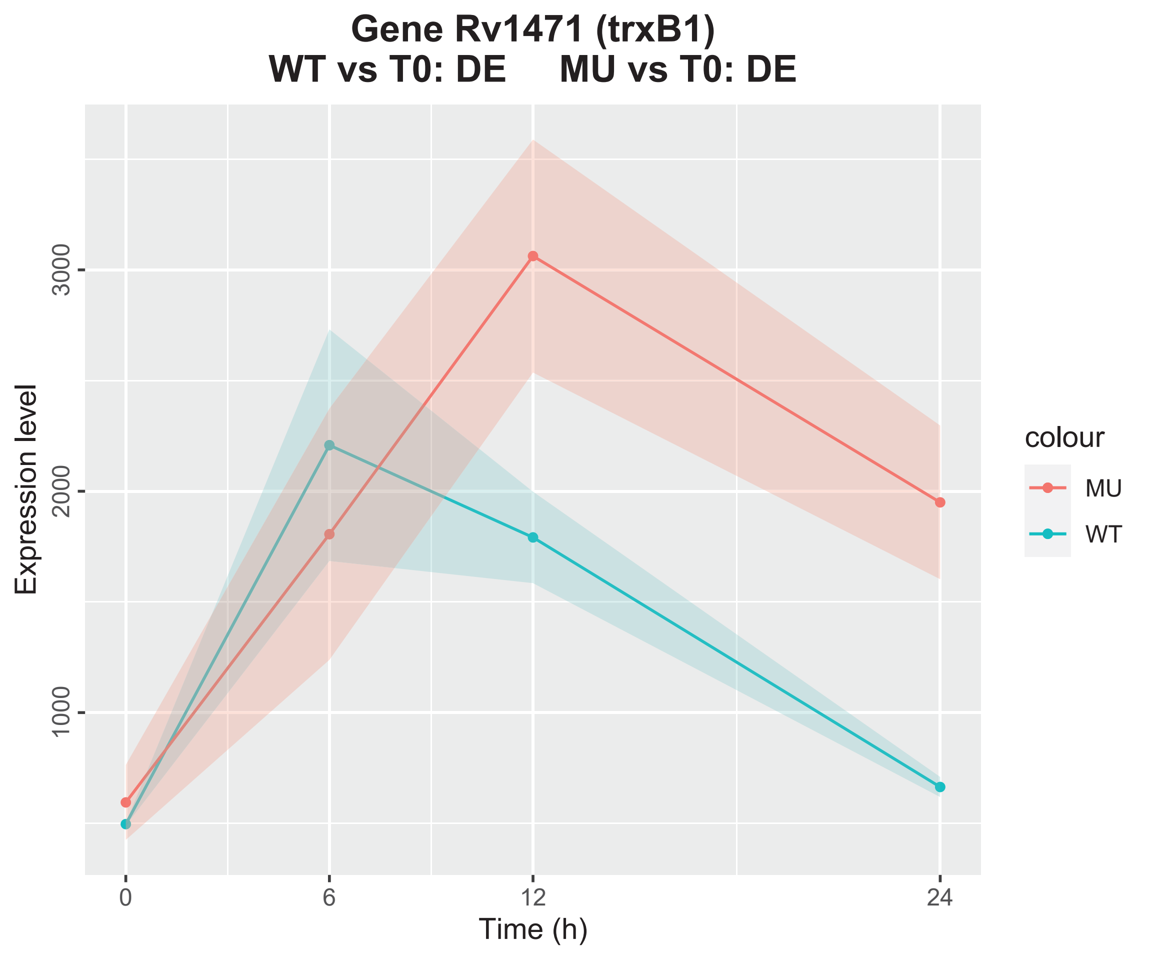
Gene Rv1471 (trxB1)
WT vs T0: DE   MU vs T0: DE
Expression level
3000
2000
1000
0
6
12
24
Time (h)
colour
MU
WT

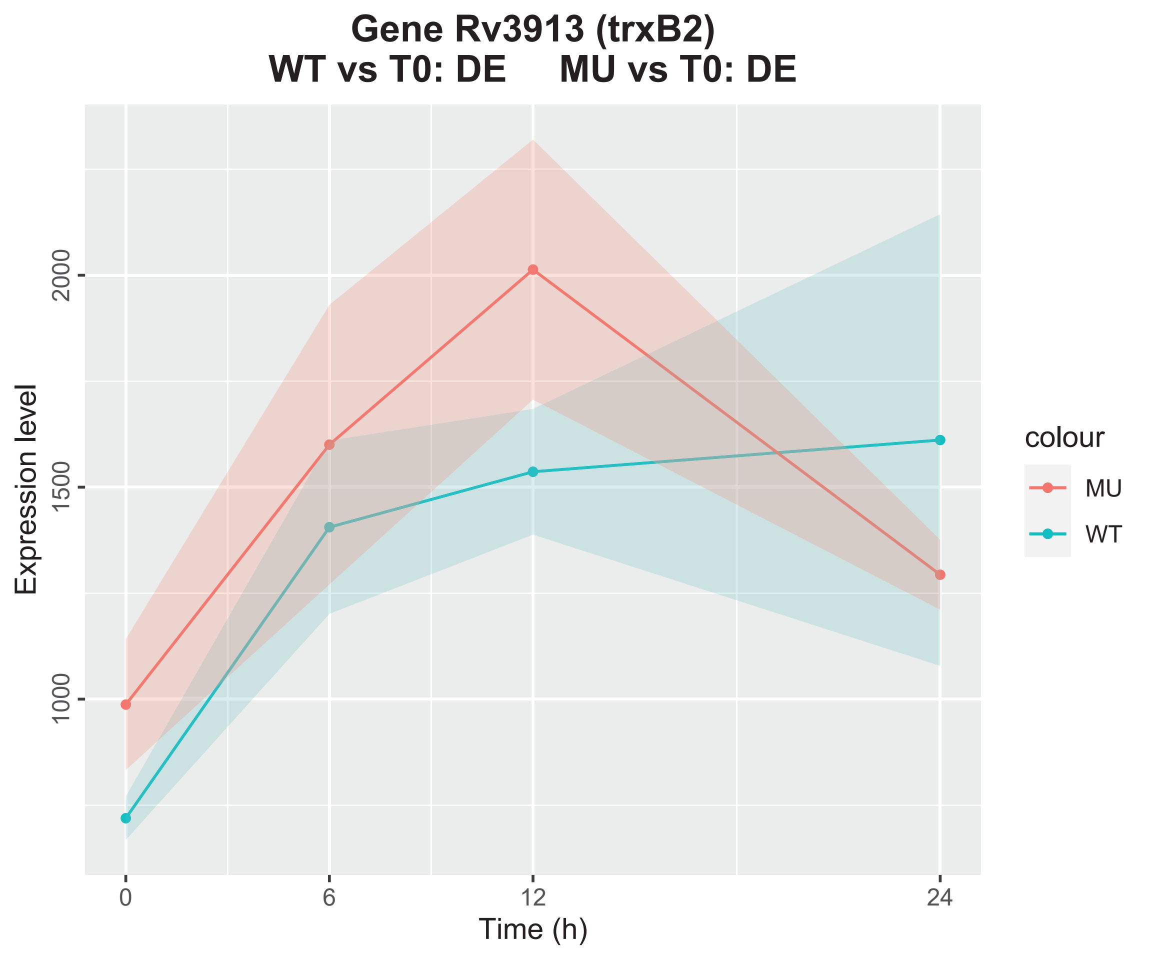
Gene Rv3913 (trxB2)
WT vs T0: DE MU vs T0: DE
Expression level
2000
1500
1000
0
6
12
24
Time (h)
colour
MU
WT

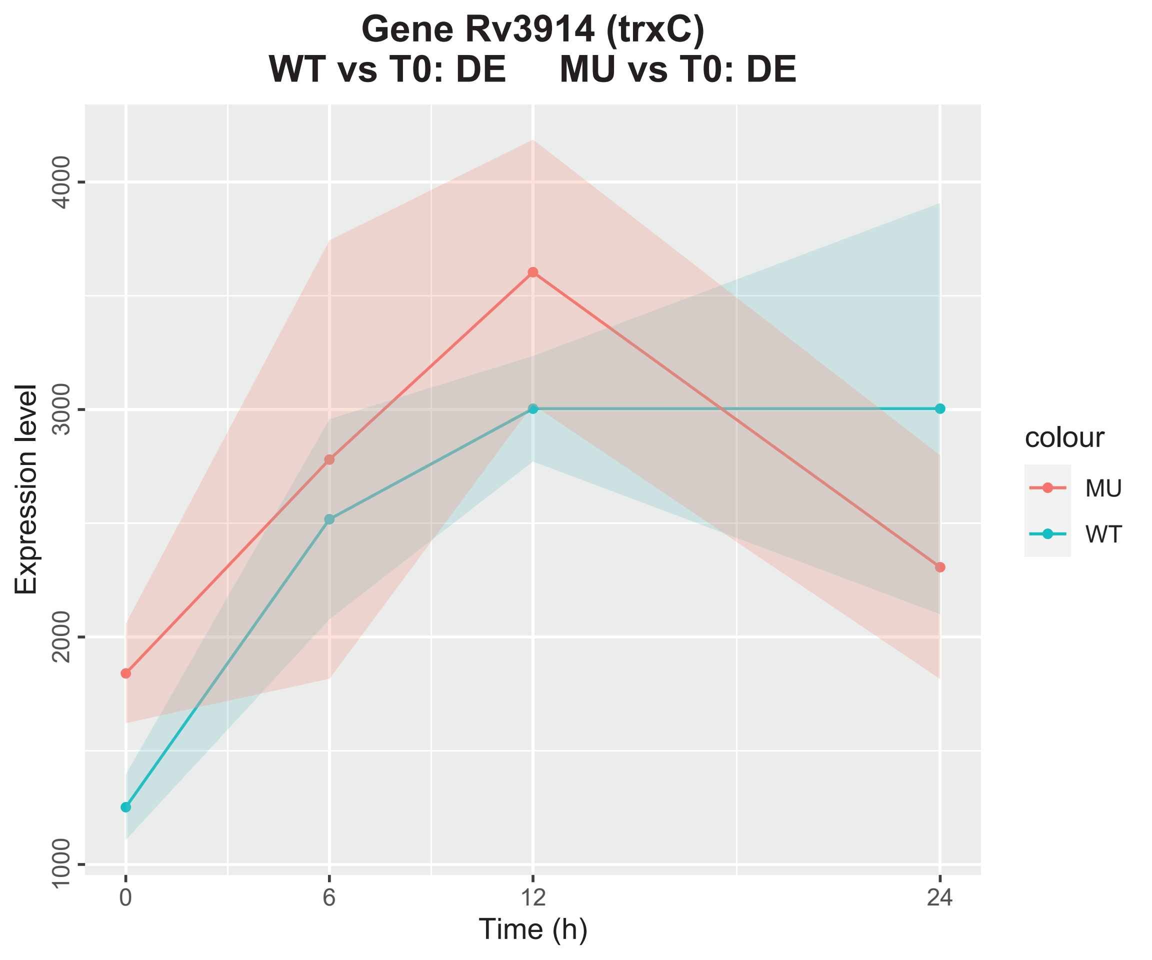
Gene Rv3914 (trxC)
WT vs T0: DE   MU vs T0: DE
Expression level
4000
3000
2000
1000
0
6
12
24
Time (h)
colour
MU
WT

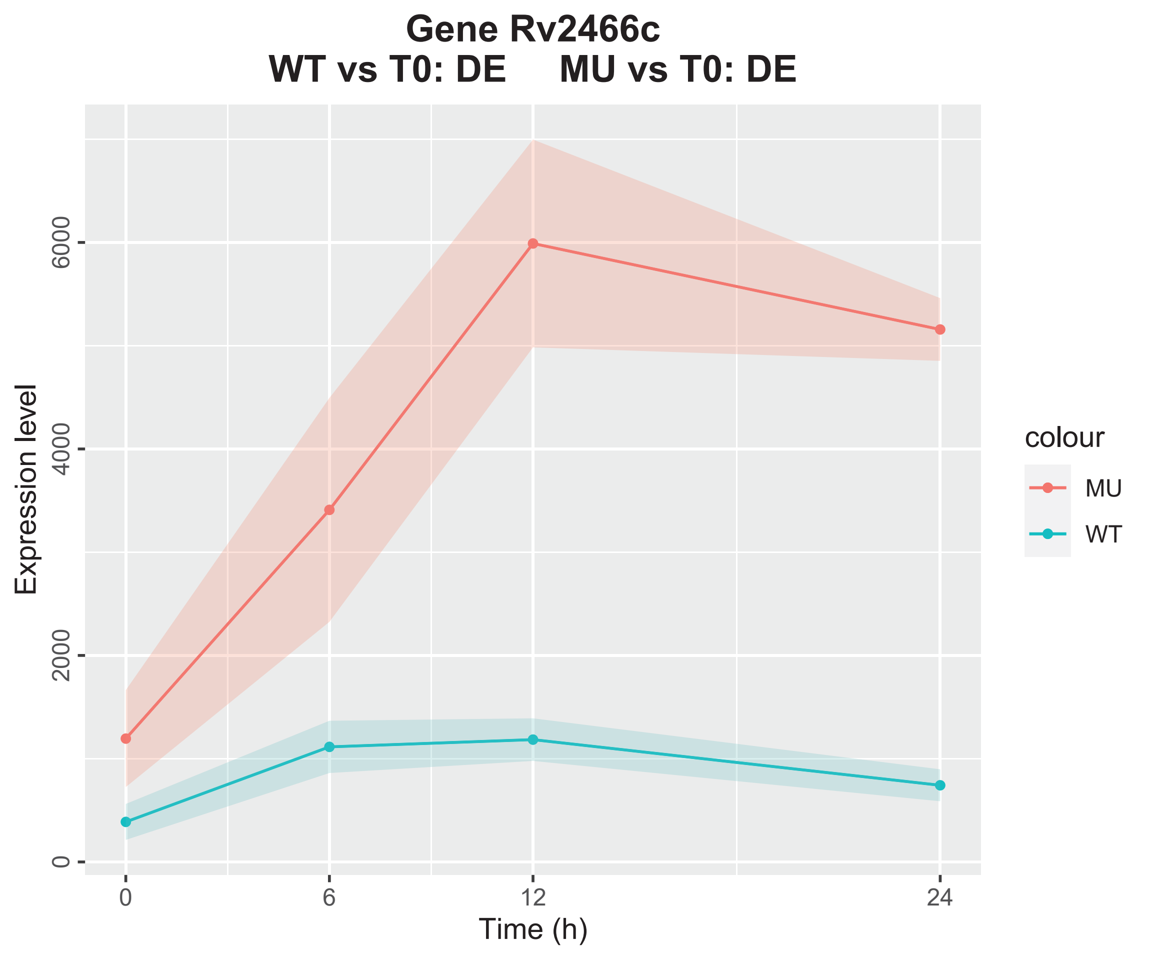
Gene Rv2466c
WT vs T0: DE MU vs T0: DE
Expression level
0
2000
4000
6000
0
6
12
24
Time (h)
colour
MU
WT

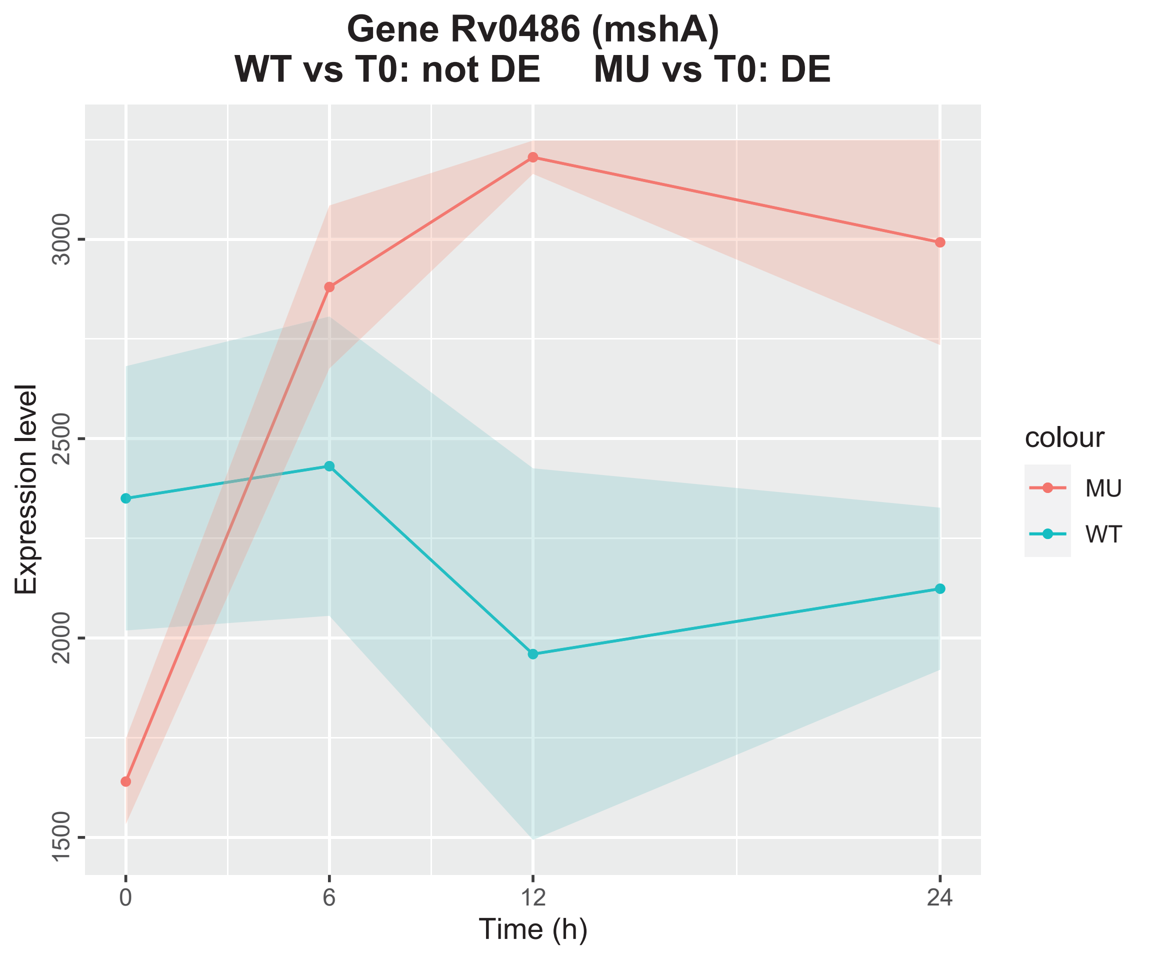

Gene Rv0486 (mshA)
WT vs T0: not DE   MU vs T0: DE
Expression level
1500
2000
2500
3000
0
6
12
24
Time (h)
colour
MU
WT

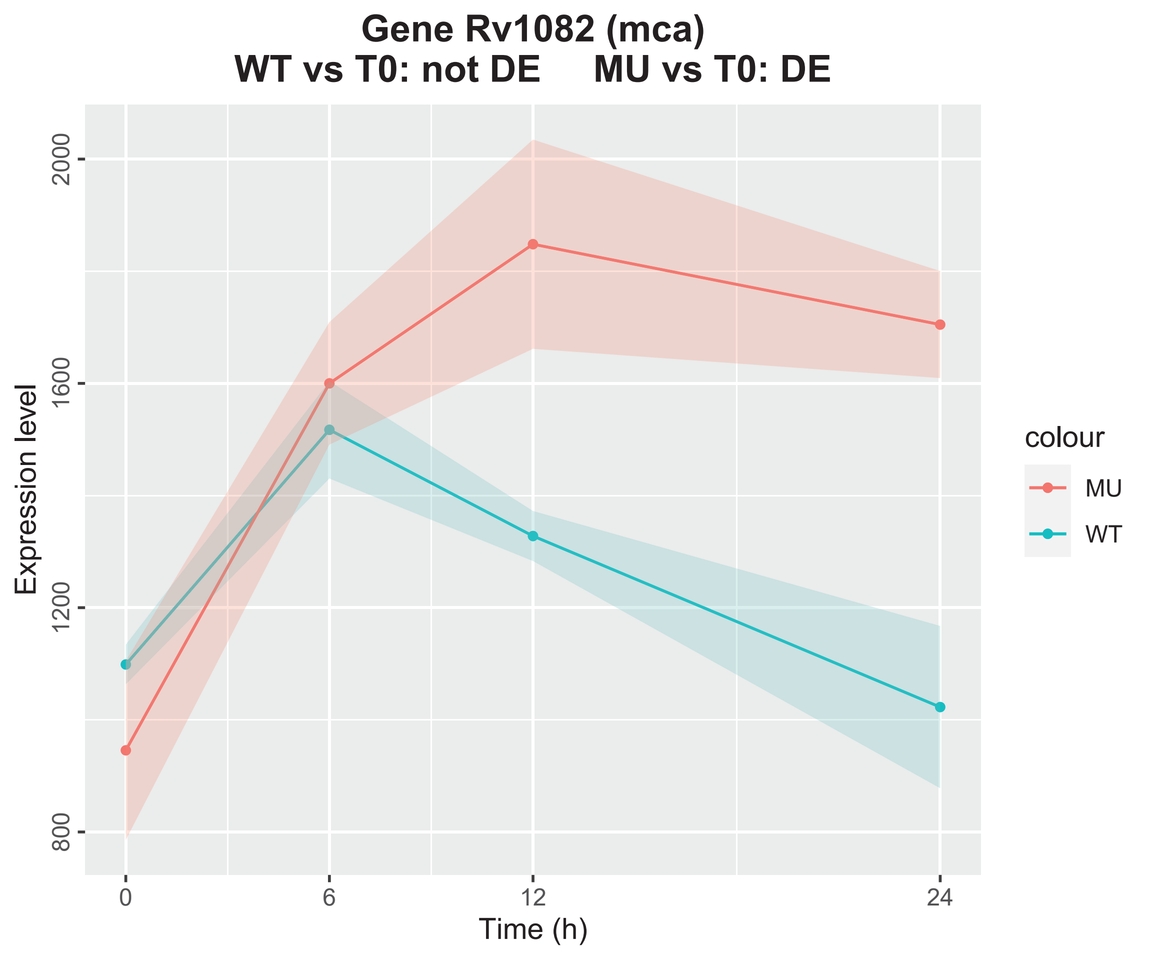

Gene Rv1082 (mca)
WT vs T0: not DE MU vs T0: DE
Expression level
2000
1600
1200
800
0
6
12
24
Time (h)
colour
MU
WT

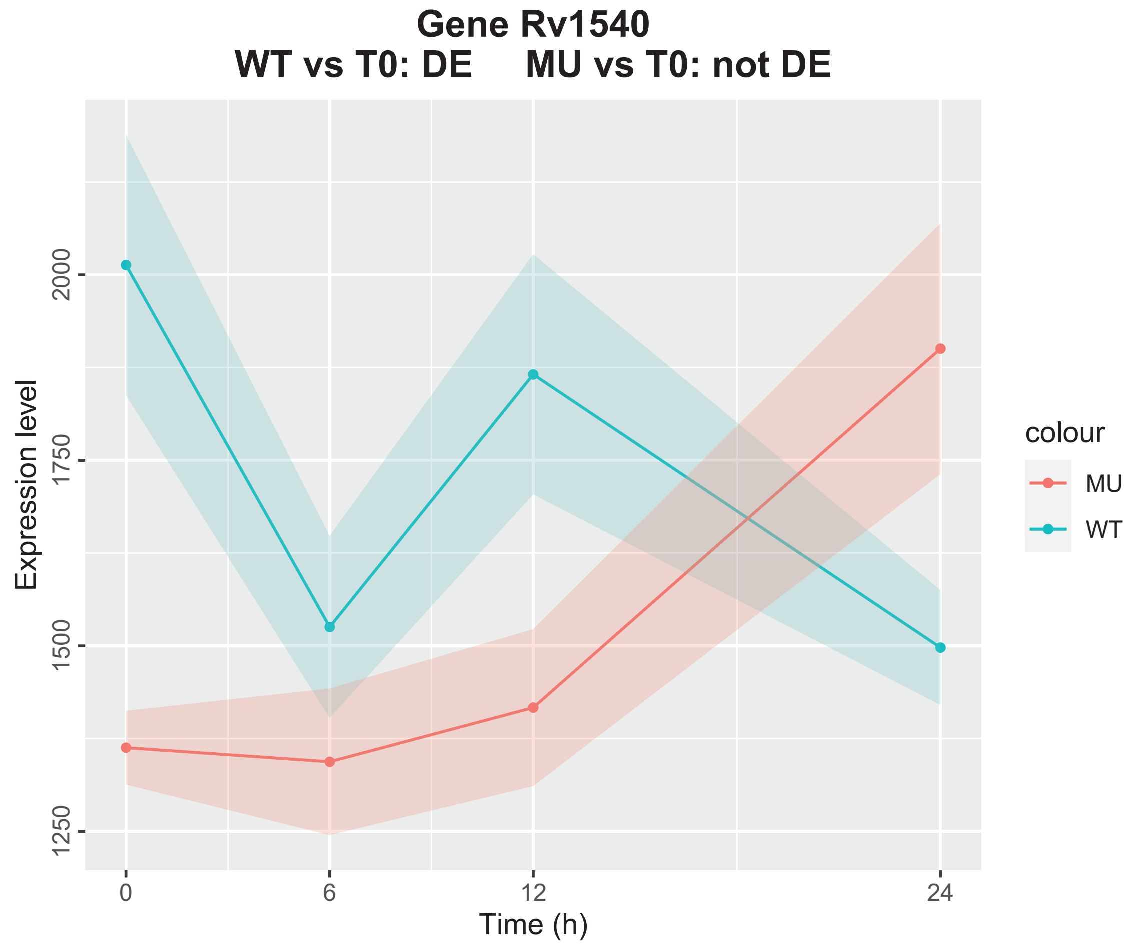

Gene Rv1540
WT vs T0: DE MU vs T0: not DE
Expression level
2000
1750
1500
1250
0
6
12
24
Time (h)
colour
MU
WT

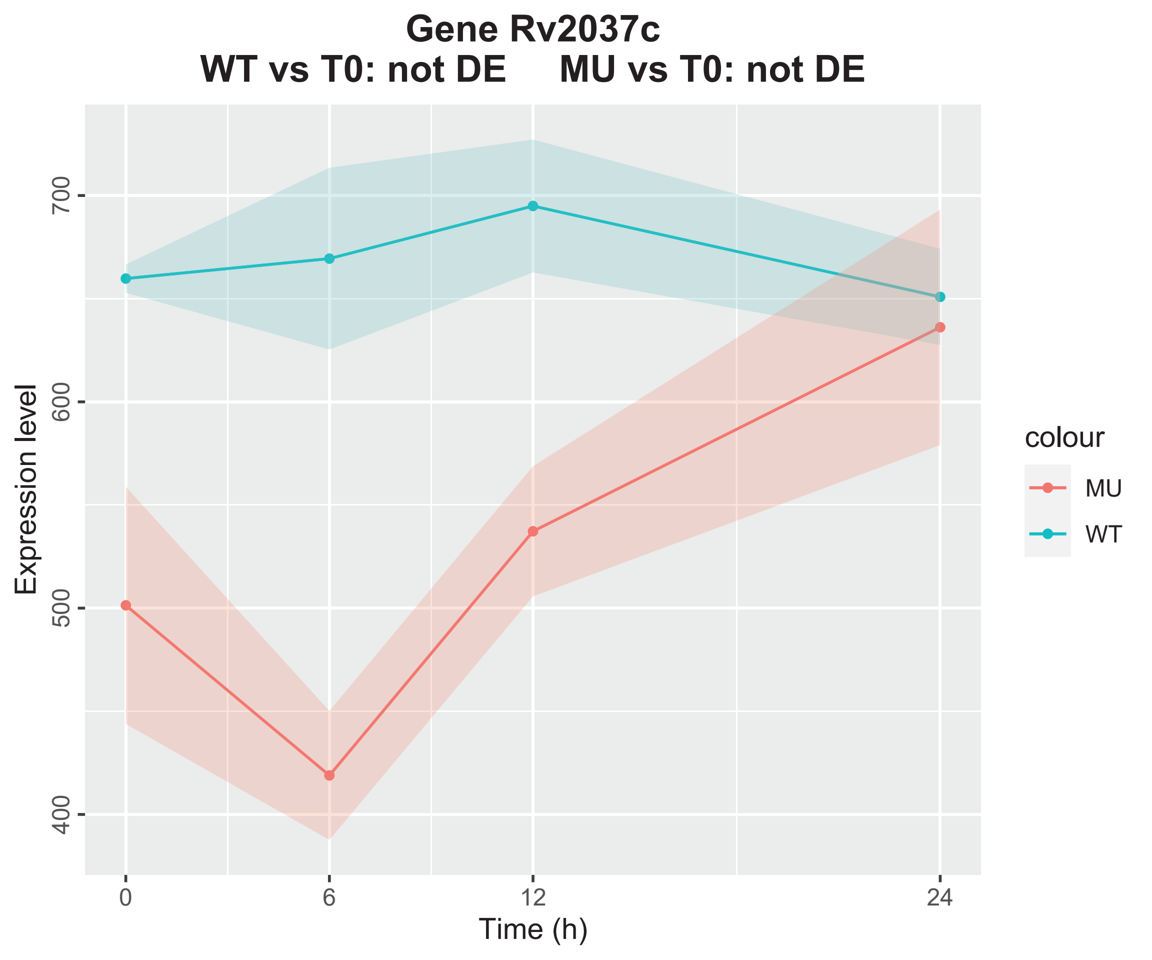
Gene Rv2037c
WT vs T0: not DE   MU vs T0: not DE
Expression level
700
600
500
400
0
6
12
24
Time (h)
colour
MU
WT
