# Supplementary material for: Role of the Extracytoplasmic Function Sigma Factor SigE in the Stringent Response of Mycobacterium tuberculosis
Source: Microbiol Spectr. 2023 Mar 22;11(2):e02944-22. doi: 10.1128/spectrum.02944-22 (PMC10100808; doi:10.1128/spectrum.02944-22)
Supplement: Supplemental file 9 — Data S9. Download spectrum.02944-22-s0010.pdf, PDF file, 0.4 MB [file spectrum.02944-22-s0010.pdf]

<u>Supplementary Data S9:</u> Gene expression profiles over time of genes discussed in section "Other alterations specific of sigE mutant", i.e. *sigE*, *whiB6*, *whiB7*, *eis*, *rv1258*, *rv0492c-rv0493c*, *mmpL5-mmpS5*, *rv2686-rv2687*, *mmr*, *rv2640c-cadI*, *rv2642-arsC*, *rv0299*, *vapB4-C4*, *vapB27-C27*, *vapB29-C29*, *mazF5-E5* and *relF-G*. For each gene, the plot shows the average expression level and the standard deviation (shaded area) for both the wild-type (cyan color) and *sigE*-mutant (salmon/pink color) strains.

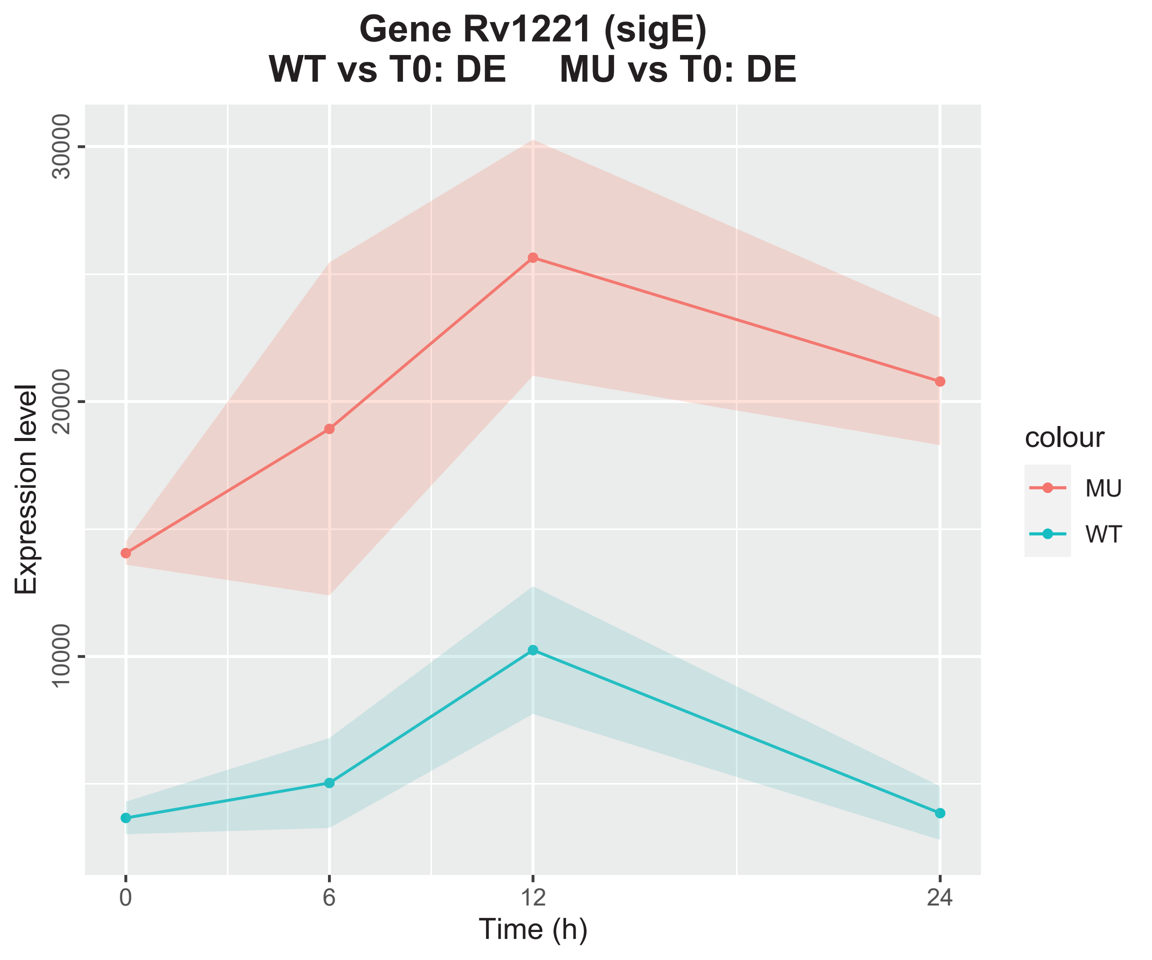

Gene Rv1221 (sigE)
WT vs T0: DE MU vs T0: DE
30000
20000
10000
Expression level
0
6
12
24
Time (h)
colour
MU
WT

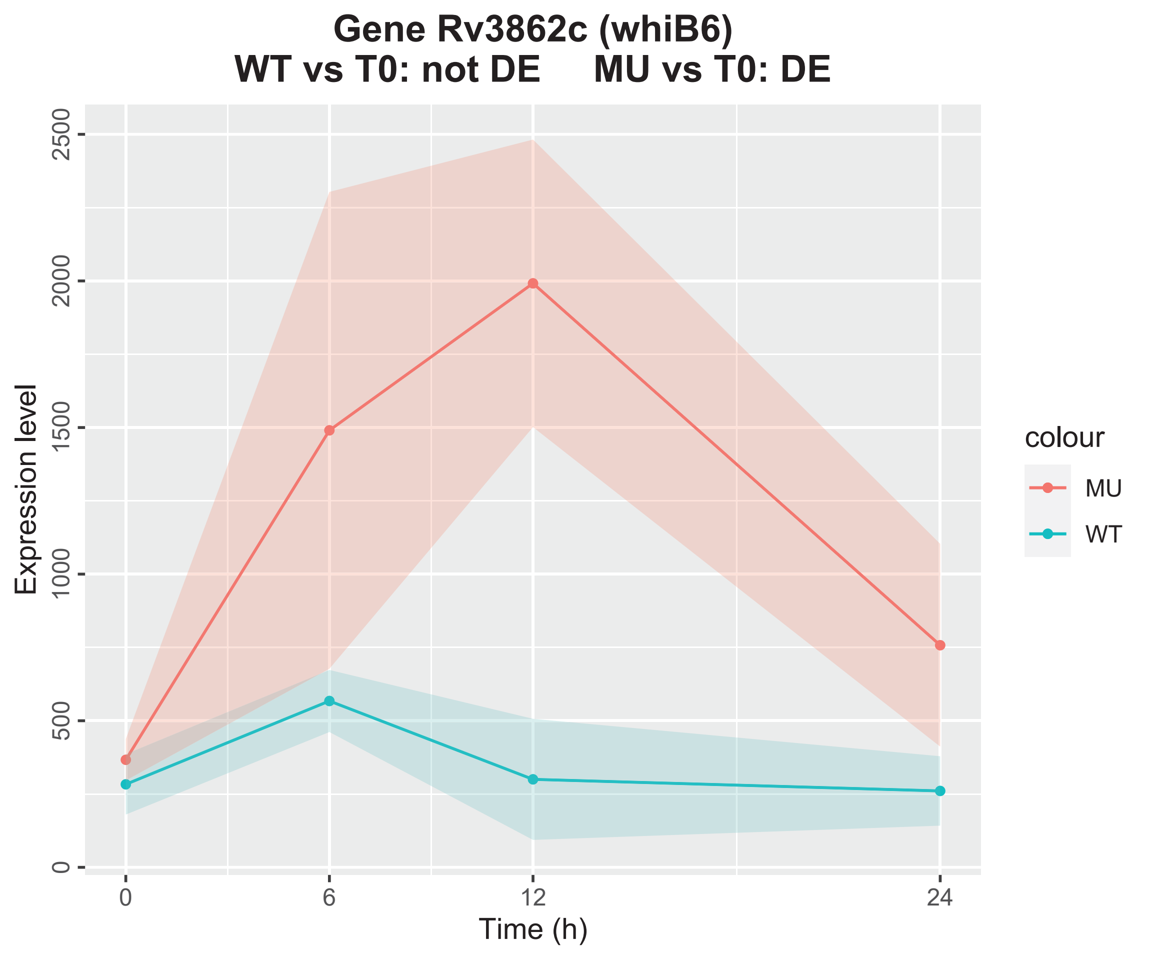
Gene Rv3862c (whiB6)
WT vs T0: not DE MU vs T0: DE
Expression level
2500
2000
1500
1000
500
0
0
6
12
24
Time (h)
colour
MU
WT

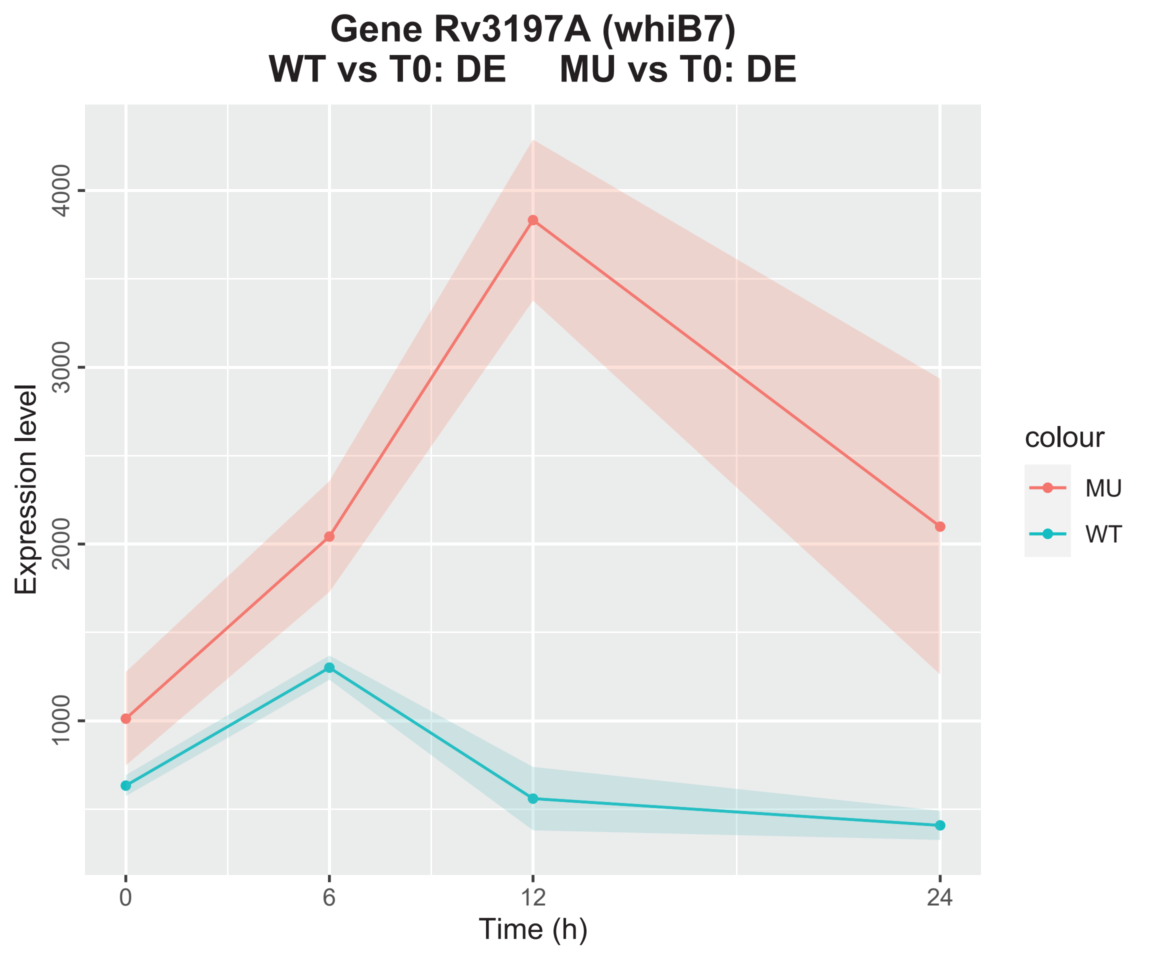
Gene Rv3197A (whiB7)
WT vs T0: DE MU vs T0: DE
Expression level
4000
3000
2000
1000
0
6
12
24
Time (h)
colour
MU
WT

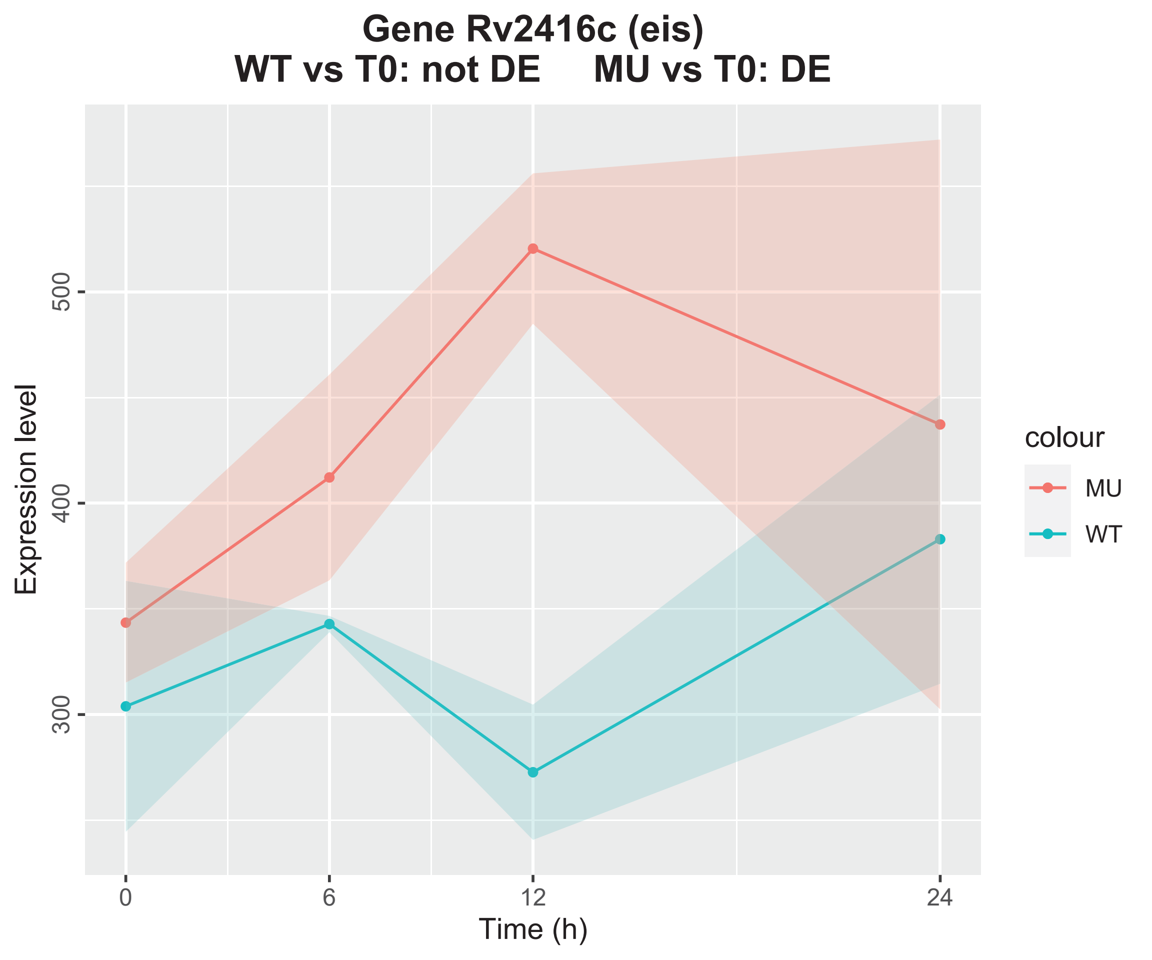
Gene Rv2416c (eis)
WT vs T0: not DE MU vs T0: DE
Expression level
500
400
300
0
6
12
24
Time (h)
colour
MU
WT

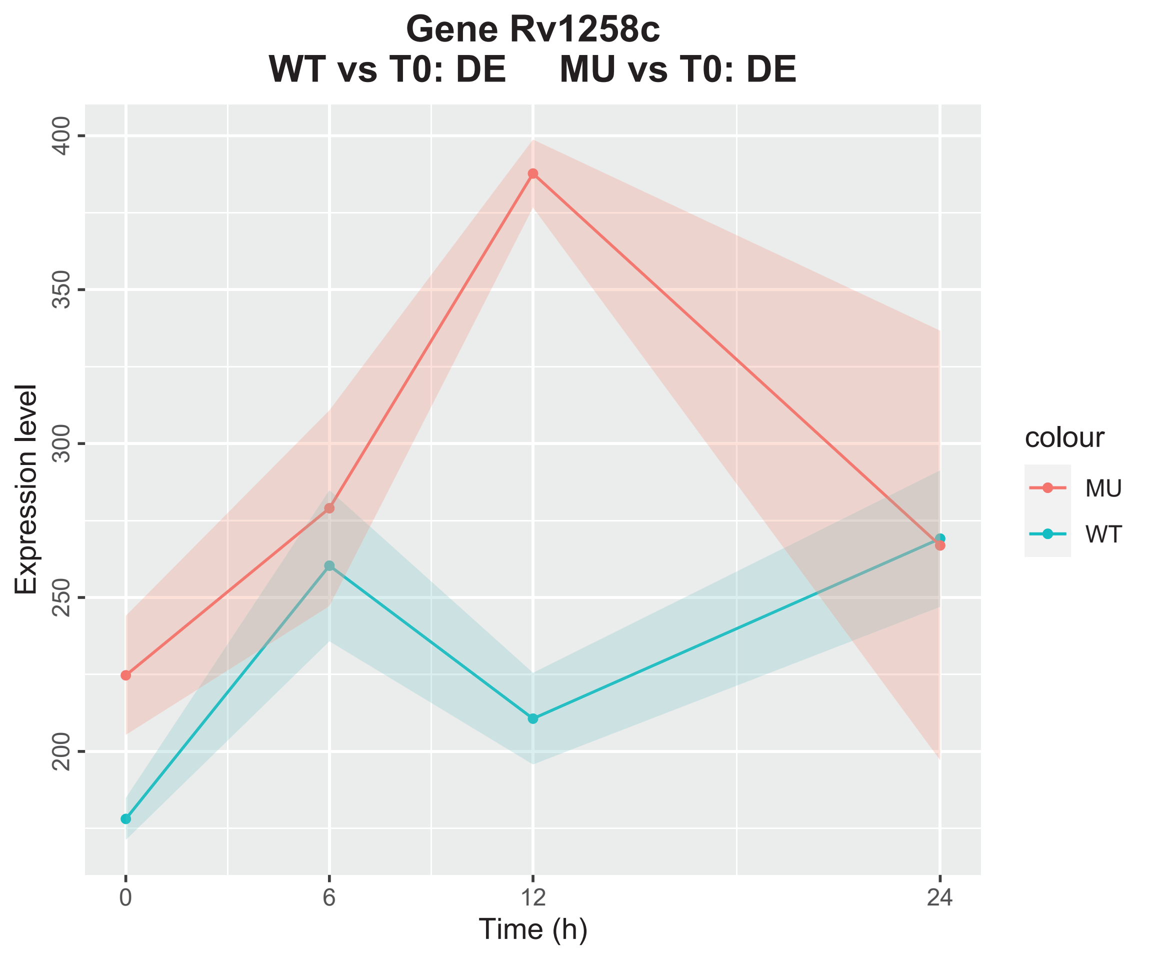

Gene Rv1258c
WT vs T0: DE   MU vs T0: DE
Expression level
400
350
300
250
200
0
6
12
24
Time (h)
colour
MU
WT

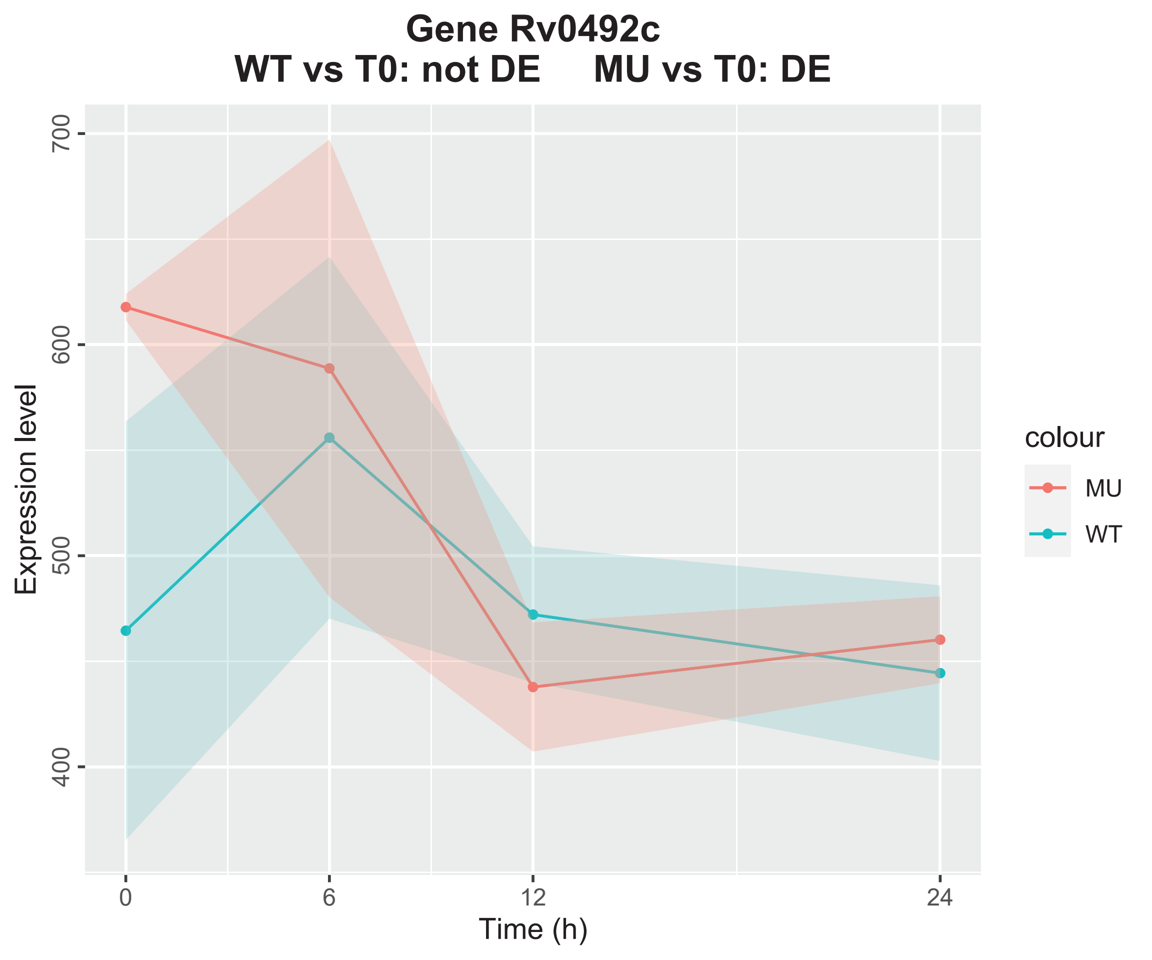
Gene Rv0492c
WT vs T0: not DE MU vs T0: DE
Expression level
700
600
500
400
0
6
12
24
Time (h)
colour
MU
WT

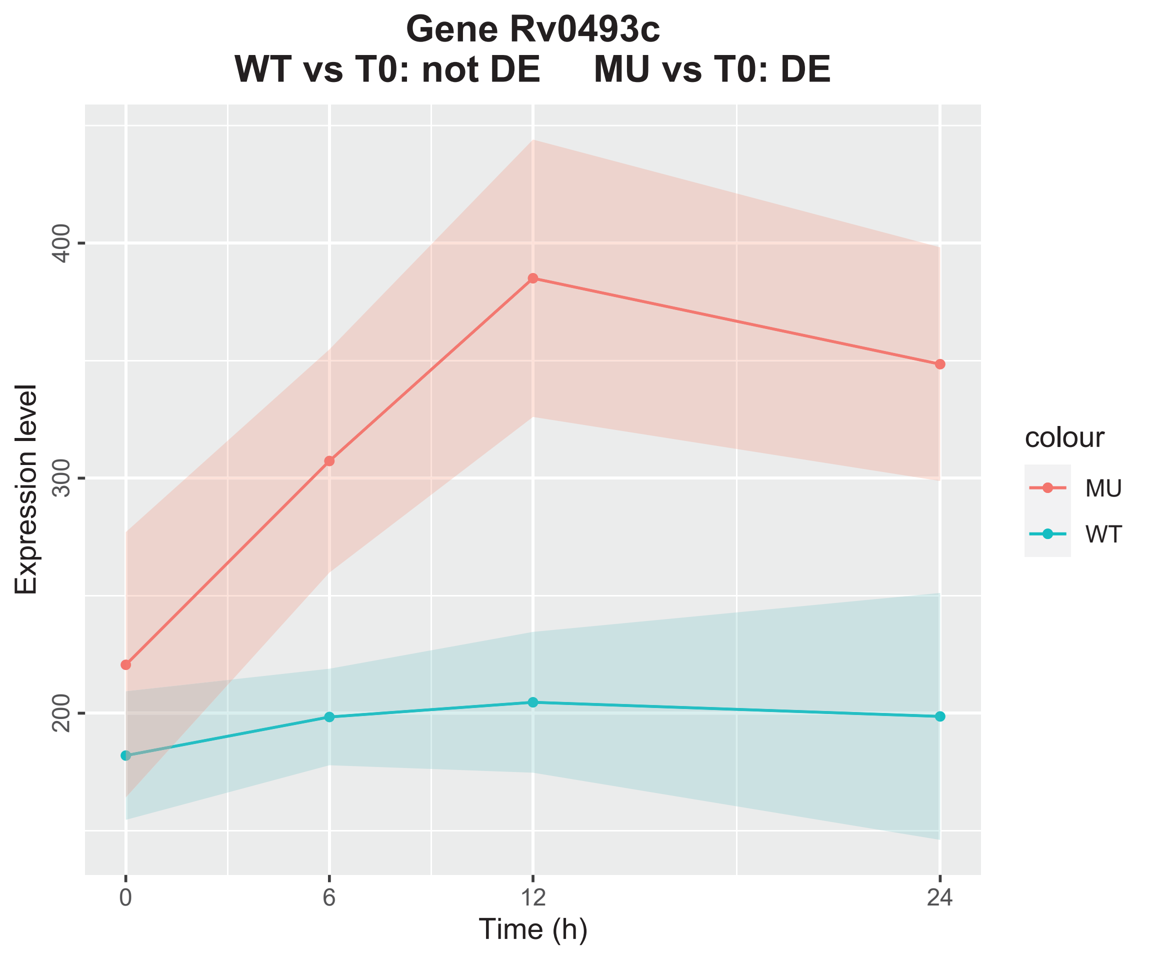

Gene Rv0493c
WT vs T0: not DE   MU vs T0: DE
Expression level
400
300
200
Time (h)
0
6
12
24
colour
MU
WT

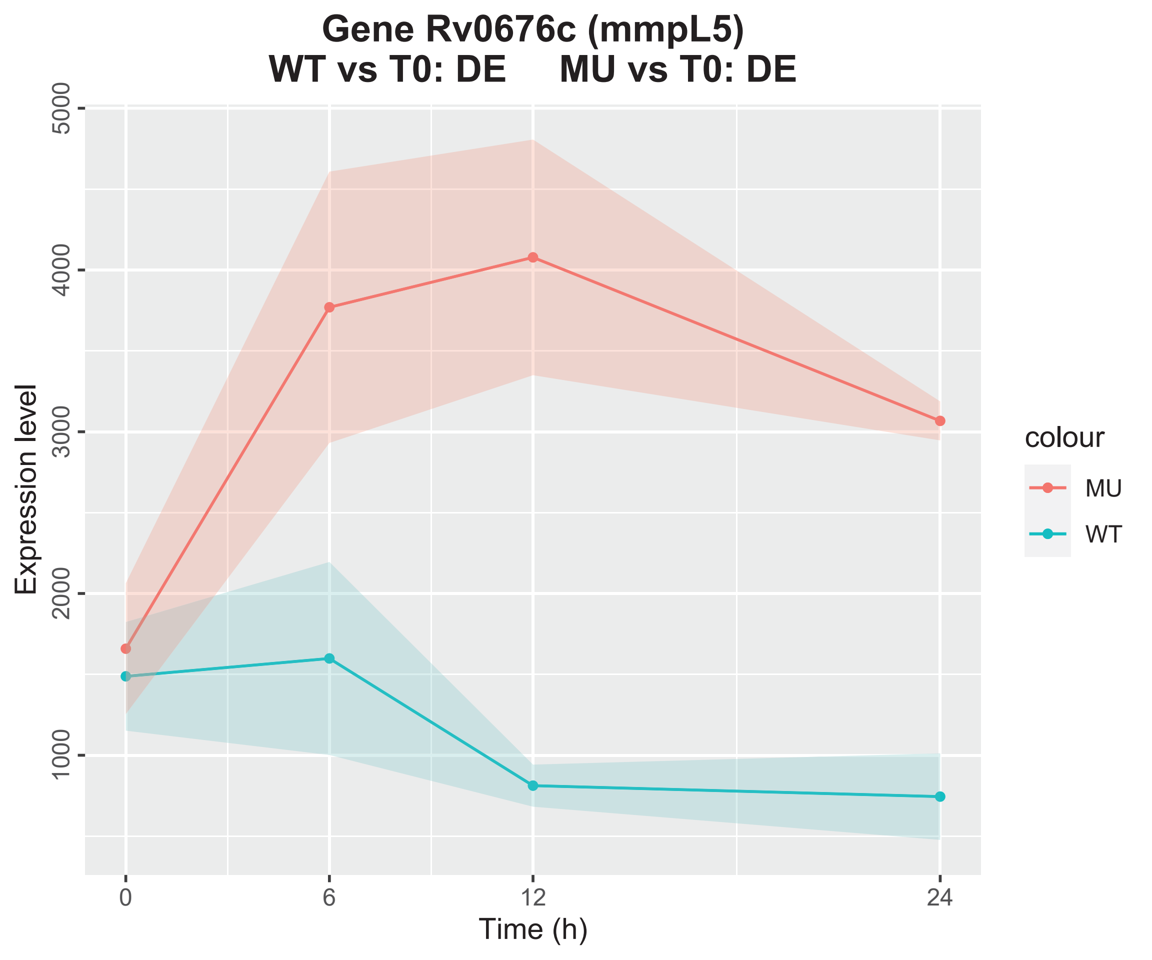

Gene Rv0676c (mmpL5)
WT vs T0: DE MU vs T0: DE
Expression level
5000
4000
3000
2000
1000
0
6
12
24
Time (h)
colour
MU
WT

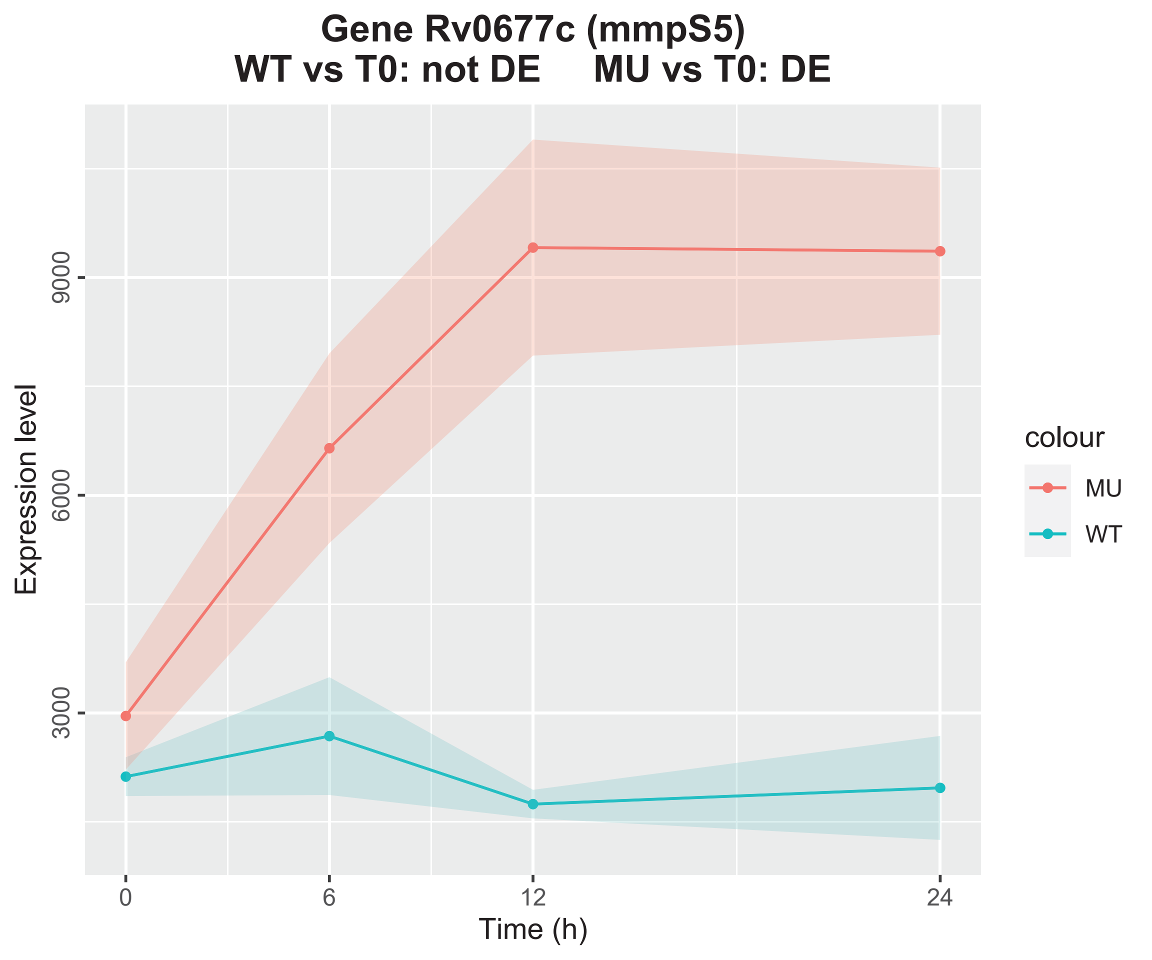
Gene Rv0677c (mmpS5)
WT vs T0: not DE   MU vs T0: DE
Expression level
9000
6000
3000
0
6
12
24
Time (h)
colour
MU
WT

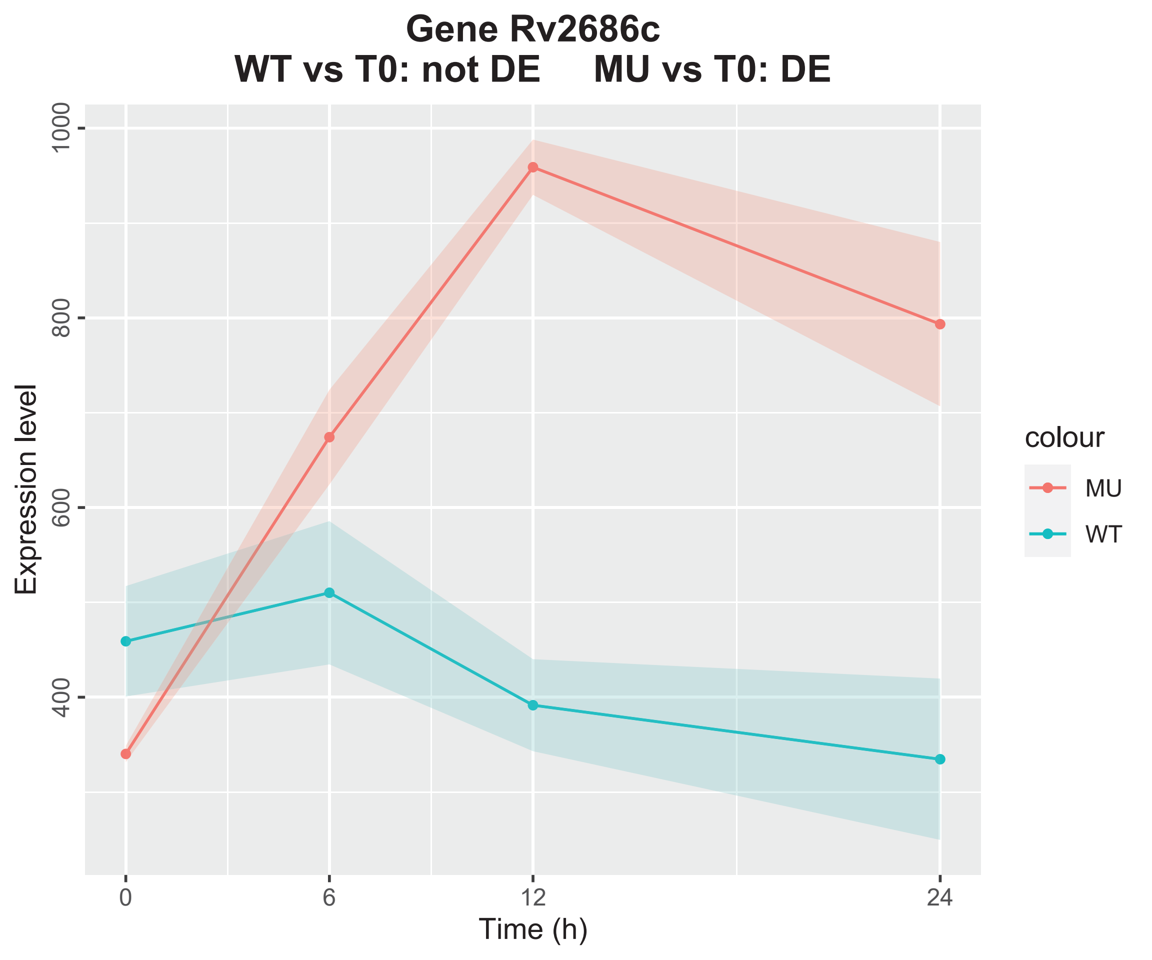
Gene Rv2686c
WT vs T0: not DE MU vs T0: DE
1000
800
600
400
Expression level
0
6
12
24
Time (h)
colour
MU
WT

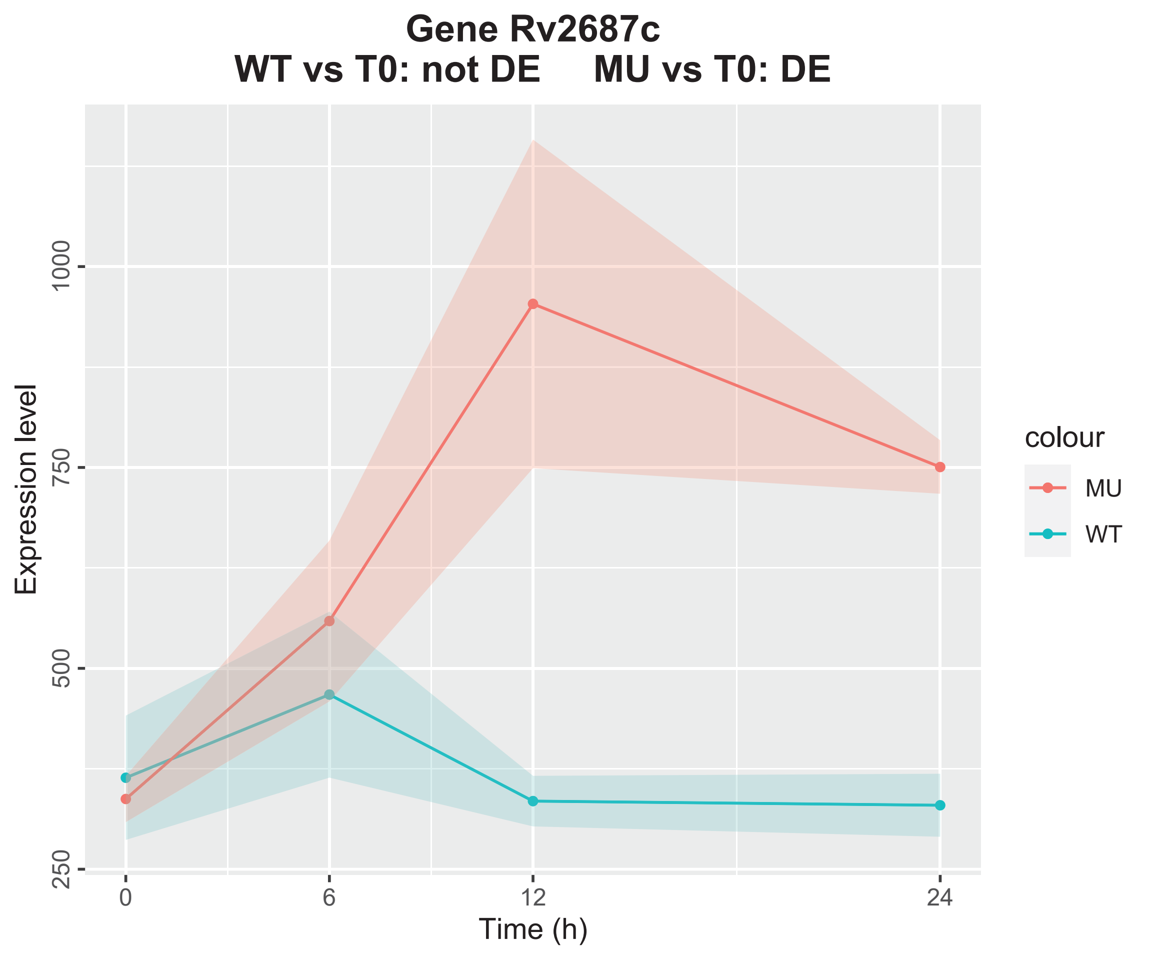
Gene Rv2687c
WT vs T0: not DE MU vs T0: DE
Expression level
1000
750
500
250
0
6
12
24
Time (h)
colour
MU
WT

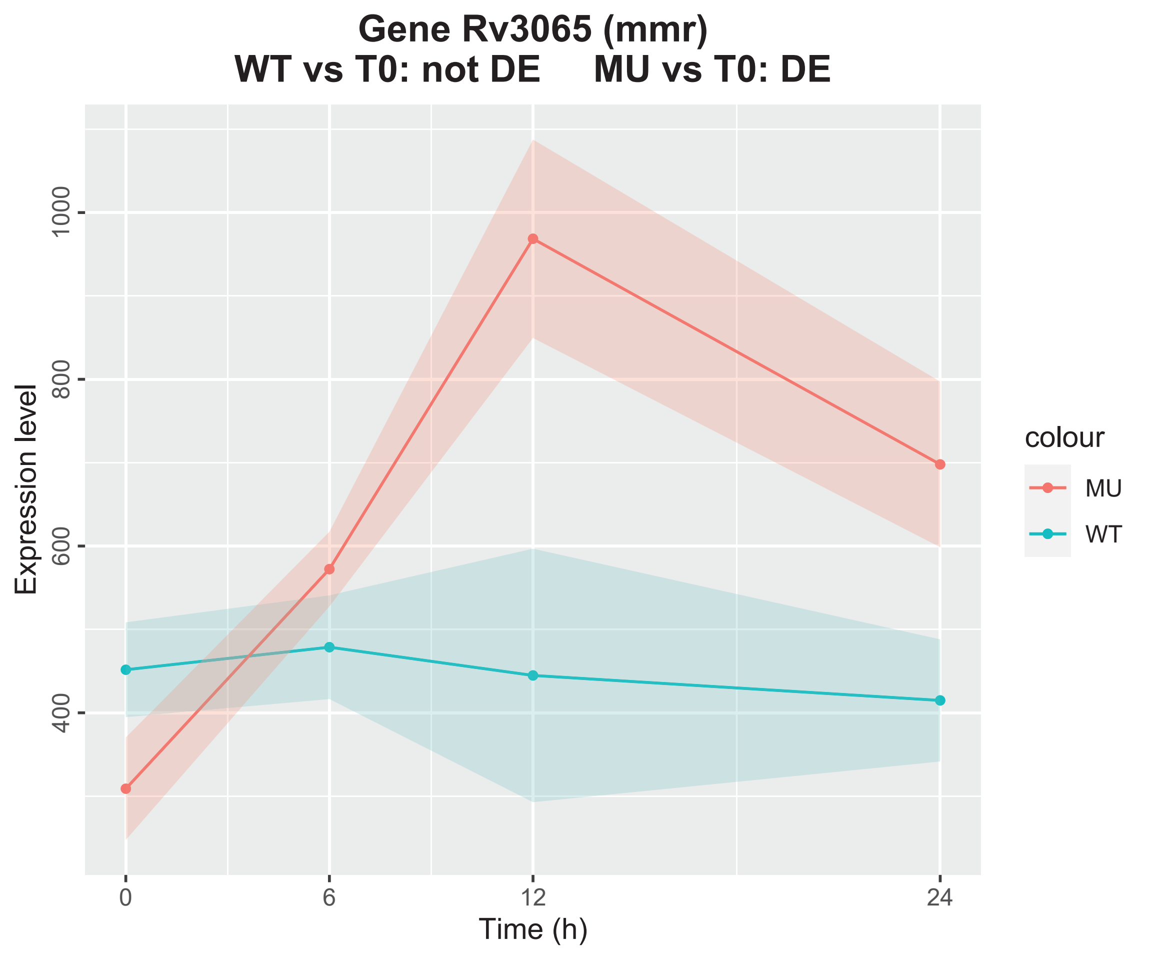
Gene Rv3065 (mmr)
WT vs T0: not DE   MU vs T0: DE
1000
800
600
400
Expression level
0
6
12
24
Time (h)
colour
MU
WT

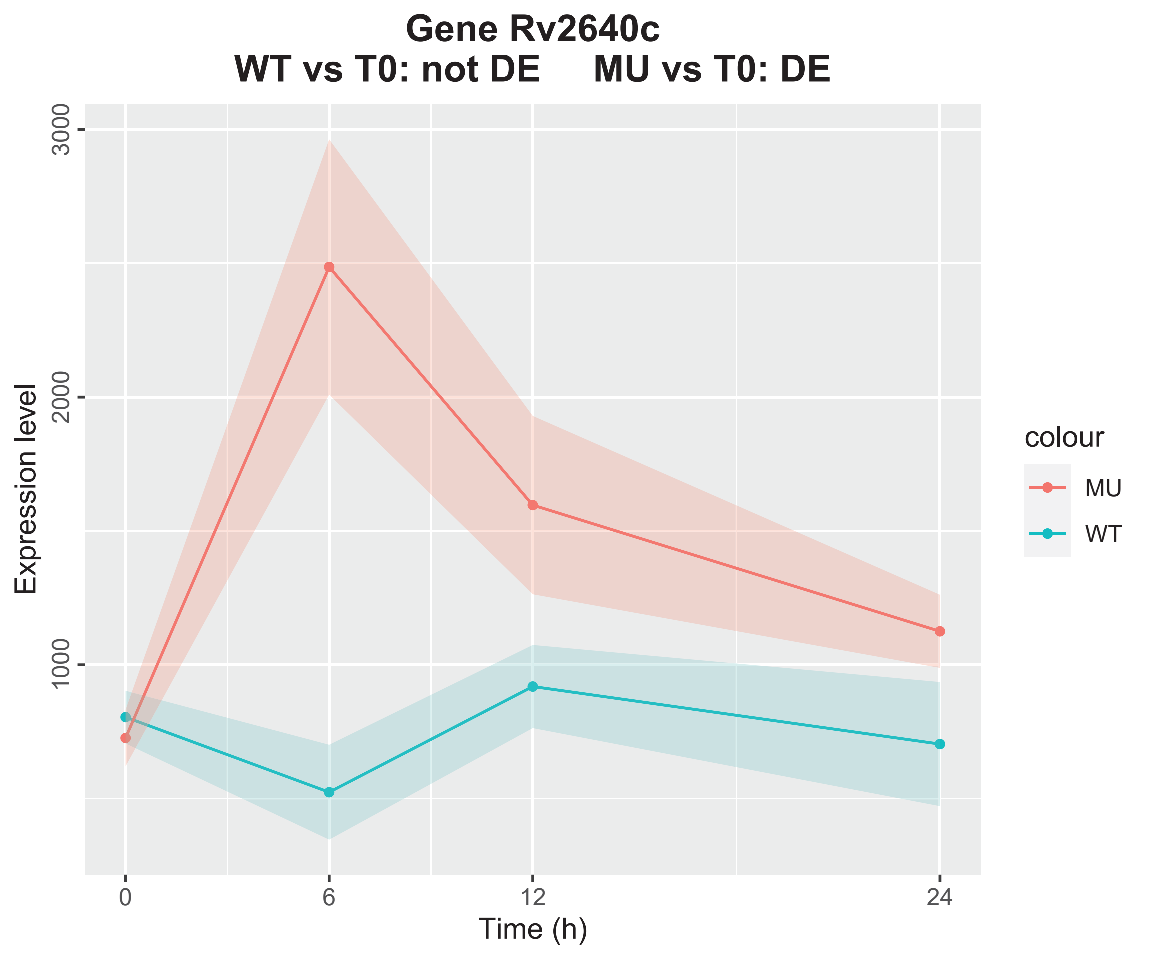

Gene Rv2640c
WT vs T0: not DE   MU vs T0: DE
3000
2000
1000
Expression level
0
6
12
24
Time (h)
colour
MU
WT

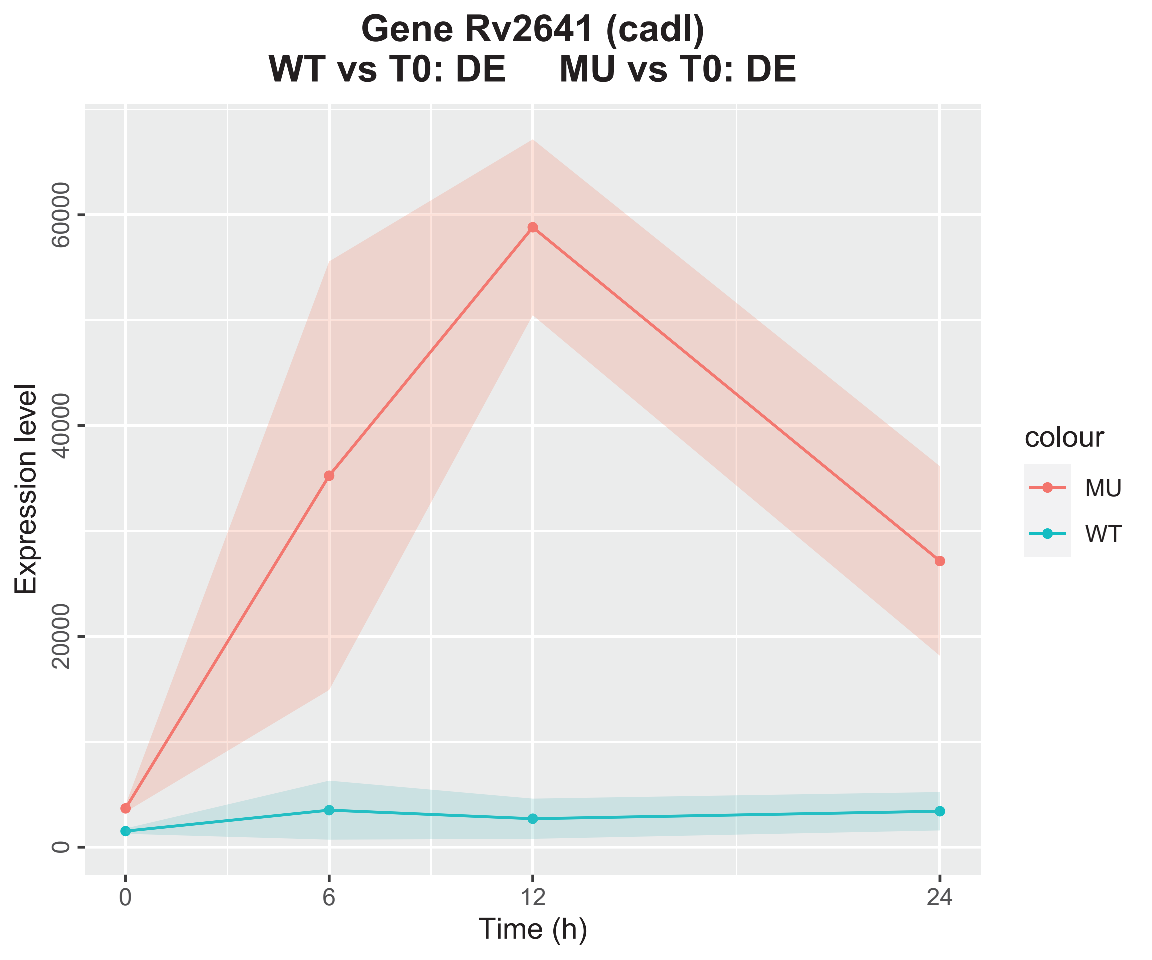

Gene Rv2641 (cadI)
WT vs T0: DE   MU vs T0: DE
Expression level
0
20000
40000
60000
0
6
12
24
Time (h)
colour
MU
WT

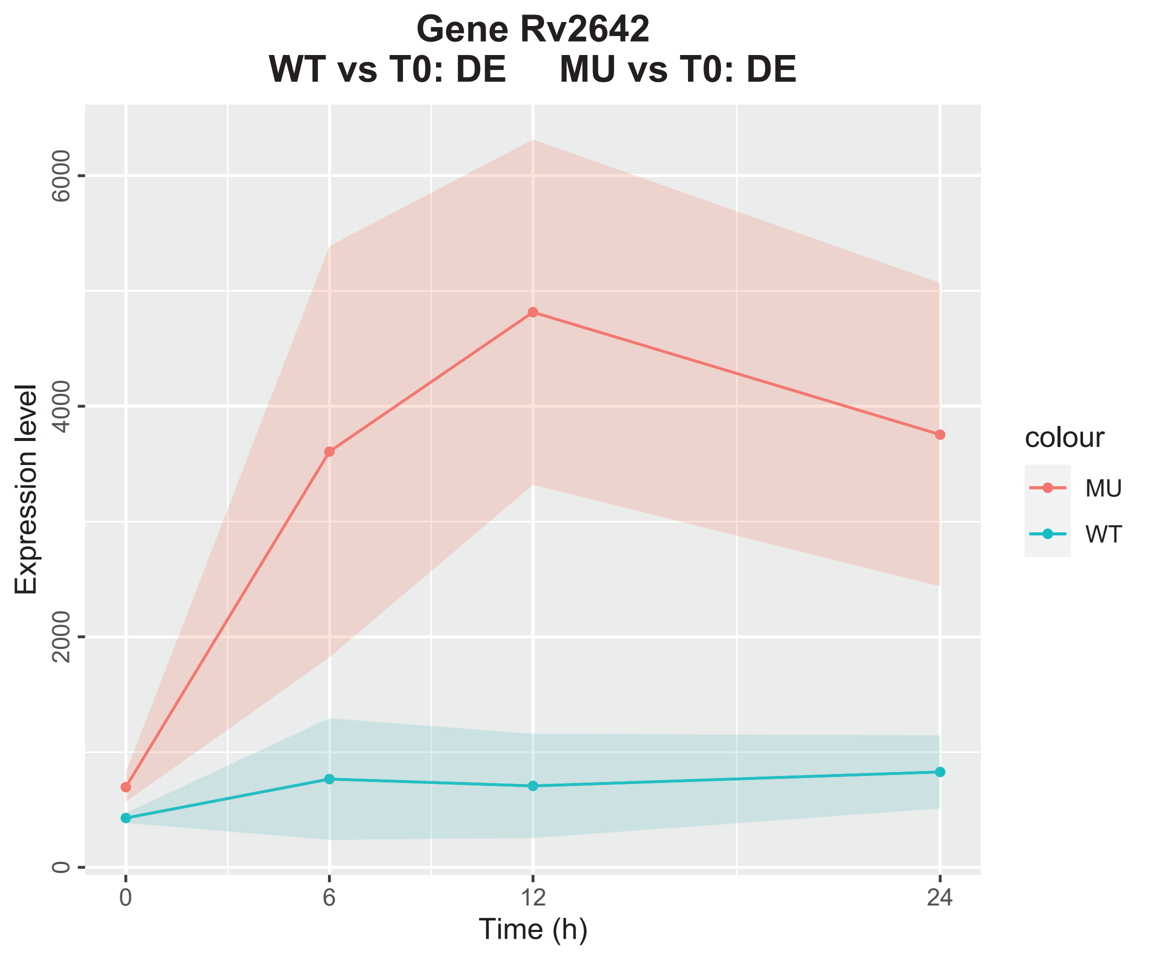
Gene Rv2642
WT vs T0: DE MU vs T0: DE
Expression level
0
2000
4000
6000
Time (h)
0
6
12
24
colour
MU
WT

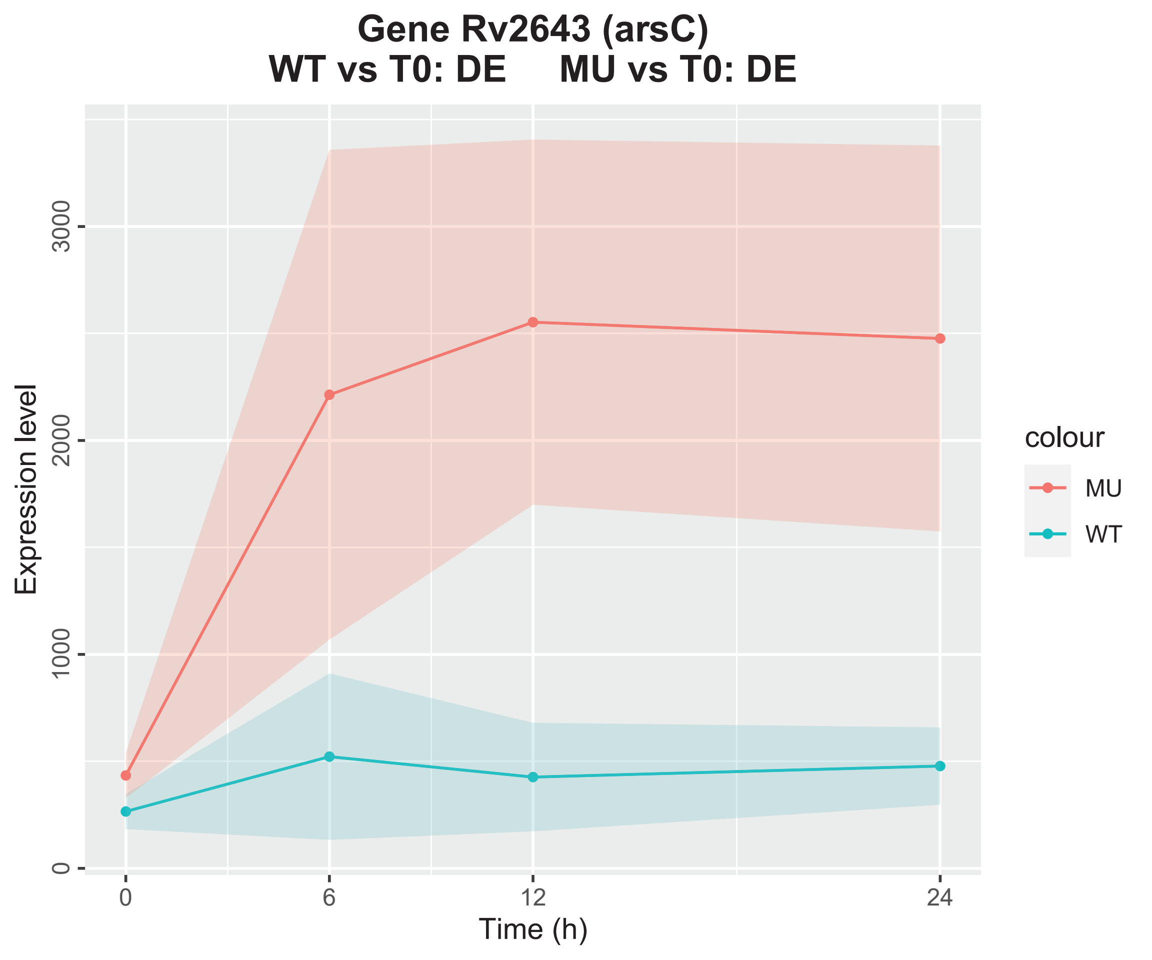

Gene Rv2643 (arsC)
WT vs T0: DE MU vs T0: DE
Expression level
3000
2000
1000
0
0
6
12
24
Time (h)
colour
MU
WT

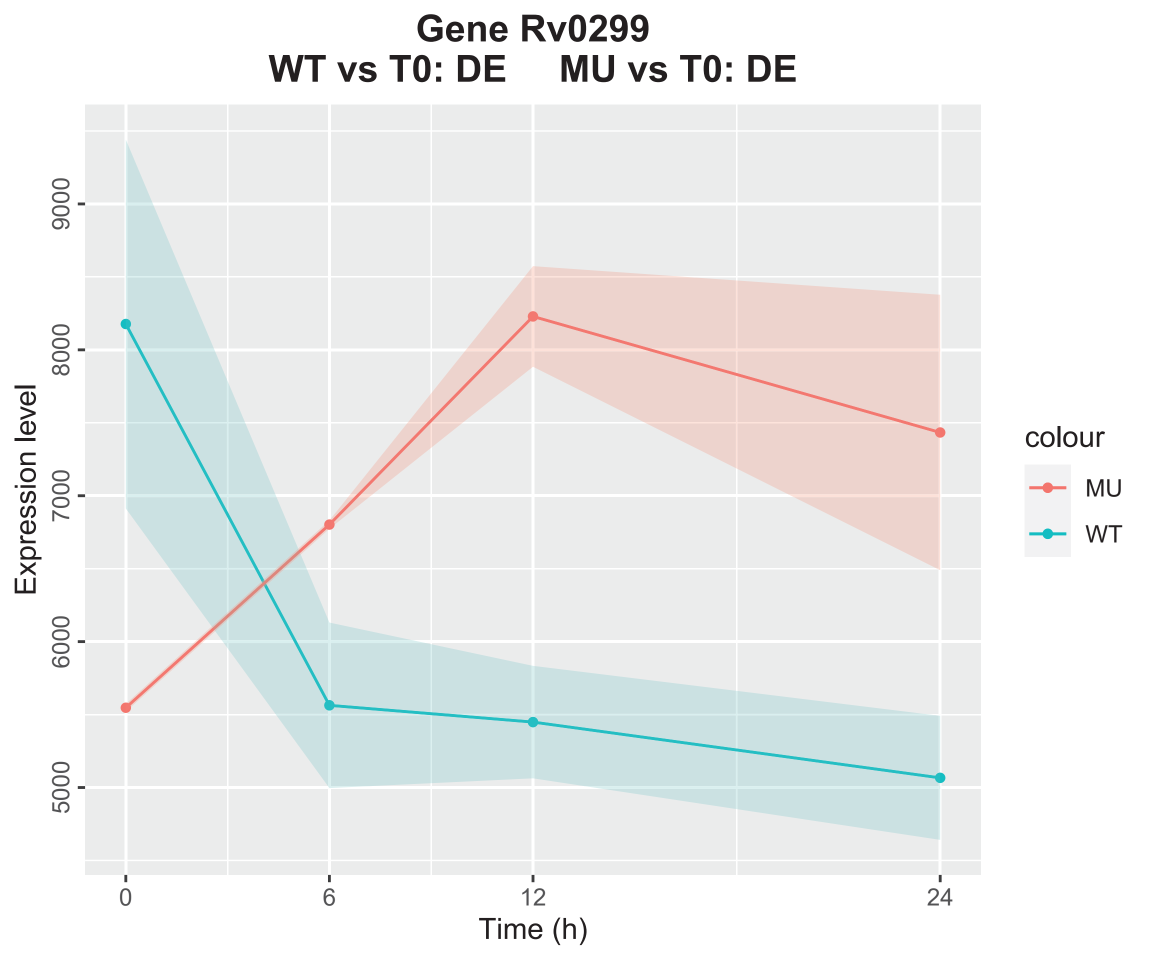
Gene Rv0299
WT vs T0: DE MU vs T0: DE
Expression level
9000
8000
7000
6000
5000
0
6
12
24
Time (h)
colour
MU
WT

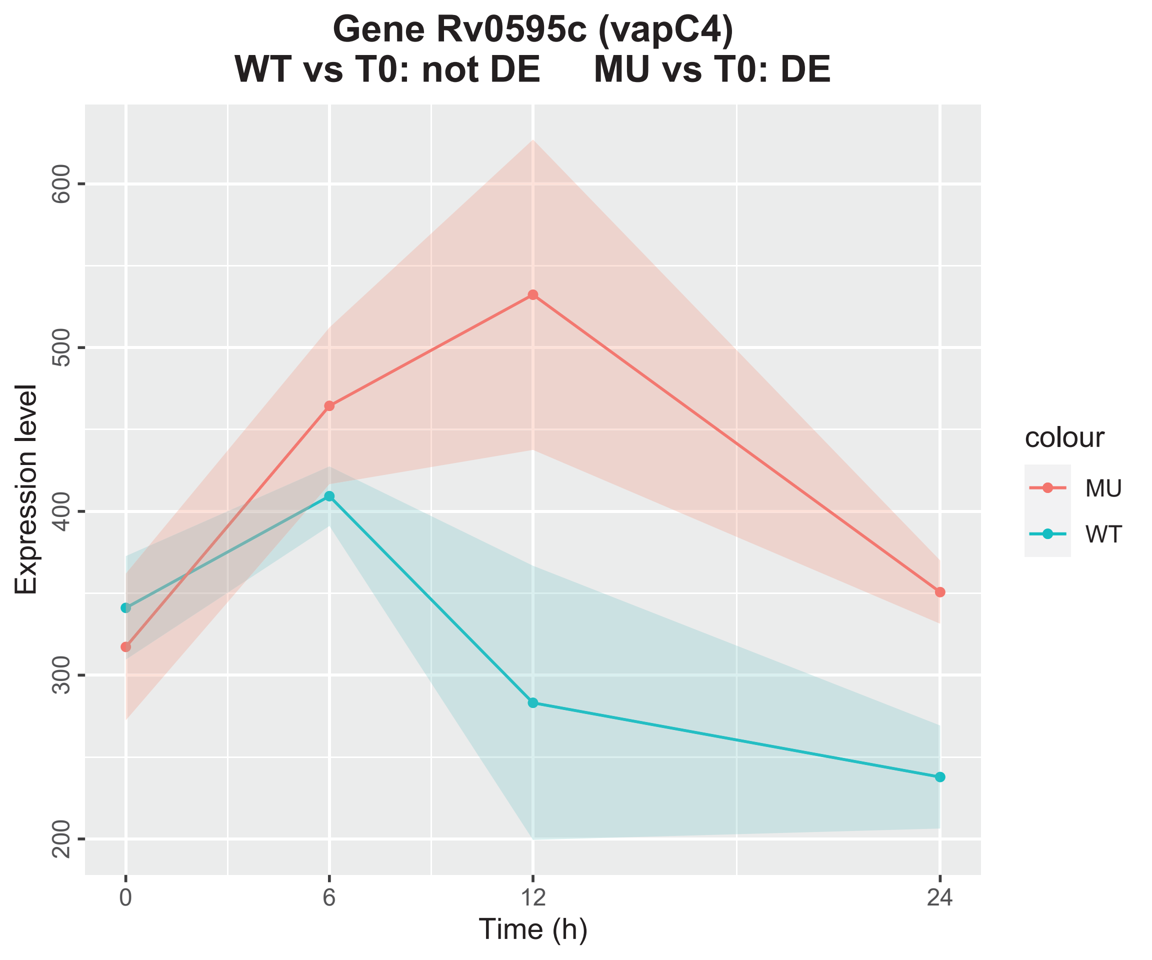

Gene Rv0595c (vapC4)
WT vs T0: not DE MU vs T0: DE
Expression level
600
500
400
300
200
0
6
12
24
Time (h)
colour
MU
WT

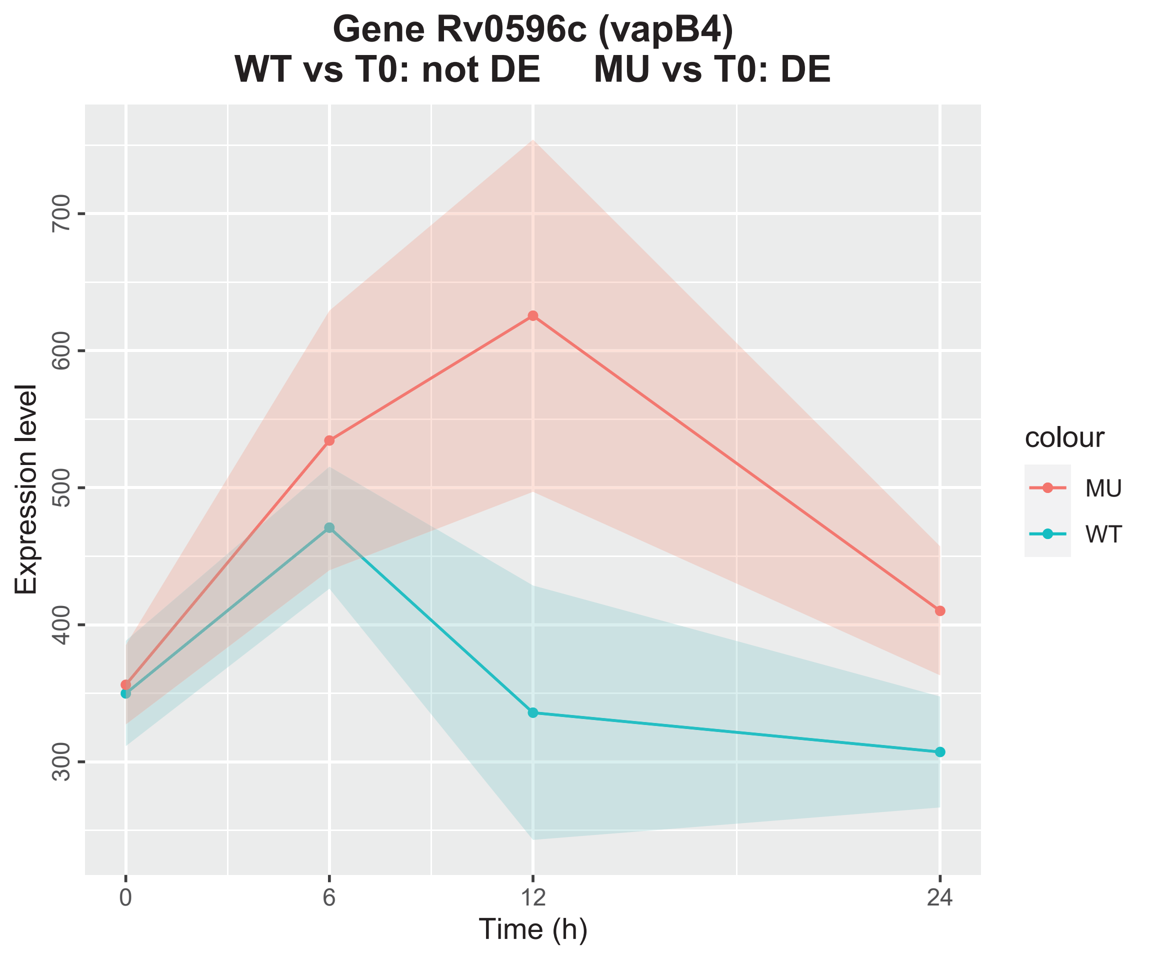

Gene Rv0596c (vapB4)
WT vs T0: not DE   MU vs T0: DE
Expression level
300
400
500
600
700
0
6
12
24
Time (h)
colour
MU
WT

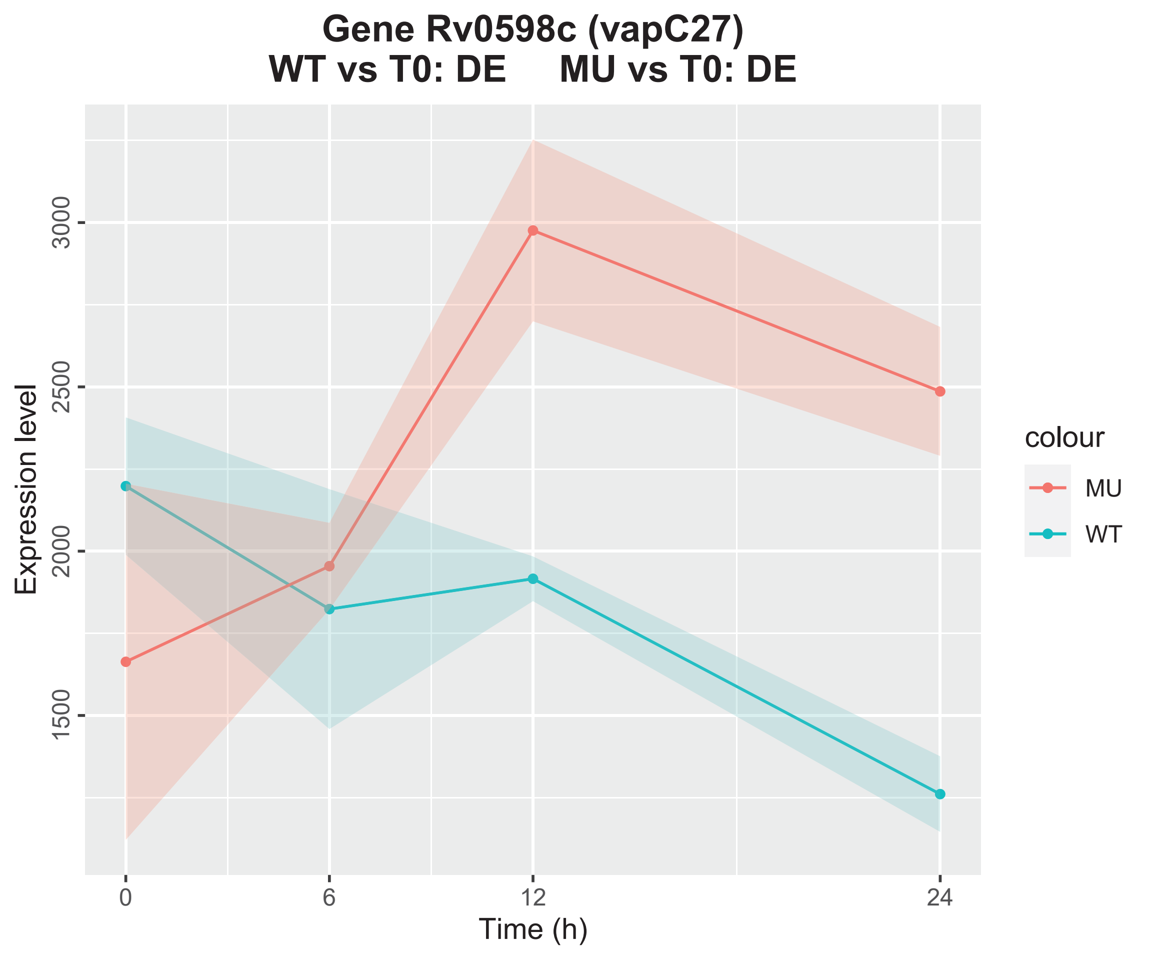
Gene Rv0598c (vapC27)
WT vs T0: DE MU vs T0: DE
Expression level
3000
2500
2000
1500
0
6
12
24
Time (h)
colour
MU
WT

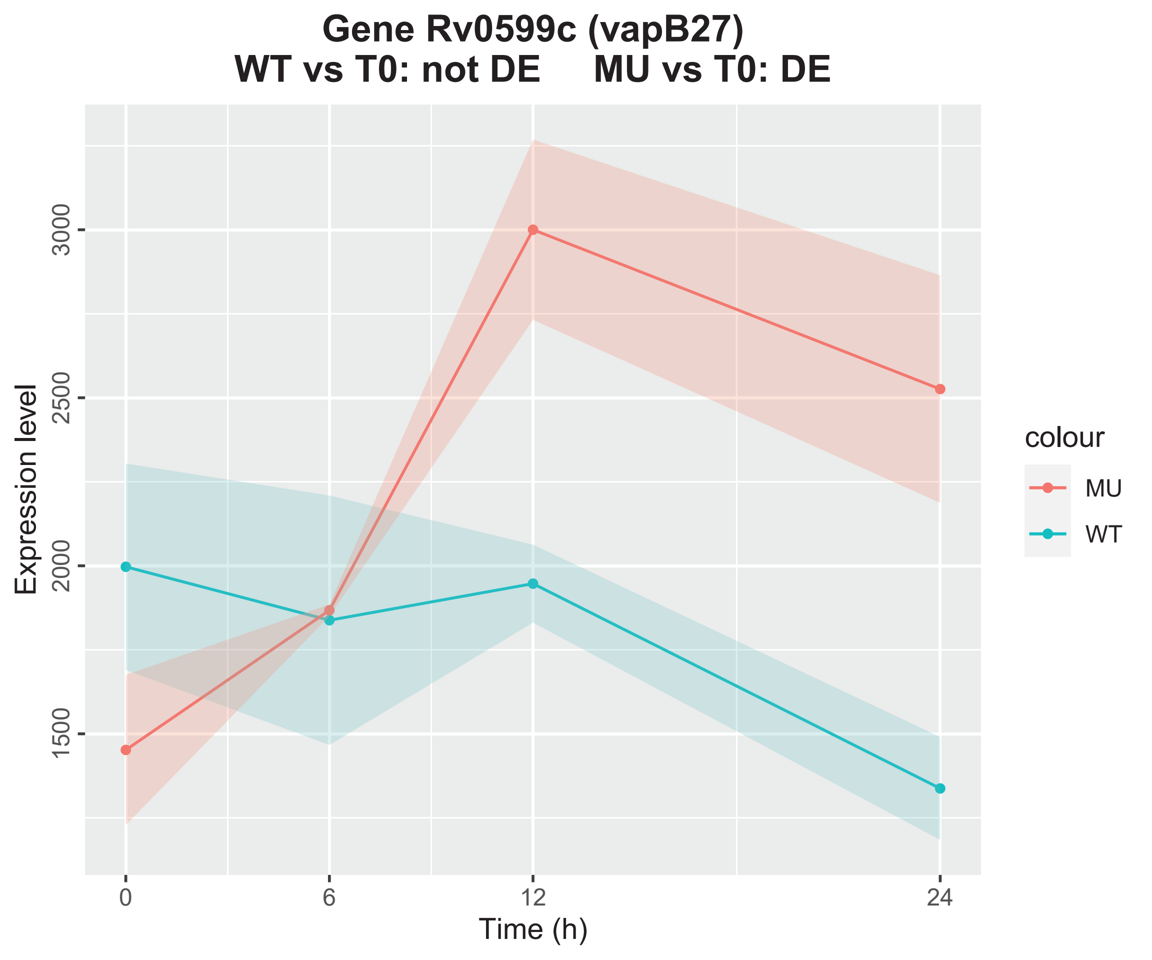

Gene Rv0599c (vapB27)
WT vs T0: not DE   MU vs T0: DE
Expression level
3000
2500
2000
1500
0
6
12
24
Time (h)
colour
MU
WT

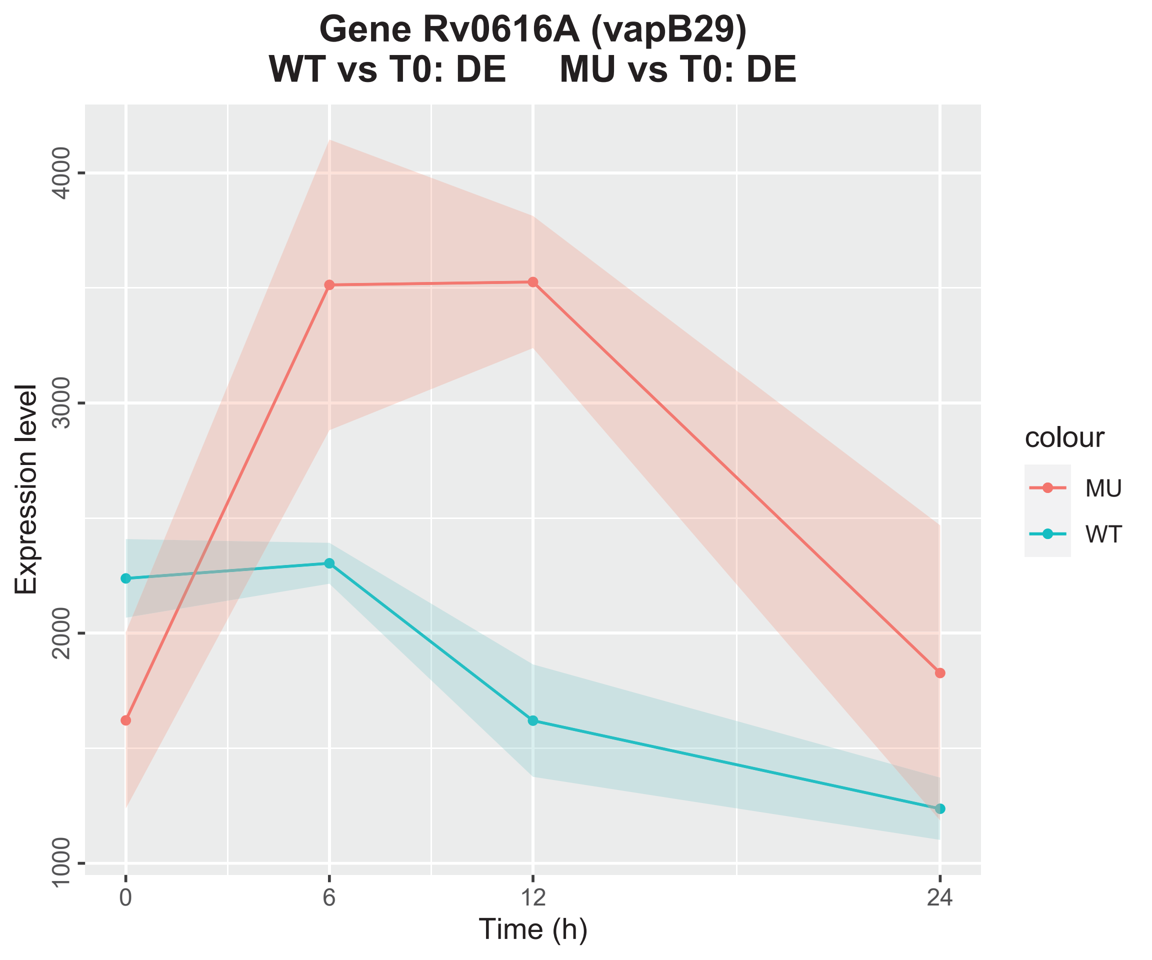

Gene Rv0616A (vapB29)
WT vs T0: DE   MU vs T0: DE
Expression level
4000
3000
2000
1000
0
6
12
24
Time (h)
colour
MU
WT

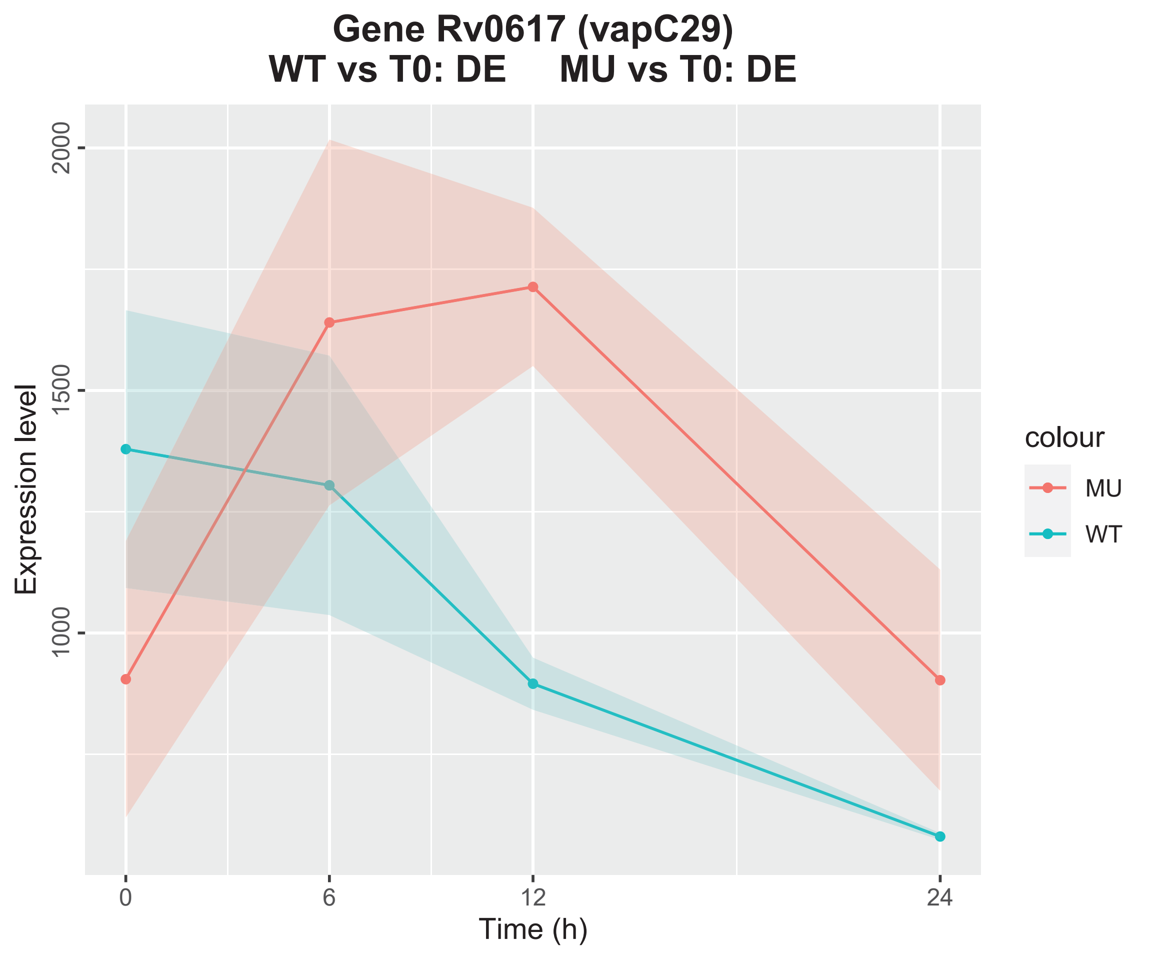

Gene Rv0617 (vapC29)
WT vs T0: DE MU vs T0: DE
2000
1500
1000
0
6
12
24
Expression level
Time (h)
colour
MU
WT

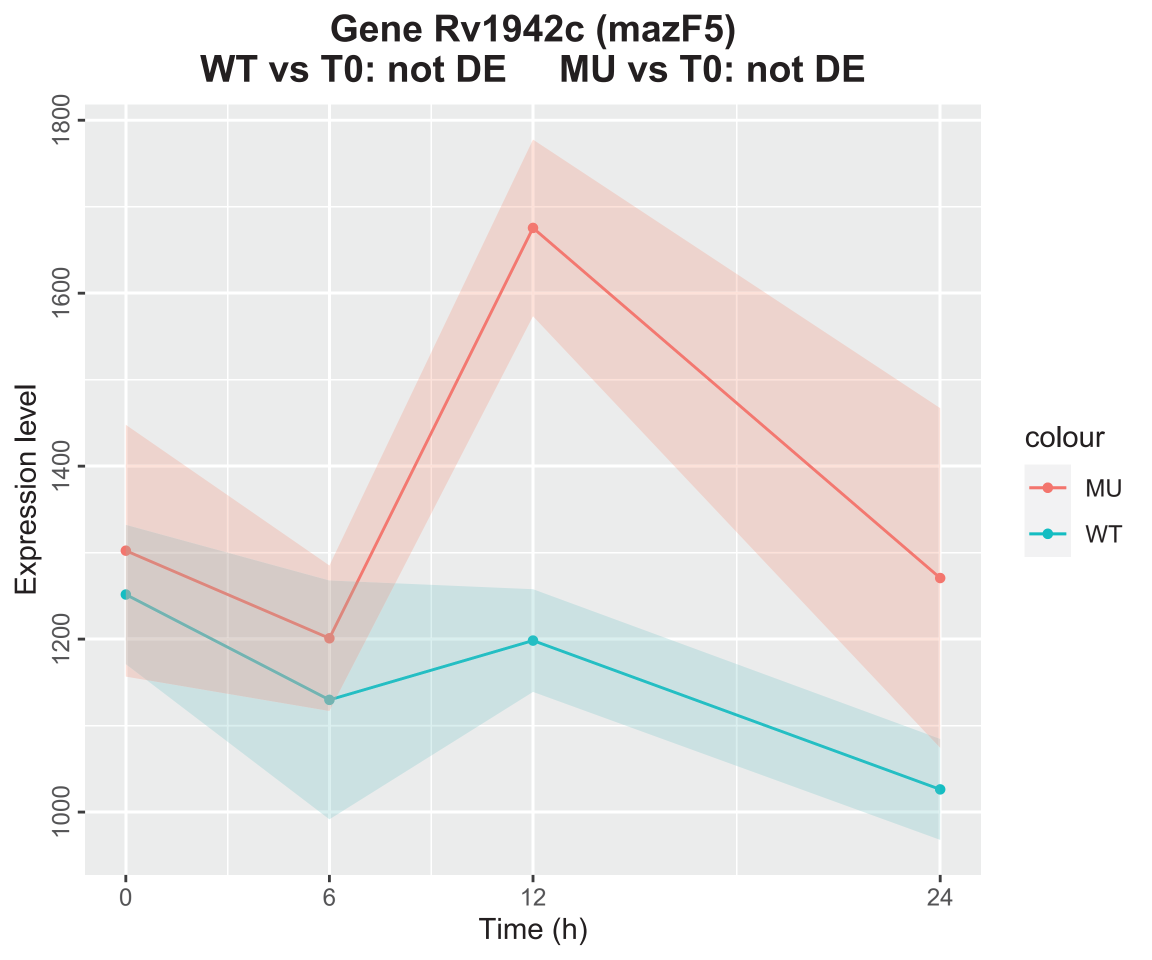
Gene Rv1942c (mazF5)
WT vs T0: not DE   MU vs T0: not DE
Expression level
1000
1200
1400
1600
1800
Time (h)
0
6
12
24
colour
MU
WT

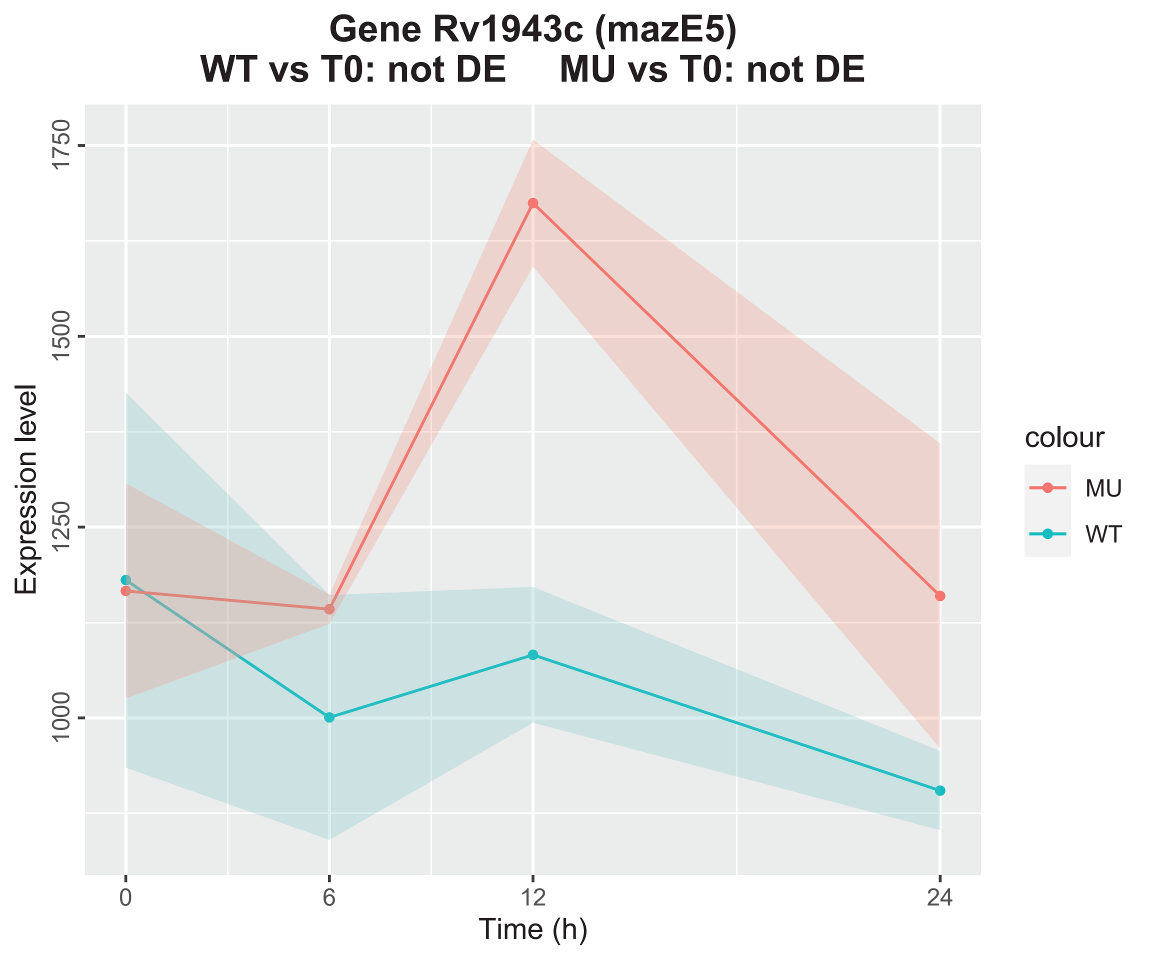
Gene Rv1943c (mazE5)
WT vs T0: not DE   MU vs T0: not DE
Expression level
1750
1500
1250
1000
Time (h)
0
6
12
24
colour
MU
WT

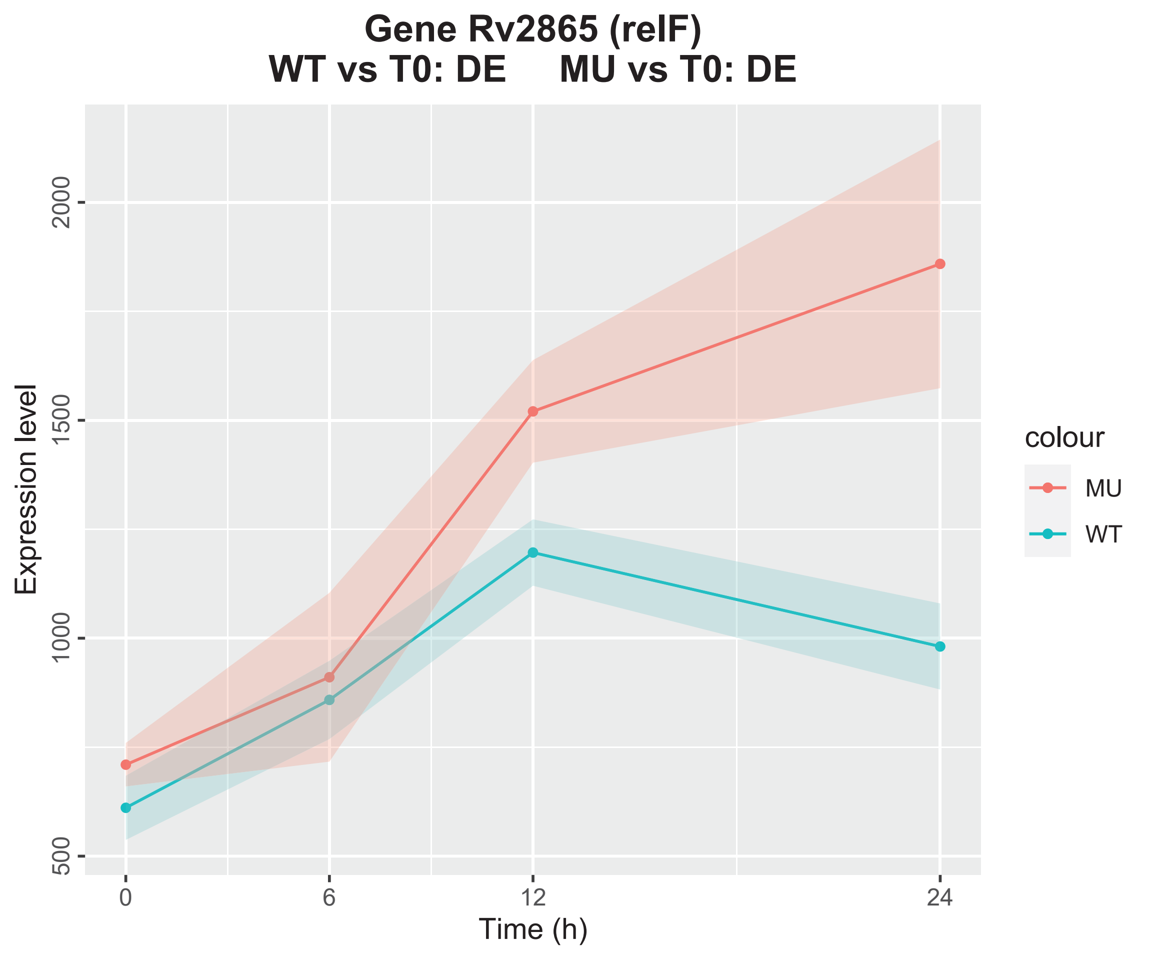
Gene Rv2865 (relF)
WT vs T0: DE   MU vs T0: DE
Expression level
500
1000
1500
2000
0
6
12
24
Time (h)
colour
MU
WT

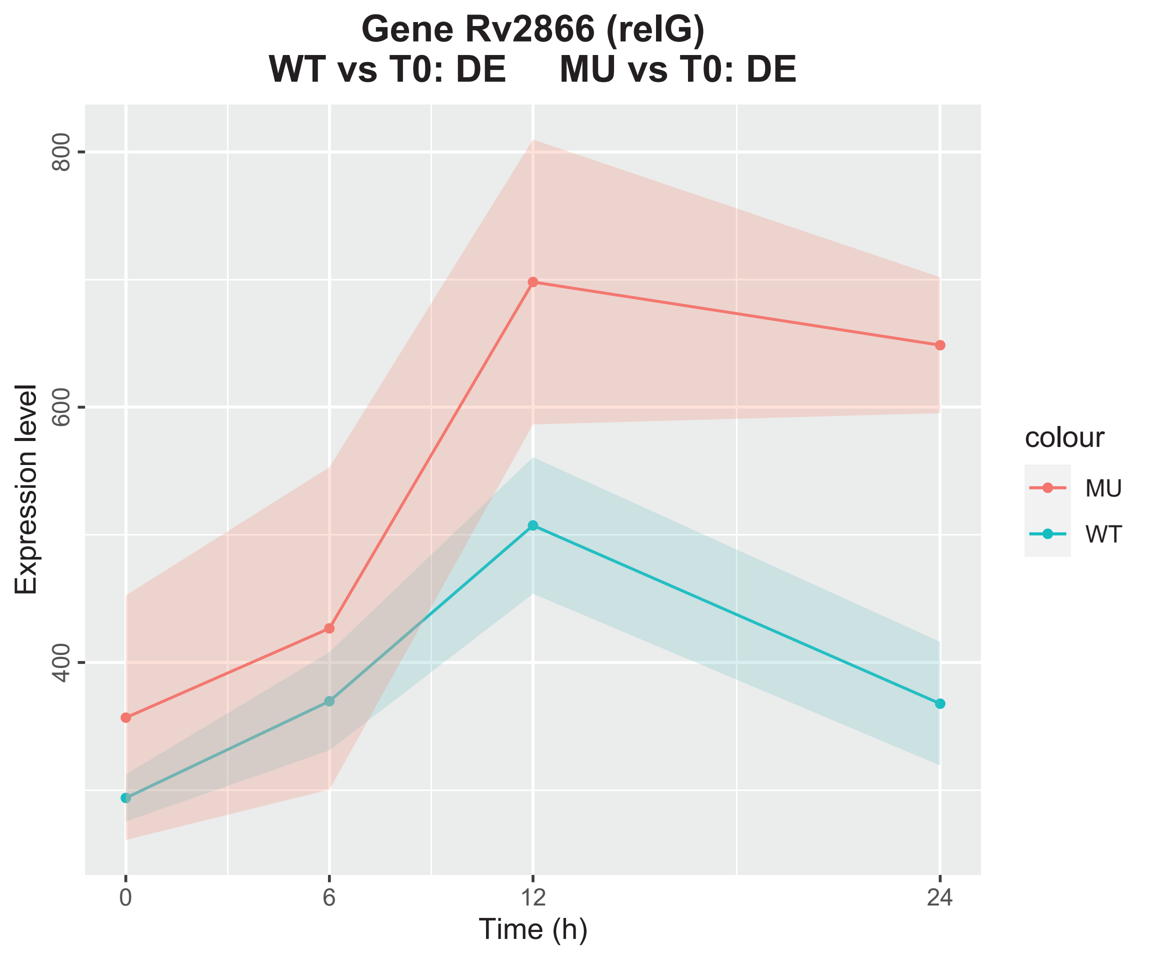
Gene Rv2866 (relG)
WT vs T0: DE   MU vs T0: DE
Expression level
800
600
400
0
6
12
24
Time (h)
colour
MU
WT
