# Supplementary material for: Role of the Extracytoplasmic Function Sigma Factor SigE in the Stringent Response of Mycobacterium tuberculosis
Source: Microbiol Spectr. 2023 Mar 22;11(2):e02944-22. doi: 10.1128/spectrum.02944-22 (PMC10100808; doi:10.1128/spectrum.02944-22)
Supplement: Supplemental file 3 — Data S3. Download spectrum.02944-22-s0004.pdf, PDF file, 7.6 MB [file spectrum.02944-22-s0004.pdf]

<u>Supplementary Data S3:</u> Gene expression profiles over time of all *M.tuberculosis* genes. For each gene, the plot shows the average expression level and the standard deviation (shaded area) for both the wild-type (cyan color) and *sigE*-mutant (salmon/pink color) strains.

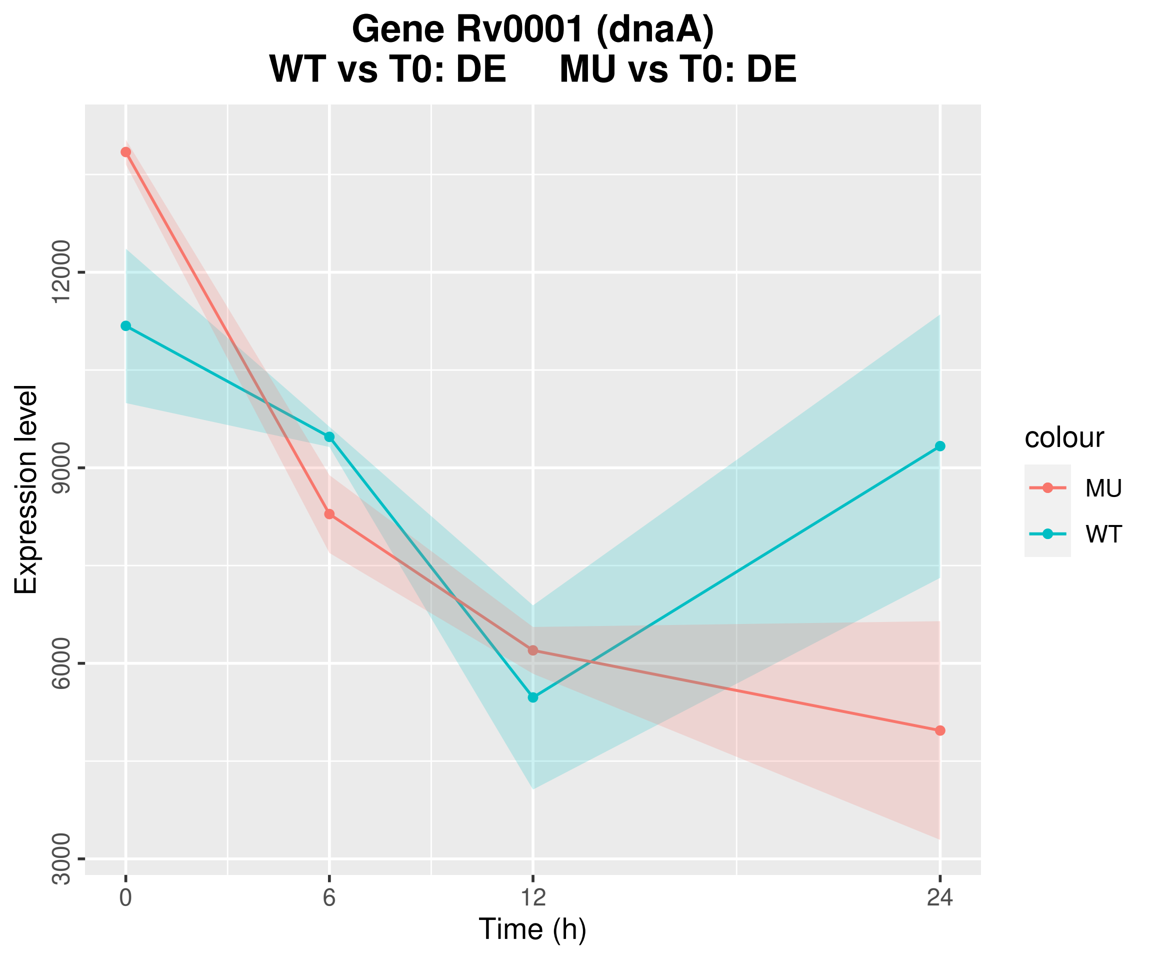
Gene Rv0001 (dnaA)
WT vs T0: DE MU vs T0: DE
Expression level
12000
9000
6000
3000
0
6
12
24
Time (h)
colour
MU
WT

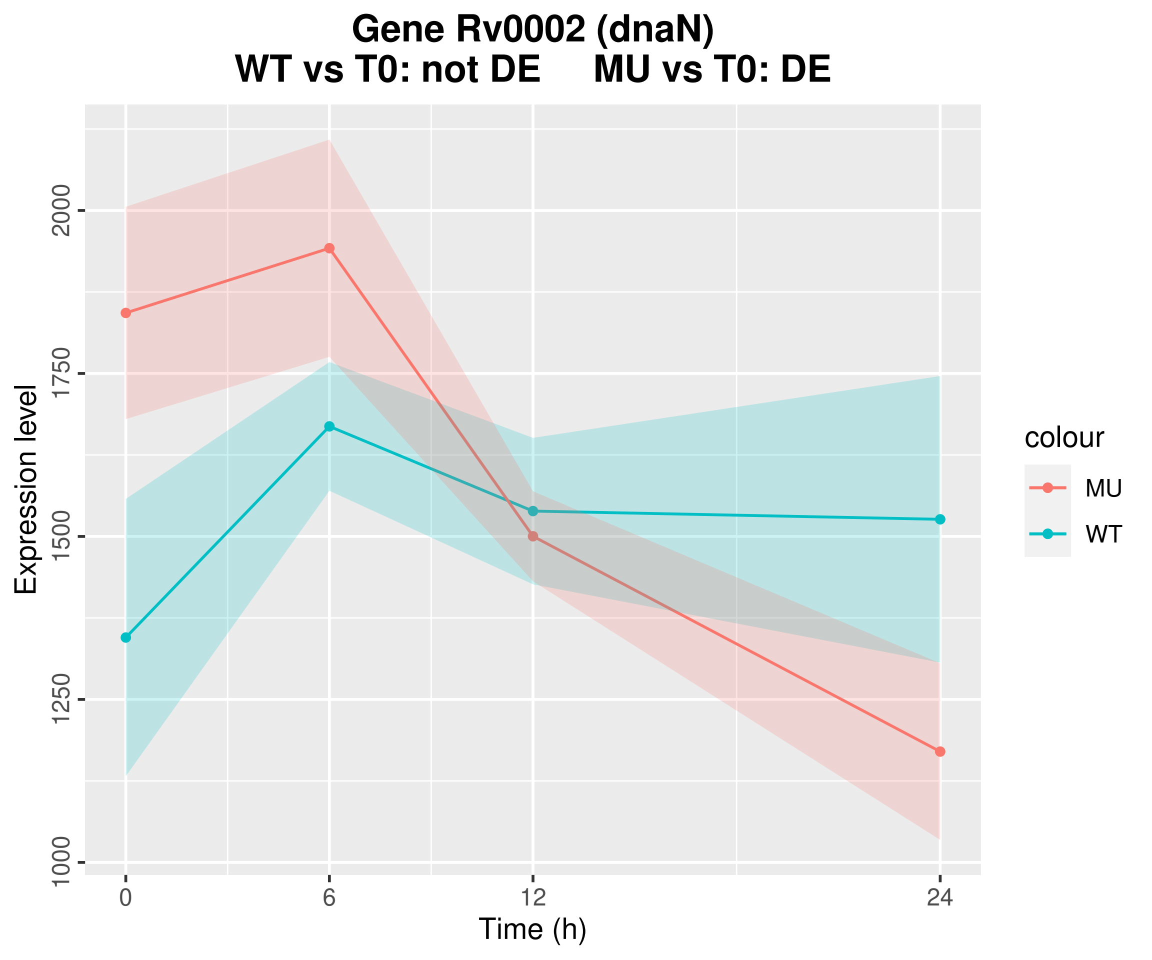
Gene Rv0002 (dnaN)
WT vs T0: not DE MU vs T0: DE
Expression level
2000
1750
1500
1250
1000
0
6
12
24
Time (h)
colour
MU
WT

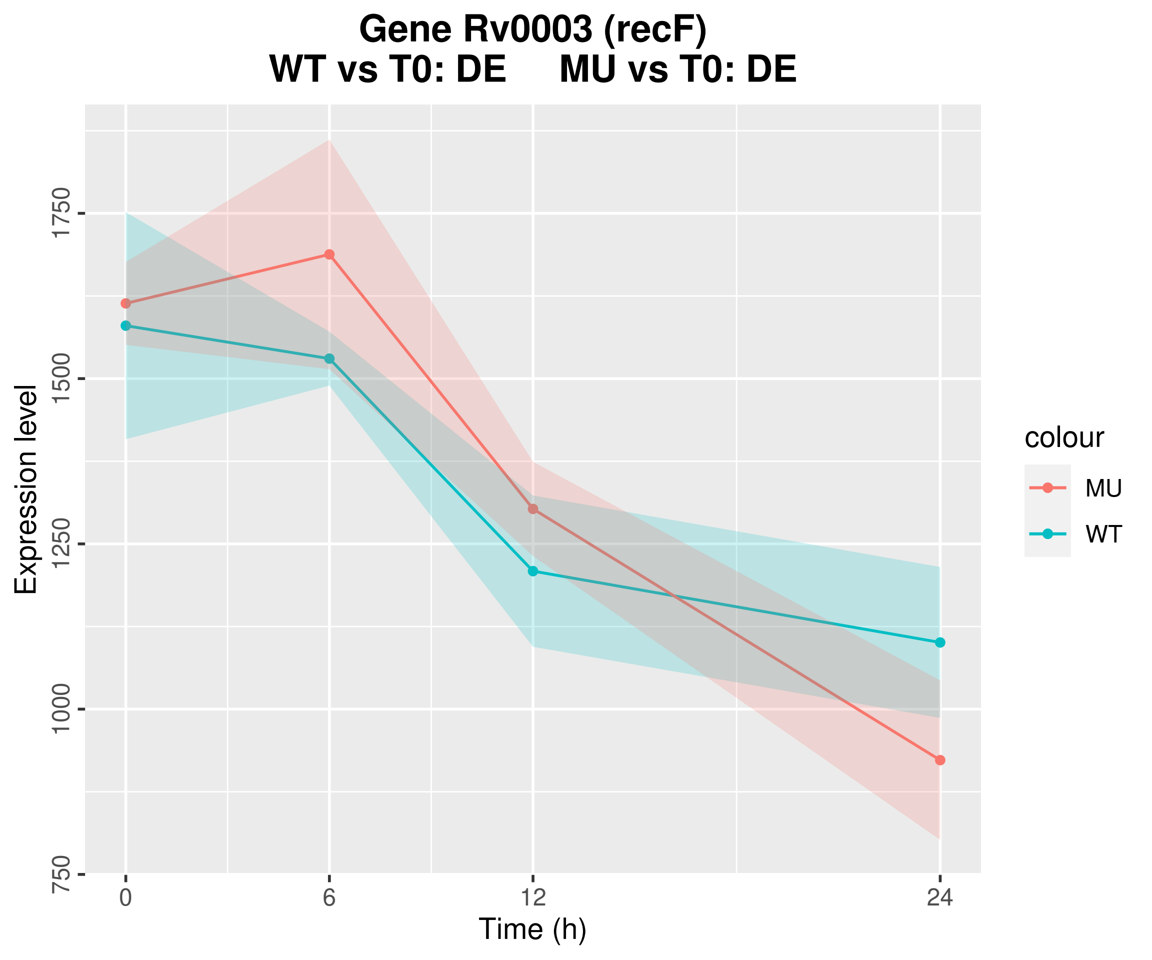
Gene Rv0003 (recF)
WT vs T0: DE   MU vs T0: DE
Expression level
1750
1500
1250
1000
750
0
6
12
24
Time (h)
colour
MU
WT

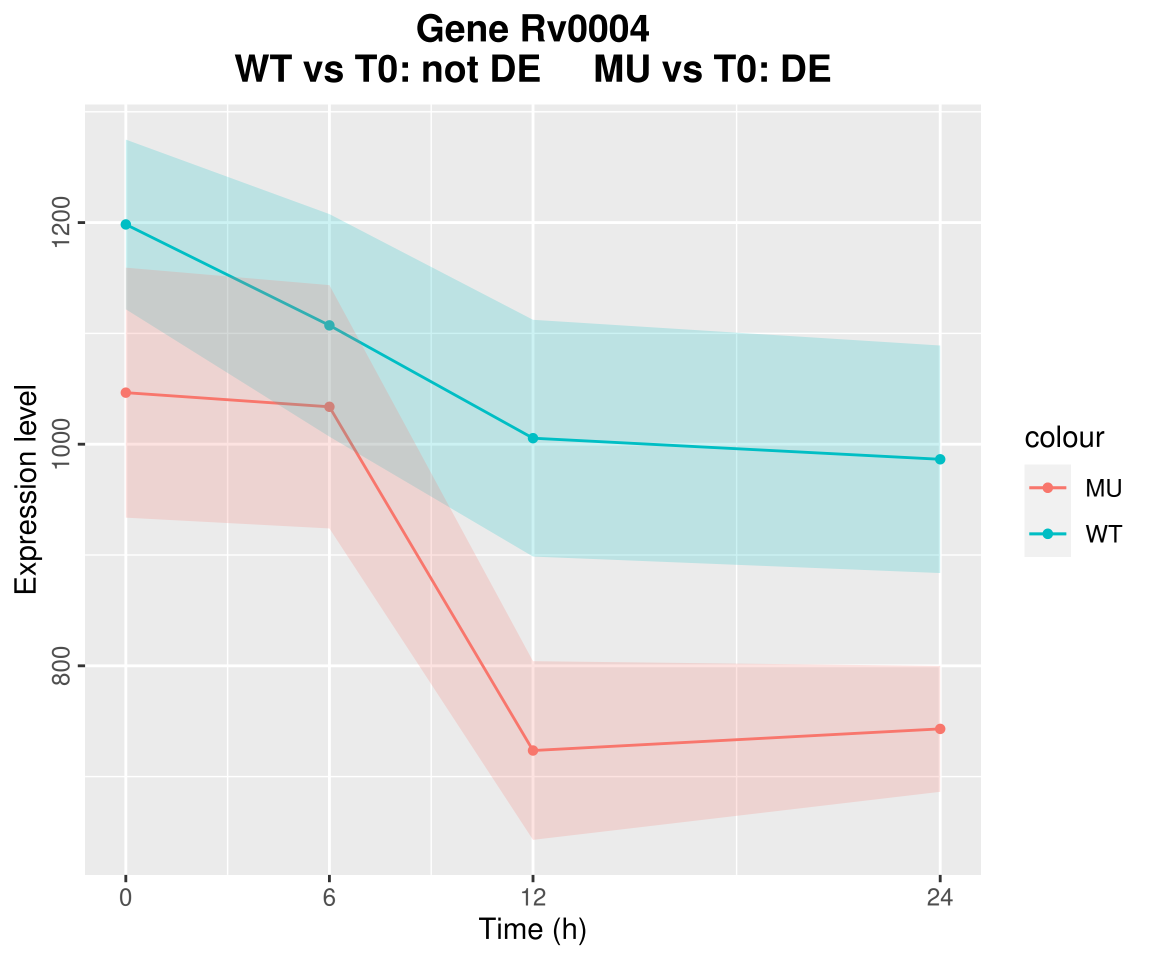
Gene Rv0004
WT vs T0: not DE   MU vs T0: DE
1200
1000
800
Expression level
0
6
12
24
Time (h)
colour
MU
WT

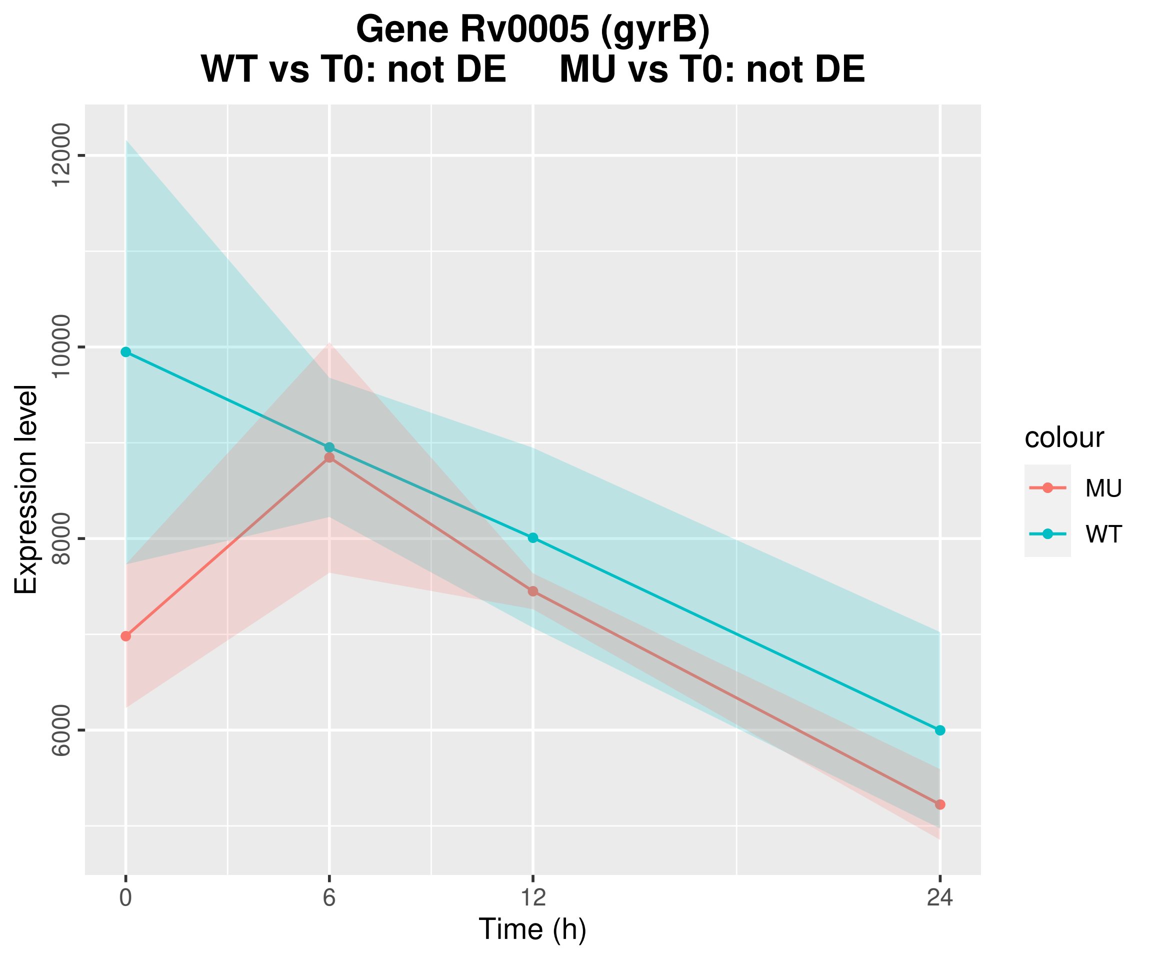

Gene Rv0005 (gyrB)
WT vs T0: not DE   MU vs T0: not DE
Expression level
12000
10000
8000
6000
0
6
12
24
Time (h)
colour
MU
WT

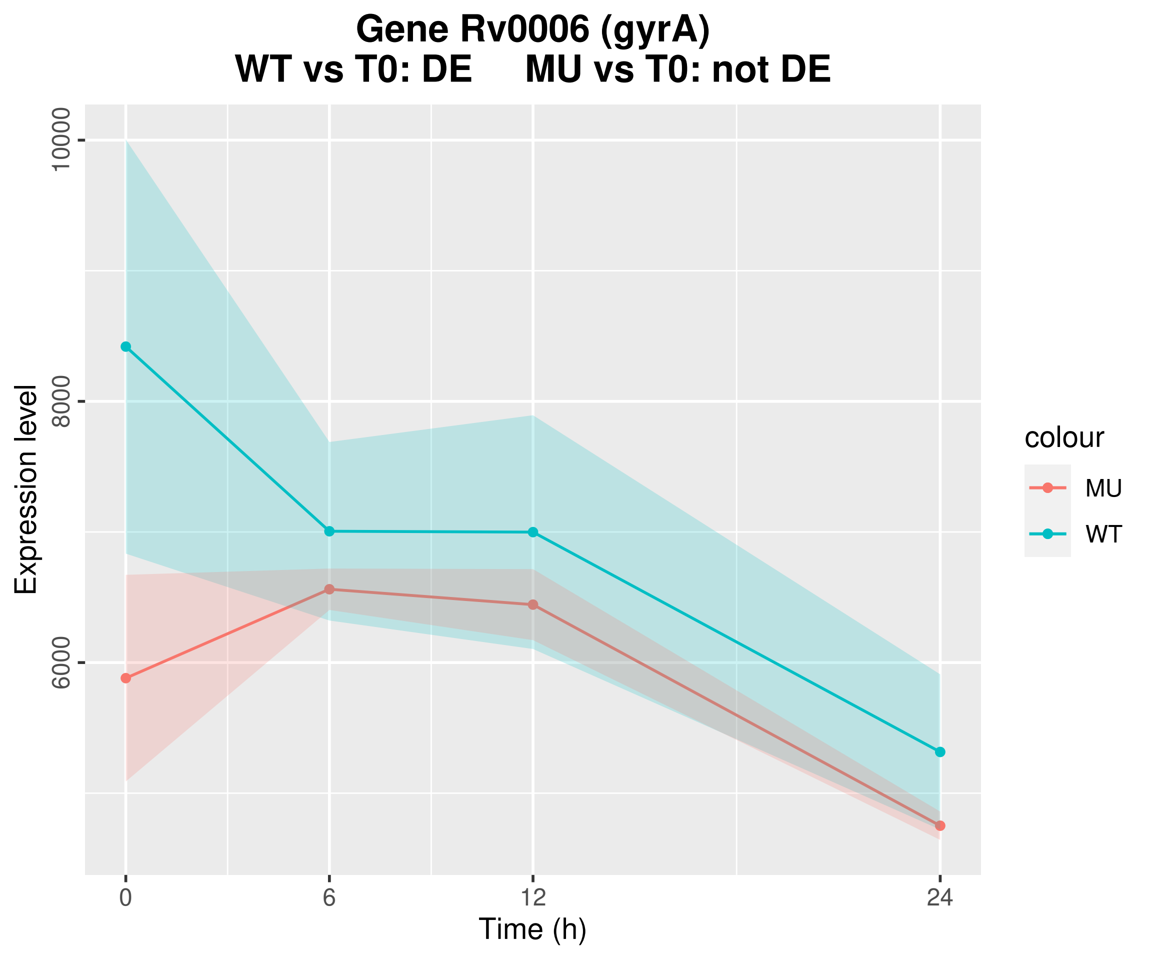
Gene Rv0006 (gyrA)
WT vs T0: DE MU vs T0: not DE
Expression level
10000
8000
6000
0
6
12
24
Time (h)
colour
MU
WT

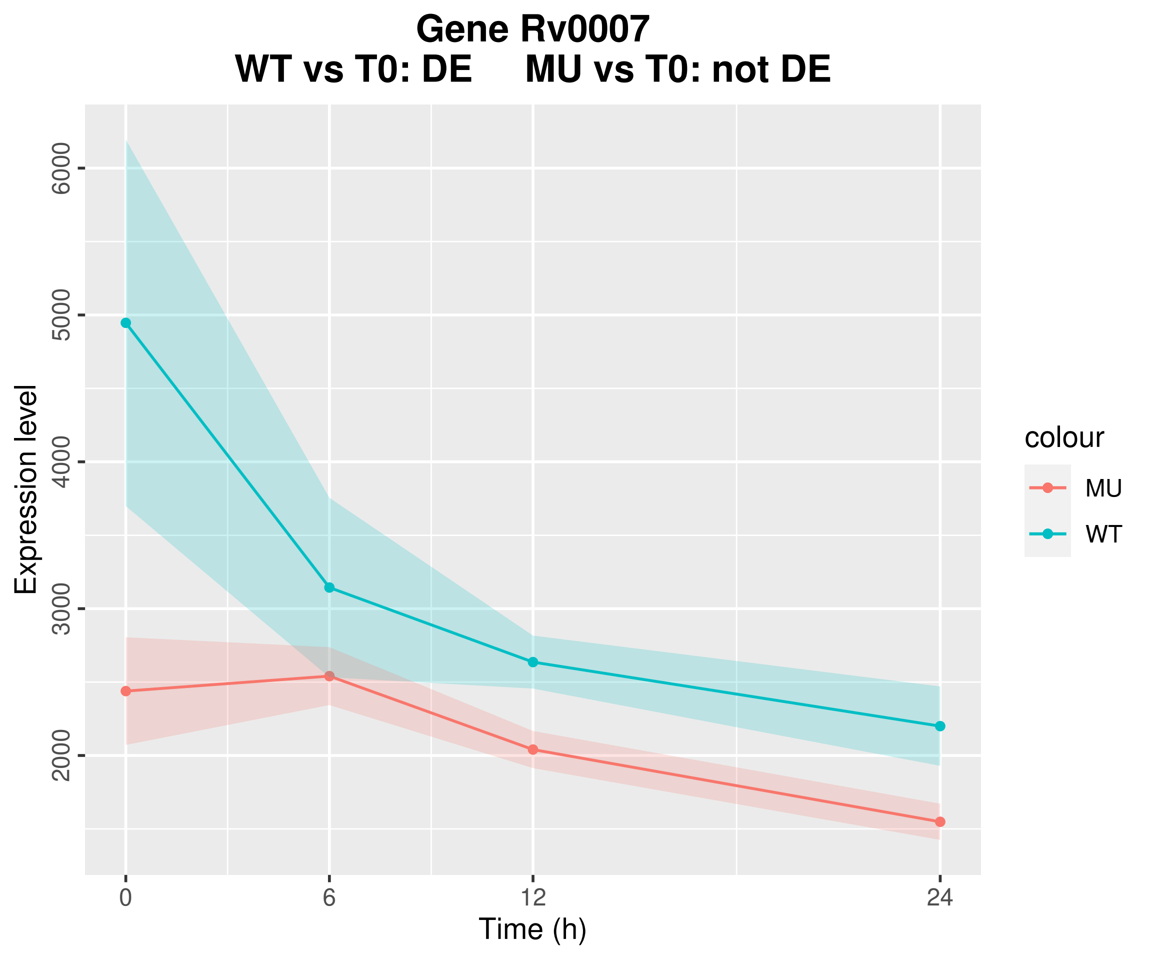

Gene Rv0007
WT vs T0: DE   MU vs T0: not DE
Expression level
6000
5000
4000
3000
2000
0
6
12
24
Time (h)
colour
MU
WT

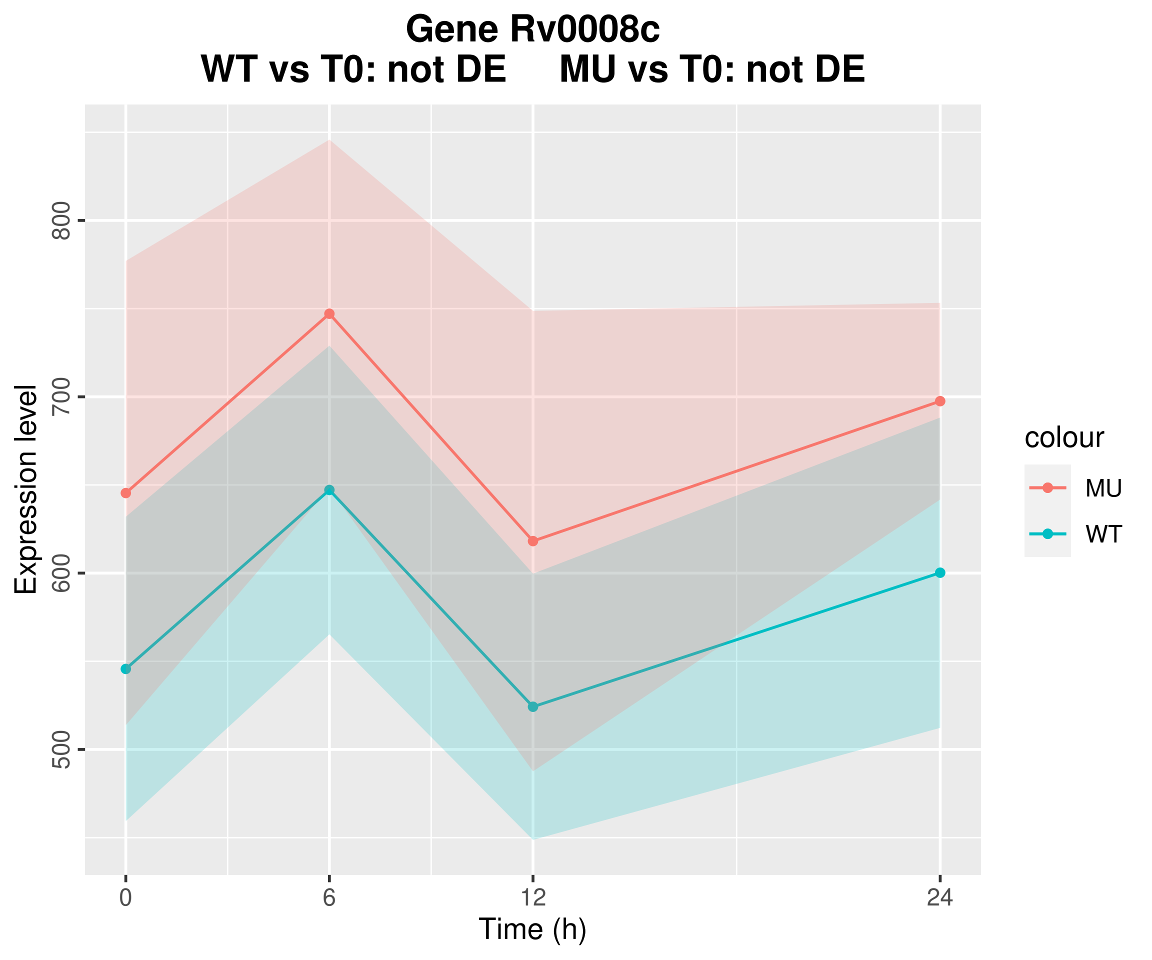

Gene Rv0008c
WT vs T0: not DE MU vs T0: not DE
Expression level
800
700
600
500
0
6
12
24
Time (h)
colour
MU
WT

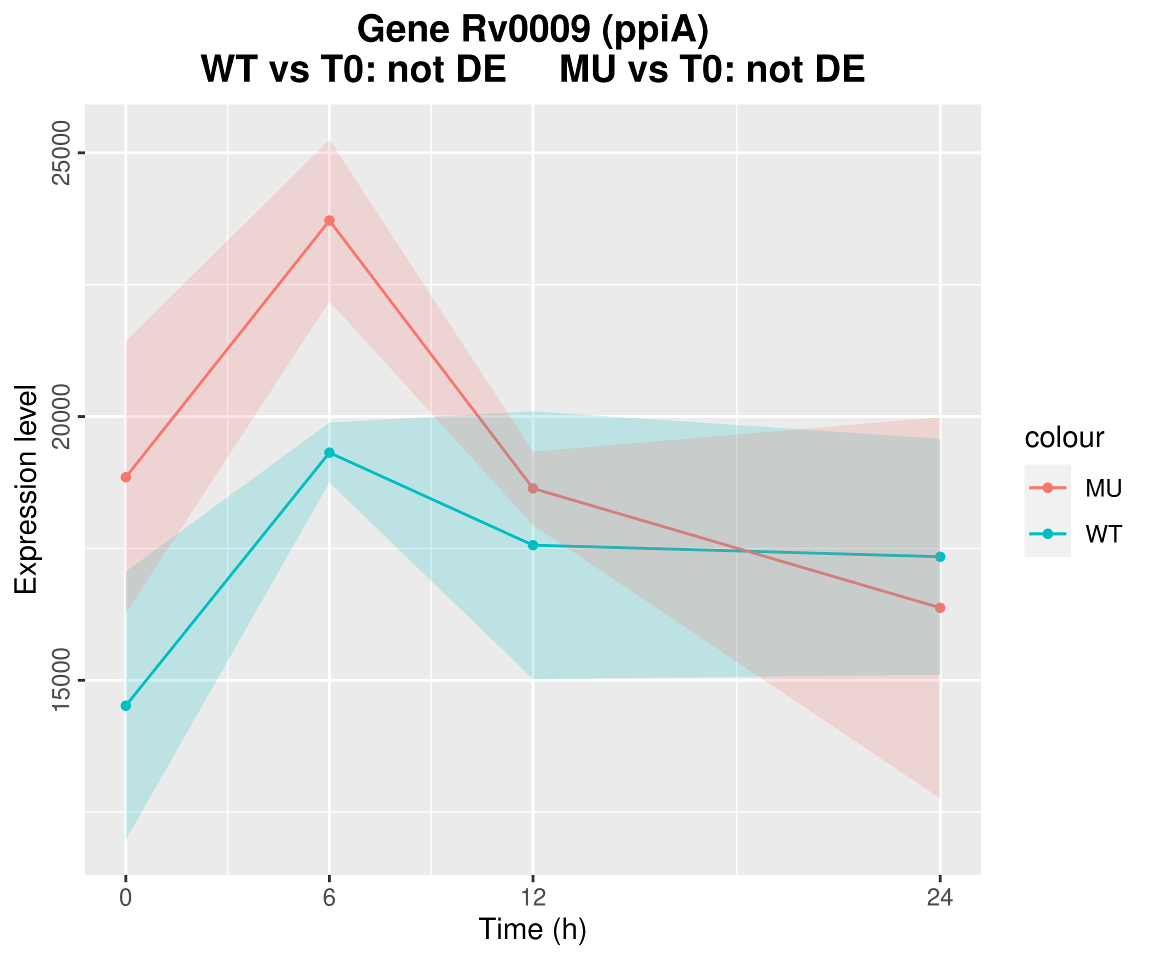

Gene Rv0009 (ppiA)
WT vs T0: not DE   MU vs T0: not DE
25000
20000
15000
Expression level
0
6
12
24
Time (h)
colour
MU
WT

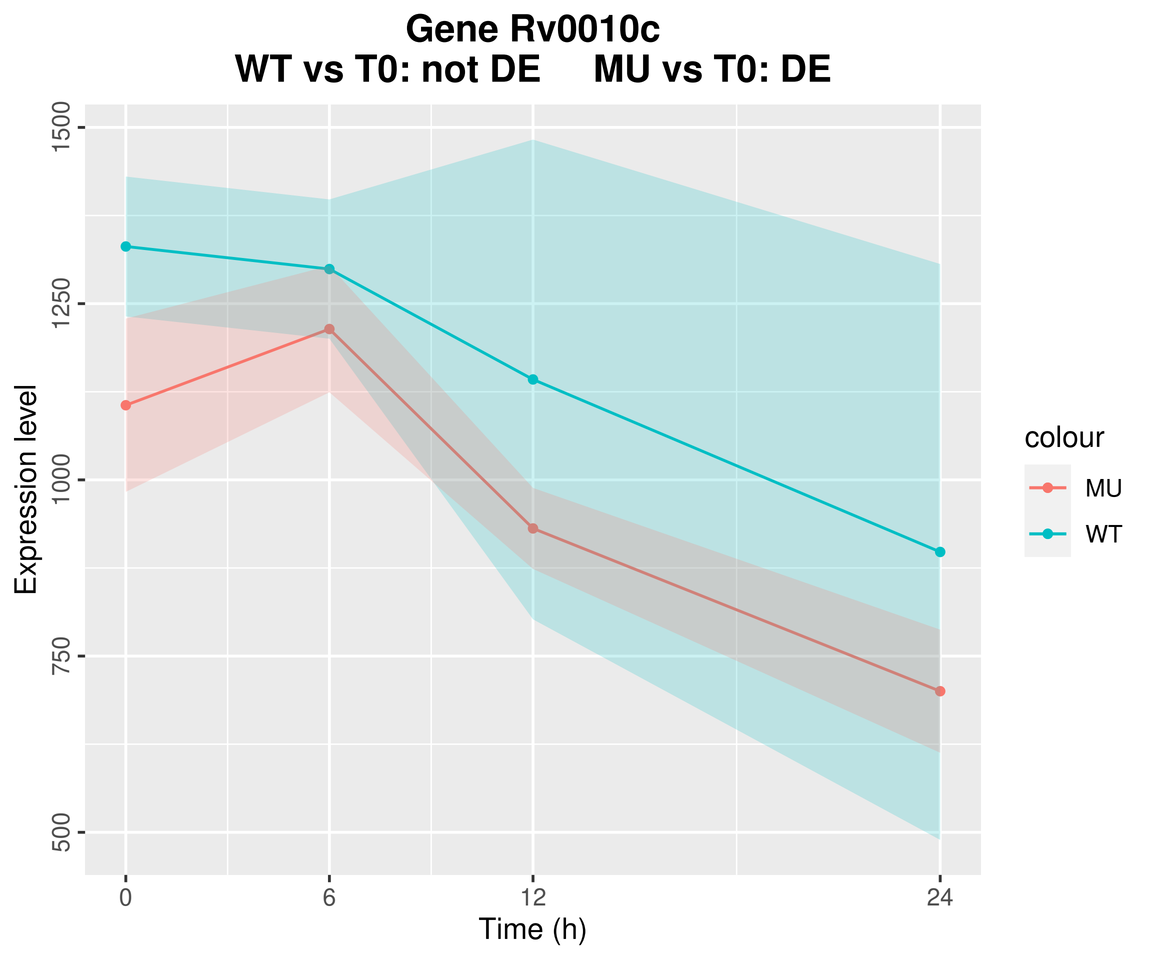

Gene Rv0010c
WT vs T0: not DE MU vs T0: DE
Expression level
1500
1250
1000
750
500
0
6
12
24
Time (h)
colour
MU
WT

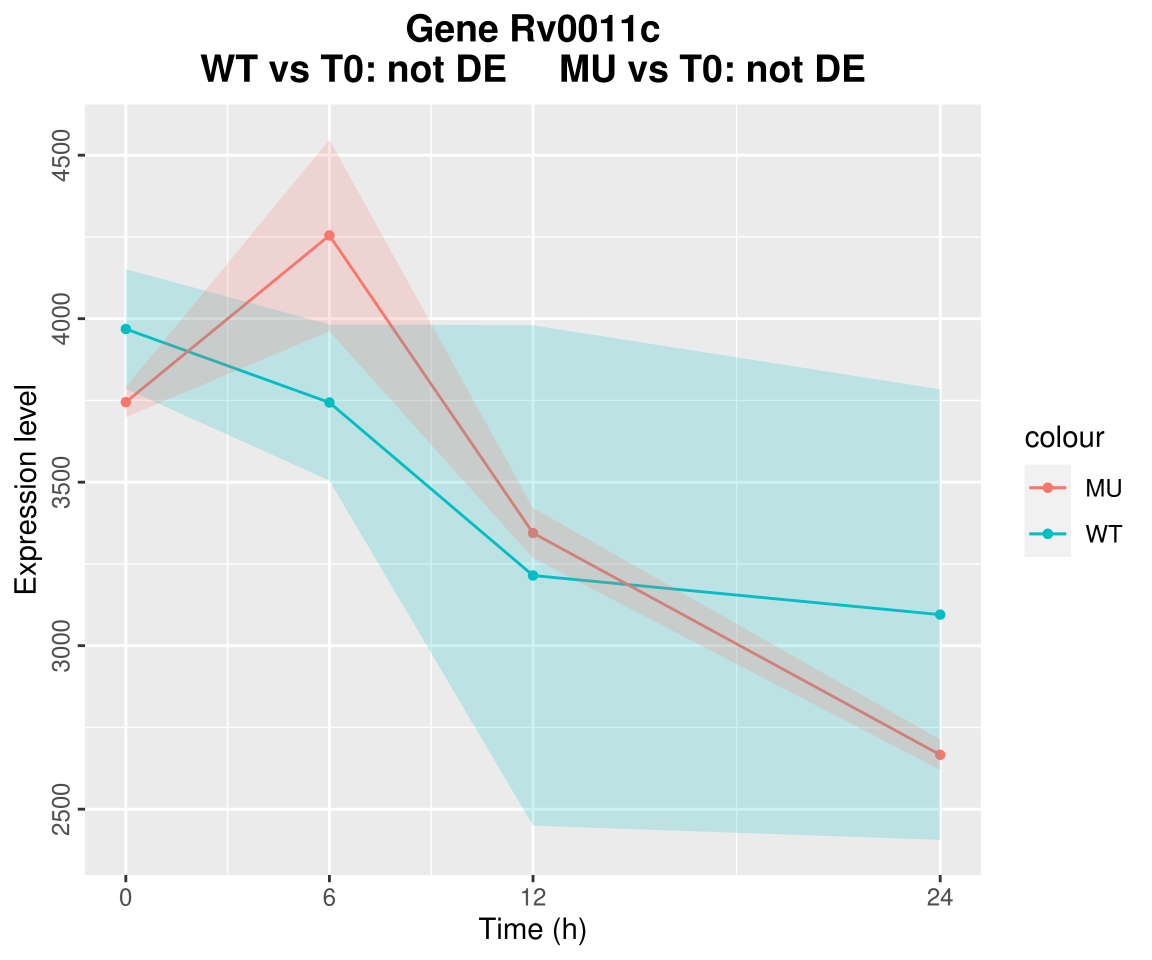

Gene Rv0011c
WT vs T0: not DE MU vs T0: not DE
Expression level
4500
4000
3500
3000
2500
0
6
12
24
Time (h)
colour
MU
WT

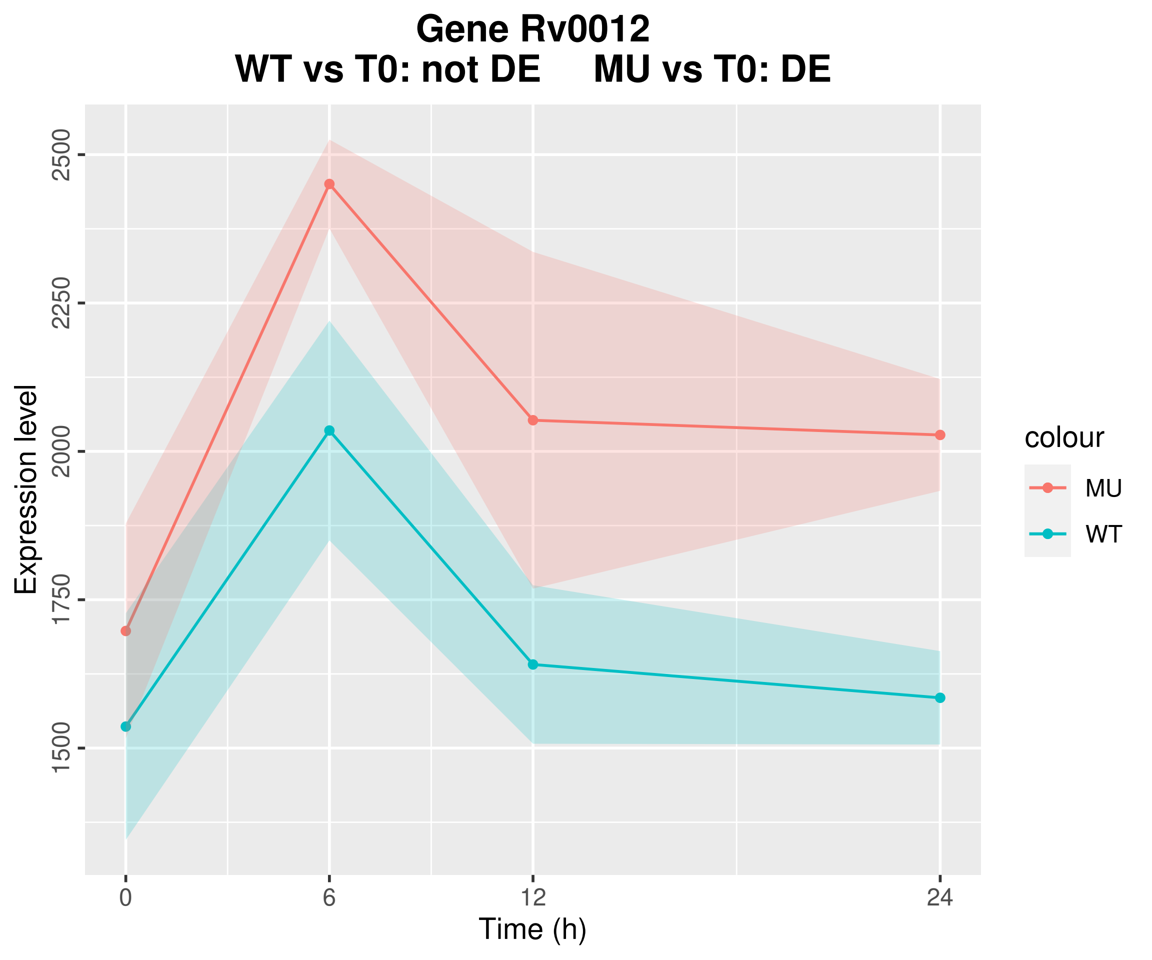

Gene Rv0012
WT vs T0: not DE MU vs T0: DE
Expression level
2500
2250
2000
1750
1500
Time (h)
0
6
12
24
colour
MU
WT

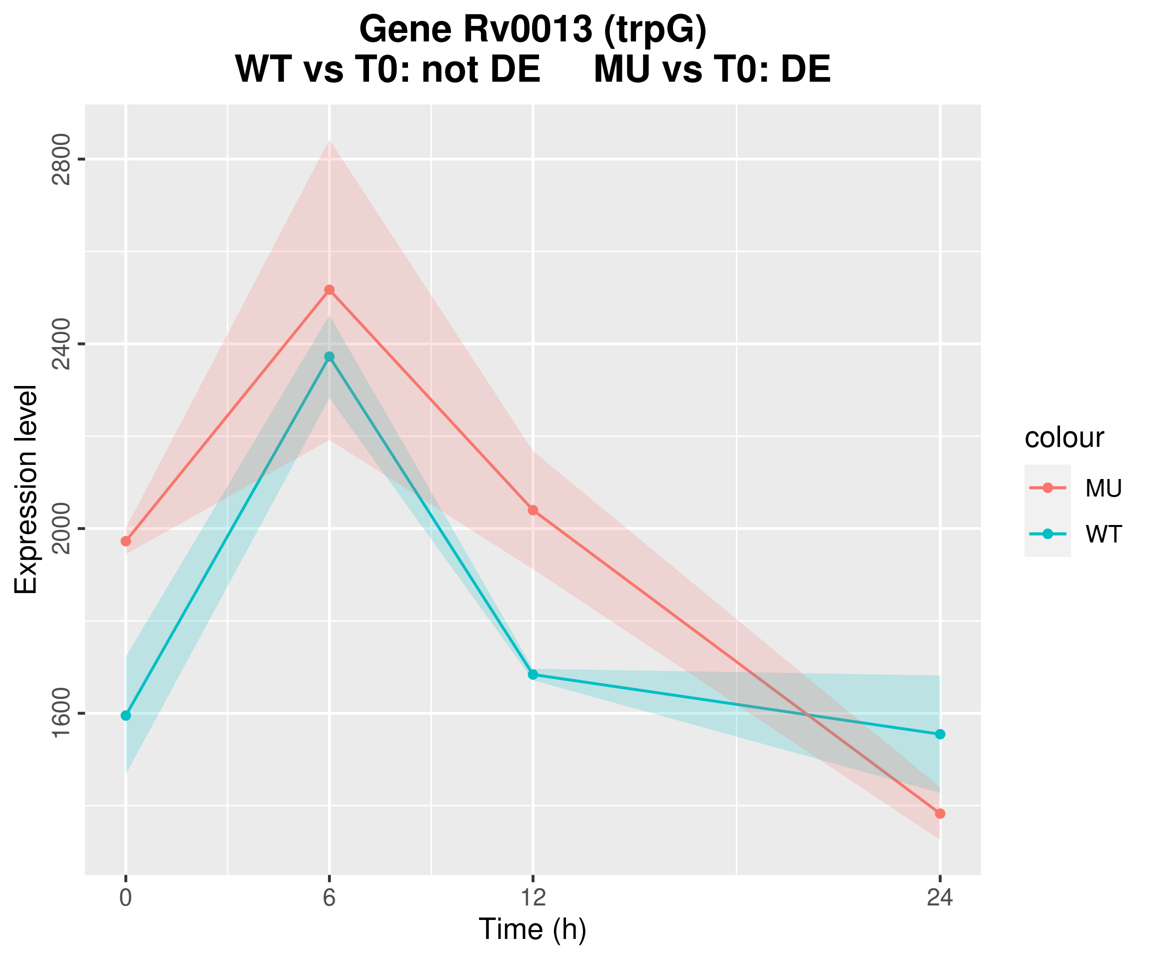
Gene Rv0013 (trpG)
WT vs T0: not DE MU vs T0: DE
Expression level
2800
2400
2000
1600
0
6
12
24
Time (h)
colour
MU
WT

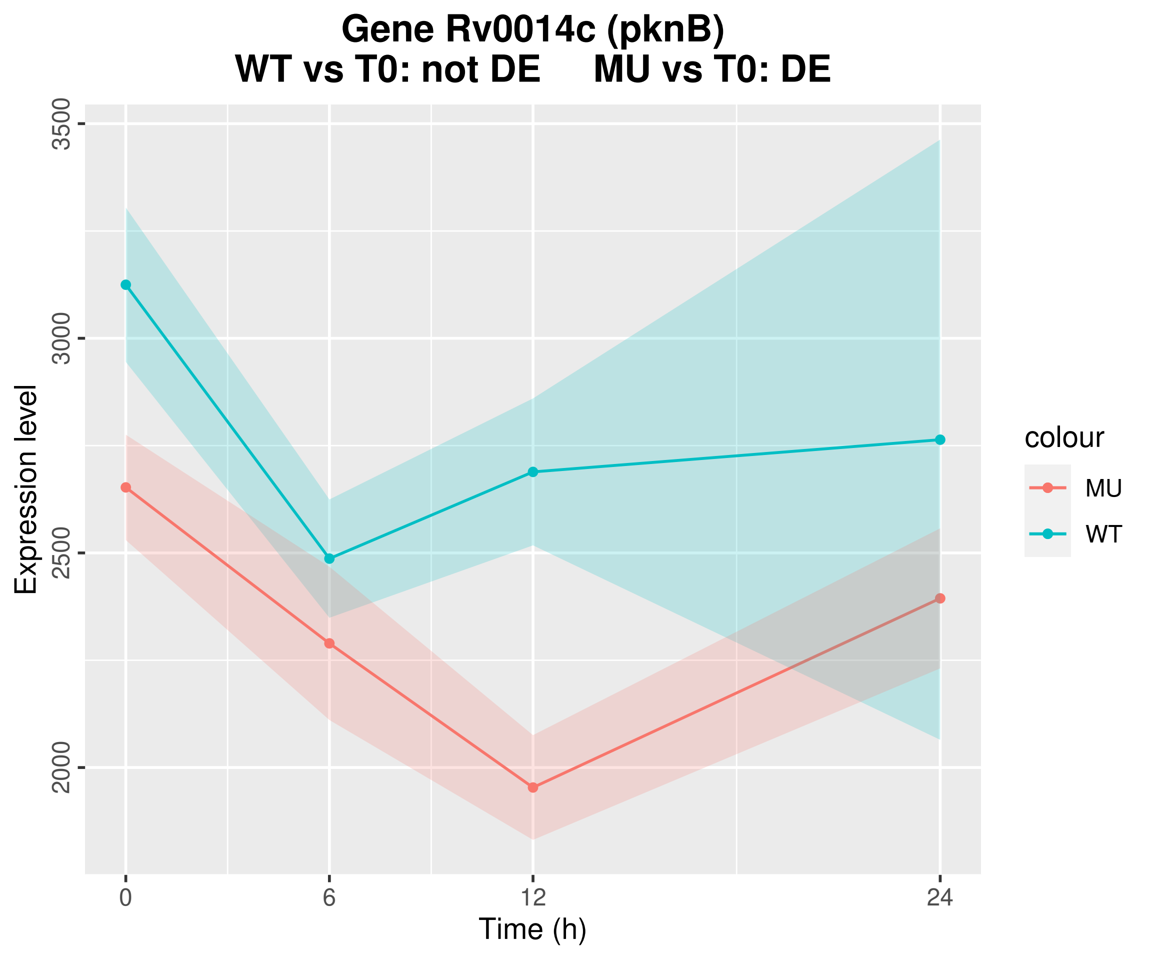
Gene Rv0014c (pknB)
WT vs T0: not DE   MU vs T0: DE
Expression level
3500
3000
2500
2000
0
6
12
24
Time (h)
colour
MU
WT

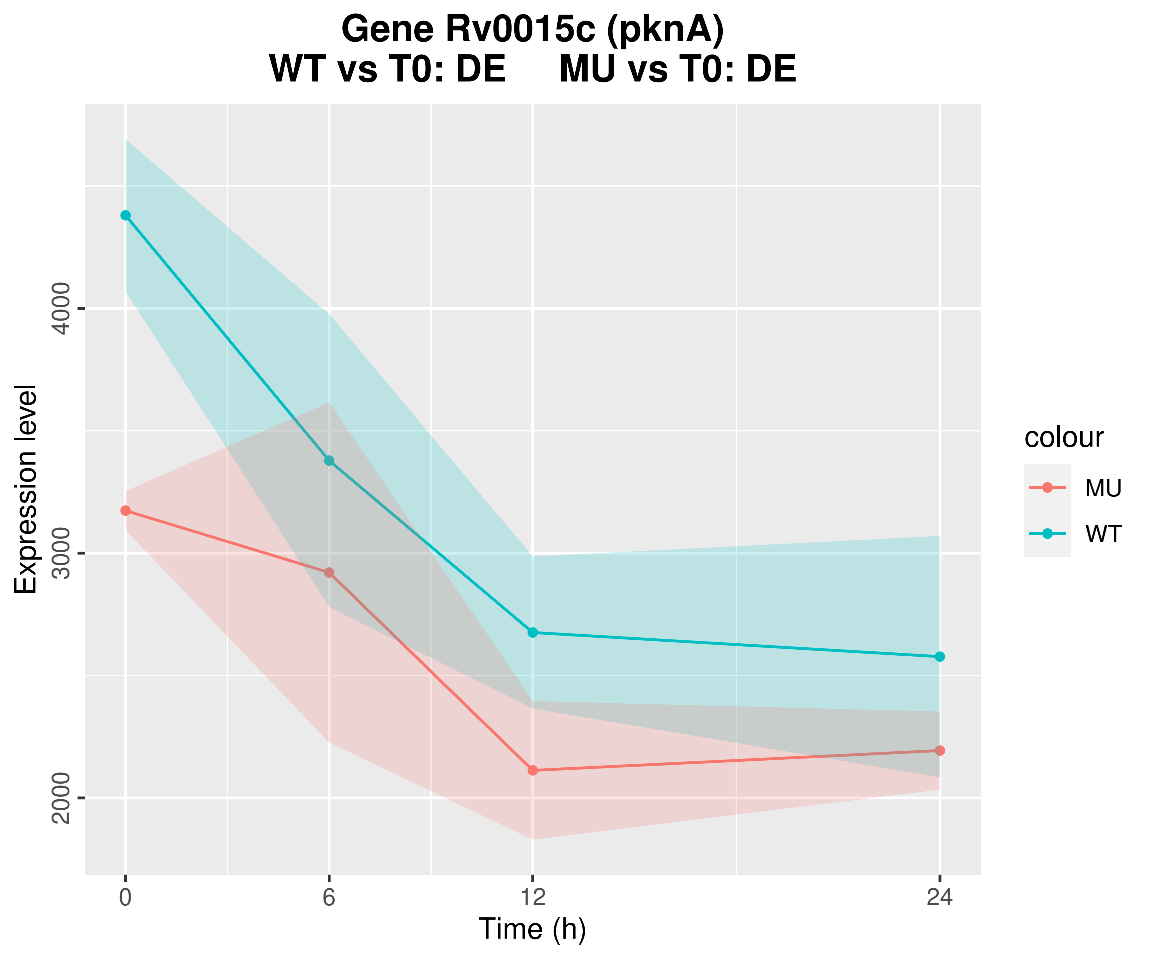
Gene Rv0015c (pknA)
WT vs T0: DE   MU vs T0: DE
Expression level
2000
3000
4000
Time (h)
0
6
12
24
colour
MU
WT

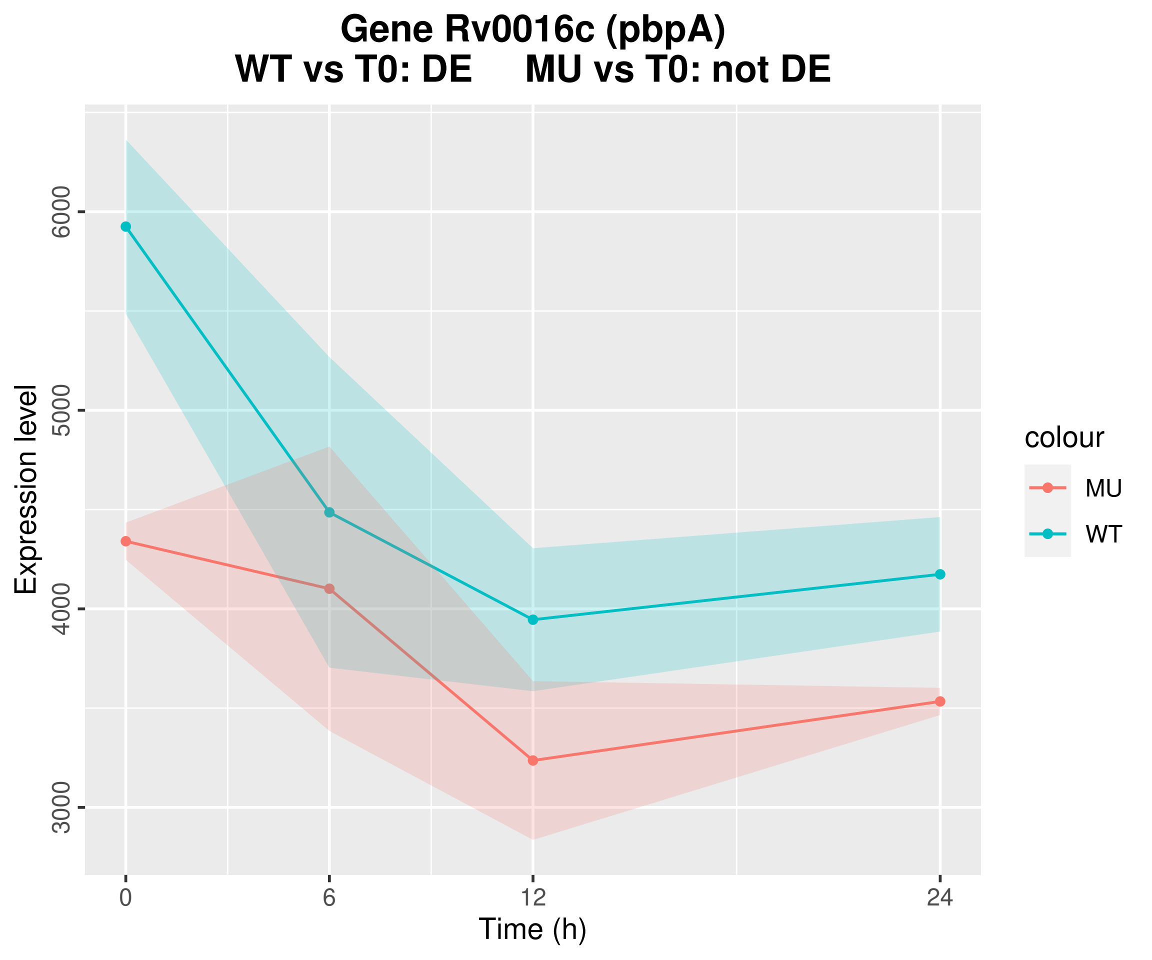

Gene Rv0016c (pbpA)
WT vs T0: DE   MU vs T0: not DE
Expression level
6000
5000
4000
3000
0
6
12
24
Time (h)
colour
MU
WT

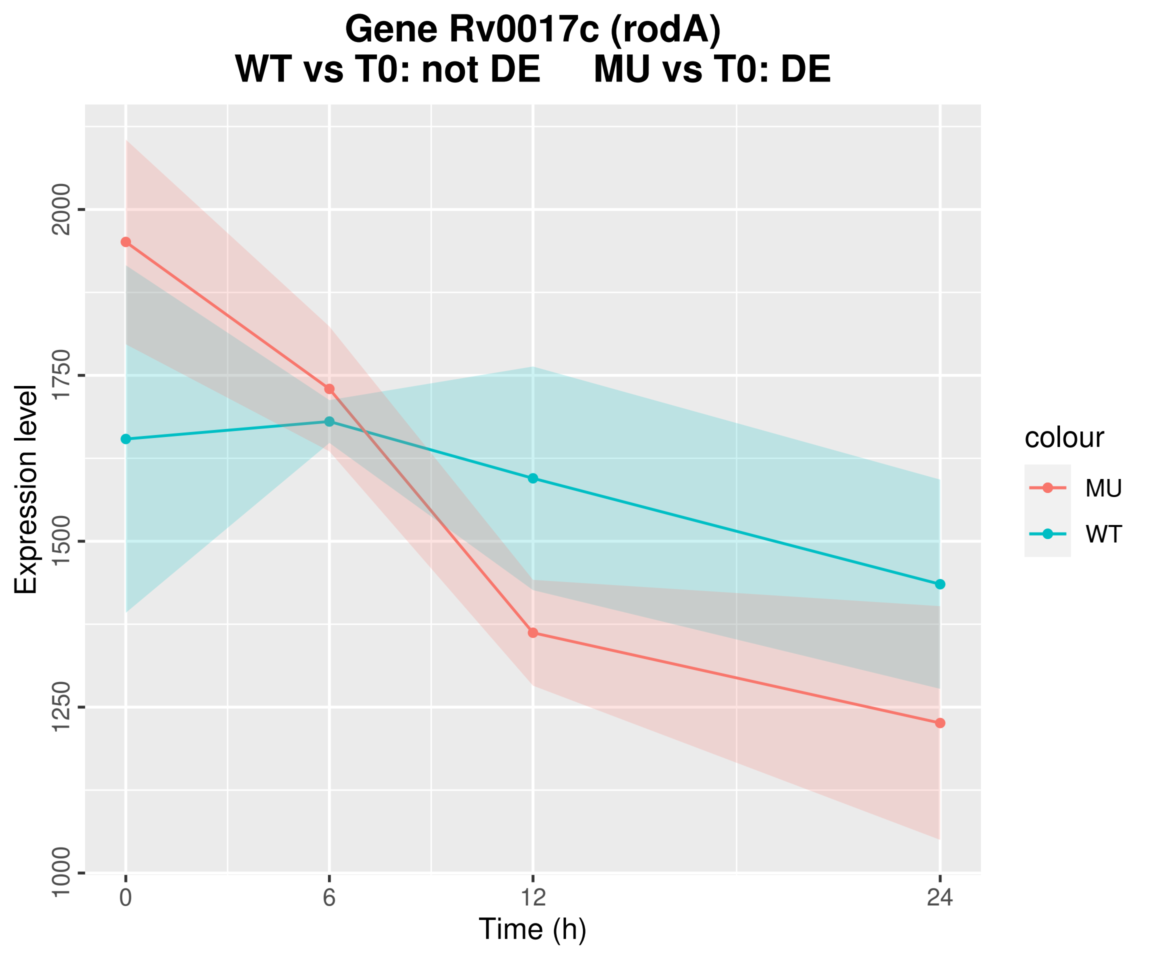

Gene Rv0017c (rodA)
WT vs T0: not DE   MU vs T0: DE
Expression level
2000
1750
1500
1250
1000
0
6
12
24
Time (h)
colour
MU
WT

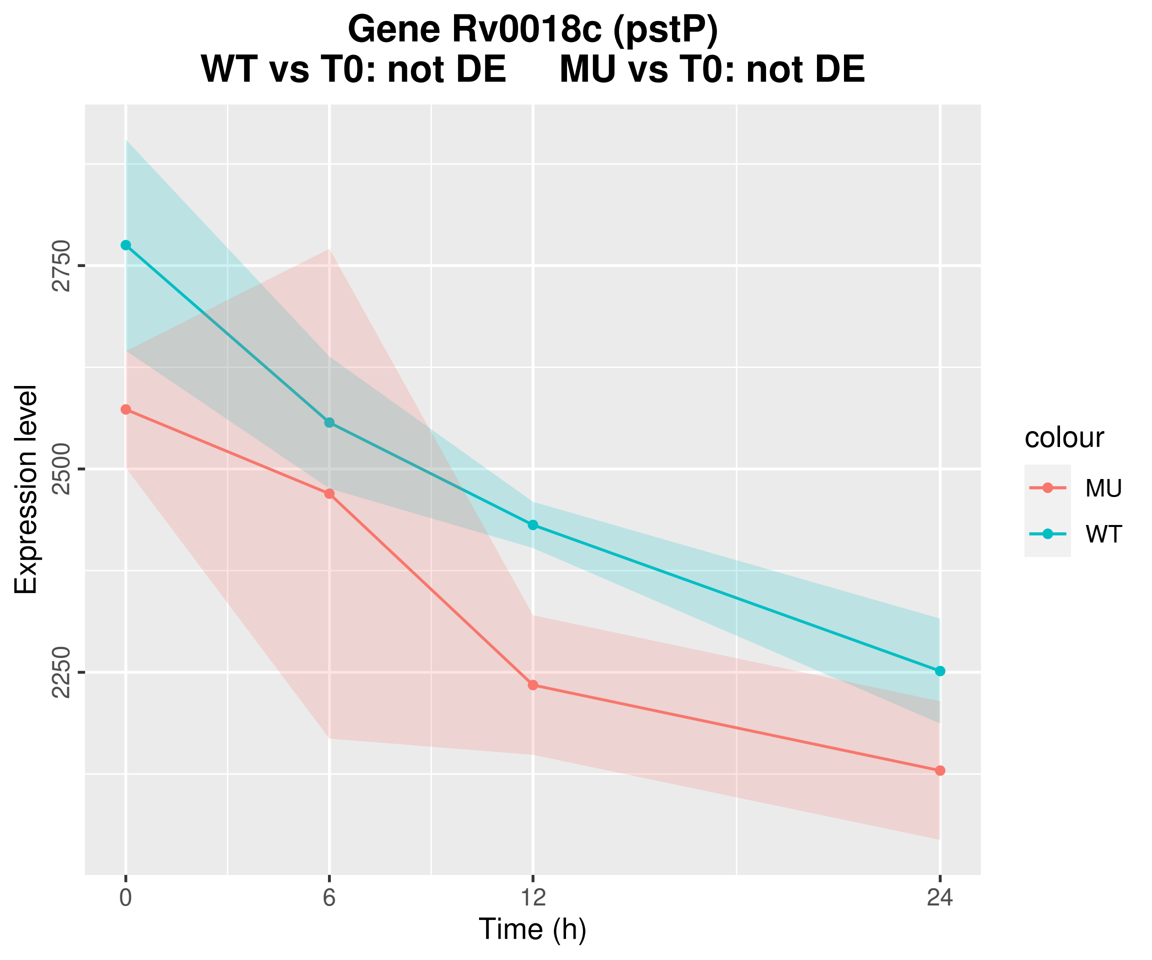
Gene Rv0018c (pstP)
WT vs T0: not DE   MU vs T0: not DE
Expression level
2750
2500
2250
Time (h)
0
6
12
24
colour
MU
WT

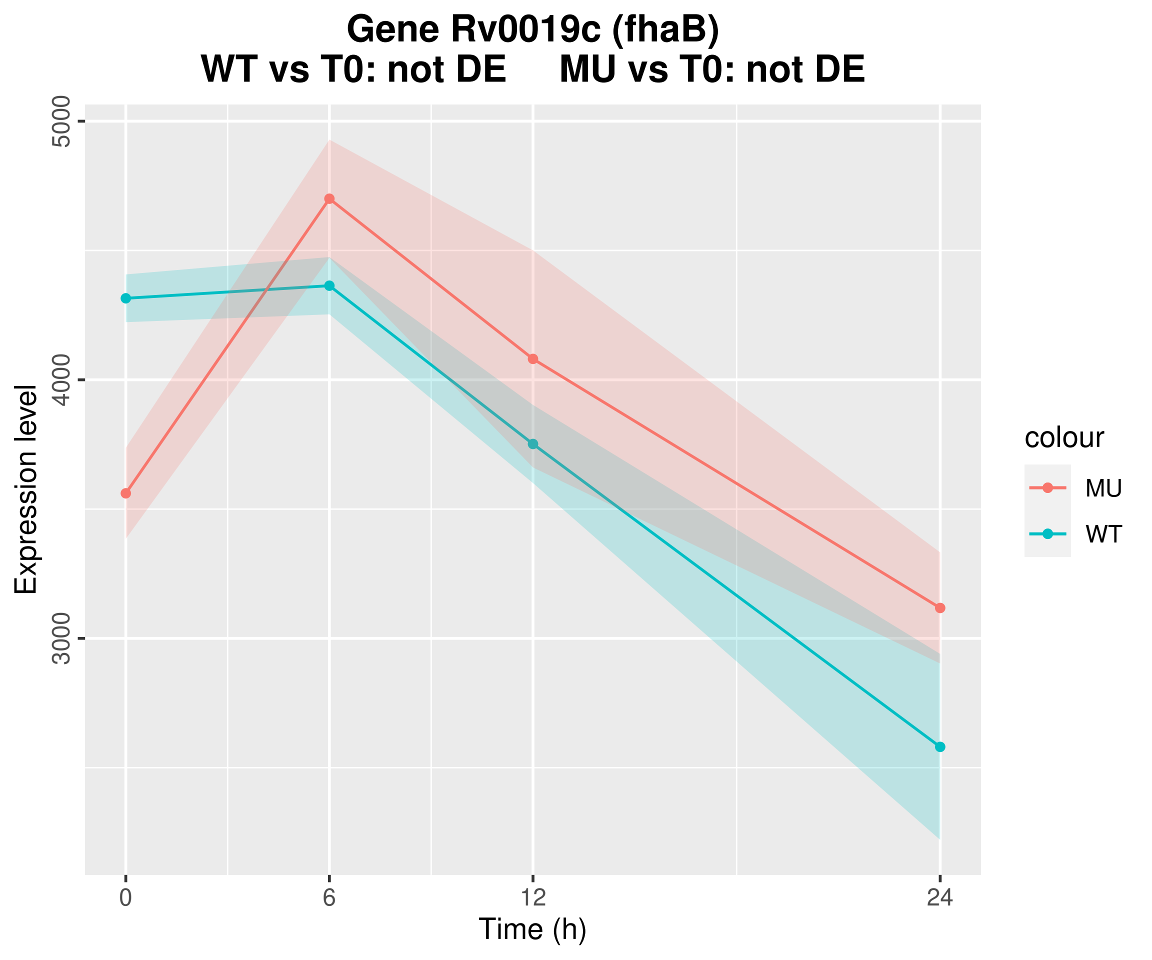

Gene Rv0019c (fhaB)
WT vs T0: not DE   MU vs T0: not DE
Expression level
5000
4000
3000
0
6
12
24
Time (h)
colour
MU
WT

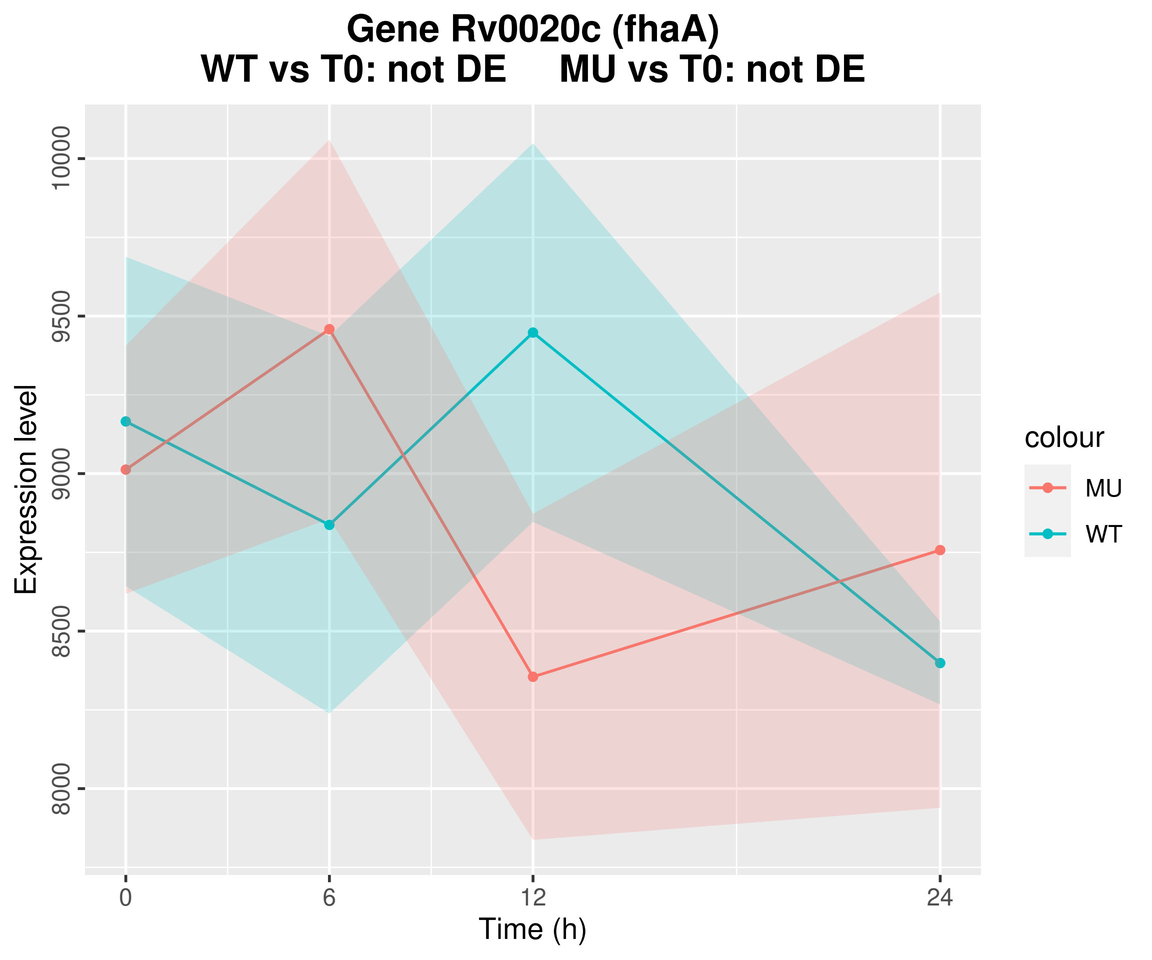

Gene Rv0020c (fhaA)
WT vs T0: not DE   MU vs T0: not DE
Expression level
10000
9500
9000
8500
8000
0
6
12
24
Time (h)
colour
MU
WT

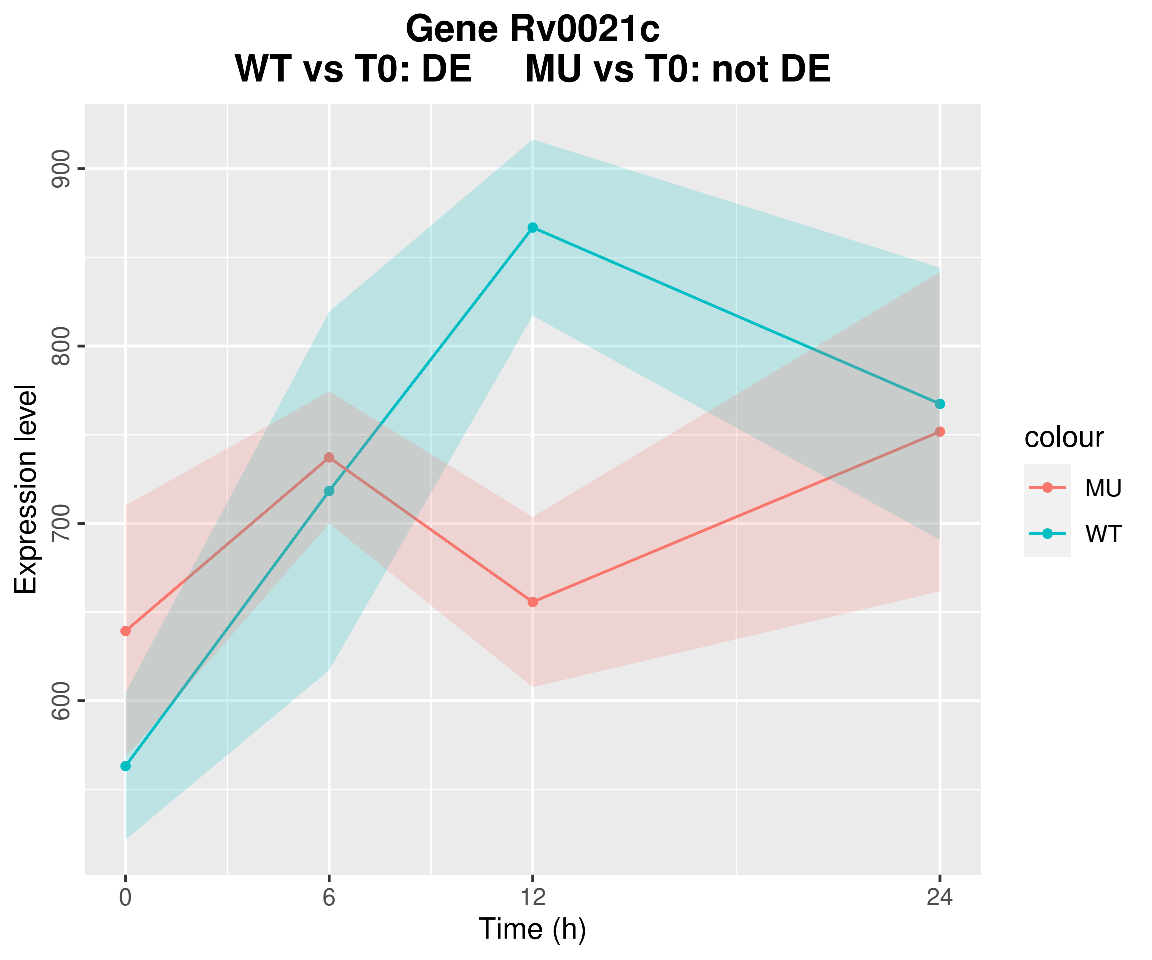
Gene Rv0021c
WT vs T0: DE MU vs T0: not DE
Expression level
900
800
700
600
0
6
12
24
Time (h)
colour
MU
WT

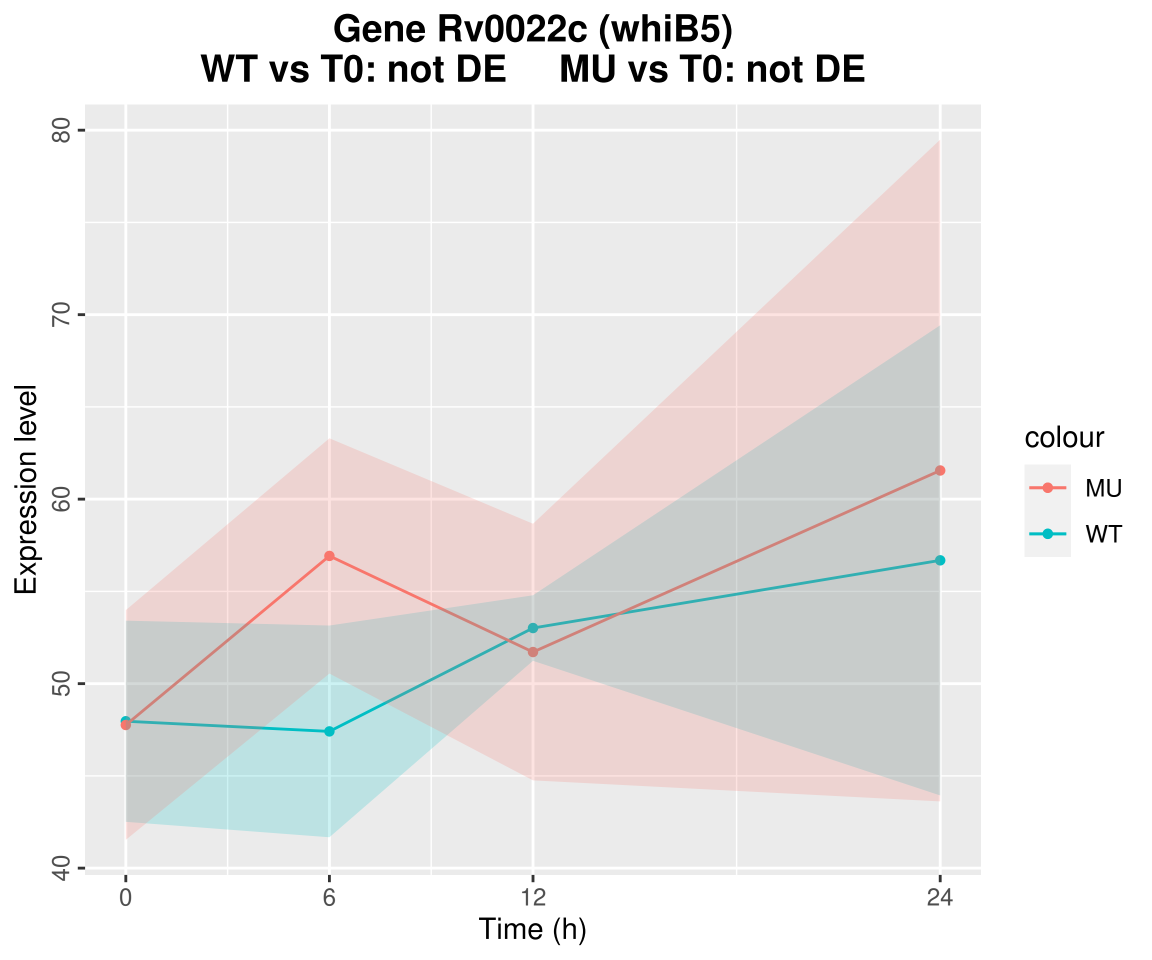
Gene Rv0022c (whiB5)
WT vs T0: not DE   MU vs T0: not DE
Expression level
80
70
60
50
40
0
6
12
24
Time (h)
colour
MU
WT

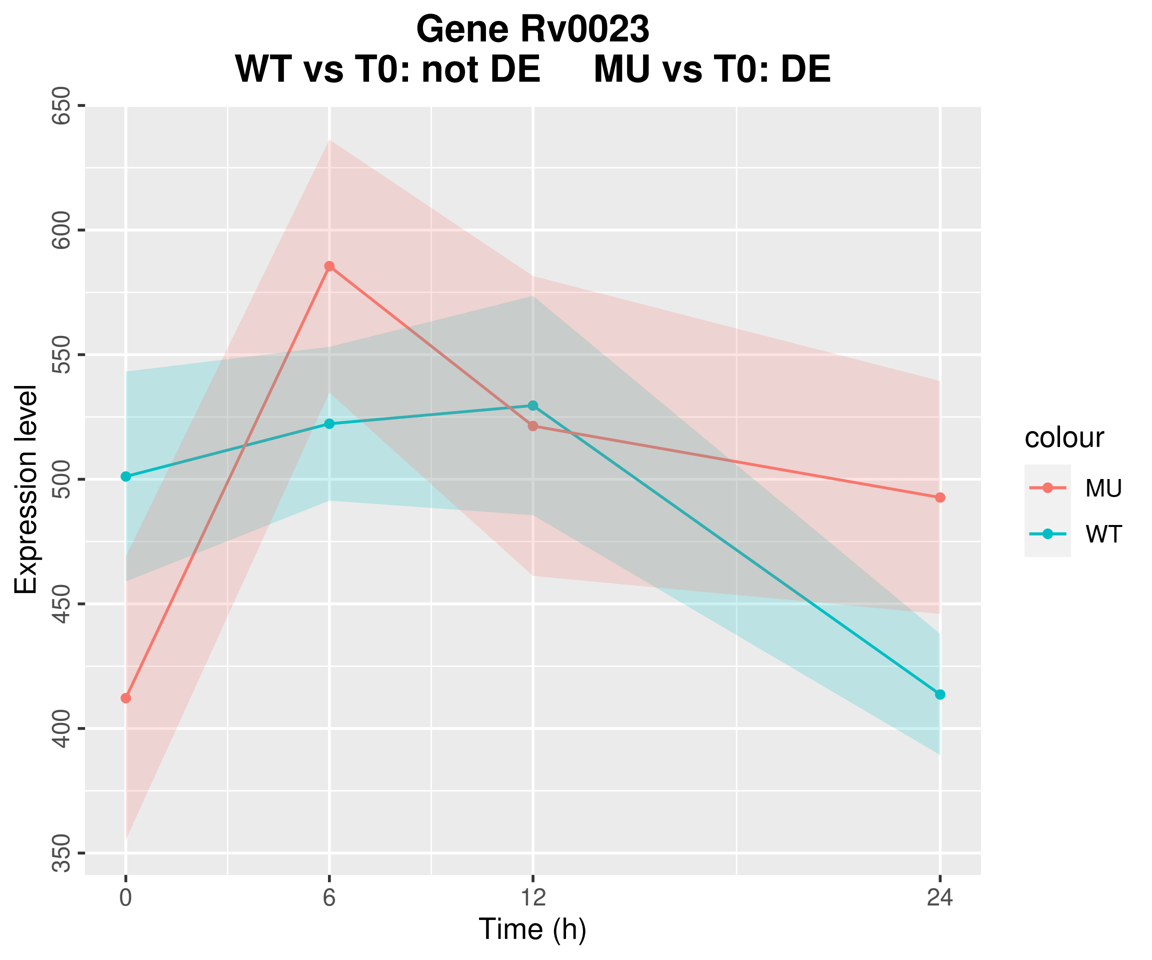
Gene Rv0023
WT vs T0: not DE MU vs T0: DE
Expression level
650
600
550
500
450
400
350
0
6
12
24
Time (h)
colour
MU
WT

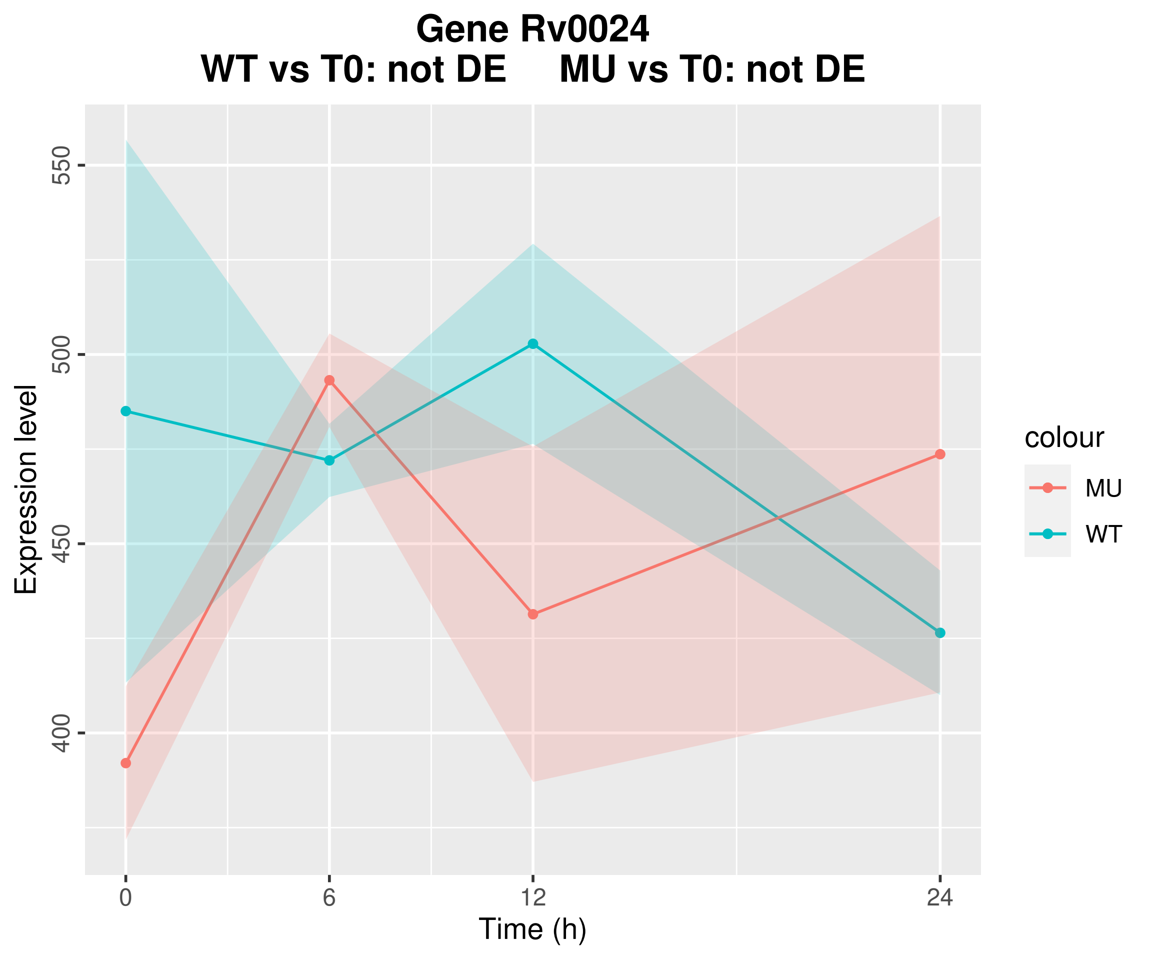
Gene Rv0024
WT vs T0: not DE MU vs T0: not DE
Expression level
550
500
450
400
0
6
12
24
Time (h)
colour
MU
WT

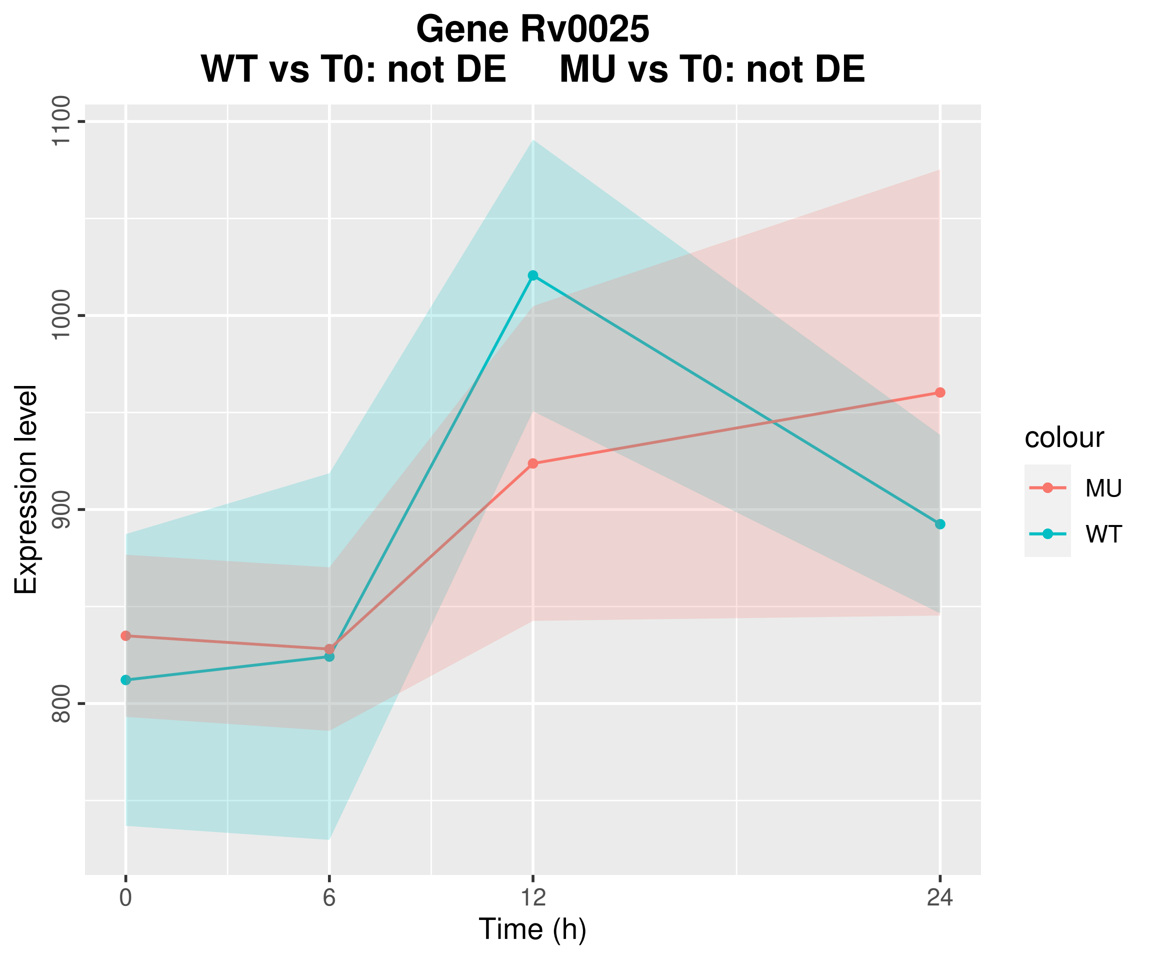

Gene Rv0025
WT vs T0: not DE   MU vs T0: not DE
Expression level
1100
1000
900
800
0
6
12
24
Time (h)
colour
MU
WT

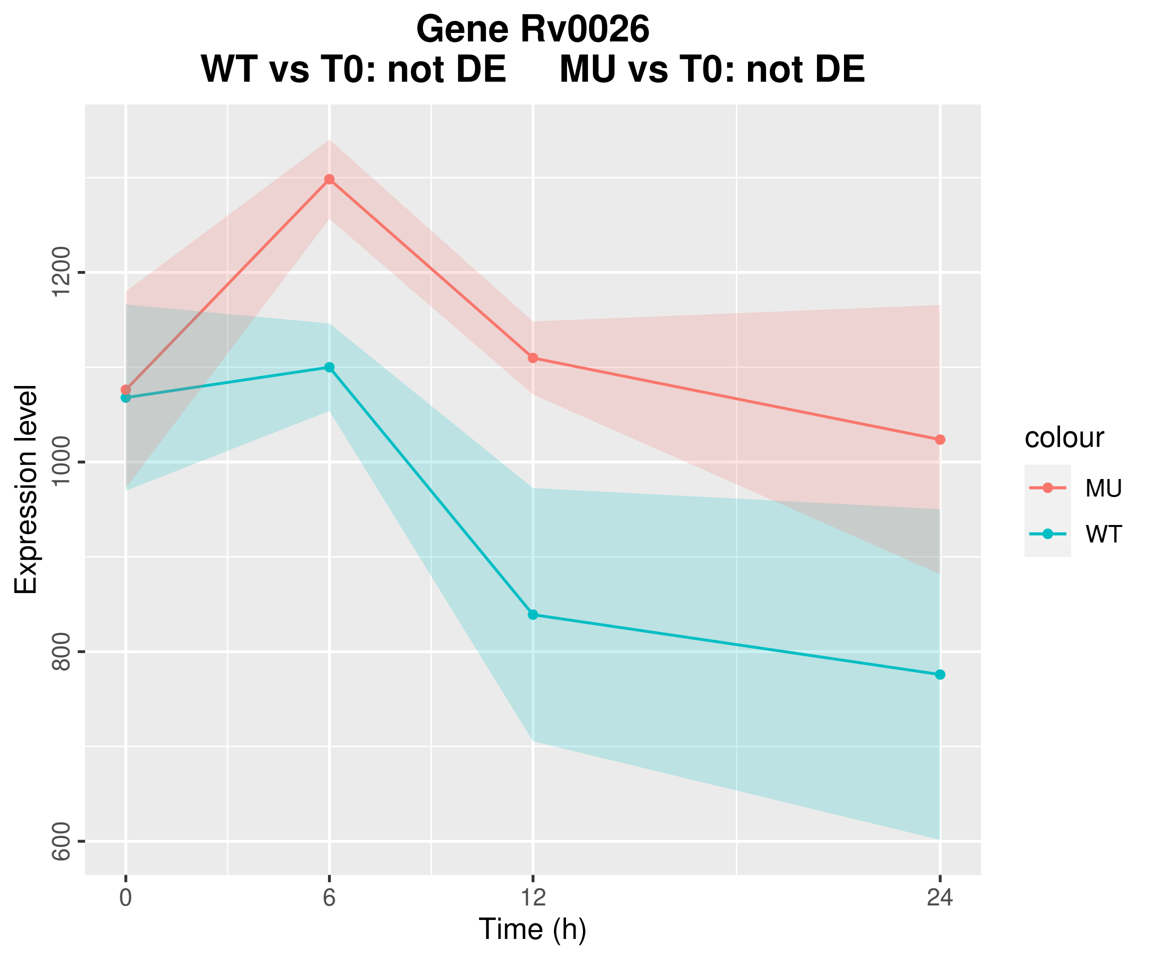
Gene Rv0026
WT vs T0: not DE MU vs T0: not DE
Expression level
1200
1000
800
600
0
6
12
24
Time (h)
colour
MU
WT

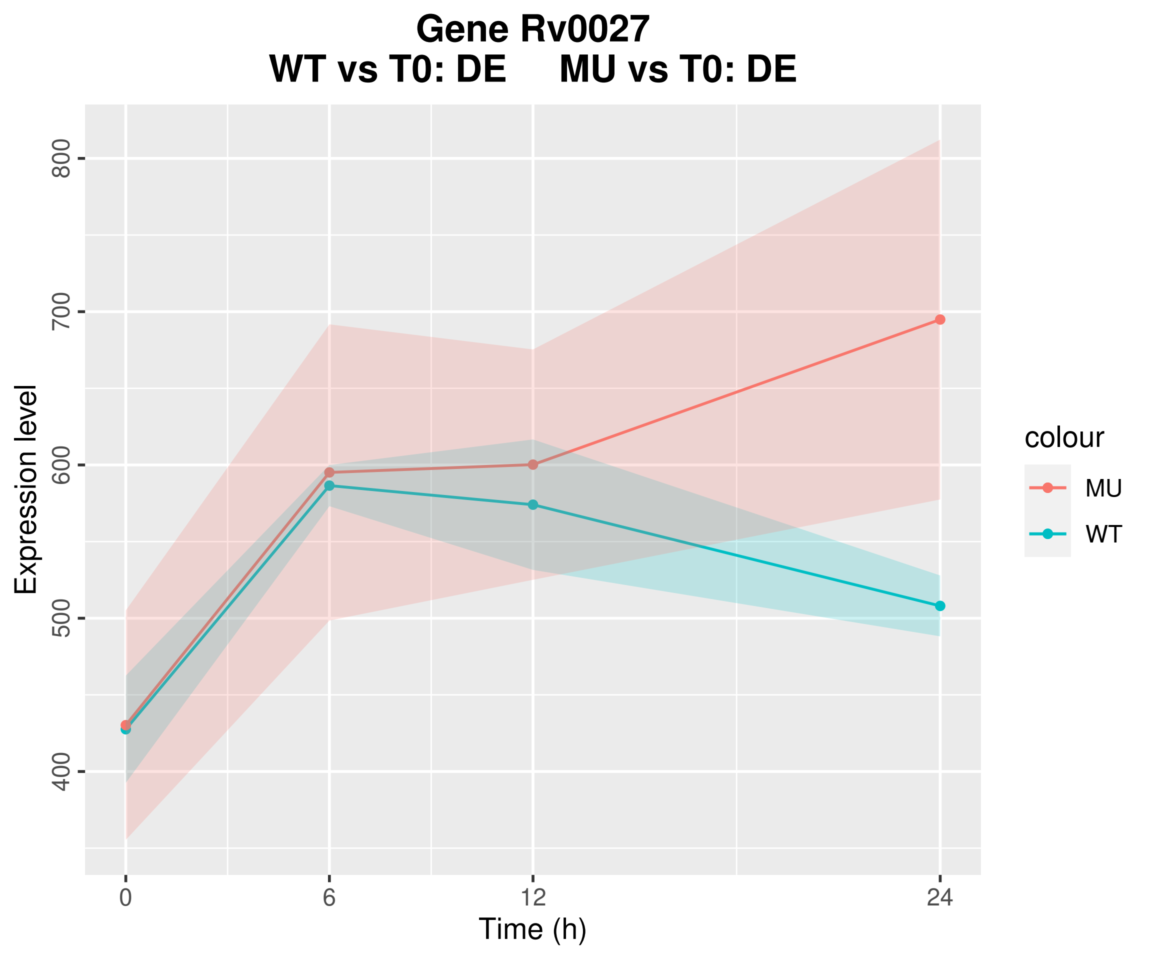
Gene Rv0027
WT vs T0: DE MU vs T0: DE
Expression level
Time (h)
400
500
600
700
800
0
6
12
24
colour
MU
WT

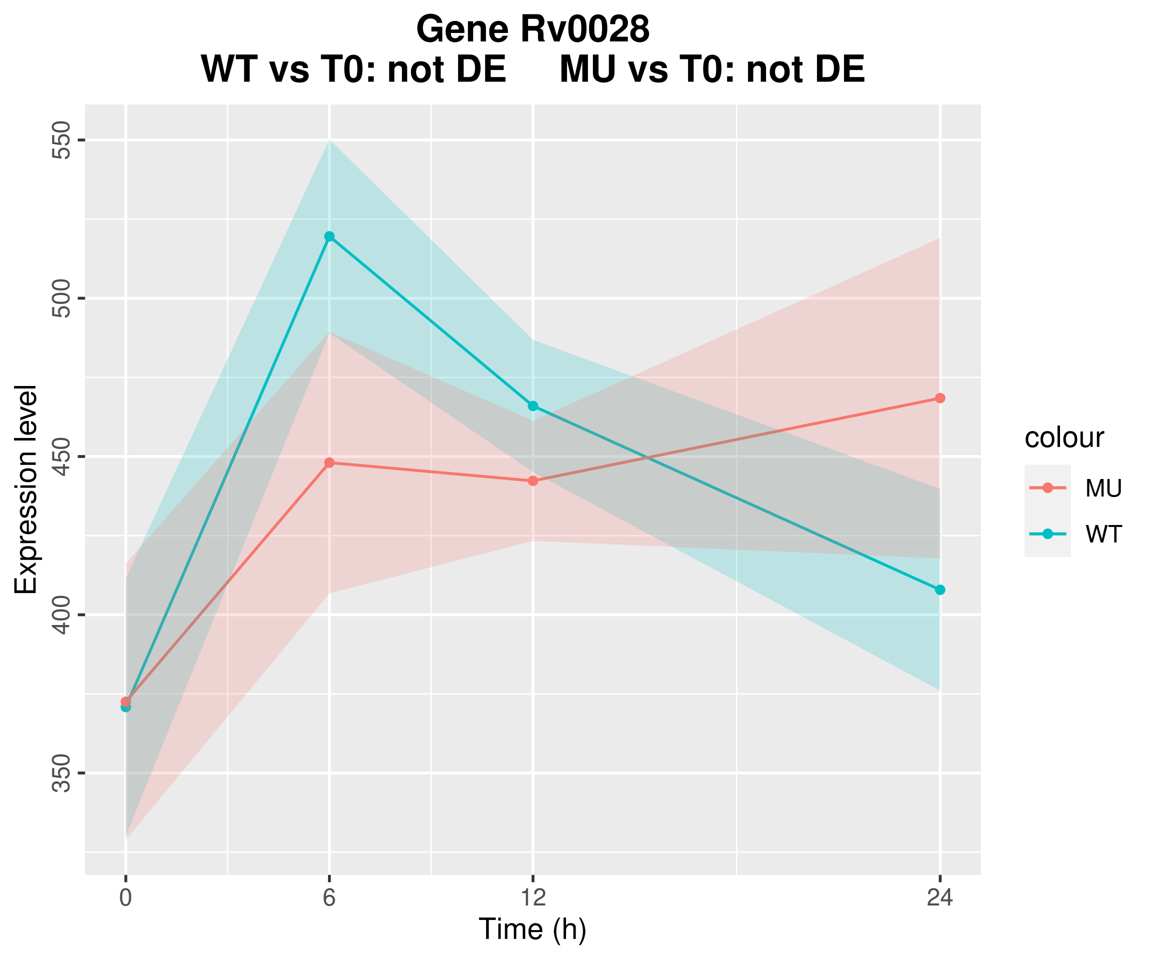

Gene Rv0028
WT vs T0: not DE MU vs T0: not DE
Expression level
550
500
450
400
350
0
6
12
24
Time (h)
colour
MU
WT

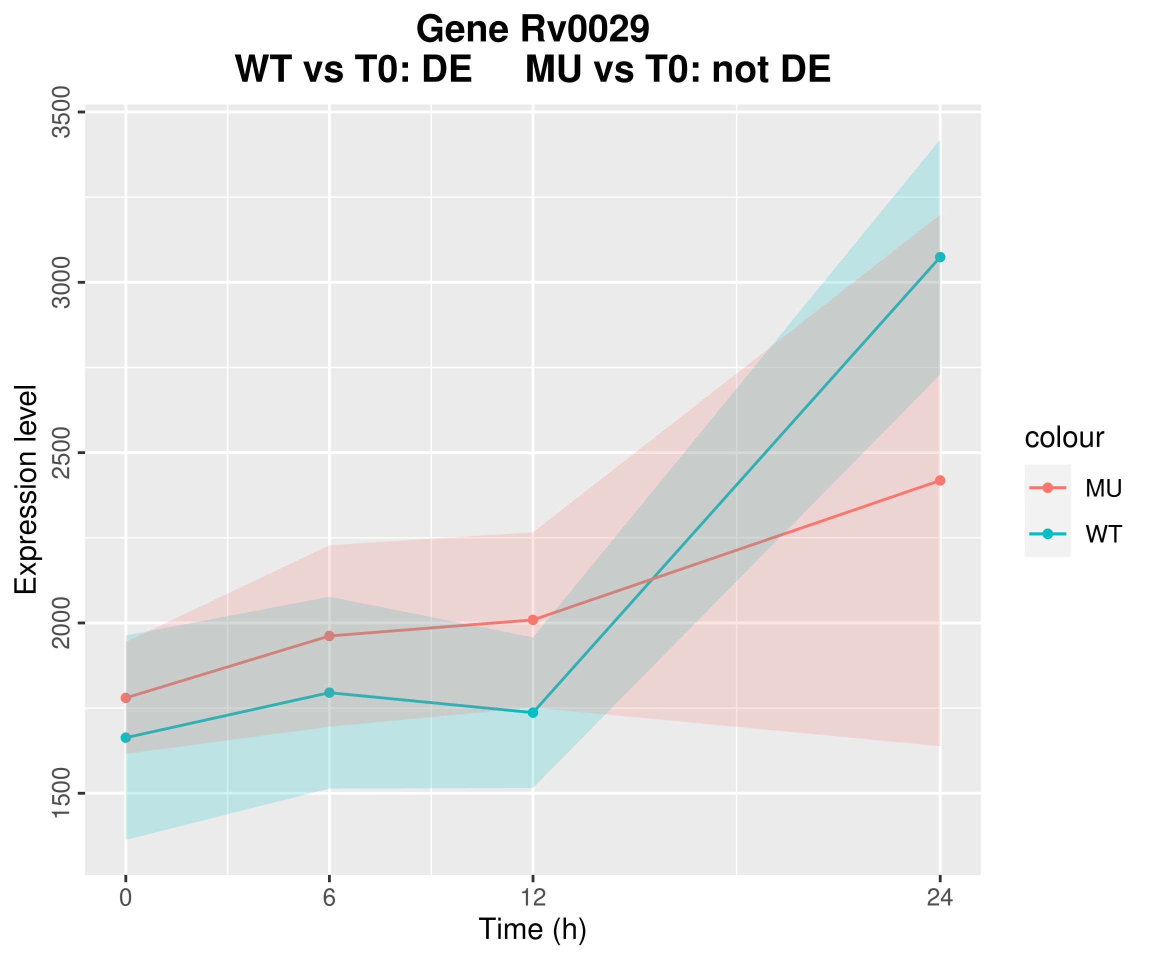

Gene Rv0029
WT vs T0: DE MU vs T0: not DE
Expression level
3500
3000
2500
2000
1500
0
6
12
24
Time (h)
colour
MU
WT

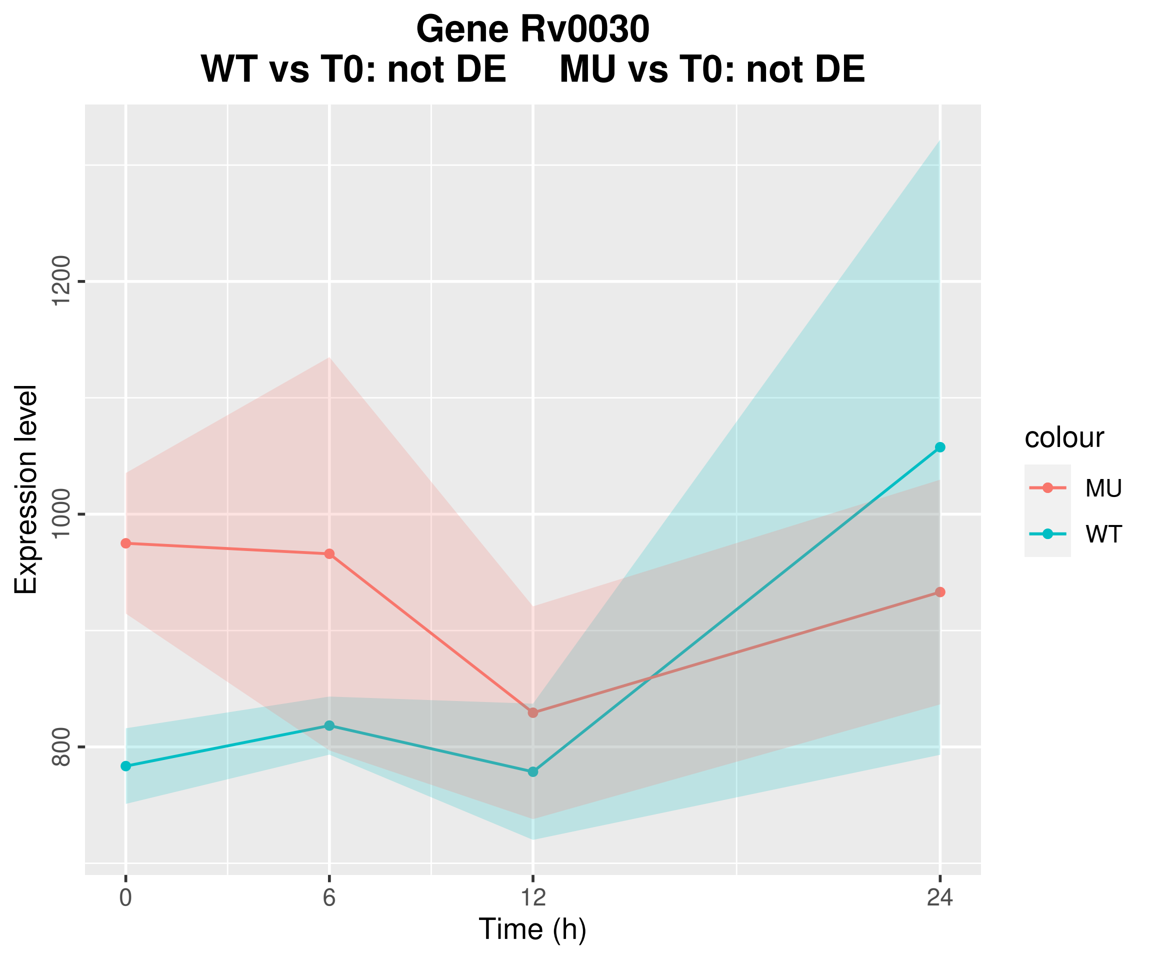
Gene Rv0030
WT vs T0: not DE   MU vs T0: not DE
Expression level
1200
1000
800
0
6
12
24
Time (h)
colour
MU
WT

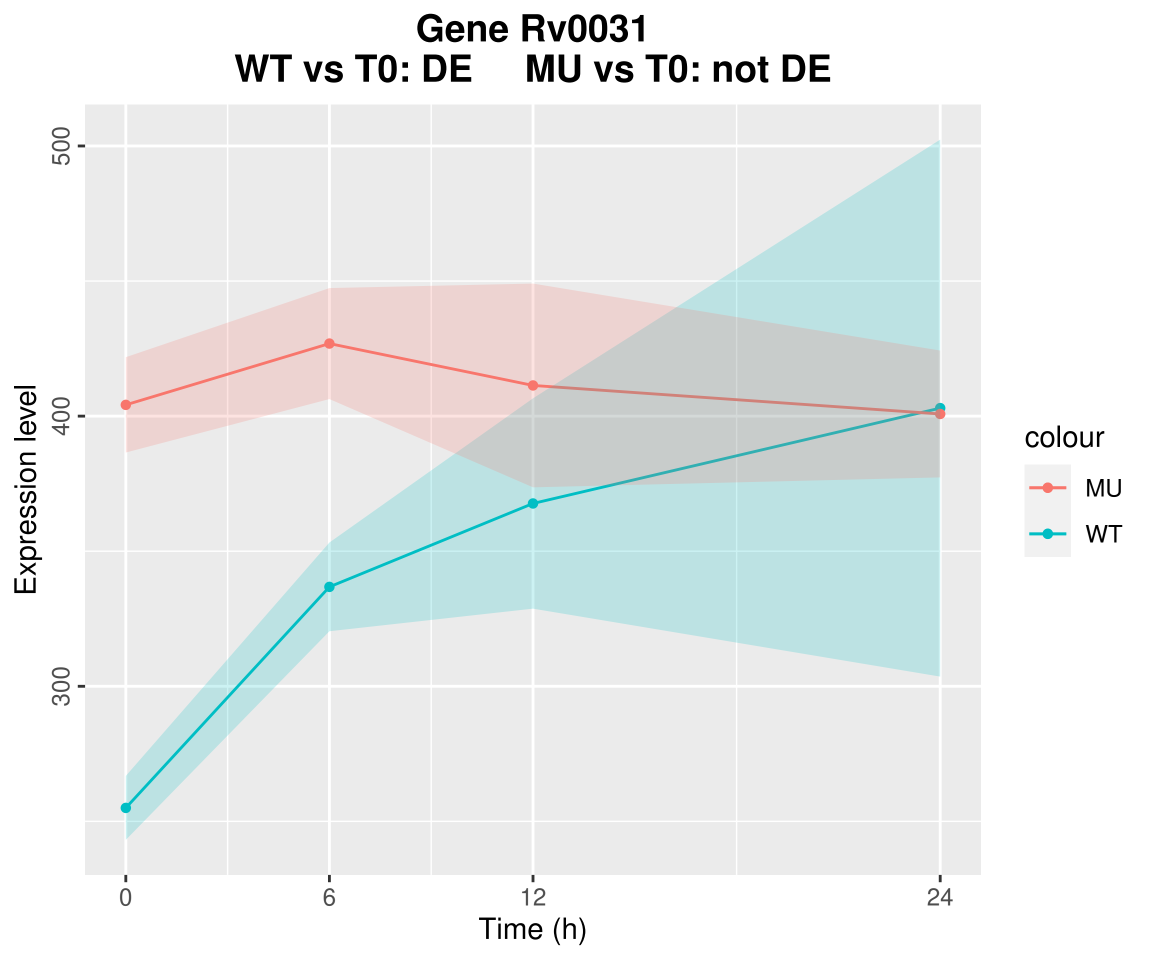

Gene Rv0031
WT vs T0: DE MU vs T0: not DE
500
400
300
Expression level
0
6
12
24
Time (h)
colour
MU
WT

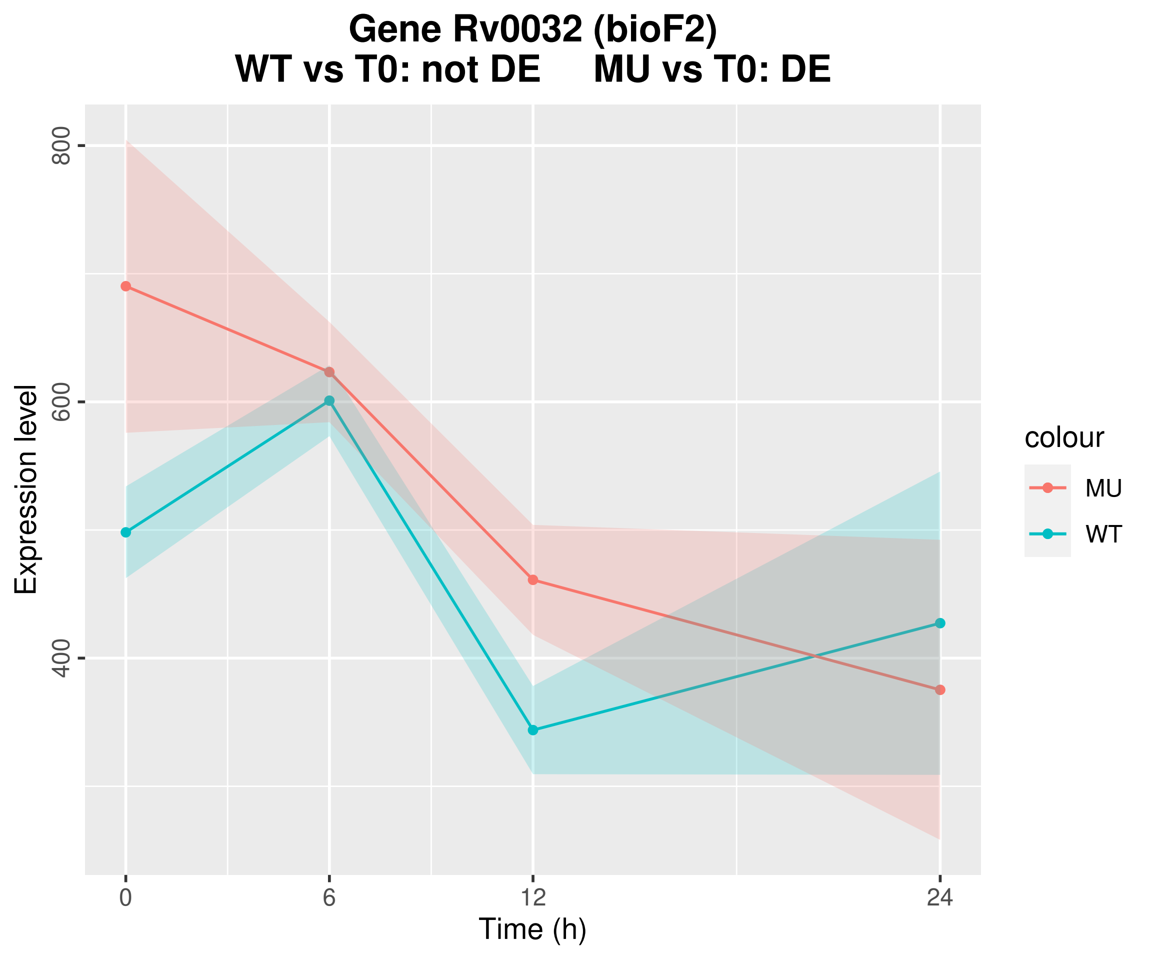

Gene Rv0032 (bioF2)
WT vs T0: not DE   MU vs T0: DE
Expression level
800
600
400
0
6
12
24
Time (h)
colour
MU
WT

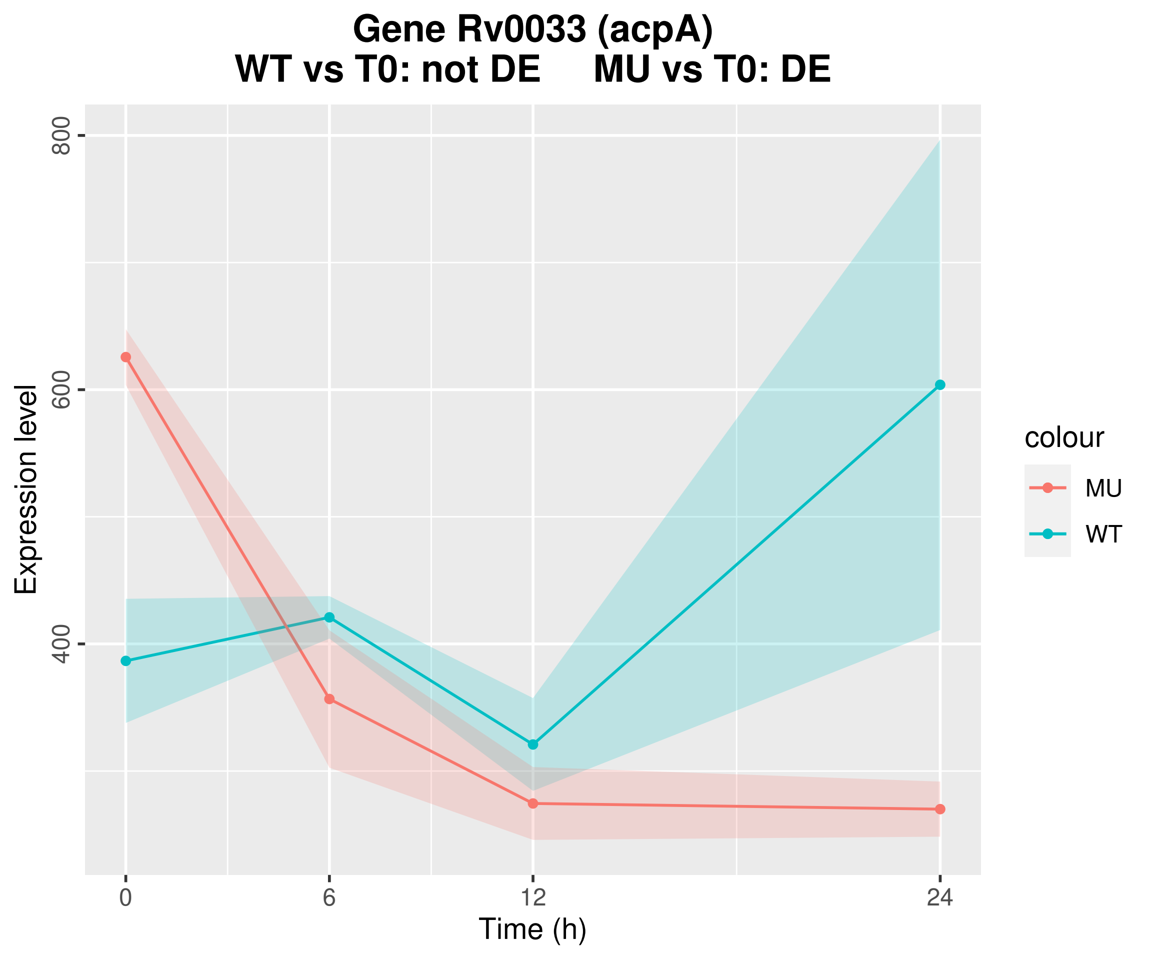

Gene Rv0033 (acpA)
WT vs T0: not DE MU vs T0: DE
800
600
400
0
6
12
24
Time (h)
Expression level
colour
MU
WT

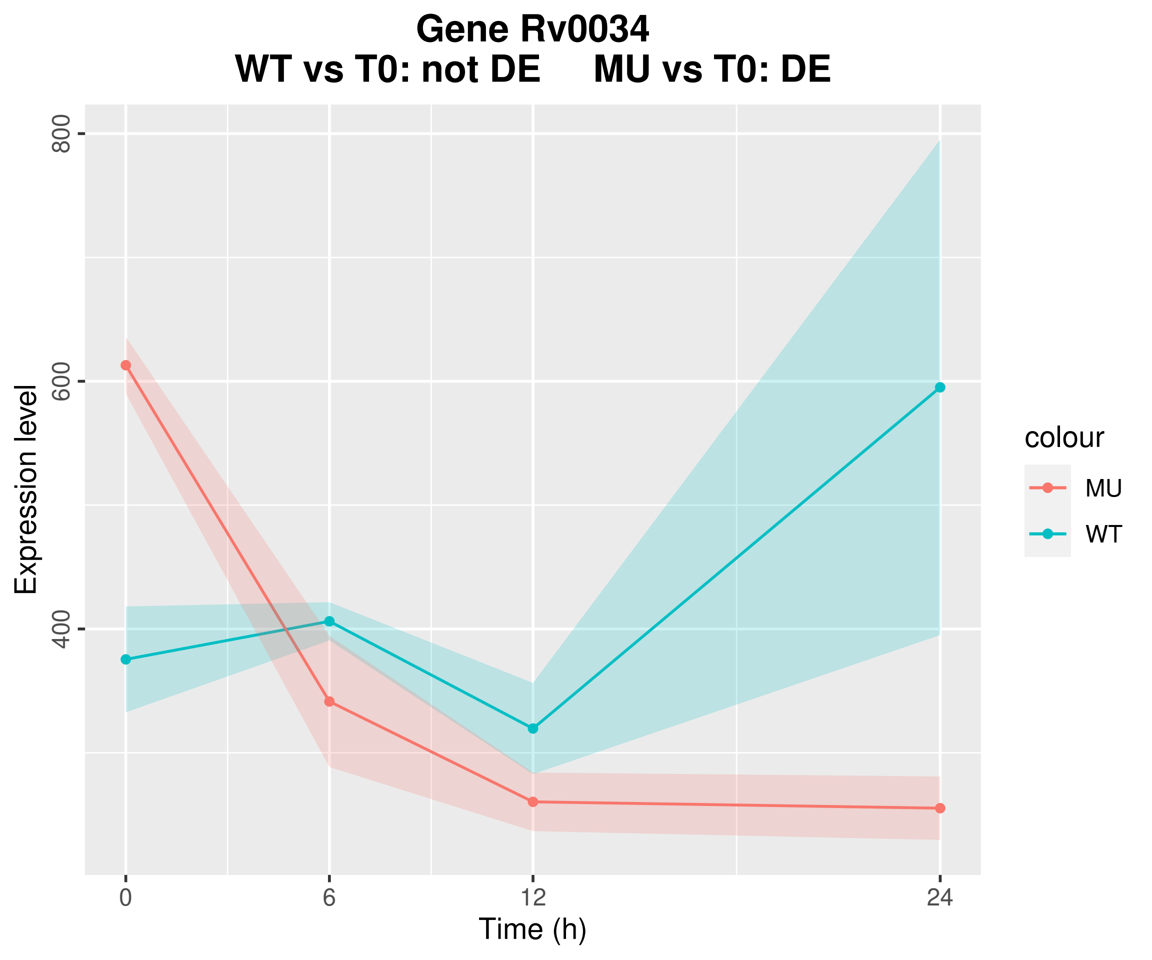
Gene Rv0034
WT vs T0: not DE MU vs T0: DE
800
600
400
Expression level
0
6
12
24
Time (h)
colour
MU
WT

Gene Rv0035 (fadD34)
WT vs T0: DE   MU vs T0: DE

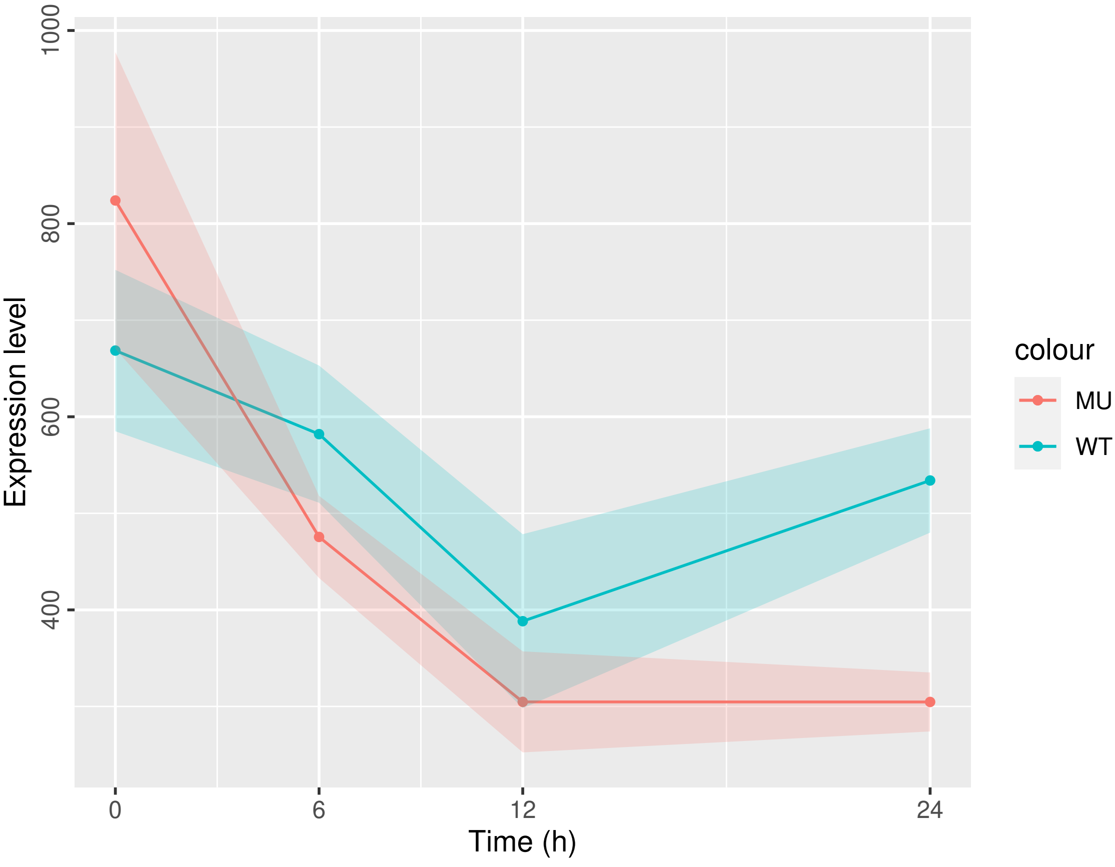

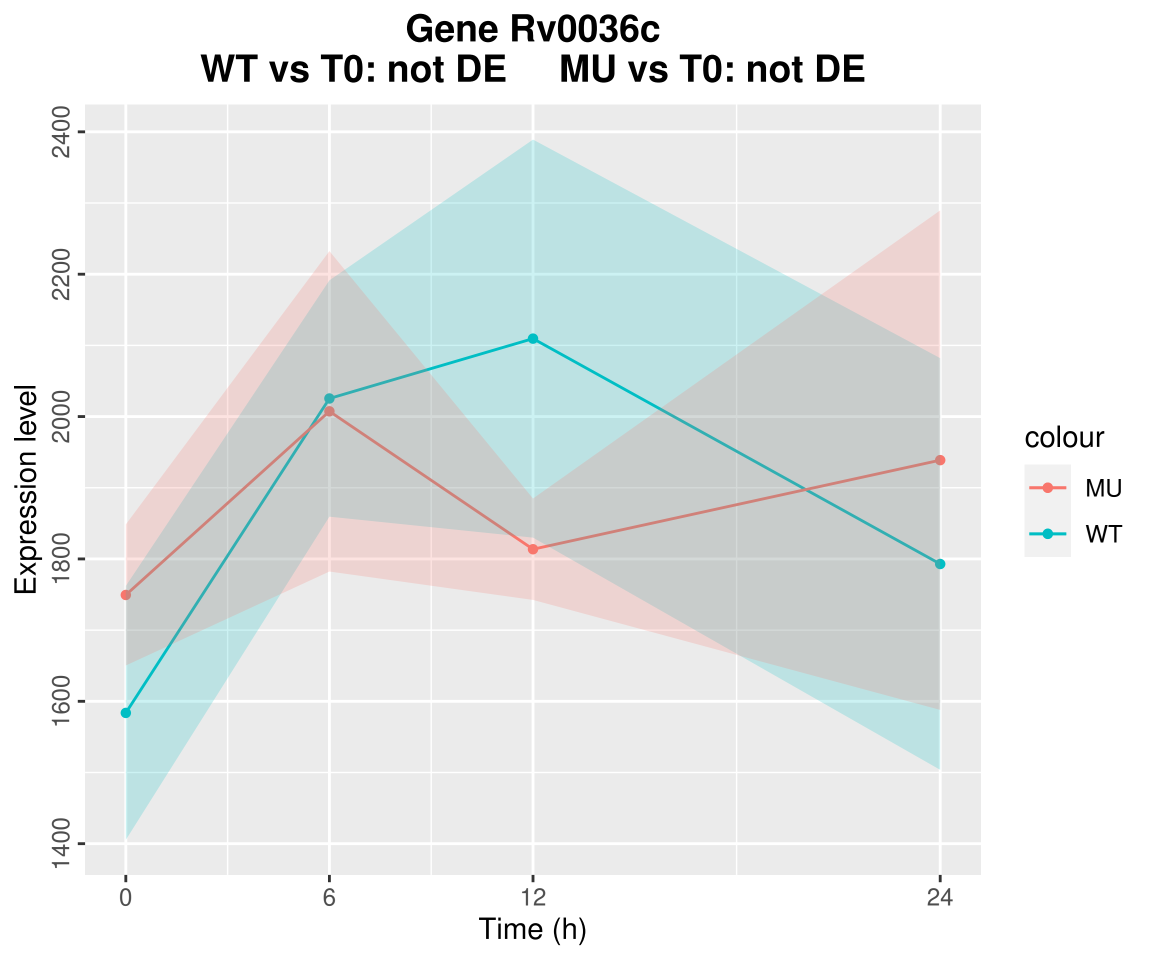

Gene Rv0036c
WT vs T0: not DE   MU vs T0: not DE
Expression level
2400
2200
2000
1800
1600
1400
0
6
12
24
Time (h)
colour
MU
WT

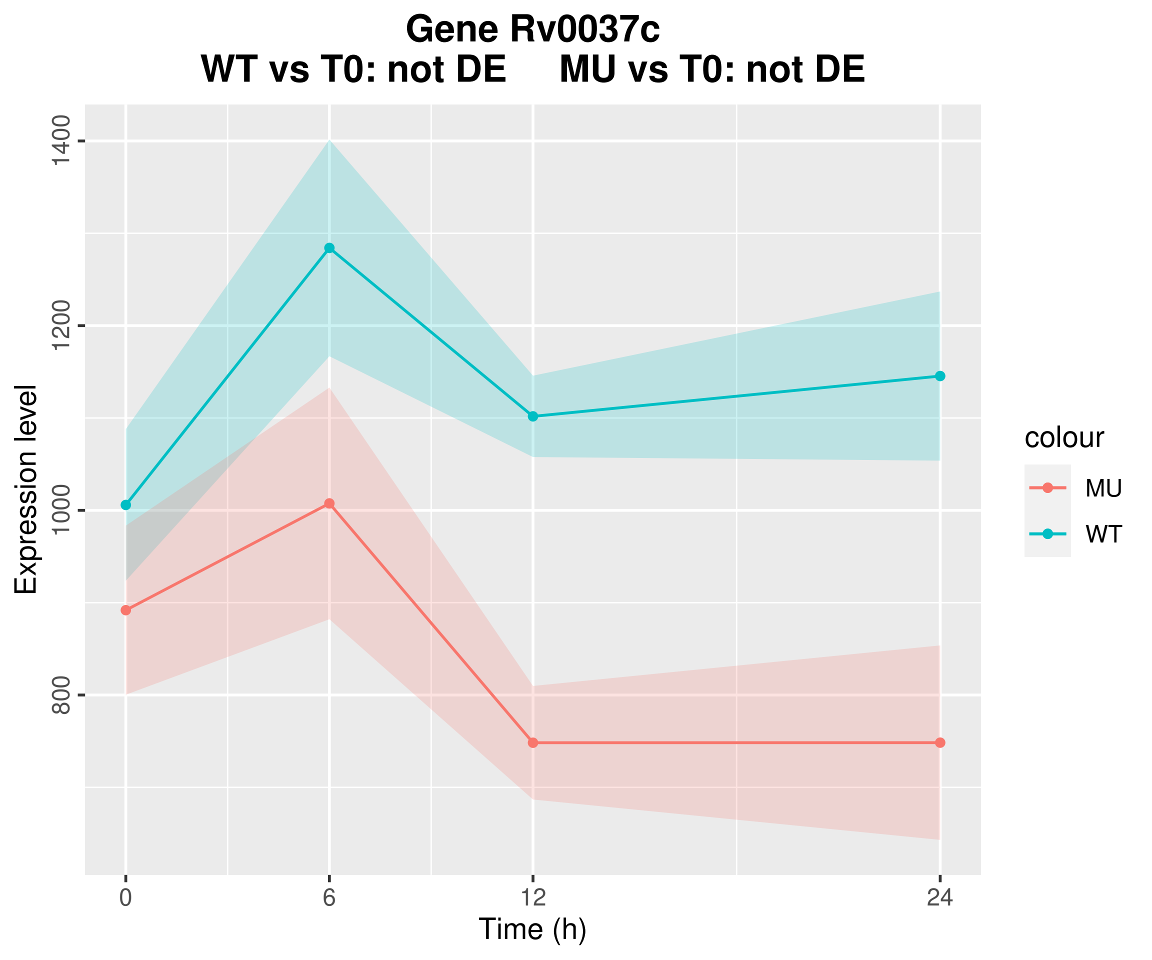
Gene Rv0037c
WT vs T0: not DE MU vs T0: not DE
1400
1200
1000
800
Expression level
0
6
12
24
Time (h)
colour
MU
WT

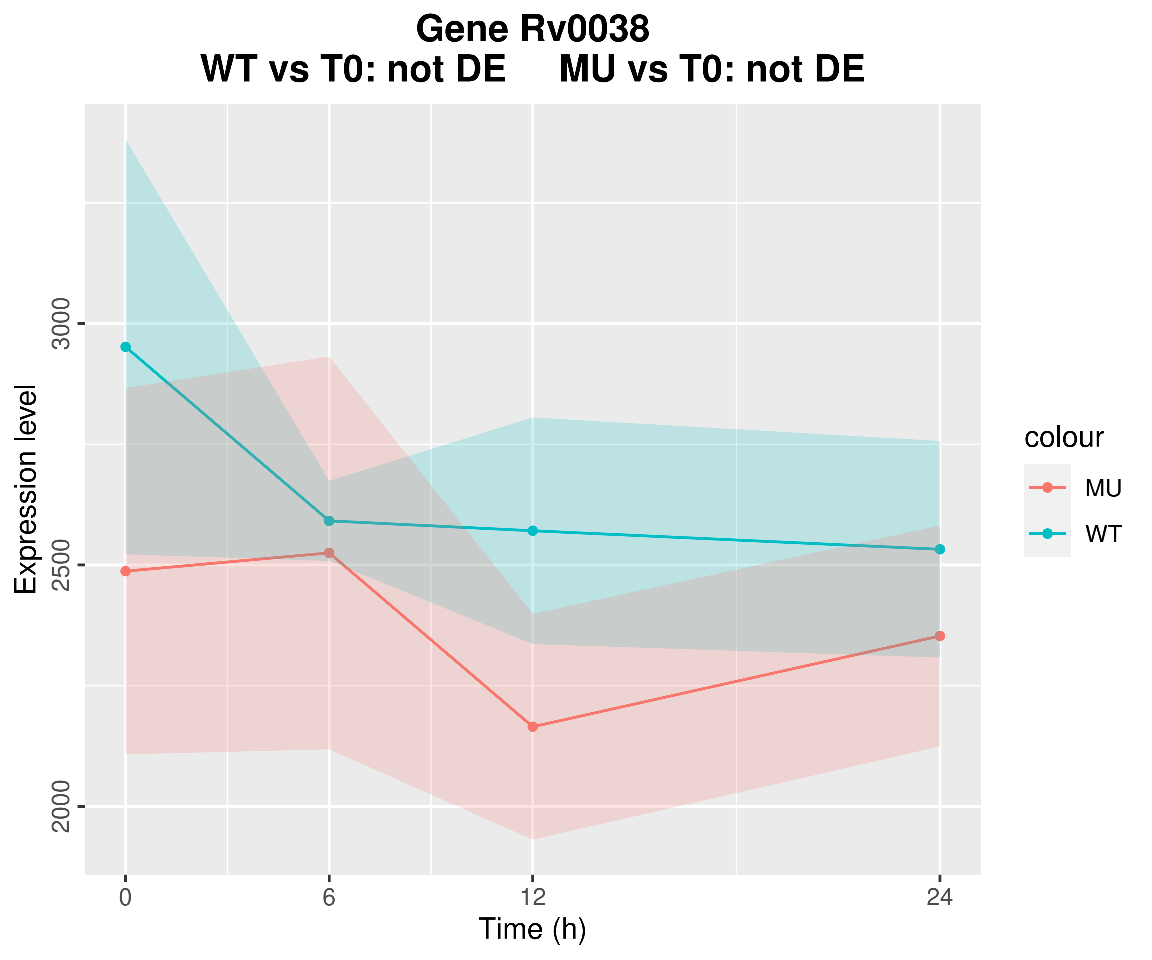

Gene Rv0038
WT vs T0: not DE   MU vs T0: not DE
Expression level
3000
2500
2000
0
6
12
24
Time (h)
colour
MU
WT

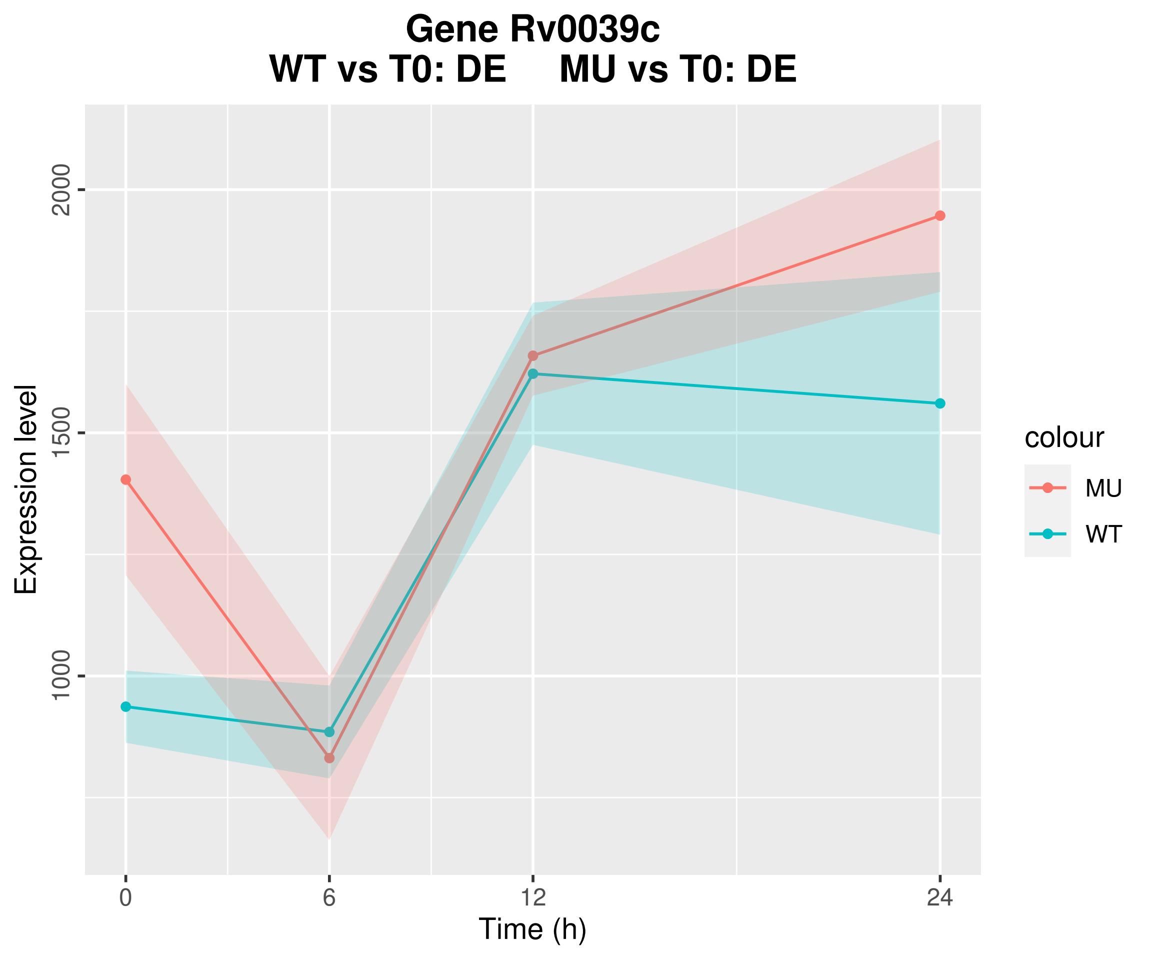

Gene Rv0039c
WT vs T0: DE MU vs T0: DE
2000
1500
1000
Expression level
0
6
12
24
Time (h)
colour
MU
WT

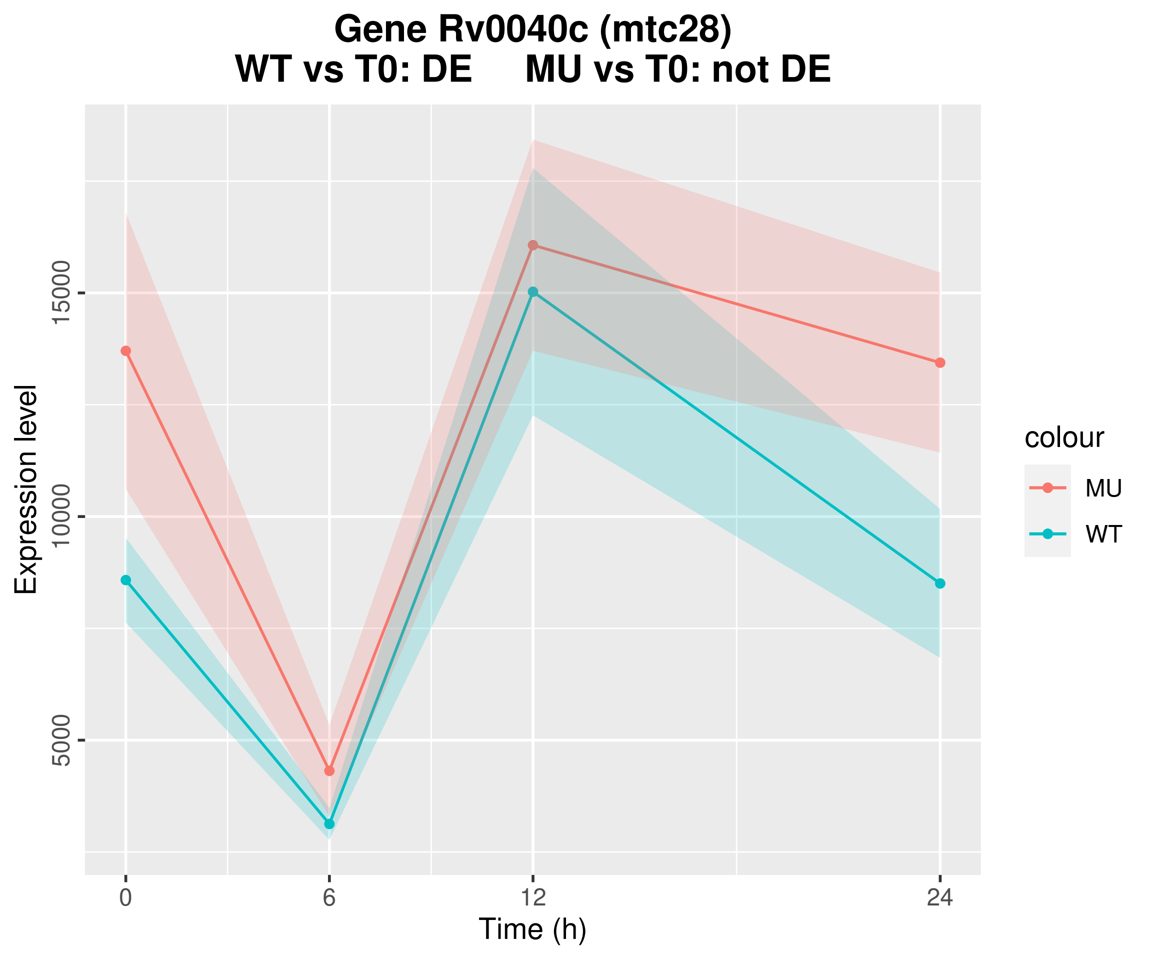

Gene Rv0040c (mtc28)
WT vs T0: DE   MU vs T0: not DE
Expression level
15000
10000
5000
0
6
12
24
Time (h)
colour
MU
WT

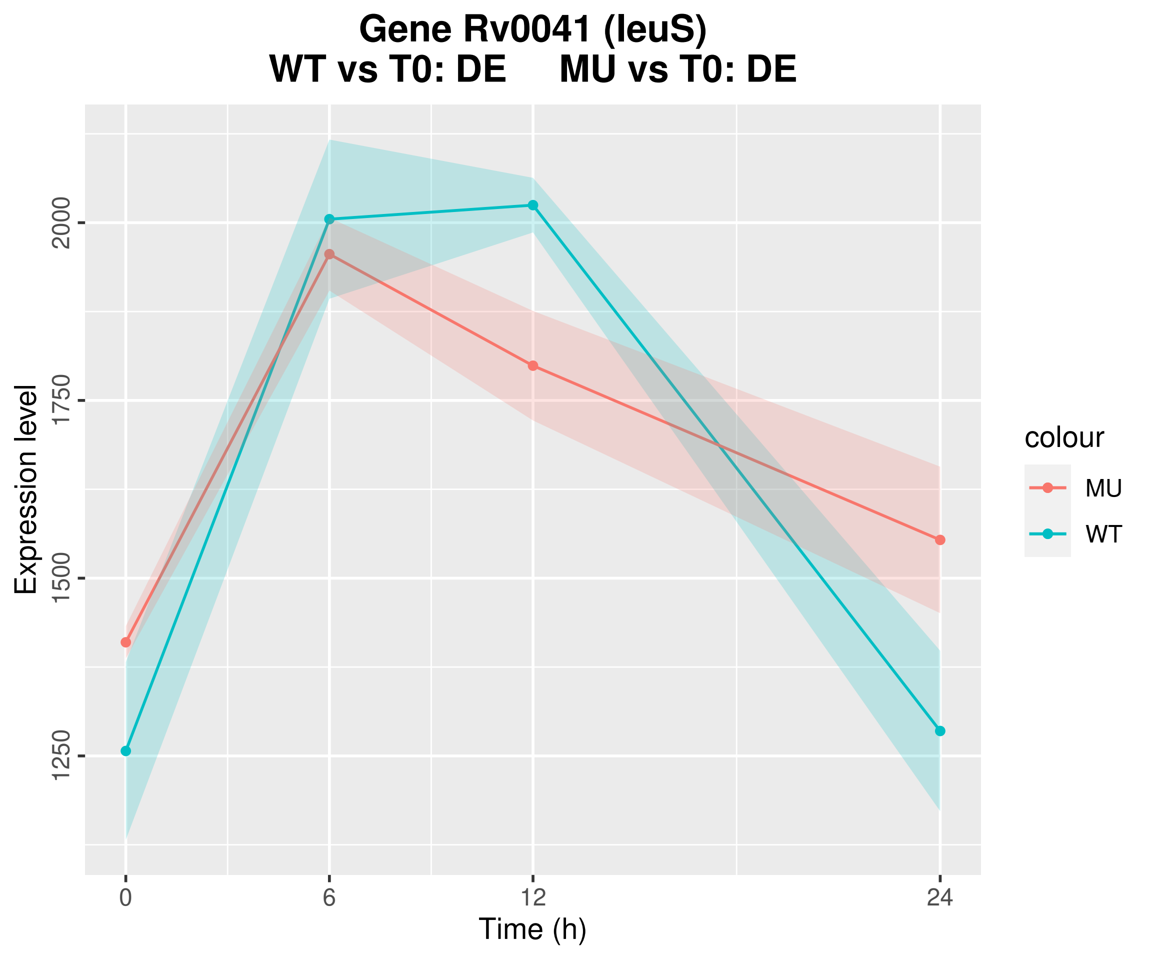

Gene Rv0041 (leuS)
WT vs T0: DE   MU vs T0: DE
Expression level
1250
1500
1750
2000
0
6
12
24
Time (h)
colour
MU
WT

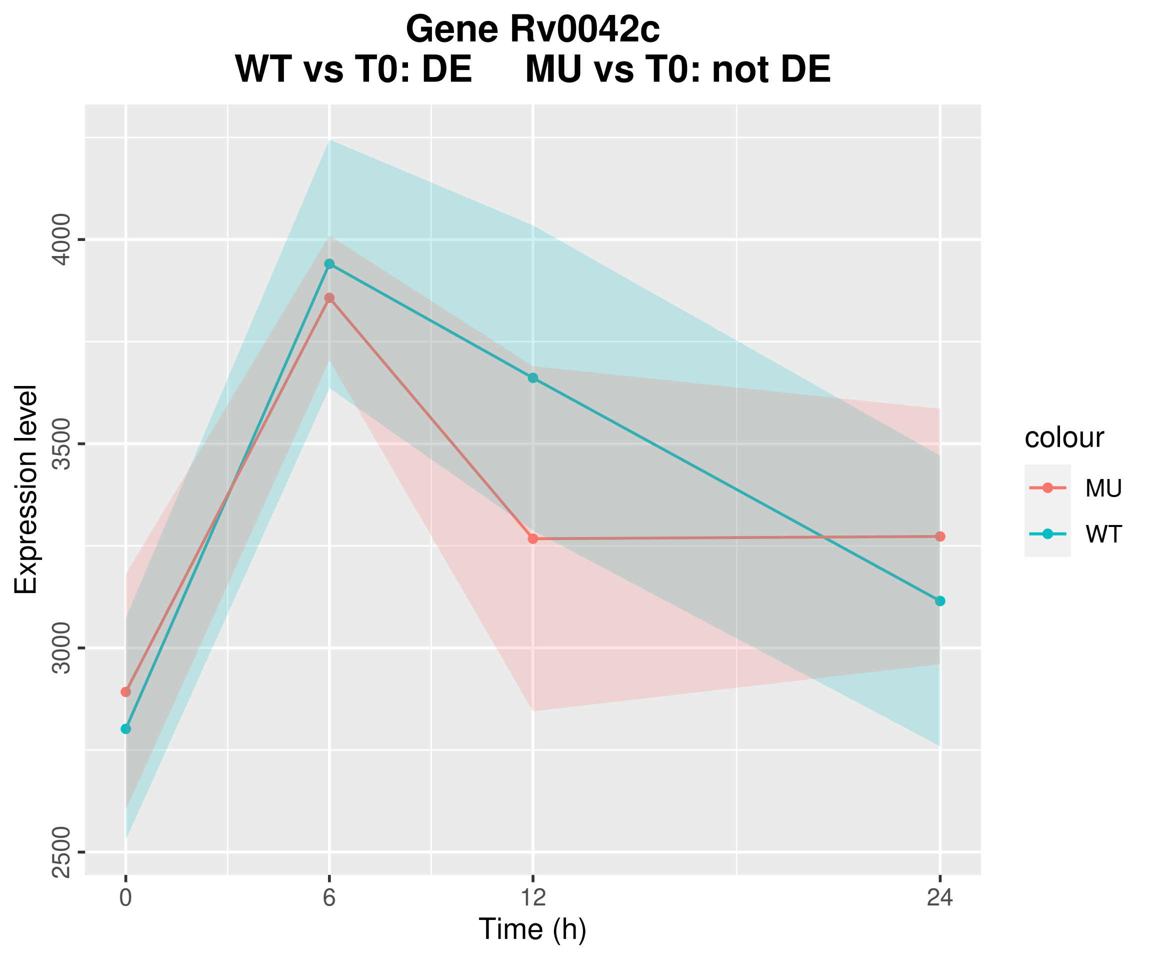

Gene Rv0042c
WT vs T0: DE   MU vs T0: not DE
Expression level
2500
3000
3500
4000
Time (h)
0
6
12
24
colour
MU
WT

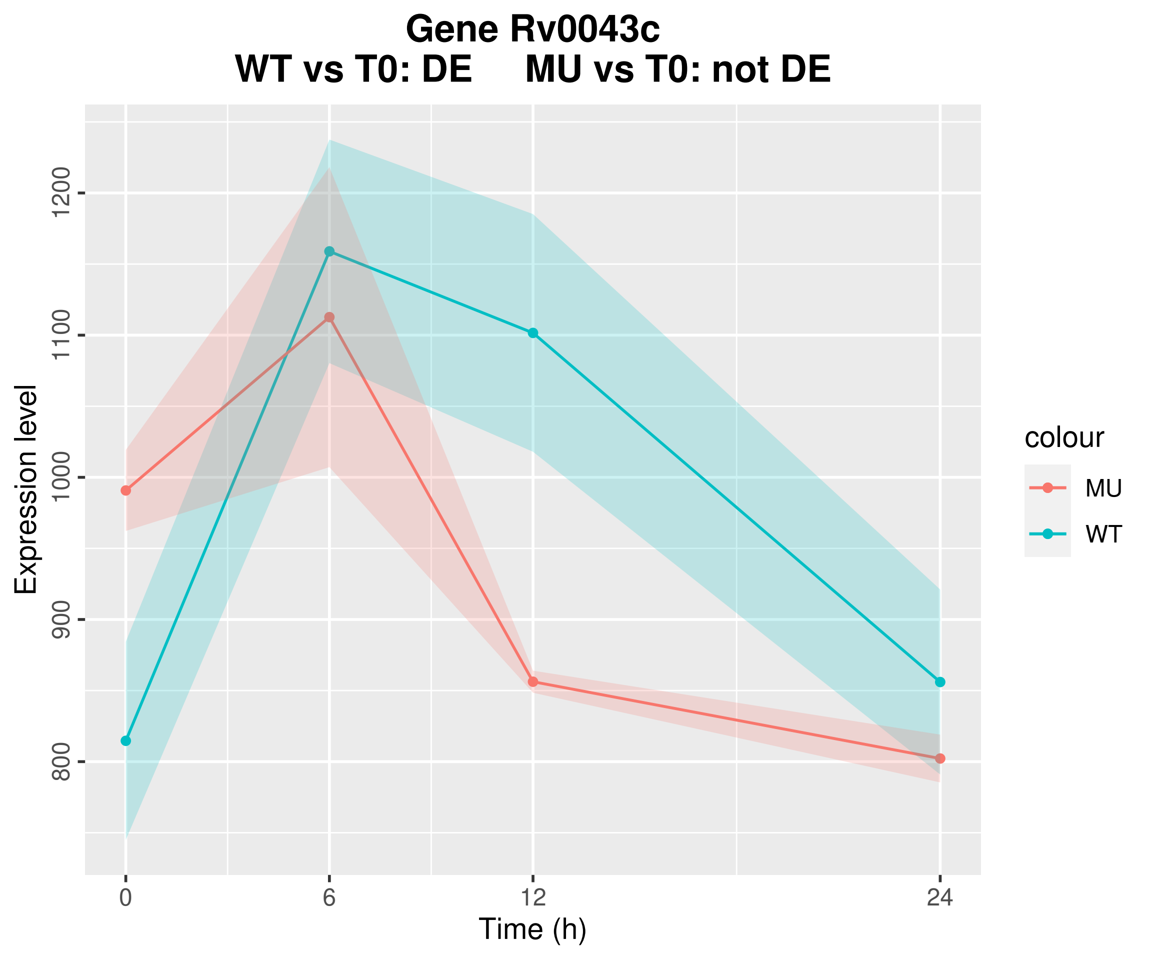
Gene Rv0043c
WT vs T0: DE MU vs T0: not DE
Expression level
1200
1100
1000
900
800
0
6
12
24
Time (h)
colour
MU
WT

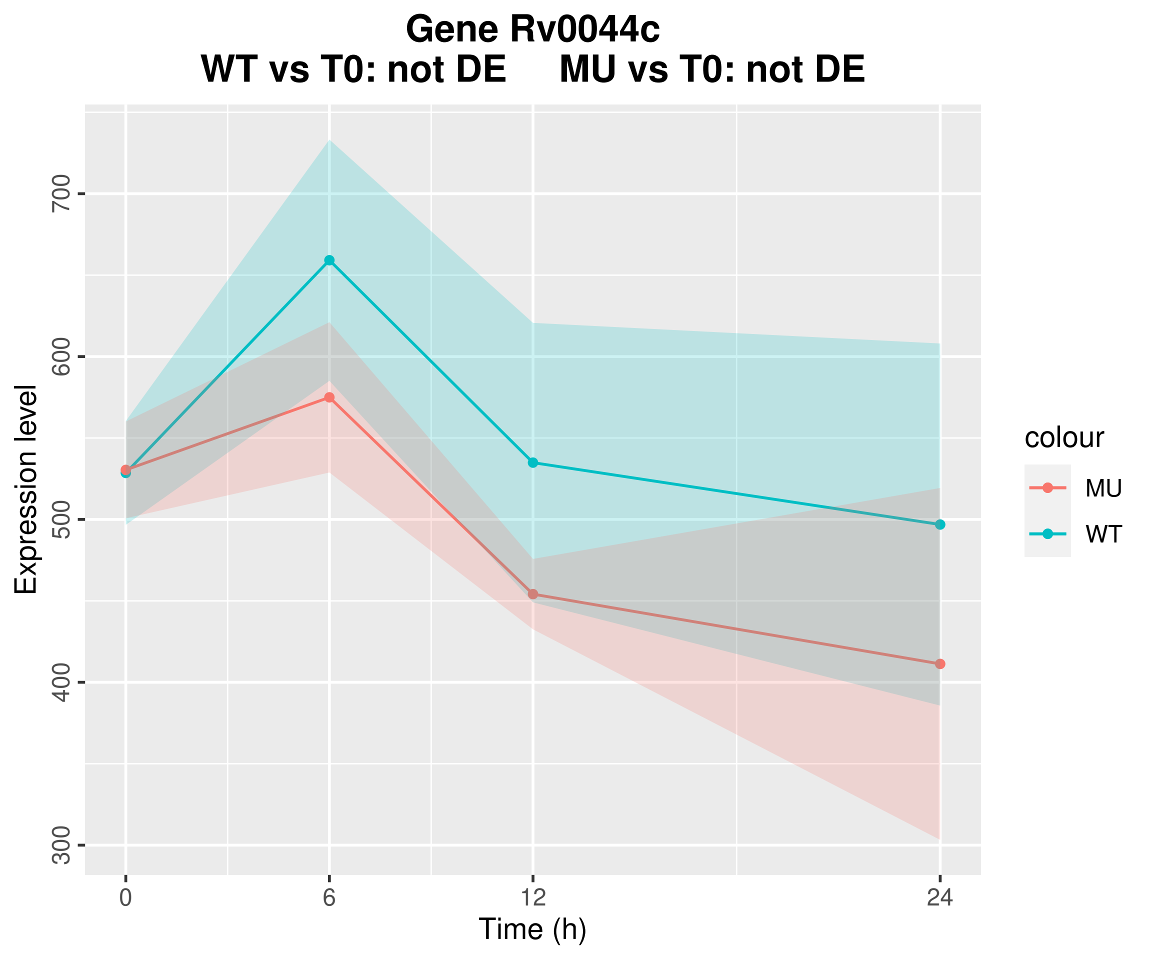

Gene Rv0044c
WT vs T0: not DE MU vs T0: not DE
Expression level
700
600
500
400
300
0
6
12
24
Time (h)
colour
MU
WT

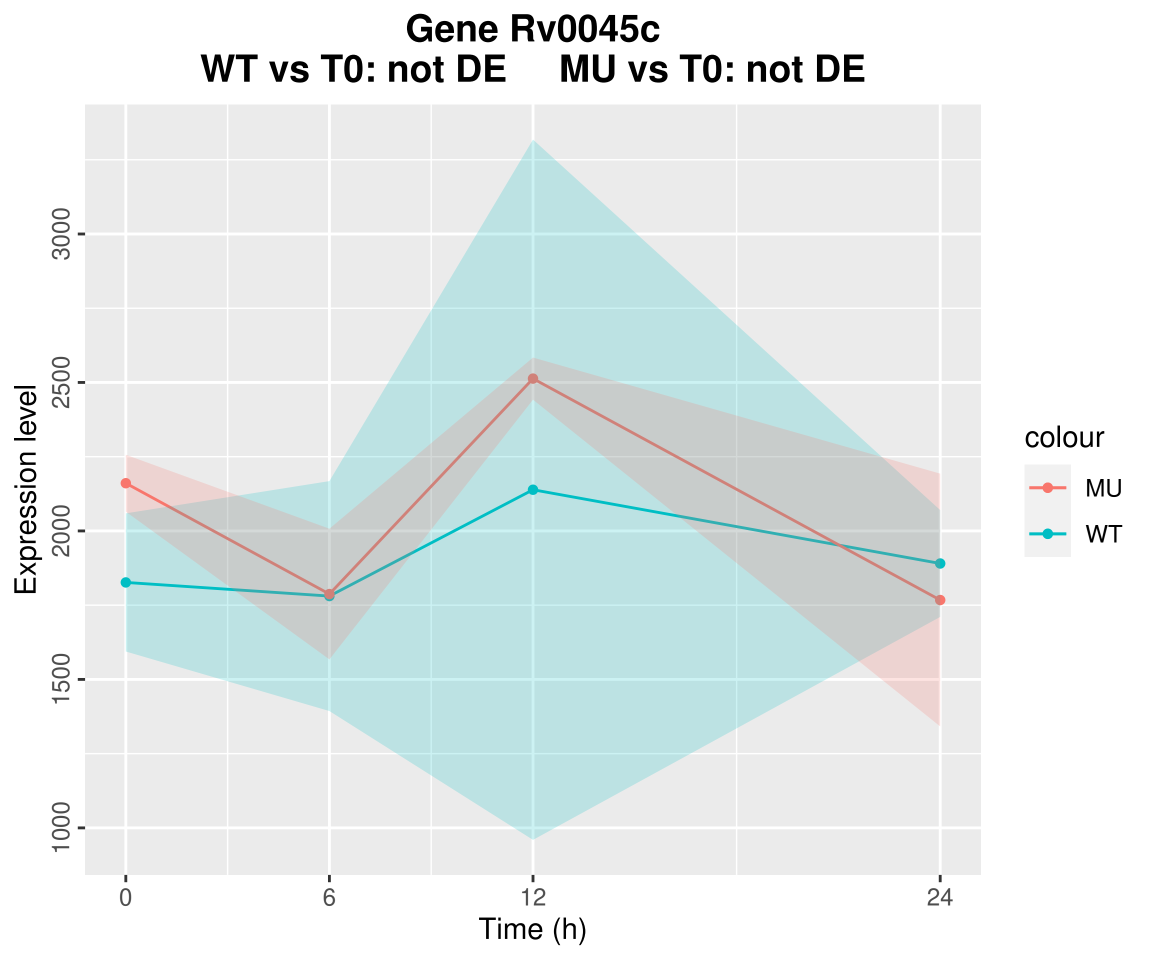

Gene Rv0045c
WT vs T0: not DE   MU vs T0: not DE
Expression level
3000
2500
2000
1500
1000
0
6
12
24
Time (h)
colour
MU
WT

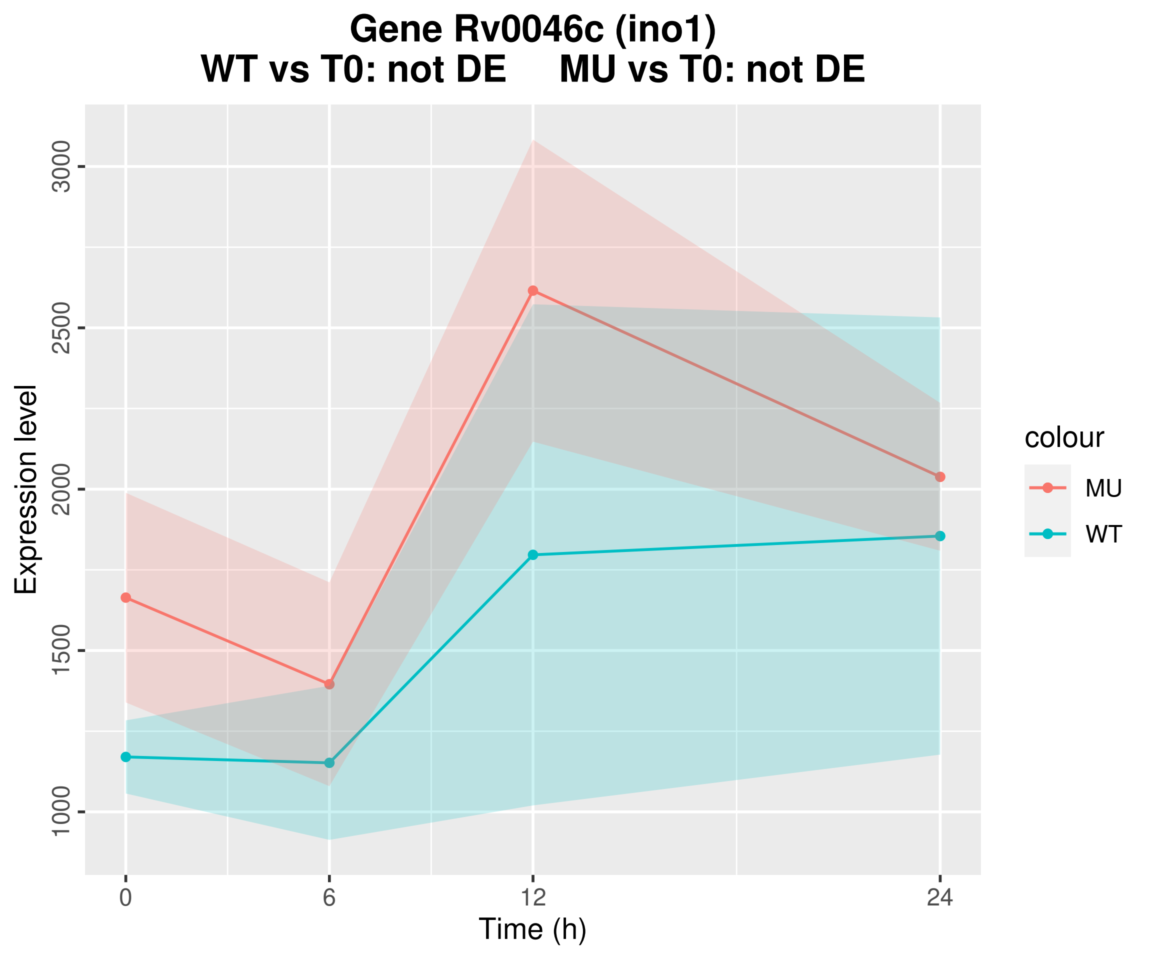
Gene Rv0046c (ino1)
WT vs T0: not DE   MU vs T0: not DE
Expression level
1000
1500
2000
2500
3000
0
6
12
24
Time (h)
colour
MU
WT

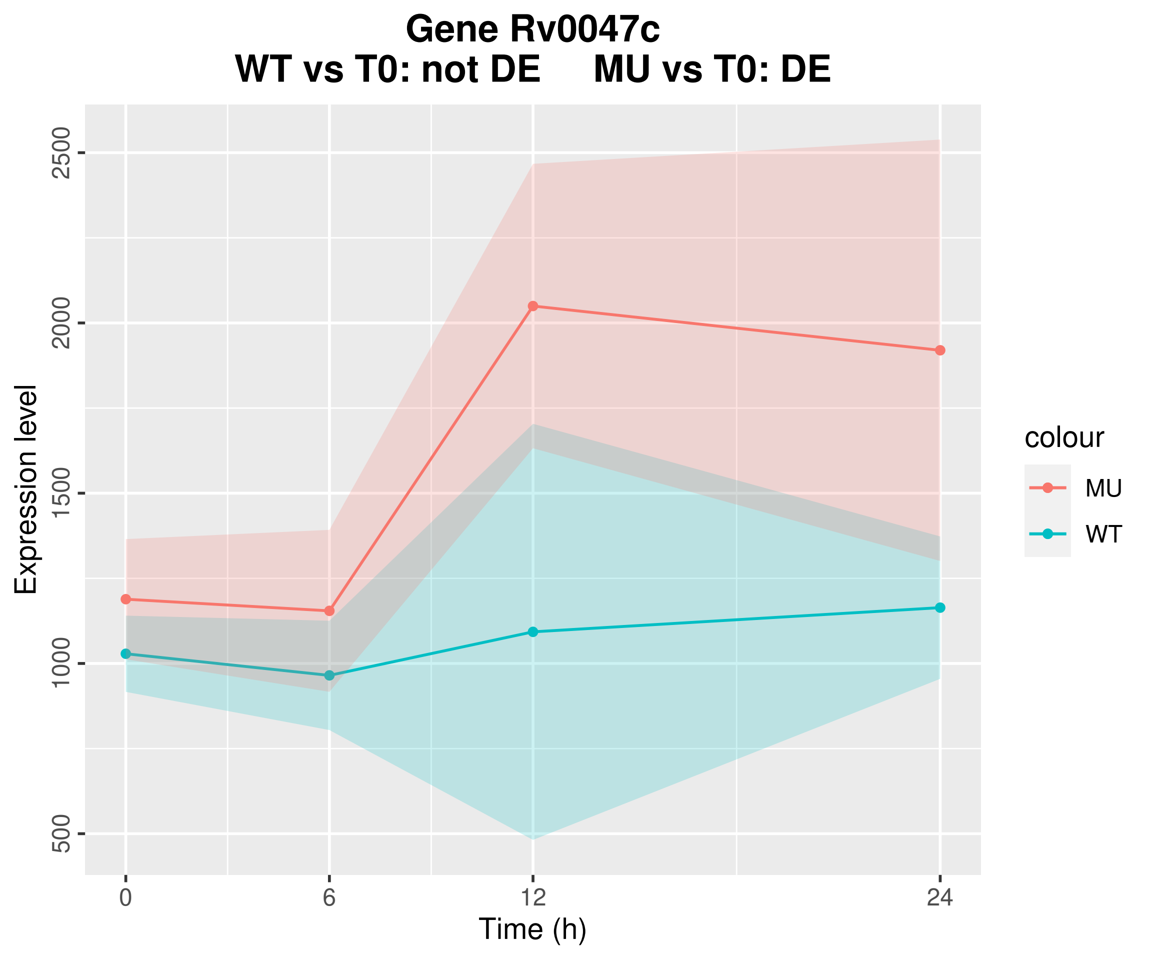

Gene Rv0047c
WT vs T0: not DE   MU vs T0: DE
Expression level
2500
2000
1500
1000
500
0
6
12
24
Time (h)
colour
MU
WT

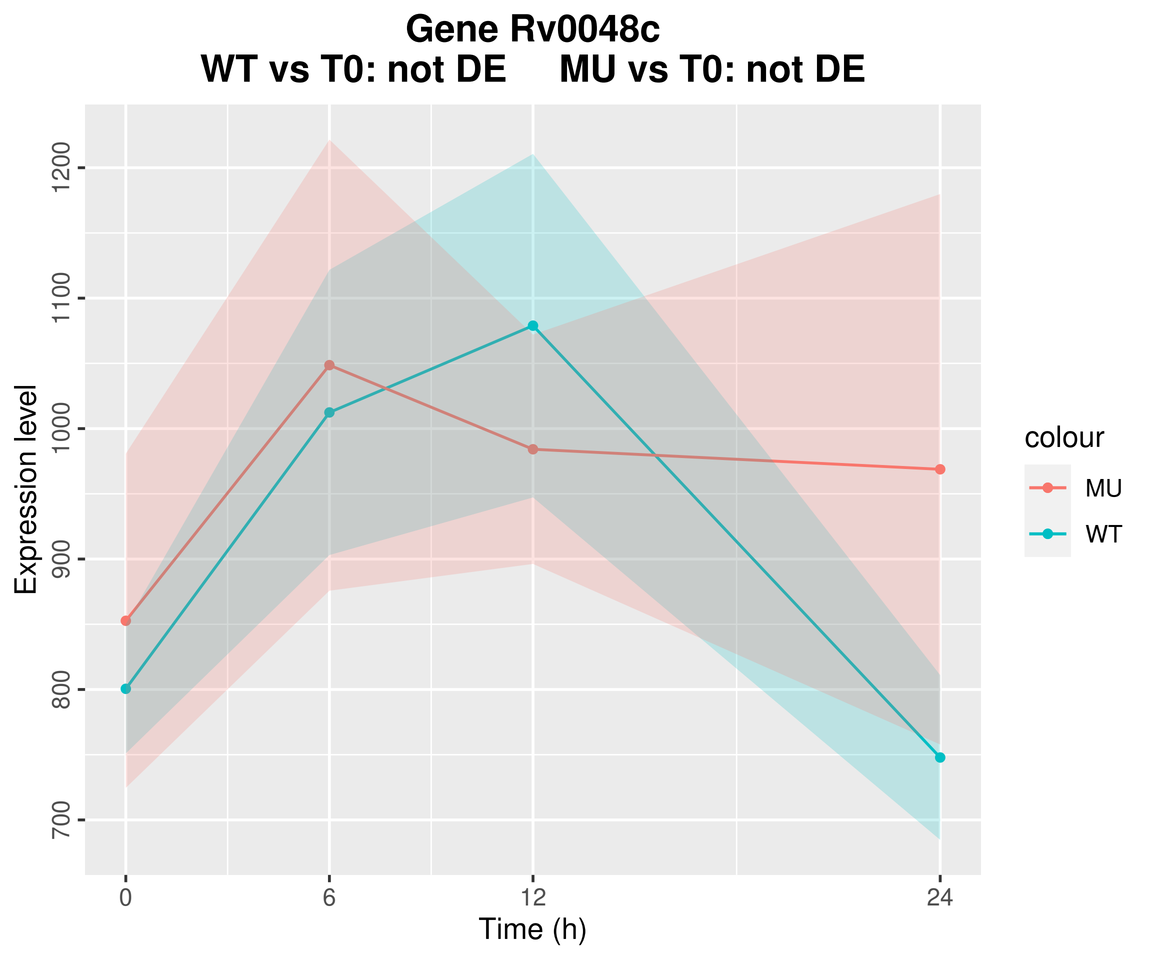

Gene Rv0048c
WT vs T0: not DE   MU vs T0: not DE
Expression level
1200
1100
1000
900
800
700
0
6
12
24
Time (h)
colour
MU
WT

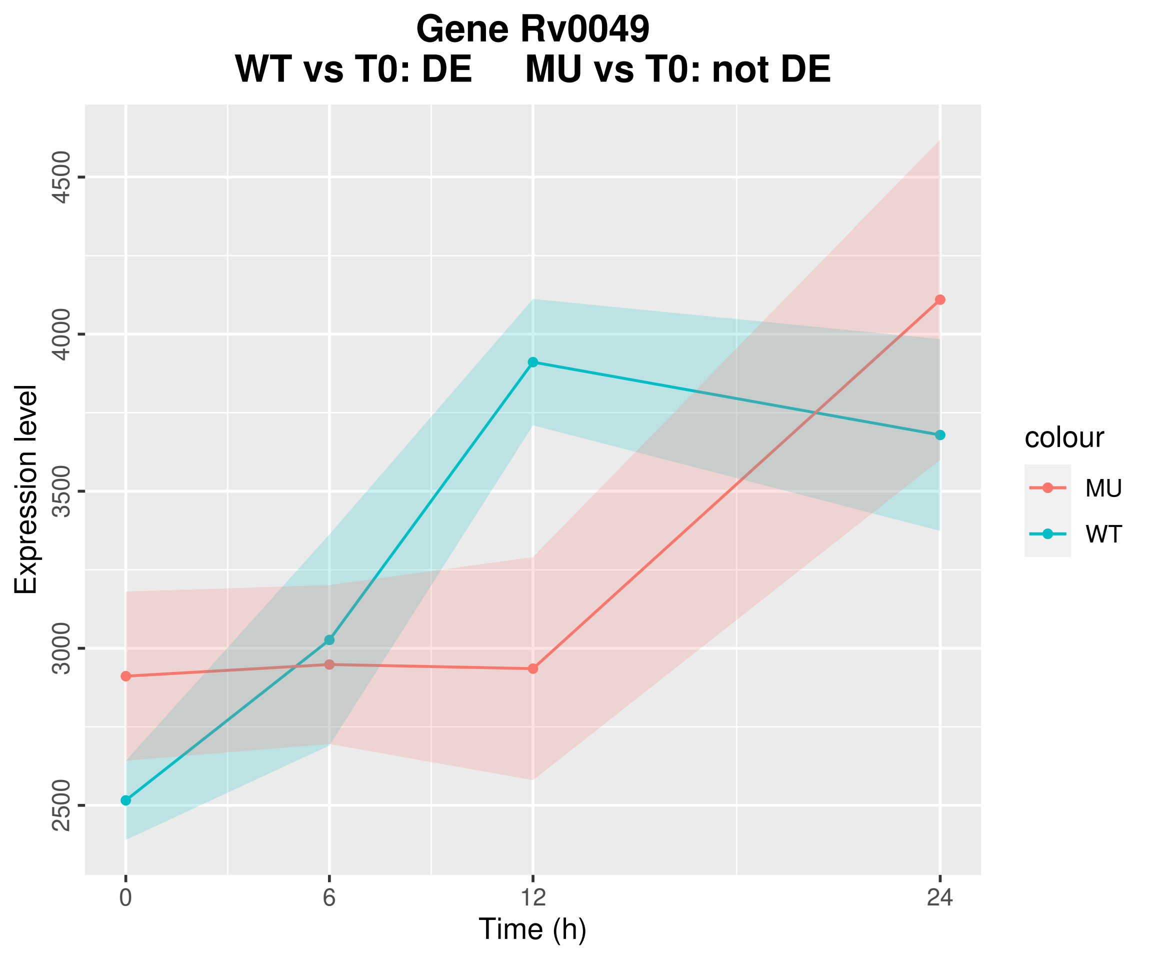

Gene Rv0049
WT vs T0: DE   MU vs T0: not DE
Expression level
Time (h)
2500
3000
3500
4000
4500
0
6
12
24
colour
MU
WT

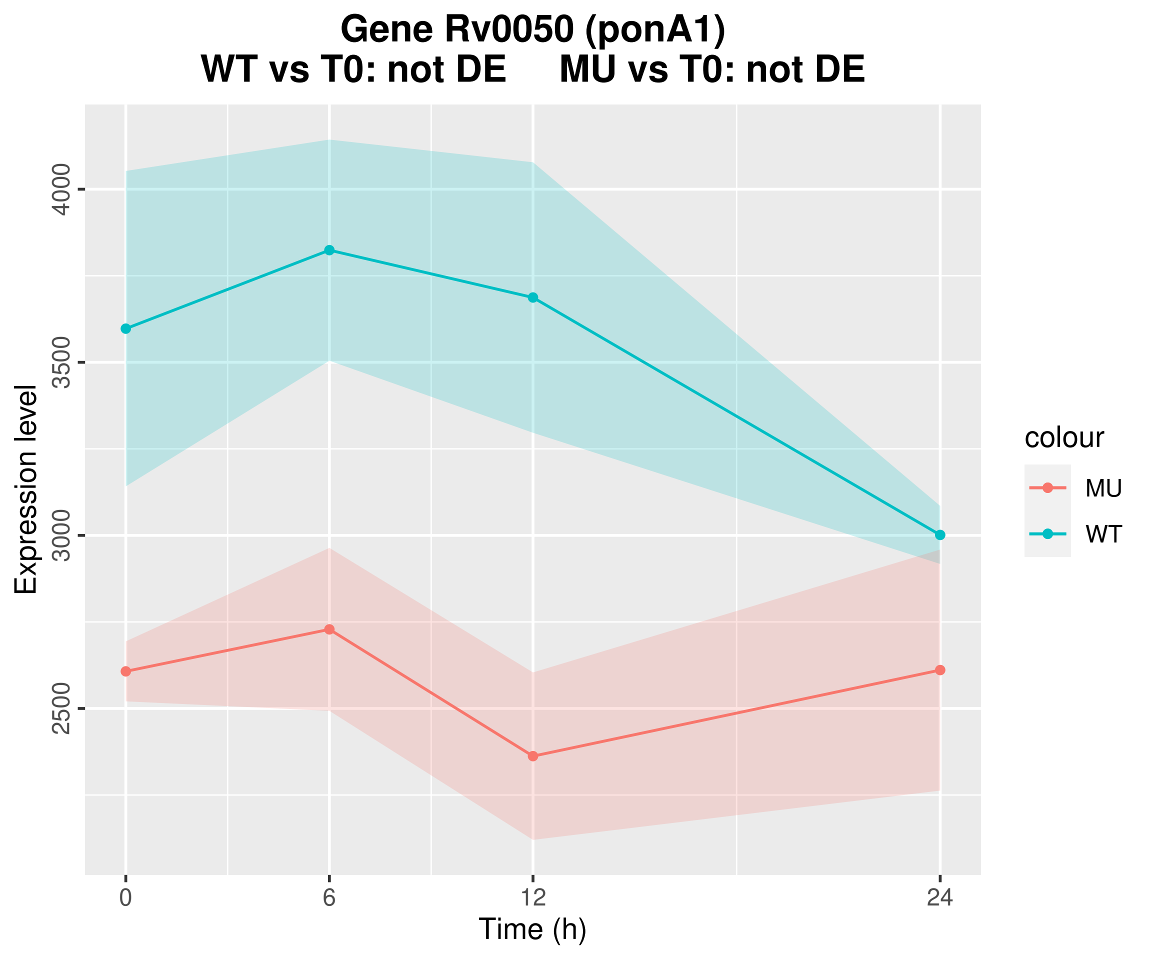
Gene Rv0050 (ponA1)
WT vs T0: not DE   MU vs T0: not DE
Expression level
4000
3500
3000
2500
0
6
12
24
Time (h)
colour
MU
WT

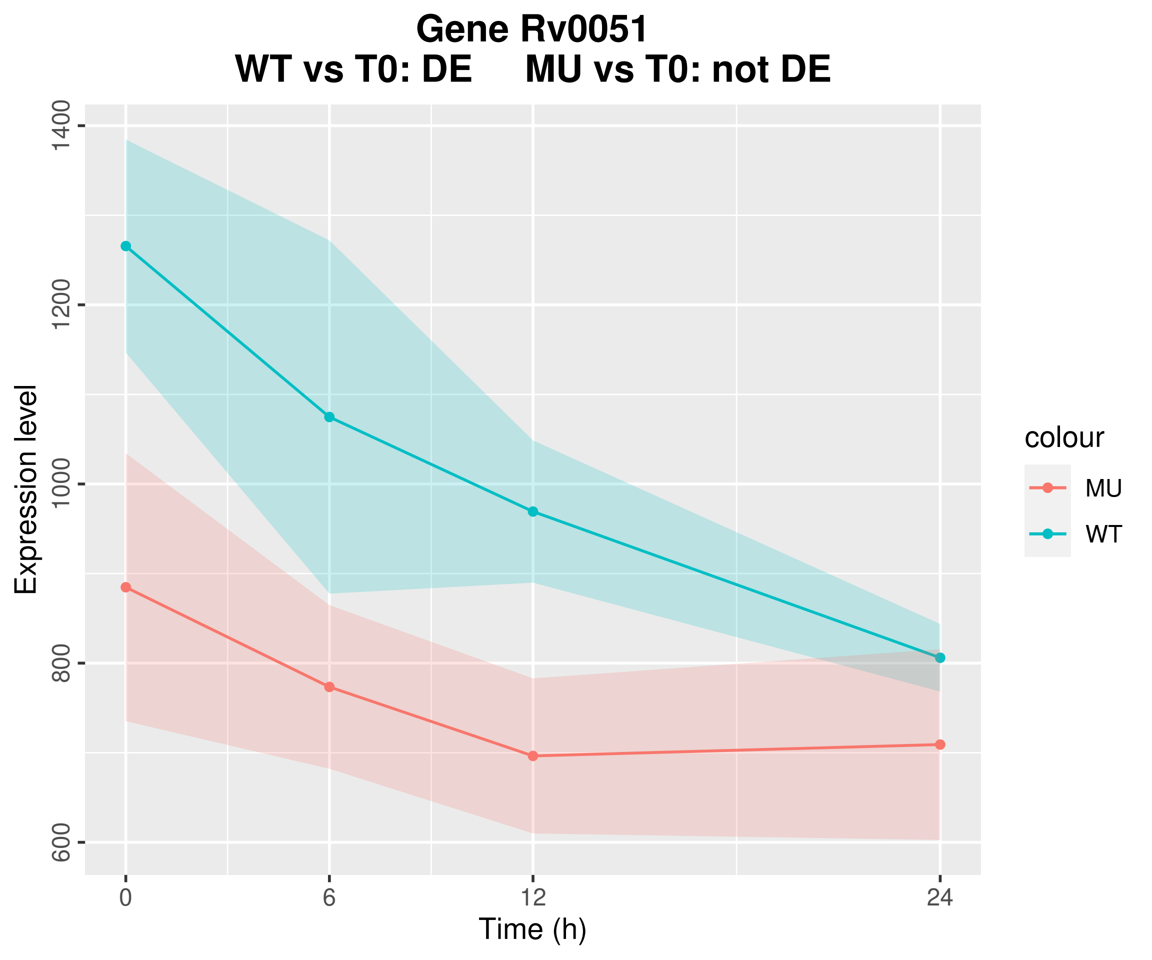

Gene Rv0051
WT vs T0: DE   MU vs T0: not DE
Expression level
1400
1200
1000
800
600
Time (h)
0
6
12
24
colour
MU
WT

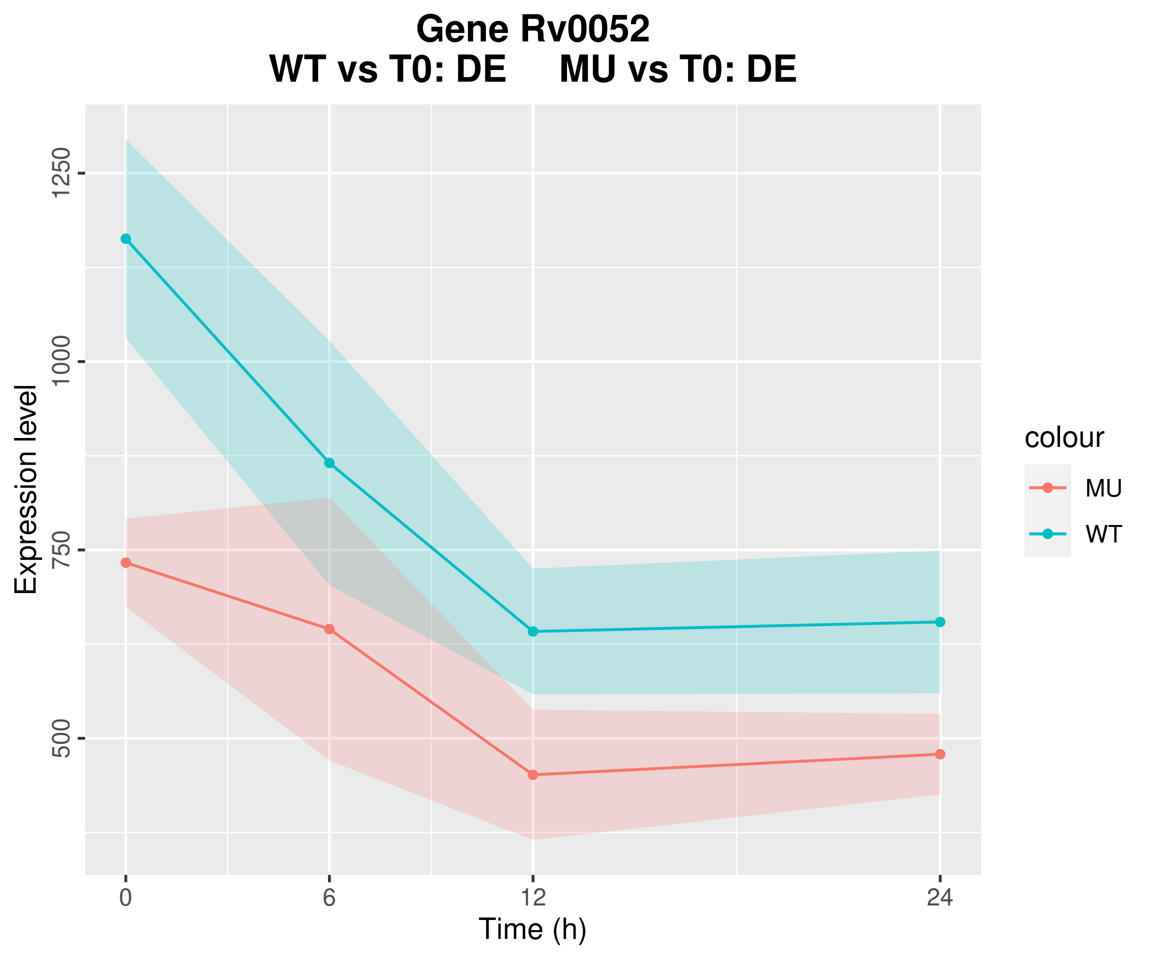
Gene Rv0052
WT vs T0: DE   MU vs T0: DE
Expression level
1250
1000
750
500
0
6
12
24
Time (h)
colour
MU
WT

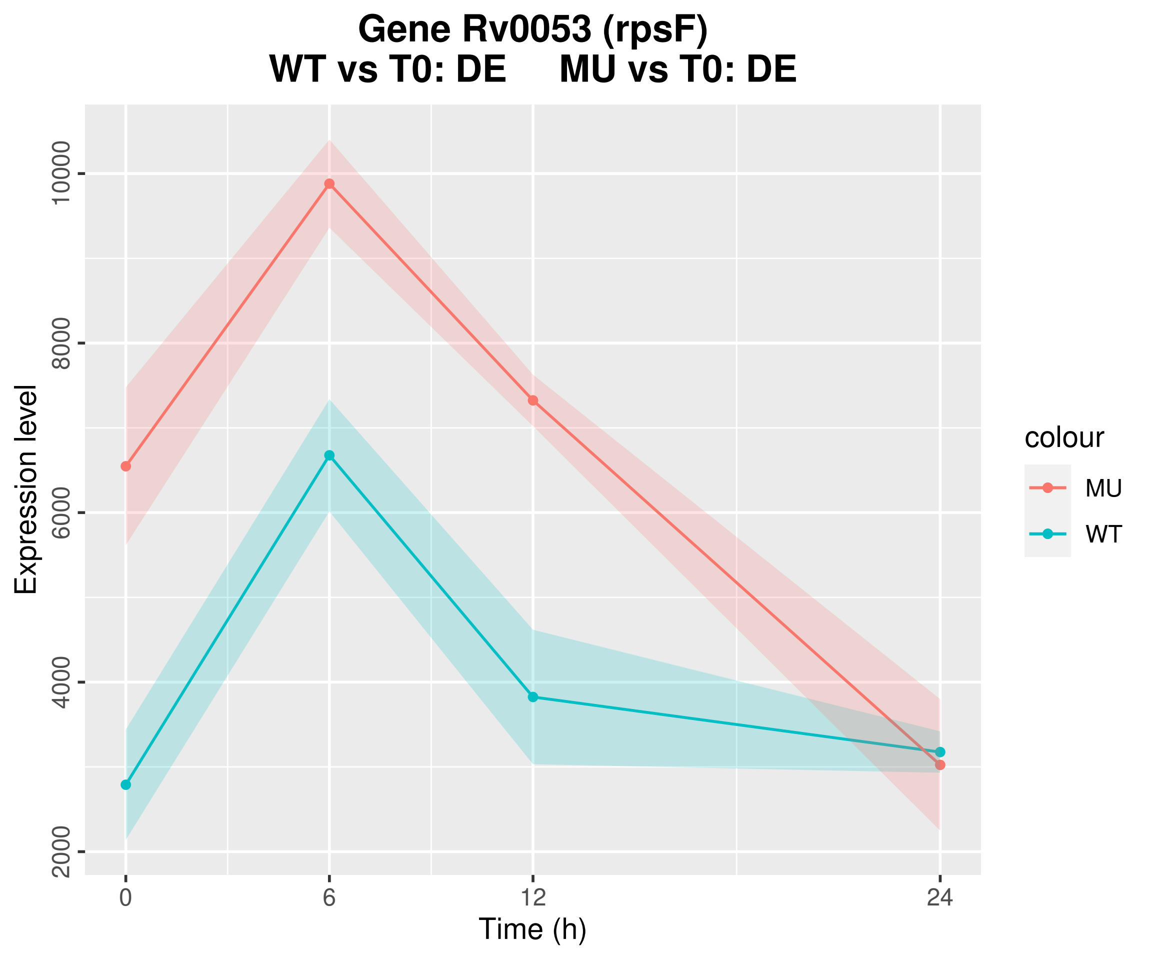

Gene Rv0053 (rpsF)
WT vs T0: DE   MU vs T0: DE
Expression level
10000
8000
6000
4000
2000
0
6
12
24
Time (h)
colour
MU
WT

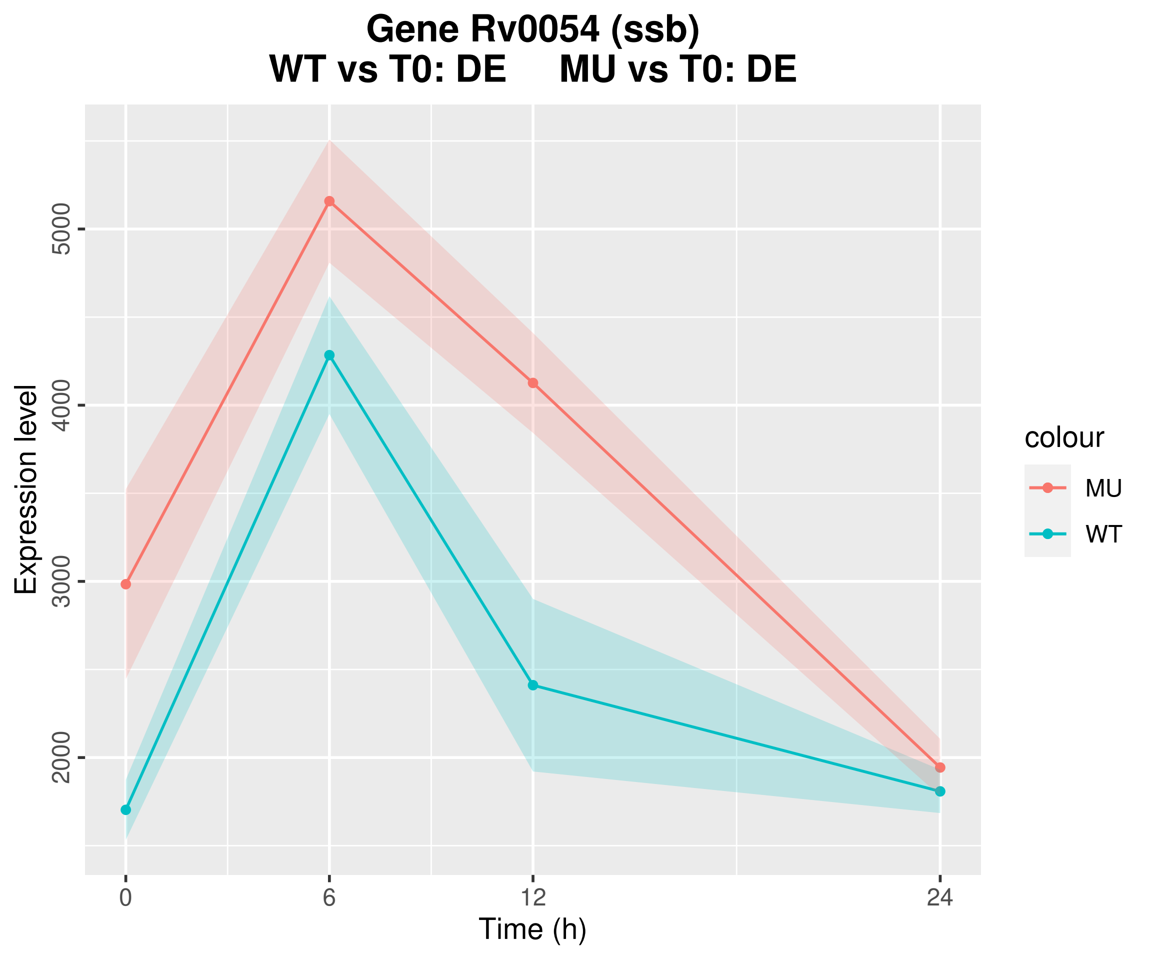
Gene Rv0054 (ssb)
WT vs T0: DE MU vs T0: DE
Expression level
5000
4000
3000
2000
0
6
12
24
Time (h)
colour
MU
WT

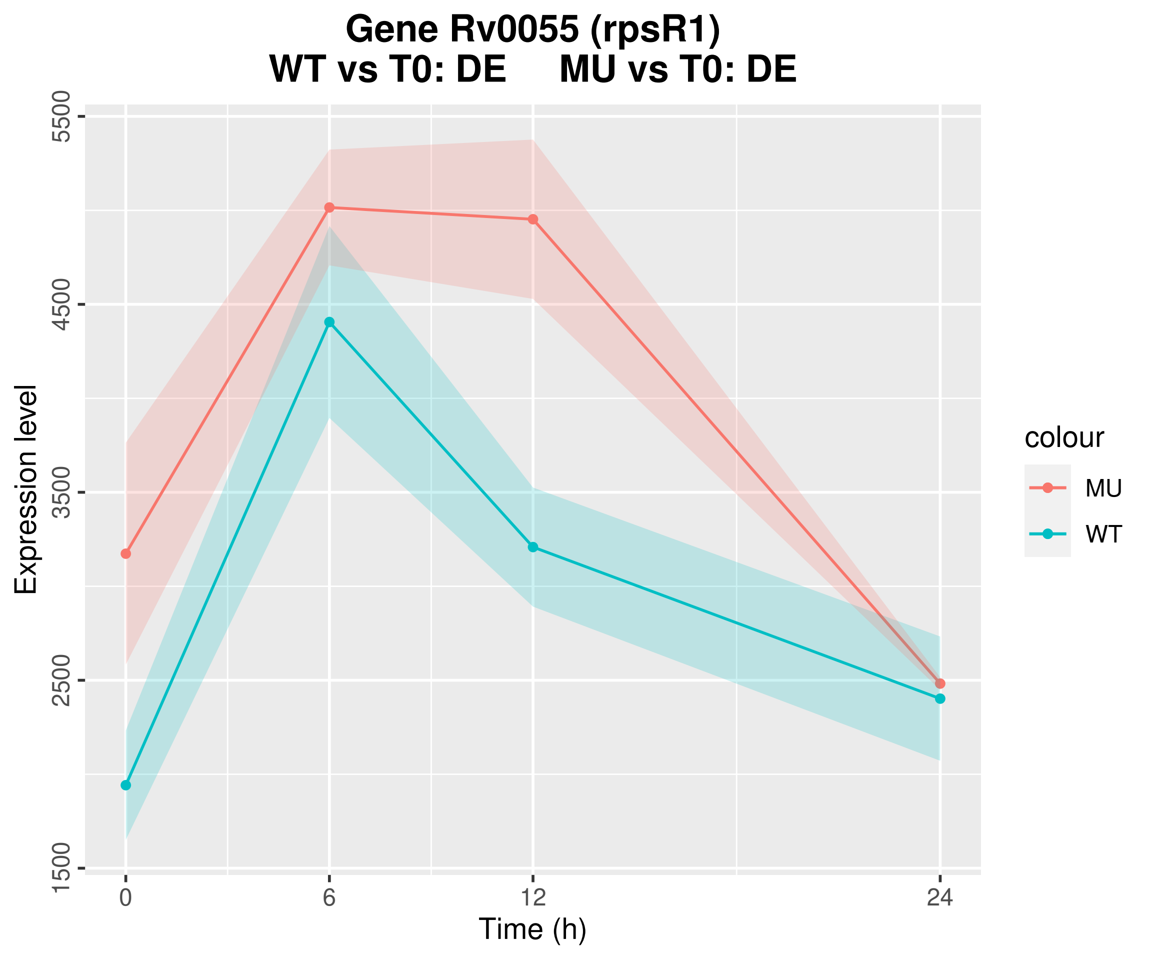

Gene Rv0055 (rpsR1)
WT vs T0: DE   MU vs T0: DE
Expression level
5500
4500
3500
2500
1500
0
6
12
24
Time (h)
colour
MU
WT

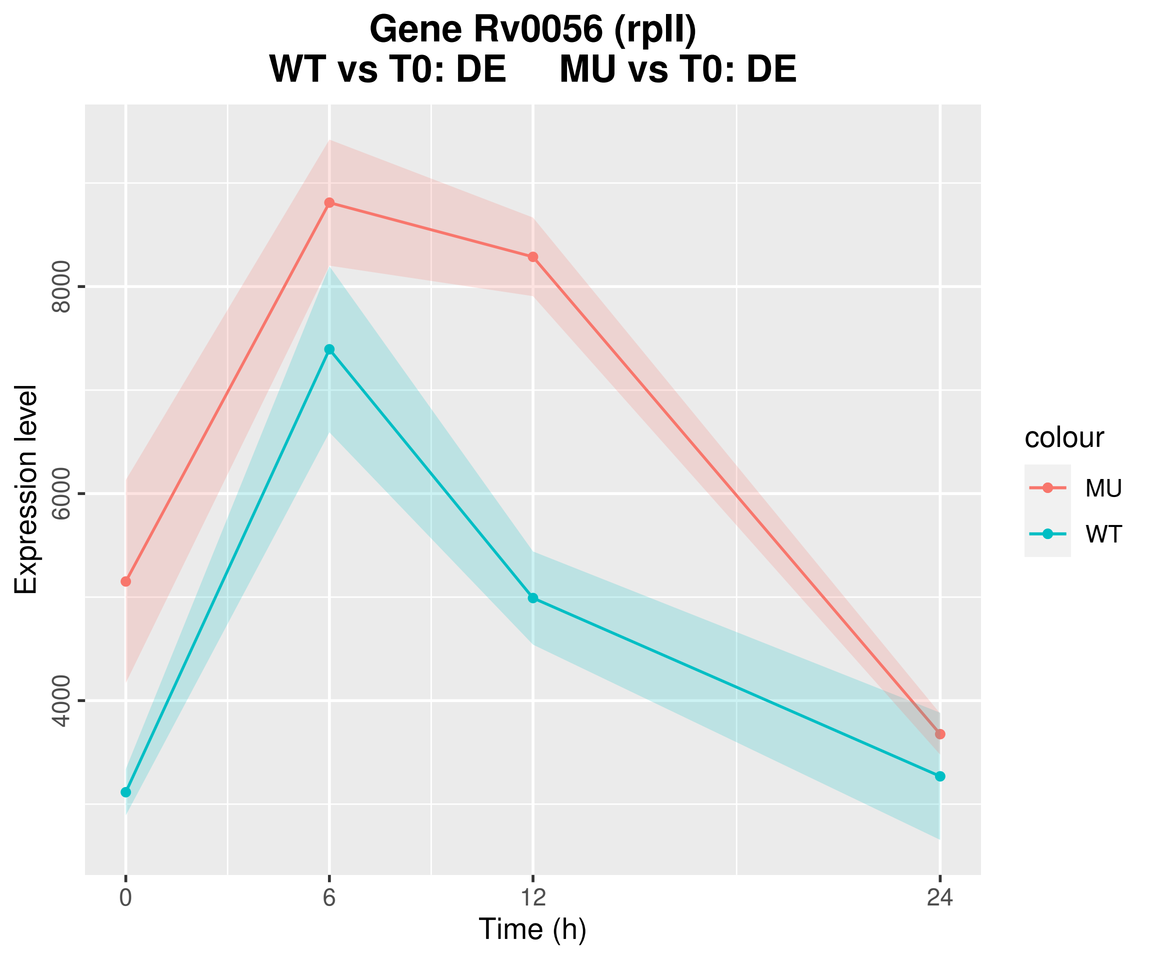

Gene Rv0056 (rplI)
WT vs T0: DE MU vs T0: DE
Expression level
8000
6000
4000
0
6
12
24
Time (h)
colour
MU
WT

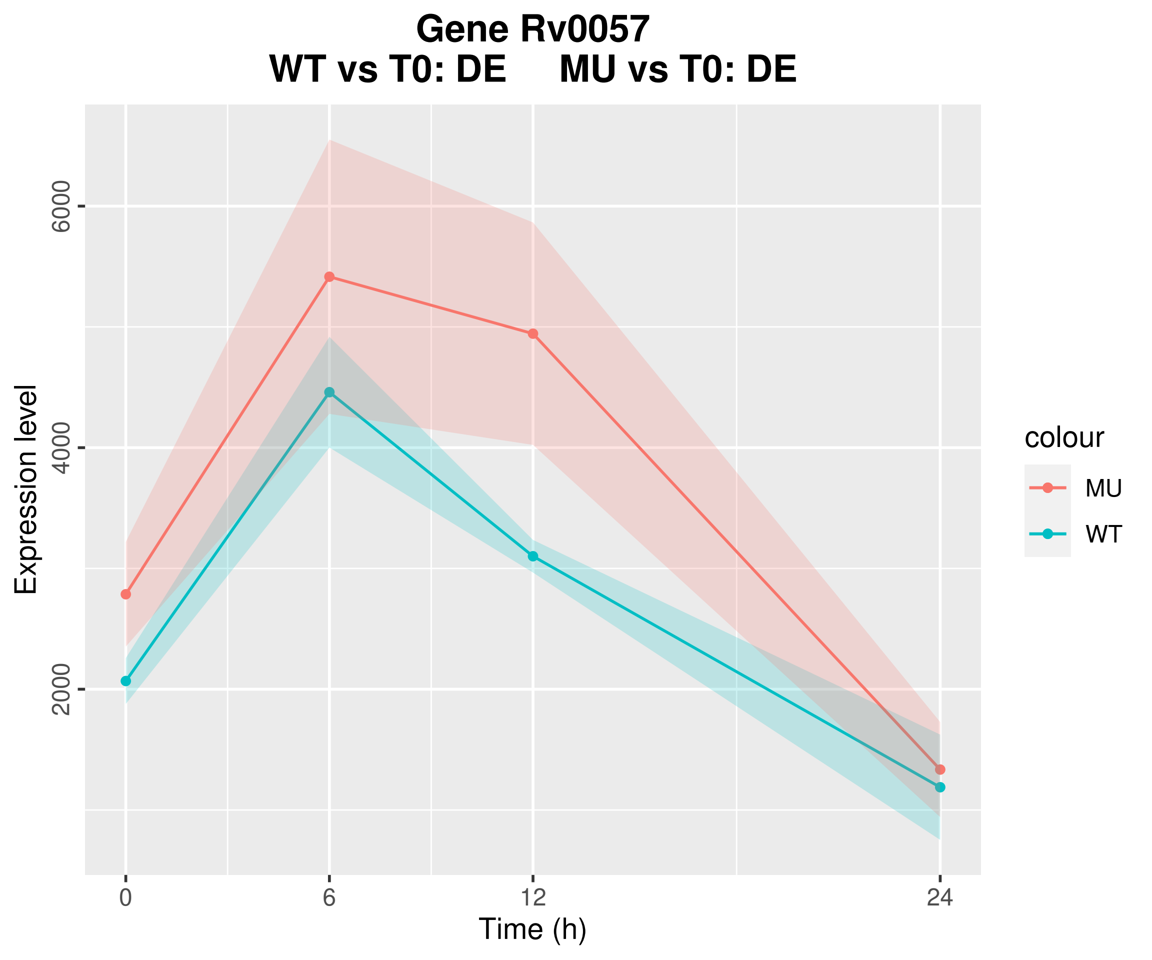
Gene Rv0057
WT vs T0: DE MU vs T0: DE
6000
4000
2000
Expression level
0
6
12
24
Time (h)
colour
MU
WT

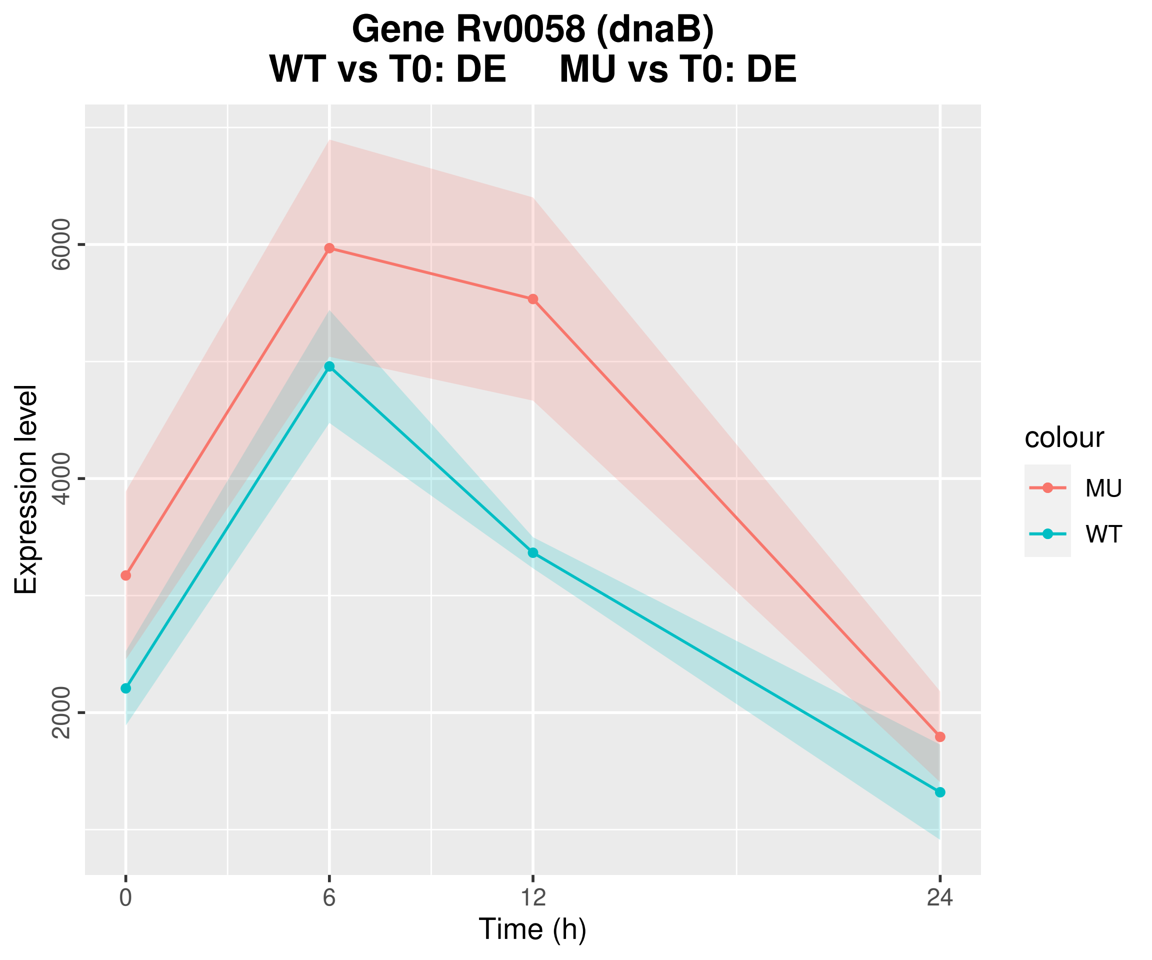

Gene Rv0058 (dnaB)
WT vs T0: DE   MU vs T0: DE
Expression level
6000
4000
2000
0
6
12
24
Time (h)
colour
MU
WT

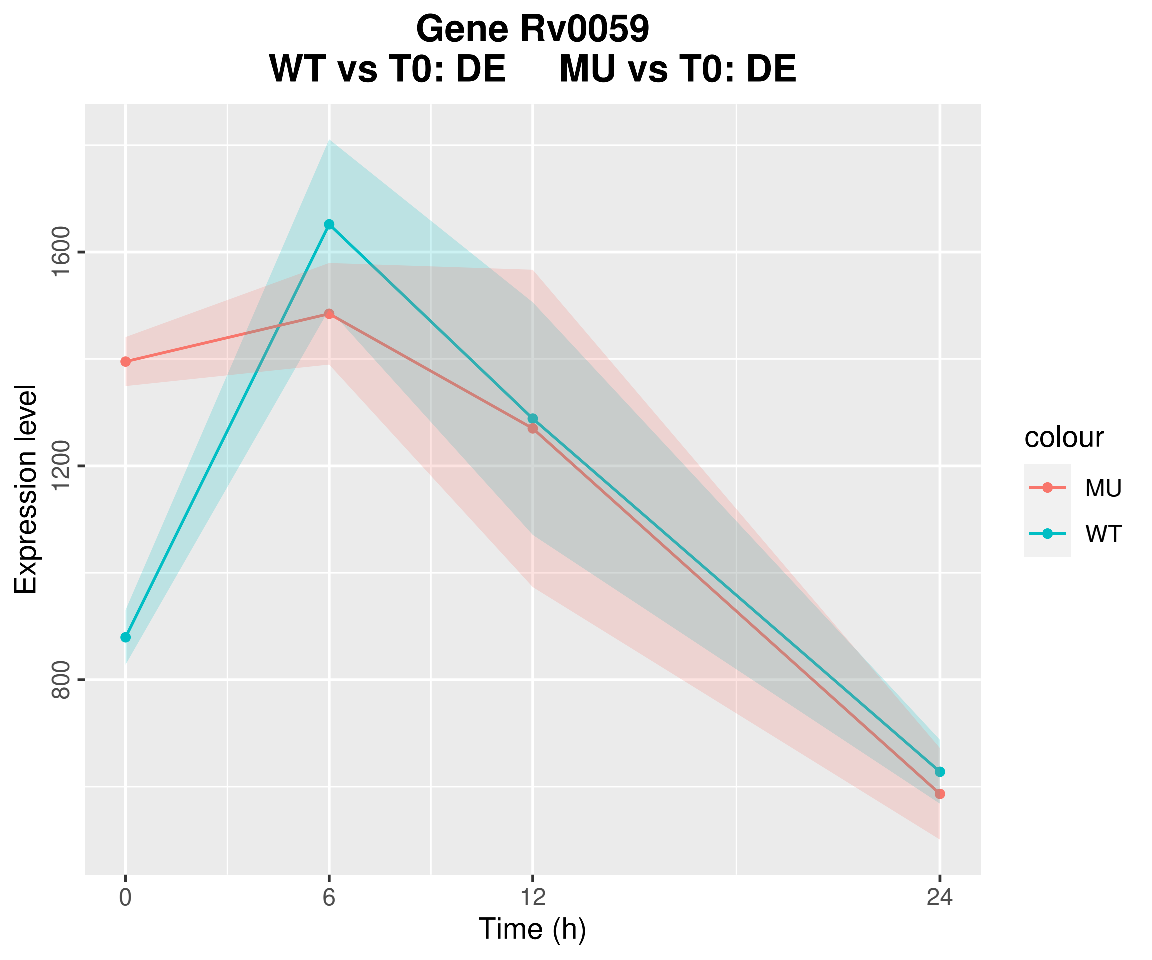

Gene Rv0059
WT vs T0: DE   MU vs T0: DE
1600
1200
800
Expression level
0
6
12
24
Time (h)
colour
MU
WT

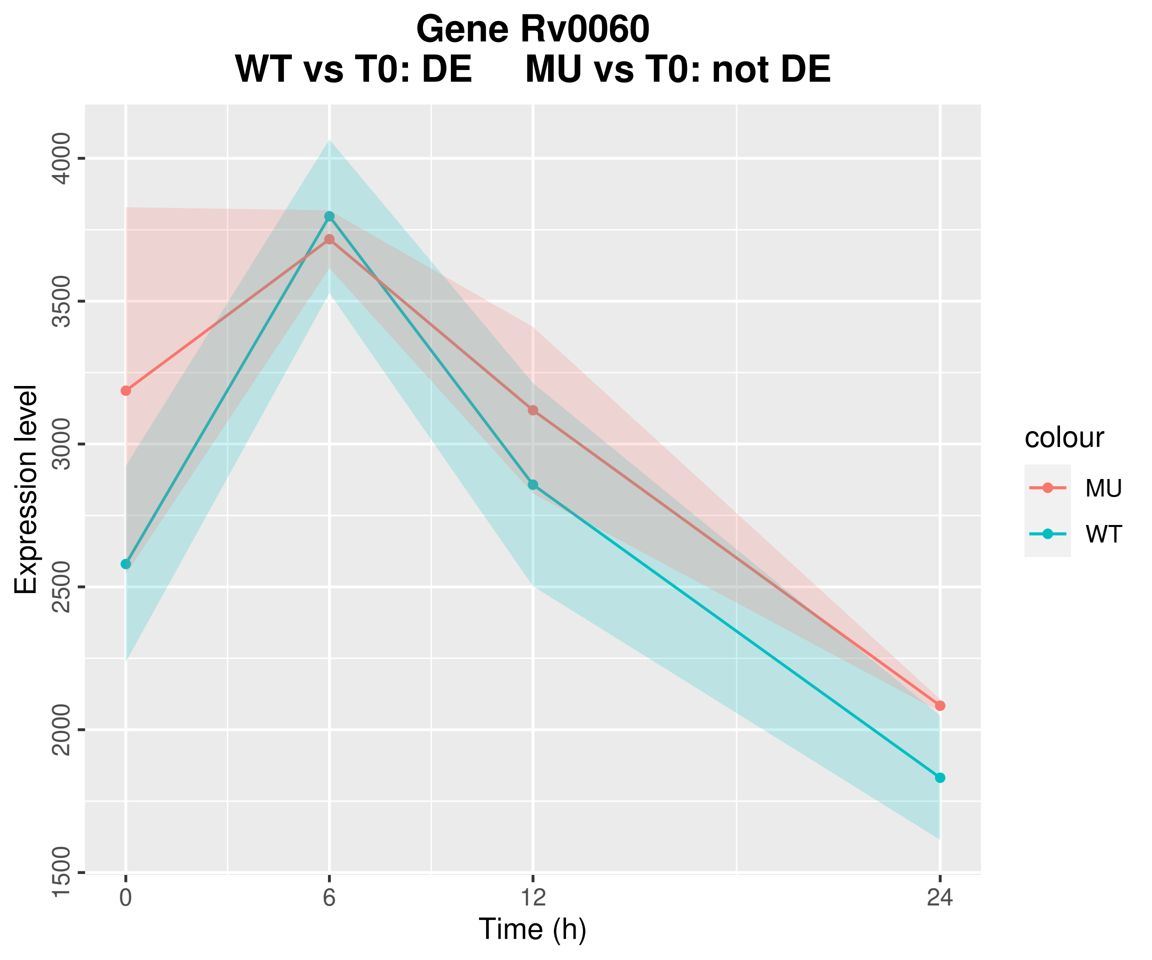
Gene Rv0060
WT vs T0: DE   MU vs T0: not DE
Expression level
4000
3500
3000
2500
2000
1500
0
6
12
24
Time (h)
colour
MU
WT

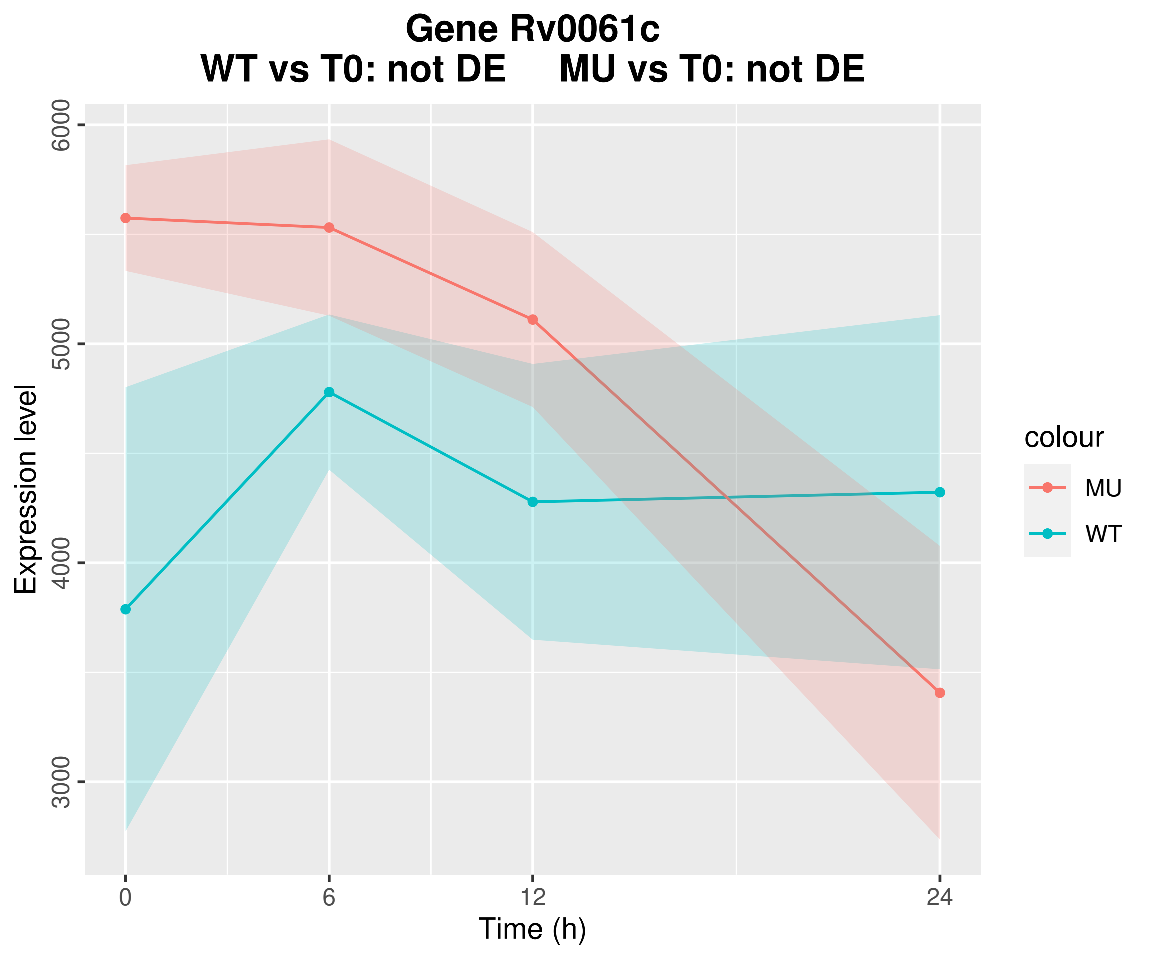
Gene Rv0061c
WT vs T0: not DE MU vs T0: not DE
Expression level
6000
5000
4000
3000
0
6
12
24
Time (h)
colour
MU
WT

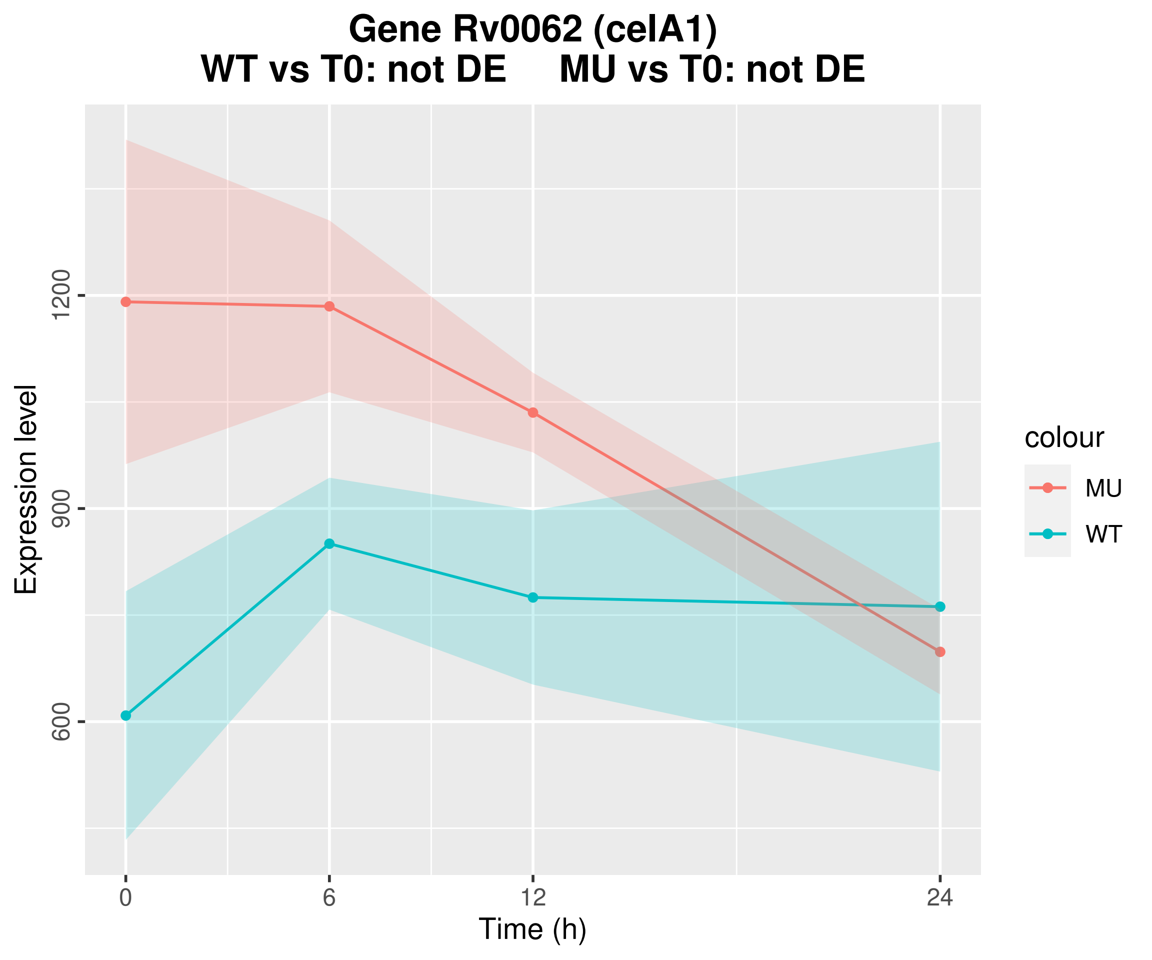

Gene Rv0062 (celA1)
WT vs T0: not DE   MU vs T0: not DE
Expression level
1200
900
600
0
6
12
24
Time (h)
colour
MU
WT

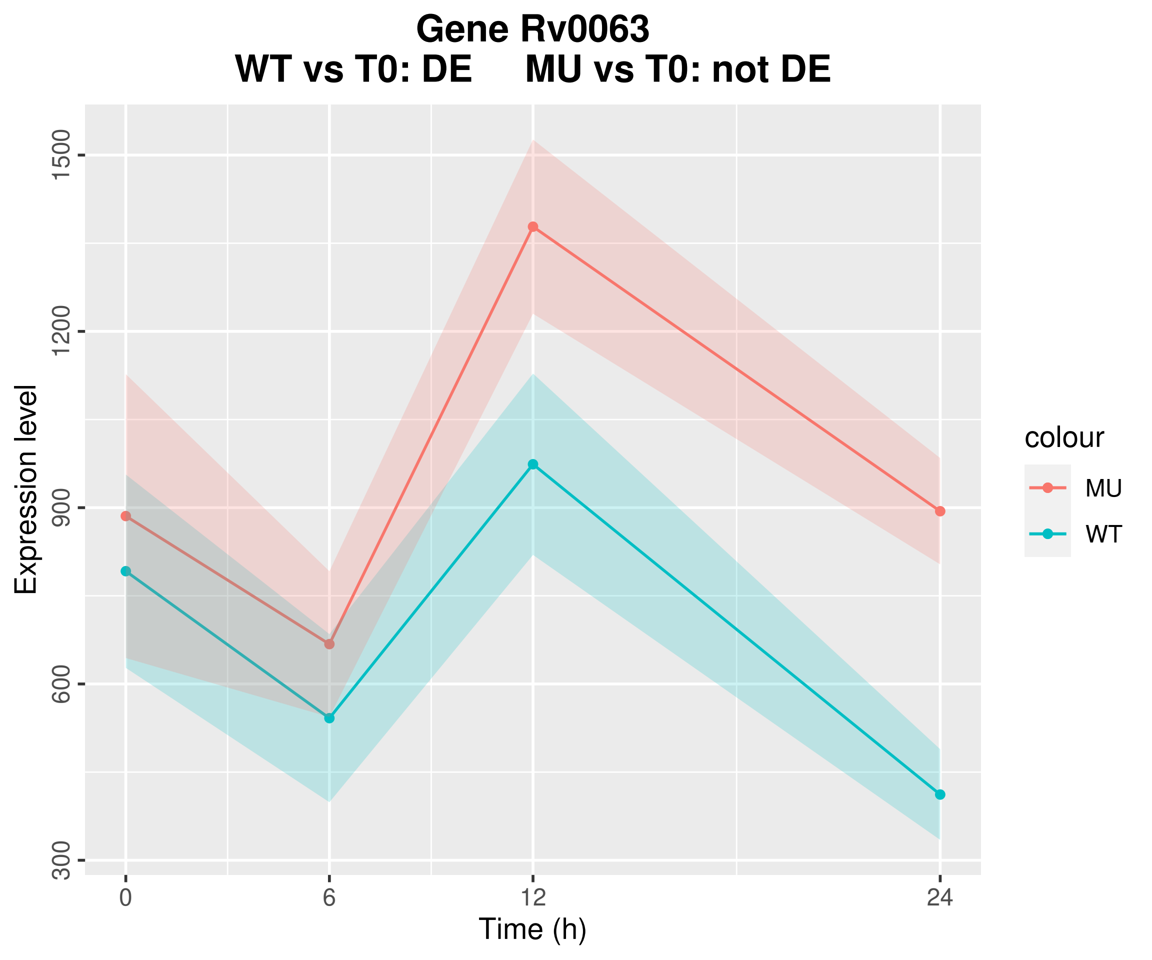

Gene Rv0063
WT vs T0: DE   MU vs T0: not DE
1500
1200
900
600
300
Expression level
0
6
12
24
Time (h)
colour
MU
WT

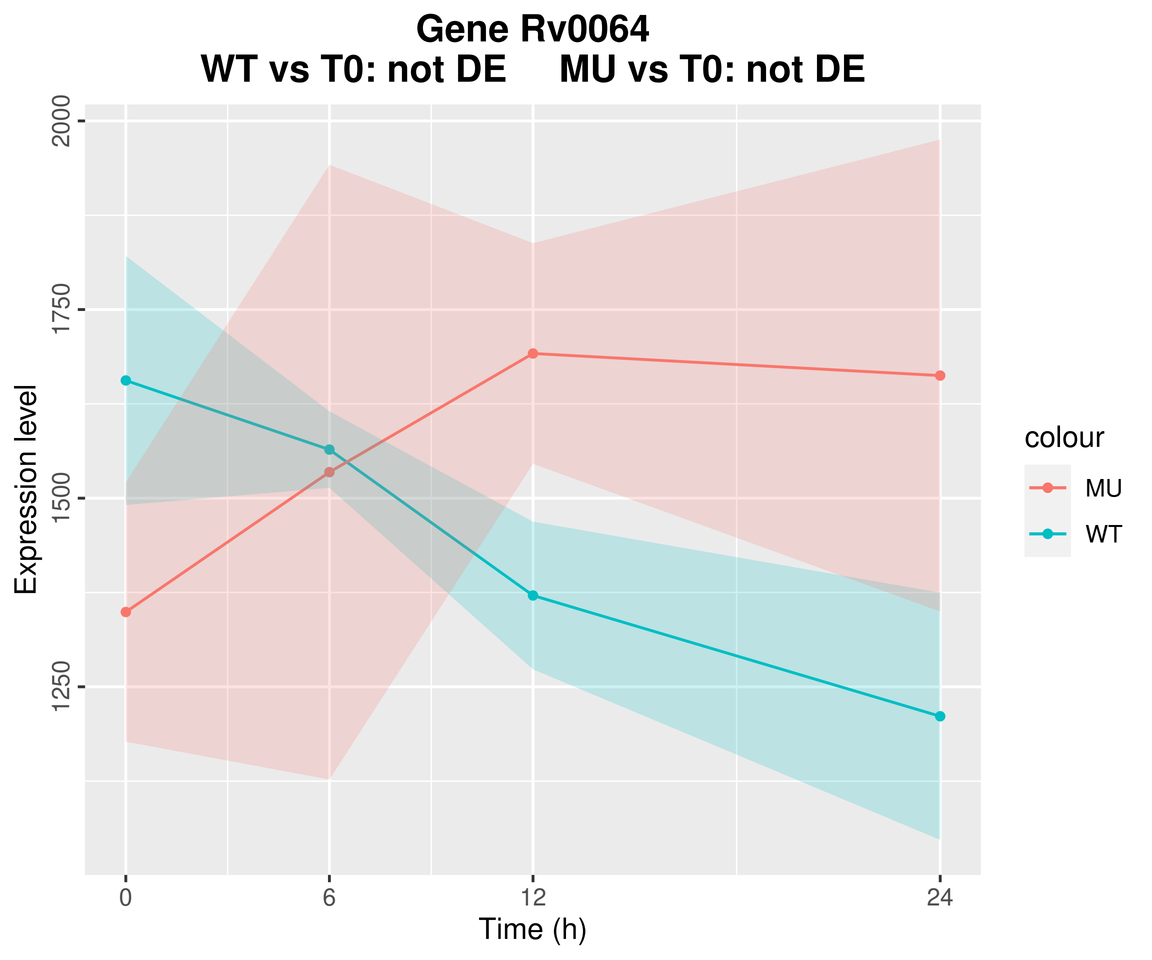
Gene Rv0064
WT vs T0: not DE MU vs T0: not DE
Expression level
2000
1750
1500
1250
0
6
12
24
Time (h)
colour
MU
WT

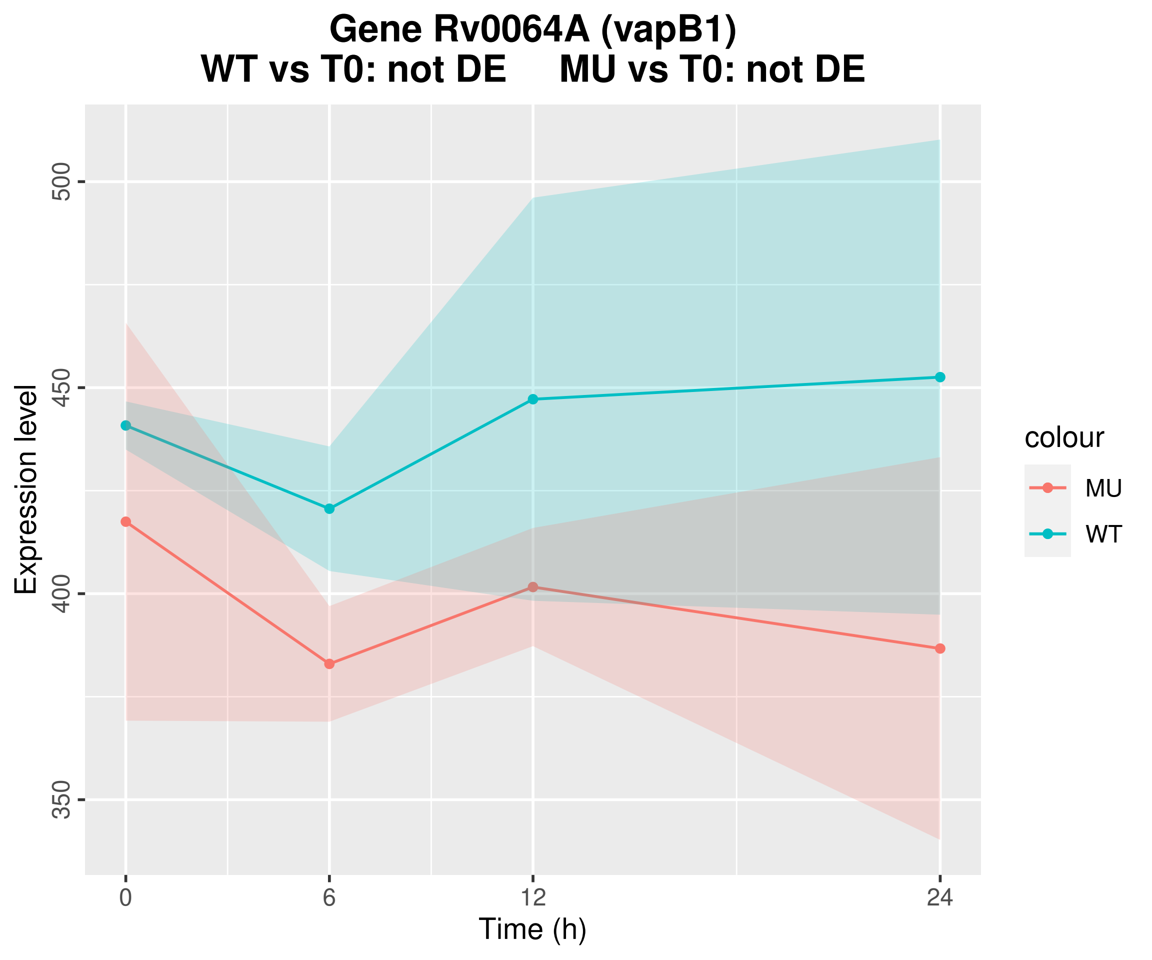

Gene Rv0064A (vapB1)
WT vs T0: not DE MU vs T0: not DE
Expression level
500
450
400
350
0
6
12
24
Time (h)
colour
MU
WT

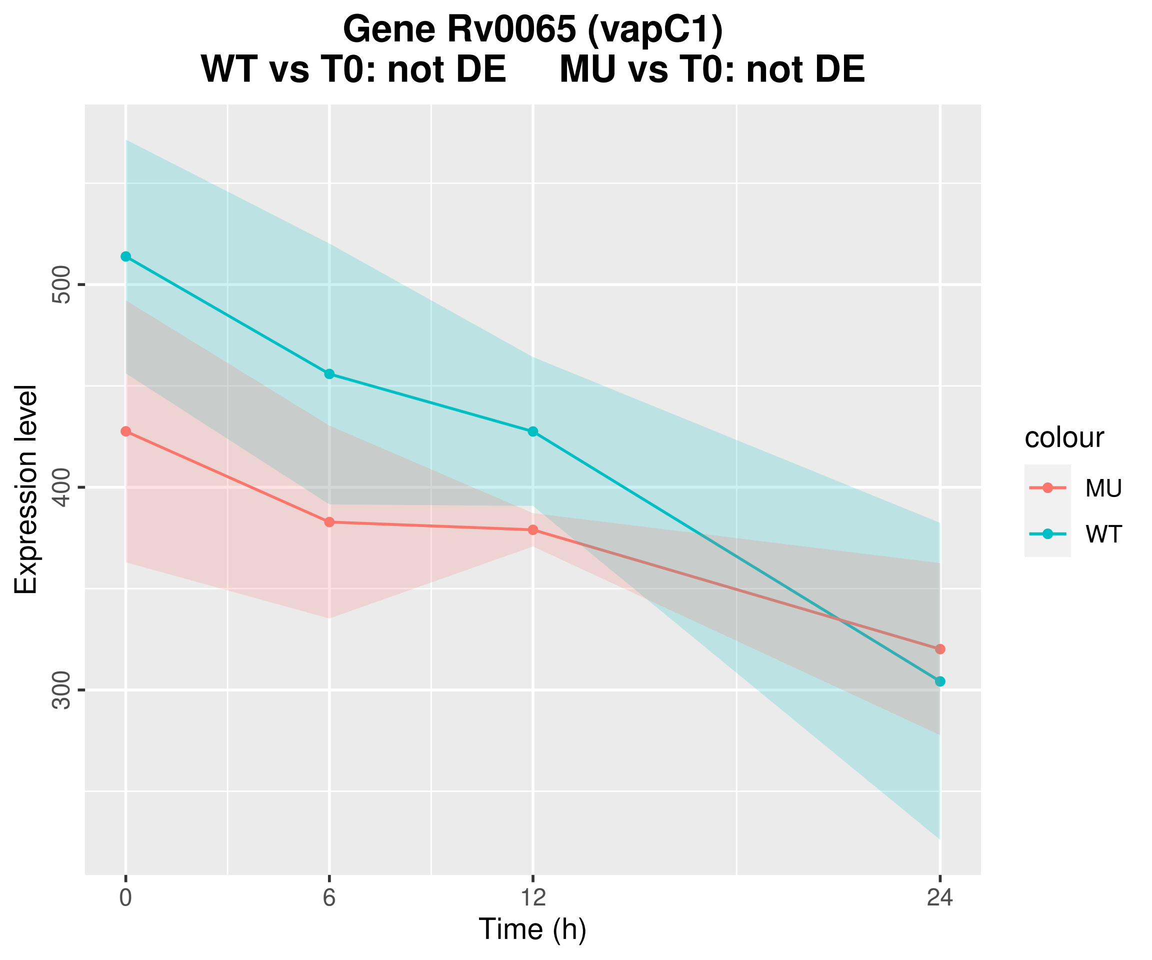

Gene Rv0065 (vapC1)
WT vs T0: not DE MU vs T0: not DE
Expression level
500
400
300
0
6
12
24
Time (h)
colour
MU
WT

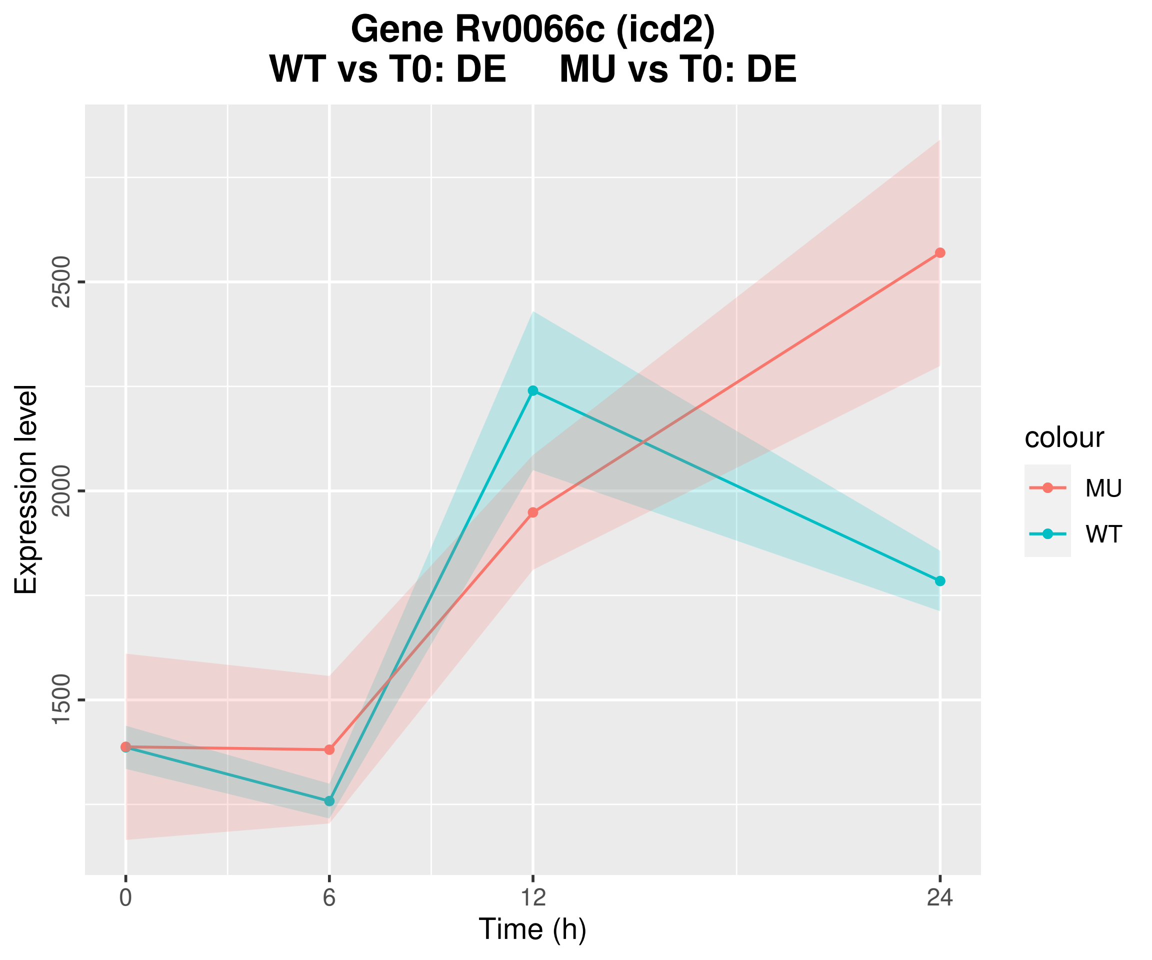

Gene Rv0066c (icd2)
WT vs T0: DE MU vs T0: DE
Expression level
2500
2000
1500
0
6
12
24
Time (h)
colour
MU
WT

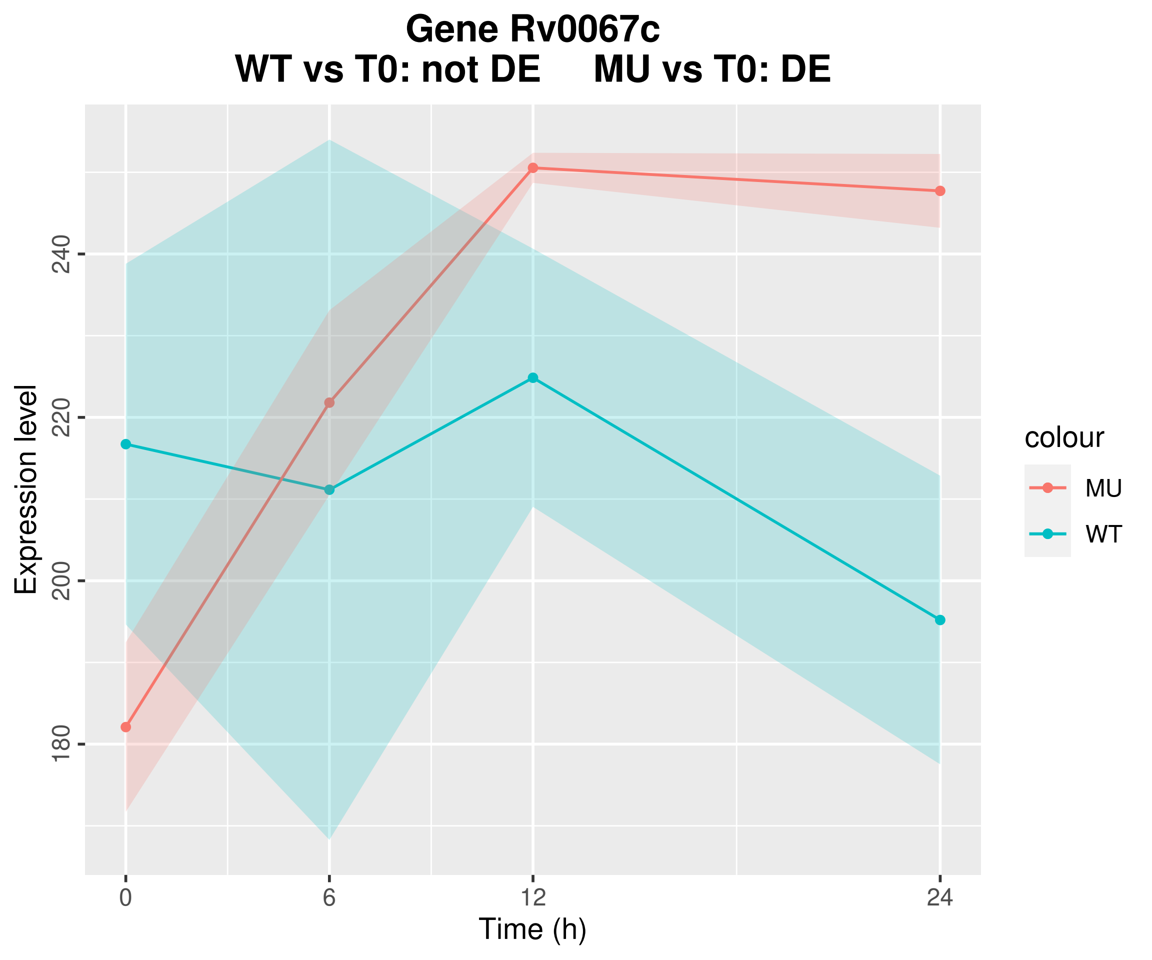

Gene Rv0067c
WT vs T0: not DE MU vs T0: DE
Expression level
180
200
220
240
0
6
12
24
Time (h)
colour
MU
WT

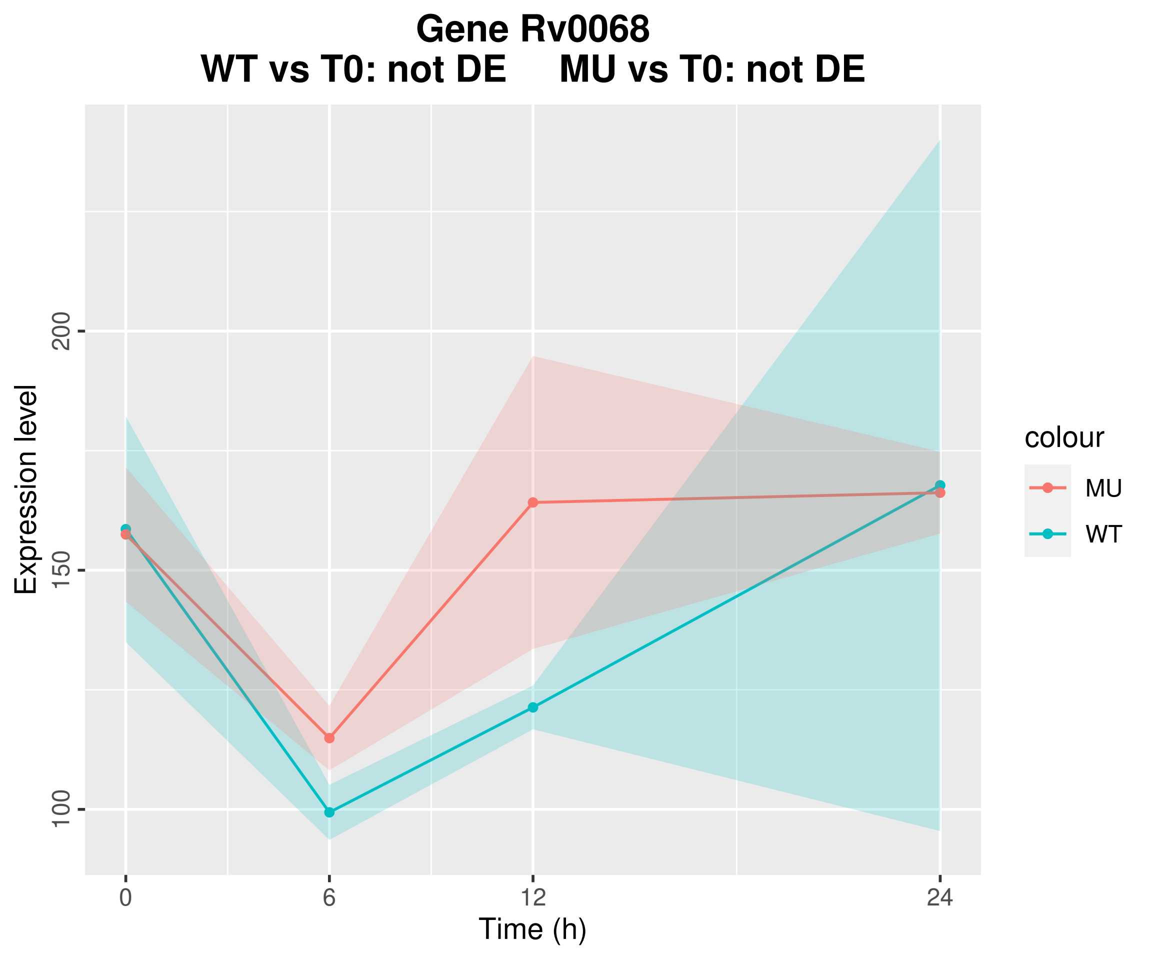

Gene Rv0068
WT vs T0: not DE   MU vs T0: not DE
Expression level
100
150
200
0
6
12
24
Time (h)
colour
MU
WT

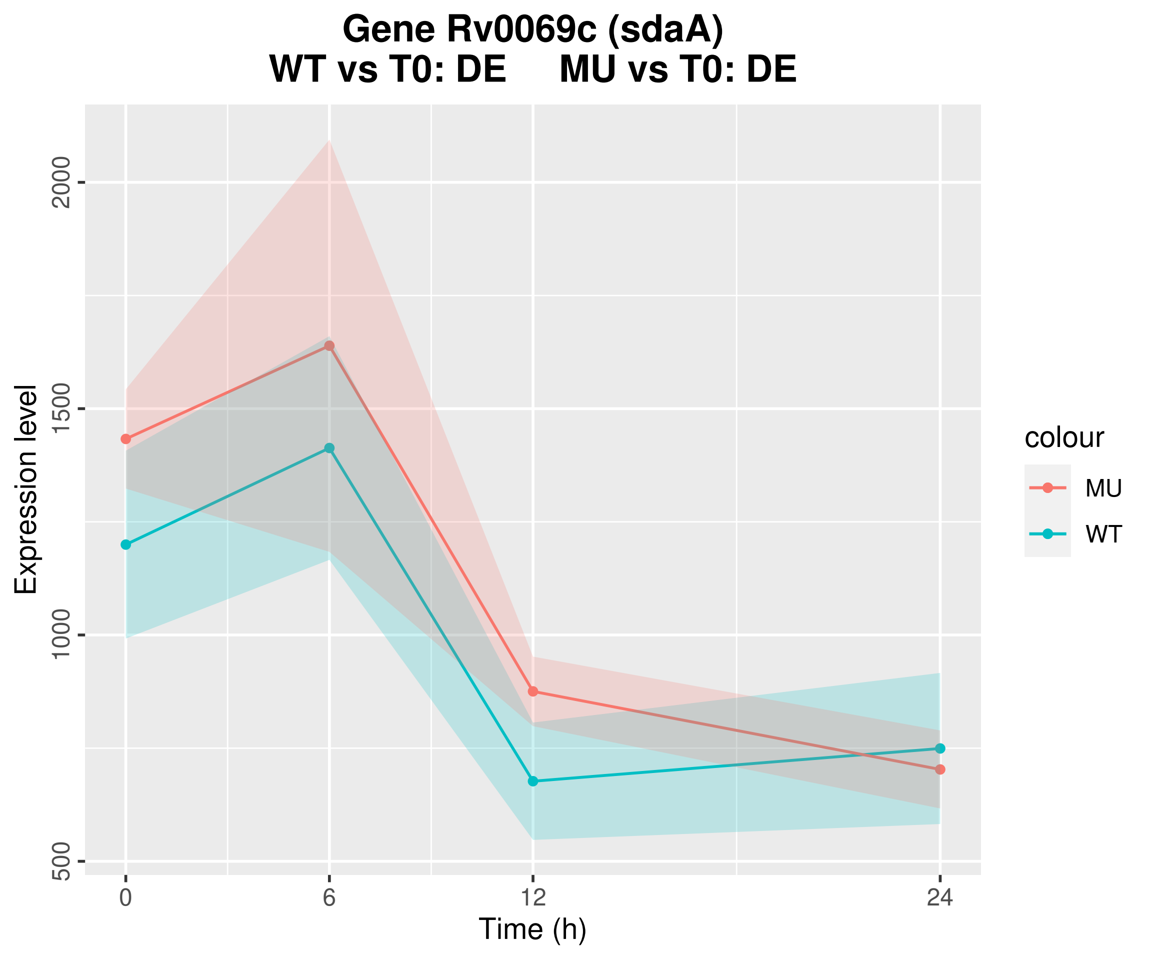

Gene Rv0069c (sdaA)
WT vs T0: DE MU vs T0: DE
2000
1500
1000
500
Expression level
0
6
12
24
Time (h)
colour
MU
WT

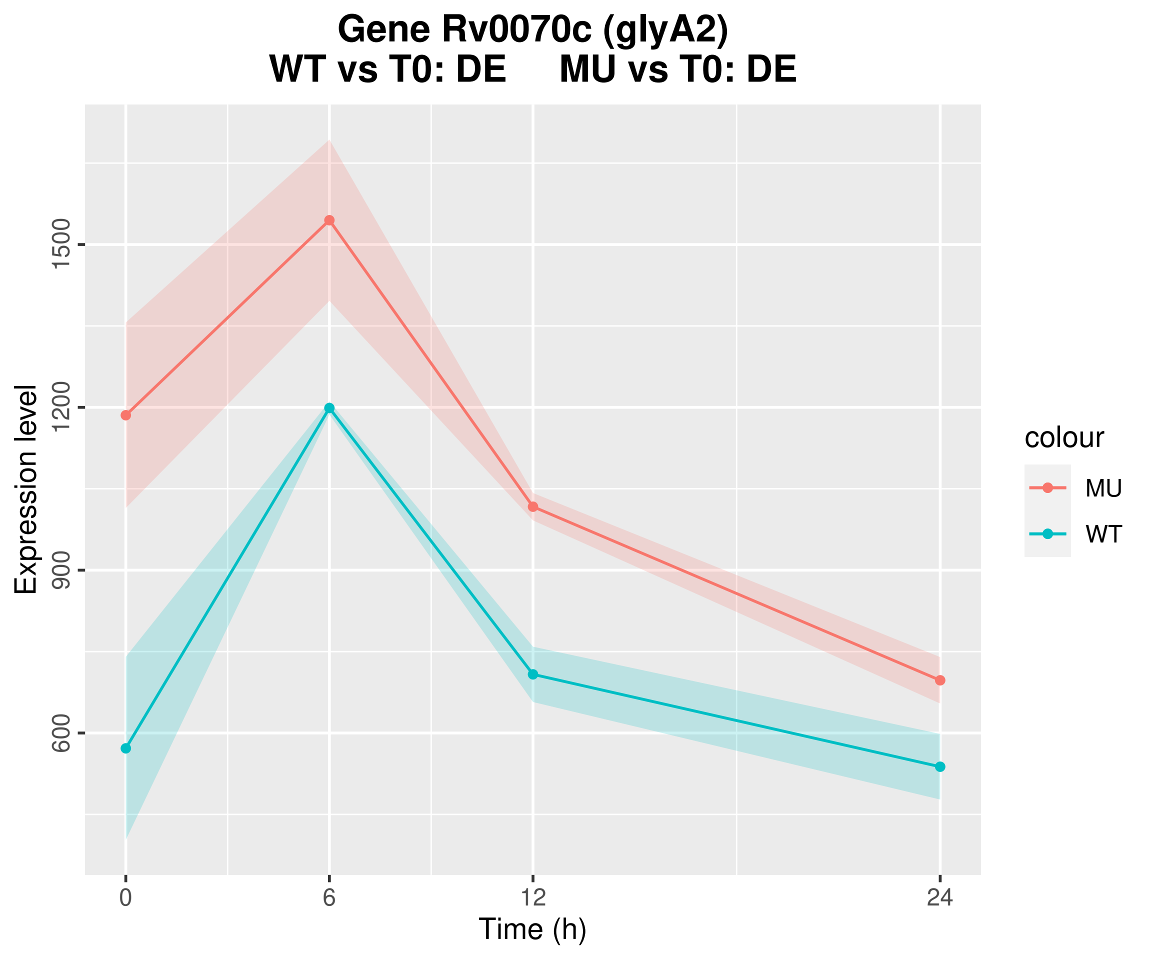

Gene Rv0070c (glyA2)
WT vs T0: DE   MU vs T0: DE
1500
1200
900
600
Expression level
0
6
12
24
Time (h)
colour
MU
WT

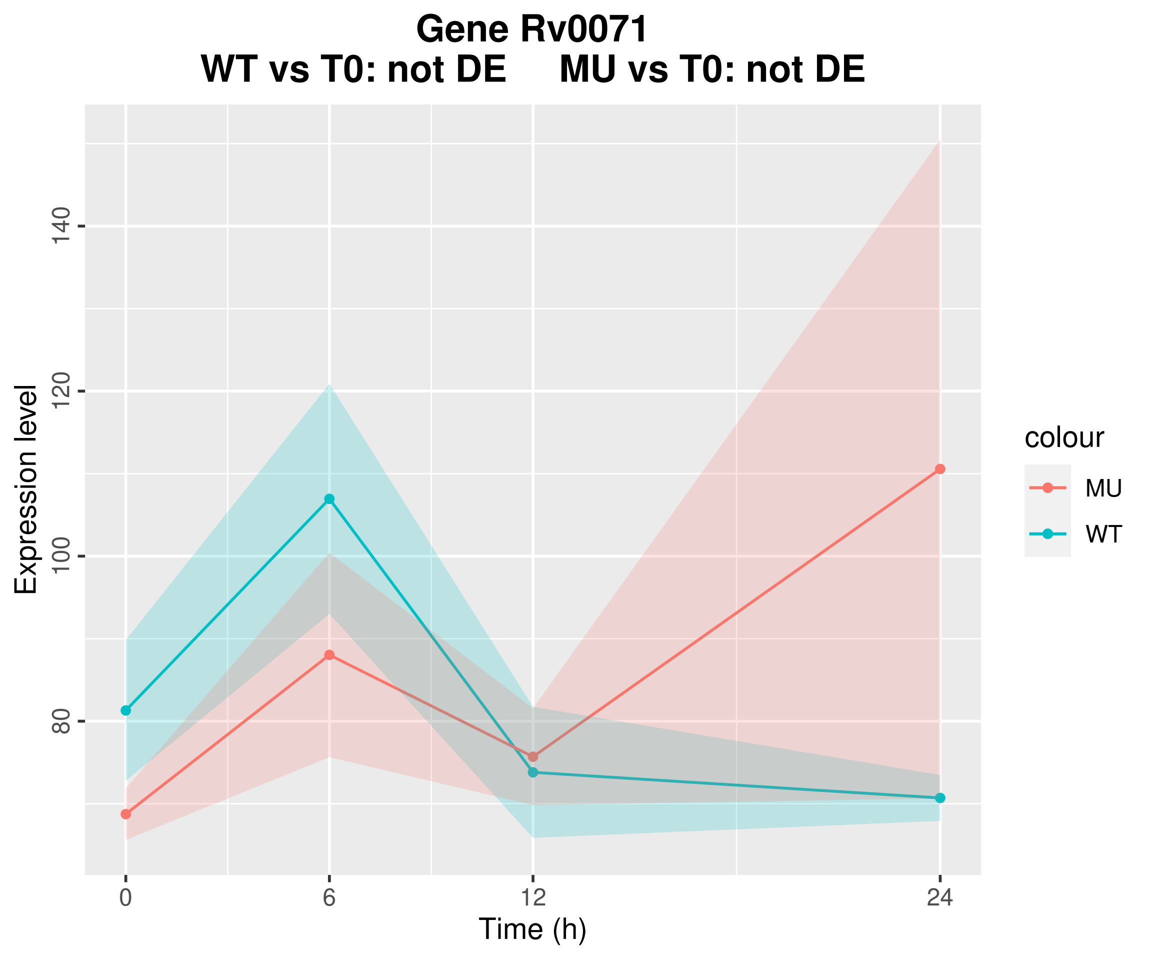

Gene Rv0071
WT vs T0: not DE MU vs T0: not DE
Expression level
140
120
100
80
0
6
12
24
Time (h)
colour
MU
WT

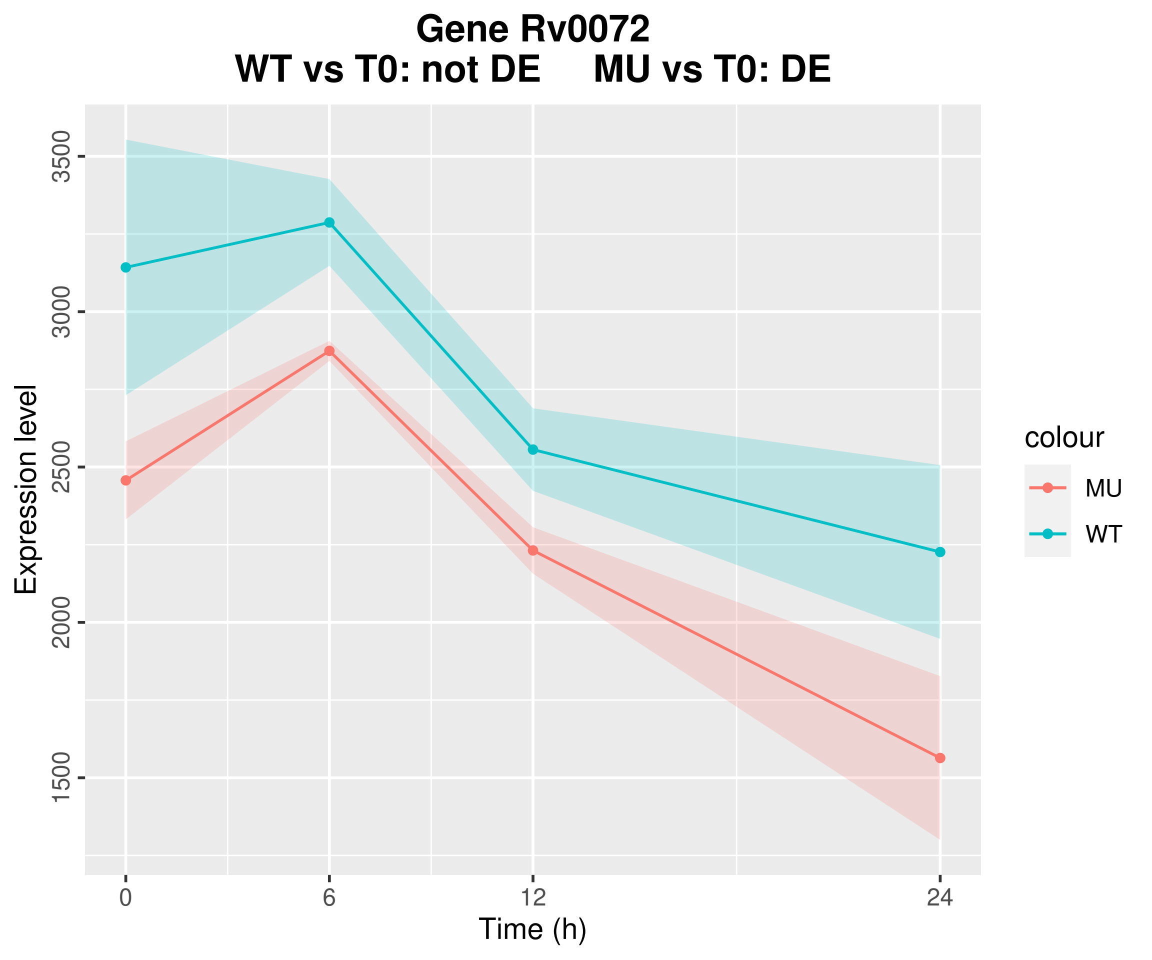

Gene Rv0072
WT vs T0: not DE MU vs T0: DE
Expression level
3500
3000
2500
2000
1500
0
6
12
24
Time (h)
colour
MU
WT

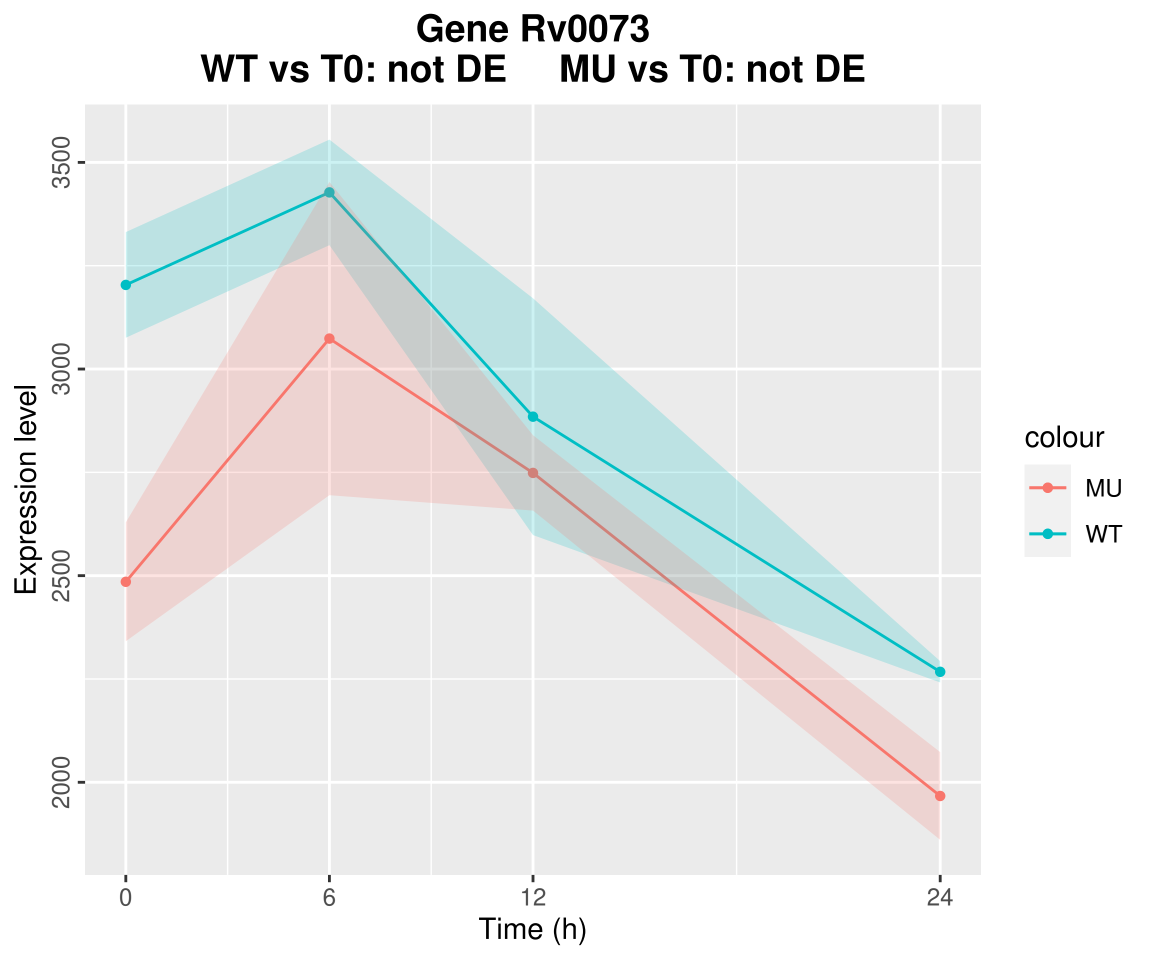
Gene Rv0073
WT vs T0: not DE MU vs T0: not DE
Expression level
3500
3000
2500
2000
0
6
12
24
Time (h)
colour
MU
WT

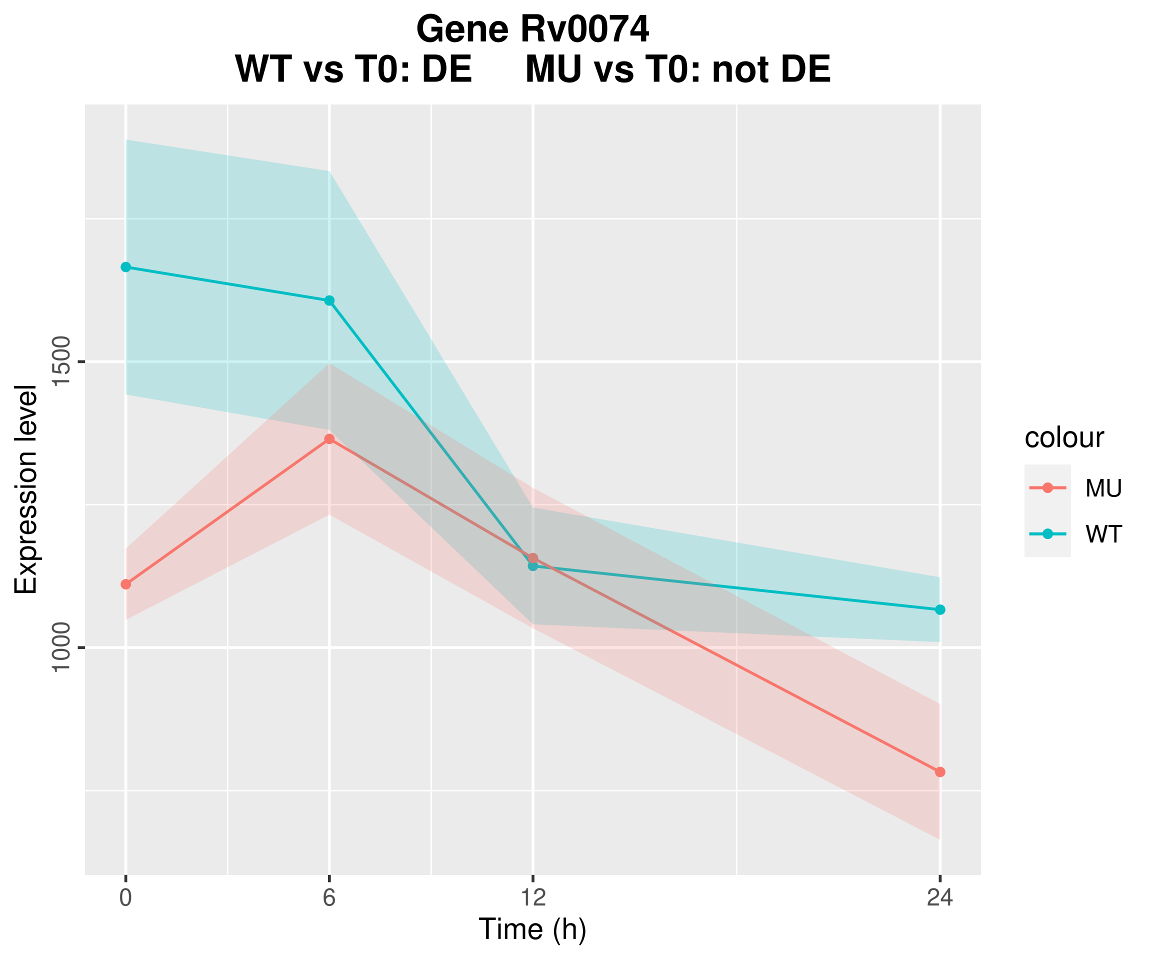

Gene Rv0074
WT vs T0: DE   MU vs T0: not DE
Expression level
1500
1000
0
6
12
24
Time (h)
colour
MU
WT

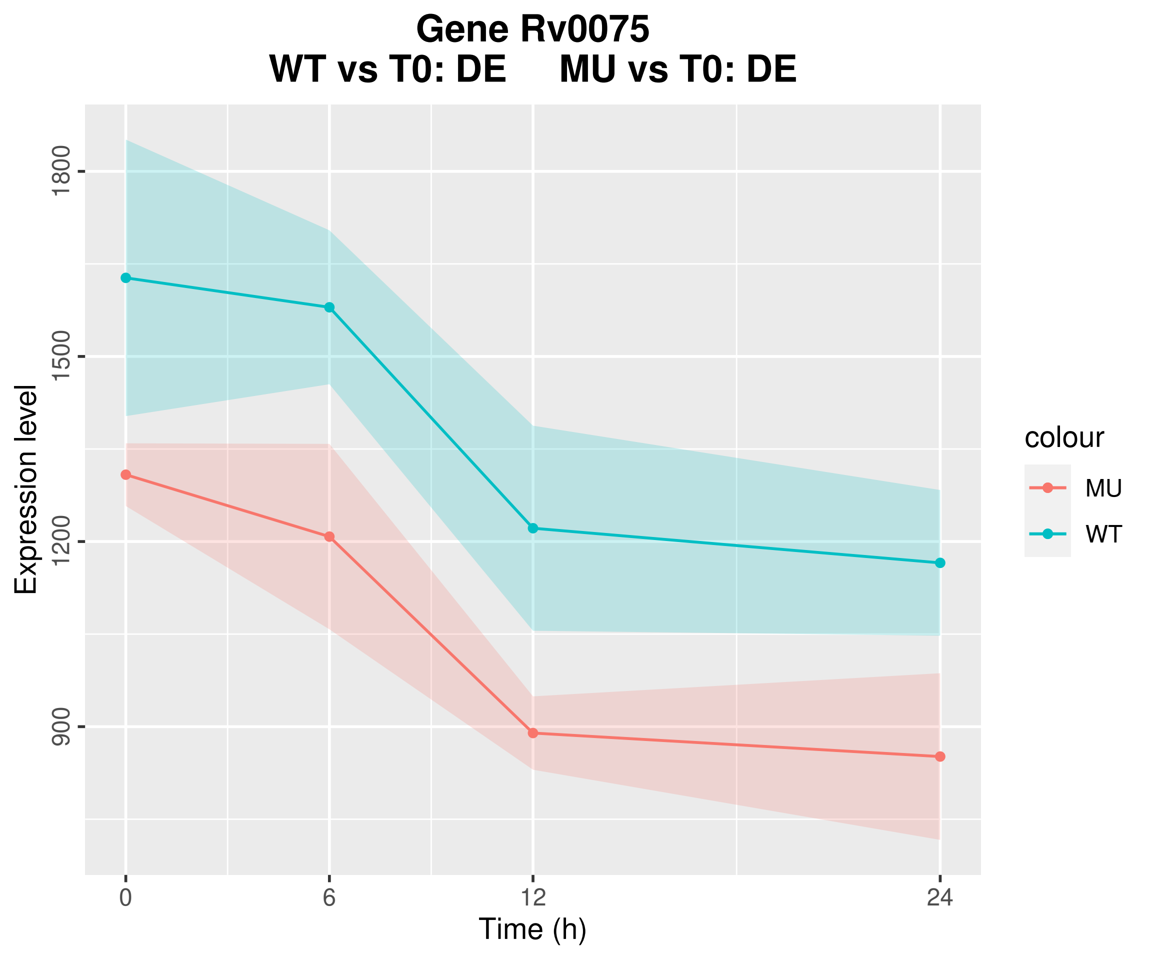

Gene Rv0075
WT vs T0: DE MU vs T0: DE
Expression level
1800
1500
1200
900
0
6
12
24
Time (h)
colour
MU
WT

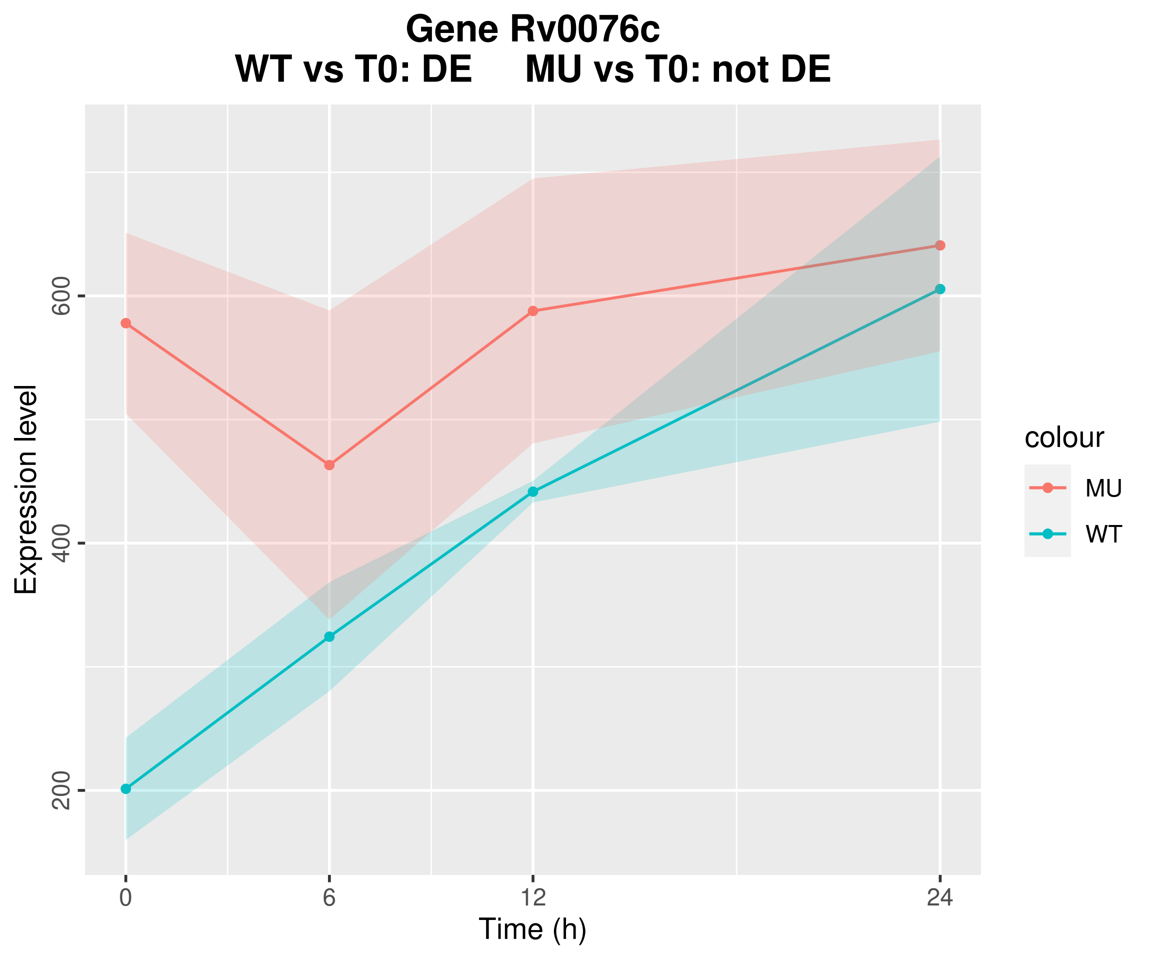

Gene Rv0076c
WT vs T0: DE MU vs T0: not DE
Expression level
200
400
600
0
6
12
24
Time (h)
colour
MU
WT

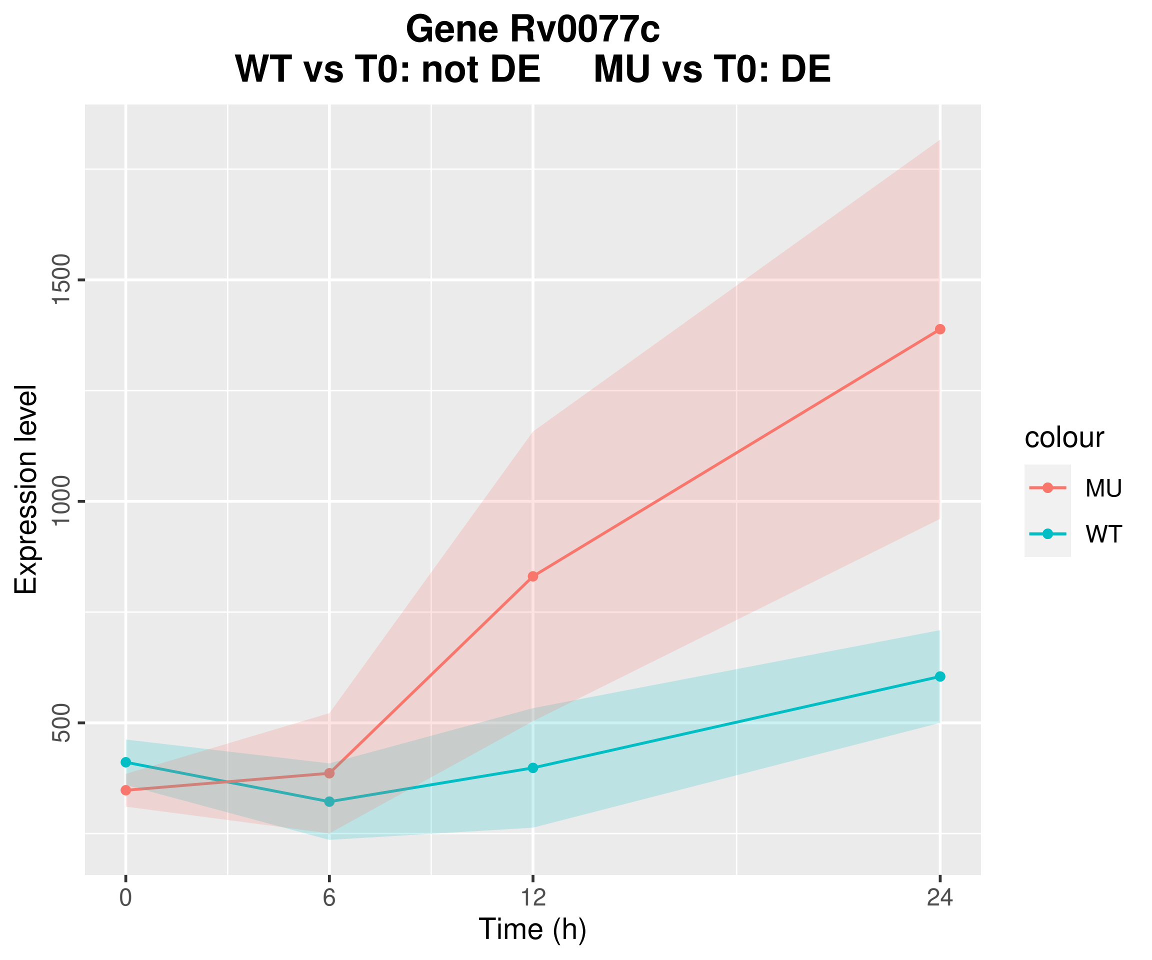
Gene Rv0077c
WT vs T0: not DE MU vs T0: DE
Expression level
1500
1000
500
0
6
12
24
Time (h)
colour
MU
WT

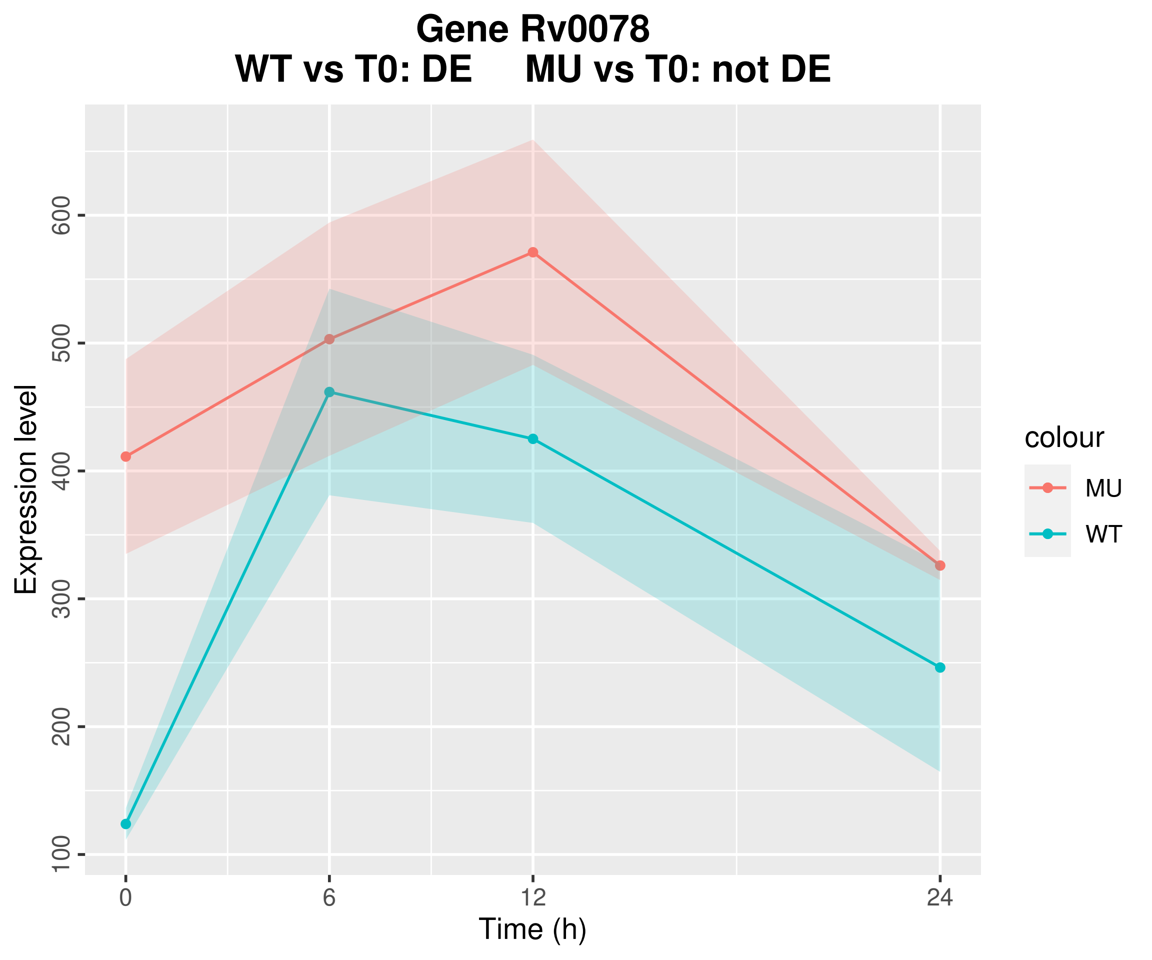
Gene Rv0078
WT vs T0: DE   MU vs T0: not DE
Expression level
600
500
400
300
200
100
0
6
12
24
Time (h)
colour
MU
WT

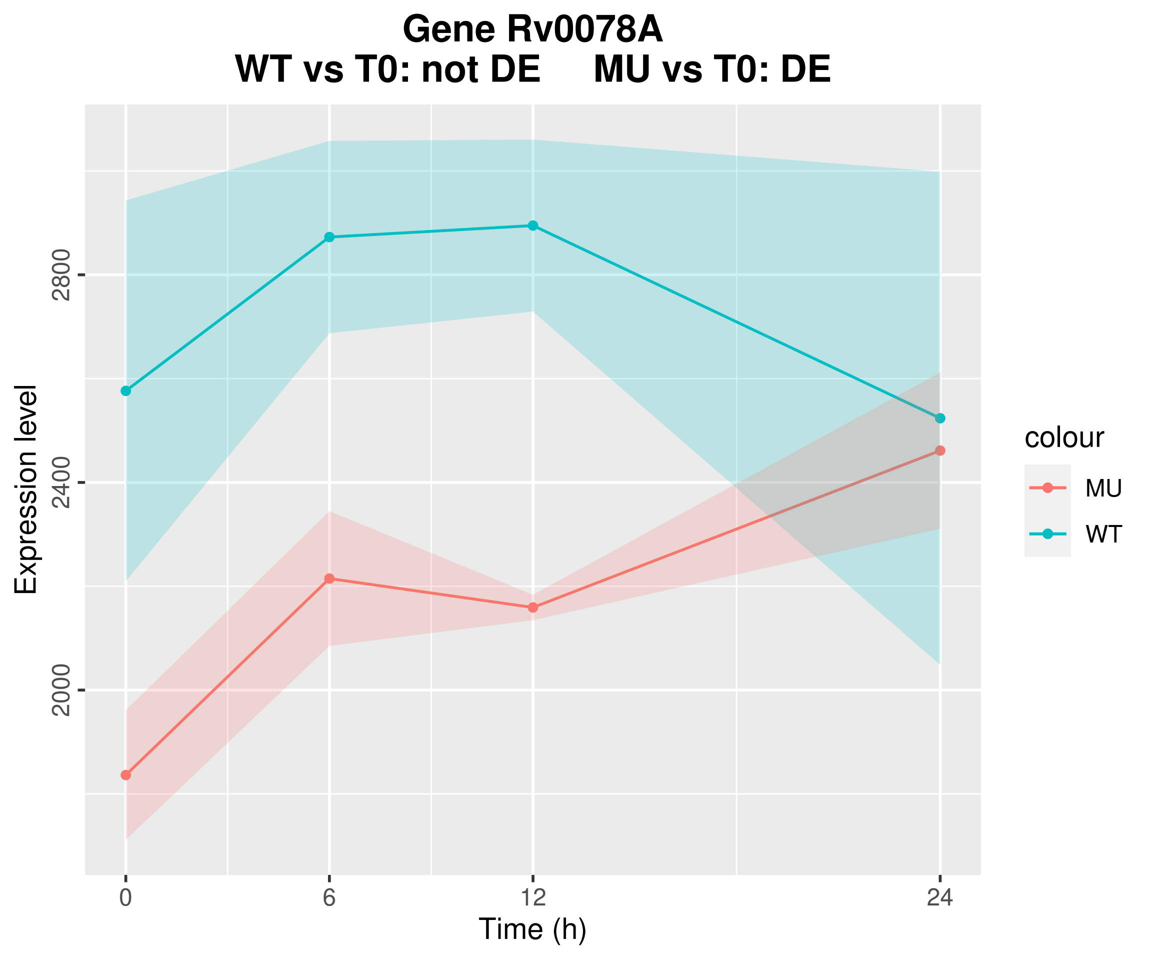

Gene Rv0078A
WT vs T0: not DE MU vs T0: DE
Expression level
2000
2400
2800
Time (h)
0
6
12
24
colour
MU
WT

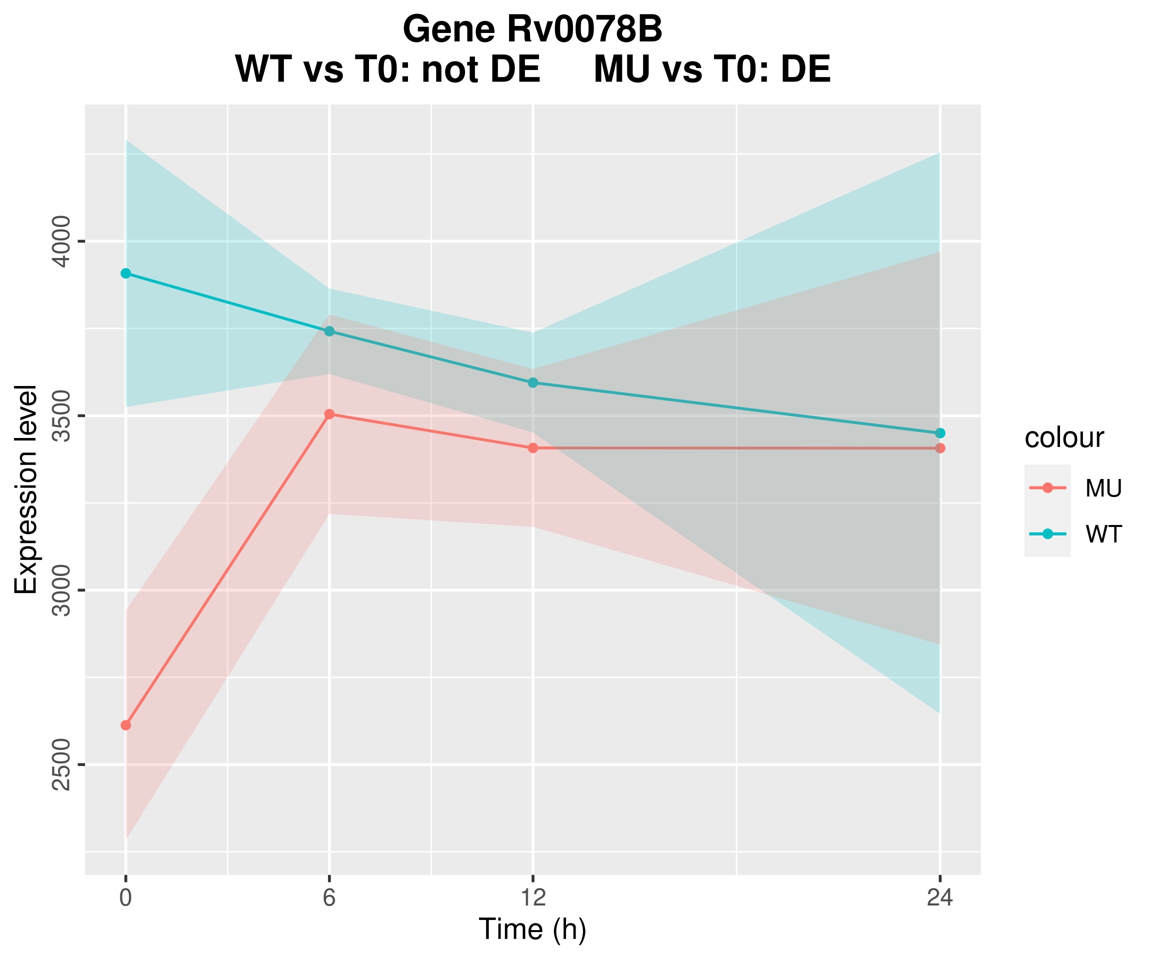
Gene Rv0078B
WT vs T0: not DE MU vs T0: DE
Expression level
4000
3500
3000
2500
0
6
12
24
Time (h)
colour
MU
WT

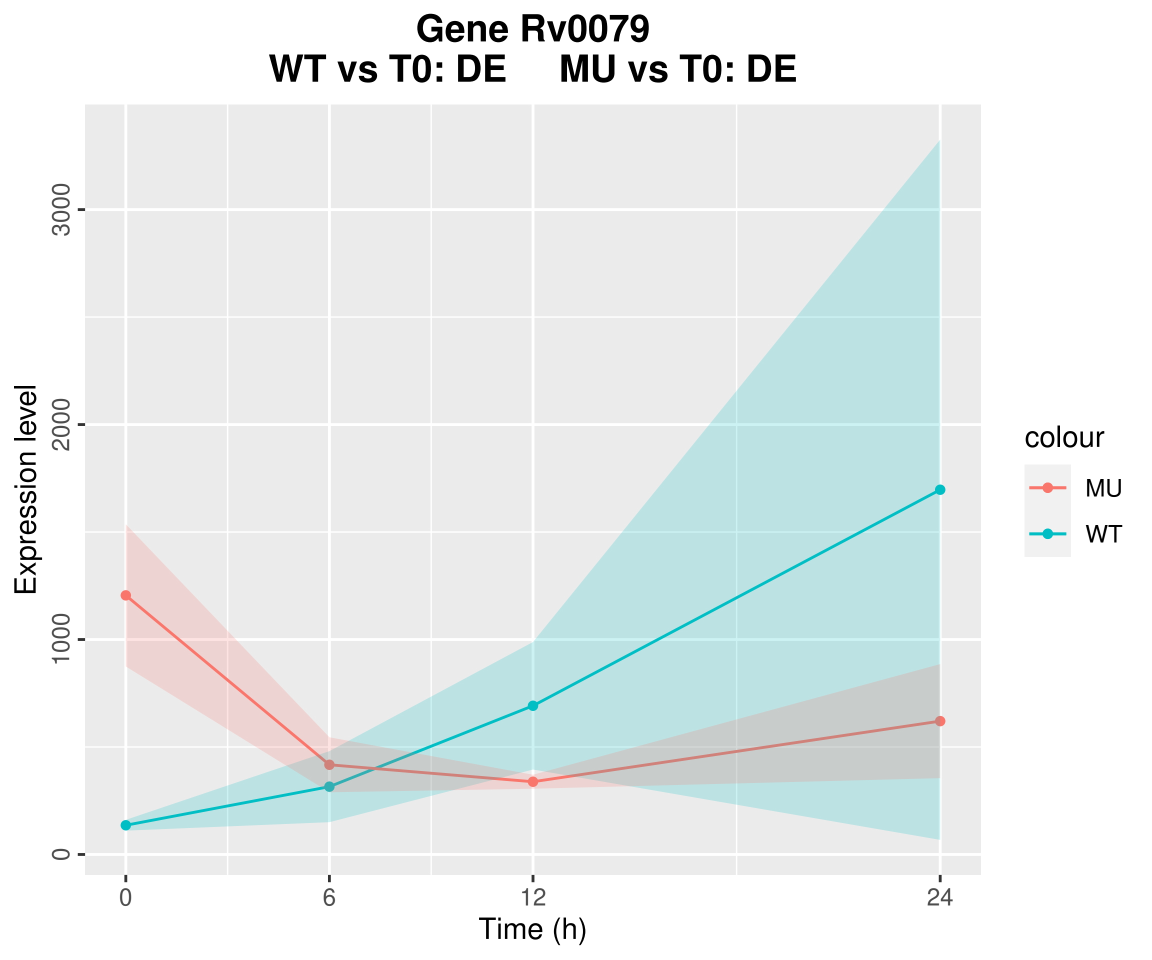

Gene Rv0079
WT vs T0: DE MU vs T0: DE
Expression level
3000
2000
1000
0
0
6
12
24
Time (h)
colour
MU
WT

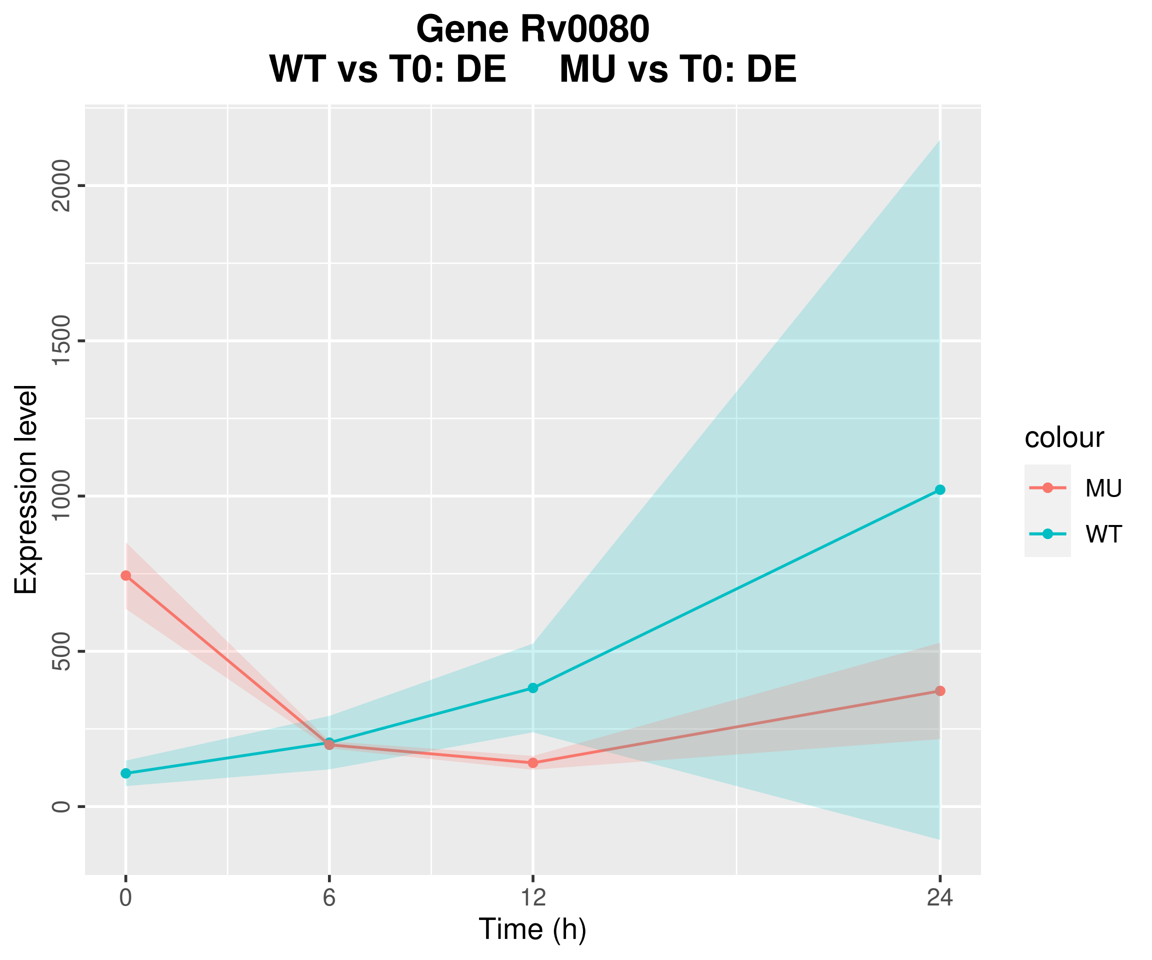
Gene Rv0080
WT vs T0: DE MU vs T0: DE
Expression level
0
500
1000
1500
2000
0
6
12
24
Time (h)
colour
MU
WT

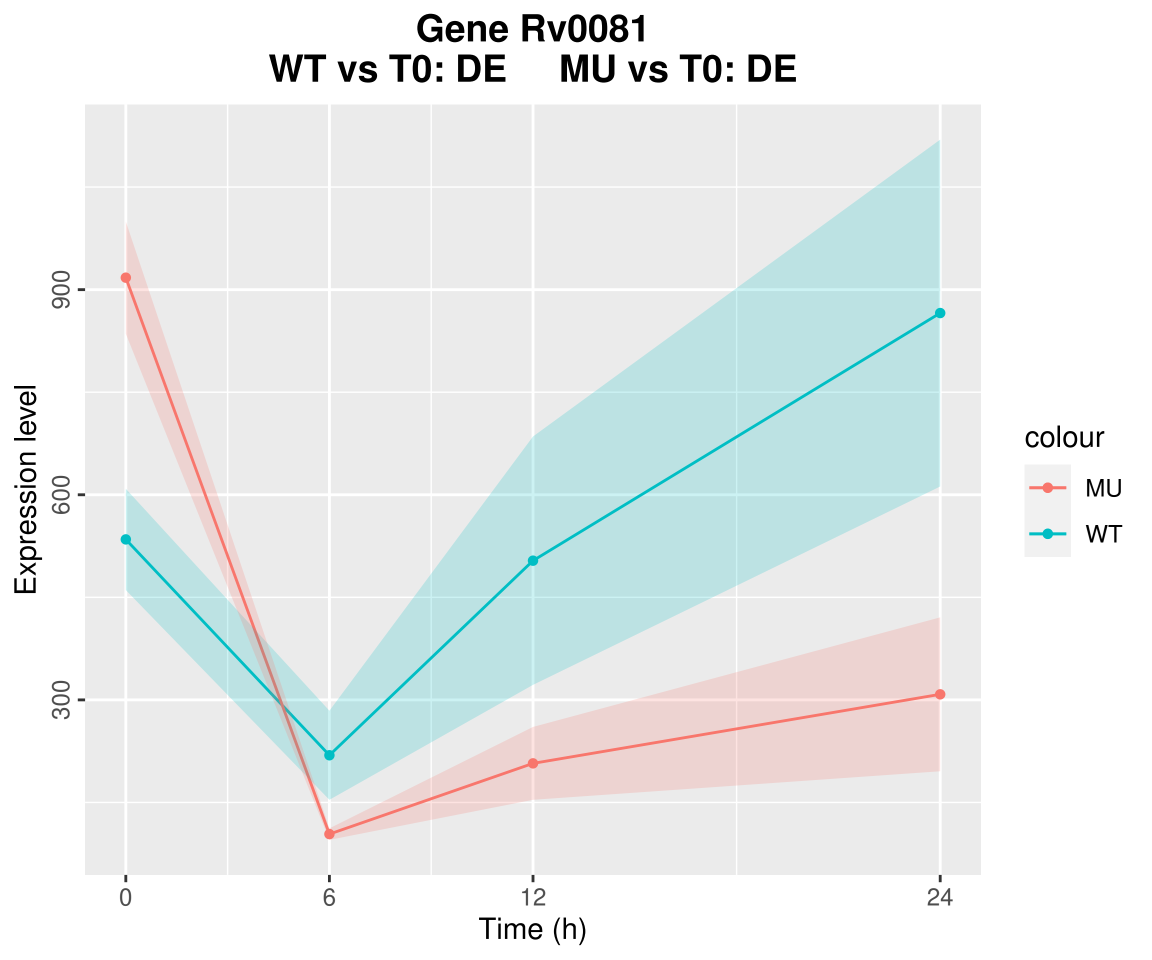

Gene Rv0081
WT vs T0: DE MU vs T0: DE
Expression level
900
600
300
0
6
12
24
Time (h)
colour
MU
WT

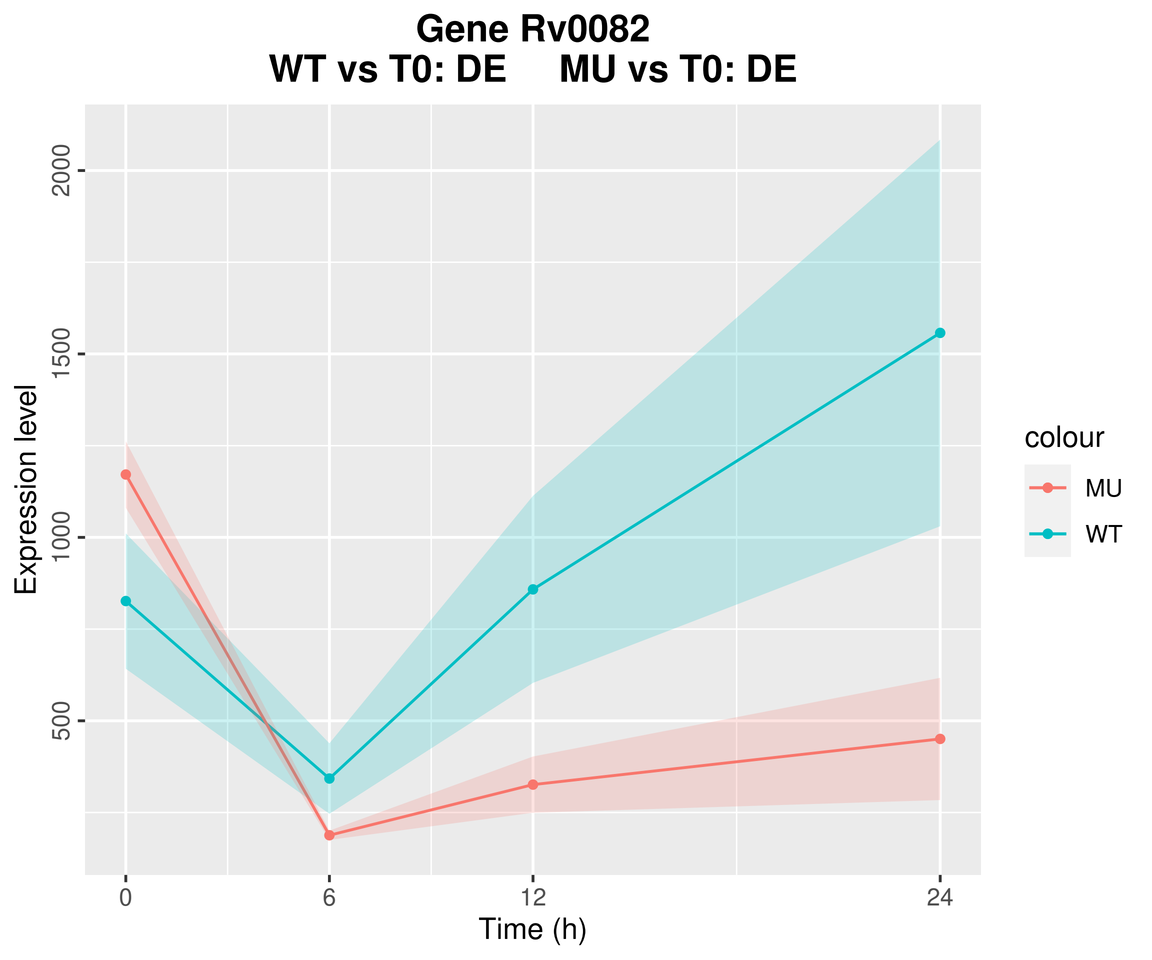
Gene Rv0082
WT vs T0: DE MU vs T0: DE
Expression level
2000
1500
1000
500
0
6
12
24
Time (h)
colour
MU
WT

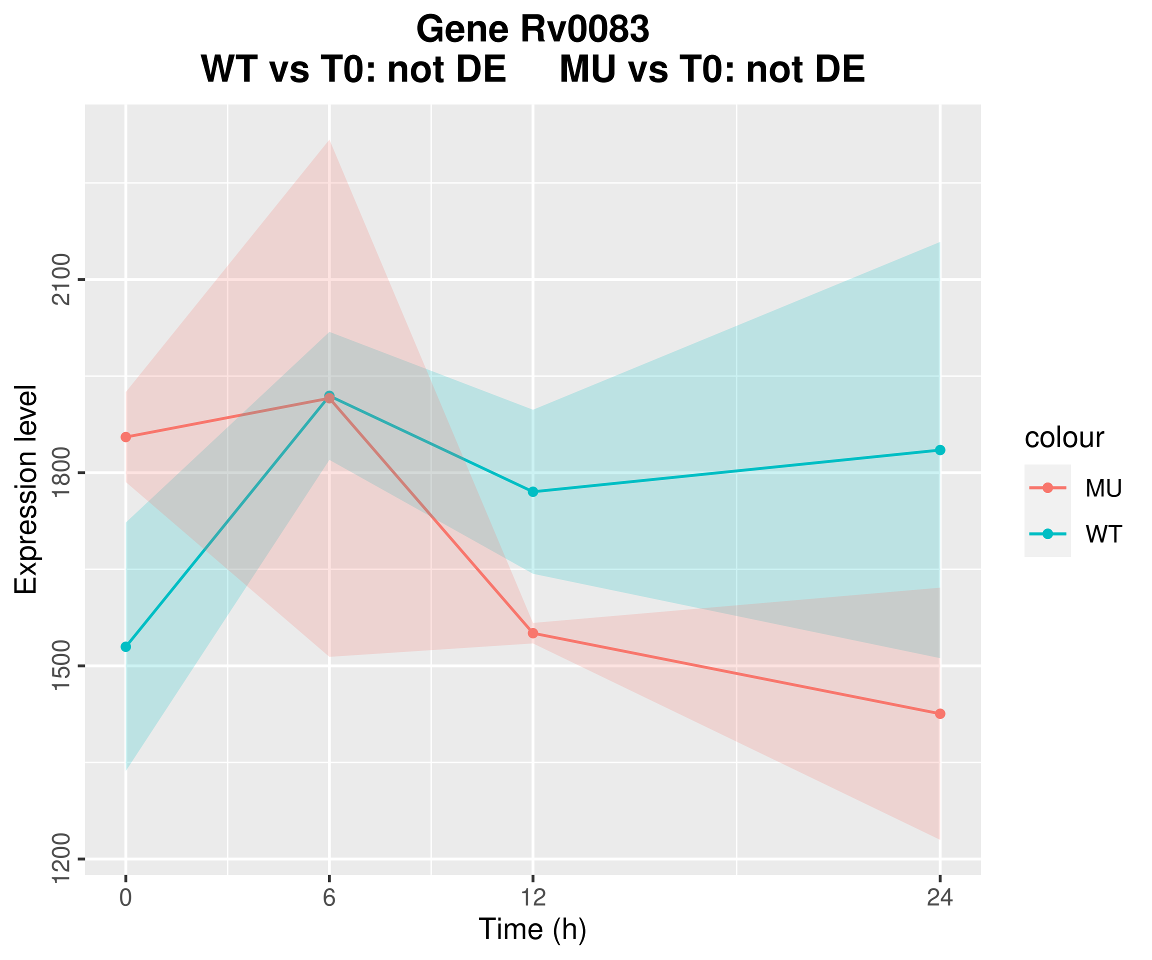

Gene Rv0083
WT vs T0: not DE MU vs T0: not DE
Expression level
2100
1800
1500
1200
0
6
12
24
Time (h)
colour
MU
WT

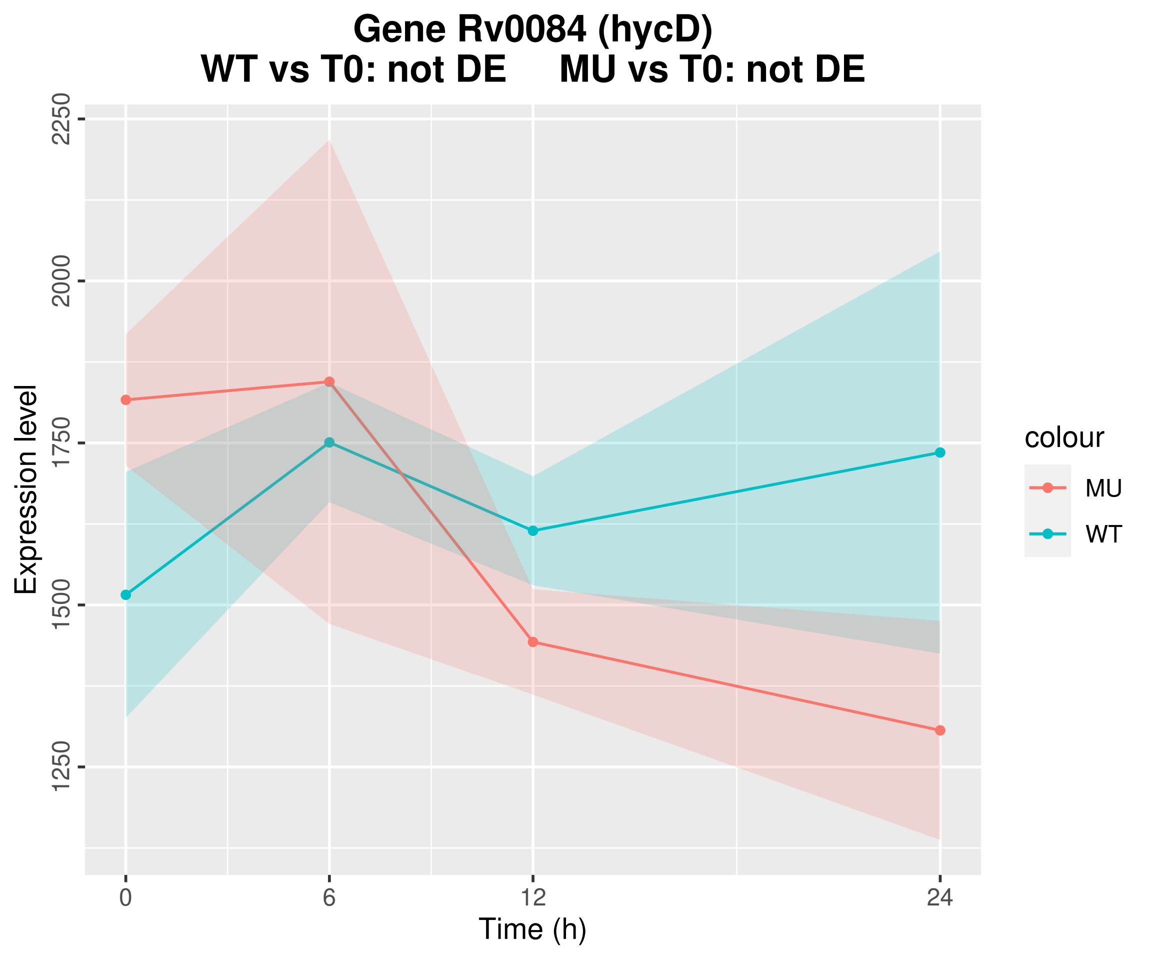

Gene Rv0084 (hycD)
WT vs T0: not DE MU vs T0: not DE
Expression level
2250
2000
1750
1500
1250
0
6
12
24
Time (h)
colour
MU
WT

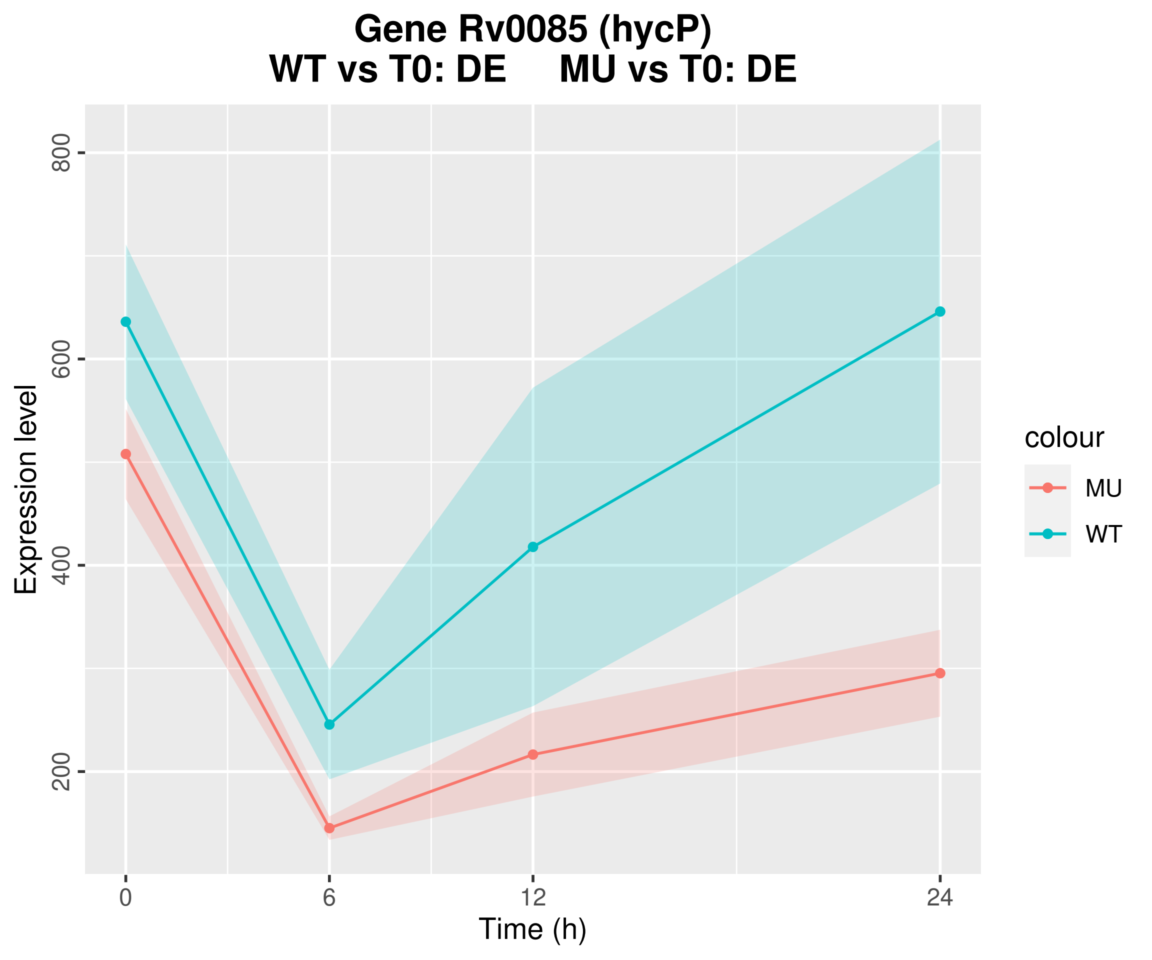
Gene Rv0085 (hycP)
WT vs T0: DE MU vs T0: DE
Expression level
800
600
400
200
0
6
12
24
Time (h)
colour
MU
WT

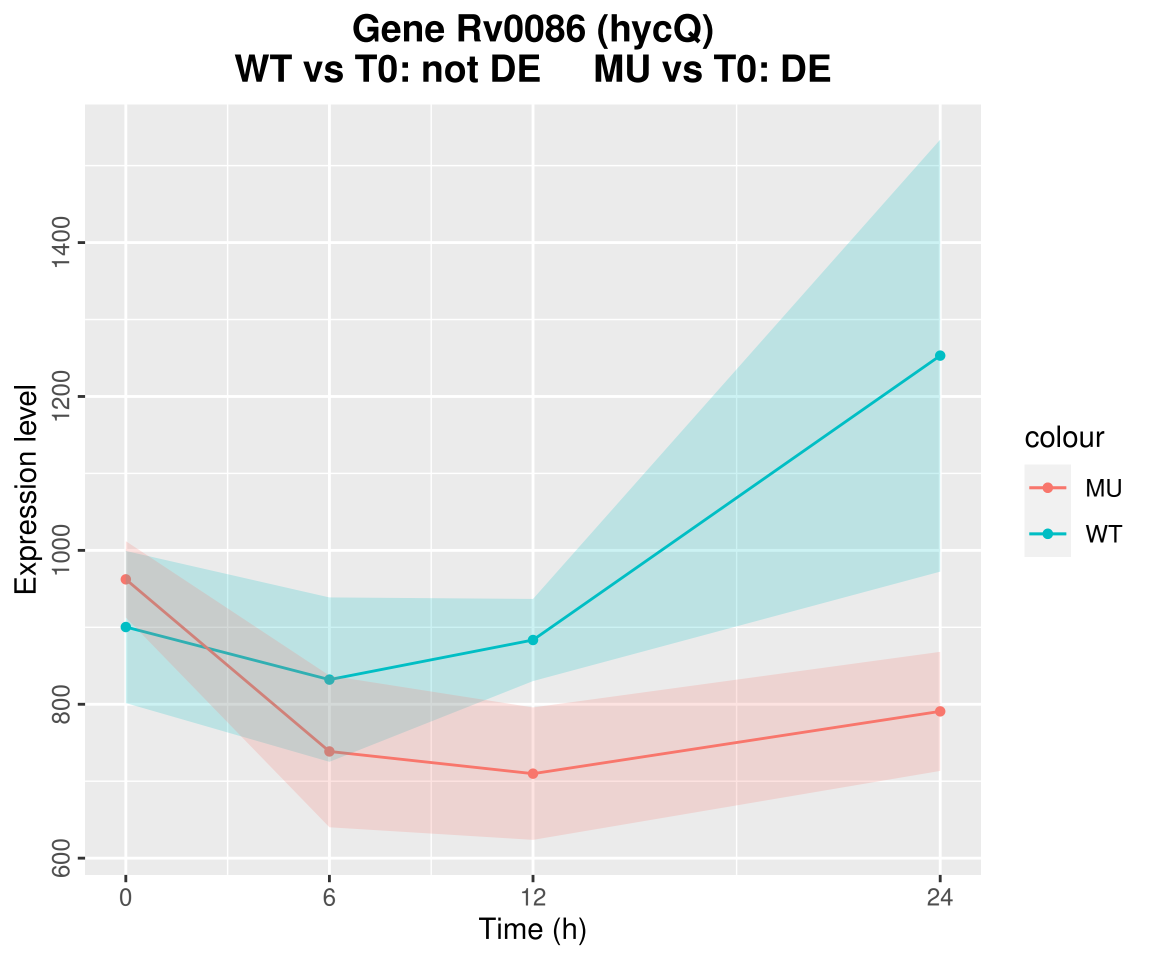

Gene Rv0086 (hycQ)
WT vs T0: not DE MU vs T0: DE
Expression level
1400
1200
1000
800
600
0
6
12
24
Time (h)
colour
MU
WT

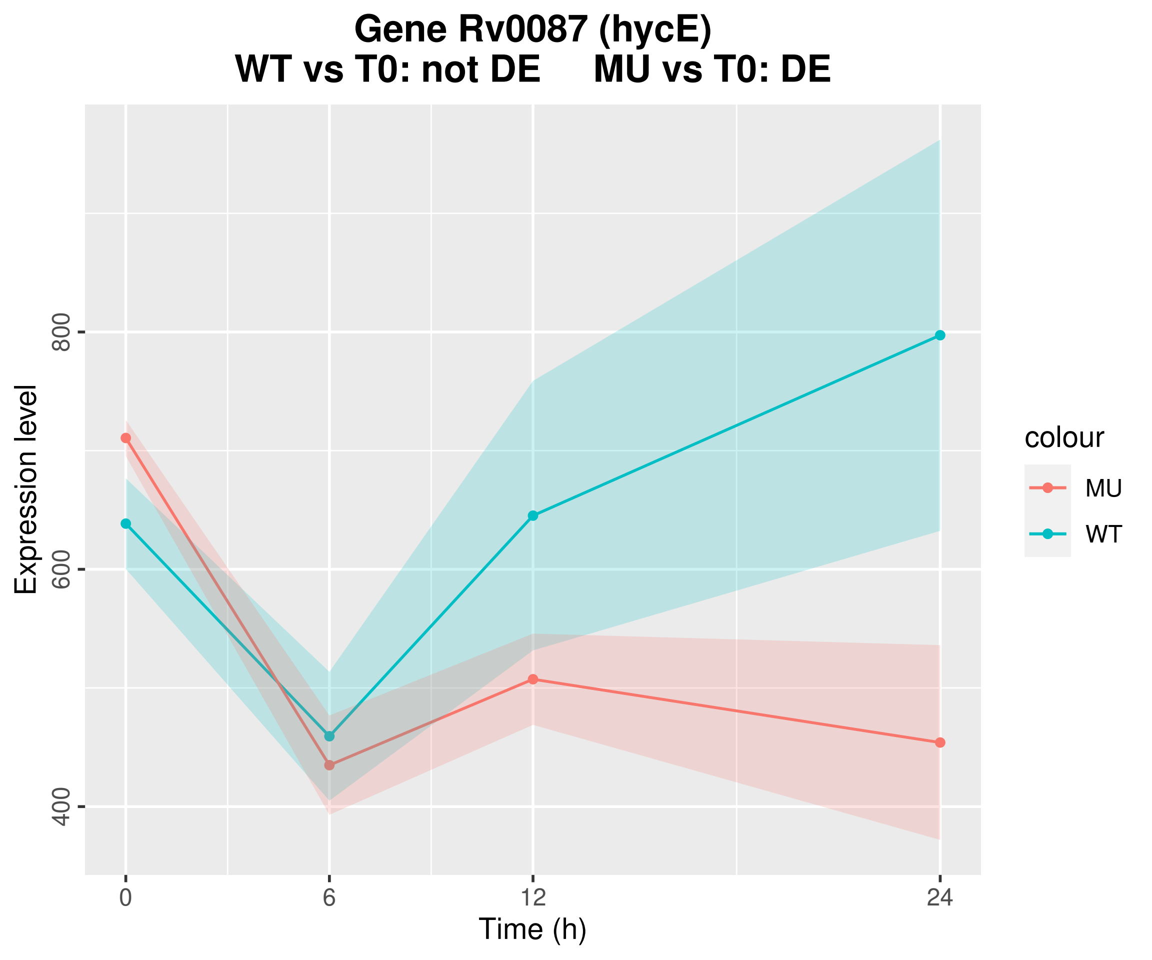

Gene Rv0087 (hycE)
WT vs T0: not DE   MU vs T0: DE
Expression level
800
600
400
0
6
12
24
Time (h)
colour
MU
WT

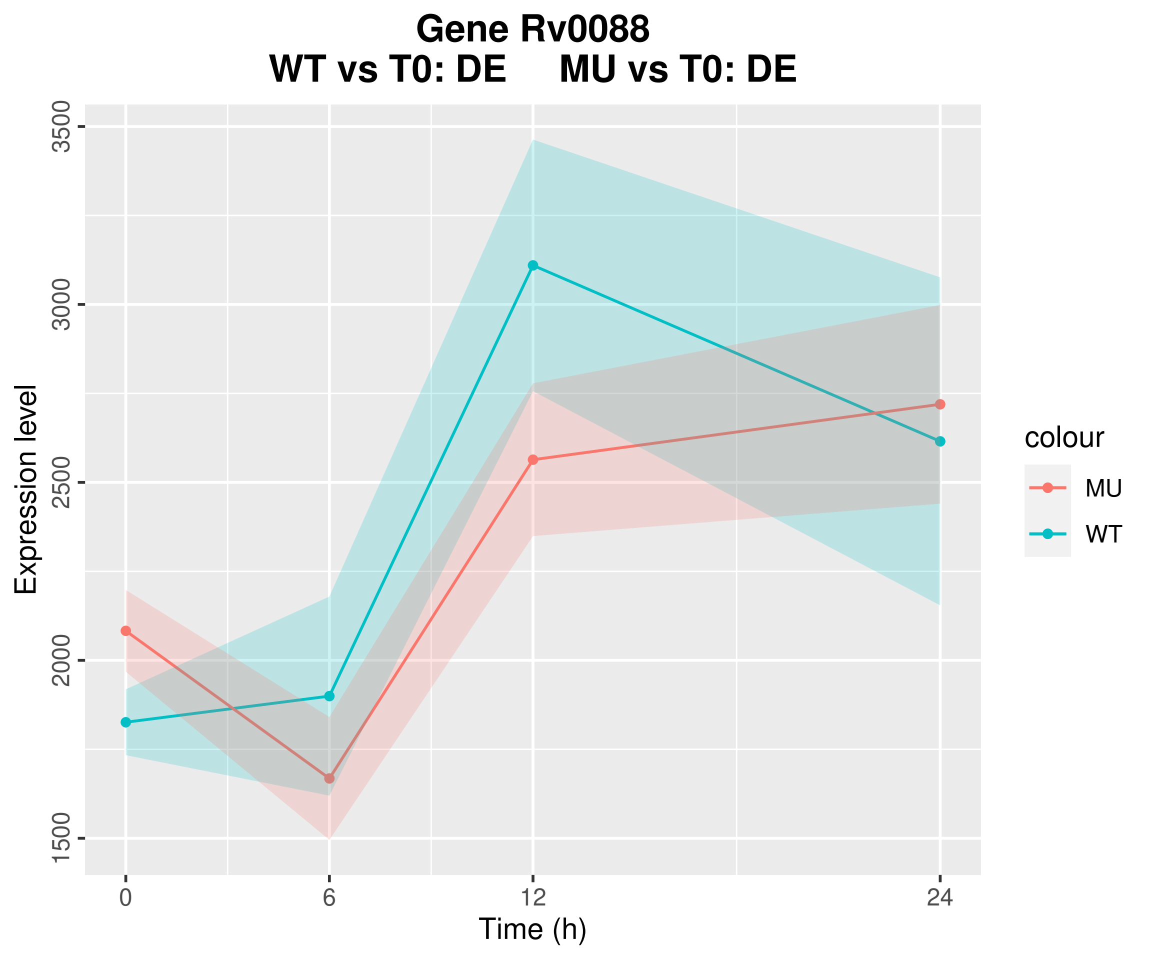

Gene Rv0088
WT vs T0: DE   MU vs T0: DE
Expression level
3500
3000
2500
2000
1500
0
6
12
24
Time (h)
colour
MU
WT

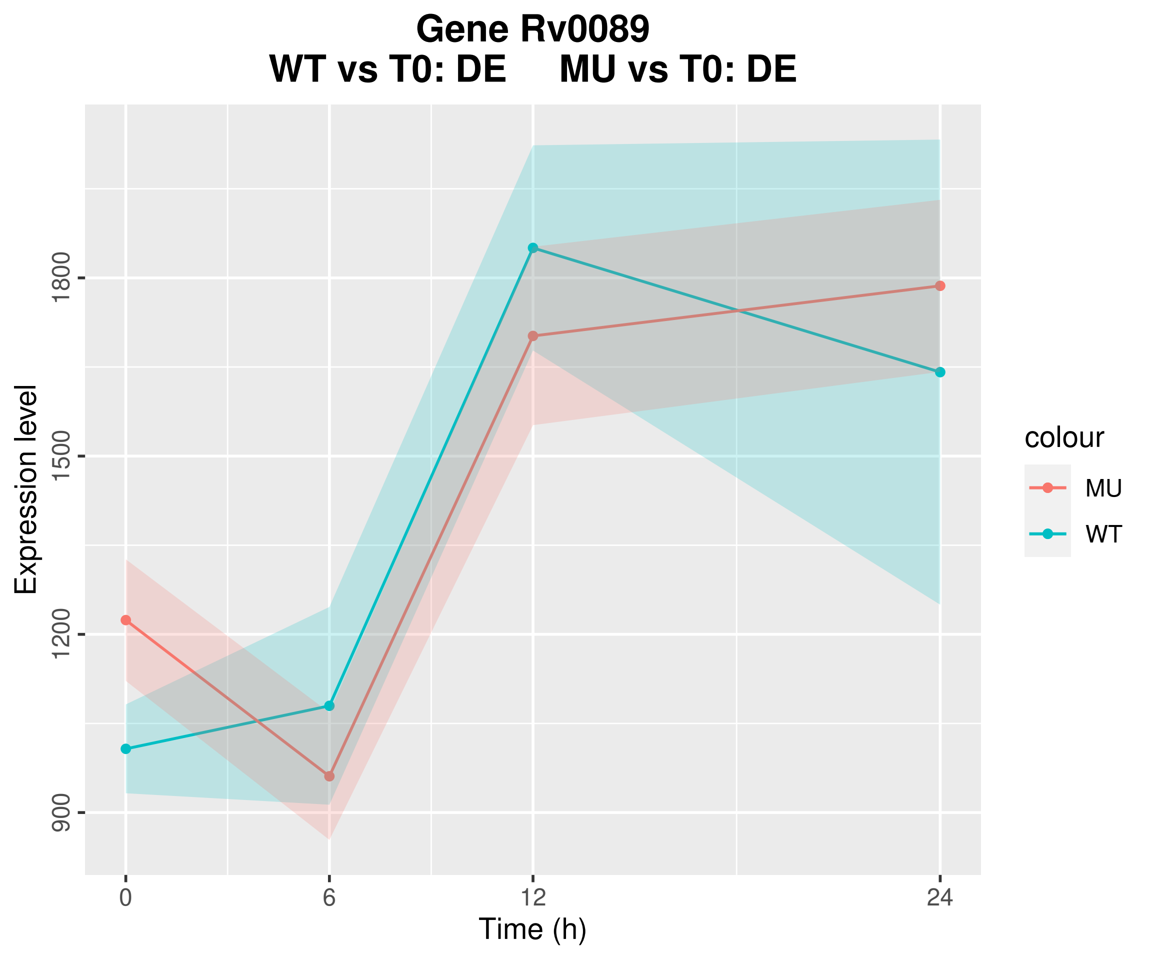
Gene Rv0089
WT vs T0: DE   MU vs T0: DE
Expression level
900
1200
1500
1800
0
6
12
24
Time (h)
colour
MU
WT

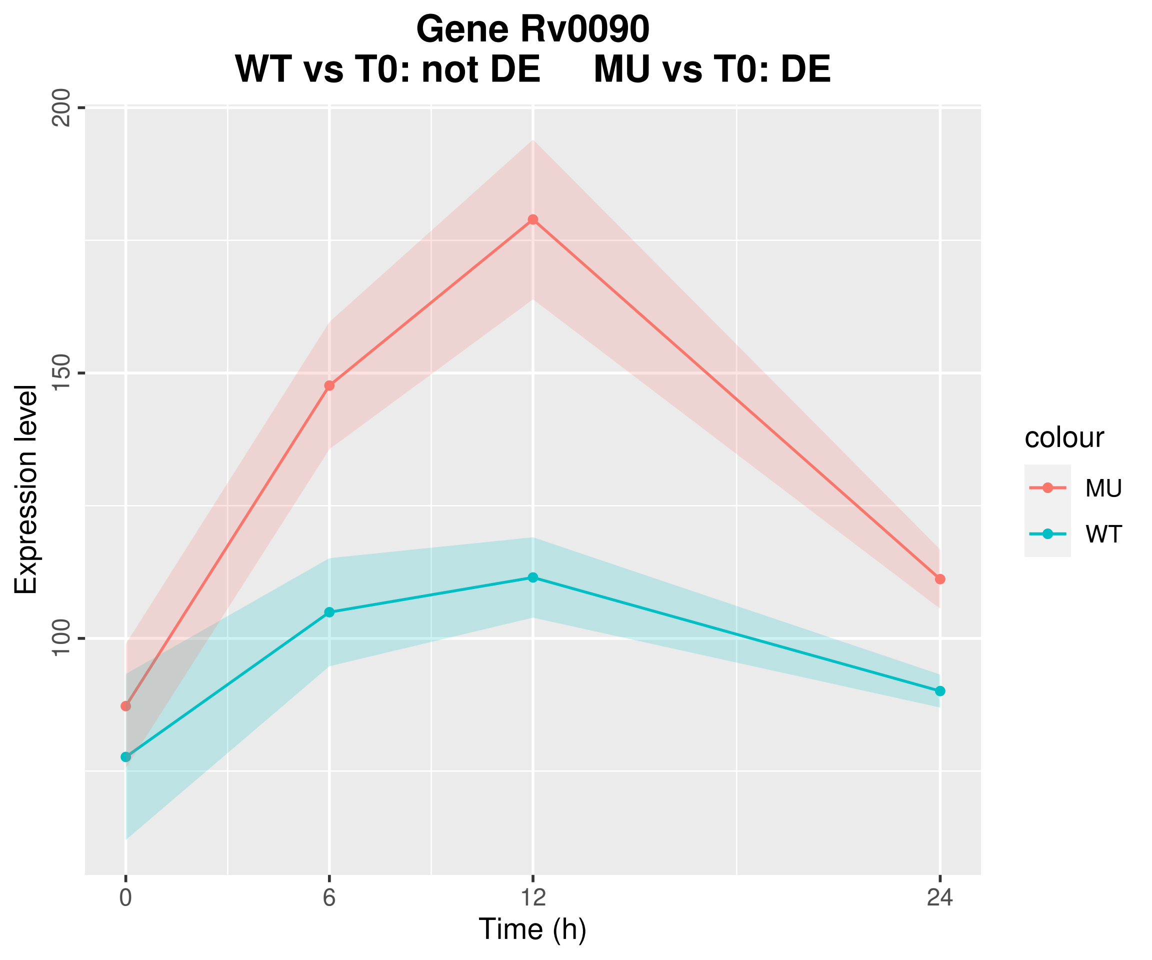

Gene Rv0090
WT vs T0: not DE MU vs T0: DE
200
150
100
Expression level
0
6
12
24
Time (h)
colour
MU
WT

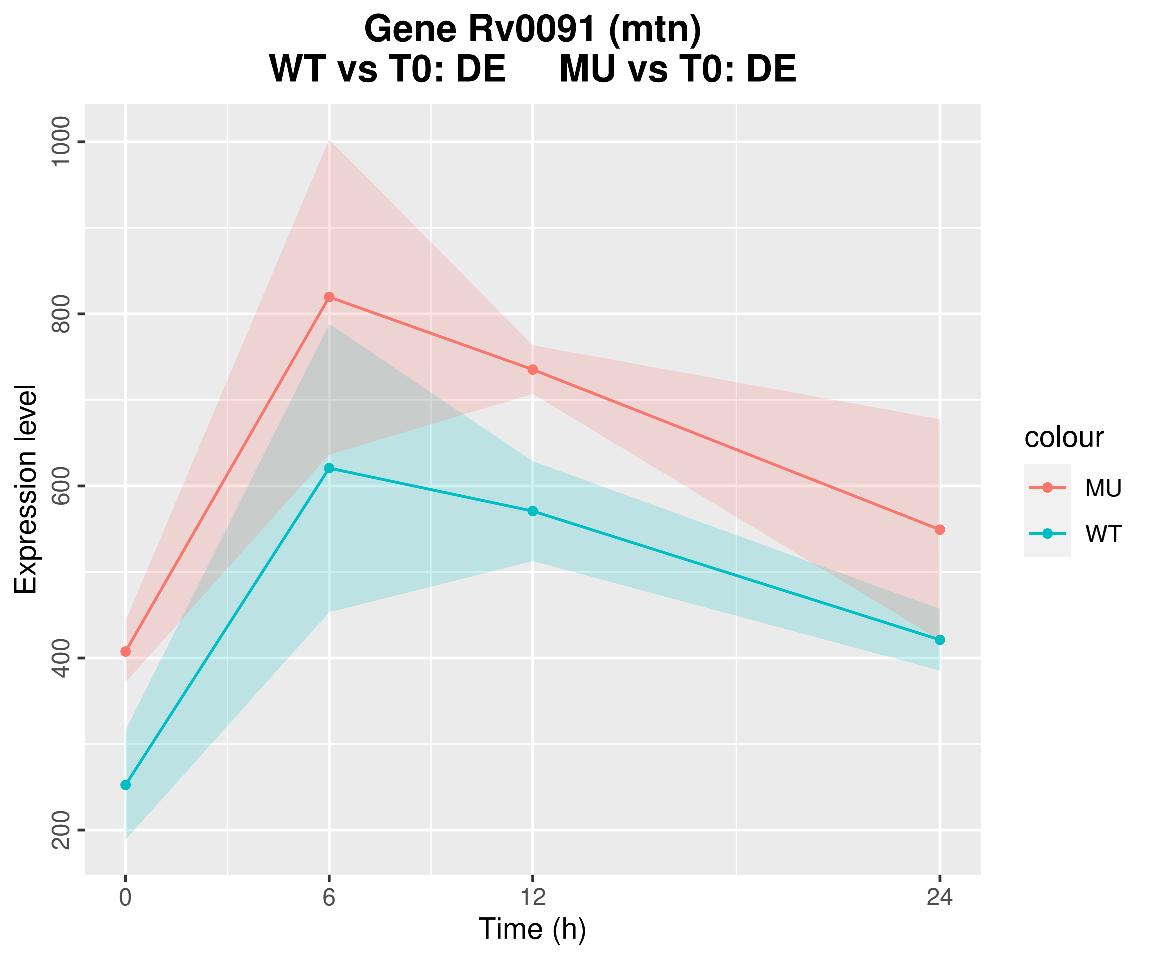
Gene Rv0091 (mtn)
WT vs T0: DE   MU vs T0: DE
Expression level
1000
800
600
400
200
0
6
12
24
Time (h)
colour
MU
WT

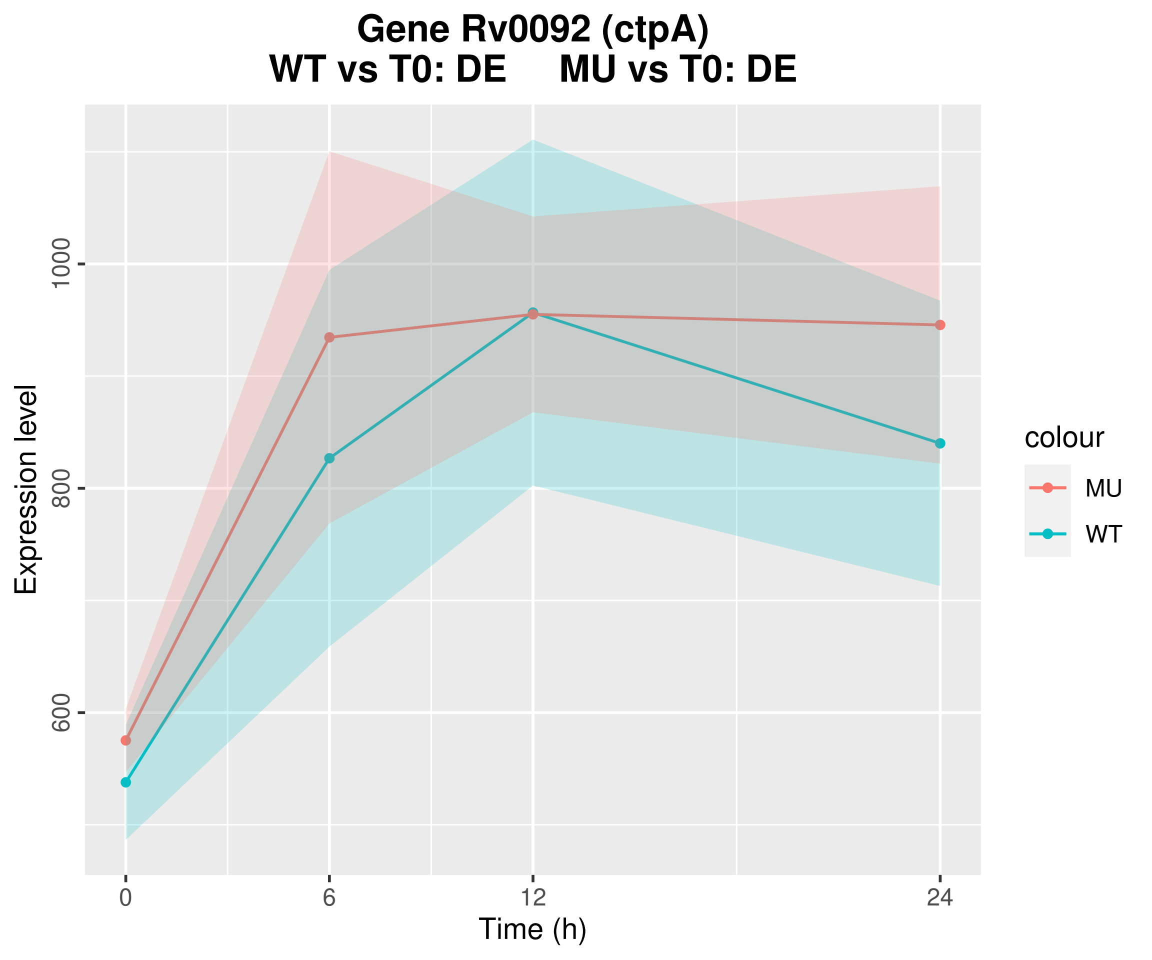

Gene Rv0092 (ctpA)
WT vs T0: DE MU vs T0: DE
Expression level
600
800
1000
0
6
12
24
Time (h)
colour
MU
WT

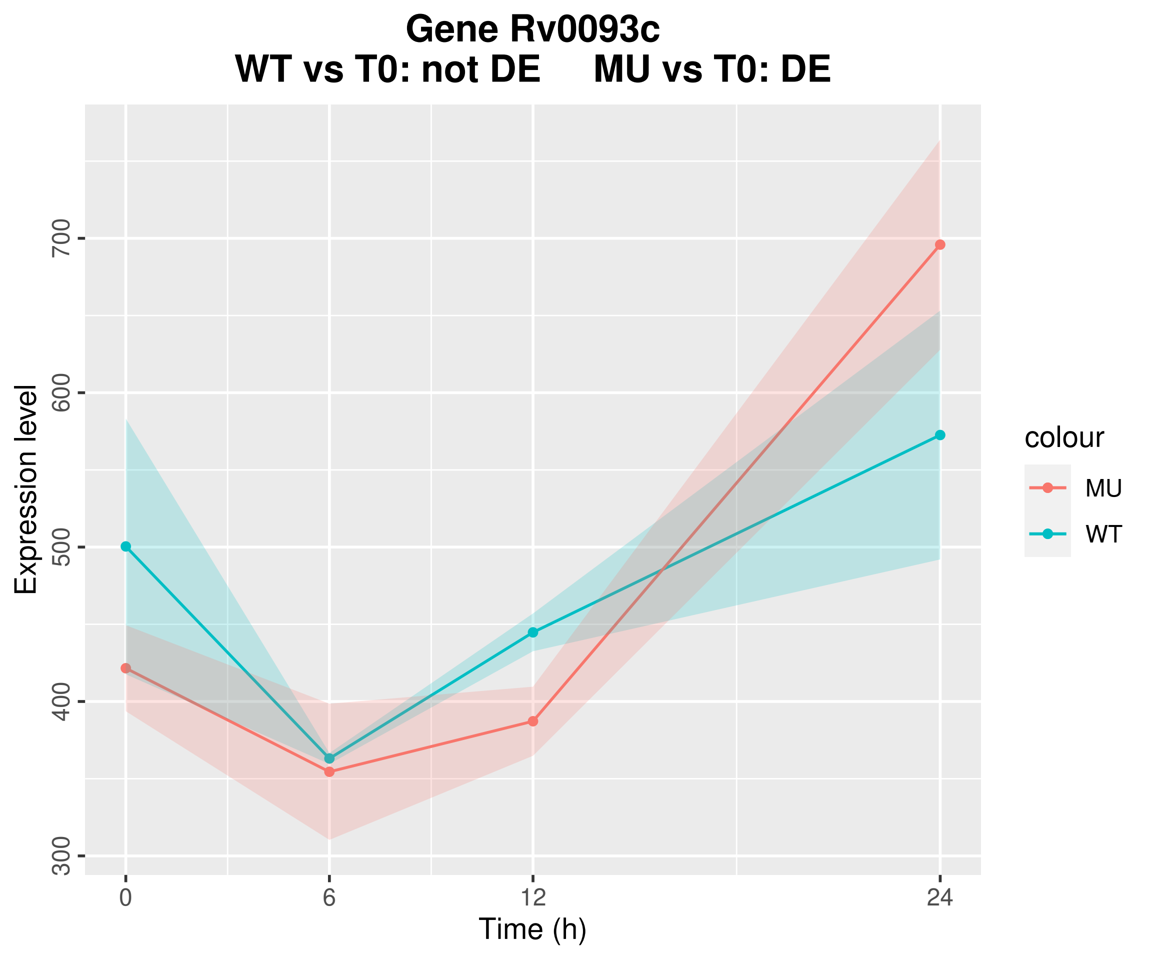
Gene Rv0093c
WT vs T0: not DE MU vs T0: DE
Expression level
700
600
500
400
300
0
6
12
24
Time (h)
colour
MU
WT

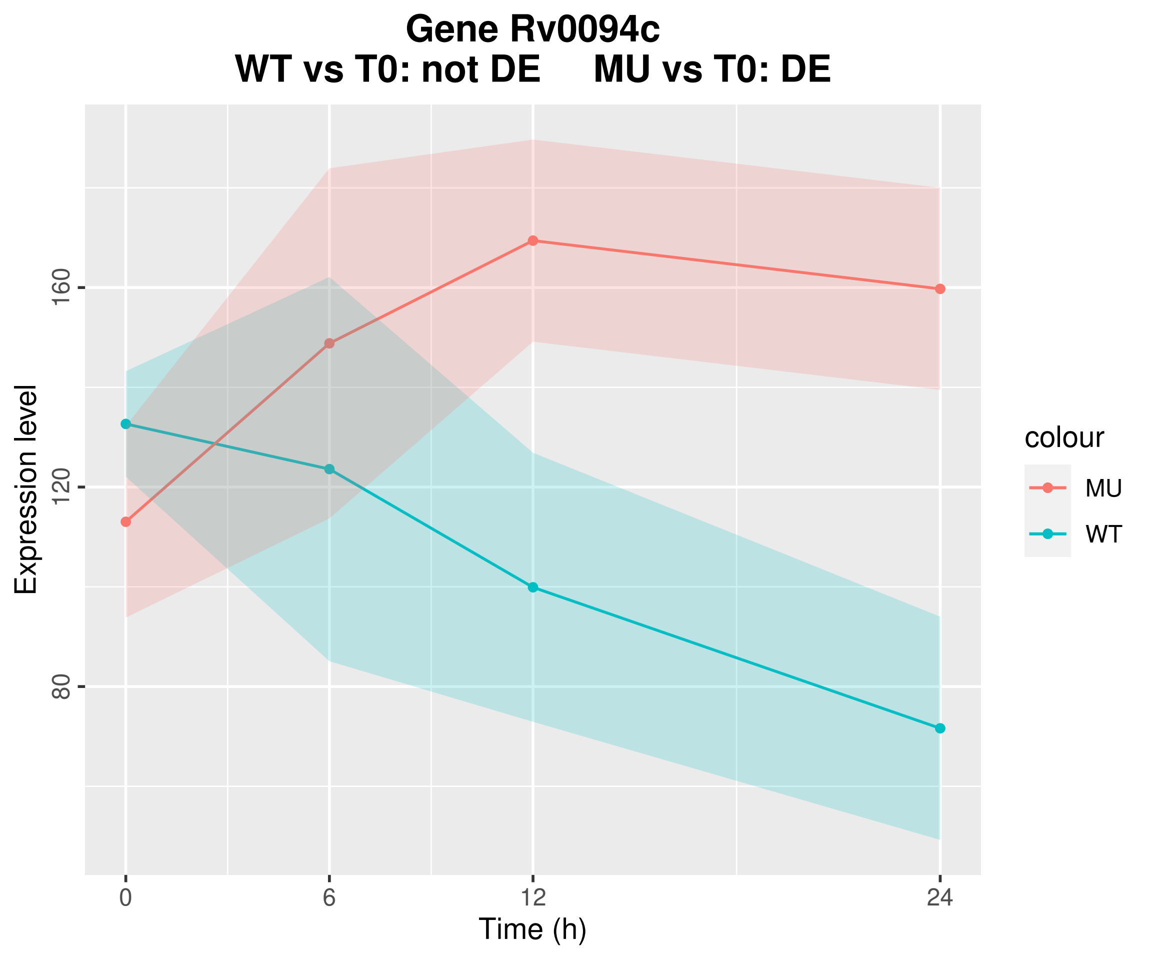

Gene Rv0094c
WT vs T0: not DE MU vs T0: DE
Expression level
160
120
80
0
6
12
24
Time (h)
colour
MU
WT

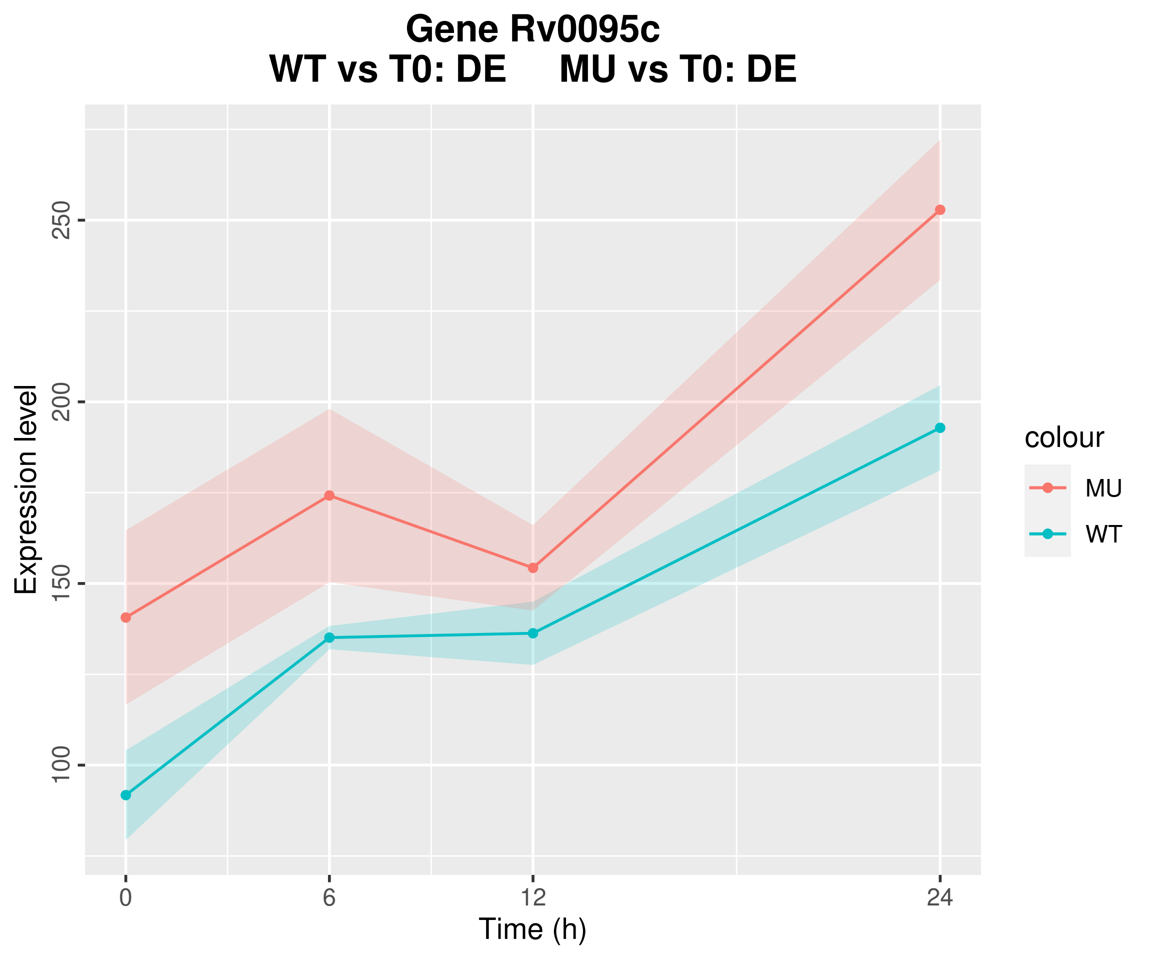

Gene Rv0095c
WT vs T0: DE MU vs T0: DE
Expression level
250
200
150
100
0
6
12
24
Time (h)
colour
MU
WT

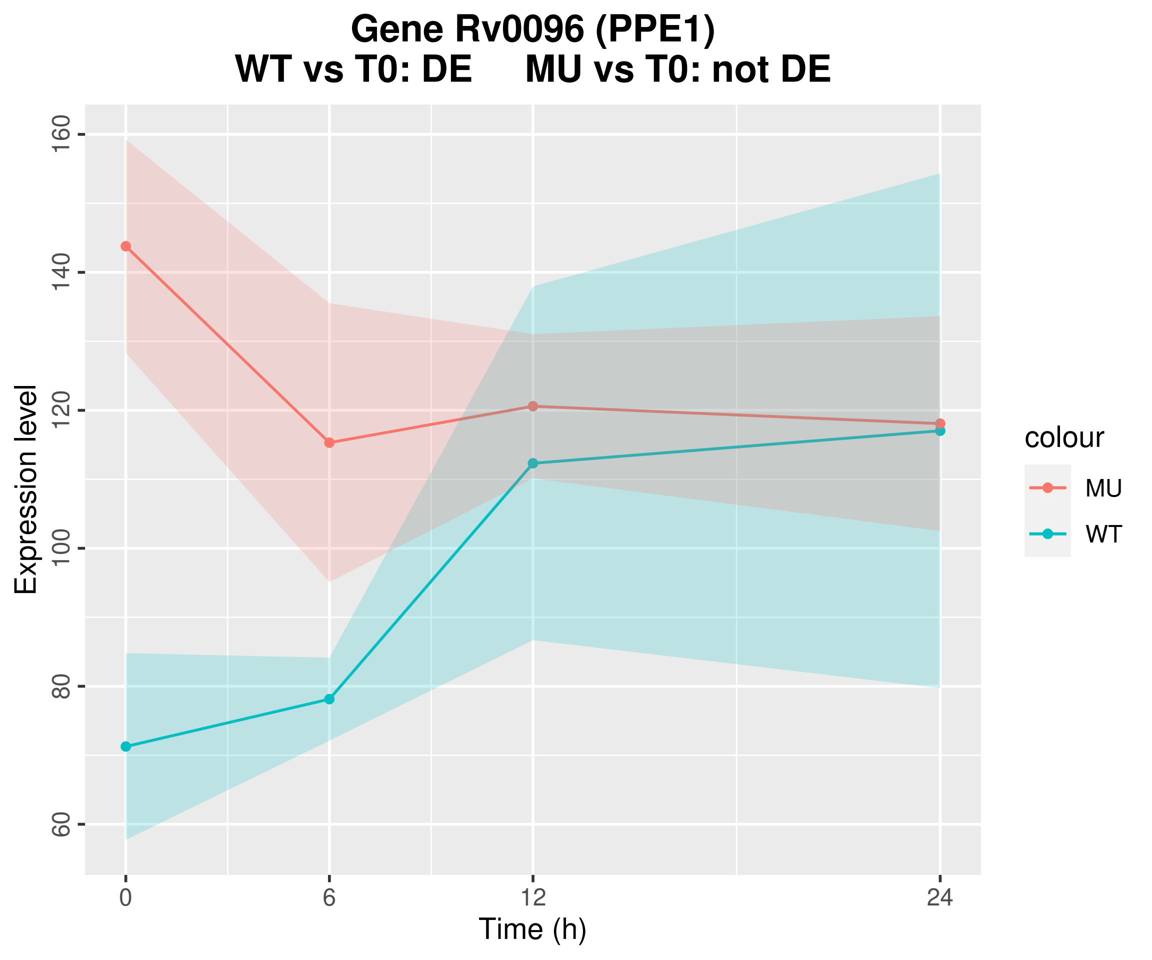

Gene Rv0096 (PPE1)
WT vs T0: DE MU vs T0: not DE
Expression level
160
140
120
100
80
60
0
6
12
24
Time (h)
colour
MU
WT

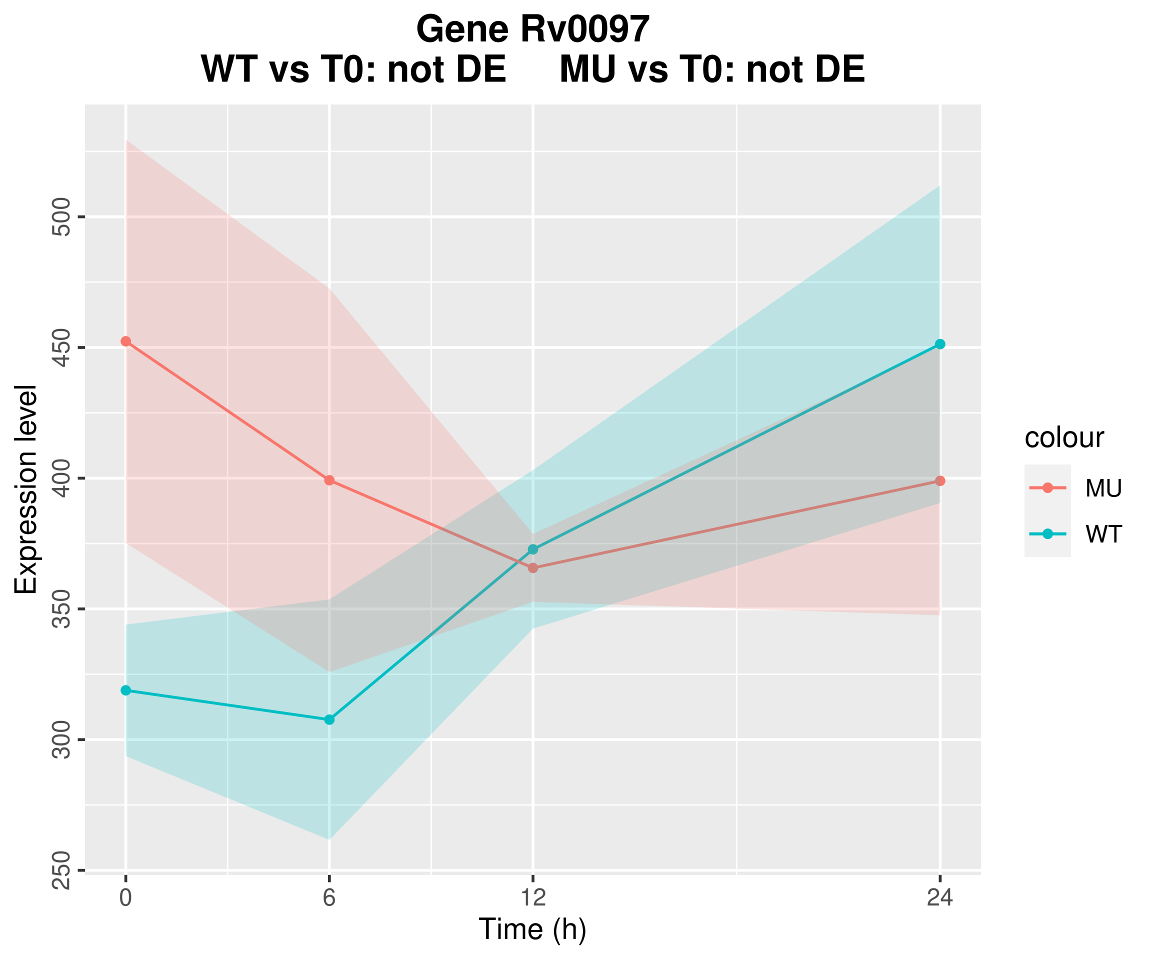

Gene Rv0097
WT vs T0: not DE   MU vs T0: not DE
Expression level
500
450
400
350
300
250
0
6
12
24
Time (h)
colour
MU
WT

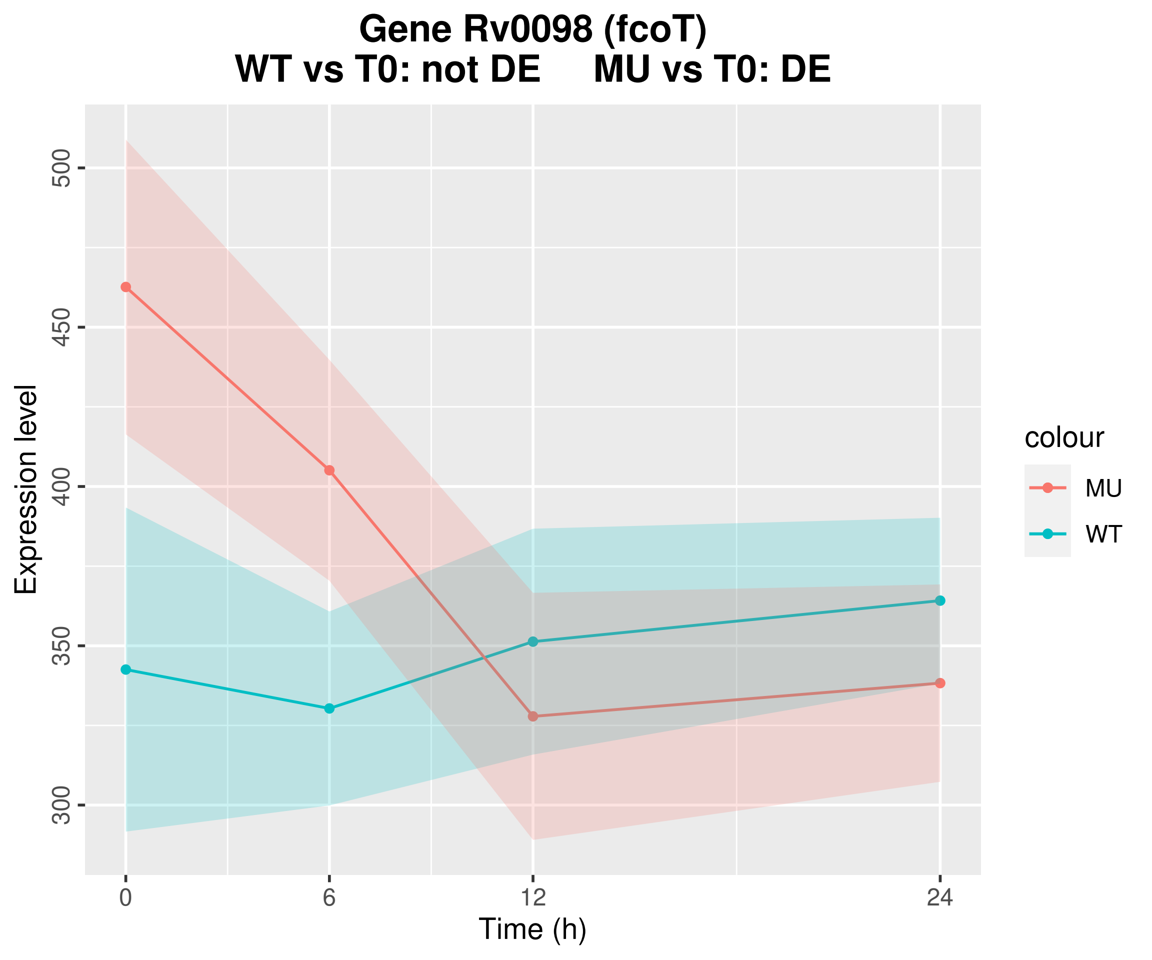

Gene Rv0098 (fcoT)
WT vs T0: not DE   MU vs T0: DE
Expression level
500
450
400
350
300
0
6
12
24
Time (h)
colour
MU
WT

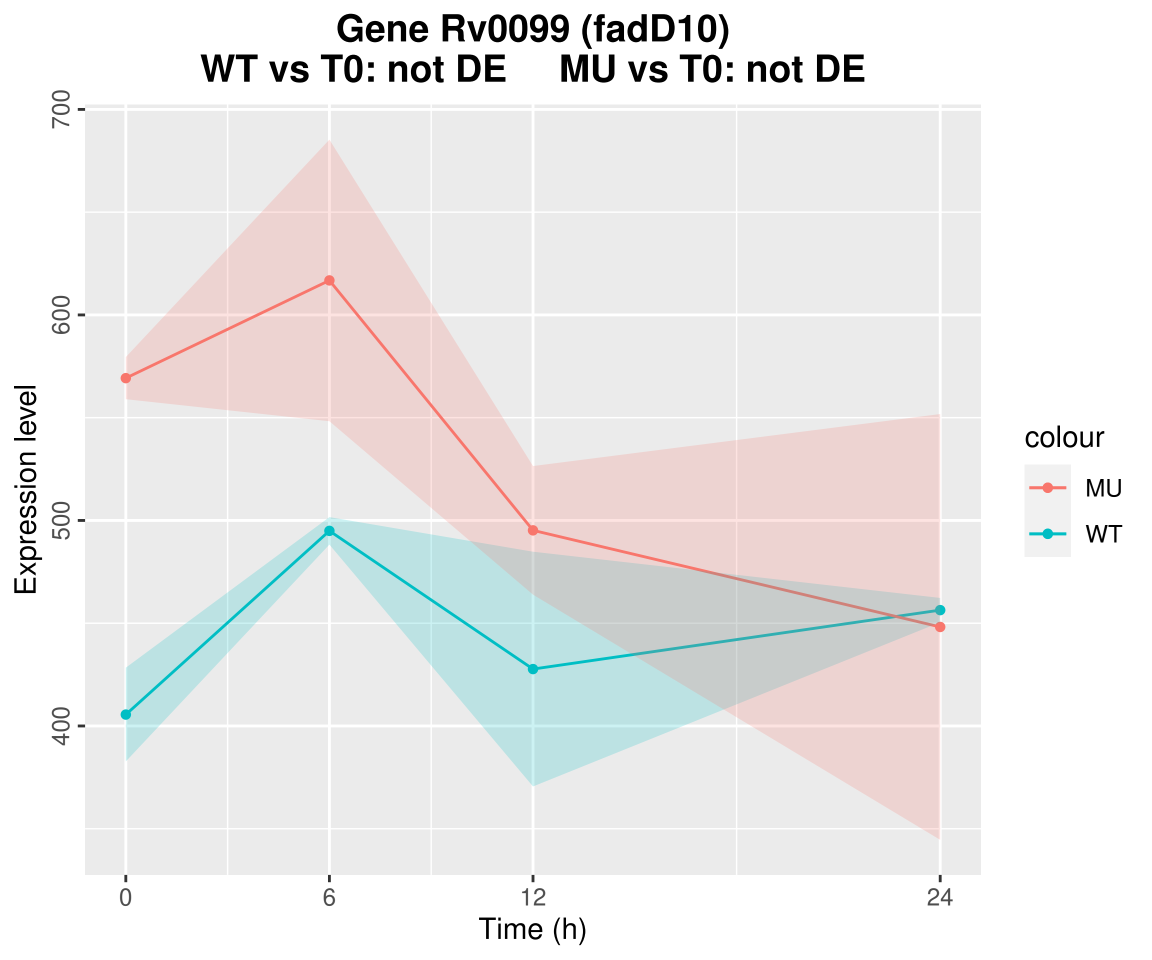
Gene Rv0099 (fadD10)
WT vs T0: not DE   MU vs T0: not DE
700
600
500
400
Expression level
0
6
12
24
Time (h)
colour
MU
WT

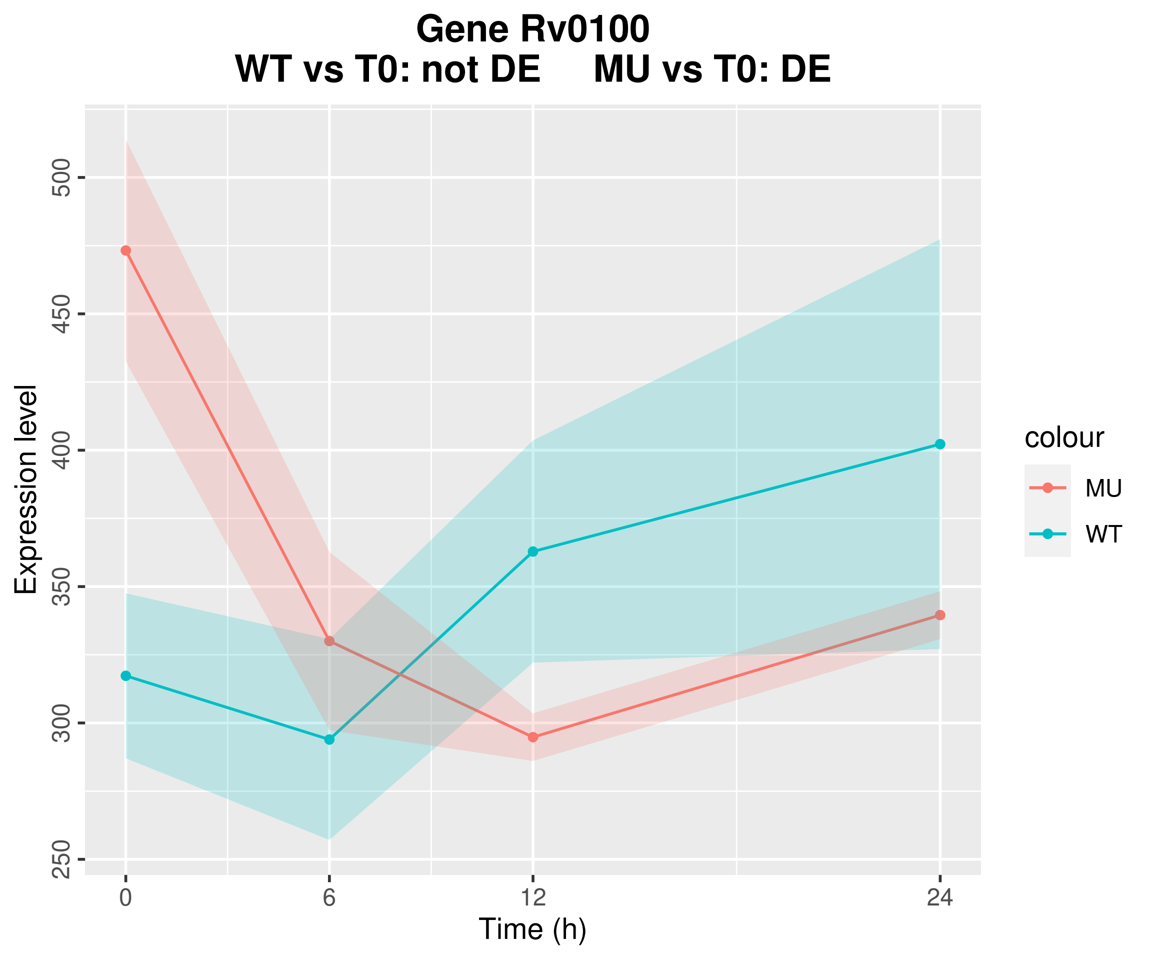

Gene Rv0100
WT vs T0: not DE MU vs T0: DE
Expression level
500
450
400
350
300
250
0
6
12
24
Time (h)
colour
MU
WT

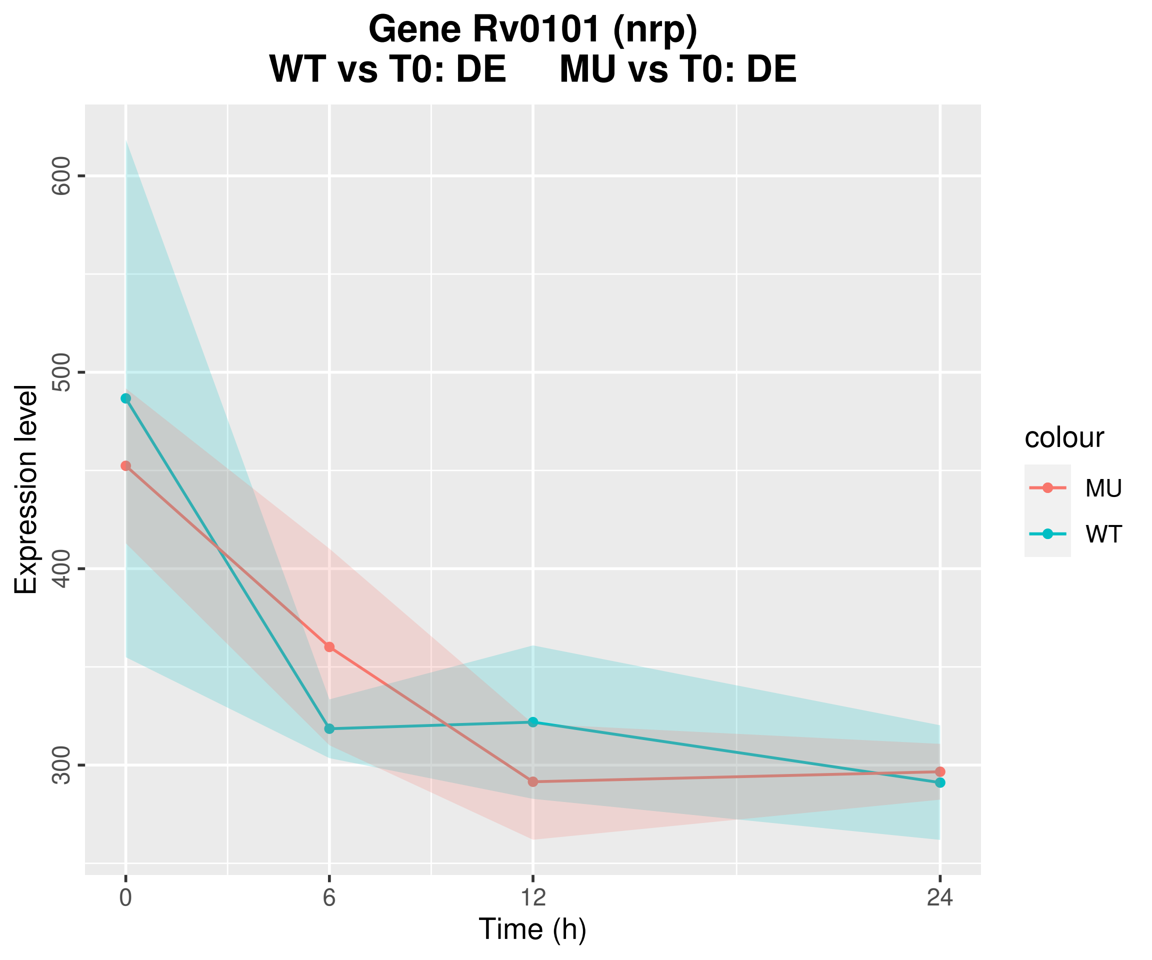

Gene Rv0101 (nrp)
WT vs T0: DE MU vs T0: DE
Expression level
600
500
400
300
0
6
12
24
Time (h)
colour
MU
WT

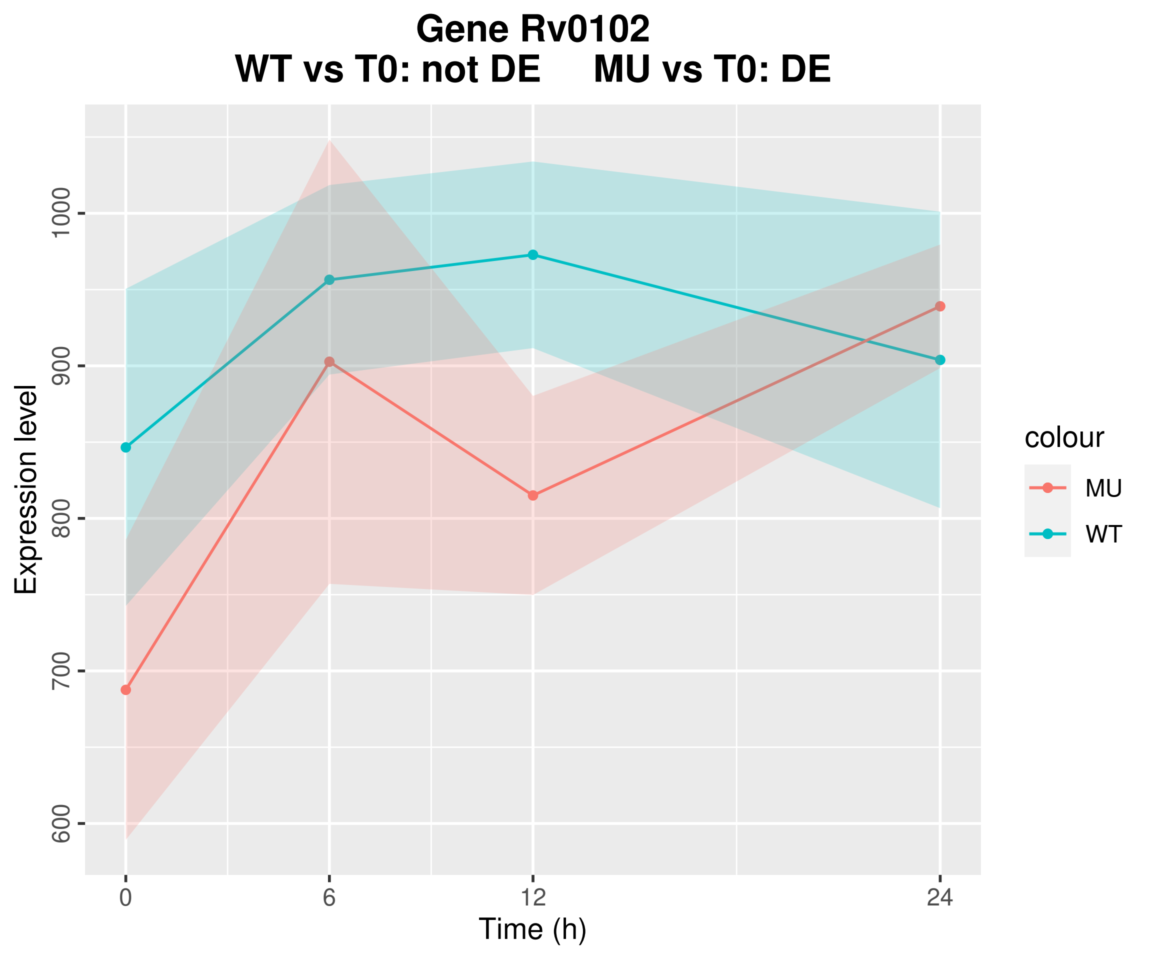

Gene Rv0102
WT vs T0: not DE   MU vs T0: DE
Expression level
1000
900
800
700
600
0
6
12
24
Time (h)
colour
MU
WT

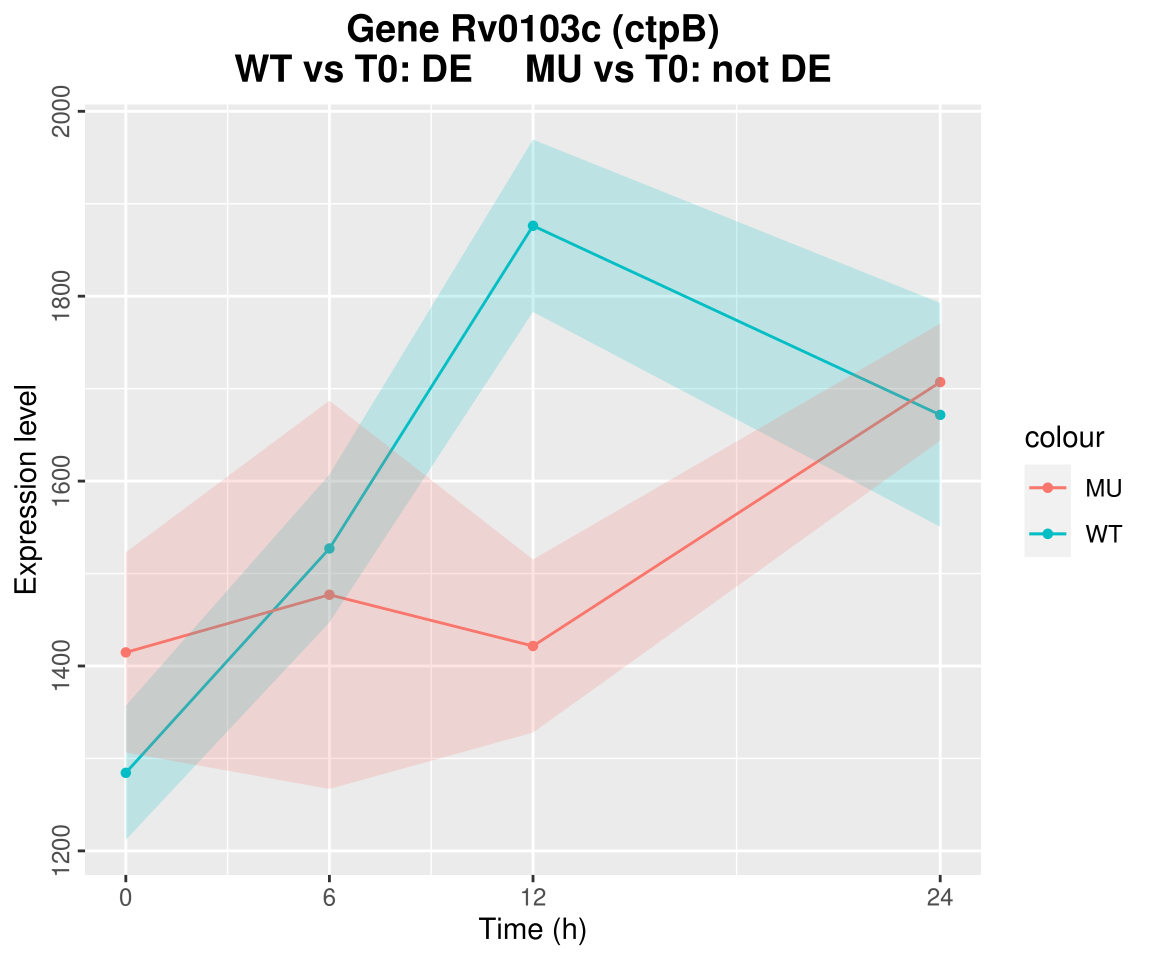

Gene Rv0103c (ctpB)
WT vs T0: DE MU vs T0: not DE
Expression level
2000
1800
1600
1400
1200
0
6
12
24
Time (h)
colour
MU
WT

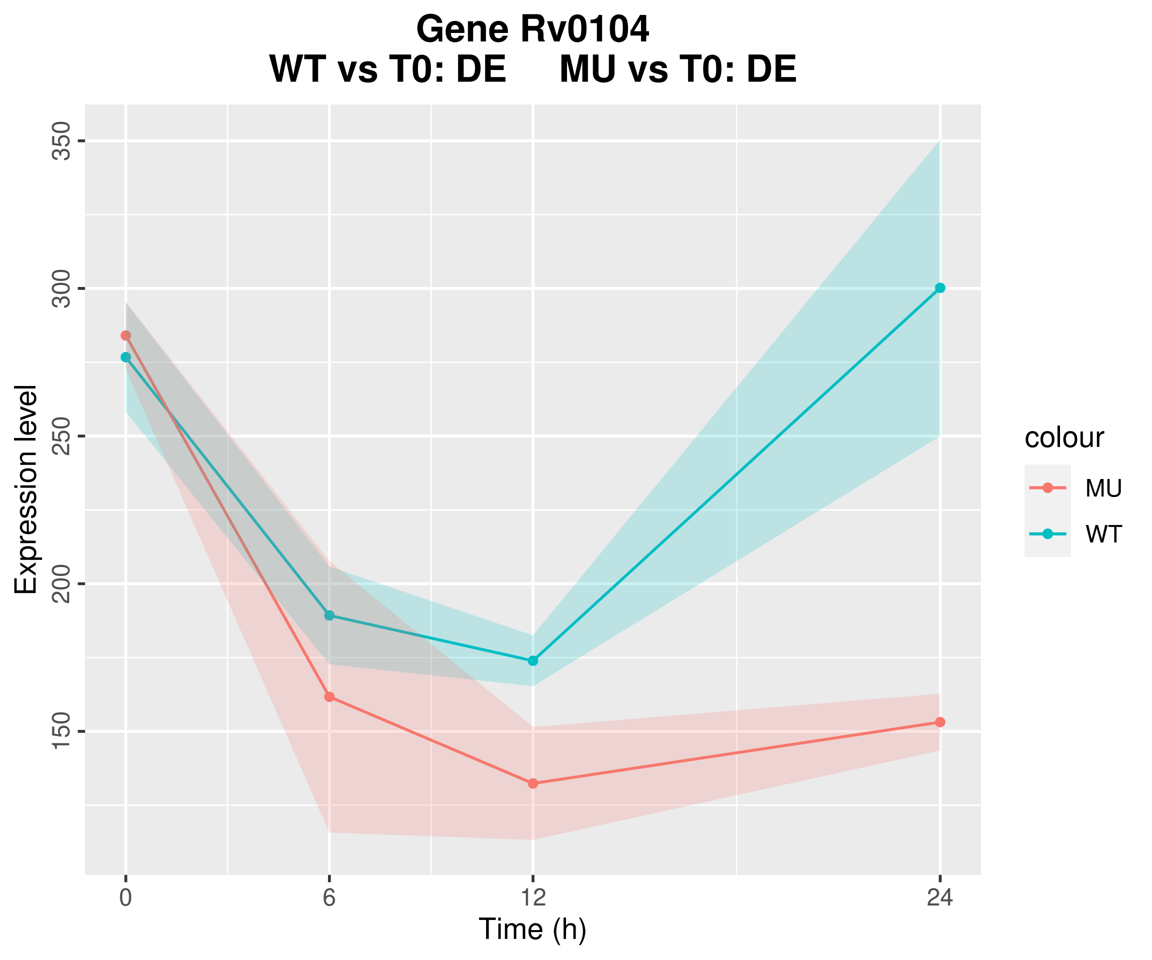

Gene Rv0104
WT vs T0: DE   MU vs T0: DE
Expression level
350
300
250
200
150
0
6
12
24
Time (h)
colour
MU
WT

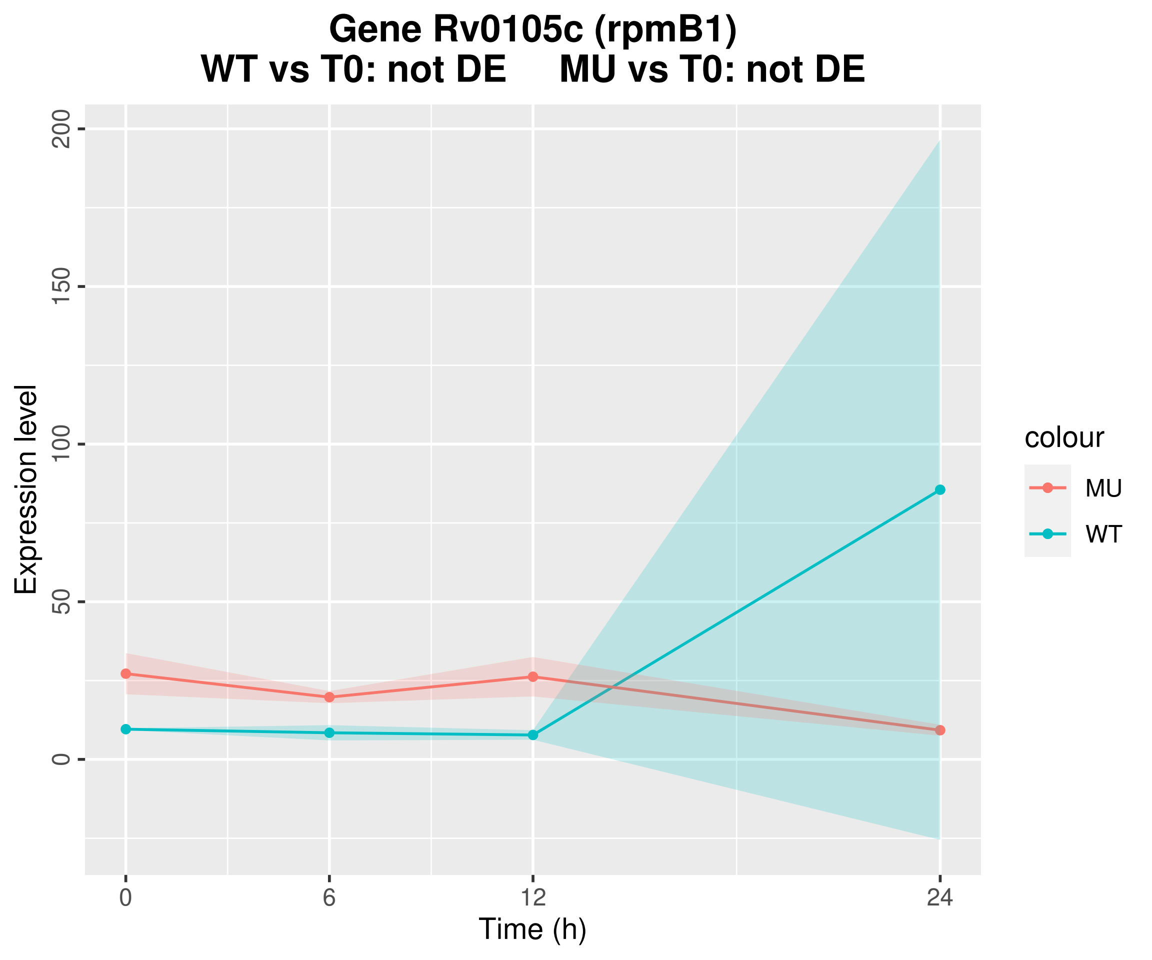
Gene Rv0105c (rpmB1)
WT vs T0: not DE MU vs T0: not DE
Expression level
200
150
100
50
0
0
6
12
24
Time (h)
colour
MU
WT

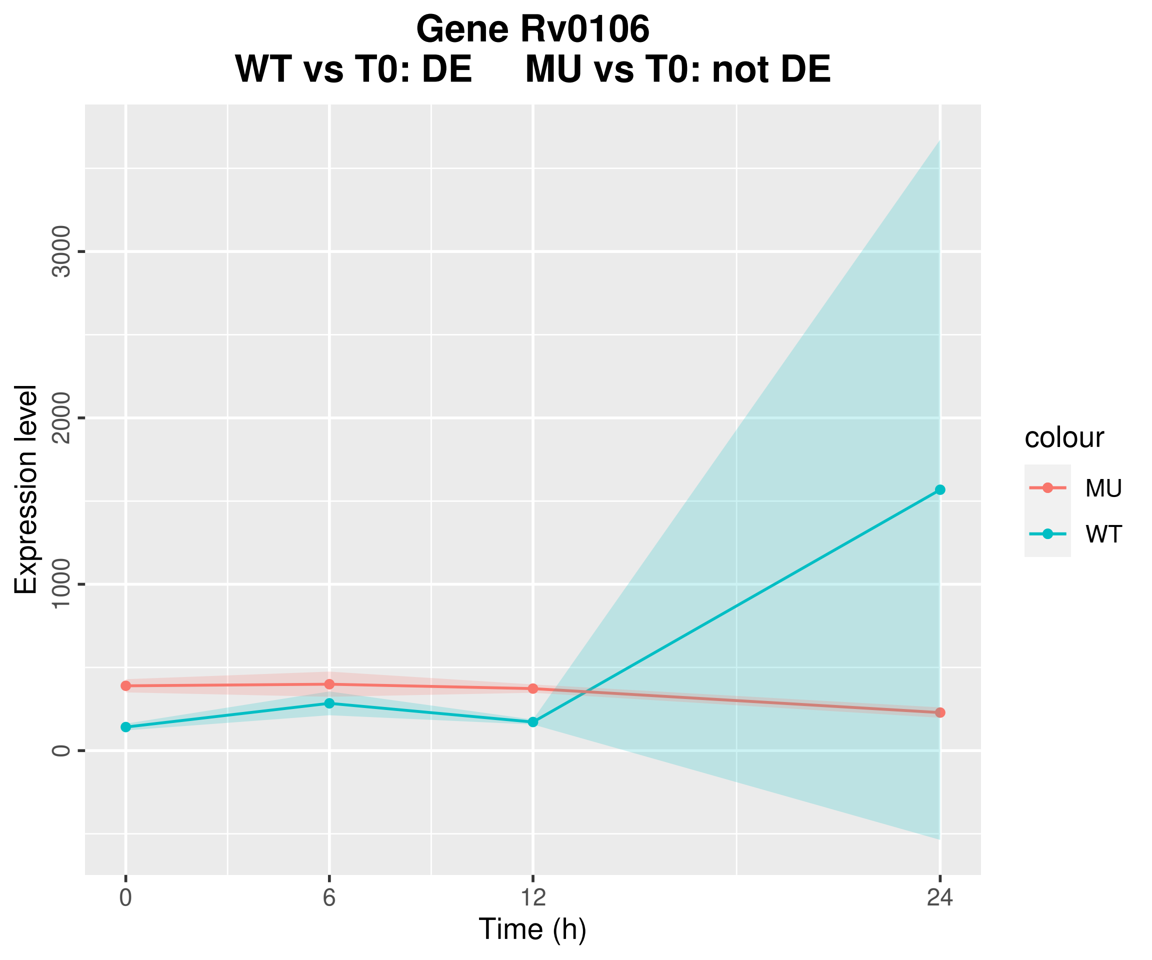

Gene Rv0106
WT vs T0: DE MU vs T0: not DE
Expression level
3000
2000
1000
0
0
6
12
24
Time (h)
colour
MU
WT

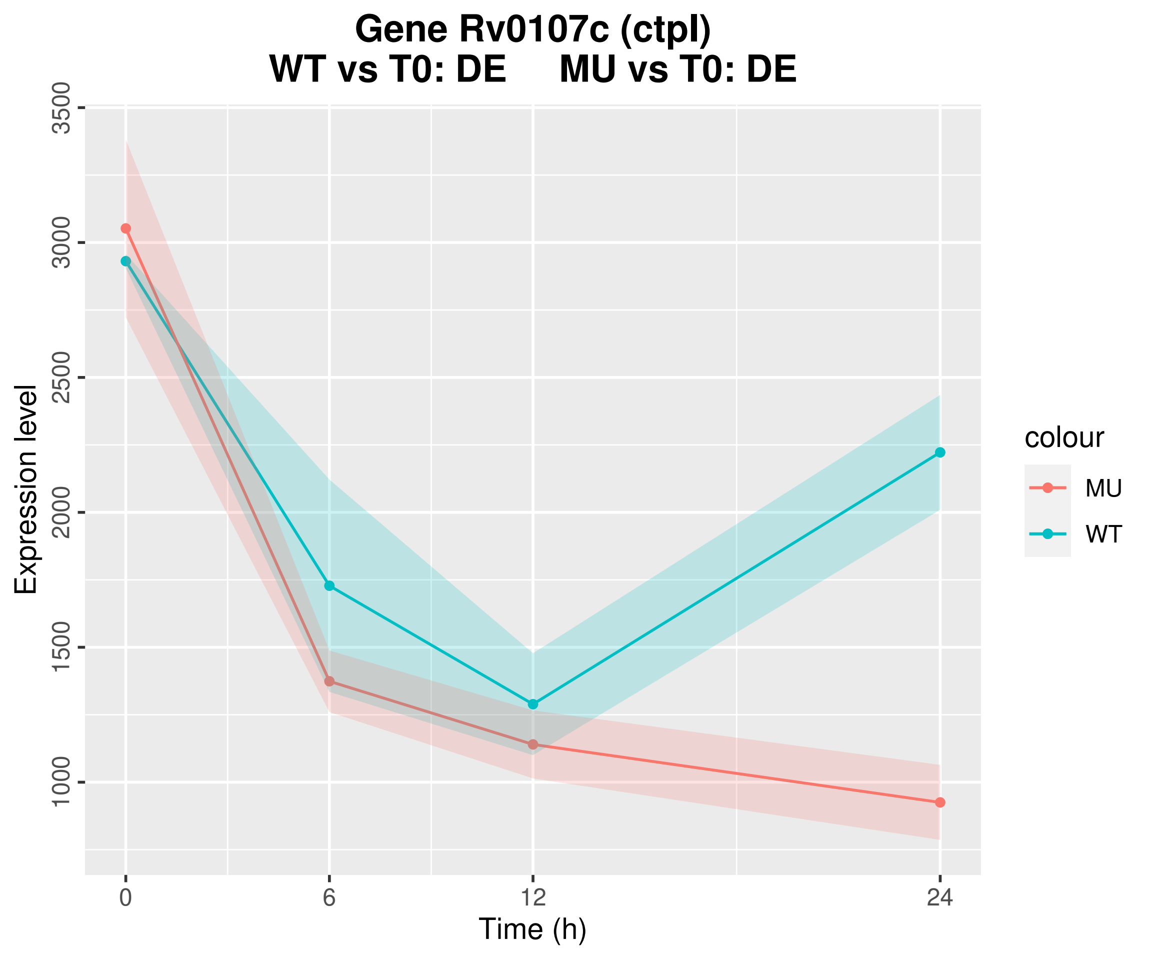

Gene Rv0107c (ctpI)
WT vs T0: DE   MU vs T0: DE
Expression level
3500
3000
2500
2000
1500
1000
0
6
12
24
Time (h)
colour
MU
WT

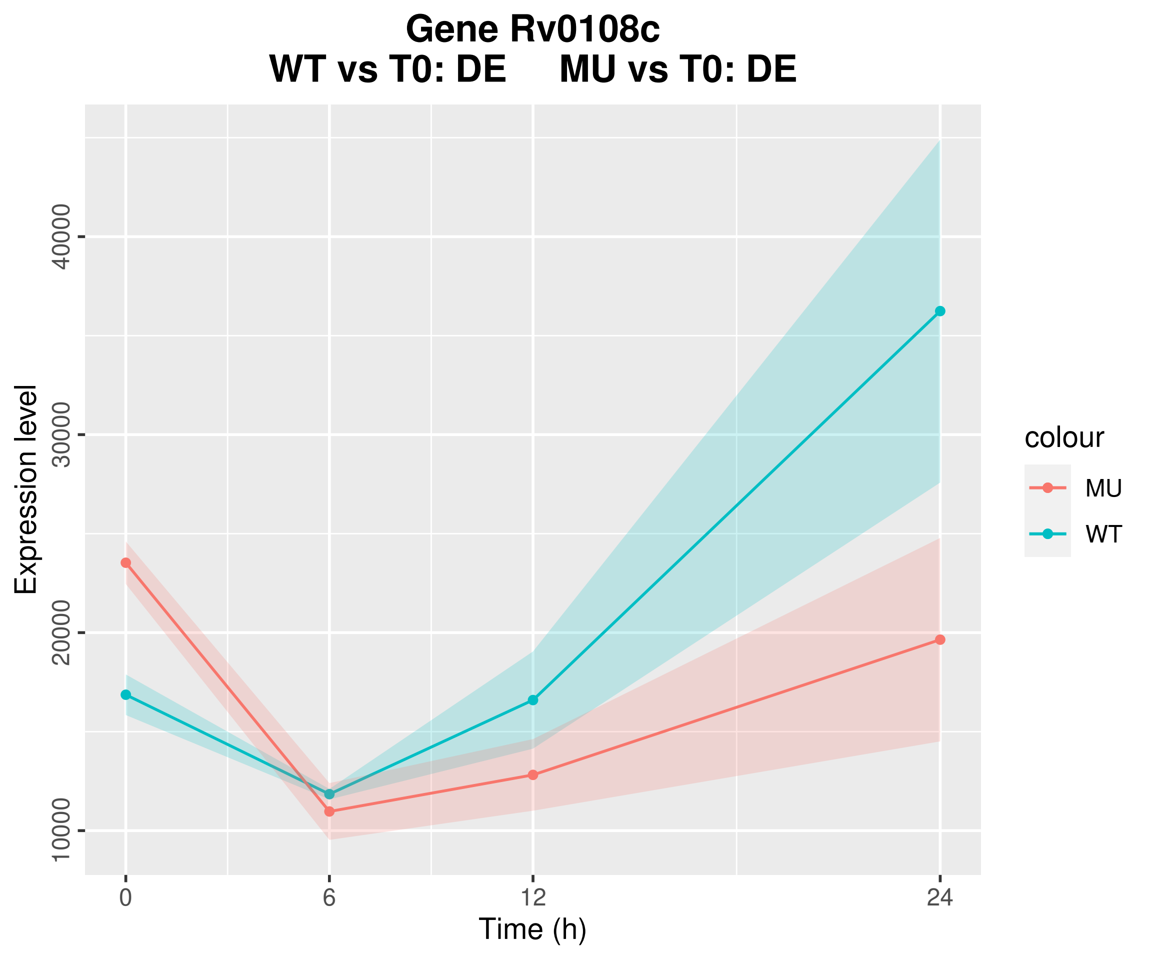
Gene Rv0108c
WT vs T0: DE MU vs T0: DE
Expression level
10000
20000
30000
40000
0
6
12
24
Time (h)
colour
MU
WT

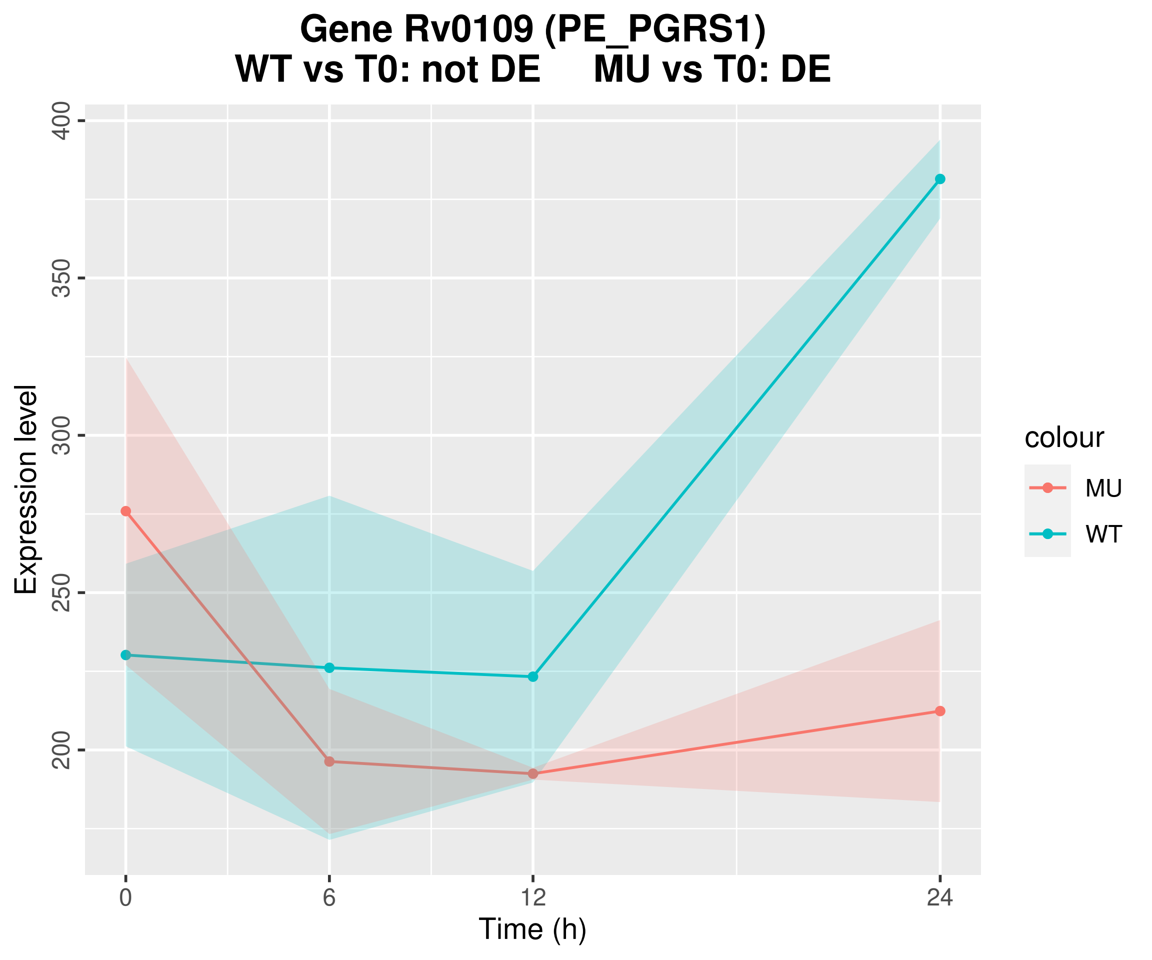

Gene Rv0109 (PE_PGRS1)
WT vs T0: not DE   MU vs T0: DE
Expression level
400
350
300
250
200
0
6
12
24
Time (h)
colour
MU
WT

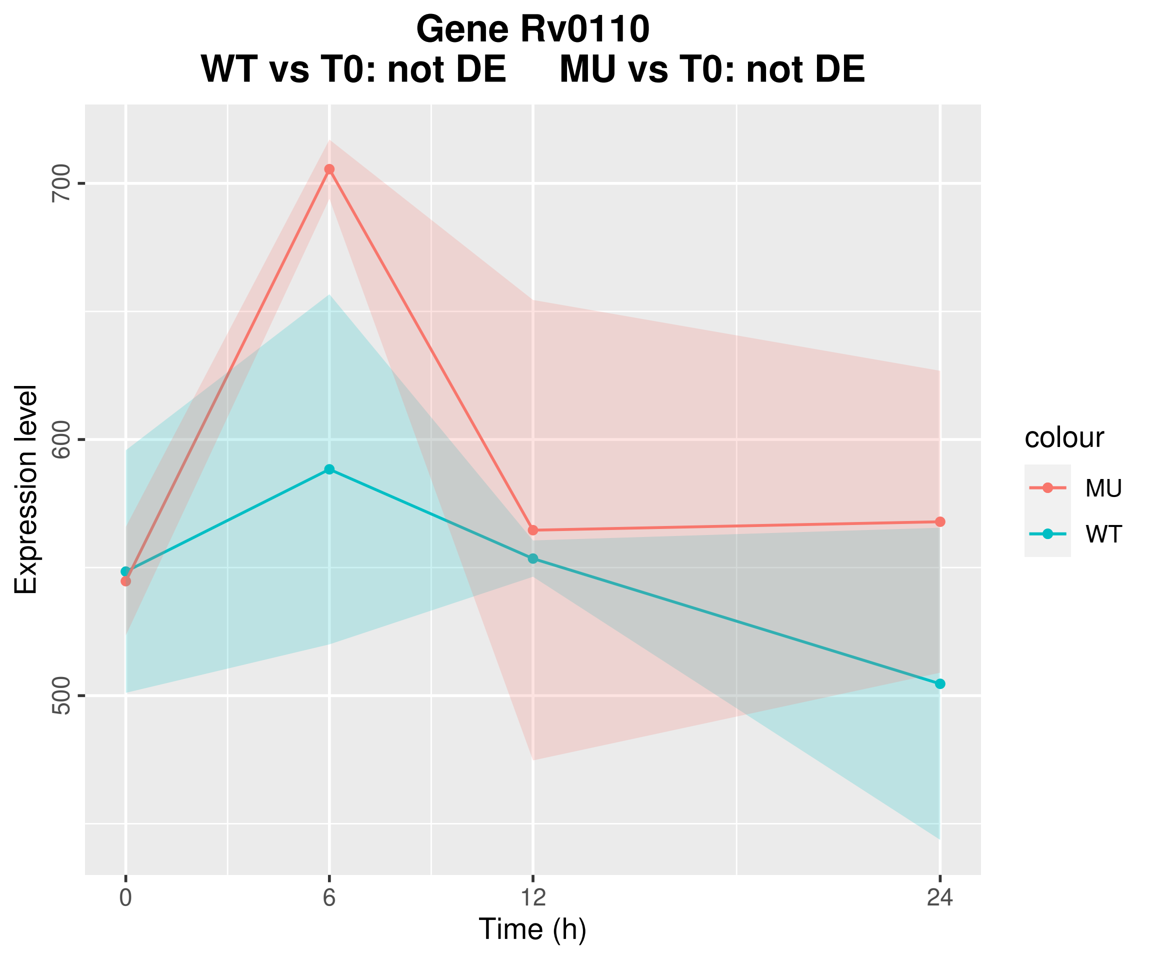
Gene Rv0110
WT vs T0: not DE   MU vs T0: not DE
700
600
500
Expression level
0
6
12
24
Time (h)
colour
MU
WT

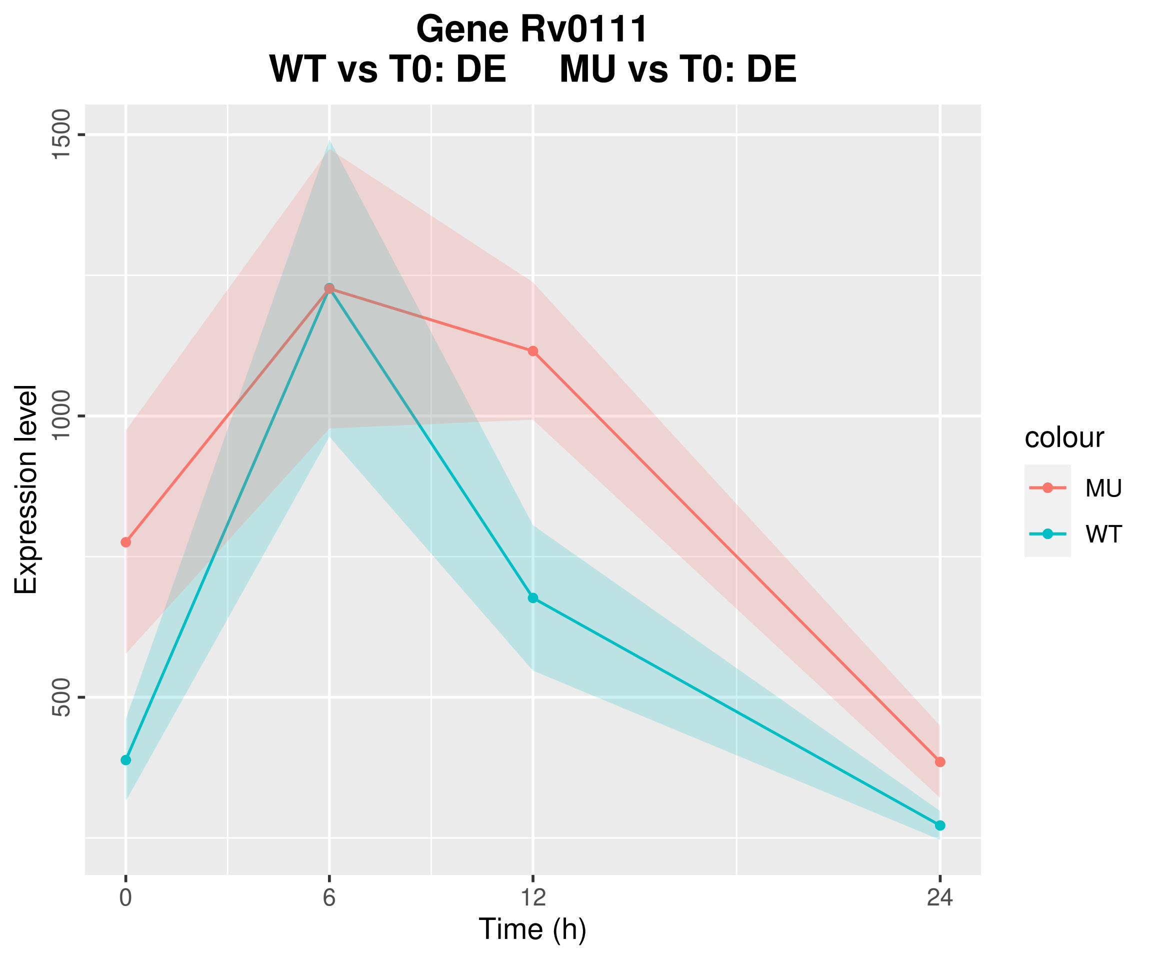

Gene Rv0111
WT vs T0: DE   MU vs T0: DE
Expression level
1500
1000
500
0
6
12
24
Time (h)
colour
MU
WT

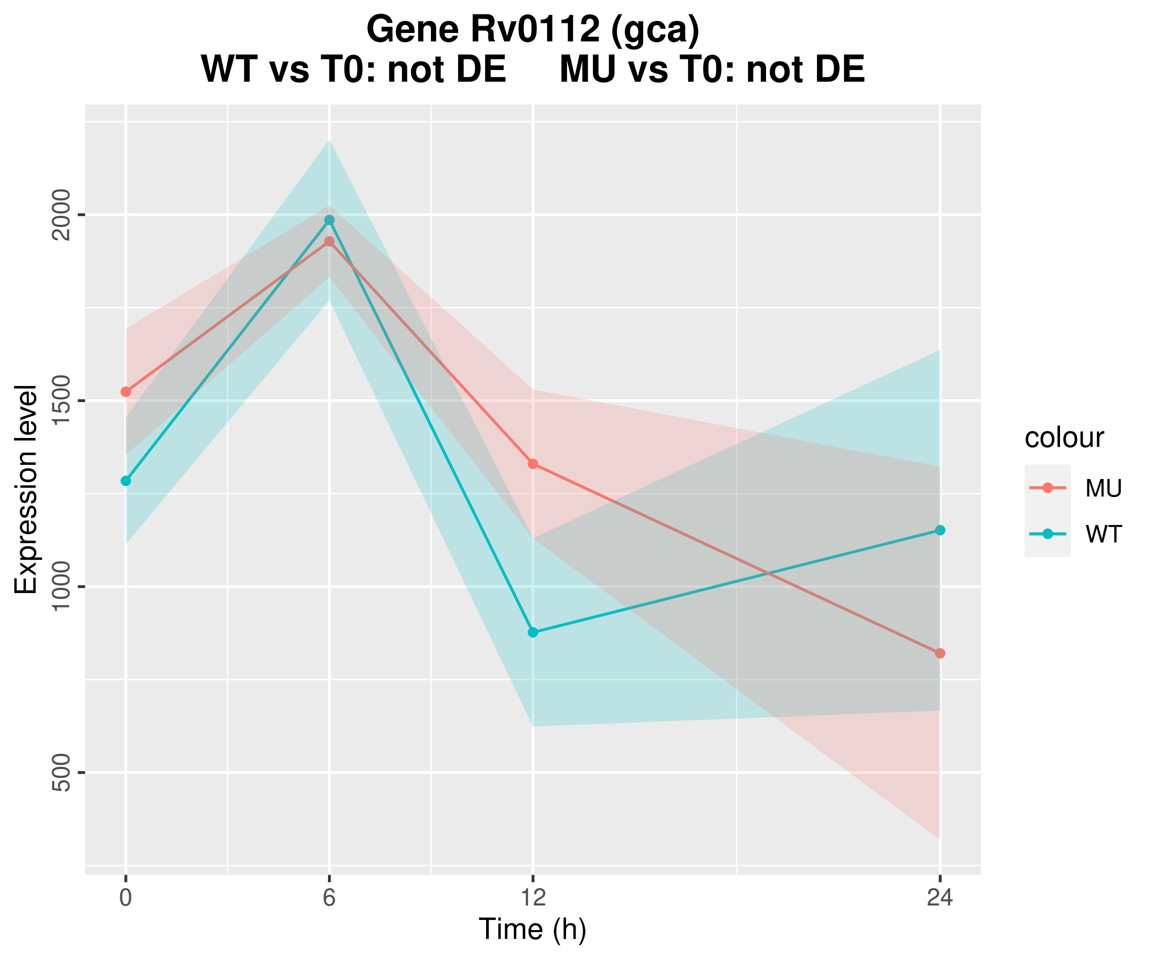
Gene Rv0112 (gca)
WT vs T0: not DE   MU vs T0: not DE
Expression level
2000
1500
1000
500
0
6
12
24
Time (h)
colour
MU
WT

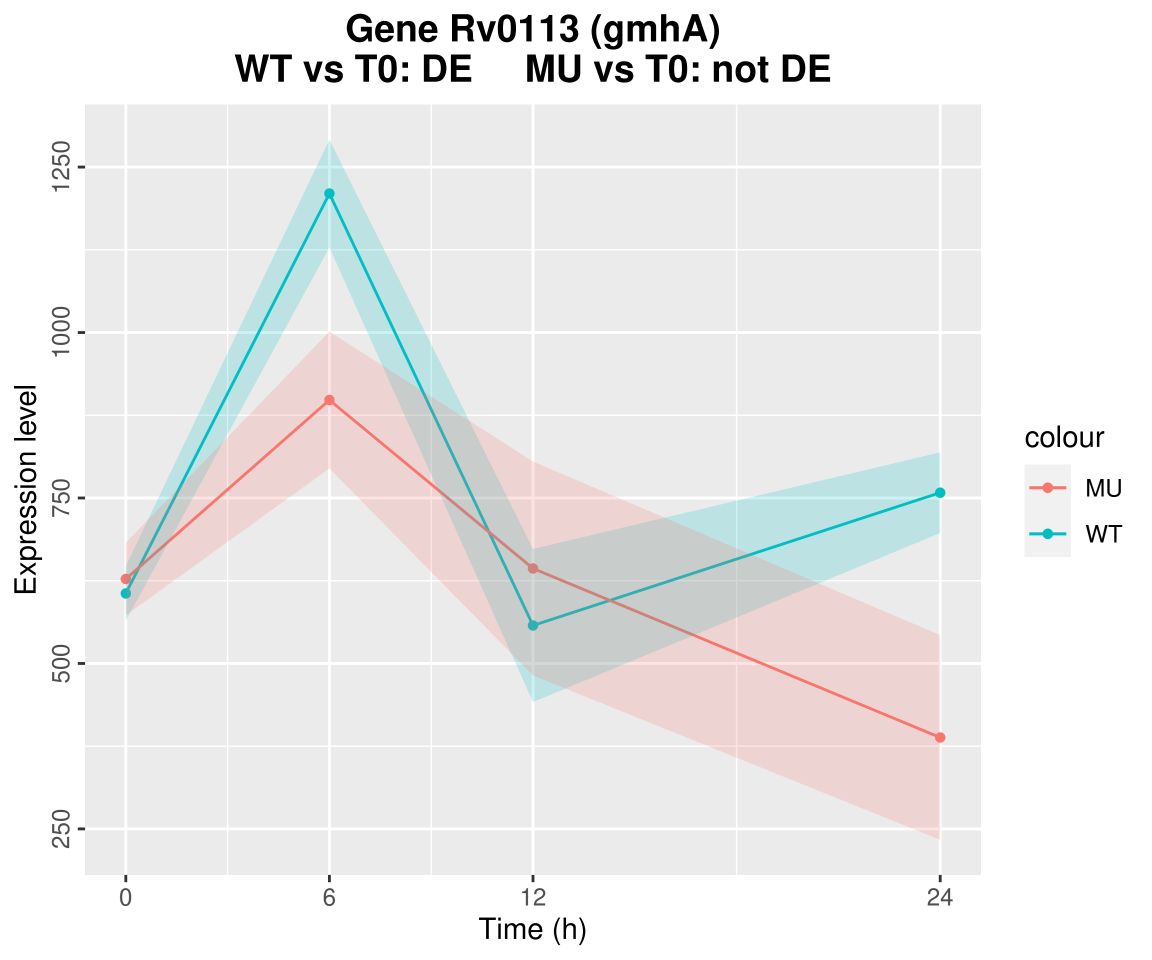

Gene Rv0113 (gmhA)
WT vs T0: DE MU vs T0: not DE
Expression level
1250
1000
750
500
250
0
6
12
24
Time (h)
colour
MU
WT

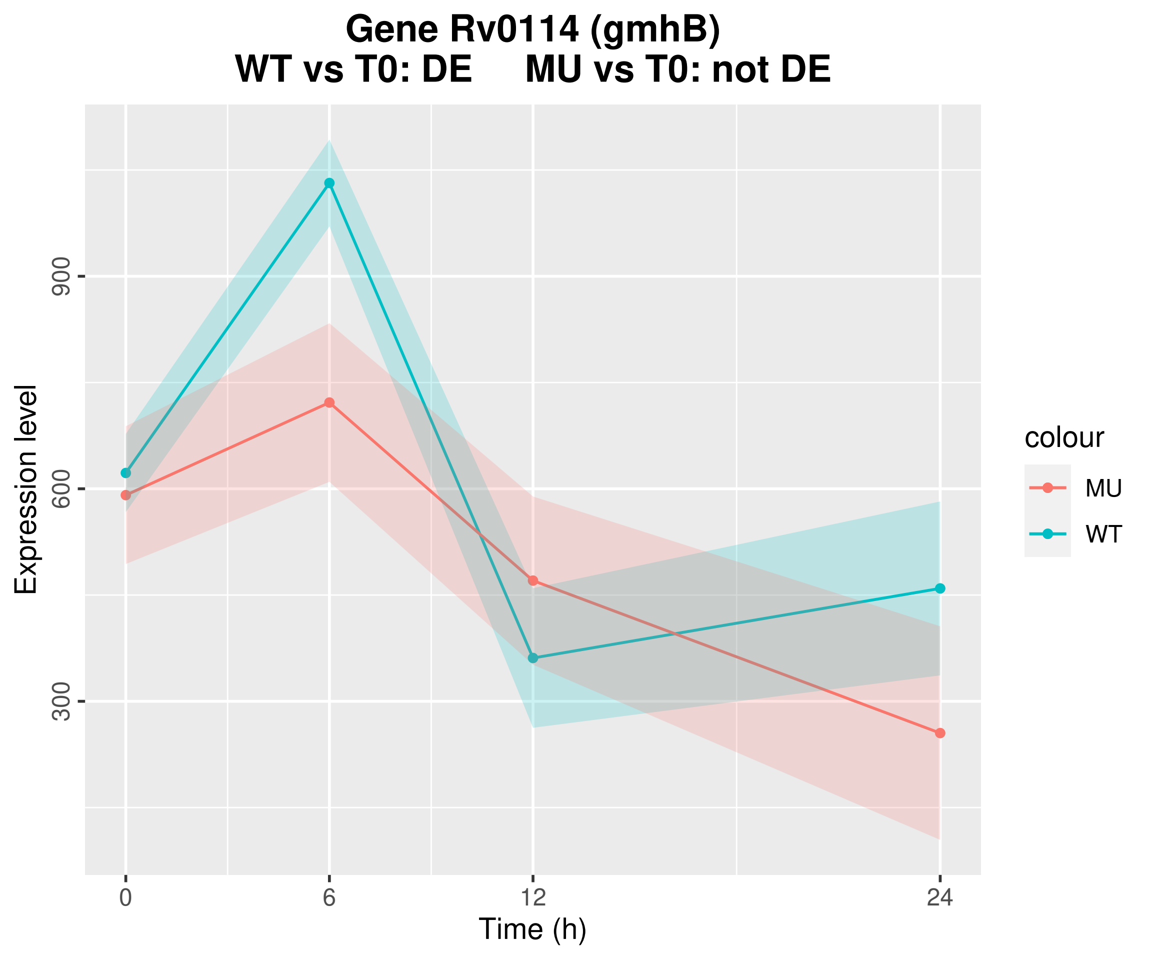

Gene Rv0114 (gmhB)
WT vs T0: DE MU vs T0: not DE
Expression level
900
600
300
0
6
12
24
Time (h)
colour
MU
WT

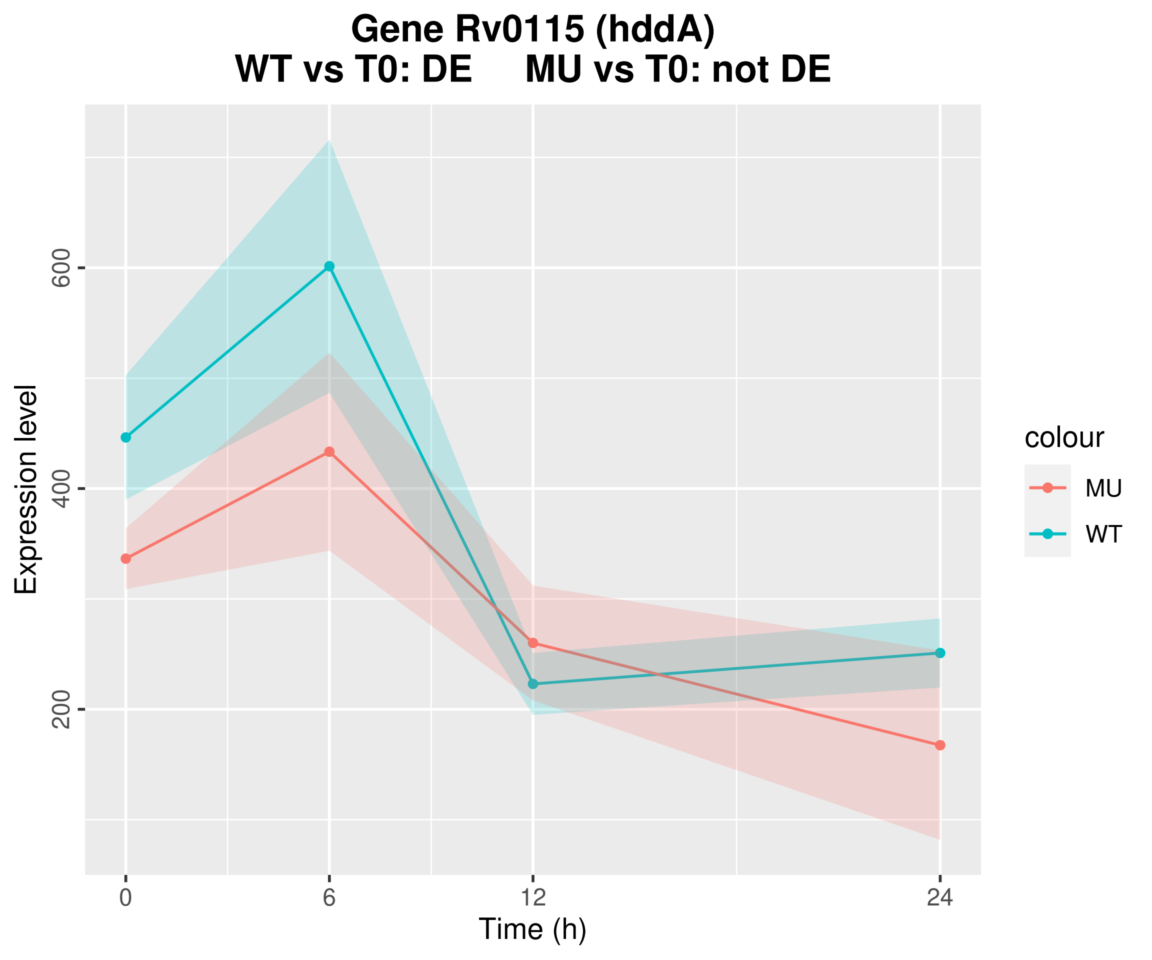
Gene Rv0115 (hddA)
WT vs T0: DE   MU vs T0: not DE
Expression level
Time (h)
200
400
600
0
6
12
24
colour
MU
WT

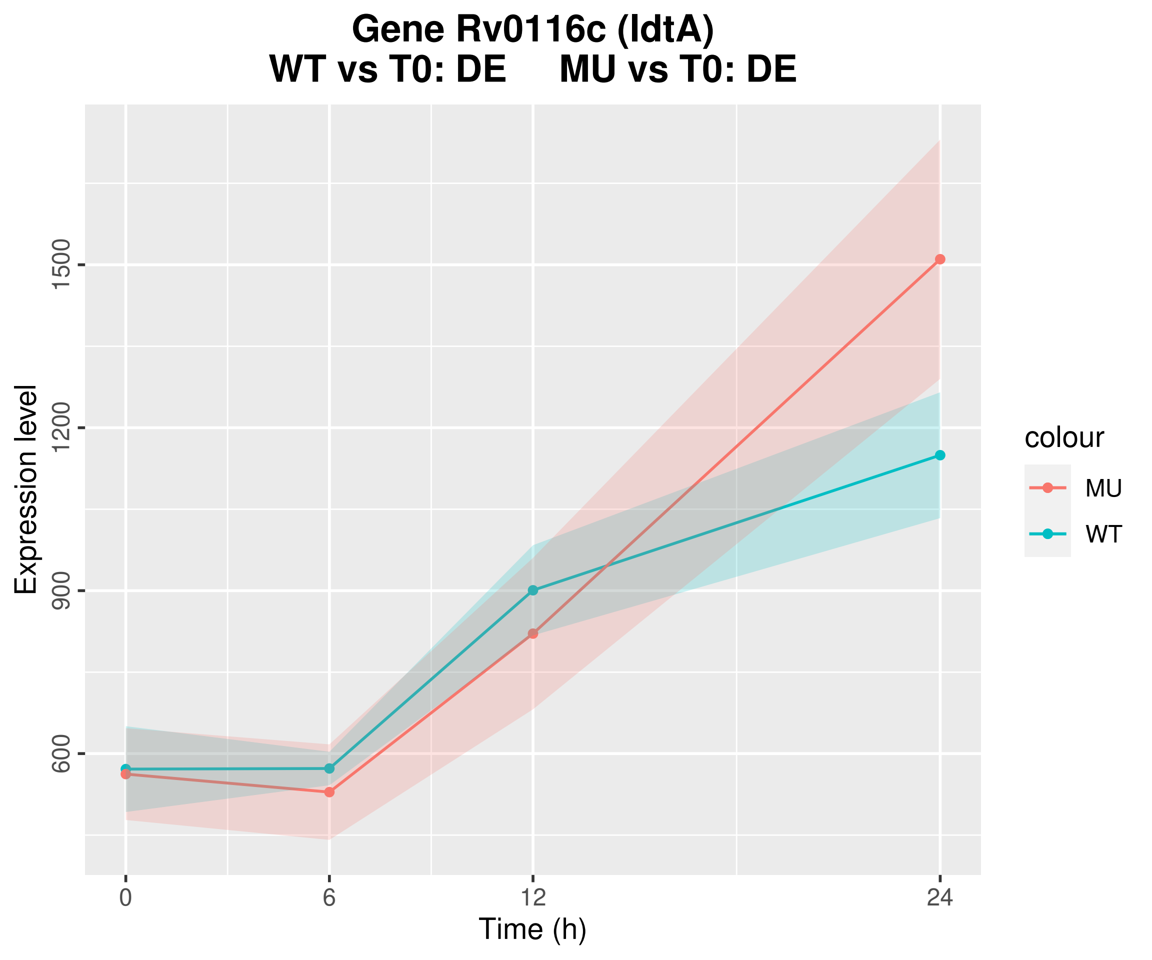

Gene Rv0116c (ldtA)
WT vs T0: DE MU vs T0: DE
Expression level
1500
1200
900
600
0
6
12
24
Time (h)
colour
MU
WT

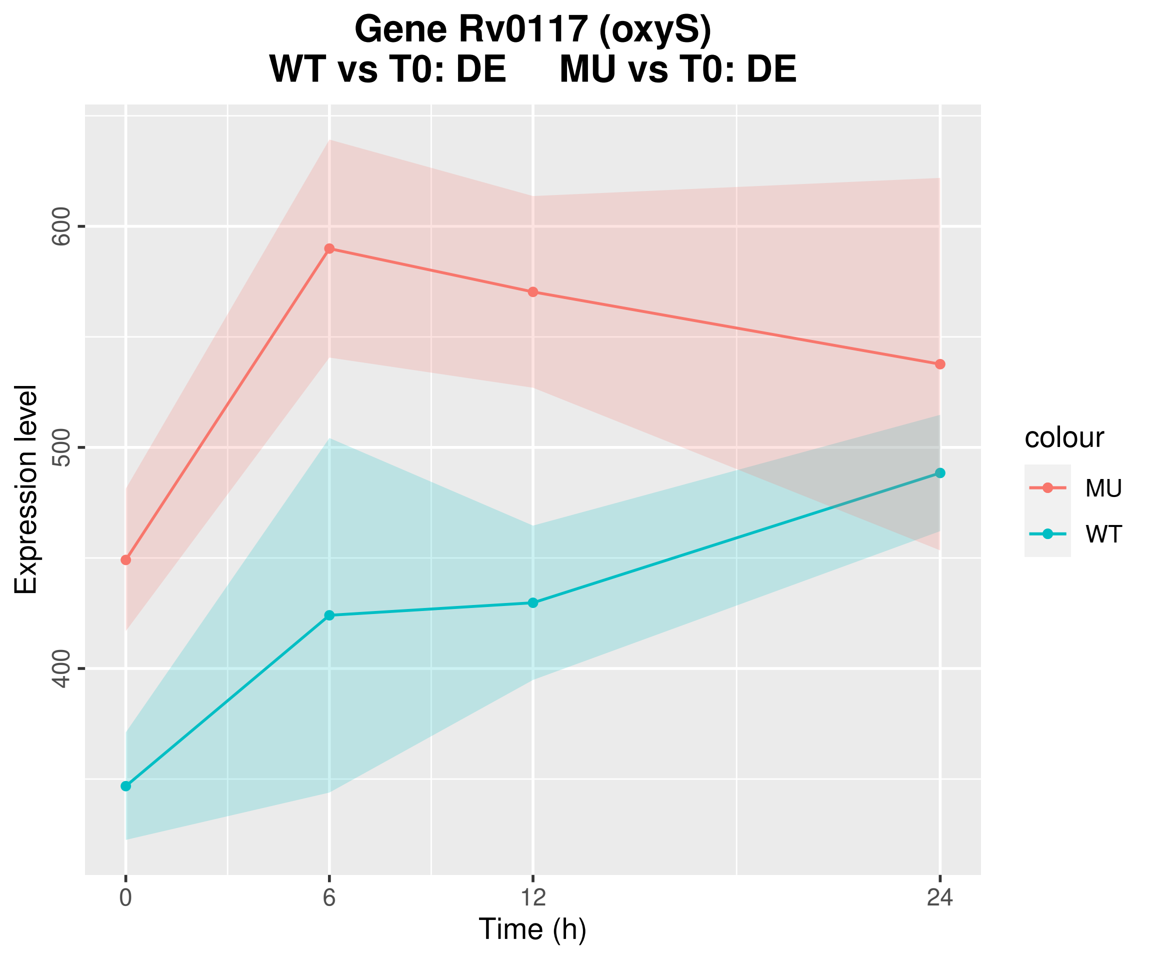

Gene Rv0117 (oxyS)
WT vs T0: DE MU vs T0: DE
600
500
400
Expression level
0
6
12
24
Time (h)
colour
MU
WT

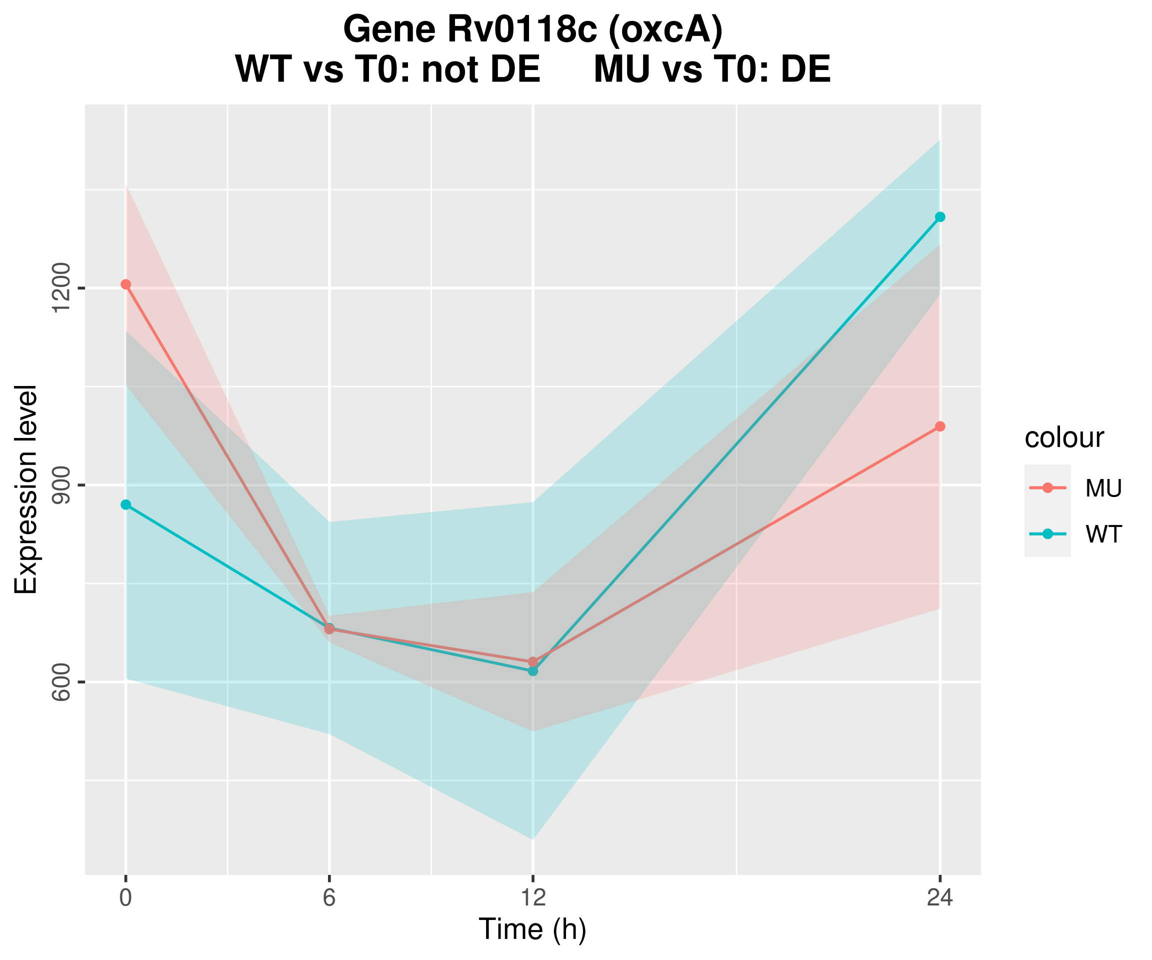

Gene Rv0118c (oxcA)
WT vs T0: not DE MU vs T0: DE
Expression level
1200
900
600
0
6
12
24
Time (h)
colour
MU
WT

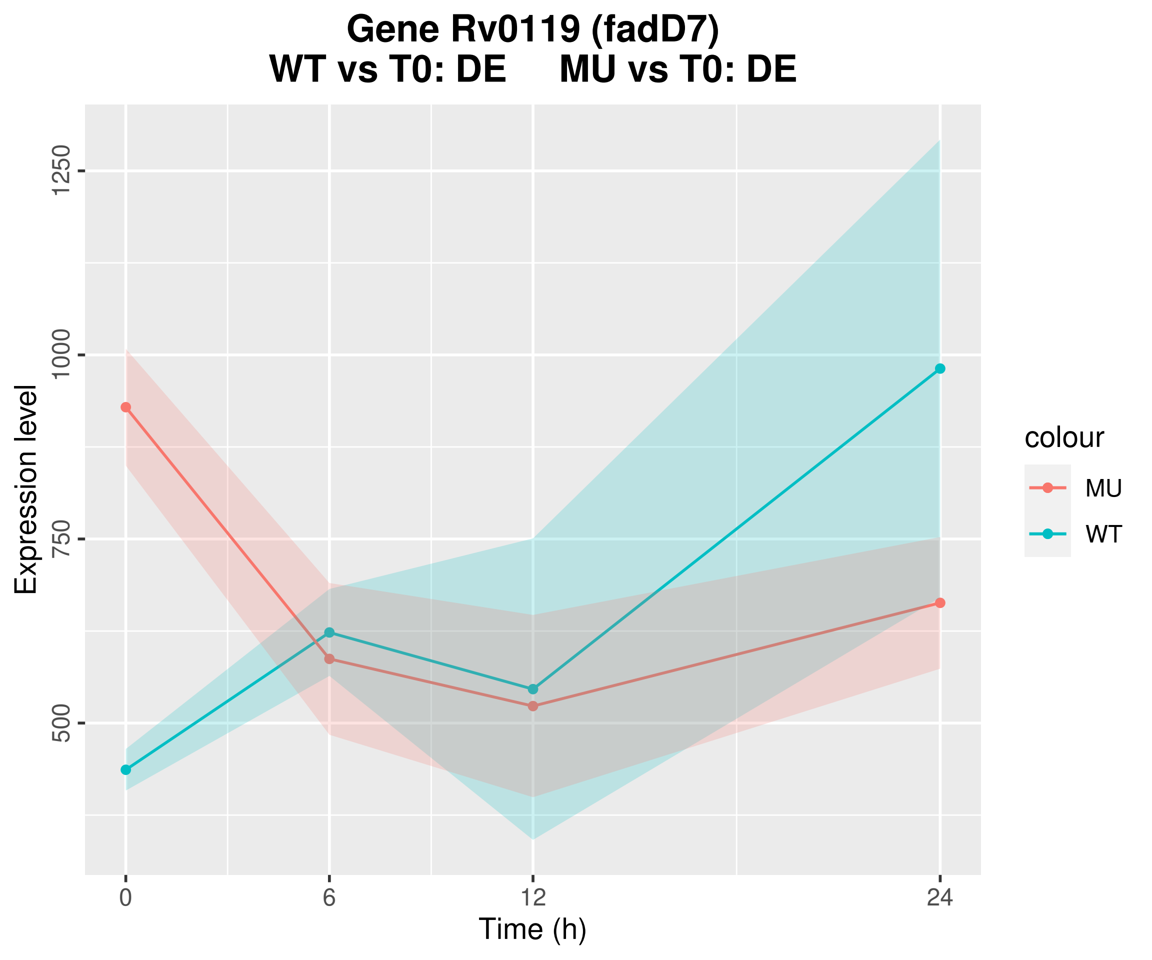

Gene Rv0119 (fadD7)
WT vs T0: DE   MU vs T0: DE
1250
1000
750
500
Expression level
0
6
12
24
Time (h)
colour
MU
WT

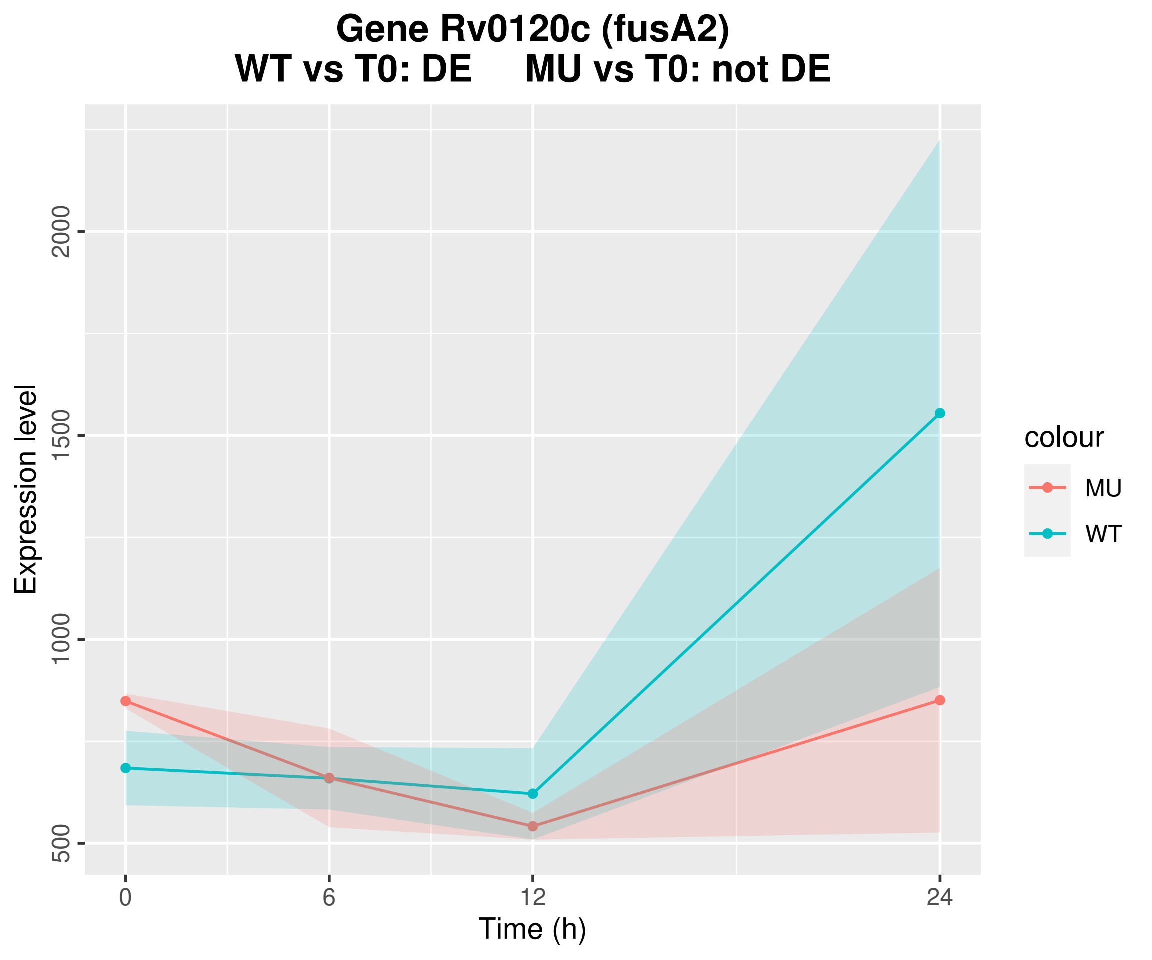
Gene Rv0120c (fusA2)
WT vs T0: DE   MU vs T0: not DE
Expression level
2000
1500
1000
500
0
6
12
24
Time (h)
colour
MU
WT

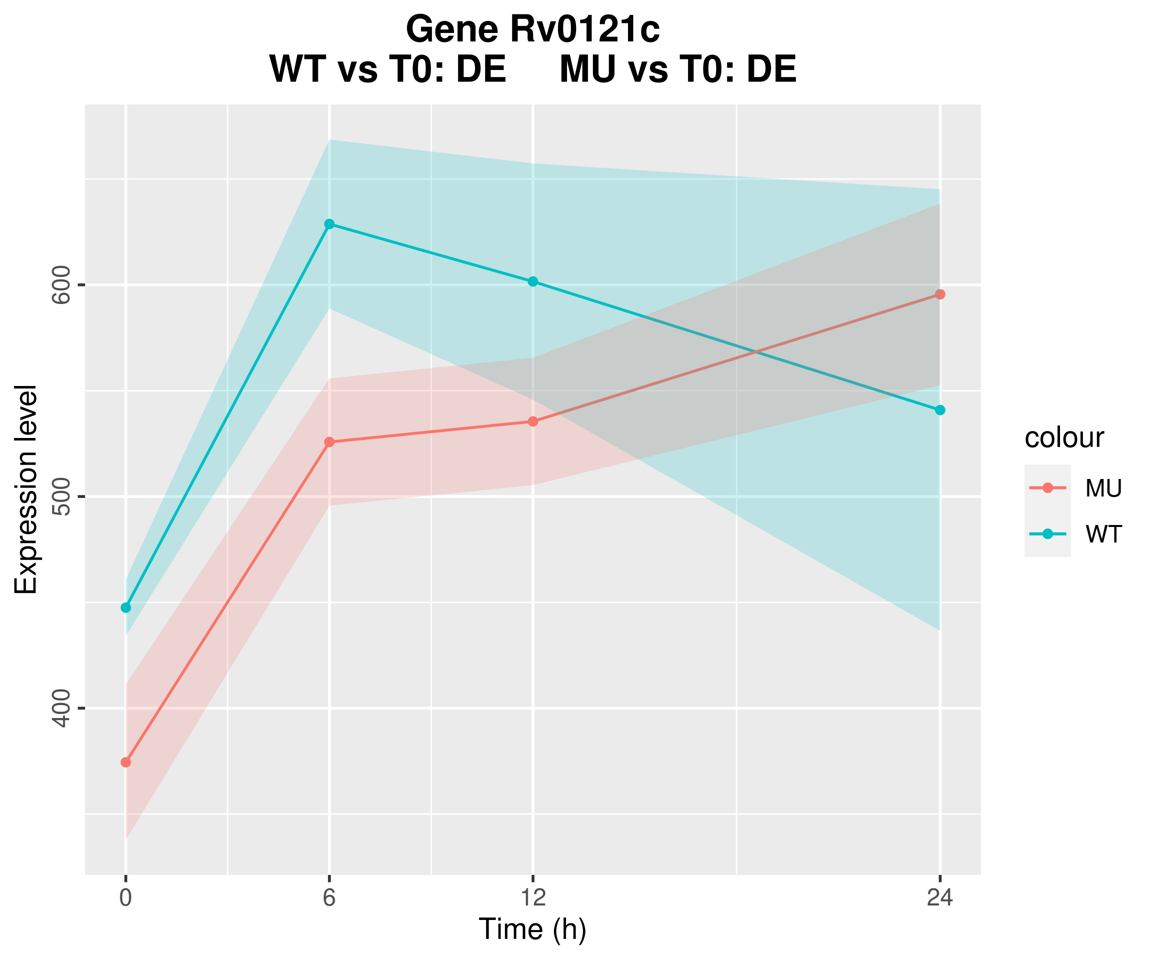

Gene Rv0121c
WT vs T0: DE MU vs T0: DE
Expression level
600
500
400
0
6
12
24
Time (h)
colour
MU
WT

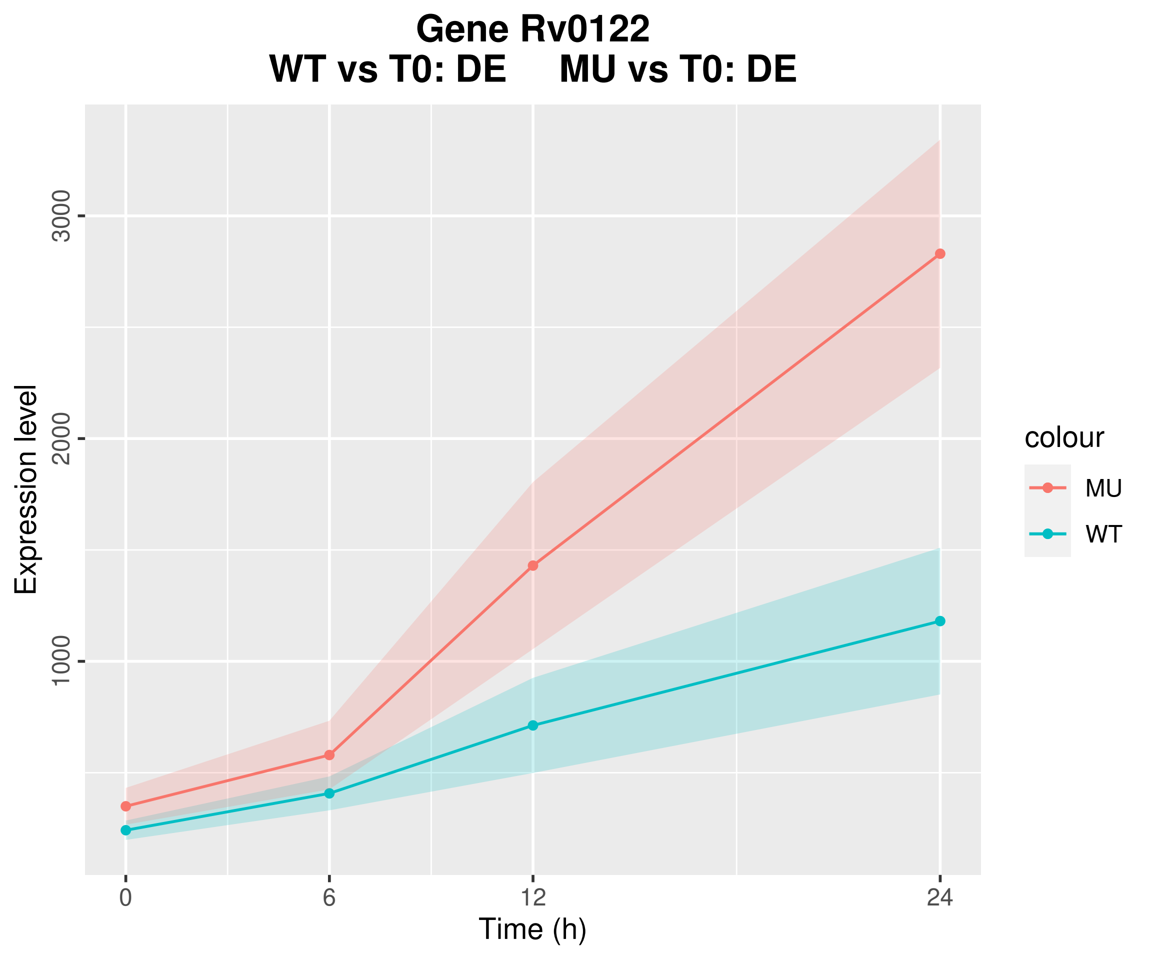
Gene Rv0122
WT vs T0: DE MU vs T0: DE
Expression level
3000
2000
1000
0
6
12
24
Time (h)
colour
MU
WT

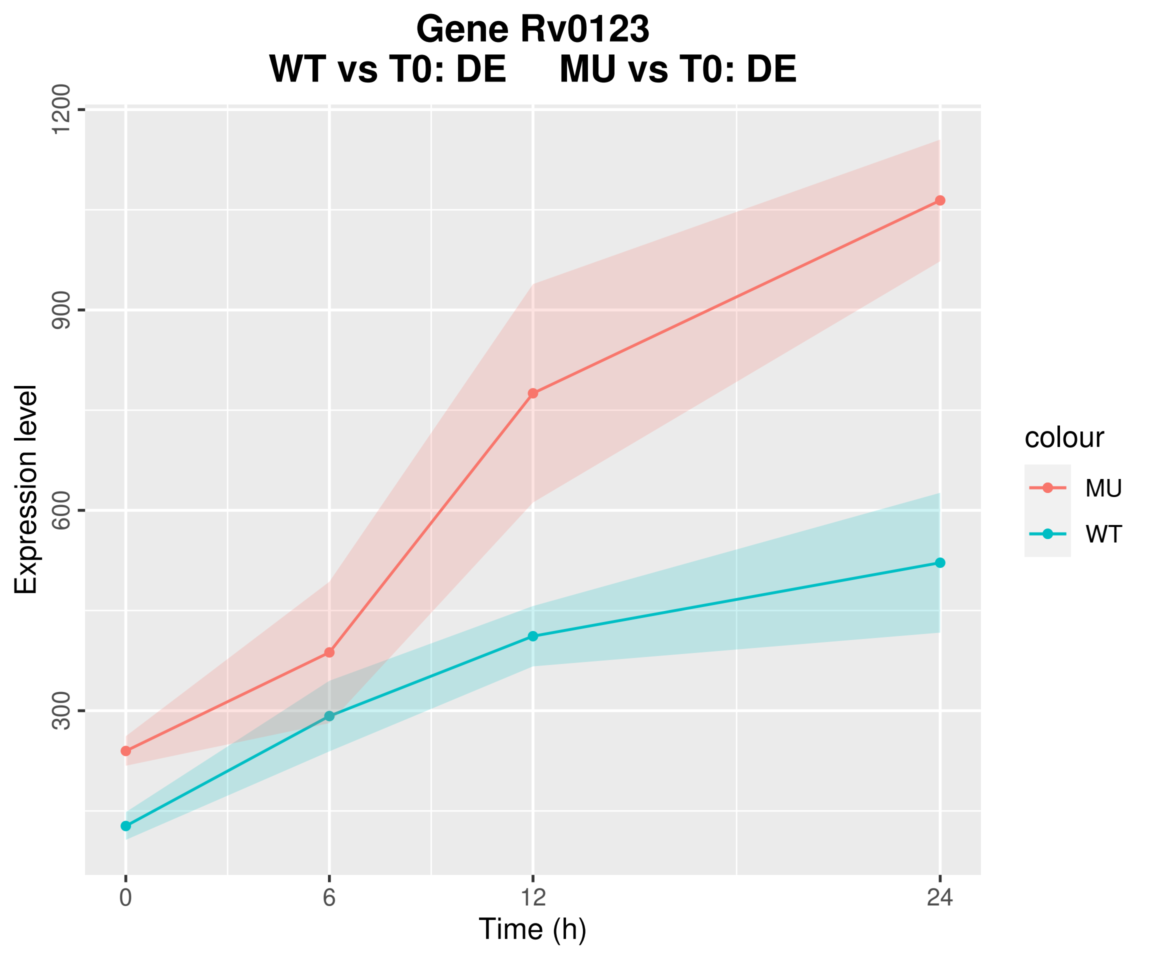

Gene Rv0123
WT vs T0: DE MU vs T0: DE
Expression level
1200
900
600
300
0
6
12
24
Time (h)
colour
MU
WT

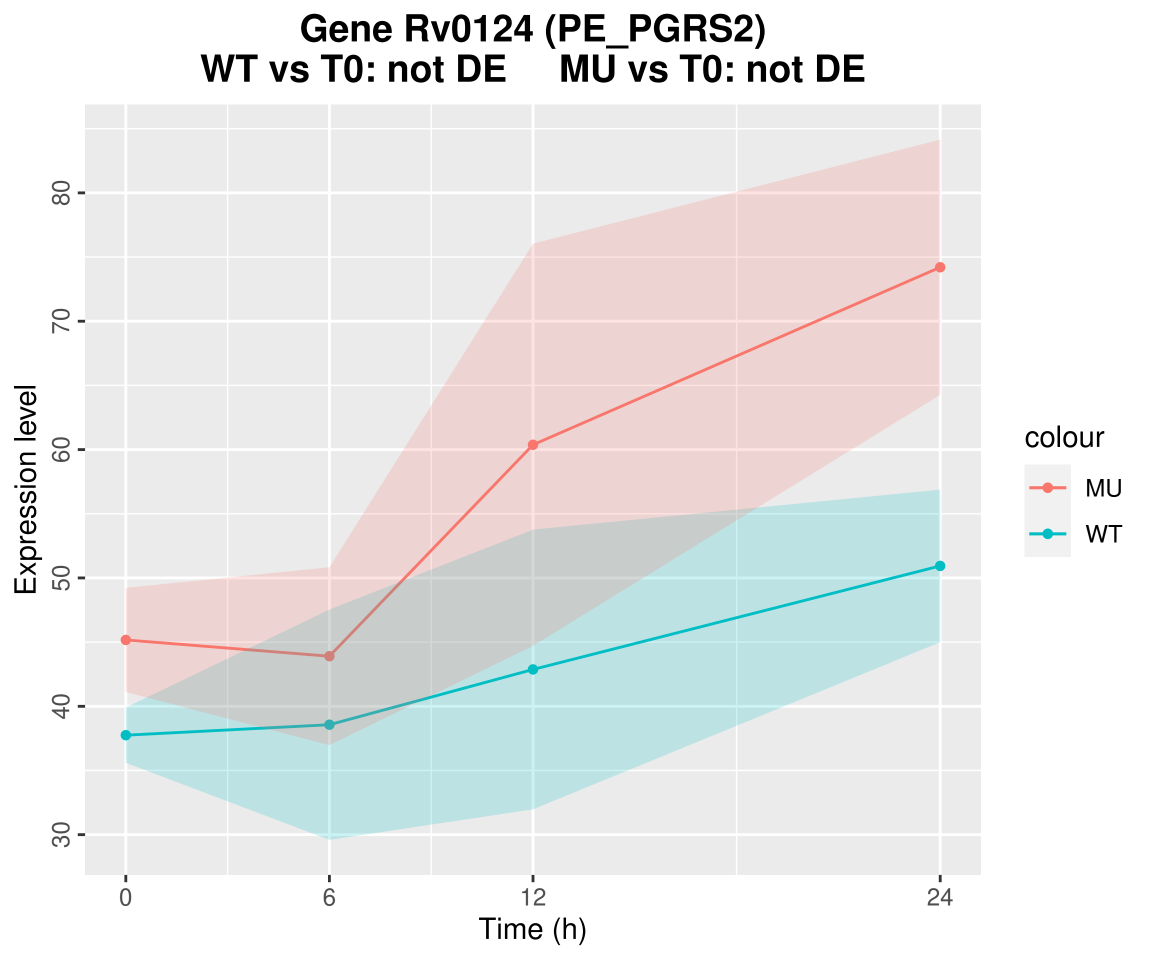

Gene Rv0124 (PE_PGRS2)
WT vs T0: not DE MU vs T0: not DE
Expression level
80
70
60
50
40
30
0
6
12
24
Time (h)
colour
MU
WT

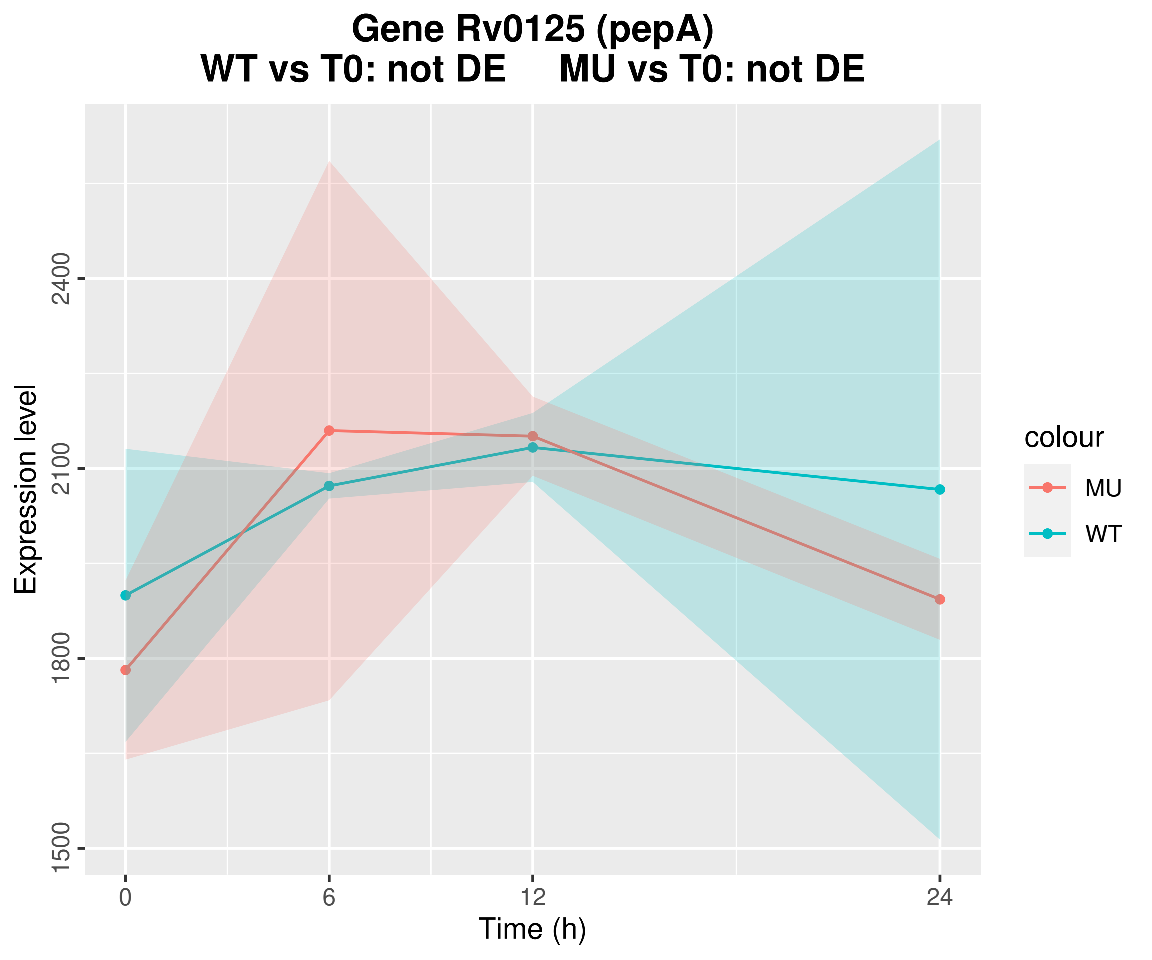
Gene Rv0125 (pepA)
WT vs T0: not DE   MU vs T0: not DE
Expression level
2400
2100
1800
1500
0
6
12
24
Time (h)
colour
MU
WT

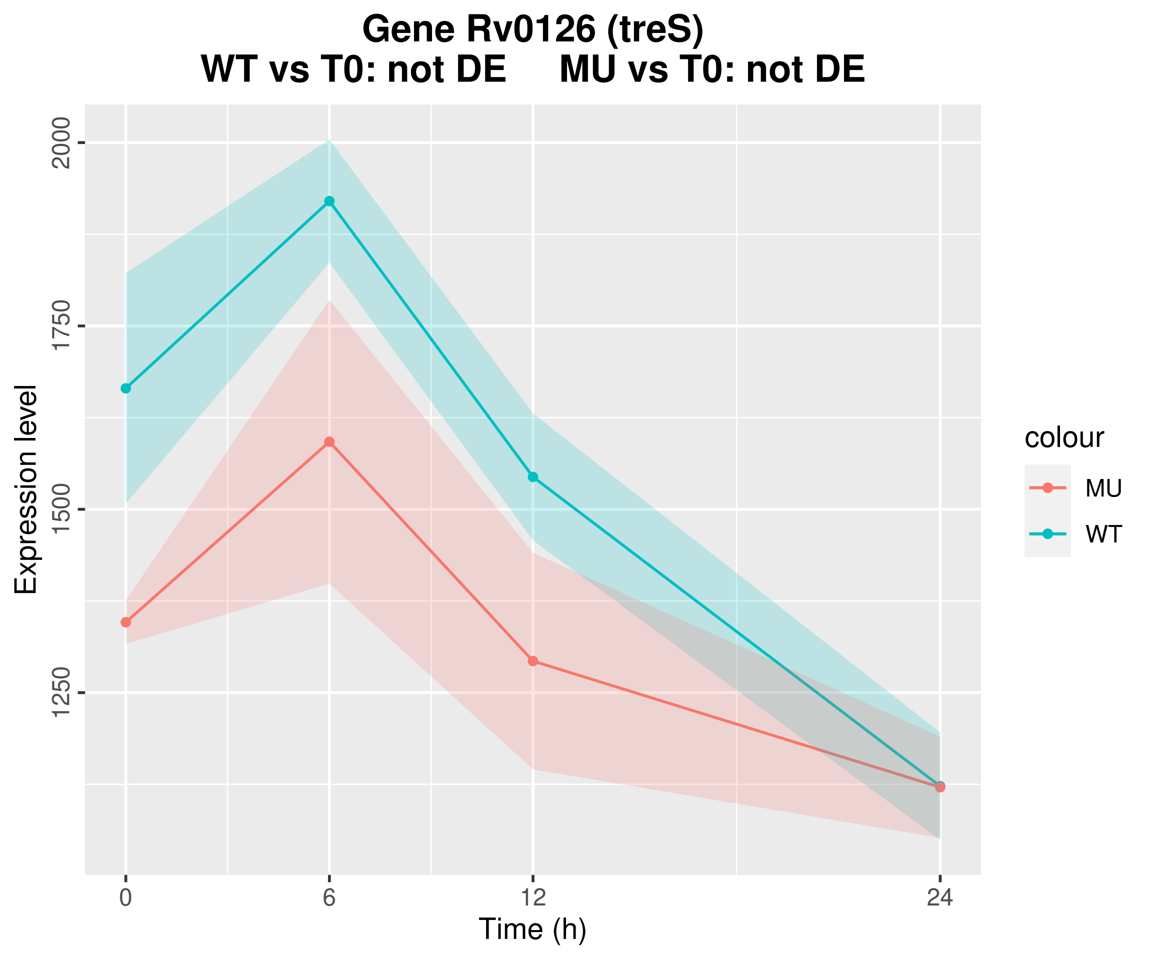
Gene Rv0126 (treS)
WT vs T0: not DE   MU vs T0: not DE
Expression level
2000
1750
1500
1250
Time (h)
0
6
12
24
colour
MU
WT

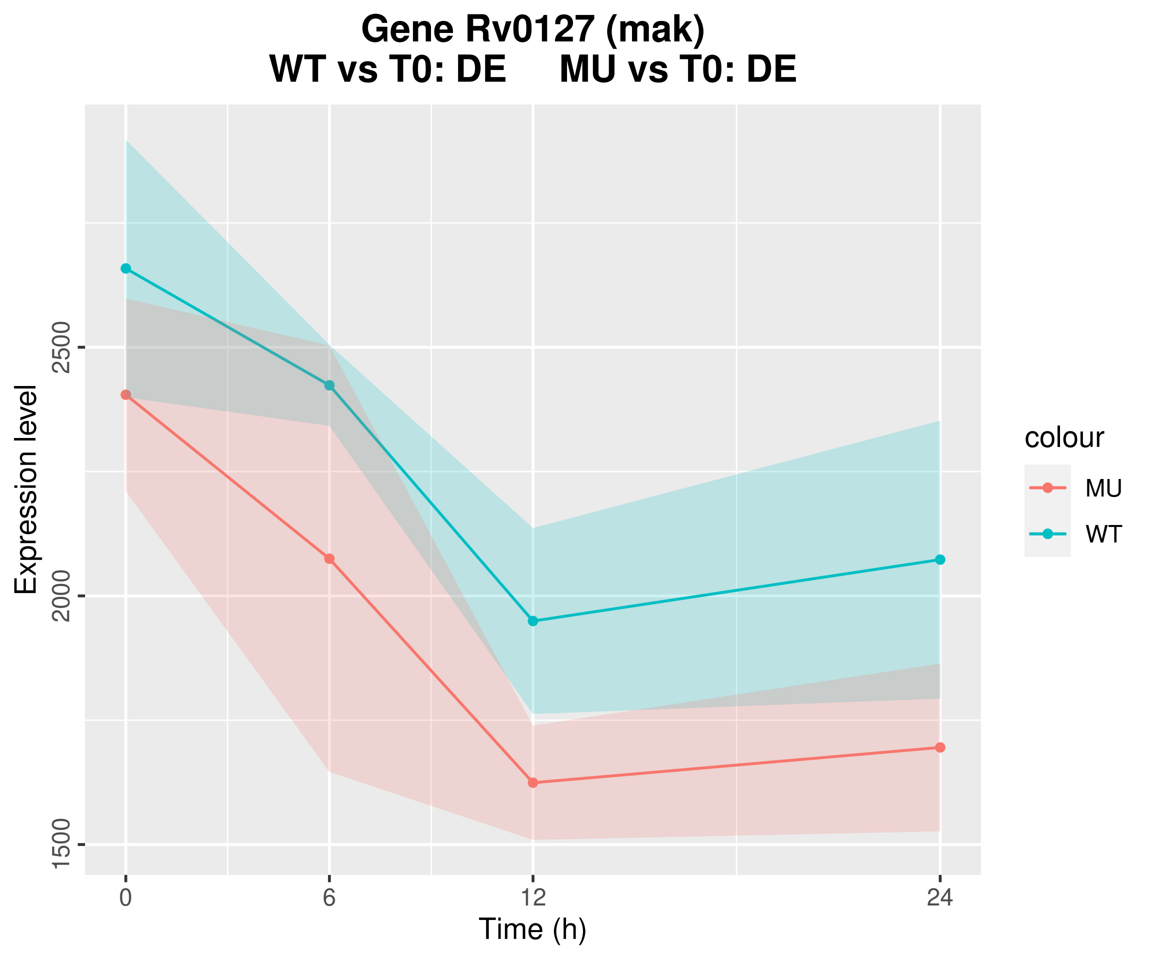

Gene Rv0127 (mak)
WT vs T0: DE MU vs T0: DE
Expression level
2500
2000
1500
0
6
12
24
Time (h)
colour
MU
WT

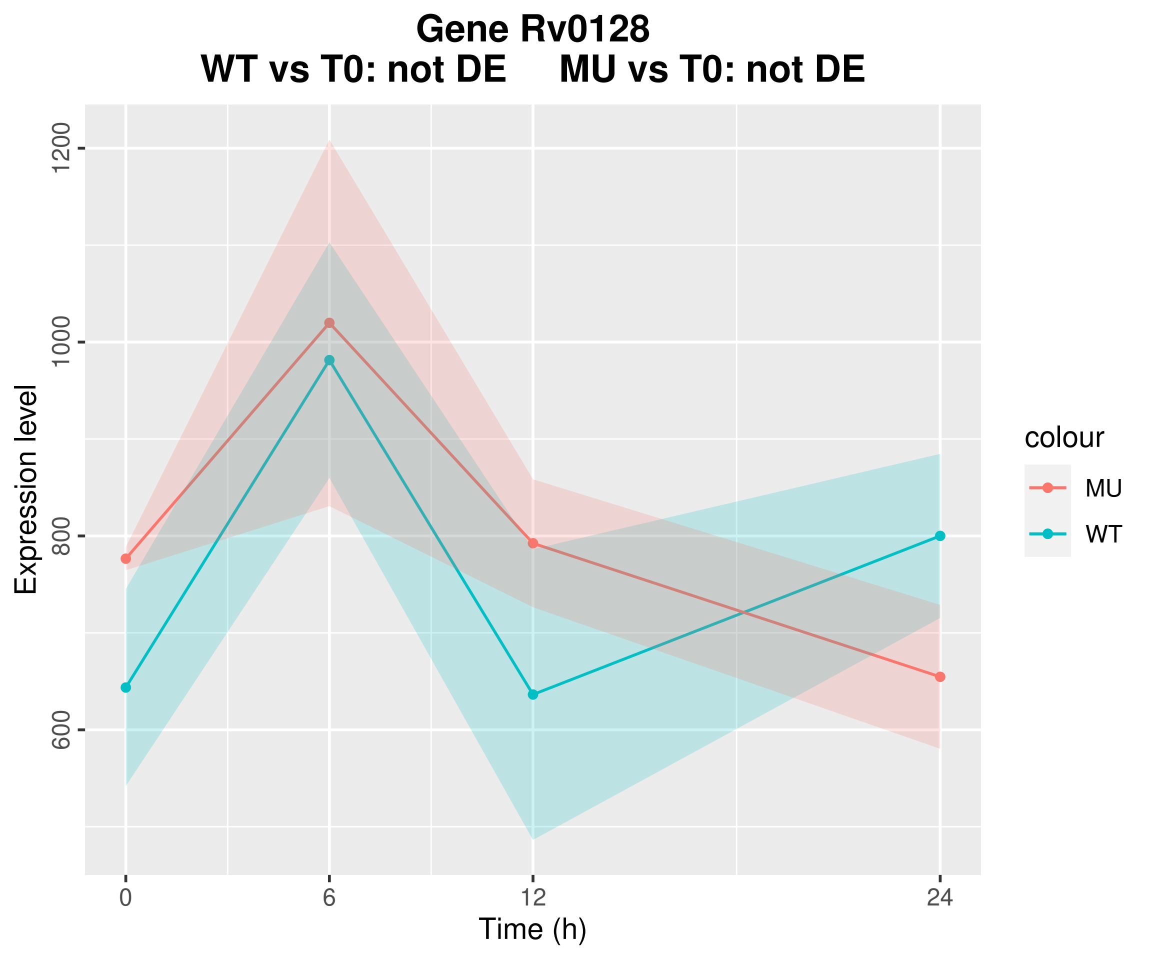

Gene Rv0128
WT vs T0: not DE
MU vs T0: not DE
Expression level
1200
1000
800
600
0
6
12
24
Time (h)
colour
MU
WT

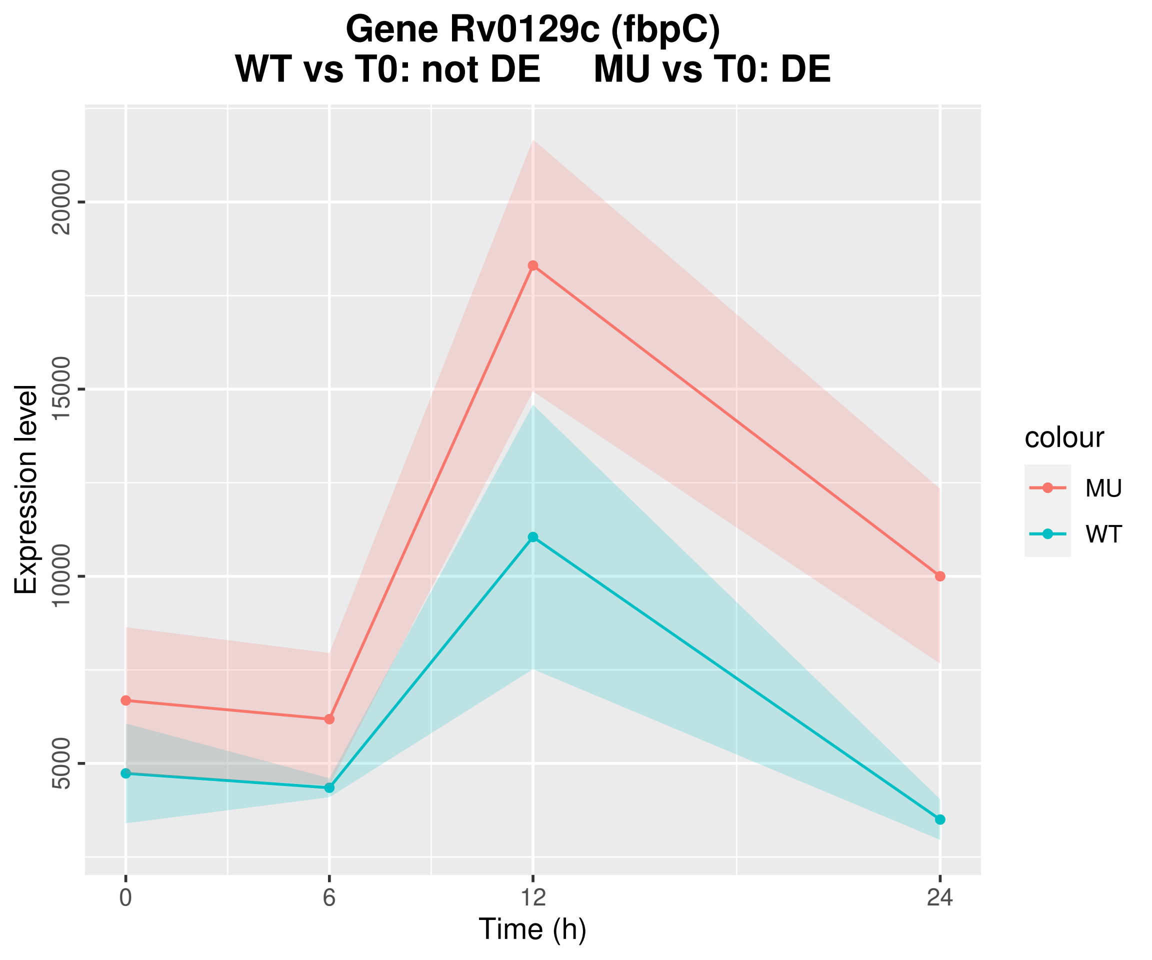

Gene Rv0129c (fbpC)
WT vs T0: not DE   MU vs T0: DE
Expression level
20000
15000
10000
5000
0
6
12
24
Time (h)
colour
MU
WT

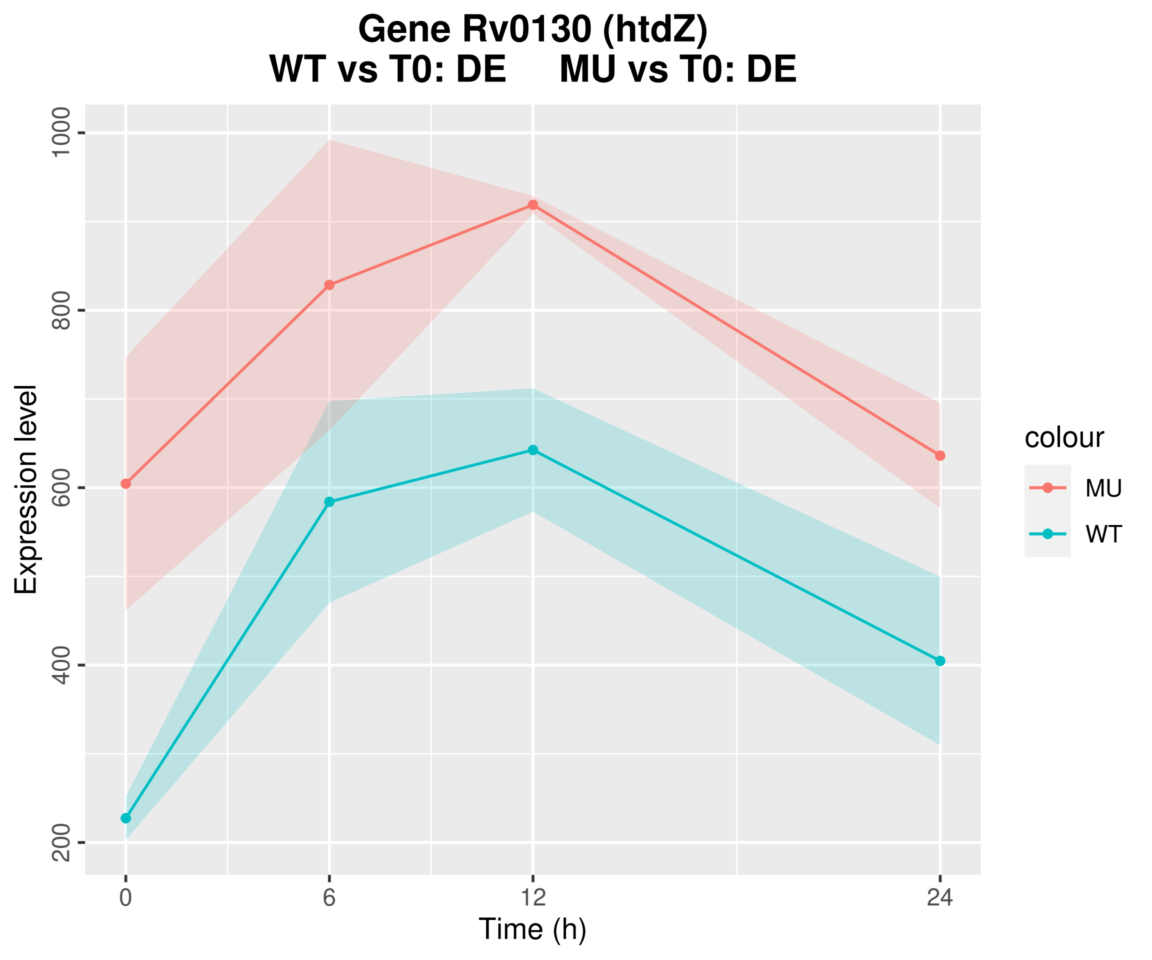

Gene Rv0130 (htdZ)
WT vs T0: DE MU vs T0: DE
Expression level
1000
800
600
400
200
0
6
12
24
Time (h)
colour
MU
WT

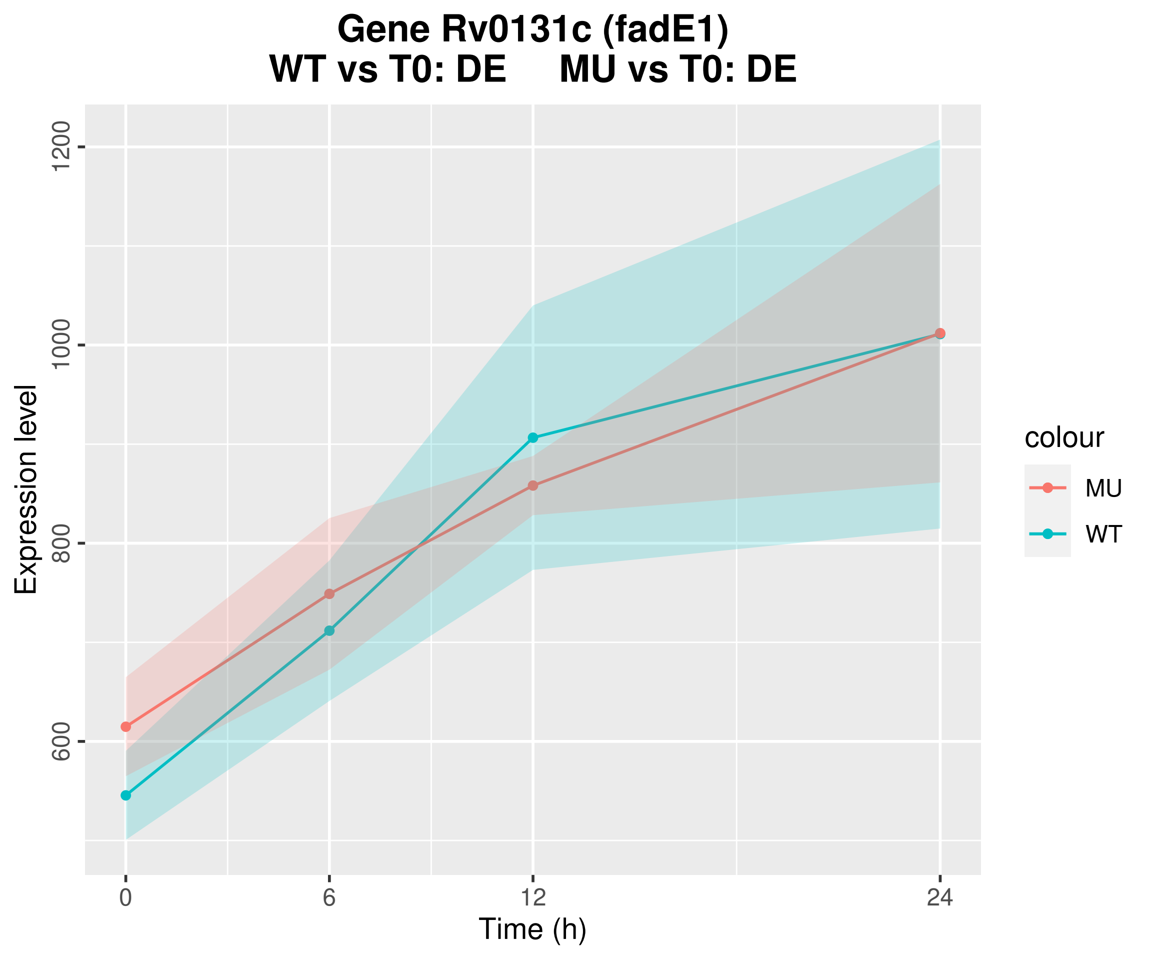

Gene Rv0131c (fadE1)
WT vs T0: DE   MU vs T0: DE
Expression level
1200
1000
800
600
0
6
12
24
Time (h)
colour
MU
WT

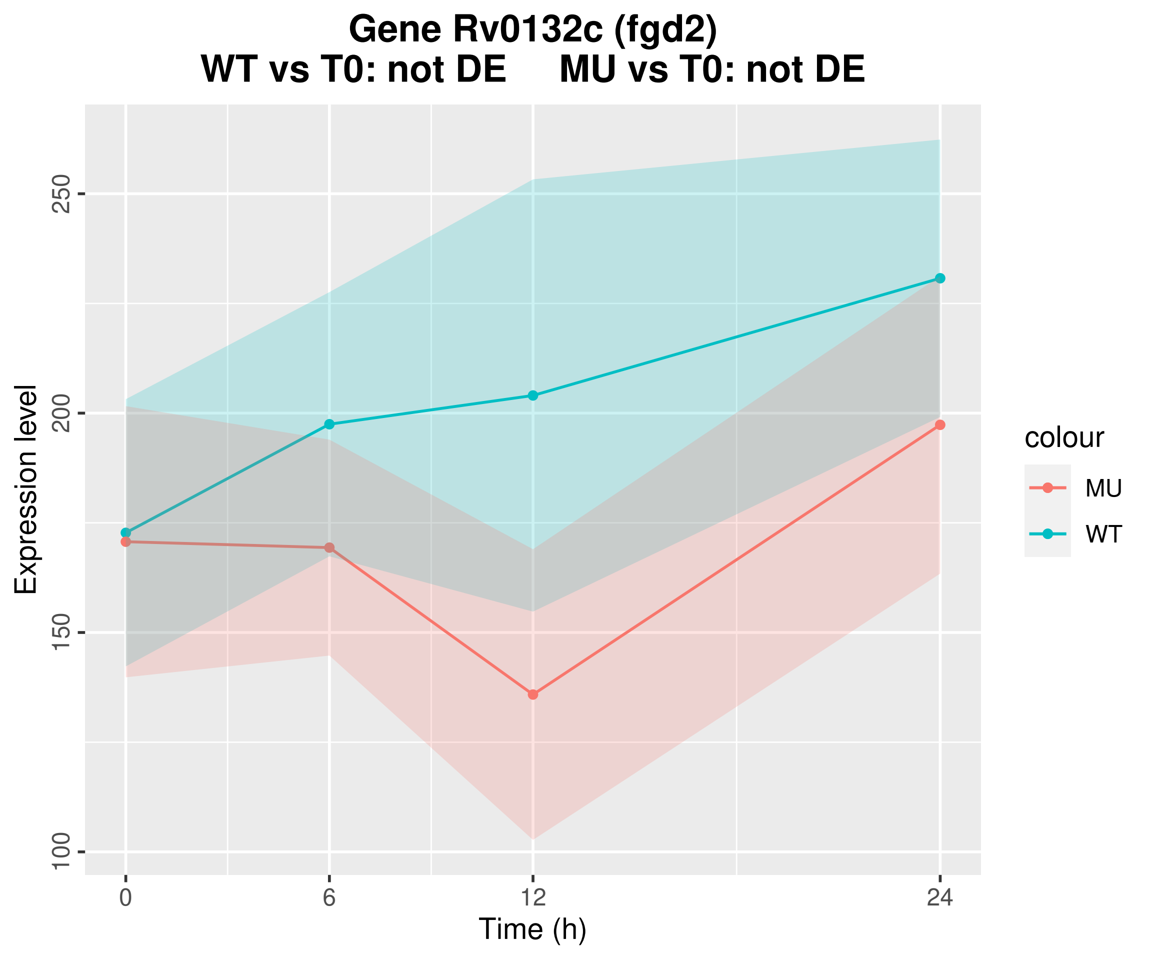

Gene Rv0132c (fgd2)
WT vs T0: not DE   MU vs T0: not DE
Expression level
250
200
150
100
0
6
12
24
Time (h)
colour
MU
WT

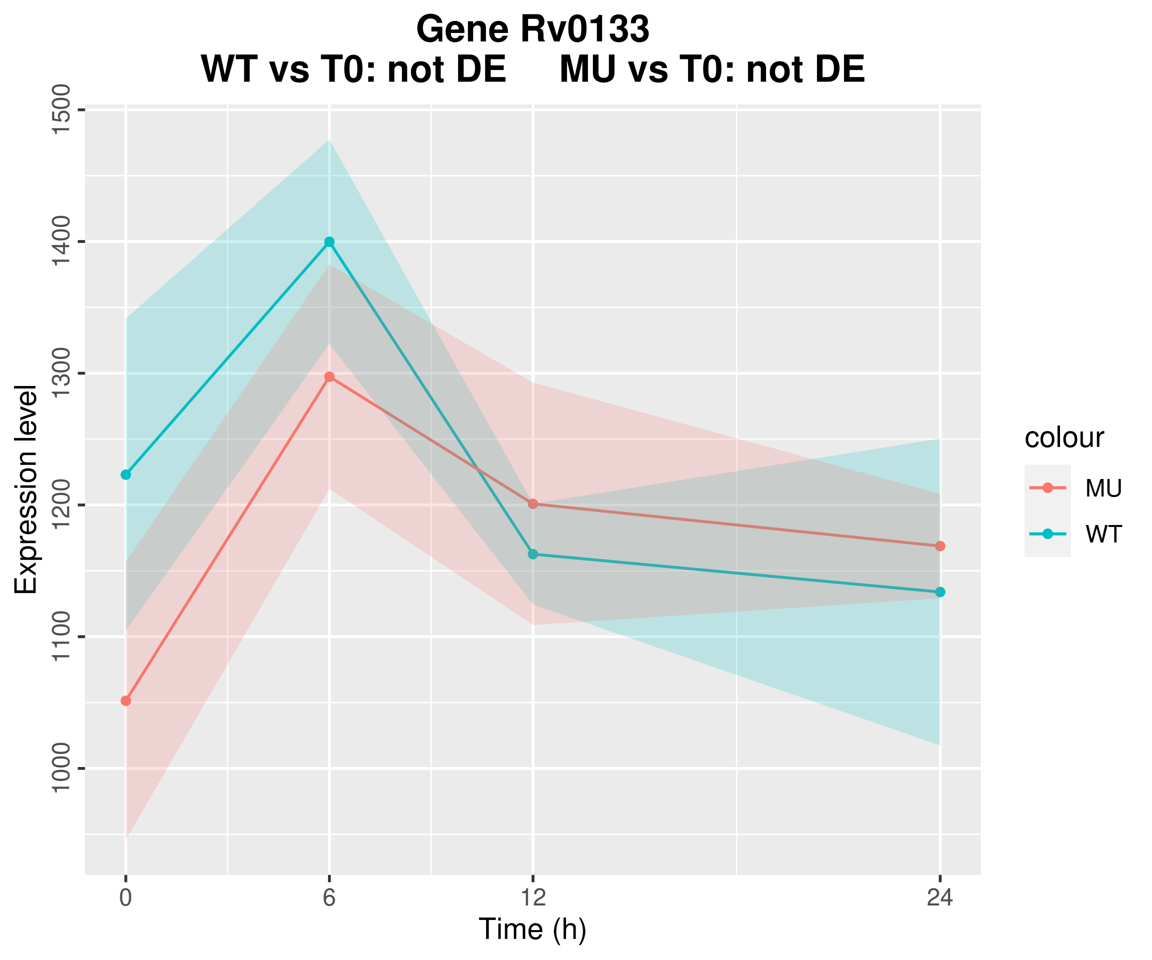
Gene Rv0133
WT vs T0: not DE   MU vs T0: not DE
Expression level
1000
1100
1200
1300
1400
1500
Time (h)
0
6
12
24
colour
MU
WT

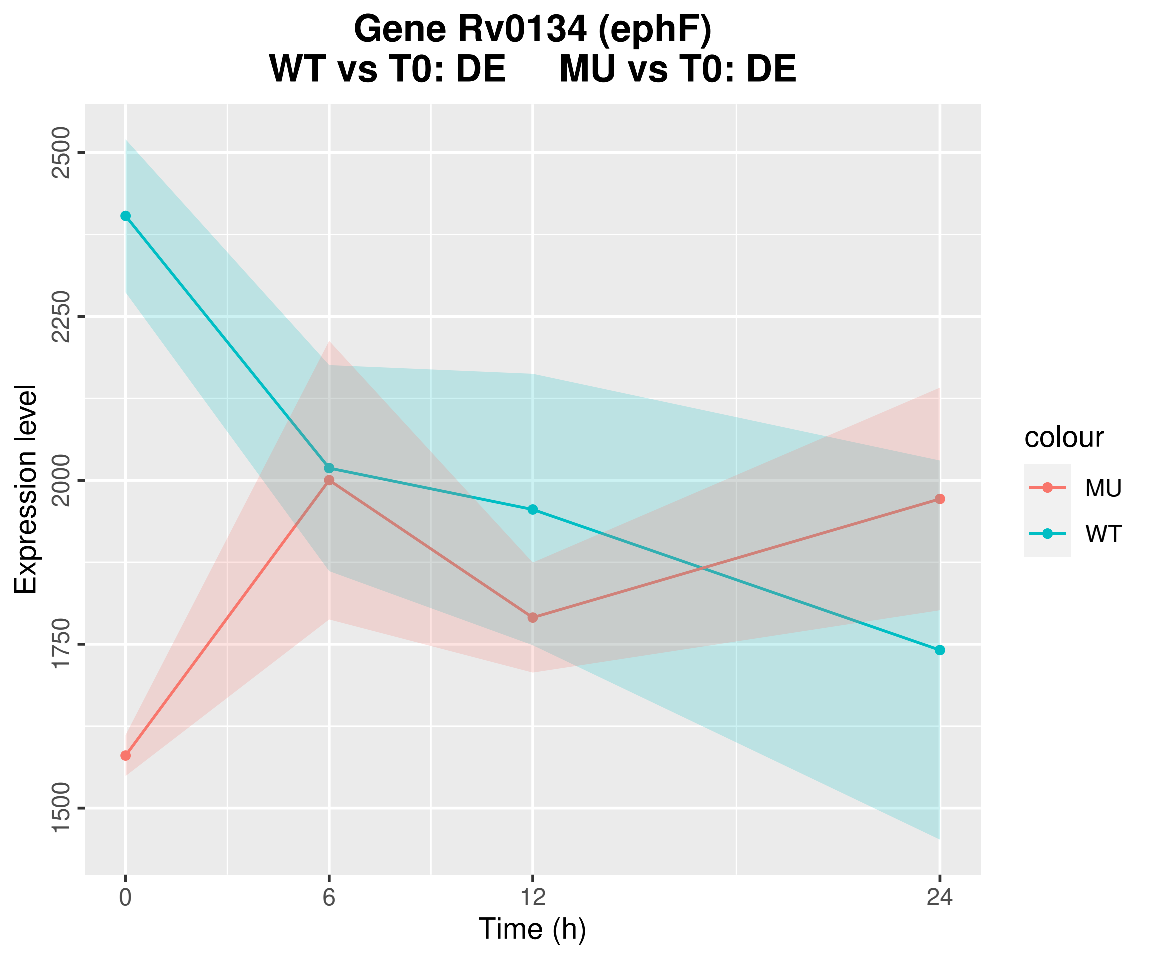

Gene Rv0134 (ephF)
WT vs T0: DE MU vs T0: DE
Expression level
2500
2250
2000
1750
1500
Time (h)
0
6
12
24
colour
MU
WT

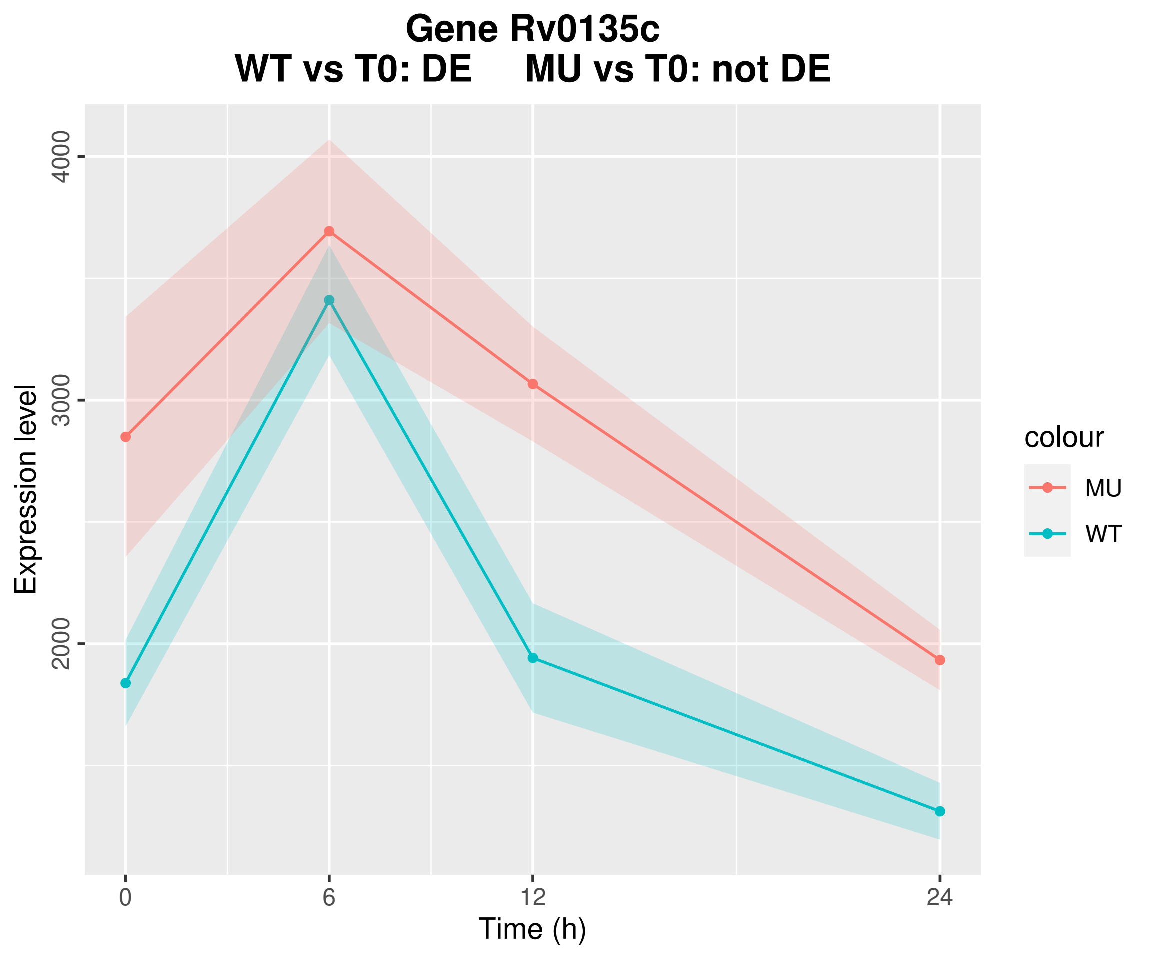

Gene Rv0135c
WT vs T0: DE MU vs T0: not DE
Expression level
4000
3000
2000
0
6
12
24
Time (h)
colour
MU
WT

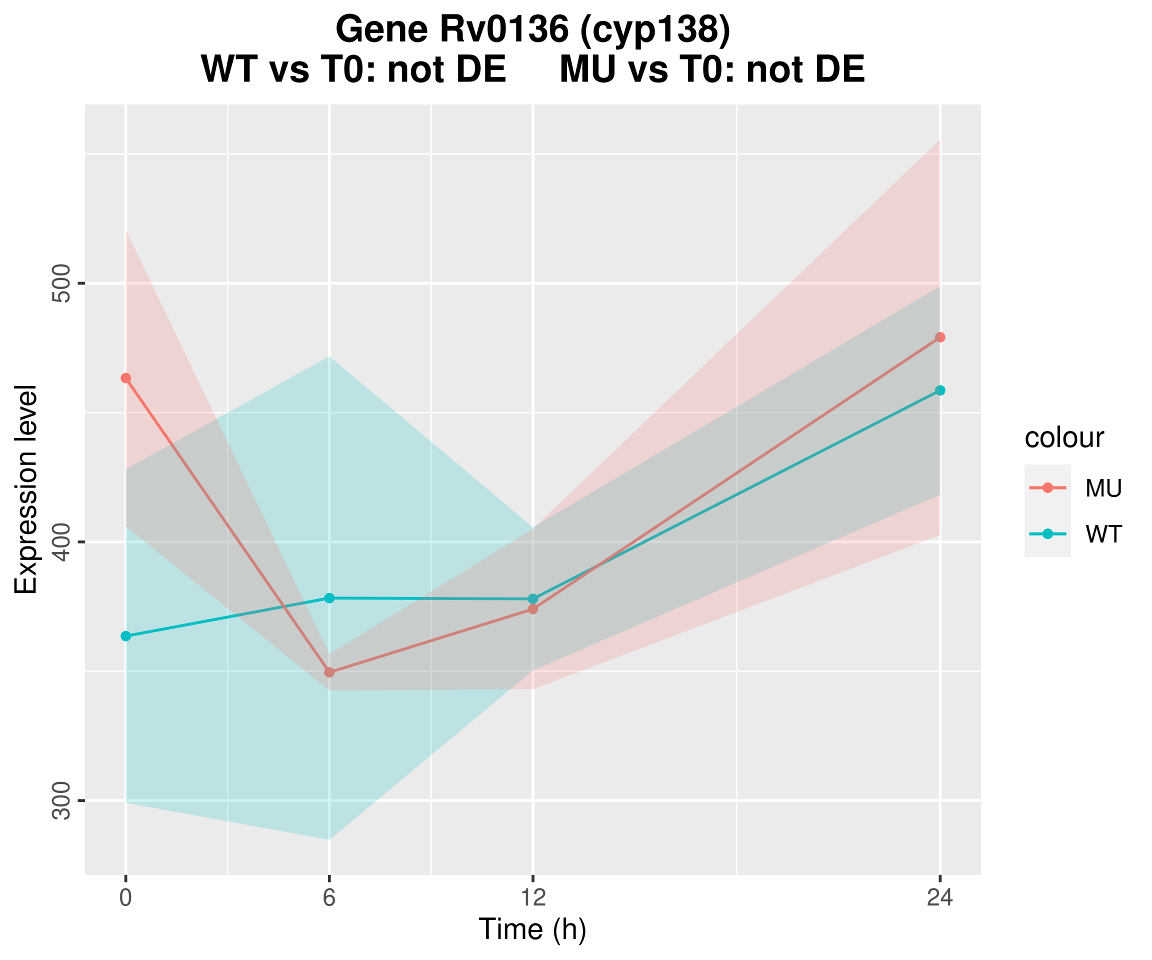

Gene Rv0136 (cyp138)
WT vs T0: not DE   MU vs T0: not DE
Expression level
500
400
300
0
6
12
24
Time (h)
colour
MU
WT

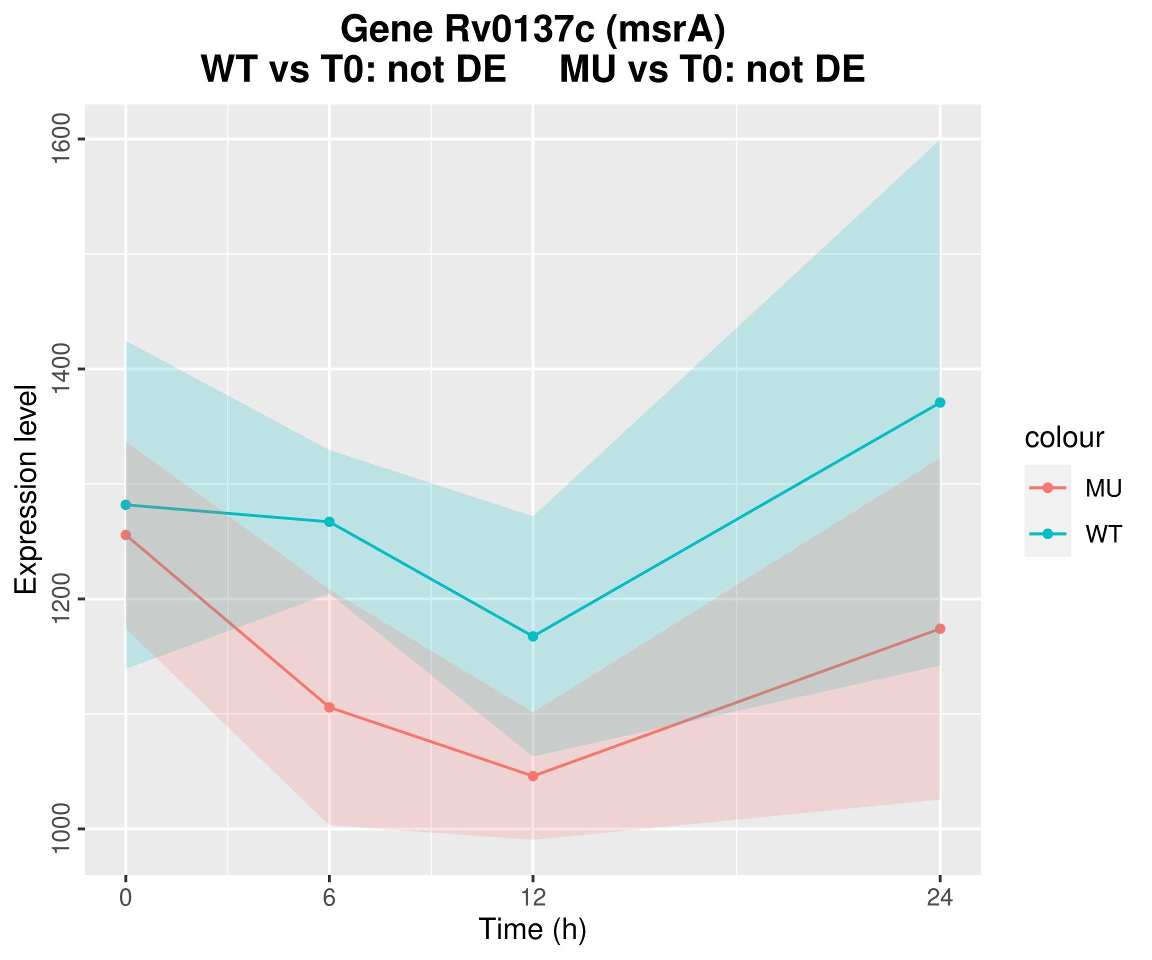

Gene Rv0137c (msrA)
WT vs T0: not DE MU vs T0: not DE
Expression level
1600
1400
1200
1000
0
6
12
24
Time (h)
colour
MU
WT

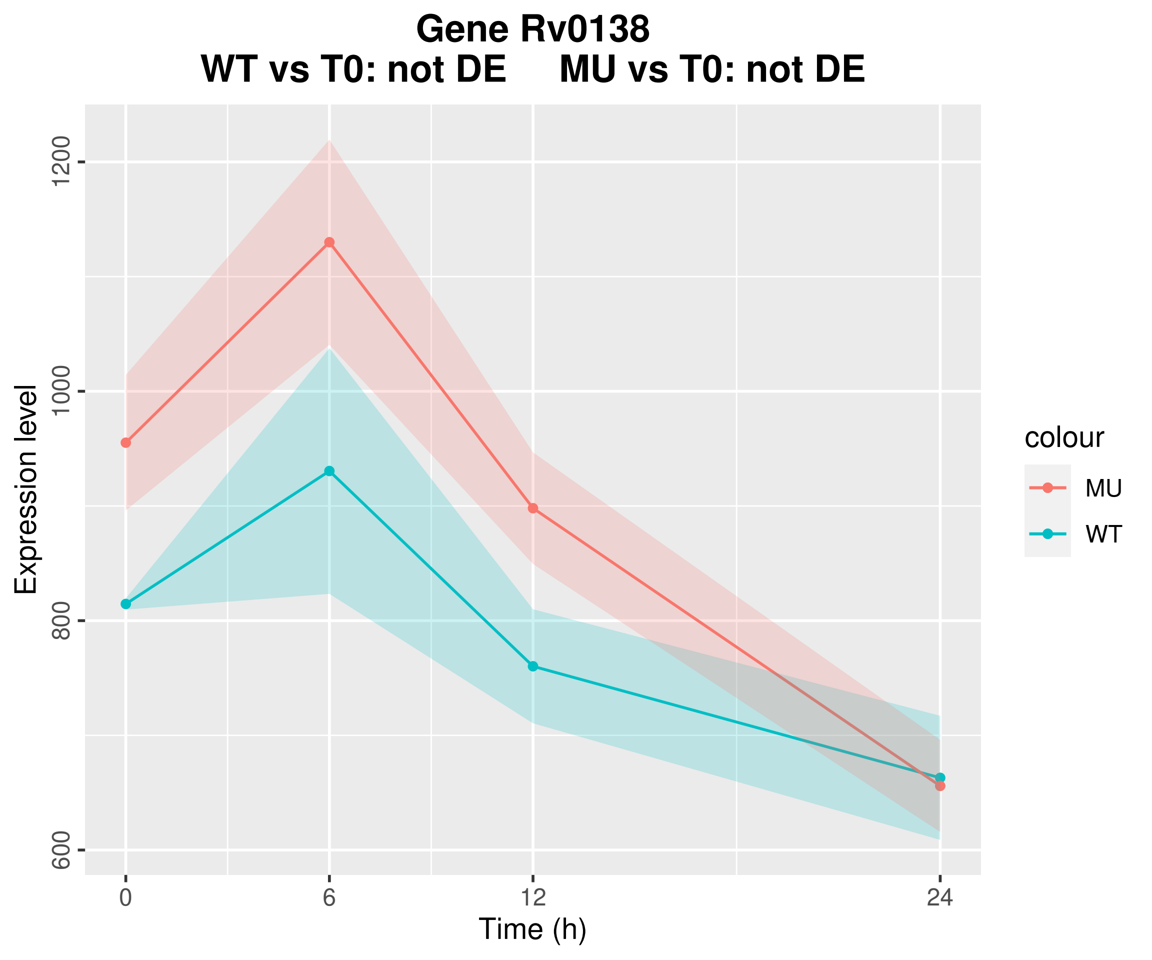
Gene Rv0138
WT vs T0: not DE   MU vs T0: not DE
Expression level
1200
1000
800
600
0
6
12
24
Time (h)
colour
MU
WT

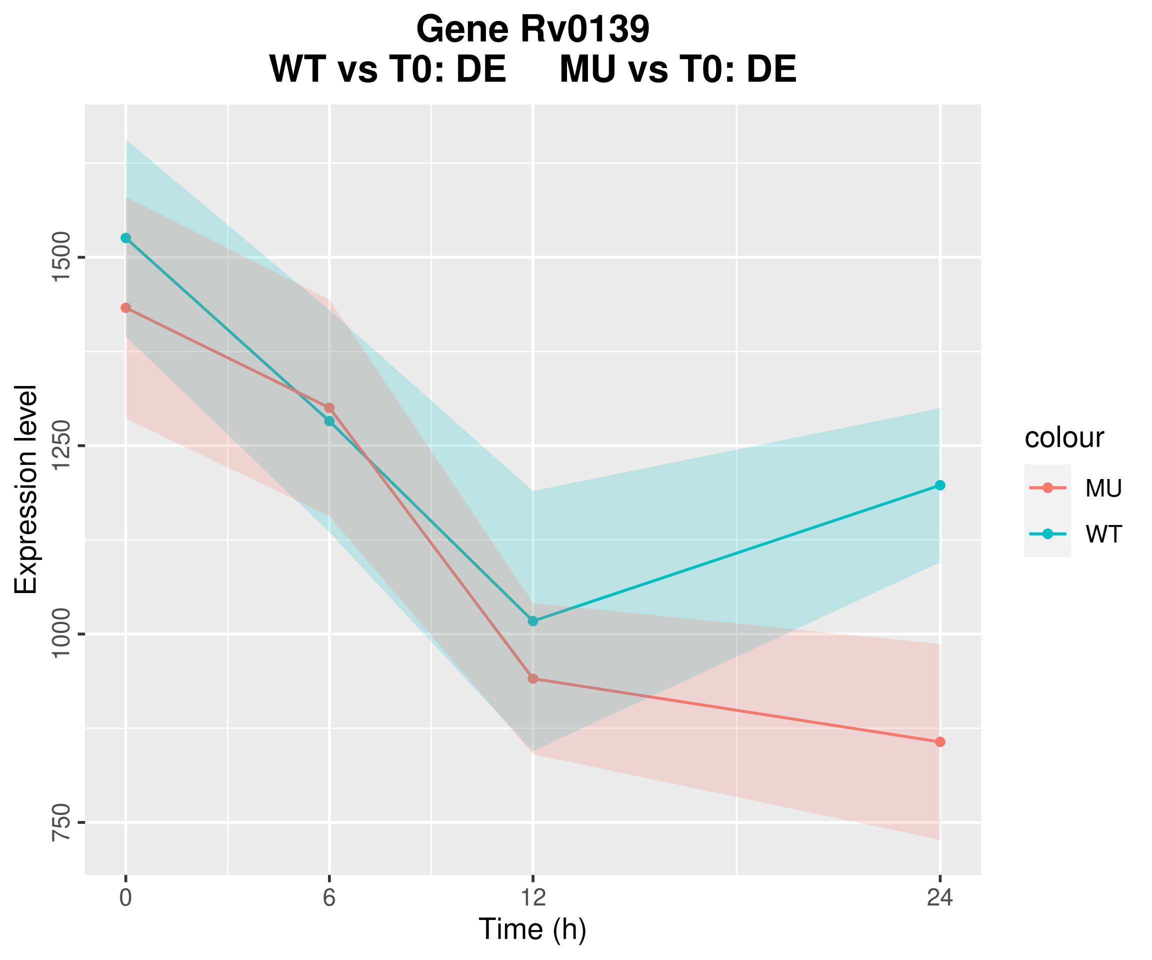

Gene Rv0139
WT vs T0: DE   MU vs T0: DE
Expression level
1500
1250
1000
750
Time (h)
0
6
12
24
colour
MU
WT

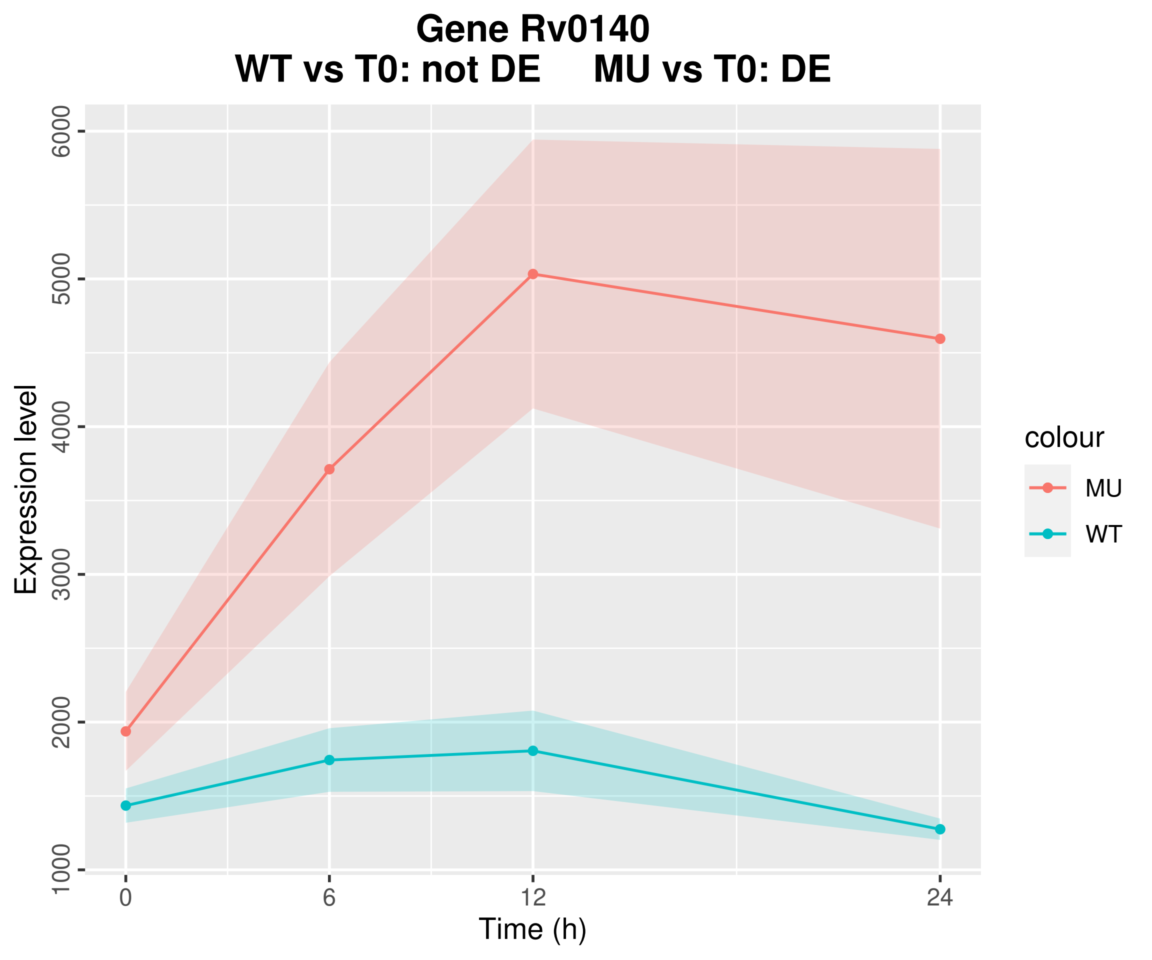

Gene Rv0140
WT vs T0: not DE MU vs T0: DE
Expression level
6000
5000
4000
3000
2000
1000
0
6
12
24
Time (h)
colour
MU
WT

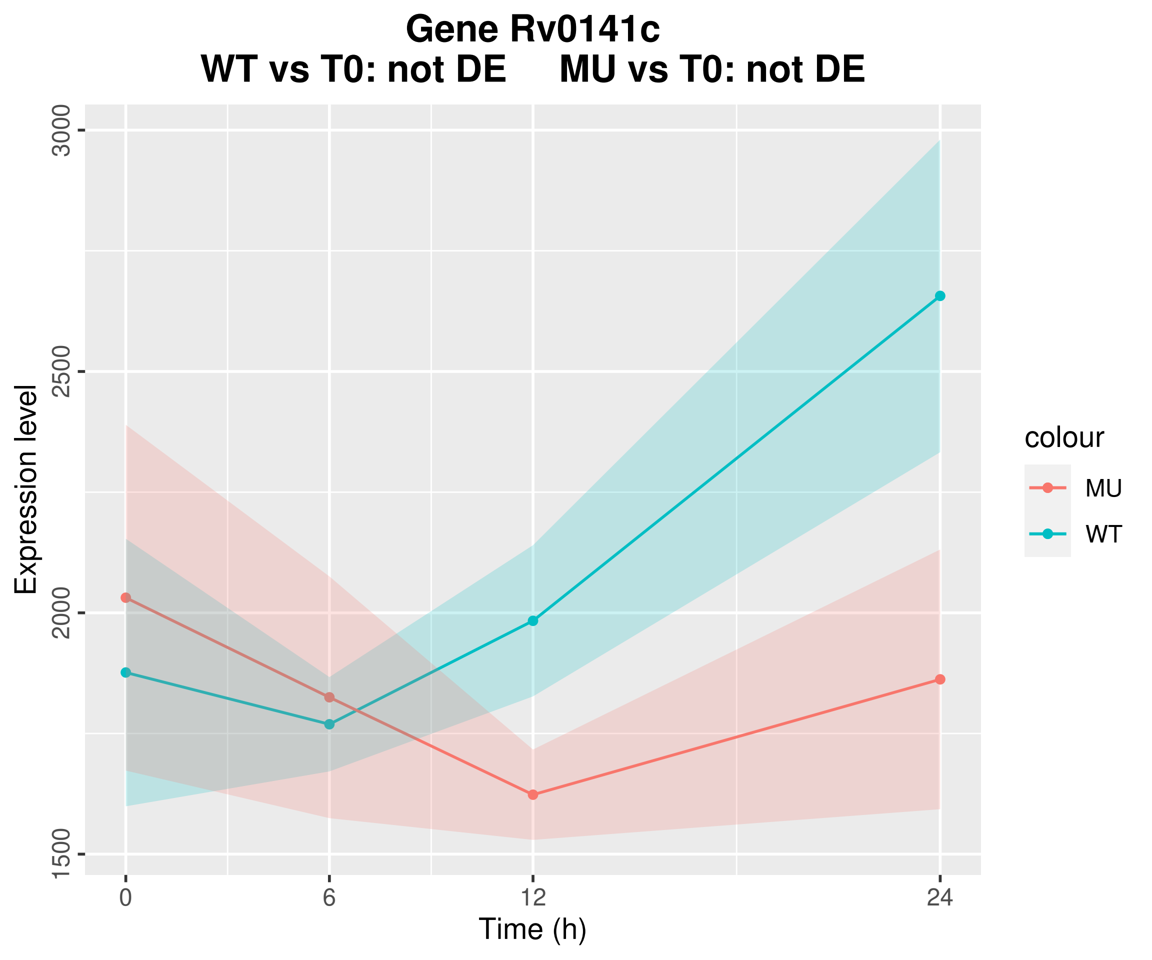
Gene Rv0141c
WT vs T0: not DE   MU vs T0: not DE
3000
2500
2000
1500
Expression level
0
6
12
24
Time (h)
colour
MU
WT

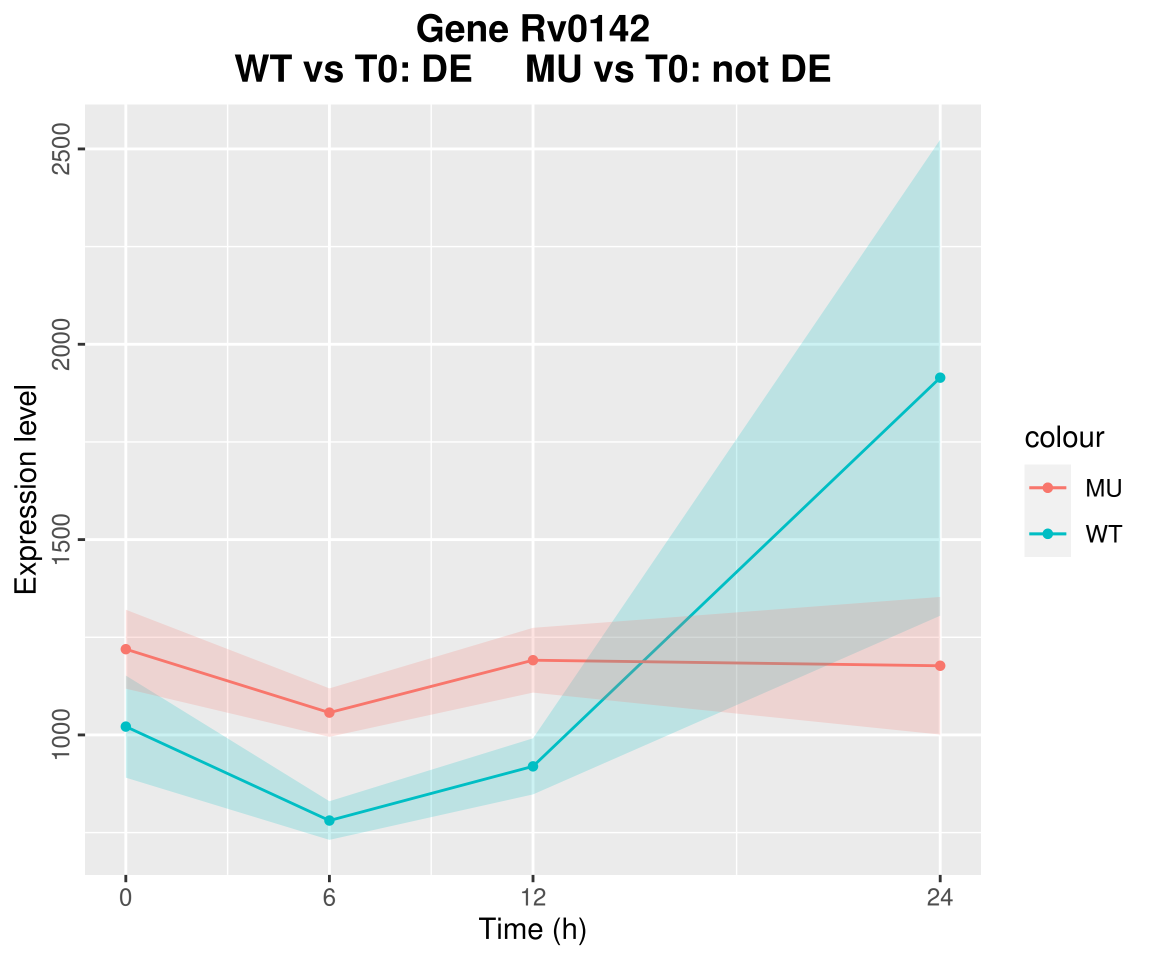
Gene Rv0142
WT vs T0: DE   MU vs T0: not DE
Expression level
2500
2000
1500
1000
0
6
12
24
Time (h)
colour
MU
WT

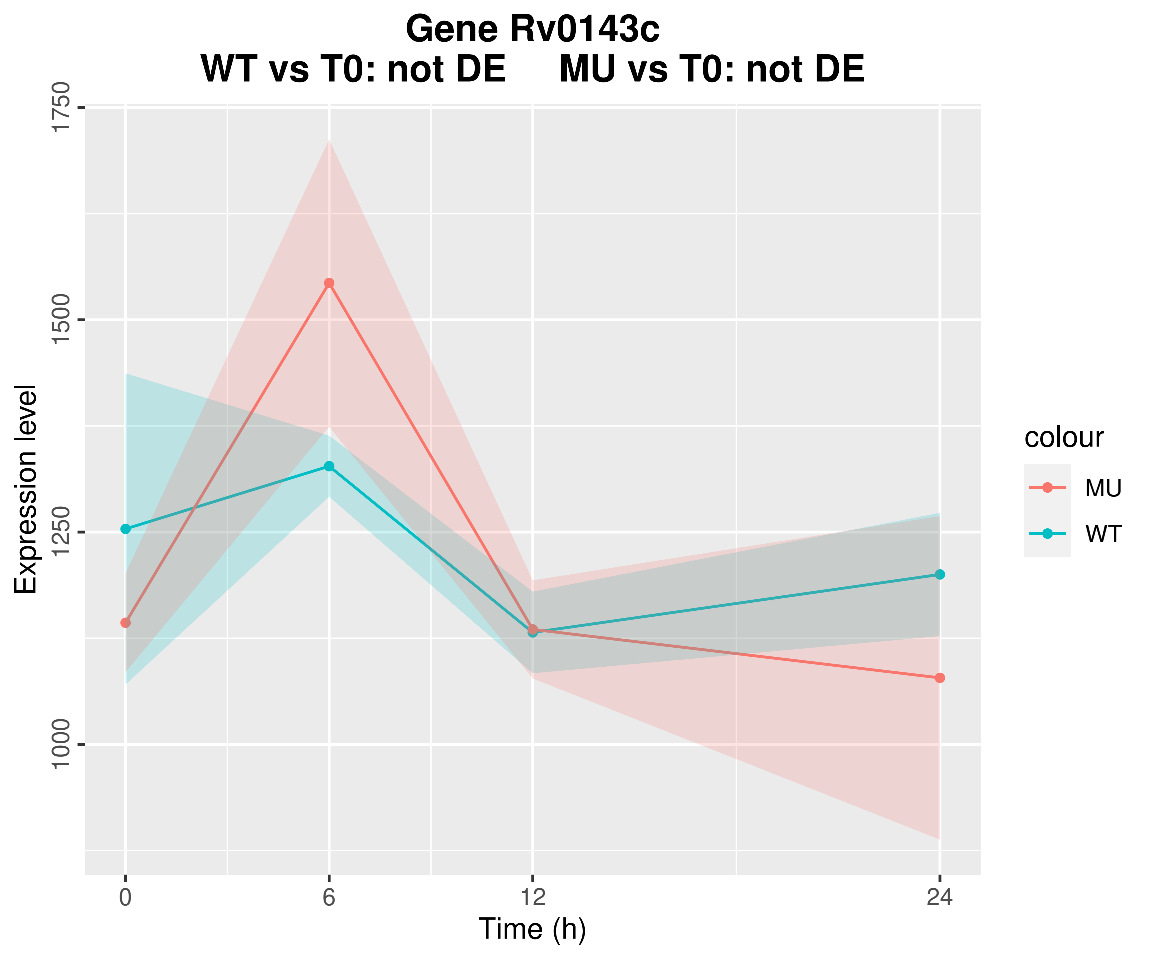
Gene Rv0143c
WT vs T0: not DE   MU vs T0: not DE
1750
1500
1250
1000
Expression level
0
6
12
24
Time (h)
colour
MU
WT

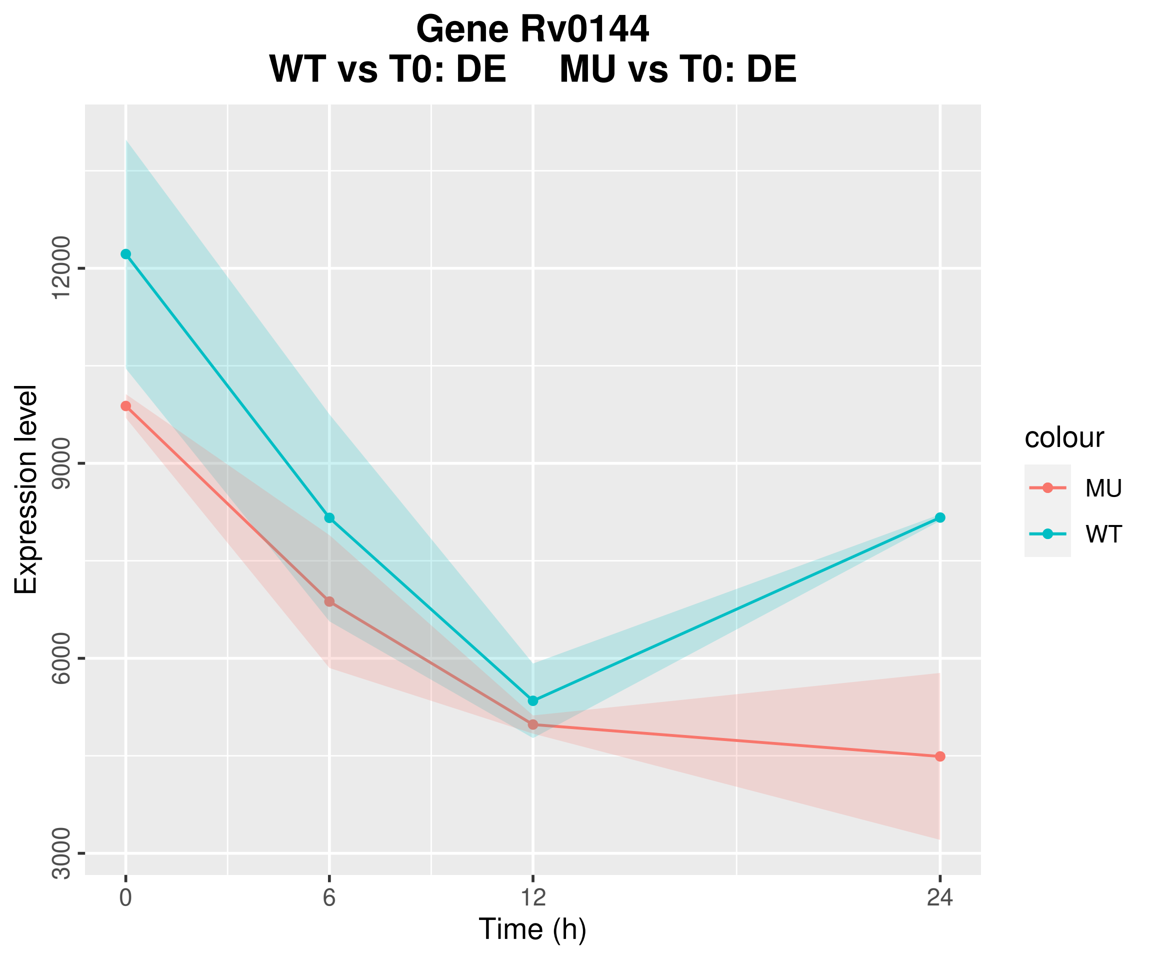

Gene Rv0144
WT vs T0: DE   MU vs T0: DE
Expression level
12000
9000
6000
3000
0
6
12
24
Time (h)
colour
MU
WT

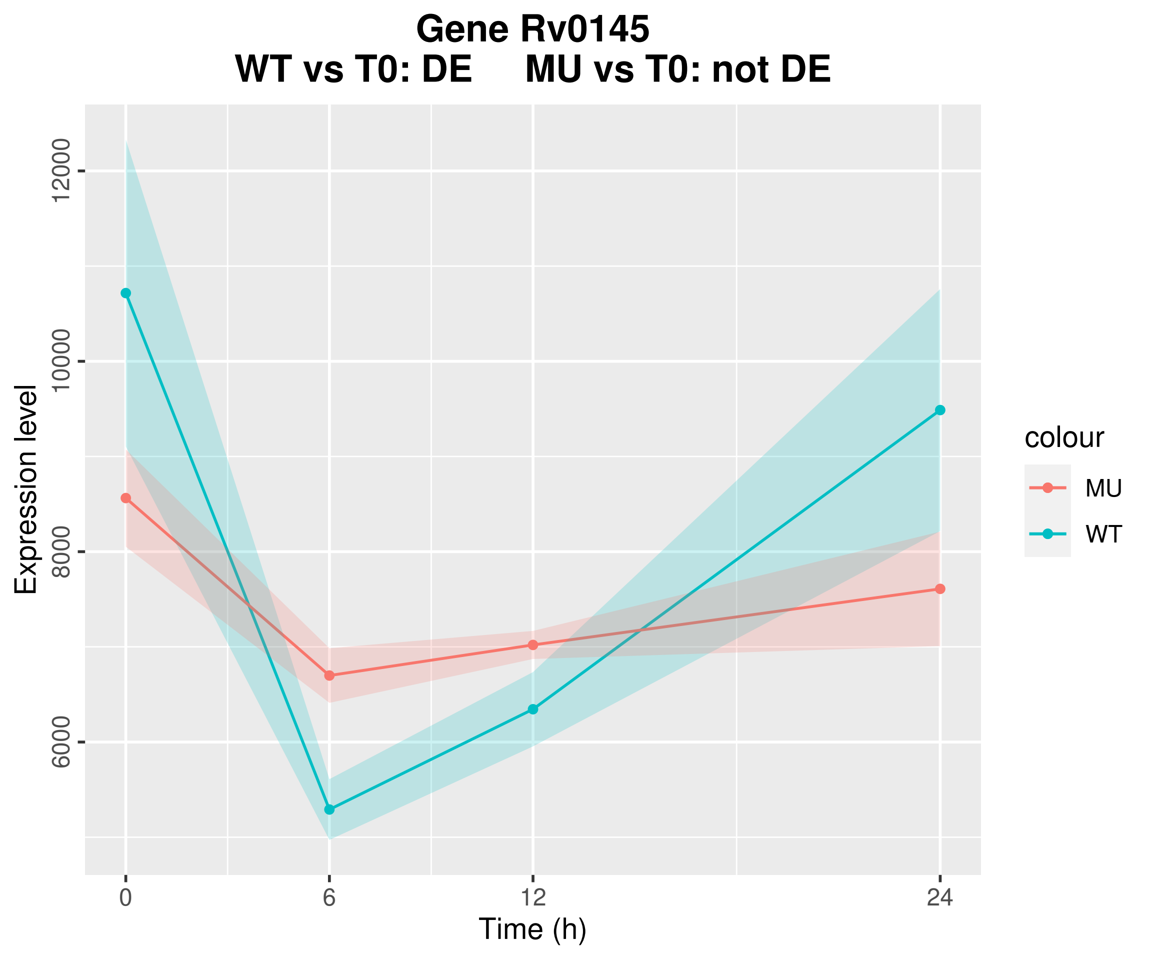

Gene Rv0145
WT vs T0: DE MU vs T0: not DE
Expression level
12000
10000
8000
6000
0
6
12
24
Time (h)
colour
MU
WT

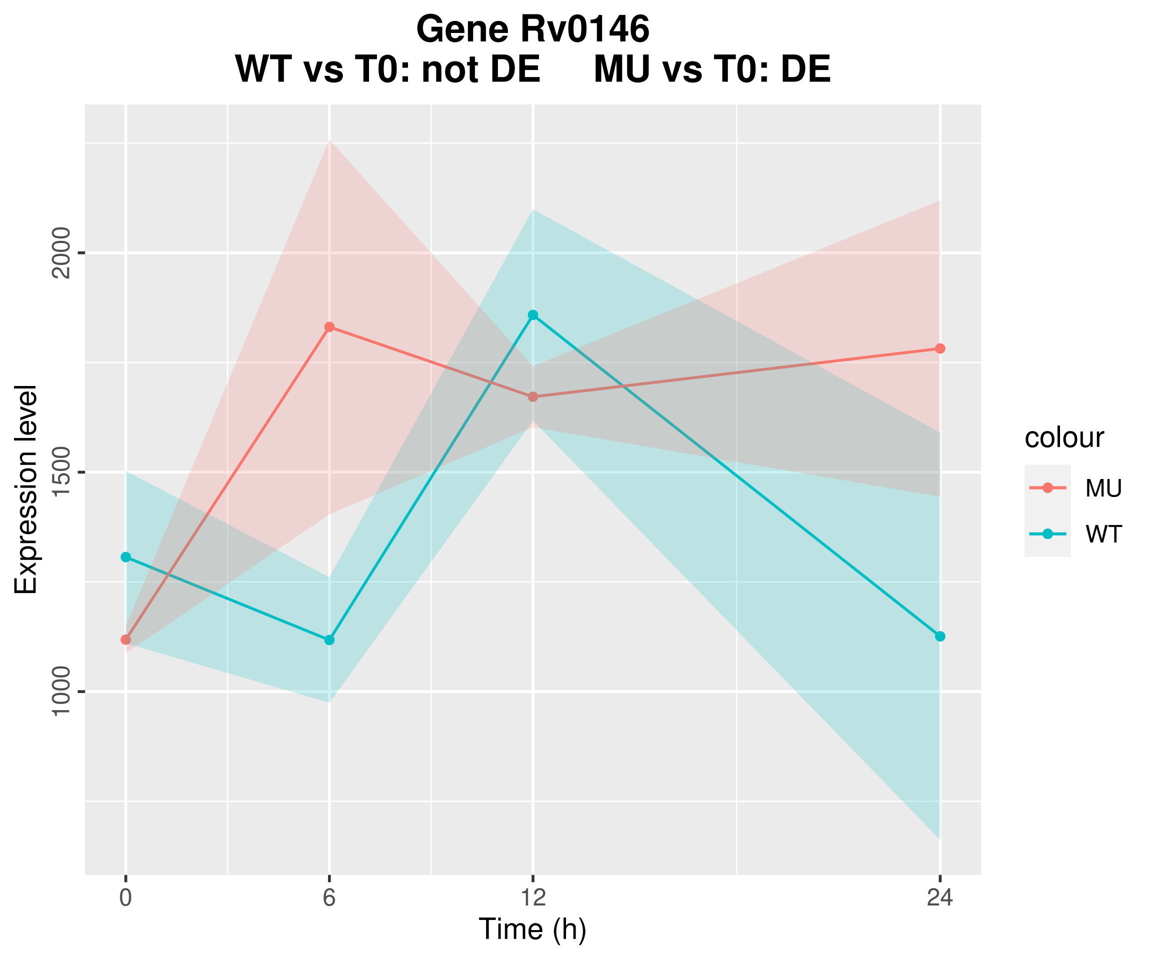
Gene Rv0146
WT vs T0: not DE MU vs T0: DE
Expression level
2000
1500
1000
0
6
12
24
Time (h)
colour
MU
WT

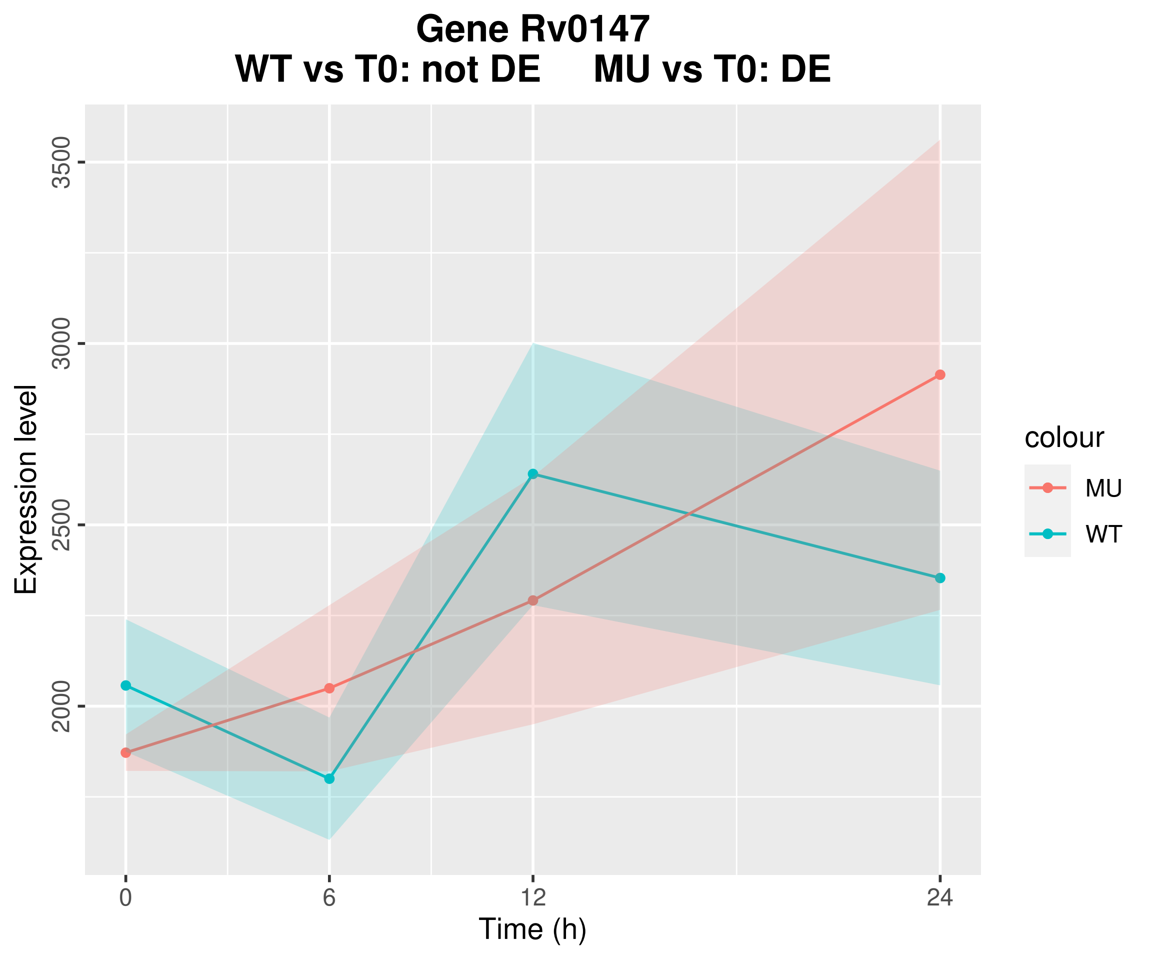
Gene Rv0147
WT vs T0: not DE MU vs T0: DE
Expression level
3500
3000
2500
2000
0
6
12
24
Time (h)
colour
MU
WT

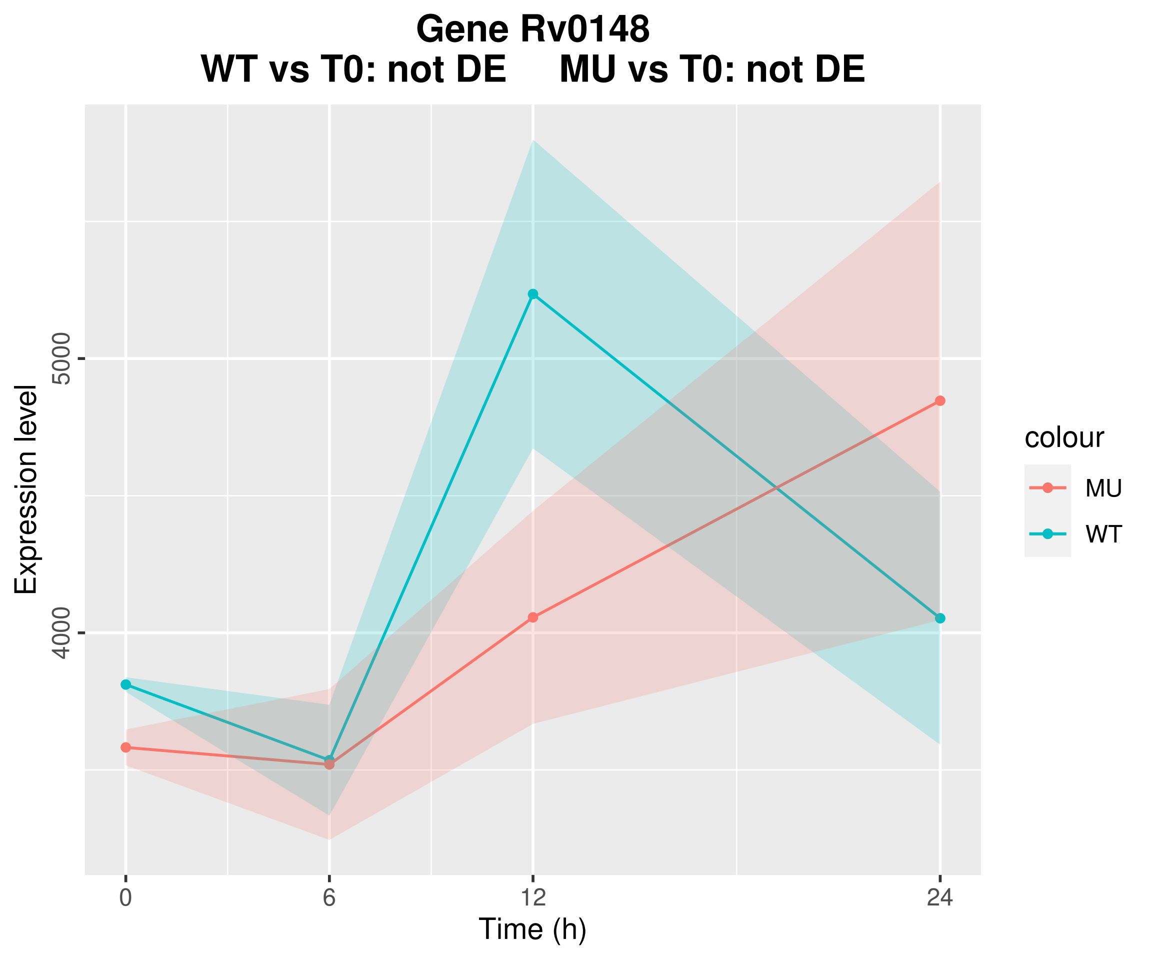

Gene Rv0148
WT vs T0: not DE   MU vs T0: not DE
Expression level
5000
4000
0
6
12
24
Time (h)
colour
MU
WT

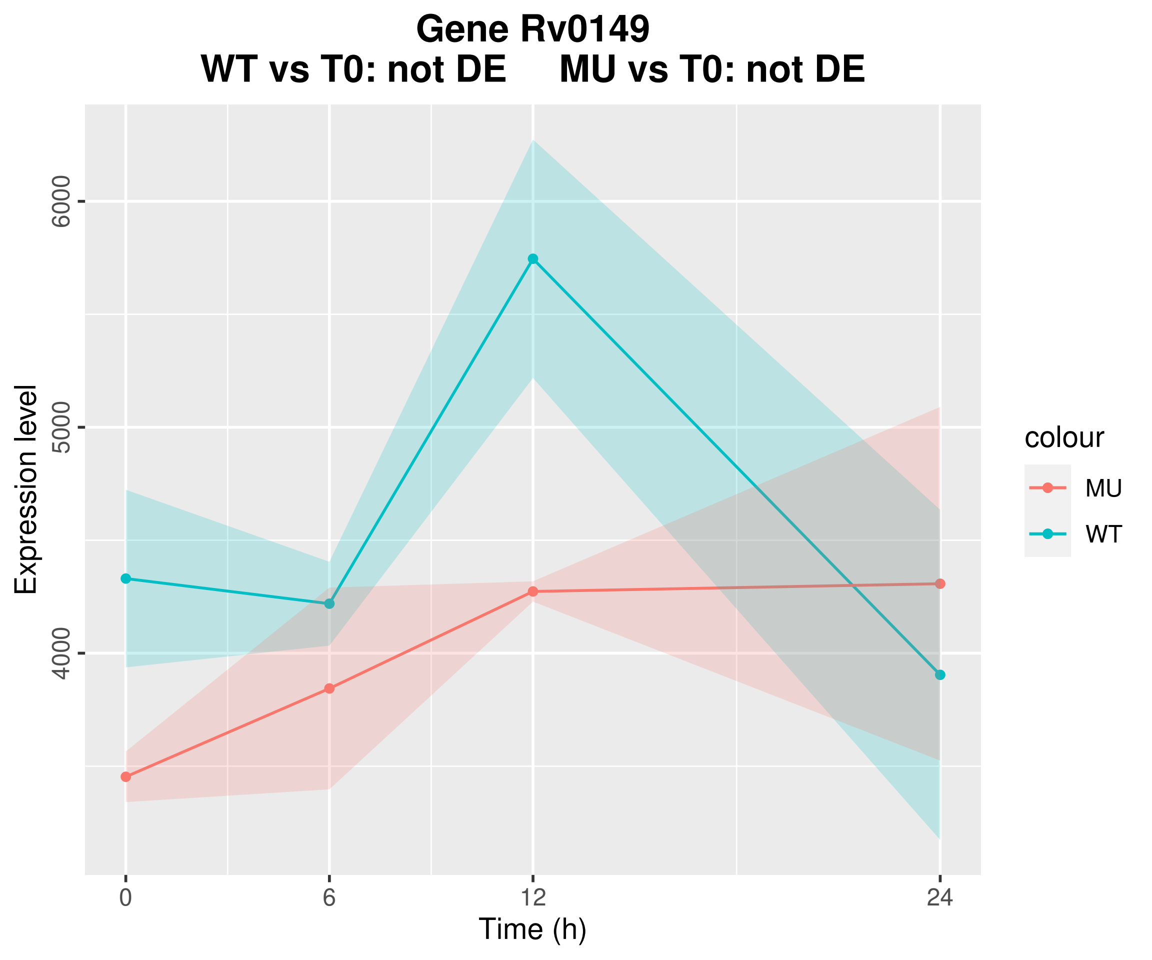
Gene Rv0149
WT vs T0: not DE MU vs T0: not DE
Expression level
6000
5000
4000
0
6
12
24
Time (h)
colour
MU
WT

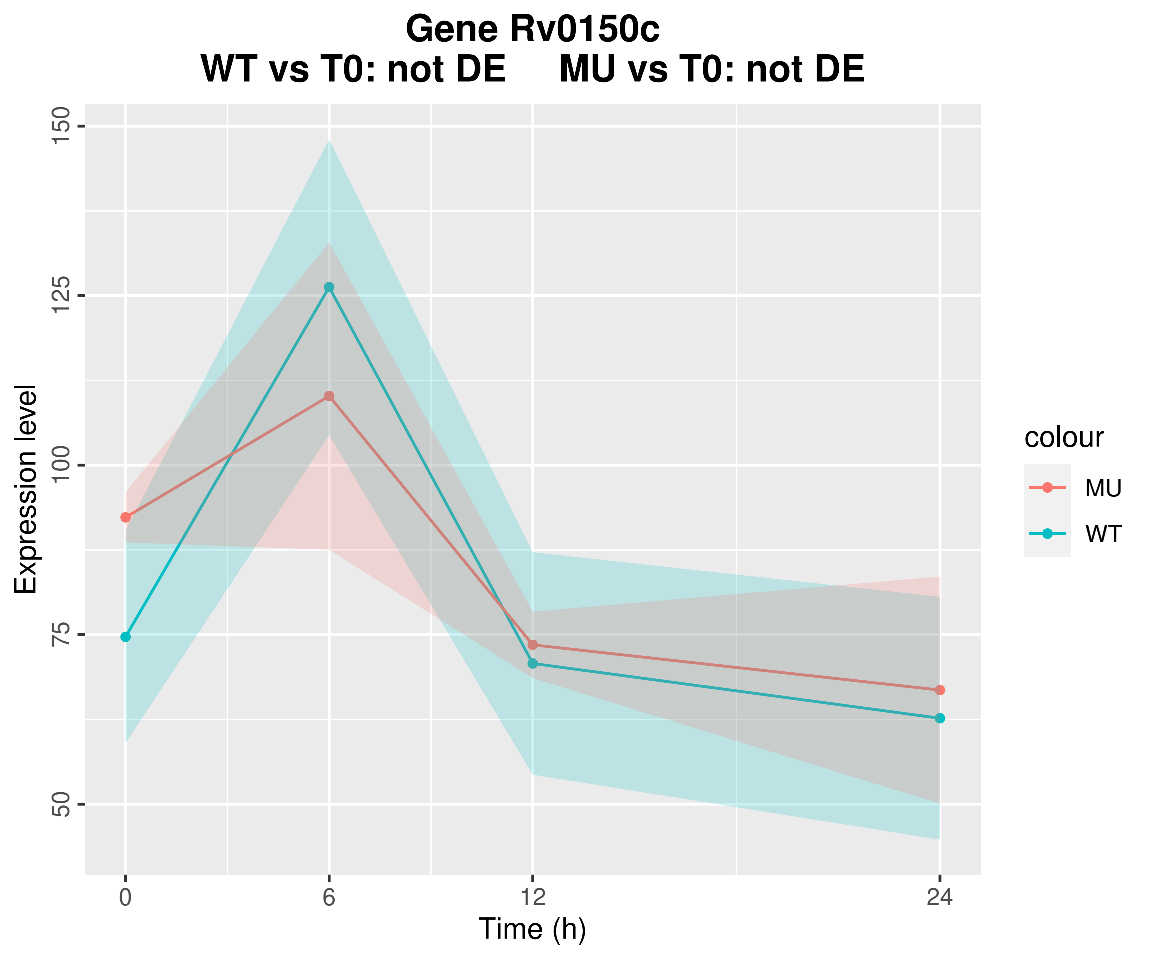
Gene Rv0150c
WT vs T0: not DE MU vs T0: not DE
Expression level
150
125
100
75
50
0
6
12
24
Time (h)
colour
MU
WT

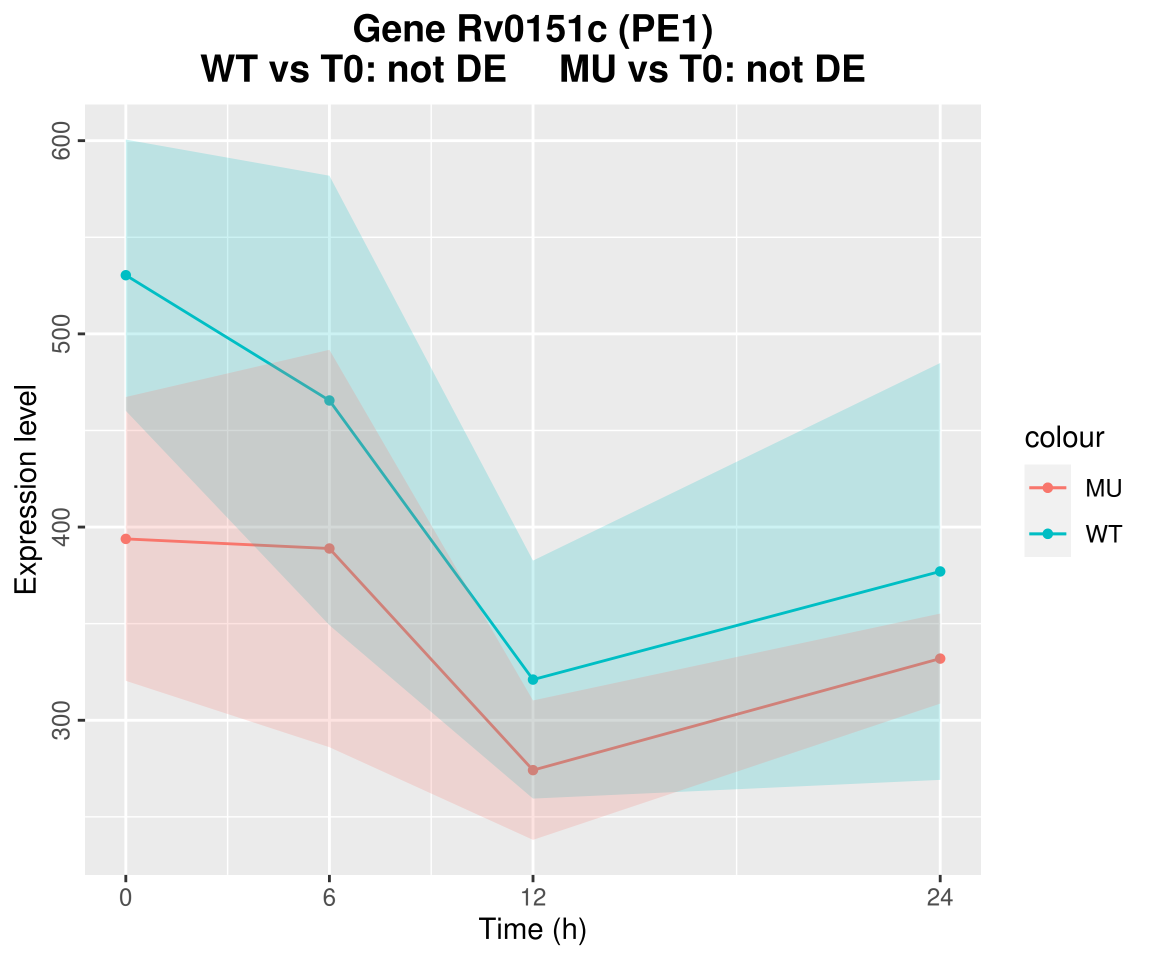
Gene Rv0151c (PE1)
WT vs T0: not DE   MU vs T0: not DE
Expression level
600
500
400
300
0
6
12
24
Time (h)
colour
MU
WT

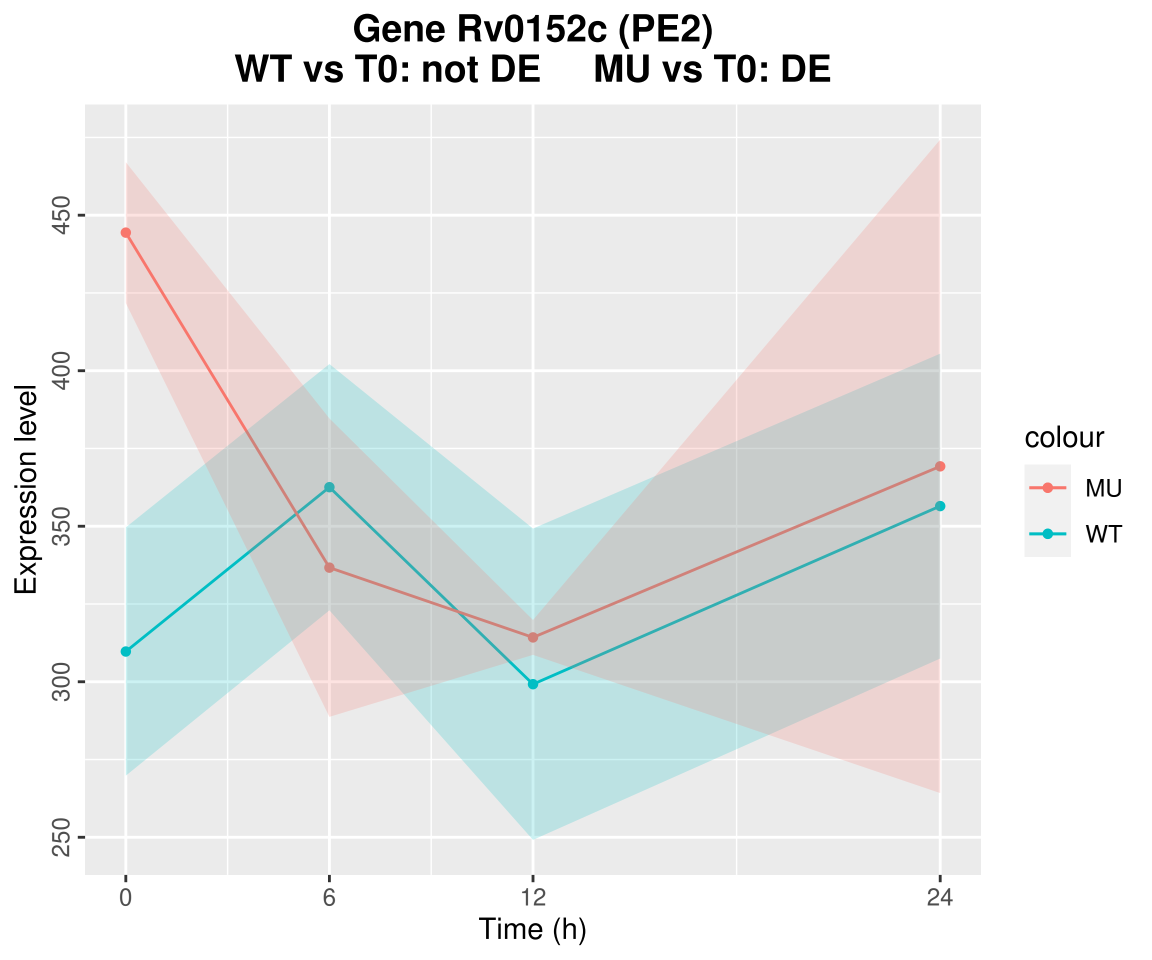

Gene Rv0152c (PE2)
WT vs T0: not DE   MU vs T0: DE
Expression level
450
400
350
300
250
0
6
12
24
Time (h)
colour
MU
WT

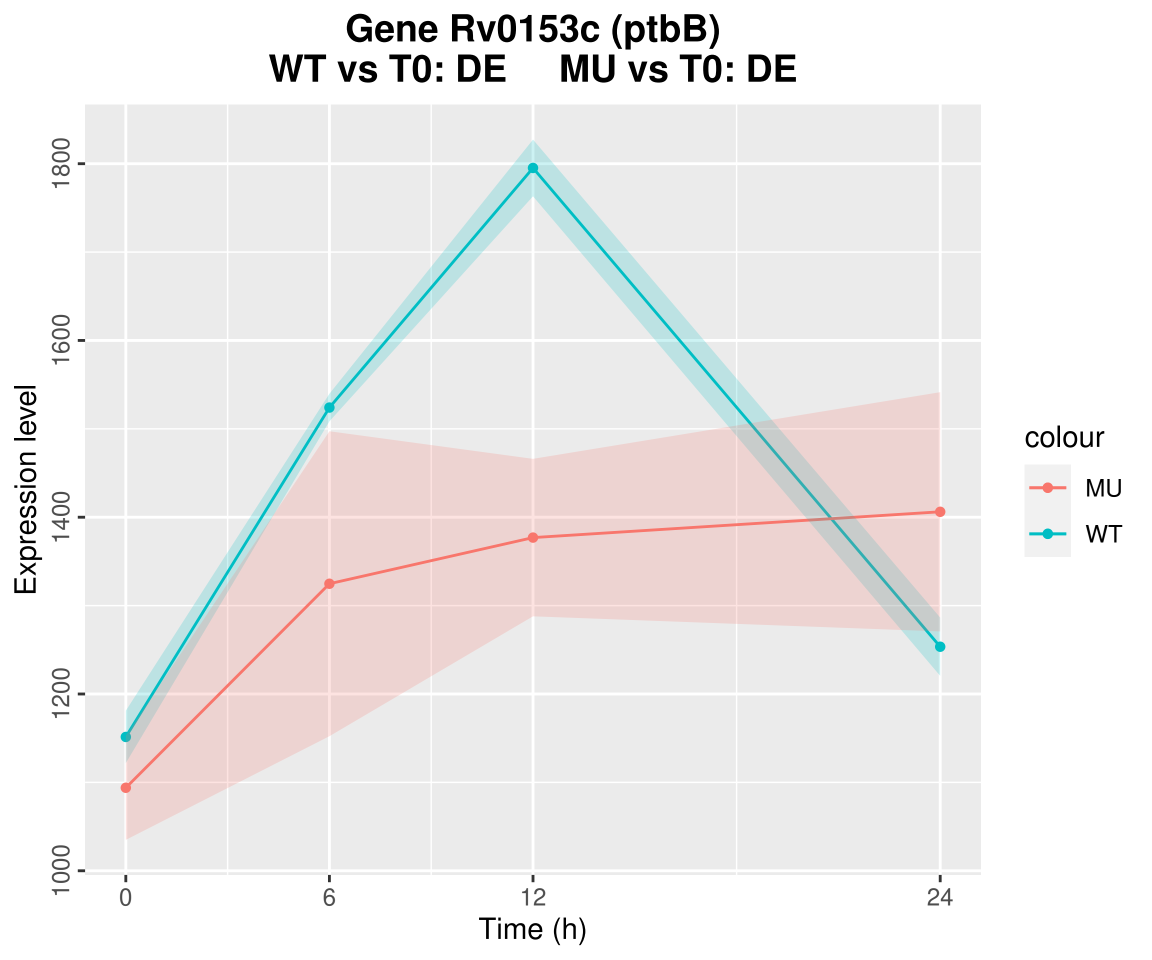
Gene Rv0153c (ptbB)
WT vs T0: DE   MU vs T0: DE
Expression level
1000
1200
1400
1600
1800
0
6
12
24
Time (h)
colour
MU
WT

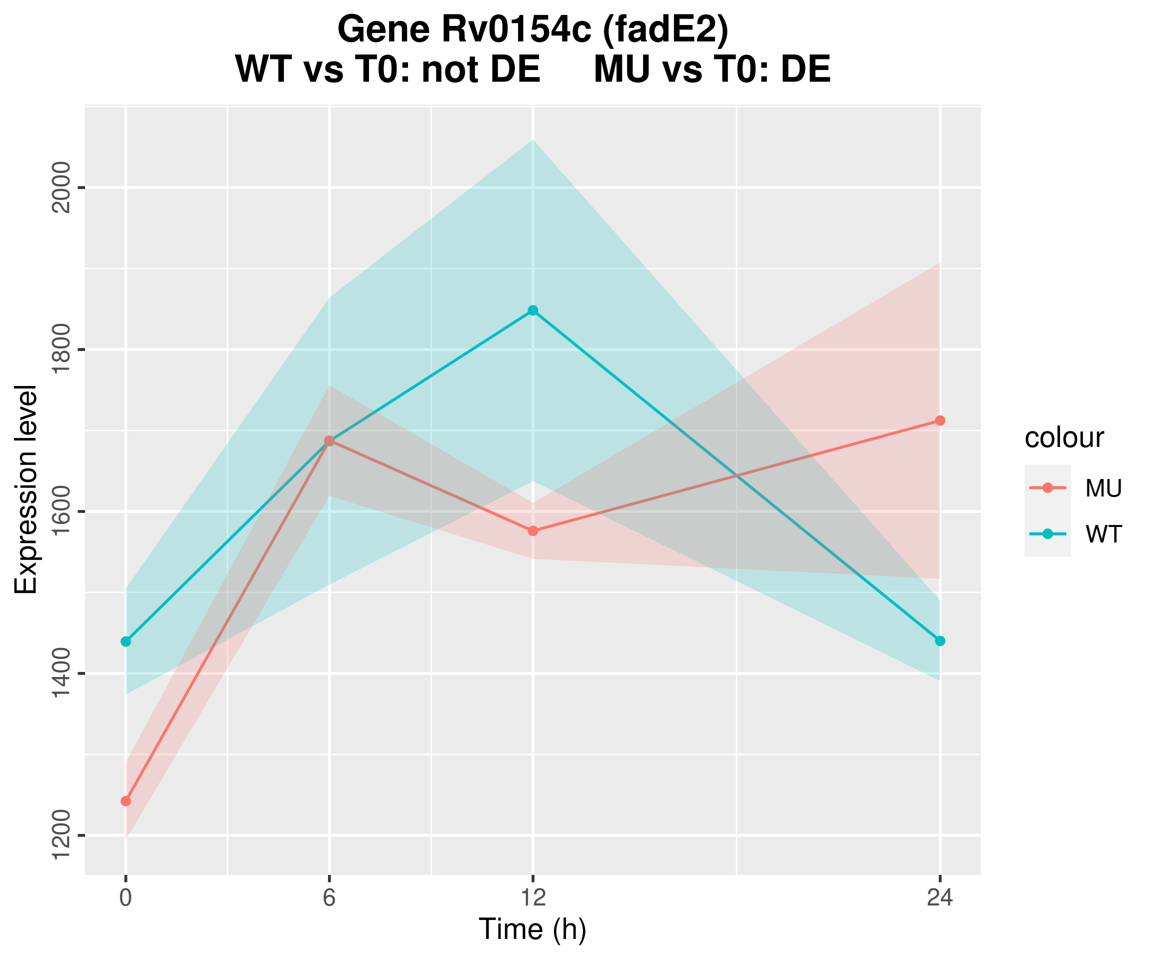

Gene Rv0154c (fadE2)
WT vs T0: not DE MU vs T0: DE
Expression level
1200
1400
1600
1800
2000
Time (h)
0
6
12
24
colour
MU
WT

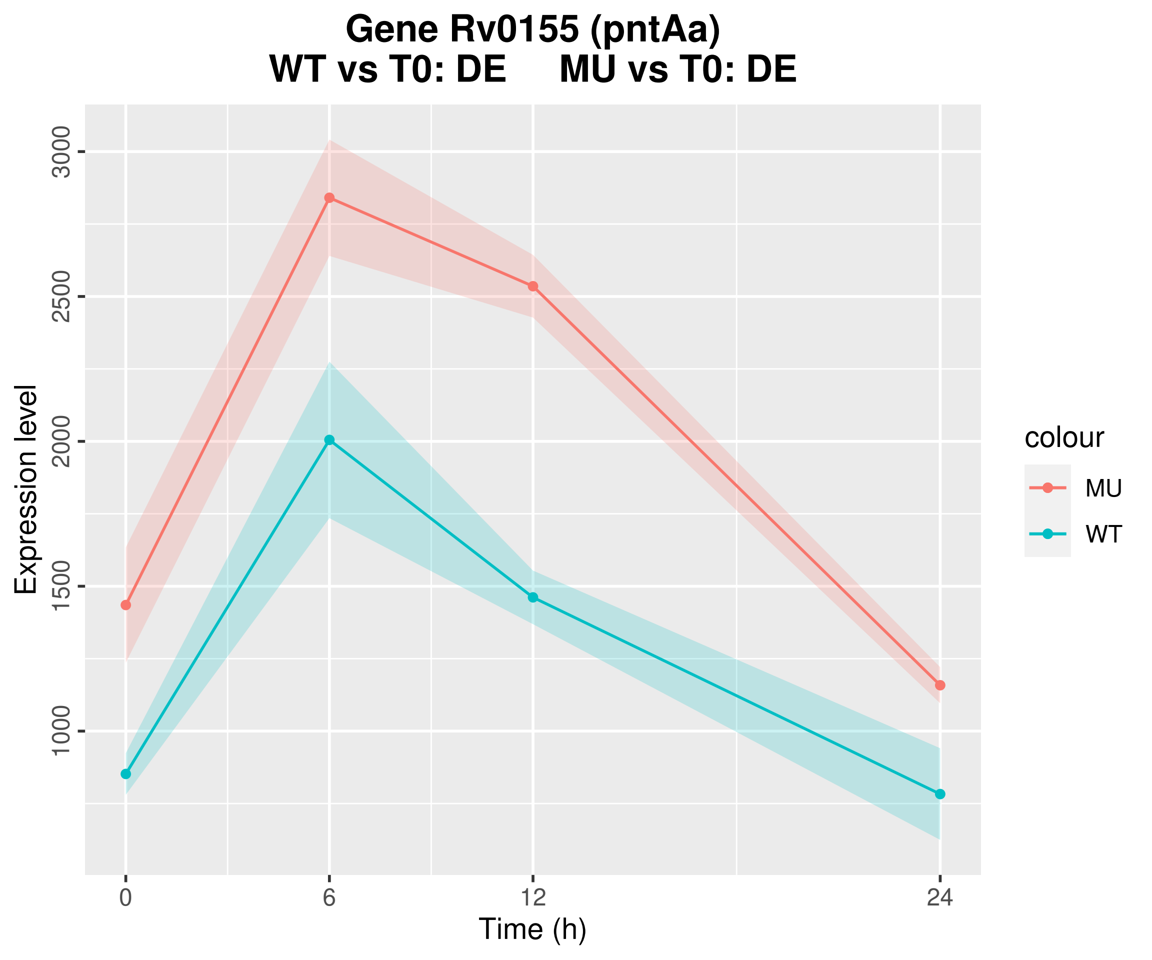

Gene Rv0155 (pntAa)
WT vs T0: DE MU vs T0: DE
Expression level
3000
2500
2000
1500
1000
0
6
12
24
Time (h)
colour
MU
WT

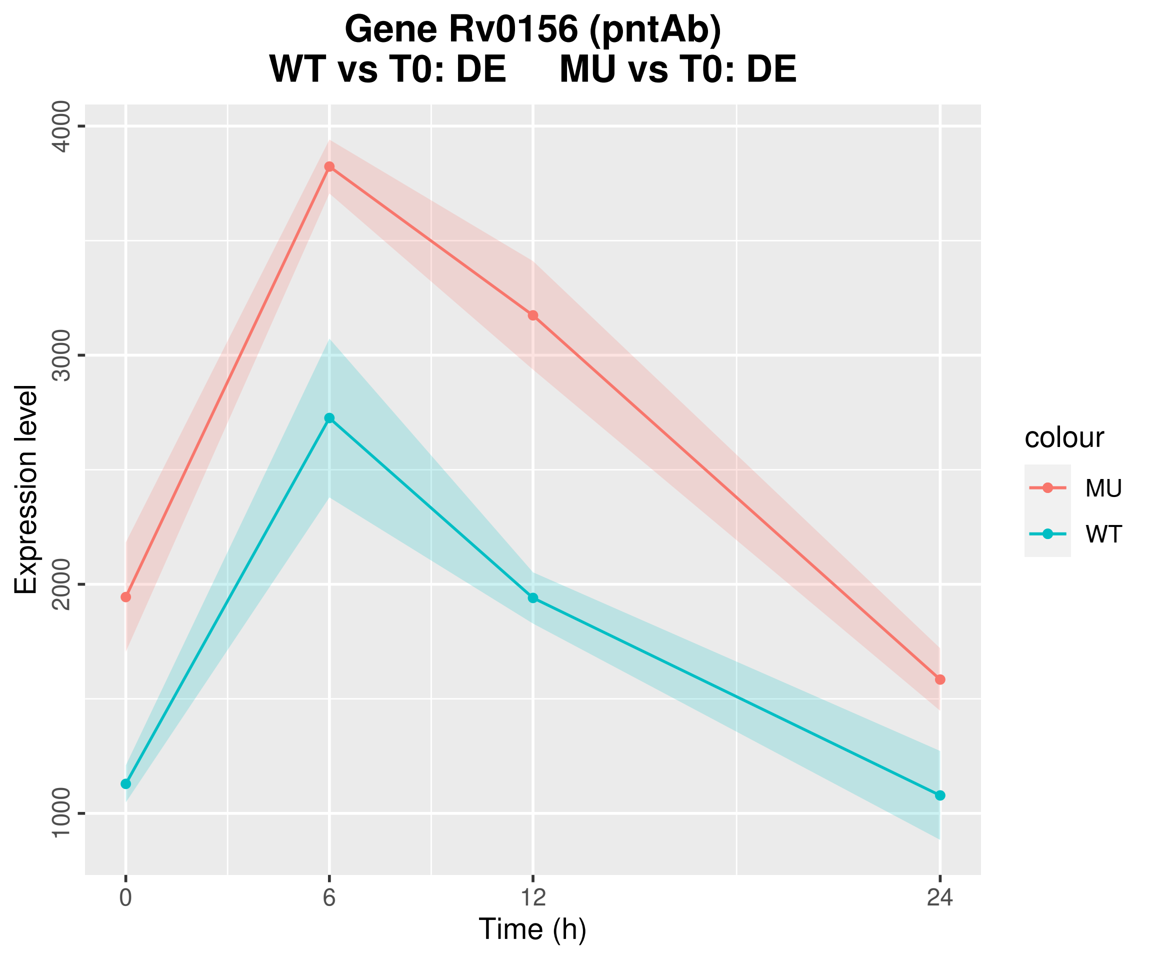

Gene Rv0156 (pntAb)
WT vs T0: DE MU vs T0: DE
Expression level
4000
3000
2000
1000
0
6
12
24
Time (h)
colour
MU
WT

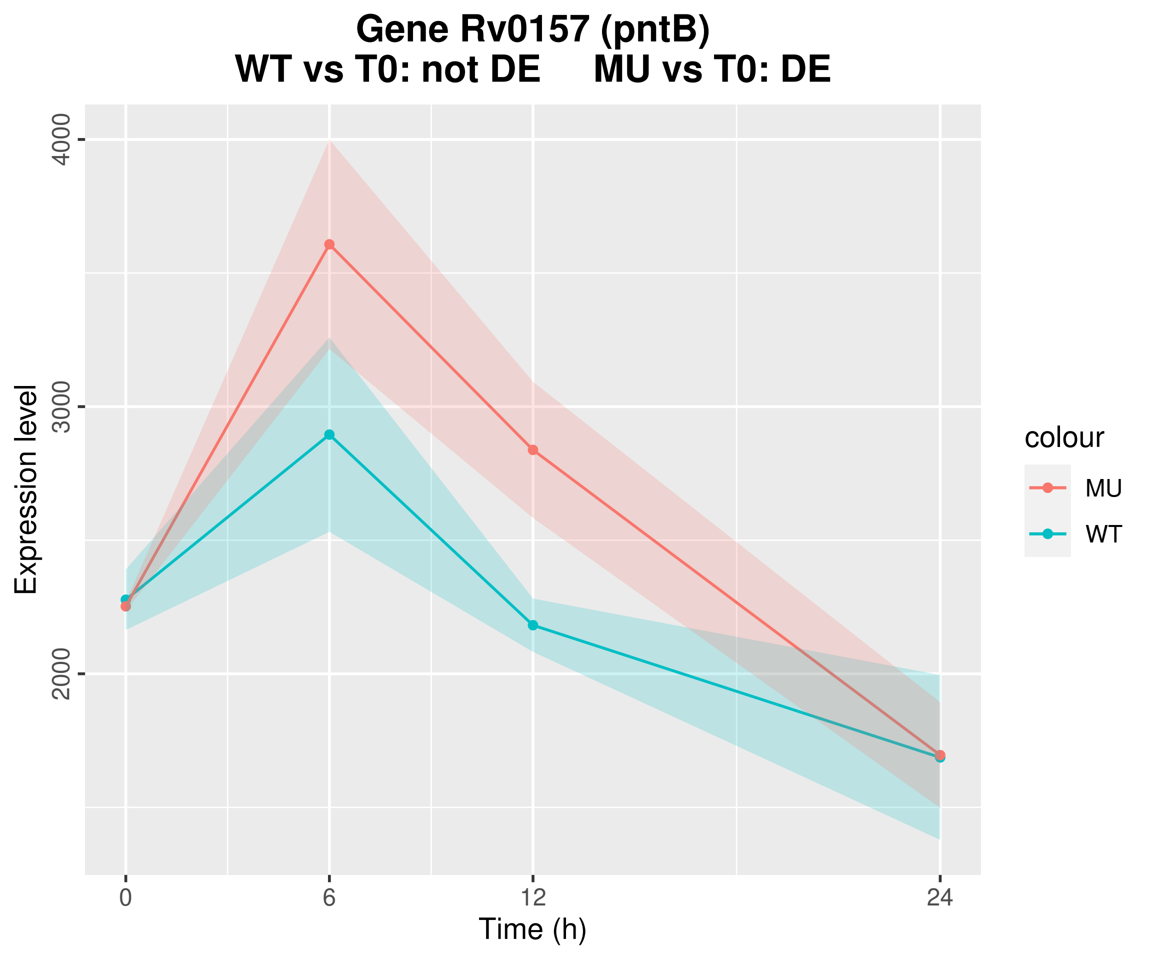

Gene Rv0157 (pntB)
WT vs T0: not DE   MU vs T0: DE
4000
3000
2000
Expression level
0
6
12
24
Time (h)
colour
MU
WT

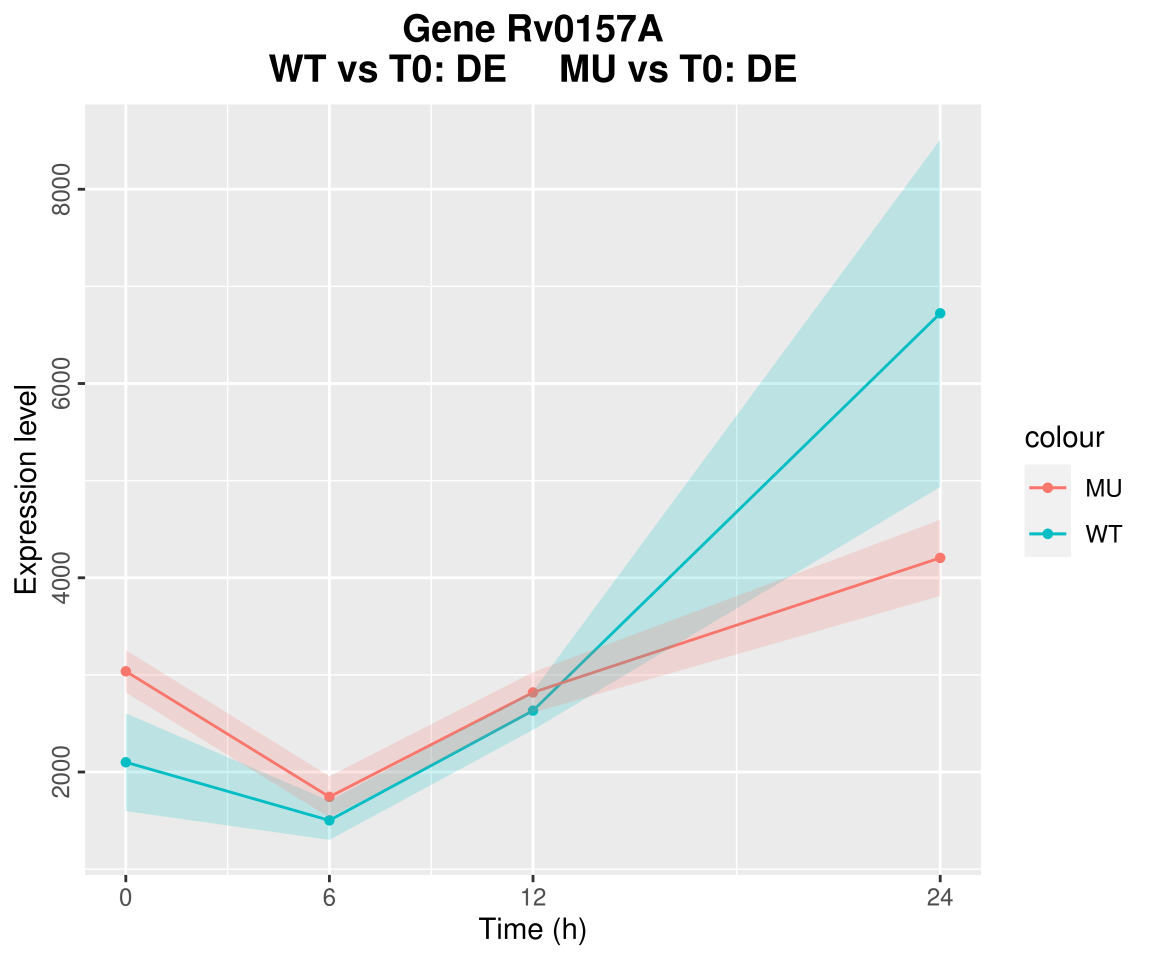

Gene Rv0157A
WT vs T0: DE   MU vs T0: DE
Expression level
8000
6000
4000
2000
0
6
12
24
Time (h)
colour
MU
WT

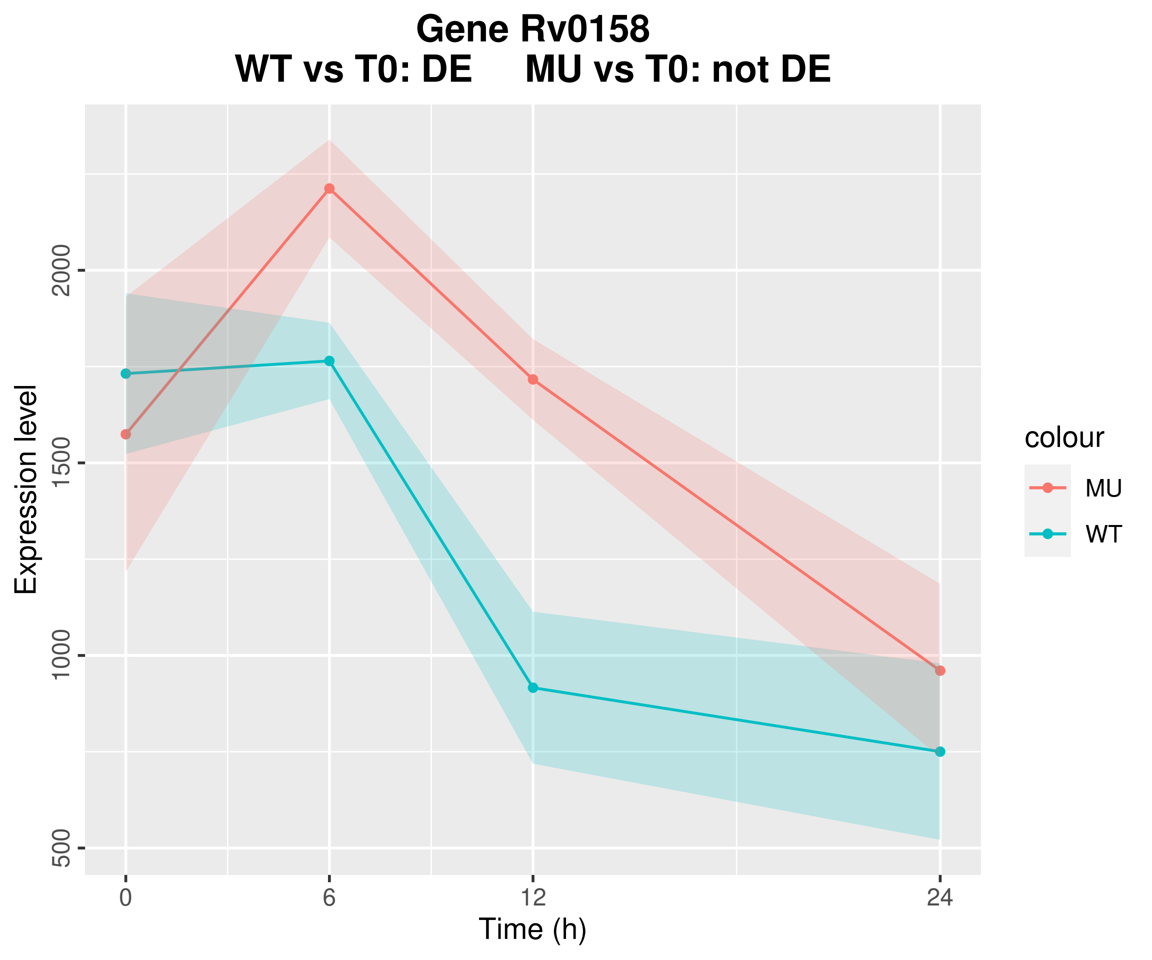

Gene Rv0158
WT vs T0: DE   MU vs T0: not DE
Expression level
2000
1500
1000
500
0
6
12
24
Time (h)
colour
MU
WT

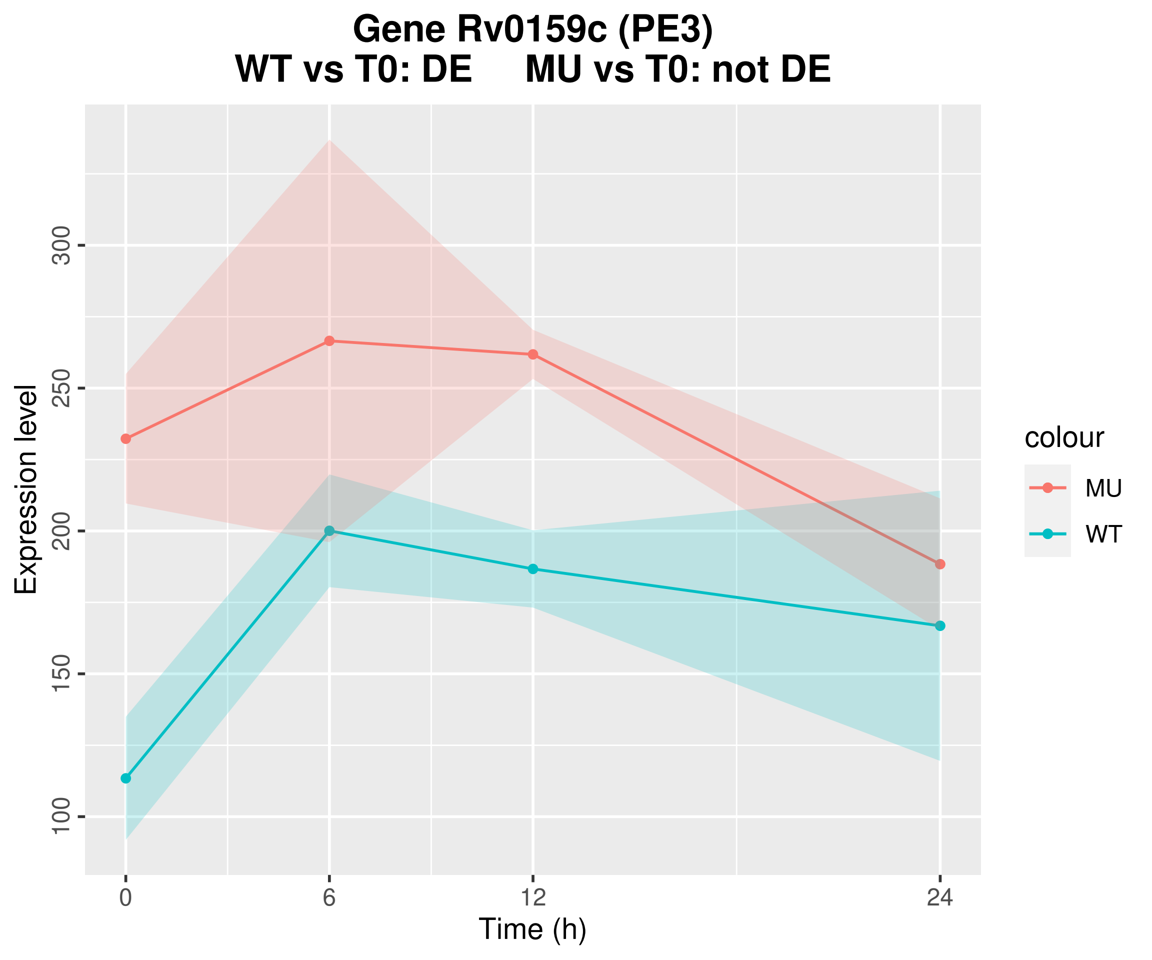

Gene Rv0159c (PE3)
WT vs T0: DE MU vs T0: not DE
Expression level
100
150
200
250
300
0
6
12
24
Time (h)
colour
MU
WT

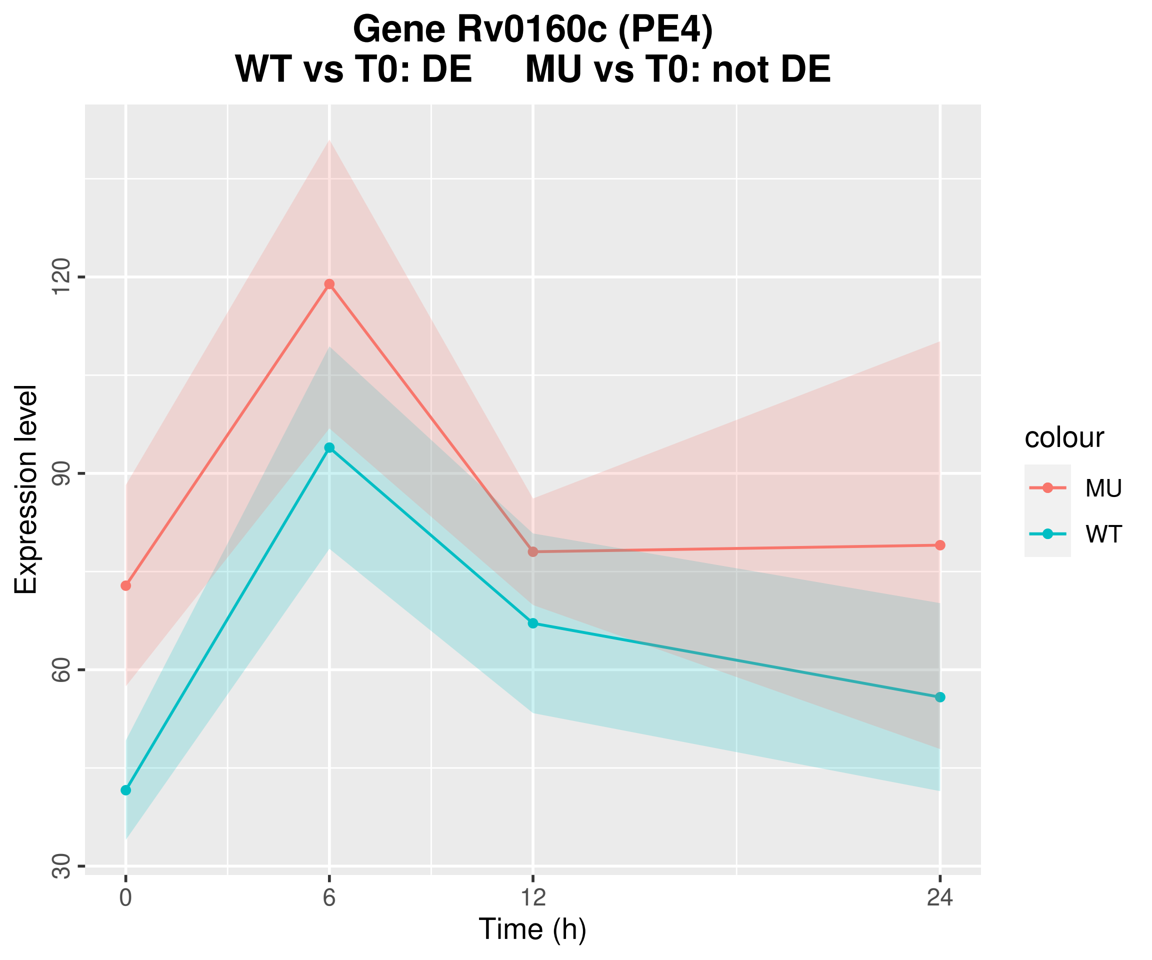

Gene Rv0160c (PE4)
WT vs T0: DE   MU vs T0: not DE
Expression level
120
90
60
30
0
6
12
24
Time (h)
colour
MU
WT

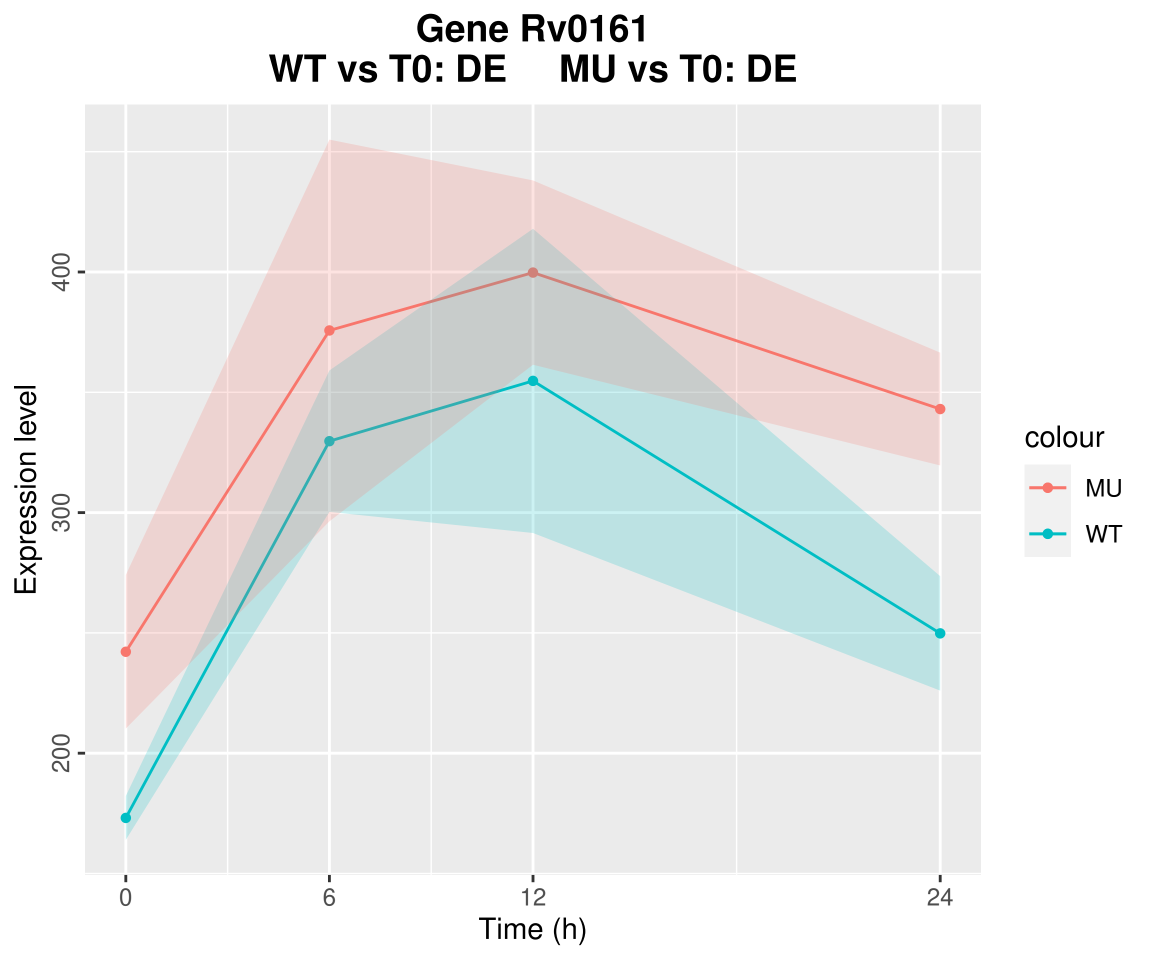

Gene Rv0161
WT vs T0: DE MU vs T0: DE
Expression level
400
300
200
0
6
12
24
Time (h)
colour
MU
WT

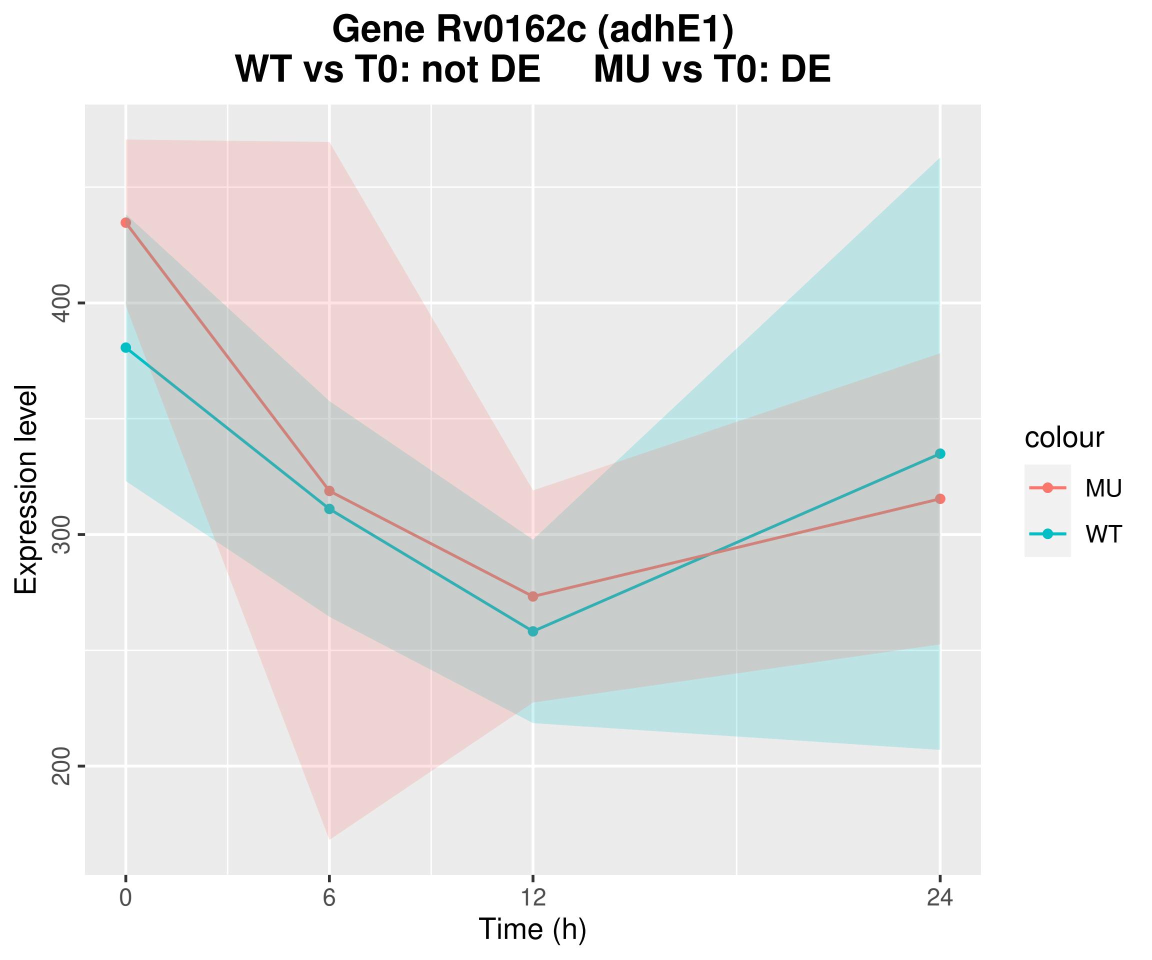

Gene Rv0162c (adhE1)
WT vs T0: not DE MU vs T0: DE
Expression level
400
300
200
0
6
12
24
Time (h)
colour
MU
WT

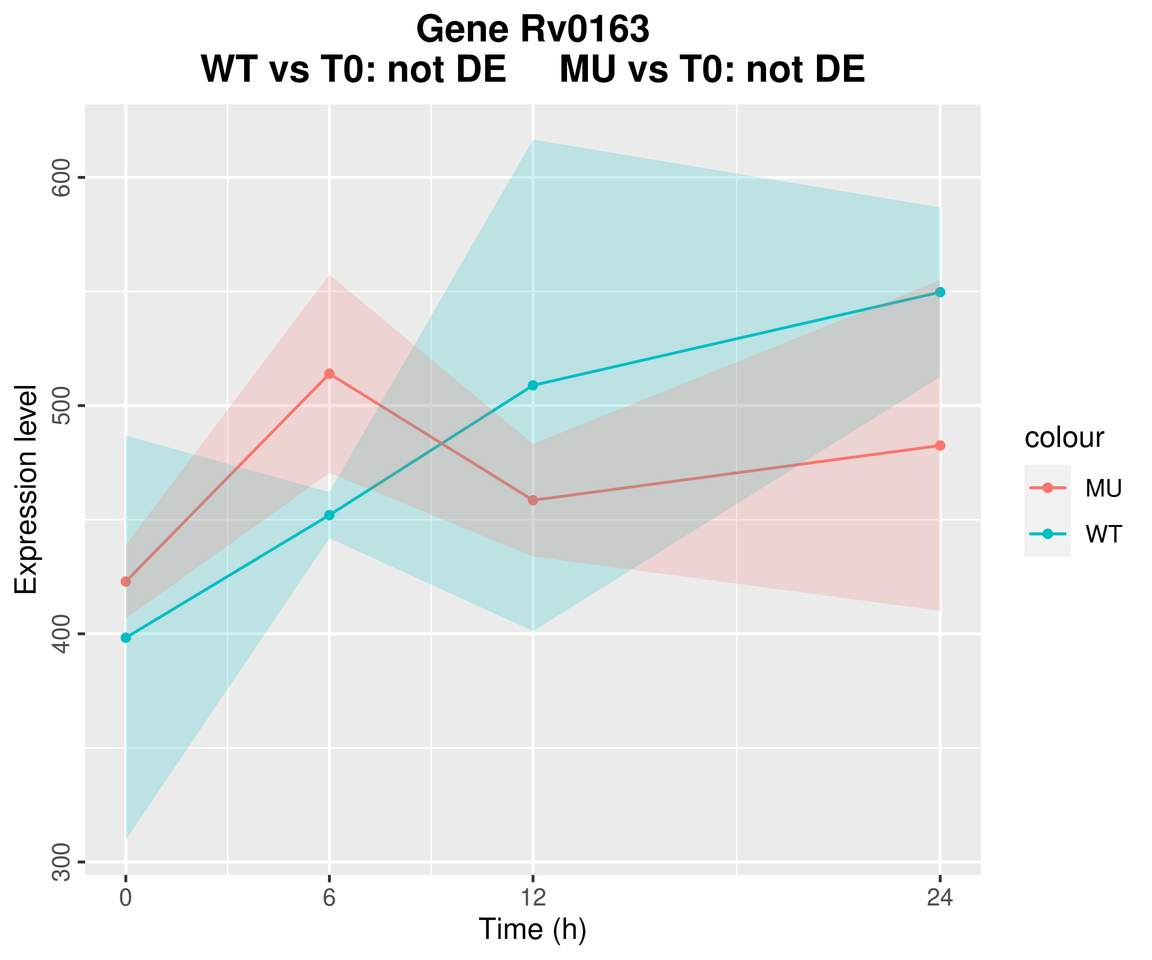
Gene Rv0163
WT vs T0: not DE MU vs T0: not DE
Expression level
600
500
400
300
0
6
12
24
Time (h)
colour
MU
WT

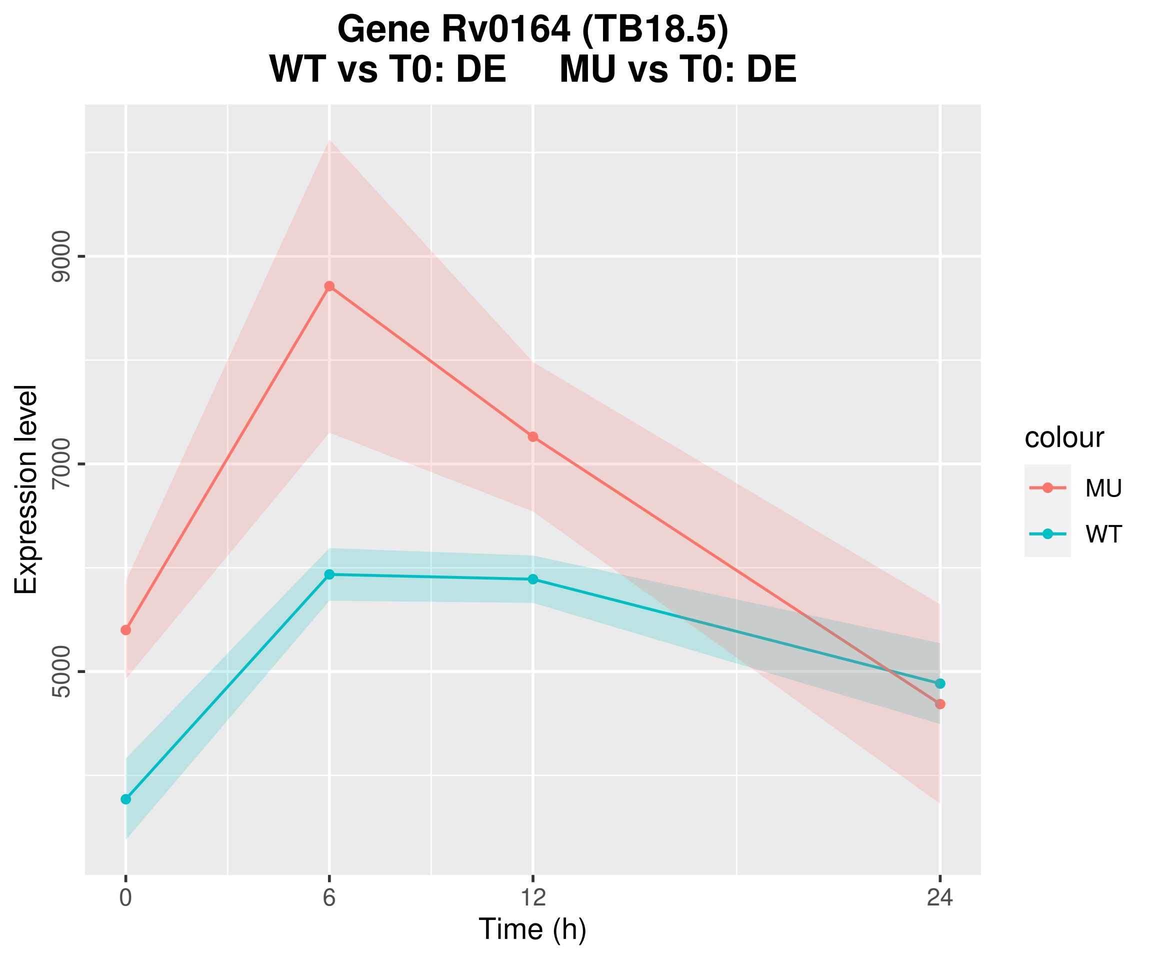
Gene Rv0164 (TB18.5)
WT vs T0: DE   MU vs T0: DE
Expression level
9000
7000
5000
0
6
12
24
Time (h)
colour
MU
WT

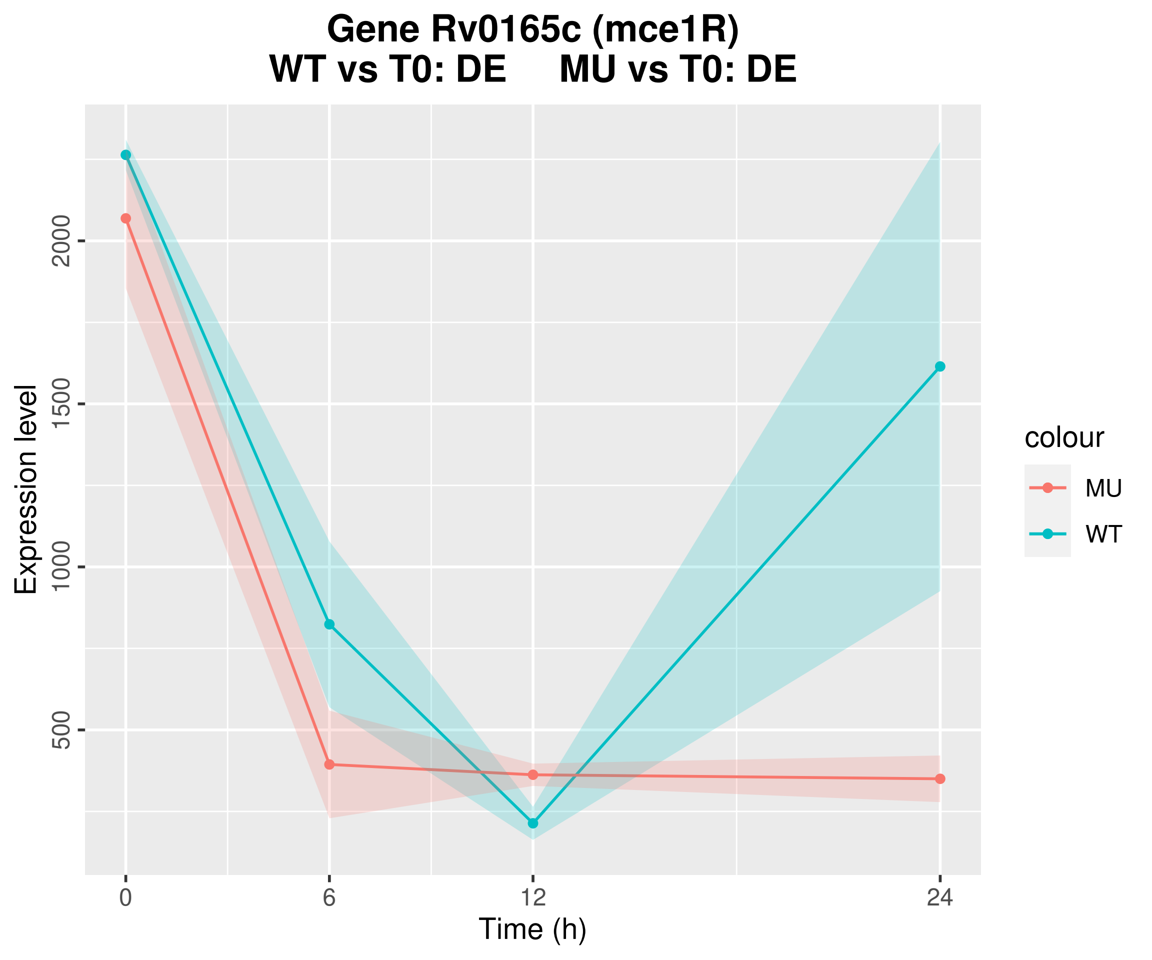

Gene Rv0165c (mce1R)
WT vs T0: DE   MU vs T0: DE
Expression level
2000
1500
1000
500
0
6
12
24
Time (h)
colour
MU
WT

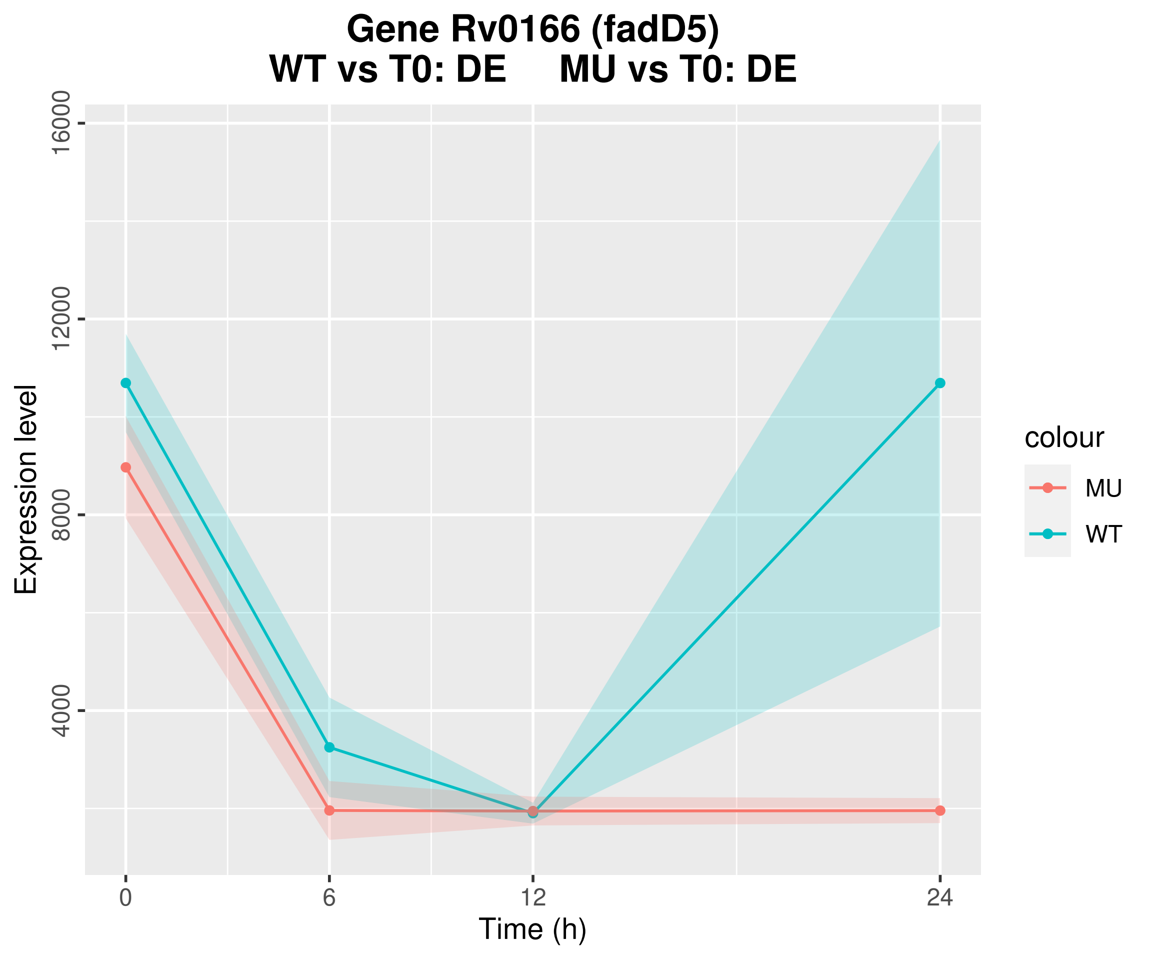
Gene Rv0166 (fadD5)
WT vs T0: DE MU vs T0: DE
Expression level
16000
12000
8000
4000
0
6
12
24
Time (h)
colour
MU
WT

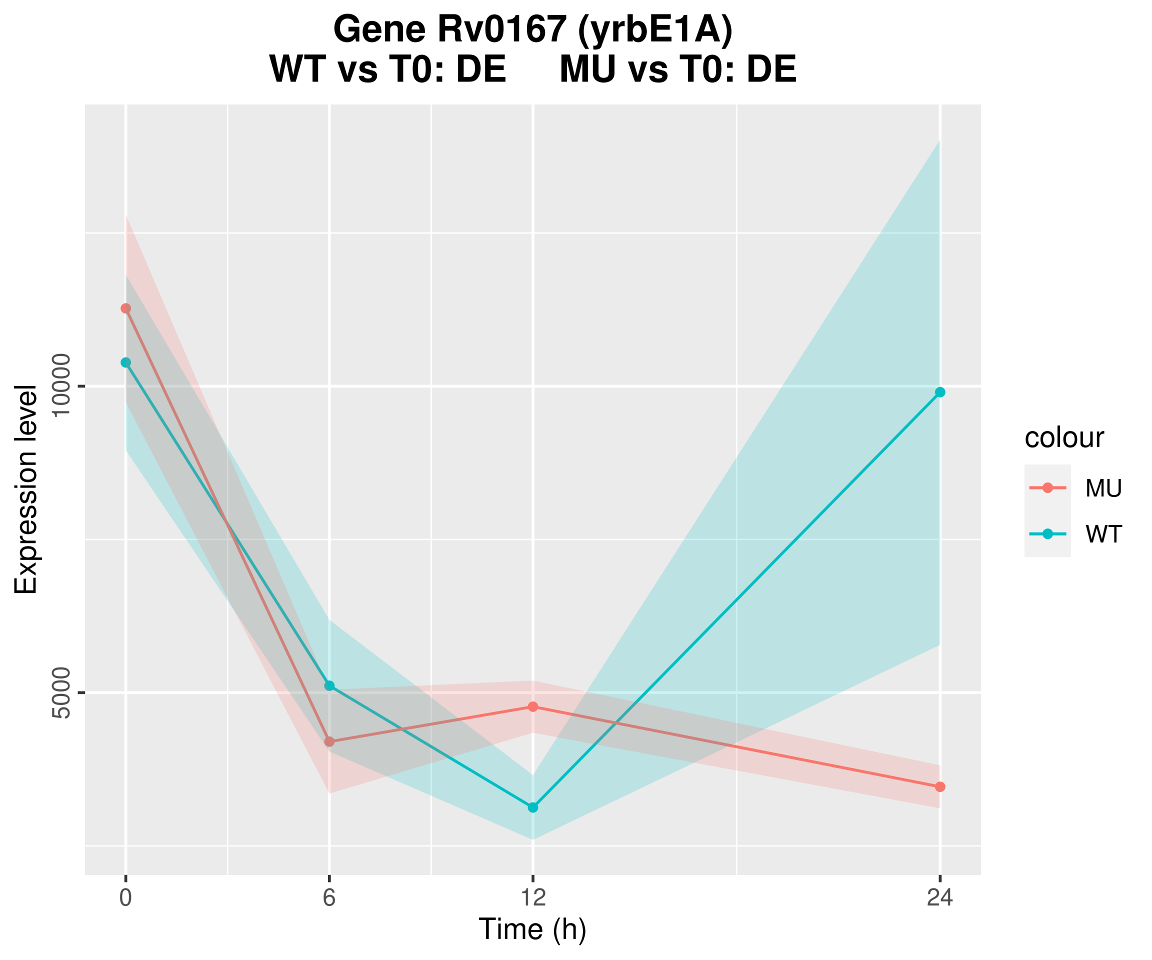

Gene Rv0167 (yrbE1A)
WT vs T0: DE MU vs T0: DE
Expression level
10000
5000
0
6
12
24
Time (h)
colour
MU
WT

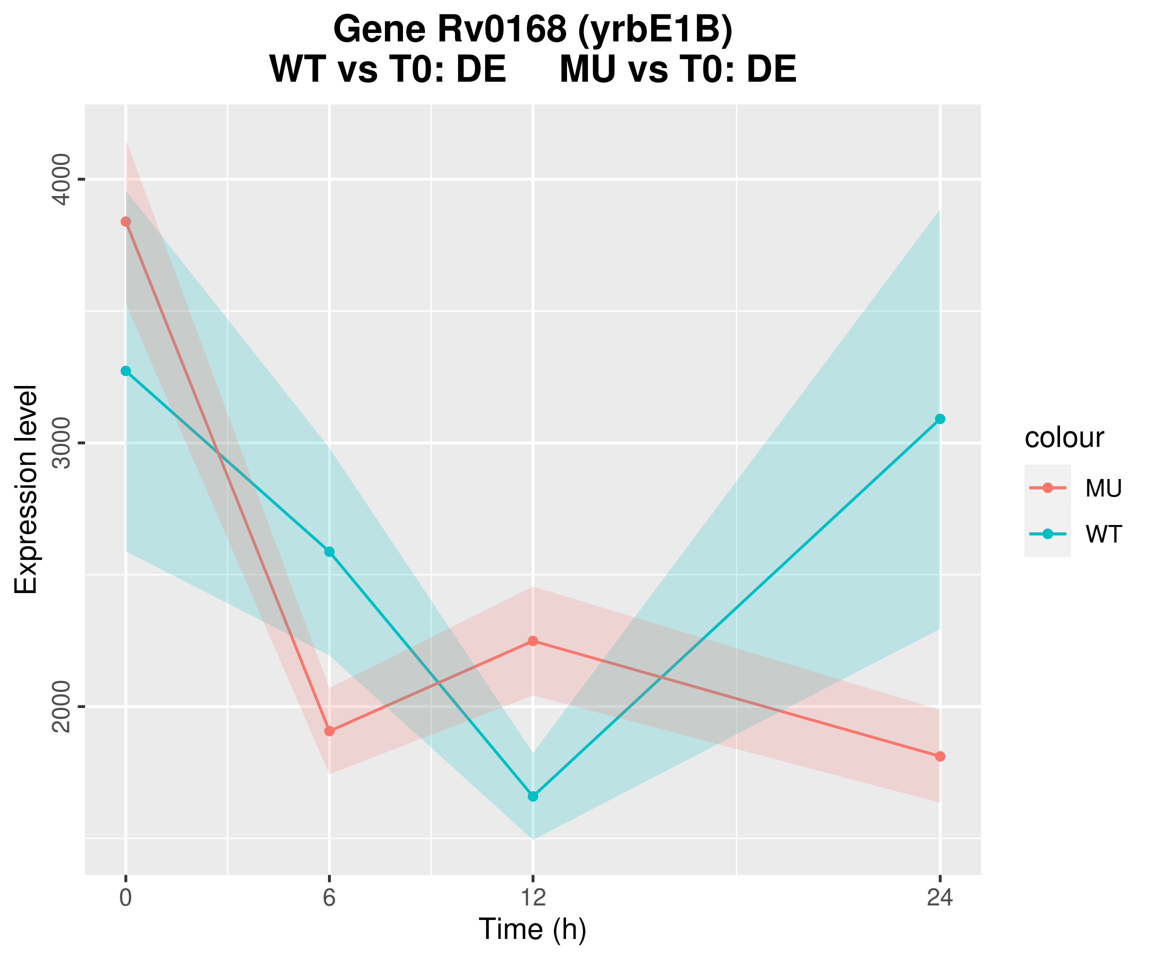

Gene Rv0168 (yrbE1B)
WT vs T0: DE MU vs T0: DE
Expression level
4000
3000
2000
0
6
12
24
Time (h)
colour
MU
WT

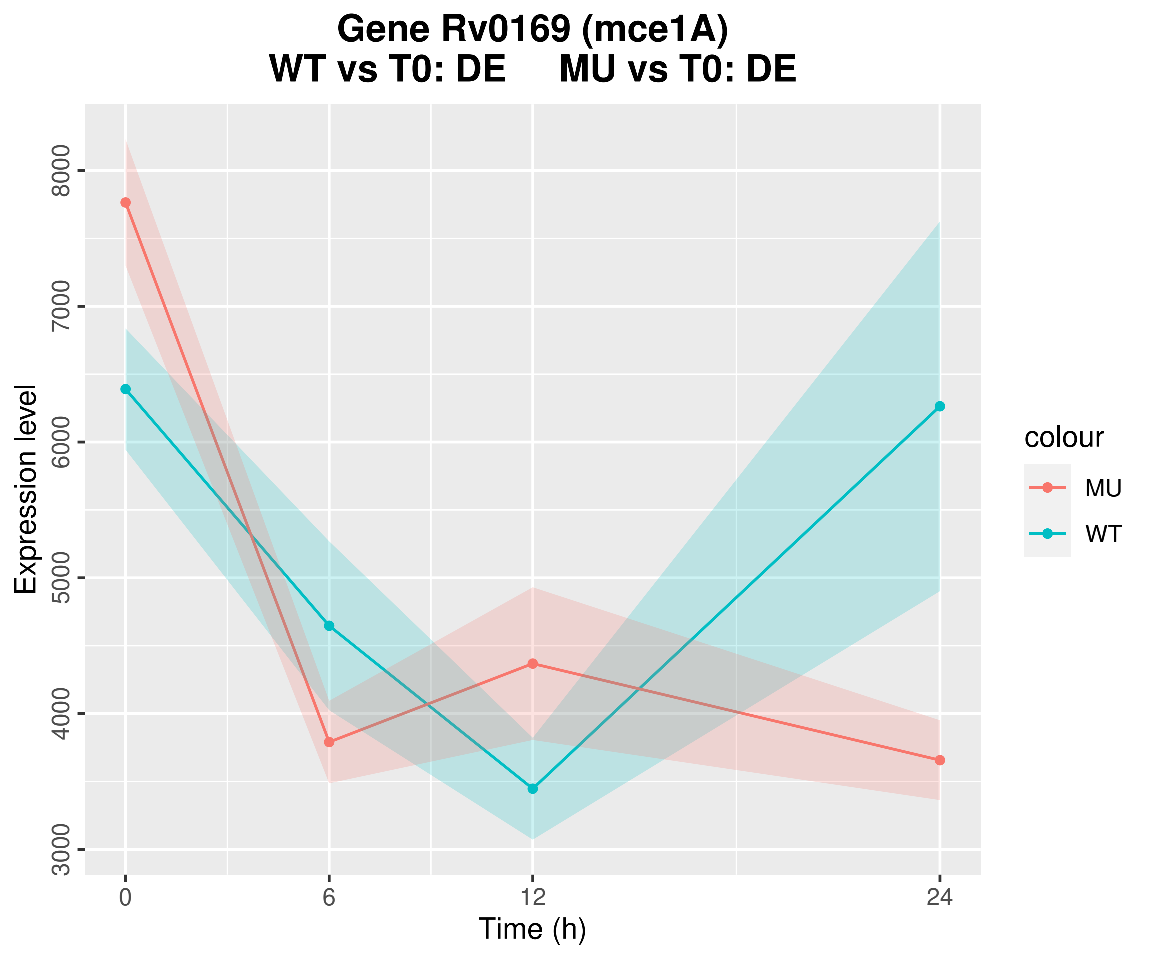
Gene Rv0169 (mce1A)
WT vs T0: DE MU vs T0: DE
Expression level
3000
4000
5000
6000
7000
8000
0
6
12
24
Time (h)
colour
MU
WT

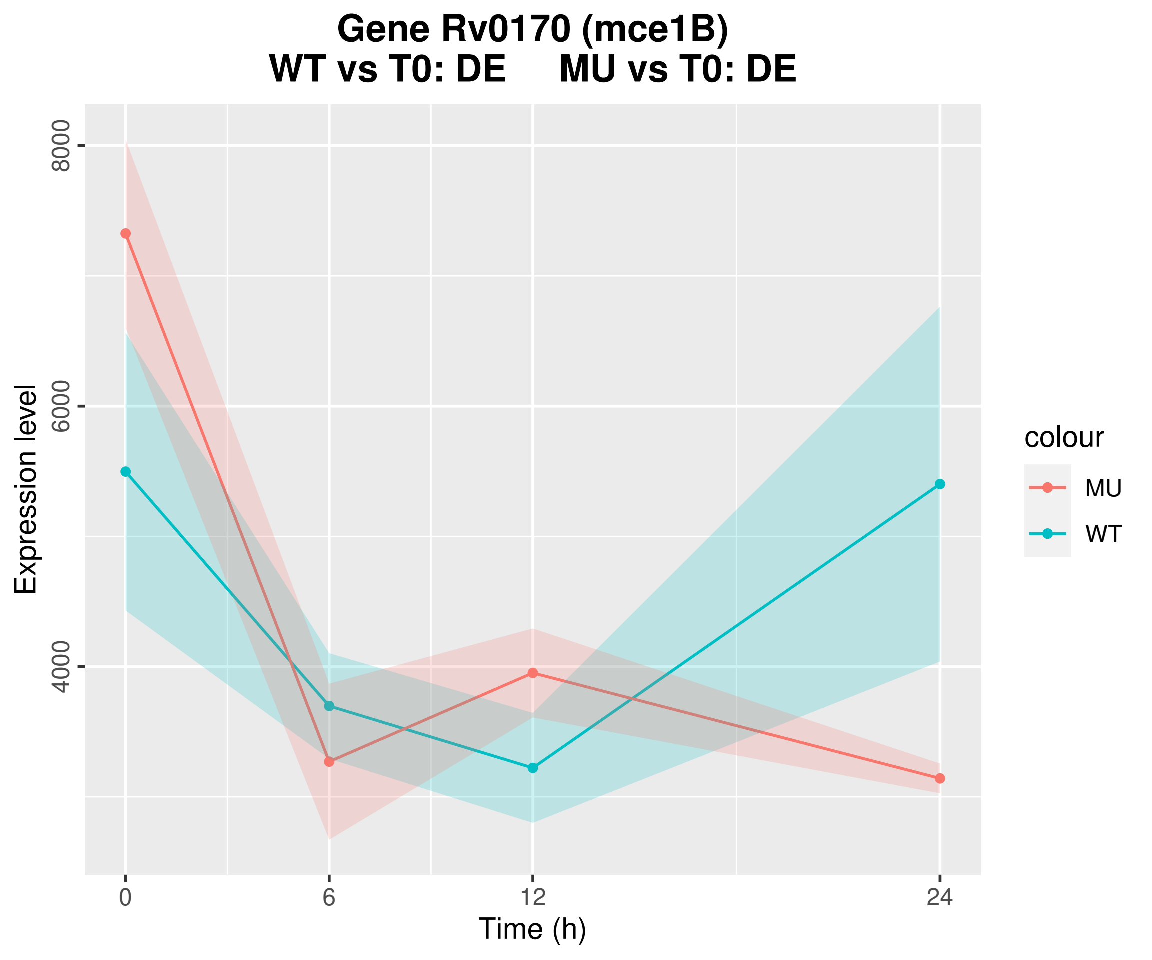
Gene Rv0170 (mce1B)
WT vs T0: DE   MU vs T0: DE
Expression level
8000
6000
4000
0
6
12
24
Time (h)
colour
MU
WT

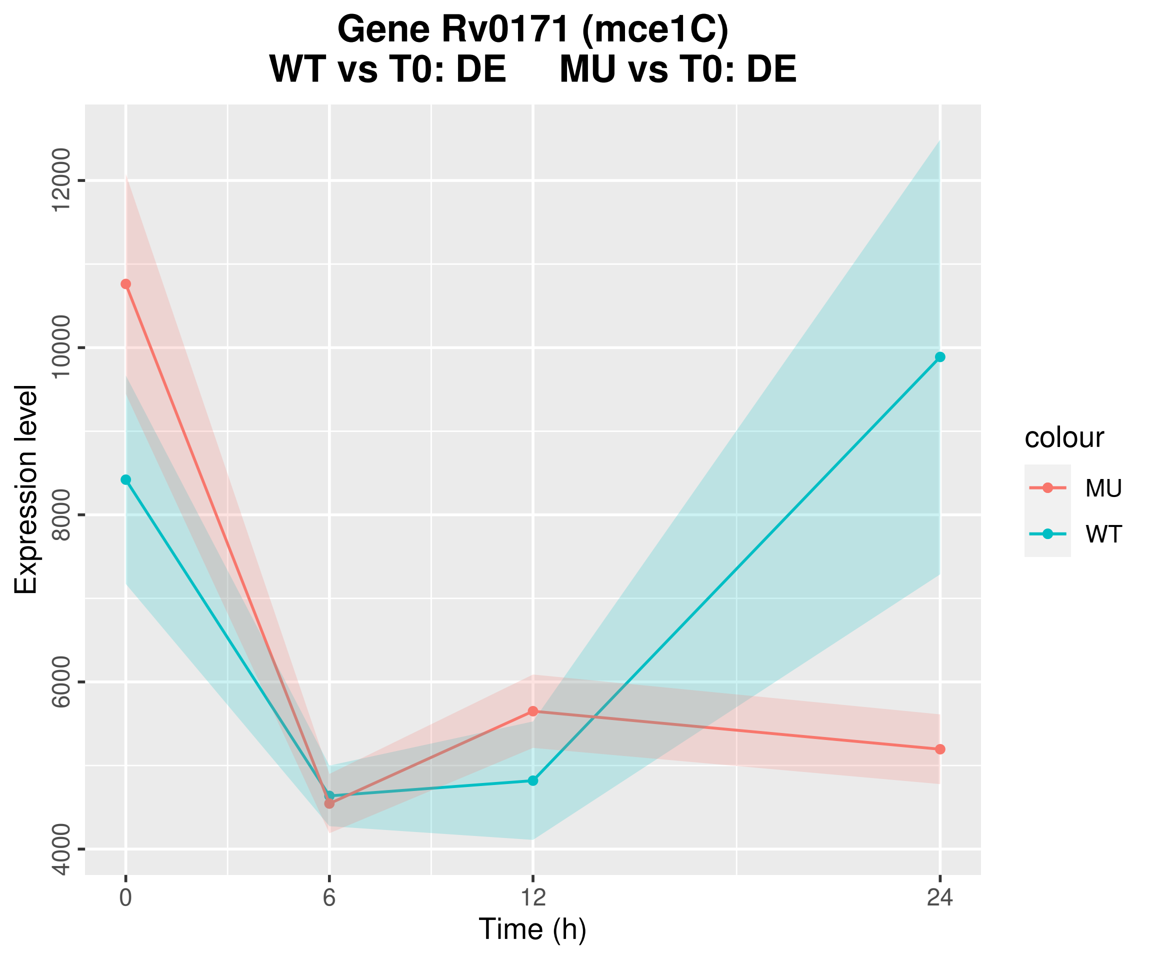
Gene Rv0171 (mce1C)
WT vs T0: DE MU vs T0: DE
Expression level
4000
6000
8000
10000
12000
0
6
12
24
Time (h)
colour
MU
WT

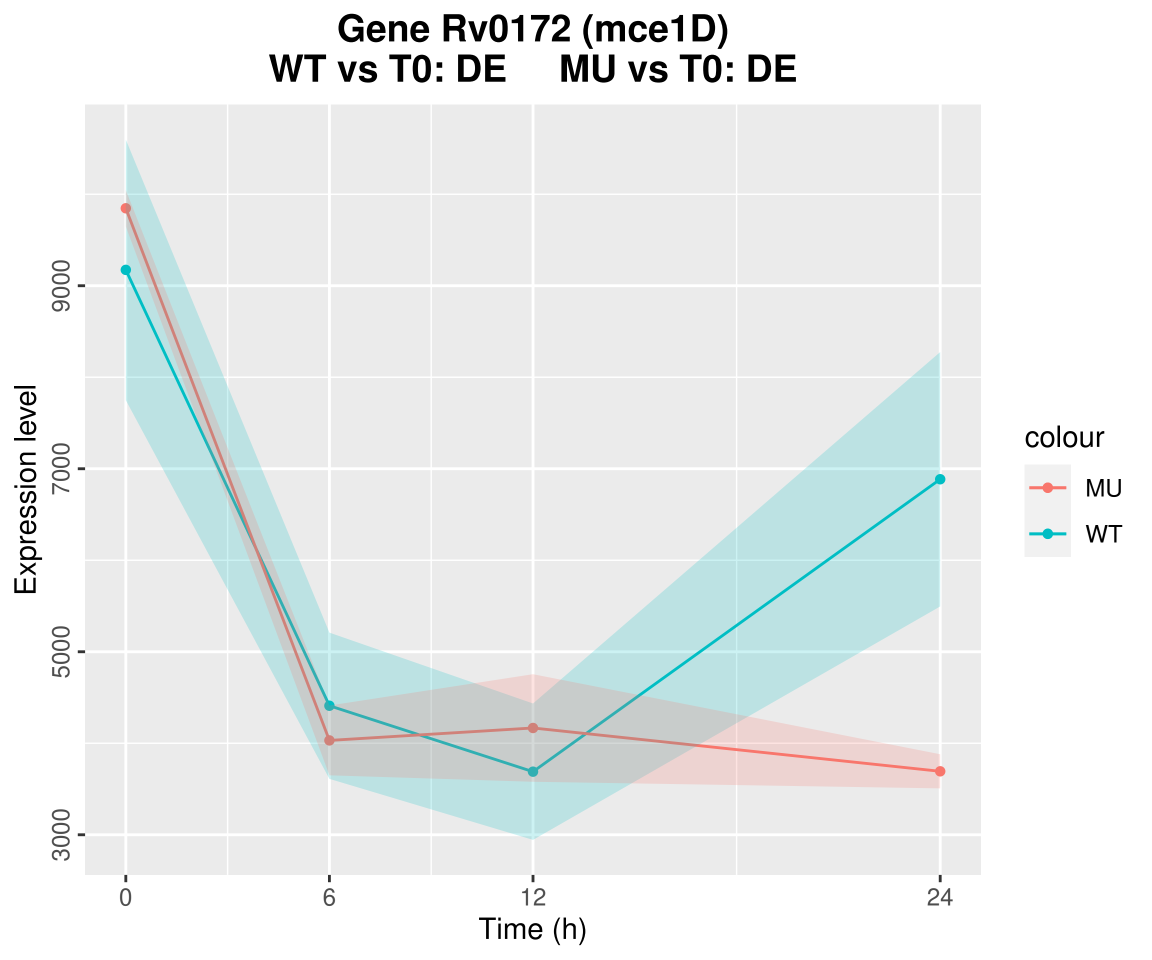

Gene Rv0172 (mce1D)
WT vs T0: DE MU vs T0: DE
9000
7000
5000
3000
Expression level
0
6
12
24
Time (h)
colour
MU
WT

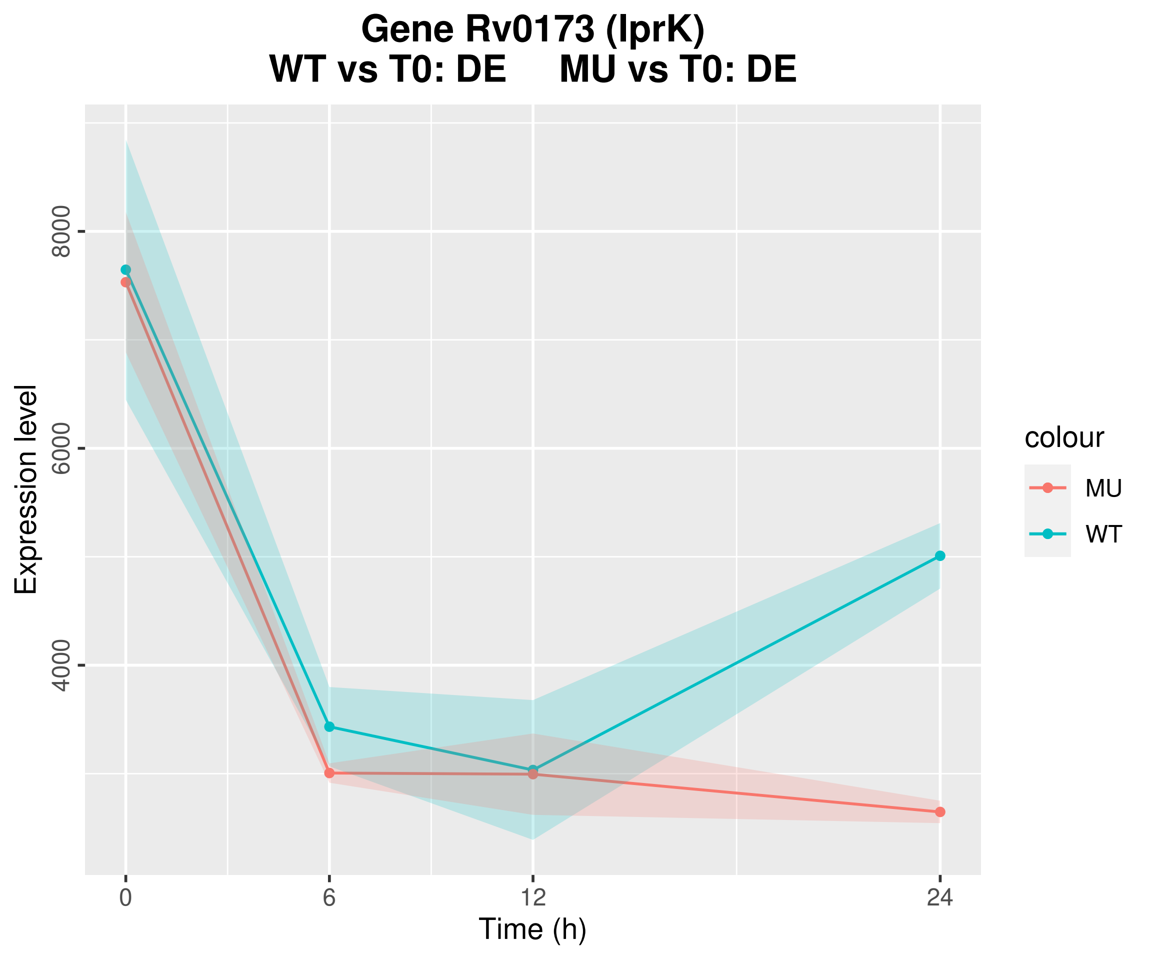
Gene Rv0173 (lprK)
WT vs T0: DE   MU vs T0: DE
Expression level
8000
6000
4000
0
6
12
24
Time (h)
colour
MU
WT

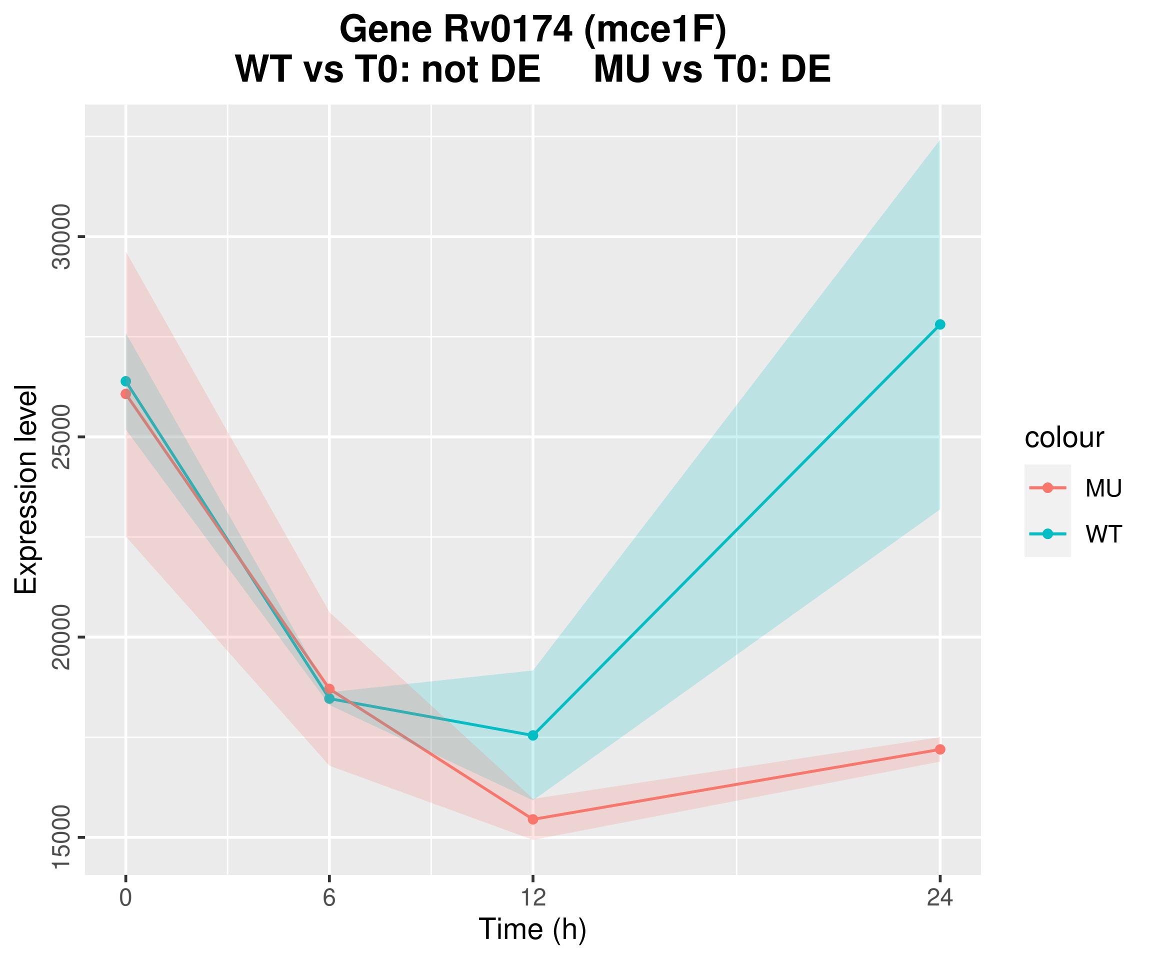

Gene Rv0174 (mce1F)
WT vs T0: not DE   MU vs T0: DE
Expression level
30000
25000
20000
15000
0
6
12
24
Time (h)
colour
MU
WT

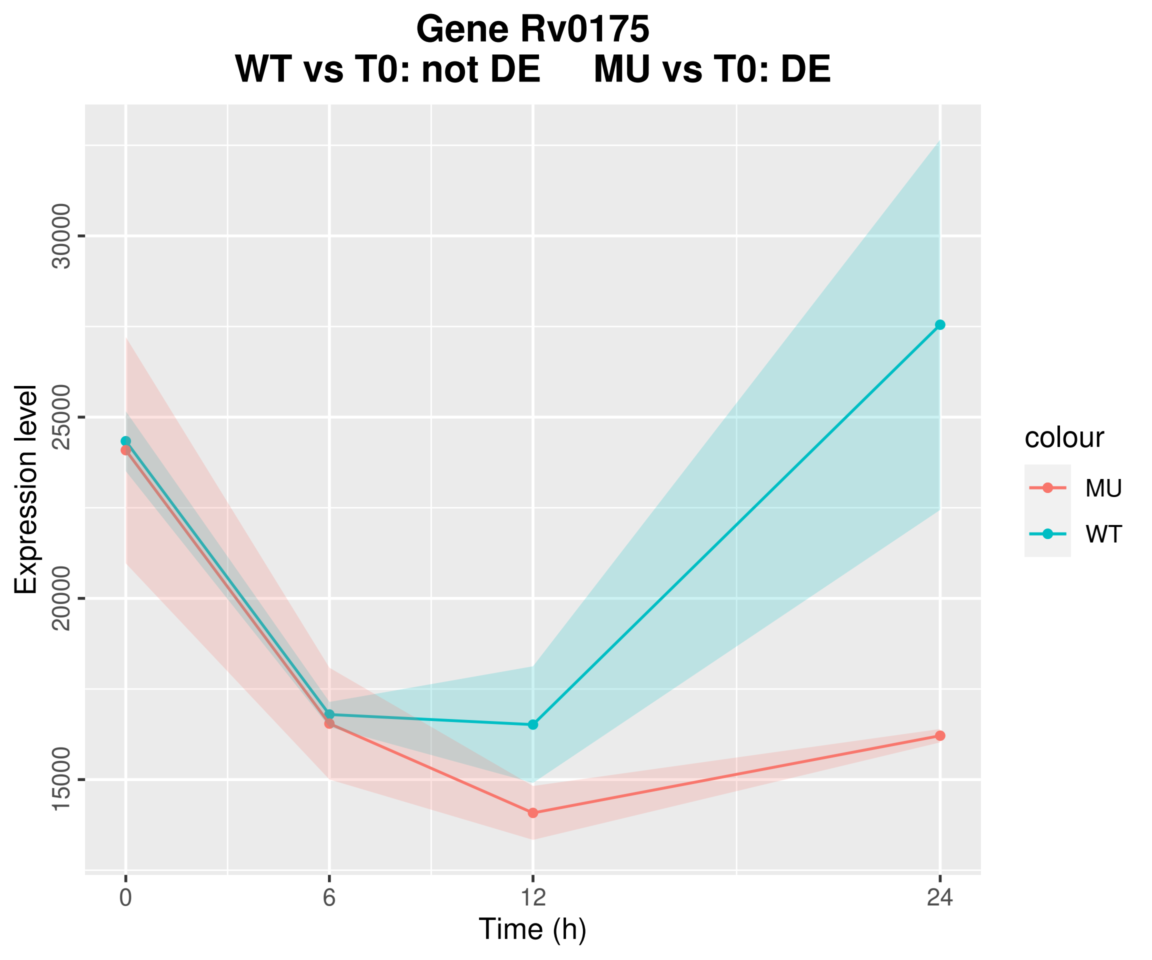

Gene Rv0175
WT vs T0: not DE   MU vs T0: DE
Expression level
15000
20000
25000
30000
Time (h)
0
6
12
24
colour
MU
WT

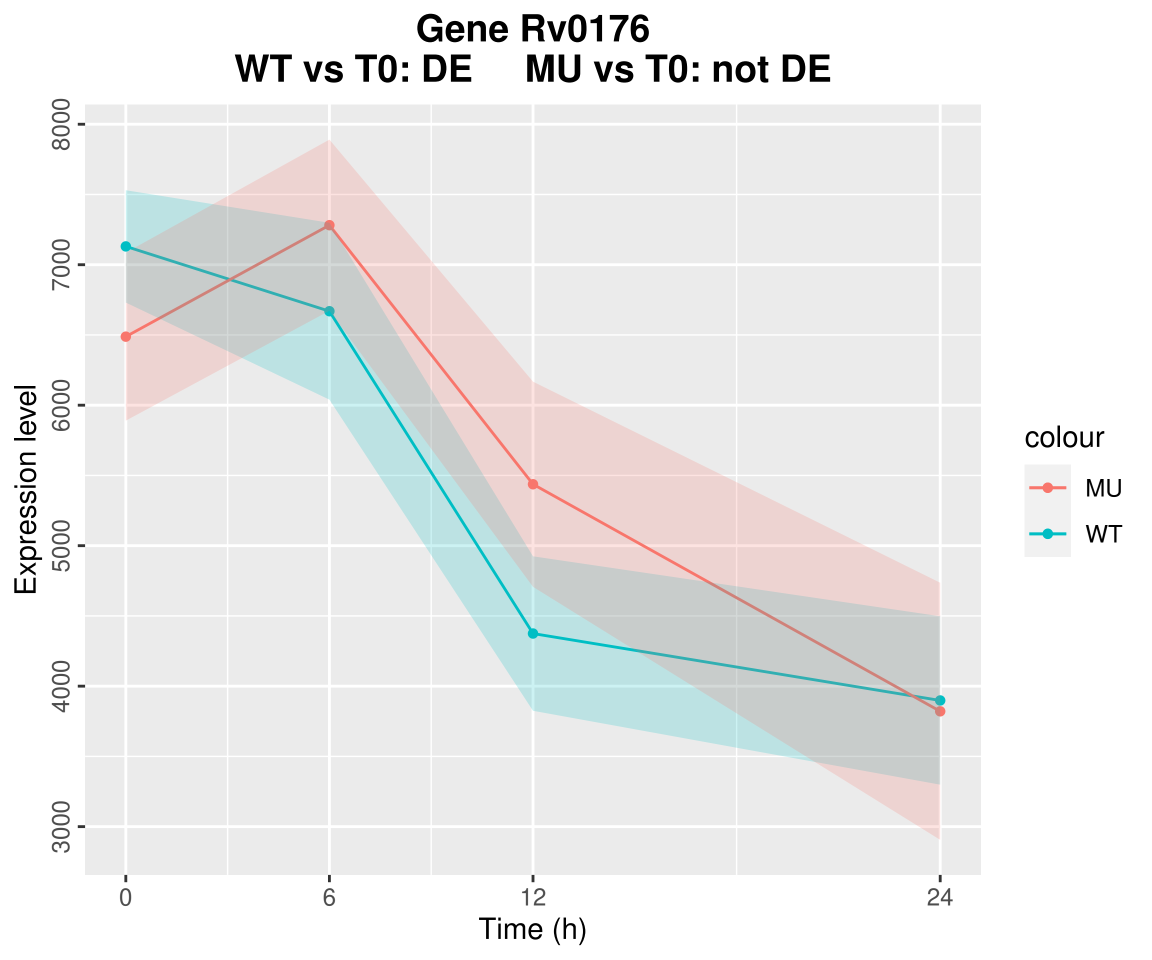

Gene Rv0176
WT vs T0: DE   MU vs T0: not DE
Expression level
8000
7000
6000
5000
4000
3000
0
6
12
24
Time (h)
colour
MU
WT

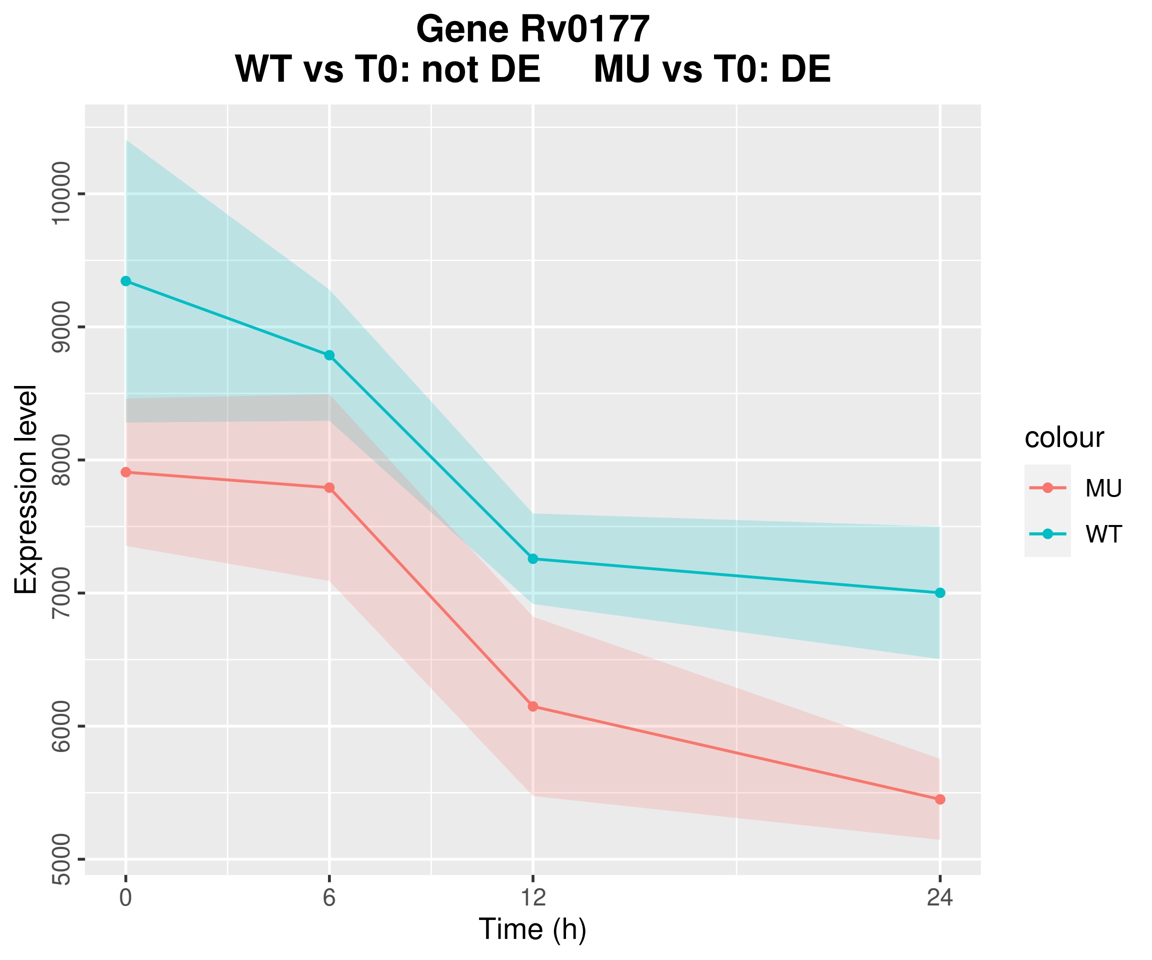

Gene Rv0177
WT vs T0: not DE MU vs T0: DE
Expression level
10000
9000
8000
7000
6000
5000
0
6
12
24
Time (h)
colour
MU
WT

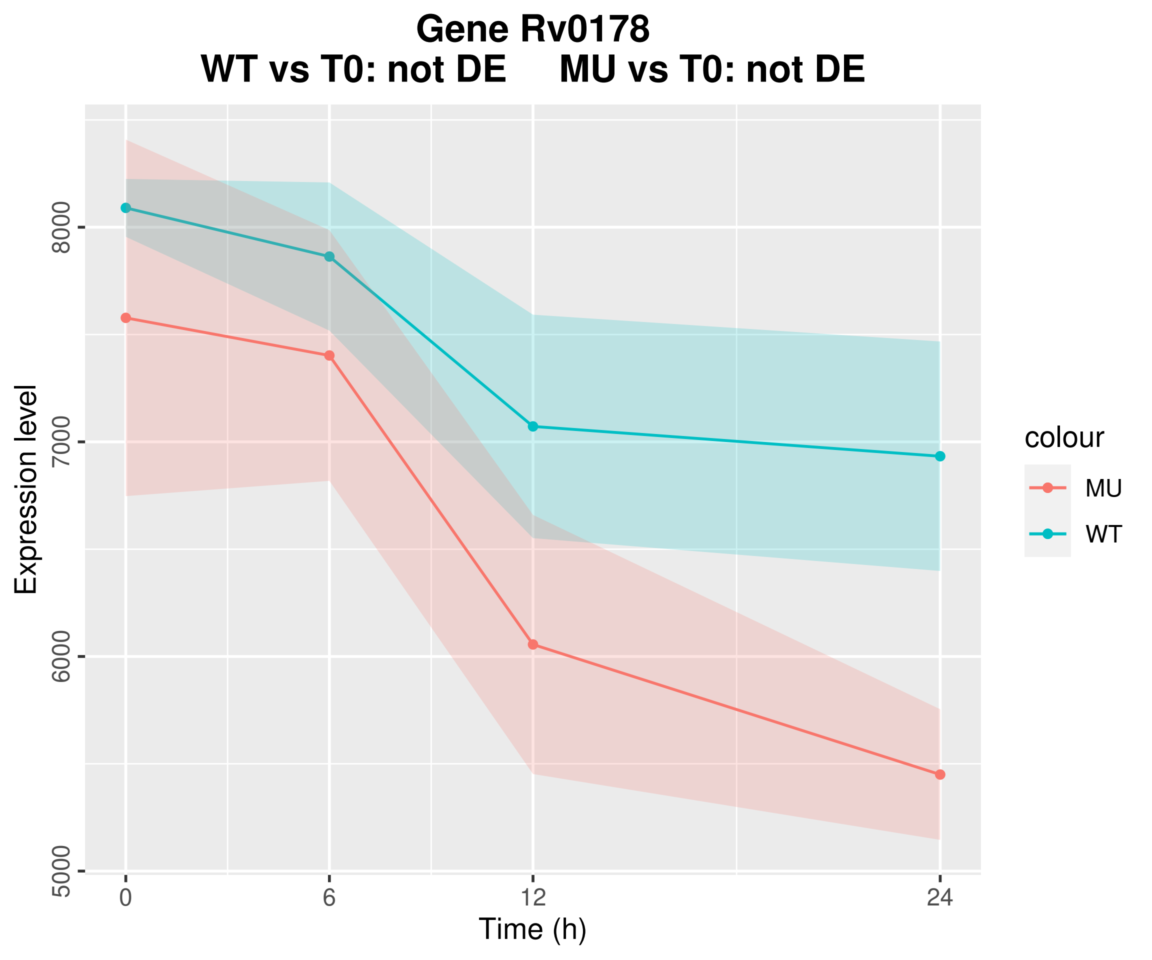

Gene Rv0178
WT vs T0: not DE   MU vs T0: not DE
Expression level
5000
6000
7000
8000
0
6
12
24
Time (h)
colour
MU
WT

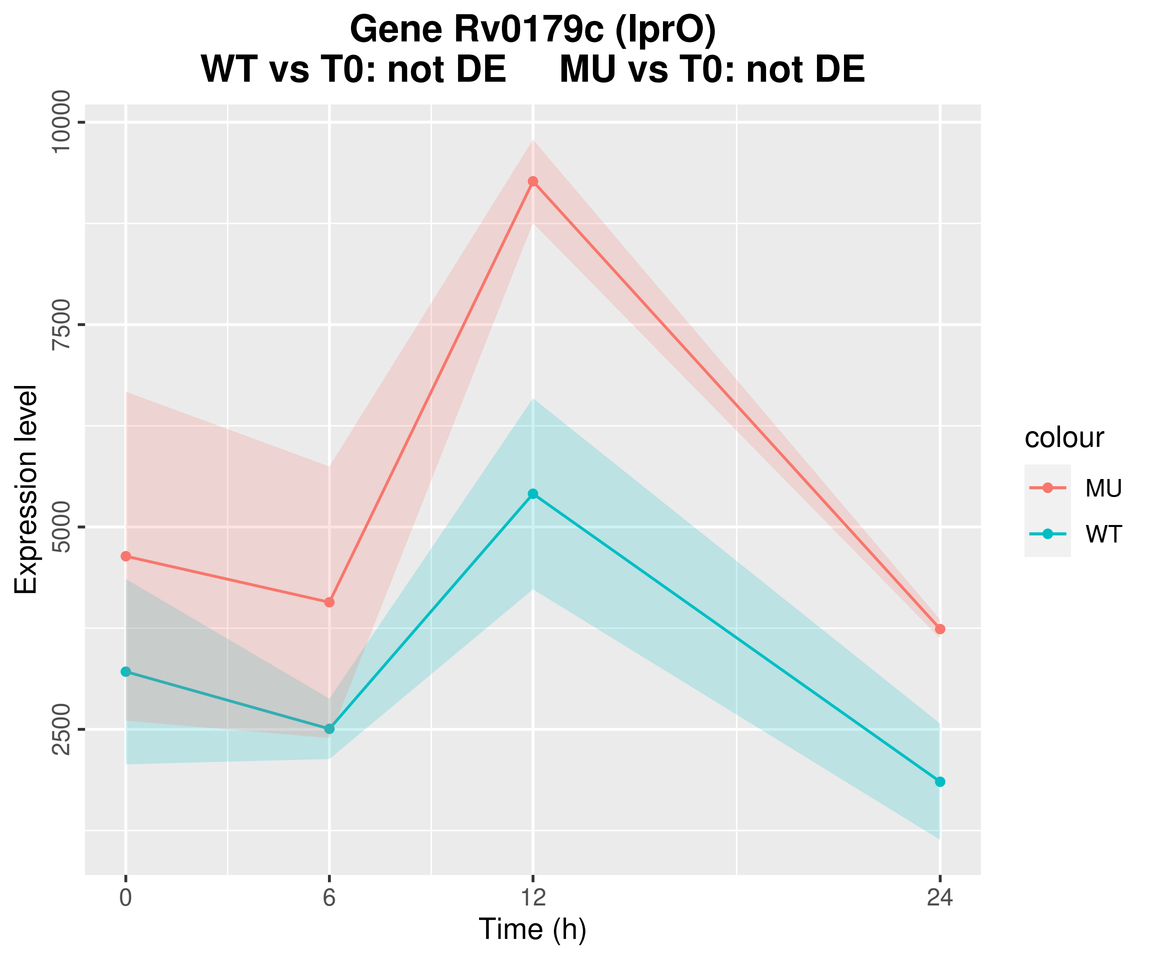
Gene Rv0179c (lprO)
WT vs T0: not DE   MU vs T0: not DE
Expression level
10000
7500
5000
2500
0
6
12
24
Time (h)
colour
MU
WT

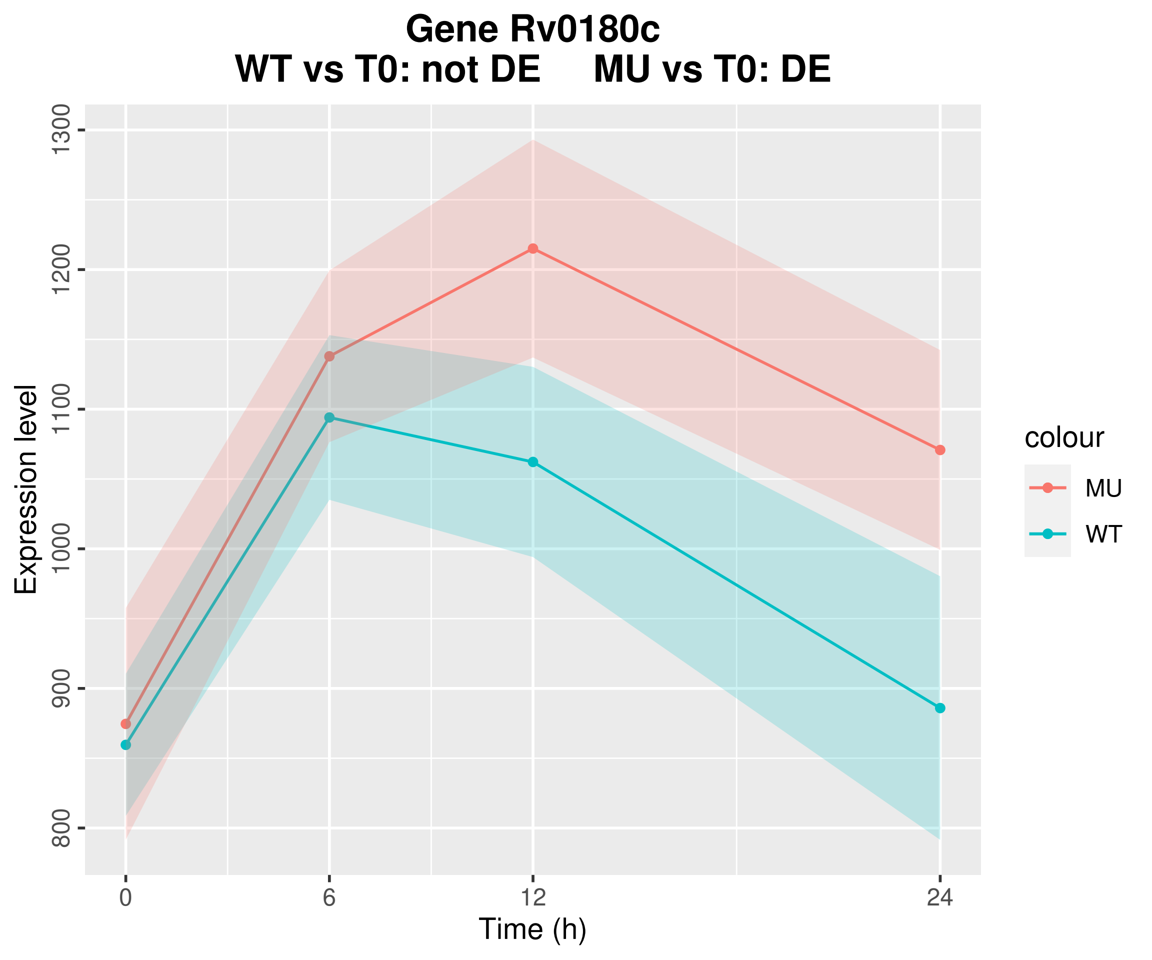

Gene Rv0180c
WT vs T0: not DE MU vs T0: DE
Expression level
1300
1200
1100
1000
900
800
0
6
12
24
Time (h)
colour
MU
WT

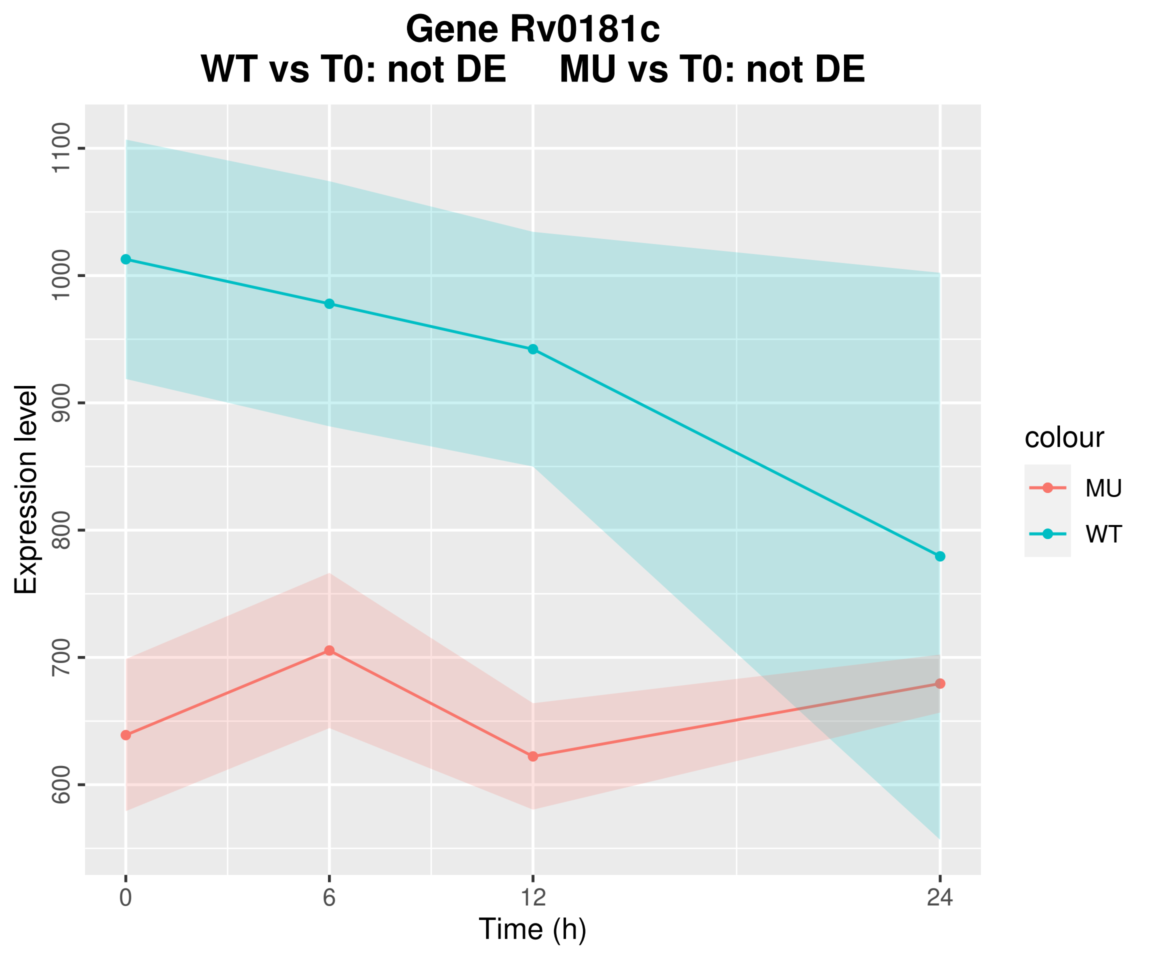
Gene Rv0181c
WT vs T0: not DE   MU vs T0: not DE
Expression level
1100
1000
900
800
700
600
0
6
12
24
Time (h)
colour
MU
WT

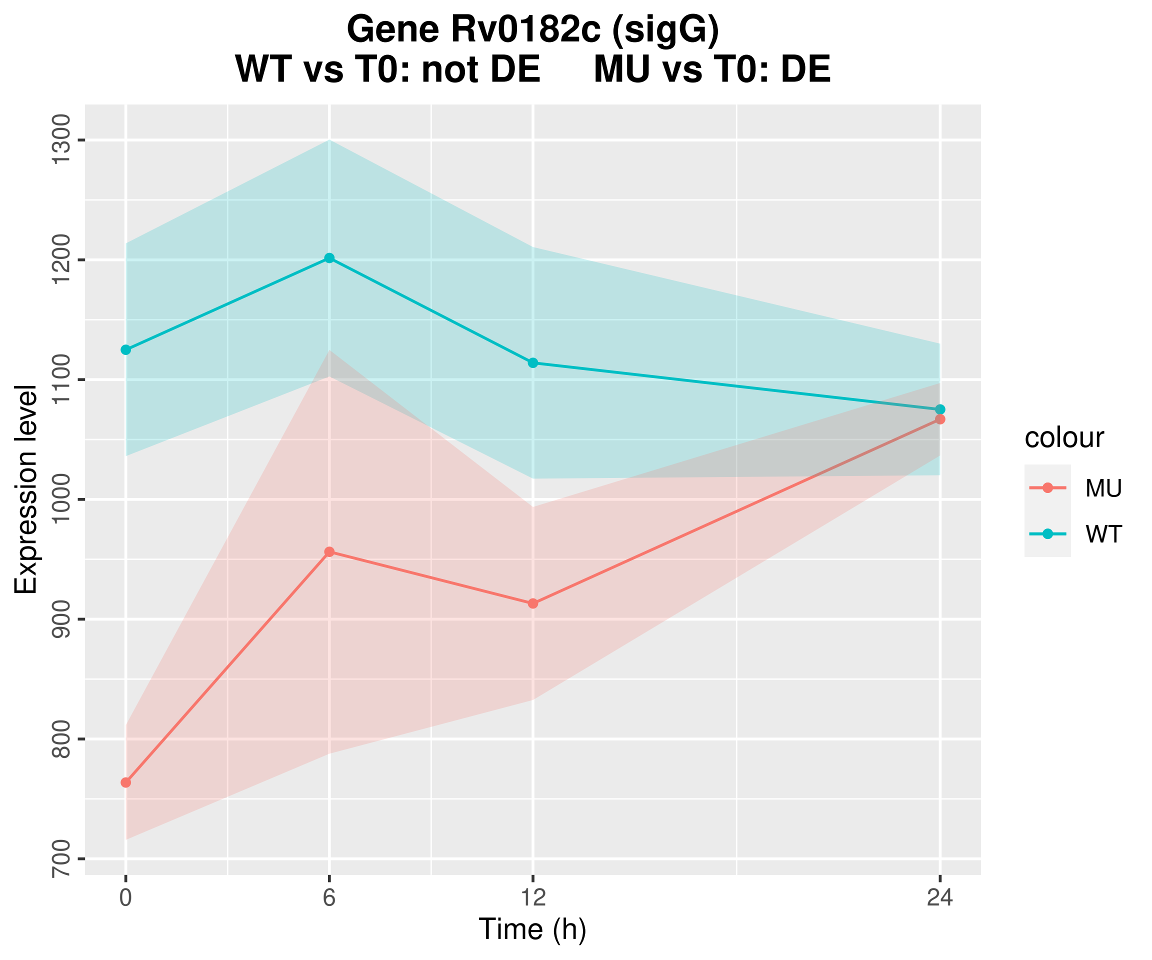

Gene Rv0182c (sigG)
WT vs T0: not DE MU vs T0: DE
Expression level
1300
1200
1100
1000
900
800
700
0
6
12
24
Time (h)
colour
MU
WT

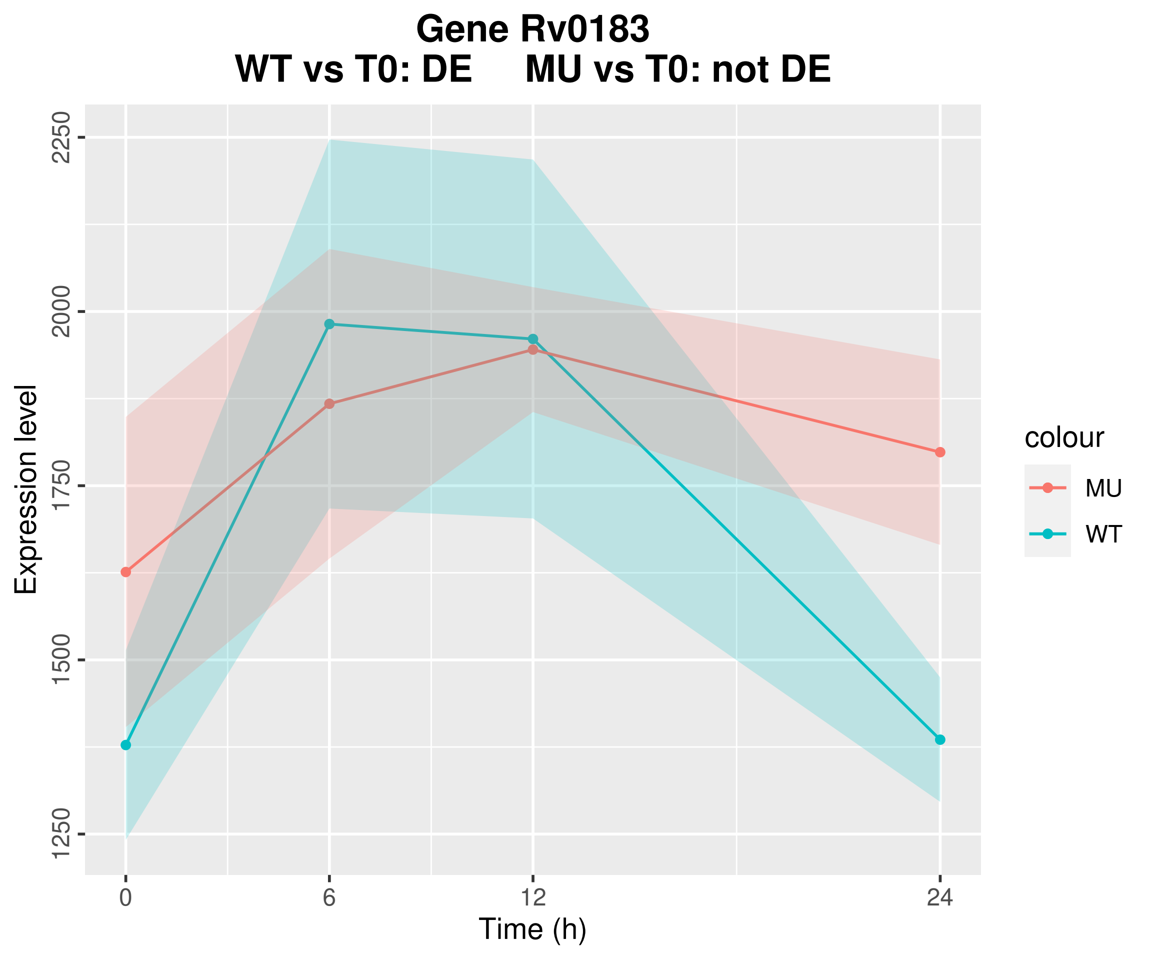

Gene Rv0183
WT vs T0: DE   MU vs T0: not DE
Expression level
2250
2000
1750
1500
1250
0
6
12
24
Time (h)
colour
MU
WT

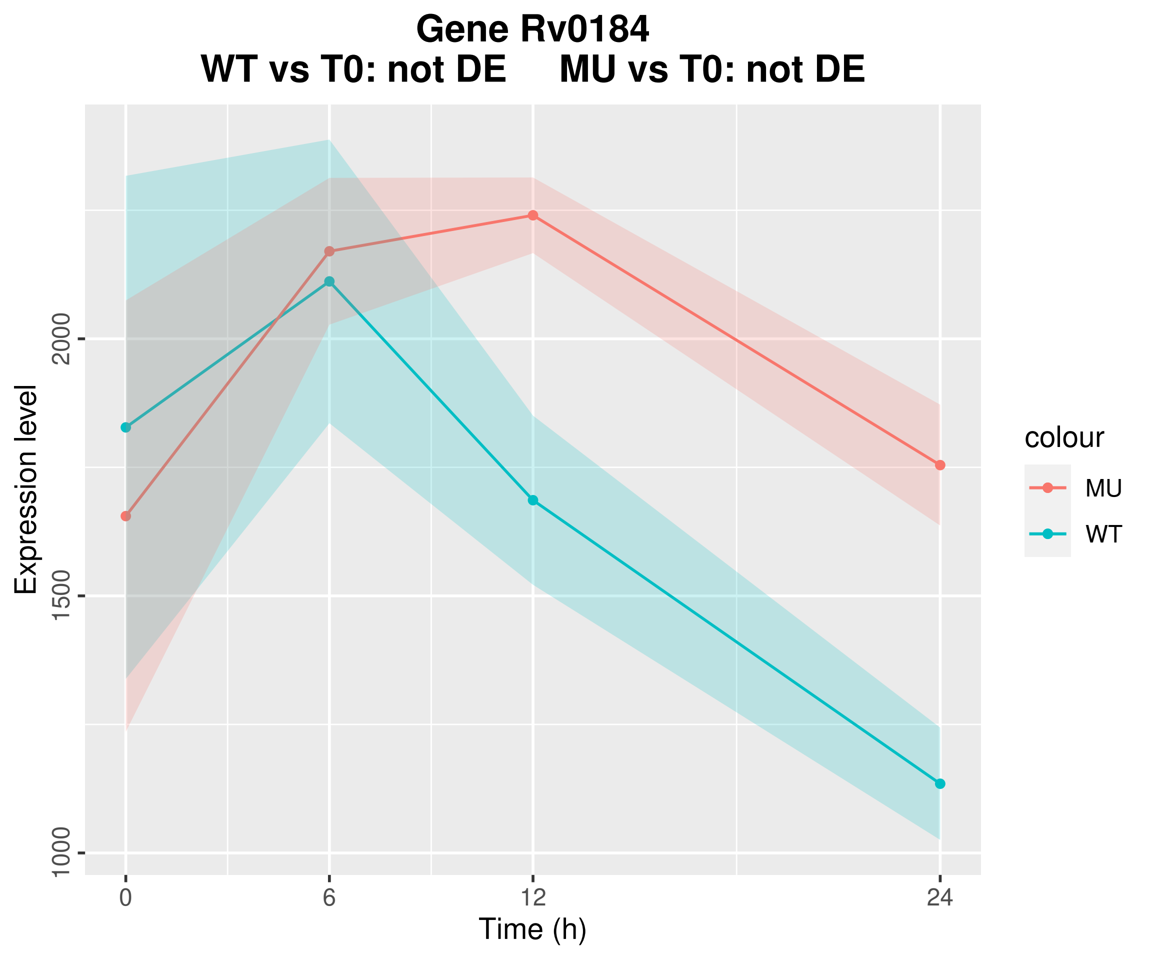
Gene Rv0184
WT vs T0: not DE MU vs T0: not DE
Expression level
1000
1500
2000
0
6
12
24
Time (h)
colour
MU
WT

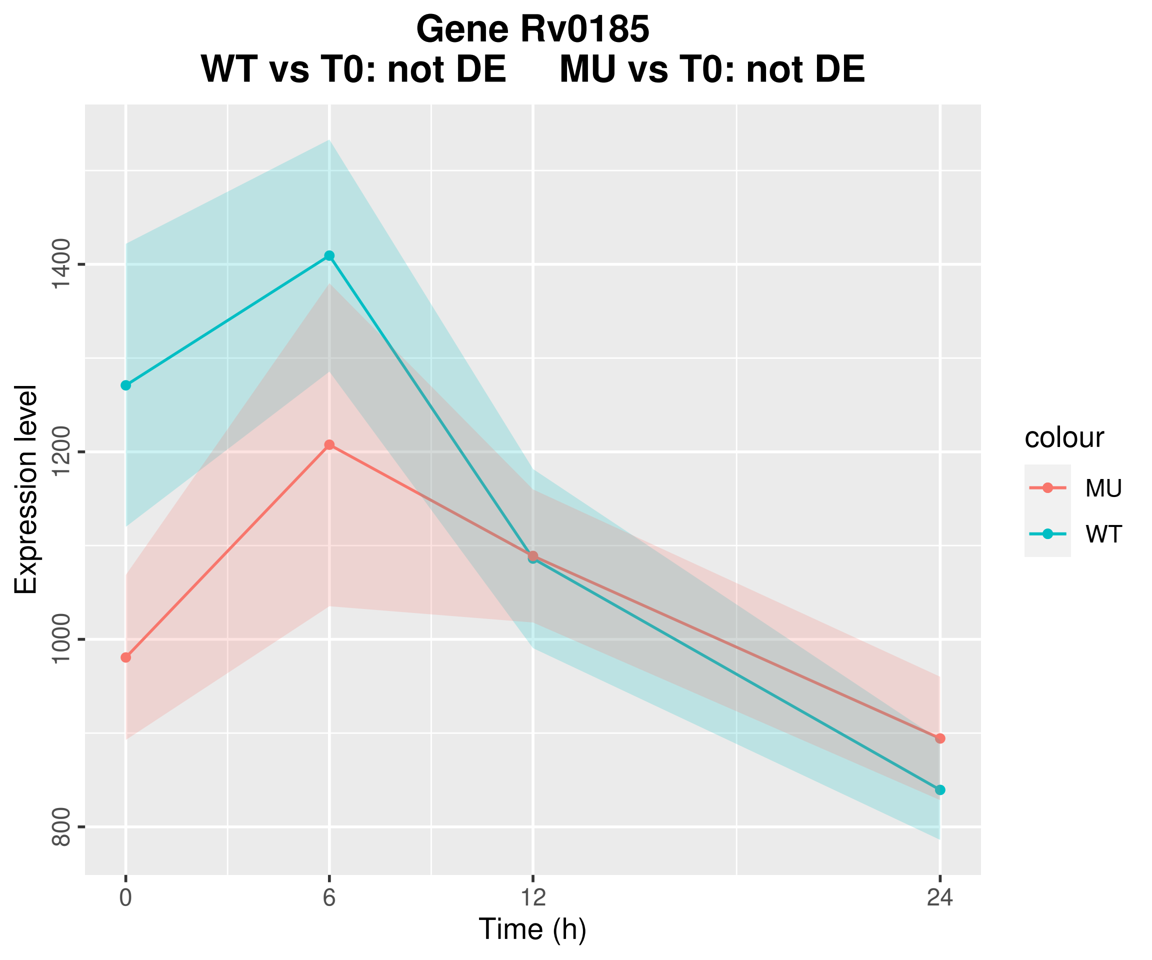
Gene Rv0185
WT vs T0: not DE MU vs T0: not DE
Expression level
800
1000
1200
1400
0
6
12
24
Time (h)
colour
MU
WT

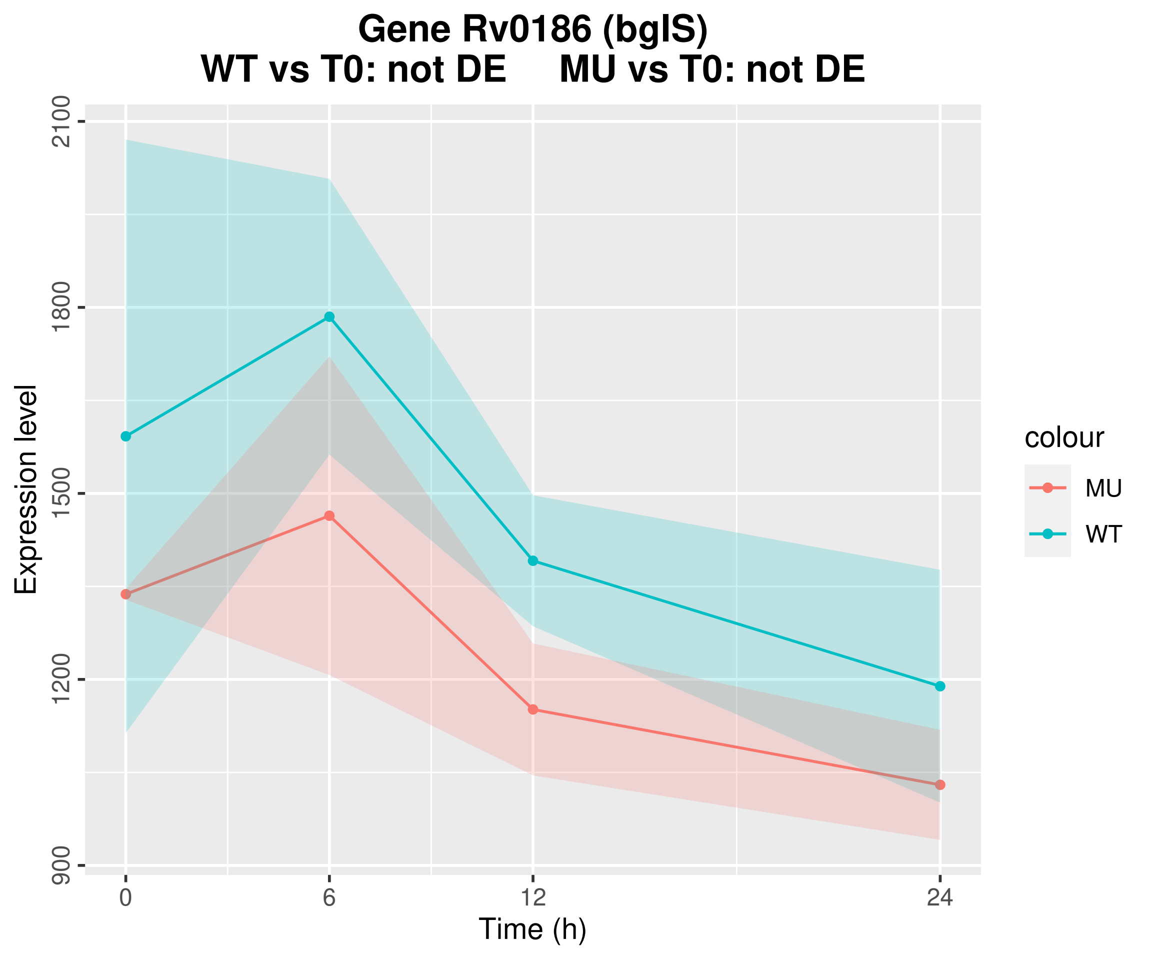

Gene Rv0186 (bglS)
WT vs T0: not DE   MU vs T0: not DE
Expression level
2100
1800
1500
1200
900
0
6
12
24
Time (h)
colour
MU
WT

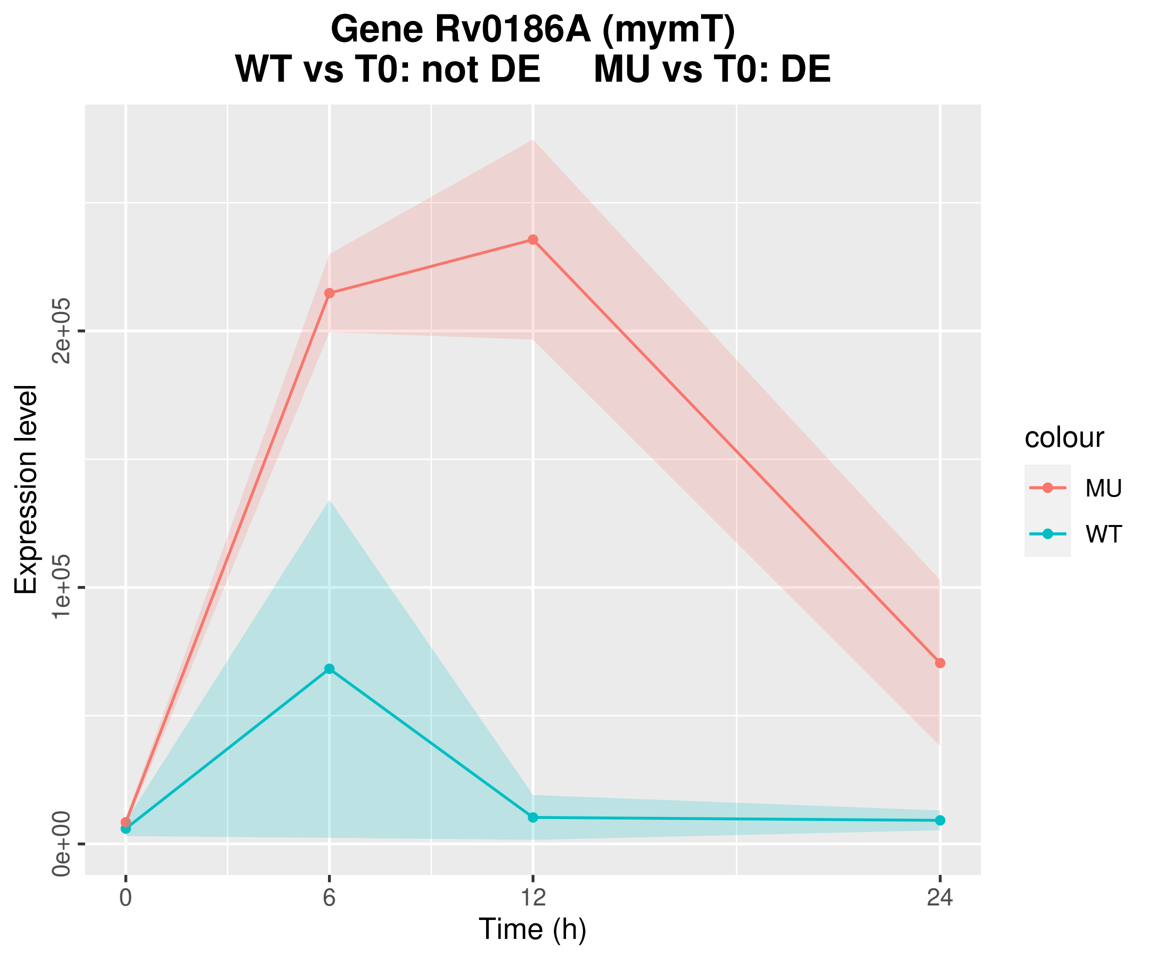
Gene Rv0186A (mymT)
WT vs T0: not DE   MU vs T0: DE
Expression level
2e+05
1e+05
0e+00
0
6
12
24
Time (h)
colour
MU
WT

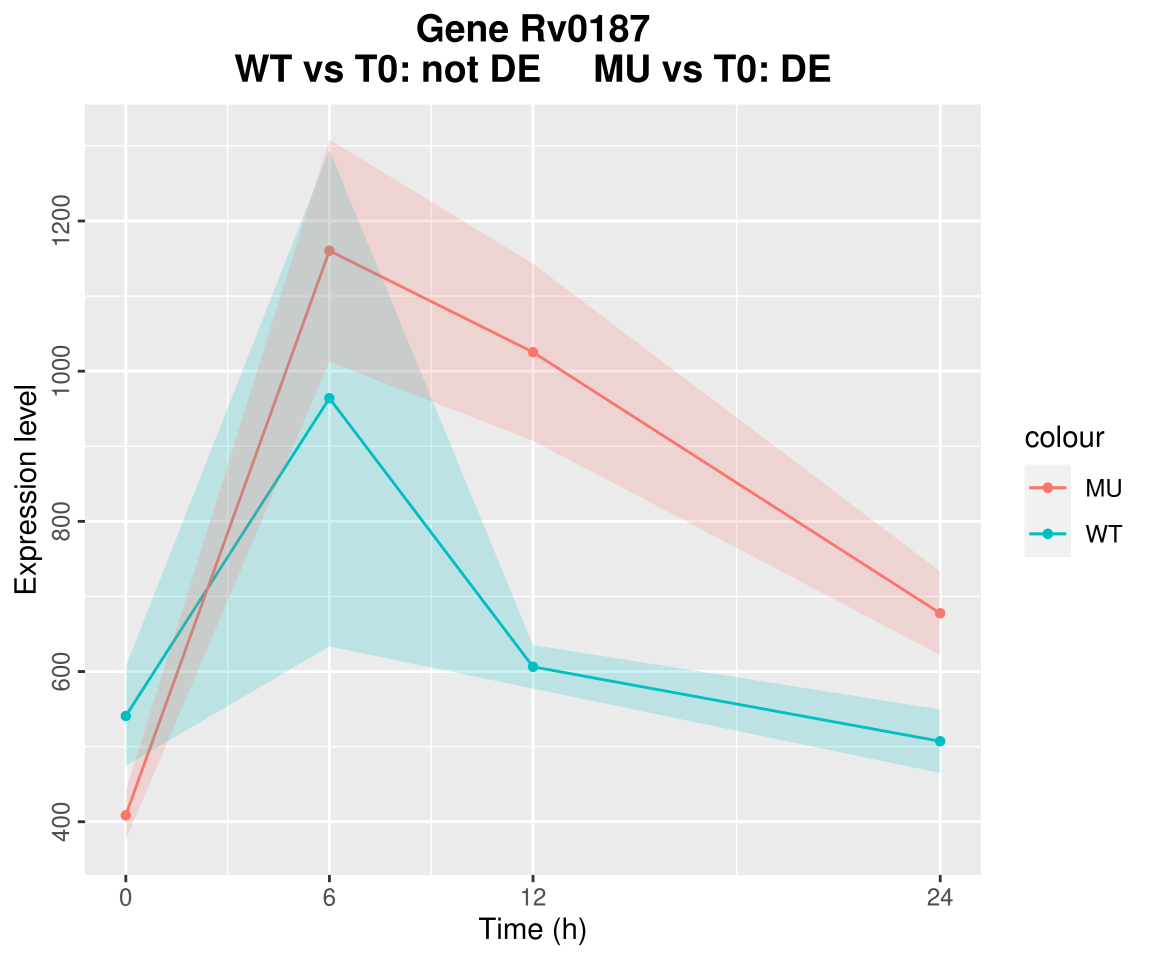
Gene Rv0187
WT vs T0: not DE   MU vs T0: DE
Expression level
400
600
800
1000
1200
0
6
12
24
Time (h)
colour
MU
WT

Gene Rv0188
WT vs T0: DE MU vs T0: DE

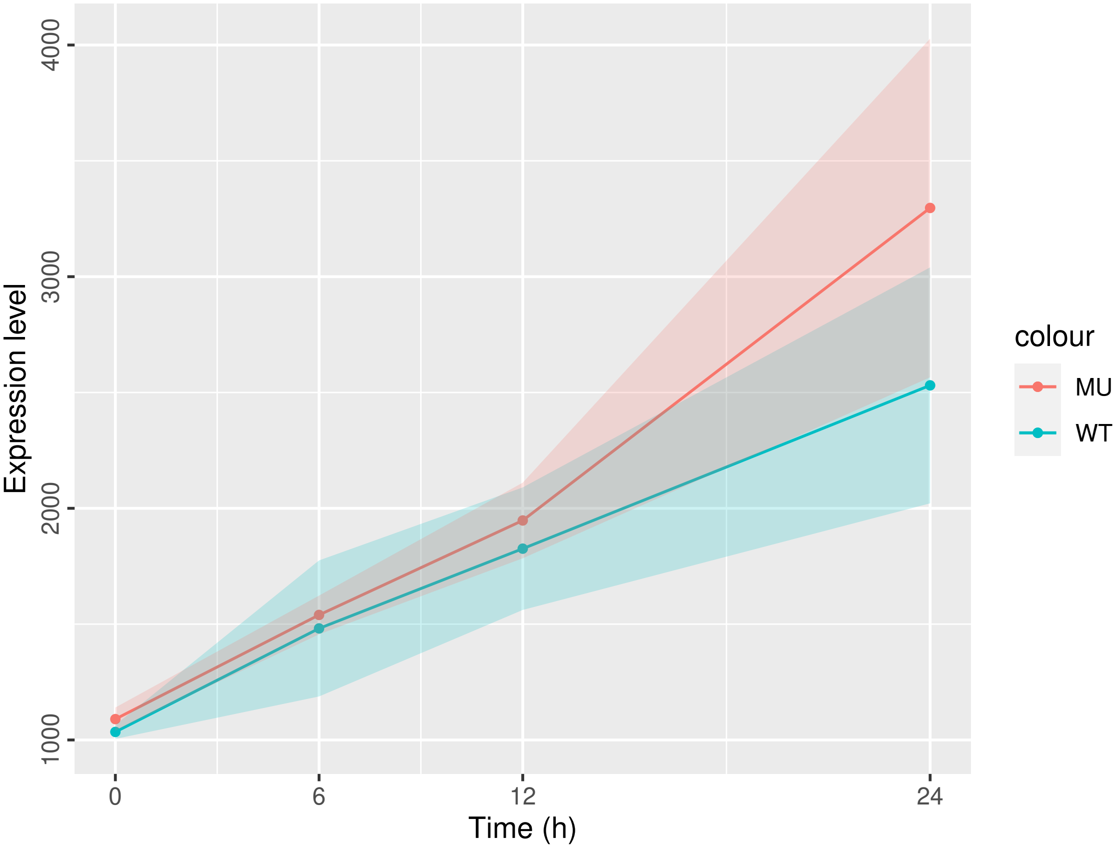

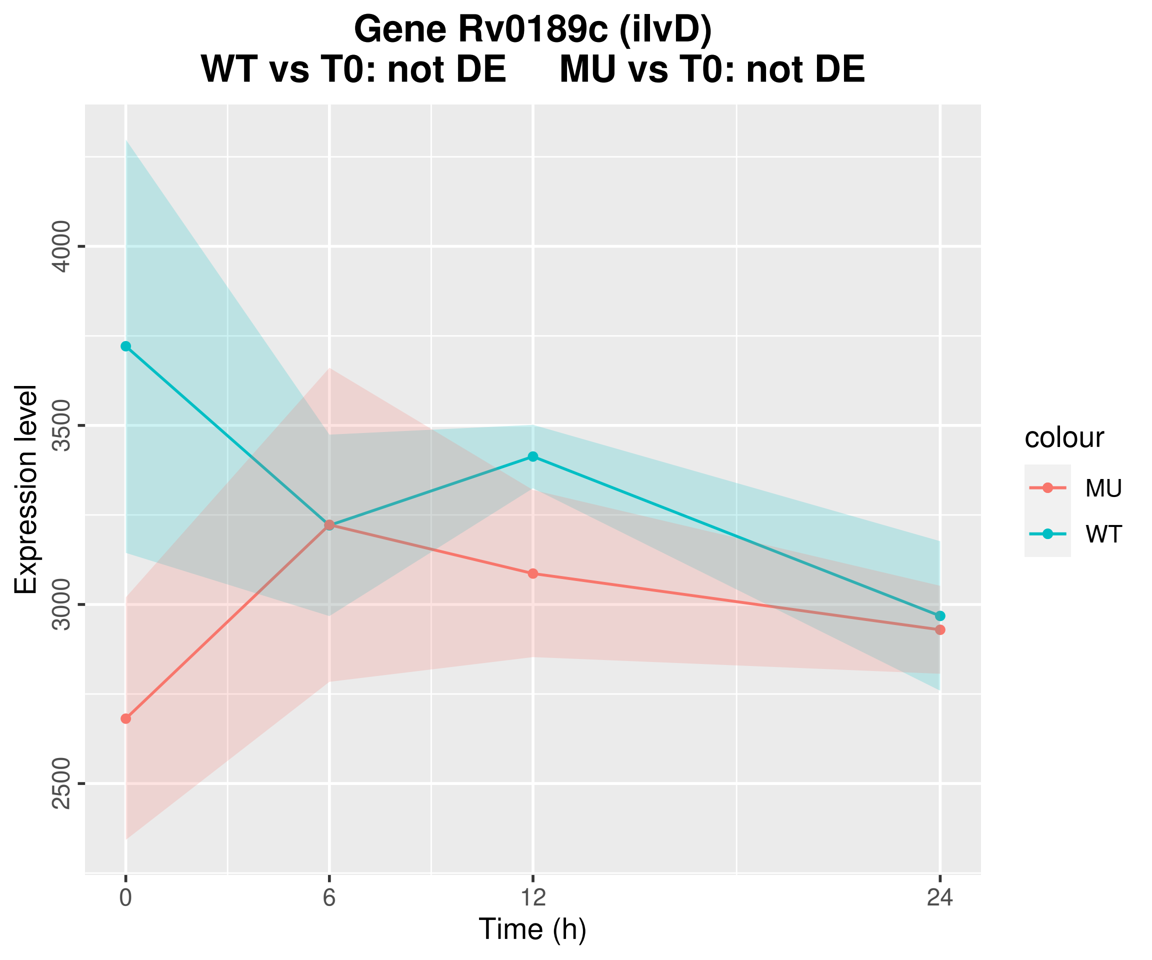

Gene Rv0189c (ilvD)
WT vs T0: not DE   MU vs T0: not DE
Expression level
4000
3500
3000
2500
0
6
12
24
Time (h)
colour
MU
WT

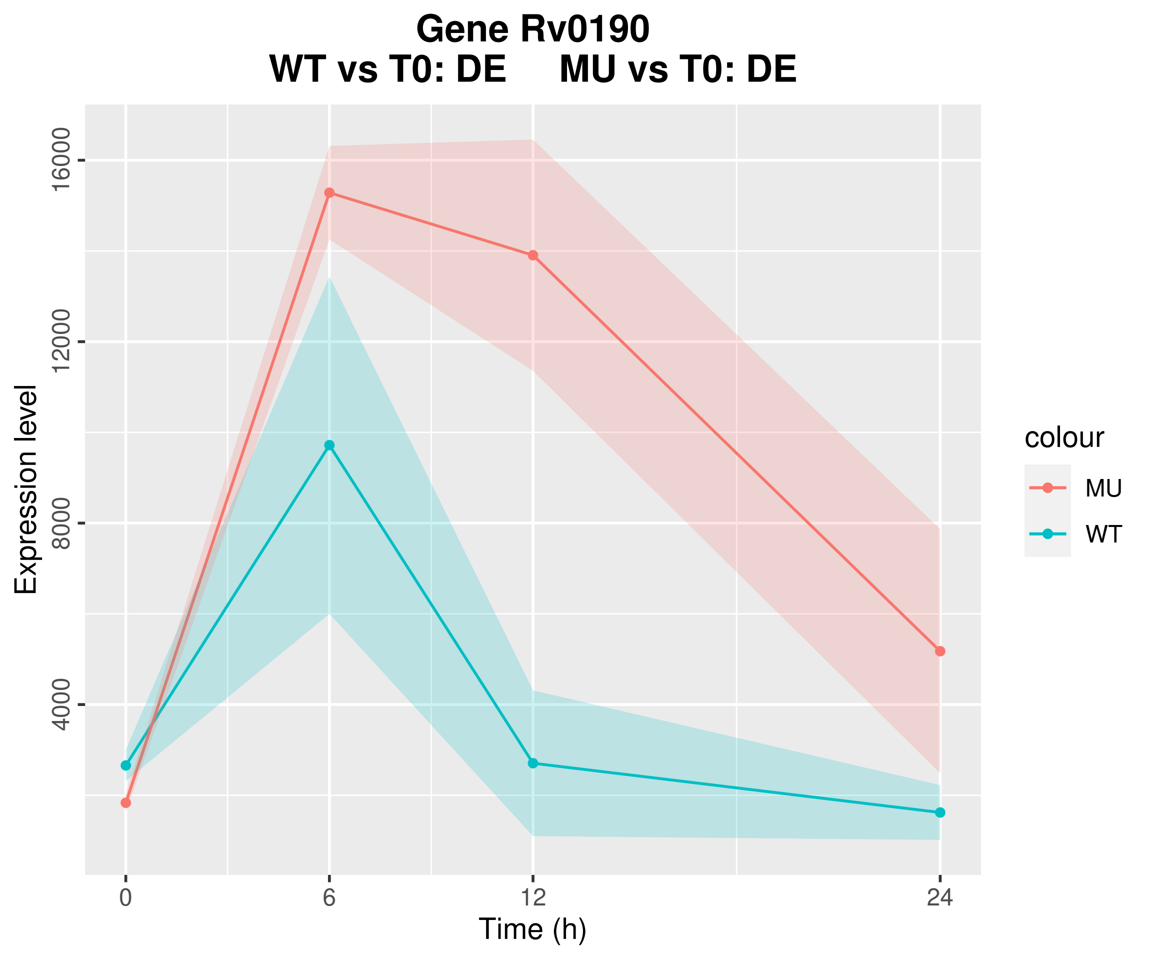
Gene Rv0190
WT vs T0: DE   MU vs T0: DE
Expression level
16000
12000
8000
4000
0
6
12
24
Time (h)
colour
MU
WT

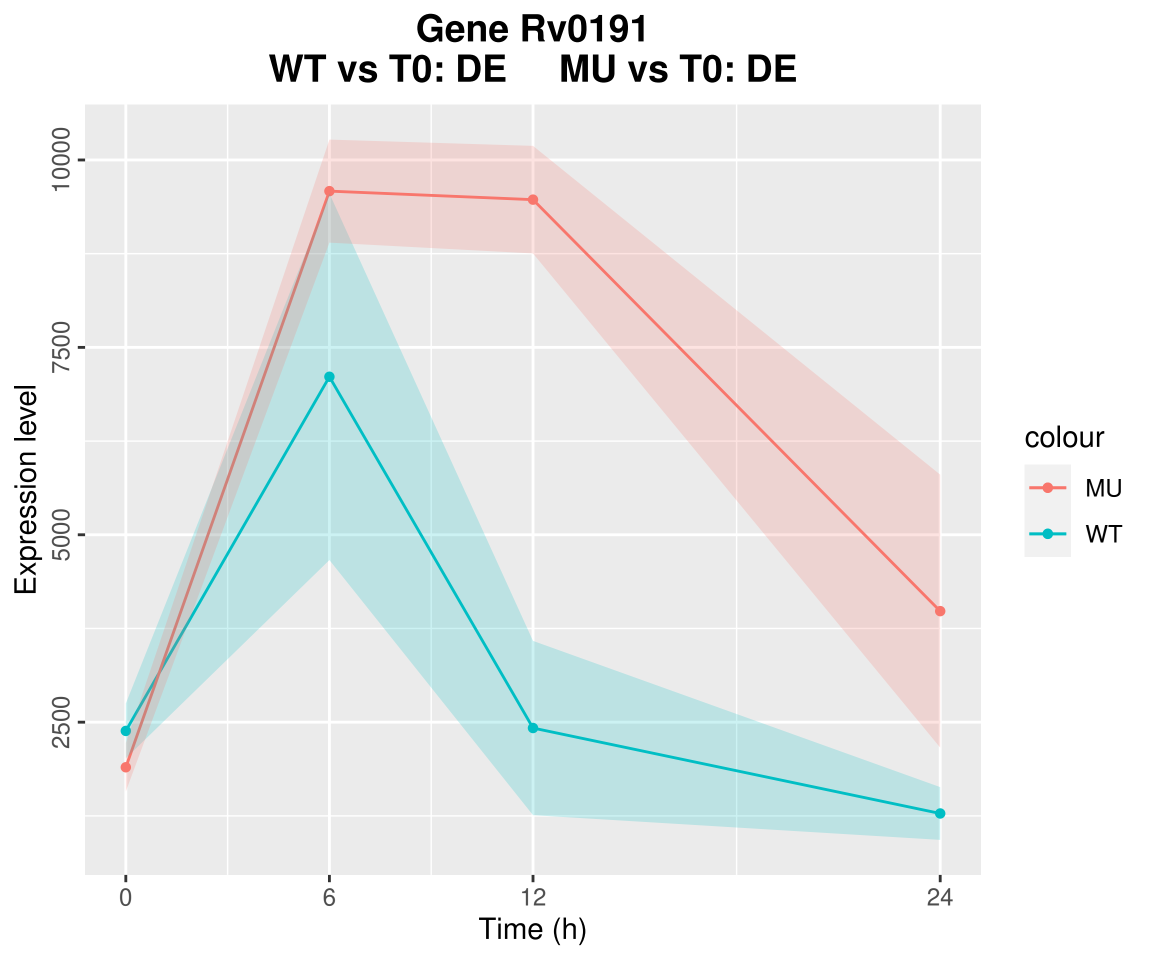
Gene Rv0191
WT vs T0: DE   MU vs T0: DE
Expression level
10000
7500
5000
2500
0
6
12
24
Time (h)
colour
MU
WT

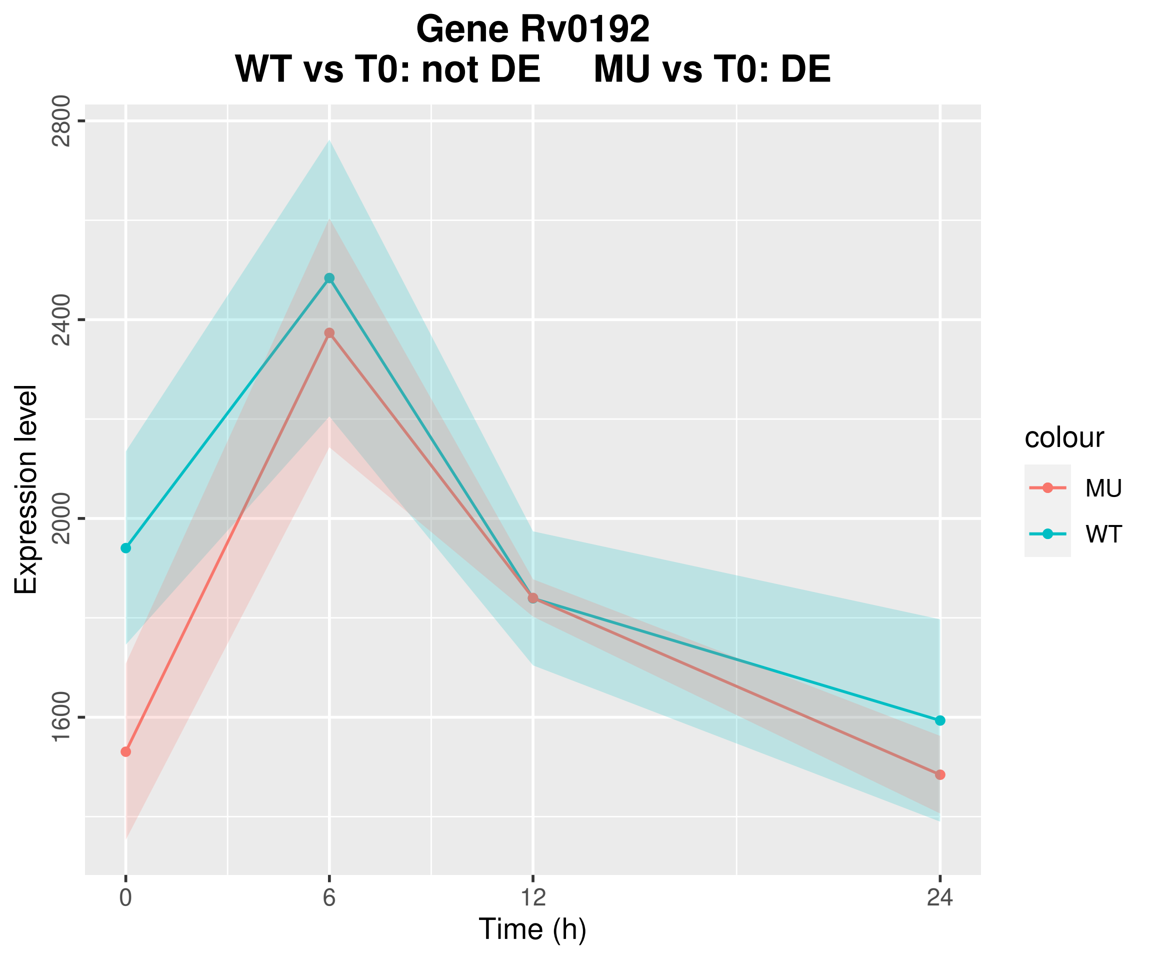

Gene Rv0192
WT vs T0: not DE MU vs T0: DE
Expression level
2800
2400
2000
1600
0
6
12
24
Time (h)
colour
MU
WT

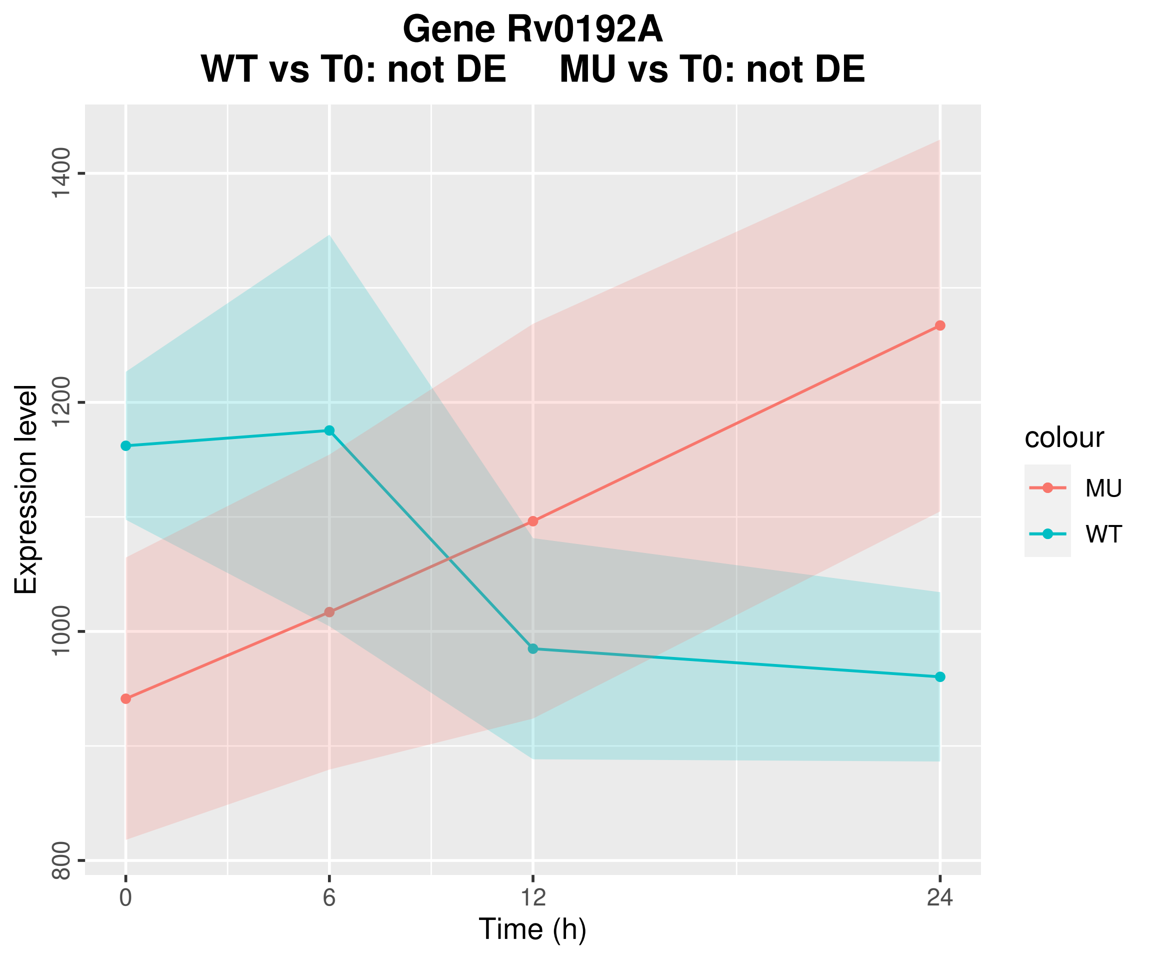

Gene Rv0192A
WT vs T0: not DE   MU vs T0: not DE
1400
1200
1000
800
Expression level
0
6
12
24
Time (h)
colour
MU
WT

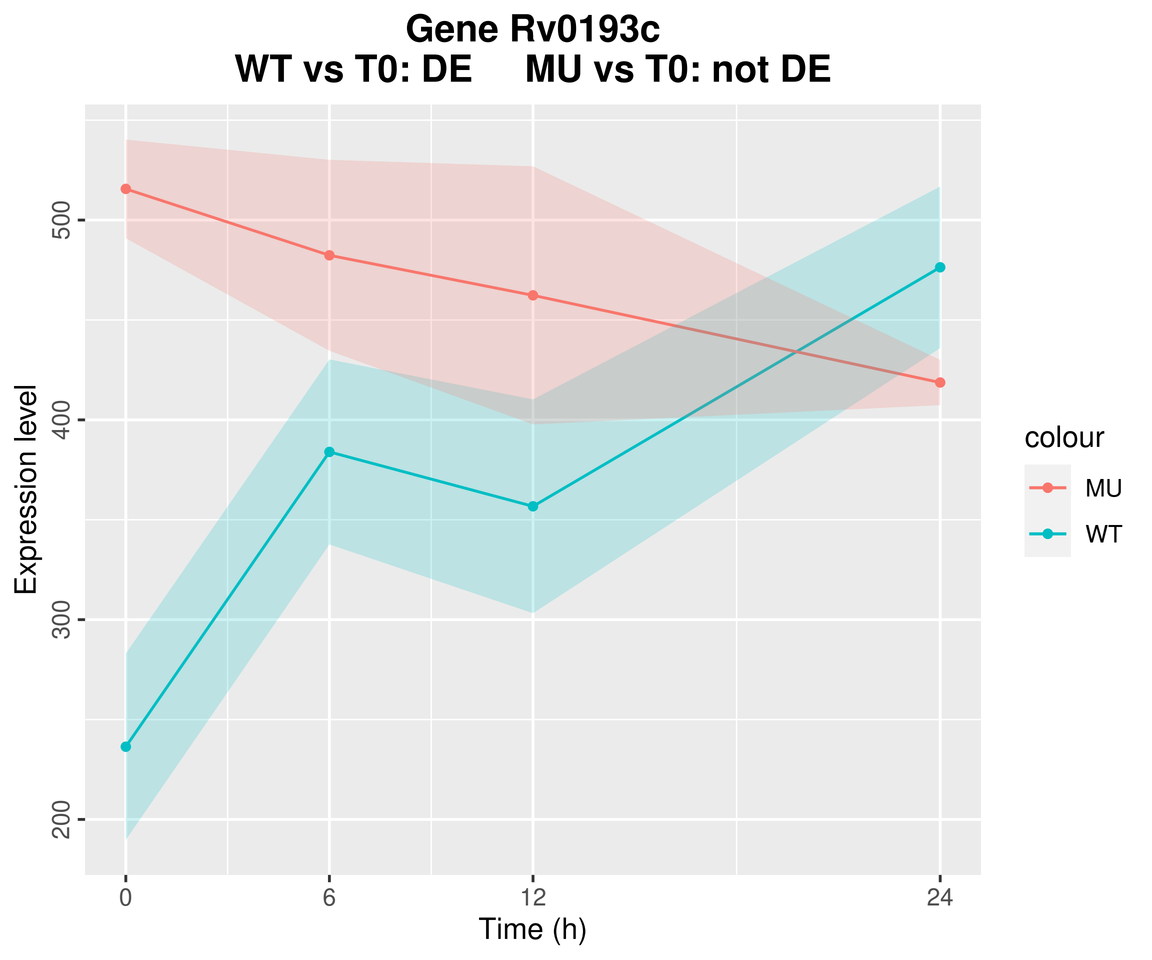

Gene Rv0193c
WT vs T0: DE MU vs T0: not DE
Expression level
500
400
300
200
0
6
12
24
Time (h)
colour
MU
WT

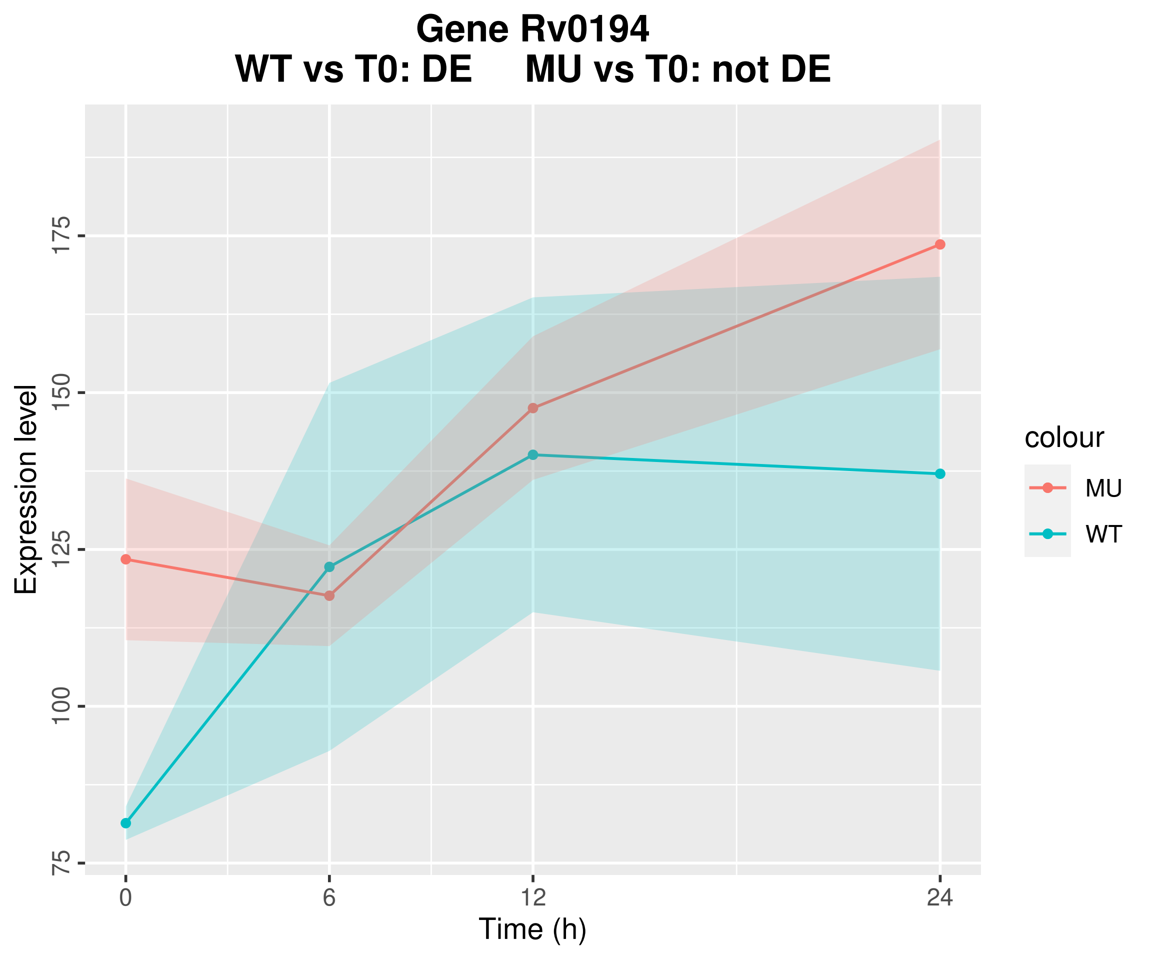
Gene Rv0194
WT vs T0: DE   MU vs T0: not DE
Expression level
175
150
125
100
75
0
6
12
24
Time (h)
colour
MU
WT

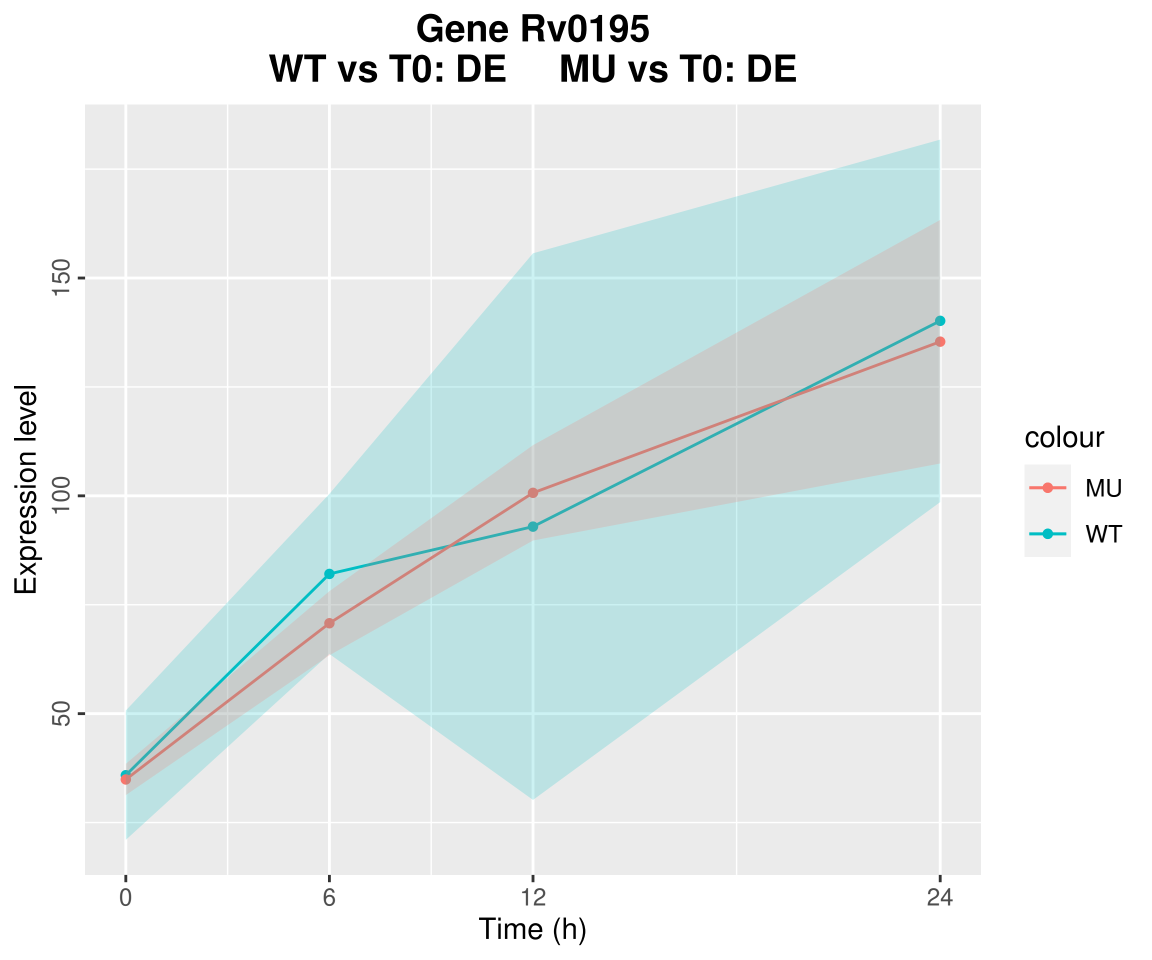

Gene Rv0195
WT vs T0: DE MU vs T0: DE
Expression level
150
100
50
0
6
12
24
Time (h)
colour
MU
WT

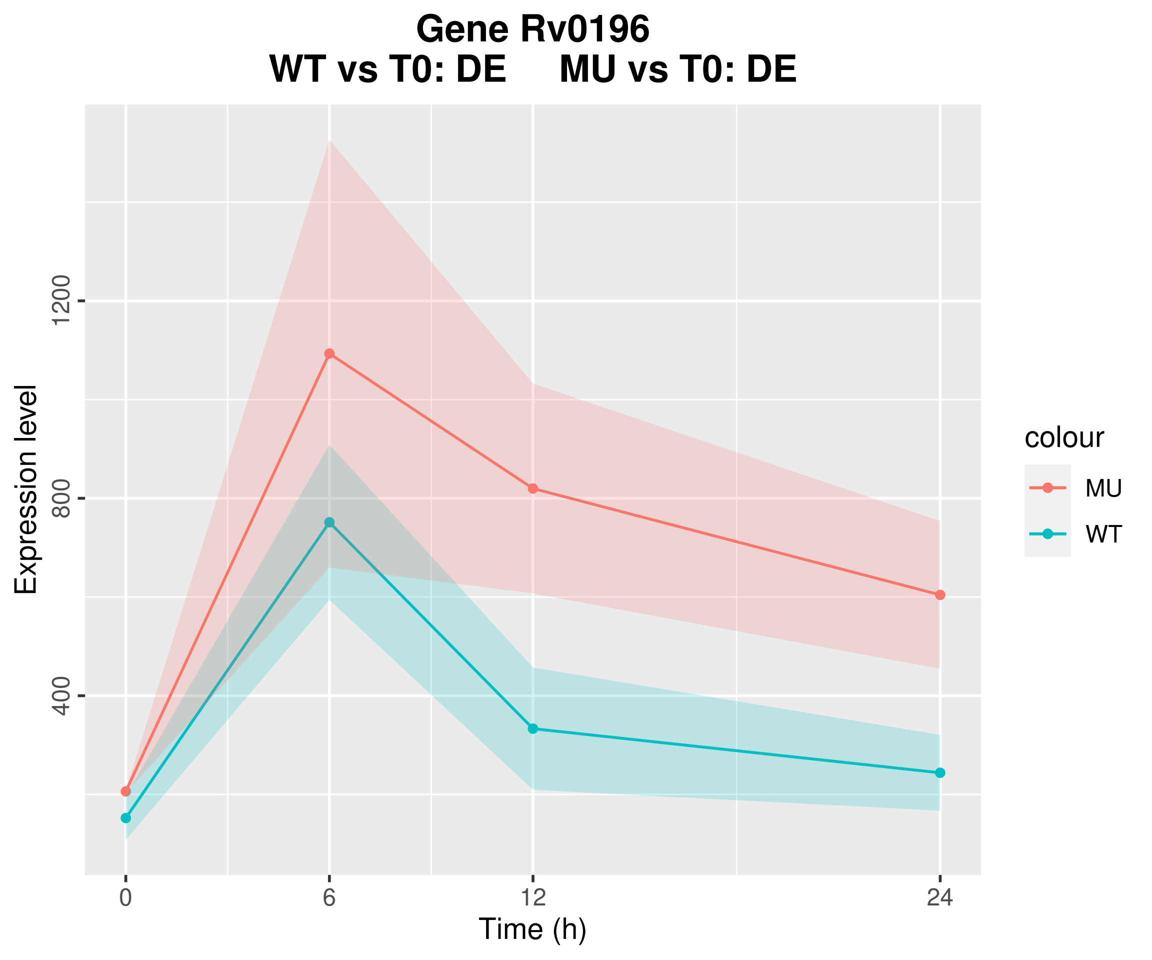
Gene Rv0196
WT vs T0: DE MU vs T0: DE
Expression level
1200
800
400
0
6
12
24
Time (h)
colour
MU
WT

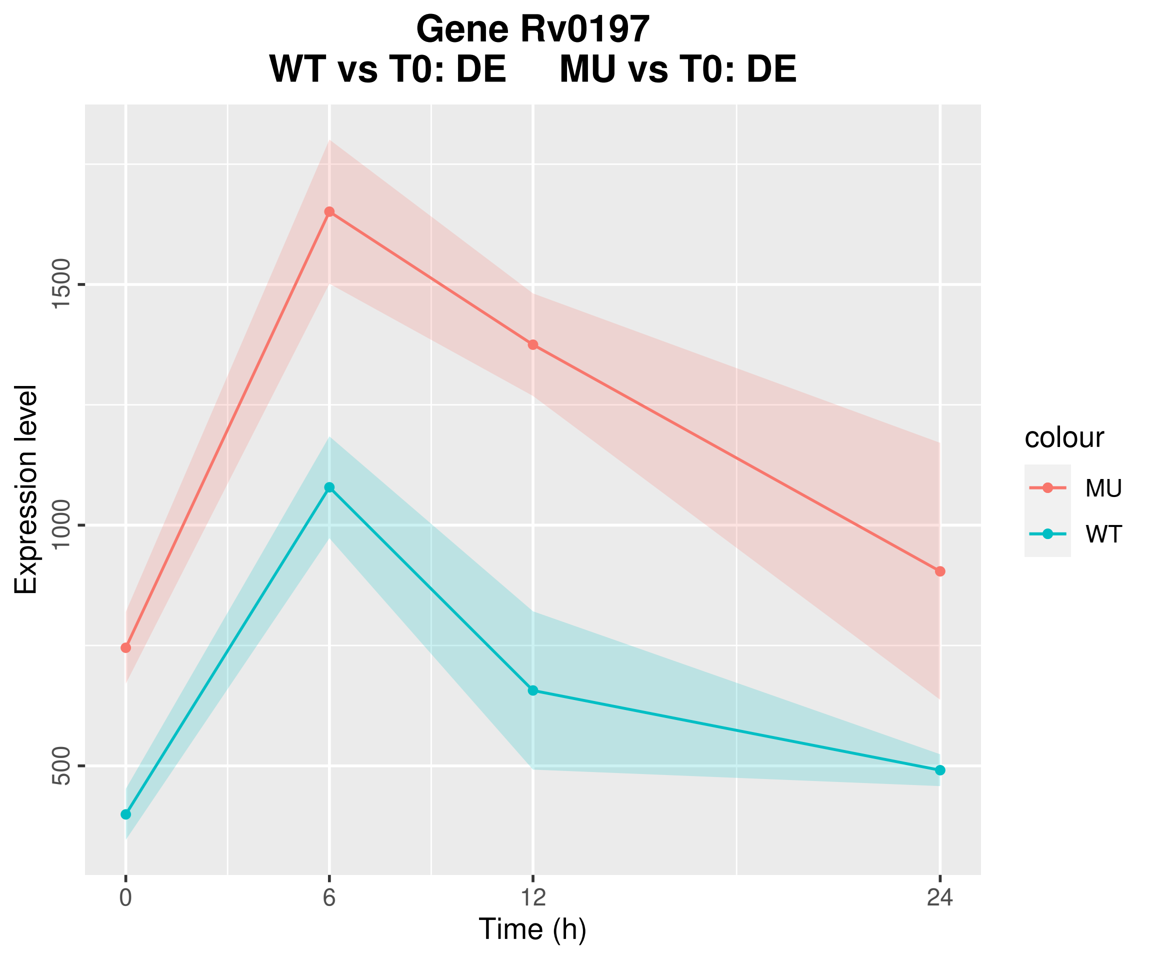
Gene Rv0197
WT vs T0: DE MU vs T0: DE
Expression level
1500
1000
500
0
6
12
24
Time (h)
colour
MU
WT

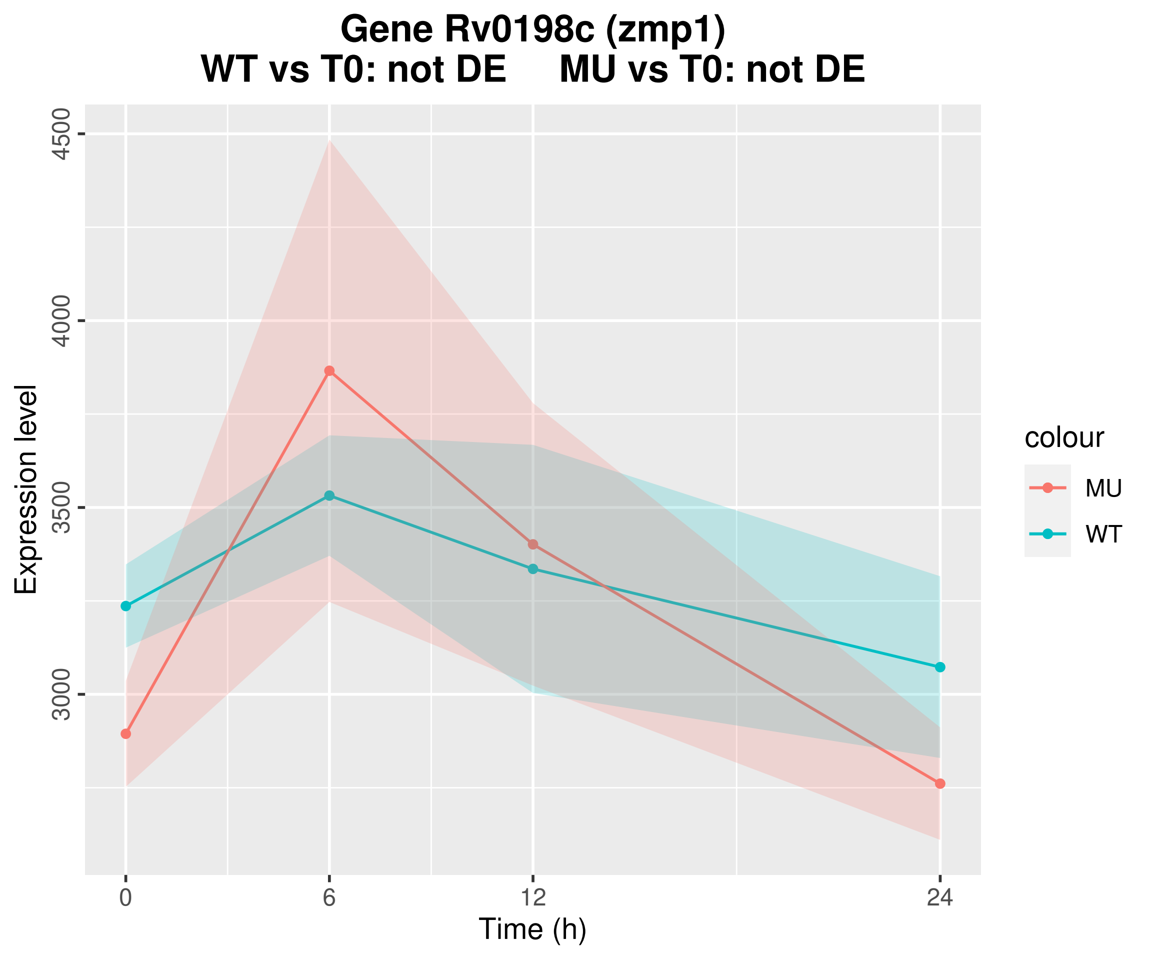

Gene Rv0198c (zmp1)
WT vs T0: not DE MU vs T0: not DE
Expression level
4500
4000
3500
3000
0
6
12
24
Time (h)
colour
MU
WT

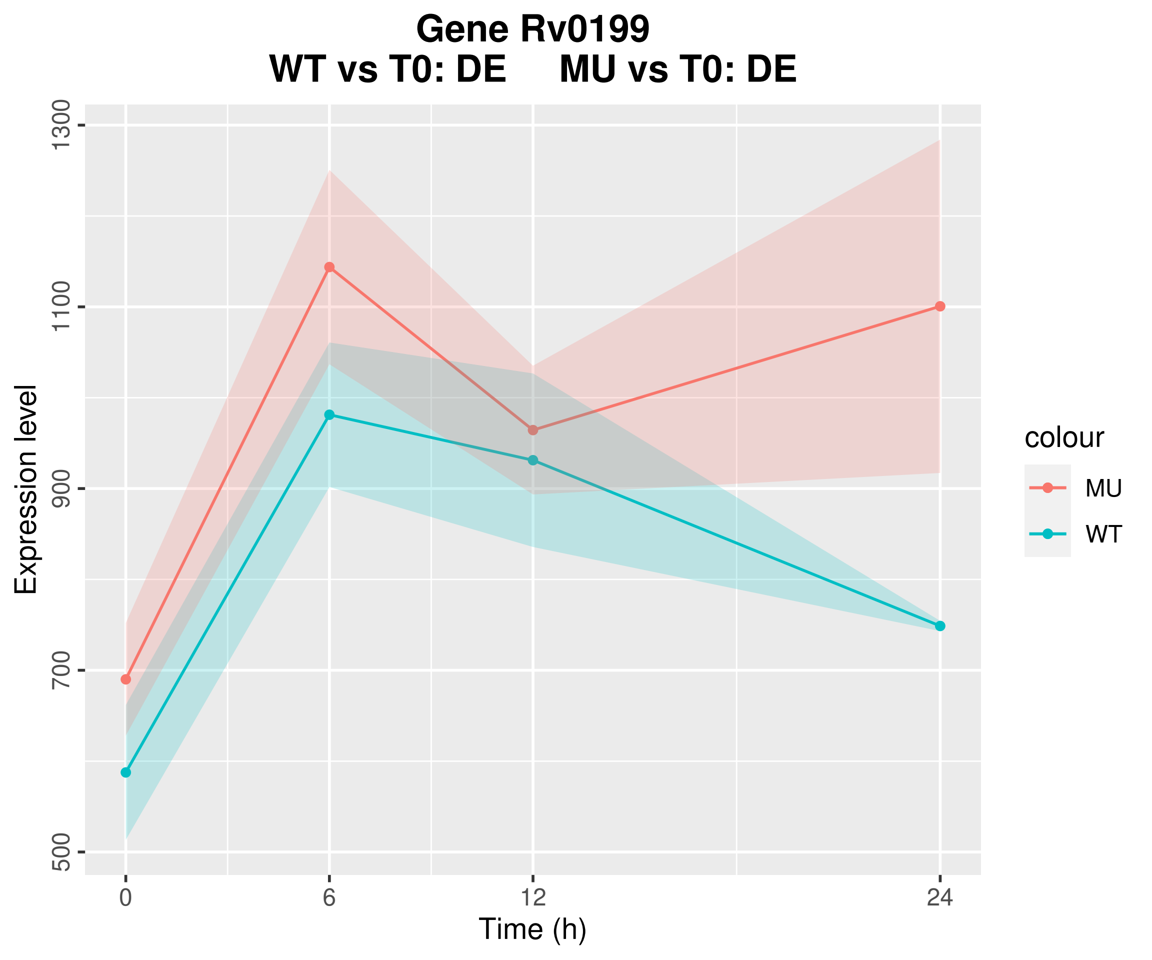

Gene Rv0199
WT vs T0: DE   MU vs T0: DE
1300
1100
900
700
500
Expression level
0
6
12
24
Time (h)
colour
MU
WT

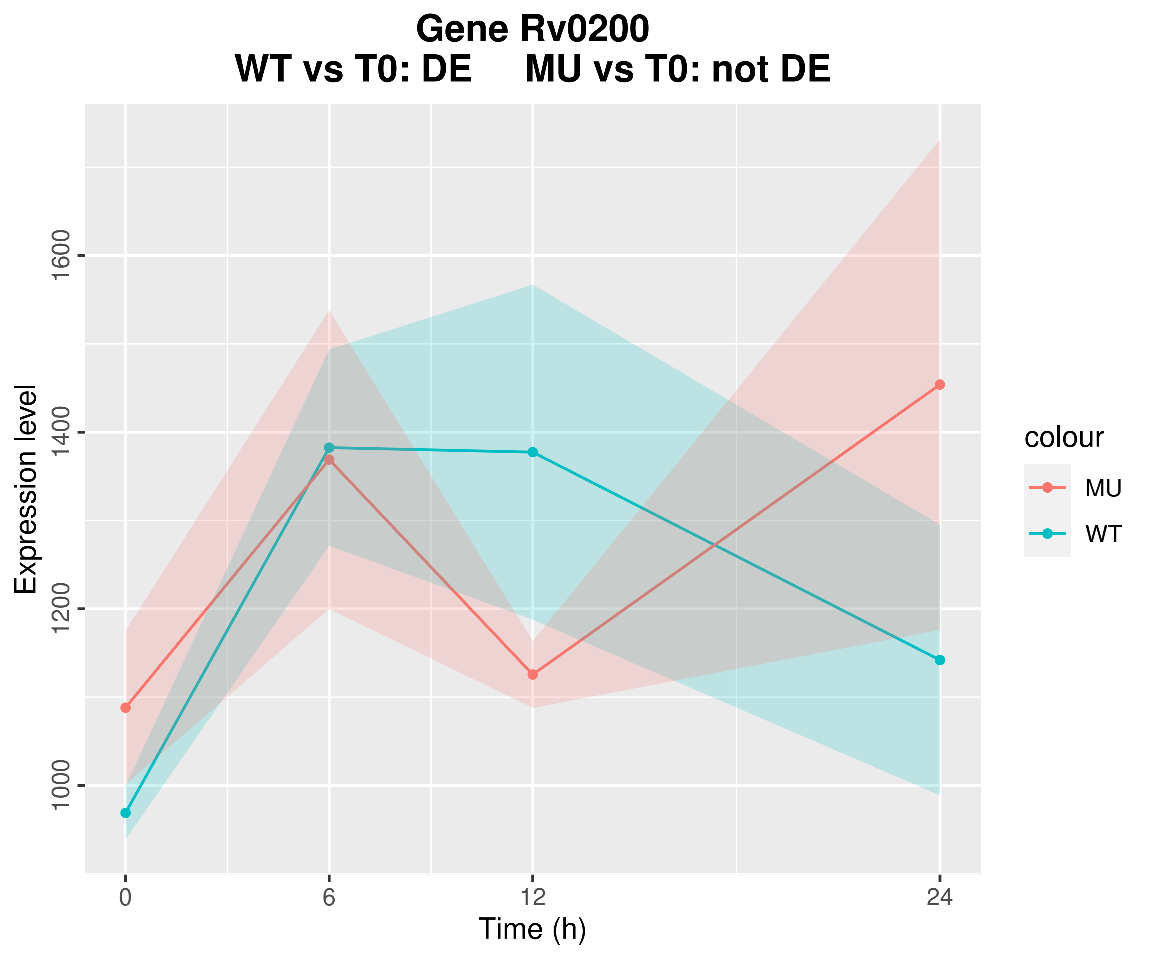
Gene Rv0200
WT vs T0: DE MU vs T0: not DE
Expression level
1000
1200
1400
1600
0
6
12
24
Time (h)
colour
MU
WT

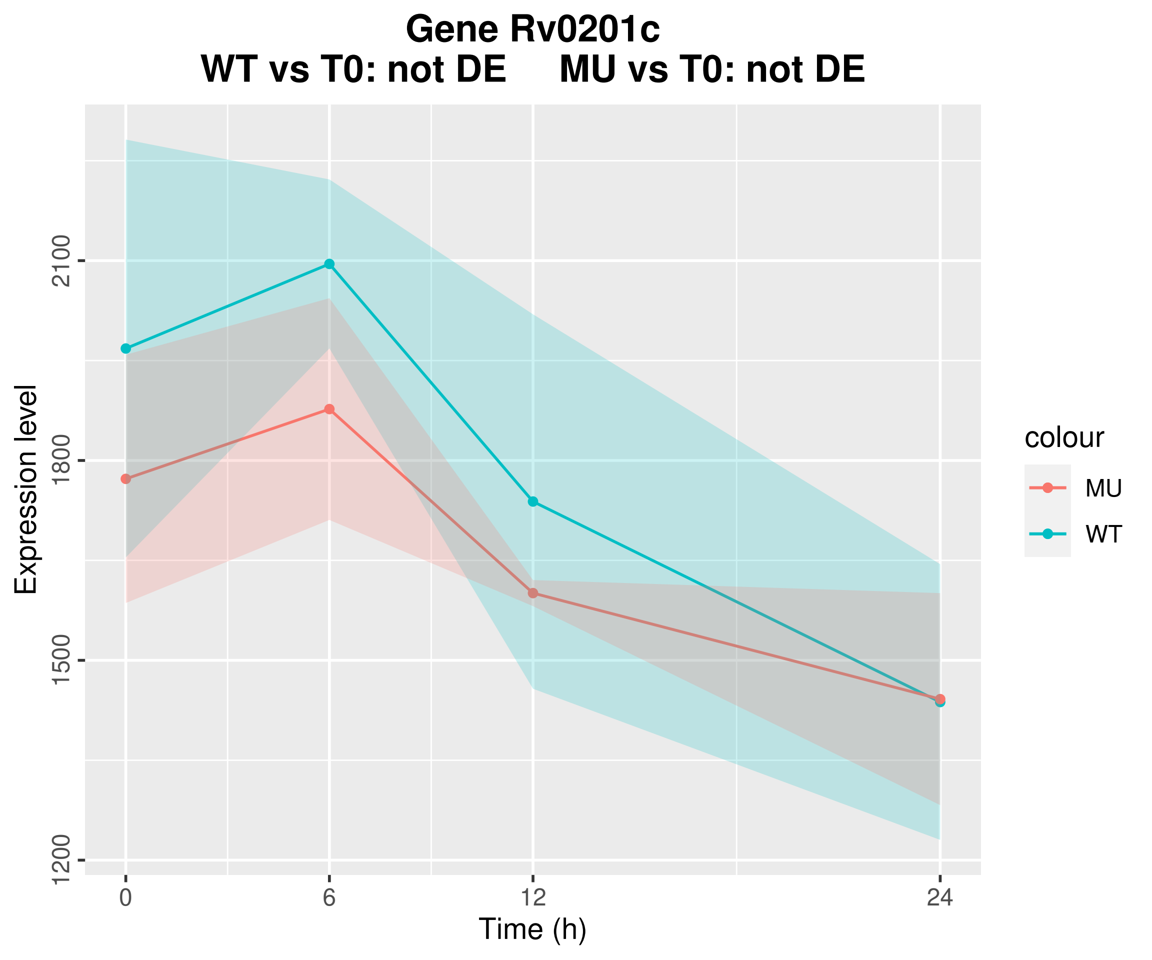

Gene Rv0201c
WT vs T0: not DE   MU vs T0: not DE
Expression level
2100
1800
1500
1200
0
6
12
24
Time (h)
colour
MU
WT

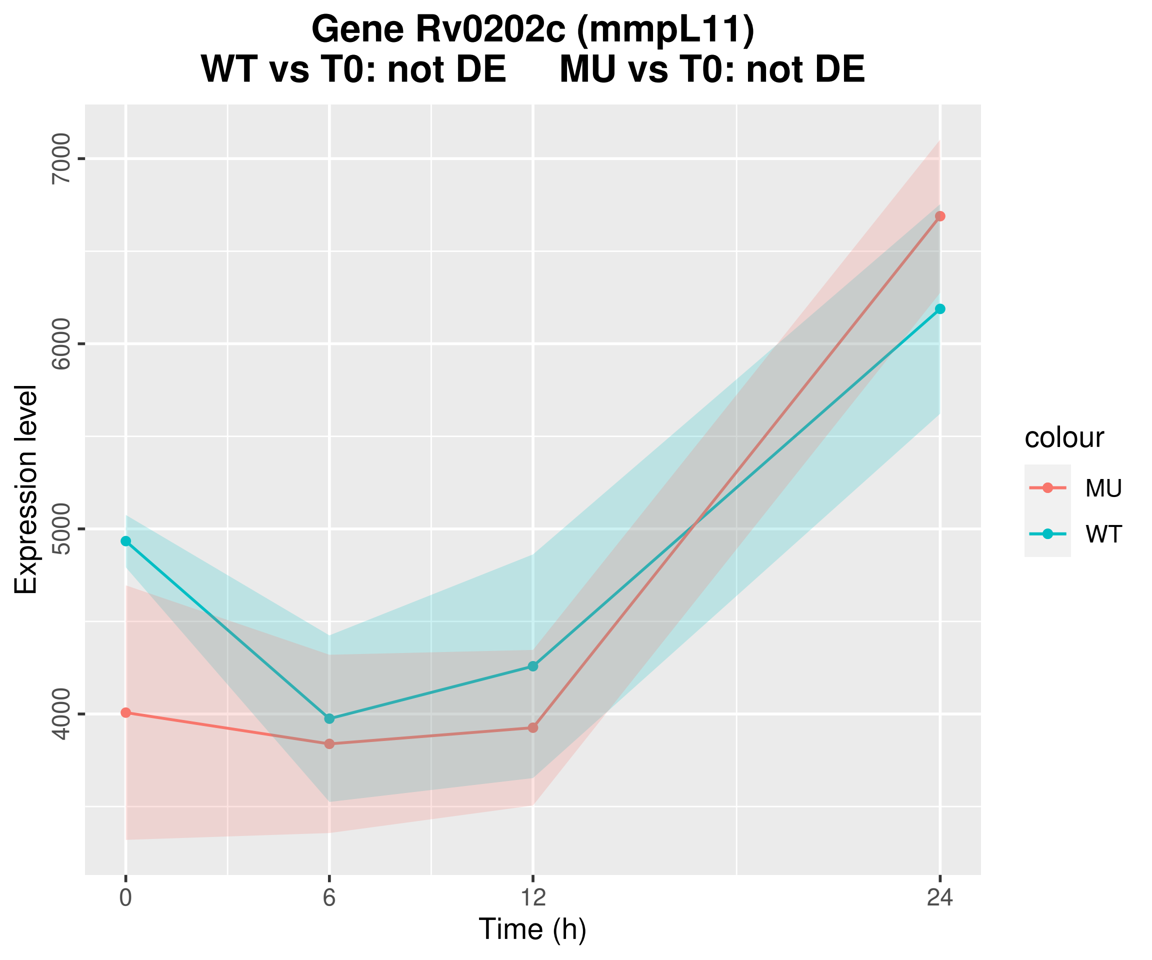

Gene Rv0202c (mmpL11)
WT vs T0: not DE   MU vs T0: not DE
Expression level
7000
6000
5000
4000
0
6
12
24
Time (h)
colour
MU
WT

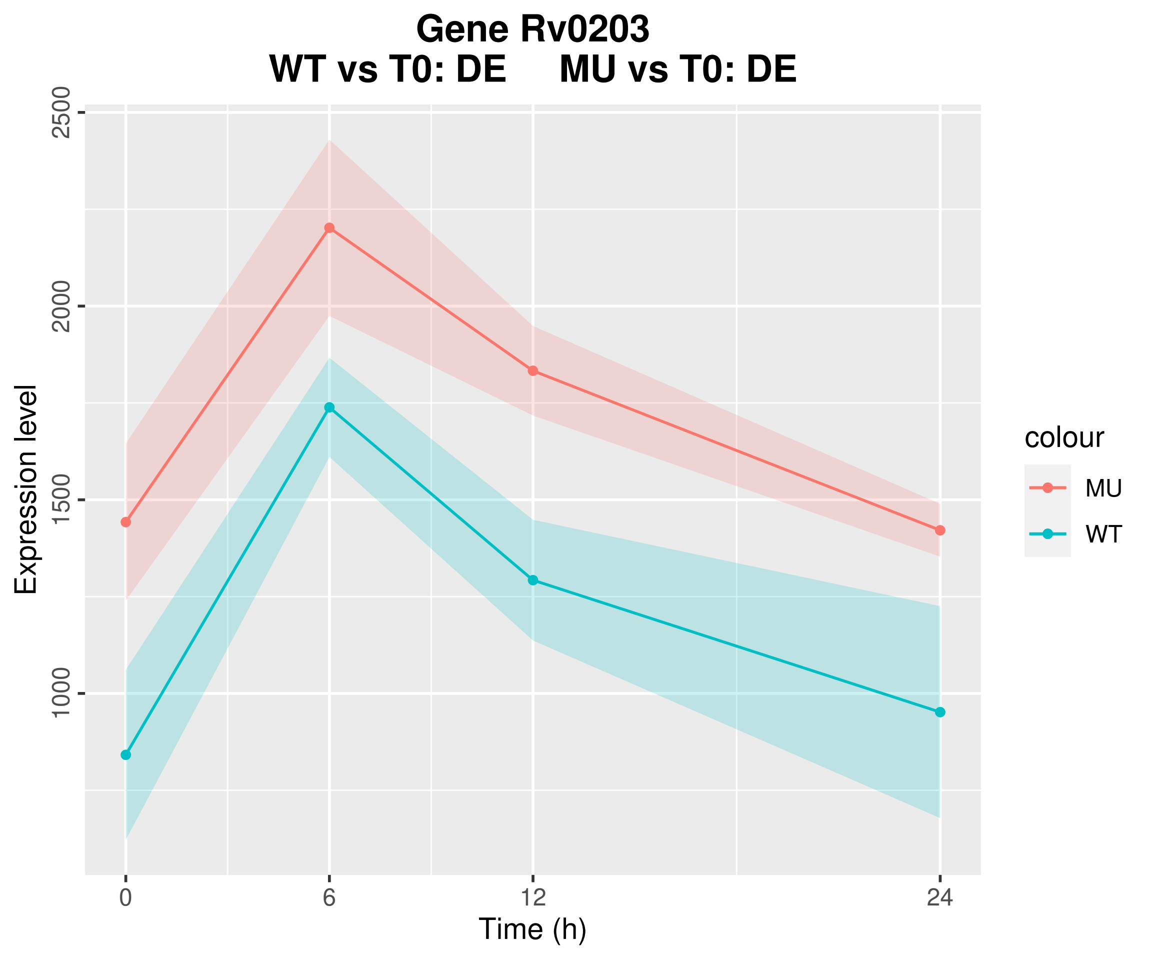

Gene Rv0203
WT vs T0: DE   MU vs T0: DE
2500
2000
1500
1000
Expression level
0
6
12
24
Time (h)
colour
MU
WT

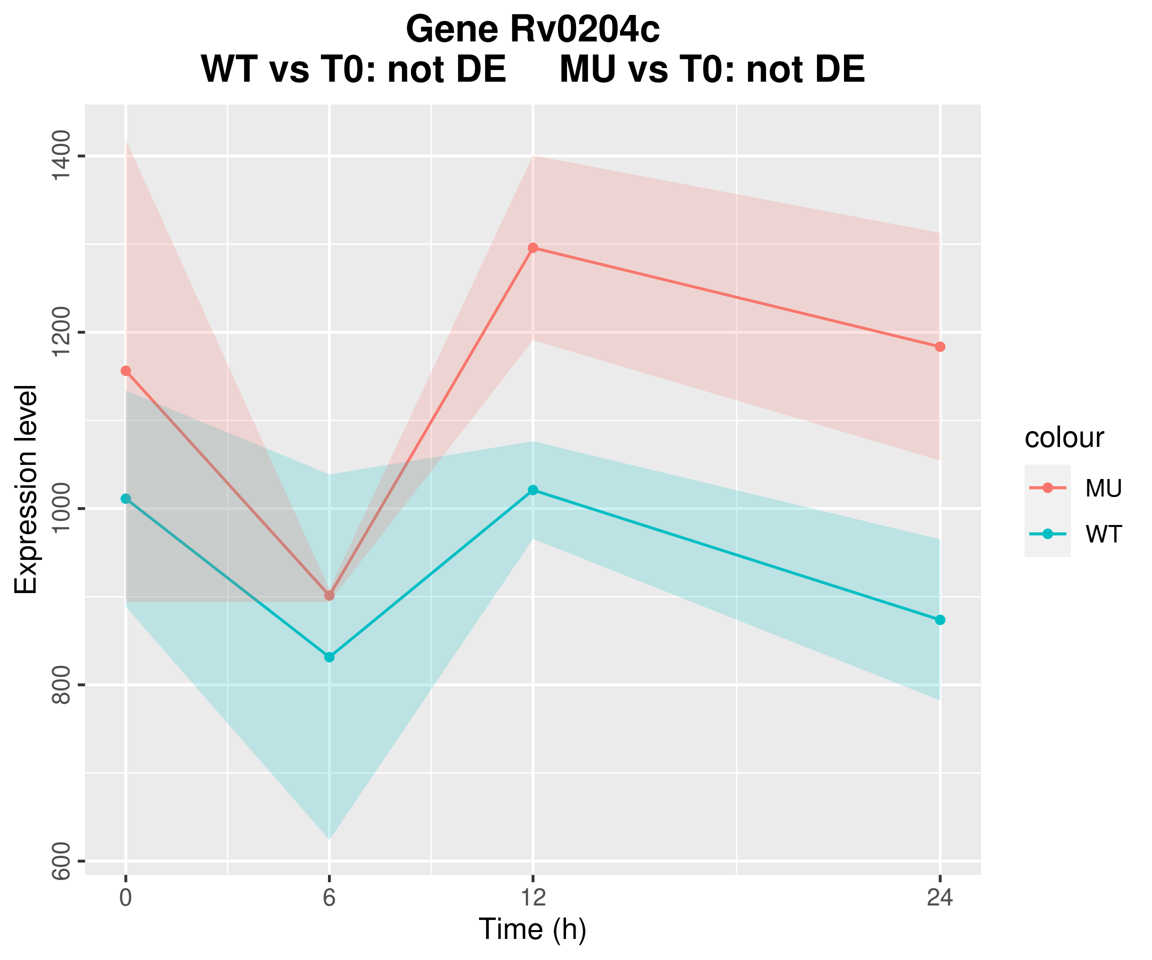
Gene Rv0204c
WT vs T0: not DE   MU vs T0: not DE
1400
1200
1000
800
600
Expression level
0
6
12
24
Time (h)
colour
MU
WT

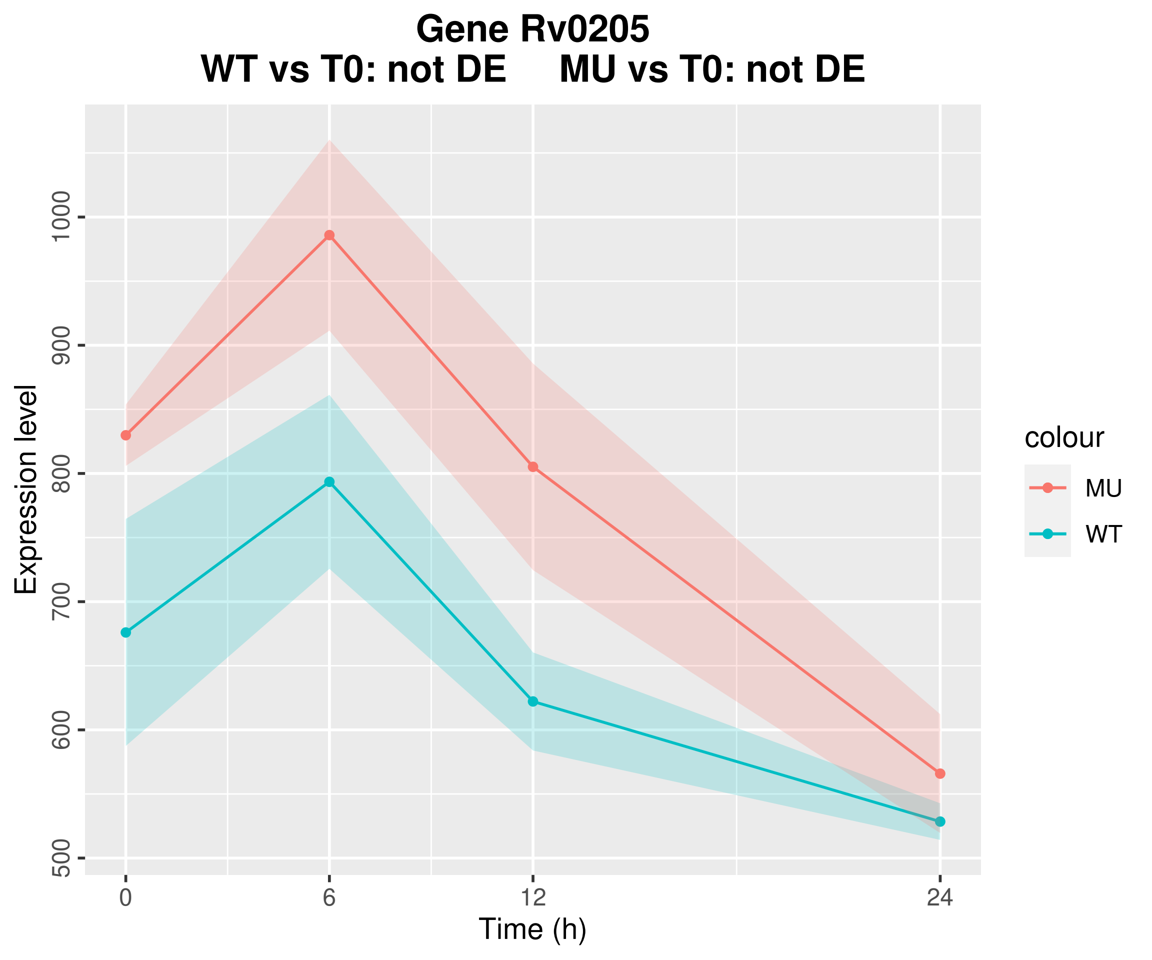

Gene Rv0205
WT vs T0: not DE MU vs T0: not DE
Expression level
1000
900
800
700
600
500
0
6
12
24
Time (h)
colour
MU
WT

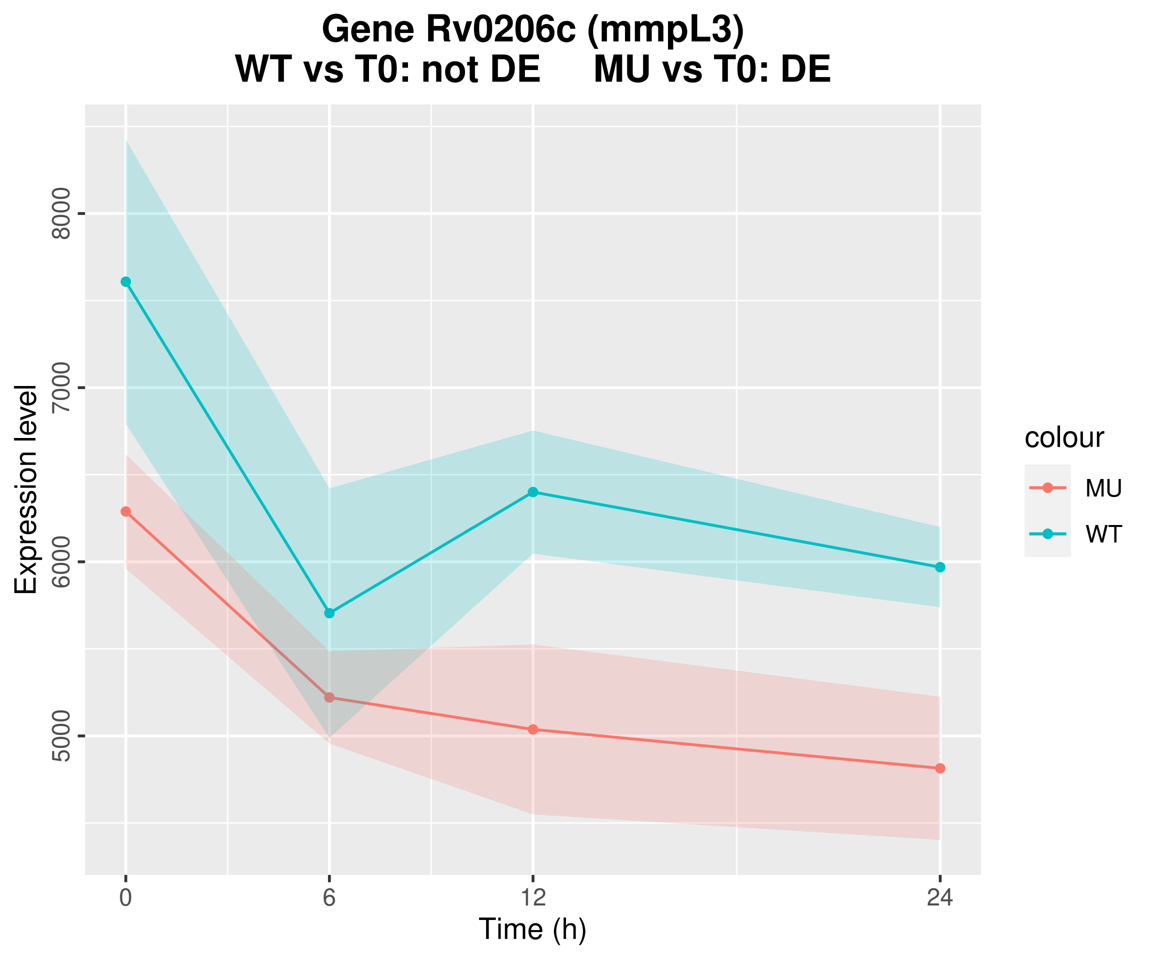

Gene Rv0206c (mmpL3)
WT vs T0: not DE MU vs T0: DE
Expression level
8000
7000
6000
5000
0
6
12
24
Time (h)
colour
MU
WT

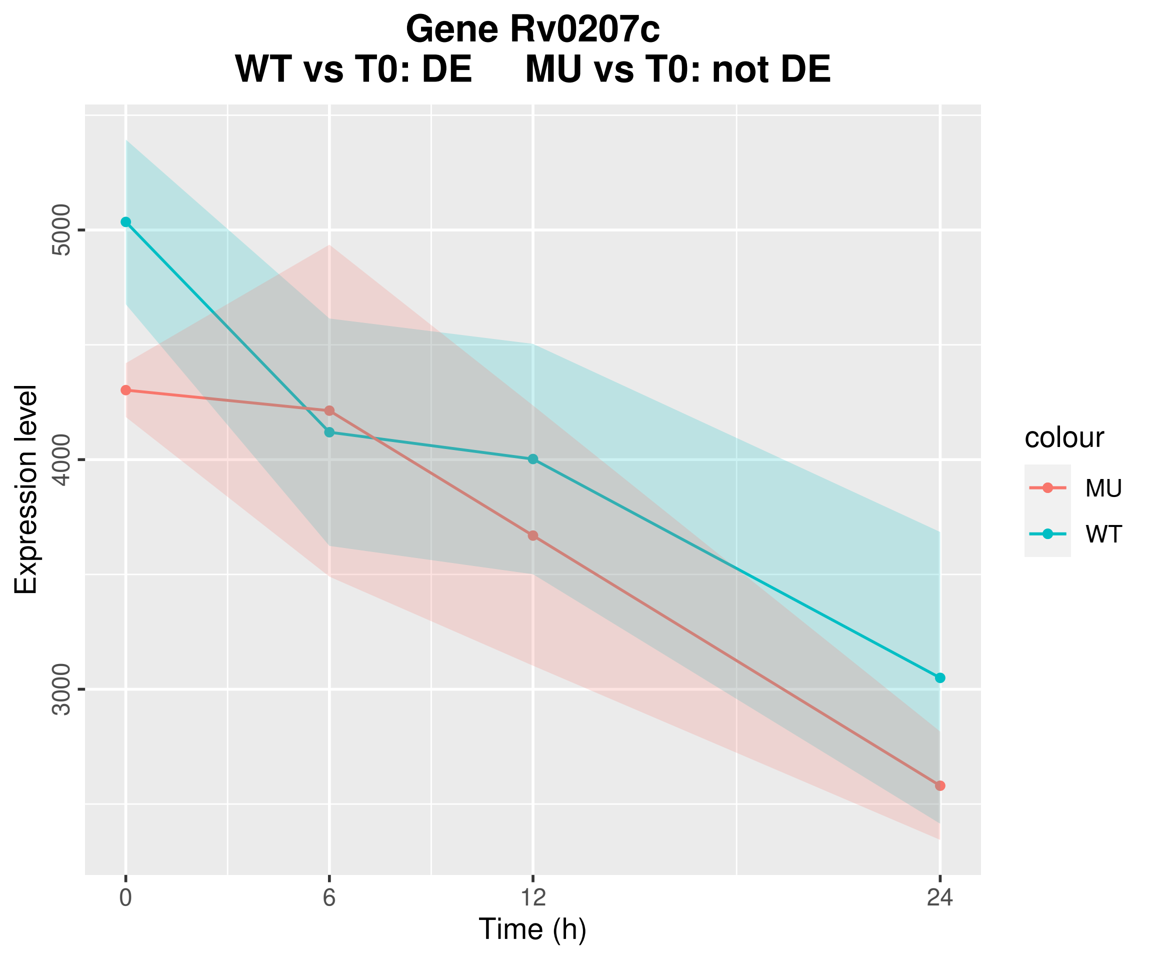

Gene Rv0207c
WT vs T0: DE MU vs T0: not DE
Expression level
5000
4000
3000
0
6
12
24
Time (h)
colour
MU
WT

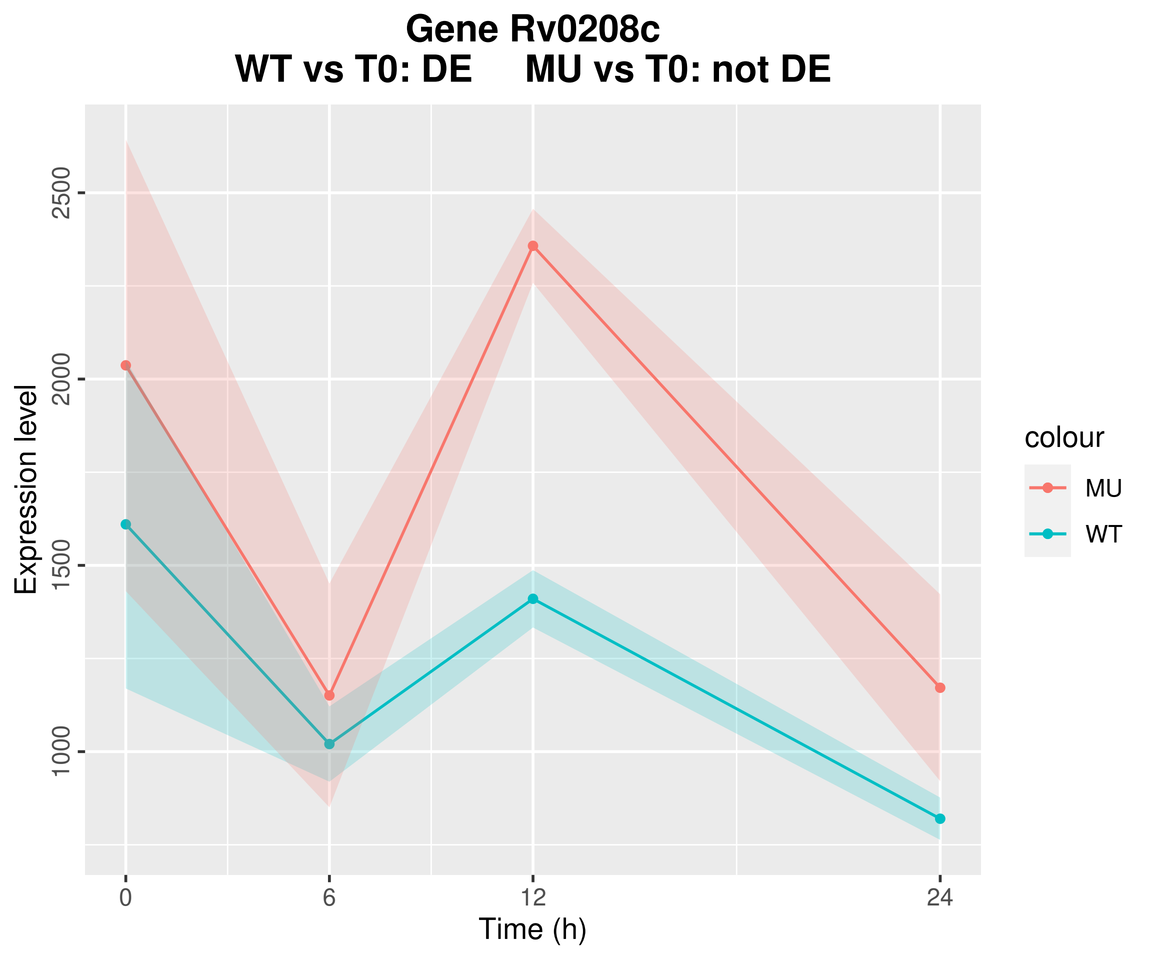

Gene Rv0208c
WT vs T0: DE   MU vs T0: not DE
Expression level
2500
2000
1500
1000
0
6
12
24
Time (h)
colour
MU
WT

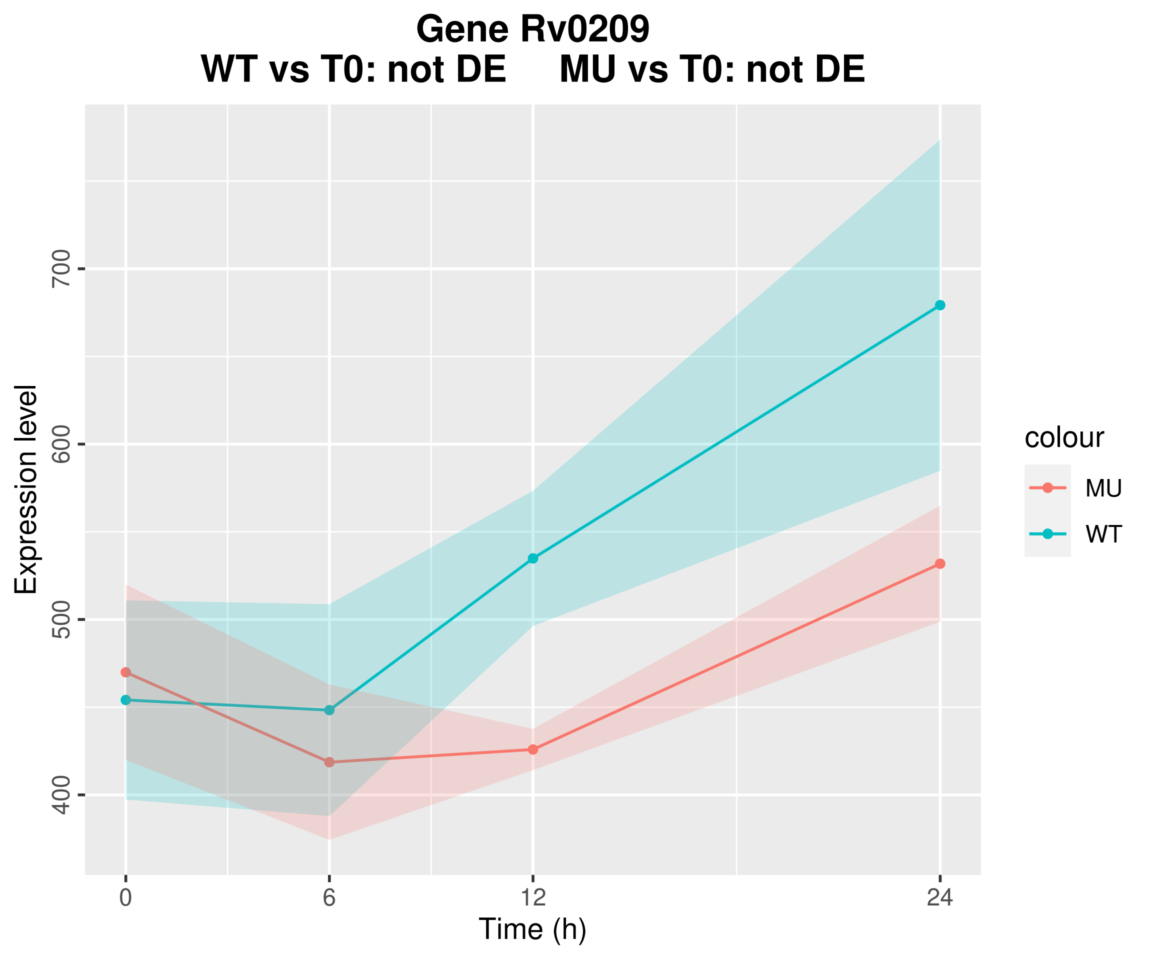
Gene Rv0209
WT vs T0: not DE MU vs T0: not DE
Expression level
400
500
600
700
0
6
12
24
Time (h)
colour
MU
WT

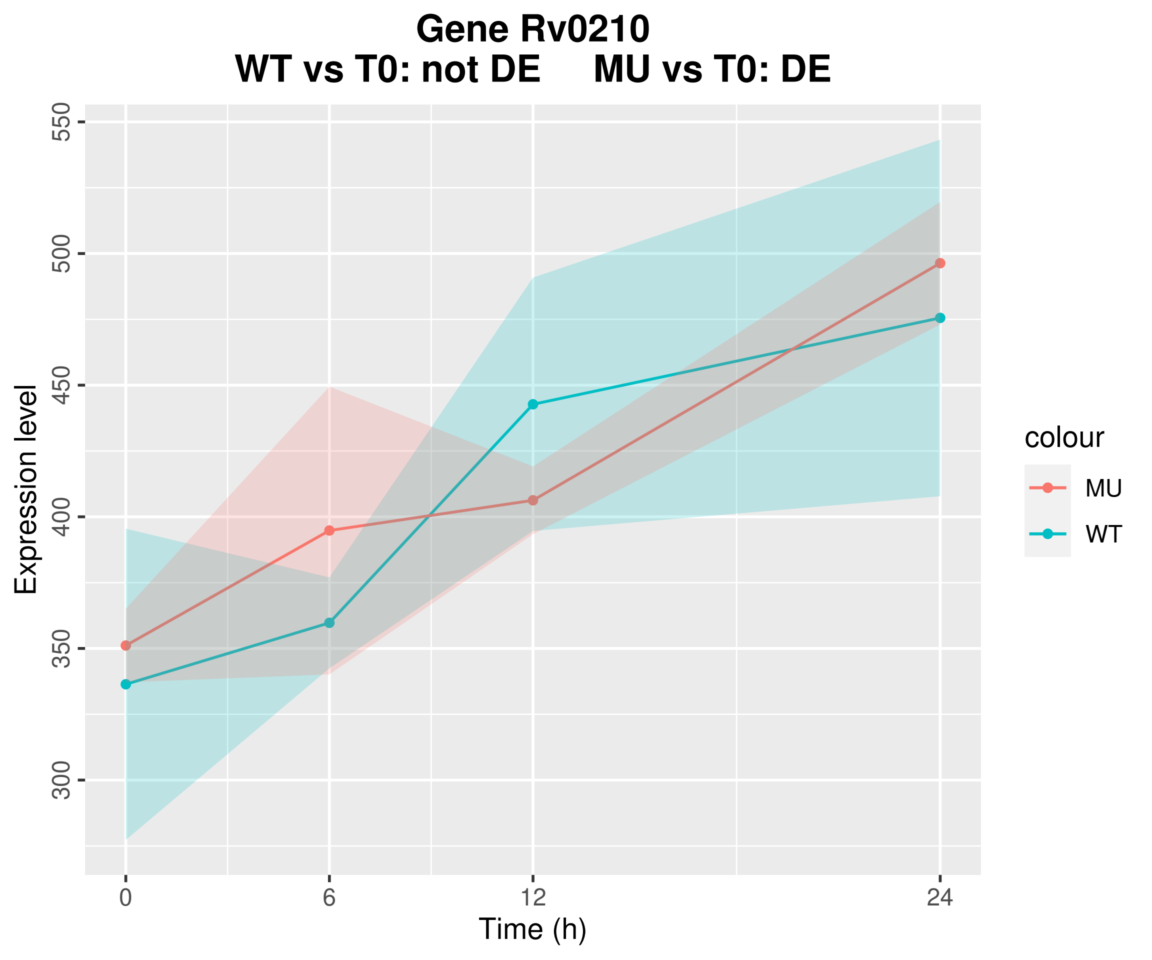

Gene Rv0210
WT vs T0: not DE   MU vs T0: DE
550
500
450
400
350
300
Expression level
0
6
12
24
Time (h)
colour
MU
WT

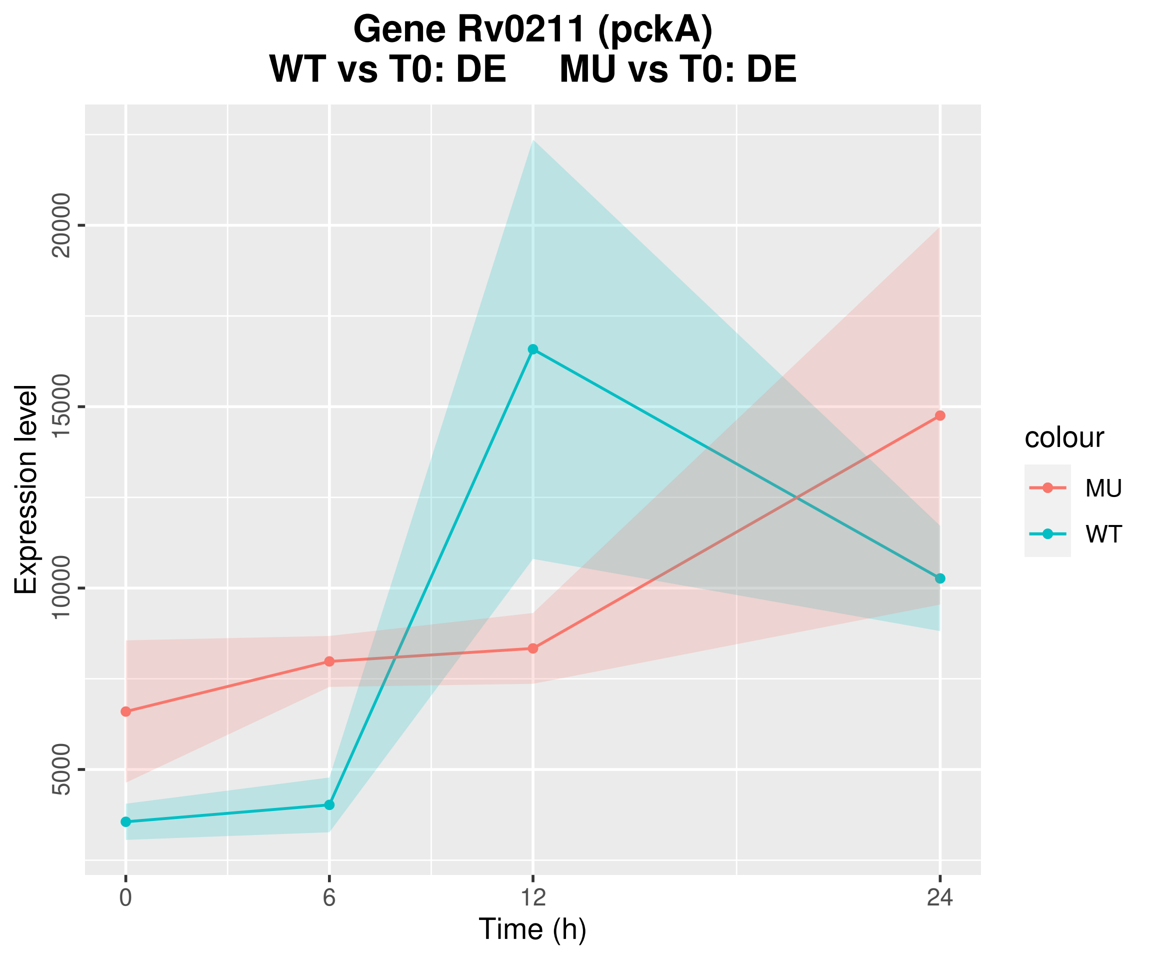

Gene Rv0211 (pckA)
WT vs T0: DE MU vs T0: DE
Expression level
20000
15000
10000
5000
0
6
12
24
Time (h)
colour
MU
WT

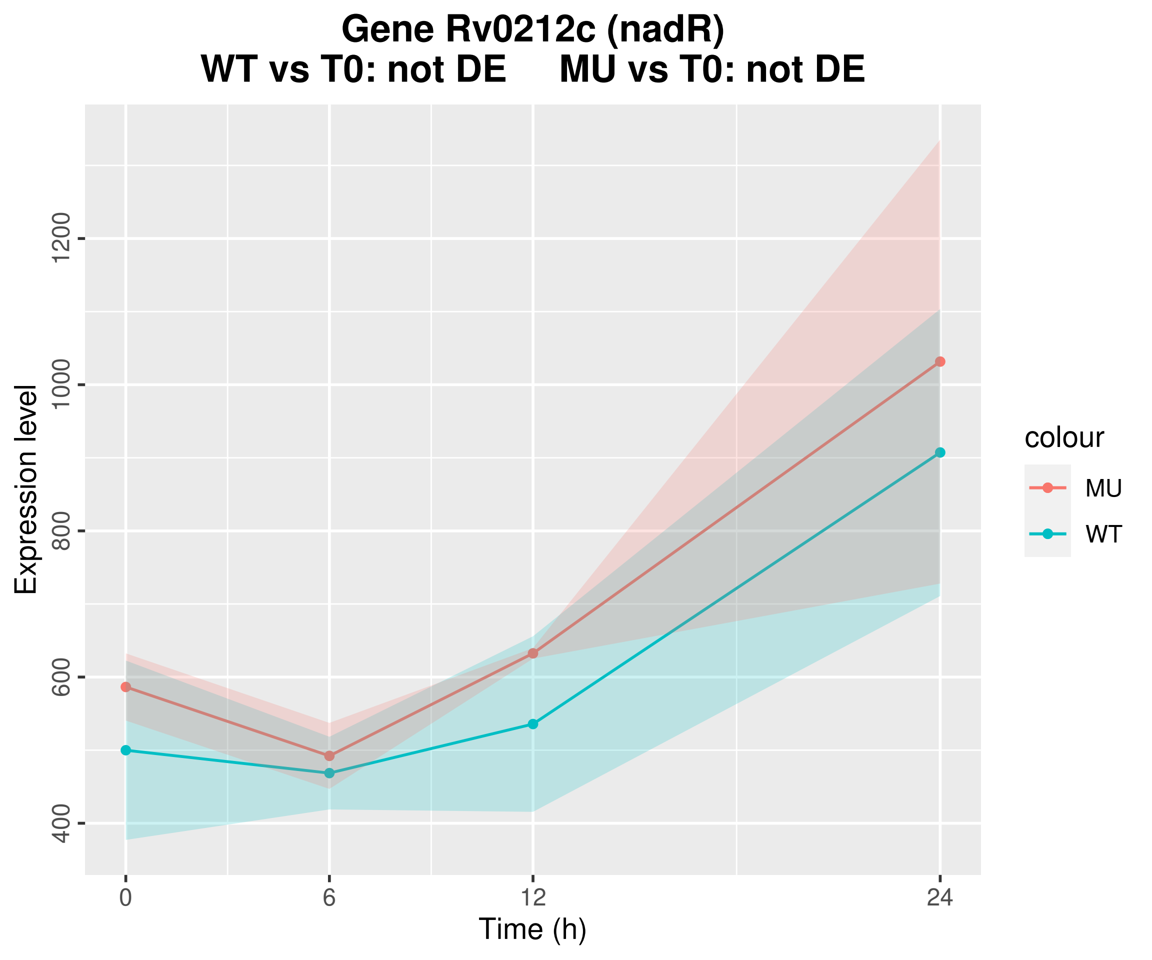
Gene Rv0212c (nadR)
WT vs T0: not DE   MU vs T0: not DE
Expression level
400
600
800
1000
1200
0
6
12
24
Time (h)
colour
MU
WT

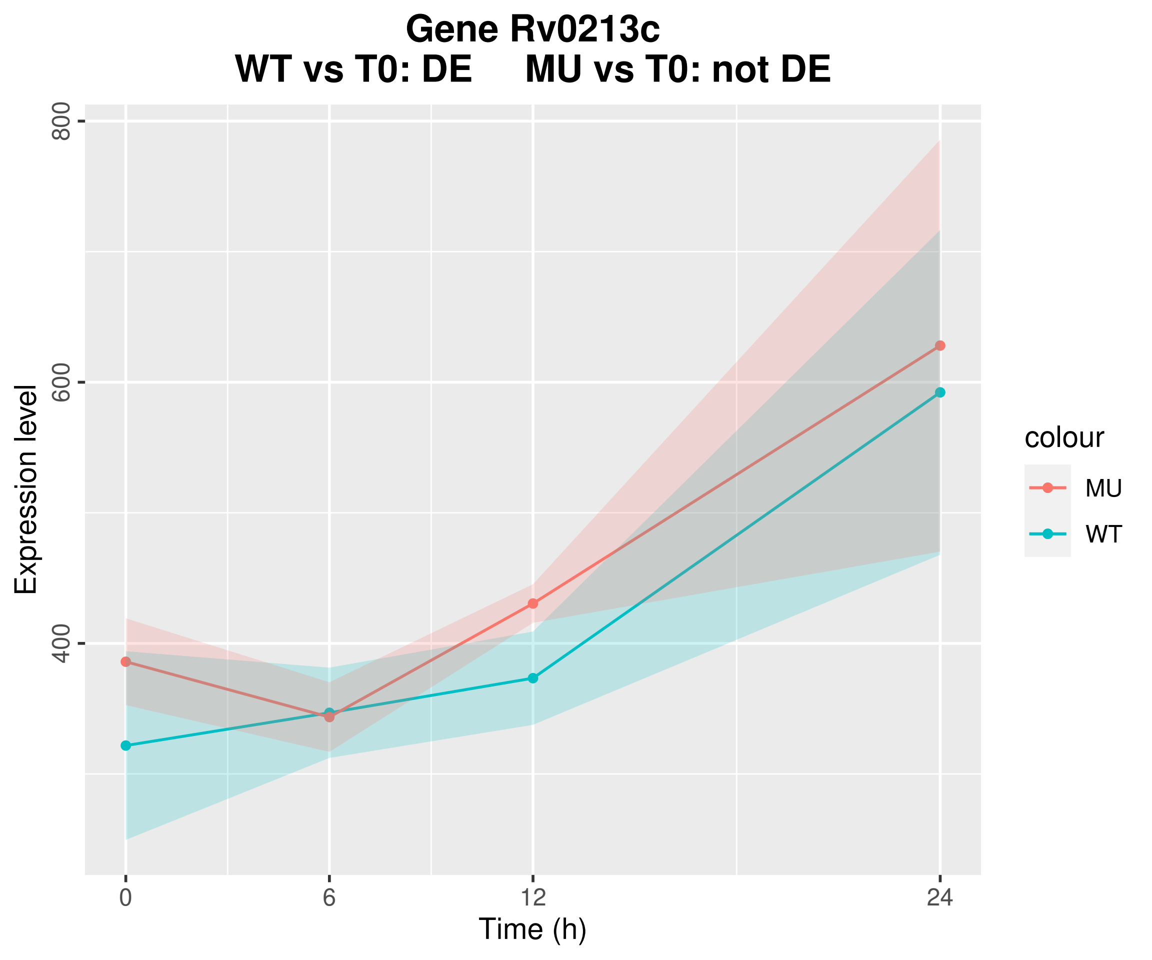
Gene Rv0213c
WT vs T0: DE   MU vs T0: not DE
800
600
400
Expression level
0
6
12
24
Time (h)
colour
MU
WT

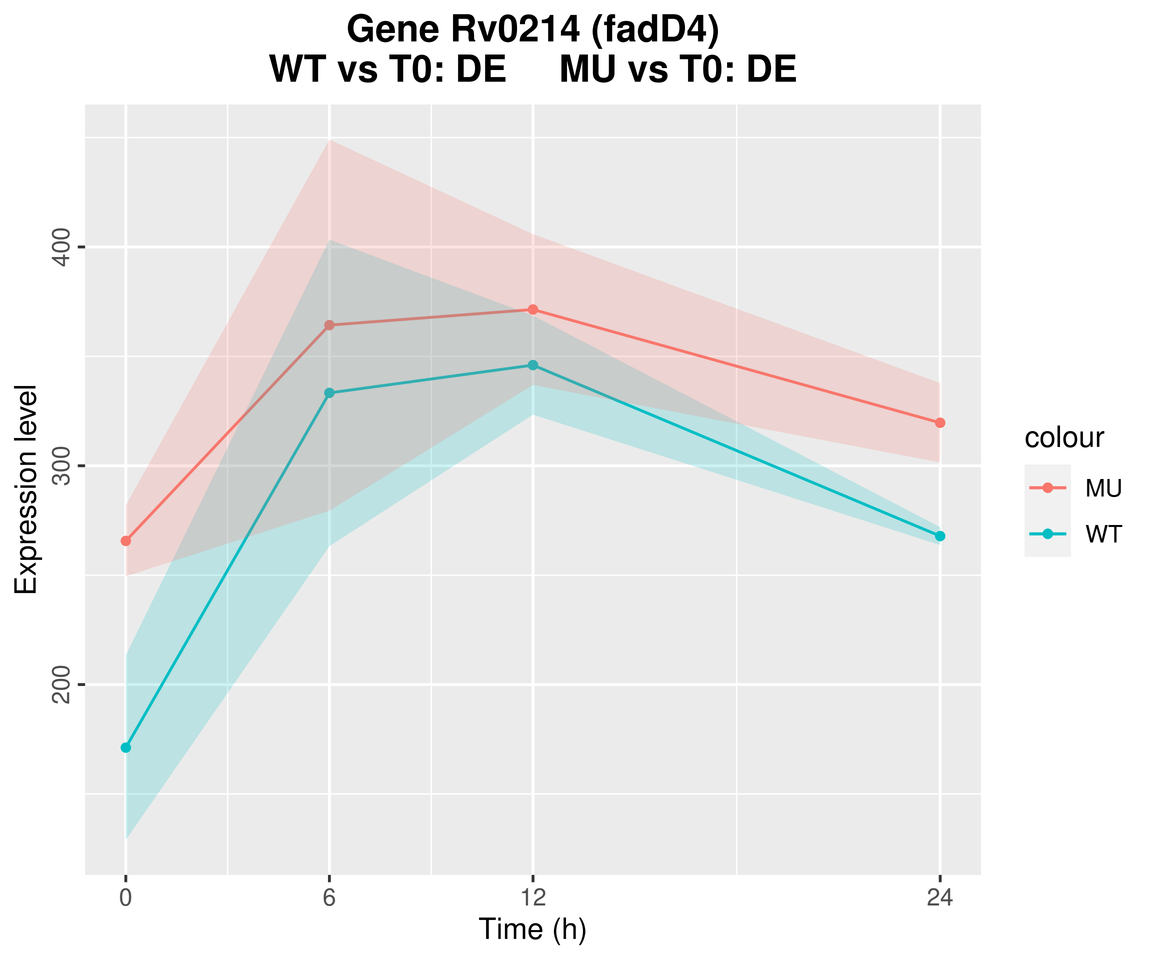
Gene Rv0214 (fadD4)
WT vs T0: DE MU vs T0: DE
Expression level
400
300
200
0
6
12
24
Time (h)
colour
MU
WT

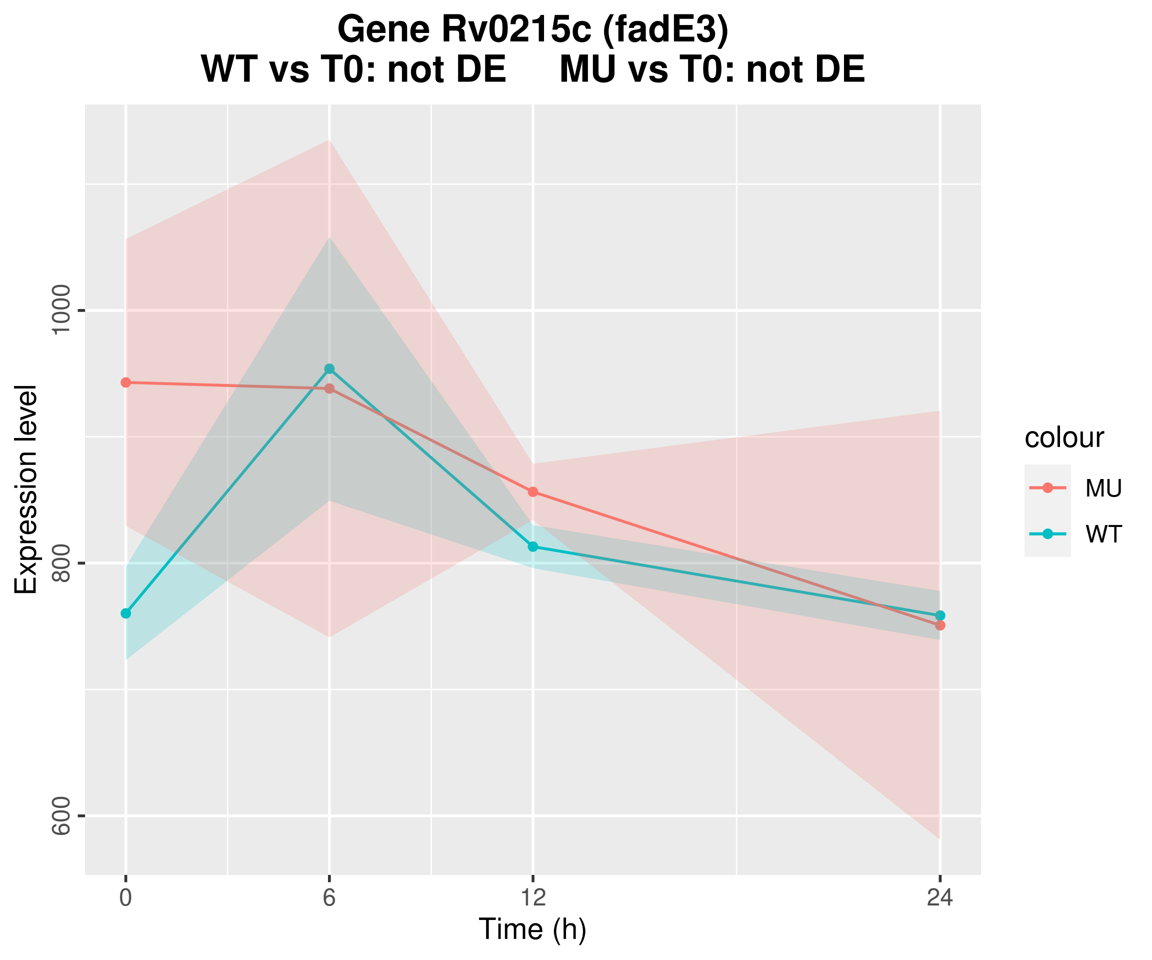

Gene Rv0215c (fadE3)
WT vs T0: not DE   MU vs T0: not DE
Expression level
1000
800
600
Time (h)
0
6
12
24
colour
MU
WT

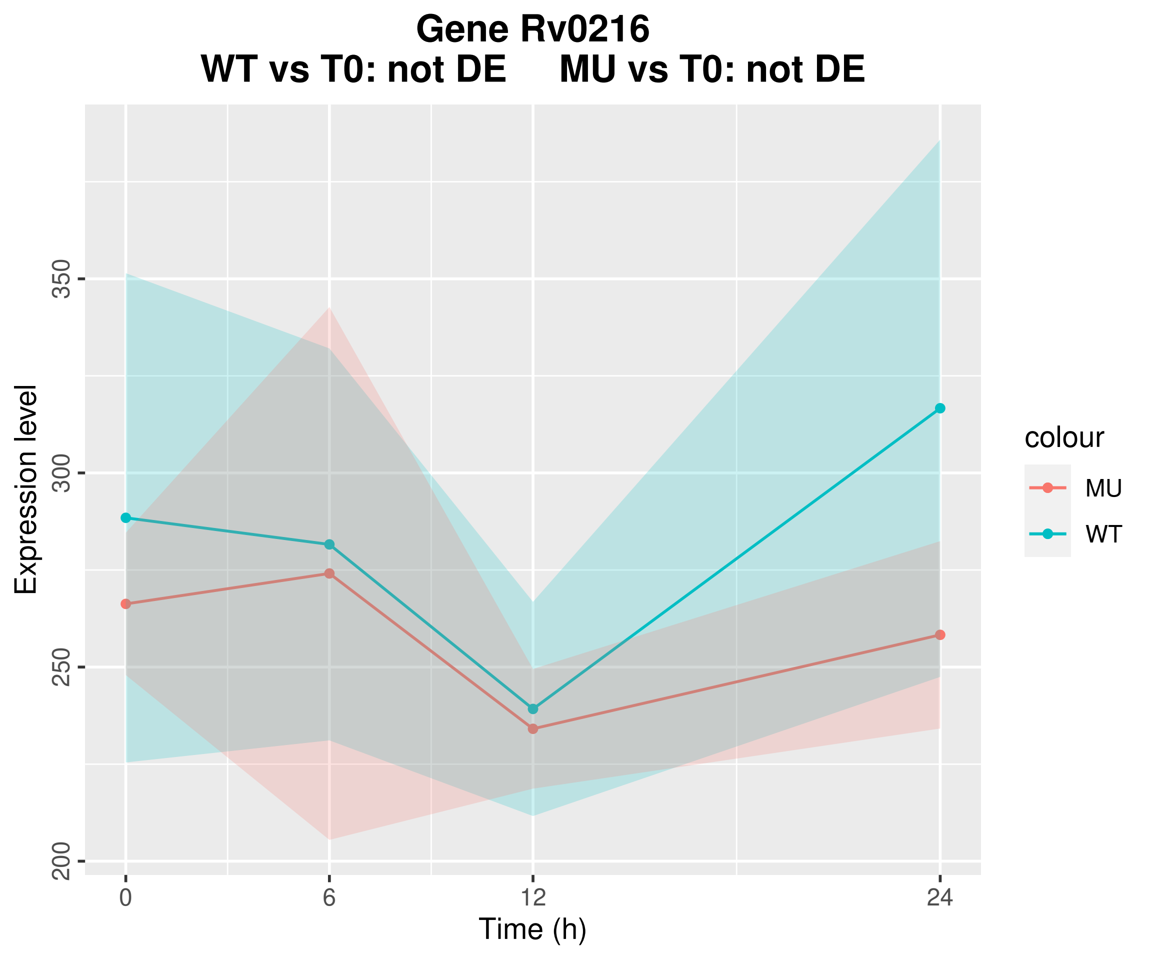
Gene Rv0216
WT vs T0: not DE   MU vs T0: not DE
Expression level
350
300
250
200
0
6
12
24
Time (h)
colour
MU
WT

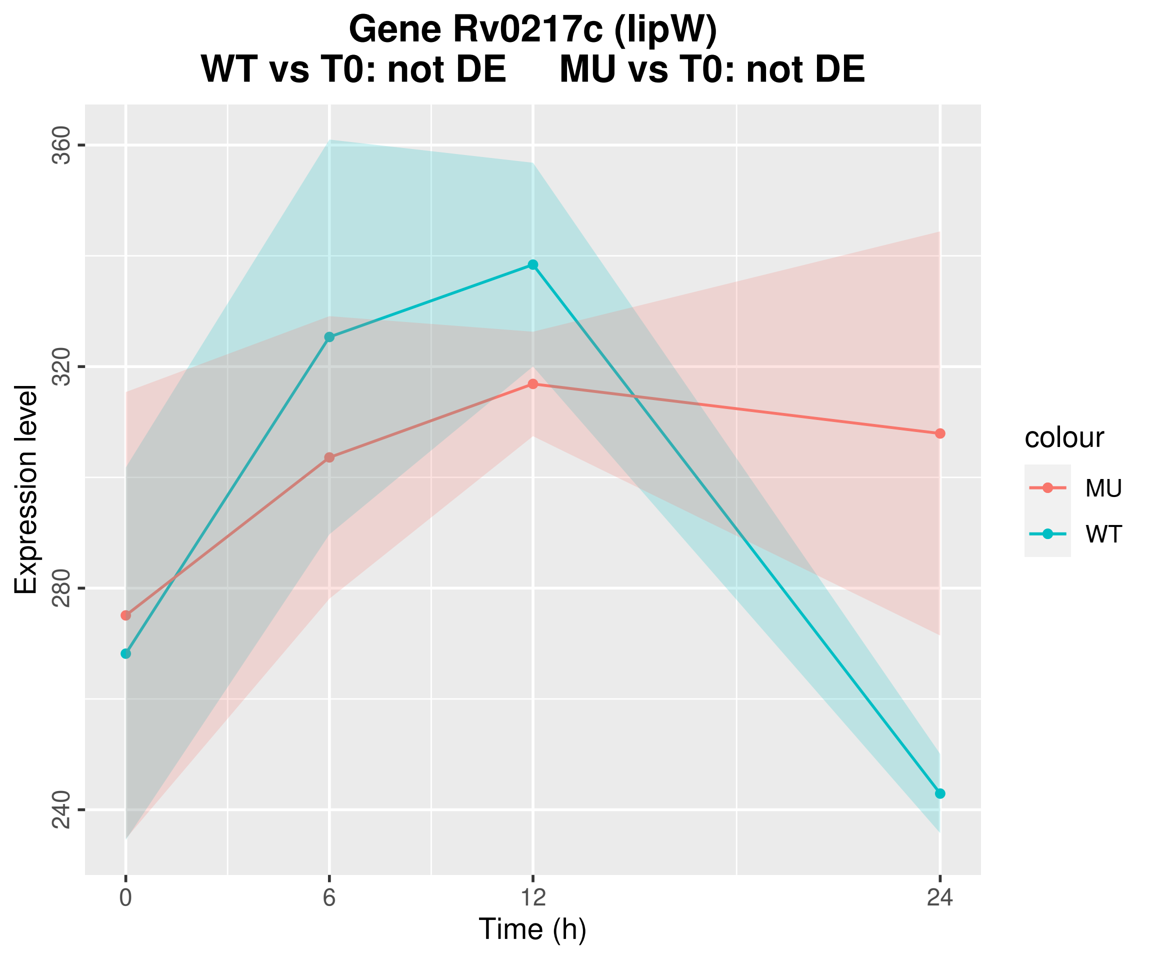

Gene Rv0217c (lipW)
WT vs T0: not DE MU vs T0: not DE
Expression level
360
320
280
240
0
6
12
24
Time (h)
colour
MU
WT

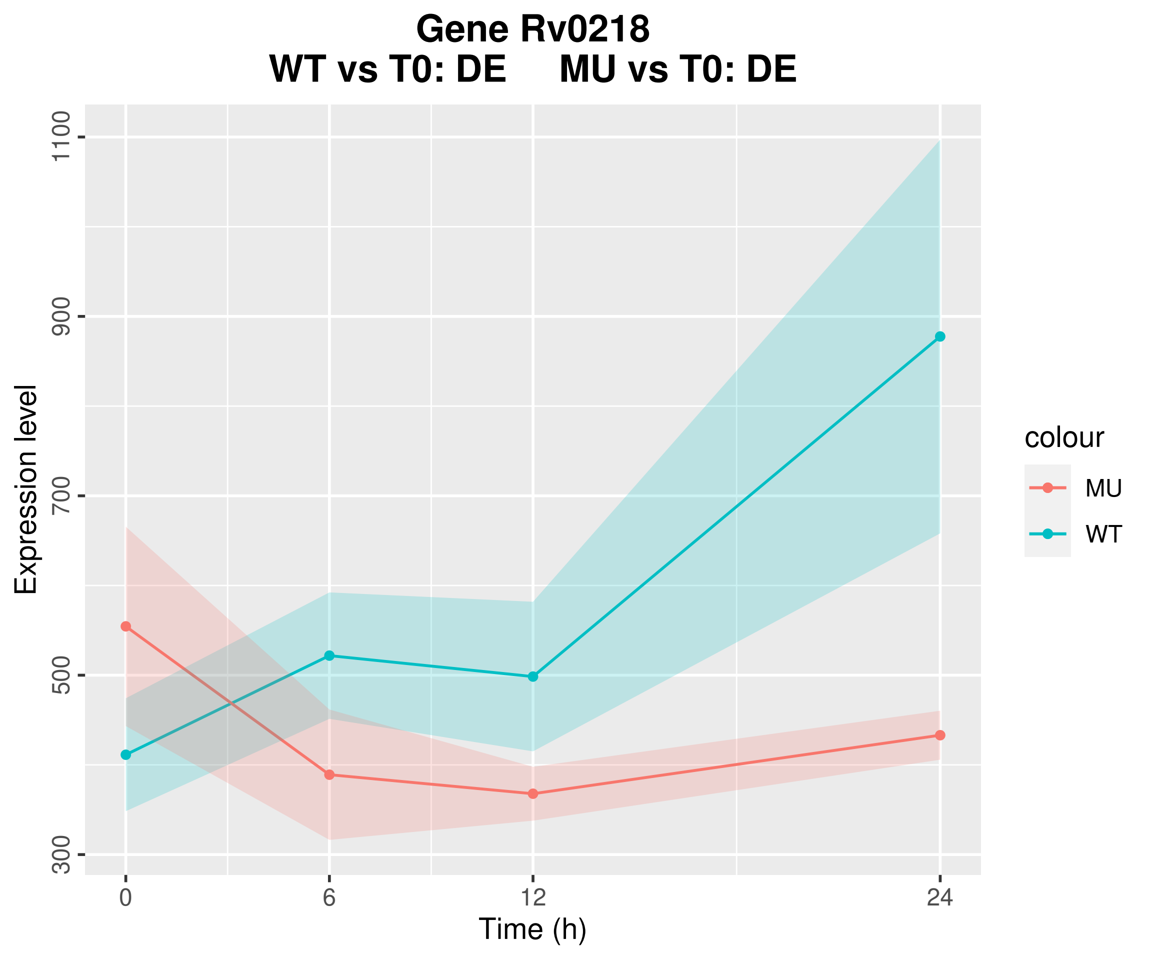
Gene Rv0218
WT vs T0: DE   MU vs T0: DE
Expression level
300
500
700
900
1100
0
6
12
24
Time (h)
colour
MU
WT

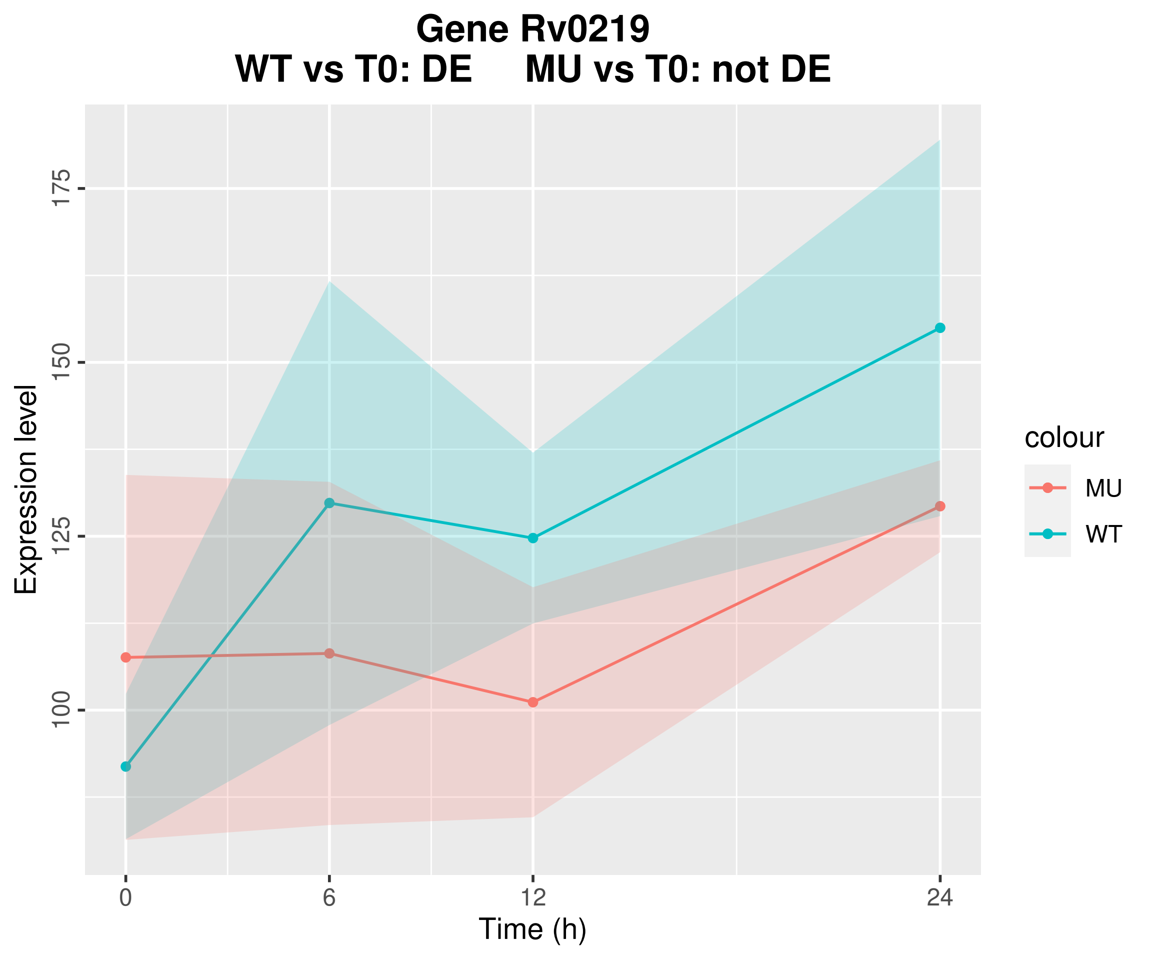

Gene Rv0219
WT vs T0: DE MU vs T0: not DE
Expression level
175
150
125
100
0
6
12
24
Time (h)
colour
MU
WT

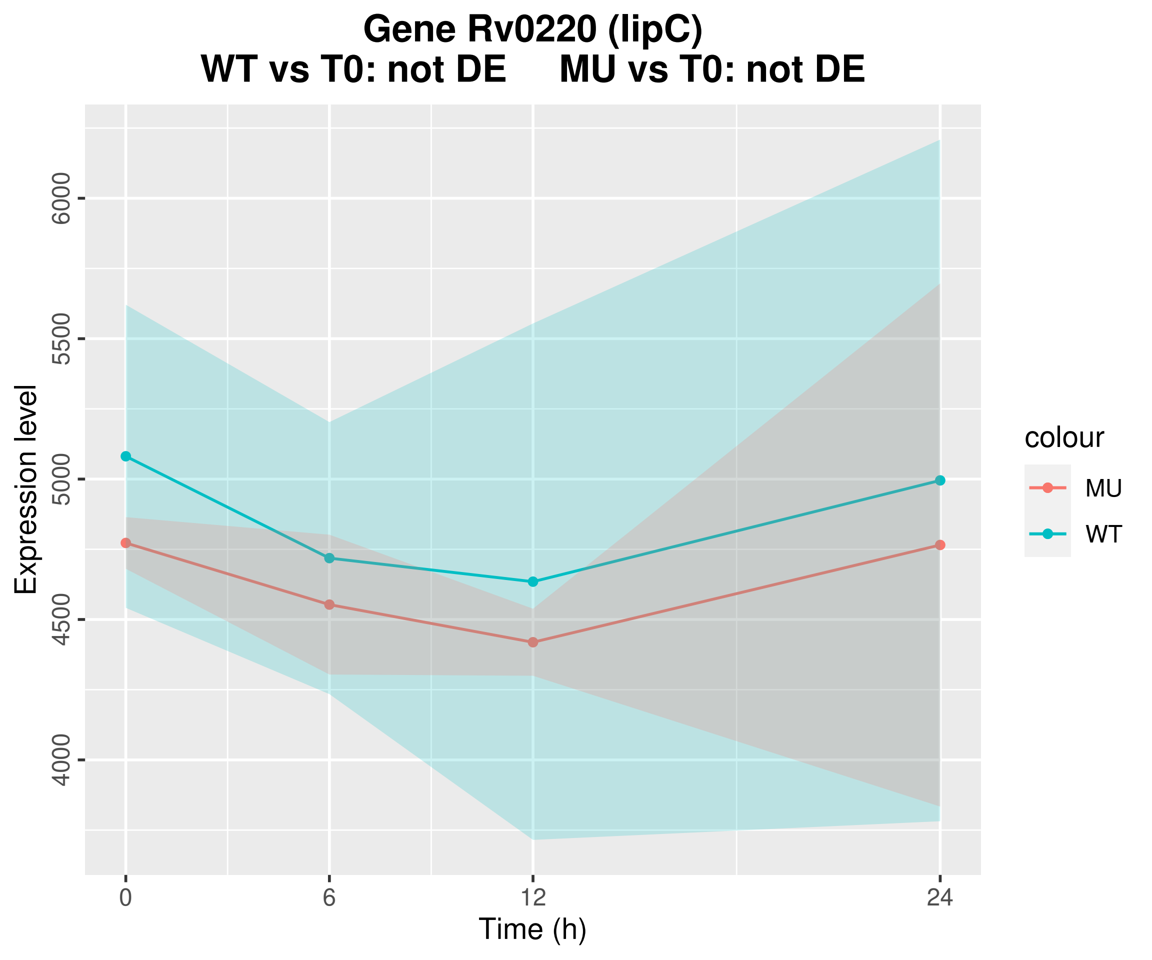
Gene Rv0220 (lipC)
WT vs T0: not DE MU vs T0: not DE
Expression level
6000
5500
5000
4500
4000
0
6
12
24
Time (h)
colour
MU
WT

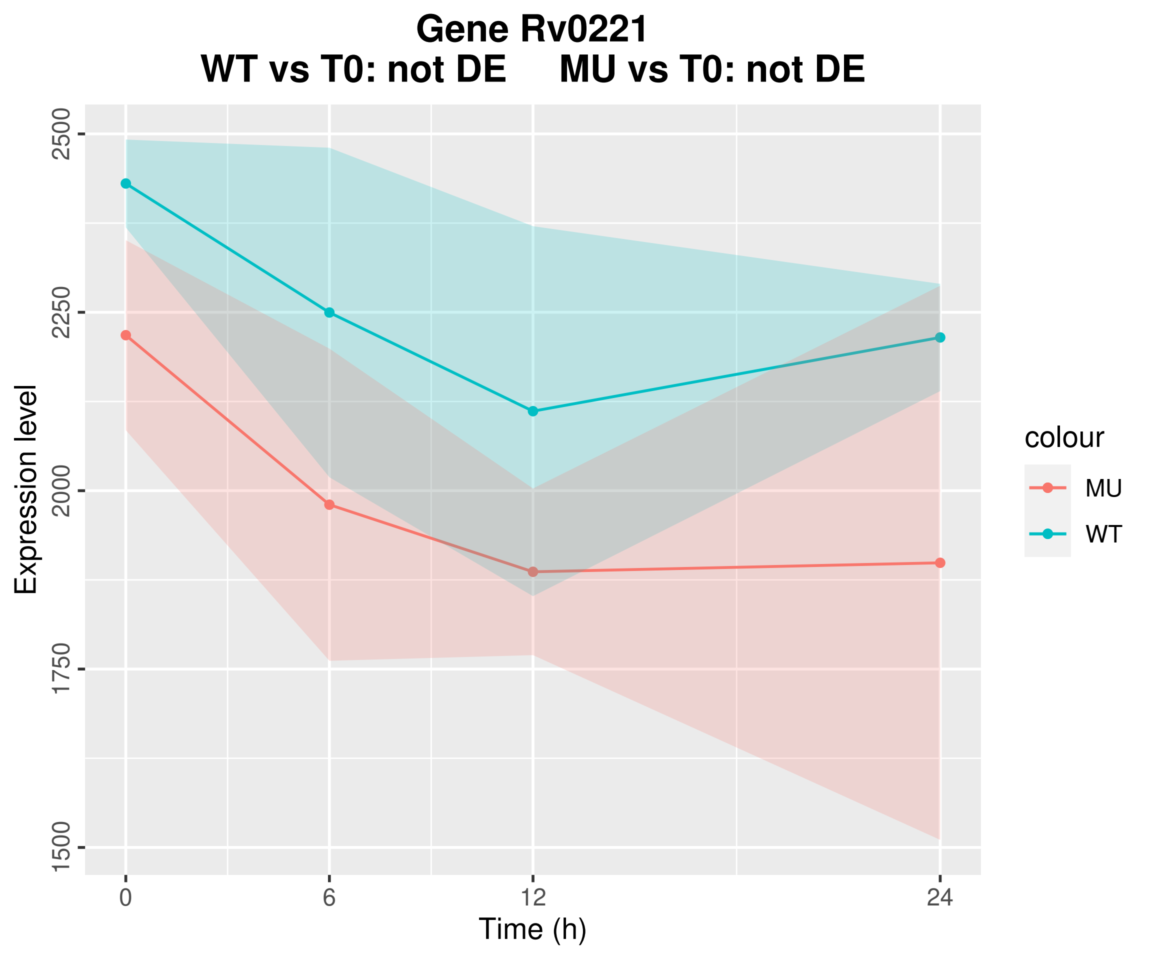

Gene Rv0221
WT vs T0: not DE MU vs T0: not DE
Expression level
2500
2250
2000
1750
1500
0
6
12
24
Time (h)
colour
MU
WT

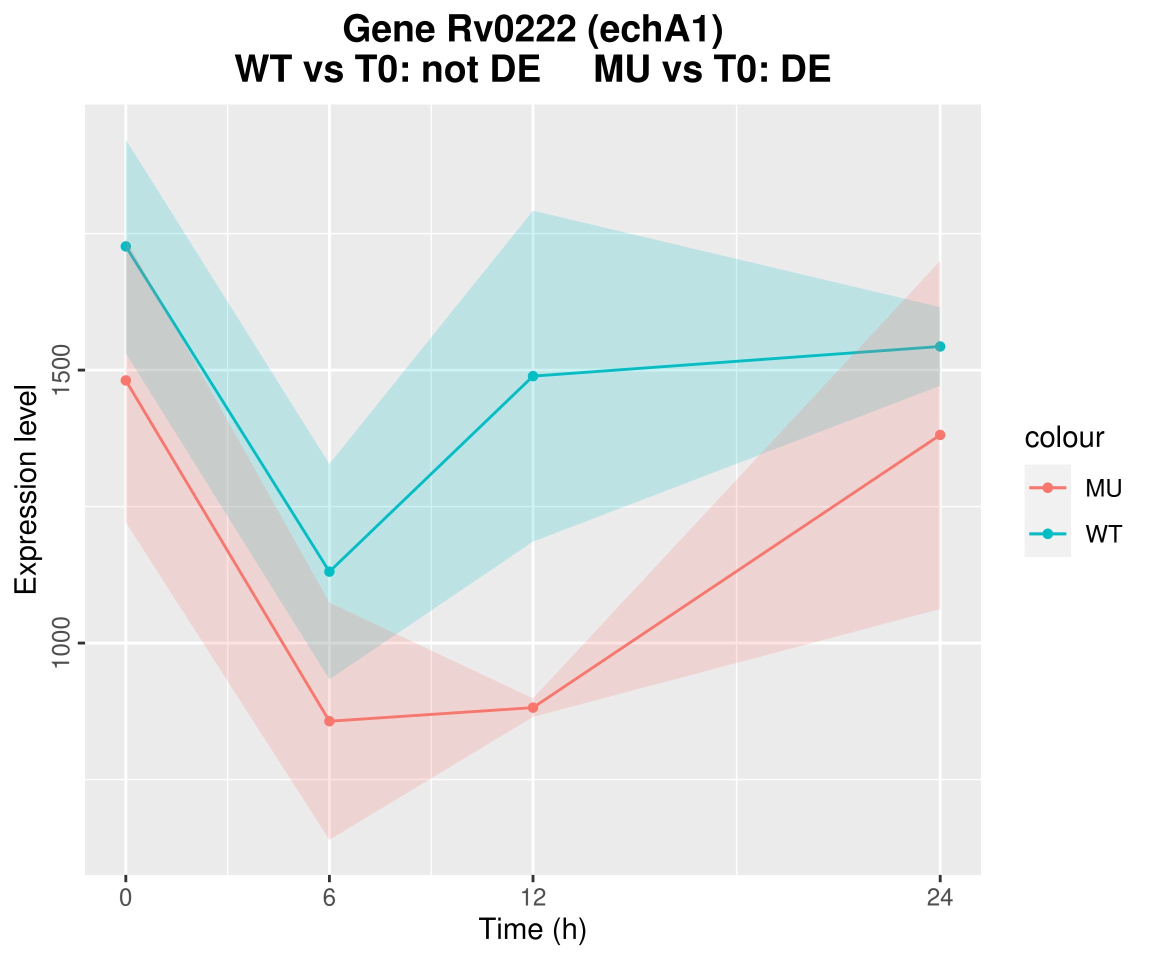

Gene Rv0222 (echA1)
WT vs T0: not DE   MU vs T0: DE
Expression level
1000
1500
Time (h)
0
6
12
24
colour
MU
WT

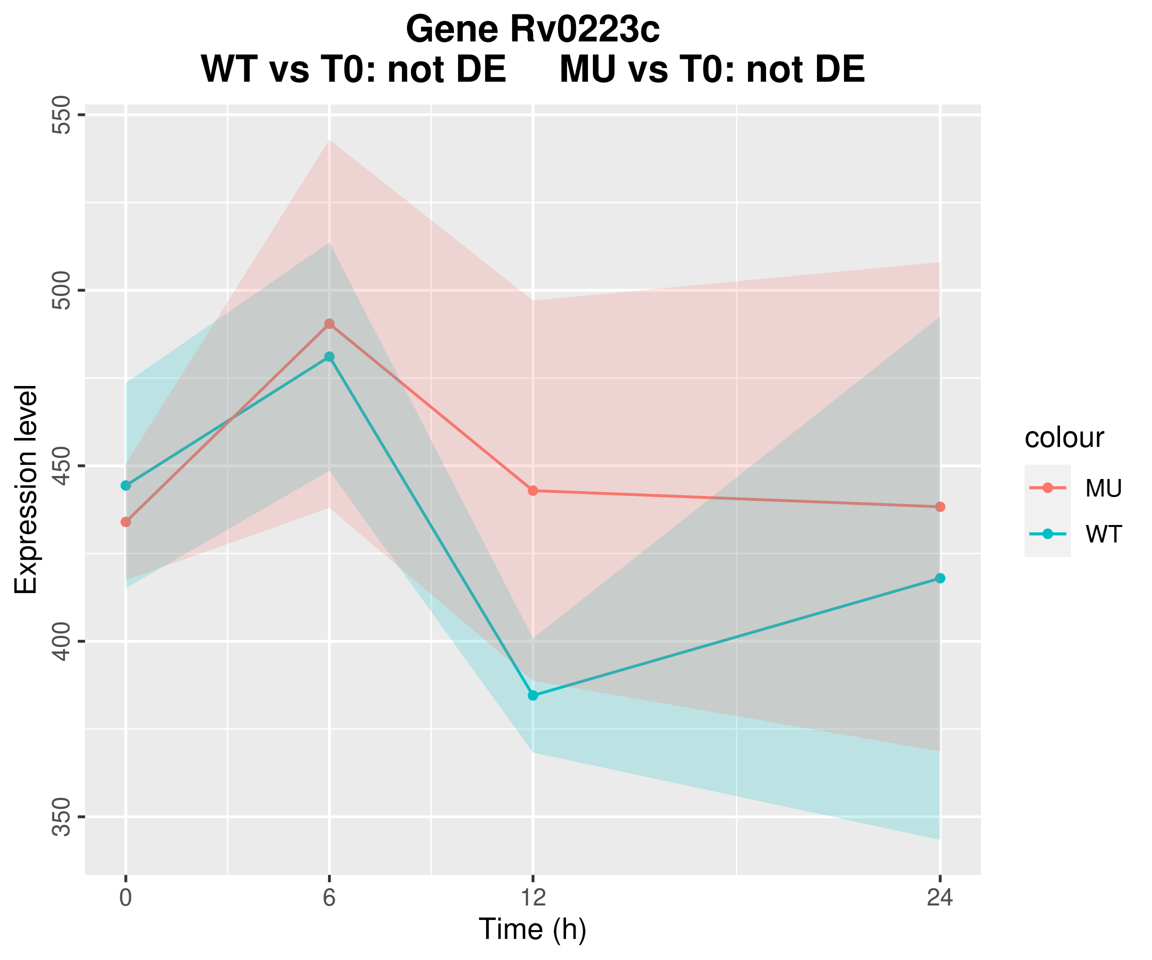

Gene Rv0223c
WT vs T0: not DE   MU vs T0: not DE
Expression level
550
500
450
400
350
0
6
12
24
Time (h)
colour
MU
WT

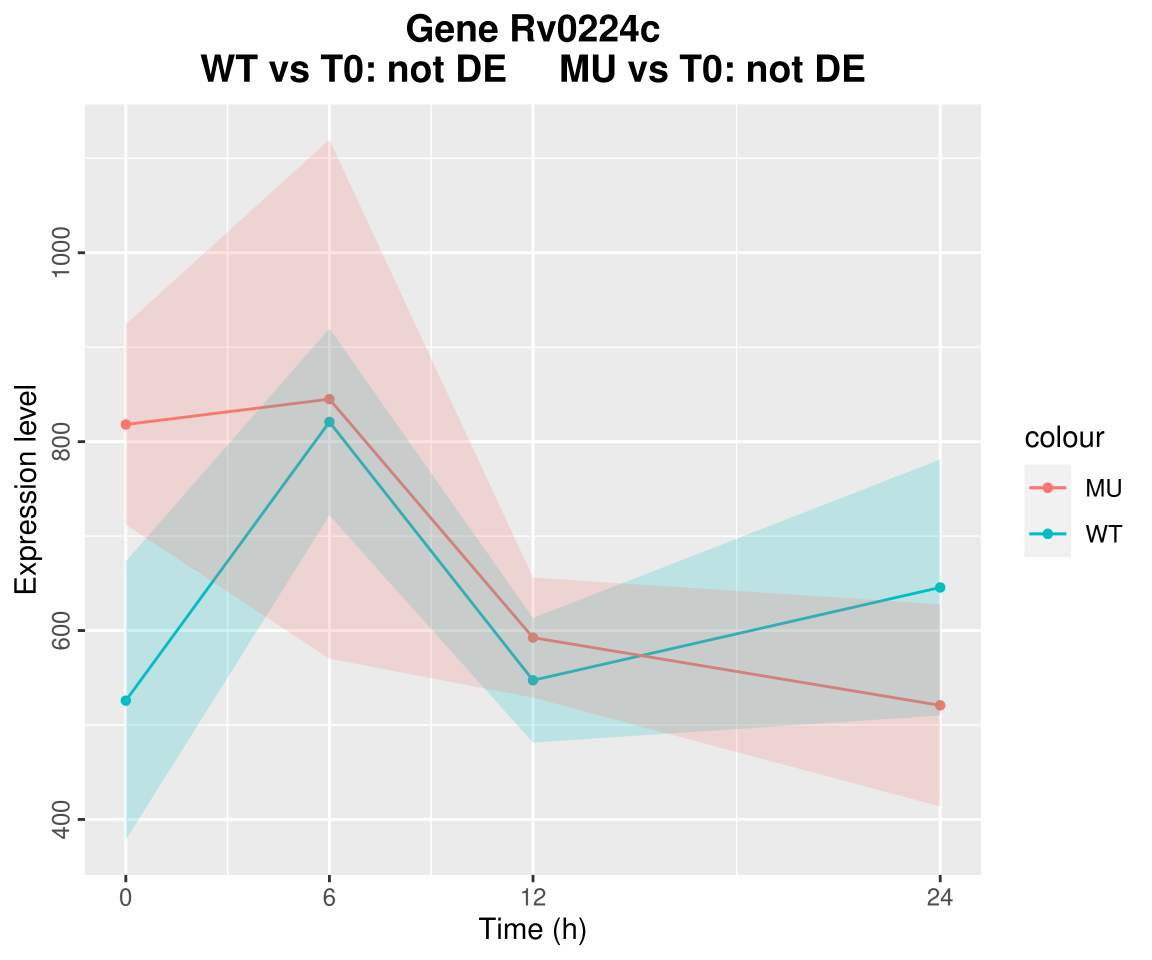

Gene Rv0224c
WT vs T0: not DE MU vs T0: not DE
Expression level
400
600
800
1000
0
6
12
24
Time (h)
colour
MU
WT

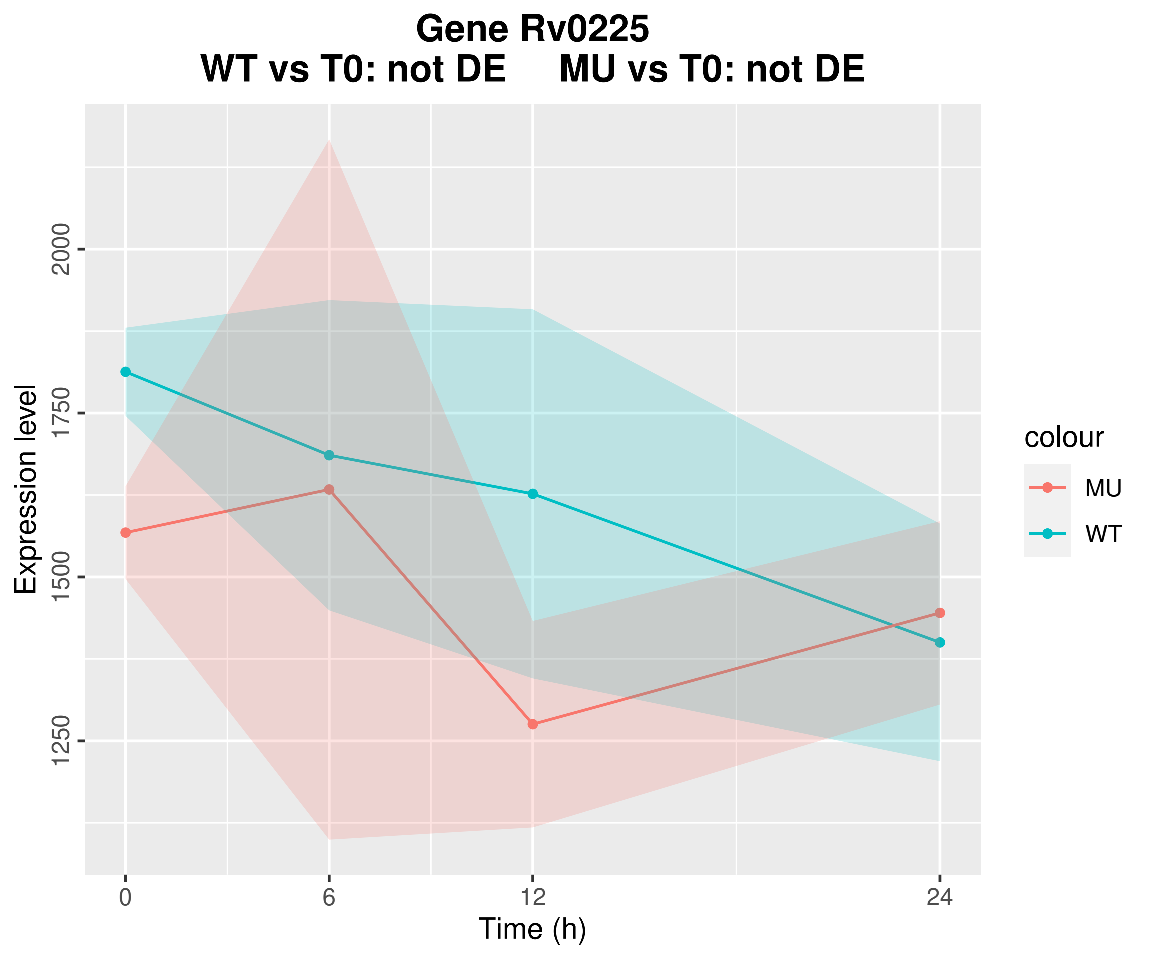
Gene Rv0225
WT vs T0: not DE MU vs T0: not DE
Expression level
2000
1750
1500
1250
0
6
12
24
Time (h)
colour
MU
WT

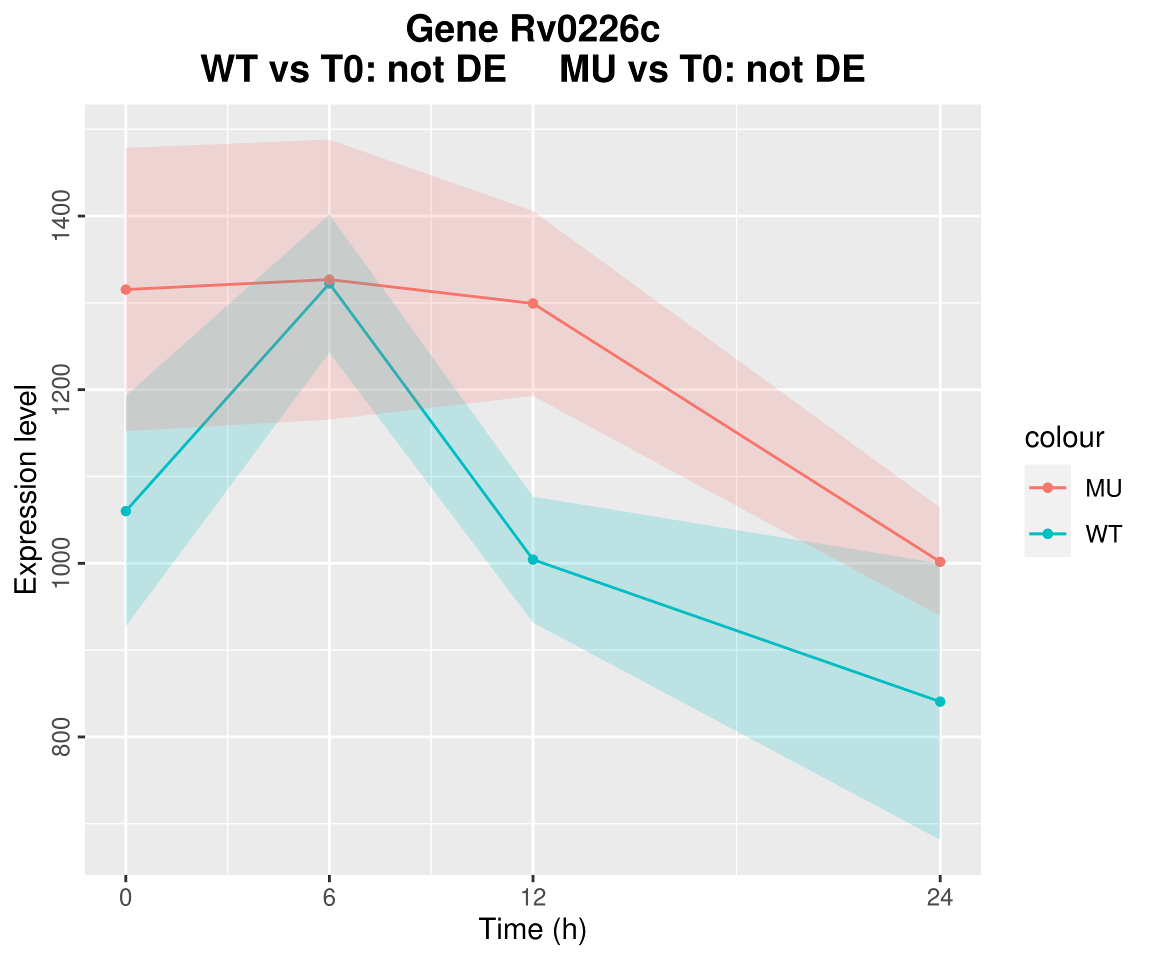

Gene Rv0226c
WT vs T0: not DE   MU vs T0: not DE
Expression level
1400
1200
1000
800
0
6
12
24
Time (h)
colour
MU
WT

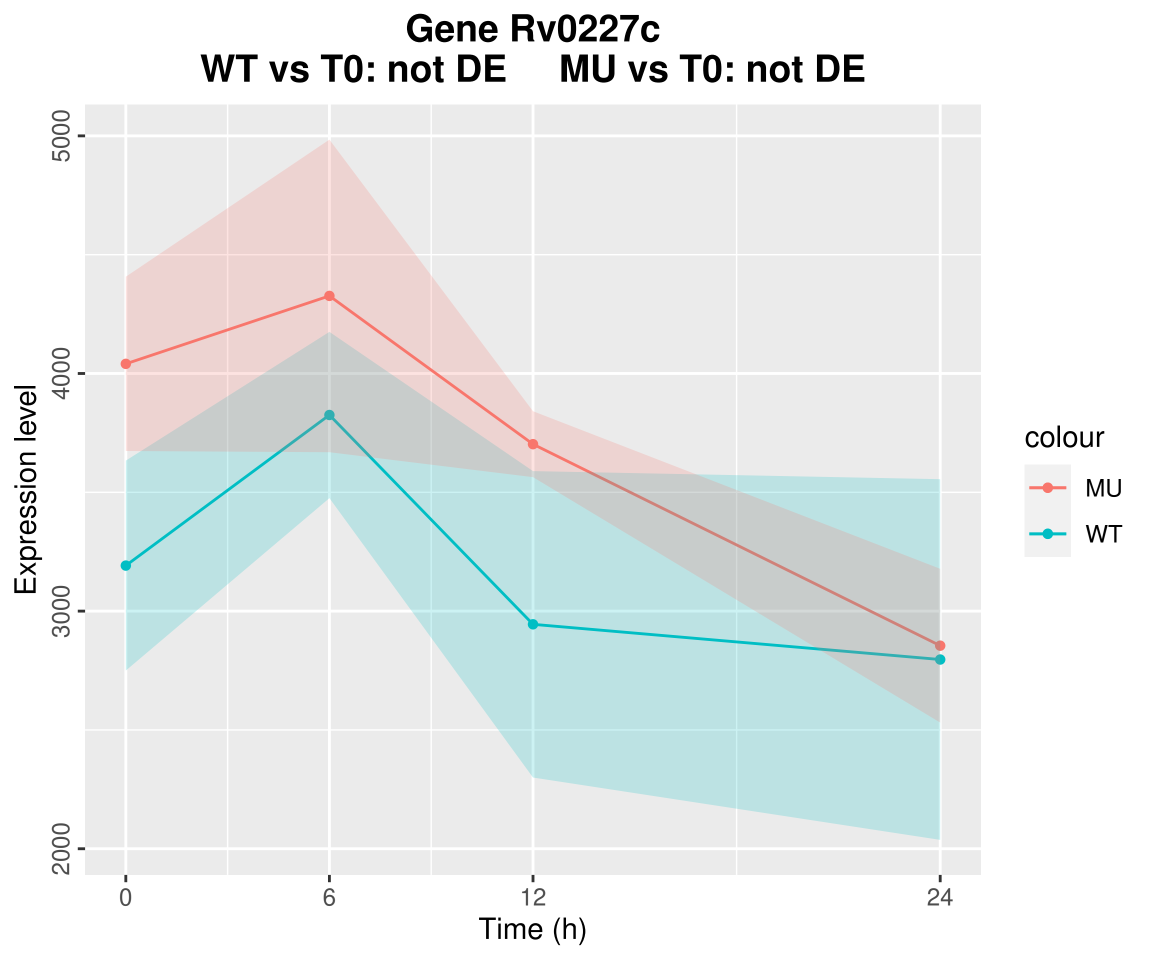

Gene Rv0227c
WT vs T0: not DE   MU vs T0: not DE
Expression level
5000
4000
3000
2000
0
6
12
24
Time (h)
colour
MU
WT

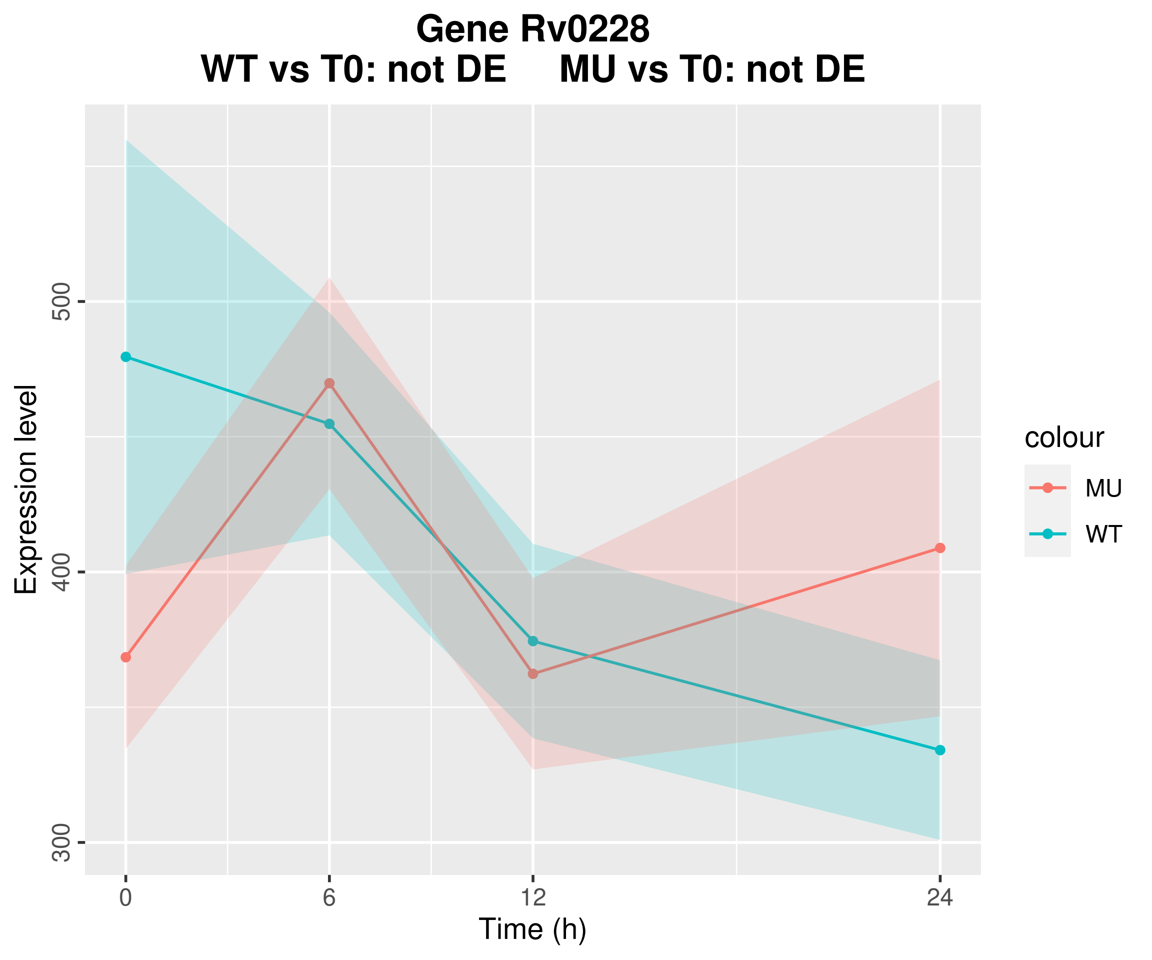

Gene Rv0228
WT vs T0: not DE   MU vs T0: not DE
Expression level
500
400
300
0
6
12
24
Time (h)
colour
MU
WT

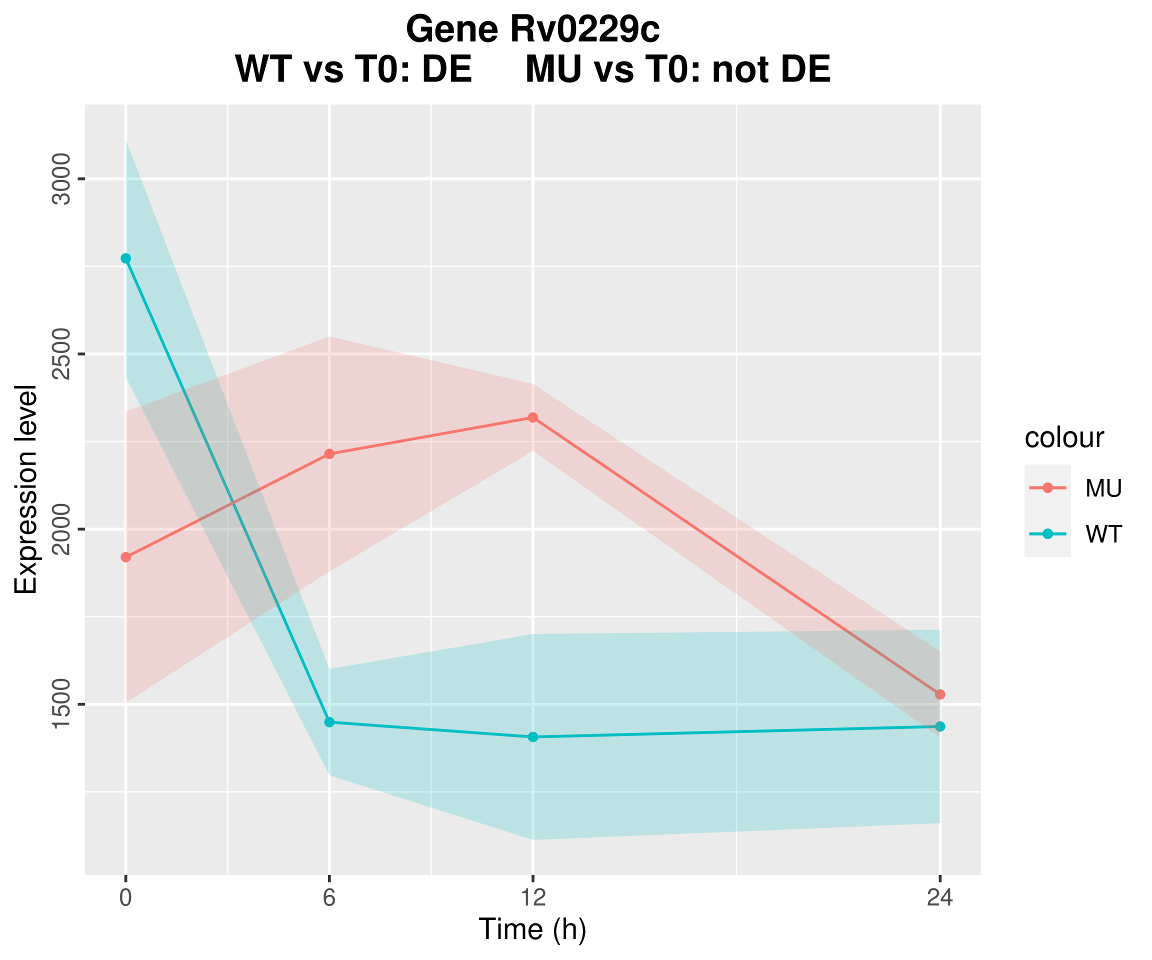

Gene Rv0229c
WT vs T0: DE   MU vs T0: not DE
Expression level
3000
2500
2000
1500
0
6
12
24
Time (h)
colour
MU
WT

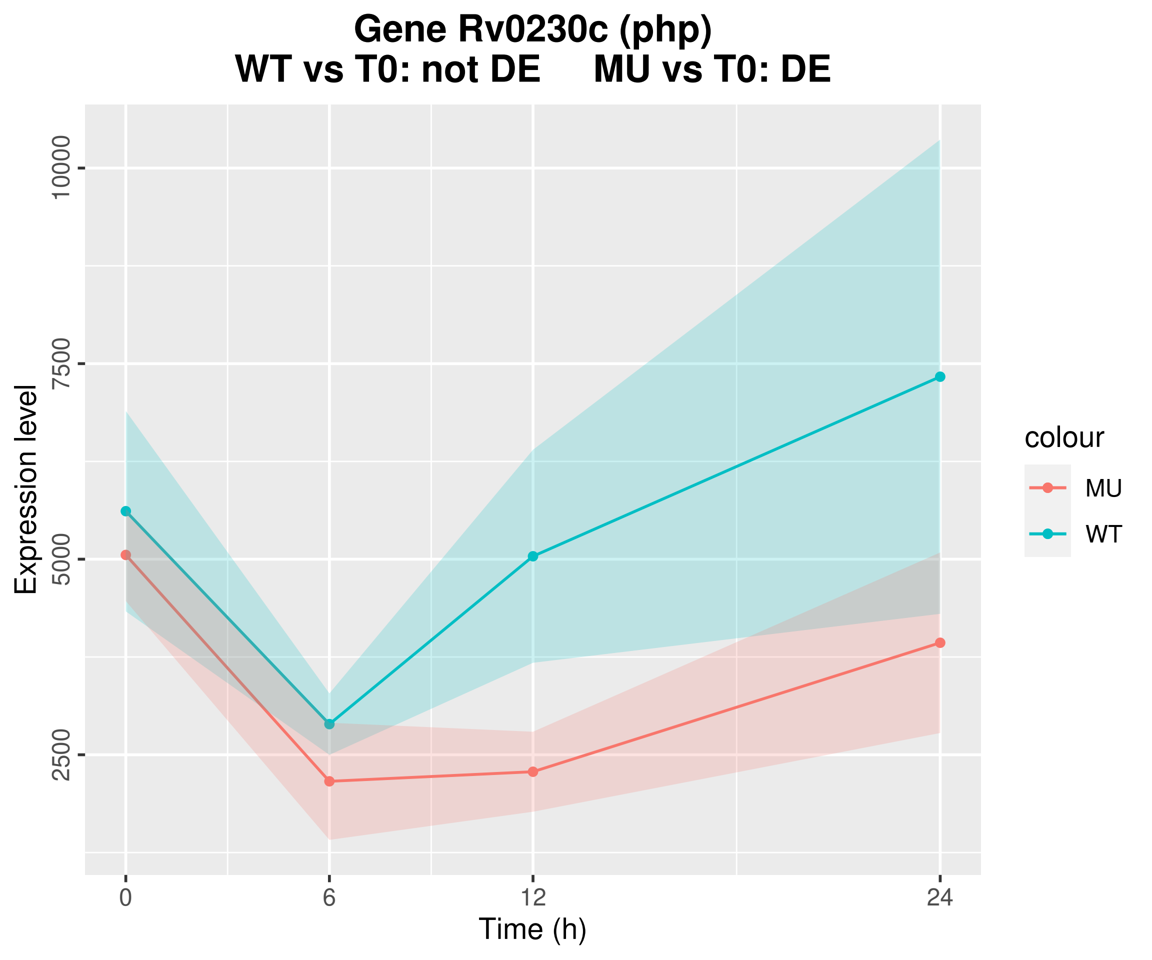
Gene Rv0230c (php)
WT vs T0: not DE   MU vs T0: DE
Expression level
10000
7500
5000
2500
0
6
12
24
Time (h)
colour
MU
WT

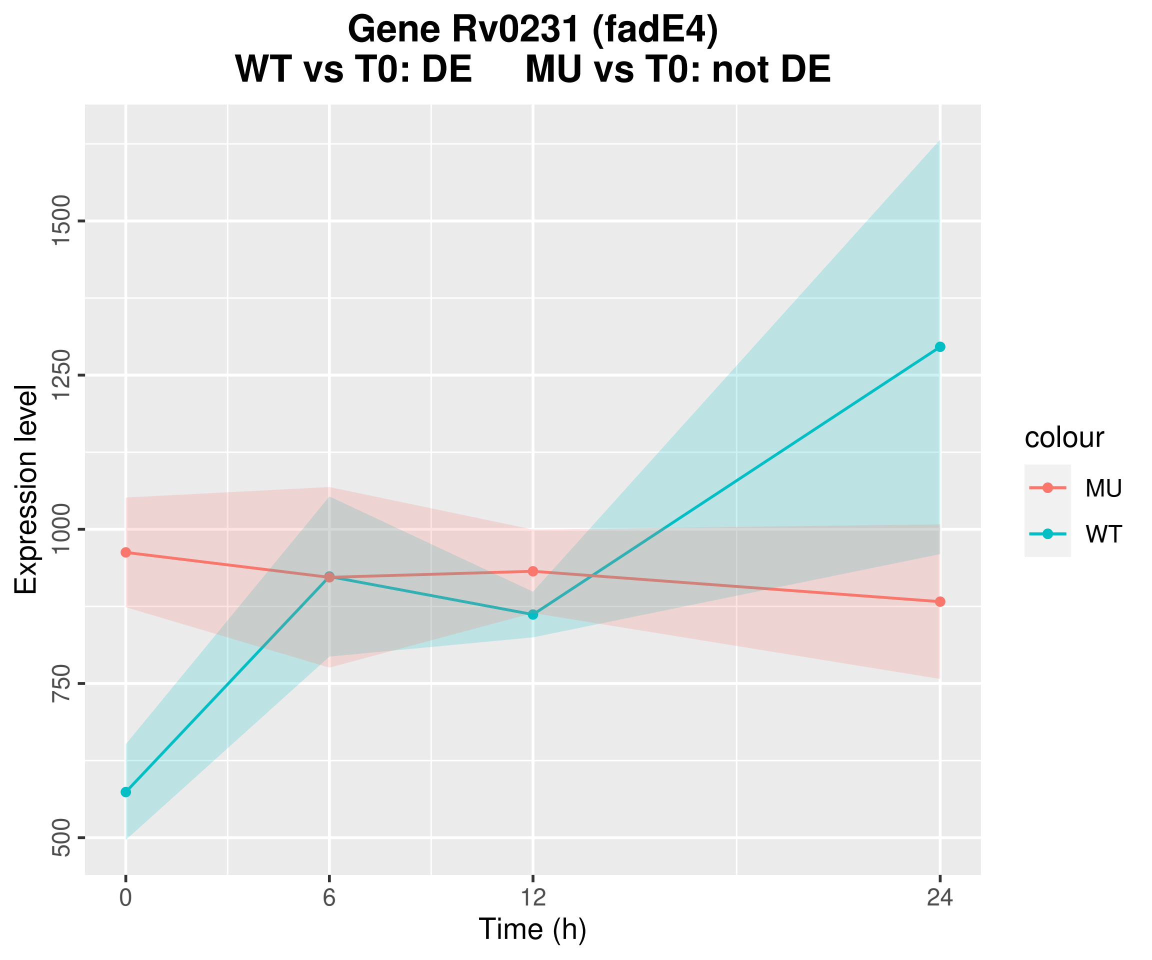

Gene Rv0231 (fadE4)
WT vs T0: DE MU vs T0: not DE
Expression level
1500
1250
1000
750
500
0
6
12
24
Time (h)
colour
MU
WT

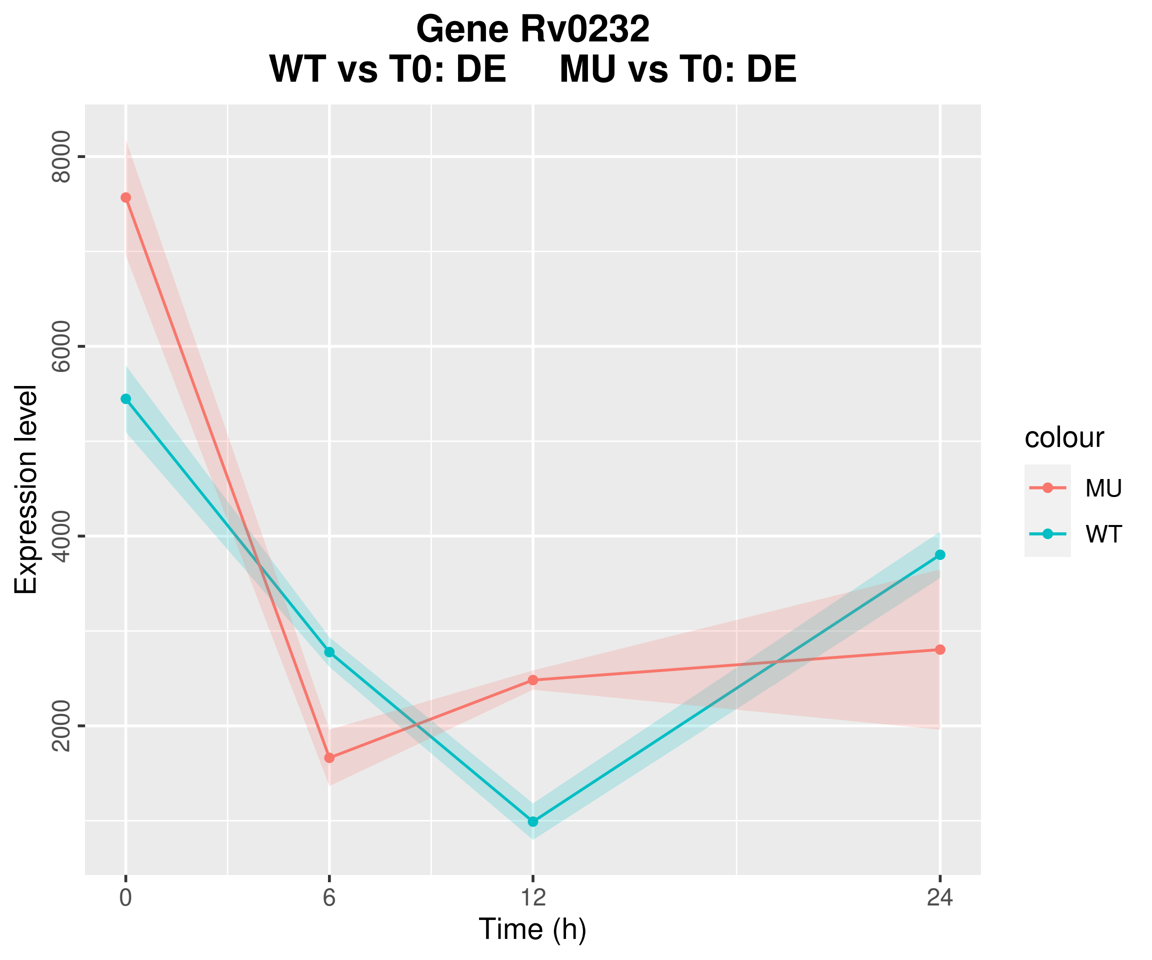

Gene Rv0232
WT vs T0: DE MU vs T0: DE
Expression level
8000
6000
4000
2000
0
6
12
24
Time (h)
colour
MU
WT

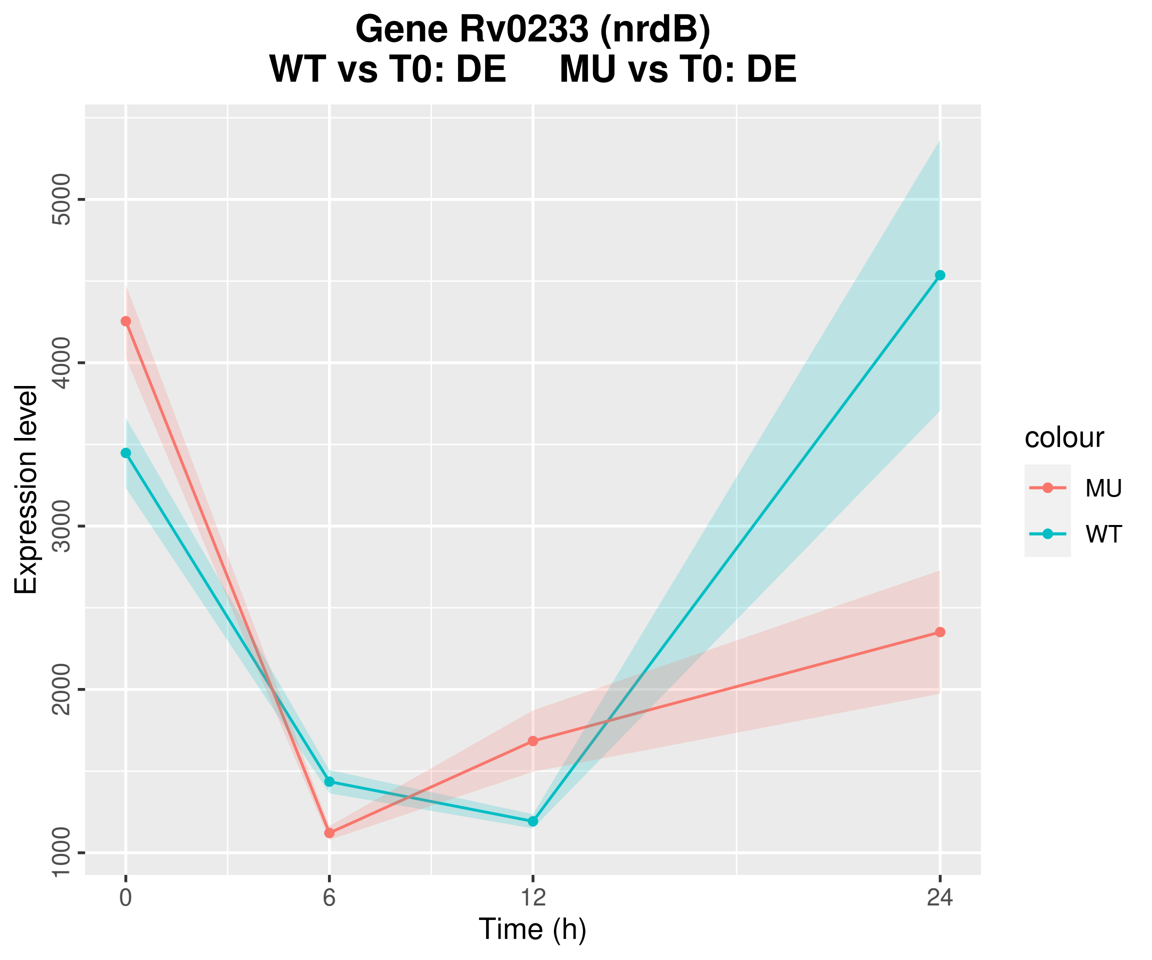

Gene Rv0233 (nrdB)
WT vs T0: DE   MU vs T0: DE
Expression level
5000
4000
3000
2000
1000
0
6
12
24
Time (h)
colour
MU
WT

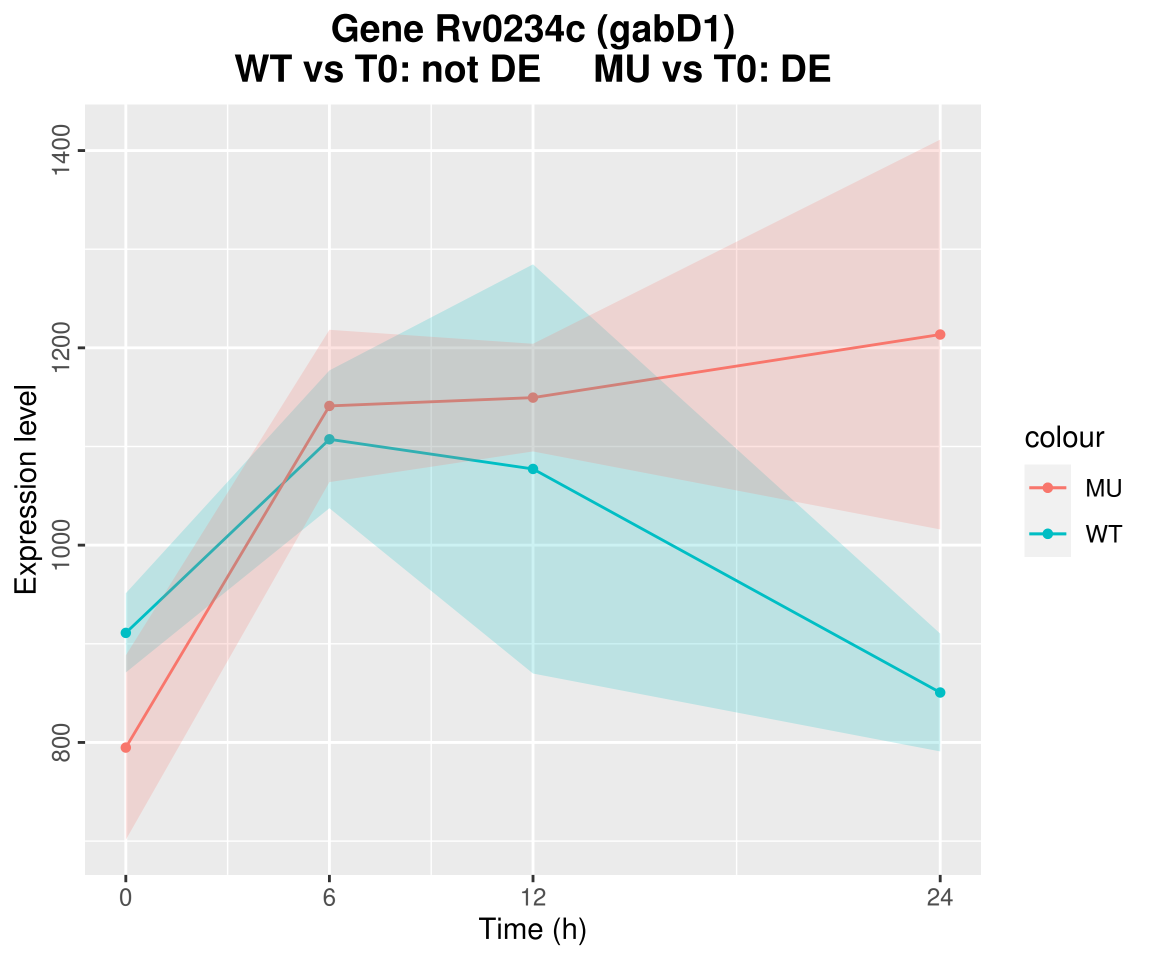

Gene Rv0234c (gabD1)
WT vs T0: not DE   MU vs T0: DE
Expression level
1400
1200
1000
800
0
6
12
24
Time (h)
colour
MU
WT

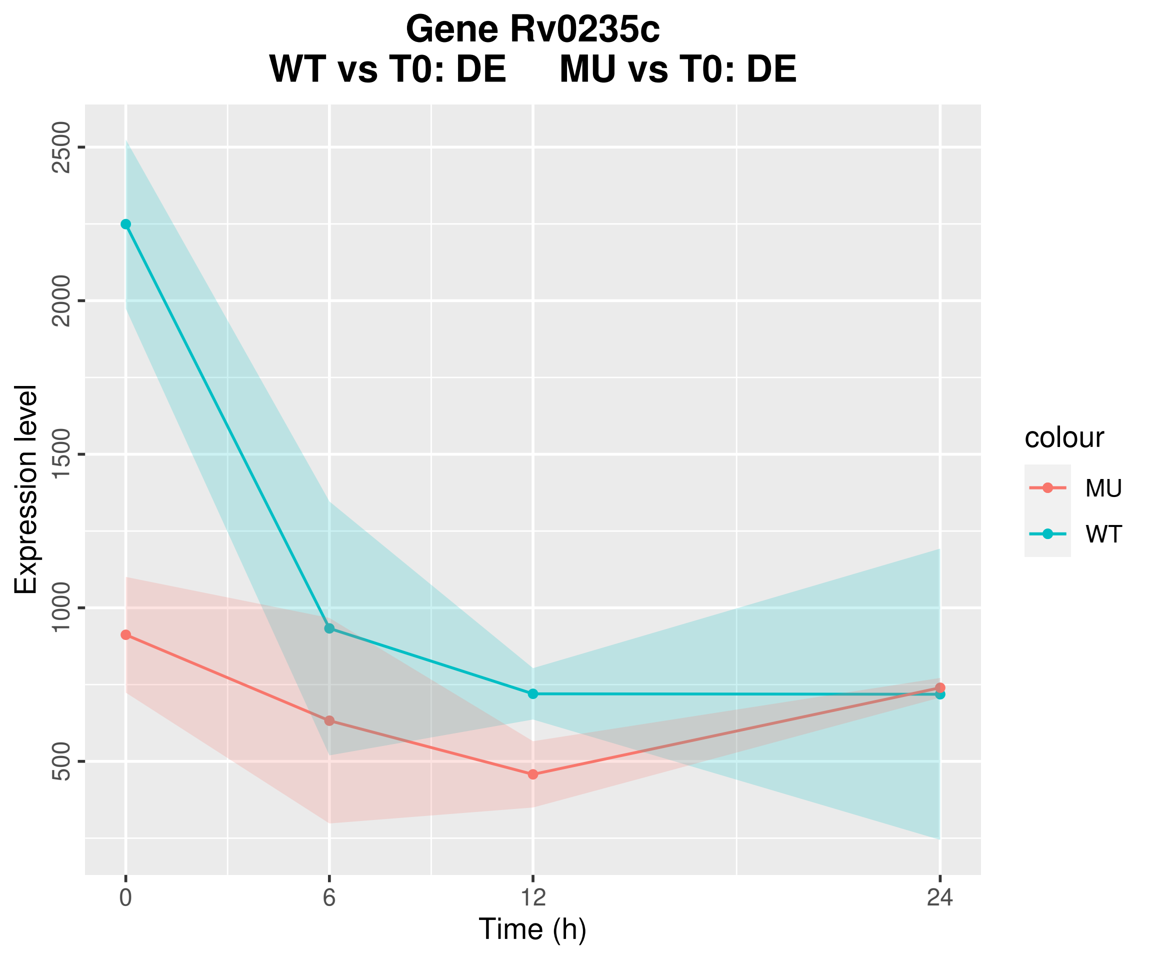

Gene Rv0235c
WT vs T0: DE MU vs T0: DE
Expression level
2500
2000
1500
1000
500
0
6
12
24
Time (h)
colour
MU
WT

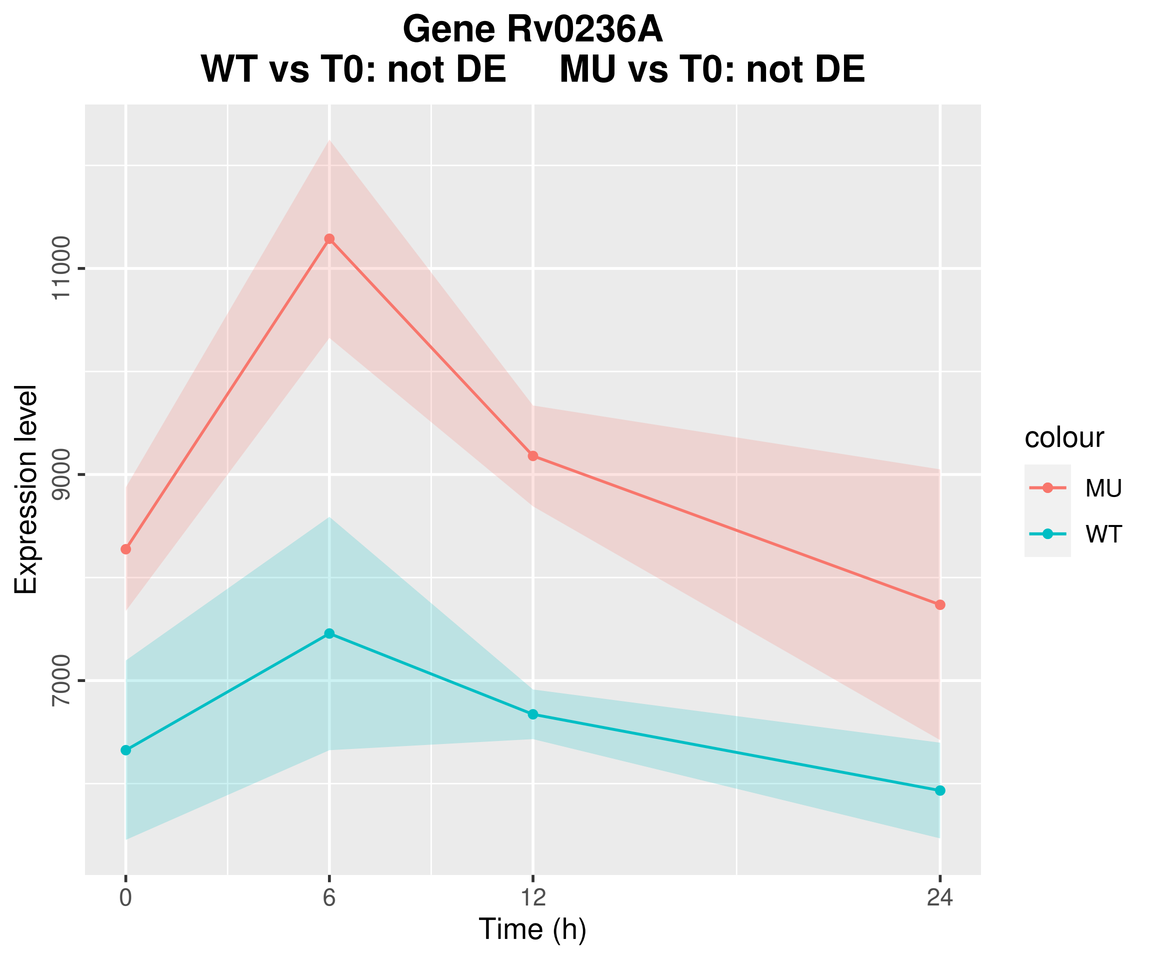

Gene Rv0236A
WT vs T0: not DE   MU vs T0: not DE
Expression level
11000
9000
7000
0
6
12
24
Time (h)
colour
MU
WT

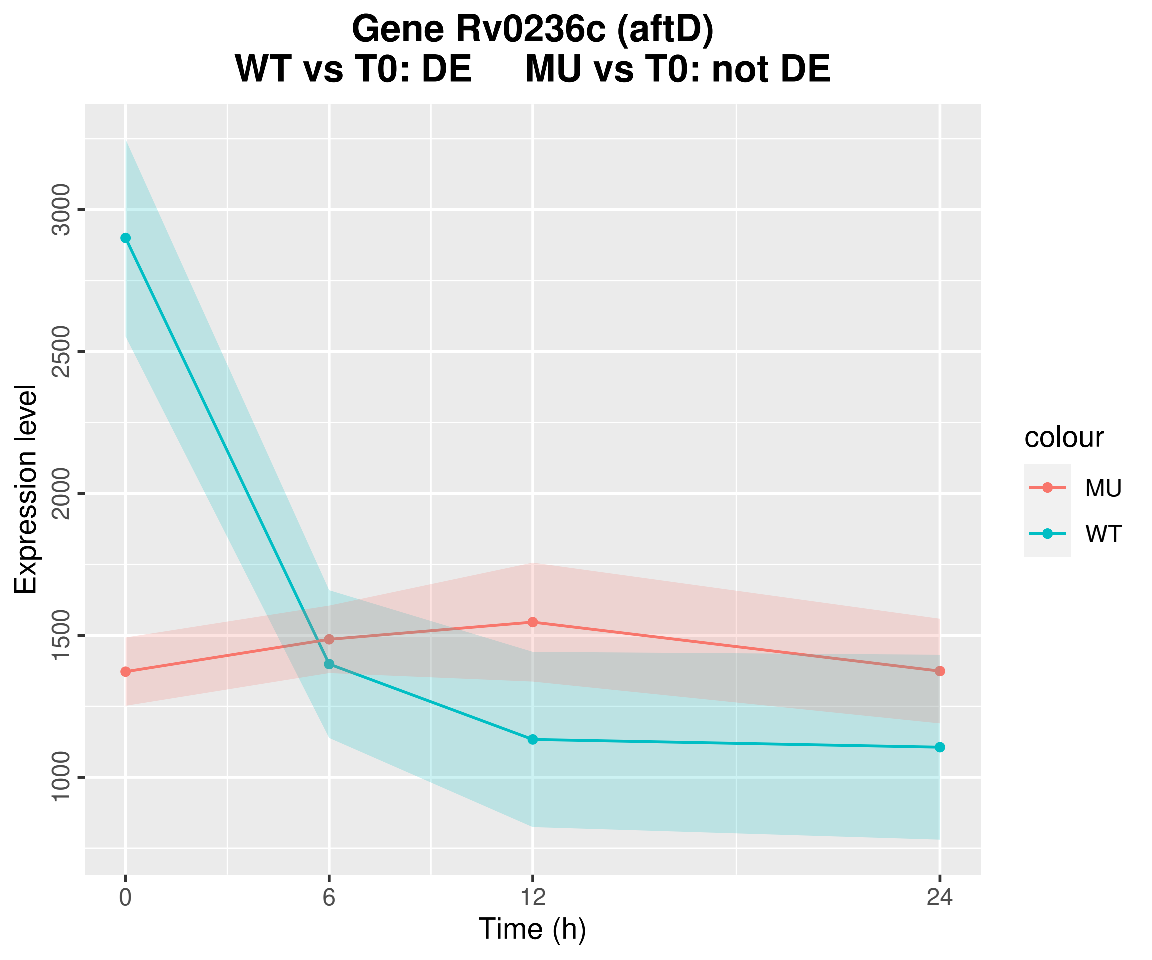
Gene Rv0236c (aftD)
WT vs T0: DE   MU vs T0: not DE
Expression level
3000
2500
2000
1500
1000
0
6
12
24
Time (h)
colour
MU
WT

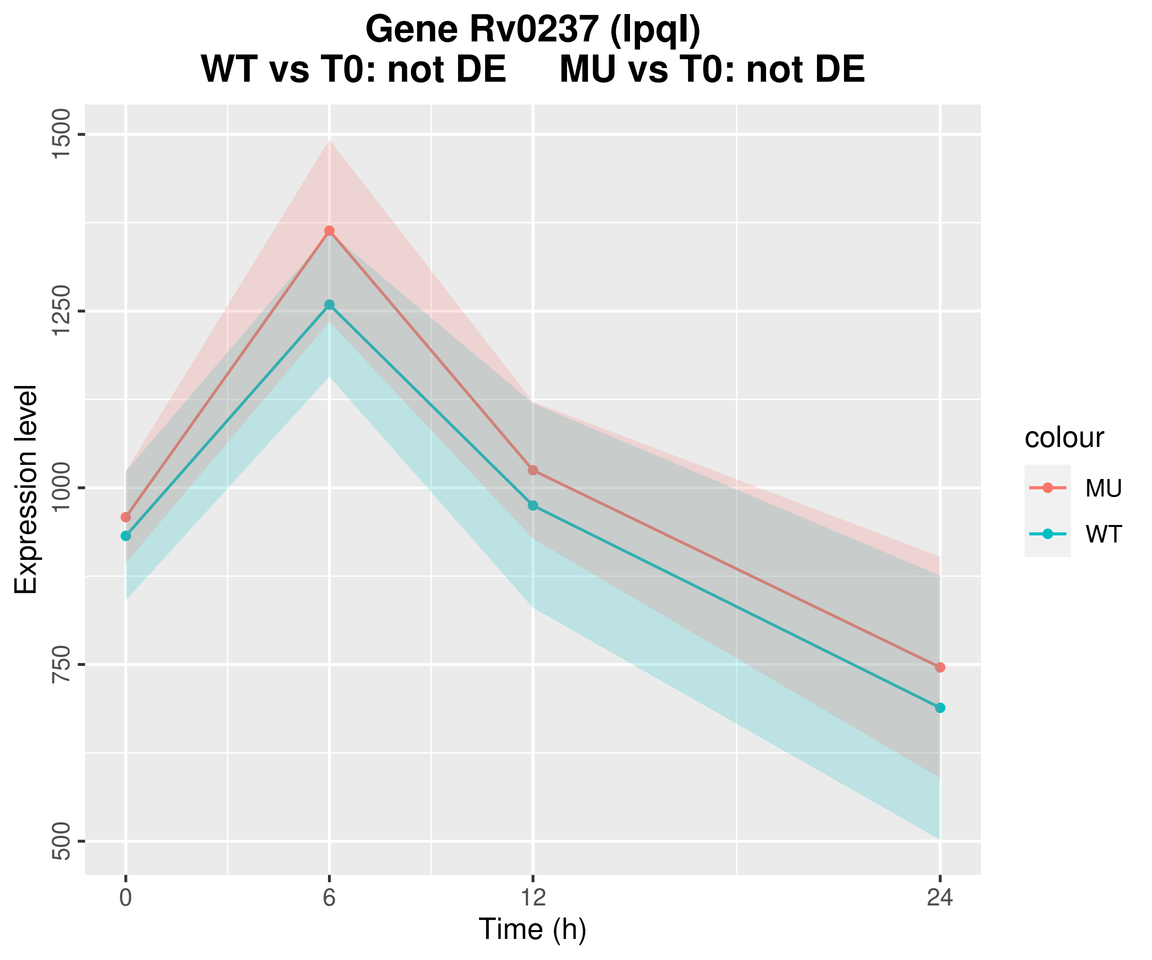
Gene Rv0237 (lpqI)
WT vs T0: not DE MU vs T0: not DE
Expression level
1500
1250
1000
750
500
0
6
12
24
Time (h)
colour
MU
WT

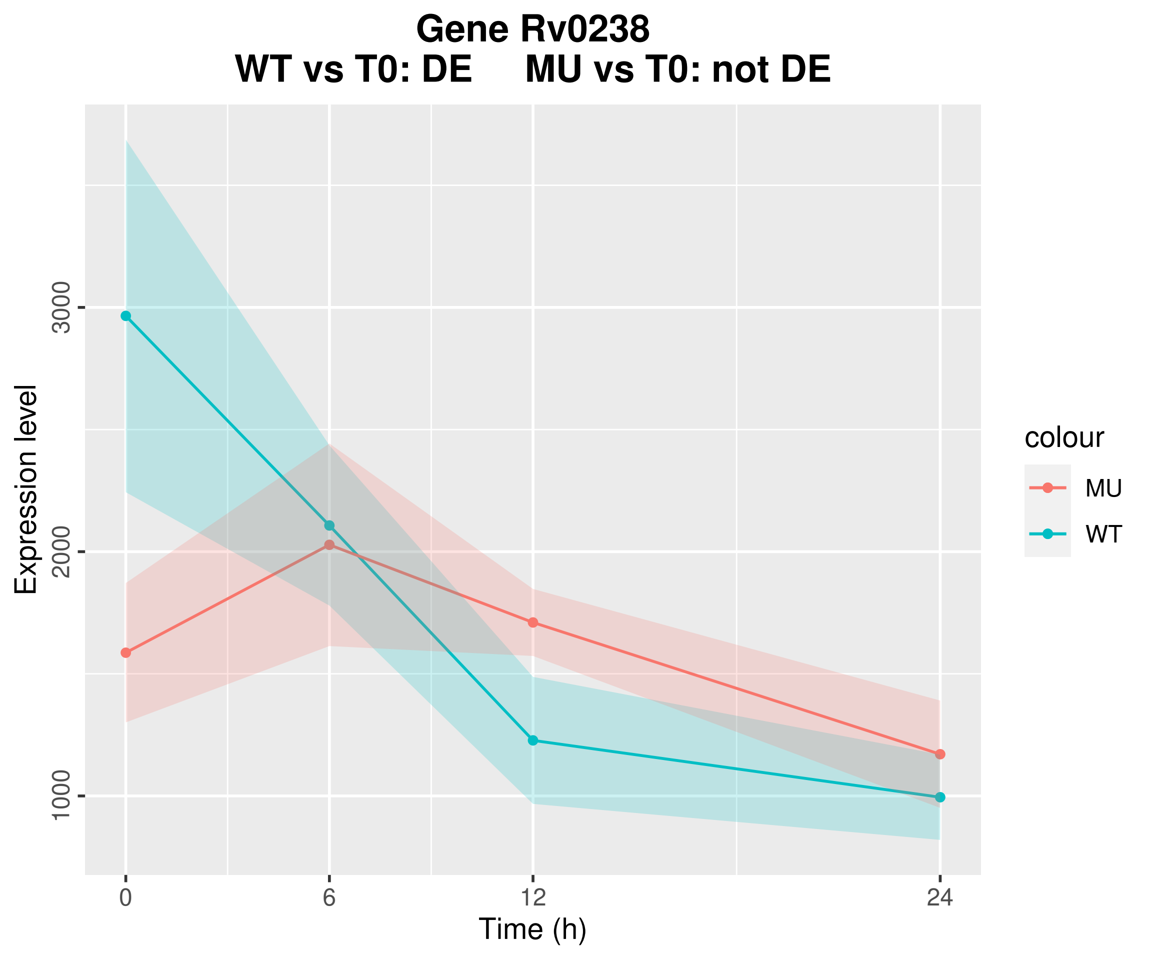

Gene Rv0238
WT vs T0: DE MU vs T0: not DE
Expression level
3000
2000
1000
0
6
12
24
Time (h)
colour
MU
WT

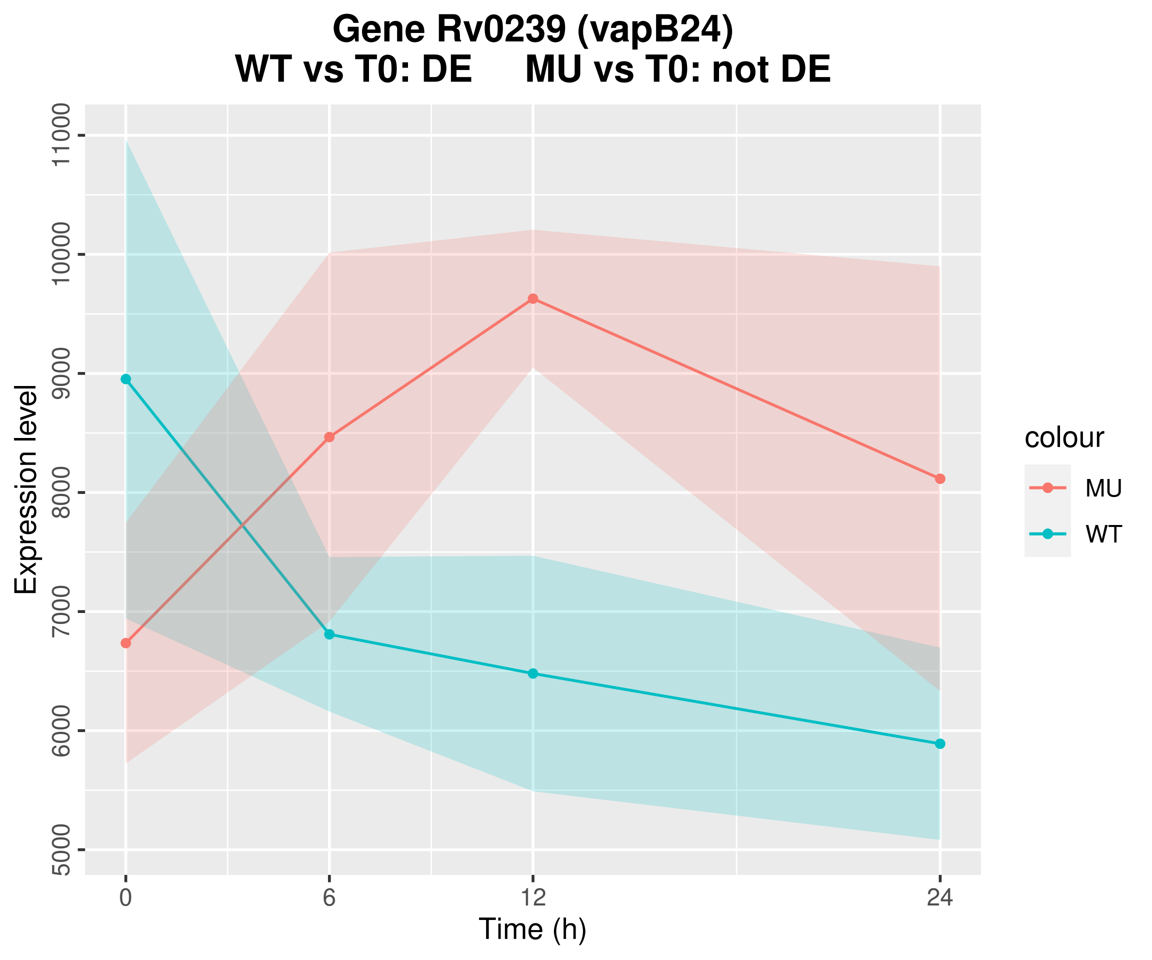

Gene Rv0239 (vapB24)
WT vs T0: DE   MU vs T0: not DE
Expression level
11000
10000
9000
8000
7000
6000
5000
0
6
12
24
Time (h)
colour
MU
WT

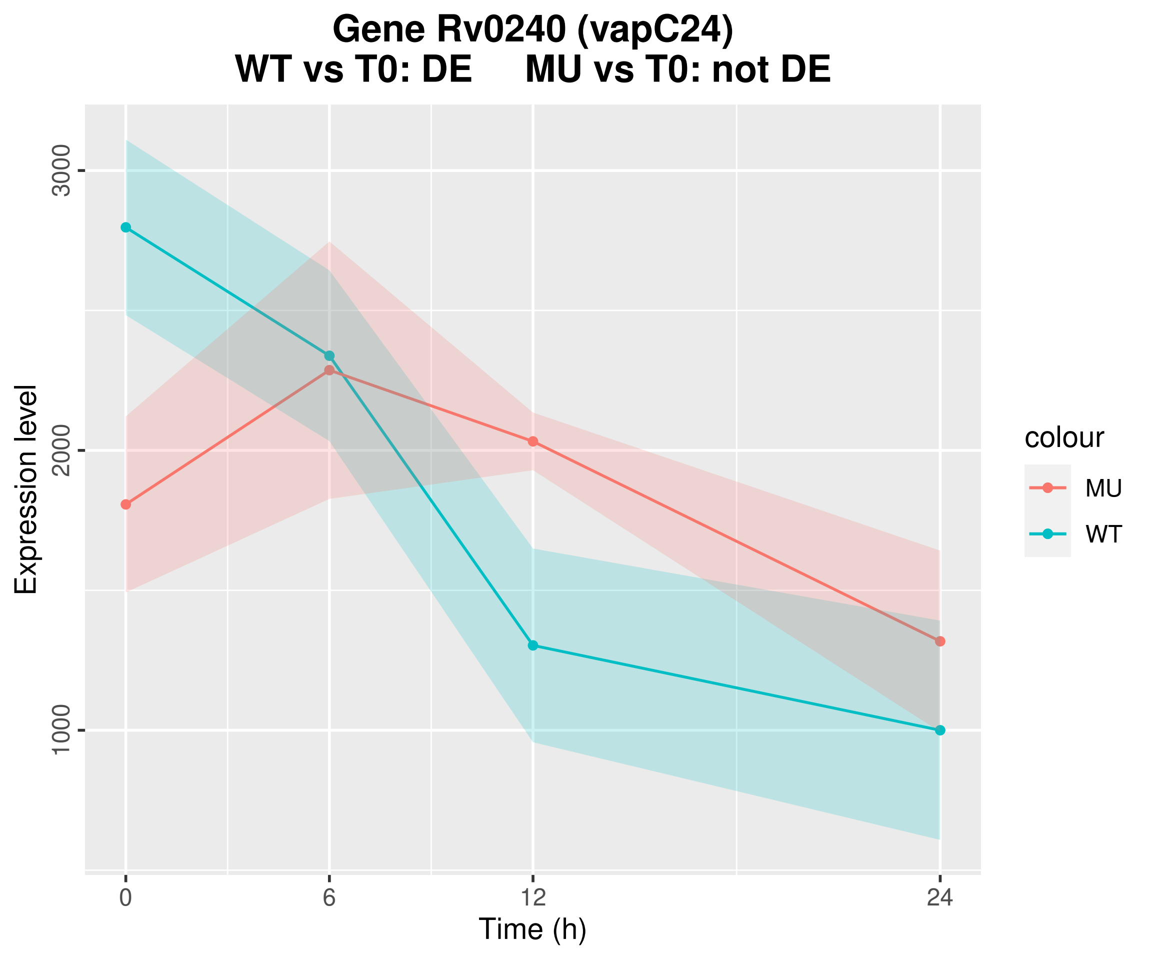

Gene Rv0240 (vapC24)
WT vs T0: DE   MU vs T0: not DE
3000
2000
1000
Expression level
0
6
12
24
Time (h)
colour
MU
WT

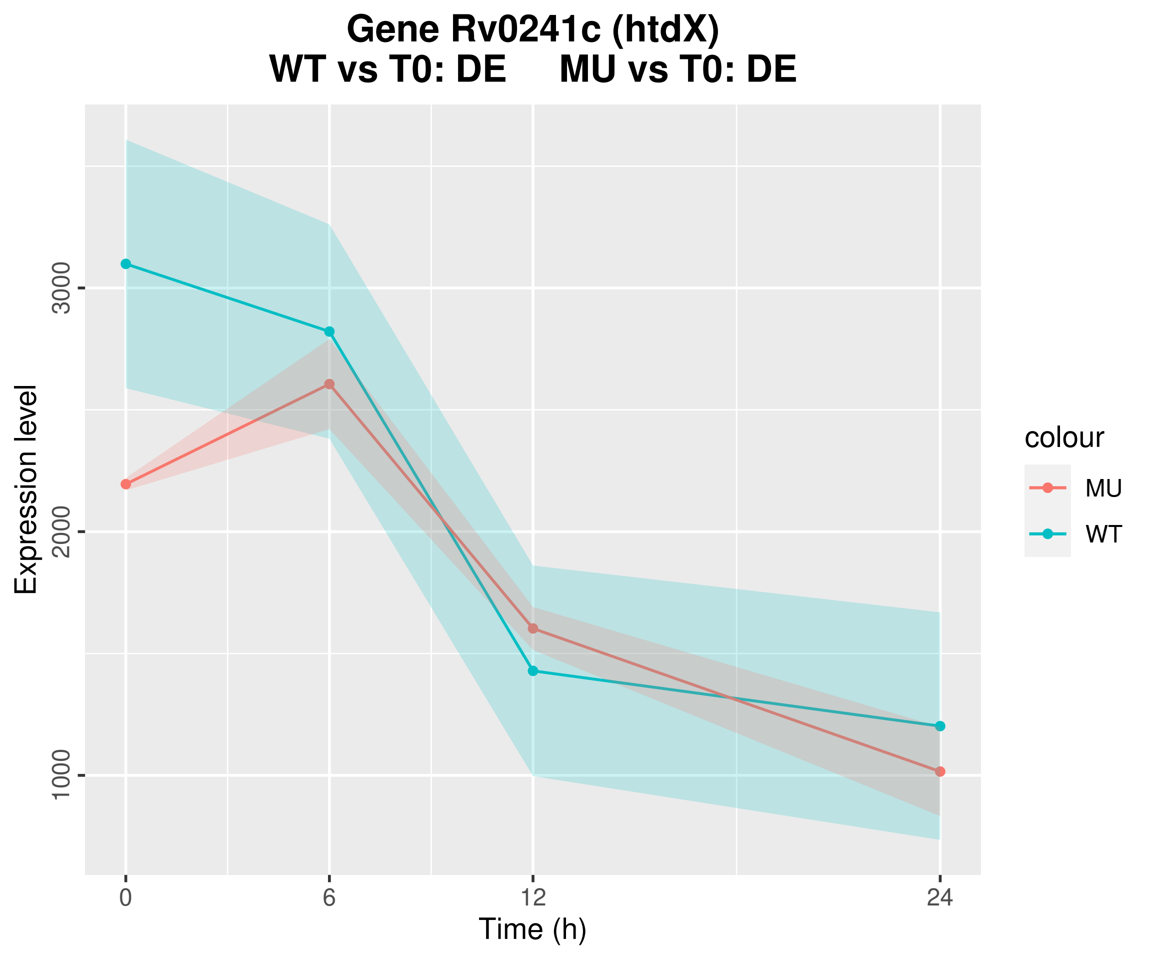

Gene Rv0241c (htdX)
WT vs T0: DE MU vs T0: DE
Expression level
3000
2000
1000
0
6
12
24
Time (h)
colour
MU
WT

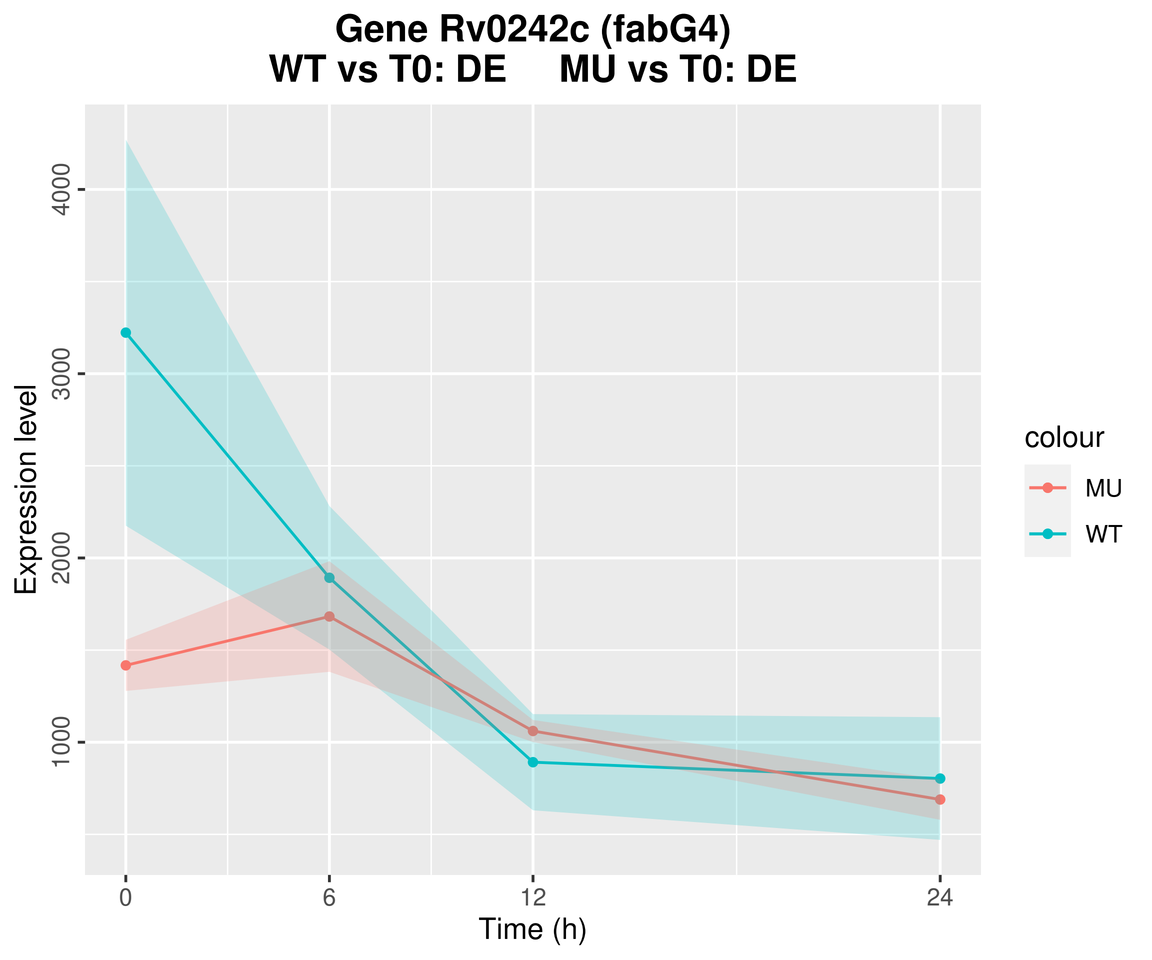

Gene Rv0242c (fabG4)
WT vs T0: DE MU vs T0: DE
Expression level
4000
3000
2000
1000
0
6
12
24
Time (h)
colour
MU
WT

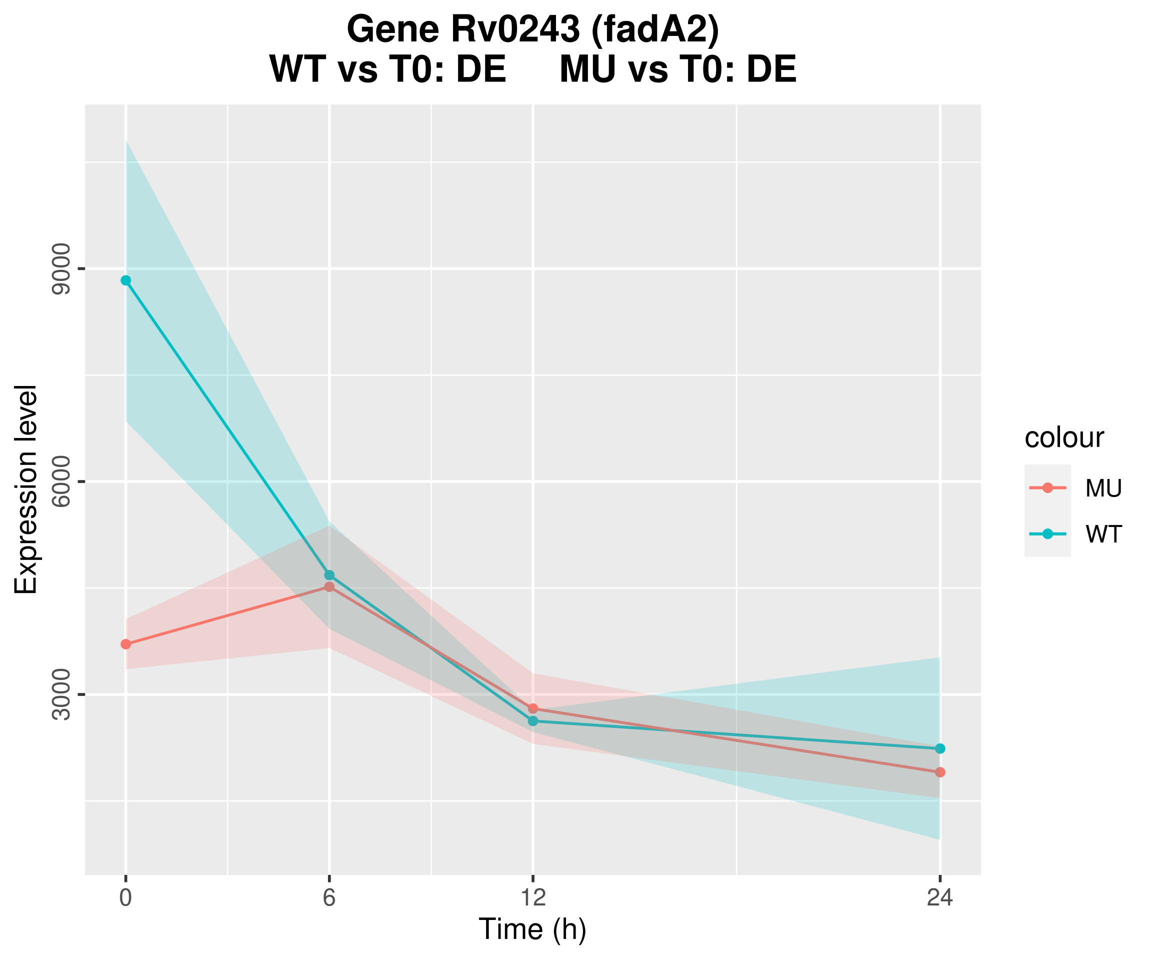

Gene Rv0243 (fadA2)
WT vs T0: DE   MU vs T0: DE
Expression level
9000
6000
3000
0
6
12
24
Time (h)
colour
MU
WT

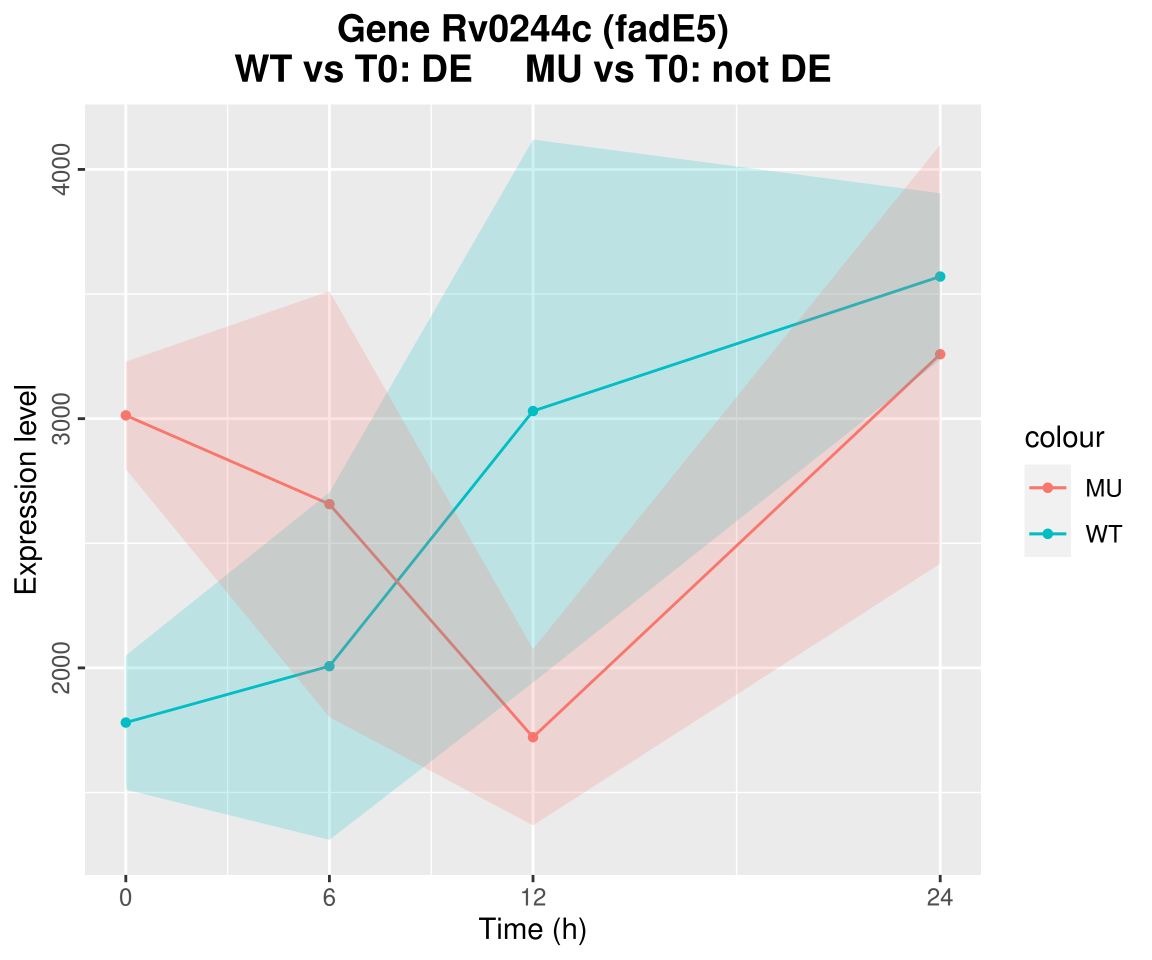
Gene Rv0244c (fadE5)
WT vs T0: DE MU vs T0: not DE
Expression level
4000
3000
2000
0
6
12
24
Time (h)
colour
MU
WT

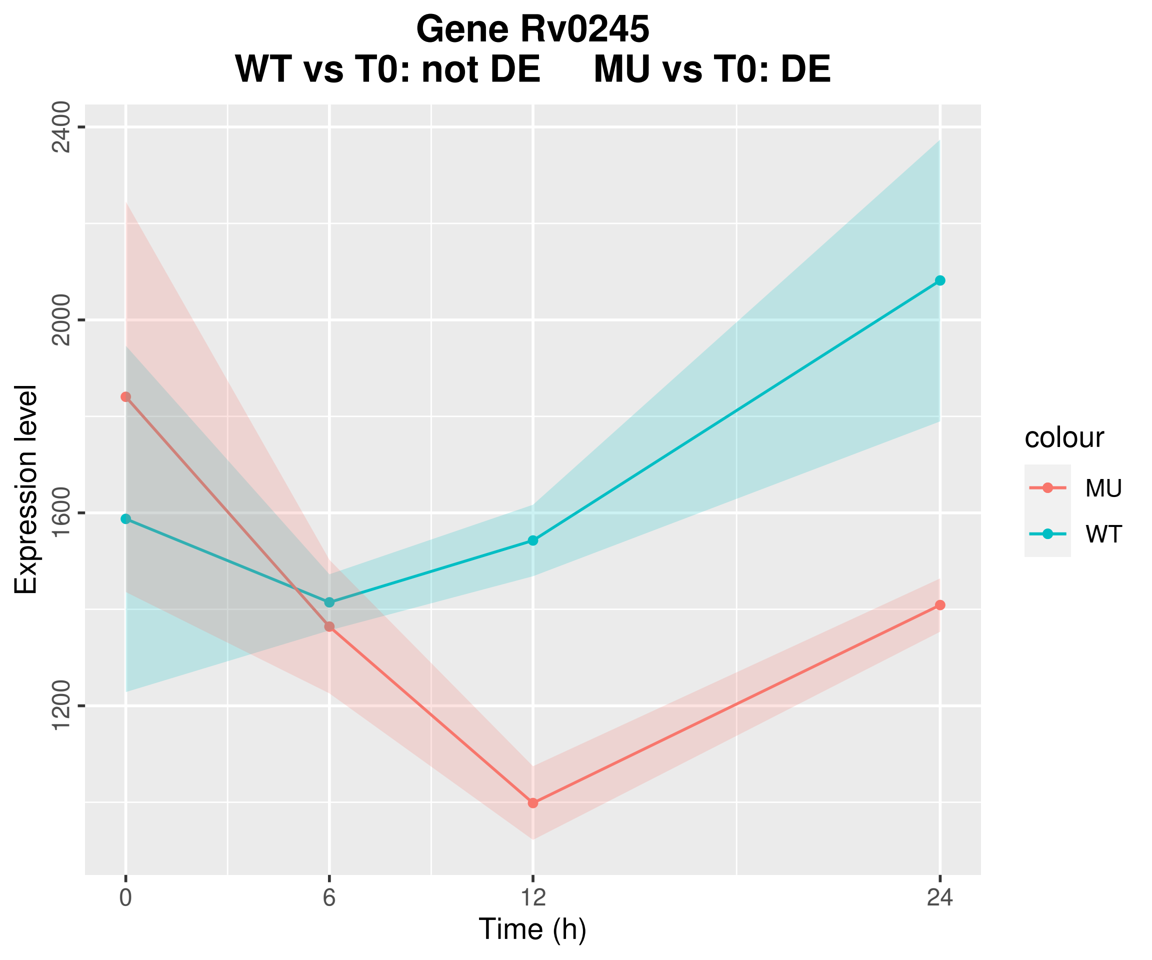
Gene Rv0245
WT vs T0: not DE MU vs T0: DE
Expression level
2400
2000
1600
1200
0
6
12
24
Time (h)
colour
MU
WT

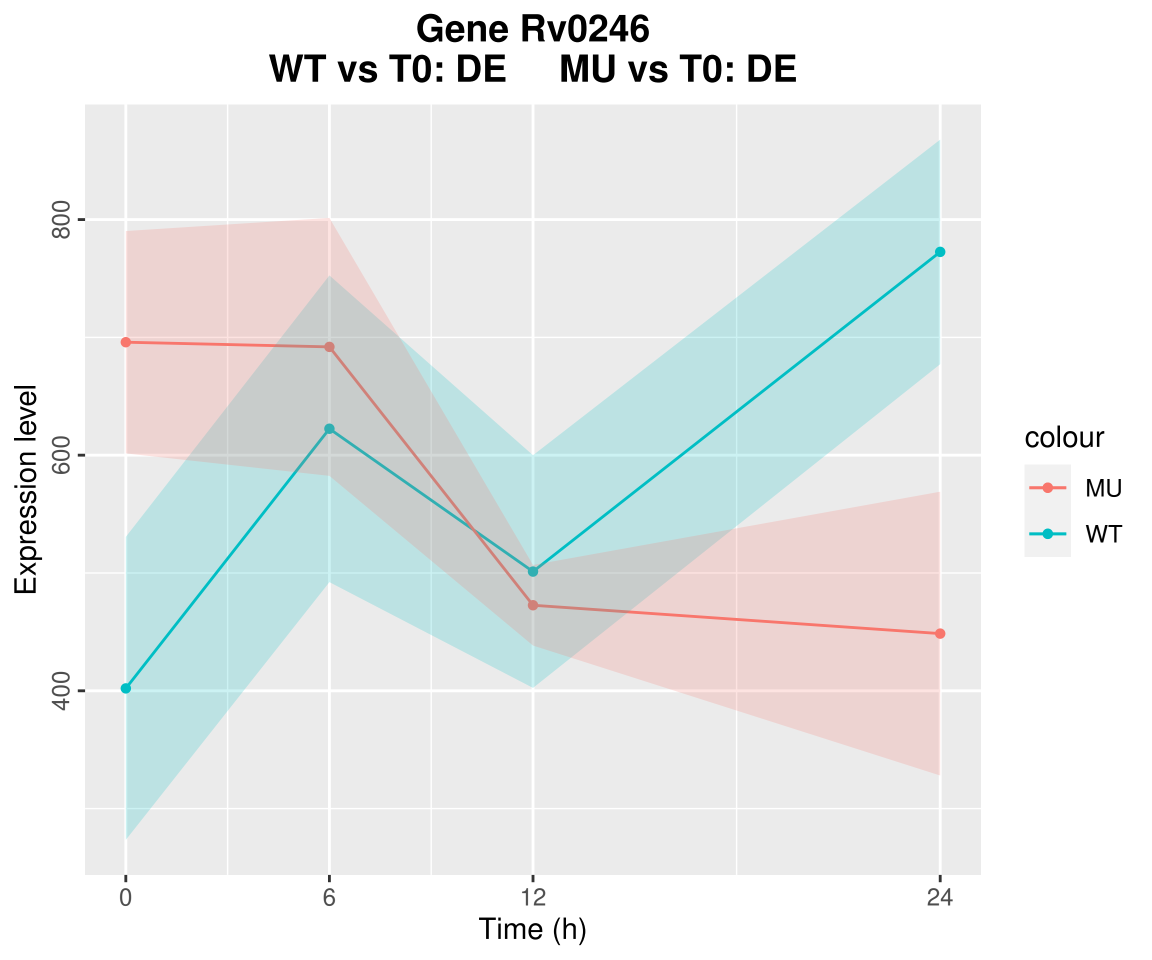
Gene Rv0246
WT vs T0: DE MU vs T0: DE
Expression level
800
600
400
0
6
12
24
Time (h)
colour
MU
WT

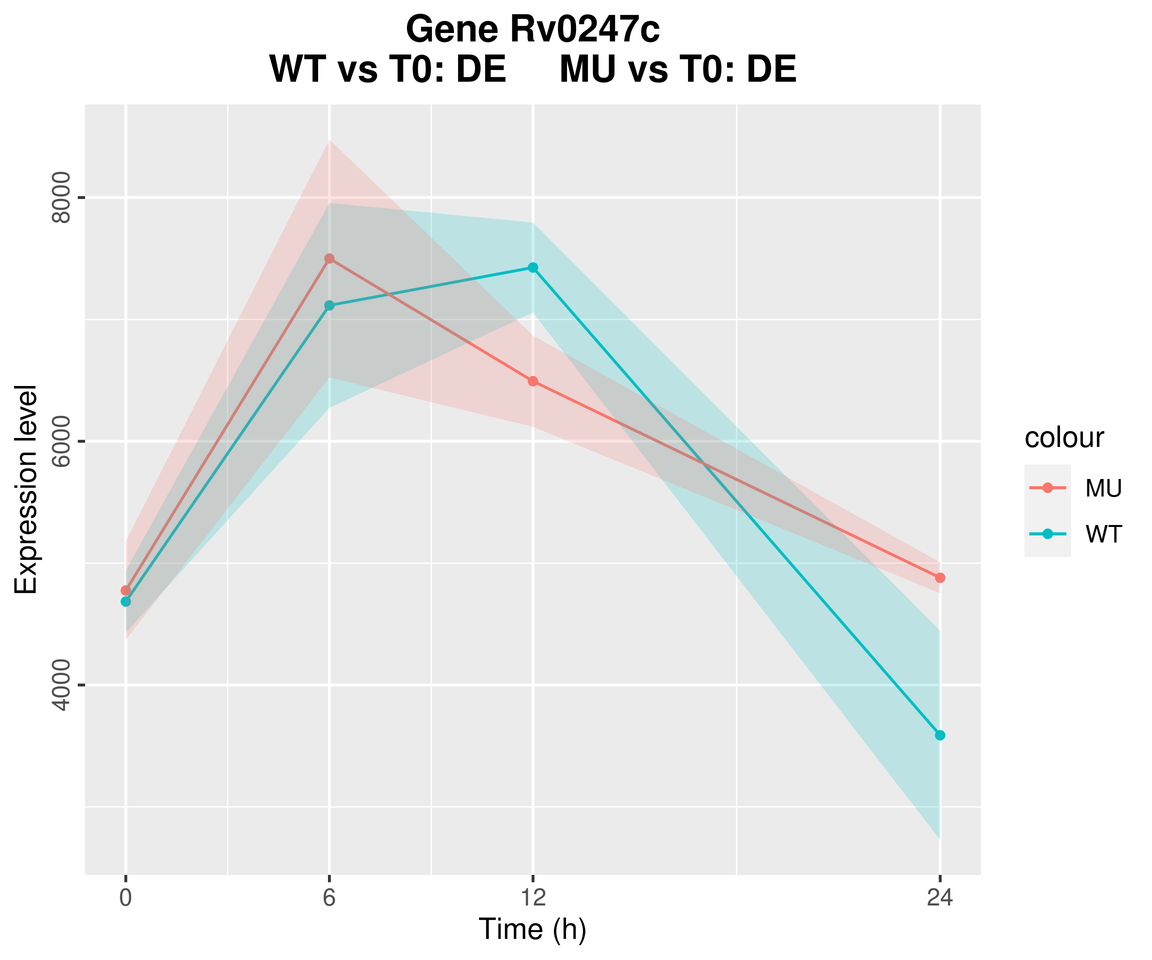

Gene Rv0247c
WT vs T0: DE   MU vs T0: DE
Expression level
8000
6000
4000
0
6
12
24
Time (h)
colour
MU
WT

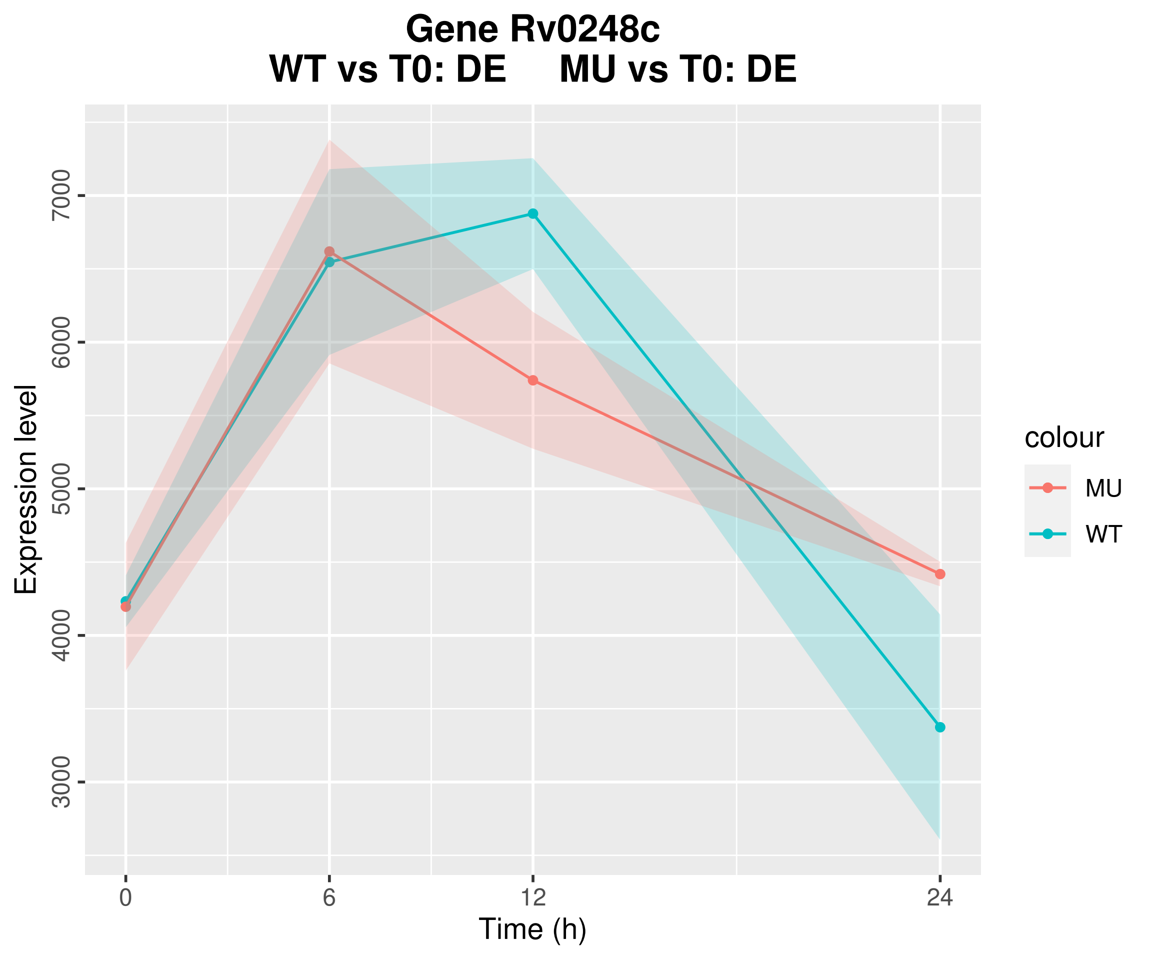

Gene Rv0248c
WT vs T0: DE MU vs T0: DE
Expression level
7000
6000
5000
4000
3000
0
6
12
24
Time (h)
colour
MU
WT

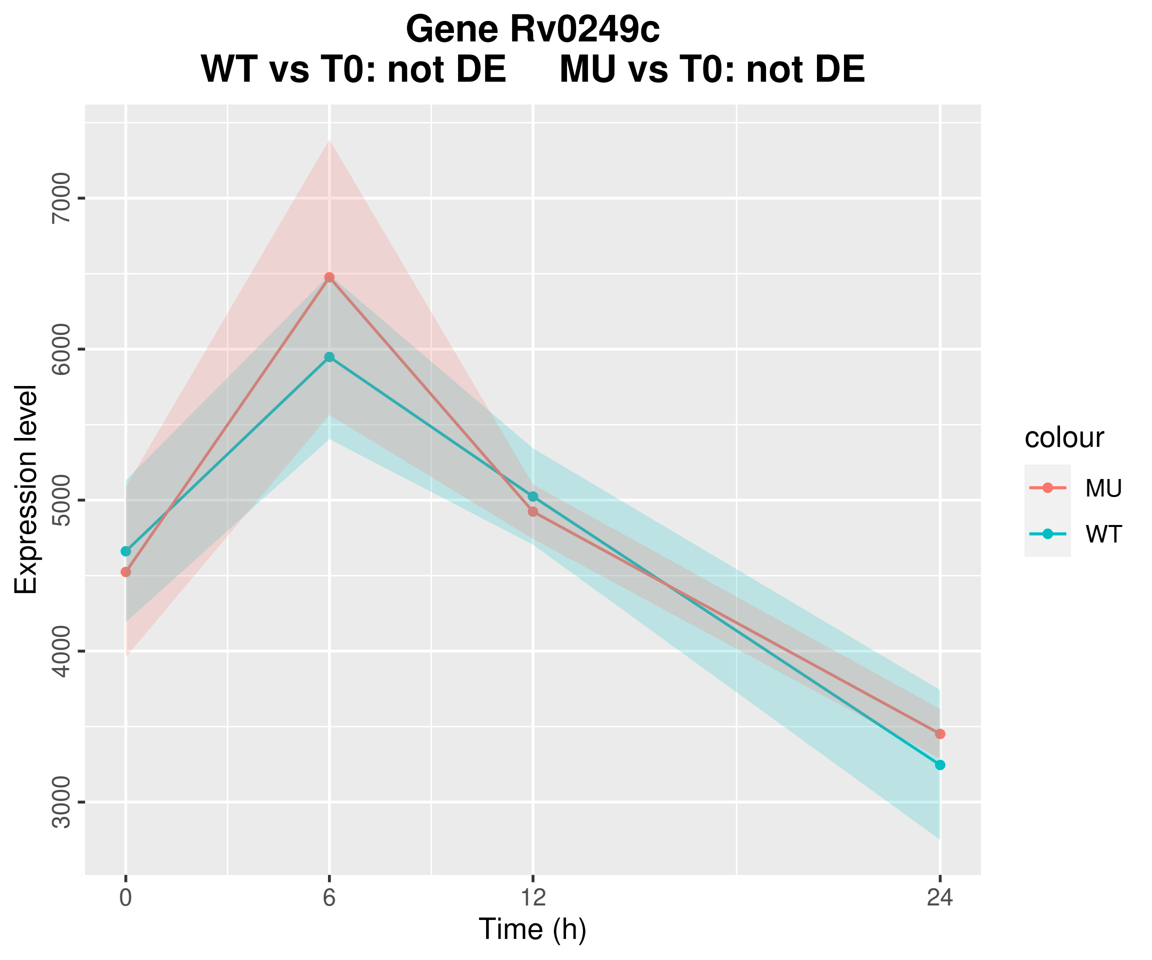
Gene Rv0249c
WT vs T0: not DE   MU vs T0: not DE
Expression level
7000
6000
5000
4000
3000
0
6
12
24
Time (h)
colour
MU
WT

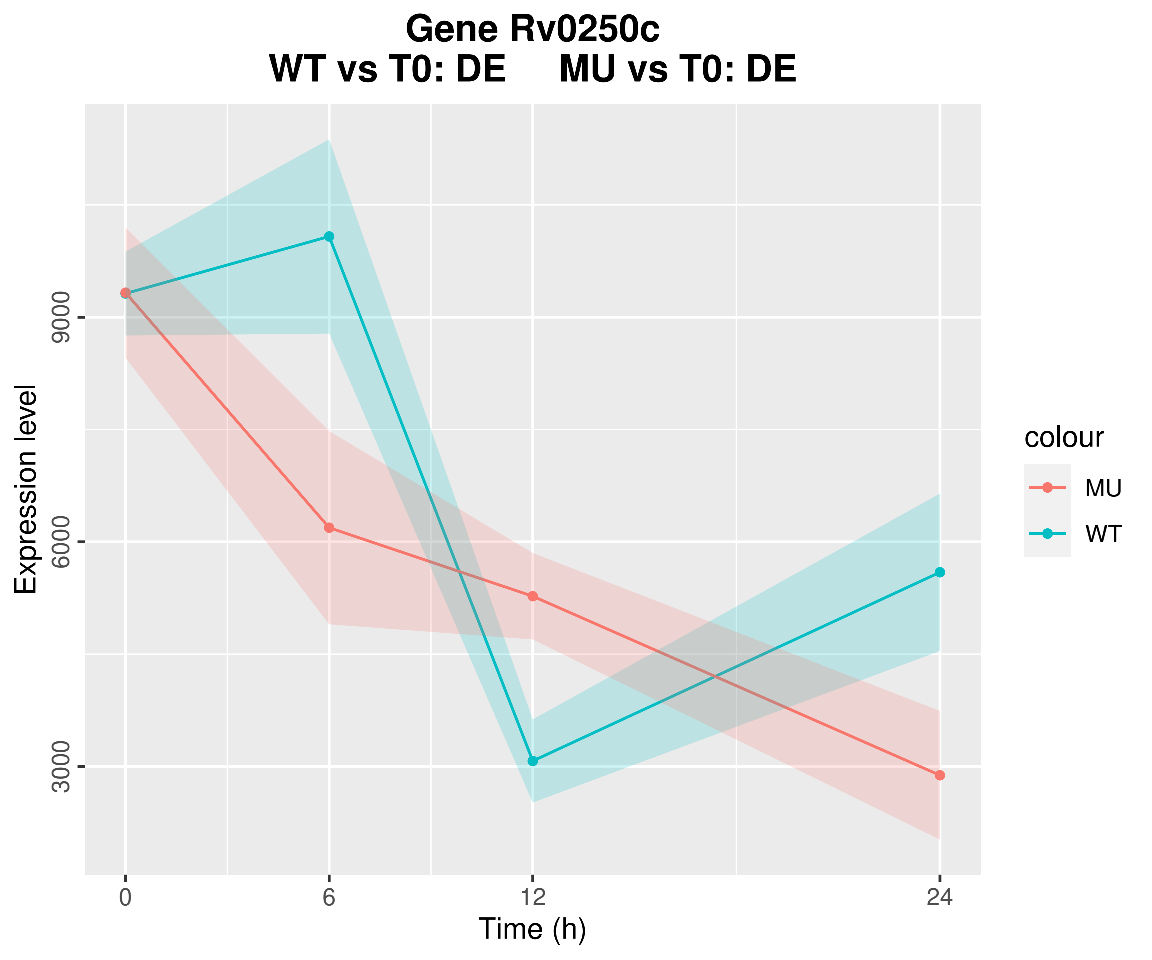
Gene Rv0250c
WT vs T0: DE   MU vs T0: DE
Expression level
9000
6000
3000
0
6
12
24
Time (h)
colour
MU
WT

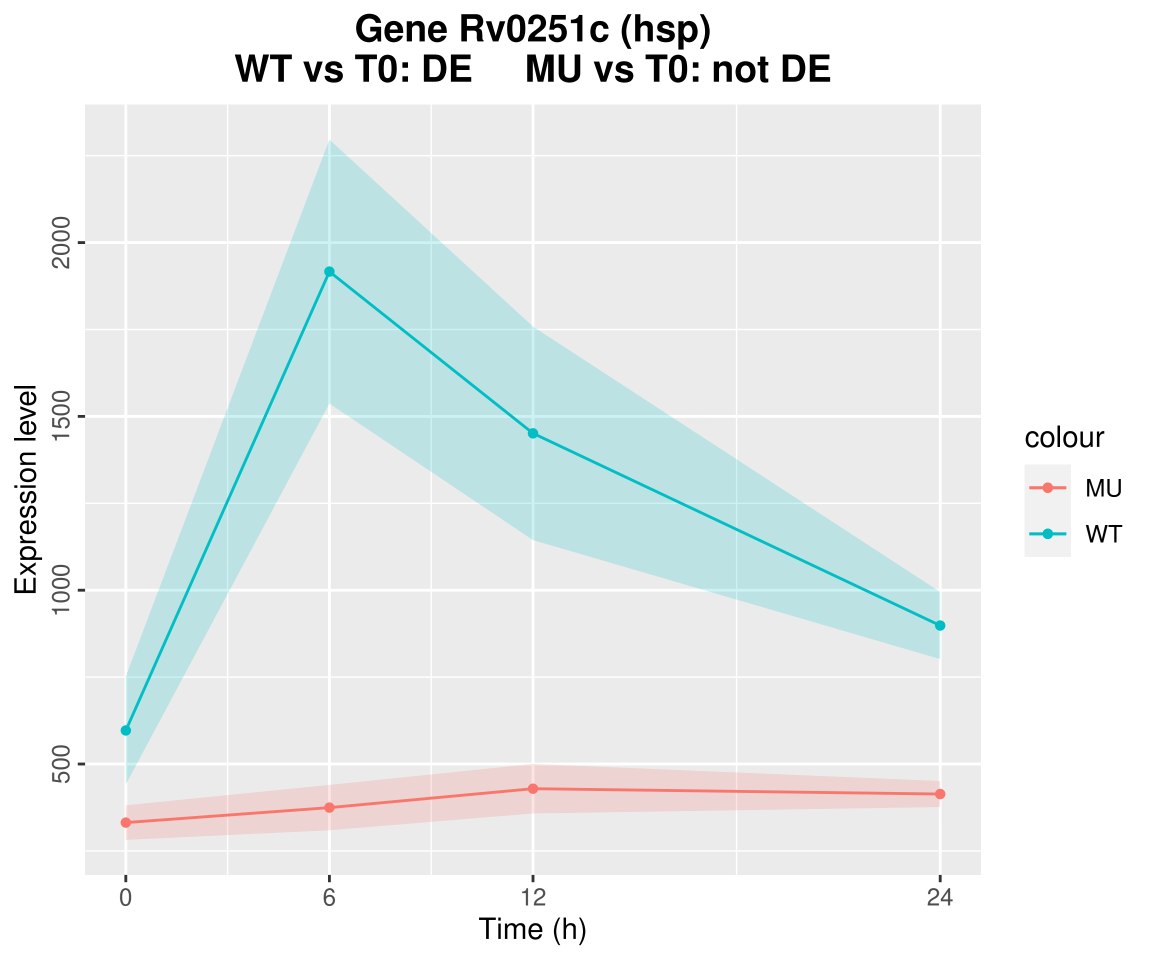

Gene Rv0251c (hsp)
WT vs T0: DE   MU vs T0: not DE
Expression level
2000
1500
1000
500
0
6
12
24
Time (h)
colour
MU
WT

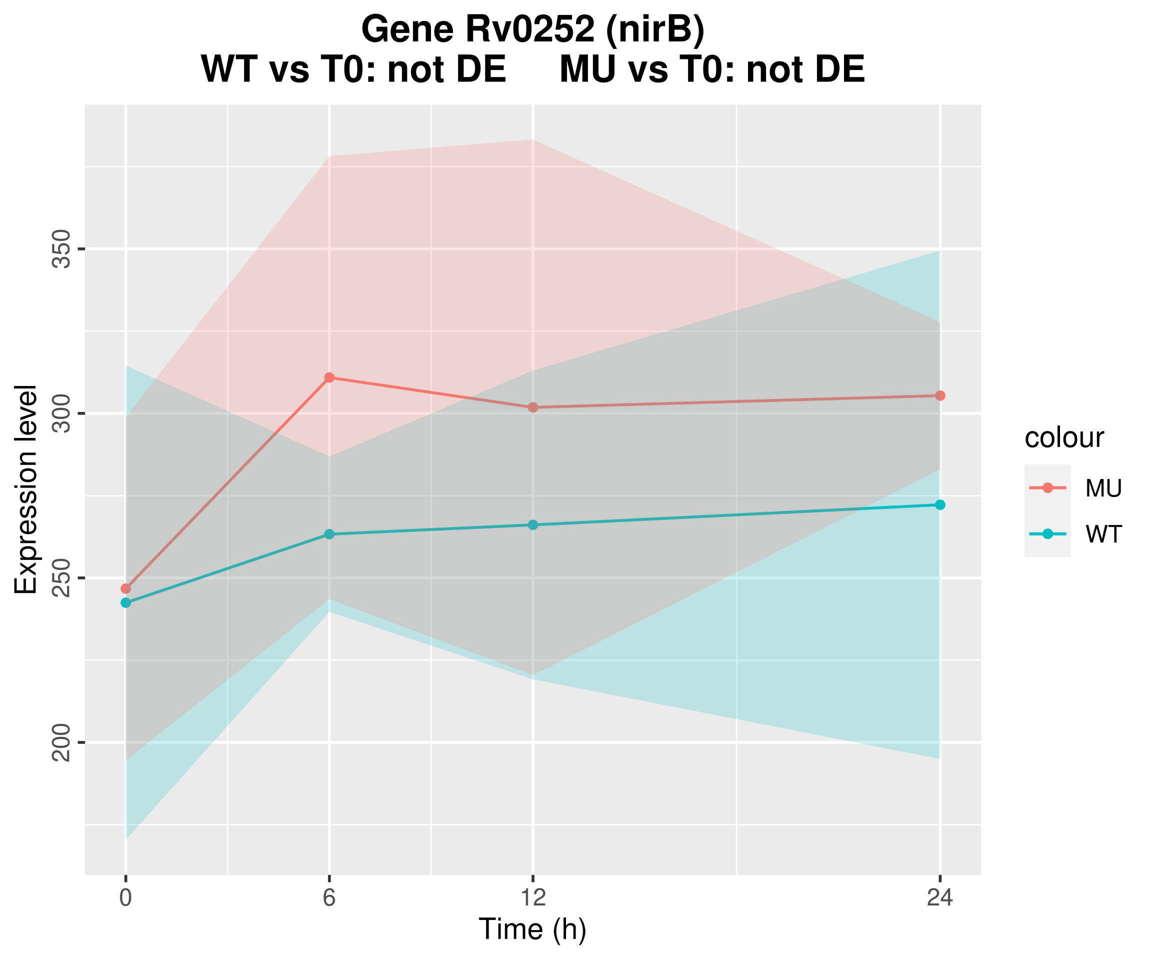

Gene Rv0252 (nirB)
WT vs T0: not DE   MU vs T0: not DE
Expression level
350
300
250
200
0
6
12
24
Time (h)
colour
MU
WT

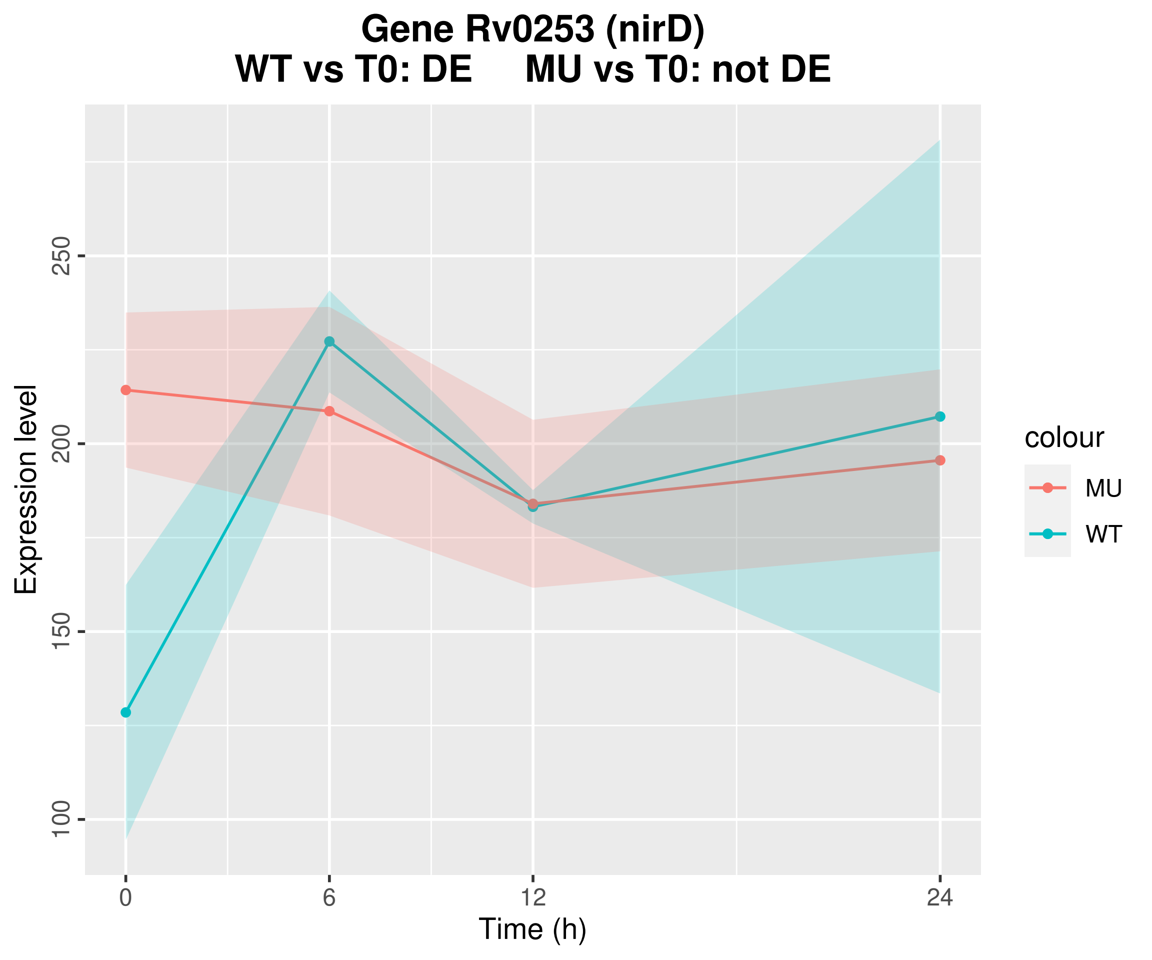
Gene Rv0253 (nirD)
WT vs T0: DE   MU vs T0: not DE
Expression level
250
200
150
100
0
6
12
24
Time (h)
colour
MU
WT

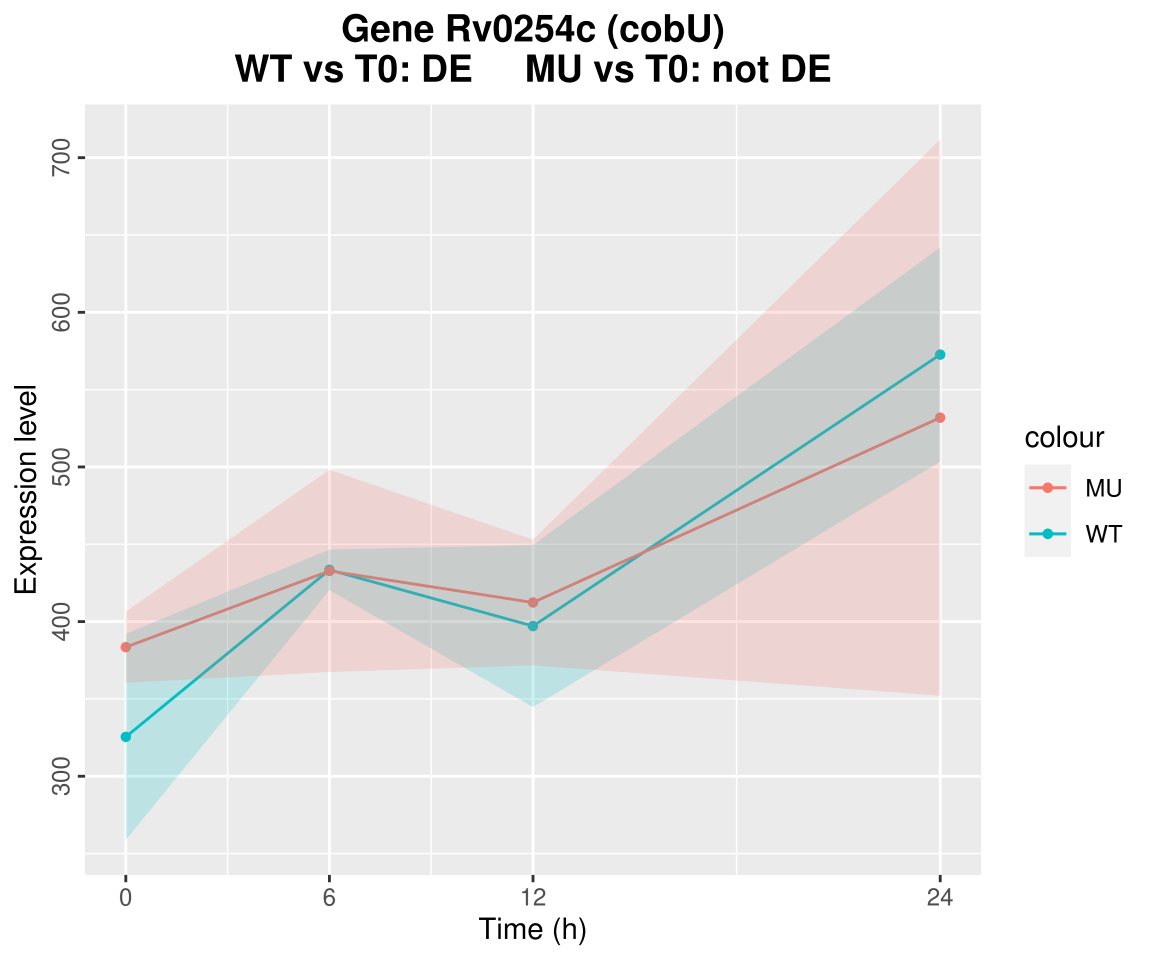

Gene Rv0254c (cobU)
WT vs T0: DE   MU vs T0: not DE
Expression level
700
600
500
400
300
0
6
12
24
Time (h)
colour
MU
WT

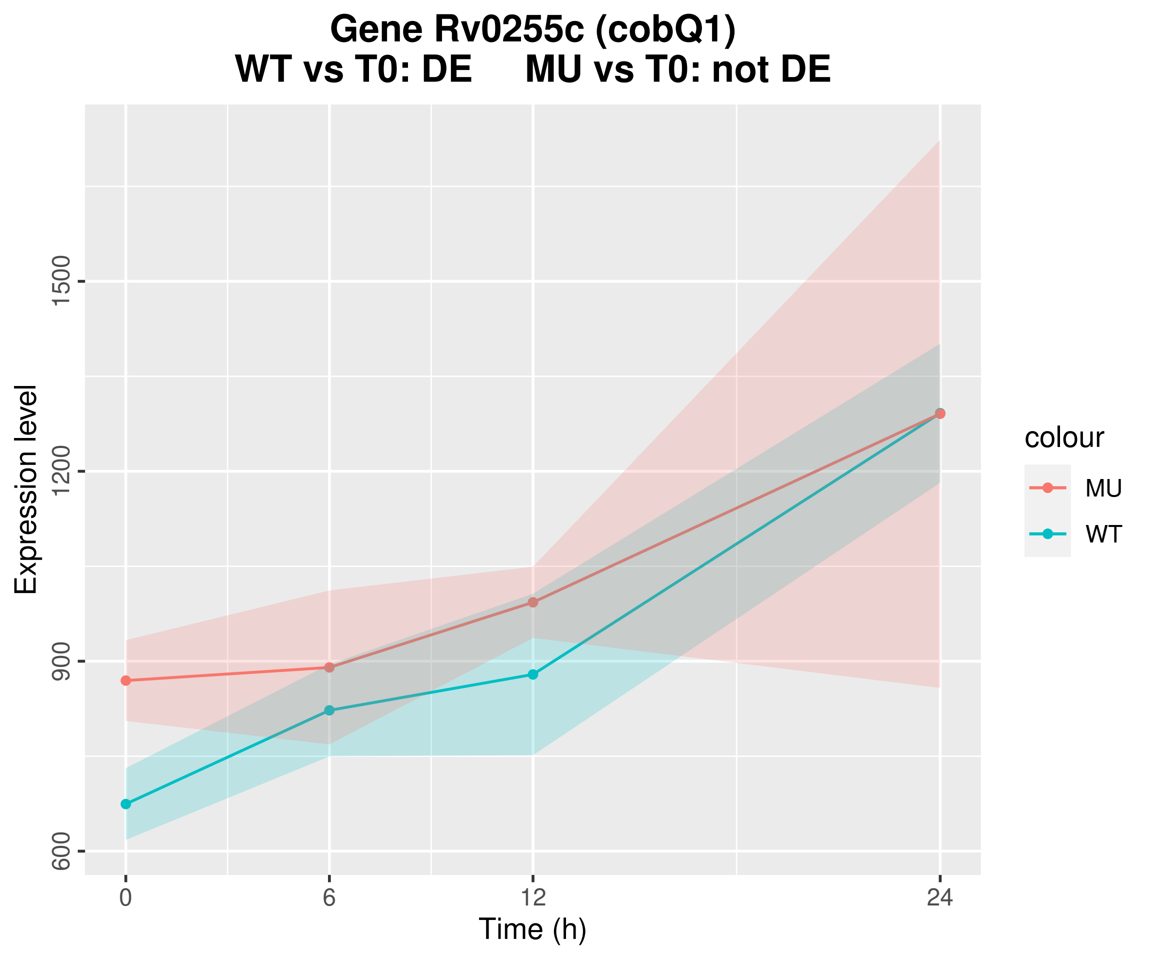
Gene Rv0255c (cobQ1)
WT vs T0: DE   MU vs T0: not DE
Expression level
1500
1200
900
600
0
6
12
24
Time (h)
colour
MU
WT

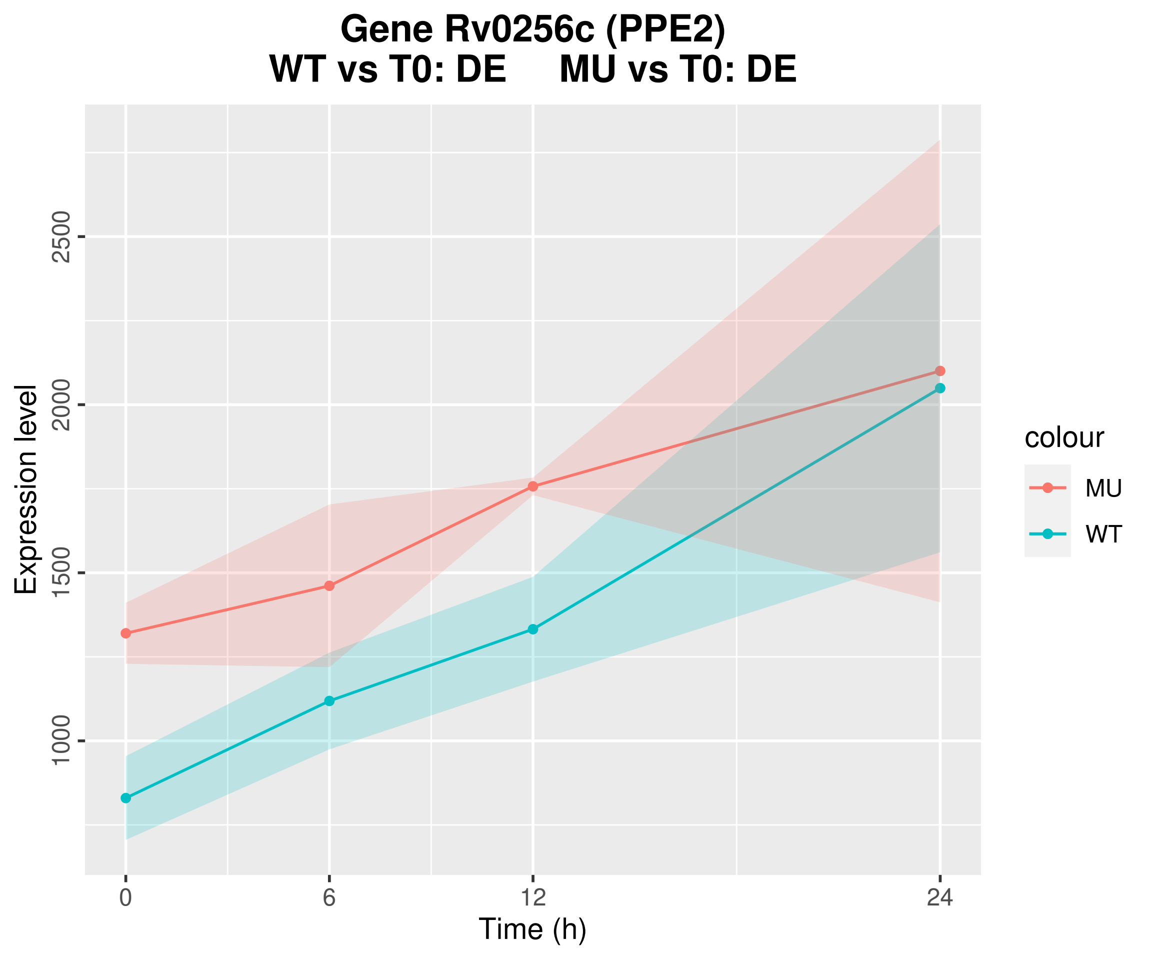
Gene Rv0256c (PPE2)
WT vs T0: DE MU vs T0: DE
Expression level
2500
2000
1500
1000
0
6
12
24
Time (h)
colour
MU
WT

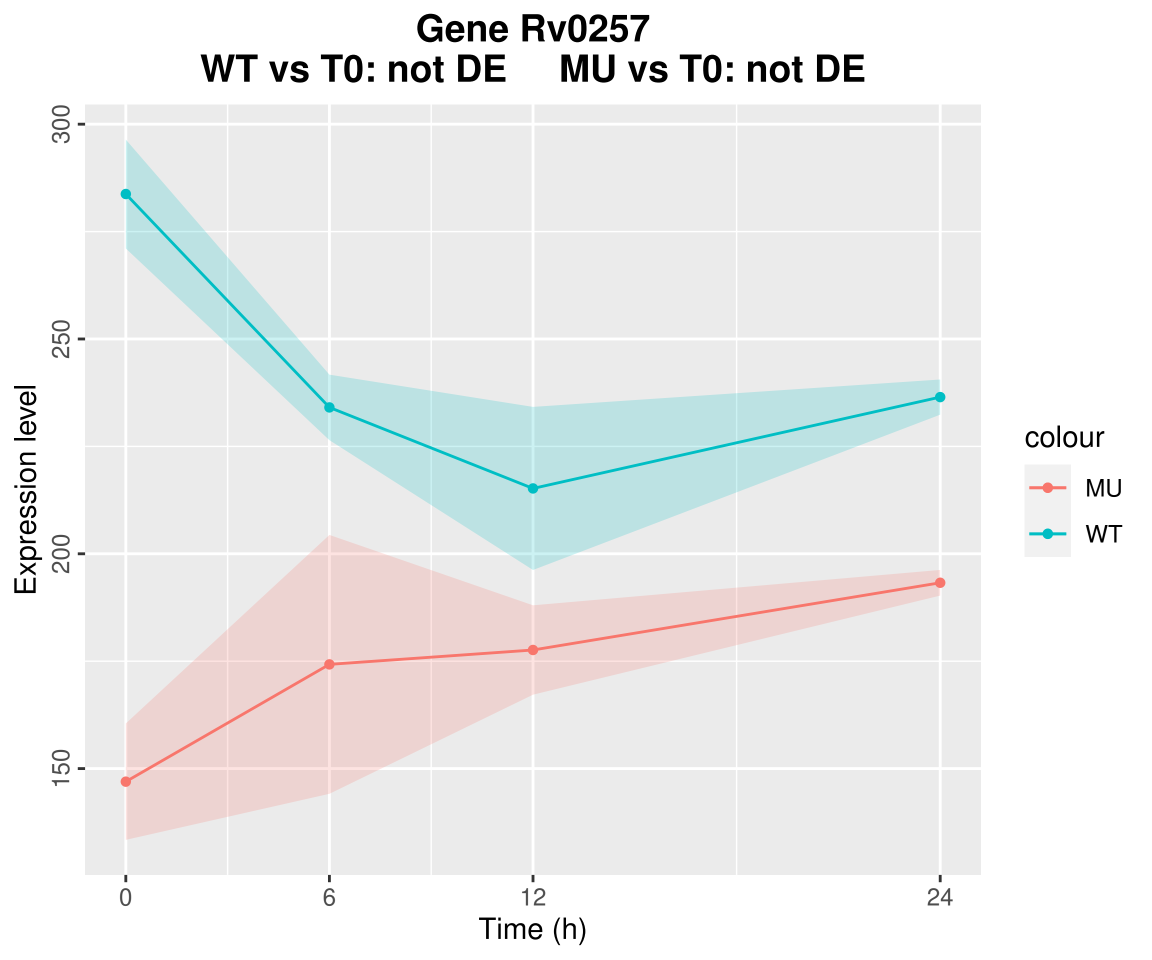
Gene Rv0257
WT vs T0: not DE   MU vs T0: not DE
Expression level
300
250
200
150
0
6
12
24
Time (h)
colour
MU
WT

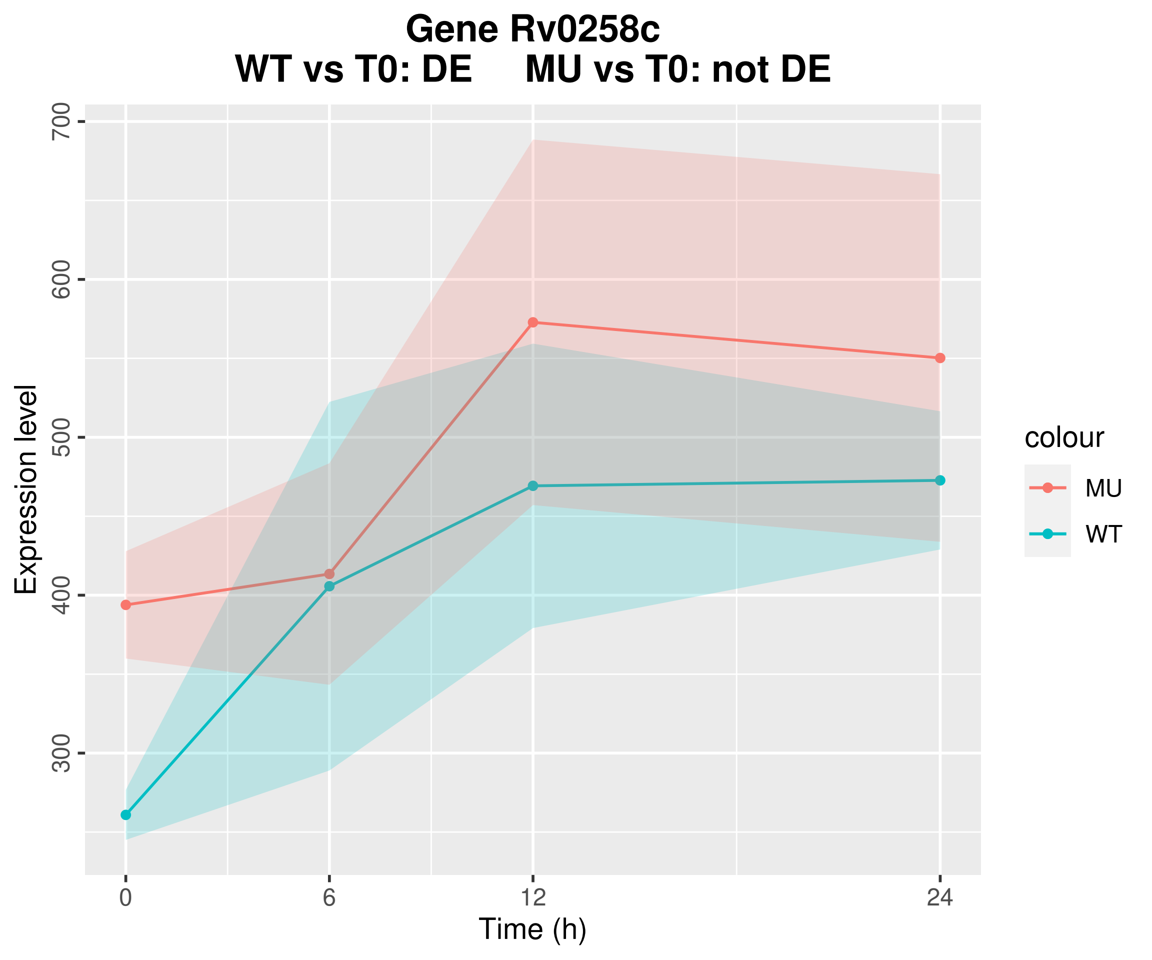
Gene Rv0258c
WT vs T0: DE MU vs T0: not DE
Expression level
700
600
500
400
300
0
6
12
24
Time (h)
colour
MU
WT

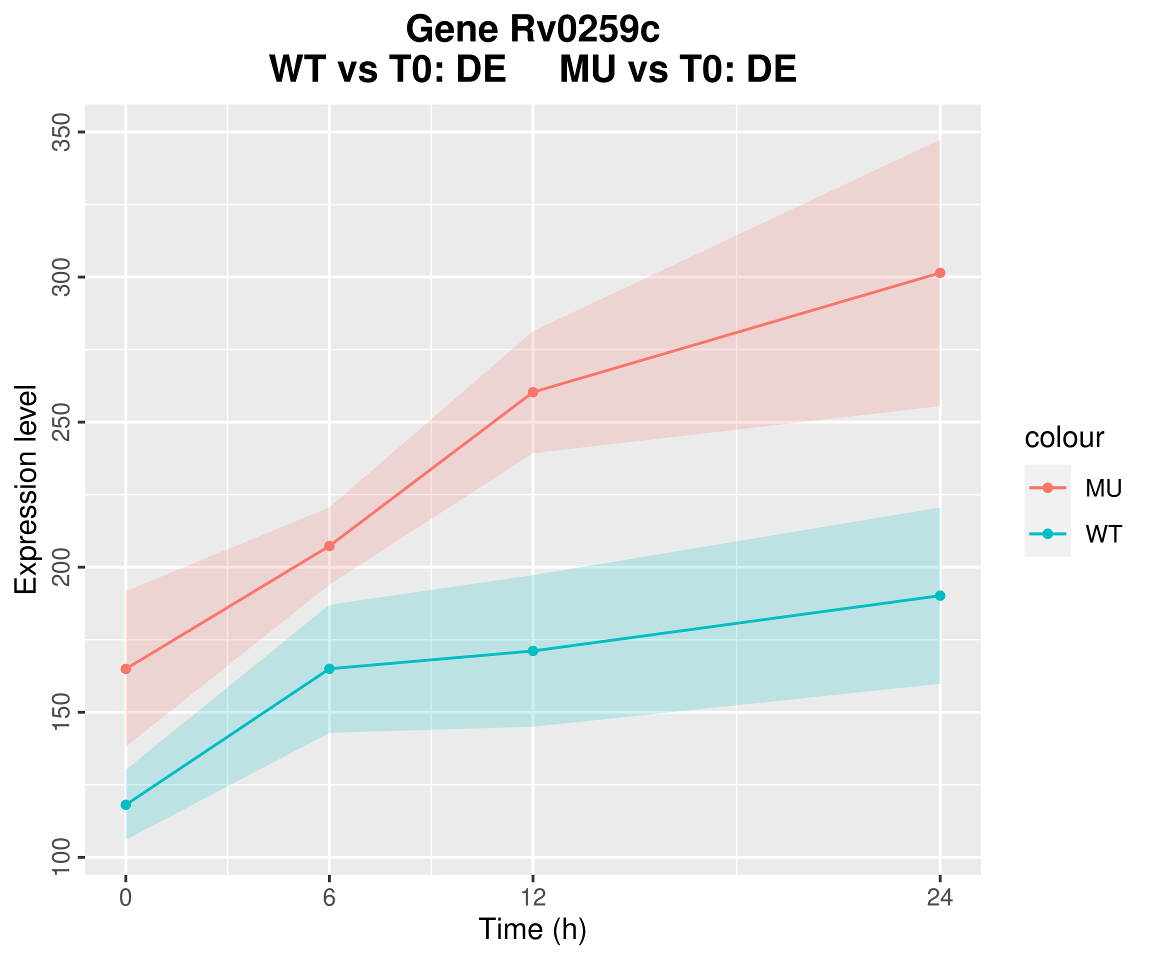
Gene Rv0259c
WT vs T0: DE   MU vs T0: DE
Expression level
Time (h)
100
150
200
250
300
350
0
6
12
24
colour
MU
WT

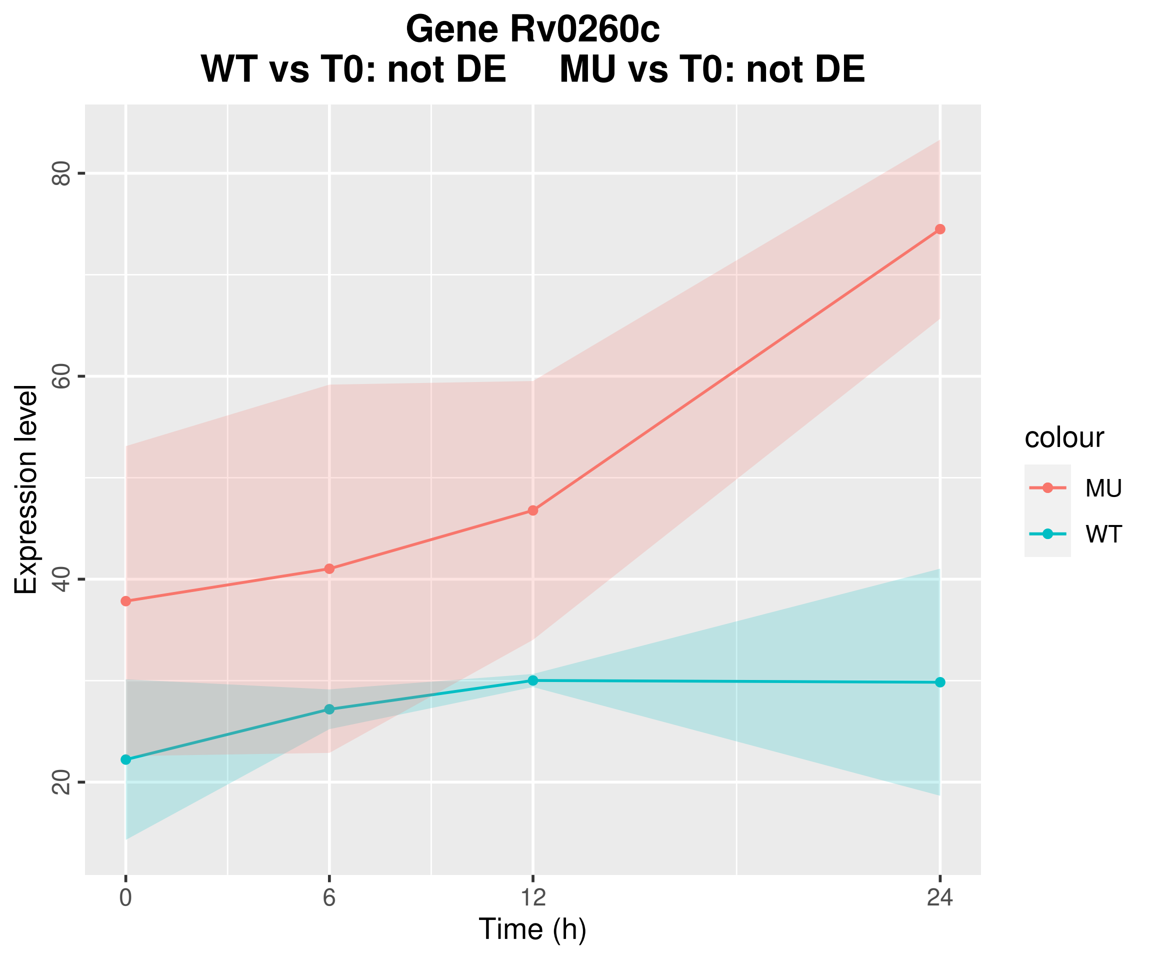

Gene Rv0260c
WT vs T0: not DE   MU vs T0: not DE
Expression level
80
60
40
20
0
6
12
24
Time (h)
colour
MU
WT

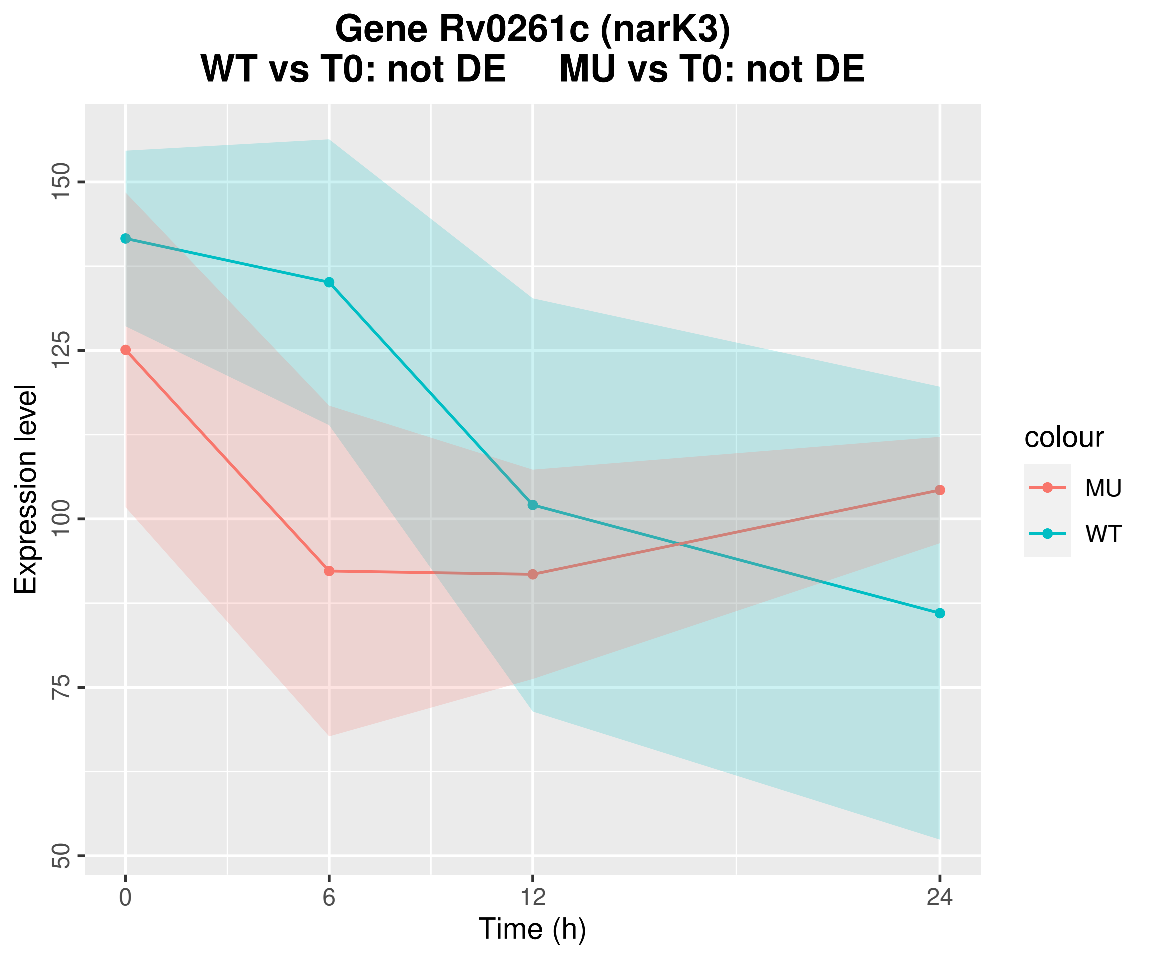
Gene Rv0261c (narK3)
WT vs T0: not DE MU vs T0: not DE
150
125
100
75
50
Expression level
0
6
12
24
Time (h)
colour
MU
WT

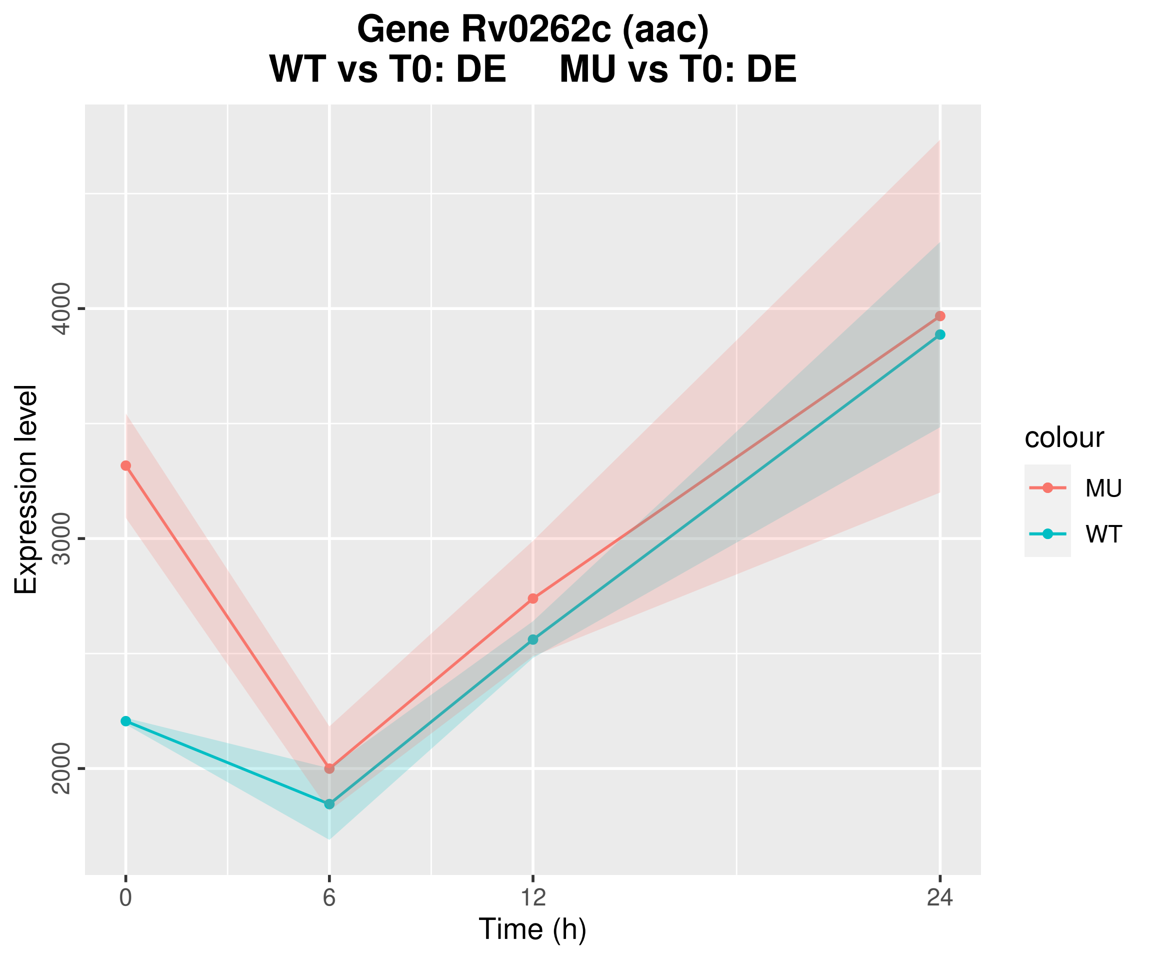
Gene Rv0262c (aac)
WT vs T0: DE MU vs T0: DE
Expression level
4000
3000
2000
0
6
12
24
Time (h)
colour
MU
WT

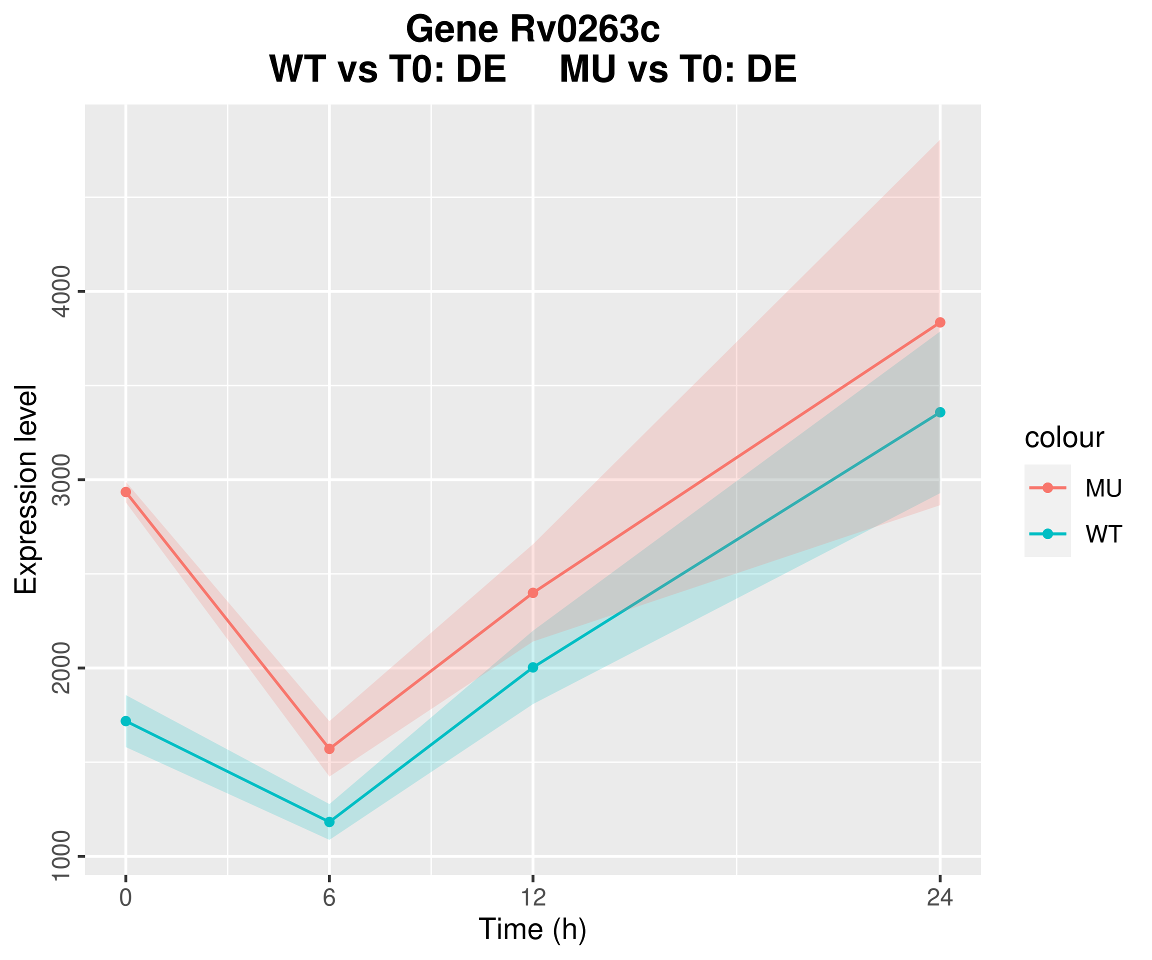
Gene Rv0263c
WT vs T0: DE   MU vs T0: DE
Expression level
1000
2000
3000
4000
0
6
12
24
Time (h)
colour
MU
WT

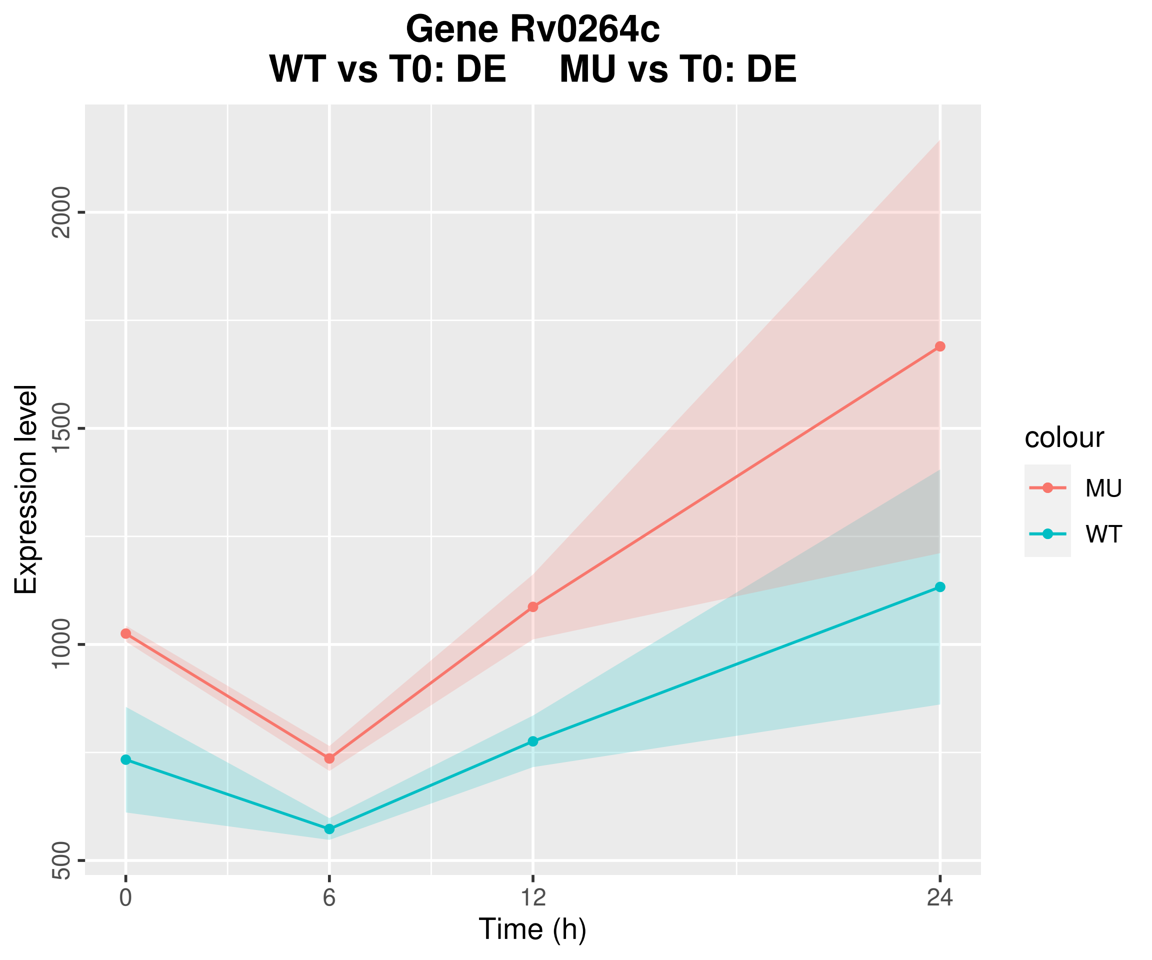

Gene Rv0264c
WT vs T0: DE MU vs T0: DE
Expression level
2000
1500
1000
500
0
6
12
24
Time (h)
colour
MU
WT

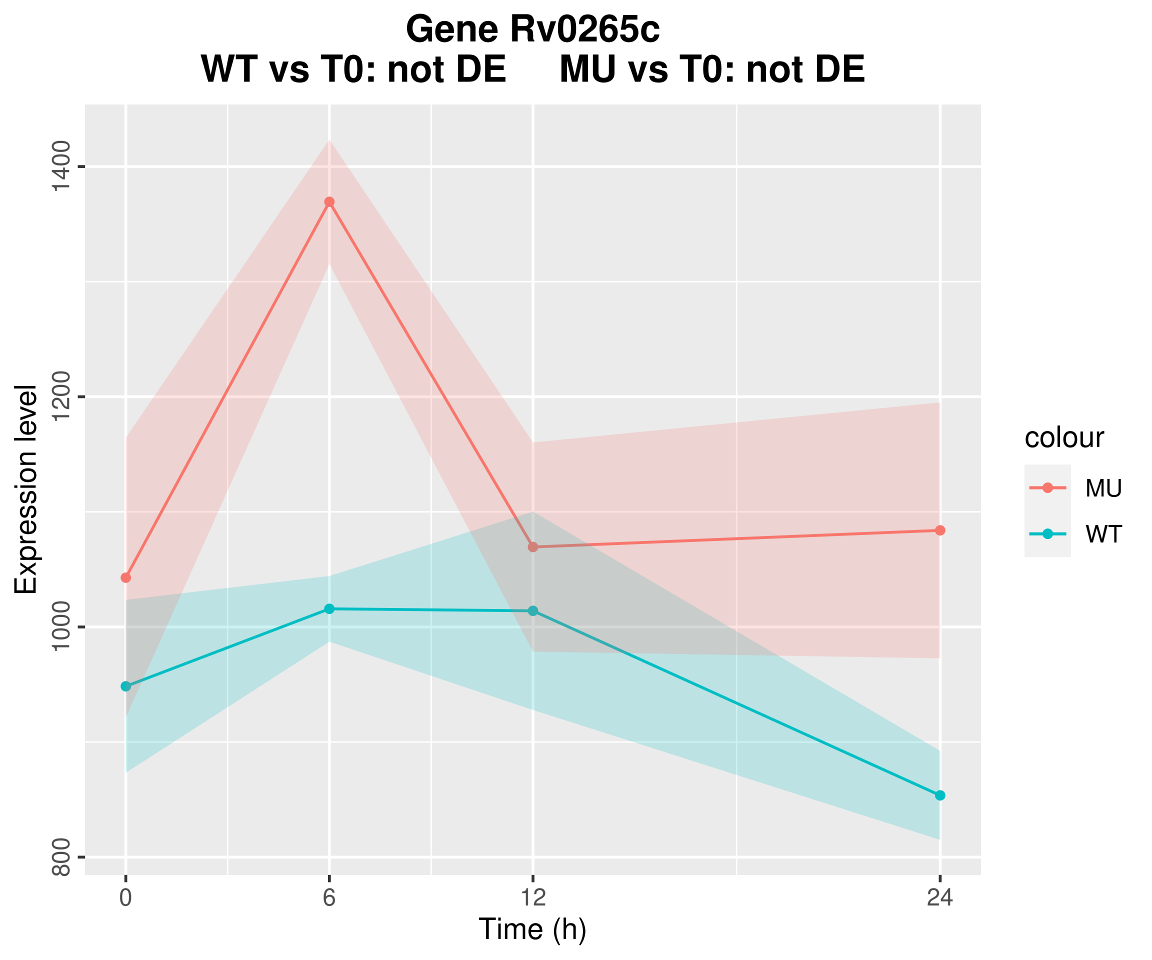

Gene Rv0265c
WT vs T0: not DE   MU vs T0: not DE
Expression level
1400
1200
1000
800
0
6
12
24
Time (h)
colour
MU
WT

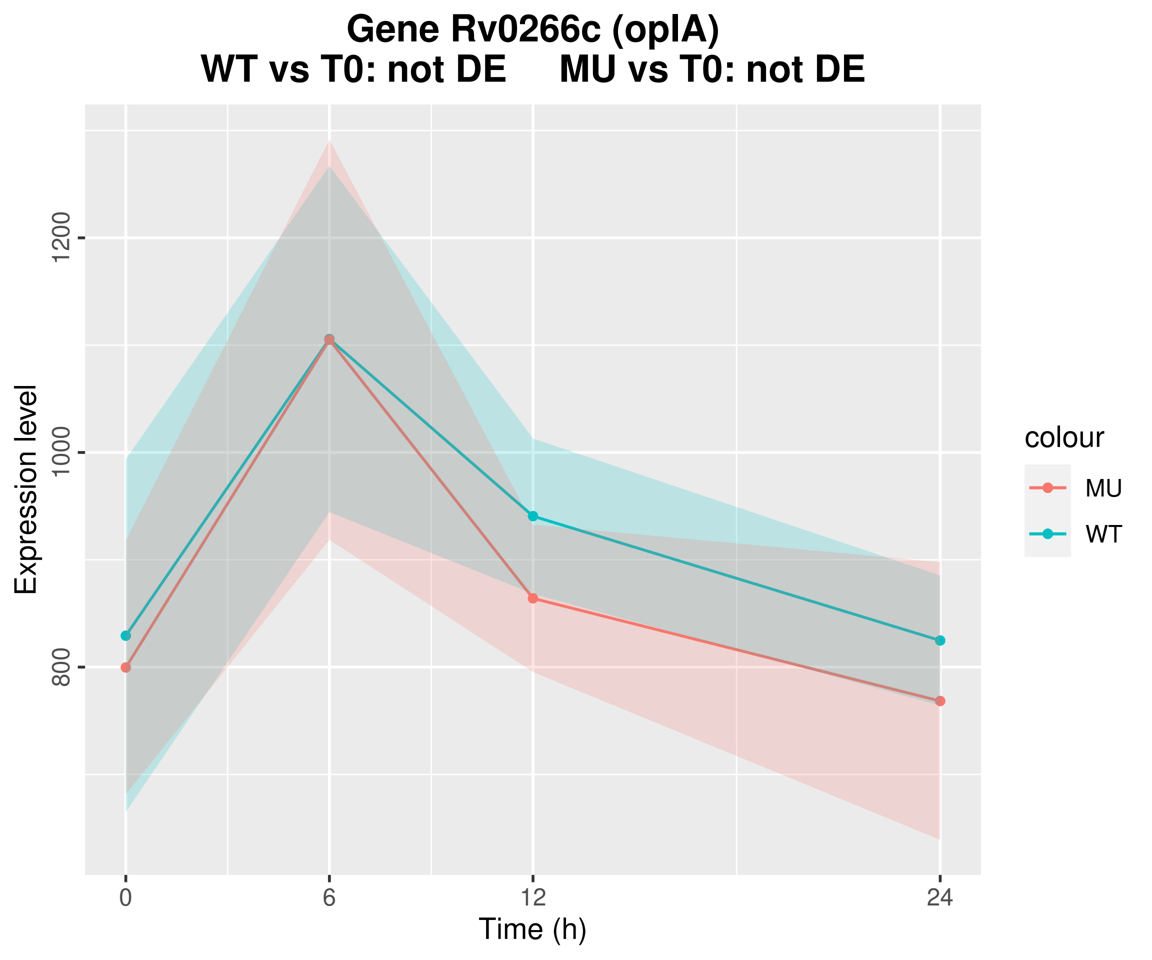

Gene Rv0266c (oplA)
WT vs T0: not DE   MU vs T0: not DE
Expression level
1200
1000
800
0
6
12
24
Time (h)
colour
MU
WT

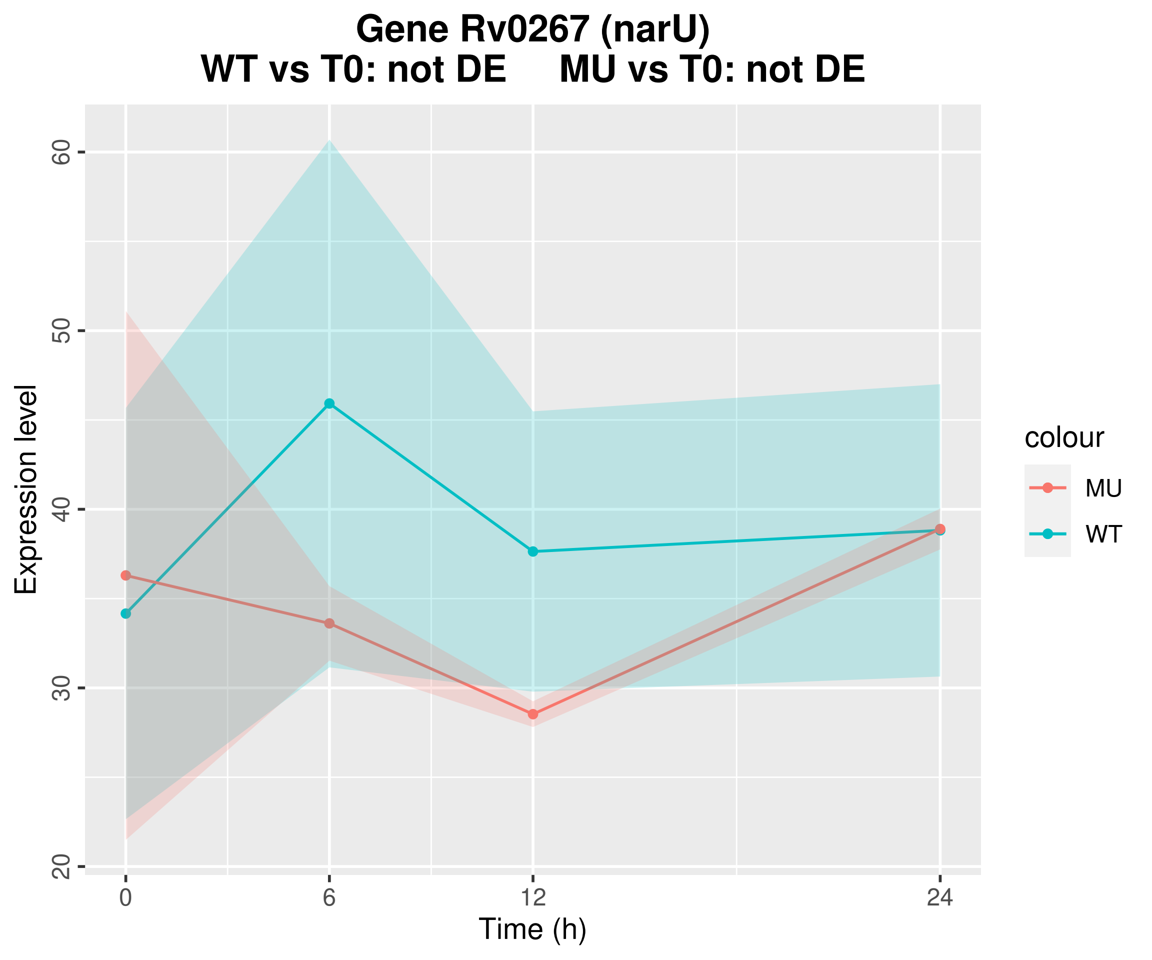

Gene Rv0267 (narU)
WT vs T0: not DE   MU vs T0: not DE
Expression level
60
50
40
30
20
0
6
12
24
Time (h)
colour
MU
WT

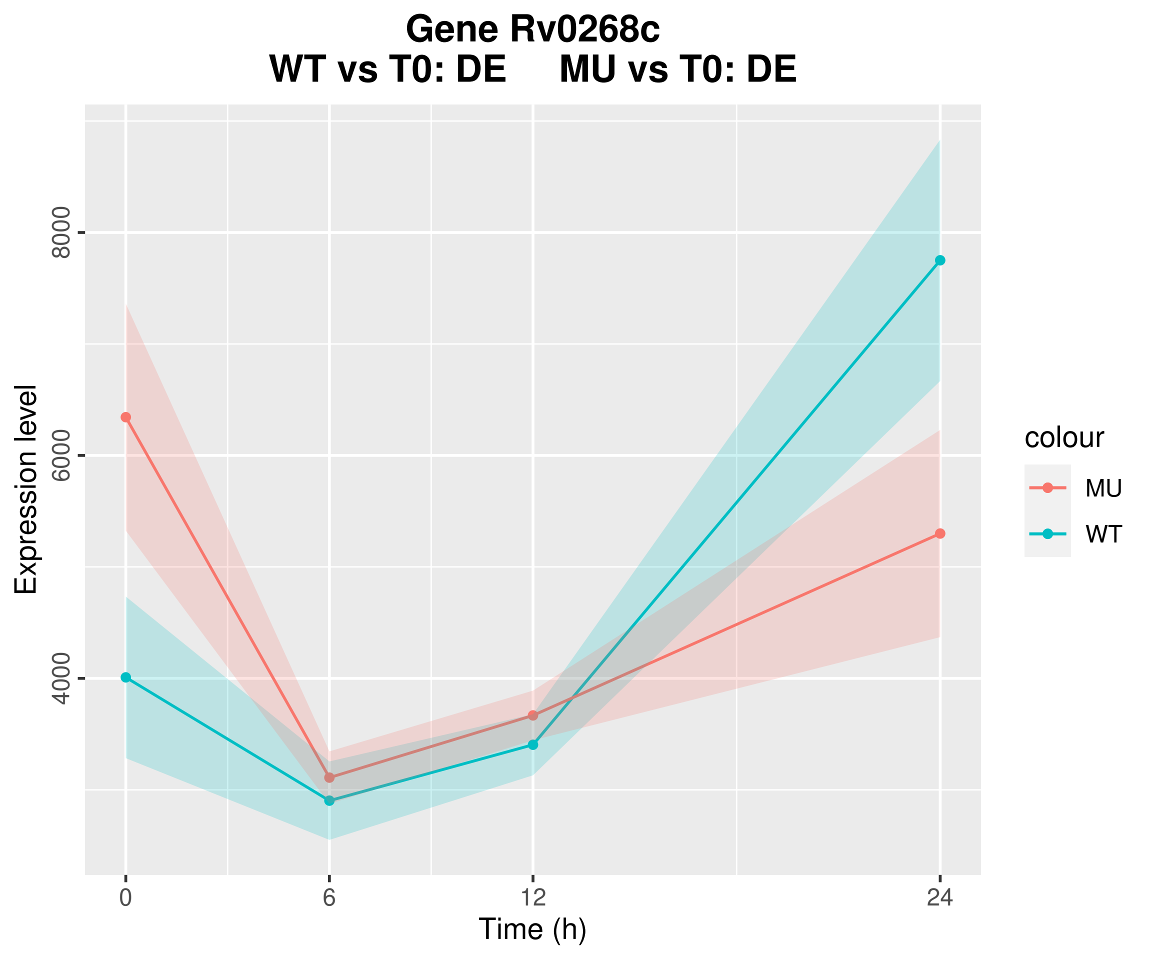

Gene Rv0268c
WT vs T0: DE   MU vs T0: DE
8000
6000
4000
Expression level
0
6
12
24
Time (h)
colour
MU
WT

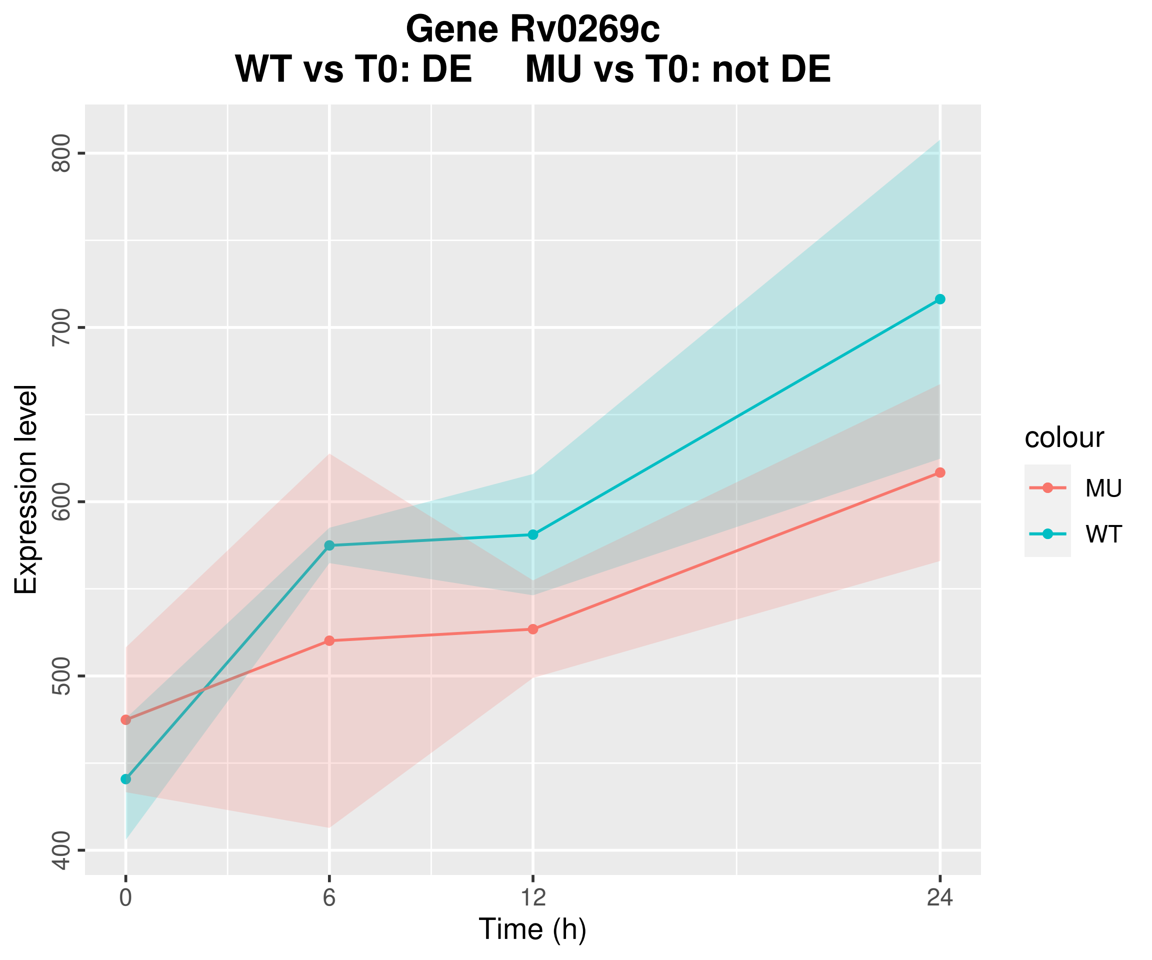
Gene Rv0269c
WT vs T0: DE   MU vs T0: not DE
Expression level
800
700
600
500
400
0
6
12
24
Time (h)
colour
MU
WT

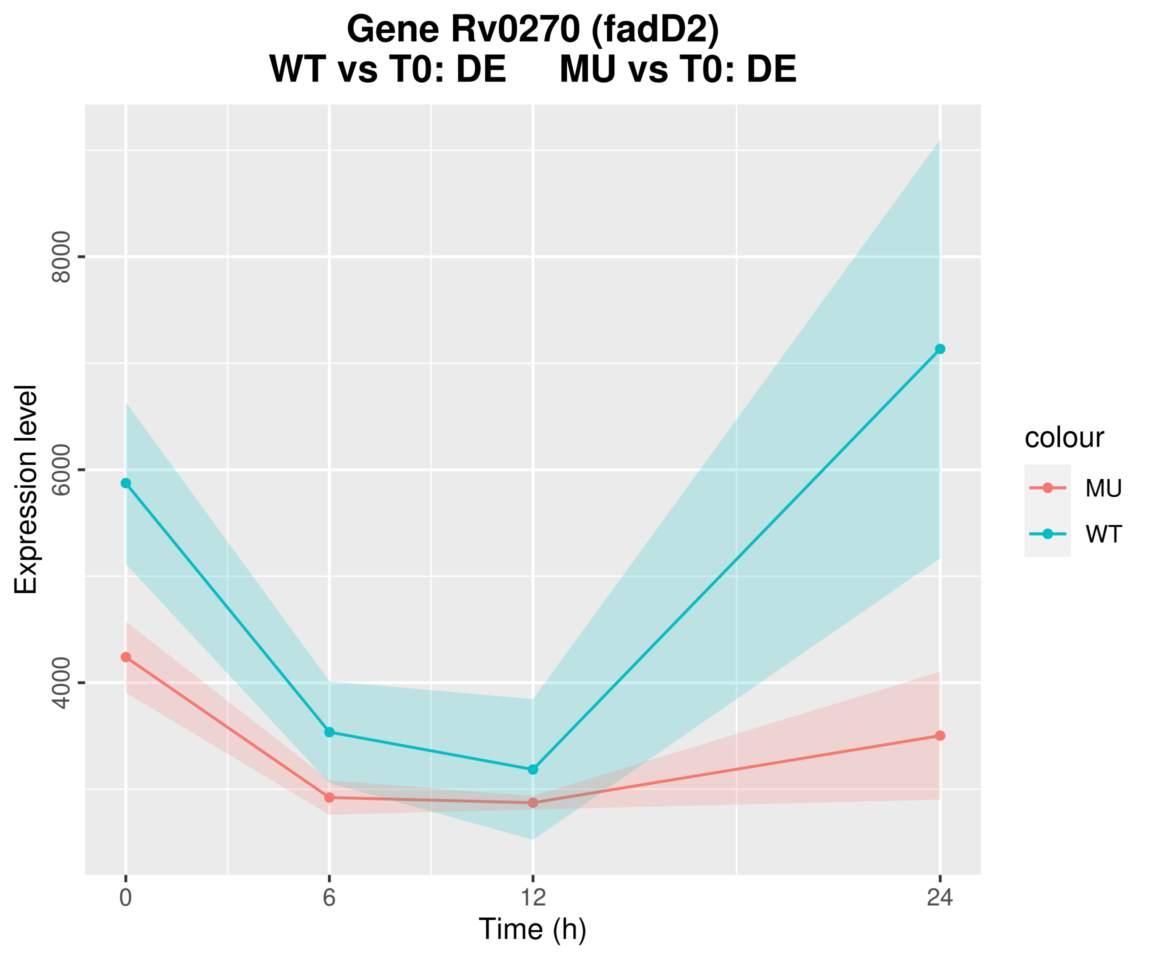
Gene Rv0270 (fadD2)
WT vs T0: DE MU vs T0: DE
Expression level
8000
6000
4000
0
6
12
24
Time (h)
colour
MU
WT

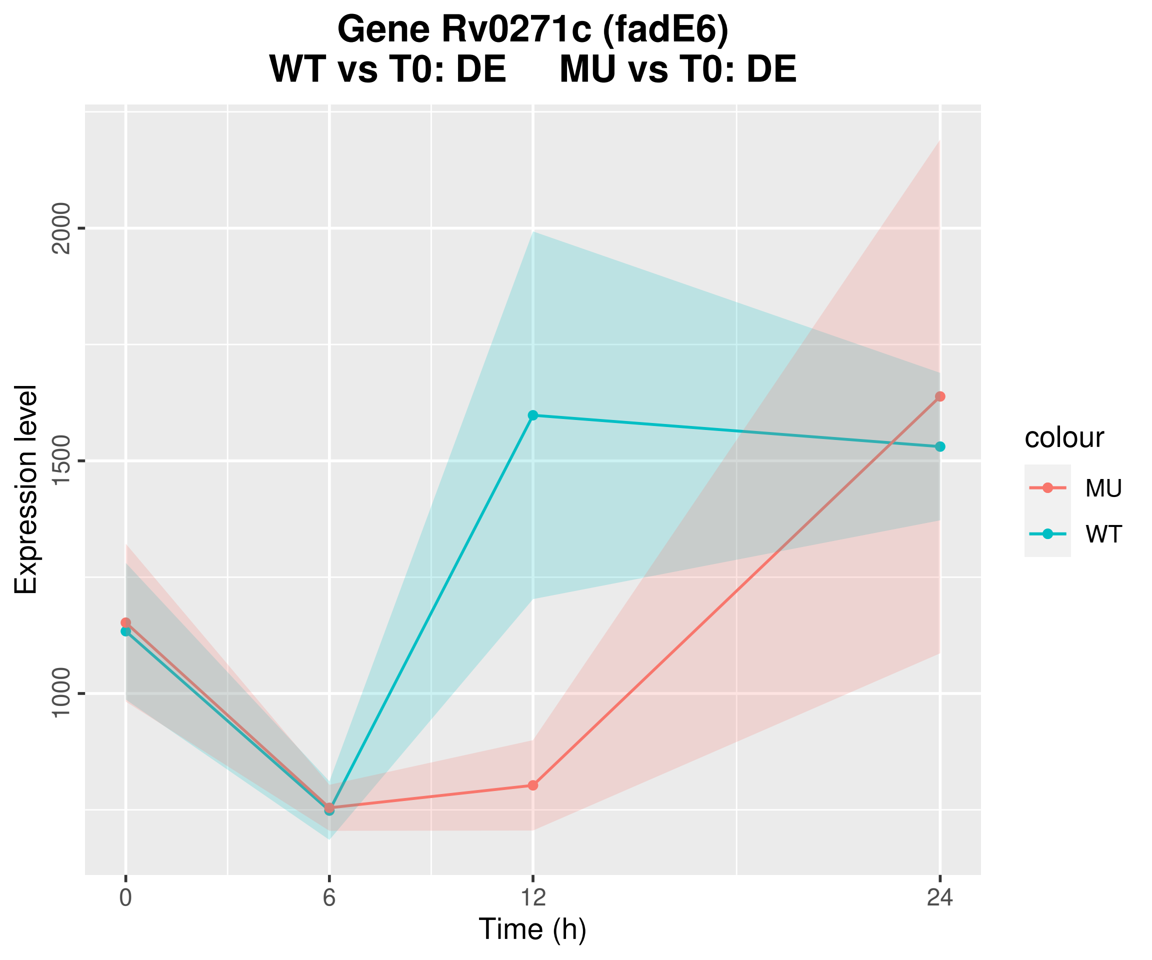

Gene Rv0271c (fadE6)
WT vs T0: DE MU vs T0: DE
Expression level
2000
1500
1000
0
6
12
24
Time (h)
colour
MU
WT

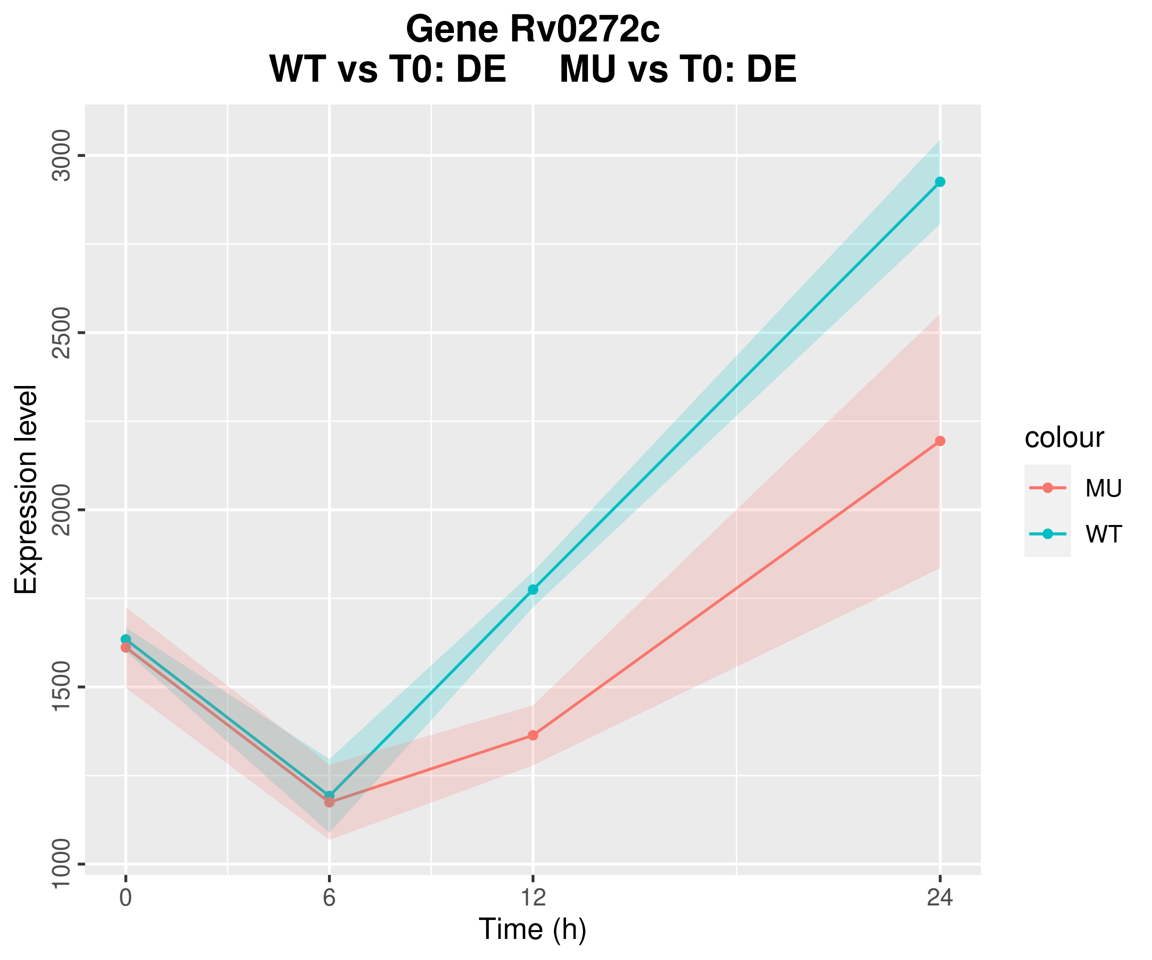

Gene Rv0272c
WT vs T0: DE MU vs T0: DE
Expression level
3000
2500
2000
1500
1000
0
6
12
24
Time (h)
colour
MU
WT

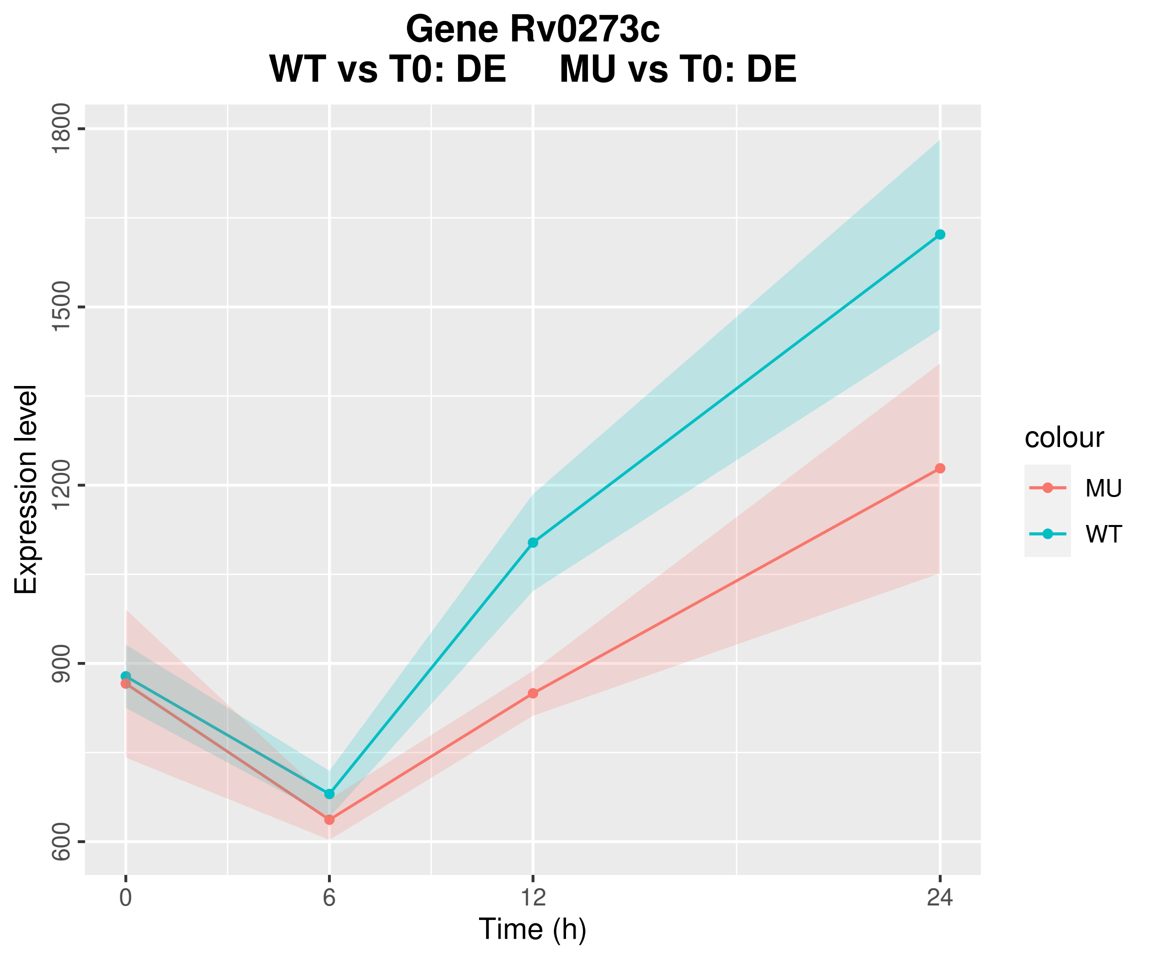
Gene Rv0273c
WT vs T0: DE   MU vs T0: DE
1800
1500
1200
900
600
Expression level
0
6
12
24
Time (h)
colour
MU
WT

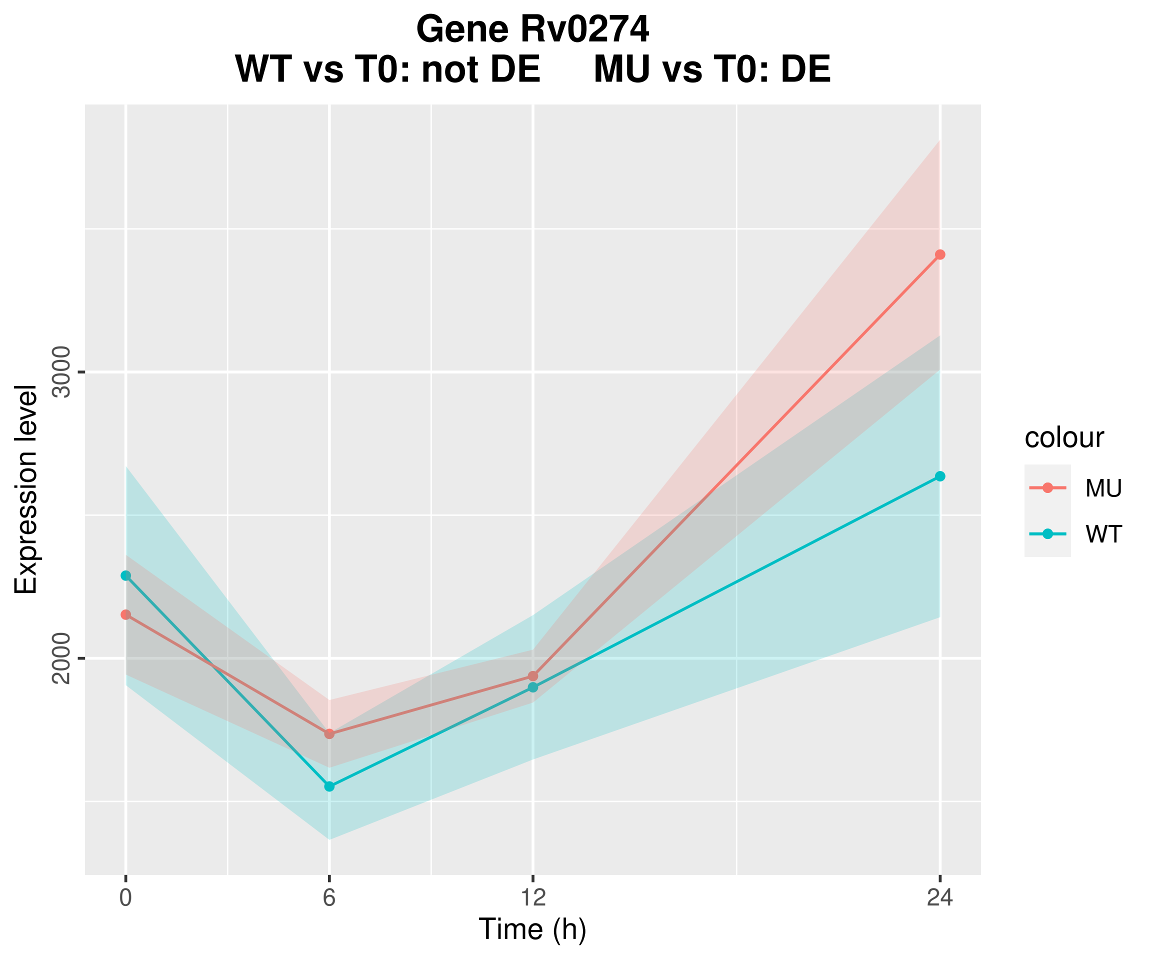
Gene Rv0274
WT vs T0: not DE   MU vs T0: DE
Expression level
3000
2000
0
6
12
24
Time (h)
colour
MU
WT

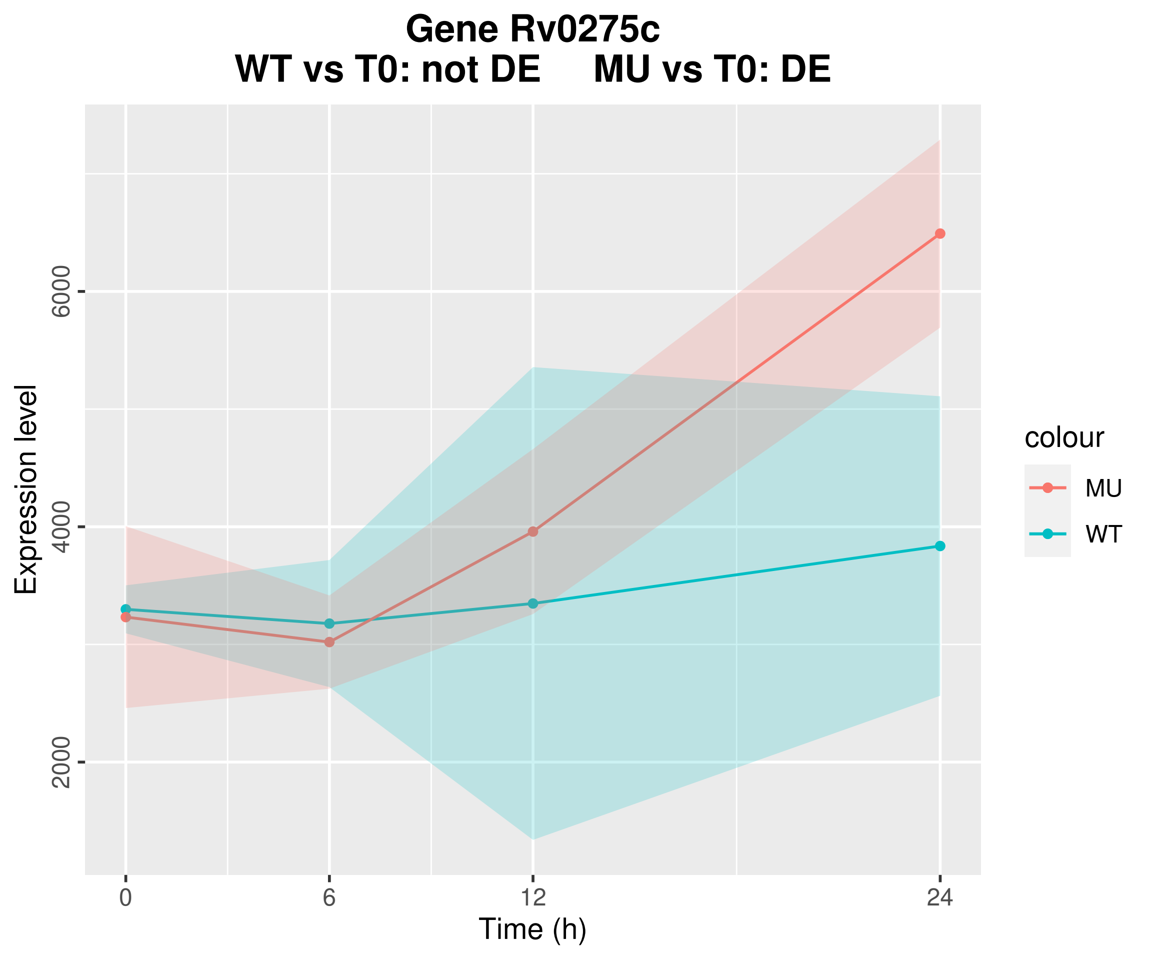

Gene Rv0275c
WT vs T0: not DE MU vs T0: DE
Expression level
6000
4000
2000
0
6
12
24
Time (h)
colour
MU
WT

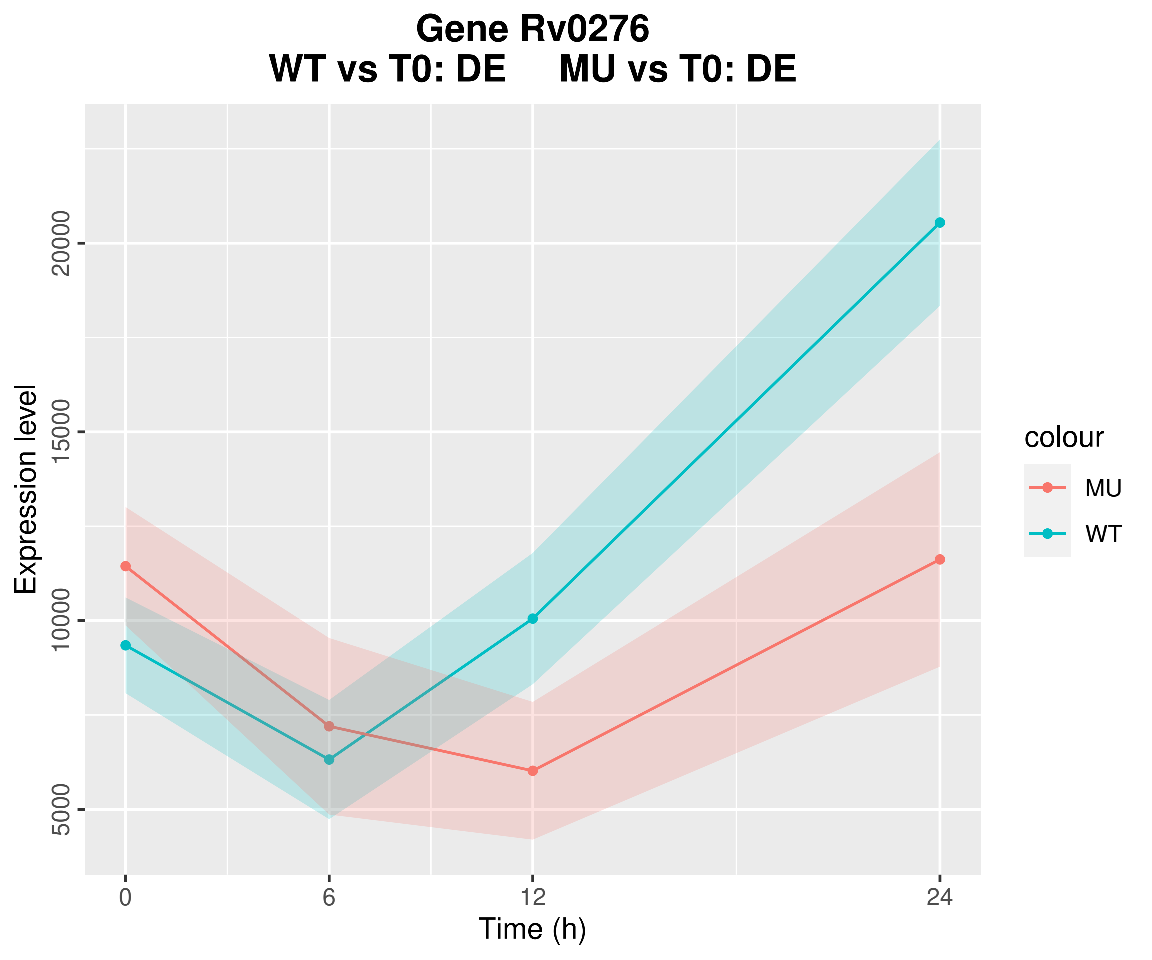

Gene Rv0276
WT vs T0: DE   MU vs T0: DE
Expression level
20000
15000
10000
5000
0
6
12
24
Time (h)
colour
MU
WT

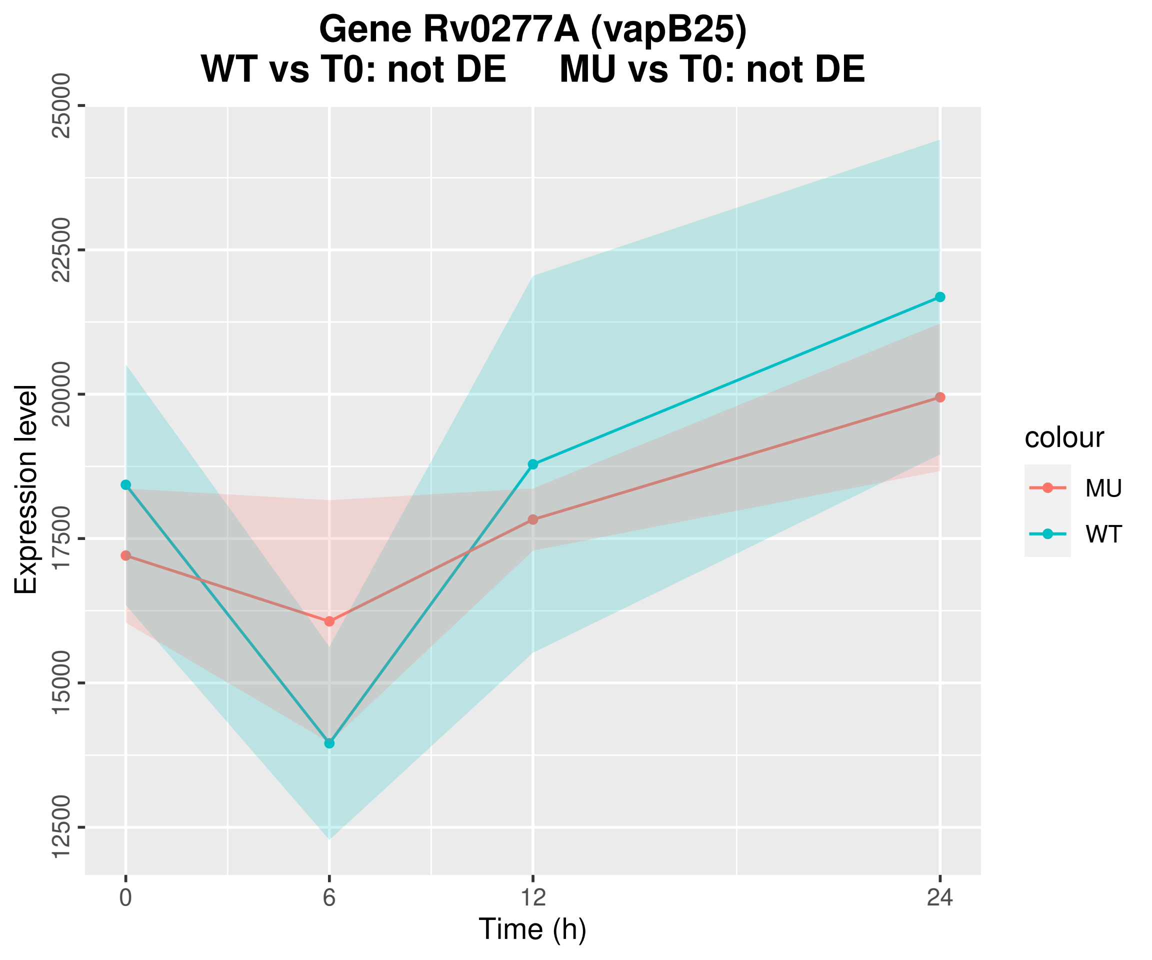

Gene Rv0277A (vapB25)
WT vs T0: not DE   MU vs T0: not DE
25000
22500
20000
17500
15000
12500
Expression level
0
6
12
24
Time (h)
colour
MU
WT

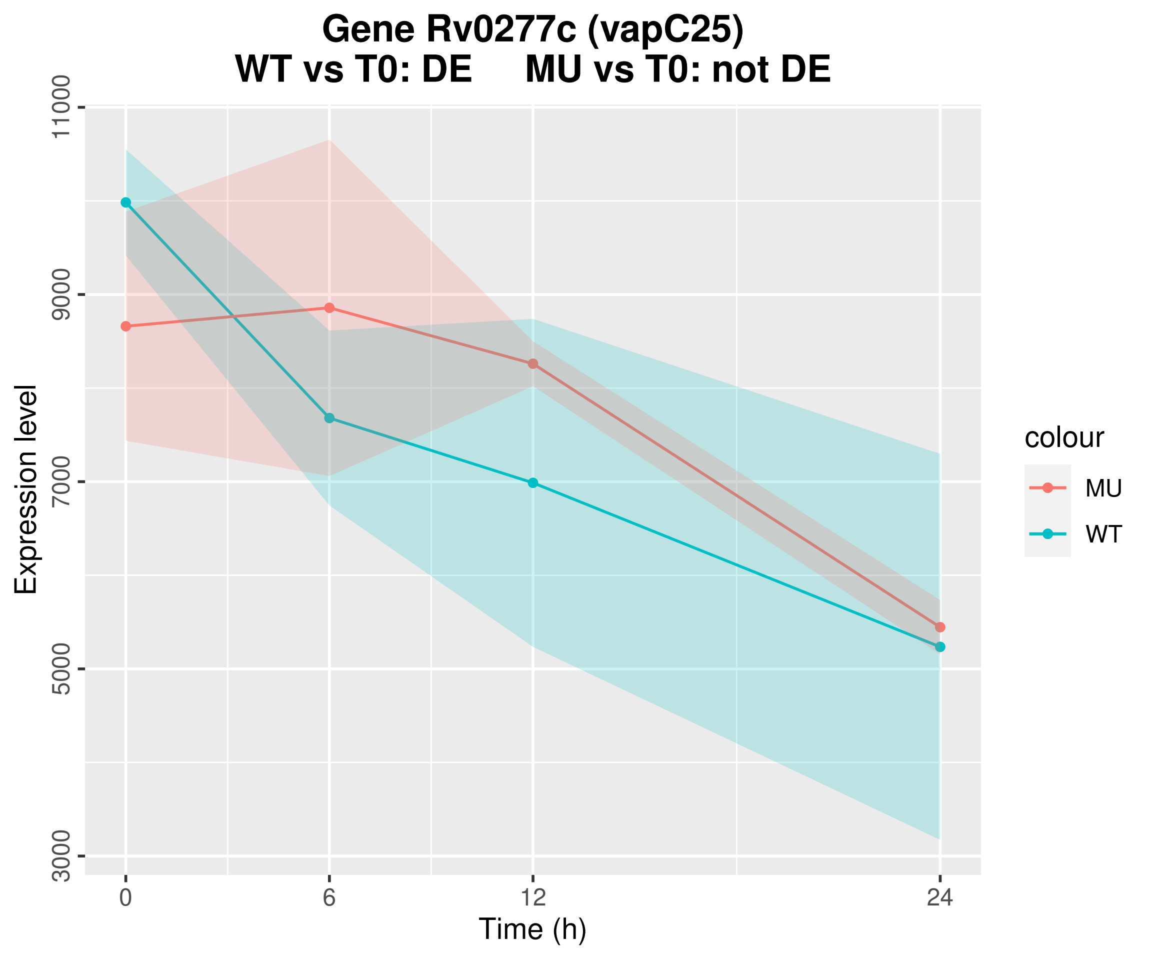
Gene Rv0277c (vapC25)
WT vs T0: DE   MU vs T0: not DE
Expression level
11000
9000
7000
5000
3000
0
6
12
24
Time (h)
colour
MU
WT

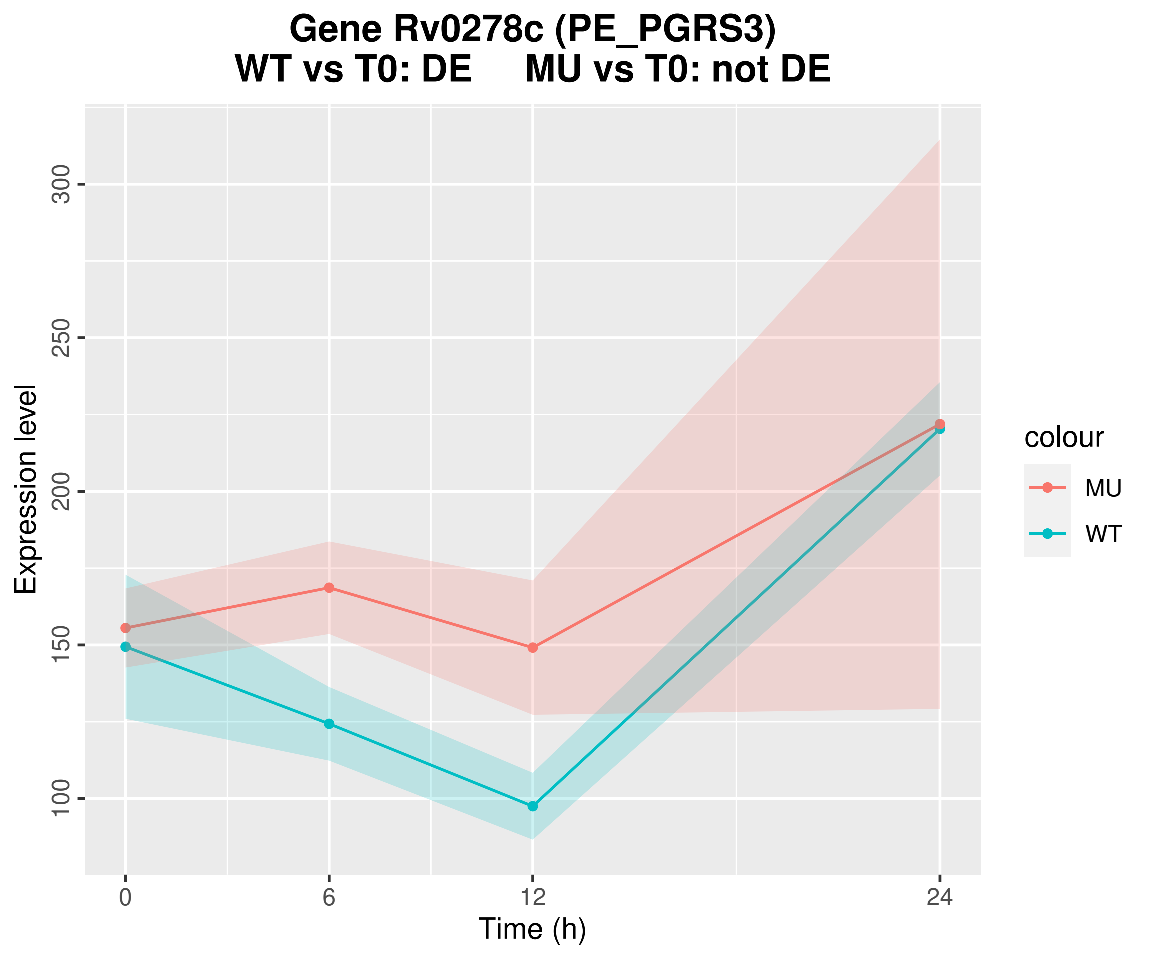
Gene Rv0278c (PE_PGRS3)
WT vs T0: DE MU vs T0: not DE
Expression level
300
250
200
150
100
0
6
12
24
Time (h)
colour
MU
WT

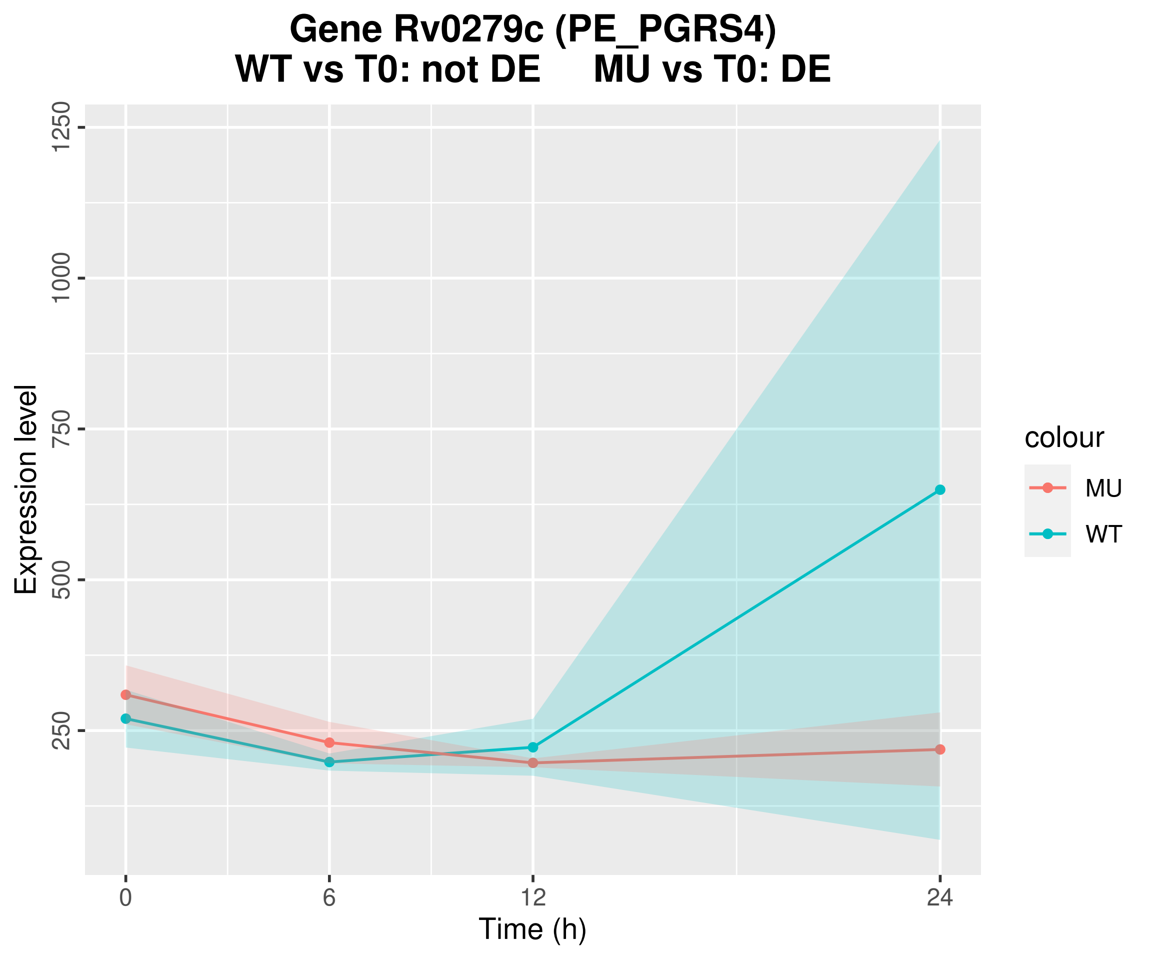

Gene Rv0279c (PE_PGRS4)
WT vs T0: not DE   MU vs T0: DE
Expression level
1250
1000
750
500
250
0
6
12
24
Time (h)
colour
MU
WT

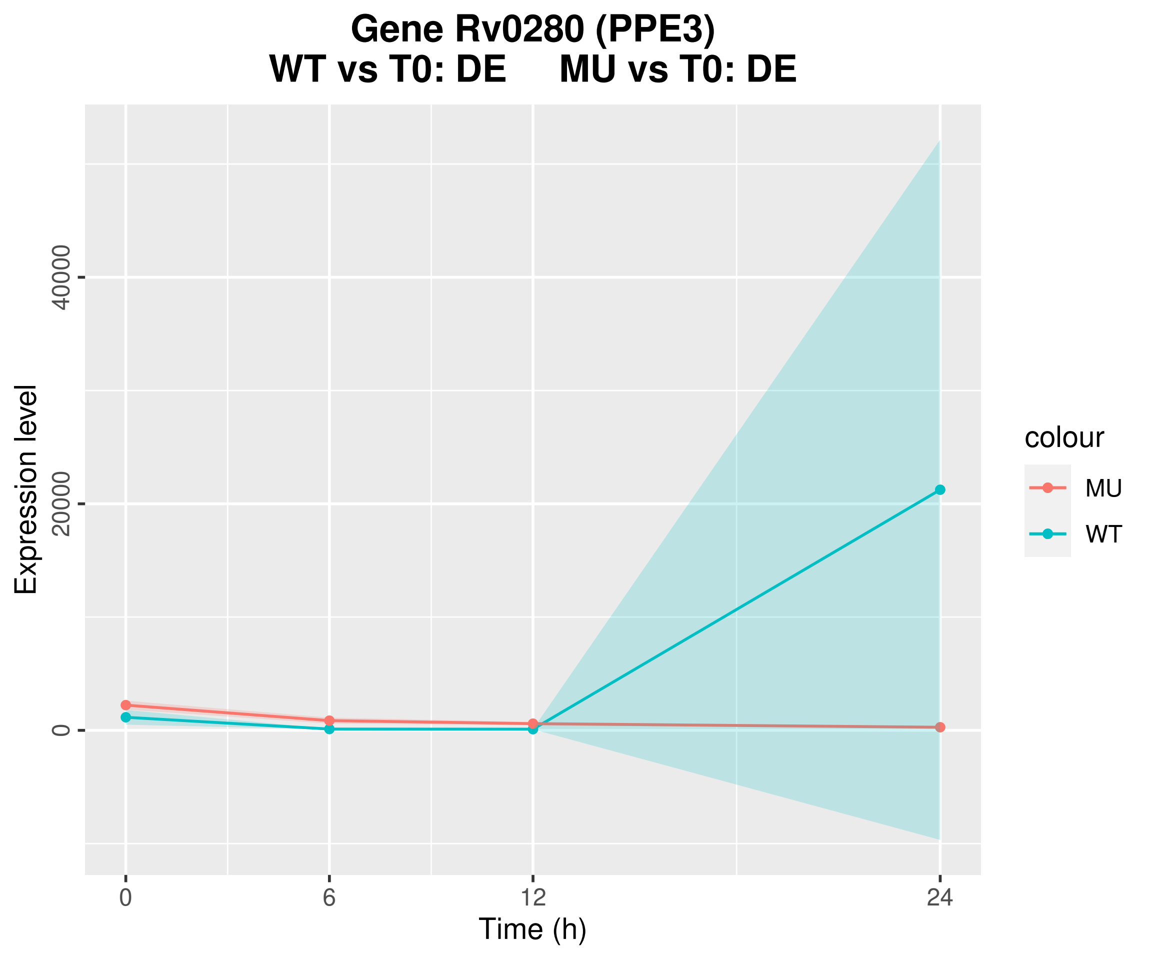
Gene Rv0280 (PPE3)
WT vs T0: DE   MU vs T0: DE
Expression level
40000
20000
0
0
6
12
24
Time (h)
colour
MU
WT

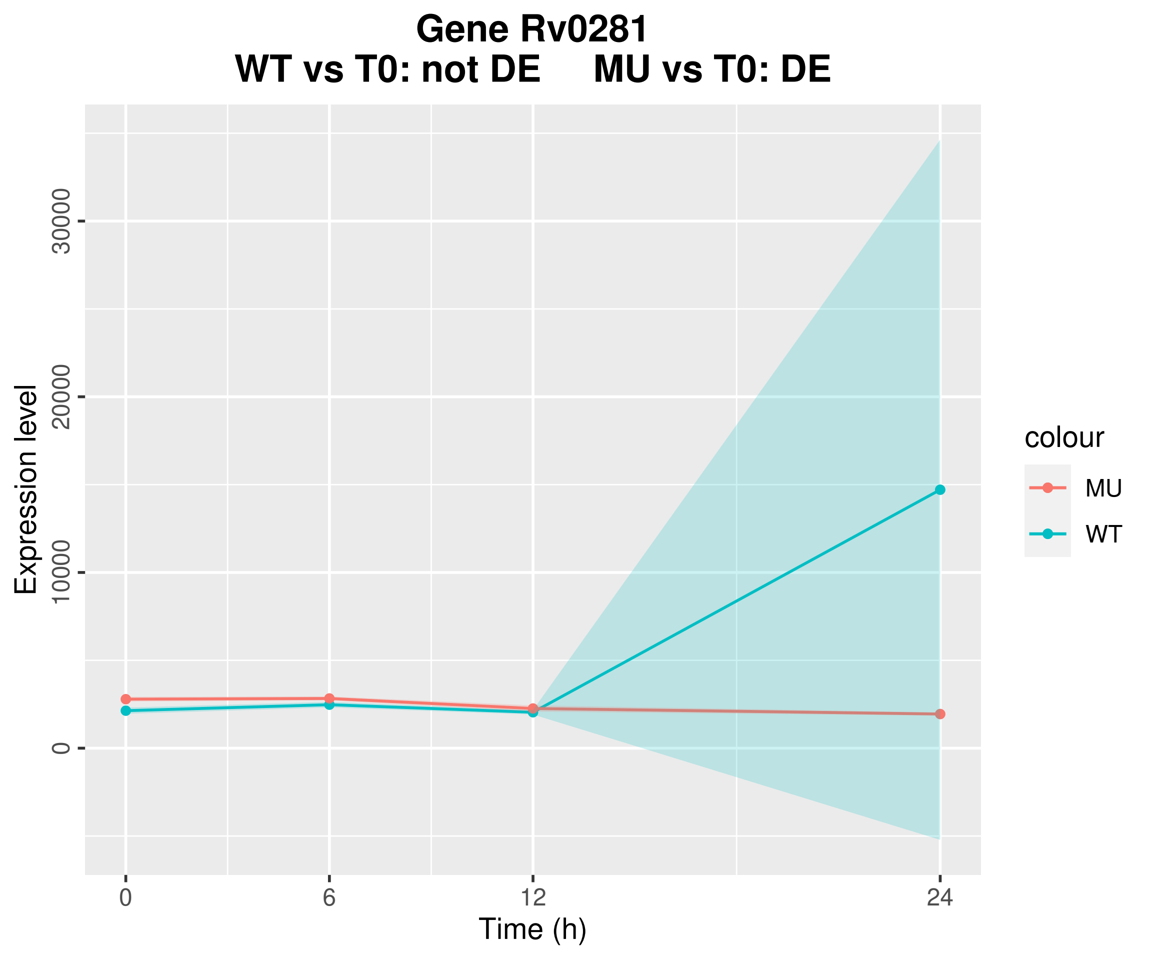
Gene Rv0281
WT vs T0: not DE   MU vs T0: DE
Expression level
30000
20000
10000
0
0
6
12
24
Time (h)
colour
MU
WT

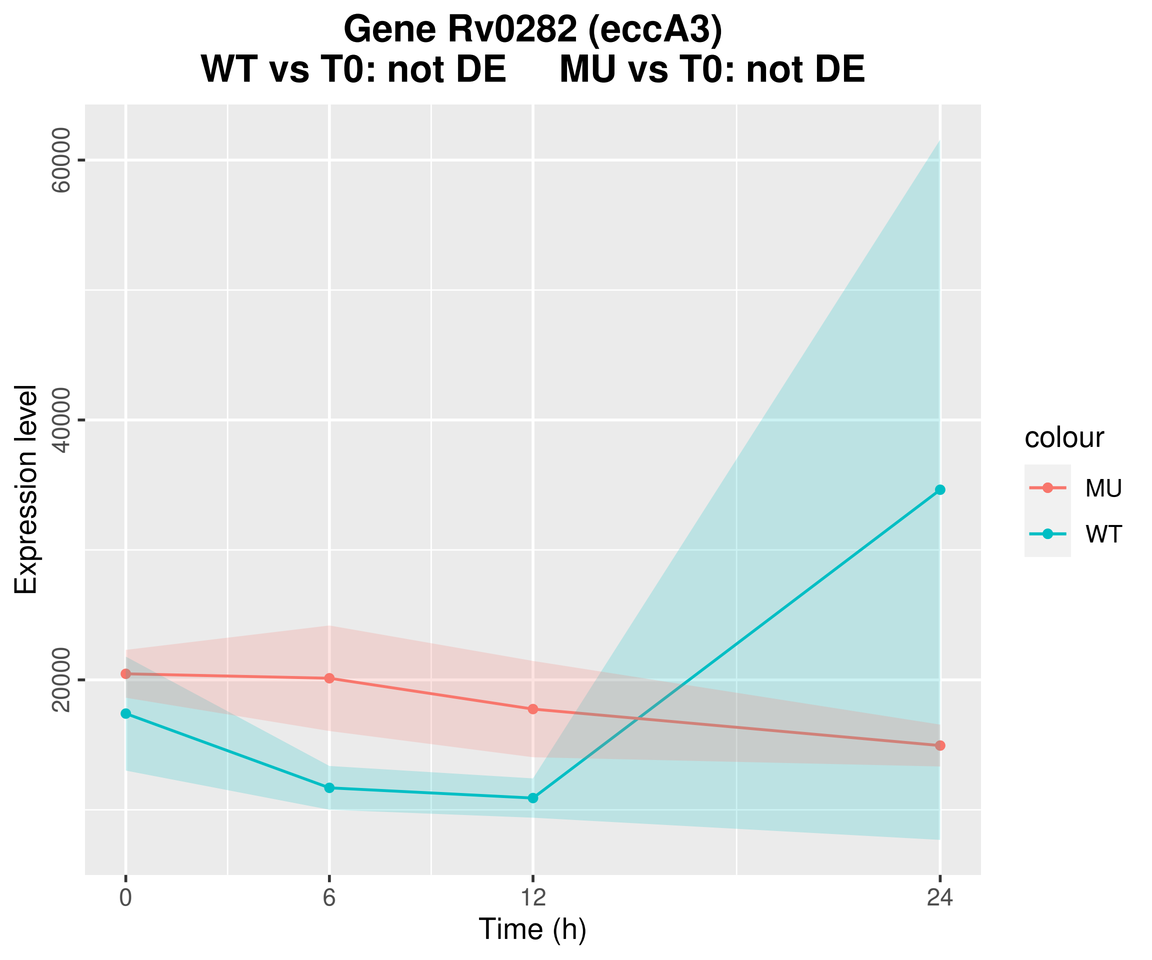
Gene Rv0282 (eccA3)
WT vs T0: not DE MU vs T0: not DE
60000
40000
20000
Expression level
0
6
12
24
Time (h)
colour
MU
WT

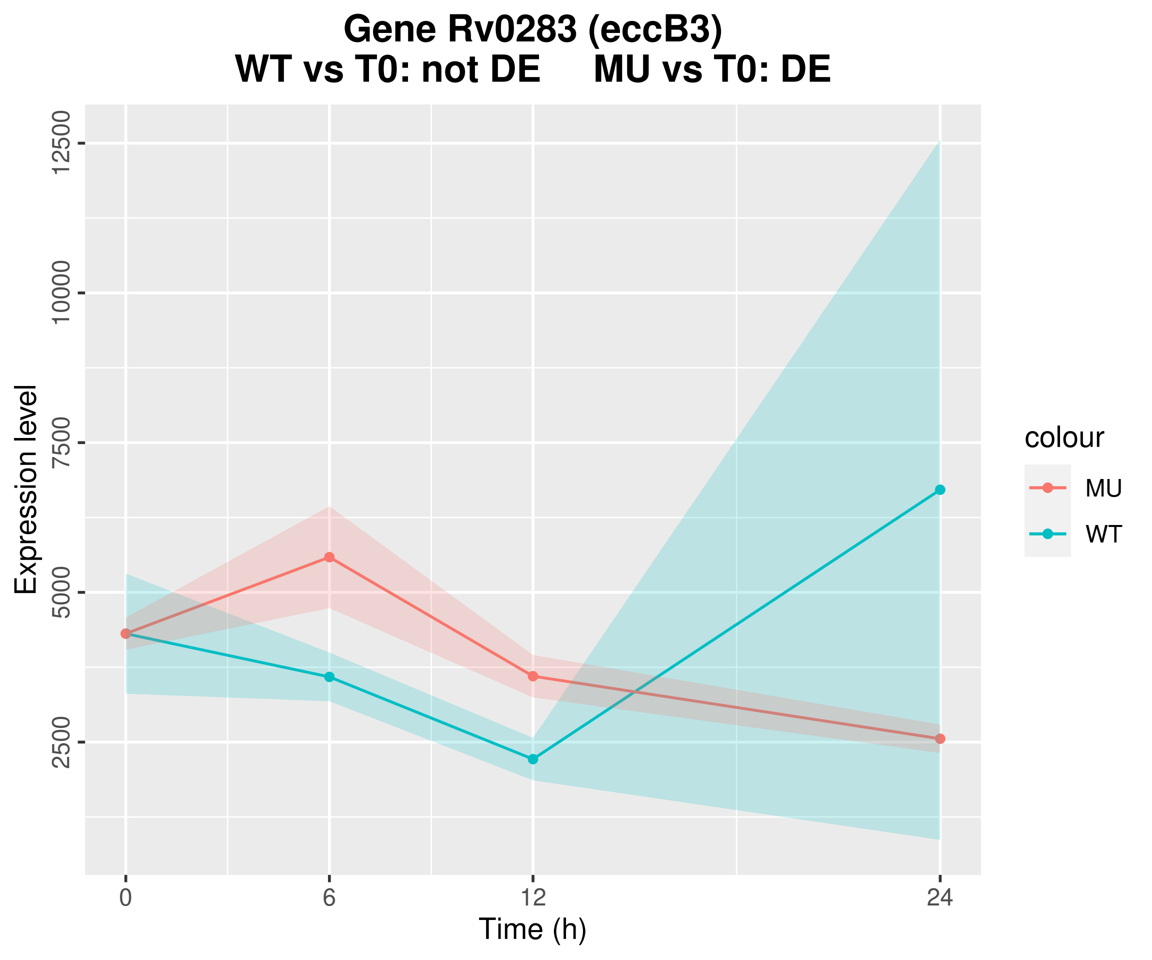
Gene Rv0283 (eccB3)
WT vs T0: not DE   MU vs T0: DE
12500
10000
7500
5000
2500
Expression level
0
6
12
24
Time (h)
colour
MU
WT

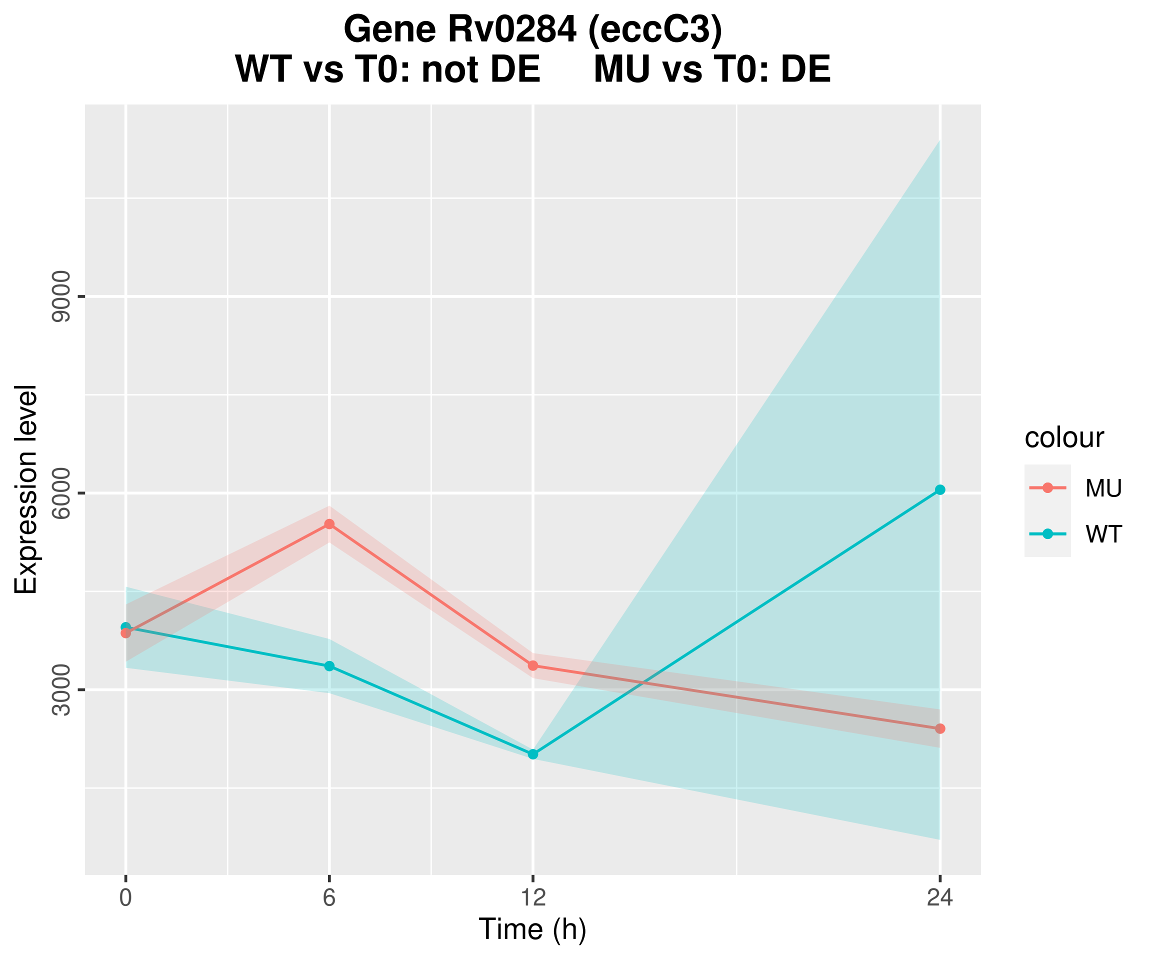

Gene Rv0284 (eccC3)
WT vs T0: not DE MU vs T0: DE
Expression level
9000
6000
3000
0
6
12
24
Time (h)
colour
MU
WT

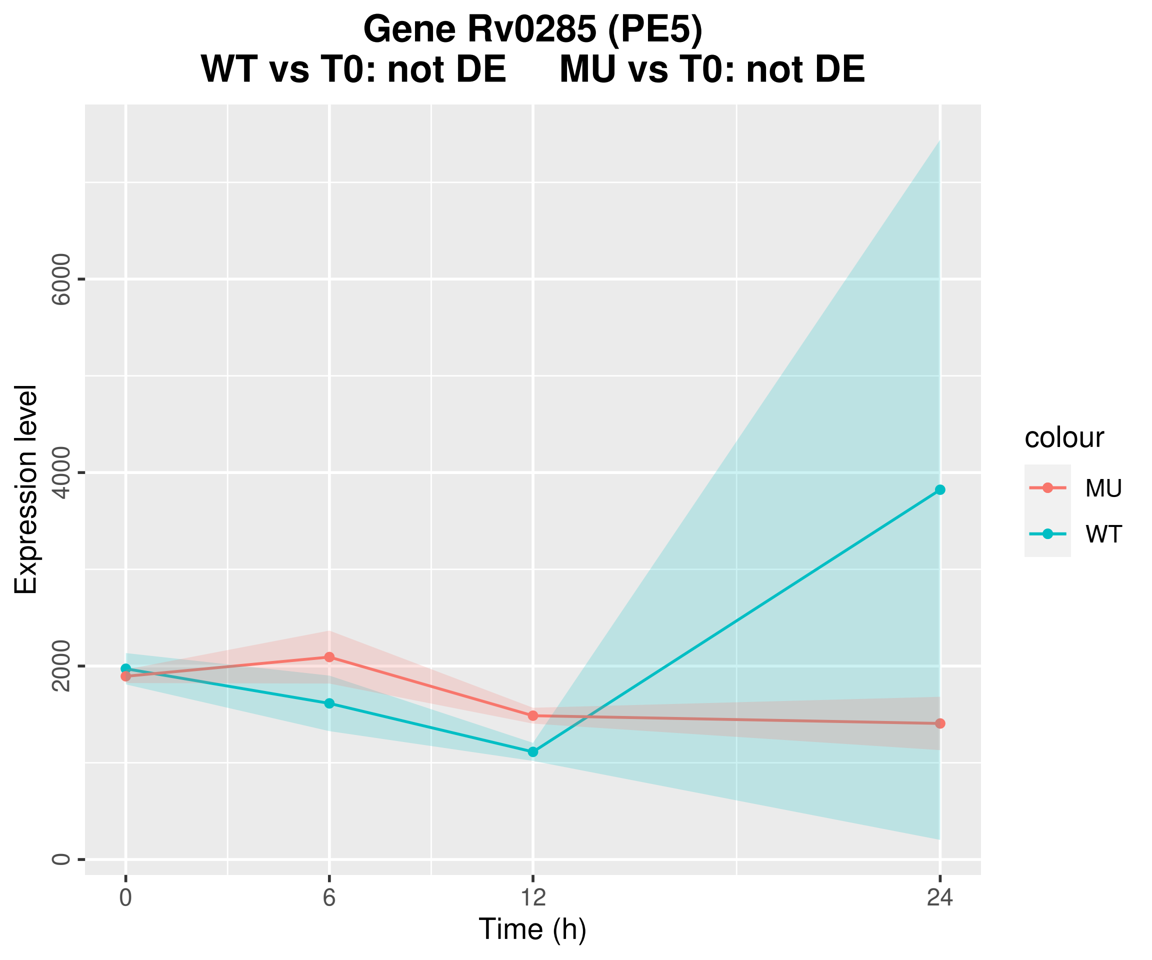

Gene Rv0285 (PE5)
WT vs T0: not DE   MU vs T0: not DE
Expression level
6000
4000
2000
0
0
6
12
24
Time (h)
colour
MU
WT

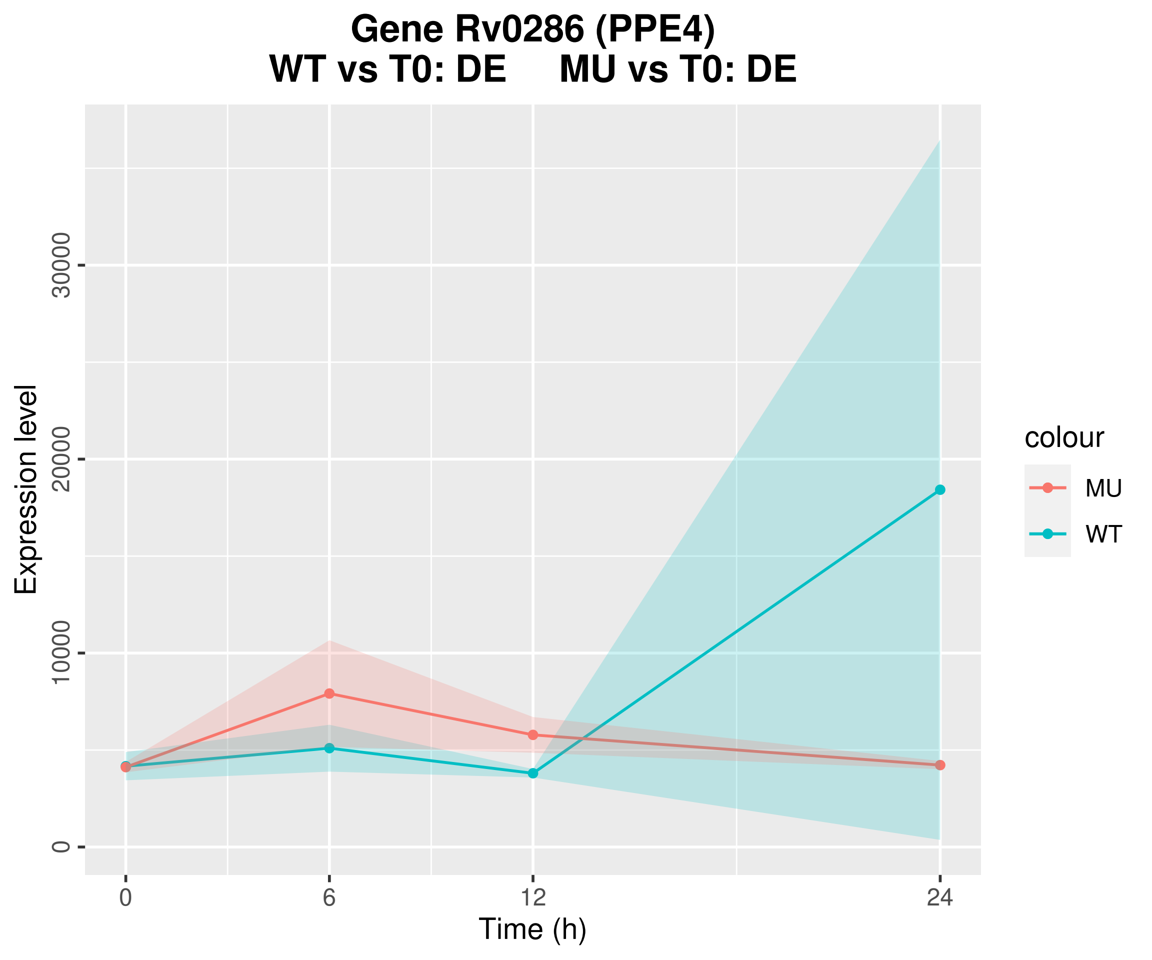
Gene Rv0286 (PPE4)
WT vs T0: DE MU vs T0: DE
Expression level
30000
20000
10000
0
0
6
12
24
Time (h)
colour
MU
WT

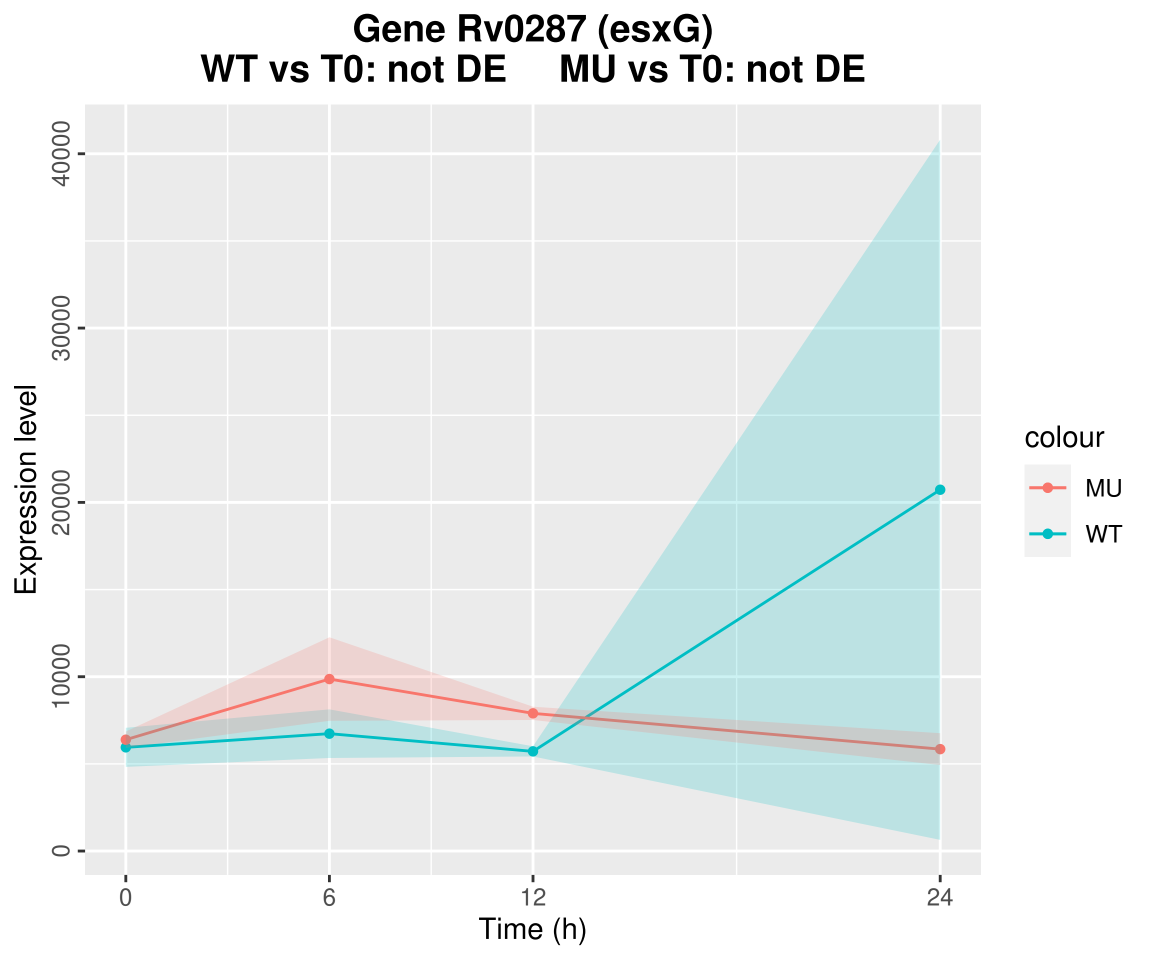

Gene Rv0287 (esxG)
WT vs T0: not DE MU vs T0: not DE
Expression level
40000
30000
20000
10000
0
Time (h)
0
6
12
24
colour
MU
WT

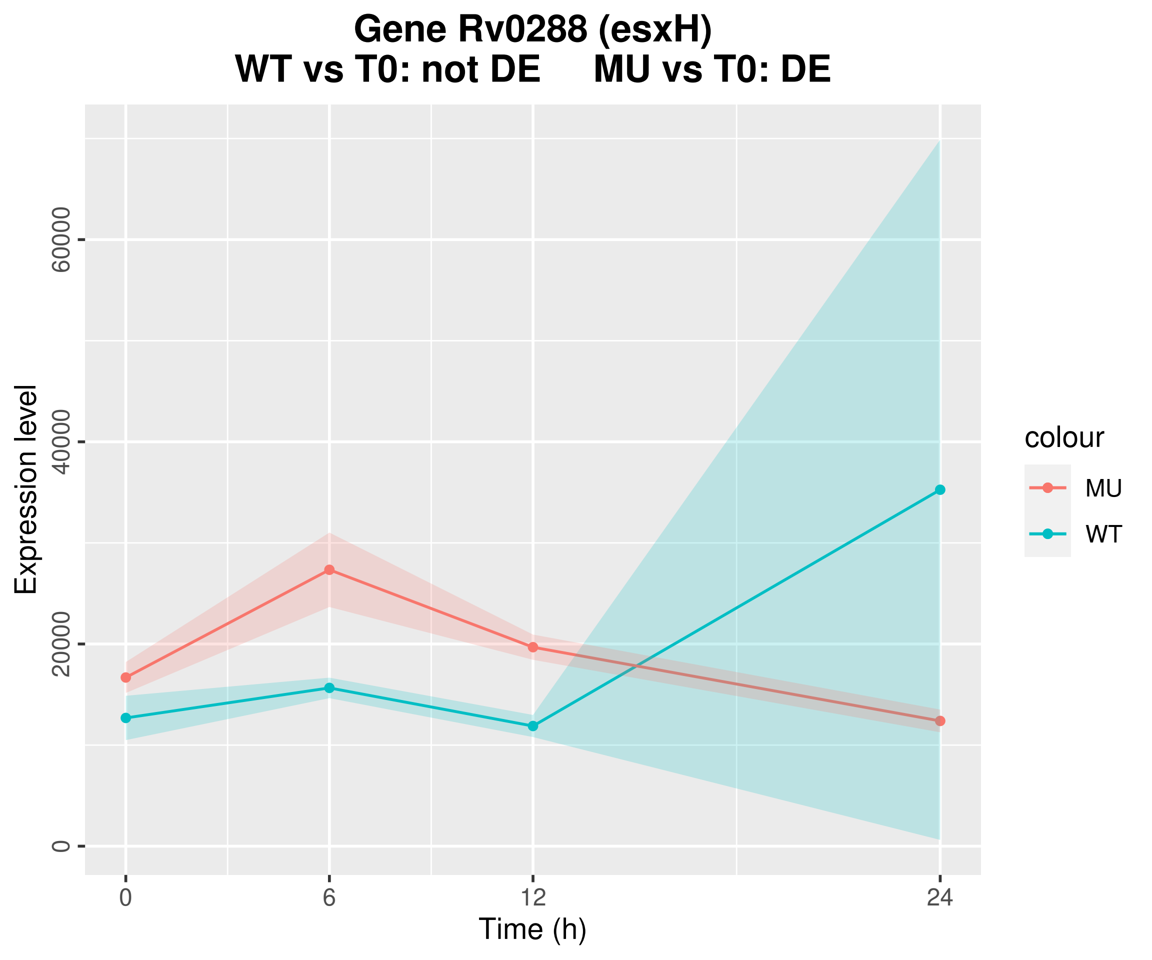

Gene Rv0288 (esxH)
WT vs T0: not DE   MU vs T0: DE
Expression level
0
20000
40000
60000
0
6
12
24
Time (h)
colour
MU
WT

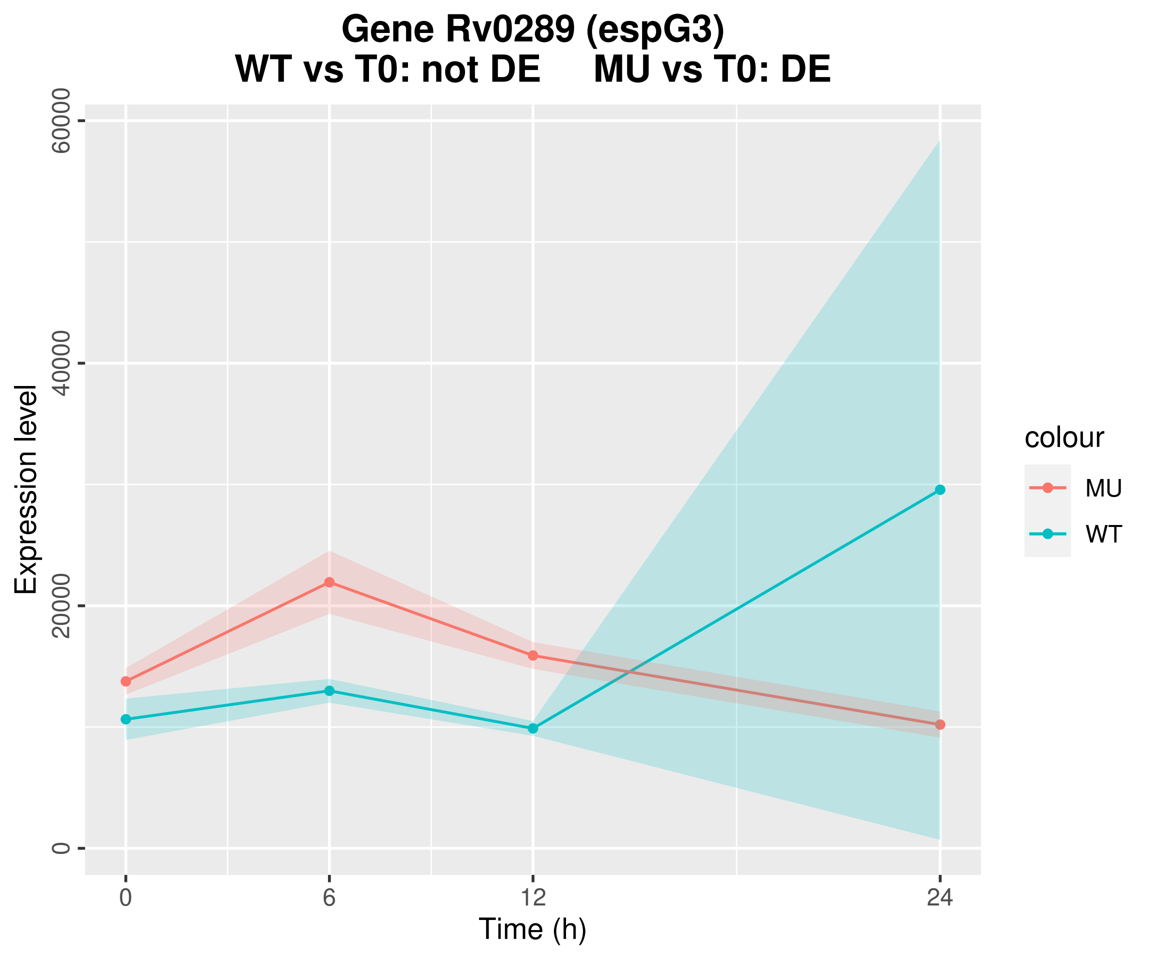

Gene Rv0289 (espG3)
WT vs T0: not DE   MU vs T0: DE
Expression level
60000
40000
20000
0
0
6
12
24
Time (h)
colour
MU
WT

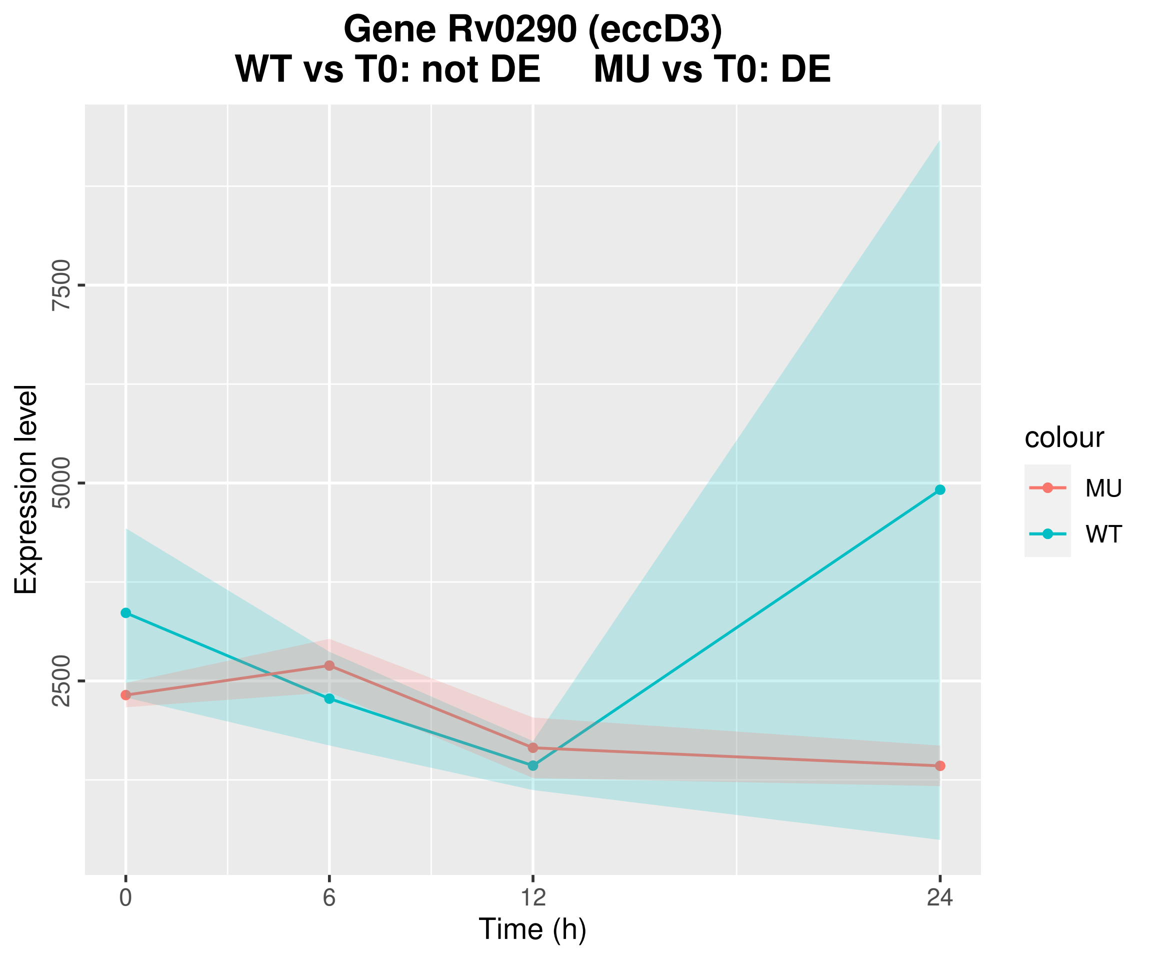

Gene Rv0290 (eccD3)
WT vs T0: not DE   MU vs T0: DE
Expression level
7500
5000
2500
0
6
12
24
Time (h)
colour
MU
WT

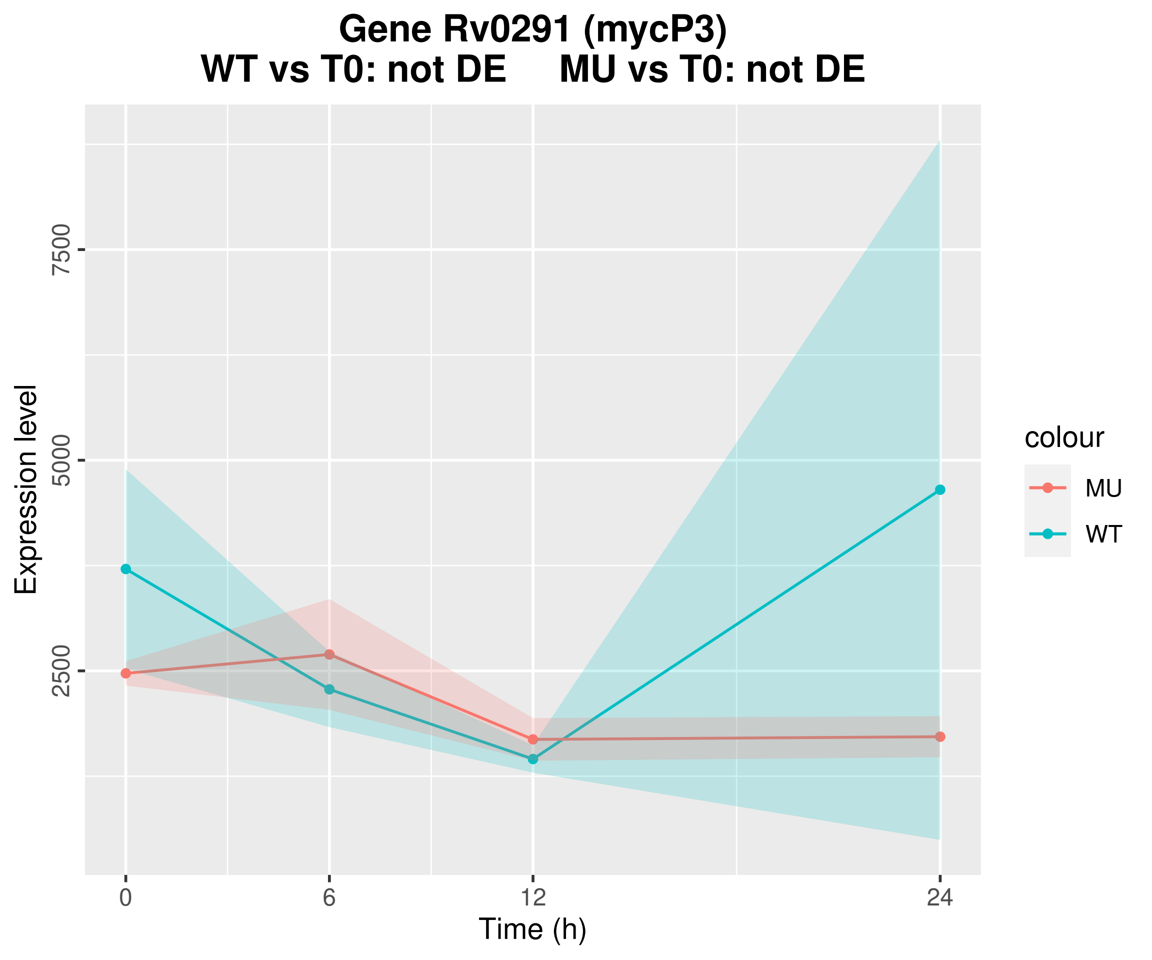

Gene Rv0291 (mycP3)
WT vs T0: not DE MU vs T0: not DE
Expression level
7500
5000
2500
0
6
12
24
Time (h)
colour
MU
WT

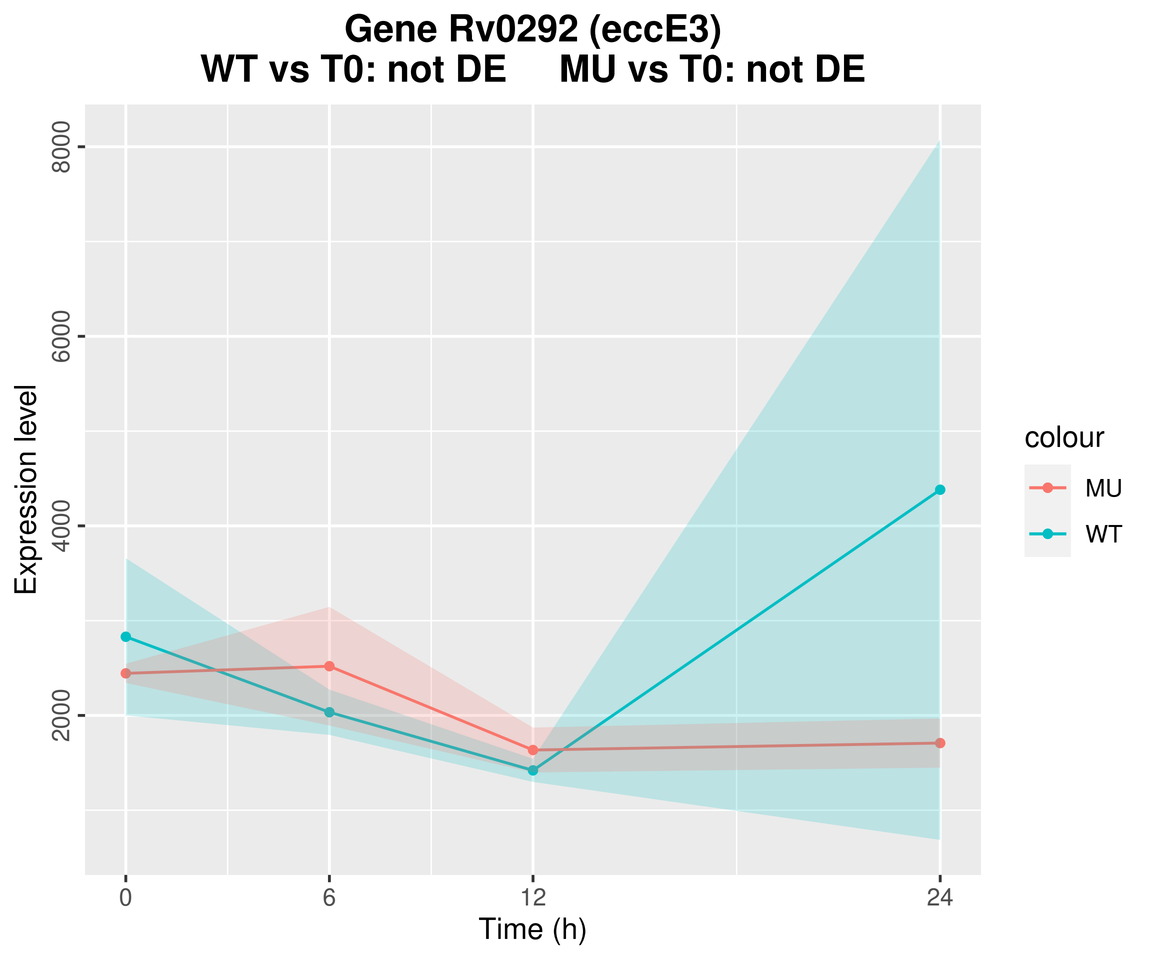
Gene Rv0292 (eccE3)
WT vs T0: not DE   MU vs T0: not DE
Expression level
8000
6000
4000
2000
0
6
12
24
Time (h)
colour
MU
WT

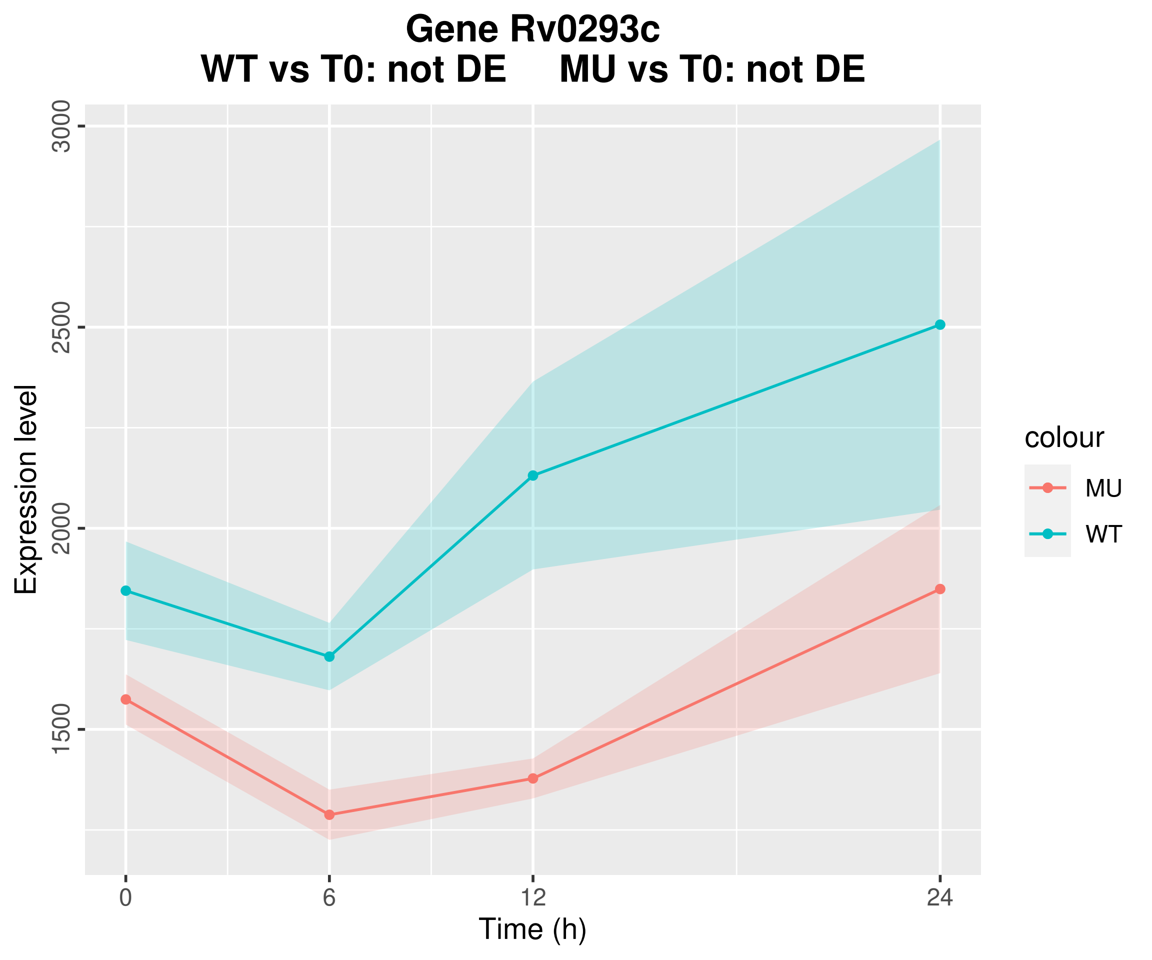

Gene Rv0293c
WT vs T0: not DE MU vs T0: not DE
Expression level
3000
2500
2000
1500
0
6
12
24
Time (h)
colour
MU
WT

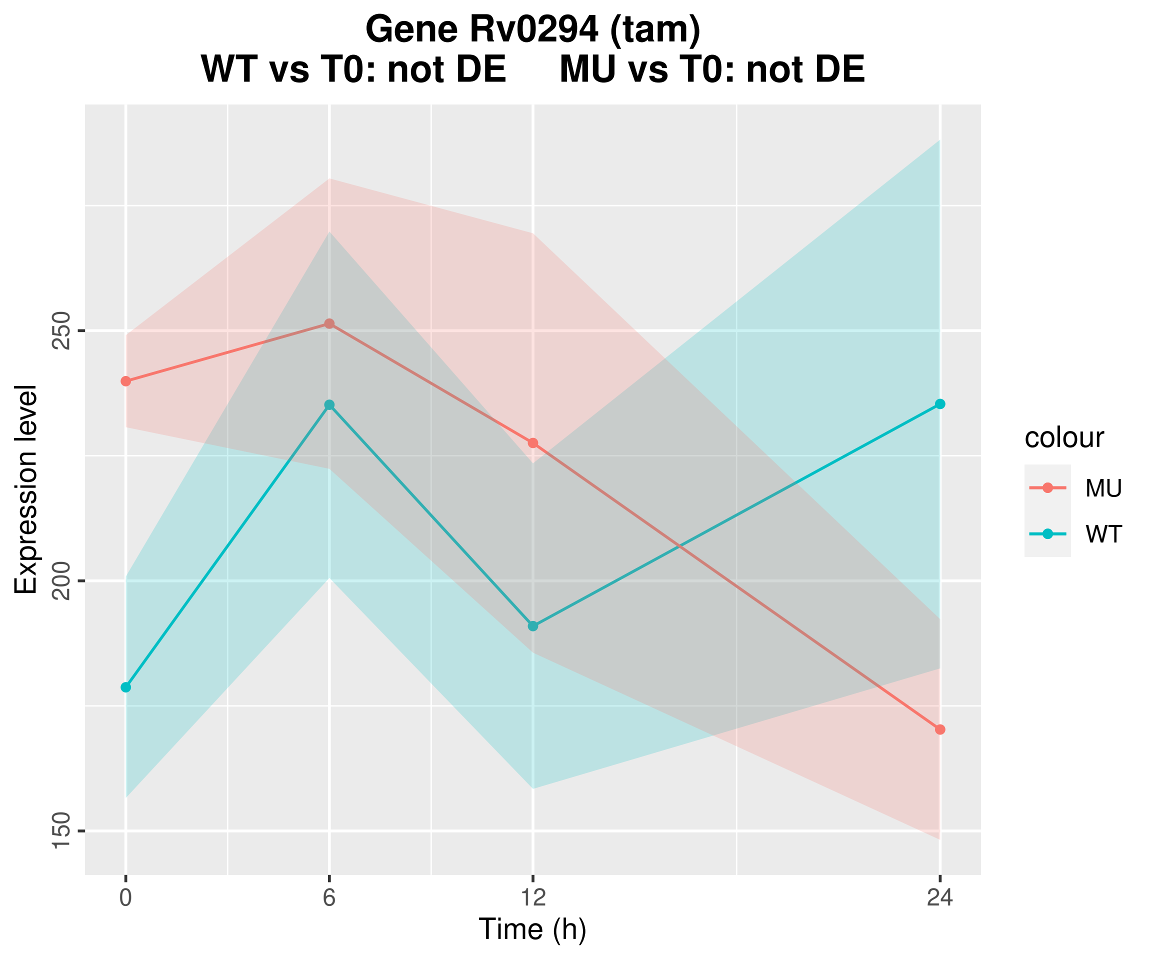
Gene Rv0294 (tam)
WT vs T0: not DE MU vs T0: not DE
Expression level
250
200
150
0
6
12
24
Time (h)
colour
MU
WT

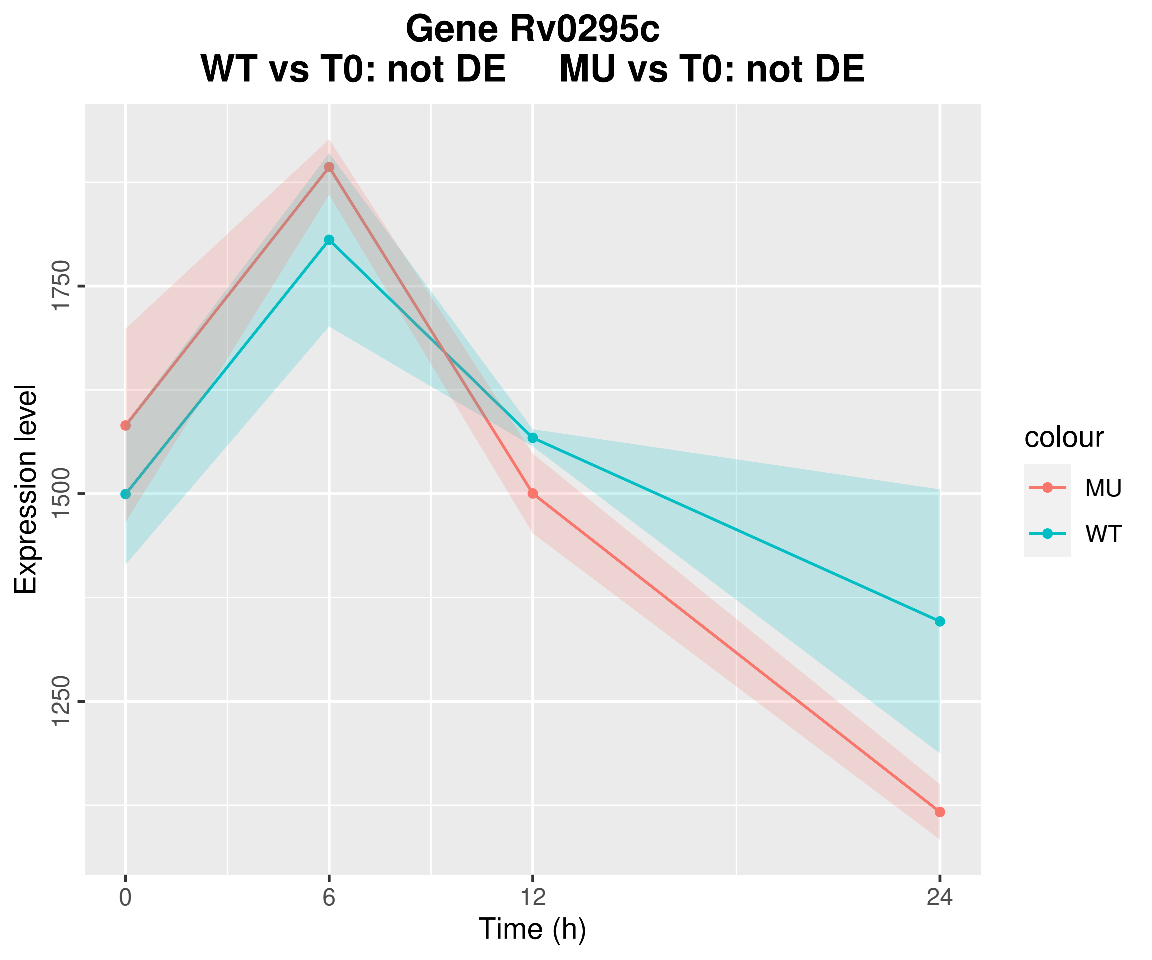

Gene Rv0295c
WT vs T0: not DE MU vs T0: not DE
Expression level
1250
1500
1750
Time (h)
0
6
12
24
colour
MU
WT

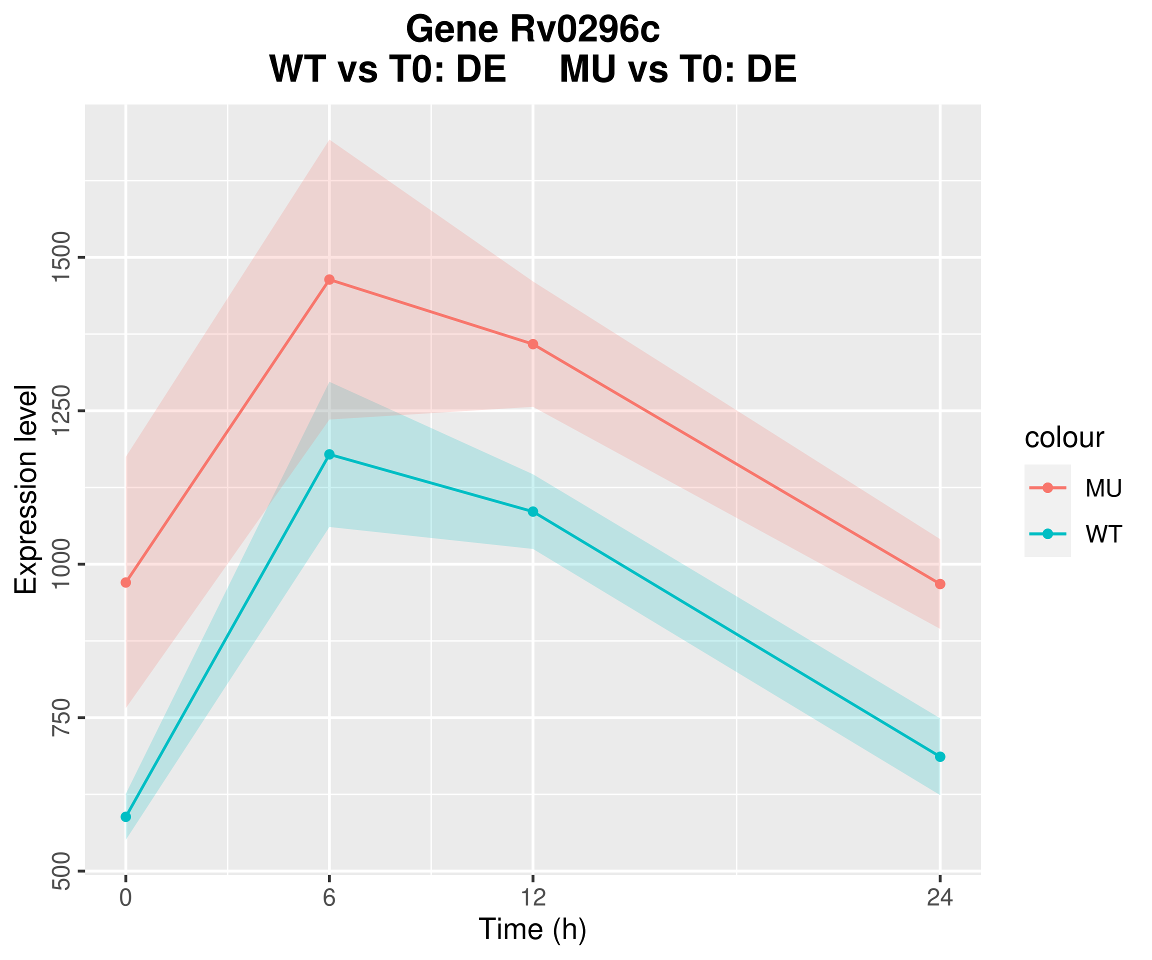

Gene Rv0296c
WT vs T0: DE   MU vs T0: DE
Expression level
500
750
1000
1250
1500
0
6
12
24
Time (h)
colour
MU
WT

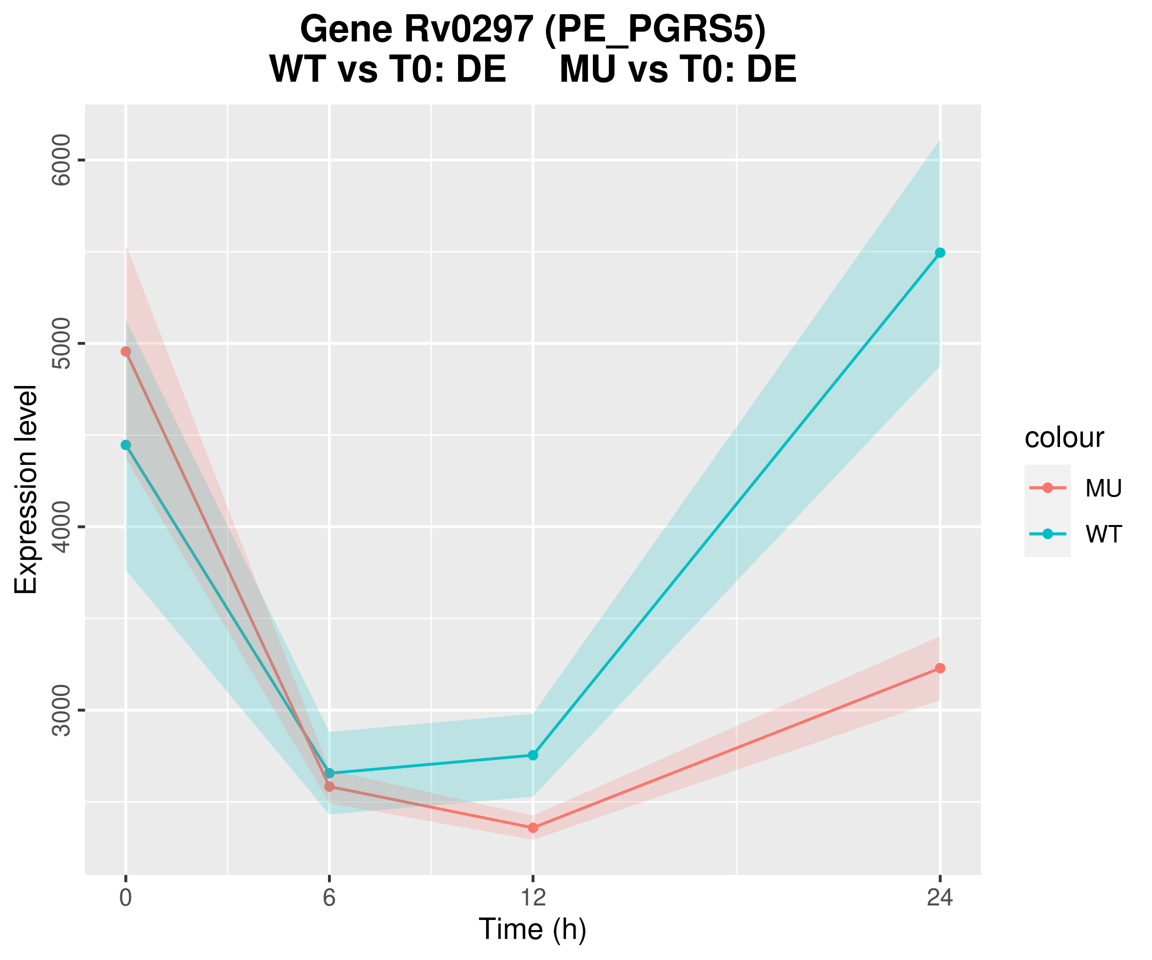

Gene Rv0297 (PE_PGRS5)
WT vs T0: DE   MU vs T0: DE
Expression level
6000
5000
4000
3000
0
6
12
24
Time (h)
colour
MU
WT

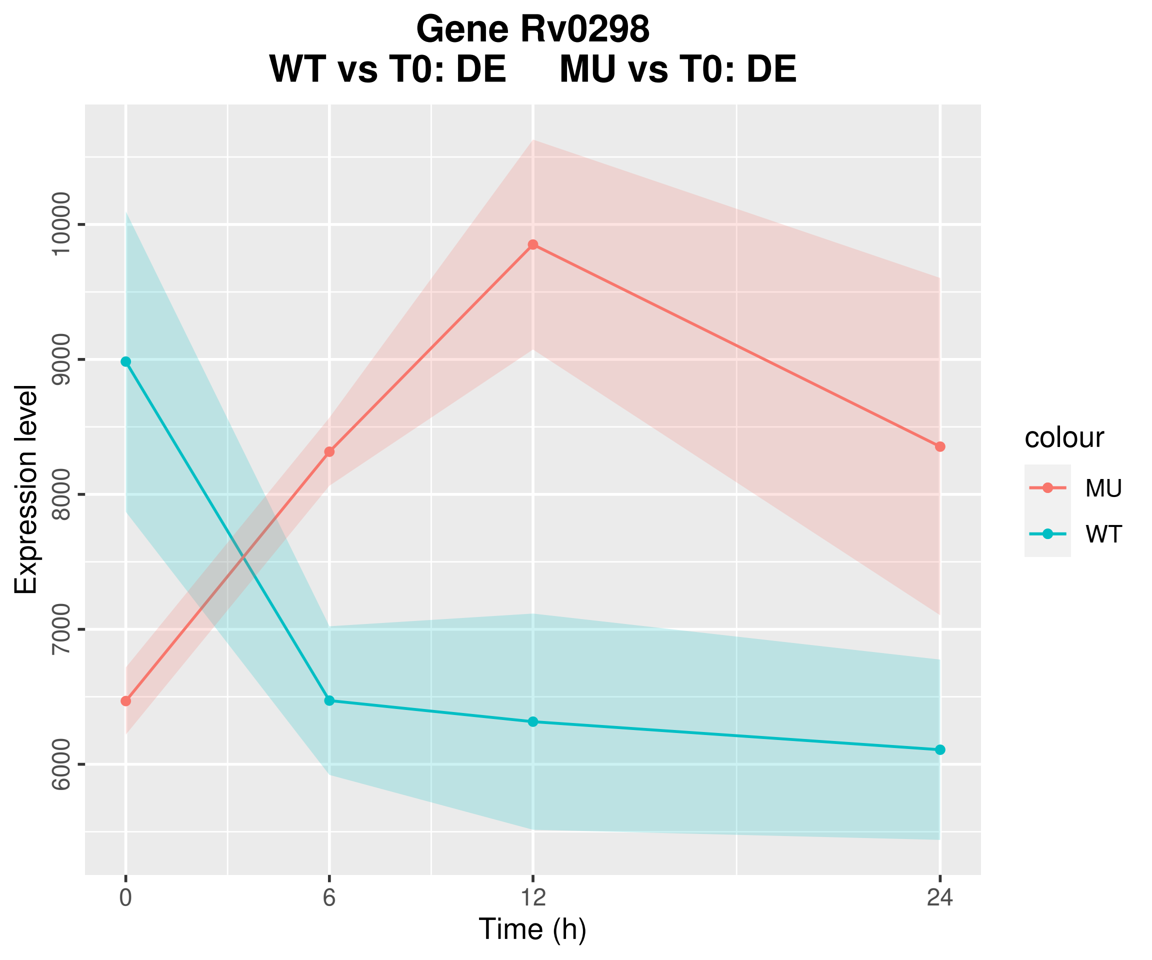
Gene Rv0298
WT vs T0: DE MU vs T0: DE
Expression level
10000
9000
8000
7000
6000
0
6
12
24
Time (h)
colour
MU
WT

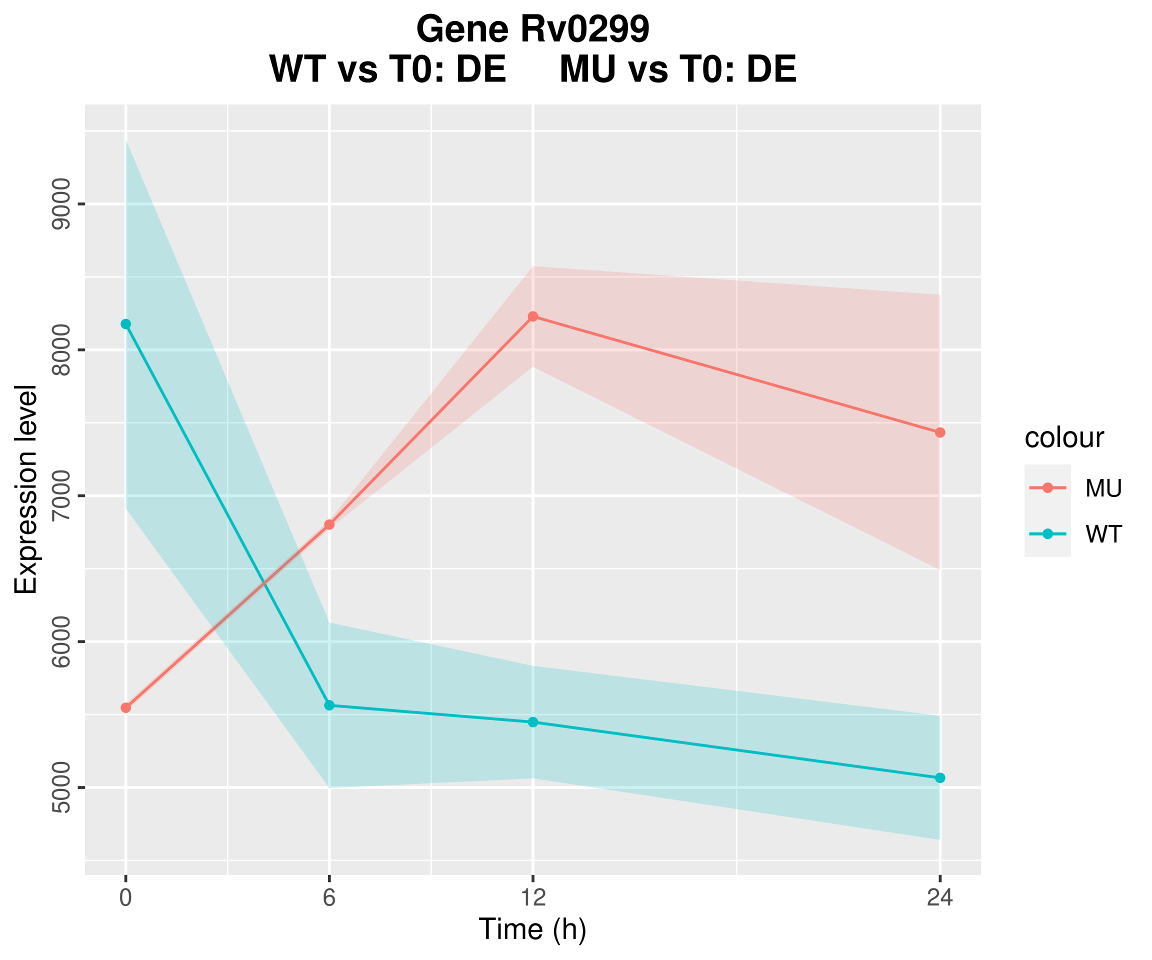

Gene Rv0299
WT vs T0: DE MU vs T0: DE
Expression level
9000
8000
7000
6000
5000
0
6
12
24
Time (h)
colour
MU
WT

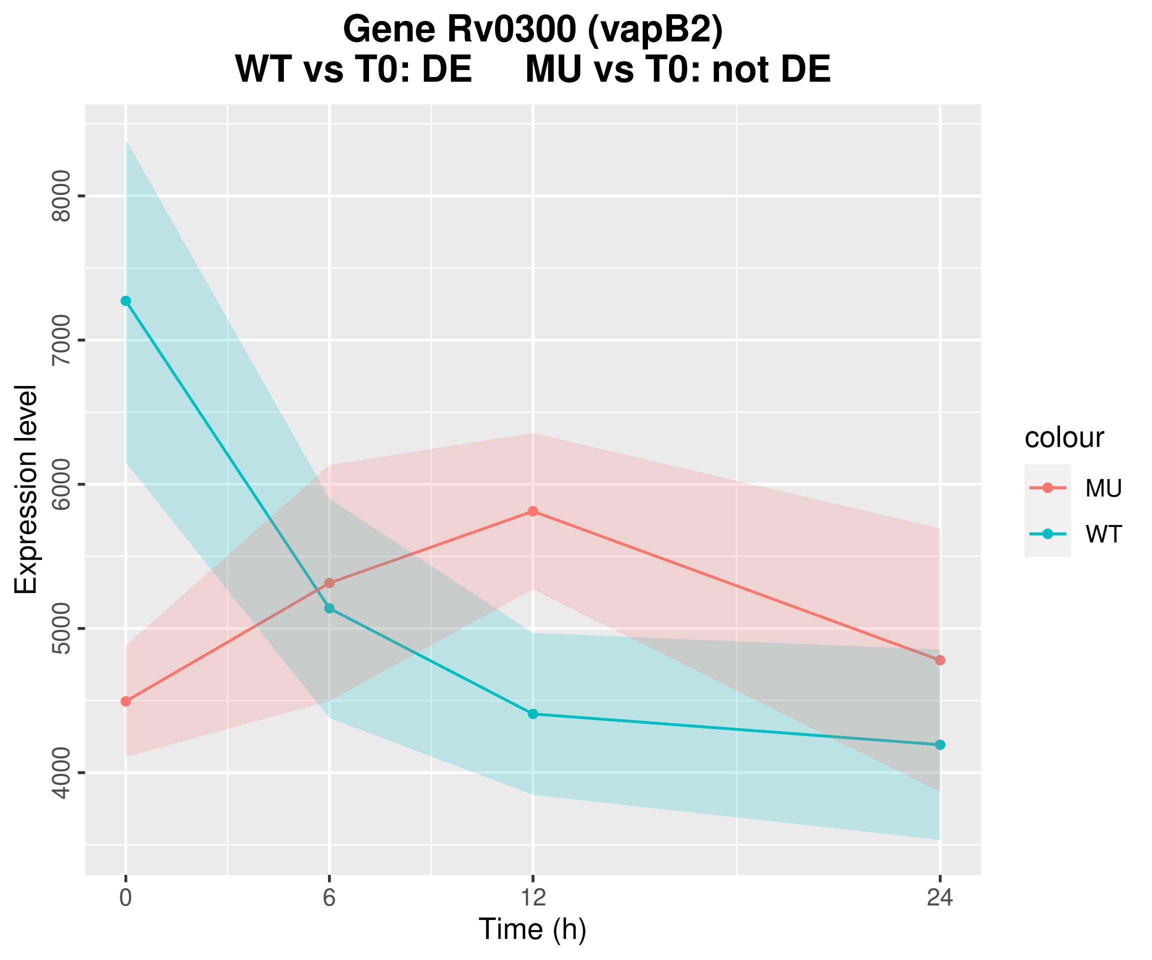
Gene Rv0300 (vapB2)
WT vs T0: DE   MU vs T0: not DE
Expression level
8000
7000
6000
5000
4000
0
6
12
24
Time (h)
colour
MU
WT

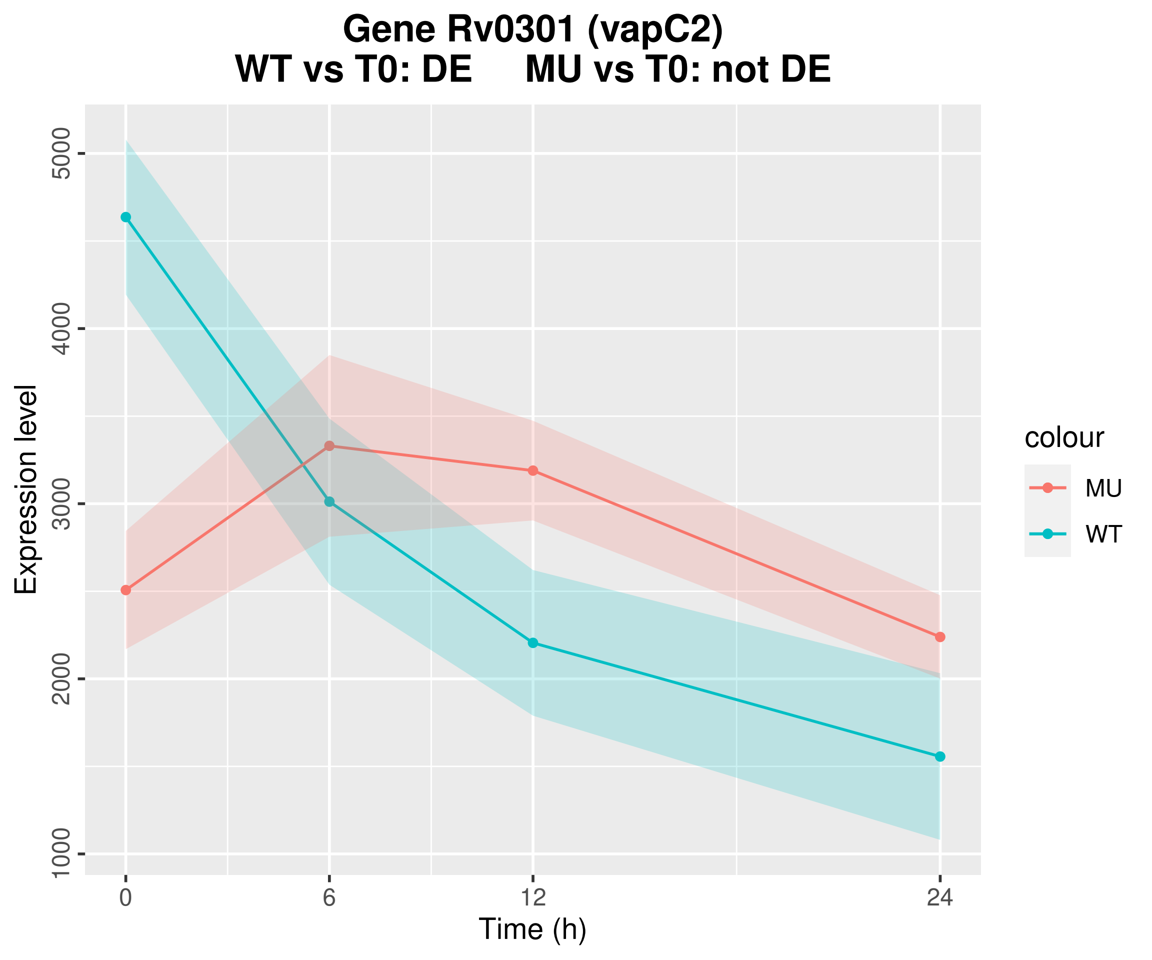

Gene Rv0301 (vapC2)
WT vs T0: DE   MU vs T0: not DE
Expression level
5000
4000
3000
2000
1000
0
6
12
24
Time (h)
colour
MU
WT

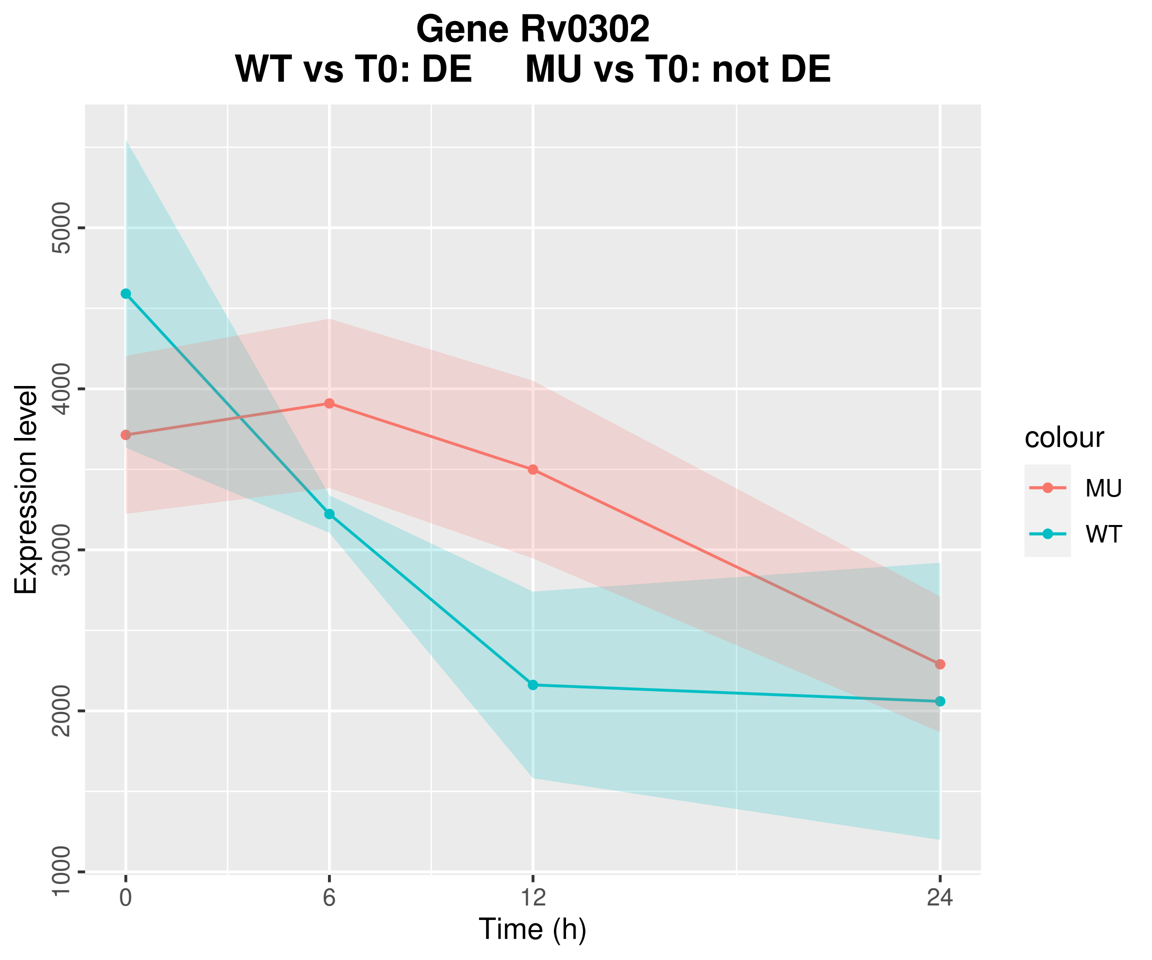

Gene Rv0302
WT vs T0: DE MU vs T0: not DE
Expression level
1000
2000
3000
4000
5000
0
6
12
24
Time (h)
colour
MU
WT

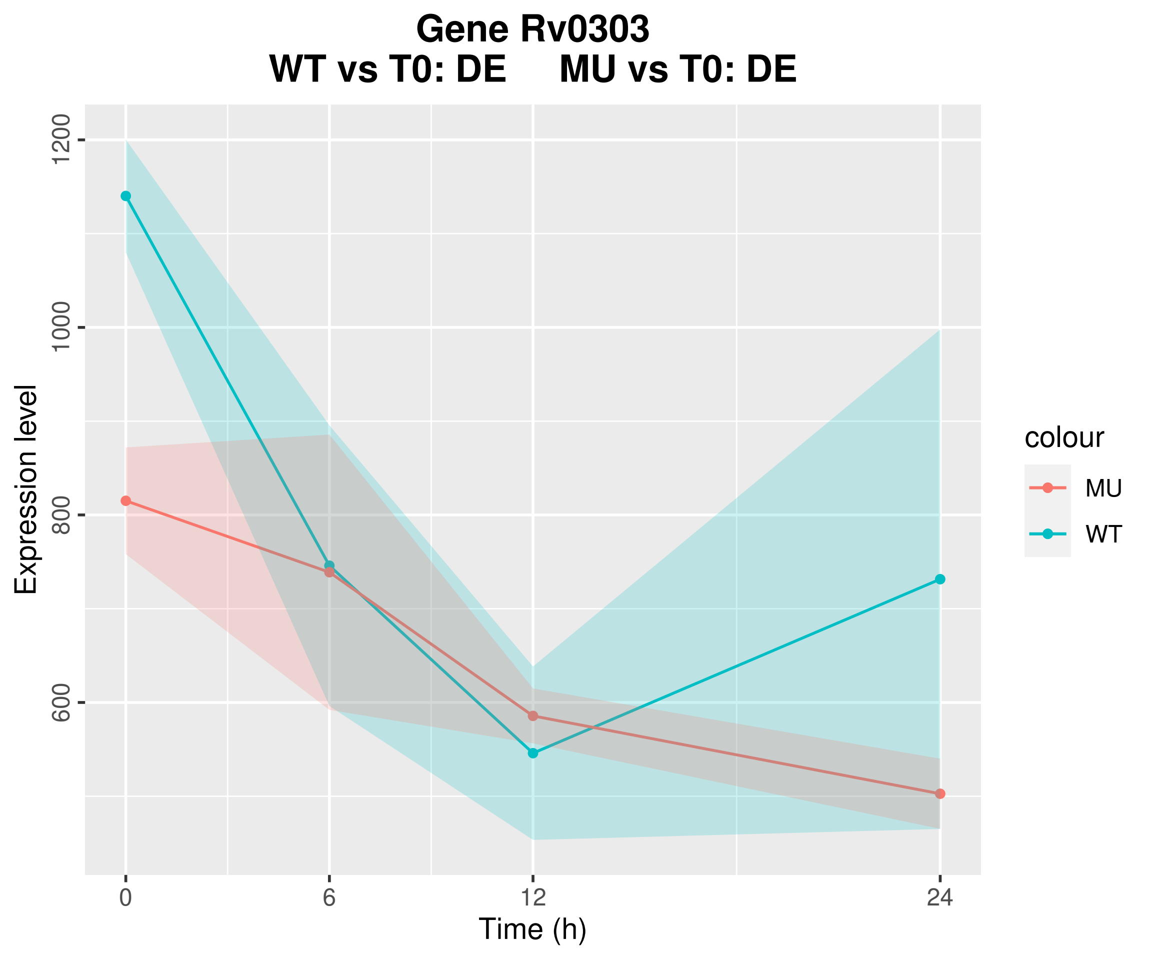
Gene Rv0303
WT vs T0: DE MU vs T0: DE
Expression level
1200
1000
800
600
0
6
12
24
Time (h)
colour
MU
WT

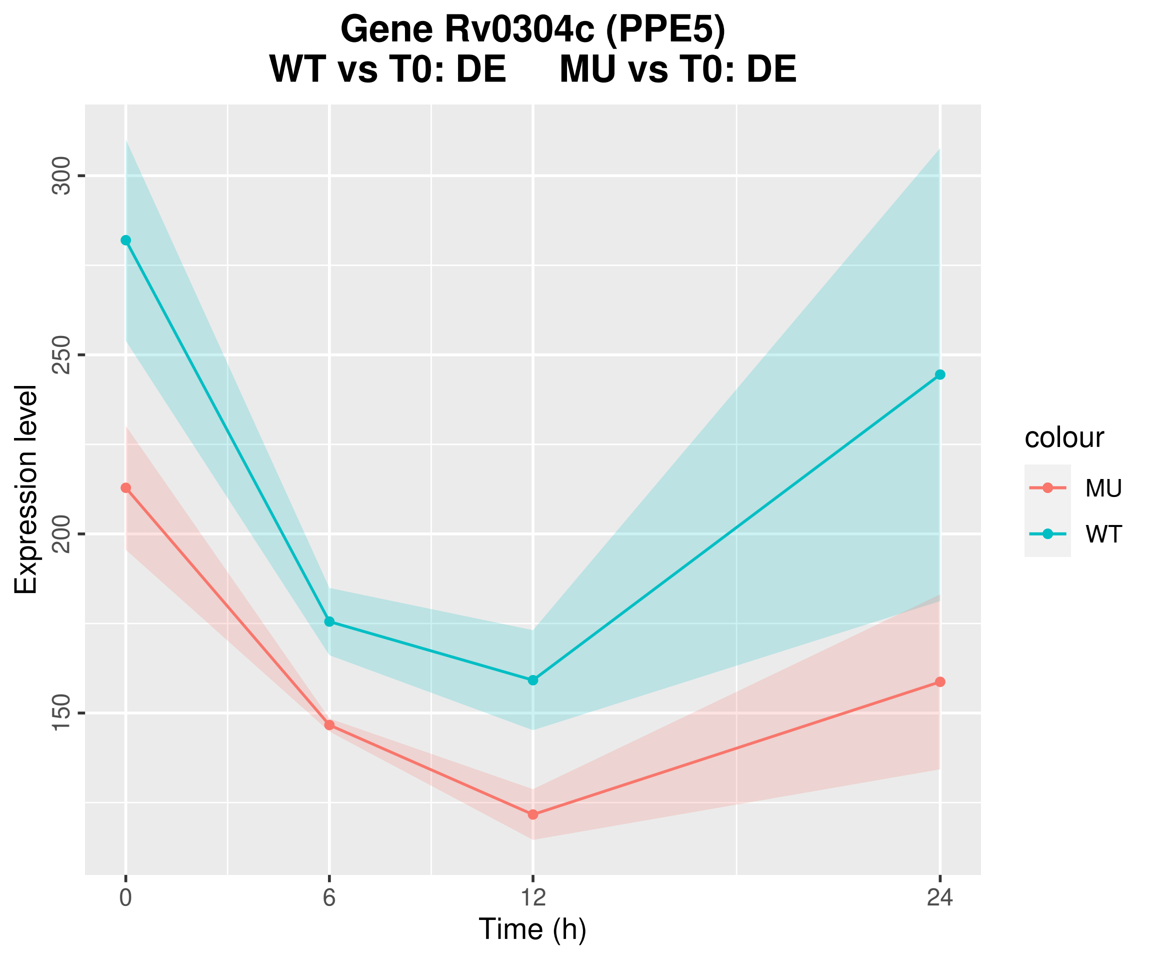
Gene Rv0304c (PPE5)
WT vs T0: DE   MU vs T0: DE
Expression level
300
250
200
150
0
6
12
24
Time (h)
colour
MU
WT

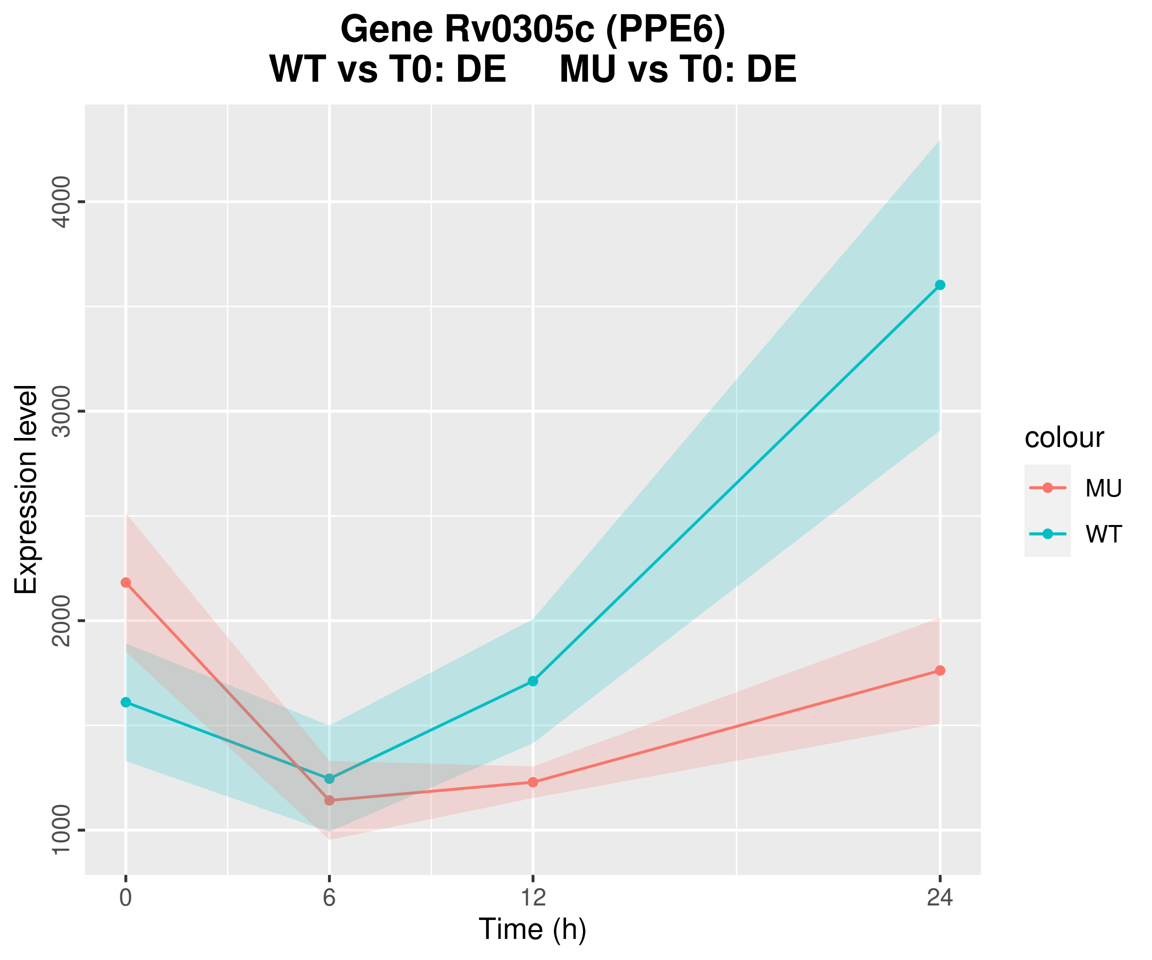

Gene Rv0305c (PPE6)
WT vs T0: DE MU vs T0: DE
Expression level
4000
3000
2000
1000
0
6
12
24
Time (h)
colour
MU
WT

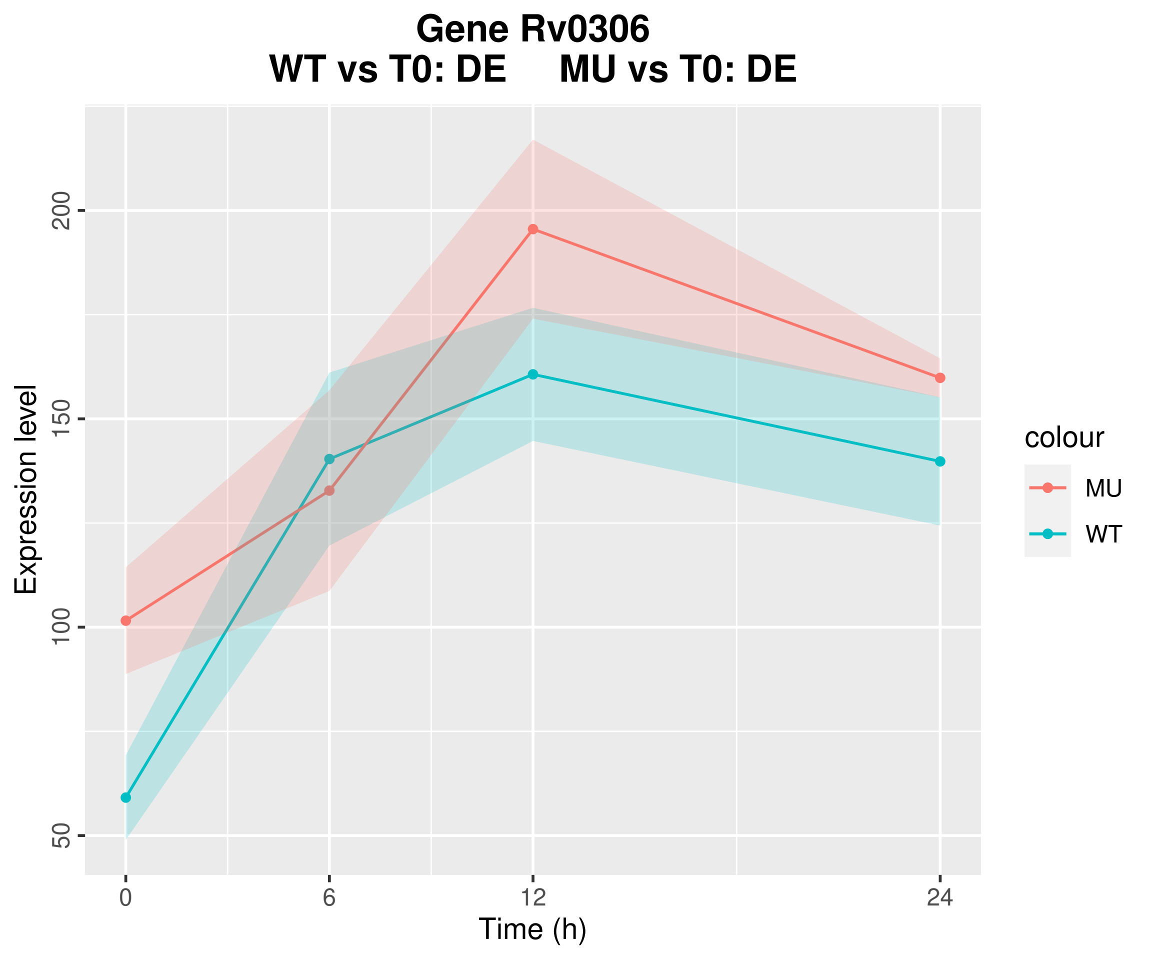

Gene Rv0306
WT vs T0: DE MU vs T0: DE
Expression level
200
150
100
50
0
6
12
24
Time (h)
colour
MU
WT

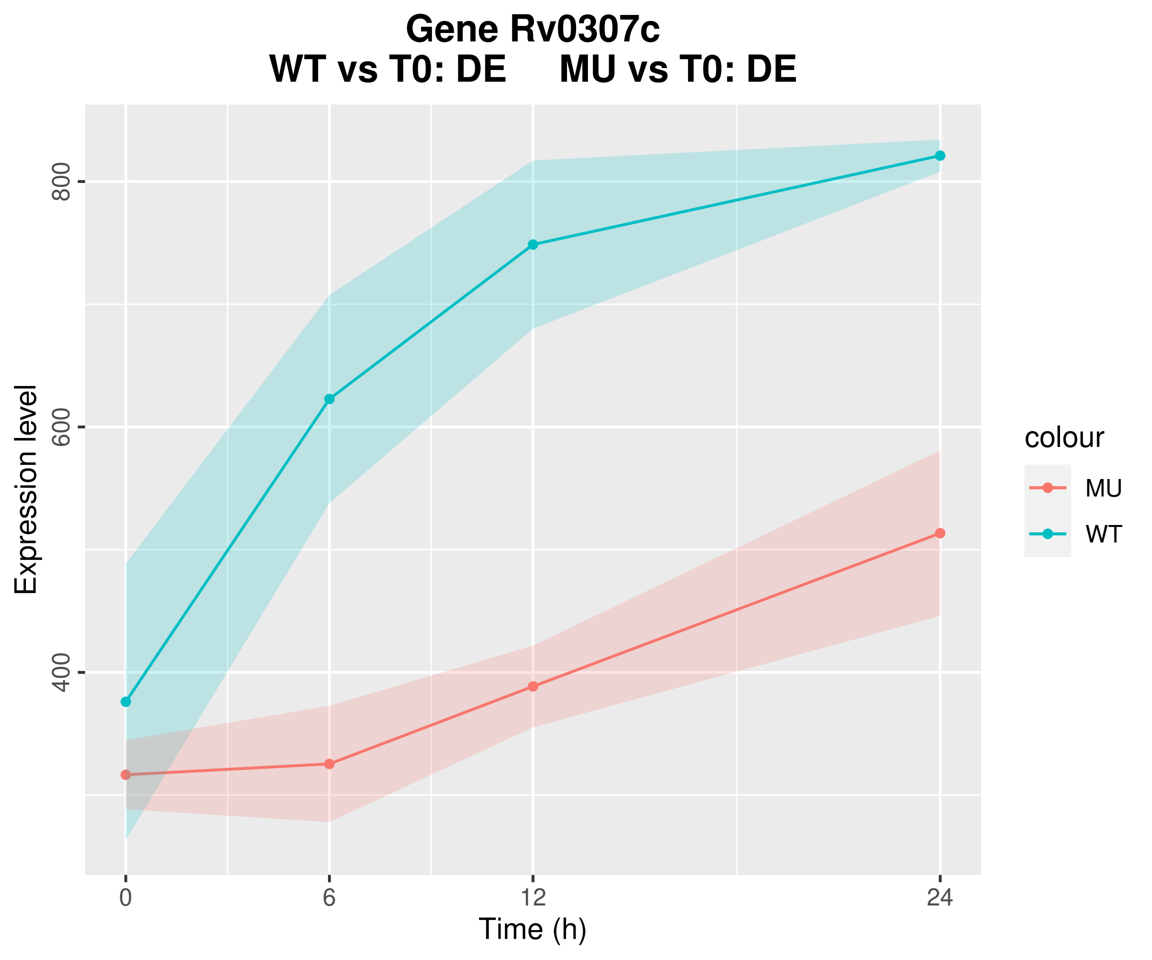
Gene Rv0307c
WT vs T0: DE MU vs T0: DE
800
600
400
Expression level
0
6
12
24
Time (h)
colour
MU
WT

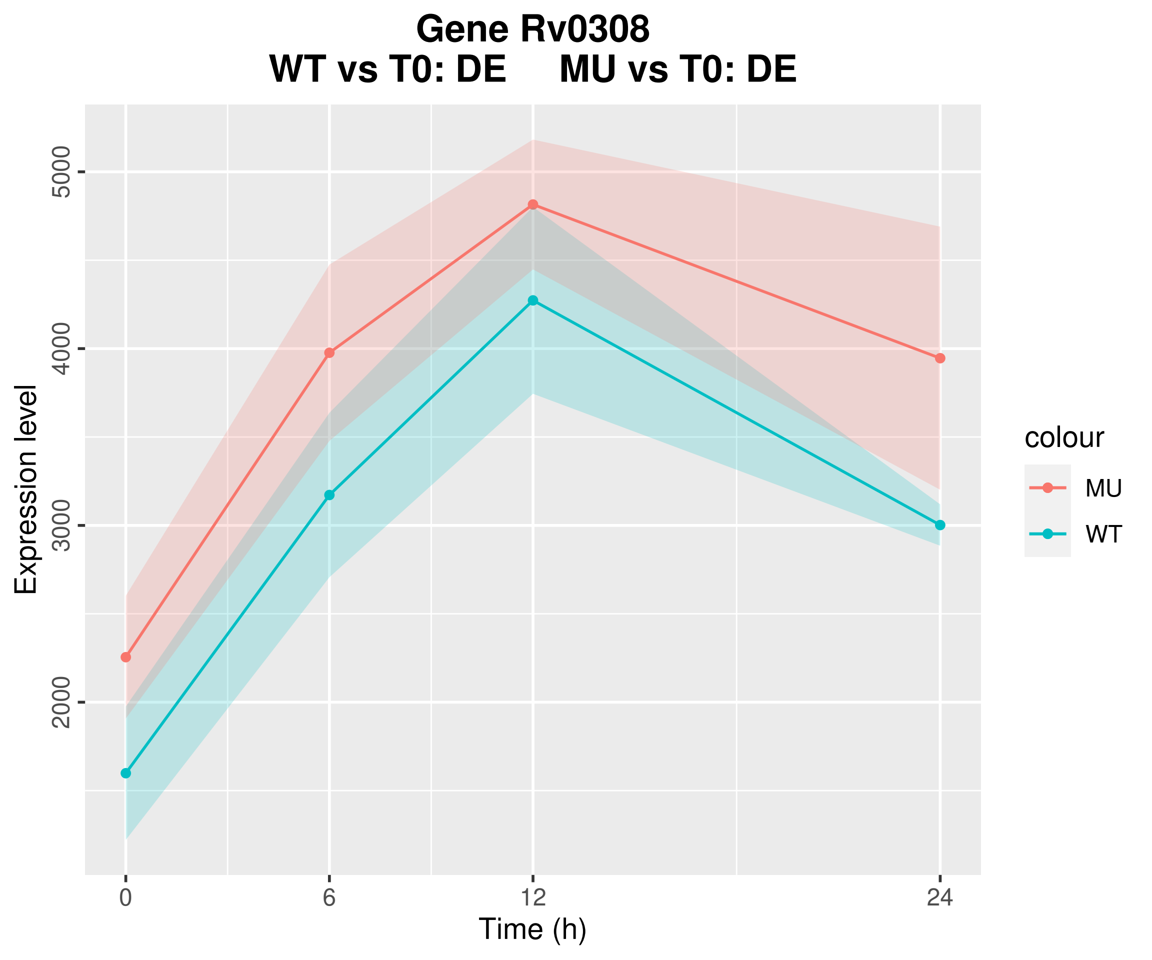

Gene Rv0308
WT vs T0: DE   MU vs T0: DE
Expression level
5000
4000
3000
2000
0
6
12
24
Time (h)
colour
MU
WT

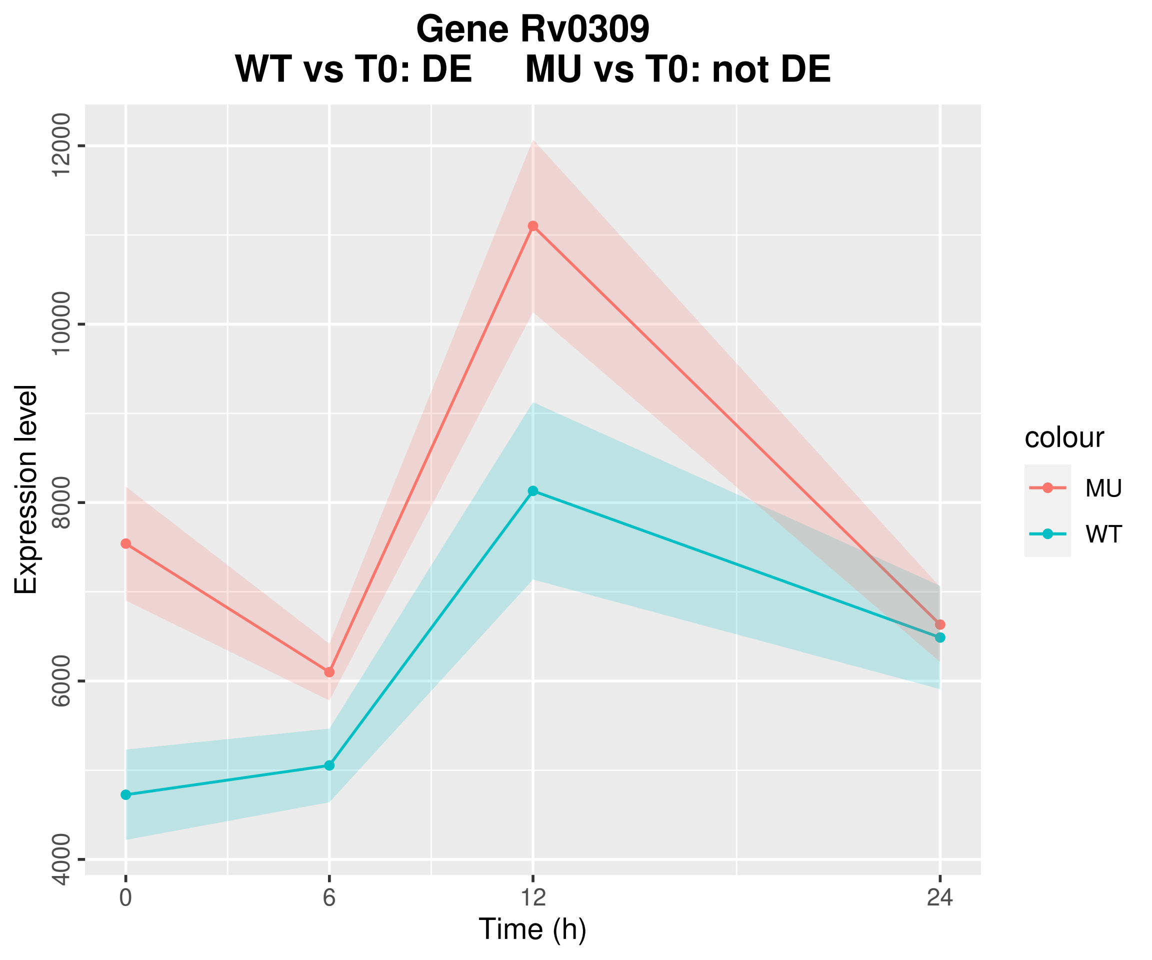
Gene Rv0309
WT vs T0: DE MU vs T0: not DE
Expression level
12000
10000
8000
6000
4000
0
6
12
24
Time (h)
colour
MU
WT

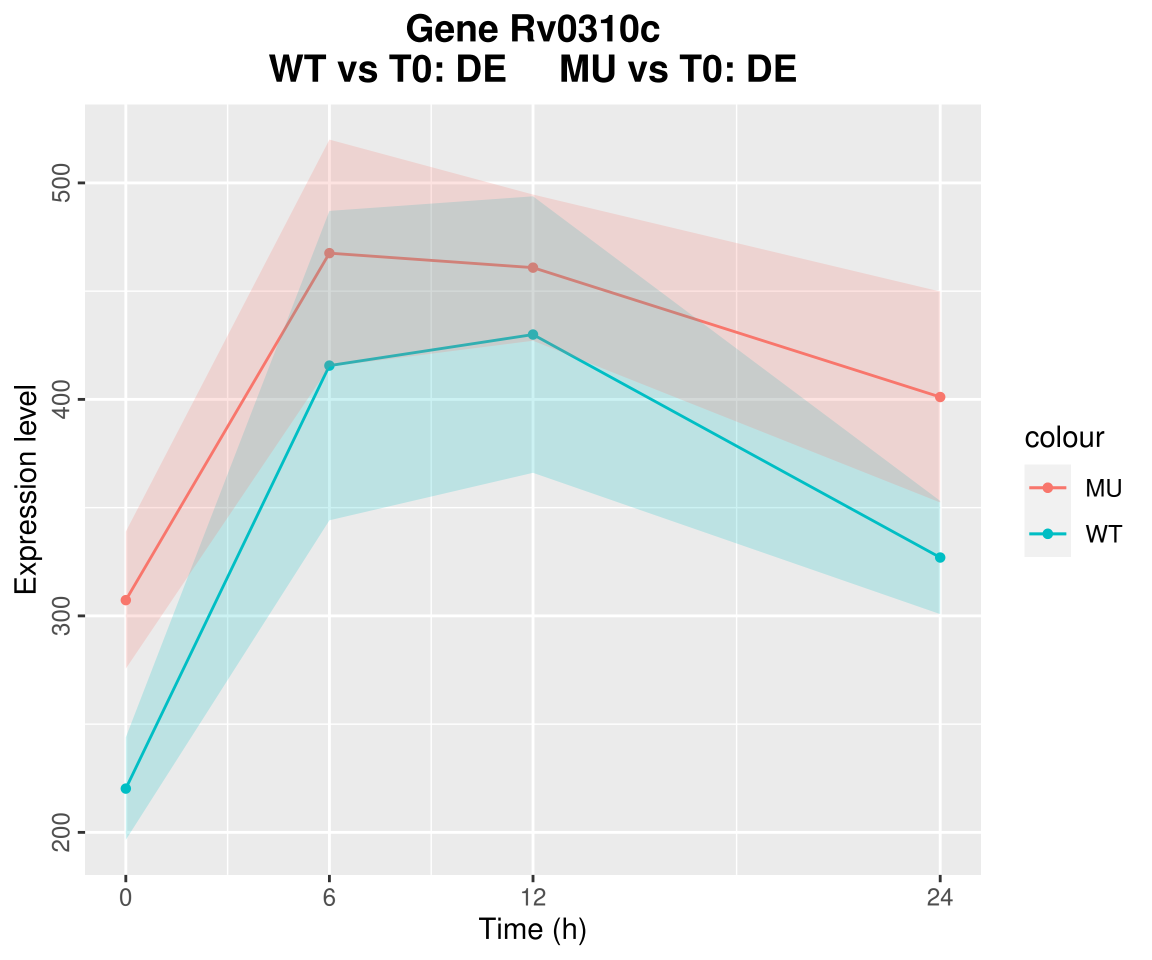
Gene Rv0310c
WT vs T0: DE   MU vs T0: DE
Expression level
500
400
300
200
0
6
12
24
Time (h)
colour
MU
WT

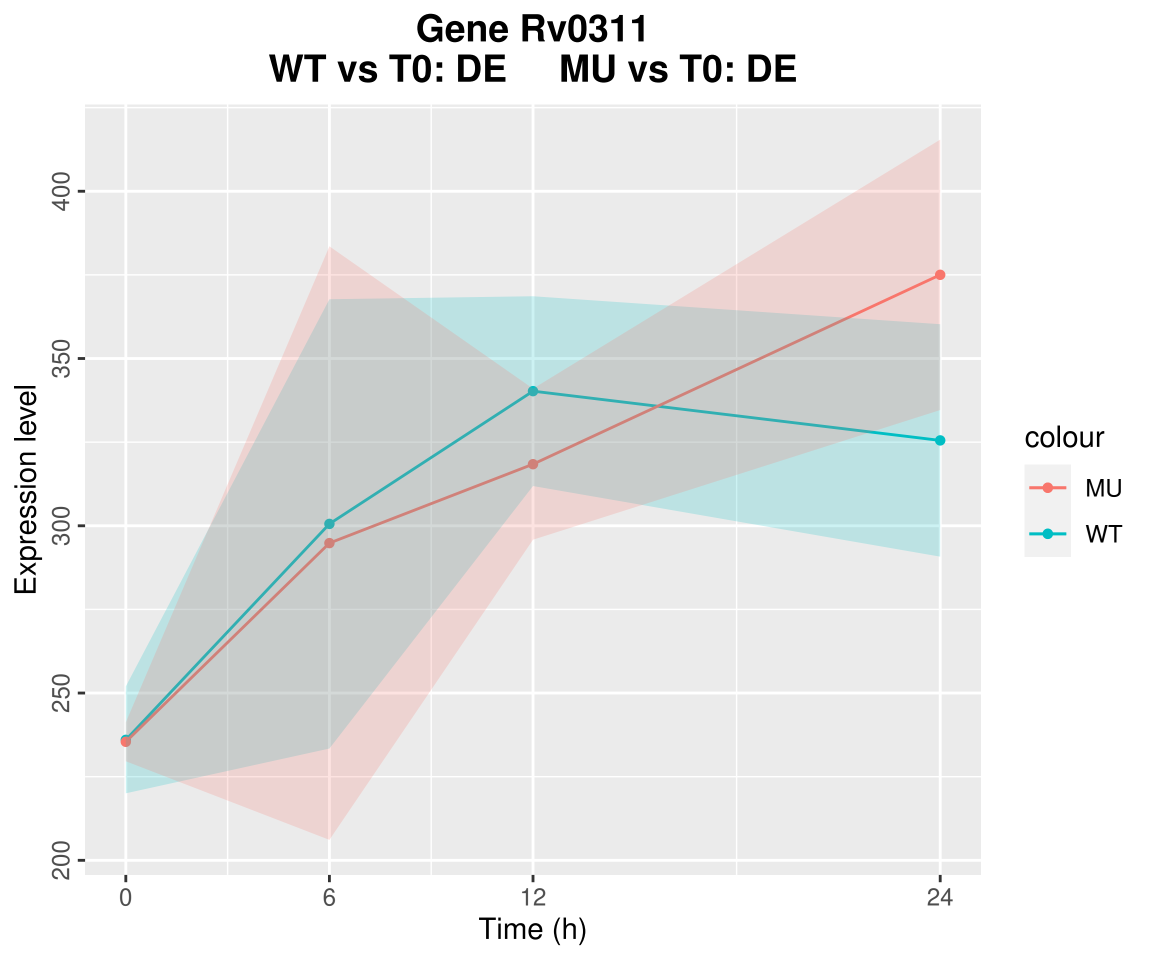

Gene Rv0311
WT vs T0: DE   MU vs T0: DE
Expression level
400
350
300
250
200
0
6
12
24
Time (h)
colour
MU
WT

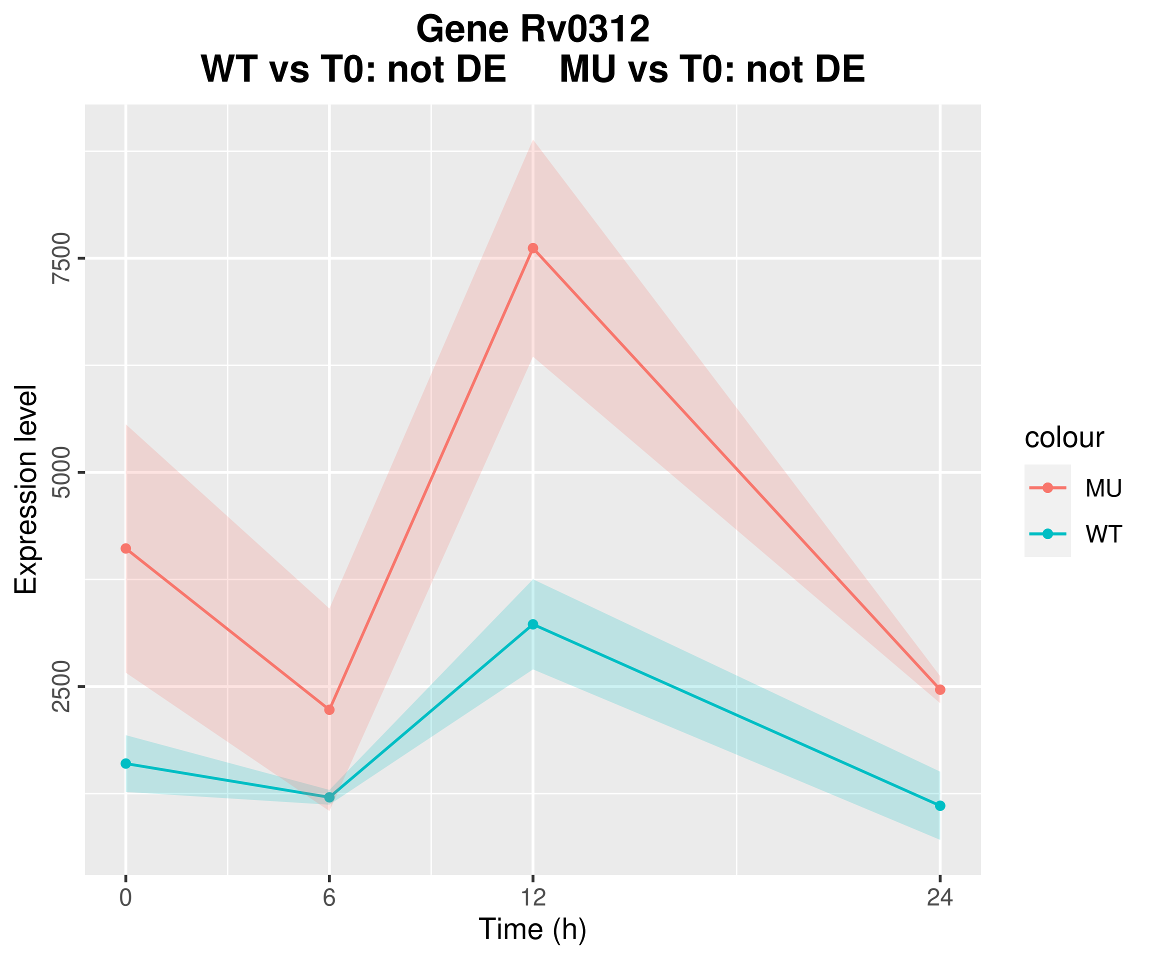
Gene Rv0312
WT vs T0: not DE MU vs T0: not DE
Expression level
7500
5000
2500
0
6
12
24
Time (h)
colour
MU
WT

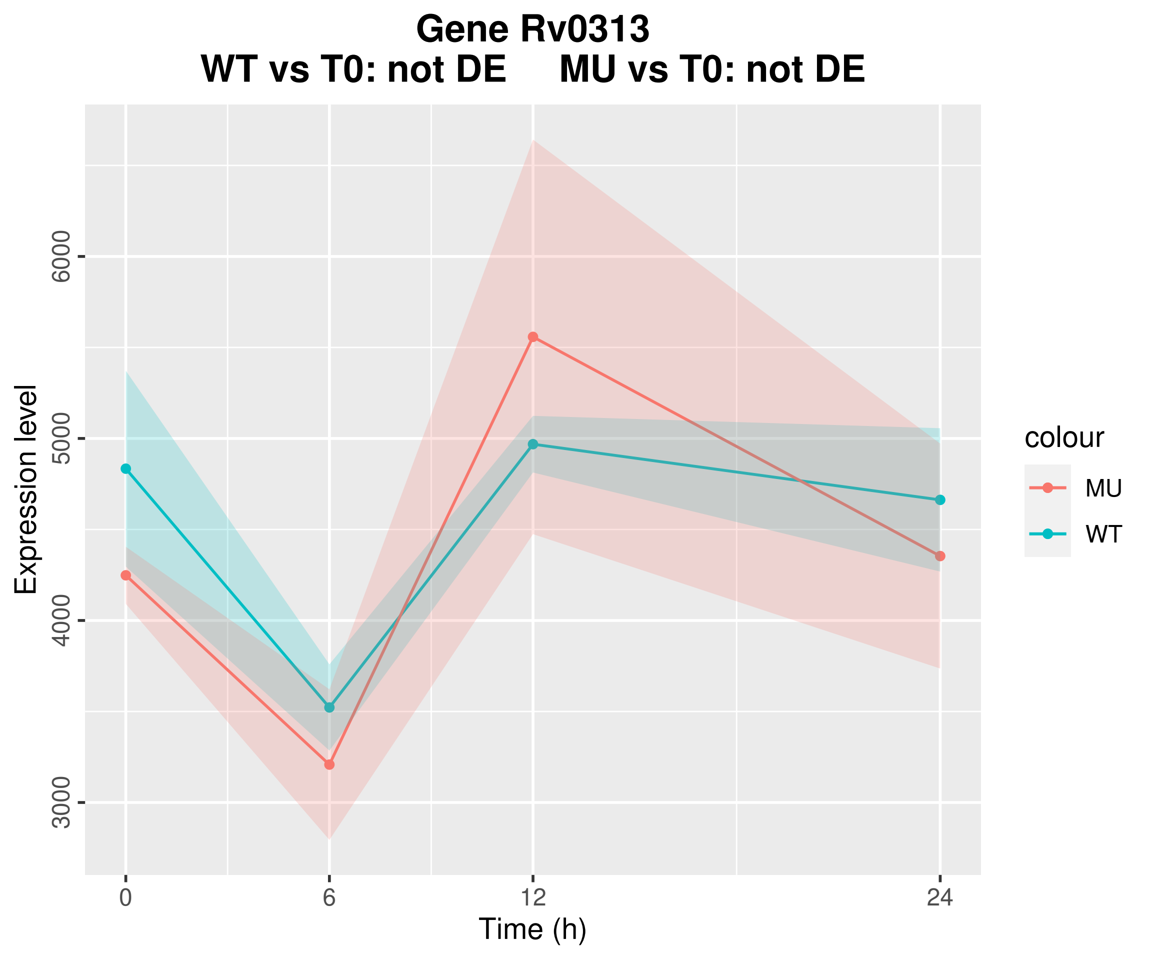

Gene Rv0313
WT vs T0: not DE   MU vs T0: not DE
Expression level
6000
5000
4000
3000
0
6
12
24
Time (h)
colour
MU
WT

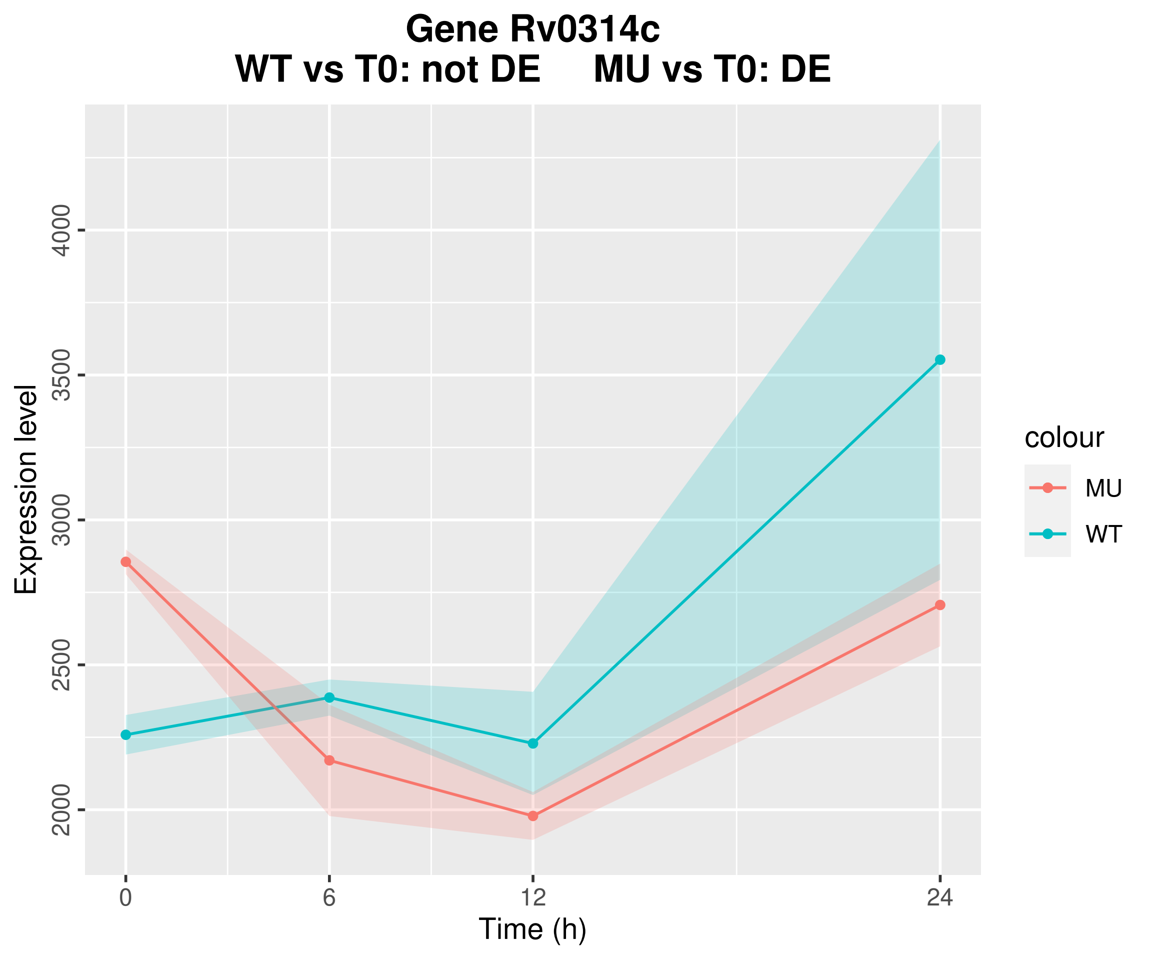

Gene Rv0314c
WT vs T0: not DE   MU vs T0: DE
Expression level
4000
3500
3000
2500
2000
0
6
12
24
Time (h)
colour
MU
WT

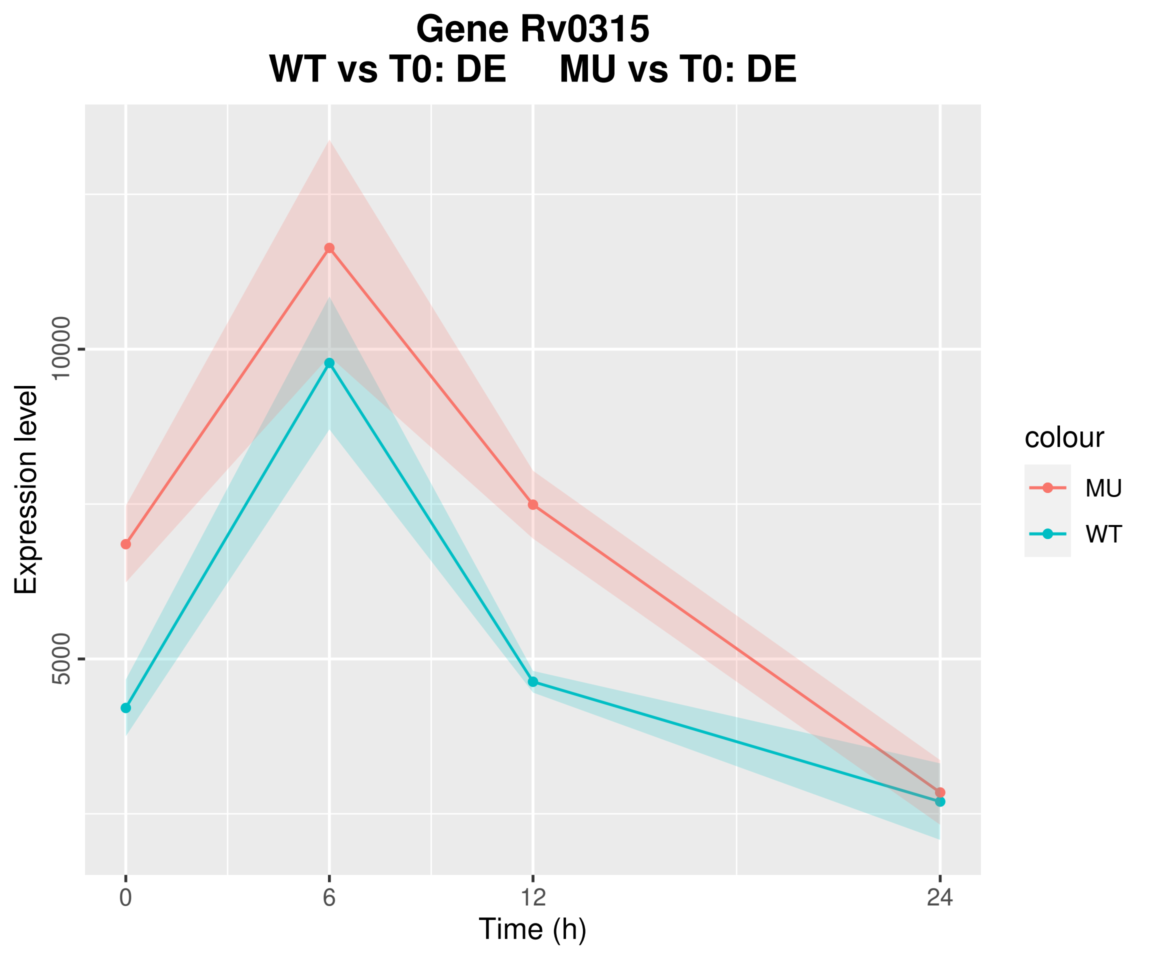

Gene Rv0315
WT vs T0: DE MU vs T0: DE
Expression level
10000
5000
0
6
12
24
Time (h)
colour
MU
WT

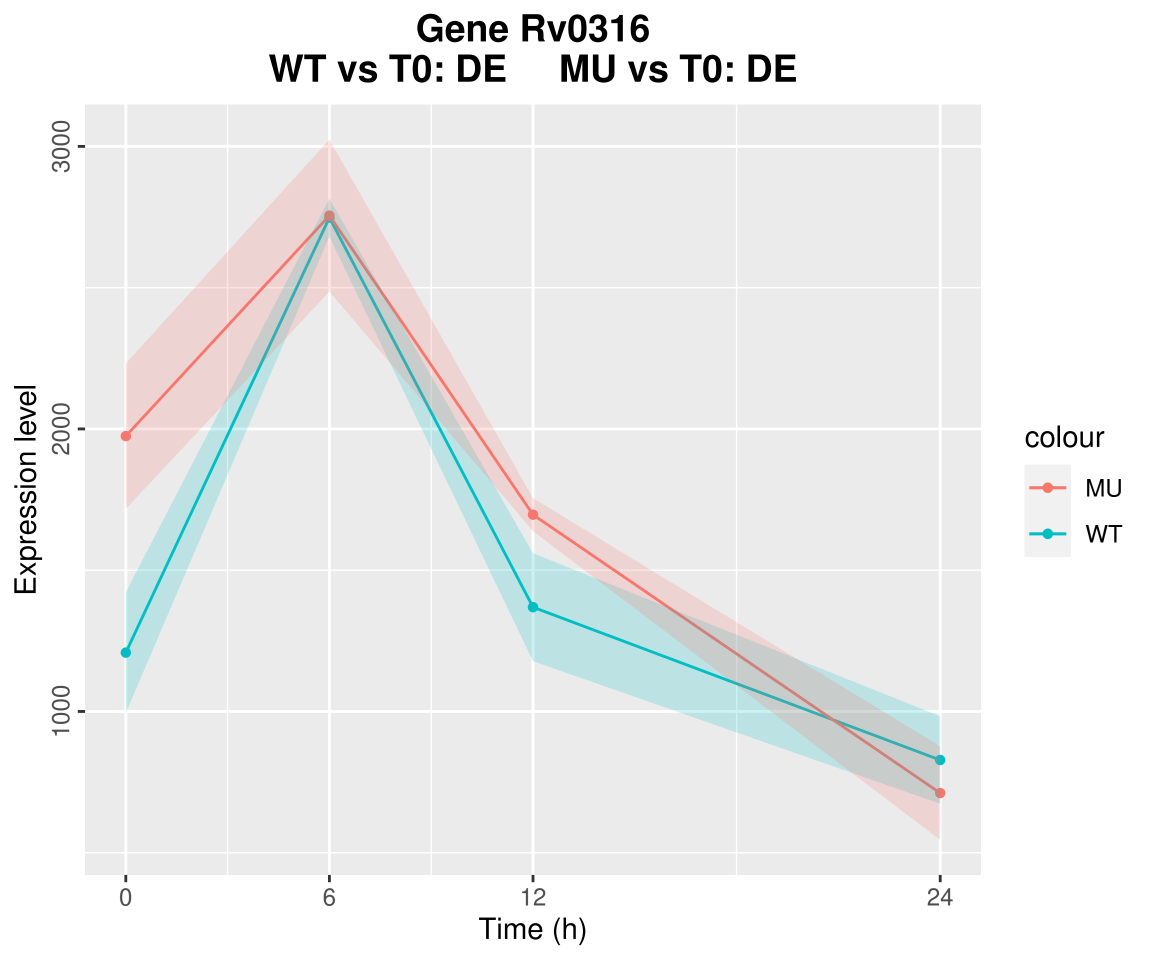

Gene Rv0316
WT vs T0: DE MU vs T0: DE
3000
2000
1000
Expression level
0
6
12
24
Time (h)
colour
MU
WT

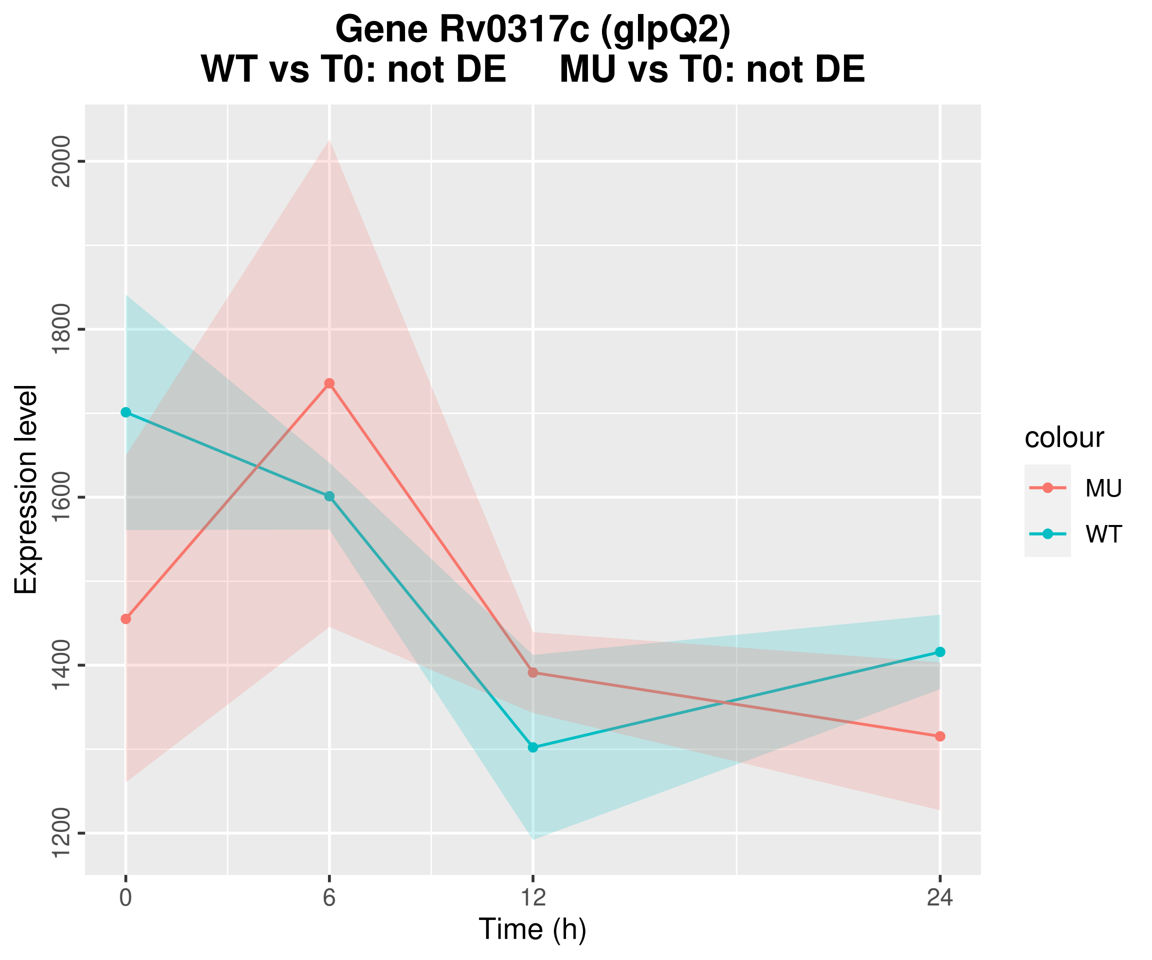

Gene Rv0317c (glpQ2)
WT vs T0: not DE   MU vs T0: not DE
Expression level
2000
1800
1600
1400
1200
0
6
12
24
Time (h)
colour
MU
WT

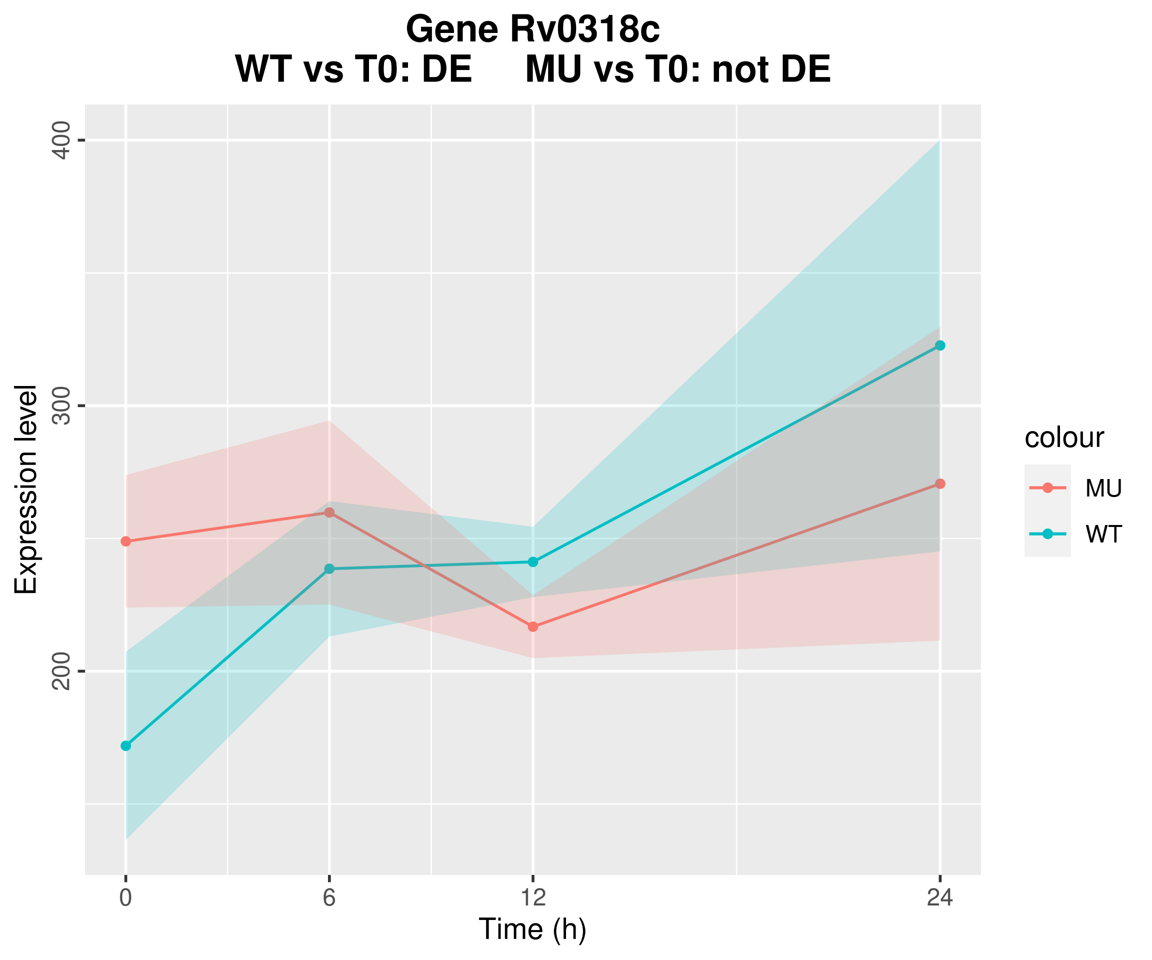

Gene Rv0318c
WT vs T0: DE MU vs T0: not DE
Expression level
400
300
200
0
6
12
24
Time (h)
colour
MU
WT

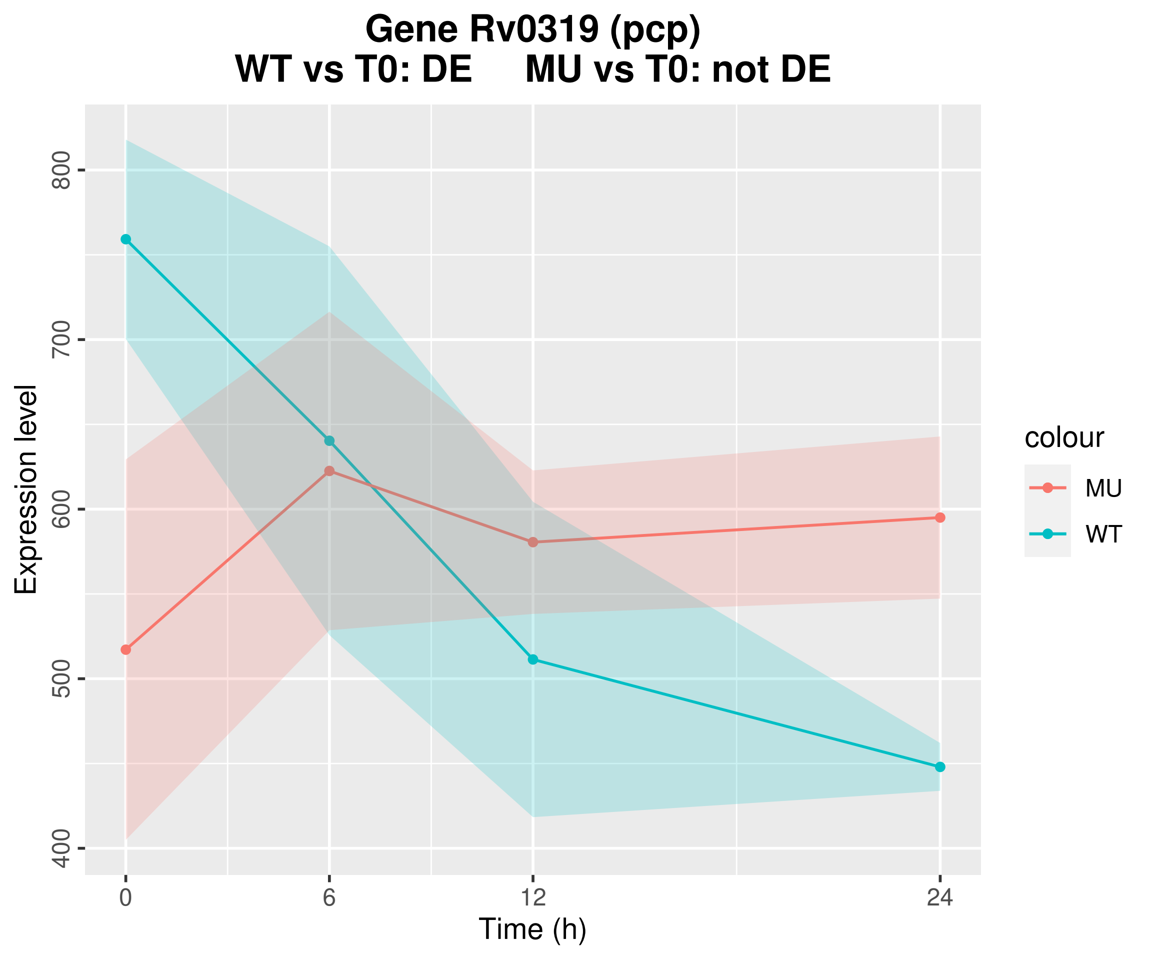

Gene Rv0319 (pcp)
WT vs T0: DE   MU vs T0: not DE
Expression level
800
700
600
500
400
0
6
12
24
Time (h)
colour
MU
WT

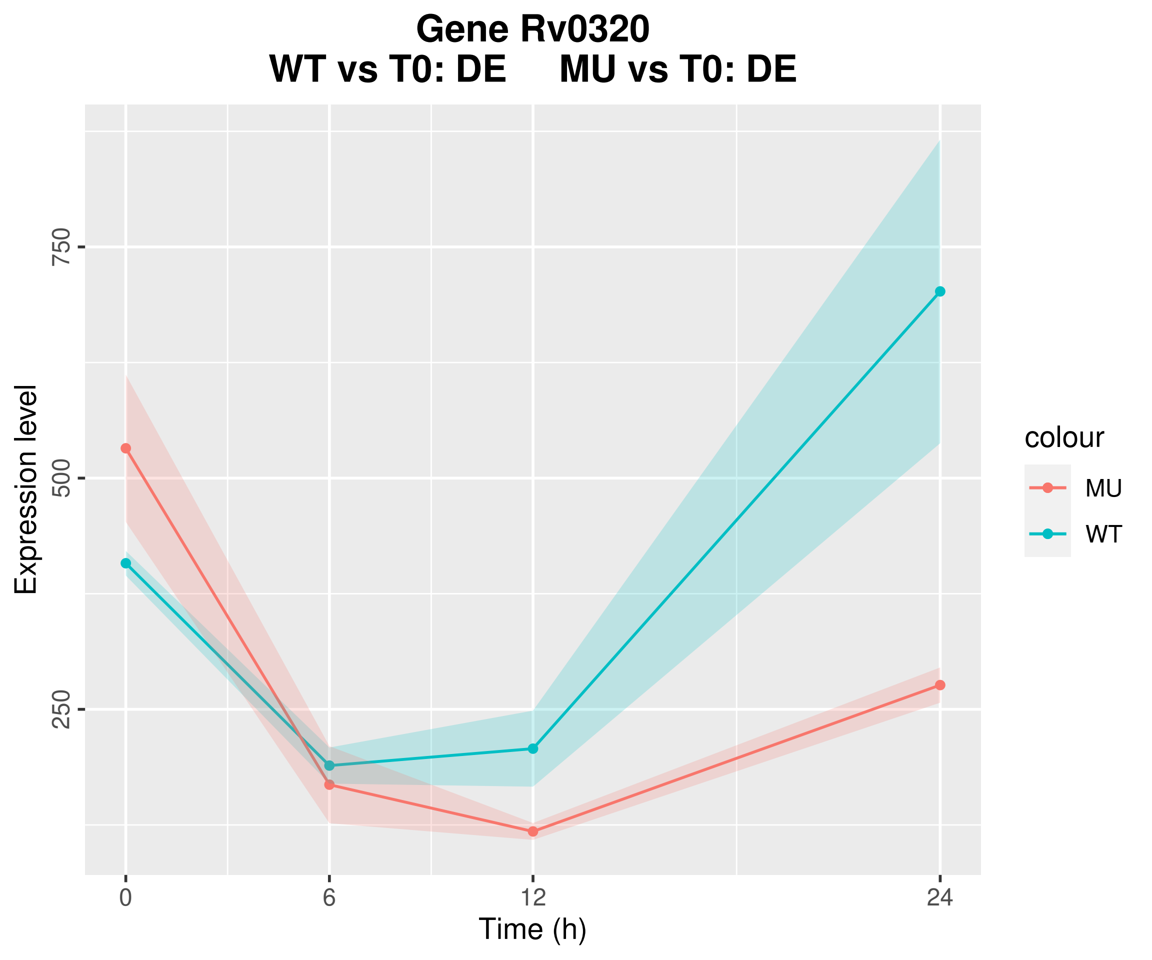
Gene Rv0320
WT vs T0: DE MU vs T0: DE
Expression level
750
500
250
0
6
12
24
Time (h)
colour
MU
WT

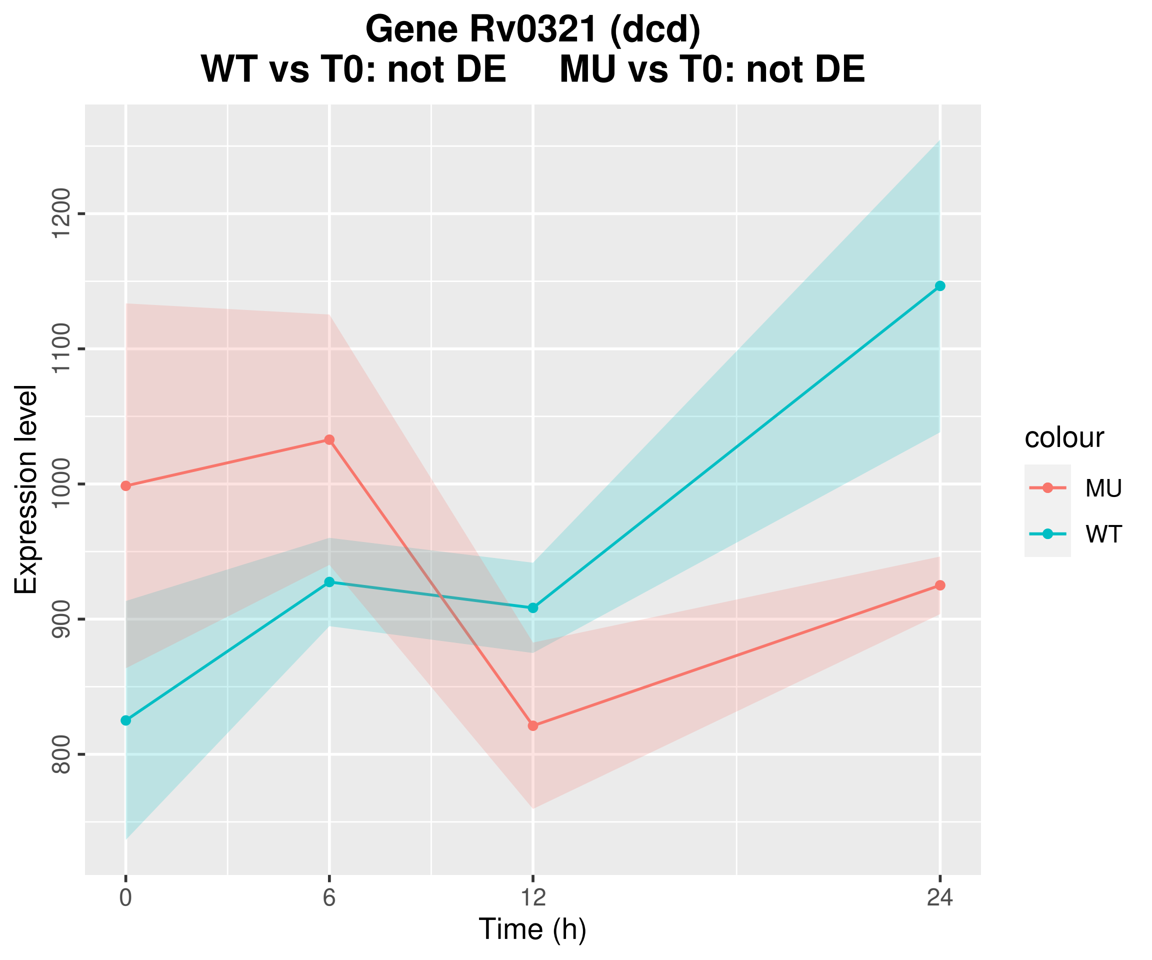

Gene Rv0321 (dcd)
WT vs T0: not DE MU vs T0: not DE
Expression level
1200
1100
1000
900
800
0
6
12
24
Time (h)
colour
MU
WT

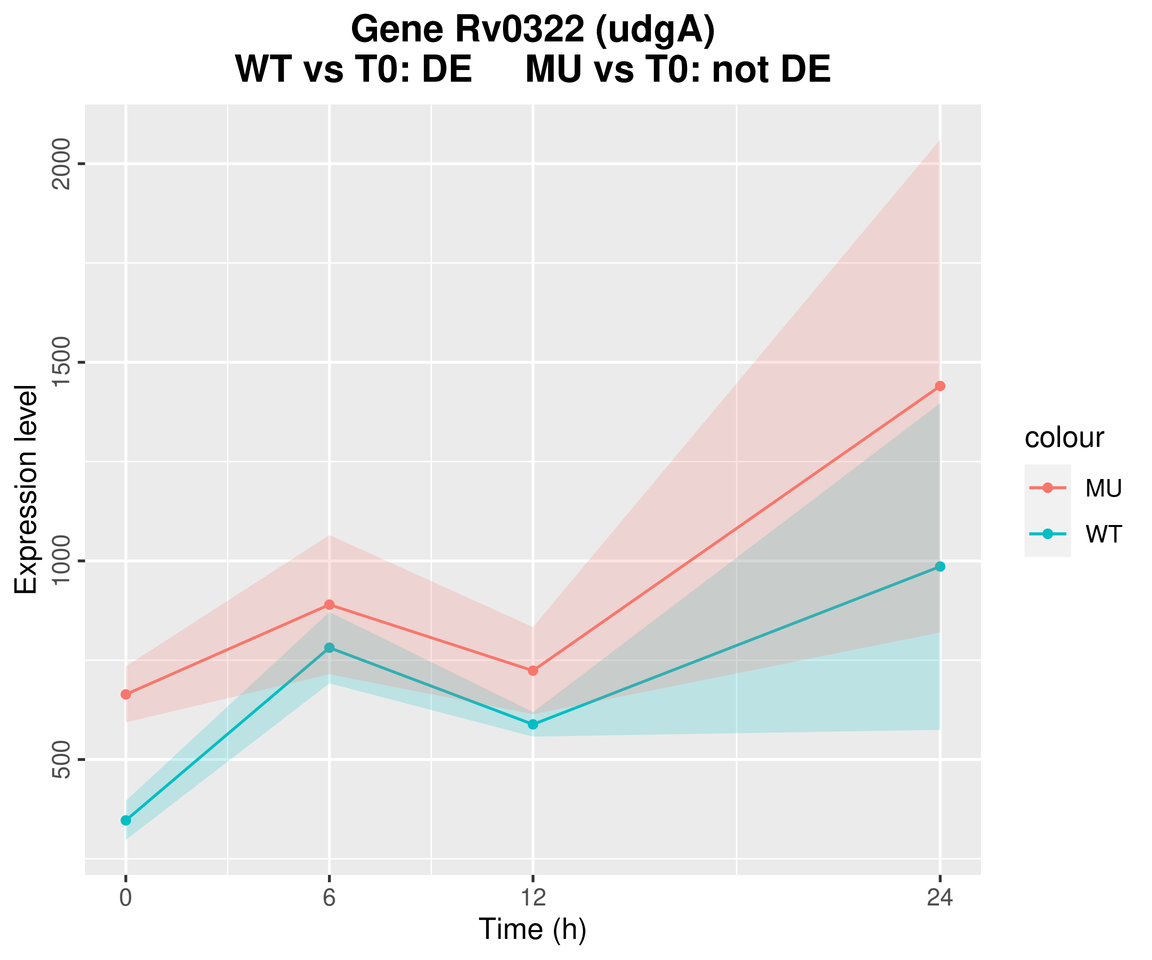

Gene Rv0322 (udgA)
WT vs T0: DE MU vs T0: not DE
Expression level
2000
1500
1000
500
0
6
12
24
Time (h)
colour
MU
WT

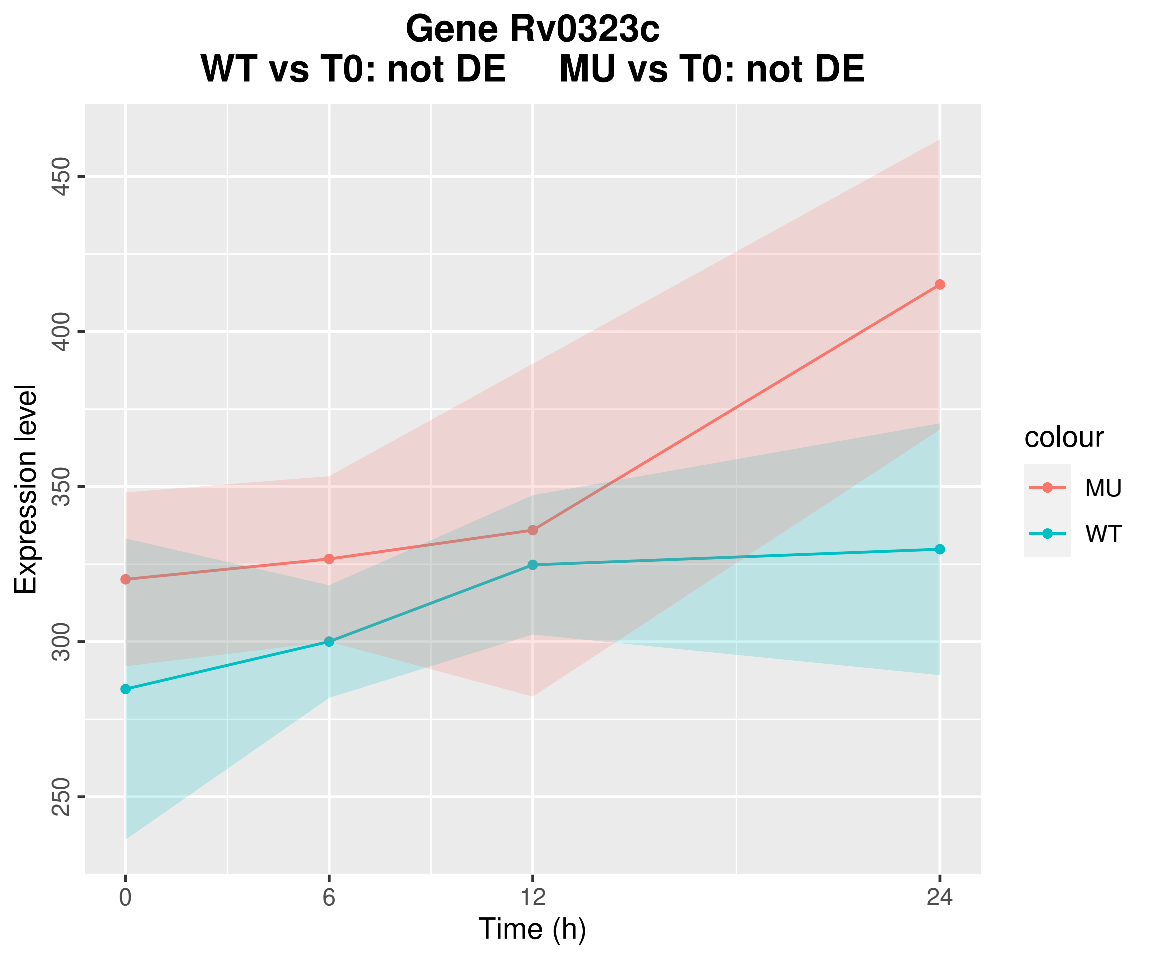
Gene Rv0323c
WT vs T0: not DE   MU vs T0: not DE
Expression level
450
400
350
300
250
0
6
12
24
Time (h)
colour
MU
WT

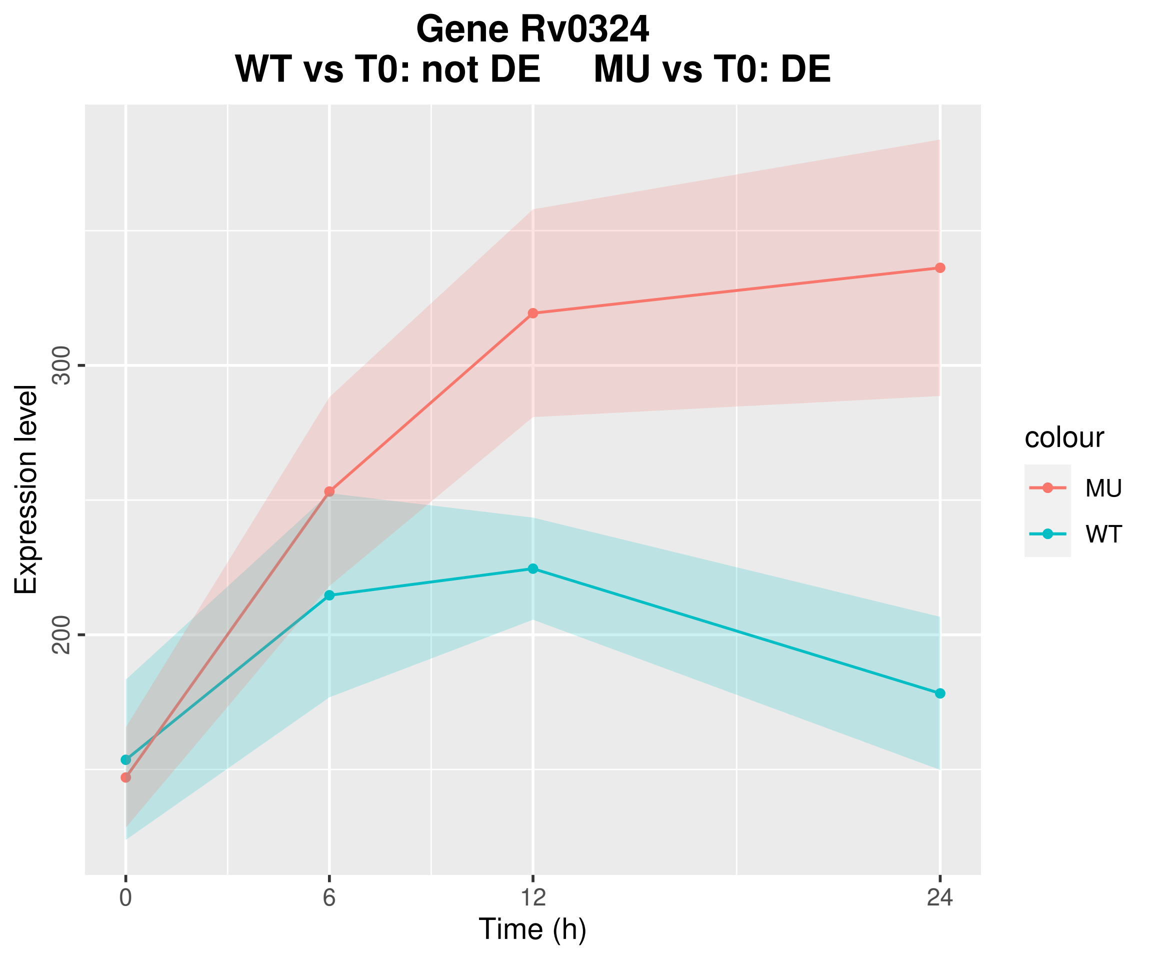
Gene Rv0324
WT vs T0: not DE   MU vs T0: DE
Expression level
300
200
0
6
12
24
Time (h)
colour
MU
WT

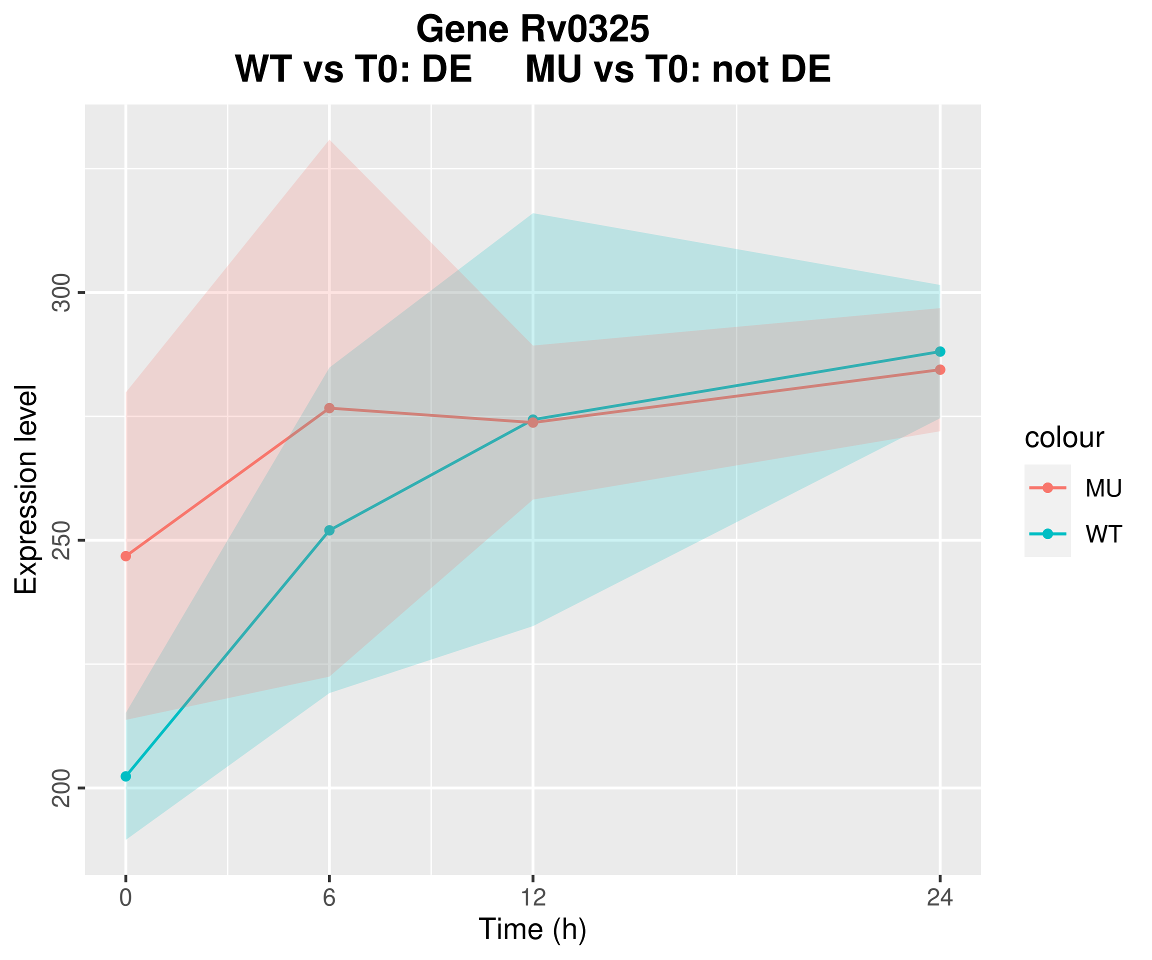

Gene Rv0325
WT vs T0: DE MU vs T0: not DE
Expression level
300
250
200
0
6
12
24
Time (h)
colour
MU
WT

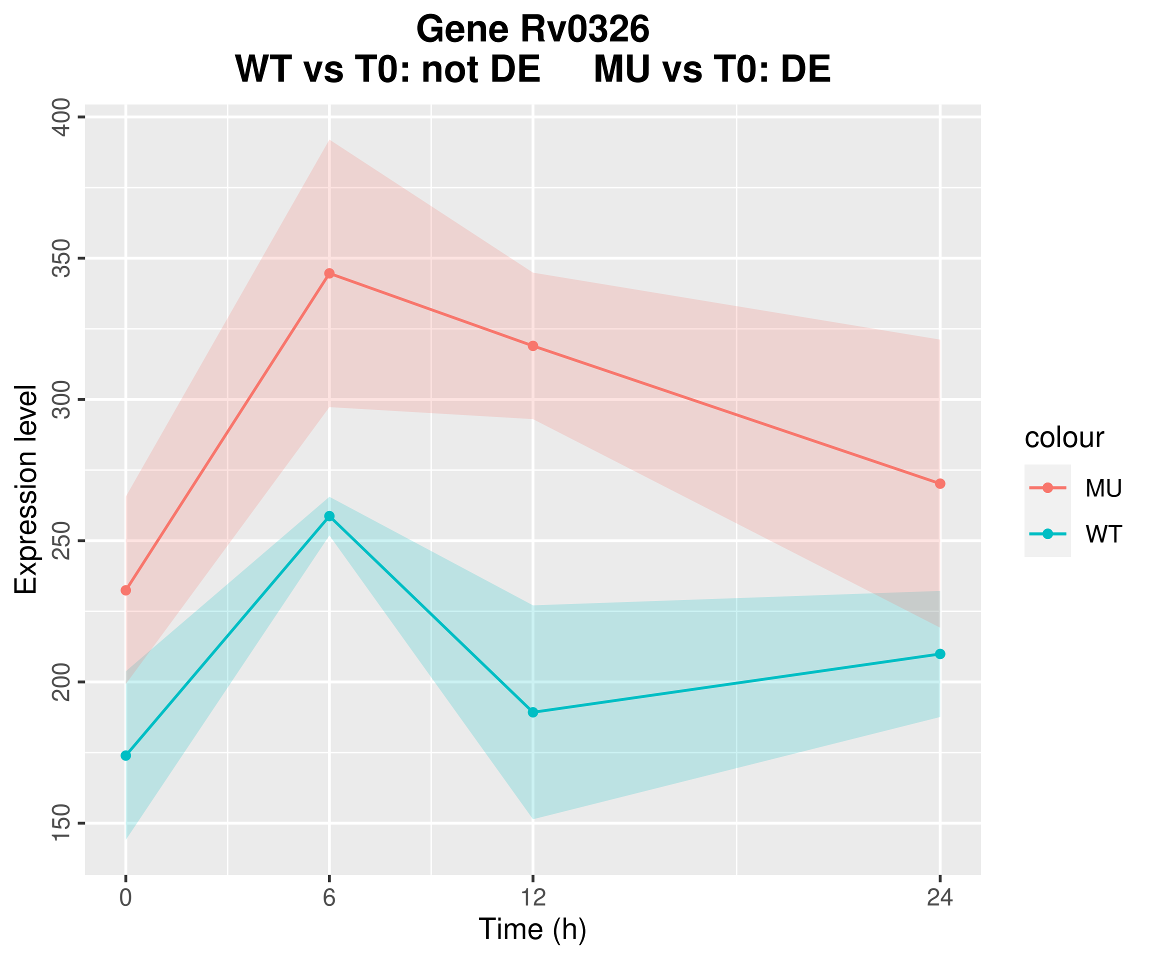

Gene Rv0326
WT vs T0: not DE MU vs T0: DE
Expression level
400
350
300
250
200
150
0
6
12
24
Time (h)
colour
MU
WT

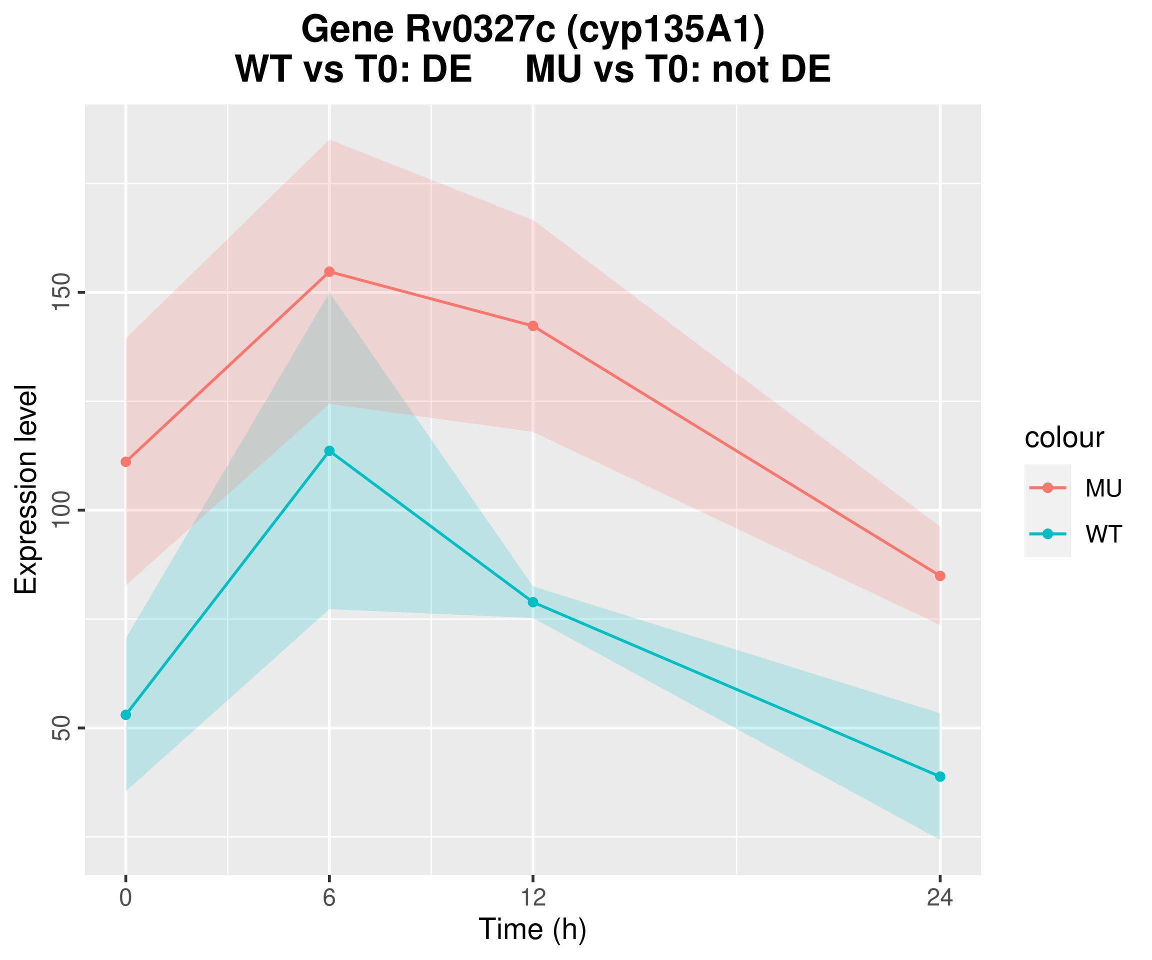
Gene Rv0327c (cyp135A1)
WT vs T0: DE   MU vs T0: not DE
Expression level
150
100
50
0
6
12
24
Time (h)
colour
MU
WT

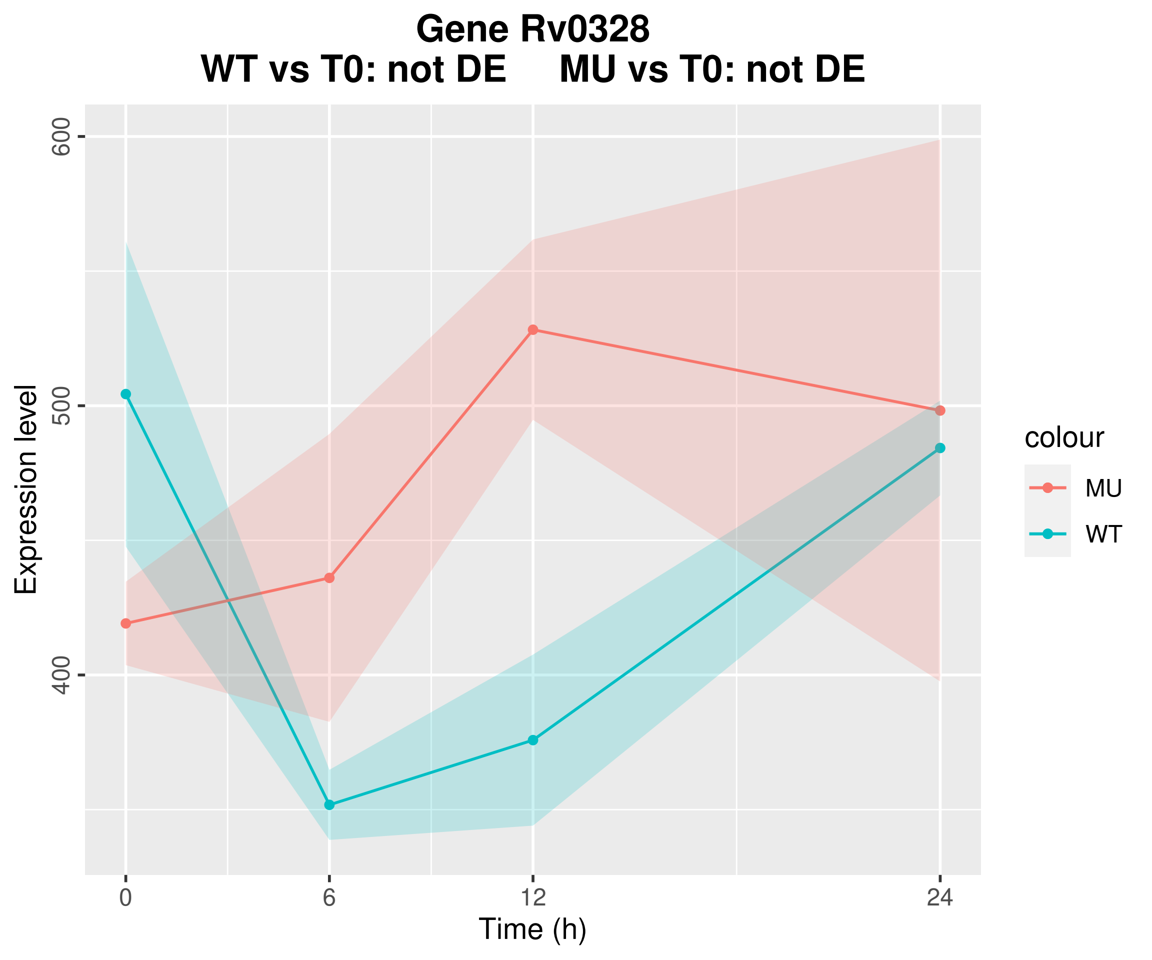
Gene Rv0328
WT vs T0: not DE MU vs T0: not DE
Expression level
400
500
600
0
6
12
24
Time (h)
colour
MU
WT

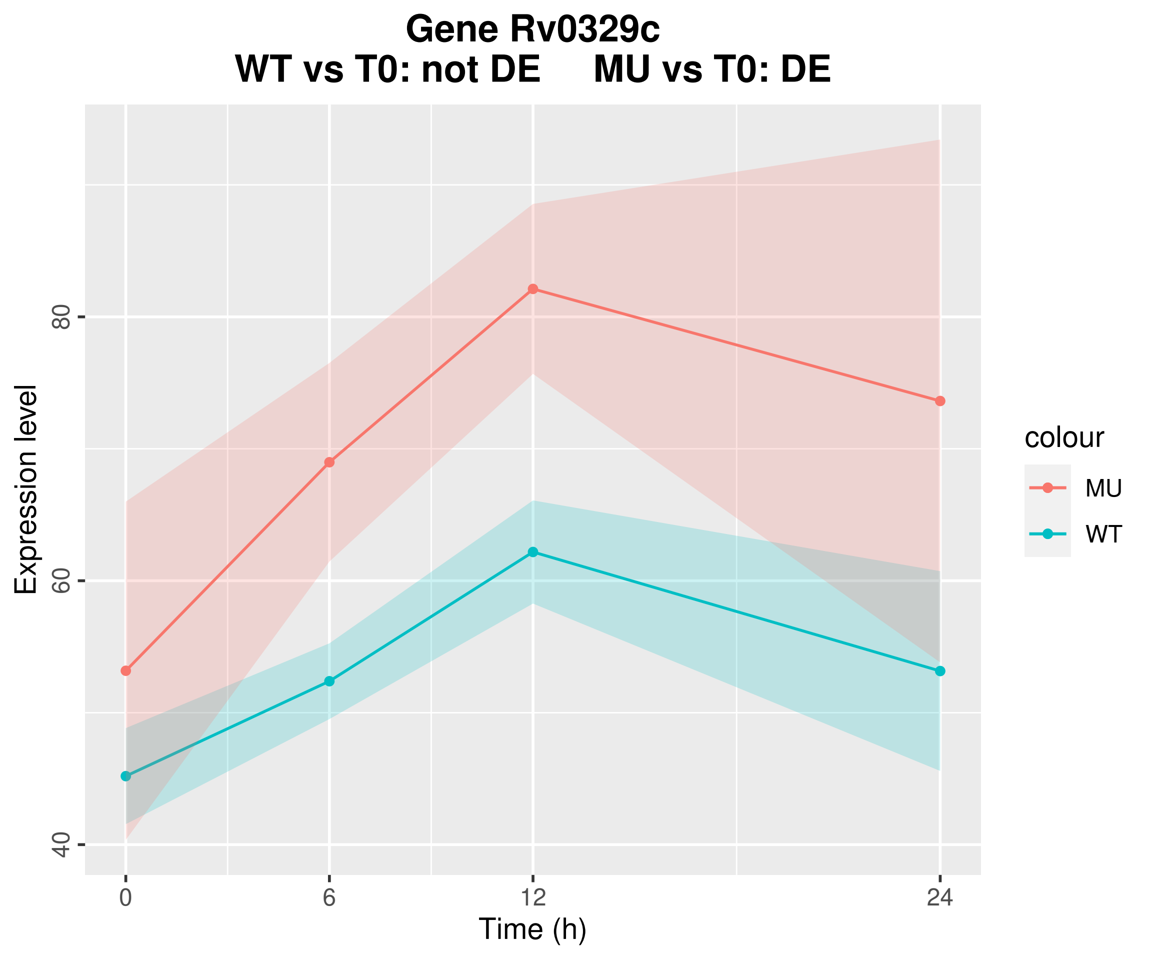

Gene Rv0329c
WT vs T0: not DE   MU vs T0: DE
Expression level
80
60
40
0
6
12
24
Time (h)
colour
MU
WT

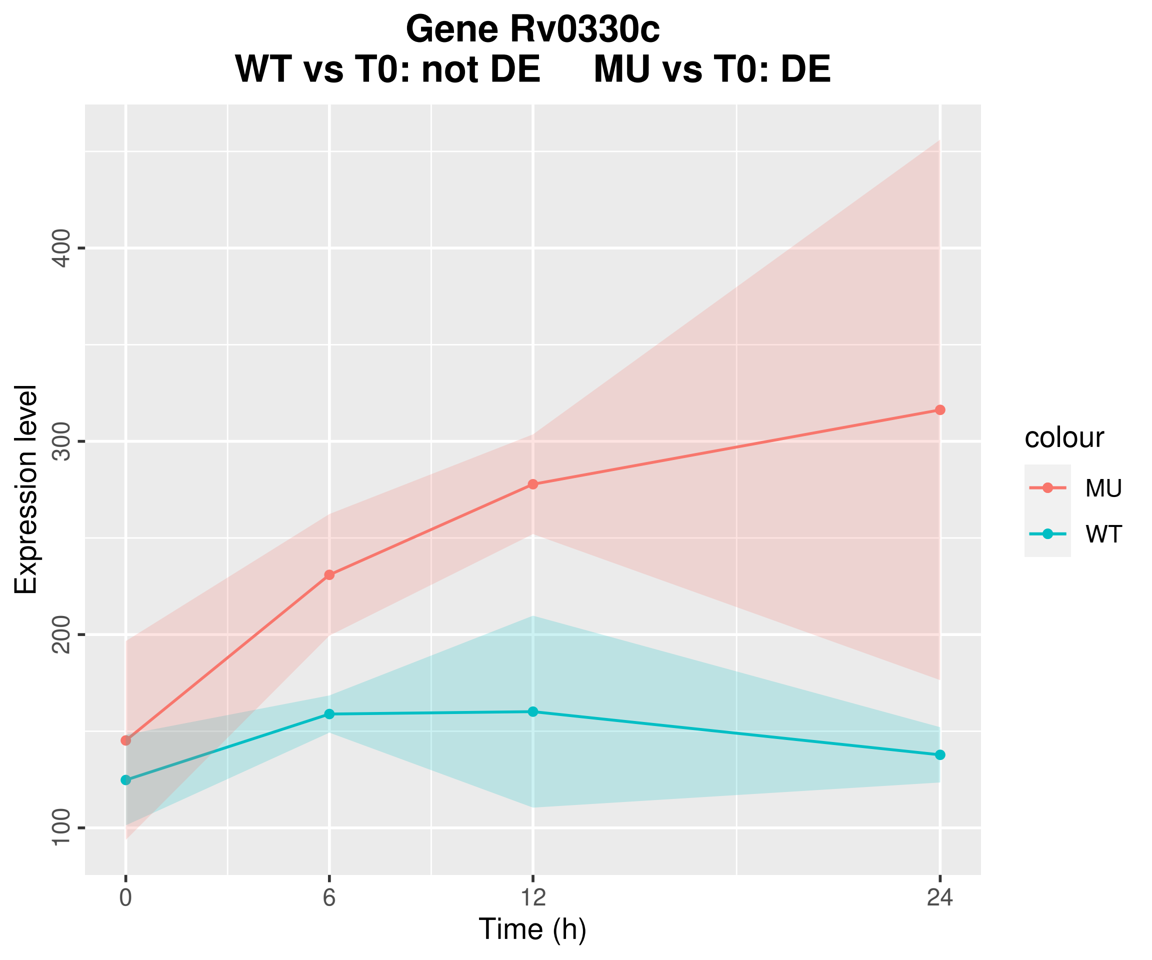

Gene Rv0330c
WT vs T0: not DE MU vs T0: DE
Expression level
100
200
300
400
Time (h)
0
6
12
24
colour
MU
WT

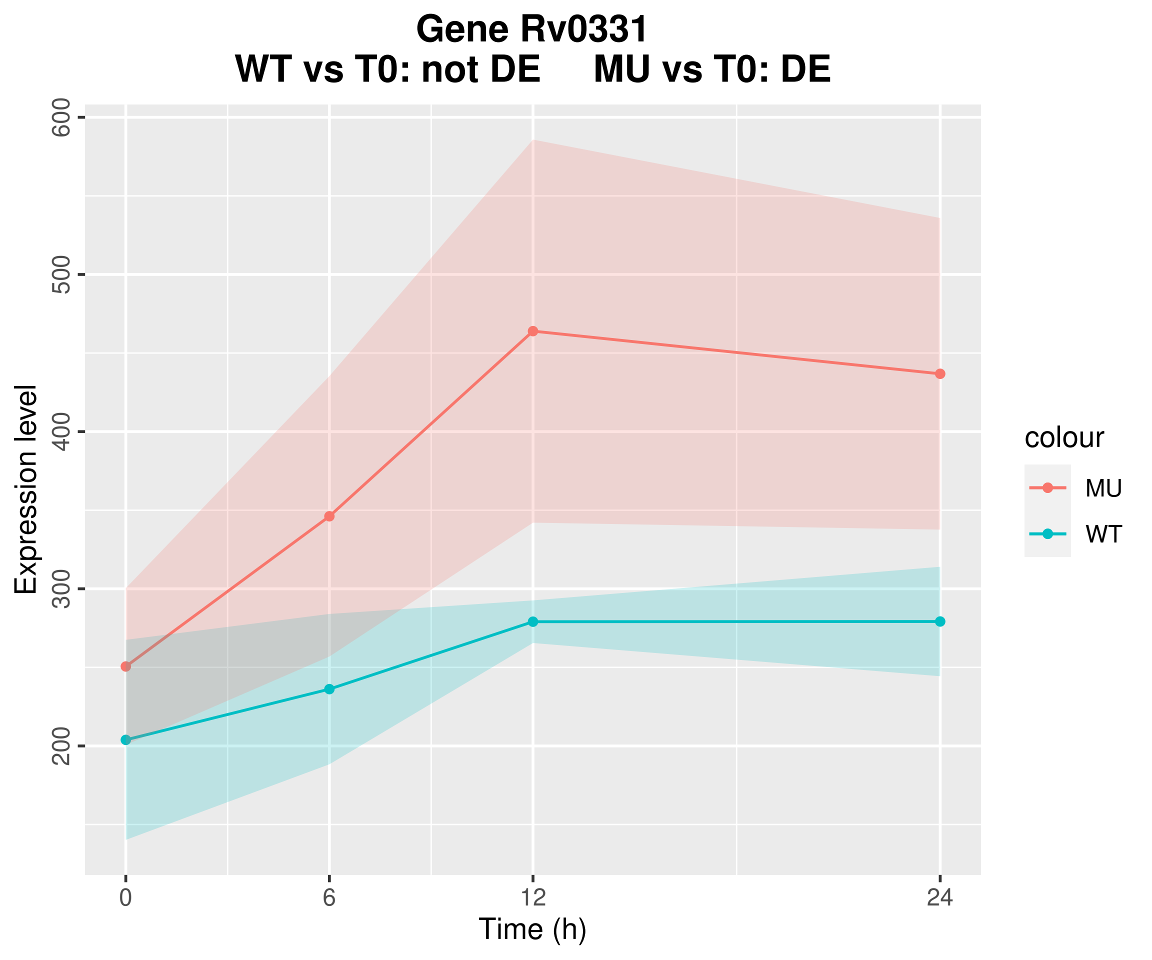
Gene Rv0331
WT vs T0: not DE MU vs T0: DE
Expression level
600
500
400
300
200
0
6
12
24
Time (h)
colour
MU
WT

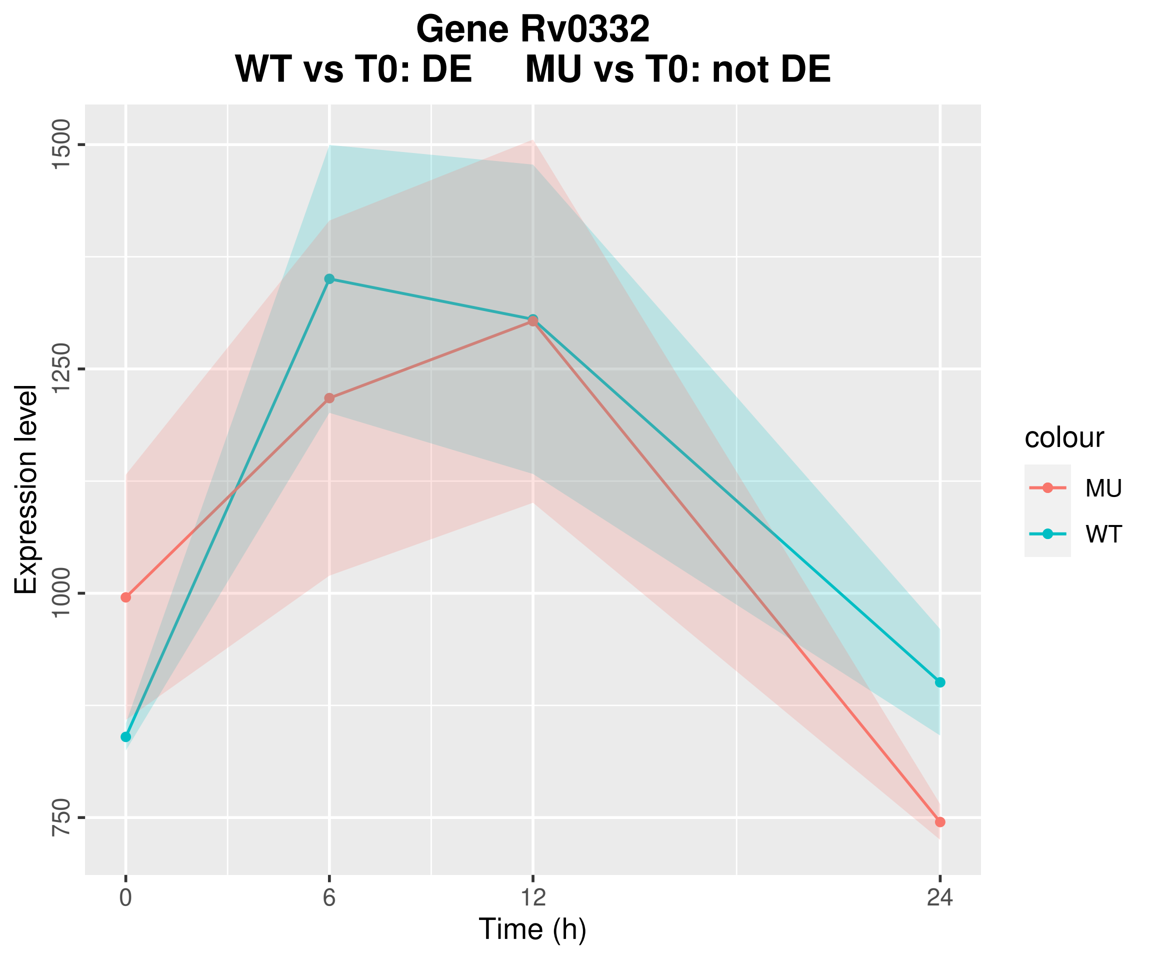
Gene Rv0332
WT vs T0: DE MU vs T0: not DE
Expression level
1500
1250
1000
750
0
6
12
24
Time (h)
colour
MU
WT

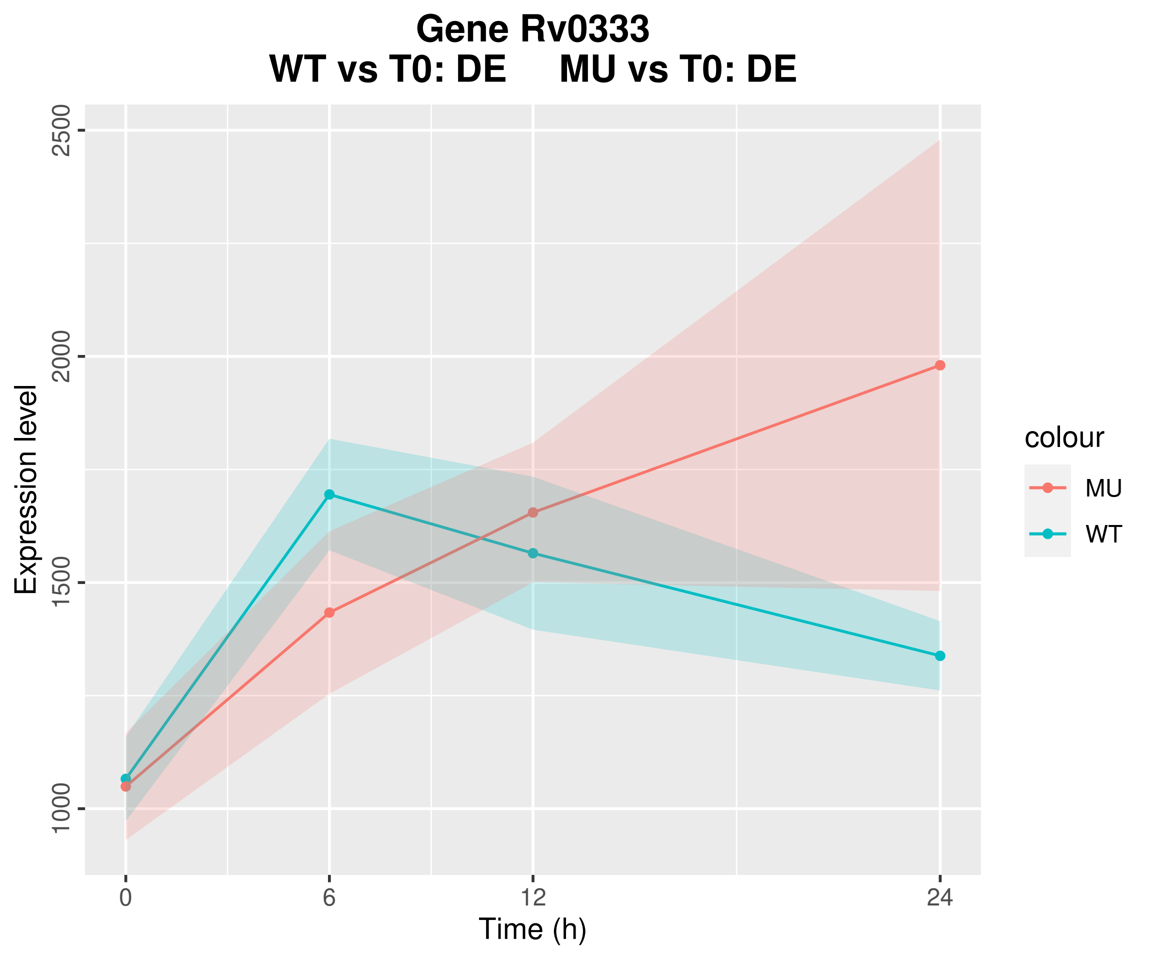
Gene Rv0333
WT vs T0: DE   MU vs T0: DE
Expression level
2500
2000
1500
1000
0
6
12
24
Time (h)
colour
MU
WT

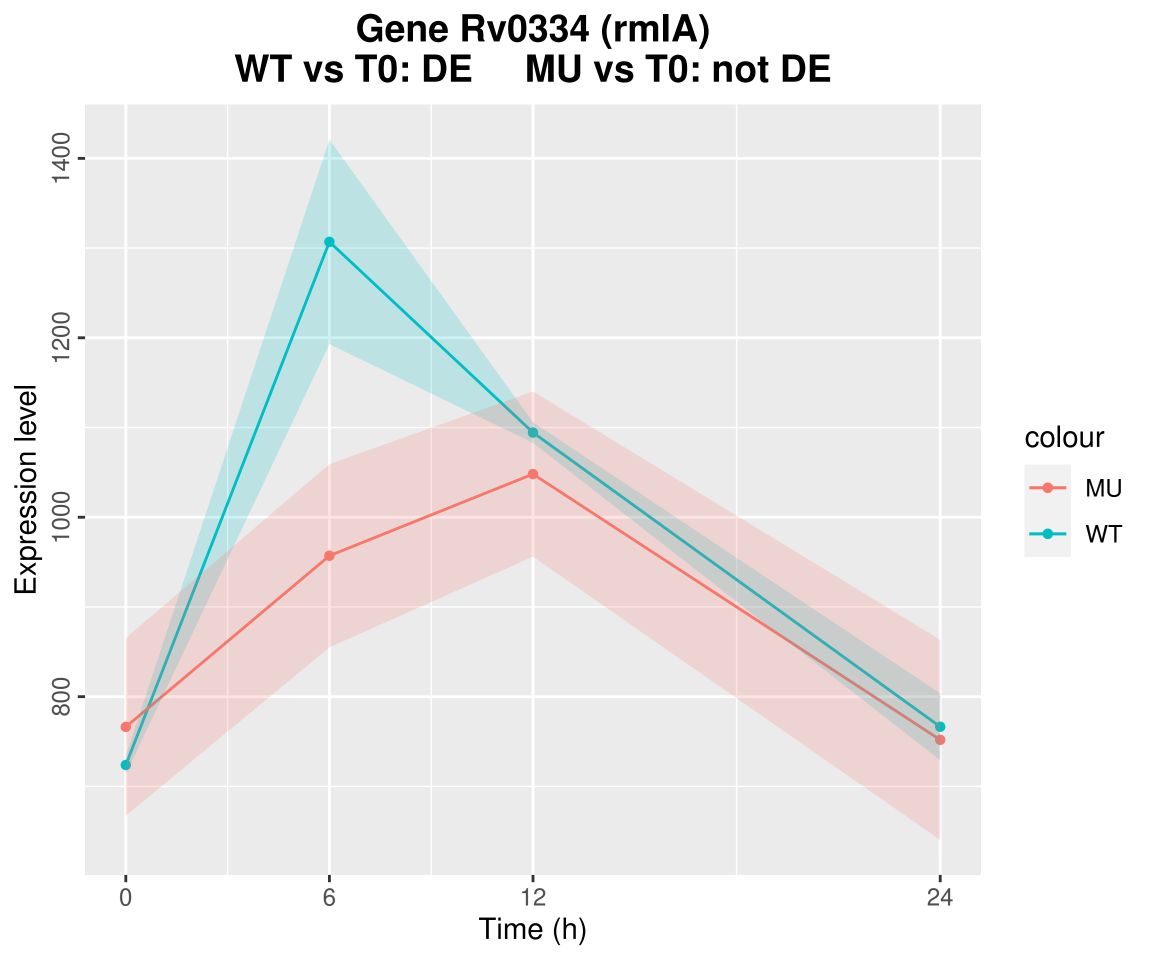
Gene Rv0334 (rmlA)
WT vs T0: DE   MU vs T0: not DE
Expression level
1400
1200
1000
800
0
6
12
24
Time (h)
colour
MU
WT

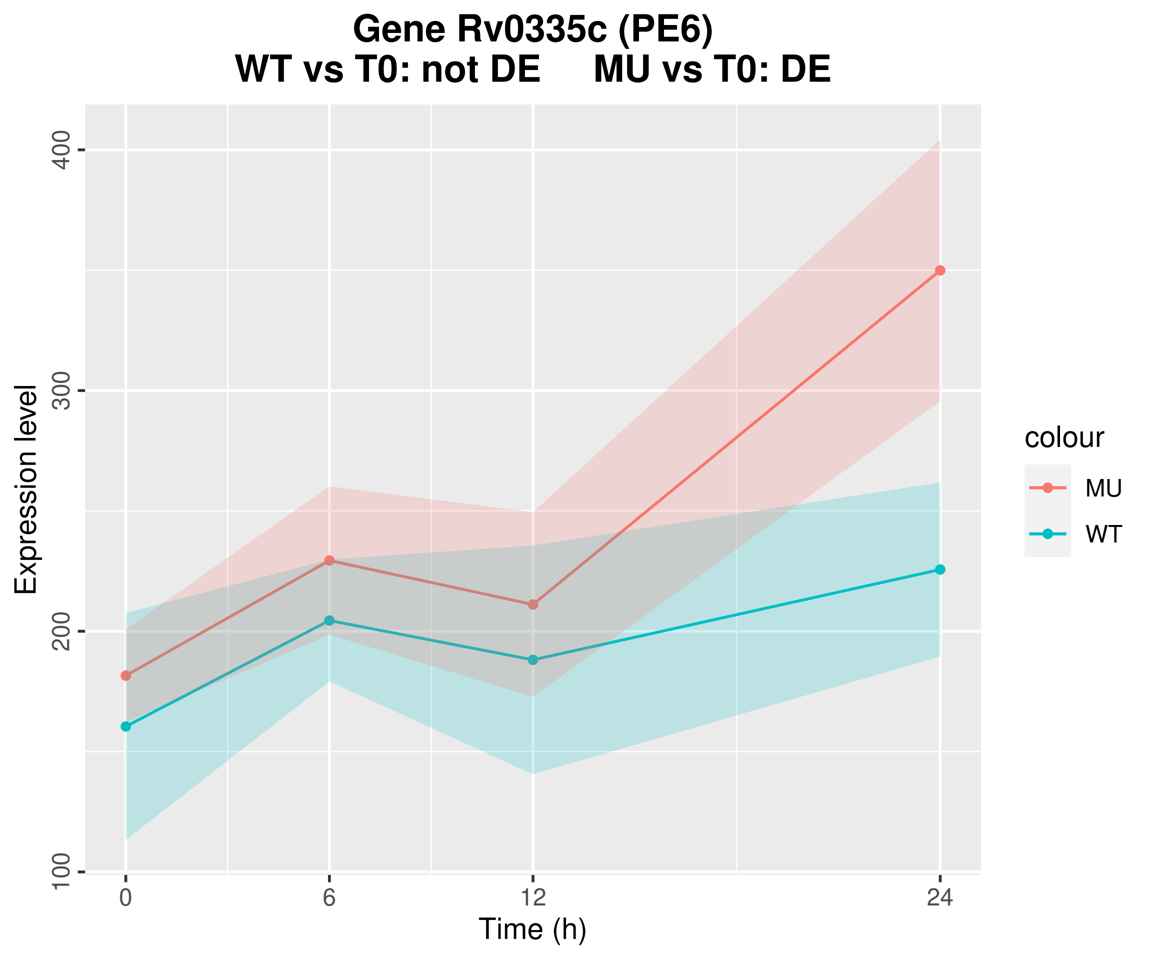

Gene Rv0335c (PE6)
WT vs T0: not DE   MU vs T0: DE
Expression level
400
300
200
100
0
6
12
24
Time (h)
colour
MU
WT

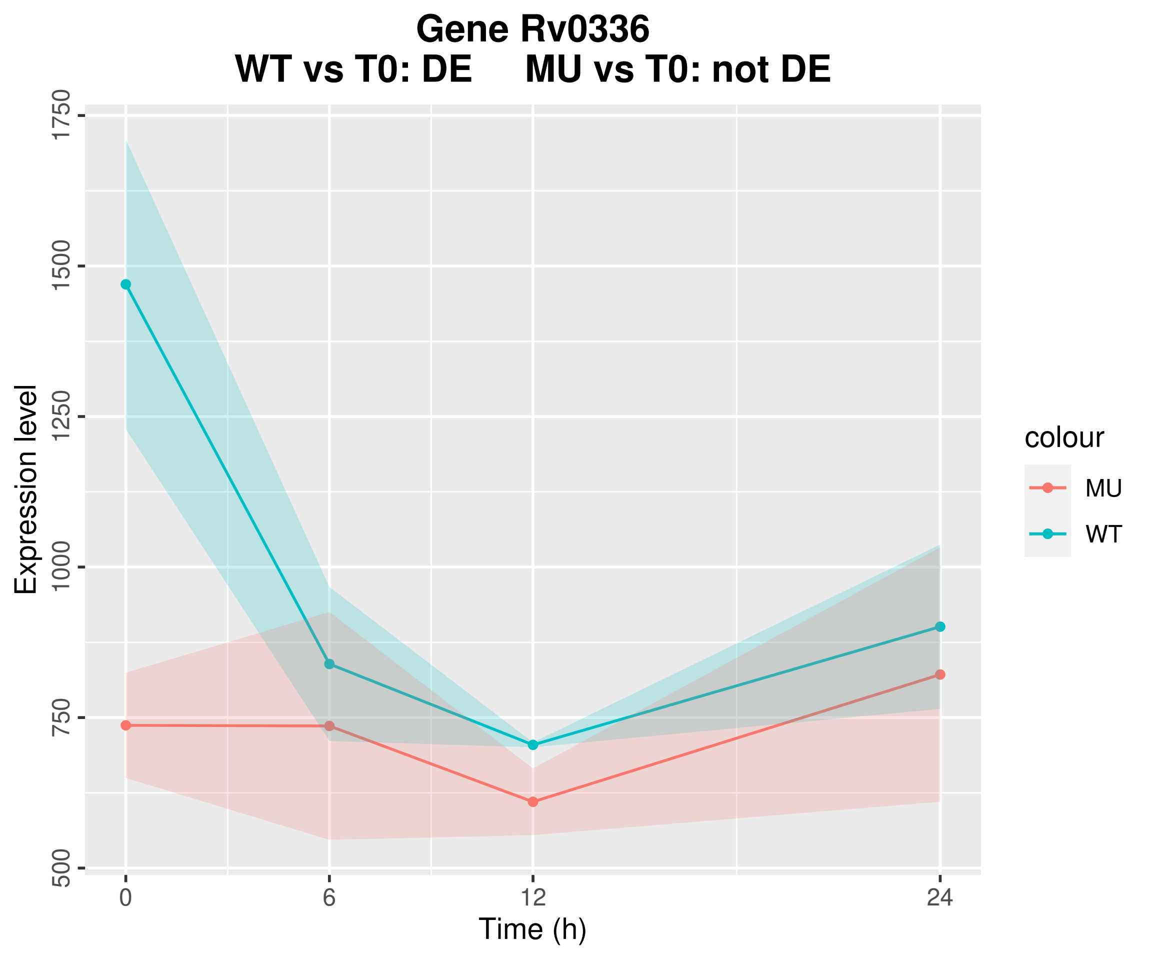
Gene Rv0336
WT vs T0: DE   MU vs T0: not DE
Expression level
1750
1500
1250
1000
750
500
0
6
12
24
Time (h)
colour
MU
WT

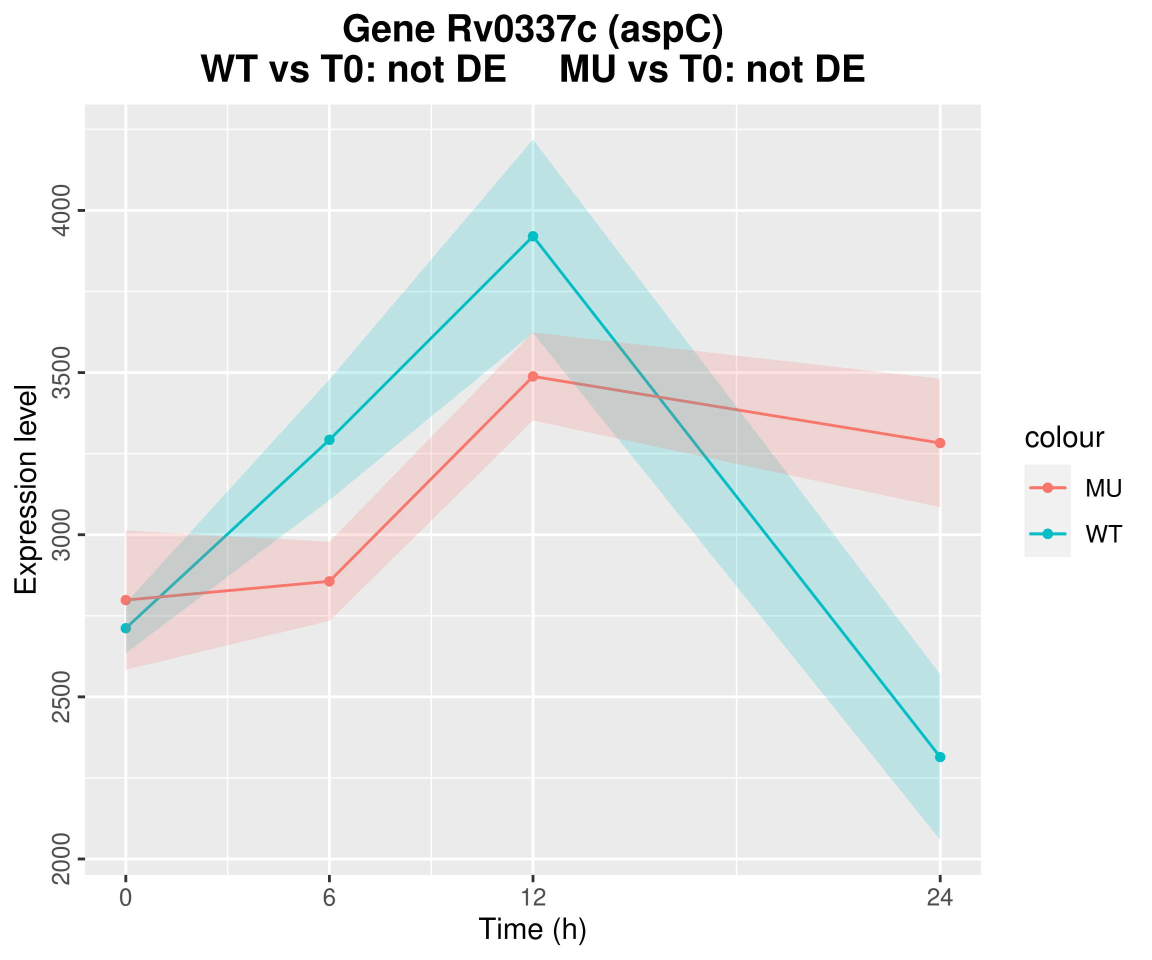

Gene Rv0337c (aspC)
WT vs T0: not DE MU vs T0: not DE
Expression level
4000
3500
3000
2500
2000
0
6
12
24
Time (h)
colour
MU
WT

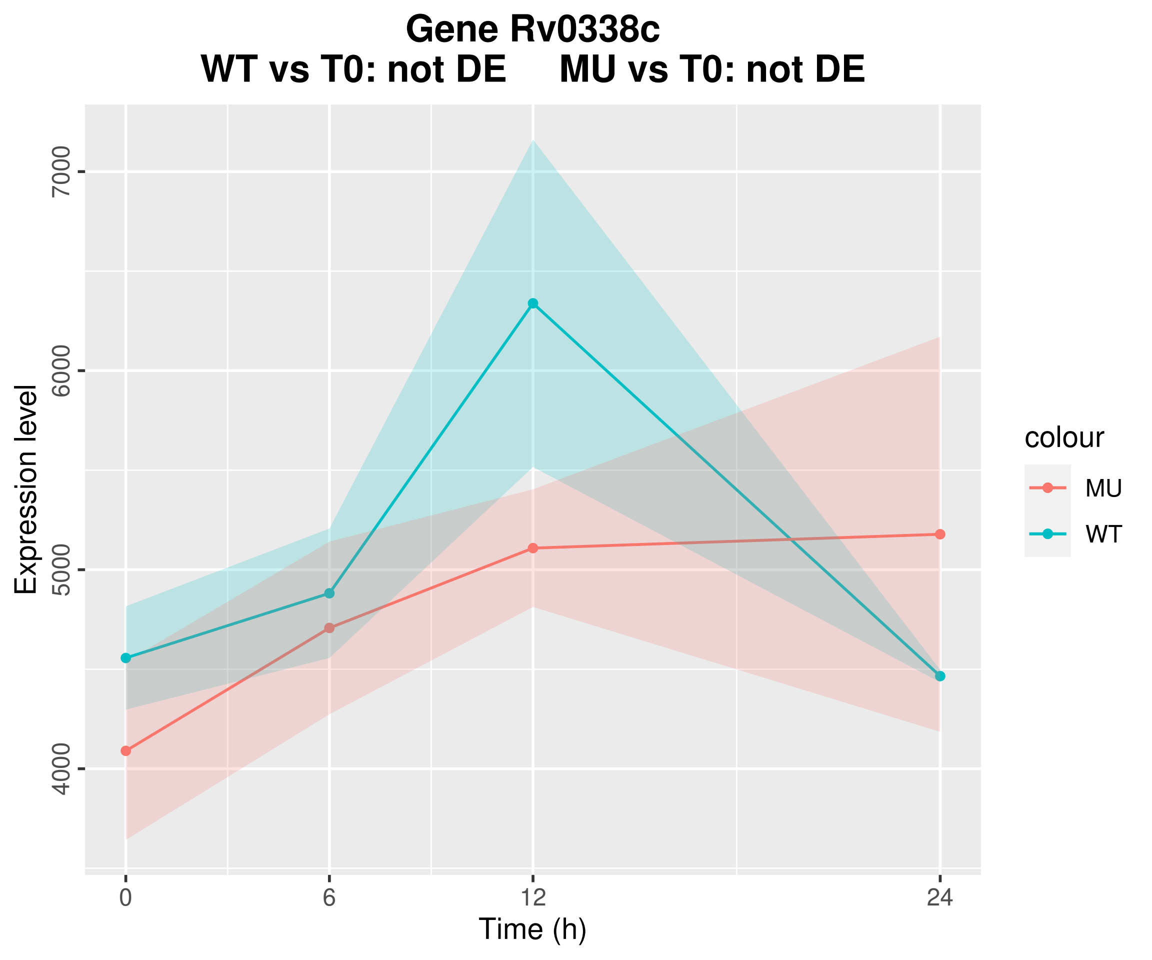

Gene Rv0338c
WT vs T0: not DE MU vs T0: not DE
Expression level
7000
6000
5000
4000
0
6
12
24
Time (h)
colour
MU
WT

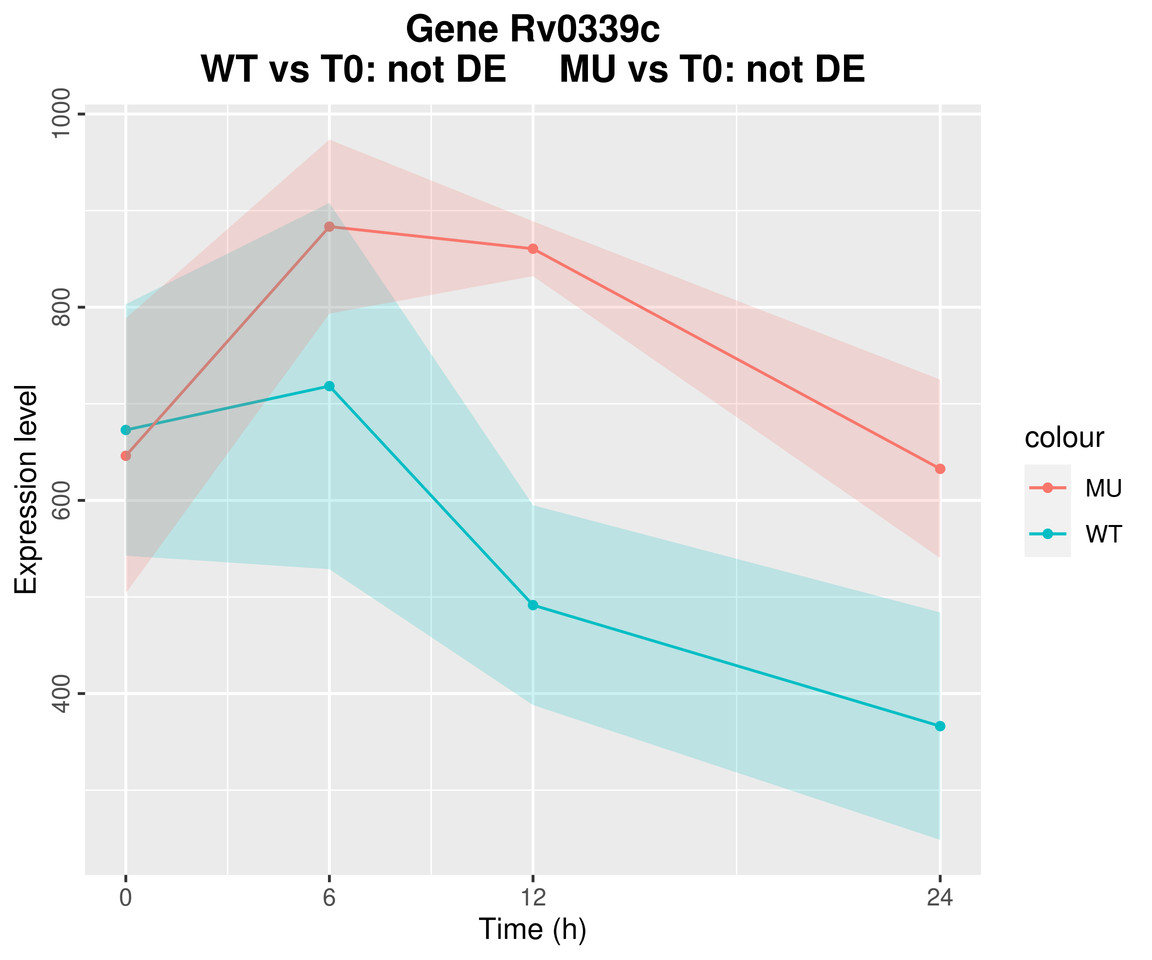

Gene Rv0339c
WT vs T0: not DE MU vs T0: not DE
Expression level
1000
800
600
400
0
6
12
24
Time (h)
colour
MU
WT

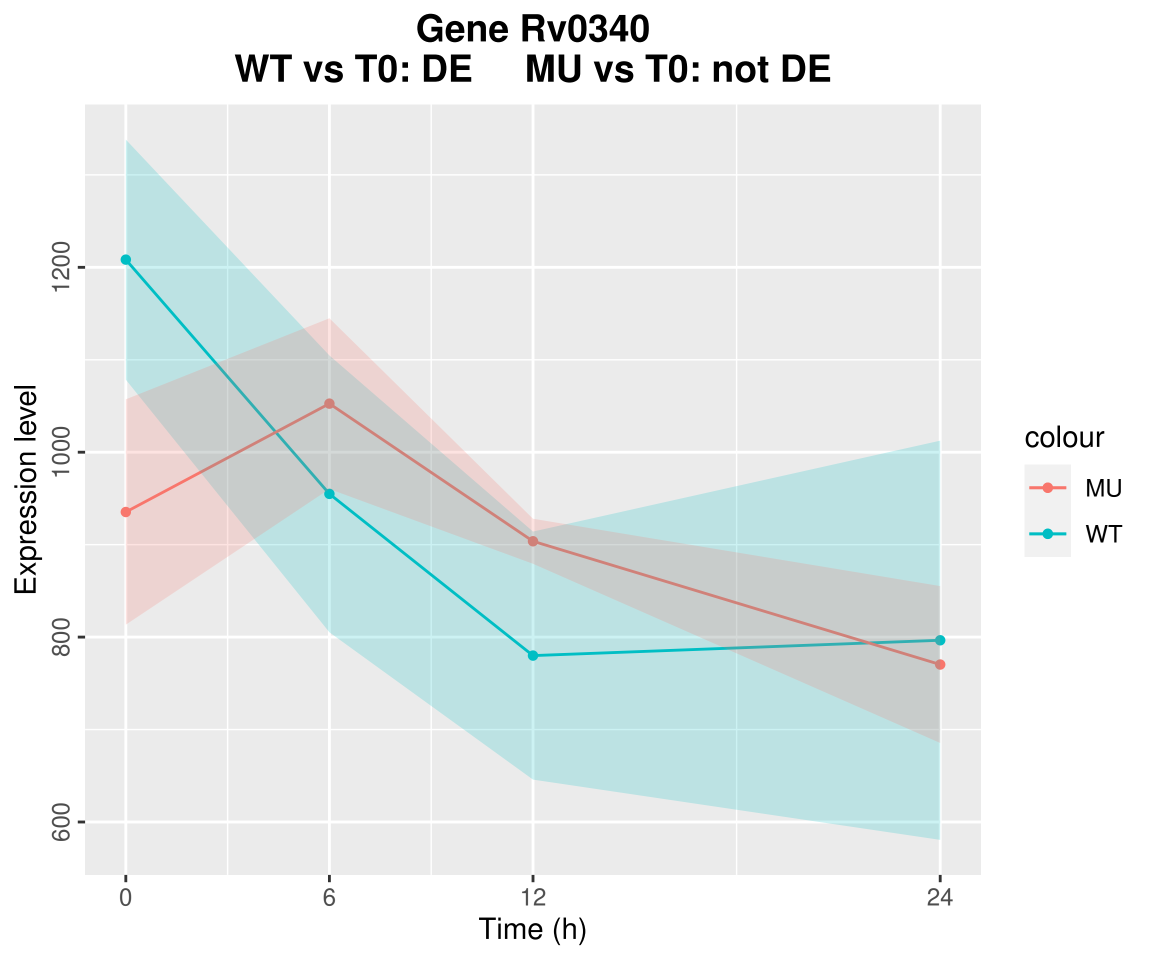
Gene Rv0340
WT vs T0: DE MU vs T0: not DE
Expression level
1200
1000
800
600
0
6
12
24
Time (h)
colour
MU
WT

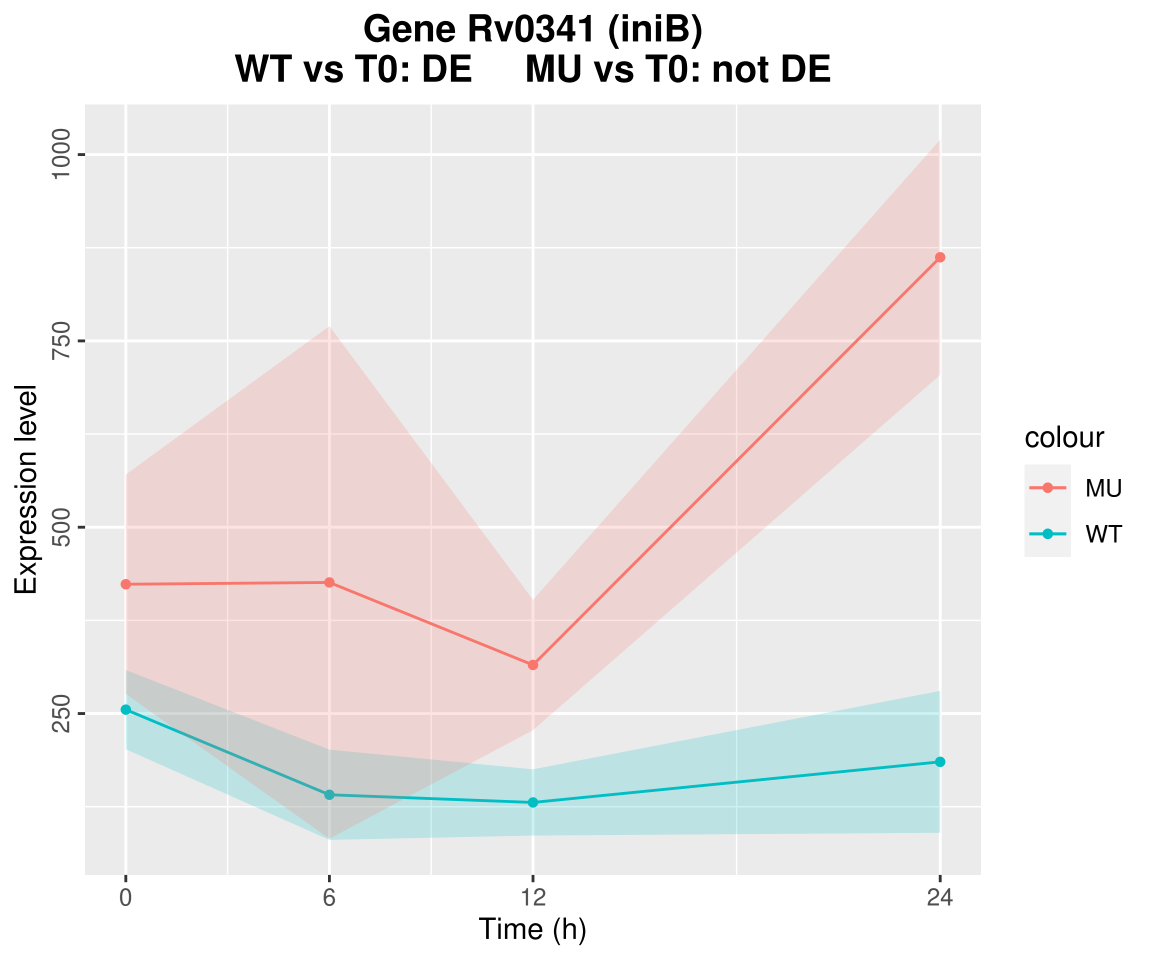

Gene Rv0341 (iniB)
WT vs T0: DE MU vs T0: not DE
Expression level
1000
750
500
250
0
6
12
24
Time (h)
colour
MU
WT

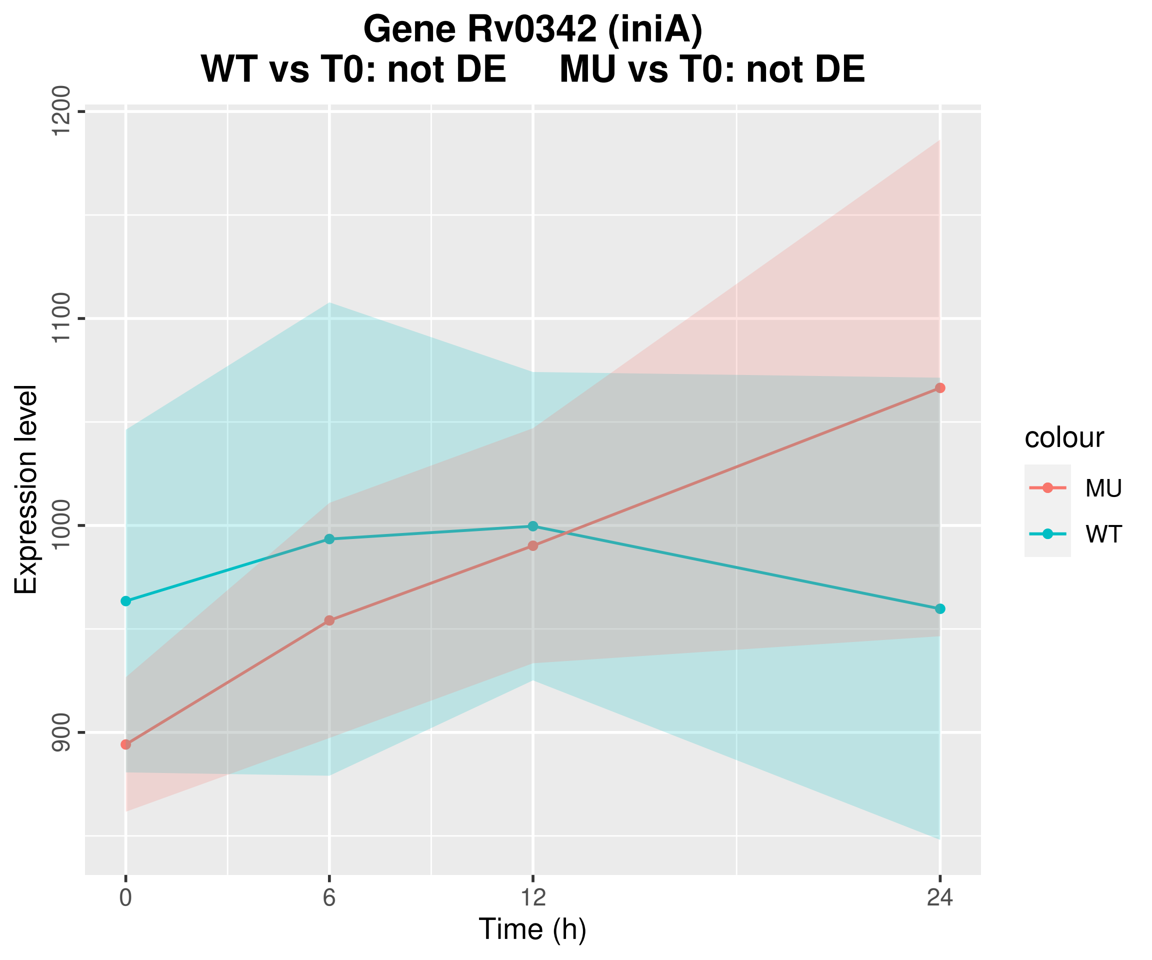
Gene Rv0342 (iniA)
WT vs T0: not DE   MU vs T0: not DE
Expression level
1200
1100
1000
900
0
6
12
24
Time (h)
colour
MU
WT

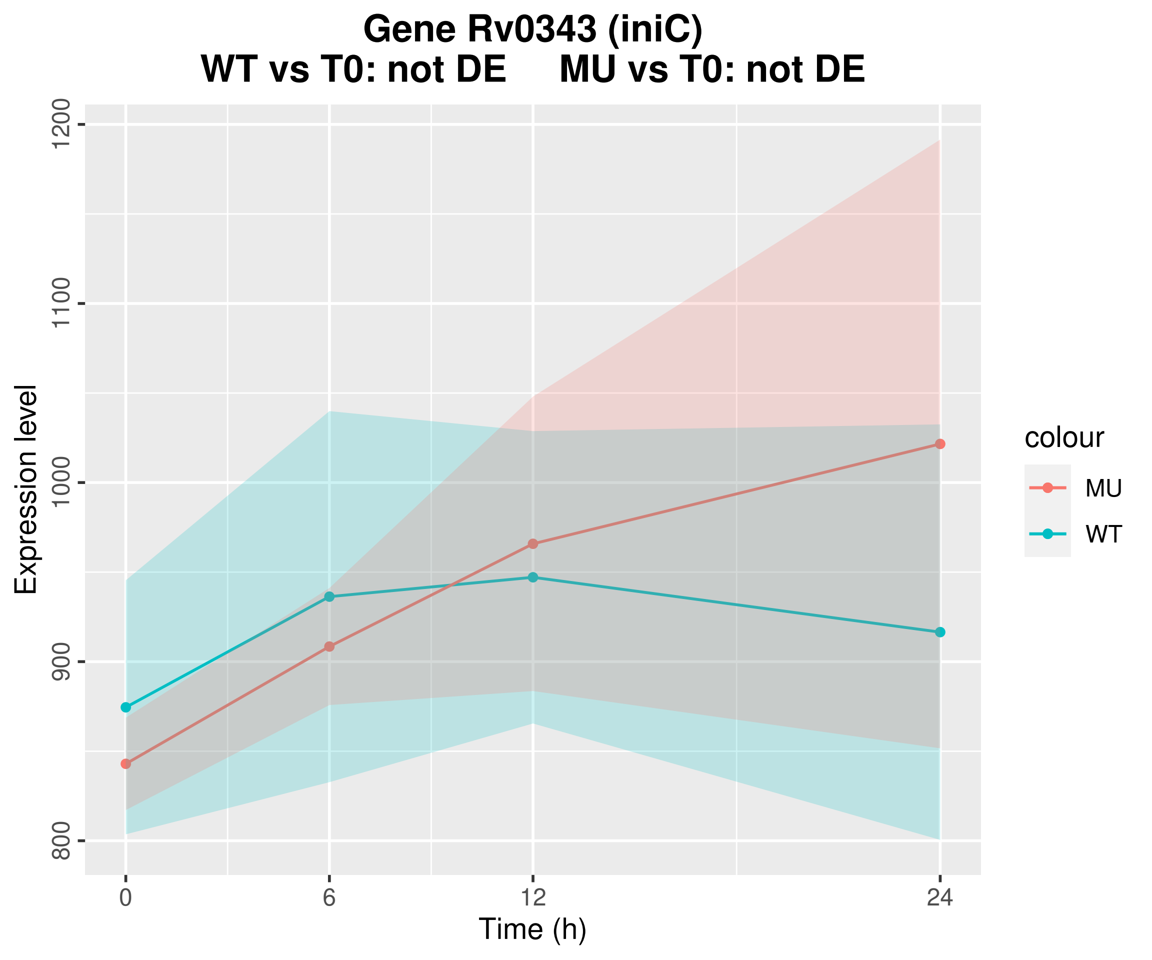

Gene Rv0343 (iniC)
WT vs T0: not DE MU vs T0: not DE
Expression level
1200
1100
1000
900
800
0
6
12
24
Time (h)
colour
MU
WT

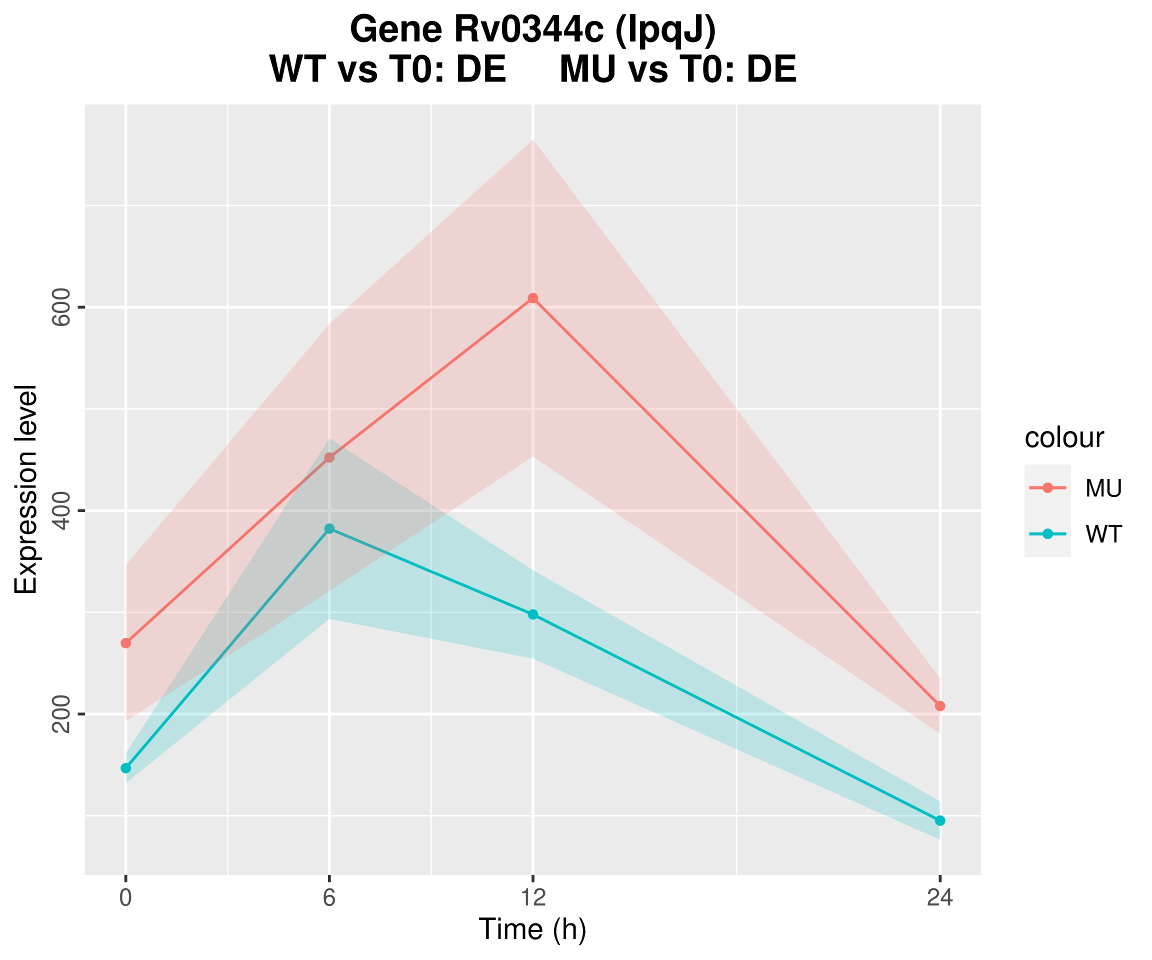

Gene Rv0344c (lpqJ)
WT vs T0: DE   MU vs T0: DE
Expression level
200
400
600
0
6
12
24
Time (h)
colour
MU
WT

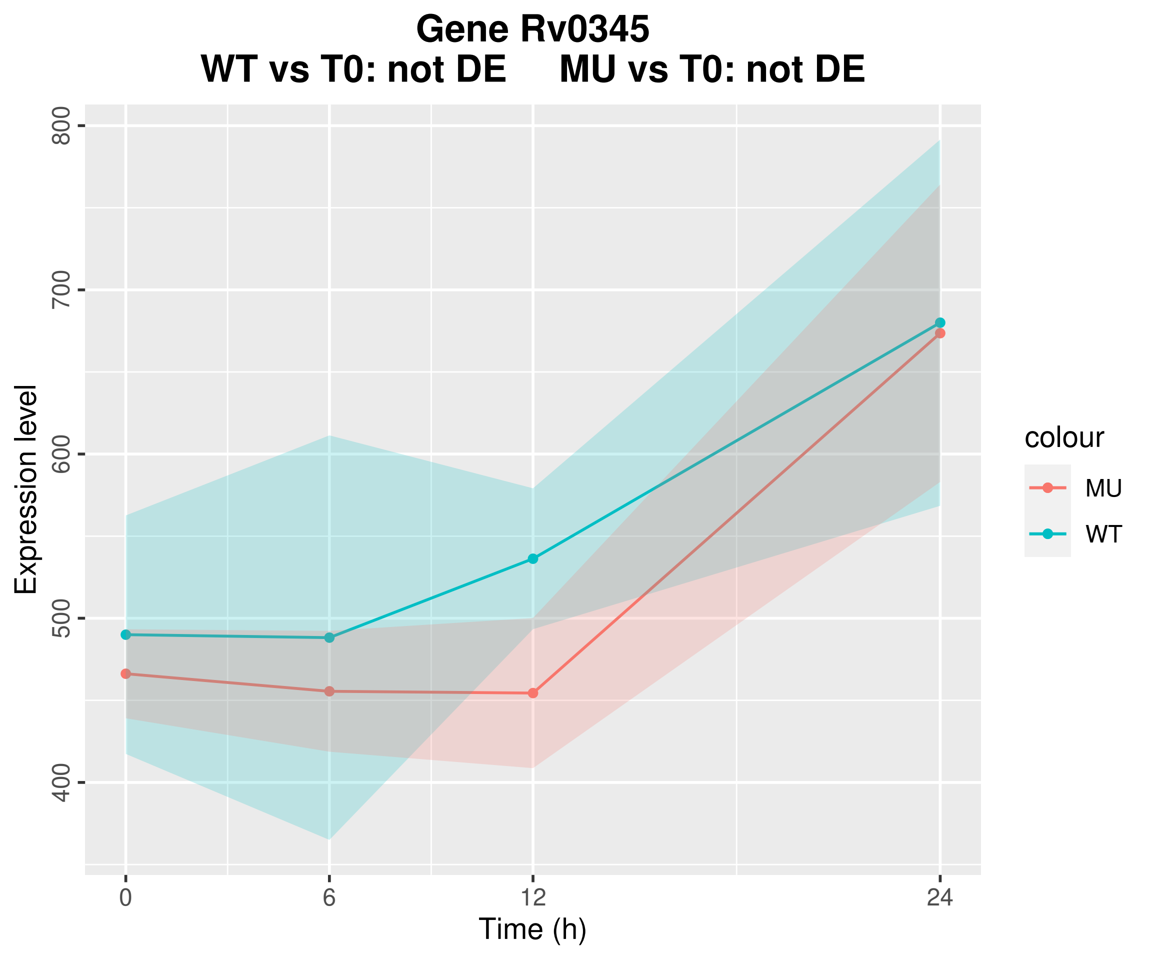
Gene Rv0345
WT vs T0: not DE MU vs T0: not DE
800
700
600
500
400
Expression level
0
6
12
24
Time (h)
colour
MU
WT

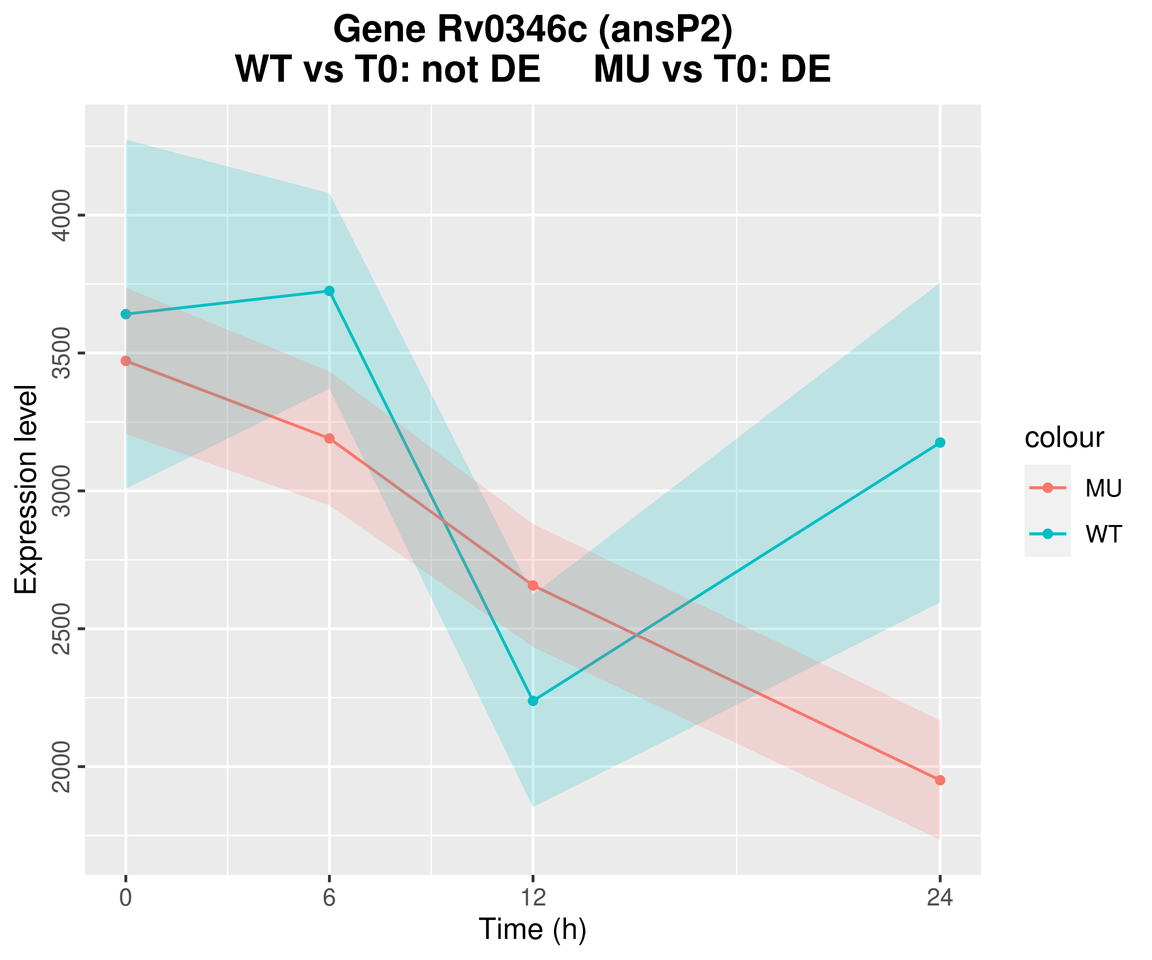

Gene Rv0346c (ansP2)
WT vs T0: not DE MU vs T0: DE
Expression level
4000
3500
3000
2500
2000
0
6
12
24
Time (h)
colour
MU
WT

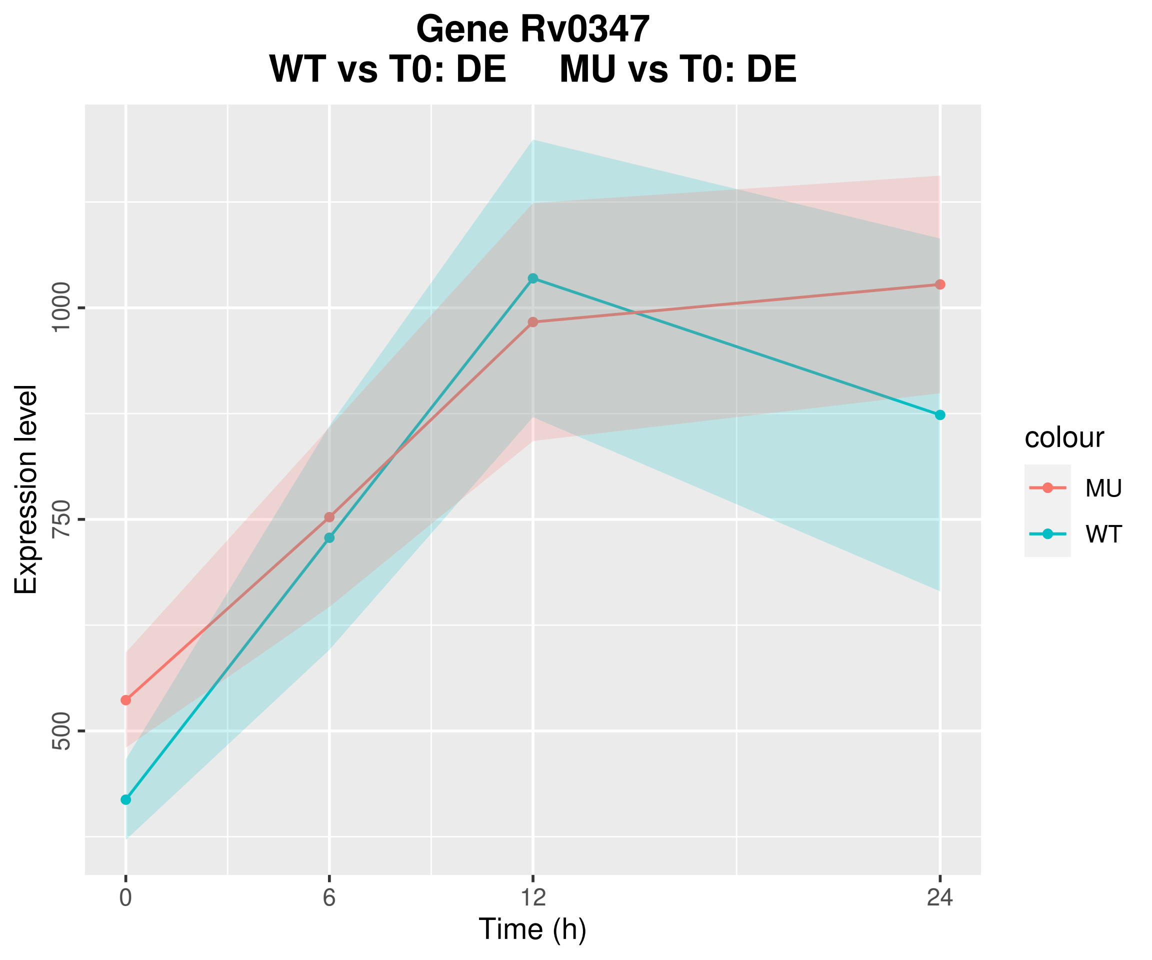

Gene Rv0347
WT vs T0: DE MU vs T0: DE
Expression level
1000
750
500
0
6
12
24
Time (h)
colour
MU
WT

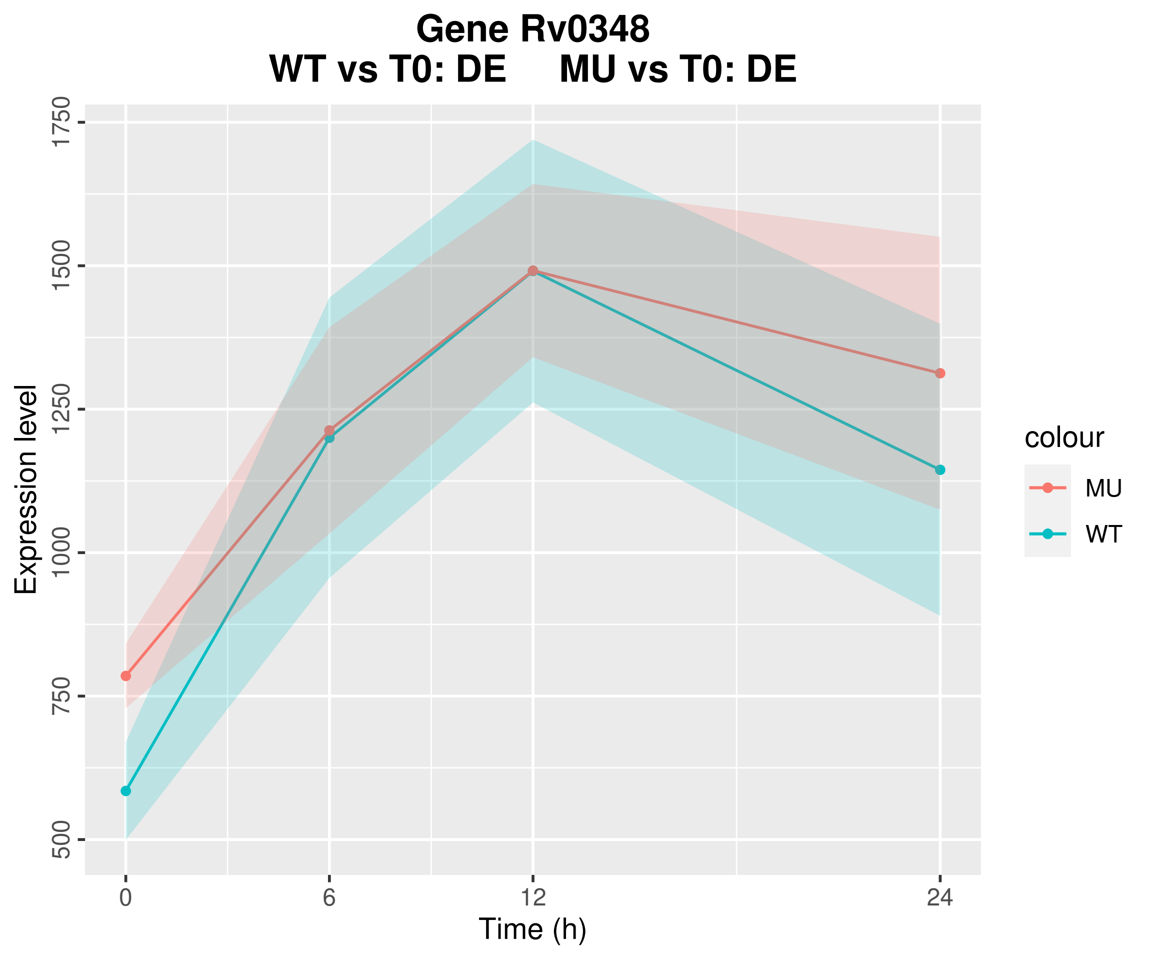

Gene Rv0348
WT vs T0: DE   MU vs T0: DE
Expression level
1750
1500
1250
1000
750
500
0
6
12
24
Time (h)
colour
MU
WT

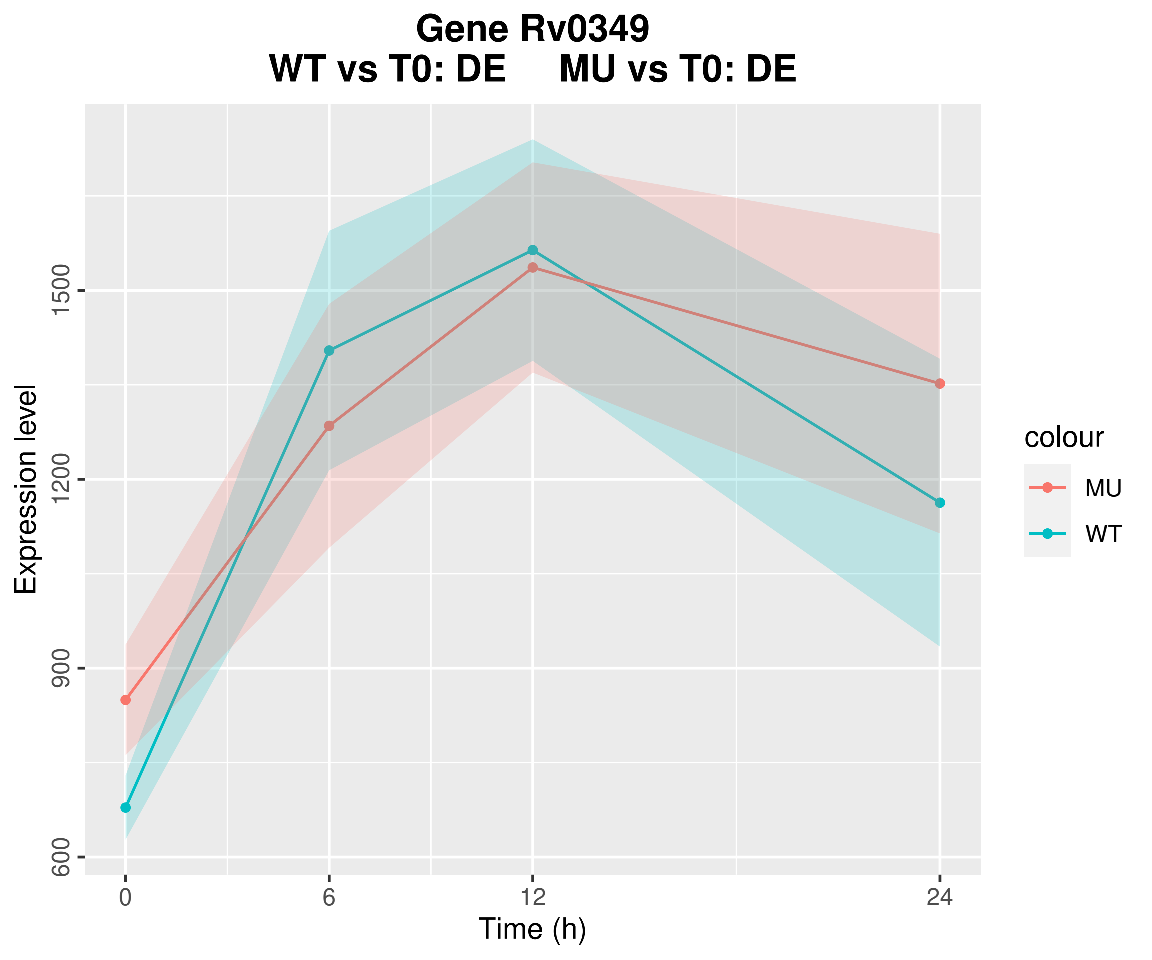
Gene Rv0349
WT vs T0: DE MU vs T0: DE
Expression level
1500
1200
900
600
0
6
12
24
Time (h)
colour
MU
WT

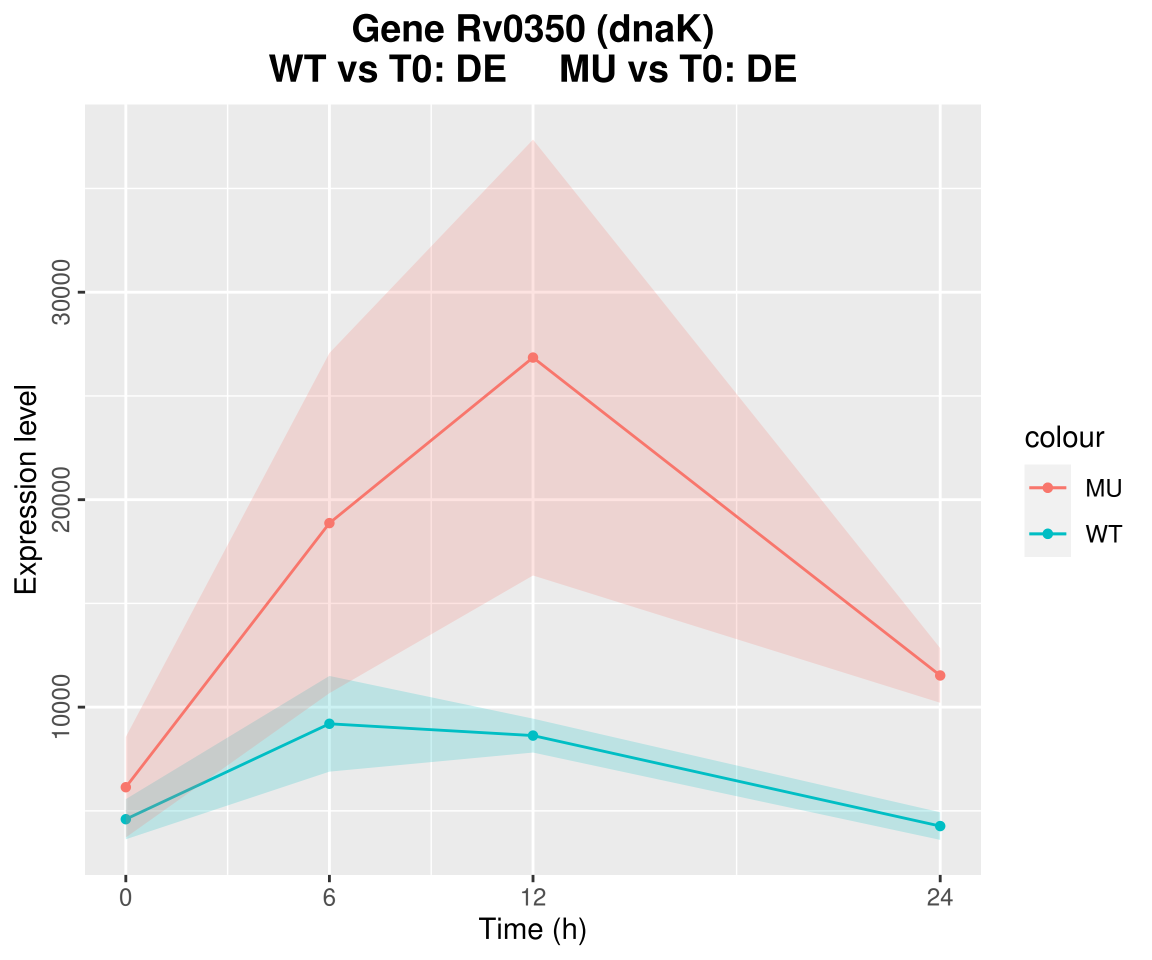
Gene Rv0350 (dnaK)
WT vs T0: DE MU vs T0: DE
Expression level
30000
20000
10000
0
6
12
24
Time (h)
colour
MU
WT

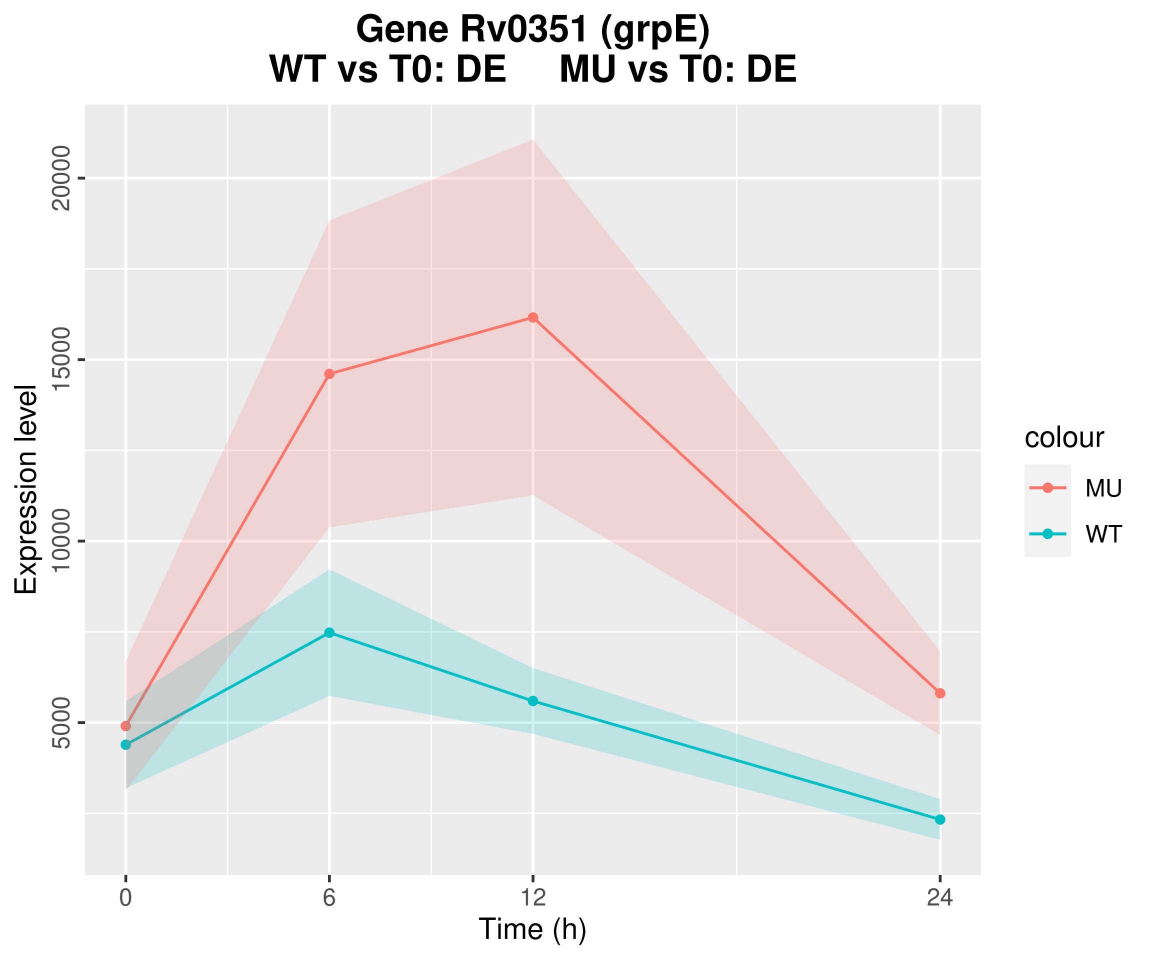

Gene Rv0351 (grpE)
WT vs T0: DE MU vs T0: DE
Expression level
20000
15000
10000
5000
0
6
12
24
Time (h)
colour
MU
WT

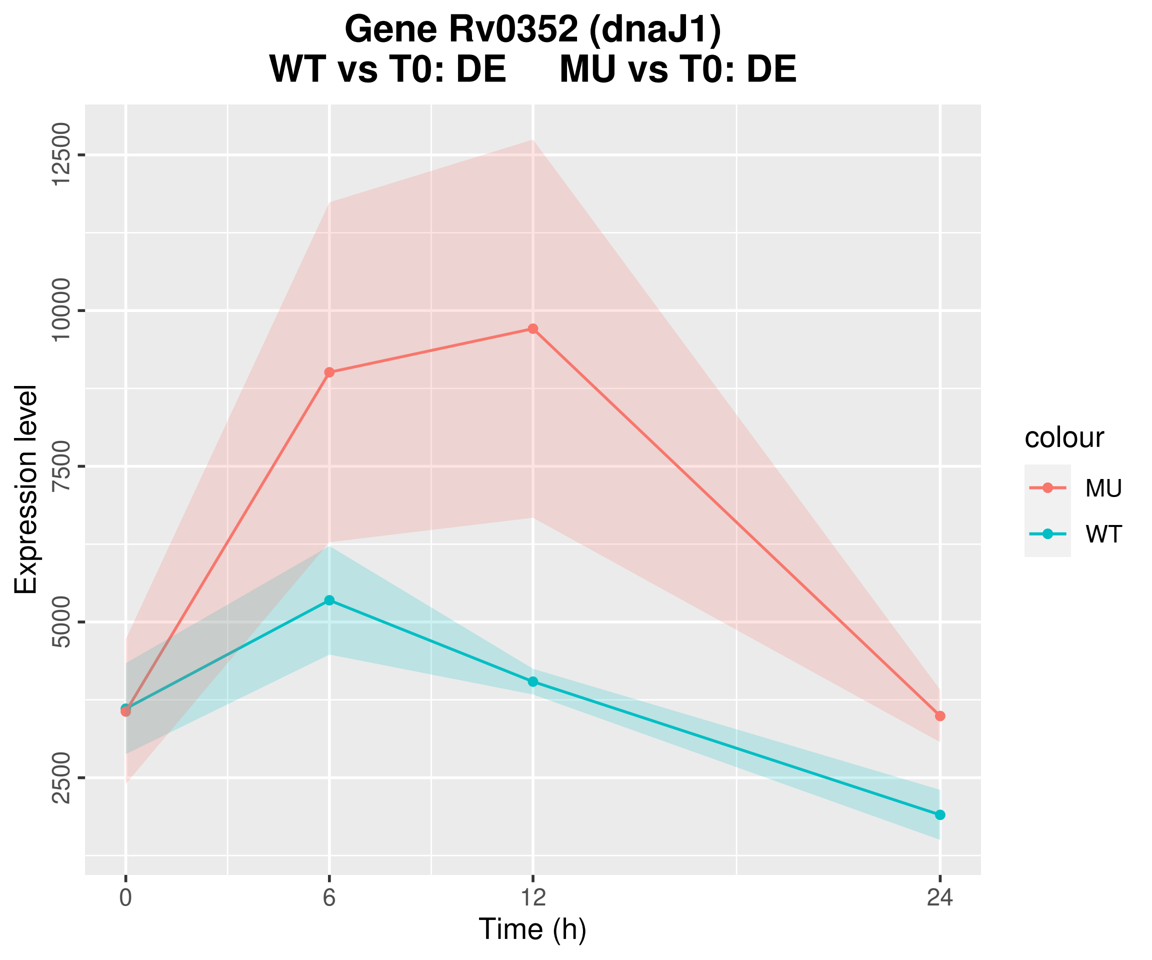

Gene Rv0352 (dnaJ1)
WT vs T0: DE MU vs T0: DE
Expression level
12500
10000
7500
5000
2500
0
6
12
24
Time (h)
colour
MU
WT

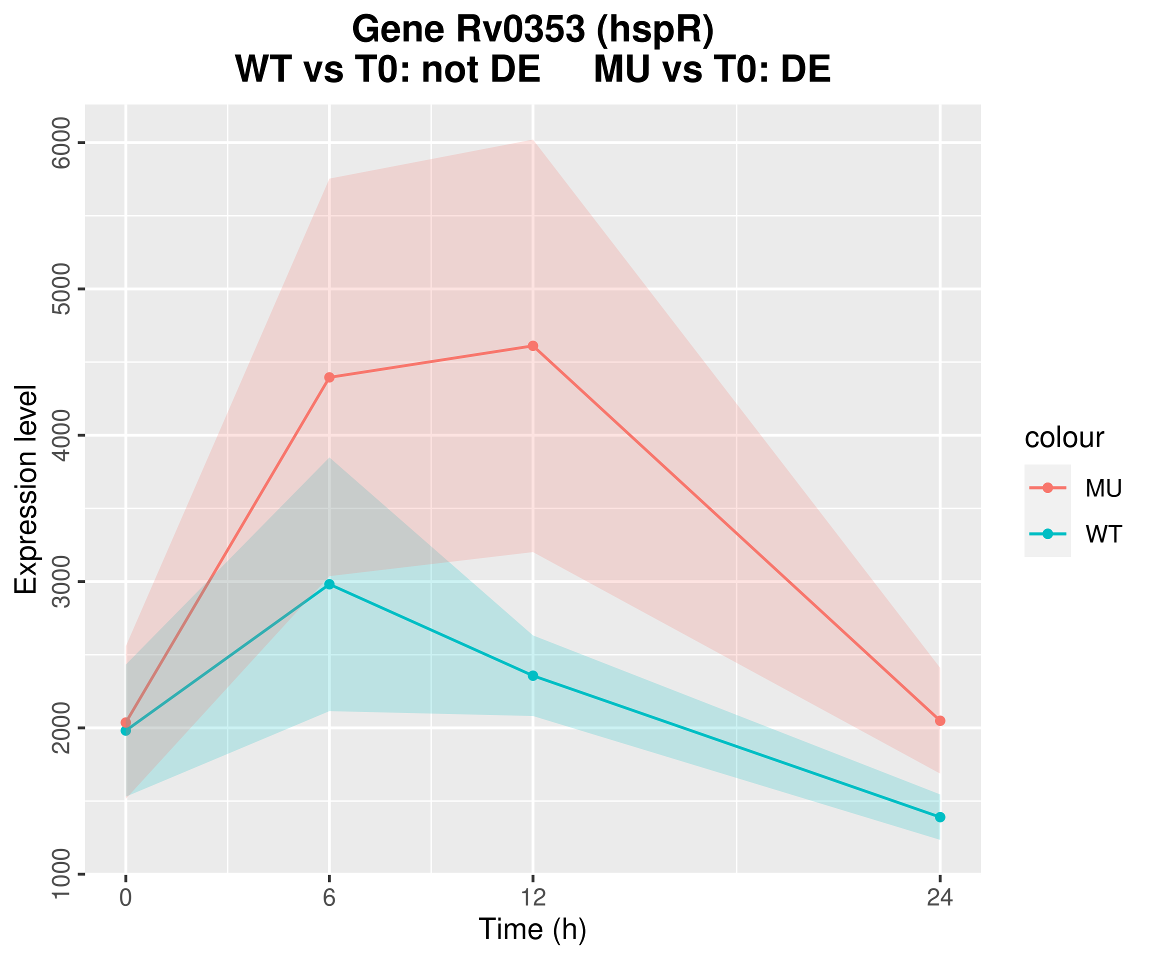

Gene Rv0353 (hspR)
WT vs T0: not DE   MU vs T0: DE
Expression level
6000
5000
4000
3000
2000
1000
0
6
12
24
Time (h)
colour
MU
WT

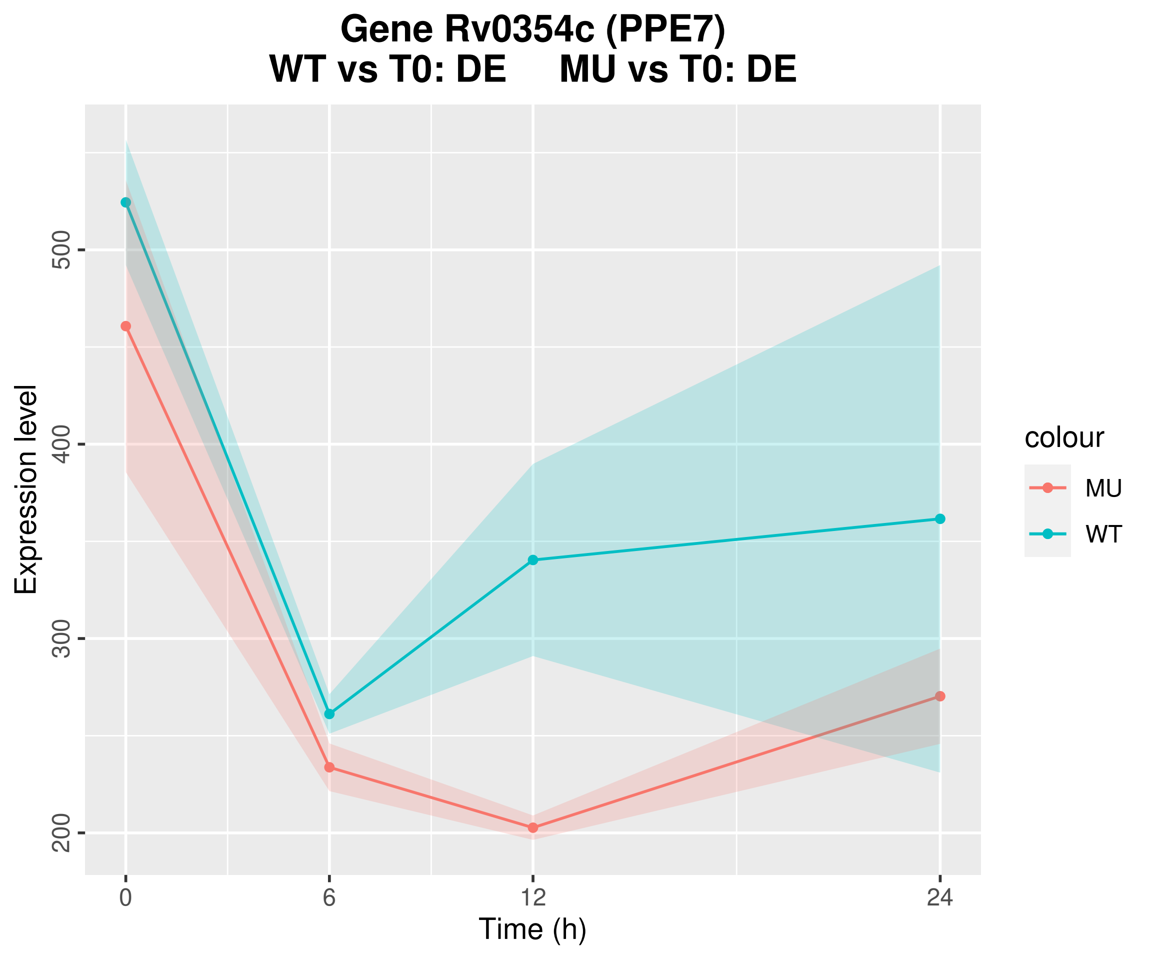

Gene Rv0354c (PPE7)
WT vs T0: DE   MU vs T0: DE
Expression level
500
400
300
200
0
6
12
24
Time (h)
colour
MU
WT

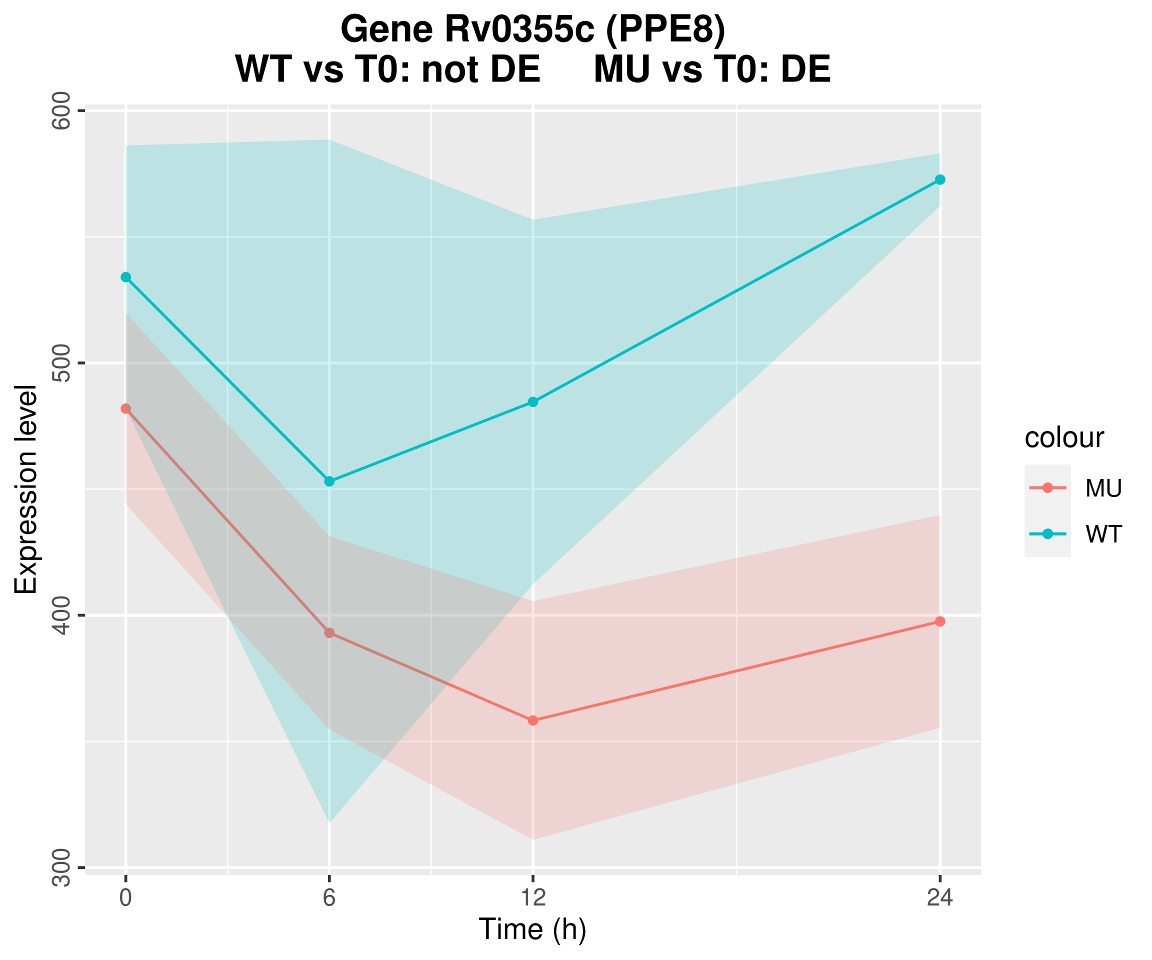

Gene Rv0355c (PPE8)
WT vs T0: not DE   MU vs T0: DE
600
500
400
300
Expression level
0
6
12
24
Time (h)
colour
MU
WT

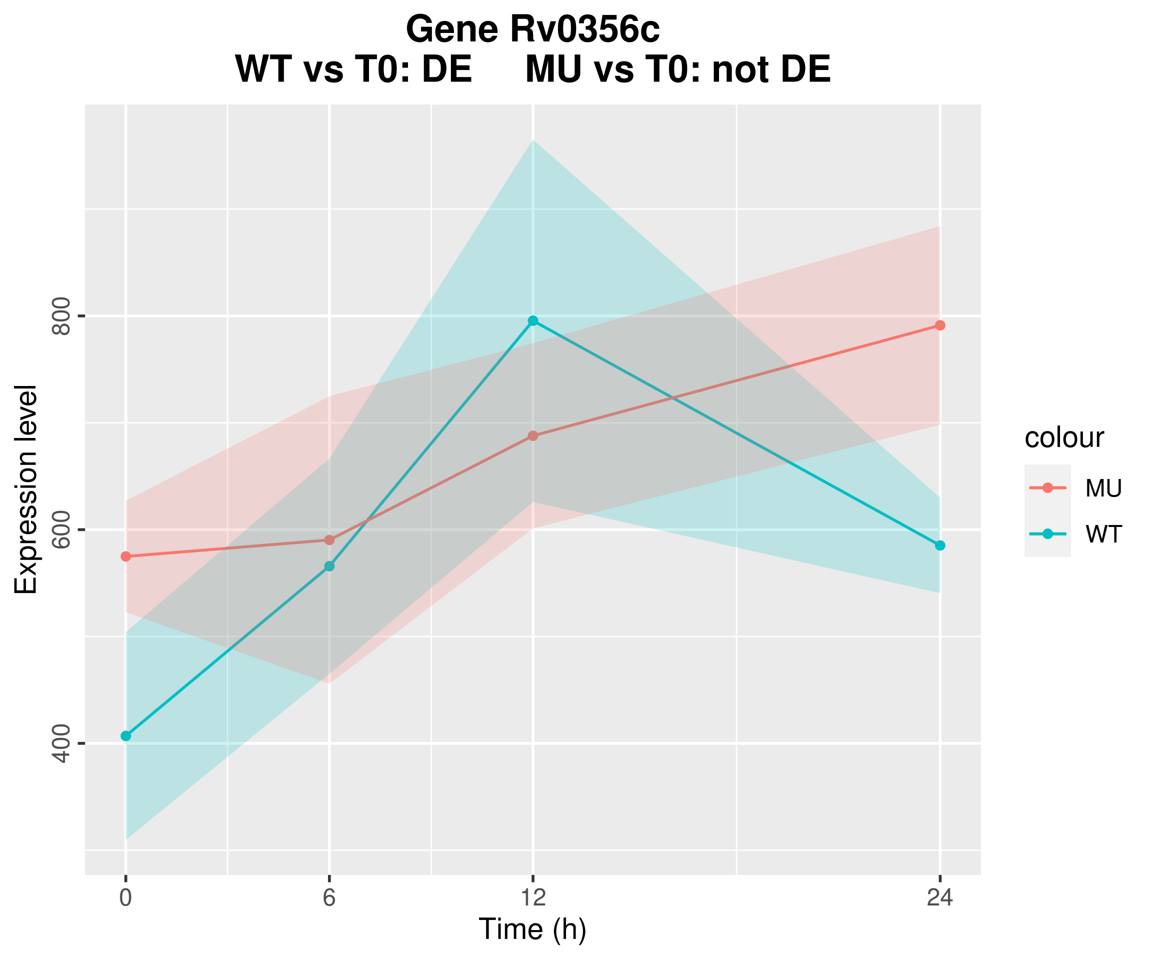
Gene Rv0356c
WT vs T0: DE   MU vs T0: not DE
Expression level
800
600
400
0
6
12
24
Time (h)
colour
MU
WT

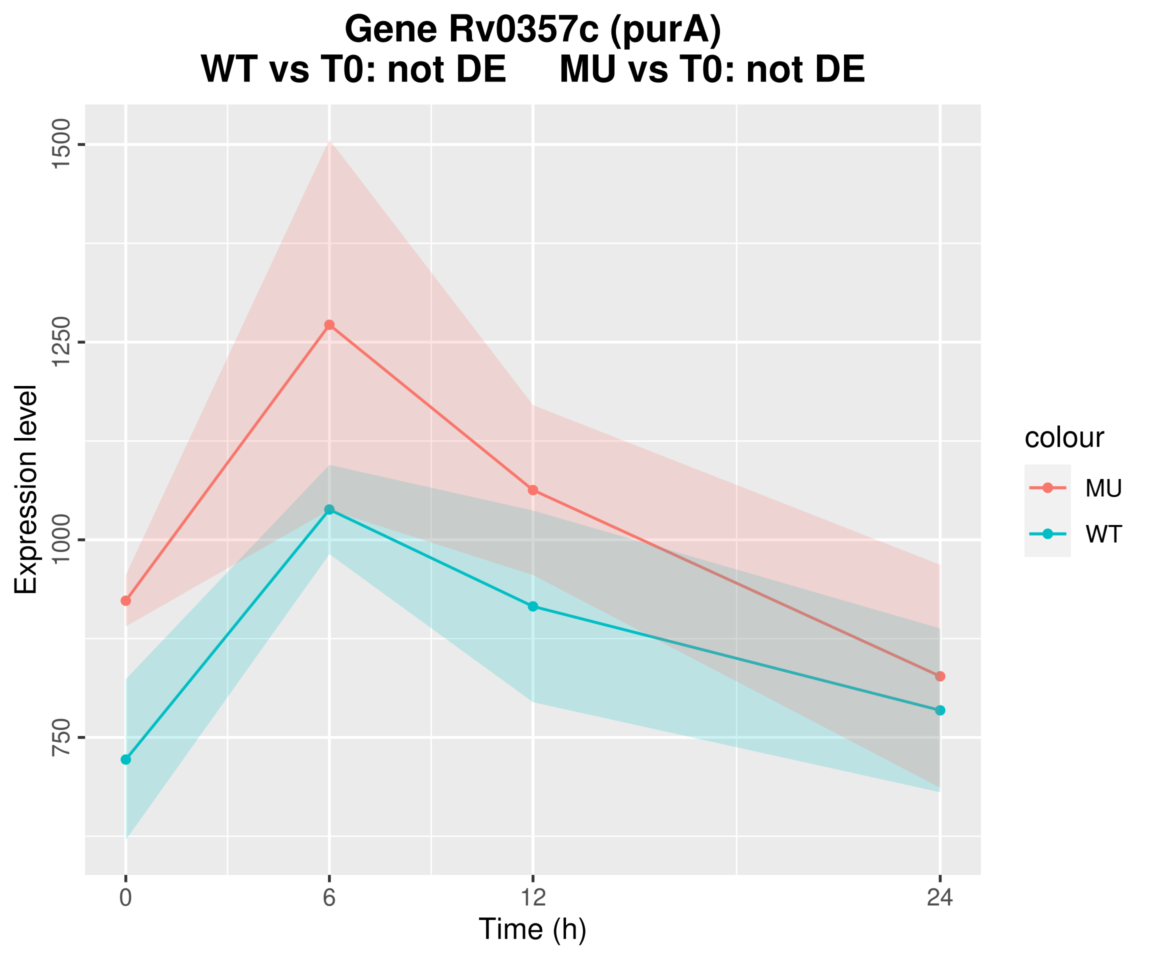

Gene Rv0357c (purA)
WT vs T0: not DE   MU vs T0: not DE
Expression level
1500
1250
1000
750
0
6
12
24
Time (h)
colour
MU
WT

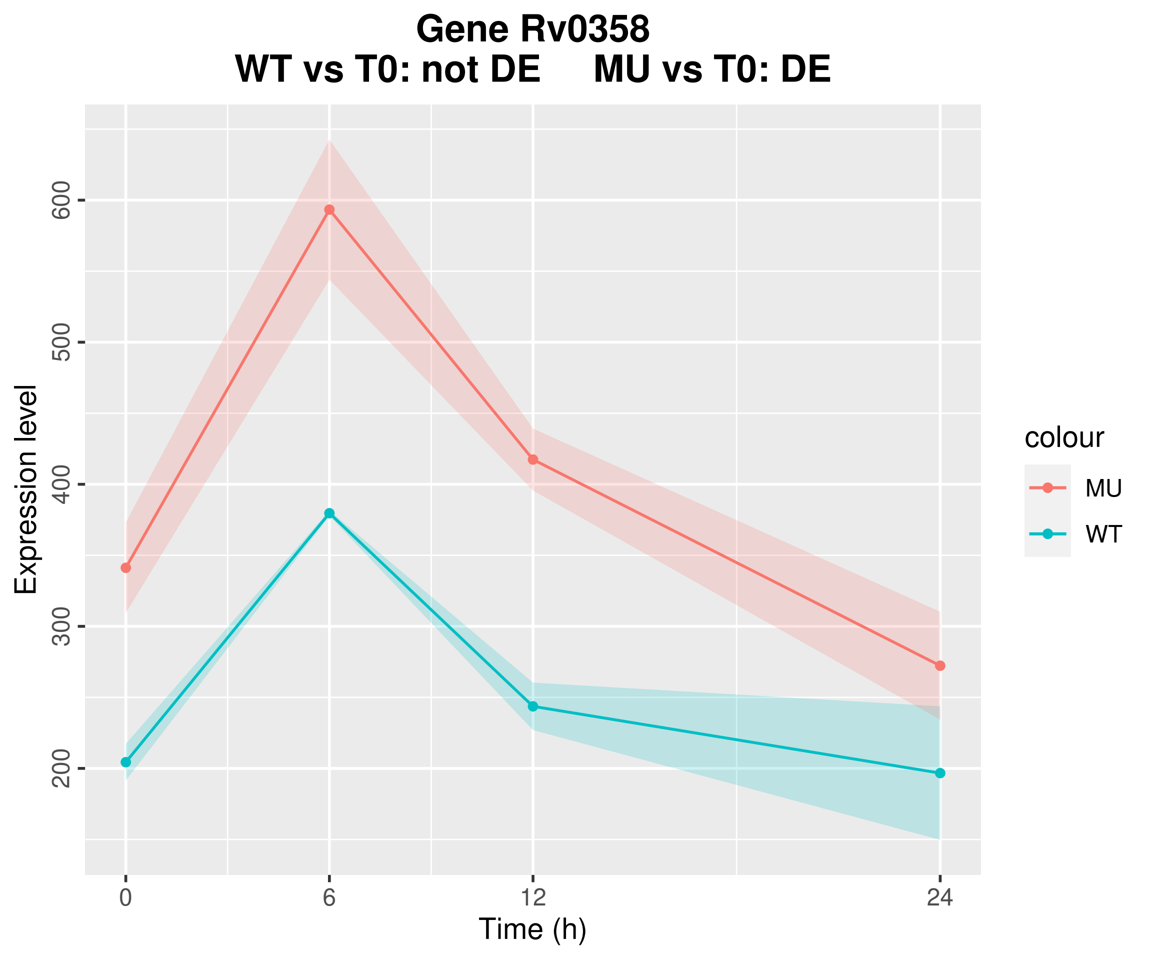

Gene Rv0358
WT vs T0: not DE MU vs T0: DE
Expression level
600
500
400
300
200
0
6
12
24
Time (h)
colour
MU
WT

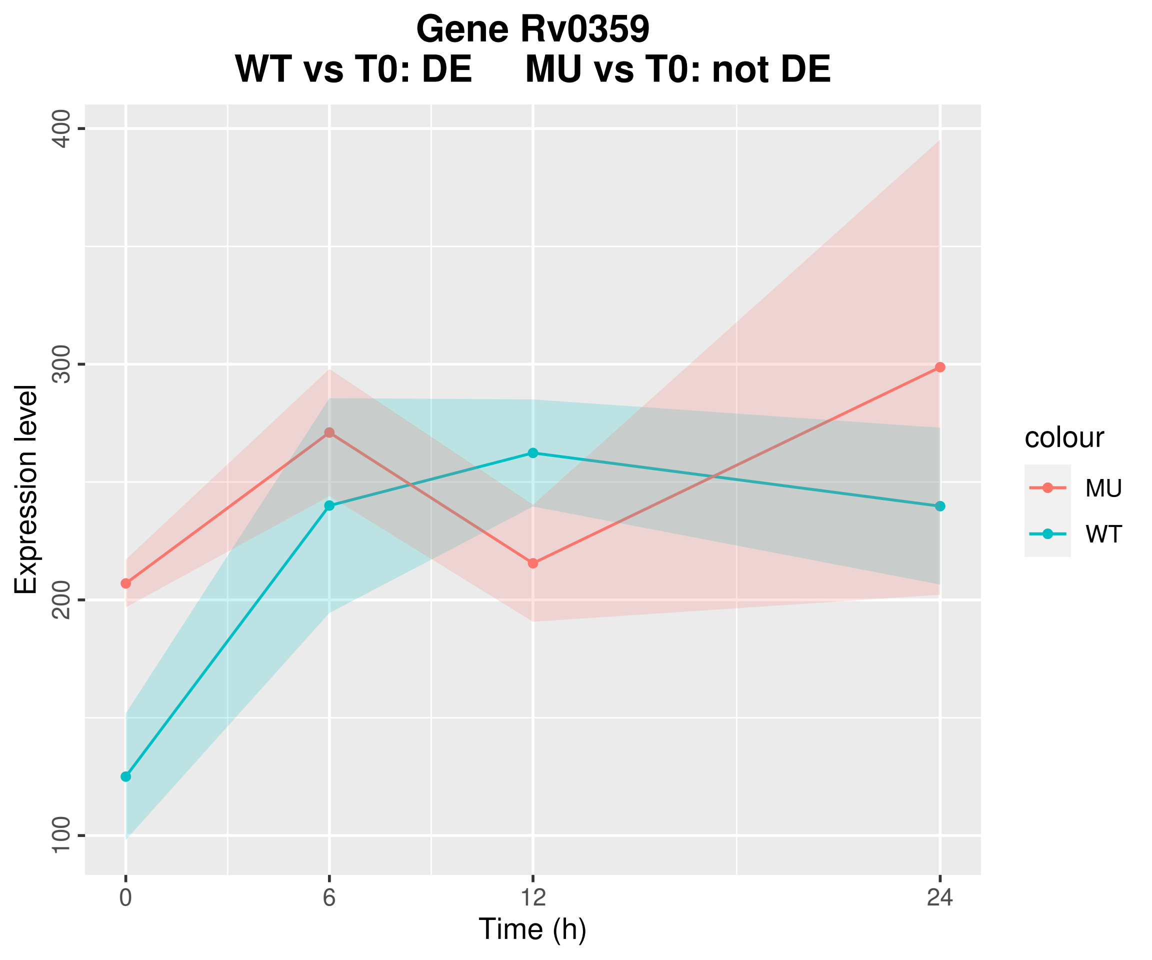

Gene Rv0359
WT vs T0: DE MU vs T0: not DE
Expression level
100
200
300
400
0
6
12
24
Time (h)
colour
MU
WT

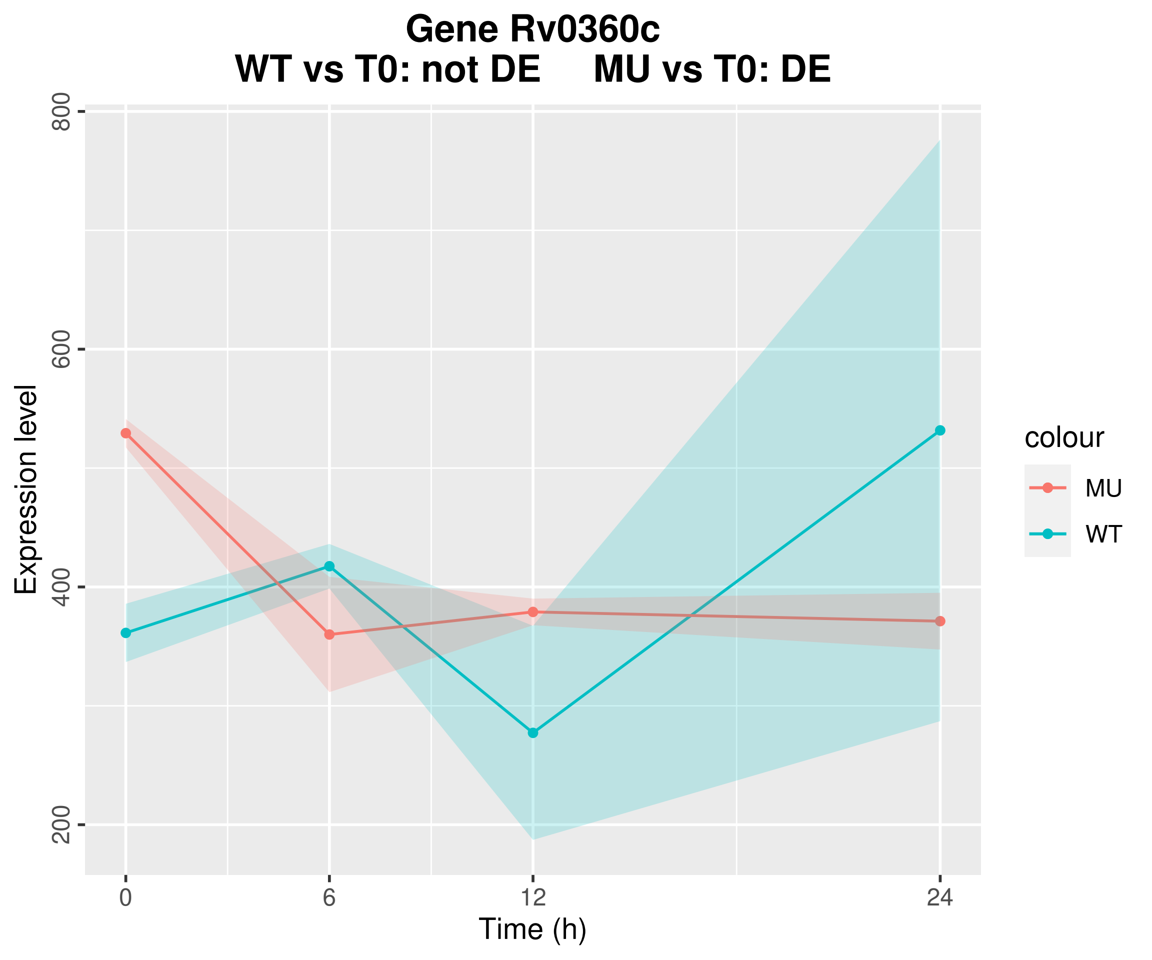

Gene Rv0360c
WT vs T0: not DE MU vs T0: DE
Expression level
800
600
400
200
0
6
12
24
Time (h)
colour
MU
WT

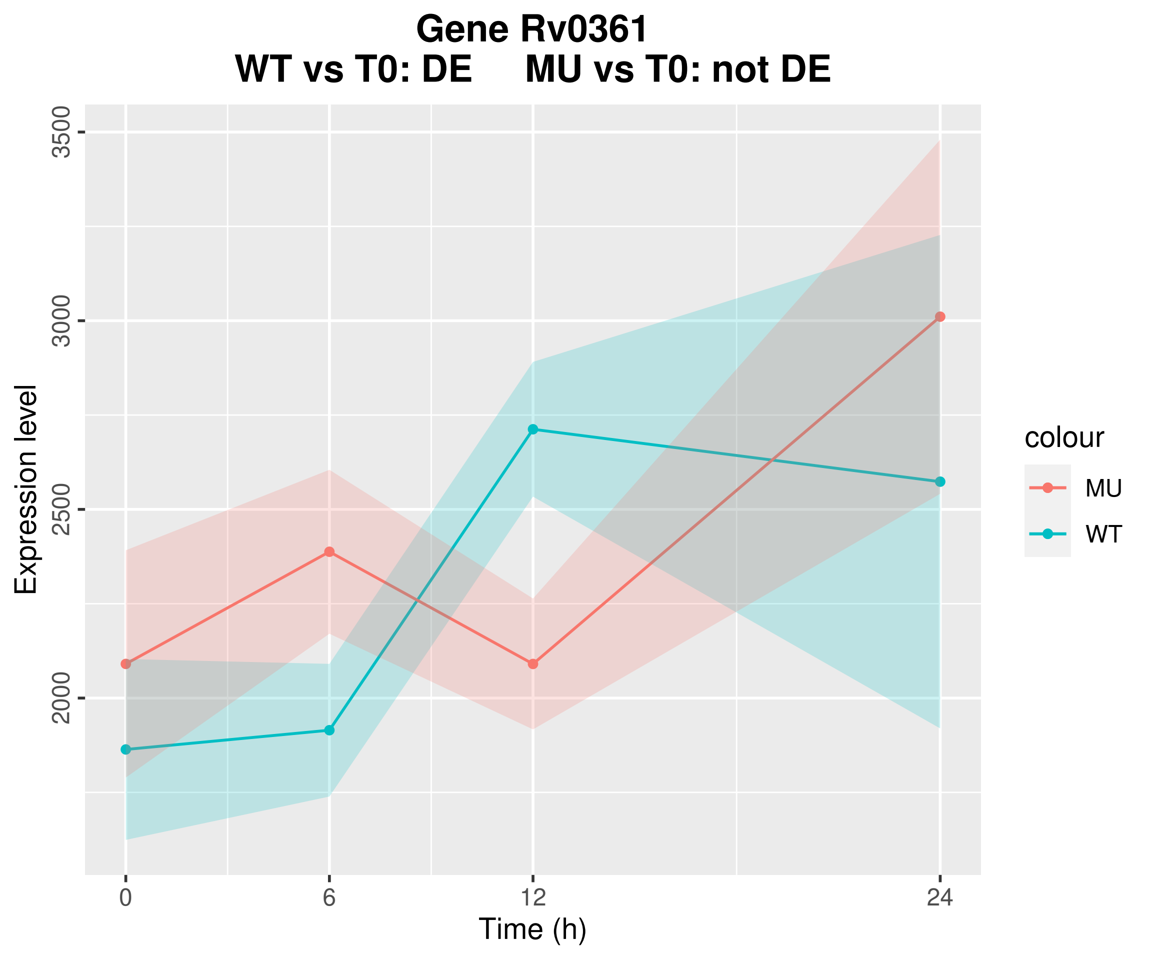

Gene Rv0361
WT vs T0: DE   MU vs T0: not DE
3500
3000
2500
2000
Expression level
0
6
12
24
Time (h)
colour
MU
WT

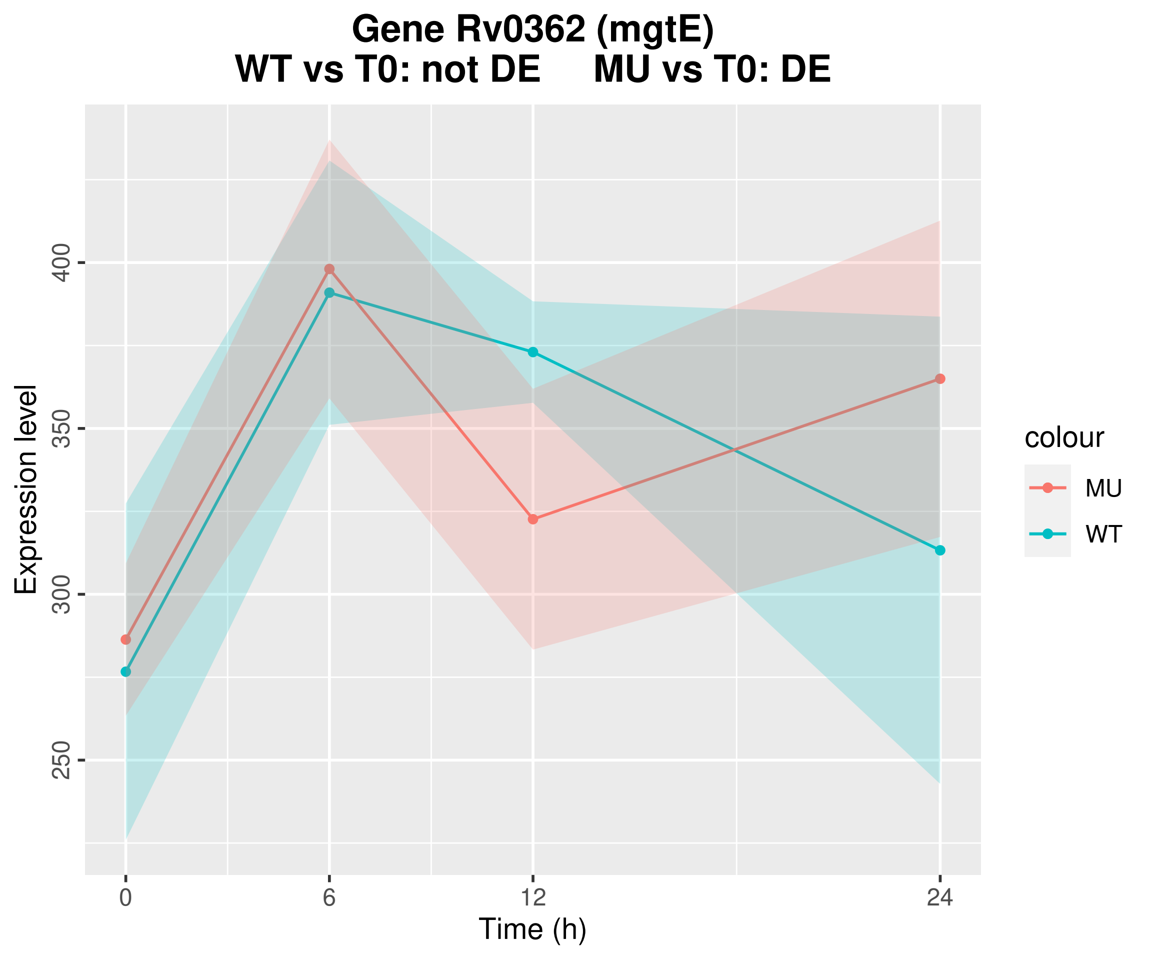

Gene Rv0362 (mgtE)
WT vs T0: not DE MU vs T0: DE
Expression level
250
300
350
400
0
6
12
24
Time (h)
colour
MU
WT

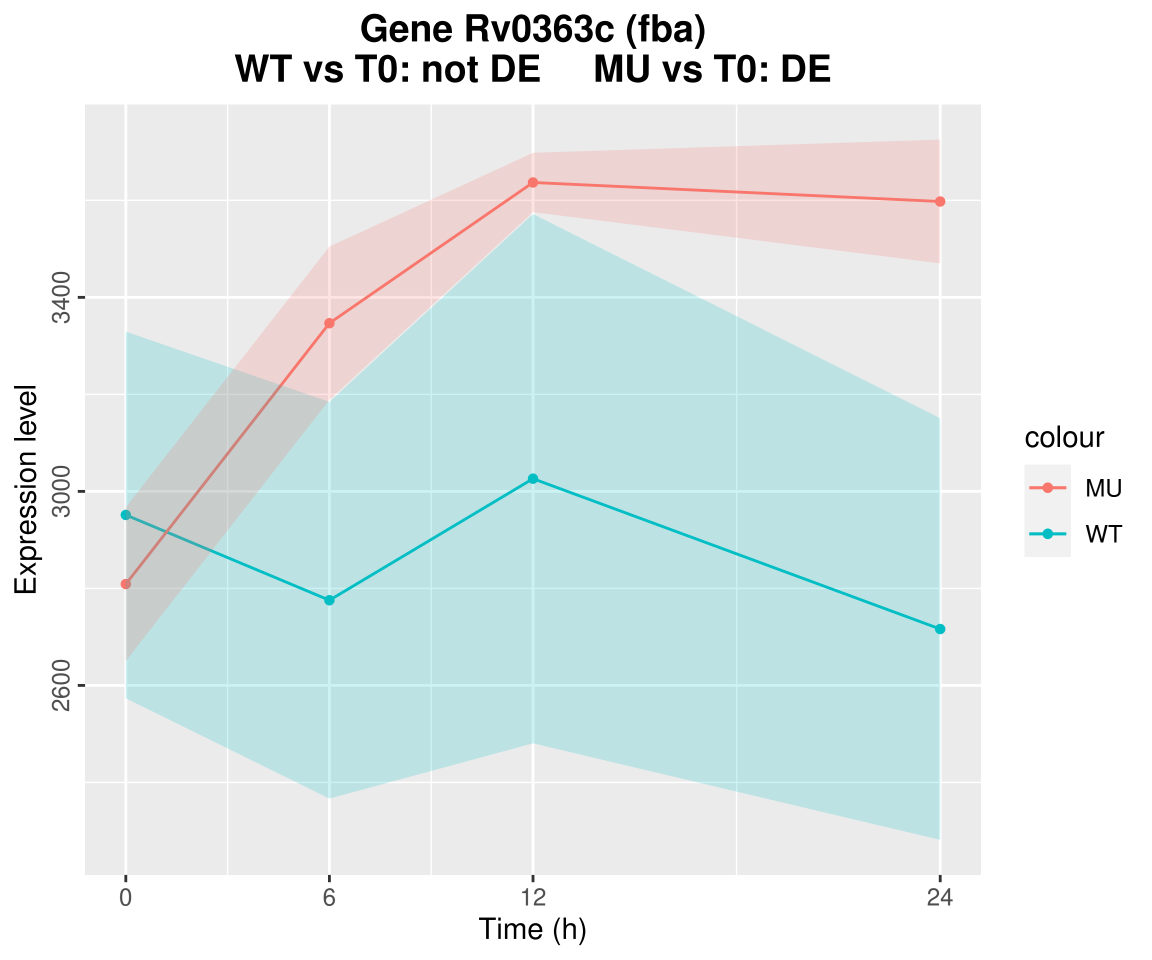

Gene Rv0363c (fba)
WT vs T0: not DE   MU vs T0: DE
Expression level
3400
3000
2600
0
6
12
24
Time (h)
colour
MU
WT

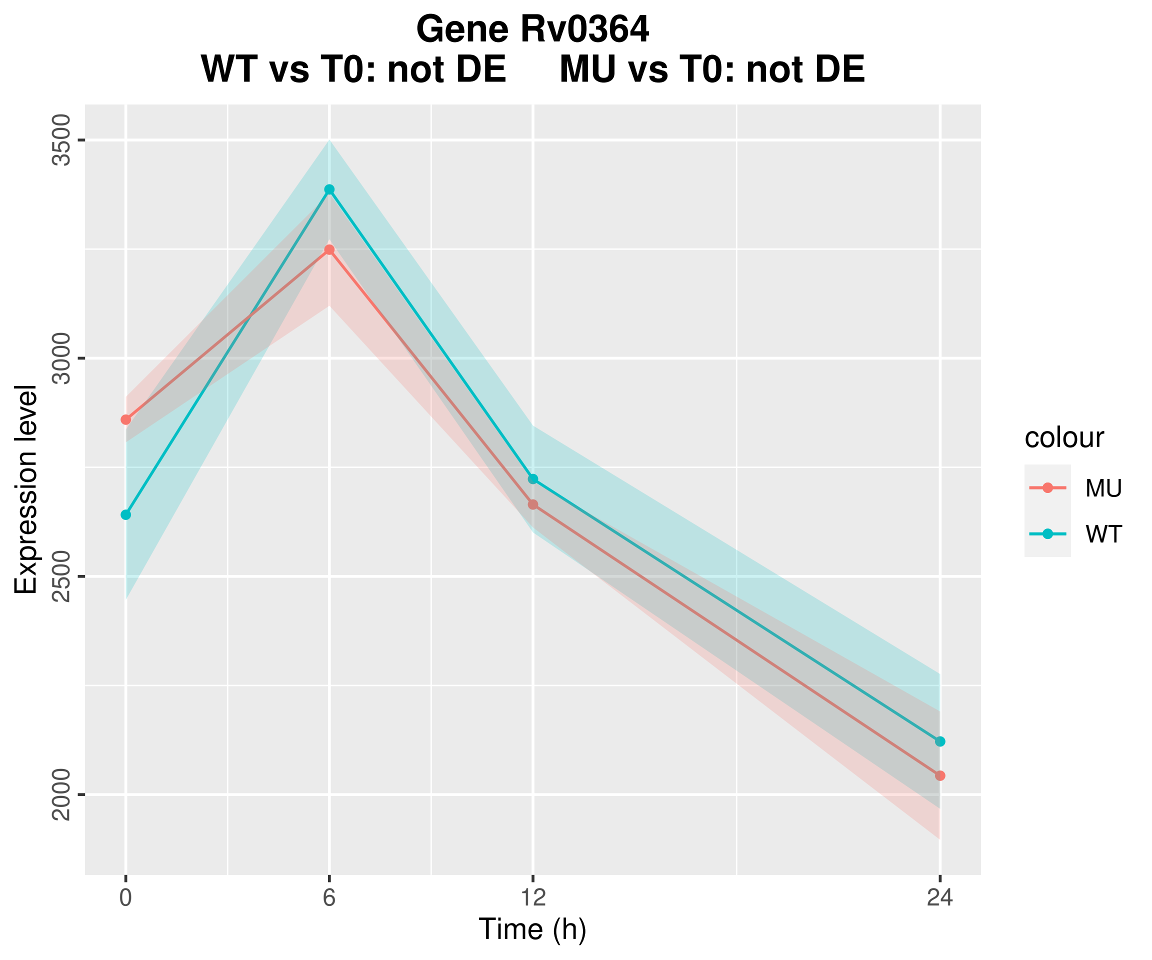

Gene Rv0364
WT vs T0: not DE   MU vs T0: not DE
Expression level
2000
2500
3000
3500
0
6
12
24
Time (h)
colour
MU
WT

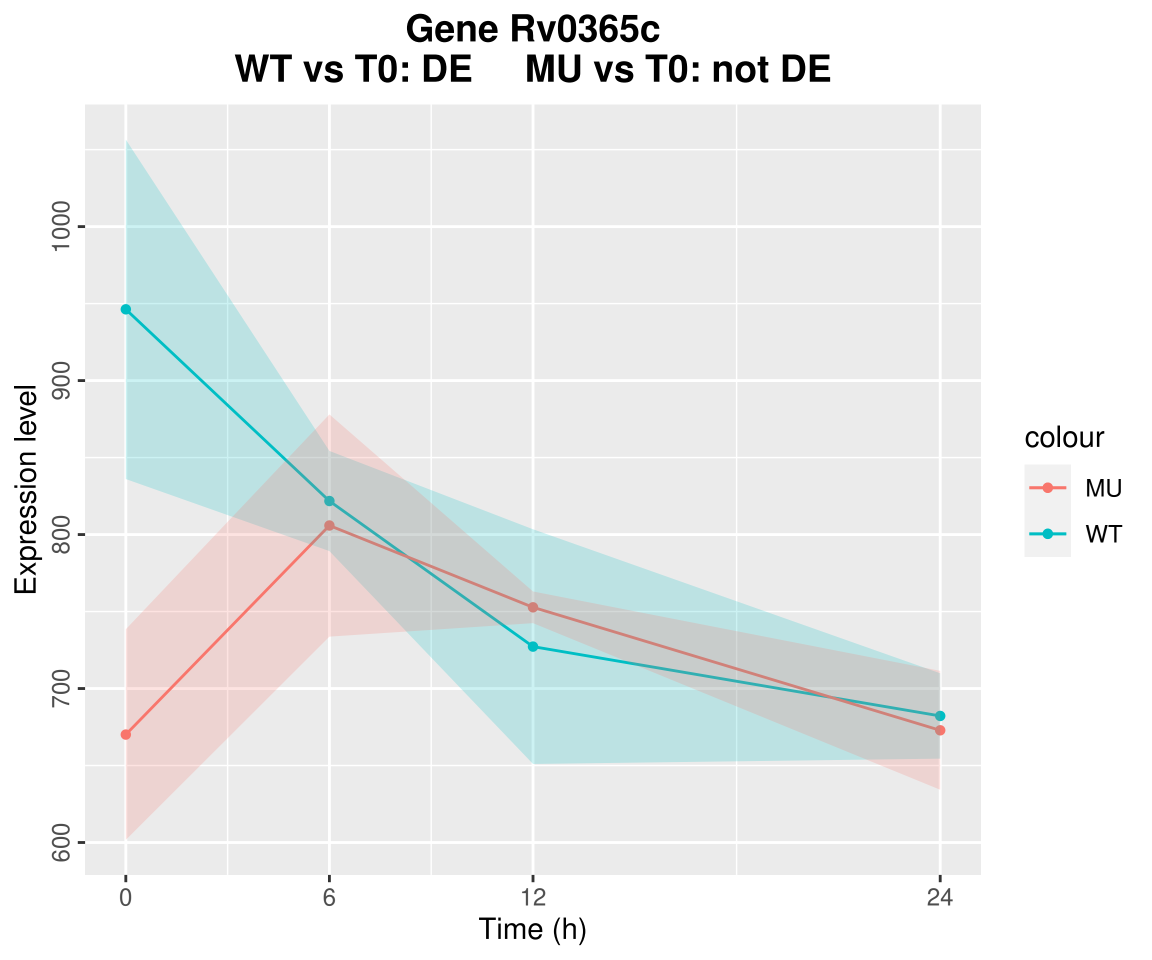
Gene Rv0365c
WT vs T0: DE   MU vs T0: not DE
Expression level
1000
900
800
700
600
0
6
12
24
Time (h)
colour
MU
WT

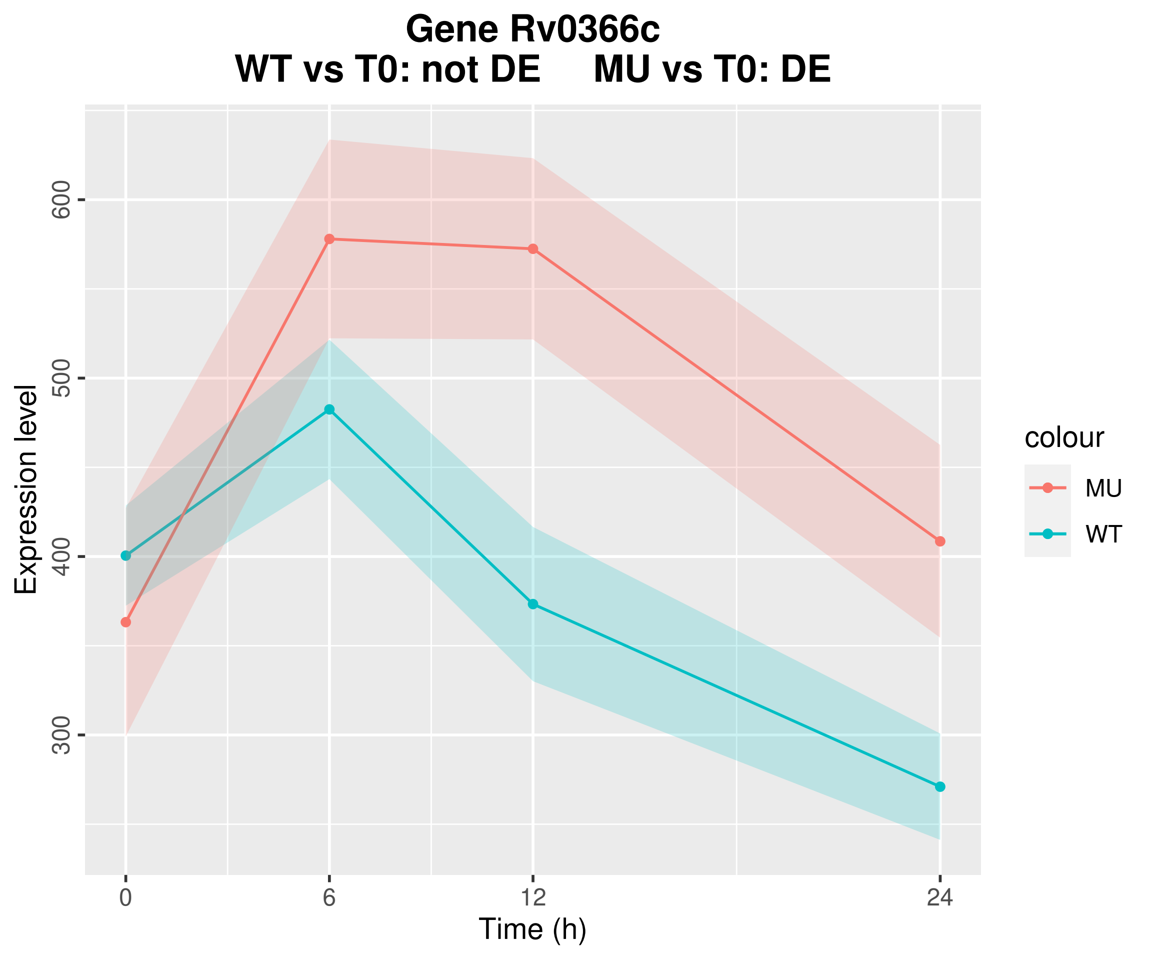

Gene Rv0366c
WT vs T0: not DE   MU vs T0: DE
Expression level
600
500
400
300
0
6
12
24
Time (h)
colour
MU
WT

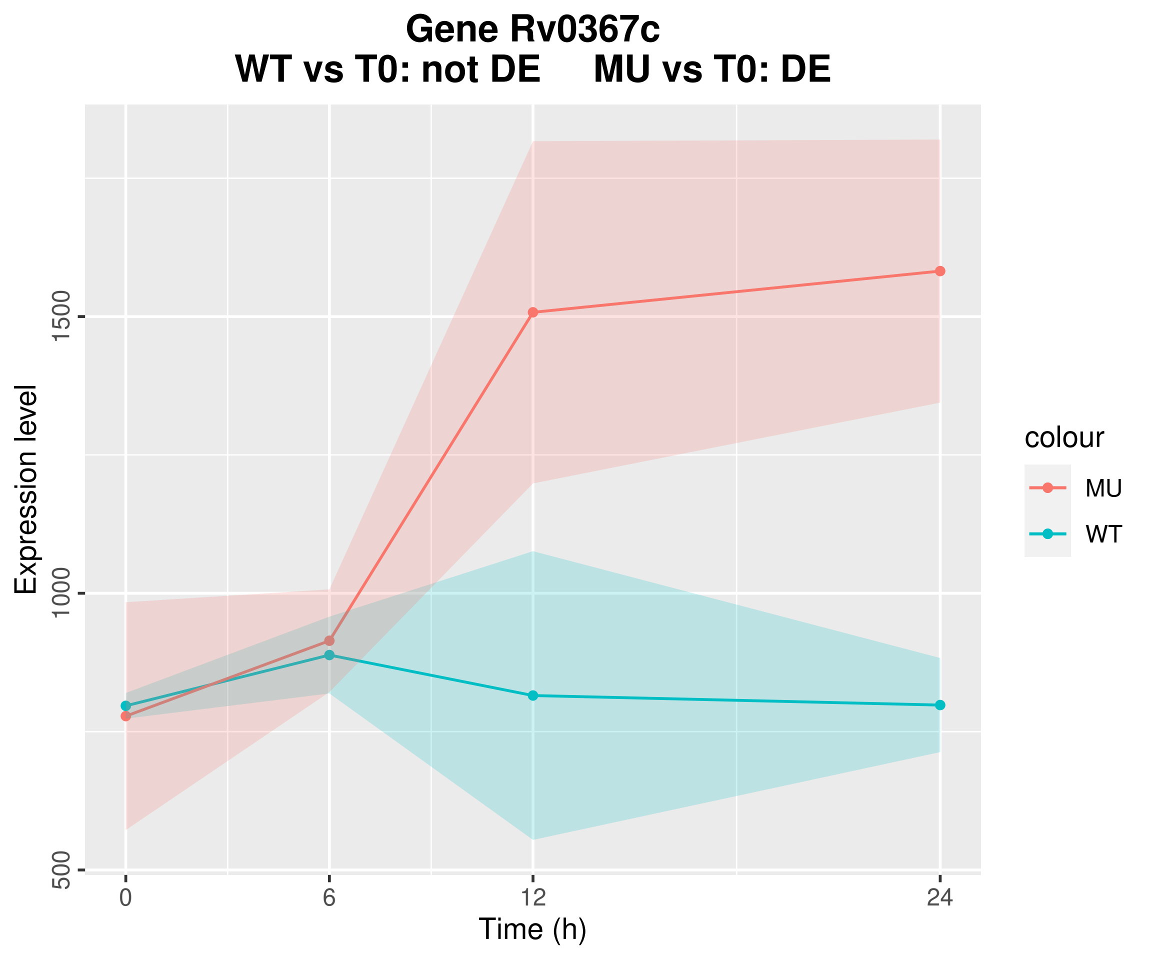
Gene Rv0367c
WT vs T0: not DE   MU vs T0: DE
Expression level
500
1000
1500
0
6
12
24
Time (h)
colour
MU
WT

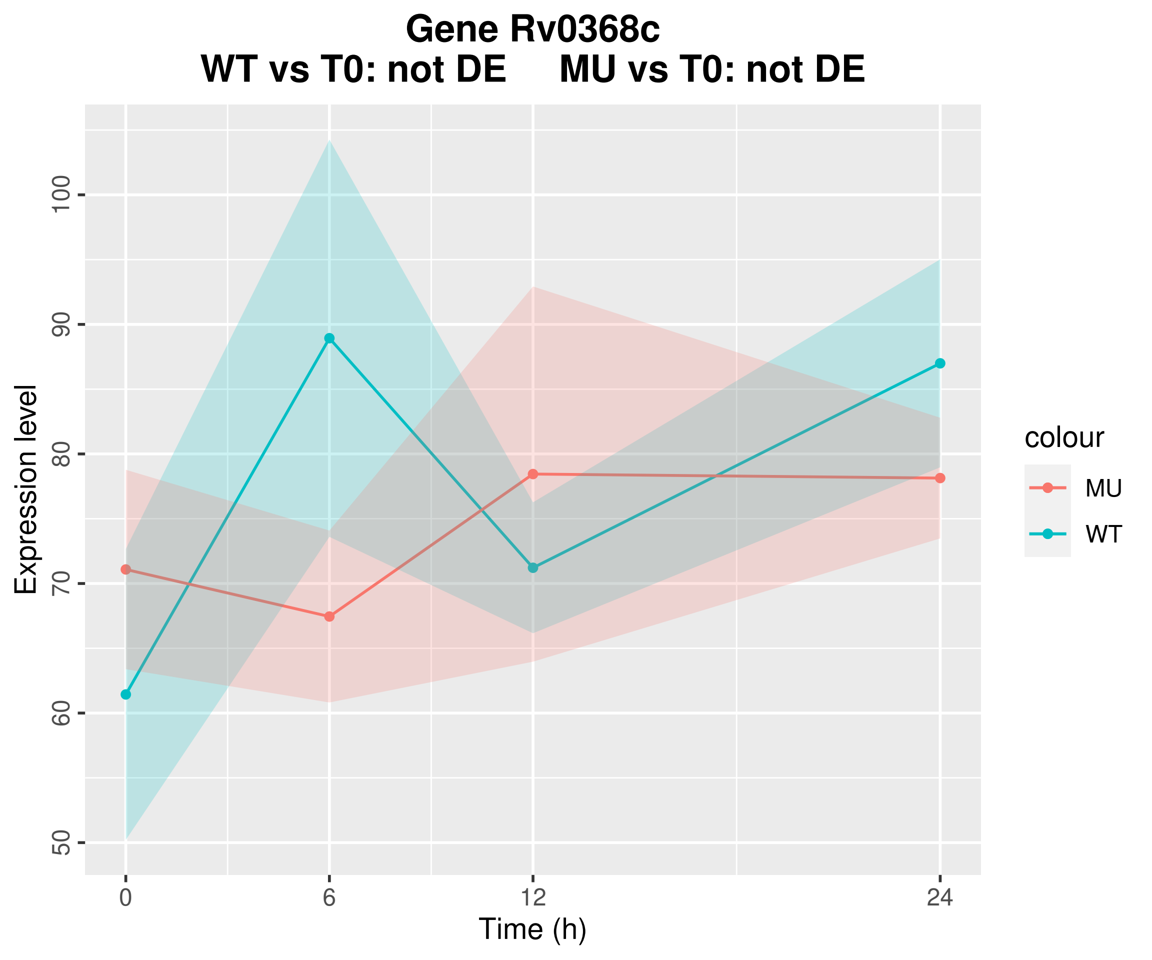

Gene Rv0368c
WT vs T0: not DE   MU vs T0: not DE
Expression level
100
90
80
70
60
50
0
6
12
24
Time (h)
colour
MU
WT

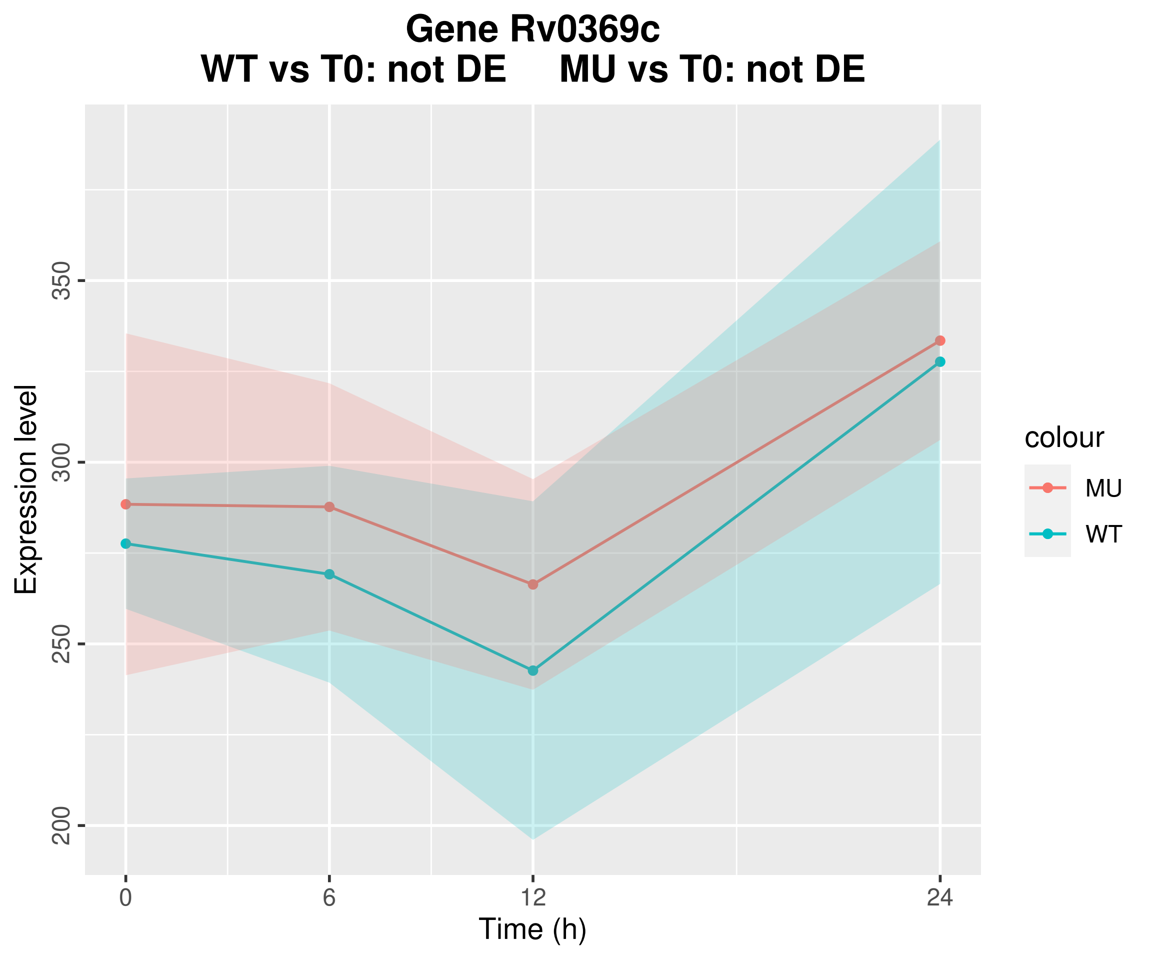

Gene Rv0369c
WT vs T0: not DE MU vs T0: not DE
Expression level
350
300
250
200
0
6
12
24
Time (h)
colour
MU
WT

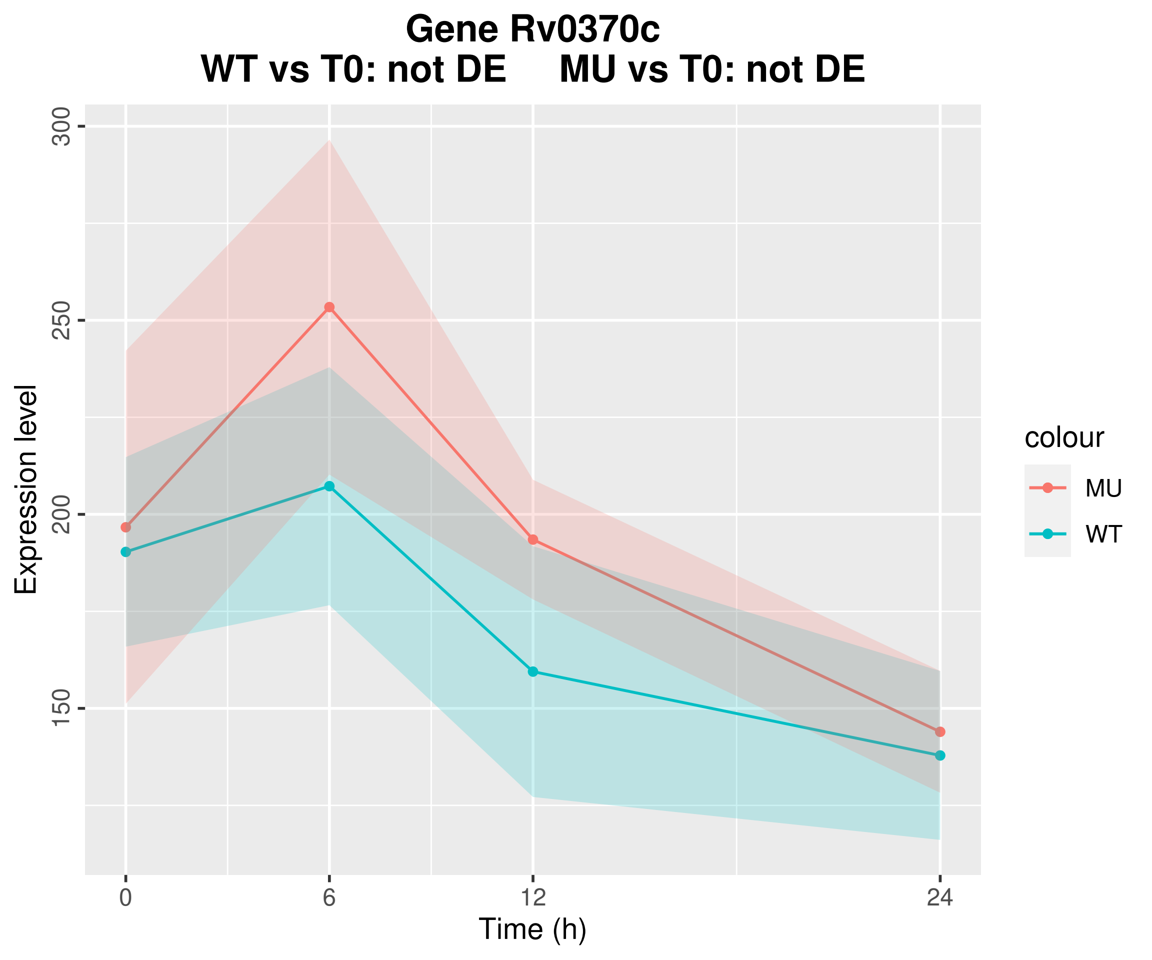
Gene Rv0370c
WT vs T0: not DE MU vs T0: not DE
Expression level
300
250
200
150
0
6
12
24
Time (h)
colour
MU
WT

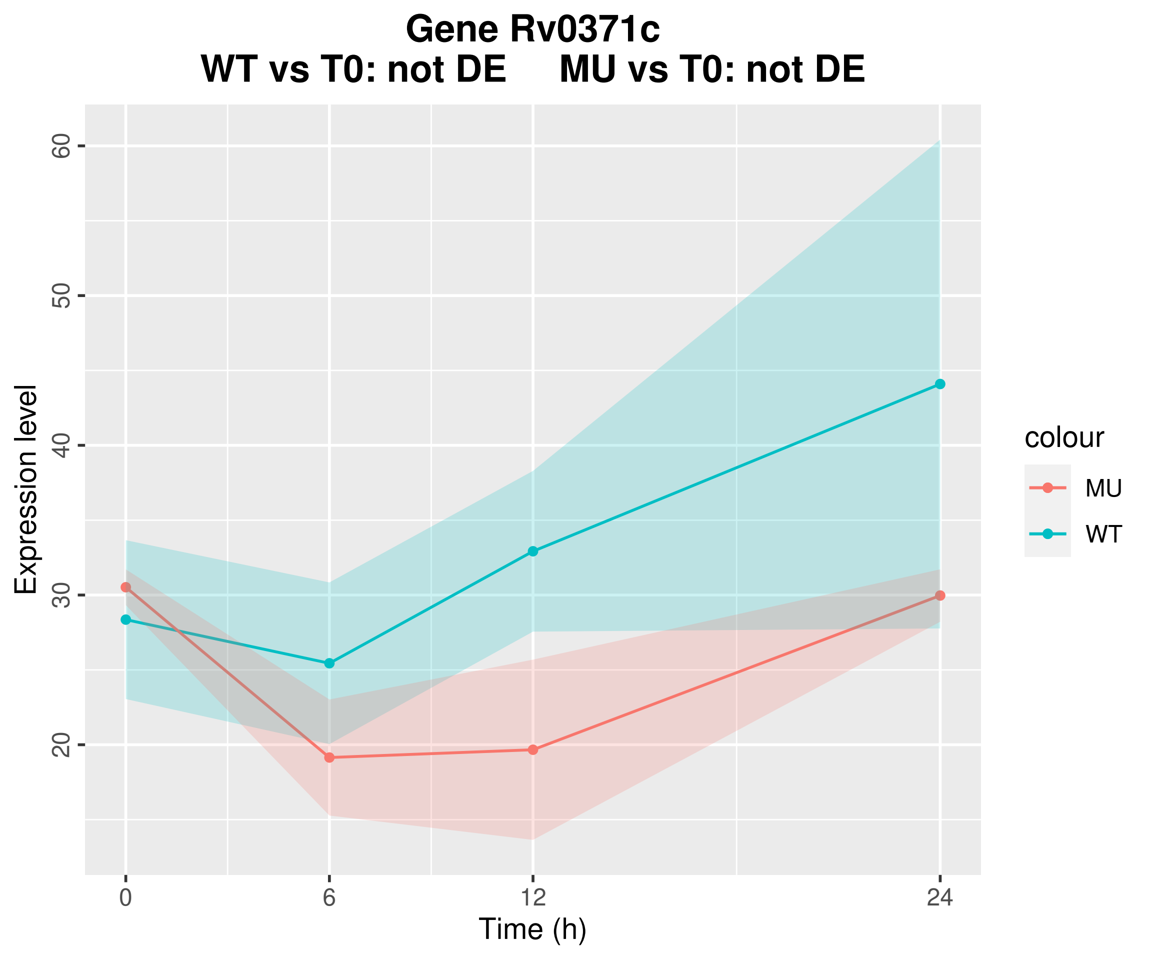
Gene Rv0371c
WT vs T0: not DE MU vs T0: not DE
Expression level
60
50
40
30
20
0
6
12
24
Time (h)
colour
MU
WT

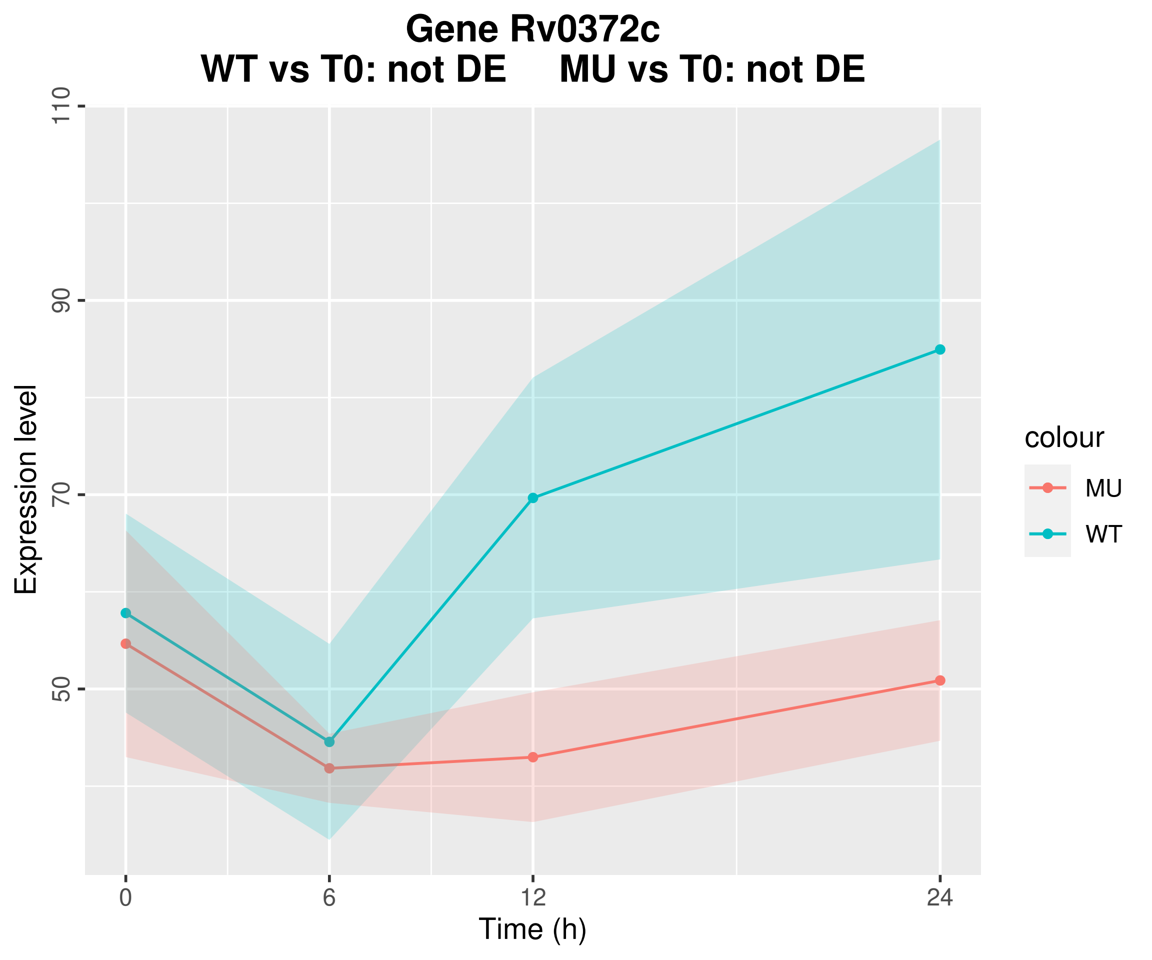
Gene Rv0372c
WT vs T0: not DE MU vs T0: not DE
110
90
70
50
Expression level
0
6
12
24
Time (h)
colour
MU
WT

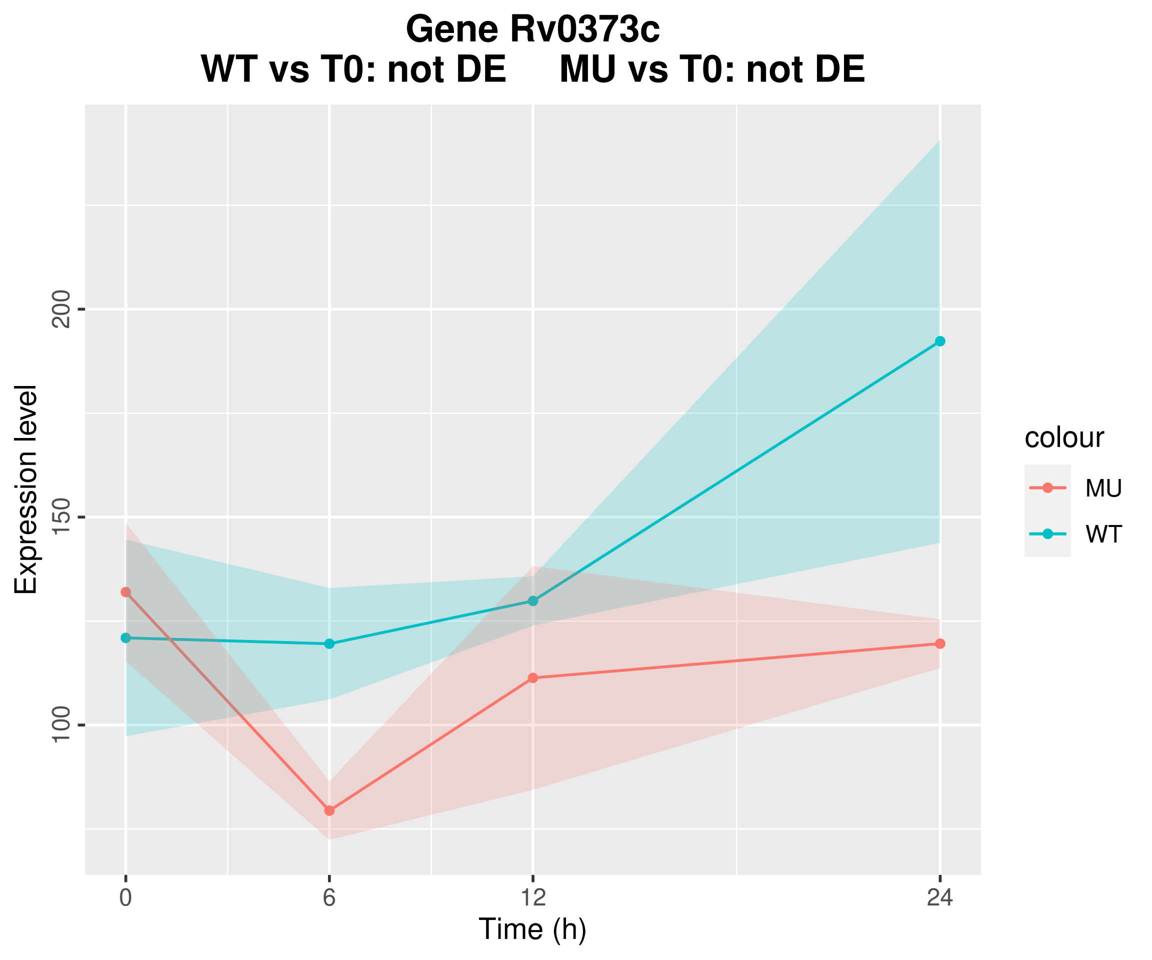

Gene Rv0373c
WT vs T0: not DE MU vs T0: not DE
Expression level
200
150
100
0
6
12
24
Time (h)
colour
MU
WT

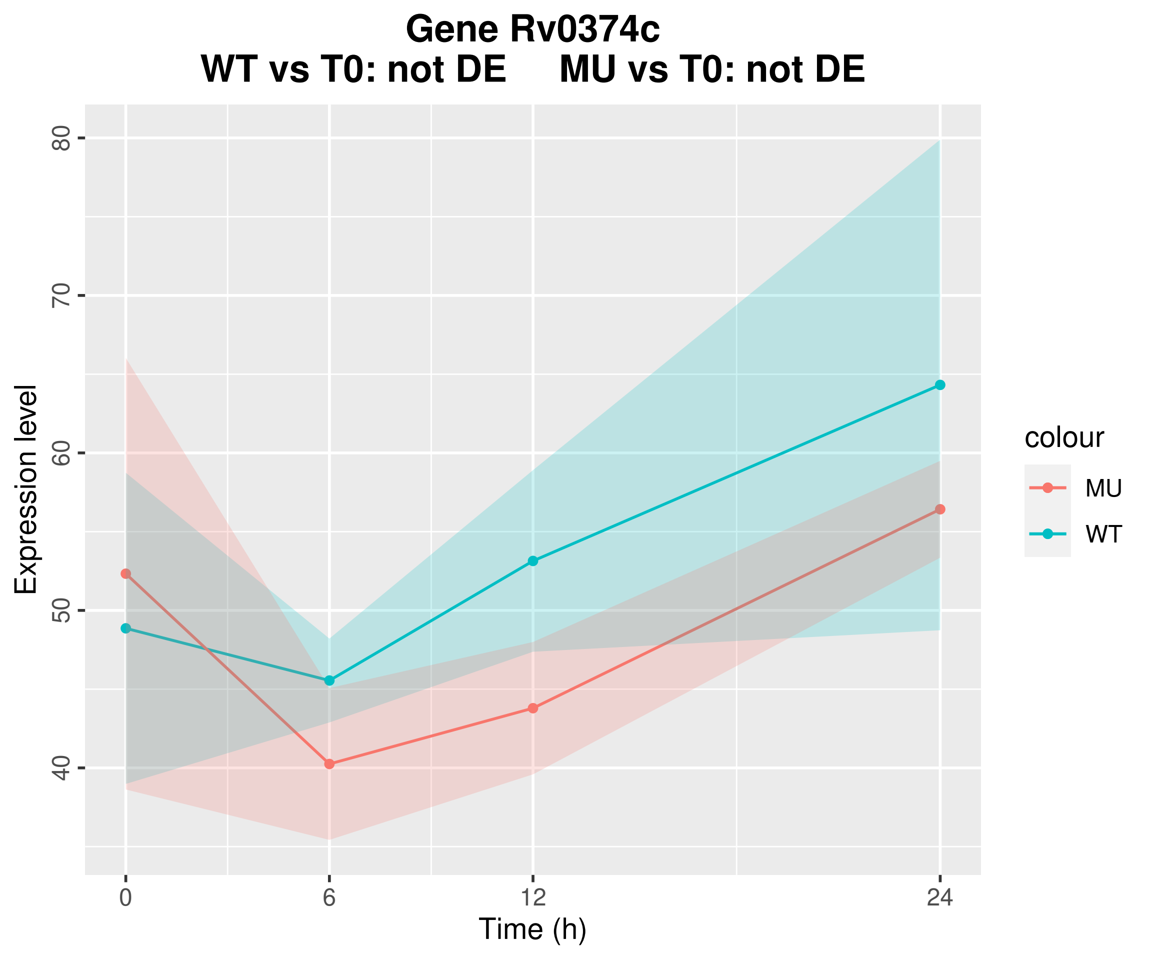

Gene Rv0374c
WT vs T0: not DE MU vs T0: not DE
Expression level
80
70
60
50
40
0
6
12
24
Time (h)
colour
MU
WT

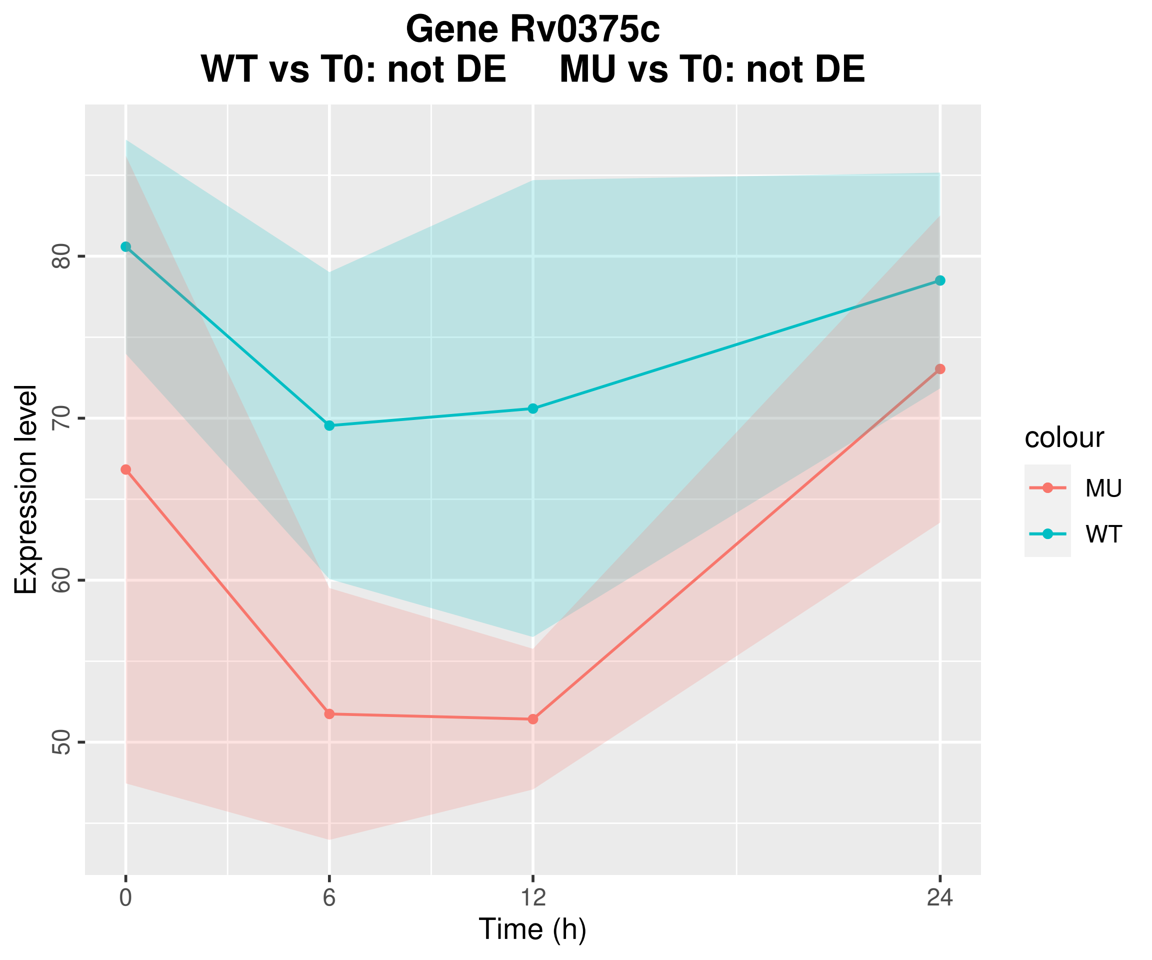

Gene Rv0375c
WT vs T0: not DE MU vs T0: not DE
Expression level
80
70
60
50
0
6
12
24
Time (h)
colour
MU
WT

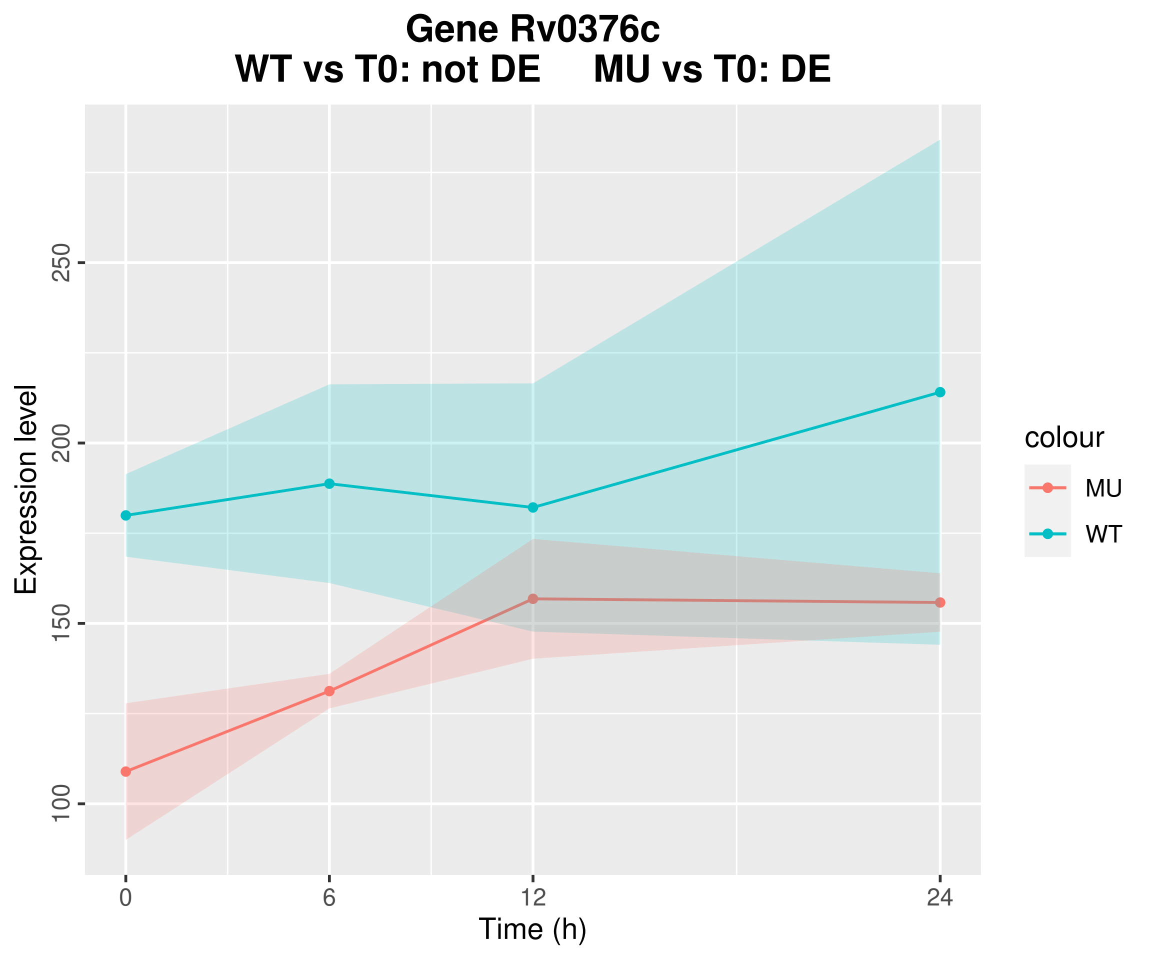
Gene Rv0376c
WT vs T0: not DE MU vs T0: DE
Expression level
250
200
150
100
0
6
12
24
Time (h)
colour
MU
WT

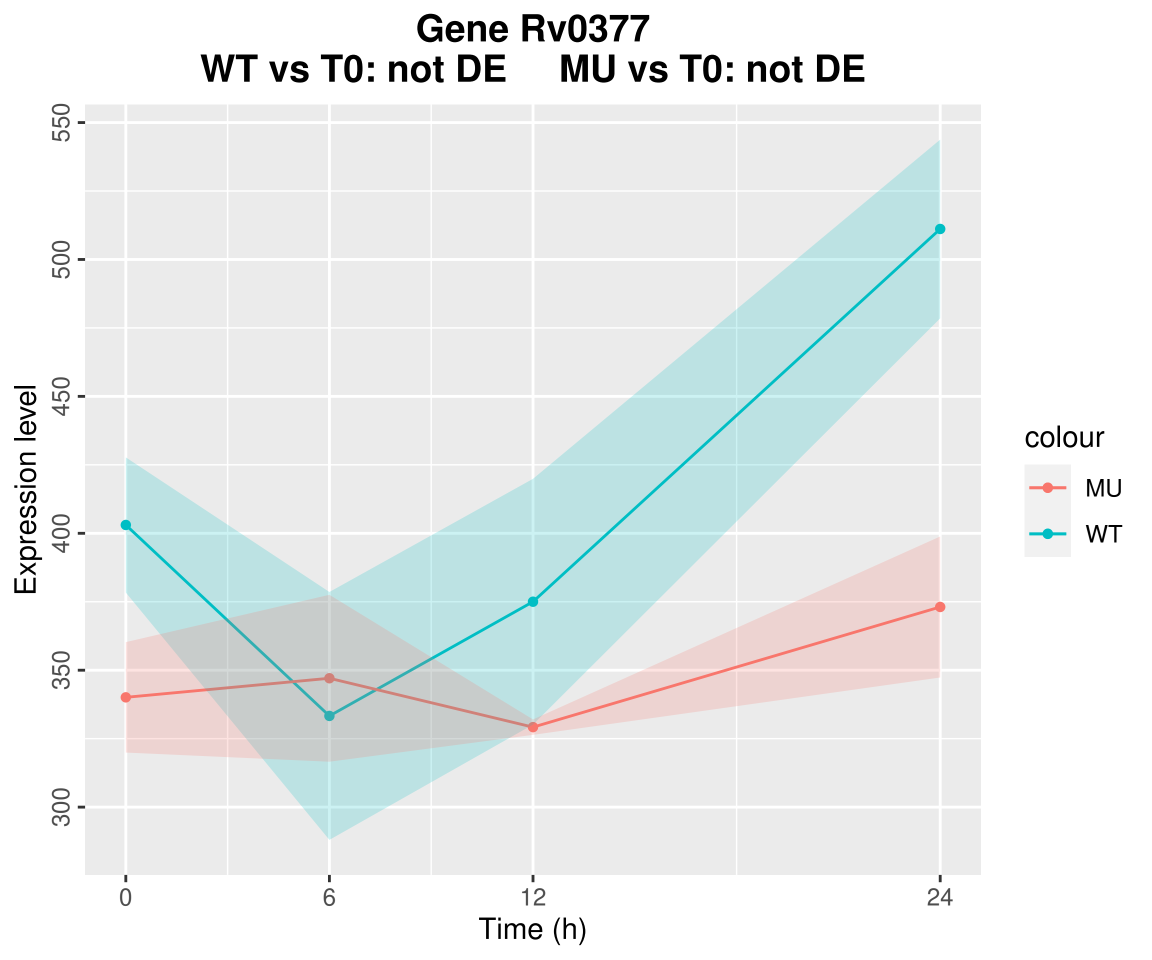

Gene Rv0377
WT vs T0: not DE MU vs T0: not DE
Expression level
300
350
400
450
500
550
0
6
12
24
Time (h)
colour
MU
WT

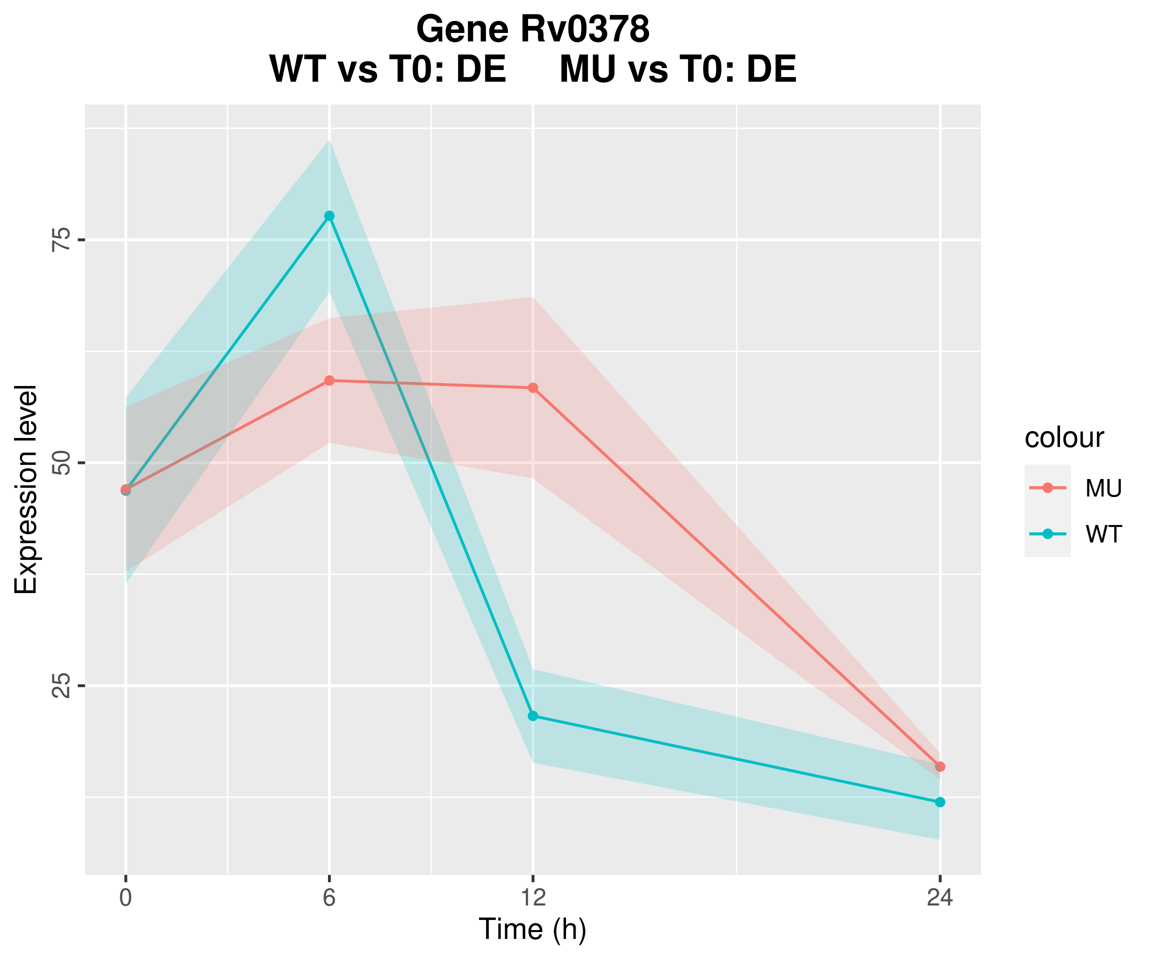

Gene Rv0378
WT vs T0: DE MU vs T0: DE
Expression level
75
50
25
0
6
12
24
Time (h)
colour
MU
WT

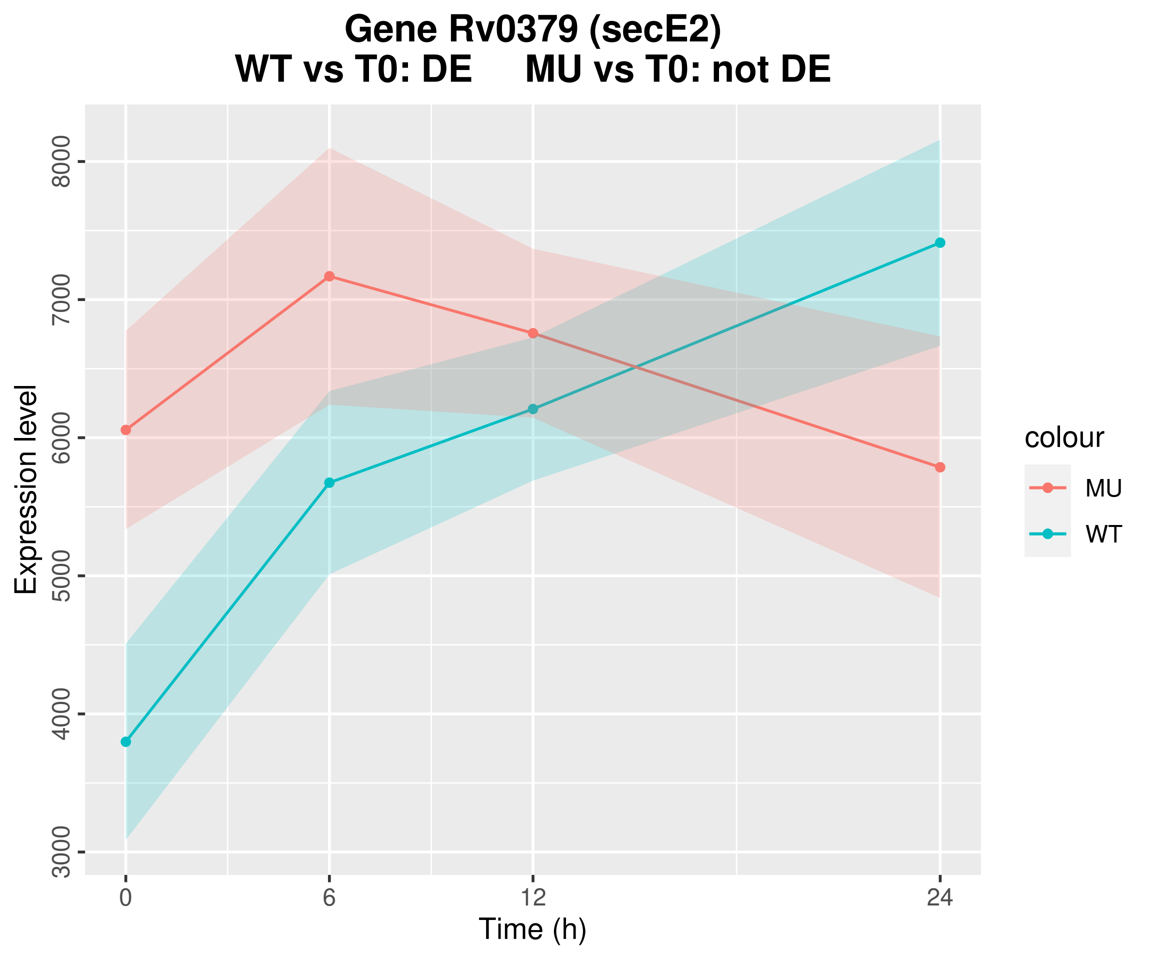

Gene Rv0379 (secE2)
WT vs T0: DE MU vs T0: not DE
Expression level
8000
7000
6000
5000
4000
3000
0
6
12
24
Time (h)
colour
MU
WT

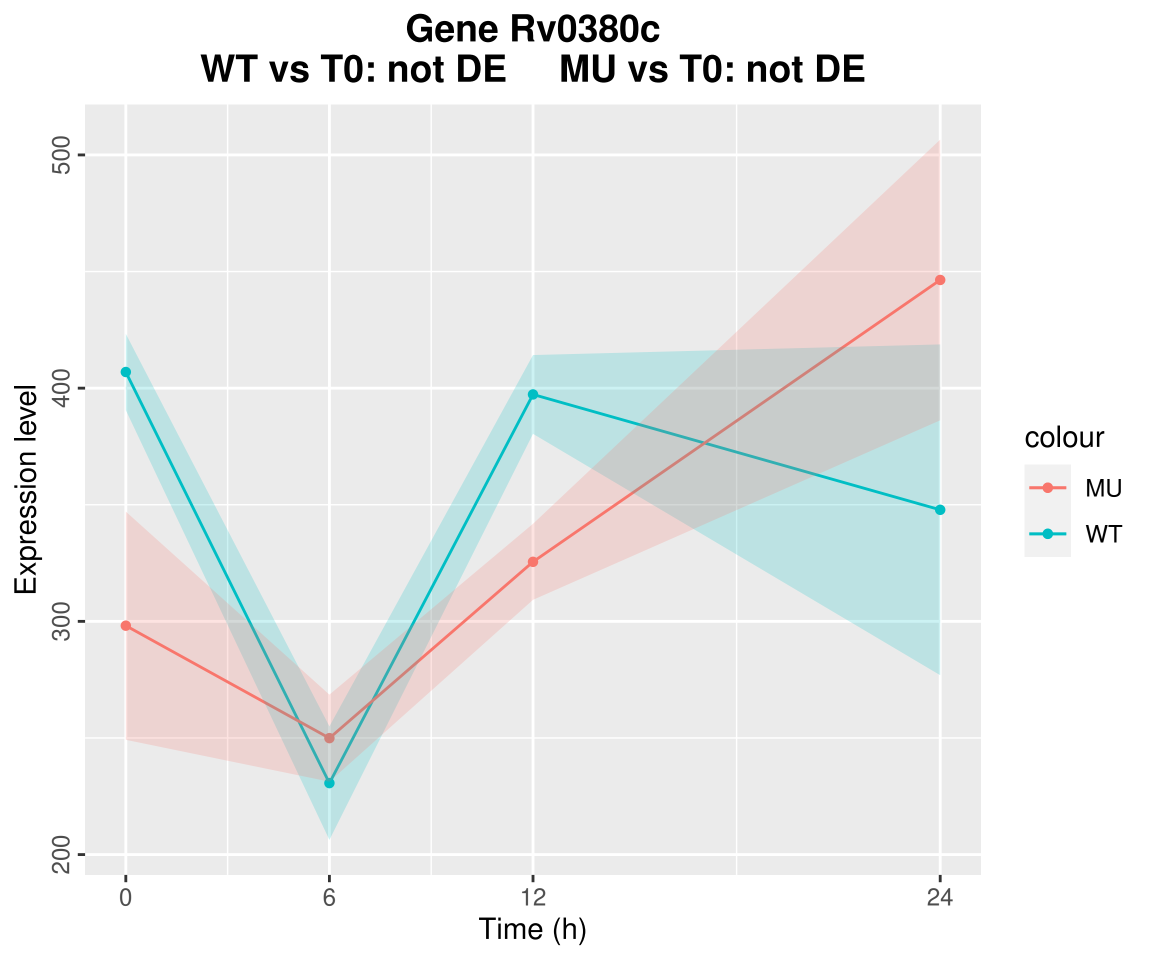
Gene Rv0380c
WT vs T0: not DE MU vs T0: not DE
Expression level
500
400
300
200
0
6
12
24
Time (h)
colour
MU
WT

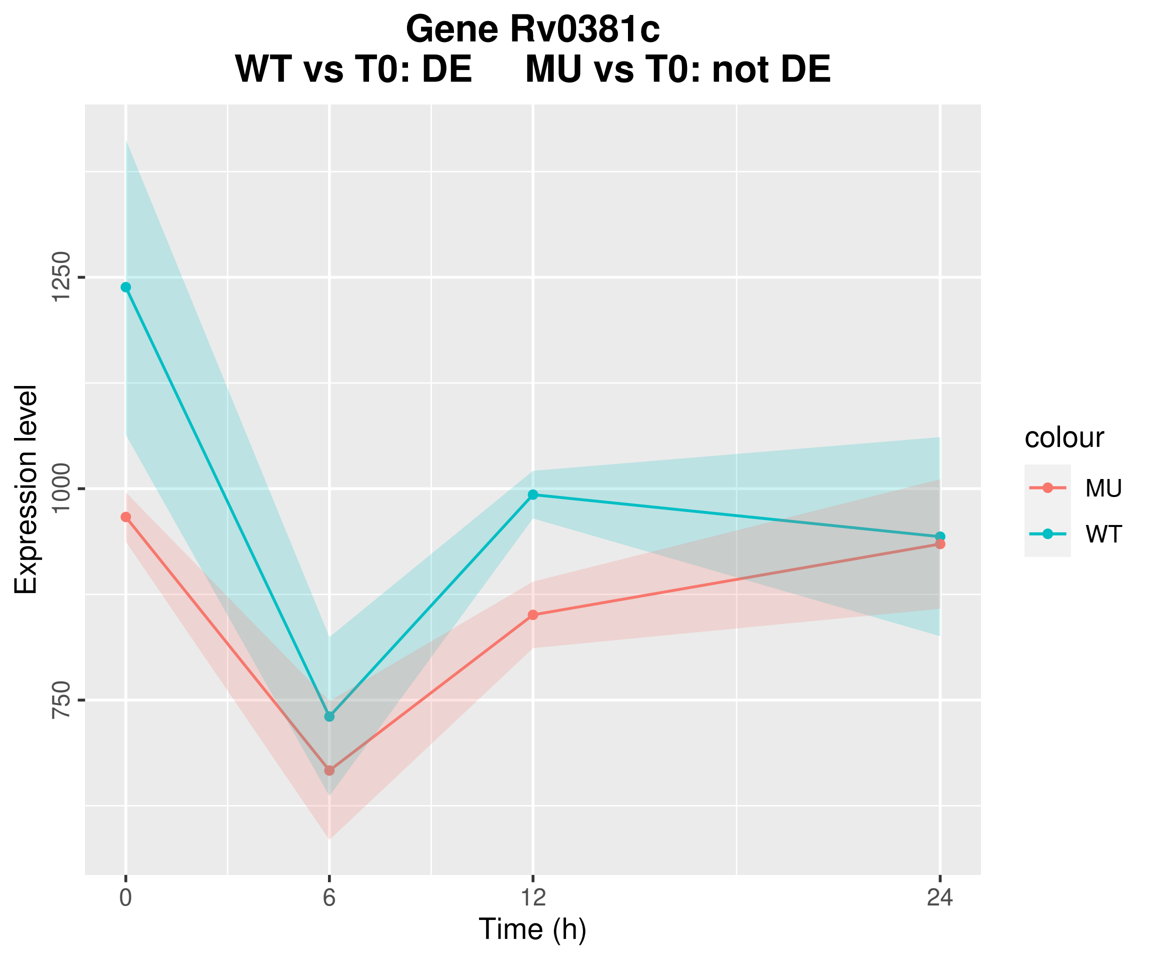
Gene Rv0381c
WT vs T0: DE   MU vs T0: not DE
Expression level
1250
1000
750
0
6
12
24
Time (h)
colour
MU
WT

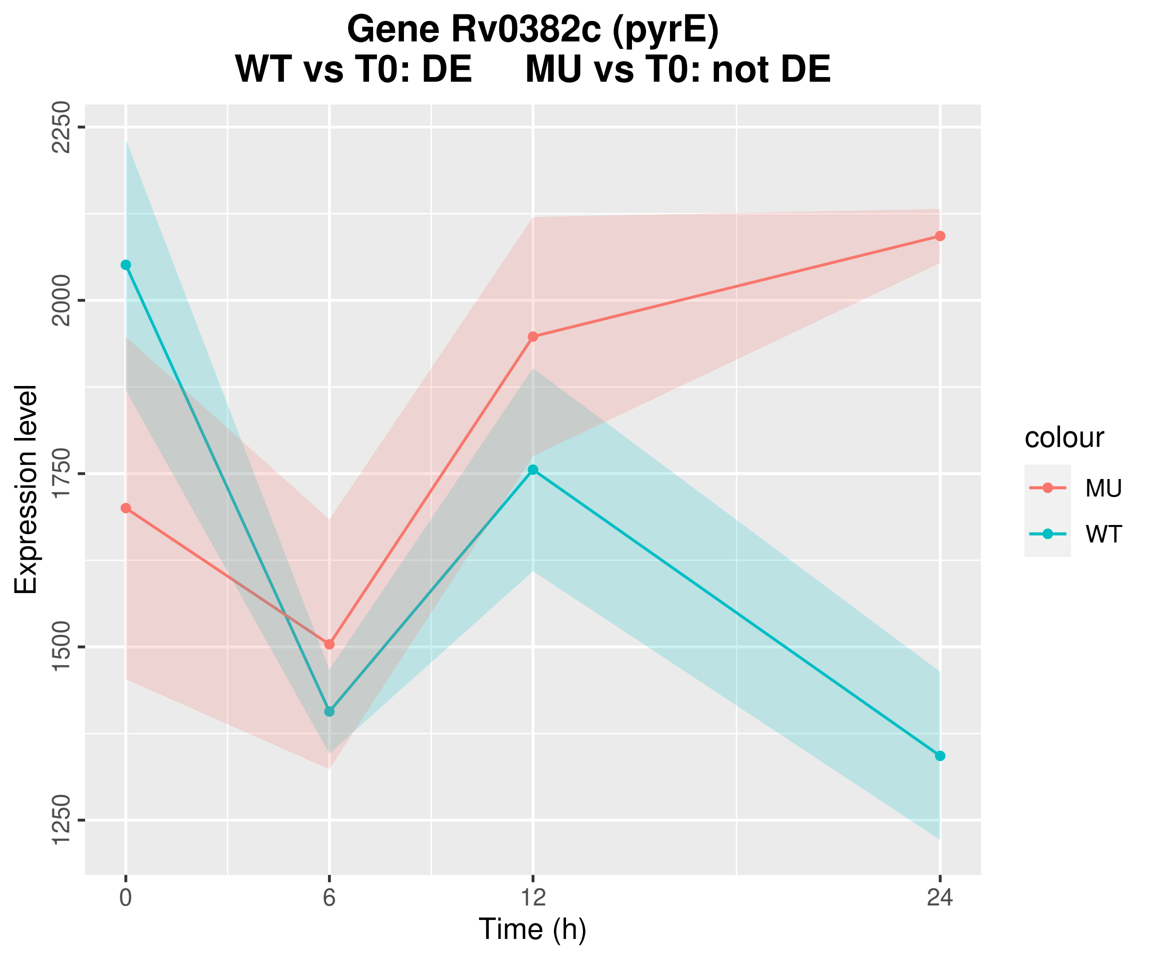

Gene Rv0382c (pyrE)
WT vs T0: DE MU vs T0: not DE
Expression level
2250
2000
1750
1500
1250
0
6
12
24
Time (h)
colour
MU
WT

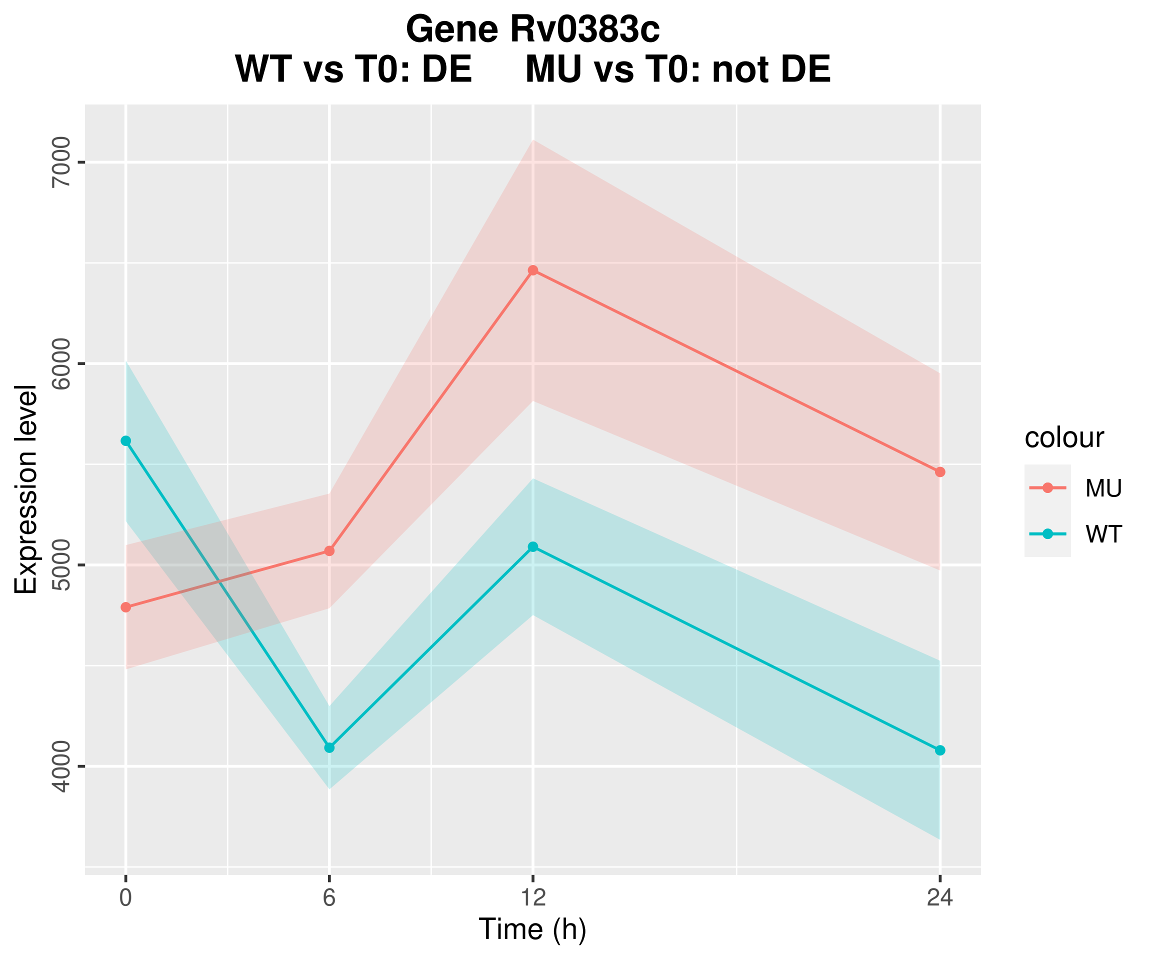
Gene Rv0383c
WT vs T0: DE MU vs T0: not DE
7000
6000
5000
4000
Expression level
0
6
12
24
Time (h)
colour
MU
WT

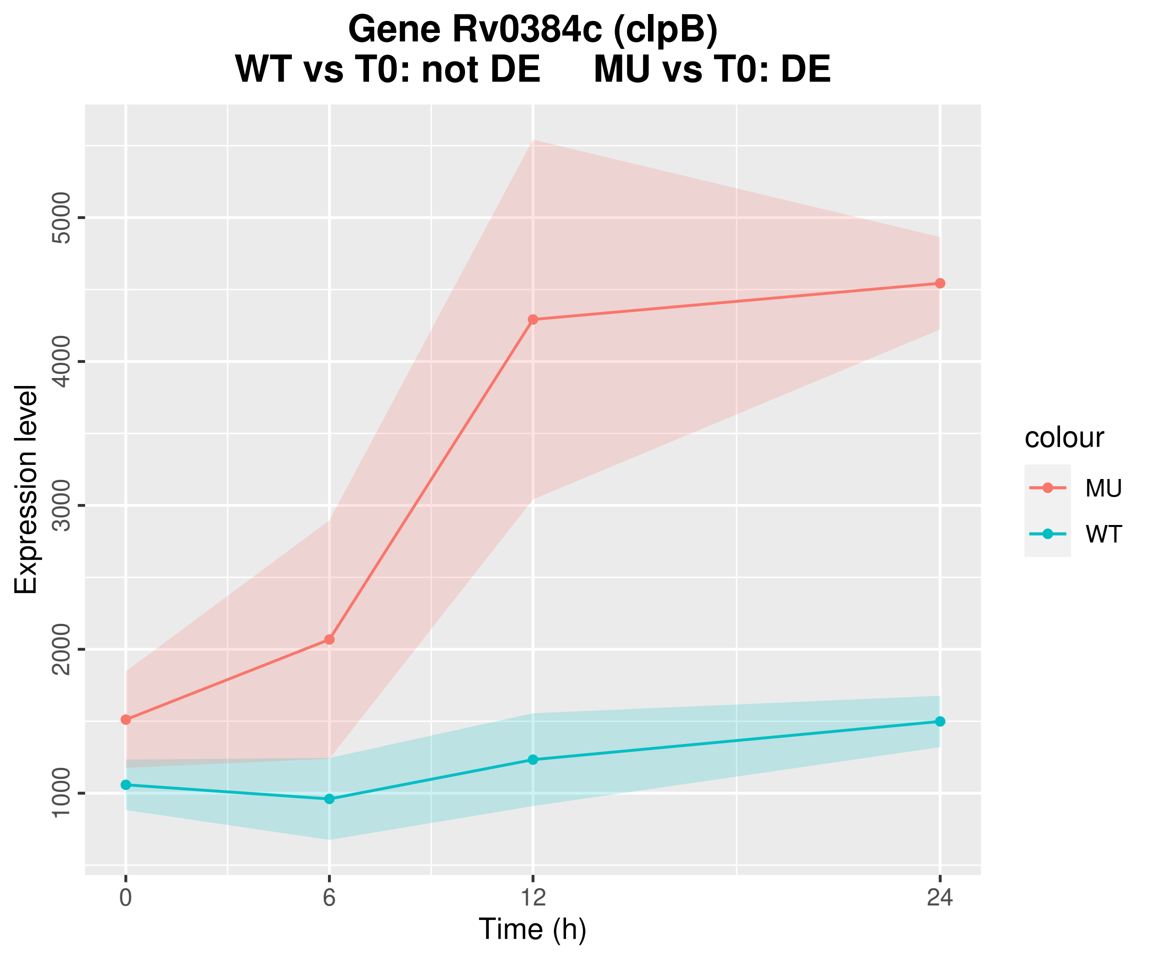

Gene Rv0384c (clpB)
WT vs T0: not DE   MU vs T0: DE
Expression level
5000
4000
3000
2000
1000
0
6
12
24
Time (h)
colour
MU
WT

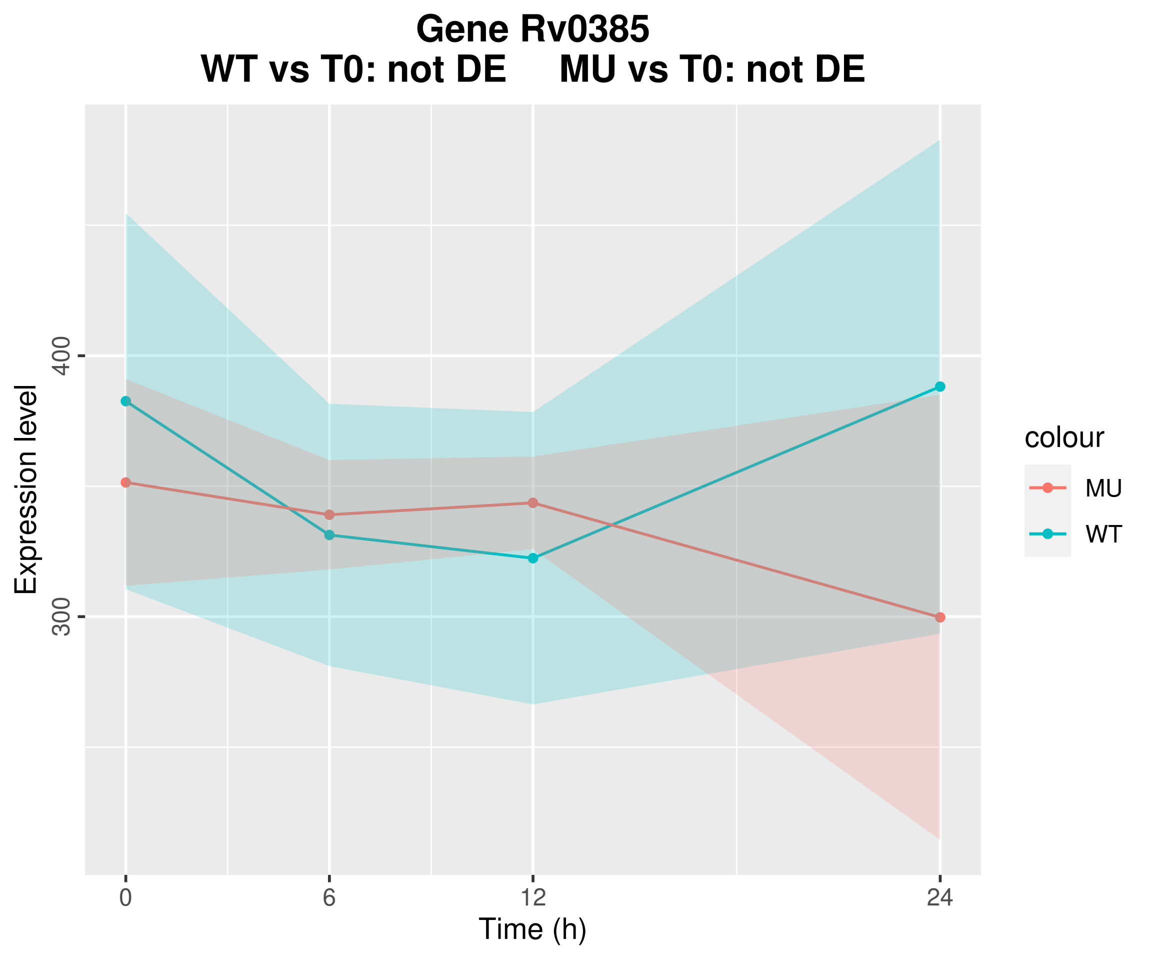

Gene Rv0385
WT vs T0: not DE MU vs T0: not DE
Expression level
400
300
0
6
12
24
Time (h)
colour
MU
WT

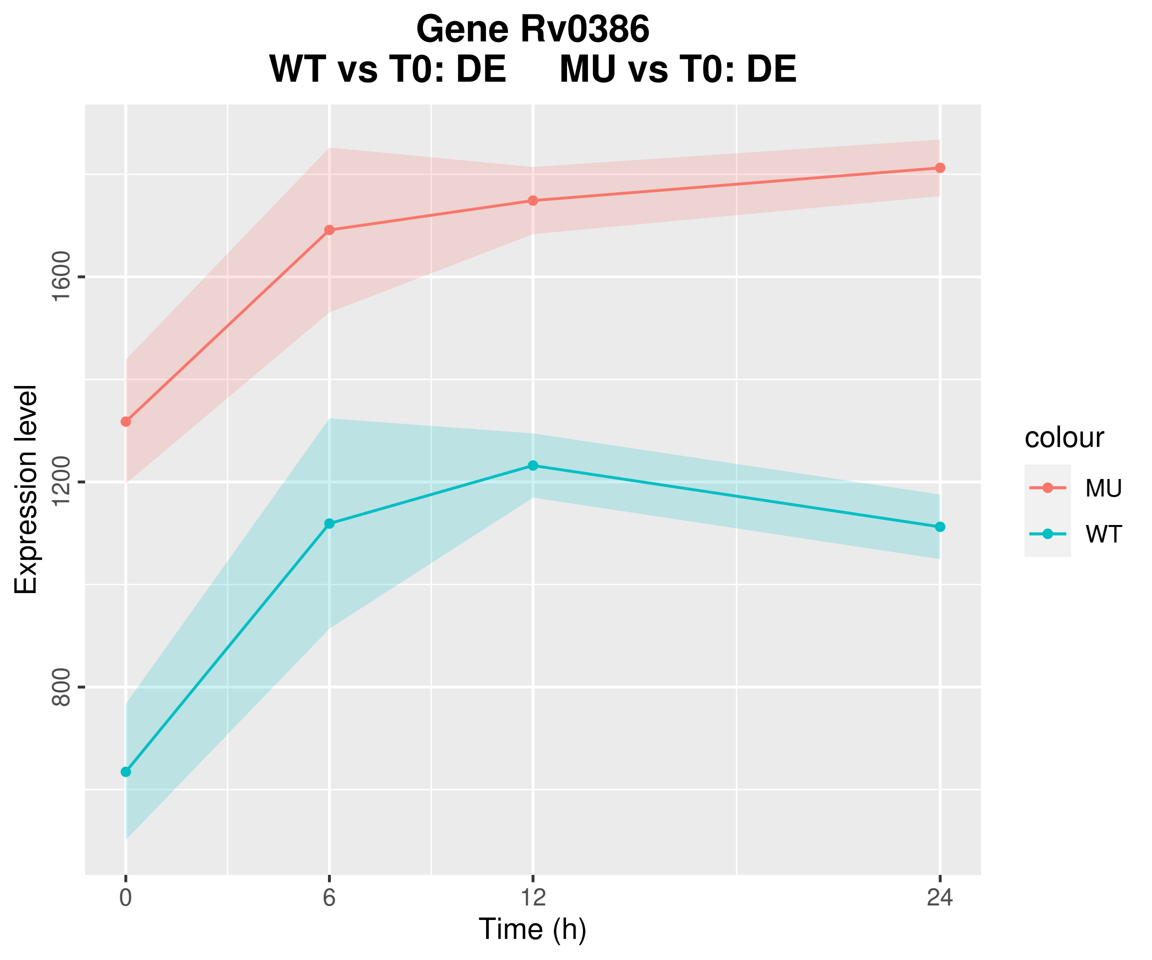

Gene Rv0386
WT vs T0: DE MU vs T0: DE
Expression level
1600
1200
800
0
6
12
24
Time (h)
colour
MU
WT

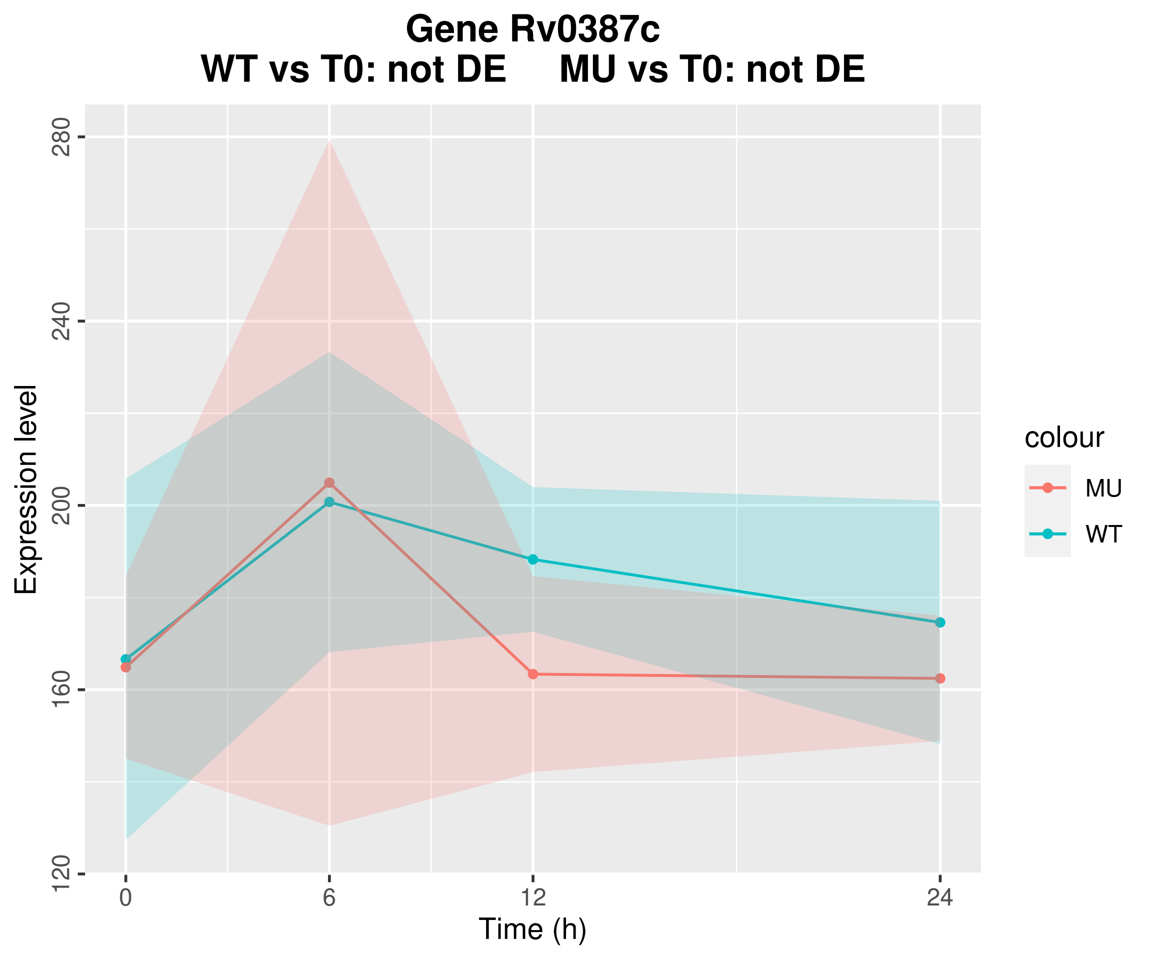
Gene Rv0387c
WT vs T0: not DE MU vs T0: not DE
Expression level
280
240
200
160
120
0
6
12
24
Time (h)
colour
MU
WT

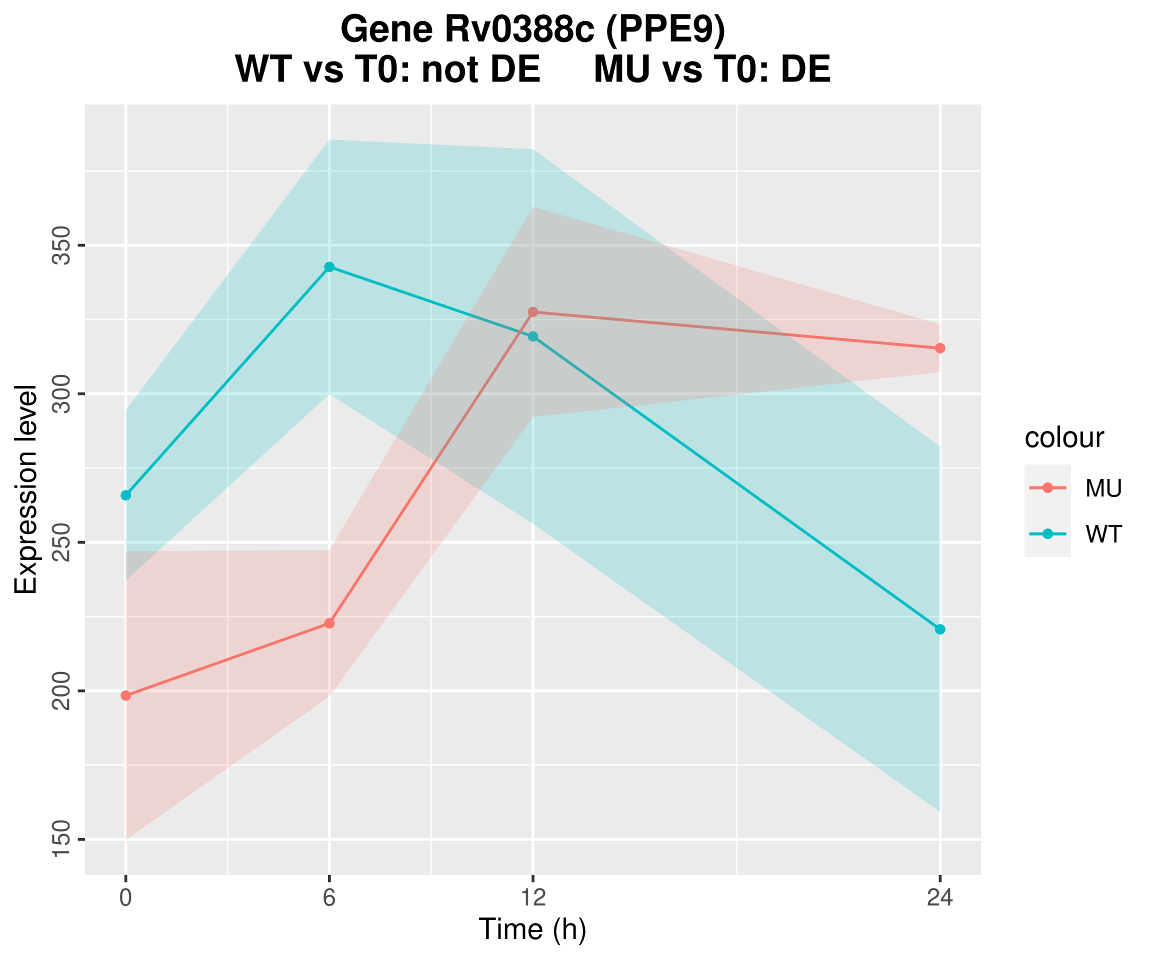

Gene Rv0388c (PPE9)
WT vs T0: not DE MU vs T0: DE
Expression level
350
300
250
200
150
0
6
12
24
Time (h)
colour
MU
WT

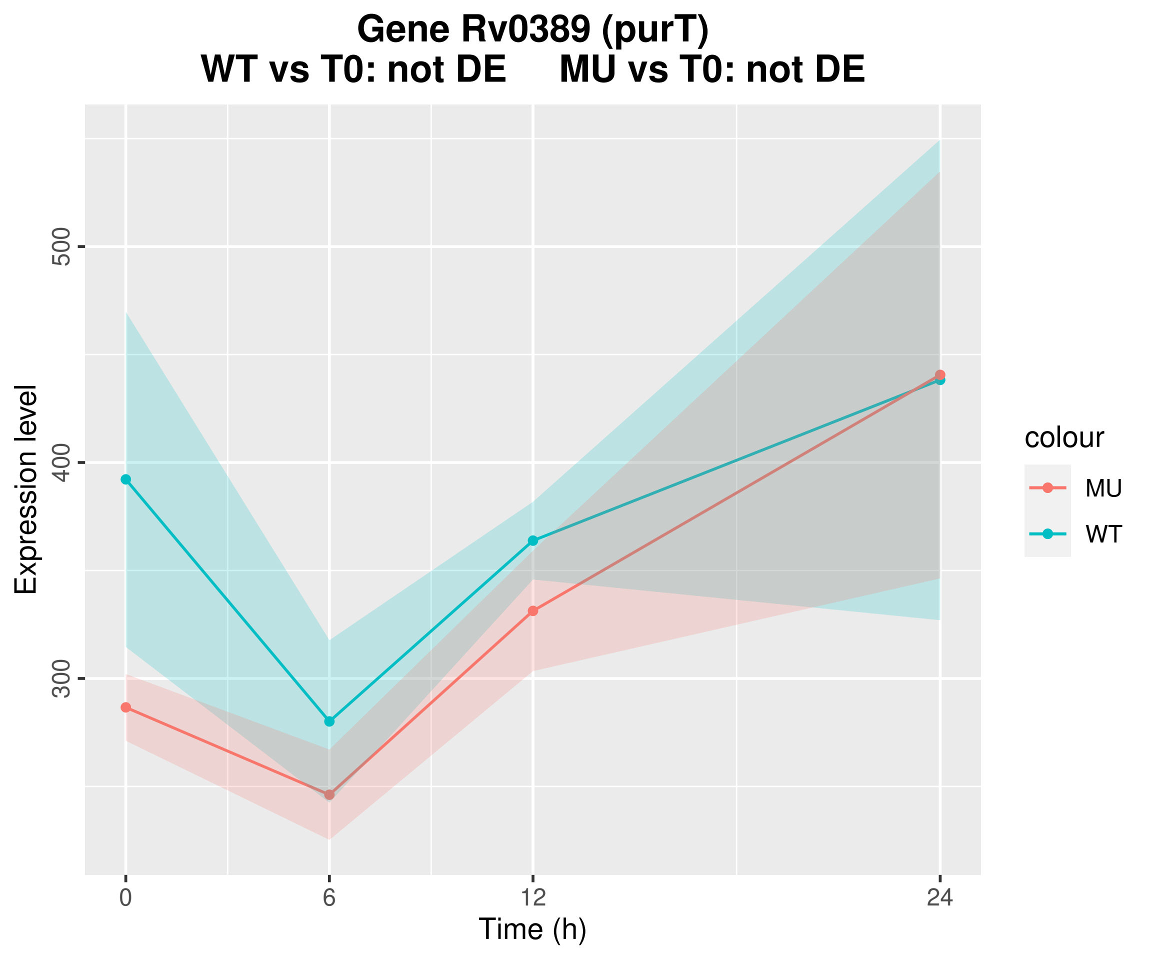
Gene Rv0389 (purT)
WT vs T0: not DE   MU vs T0: not DE
Expression level
300
400
500
0
6
12
24
Time (h)
colour
MU
WT

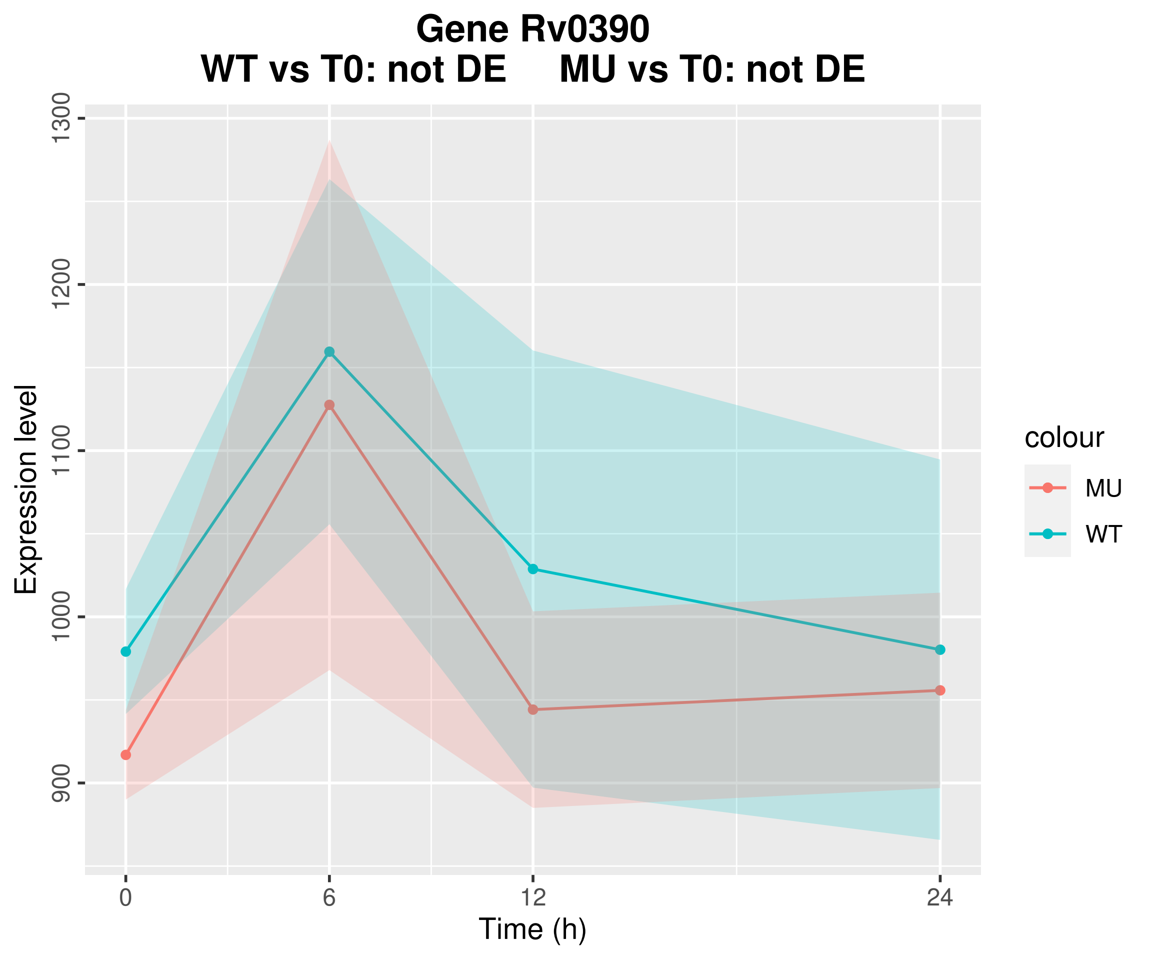
Gene Rv0390
WT vs T0: not DE MU vs T0: not DE
Expression level
1300
1200
1100
1000
900
0
6
12
24
Time (h)
colour
MU
WT

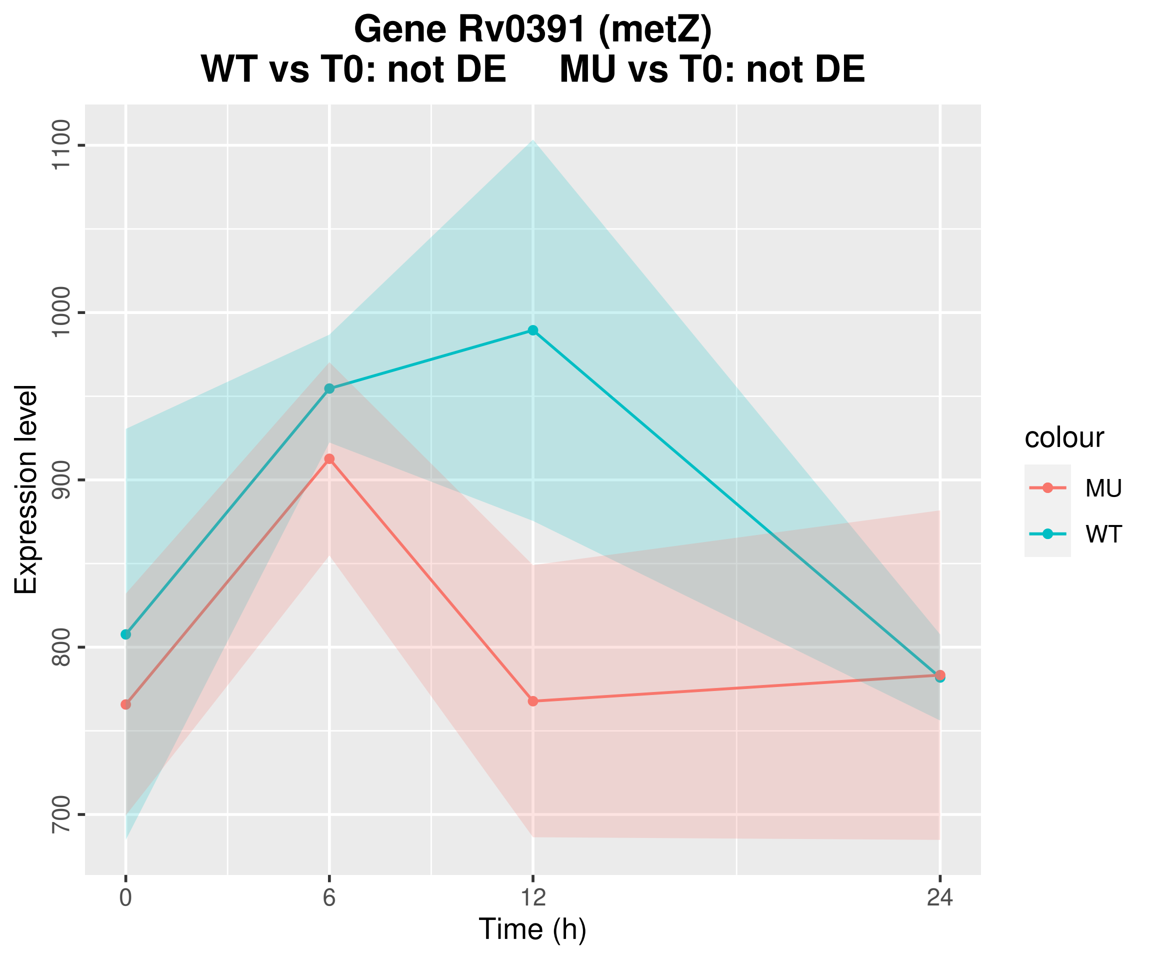
Gene Rv0391 (metZ)
WT vs T0: not DE   MU vs T0: not DE
Expression level
1100
1000
900
800
700
0
6
12
24
Time (h)
colour
MU
WT

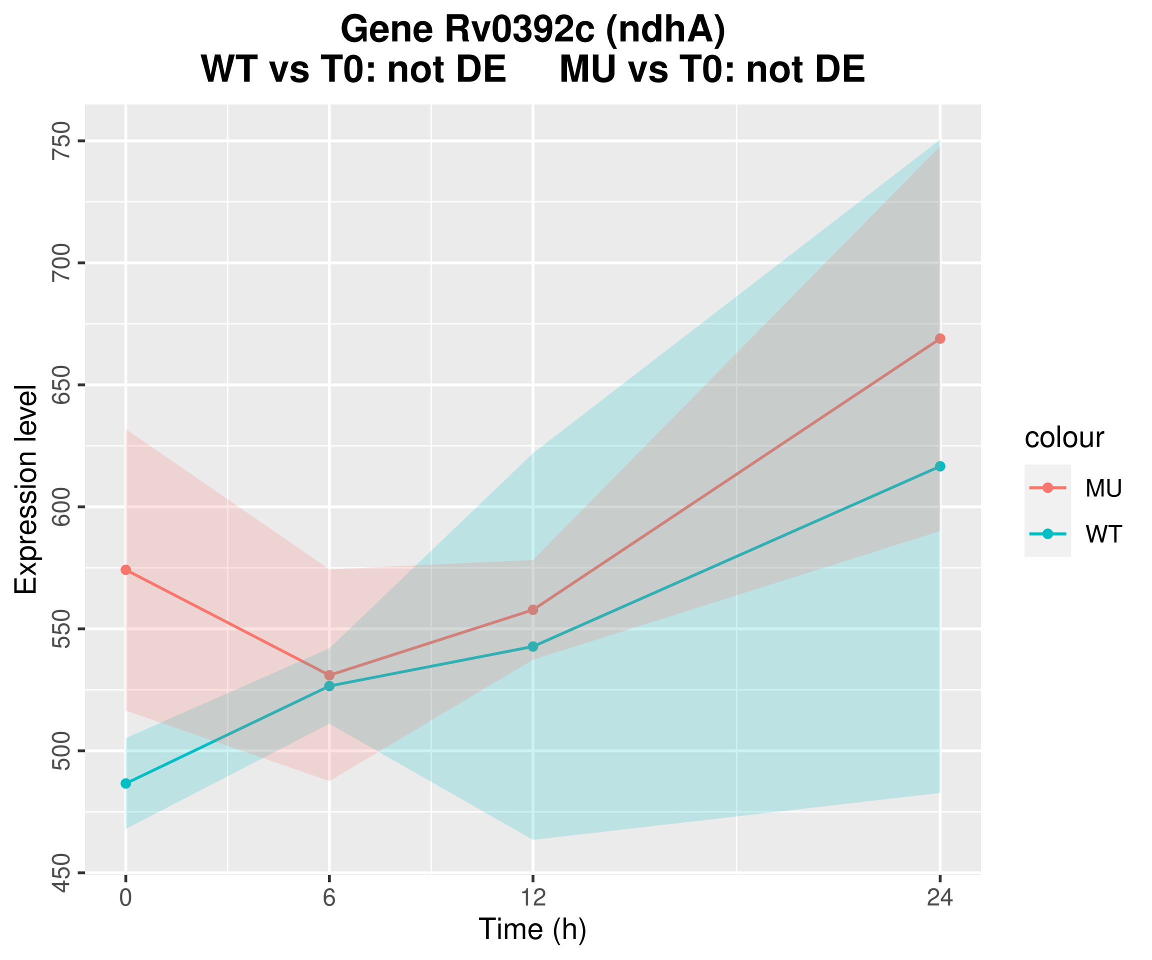
Gene Rv0392c (ndhA)
WT vs T0: not DE   MU vs T0: not DE
Expression level
750
700
650
600
550
500
450
0
6
12
24
Time (h)
colour
MU
WT

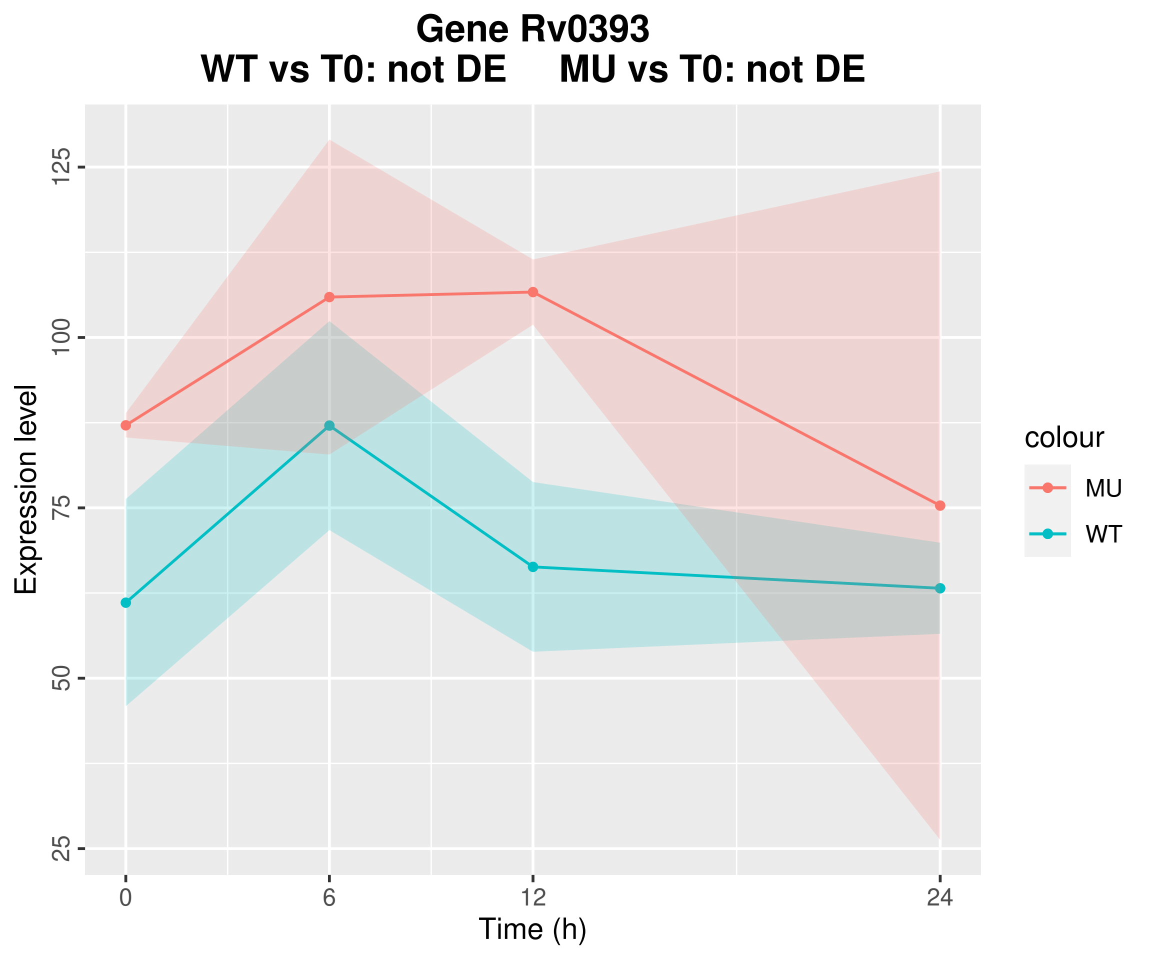
Gene Rv0393
WT vs T0: not DE   MU vs T0: not DE
Expression level
125
100
75
50
25
0
6
12
24
Time (h)
colour
MU
WT

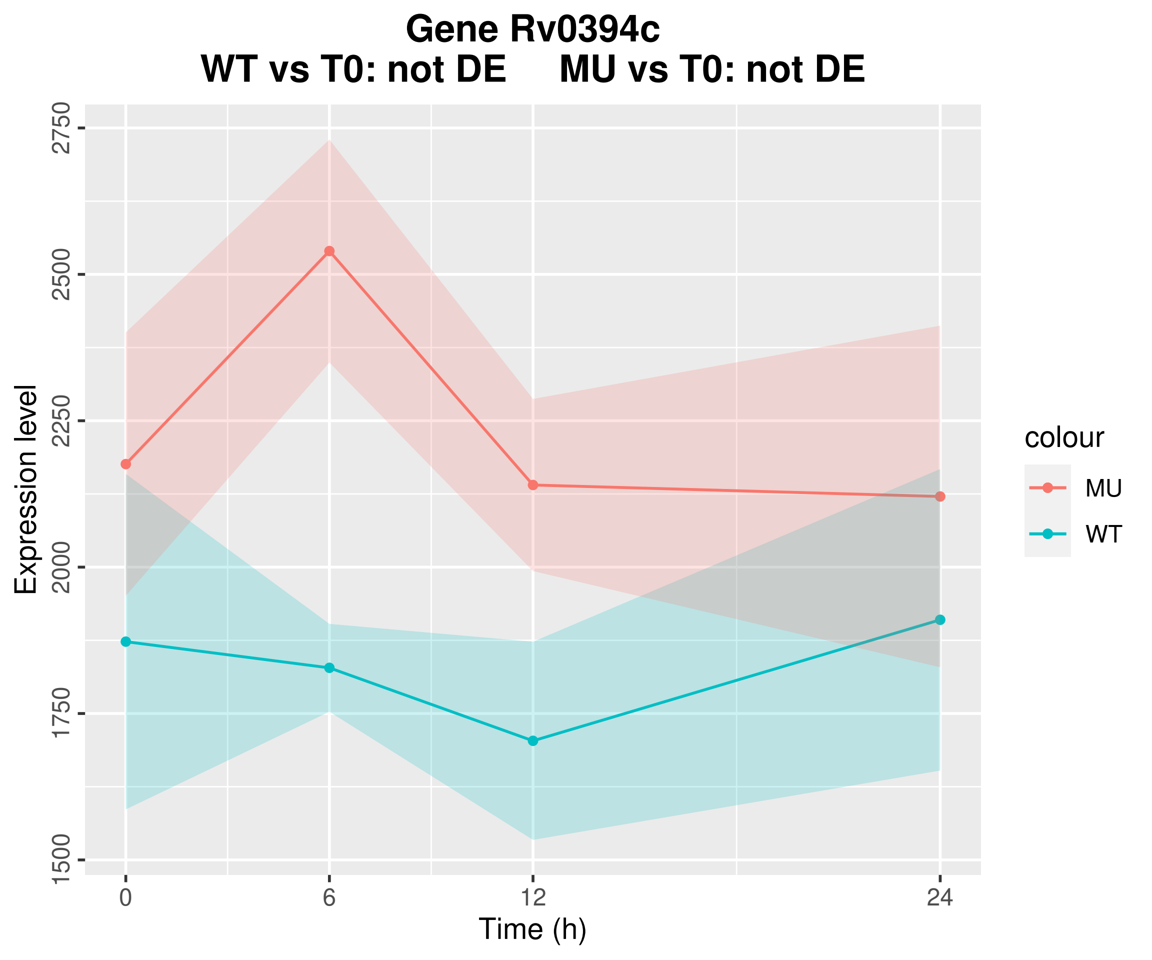

Gene Rv0394c
WT vs T0: not DE MU vs T0: not DE
Expression level
2750
2500
2250
2000
1750
1500
0
6
12
24
Time (h)
colour
MU
WT

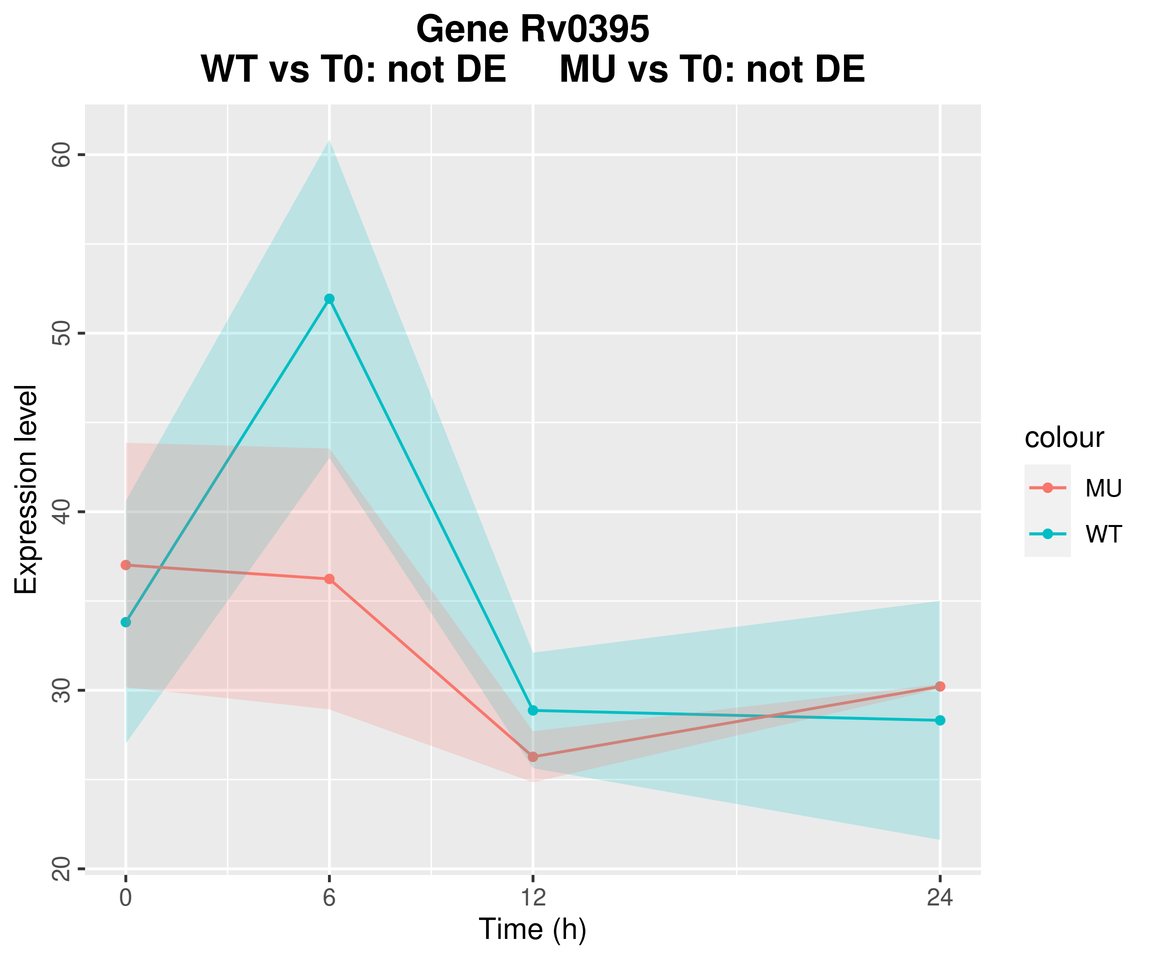

Gene Rv0395
WT vs T0: not DE MU vs T0: not DE
Expression level
60
50
40
30
20
0
6
12
24
Time (h)
colour
MU
WT

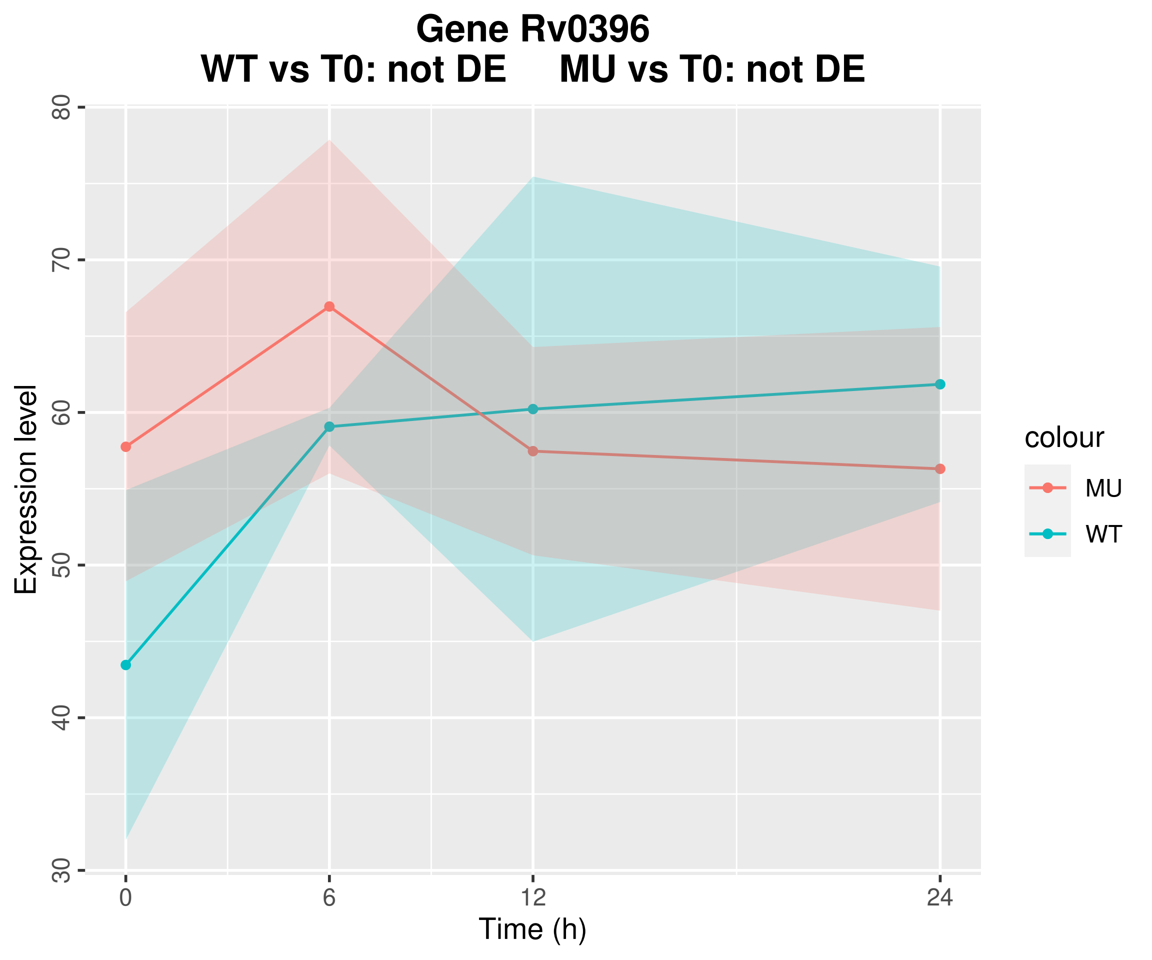
Gene Rv0396
WT vs T0: not DE MU vs T0: not DE
Expression level
80
70
60
50
40
30
0
6
12
24
Time (h)
colour
MU
WT

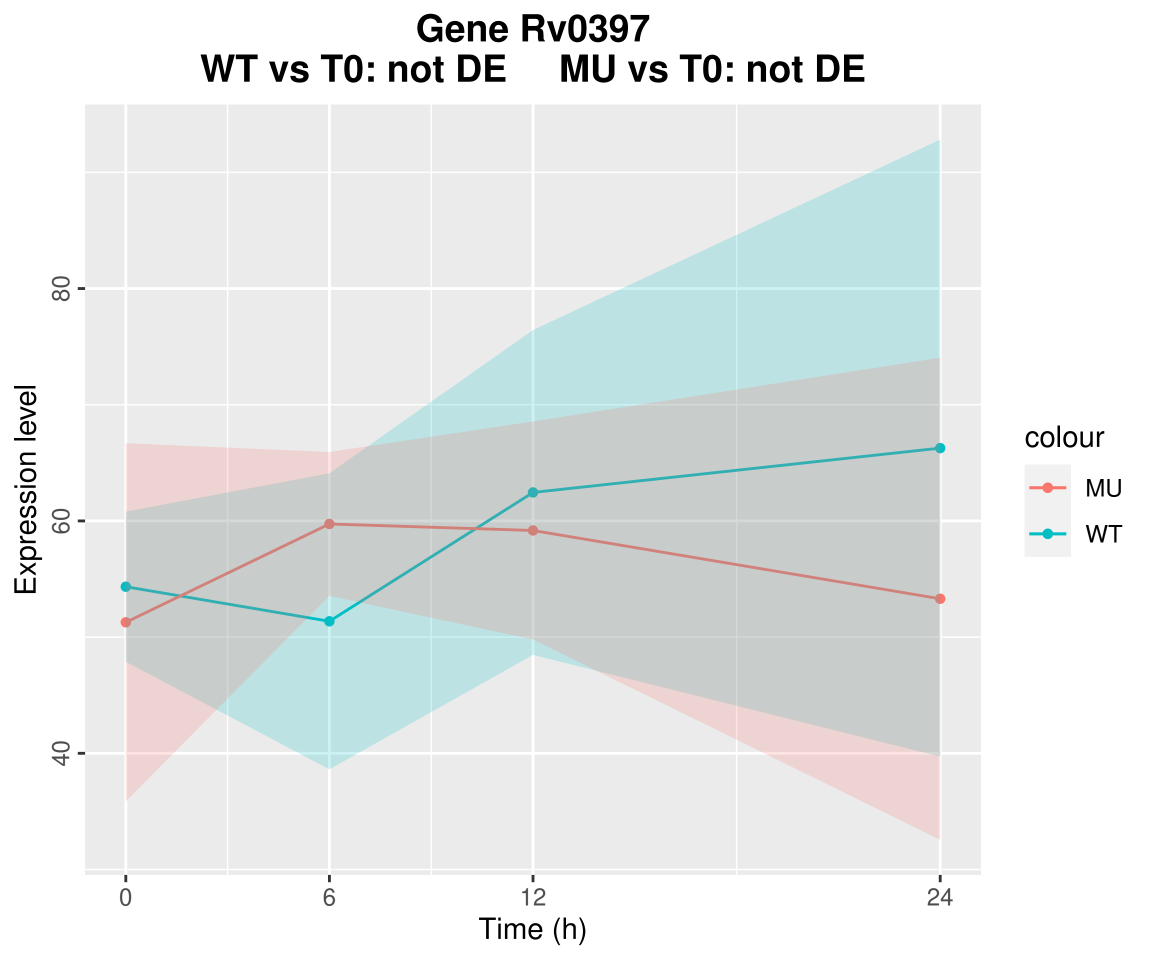
Gene Rv0397
WT vs T0: not DE MU vs T0: not DE
Expression level
80
60
40
0
6
12
24
Time (h)
colour
MU
WT

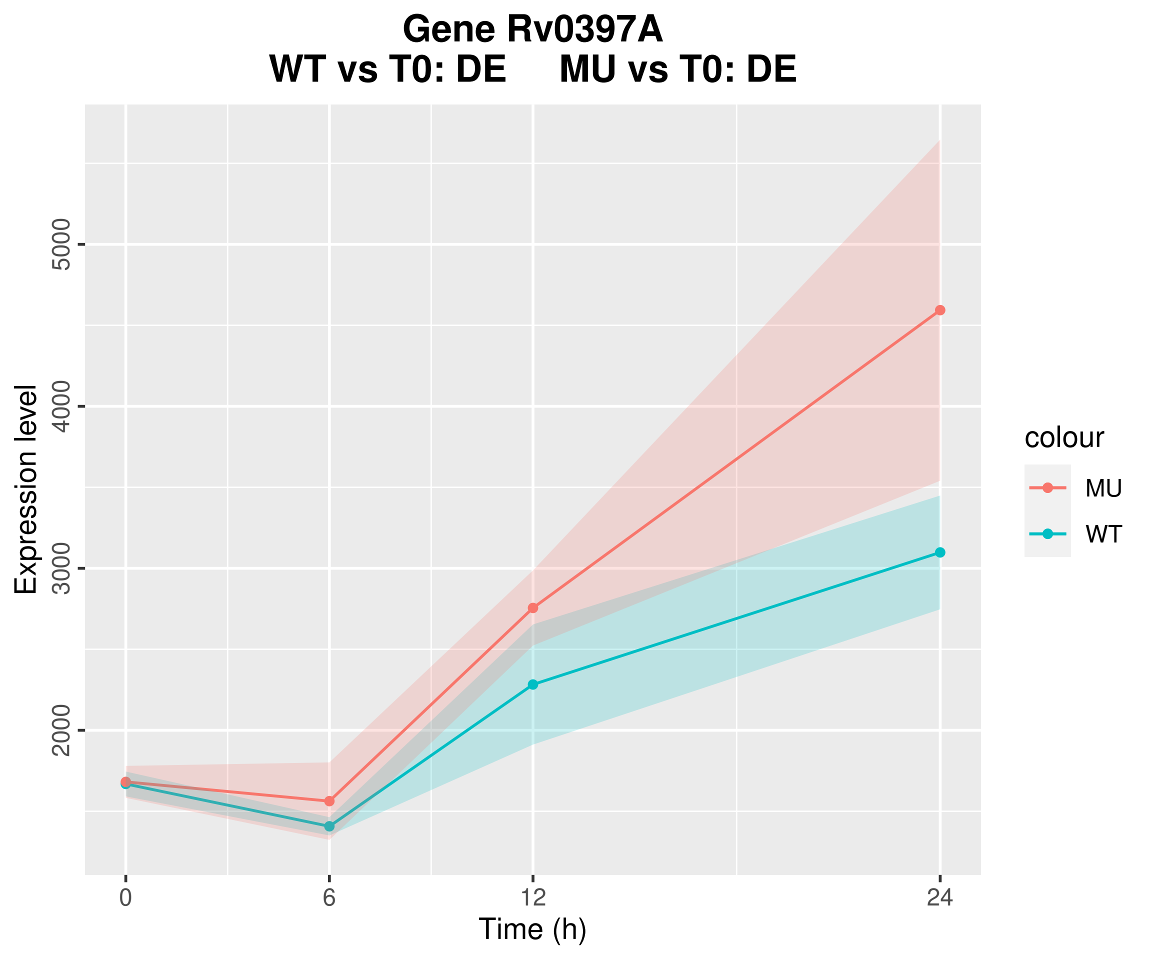

Gene Rv0397A
WT vs T0: DE MU vs T0: DE
Expression level
5000
4000
3000
2000
0
6
12
24
Time (h)
colour
MU
WT

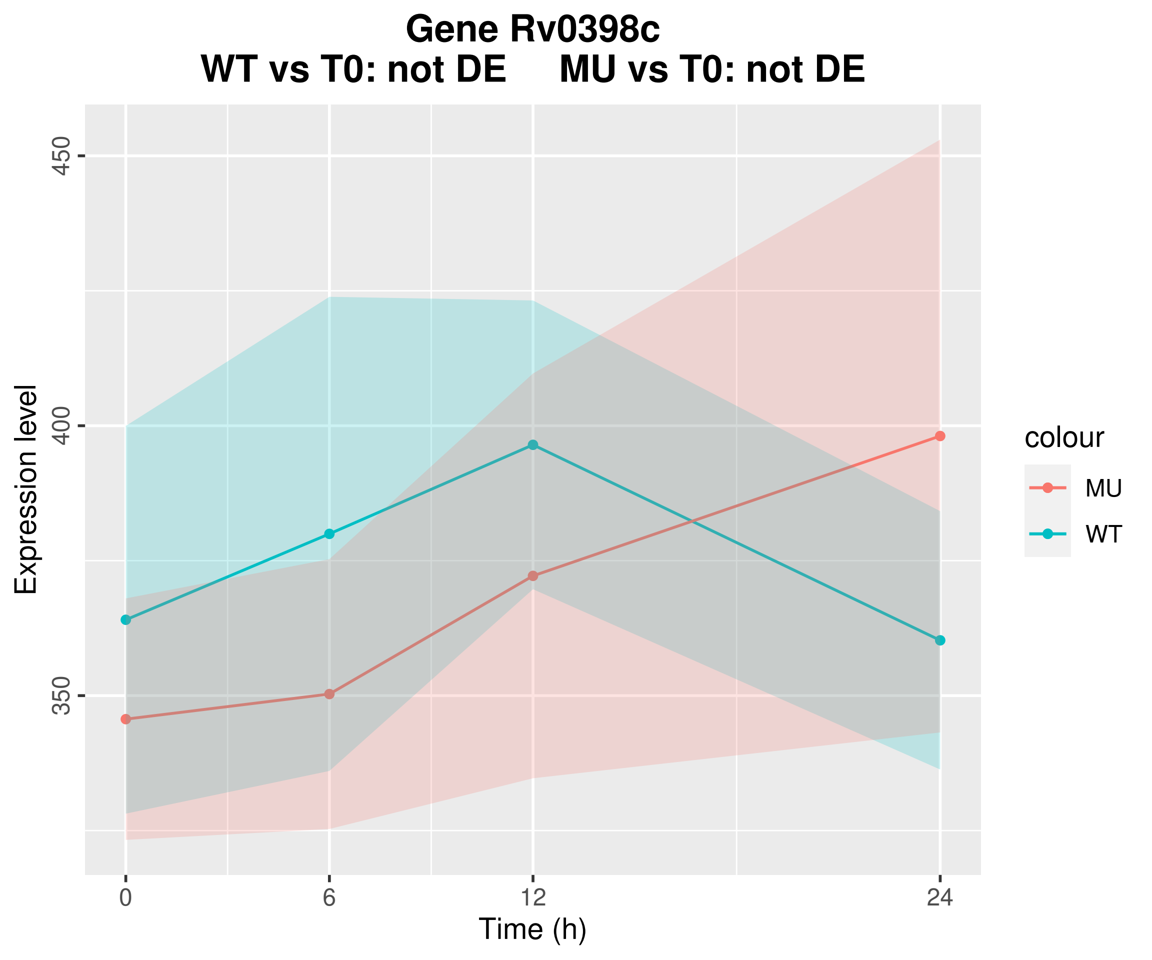
Gene Rv0398c
WT vs T0: not DE   MU vs T0: not DE
Expression level
450
400
350
0
6
12
24
Time (h)
colour
MU
WT

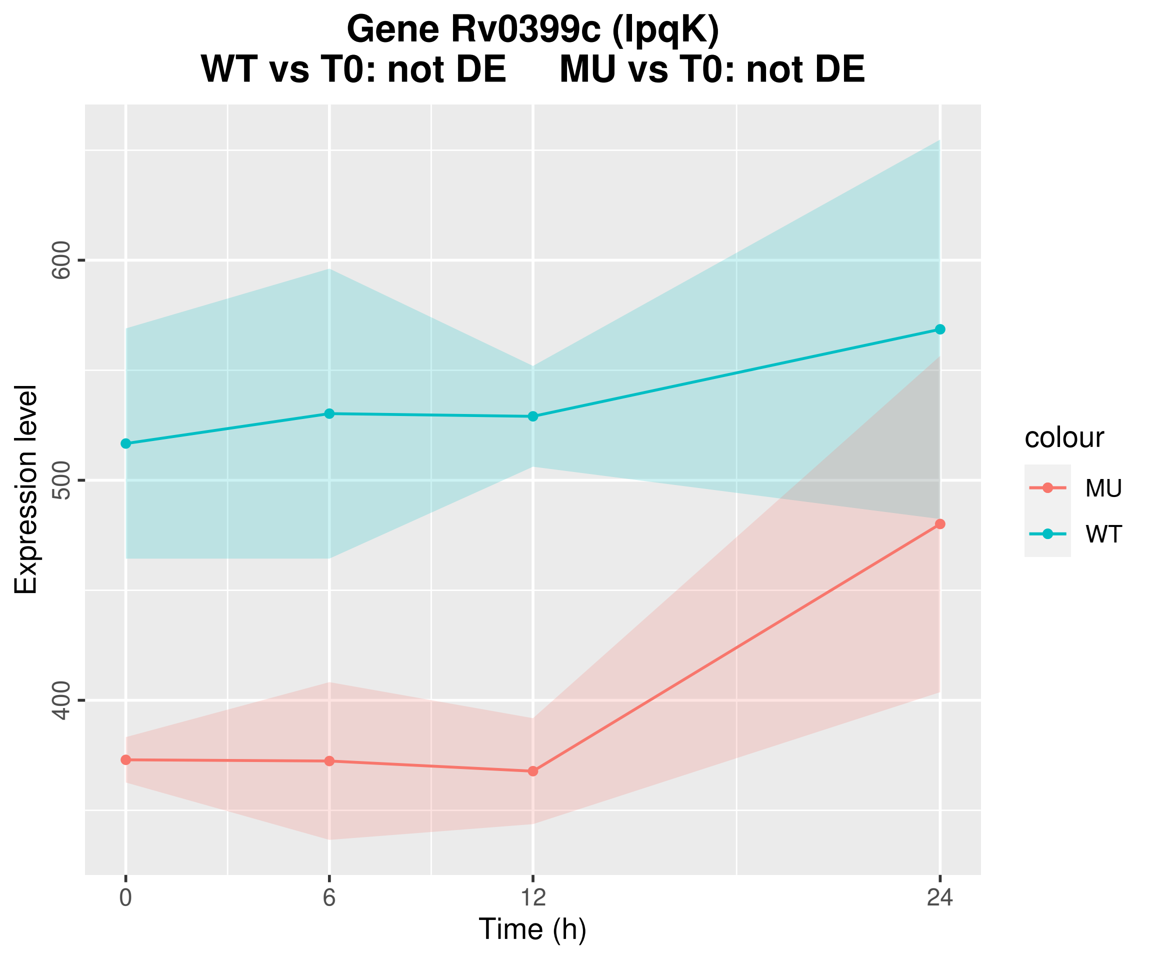
Gene Rv0399c (lpqK)
WT vs T0: not DE MU vs T0: not DE
600
500
400
0
6
12
24
Time (h)
Expression level
colour
MU
WT

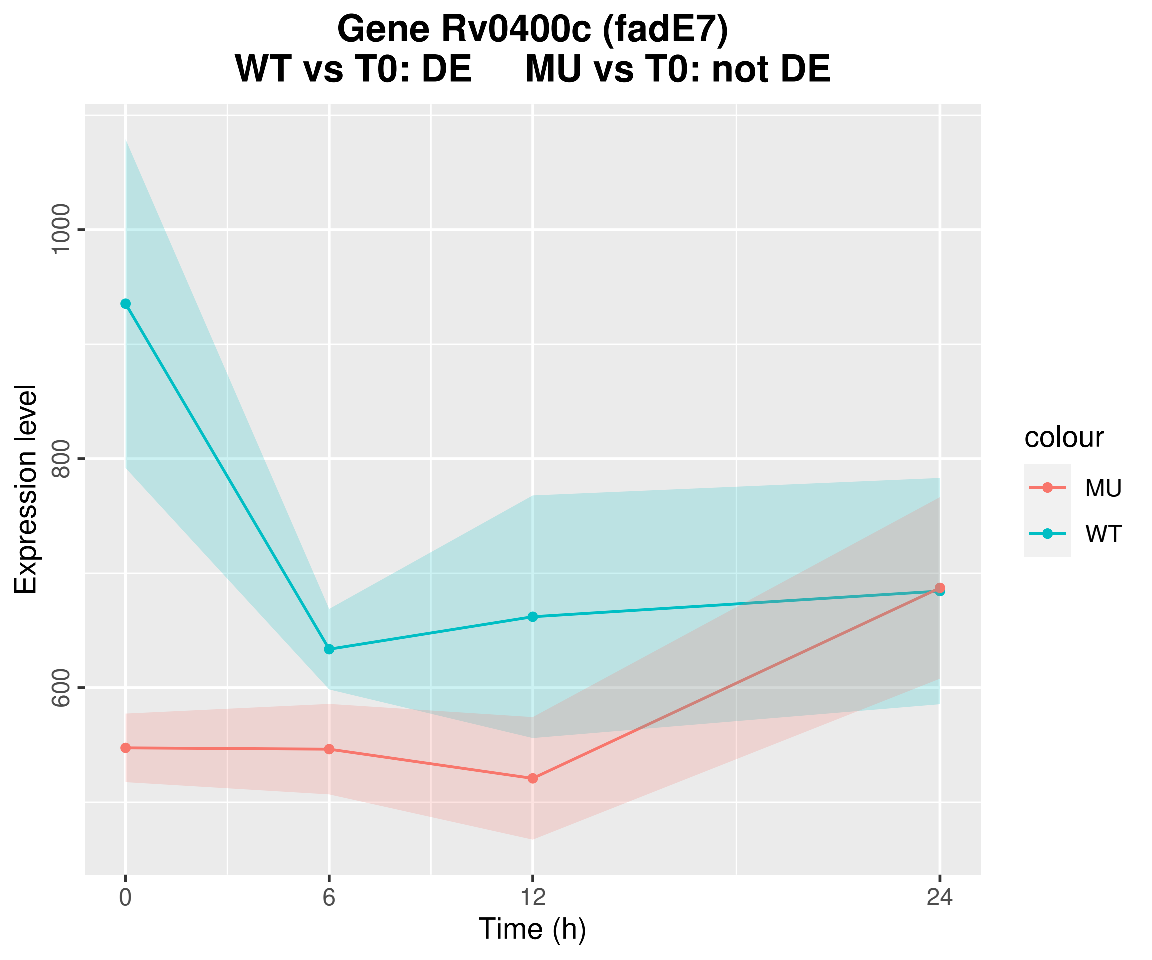

Gene Rv0400c (fadE7)
WT vs T0: DE MU vs T0: not DE
Expression level
1000
800
600
0
6
12
24
Time (h)
colour
MU
WT

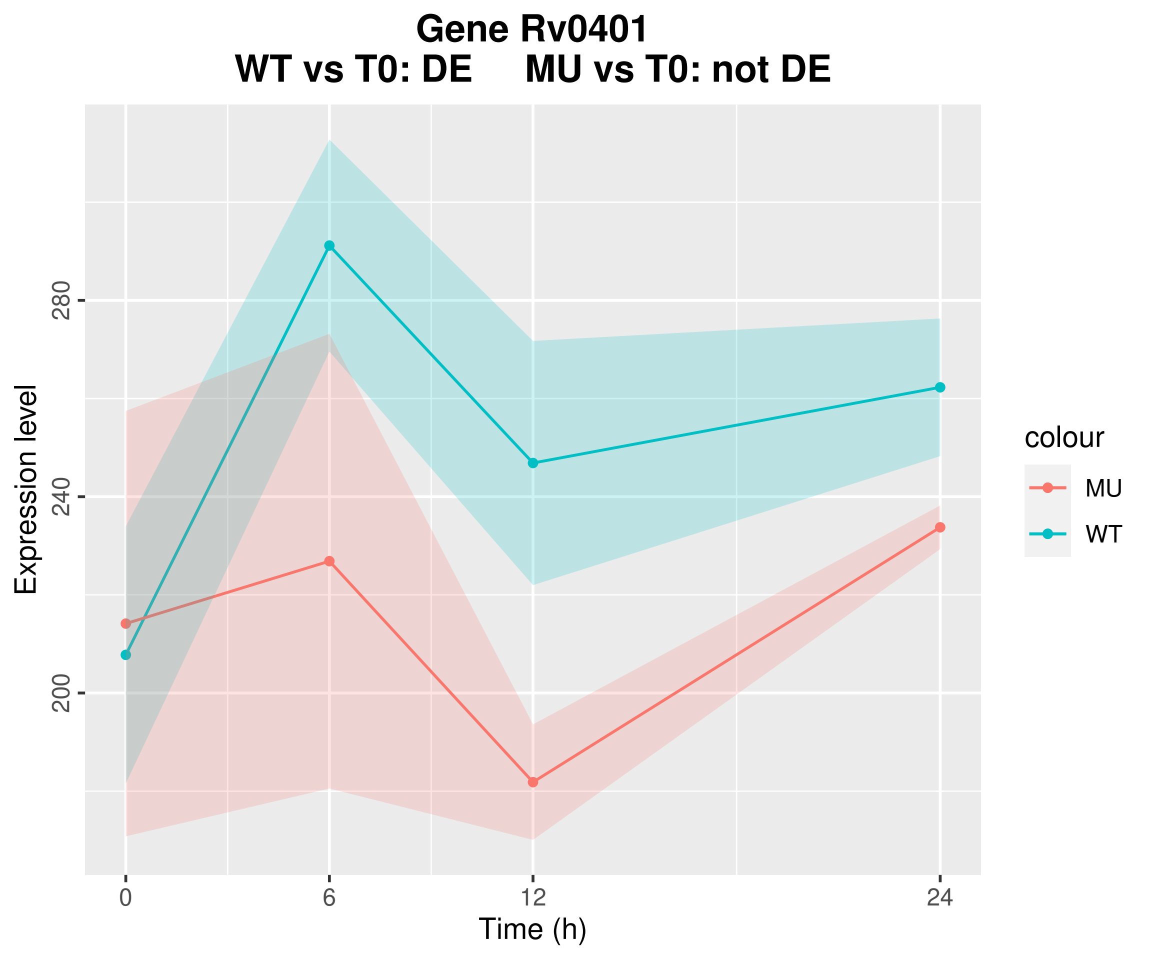
Gene Rv0401
WT vs T0: DE   MU vs T0: not DE
Expression level
280
240
200
0
6
12
24
Time (h)
colour
MU
WT

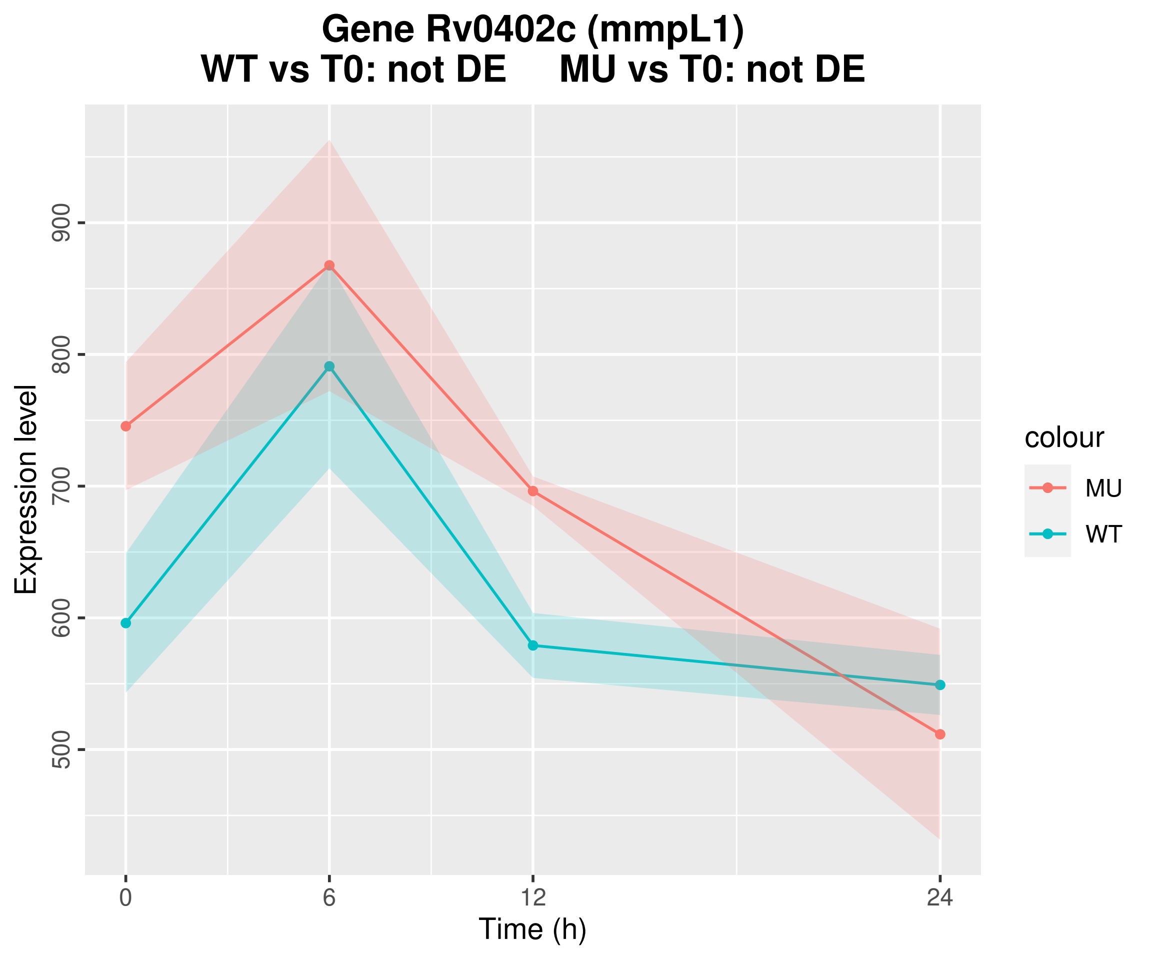

Gene Rv0402c (mmpL1)
WT vs T0: not DE MU vs T0: not DE
900
800
700
600
500
Expression level
0
6
12
24
Time (h)
colour
MU
WT

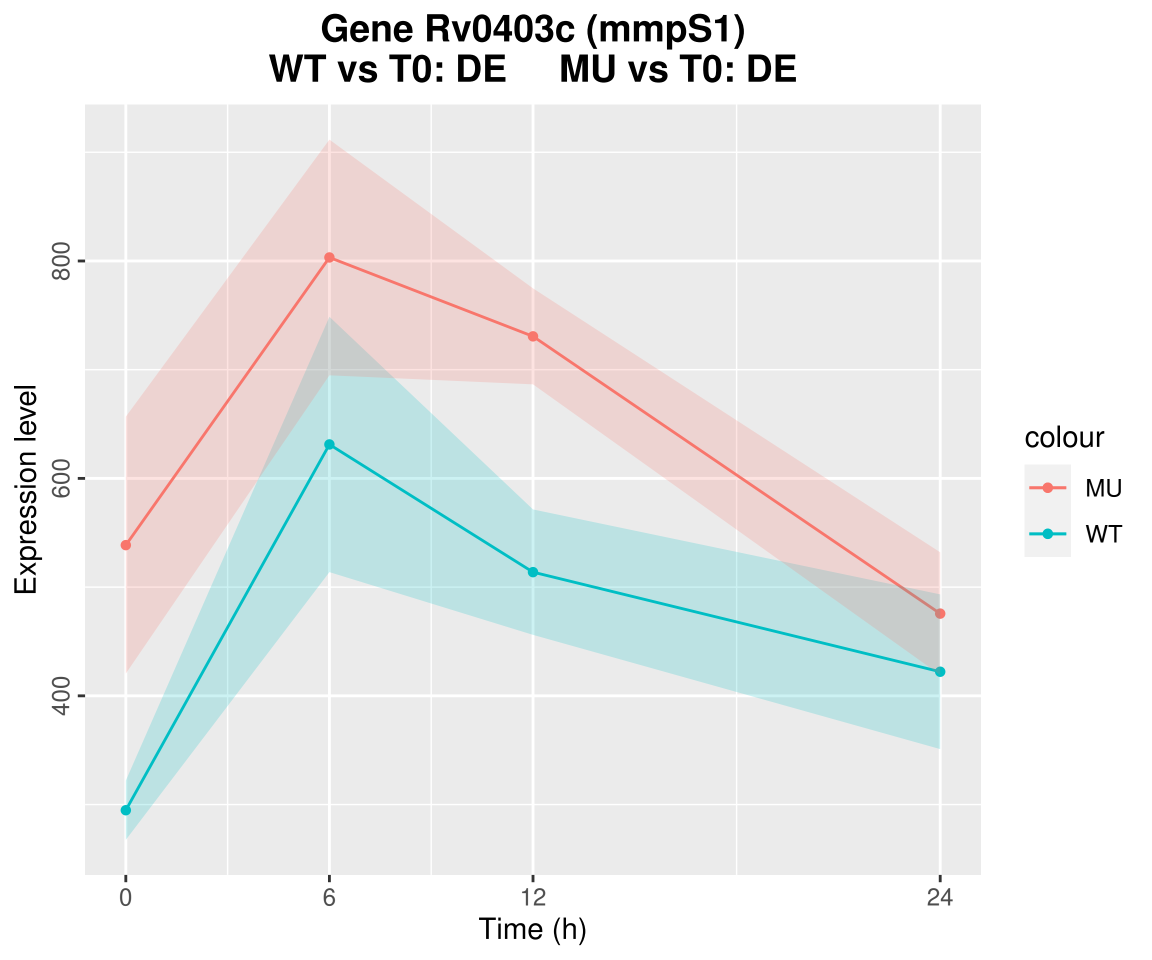

Gene Rv0403c (mmpS1)
WT vs T0: DE MU vs T0: DE
Expression level
800
600
400
0
6
12
24
Time (h)
colour
MU
WT

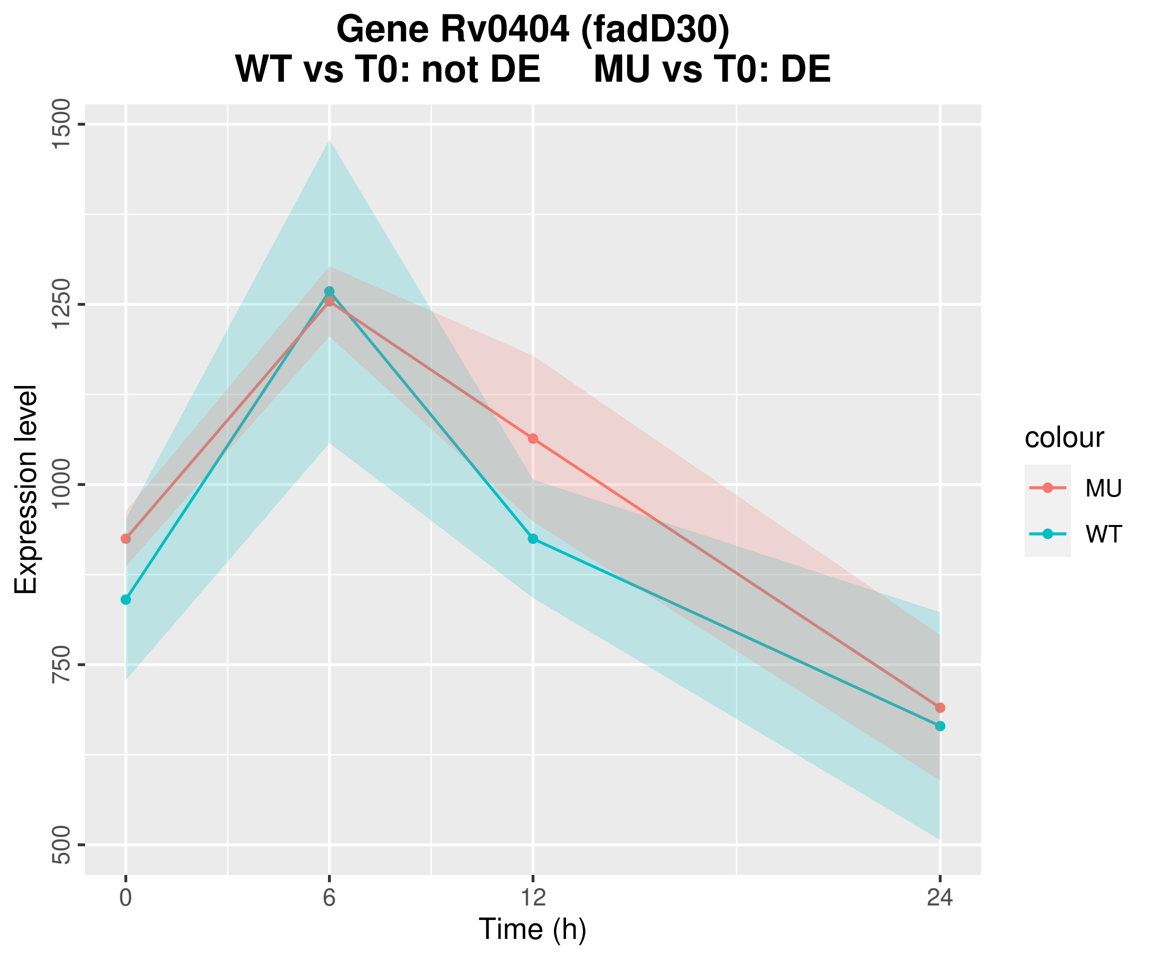
Gene Rv0404 (fadD30)
WT vs T0: not DE MU vs T0: DE
Expression level
1500
1250
1000
750
500
Time (h)
0
6
12
24
colour
MU
WT

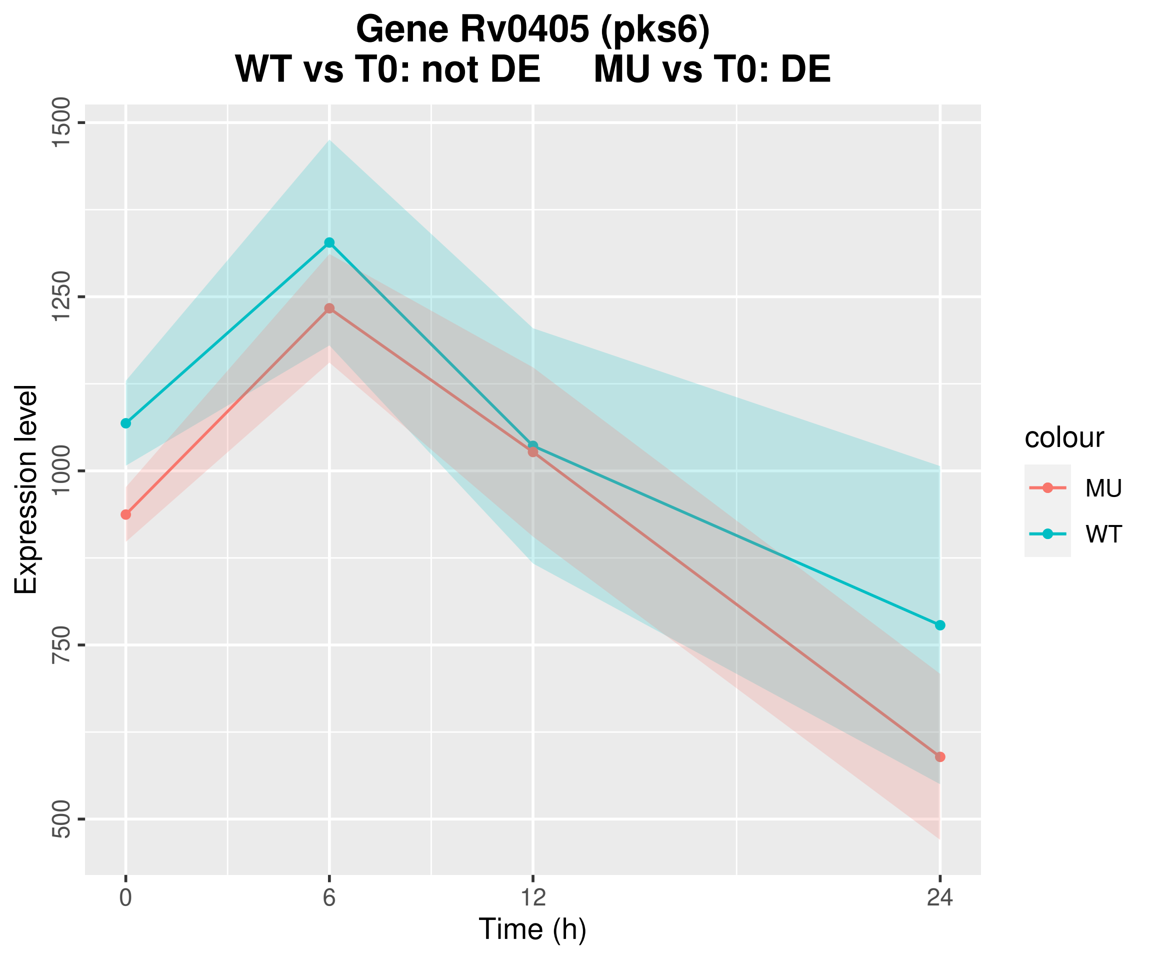
Gene Rv0405 (pks6)
WT vs T0: not DE   MU vs T0: DE
Expression level
1500
1250
1000
750
500
0
6
12
24
Time (h)
colour
MU
WT

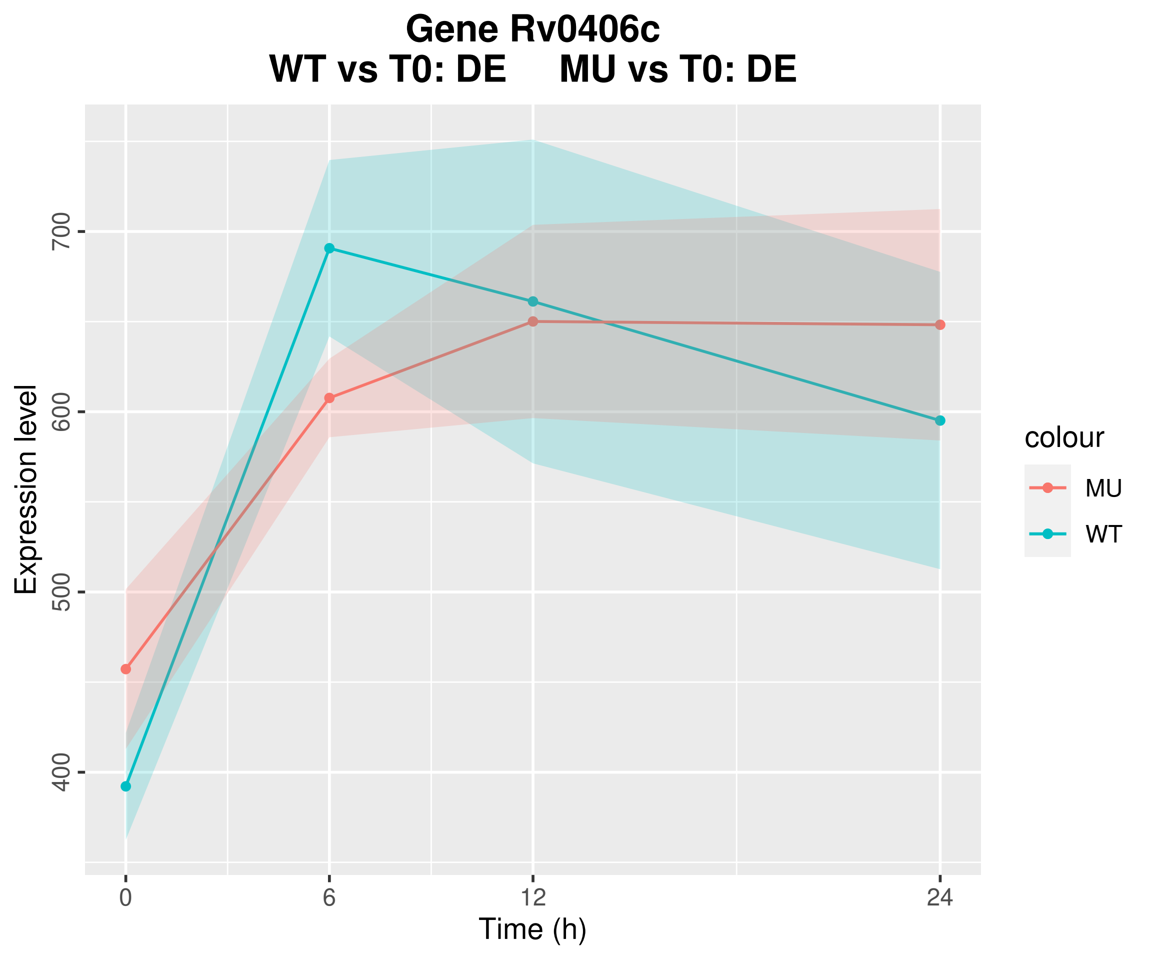
Gene Rv0406c
WT vs T0: DE   MU vs T0: DE
Expression level
700
600
500
400
0
6
12
24
Time (h)
colour
MU
WT

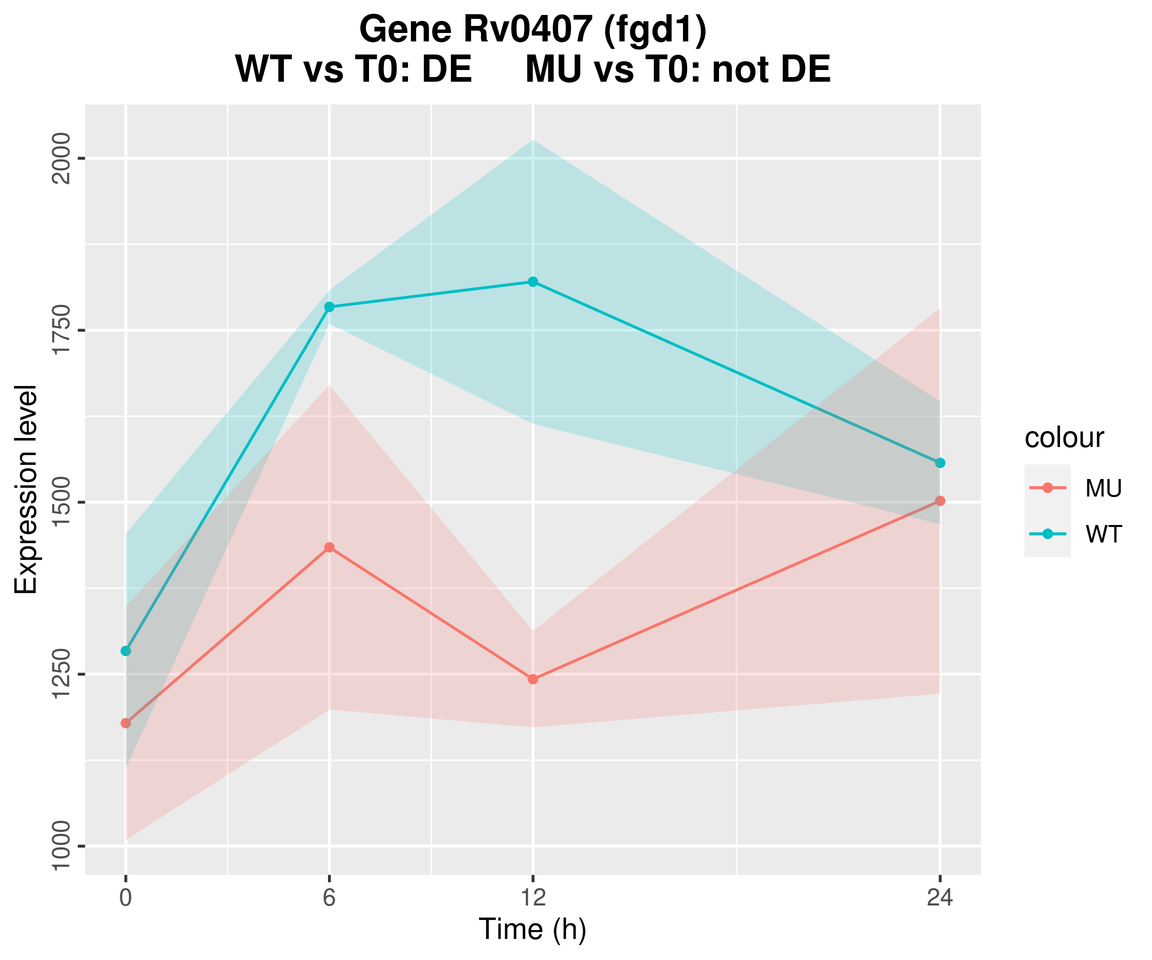

Gene Rv0407 (fgd1)
WT vs T0: DE MU vs T0: not DE
Expression level
2000
1750
1500
1250
1000
0
6
12
24
Time (h)
colour
MU
WT

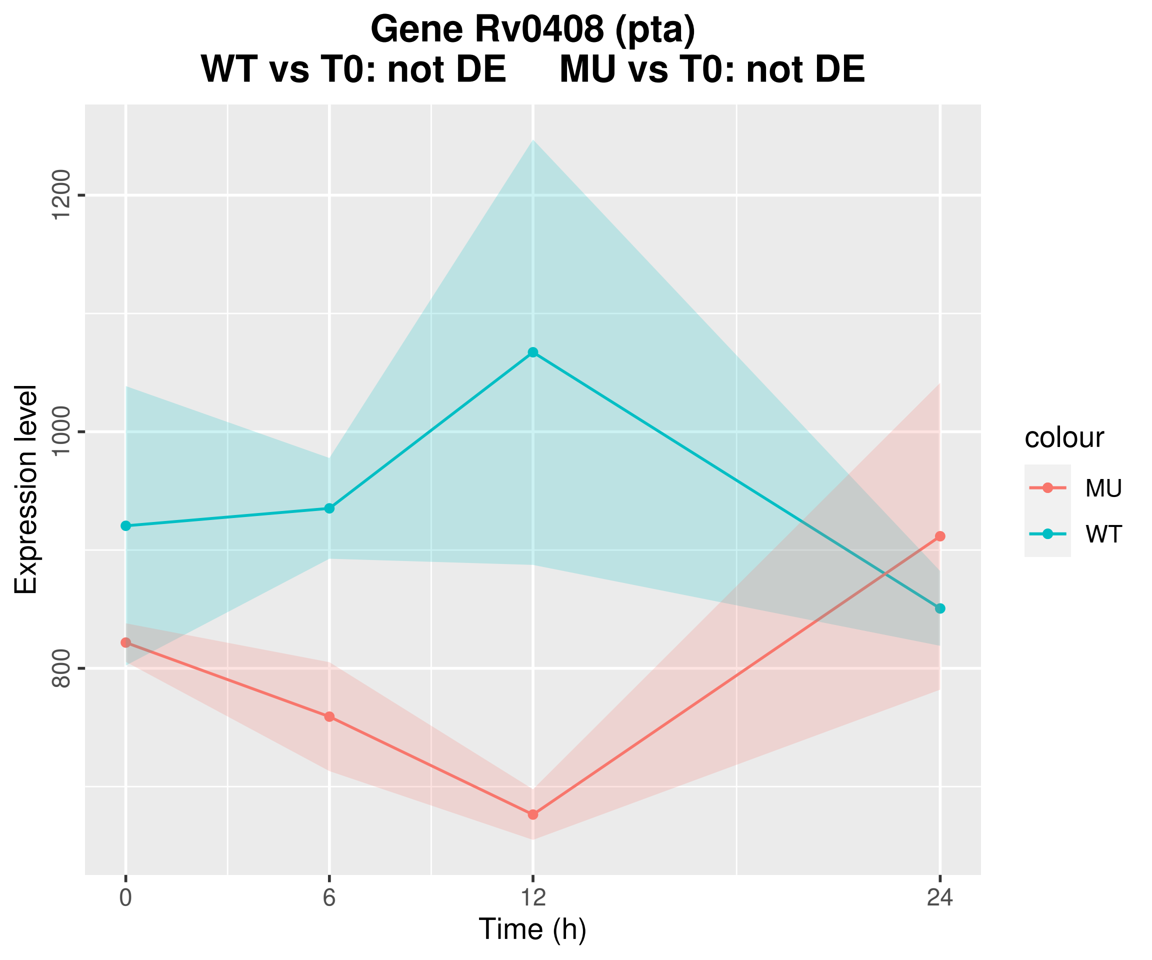

Gene Rv0408 (pta)
WT vs T0: not DE   MU vs T0: not DE
Expression level
1200
1000
800
0
6
12
24
Time (h)
colour
MU
WT

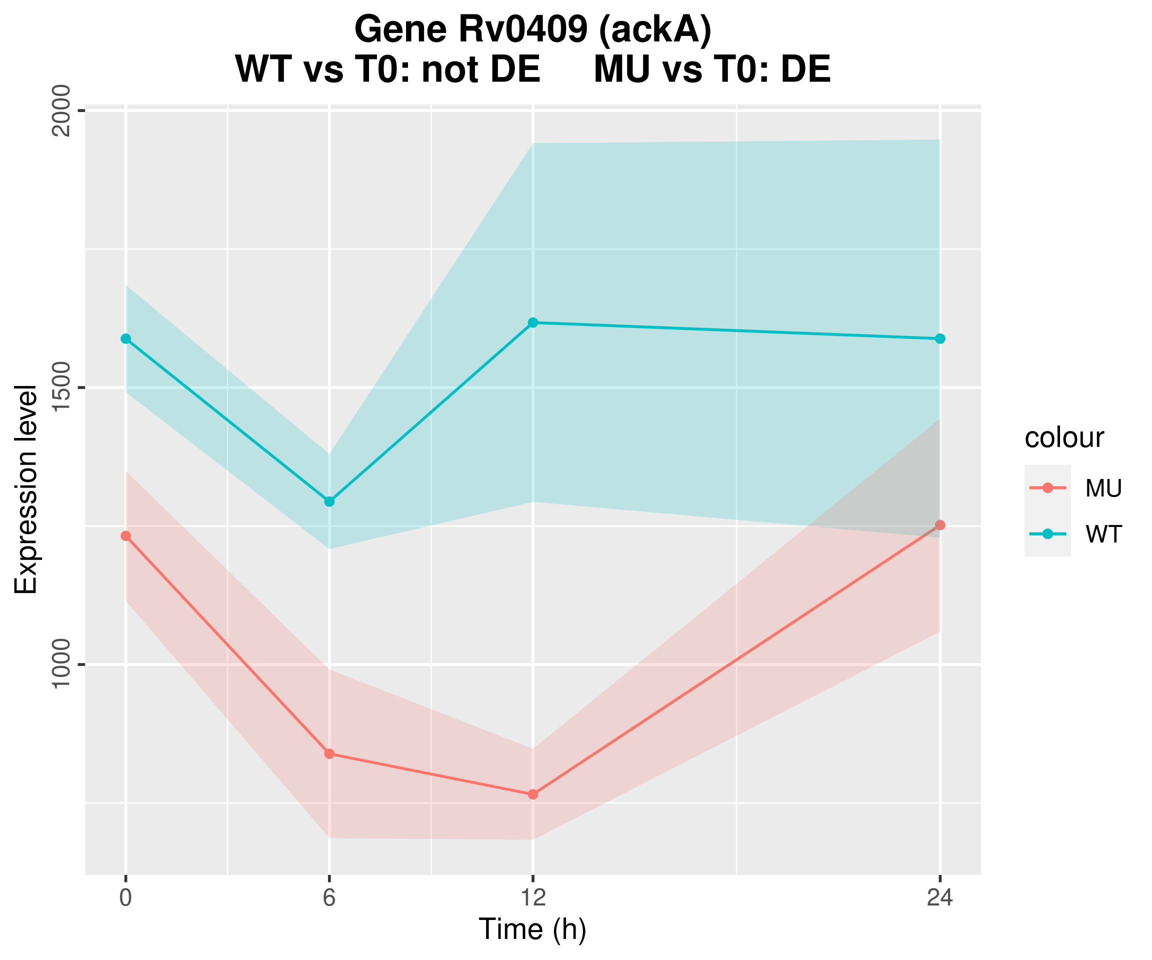

Gene Rv0409 (ackA)
WT vs T0: not DE MU vs T0: DE
2000
1500
1000
Expression level
0
6
12
24
Time (h)
colour
MU
WT

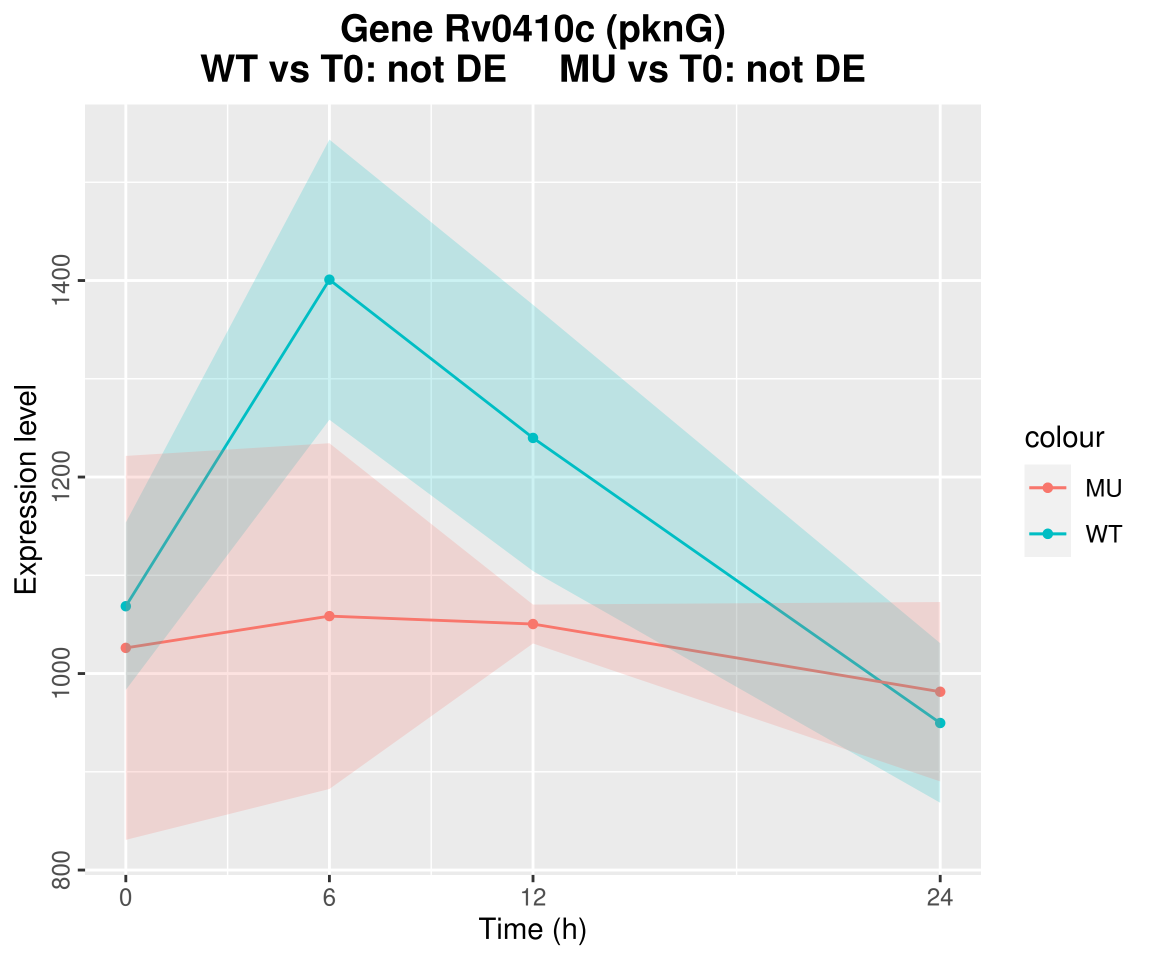

Gene Rv0410c (pknG)
WT vs T0: not DE MU vs T0: not DE
Expression level
1400
1200
1000
800
0
6
12
24
Time (h)
colour
MU
WT

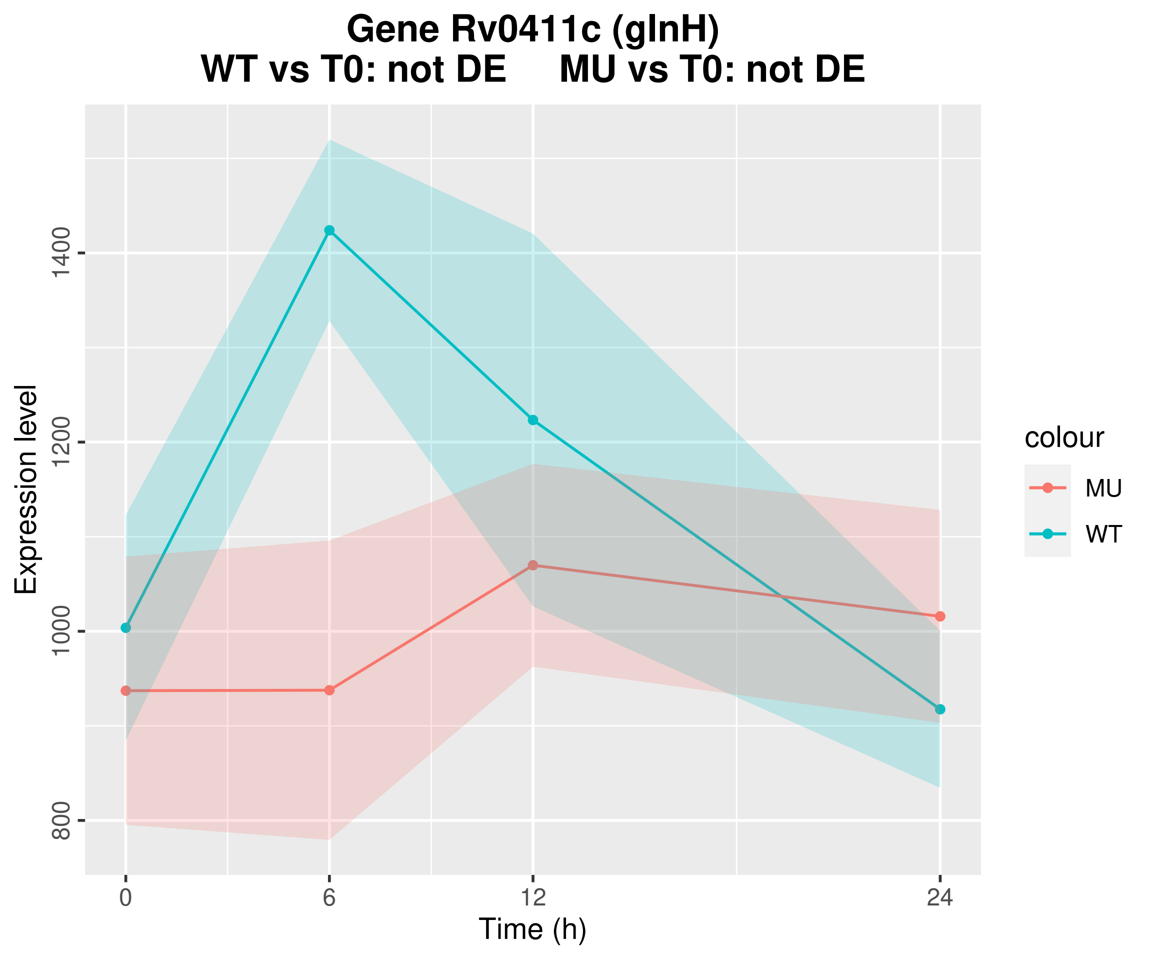
Gene Rv0411c (glnH)
WT vs T0: not DE   MU vs T0: not DE
Expression level
1400
1200
1000
800
0
6
12
24
Time (h)
colour
MU
WT

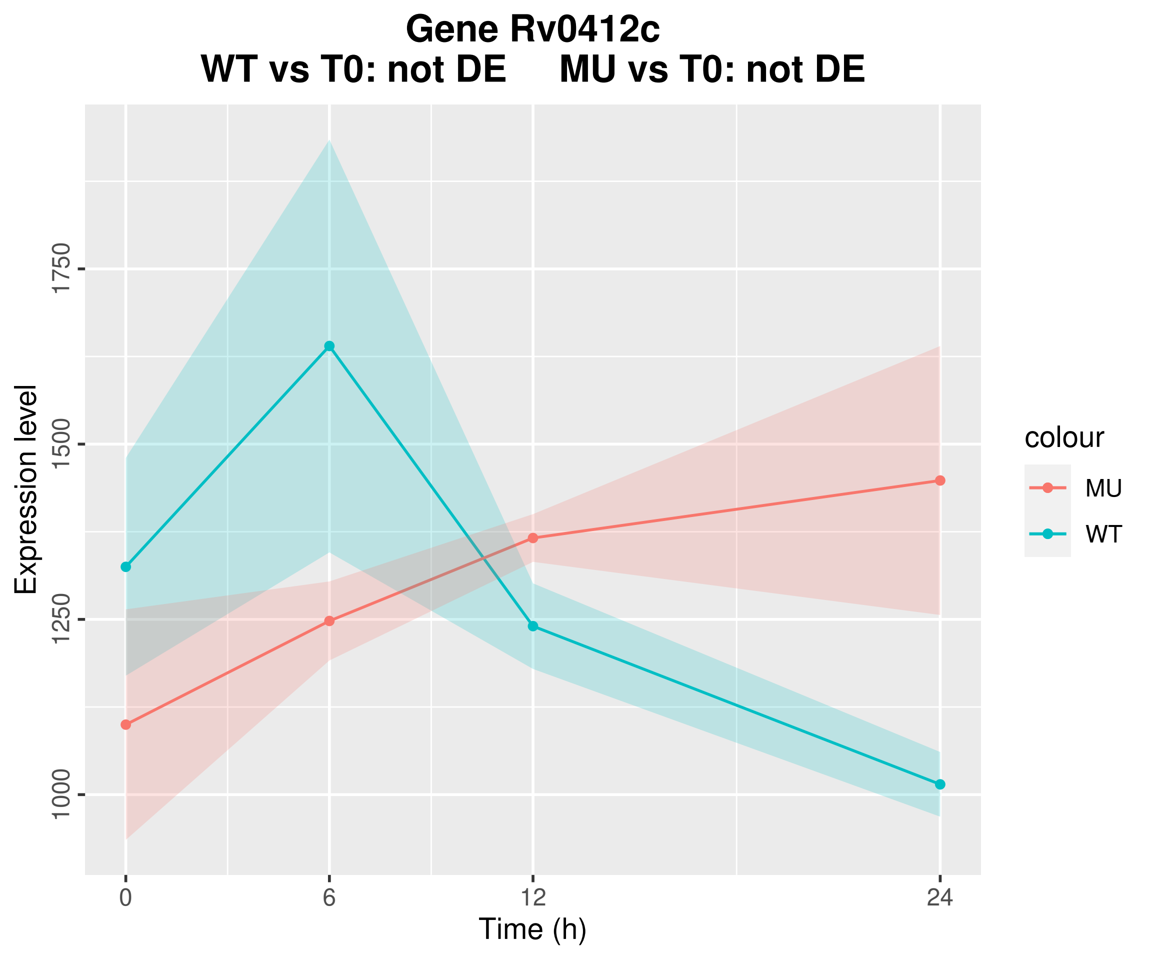
Gene Rv0412c
WT vs T0: not DE
MU vs T0: not DE
Expression level
1000
1250
1500
1750
Time (h)
0
6
12
24
colour
MU
WT

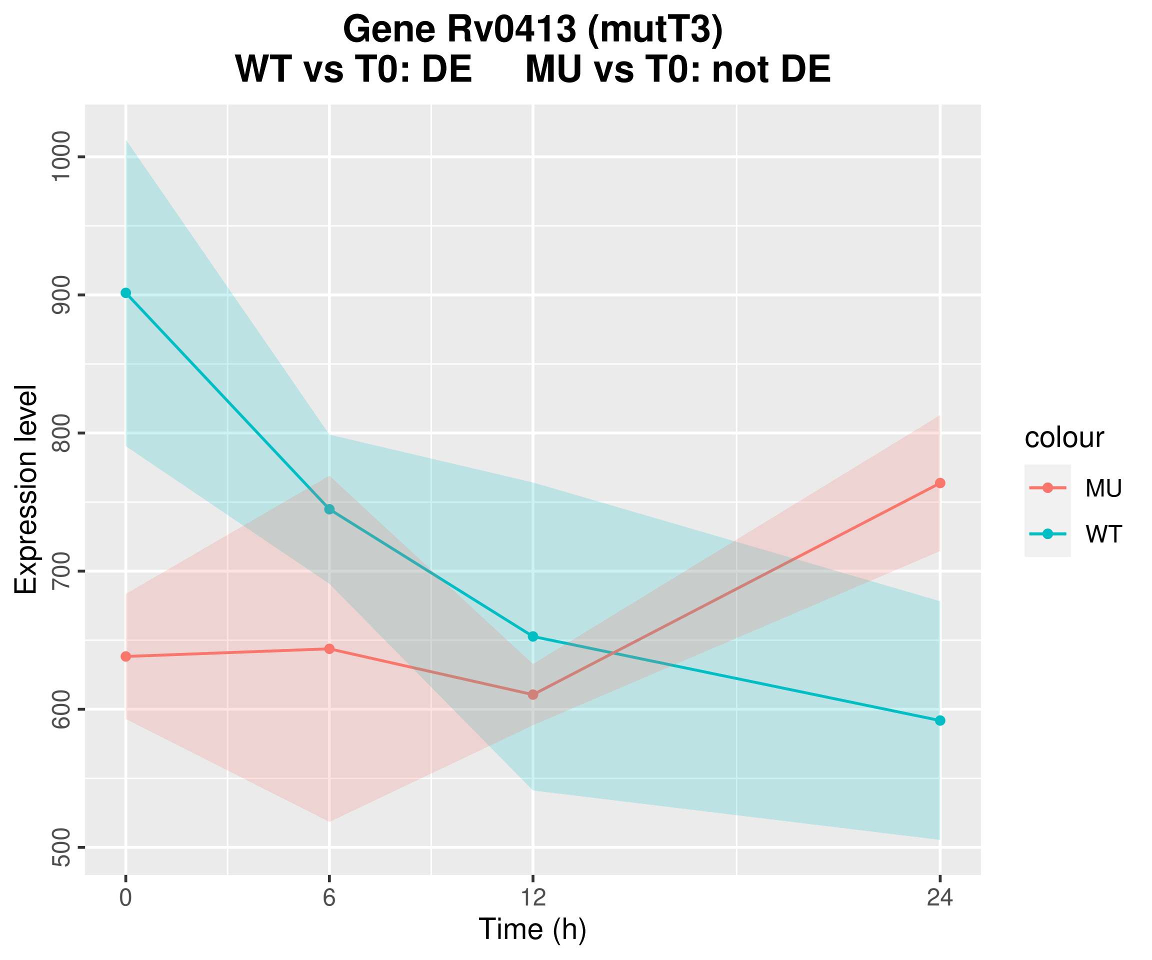

Gene Rv0413 (mutT3)
WT vs T0: DE   MU vs T0: not DE
Expression level
1000
900
800
700
600
500
0
6
12
24
Time (h)
colour
MU
WT

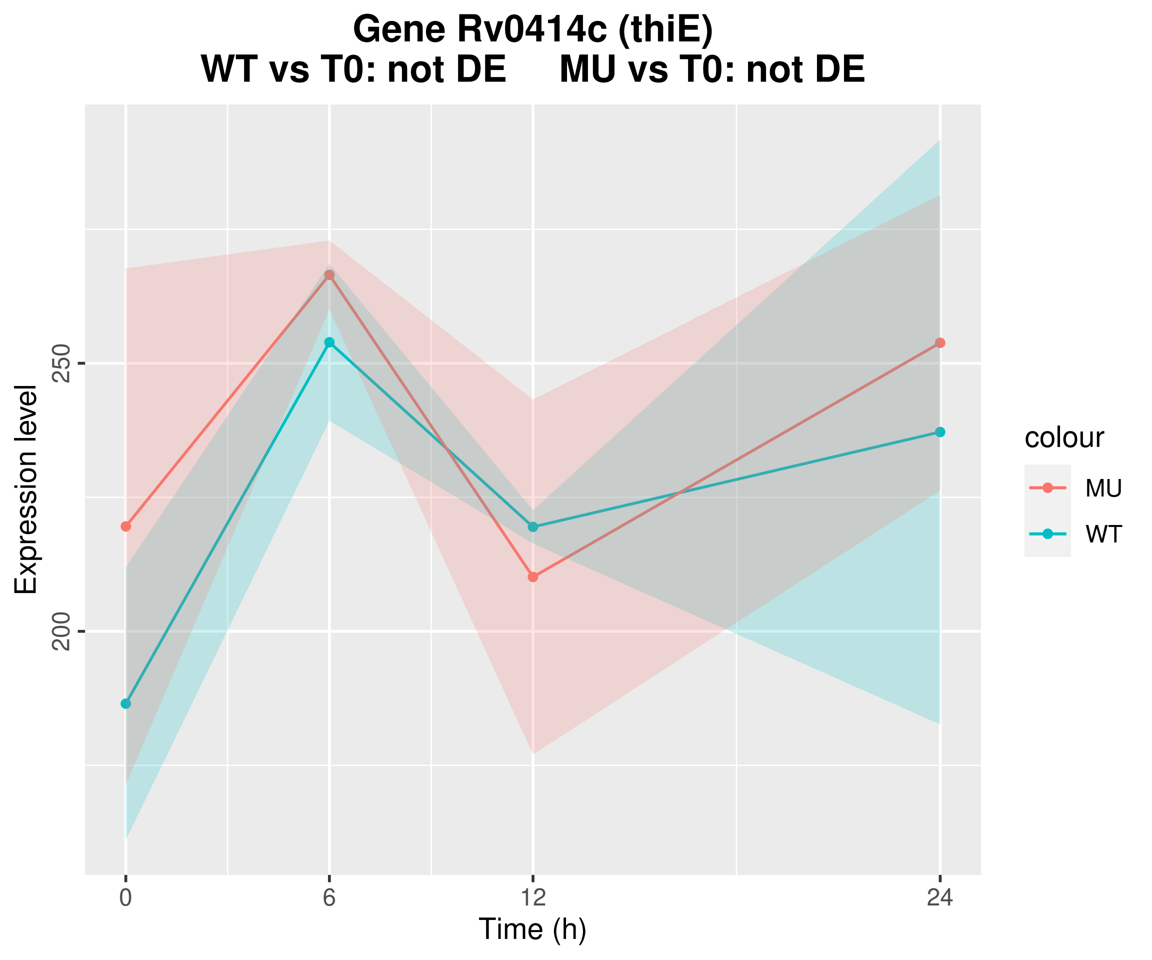
Gene Rv0414c (thiE)
WT vs T0: not DE   MU vs T0: not DE
Expression level
250
200
0
6
12
24
Time (h)
colour
MU
WT

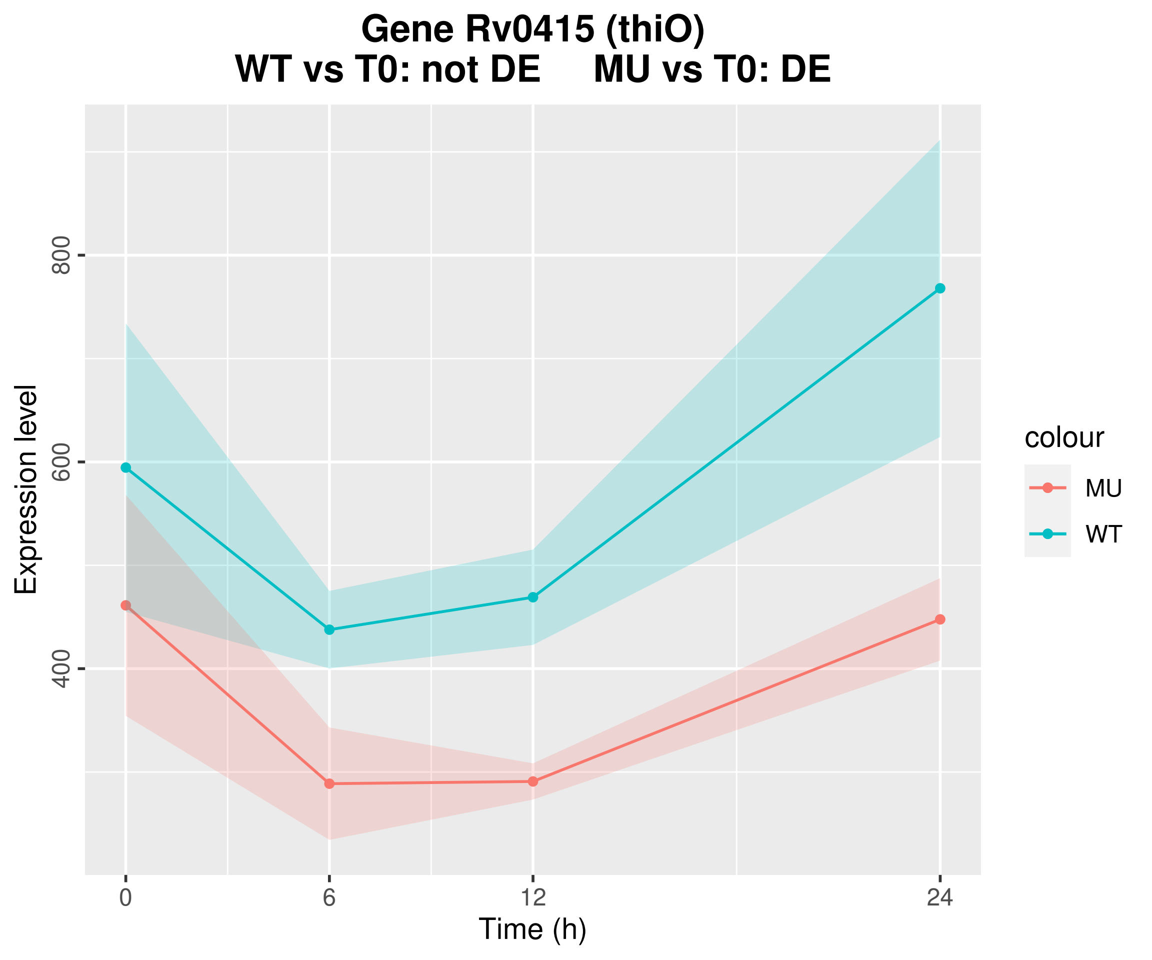

Gene Rv0415 (thiO)
WT vs T0: not DE MU vs T0: DE
Expression level
800
600
400
0
6
12
24
Time (h)
colour
MU
WT

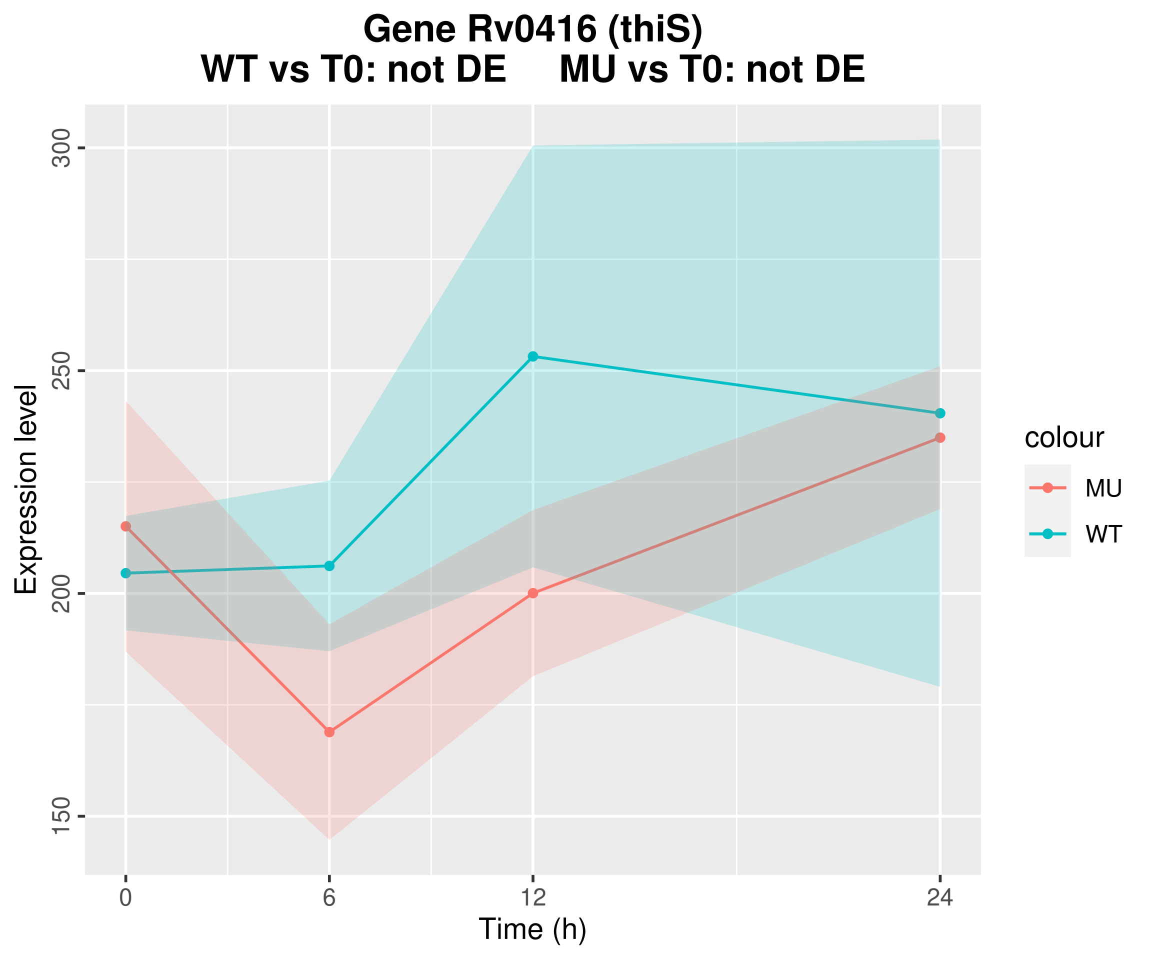

Gene Rv0416 (thiS)
WT vs T0: not DE MU vs T0: not DE
Expression level
300
250
200
150
0
6
12
24
Time (h)
colour
MU
WT

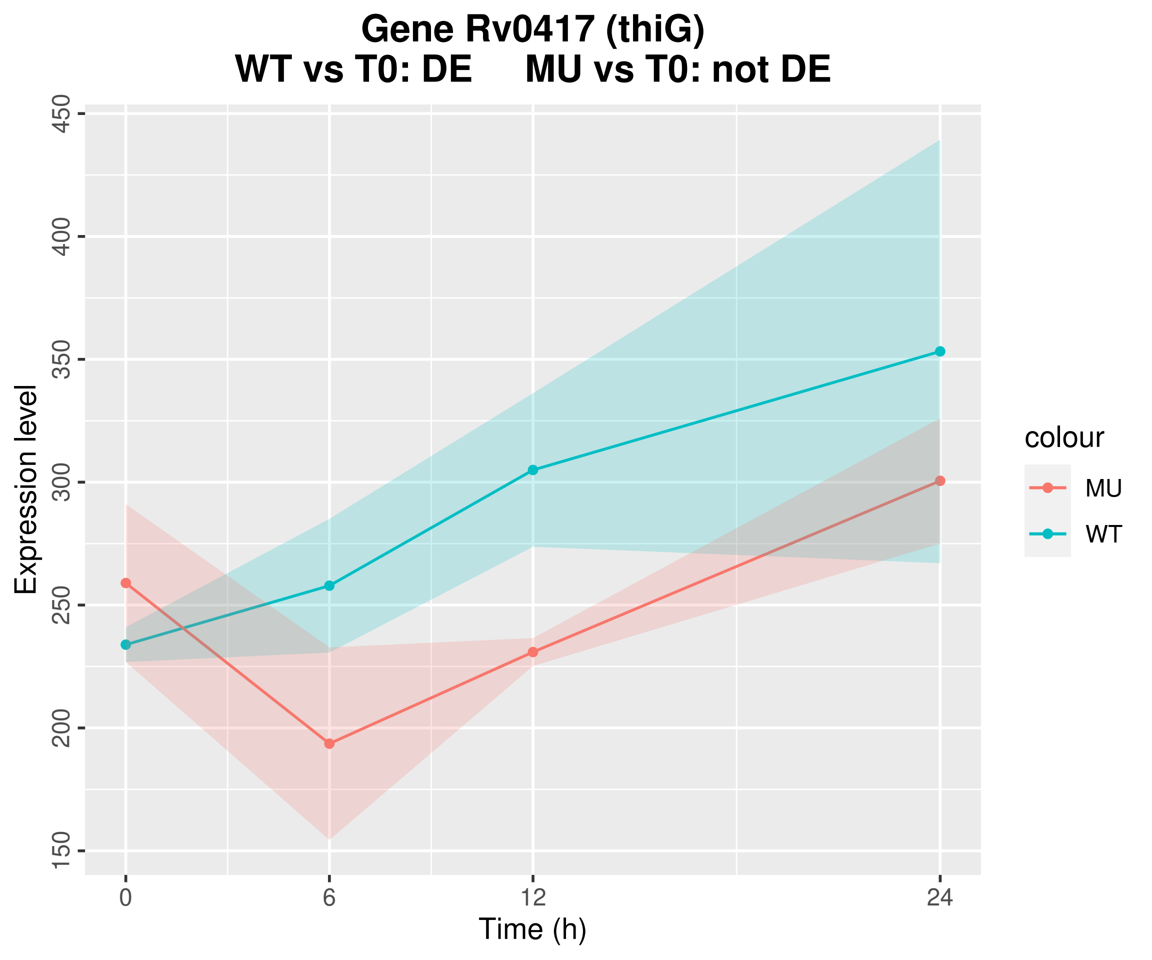

Gene Rv0417 (thiG)
WT vs T0: DE MU vs T0: not DE
Expression level
450
400
350
300
250
200
150
0
6
12
24
Time (h)
colour
MU
WT

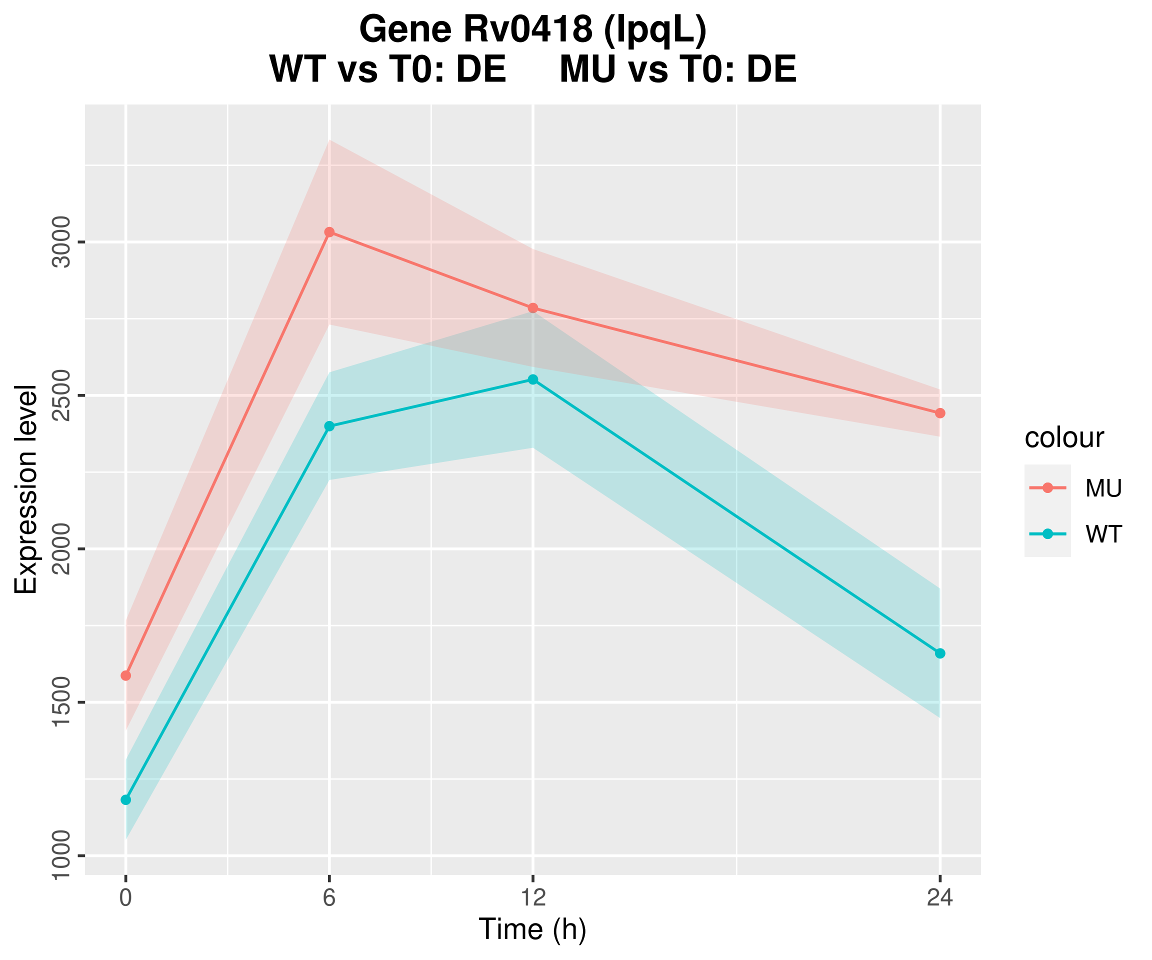
Gene Rv0418 (lpqL)
WT vs T0: DE   MU vs T0: DE
Expression level
3000
2500
2000
1500
1000
0
6
12
24
Time (h)
colour
MU
WT

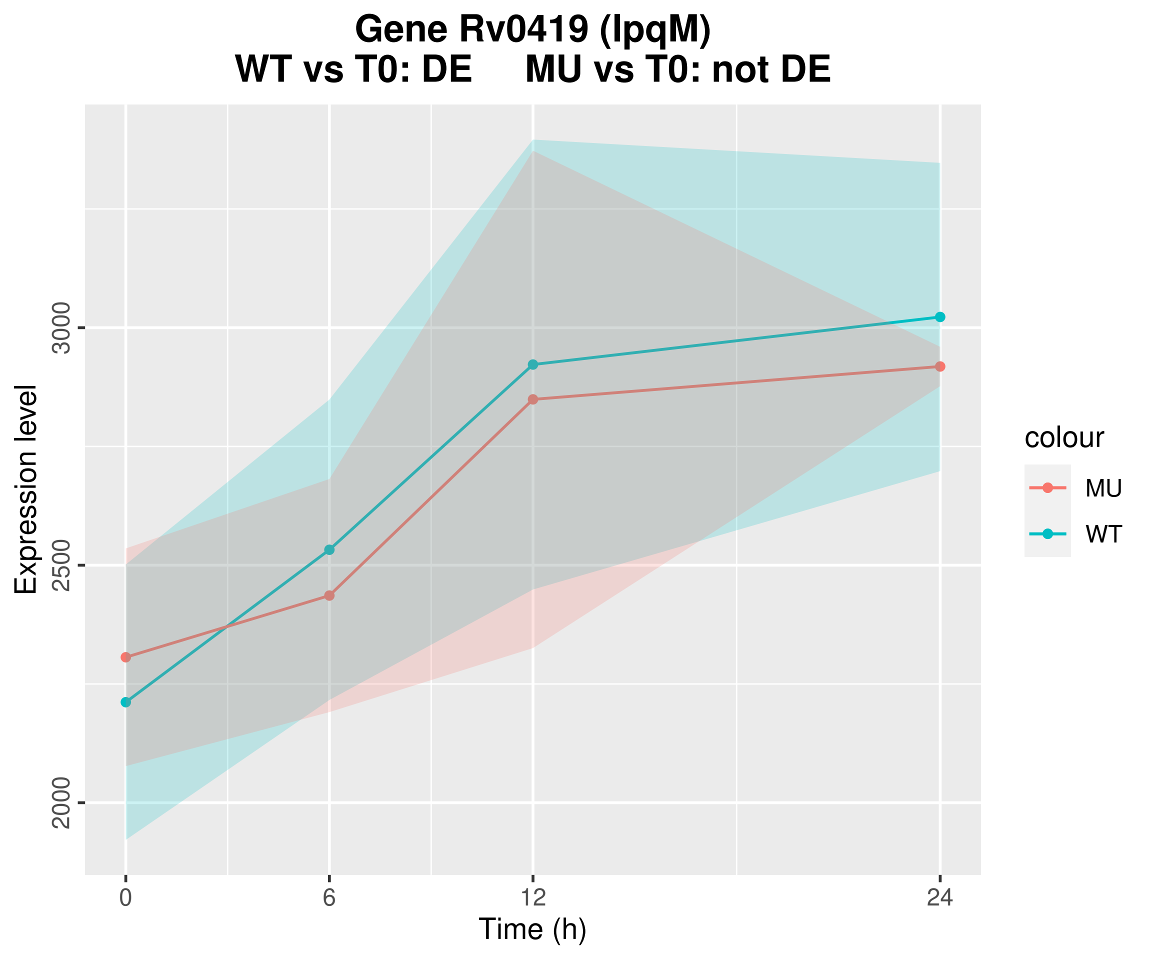
Gene Rv0419 (lpqM)
WT vs T0: DE   MU vs T0: not DE
Expression level
3000
2500
2000
0
6
12
24
Time (h)
colour
MU
WT

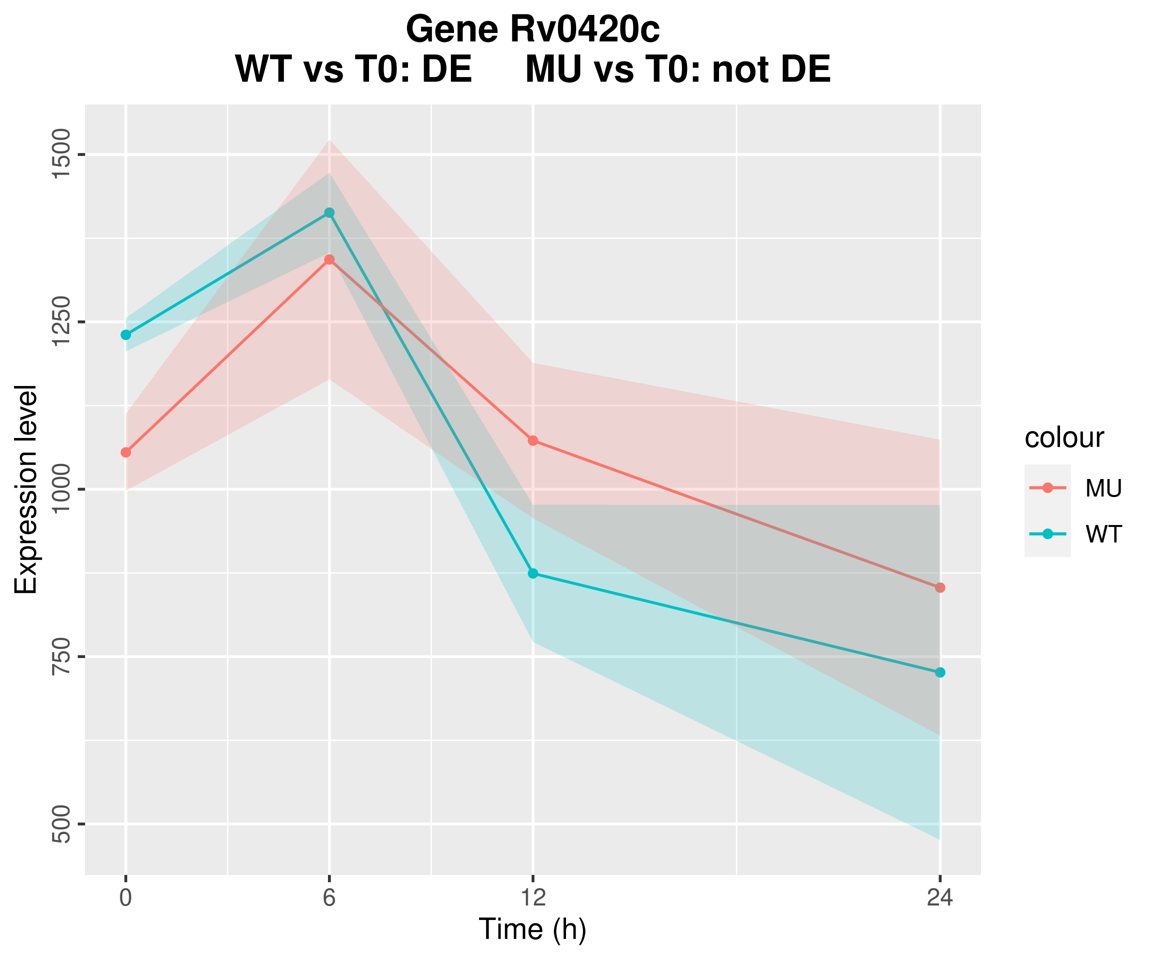

Gene Rv0420c
WT vs T0: DE MU vs T0: not DE
Expression level
1500
1250
1000
750
500
0
6
12
24
Time (h)
colour
MU
WT

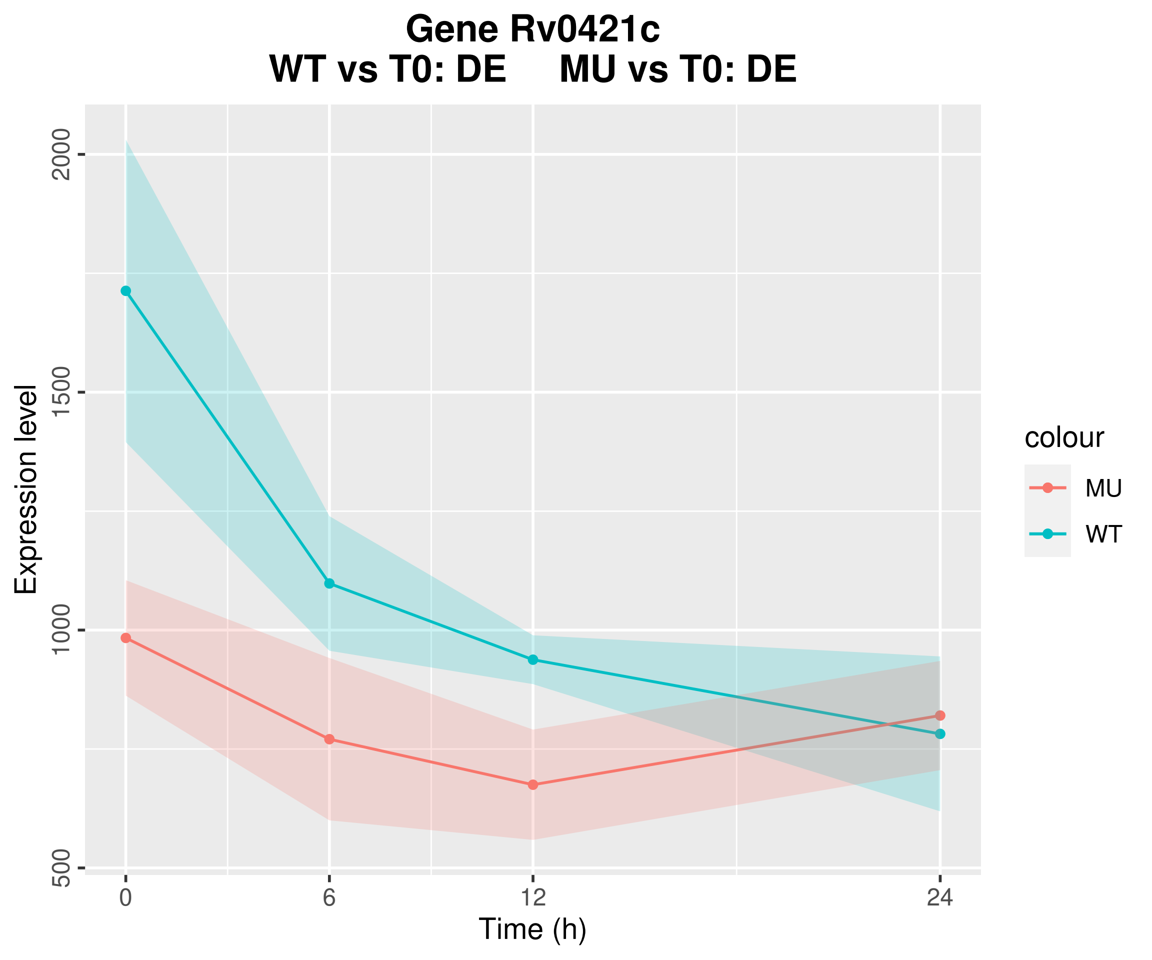

Gene Rv0421c
WT vs T0: DE MU vs T0: DE
Expression level
2000
1500
1000
500
0
6
12
24
Time (h)
colour
MU
WT

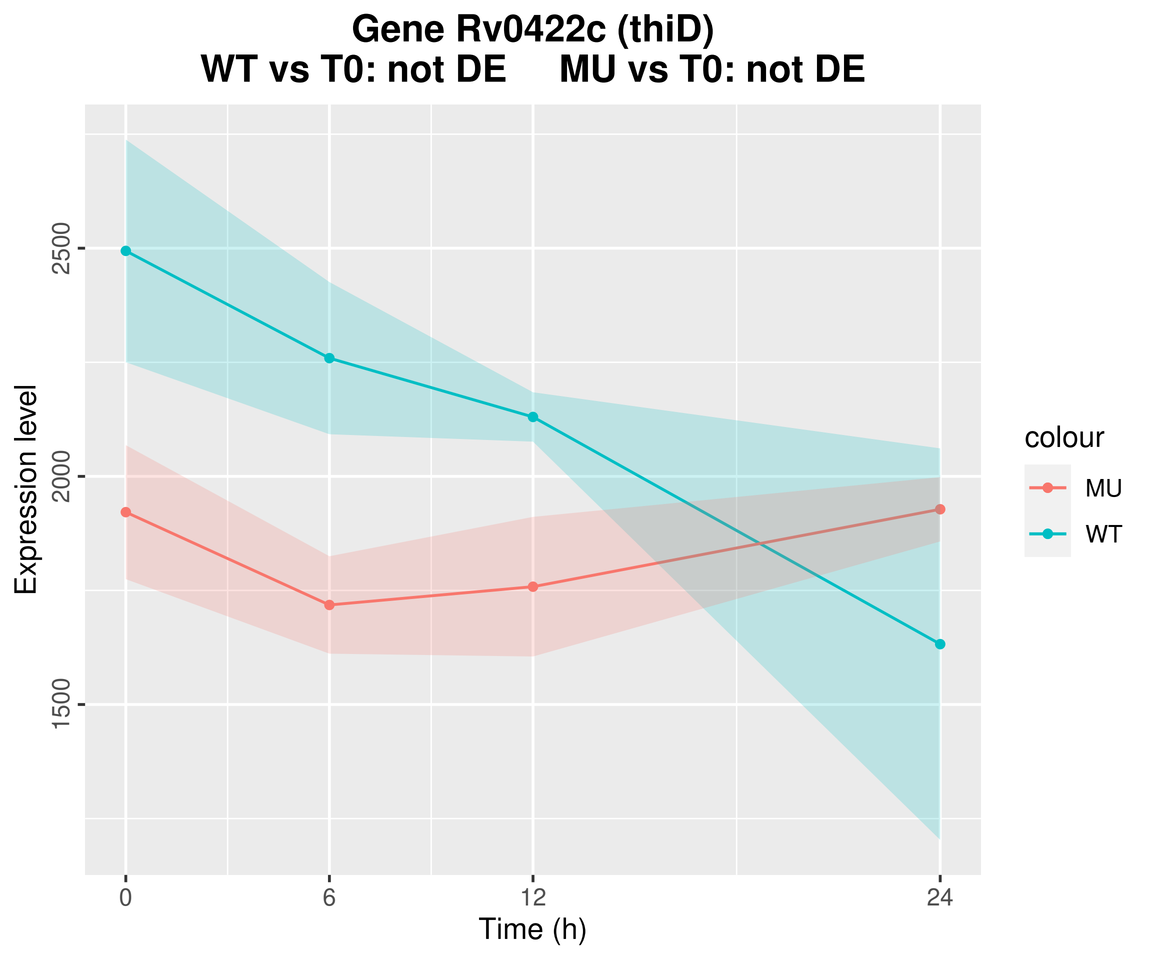

Gene Rv0422c (thiD)
WT vs T0: not DE MU vs T0: not DE
Expression level
2500
2000
1500
Time (h)
0
6
12
24
colour
MU
WT

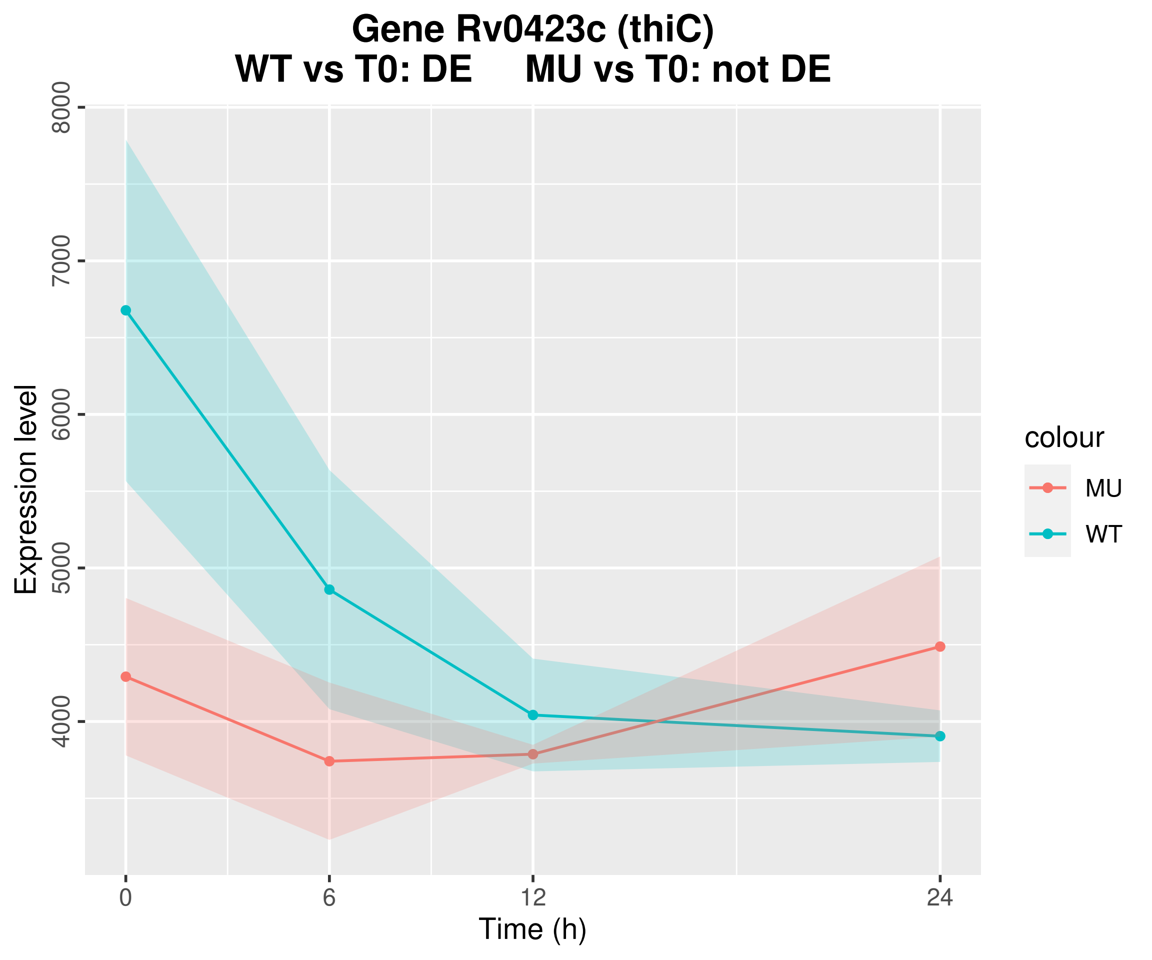

Gene Rv0423c (thiC)
WT vs T0: DE MU vs T0: not DE
Expression level
8000
7000
6000
5000
4000
0
6
12
24
Time (h)
colour
MU
WT

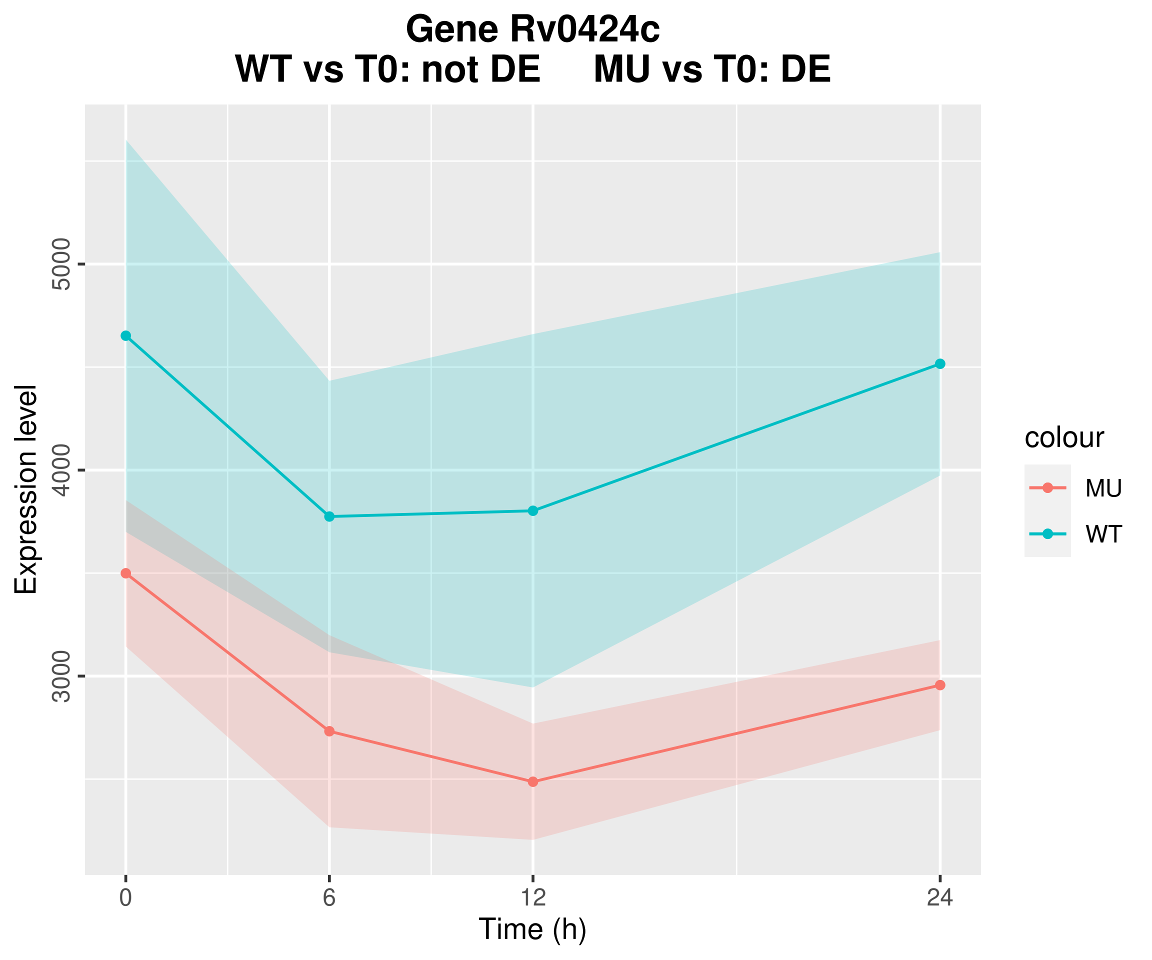

Gene Rv0424c
WT vs T0: not DE   MU vs T0: DE
Expression level
5000
4000
3000
Time (h)
0
6
12
24
colour
MU
WT

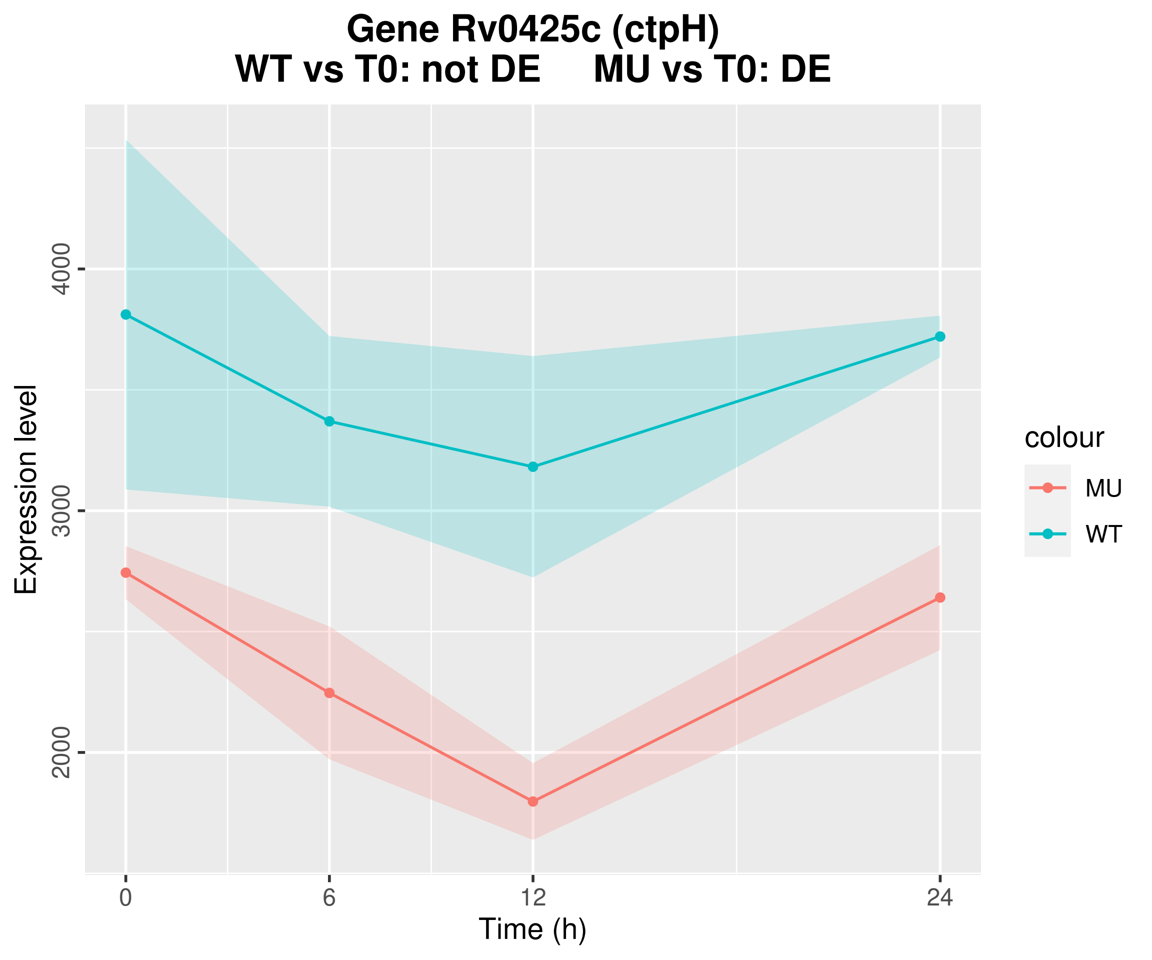
Gene Rv0425c (ctpH)
WT vs T0: not DE MU vs T0: DE
Expression level
4000
3000
2000
Time (h)
0
6
12
24
colour
MU
WT

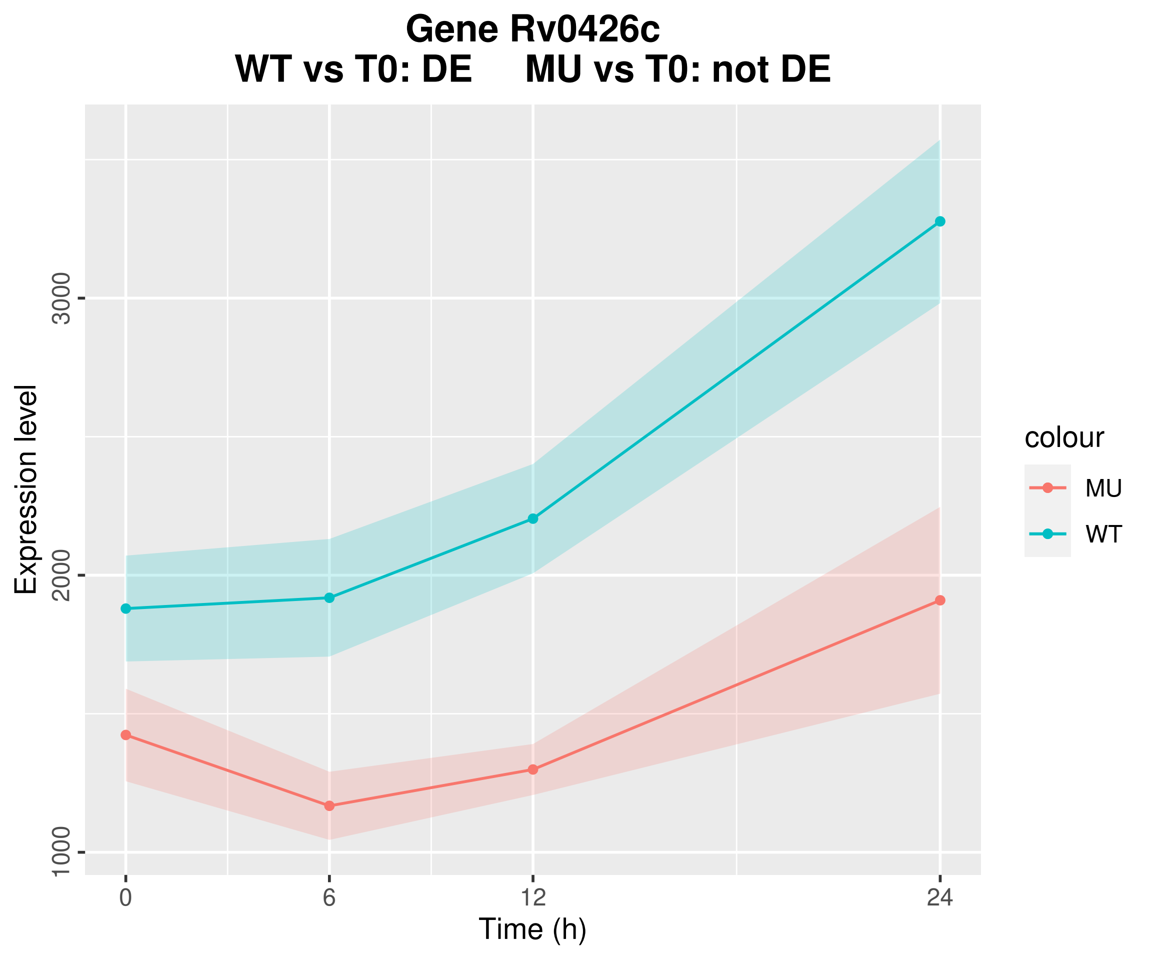

Gene Rv0426c
WT vs T0: DE MU vs T0: not DE
Expression level
3000
2000
1000
0
6
12
24
Time (h)
colour
MU
WT

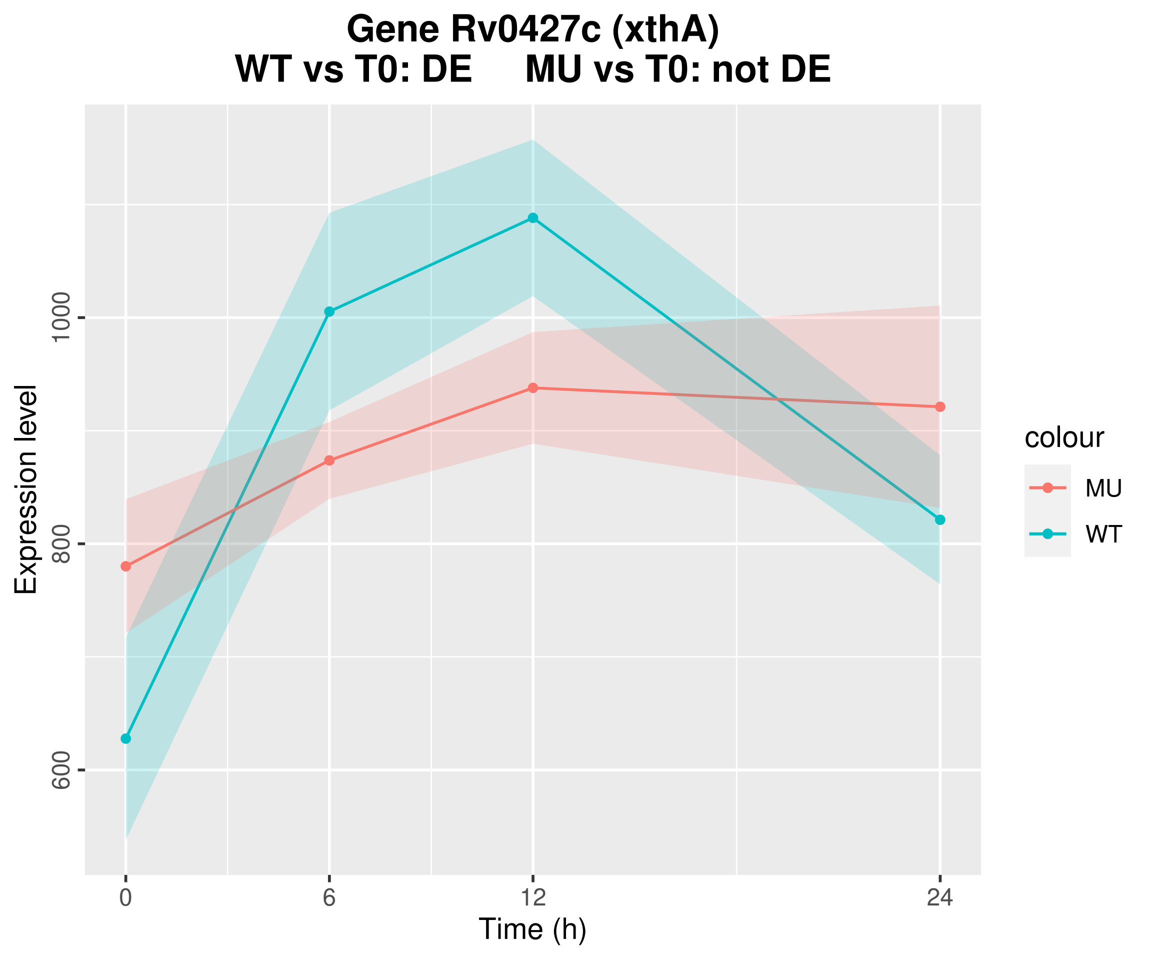

Gene Rv0427c (xthA)
WT vs T0: DE   MU vs T0: not DE
Expression level
1000
800
600
0
6
12
24
Time (h)
colour
MU
WT

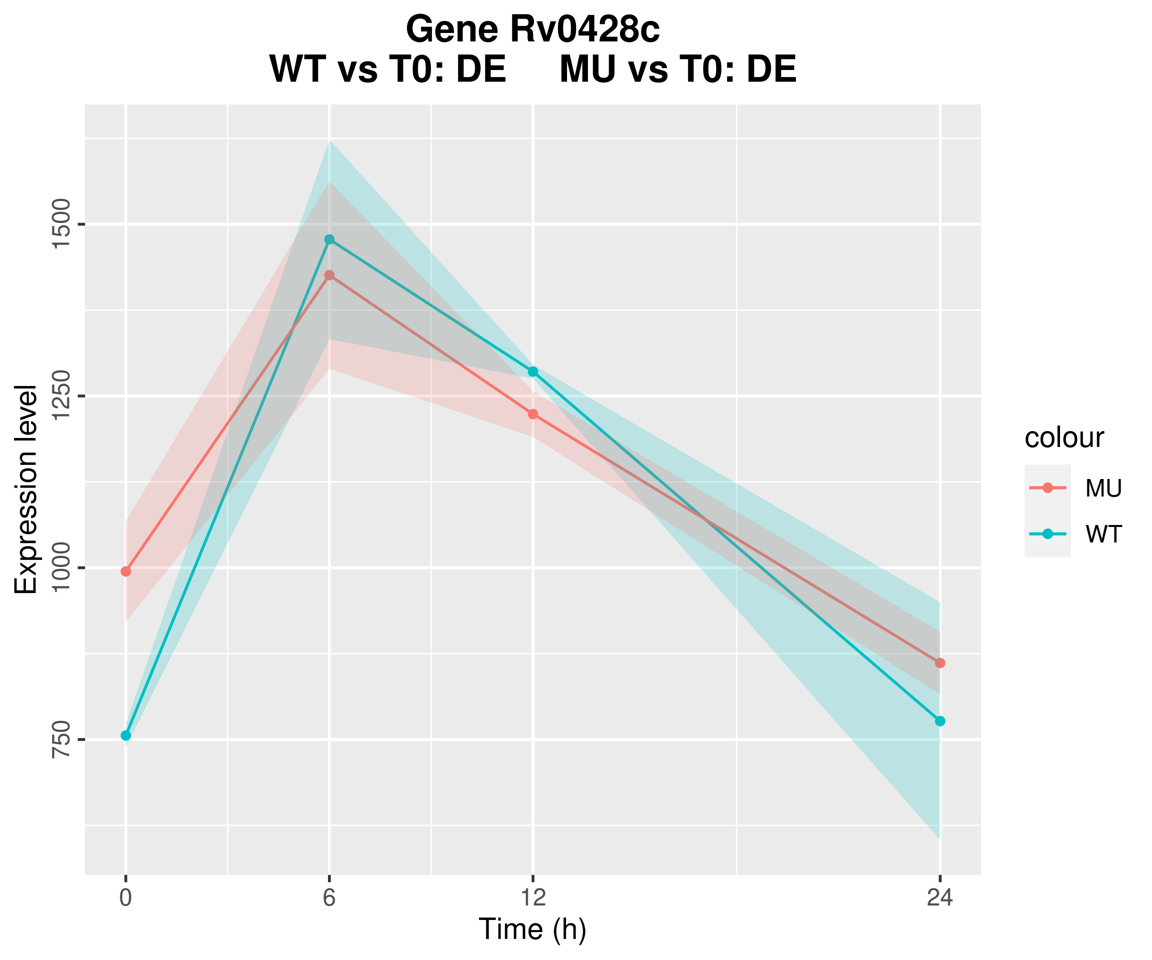
Gene Rv0428c
WT vs T0: DE MU vs T0: DE
Expression level
1500
1250
1000
750
0
6
12
24
Time (h)
colour
MU
WT

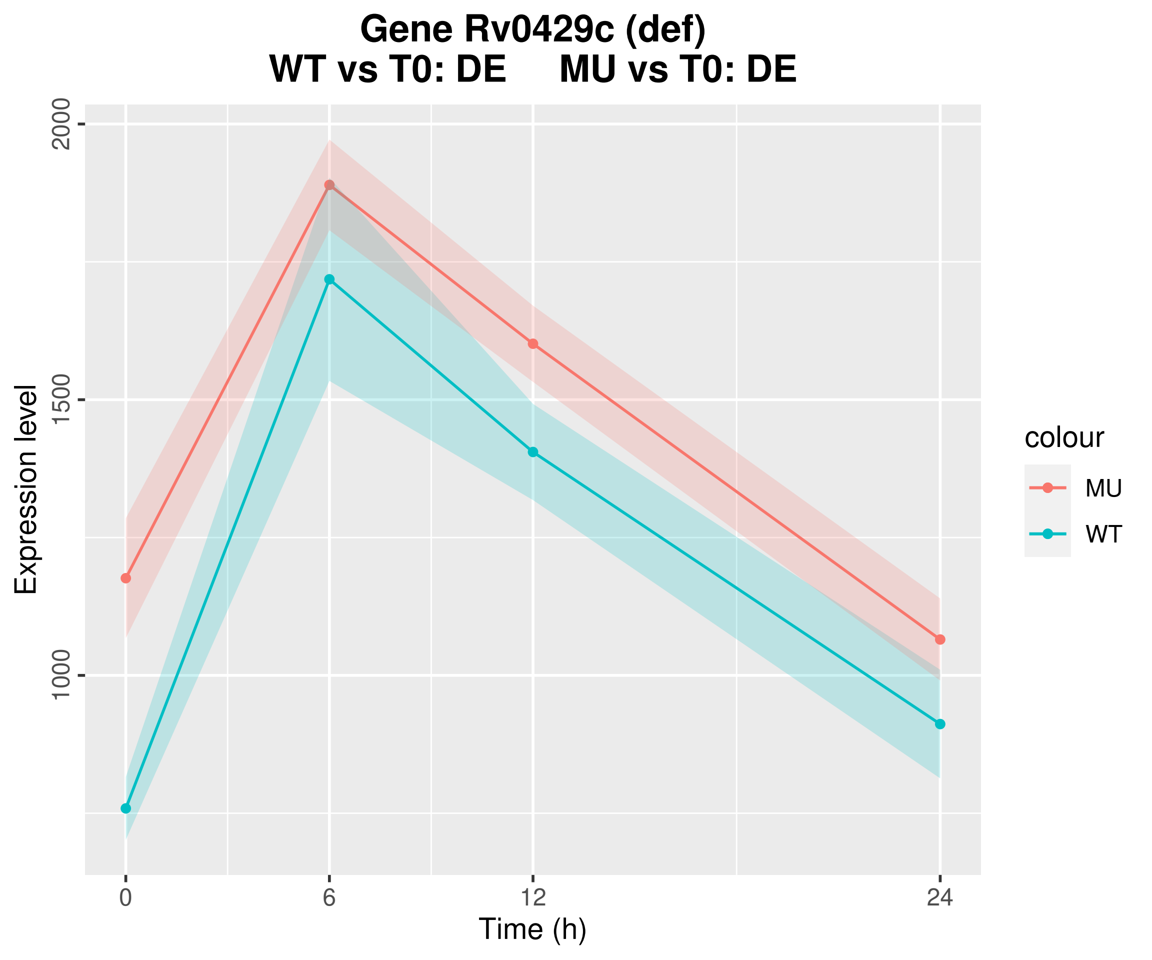

Gene Rv0429c (def)
WT vs T0: DE MU vs T0: DE
2000
1500
1000
Expression level
0
6
12
24
Time (h)
colour
MU
WT

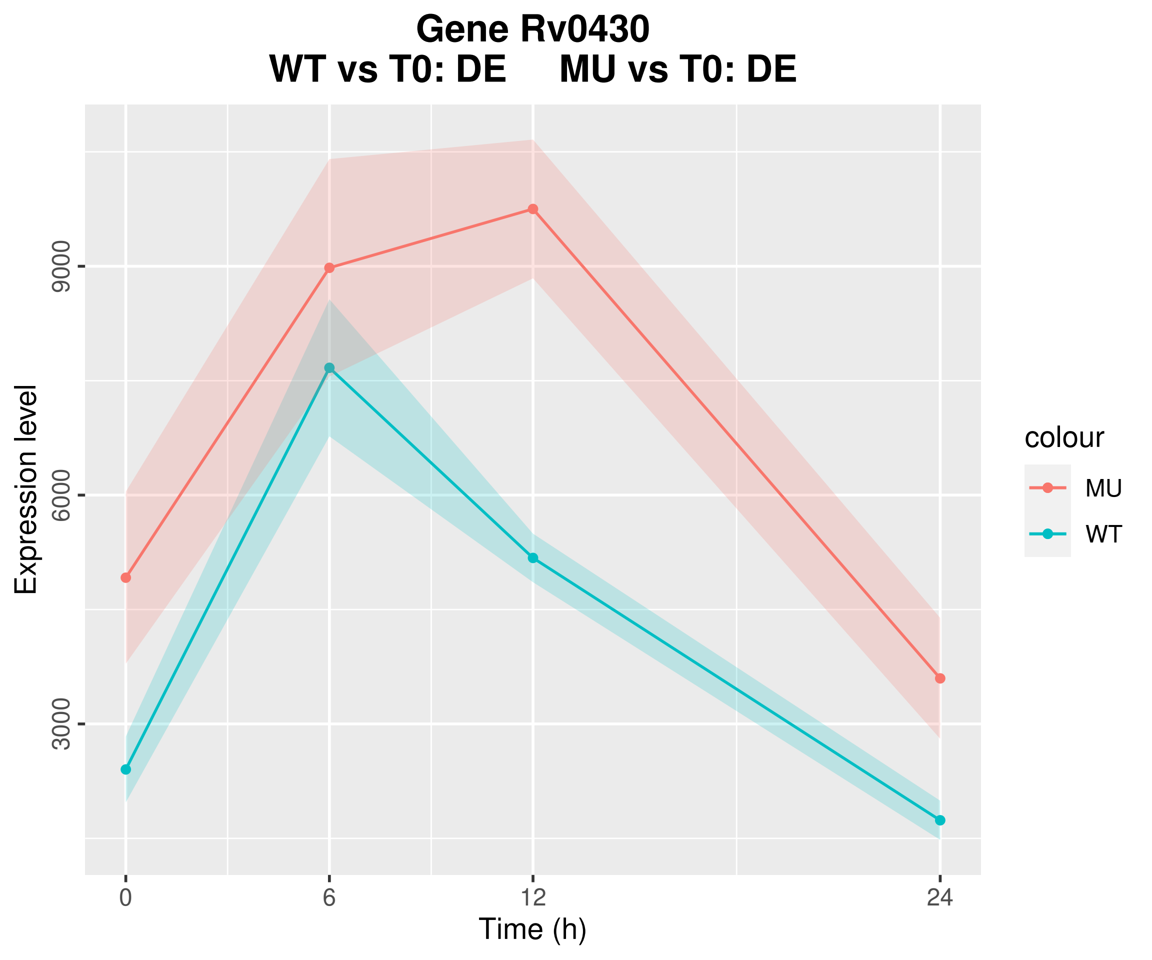

Gene Rv0430
WT vs T0: DE MU vs T0: DE
Expression level
9000
6000
3000
0
6
12
24
Time (h)
colour
MU
WT

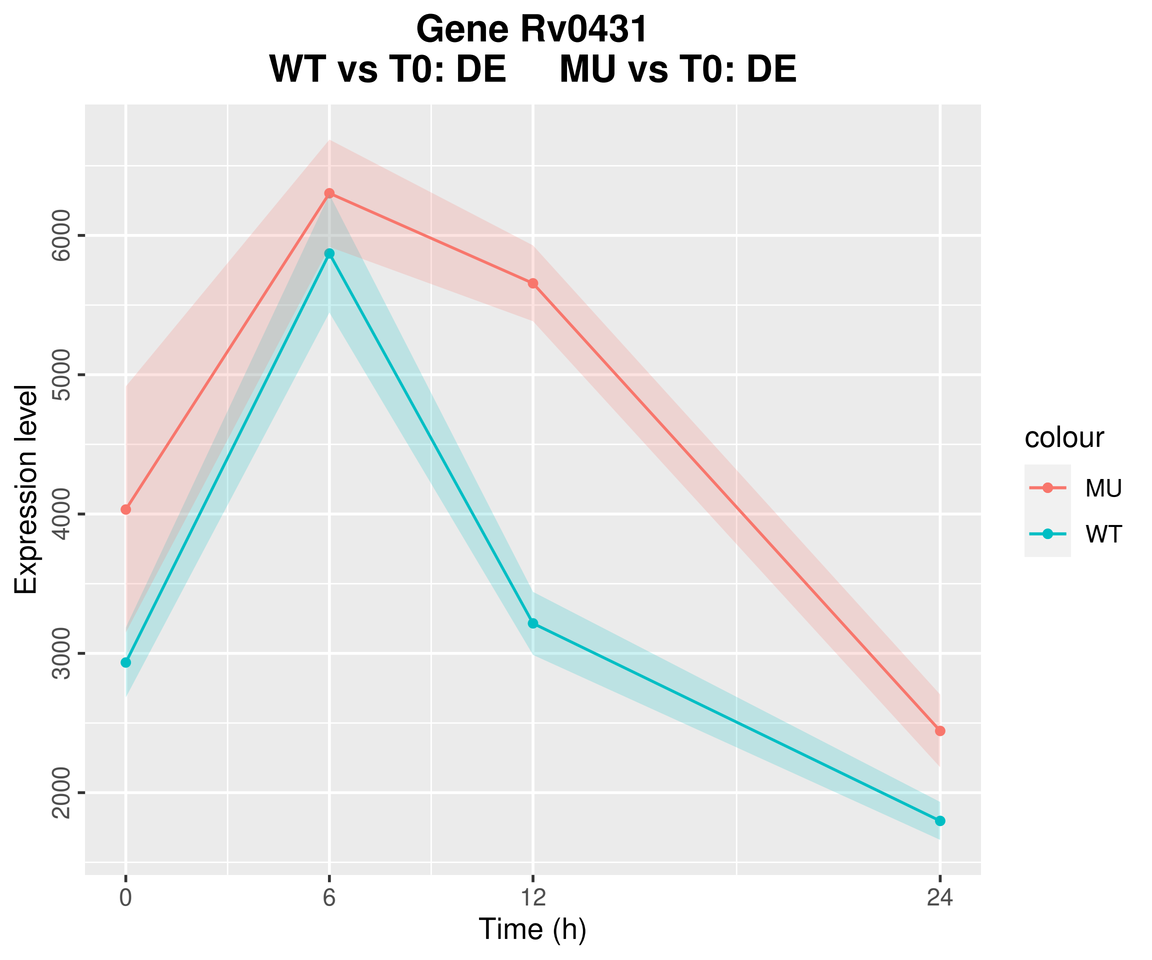
Gene Rv0431
WT vs T0: DE MU vs T0: DE
Expression level
6000
5000
4000
3000
2000
0
6
12
24
Time (h)
colour
MU
WT

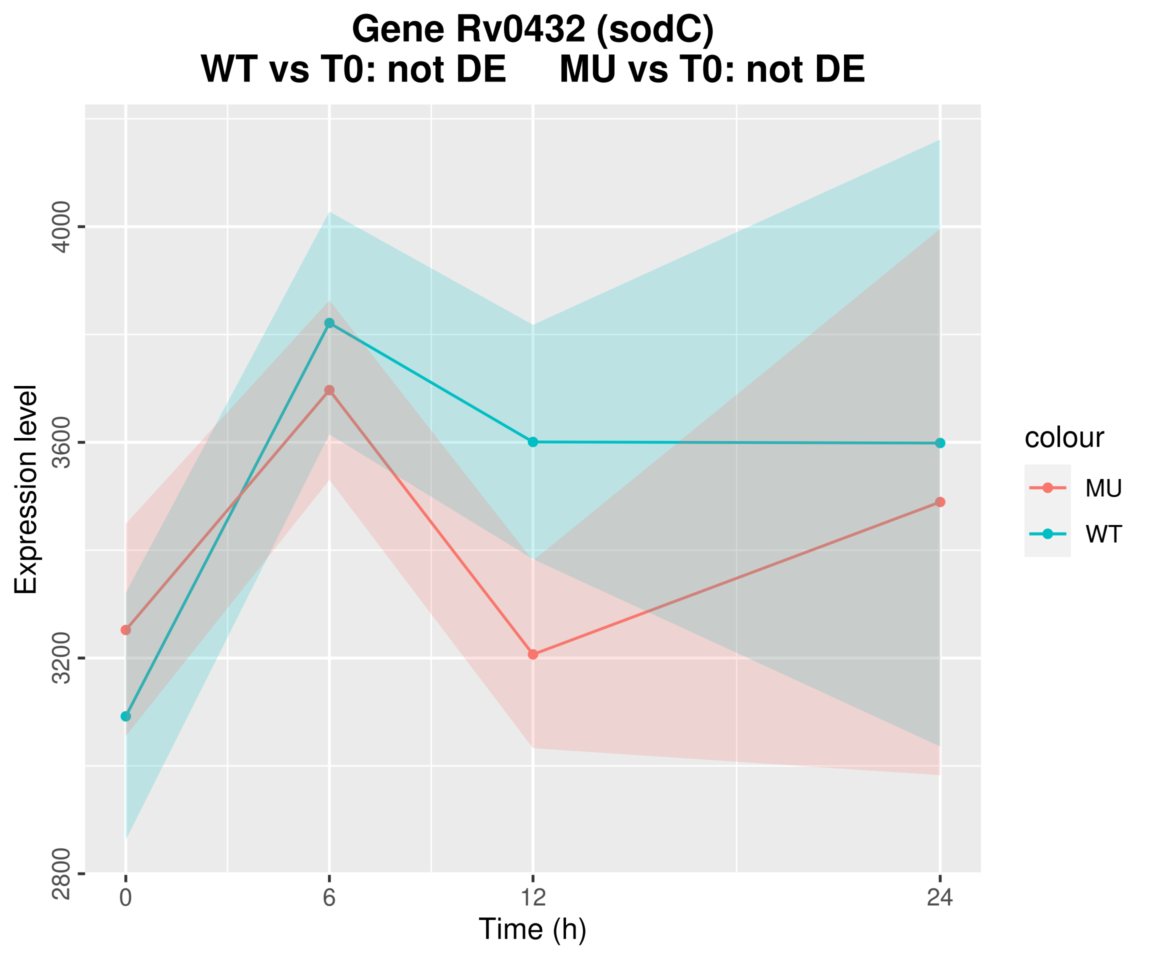

Gene Rv0432 (sodC)
WT vs T0: not DE   MU vs T0: not DE
Expression level
4000
3600
3200
2800
0
6
12
24
Time (h)
colour
MU
WT

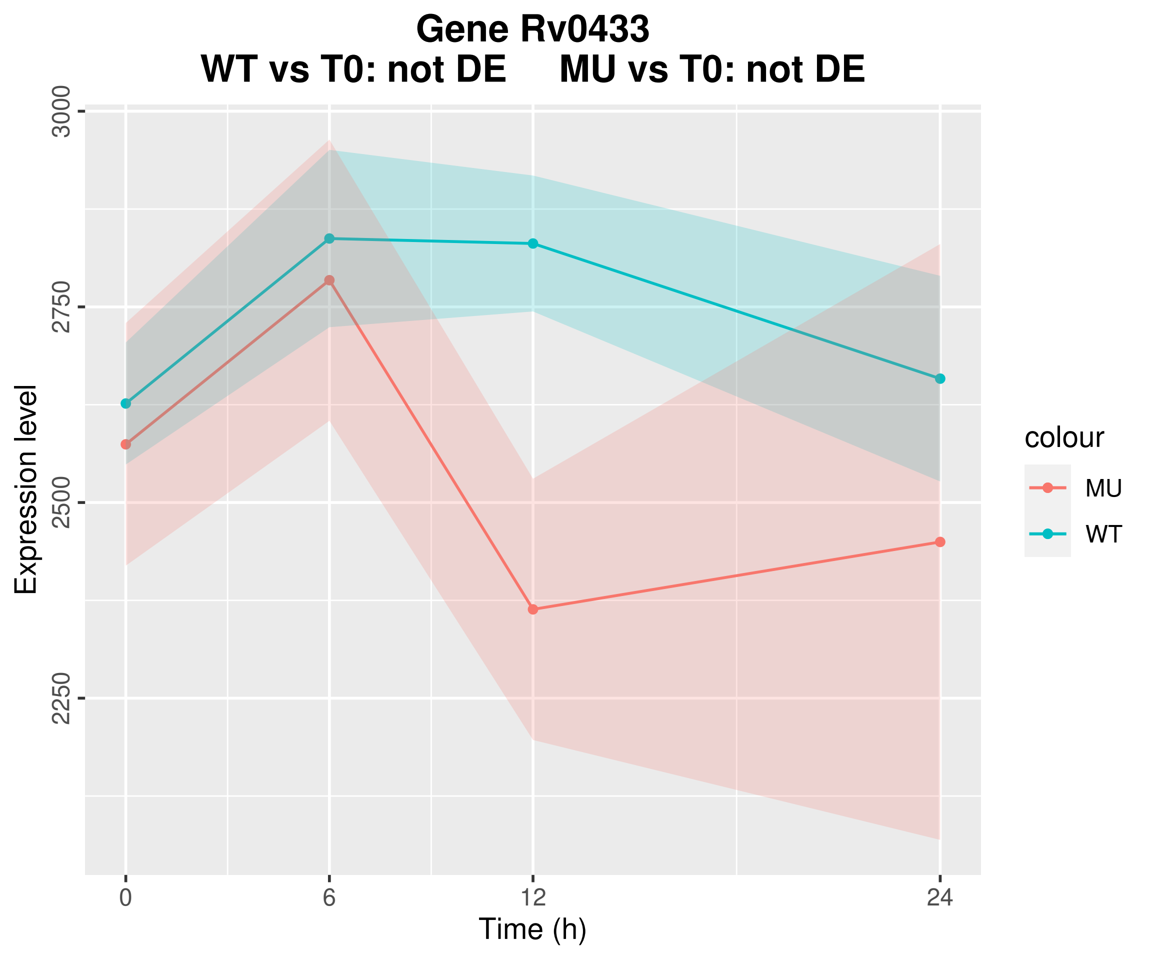

Gene Rv0433
WT vs T0: not DE MU vs T0: not DE
Expression level
3000
2750
2500
2250
0
6
12
24
Time (h)
colour
MU
WT

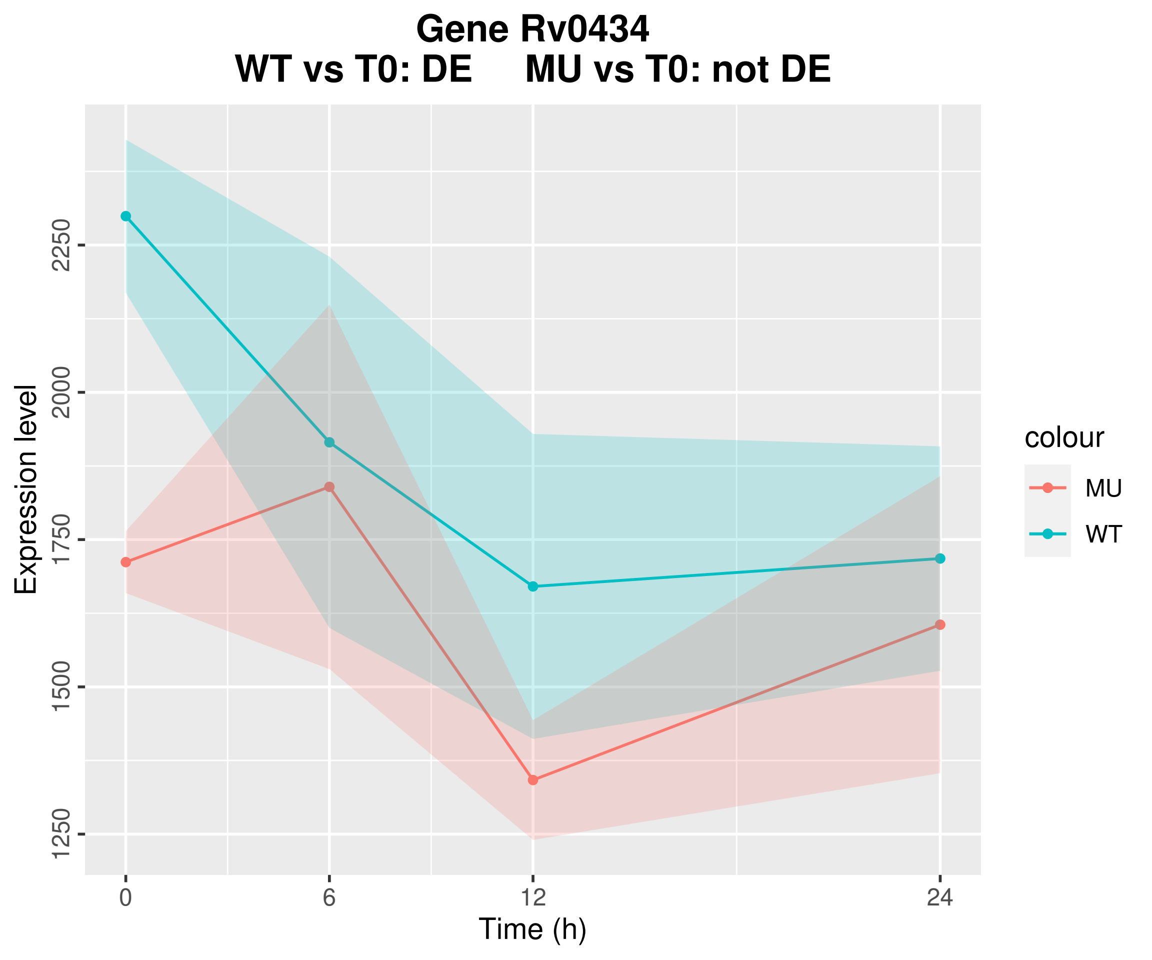

Gene Rv0434
WT vs T0: DE   MU vs T0: not DE
Expression level
2250
2000
1750
1500
1250
0
6
12
24
Time (h)
colour
MU
WT

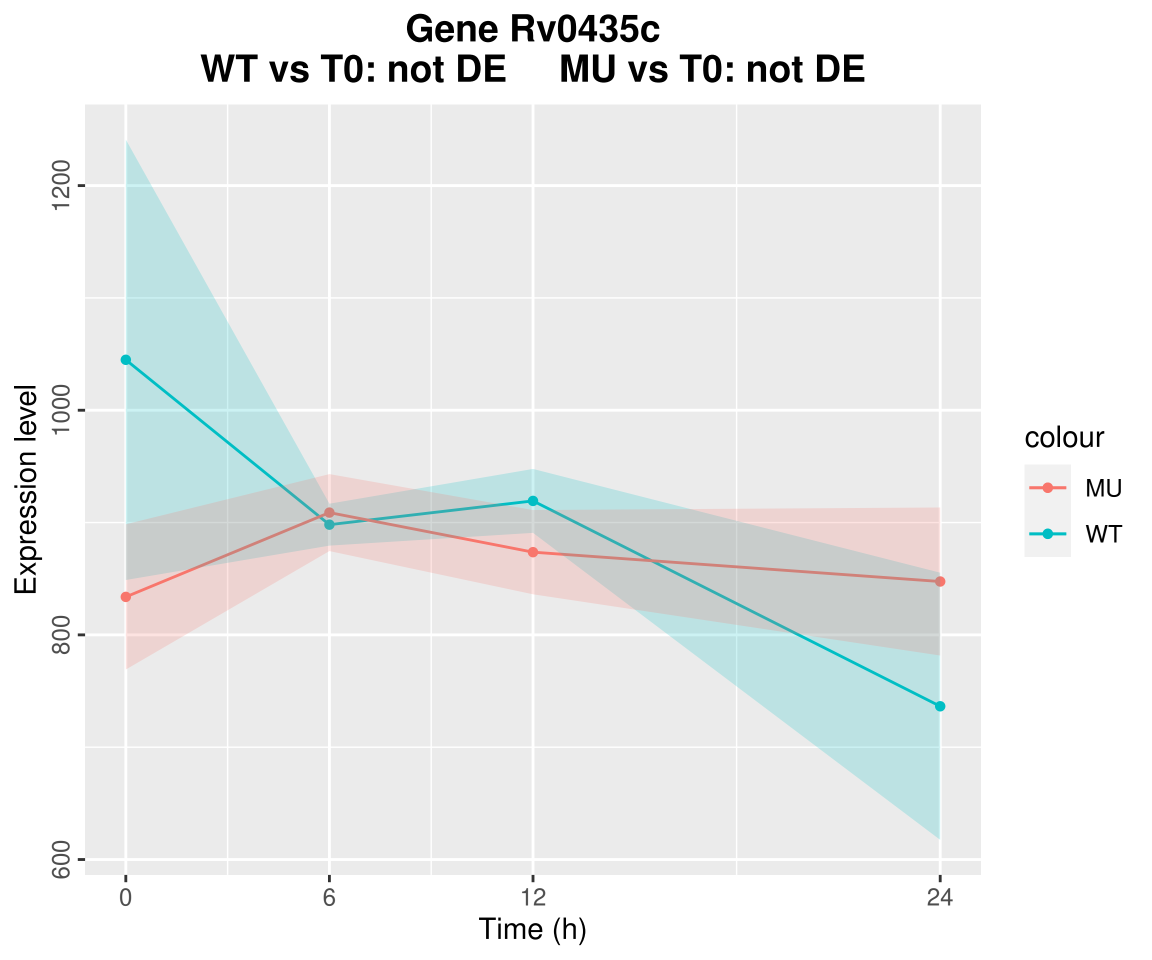

Gene Rv0435c
WT vs T0: not DE   MU vs T0: not DE
Expression level
1200
1000
800
600
0
6
12
24
Time (h)
colour
MU
WT

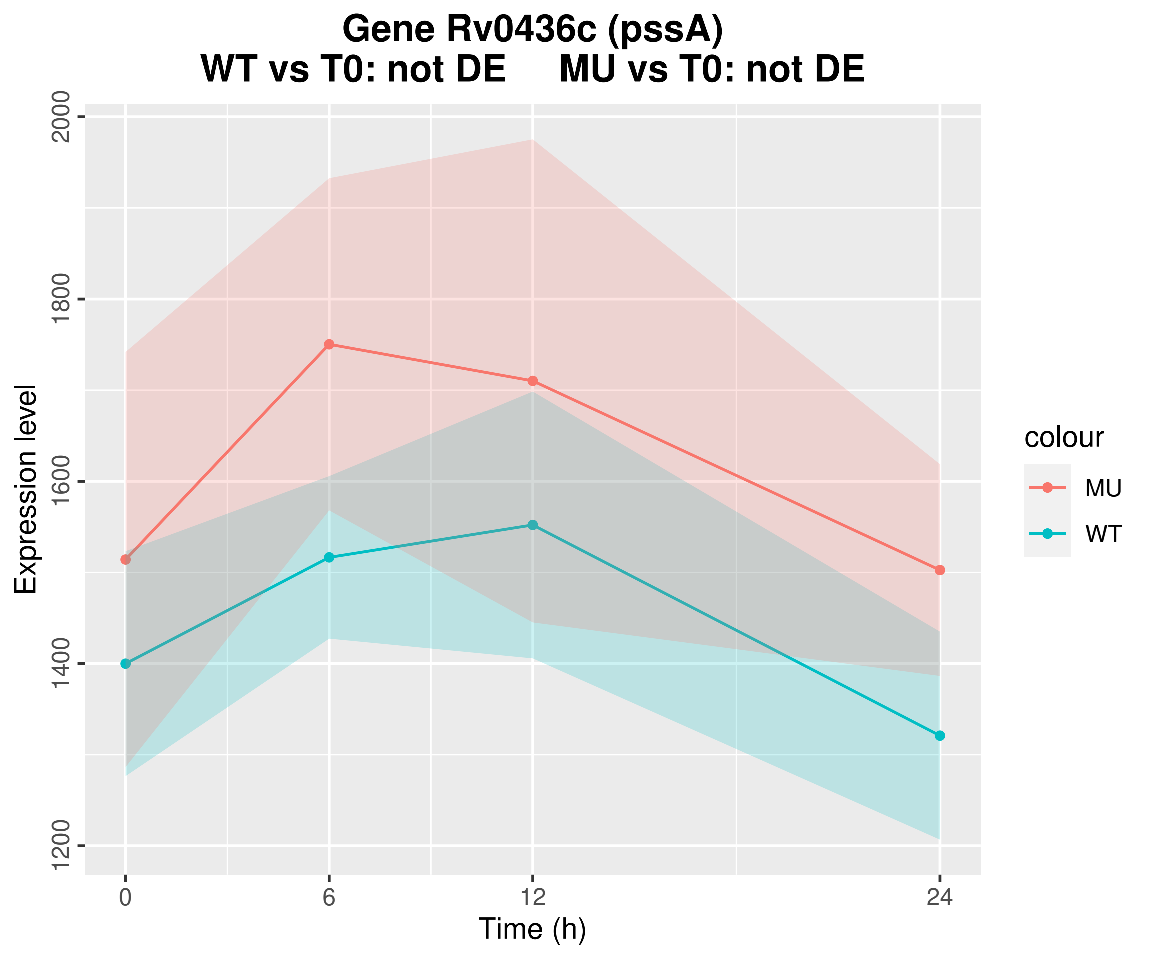

Gene Rv0436c (pssA)
WT vs T0: not DE MU vs T0: not DE
Expression level
2000
1800
1600
1400
1200
0
6
12
24
Time (h)
colour
MU
WT

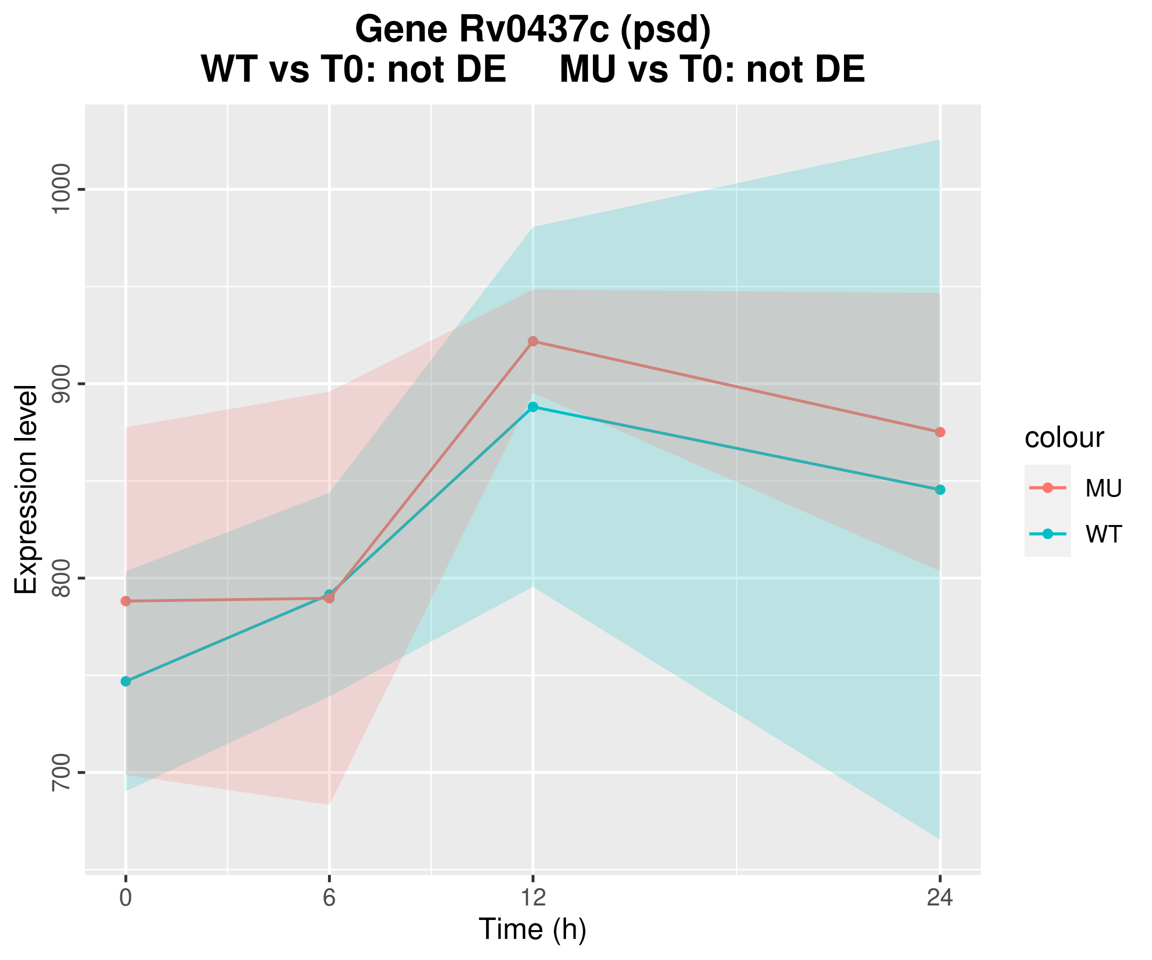

Gene Rv0437c (psd)
WT vs T0: not DE MU vs T0: not DE
Expression level
1000
900
800
700
0
6
12
24
Time (h)
colour
MU
WT

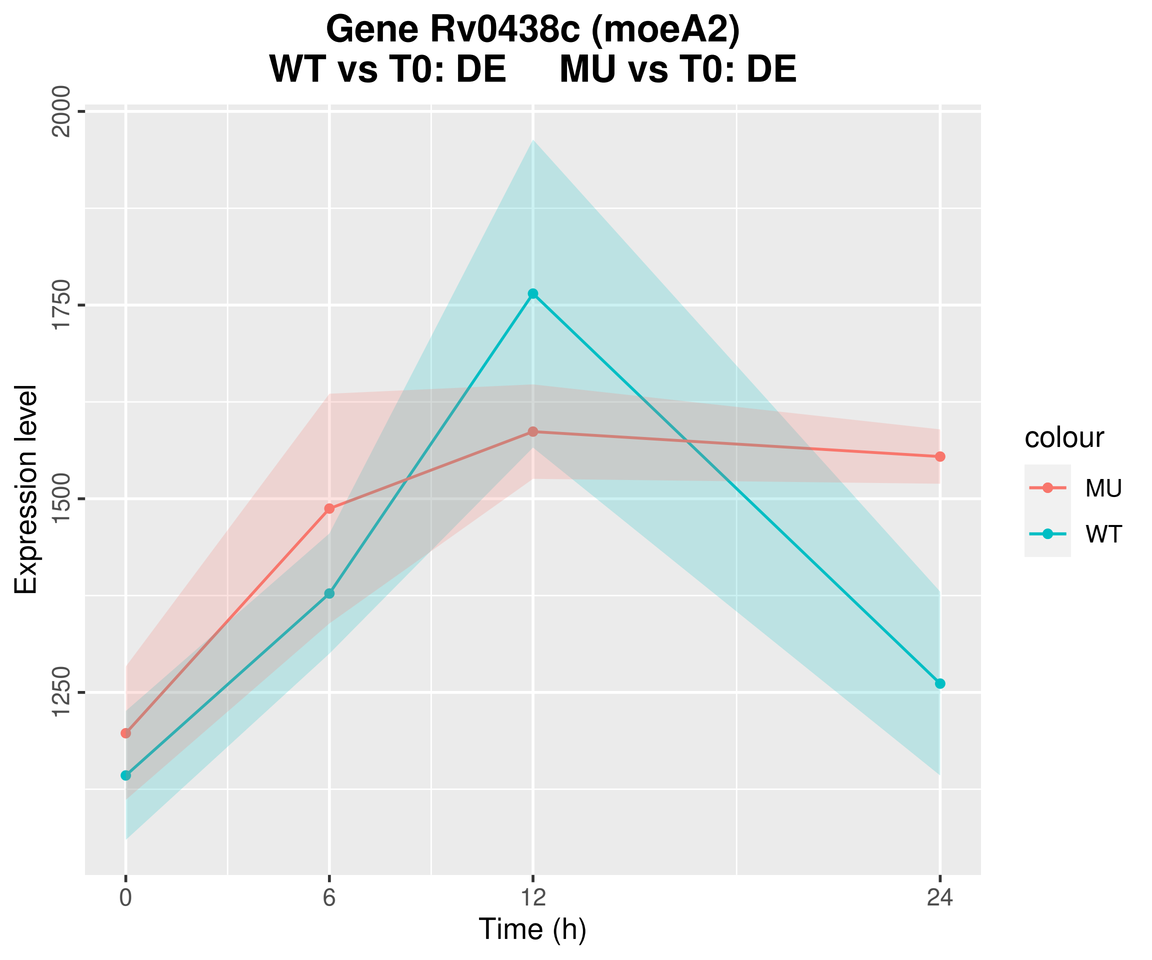
Gene Rv0438c (moeA2)
WT vs T0: DE MU vs T0: DE
Expression level
2000
1750
1500
1250
0
6
12
24
Time (h)
colour
MU
WT

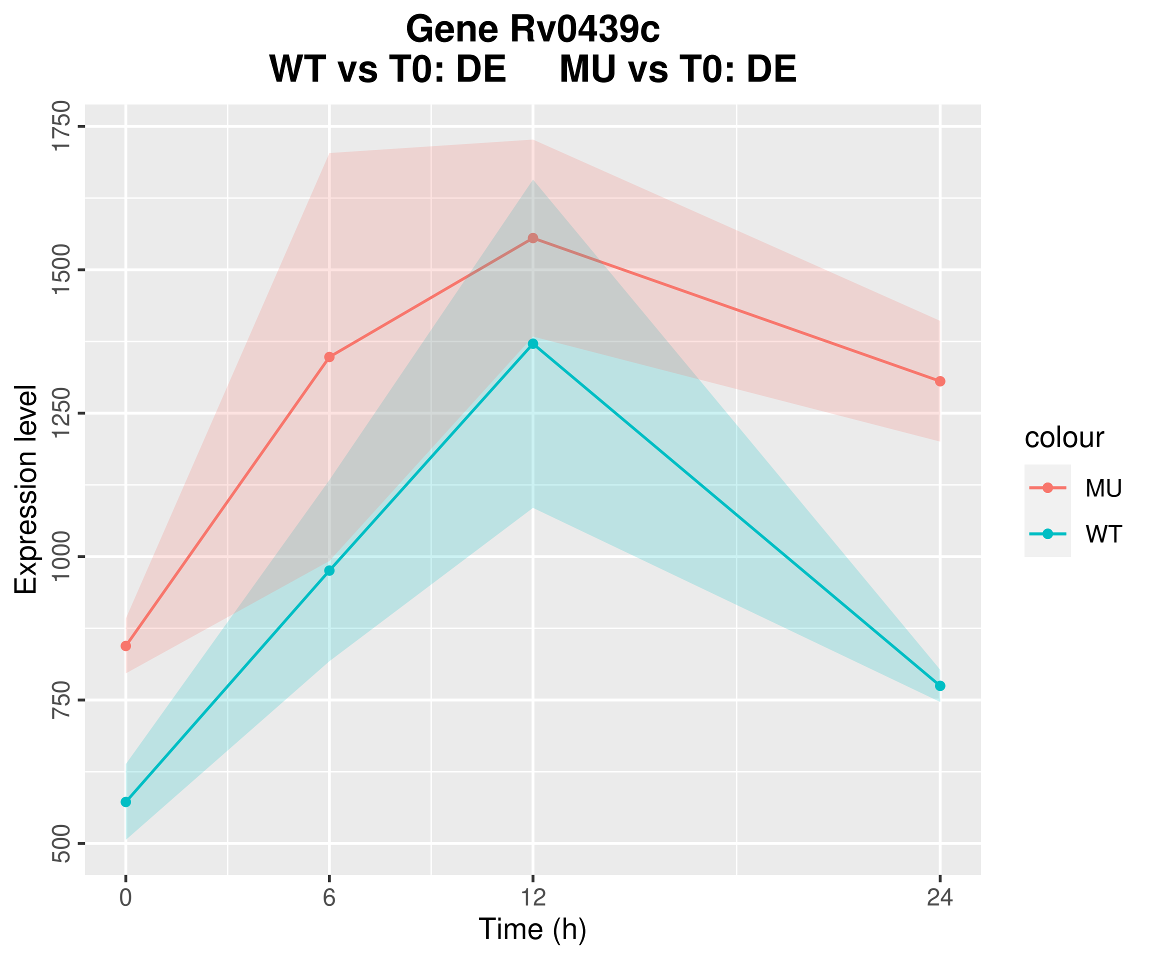

Gene Rv0439c
WT vs T0: DE   MU vs T0: DE
Expression level
1750
1500
1250
1000
750
500
0
6
12
24
Time (h)
colour
MU
WT

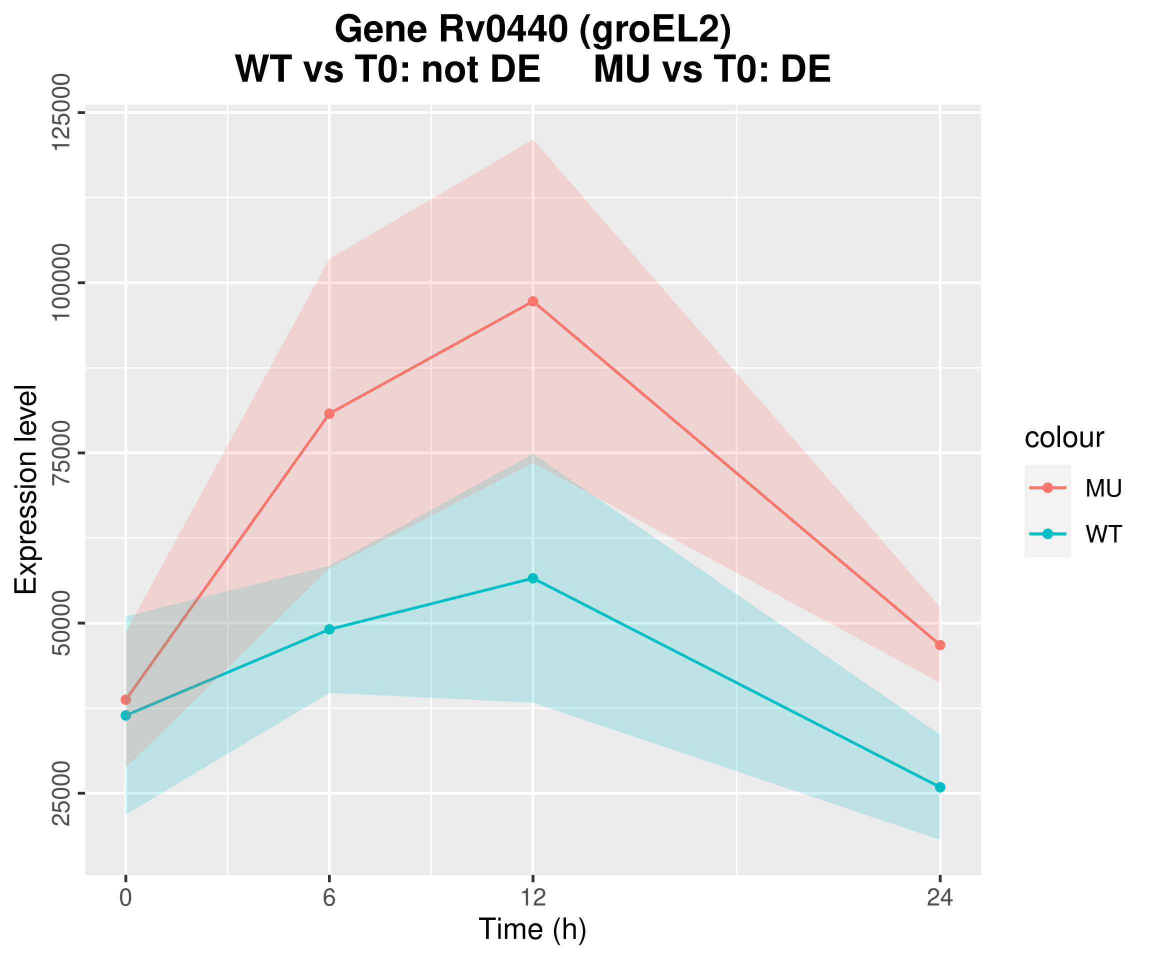
Gene Rv0440 (groEL2)
WT vs T0: not DE MU vs T0: DE
Expression level
125000
100000
75000
50000
25000
0
6
12
24
Time (h)
colour
MU
WT

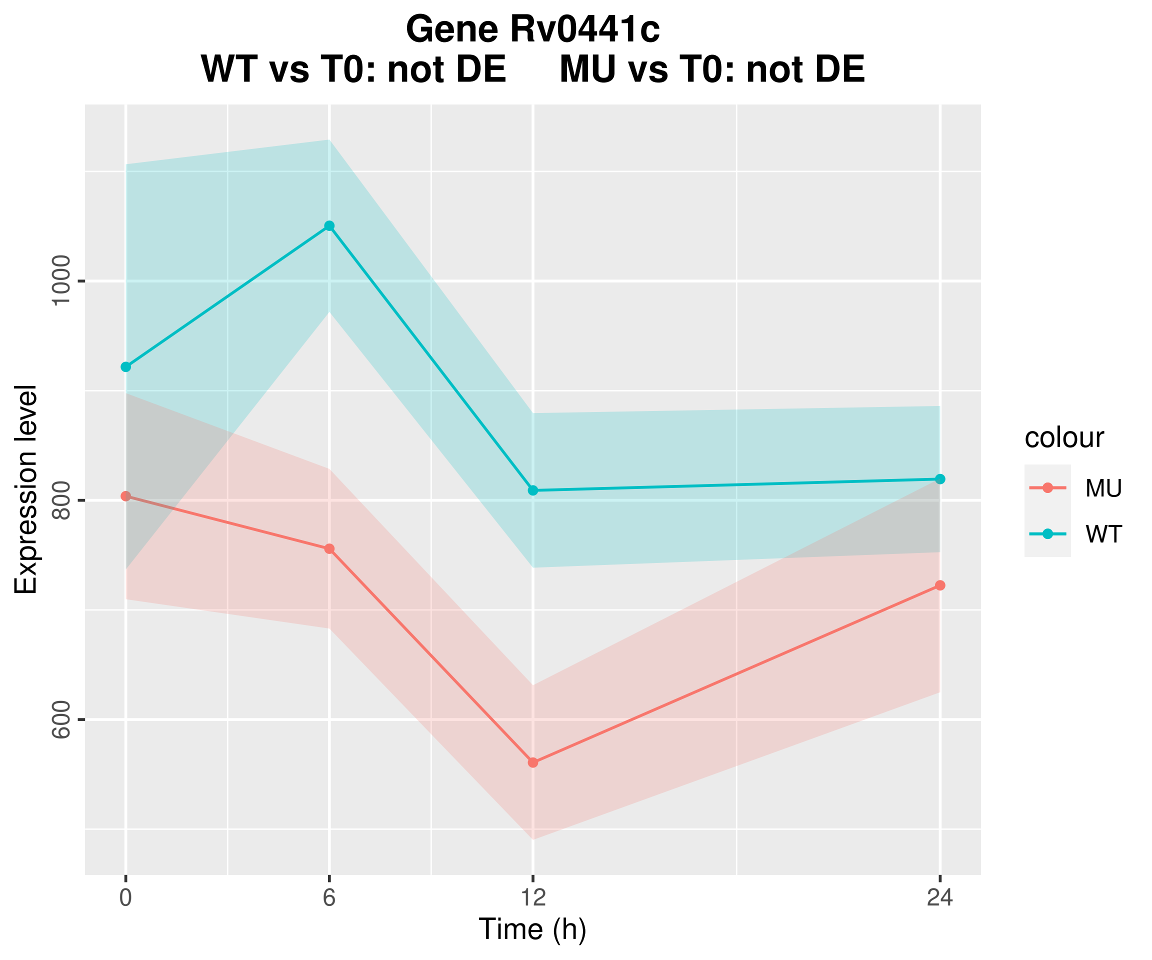
Gene Rv0441c
WT vs T0: not DE   MU vs T0: not DE
Expression level
1000
800
600
0
6
12
24
Time (h)
colour
MU
WT

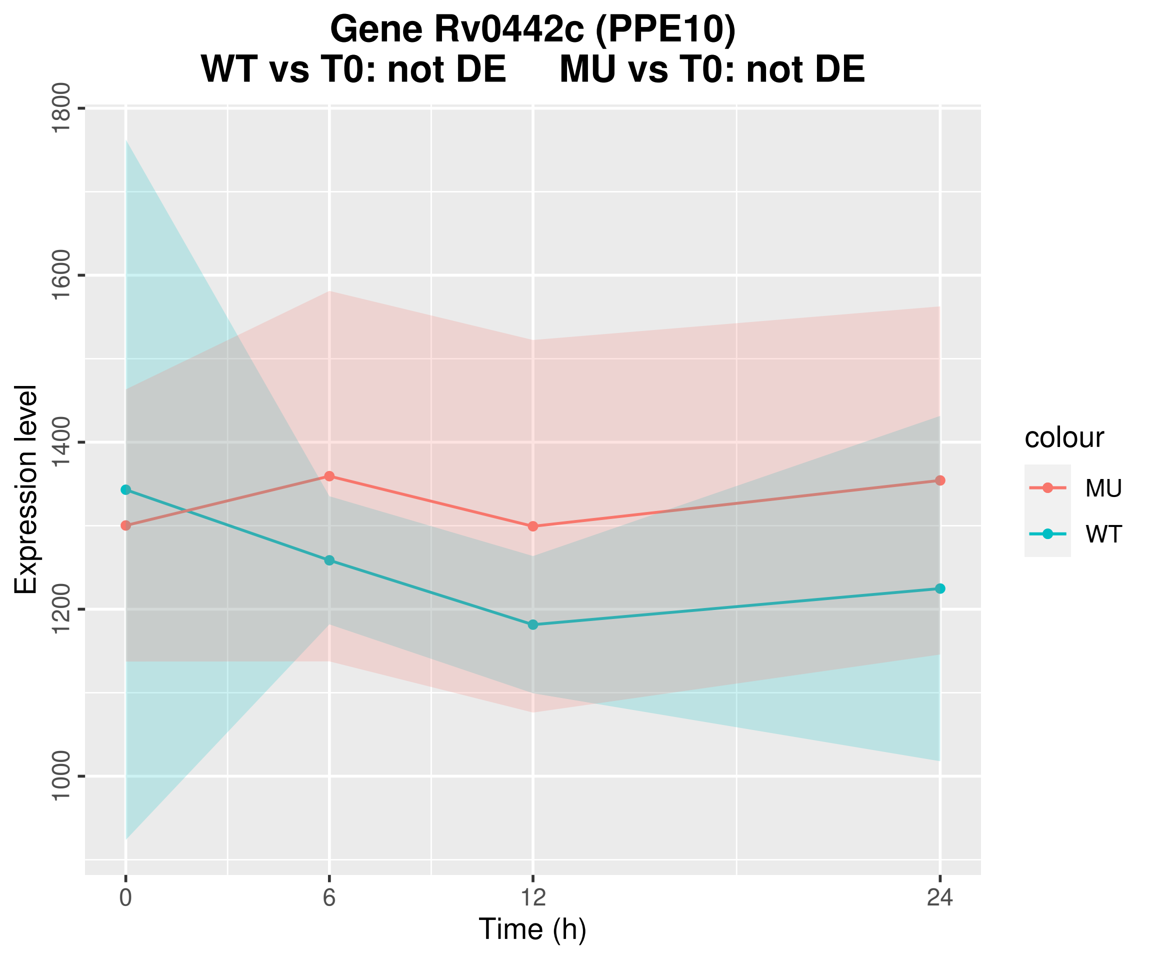

Gene Rv0442c (PPE10)
WT vs T0: not DE MU vs T0: not DE
1800
1600
1400
1200
1000
Expression level
0
6
12
24
Time (h)
colour
MU
WT

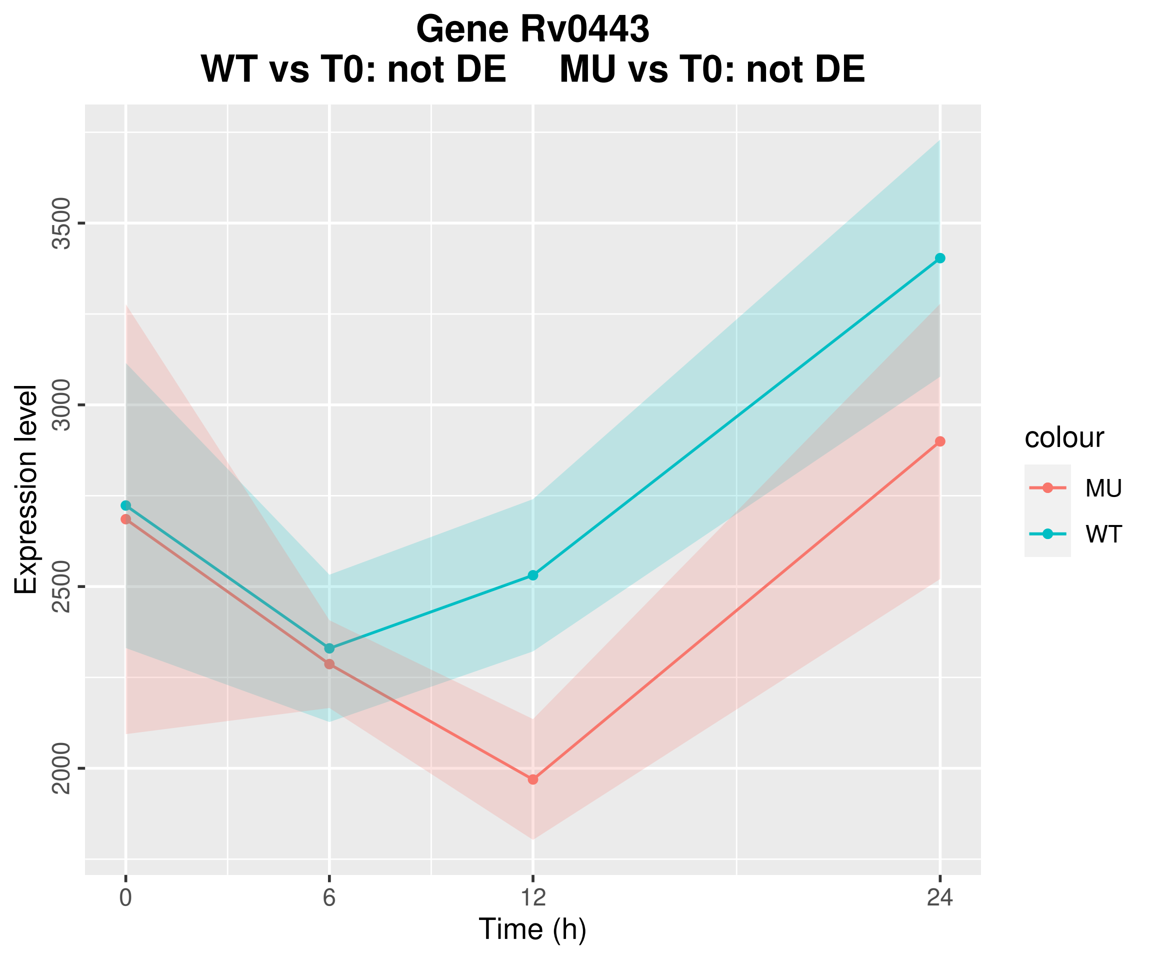

Gene Rv0443
WT vs T0: not DE MU vs T0: not DE
Expression level
2000
2500
3000
3500
0
6
12
24
Time (h)
colour
MU
WT

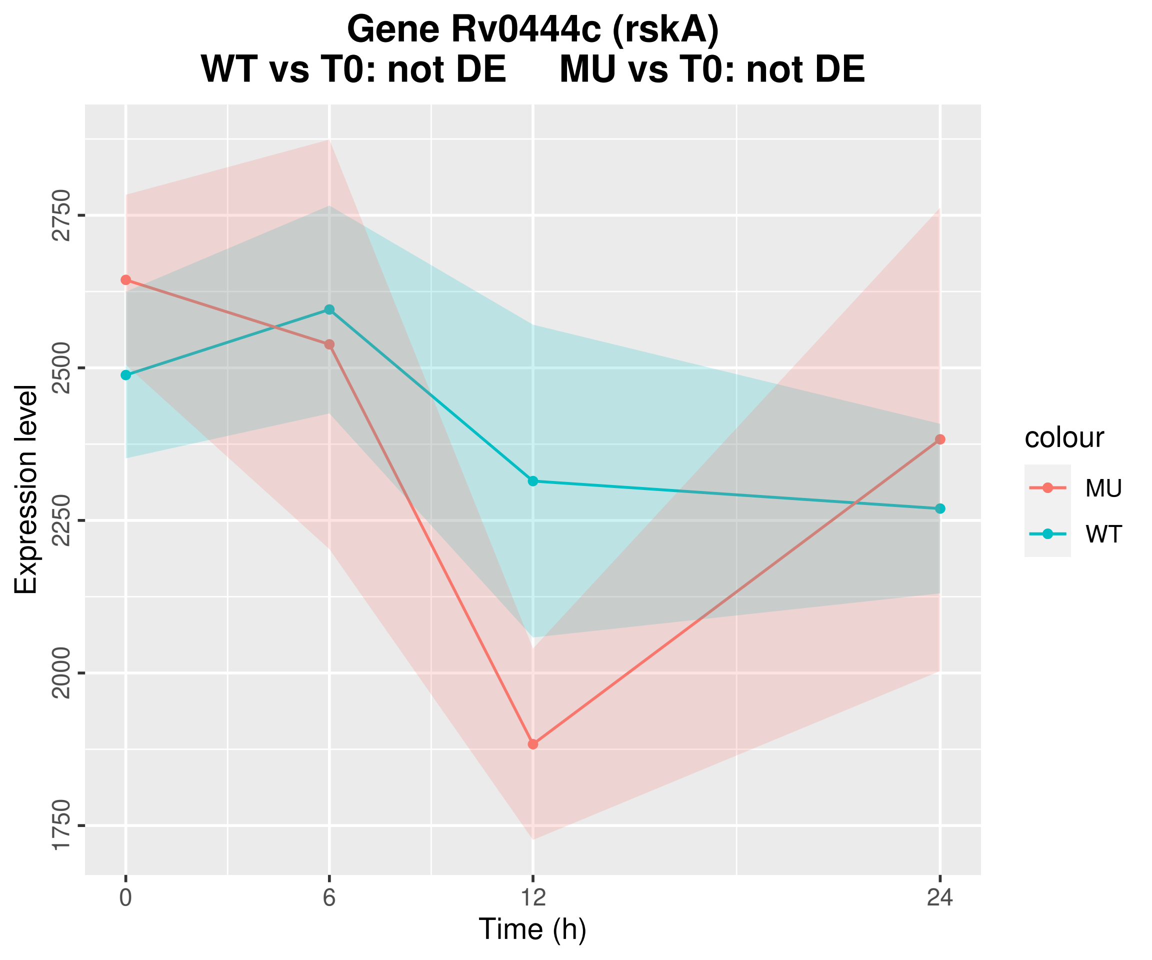

Gene Rv0444c (rskA)
WT vs T0: not DE   MU vs T0: not DE
Expression level
2750
2500
2250
2000
1750
0
6
12
24
Time (h)
colour
MU
WT

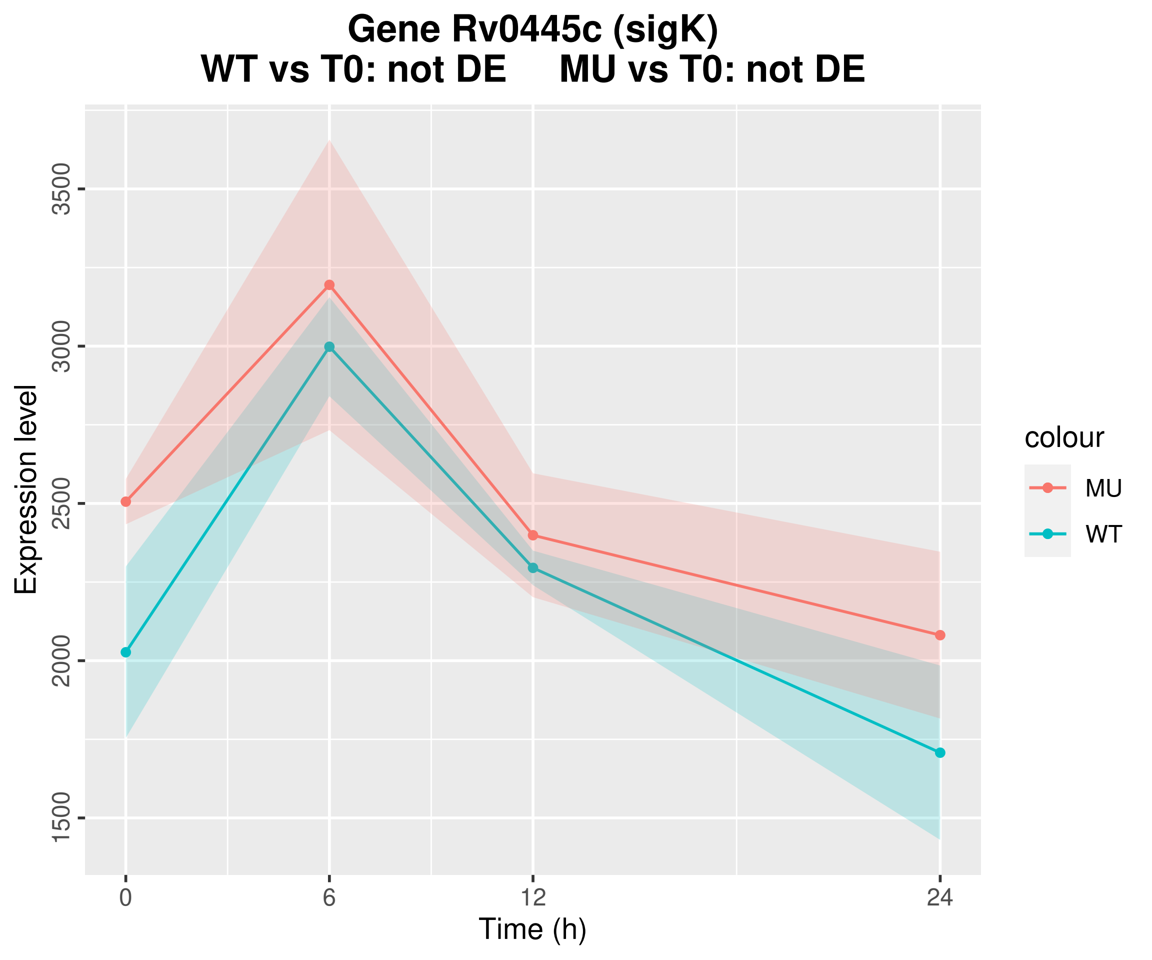

Gene Rv0445c (sigK)
WT vs T0: not DE   MU vs T0: not DE
Expression level
3500
3000
2500
2000
1500
0
6
12
24
Time (h)
colour
MU
WT

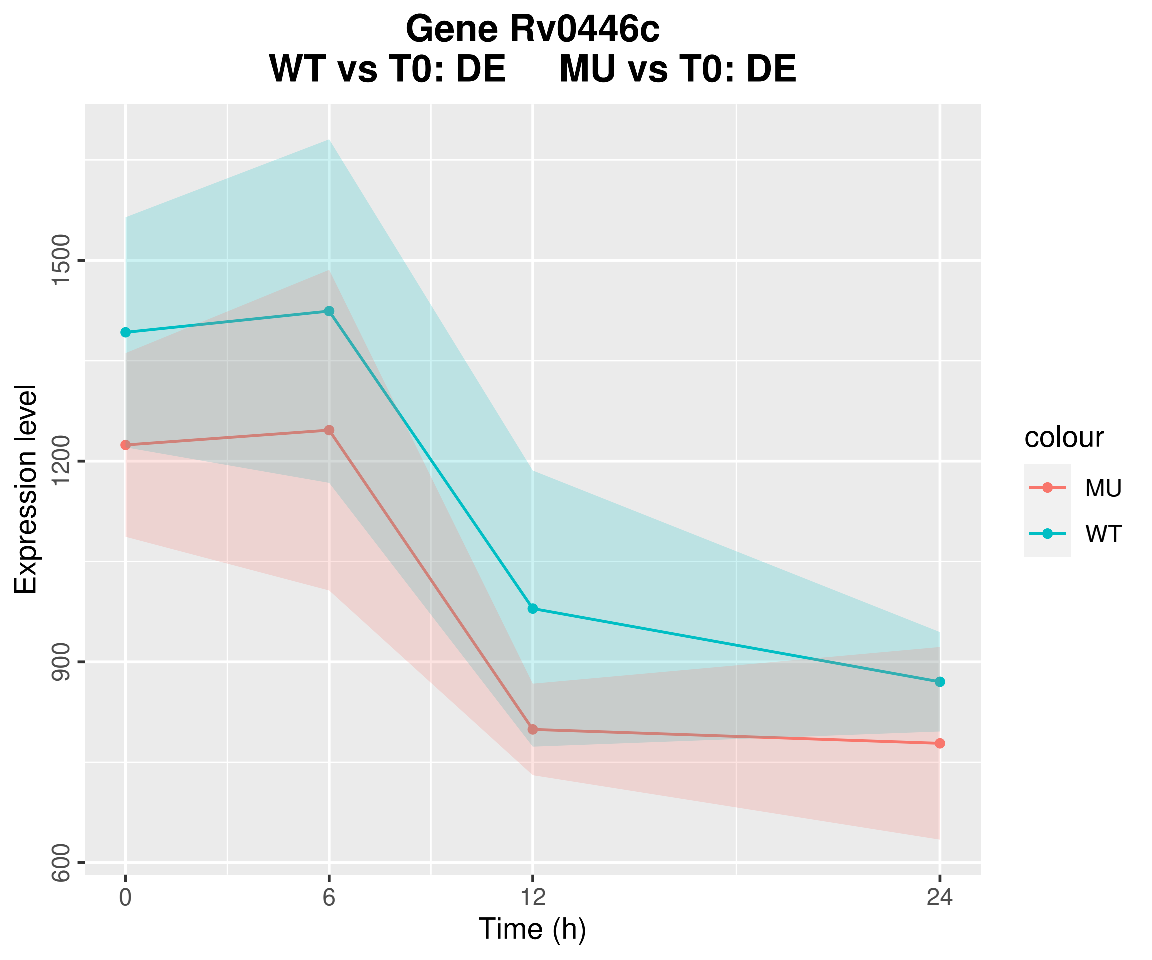

Gene Rv0446c
WT vs T0: DE   MU vs T0: DE
Expression level
1500
1200
900
600
0
6
12
24
Time (h)
colour
MU
WT

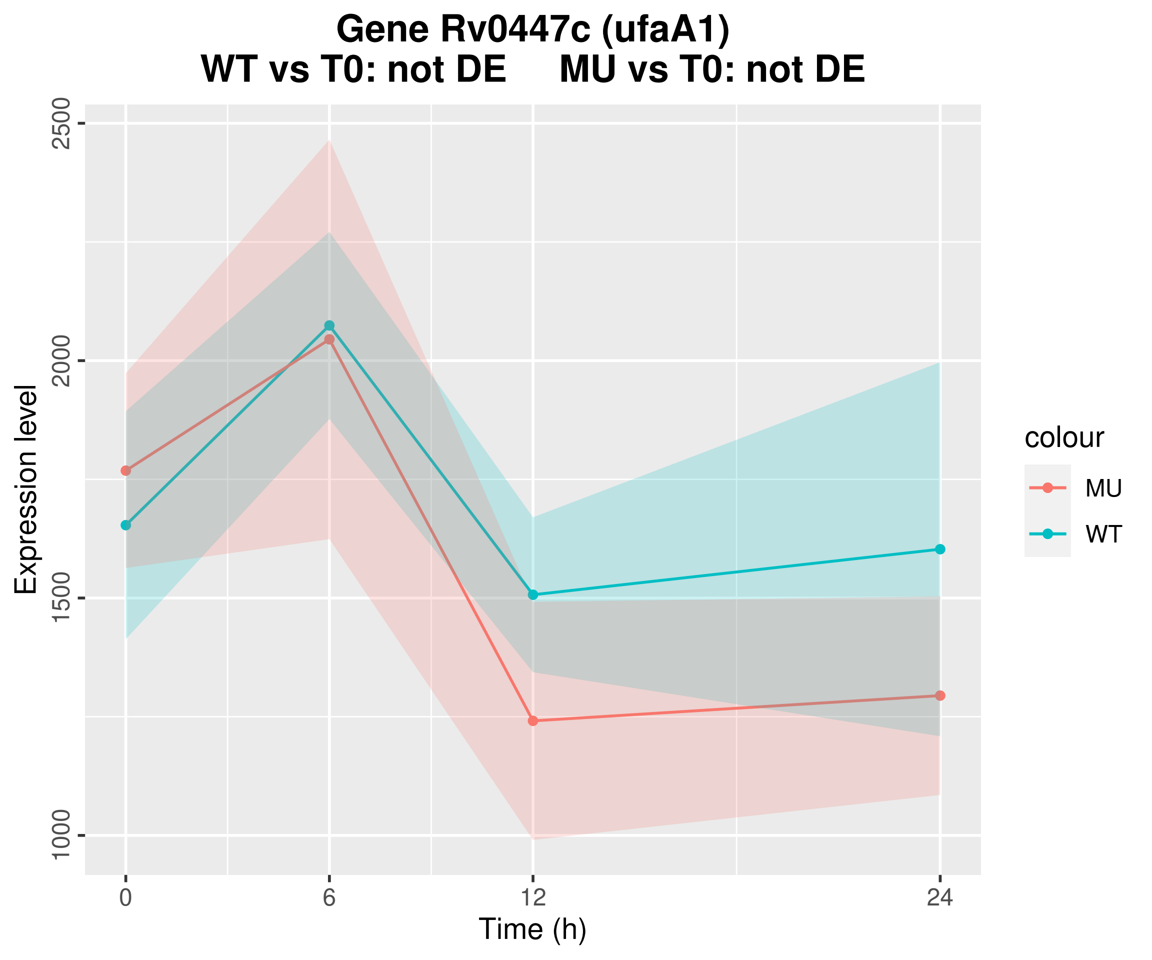
Gene Rv0447c (ufaA1)
WT vs T0: not DE   MU vs T0: not DE
Expression level
2500
2000
1500
1000
0
6
12
24
Time (h)
colour
MU
WT

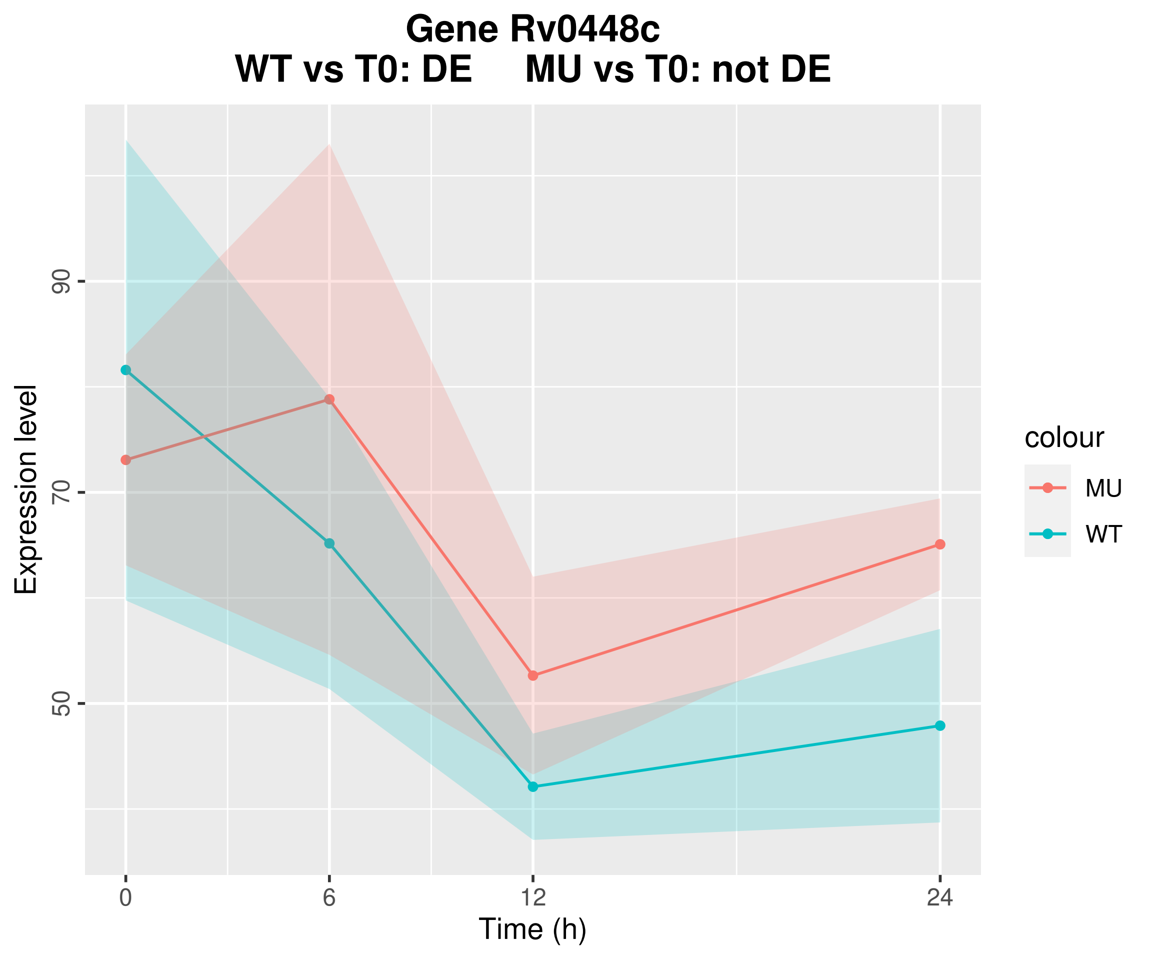

Gene Rv0448c
WT vs T0: DE   MU vs T0: not DE
Expression level
90
70
50
0
6
12
24
Time (h)
colour
MU
WT

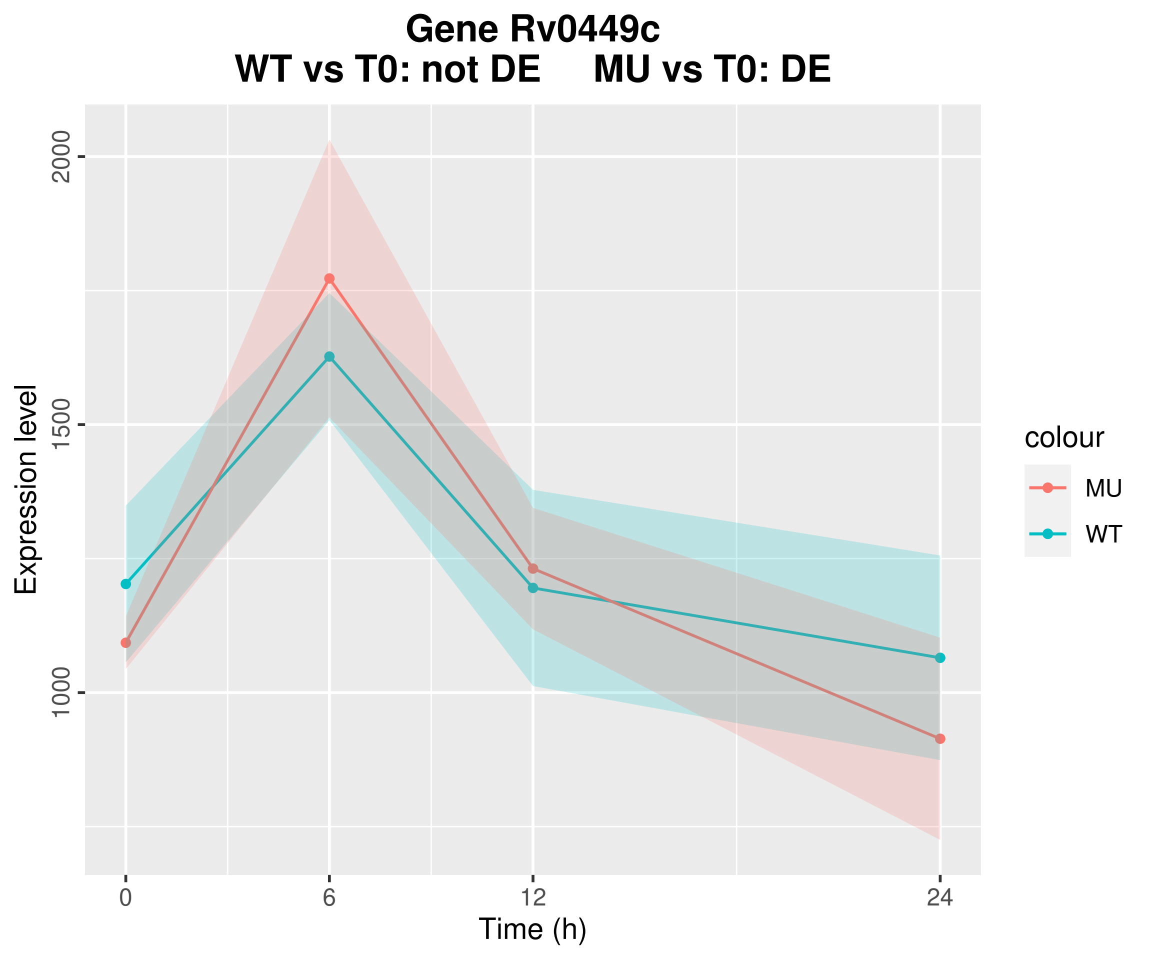
Gene Rv0449c
WT vs T0: not DE MU vs T0: DE
Expression level
2000
1500
1000
0
6
12
24
Time (h)
colour
MU
WT

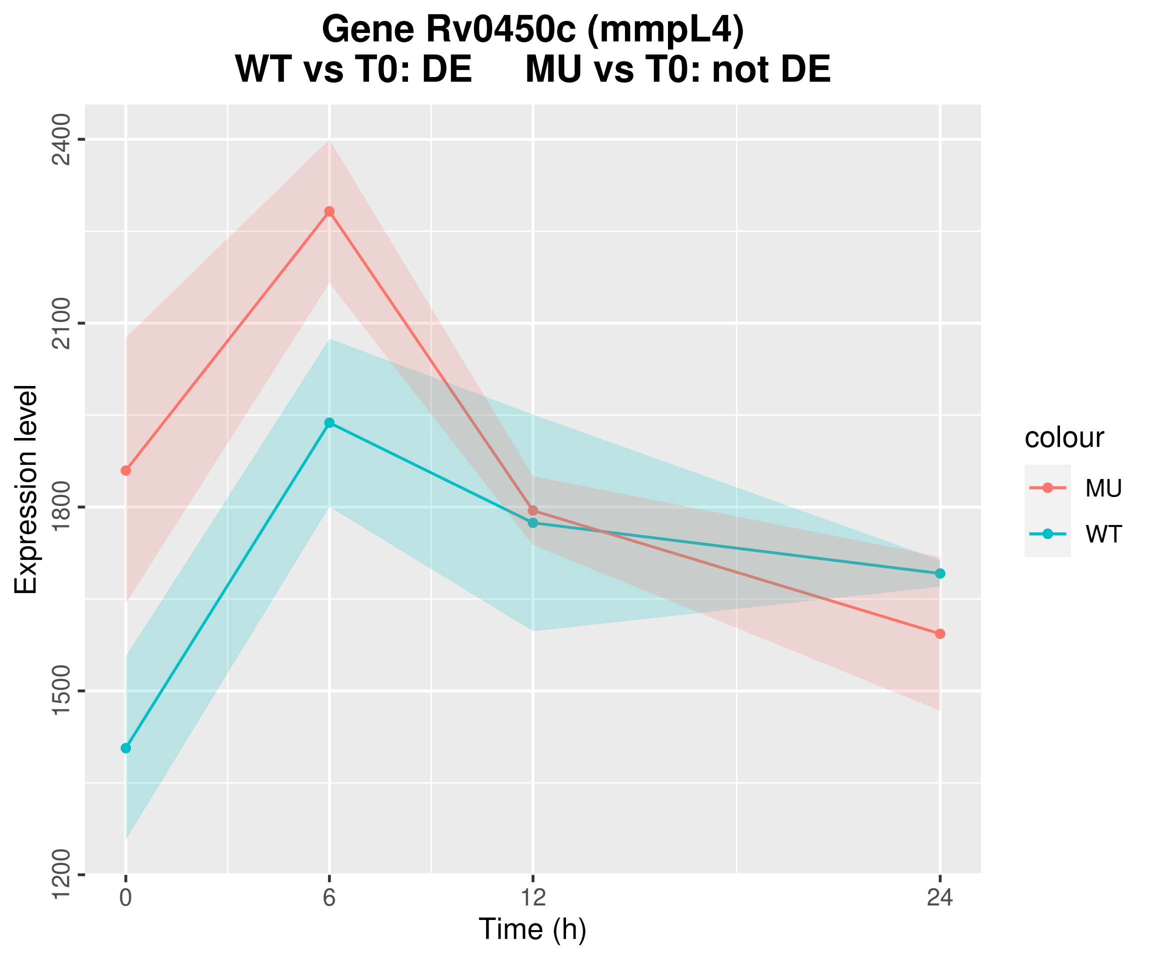

Gene Rv0450c (mmpL4)
WT vs T0: DE MU vs T0: not DE
Expression level
2400
2100
1800
1500
1200
0
6
12
24
Time (h)
colour
MU
WT

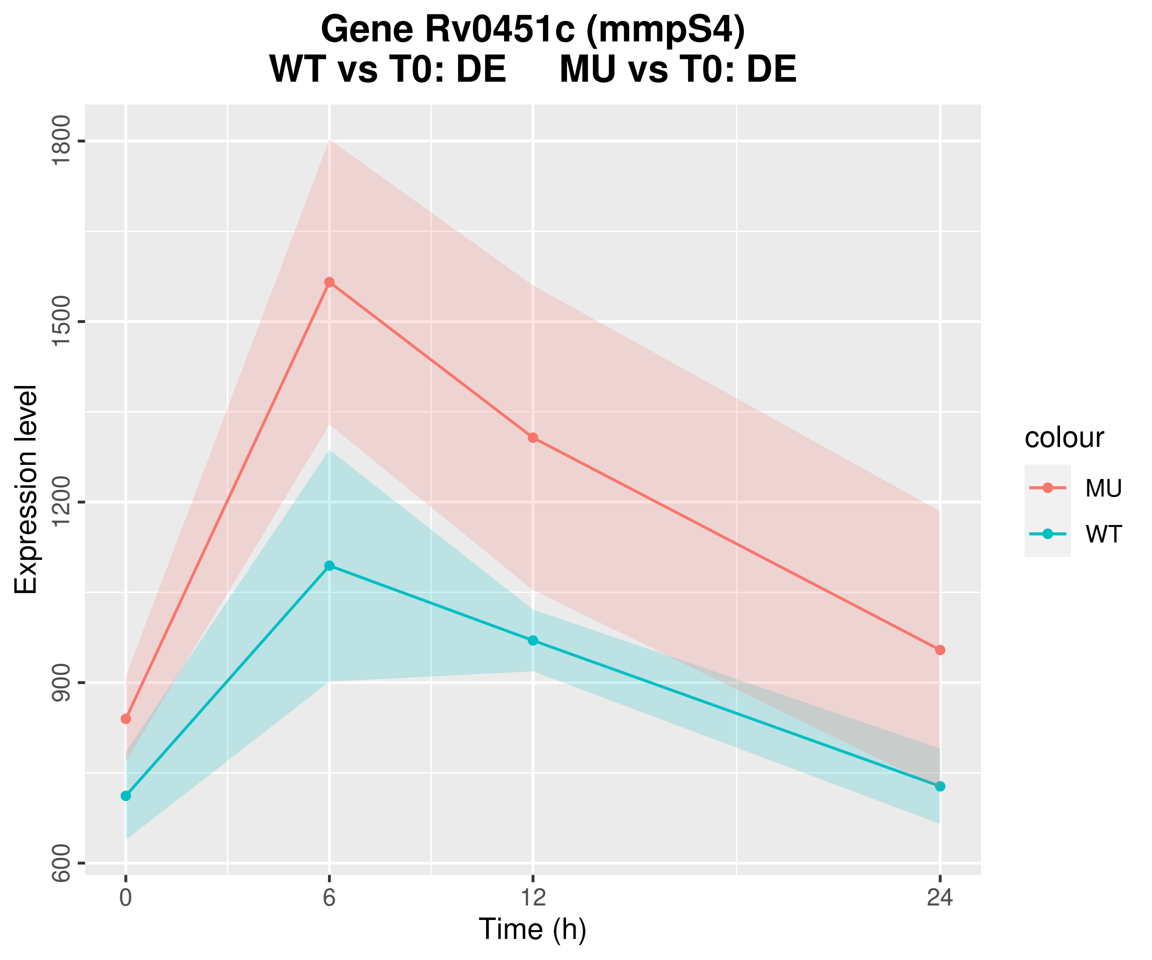

Gene Rv0451c (mmpS4)
WT vs T0: DE   MU vs T0: DE
Expression level
1800
1500
1200
900
600
0
6
12
24
Time (h)
colour
MU
WT

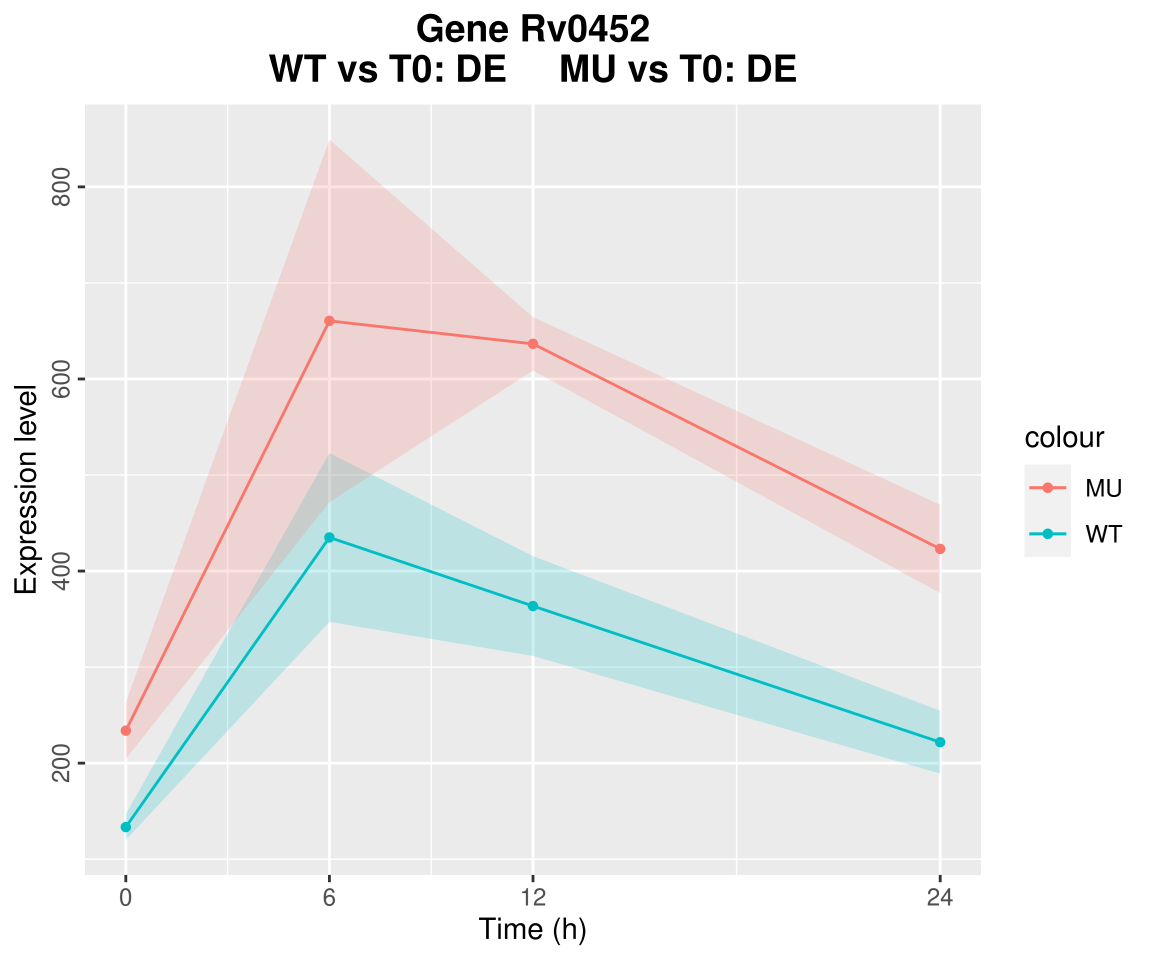
Gene Rv0452
WT vs T0: DE   MU vs T0: DE
Expression level
800
600
400
200
0
6
12
24
Time (h)
colour
MU
WT

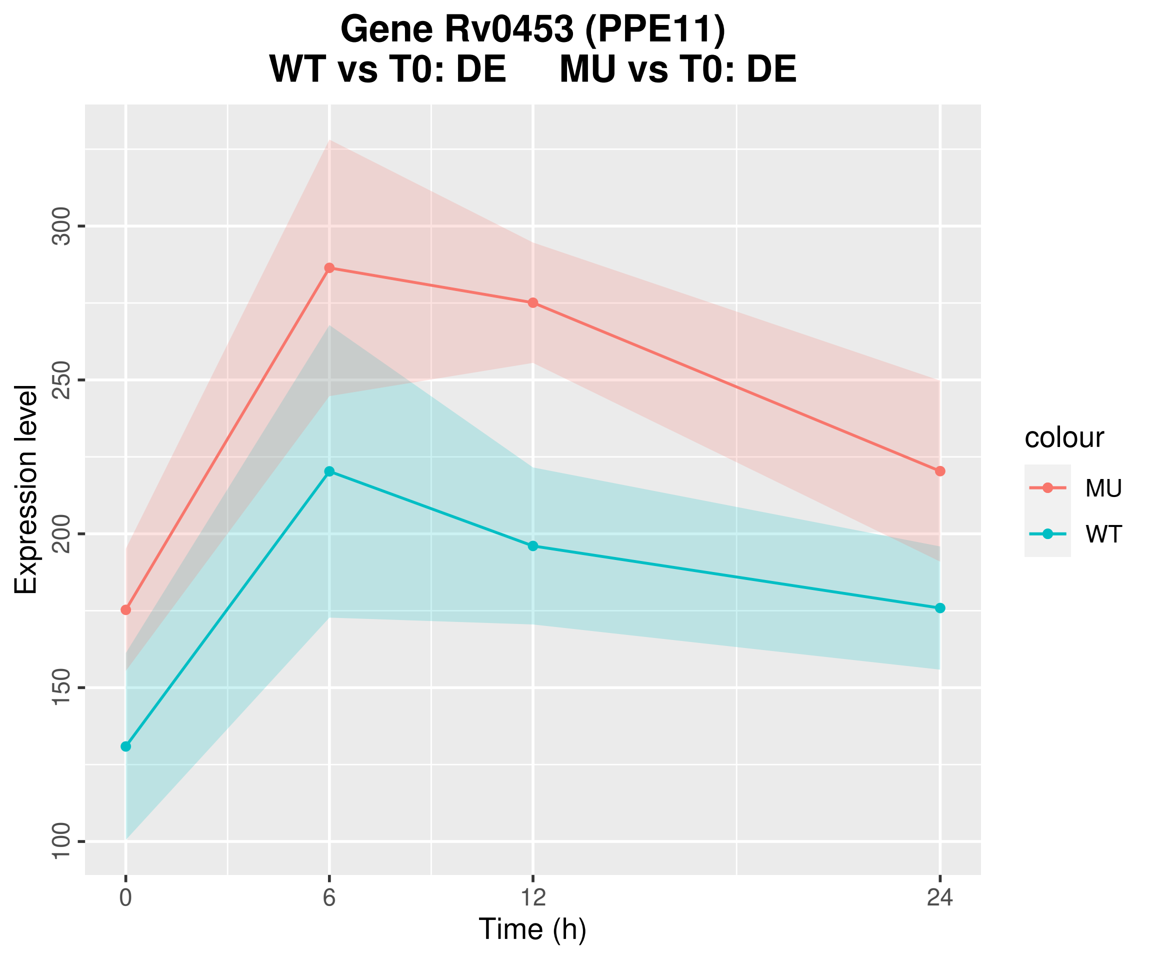
Gene Rv0453 (PPE11)
WT vs T0: DE   MU vs T0: DE
Expression level
100
150
200
250
300
0
6
12
24
Time (h)
colour
MU
WT

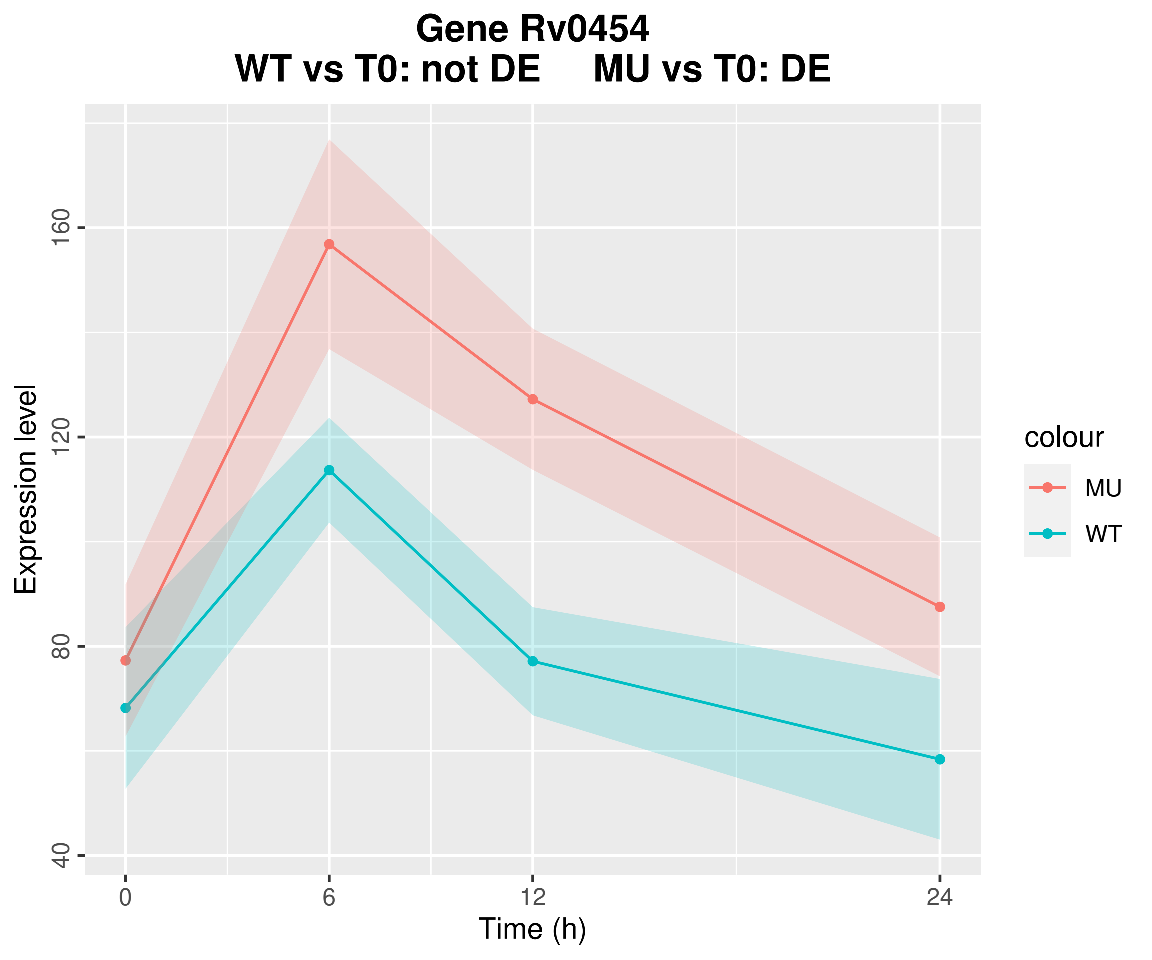

Gene Rv0454
WT vs T0: not DE MU vs T0: DE
Expression level
160
120
80
40
0
6
12
24
Time (h)
colour
MU
WT

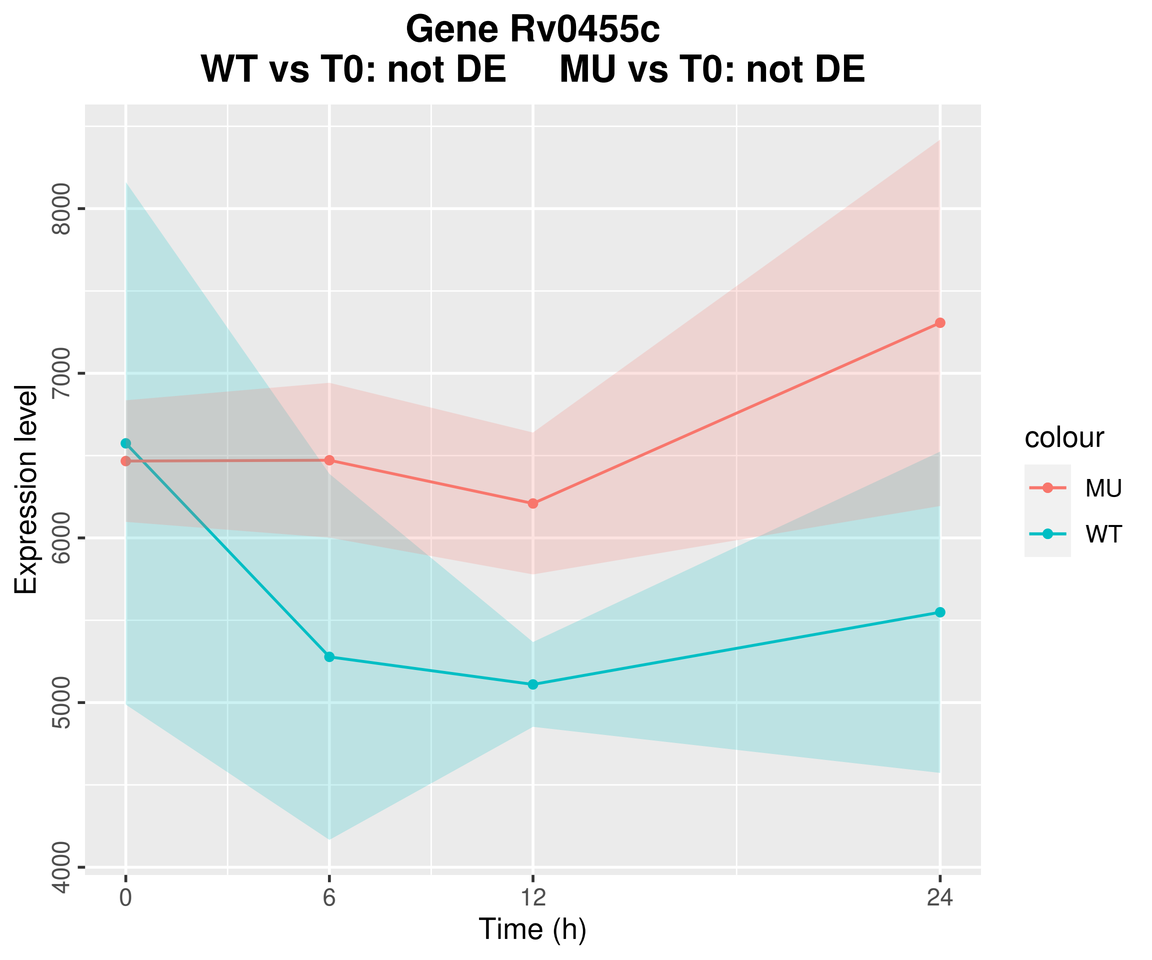
Gene Rv0455c
WT vs T0: not DE   MU vs T0: not DE
Expression level
8000
7000
6000
5000
4000
0
6
12
24
Time (h)
colour
MU
WT

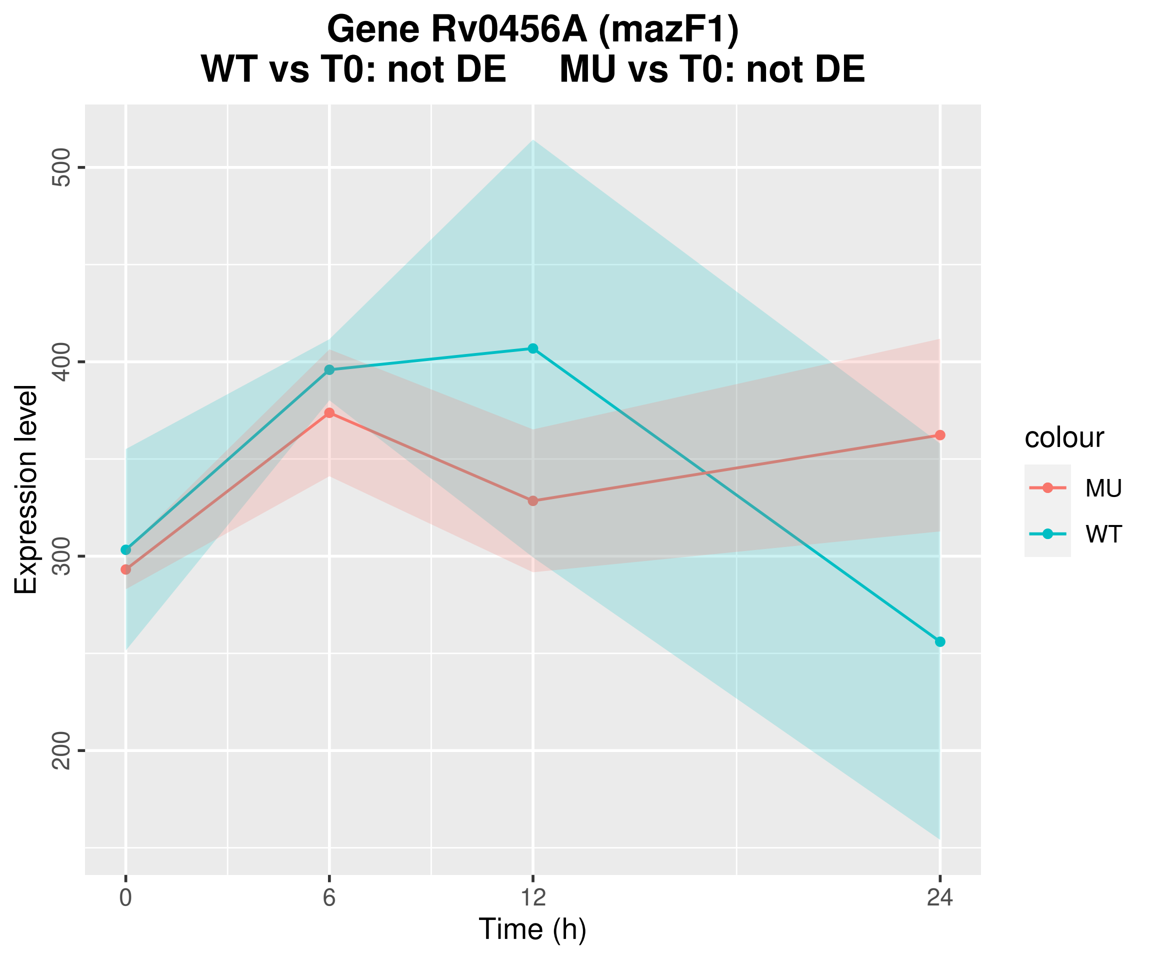

Gene Rv0456A (mazF1)
WT vs T0: not DE   MU vs T0: not DE
500
400
300
200
Expression level
0
6
12
24
Time (h)
colour
MU
WT

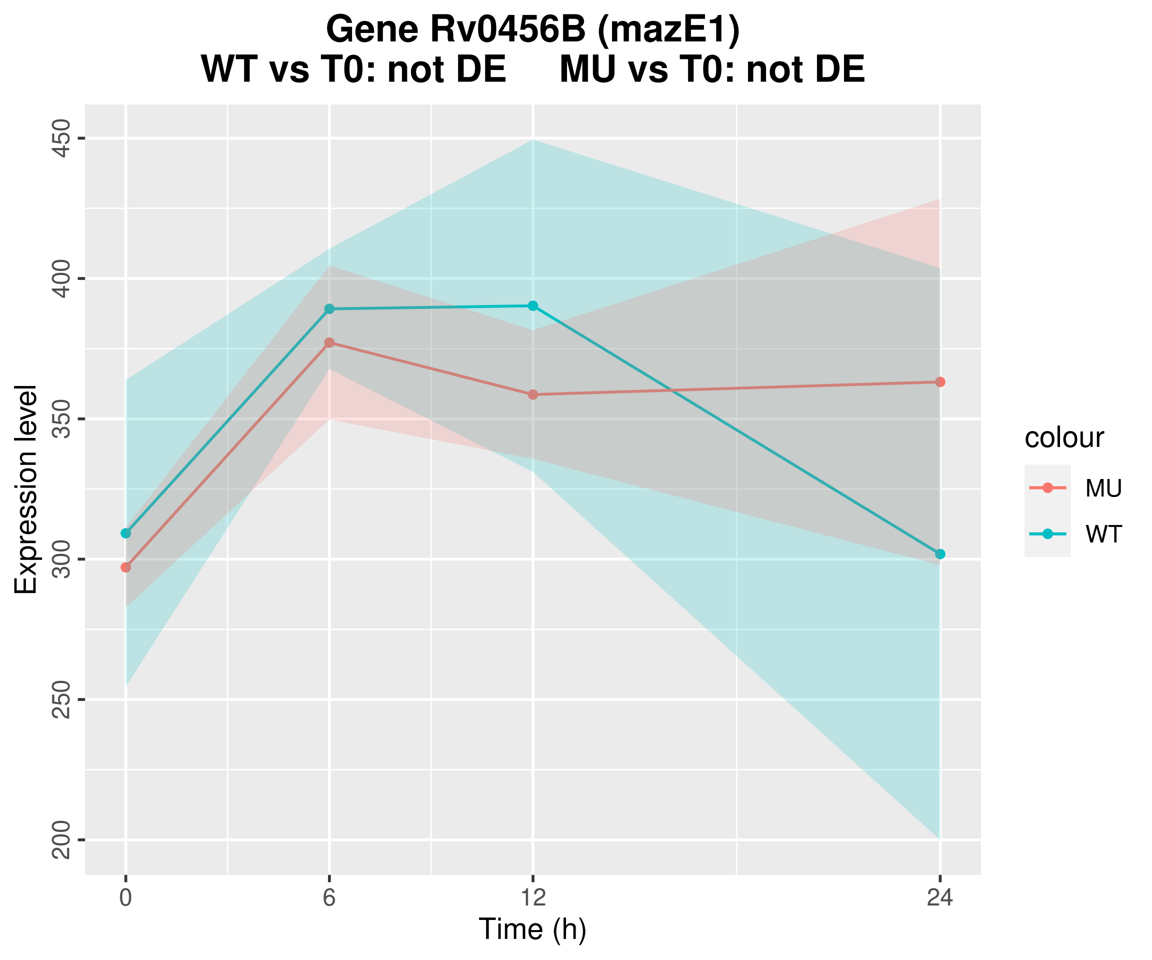

Gene Rv0456B (mazE1)
WT vs T0: not DE MU vs T0: not DE
Expression level
450
400
350
300
250
200
0
6
12
24
Time (h)
colour
MU
WT

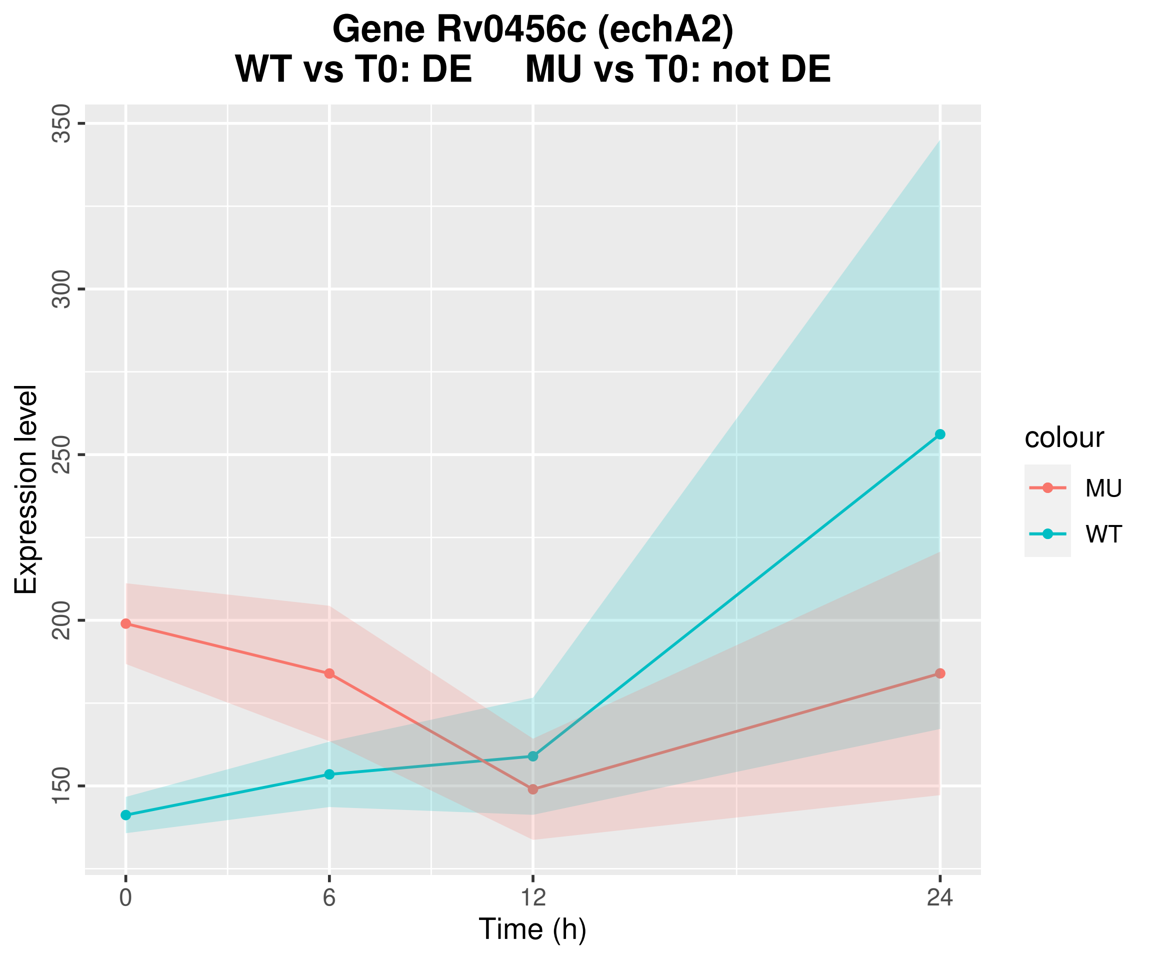

Gene Rv0456c (echA2)
WT vs T0: DE   MU vs T0: not DE
Expression level
350
300
250
200
150
0
6
12
24
Time (h)
colour
MU
WT

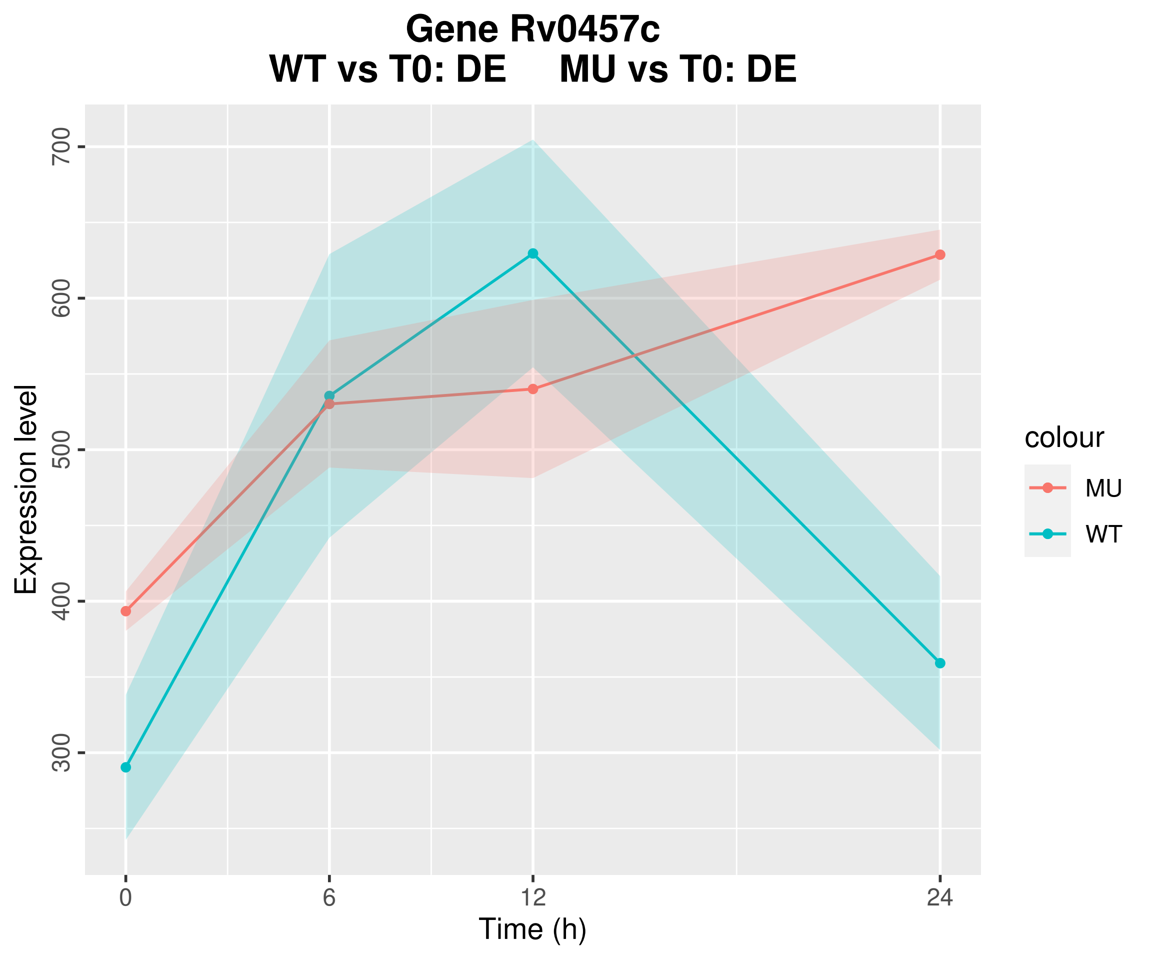
Gene Rv0457c
WT vs T0: DE MU vs T0: DE
Expression level
700
600
500
400
300
0
6
12
24
Time (h)
colour
MU
WT

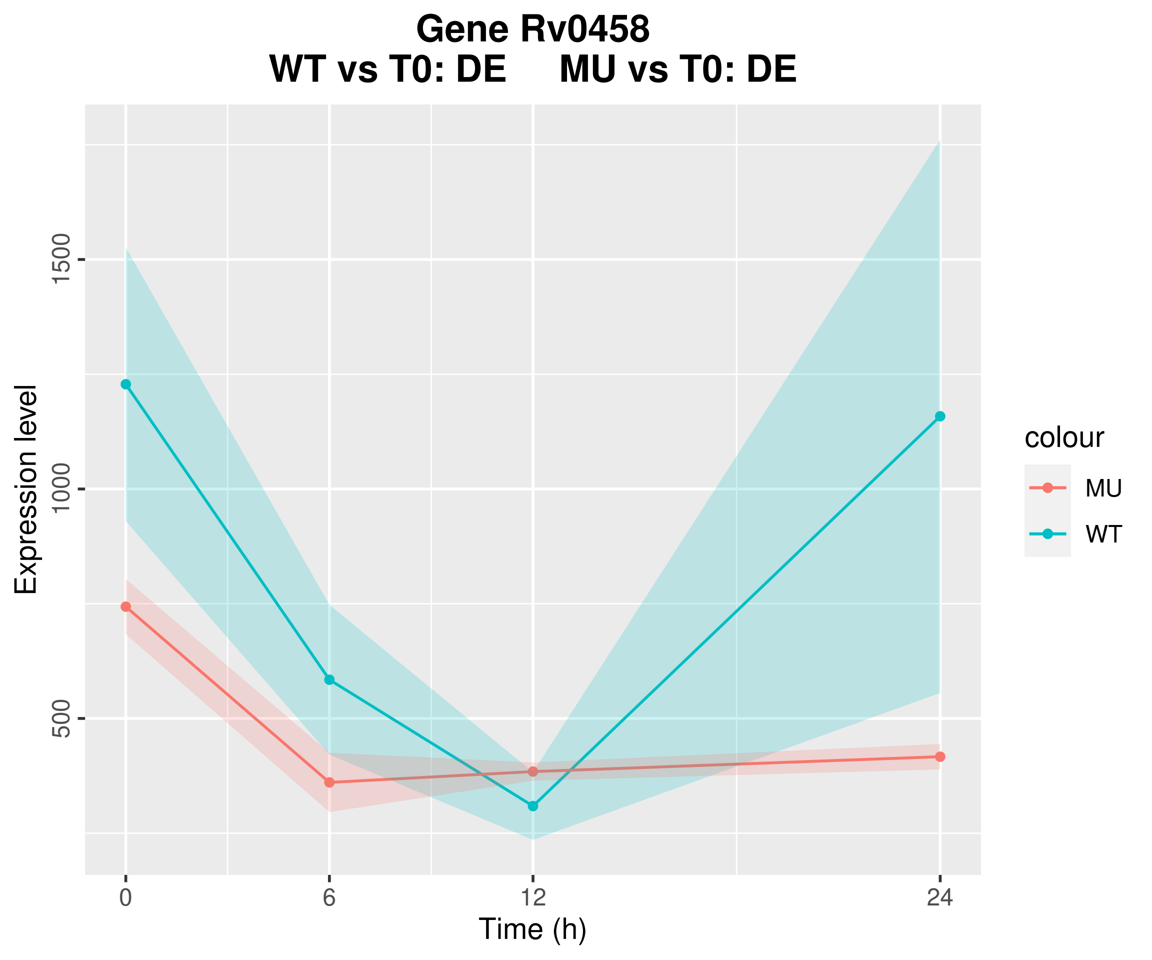

Gene Rv0458
WT vs T0: DE MU vs T0: DE
Expression level
1500
1000
500
0
6
12
24
Time (h)
colour
MU
WT

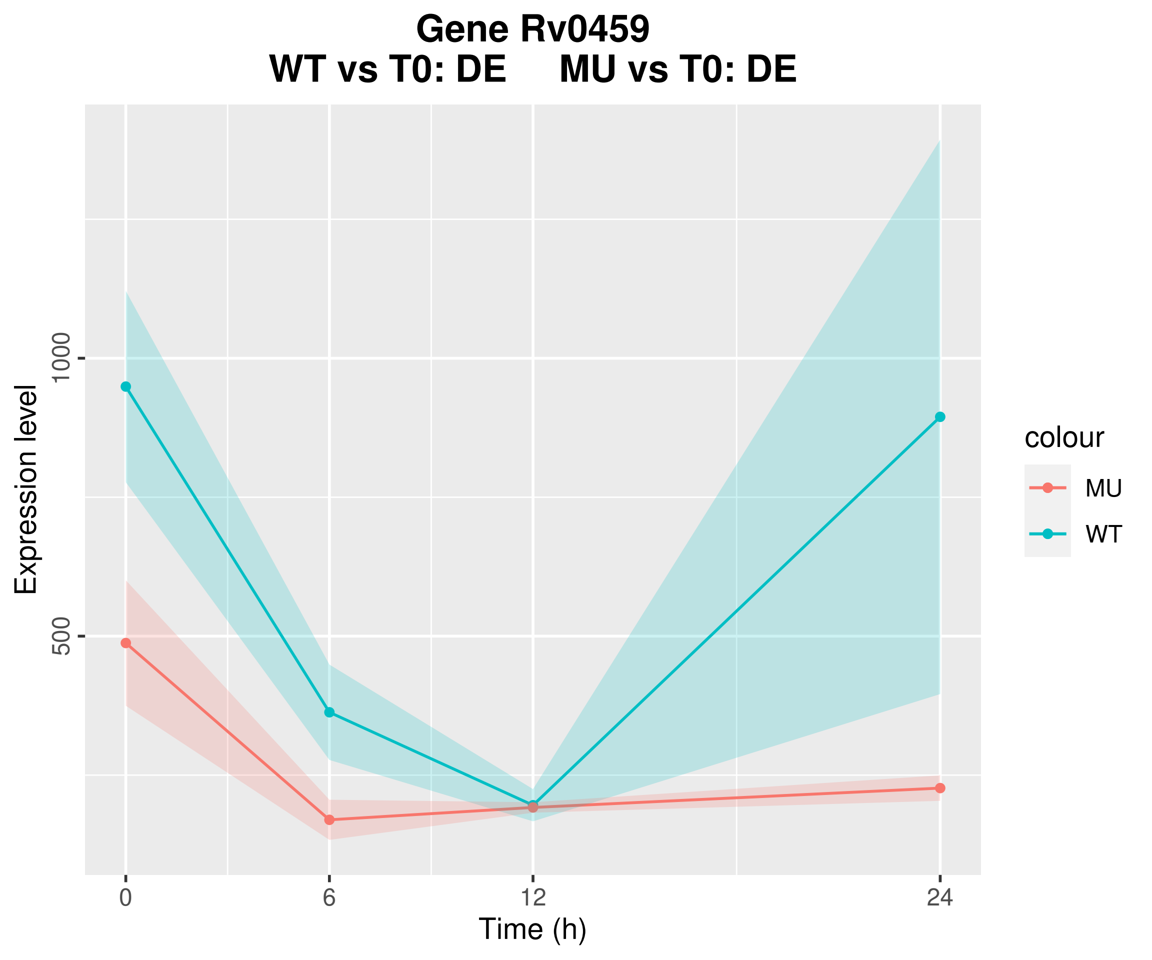

Gene Rv0459
WT vs T0: DE MU vs T0: DE
Expression level
1000
500
0
6
12
24
Time (h)
colour
MU
WT

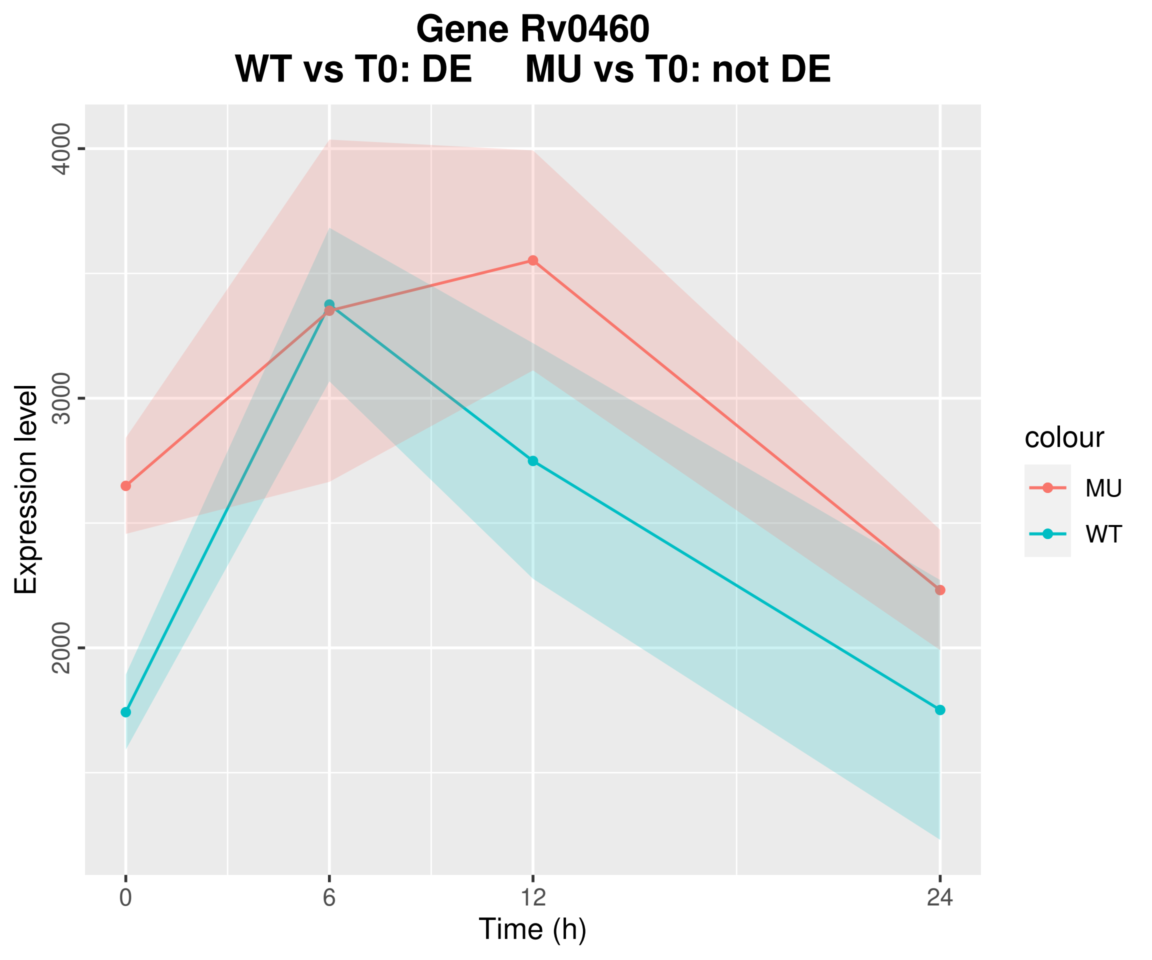
Gene Rv0460
WT vs T0: DE   MU vs T0: not DE
Expression level
4000
3000
2000
0
6
12
24
Time (h)
colour
MU
WT

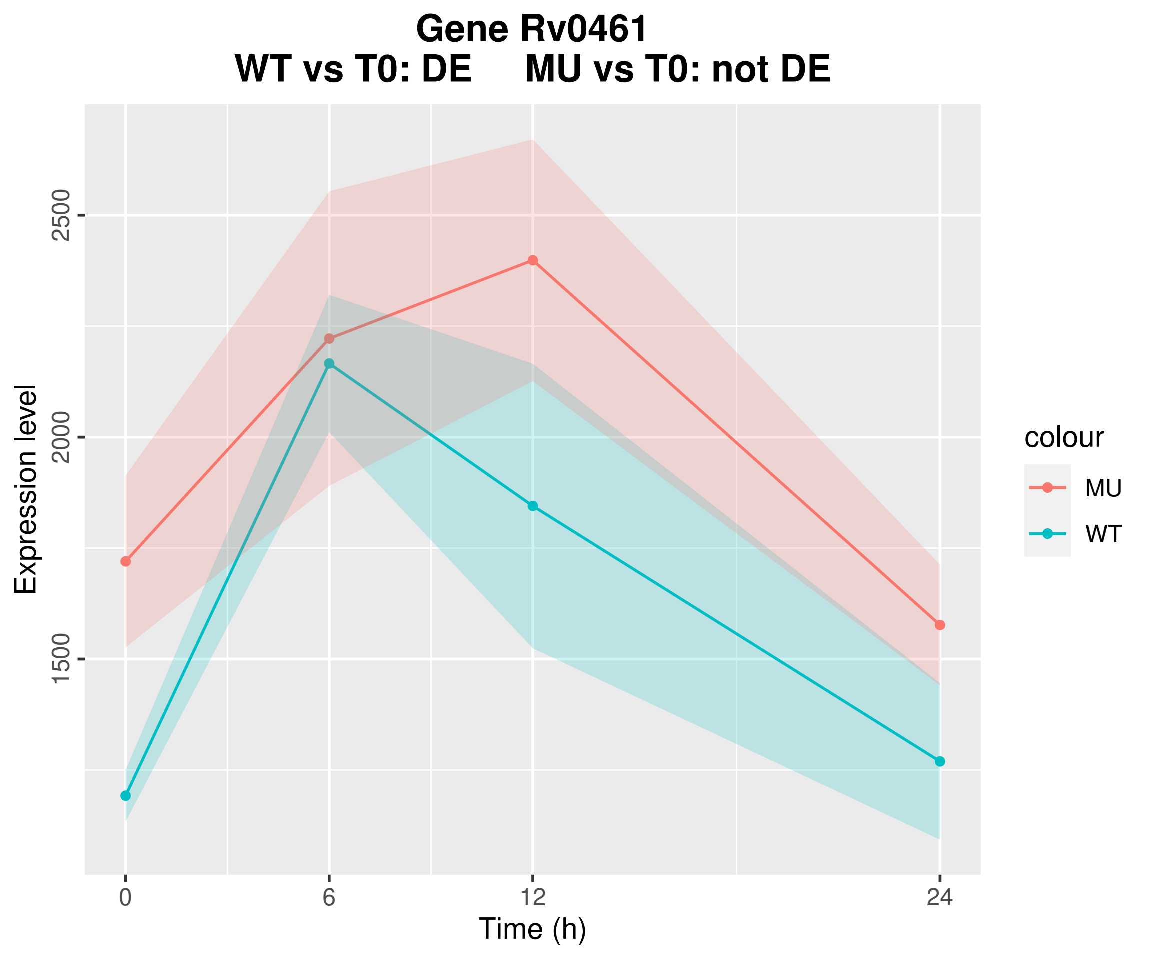
Gene Rv0461
WT vs T0: DE   MU vs T0: not DE
Expression level
2500
2000
1500
Time (h)
0
6
12
24
colour
MU
WT

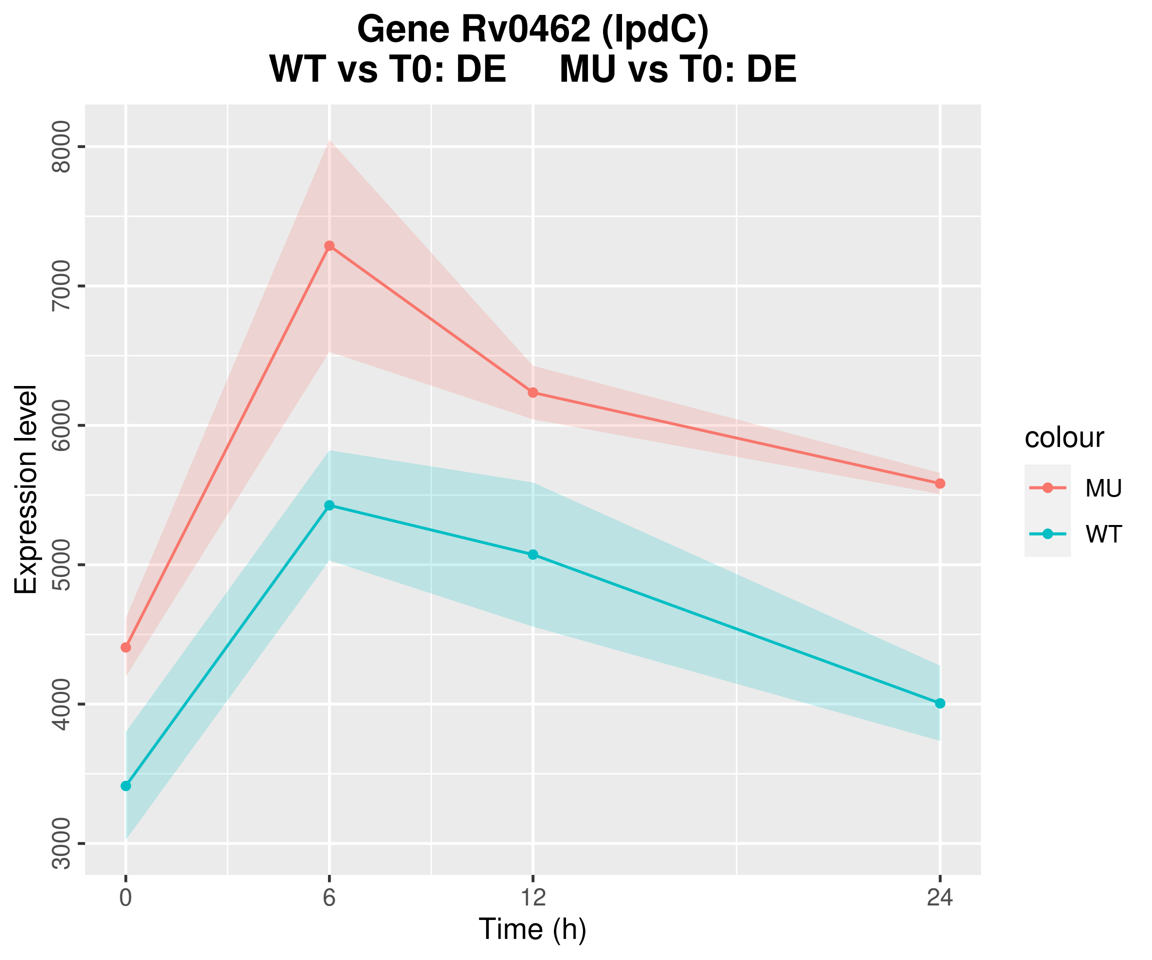
Gene Rv0462 (lpdC)
WT vs T0: DE MU vs T0: DE
Expression level
8000
7000
6000
5000
4000
3000
0
6
12
24
Time (h)
colour
MU
WT

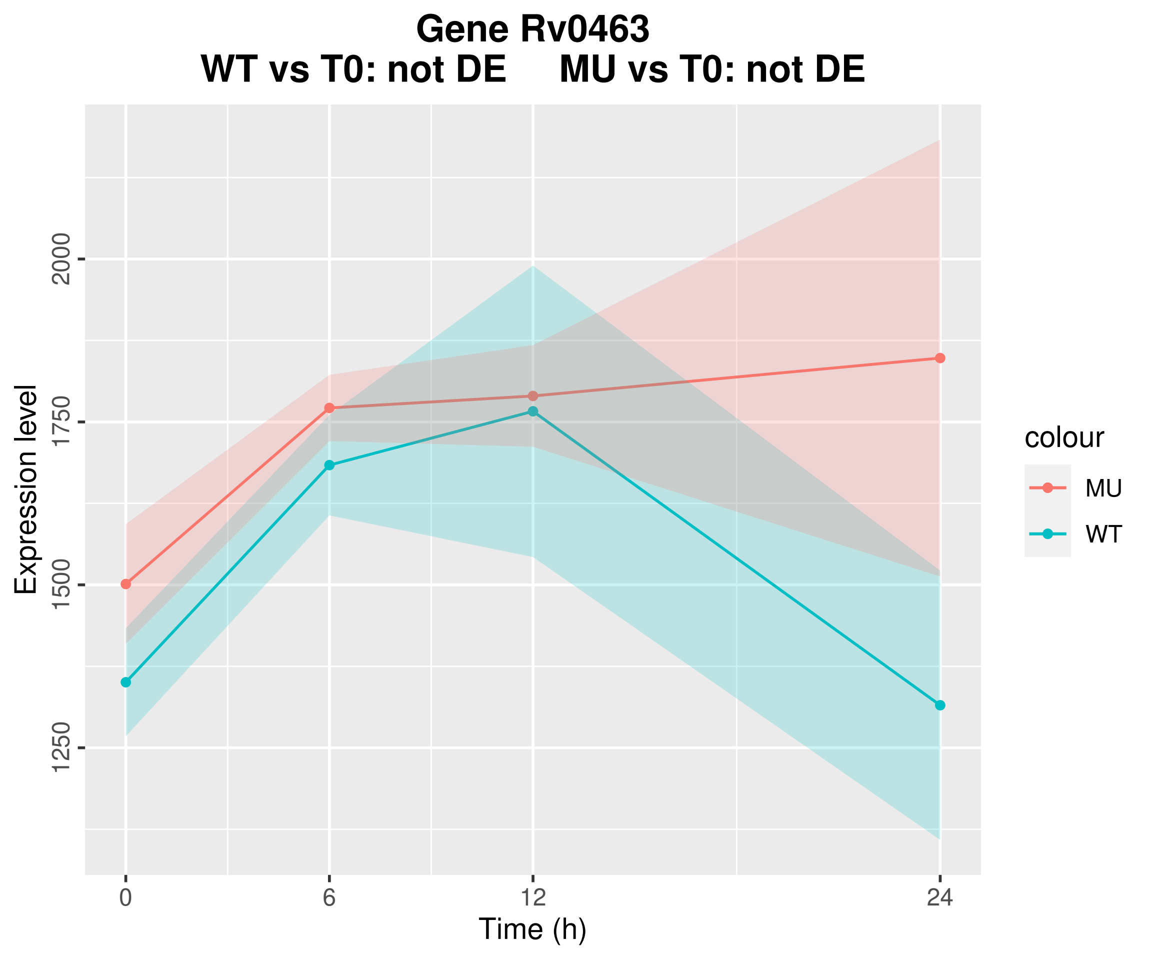

Gene Rv0463
WT vs T0: not DE MU vs T0: not DE
Expression level
2000
1750
1500
1250
0
6
12
24
Time (h)
colour
MU
WT

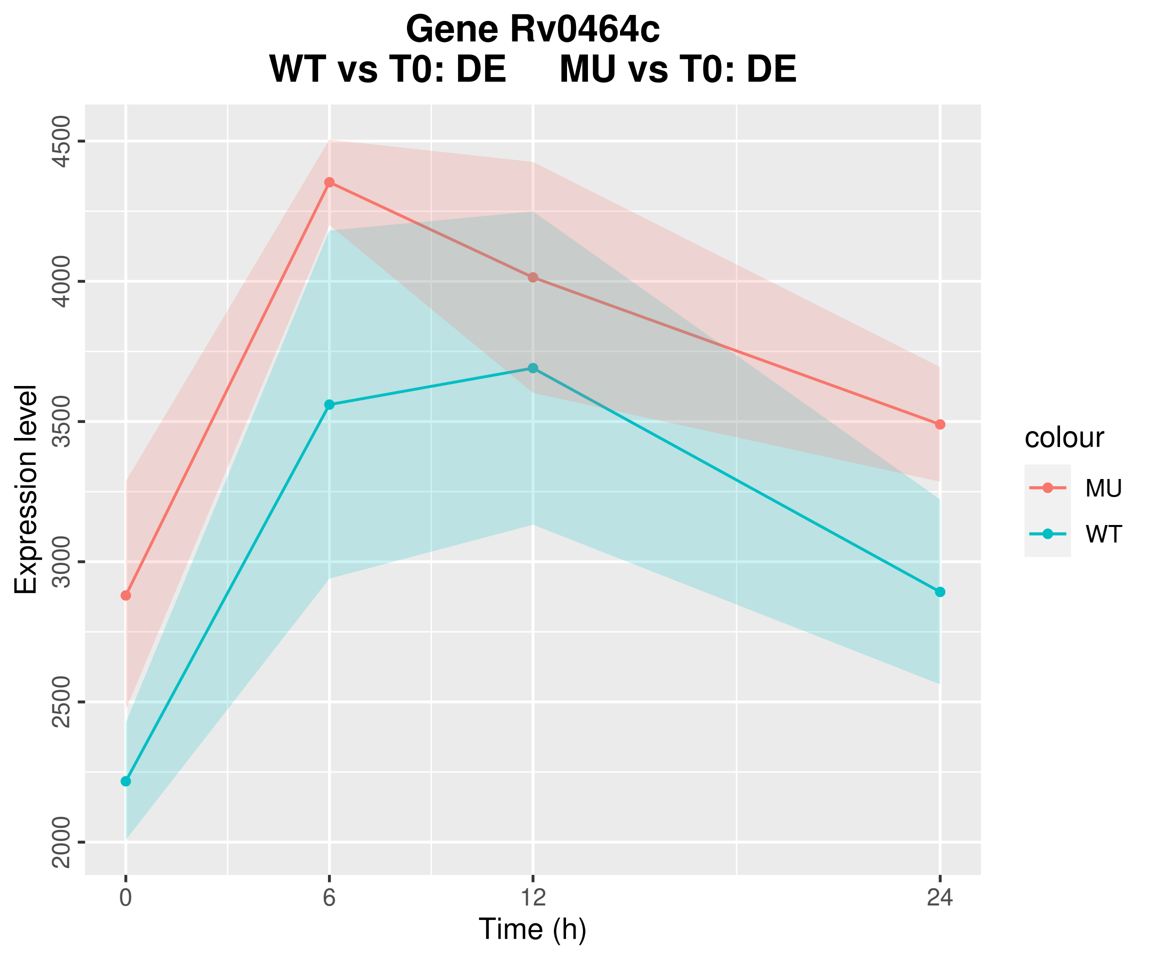

Gene Rv0464c
WT vs T0: DE   MU vs T0: DE
Expression level
4500
4000
3500
3000
2500
2000
0
6
12
24
Time (h)
colour
MU
WT

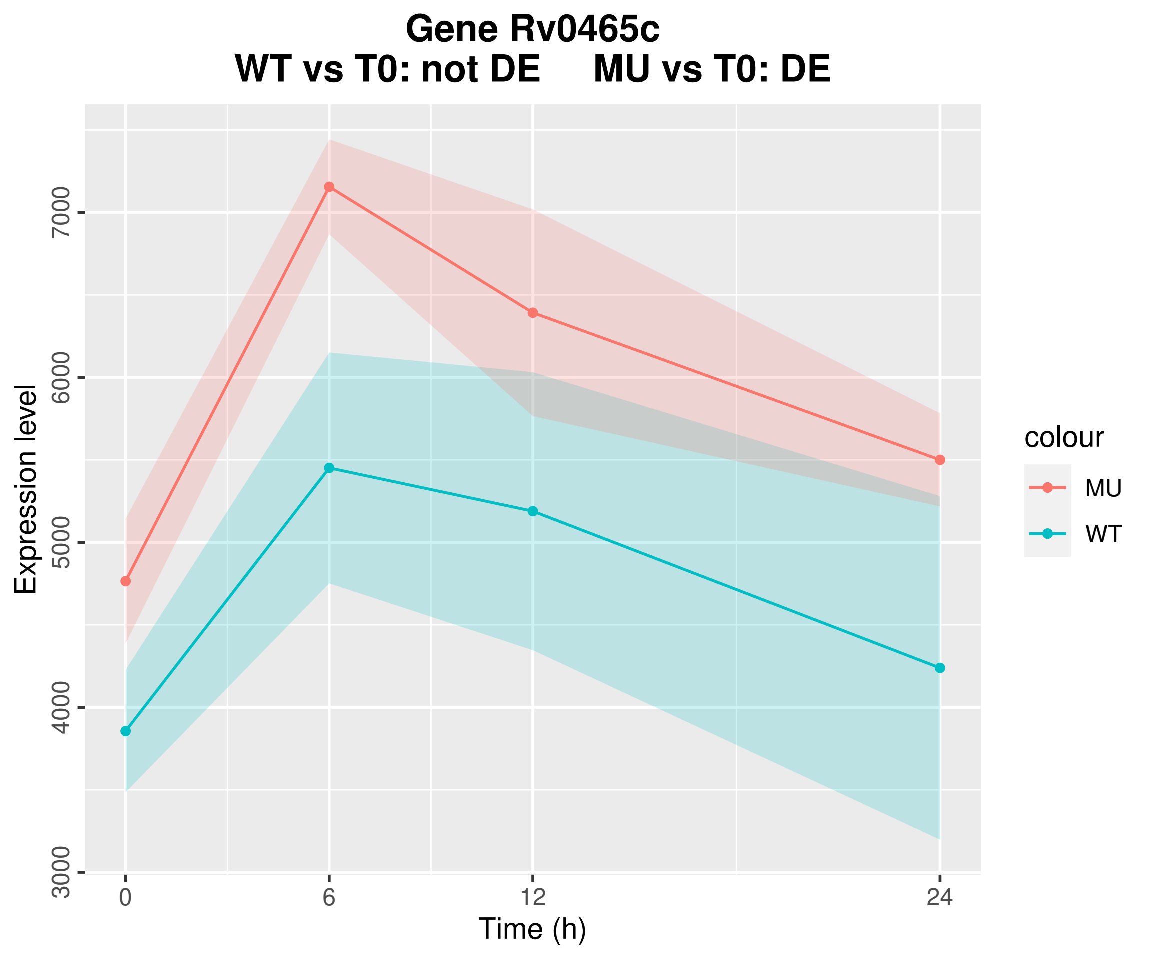
Gene Rv0465c
WT vs T0: not DE   MU vs T0: DE
Expression level
3000
4000
5000
6000
7000
0
6
12
24
Time (h)
colour
MU
WT

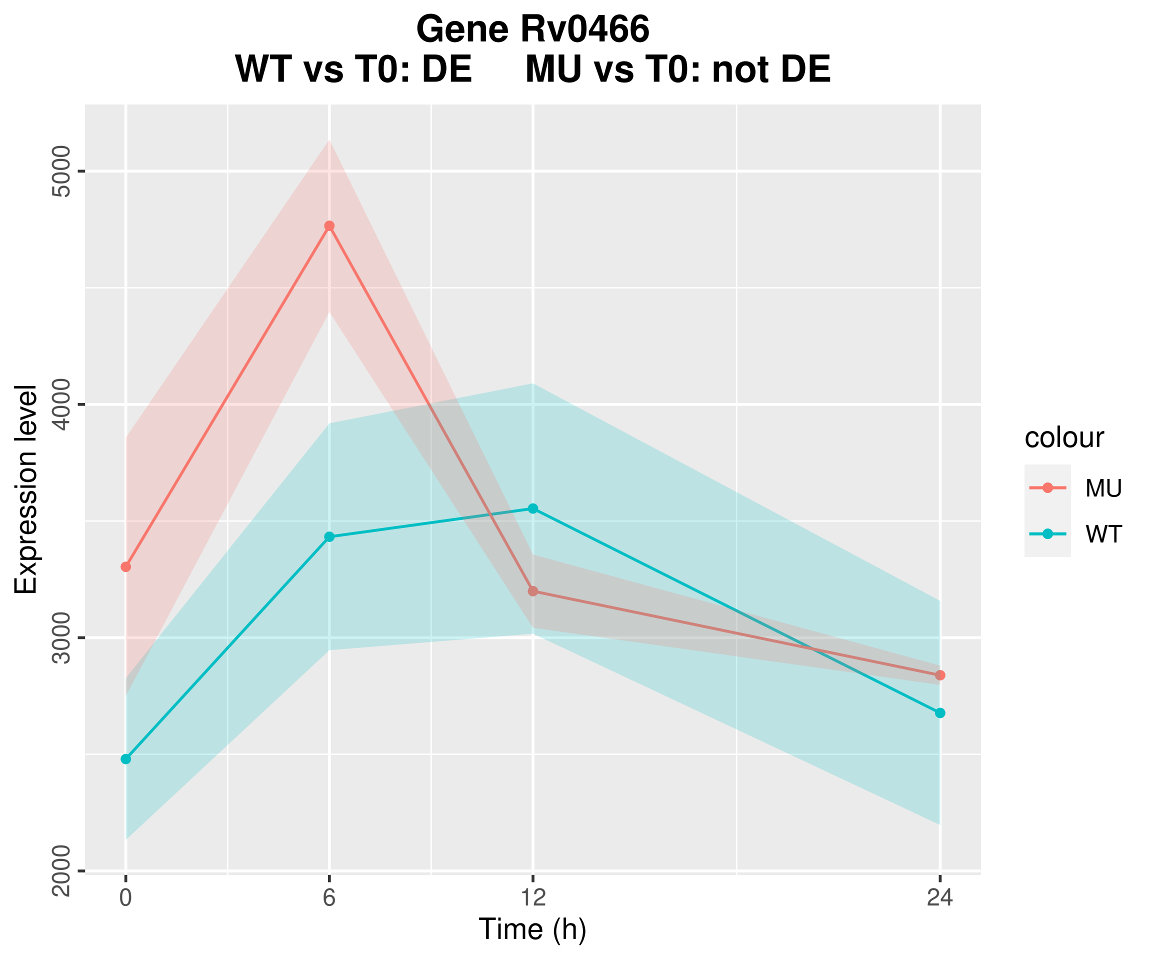
Gene Rv0466
WT vs T0: DE MU vs T0: not DE
Expression level
5000
4000
3000
2000
0
6
12
24
Time (h)
colour
MU
WT

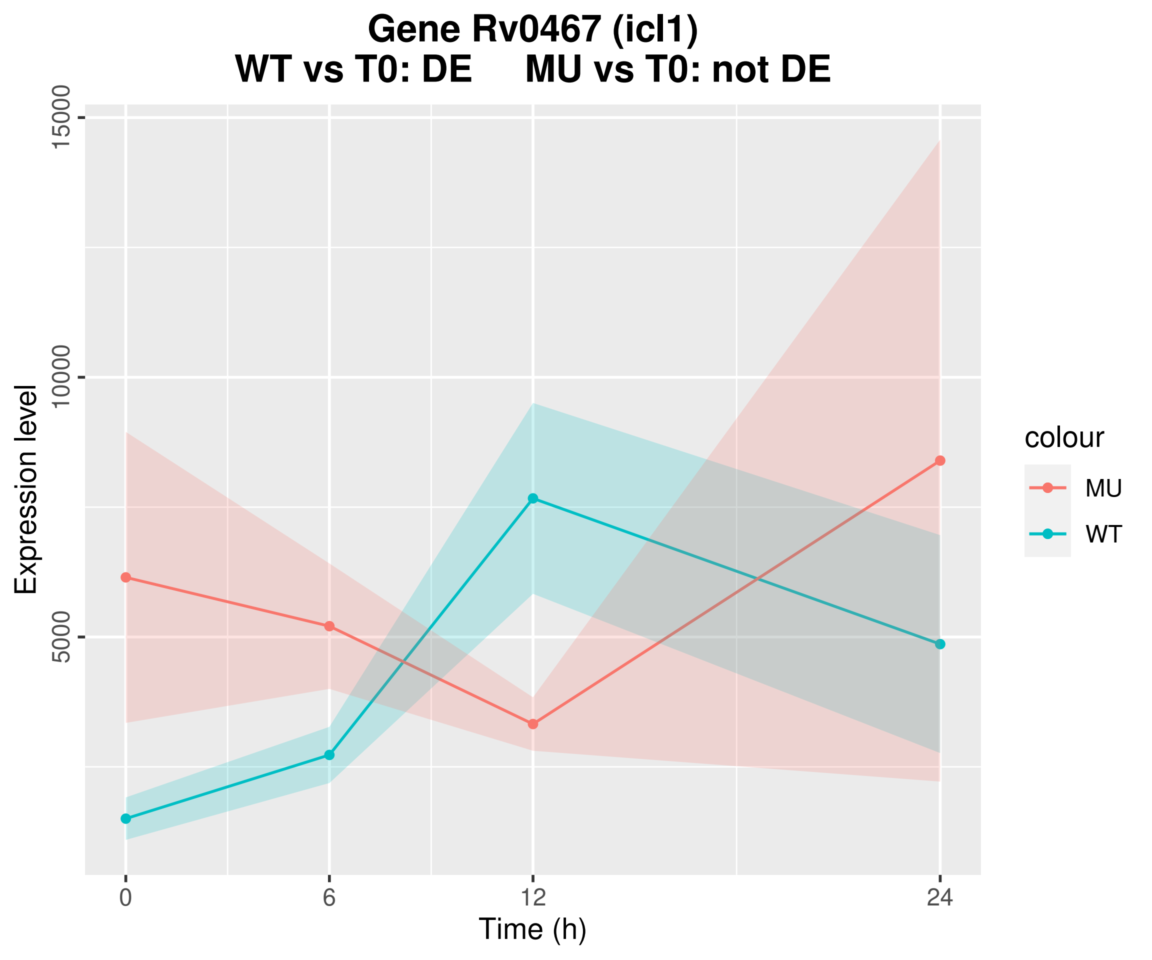

Gene Rv0467 (icl1)
WT vs T0: DE MU vs T0: not DE
15000
10000
5000
Expression level
0
6
12
24
Time (h)
colour
MU
WT

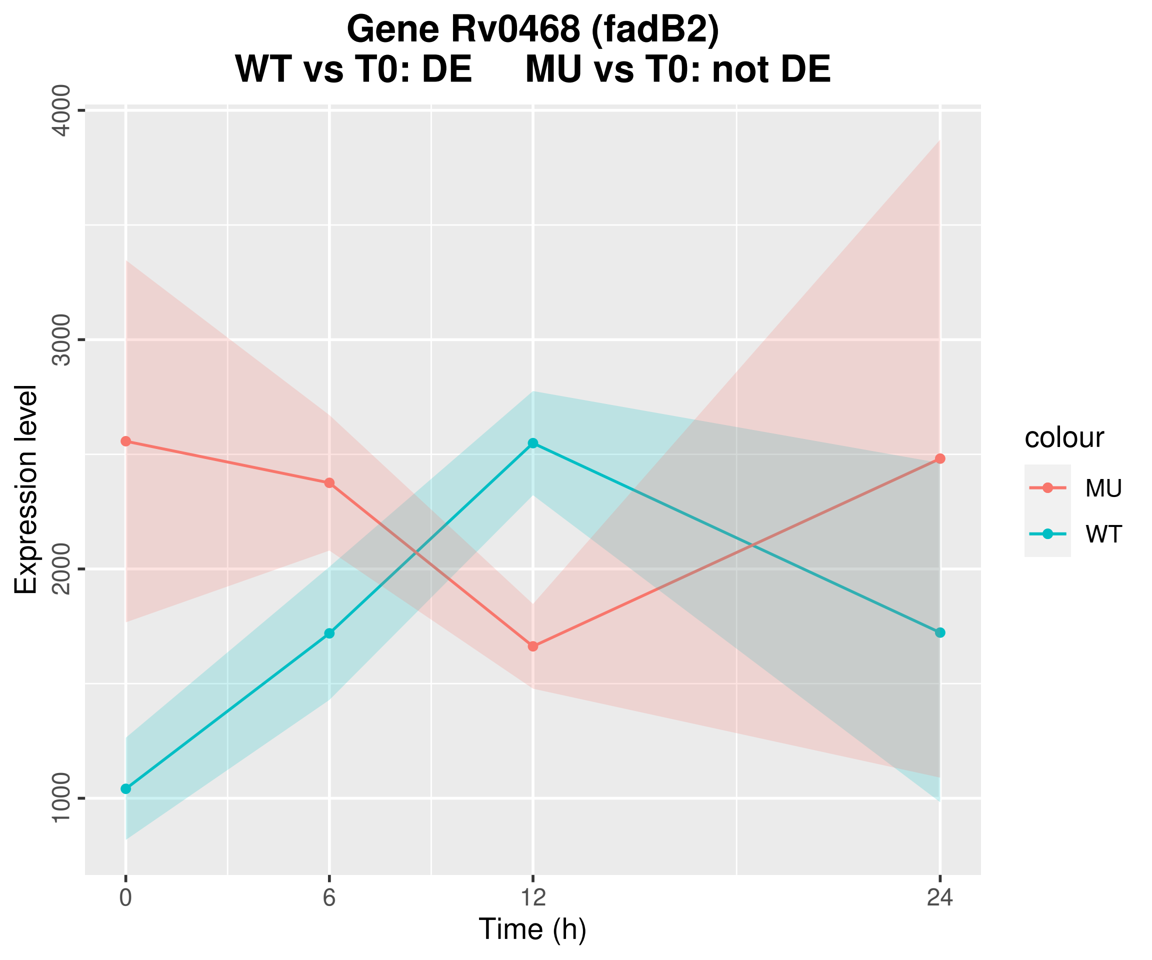

Gene Rv0468 (fadB2)
WT vs T0: DE MU vs T0: not DE
Expression level
4000
3000
2000
1000
0
6
12
24
Time (h)
colour
MU
WT

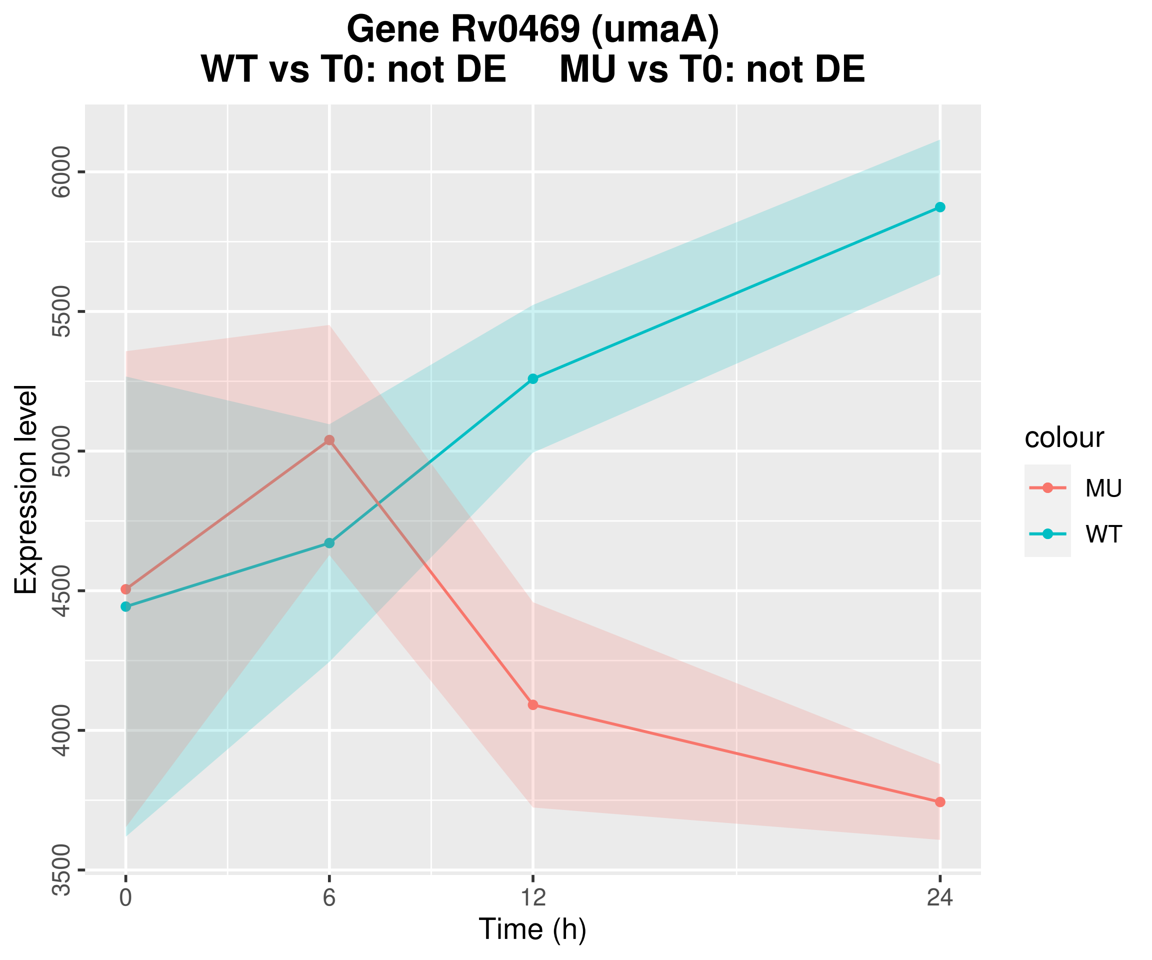

Gene Rv0469 (umaA)
WT vs T0: not DE   MU vs T0: not DE
6000
5500
5000
4500
4000
3500
Expression level
0
6
12
24
Time (h)
colour
MU
WT

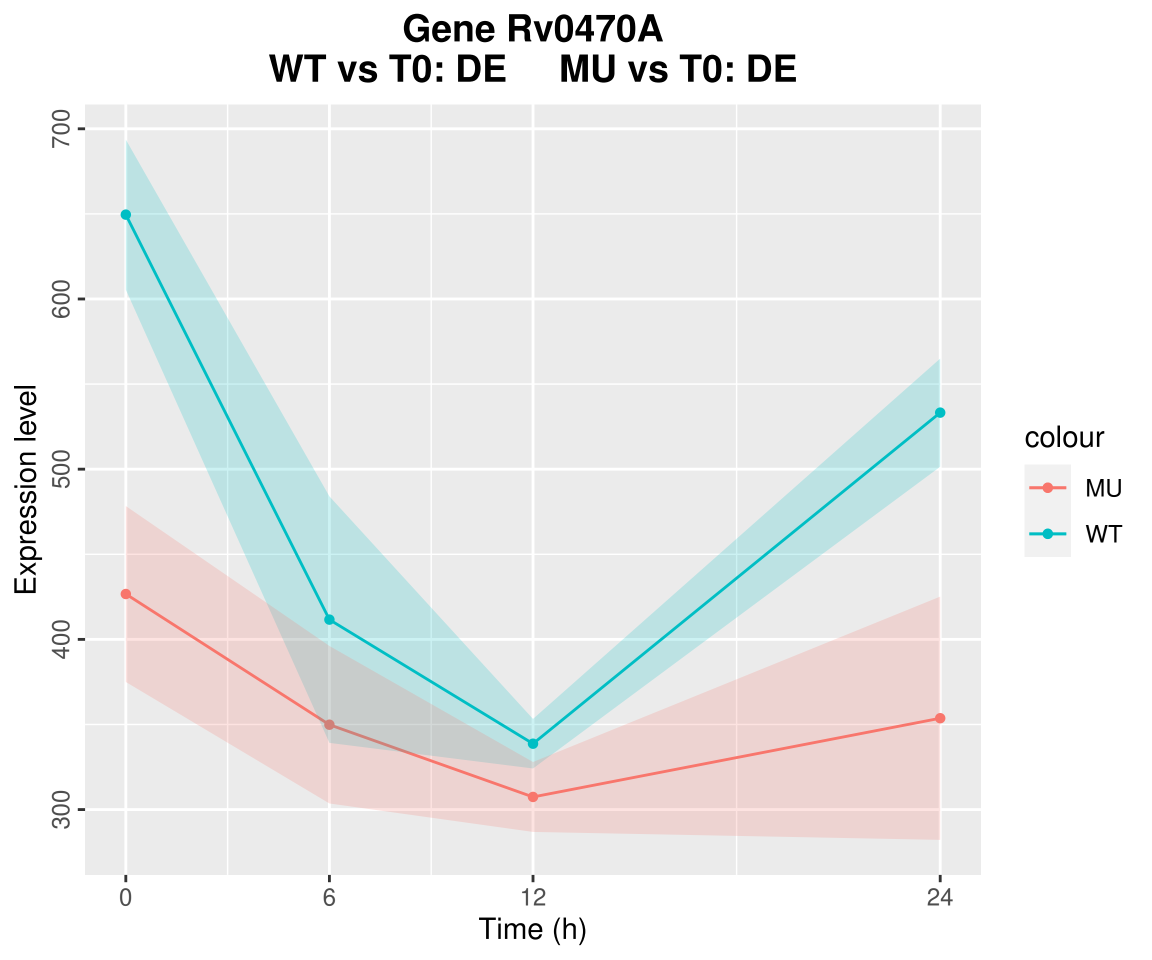
Gene Rv0470A
WT vs T0: DE MU vs T0: DE
700
600
500
400
300
Expression level
0
6
12
24
Time (h)
colour
MU
WT

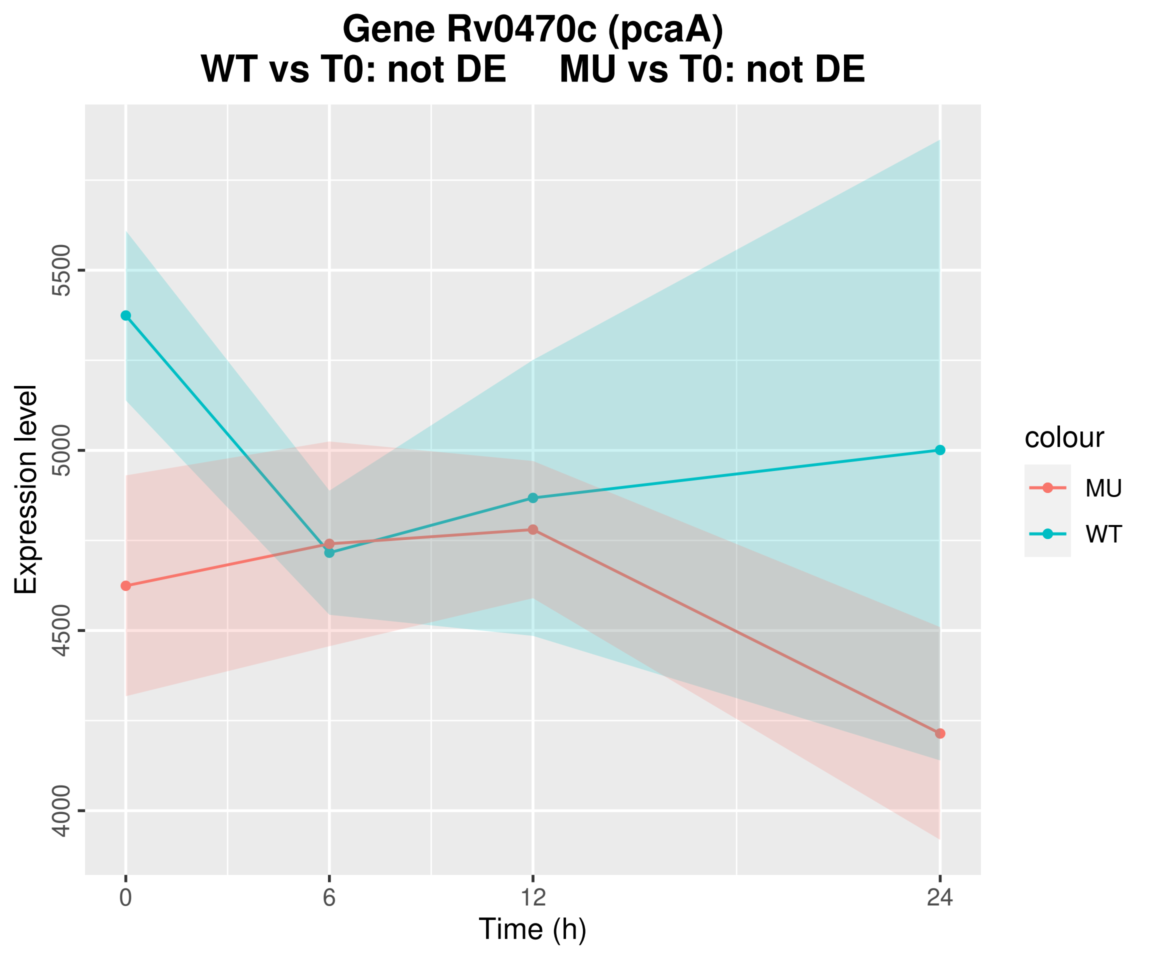
Gene Rv0470c (pcaA)
WT vs T0: not DE MU vs T0: not DE
Expression level
4000
4500
5000
5500
0
6
12
24
Time (h)
colour
MU
WT

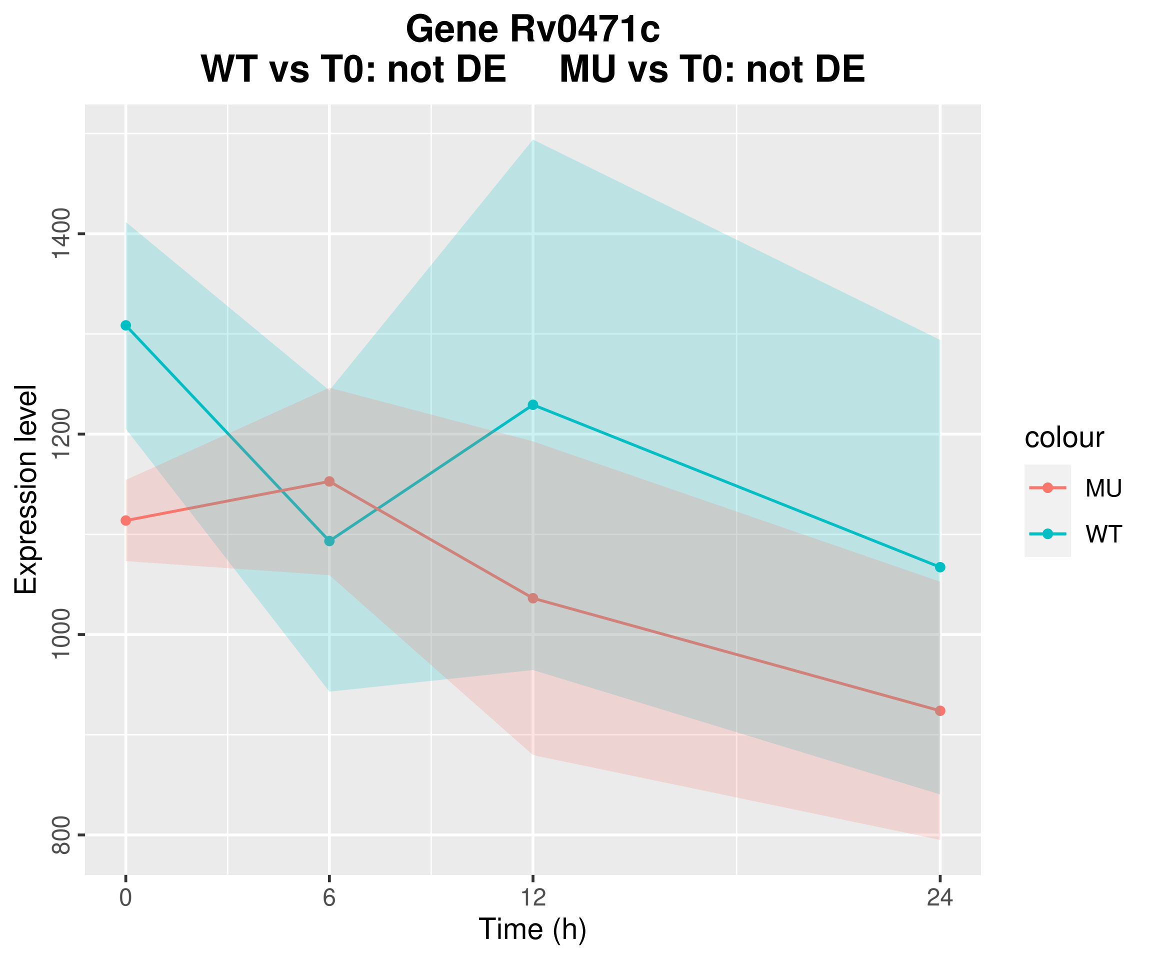

Gene Rv0471c
WT vs T0: not DE MU vs T0: not DE
Expression level
1400
1200
1000
800
0
6
12
24
Time (h)
colour
MU
WT

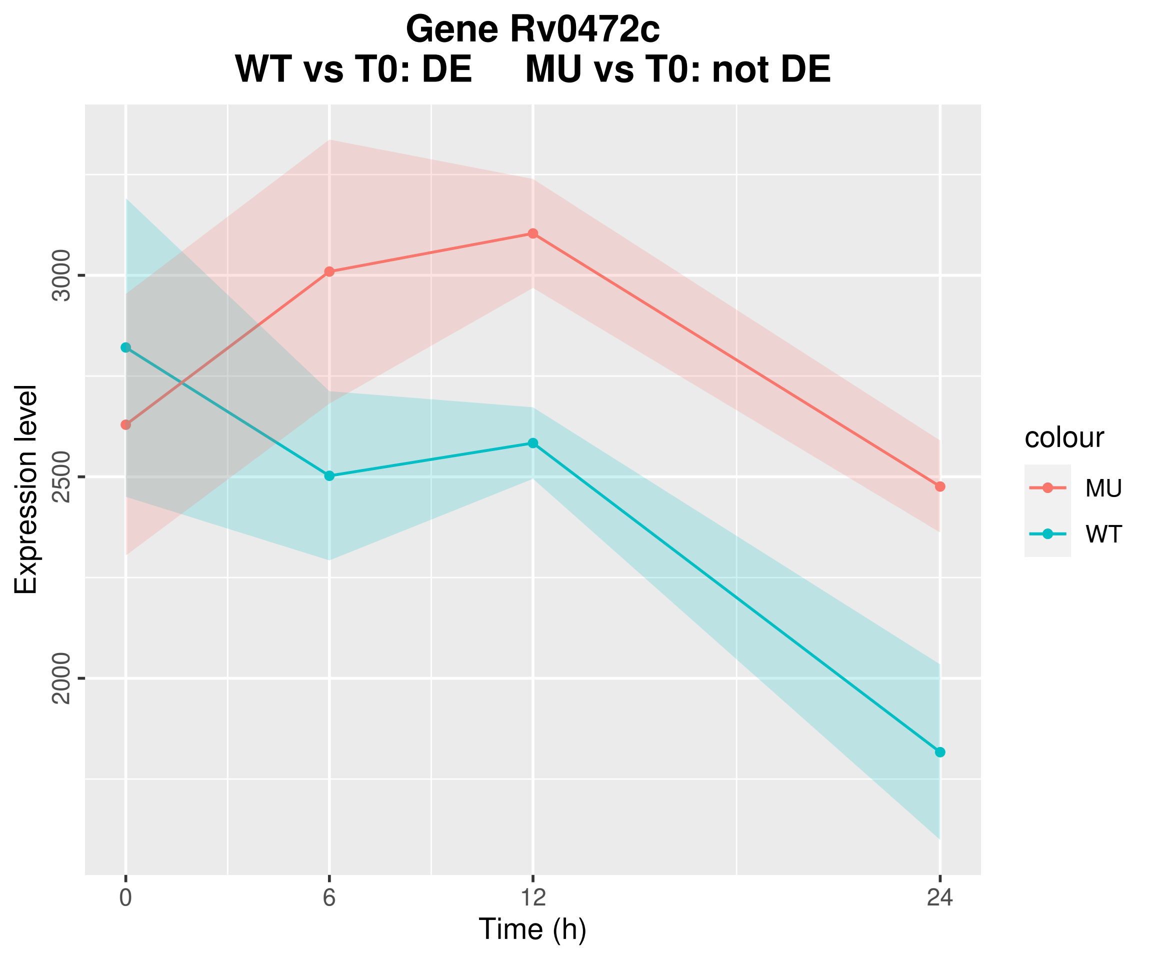
Gene Rv0472c
WT vs T0: DE   MU vs T0: not DE
Expression level
3000
2500
2000
0
6
12
24
Time (h)
colour
MU
WT

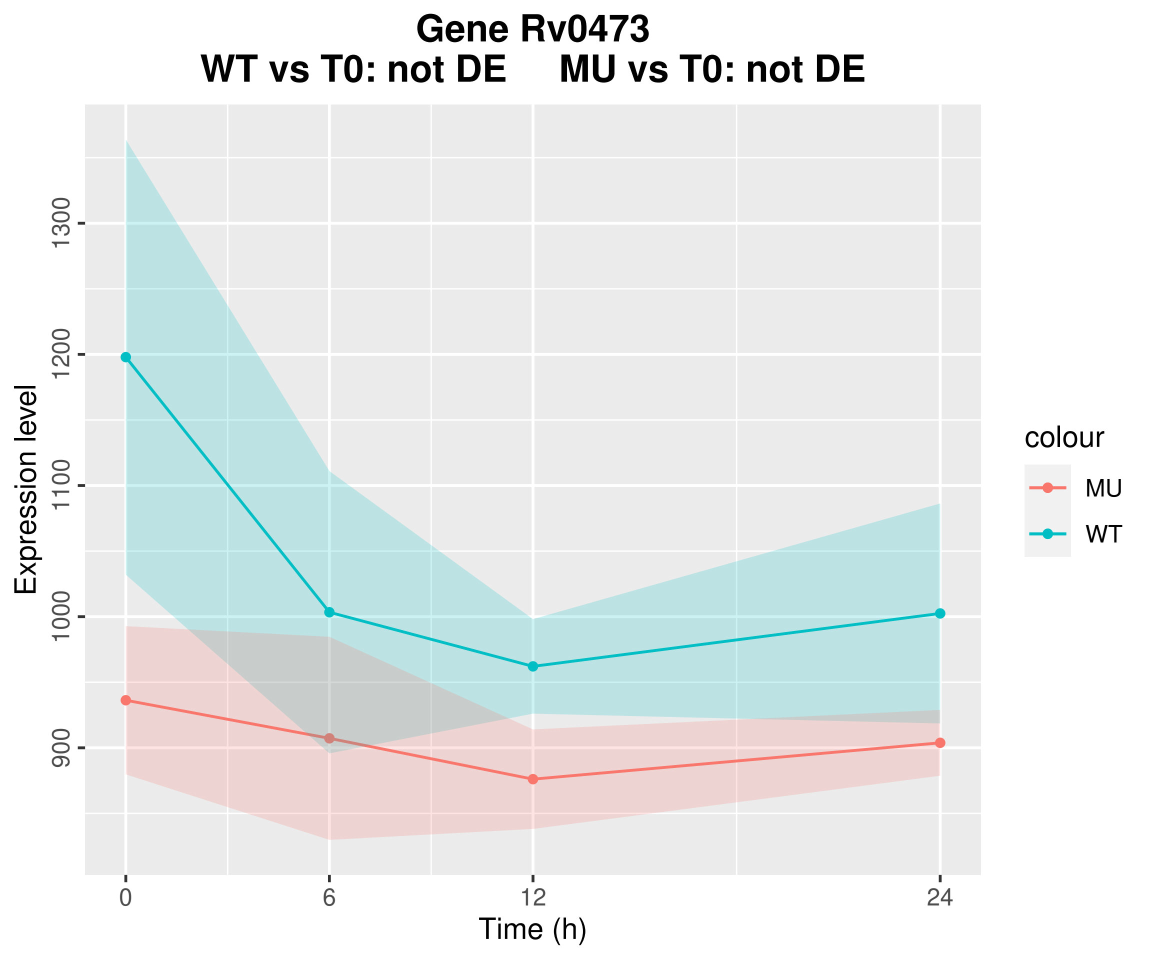
Gene Rv0473
WT vs T0: not DE MU vs T0: not DE
Expression level
1300
1200
1100
1000
900
0
6
12
24
Time (h)
colour
MU
WT

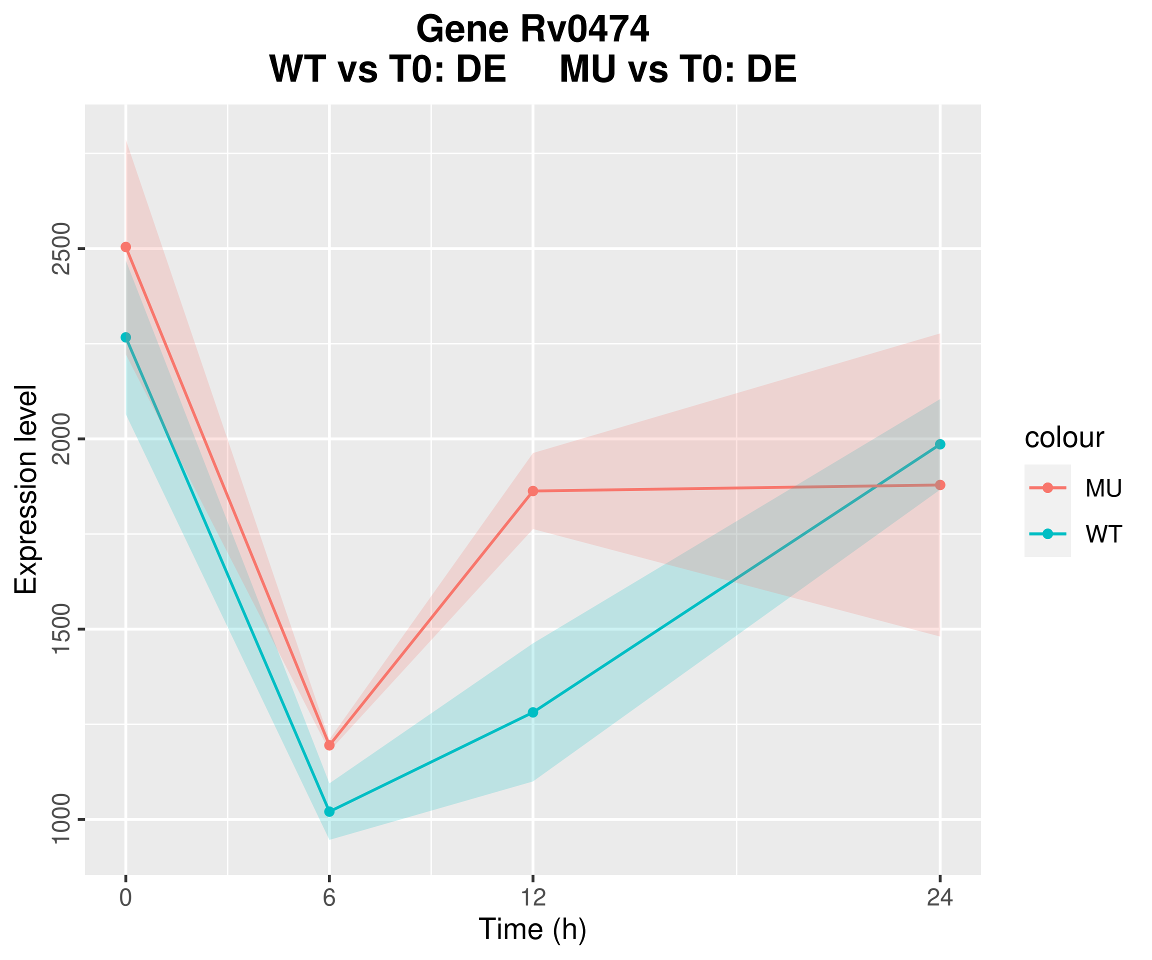
Gene Rv0474
WT vs T0: DE MU vs T0: DE
Expression level
2500
2000
1500
1000
0
6
12
24
Time (h)
colour
MU
WT

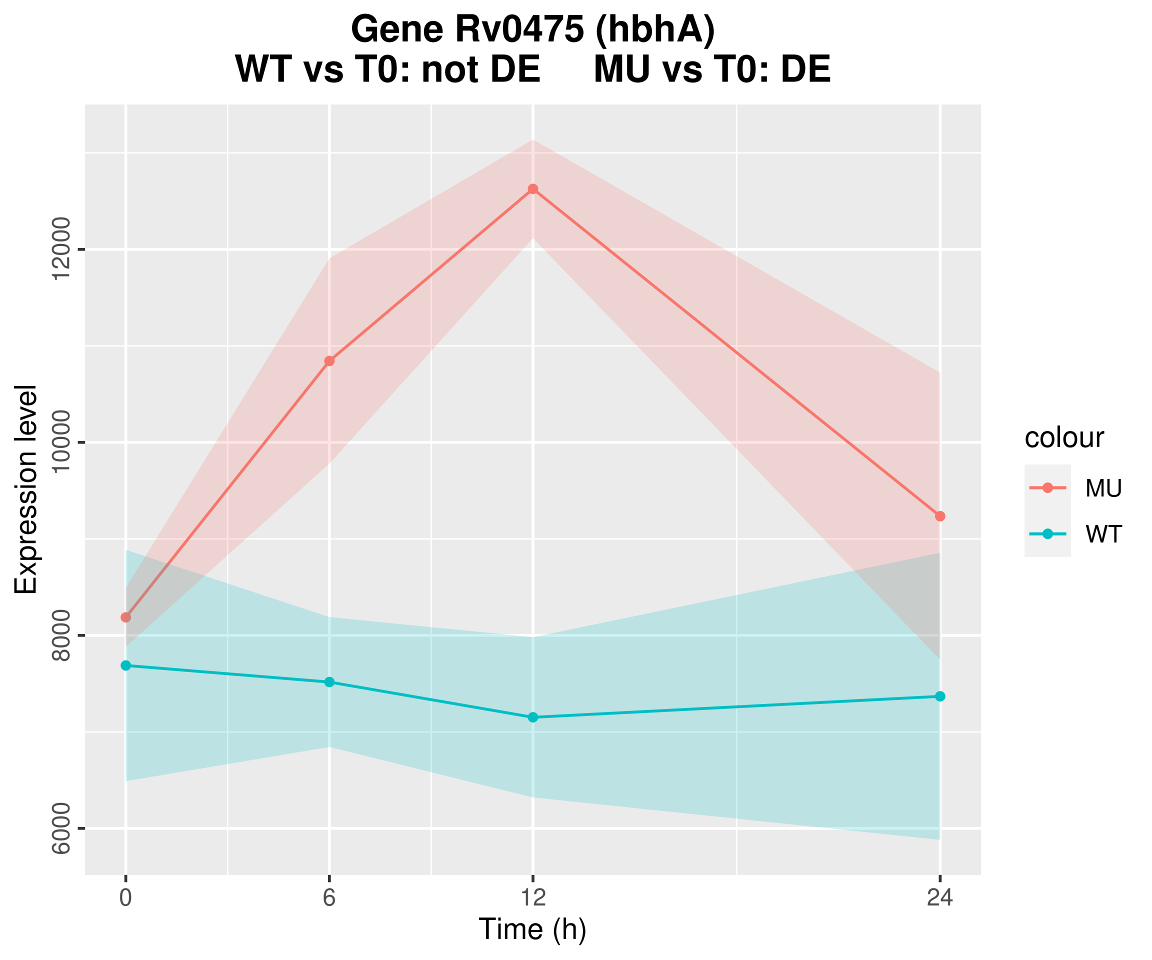

Gene Rv0475 (hbhA)
WT vs T0: not DE   MU vs T0: DE
Expression level
12000
10000
8000
6000
0
6
12
24
Time (h)
colour
MU
WT

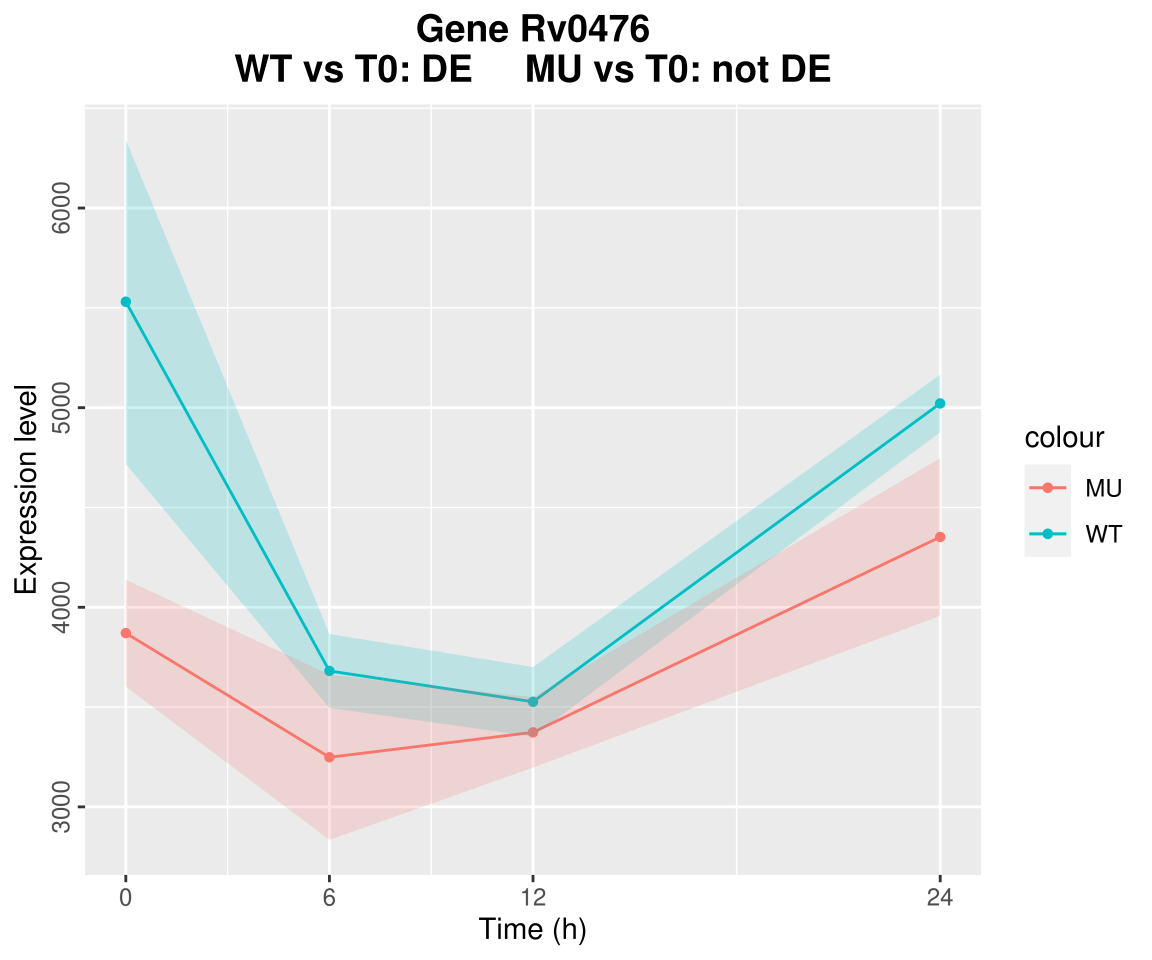
Gene Rv0476
WT vs T0: DE   MU vs T0: not DE
Expression level
6000
5000
4000
3000
0
6
12
24
Time (h)
colour
MU
WT

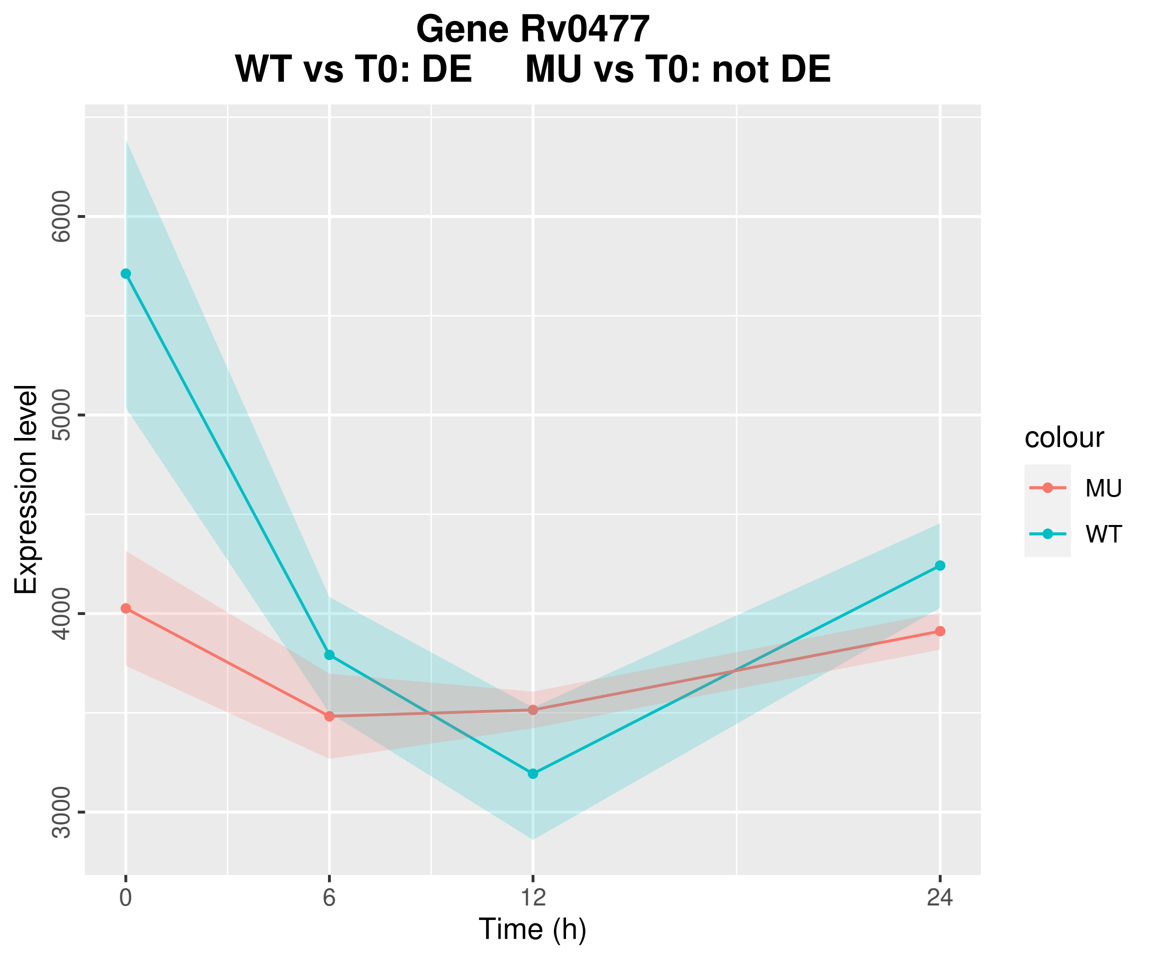

Gene Rv0477
WT vs T0: DE   MU vs T0: not DE
Expression level
6000
5000
4000
3000
0
6
12
24
Time (h)
colour
MU
WT

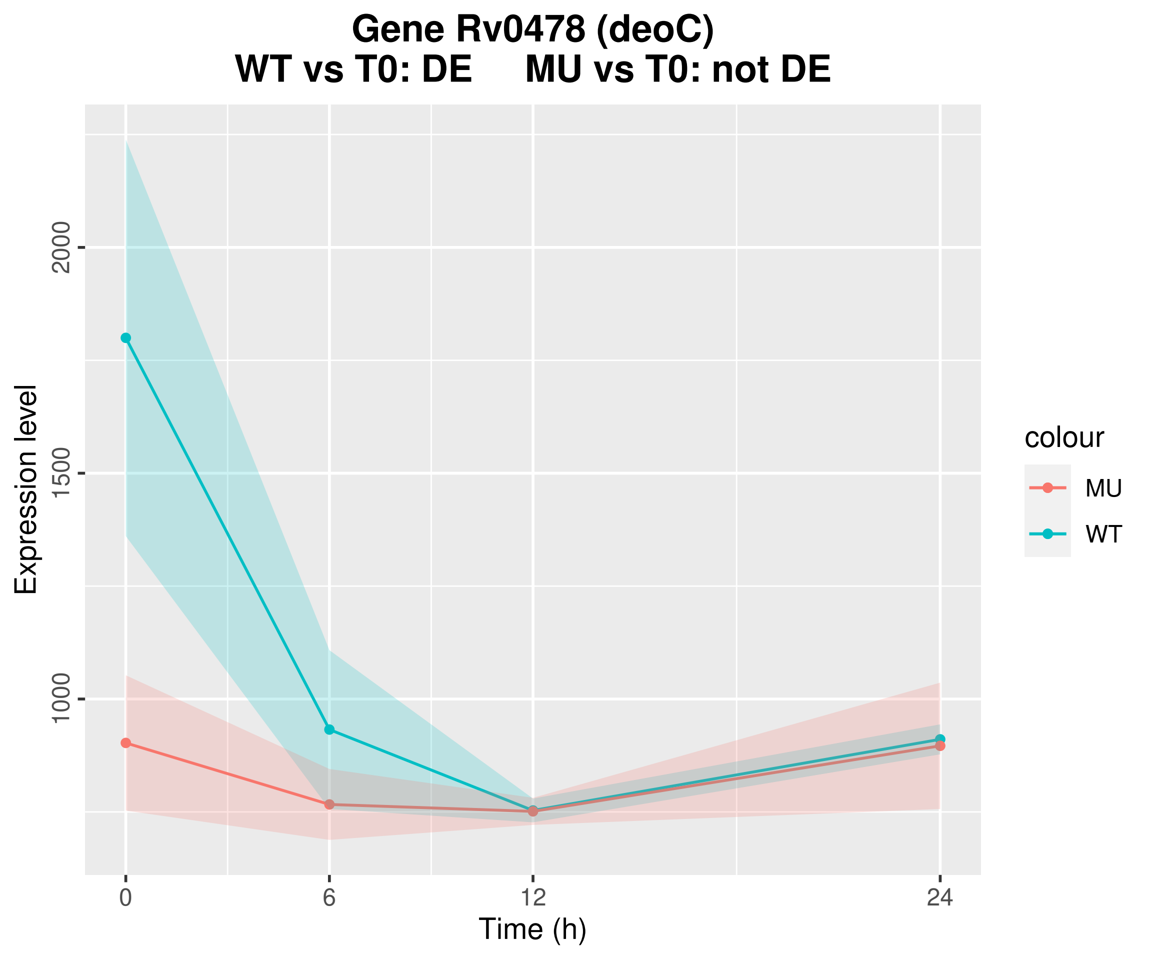
Gene Rv0478 (deoC)
WT vs T0: DE   MU vs T0: not DE
Expression level
2000
1500
1000
0
6
12
24
Time (h)
colour
MU
WT

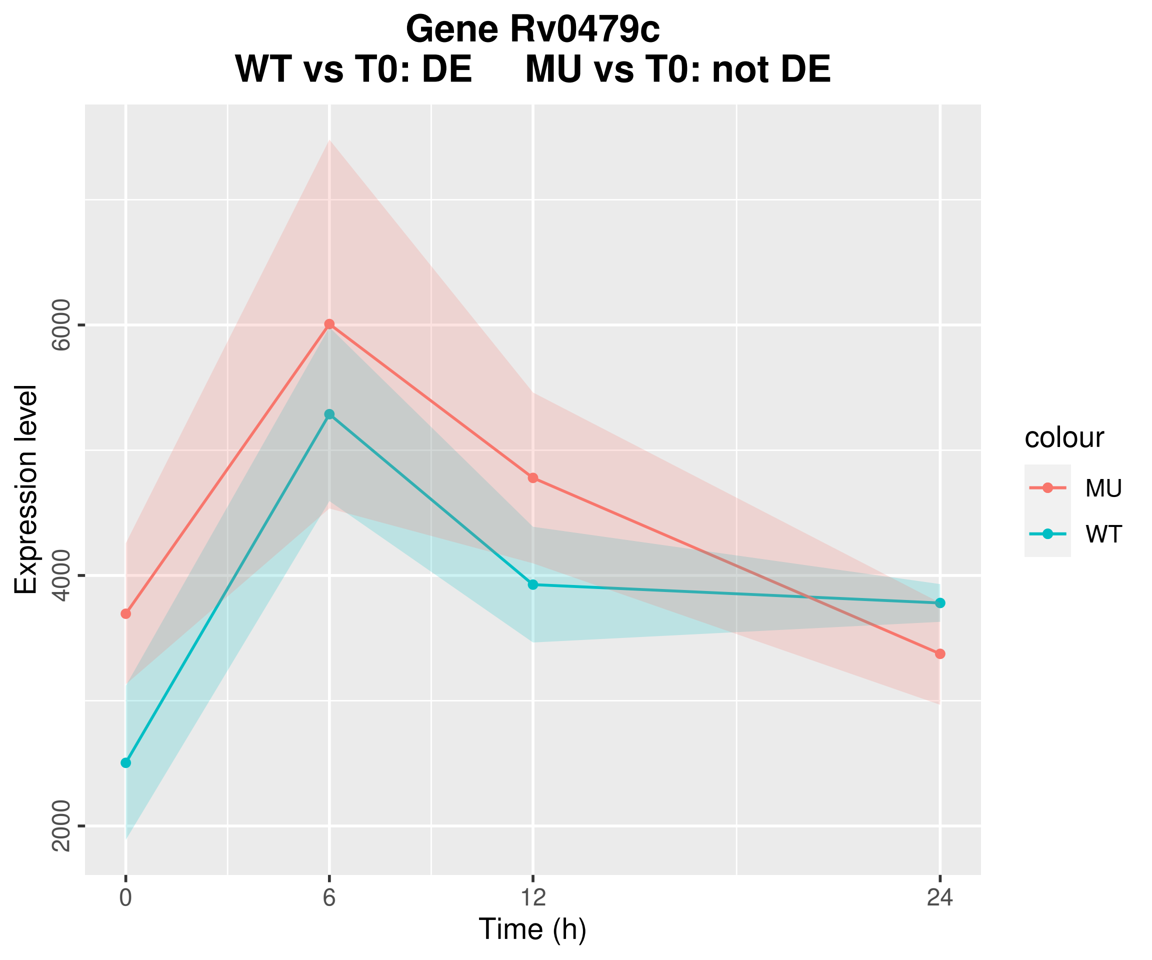
Gene Rv0479c
WT vs T0: DE MU vs T0: not DE
Expression level
2000
4000
6000
0
6
12
24
Time (h)
colour
MU
WT

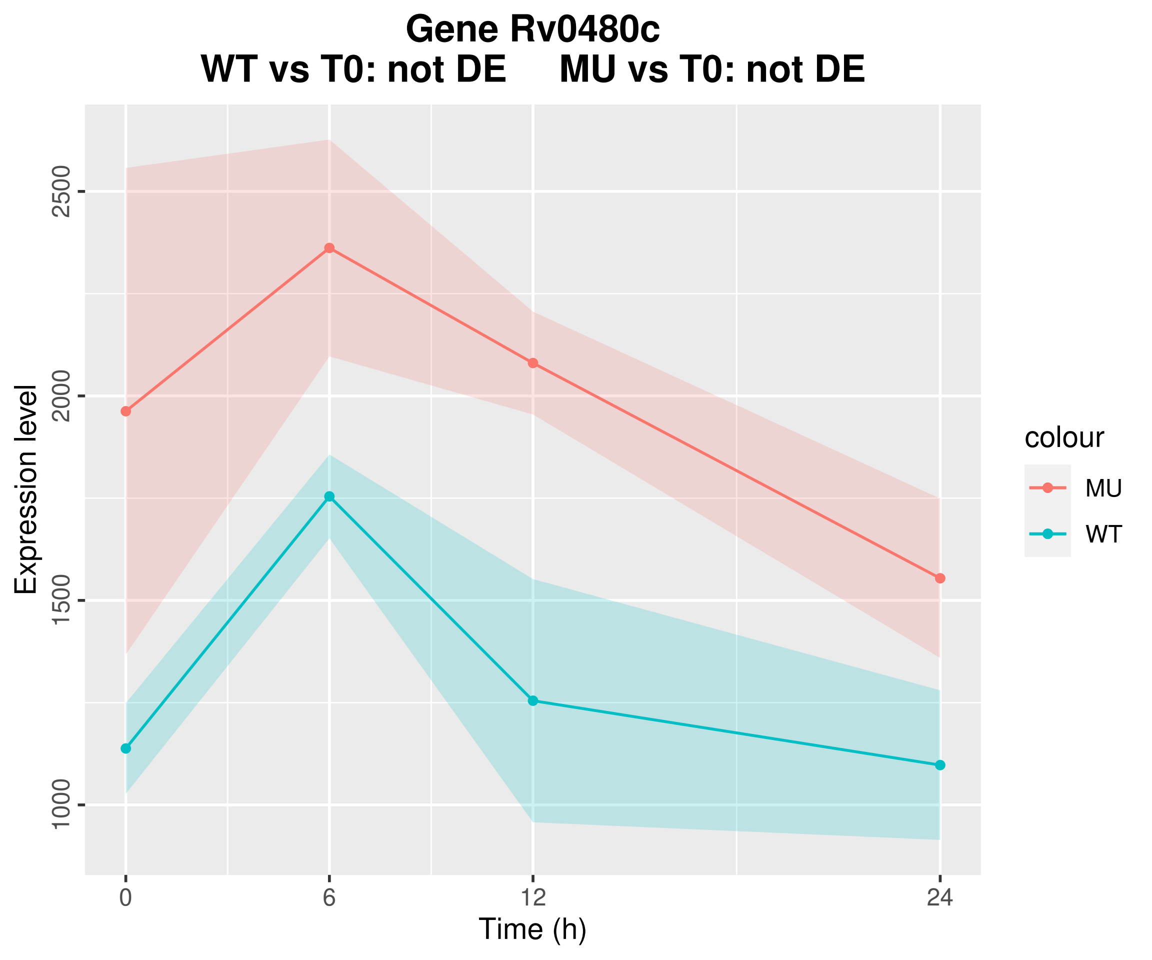

Gene Rv0480c
WT vs T0: not DE MU vs T0: not DE
Expression level
2500
2000
1500
1000
0
6
12
24
Time (h)
colour
MU
WT

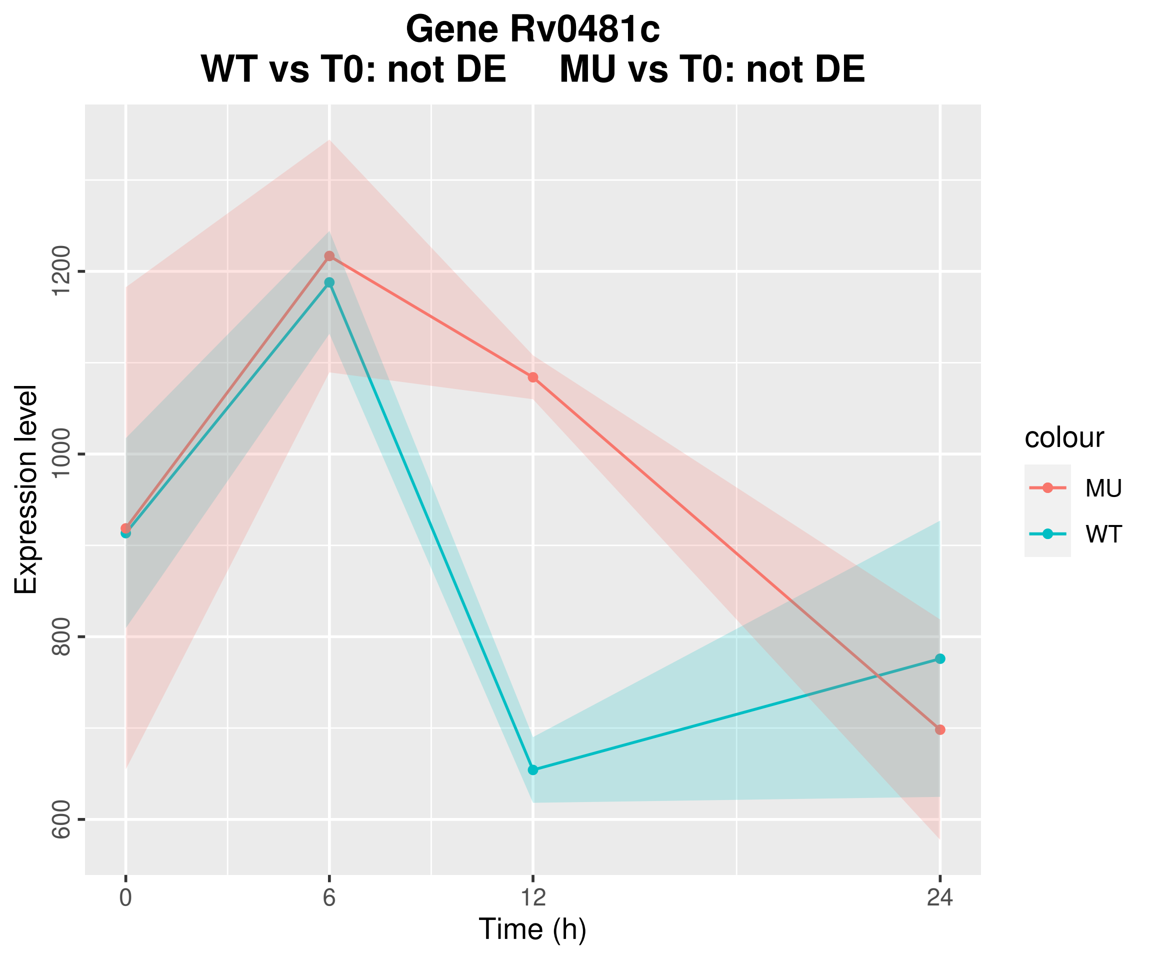

Gene Rv0481c
WT vs T0: not DE MU vs T0: not DE
Expression level
1200
1000
800
600
0
6
12
24
Time (h)
colour
MU
WT

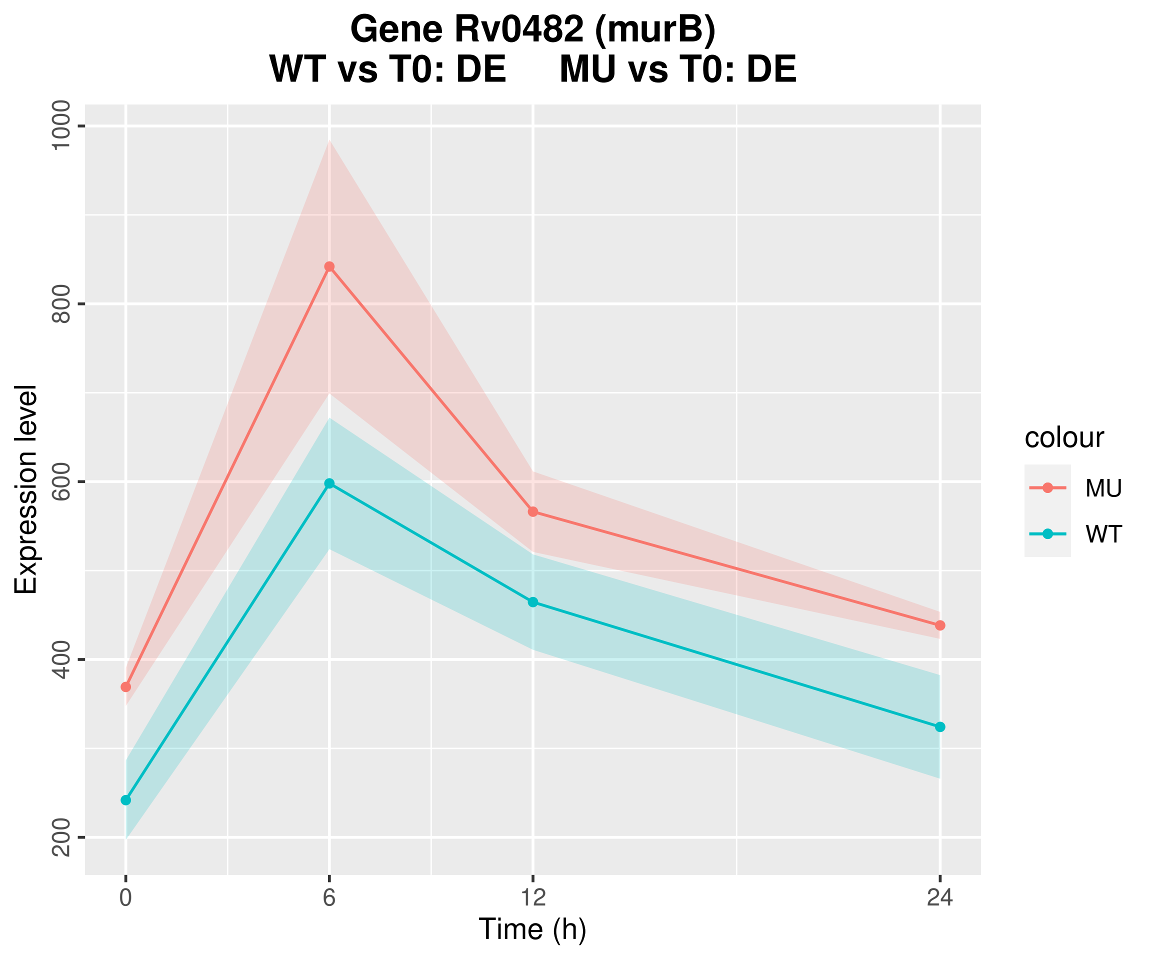

Gene Rv0482 (murB)
WT vs T0: DE MU vs T0: DE
Expression level
1000
800
600
400
200
0
6
12
24
Time (h)
colour
MU
WT

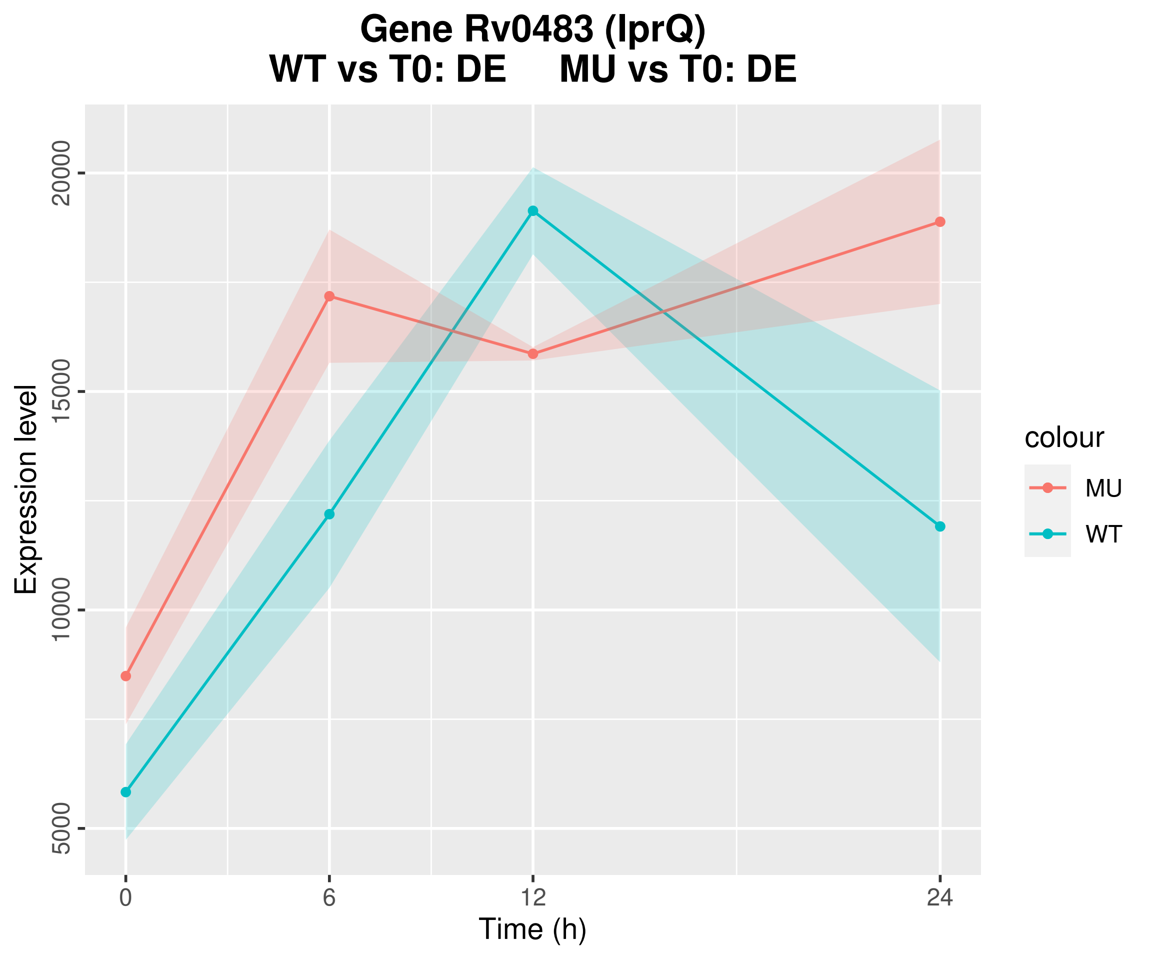

Gene Rv0483 (lprQ)
WT vs T0: DE MU vs T0: DE
Expression level
20000
15000
10000
5000
0
6
12
24
Time (h)
colour
MU
WT

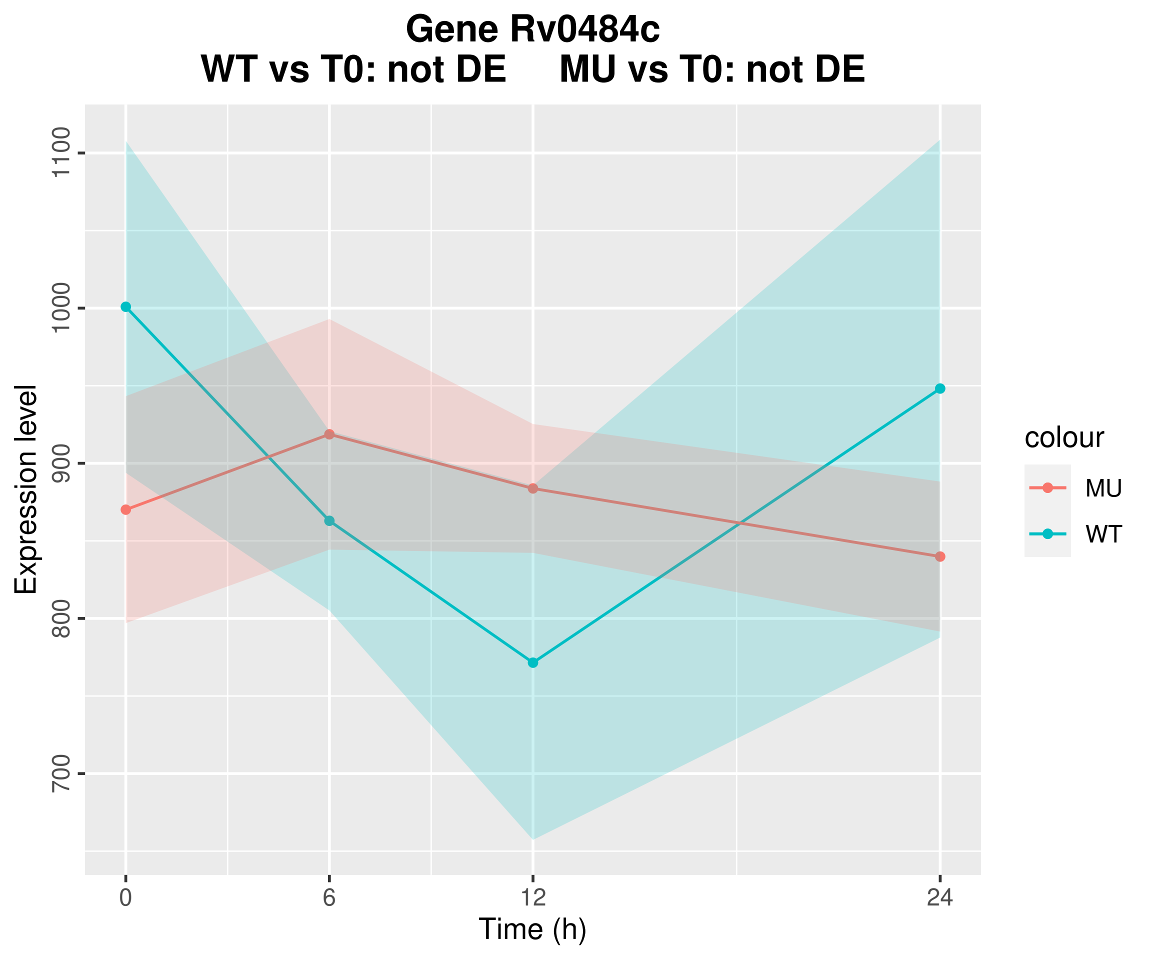

Gene Rv0484c
WT vs T0: not DE   MU vs T0: not DE
Expression level
1100
1000
900
800
700
0
6
12
24
Time (h)
colour
MU
WT

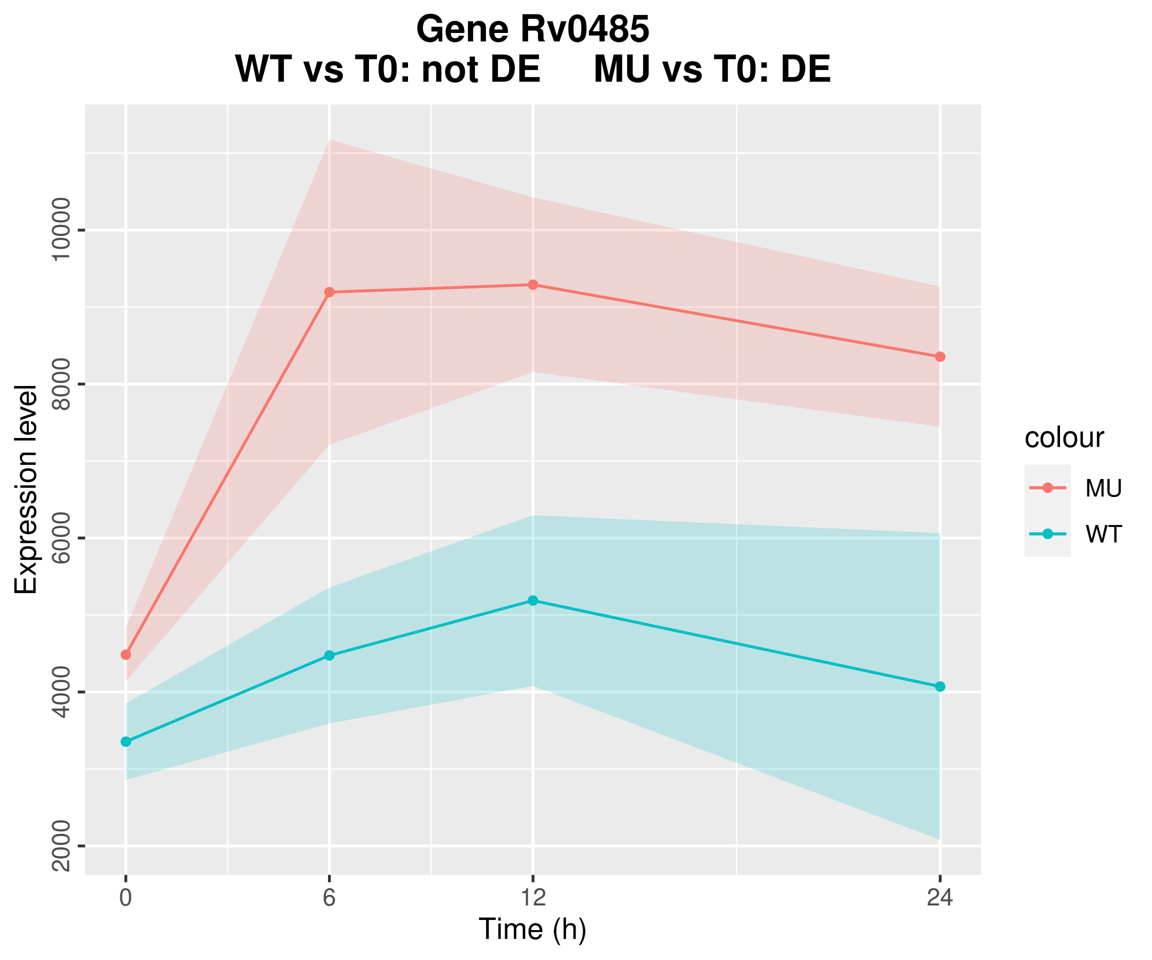

Gene Rv0485
WT vs T0: not DE MU vs T0: DE
Expression level
2000
4000
6000
8000
10000
0
6
12
24
Time (h)
colour
MU
WT

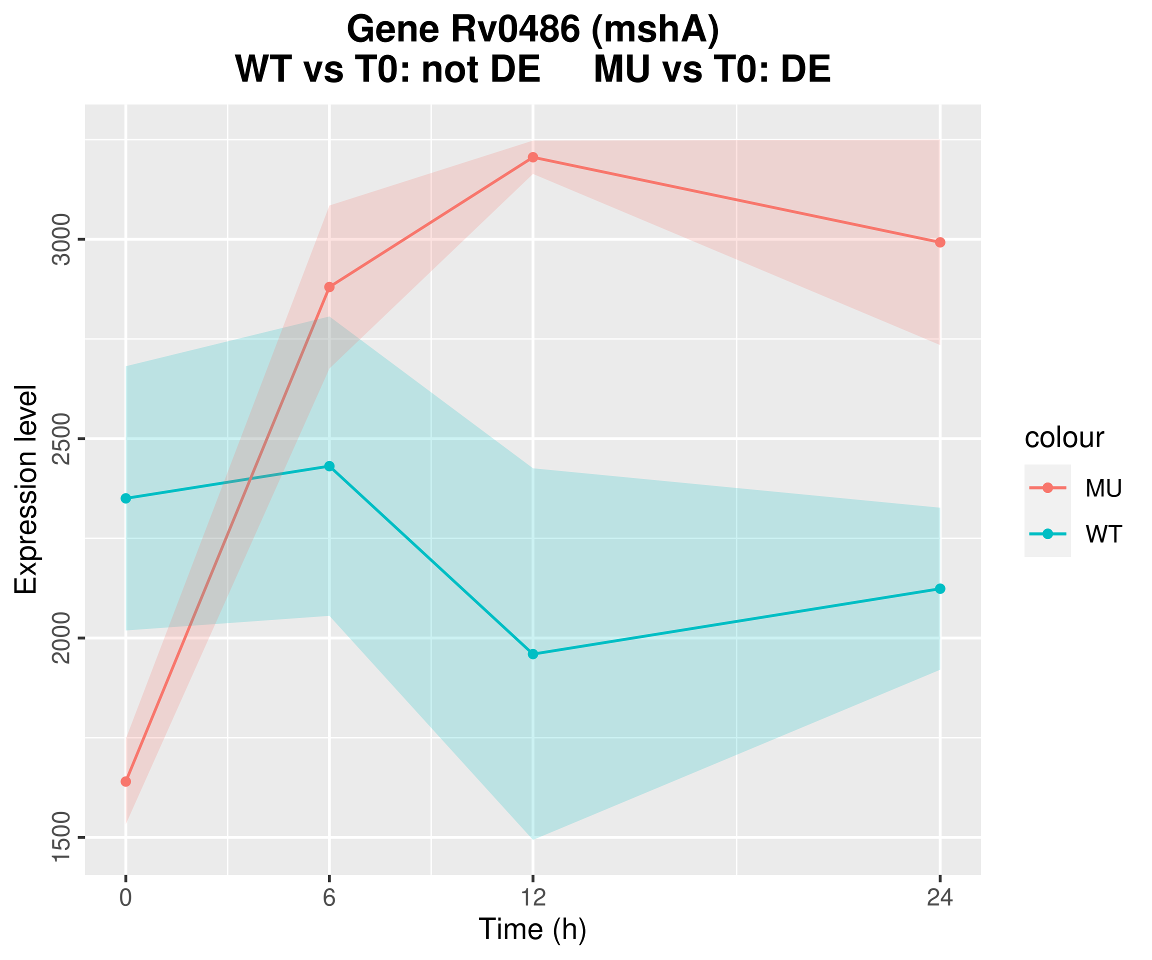
Gene Rv0486 (mshA)
WT vs T0: not DE   MU vs T0: DE
Expression level
1500
2000
2500
3000
0
6
12
24
Time (h)
colour
MU
WT

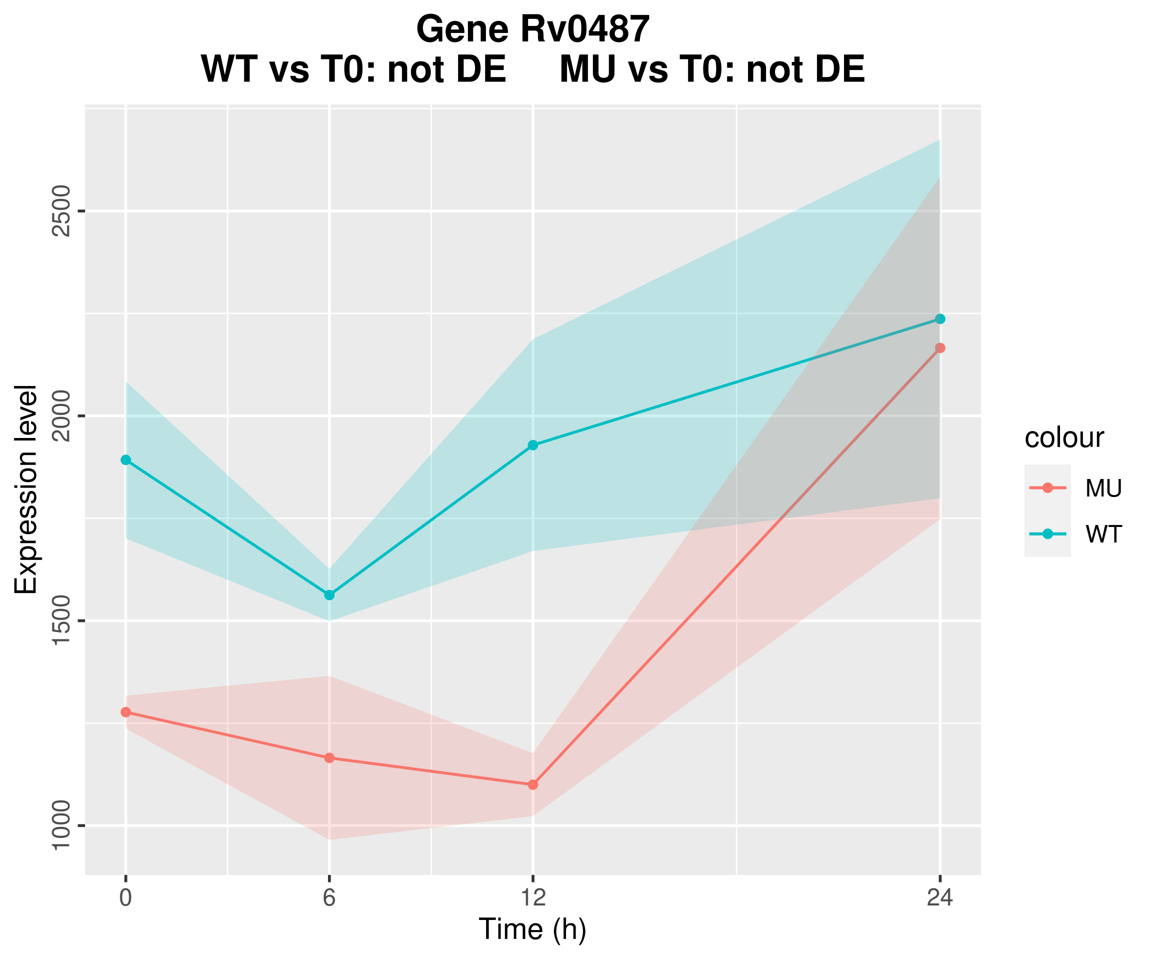

Gene Rv0487
WT vs T0: not DE   MU vs T0: not DE
Expression level
2500
2000
1500
1000
Time (h)
0
6
12
24
colour
MU
WT

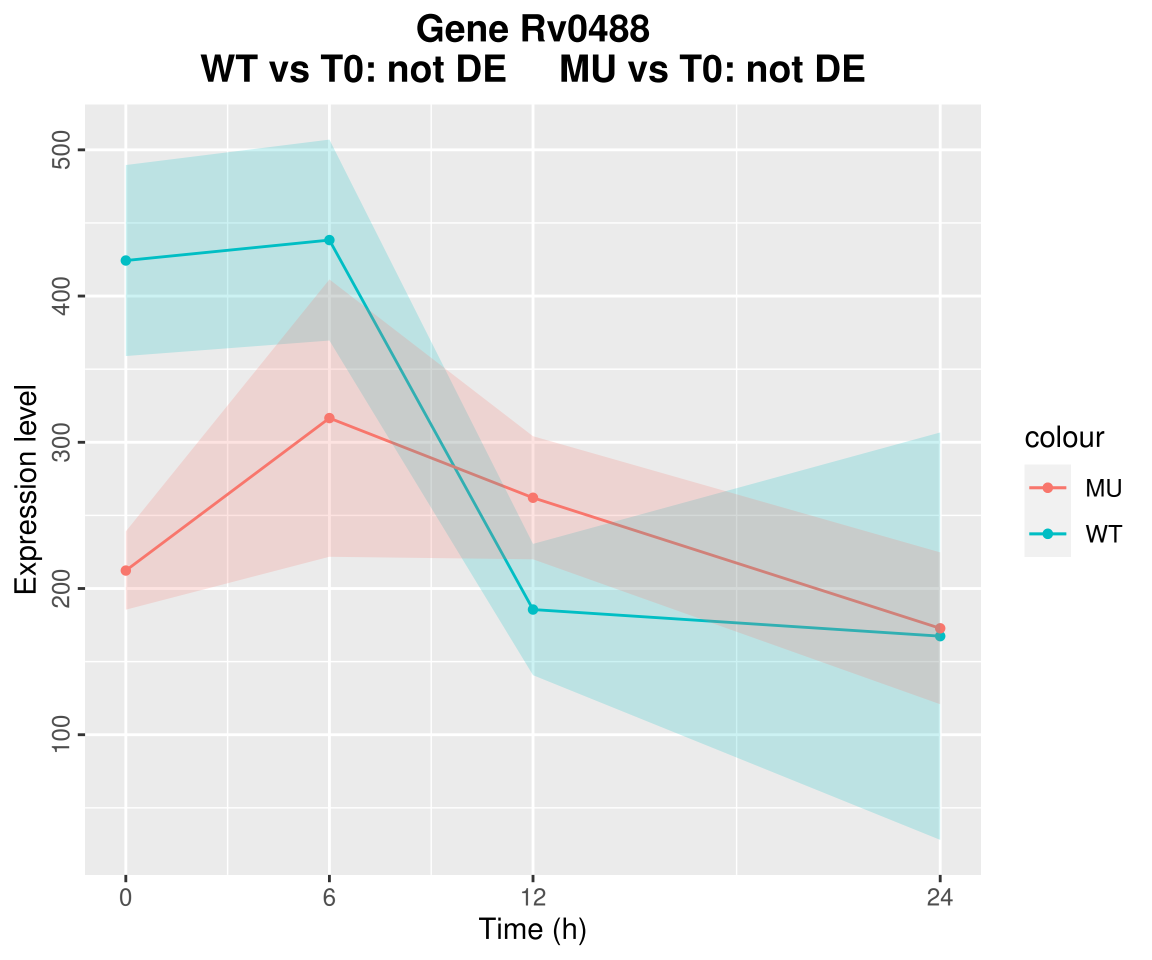

Gene Rv0488
WT vs T0: not DE MU vs T0: not DE
Expression level
100
200
300
400
500
0
6
12
24
Time (h)
colour
MU
WT

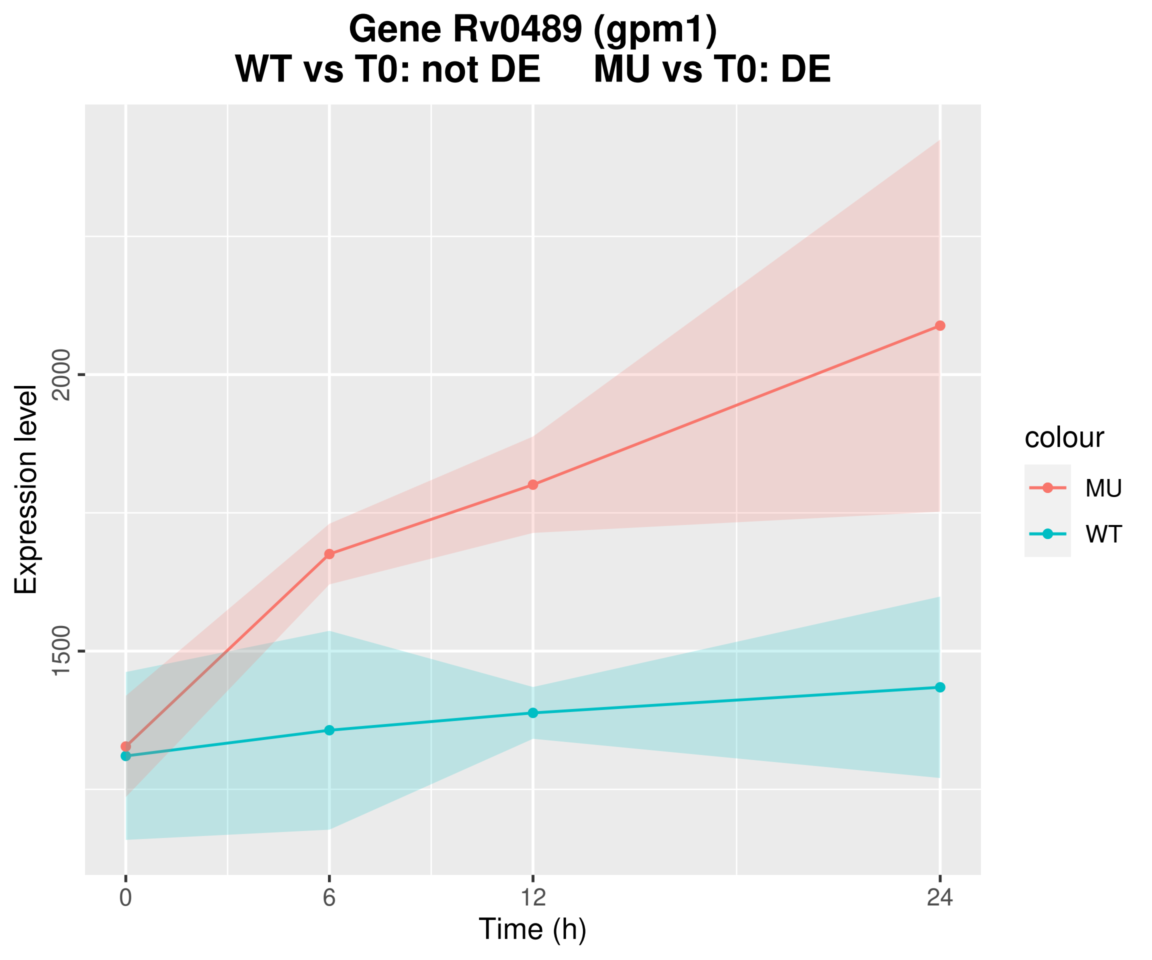

Gene Rv0489 (gpm1)
WT vs T0: not DE MU vs T0: DE
Expression level
2000
1500
0
6
12
24
Time (h)
colour
MU
WT

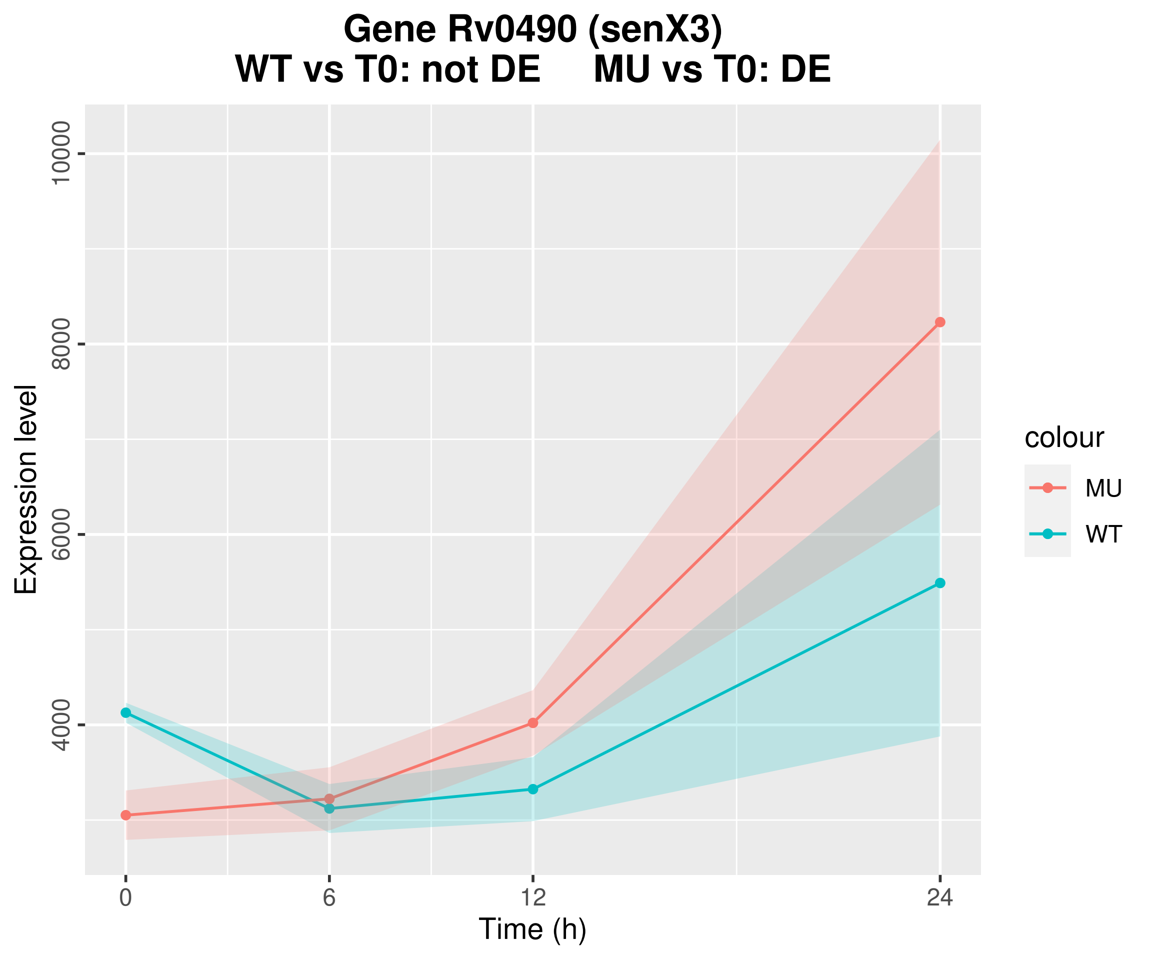

Gene Rv0490 (senX3)
WT vs T0: not DE MU vs T0: DE
Expression level
10000
8000
6000
4000
0
6
12
24
Time (h)
colour
MU
WT

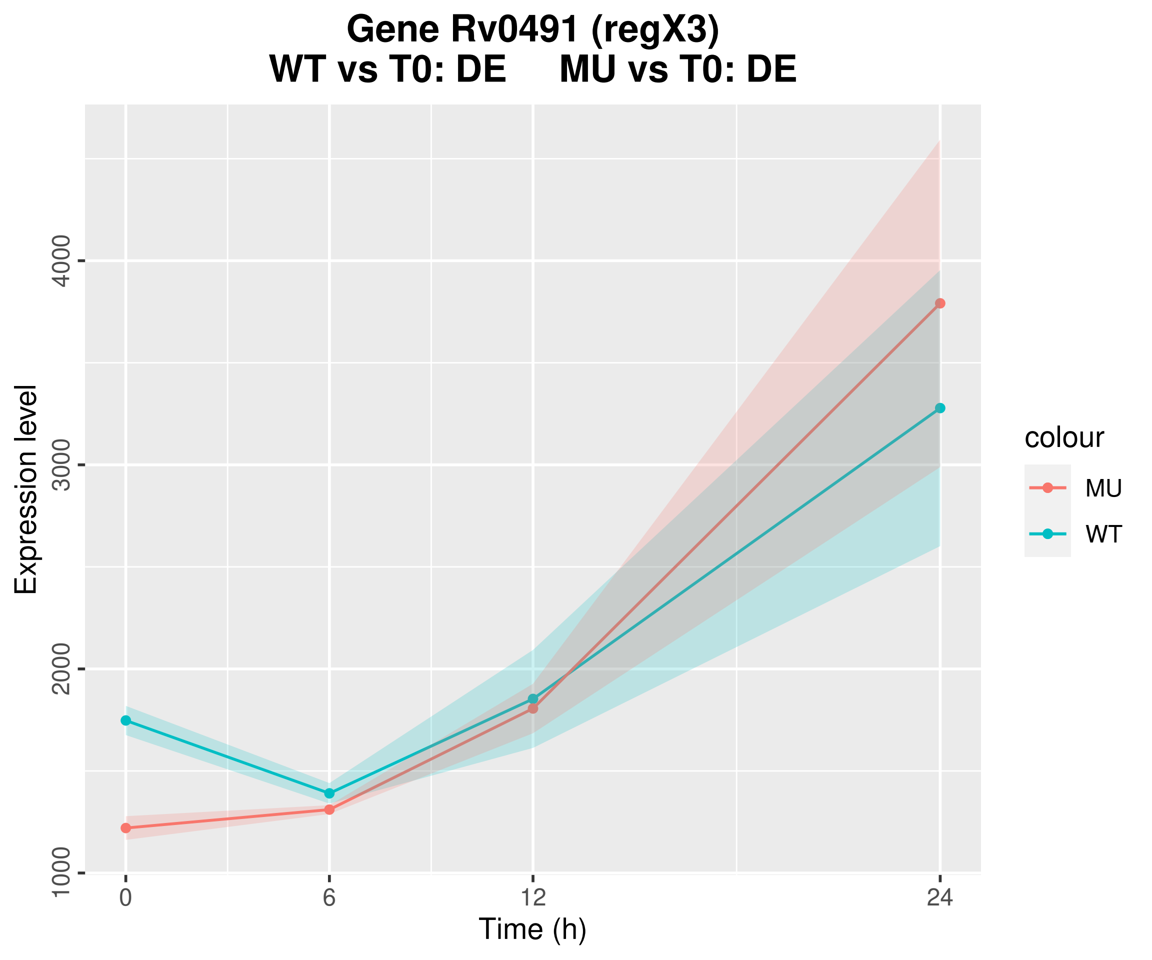
Gene Rv0491 (regX3)
WT vs T0: DE MU vs T0: DE
Expression level
1000
2000
3000
4000
Time (h)
0
6
12
24
colour
MU
WT

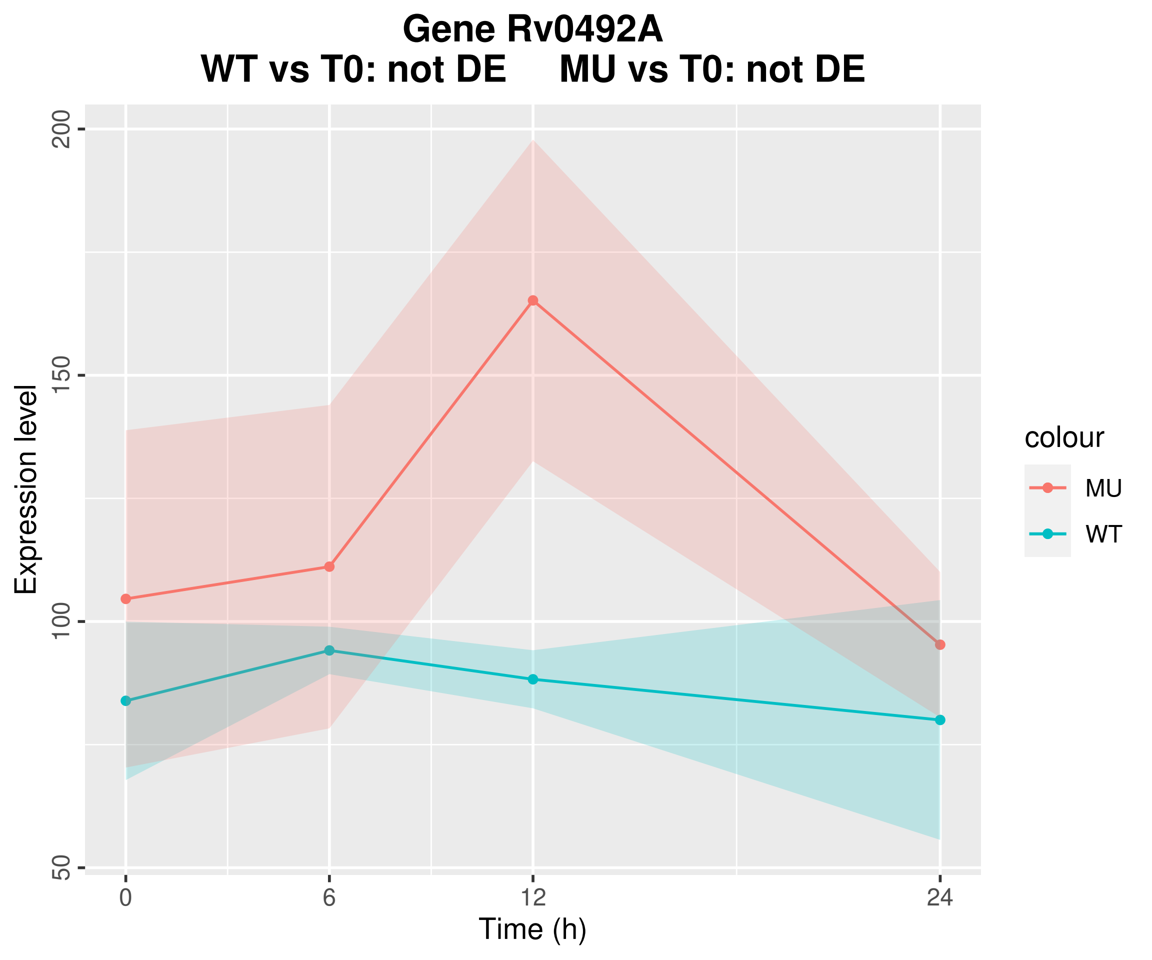

Gene Rv0492A
WT vs T0: not DE MU vs T0: not DE
Expression level
200
150
100
50
0
6
12
24
Time (h)
colour
MU
WT

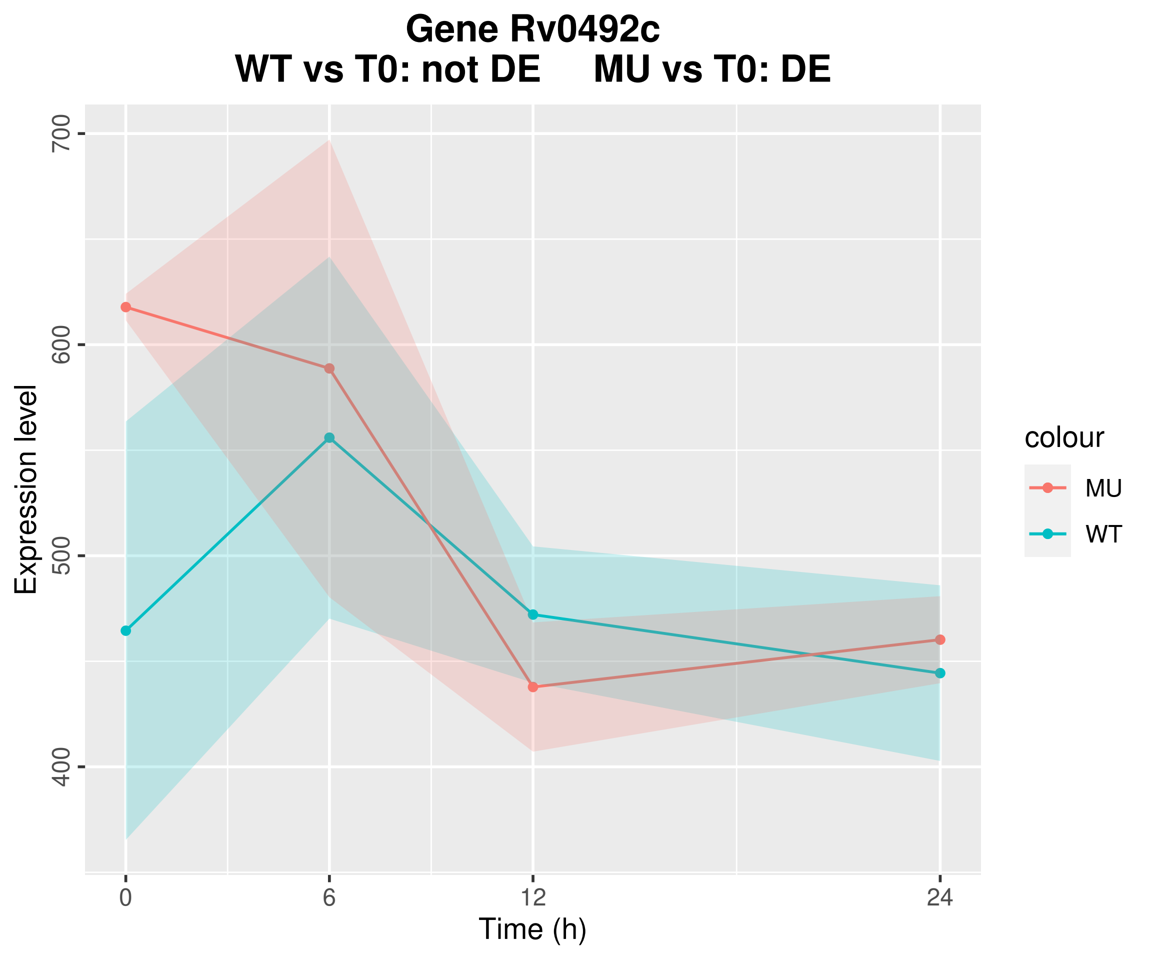

Gene Rv0492c
WT vs T0: not DE MU vs T0: DE
Expression level
700
600
500
400
0
6
12
24
Time (h)
colour
MU
WT

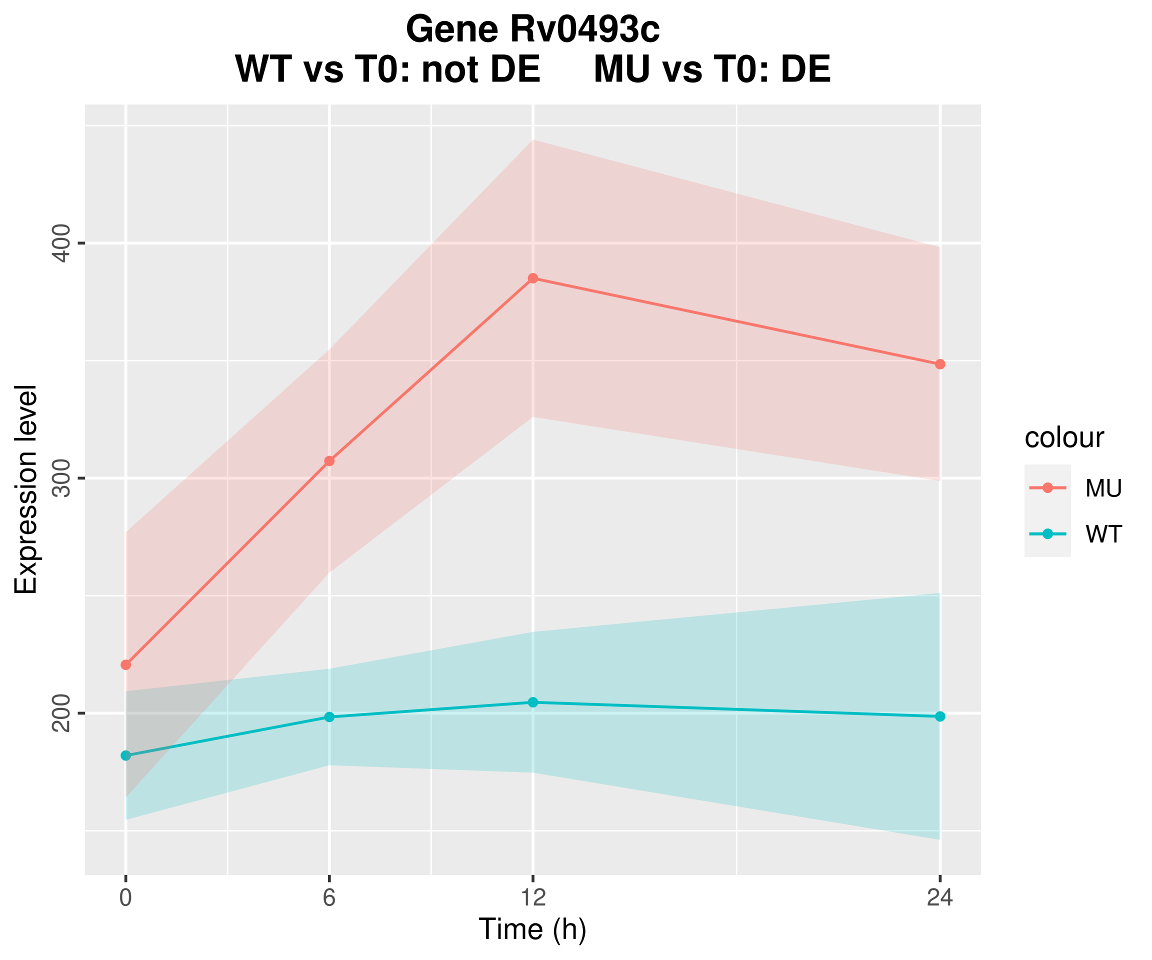
Gene Rv0493c
WT vs T0: not DE   MU vs T0: DE
Expression level
400
300
200
0
6
12
24
Time (h)
colour
MU
WT

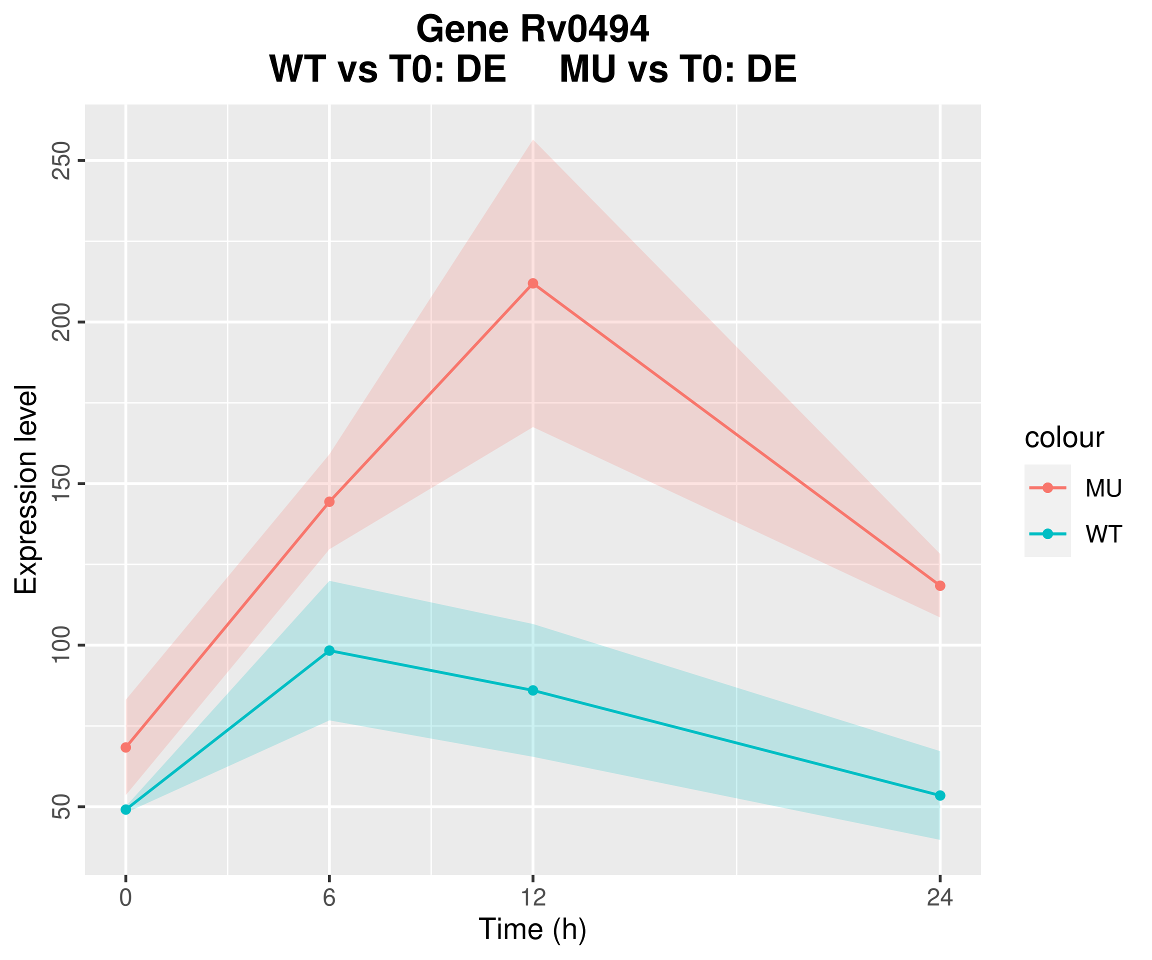
Gene Rv0494
WT vs T0: DE   MU vs T0: DE
Expression level
250
200
150
100
50
0
6
12
24
Time (h)
colour
MU
WT

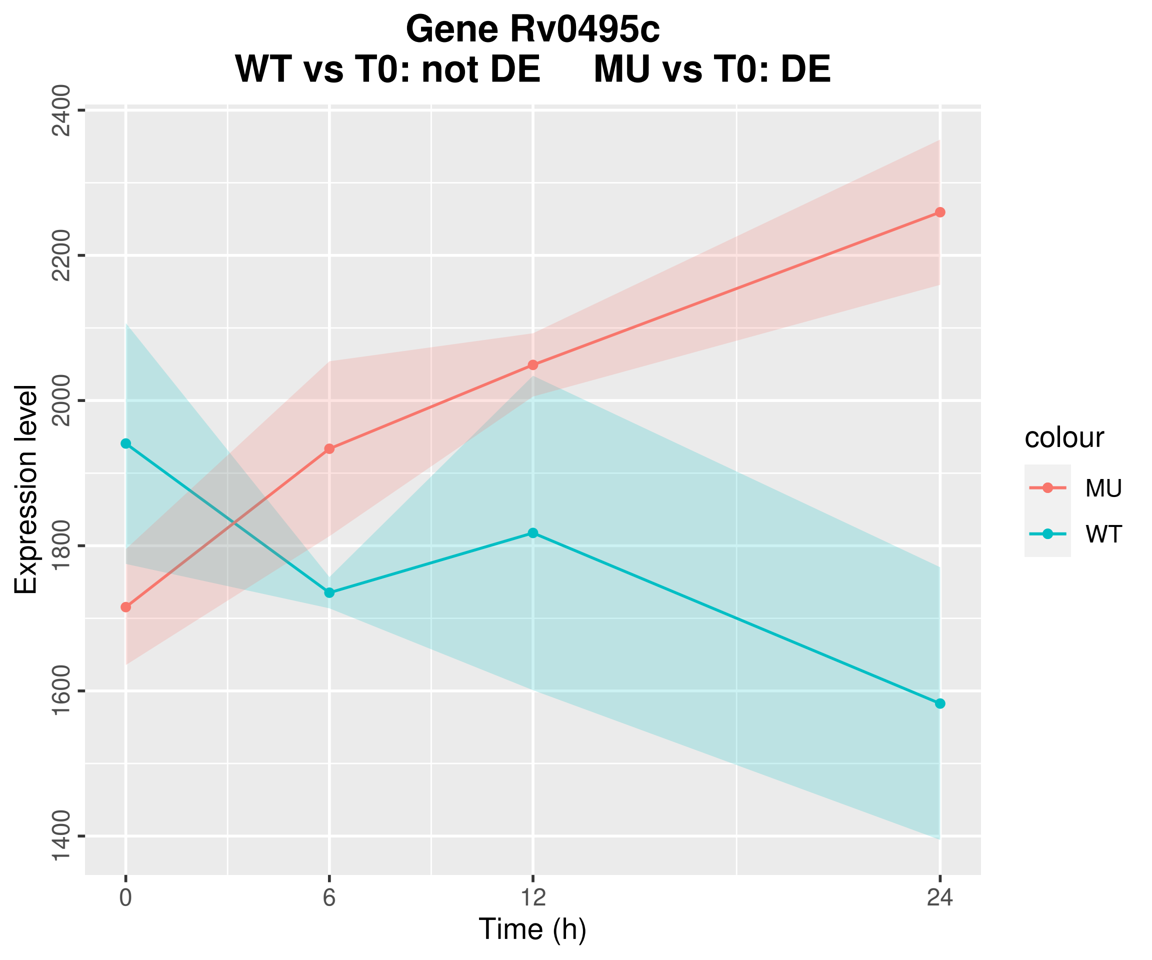

Gene Rv0495c
WT vs T0: not DE   MU vs T0: DE
Expression level
2400
2200
2000
1800
1600
1400
0
6
12
24
Time (h)
colour
MU
WT

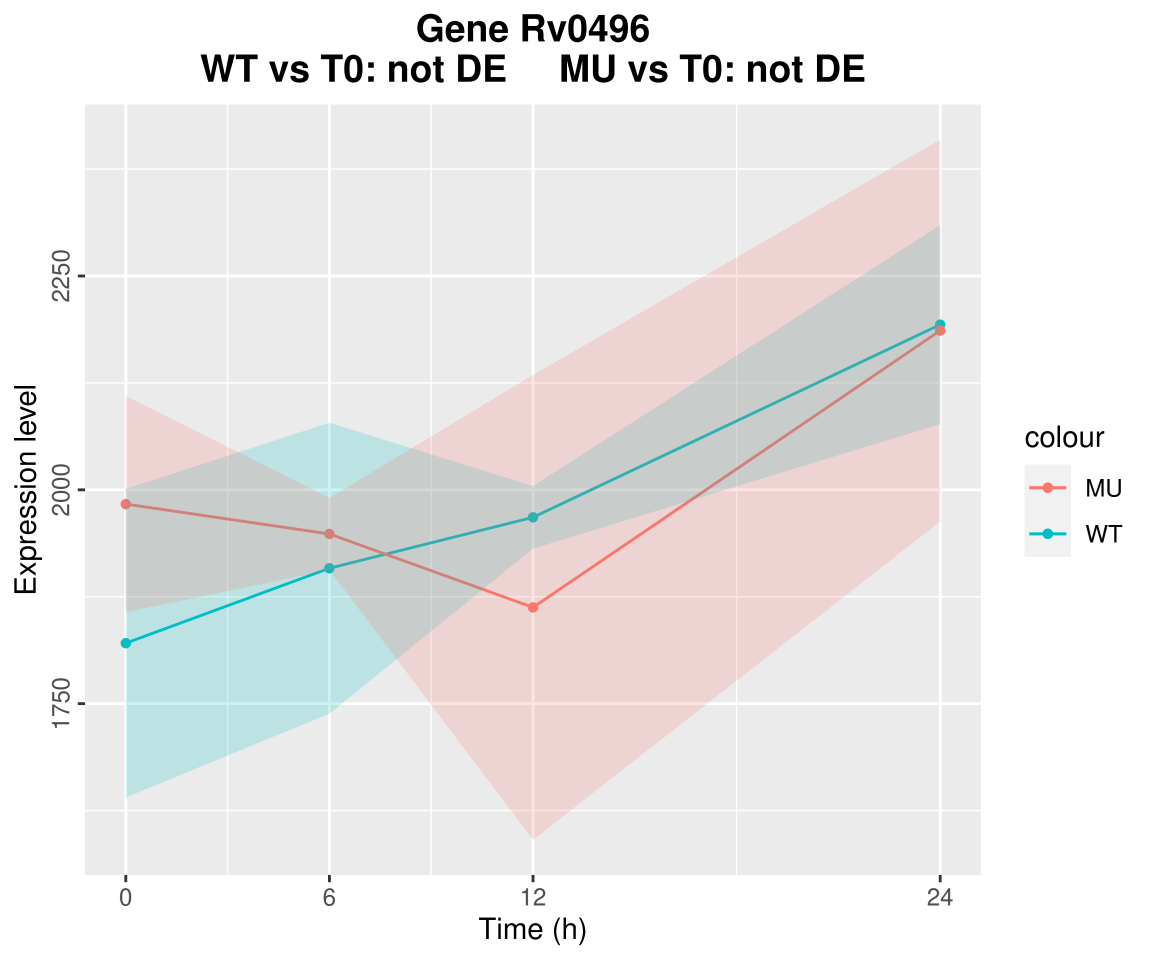
Gene Rv0496
WT vs T0: not DE MU vs T0: not DE
Expression level
2250
2000
1750
0
6
12
24
Time (h)
colour
MU
WT

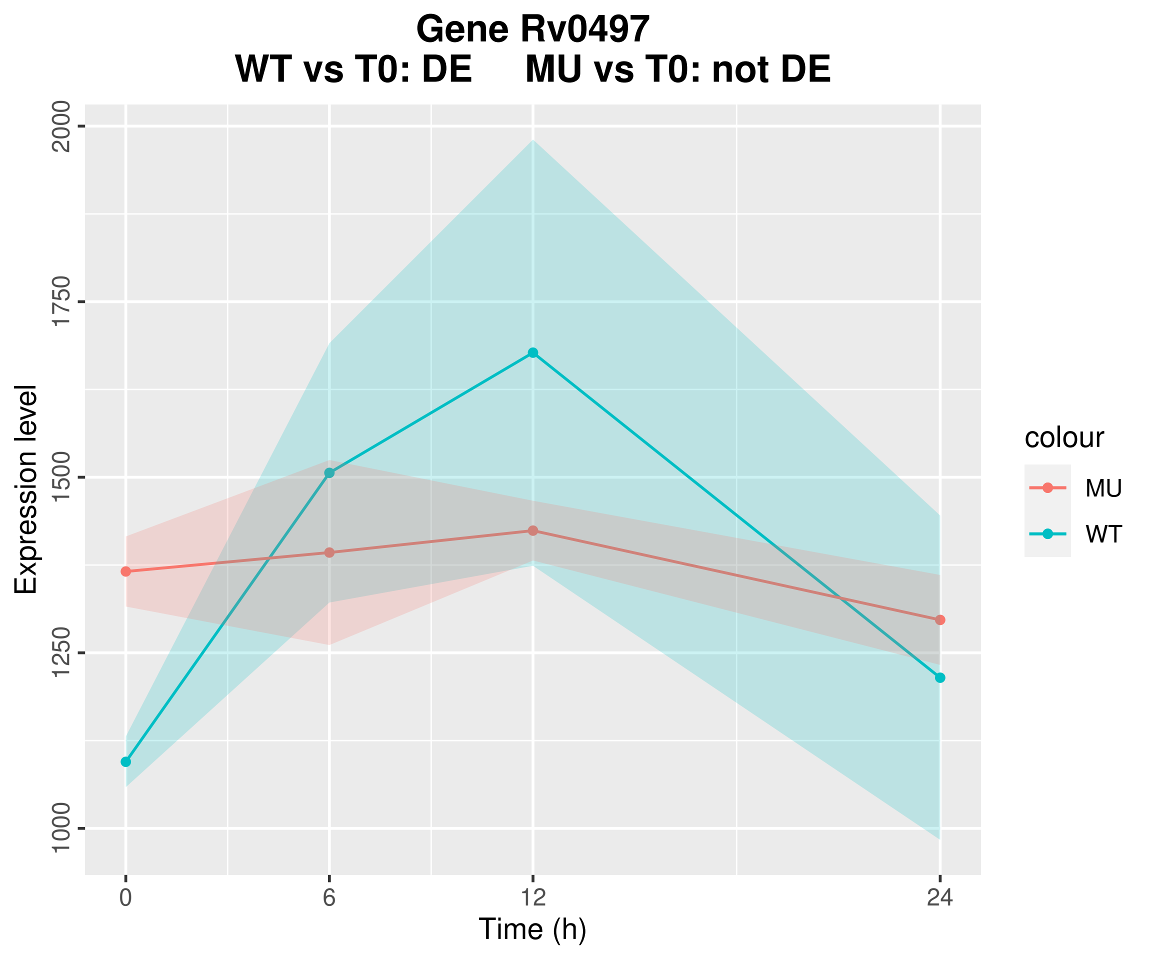
Gene Rv0497
WT vs T0: DE   MU vs T0: not DE
Expression level
2000
1750
1500
1250
1000
0
6
12
24
Time (h)
colour
MU
WT

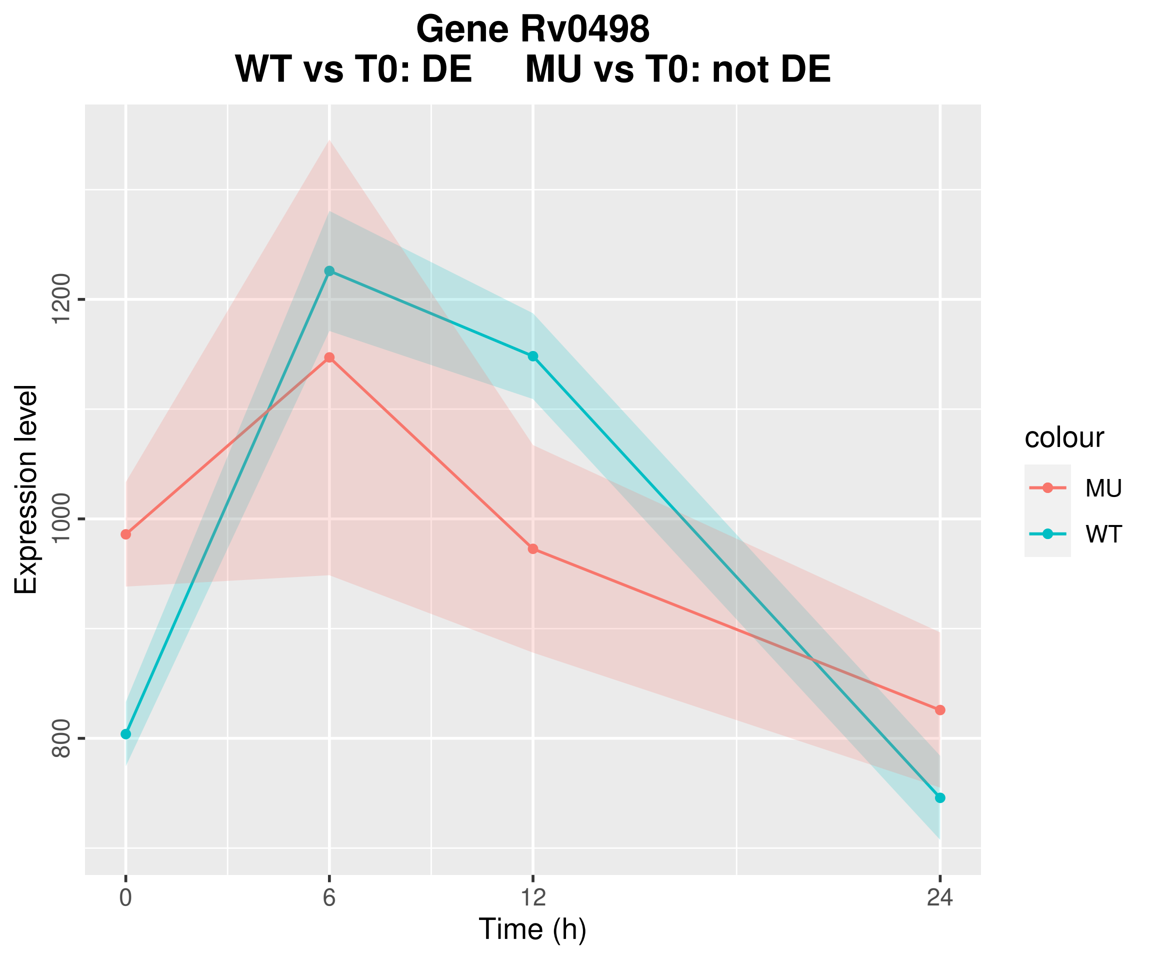

Gene Rv0498
WT vs T0: DE MU vs T0: not DE
Expression level
1200
1000
800
0
6
12
24
Time (h)
colour
MU
WT

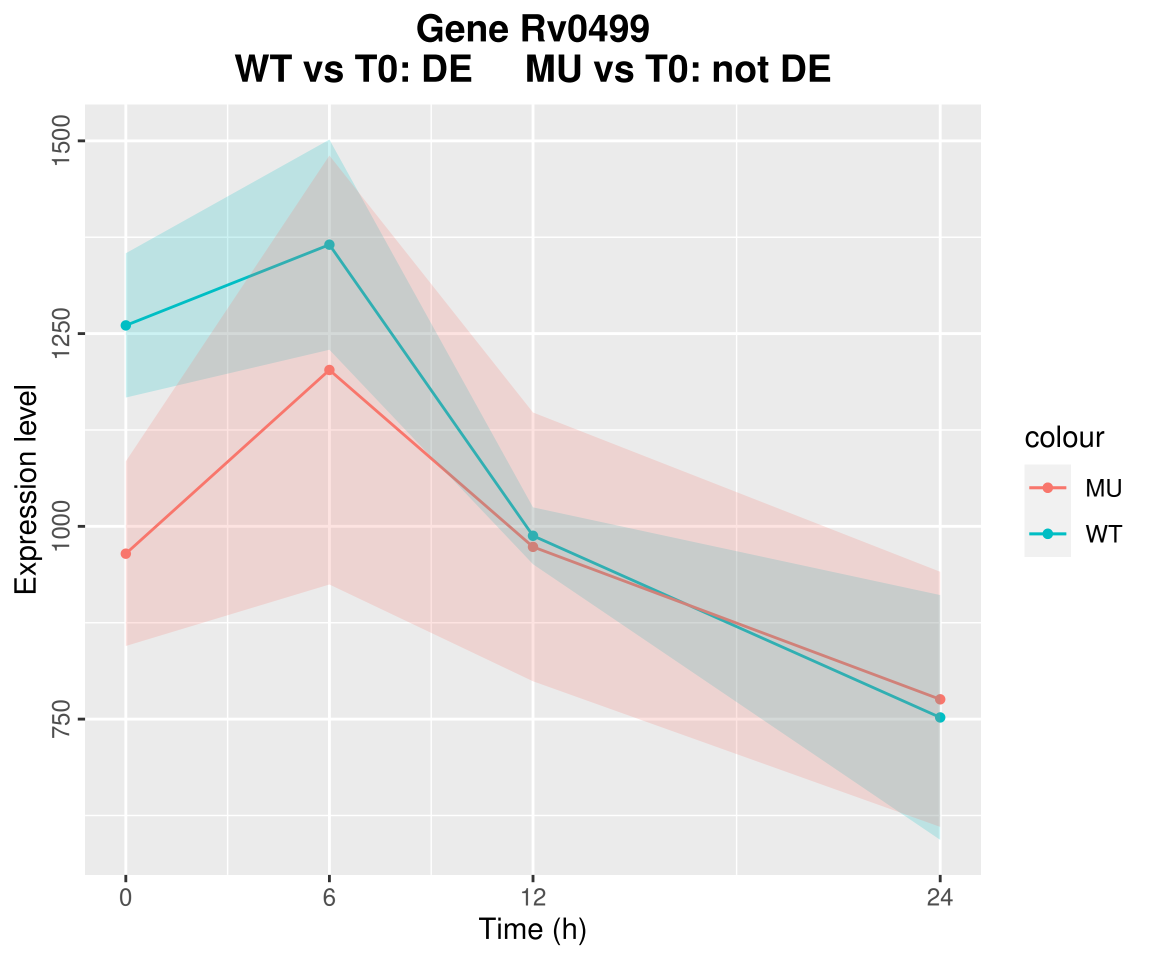

Gene Rv0499
WT vs T0: DE   MU vs T0: not DE
Expression level
1500
1250
1000
750
0
6
12
24
Time (h)
colour
MU
WT

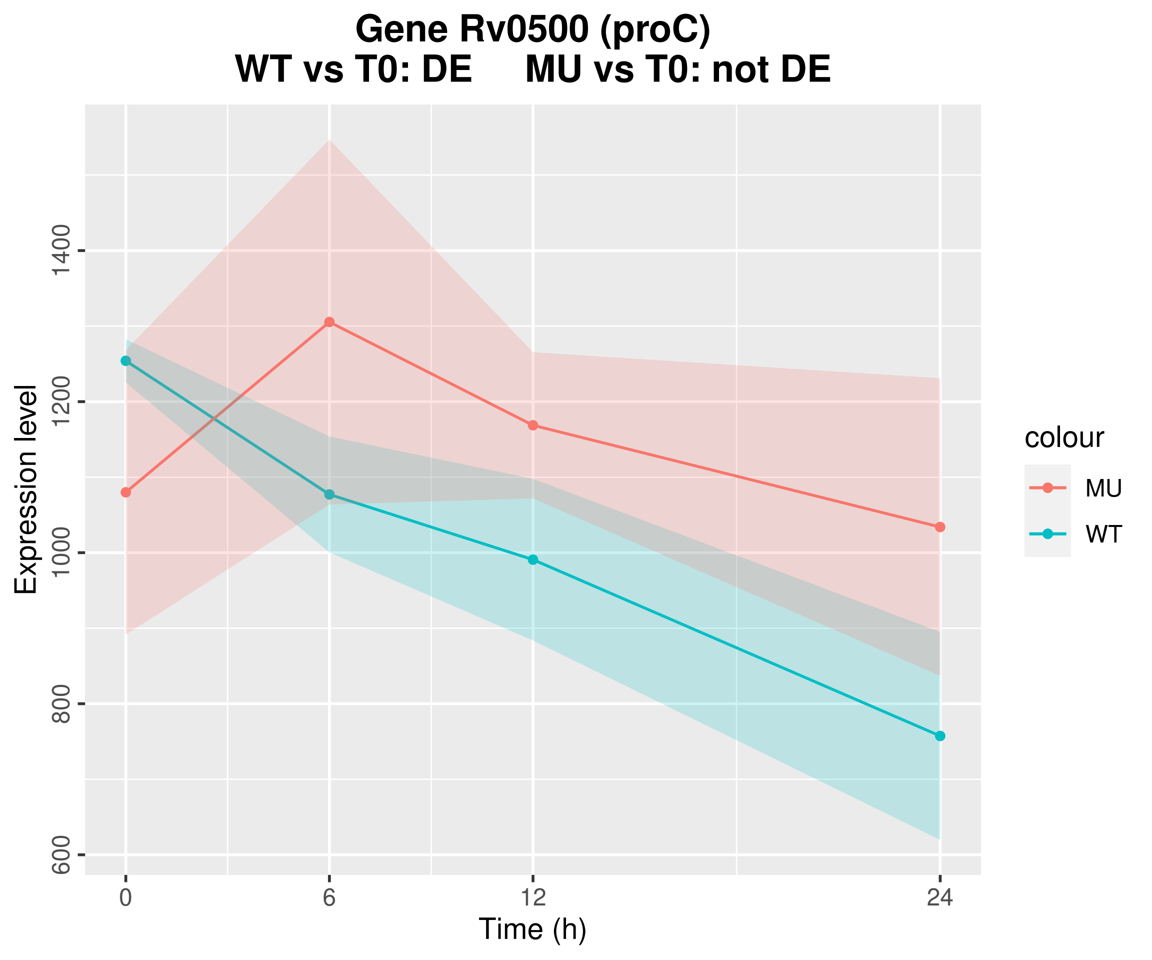

Gene Rv0500 (proC)
WT vs T0: DE   MU vs T0: not DE
Expression level
600
800
1000
1200
1400
0
6
12
24
Time (h)
colour
MU
WT

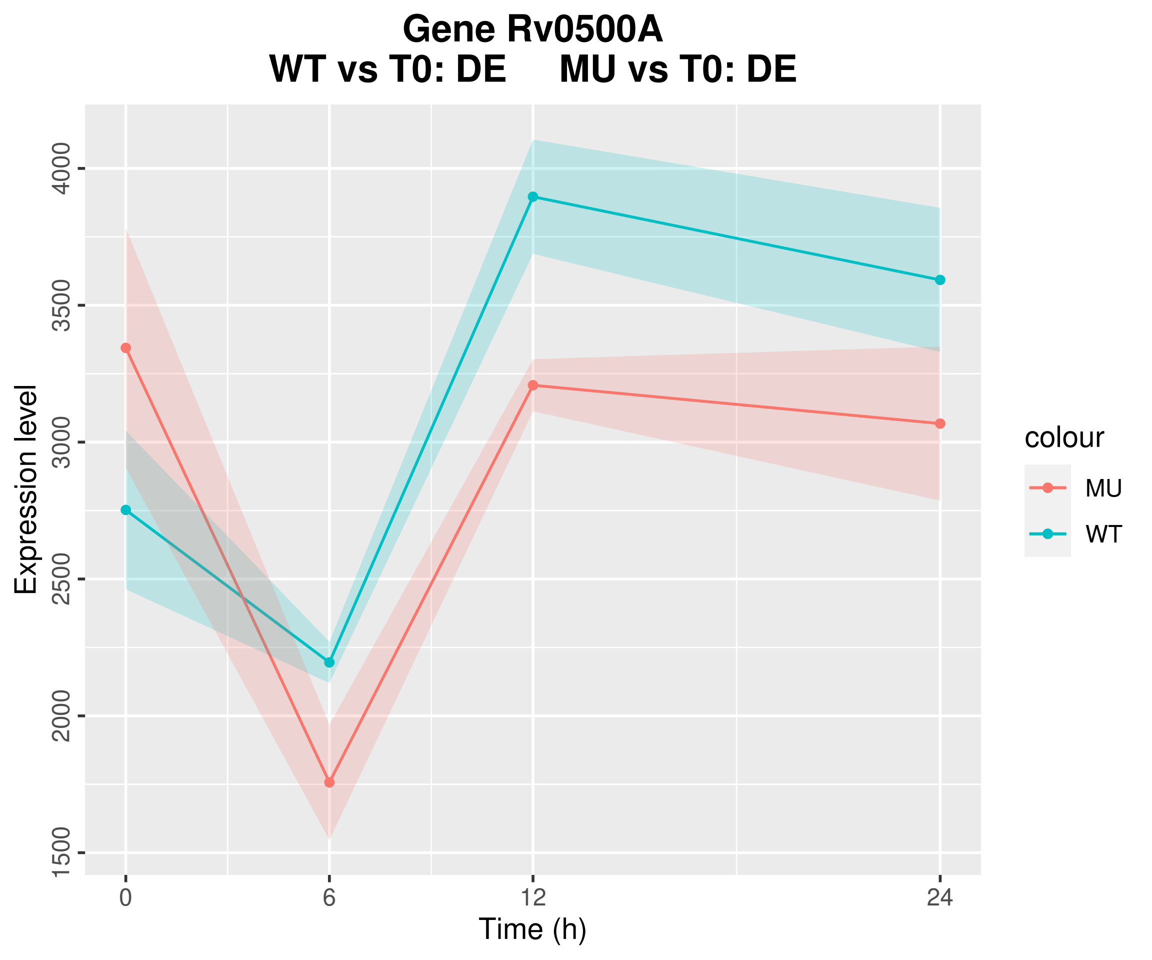

Gene Rv0500A
WT vs T0: DE   MU vs T0: DE
Expression level
4000
3500
3000
2500
2000
1500
0
6
12
24
Time (h)
colour
MU
WT

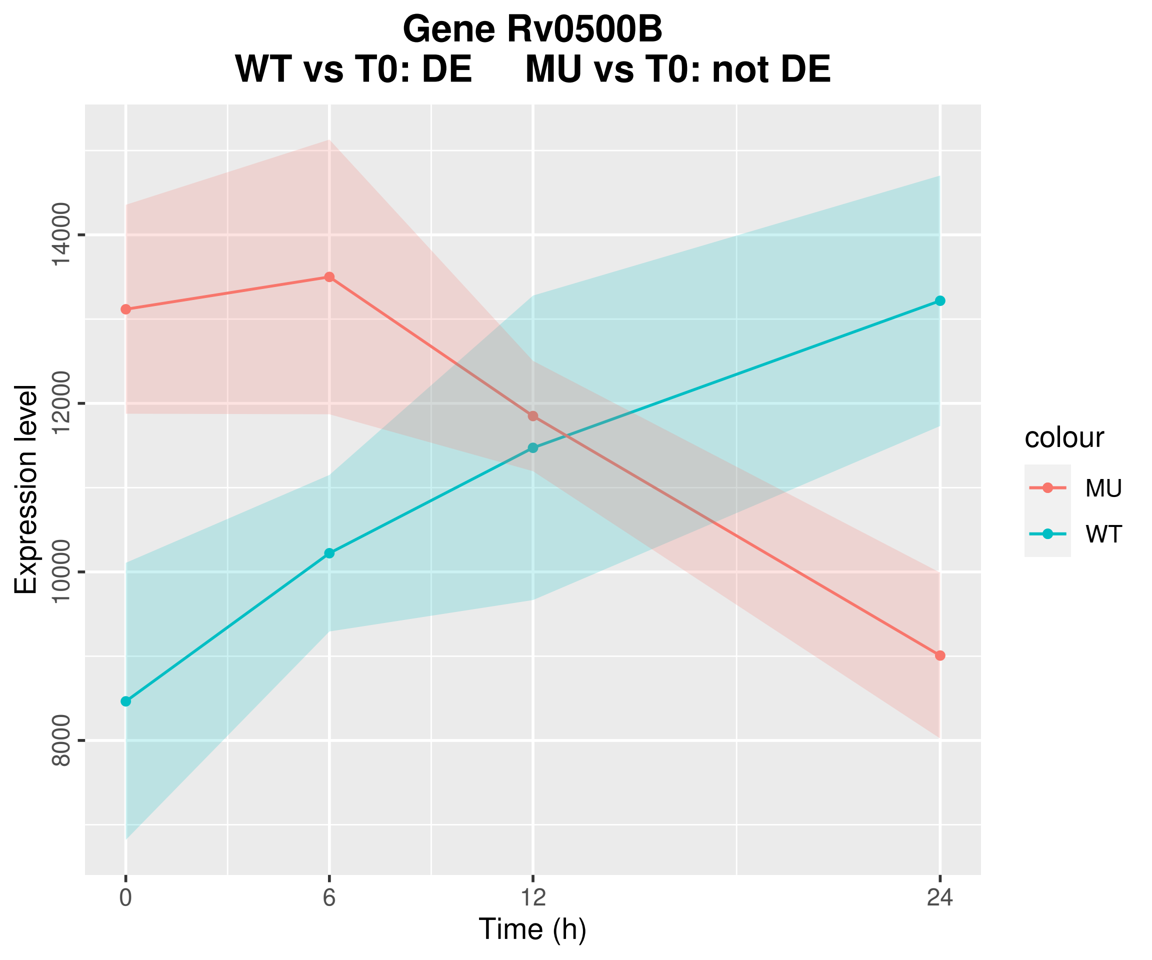
Gene Rv0500B
WT vs T0: DE MU vs T0: not DE
Expression level
14000
12000
10000
8000
0
6
12
24
Time (h)
colour
MU
WT

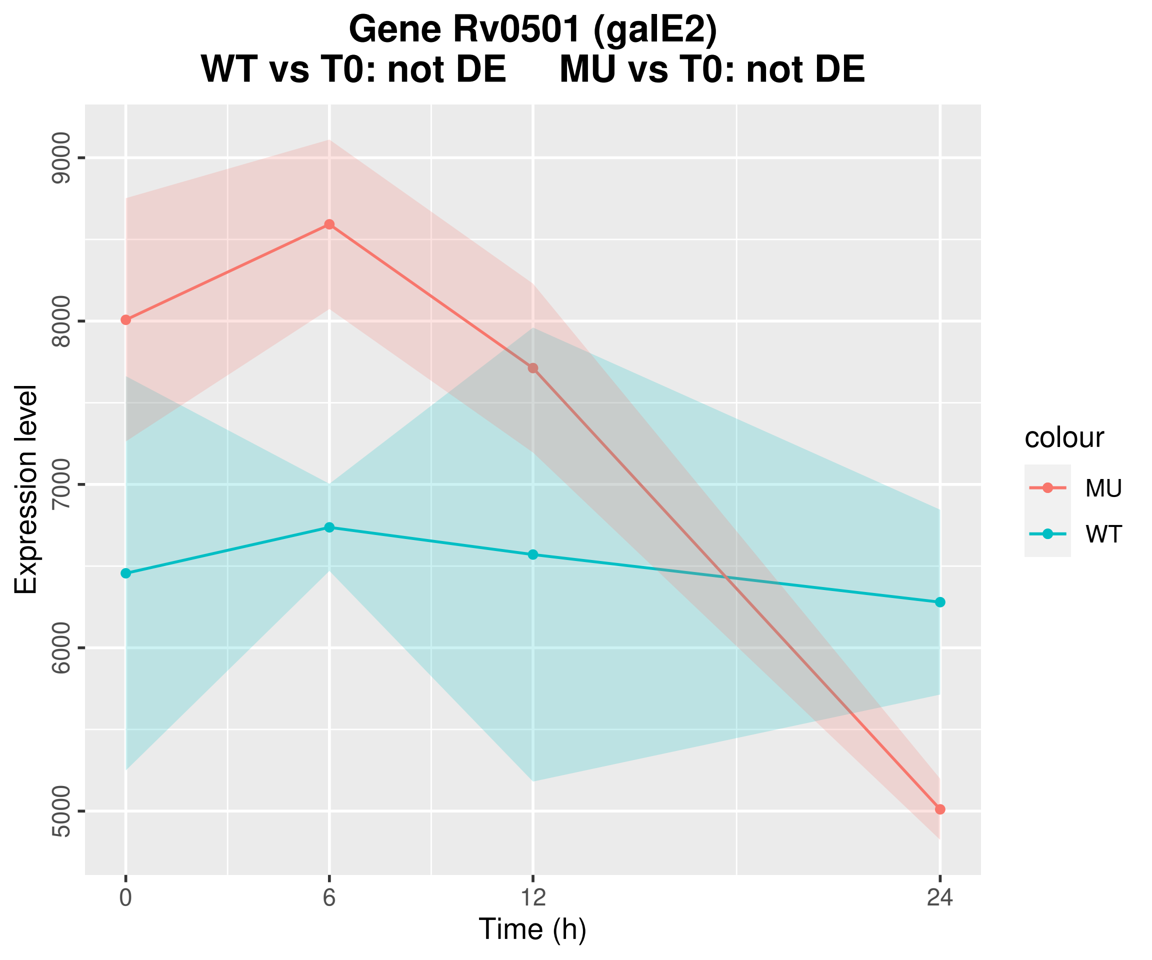
Gene Rv0501 (galE2)
WT vs T0: not DE   MU vs T0: not DE
Expression level
9000
8000
7000
6000
5000
0
6
12
24
Time (h)
colour
MU
WT

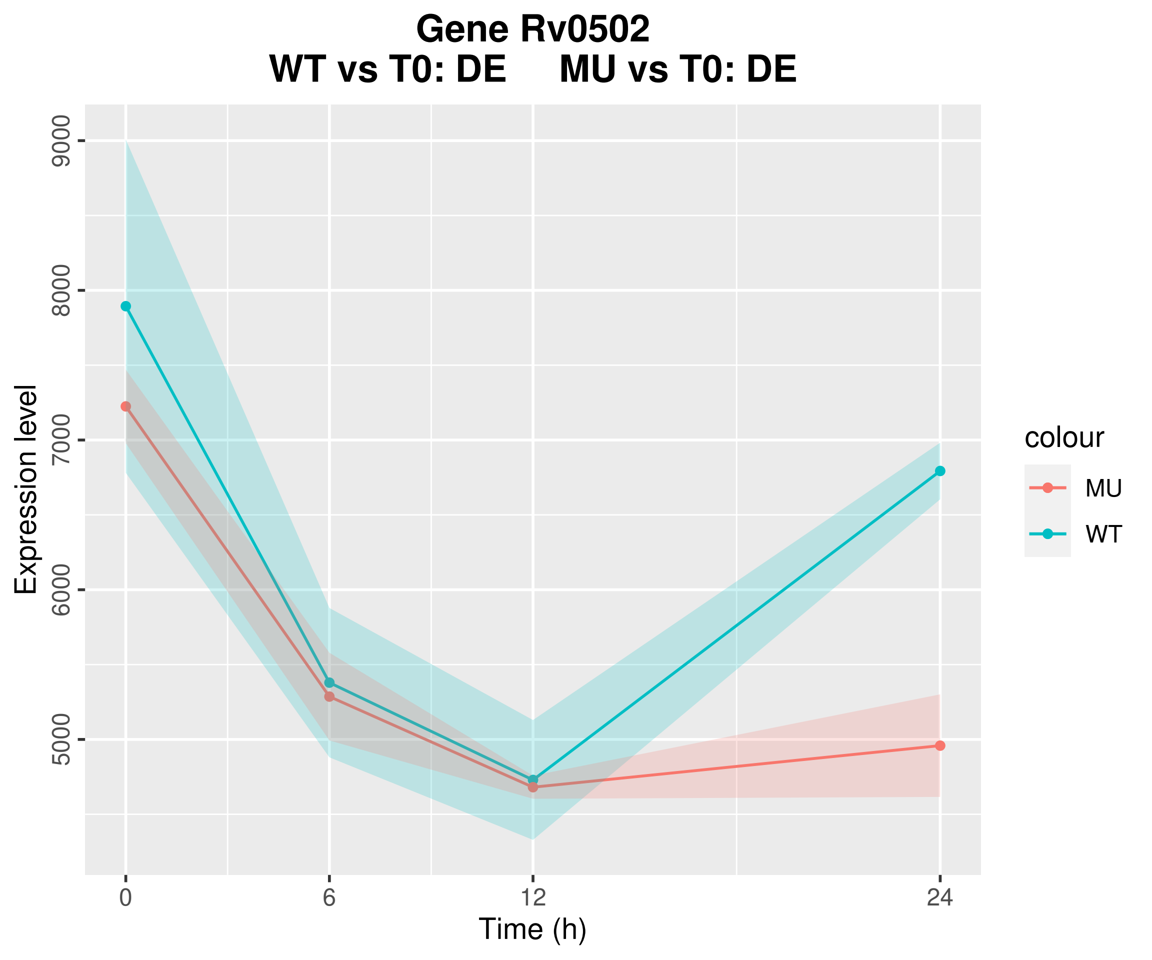

Gene Rv0502
WT vs T0: DE MU vs T0: DE
Expression level
9000
8000
7000
6000
5000
0
6
12
24
Time (h)
colour
MU
WT

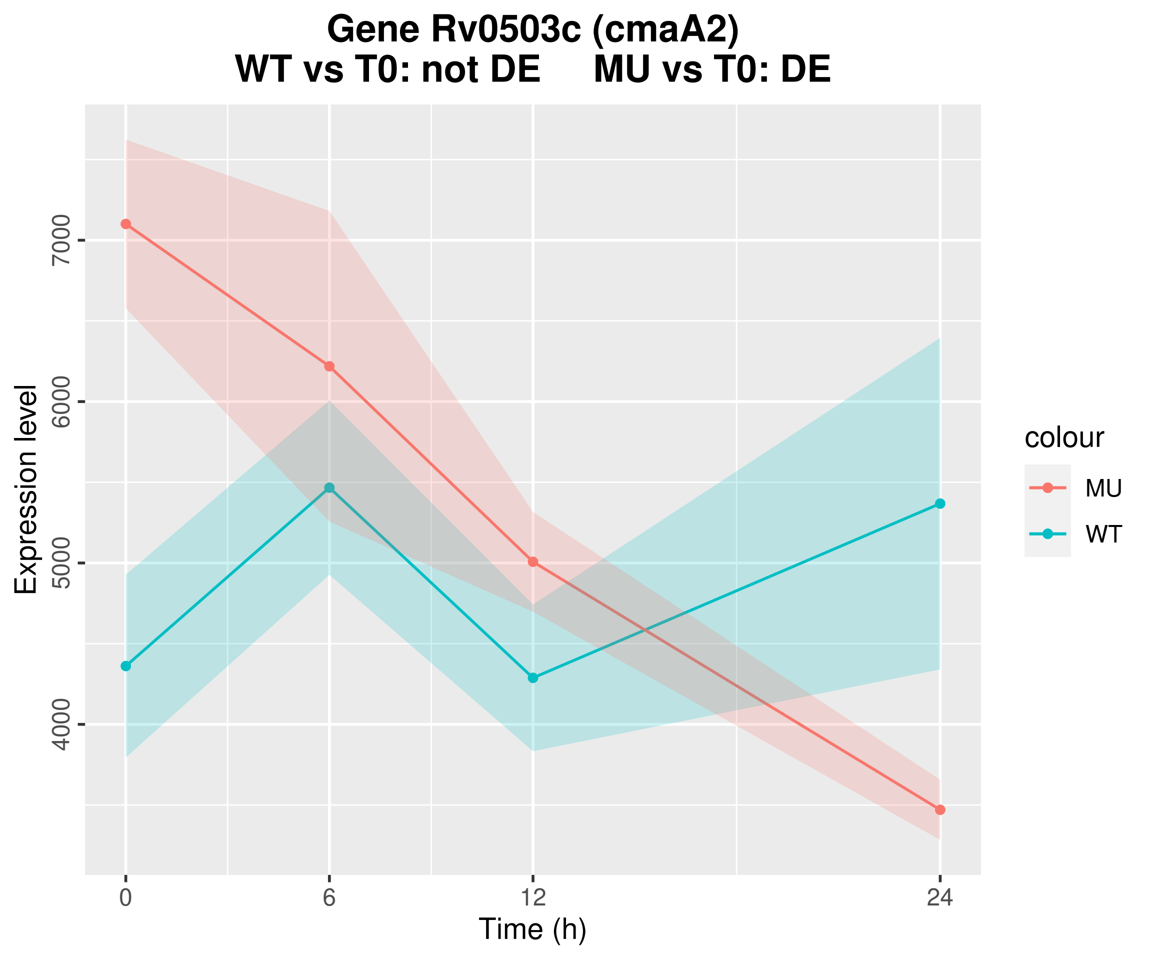
Gene Rv0503c (cmaA2)
WT vs T0: not DE   MU vs T0: DE
Expression level
7000
6000
5000
4000
0
6
12
24
Time (h)
colour
MU
WT

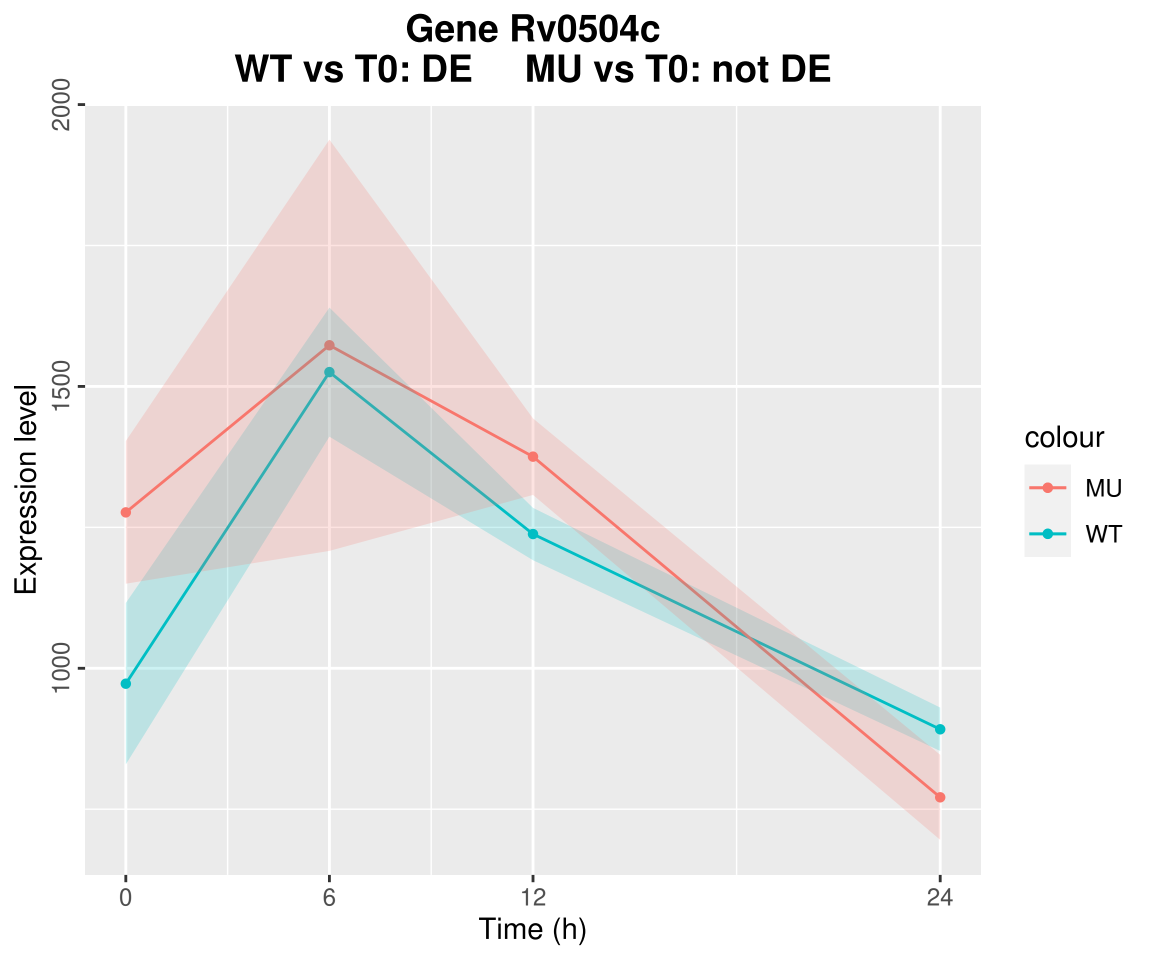
Gene Rv0504c
WT vs T0: DE   MU vs T0: not DE
2000
1500
1000
Expression level
0
6
12
24
Time (h)
colour
MU
WT

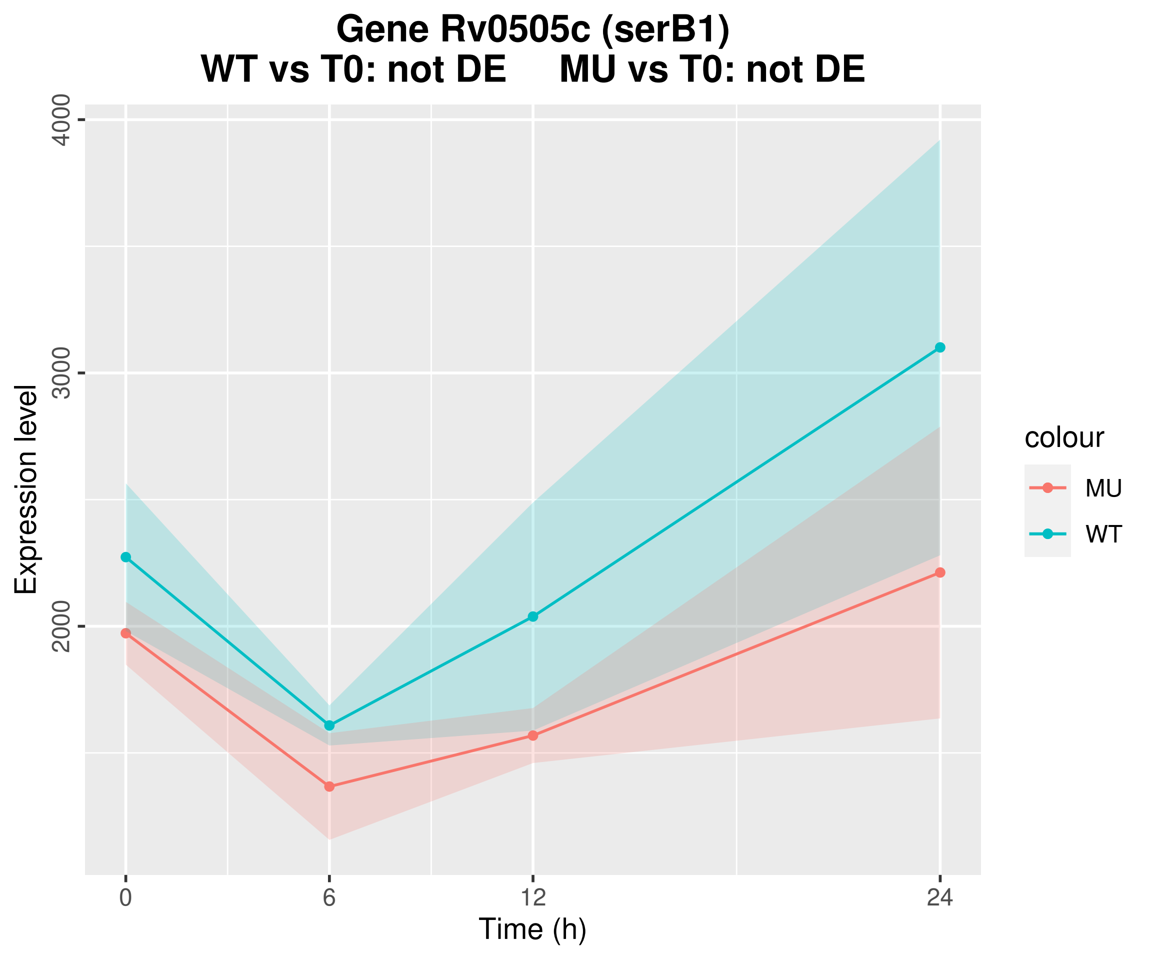

Gene Rv0505c (serB1)
WT vs T0: not DE   MU vs T0: not DE
4000
3000
2000
Expression level
0
6
12
24
Time (h)
colour
MU
WT

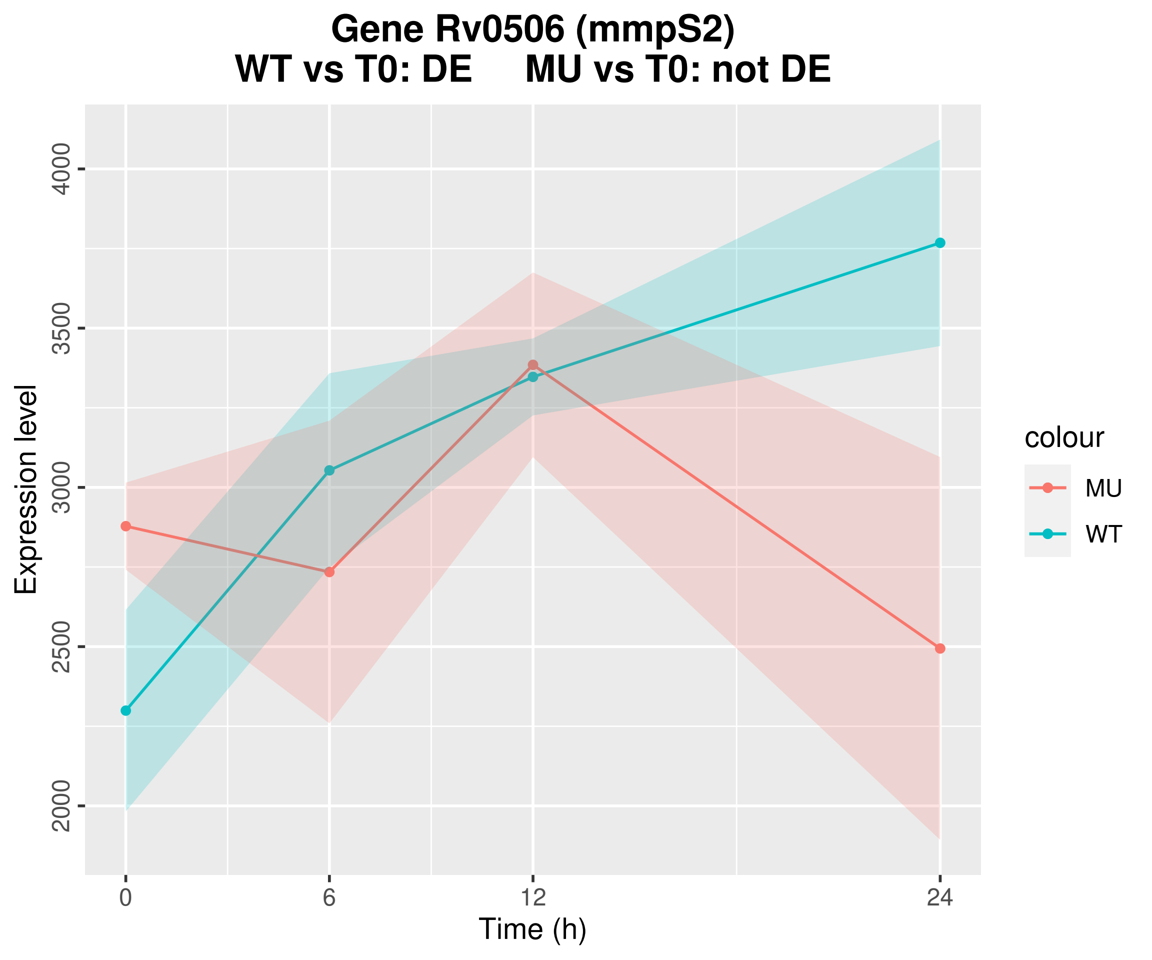

Gene Rv0506 (mmpS2)
WT vs T0: DE   MU vs T0: not DE
Expression level
4000
3500
3000
2500
2000
0
6
12
24
Time (h)
colour
MU
WT

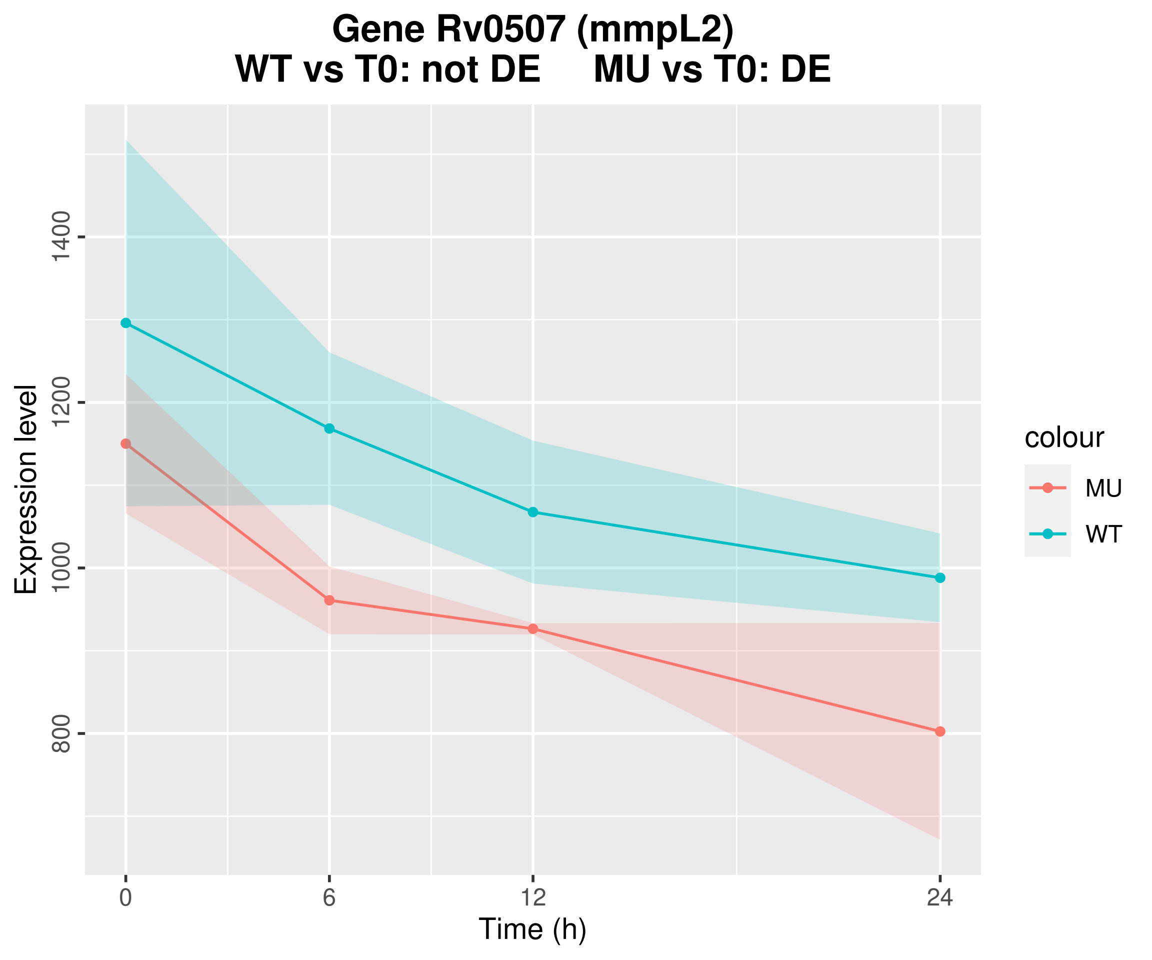
Gene Rv0507 (mmpL2)
WT vs T0: not DE MU vs T0: DE
Expression level
1400
1200
1000
800
0
6
12
24
Time (h)
colour
MU
WT

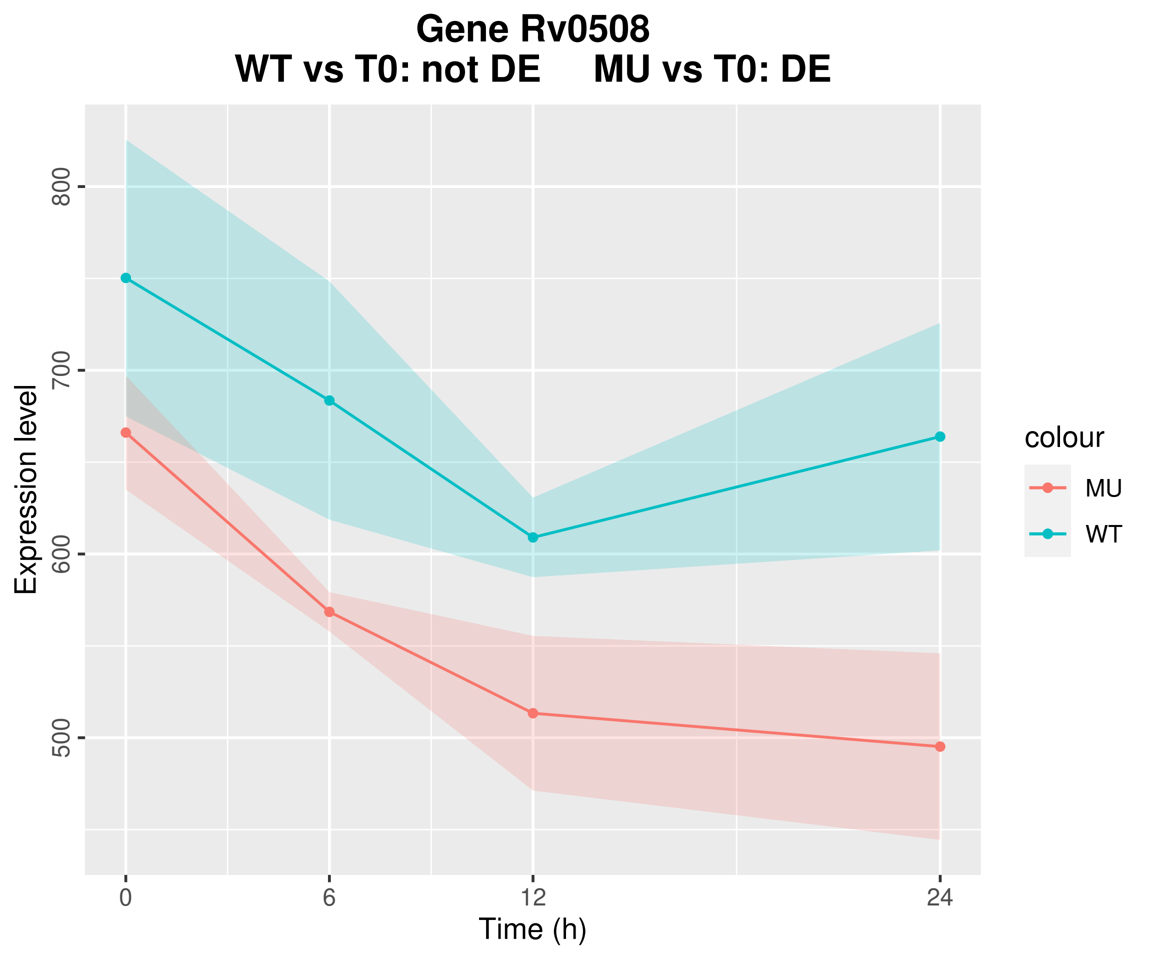

Gene Rv0508
WT vs T0: not DE MU vs T0: DE
800
700
600
500
Expression level
0
6
12
24
Time (h)
colour
MU
WT

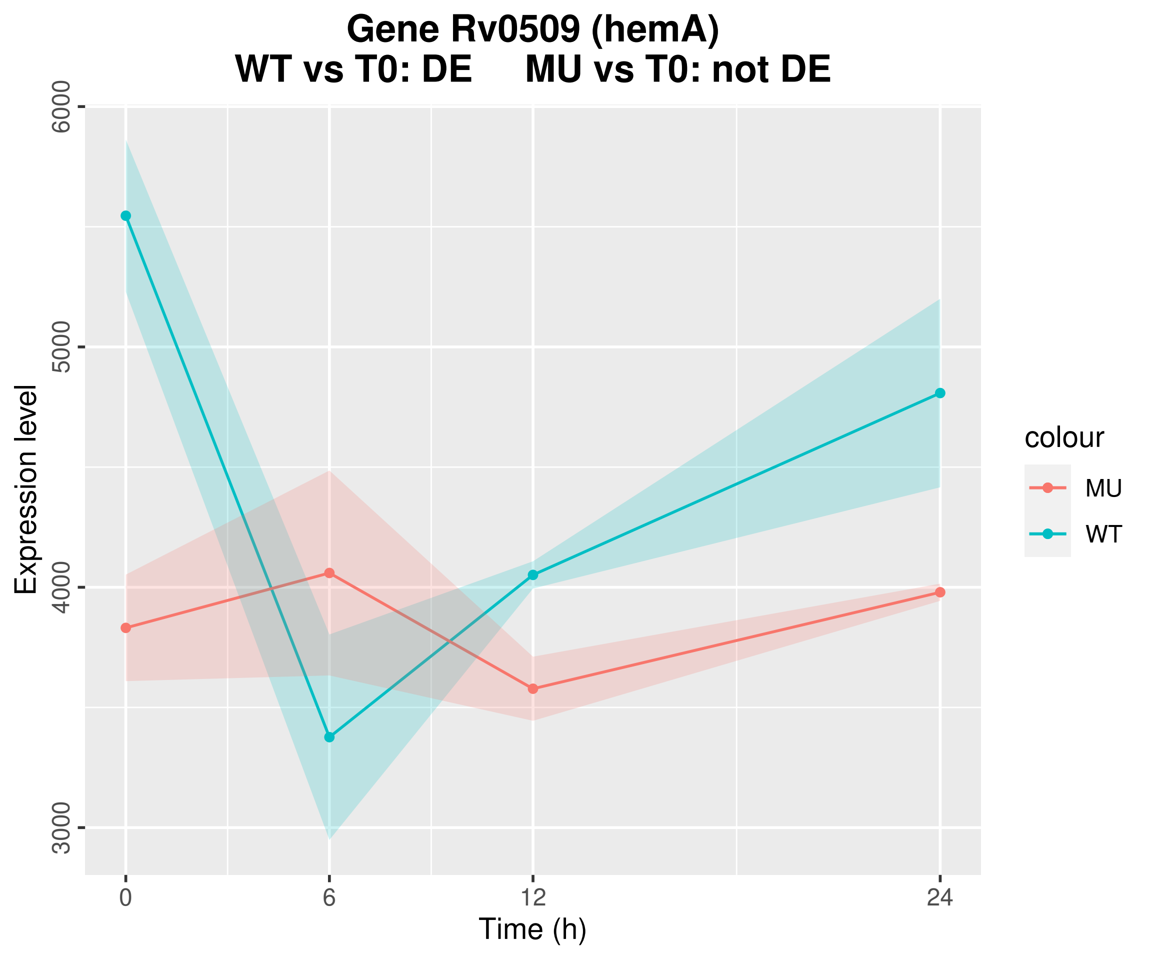
Gene Rv0509 (hemA)
WT vs T0: DE   MU vs T0: not DE
6000
5000
4000
3000
Expression level
0
6
12
24
Time (h)
colour
MU
WT

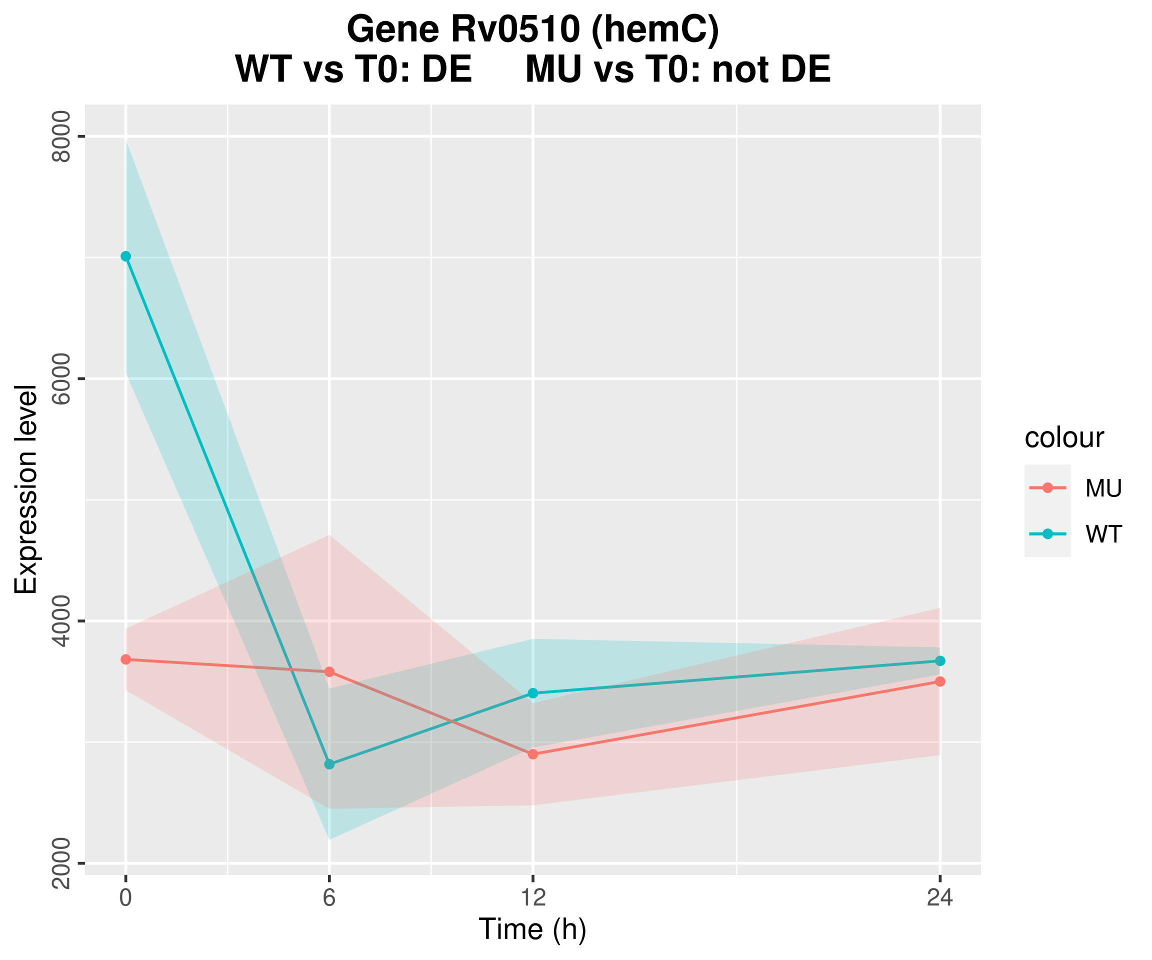
Gene Rv0510 (hemC)
WT vs T0: DE   MU vs T0: not DE
Expression level
8000
6000
4000
2000
0
6
12
24
Time (h)
colour
MU
WT

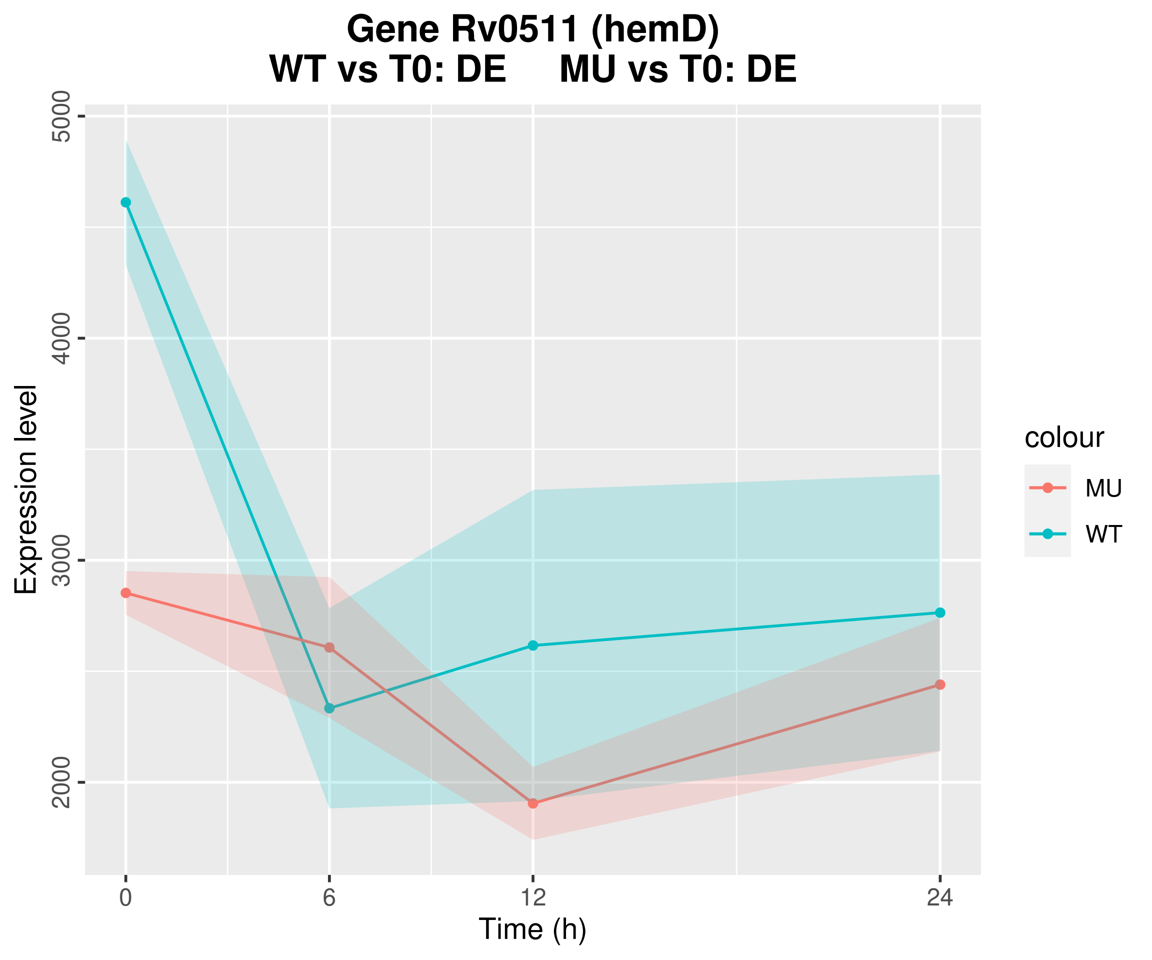

Gene Rv0511 (hemD)
WT vs T0: DE MU vs T0: DE
Expression level
5000
4000
3000
2000
0
6
12
24
Time (h)
colour
MU
WT

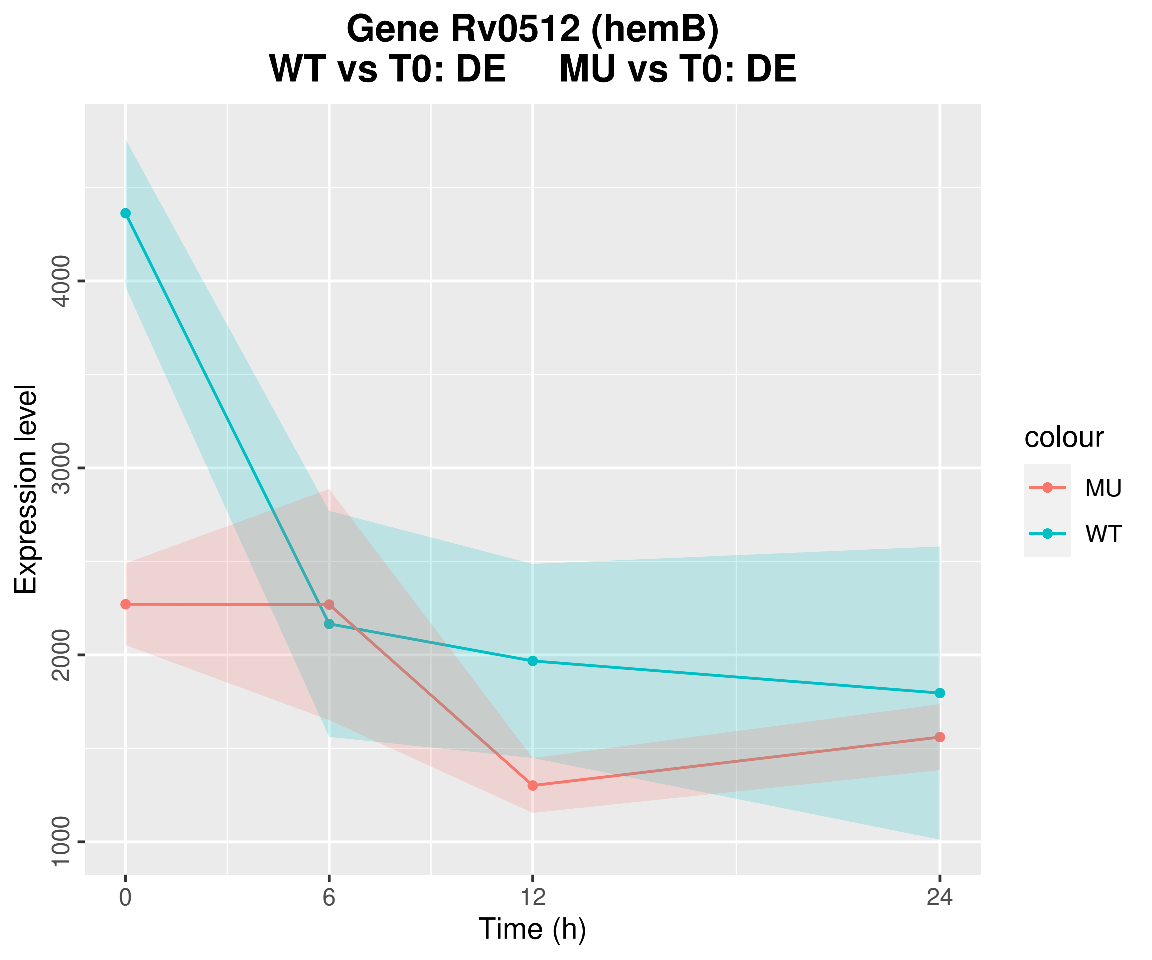

Gene Rv0512 (hemB)
WT vs T0: DE MU vs T0: DE
Expression level
1000
2000
3000
4000
0
6
12
24
Time (h)
colour
MU
WT

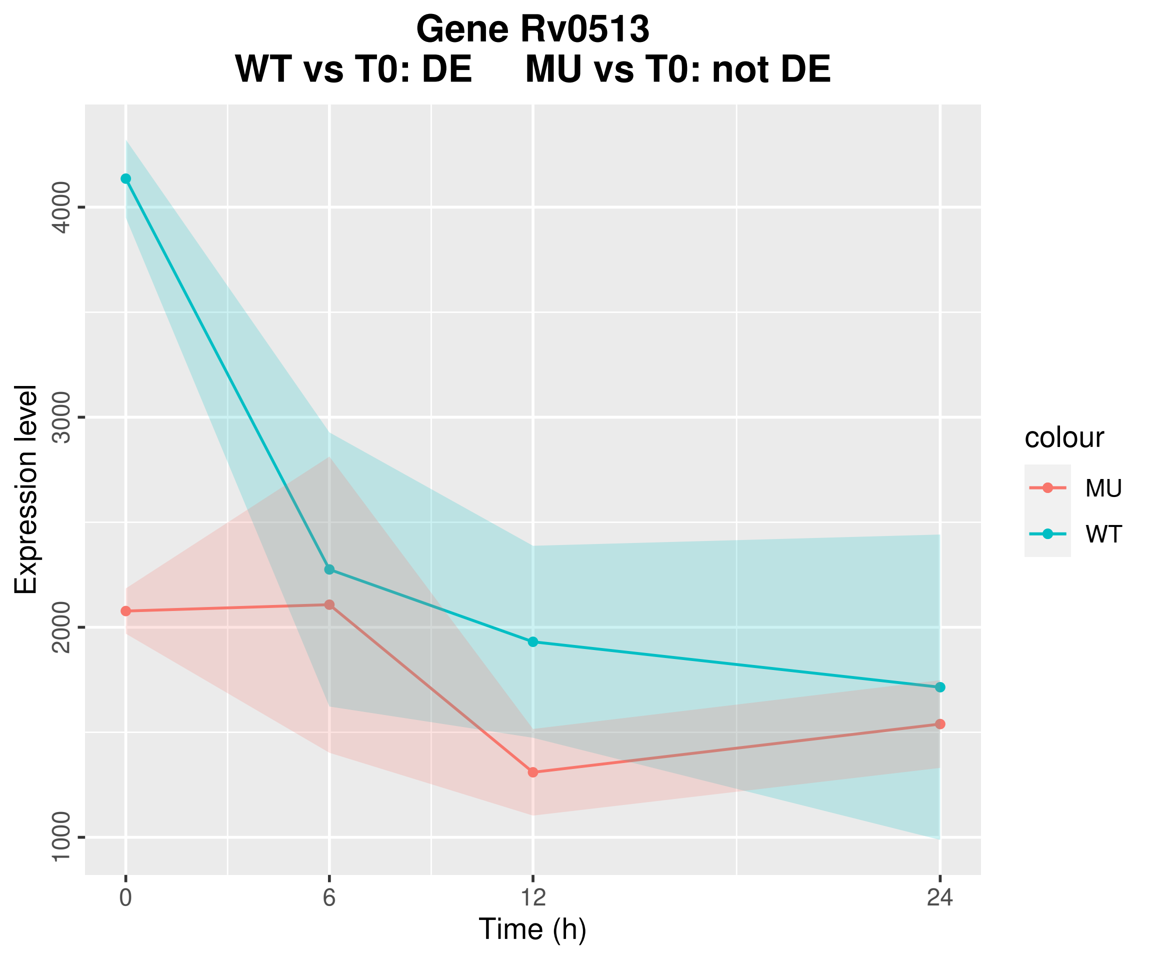
Gene Rv0513
WT vs T0: DE MU vs T0: not DE
Expression level
4000
3000
2000
1000
0
6
12
24
Time (h)
colour
MU
WT

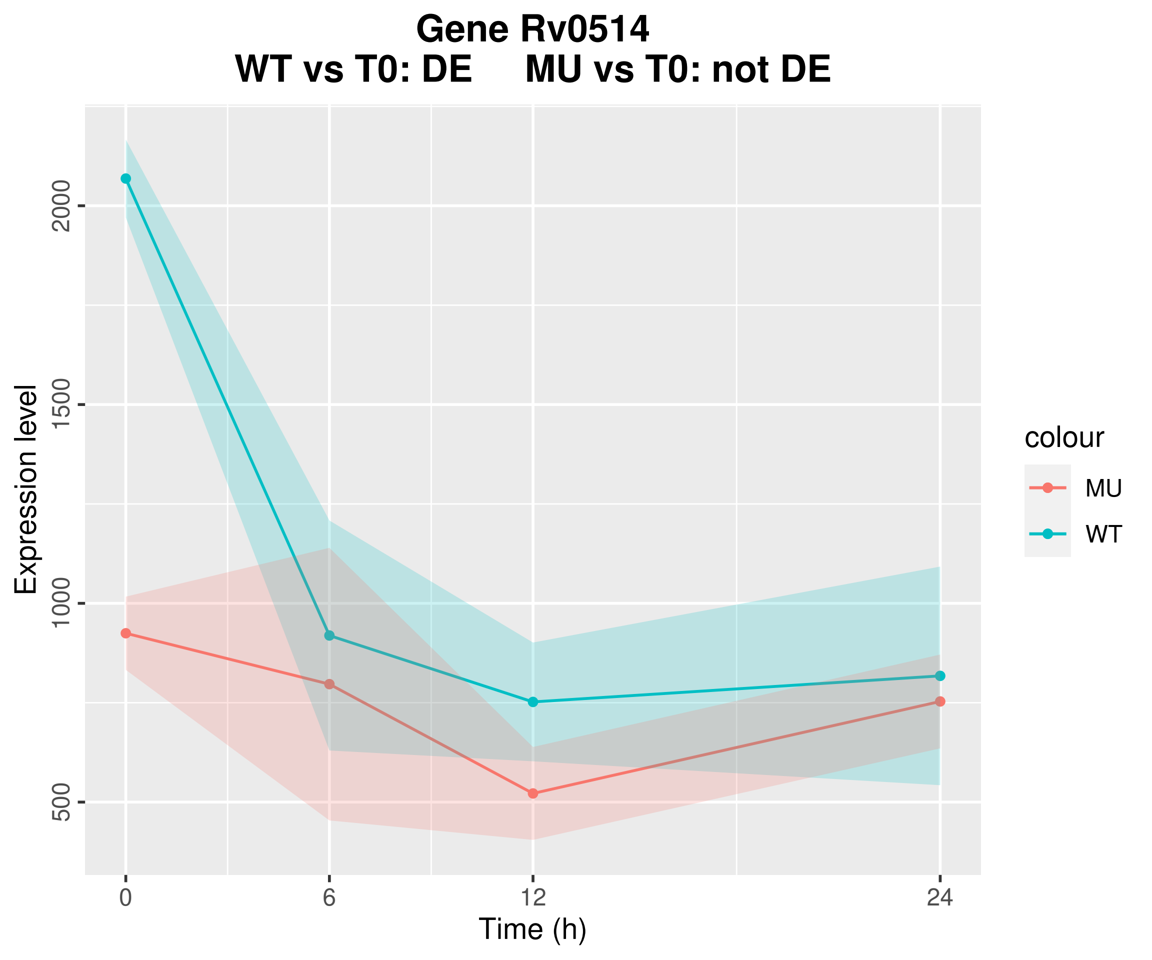
Gene Rv0514
WT vs T0: DE MU vs T0: not DE
Expression level
2000
1500
1000
500
0
6
12
24
Time (h)
colour
MU
WT

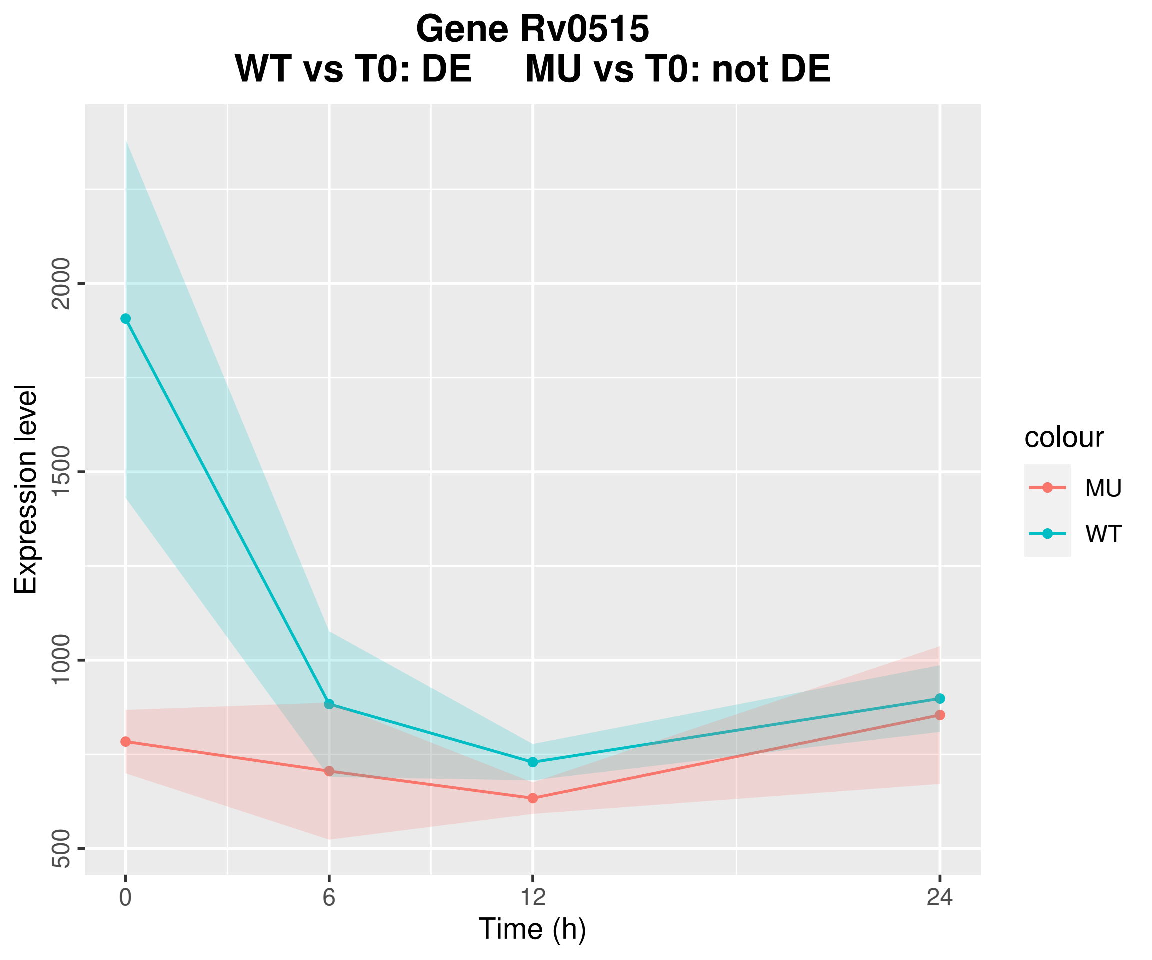

Gene Rv0515
WT vs T0: DE   MU vs T0: not DE
Expression level
2000
1500
1000
500
0
6
12
24
Time (h)
colour
MU
WT

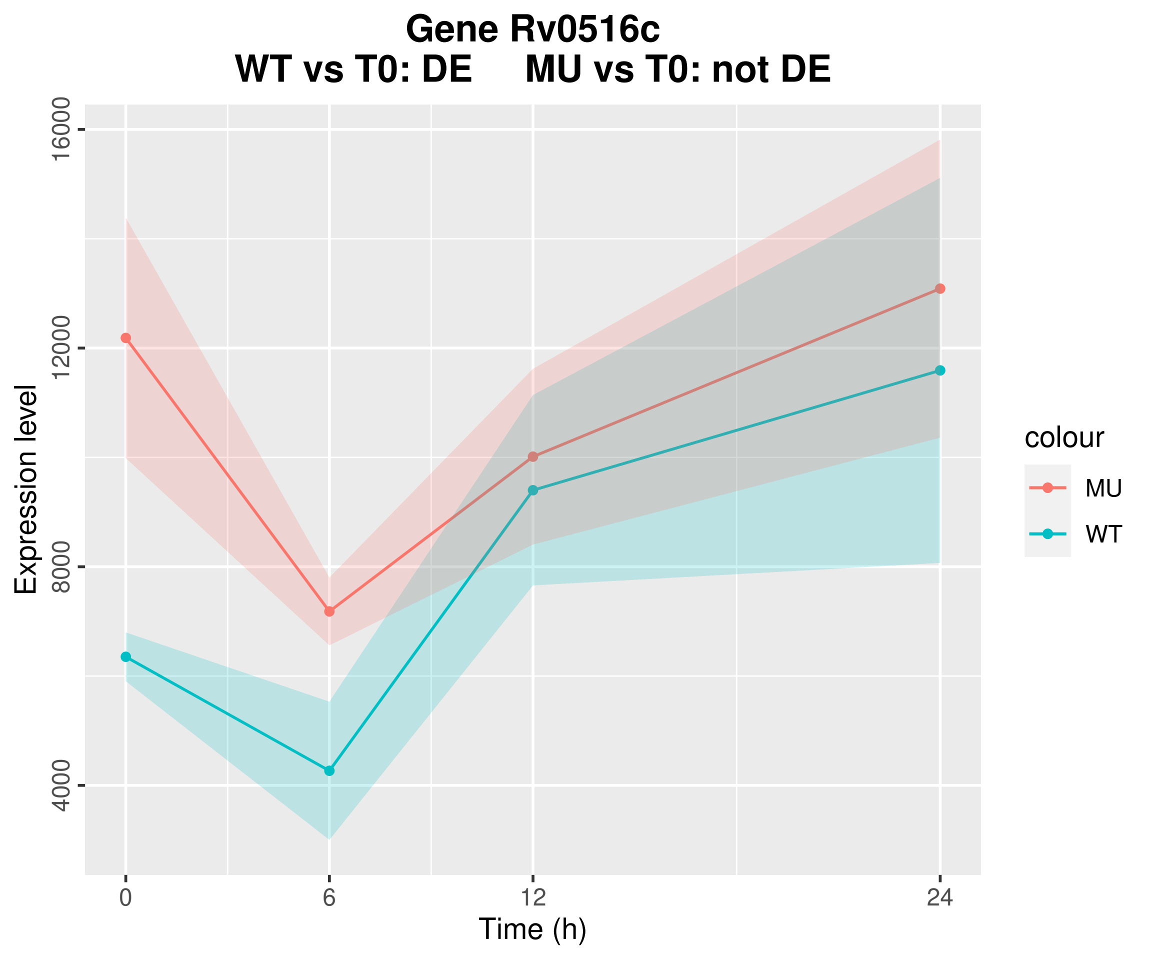

Gene Rv0516c
WT vs T0: DE   MU vs T0: not DE
Expression level
16000
12000
8000
4000
0
6
12
24
Time (h)
colour
MU
WT

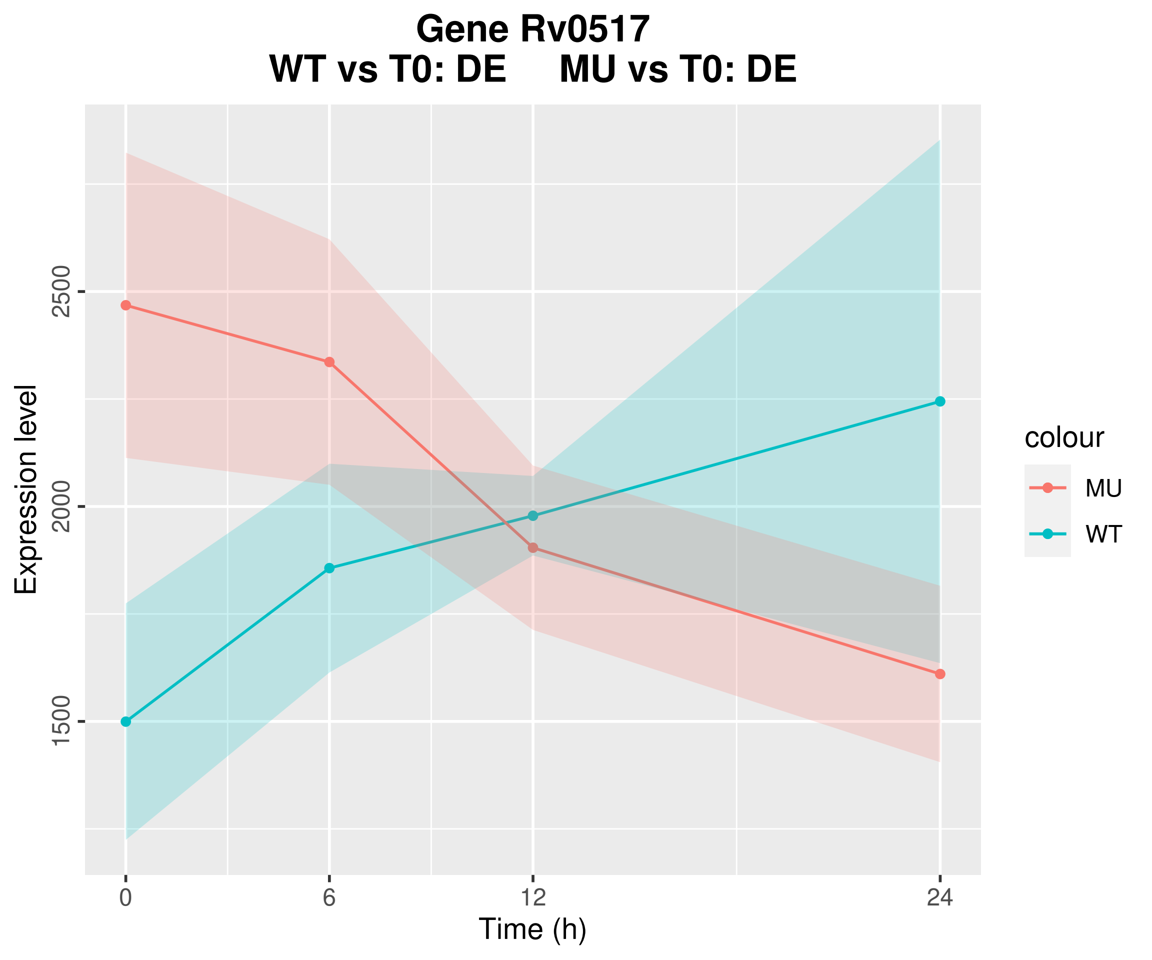

Gene Rv0517
WT vs T0: DE MU vs T0: DE
Expression level
2500
2000
1500
0
6
12
24
Time (h)
colour
MU
WT

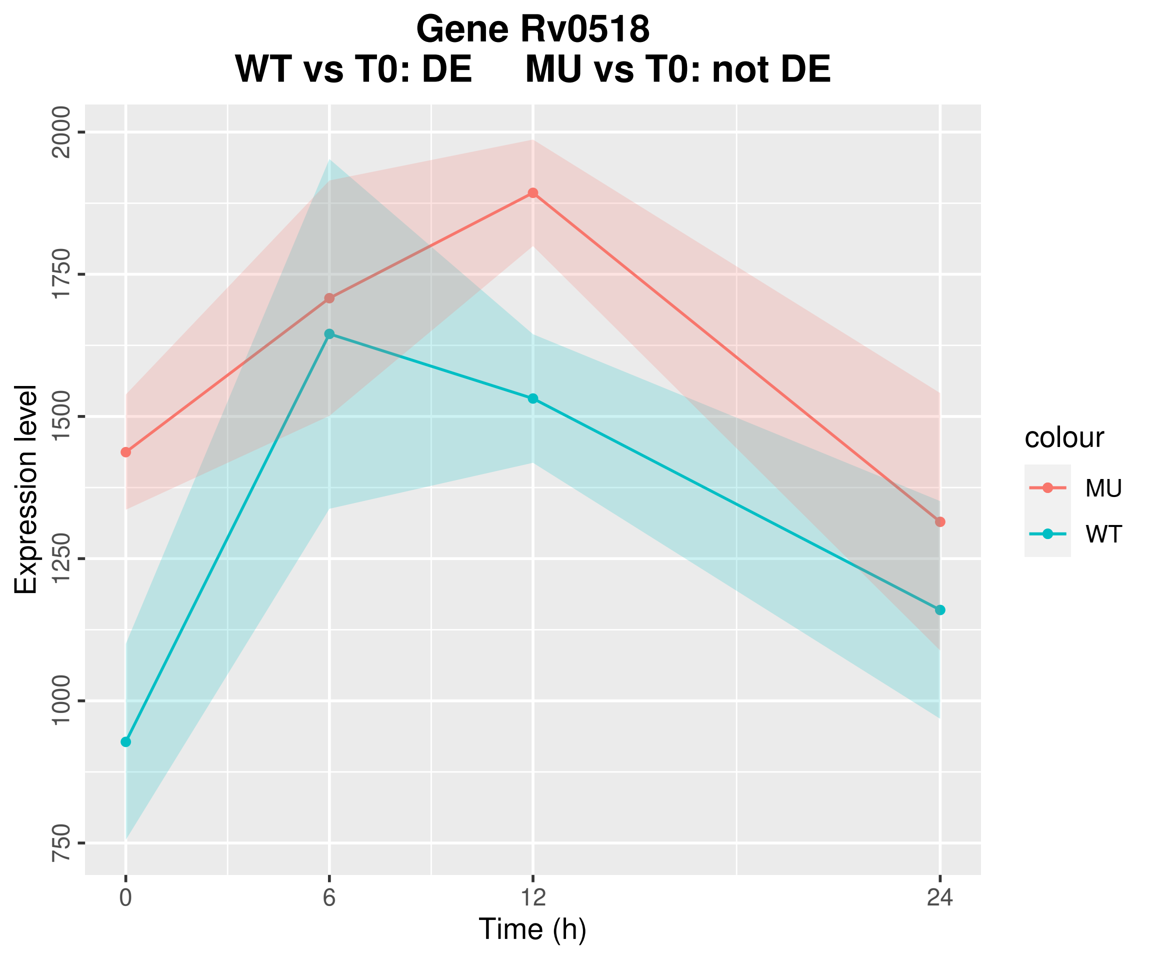

Gene Rv0518
WT vs T0: DE MU vs T0: not DE
Expression level
2000
1750
1500
1250
1000
750
0
6
12
24
Time (h)
colour
MU
WT

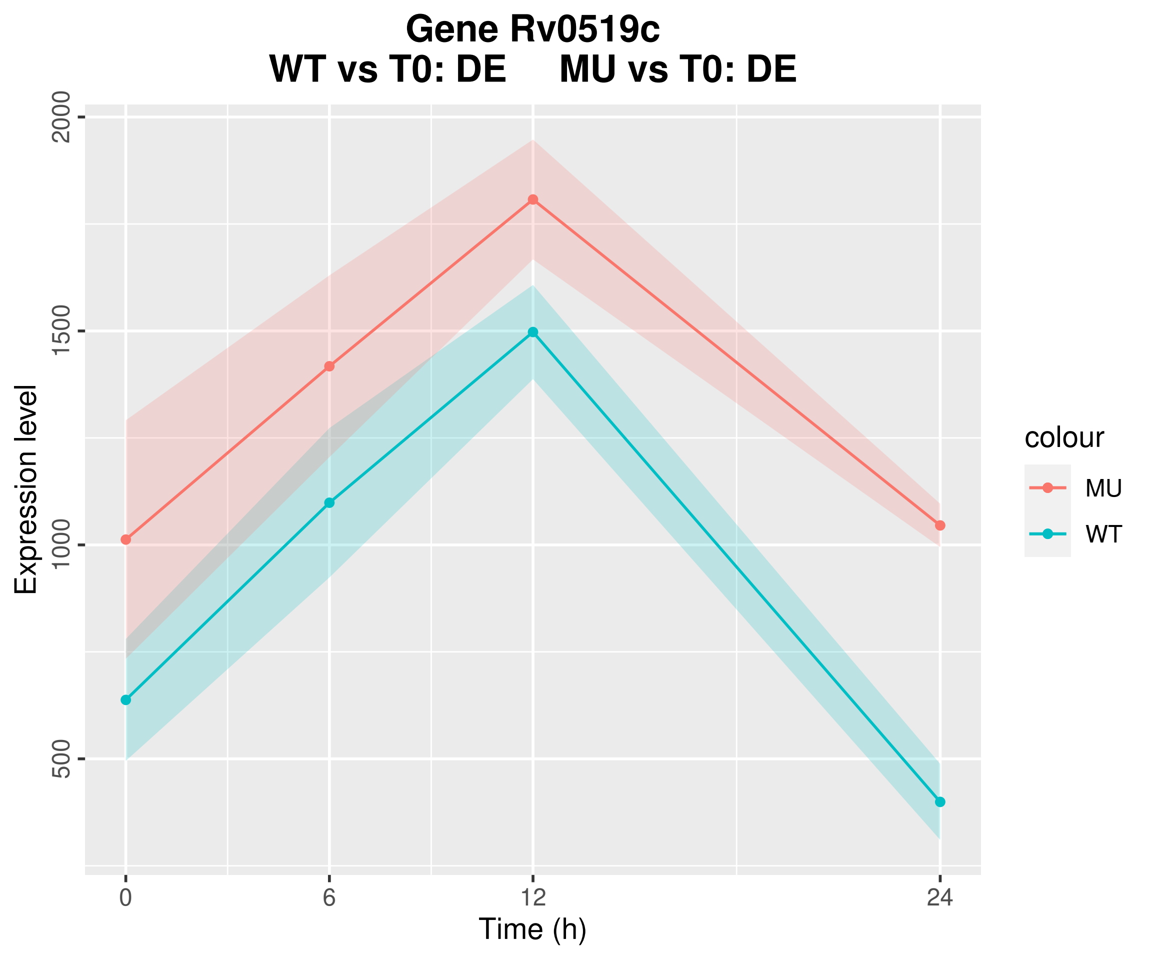
Gene Rv0519c
WT vs T0: DE MU vs T0: DE
Expression level
2000
1500
1000
500
0
6
12
24
Time (h)
colour
MU
WT

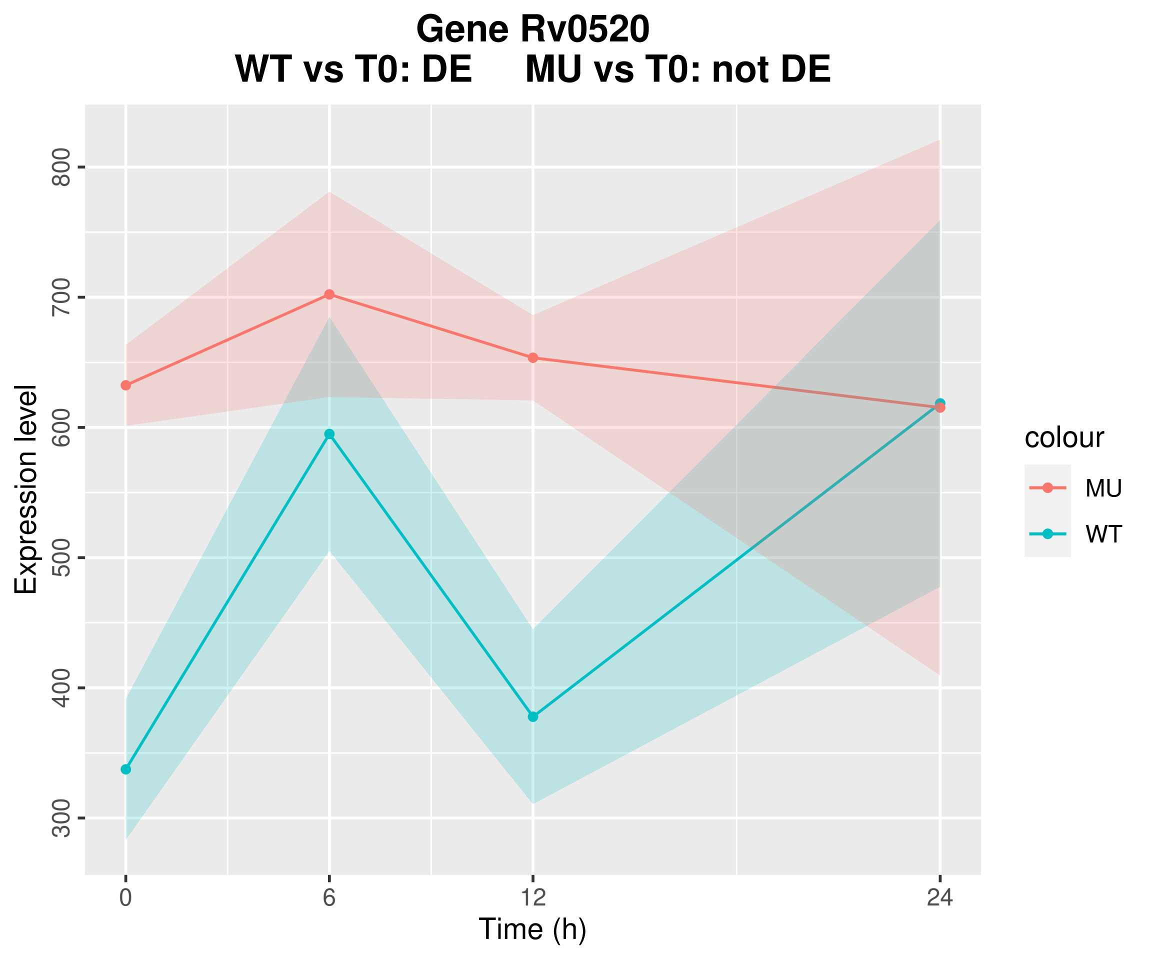

Gene Rv0520
WT vs T0: DE   MU vs T0: not DE
Expression level
800
700
600
500
400
300
0
6
12
24
Time (h)
colour
MU
WT

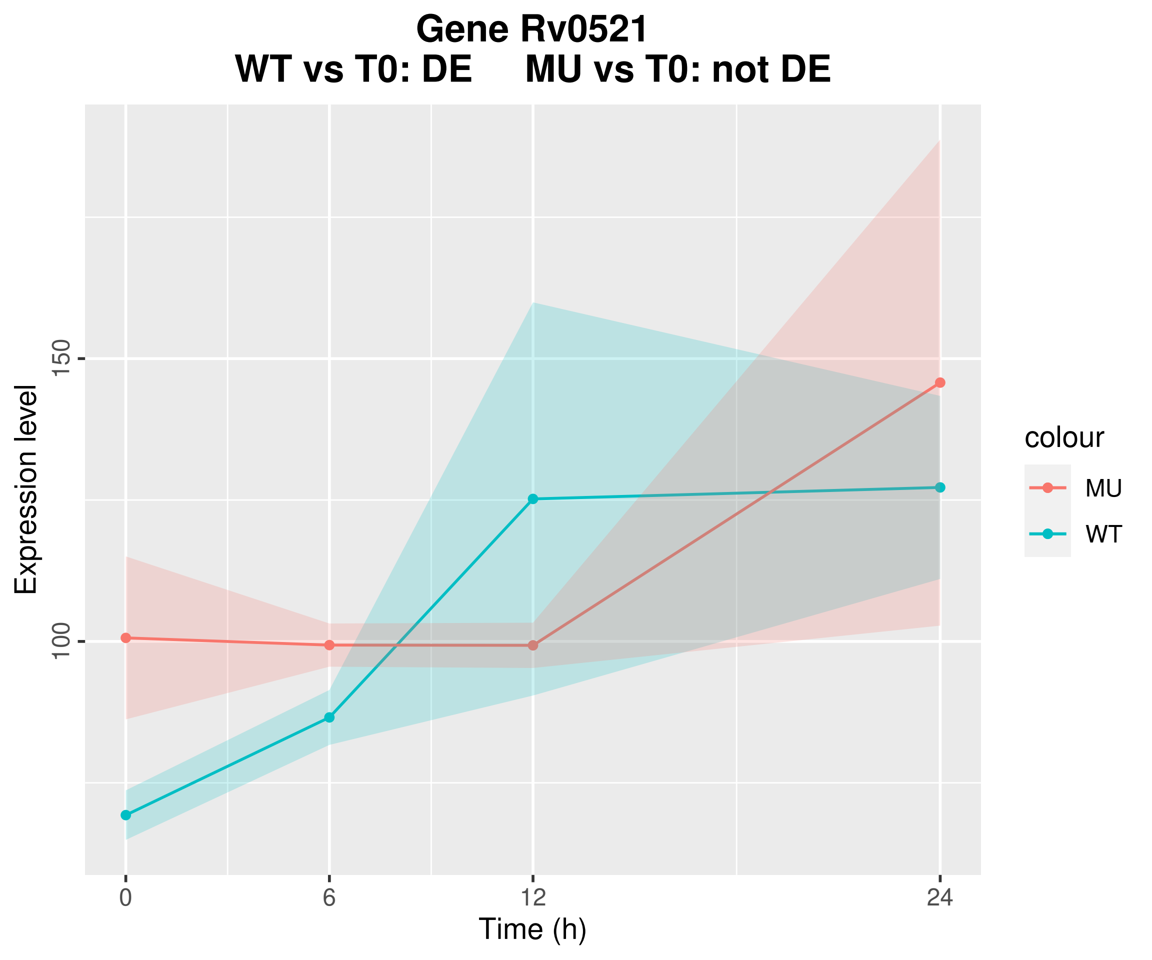
Gene Rv0521
WT vs T0: DE MU vs T0: not DE
Expression level
150
100
0
6
12
24
Time (h)
colour
MU
WT

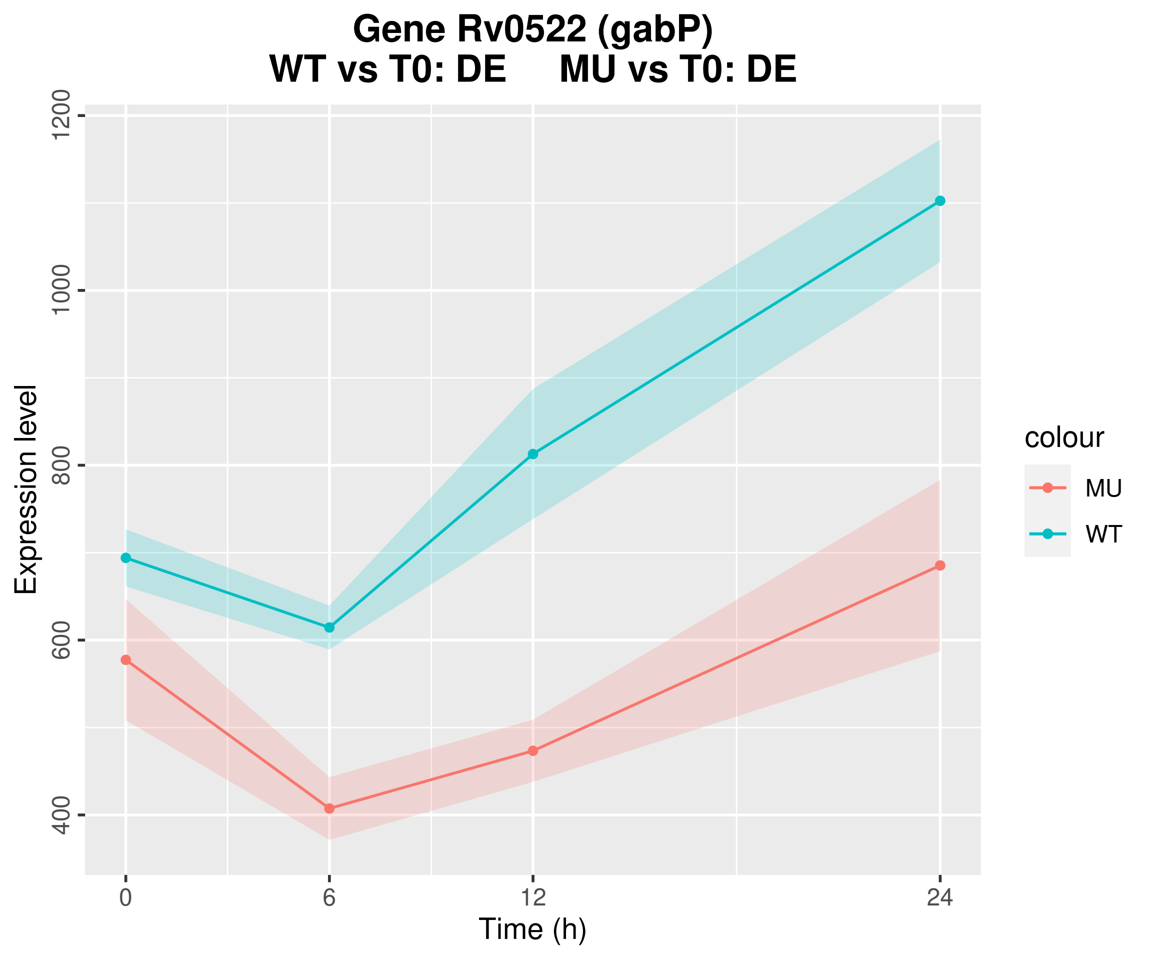

Gene Rv0522 (gabP)
WT vs T0: DE   MU vs T0: DE
Expression level
1200
1000
800
600
400
0
6
12
24
Time (h)
colour
MU
WT

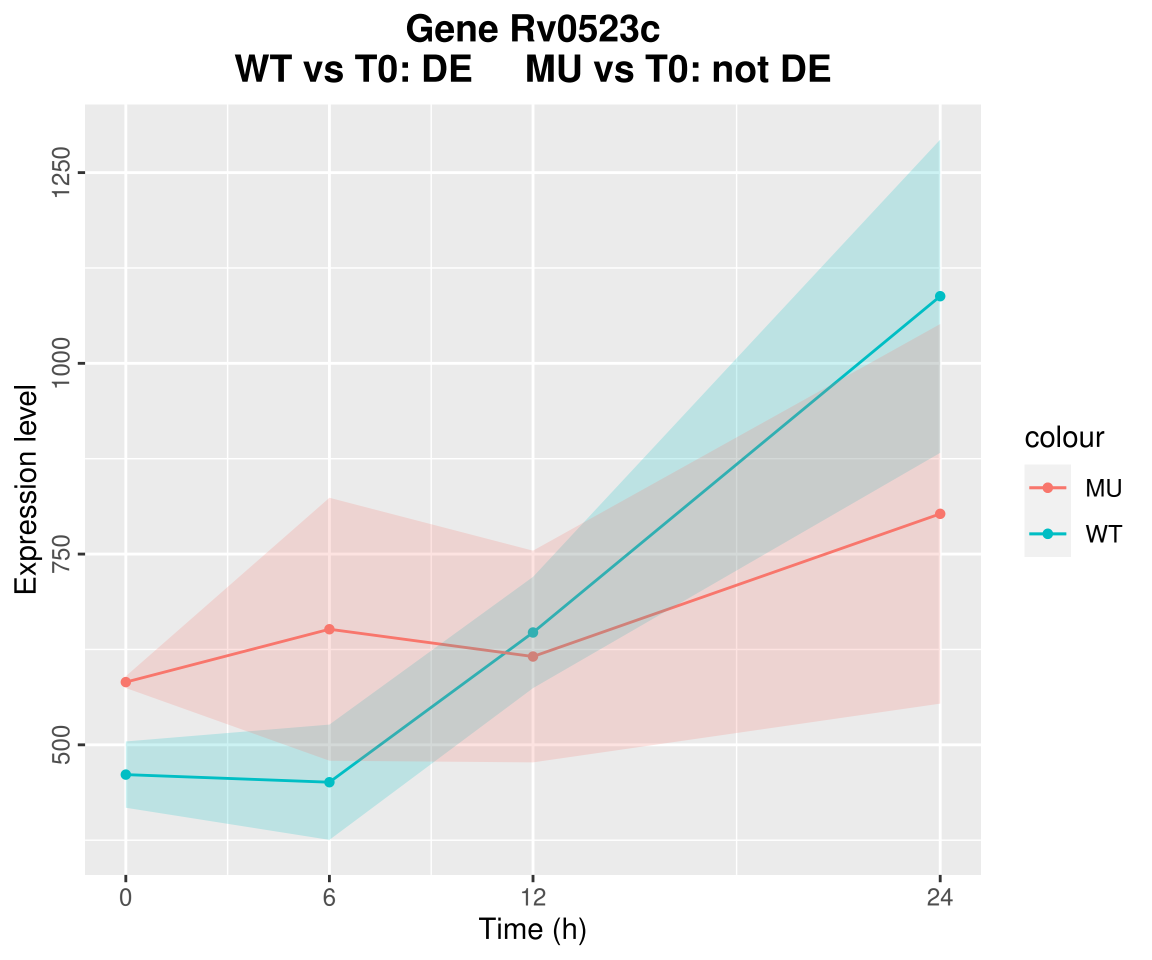

Gene Rv0523c
WT vs T0: DE   MU vs T0: not DE
1250
1000
750
500
Expression level
0
6
12
24
Time (h)
colour
MU
WT

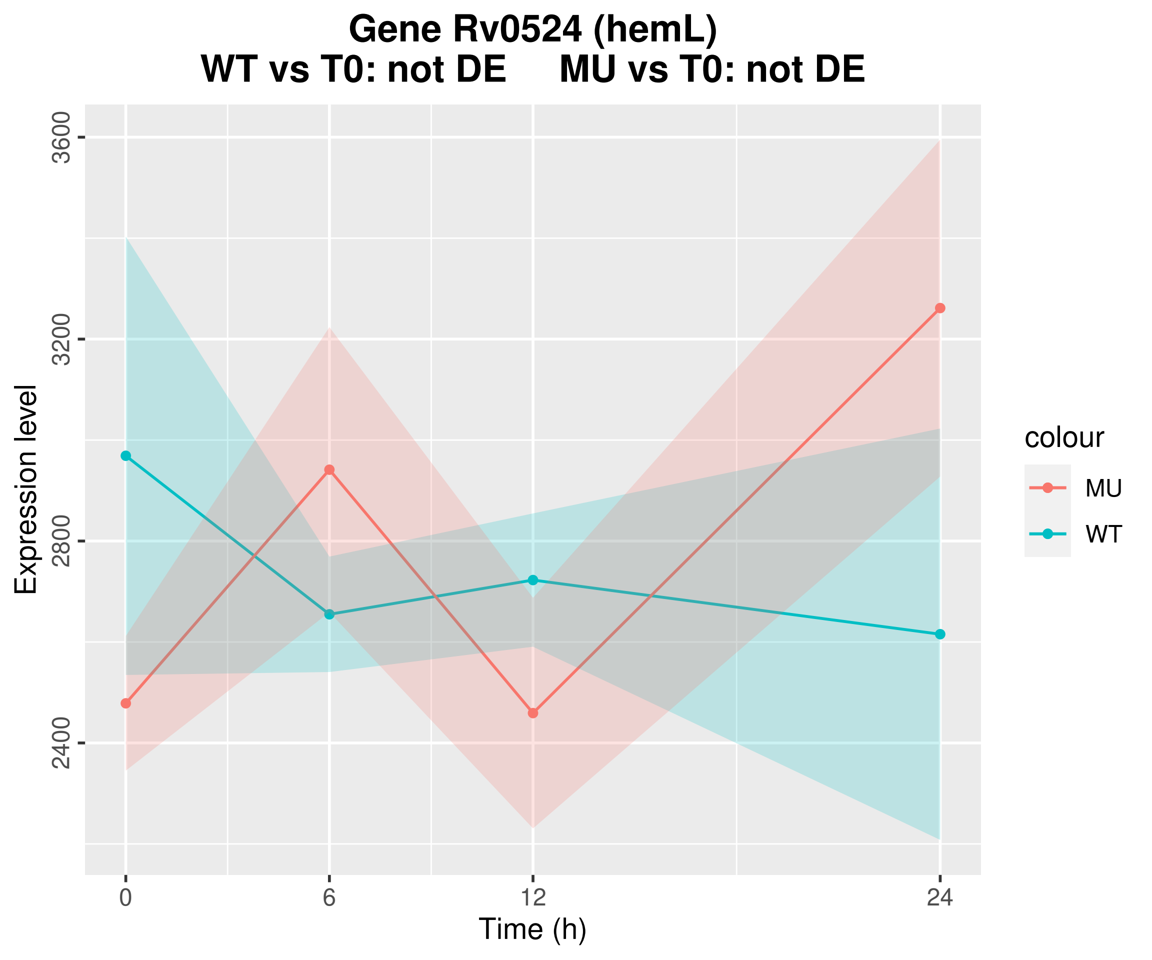
Gene Rv0524 (hemL)
WT vs T0: not DE   MU vs T0: not DE
Expression level
3600
3200
2800
2400
0
6
12
24
Time (h)
colour
MU
WT

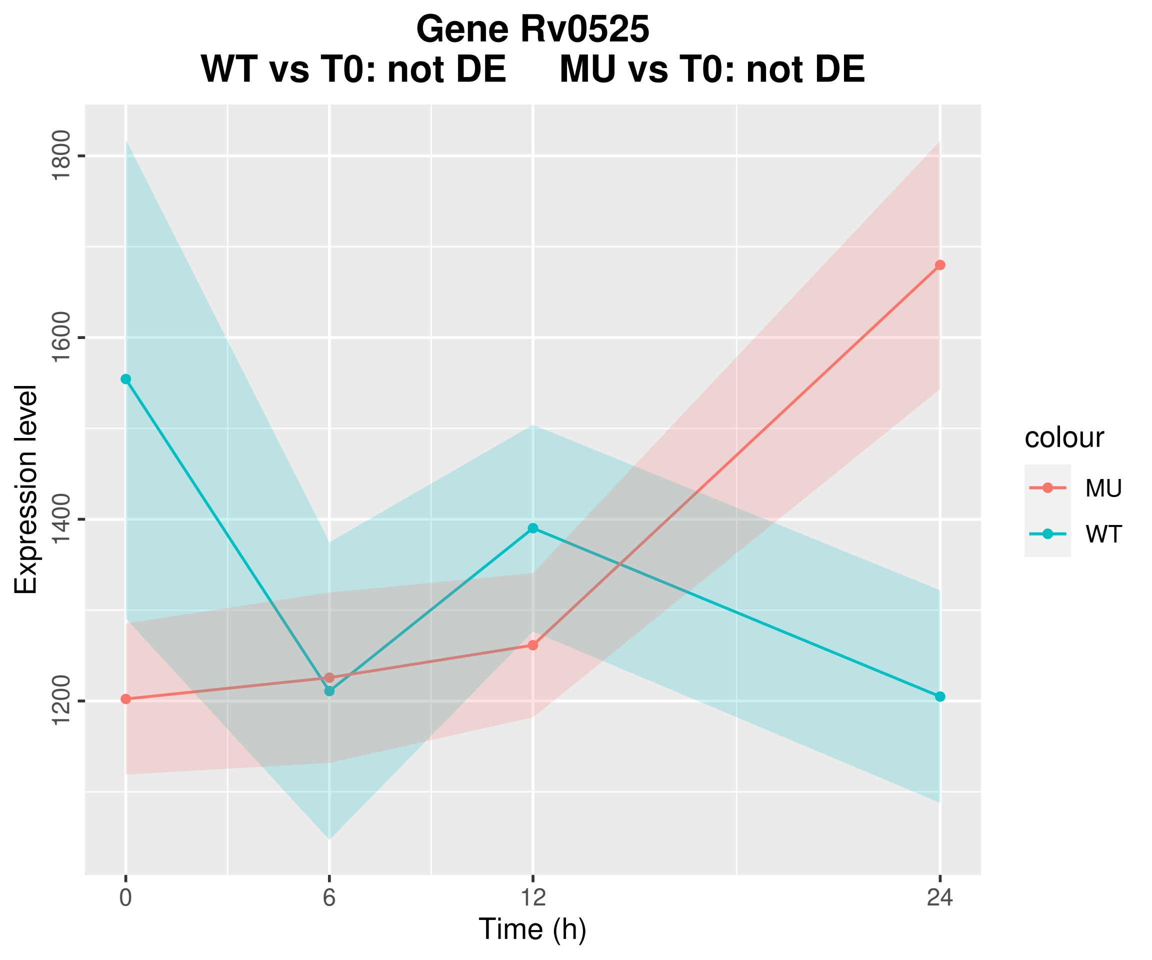

Gene Rv0525
WT vs T0: not DE   MU vs T0: not DE
Expression level
1200
1400
1600
1800
Time (h)
0
6
12
24
colour
MU
WT

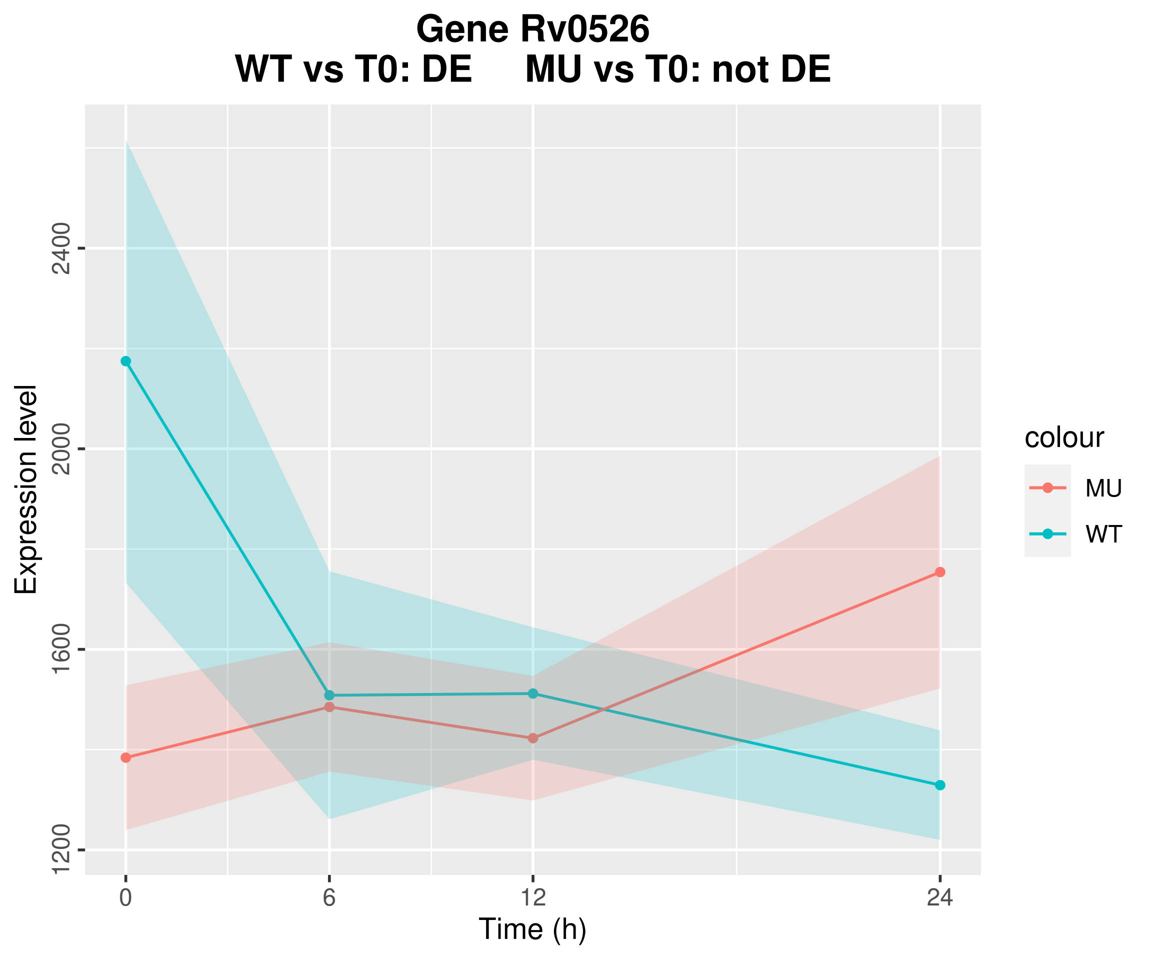

Gene Rv0526
WT vs T0: DE   MU vs T0: not DE
Expression level
2400
2000
1600
1200
0
6
12
24
Time (h)
colour
MU
WT

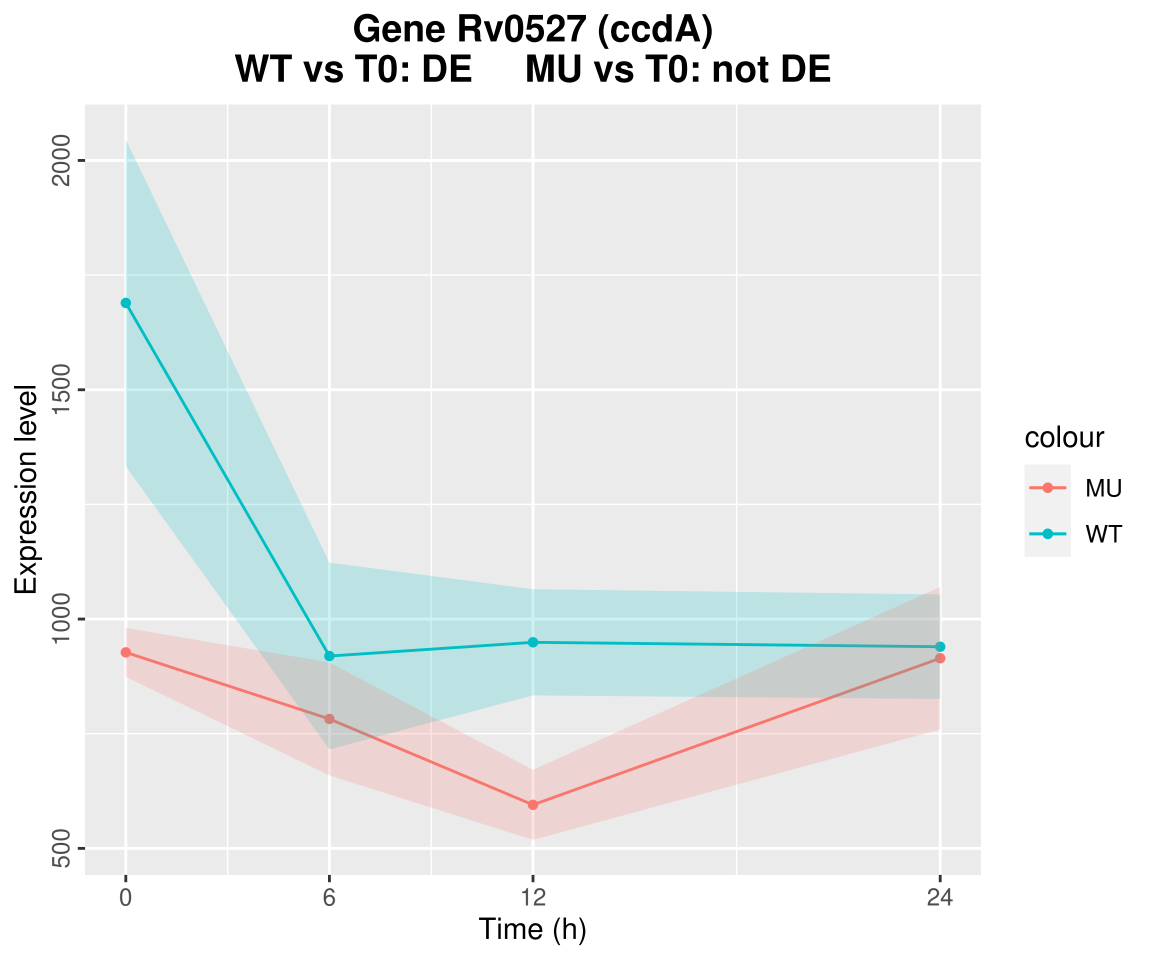

Gene Rv0527 (ccdA)
WT vs T0: DE   MU vs T0: not DE
Expression level
2000
1500
1000
500
0
6
12
24
Time (h)
colour
MU
WT

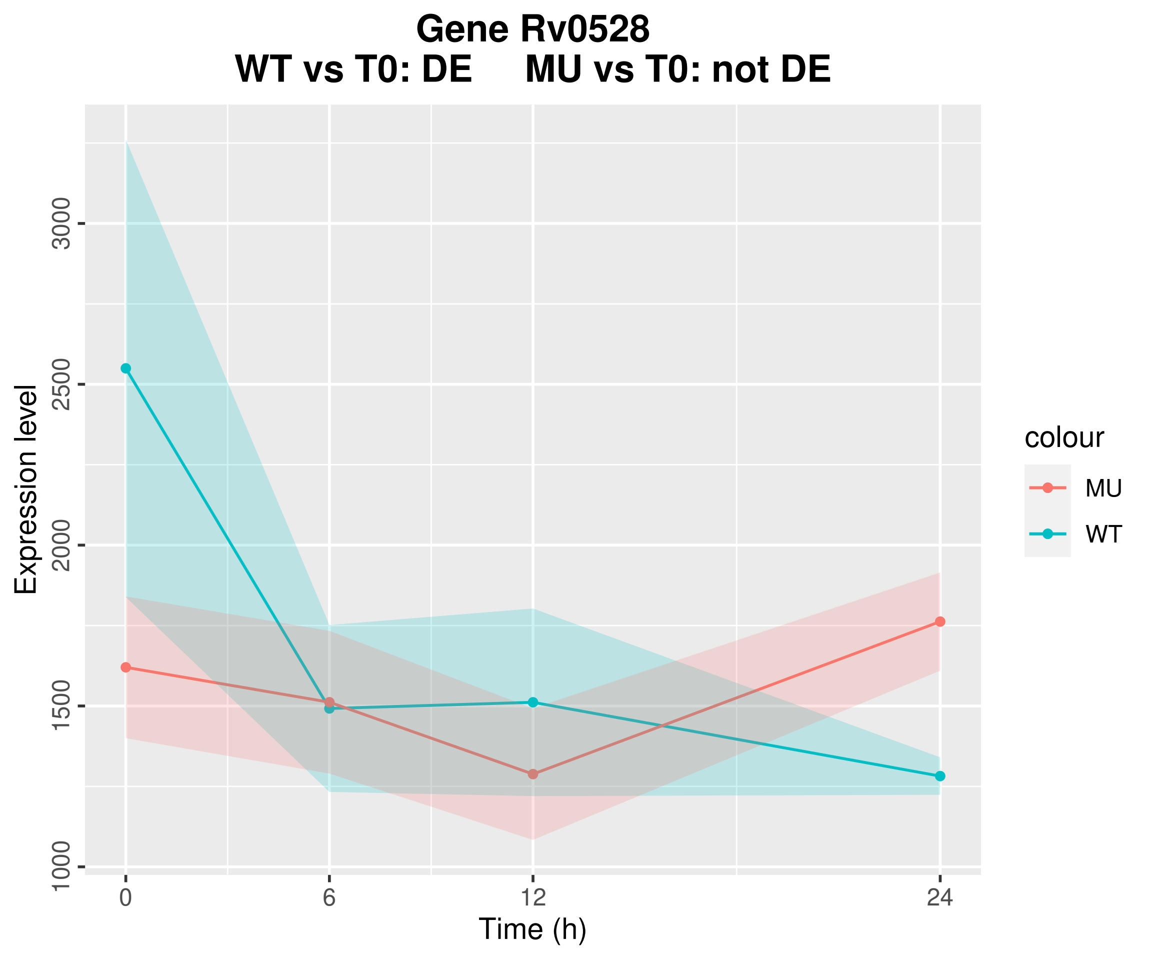

Gene Rv0528
WT vs T0: DE   MU vs T0: not DE
Expression level
3000
2500
2000
1500
1000
0
6
12
24
Time (h)
colour
MU
WT

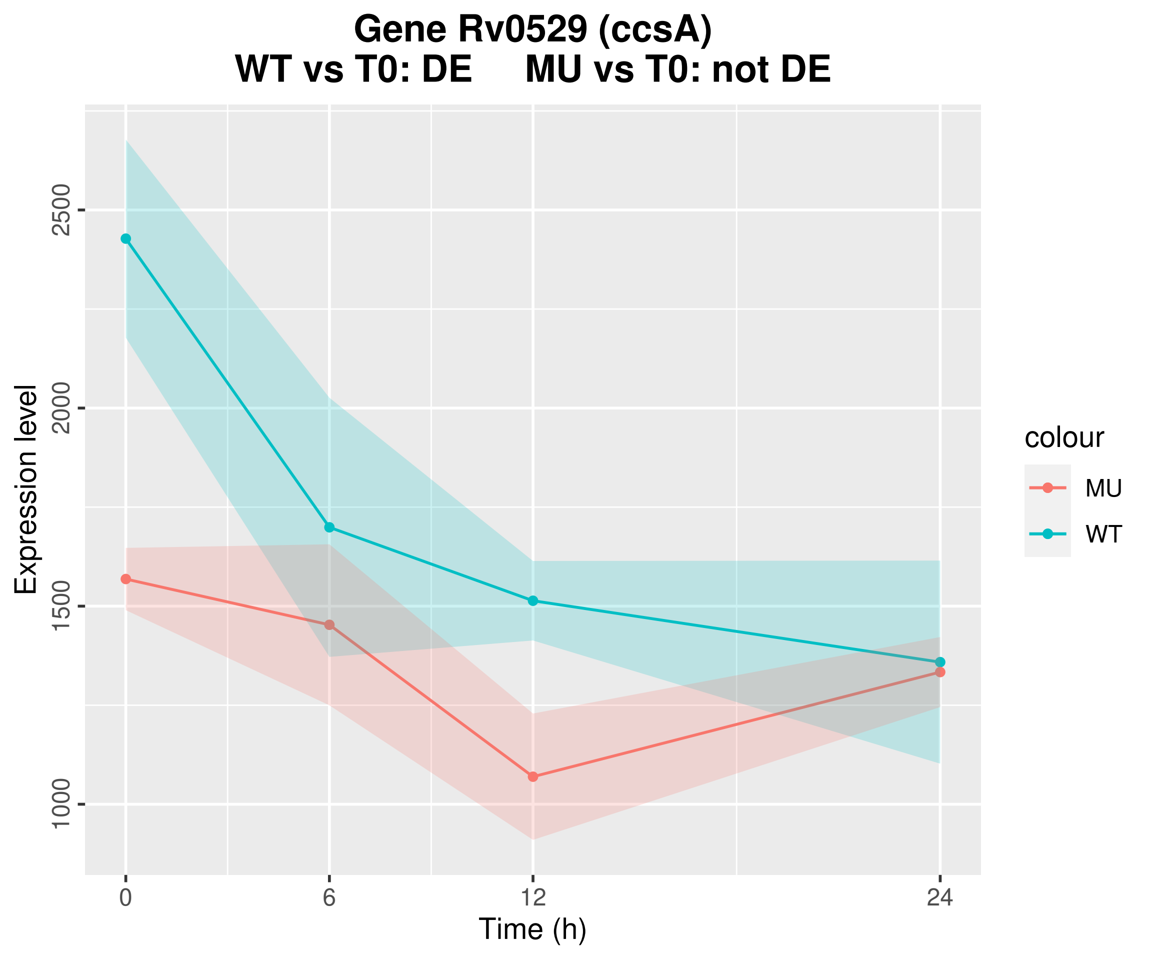

Gene Rv0529 (ccsA)
WT vs T0: DE   MU vs T0: not DE
Expression level
2500
2000
1500
1000
0
6
12
24
Time (h)
colour
MU
WT

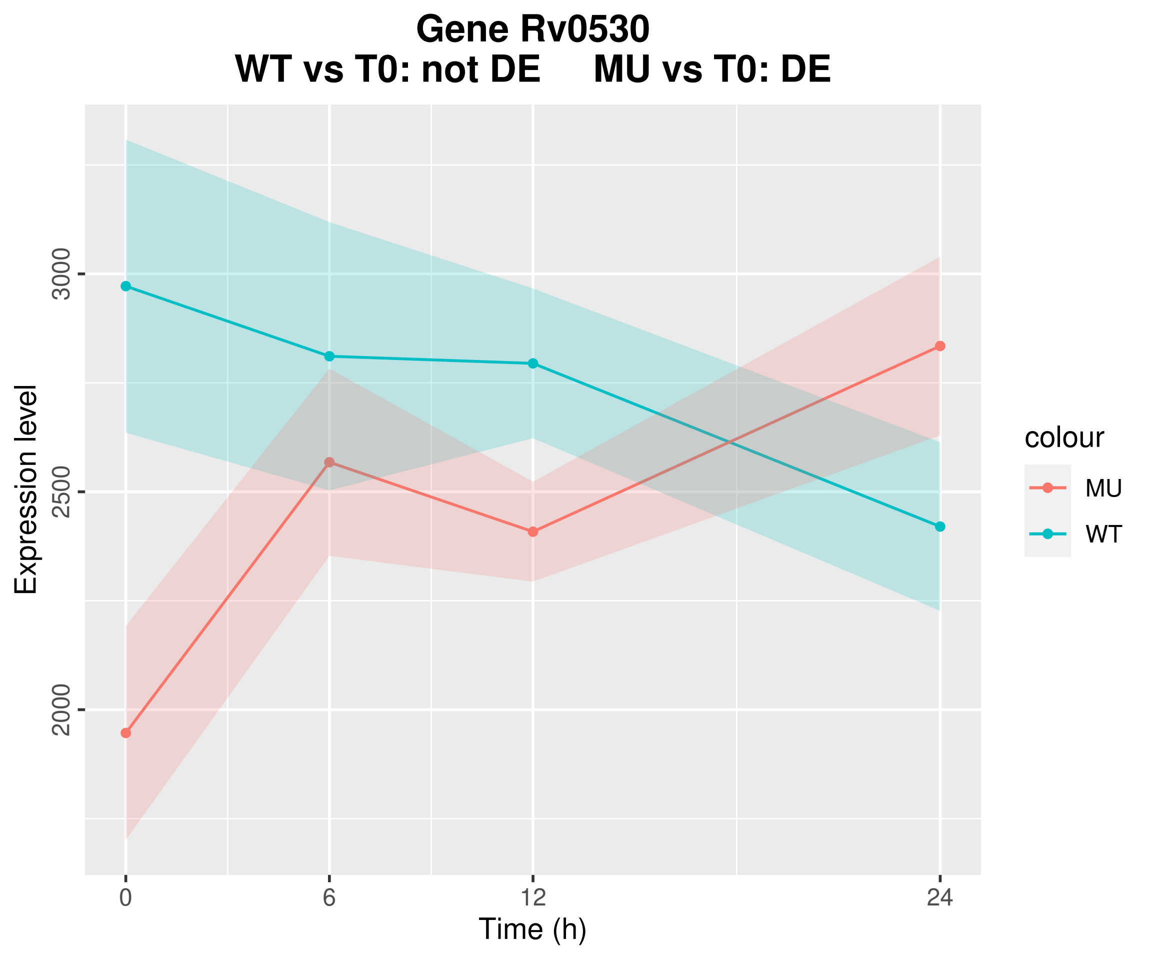
Gene Rv0530
WT vs T0: not DE MU vs T0: DE
3000
2500
2000
Expression level
0
6
12
24
Time (h)
colour
MU
WT

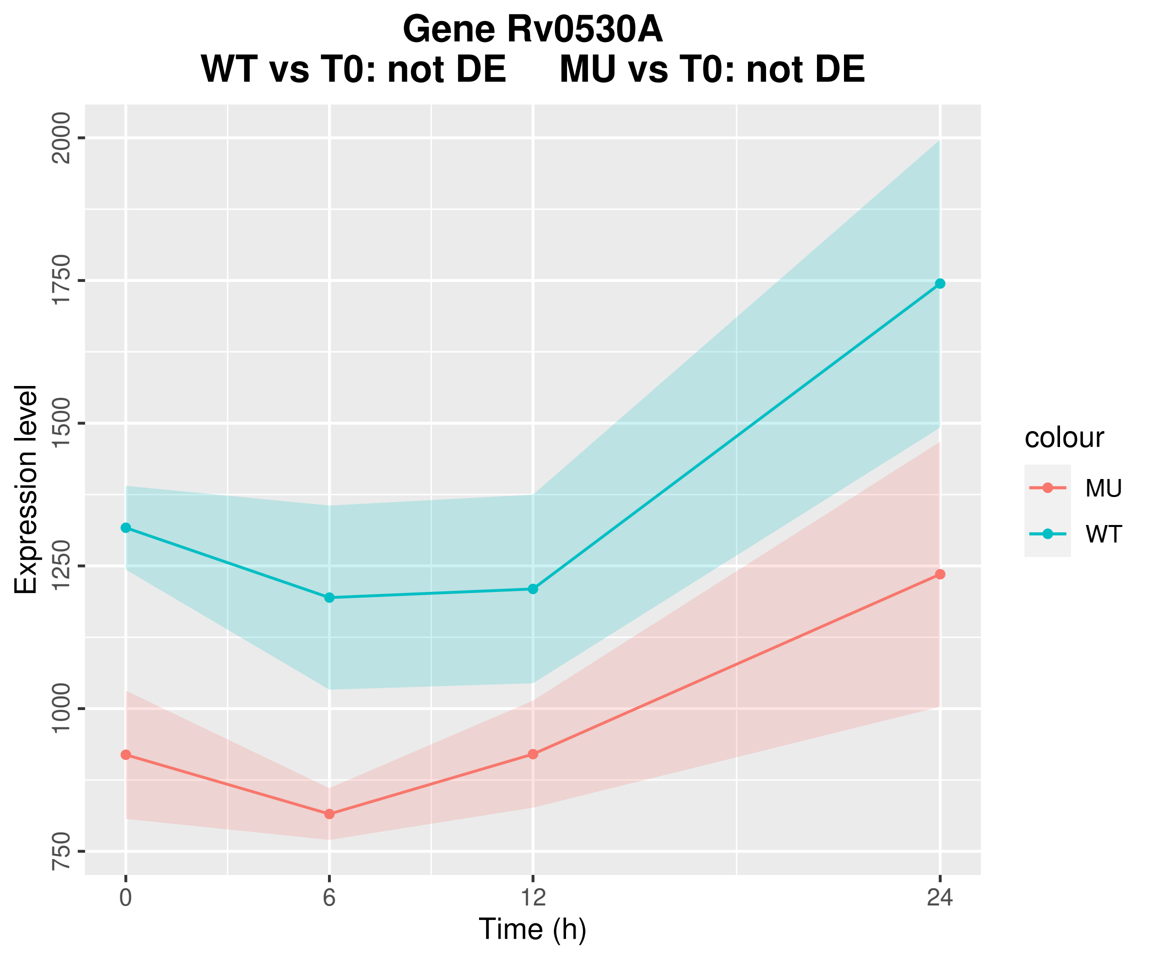
Gene Rv0530A
WT vs T0: not DE   MU vs T0: not DE
Expression level
2000
1750
1500
1250
1000
750
0
6
12
24
Time (h)
colour
MU
WT

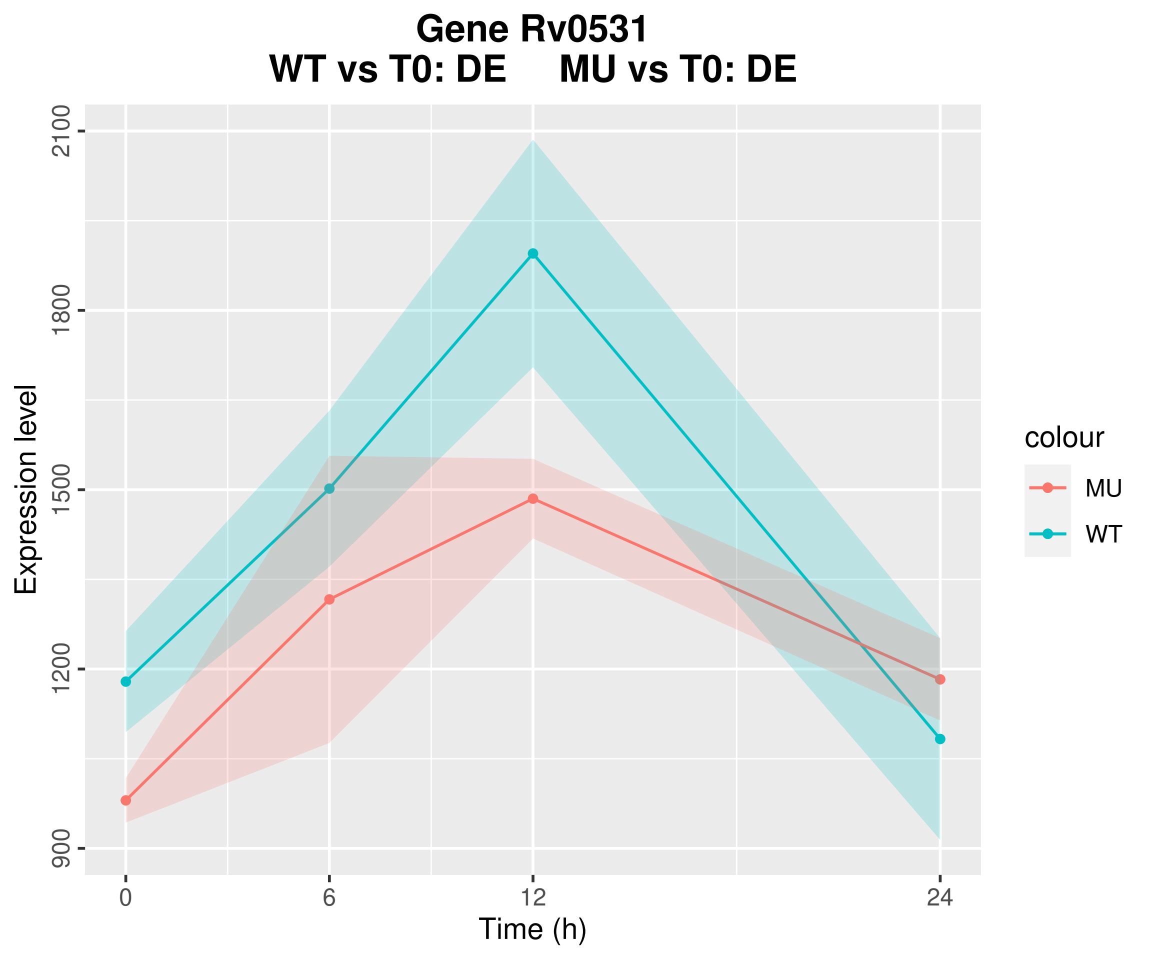

Gene Rv0531
WT vs T0: DE MU vs T0: DE
Expression level
2100
1800
1500
1200
900
0
6
12
24
Time (h)
colour
MU
WT

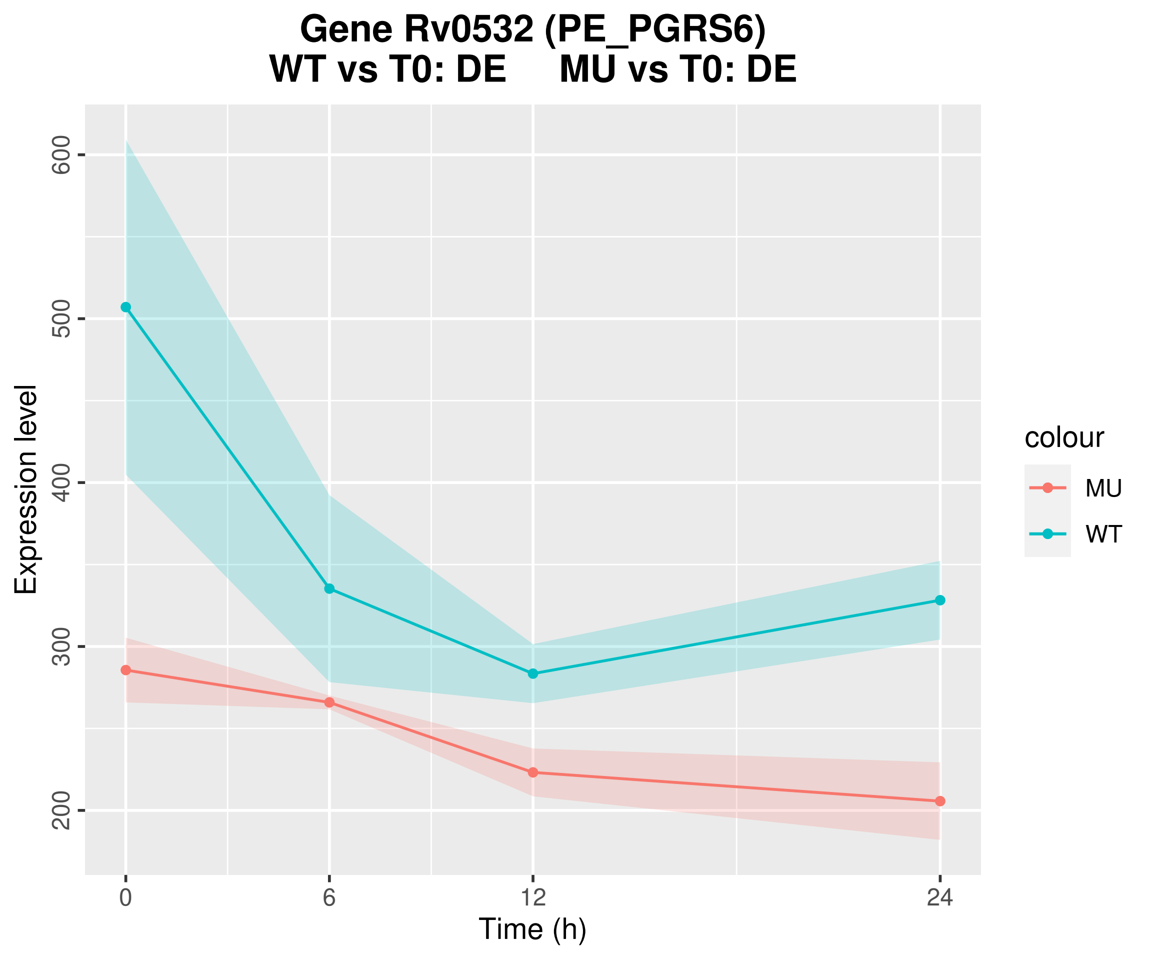

Gene Rv0532 (PE_PGRS6)
WT vs T0: DE   MU vs T0: DE
Expression level
600
500
400
300
200
0
6
12
24
Time (h)
colour
MU
WT

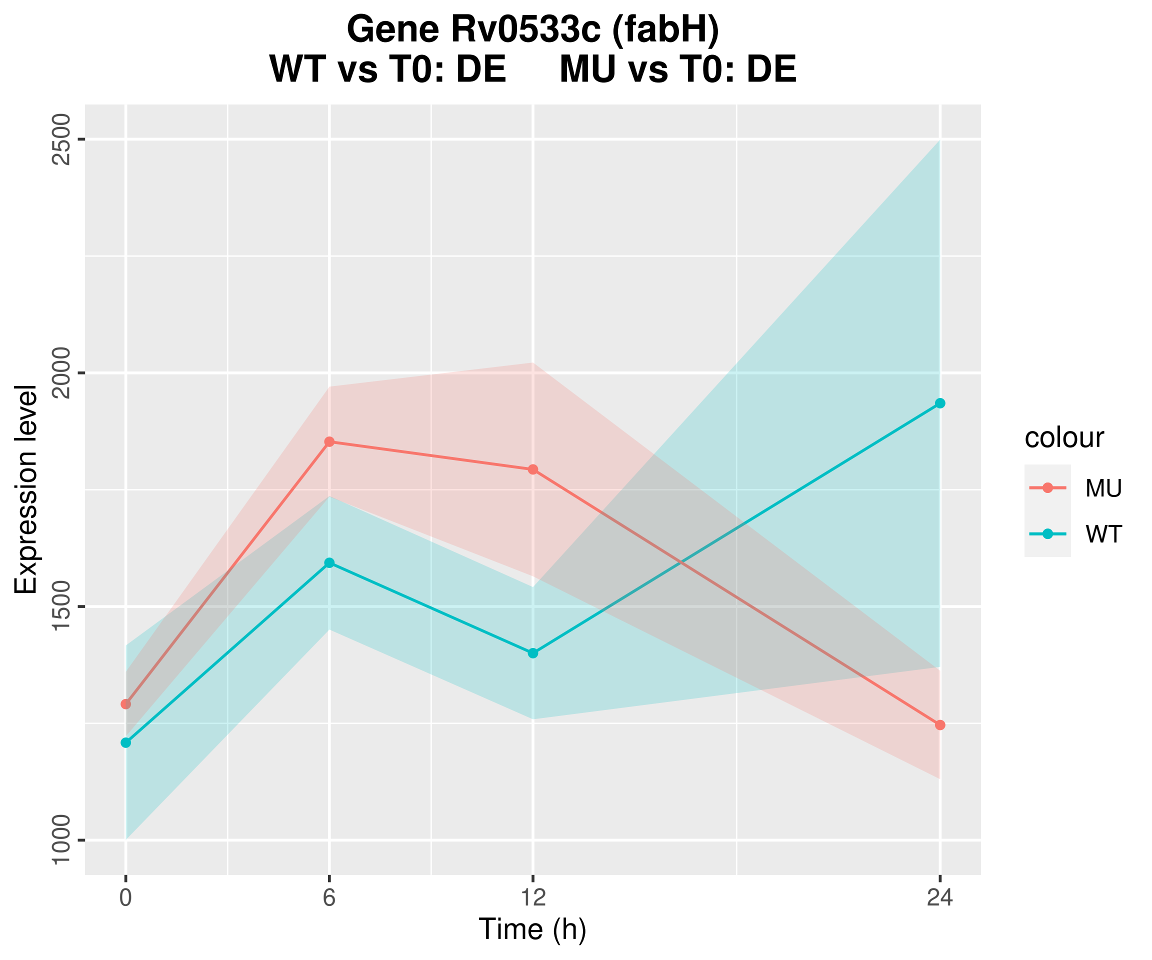

Gene Rv0533c (fabH)
WT vs T0: DE   MU vs T0: DE
Expression level
2500
2000
1500
1000
0
6
12
24
Time (h)
colour
MU
WT

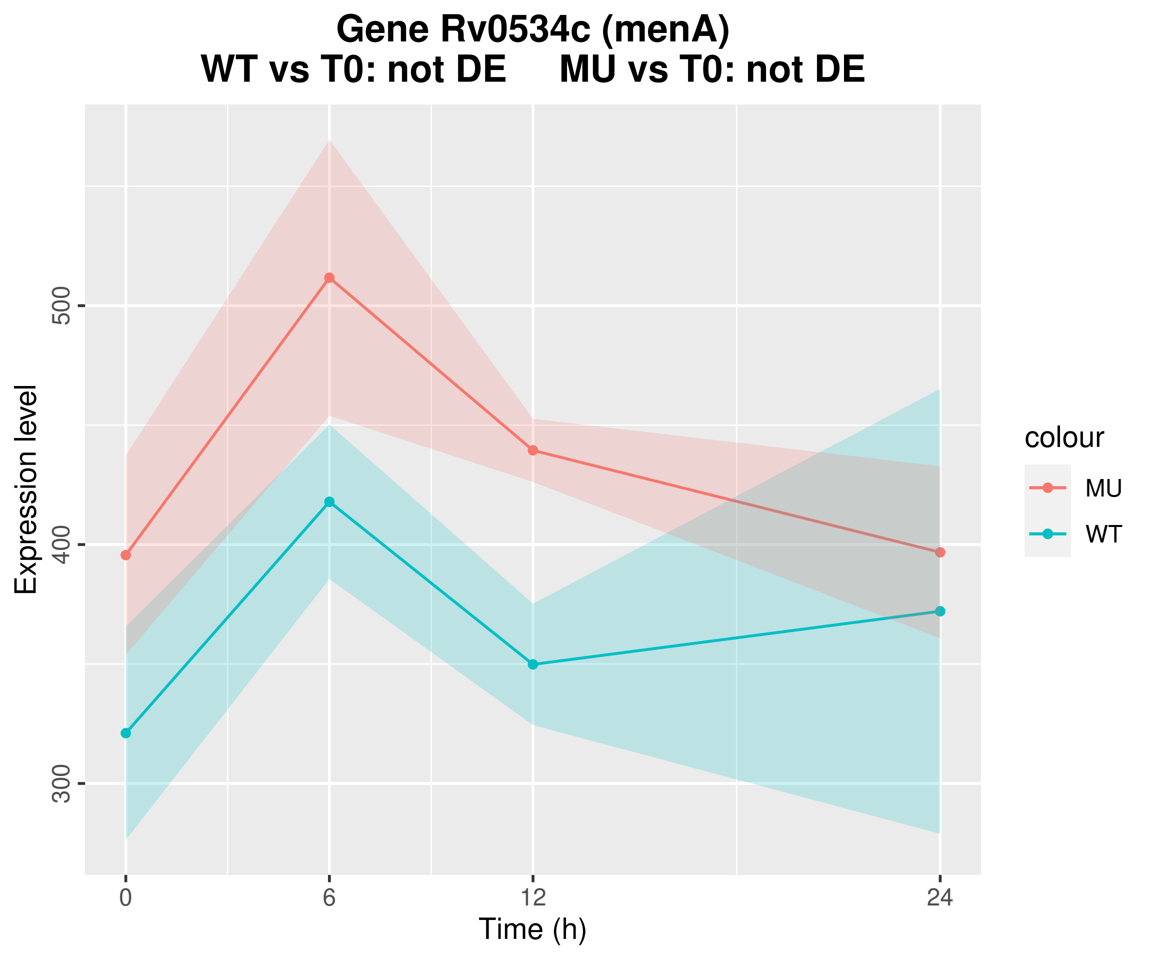

Gene Rv0534c (menA)
WT vs T0: not DE MU vs T0: not DE
Expression level
500
400
300
0
6
12
24
Time (h)
colour
MU
WT

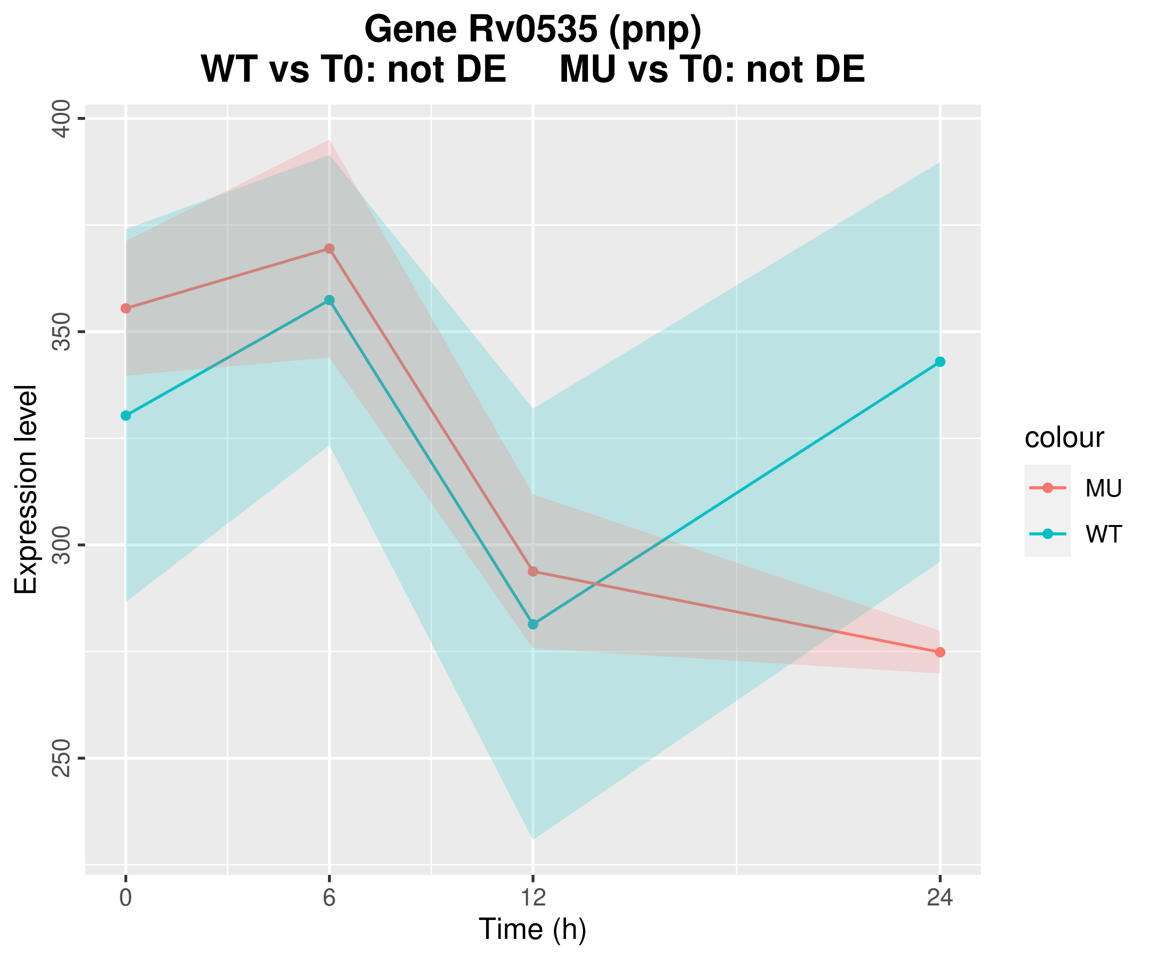

Gene Rv0535 (pnp)
WT vs T0: not DE   MU vs T0: not DE
Expression level
400
350
300
250
0
6
12
24
Time (h)
colour
MU
WT

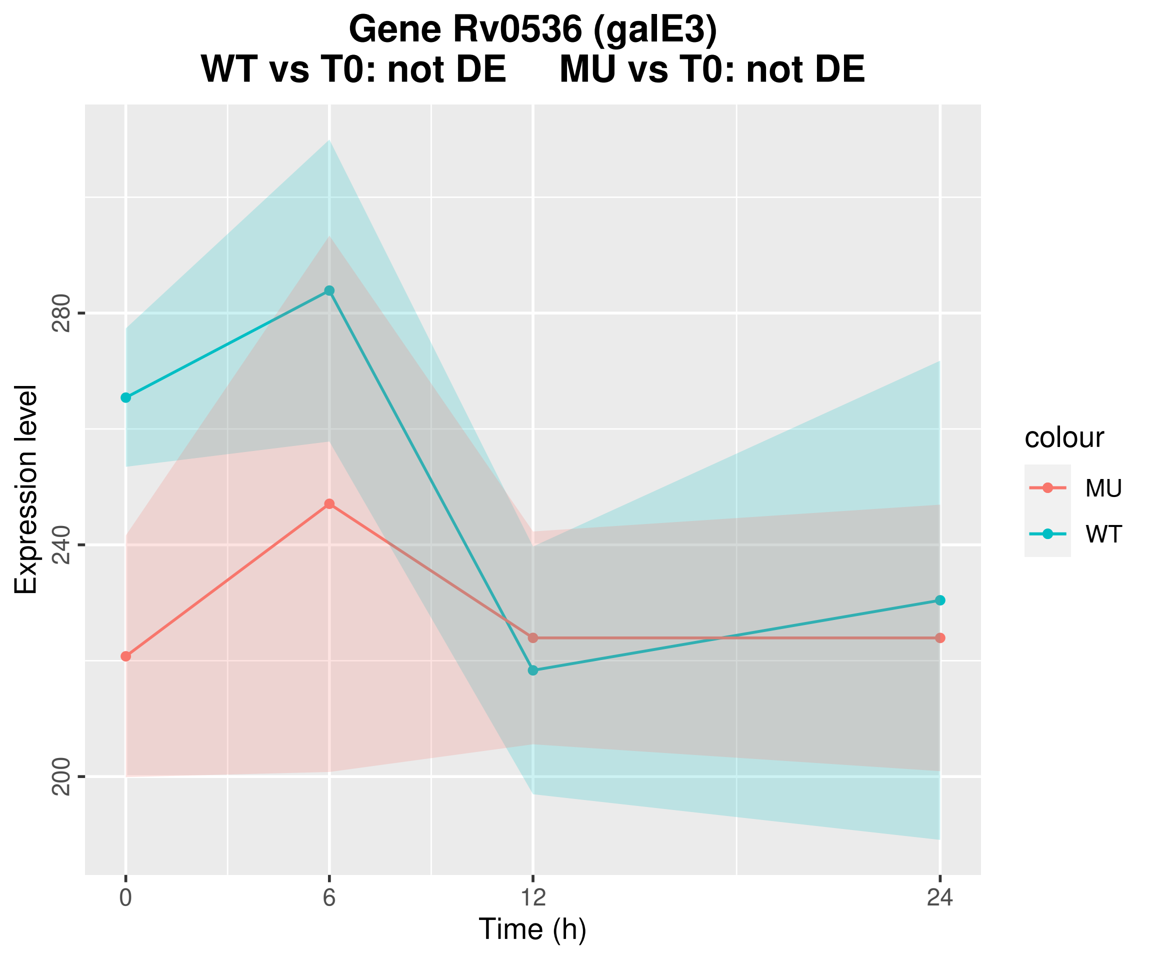

Gene Rv0536 (galE3)
WT vs T0: not DE   MU vs T0: not DE
Expression level
280
240
200
0
6
12
24
Time (h)
colour
MU
WT

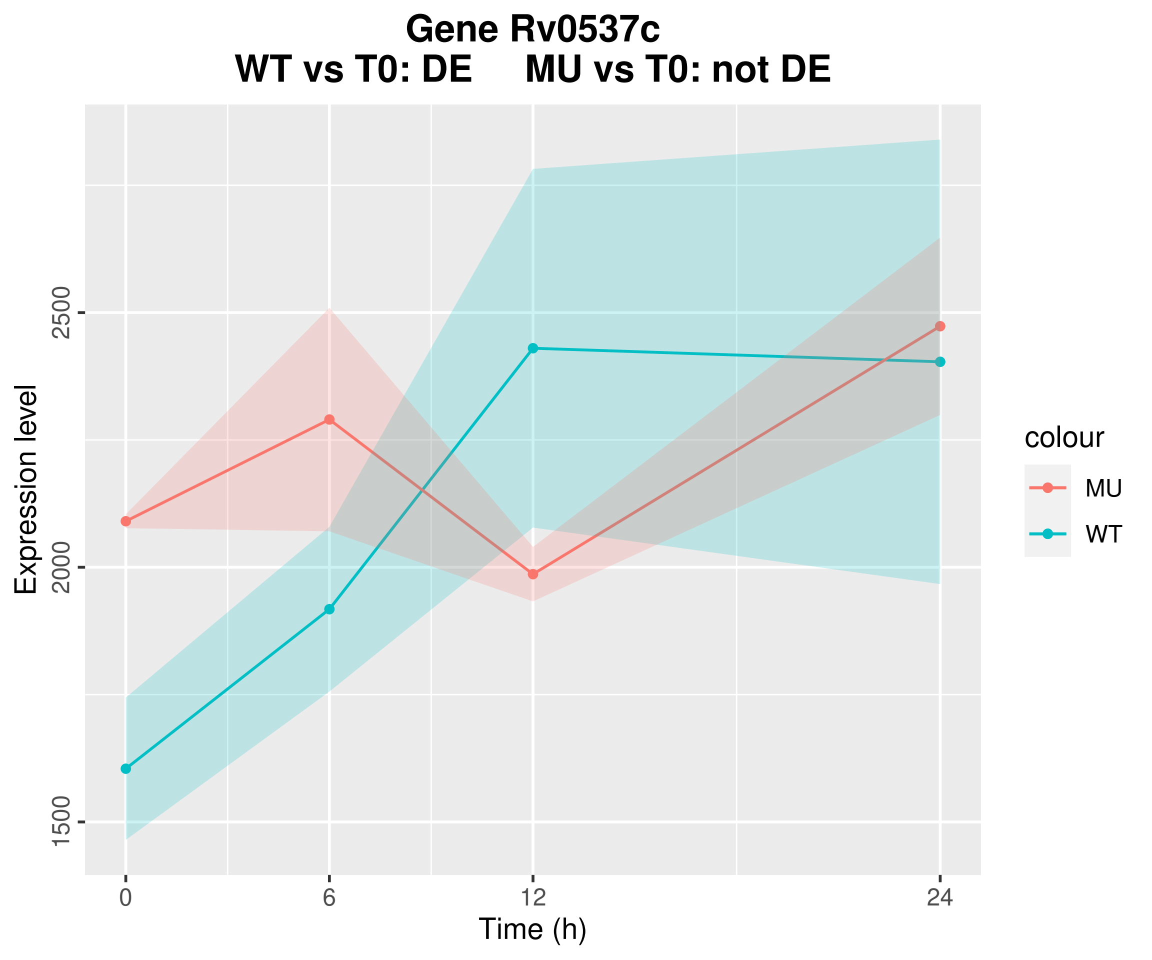

Gene Rv0537c
WT vs T0: DE   MU vs T0: not DE
Expression level
1500
2000
2500
0
6
12
24
Time (h)
colour
MU
WT

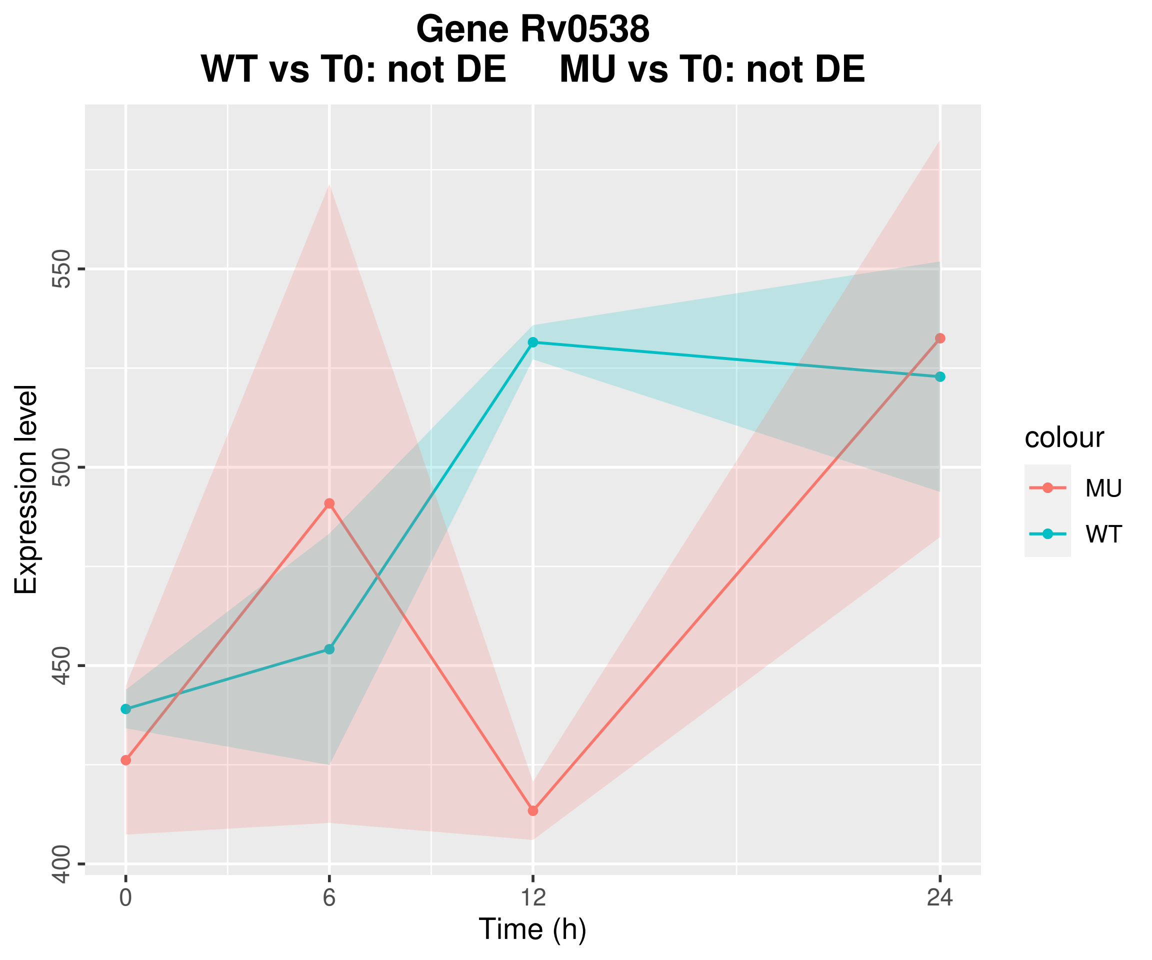

Gene Rv0538
WT vs T0: not DE MU vs T0: not DE
Expression level
400
450
500
550
0
6
12
24
Time (h)
colour
MU
WT

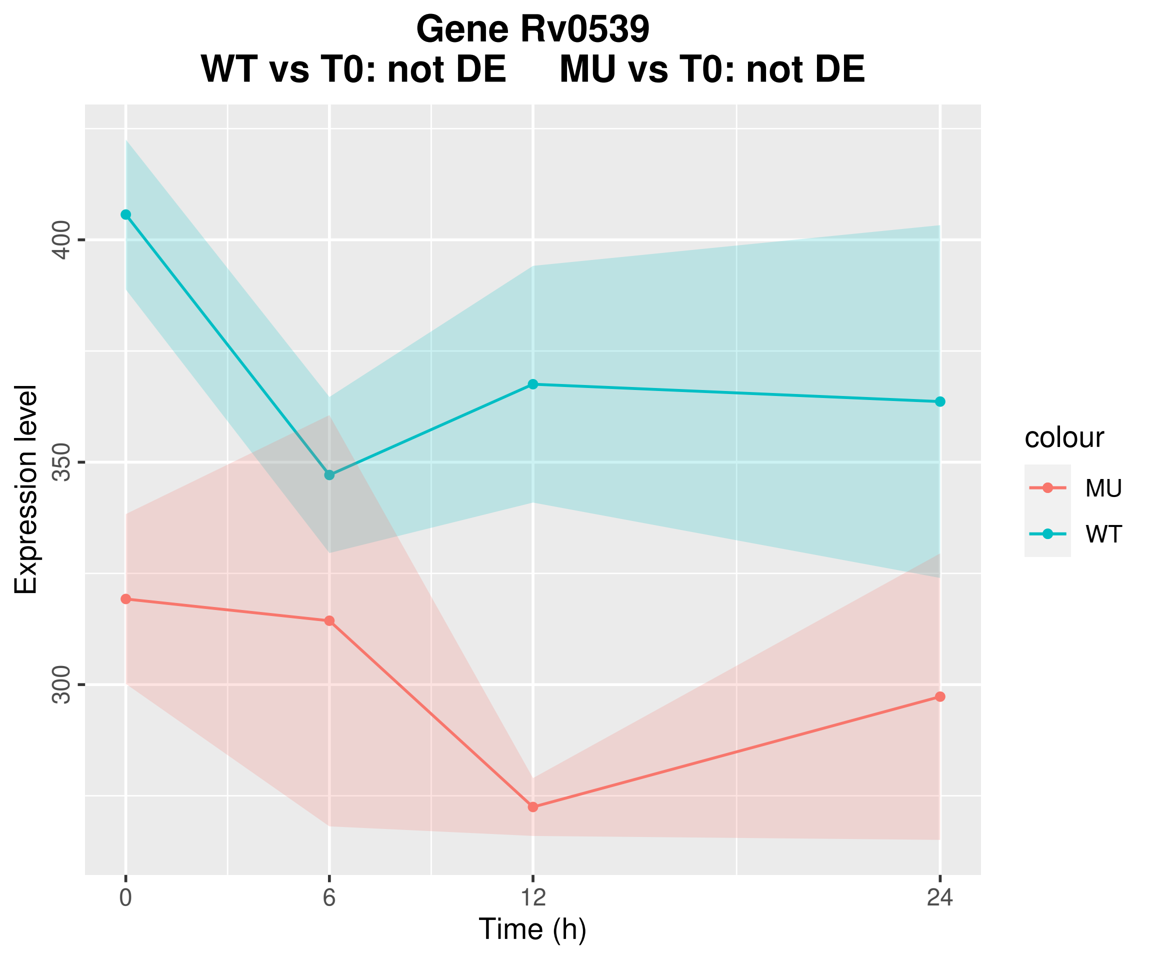

Gene Rv0539
WT vs T0: not DE   MU vs T0: not DE
Expression level
400
350
300
0
6
12
24
Time (h)
colour
MU
WT

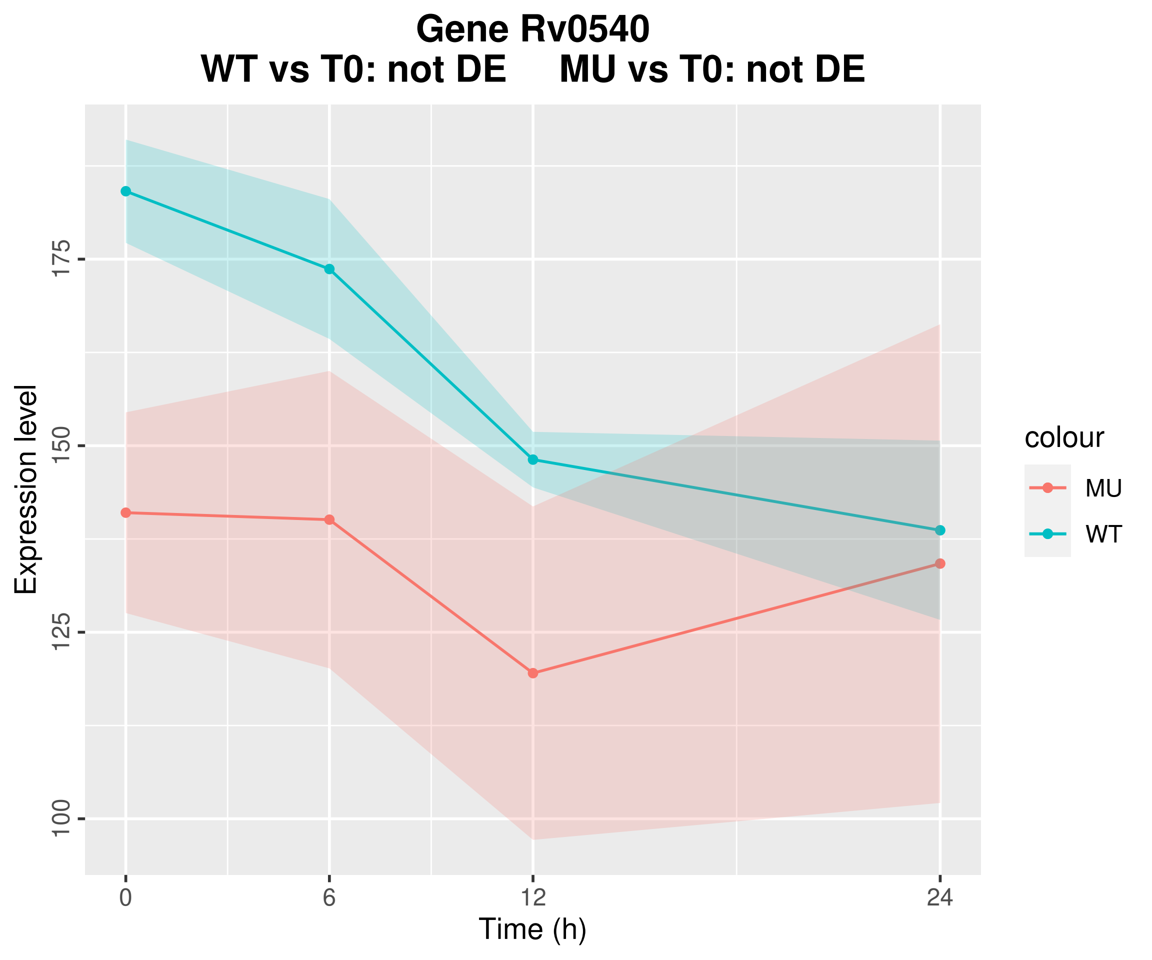

Gene Rv0540
WT vs T0: not DE MU vs T0: not DE
Expression level
175
150
125
100
0
6
12
24
Time (h)
colour
MU
WT

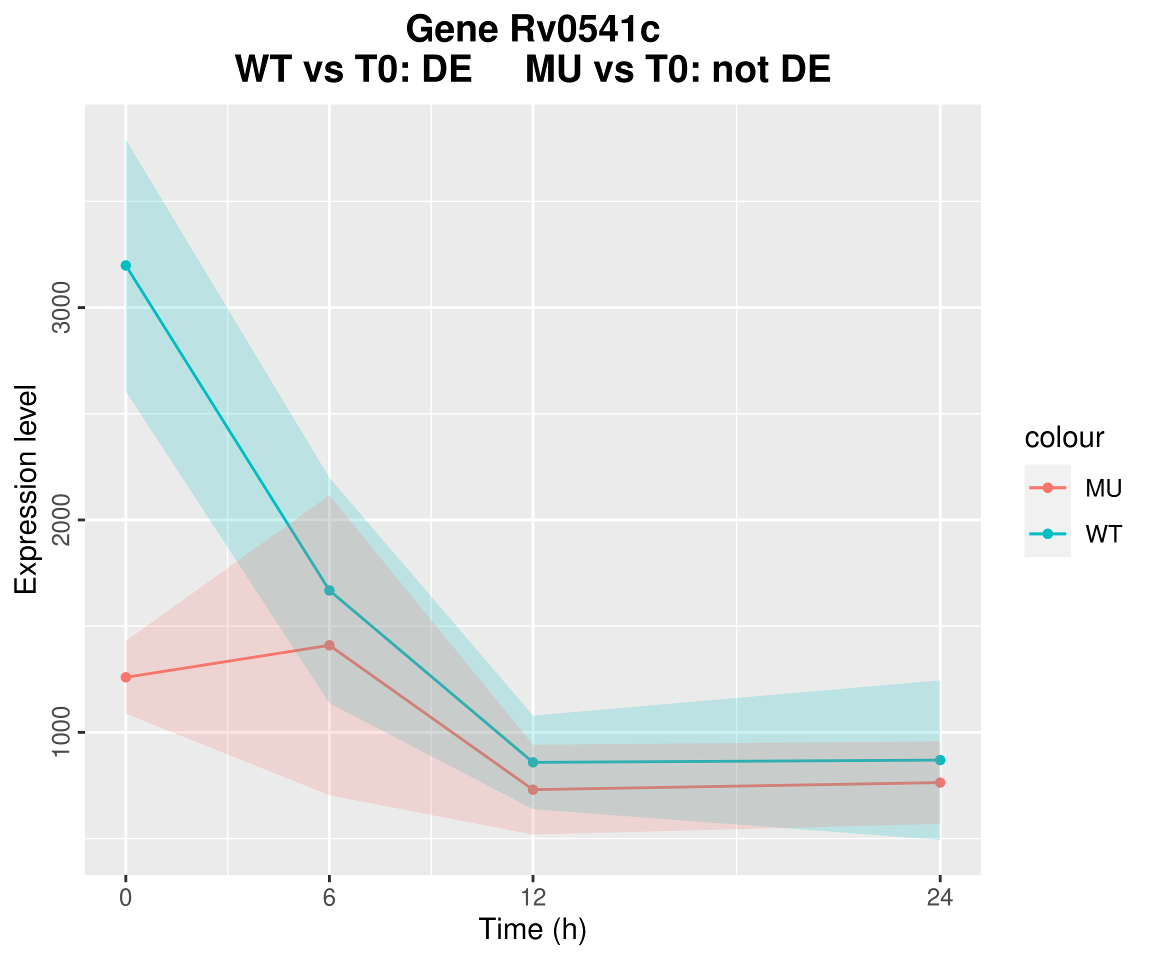

Gene Rv0541c
WT vs T0: DE   MU vs T0: not DE
Expression level
3000
2000
1000
0
6
12
24
Time (h)
colour
MU
WT

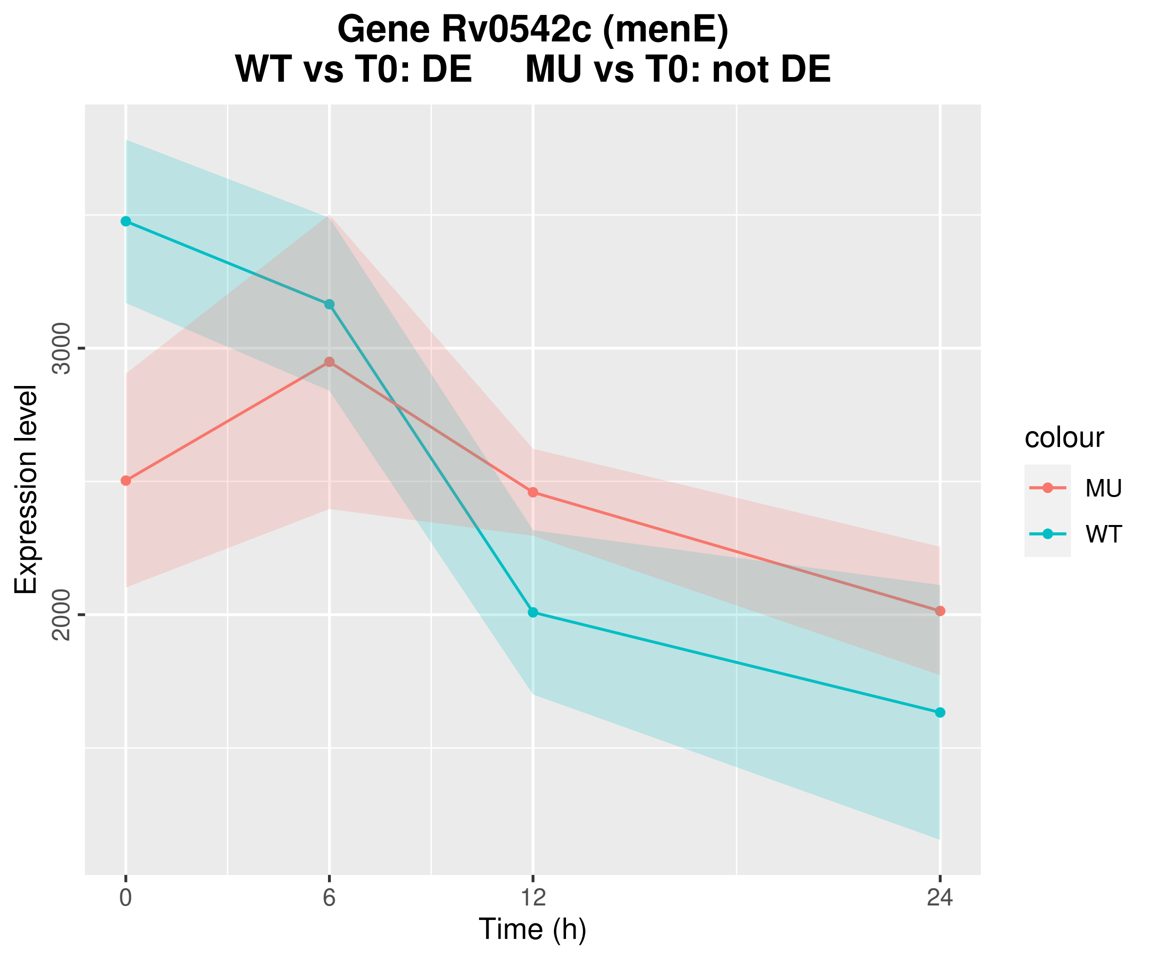

Gene Rv0542c (menE)
WT vs T0: DE   MU vs T0: not DE
Expression level
3000
2000
0
6
12
24
Time (h)
colour
MU
WT

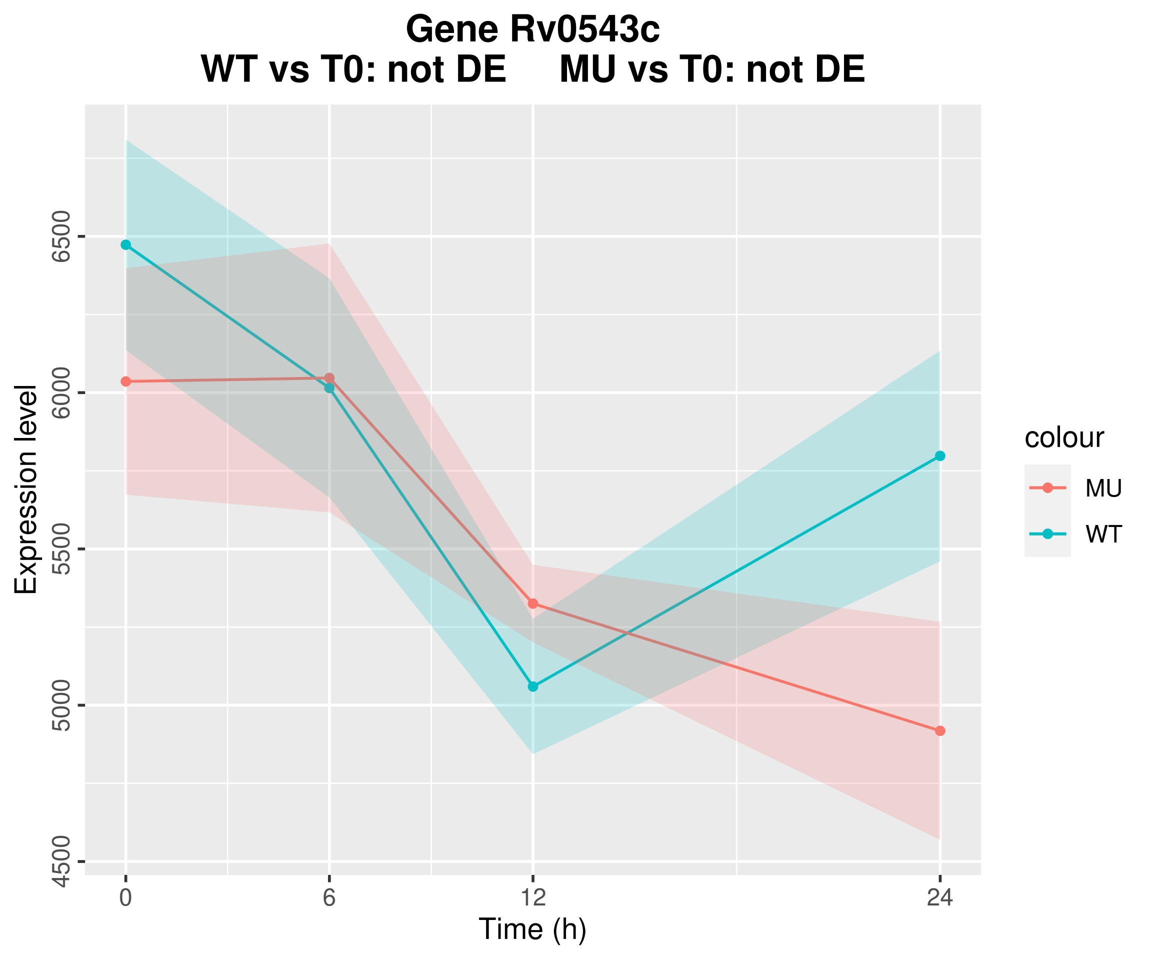

Gene Rv0543c
WT vs T0: not DE MU vs T0: not DE
Expression level
4500
5000
5500
6000
6500
0
6
12
24
Time (h)
colour
MU
WT

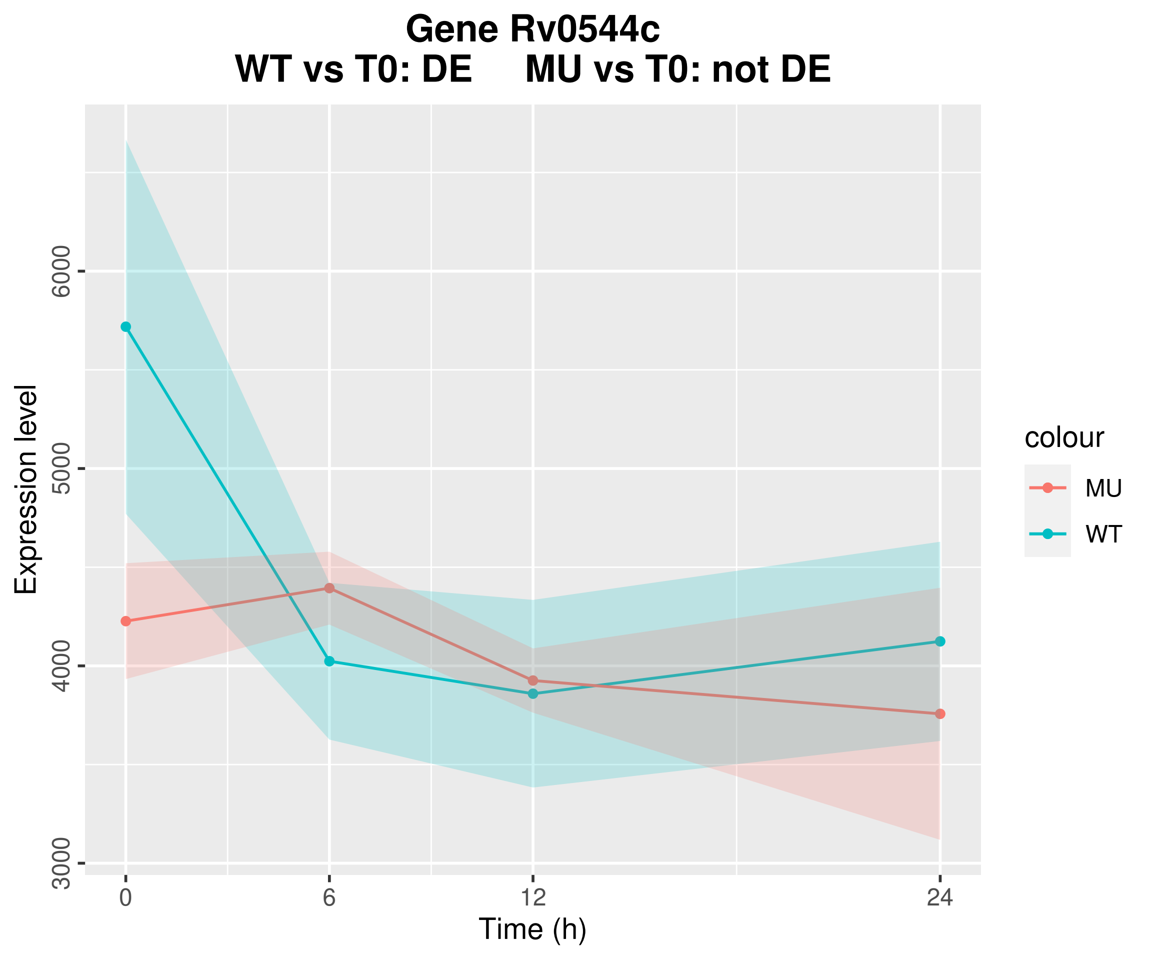

Gene Rv0544c
WT vs T0: DE MU vs T0: not DE
Expression level
6000
5000
4000
3000
0
6
12
24
Time (h)
colour
MU
WT

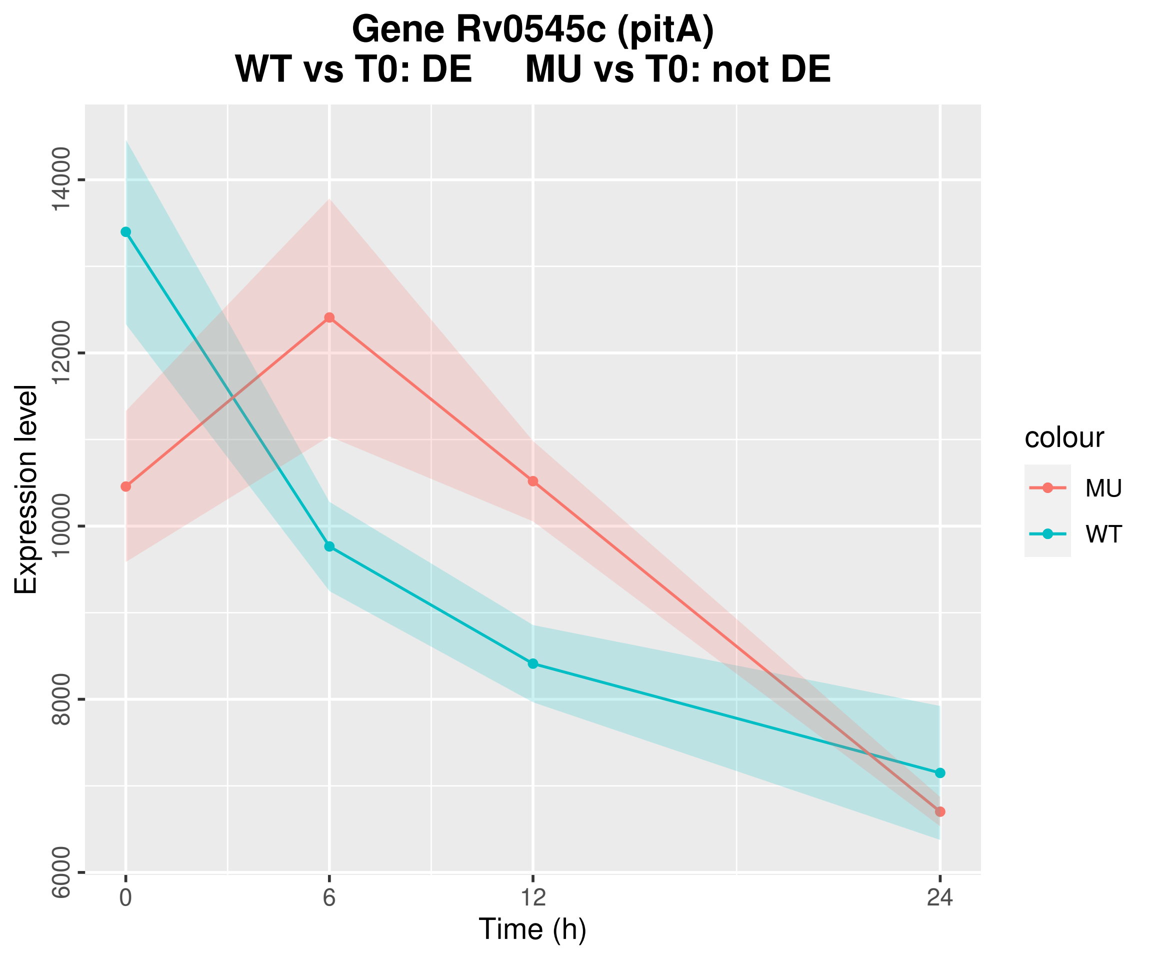

Gene Rv0545c (pitA)
WT vs T0: DE   MU vs T0: not DE
Expression level
14000
12000
10000
8000
6000
0
6
12
24
Time (h)
colour
MU
WT

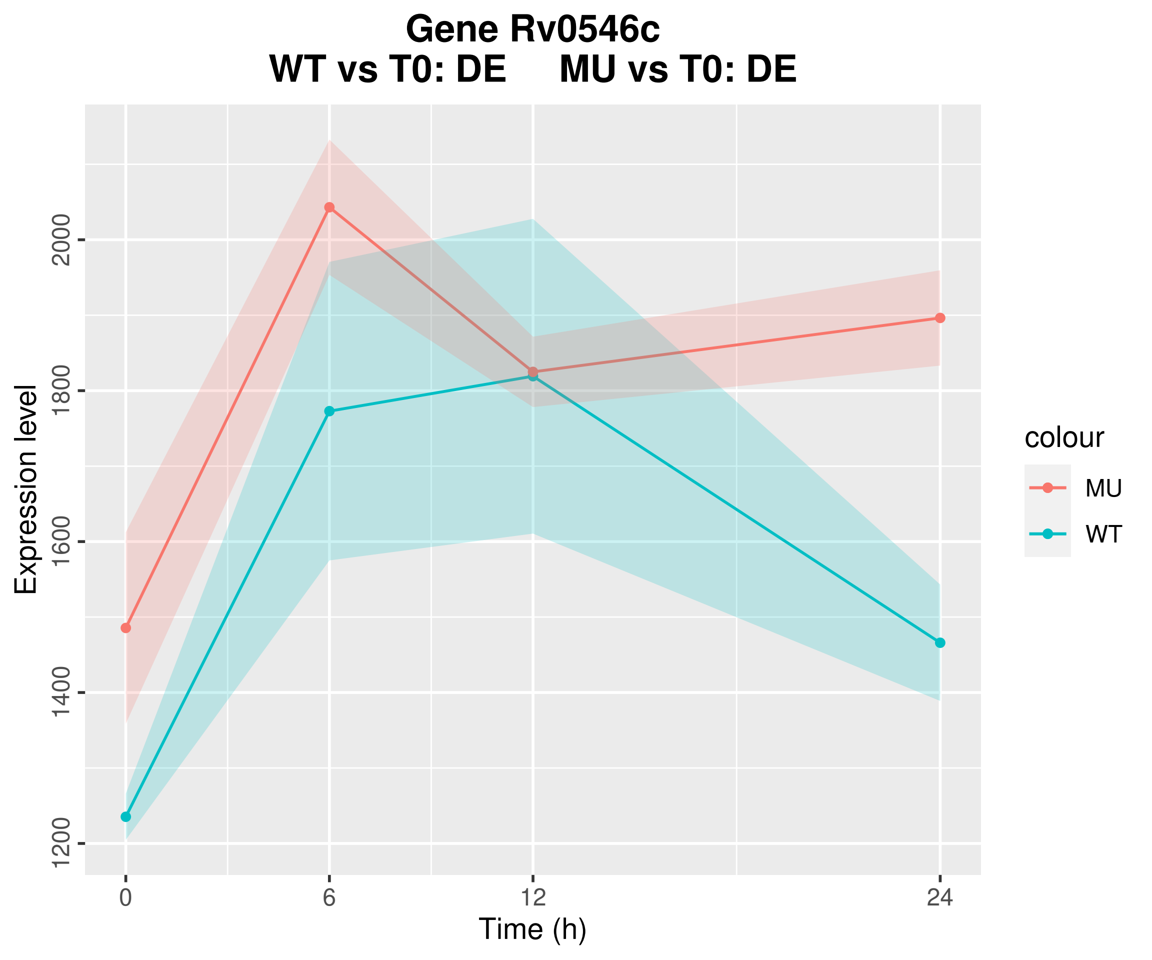

Gene Rv0546c
WT vs T0: DE   MU vs T0: DE
Expression level
1200
1400
1600
1800
2000
0
6
12
24
Time (h)
colour
MU
WT

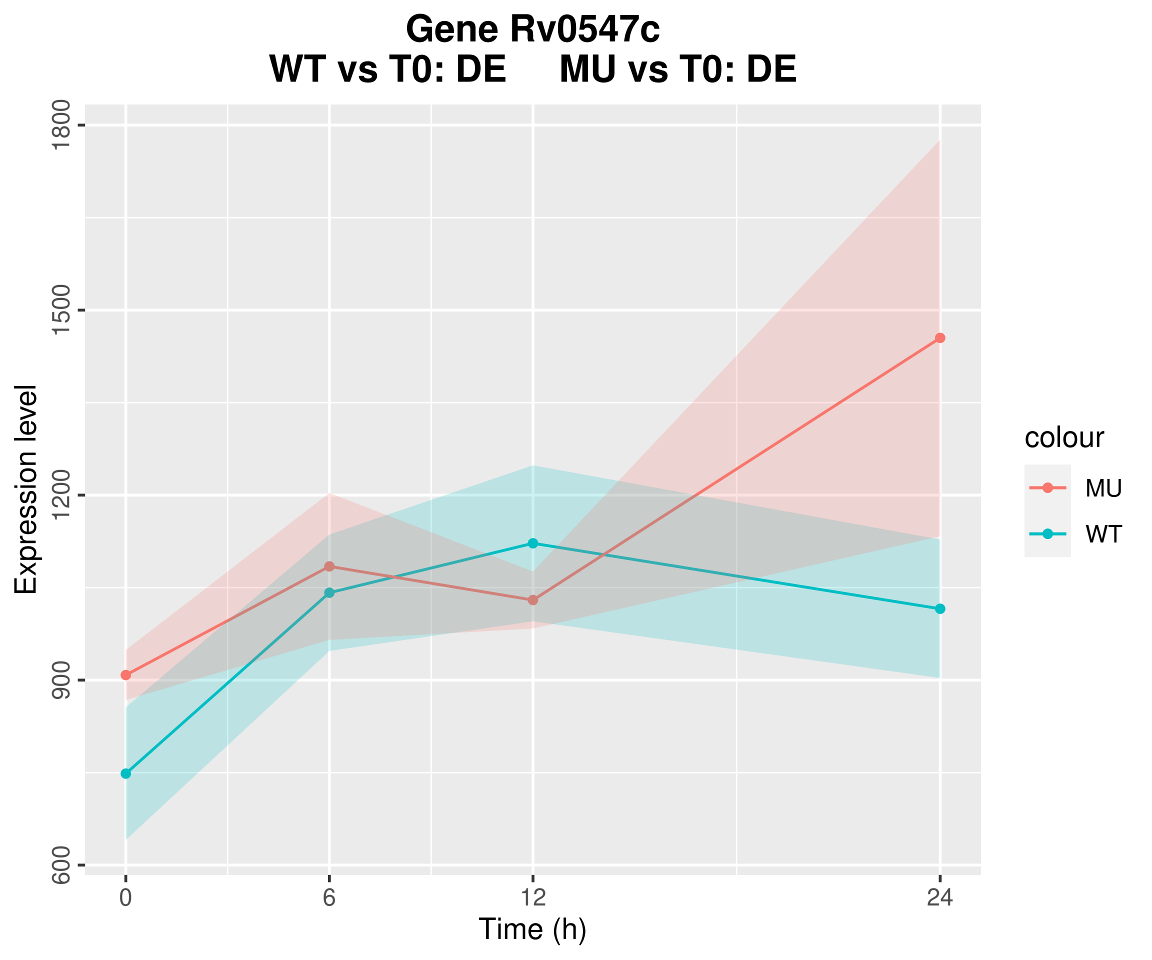
Gene Rv0547c
WT vs T0: DE   MU vs T0: DE
1800
1500
1200
900
600
Expression level
0
6
12
24
Time (h)
colour
MU
WT

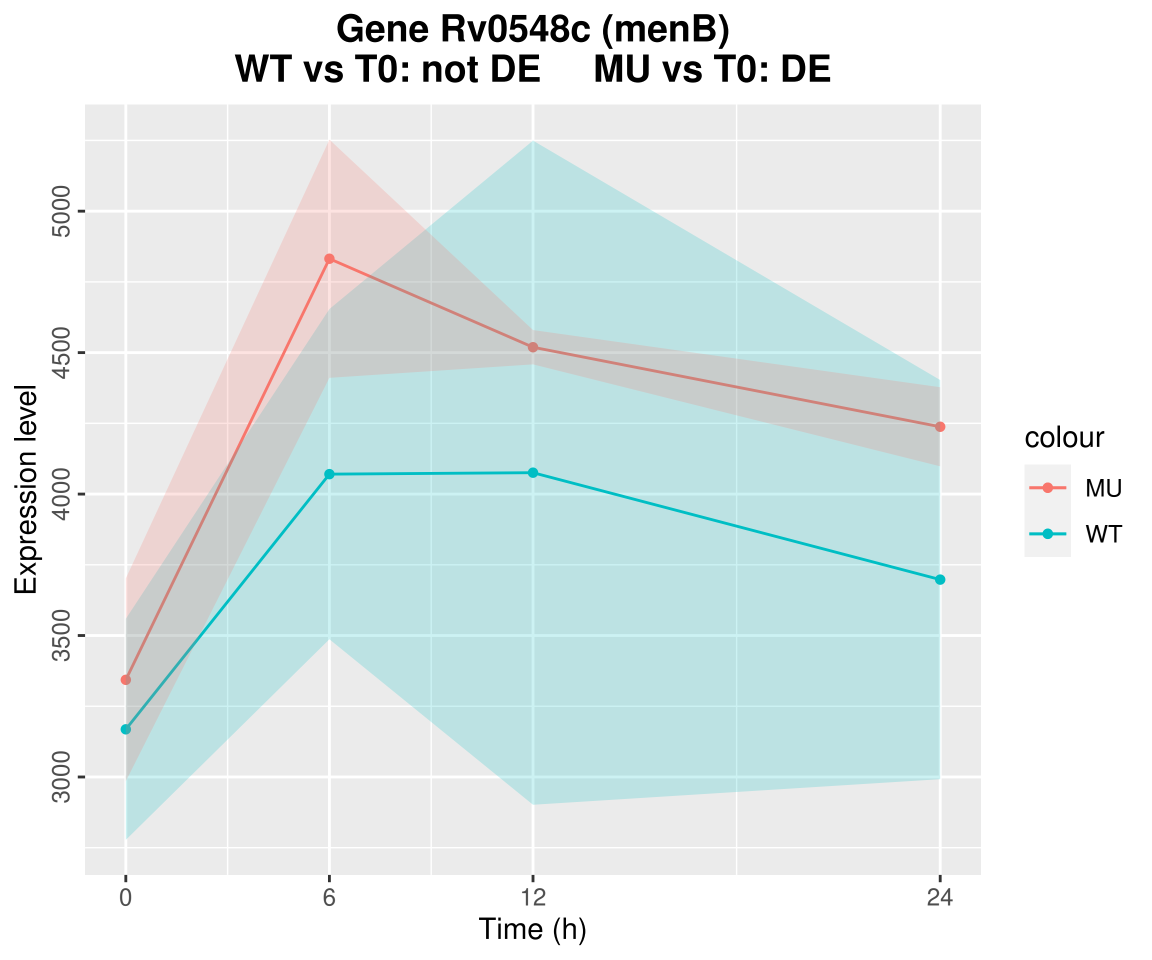

Gene Rv0548c (menB)
WT vs T0: not DE MU vs T0: DE
Expression level
5000
4500
4000
3500
3000
0
6
12
24
Time (h)
colour
MU
WT

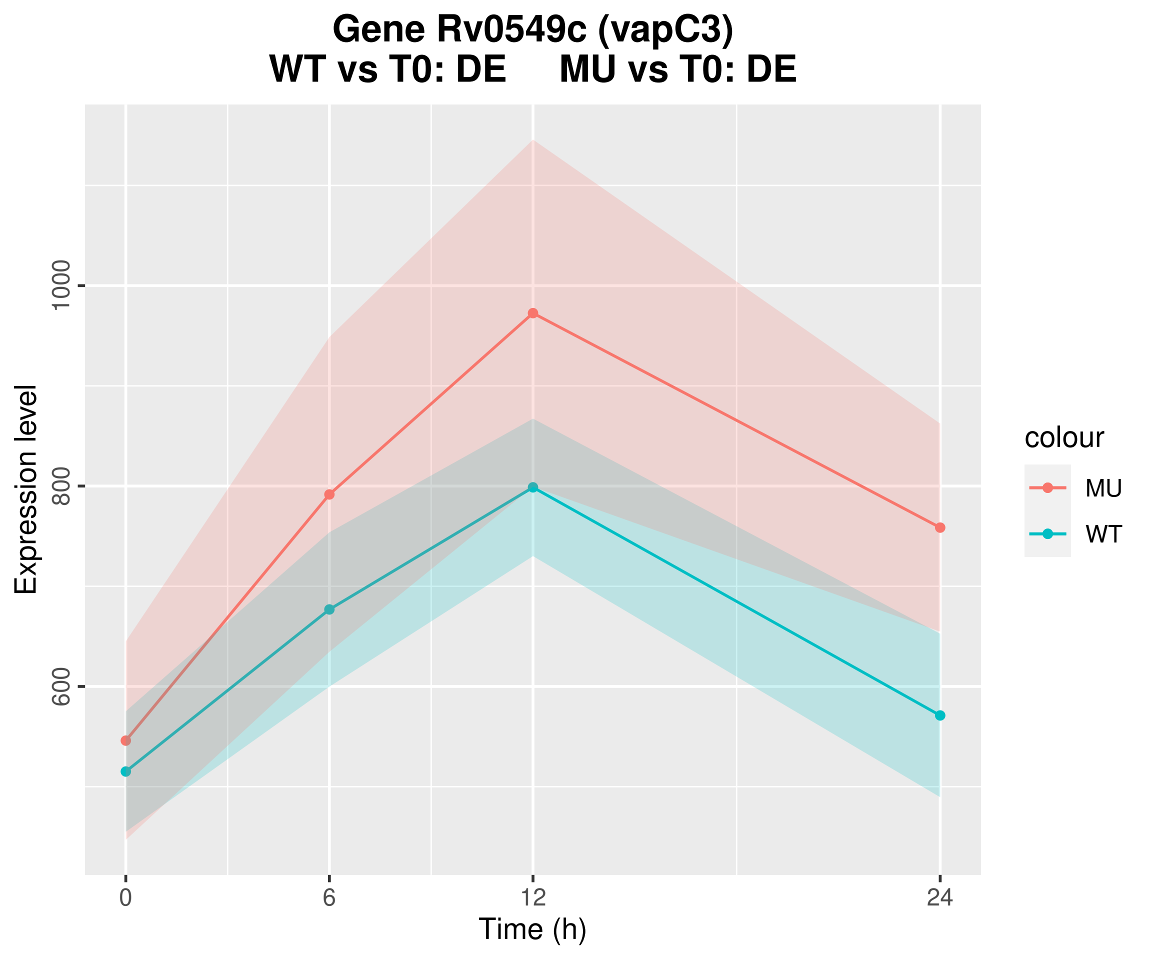
Gene Rv0549c (vapC3)
WT vs T0: DE MU vs T0: DE
Expression level
1000
800
600
0
6
12
24
Time (h)
colour
MU
WT

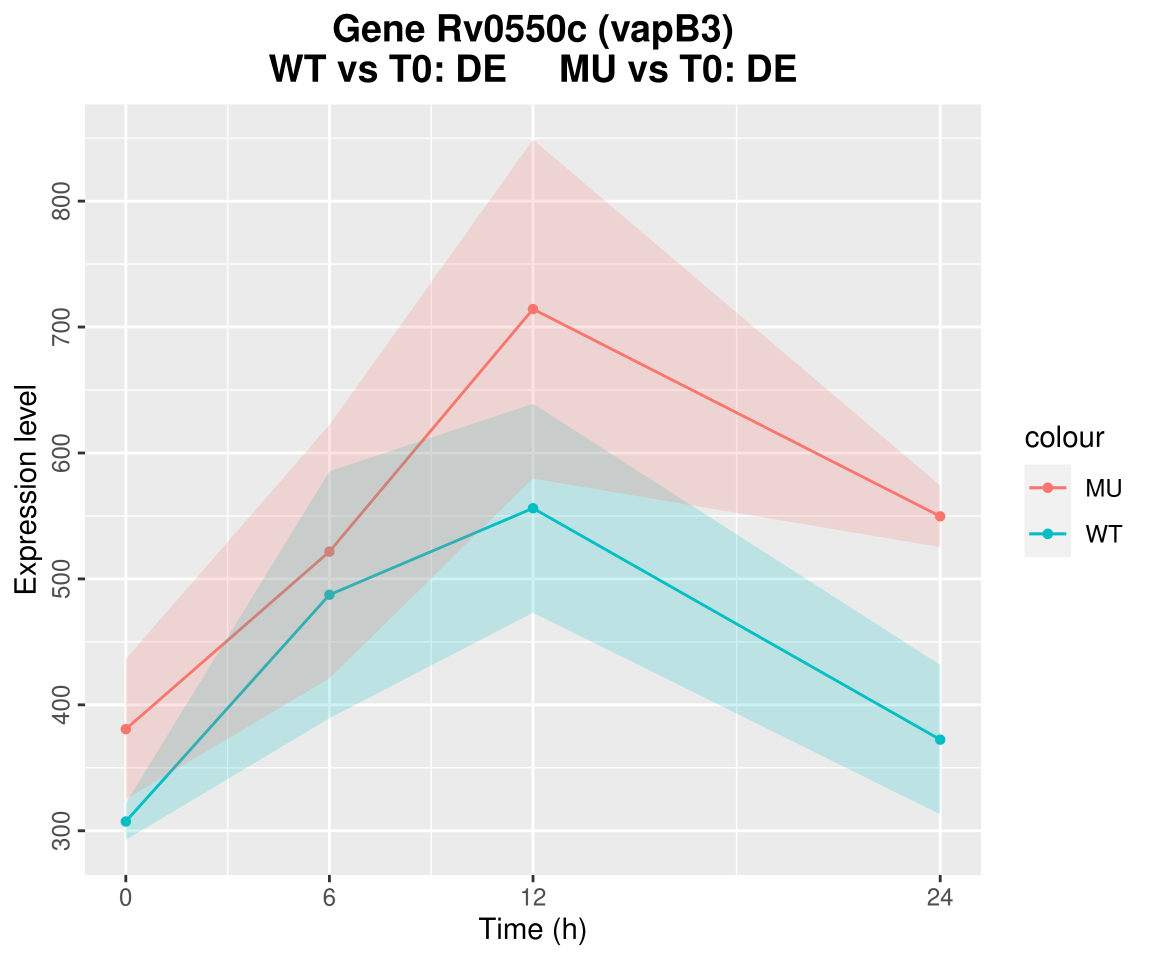
Gene Rv0550c (vapB3)
WT vs T0: DE   MU vs T0: DE
Expression level
300
400
500
600
700
800
0
6
12
24
Time (h)
colour
MU
WT

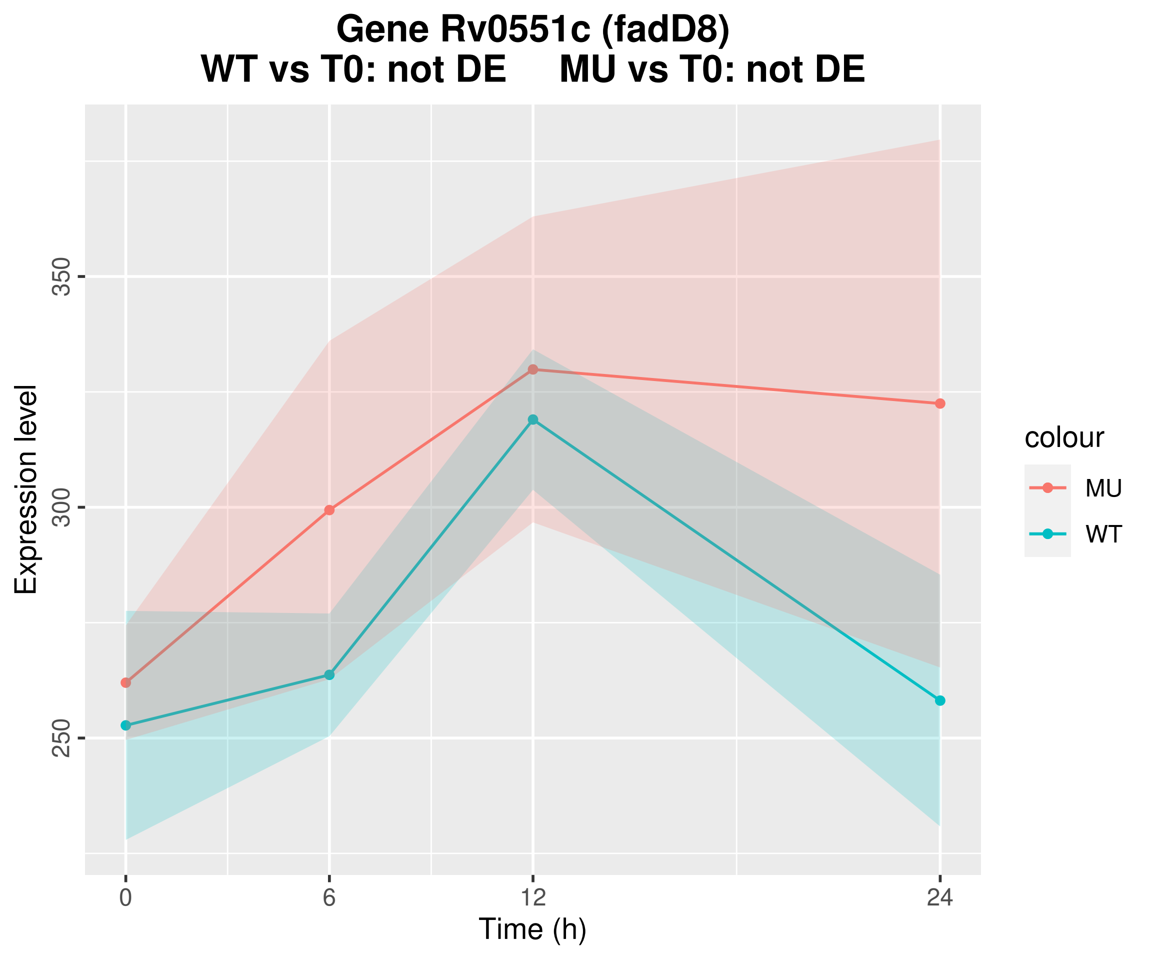
Gene Rv0551c (fadD8)
WT vs T0: not DE   MU vs T0: not DE
Expression level
250
300
350
0
6
12
24
Time (h)
colour
MU
WT

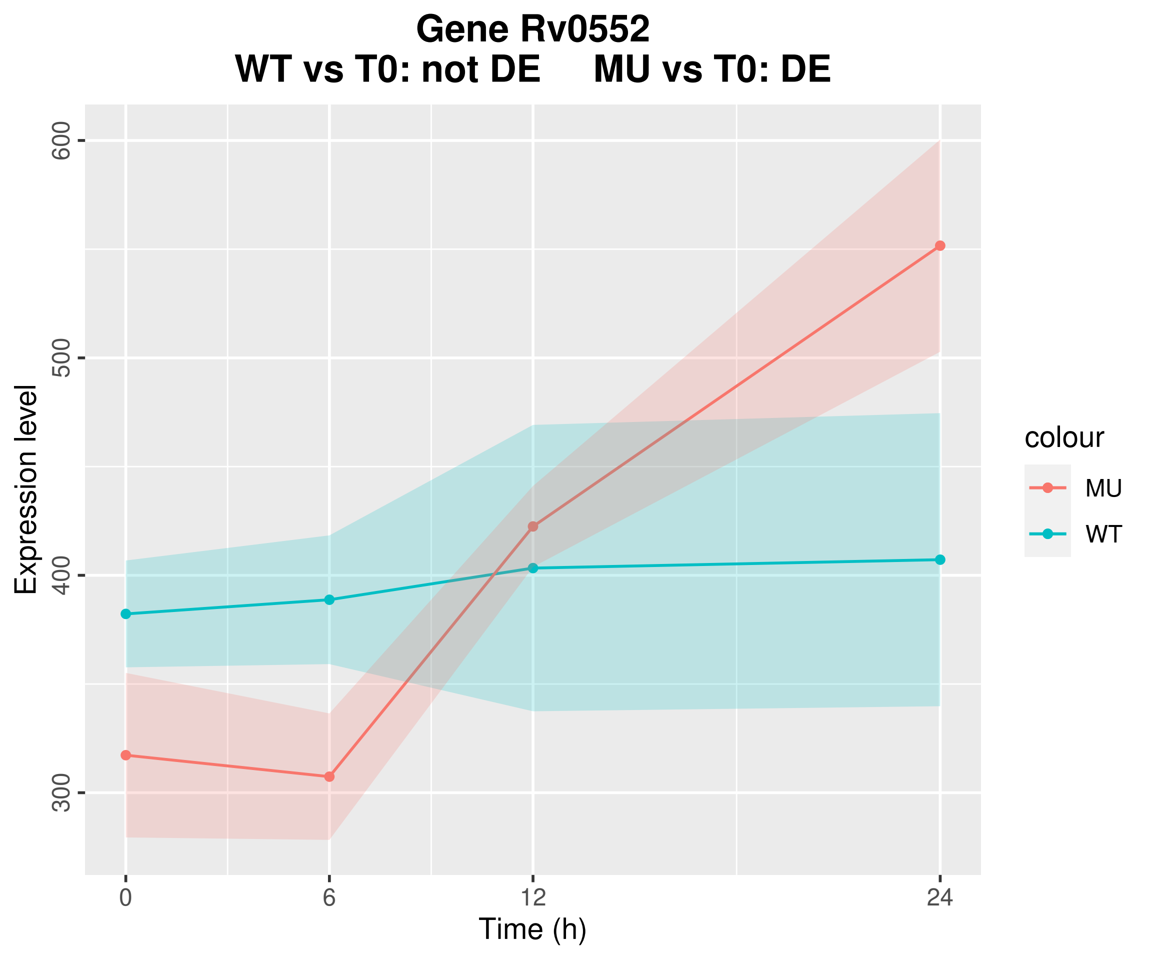

Gene Rv0552
WT vs T0: not DE MU vs T0: DE
Expression level
600
500
400
300
0
6
12
24
Time (h)
colour
MU
WT

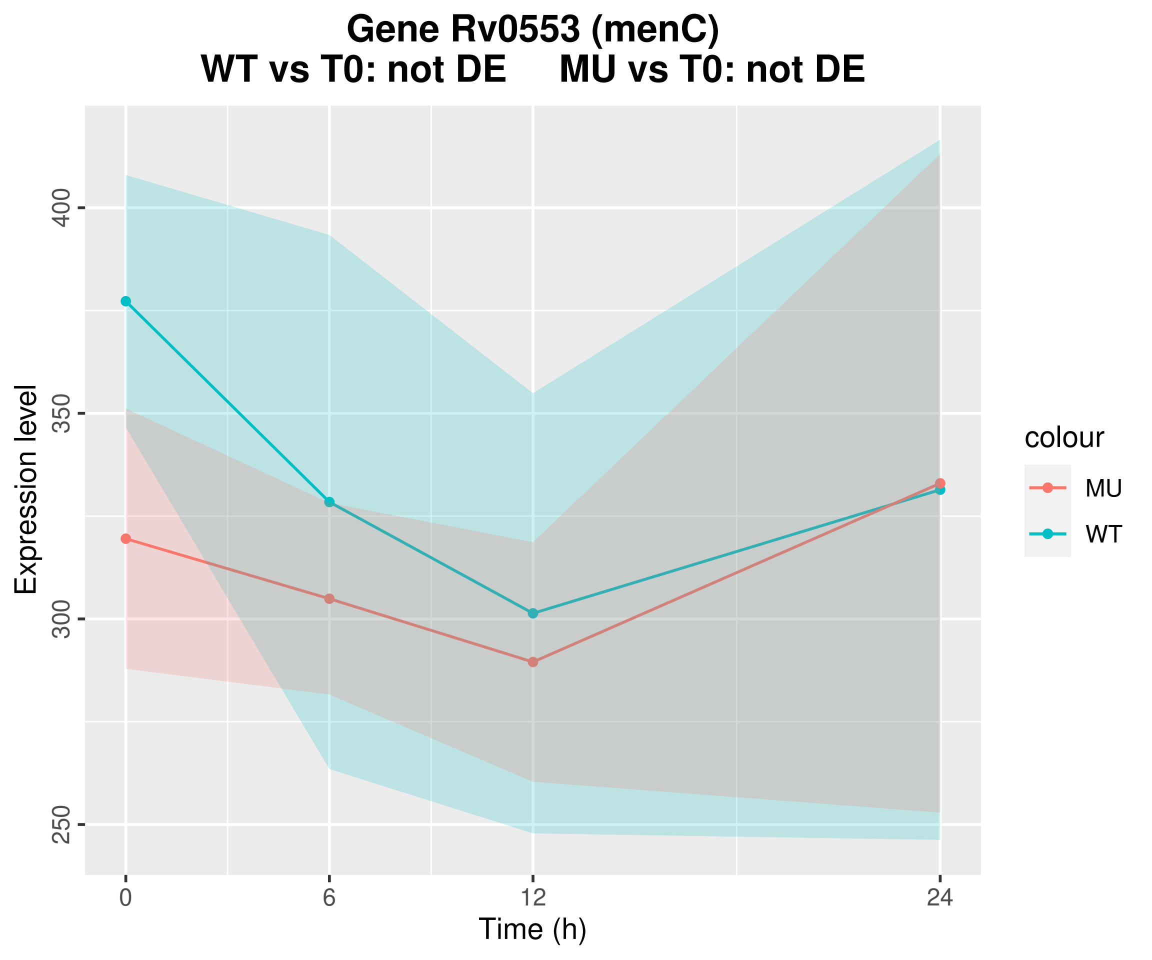

Gene Rv0553 (menC)
WT vs T0: not DE MU vs T0: not DE
Expression level
400
350
300
250
0
6
12
24
Time (h)
colour
MU
WT

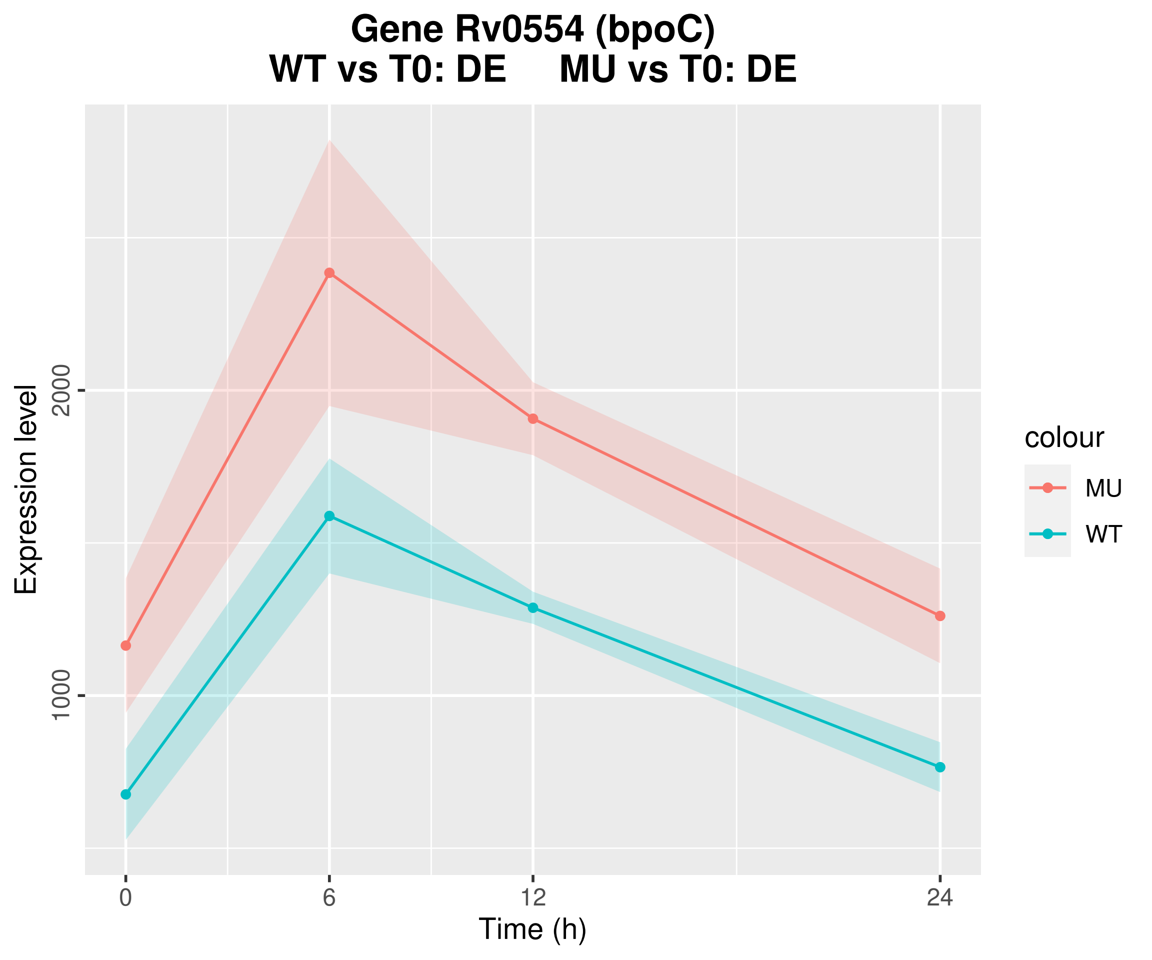

Gene Rv0554 (bpoC)
WT vs T0: DE MU vs T0: DE
Expression level
2000
1000
0
6
12
24
Time (h)
colour
MU
WT

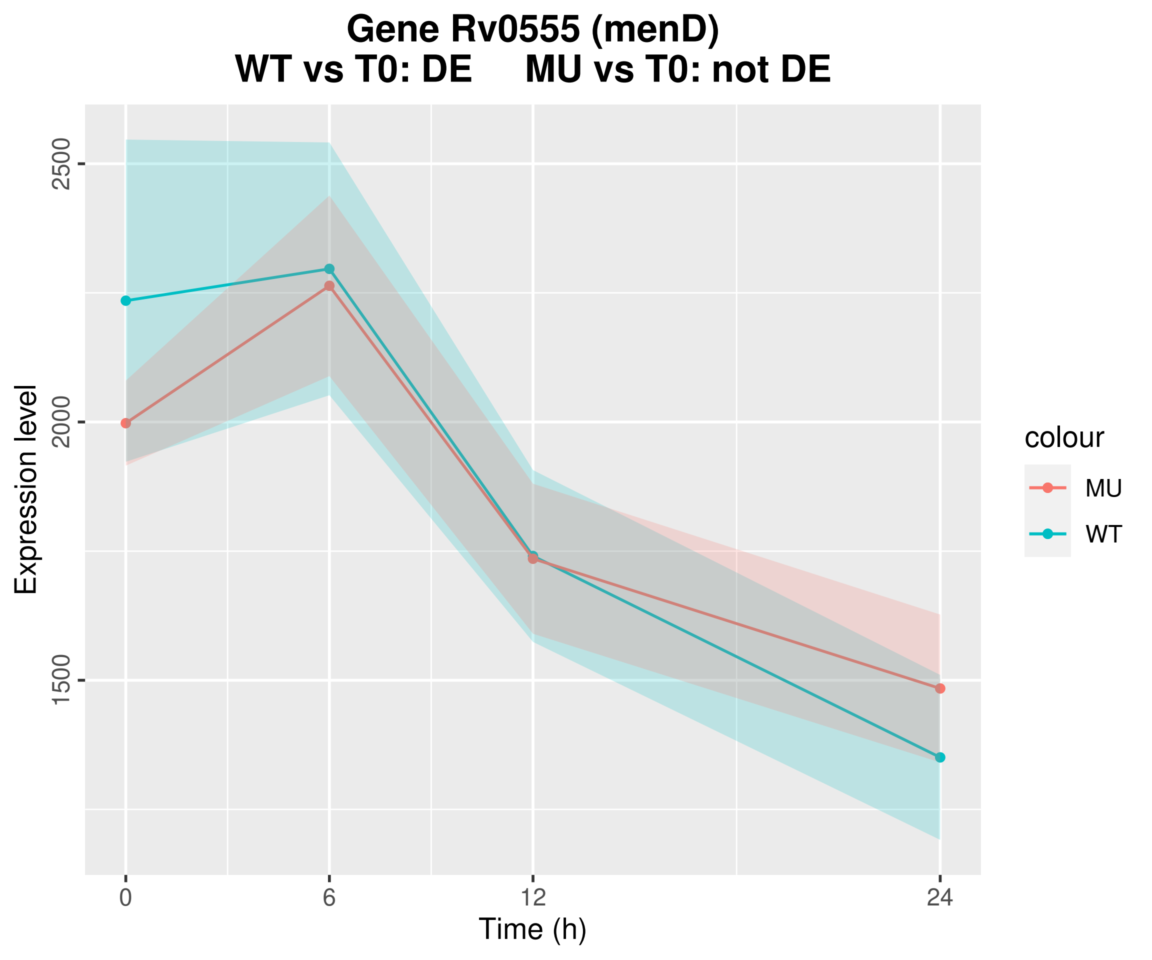

Gene Rv0555 (menD)
WT vs T0: DE MU vs T0: not DE
Expression level
2500
2000
1500
0
6
12
24
Time (h)
colour
MU
WT

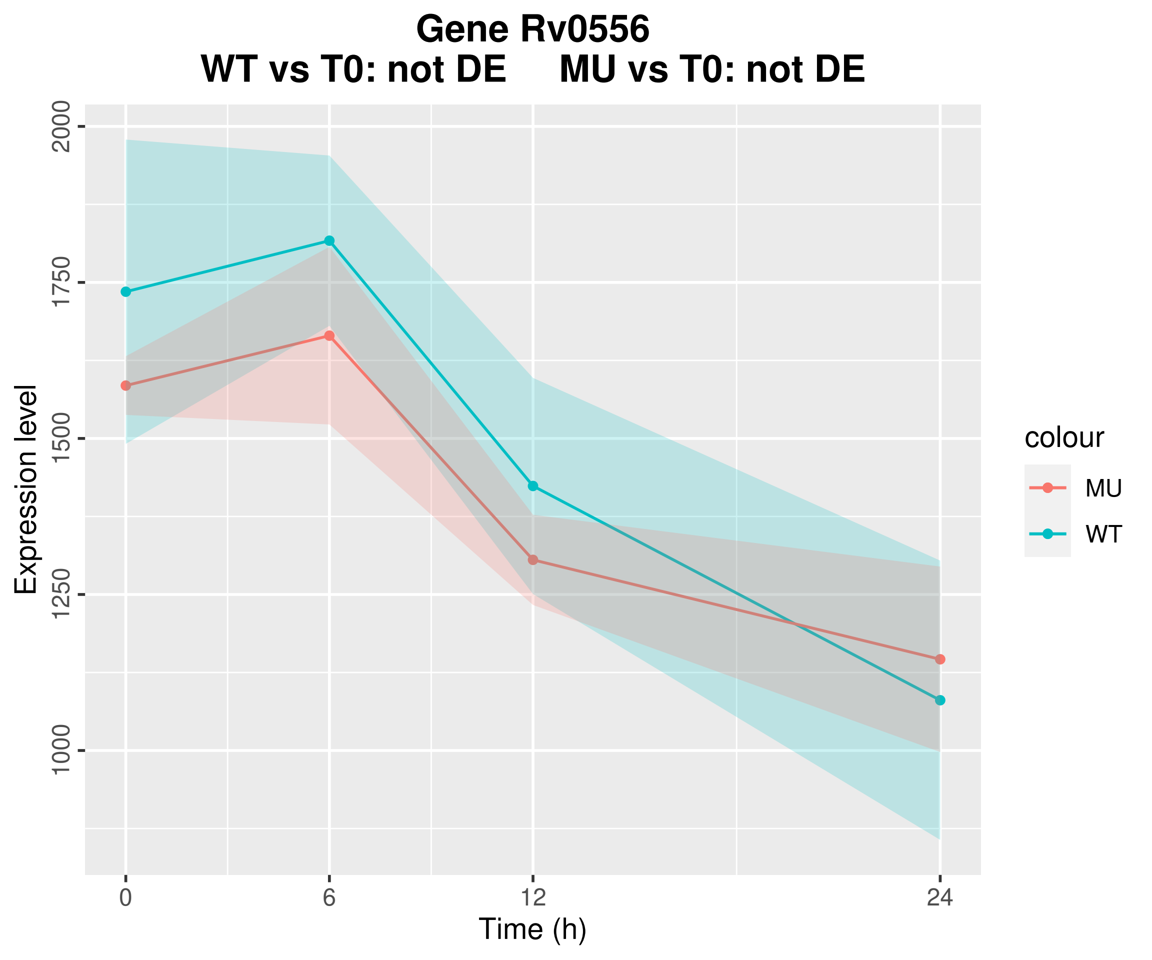
Gene Rv0556
WT vs T0: not DE   MU vs T0: not DE
Expression level
2000
1750
1500
1250
1000
0
6
12
24
Time (h)
colour
MU
WT

Gene Rv0557 (mgtA)
WT vs T0: DE   MU vs T0: DE

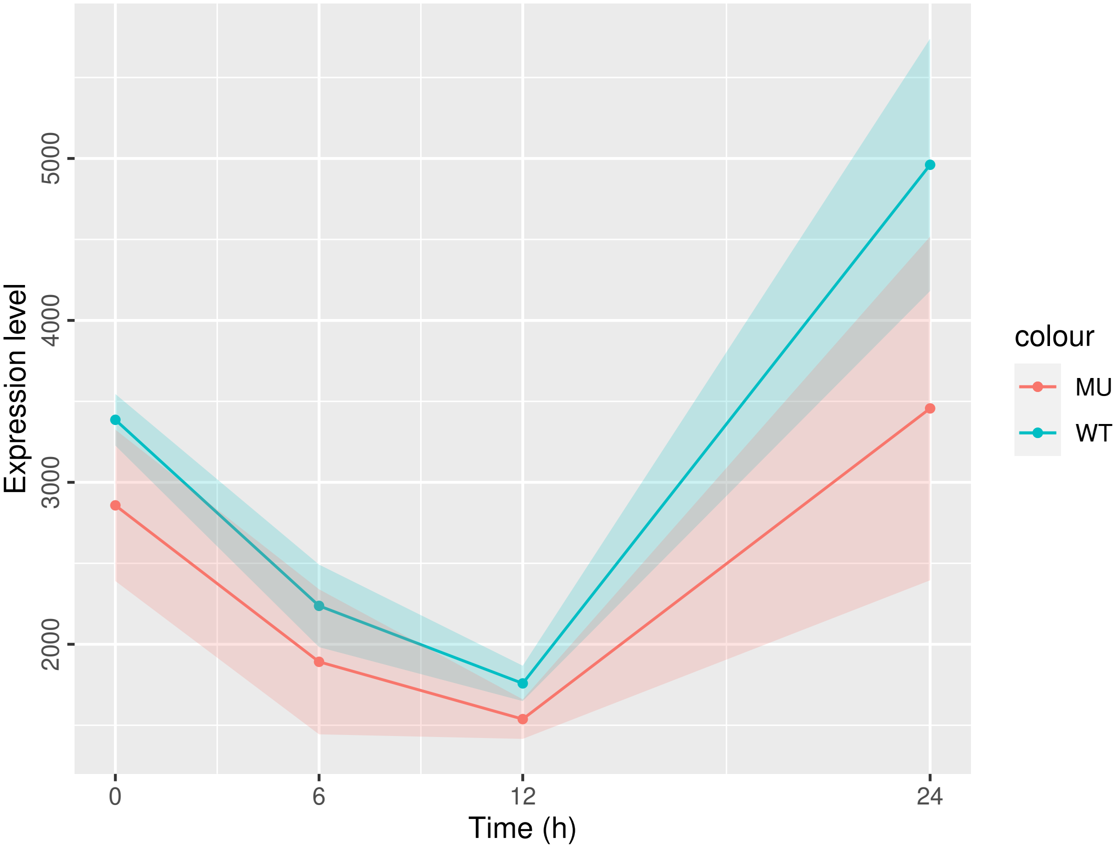

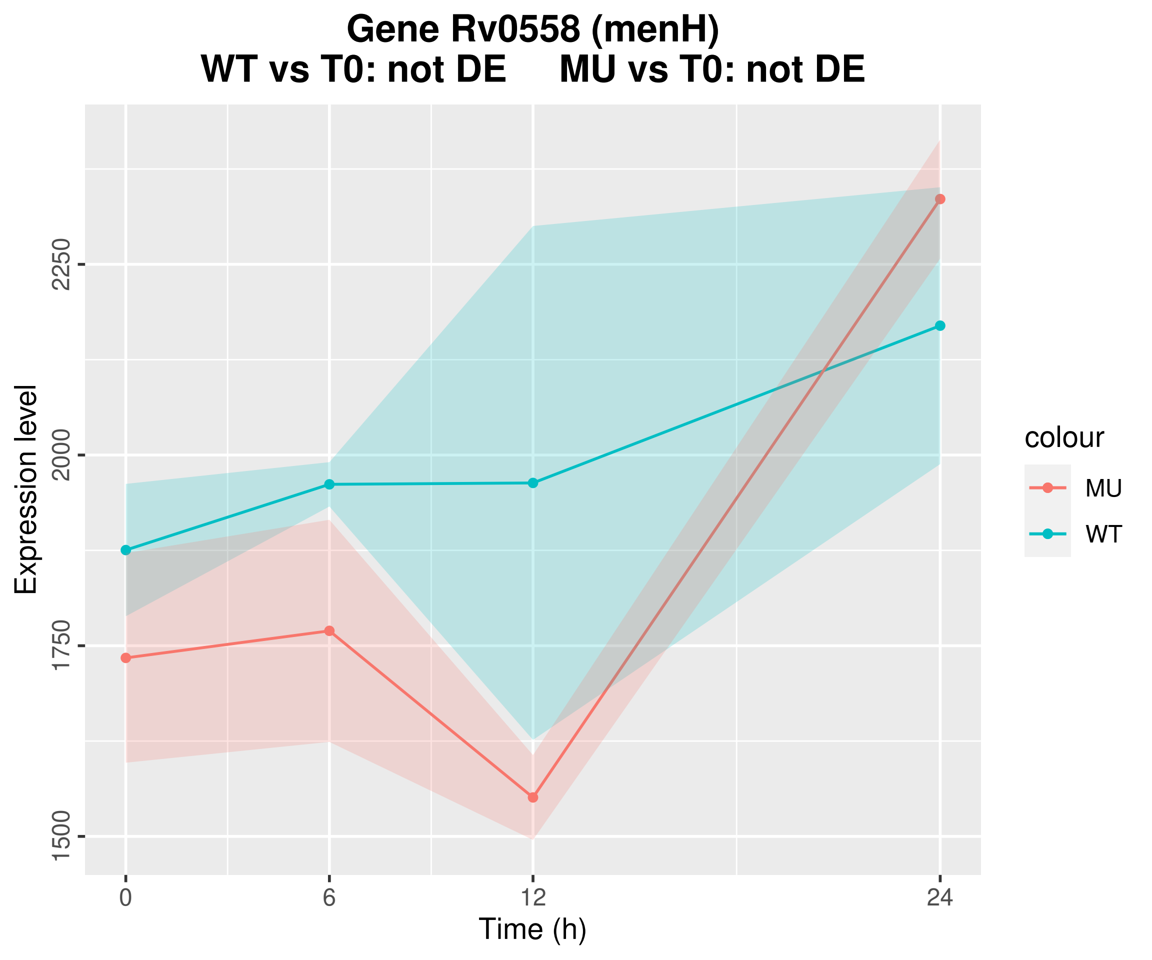

Gene Rv0558 (menH)
WT vs T0: not DE MU vs T0: not DE
Expression level
2250
2000
1750
1500
0
6
12
24
Time (h)
colour
MU
WT

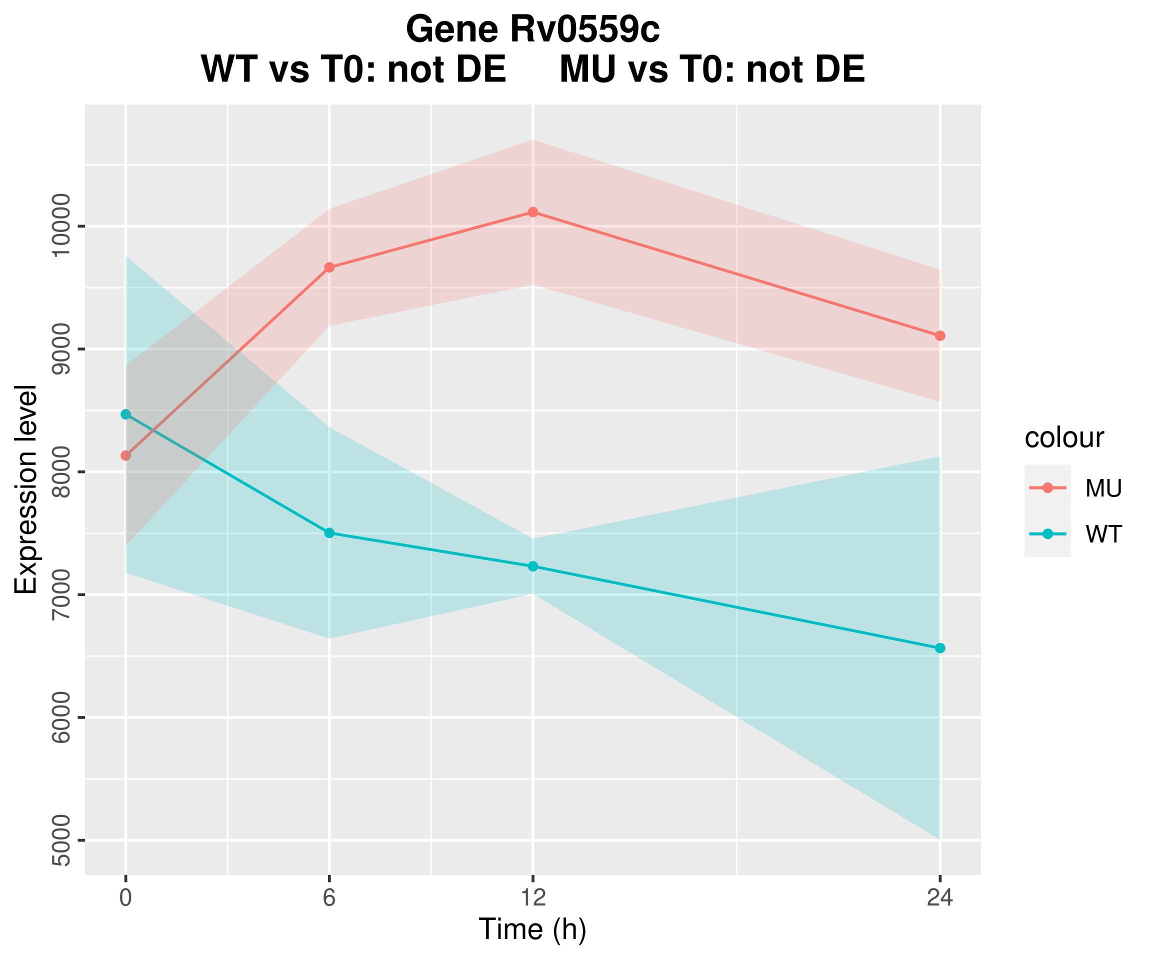

Gene Rv0559c
WT vs T0: not DE MU vs T0: not DE
Expression level
5000
6000
7000
8000
9000
10000
0
6
12
24
Time (h)
colour
MU
WT

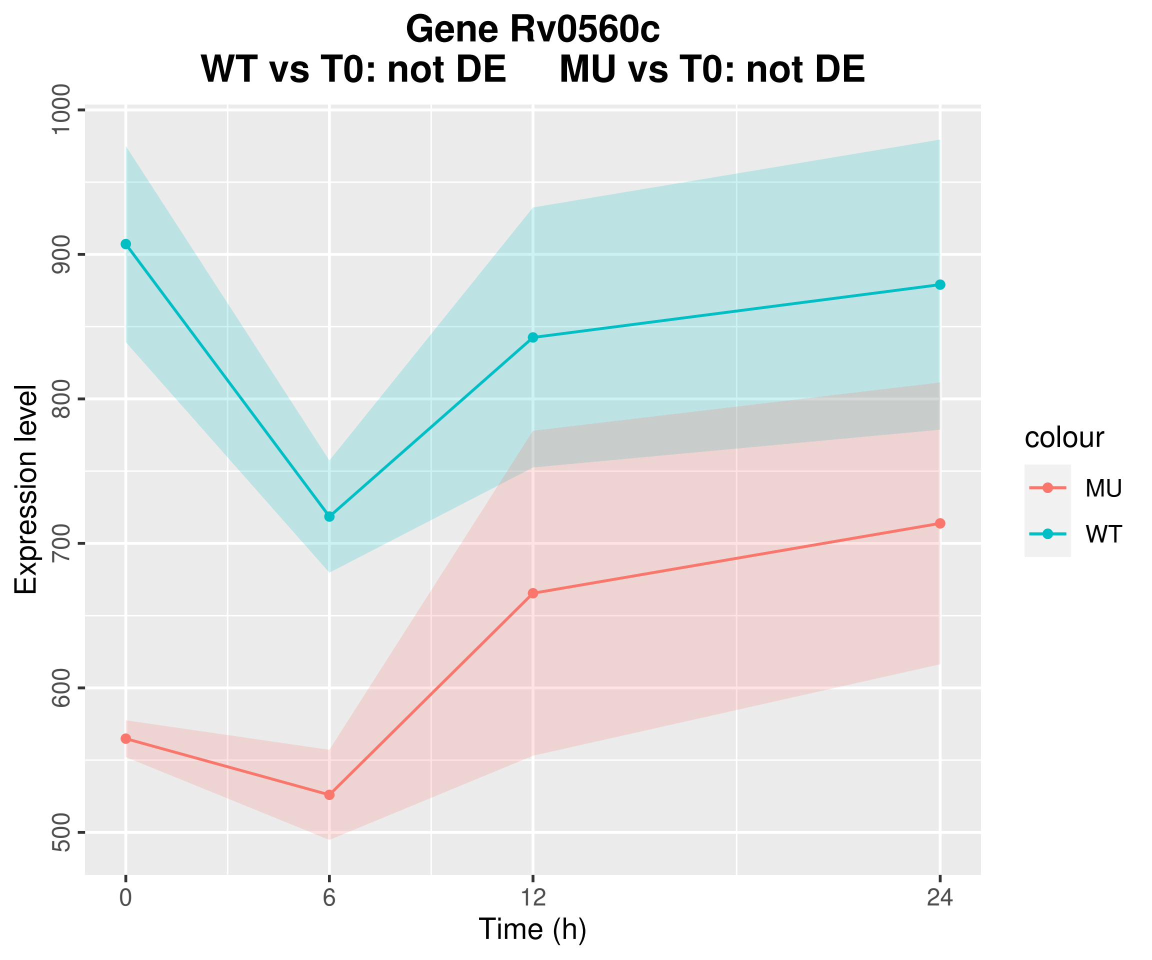

Gene Rv0560c
WT vs T0: not DE   MU vs T0: not DE
Expression level
1000
900
800
700
600
500
0
6
12
24
Time (h)
colour
MU
WT

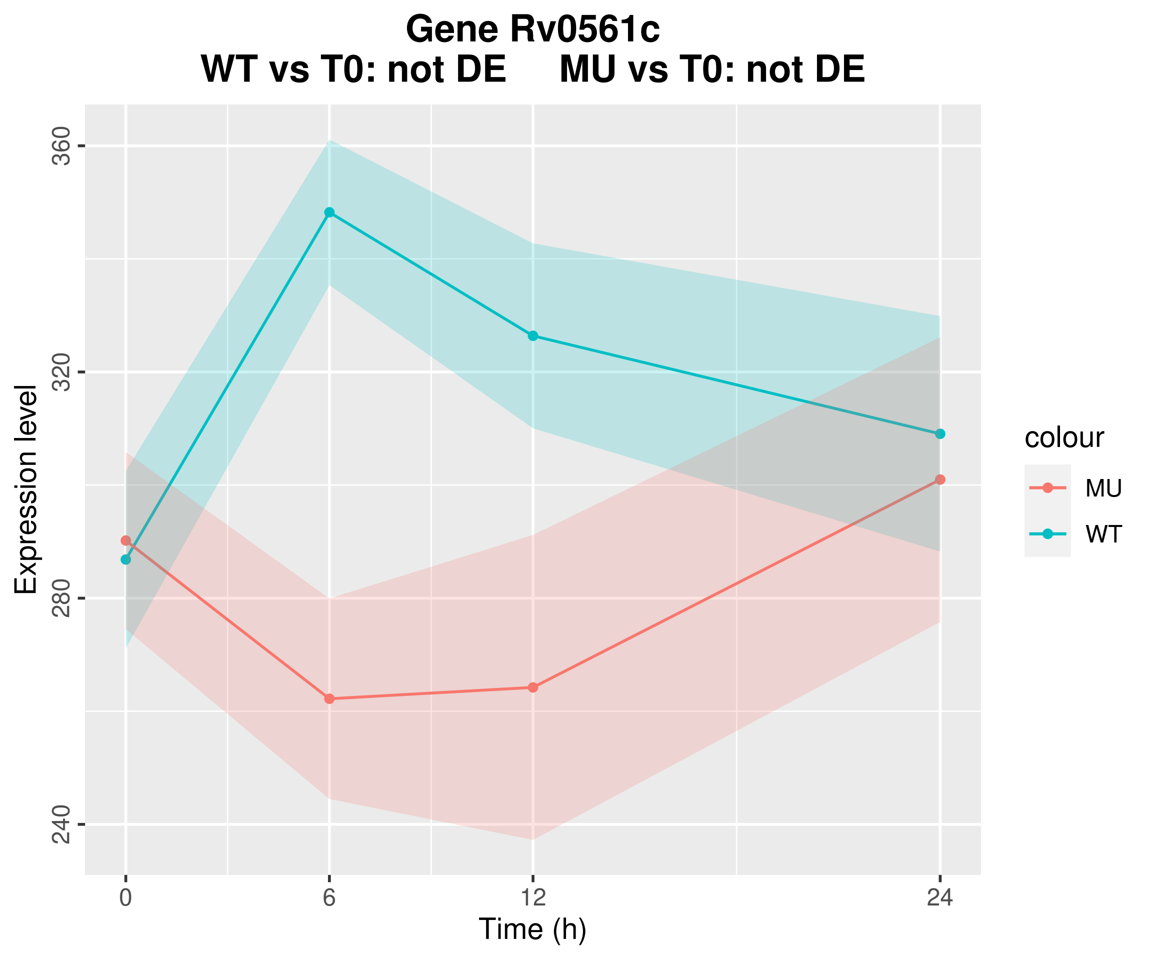

Gene Rv0561c
WT vs T0: not DE MU vs T0: not DE
Expression level
360
320
280
240
0
6
12
24
Time (h)
colour
MU
WT

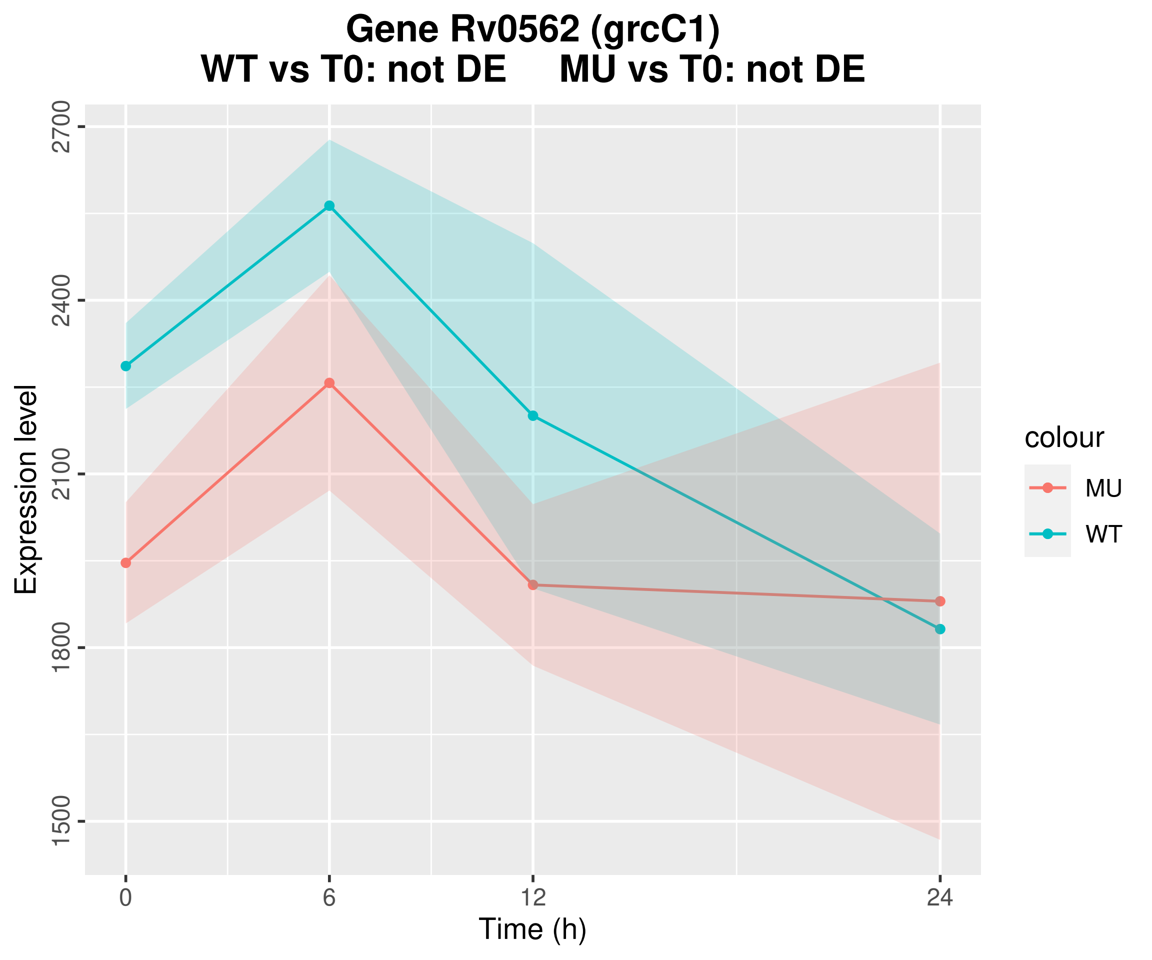

Gene Rv0562 (grcC1)
WT vs T0: not DE MU vs T0: not DE
Expression level
2700
2400
2100
1800
1500
0
6
12
24
Time (h)
colour
MU
WT

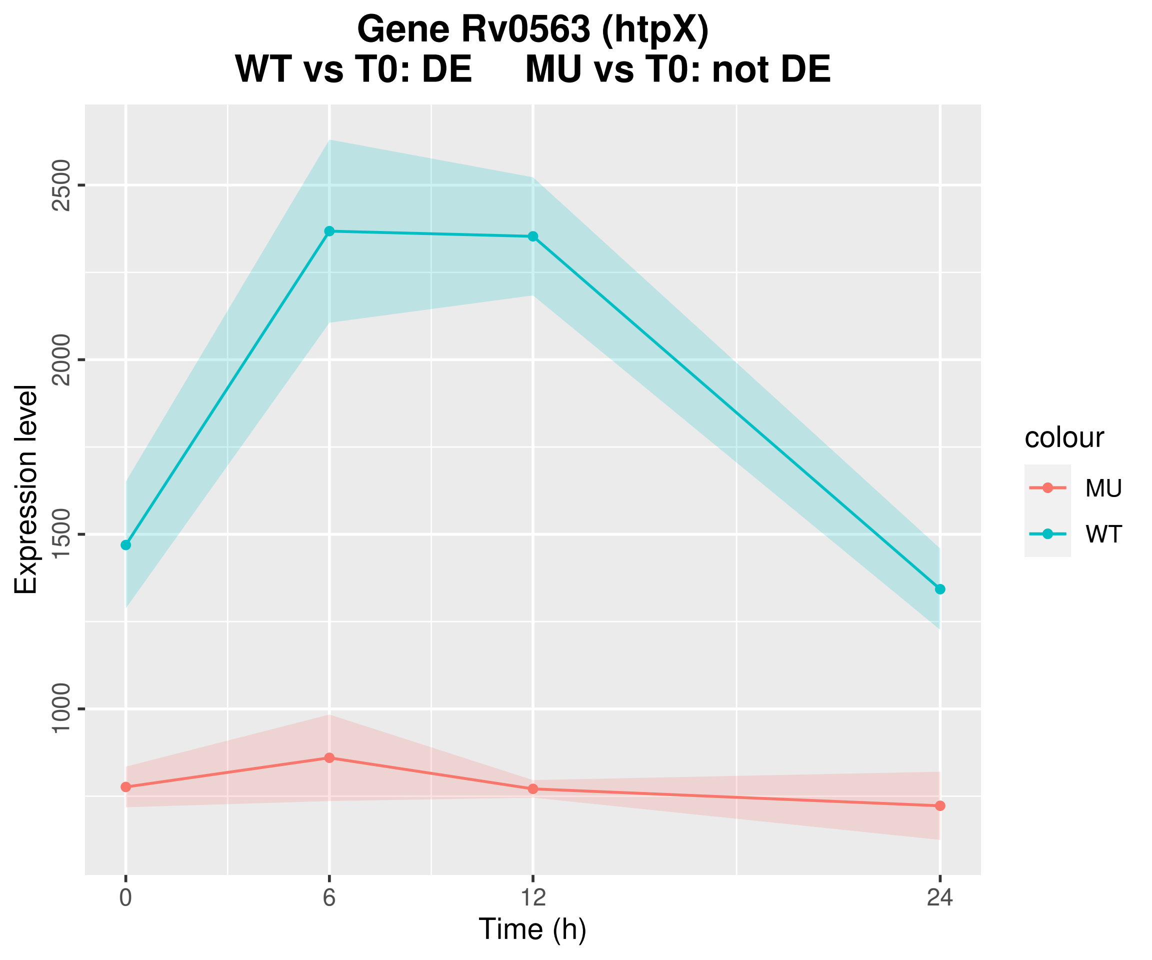
Gene Rv0563 (htpX)
WT vs T0: DE   MU vs T0: not DE
Expression level
2500
2000
1500
1000
0
6
12
24
Time (h)
colour
MU
WT

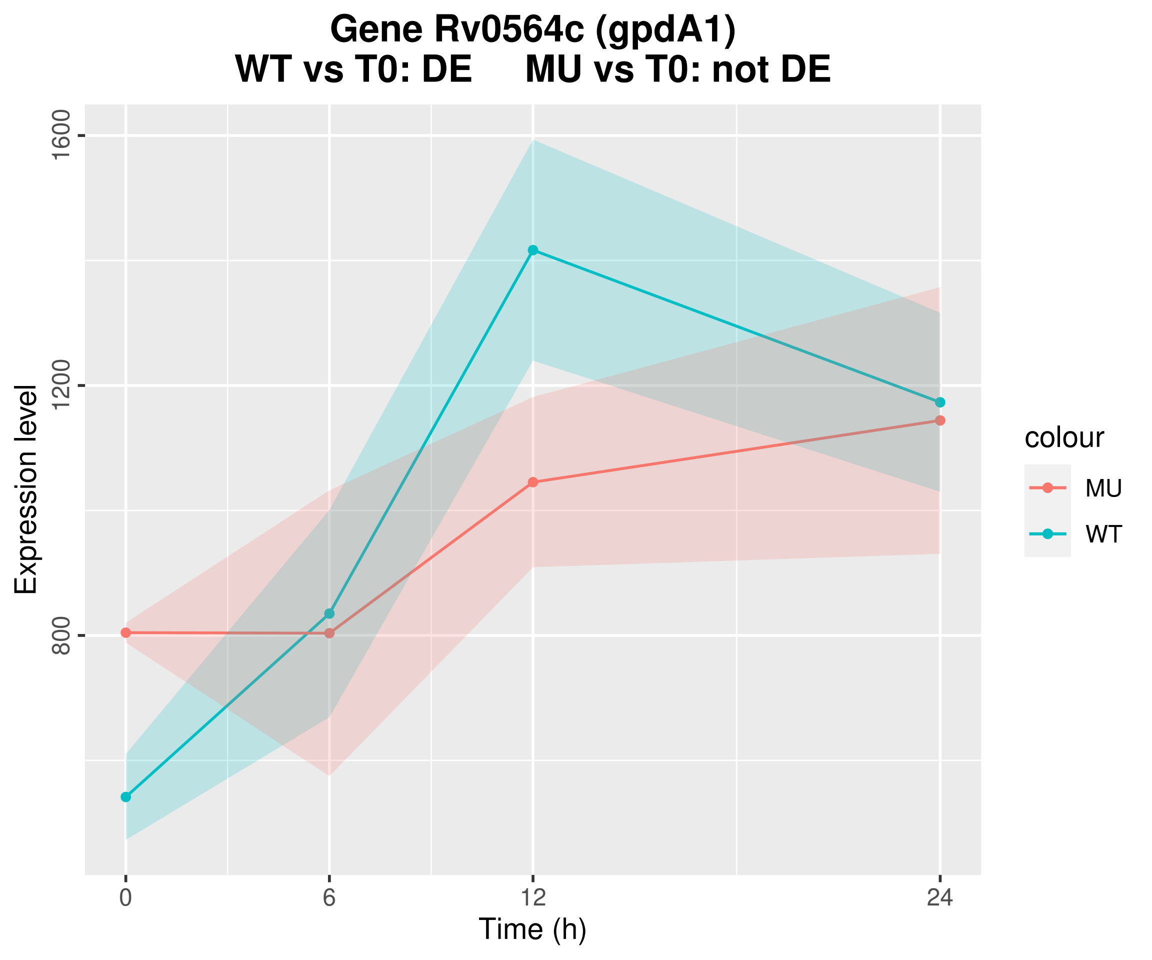

Gene Rv0564c (gpdA1)
WT vs T0: DE   MU vs T0: not DE
Expression level
1600
1200
800
Time (h)
0
6
12
24
colour
MU
WT

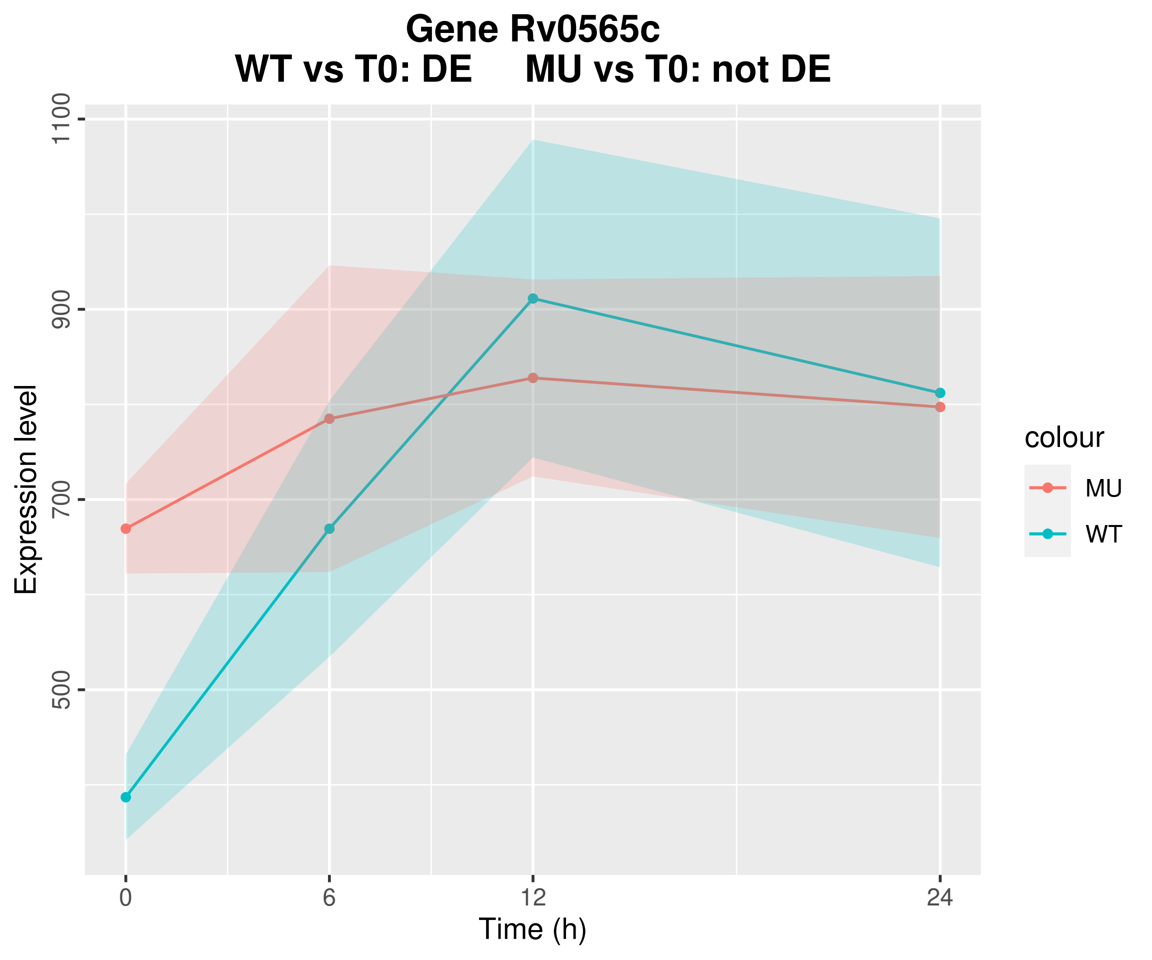
Gene Rv0565c
WT vs T0: DE MU vs T0: not DE
1100
900
700
500
Expression level
0
6
12
24
Time (h)
colour
MU
WT

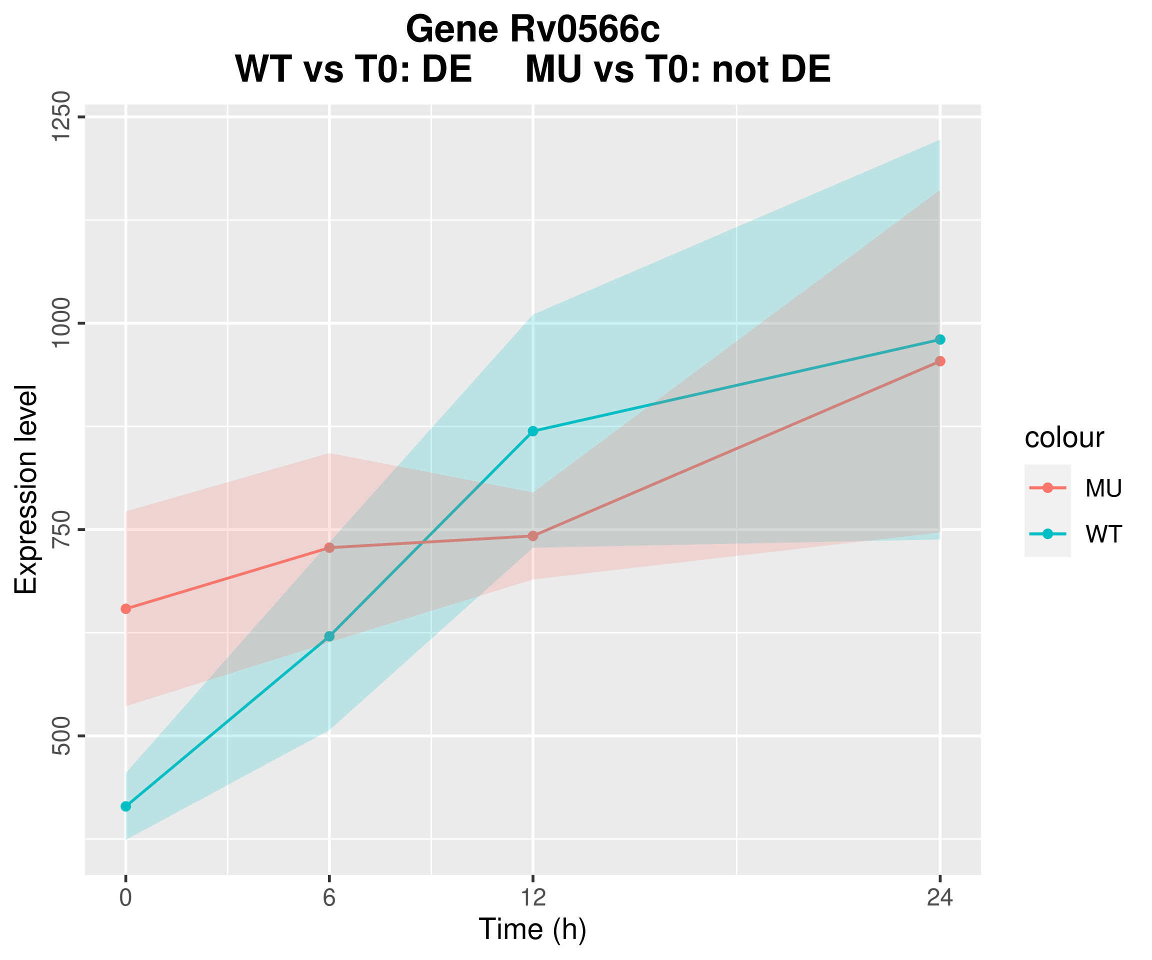
Gene Rv0566c
WT vs T0: DE   MU vs T0: not DE
Expression level
1250
1000
750
500
0
6
12
24
Time (h)
colour
MU
WT

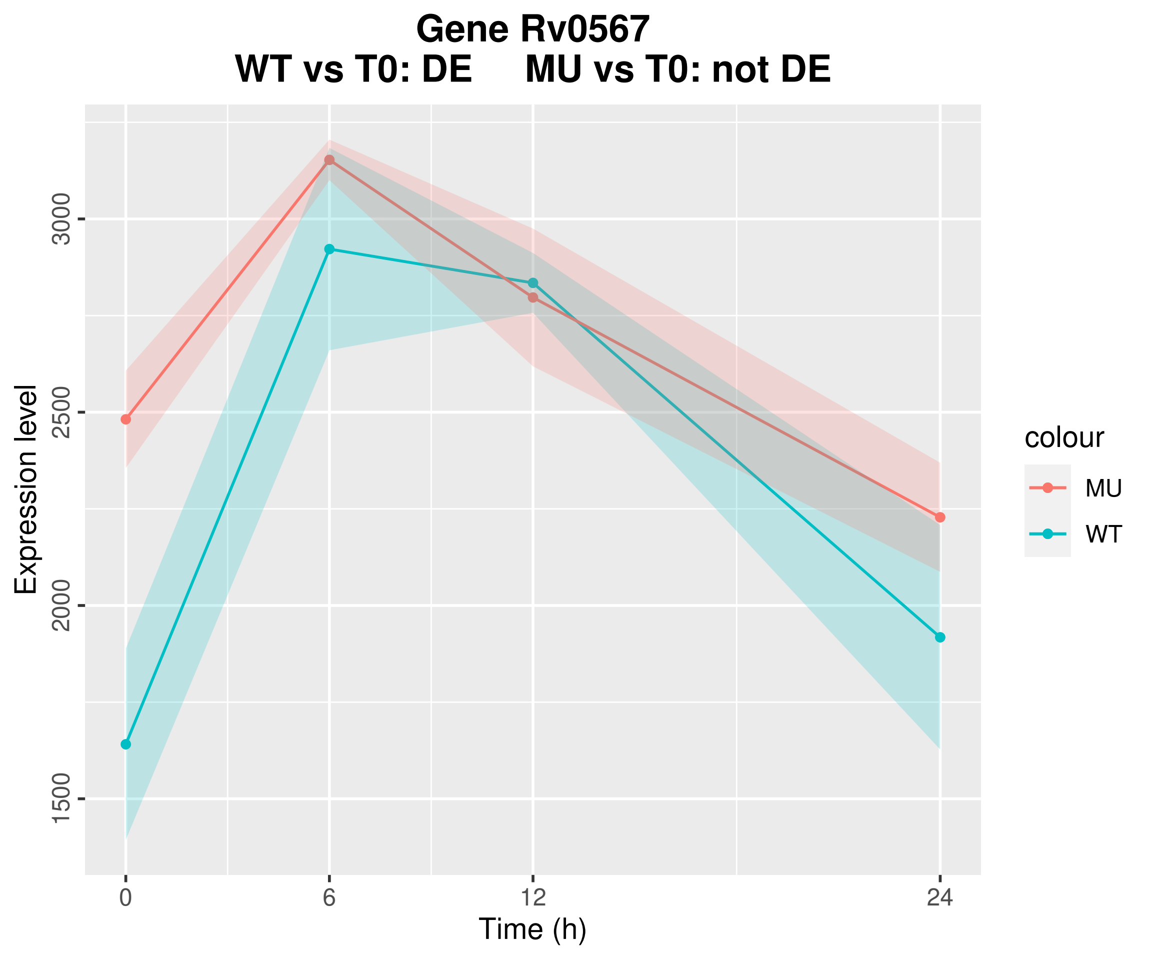
Gene Rv0567
WT vs T0: DE   MU vs T0: not DE
Expression level
3000
2500
2000
1500
0
6
12
24
Time (h)
colour
MU
WT

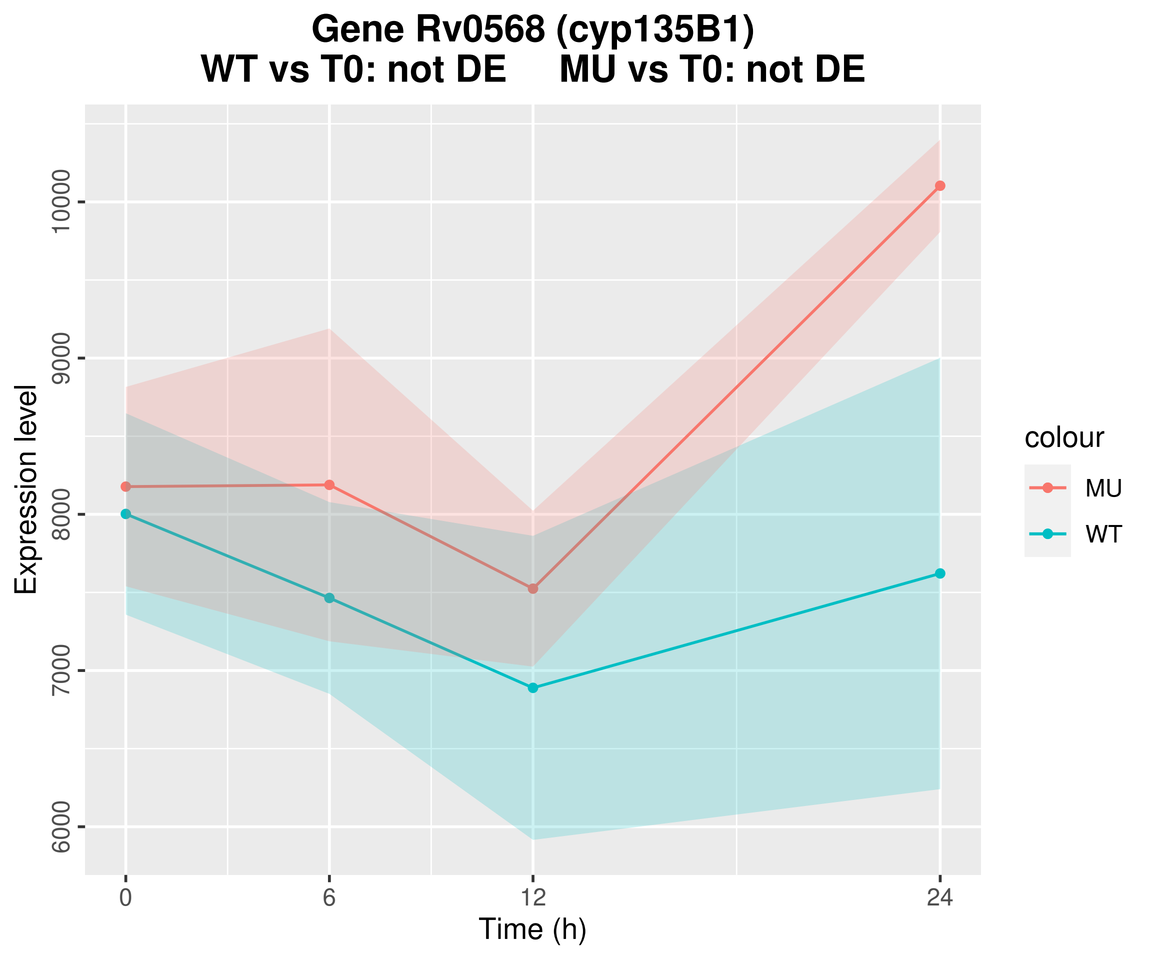
Gene Rv0568 (cyp135B1)
WT vs T0: not DE   MU vs T0: not DE
Expression level
10000
9000
8000
7000
6000
0
6
12
24
Time (h)
colour
MU
WT

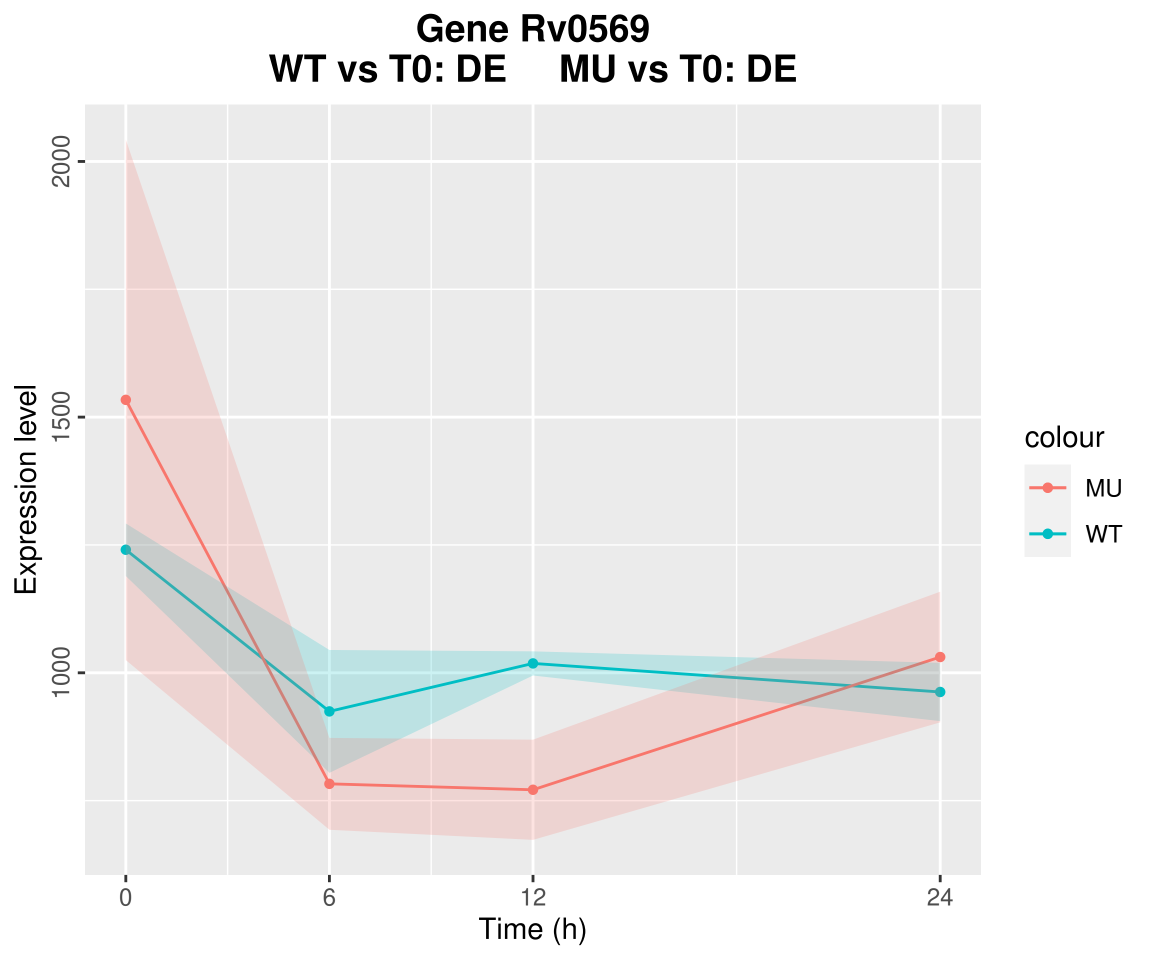

Gene Rv0569
WT vs T0: DE MU vs T0: DE
Expression level
2000
1500
1000
0
6
12
24
Time (h)
colour
MU
WT

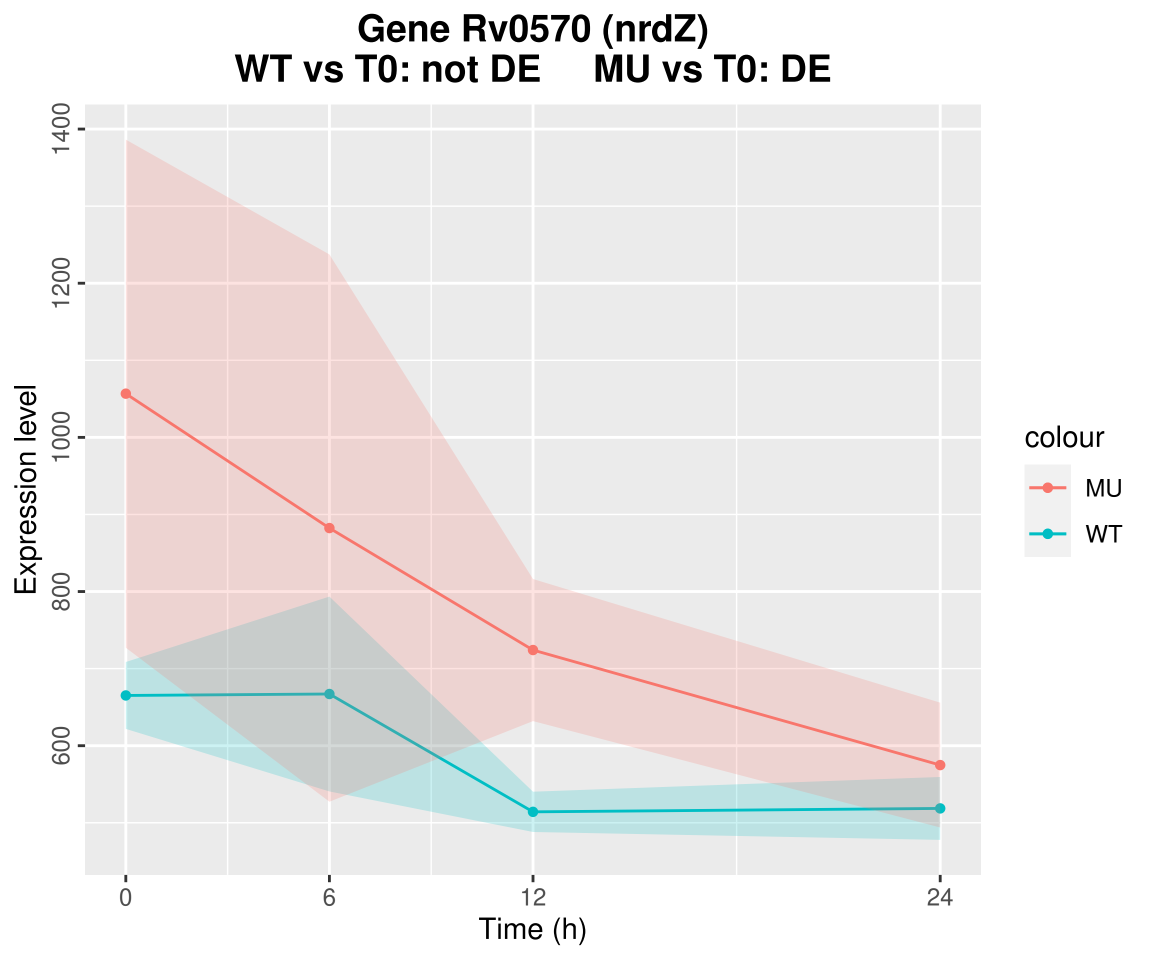
Gene Rv0570 (nrdZ)
WT vs T0: not DE MU vs T0: DE
Expression level
1400
1200
1000
800
600
0
6
12
24
Time (h)
colour
MU
WT

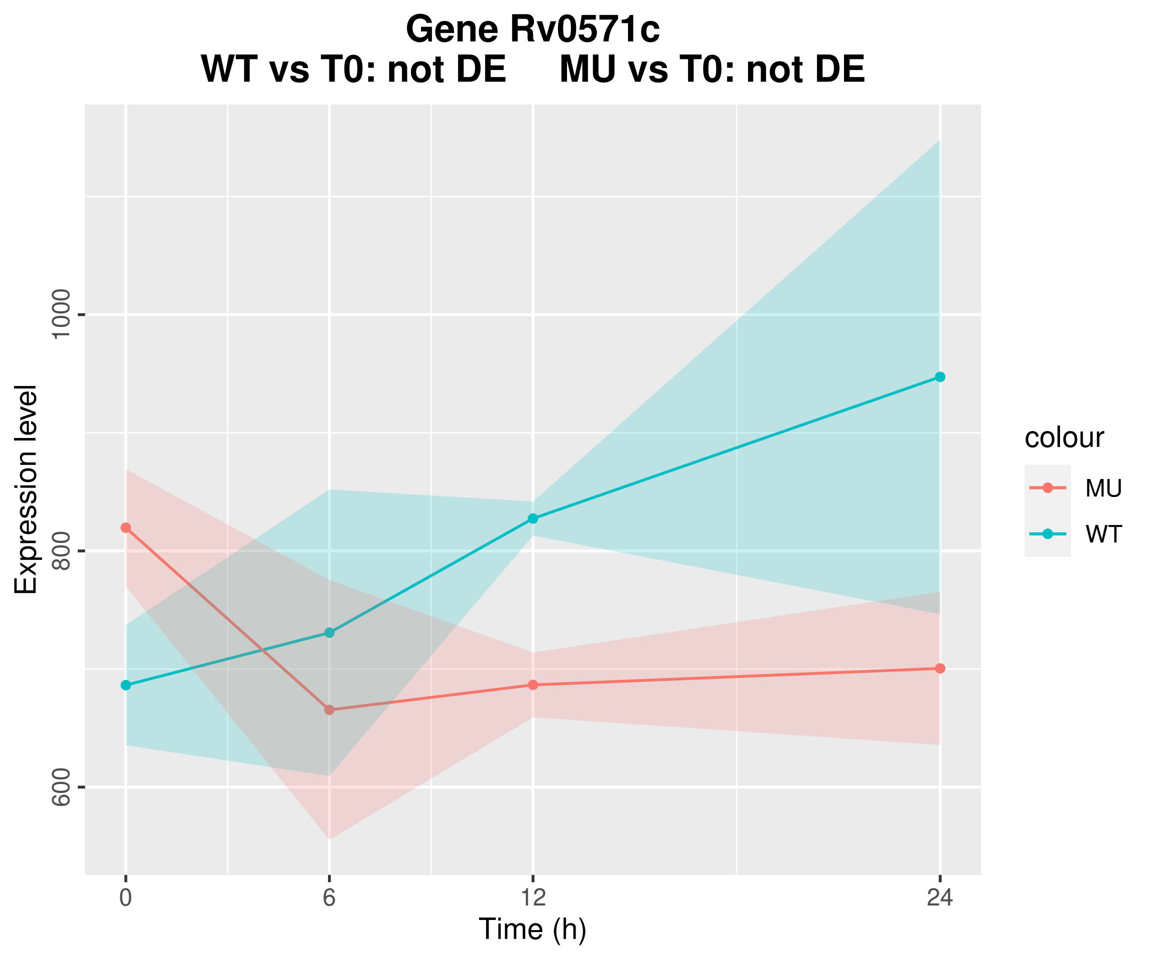

Gene Rv0571c
WT vs T0: not DE   MU vs T0: not DE
Expression level
1000
800
600
0
6
12
24
Time (h)
colour
MU
WT

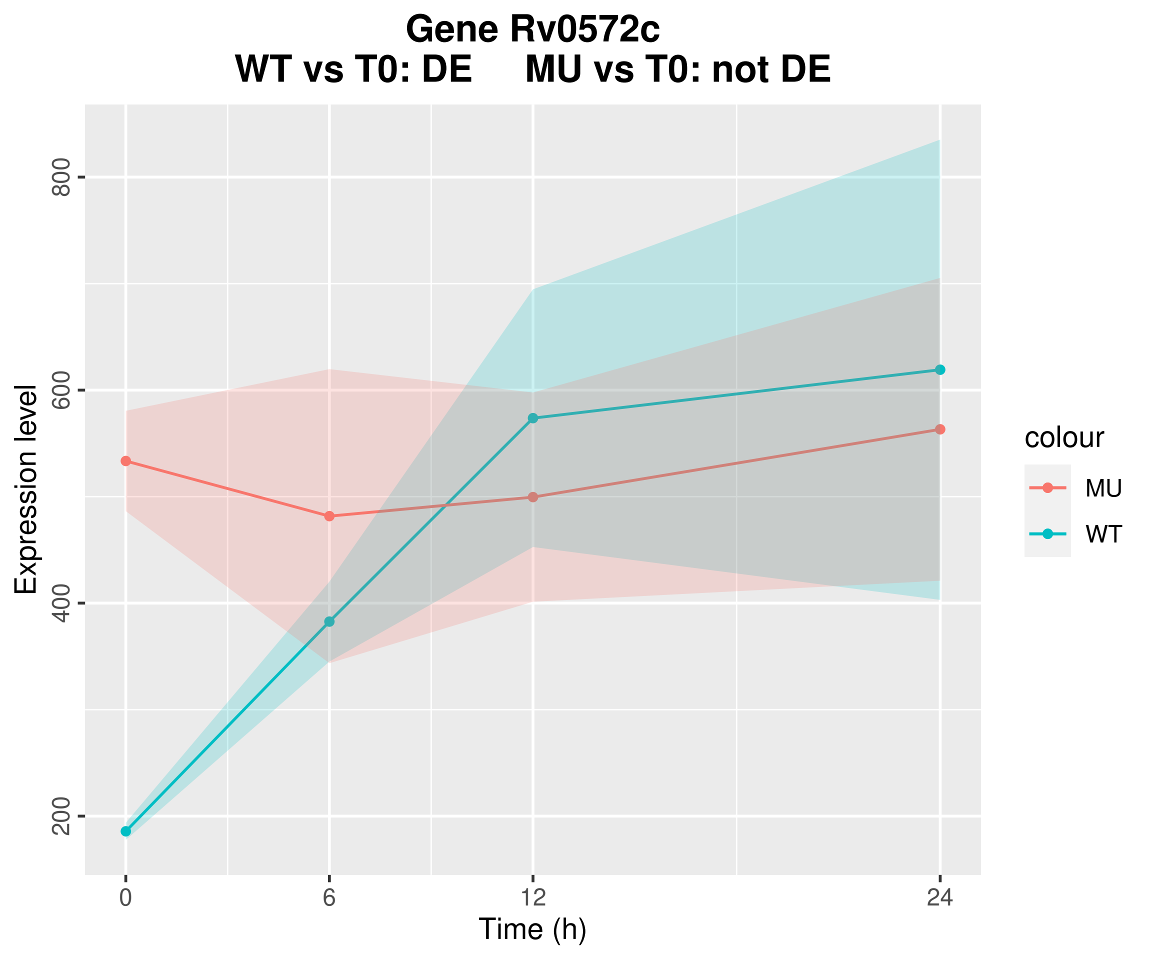

Gene Rv0572c
WT vs T0: DE MU vs T0: not DE
800
600
400
200
Expression level
0
6
12
24
Time (h)
colour
MU
WT

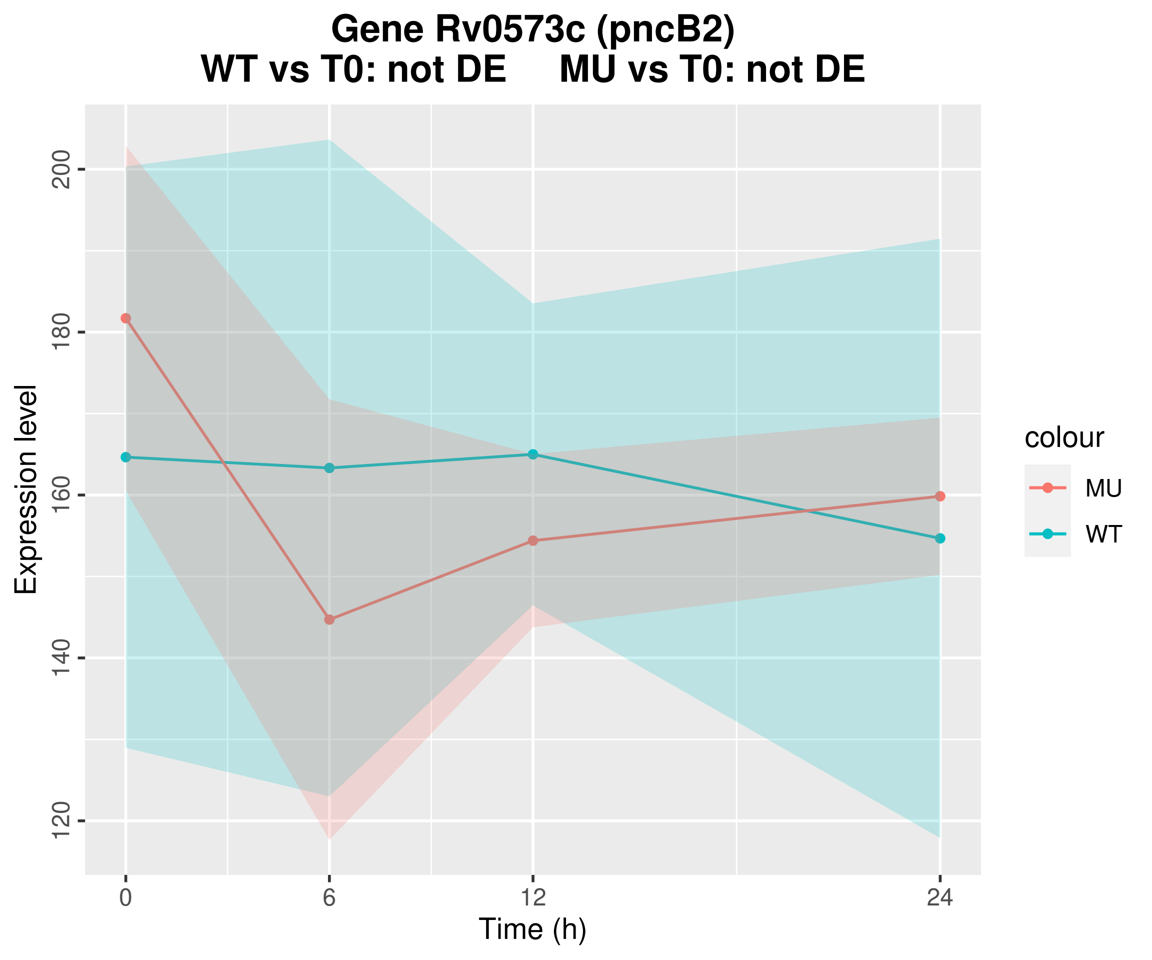
Gene Rv0573c (pncB2)
WT vs T0: not DE   MU vs T0: not DE
Expression level
200
180
160
140
120
0
6
12
24
Time (h)
colour
MU
WT

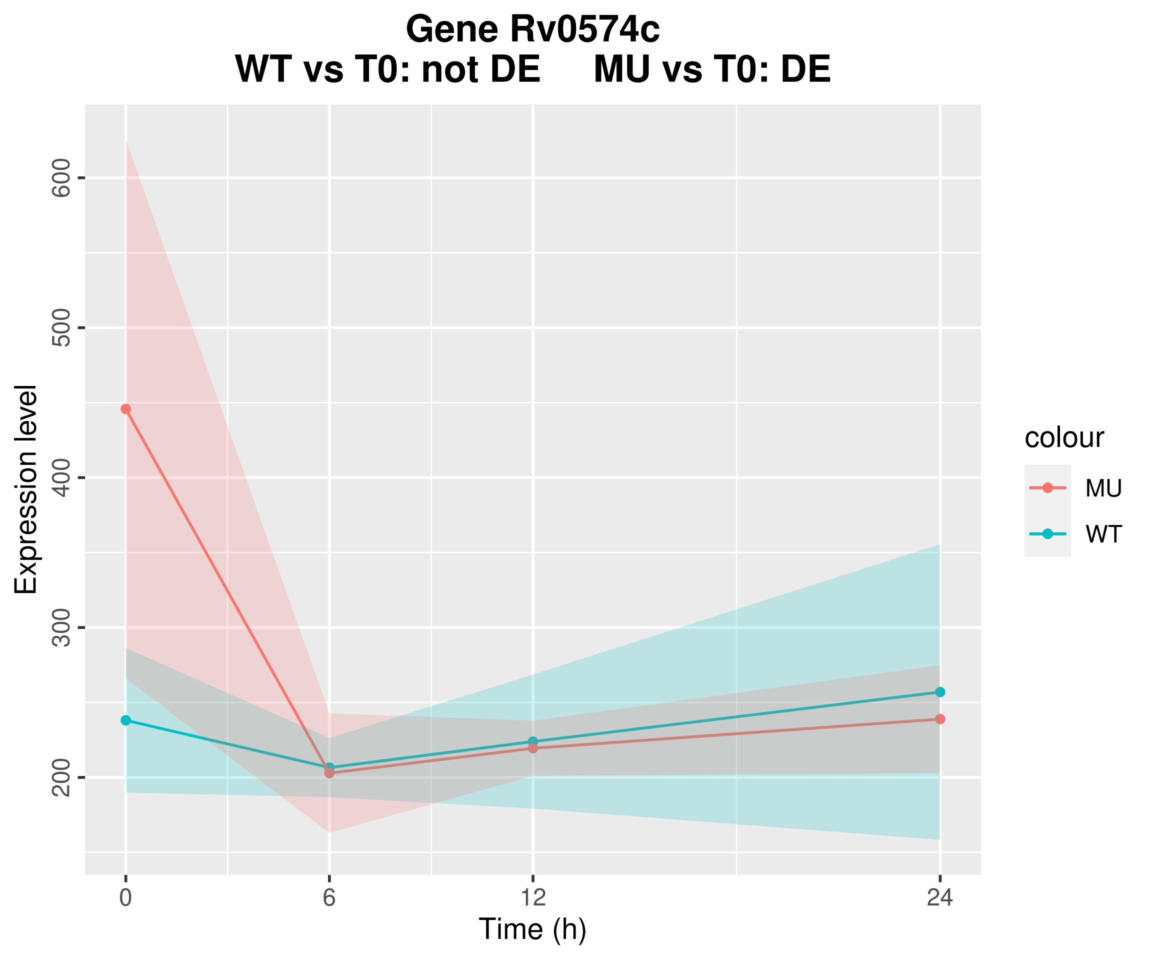
Gene Rv0574c
WT vs T0: not DE   MU vs T0: DE
Expression level
600
500
400
300
200
0
6
12
24
Time (h)
colour
MU
WT

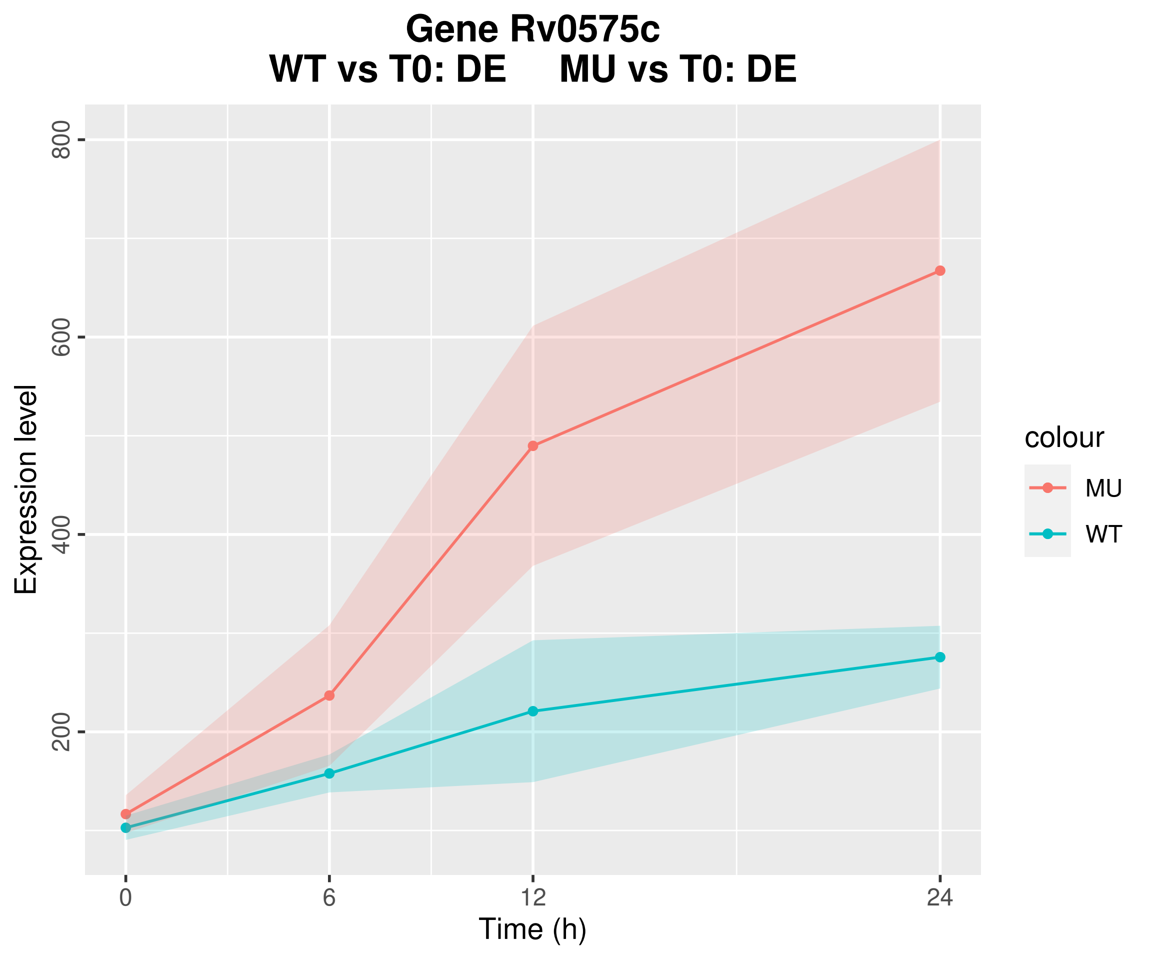

Gene Rv0575c
WT vs T0: DE   MU vs T0: DE
Expression level
800
600
400
200
0
6
12
24
Time (h)
colour
MU
WT

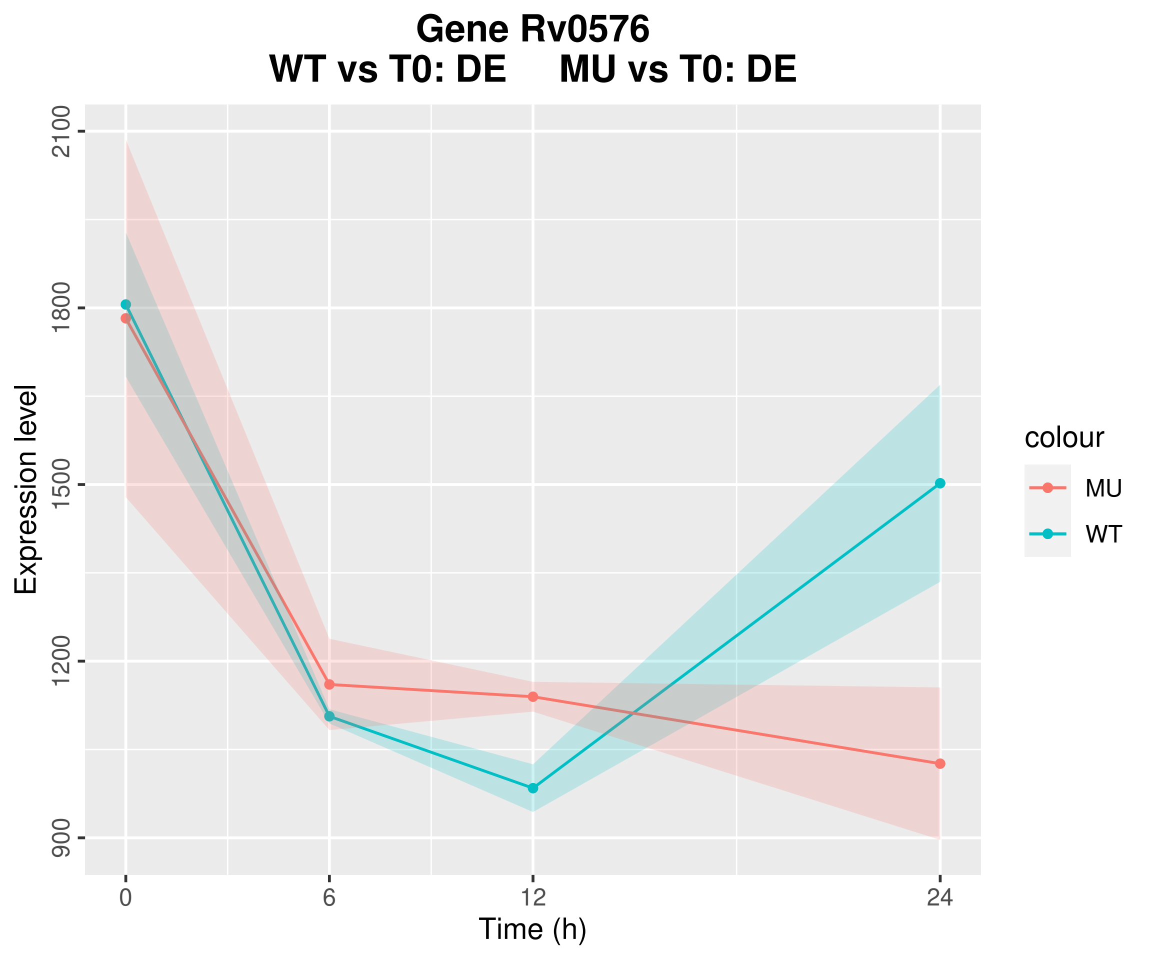

Gene Rv0576
WT vs T0: DE MU vs T0: DE
Expression level
2100
1800
1500
1200
900
0
6
12
24
Time (h)
colour
MU
WT

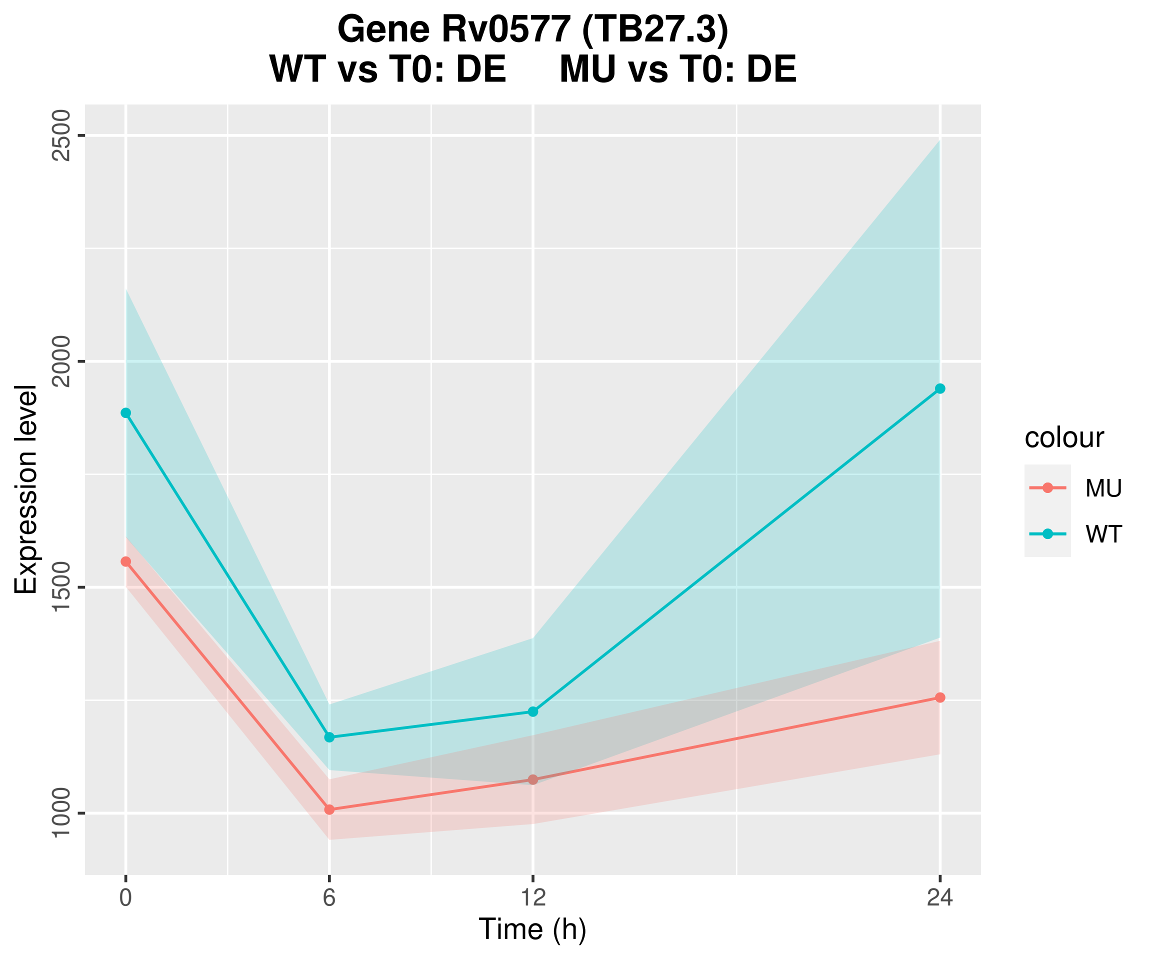

Gene Rv0577 (TB27.3)
WT vs T0: DE MU vs T0: DE
Expression level
2500
2000
1500
1000
0
6
12
24
Time (h)
colour
MU
WT

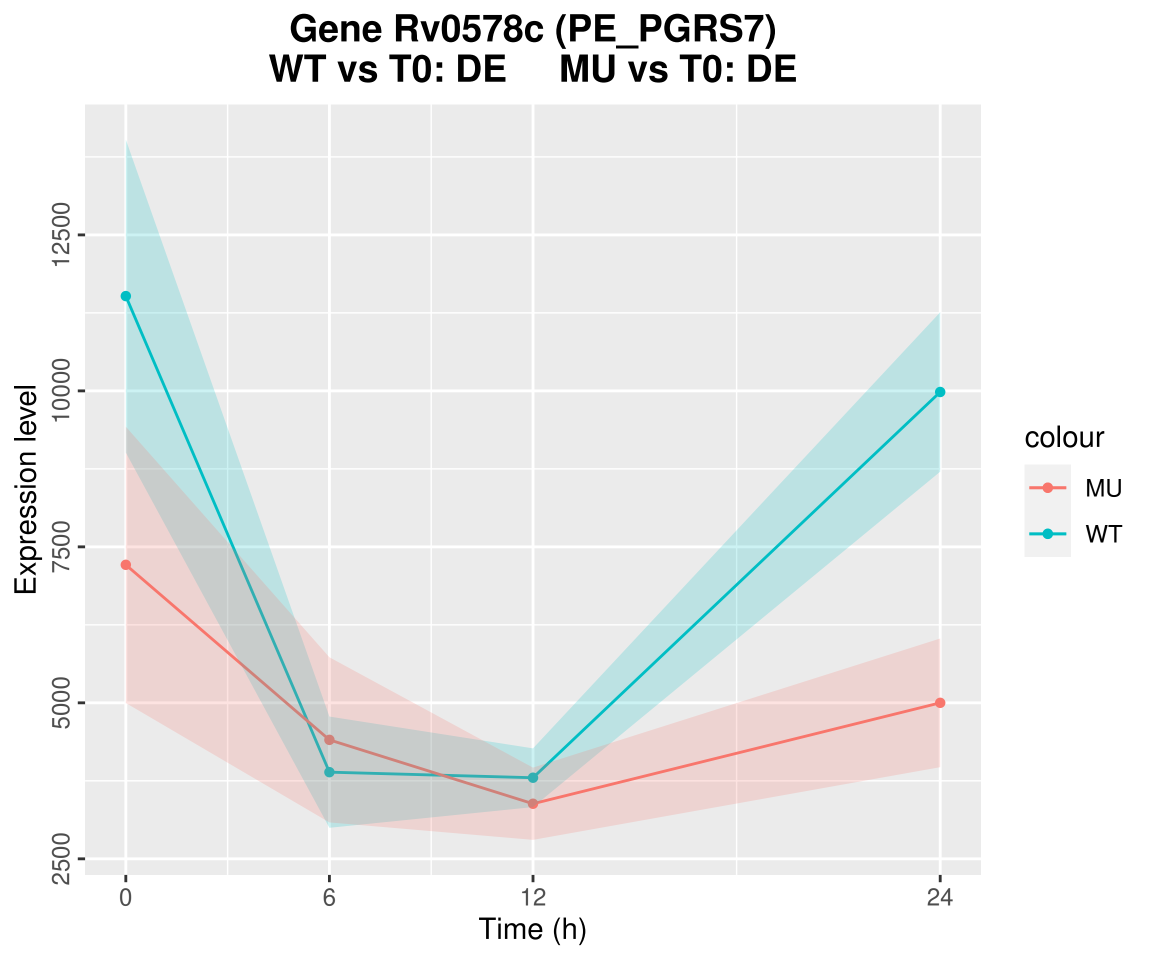

Gene Rv0578c (PE_PGRS7)
WT vs T0: DE   MU vs T0: DE
Expression level
12500
10000
7500
5000
2500
0
6
12
24
Time (h)
colour
MU
WT

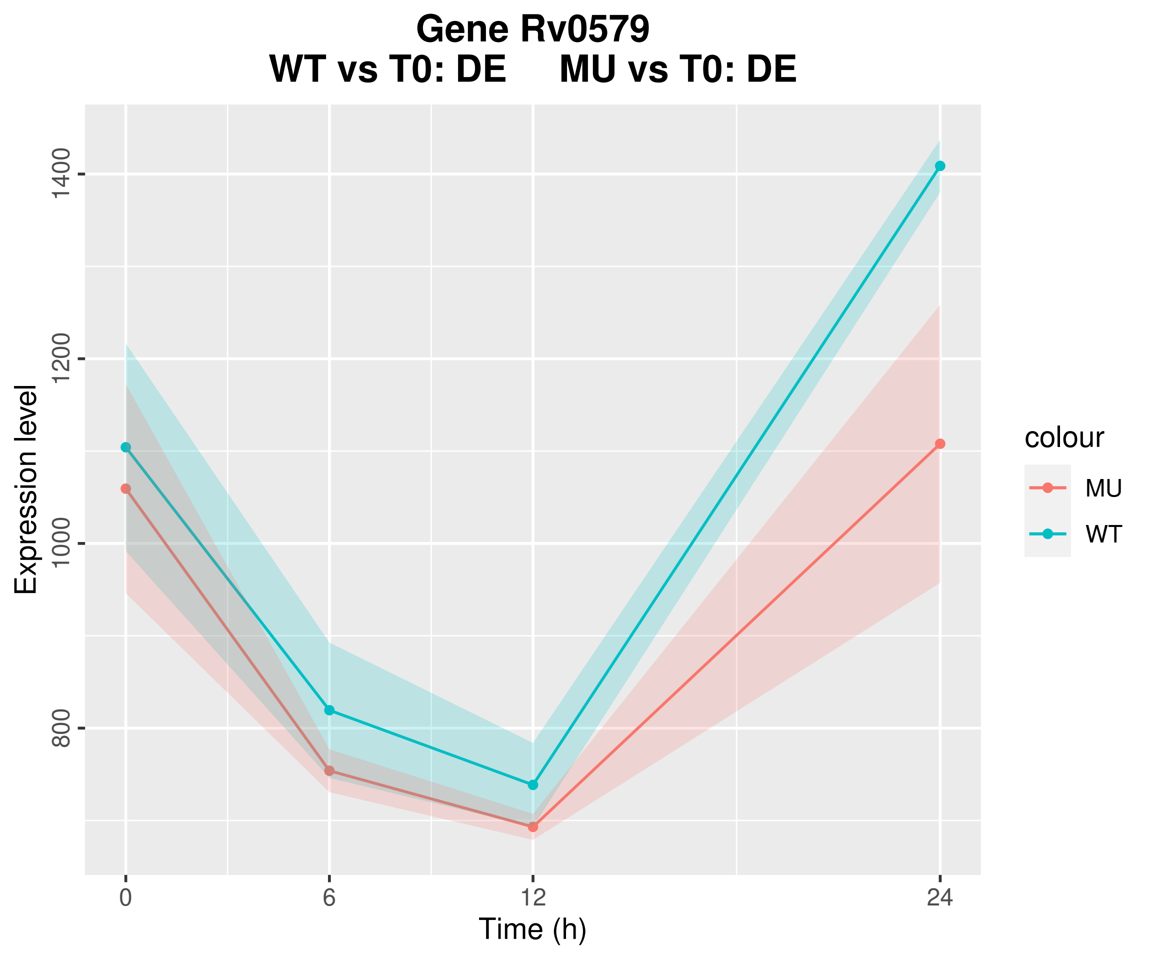
Gene Rv0579
WT vs T0: DE MU vs T0: DE
Expression level
1400
1200
1000
800
0
6
12
24
Time (h)
colour
MU
WT

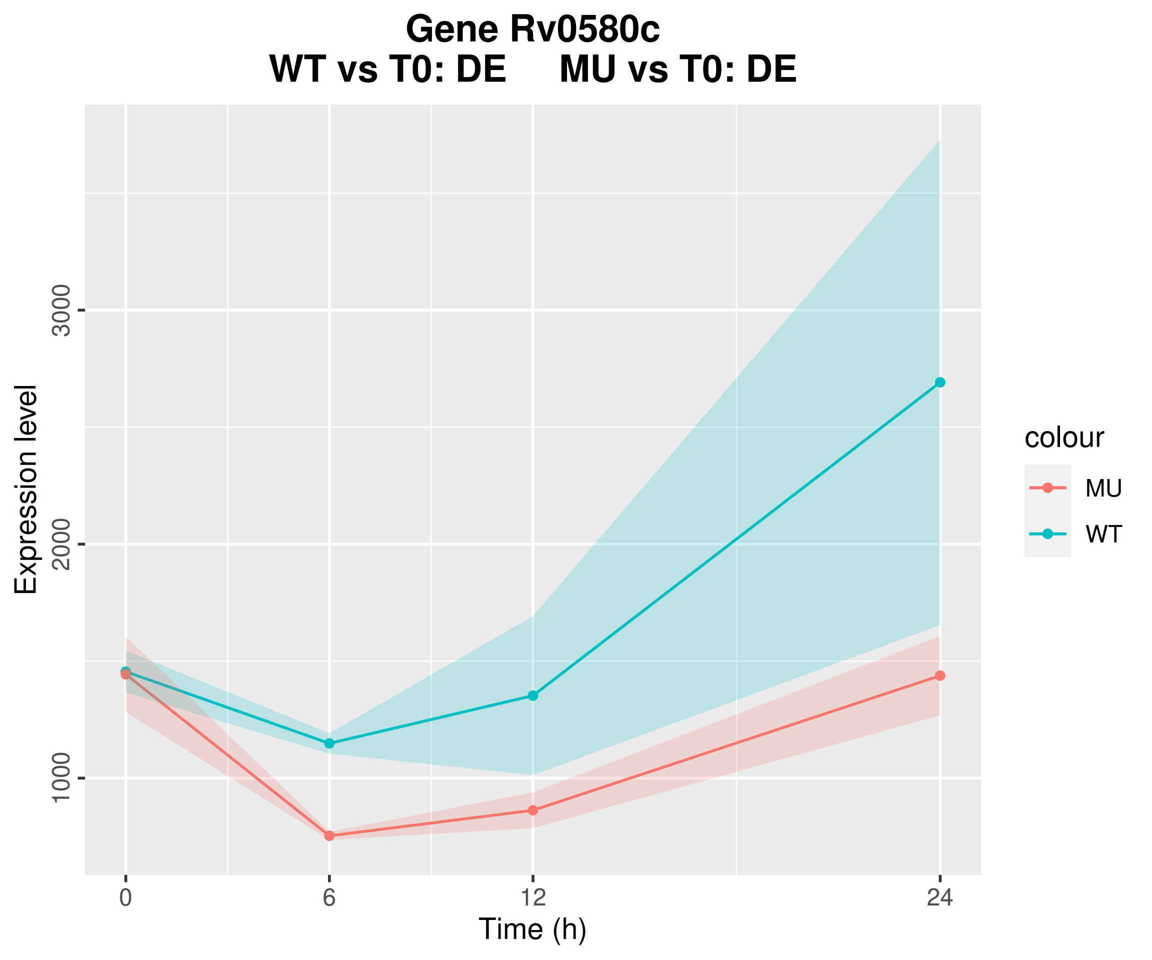

Gene Rv0580c
WT vs T0: DE   MU vs T0: DE
Expression level
3000
2000
1000
0
6
12
24
Time (h)
colour
MU
WT

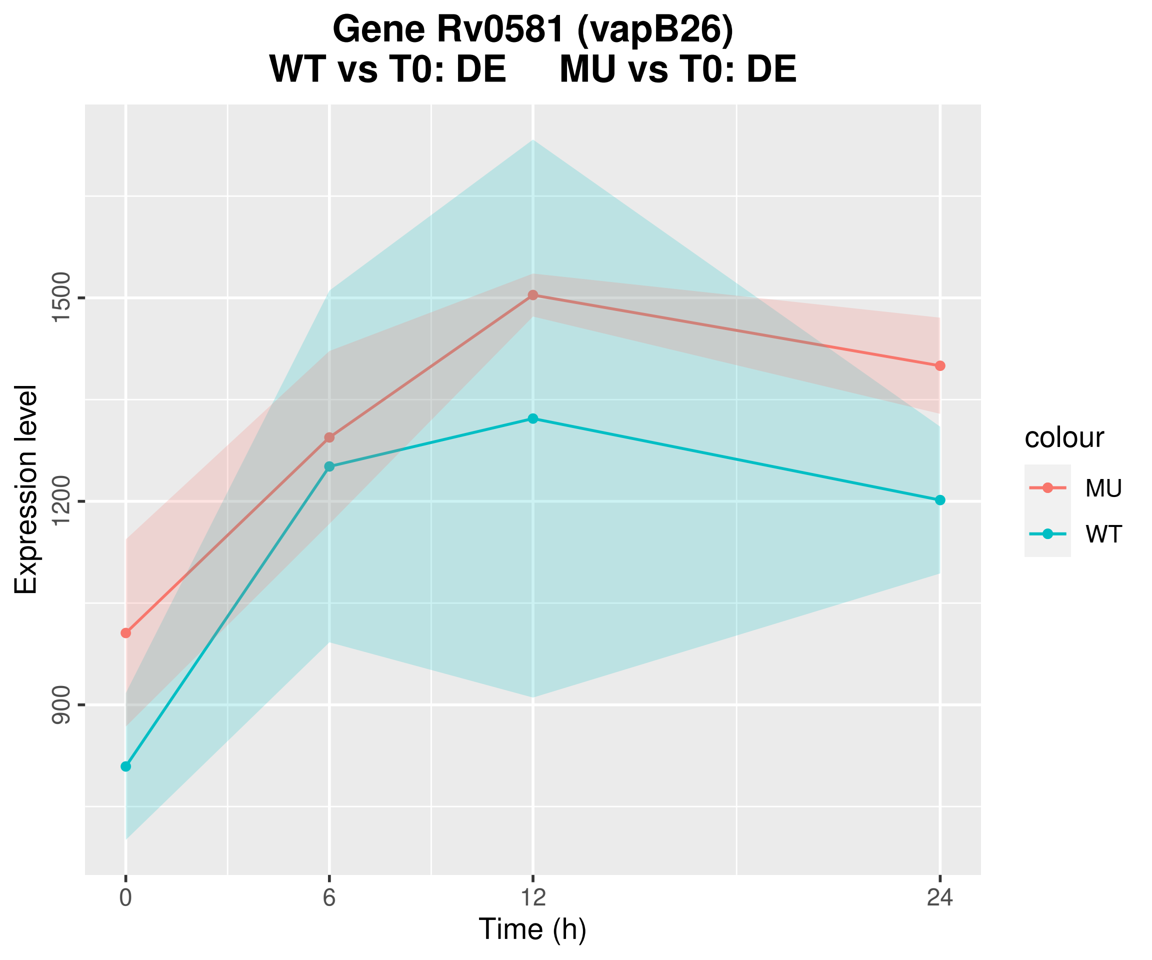

Gene Rv0581 (vapB26)
WT vs T0: DE MU vs T0: DE
Expression level
1500
1200
900
0
6
12
24
Time (h)
colour
MU
WT

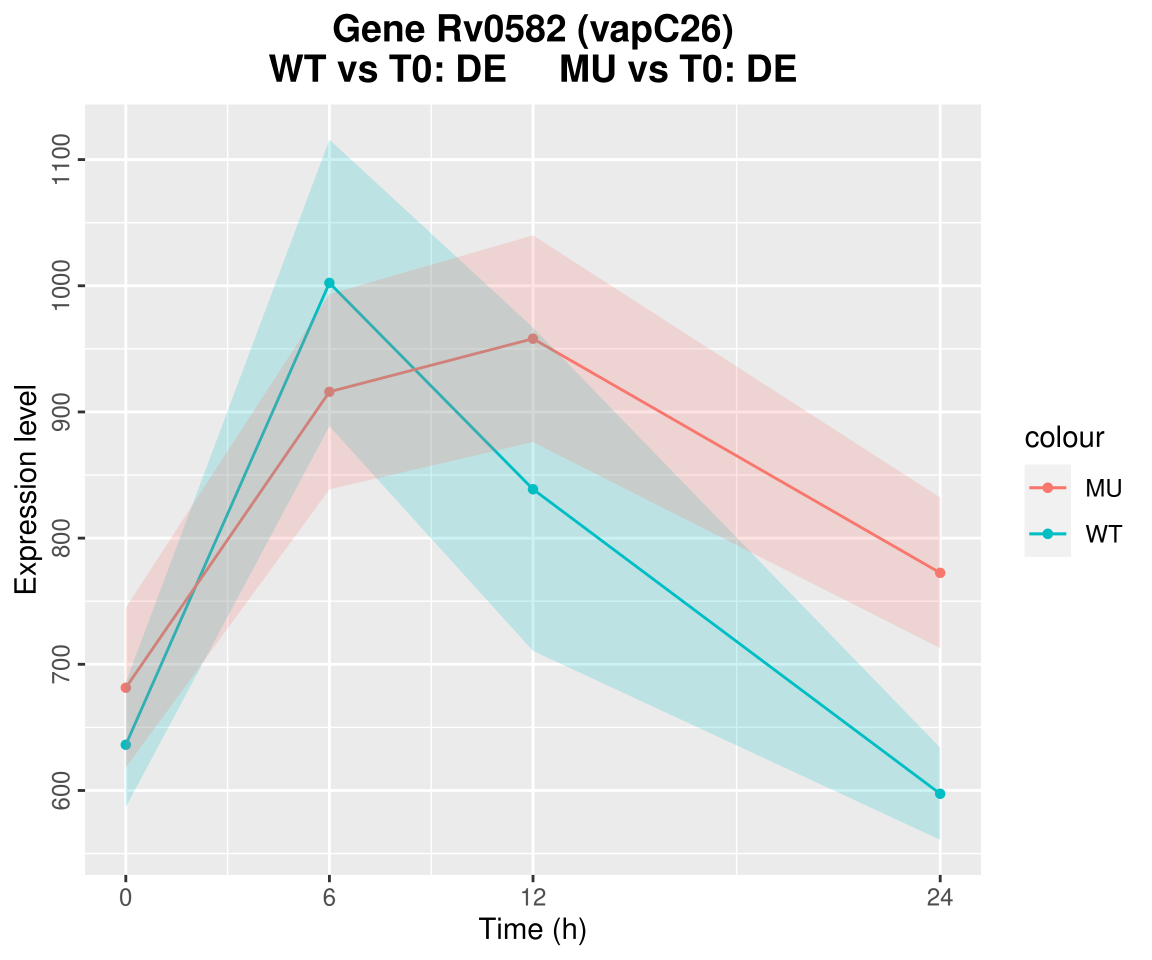
Gene Rv0582 (vapC26)
WT vs T0: DE MU vs T0: DE
Expression level
1100
1000
900
800
700
600
0
6
12
24
Time (h)
colour
MU
WT

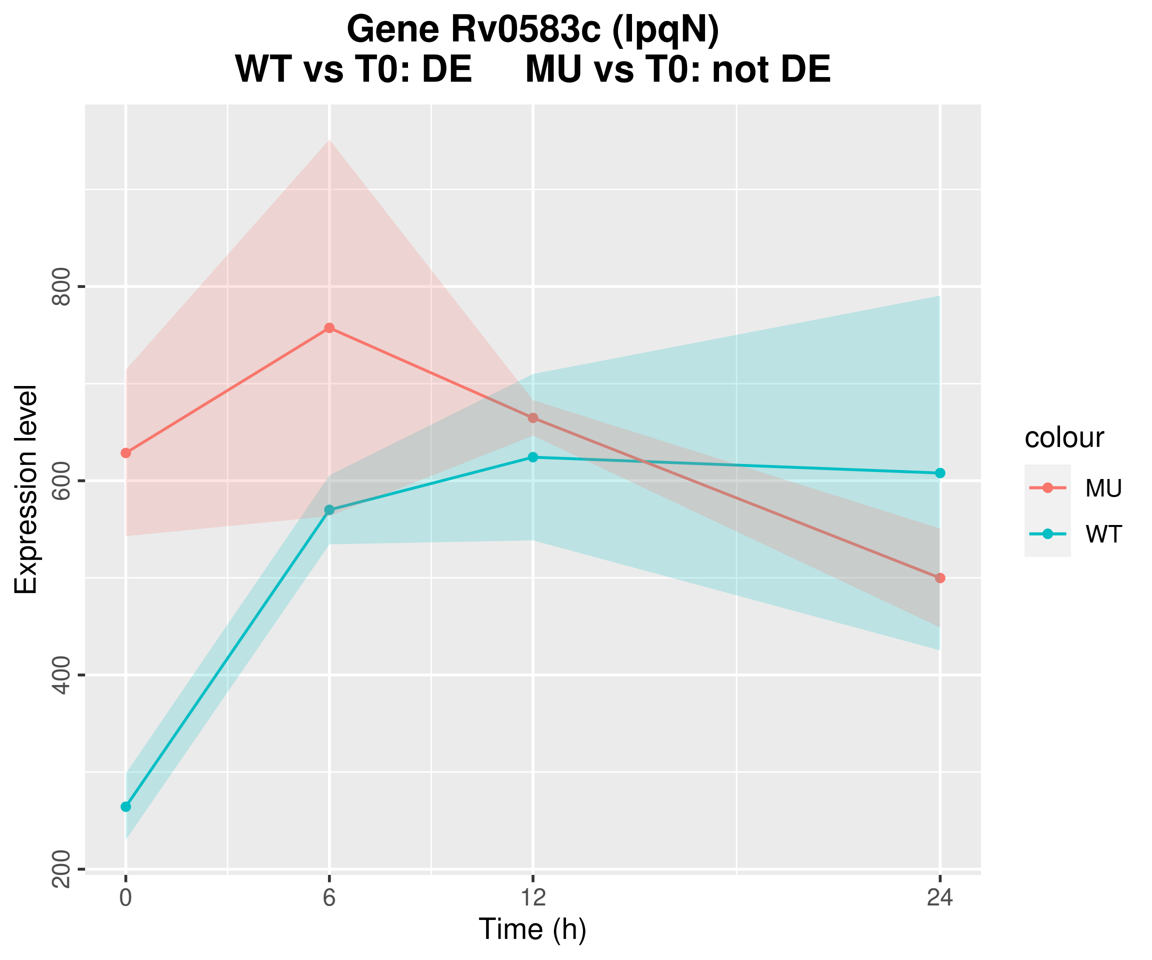

Gene Rv0583c (lpqN)
WT vs T0: DE   MU vs T0: not DE
Expression level
800
600
400
200
0
6
12
24
Time (h)
colour
MU
WT

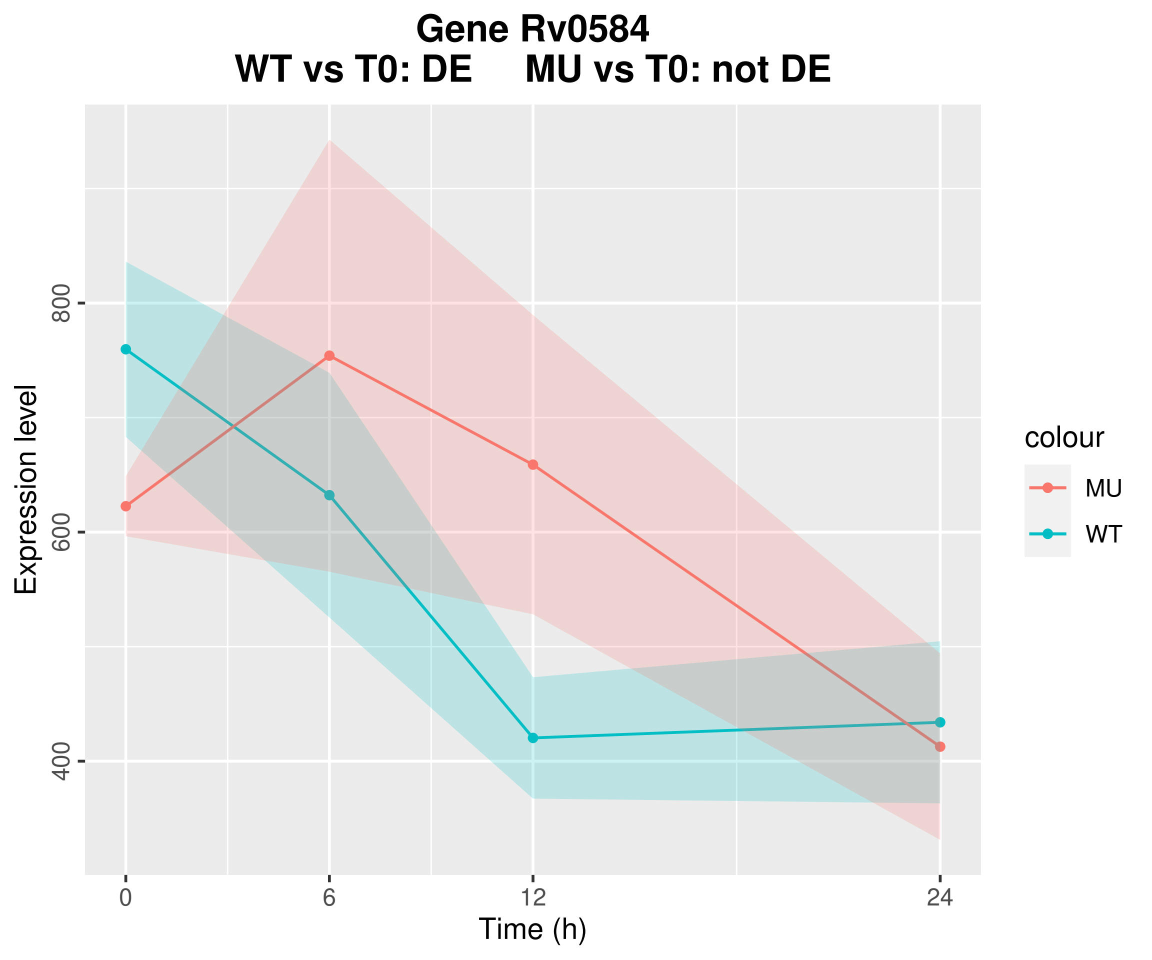
Gene Rv0584
WT vs T0: DE MU vs T0: not DE
Expression level
800
600
400
Time (h)
0
6
12
24
colour
MU
WT

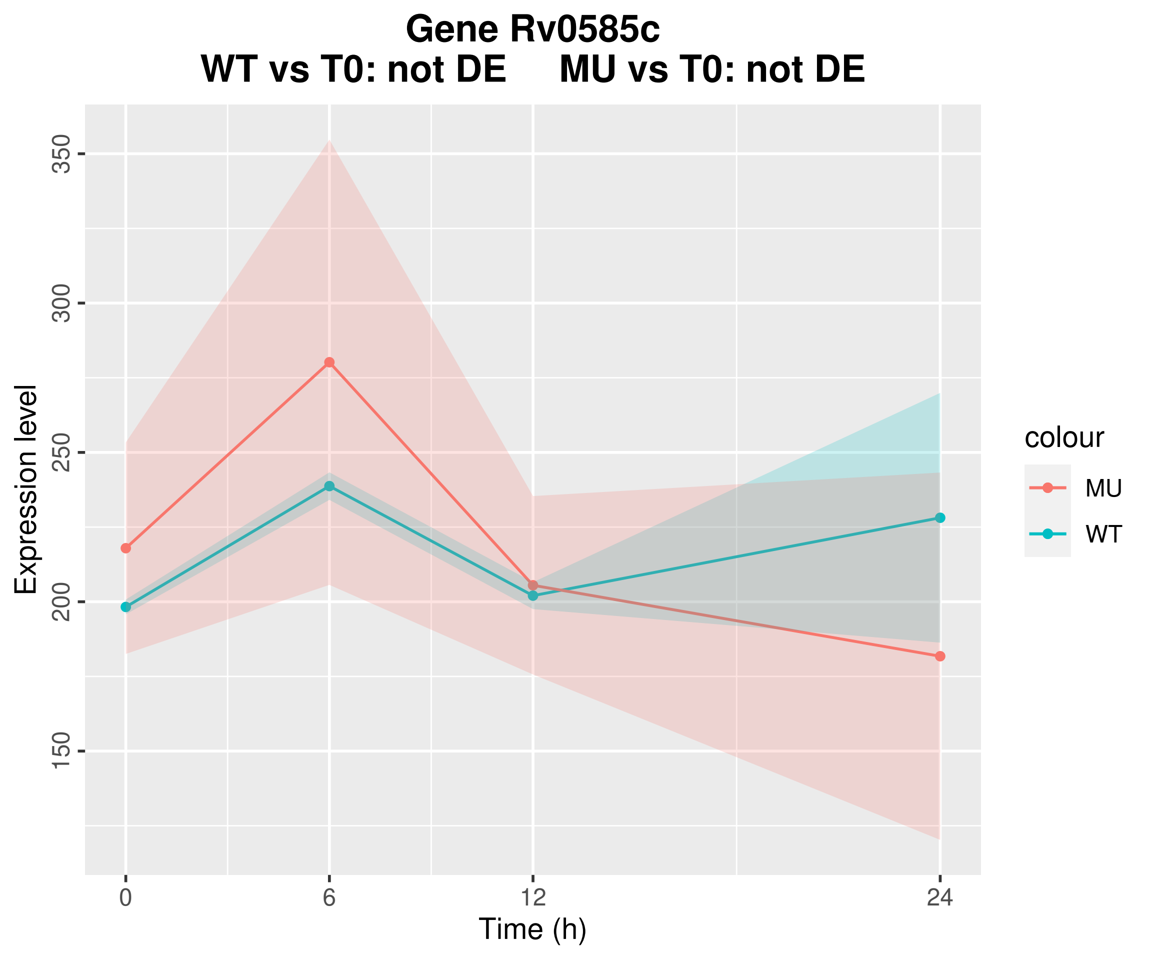

Gene Rv0585c
WT vs T0: not DE MU vs T0: not DE
Expression level
350
300
250
200
150
0
6
12
24
Time (h)
colour
MU
WT

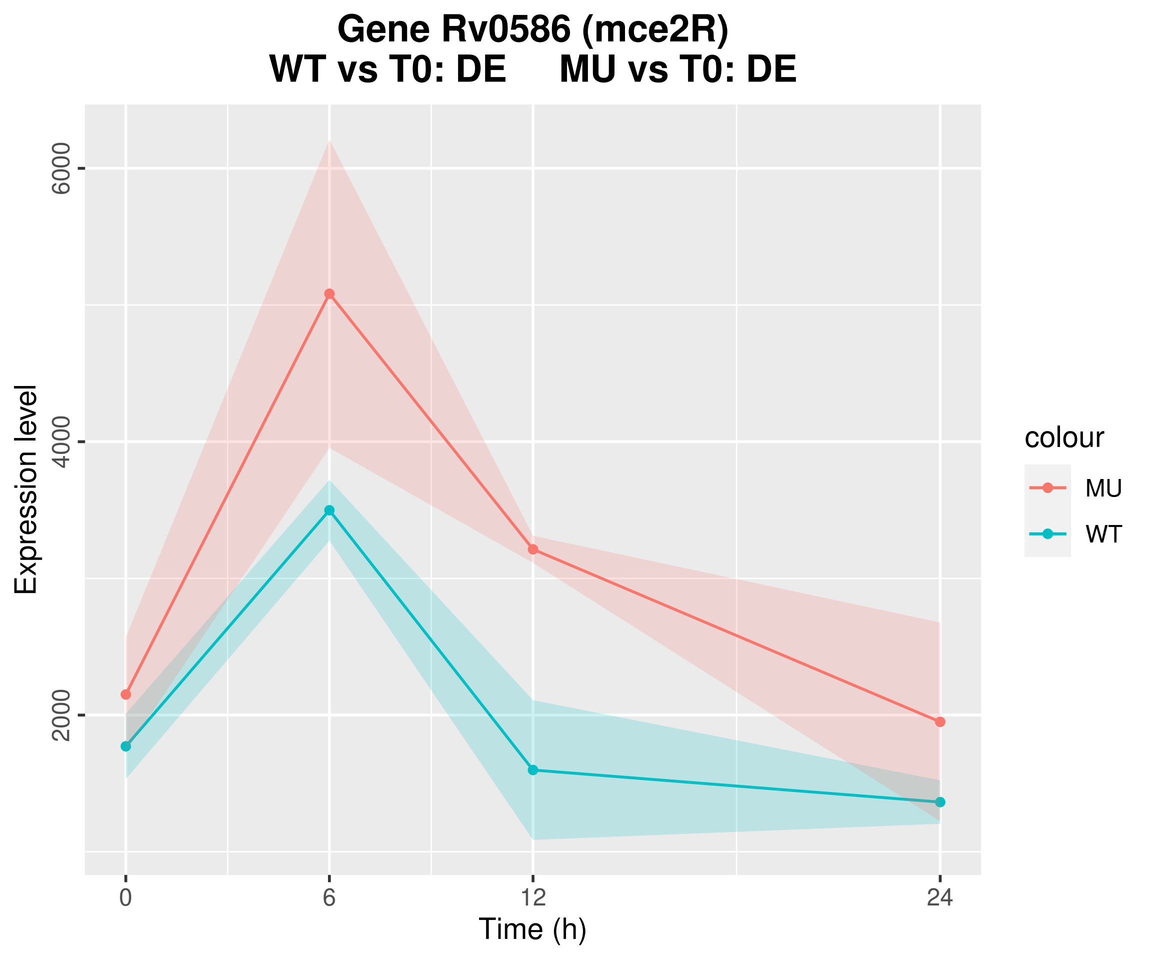

Gene Rv0586 (mce2R)
WT vs T0: DE MU vs T0: DE
Expression level
6000
4000
2000
0
6
12
24
Time (h)
colour
MU
WT

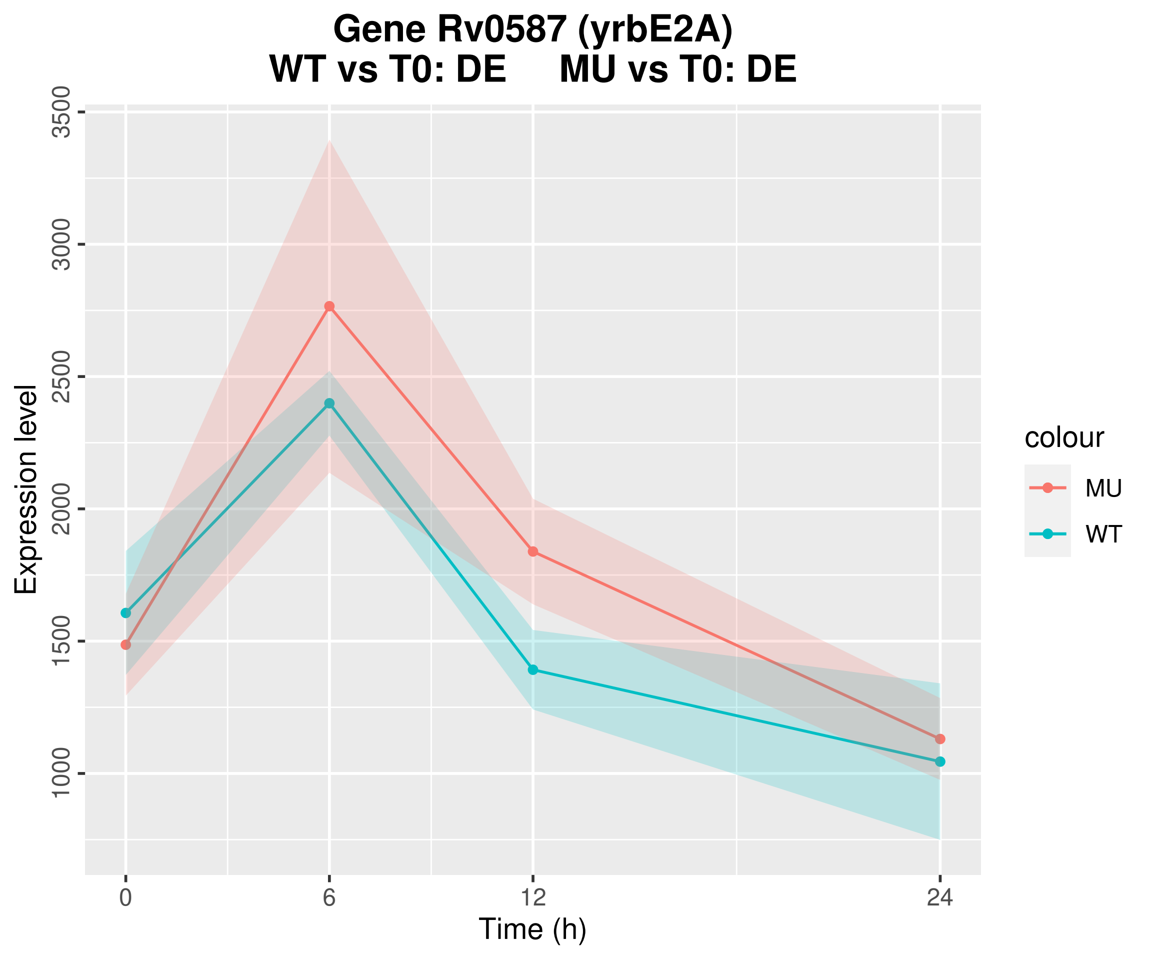

Gene Rv0587 (yrbE2A)
WT vs T0: DE MU vs T0: DE
Expression level
3500
3000
2500
2000
1500
1000
0
6
12
24
Time (h)
colour
MU
WT

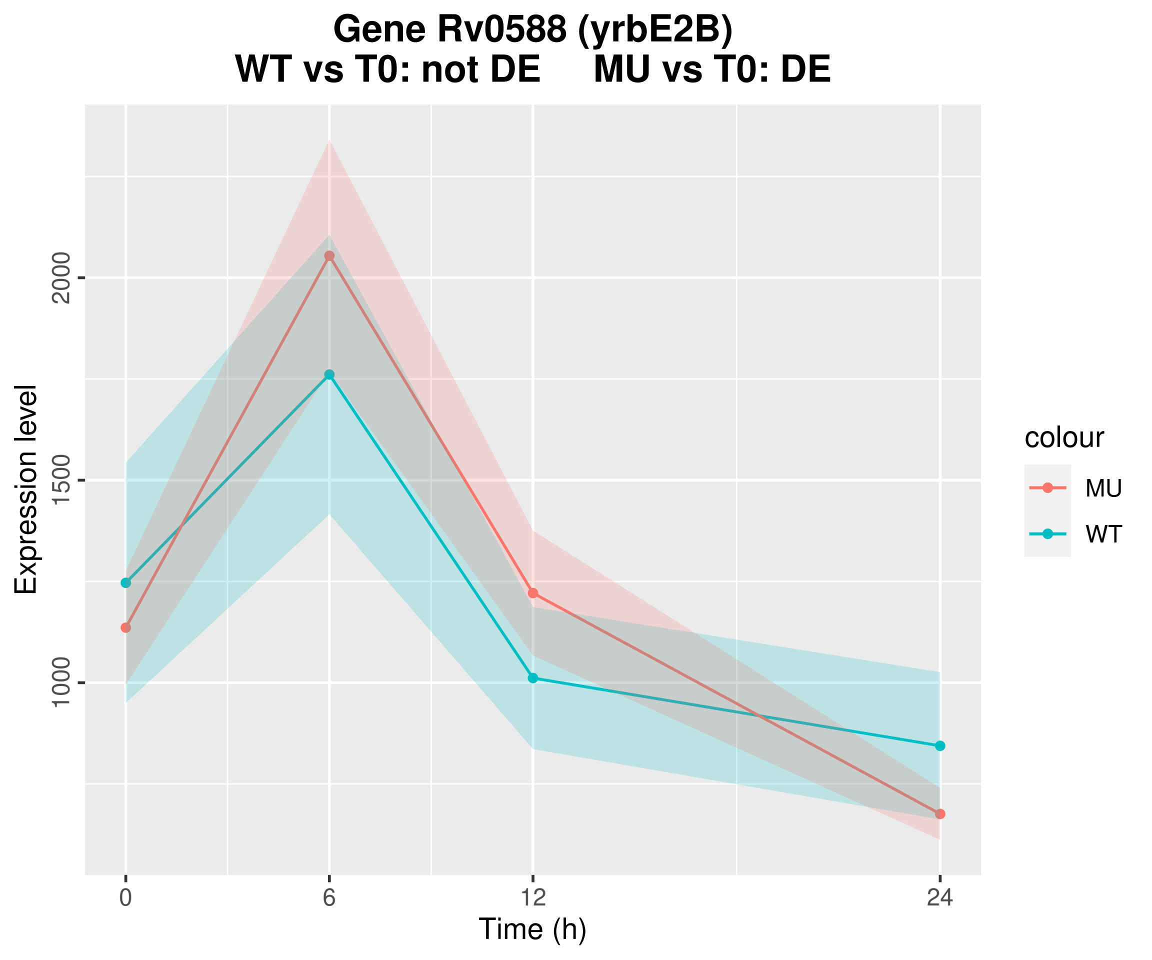

Gene Rv0588 (yrbE2B)
WT vs T0: not DE   MU vs T0: DE
Expression level
2000
1500
1000
0
6
12
24
Time (h)
colour
MU
WT

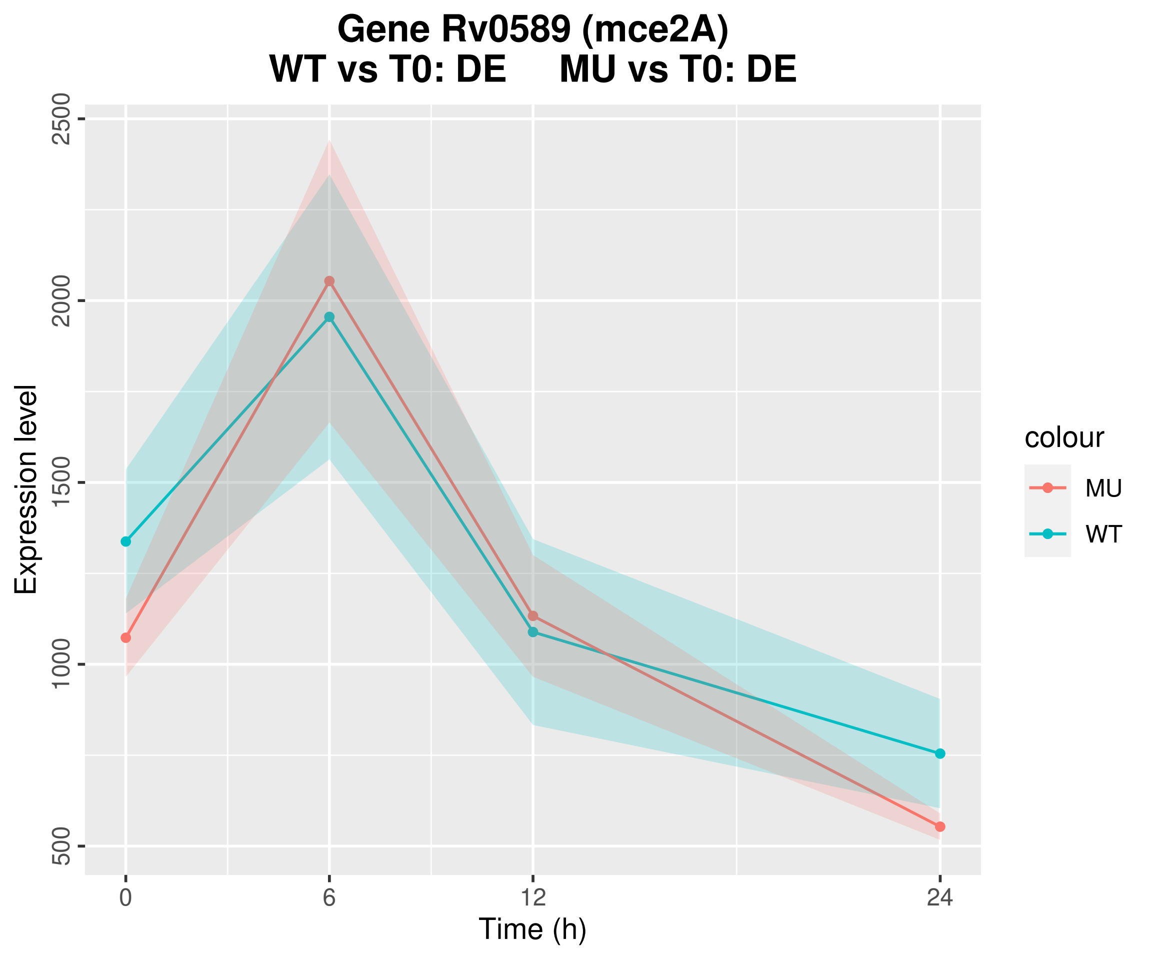
Gene Rv0589 (mce2A)
WT vs T0: DE   MU vs T0: DE
Expression level
2500
2000
1500
1000
500
0
6
12
24
Time (h)
colour
MU
WT

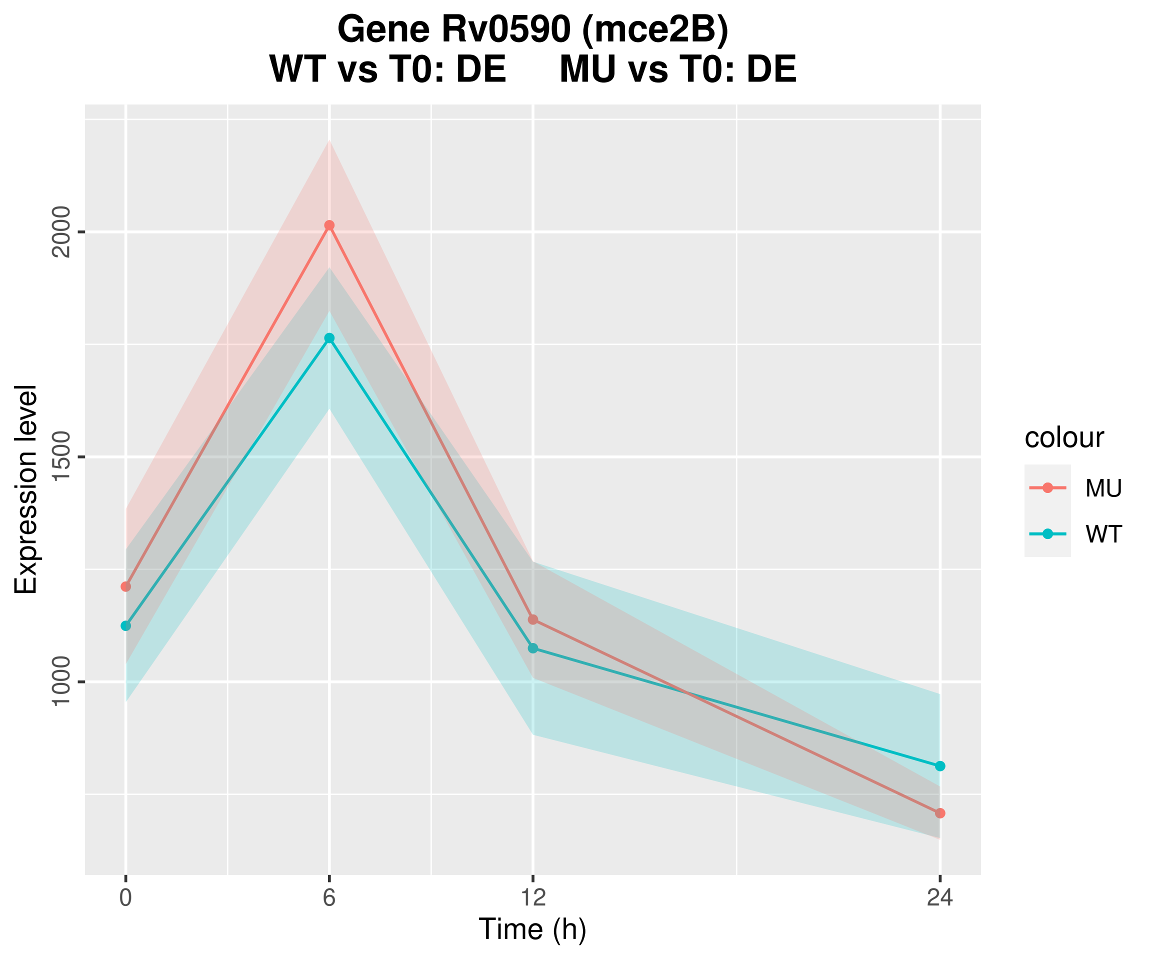
Gene Rv0590 (mce2B)
WT vs T0: DE   MU vs T0: DE
Expression level
2000
1500
1000
0
6
12
24
Time (h)
colour
MU
WT

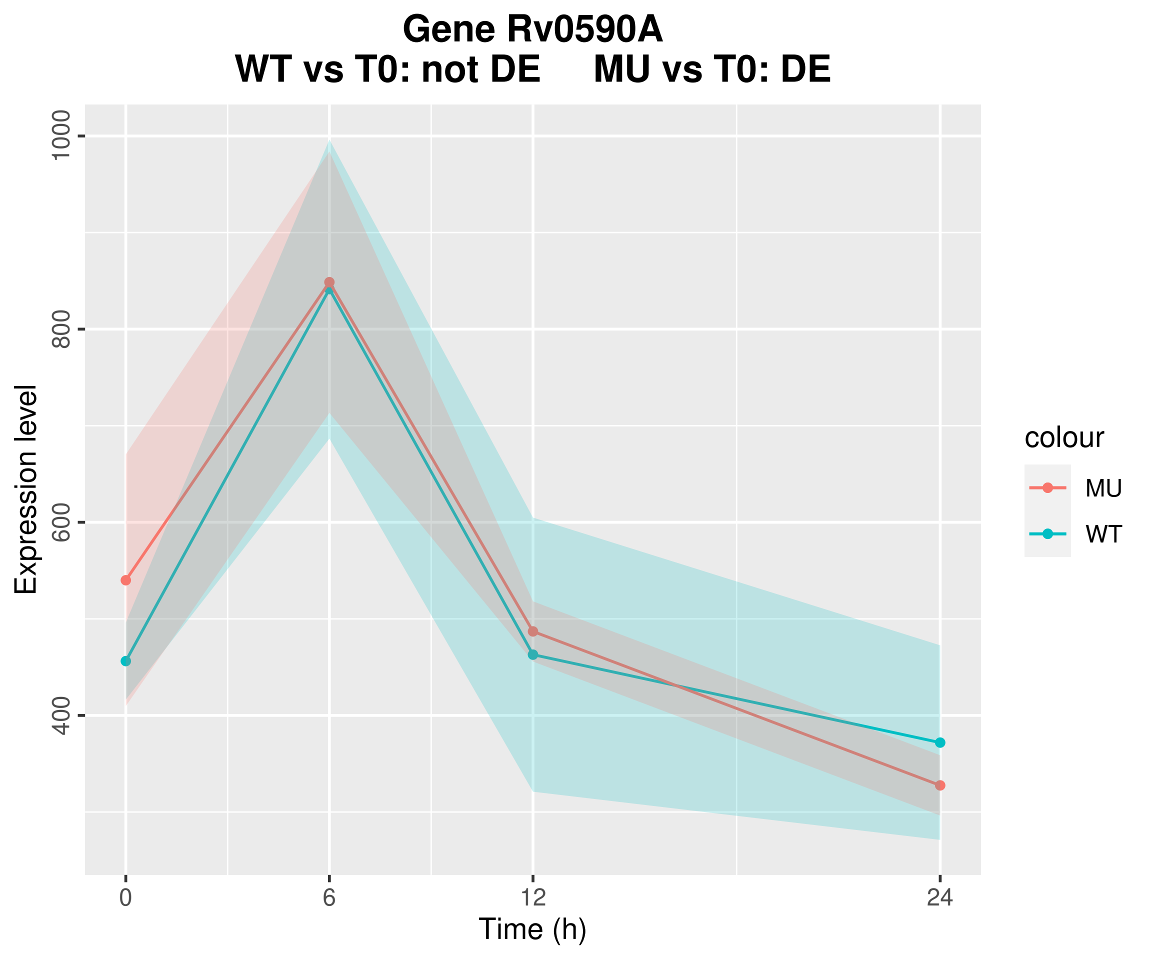
Gene Rv0590A
WT vs T0: not DE MU vs T0: DE
1000
800
600
400
Expression level
0
6
12
24
Time (h)
colour
MU
WT

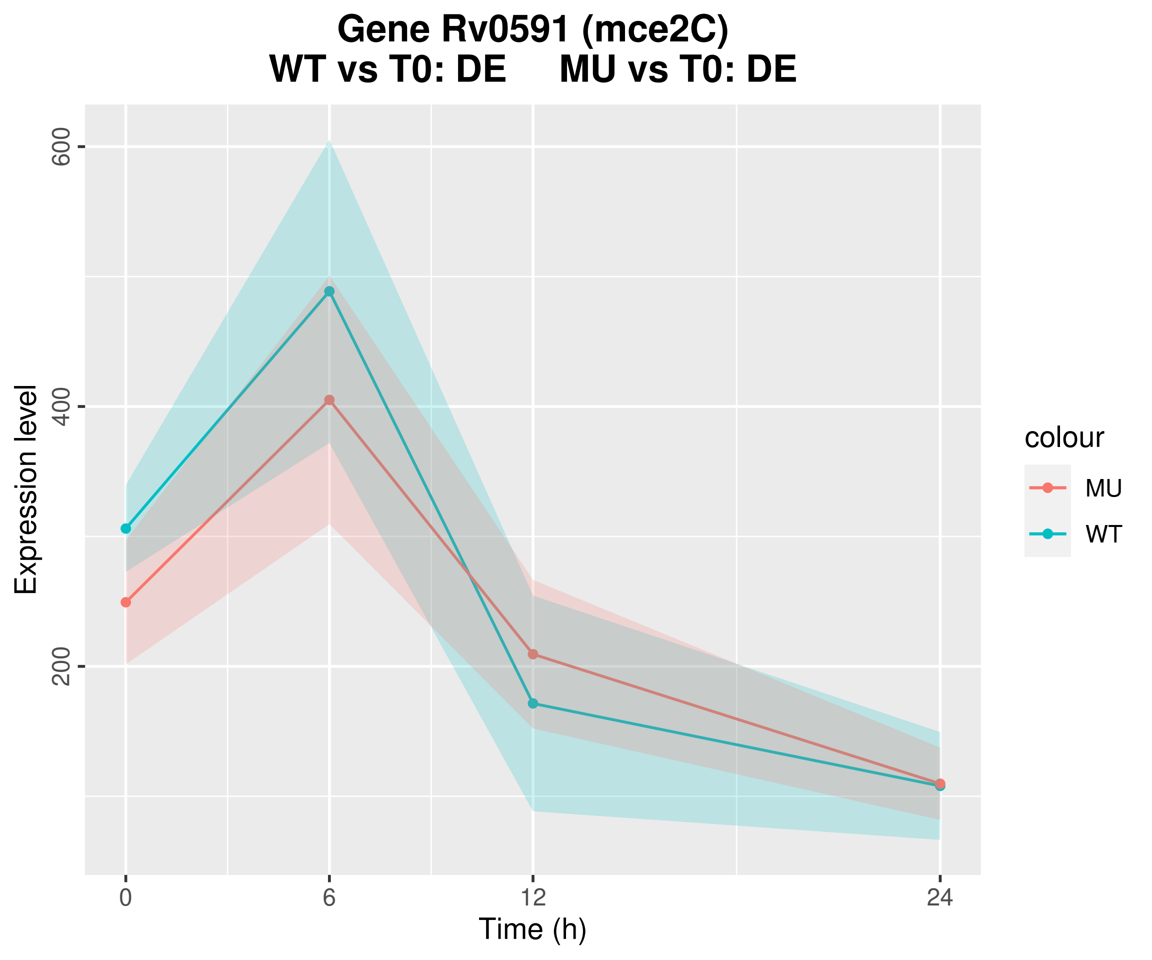

Gene Rv0591 (mce2C)
WT vs T0: DE   MU vs T0: DE
600
400
200
Expression level
0
6
12
24
Time (h)
colour
MU
WT

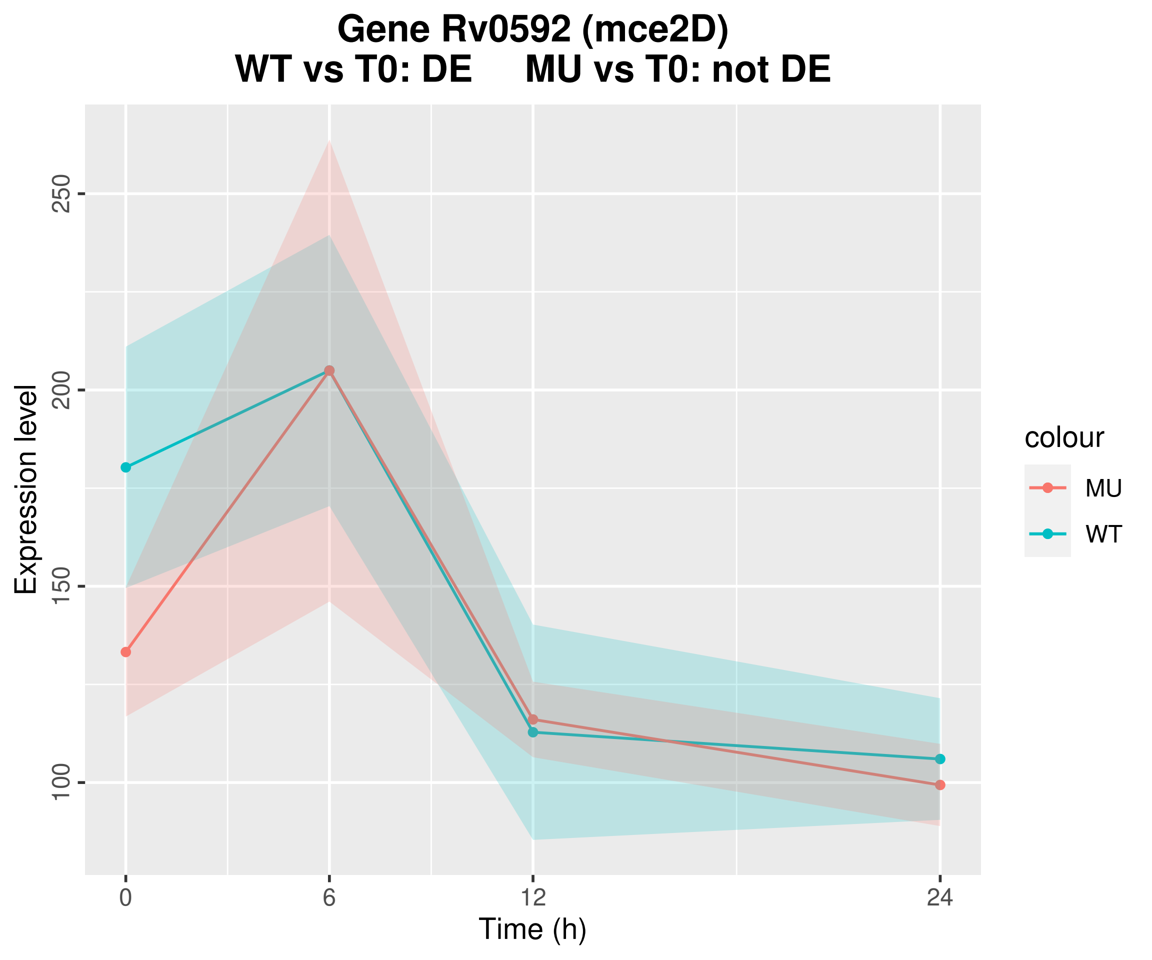

Gene Rv0592 (mce2D)
WT vs T0: DE   MU vs T0: not DE
Expression level
250
200
150
100
0
6
12
24
Time (h)
colour
MU
WT

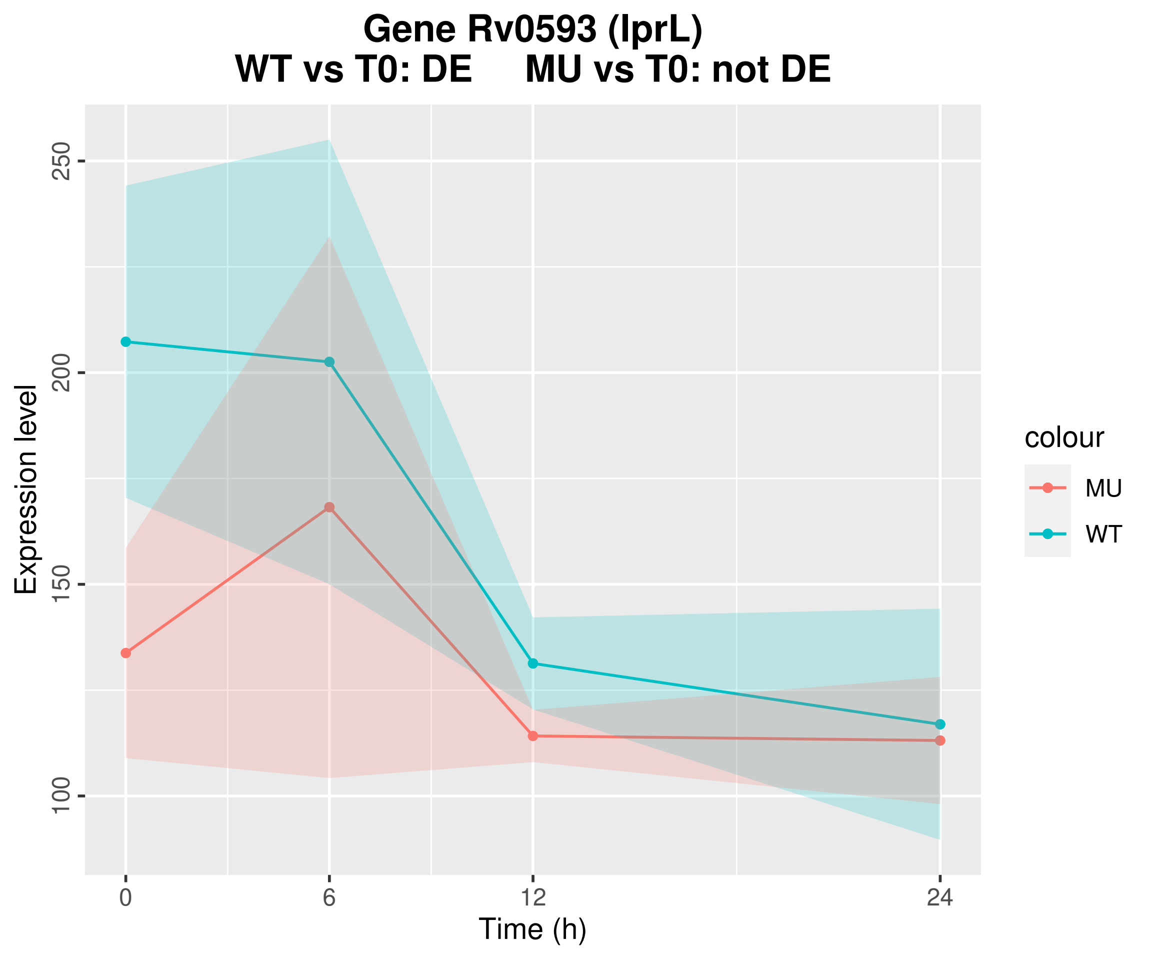

Gene Rv0593 (lprL)
WT vs T0: DE MU vs T0: not DE
Expression level
250
200
150
100
0
6
12
24
Time (h)
colour
MU
WT

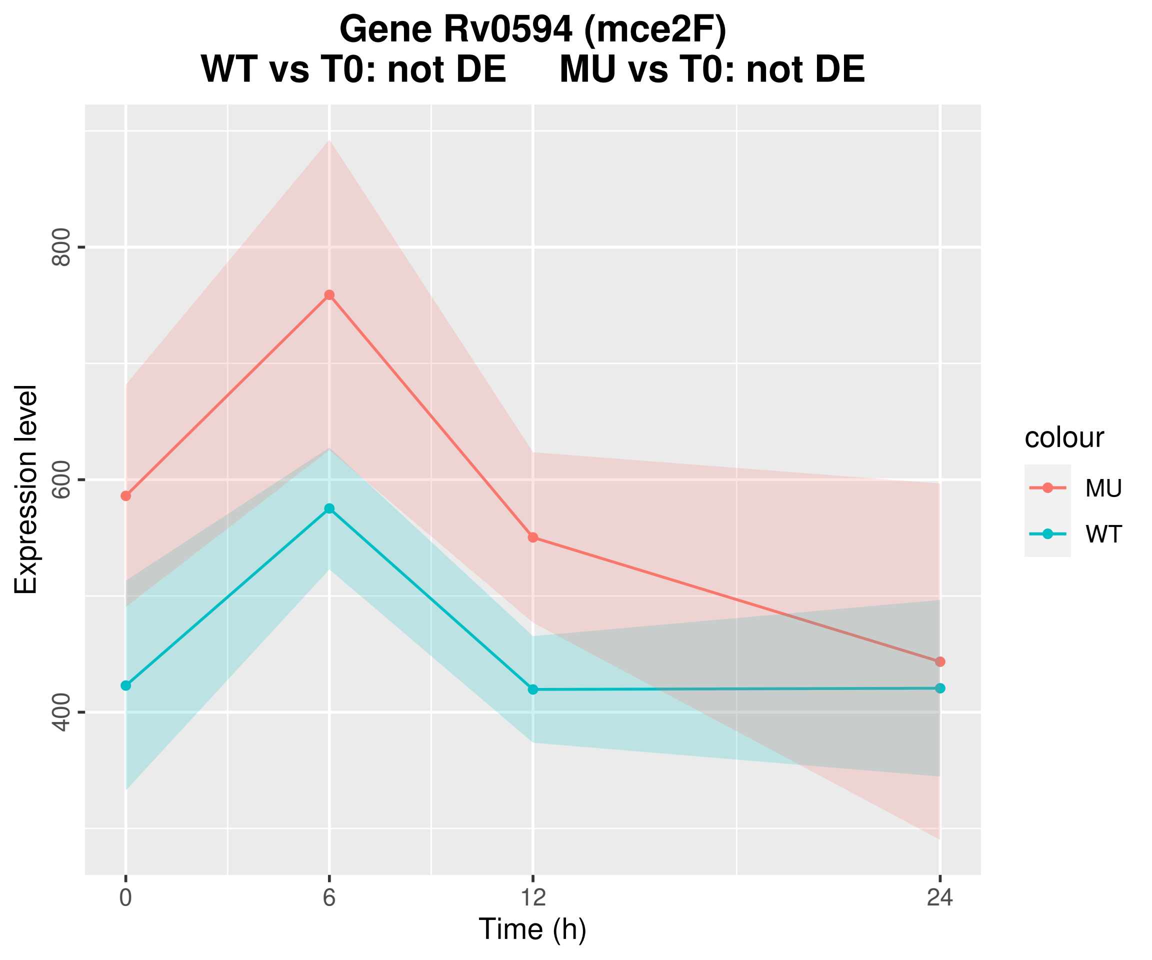
Gene Rv0594 (mce2F)
WT vs T0: not DE MU vs T0: not DE
Expression level
800
600
400
0
6
12
24
Time (h)
colour
MU
WT

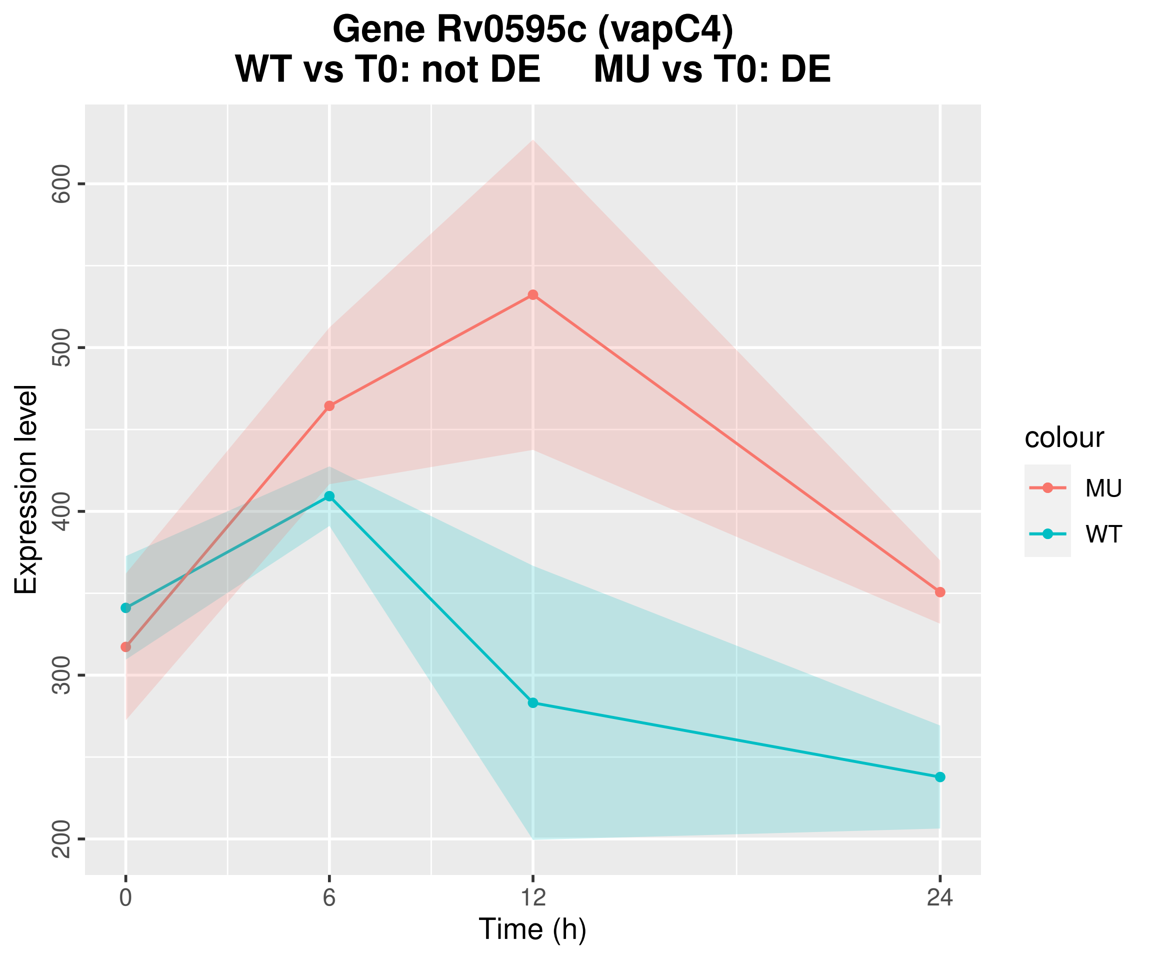

Gene Rv0595c (vapC4)
WT vs T0: not DE MU vs T0: DE
Expression level
600
500
400
300
200
0
6
12
24
Time (h)
colour
MU
WT

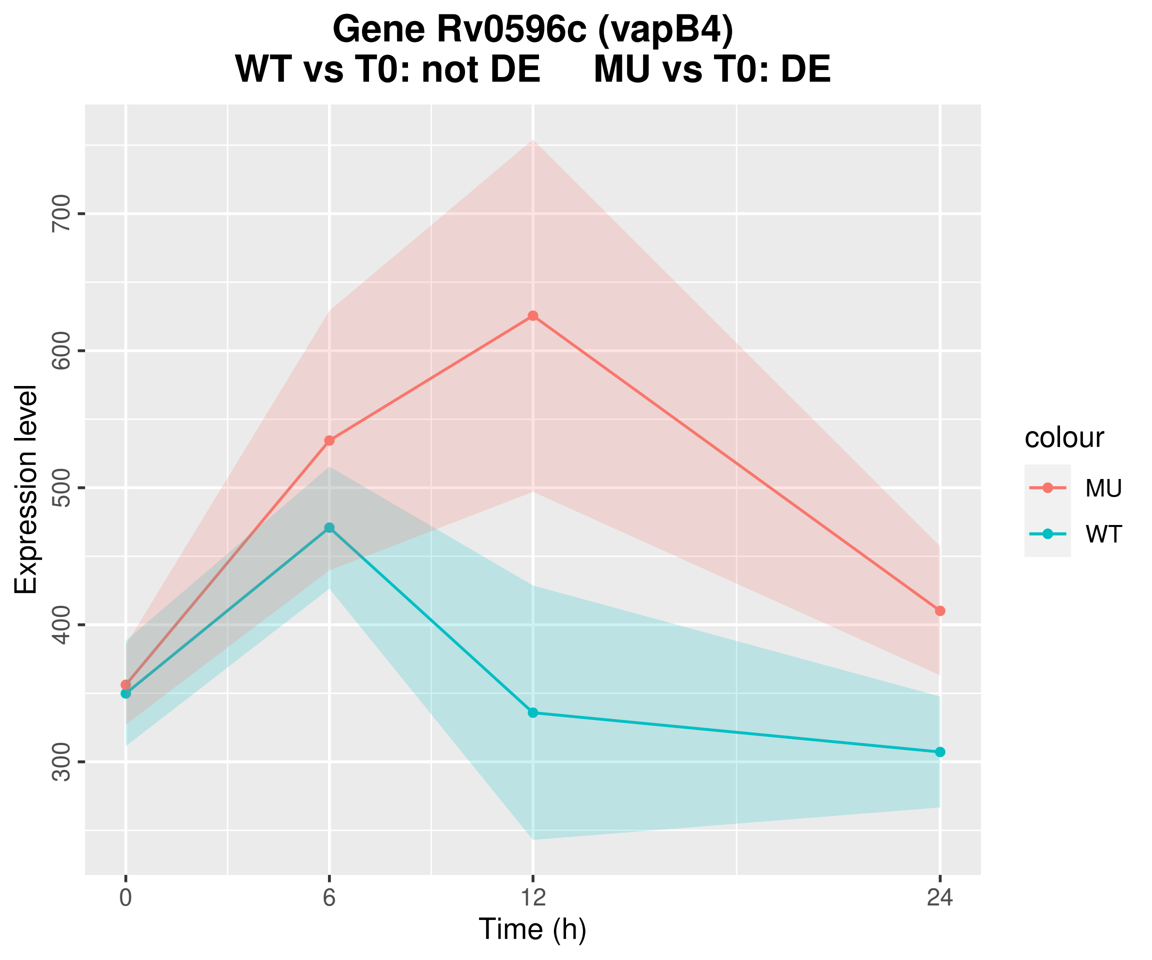

Gene Rv0596c (vapB4)
WT vs T0: not DE MU vs T0: DE
Expression level
700
600
500
400
300
0
6
12
24
Time (h)
colour
MU
WT

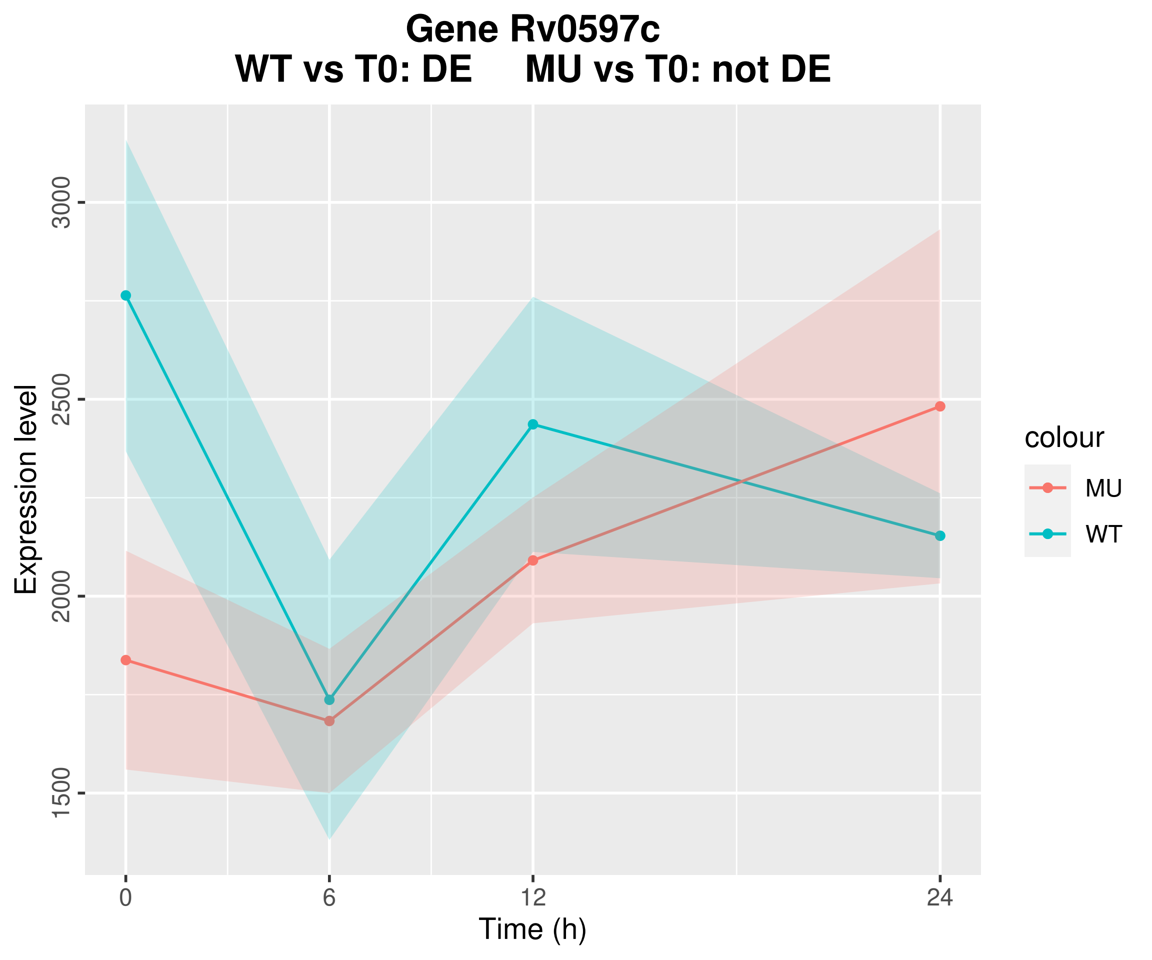

Gene Rv0597c
WT vs T0: DE MU vs T0: not DE
Expression level
3000
2500
2000
1500
0
6
12
24
Time (h)
colour
MU
WT

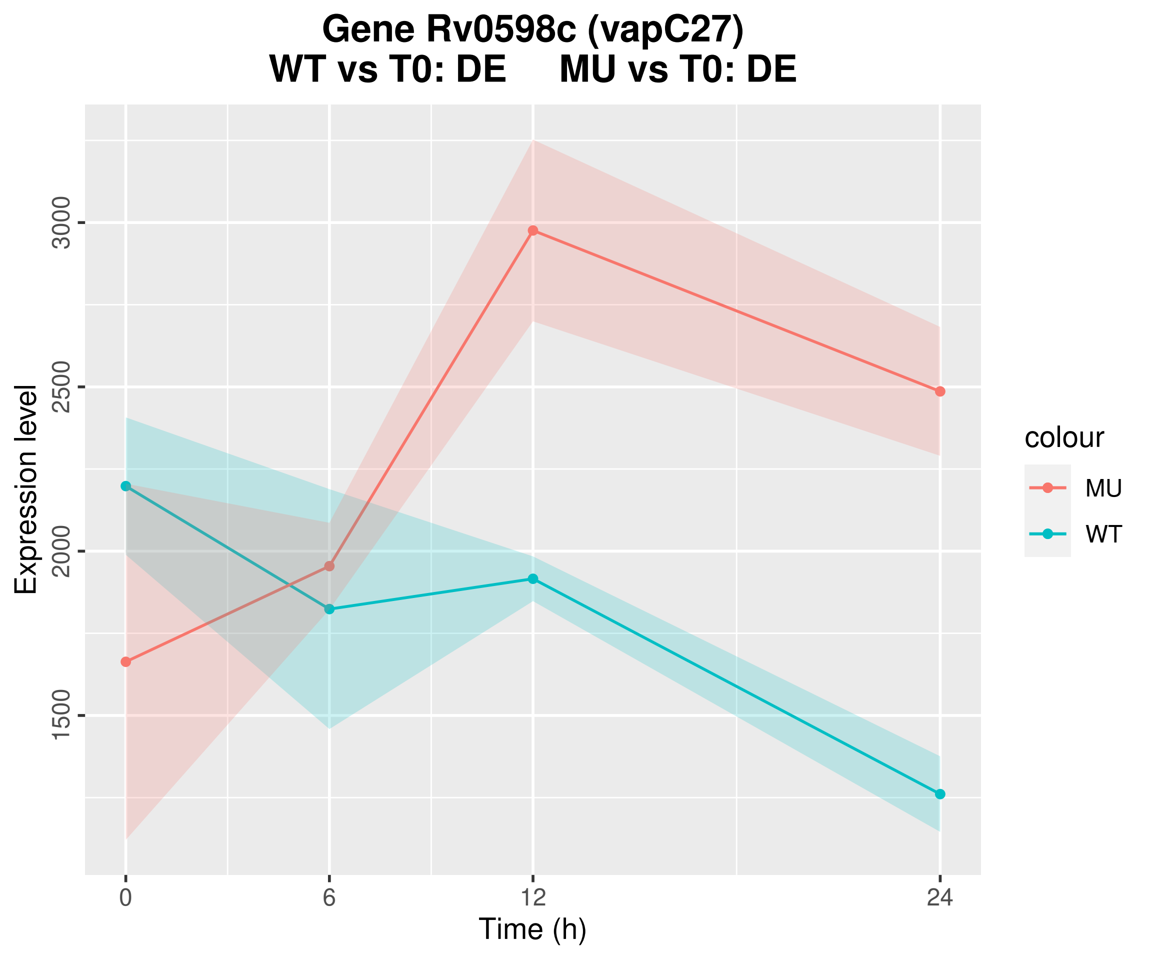

Gene Rv0598c (vapC27)
WT vs T0: DE MU vs T0: DE
Expression level
3000
2500
2000
1500
Time (h)
0
6
12
24
colour
MU
WT

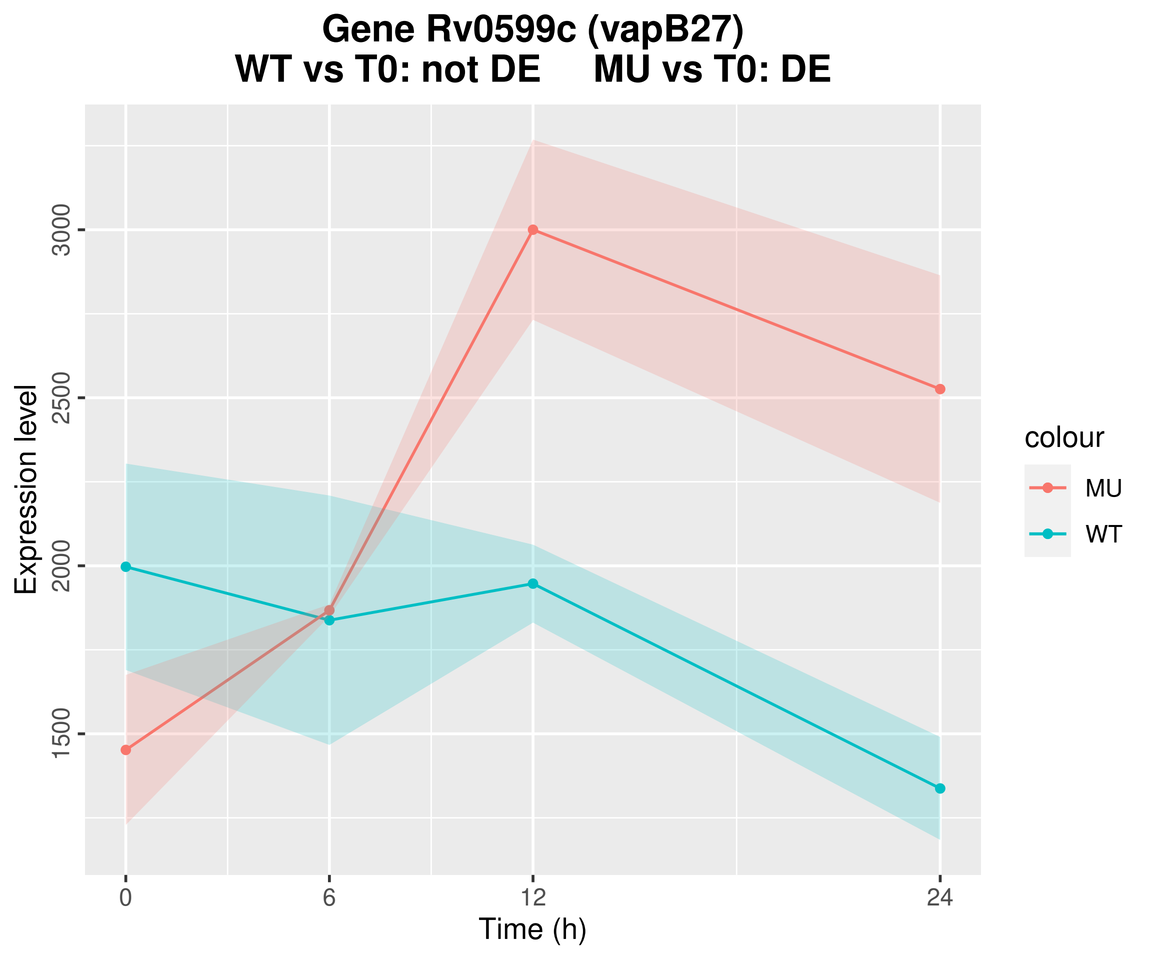
Gene Rv0599c (vapB27)
WT vs T0: not DE MU vs T0: DE
Expression level
3000
2500
2000
1500
0
6
12
24
Time (h)
colour
MU
WT

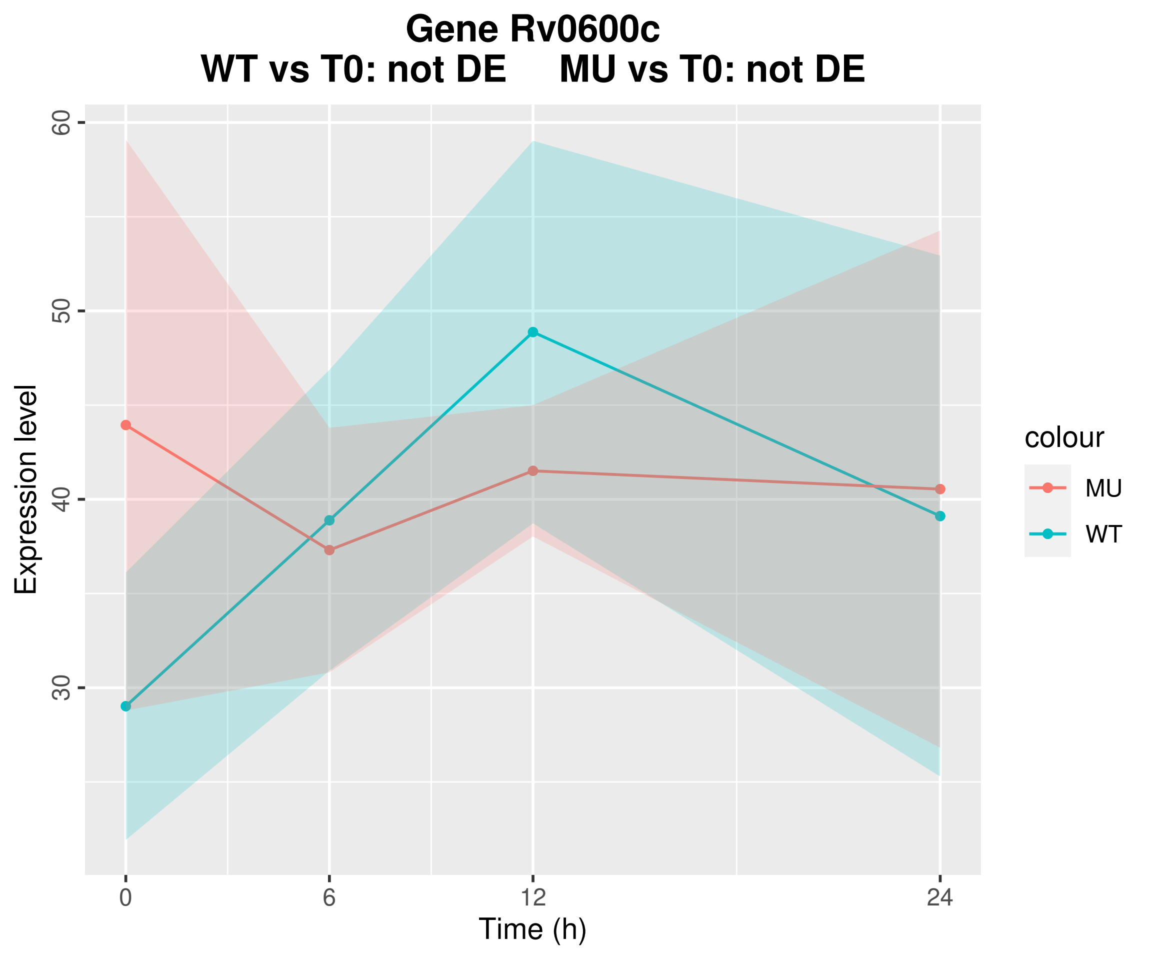

Gene Rv0600c
WT vs T0: not DE MU vs T0: not DE
Expression level
60
50
40
30
0
6
12
24
Time (h)
colour
MU
WT

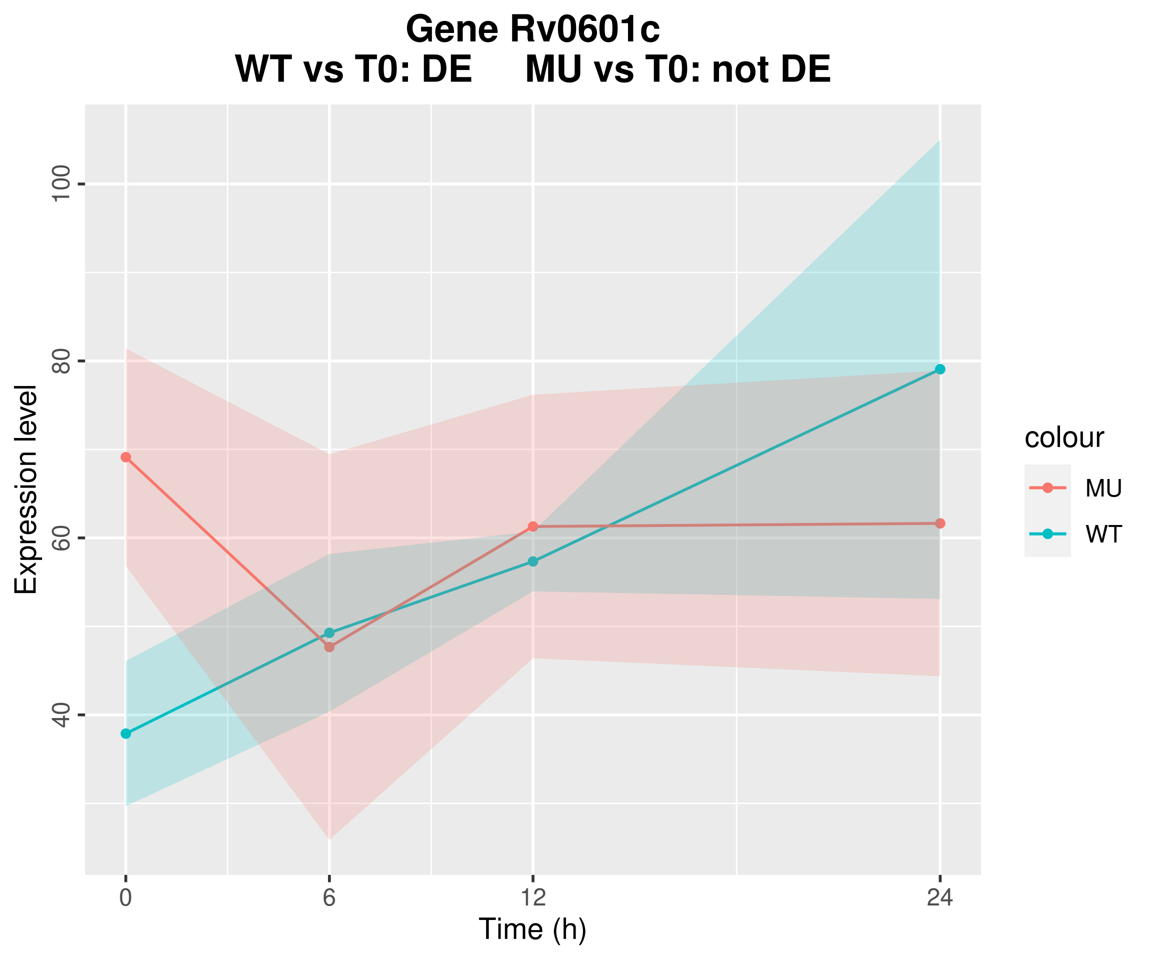
Gene Rv0601c
WT vs T0: DE   MU vs T0: not DE
Expression level
100
80
60
40
0
6
12
24
Time (h)
colour
MU
WT

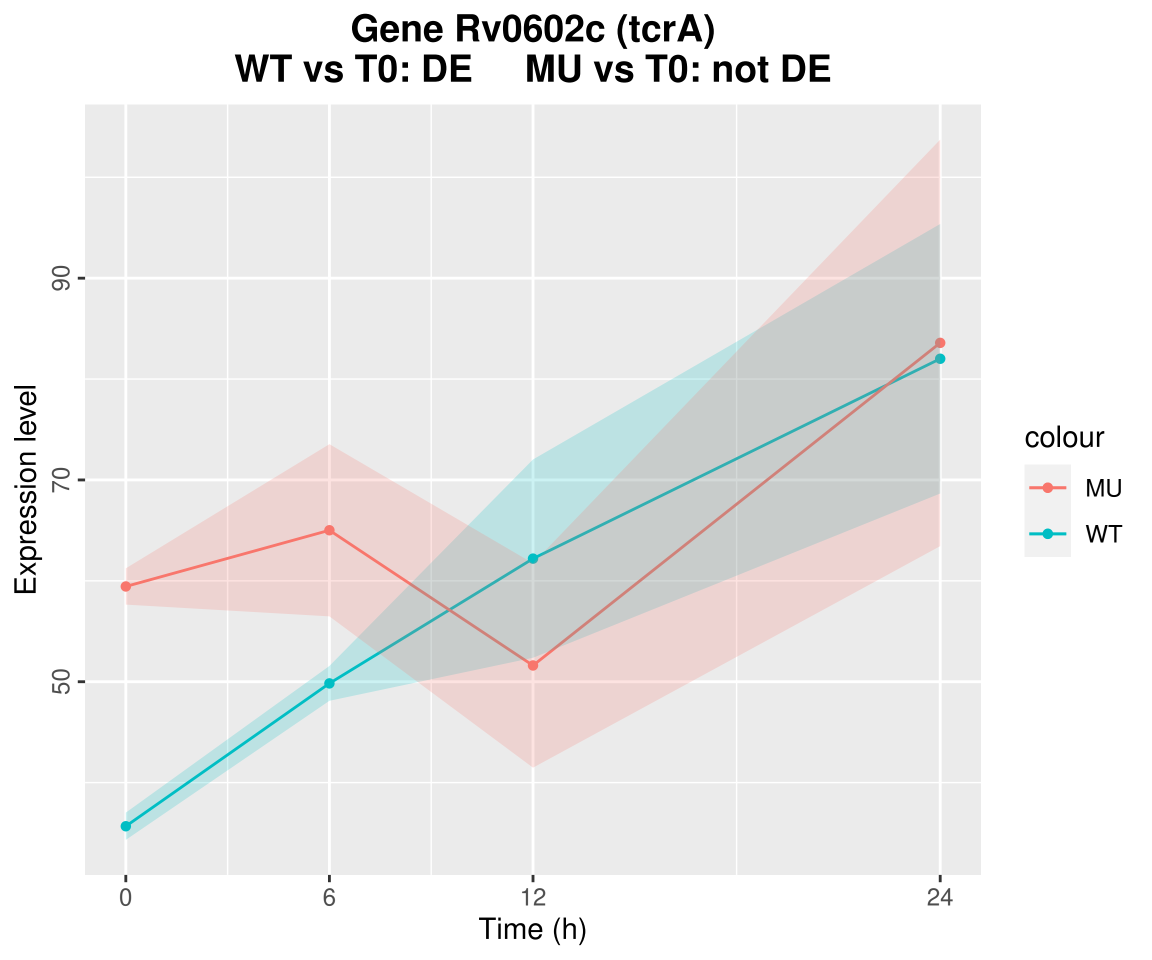

Gene Rv0602c (tcrA)
WT vs T0: DE   MU vs T0: not DE
Expression level
90
70
50
0
6
12
24
Time (h)
colour
MU
WT

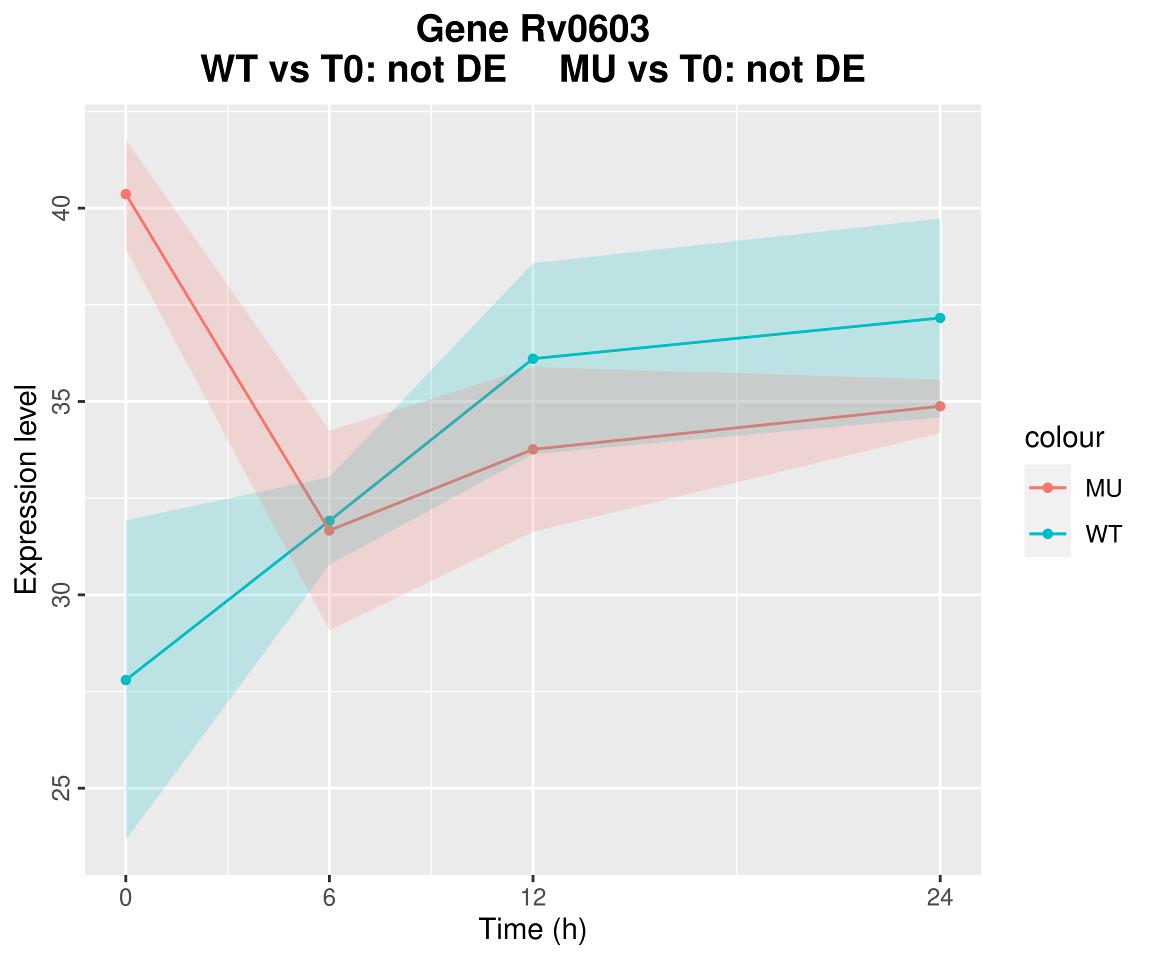

Gene Rv0603
WT vs T0: not DE MU vs T0: not DE
Expression level
25
30
35
40
0
6
12
24
Time (h)
colour
MU
WT

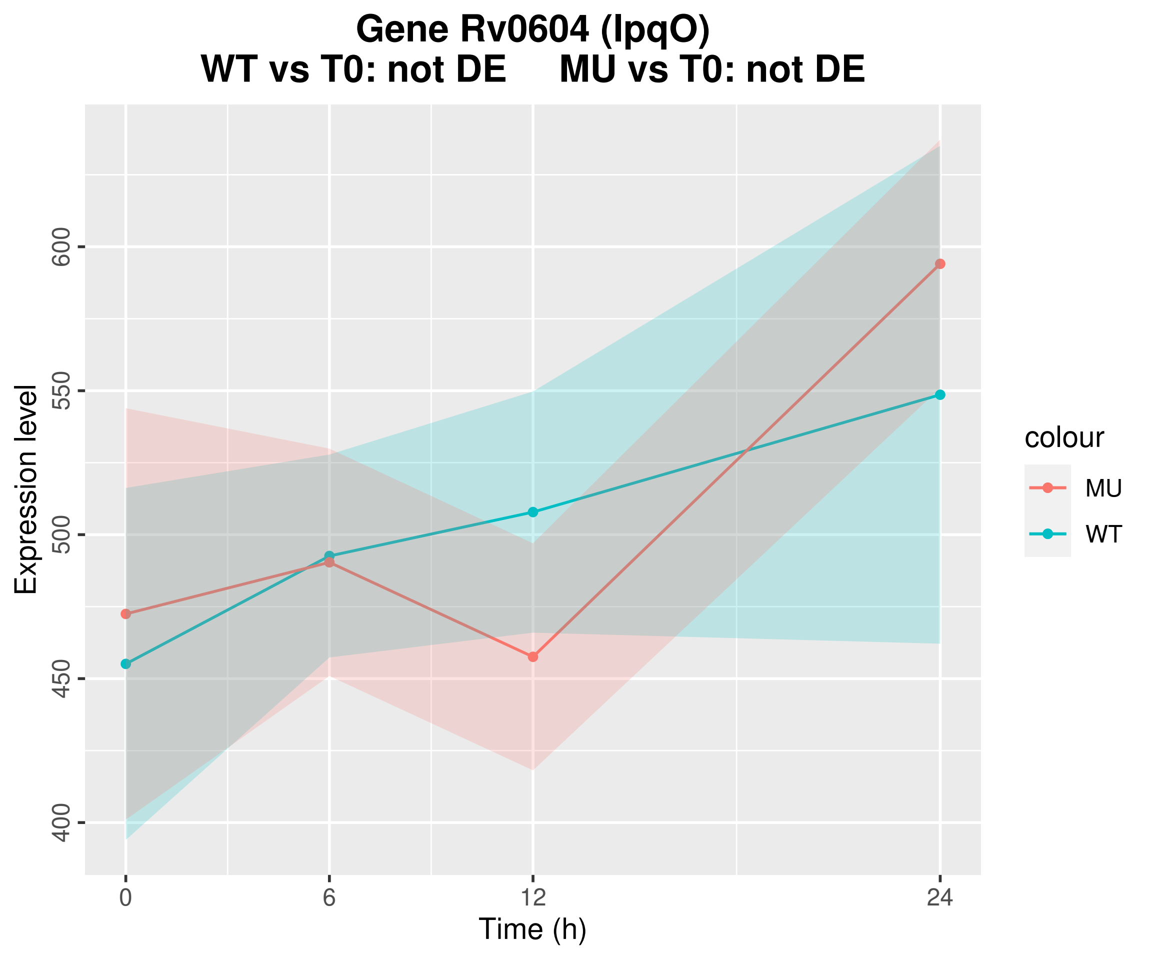
Gene Rv0604 (lpqO)
WT vs T0: not DE MU vs T0: not DE
Expression level
400
450
500
550
600
0
6
12
24
Time (h)
colour
MU
WT

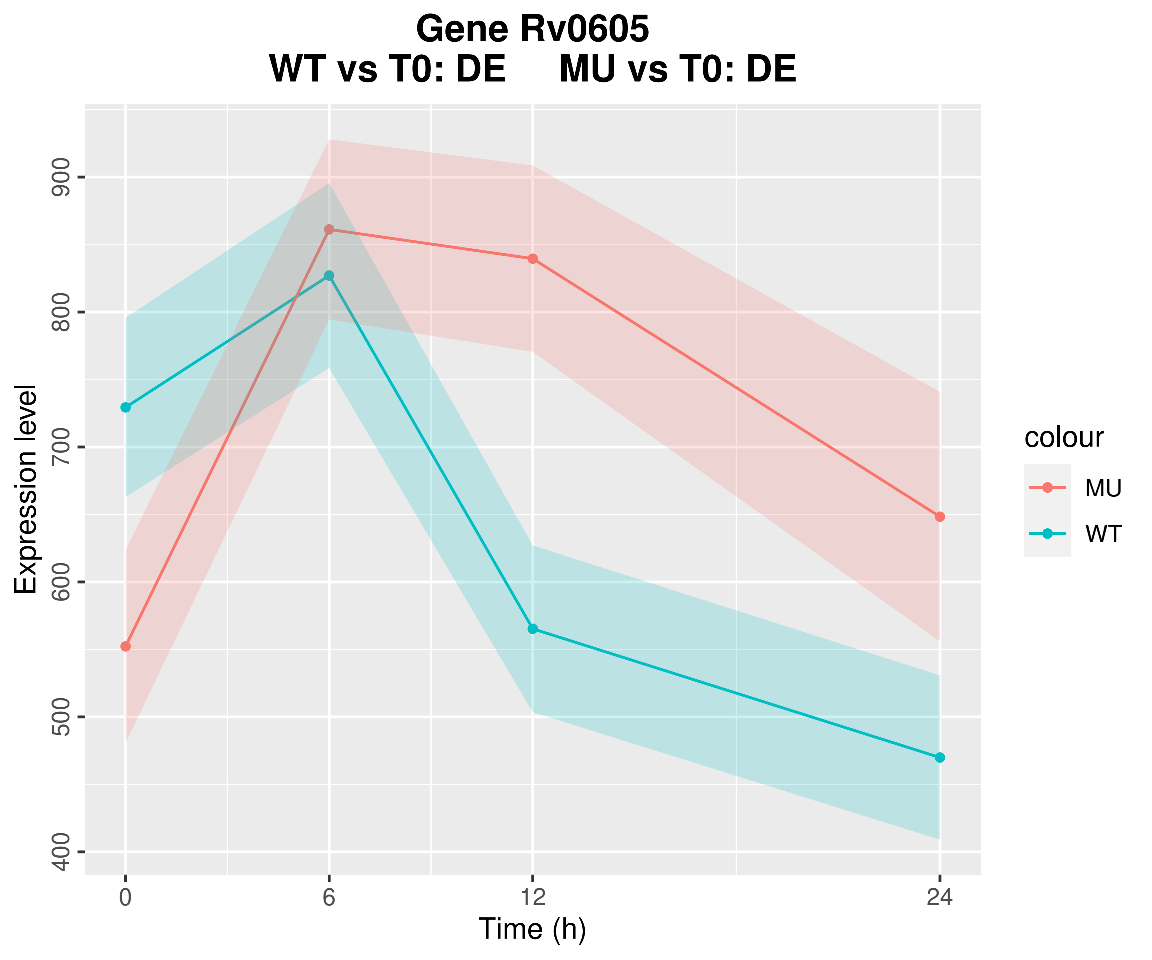
Gene Rv0605
WT vs T0: DE MU vs T0: DE
Expression level
900
800
700
600
500
400
0
6
12
24
Time (h)
colour
MU
WT

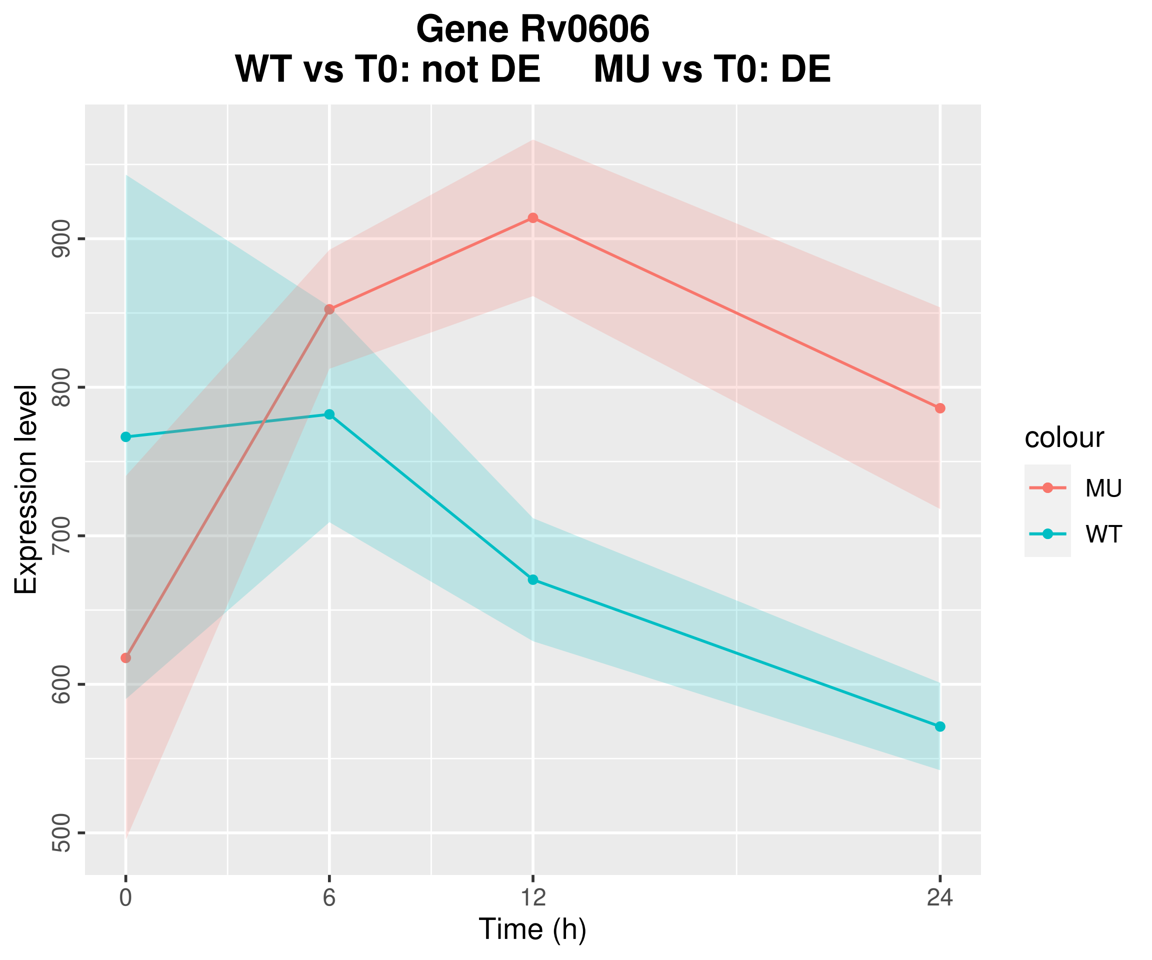

Gene Rv0606
WT vs T0: not DE   MU vs T0: DE
Expression level
900
800
700
600
500
0
6
12
24
Time (h)
colour
MU
WT

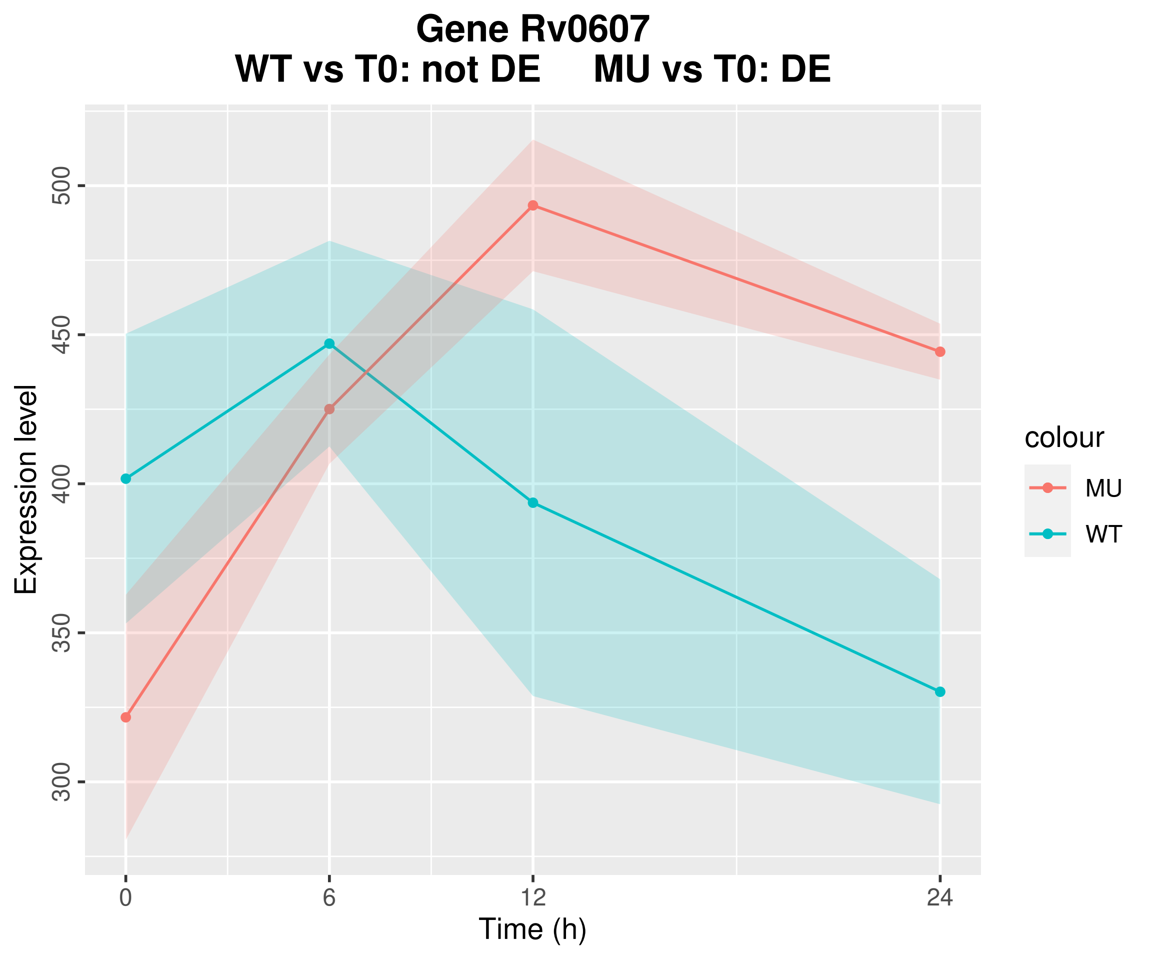
Gene Rv0607
WT vs T0: not DE   MU vs T0: DE
Expression level
500
450
400
350
300
0
6
12
24
Time (h)
colour
MU
WT

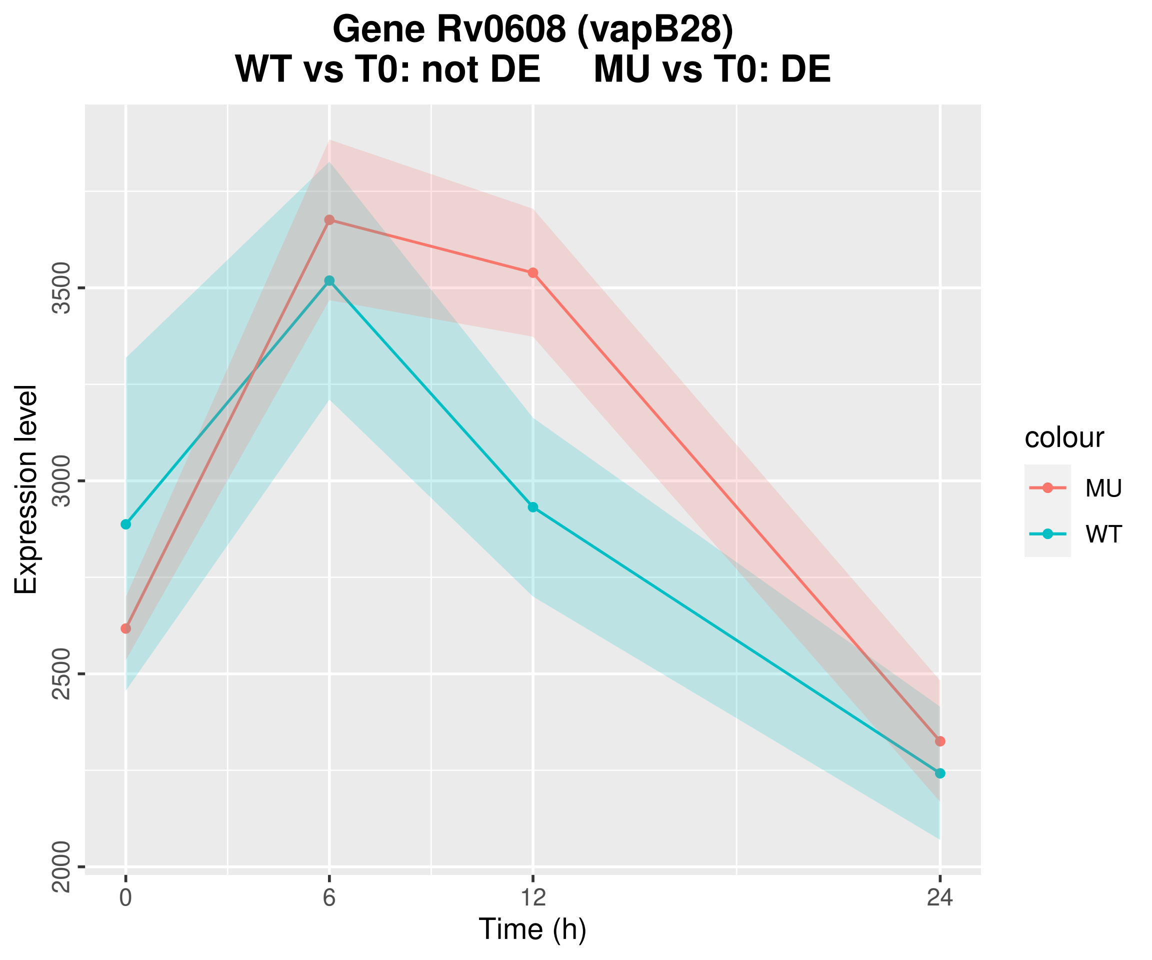

Gene Rv0608 (vapB28)
WT vs T0: not DE MU vs T0: DE
Expression level
3500
3000
2500
2000
0
6
12
24
Time (h)
colour
MU
WT

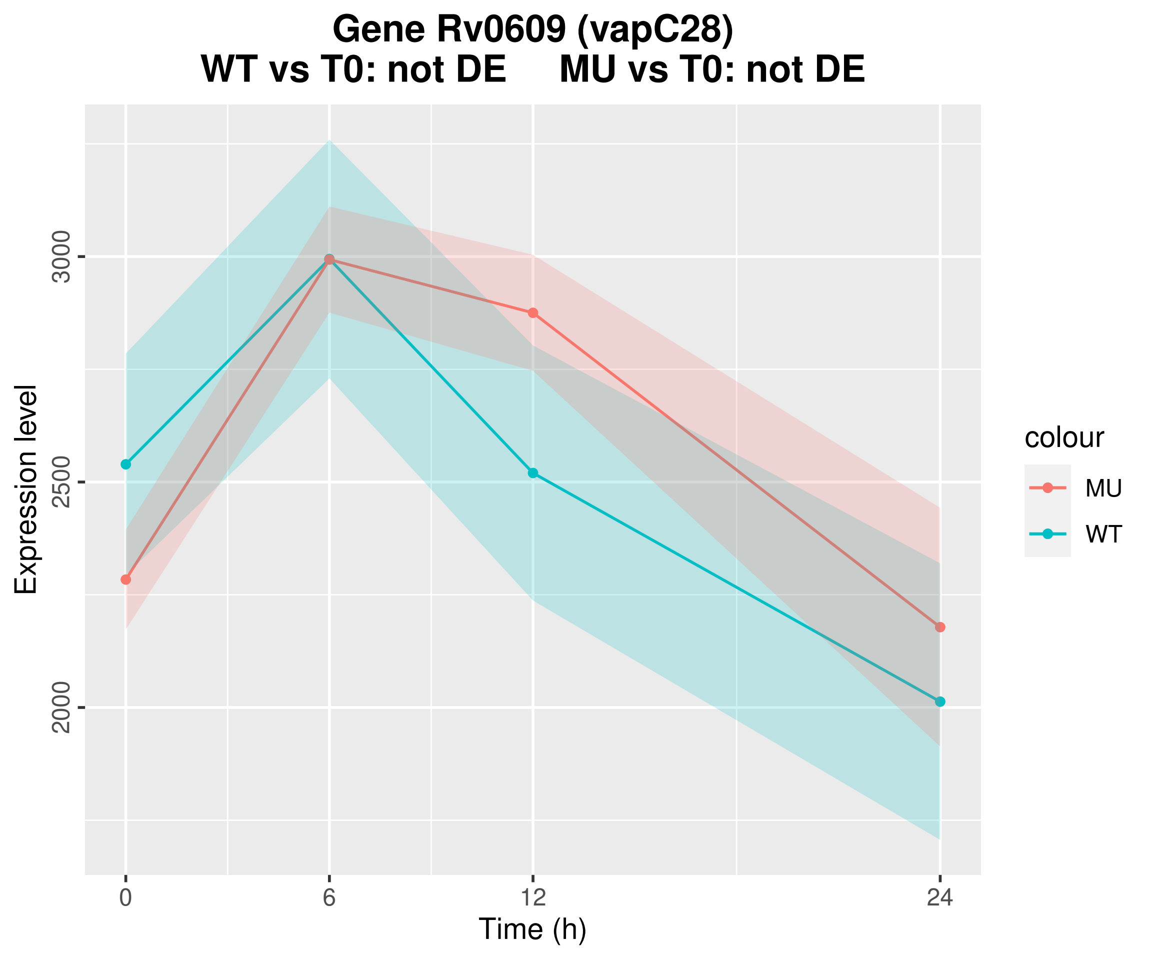

Gene Rv0609 (vapC28)
WT vs T0: not DE MU vs T0: not DE
Expression level
3000
2500
2000
0
6
12
24
Time (h)
colour
MU
WT

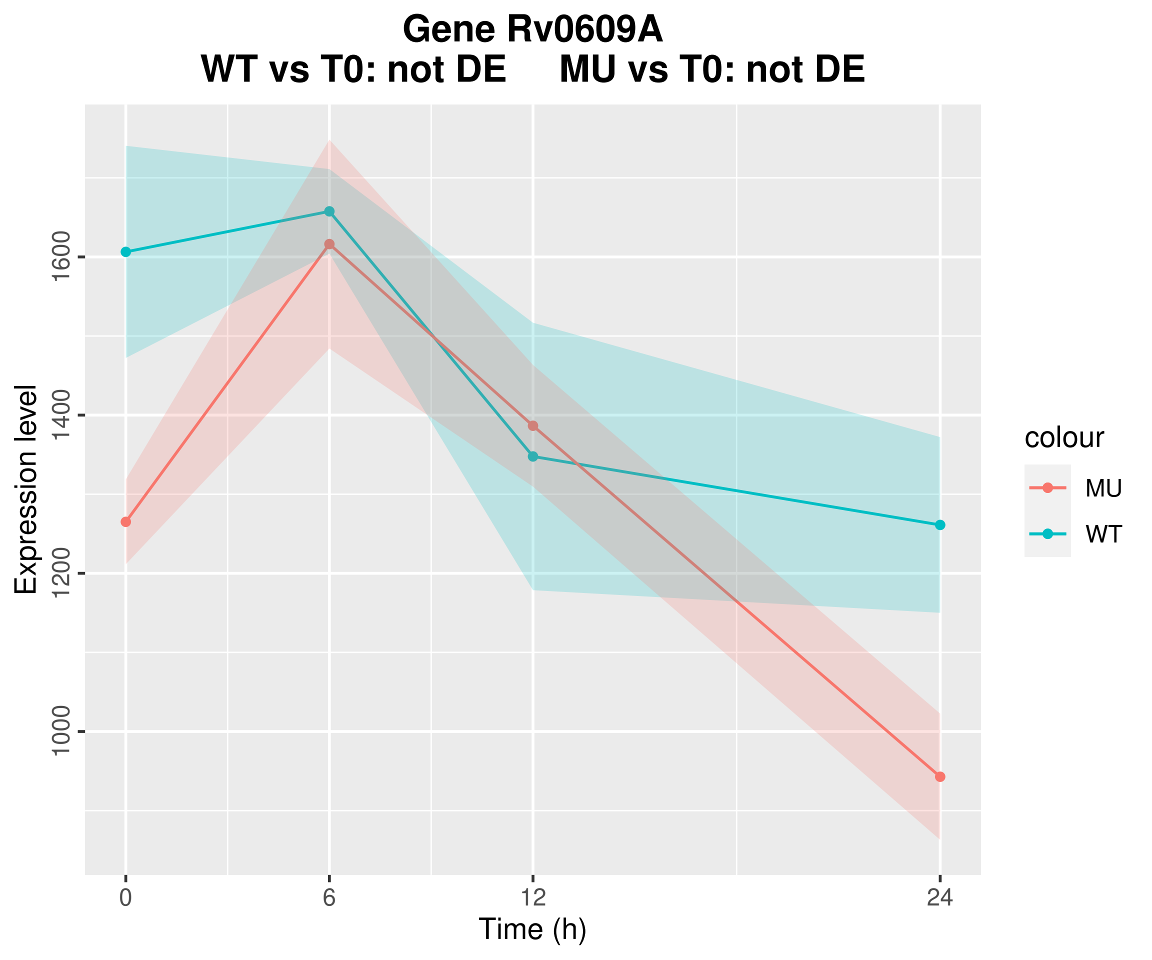

Gene Rv0609A
WT vs T0: not DE MU vs T0: not DE
Expression level
1000
1200
1400
1600
0
6
12
24
Time (h)
colour
MU
WT

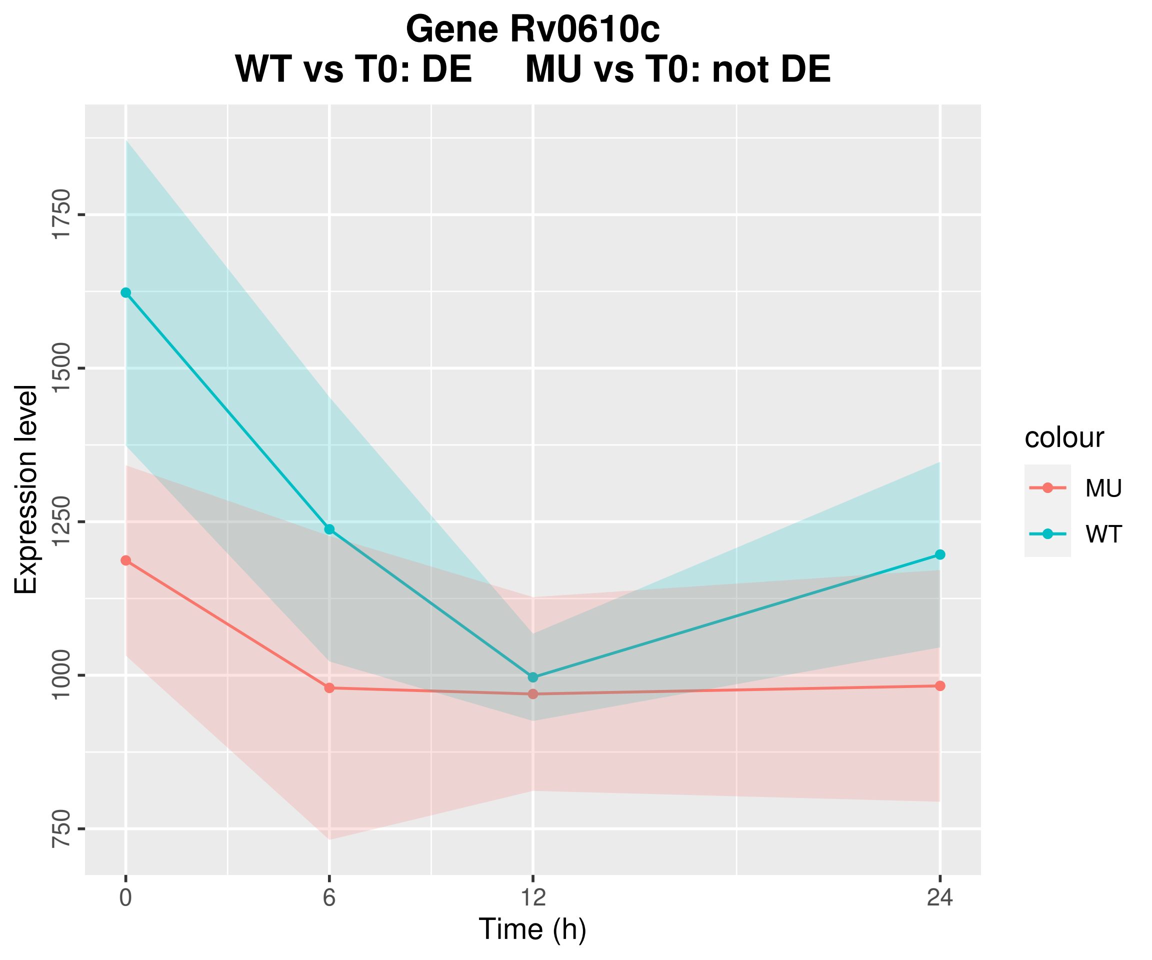

Gene Rv0610c
WT vs T0: DE MU vs T0: not DE
Expression level
750
1000
1250
1500
1750
0
6
12
24
Time (h)
colour
MU
WT

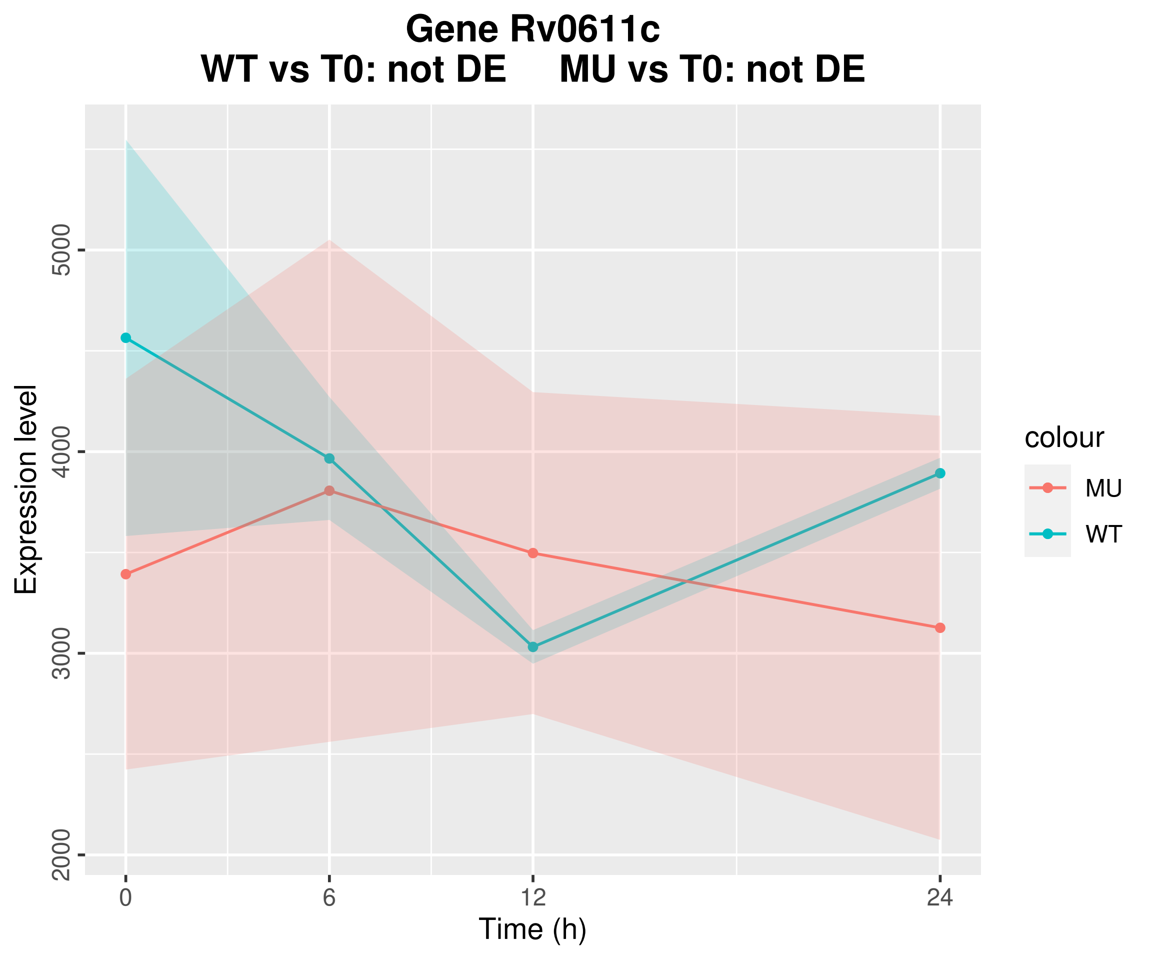
Gene Rv0611c
WT vs T0: not DE MU vs T0: not DE
Expression level
5000
4000
3000
2000
0
6
12
24
Time (h)
colour
MU
WT

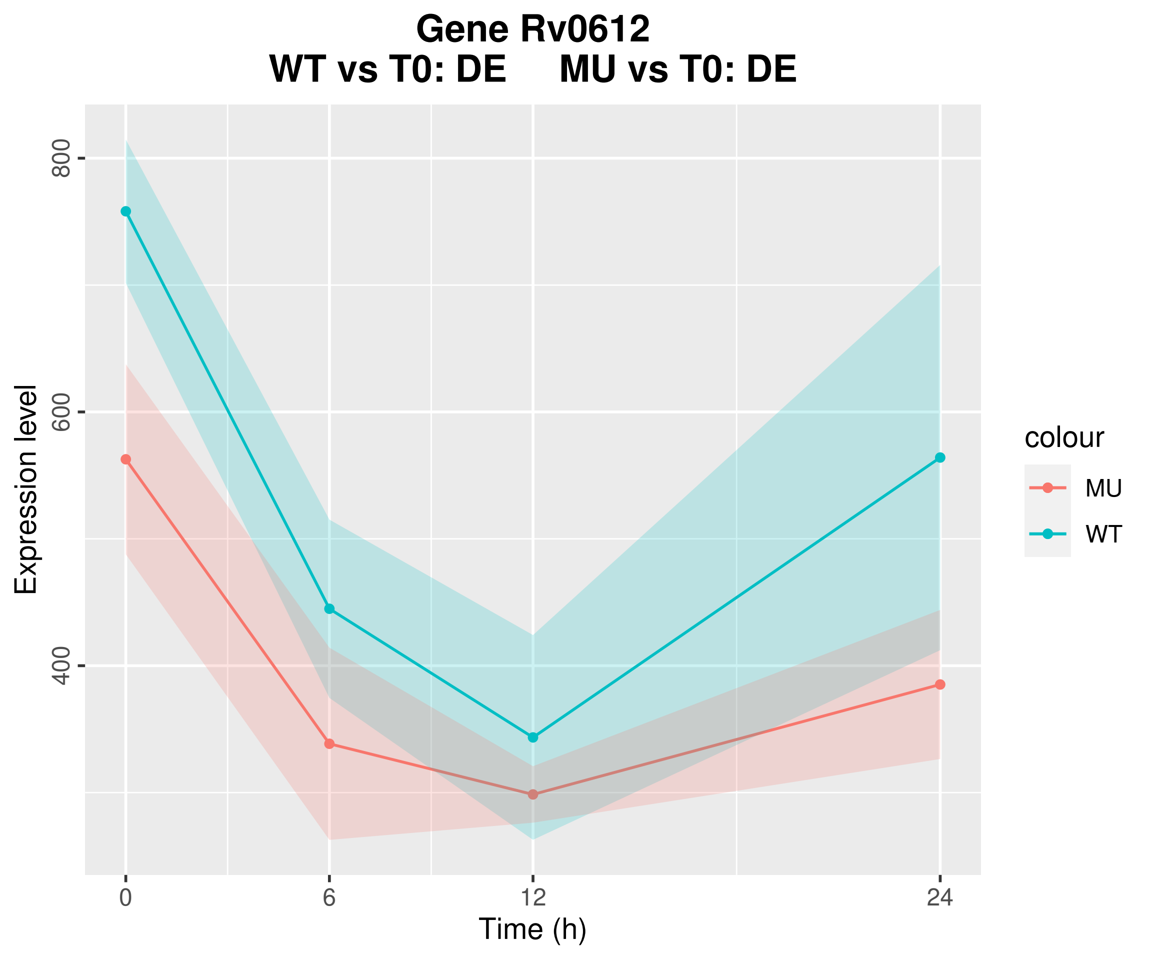

Gene Rv0612
WT vs T0: DE MU vs T0: DE
Expression level
800
600
400
0
6
12
24
Time (h)
colour
MU
WT

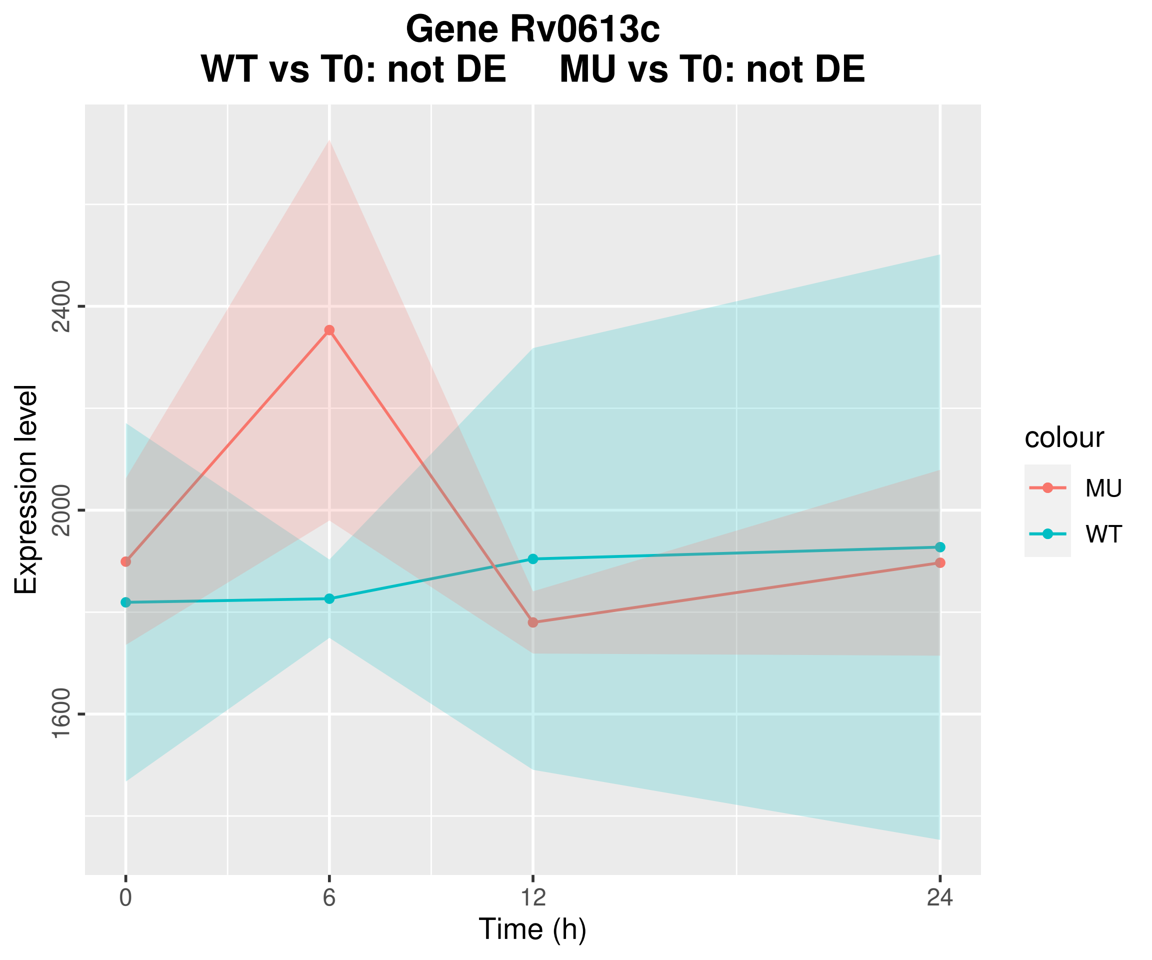

Gene Rv0613c
WT vs T0: not DE MU vs T0: not DE
Expression level
2400
2000
1600
0
6
12
24
Time (h)
colour
MU
WT

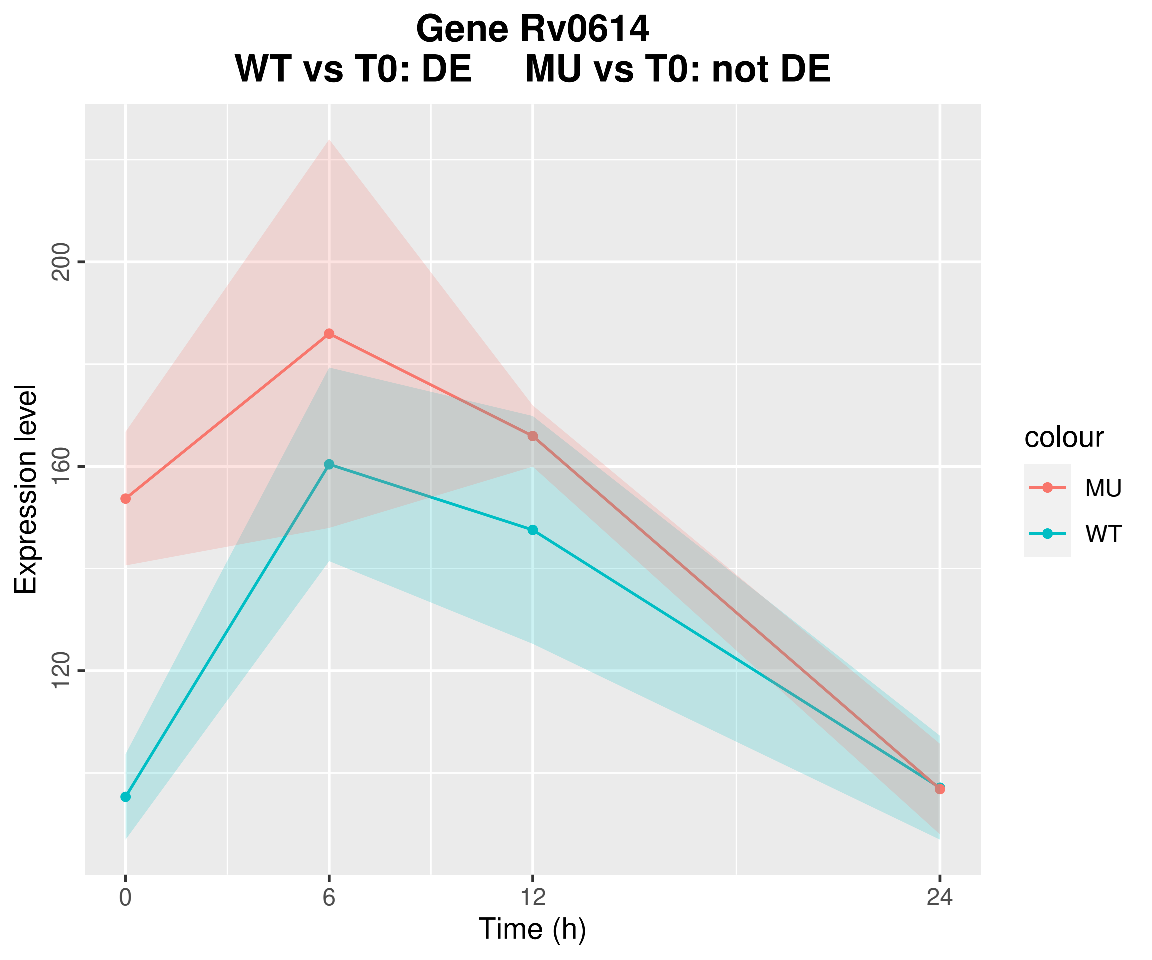

Gene Rv0614
WT vs T0: DE MU vs T0: not DE
Expression level
200
160
120
0
6
12
24
Time (h)
colour
MU
WT

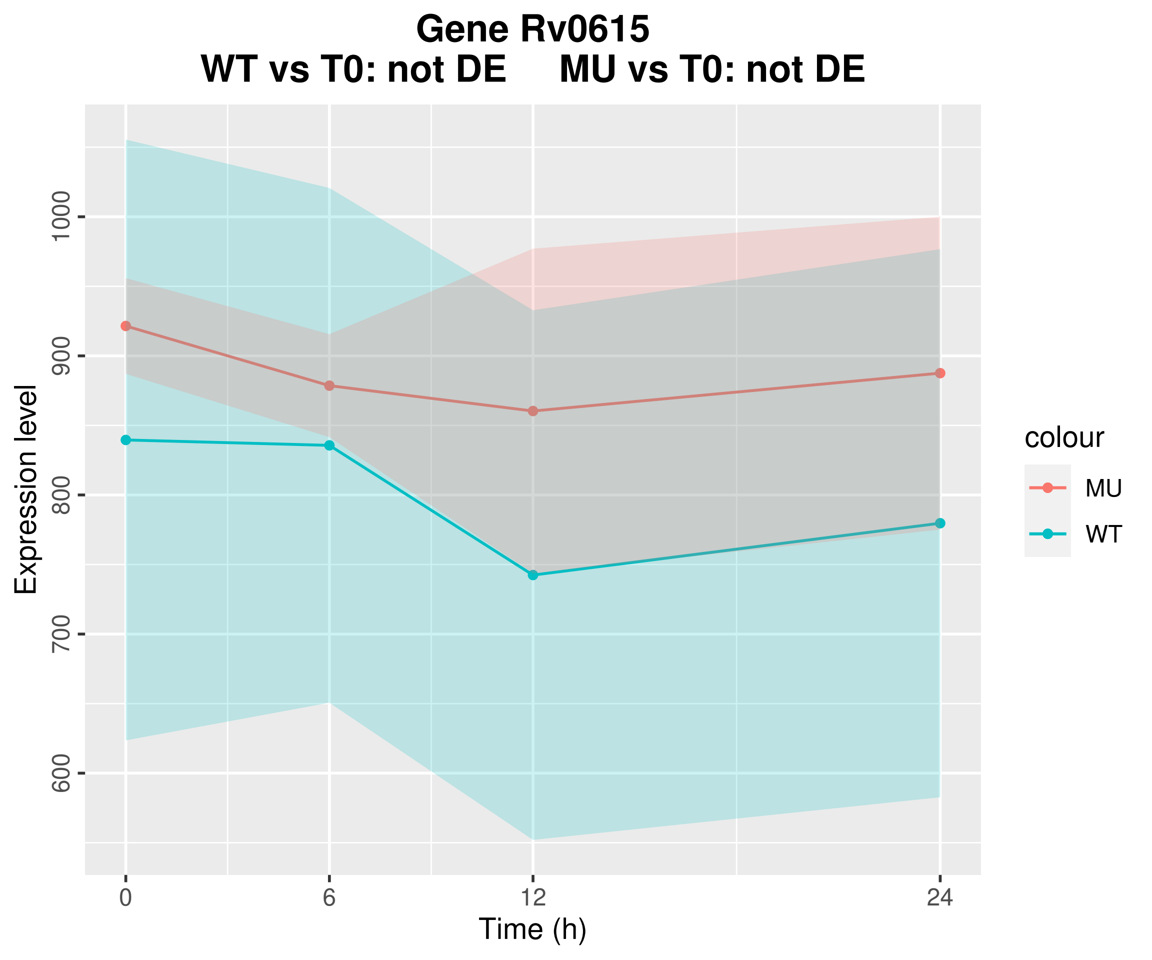

Gene Rv0615
WT vs T0: not DE MU vs T0: not DE
Expression level
1000
900
800
700
600
0
6
12
24
Time (h)
colour
MU
WT

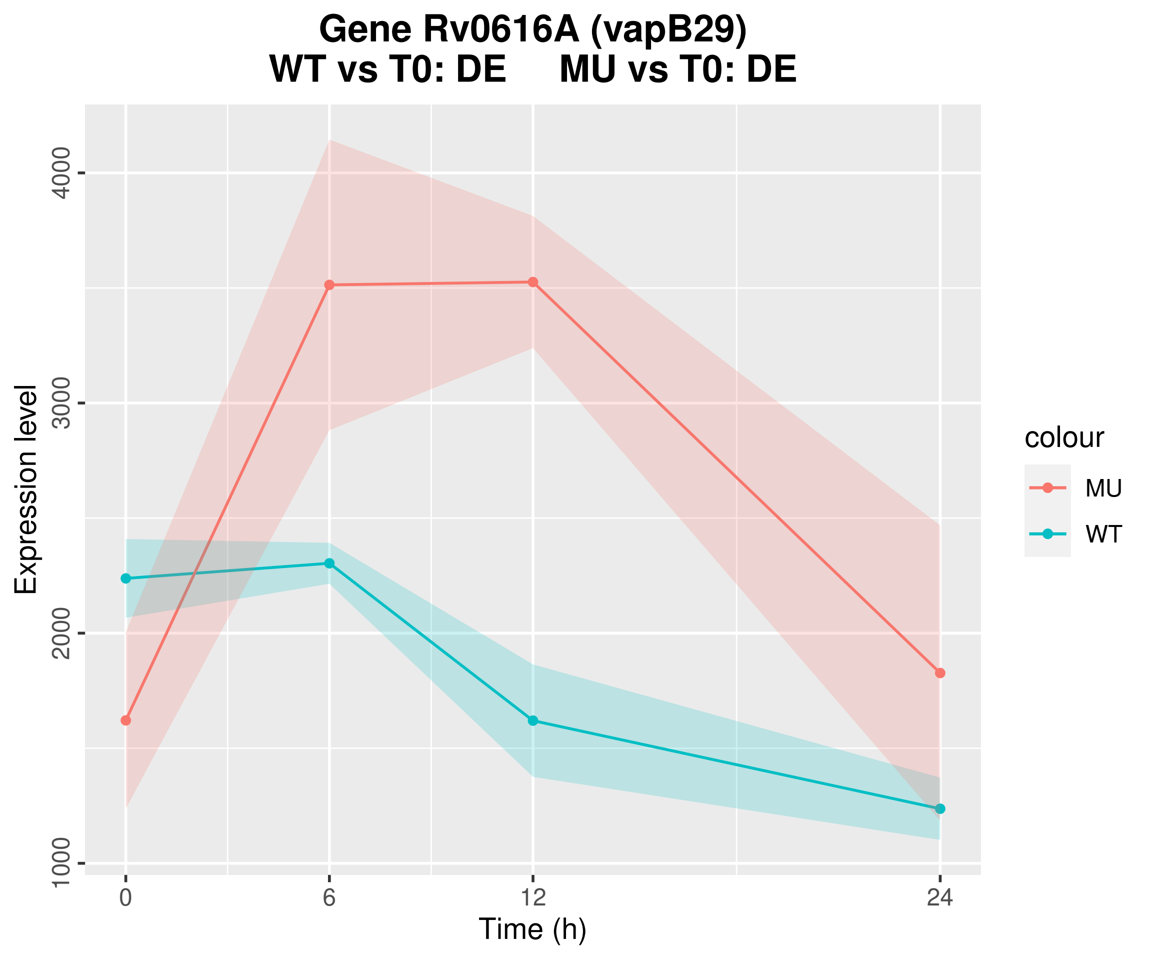

Gene Rv0616A (vapB29)
WT vs T0: DE MU vs T0: DE
Expression level
4000
3000
2000
1000
0
6
12
24
Time (h)
colour
MU
WT

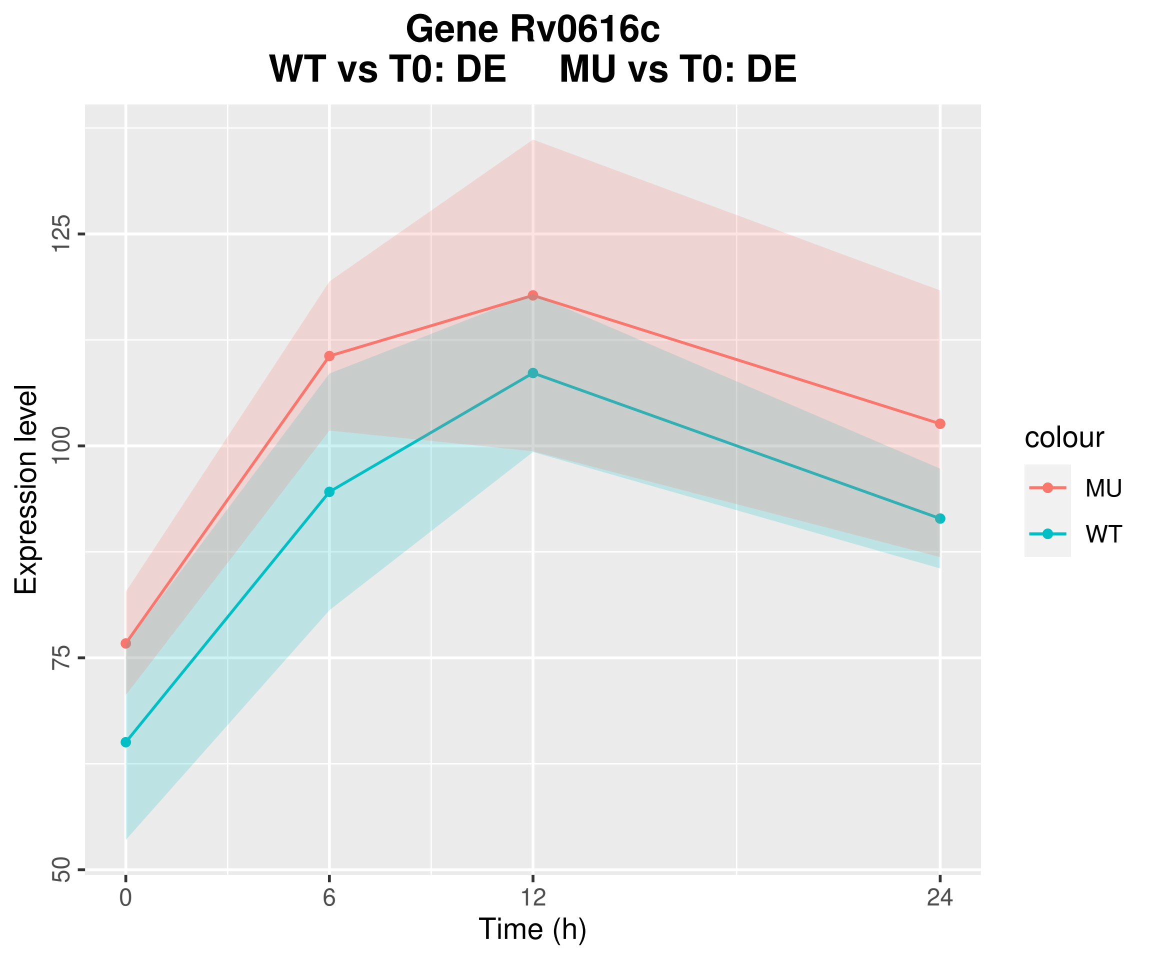

Gene Rv0616c
WT vs T0: DE MU vs T0: DE
Expression level
125
100
75
50
0
6
12
24
Time (h)
colour
MU
WT

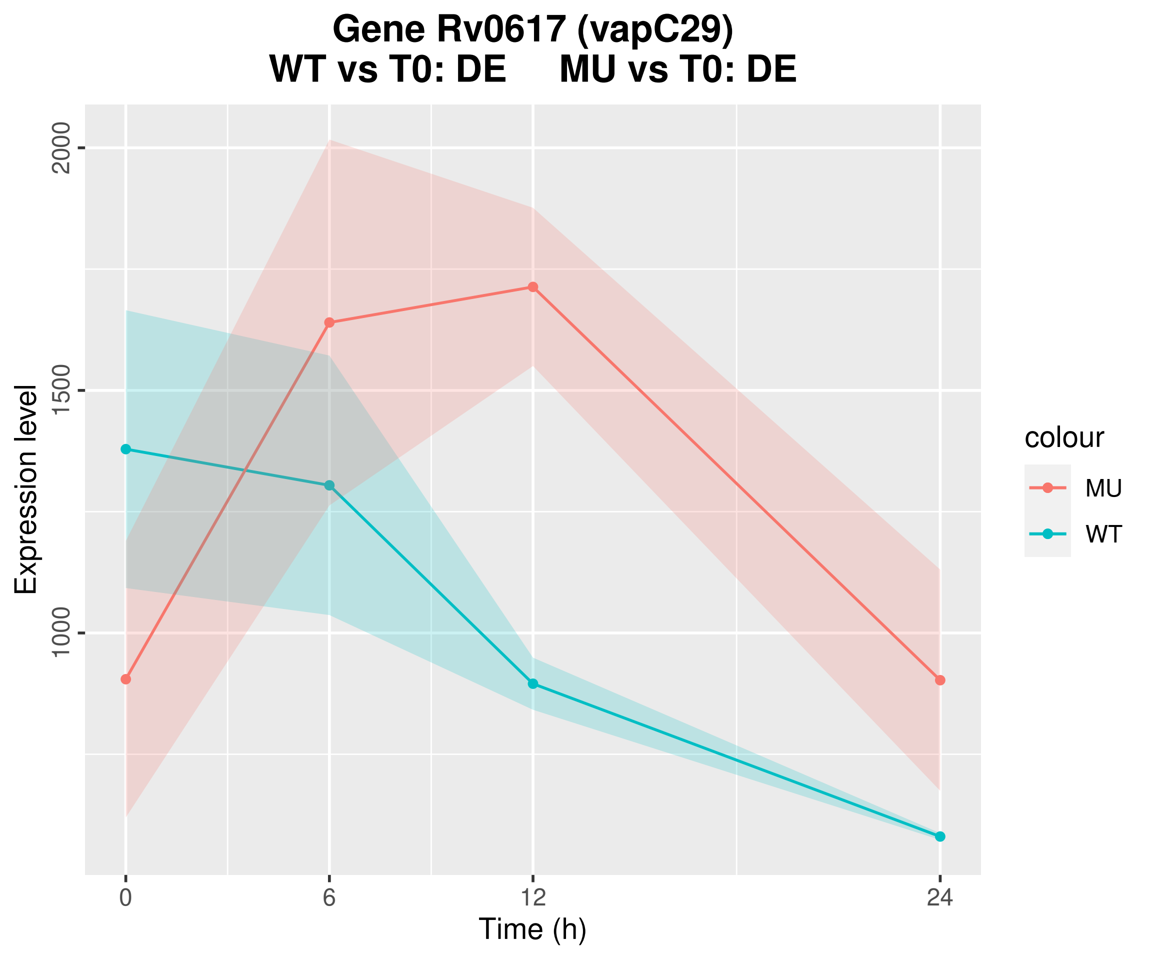

Gene Rv0617 (vapC29)
WT vs T0: DE   MU vs T0: DE
Expression level
2000
1500
1000
0
6
12
24
Time (h)
colour
MU
WT

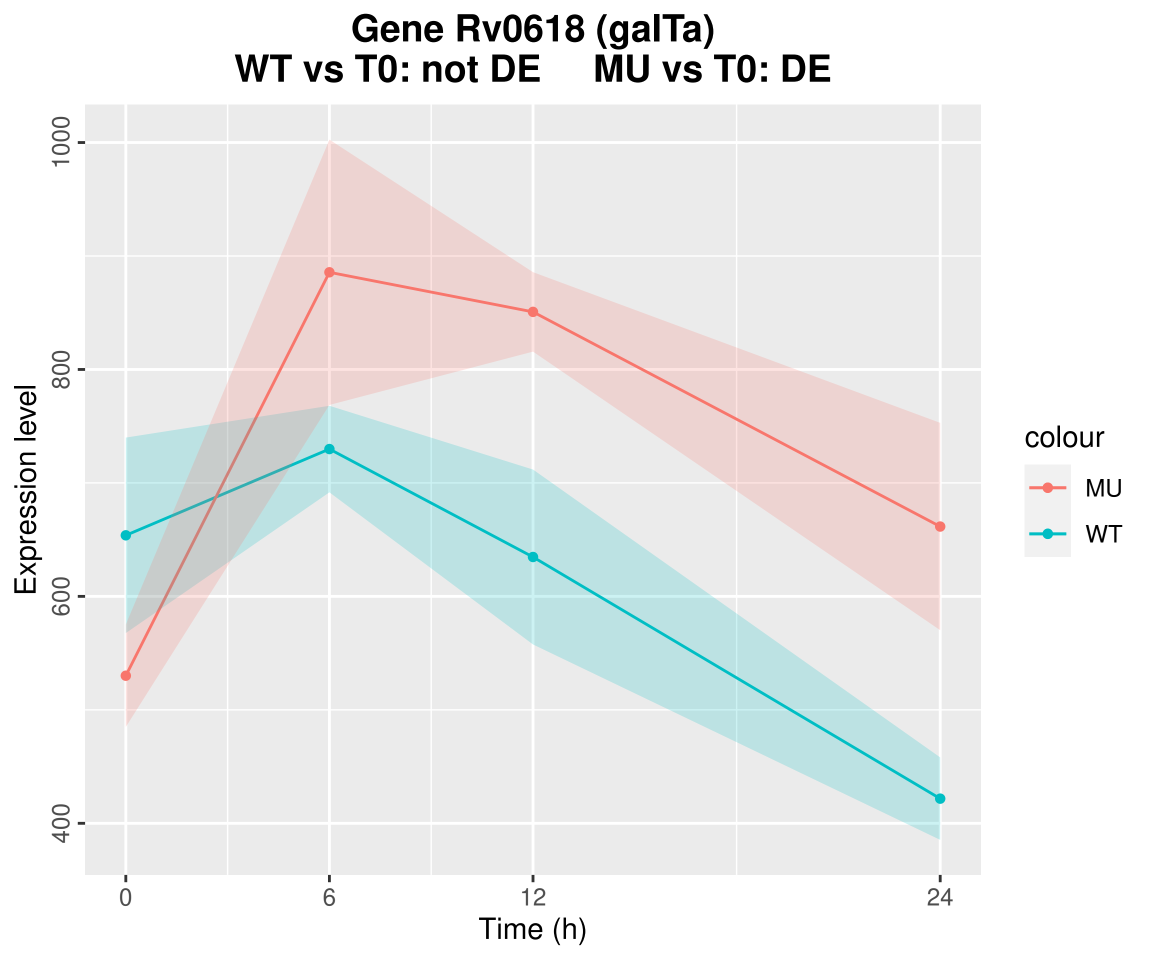

Gene Rv0618 (galTa)
WT vs T0: not DE   MU vs T0: DE
Expression level
1000
800
600
400
0
6
12
24
Time (h)
colour
MU
WT

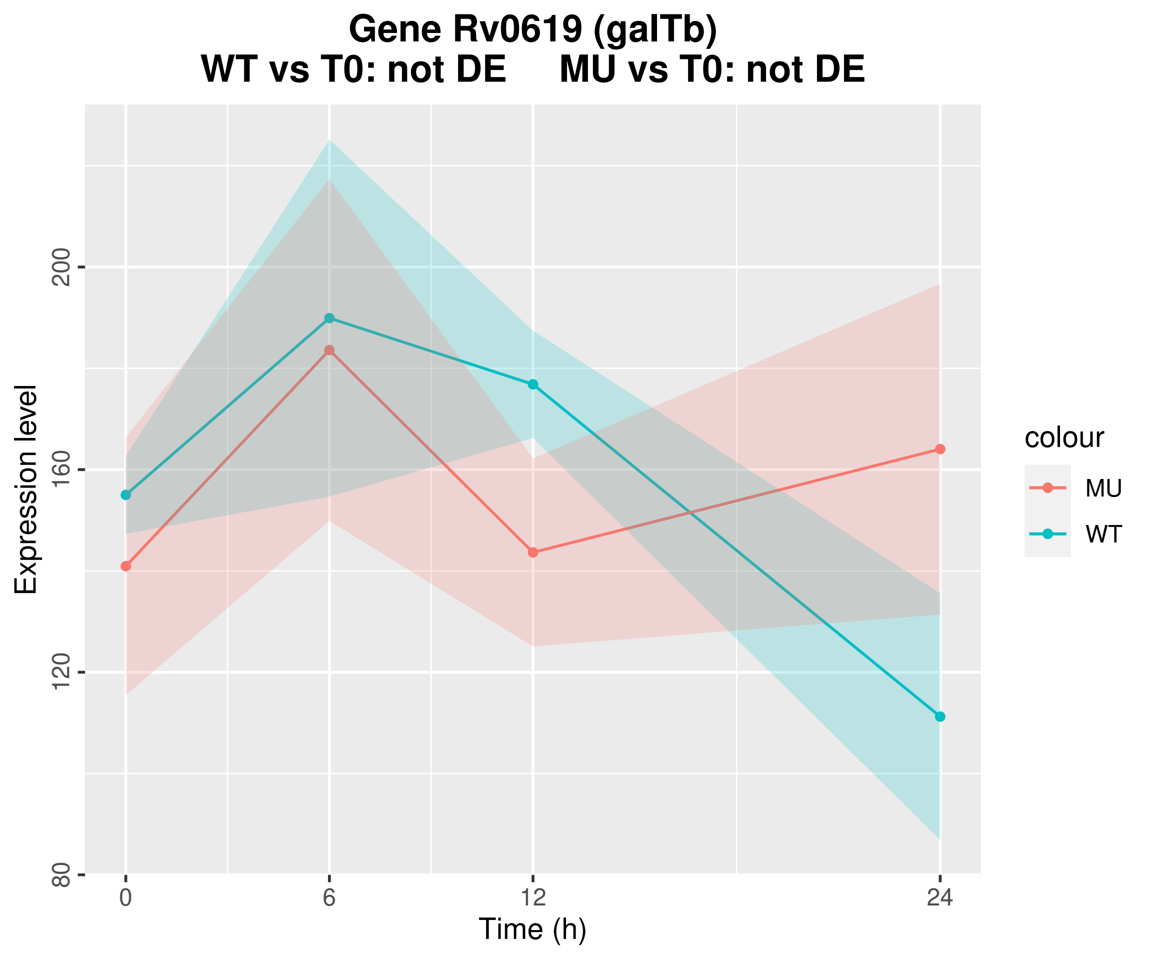
Gene Rv0619 (galTb)
WT vs T0: not DE   MU vs T0: not DE
Expression level
200
160
120
80
0
6
12
24
Time (h)
colour
MU
WT

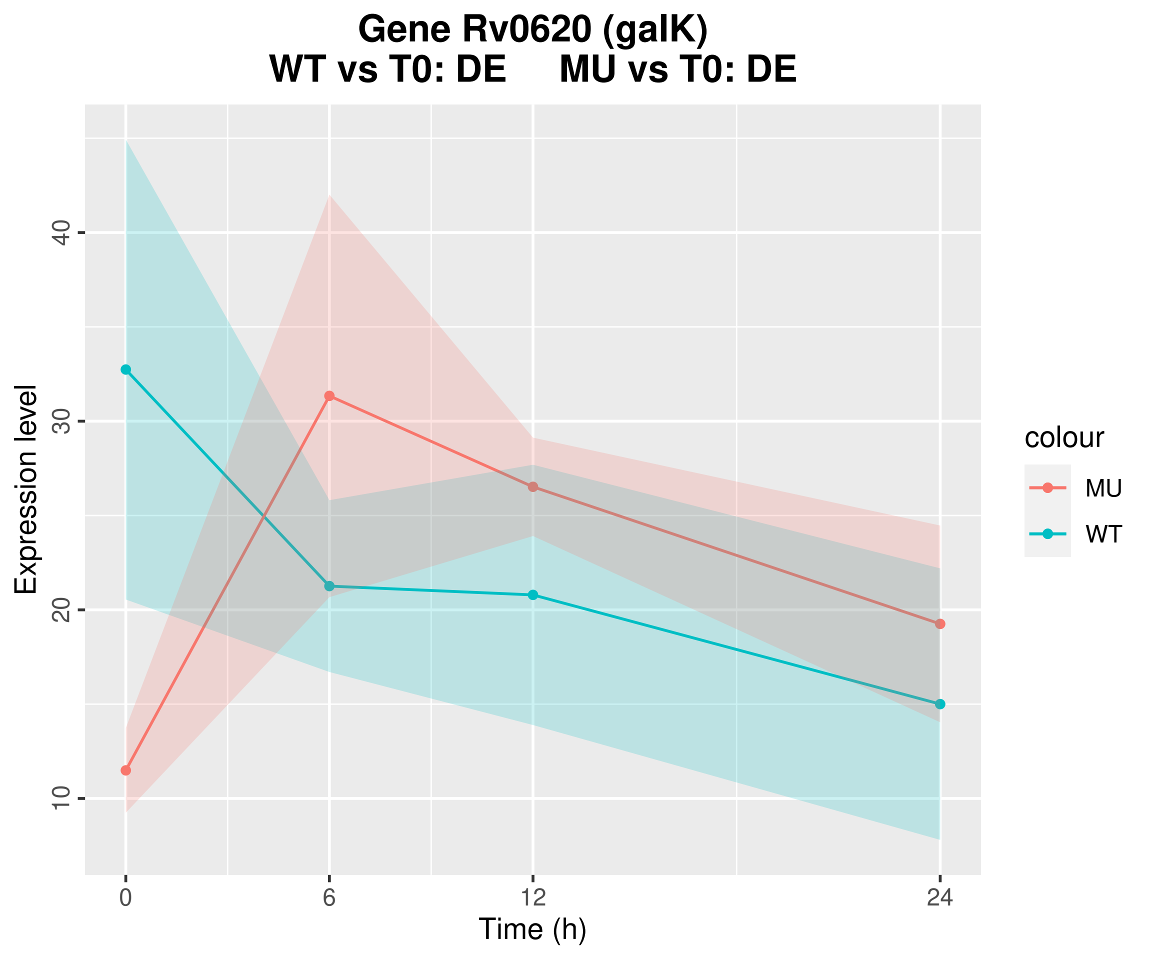

Gene Rv0620 (galK)
WT vs T0: DE MU vs T0: DE
Expression level
10
20
30
40
0
6
12
24
Time (h)
colour
MU
WT

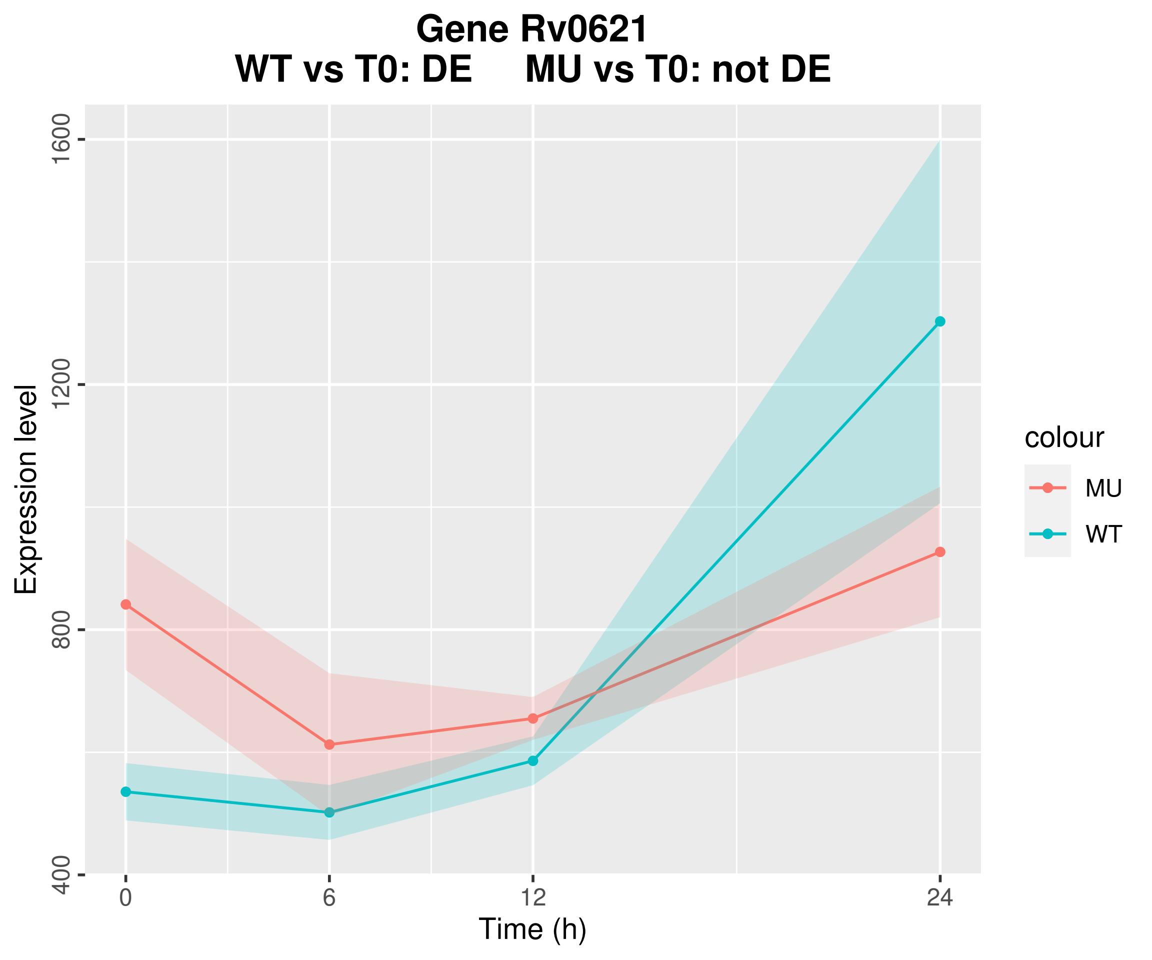

Gene Rv0621
WT vs T0: DE MU vs T0: not DE
Expression level
1600
1200
800
400
0
6
12
24
Time (h)
colour
MU
WT

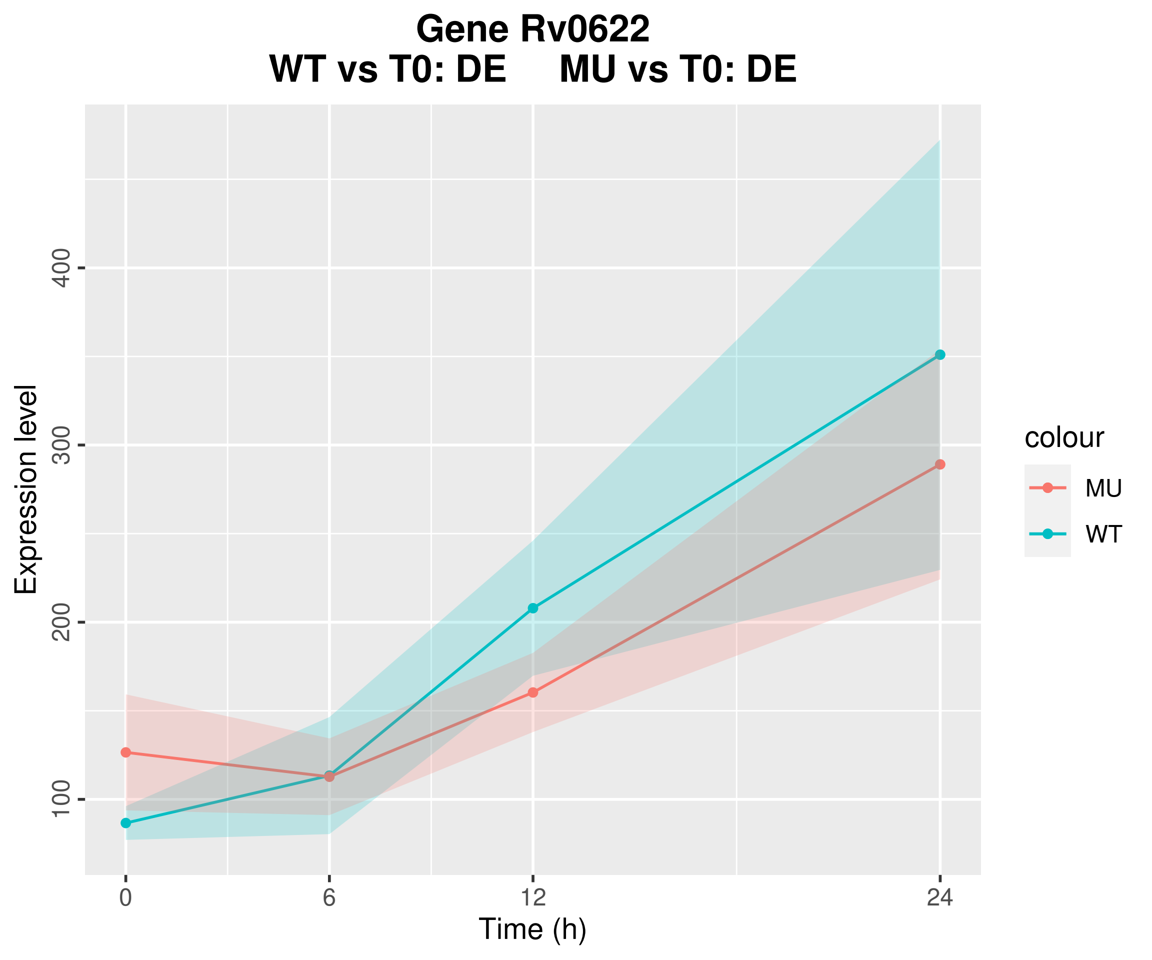

Gene Rv0622
WT vs T0: DE   MU vs T0: DE
Expression level
100
200
300
400
0
6
12
24
Time (h)
colour
MU
WT

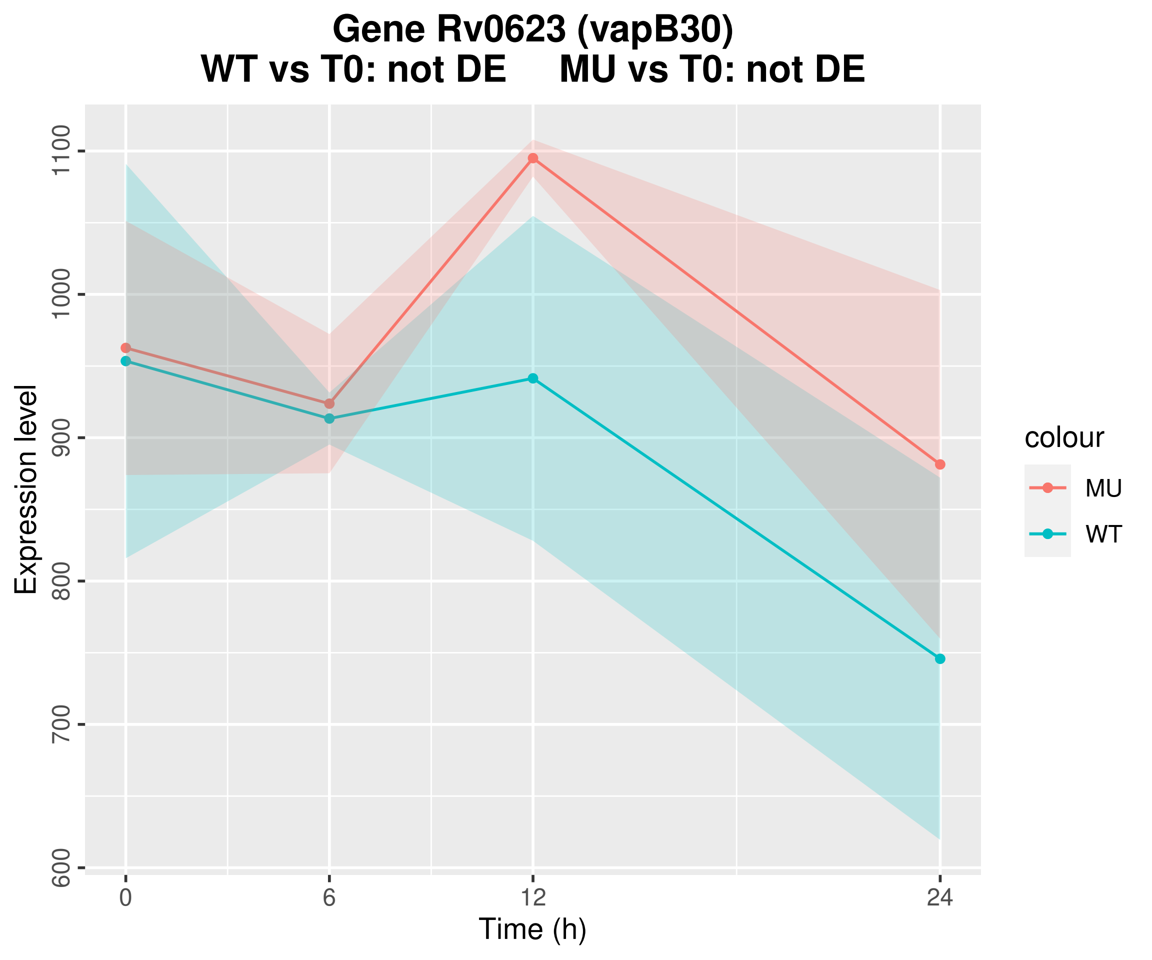
Gene Rv0623 (vapB30)
WT vs T0: not DE   MU vs T0: not DE
Expression level
1100
1000
900
800
700
600
0
6
12
24
Time (h)
colour
MU
WT

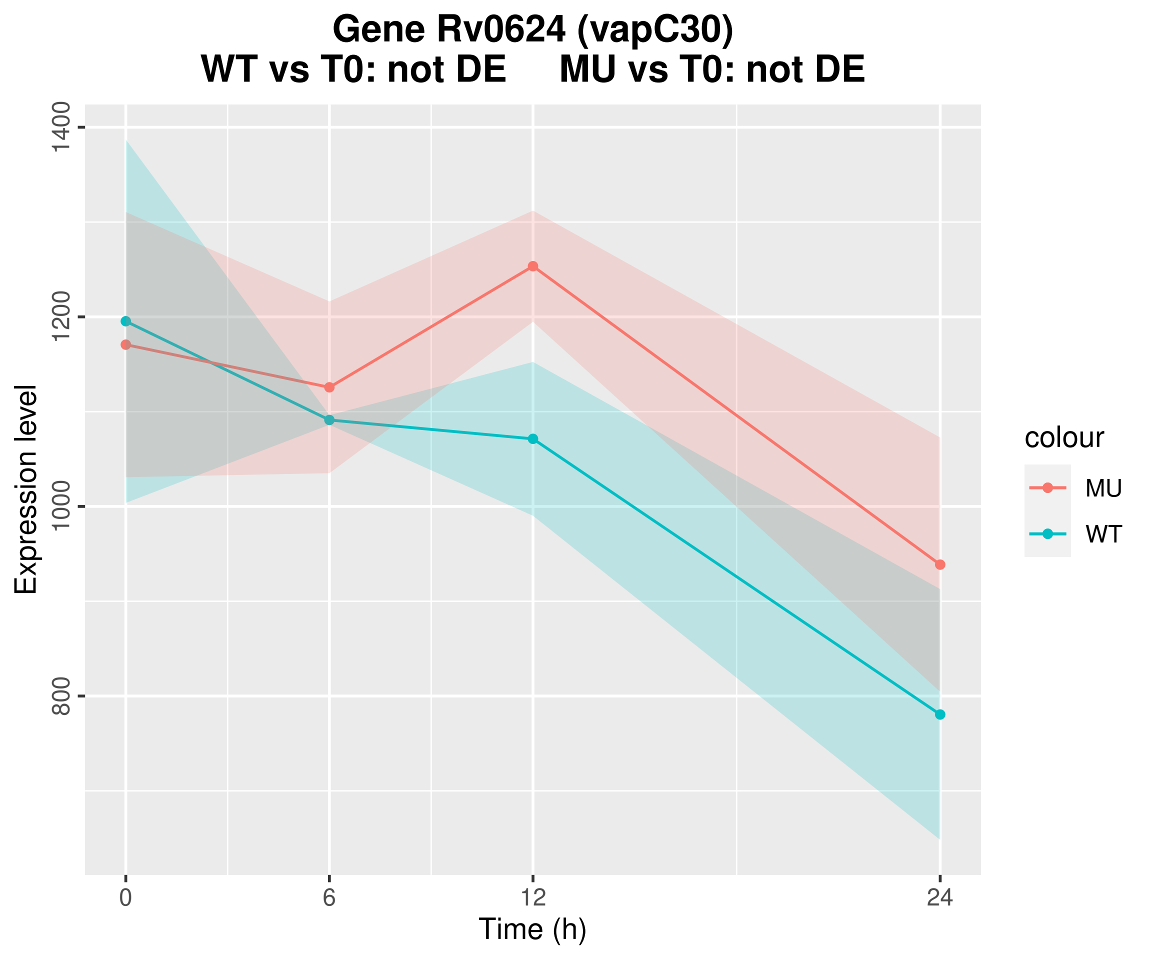

Gene Rv0624 (vapC30)
WT vs T0: not DE   MU vs T0: not DE
Expression level
1400
1200
1000
800
0
6
12
24
Time (h)
colour
MU
WT

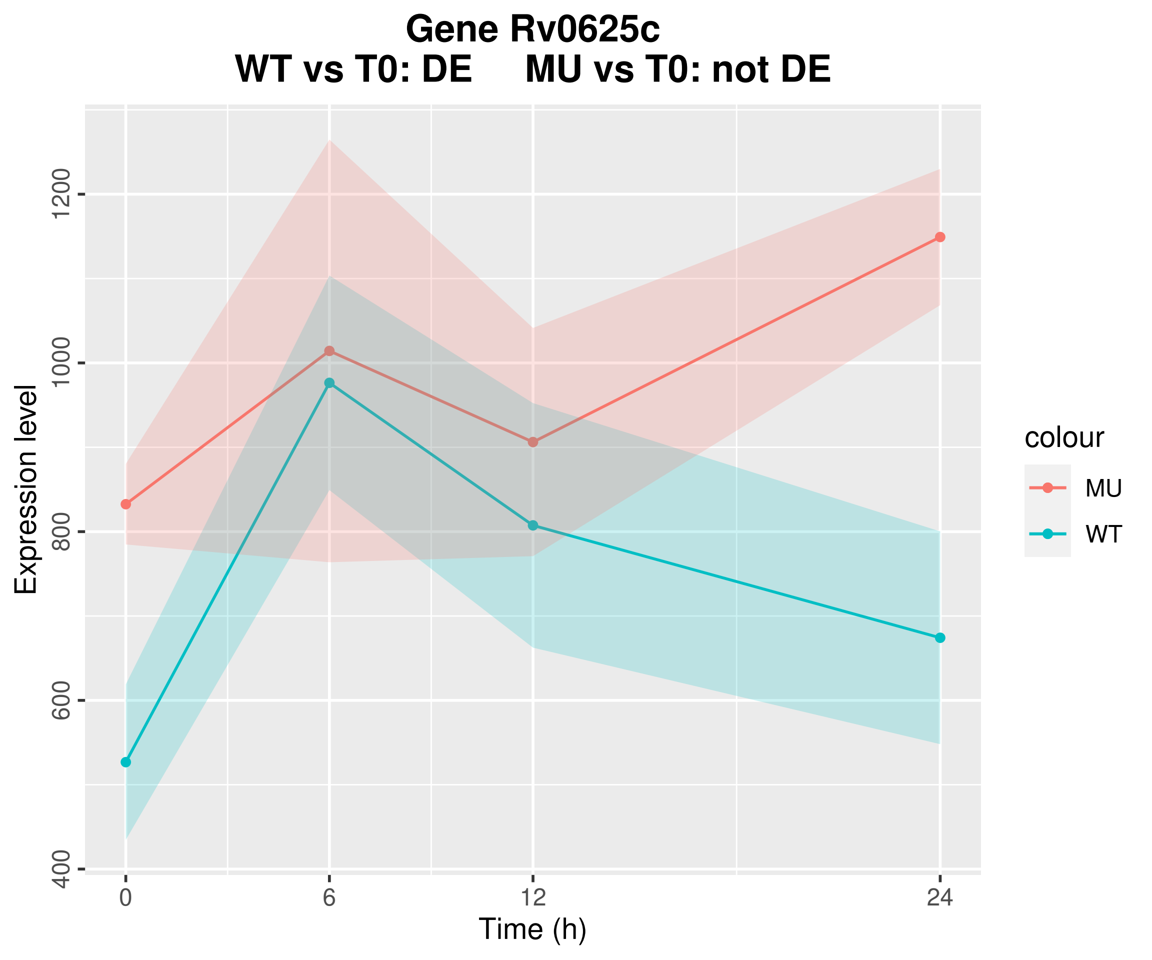
Gene Rv0625c
WT vs T0: DE MU vs T0: not DE
Expression level
1200
1000
800
600
400
0
6
12
24
Time (h)
colour
MU
WT

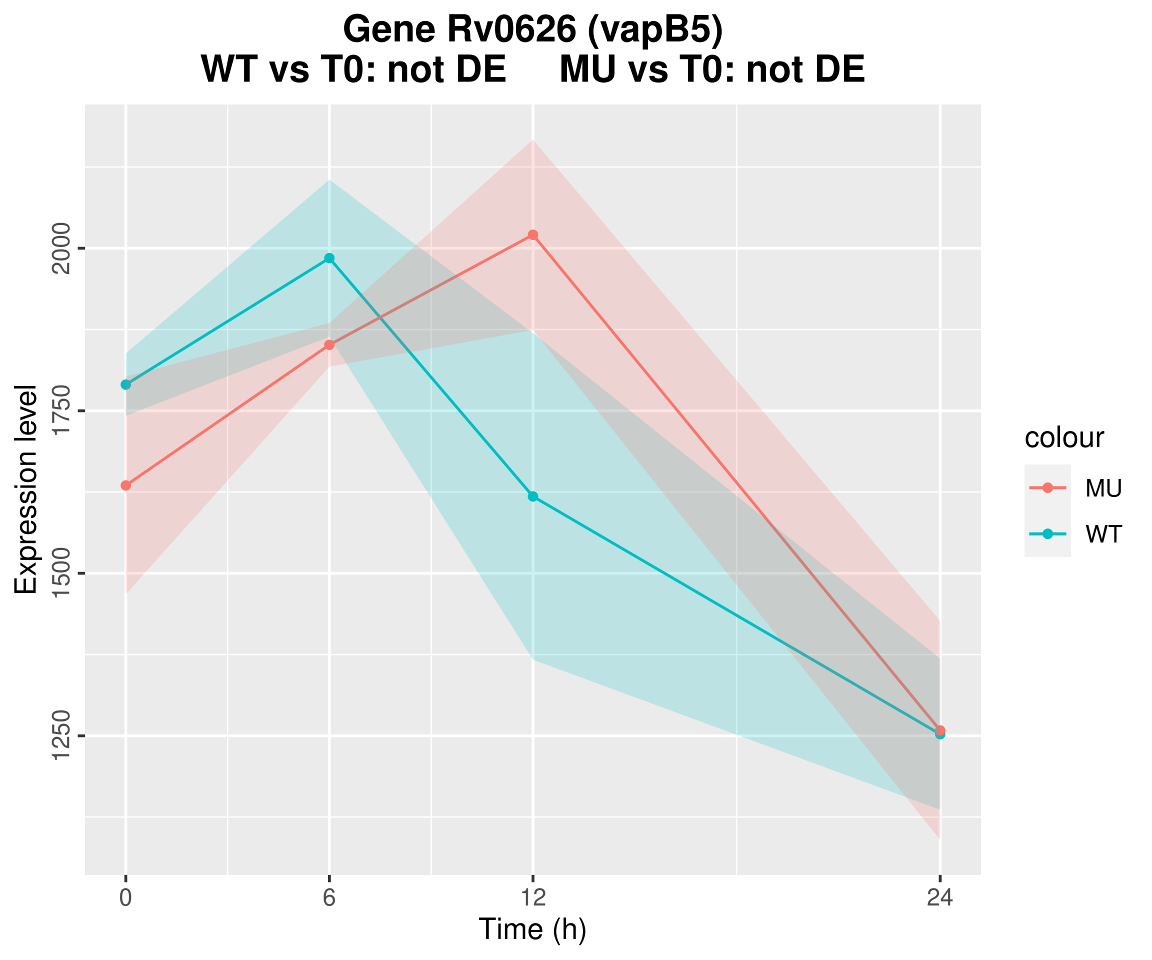
Gene Rv0626 (vapB5)
WT vs T0: not DE   MU vs T0: not DE
Expression level
2000
1750
1500
1250
0
6
12
24
Time (h)
colour
MU
WT

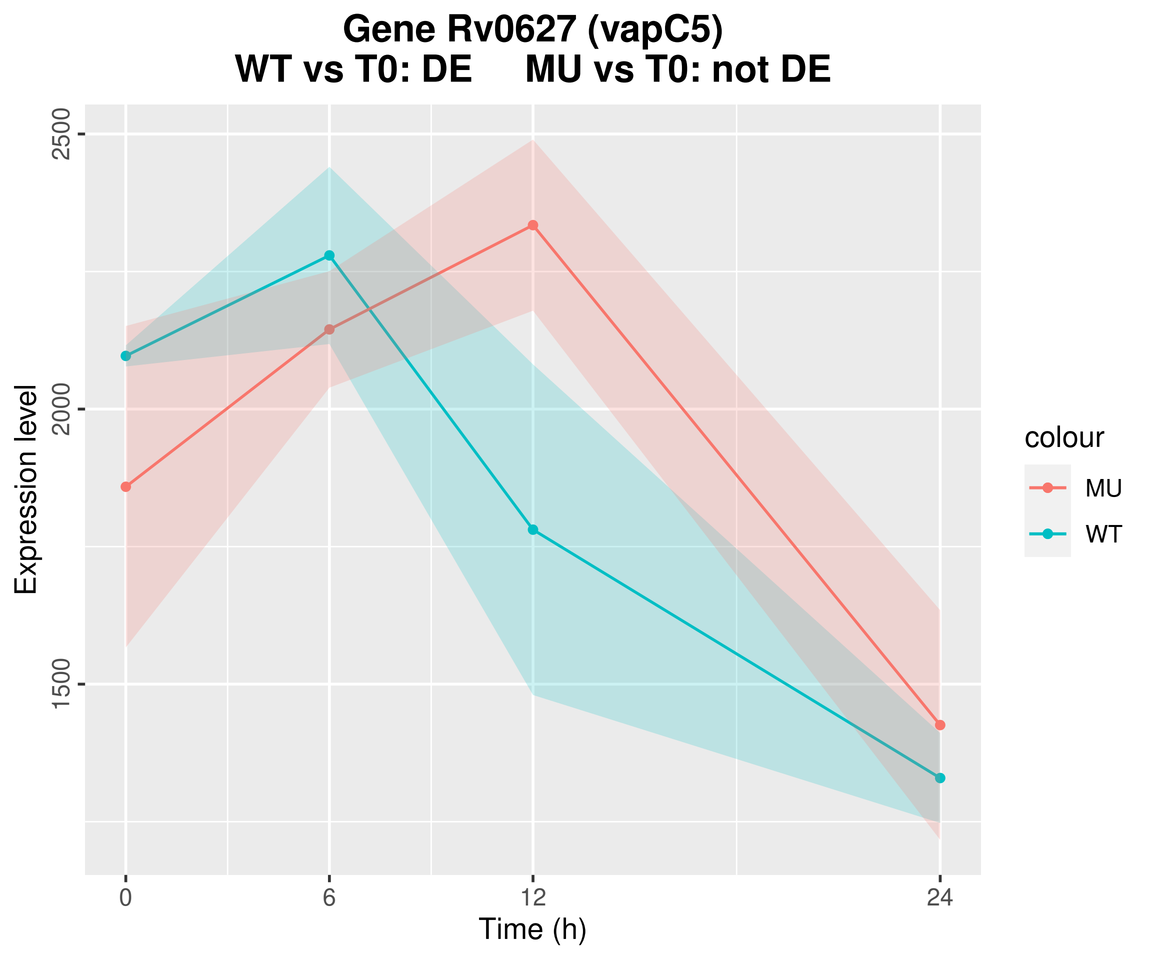

Gene Rv0627 (vapC5)
WT vs T0: DE MU vs T0: not DE
2500
2000
1500
Expression level
0
6
12
24
Time (h)
colour
MU
WT

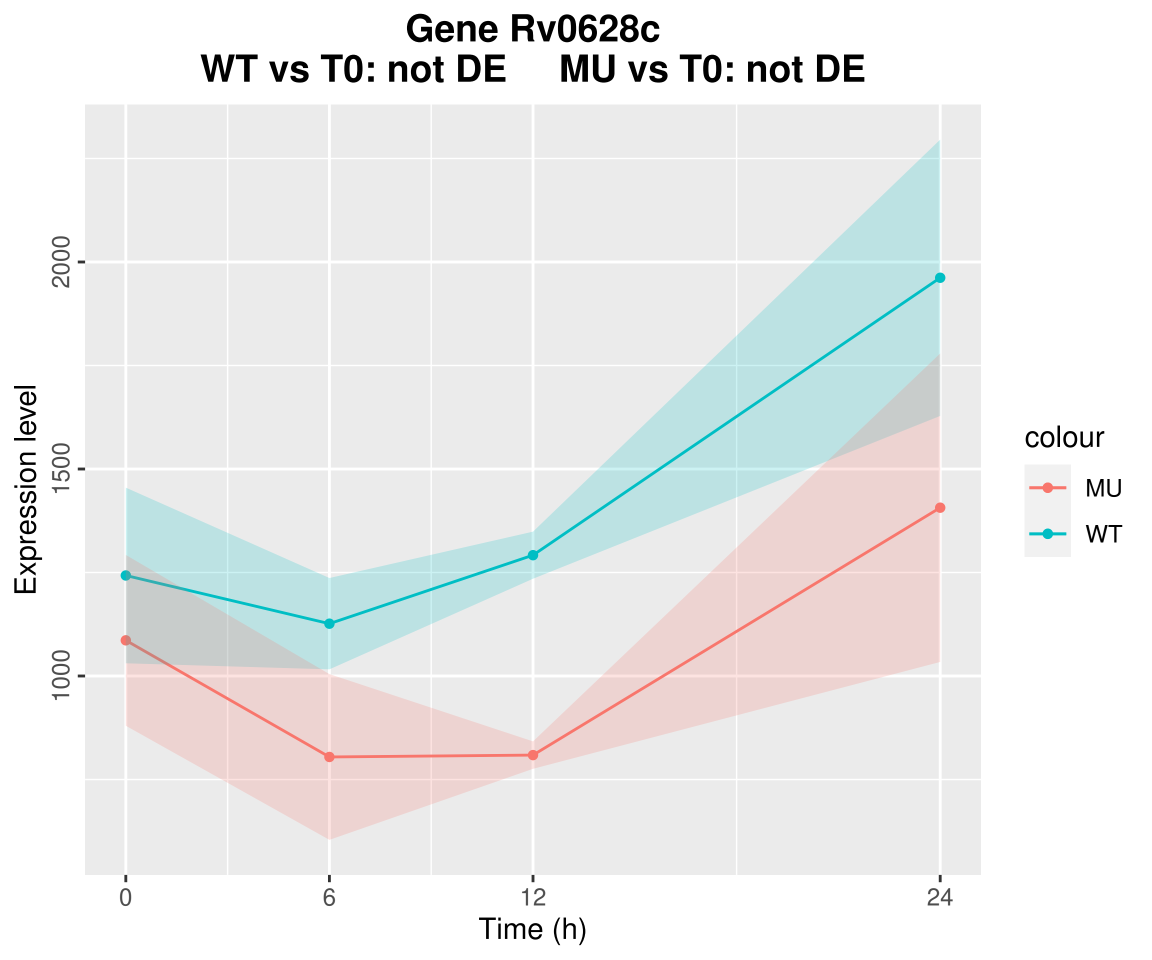
Gene Rv0628c
WT vs T0: not DE   MU vs T0: not DE
Expression level
2000
1500
1000
0
6
12
24
Time (h)
colour
MU
WT

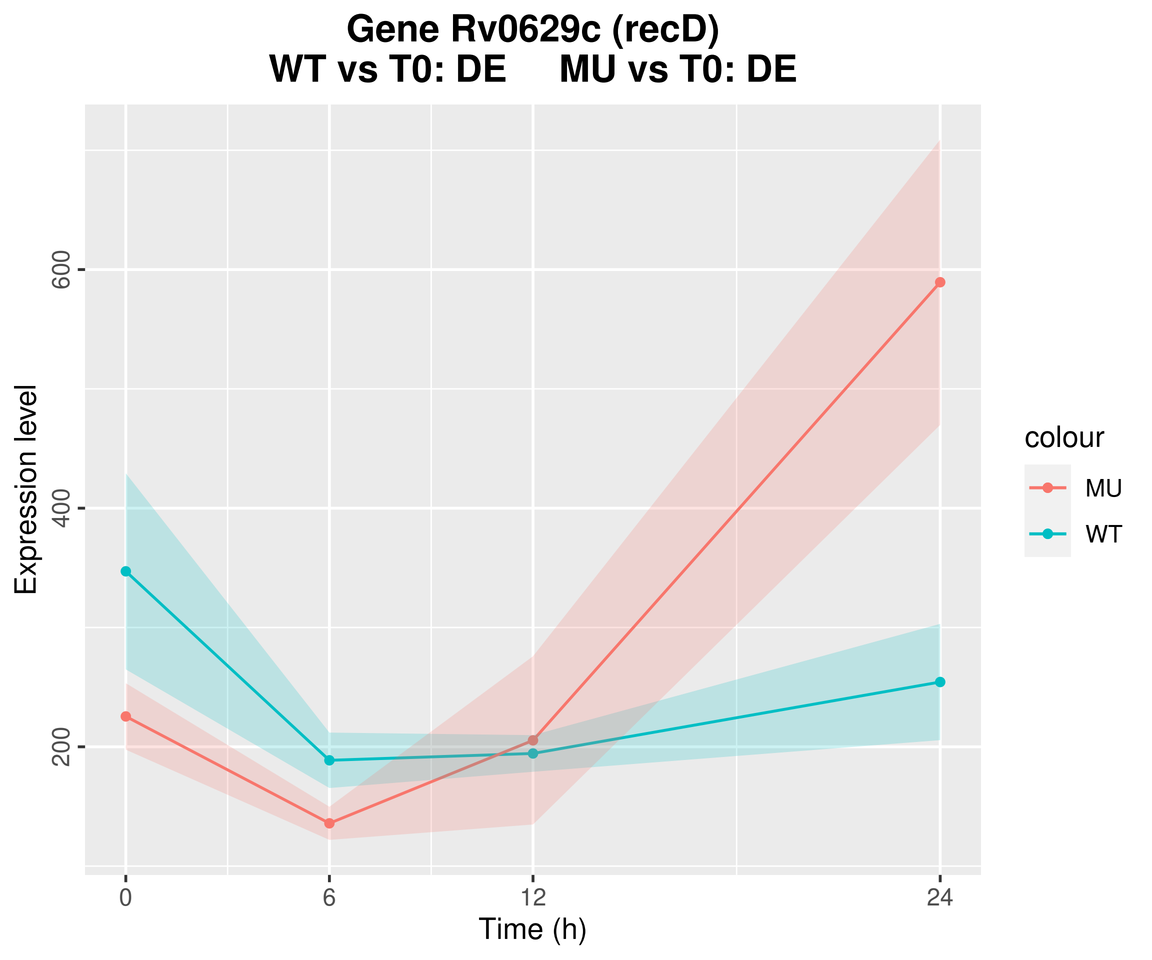

Gene Rv0629c (recD)
WT vs T0: DE MU vs T0: DE
Expression level
600
400
200
0
6
12
24
Time (h)
colour
MU
WT

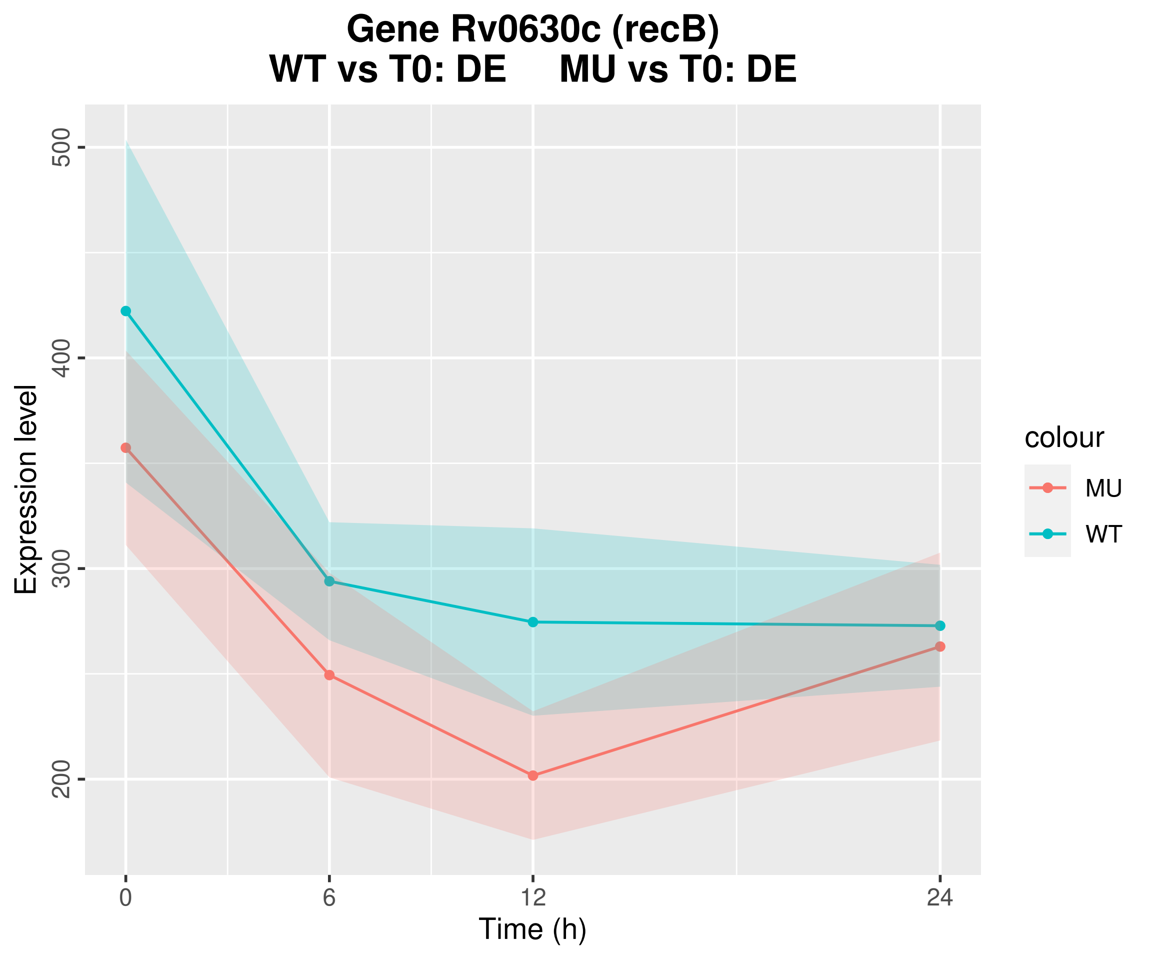

Gene Rv0630c (recB)
WT vs T0: DE MU vs T0: DE
Expression level
500
400
300
200
0
6
12
24
Time (h)
colour
MU
WT

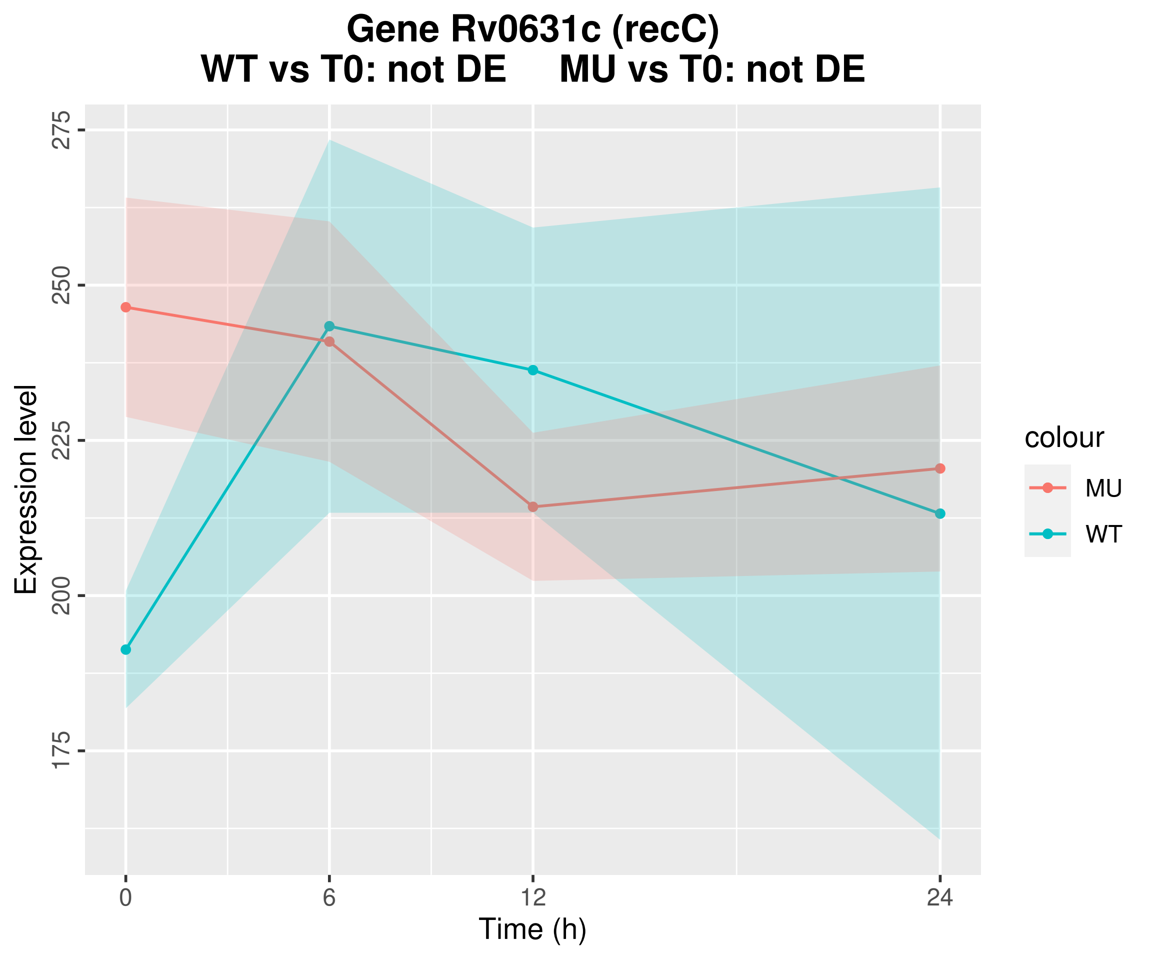

Gene Rv0631c (recC)
WT vs T0: not DE   MU vs T0: not DE
Expression level
275
250
225
200
175
0
6
12
24
Time (h)
colour
MU
WT

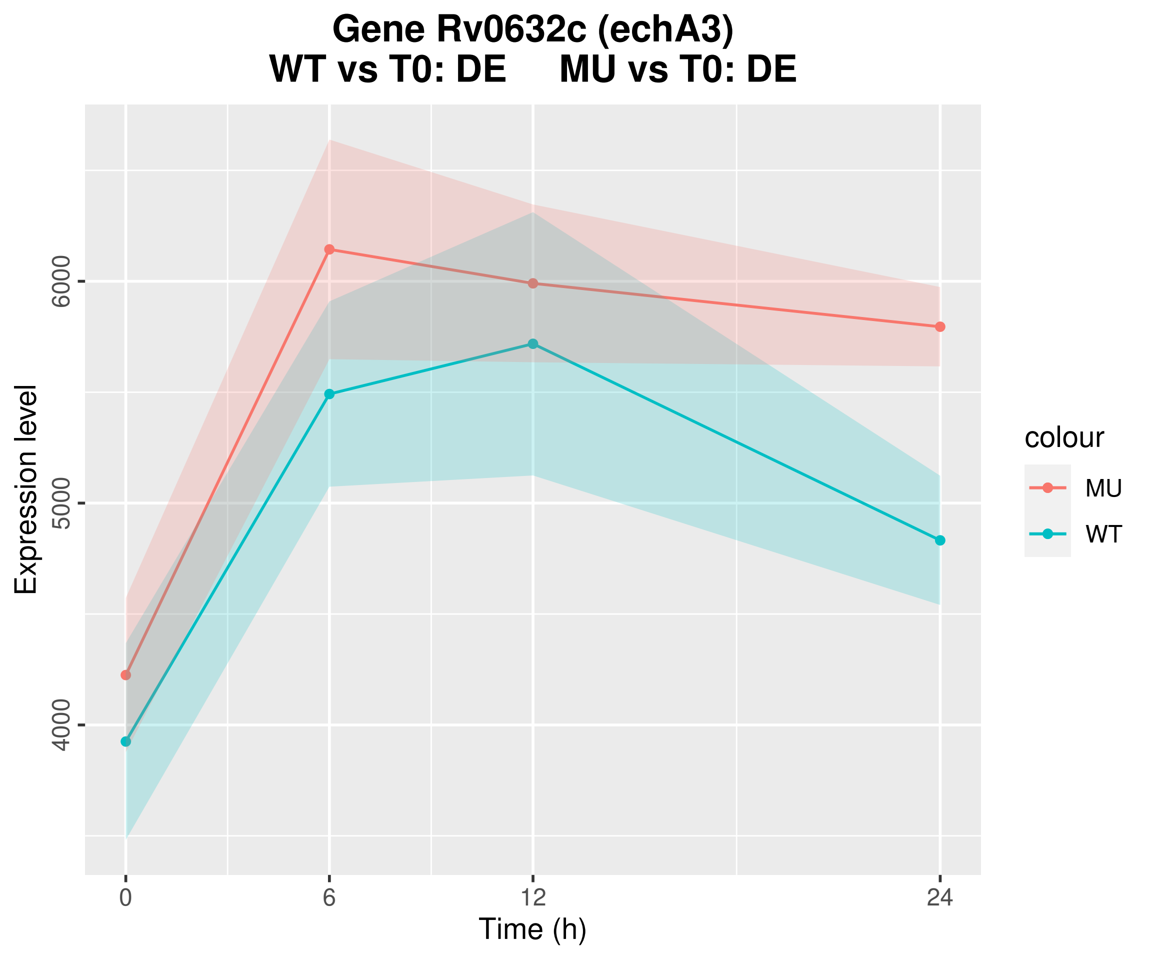

Gene Rv0632c (echA3)
WT vs T0: DE MU vs T0: DE
Expression level
6000
5000
4000
0
6
12
24
Time (h)
colour
MU
WT

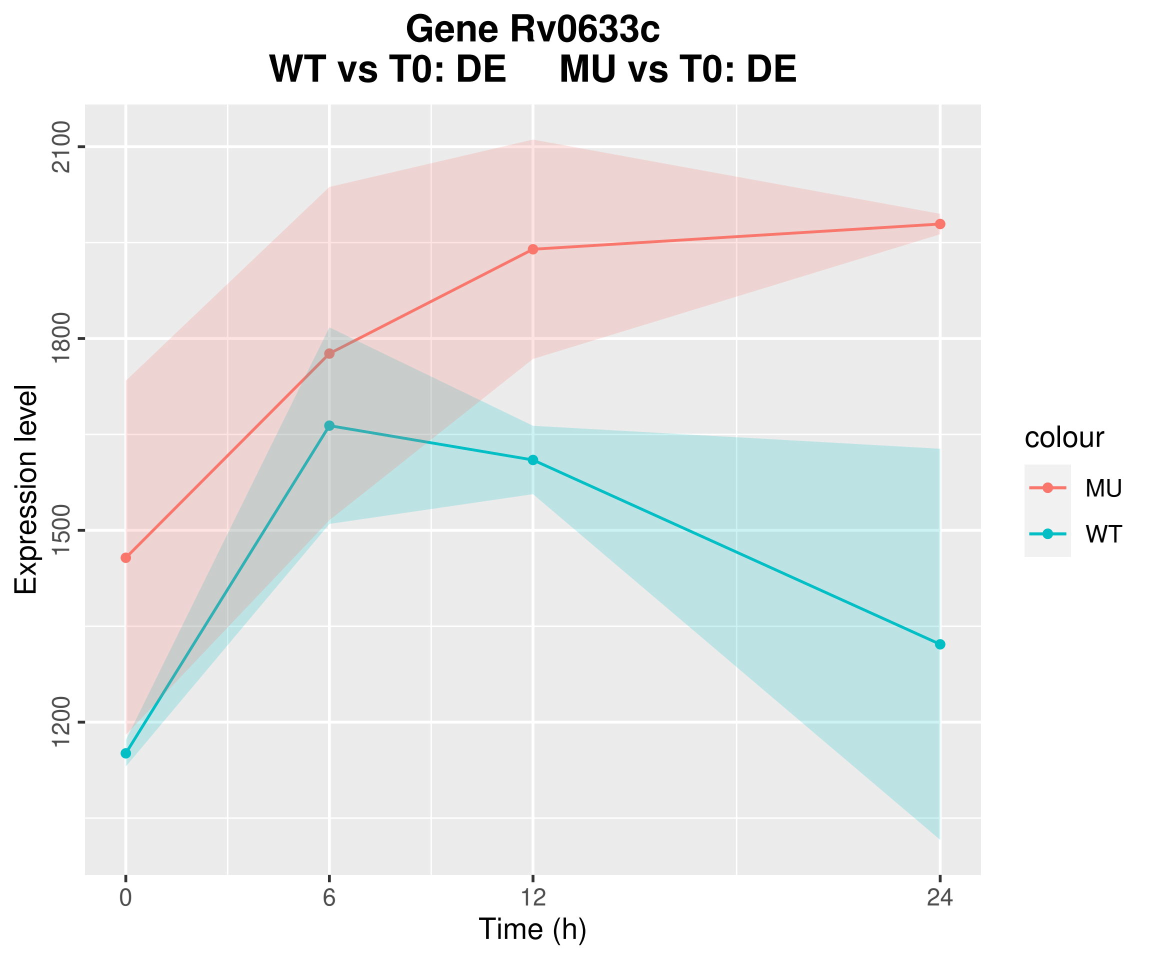

Gene Rv0633c
WT vs T0: DE   MU vs T0: DE
Expression level
2100
1800
1500
1200
Time (h)
0
6
12
24
colour
MU
WT

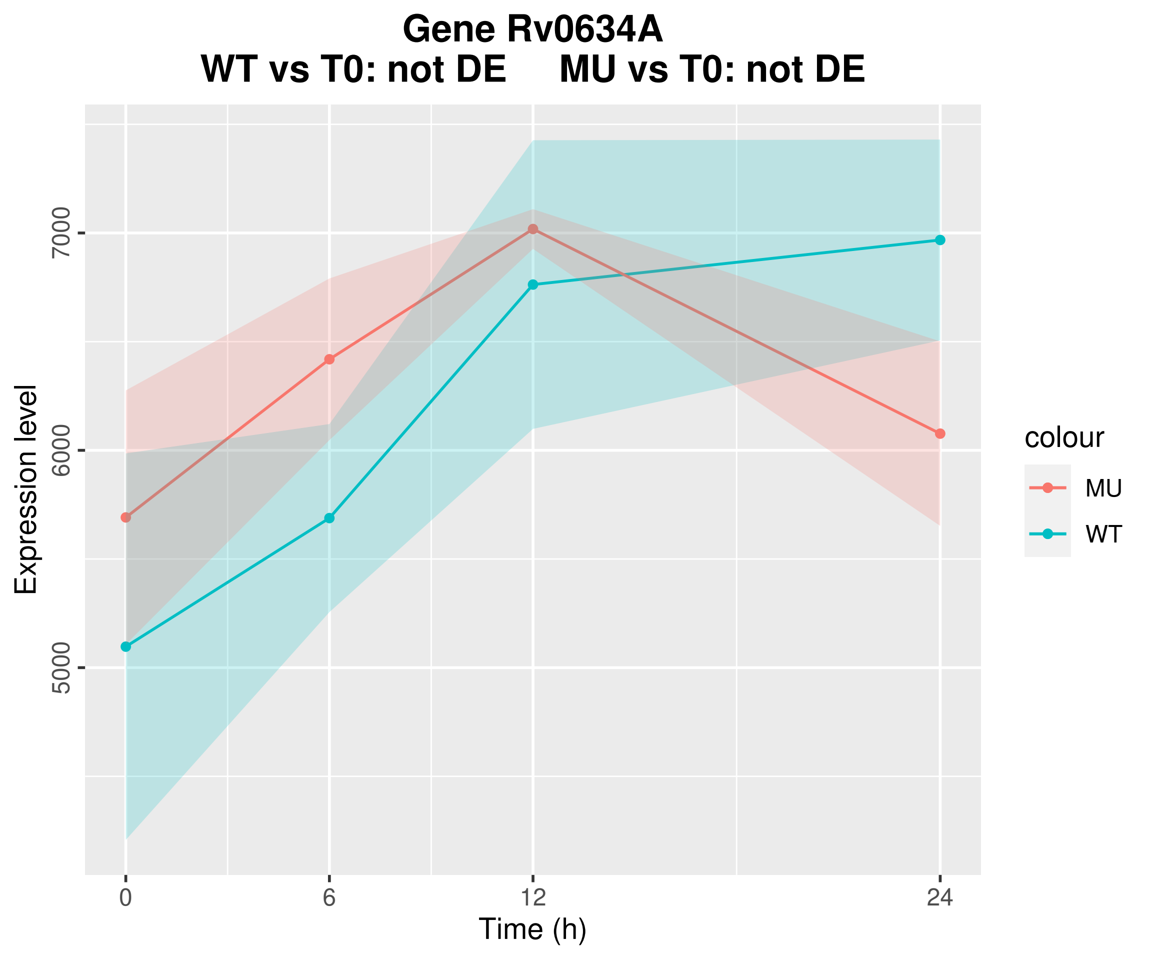
Gene Rv0634A
WT vs T0: not DE MU vs T0: not DE
Expression level
7000
6000
5000
0
6
12
24
Time (h)
colour
MU
WT

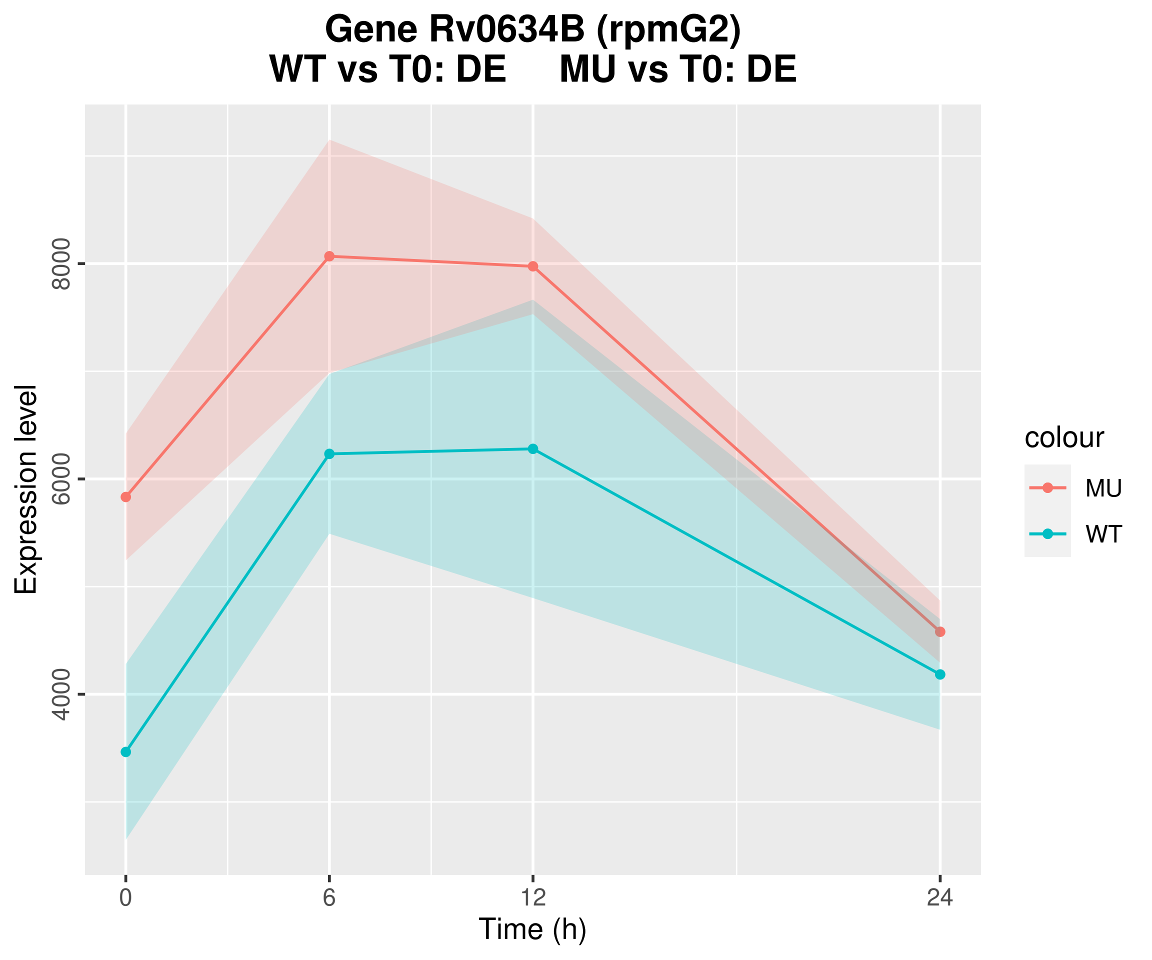
Gene Rv0634B (rpmG2)
WT vs T0: DE MU vs T0: DE
Expression level
8000
6000
4000
0
6
12
24
Time (h)
colour
MU
WT

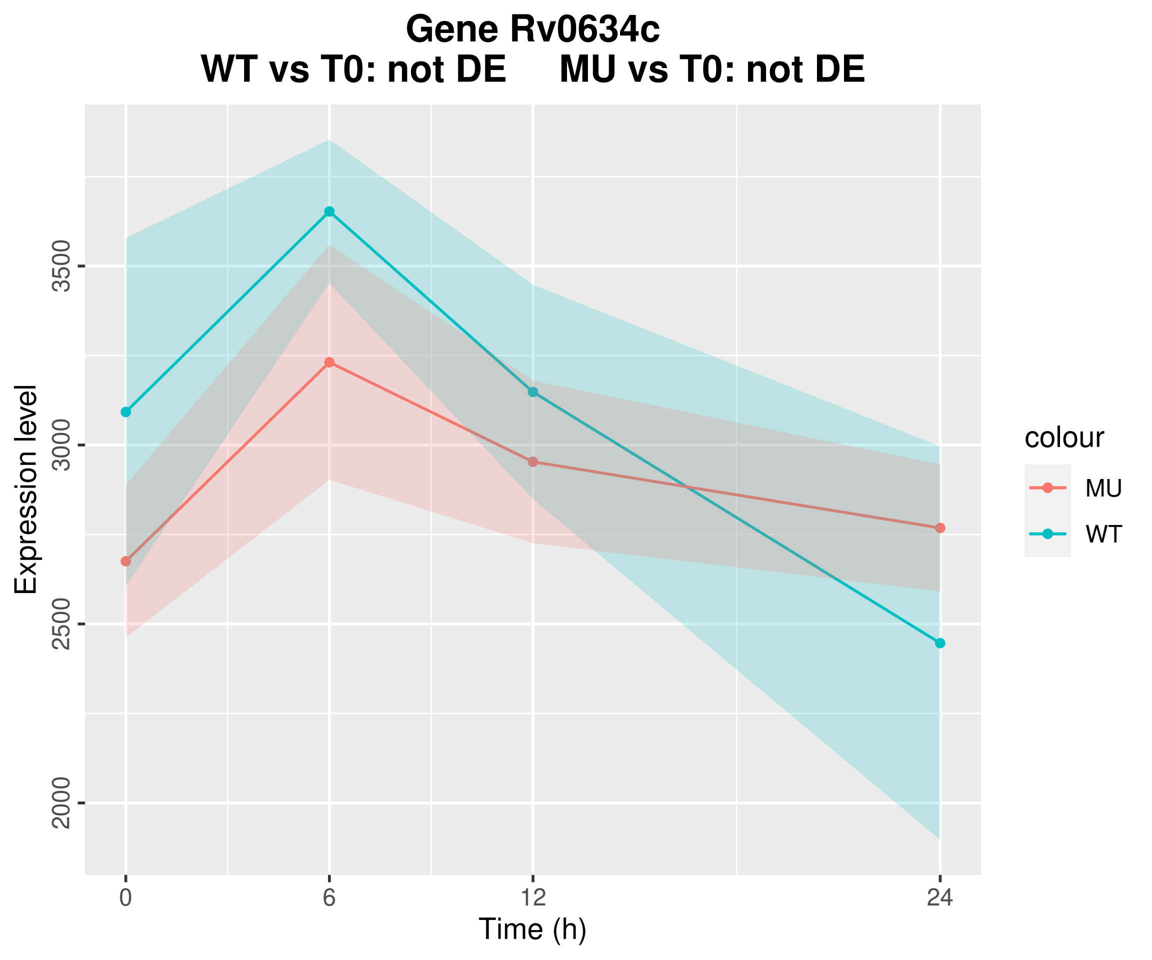

Gene Rv0634c
WT vs T0: not DE   MU vs T0: not DE
Expression level
3500
3000
2500
2000
0
6
12
24
Time (h)
colour
MU
WT

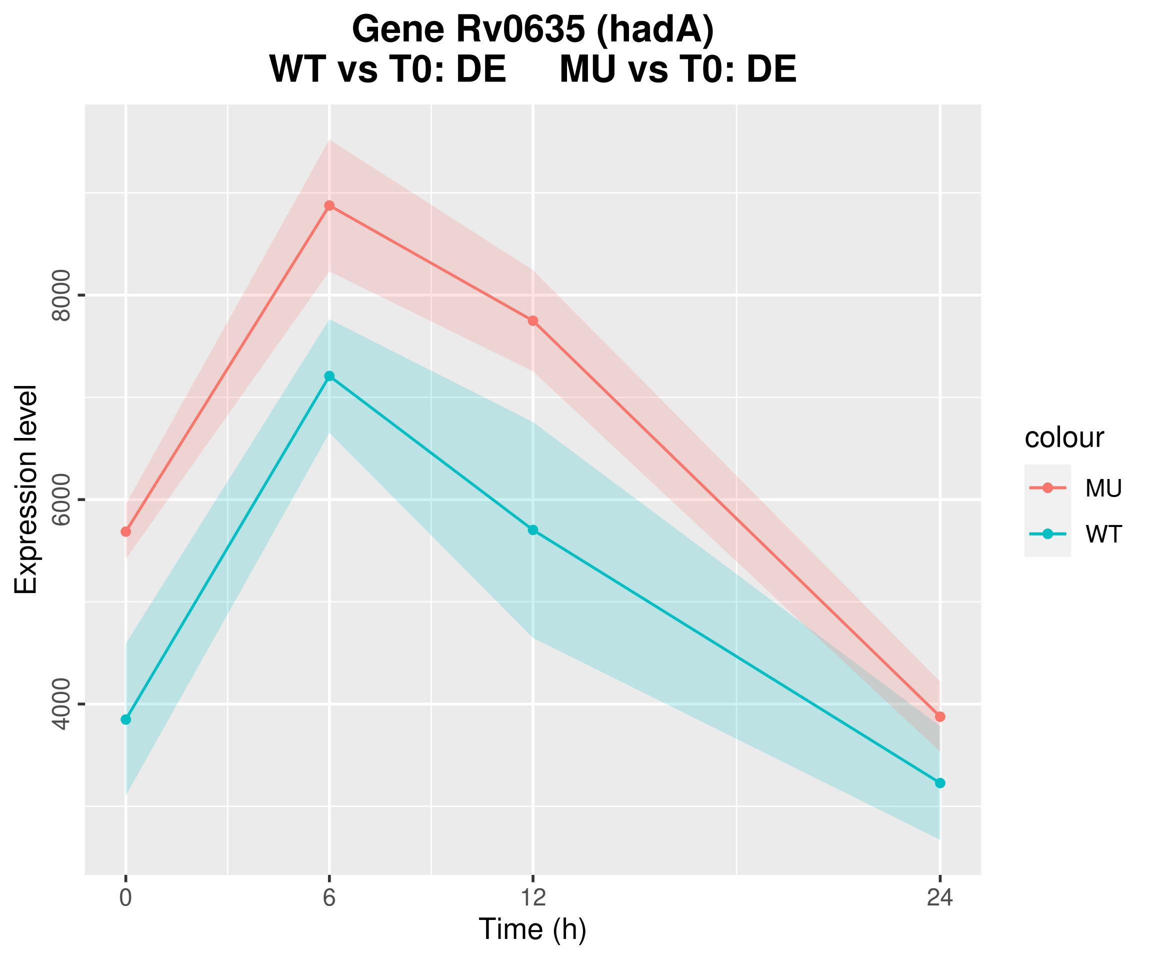

Gene Rv0635 (hadA)
WT vs T0: DE MU vs T0: DE
Expression level
8000
6000
4000
0
6
12
24
Time (h)
colour
MU
WT

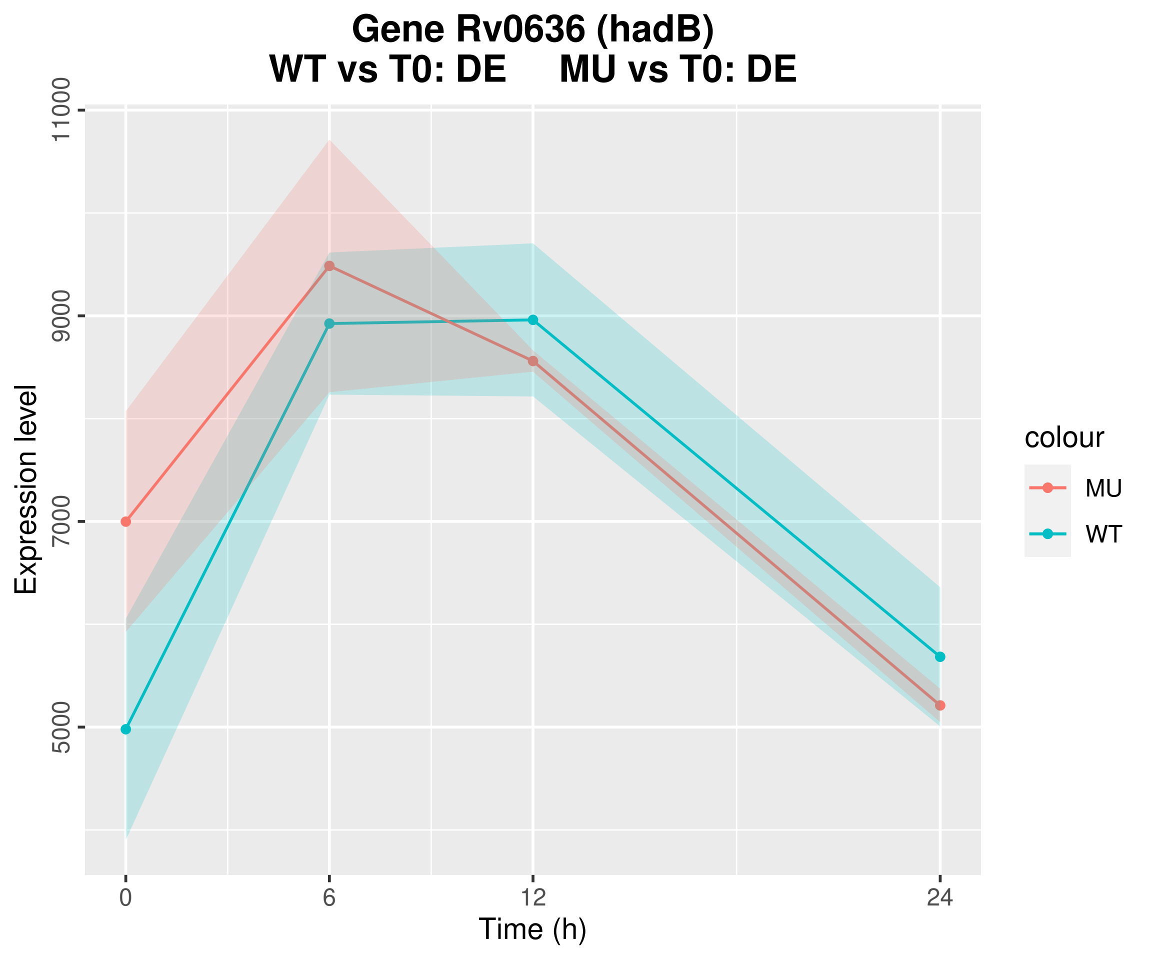

Gene Rv0636 (hadB)
WT vs T0: DE   MU vs T0: DE
Expression level
11000
9000
7000
5000
0
6
12
24
Time (h)
colour
MU
WT

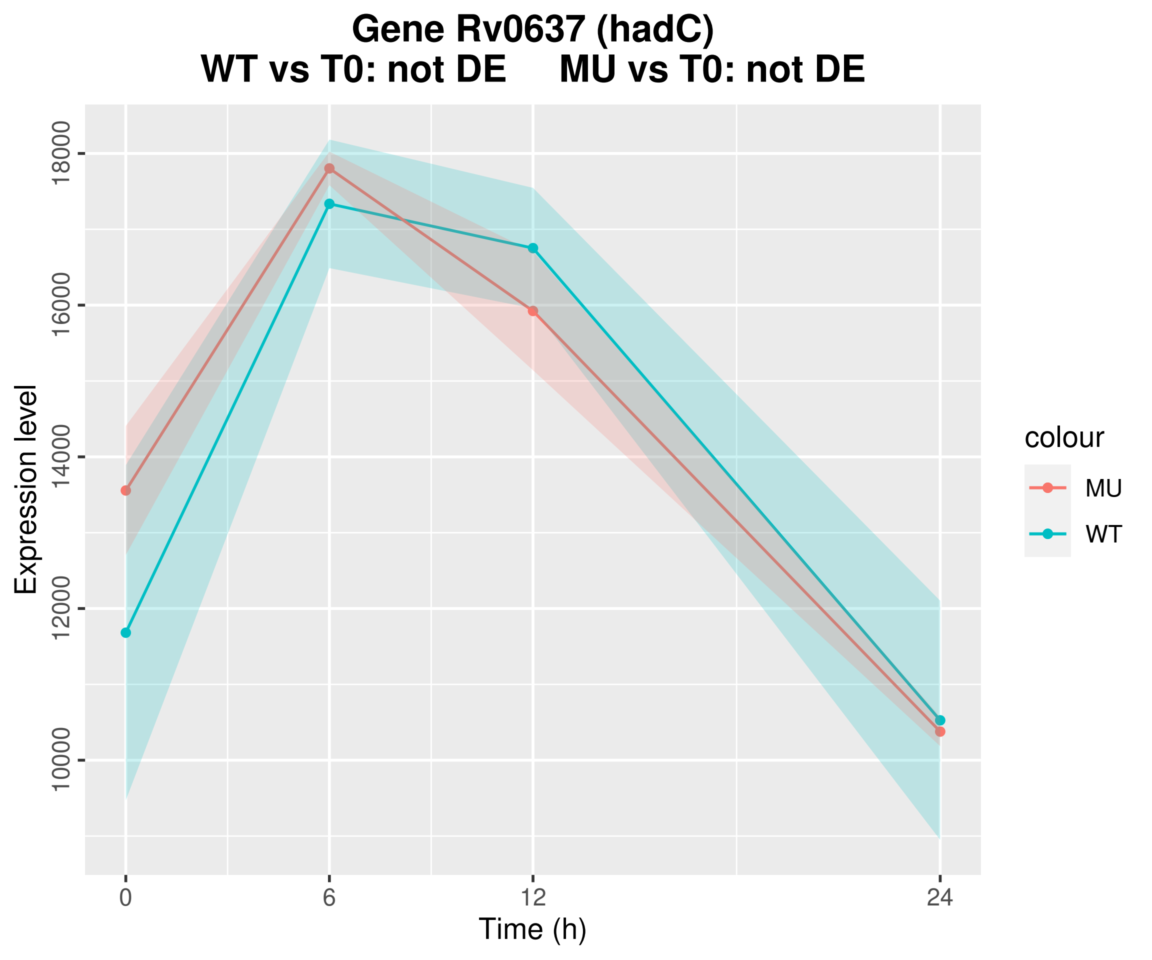
Gene Rv0637 (hadC)
WT vs T0: not DE MU vs T0: not DE
18000
16000
14000
12000
10000
Expression level
0
6
12
24
Time (h)
colour
MU
WT

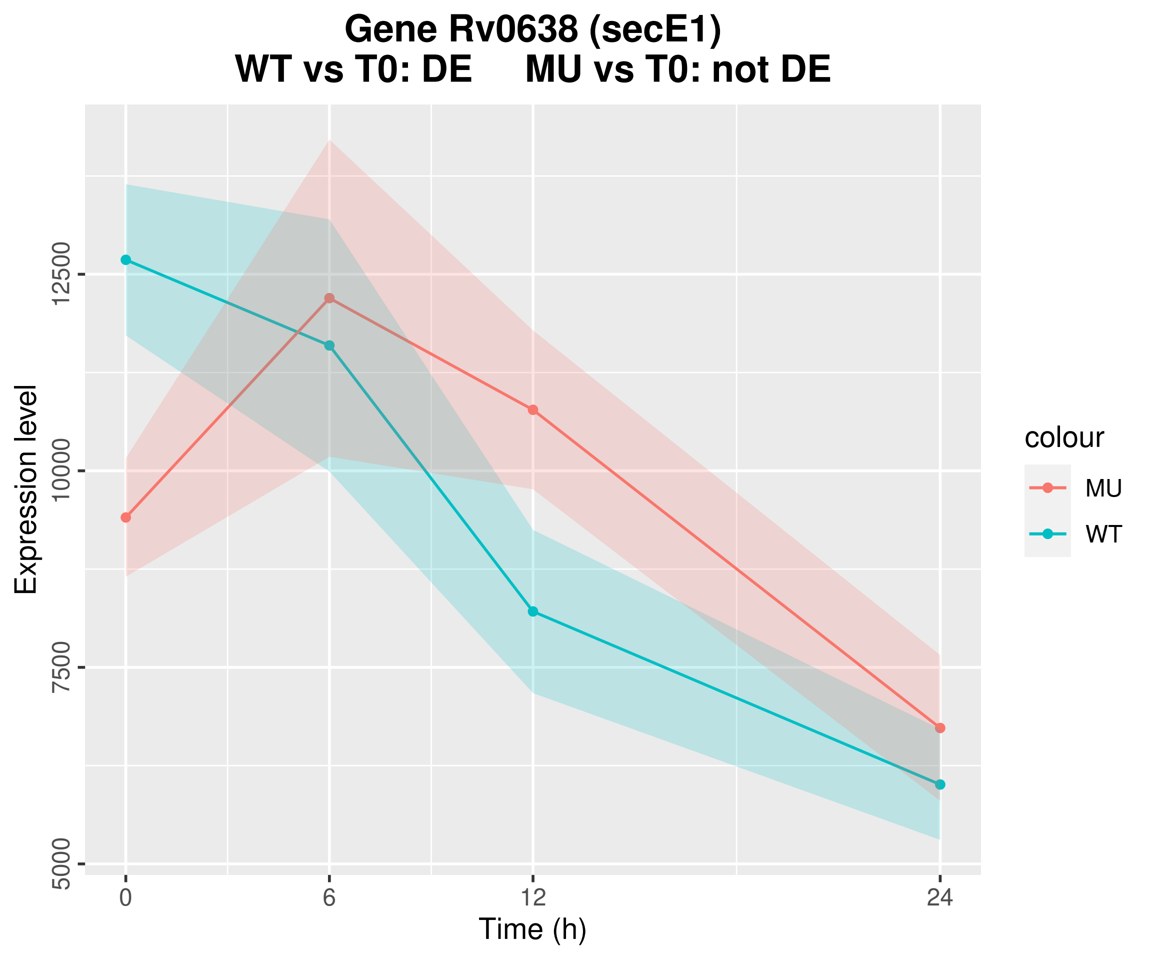
Gene Rv0638 (secE1)
WT vs T0: DE MU vs T0: not DE
Expression level
12500
10000
7500
5000
0
6
12
24
Time (h)
colour
MU
WT

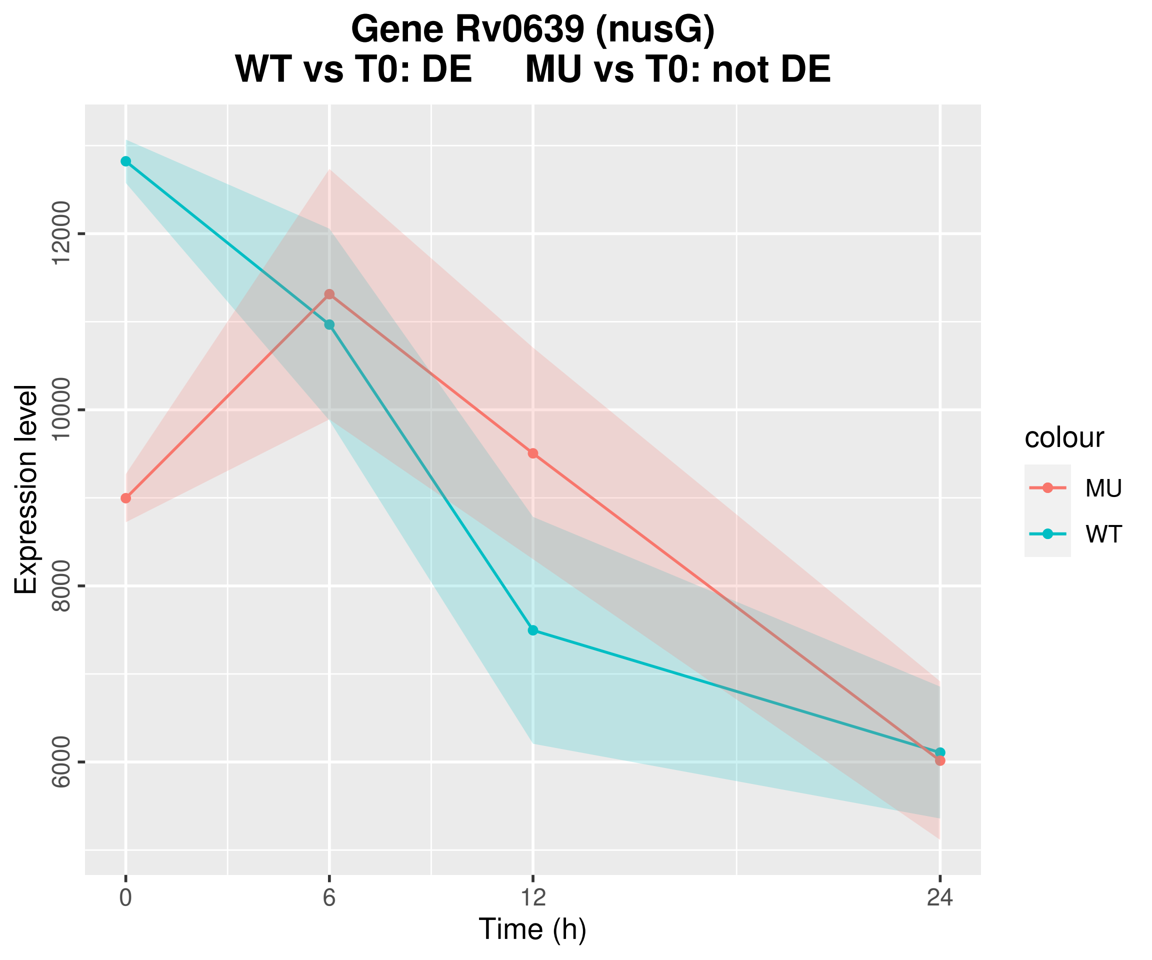

Gene Rv0639 (nusG)
WT vs T0: DE   MU vs T0: not DE
Expression level
12000
10000
8000
6000
0
6
12
24
Time (h)
colour
MU
WT

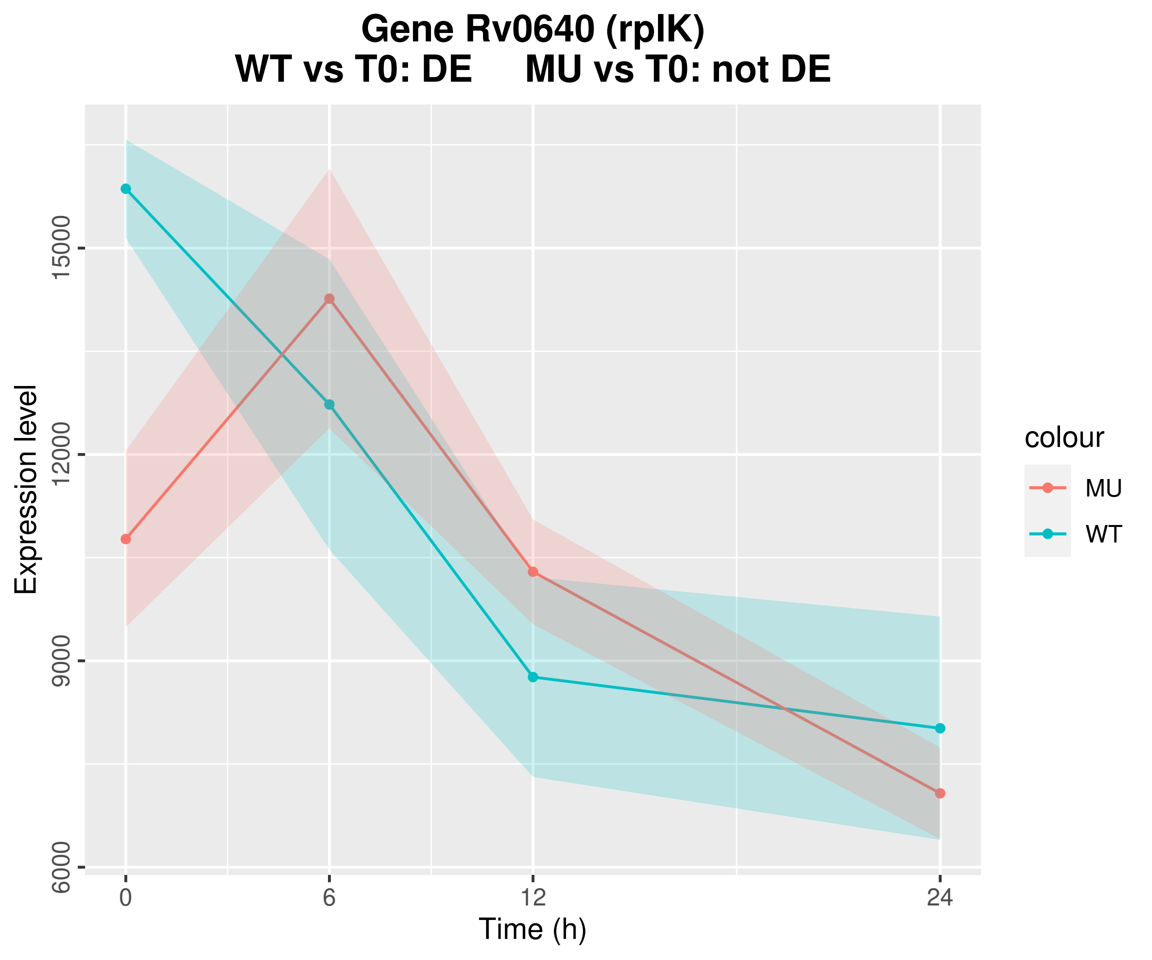

Gene Rv0640 (rplK)
WT vs T0: DE MU vs T0: not DE
Expression level
15000
12000
9000
6000
0
6
12
24
Time (h)
colour
MU
WT

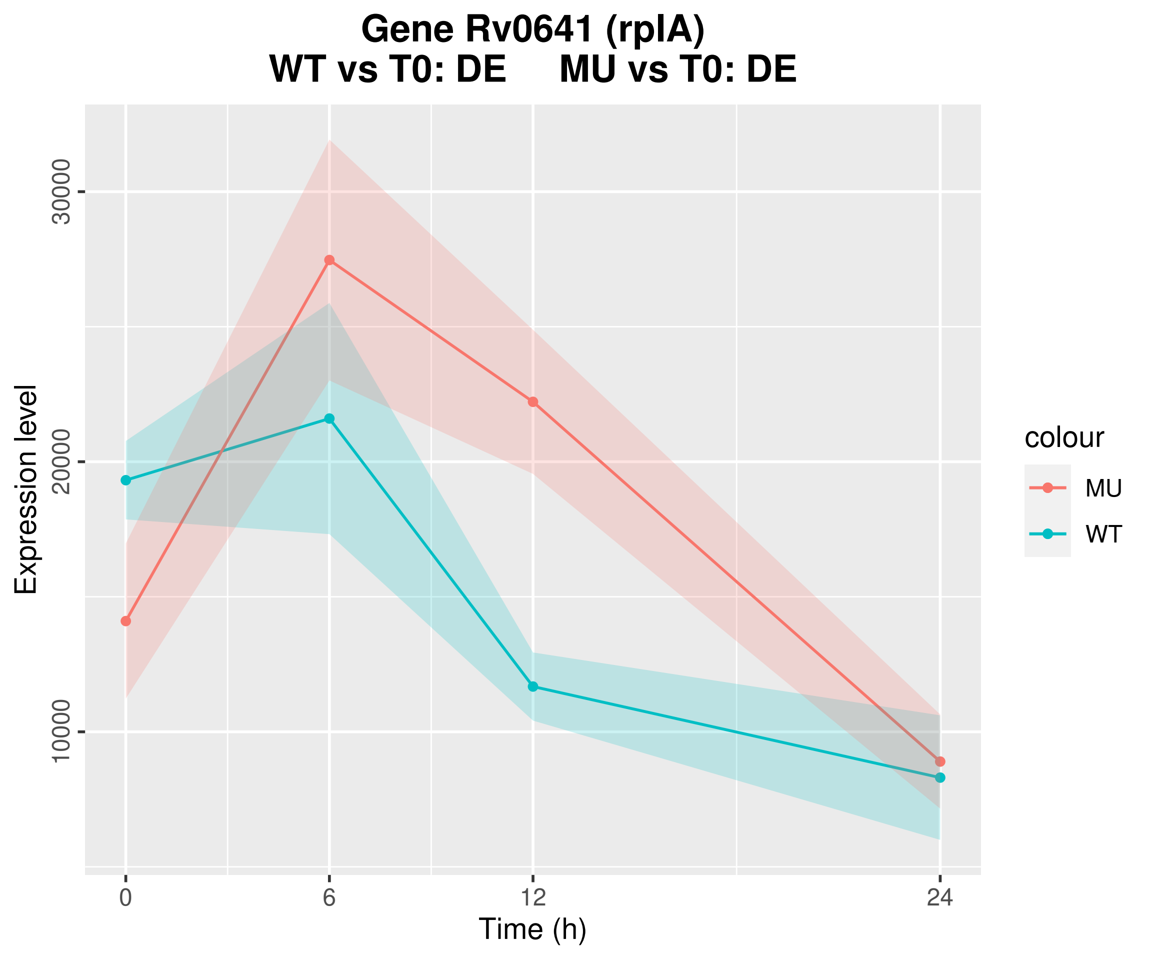

Gene Rv0641 (rplA)
WT vs T0: DE   MU vs T0: DE
30000
20000
10000
Expression level
0
6
12
24
Time (h)
colour
MU
WT

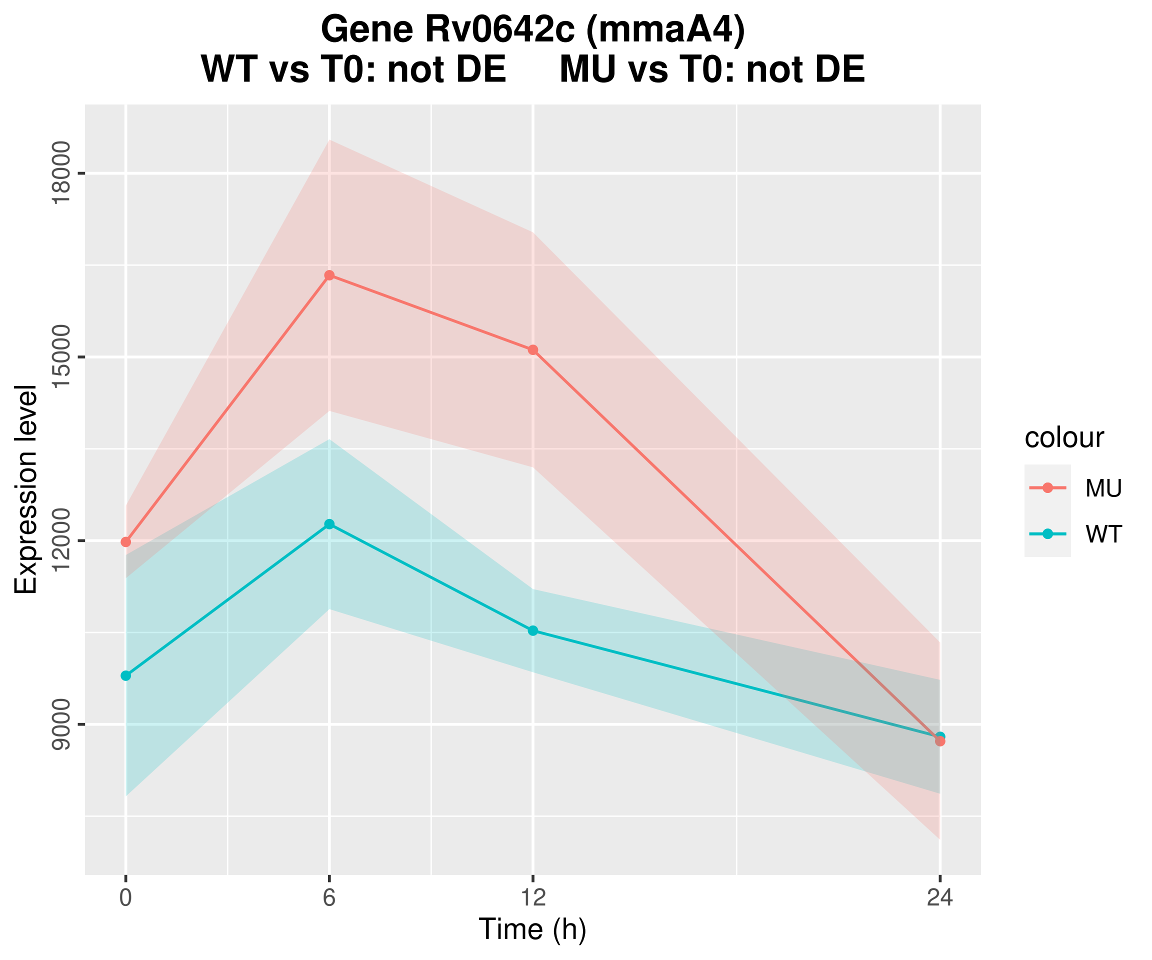

Gene Rv0642c (mmaA4)
WT vs T0: not DE MU vs T0: not DE
Expression level
18000
15000
12000
9000
0
6
12
24
Time (h)
colour
MU
WT

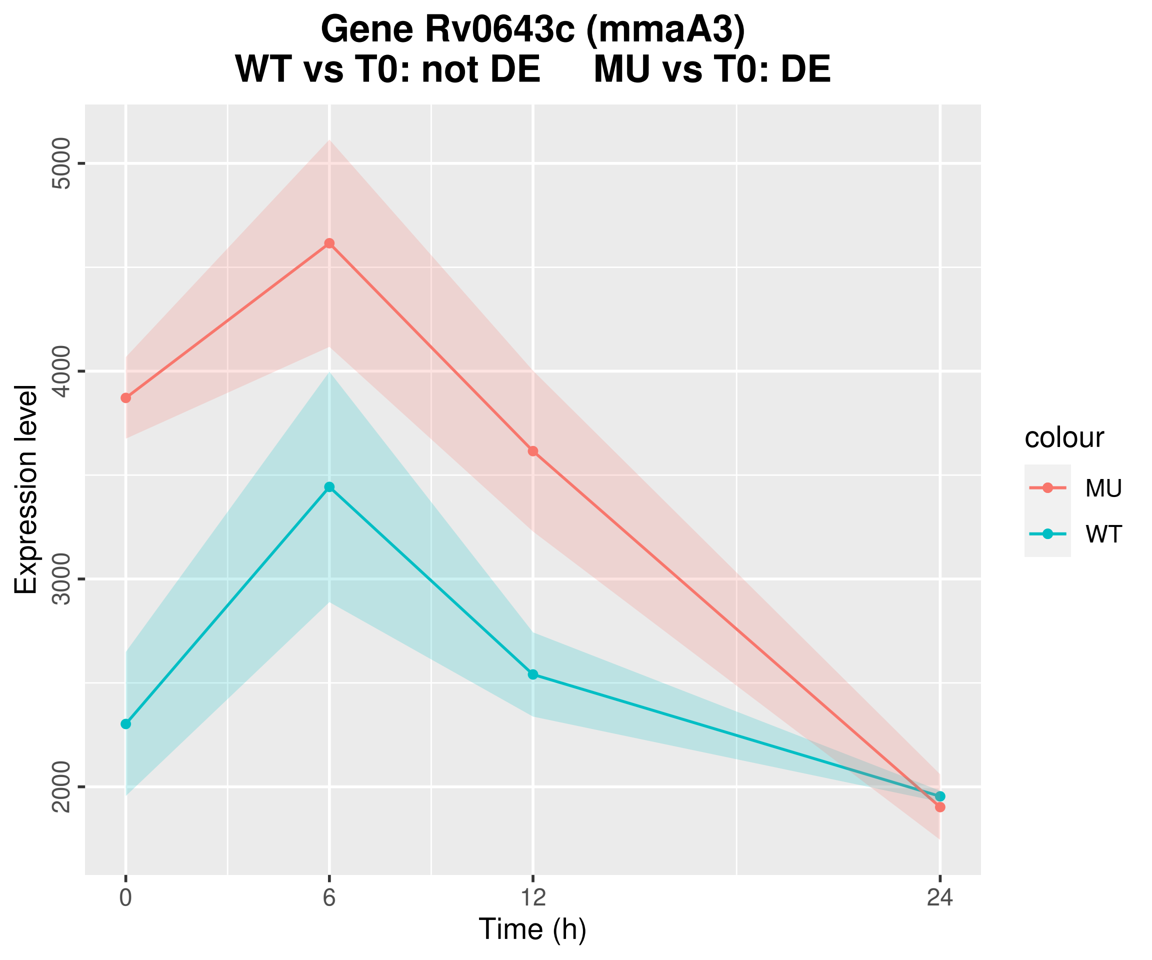
Gene Rv0643c (mmaA3)
WT vs T0: not DE   MU vs T0: DE
Expression level
5000
4000
3000
2000
0
6
12
24
Time (h)
colour
MU
WT

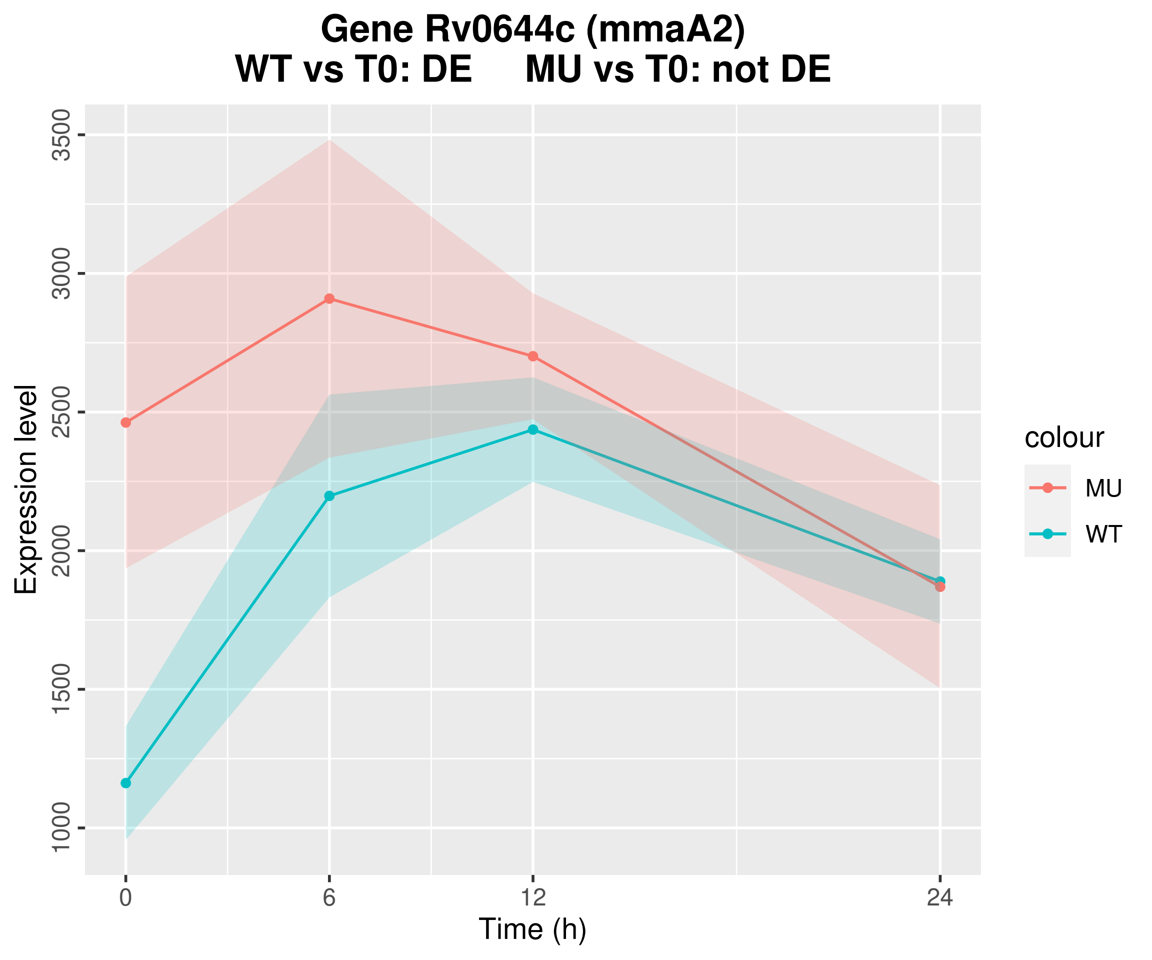
Gene Rv0644c (mmaA2)
WT vs T0: DE   MU vs T0: not DE
Expression level
Time (h)
0
6
12
24
1000
1500
2000
2500
3000
3500
colour
MU
WT

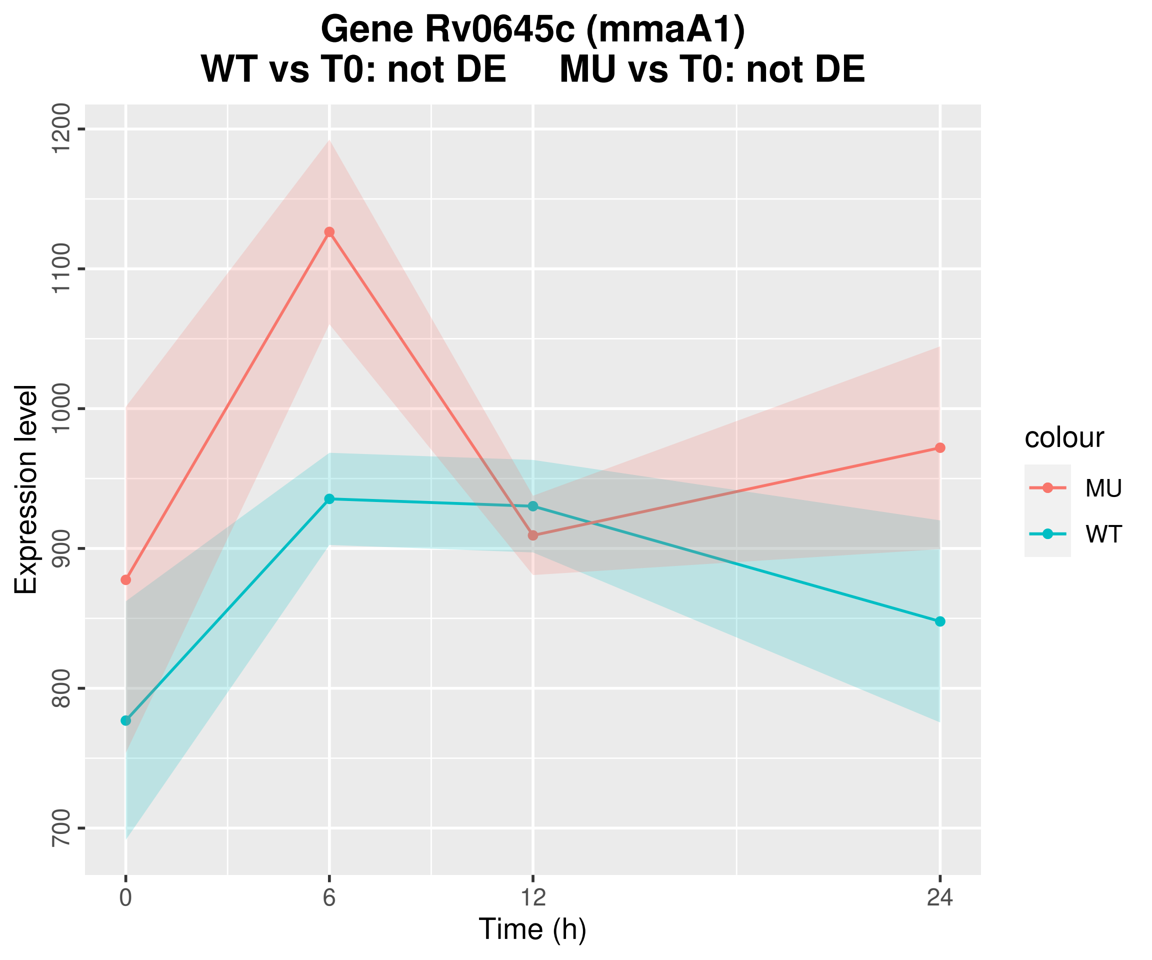
Gene Rv0645c (mmaA1)
WT vs T0: not DE   MU vs T0: not DE
Expression level
1200
1100
1000
900
800
700
0
6
12
24
Time (h)
colour
MU
WT

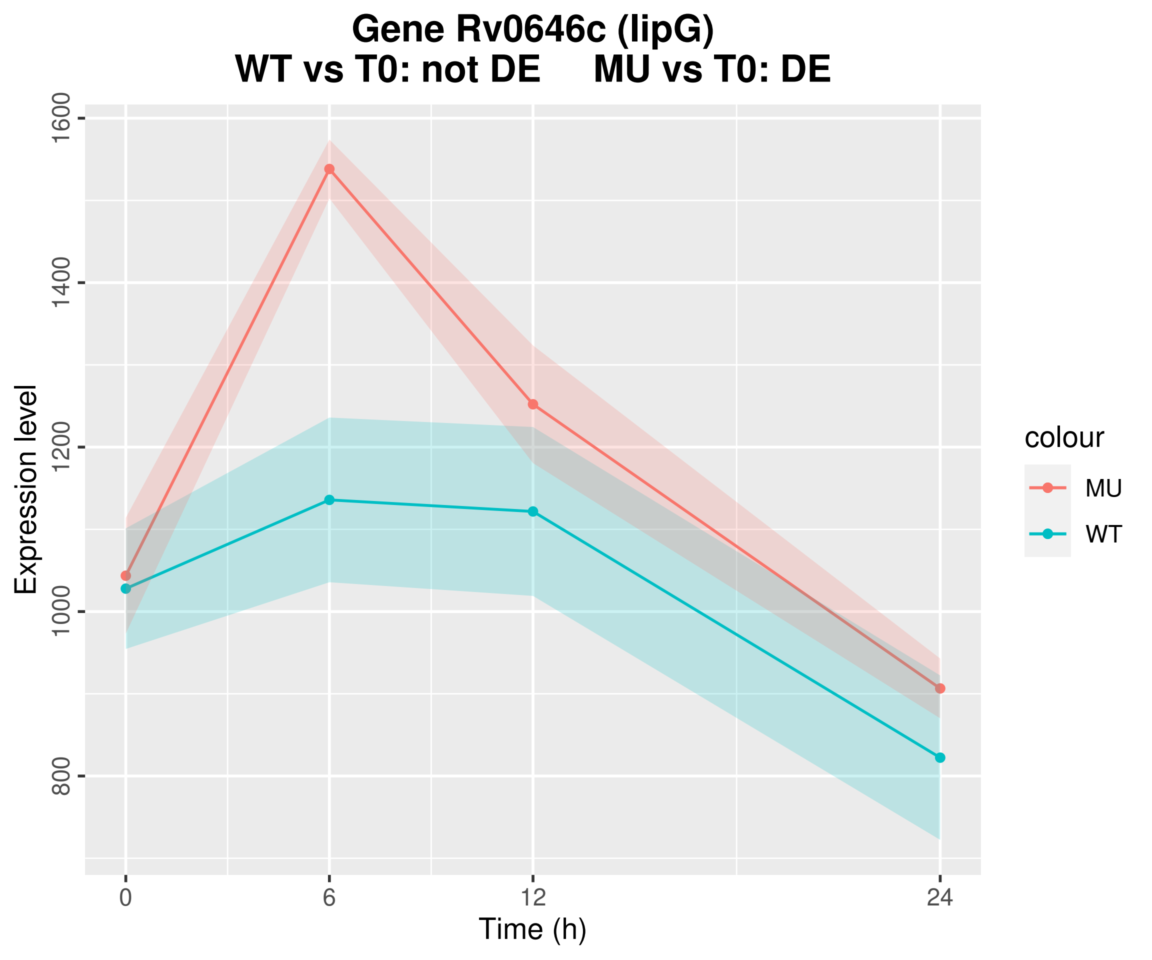

Gene Rv0646c (lipG)
WT vs T0: not DE   MU vs T0: DE
Expression level
1600
1400
1200
1000
800
0
6
12
24
Time (h)
colour
MU
WT

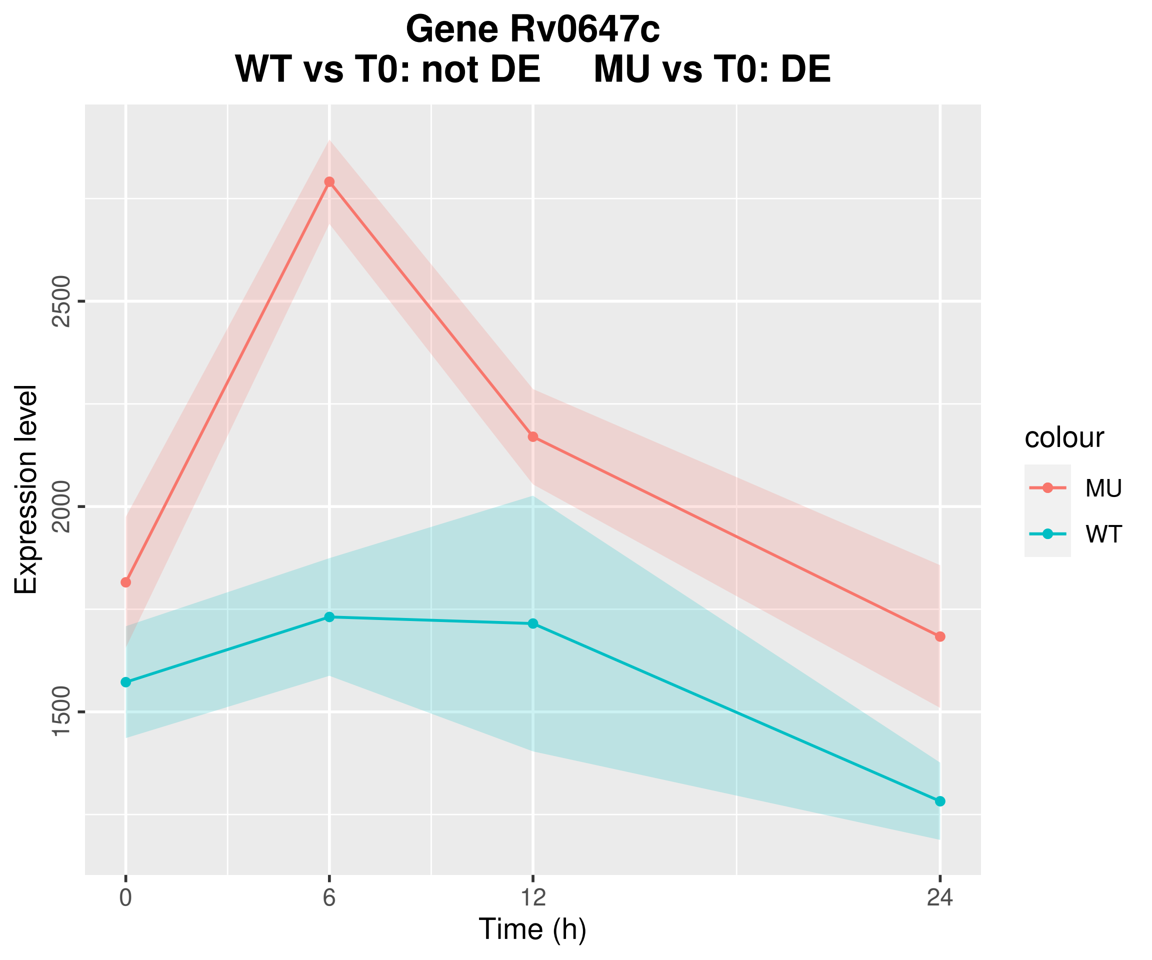
Gene Rv0647c
WT vs T0: not DE MU vs T0: DE
Expression level
2500
2000
1500
0
6
12
24
Time (h)
colour
MU
WT

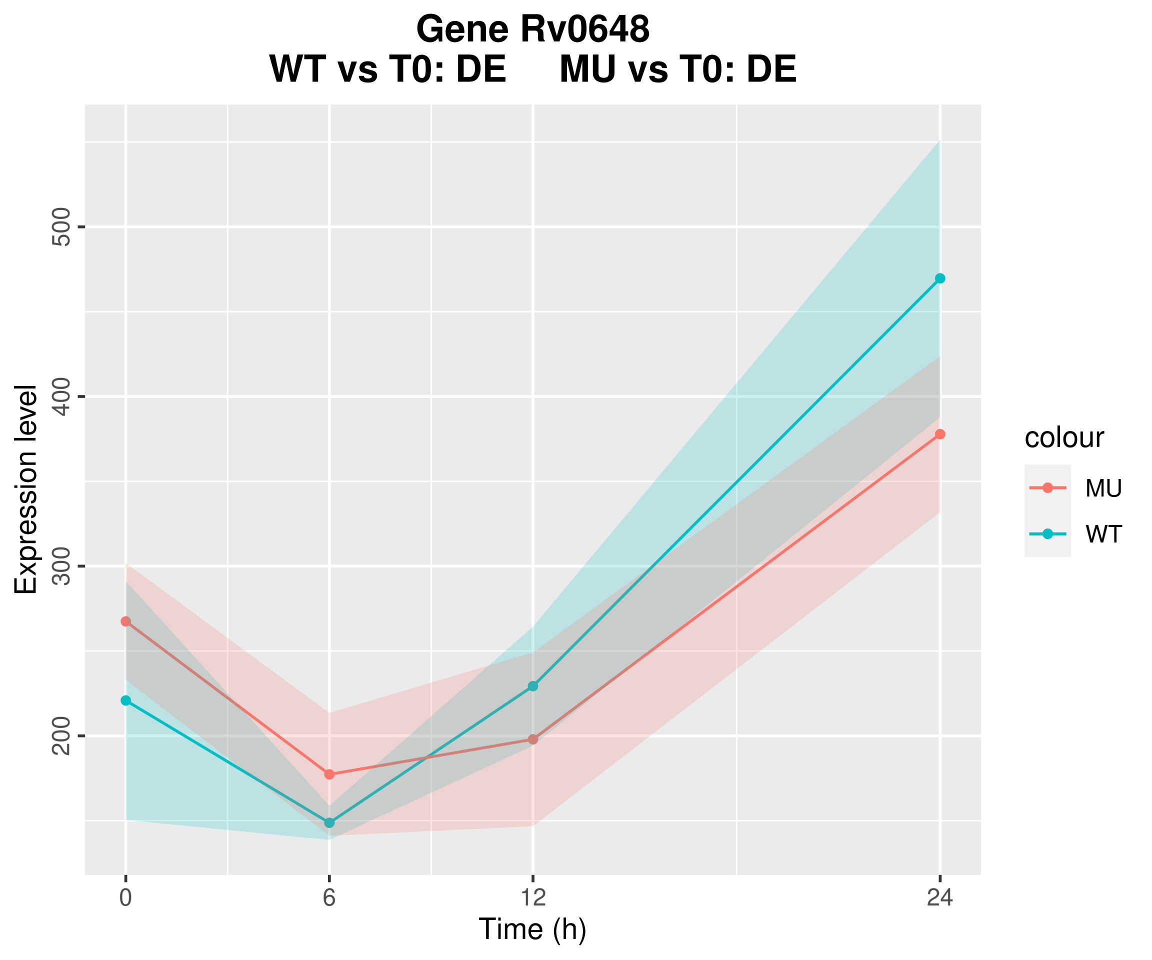
Gene Rv0648
WT vs T0: DE MU vs T0: DE
Expression level
500
400
300
200
0
6
12
24
Time (h)
colour
MU
WT

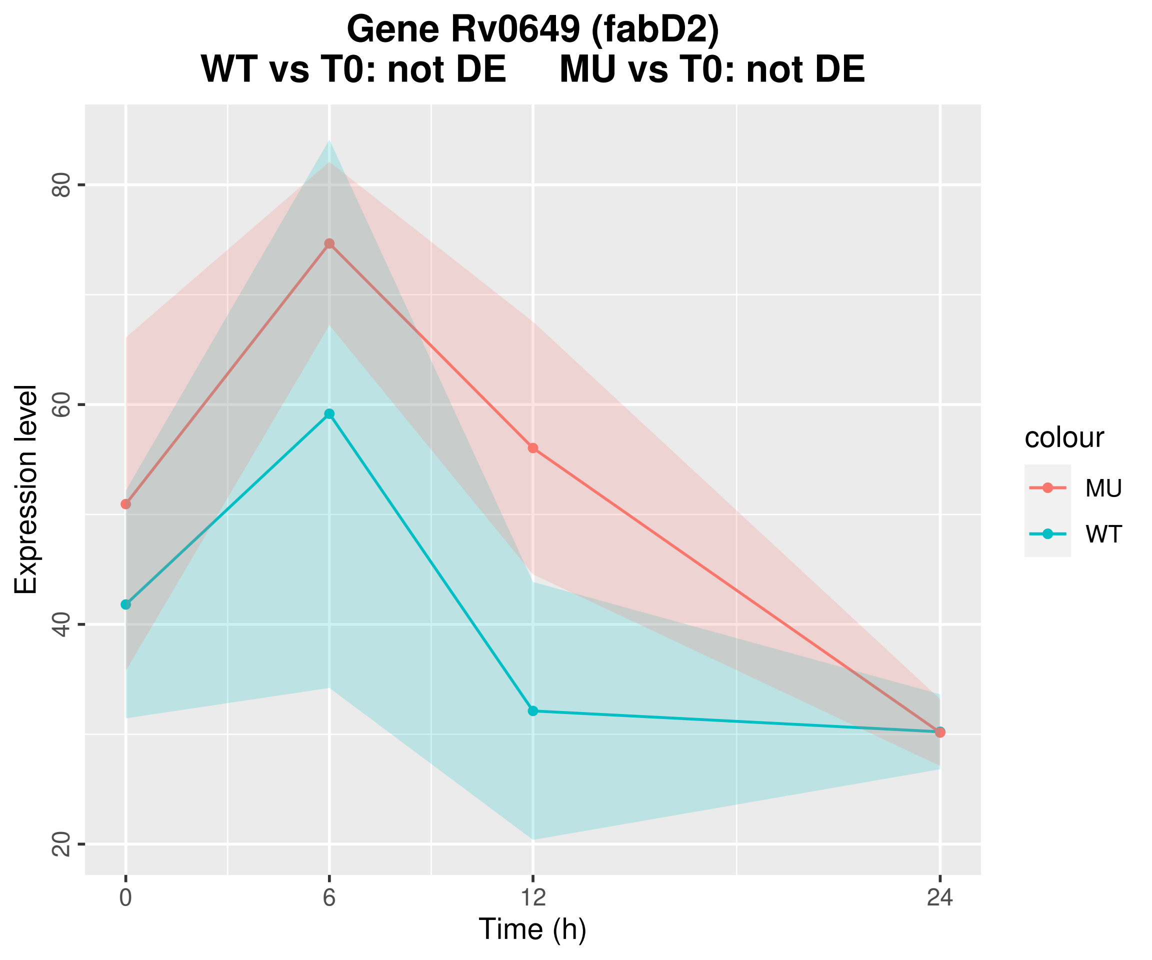
Gene Rv0649 (fabD2)
WT vs T0: not DE   MU vs T0: not DE
Expression level
80
60
40
20
0
6
12
24
Time (h)
colour
MU
WT

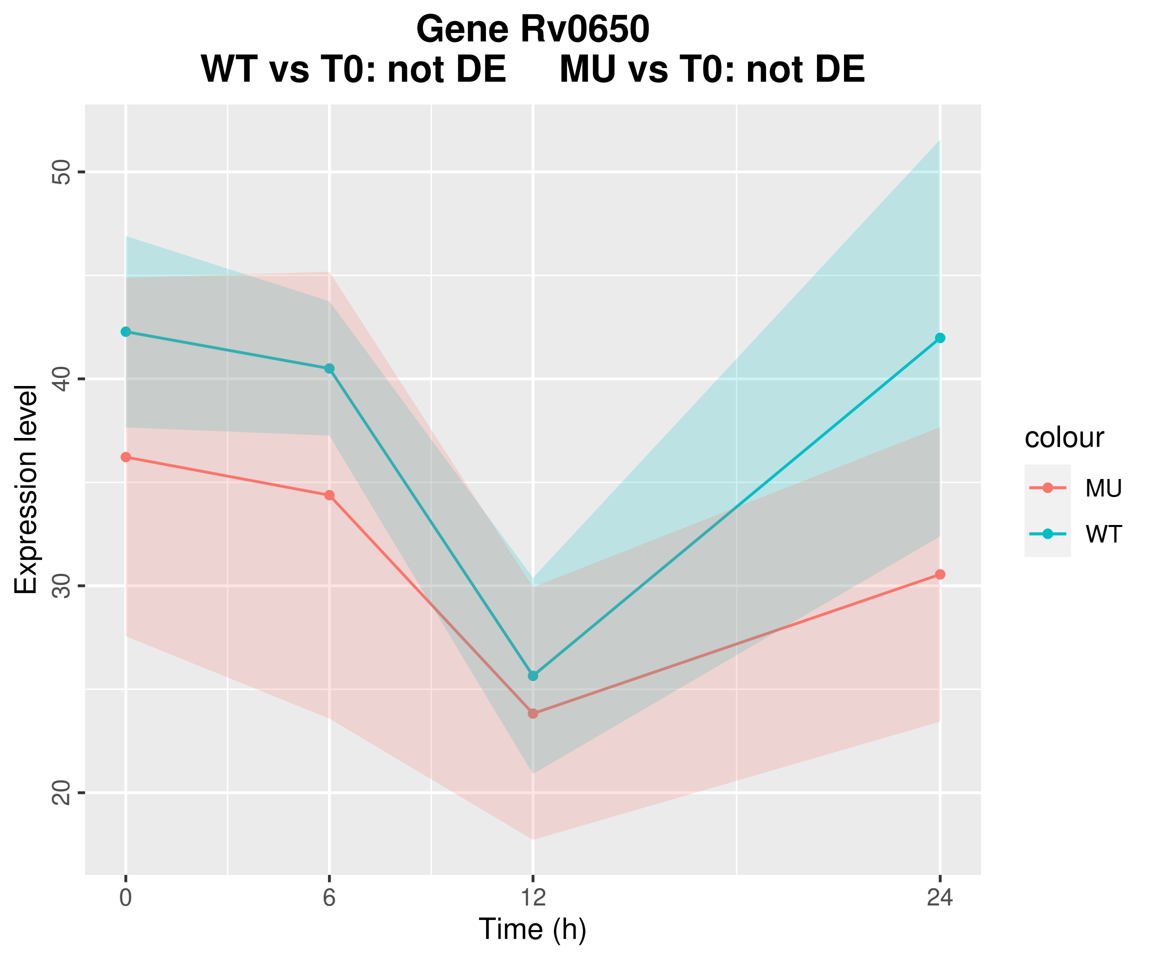

Gene Rv0650
WT vs T0: not DE MU vs T0: not DE
Expression level
50
40
30
20
0
6
12
24
Time (h)
colour
MU
WT

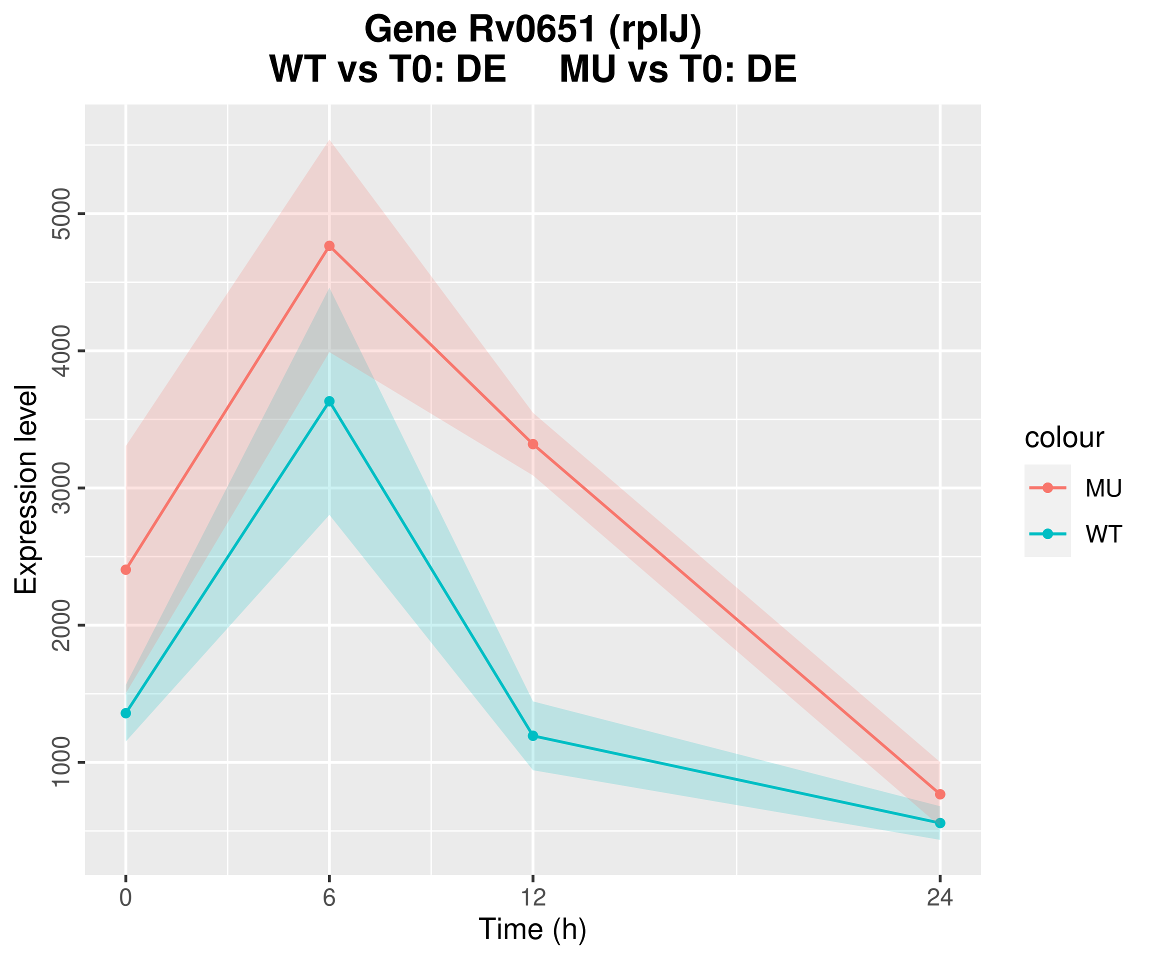

Gene Rv0651 (rplJ)
WT vs T0: DE MU vs T0: DE
Expression level
1000
2000
3000
4000
5000
0
6
12
24
Time (h)
colour
MU
WT

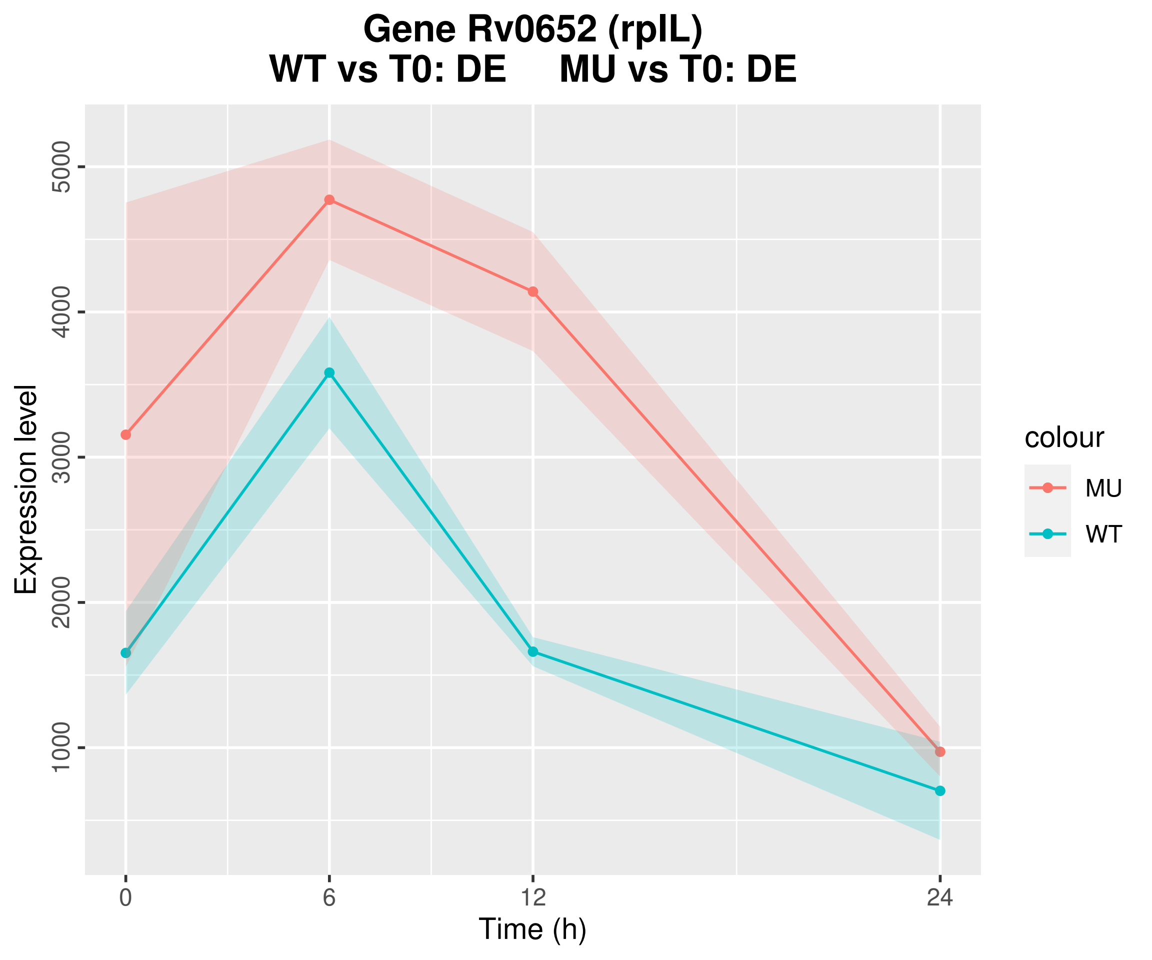

Gene Rv0652 (rplL)
WT vs T0: DE   MU vs T0: DE
Expression level
5000
4000
3000
2000
1000
0
6
12
24
Time (h)
colour
MU
WT

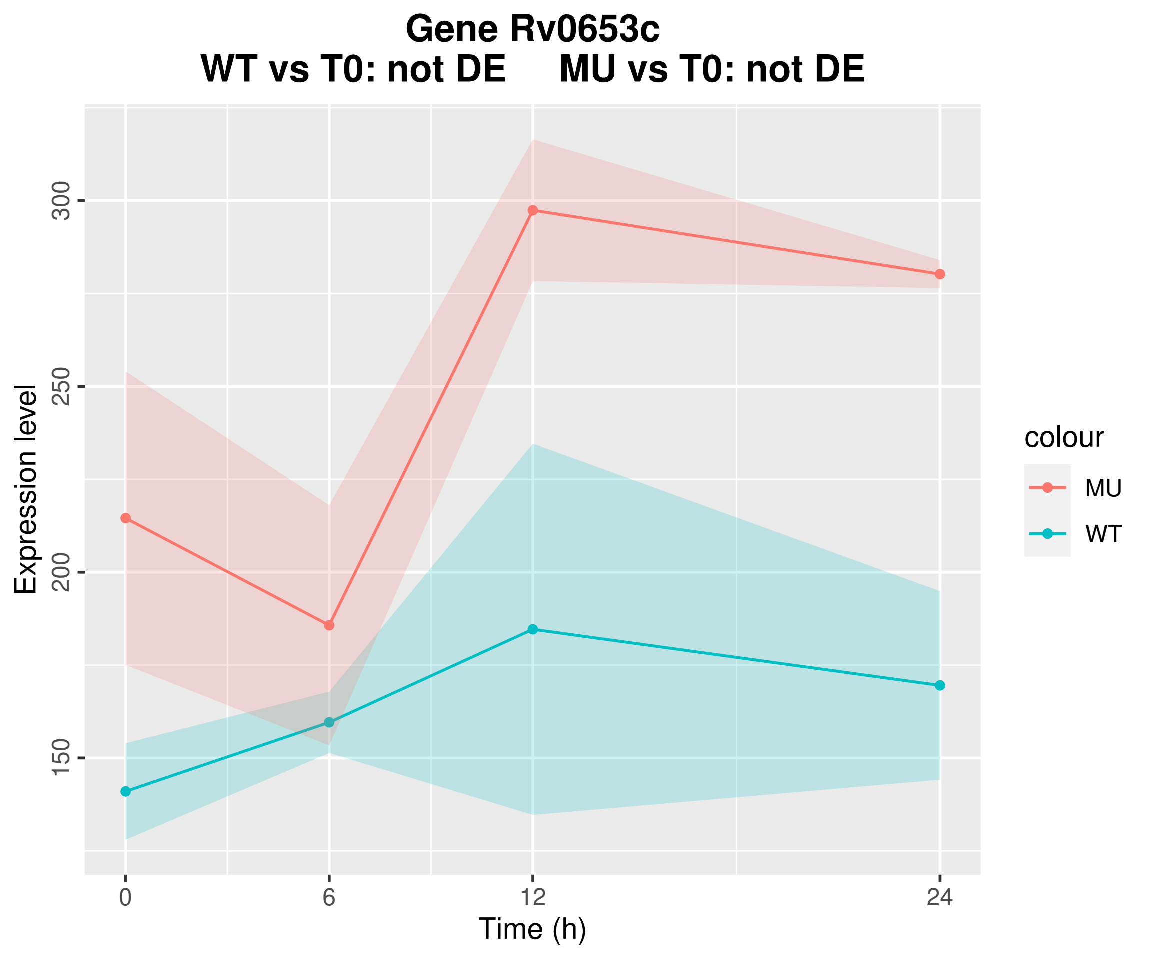
Gene Rv0653c
WT vs T0: not DE   MU vs T0: not DE
Expression level
300
250
200
150
0
6
12
24
Time (h)
colour
MU
WT

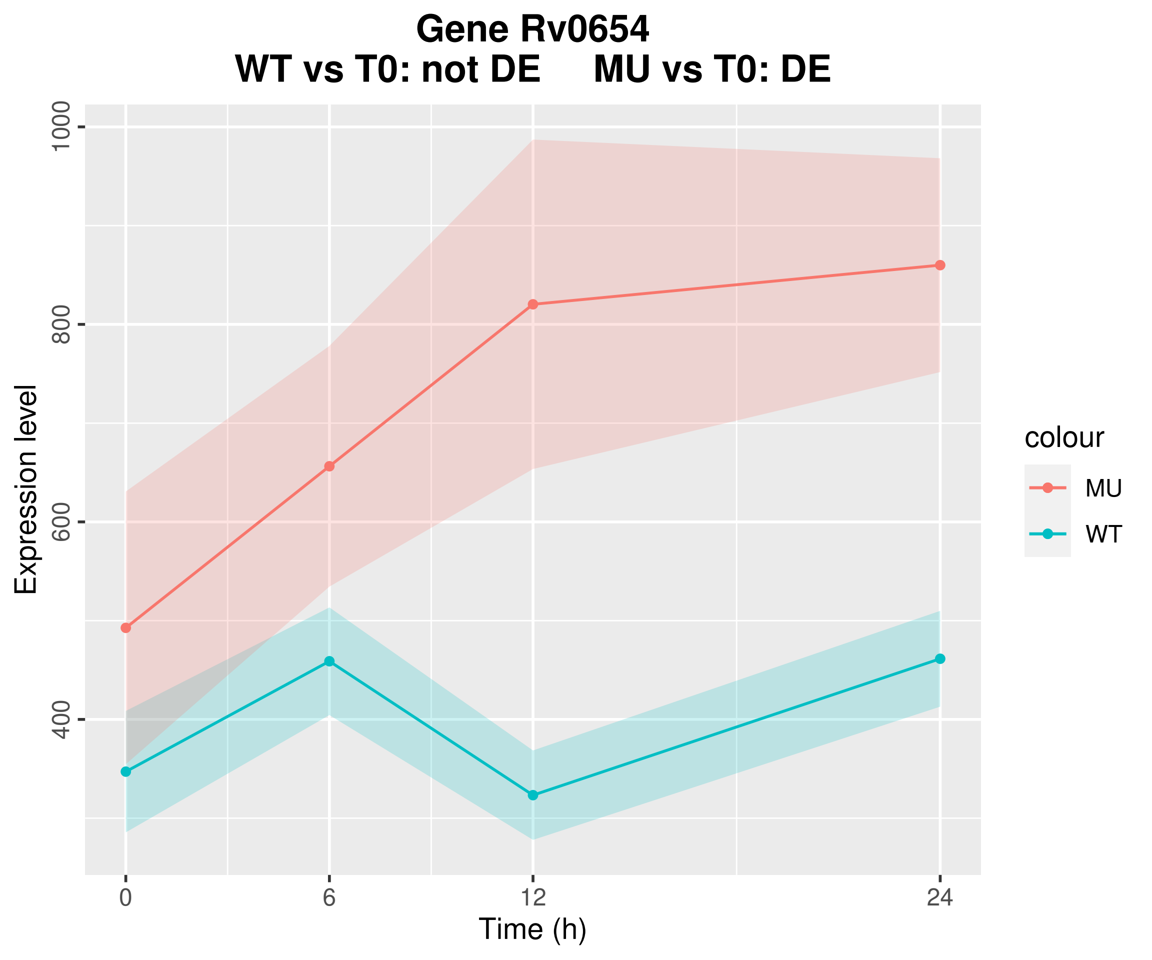

Gene Rv0654
WT vs T0: not DE MU vs T0: DE
Expression level
1000
800
600
400
0
6
12
24
Time (h)
colour
MU
WT

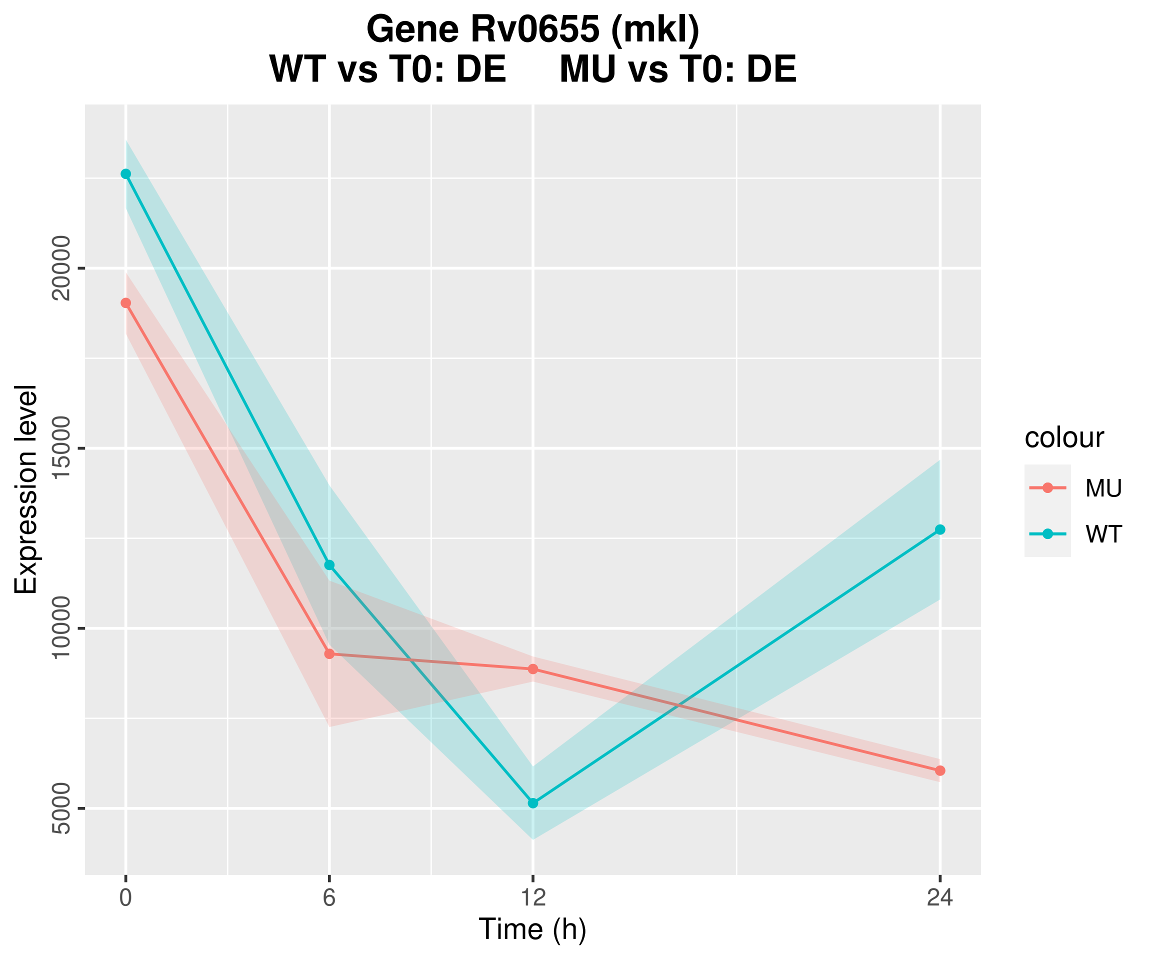

Gene Rv0655 (mkl)
WT vs T0: DE   MU vs T0: DE
Expression level
20000
15000
10000
5000
0
6
12
24
Time (h)
colour
MU
WT

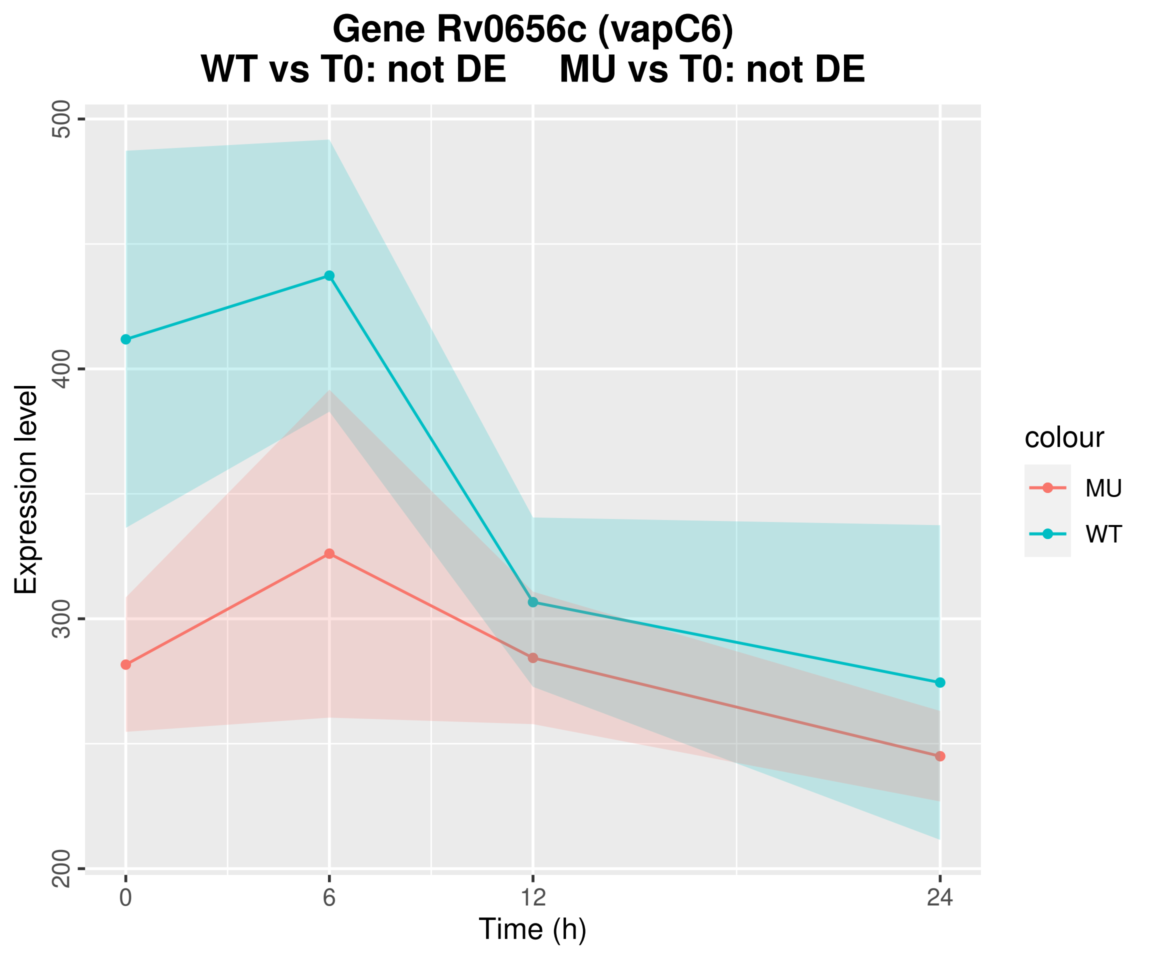
Gene Rv0656c (vapC6)
WT vs T0: not DE   MU vs T0: not DE
Expression level
500
400
300
200
0
6
12
24
Time (h)
colour
MU
WT

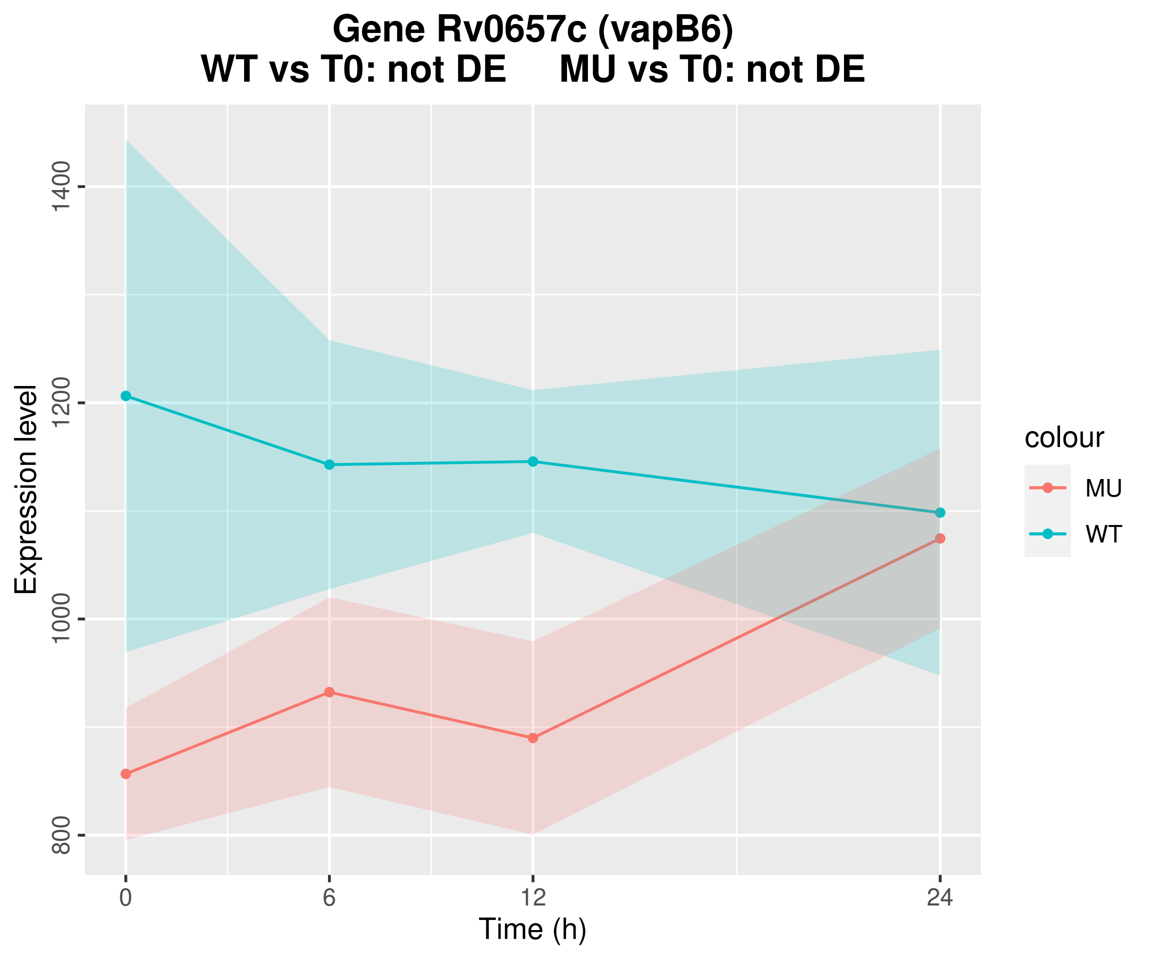

Gene Rv0657c (vapB6)
WT vs T0: not DE   MU vs T0: not DE
Expression level
1400
1200
1000
800
Time (h)
0
6
12
24
colour
MU
WT

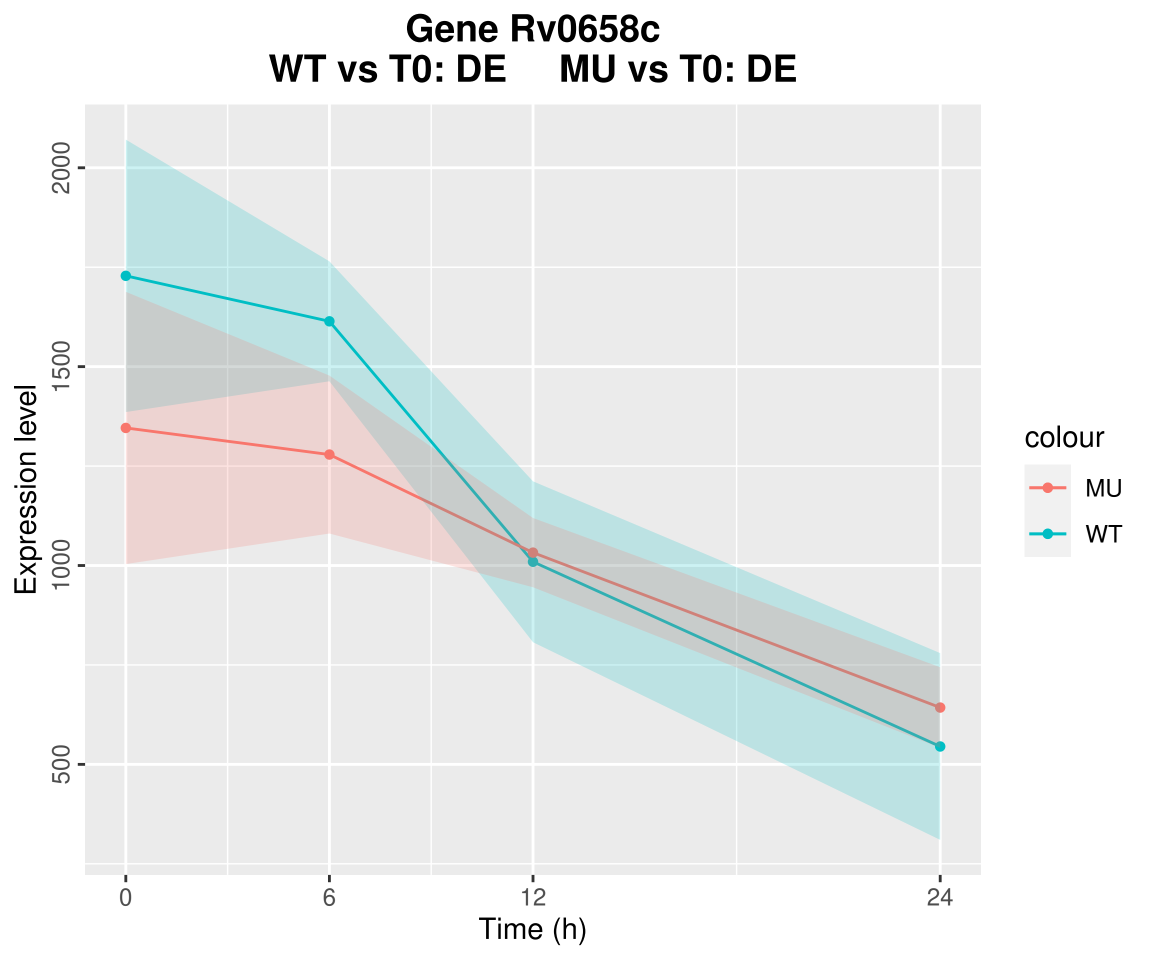

Gene Rv0658c
WT vs T0: DE   MU vs T0: DE
Expression level
2000
1500
1000
500
0
6
12
24
Time (h)
colour
MU
WT

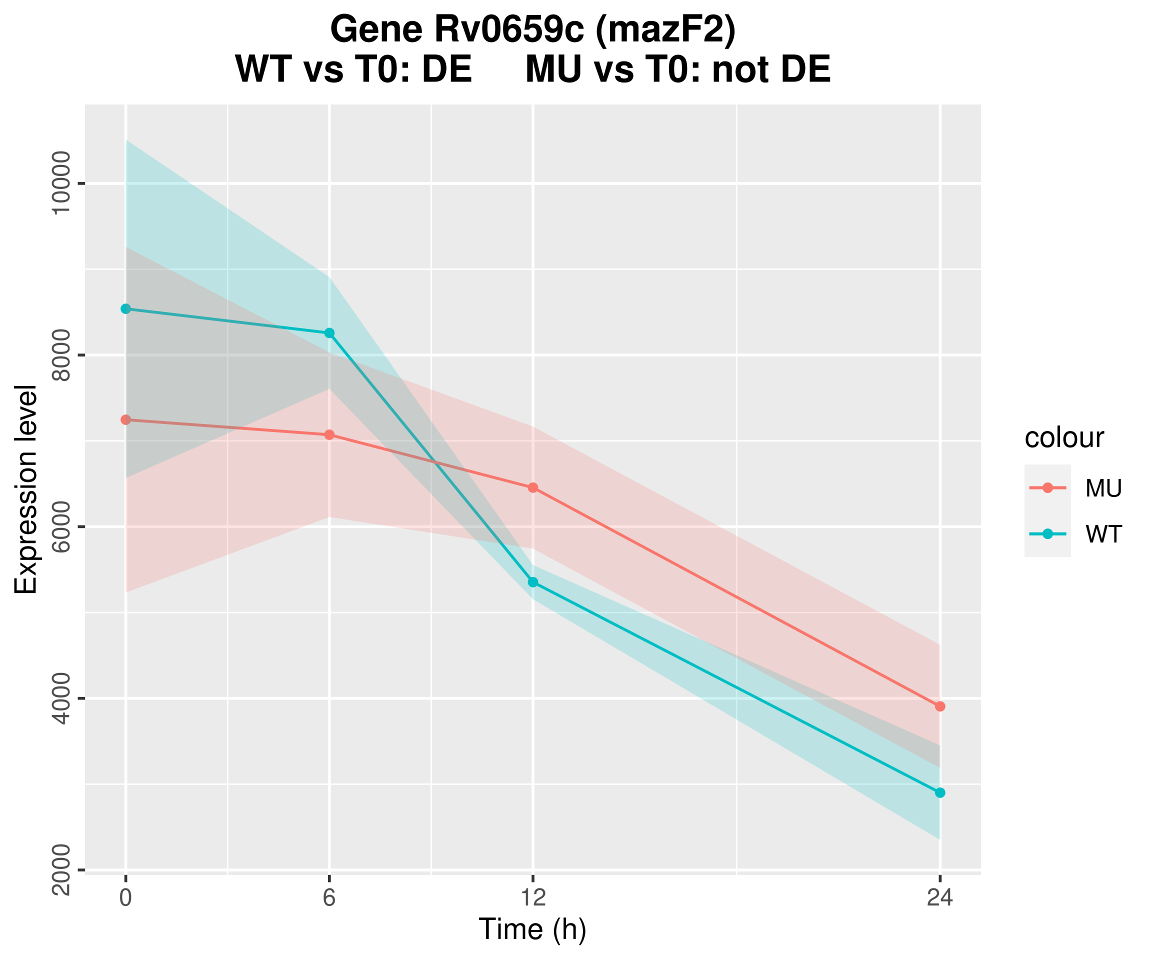
Gene Rv0659c (mazF2)
WT vs T0: DE   MU vs T0: not DE
Expression level
10000
8000
6000
4000
2000
0
6
12
24
Time (h)
colour
MU
WT

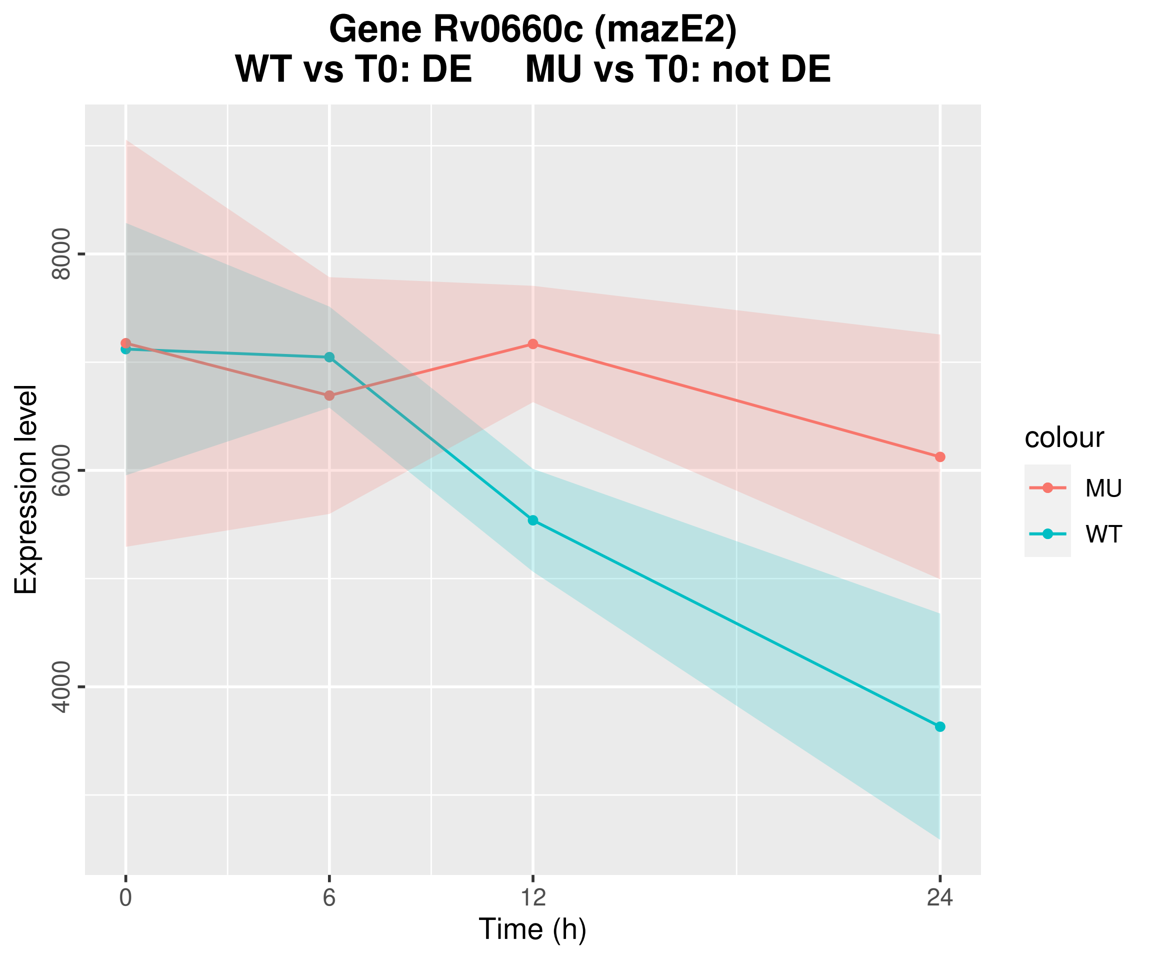
Gene Rv0660c (mazE2)
WT vs T0: DE   MU vs T0: not DE
Expression level
8000
6000
4000
0
6
12
24
Time (h)
colour
MU
WT

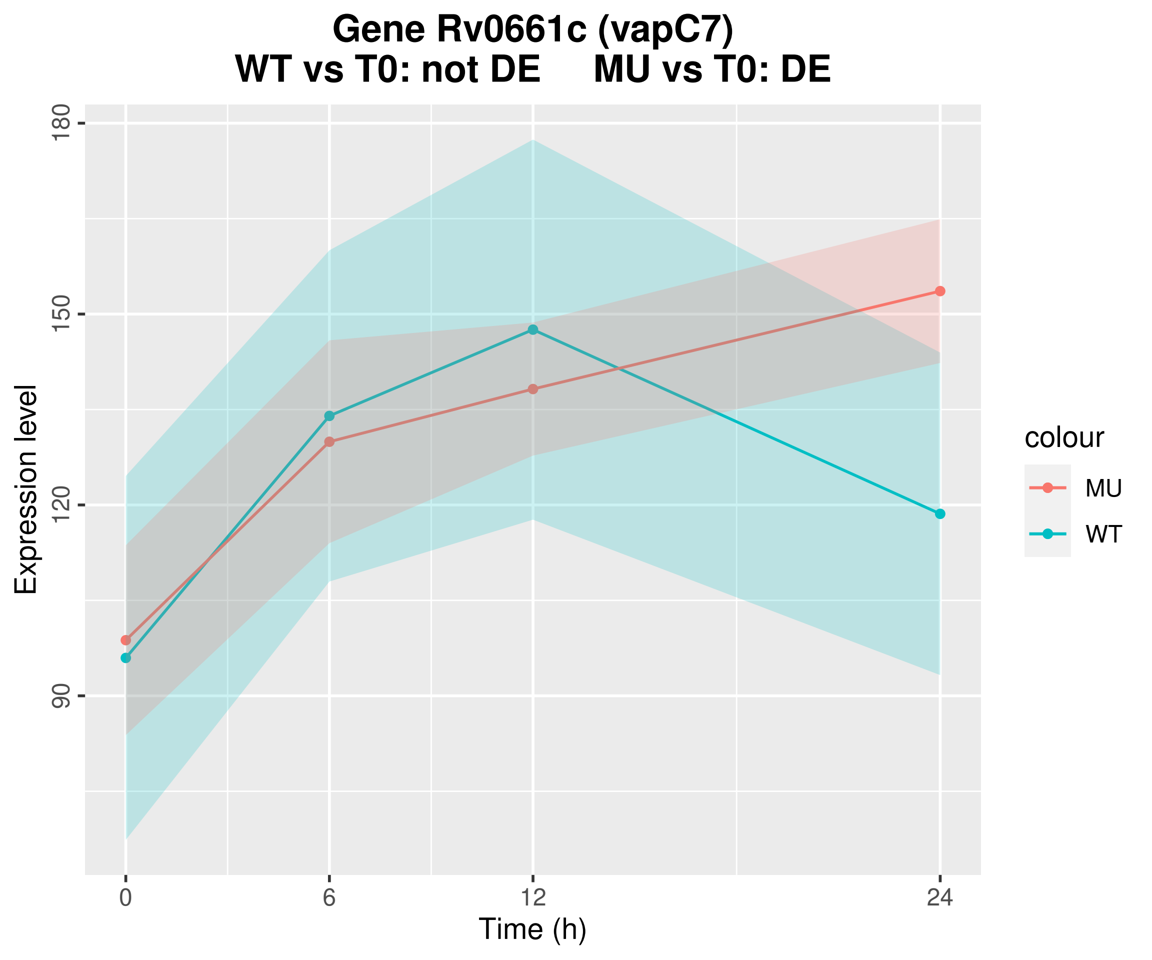

Gene Rv0661c (vapC7)
WT vs T0: not DE MU vs T0: DE
180
150
120
90
Expression level
0
6
12
24
Time (h)
colour
MU
WT

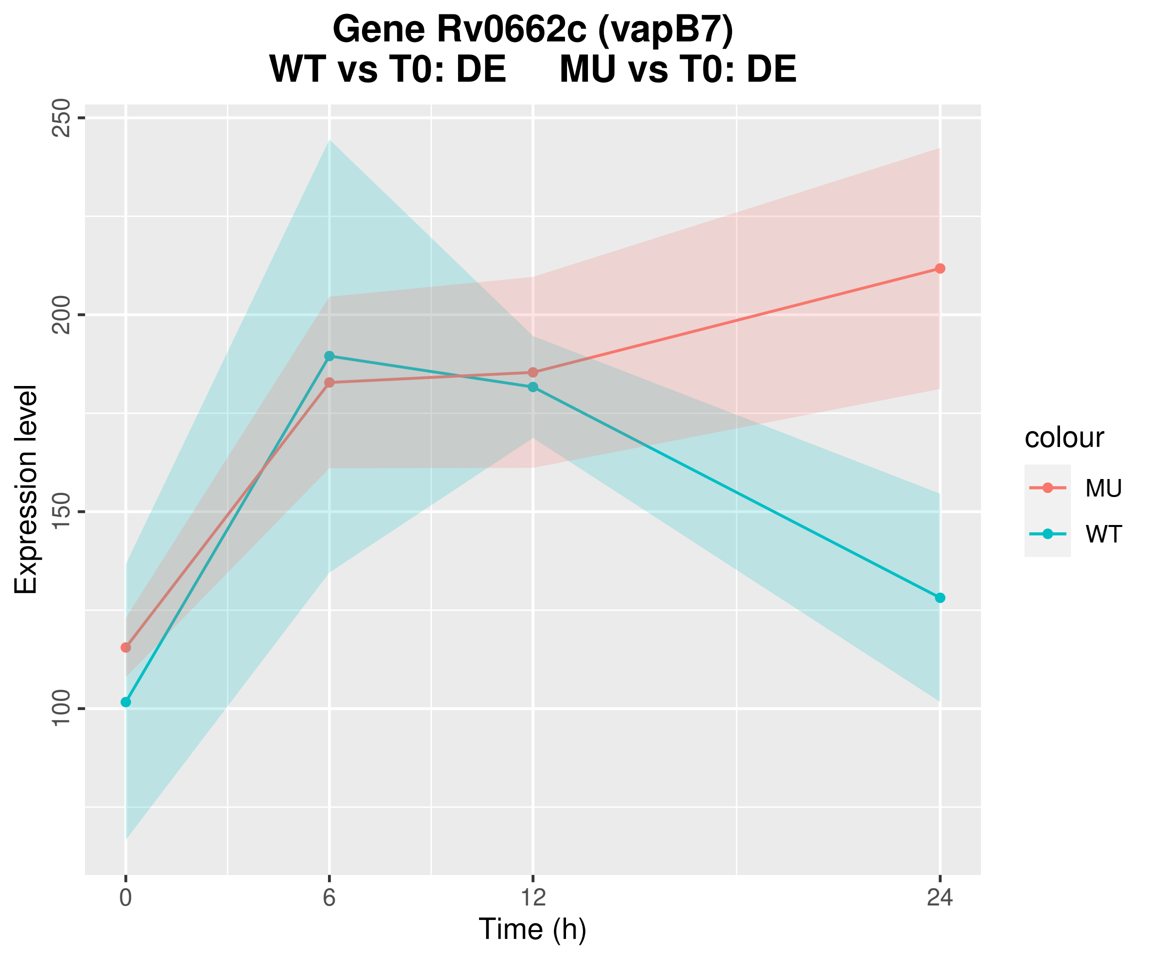
Gene Rv0662c (vapB7)
WT vs T0: DE MU vs T0: DE
250
200
150
100
Expression level
0
6
12
24
Time (h)
colour
MU
WT

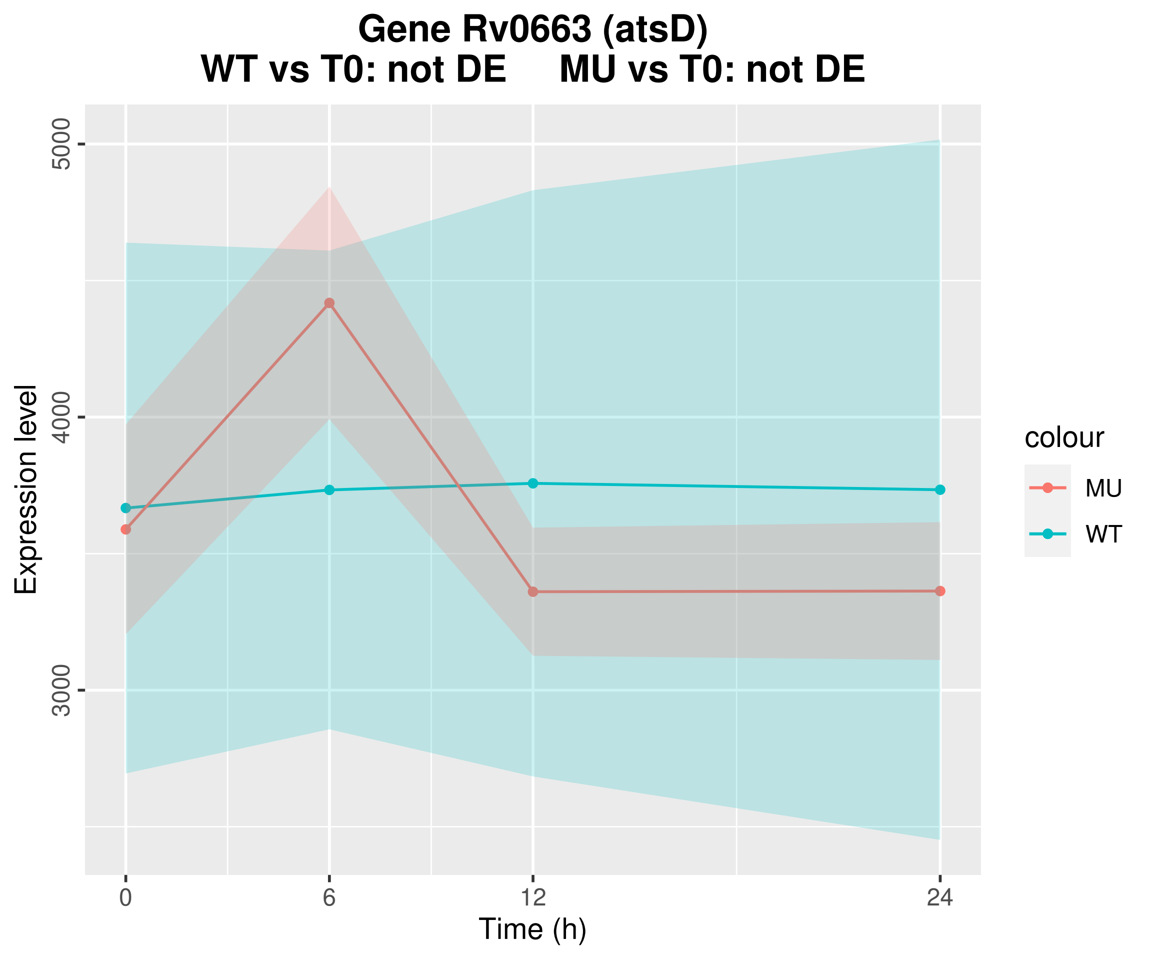

Gene Rv0663 (atsD)
WT vs T0: not DE MU vs T0: not DE
Expression level
5000
4000
3000
0
6
12
24
Time (h)
colour
MU
WT

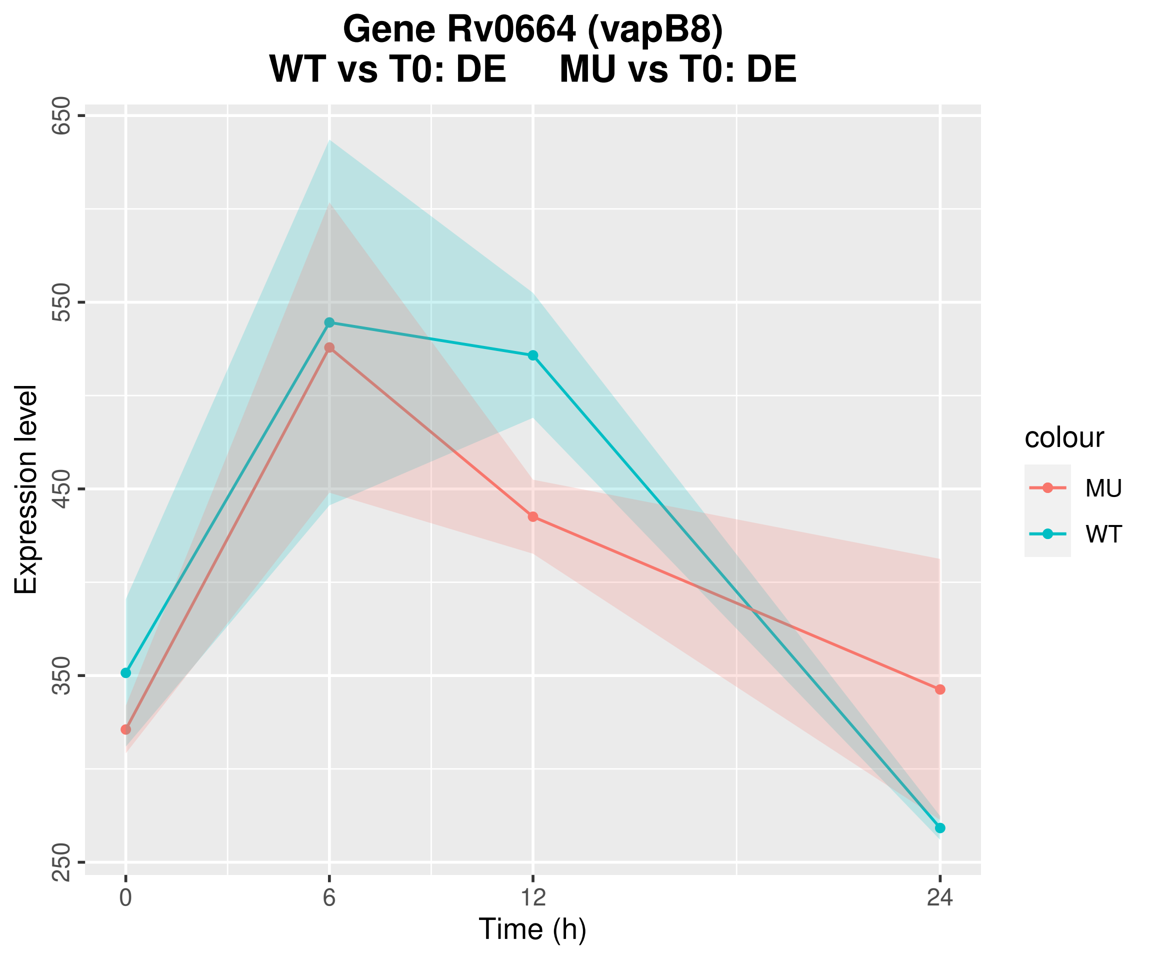

Gene Rv0664 (vapB8)
WT vs T0: DE MU vs T0: DE
Expression level
650
550
450
350
250
0
6
12
24
Time (h)
colour
MU
WT

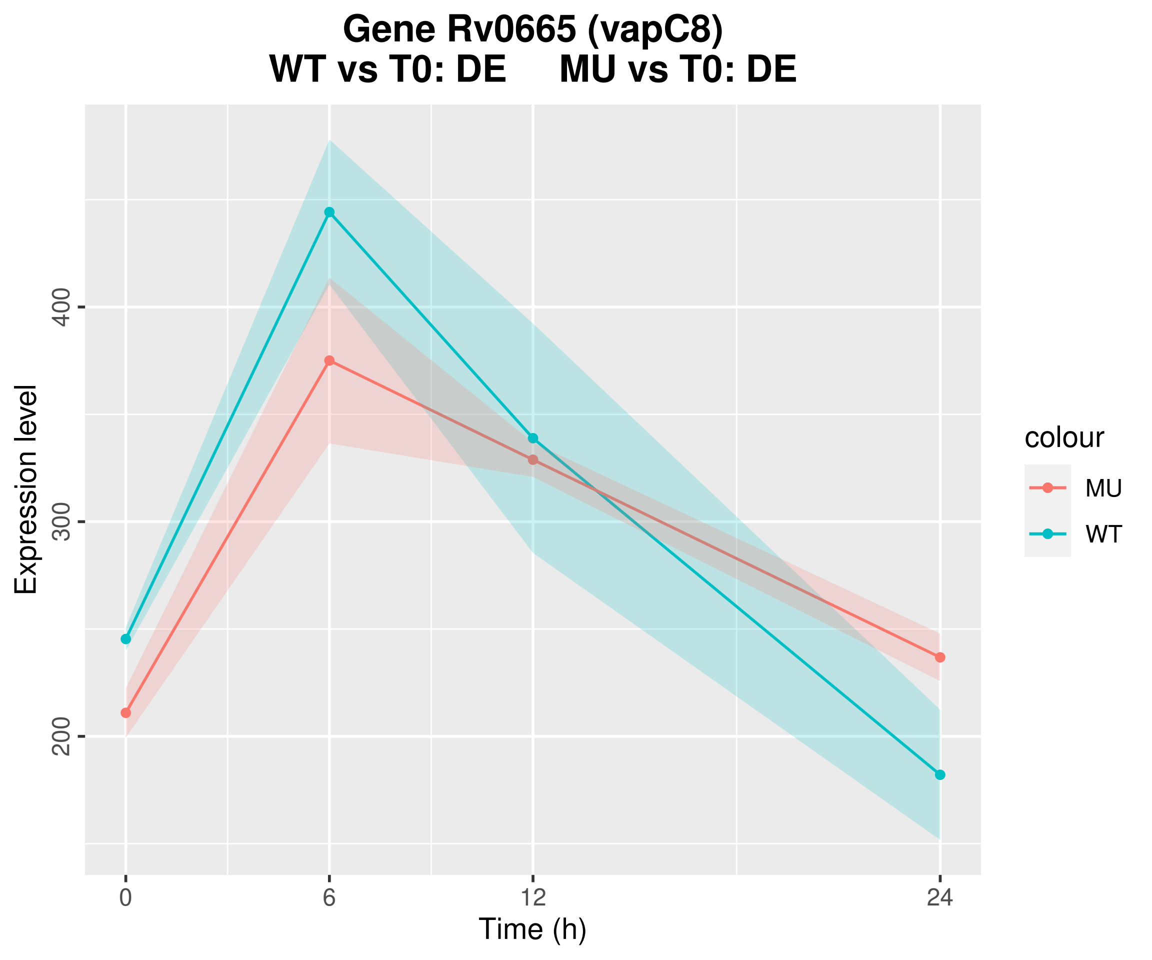

Gene Rv0665 (vapC8)
WT vs T0: DE MU vs T0: DE
Expression level
400
300
200
0
6
12
24
Time (h)
colour
MU
WT

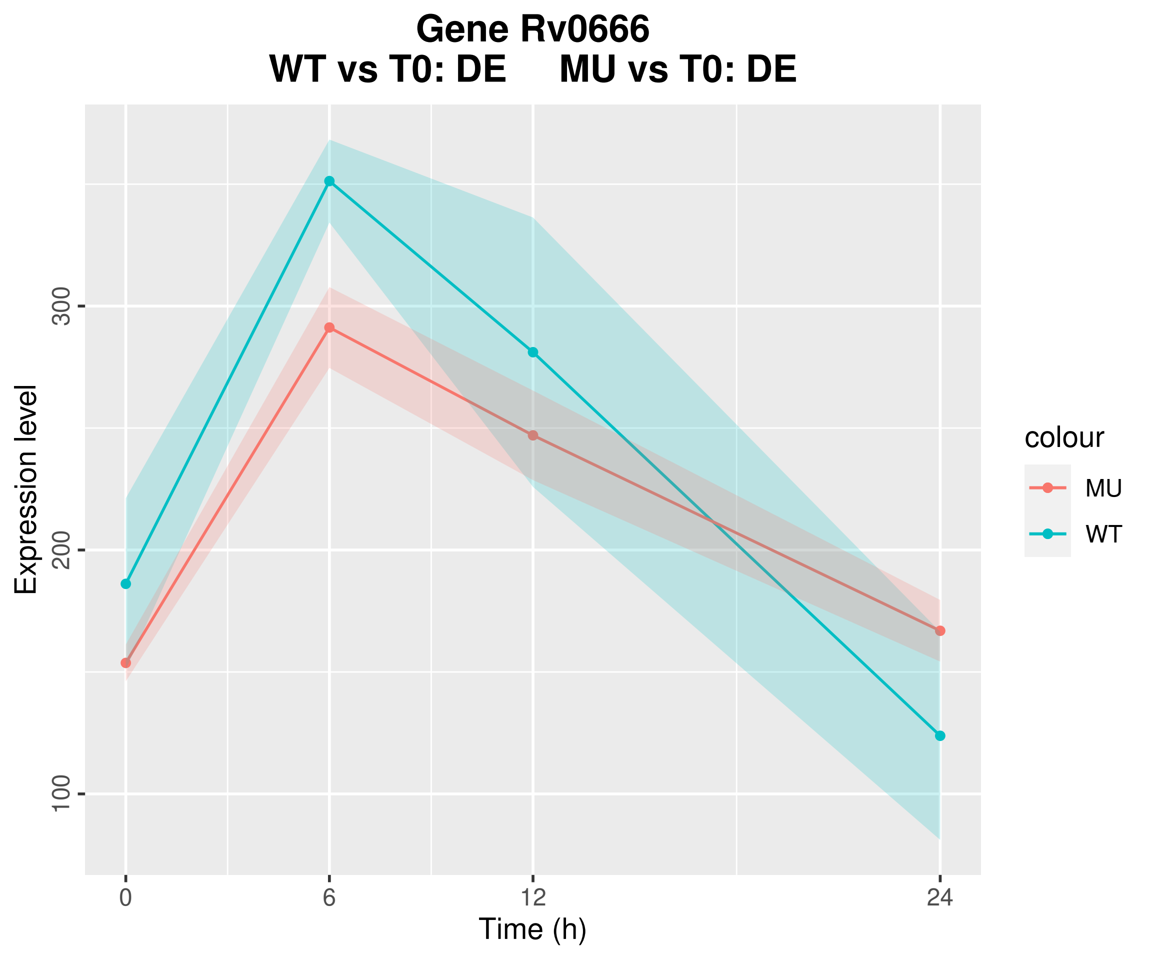
Gene Rv0666
WT vs T0: DE   MU vs T0: DE
Expression level
300
200
100
0
6
12
24
Time (h)
colour
MU
WT

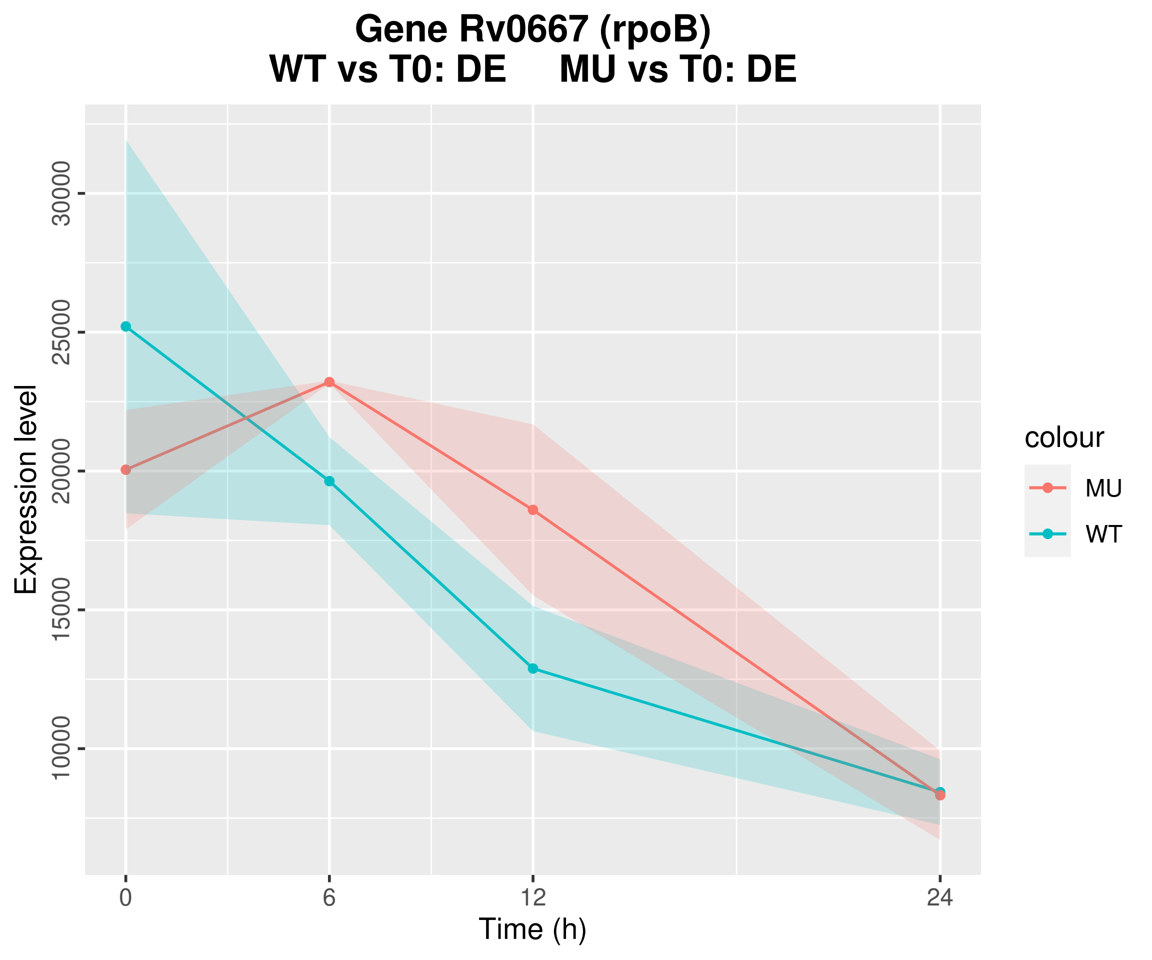
Gene Rv0667 (rpoB)
WT vs T0: DE   MU vs T0: DE
Expression level
30000
25000
20000
15000
10000
0
6
12
24
Time (h)
colour
MU
WT

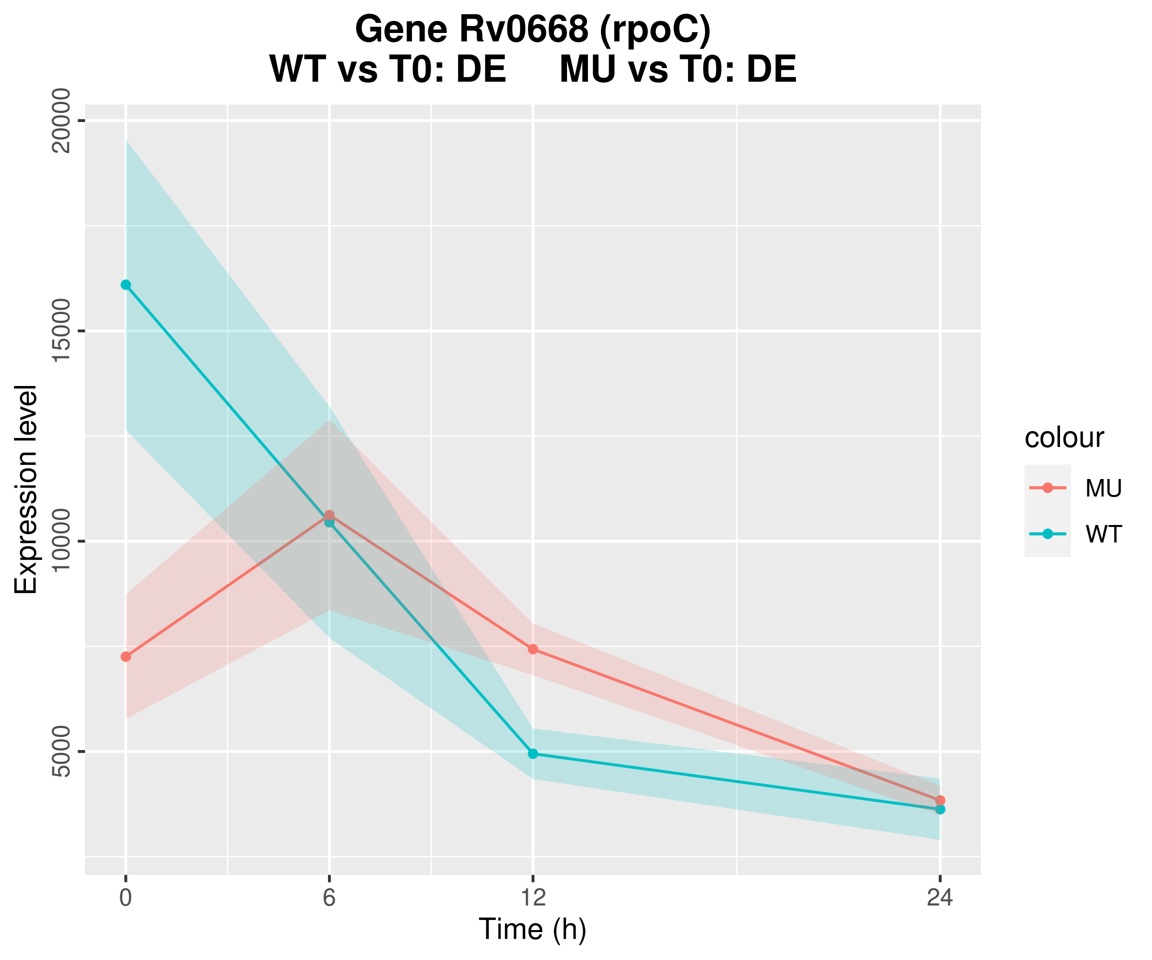

Gene Rv0668 (rpoC)
WT vs T0: DE MU vs T0: DE
Expression level
20000
15000
10000
5000
0
6
12
24
Time (h)
colour
MU
WT

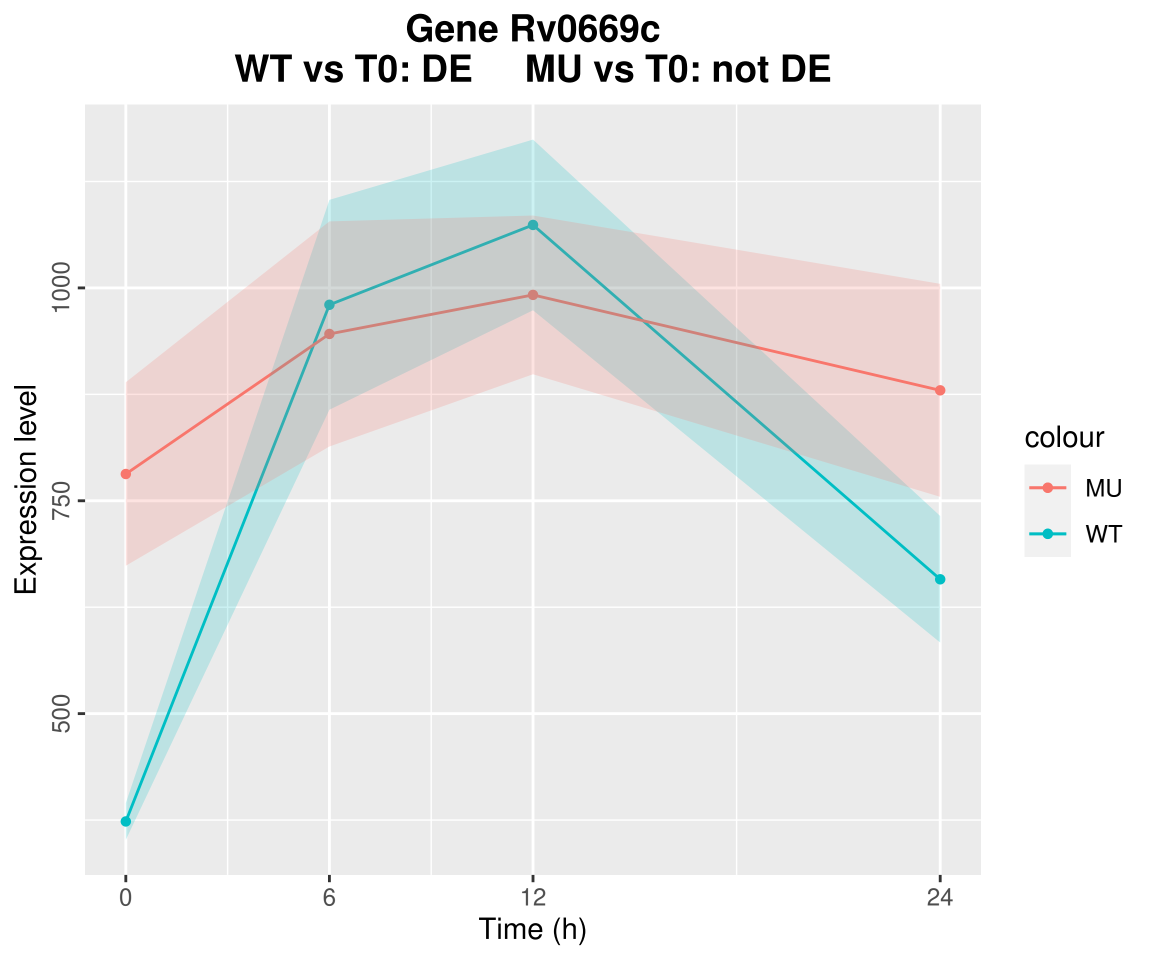
Gene Rv0669c
WT vs T0: DE MU vs T0: not DE
Expression level
1000
750
500
0
6
12
24
Time (h)
colour
MU
WT

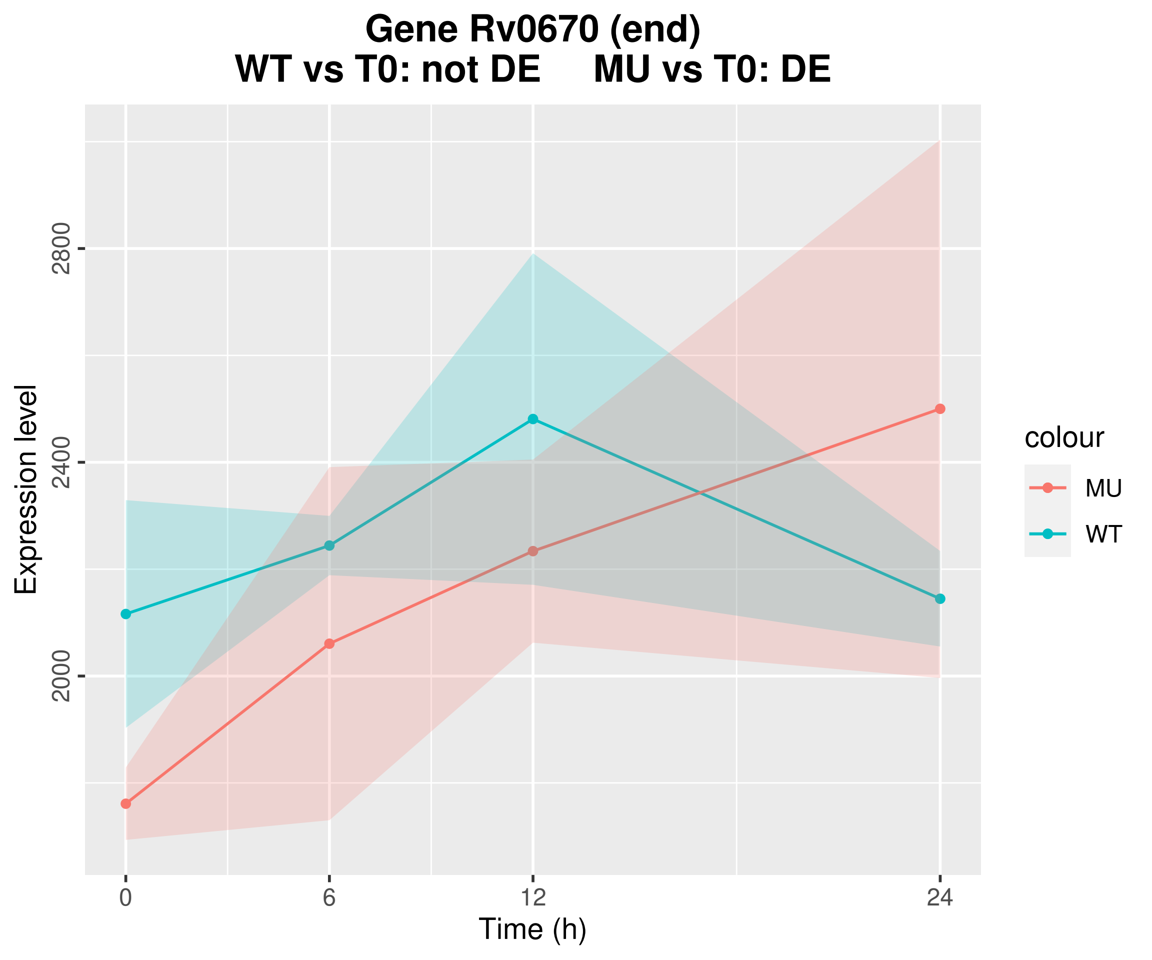
Gene Rv0670 (end)
WT vs T0: not DE
MU vs T0: DE
Expression level
2000
2400
2800
Time (h)
0
6
12
24
colour
MU
WT

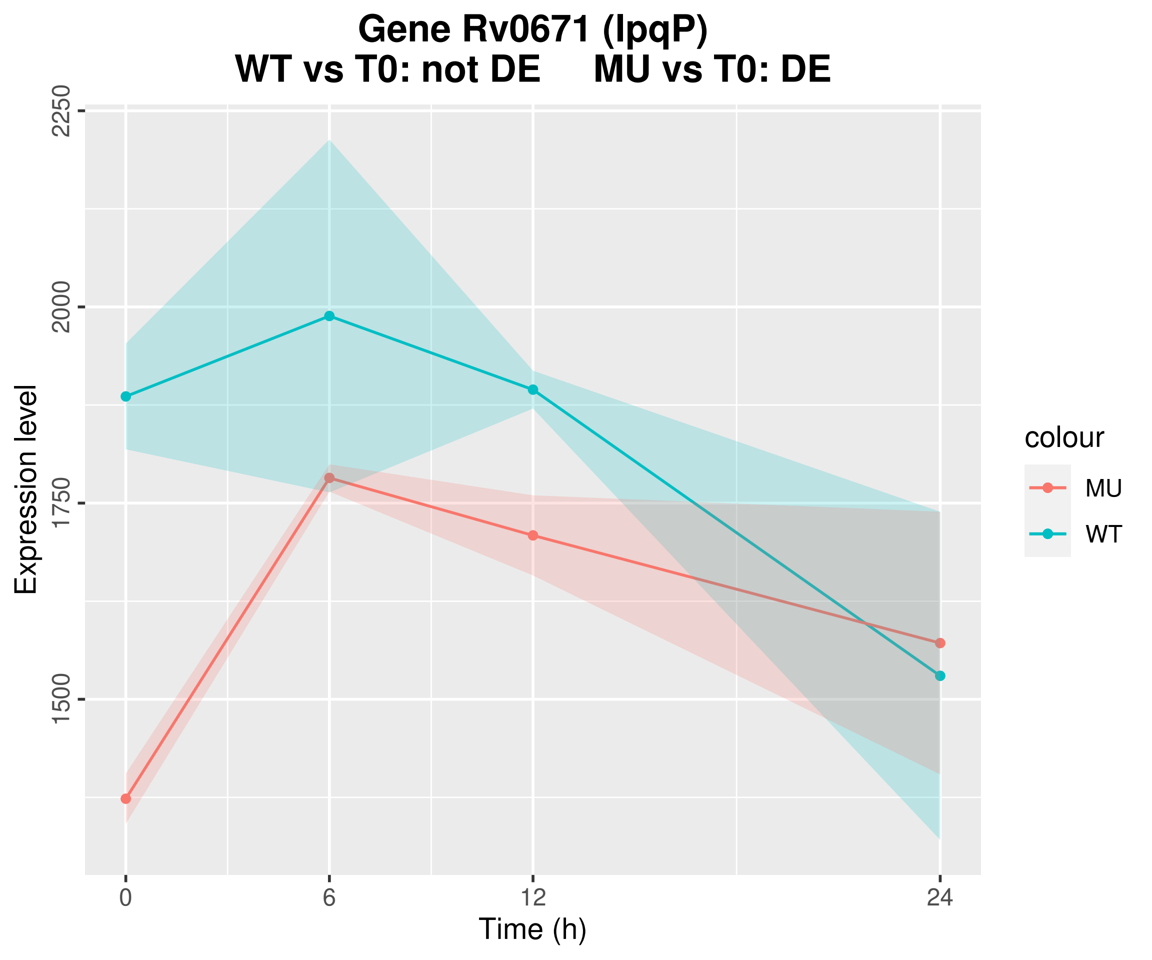

Gene Rv0671 (lpqP)
WT vs T0: not DE MU vs T0: DE
2250
2000
1750
1500
Expression level
0
6
12
24
Time (h)
colour
MU
WT

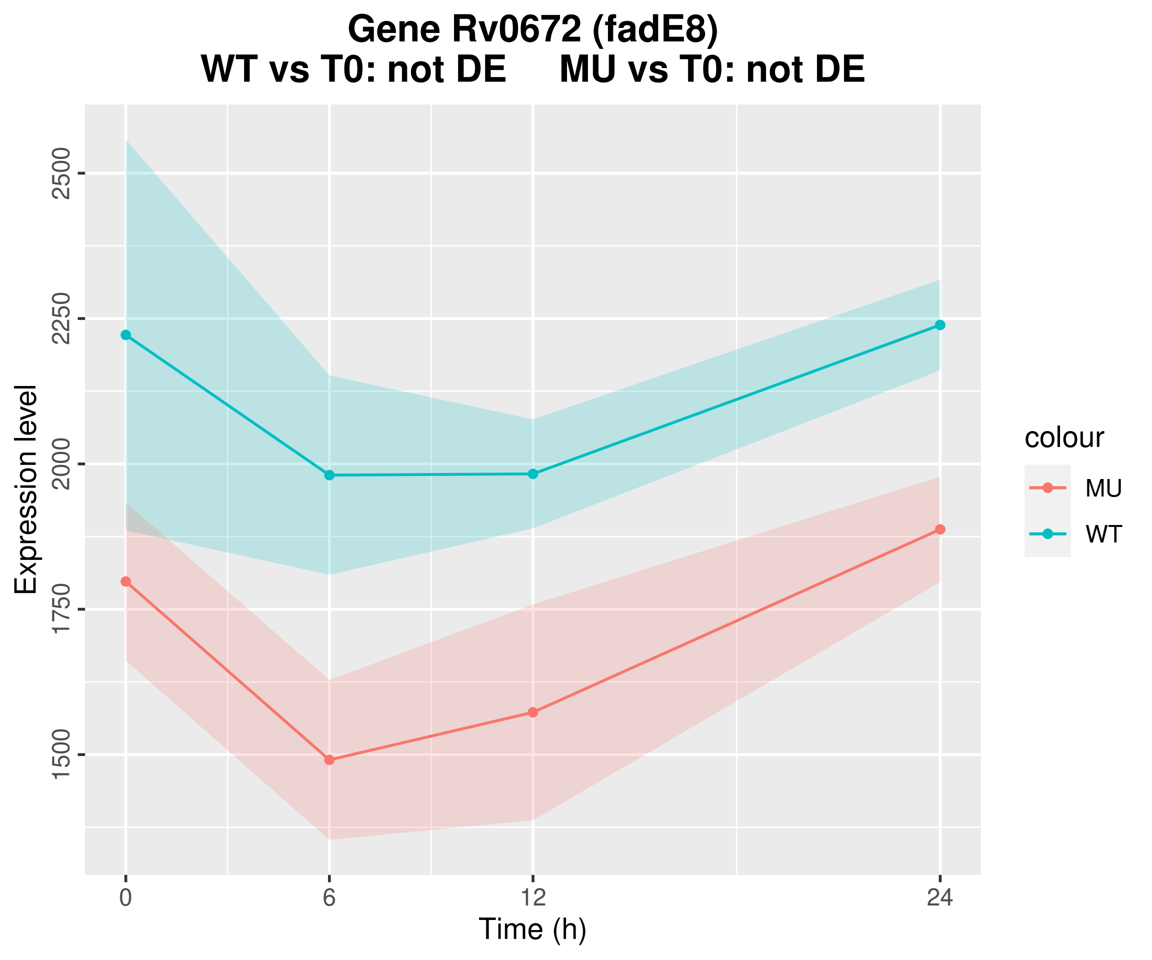

Gene Rv0672 (fadE8)
WT vs T0: not DE   MU vs T0: not DE
Expression level
2500
2250
2000
1750
1500
0
6
12
24
Time (h)
colour
MU
WT

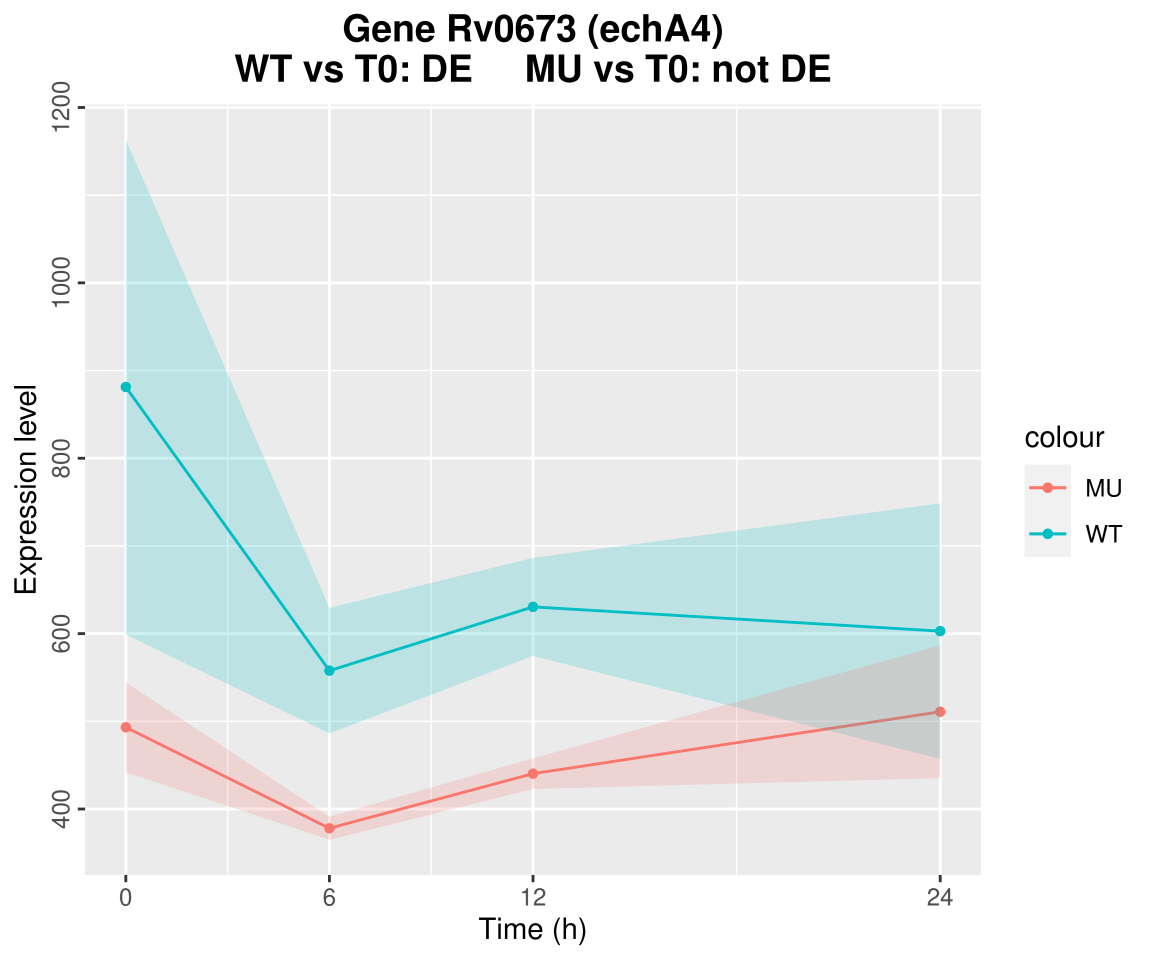
Gene Rv0673 (echA4)
WT vs T0: DE MU vs T0: not DE
Expression level
1200
1000
800
600
400
0
6
12
24
Time (h)
colour
MU
WT

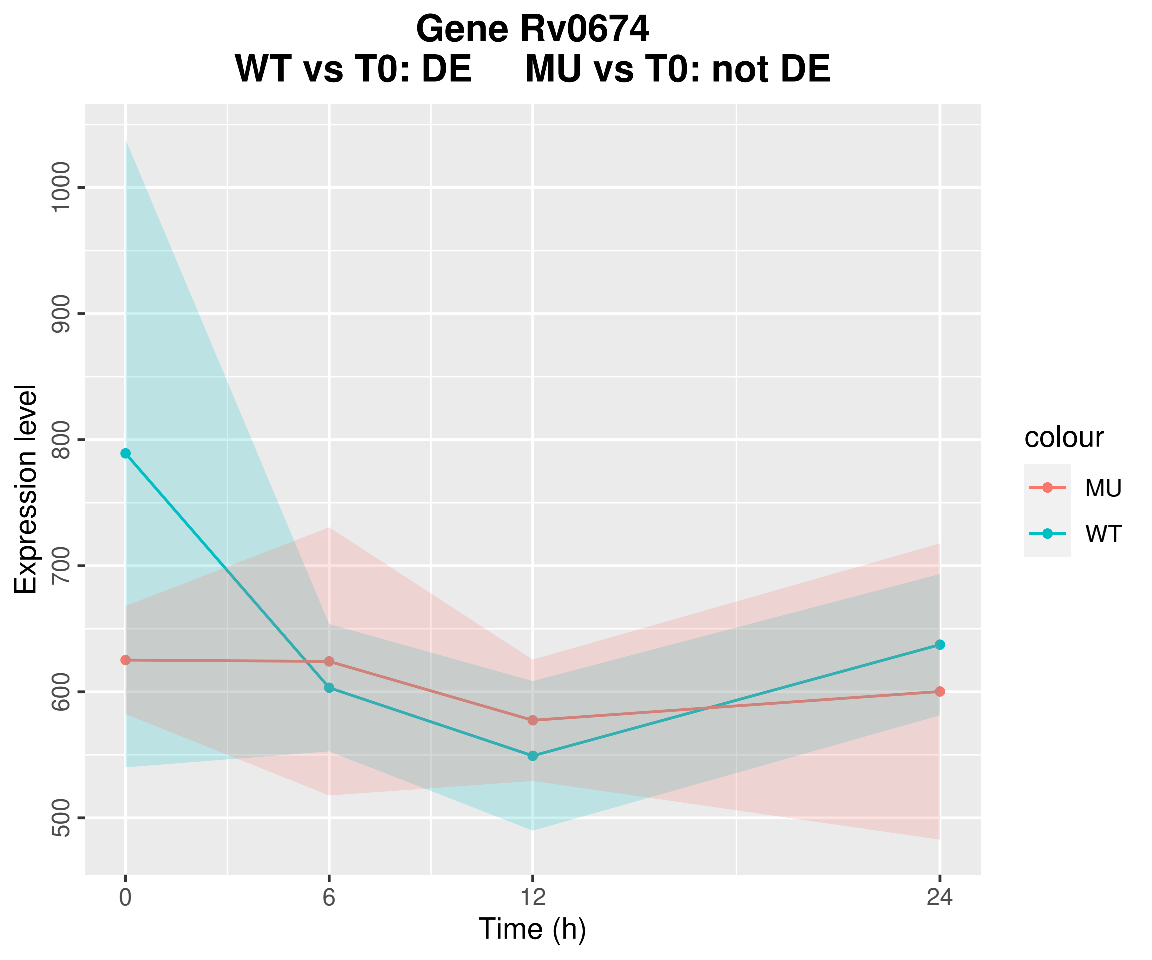
Gene Rv0674
WT vs T0: DE MU vs T0: not DE
Expression level
1000
900
800
700
600
500
0
6
12
24
Time (h)
colour
MU
WT

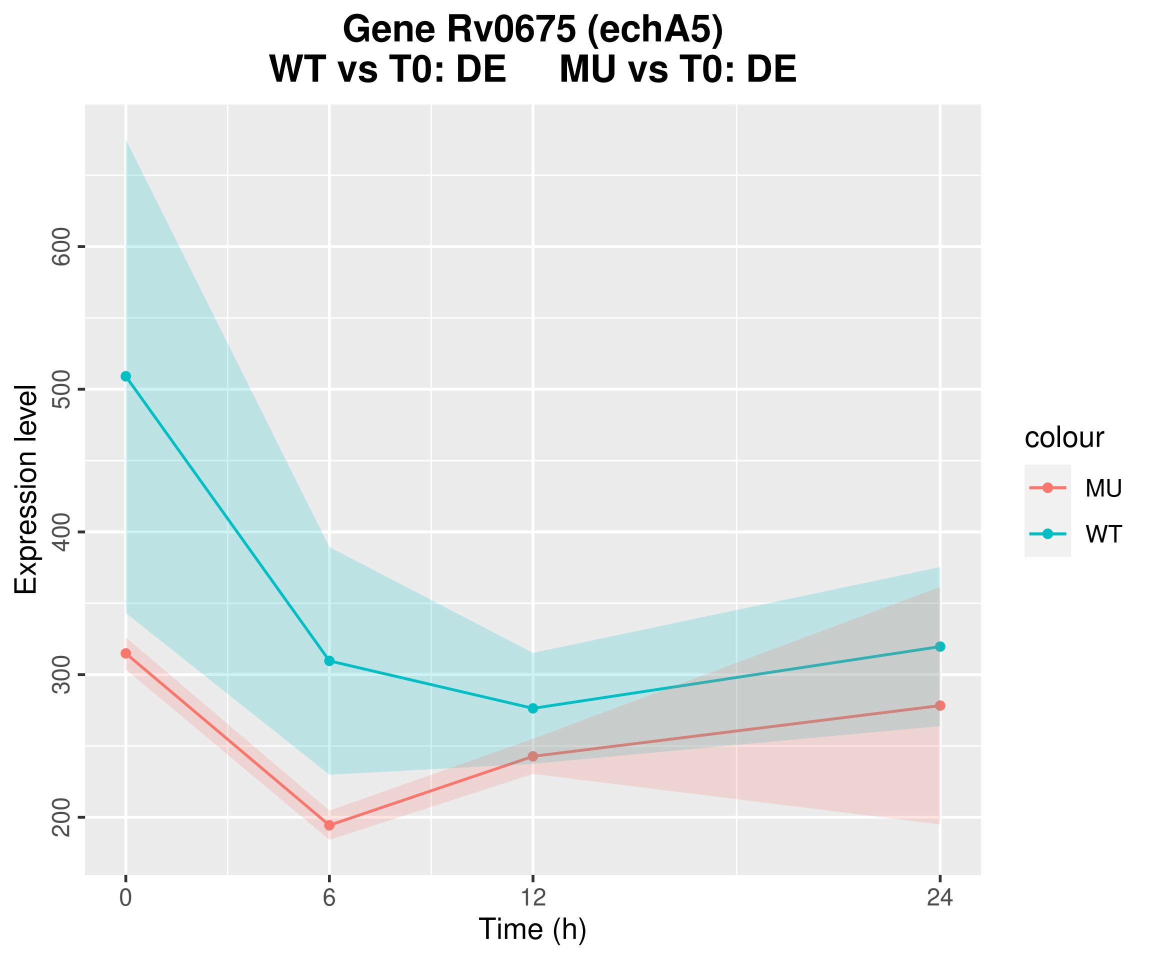

Gene Rv0675 (echA5)
WT vs T0: DE MU vs T0: DE
Expression level
600
500
400
300
200
0
6
12
24
Time (h)
colour
MU
WT

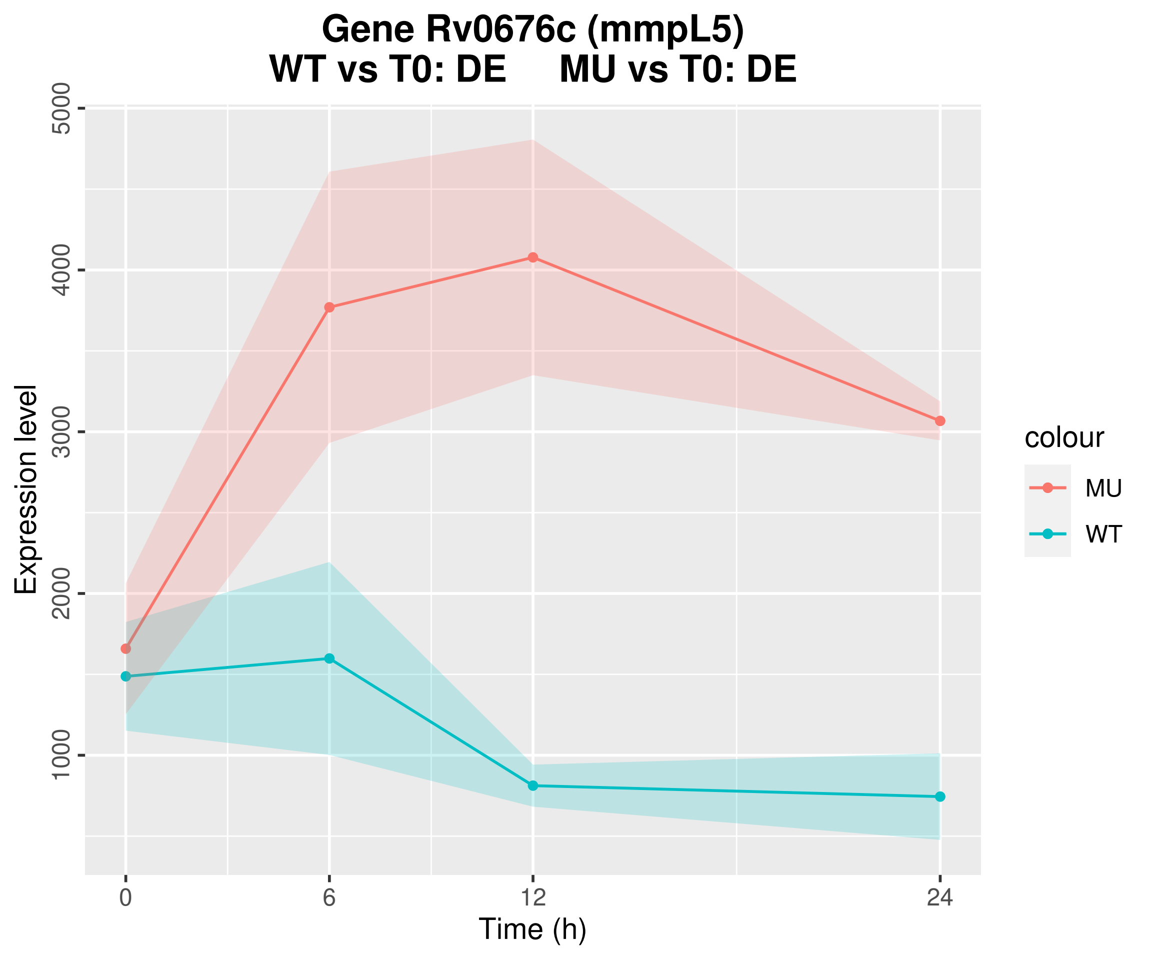
Gene Rv0676c (mmpL5)
WT vs T0: DE   MU vs T0: DE
Expression level
5000
4000
3000
2000
1000
0
6
12
24
Time (h)
colour
MU
WT

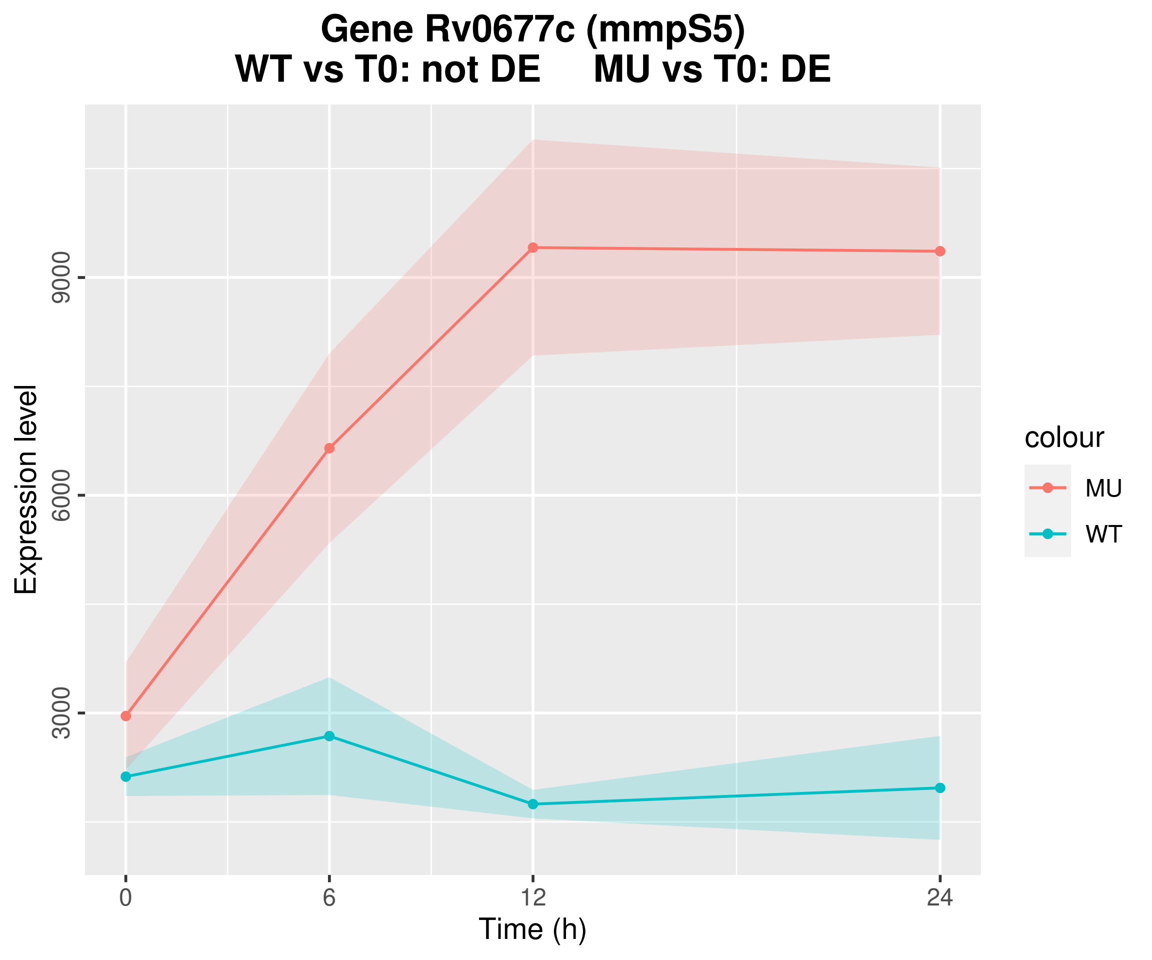
Gene Rv0677c (mmpS5)
WT vs T0: not DE MU vs T0: DE
Expression level
9000
6000
3000
0
6
12
24
Time (h)
colour
MU
WT

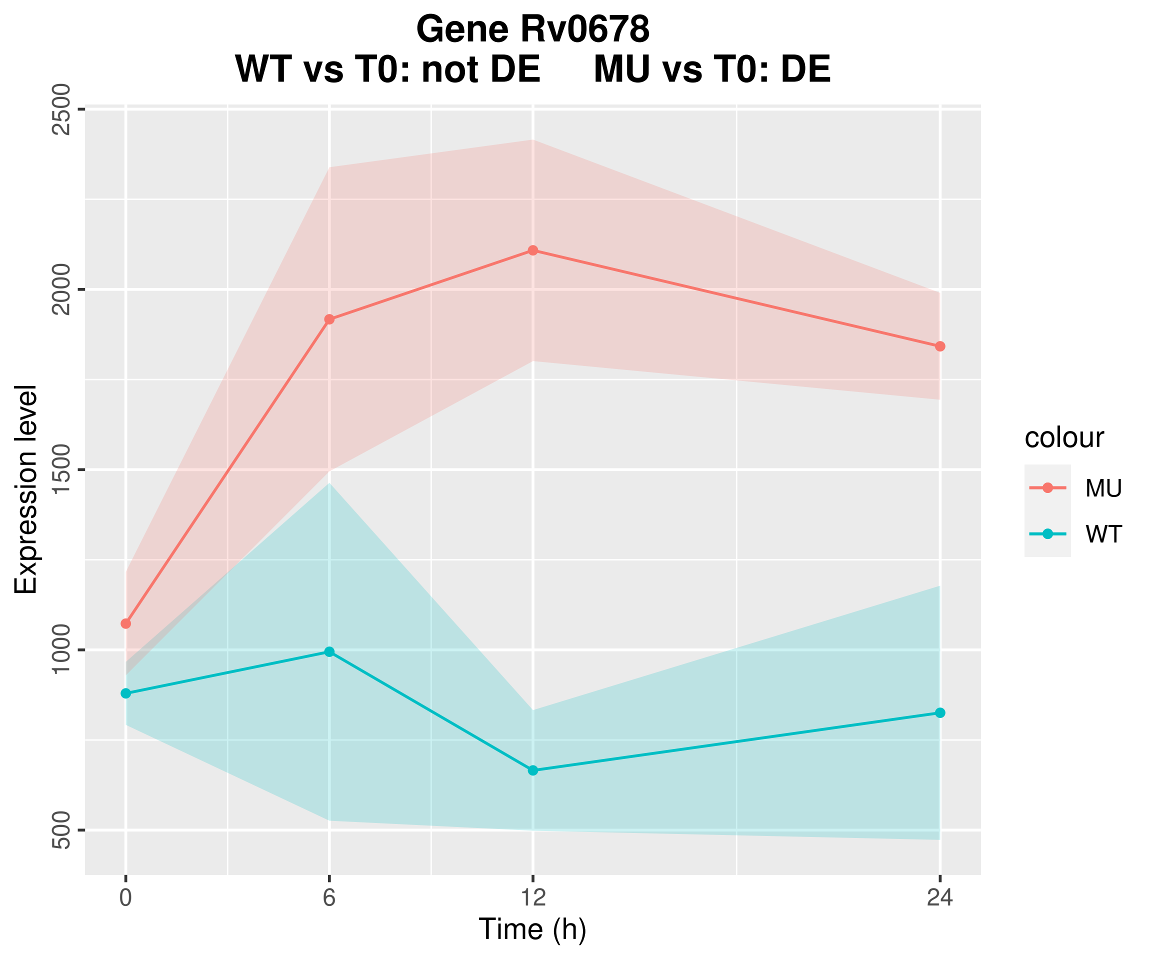

Gene Rv0678
WT vs T0: not DE MU vs T0: DE
Expression level
2500
2000
1500
1000
500
0
6
12
24
Time (h)
colour
MU
WT

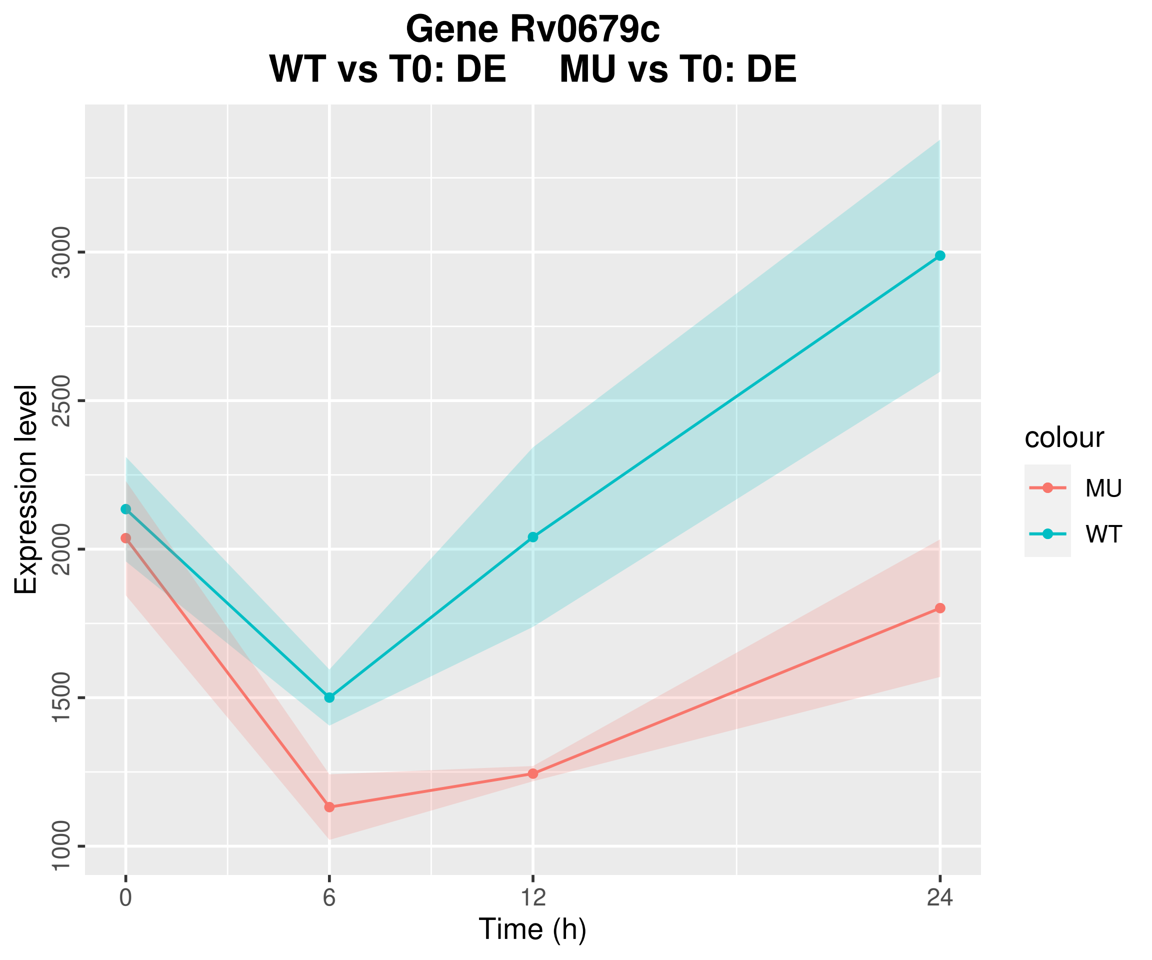

Gene Rv0679c
WT vs T0: DE   MU vs T0: DE
Expression level
3000
2500
2000
1500
1000
0
6
12
24
Time (h)
colour
MU
WT

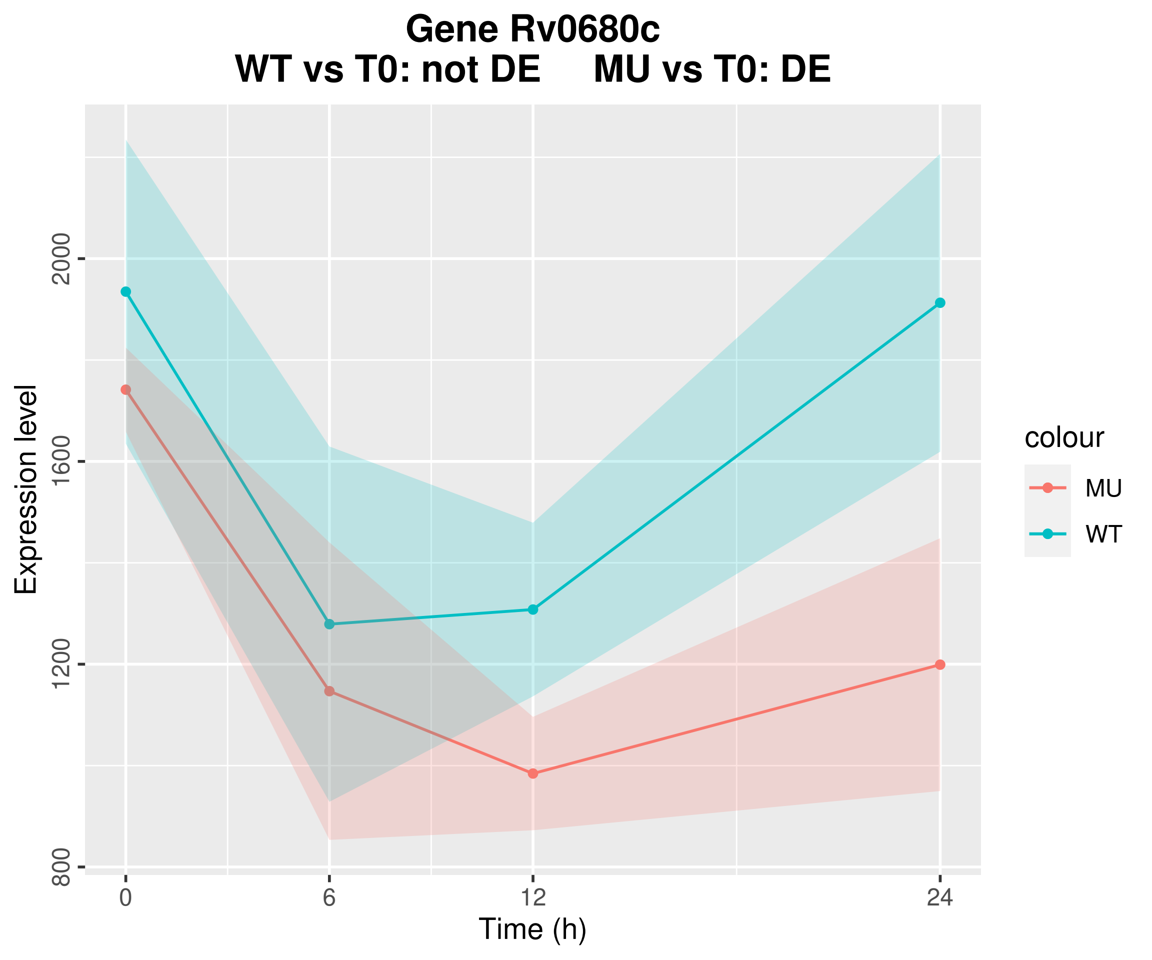

Gene Rv0680c
WT vs T0: not DE   MU vs T0: DE
Expression level
2000
1600
1200
800
0
6
12
24
Time (h)
colour
MU
WT

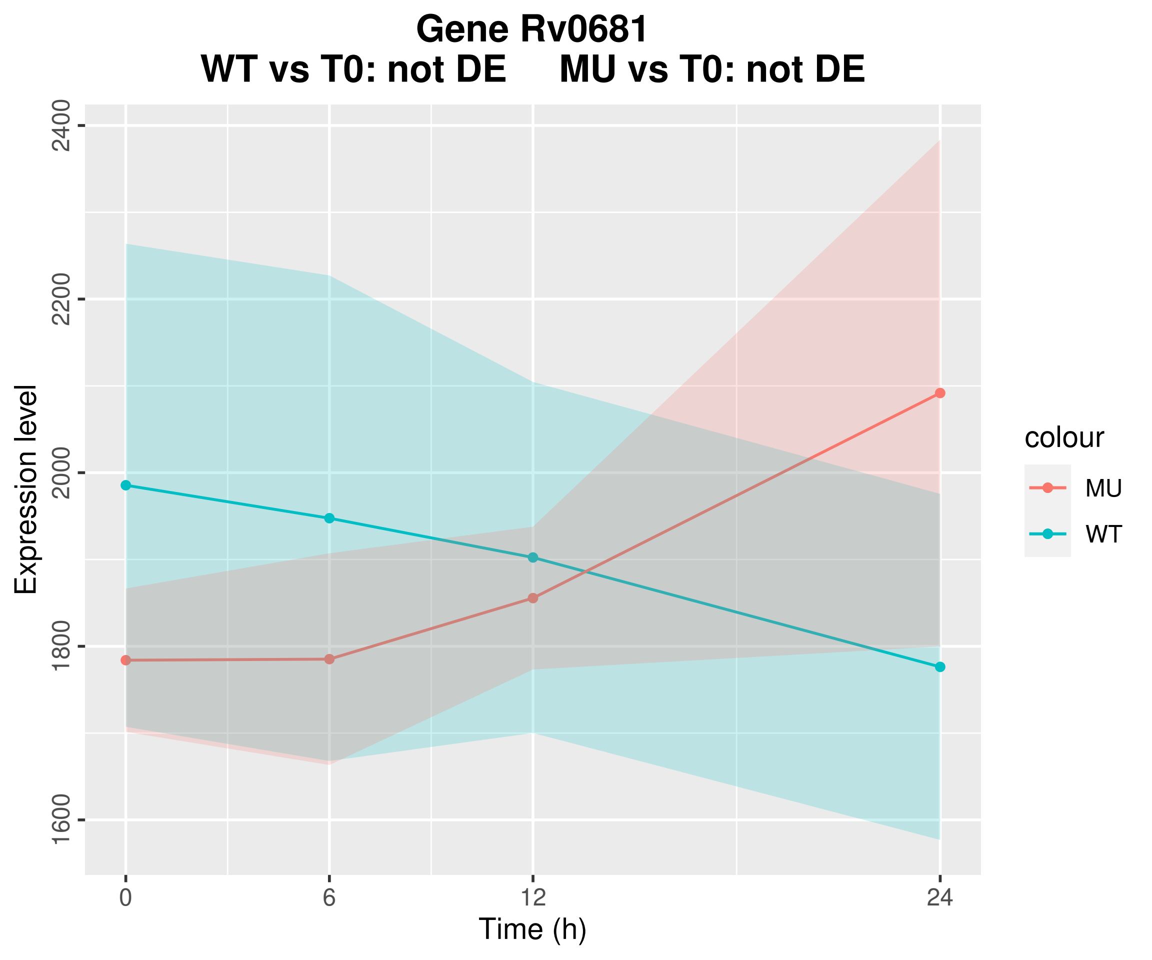

Gene Rv0681
WT vs T0: not DE MU vs T0: not DE
Expression level
2400
2200
2000
1800
1600
Time (h)
0
6
12
24
colour
MU
WT

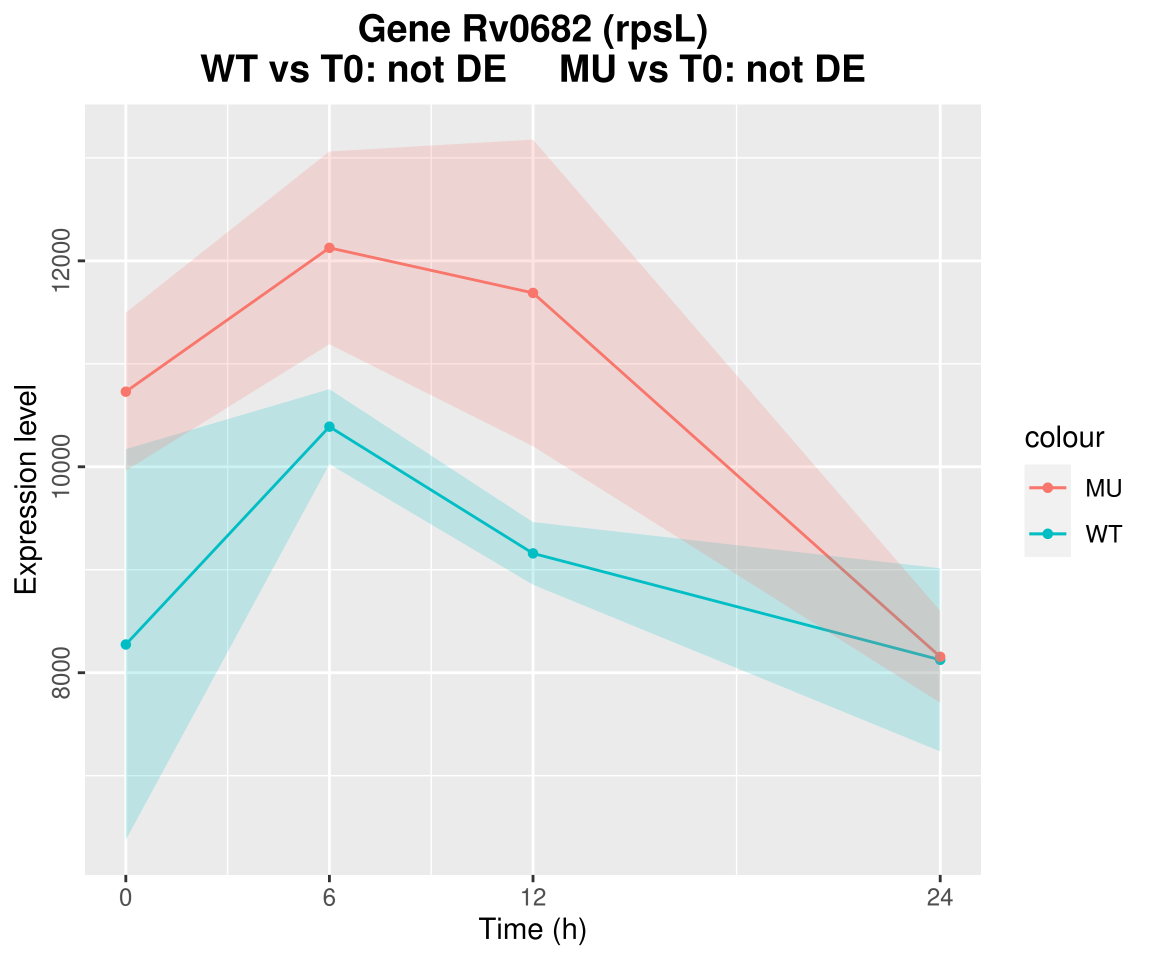

Gene Rv0682 (rpsL)
WT vs T0: not DE   MU vs T0: not DE
Expression level
12000
10000
8000
0
6
12
24
Time (h)
colour
MU
WT

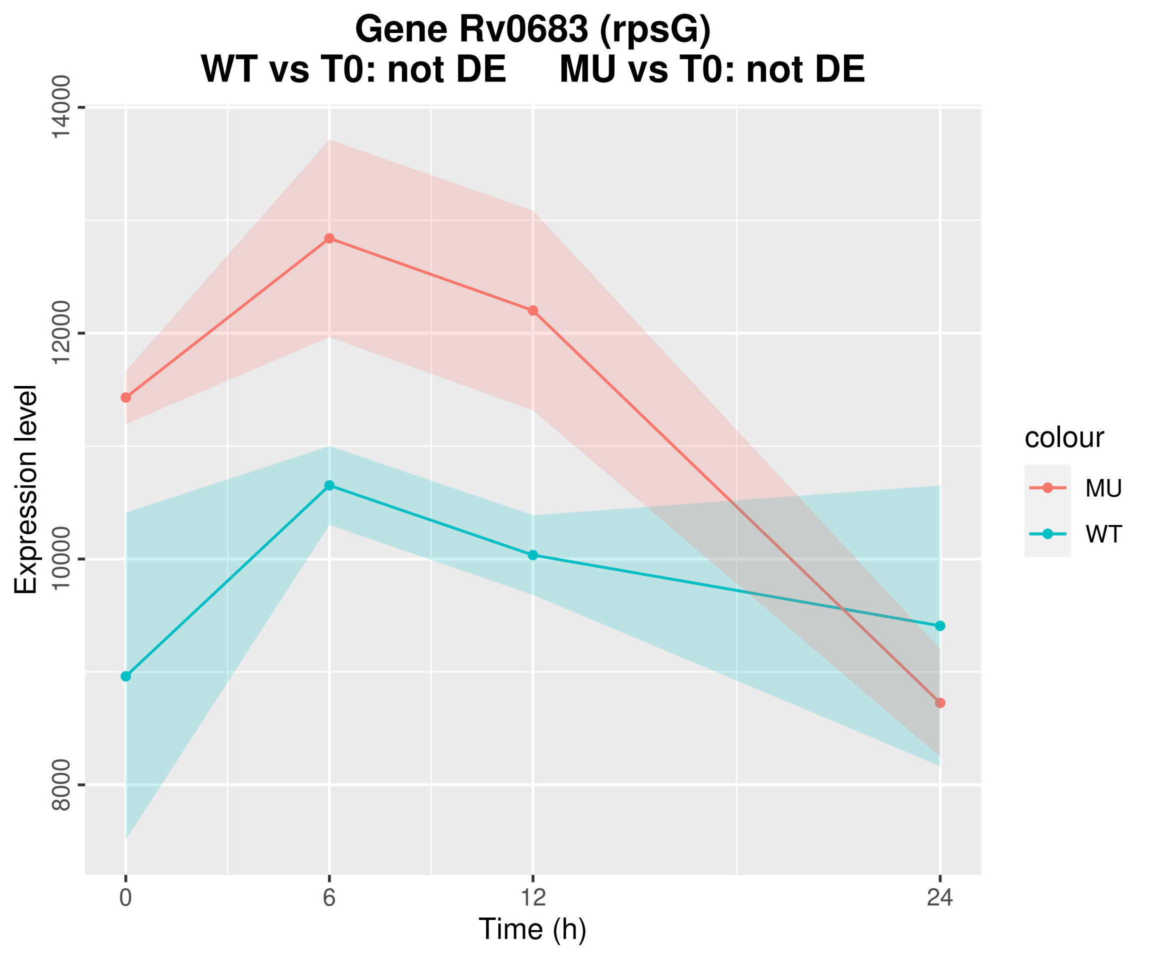

Gene Rv0683 (rpsG)
WT vs T0: not DE   MU vs T0: not DE
Expression level
14000
12000
10000
8000
0
6
12
24
Time (h)
colour
MU
WT

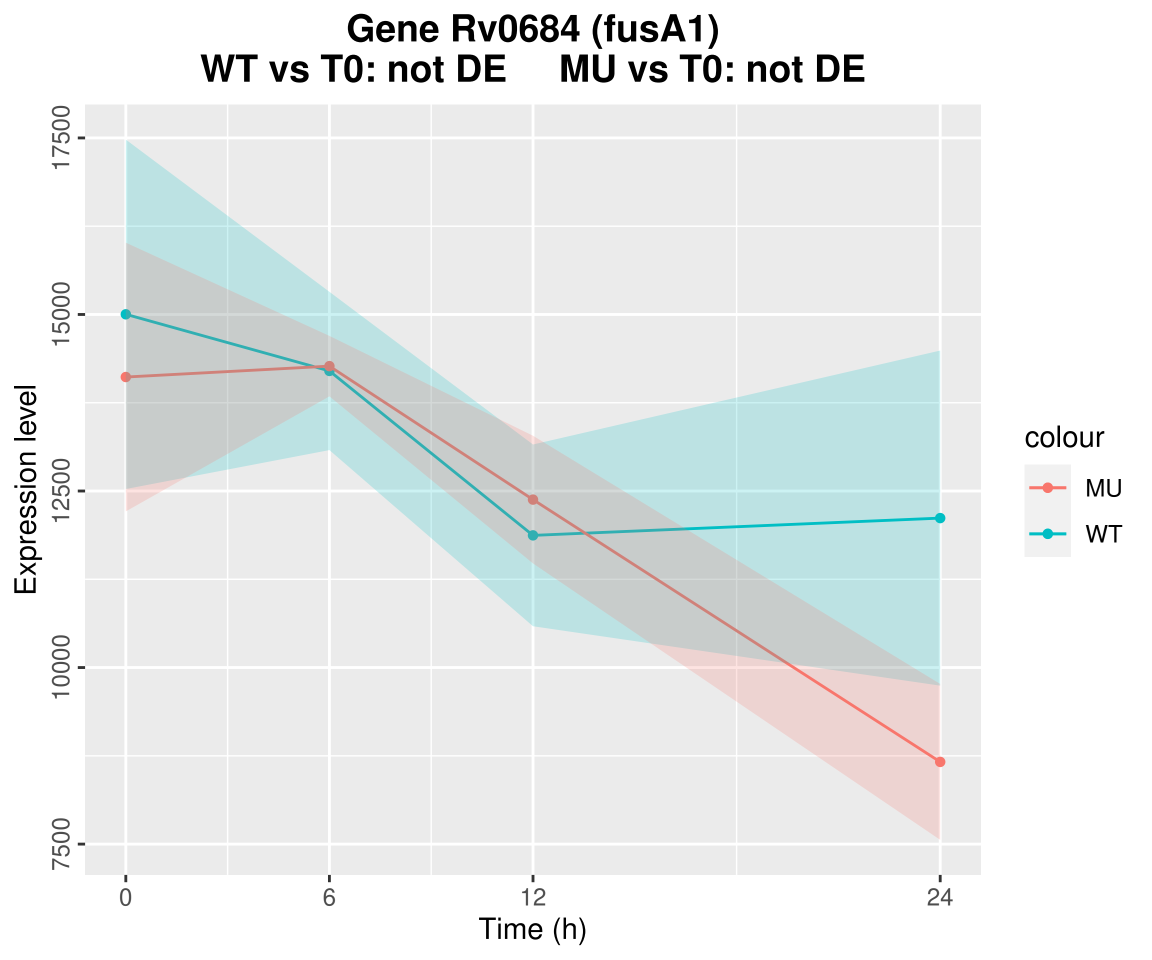

Gene Rv0684 (fusA1)
WT vs T0: not DE MU vs T0: not DE
Expression level
17500
15000
12500
10000
7500
0
6
12
24
Time (h)
colour
MU
WT

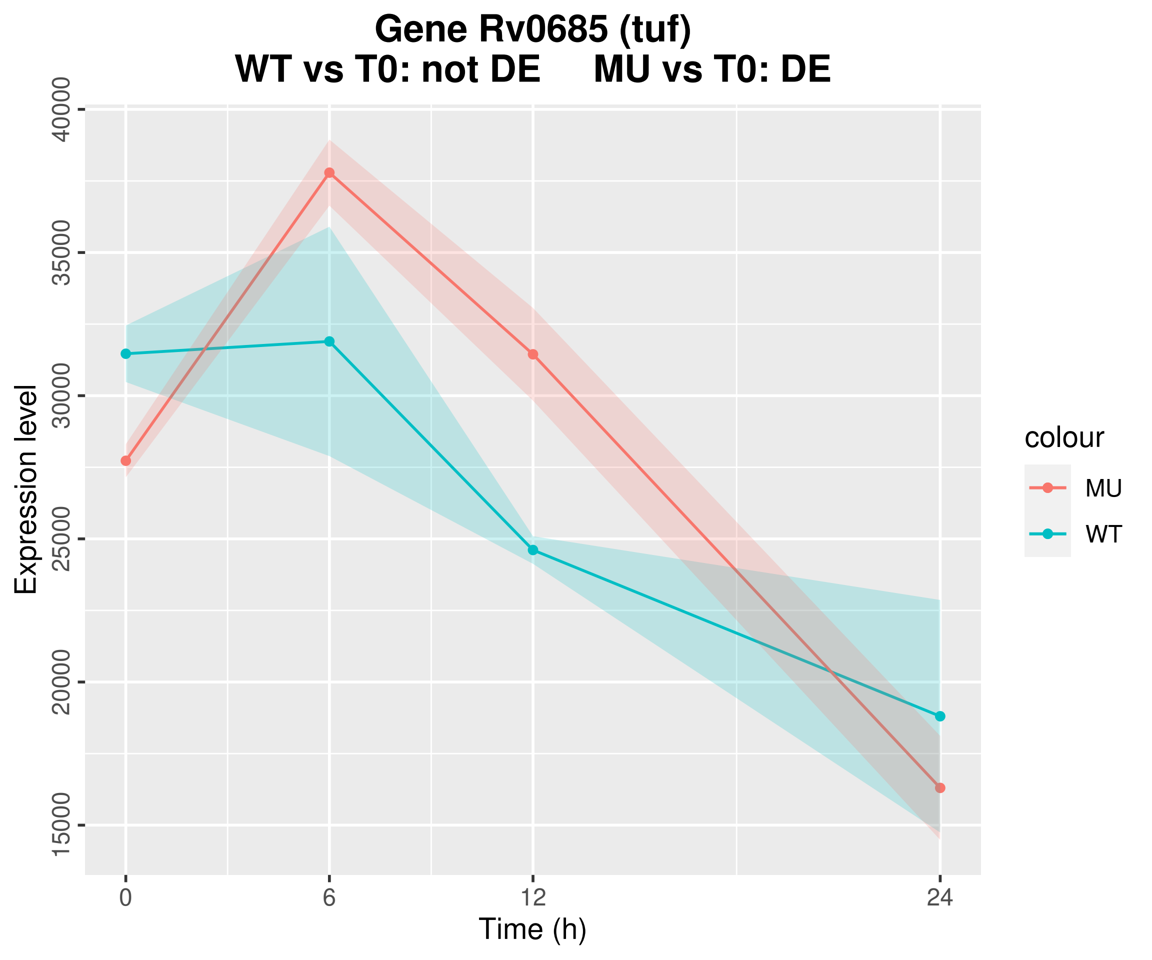

Gene Rv0685 (tuf)
WT vs T0: not DE   MU vs T0: DE
Expression level
40000
35000
30000
25000
20000
15000
0
6
12
24
Time (h)
colour
MU
WT

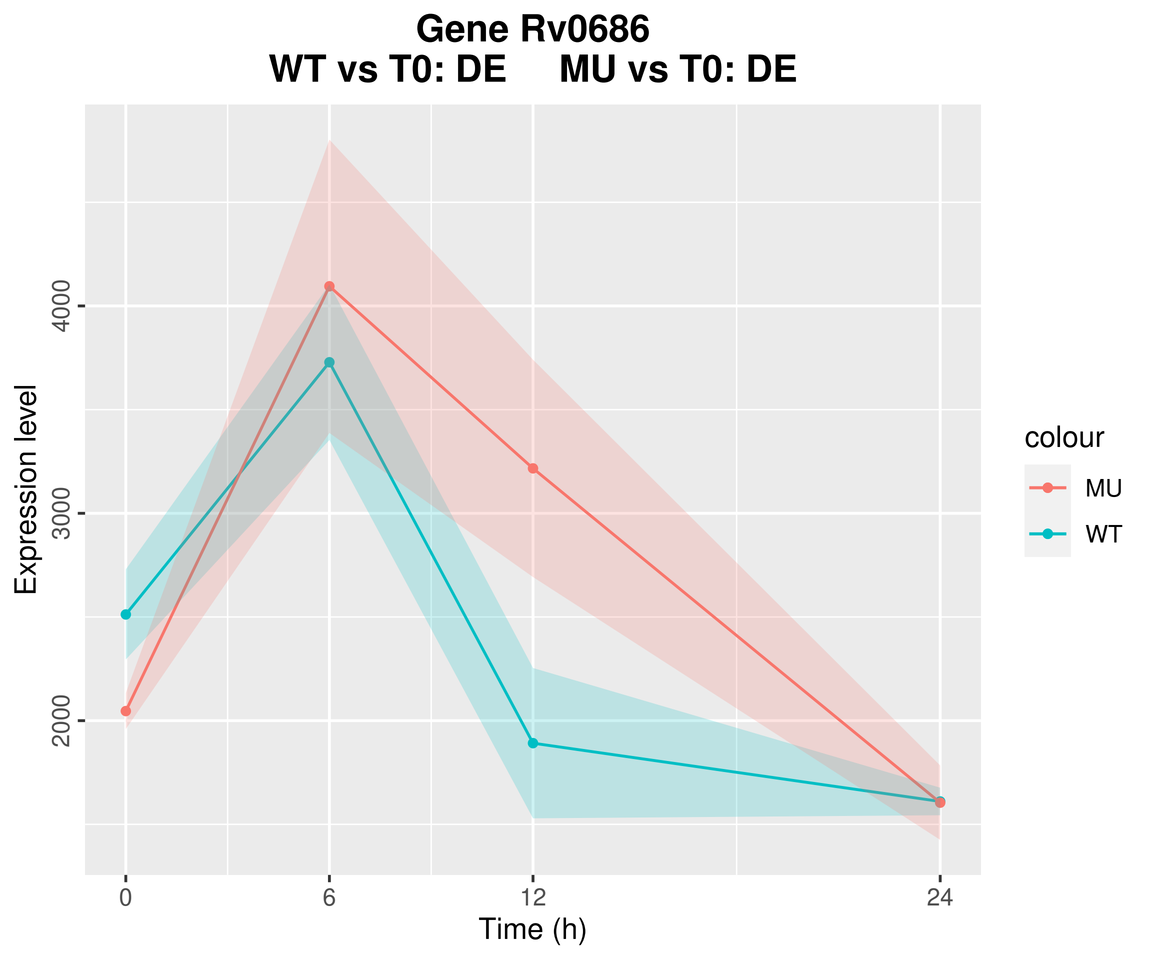

Gene Rv0686
WT vs T0: DE MU vs T0: DE
Expression level
4000
3000
2000
0
6
12
24
Time (h)
colour
MU
WT

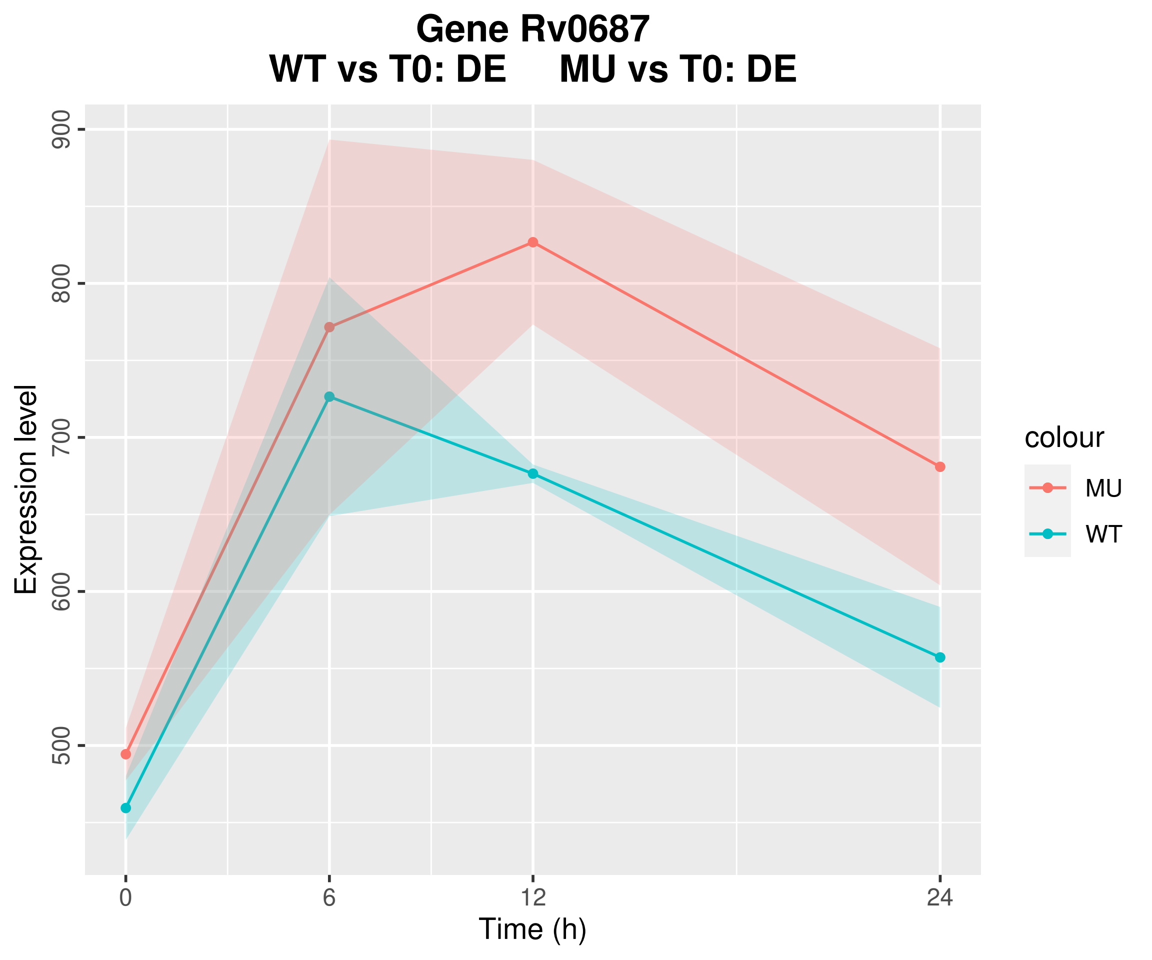

Gene Rv0687
WT vs T0: DE MU vs T0: DE
900
800
700
600
500
Expression level
0
6
12
24
Time (h)
colour
MU
WT

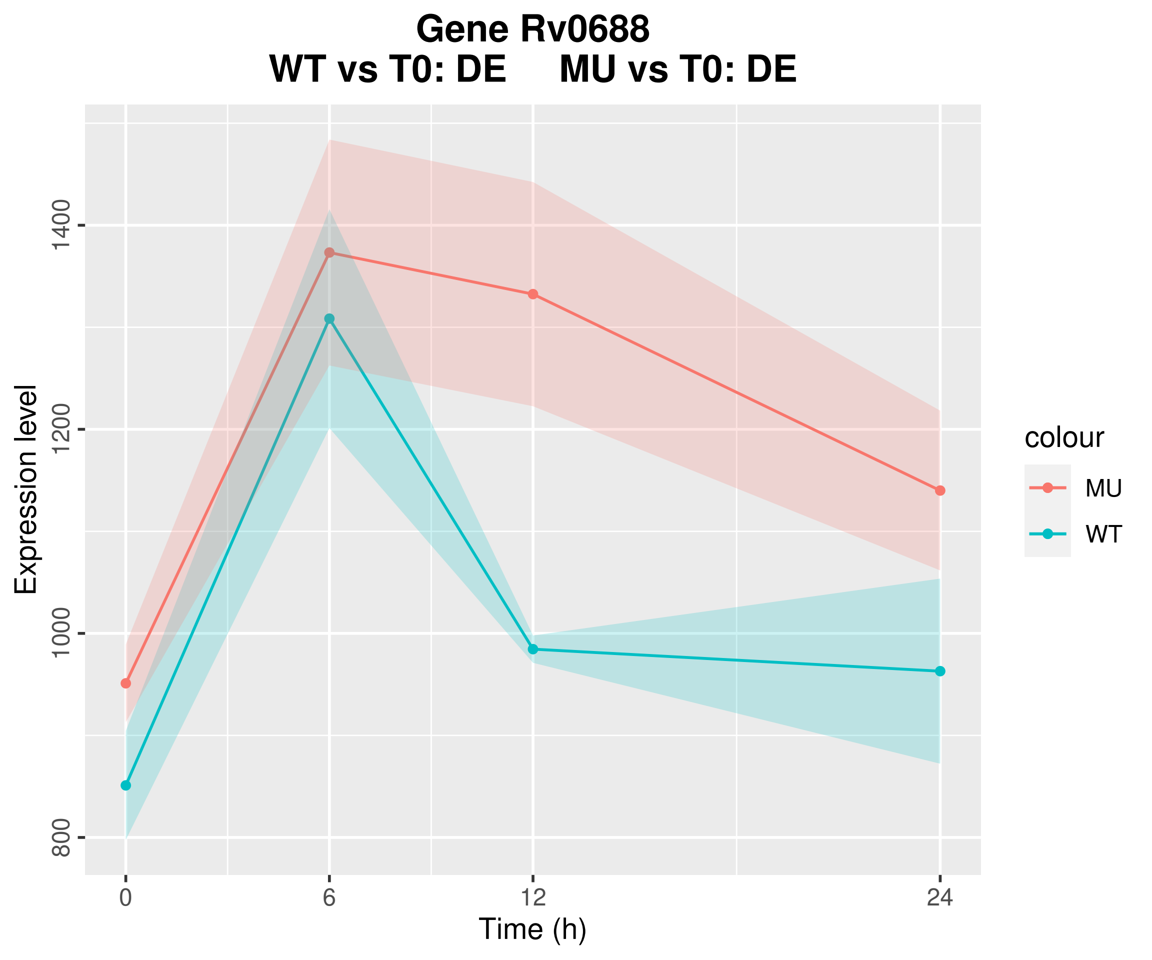
Gene Rv0688
WT vs T0: DE MU vs T0: DE
Expression level
1400
1200
1000
800
0
6
12
24
Time (h)
colour
MU
WT

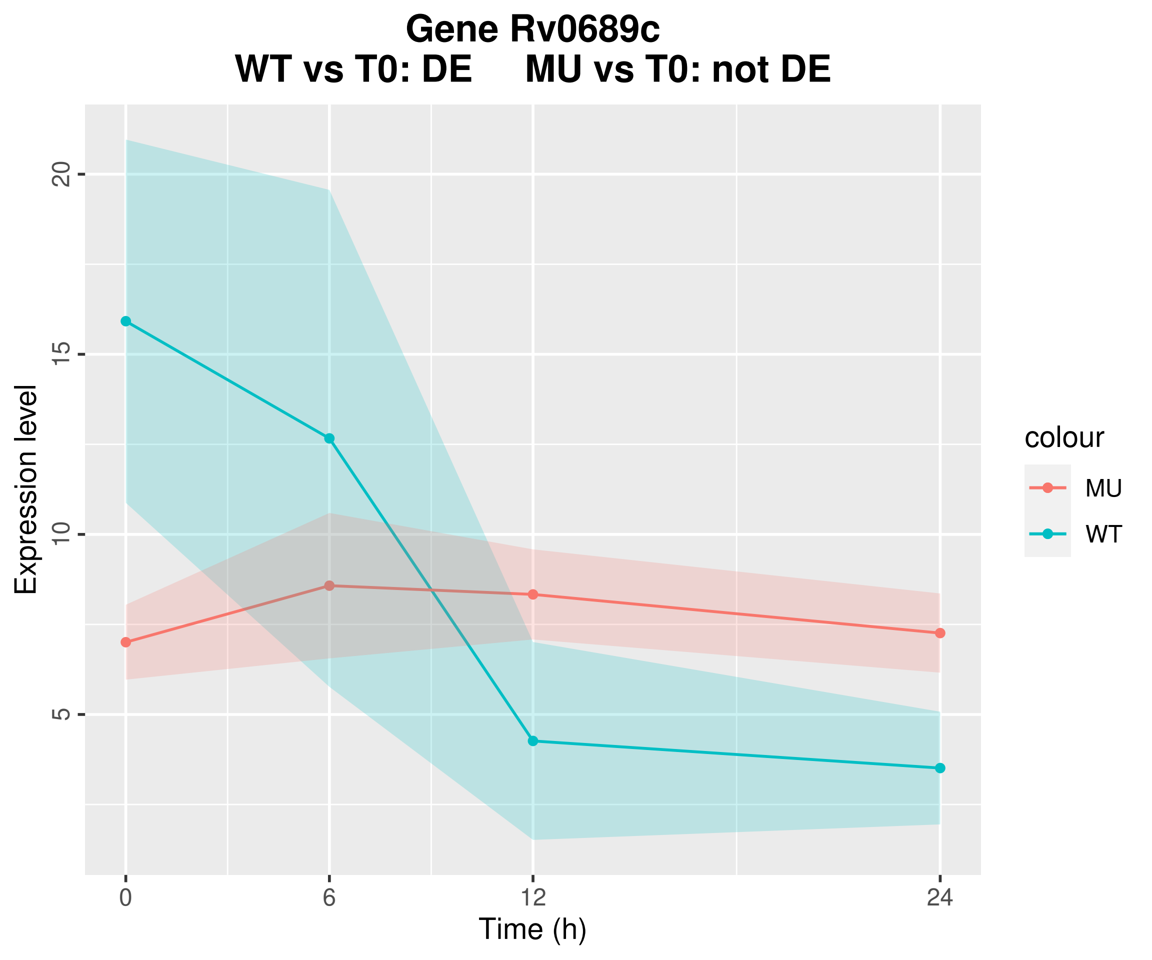
Gene Rv0689c
WT vs T0: DE   MU vs T0: not DE
Expression level
20
15
10
5
0
6
12
24
Time (h)
colour
MU
WT

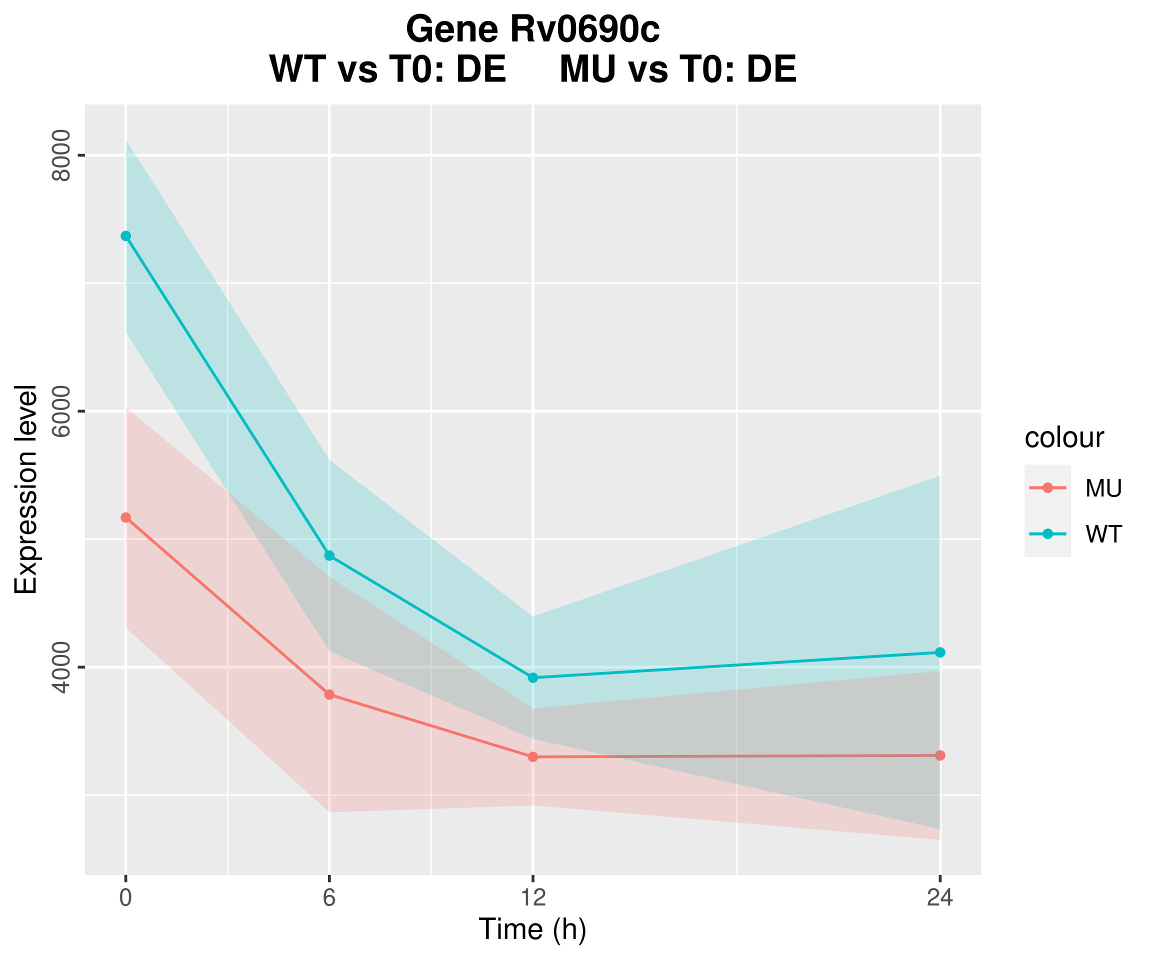

Gene Rv0690c
WT vs T0: DE MU vs T0: DE
Expression level
8000
6000
4000
0
6
12
24
Time (h)
colour
MU
WT

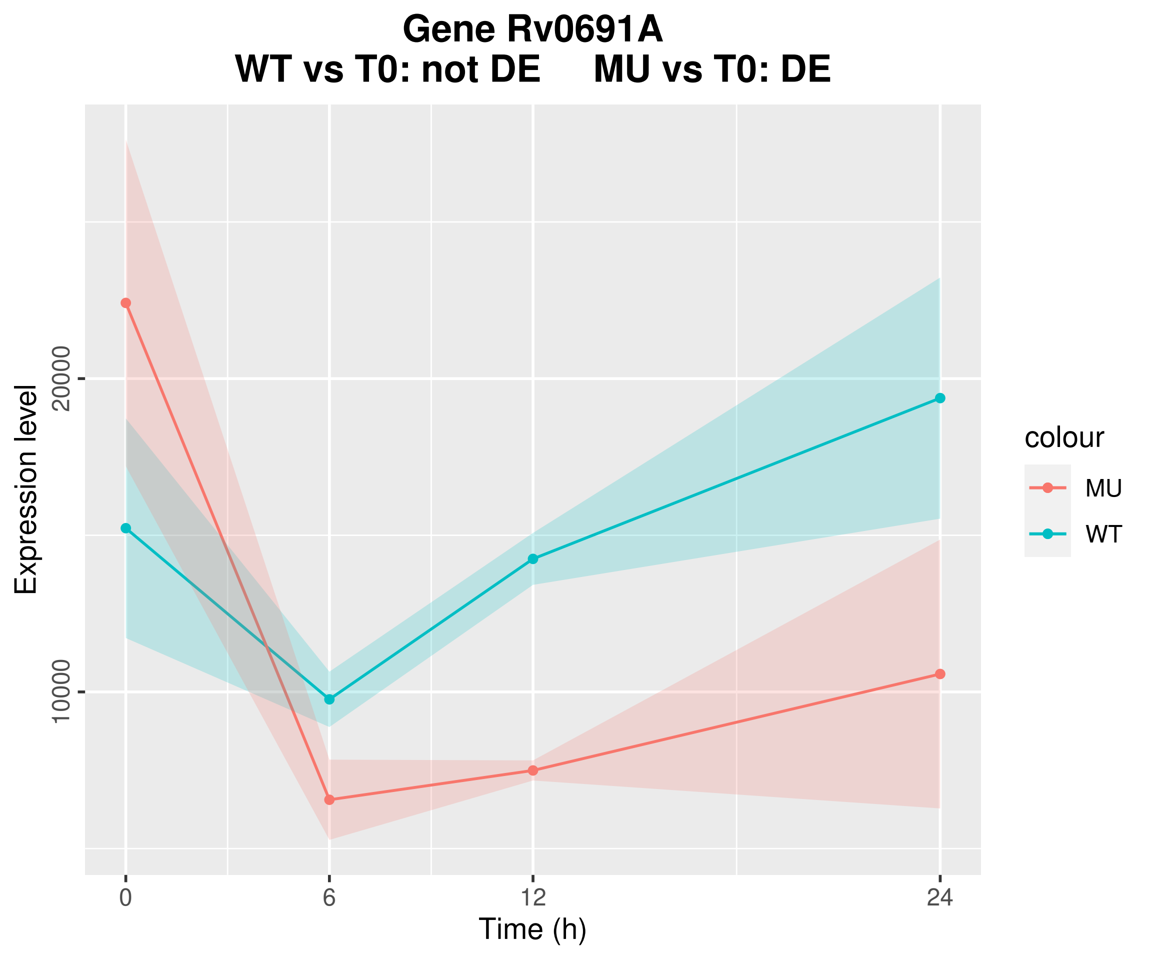
Gene Rv0691A
WT vs T0: not DE   MU vs T0: DE
Expression level
20000
10000
0
6
12
24
Time (h)
colour
MU
WT

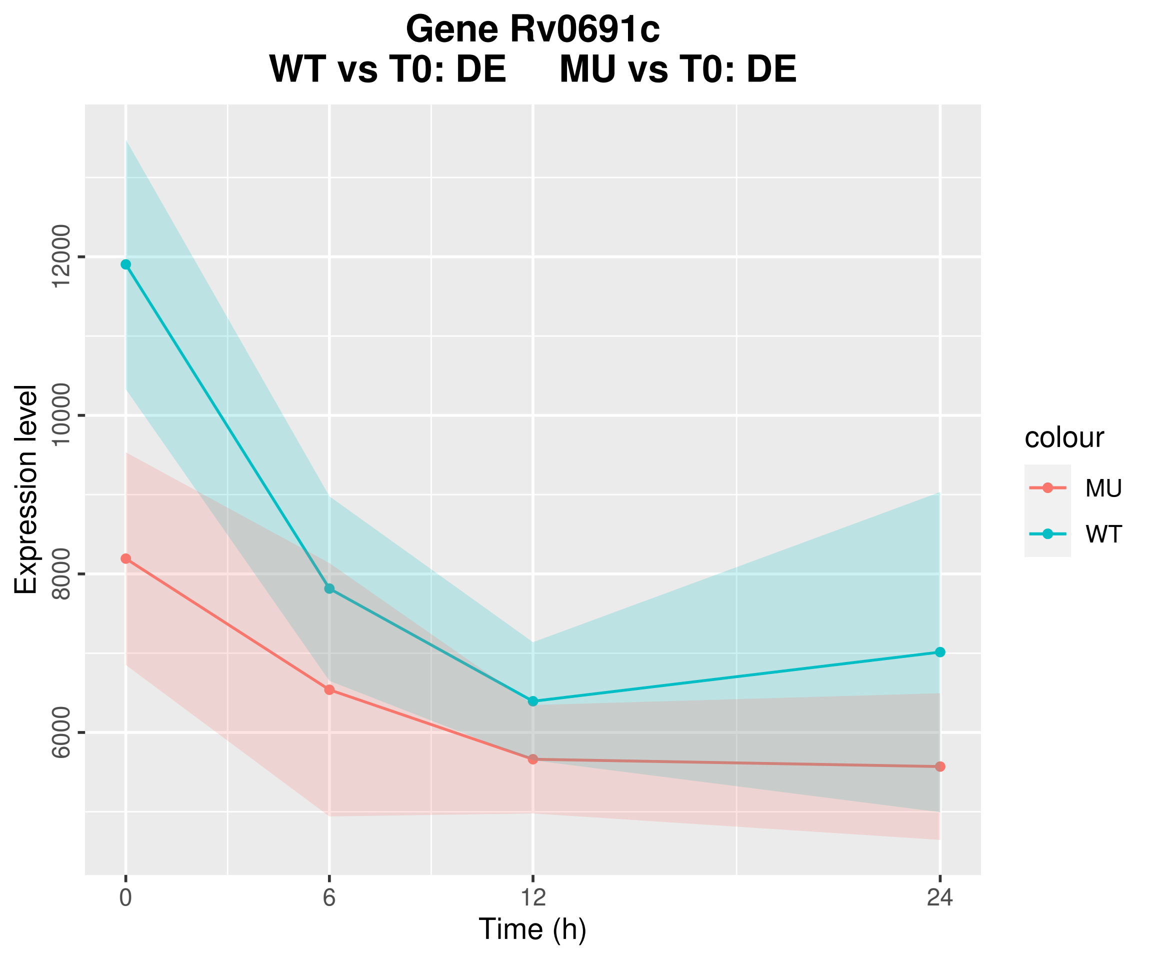

Gene Rv0691c
WT vs T0: DE MU vs T0: DE
Expression level
12000
10000
8000
6000
0
6
12
24
Time (h)
colour
MU
WT

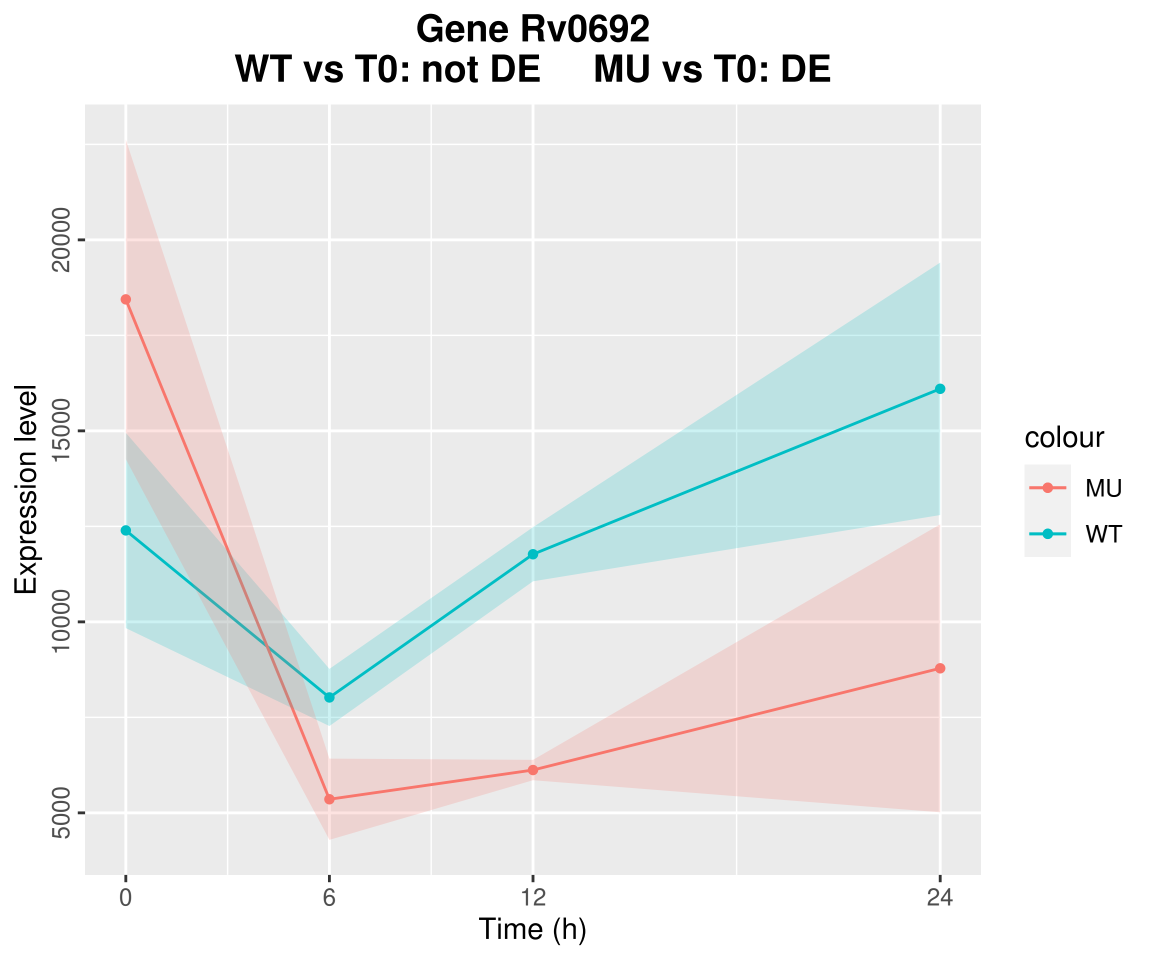

Gene Rv0692
WT vs T0: not DE   MU vs T0: DE
20000
15000
10000
5000
Expression level
0
6
12
24
Time (h)
colour
MU
WT

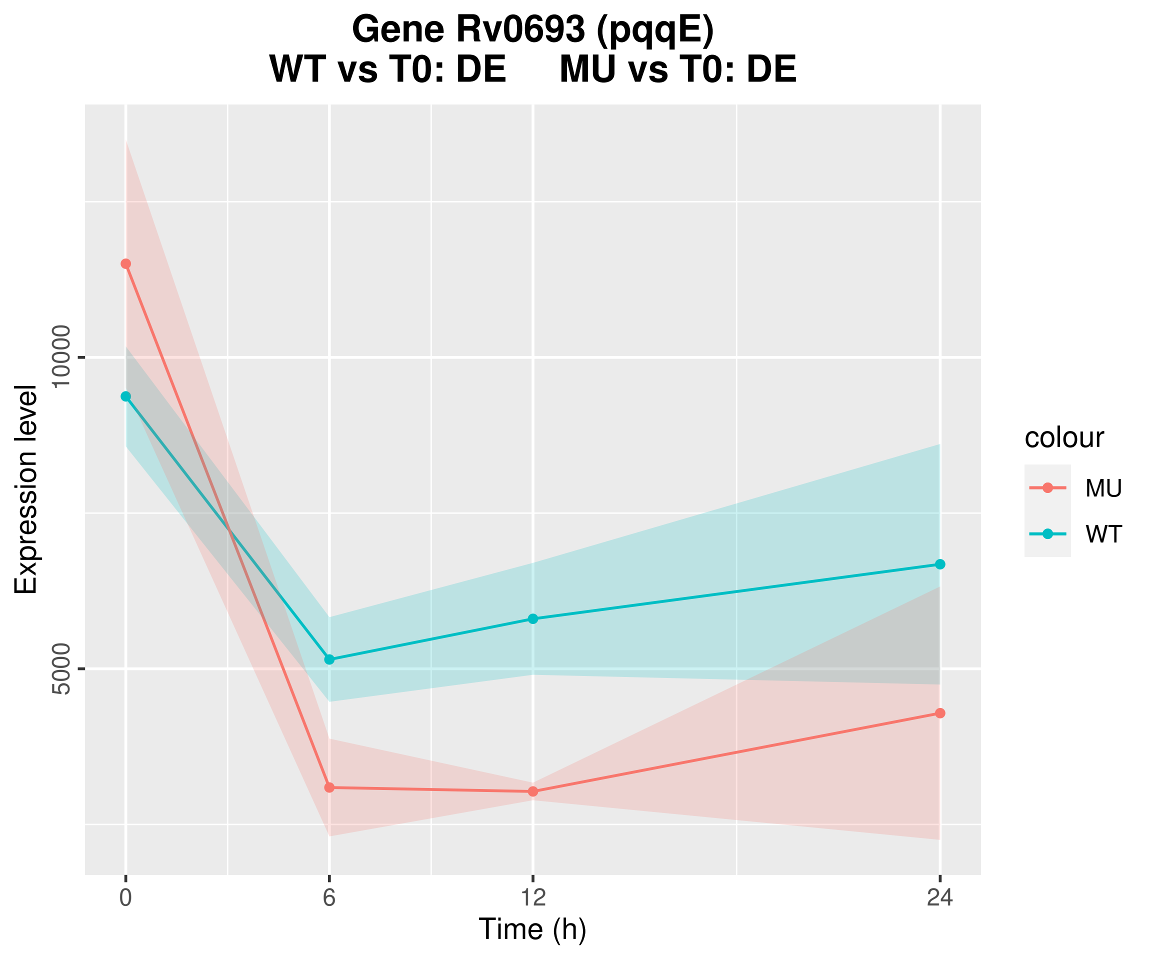

Gene Rv0693 (pqqE)
WT vs T0: DE MU vs T0: DE
Expression level
10000
5000
0
6
12
24
Time (h)
colour
MU
WT

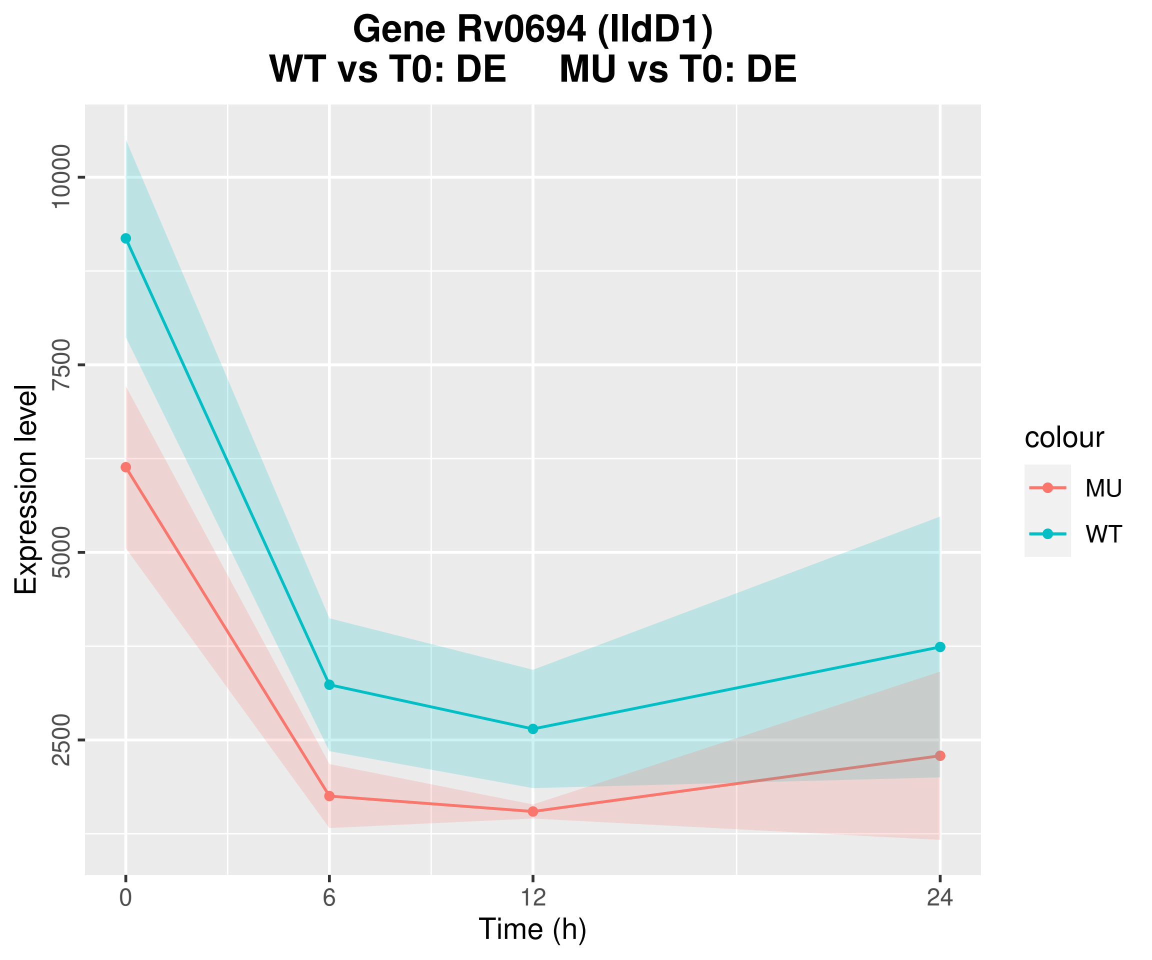

Gene Rv0694 (lldD1)
WT vs T0: DE MU vs T0: DE
10000
7500
5000
2500
Expression level
0
6
12
24
Time (h)
colour
MU
WT

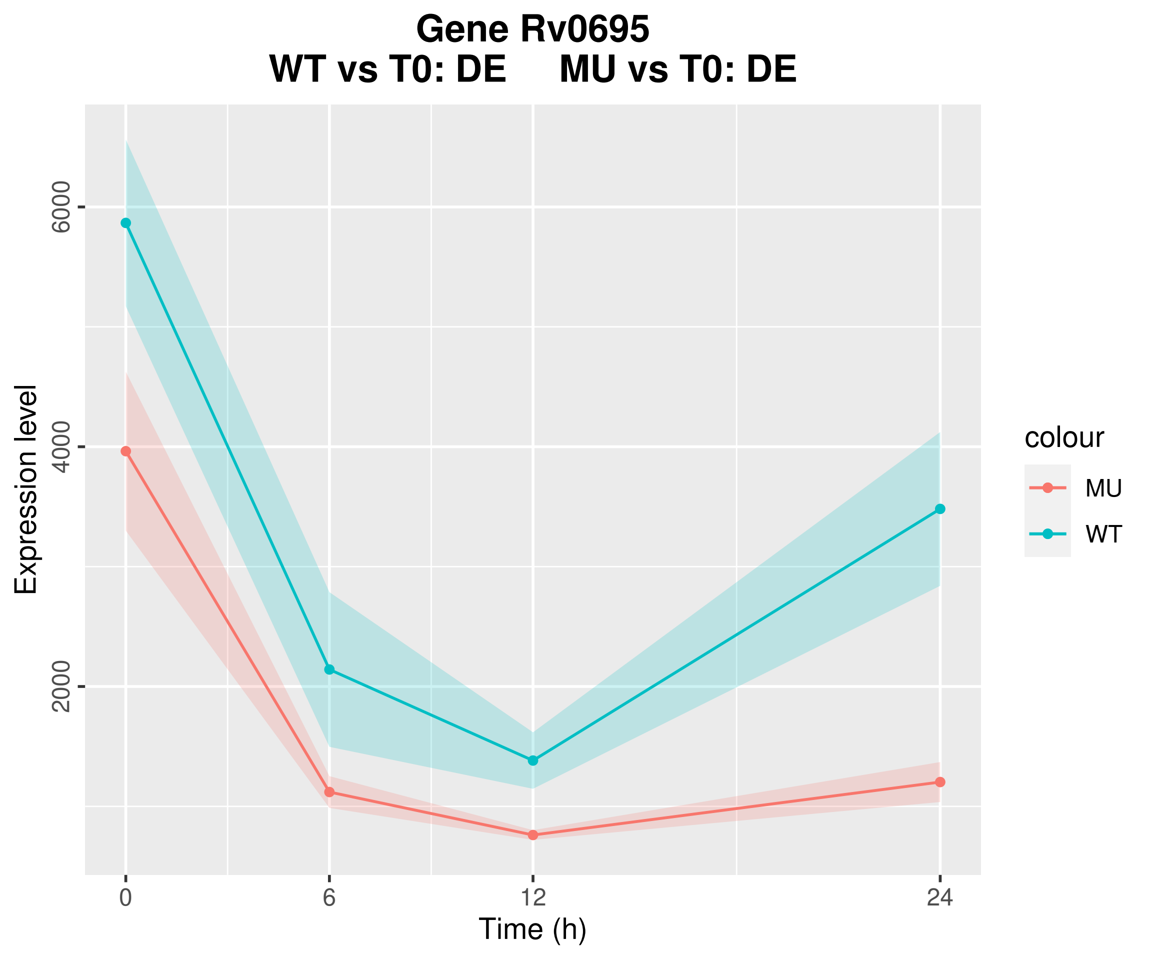
Gene Rv0695
WT vs T0: DE MU vs T0: DE
Expression level
6000
4000
2000
0
6
12
24
Time (h)
colour
MU
WT

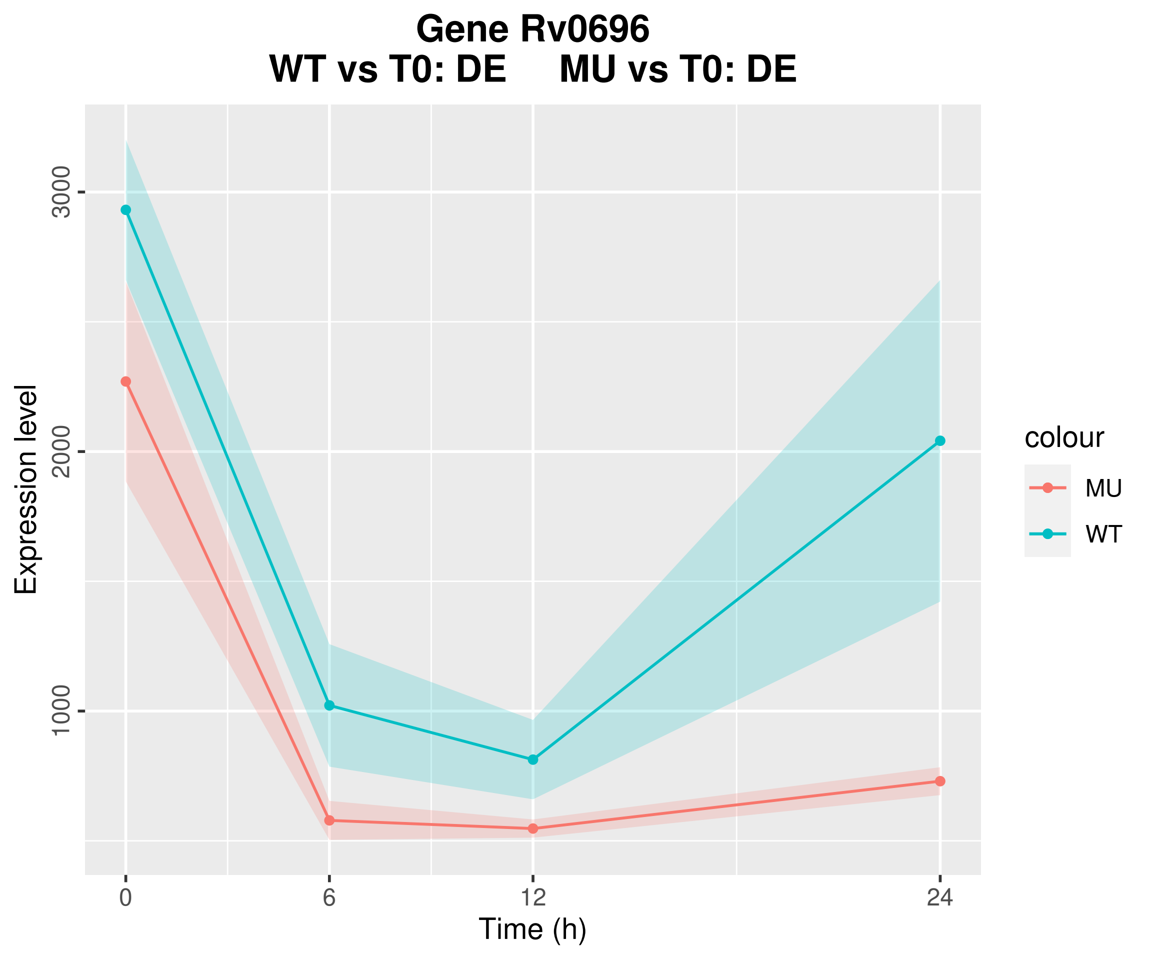

Gene Rv0696
WT vs T0: DE MU vs T0: DE
Expression level
3000
2000
1000
0
6
12
24
Time (h)
colour
MU
WT

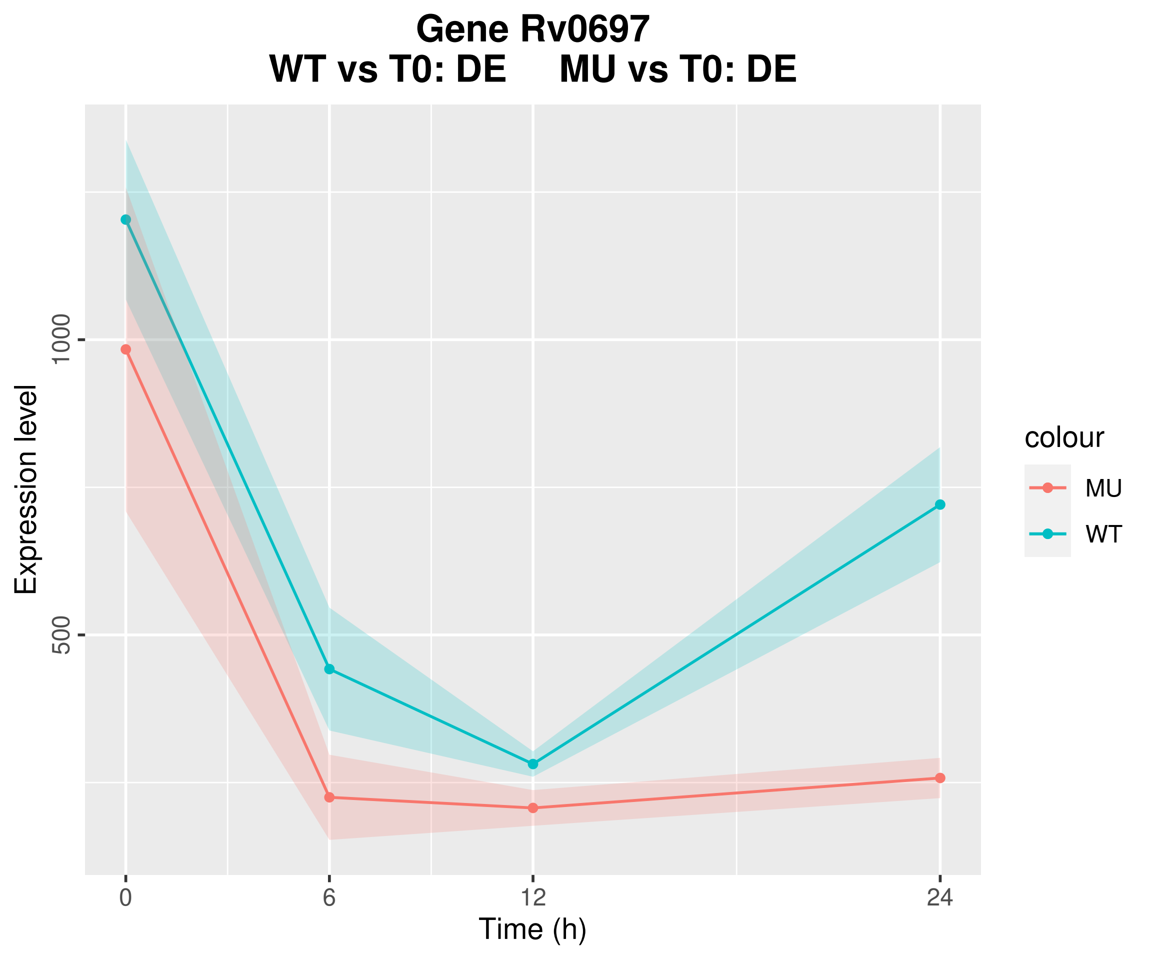
Gene Rv0697
WT vs T0: DE MU vs T0: DE
Expression level
1000
500
0
6
12
24
Time (h)
colour
MU
WT

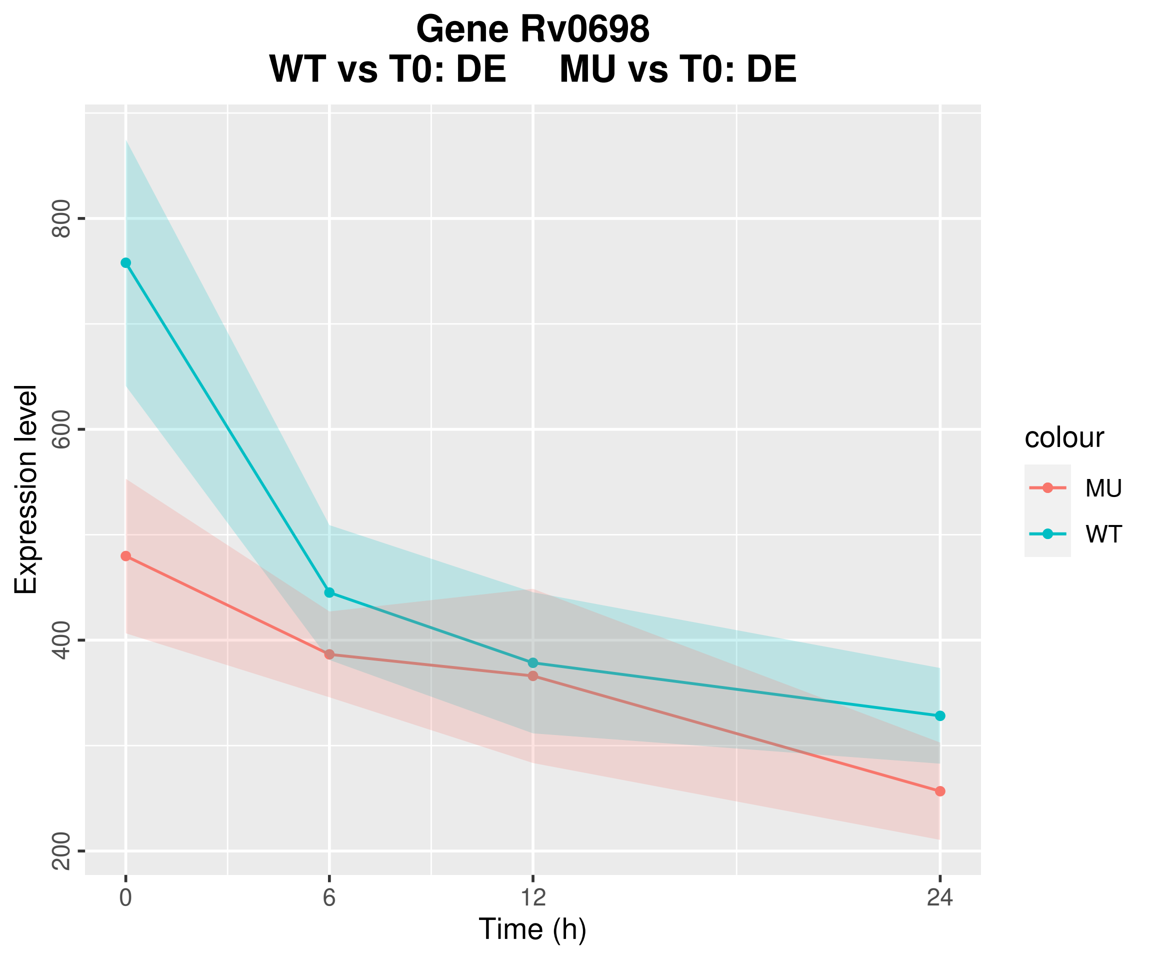
Gene Rv0698
WT vs T0: DE MU vs T0: DE
Expression level
800
600
400
200
0
6
12
24
Time (h)
colour
MU
WT

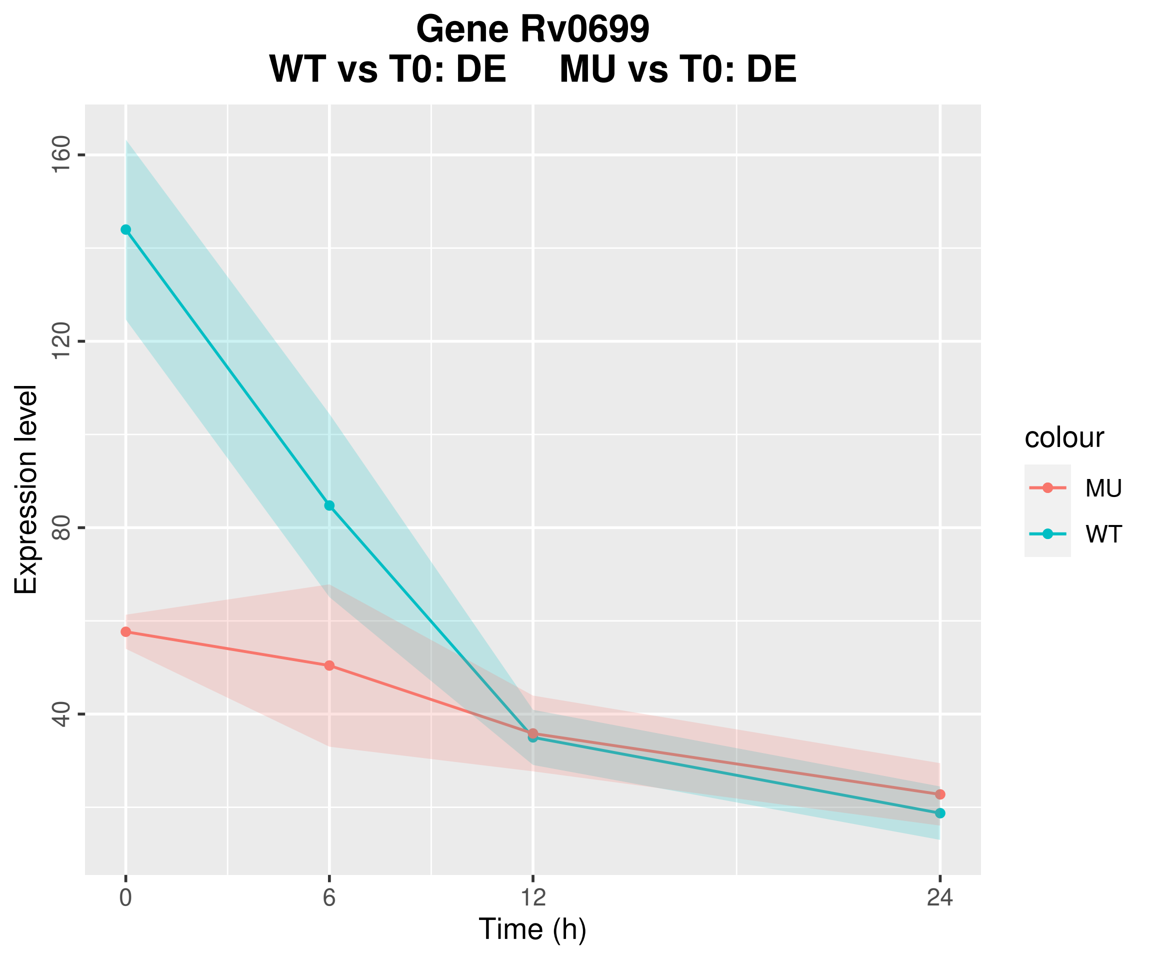
Gene Rv0699
WT vs T0: DE   MU vs T0: DE
Expression level
160
120
80
40
0
6
12
24
Time (h)
colour
MU
WT

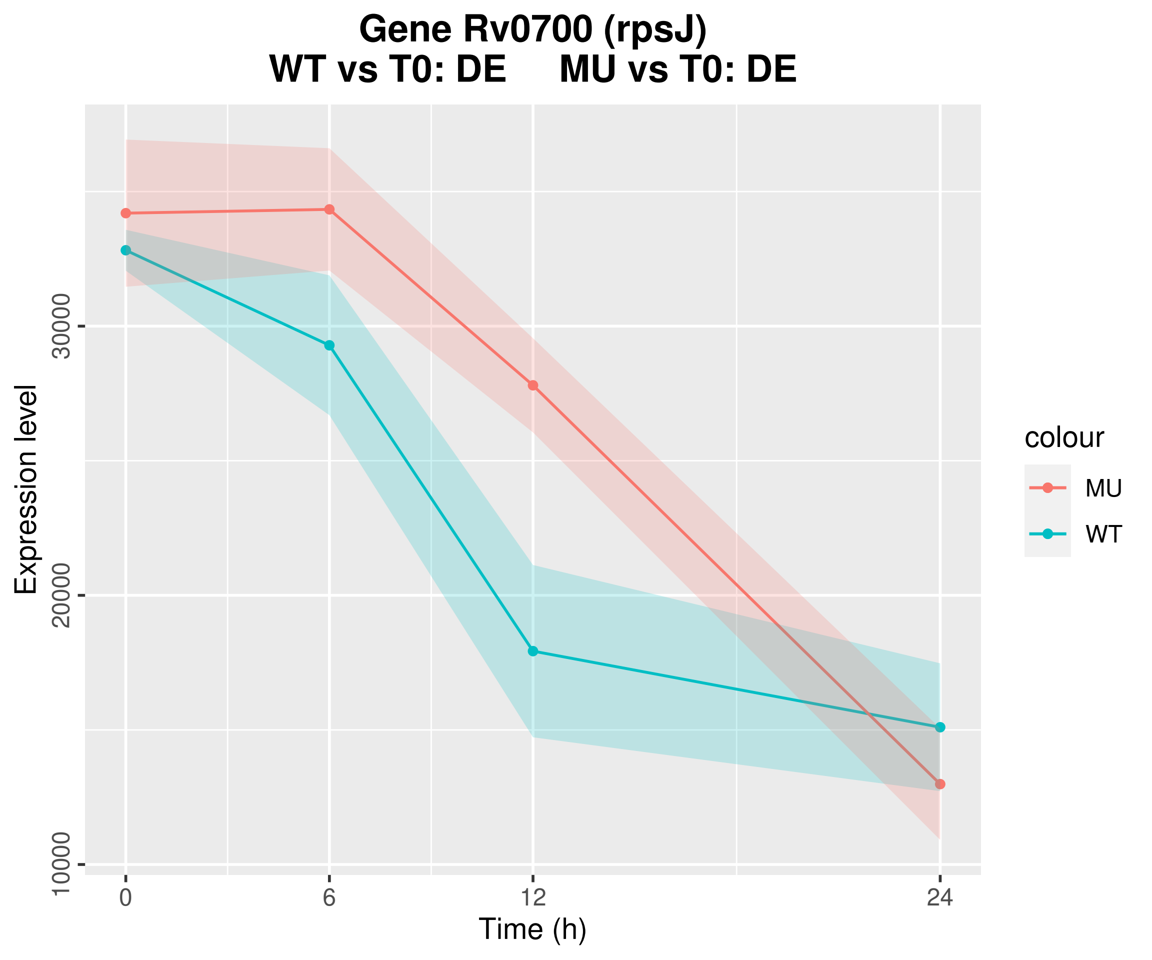
Gene Rv0700 (rpsJ)
WT vs T0: DE   MU vs T0: DE
Expression level
30000
20000
10000
0
6
12
24
Time (h)
colour
MU
WT

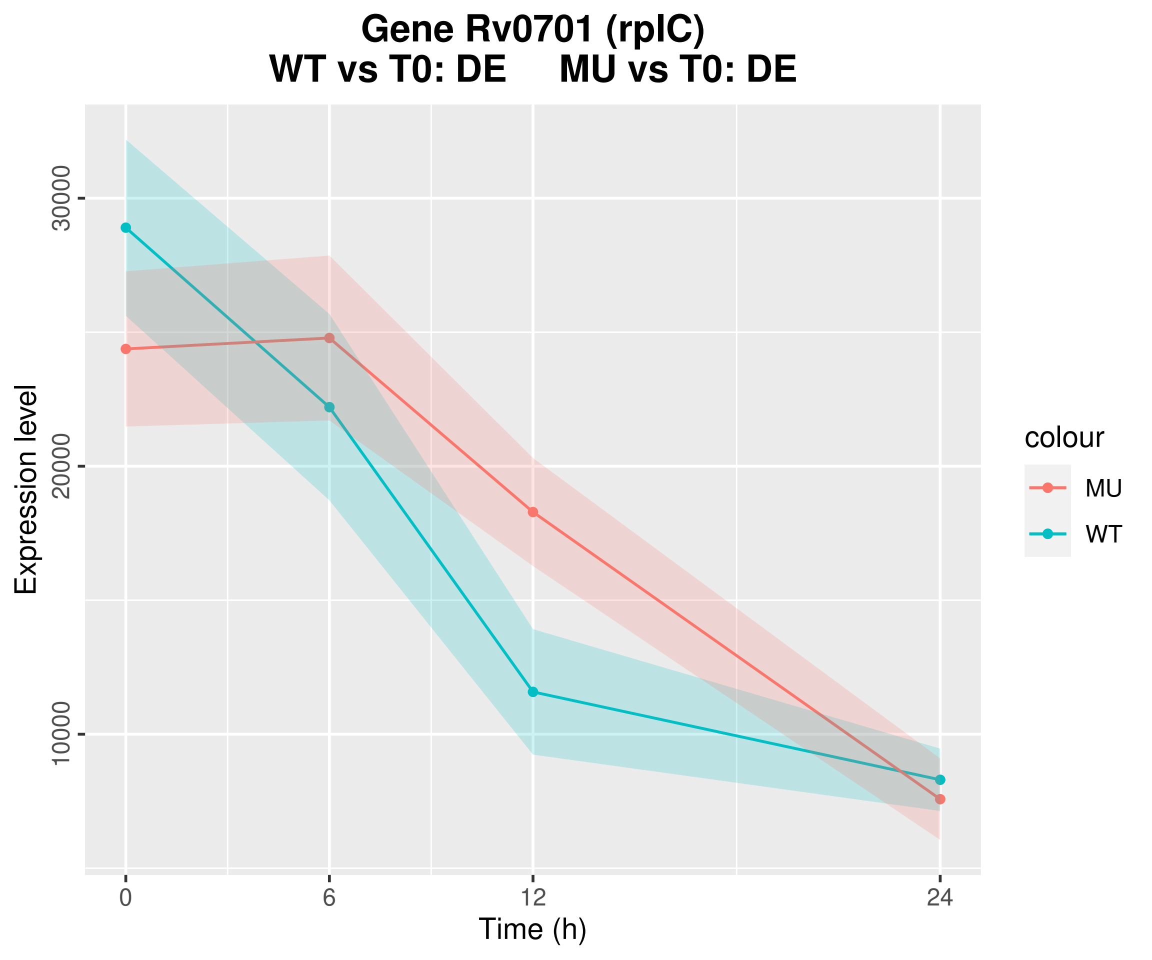

Gene Rv0701 (rplC)
WT vs T0: DE MU vs T0: DE
30000
20000
10000
Expression level
0
6
12
24
Time (h)
colour
MU
WT

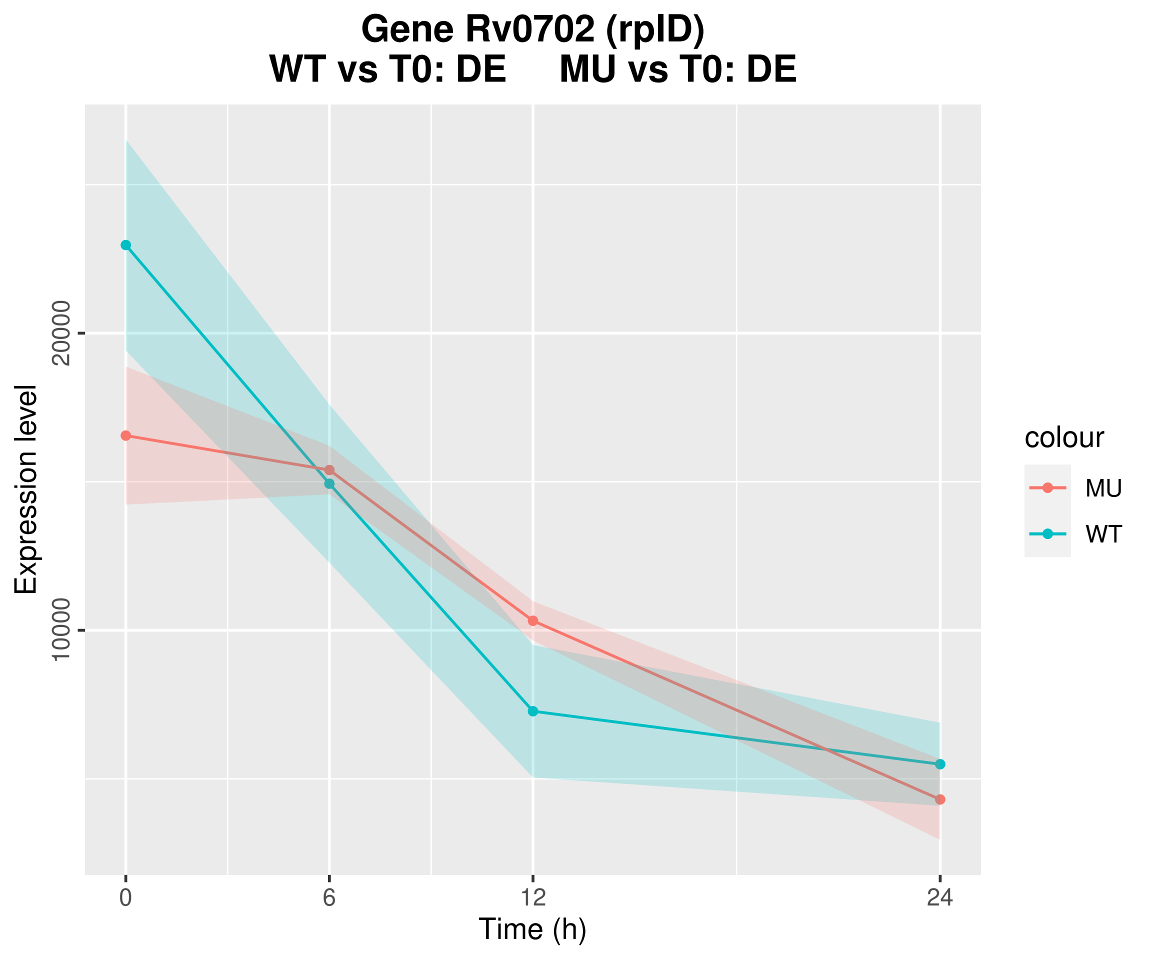
Gene Rv0702 (rplD)
WT vs T0: DE   MU vs T0: DE
Expression level
20000
10000
0
6
12
24
Time (h)
colour
MU
WT

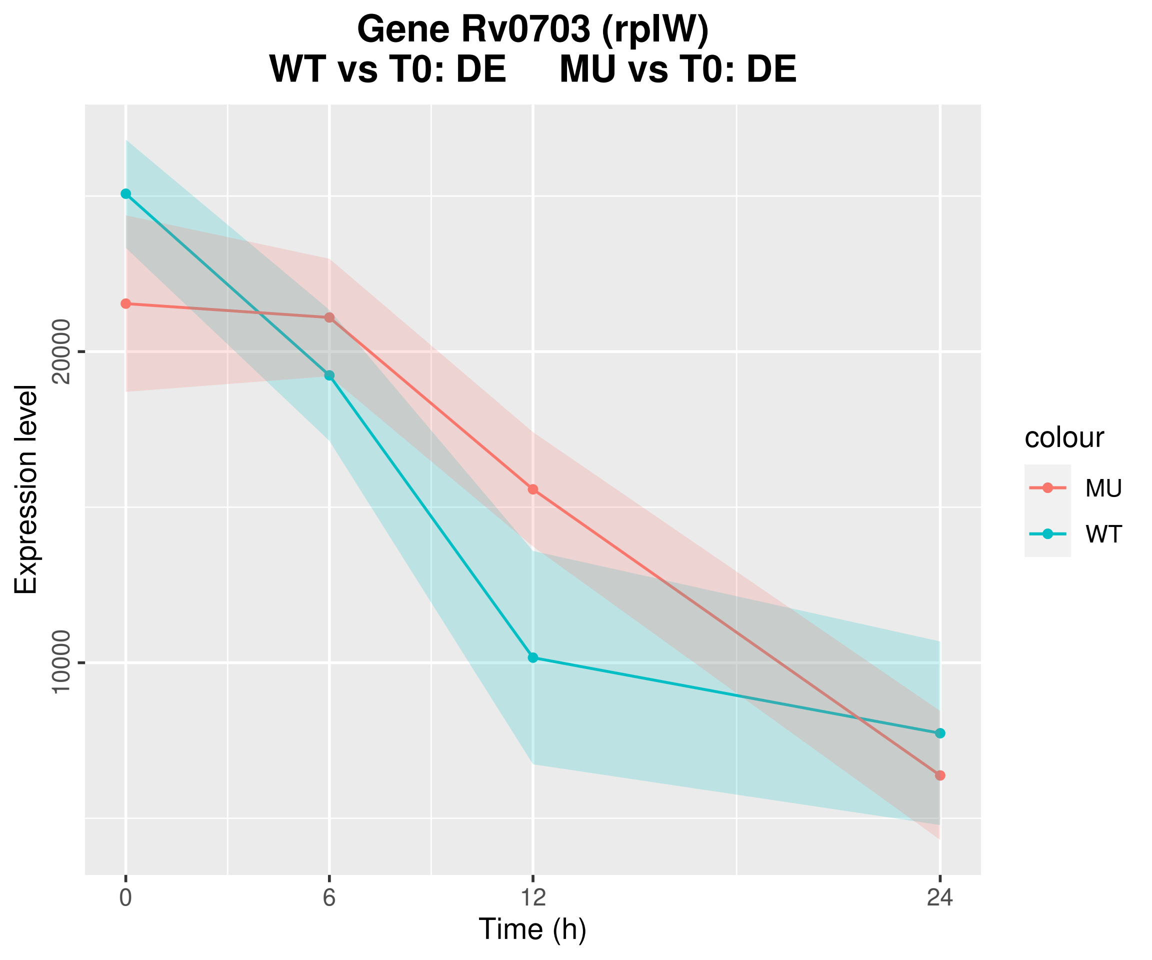
Gene Rv0703 (rplW)
WT vs T0: DE MU vs T0: DE
Expression level
20000
10000
0
6
12
24
Time (h)
colour
MU
WT

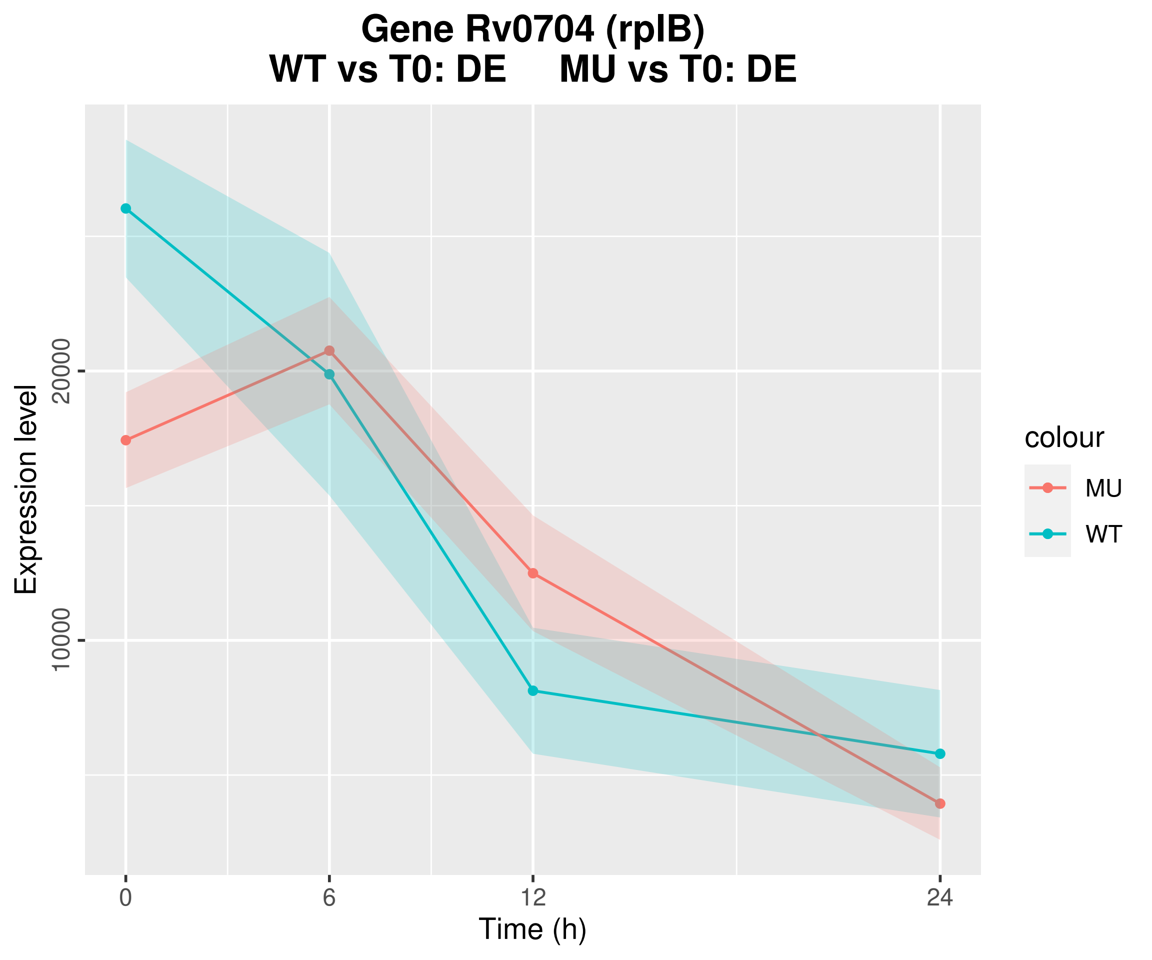
Gene Rv0704 (rplB)
WT vs T0: DE MU vs T0: DE
Expression level
20000
10000
0
6
12
24
Time (h)
colour
MU
WT

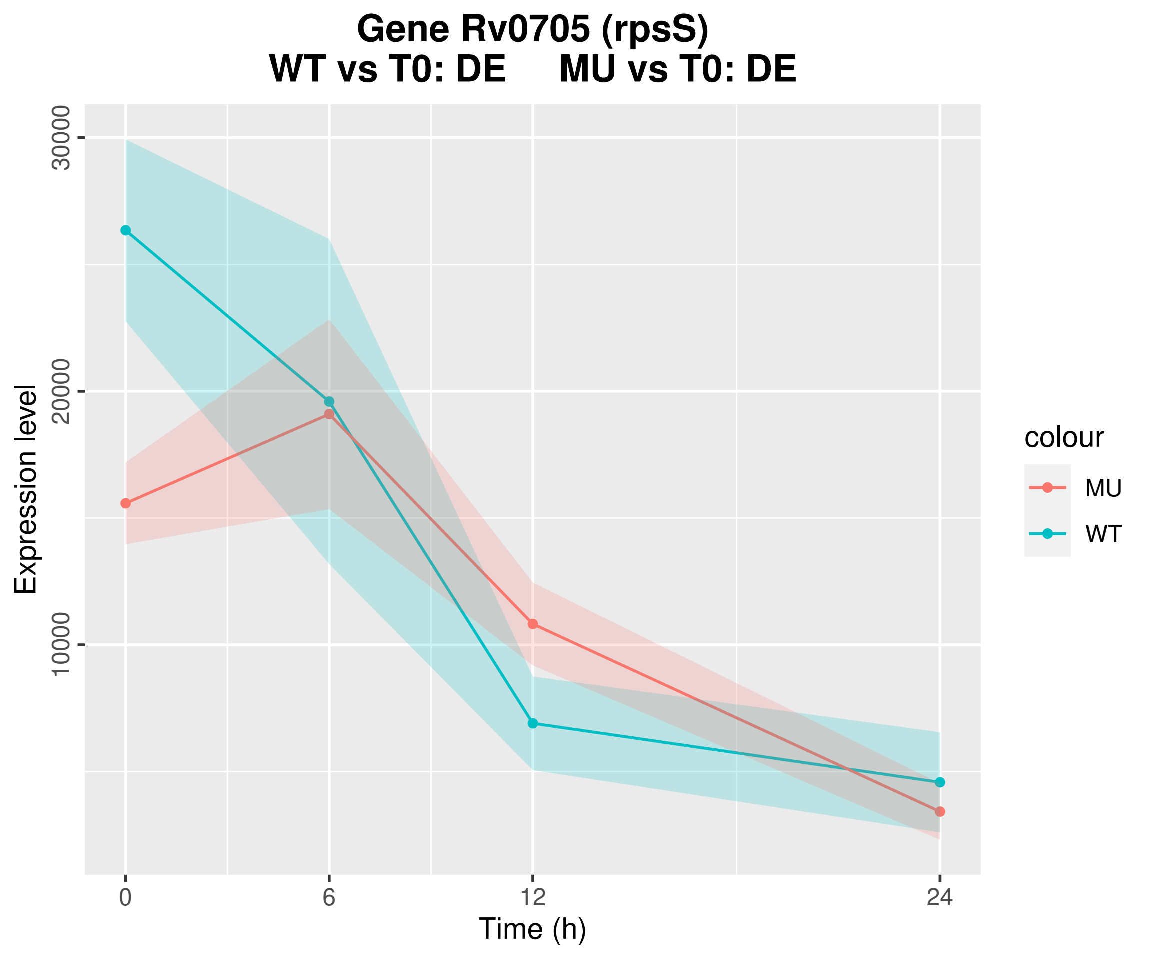

Gene Rv0705 (rpsS)
WT vs T0: DE   MU vs T0: DE
Expression level
30000
20000
10000
0
6
12
24
Time (h)
colour
MU
WT

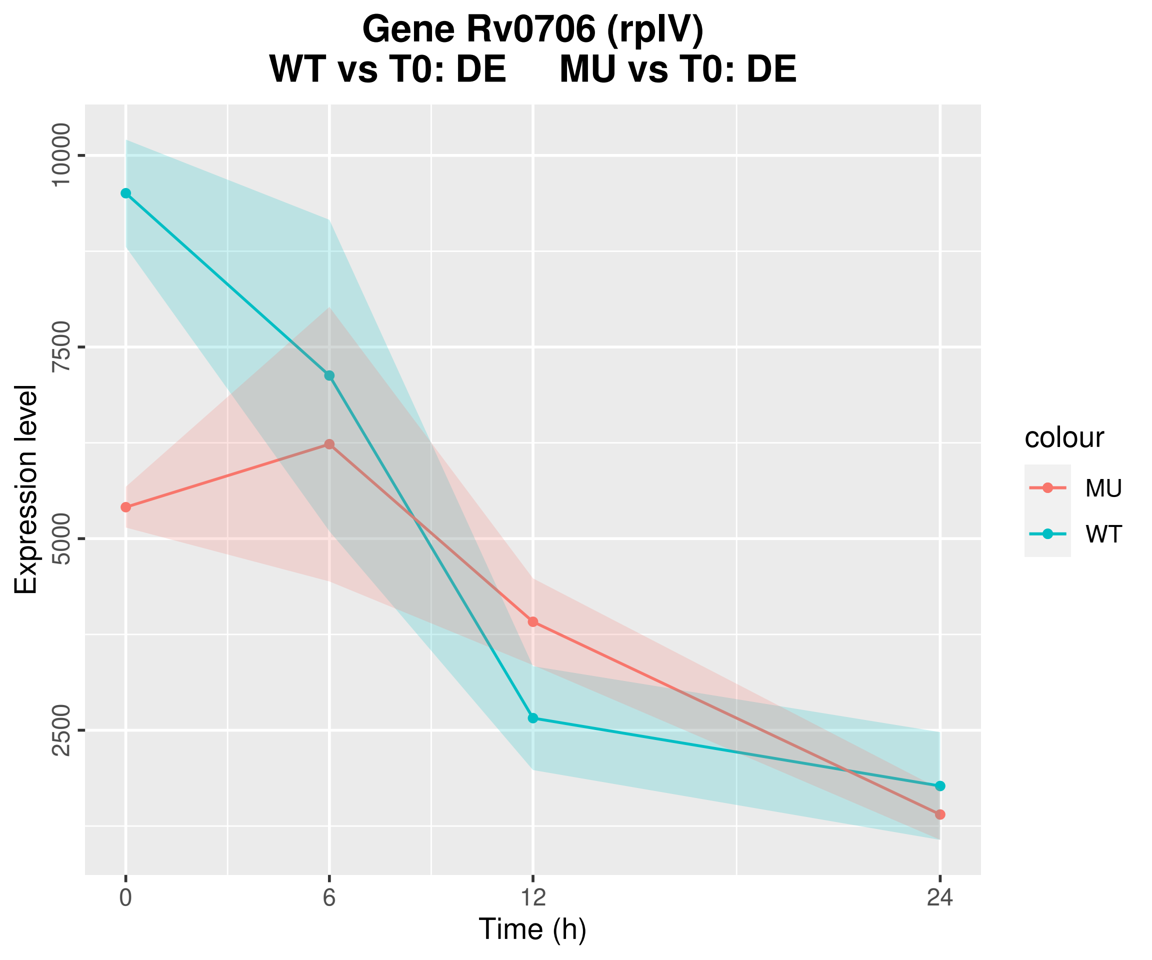

Gene Rv0706 (rplV)
WT vs T0: DE   MU vs T0: DE
Expression level
10000
7500
5000
2500
0
6
12
24
Time (h)
colour
MU
WT

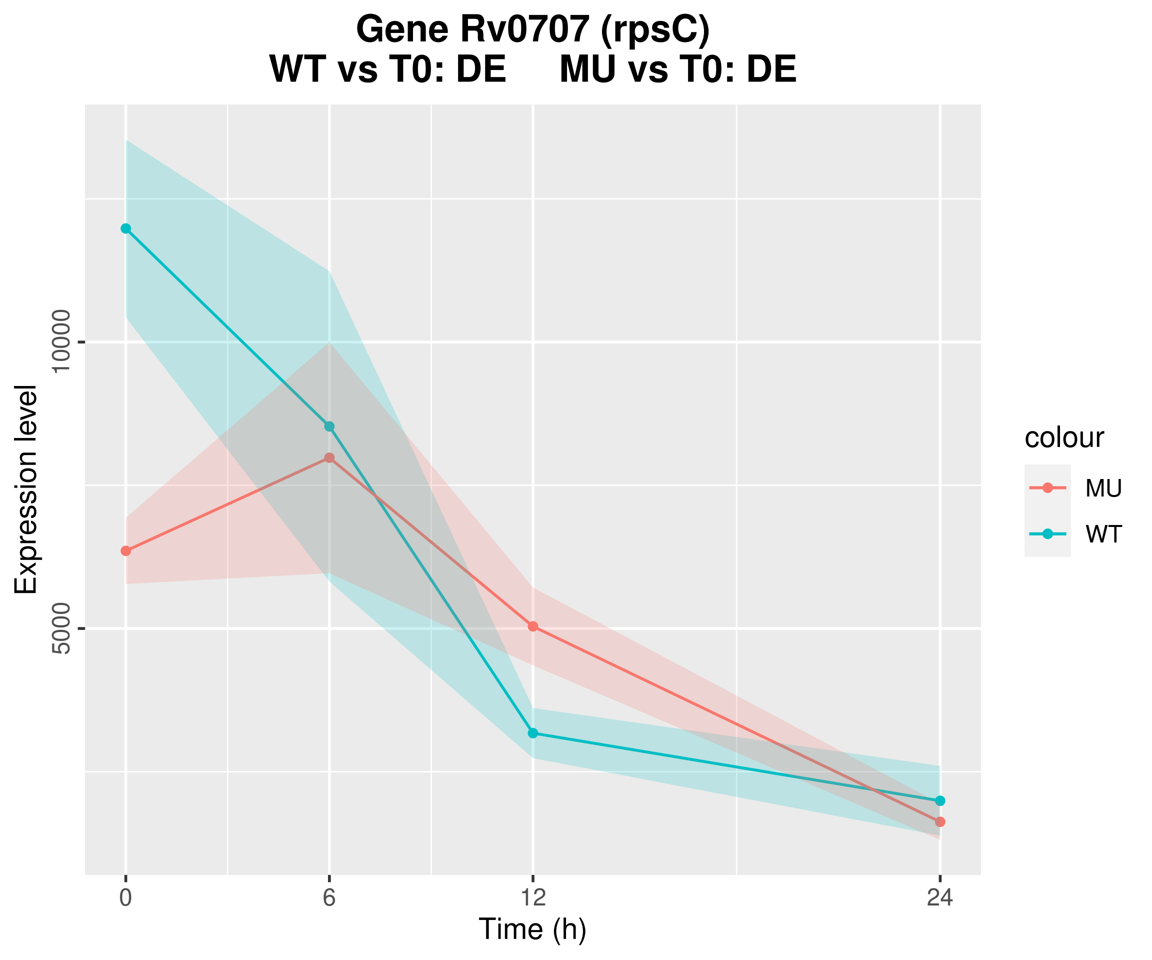
Gene Rv0707 (rpsC)
WT vs T0: DE   MU vs T0: DE
Expression level
10000
5000
0
6
12
24
Time (h)
colour
MU
WT

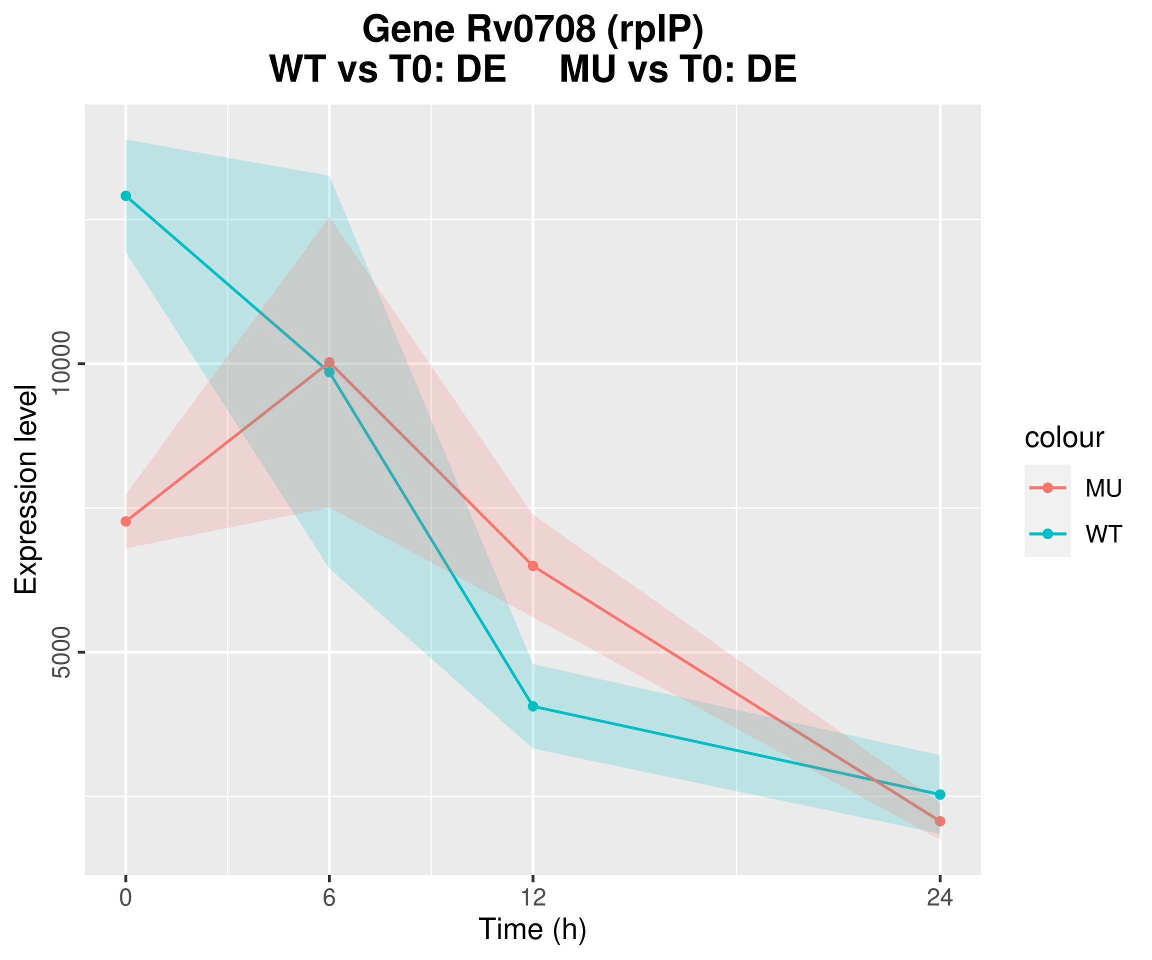

Gene Rv0708 (rplP)
WT vs T0: DE   MU vs T0: DE
Expression level
10000
5000
0
6
12
24
Time (h)
colour
MU
WT

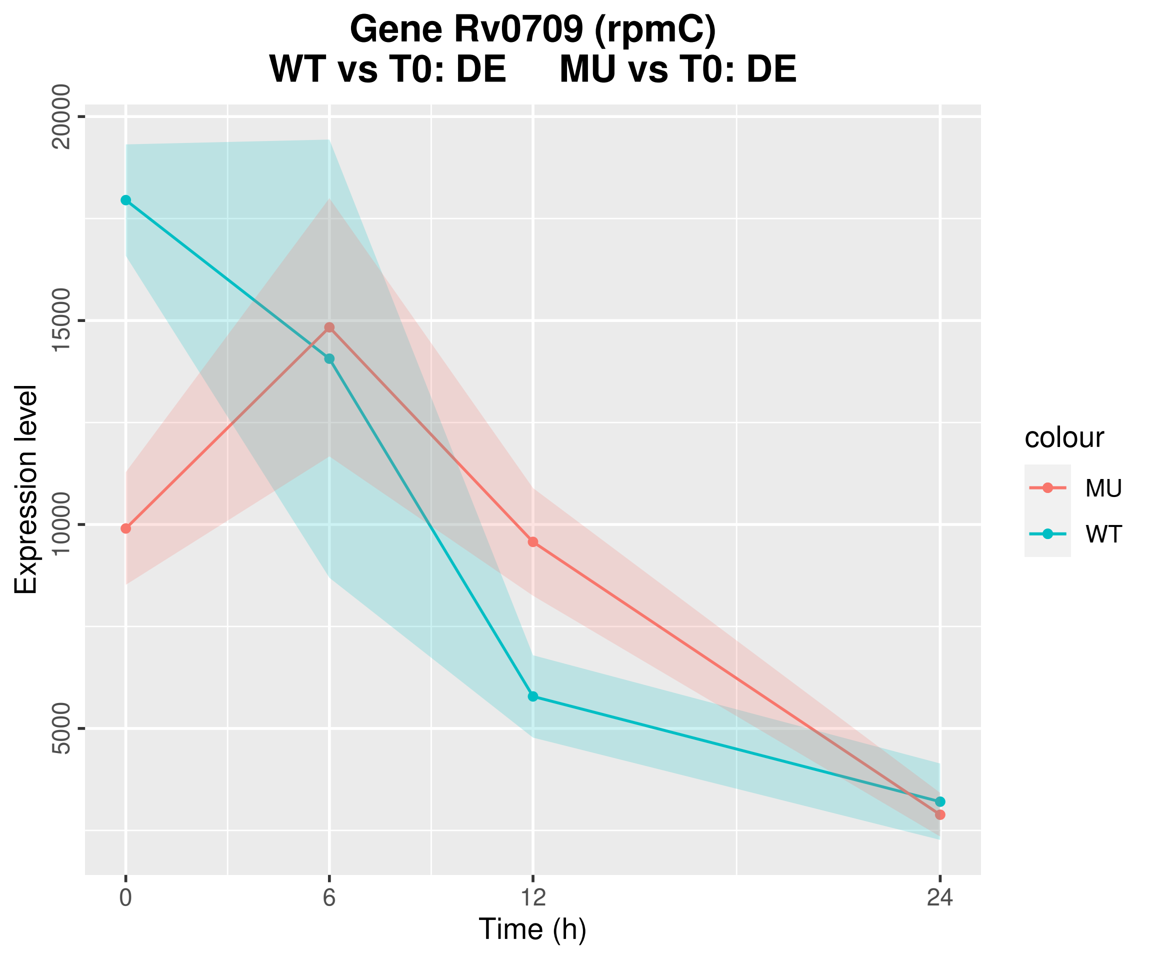

Gene Rv0709 (rpmC)
WT vs T0: DE   MU vs T0: DE
20000
15000
10000
5000
Expression level
0
6
12
24
Time (h)
colour
MU
WT

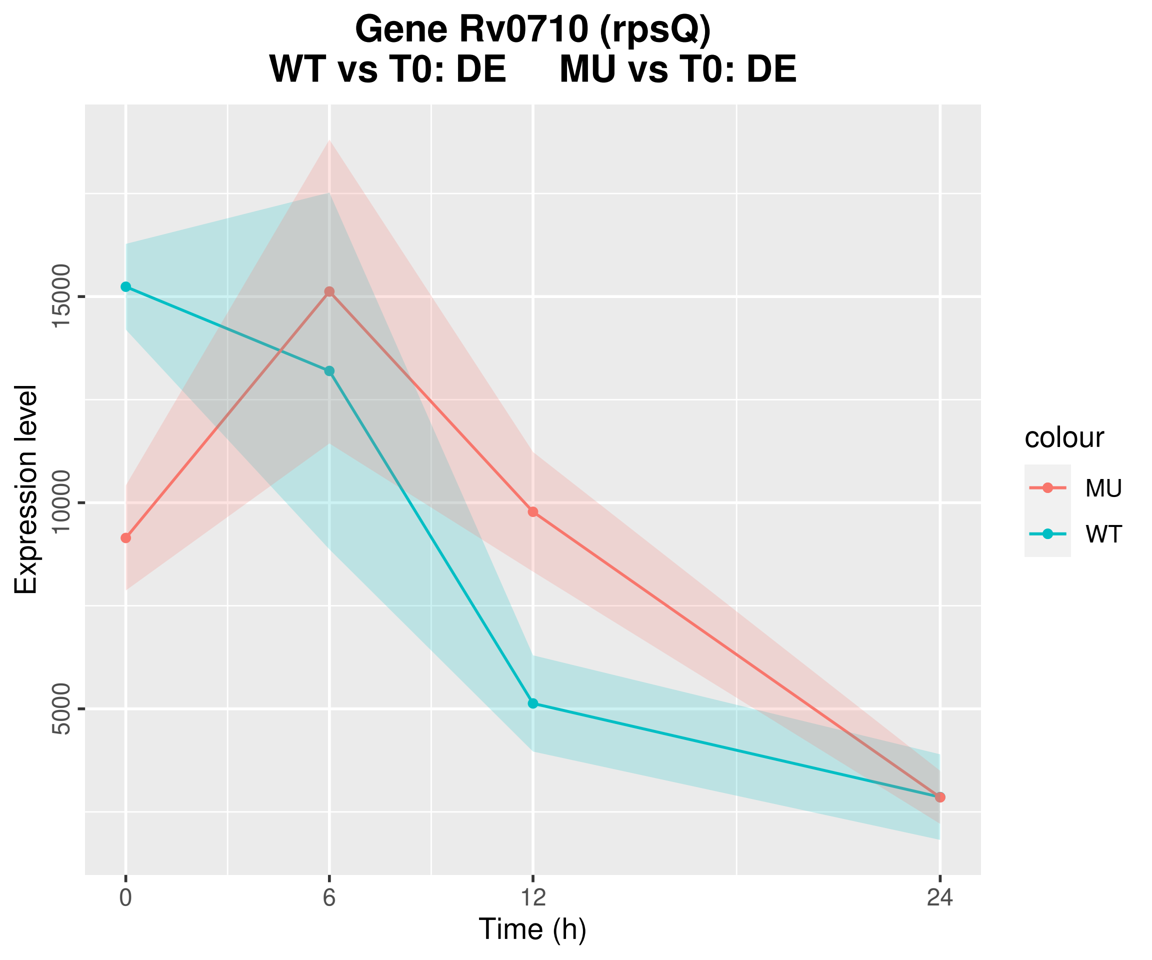

Gene Rv0710 (rpsQ)
WT vs T0: DE   MU vs T0: DE
Expression level
15000
10000
5000
0
6
12
24
Time (h)
colour
MU
WT

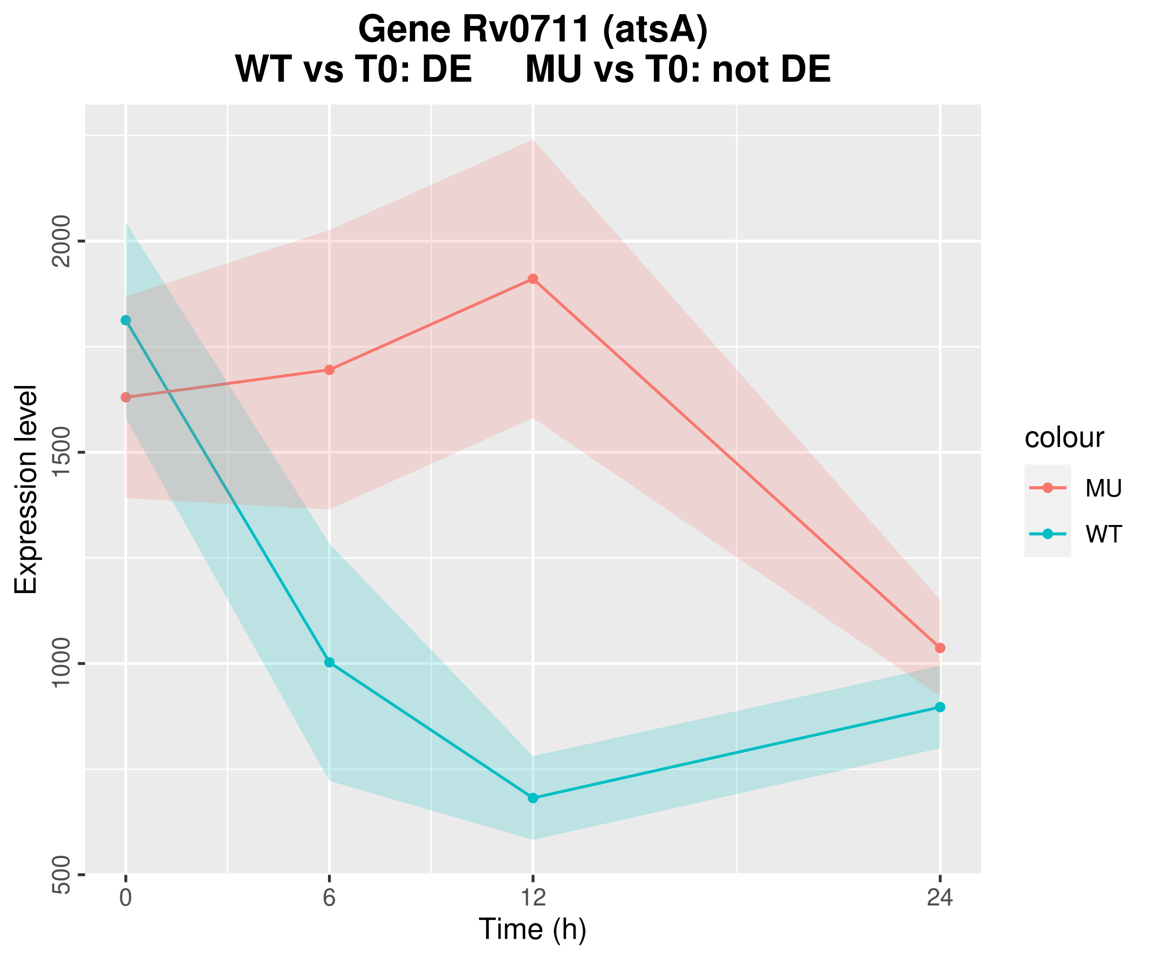

Gene Rv0711 (atsA)
WT vs T0: DE   MU vs T0: not DE
Expression level
2000
1500
1000
500
0
6
12
24
Time (h)
colour
MU
WT

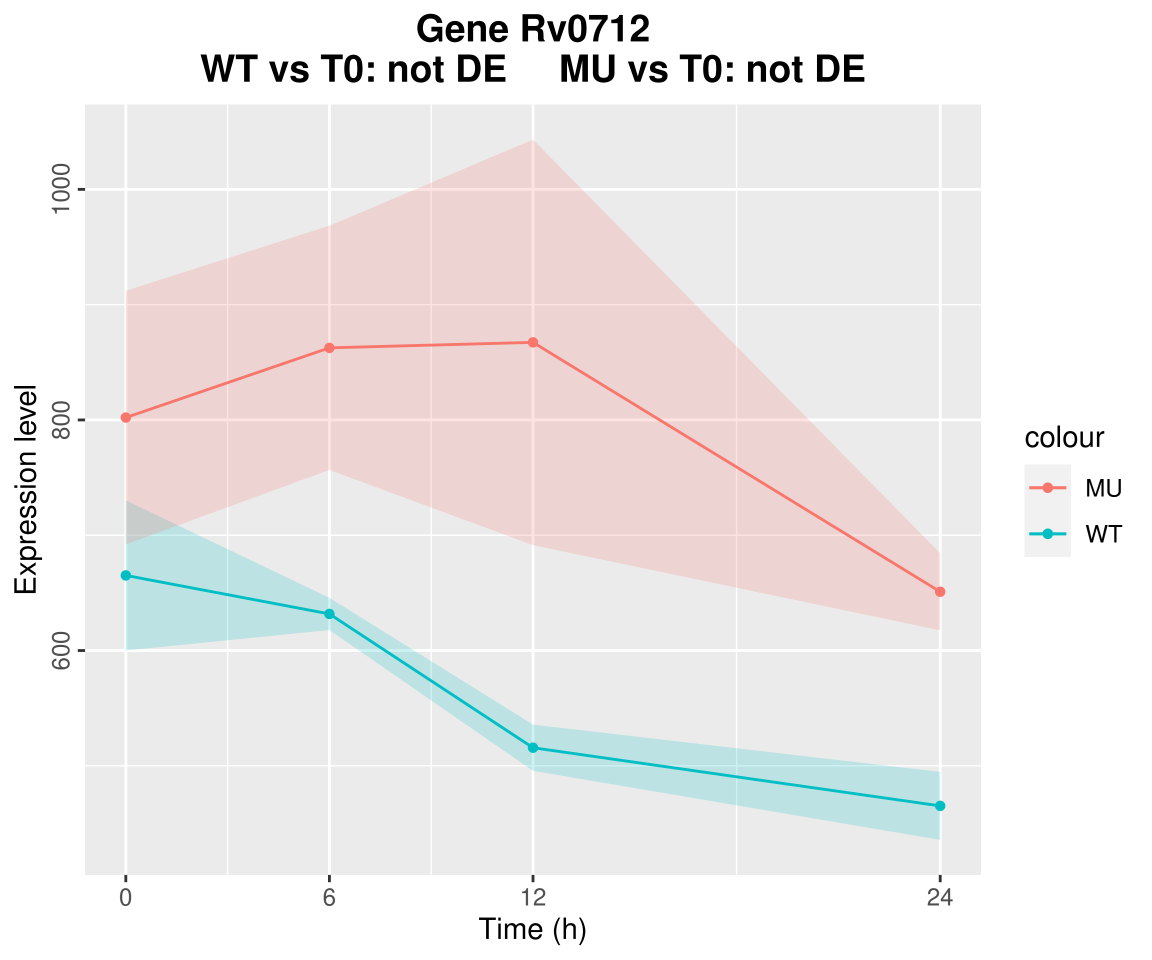

Gene Rv0712
WT vs T0: not DE MU vs T0: not DE
Expression level
1000
800
600
0
6
12
24
Time (h)
colour
MU
WT

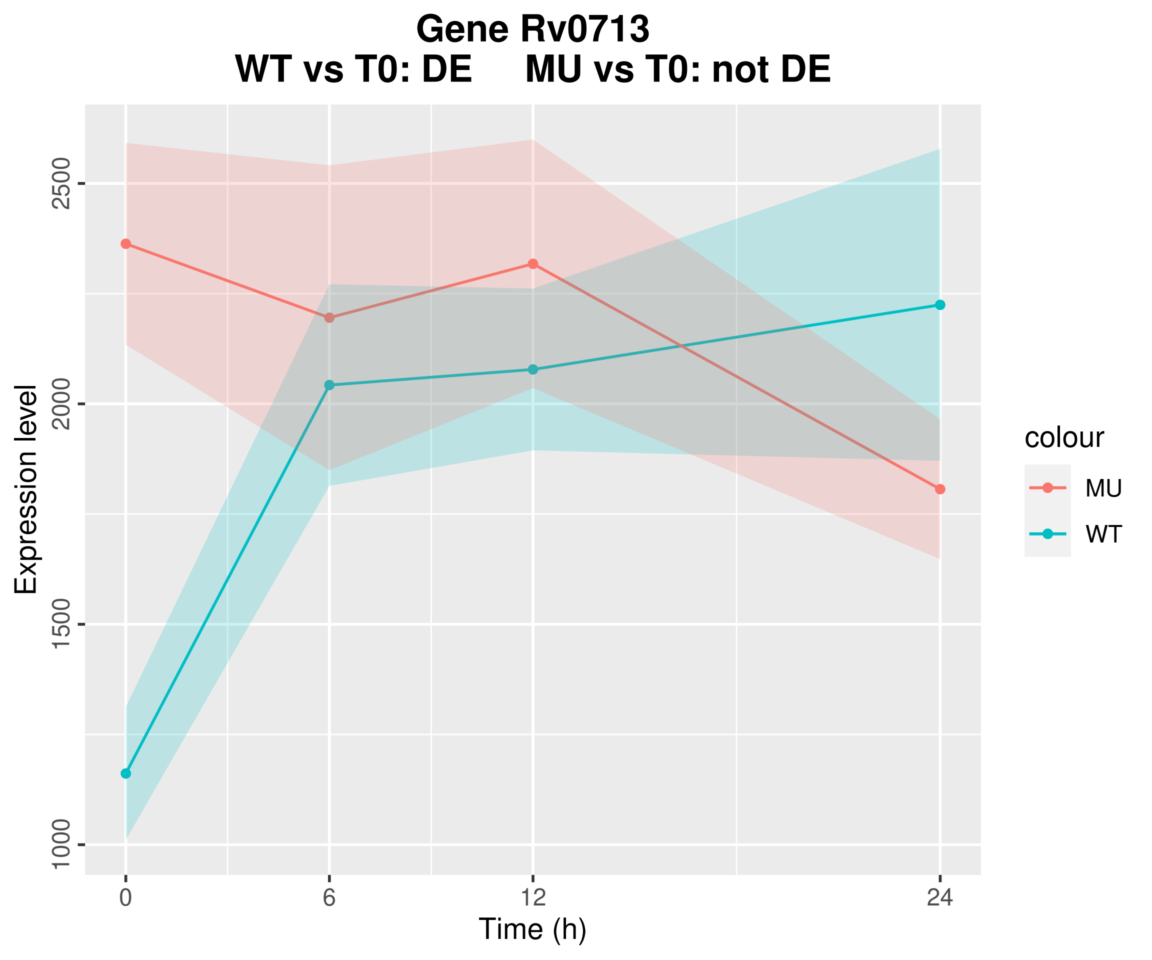
Gene Rv0713
WT vs T0: DE MU vs T0: not DE
Expression level
2500
2000
1500
1000
0
6
12
24
Time (h)
colour
MU
WT

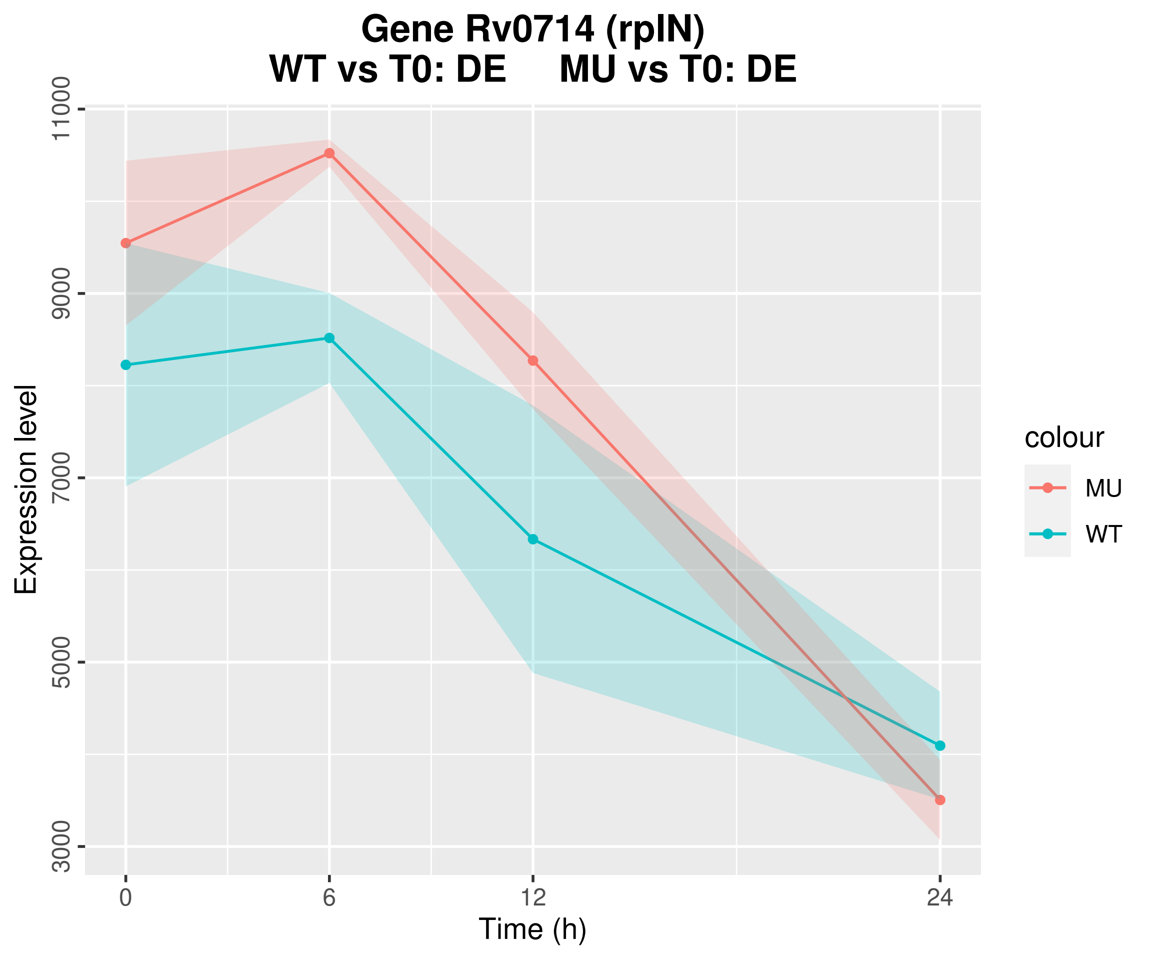

Gene Rv0714 (rplN)
WT vs T0: DE   MU vs T0: DE
Expression level
11000
9000
7000
5000
3000
0
6
12
24
Time (h)
colour
MU
WT

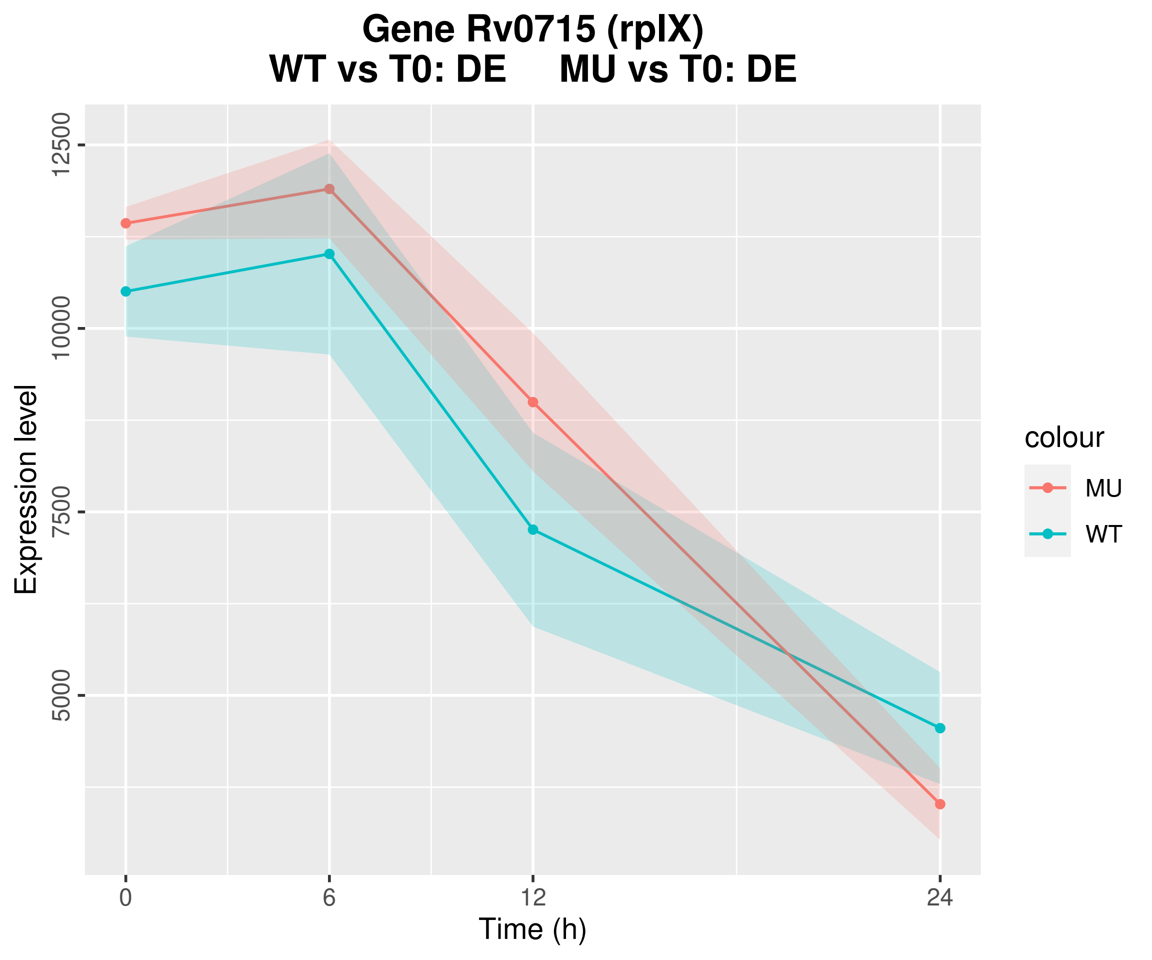
Gene Rv0715 (rplX)
WT vs T0: DE   MU vs T0: DE
Expression level
12500
10000
7500
5000
0
6
12
24
Time (h)
colour
MU
WT

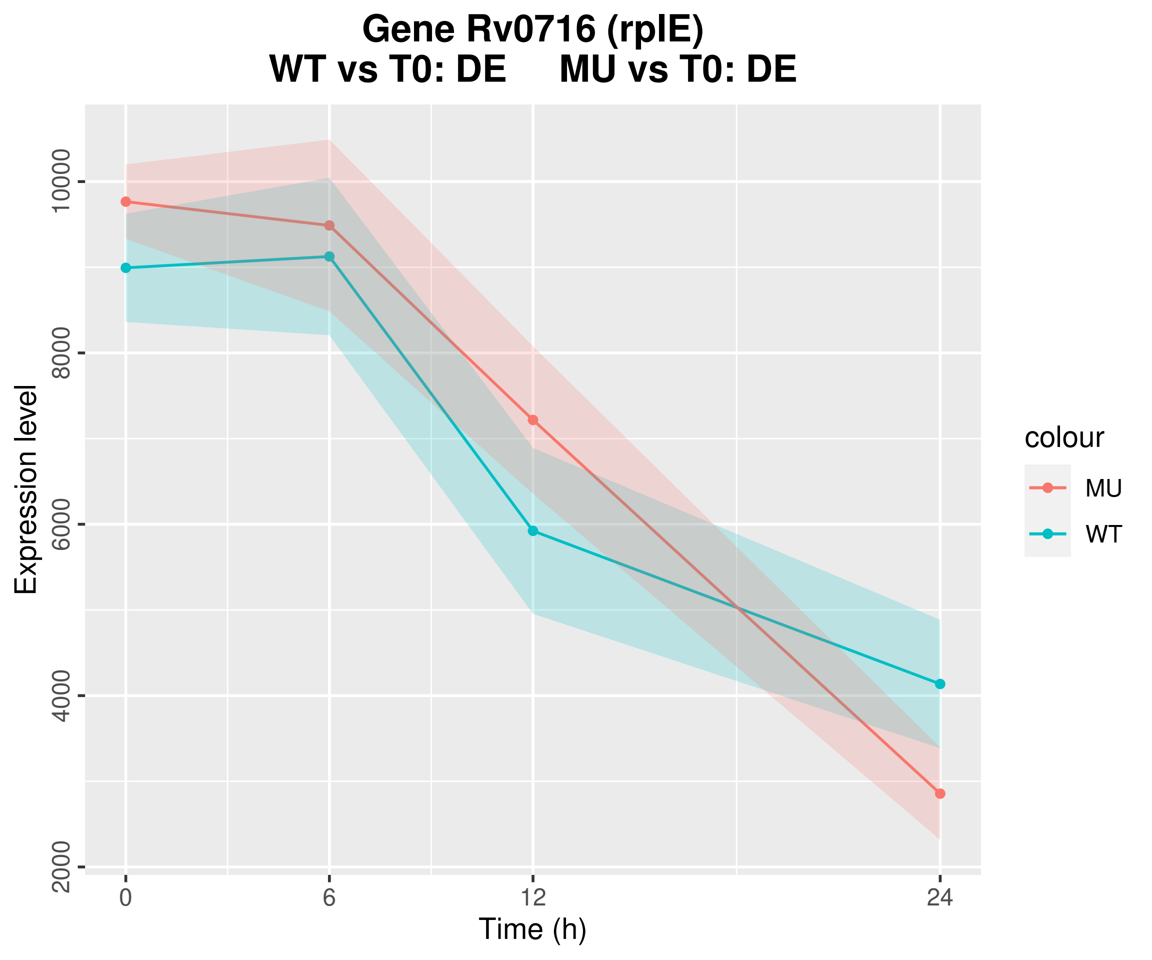

Gene Rv0716 (rplE)
WT vs T0: DE   MU vs T0: DE
Expression level
10000
8000
6000
4000
2000
0
6
12
24
Time (h)
colour
MU
WT

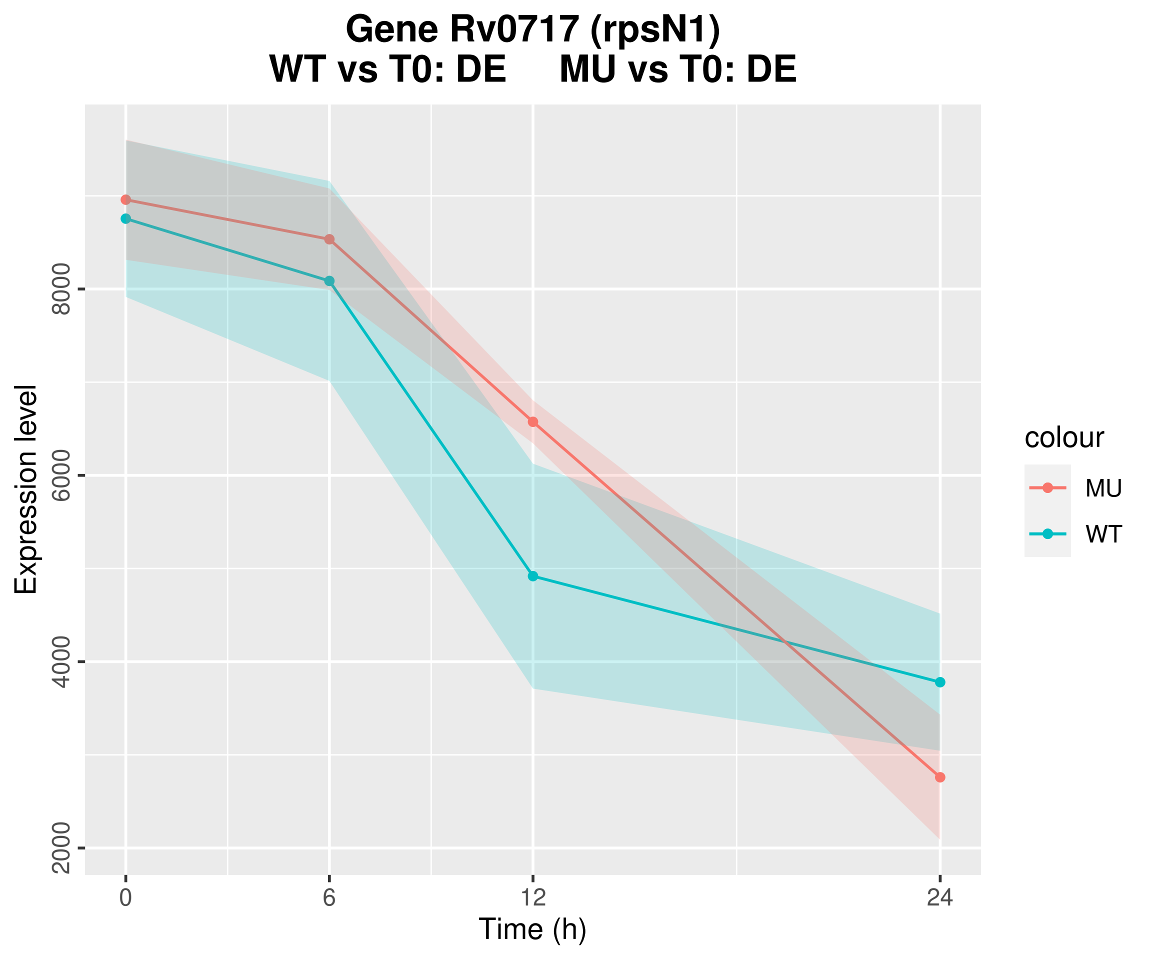

Gene Rv0717 (rpsN1)
WT vs T0: DE MU vs T0: DE
Expression level
8000
6000
4000
2000
0
6
12
24
Time (h)
colour
MU
WT

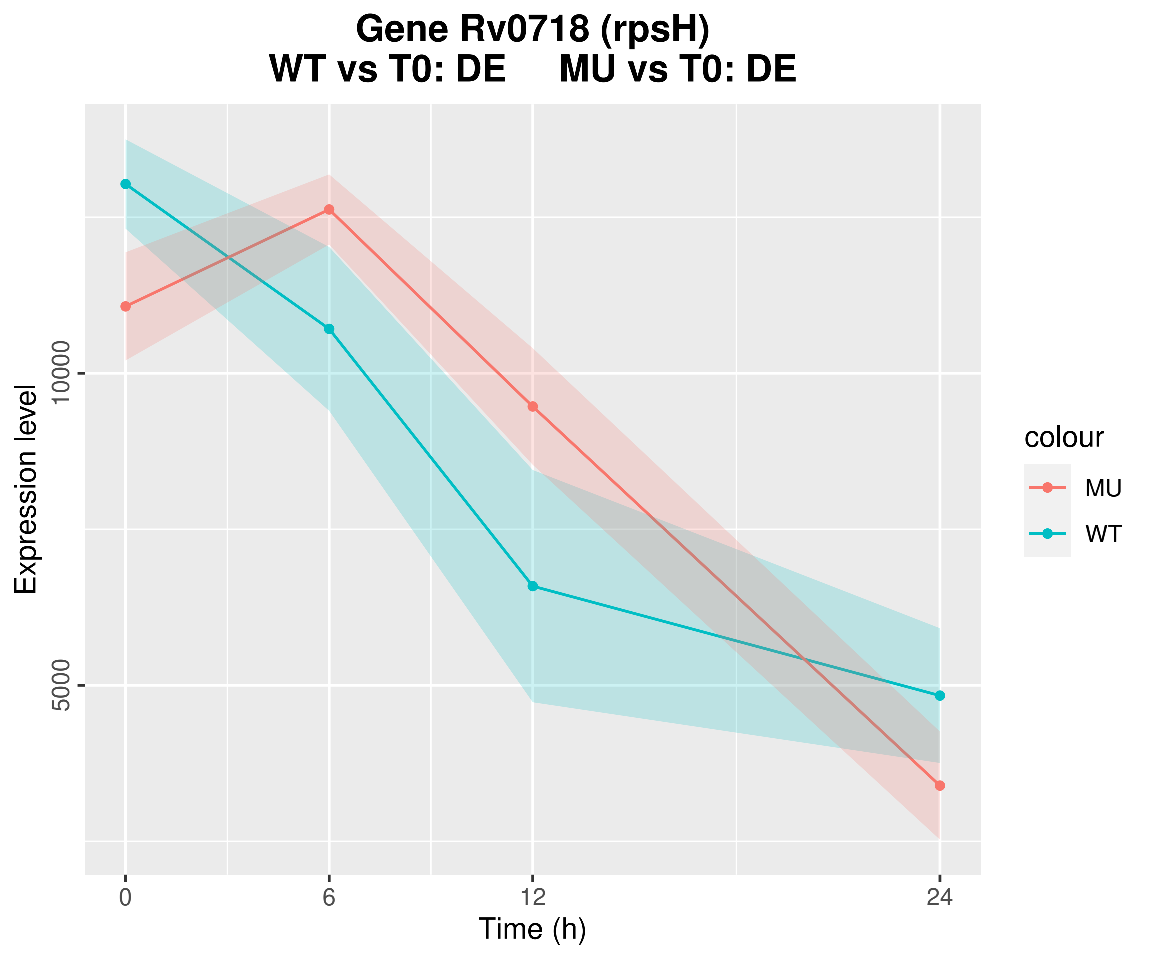

Gene Rv0718 (rpsH)
WT vs T0: DE MU vs T0: DE
Expression level
10000
5000
0
6
12
24
Time (h)
colour
MU
WT

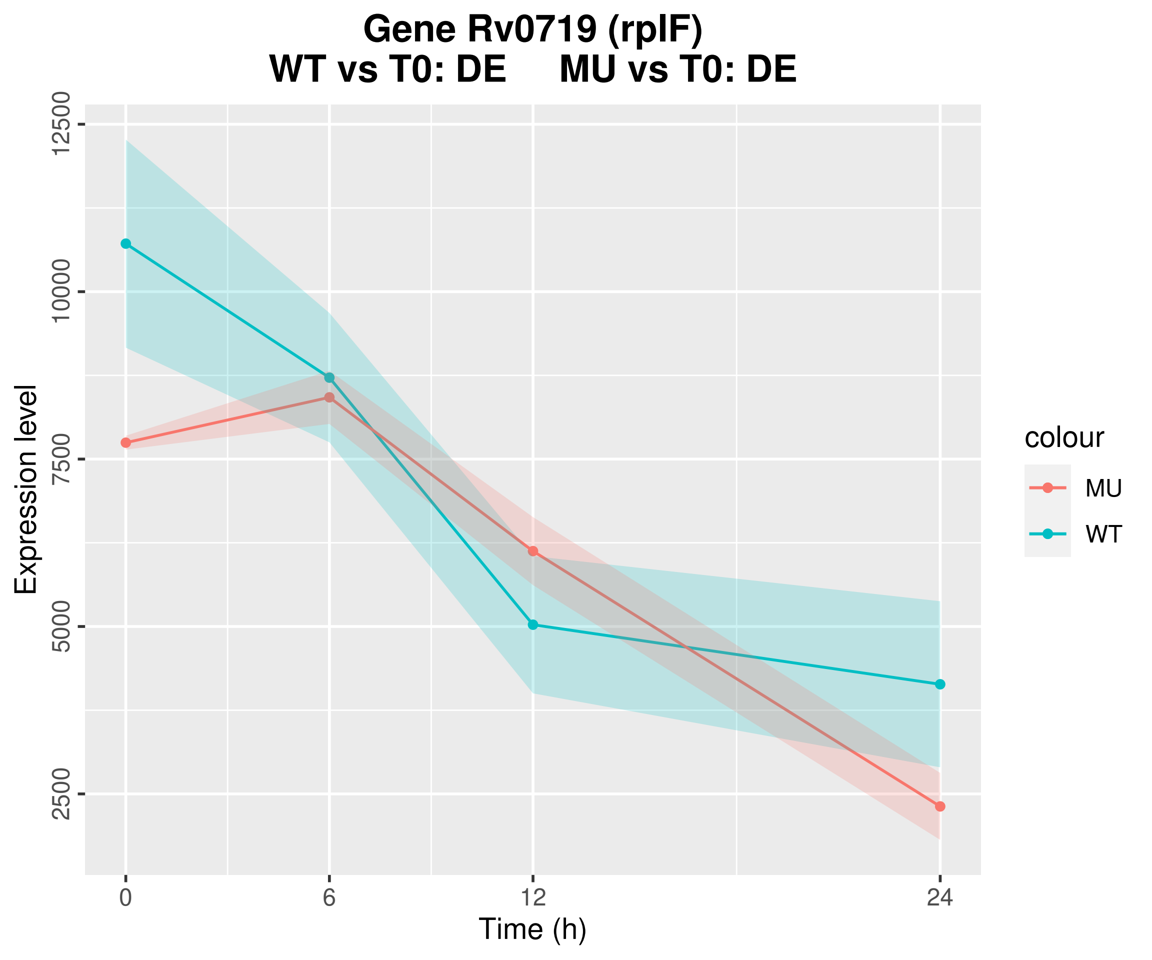

Gene Rv0719 (rplF)
WT vs T0: DE MU vs T0: DE
Expression level
12500
10000
7500
5000
2500
0
6
12
24
Time (h)
colour
MU
WT

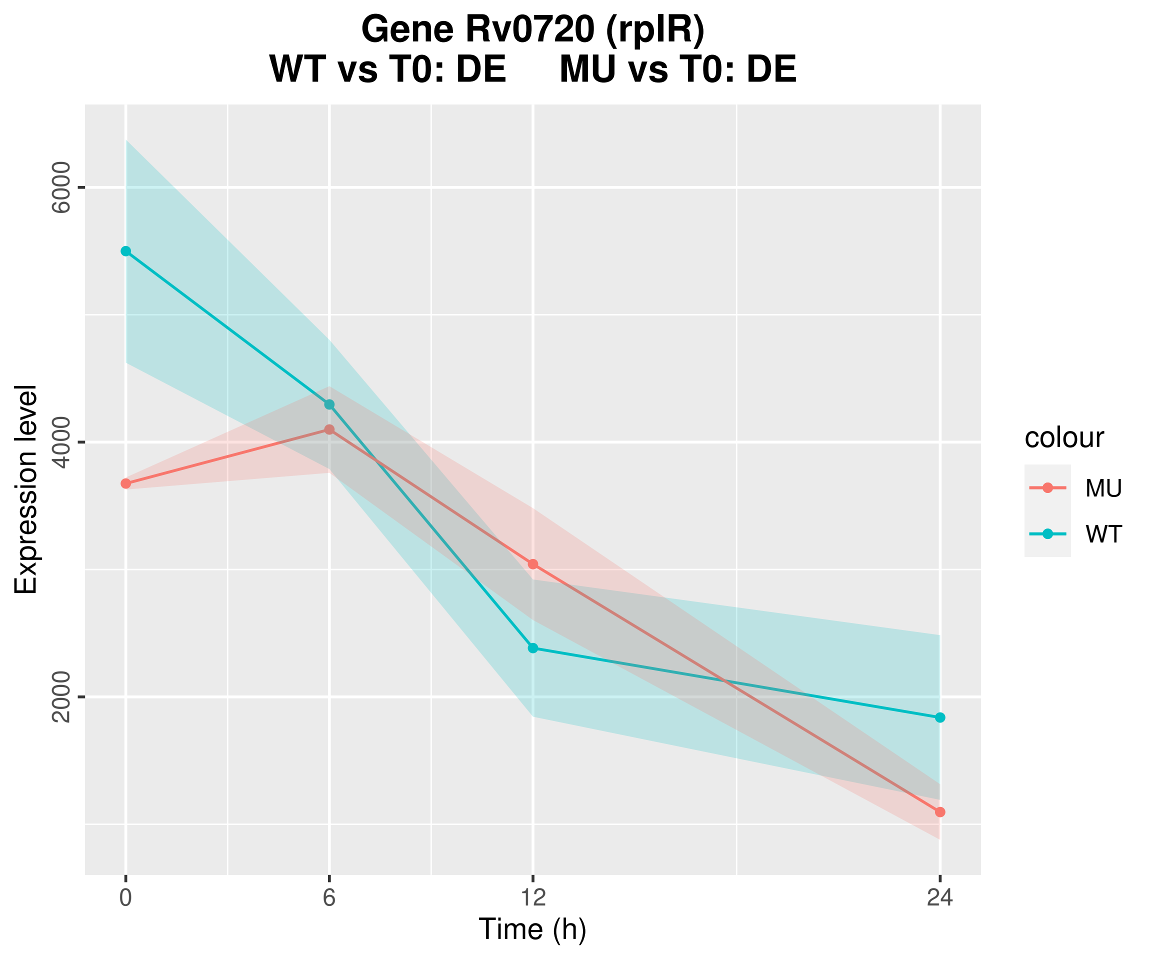
Gene Rv0720 (rplR)
WT vs T0: DE   MU vs T0: DE
Expression level
6000
4000
2000
0
6
12
24
Time (h)
colour
MU
WT

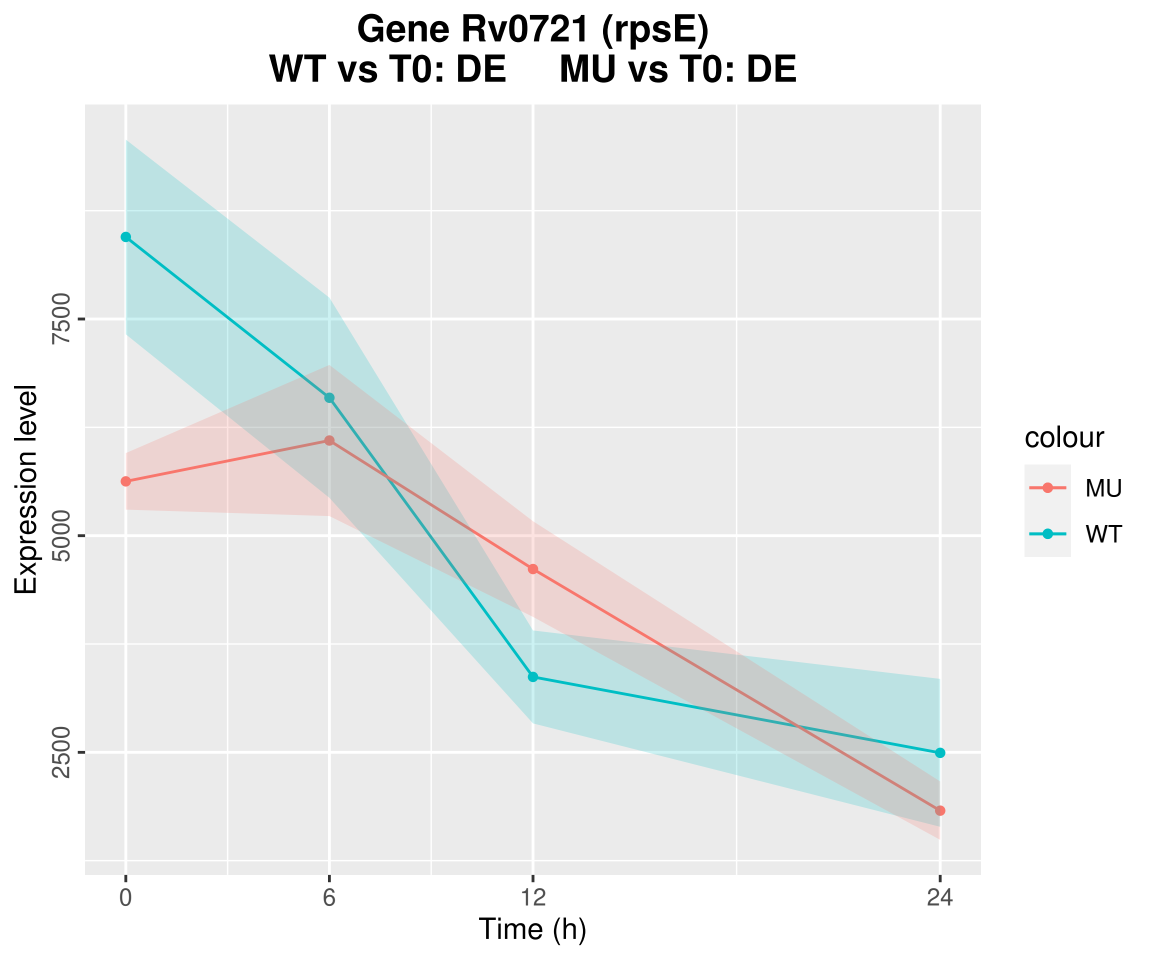

Gene Rv0721 (rpsE)
WT vs T0: DE   MU vs T0: DE
Expression level
7500
5000
2500
0
6
12
24
Time (h)
colour
MU
WT

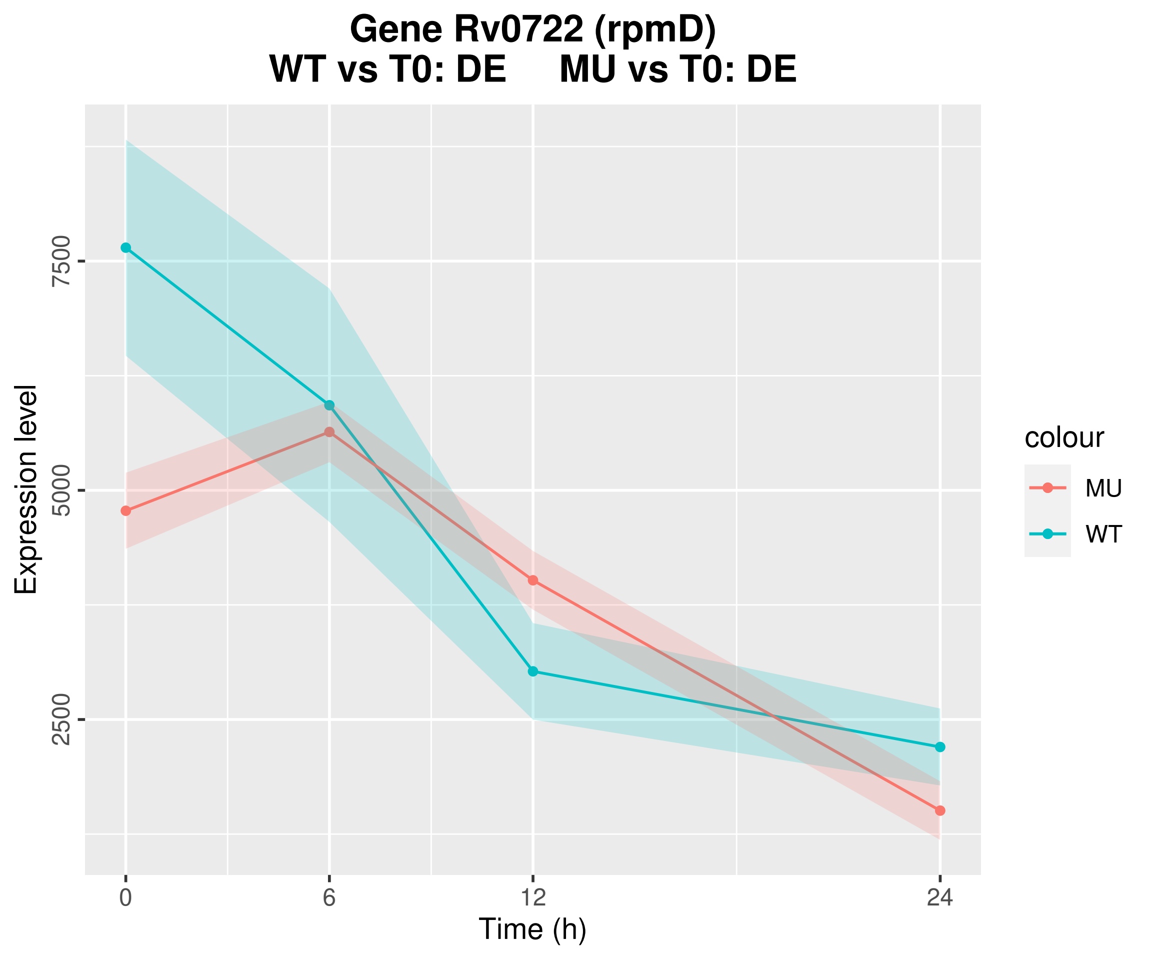

Gene Rv0722 (rpmD)
WT vs T0: DE MU vs T0: DE
Expression level
7500
5000
2500
0
6
12
24
Time (h)
colour
MU
WT

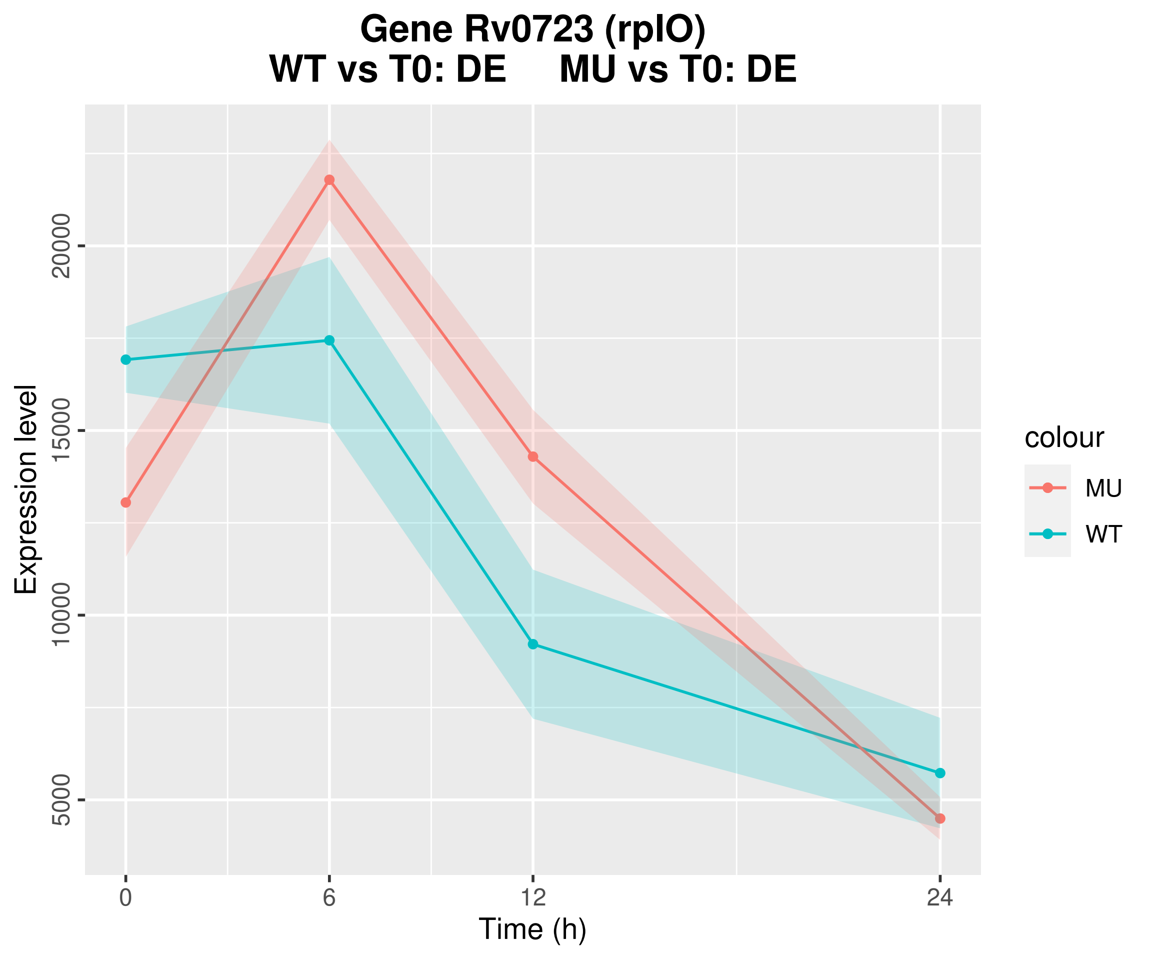

Gene Rv0723 (rplO)
WT vs T0: DE   MU vs T0: DE
Expression level
20000
15000
10000
5000
0
6
12
24
Time (h)
colour
MU
WT

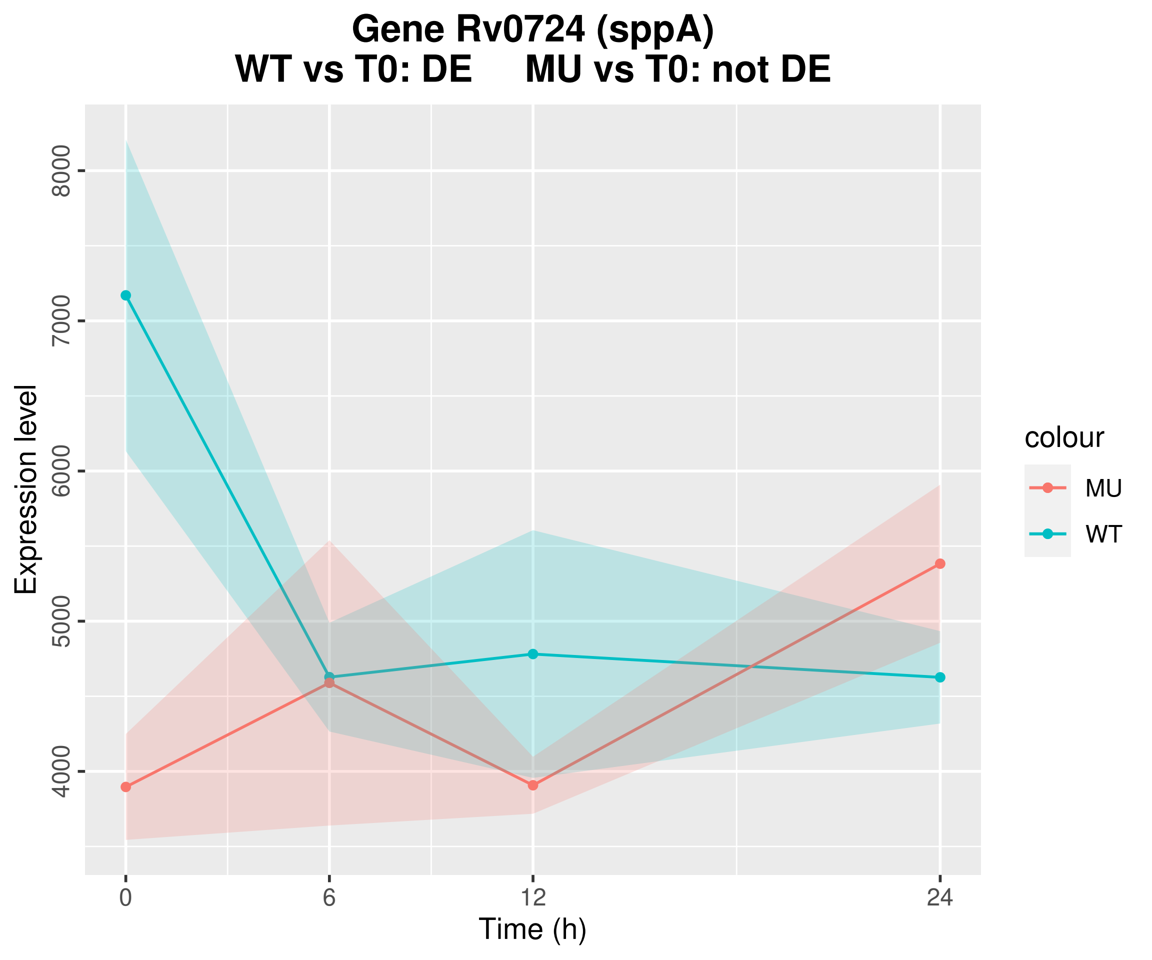

Gene Rv0724 (sppA)
WT vs T0: DE   MU vs T0: not DE
8000
7000
6000
5000
4000
Expression level
0
6
12
24
Time (h)
colour
MU
WT

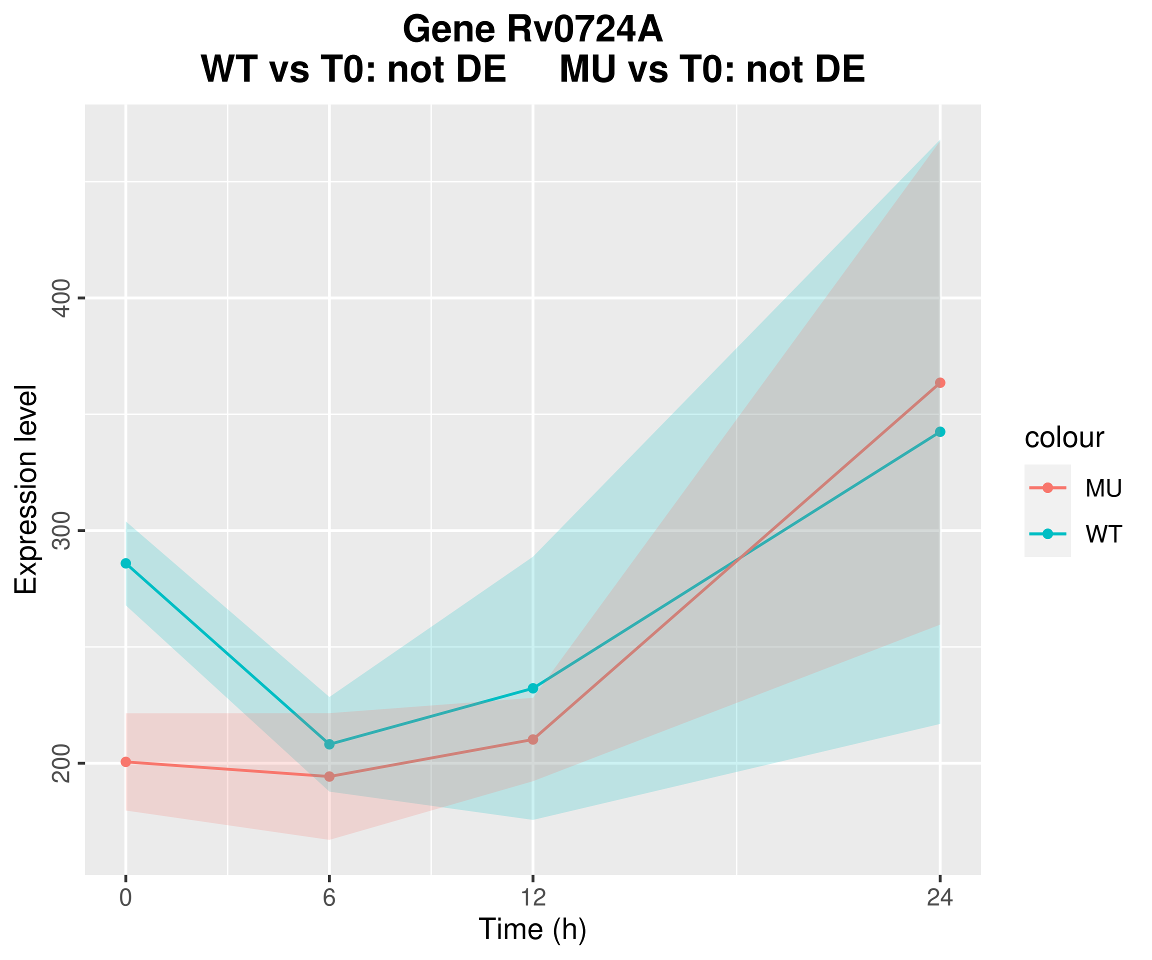

Gene Rv0724A
WT vs T0: not DE   MU vs T0: not DE
Expression level
400
300
200
0
6
12
24
Time (h)
colour
MU
WT

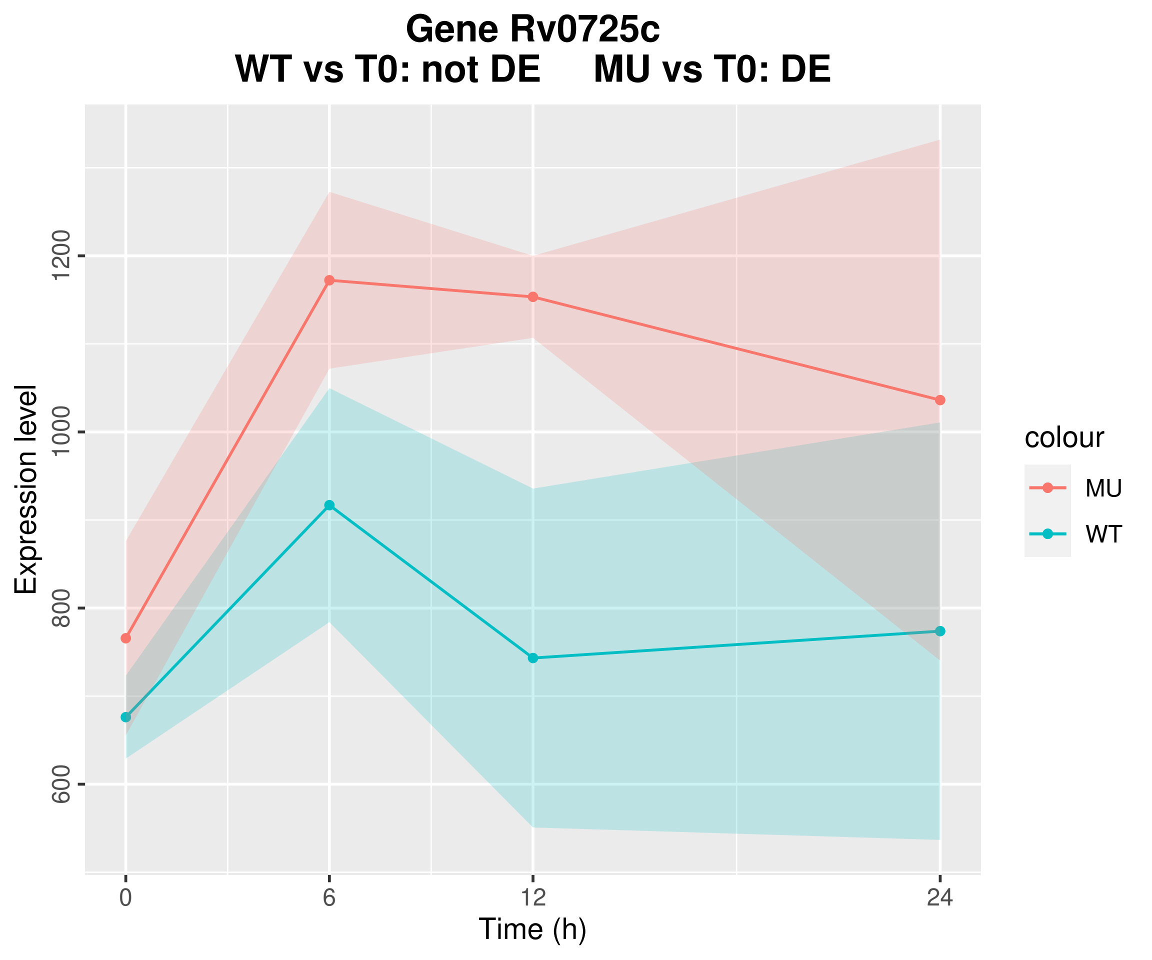
Gene Rv0725c
WT vs T0: not DE   MU vs T0: DE
Expression level
1200
1000
800
600
0
6
12
24
Time (h)
colour
MU
WT

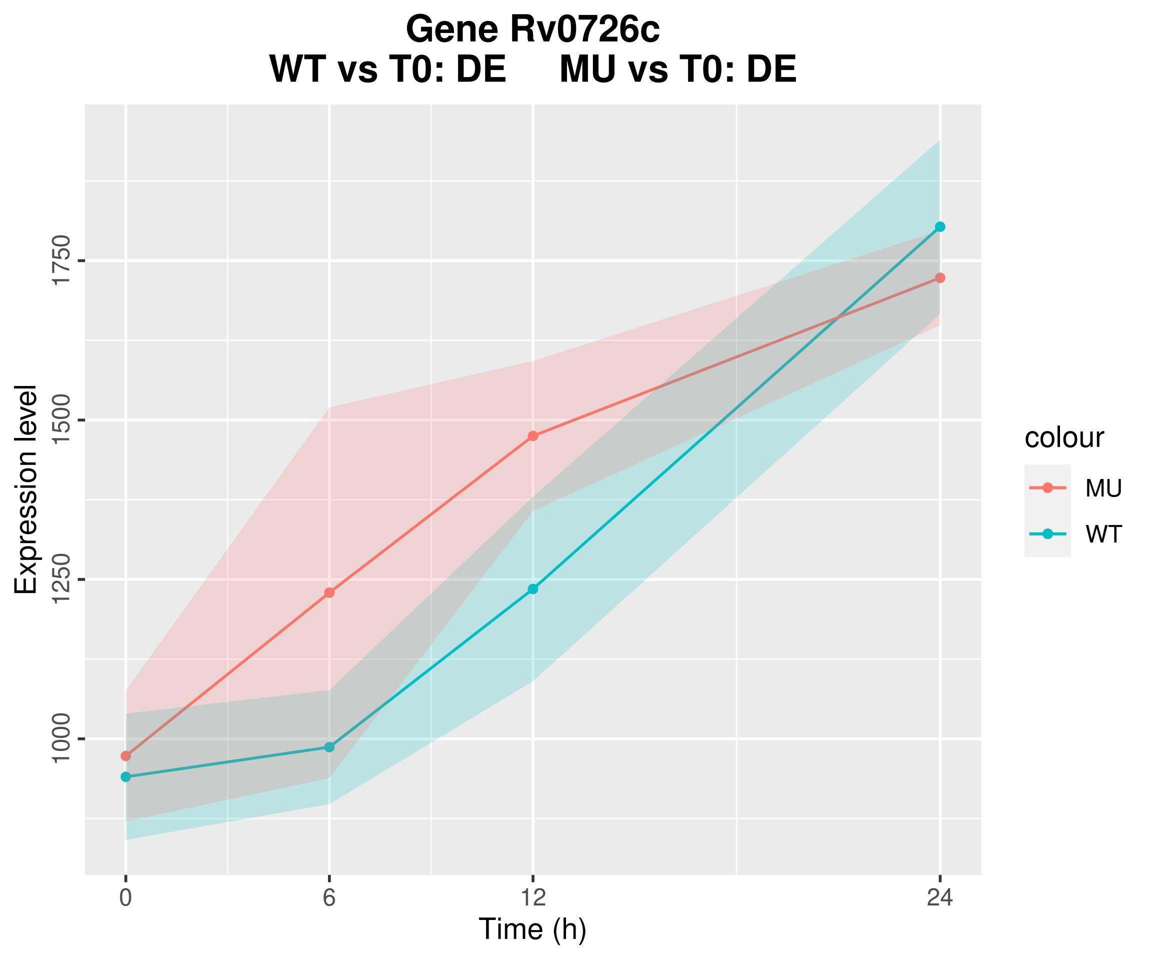
Gene Rv0726c
WT vs T0: DE   MU vs T0: DE
Expression level
1000
1250
1500
1750
0
6
12
24
Time (h)
colour
MU
WT

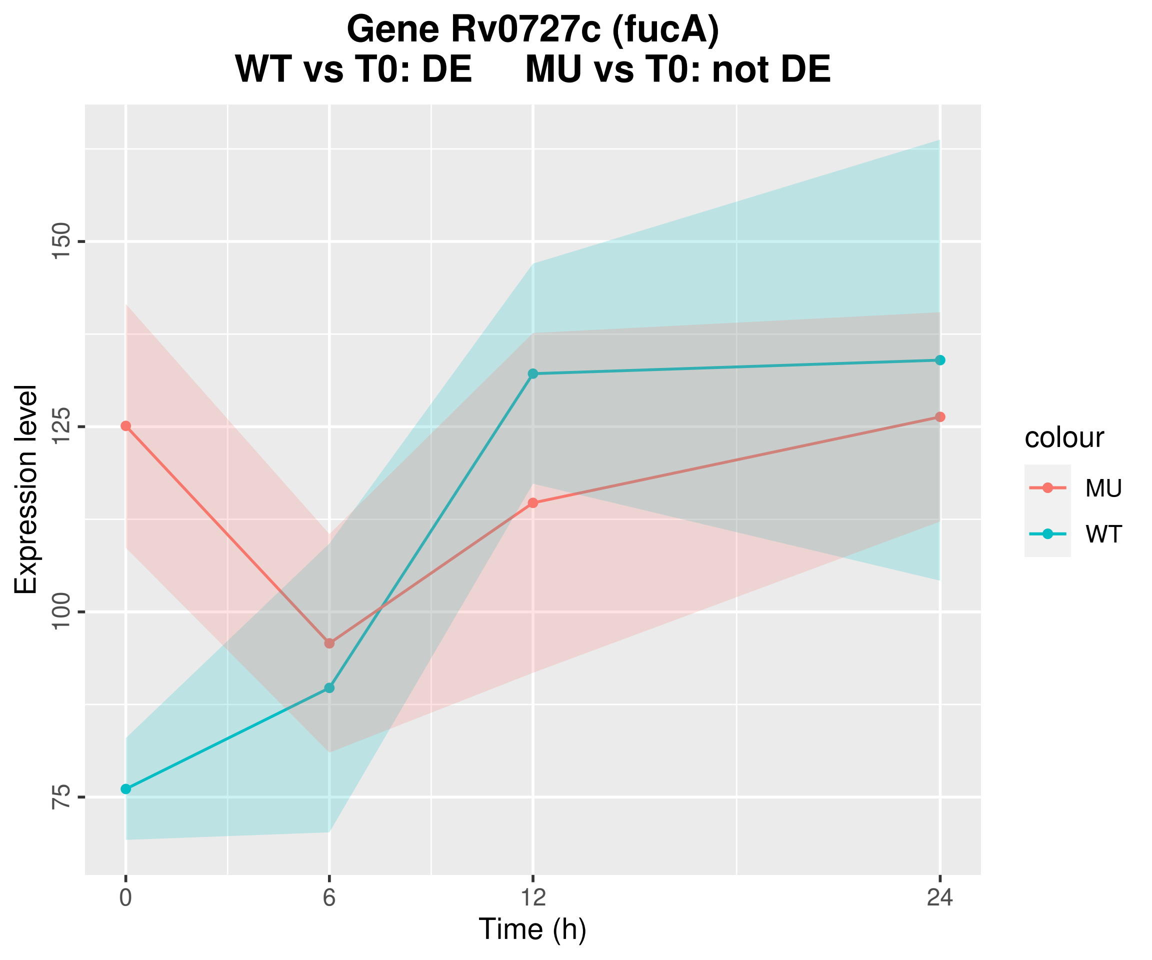

Gene Rv0727c (fucA)
WT vs T0: DE   MU vs T0: not DE
Expression level
150
125
100
75
0
6
12
24
Time (h)
colour
MU
WT

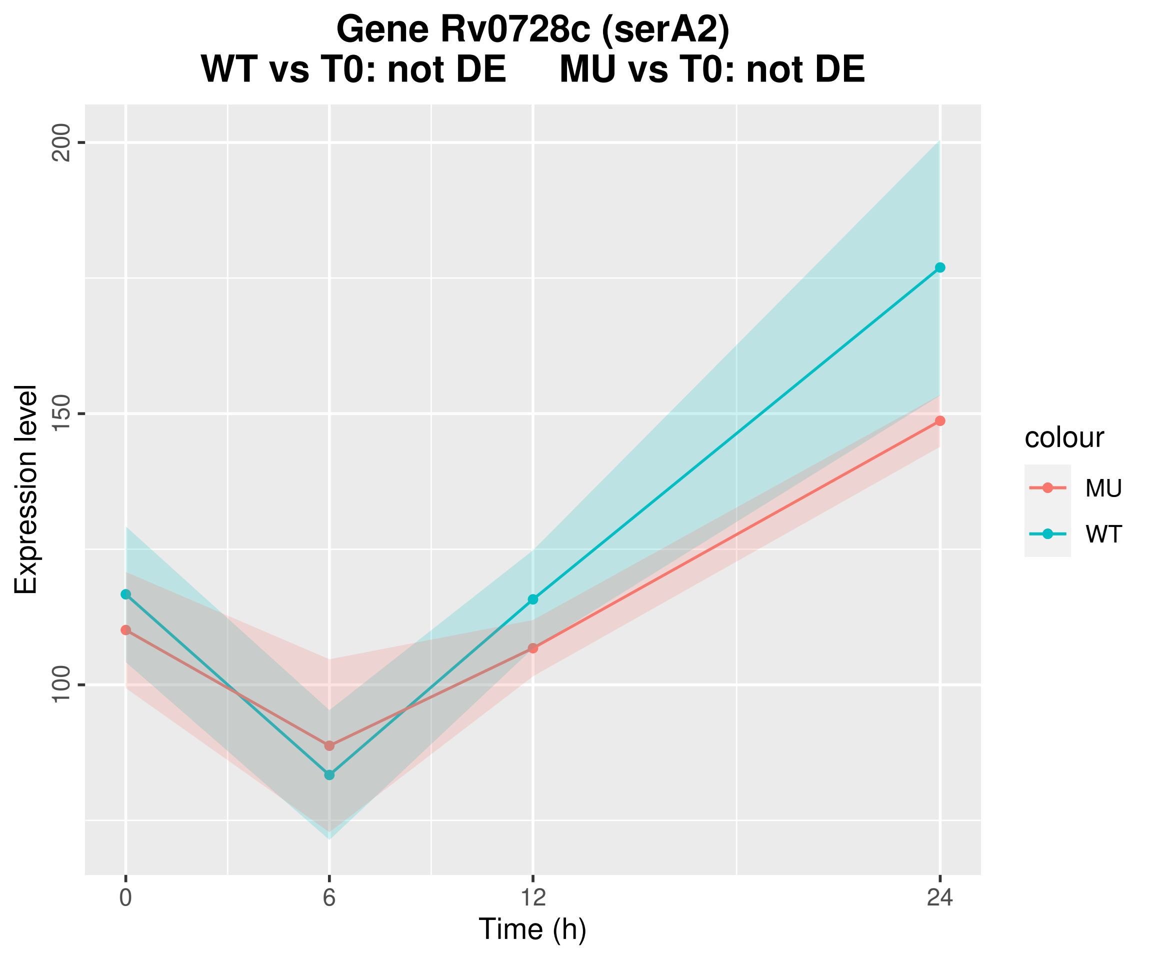
Gene Rv0728c (serA2)
WT vs T0: not DE   MU vs T0: not DE
200
150
100
Expression level
0
6
12
24
Time (h)
colour
MU
WT

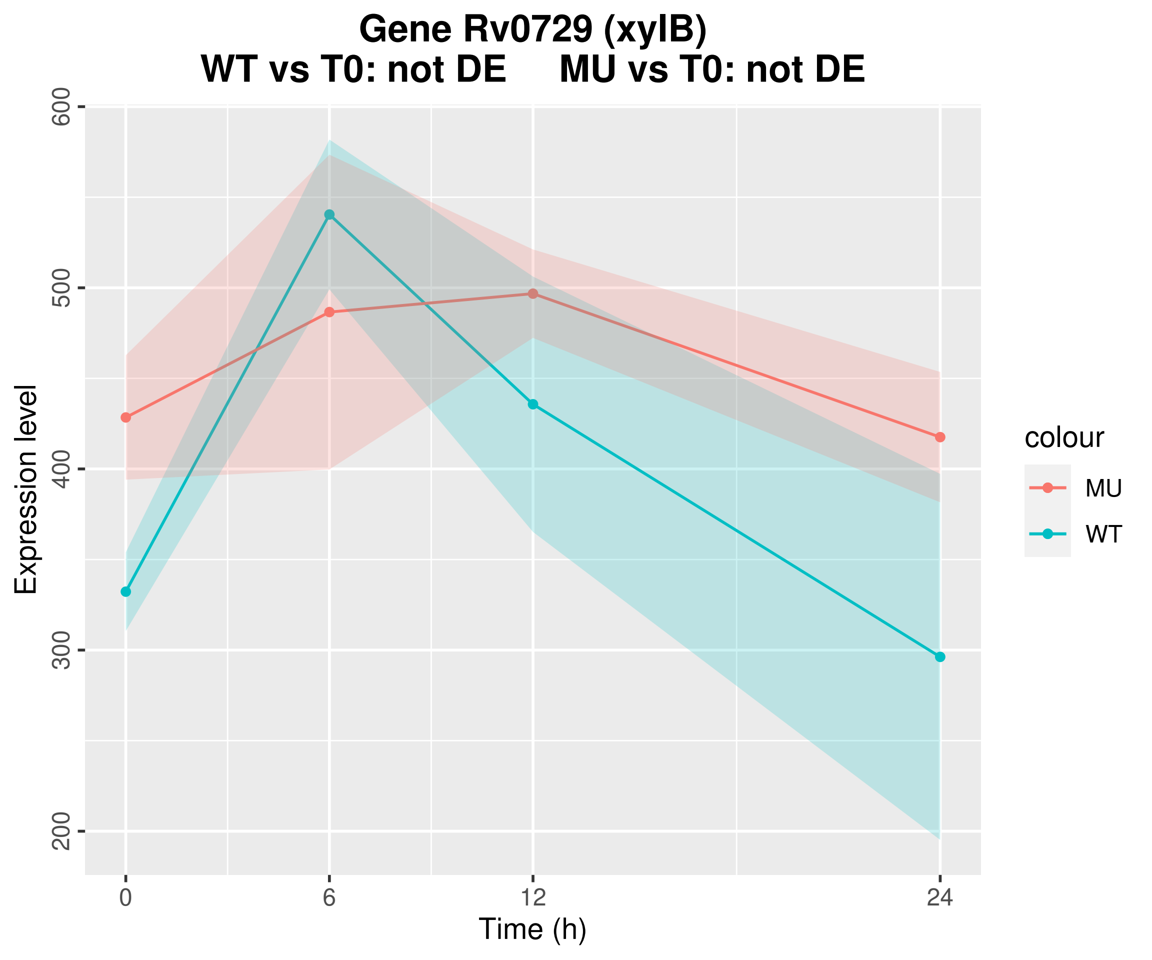

Gene Rv0729 (xylB)
WT vs T0: not DE MU vs T0: not DE
Expression level
600
500
400
300
200
0
6
12
24
Time (h)
colour
MU
WT

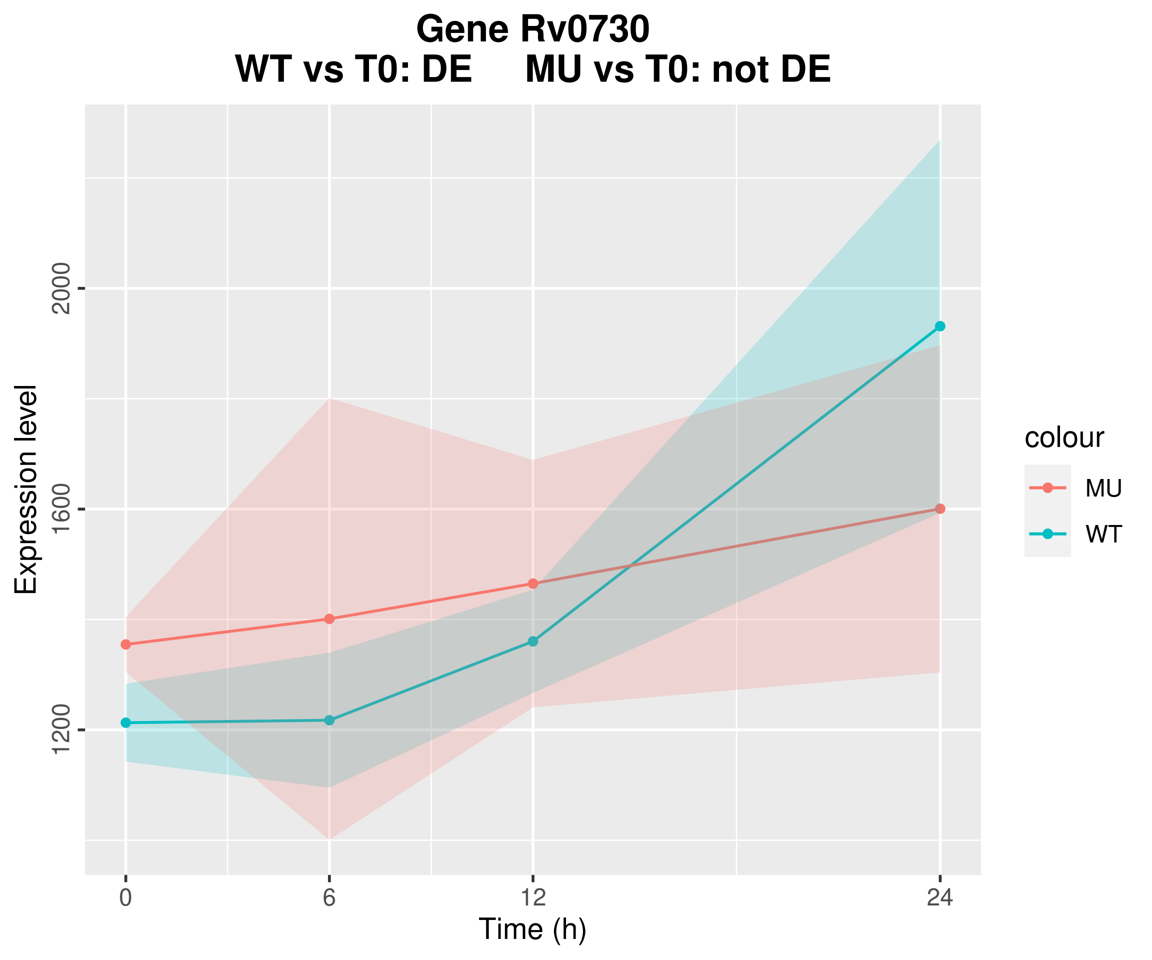

Gene Rv0730
WT vs T0: DE MU vs T0: not DE
Expression level
2000
1600
1200
0
6
12
24
Time (h)
colour
MU
WT

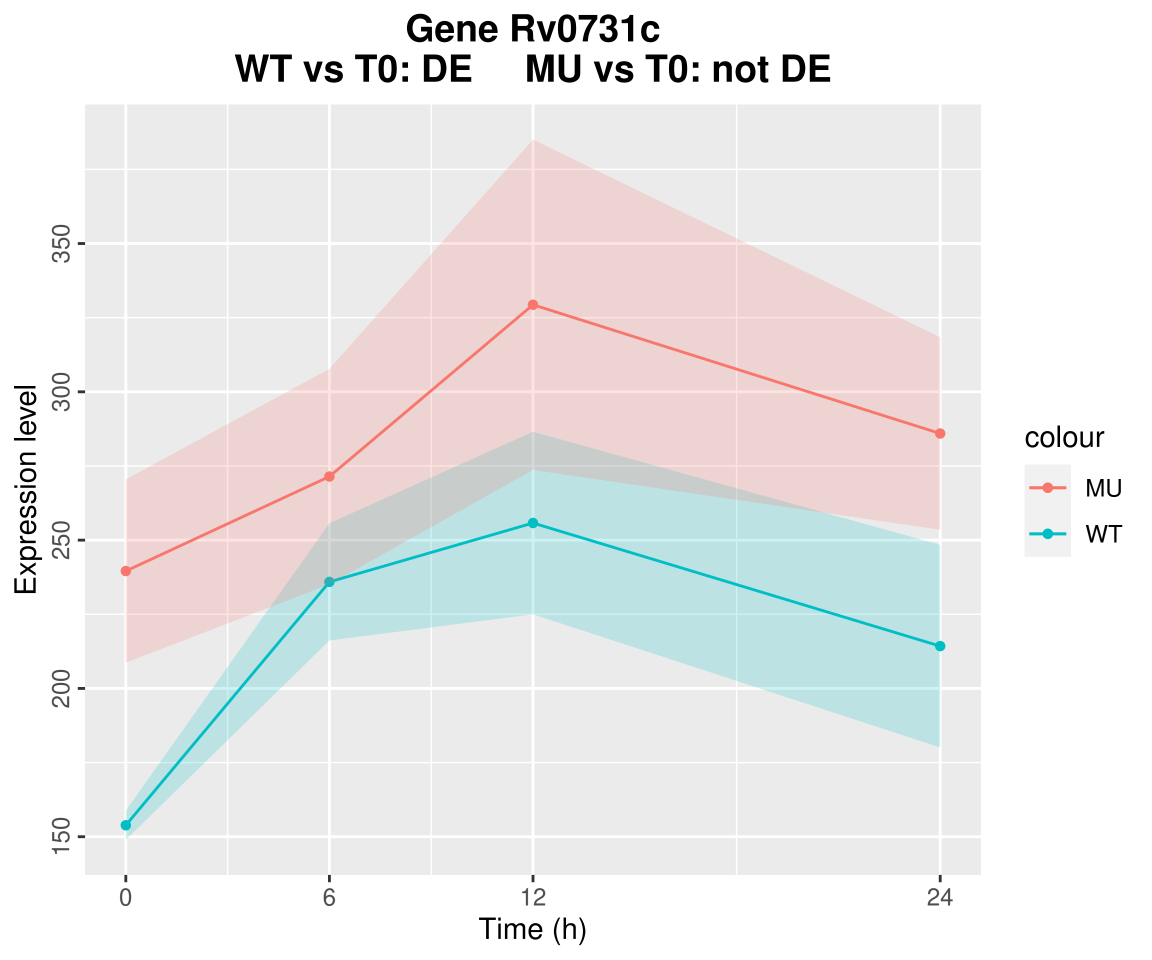

Gene Rv0731c
WT vs T0: DE   MU vs T0: not DE
Expression level
350
300
250
200
150
0
6
12
24
Time (h)
colour
MU
WT

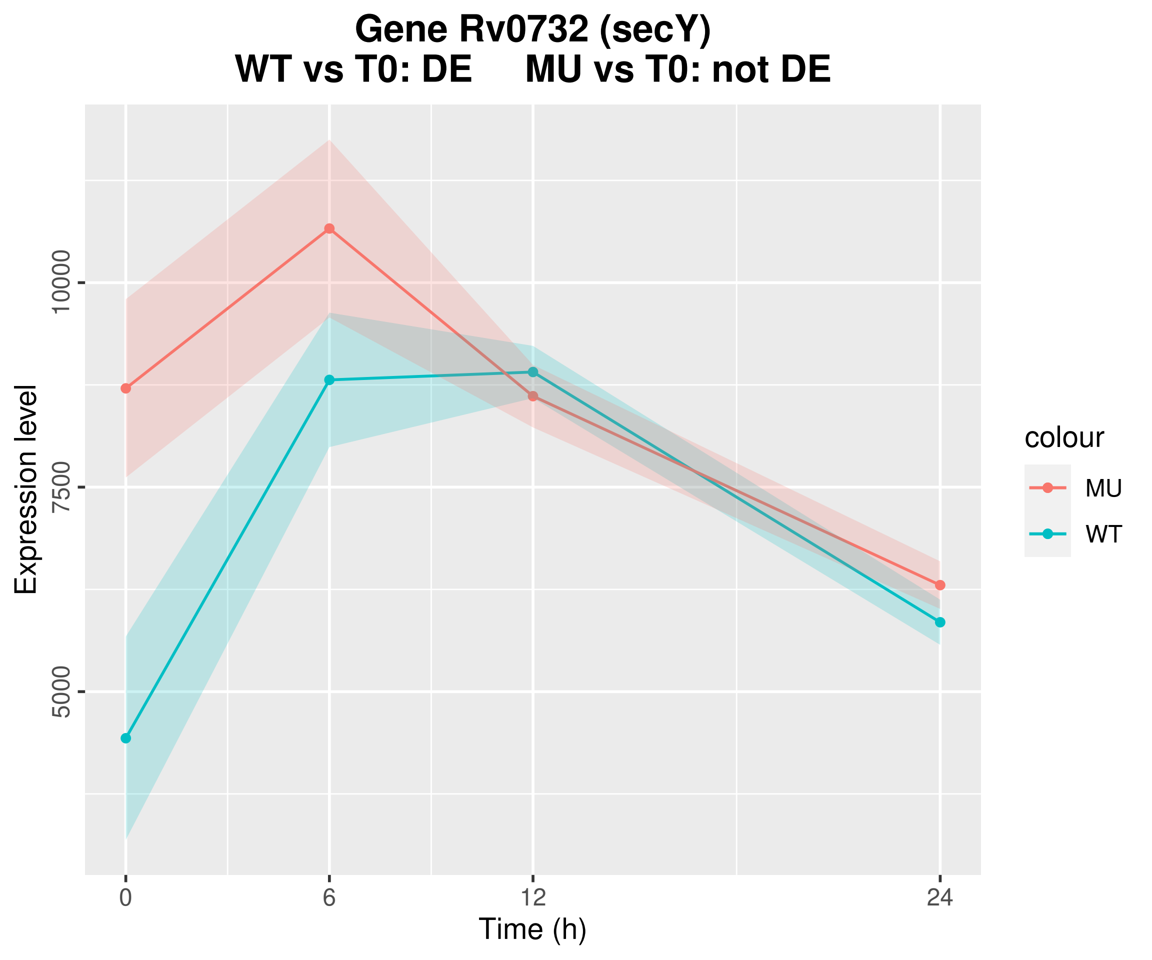

Gene Rv0732 (secY)
WT vs T0: DE MU vs T0: not DE
Expression level
10000
7500
5000
0
6
12
24
Time (h)
colour
MU
WT

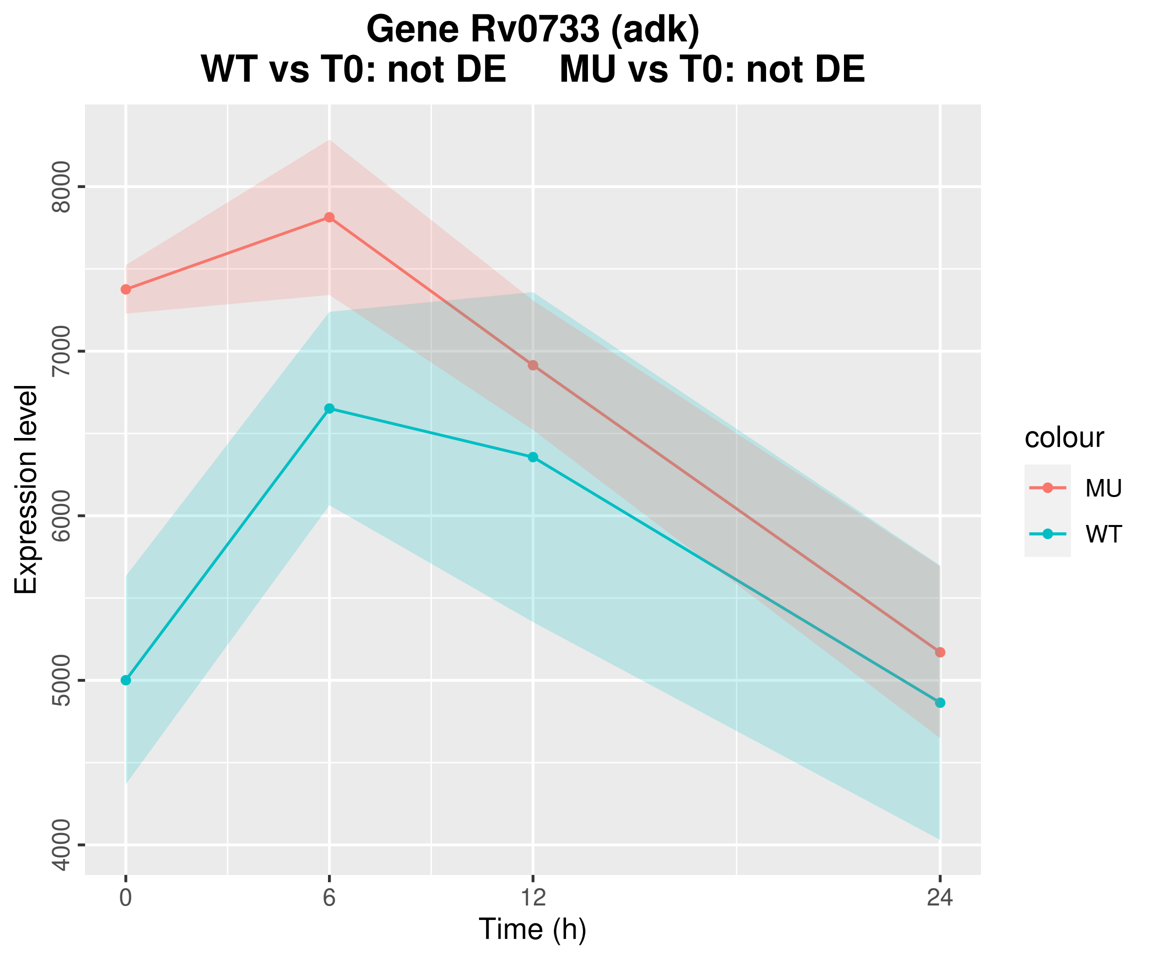

Gene Rv0733 (adk)
WT vs T0: not DE   MU vs T0: not DE
Expression level
8000
7000
6000
5000
4000
0
6
12
24
Time (h)
colour
MU
WT

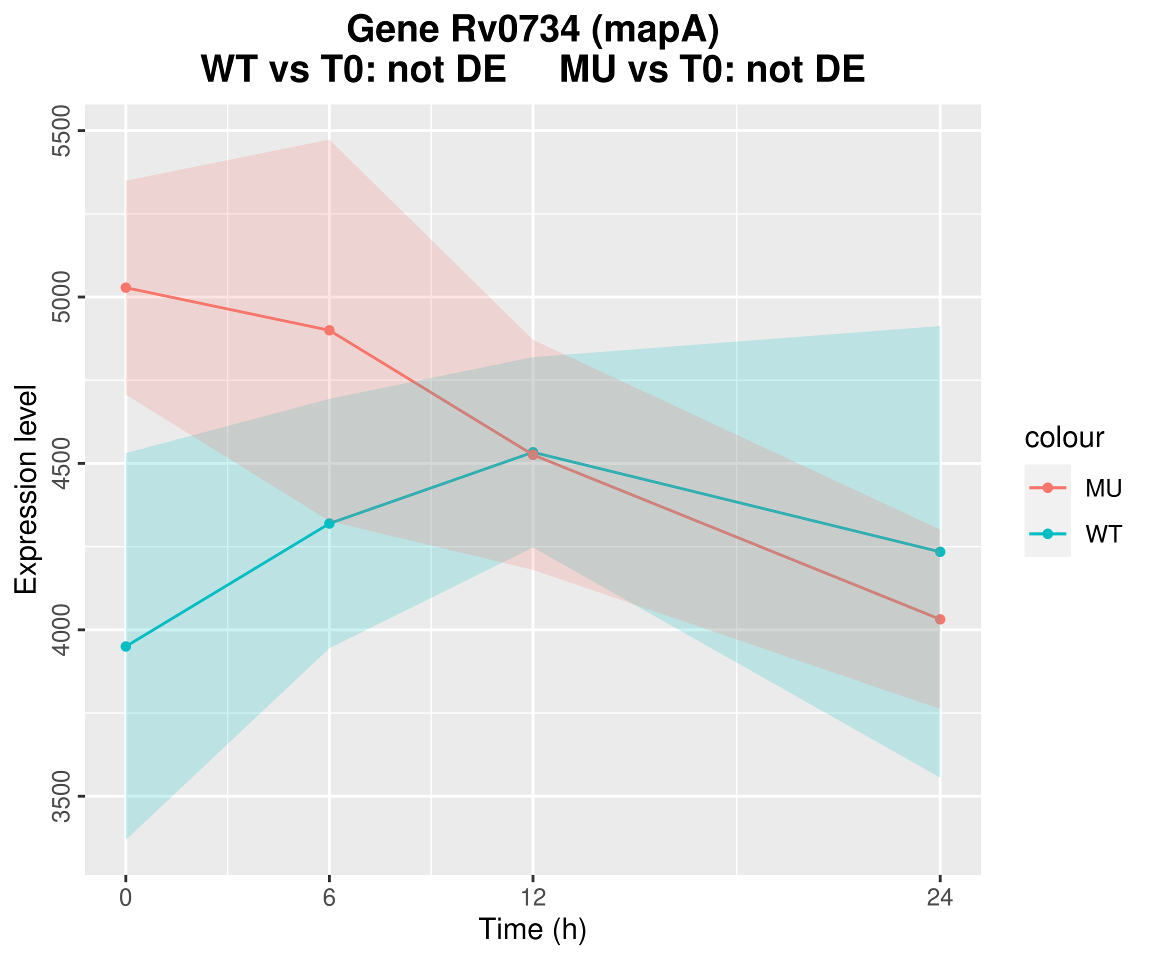
Gene Rv0734 (mapA)
WT vs T0: not DE   MU vs T0: not DE
Expression level
5500
5000
4500
4000
3500
0
6
12
24
Time (h)
colour
MU
WT

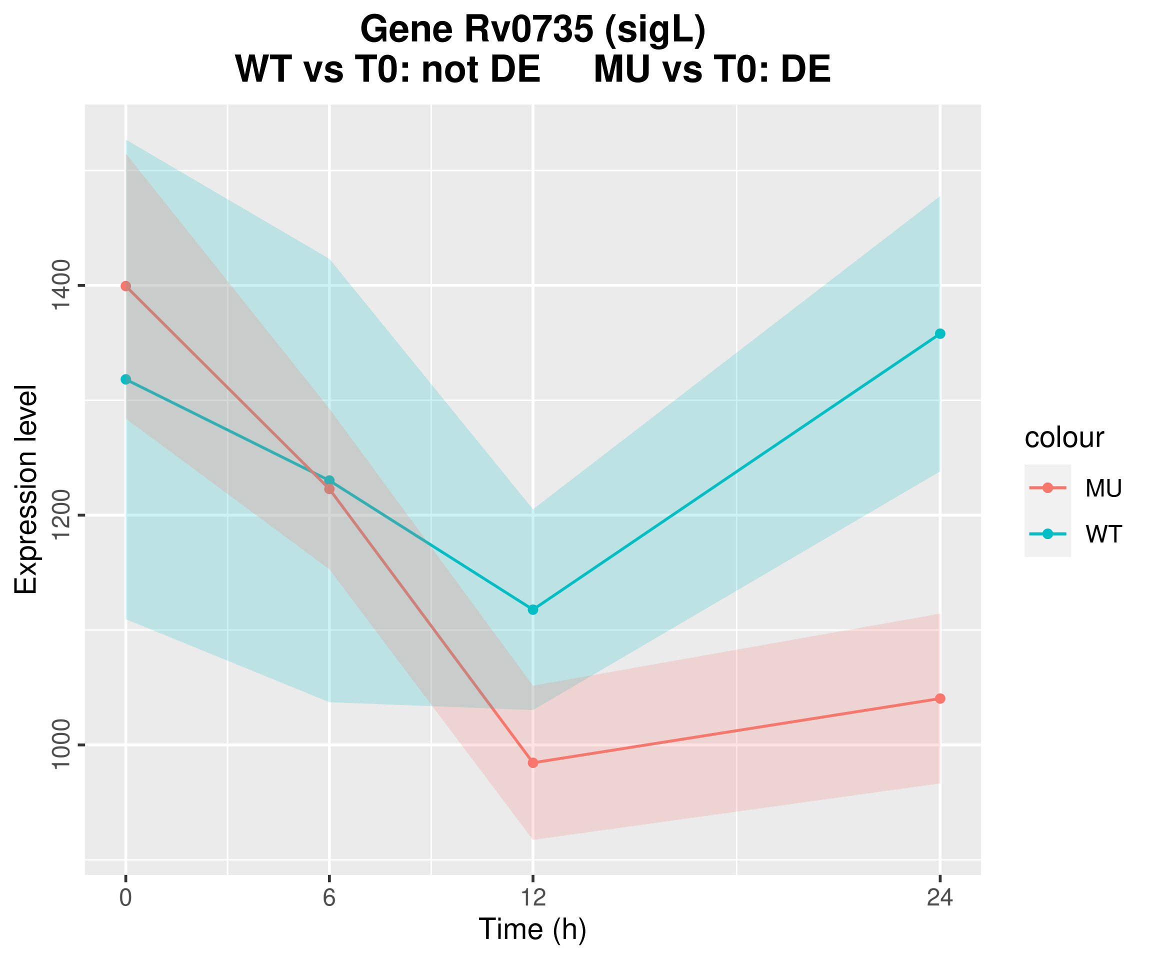

Gene Rv0735 (sigL)
WT vs T0: not DE   MU vs T0: DE
1400
1200
1000
Expression level
0
6
12
24
Time (h)
colour
MU
WT

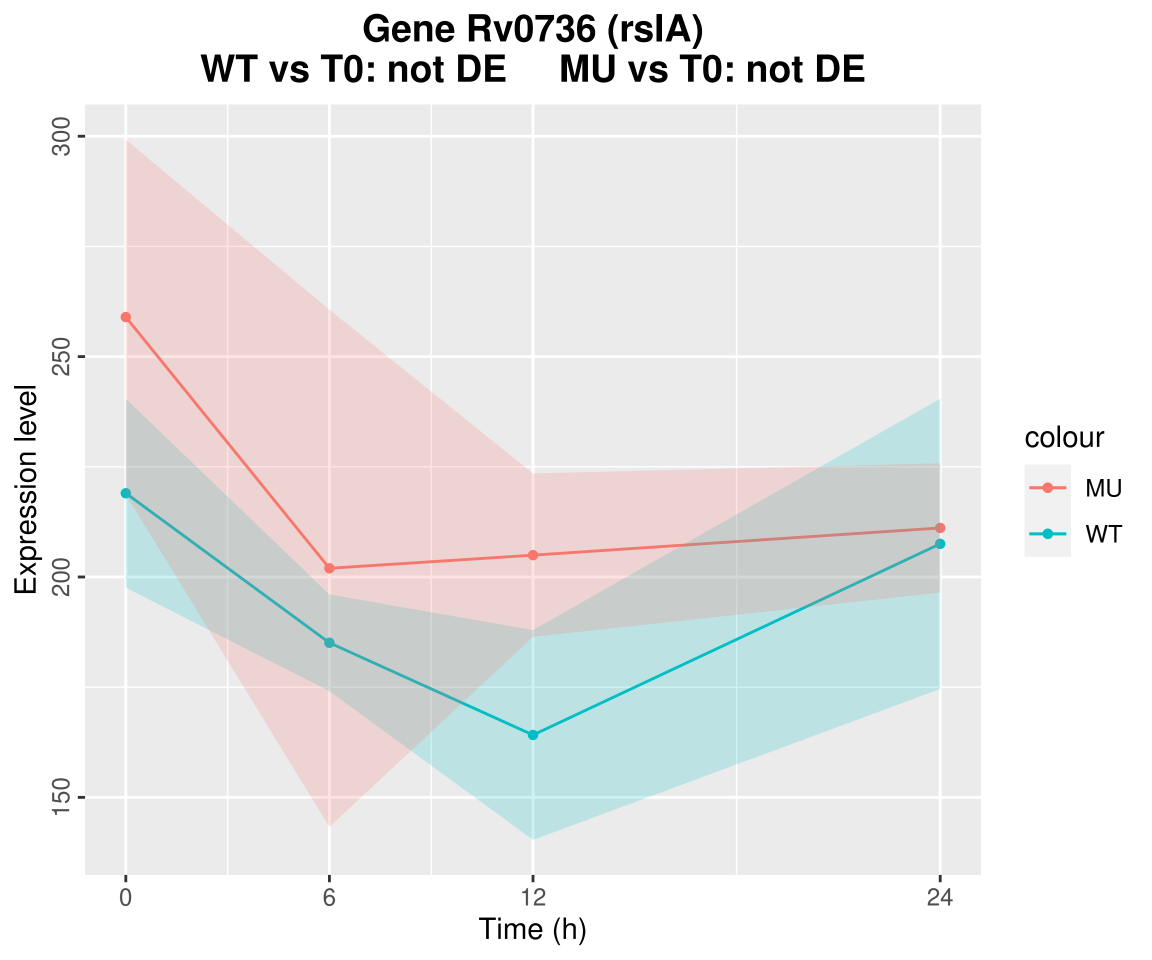

Gene Rv0736 (rslA)
WT vs T0: not DE   MU vs T0: not DE
Expression level
300
250
200
150
0
6
12
24
Time (h)
colour
MU
WT

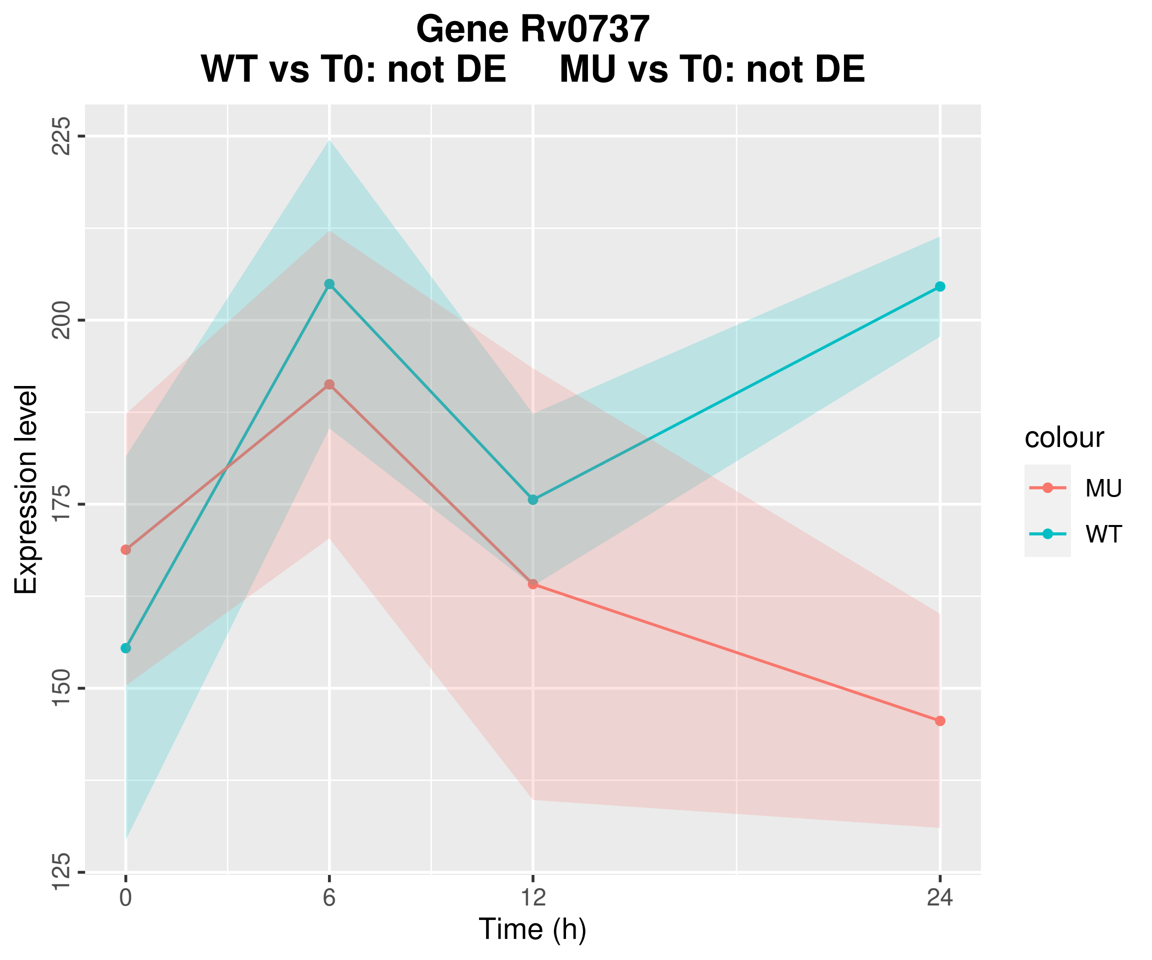
Gene Rv0737
WT vs T0: not DE   MU vs T0: not DE
Expression level
225
200
175
150
125
0
6
12
24
Time (h)
colour
MU
WT

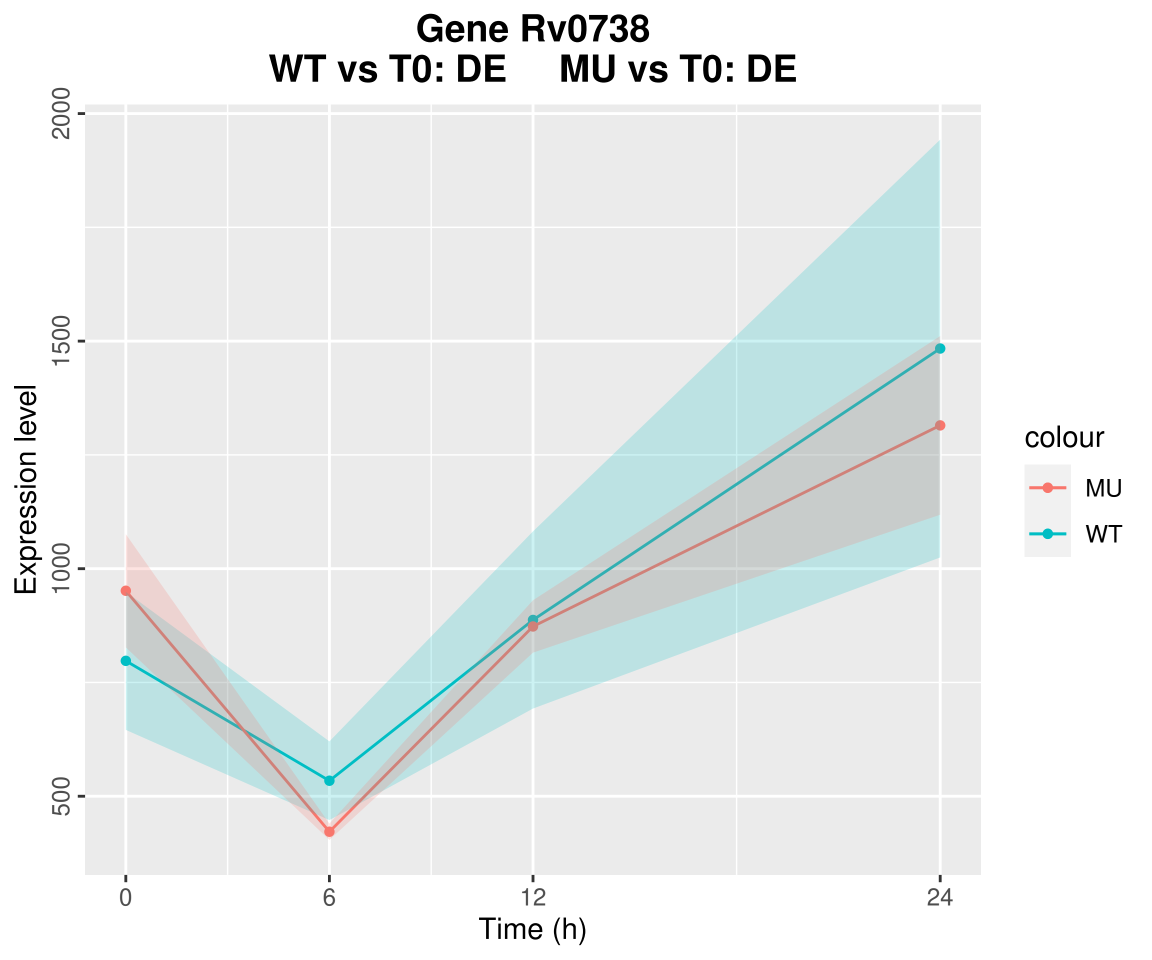

Gene Rv0738
WT vs T0: DE MU vs T0: DE
2000
1500
1000
500
Expression level
0
6
12
24
Time (h)
colour
MU
WT

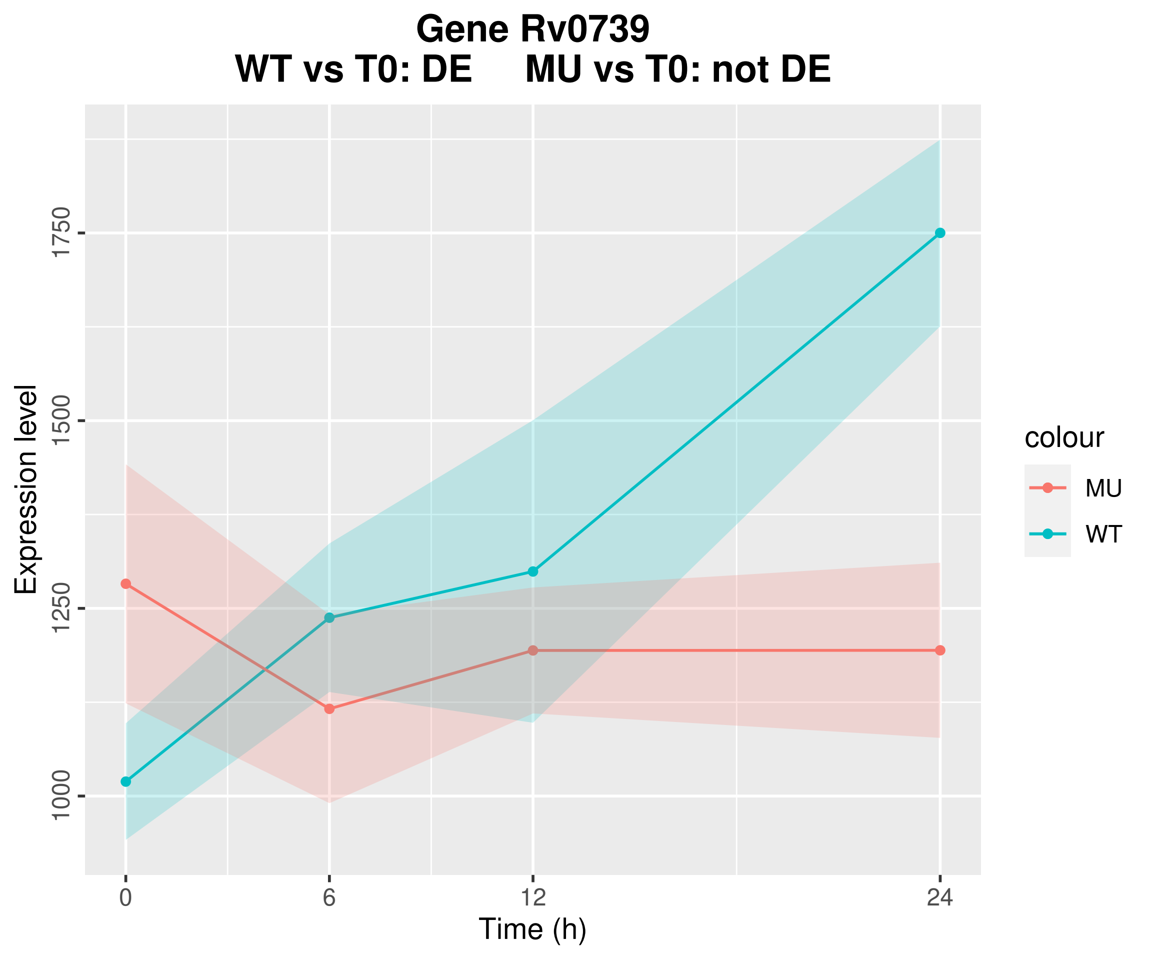
Gene Rv0739
WT vs T0: DE   MU vs T0: not DE
Expression level
1000
1250
1500
1750
0
6
12
24
Time (h)
colour
MU
WT

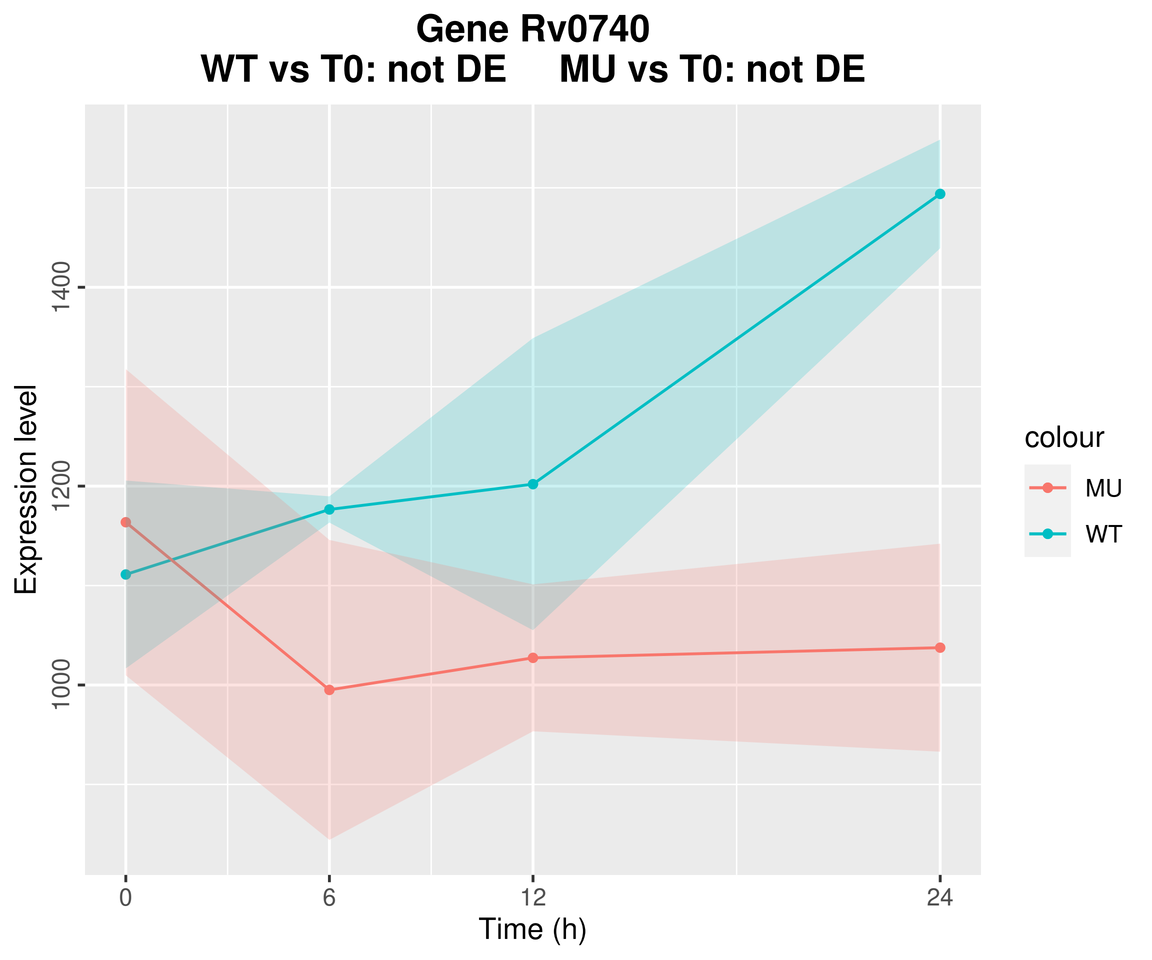

Gene Rv0740
WT vs T0: not DE   MU vs T0: not DE
Expression level
1400
1200
1000
0
6
12
24
Time (h)
colour
MU
WT

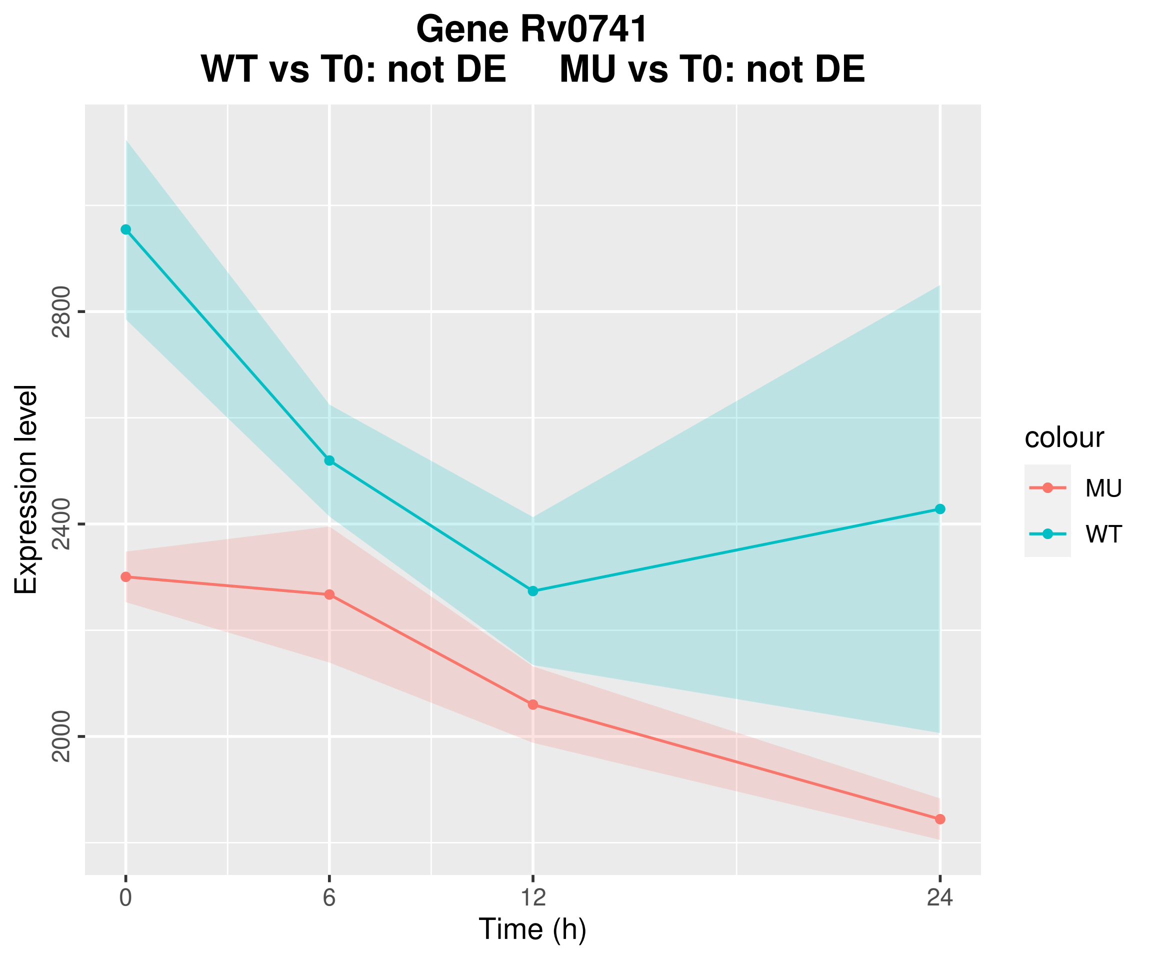

Gene Rv0741
WT vs T0: not DE MU vs T0: not DE
Expression level
2000
2400
2800
0
6
12
24
Time (h)
colour
MU
WT

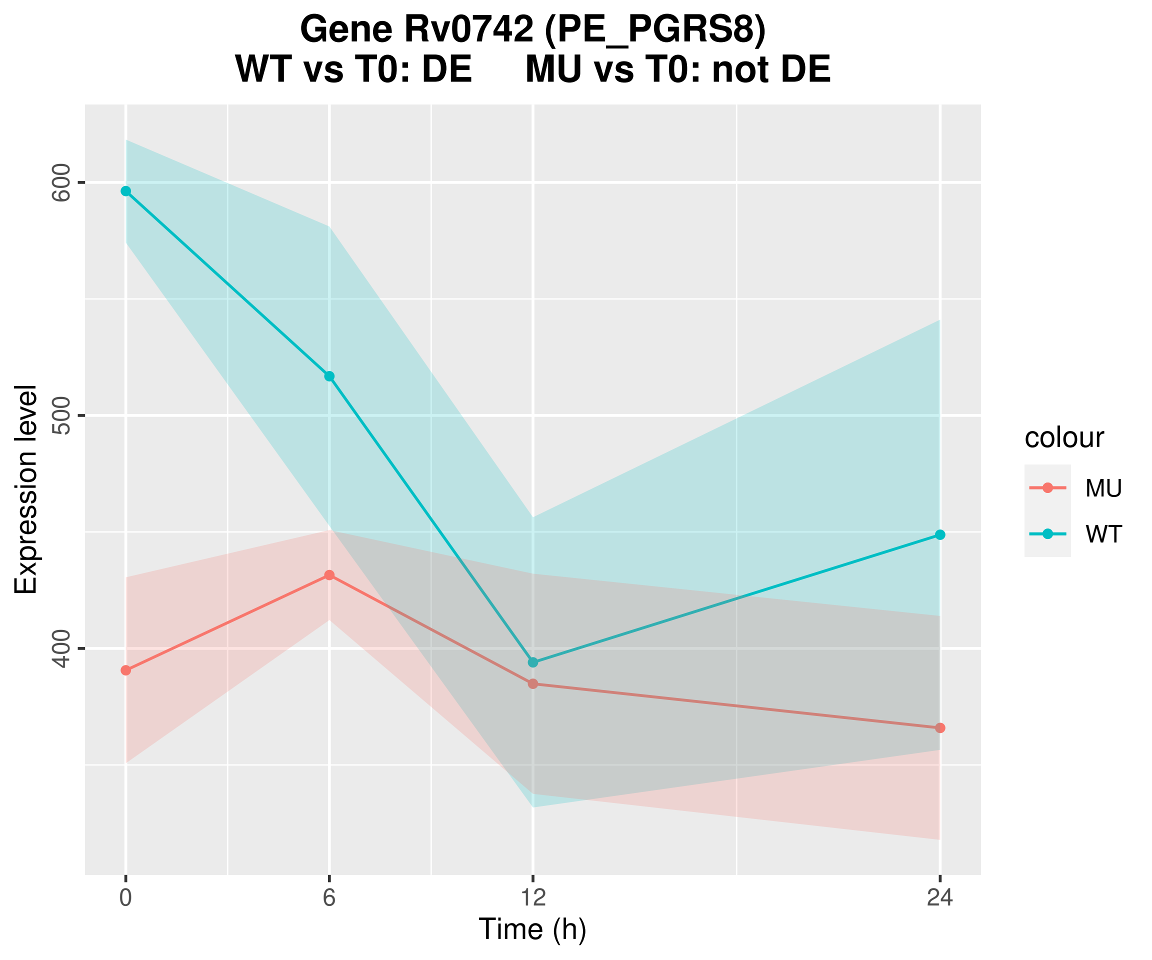
Gene Rv0742 (PE_PGRS8)
WT vs T0: DE   MU vs T0: not DE
600
500
400
Expression level
0
6
12
24
Time (h)
colour
MU
WT

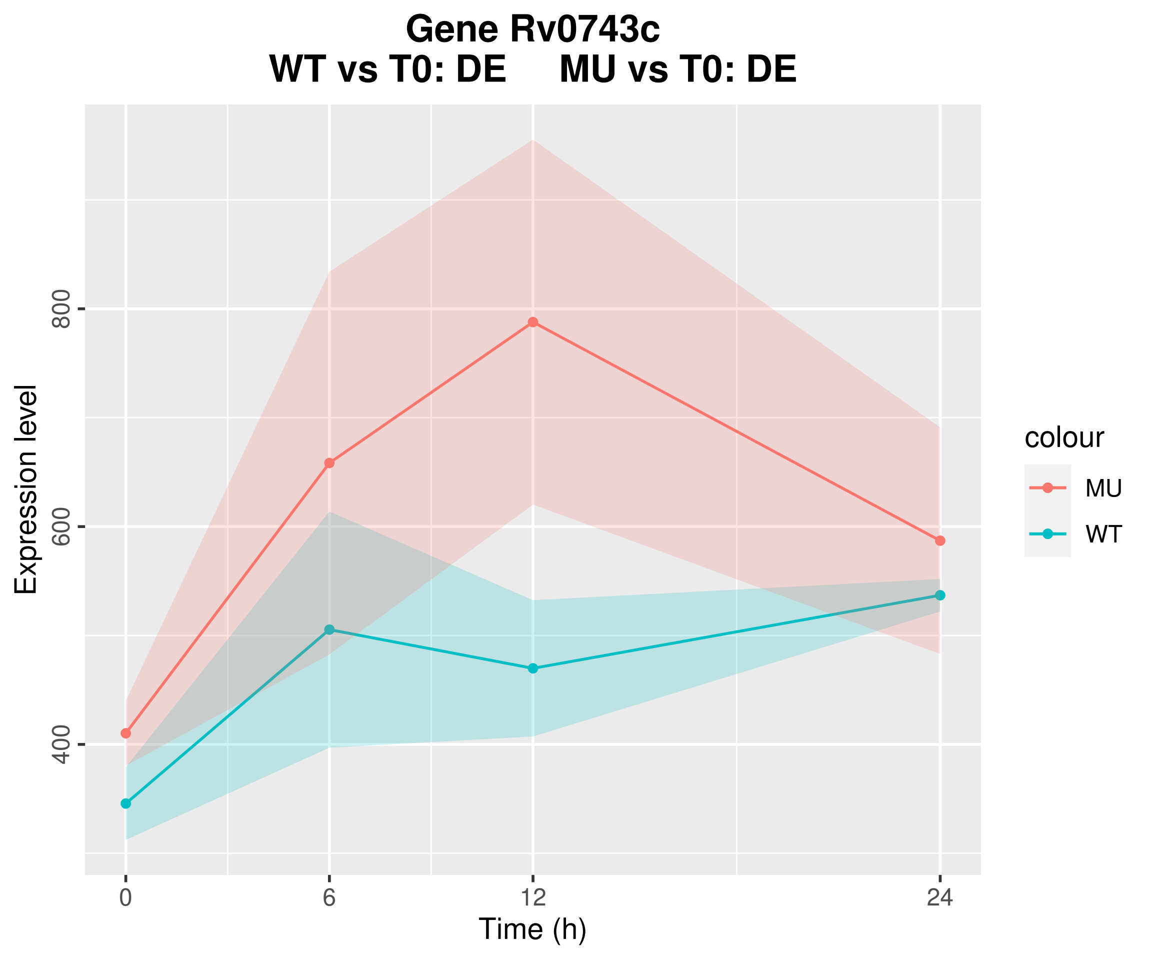

Gene Rv0743c
WT vs T0: DE   MU vs T0: DE
Expression level
800
600
400
0
6
12
24
Time (h)
colour
MU
WT

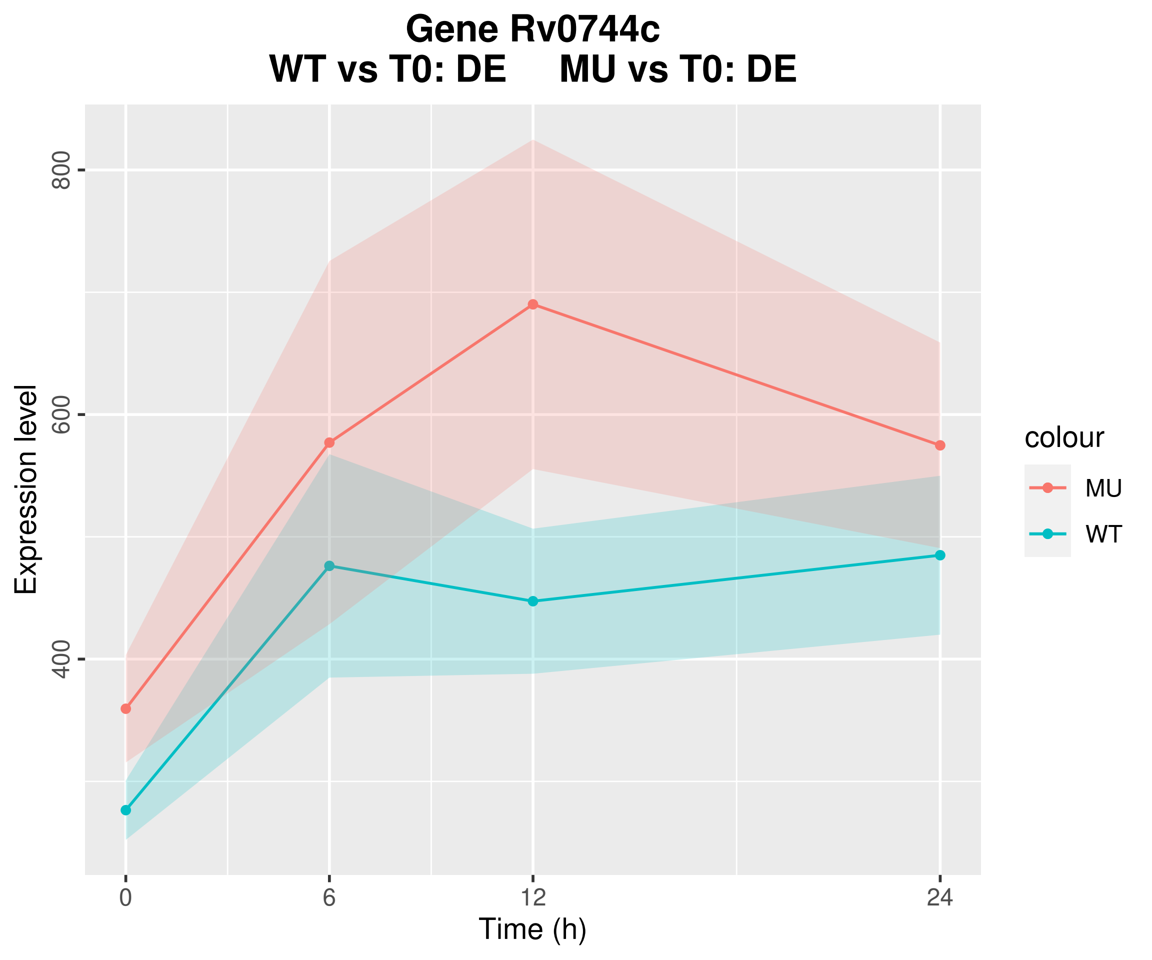
Gene Rv0744c
WT vs T0: DE   MU vs T0: DE
Expression level
800
600
400
0
6
12
24
Time (h)
colour
MU
WT

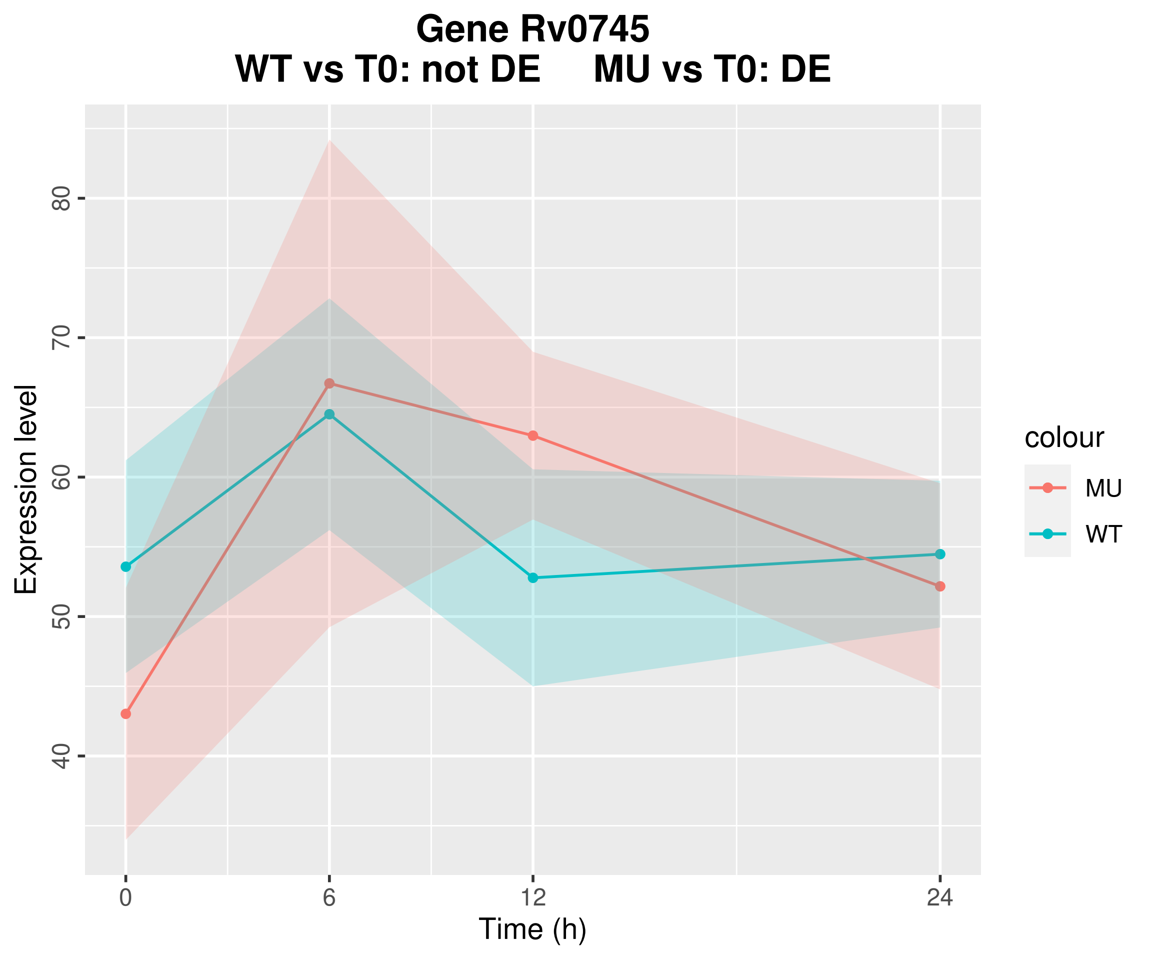
Gene Rv0745
WT vs T0: not DE   MU vs T0: DE
Expression level
80
70
60
50
40
0
6
12
24
Time (h)
colour
MU
WT

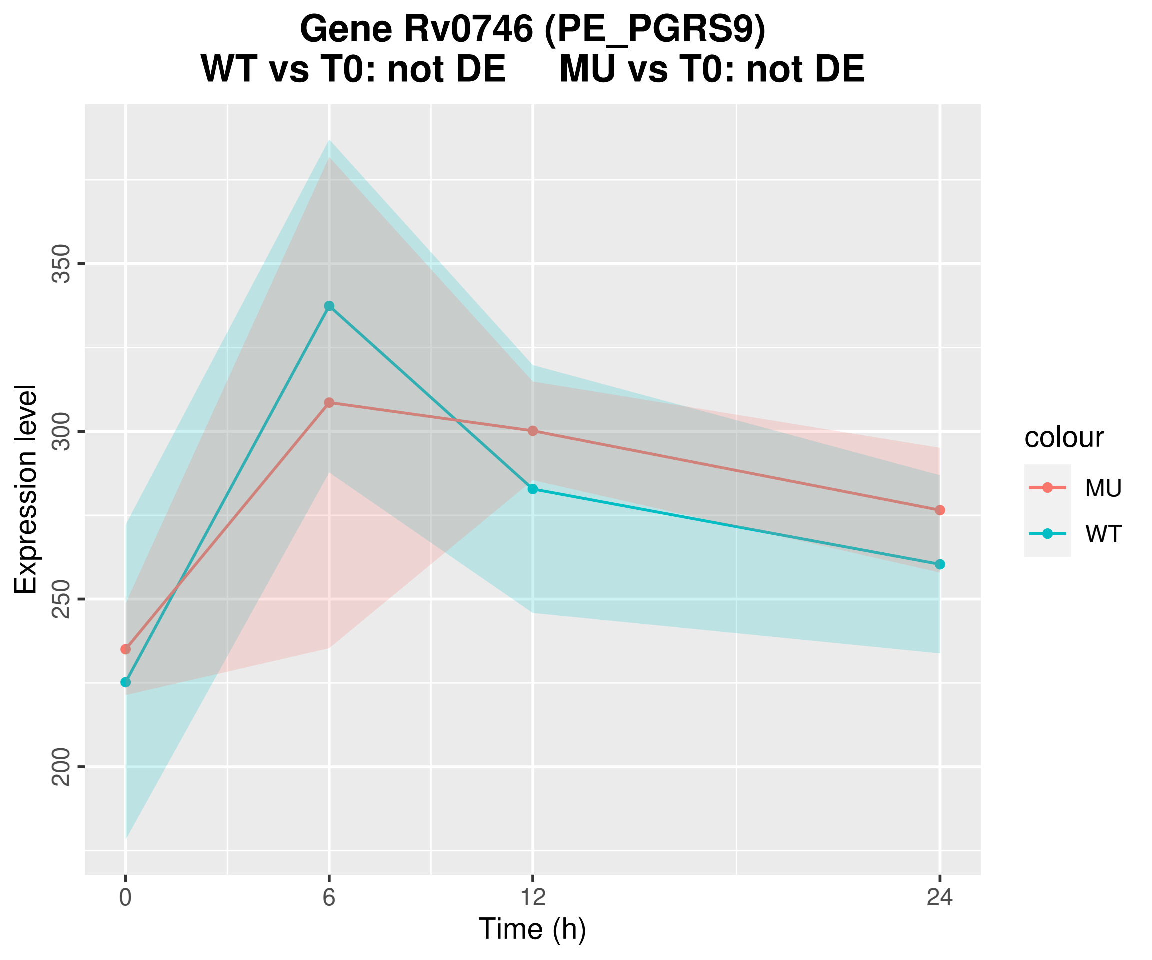
Gene Rv0746 (PE_PGRS9)
WT vs T0: not DE   MU vs T0: not DE
Expression level
350
300
250
200
0
6
12
24
Time (h)
colour
MU
WT

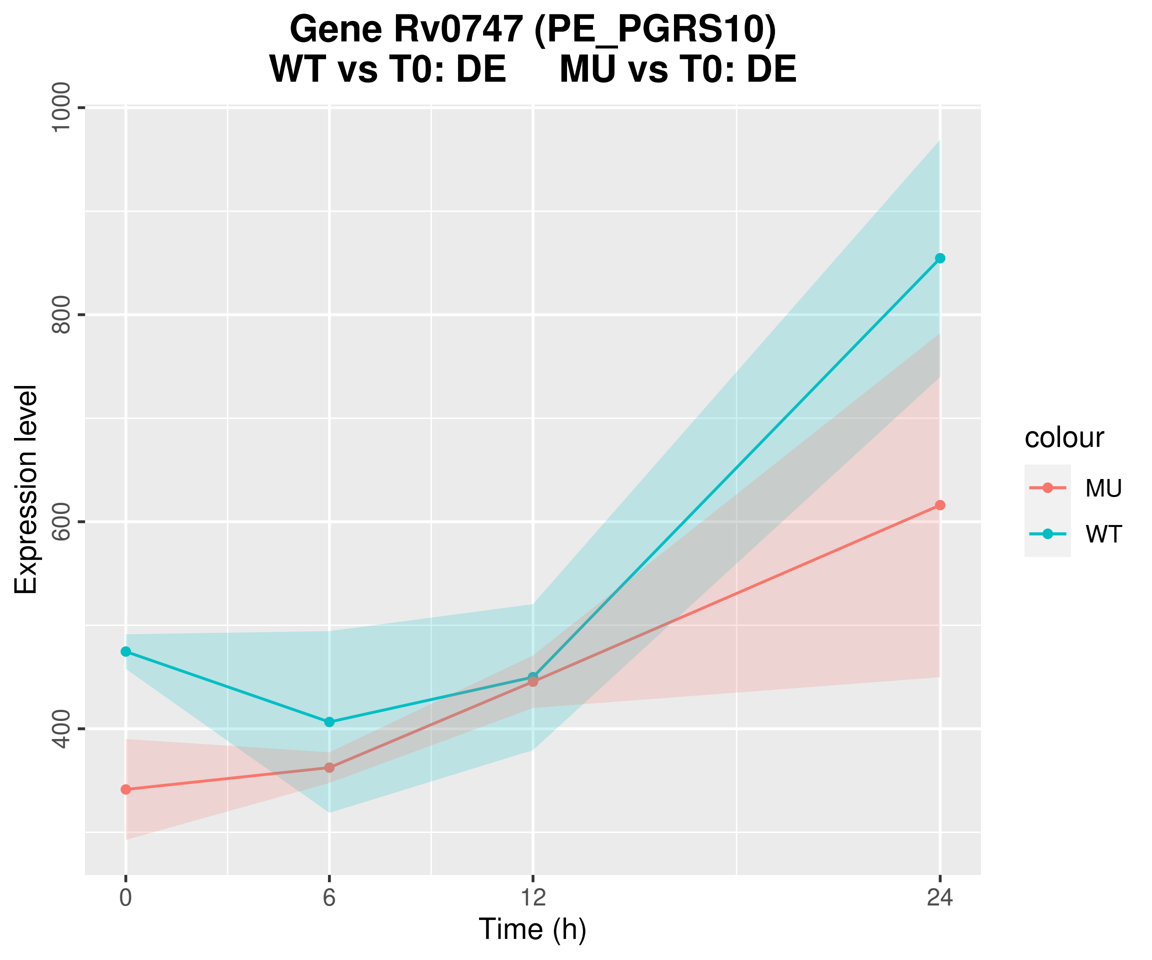
Gene Rv0747 (PE_PGRS10)
WT vs T0: DE   MU vs T0: DE
Expression level
1000
800
600
400
0
6
12
24
Time (h)
colour
MU
WT

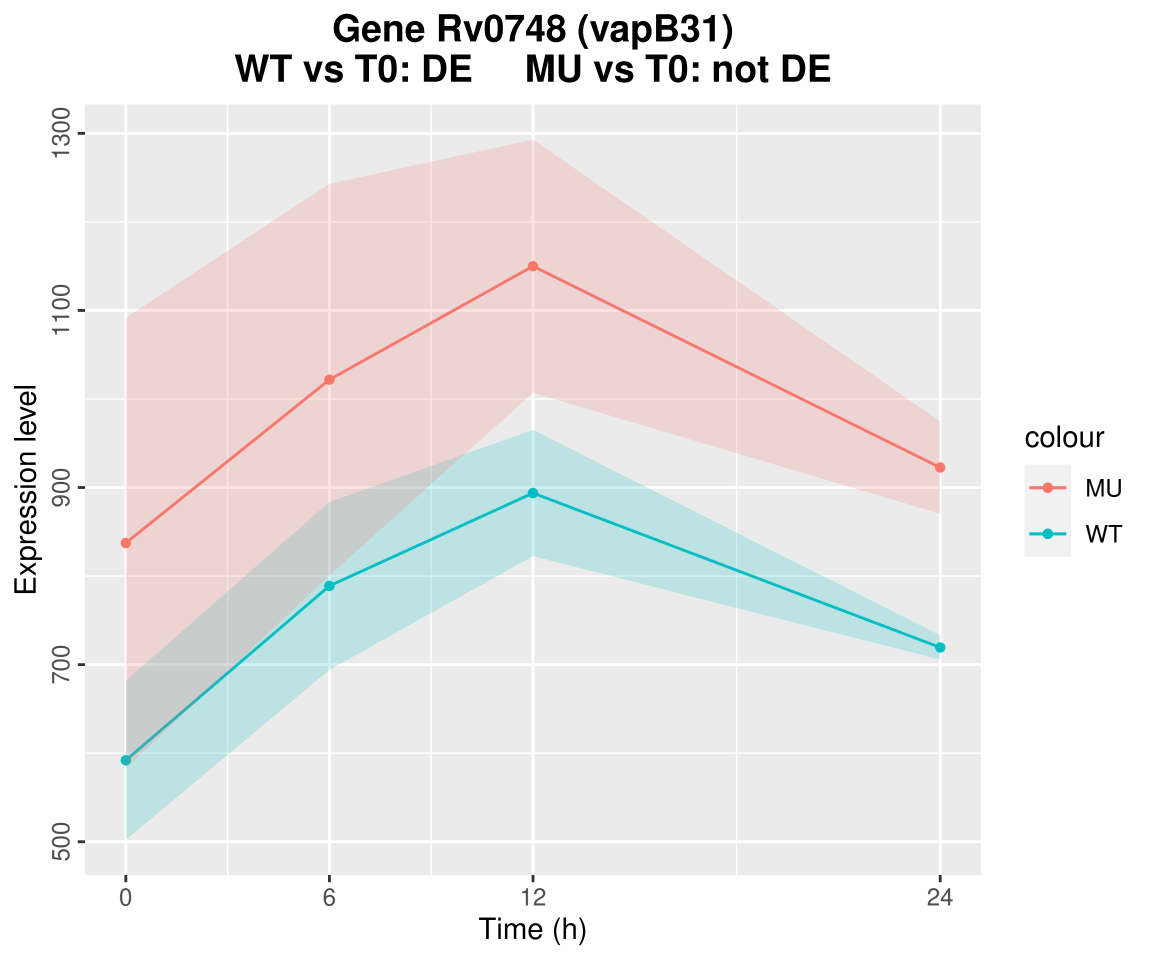
Gene Rv0748 (vapB31)
WT vs T0: DE   MU vs T0: not DE
Expression level
1300
1100
900
700
500
0
6
12
24
Time (h)
colour
MU
WT

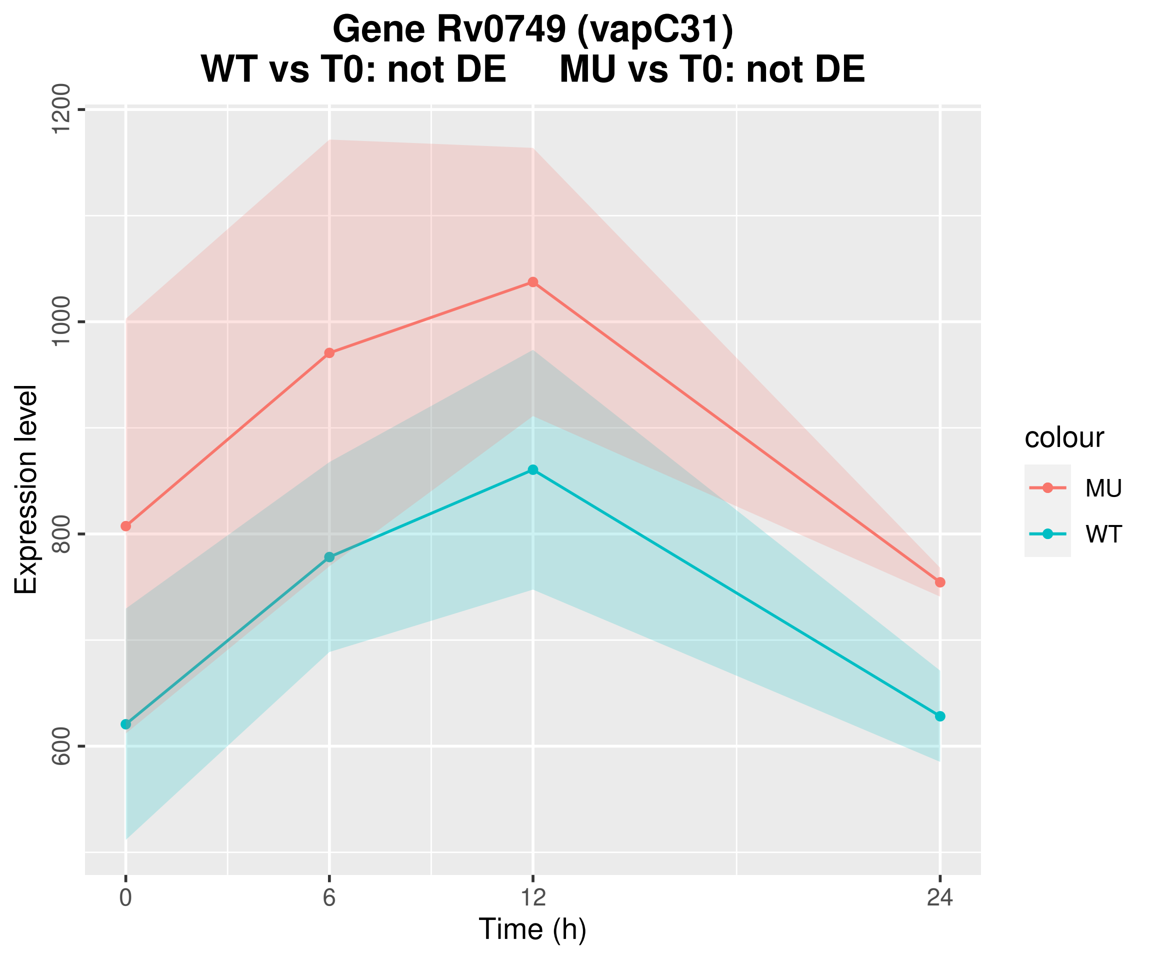

Gene Rv0749 (vapC31)
WT vs T0: not DE MU vs T0: not DE
Expression level
1200
1000
800
600
Time (h)
0
6
12
24
colour
MU
WT

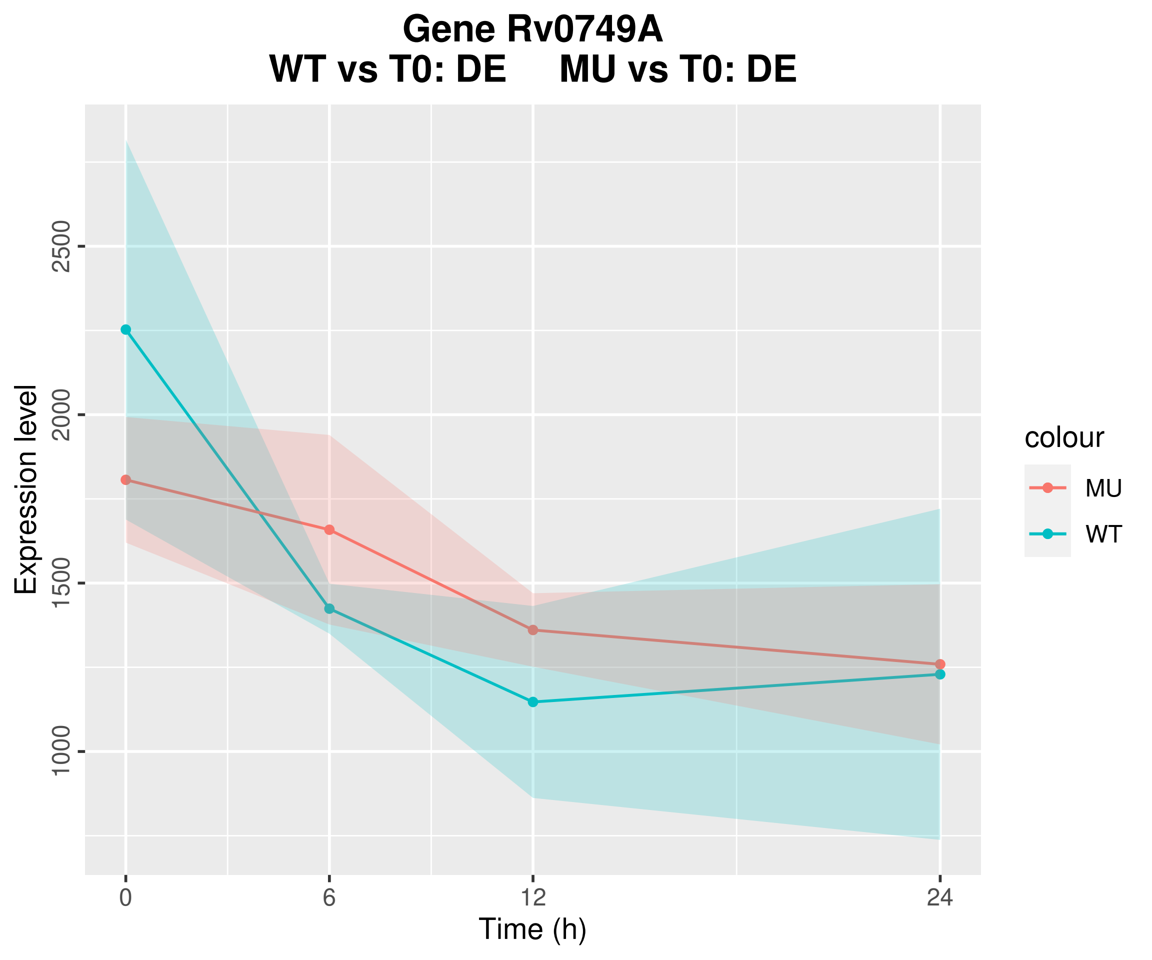

Gene Rv0749A
WT vs T0: DE   MU vs T0: DE
Expression level
2500
2000
1500
1000
0
6
12
24
Time (h)
colour
MU
WT

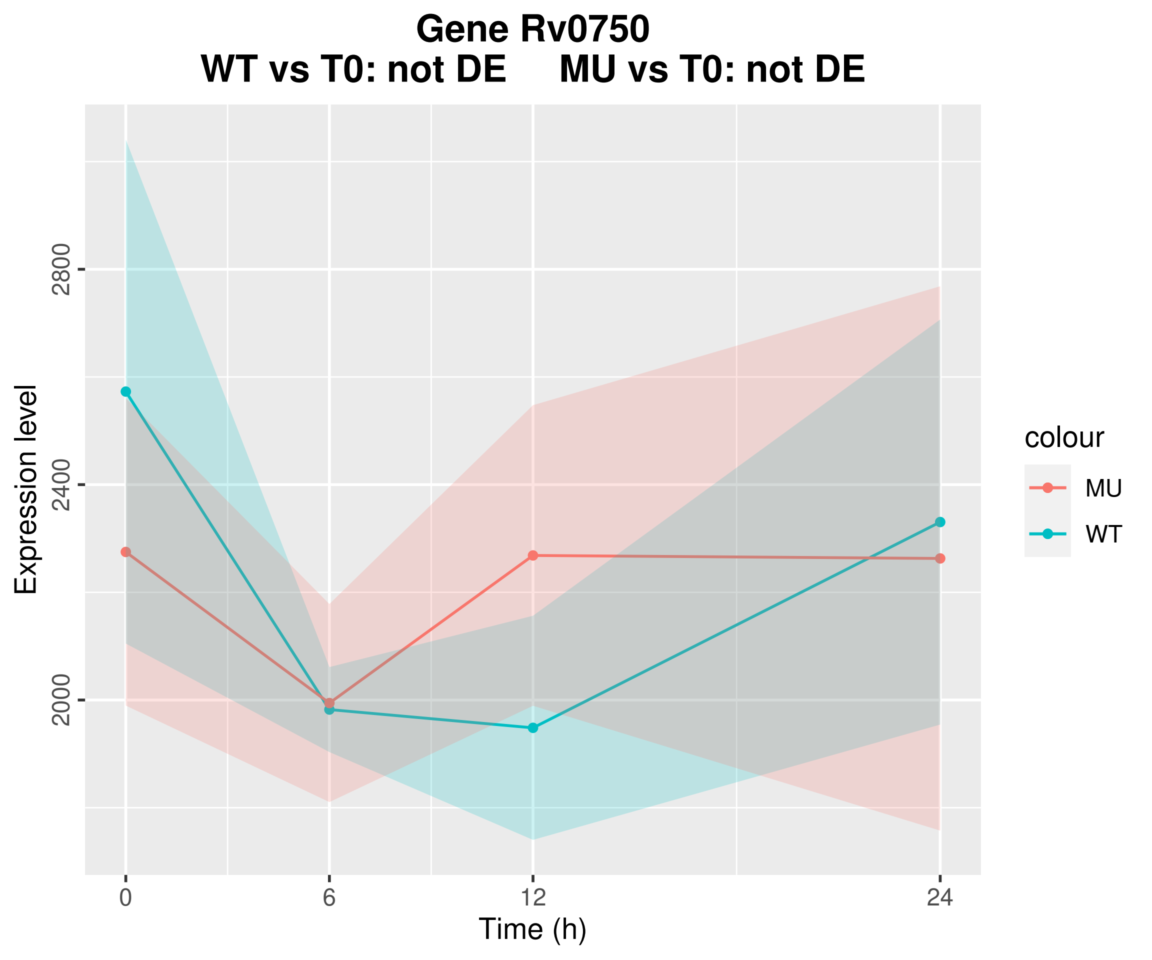
Gene Rv0750
WT vs T0: not DE MU vs T0: not DE
Expression level
2000
2400
2800
0
6
12
24
Time (h)
colour
MU
WT

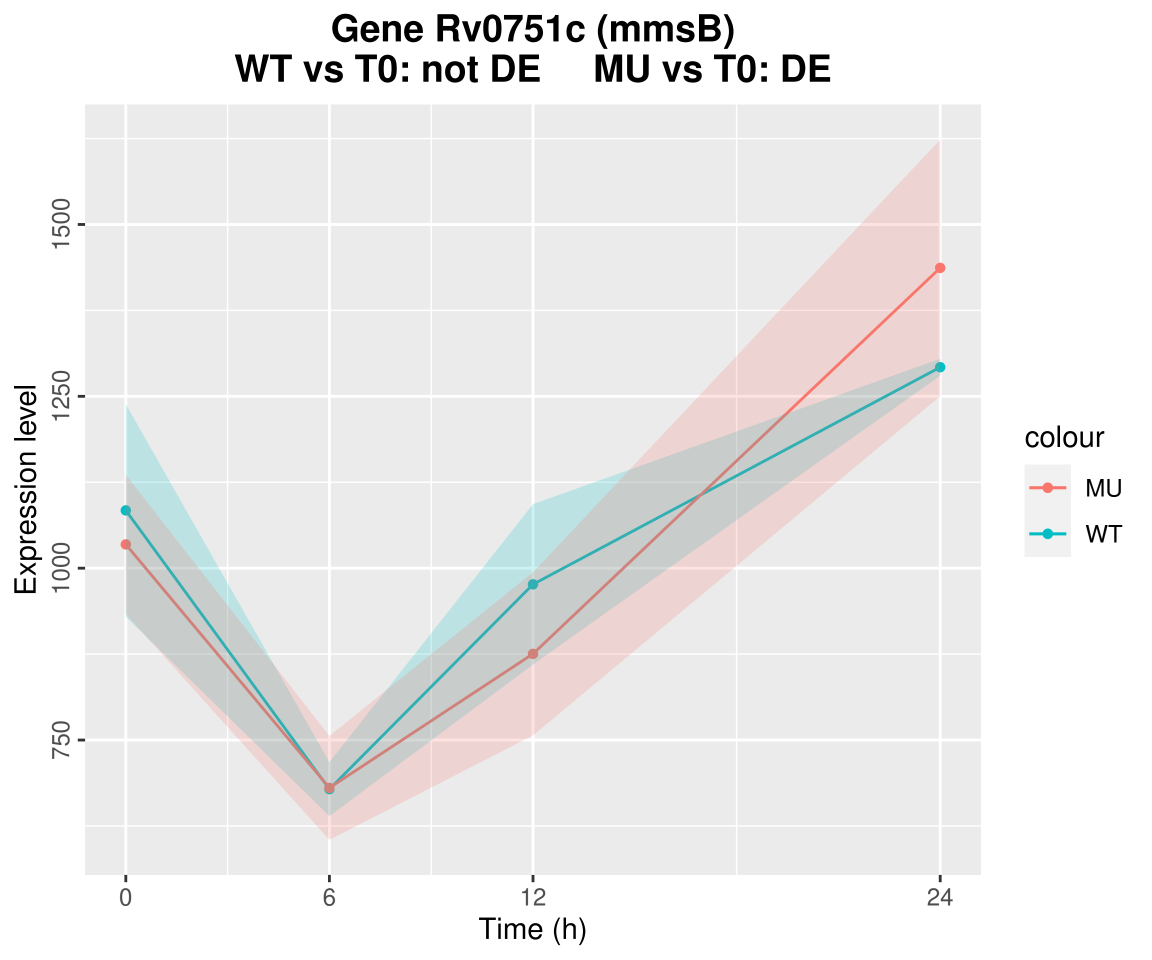
Gene Rv0751c (mmsB)
WT vs T0: not DE MU vs T0: DE
Expression level
750
1000
1250
1500
0
6
12
24
Time (h)
colour
MU
WT

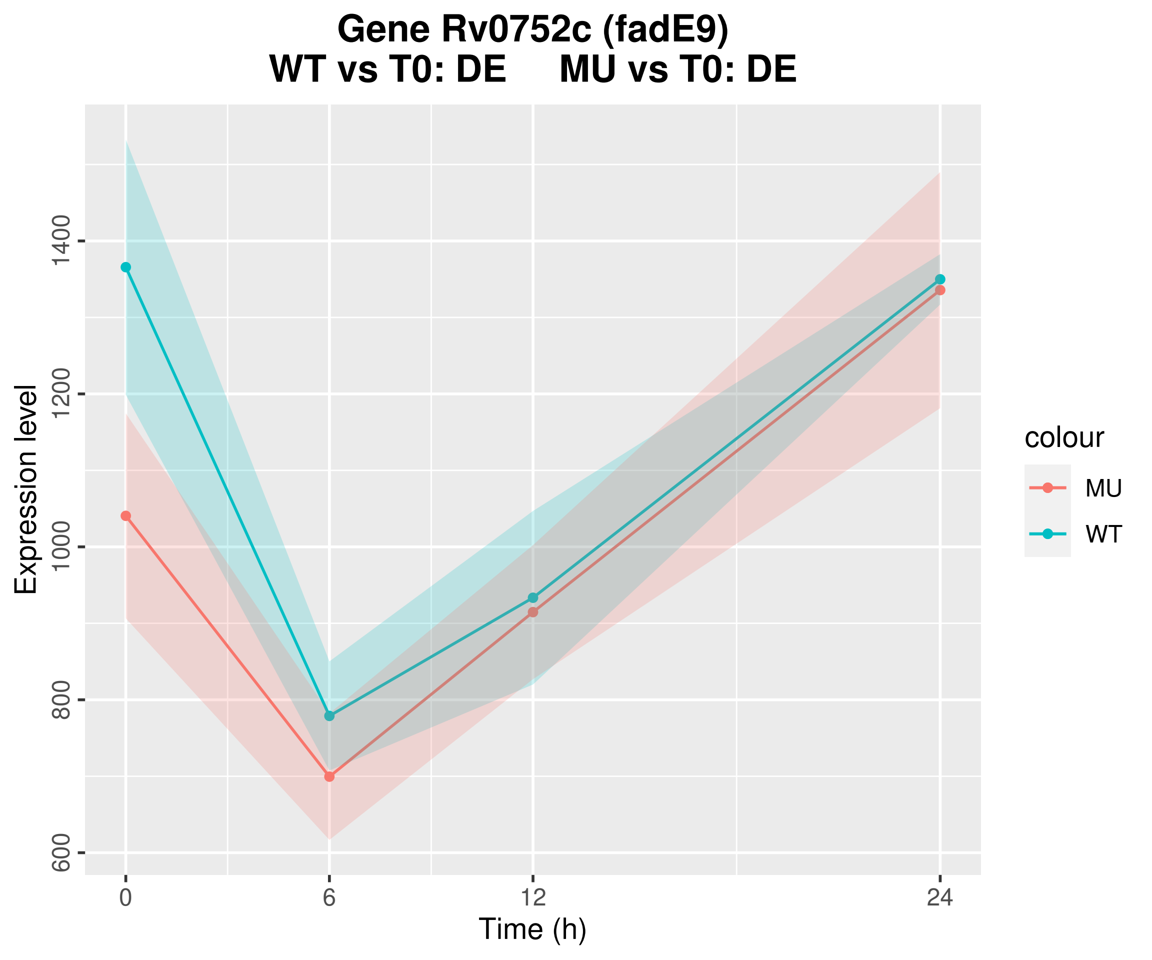
Gene Rv0752c (fadE9)
WT vs T0: DE MU vs T0: DE
Expression level
1400
1200
1000
800
600
0
6
12
24
Time (h)
colour
MU
WT

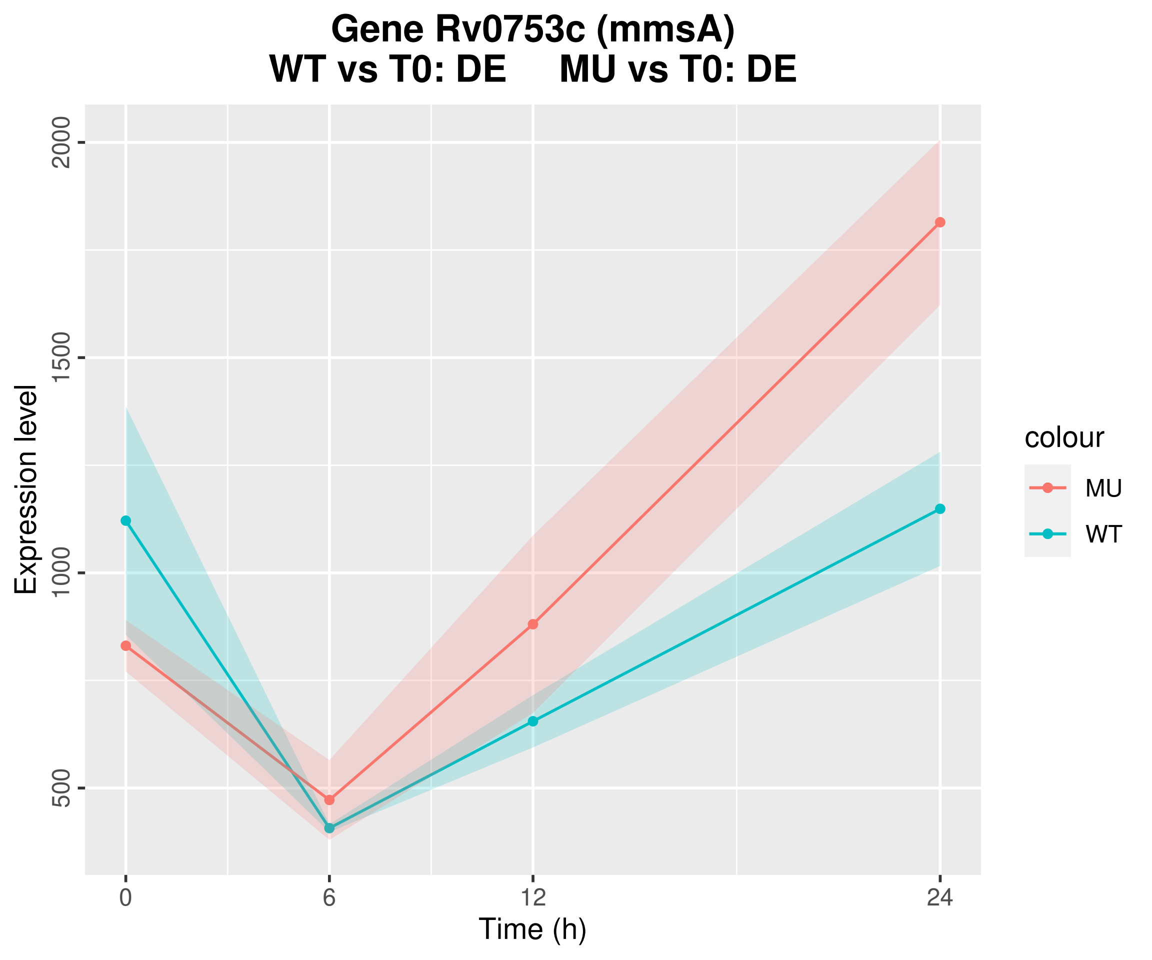

Gene Rv0753c (mmsA)
WT vs T0: DE MU vs T0: DE
Expression level
2000
1500
1000
500
0
6
12
24
Time (h)
colour
MU
WT

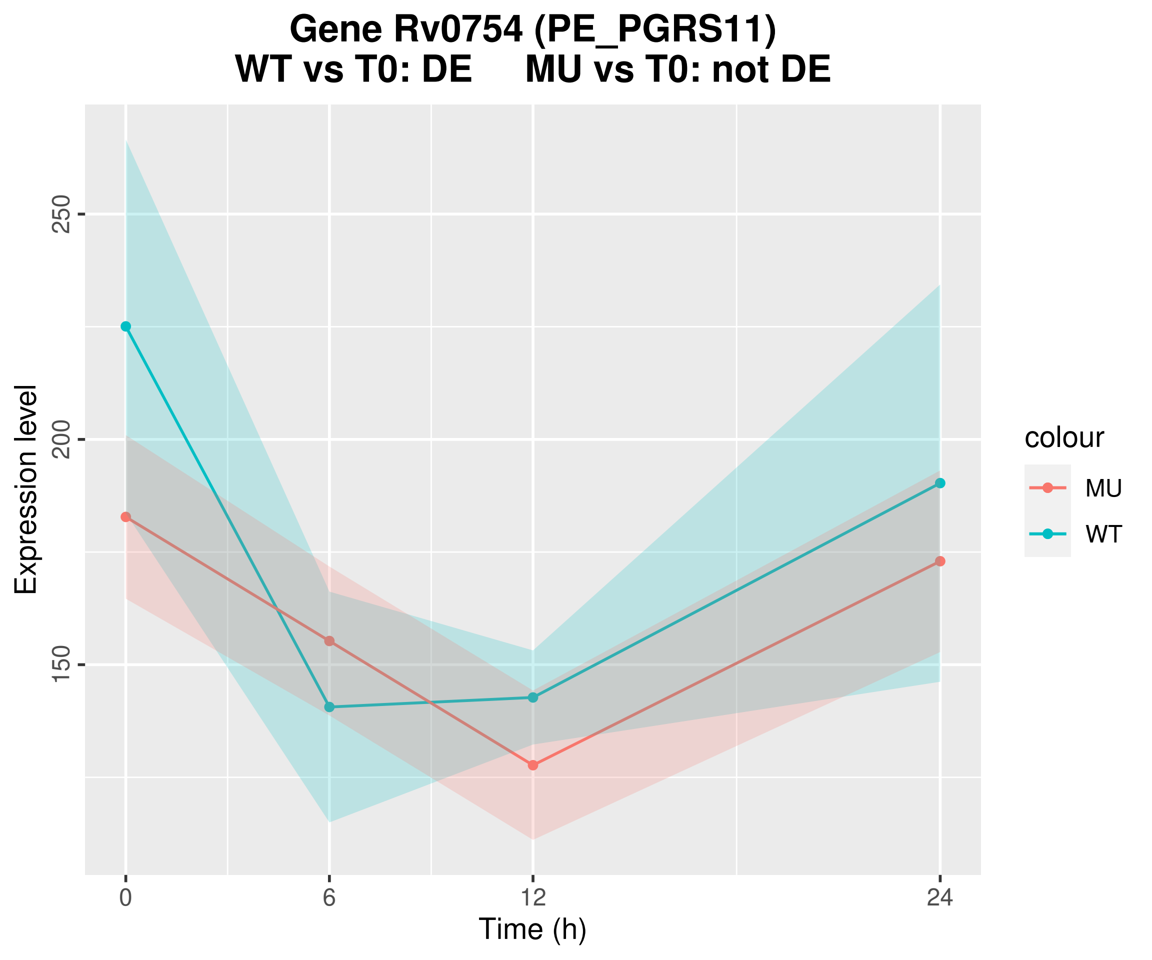

Gene Rv0754 (PE_PGRS11)
WT vs T0: DE   MU vs T0: not DE
Expression level
250
200
150
0
6
12
24
Time (h)
colour
MU
WT

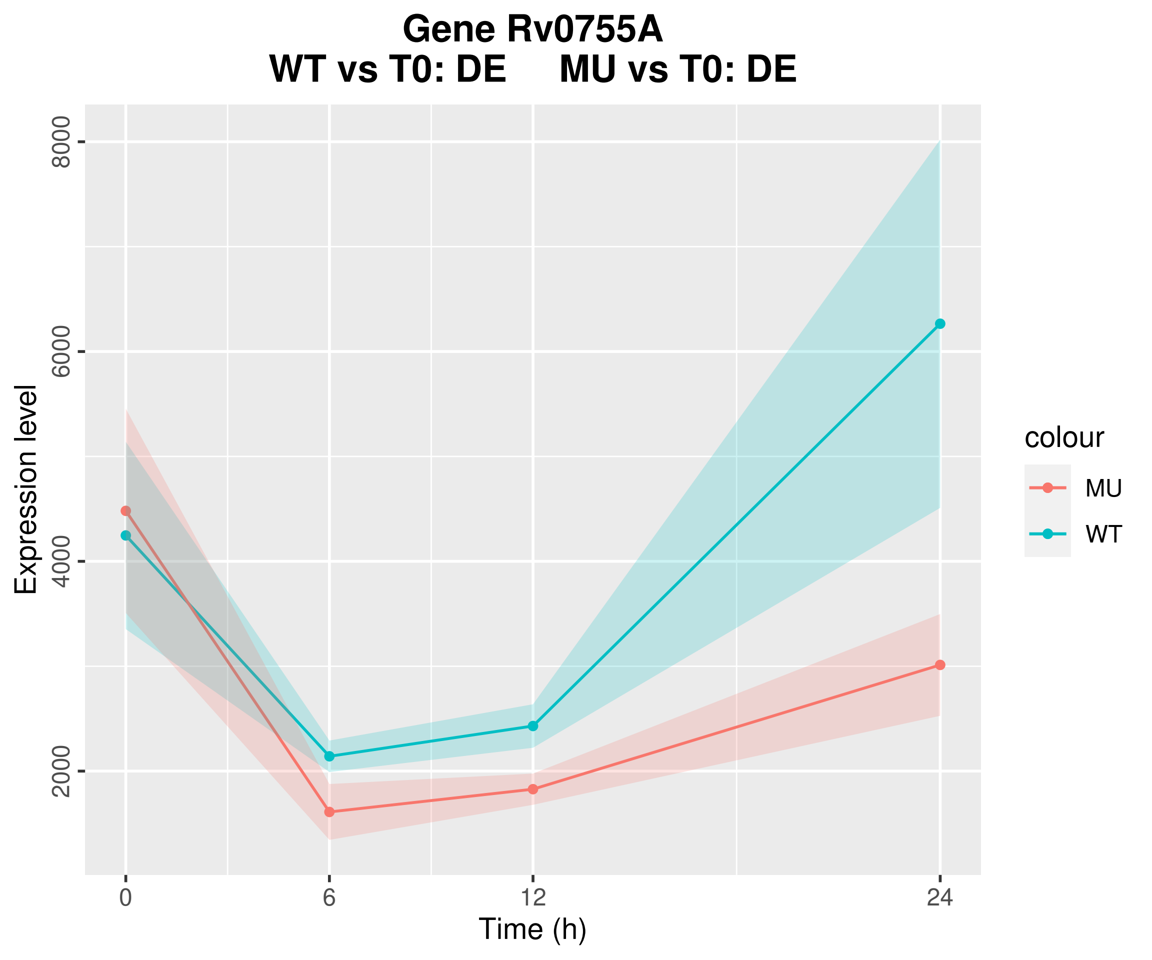

Gene Rv0755A
WT vs T0: DE MU vs T0: DE
Expression level
8000
6000
4000
2000
0
6
12
24
Time (h)
colour
MU
WT

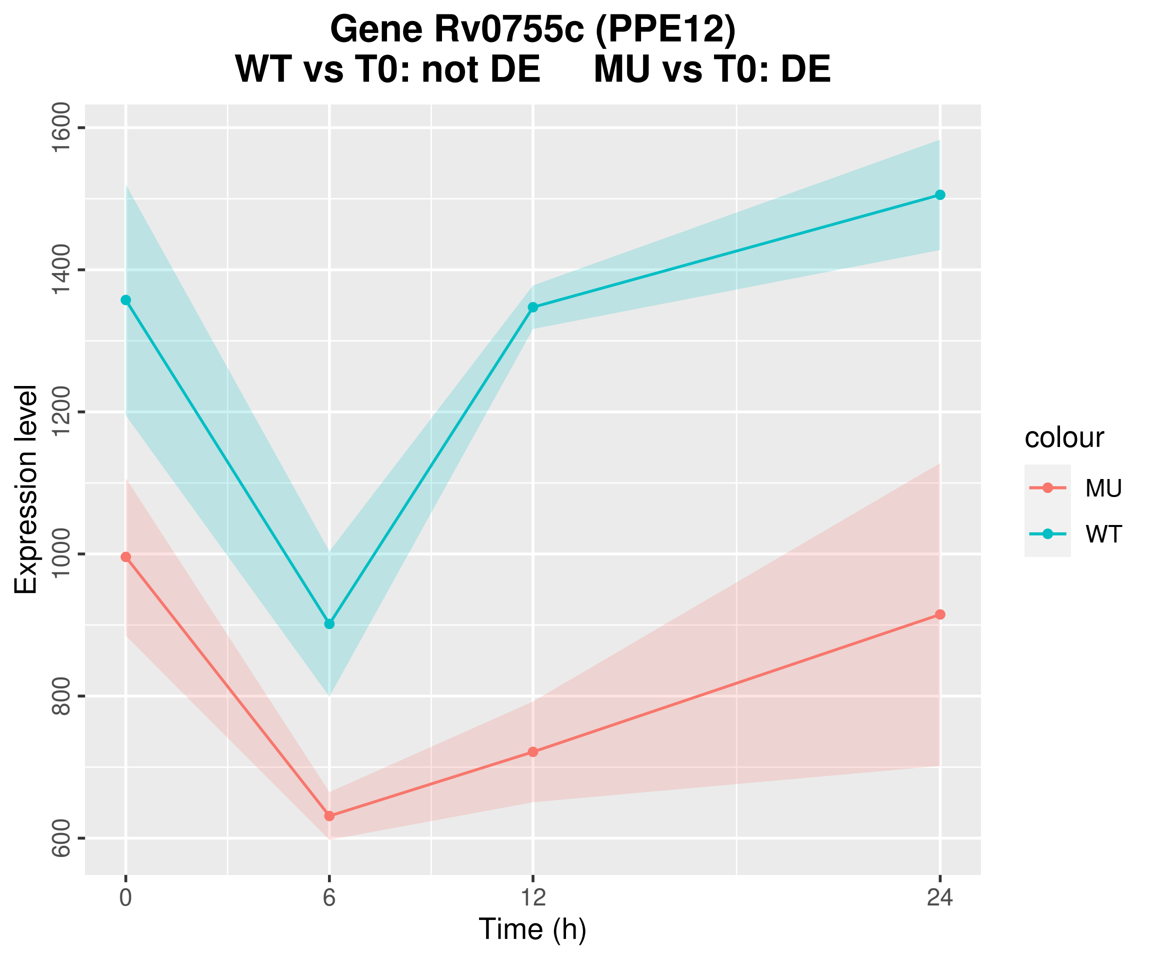

Gene Rv0755c (PPE12)
WT vs T0: not DE MU vs T0: DE
Expression level
1600
1400
1200
1000
800
600
0
6
12
24
Time (h)
colour
MU
WT

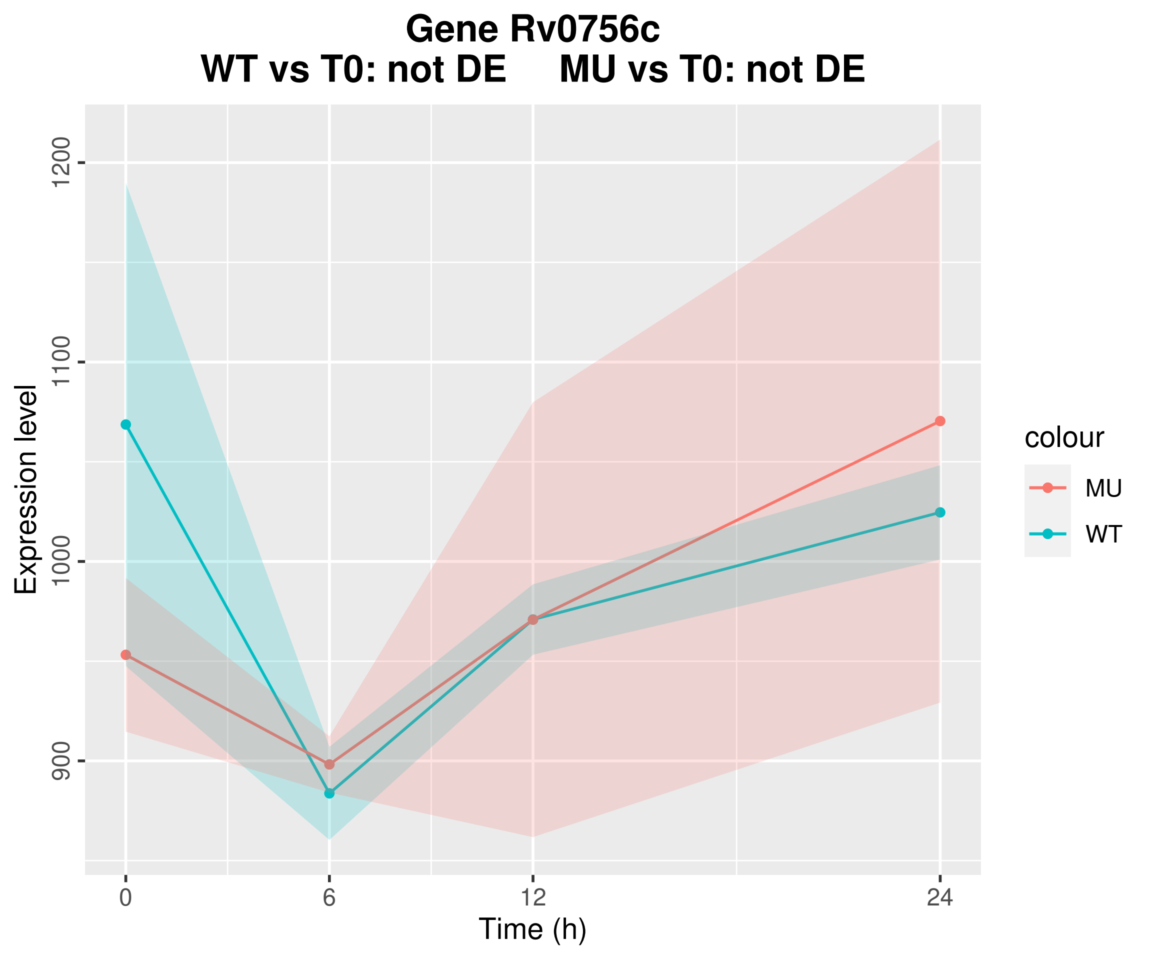

Gene Rv0756c
WT vs T0: not DE   MU vs T0: not DE
Expression level
1200
1100
1000
900
0
6
12
24
Time (h)
colour
MU
WT

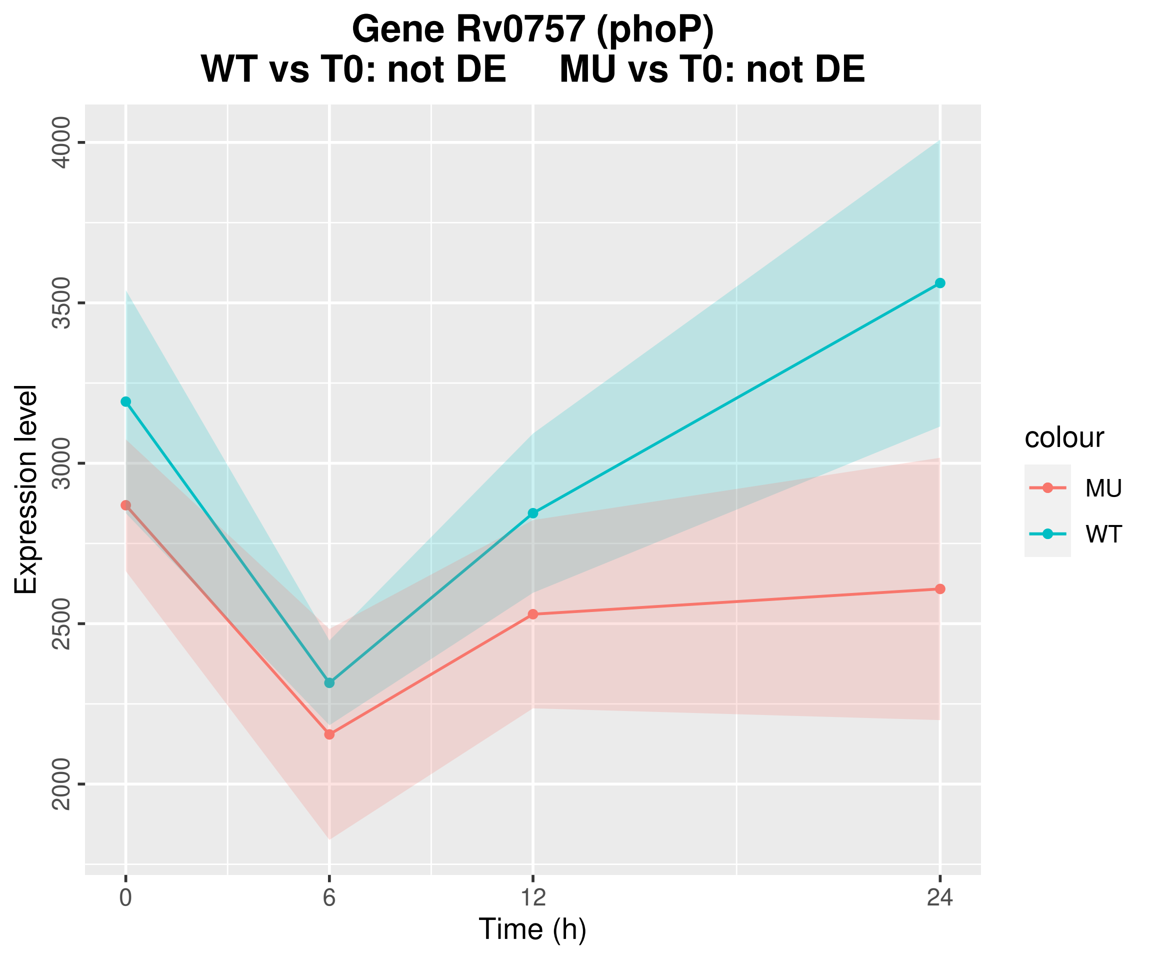

Gene Rv0757 (phoP)
WT vs T0: not DE MU vs T0: not DE
Expression level
4000
3500
3000
2500
2000
0
6
12
24
Time (h)
colour
MU
WT

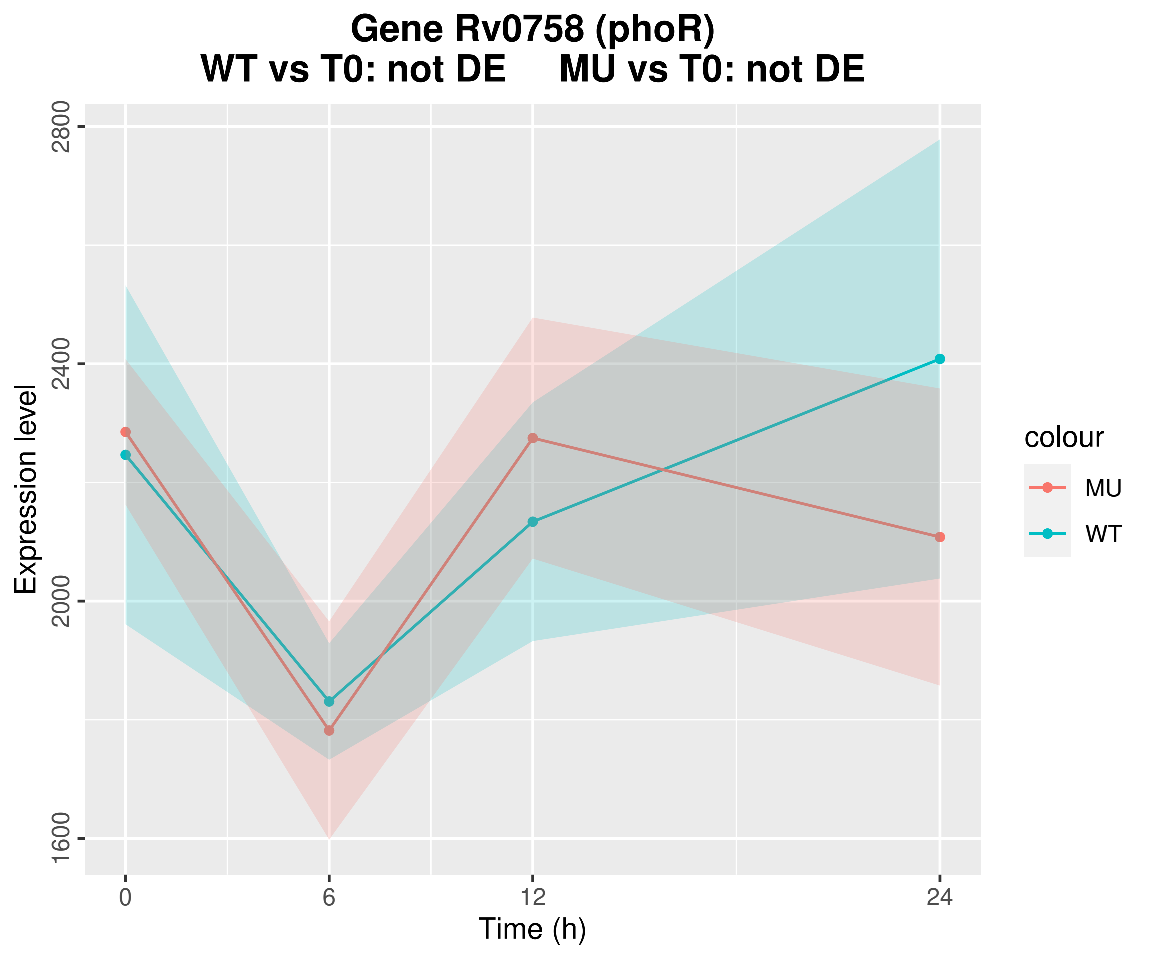
Gene Rv0758 (phoR)
WT vs T0: not DE   MU vs T0: not DE
Expression level
2800
2400
2000
1600
0
6
12
24
Time (h)
colour
MU
WT

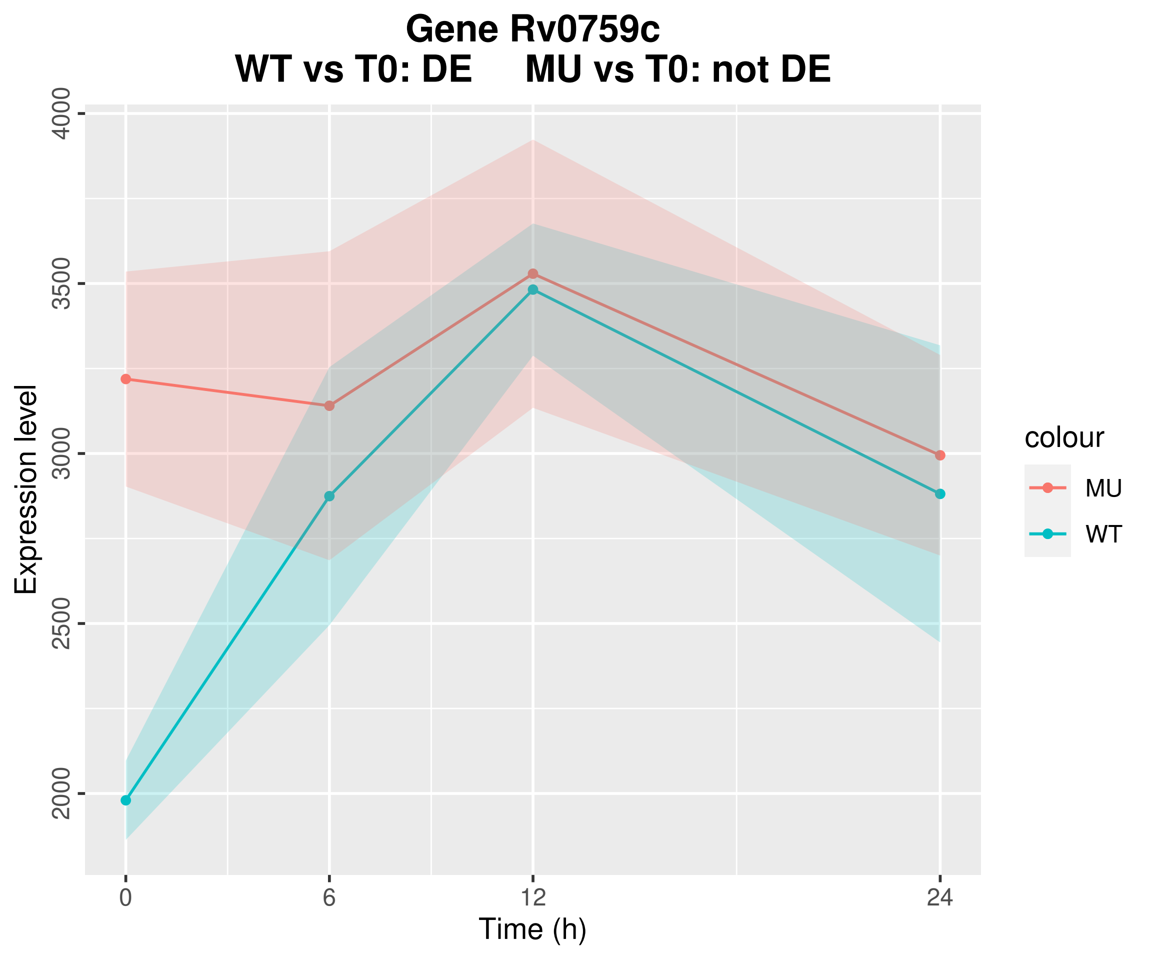

Gene Rv0759c
WT vs T0: DE   MU vs T0: not DE
Expression level
2000
2500
3000
3500
4000
0
6
12
24
Time (h)
colour
MU
WT

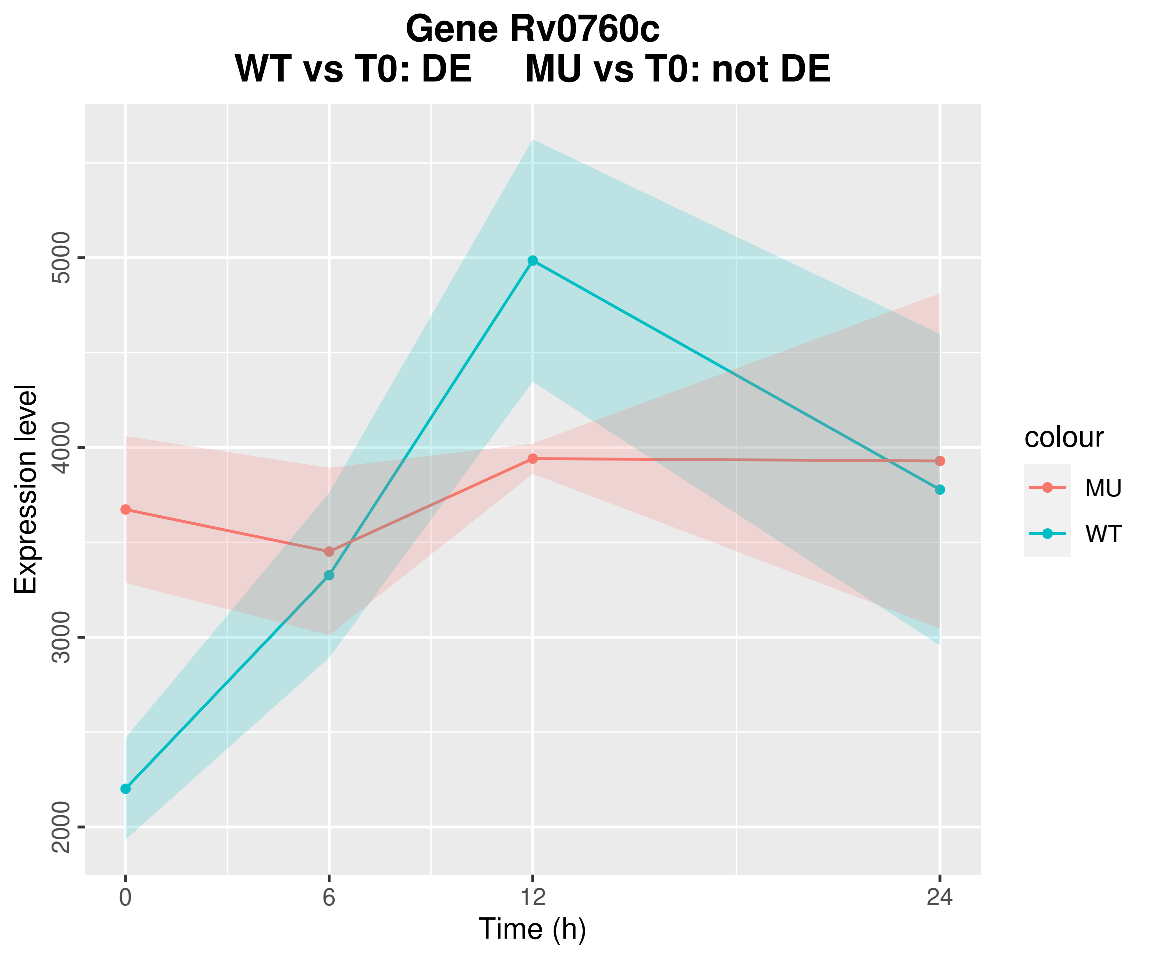
Gene Rv0760c
WT vs T0: DE   MU vs T0: not DE
Expression level
2000
3000
4000
5000
0
6
12
24
Time (h)
colour
MU
WT

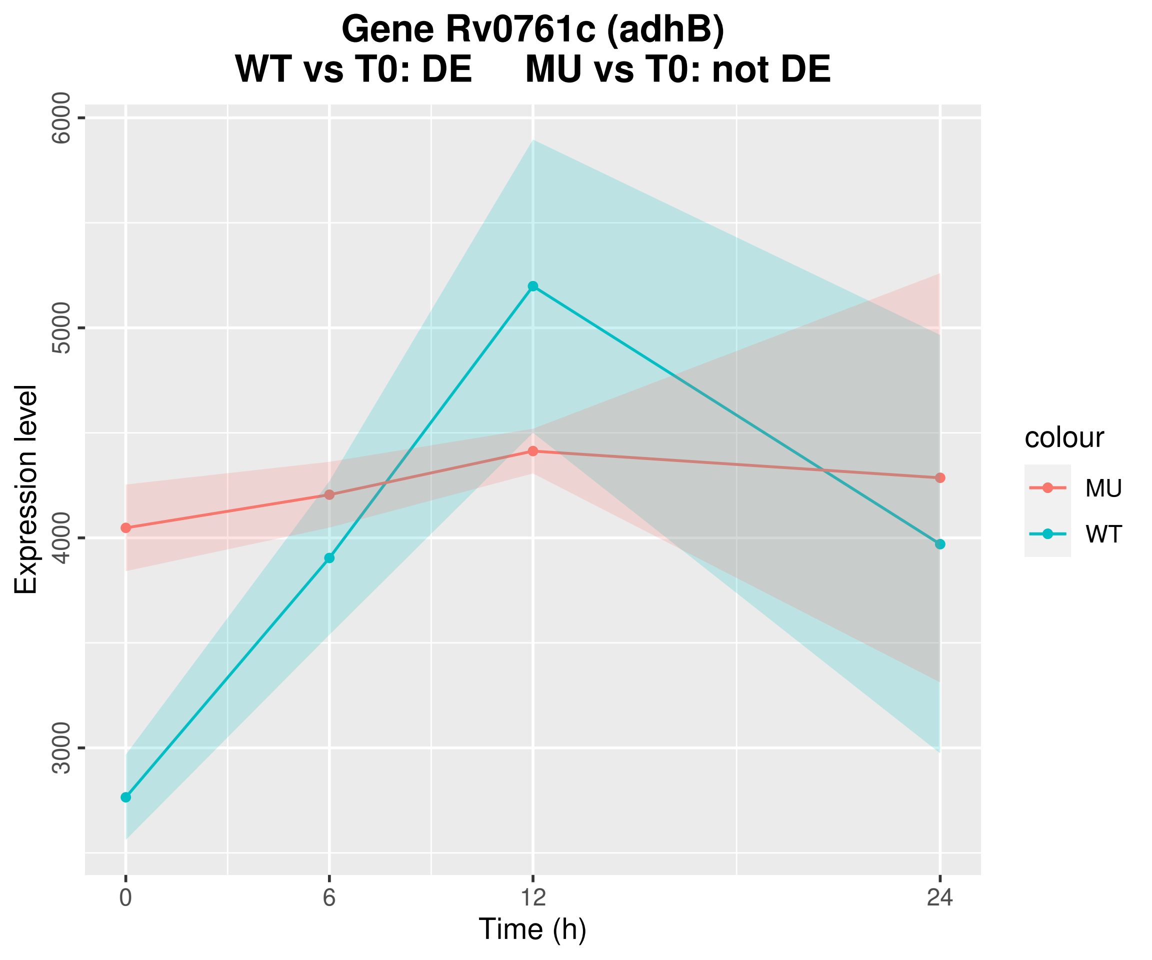
Gene Rv0761c (adhB)
WT vs T0: DE   MU vs T0: not DE
6000
5000
4000
3000
Expression level
0
6
12
24
Time (h)
colour
MU
WT

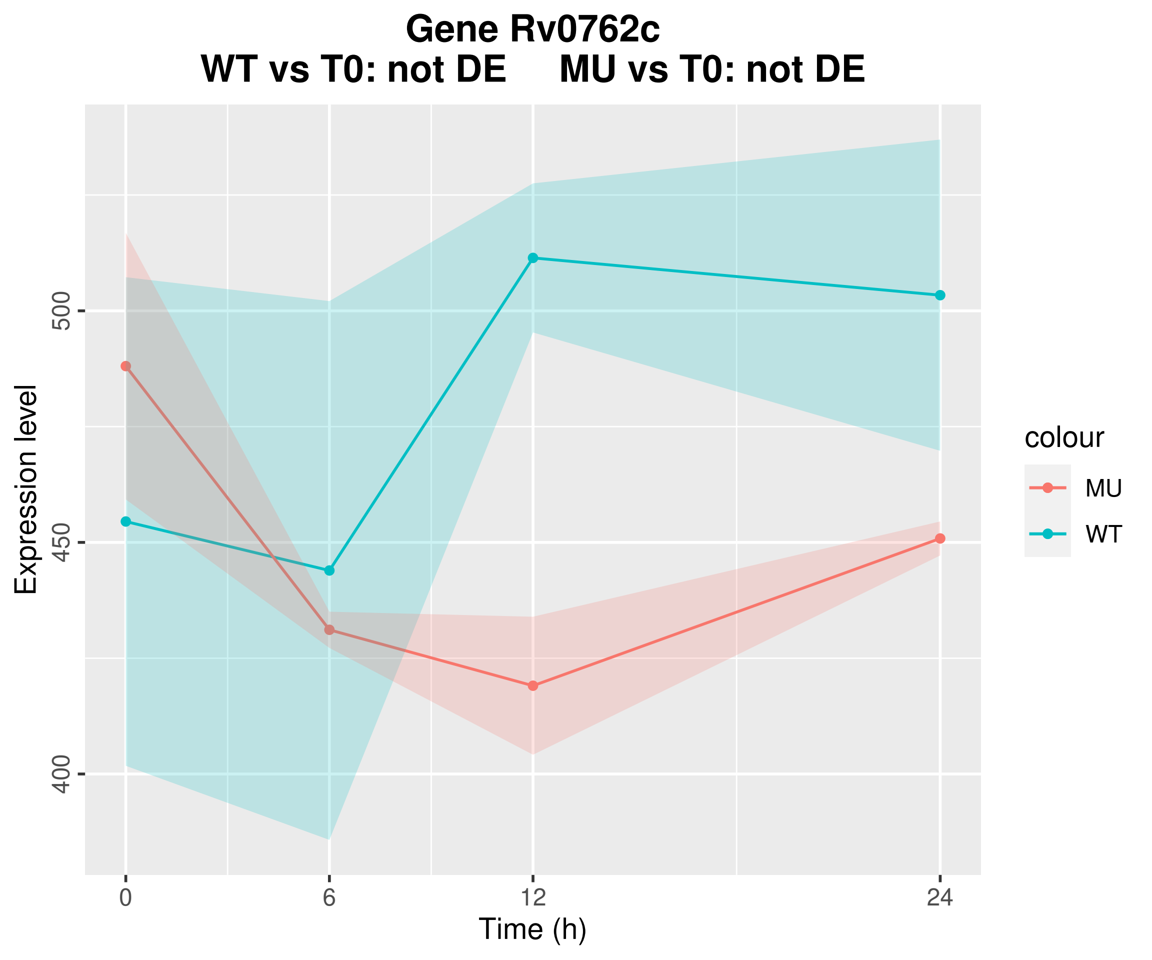

Gene Rv0762c
WT vs T0: not DE   MU vs T0: not DE
Expression level
400
450
500
0
6
12
24
Time (h)
colour
MU
WT

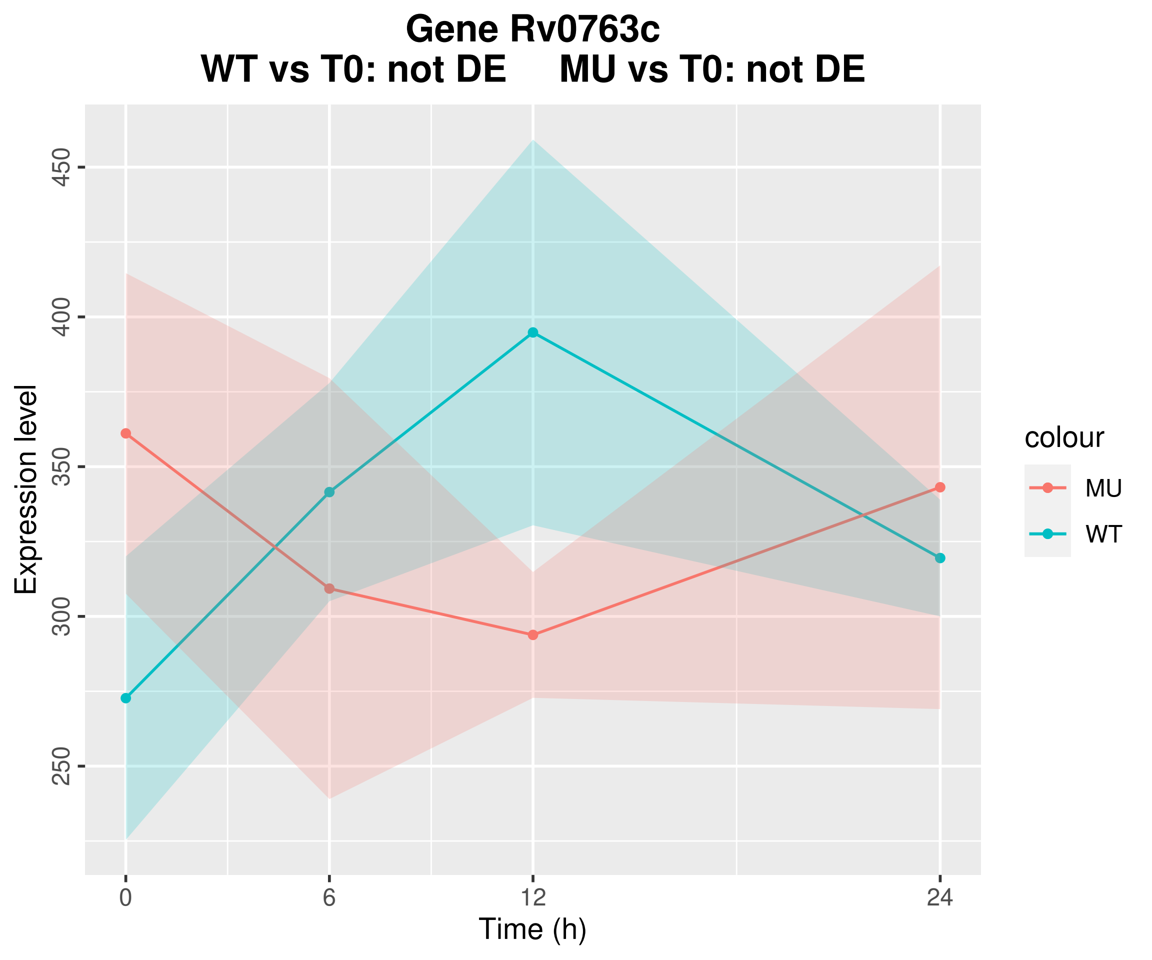

Gene Rv0763c
WT vs T0: not DE   MU vs T0: not DE
Expression level
450
400
350
300
250
0
6
12
24
Time (h)
colour
MU
WT

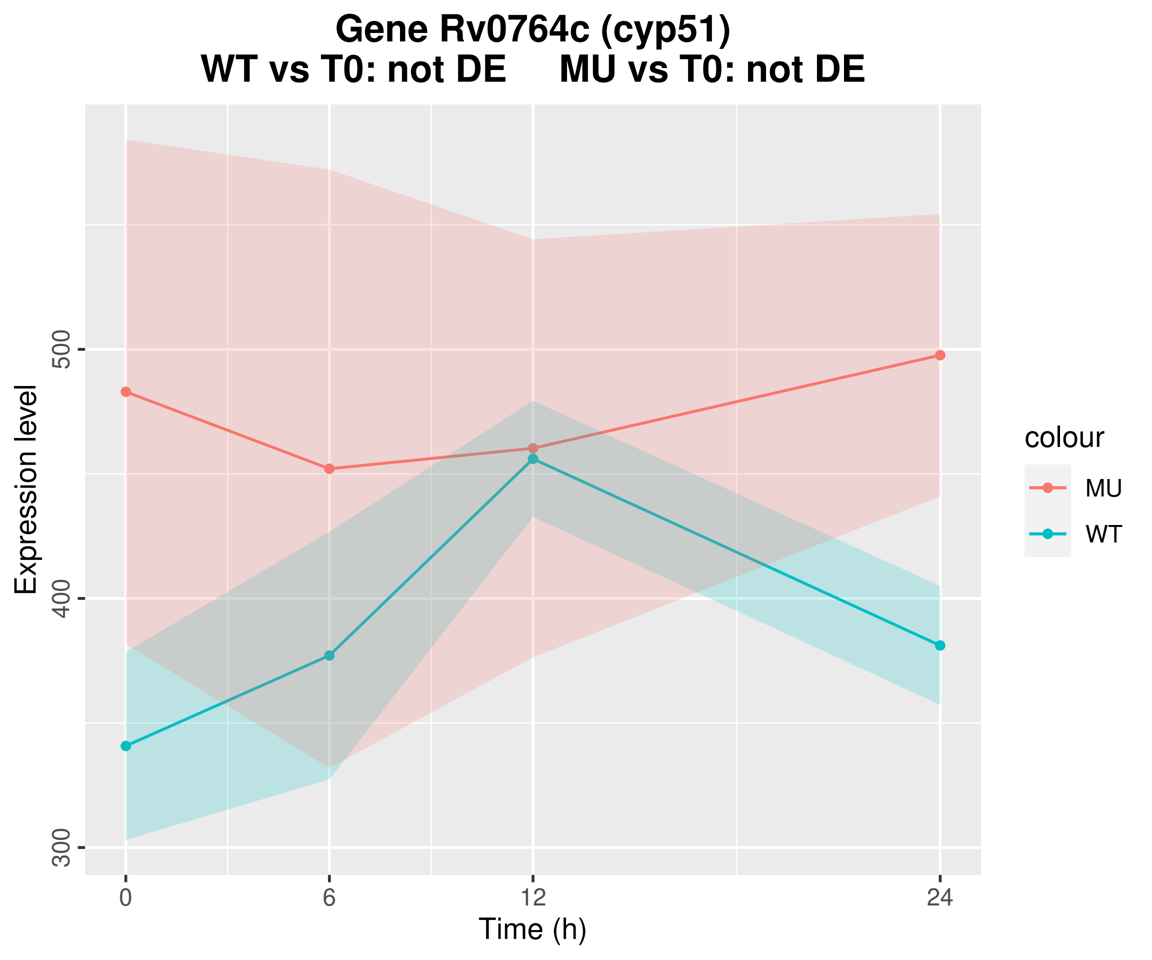
Gene Rv0764c (cyp51)
WT vs T0: not DE   MU vs T0: not DE
Expression level
500
400
300
0
6
12
24
Time (h)
colour
MU
WT

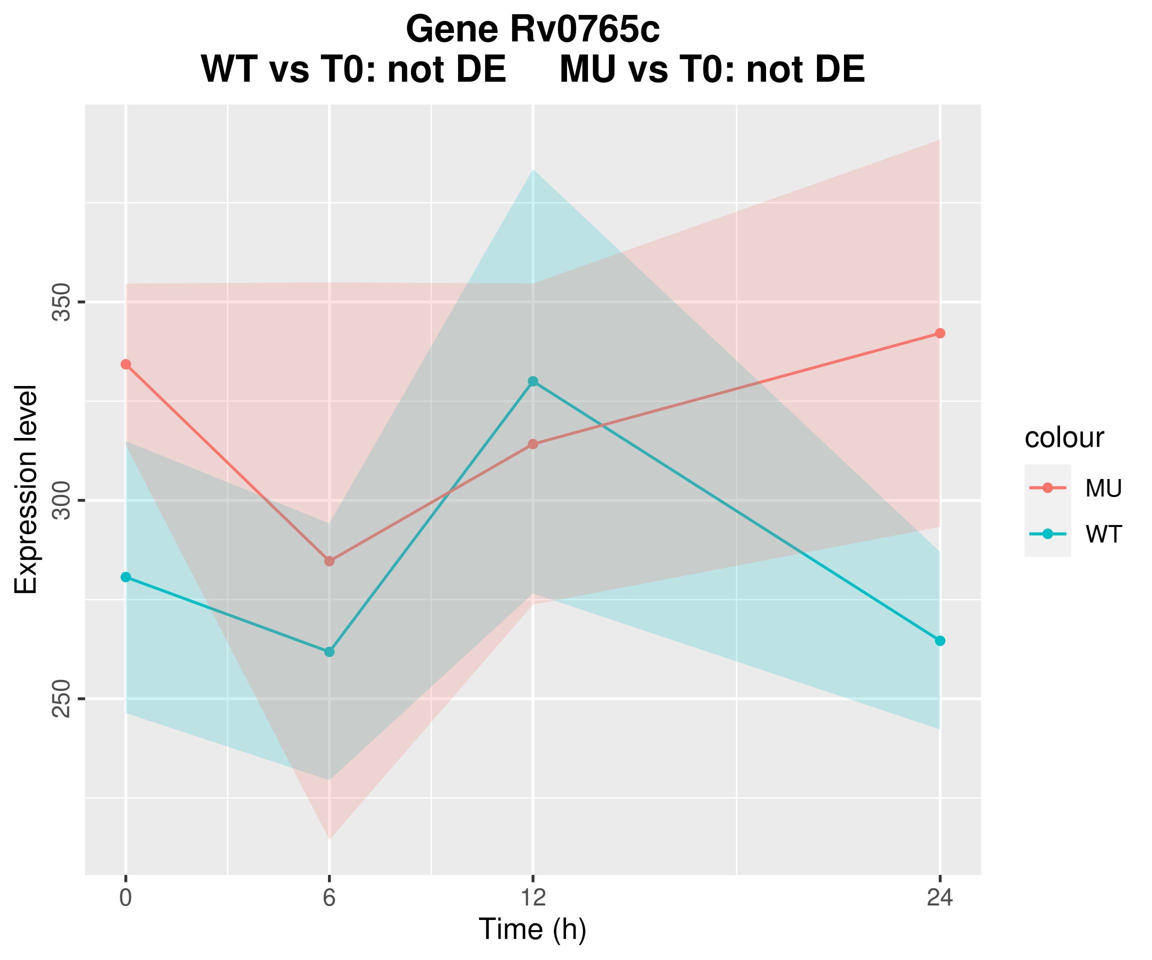
Gene Rv0765c
WT vs T0: not DE MU vs T0: not DE
350
300
250
Expression level
0
6
12
24
Time (h)
colour
MU
WT

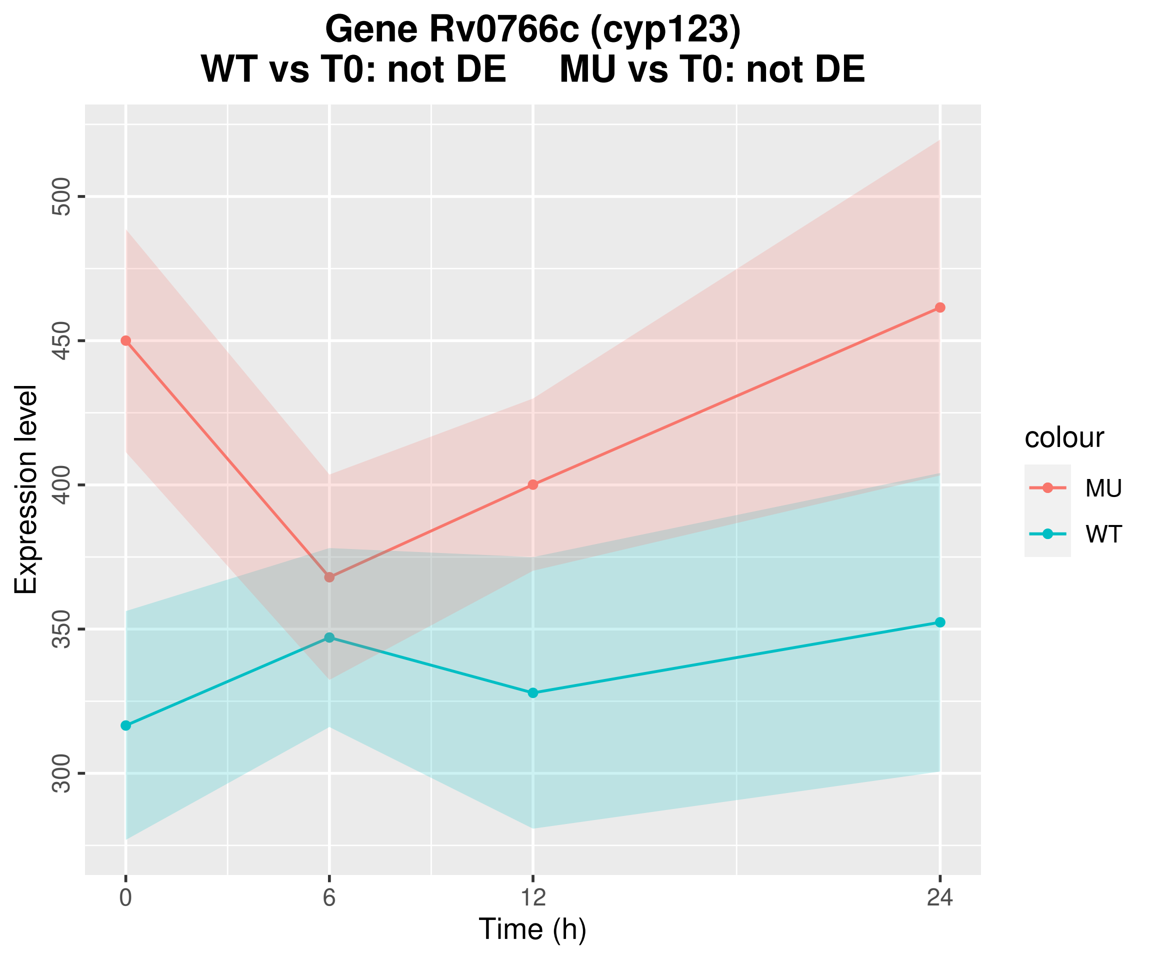

Gene Rv0766c (cyp123)
WT vs T0: not DE   MU vs T0: not DE
Expression level
500
450
400
350
300
0
6
12
24
Time (h)
colour
MU
WT

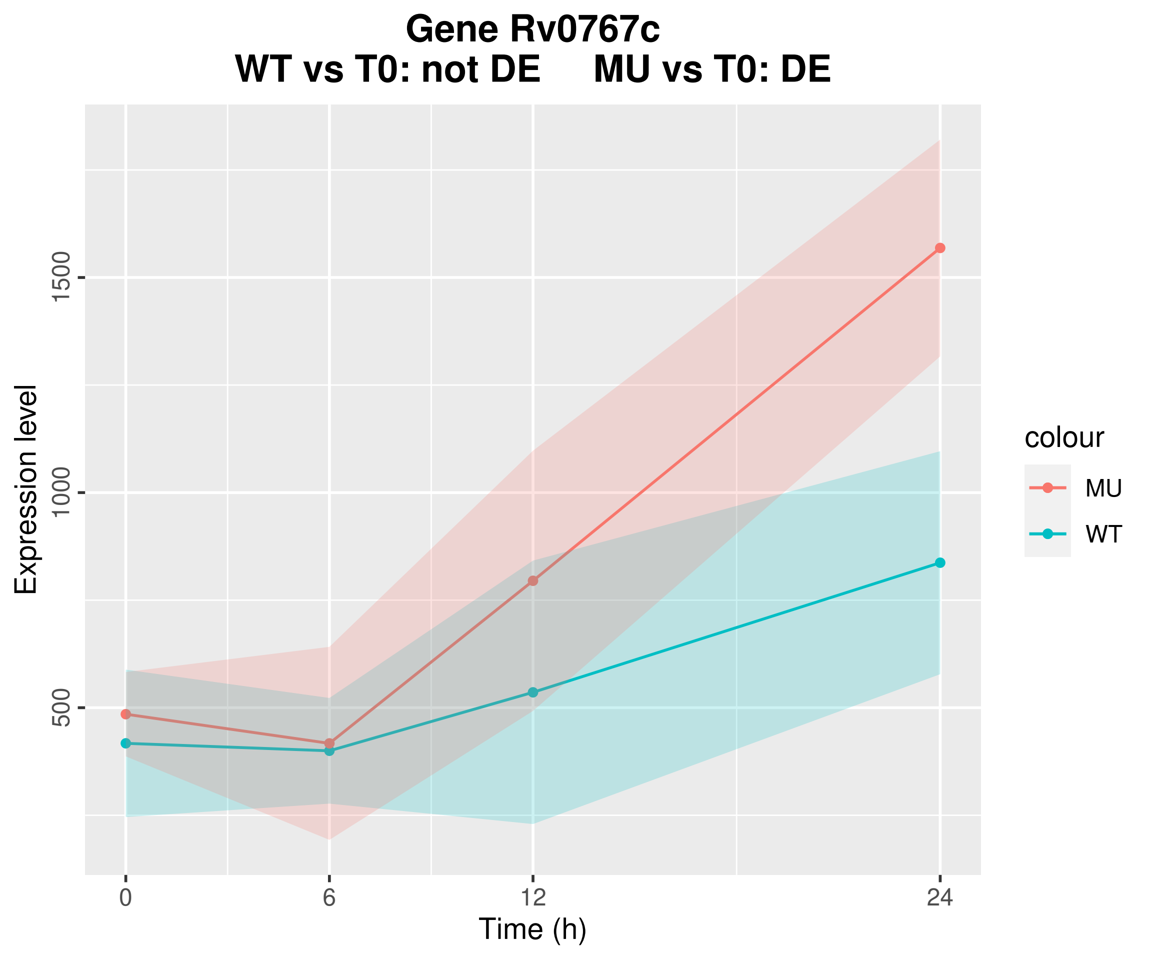
Gene Rv0767c
WT vs T0: not DE MU vs T0: DE
Expression level
500
1000
1500
0
6
12
24
Time (h)
colour
MU
WT

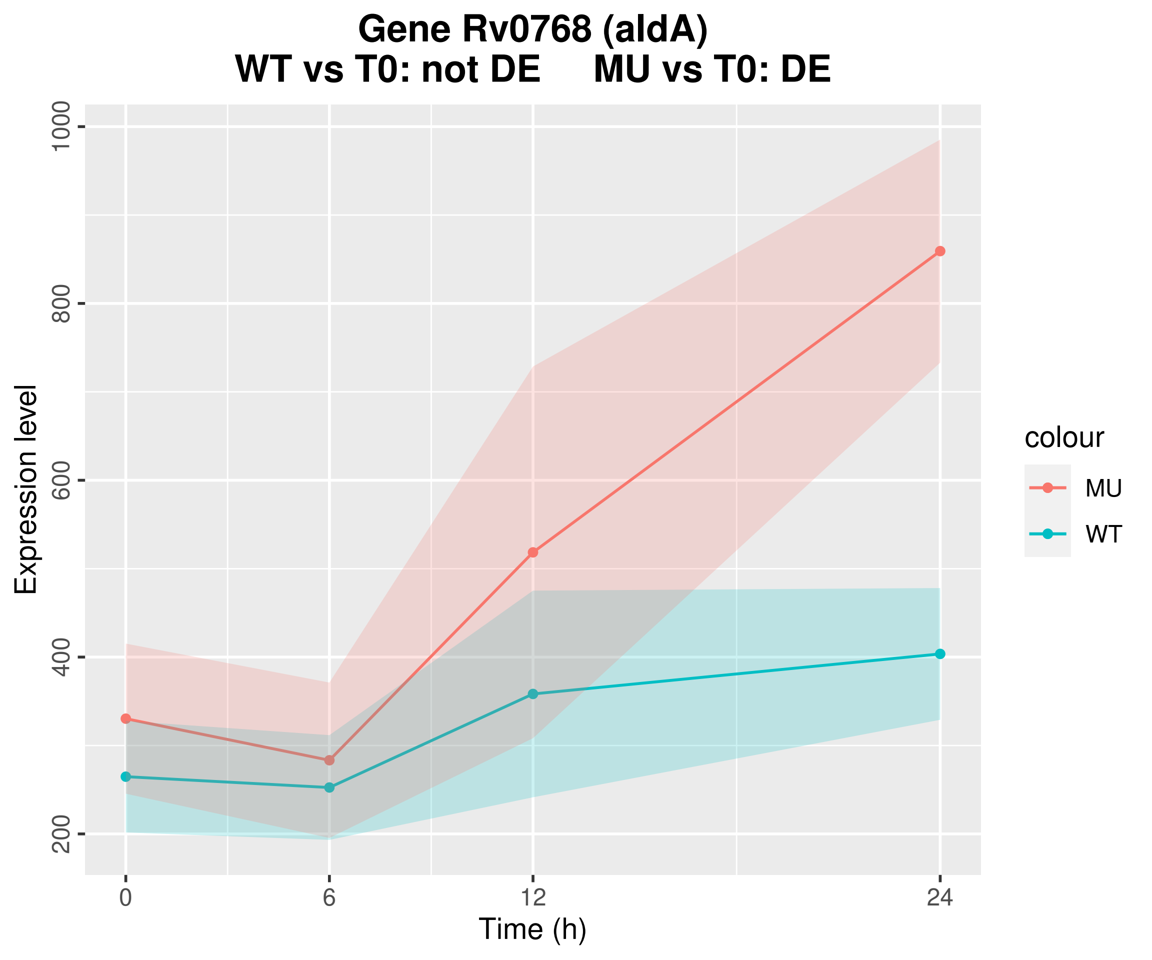

Gene Rv0768 (aldA)
WT vs T0: not DE   MU vs T0: DE
Expression level
1000
800
600
400
200
0
6
12
24
Time (h)
colour
MU
WT

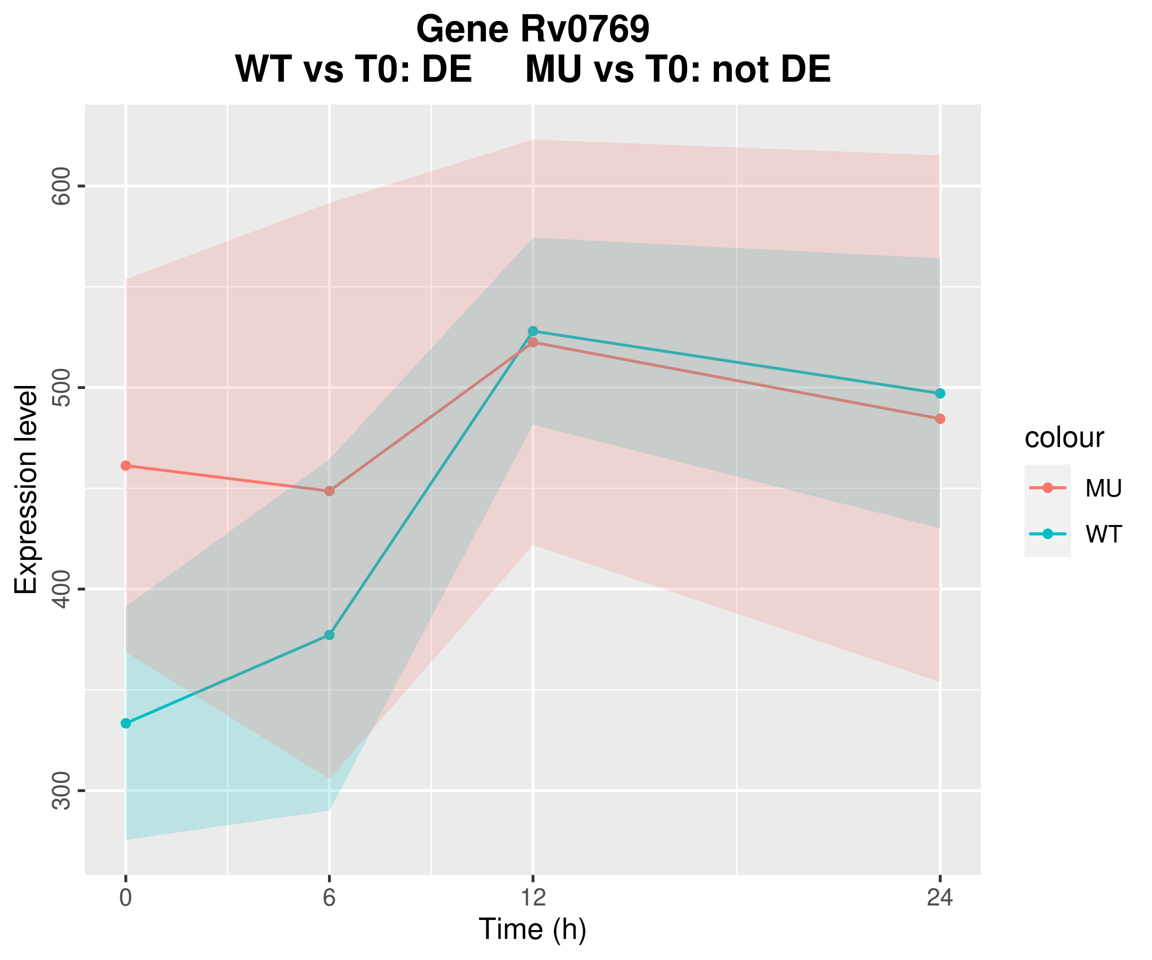
Gene Rv0769
WT vs T0: DE   MU vs T0: not DE
Expression level
600
500
400
300
0
6
12
24
Time (h)
colour
MU
WT

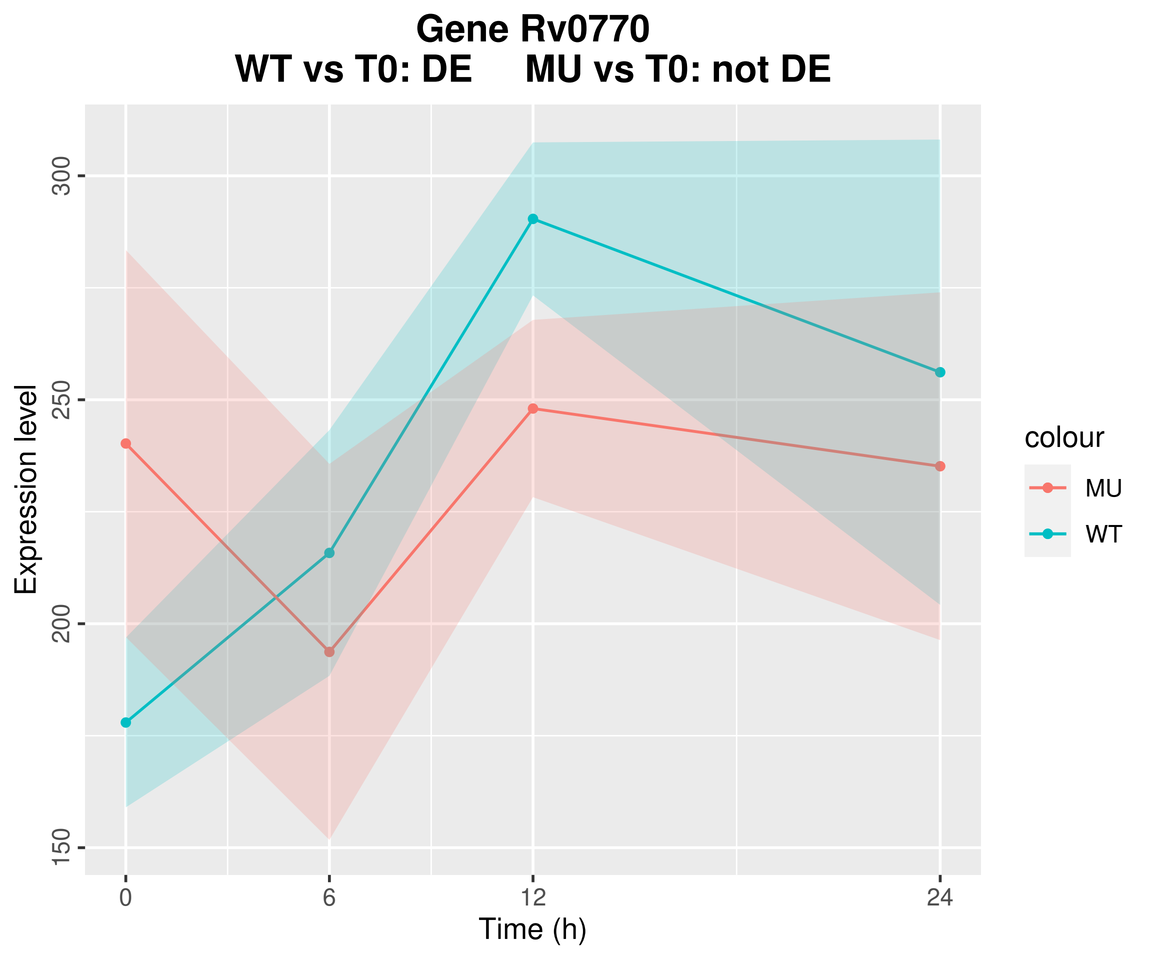
Gene Rv0770
WT vs T0: DE   MU vs T0: not DE
Expression level
300
250
200
150
0
6
12
24
Time (h)
colour
MU
WT

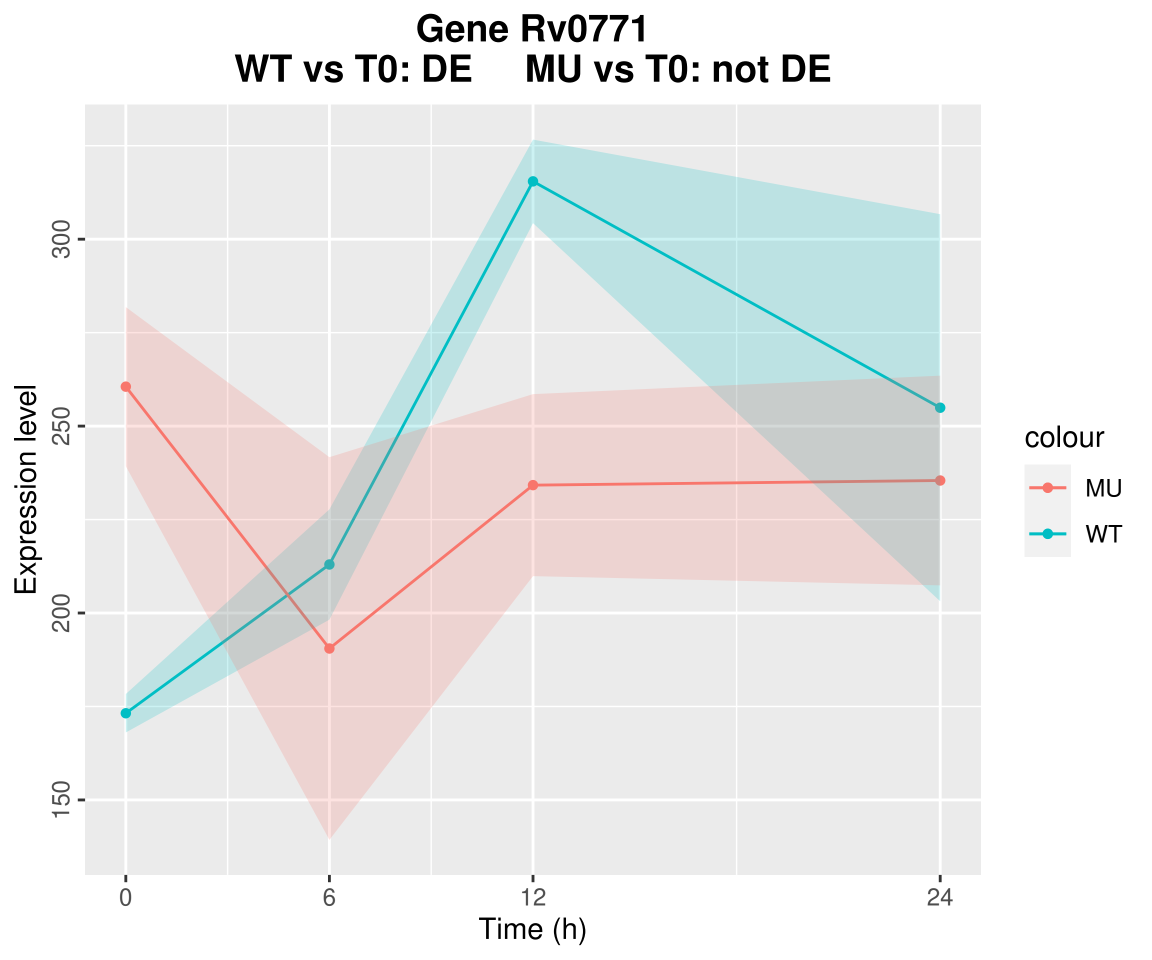
Gene Rv0771
WT vs T0: DE   MU vs T0: not DE
Expression level
300
250
200
150
0
6
12
24
Time (h)
colour
MU
WT

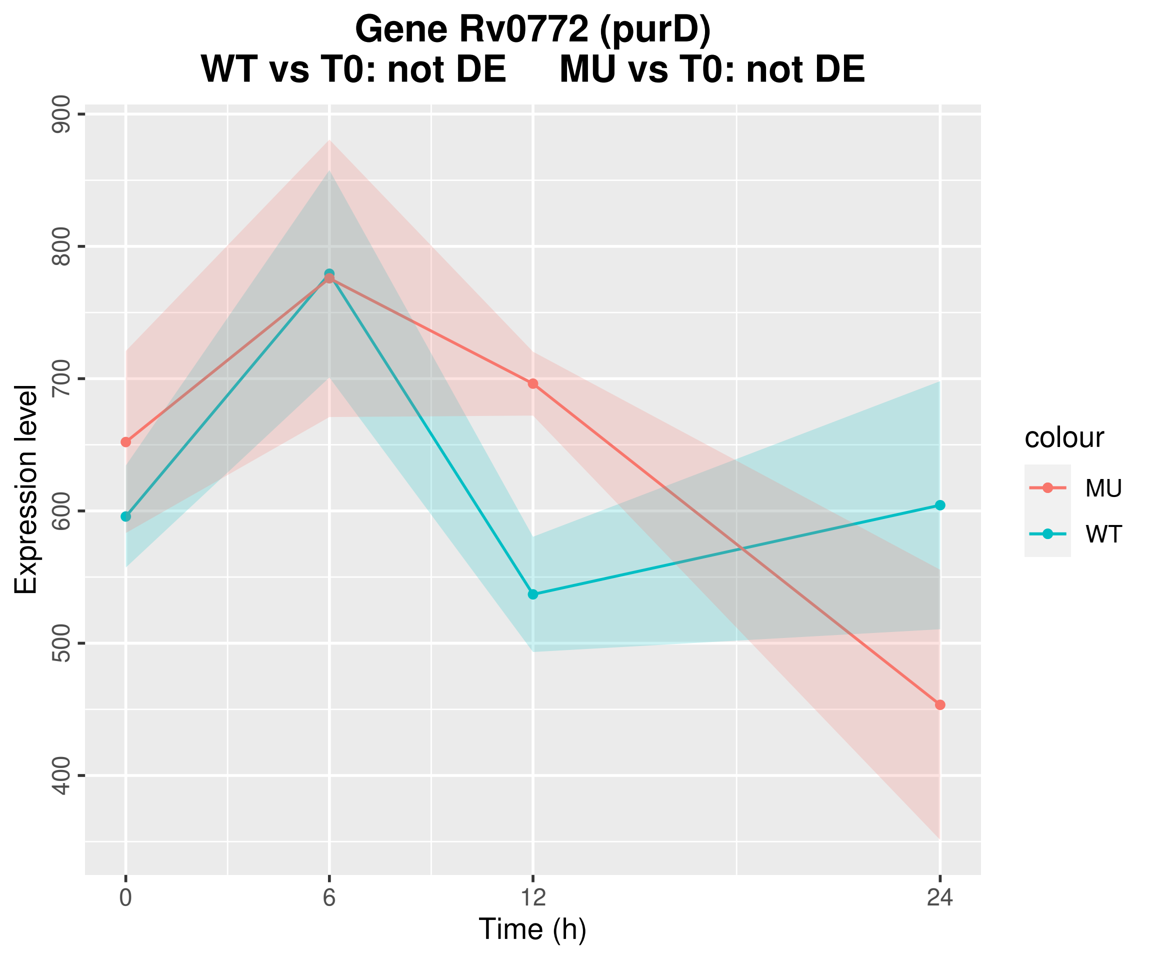
Gene Rv0772 (purD)
WT vs T0: not DE   MU vs T0: not DE
Expression level
900
800
700
600
500
400
Time (h)
0
6
12
24
colour
MU
WT

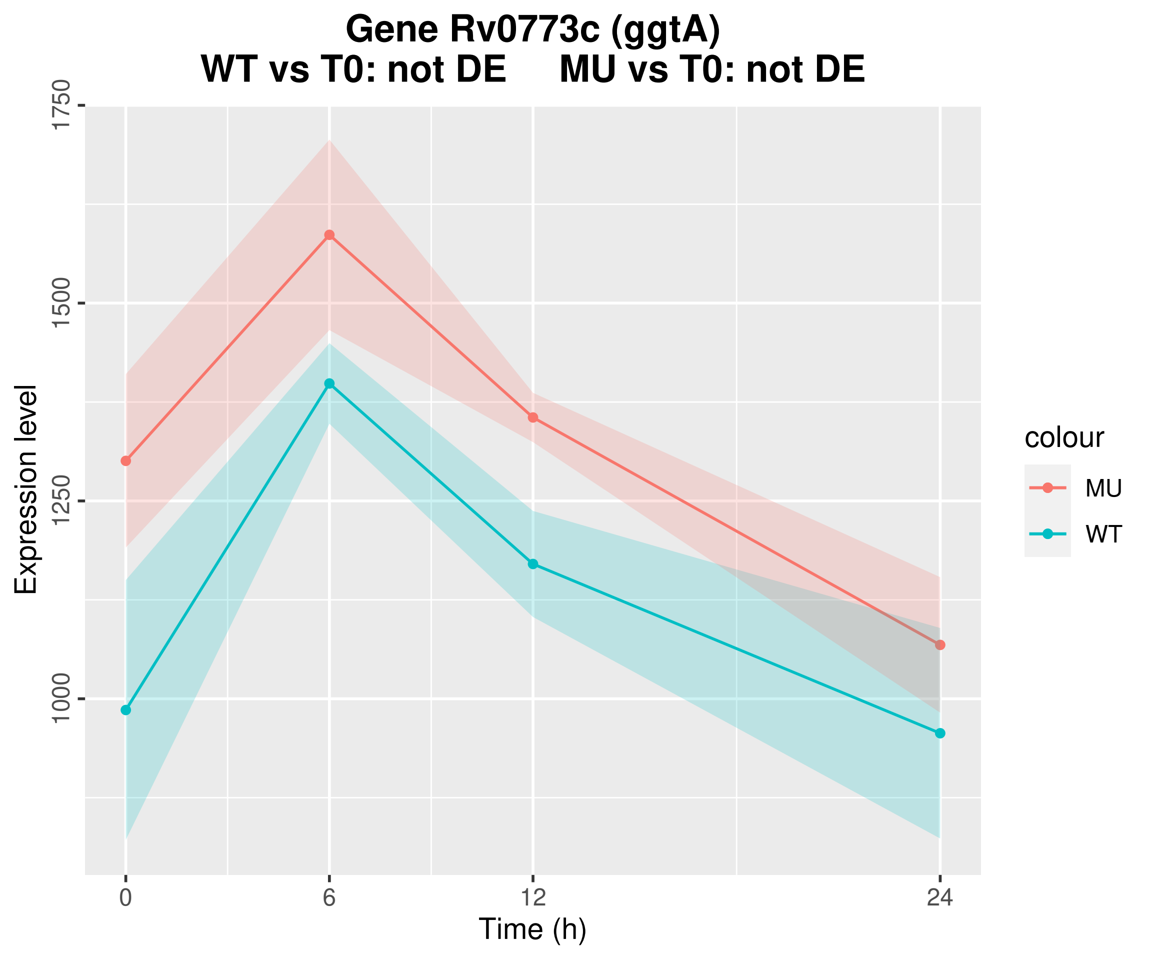
Gene Rv0773c (ggtA)
WT vs T0: not DE MU vs T0: not DE
1750
1500
1250
1000
Expression level
0
6
12
24
Time (h)
colour
MU
WT

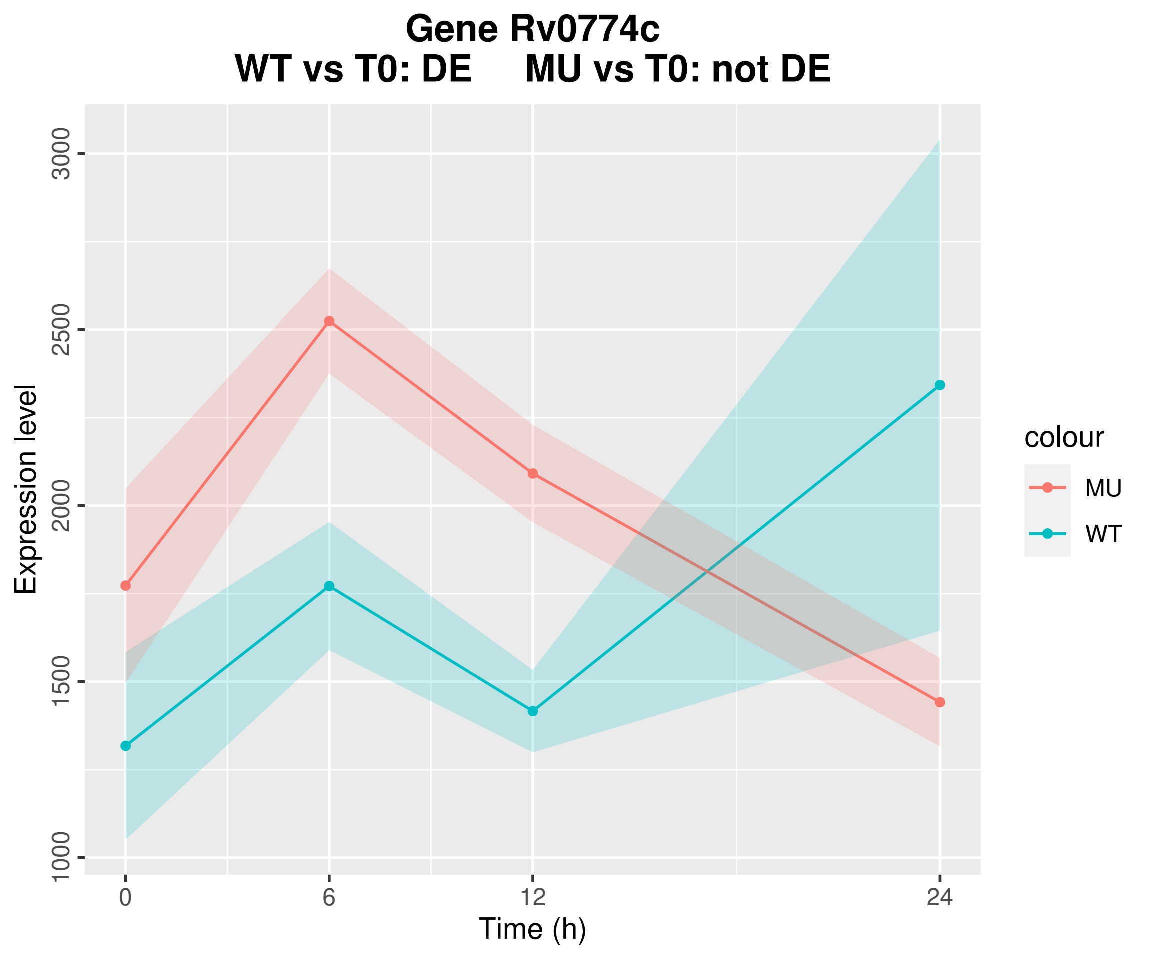

Gene Rv0774c
WT vs T0: DE   MU vs T0: not DE
Expression level
3000
2500
2000
1500
1000
0
6
12
24
Time (h)
colour
MU
WT

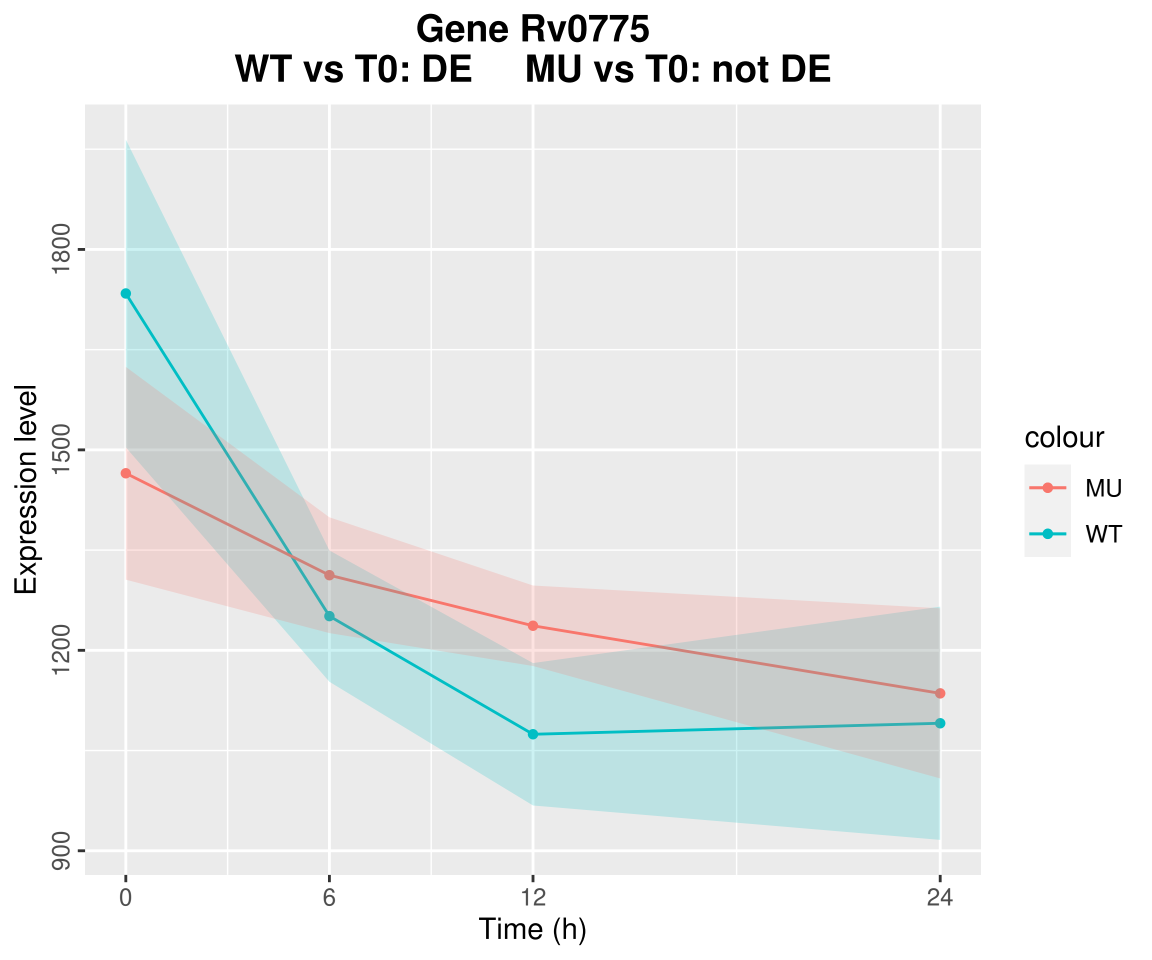
Gene Rv0775
WT vs T0: DE MU vs T0: not DE
Expression level
1800
1500
1200
900
0
6
12
24
Time (h)
colour
MU
WT

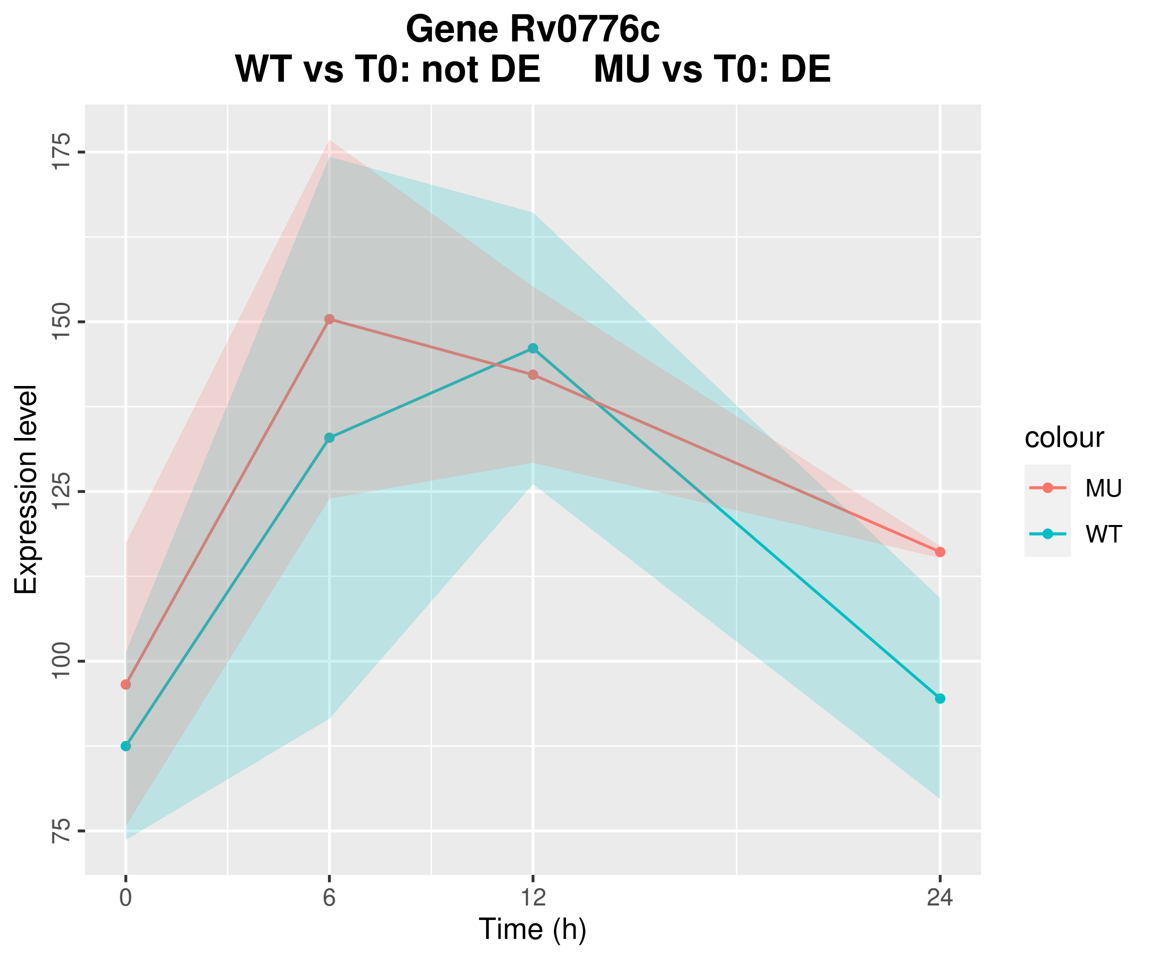

Gene Rv0776c
WT vs T0: not DE   MU vs T0: DE
175
150
125
100
75
Expression level
0
6
12
24
Time (h)
colour
MU
WT

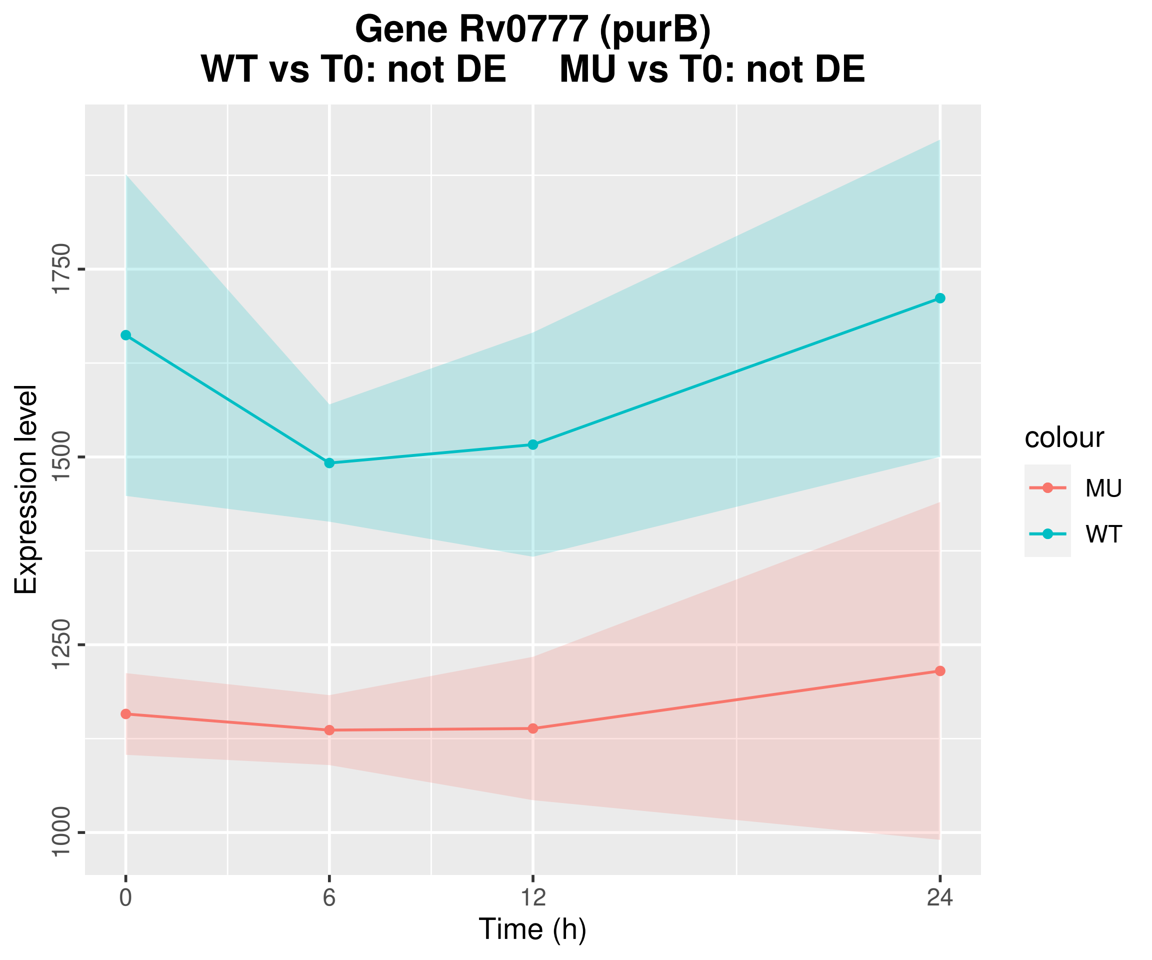
Gene Rv0777 (purB)
WT vs T0: not DE   MU vs T0: not DE
Expression level
1750
1500
1250
1000
0
6
12
24
Time (h)
colour
MU
WT

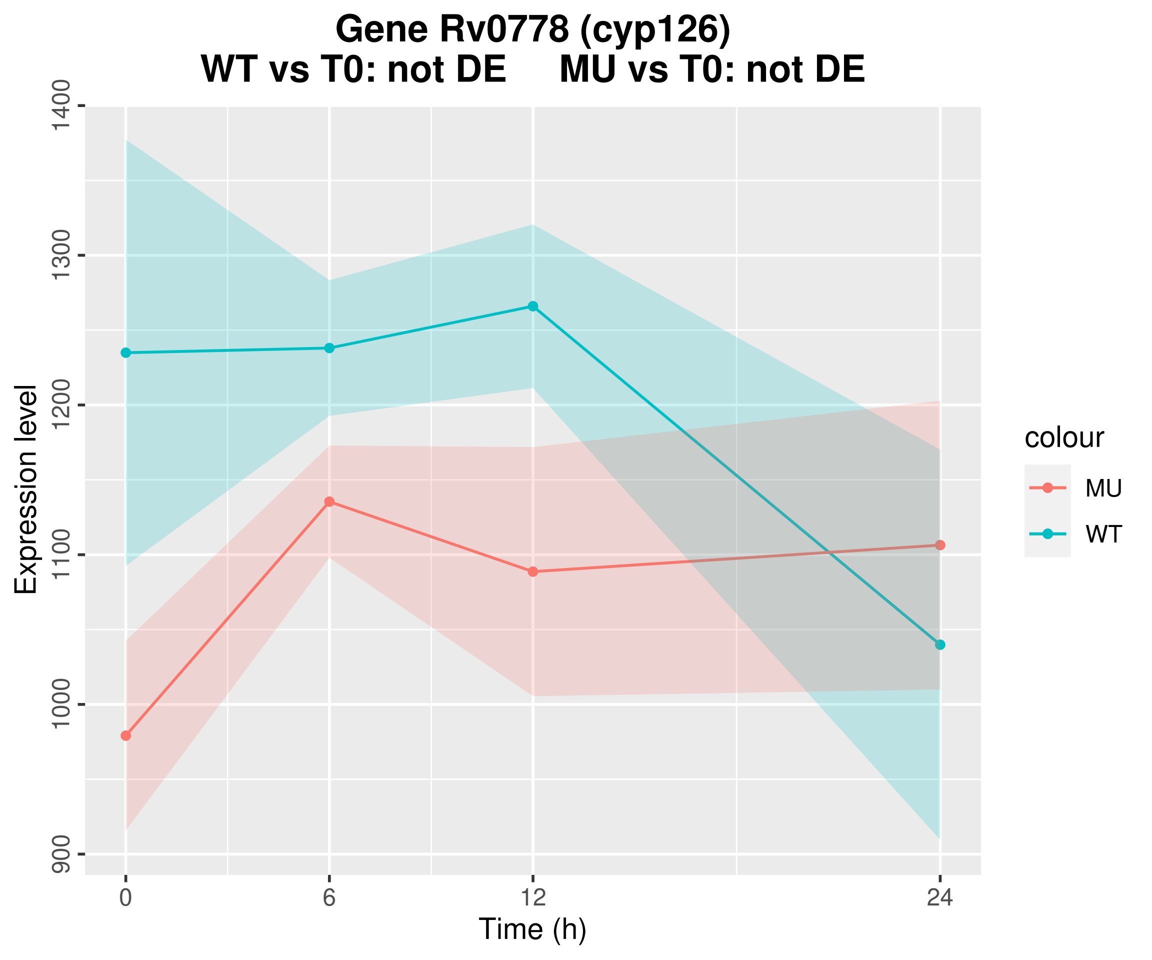

Gene Rv0778 (cyp126)
WT vs T0: not DE MU vs T0: not DE
Expression level
1400
1300
1200
1100
1000
900
0
6
12
24
Time (h)
colour
MU
WT

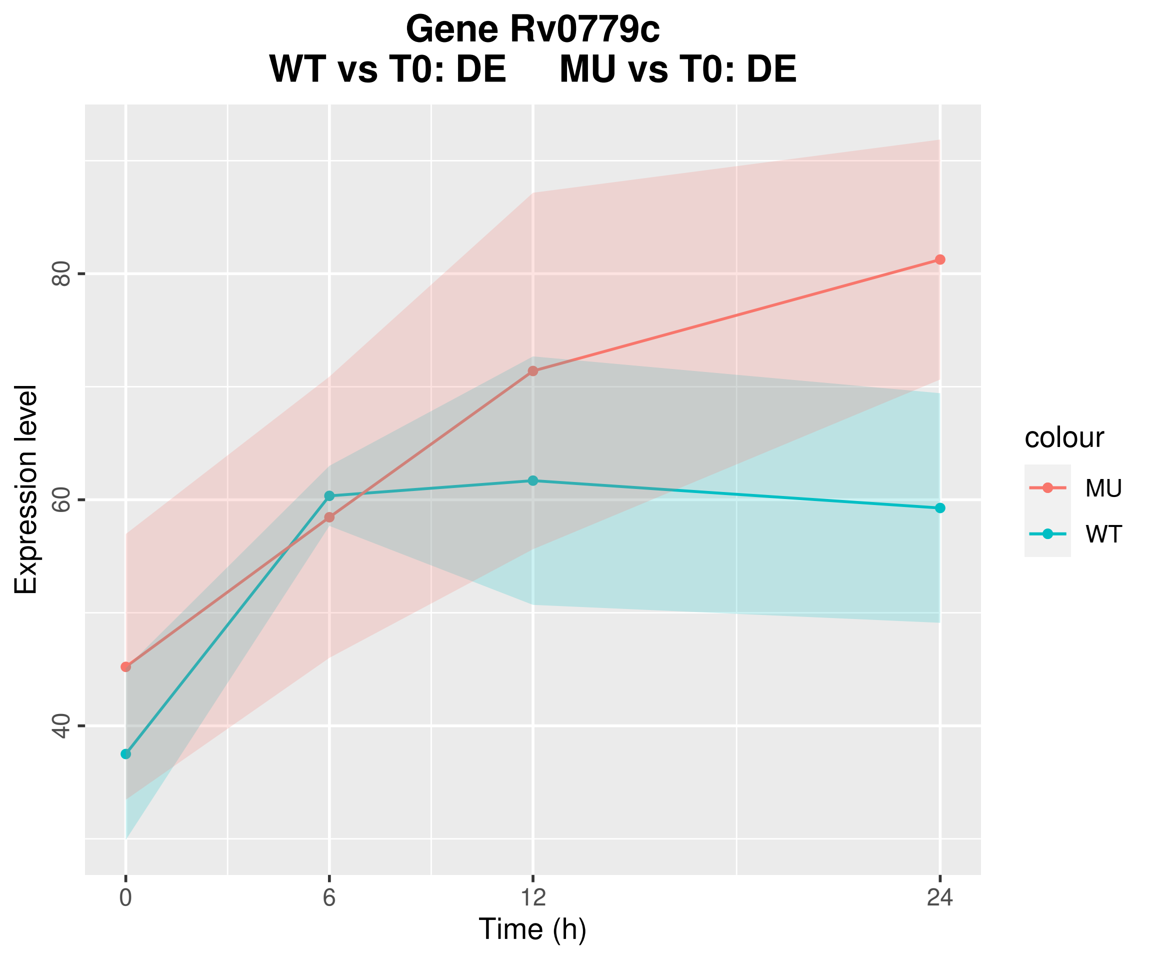

Gene Rv0779c
WT vs T0: DE   MU vs T0: DE
Expression level
80
60
40
0
6
12
24
Time (h)
colour
MU
WT

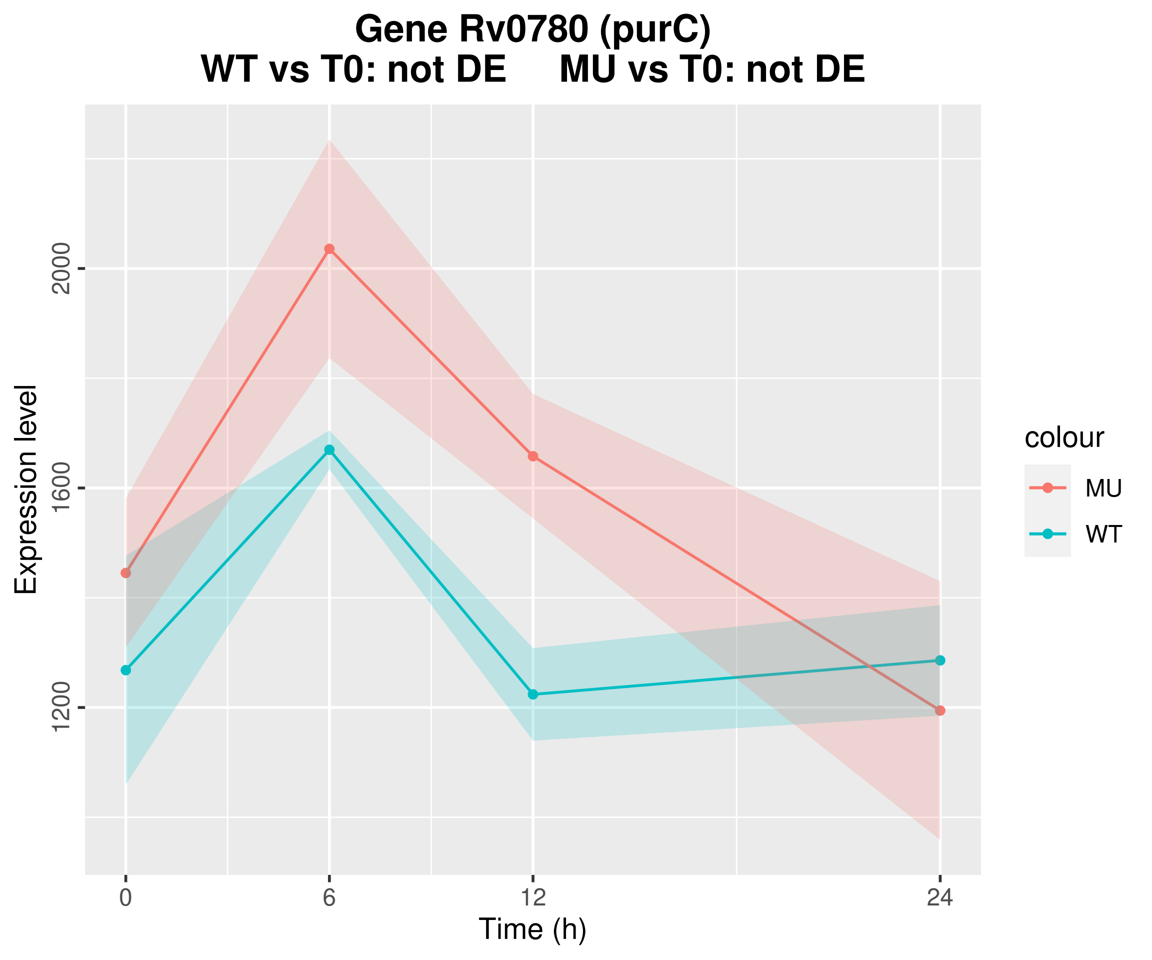
Gene Rv0780 (purC)
WT vs T0: not DE   MU vs T0: not DE
Expression level
2000
1600
1200
0
6
12
24
Time (h)
colour
MU
WT

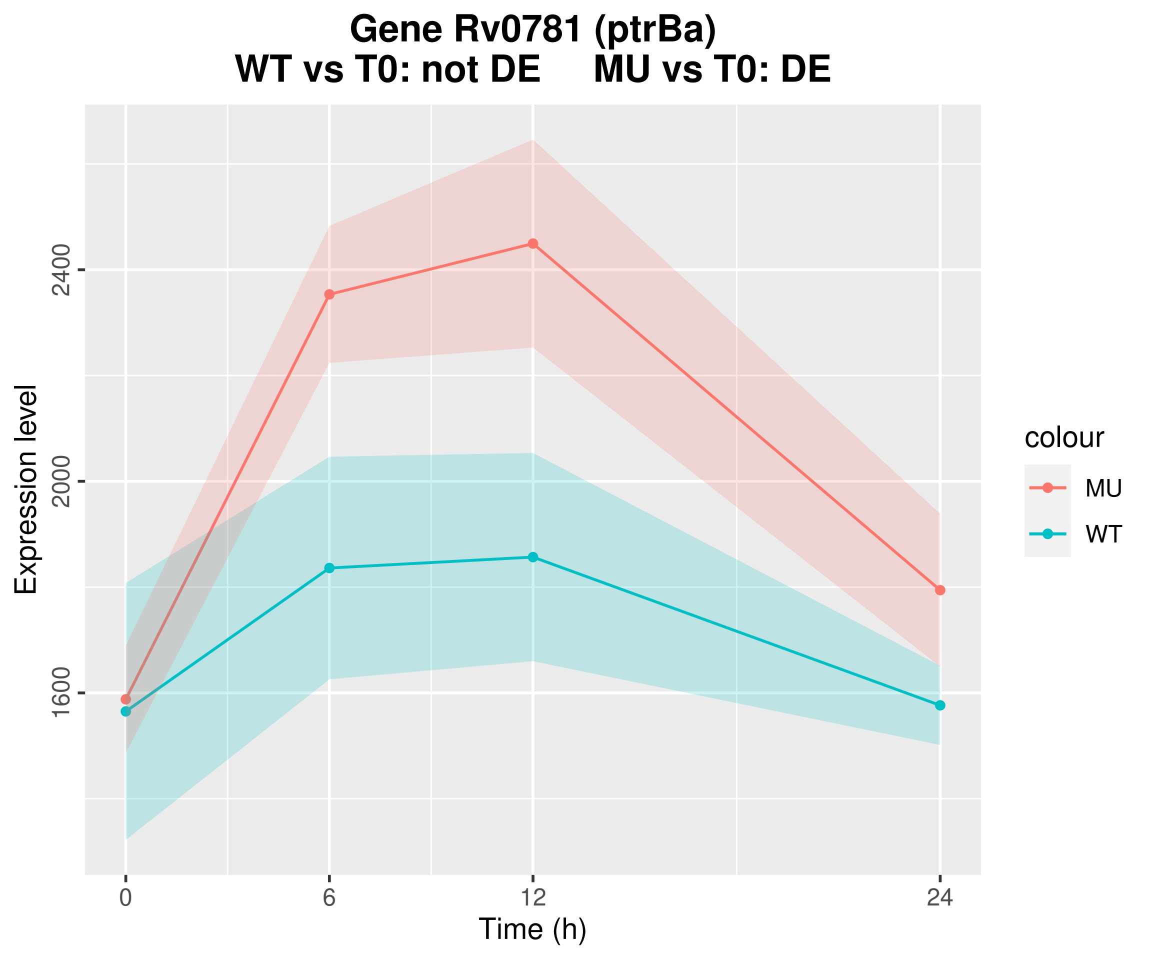
Gene Rv0781 (ptrBa)
WT vs T0: not DE   MU vs T0: DE
Expression level
2400
2000
1600
0
6
12
24
Time (h)
colour
MU
WT

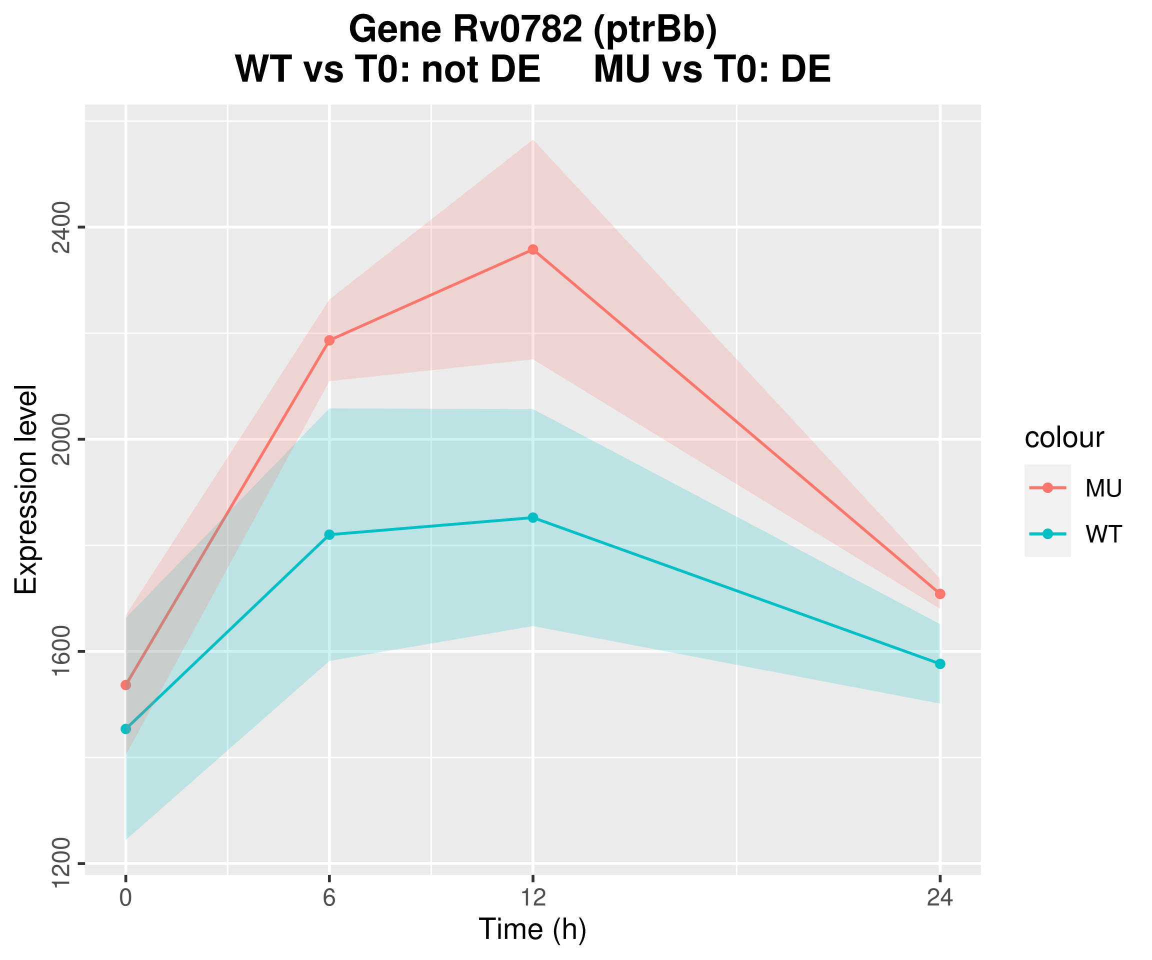

Gene Rv0782 (ptrBb)
WT vs T0: not DE   MU vs T0: DE
Expression level
2400
2000
1600
1200
0
6
12
24
Time (h)
colour
MU
WT

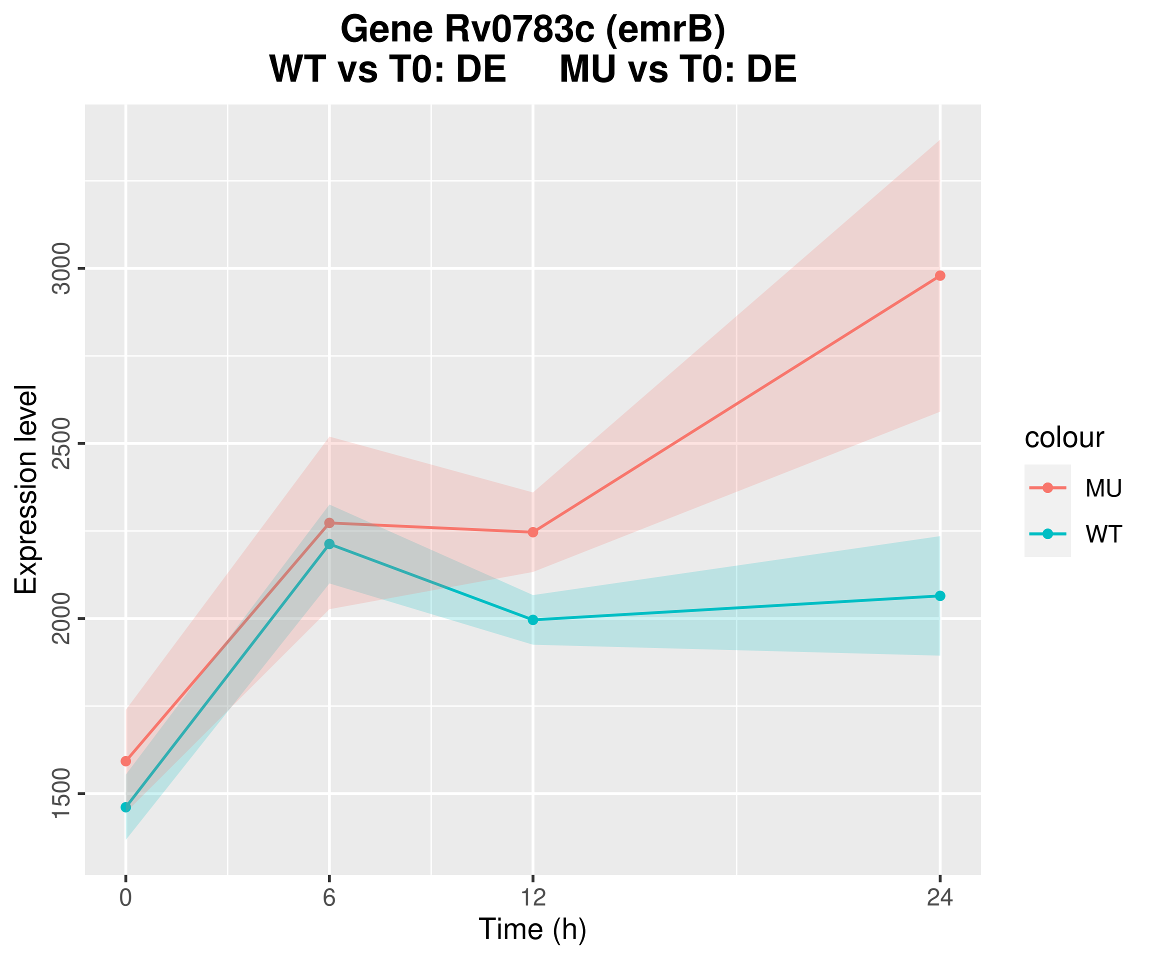

Gene Rv0783c (emrB)
WT vs T0: DE MU vs T0: DE
Expression level
3000
2500
2000
1500
0
6
12
24
Time (h)
colour
MU
WT

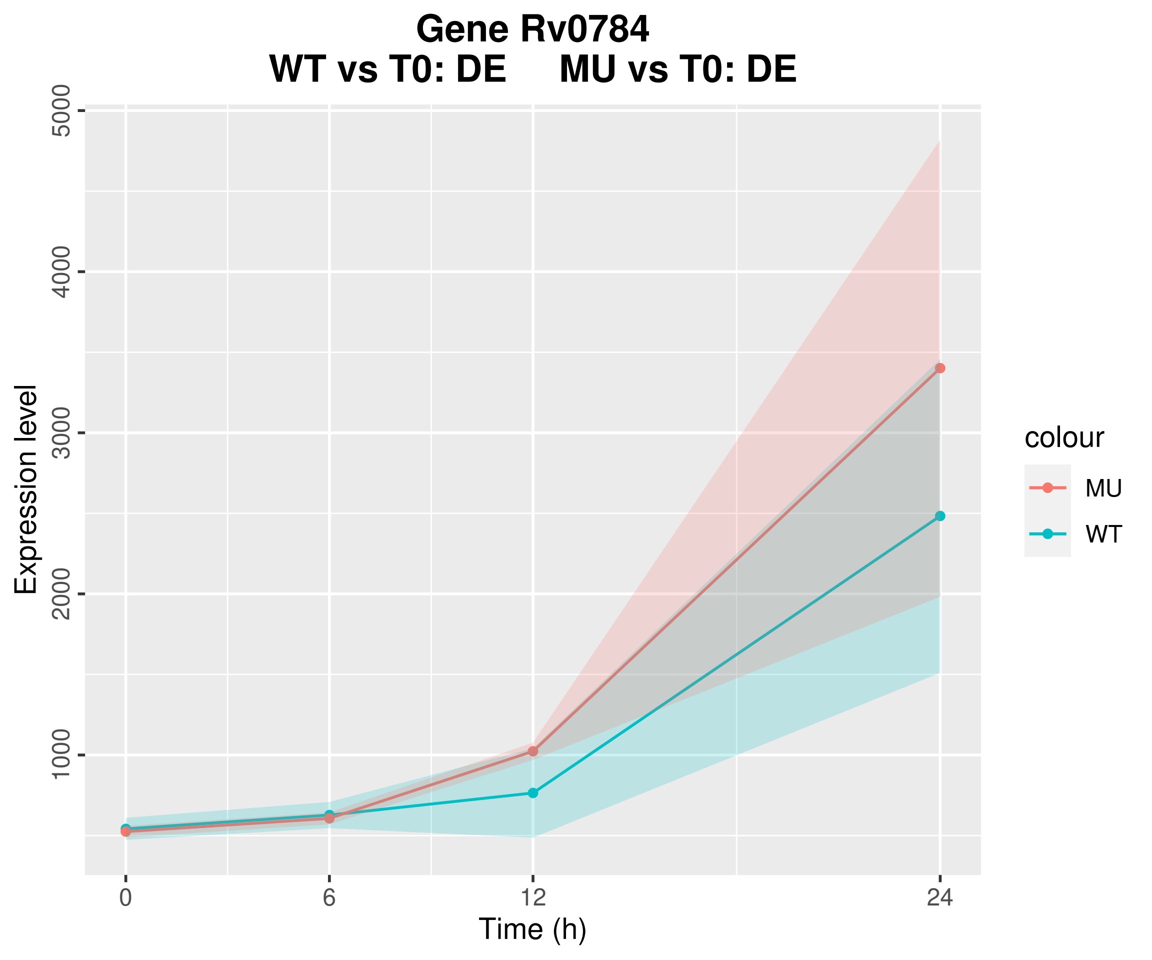

Gene Rv0784
WT vs T0: DE MU vs T0: DE
Expression level
5000
4000
3000
2000
1000
0
6
12
24
Time (h)
colour
MU
WT

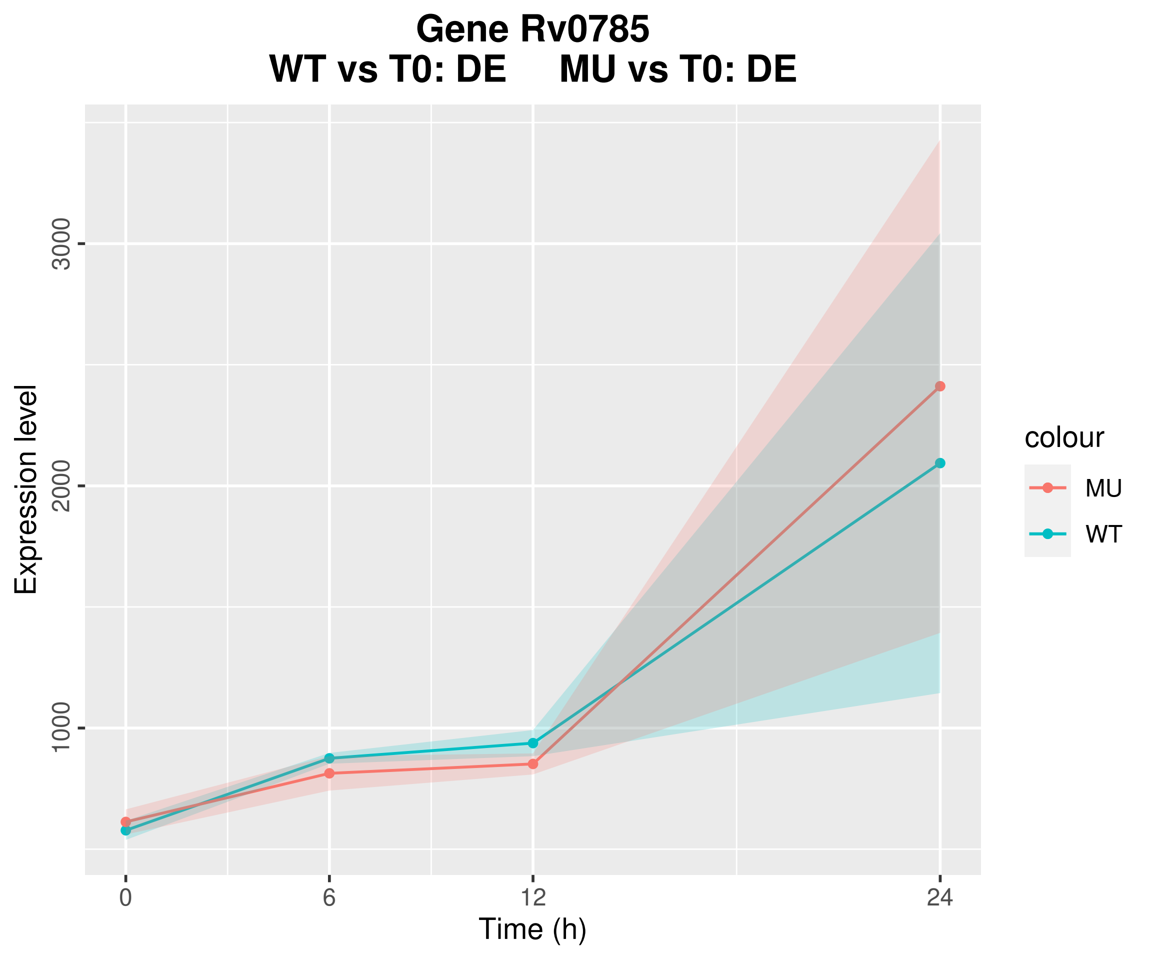
Gene Rv0785
WT vs T0: DE MU vs T0: DE
Expression level
3000
2000
1000
0
6
12
24
Time (h)
colour
MU
WT

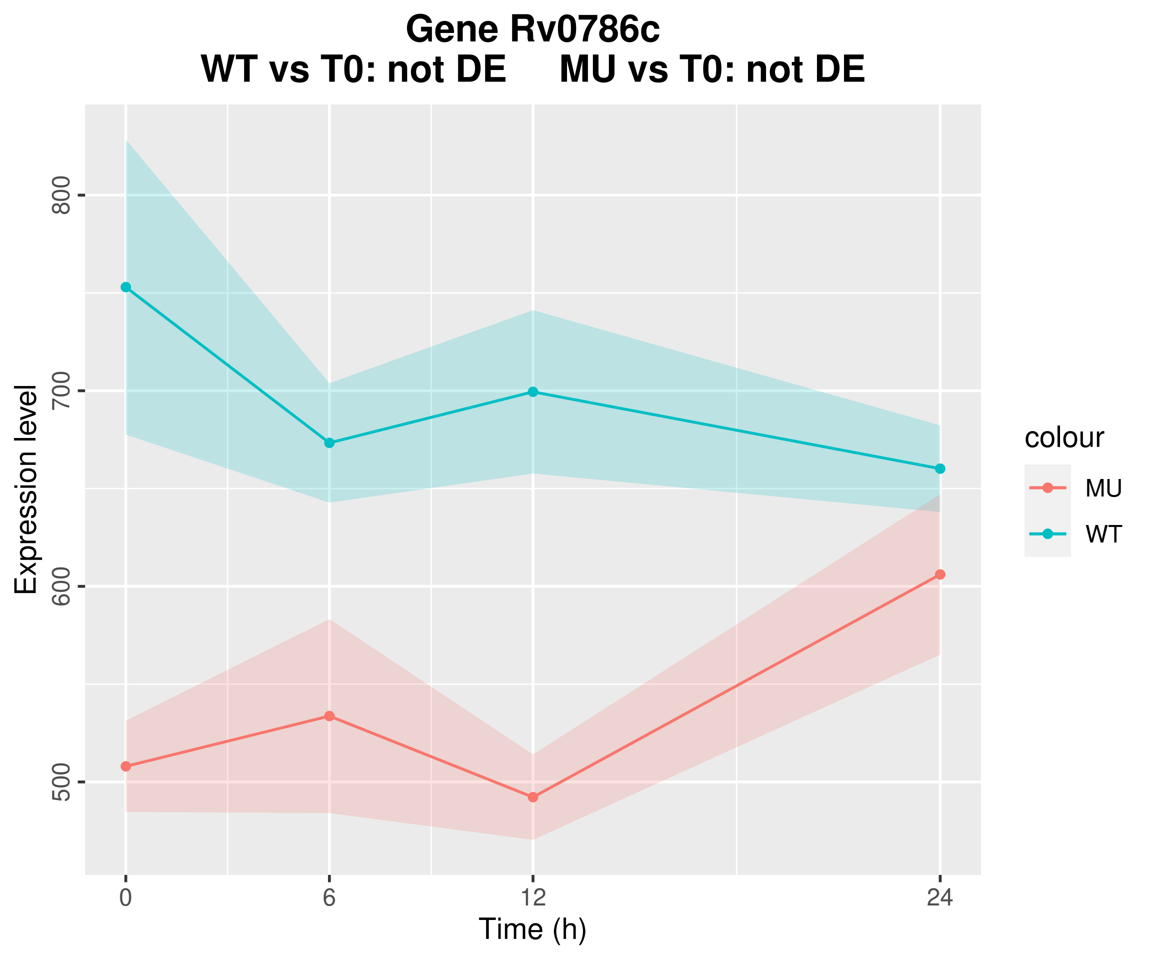
Gene Rv0786c
WT vs T0: not DE MU vs T0: not DE
Expression level
800
700
600
500
0
6
12
24
Time (h)
colour
MU
WT

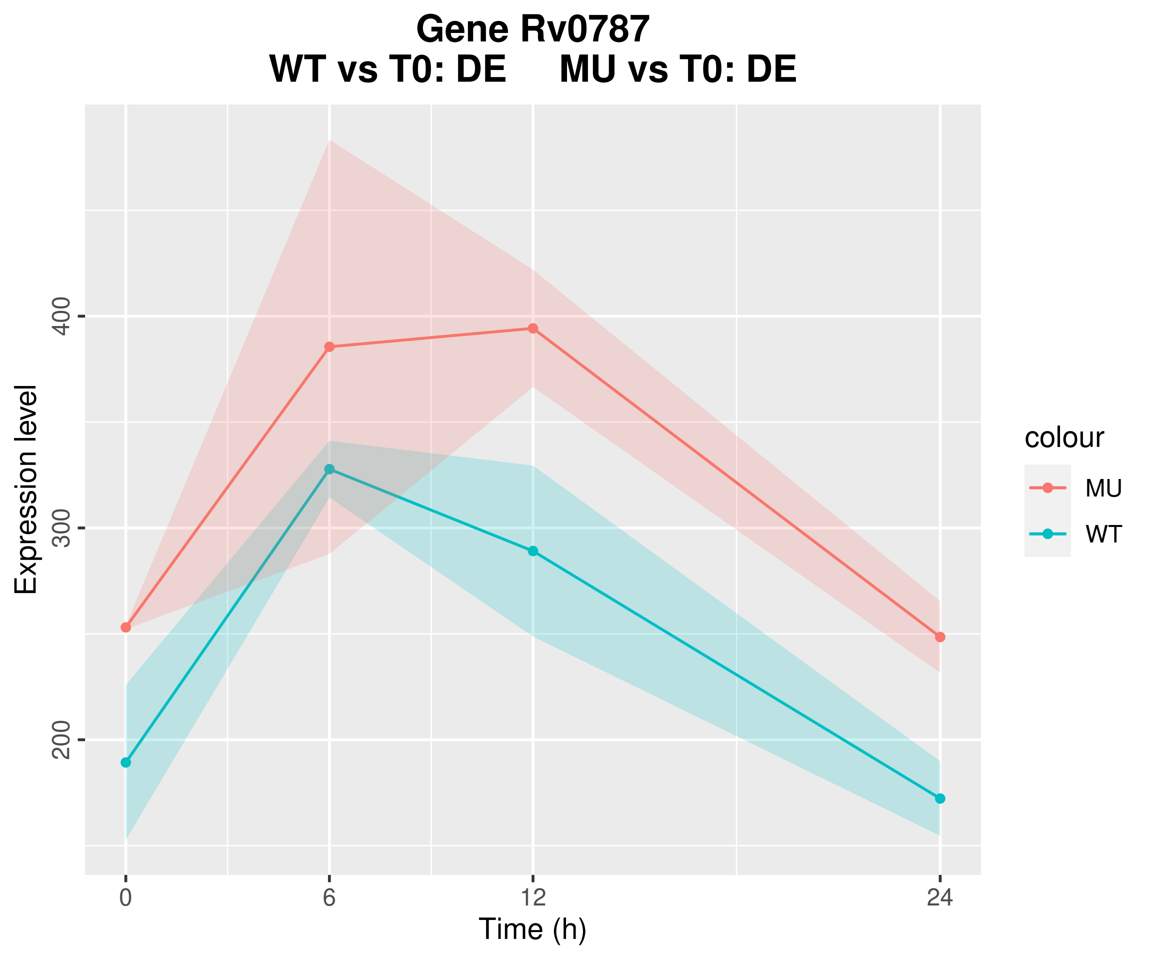

Gene Rv0787
WT vs T0: DE MU vs T0: DE
Expression level
400
300
200
0
6
12
24
Time (h)
colour
MU
WT

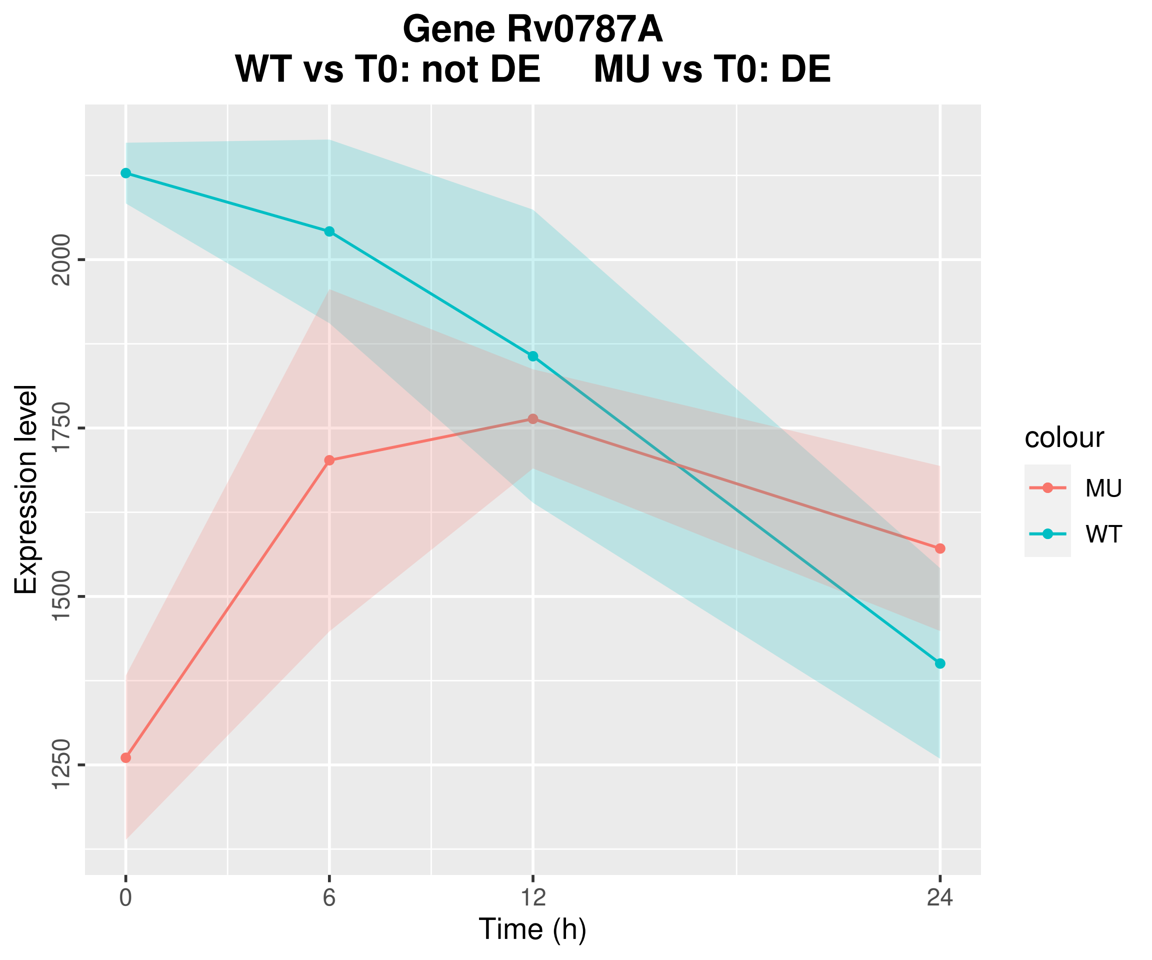

Gene Rv0787A
WT vs T0: not DE MU vs T0: DE
Expression level
2000
1750
1500
1250
0
6
12
24
Time (h)
colour
MU
WT

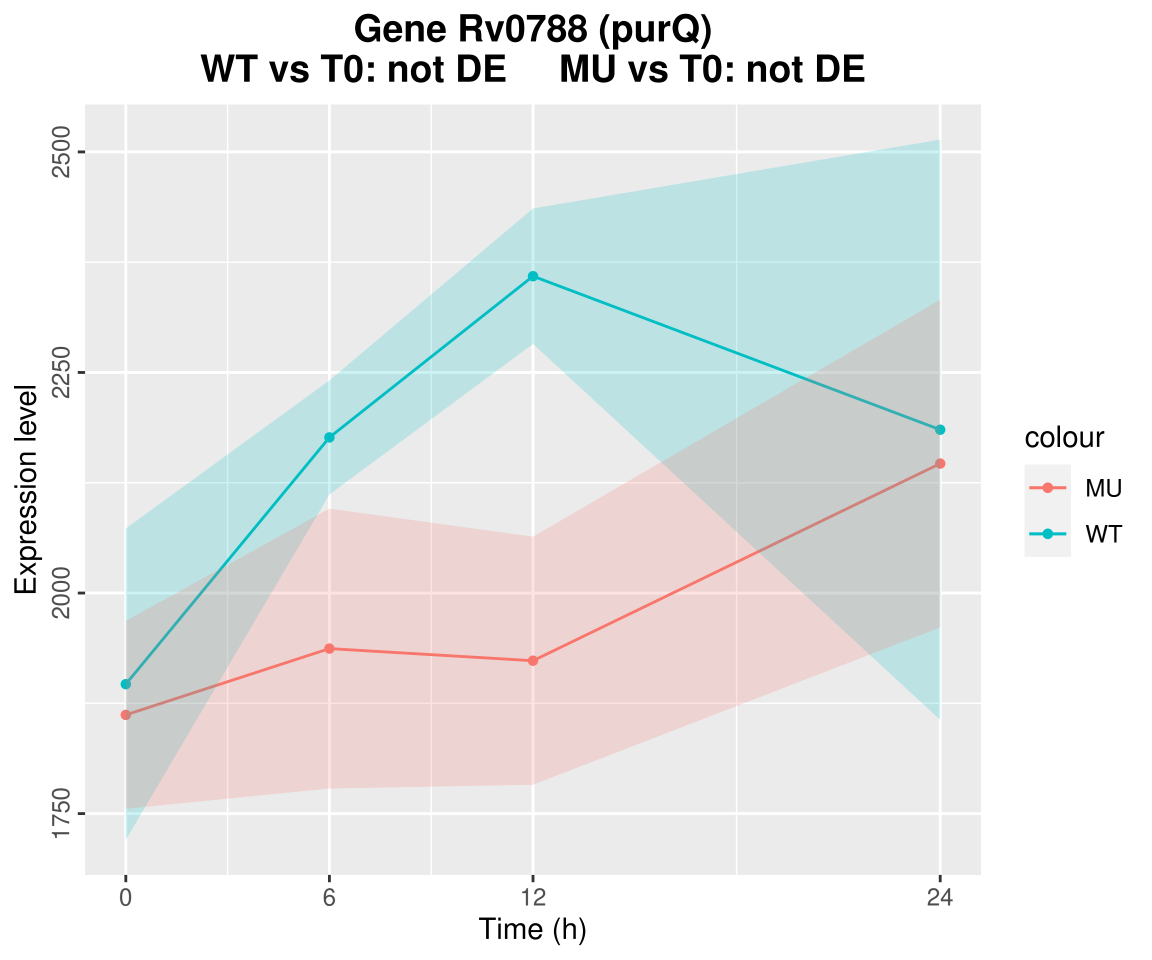

Gene Rv0788 (purQ)
WT vs T0: not DE   MU vs T0: not DE
Expression level
2500
2250
2000
1750
0
6
12
24
Time (h)
colour
MU
WT

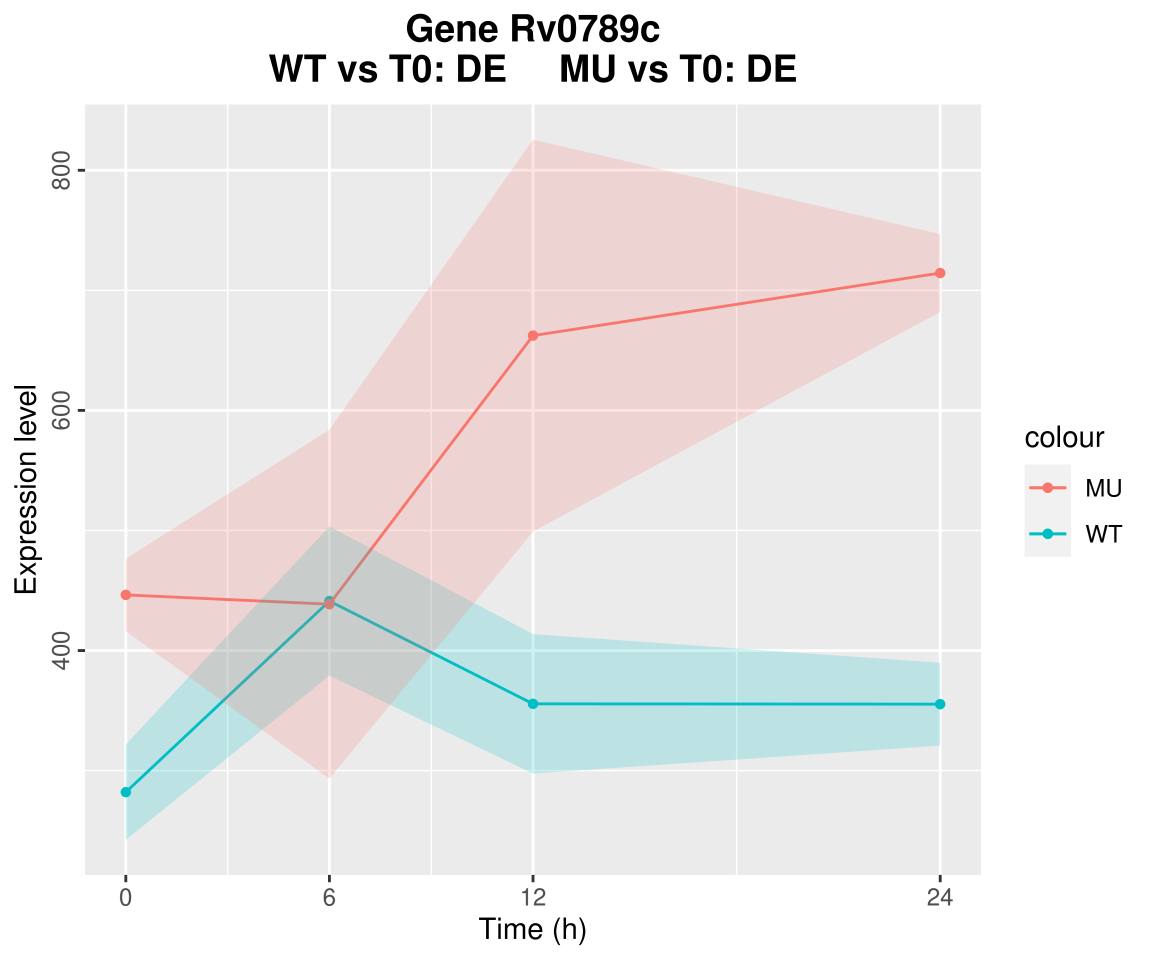

Gene Rv0789c
WT vs T0: DE MU vs T0: DE
800
600
400
Expression level
0
6
12
24
Time (h)
colour
MU
WT

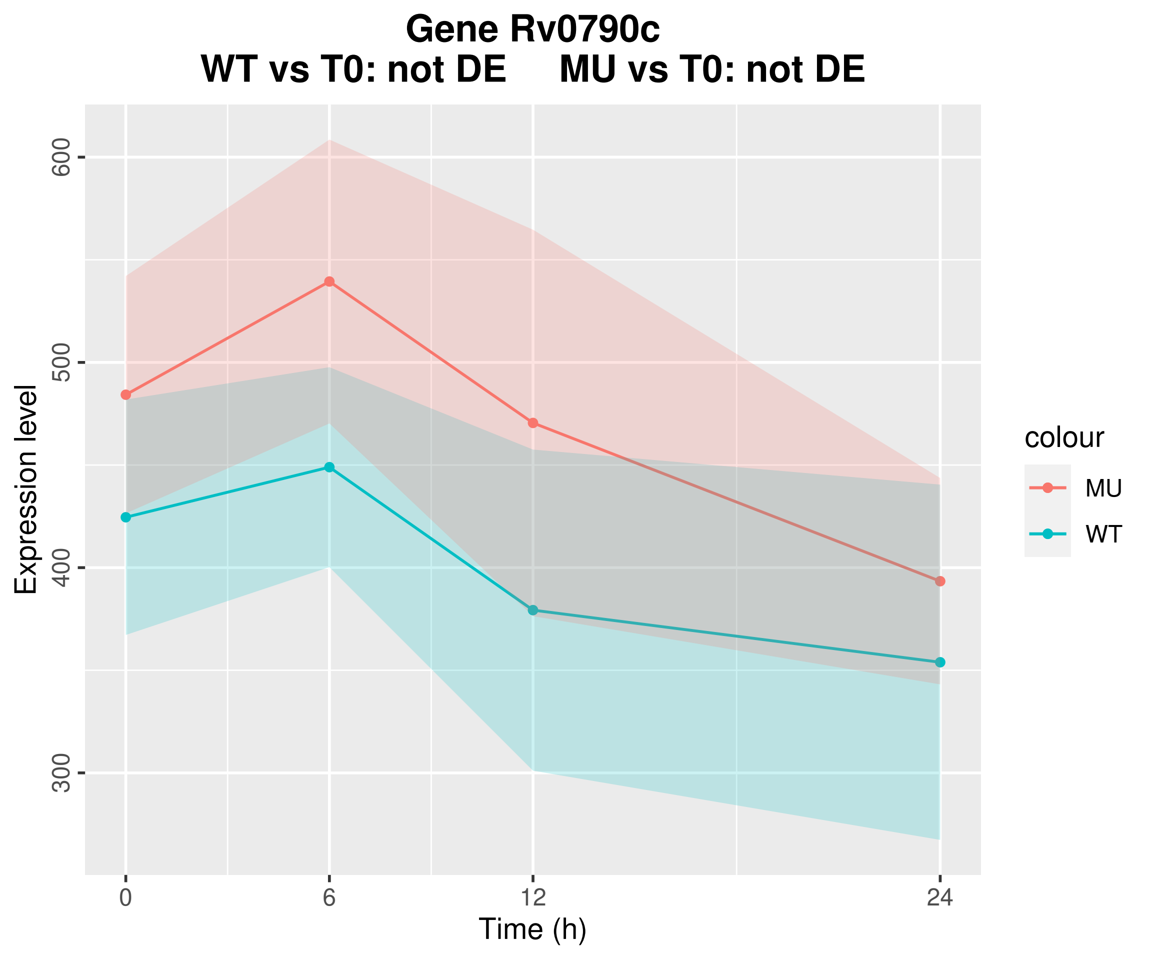
Gene Rv0790c
WT vs T0: not DE   MU vs T0: not DE
Expression level
600
500
400
300
0
6
12
24
Time (h)
colour
MU
WT

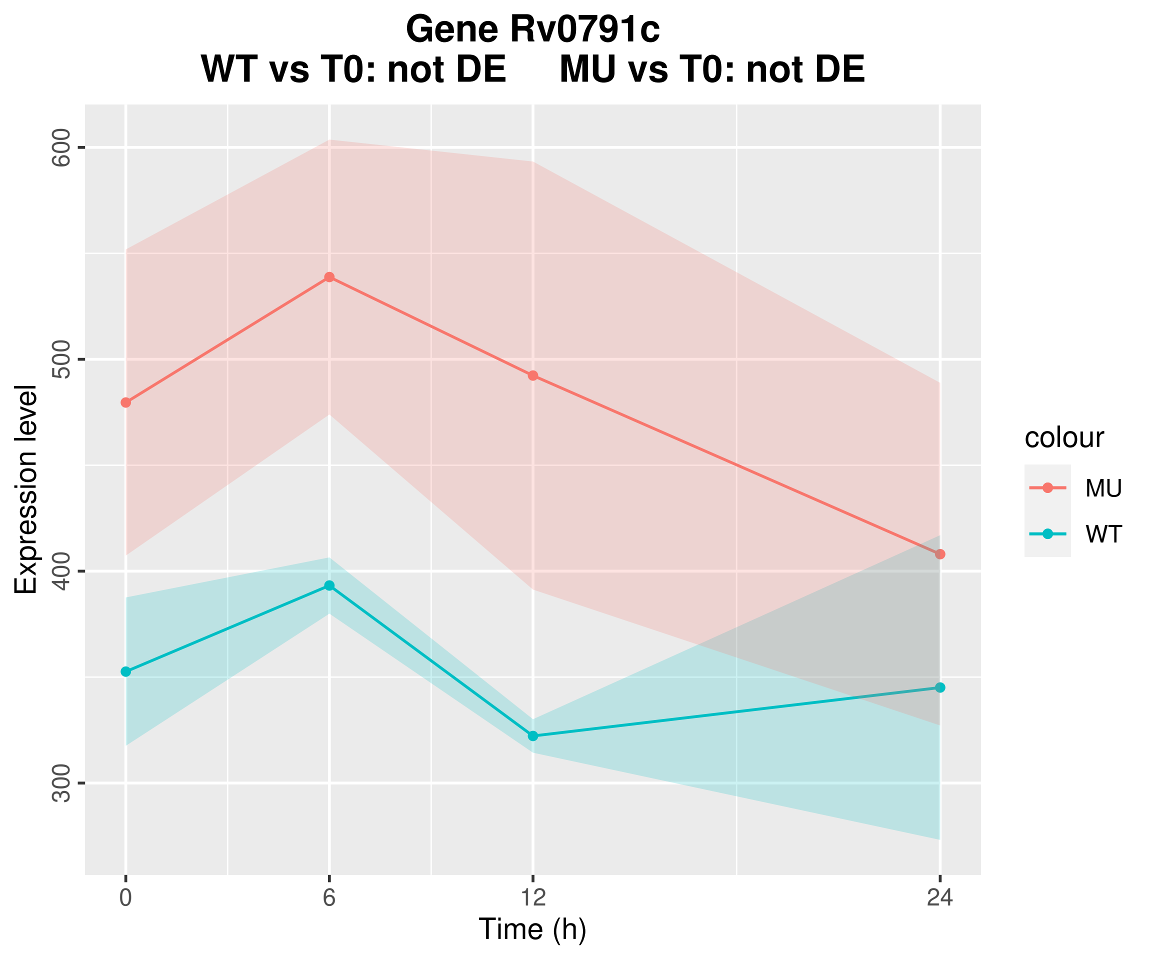

Gene Rv0791c
WT vs T0: not DE   MU vs T0: not DE
Expression level
600
500
400
300
Time (h)
0
6
12
24
colour
MU
WT

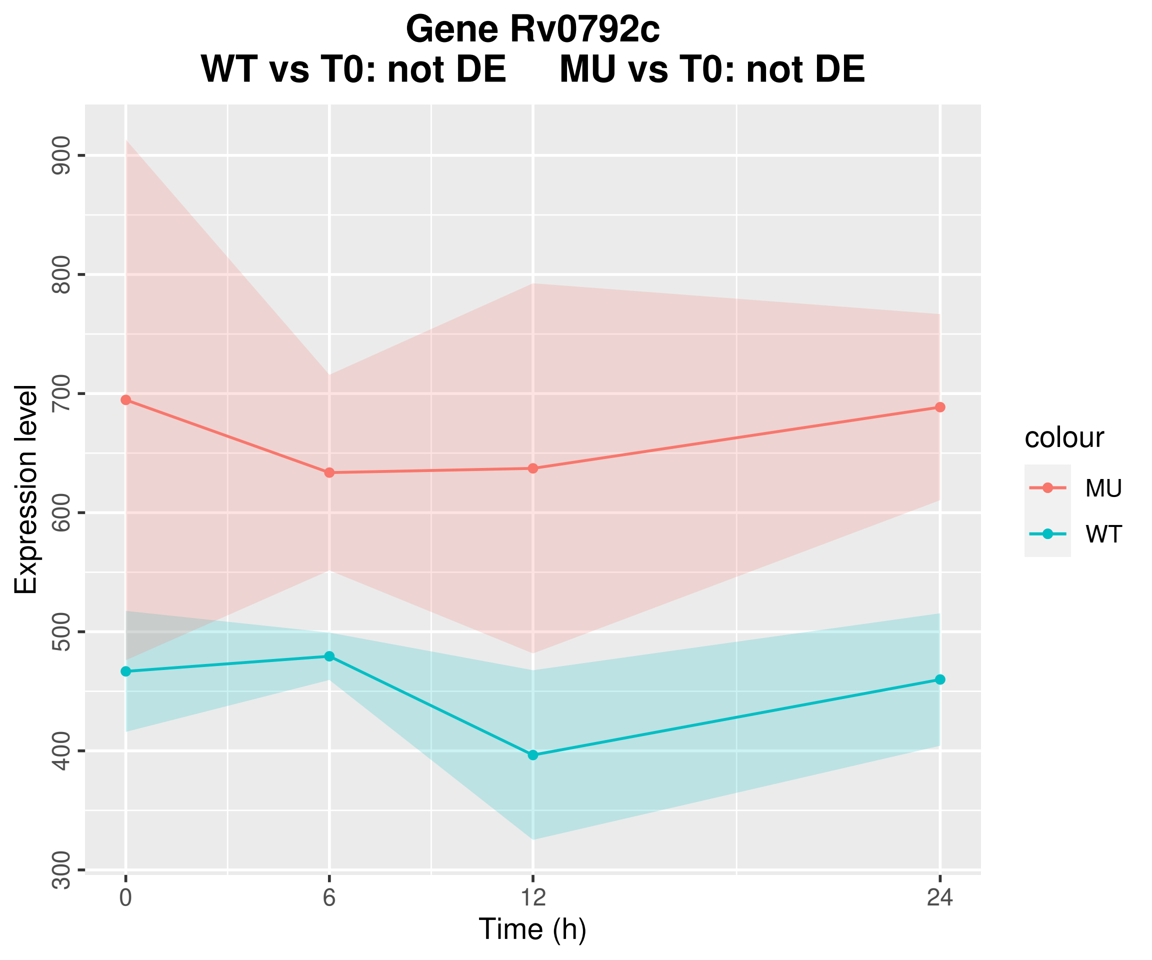

Gene Rv0792c
WT vs T0: not DE MU vs T0: not DE
Expression level
300
400
500
600
700
800
900
0
6
12
24
Time (h)
colour
MU
WT

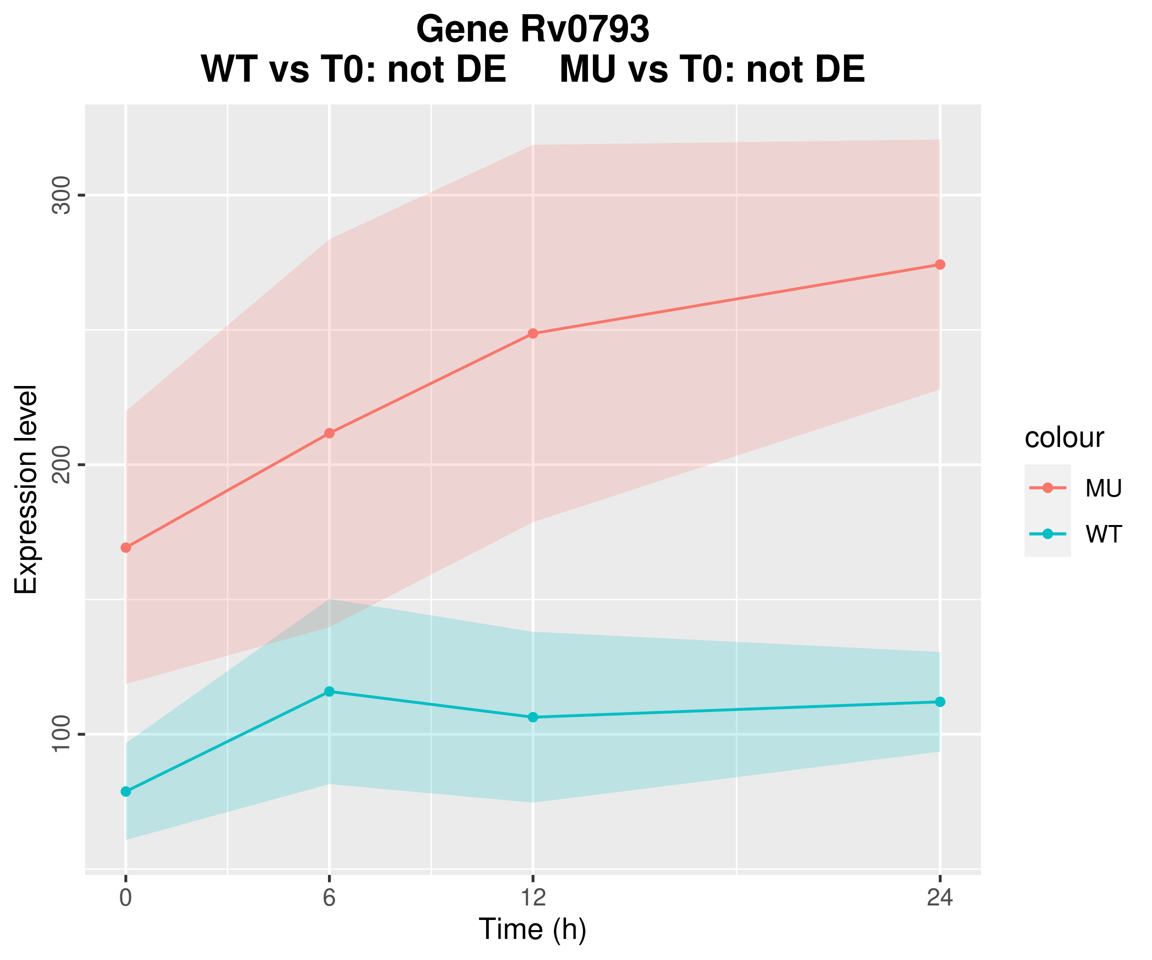

Gene Rv0793
WT vs T0: not DE MU vs T0: not DE
Expression level
300
200
100
0
6
12
24
Time (h)
colour
MU
WT

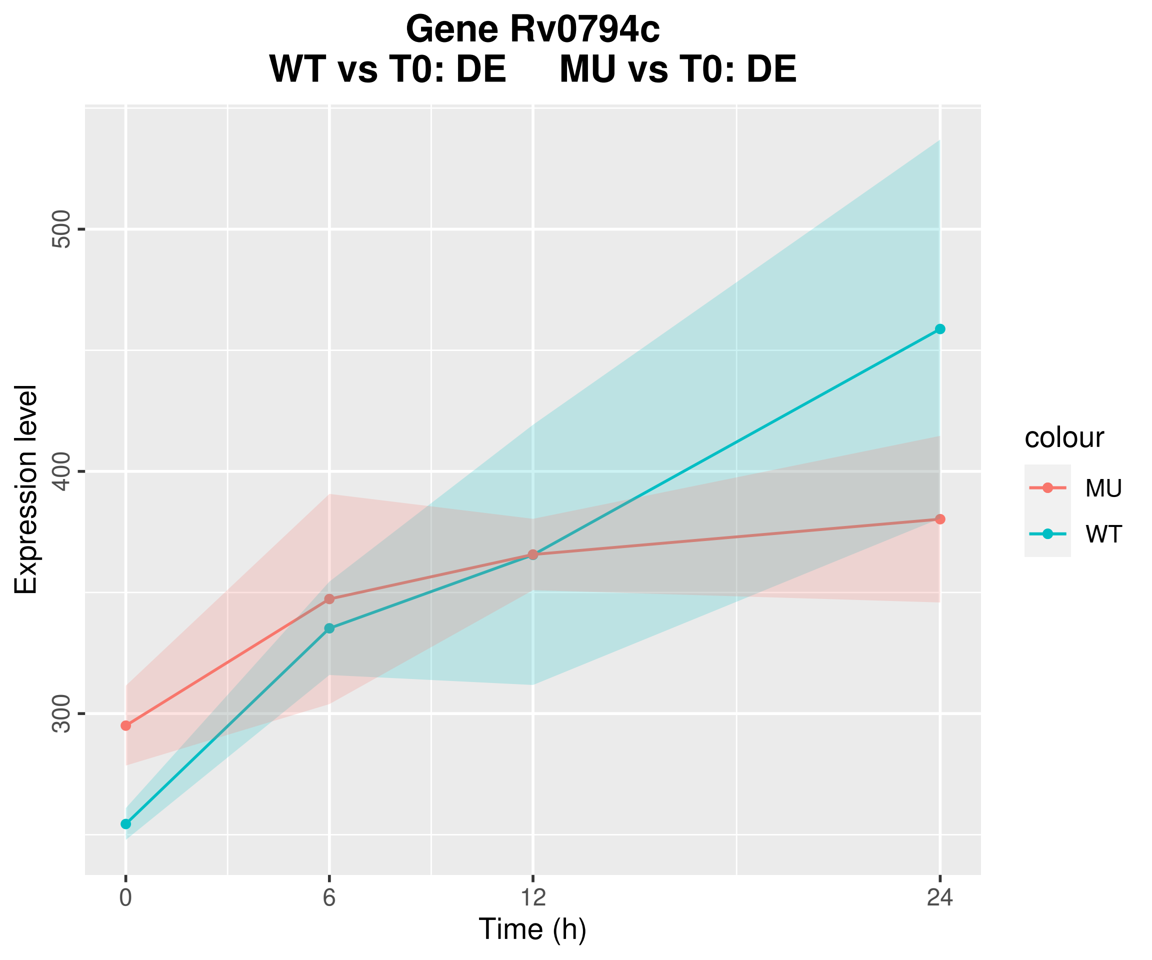

Gene Rv0794c
WT vs T0: DE   MU vs T0: DE
Expression level
500
400
300
0
6
12
24
Time (h)
colour
MU
WT

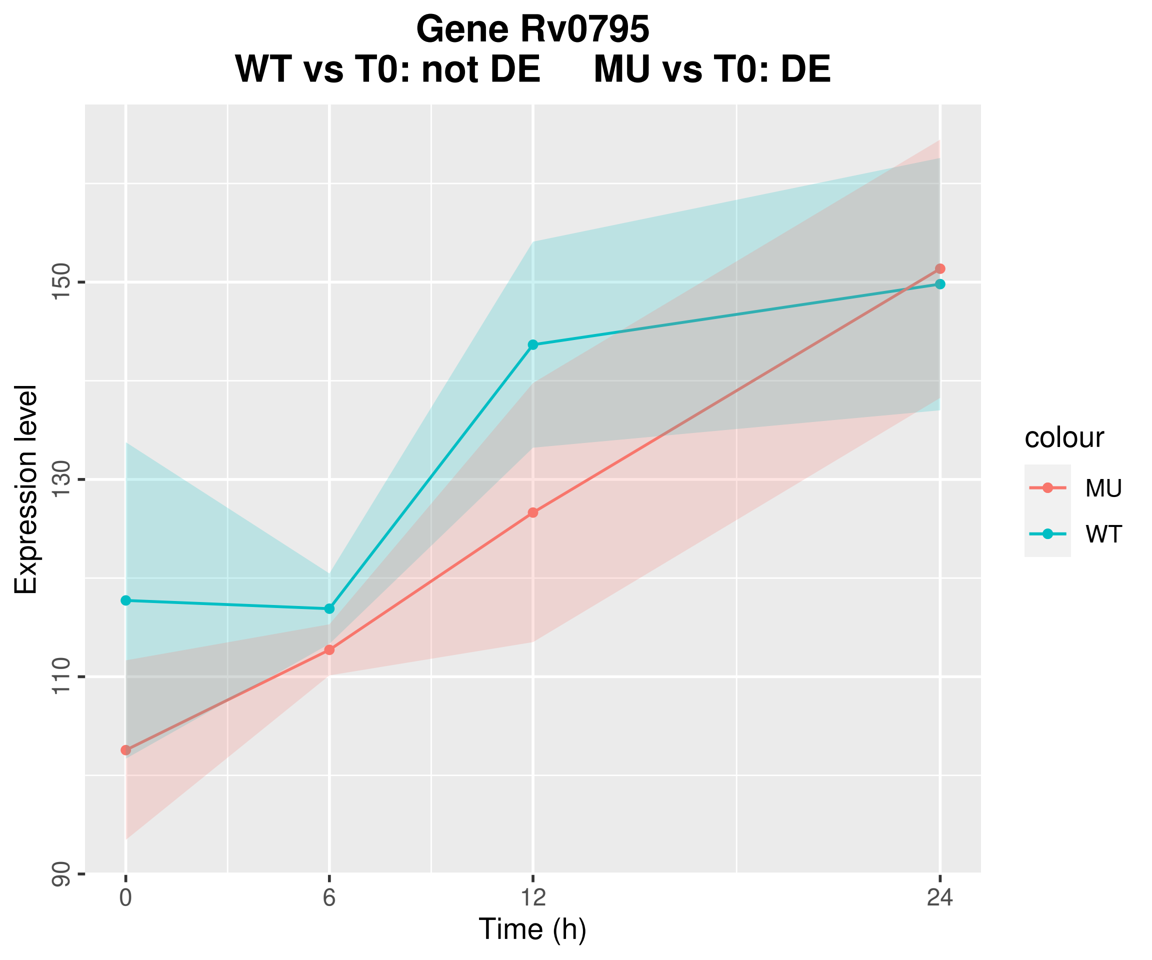

Gene Rv0795
WT vs T0: not DE   MU vs T0: DE
Expression level
90
110
130
150
0
6
12
24
Time (h)
colour
MU
WT

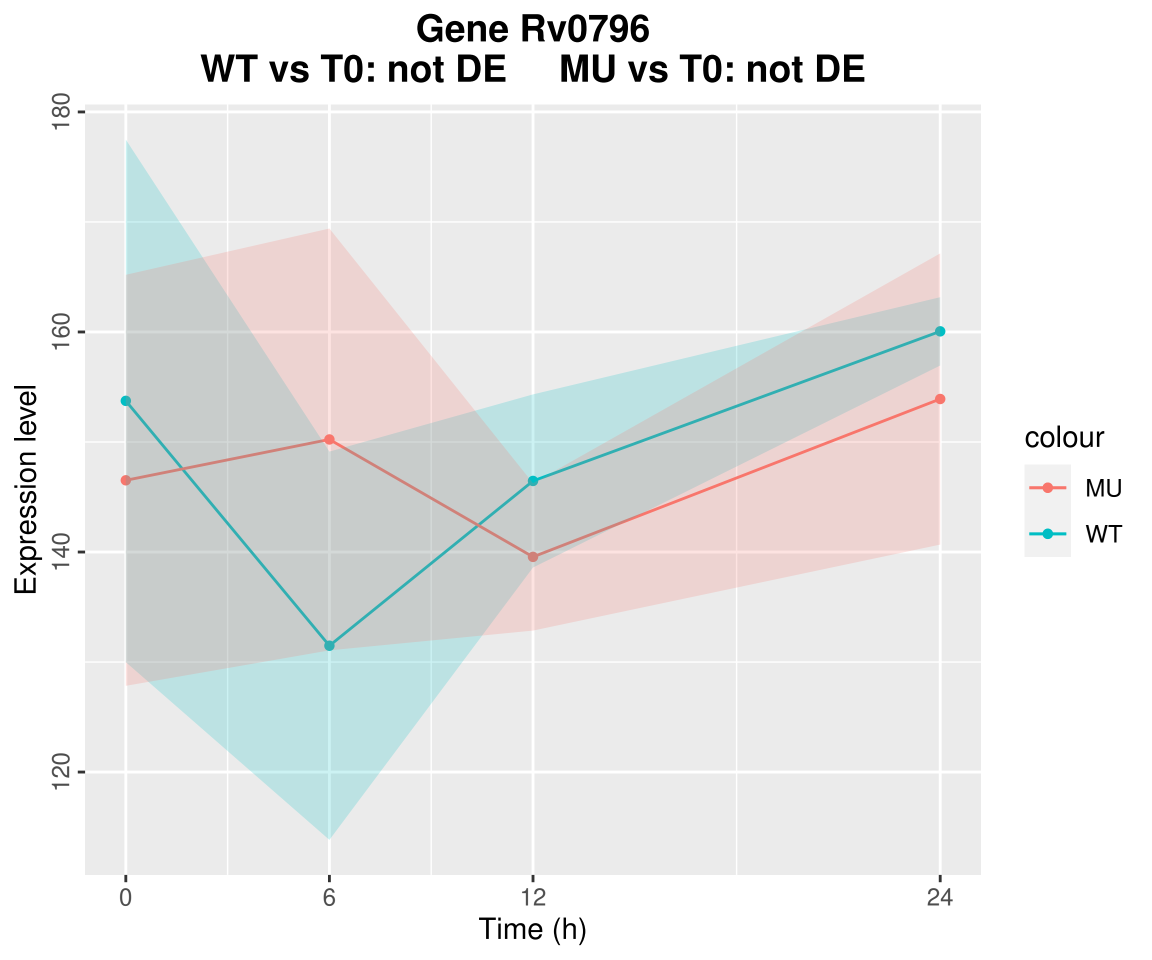
Gene Rv0796
WT vs T0: not DE MU vs T0: not DE
Expression level
180
160
140
120
0
6
12
24
Time (h)
colour
MU
WT

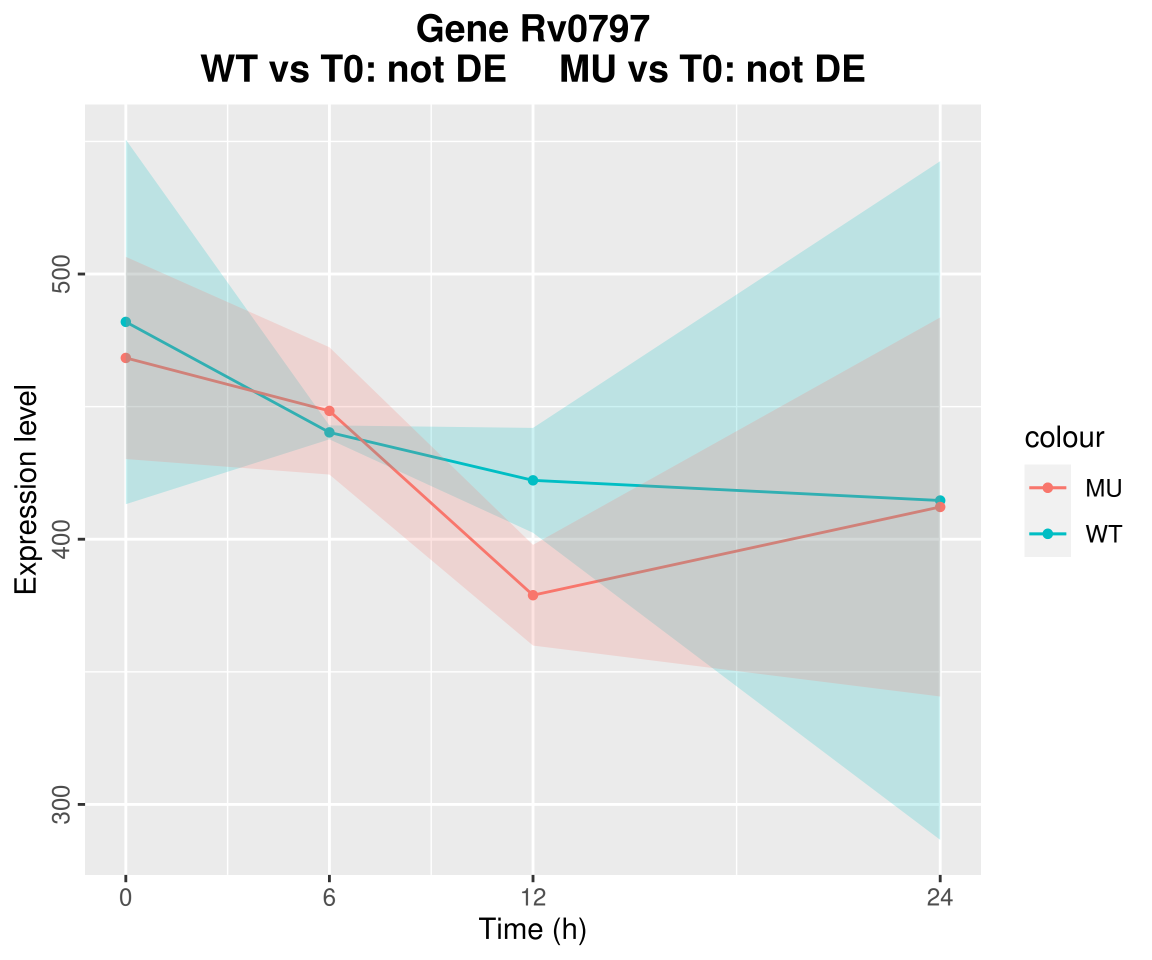

Gene Rv0797
WT vs T0: not DE   MU vs T0: not DE
Expression level
500
400
300
0
6
12
24
Time (h)
colour
MU
WT

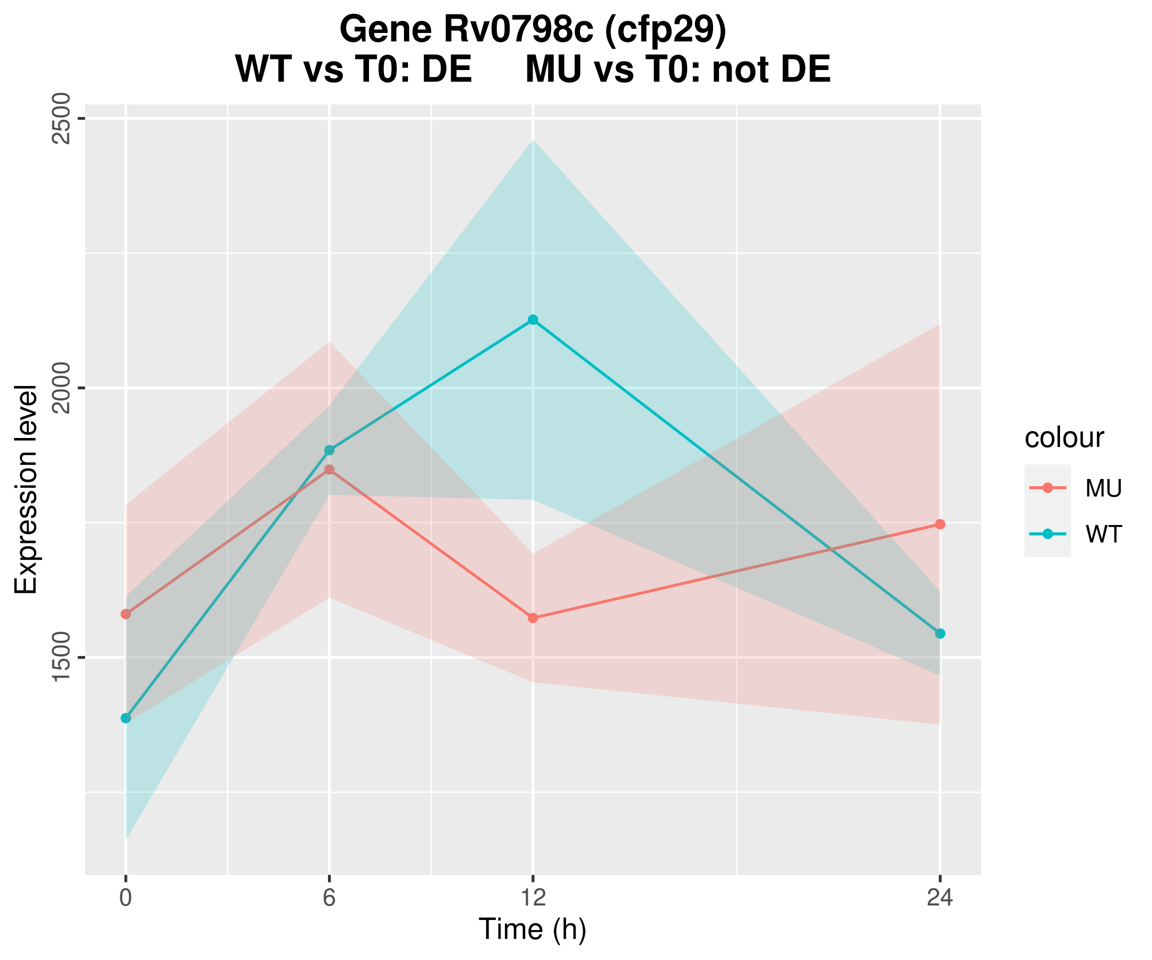
Gene Rv0798c (cfp29)
WT vs T0: DE   MU vs T0: not DE
2500
2000
1500
Expression level
0
6
12
24
Time (h)
colour
MU
WT

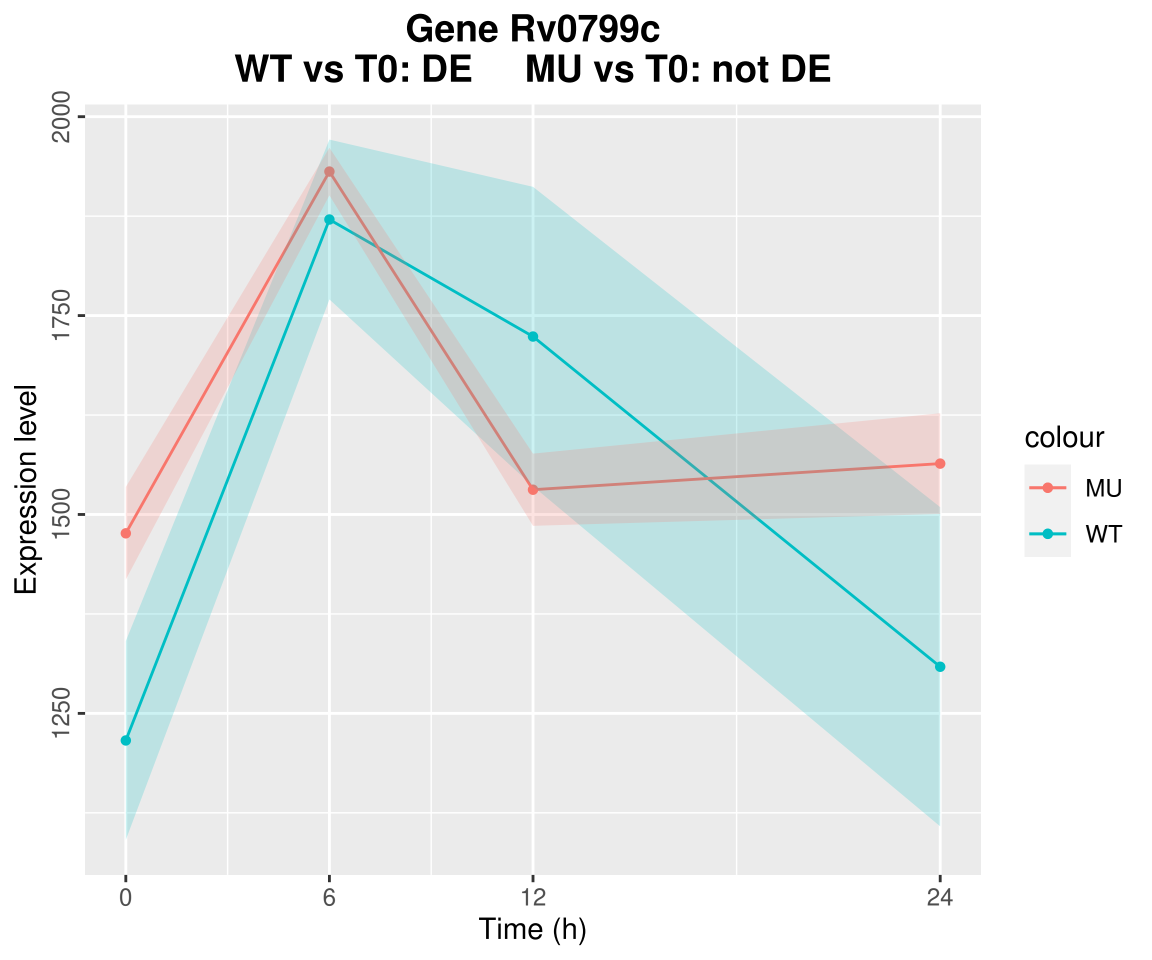
Gene Rv0799c
WT vs T0: DE MU vs T0: not DE
Expression level
2000
1750
1500
1250
0
6
12
24
Time (h)
colour
MU
WT

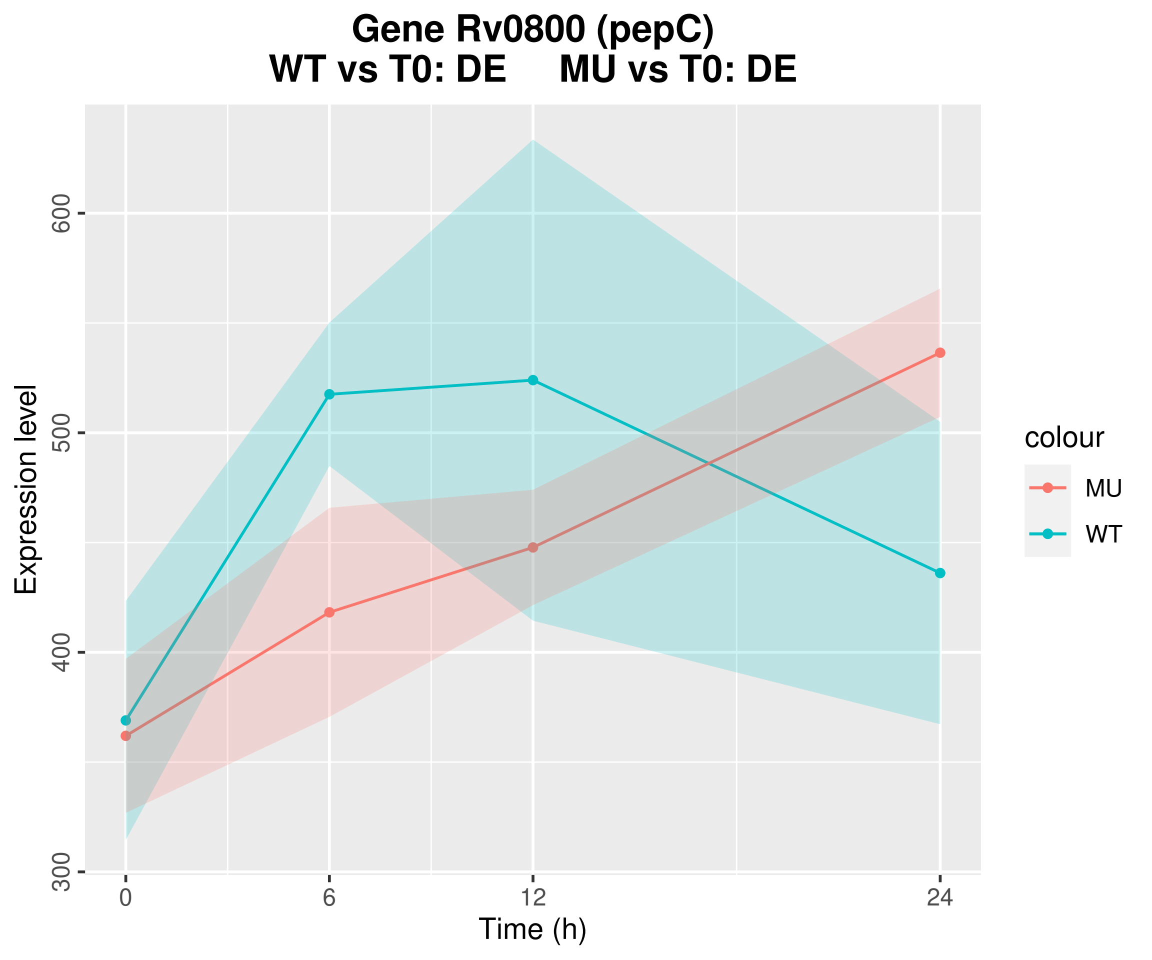
Gene Rv0800 (pepC)
WT vs T0: DE MU vs T0: DE
Expression level
600
500
400
300
0
6
12
24
Time (h)
colour
MU
WT

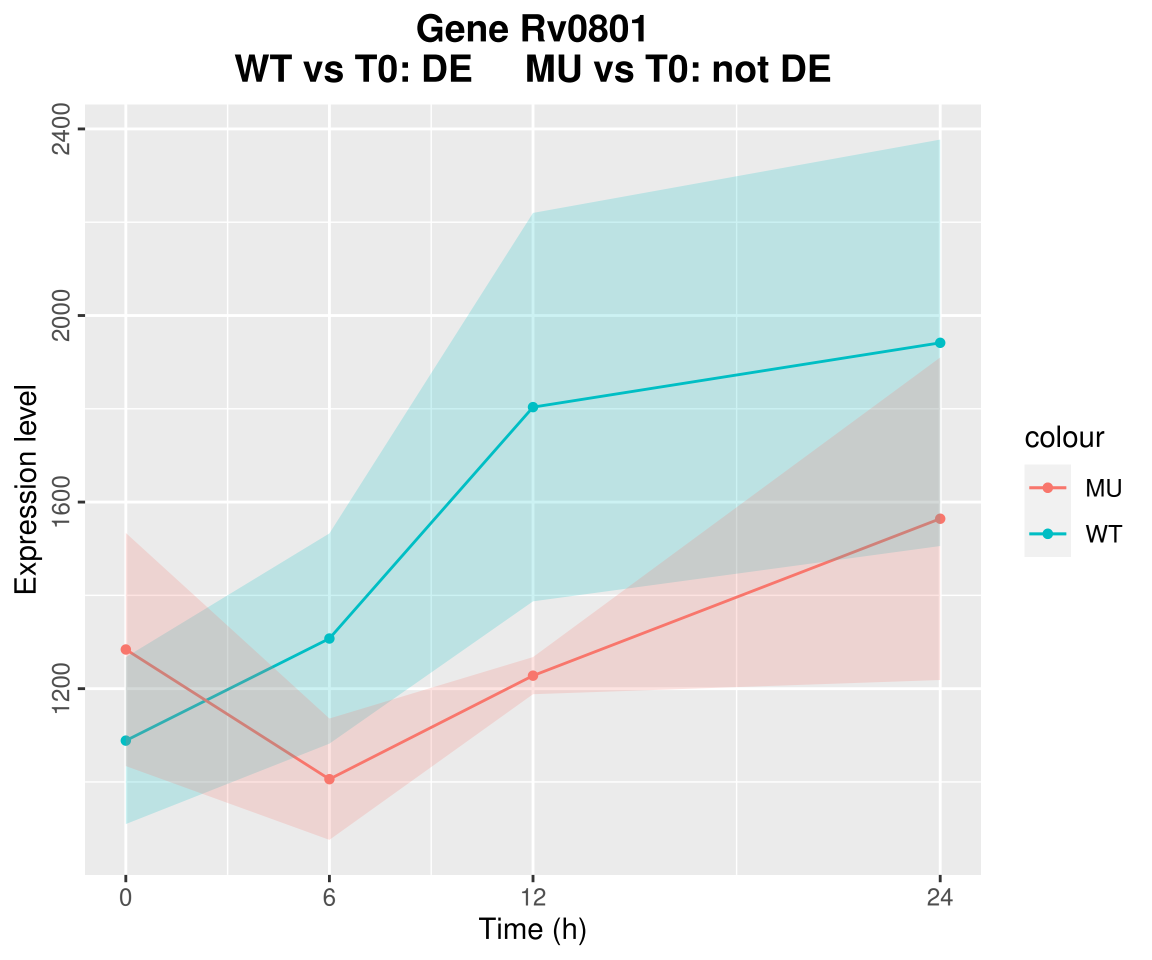

Gene Rv0801
WT vs T0: DE   MU vs T0: not DE
Expression level
2400
2000
1600
1200
0
6
12
24
Time (h)
colour
MU
WT

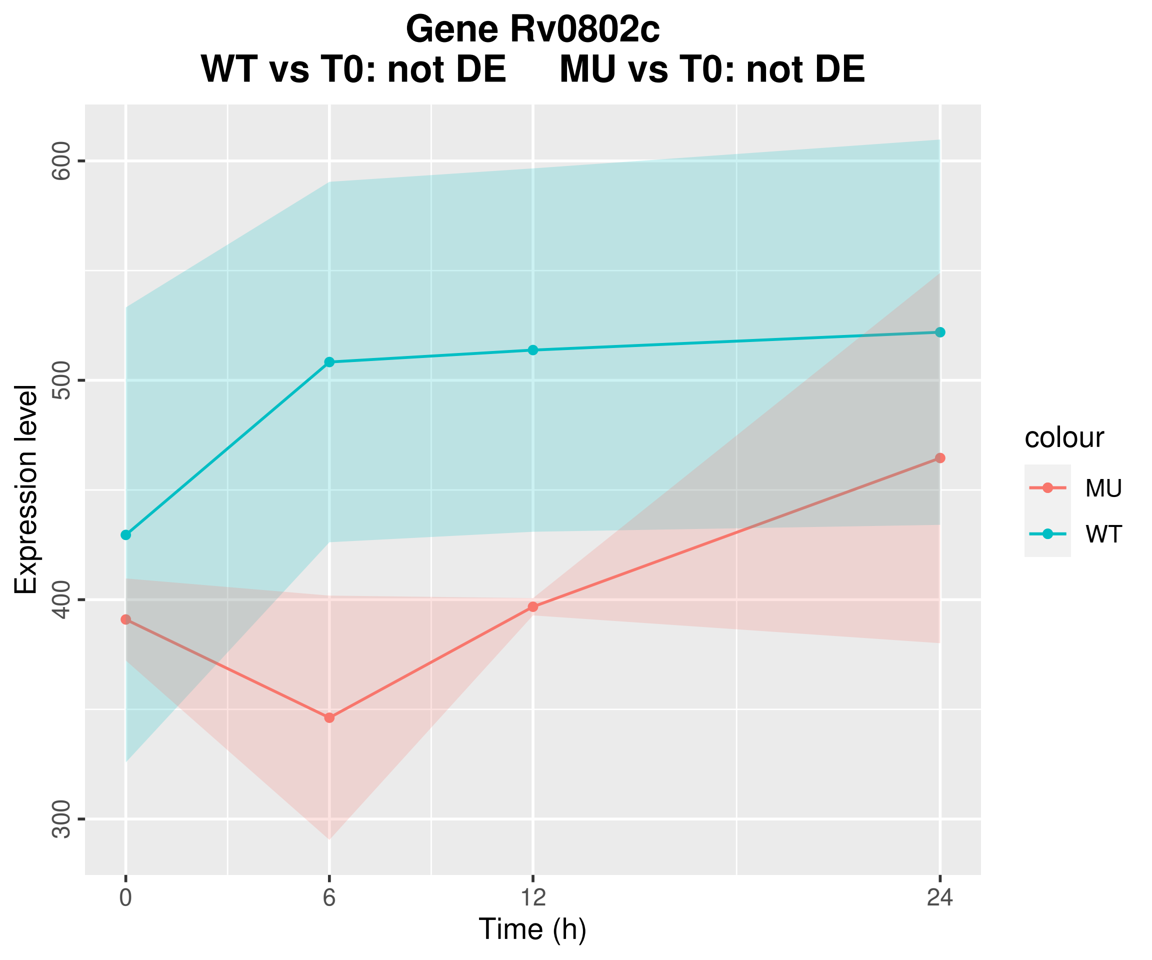

Gene Rv0802c
WT vs T0: not DE   MU vs T0: not DE
Expression level
600
500
400
300
0
6
12
24
Time (h)
colour
MU
WT

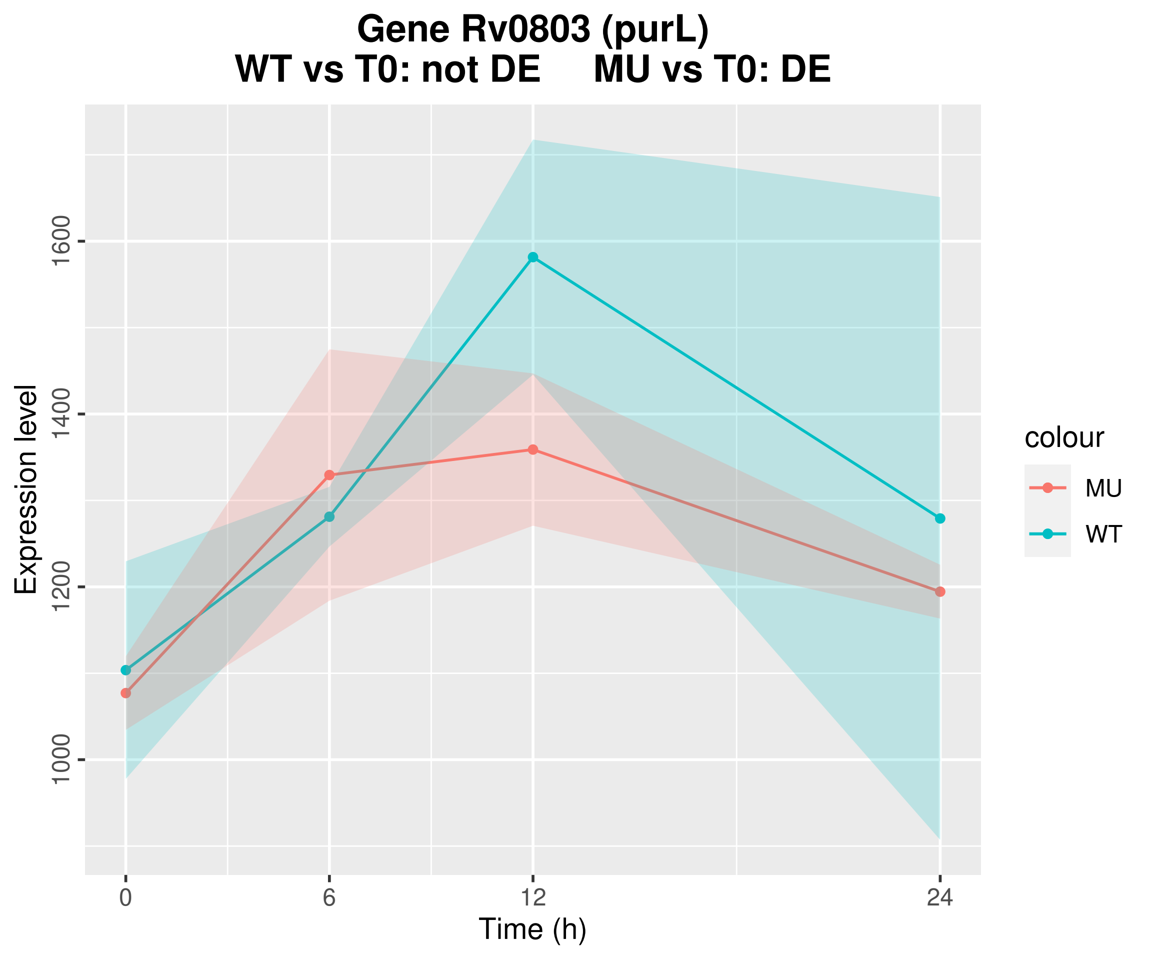

Gene Rv0803 (purL)
WT vs T0: not DE MU vs T0: DE
Expression level
1000
1200
1400
1600
0
6
12
24
Time (h)
colour
MU
WT

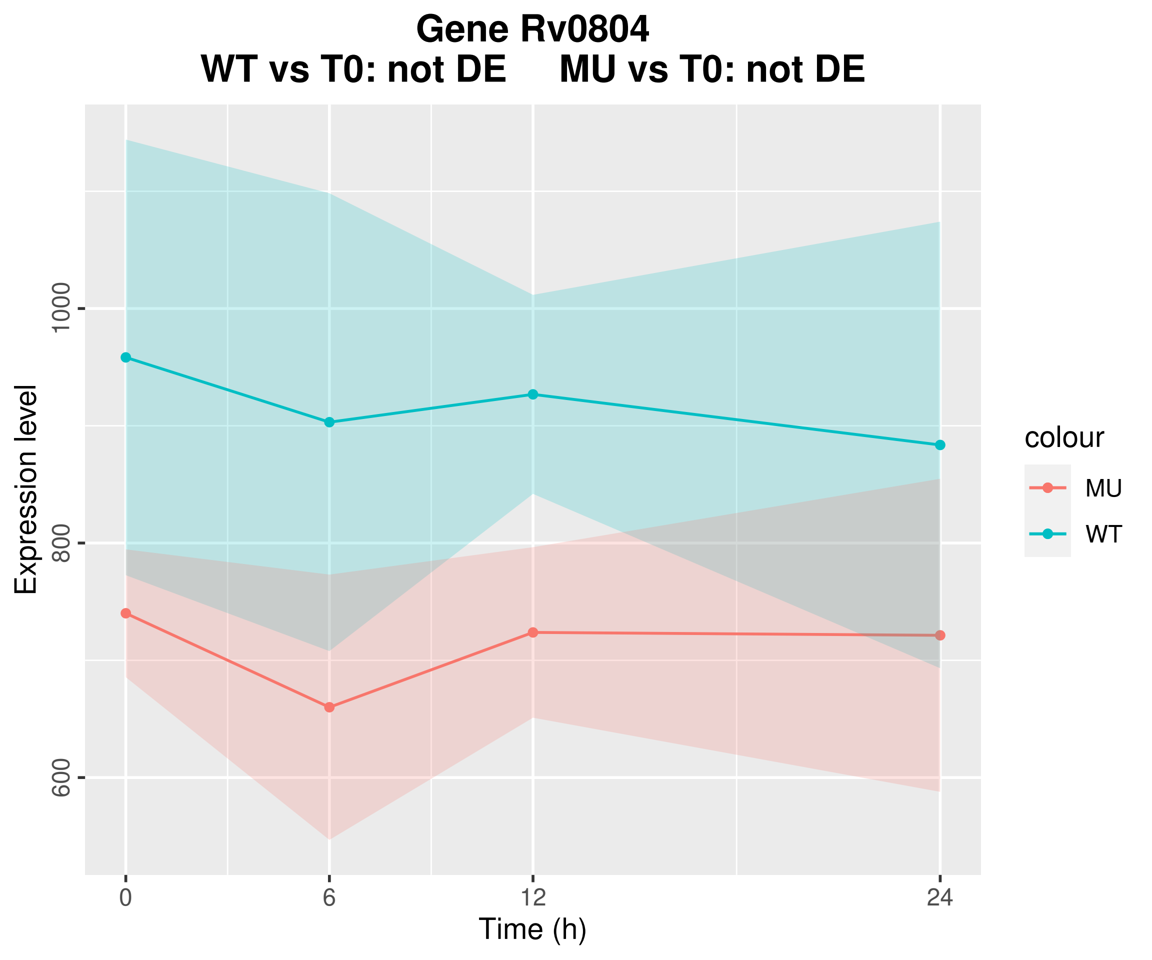

Gene Rv0804
WT vs T0: not DE   MU vs T0: not DE
Expression level
1000
800
600
Time (h)
0
6
12
24
colour
MU
WT

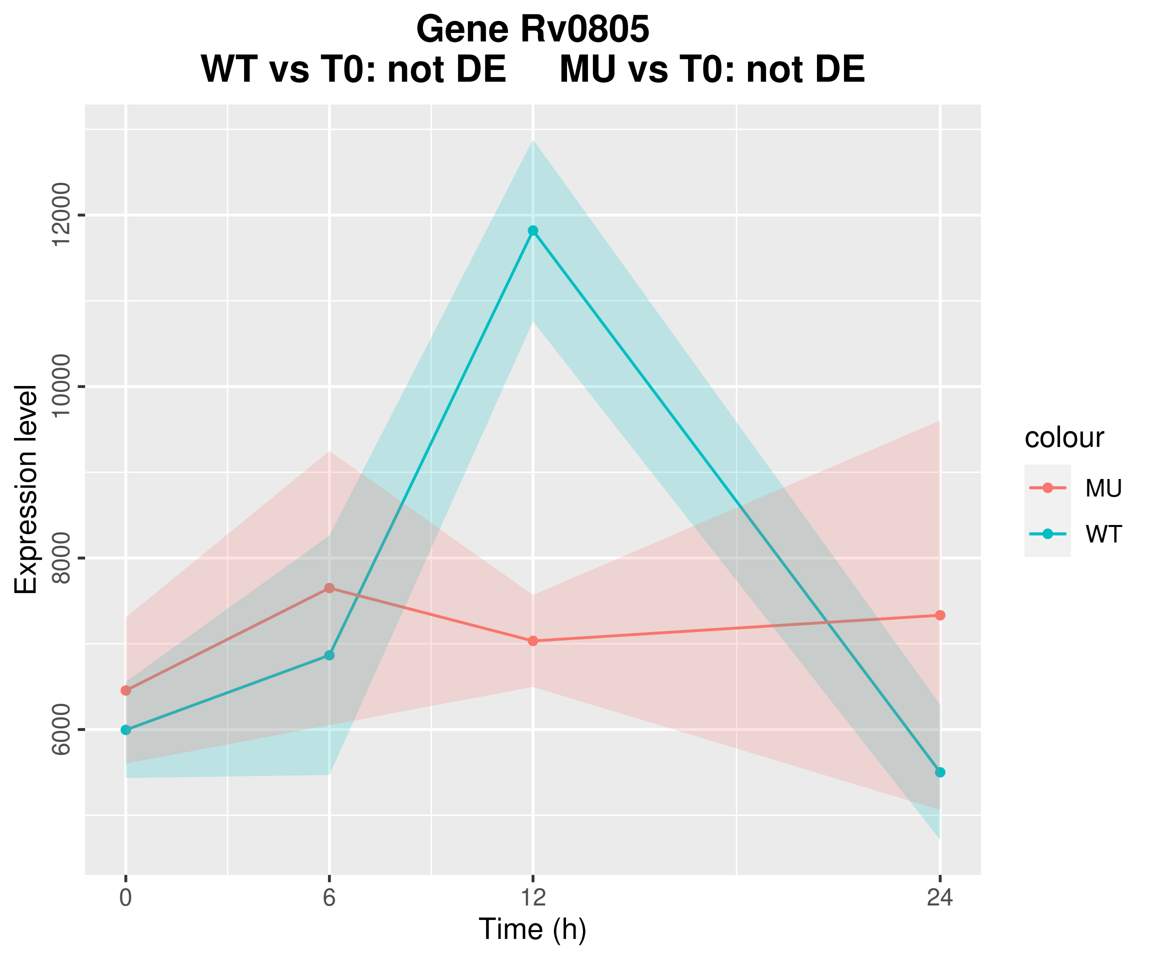

Gene Rv0805
WT vs T0: not DE   MU vs T0: not DE
Expression level
6000
8000
10000
12000
0
6
12
24
Time (h)
colour
MU
WT

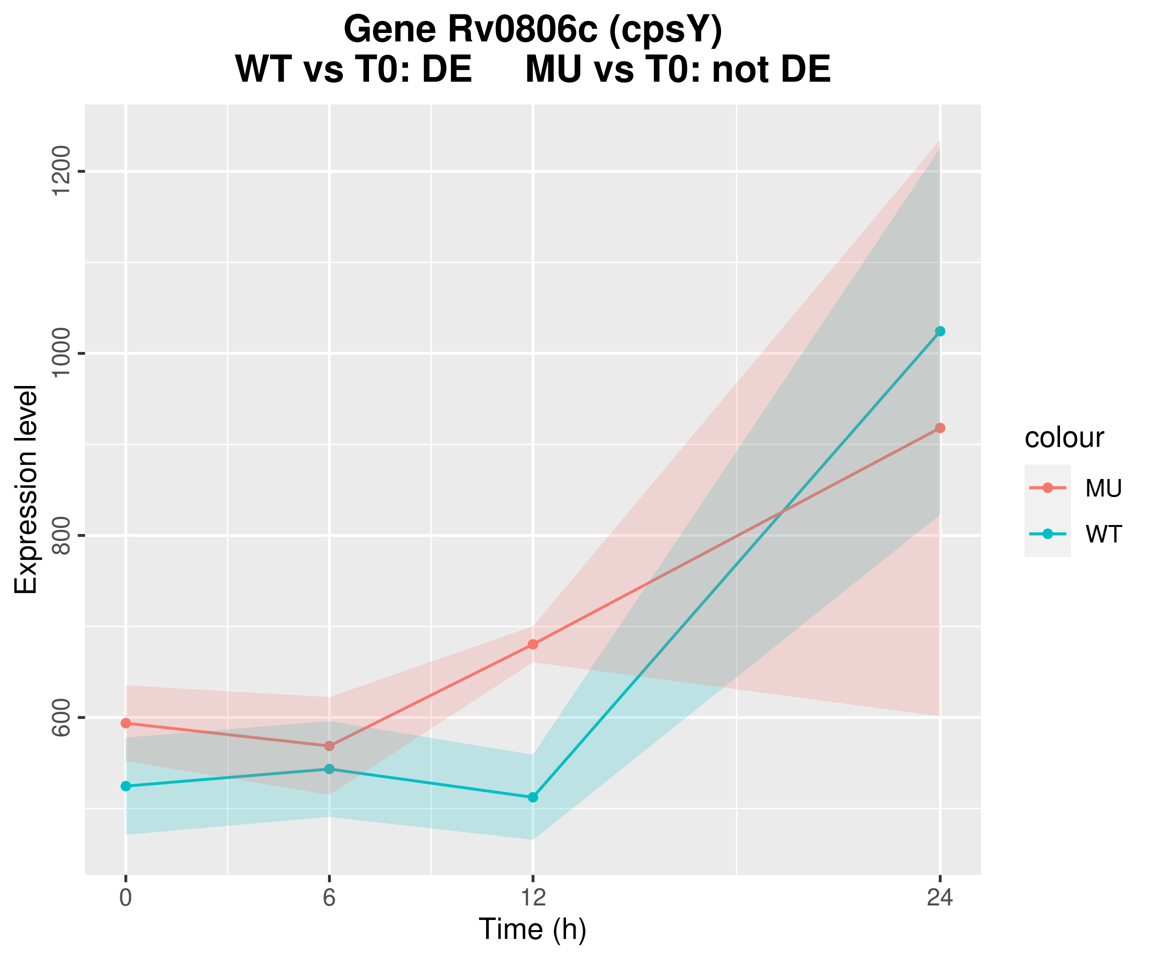
Gene Rv0806c (cpsY)
WT vs T0: DE   MU vs T0: not DE
Expression level
1200
1000
800
600
0
6
12
24
Time (h)
colour
MU
WT

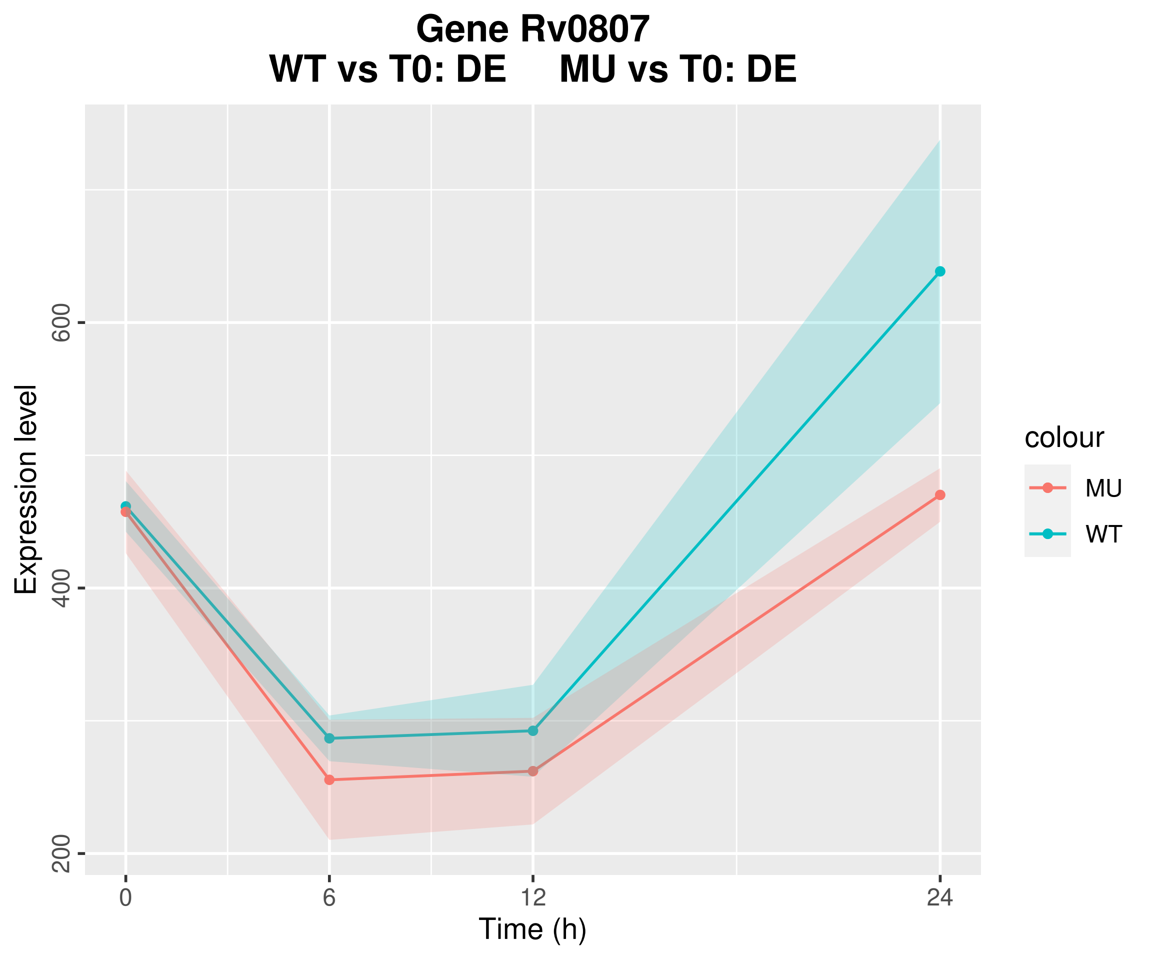
Gene Rv0807
WT vs T0: DE MU vs T0: DE
Expression level
600
400
200
0
6
12
24
Time (h)
colour
MU
WT

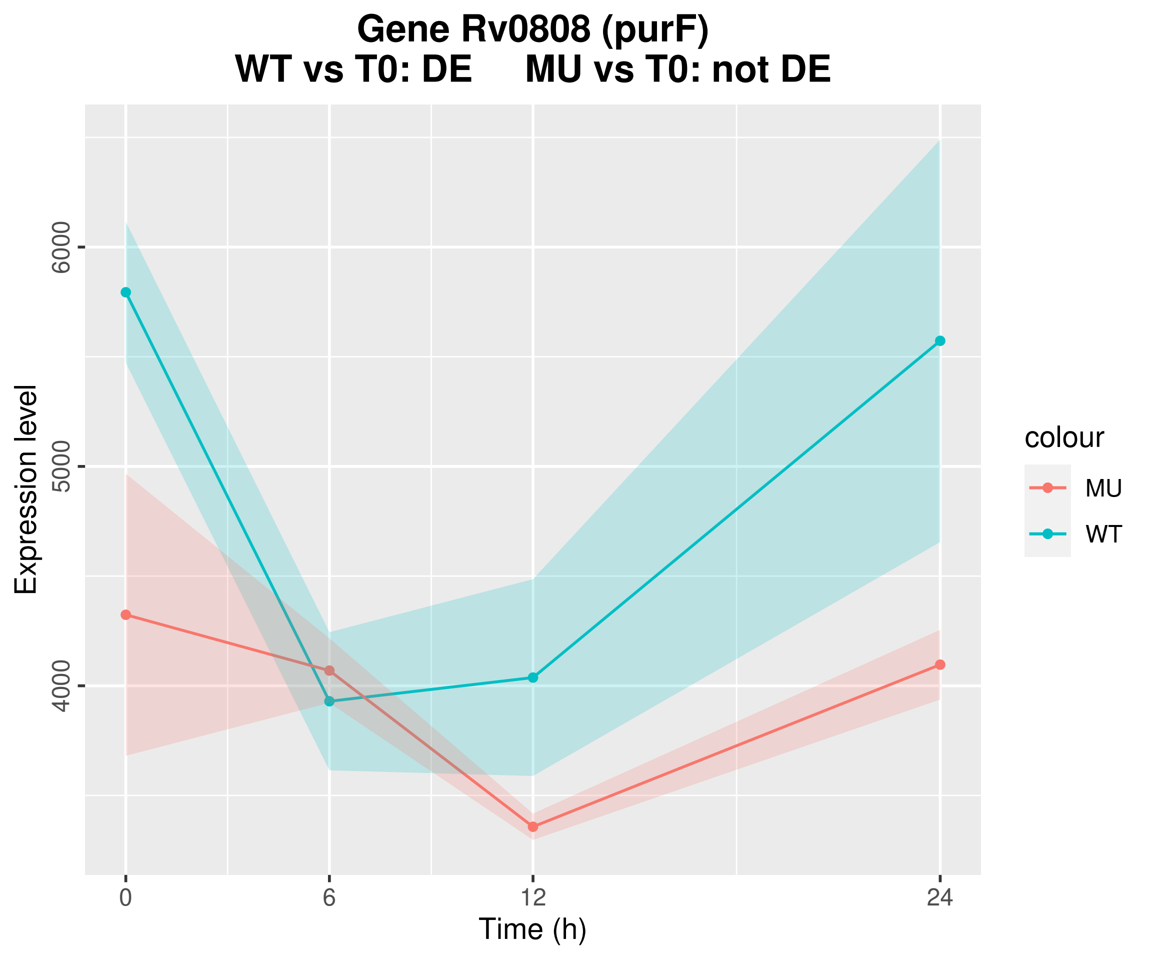

Gene Rv0808 (purF)
WT vs T0: DE   MU vs T0: not DE
Expression level
6000
5000
4000
0
6
12
24
Time (h)
colour
MU
WT

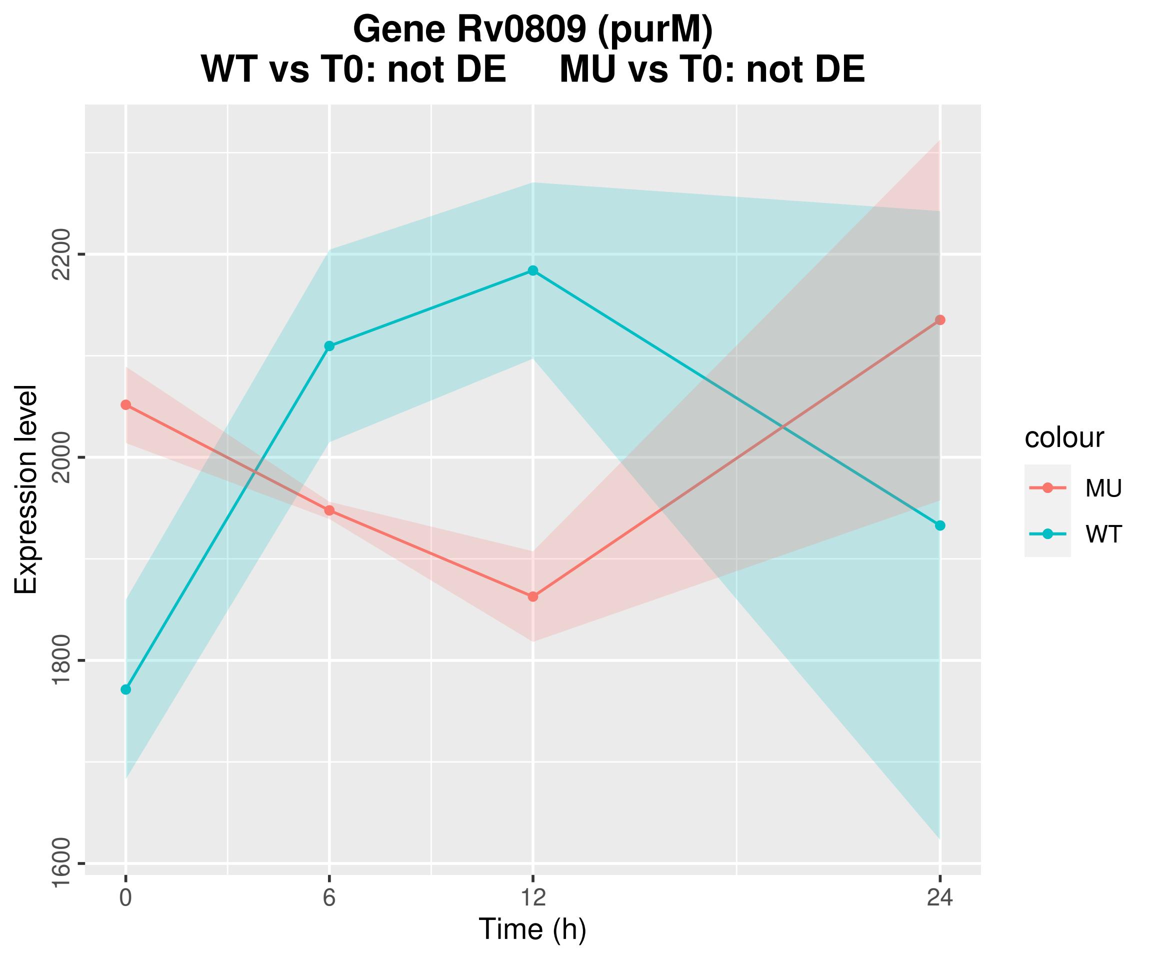
Gene Rv0809 (purM)
WT vs T0: not DE   MU vs T0: not DE
Expression level
1600
1800
2000
2200
0
6
12
24
Time (h)
colour
MU
WT

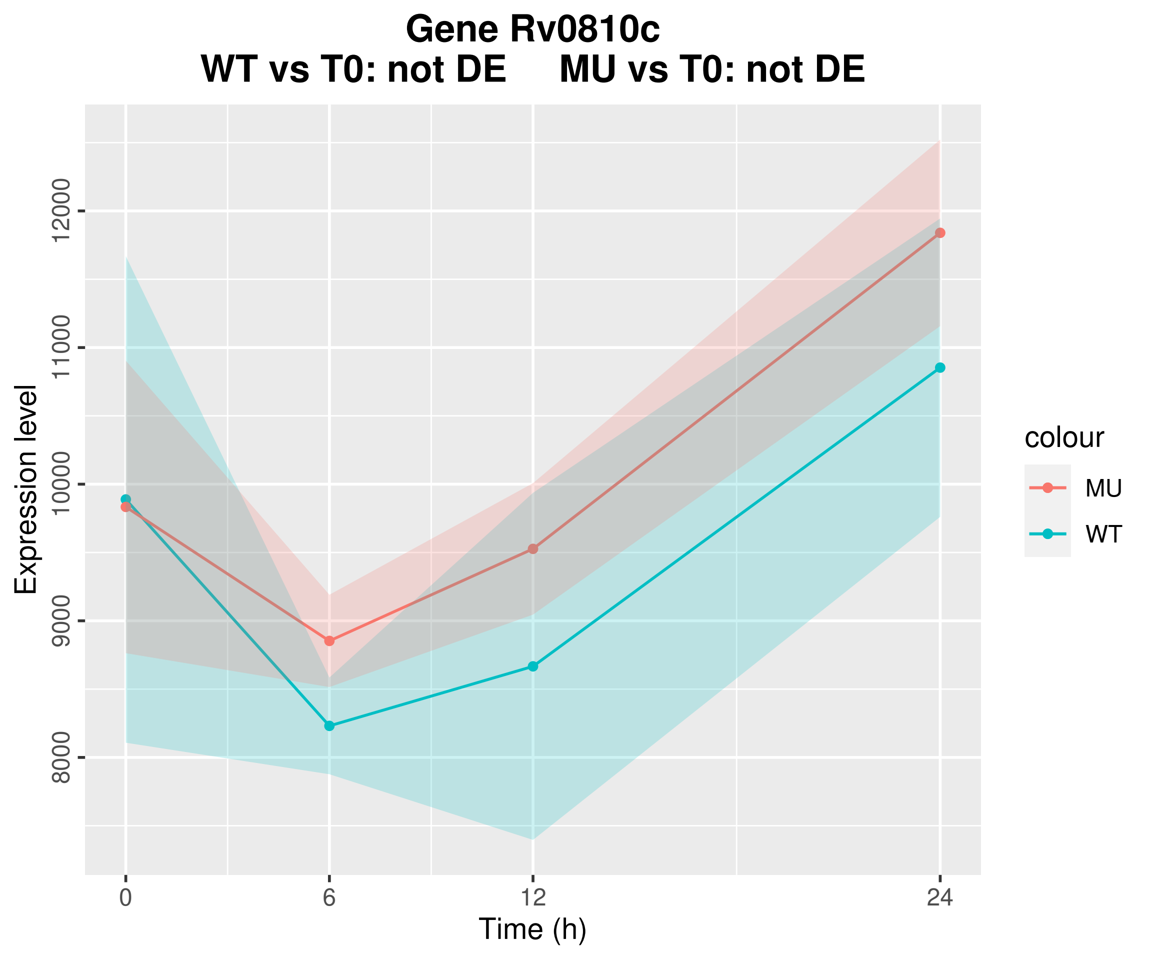

Gene Rv0810c
WT vs T0: not DE MU vs T0: not DE
Expression level
12000
11000
10000
9000
8000
0
6
12
24
Time (h)
colour
MU
WT

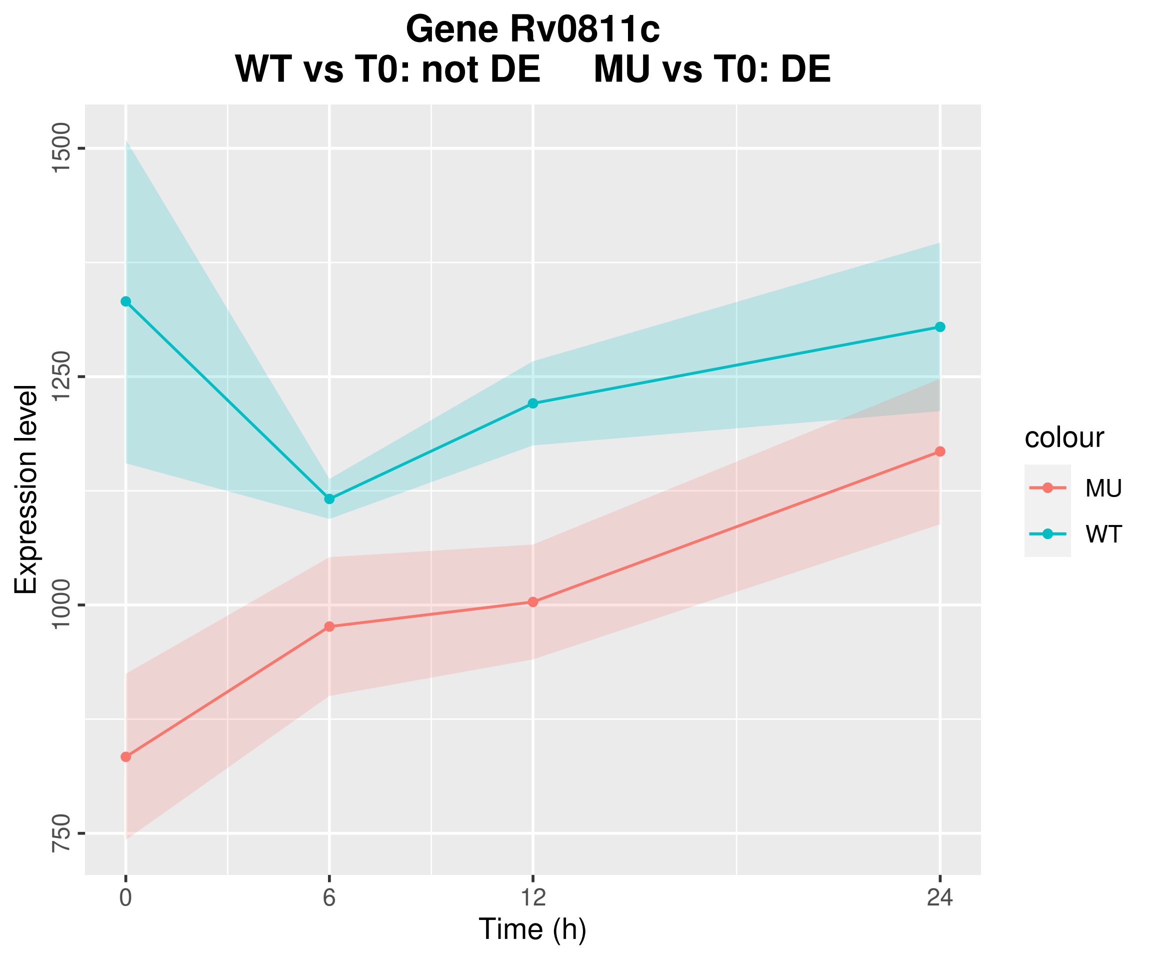

Gene Rv0811c
WT vs T0: not DE   MU vs T0: DE
Expression level
1500
1250
1000
750
0
6
12
24
Time (h)
colour
MU
WT

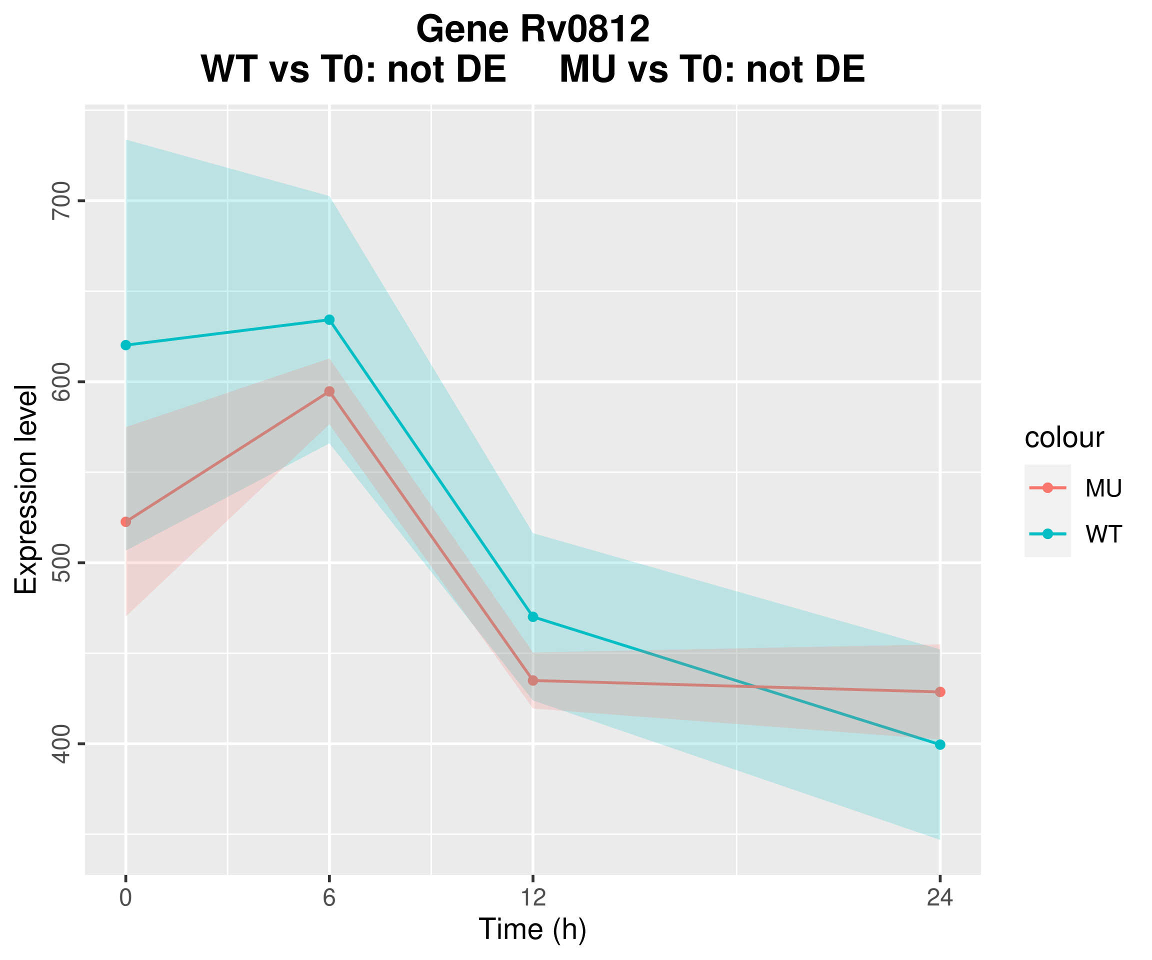
Gene Rv0812
WT vs T0: not DE   MU vs T0: not DE
Expression level
700
600
500
400
0
6
12
24
Time (h)
colour
MU
WT

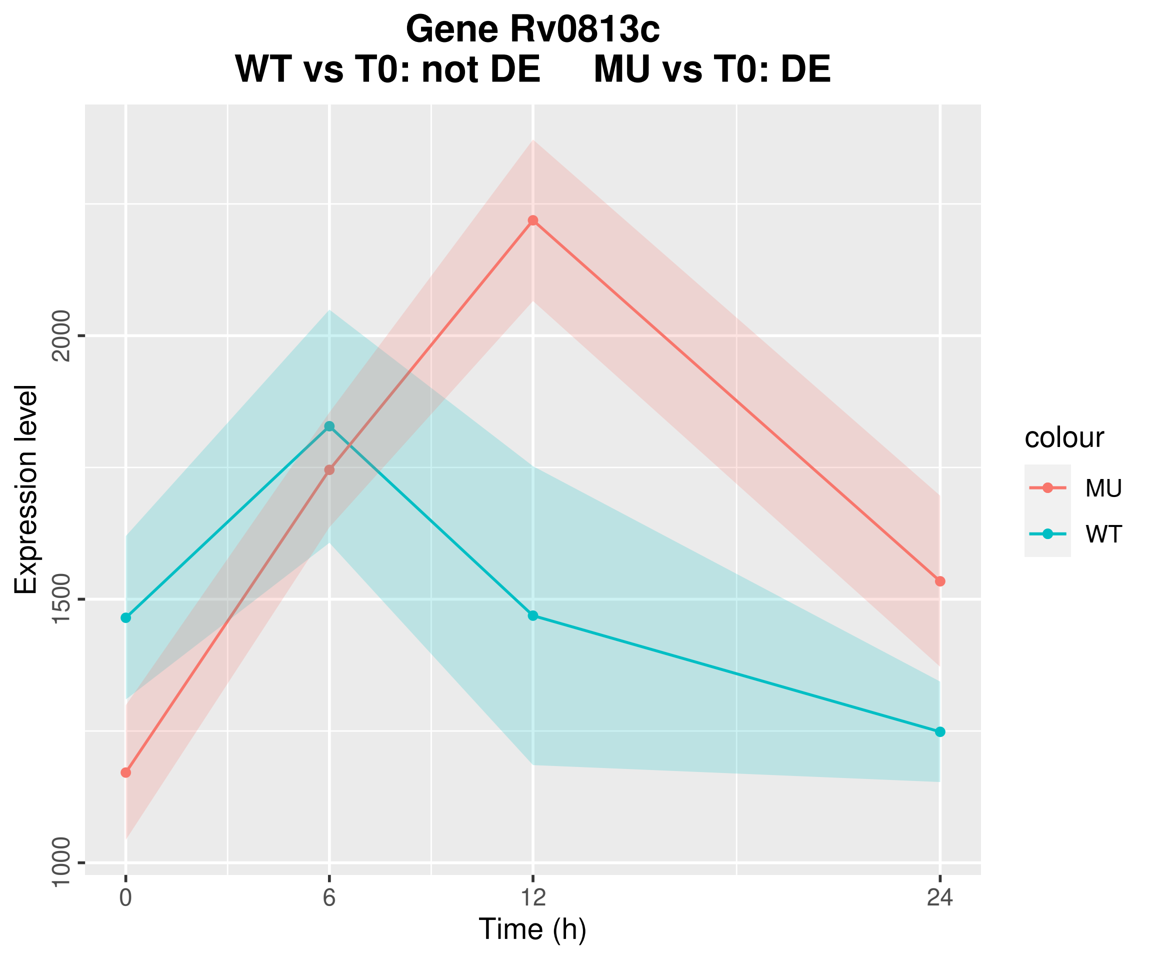

Gene Rv0813c
WT vs T0: not DE MU vs T0: DE
Expression level
1000
1500
2000
0
6
12
24
Time (h)
colour
MU
WT

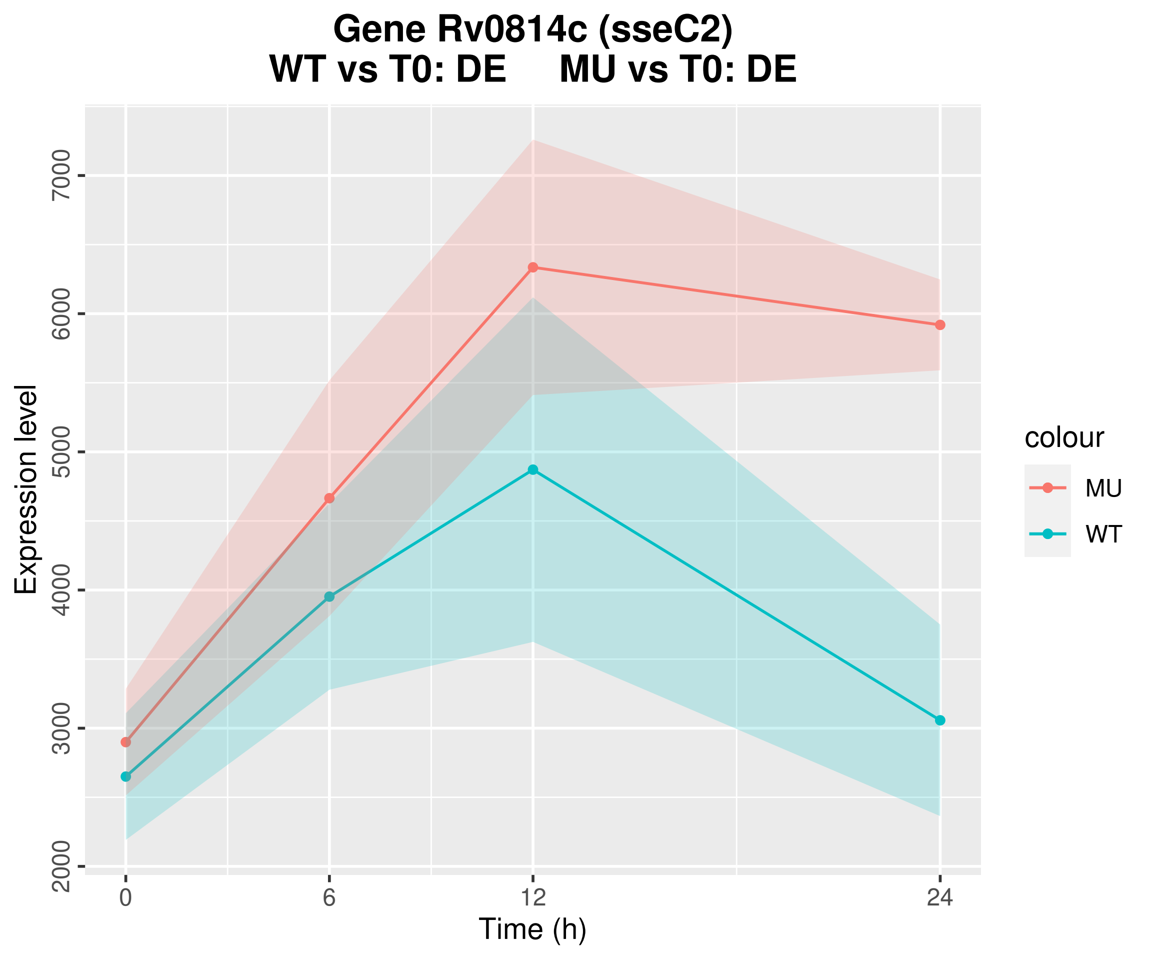

Gene Rv0814c (sseC2)
WT vs T0: DE MU vs T0: DE
Expression level
7000
6000
5000
4000
3000
2000
0
6
12
24
Time (h)
colour
MU
WT

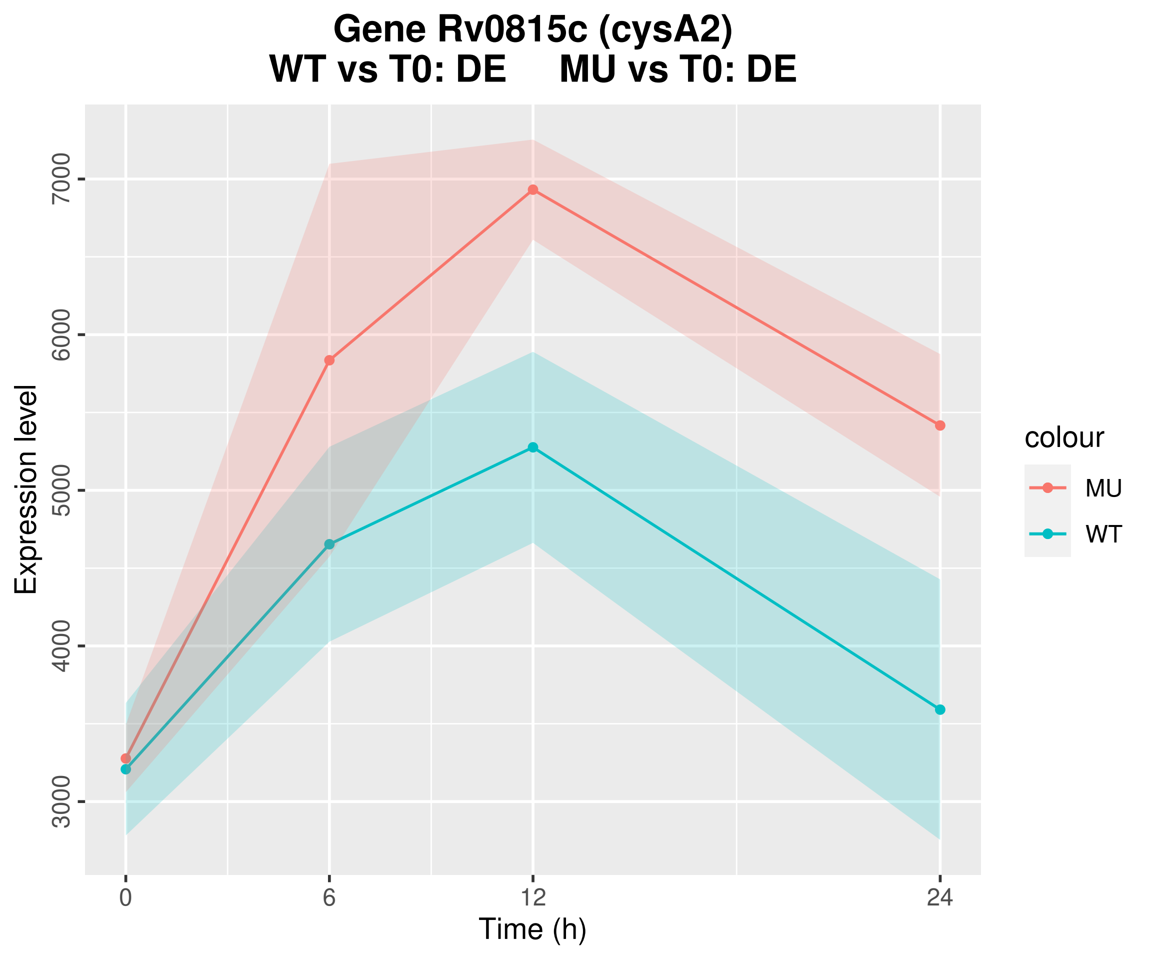

Gene Rv0815c (cysA2)
WT vs T0: DE MU vs T0: DE
Expression level
3000
4000
5000
6000
7000
0
6
12
24
Time (h)
colour
MU
WT

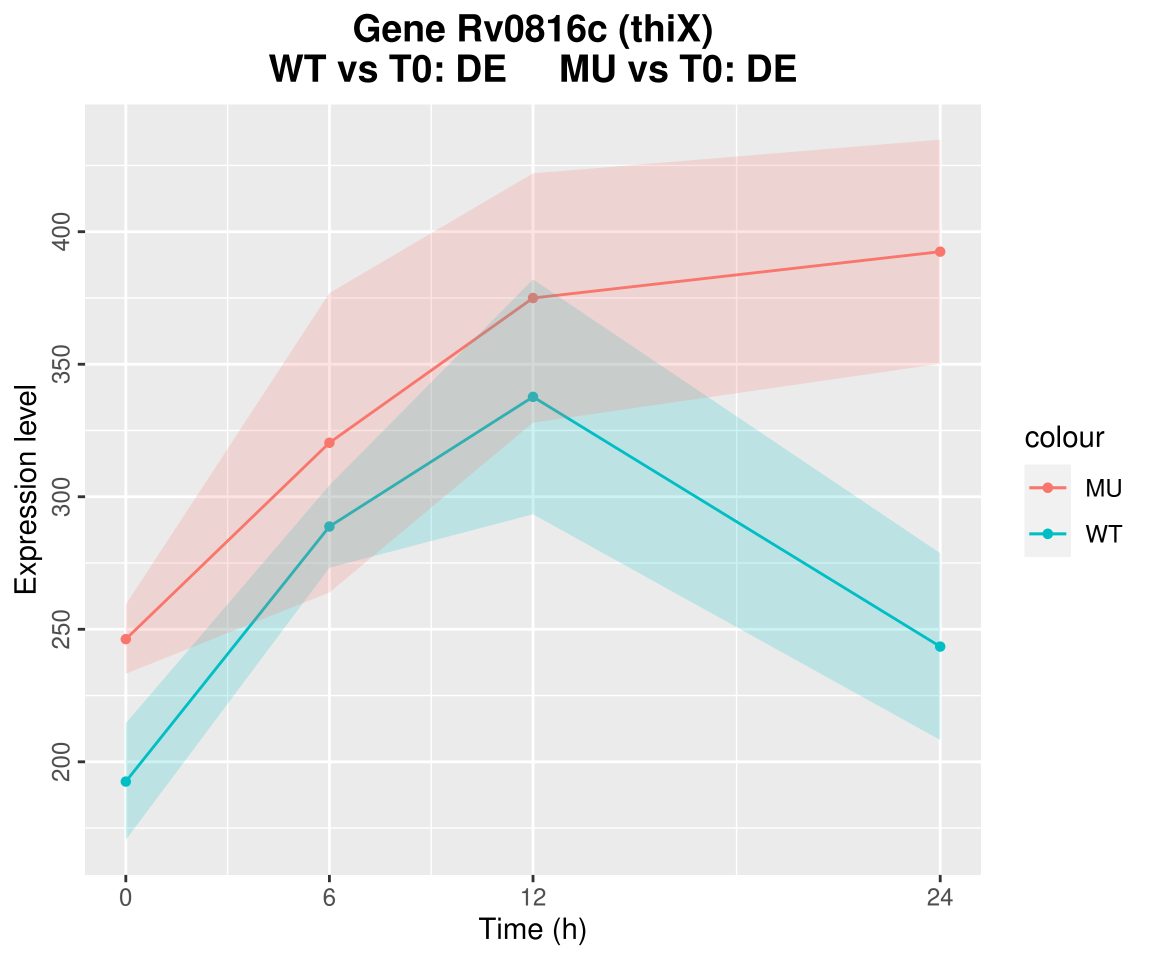

Gene Rv0816c (thiX)
WT vs T0: DE MU vs T0: DE
Expression level
400
350
300
250
200
0
6
12
24
Time (h)
colour
MU
WT

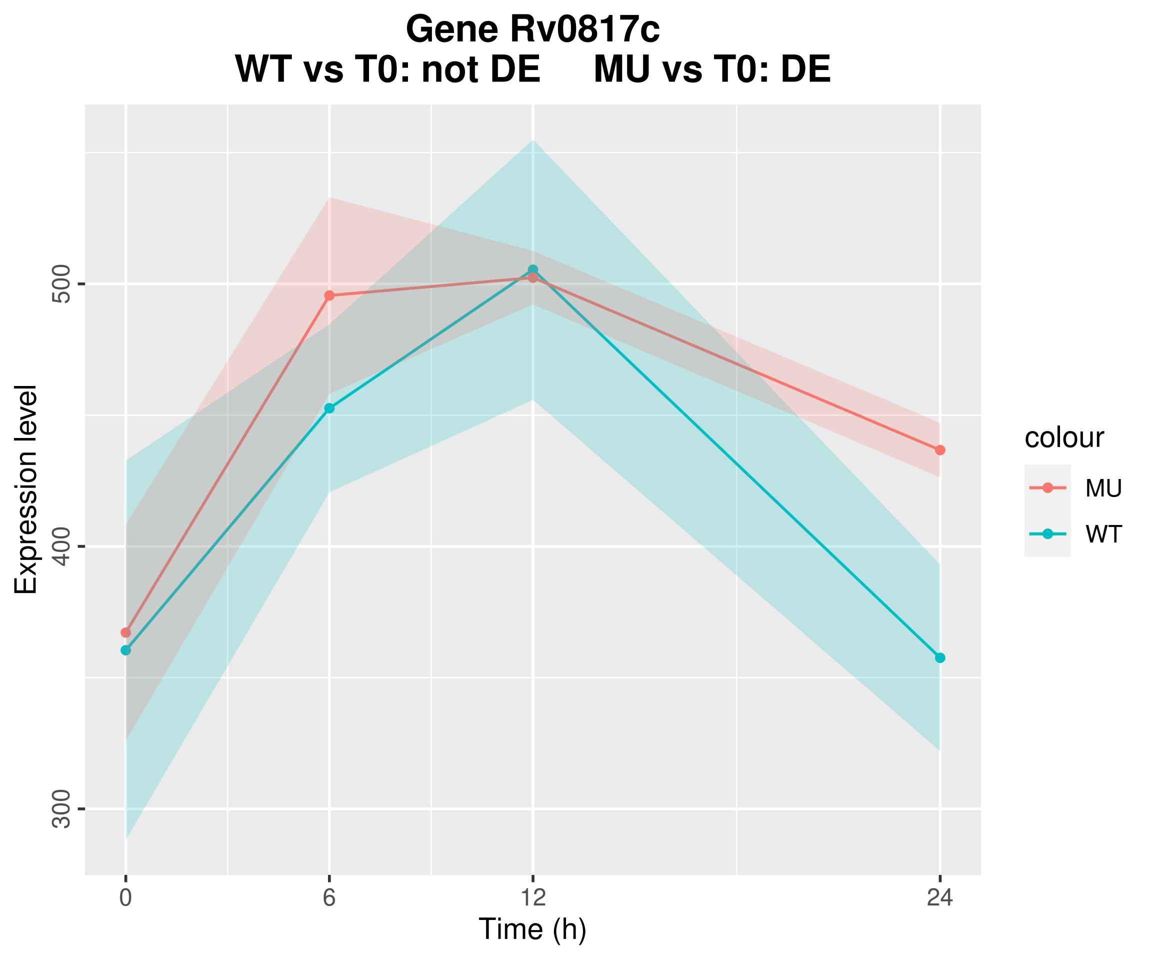

Gene Rv0817c
WT vs T0: not DE   MU vs T0: DE
Expression level
300
400
500
0
6
12
24
Time (h)
colour
MU
WT

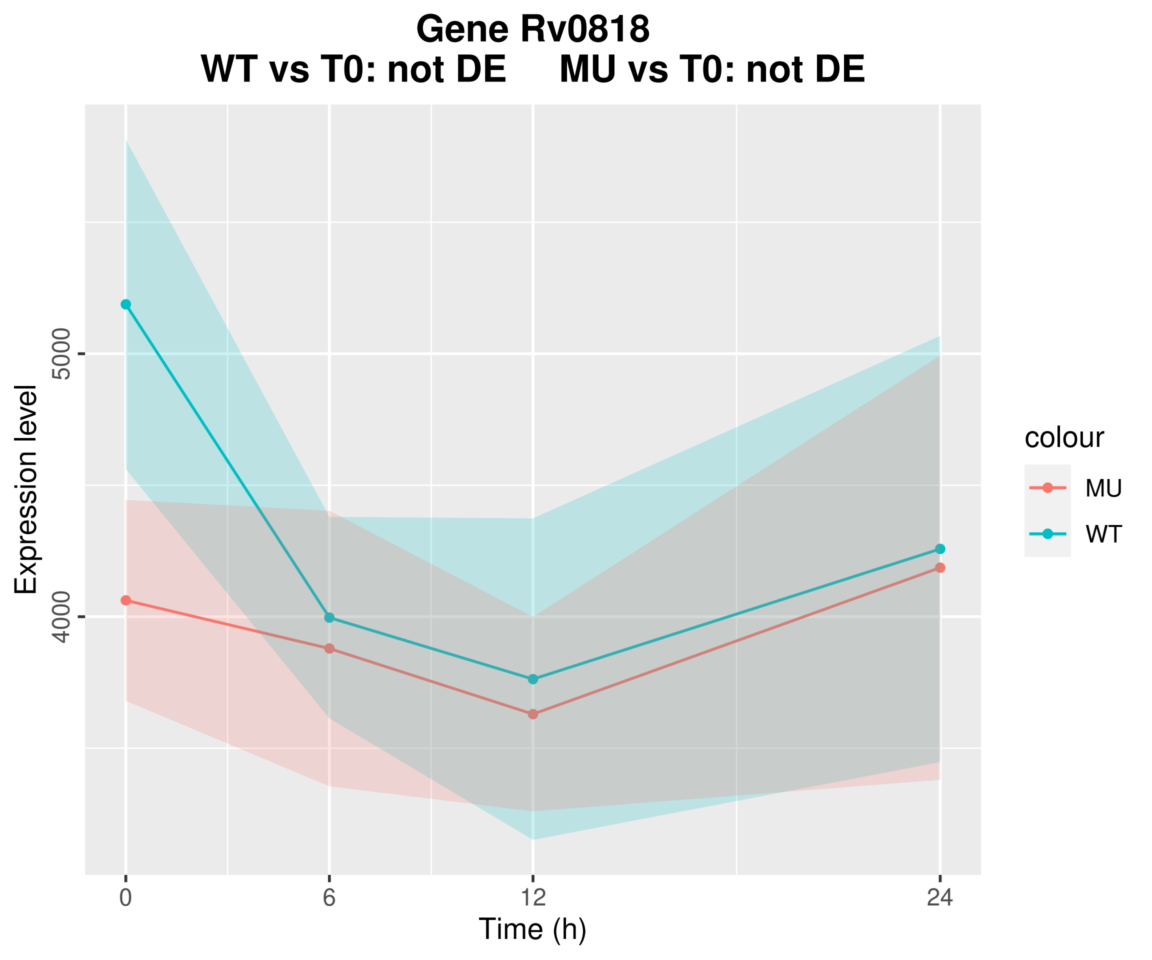

Gene Rv0818
WT vs T0: not DE   MU vs T0: not DE
Expression level
5000
4000
0
6
12
24
Time (h)
colour
MU
WT

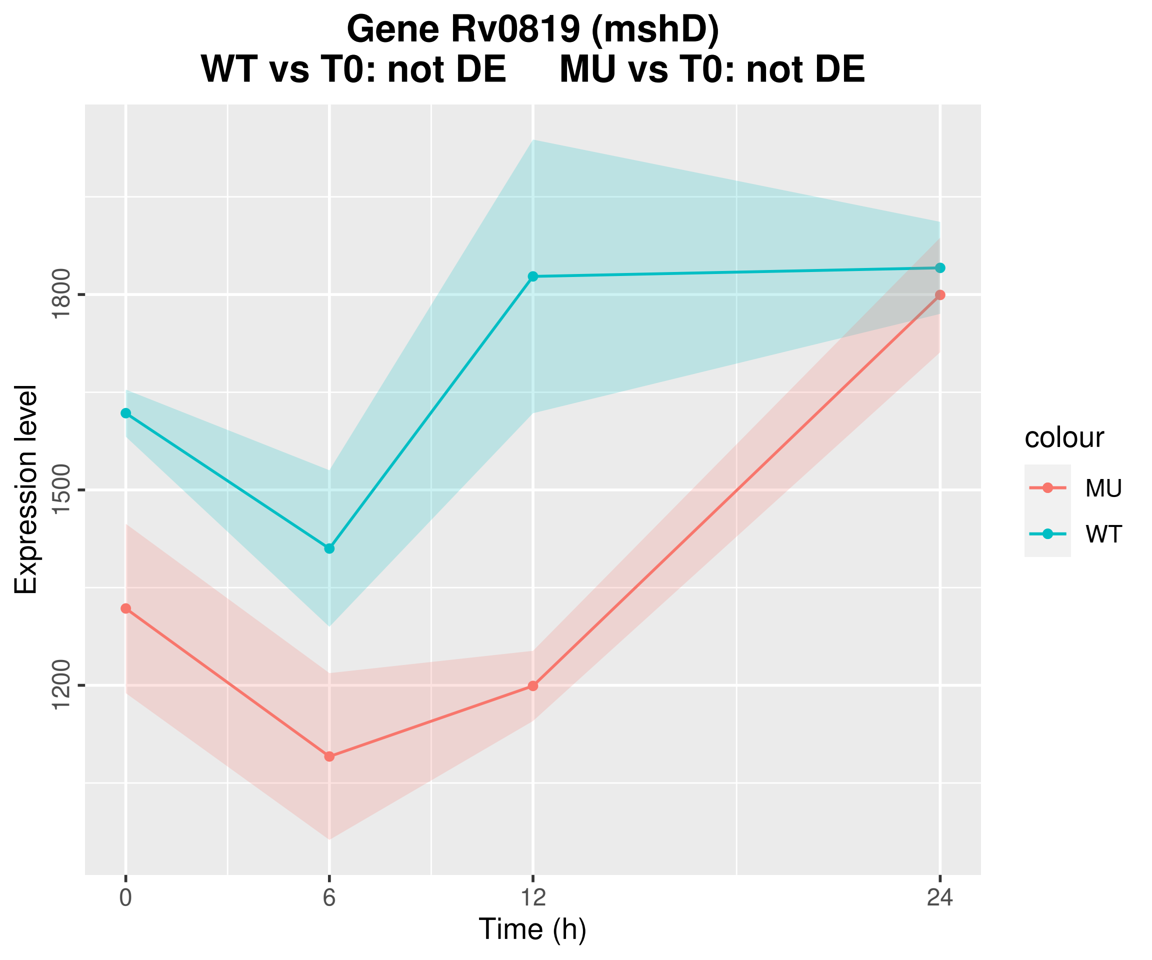

Gene Rv0819 (mshD)
WT vs T0: not DE   MU vs T0: not DE
Expression level
1200
1500
1800
0
6
12
24
Time (h)
colour
MU
WT

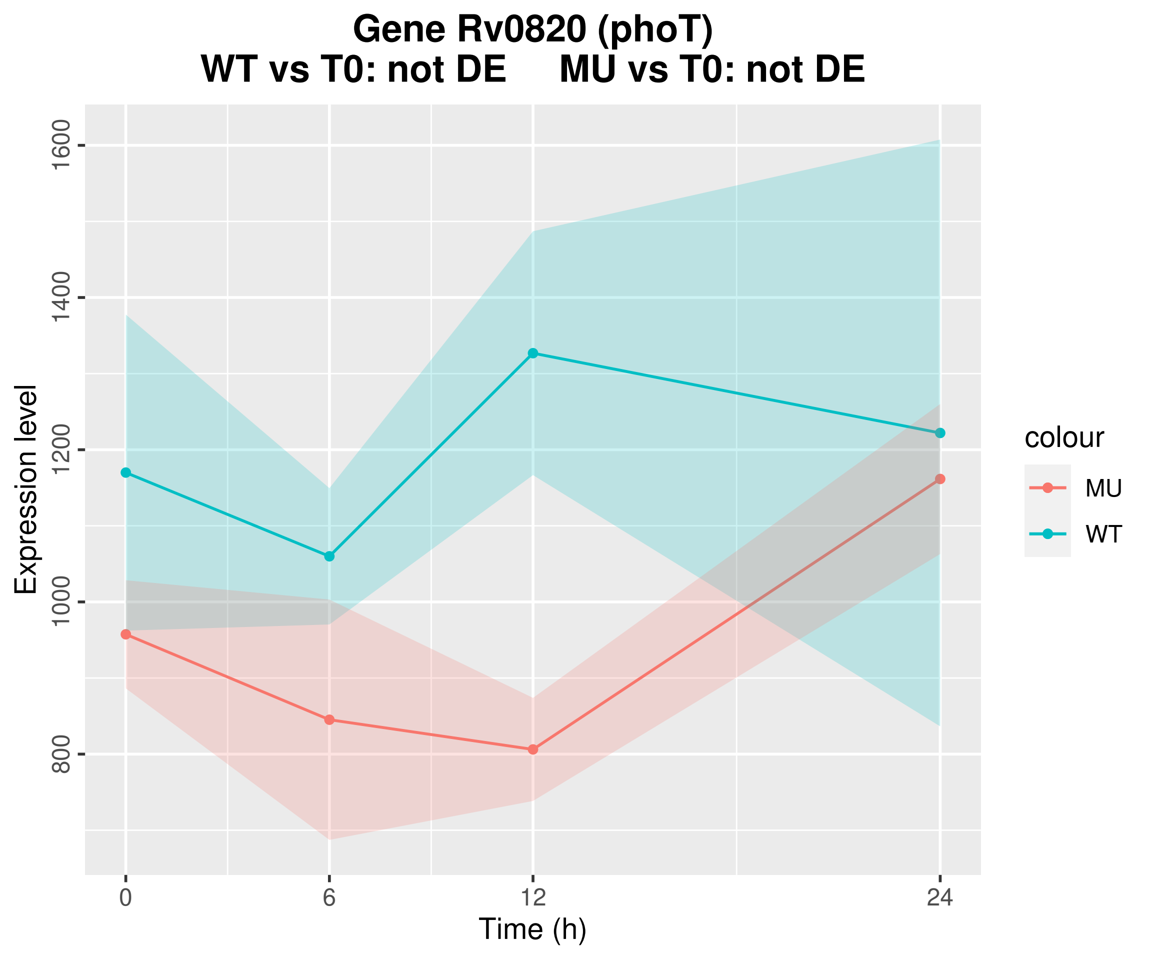
Gene Rv0820 (phoT)
WT vs T0: not DE   MU vs T0: not DE
Expression level
1600
1400
1200
1000
800
0
6
12
24
Time (h)
colour
MU
WT

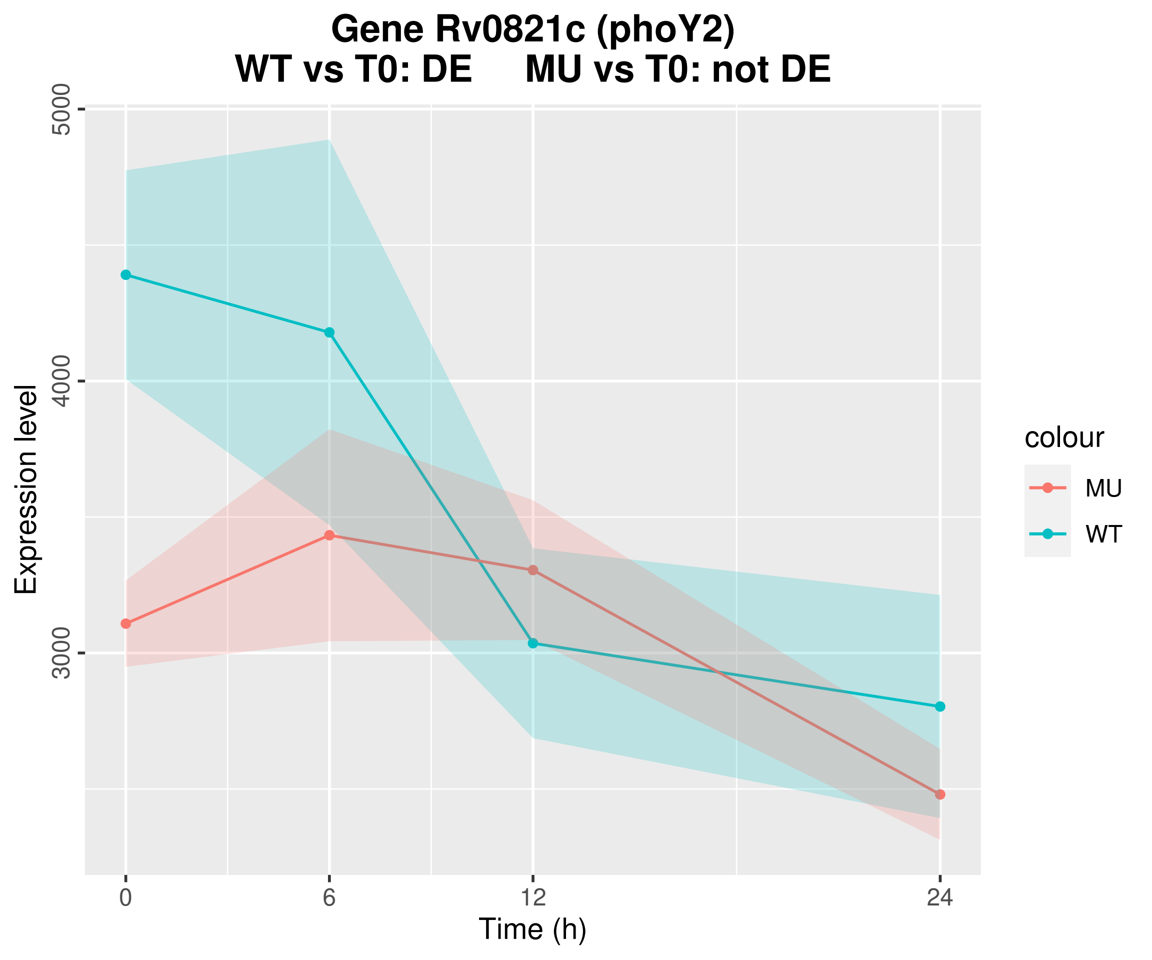

Gene Rv0821c (phoY2)
WT vs T0: DE   MU vs T0: not DE
Expression level
5000
4000
3000
Time (h)
0
6
12
24
colour
MU
WT

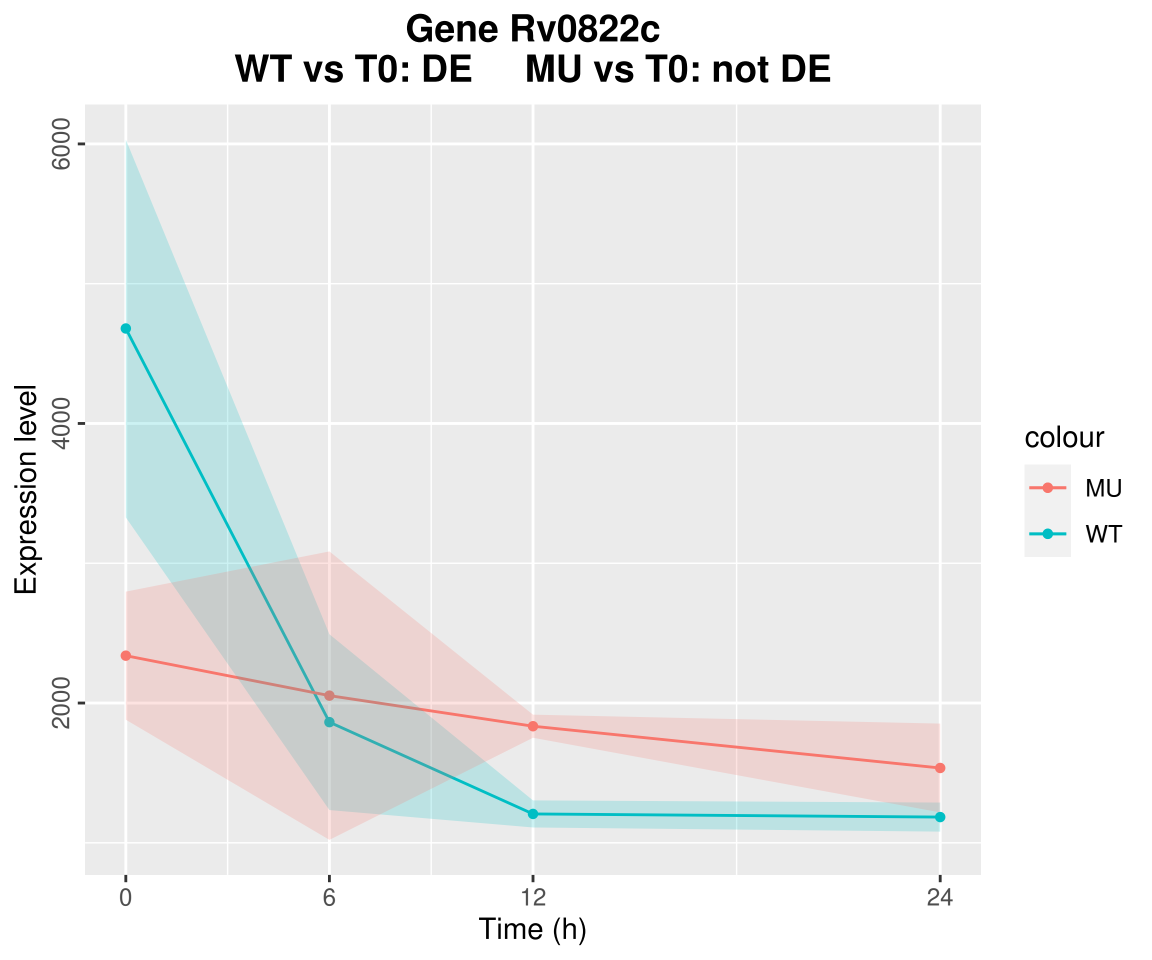
Gene Rv0822c
WT vs T0: DE MU vs T0: not DE
Expression level
6000
4000
2000
0
6
12
24
Time (h)
colour
MU
WT

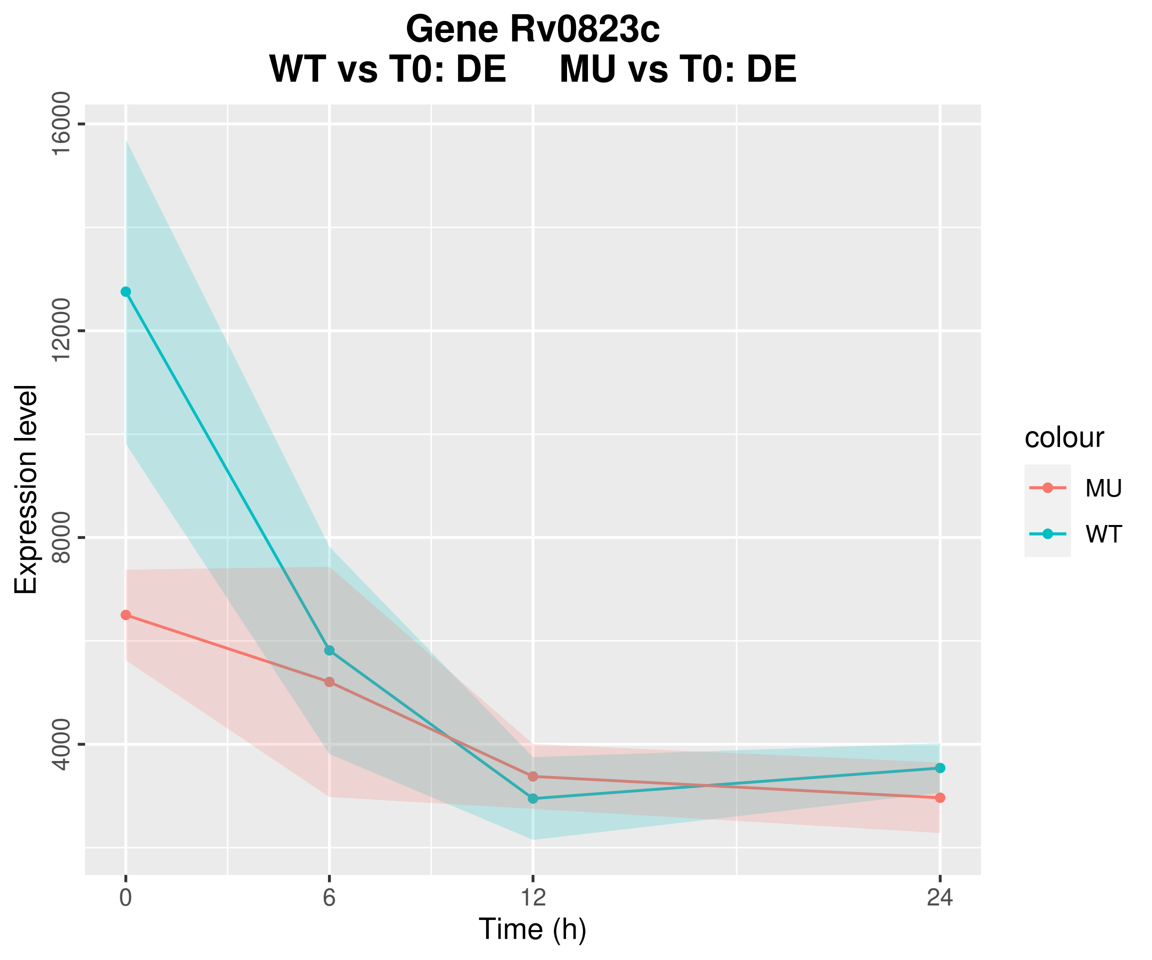

Gene Rv0823c
WT vs T0: DE   MU vs T0: DE
Expression level
16000
12000
8000
4000
0
6
12
24
Time (h)
colour
MU
WT

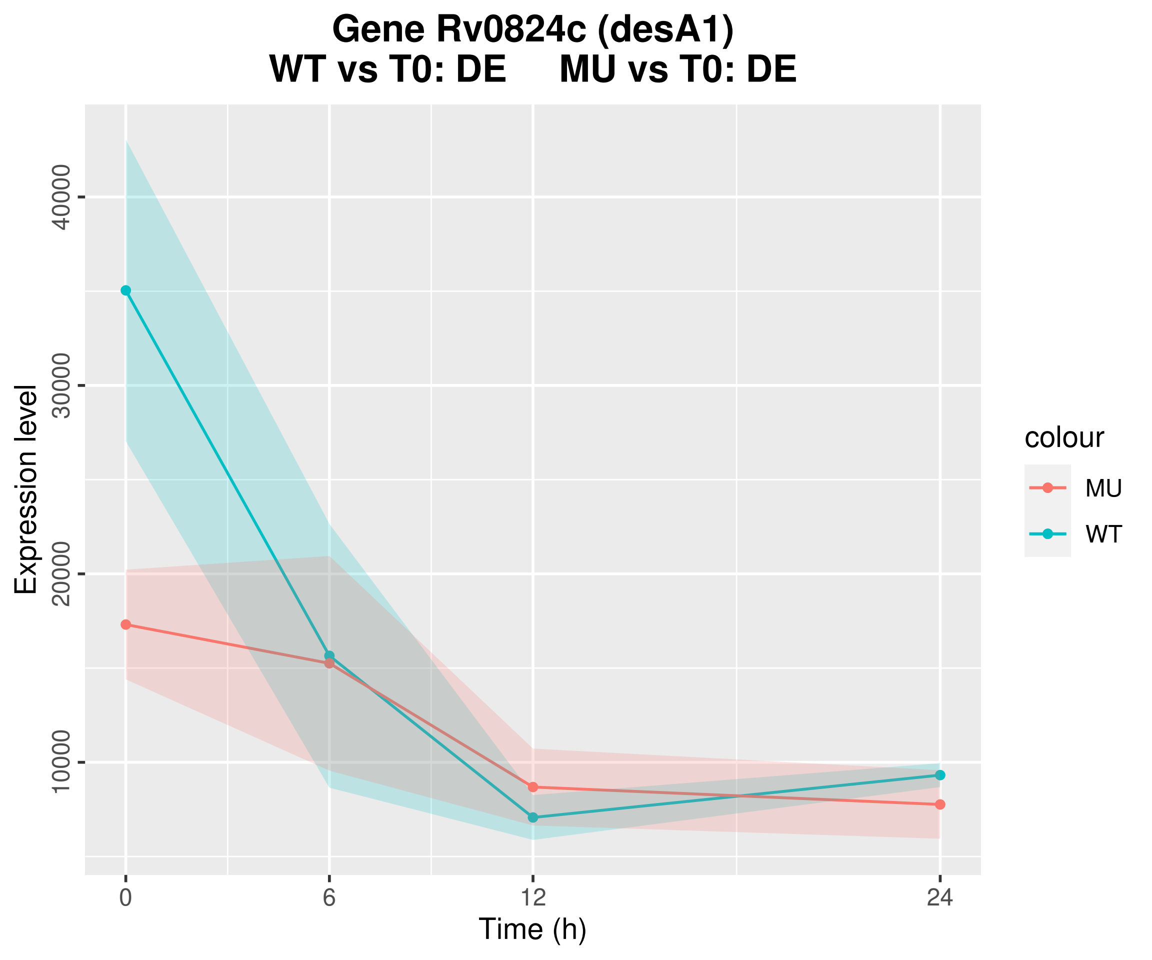

Gene Rv0824c (desA1)
WT vs T0: DE   MU vs T0: DE
Expression level
40000
30000
20000
10000
0
6
12
24
Time (h)
colour
MU
WT

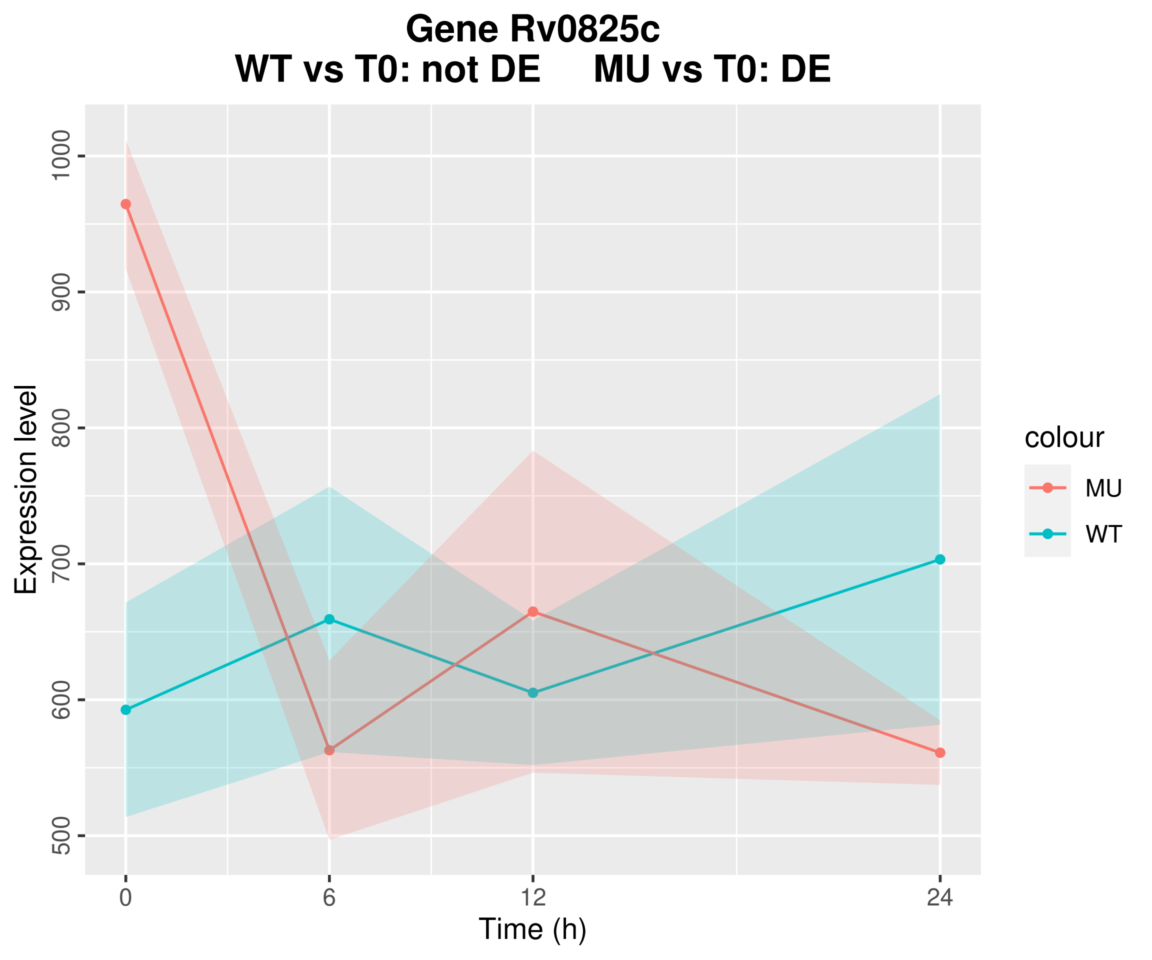

Gene Rv0825c
WT vs T0: not DE   MU vs T0: DE
Expression level
1000
900
800
700
600
500
0
6
12
24
Time (h)
colour
MU
WT

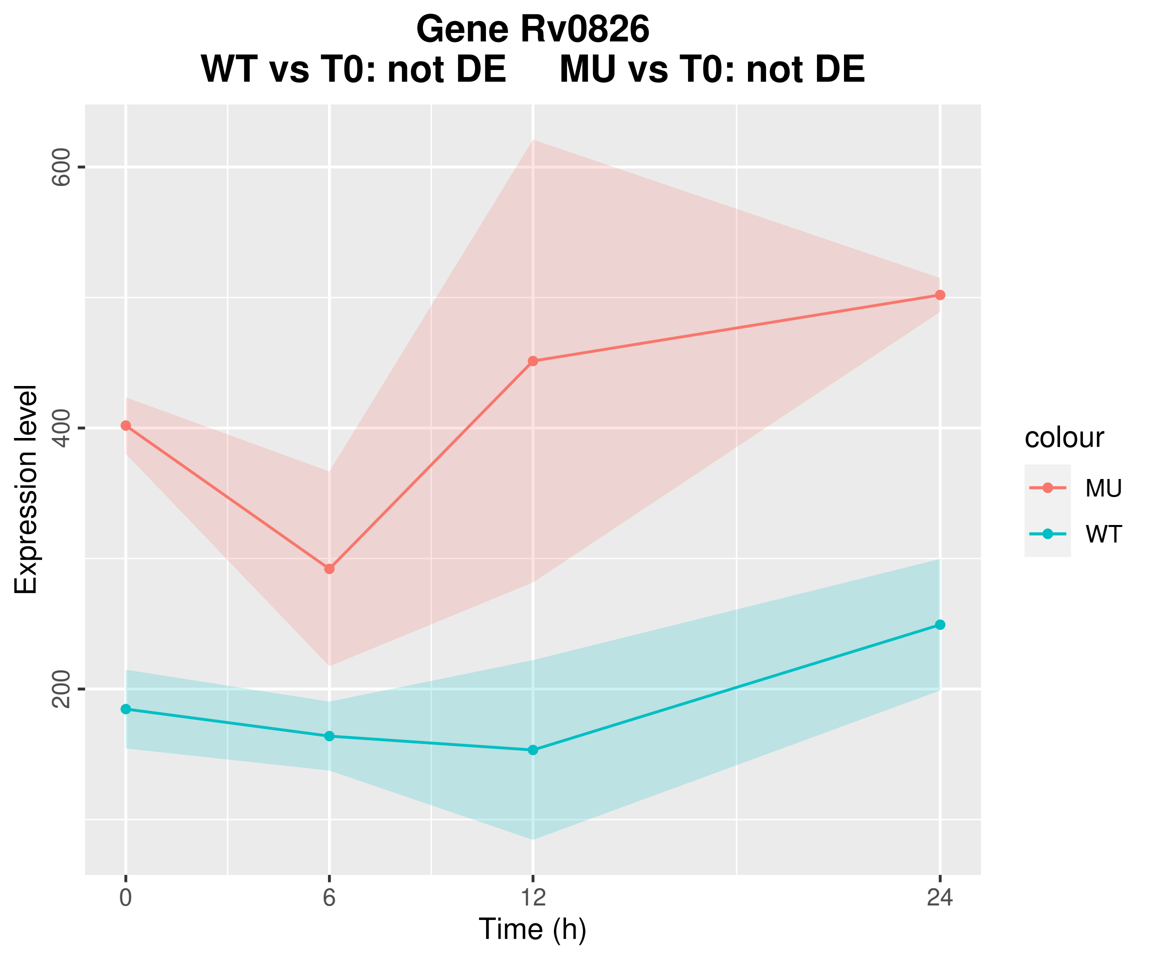
Gene Rv0826
WT vs T0: not DE   MU vs T0: not DE
Expression level
600
400
200
0
6
12
24
Time (h)
colour
MU
WT

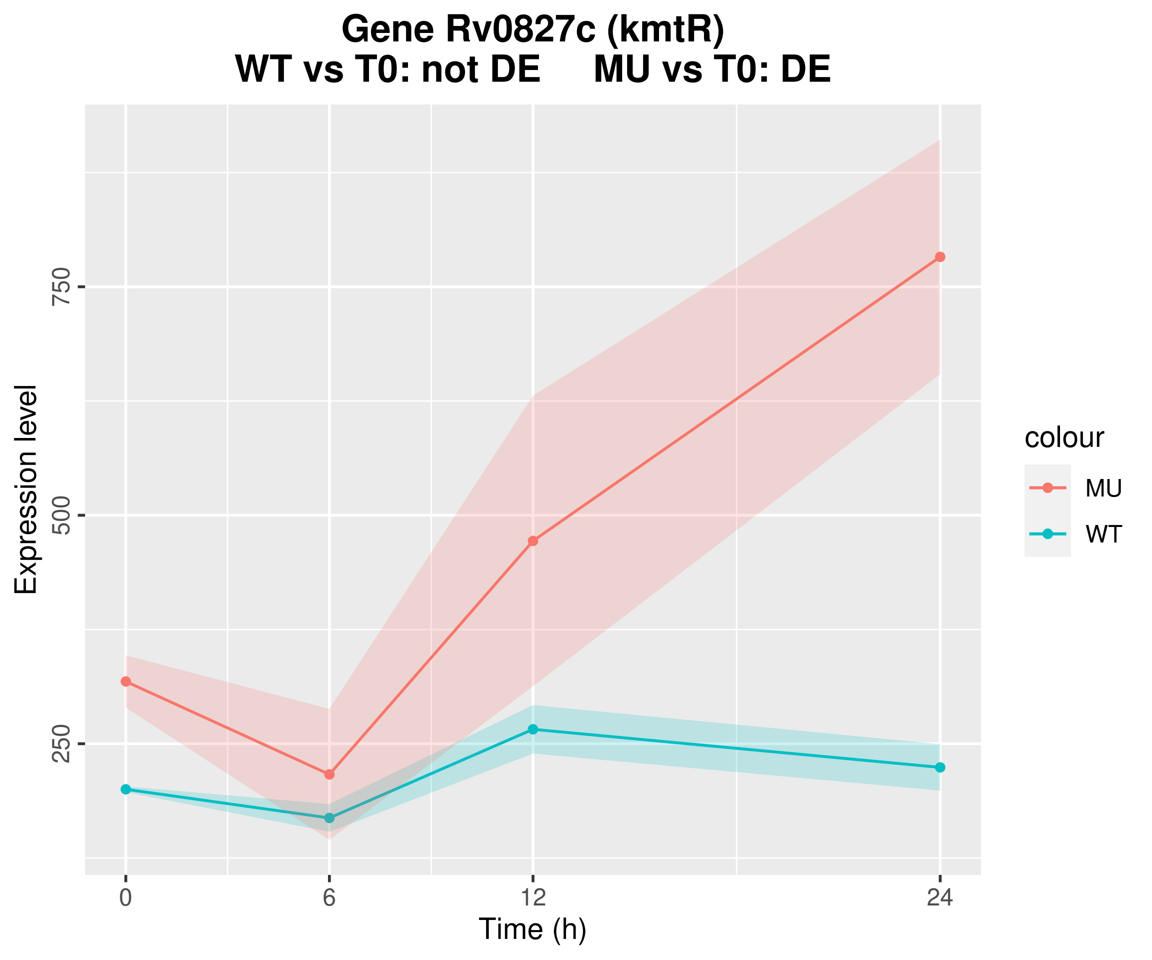
Gene Rv0827c (kmtR)
WT vs T0: not DE MU vs T0: DE
Expression level
750
500
250
0
6
12
24
Time (h)
colour
MU
WT

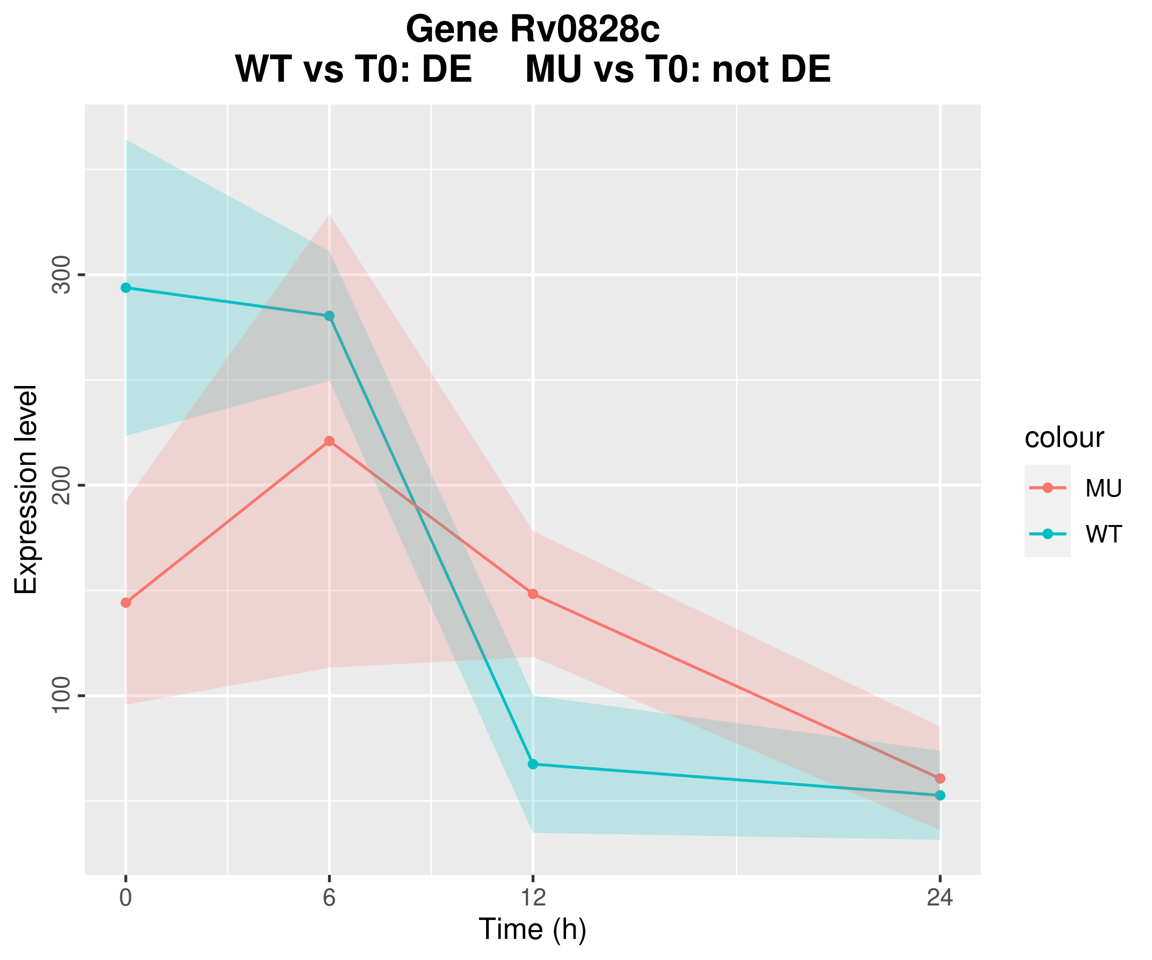

Gene Rv0828c
WT vs T0: DE   MU vs T0: not DE
Expression level
300
200
100
0
6
12
24
Time (h)
colour
MU
WT

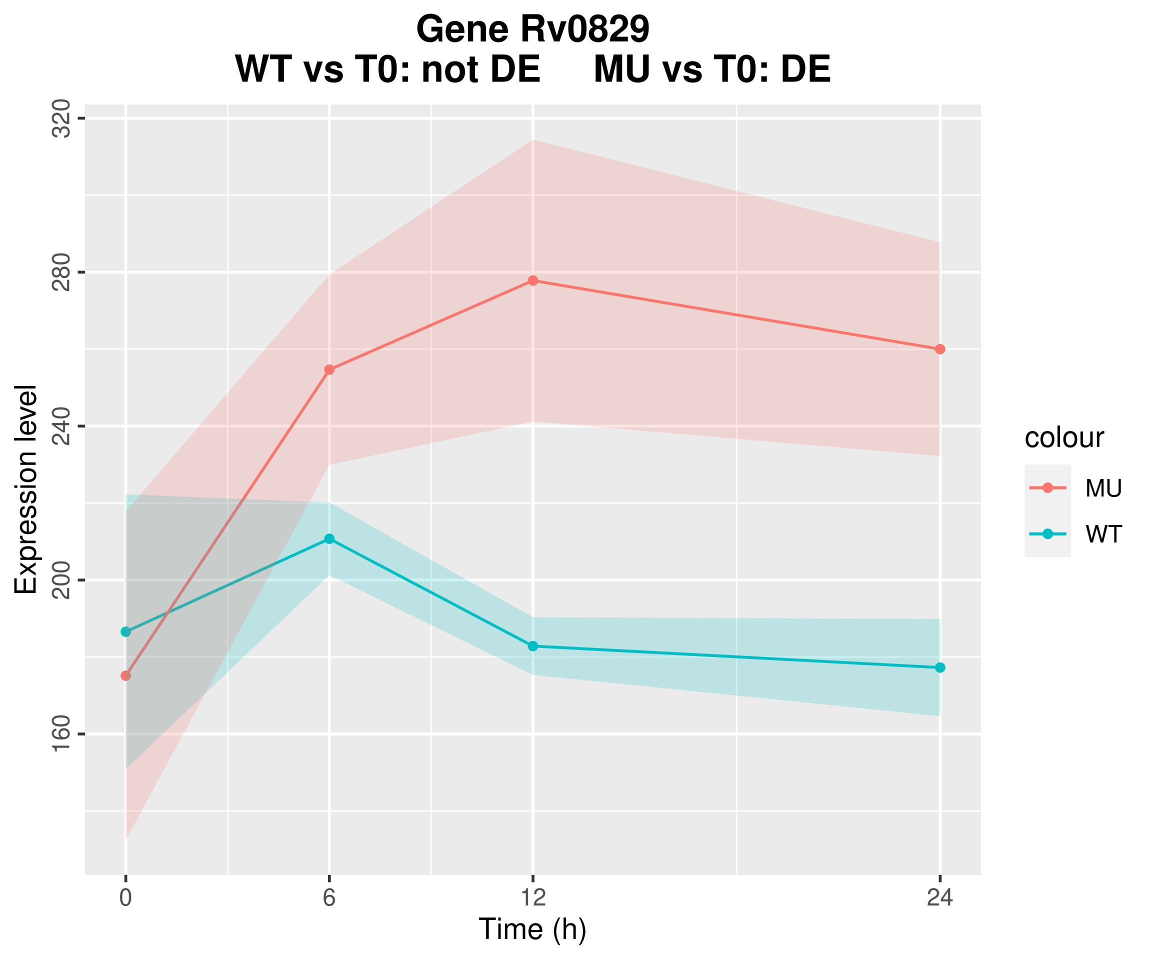

Gene Rv0829
WT vs T0: not DE MU vs T0: DE
Expression level
160
200
240
280
320
Time (h)
0
6
12
24
colour
MU
WT

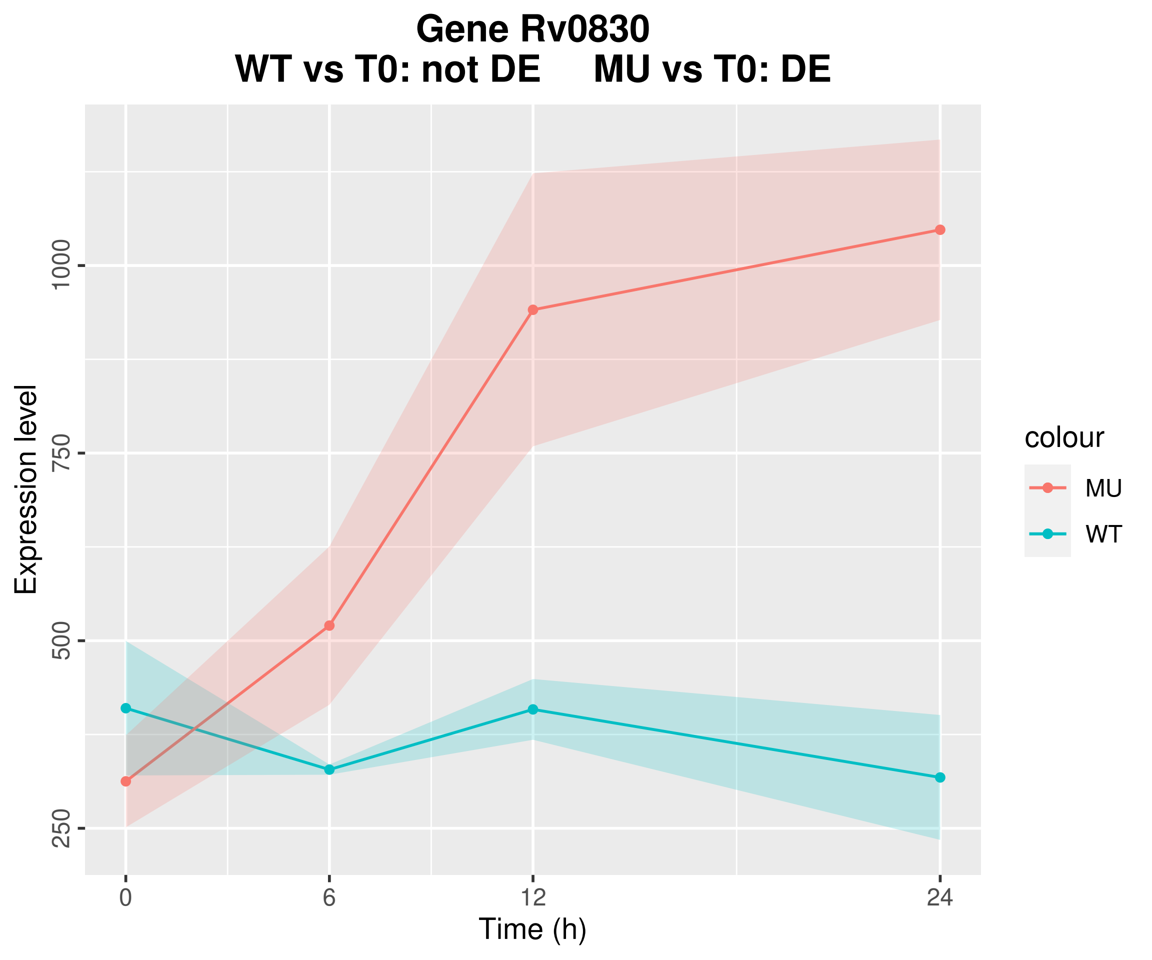
Gene Rv0830
WT vs T0: not DE   MU vs T0: DE
Expression level
1000
750
500
250
0
6
12
24
Time (h)
colour
MU
WT

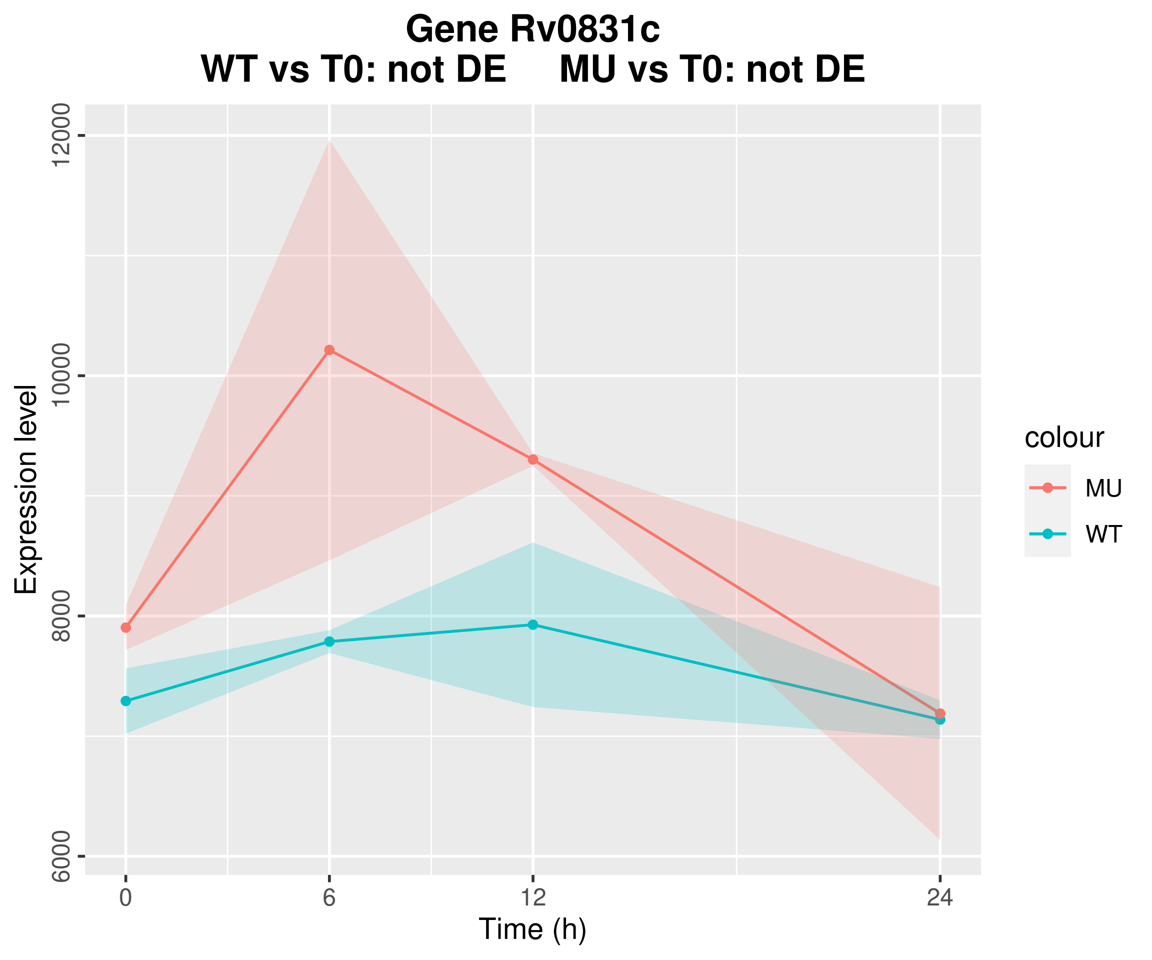

Gene Rv0831c
WT vs T0: not DE   MU vs T0: not DE
Expression level
12000
10000
8000
6000
0
6
12
24
Time (h)
colour
MU
WT

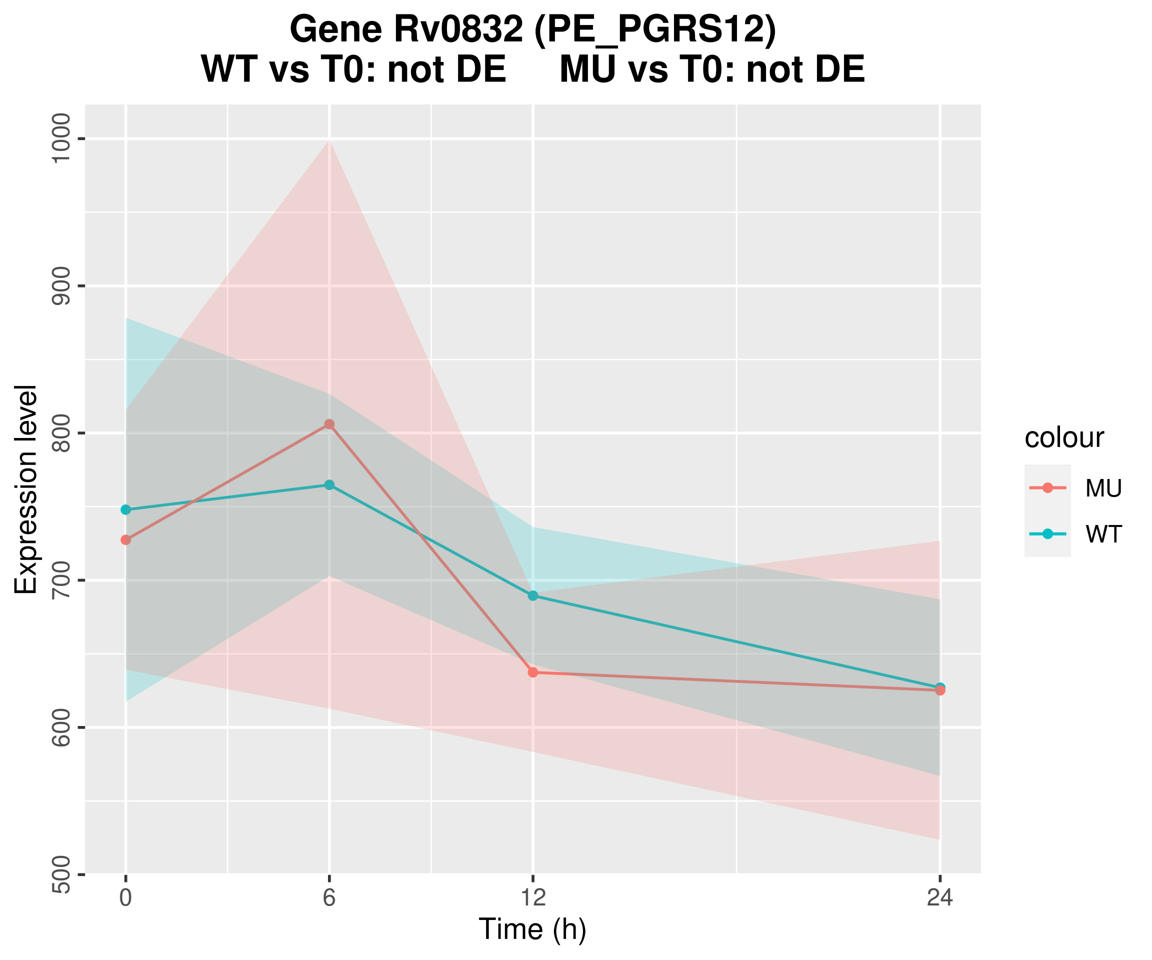

Gene Rv0832 (PE_PGRS12)
WT vs T0: not DE MU vs T0: not DE
Expression level
1000
900
800
700
600
500
0
6
12
24
Time (h)
colour
MU
WT

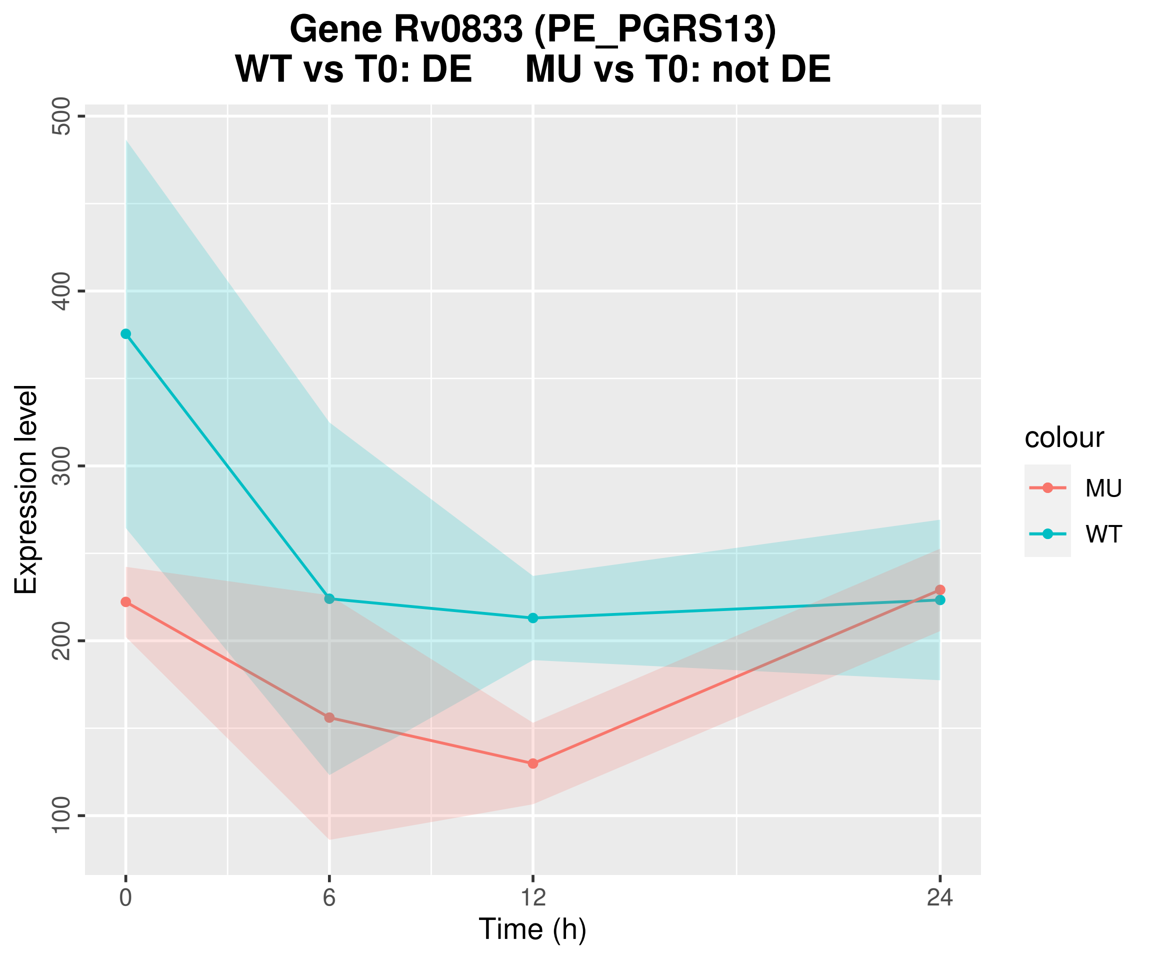

Gene Rv0833 (PE_PGRS13)
WT vs T0: DE MU vs T0: not DE
500
400
300
200
100
Expression level
0
6
12
24
Time (h)
colour
MU
WT

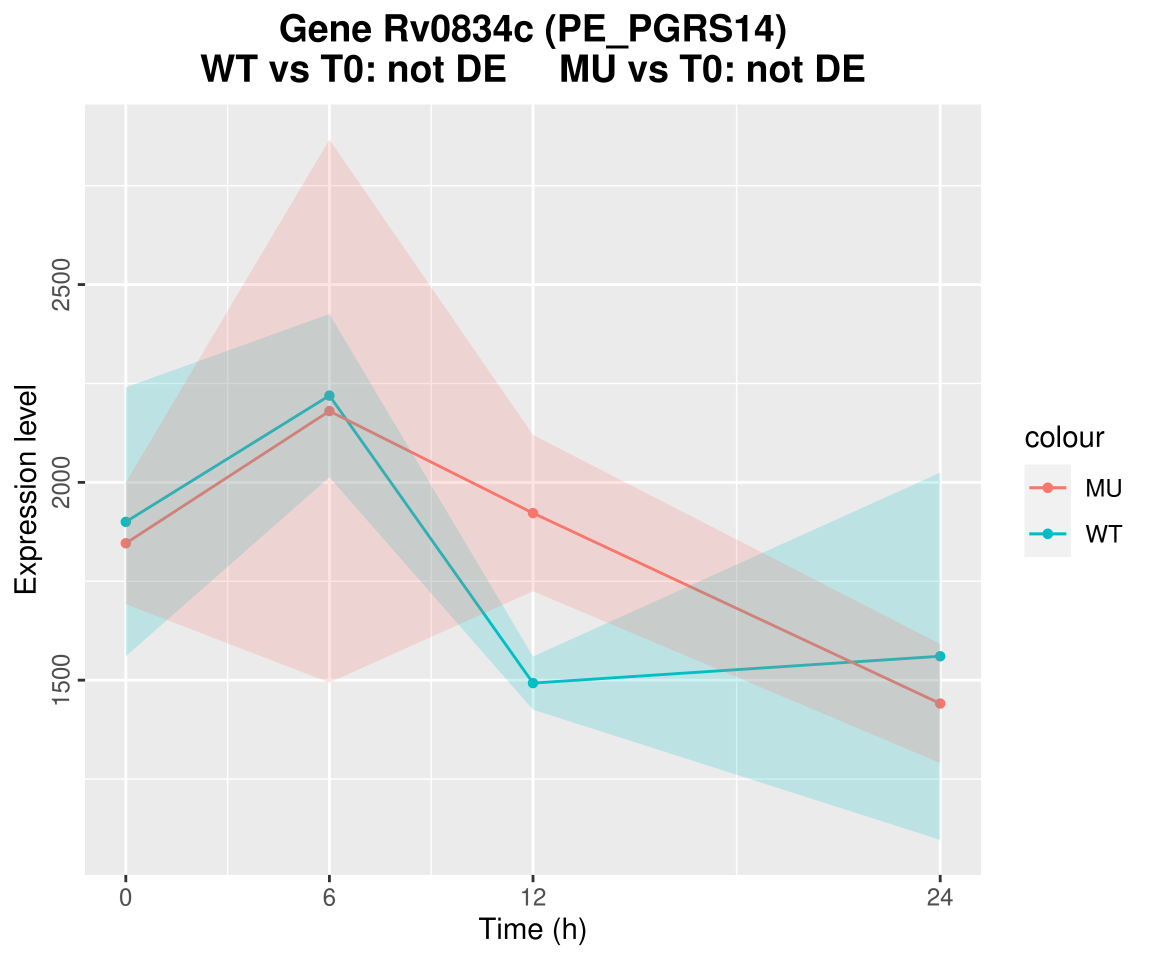

Gene Rv0834c (PE_PGRS14)
WT vs T0: not DE   MU vs T0: not DE
Expression level
2500
2000
1500
0
6
12
24
Time (h)
colour
MU
WT

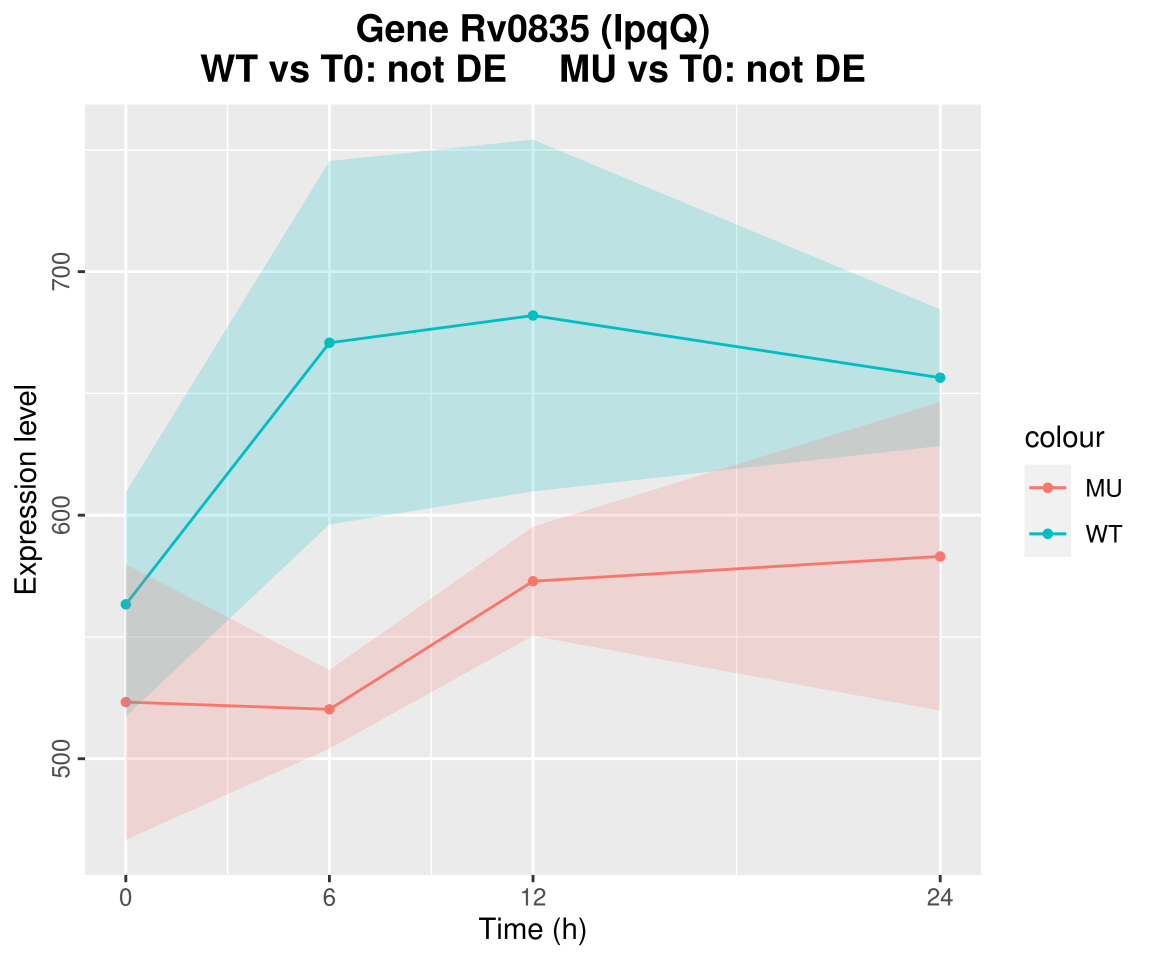

Gene Rv0835 (lpqQ)
WT vs T0: not DE   MU vs T0: not DE
700
600
500
Expression level
0
6
12
24
Time (h)
colour
MU
WT

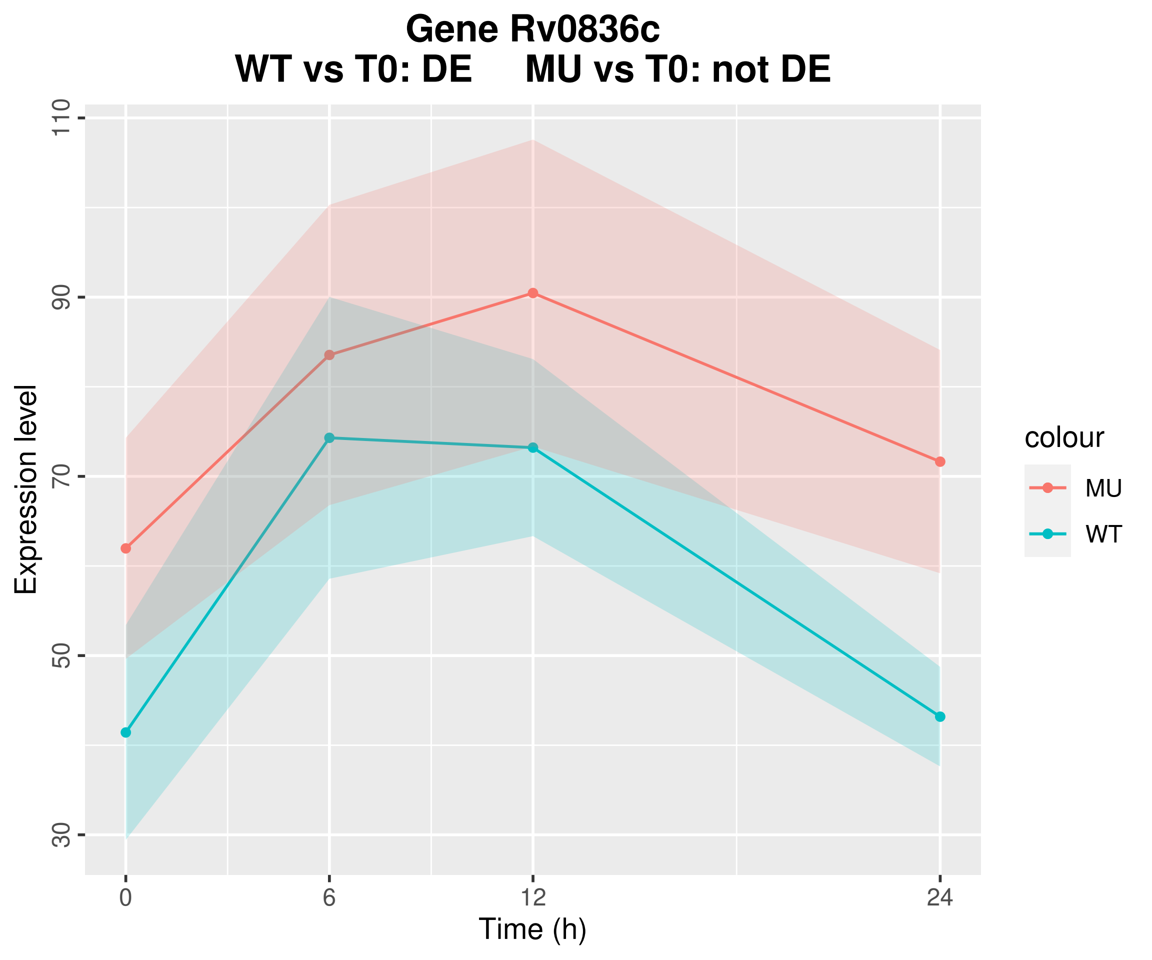
Gene Rv0836c
WT vs T0: DE MU vs T0: not DE
Expression level
110
90
70
50
30
0
6
12
24
Time (h)
colour
MU
WT

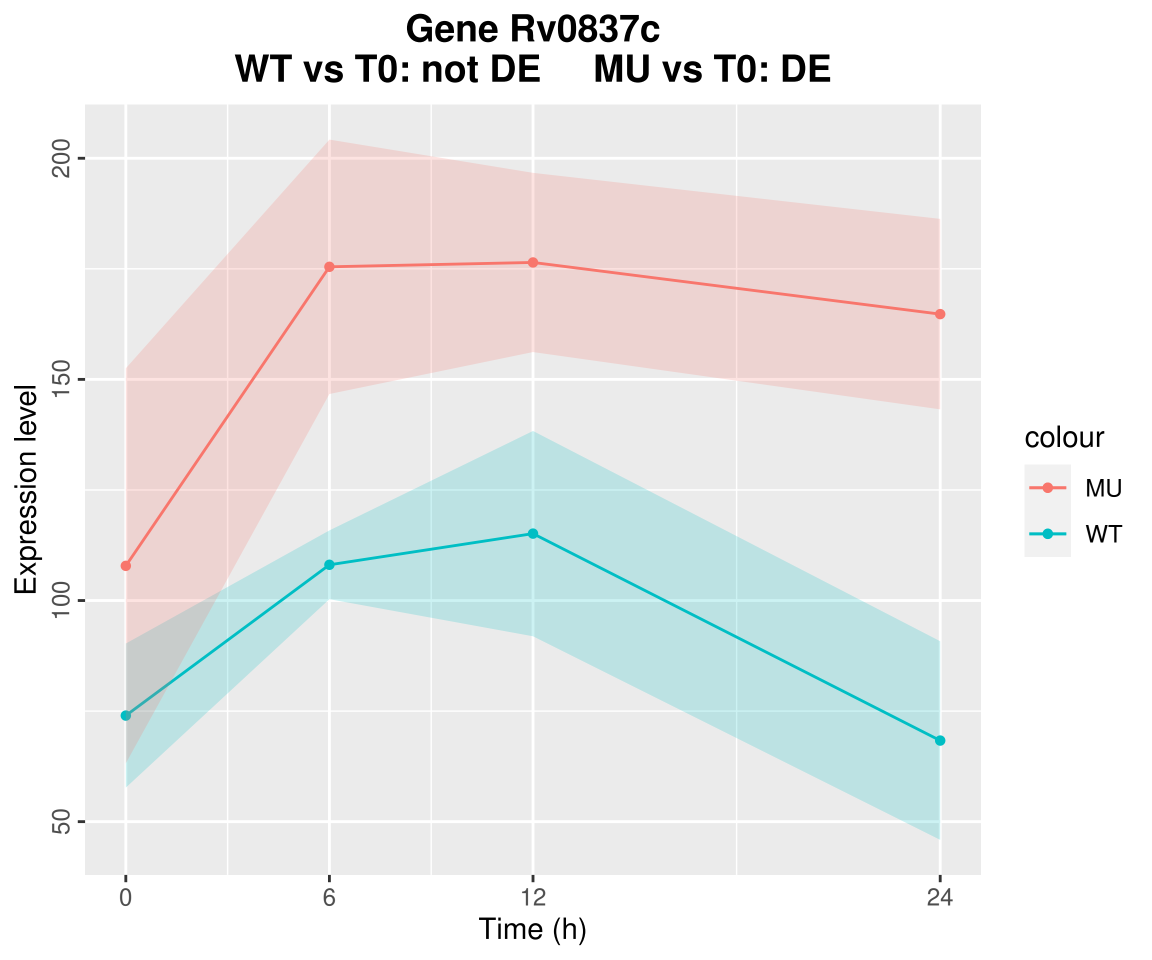

Gene Rv0837c
WT vs T0: not DE MU vs T0: DE
Expression level
200
150
100
50
0
6
12
24
Time (h)
colour
MU
WT

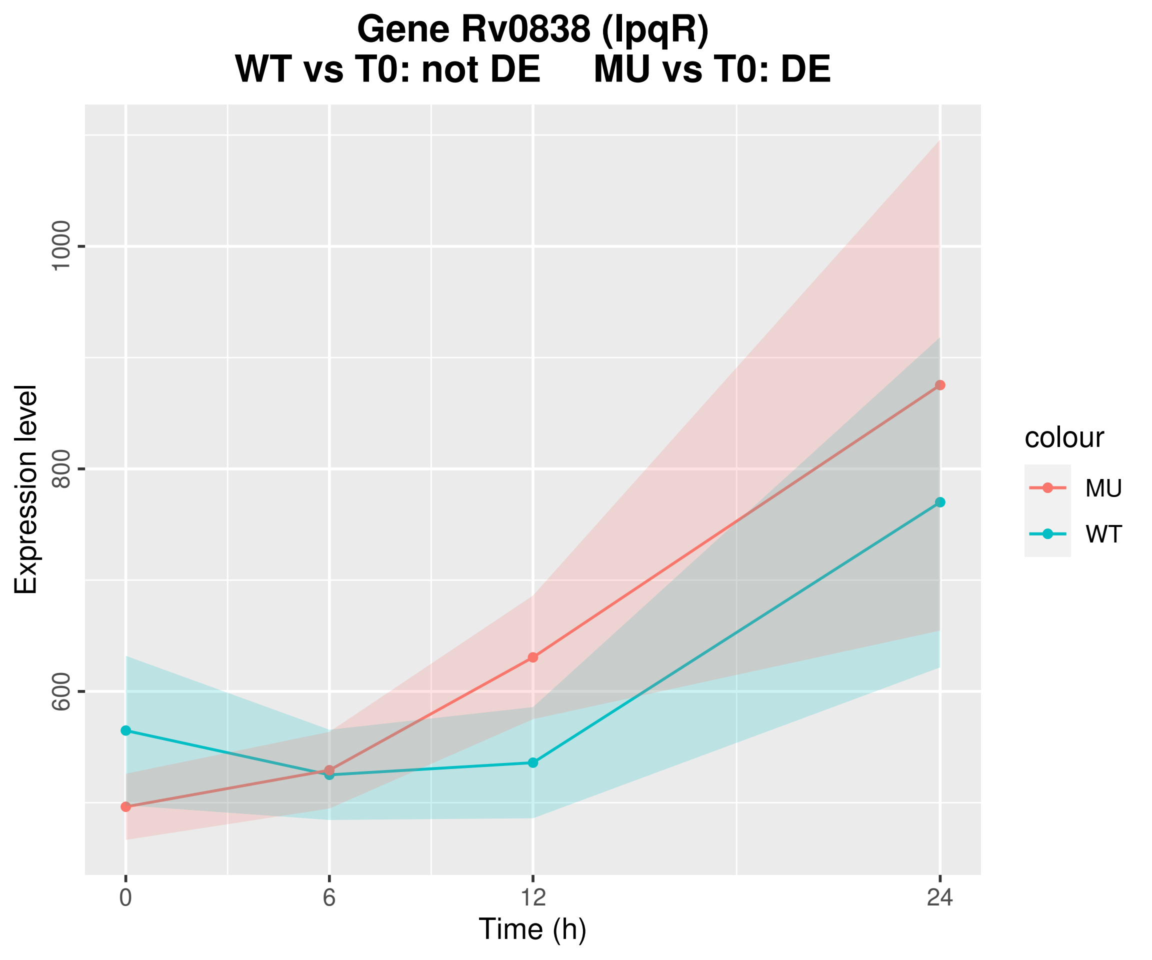

Gene Rv0838 (lpqR)
WT vs T0: not DE   MU vs T0: DE
Expression level
1000
800
600
0
6
12
24
Time (h)
colour
MU
WT

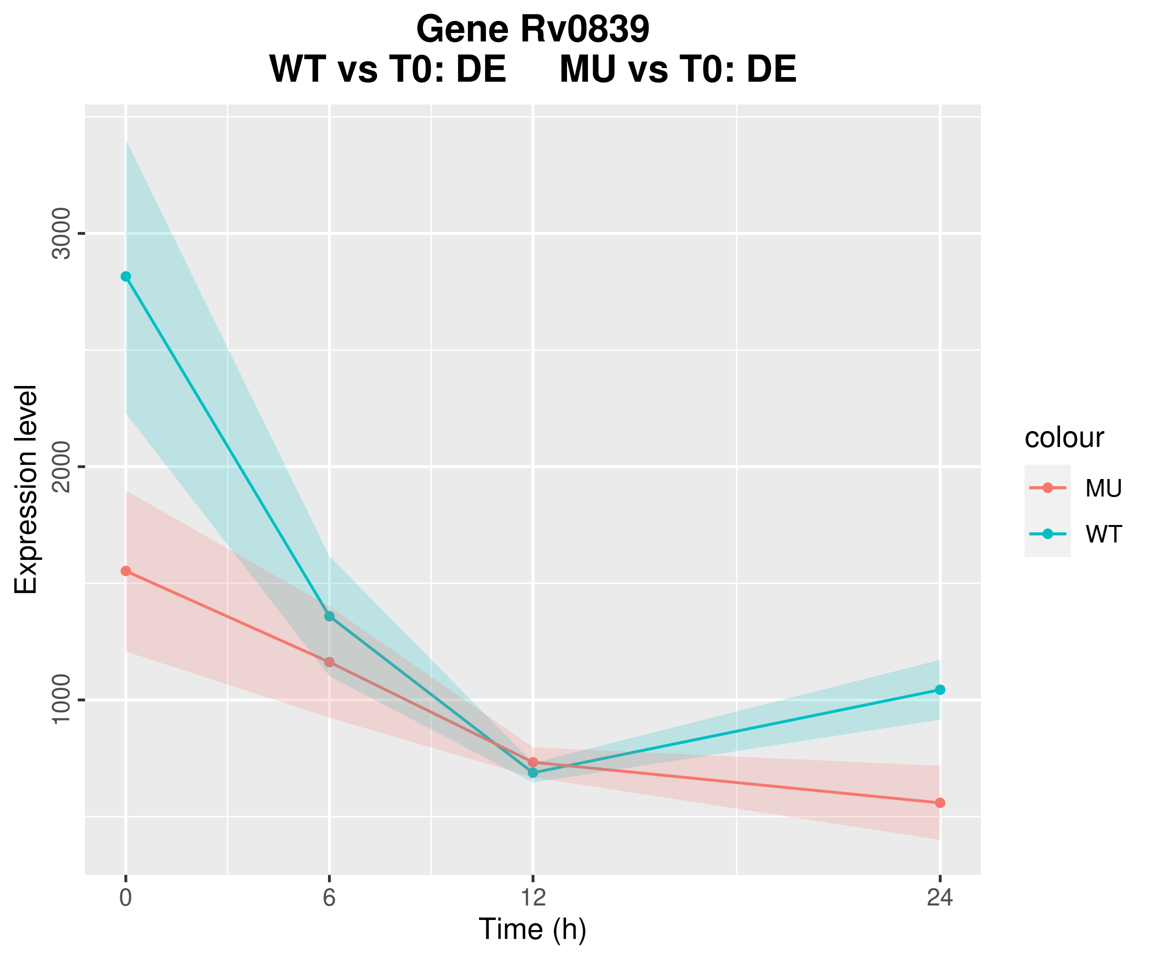

Gene Rv0839
WT vs T0: DE MU vs T0: DE
Expression level
3000
2000
1000
0
6
12
24
Time (h)
colour
MU
WT

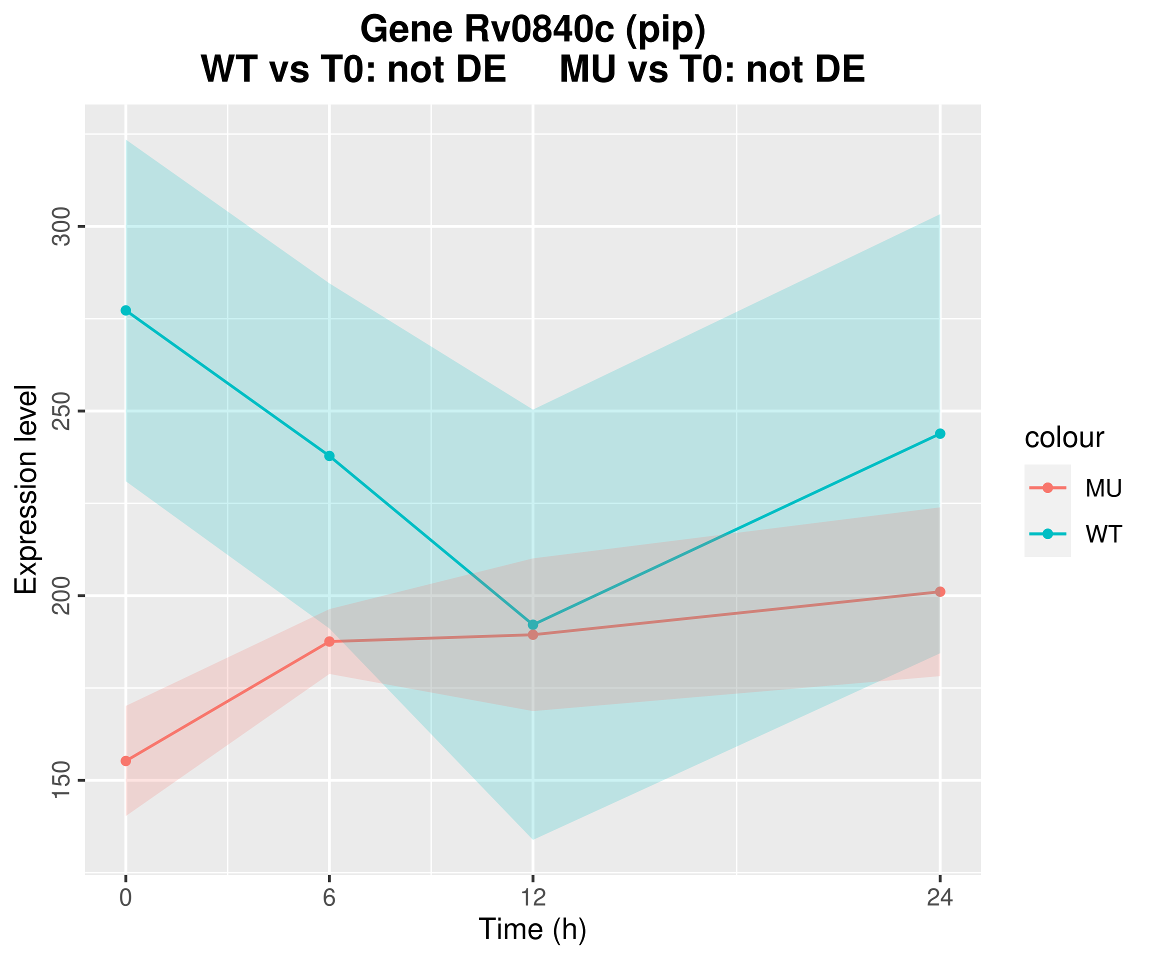

Gene Rv0840c (pip)
WT vs T0: not DE   MU vs T0: not DE
Expression level
300
250
200
150
0
6
12
24
Time (h)
colour
MU
WT

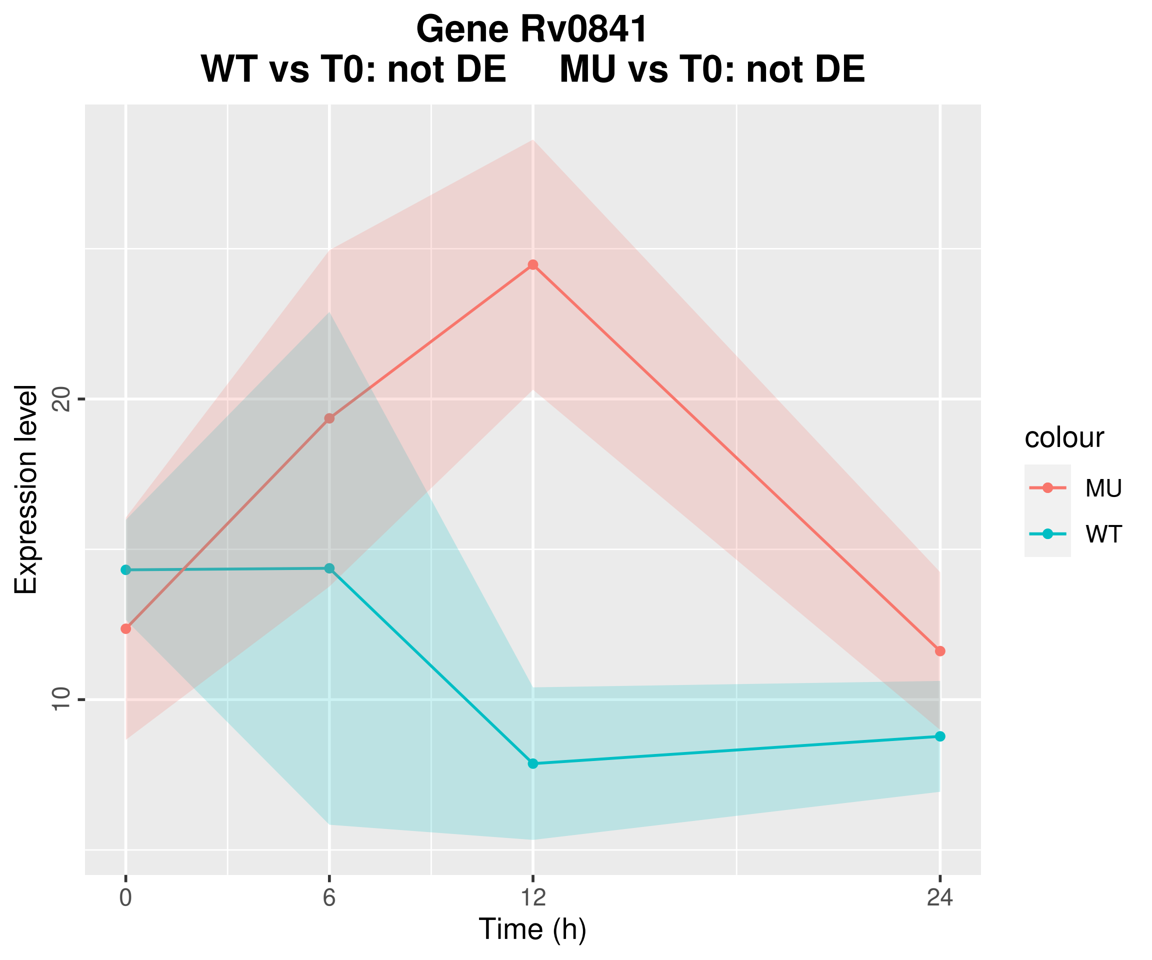

Gene Rv0841
WT vs T0: not DE   MU vs T0: not DE
Expression level
20
10
0
6
12
24
Time (h)
colour
MU
WT

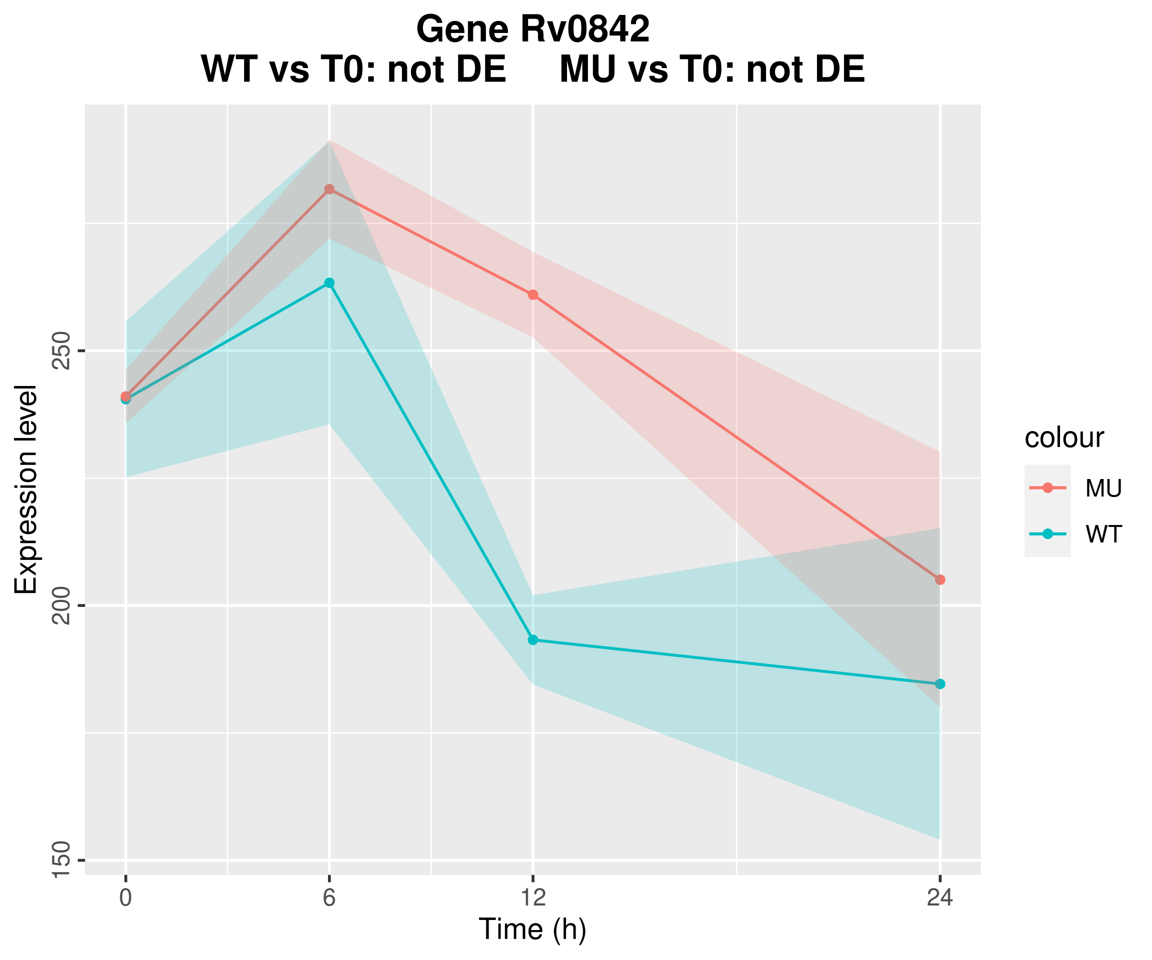
Gene Rv0842
WT vs T0: not DE MU vs T0: not DE
Expression level
150
200
250
0
6
12
24
Time (h)
colour
MU
WT

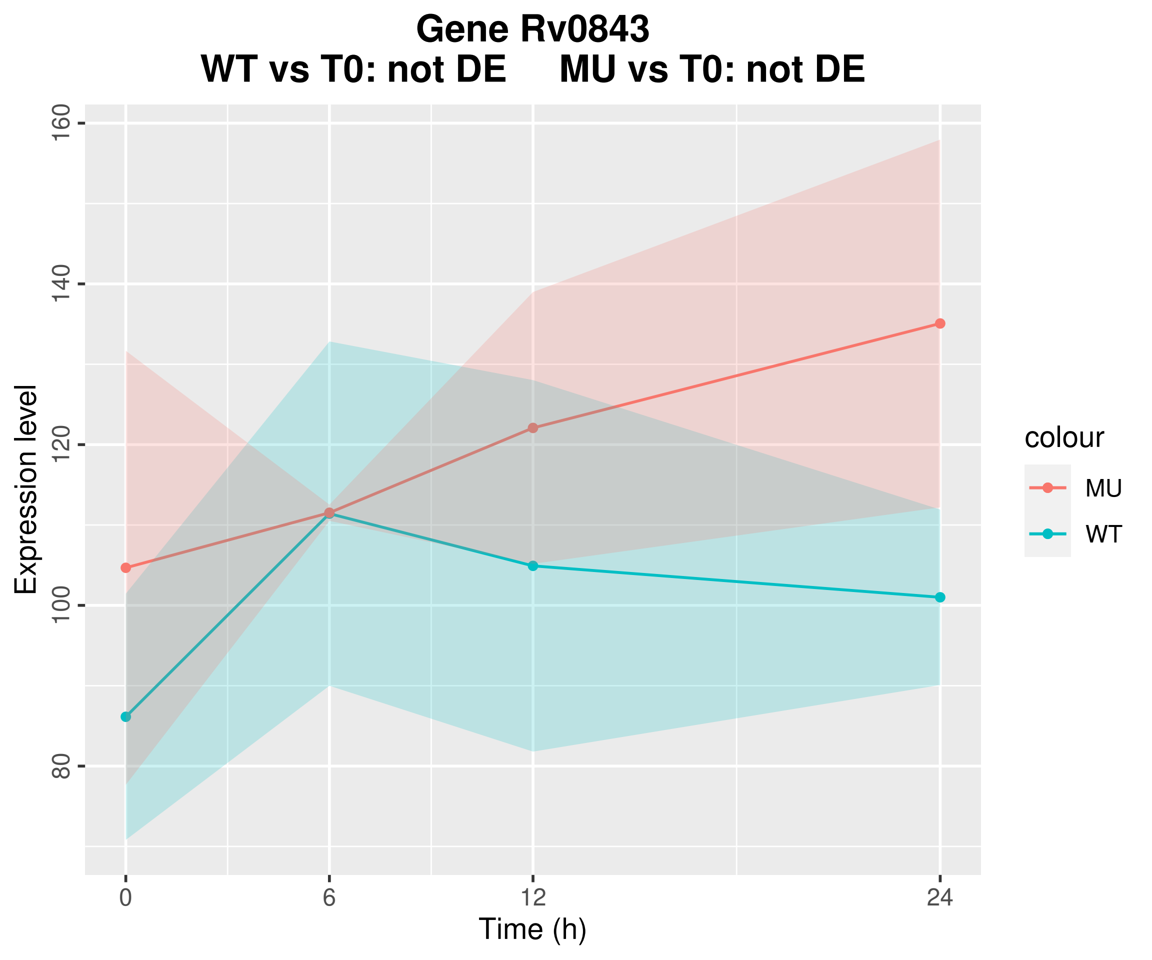

Gene Rv0843
WT vs T0: not DE   MU vs T0: not DE
160
140
120
100
80
Expression level
0
6
12
24
Time (h)
colour
MU
WT

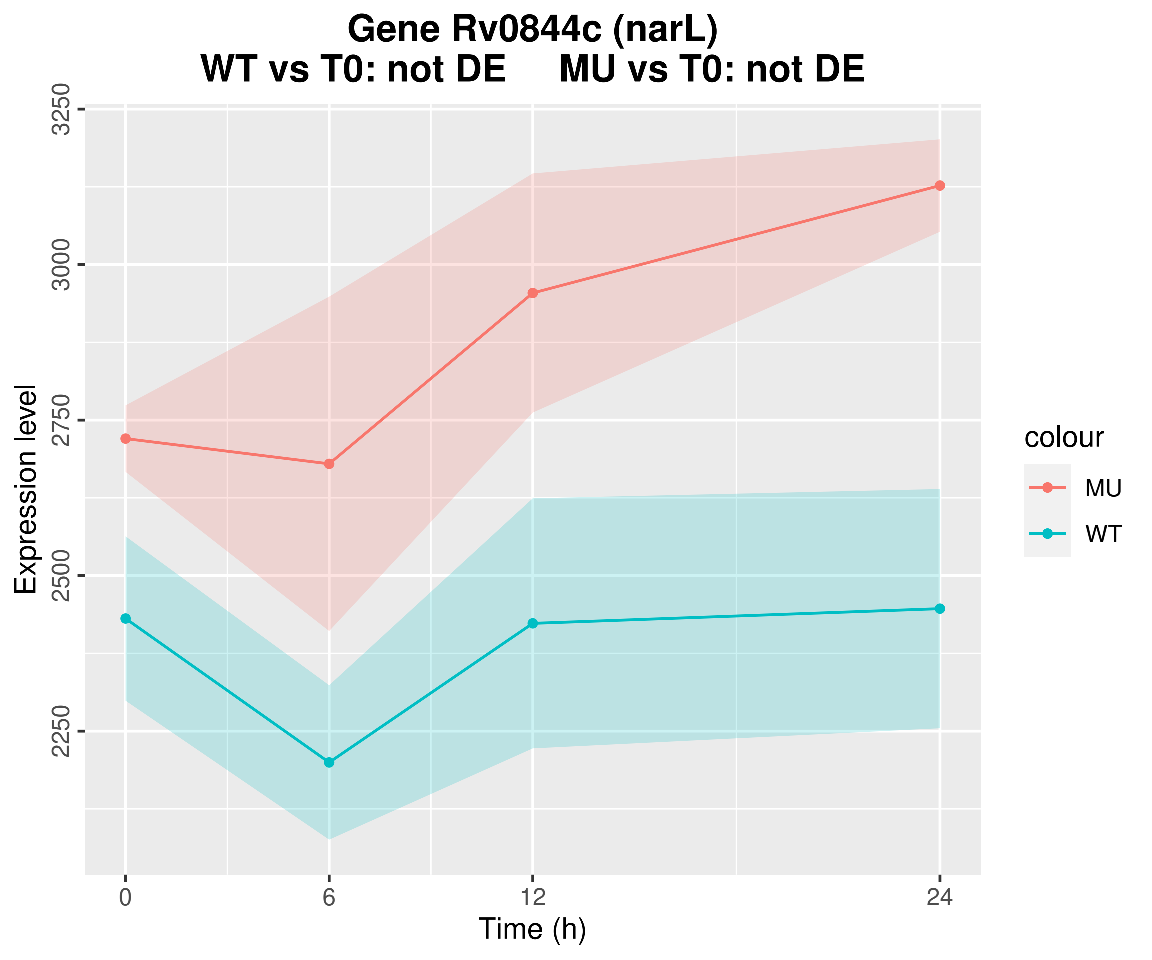
Gene Rv0844c (narL)
WT vs T0: not DE MU vs T0: not DE
Expression level
3250
3000
2750
2500
2250
0
6
12
24
Time (h)
colour
MU
WT

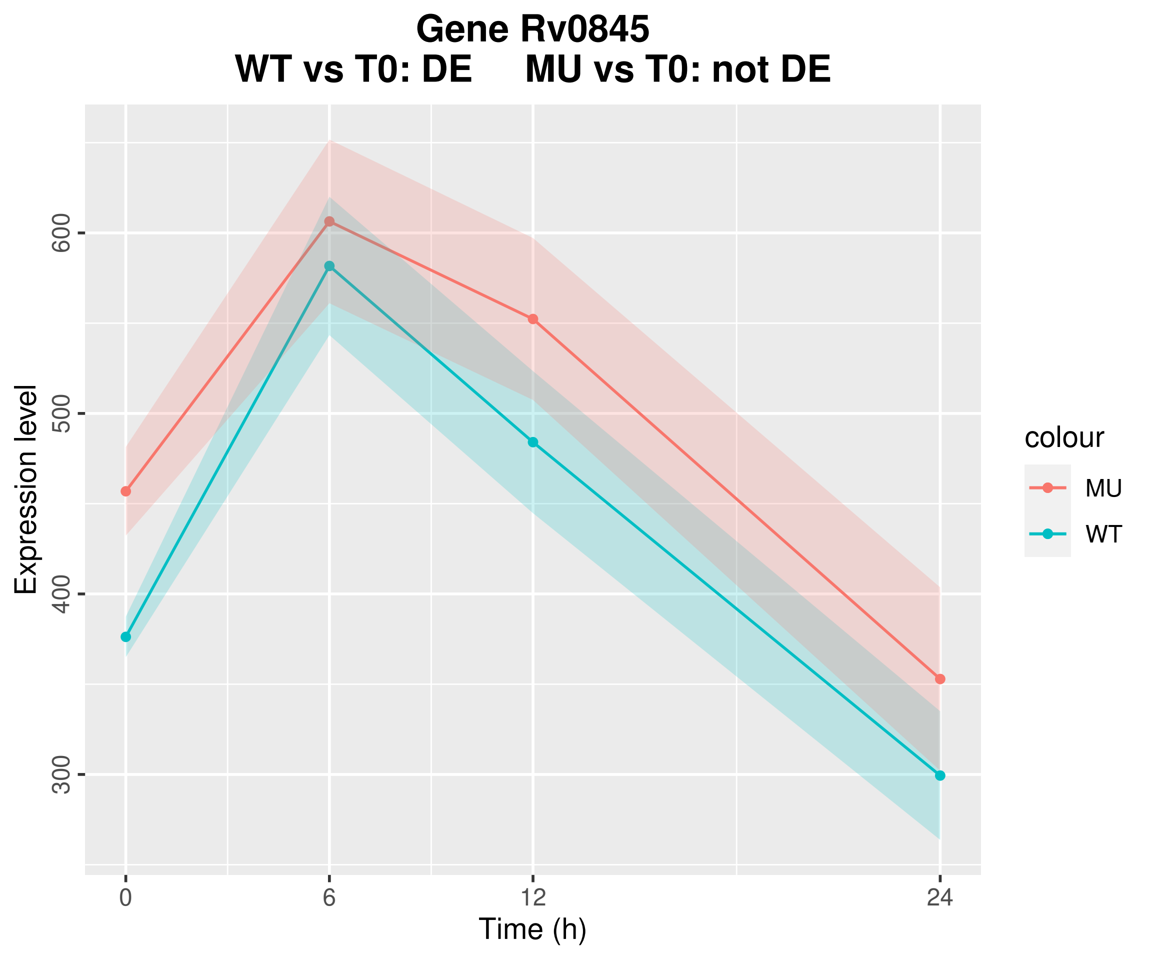

Gene Rv0845
WT vs T0: DE   MU vs T0: not DE
Expression level
600
500
400
300
0
6
12
24
Time (h)
colour
MU
WT

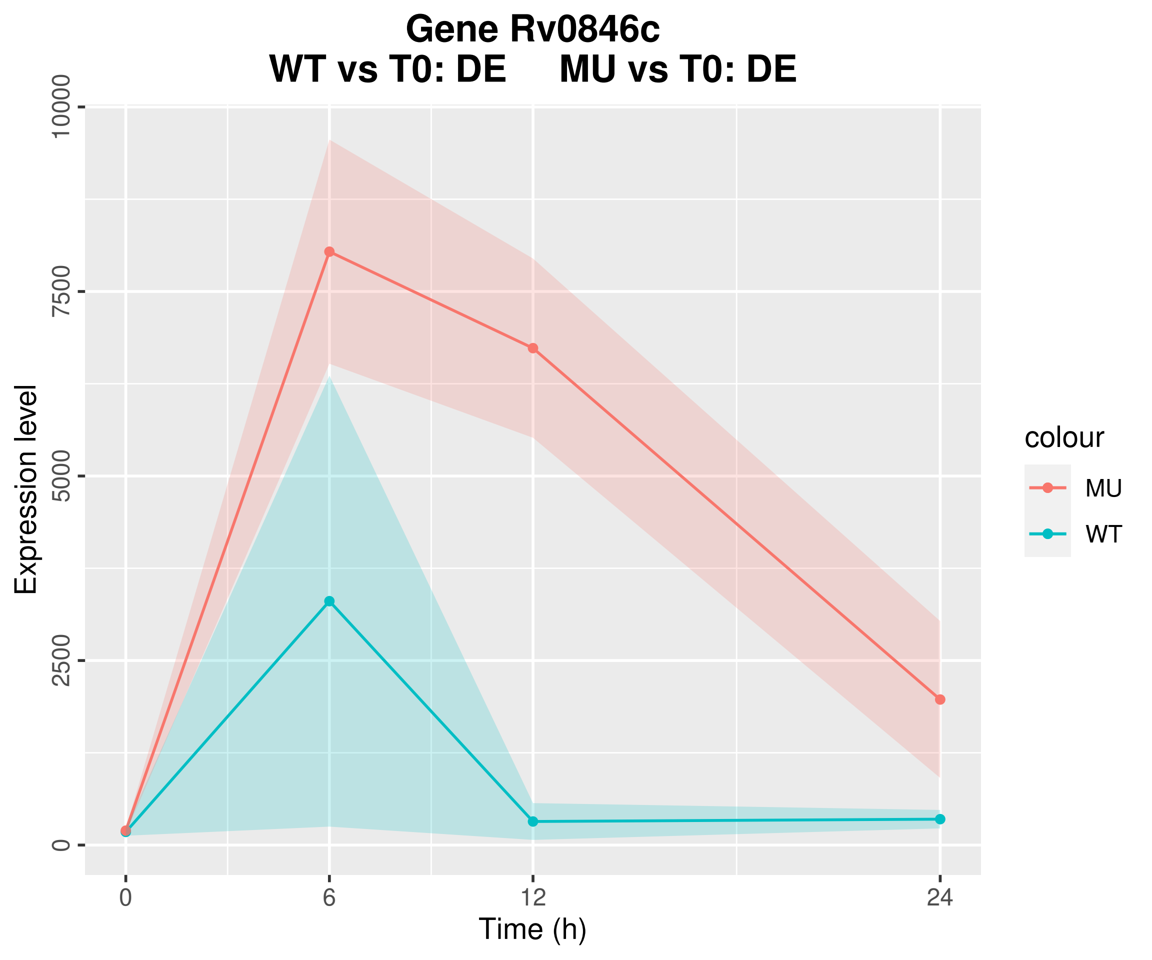
Gene Rv0846c
WT vs T0: DE   MU vs T0: DE
Expression level
10000
7500
5000
2500
0
0
6
12
24
Time (h)
colour
MU
WT

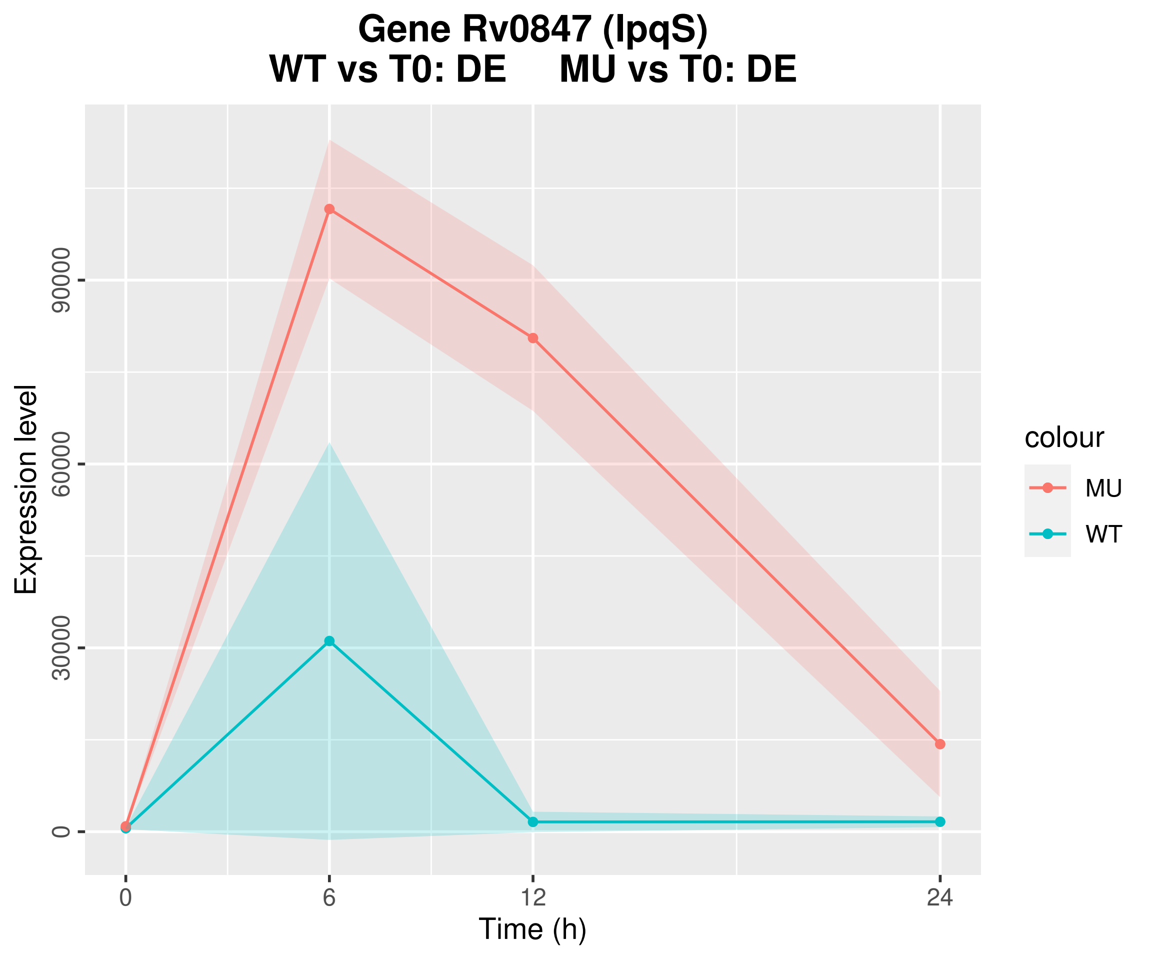

Gene Rv0847 (lpqS)
WT vs T0: DE   MU vs T0: DE
Expression level
90000
60000
30000
0
0
6
12
24
Time (h)
colour
MU
WT

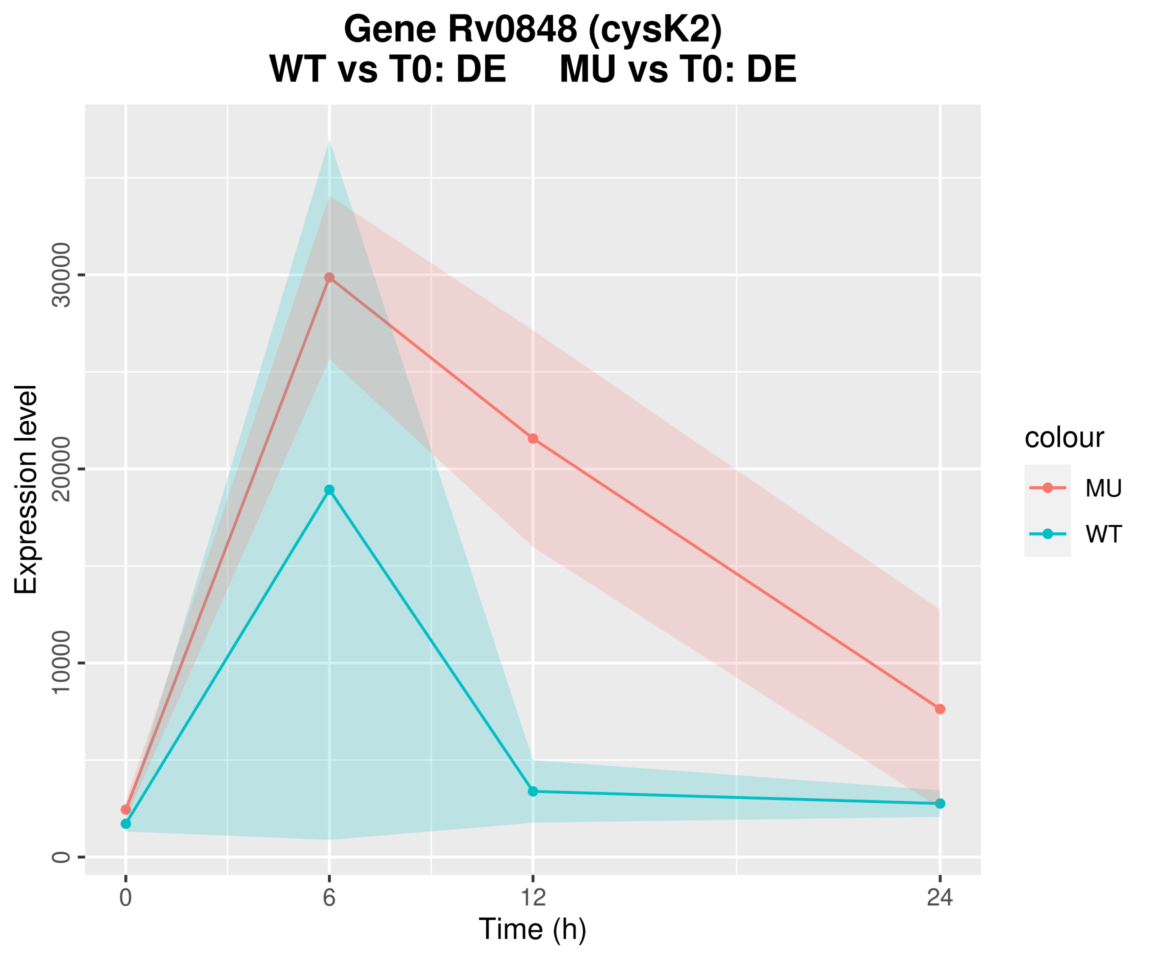

Gene Rv0848 (cysK2)
WT vs T0: DE MU vs T0: DE
Expression level
0
10000
20000
30000
0
6
12
24
Time (h)
colour
MU
WT

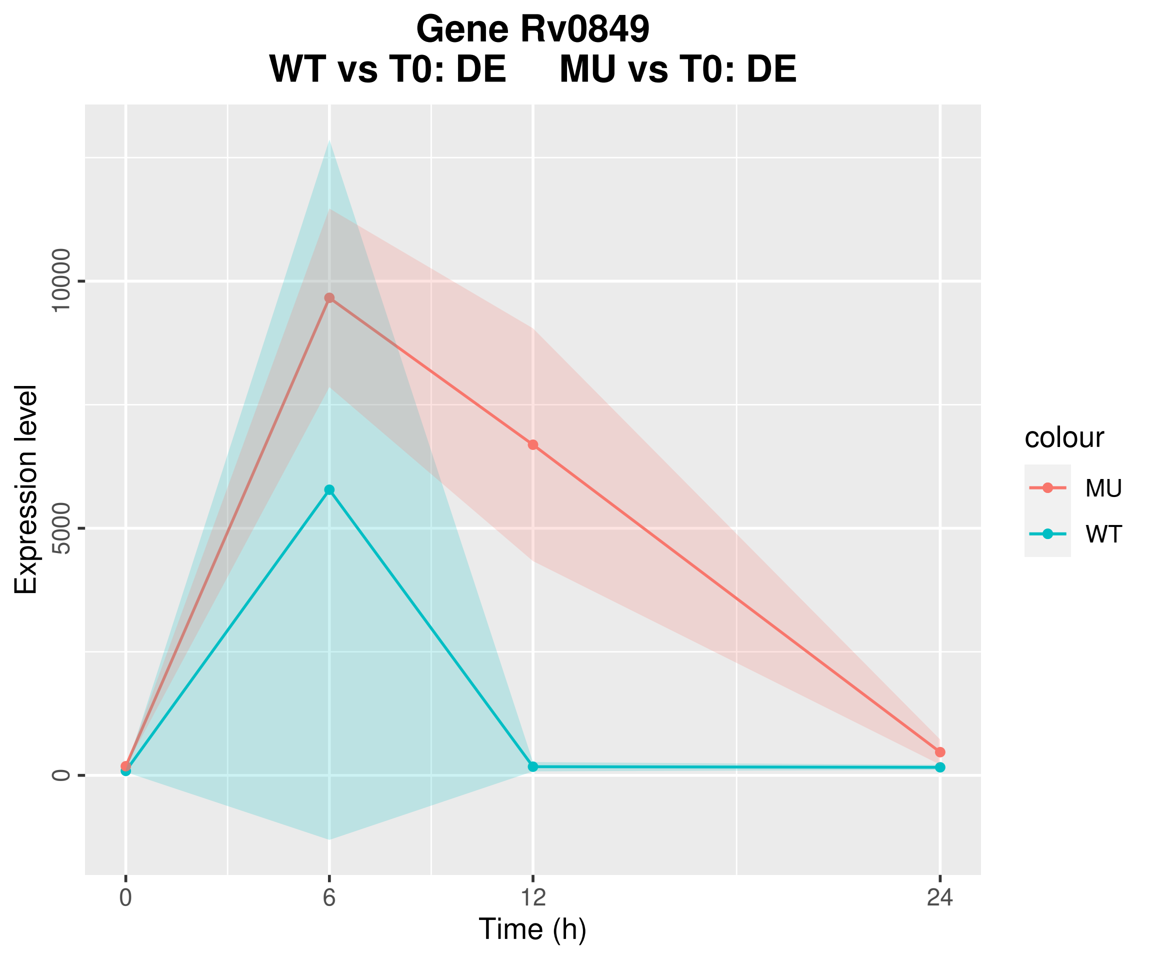
Gene Rv0849
WT vs T0: DE   MU vs T0: DE
Expression level
10000
5000
0
0
6
12
24
Time (h)
colour
MU
WT

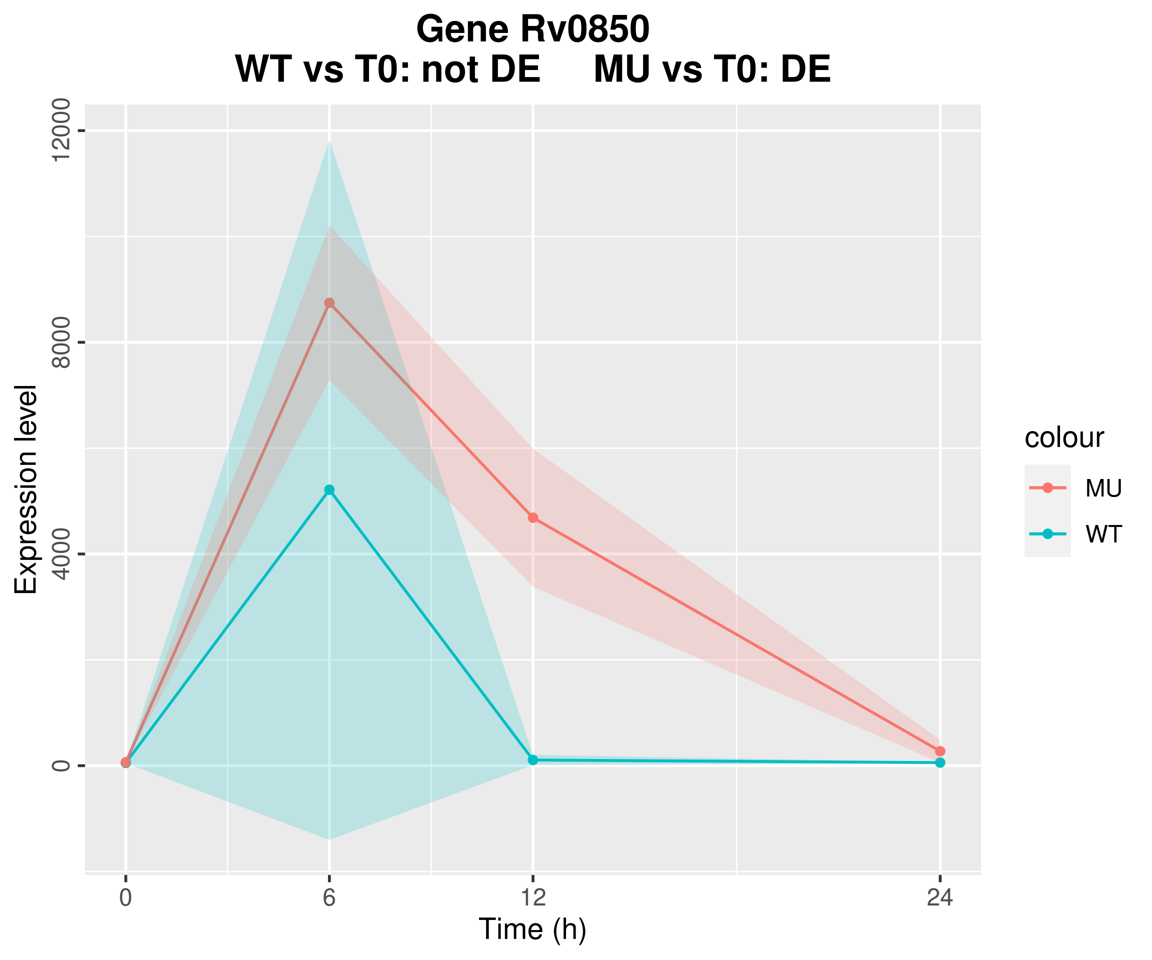
Gene Rv0850
WT vs T0: not DE MU vs T0: DE
Expression level
12000
8000
4000
0
0
6
12
24
Time (h)
colour
MU
WT

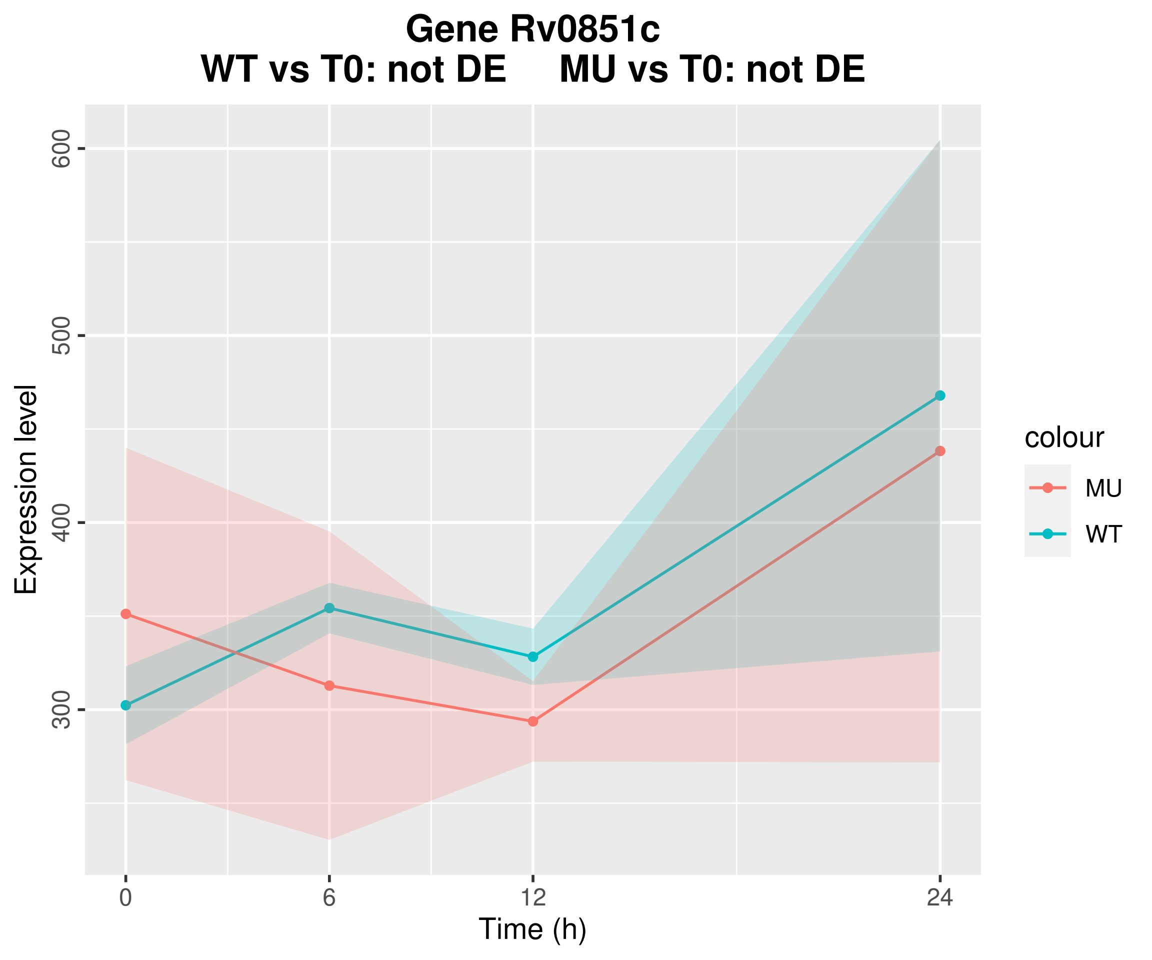
Gene Rv0851c
WT vs T0: not DE   MU vs T0: not DE
Expression level
600
500
400
300
0
6
12
24
Time (h)
colour
MU
WT

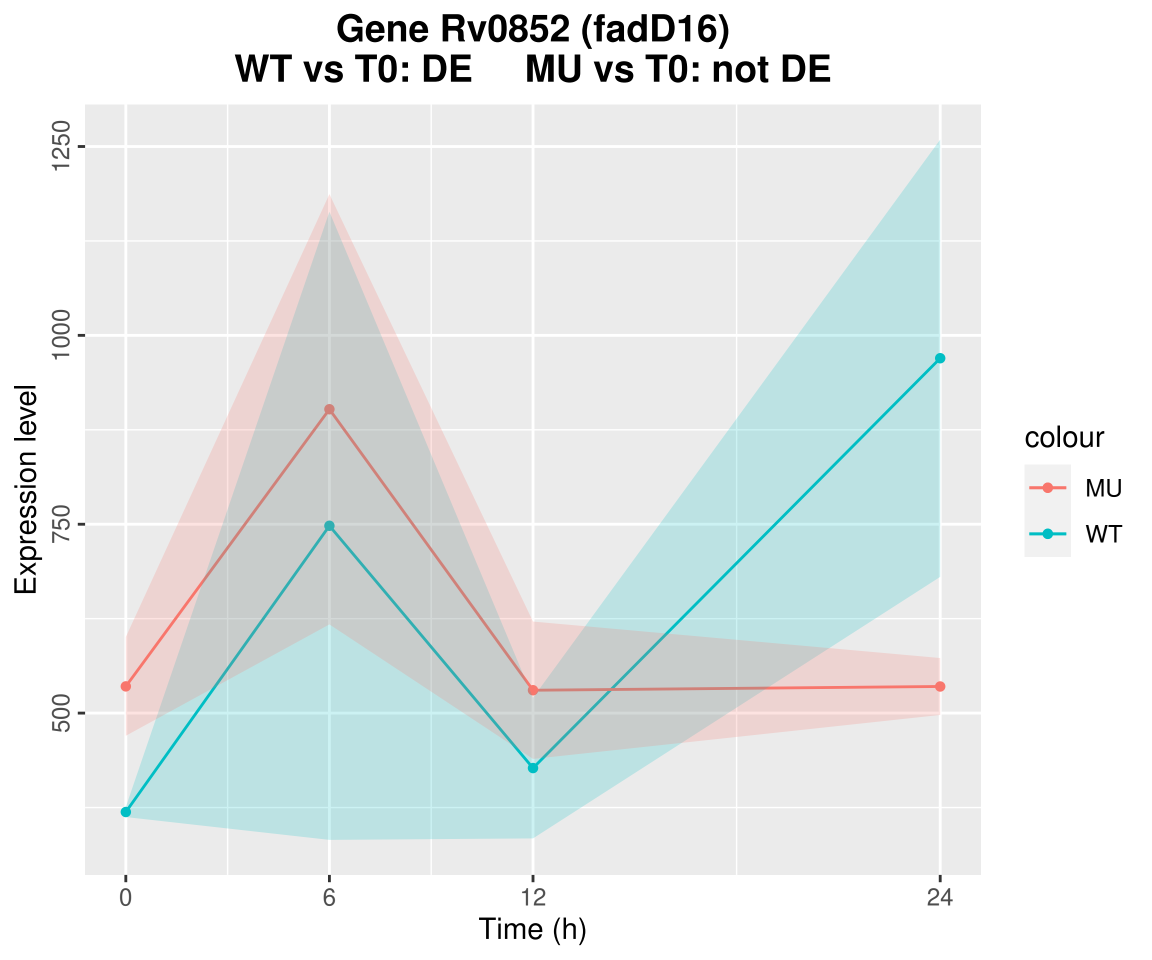

Gene Rv0852 (fadD16)
WT vs T0: DE   MU vs T0: not DE
1250
1000
750
500
Expression level
0
6
12
24
Time (h)
colour
MU
WT

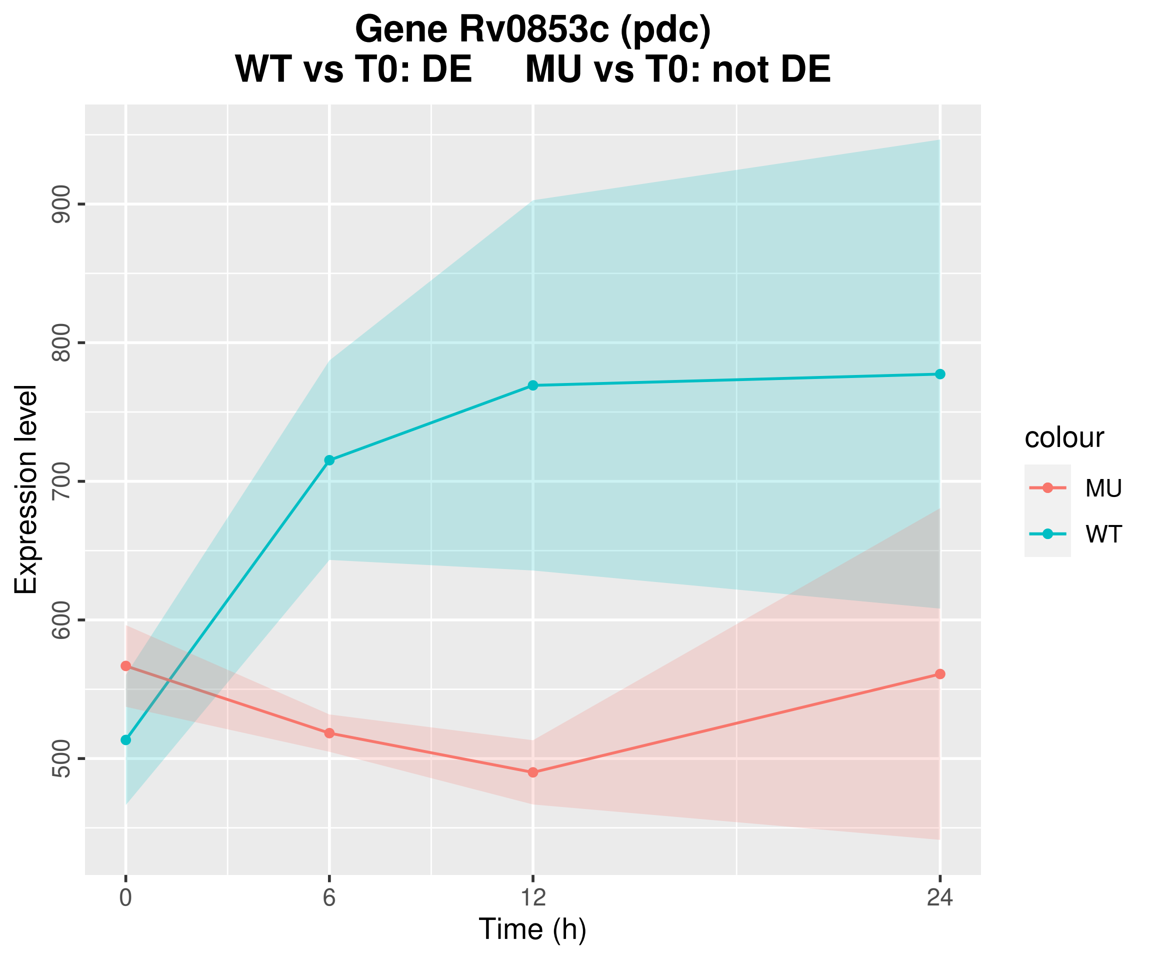
Gene Rv0853c (pdc)
WT vs T0: DE   MU vs T0: not DE
Expression level
900
800
700
600
500
0
6
12
24
Time (h)
colour
MU
WT

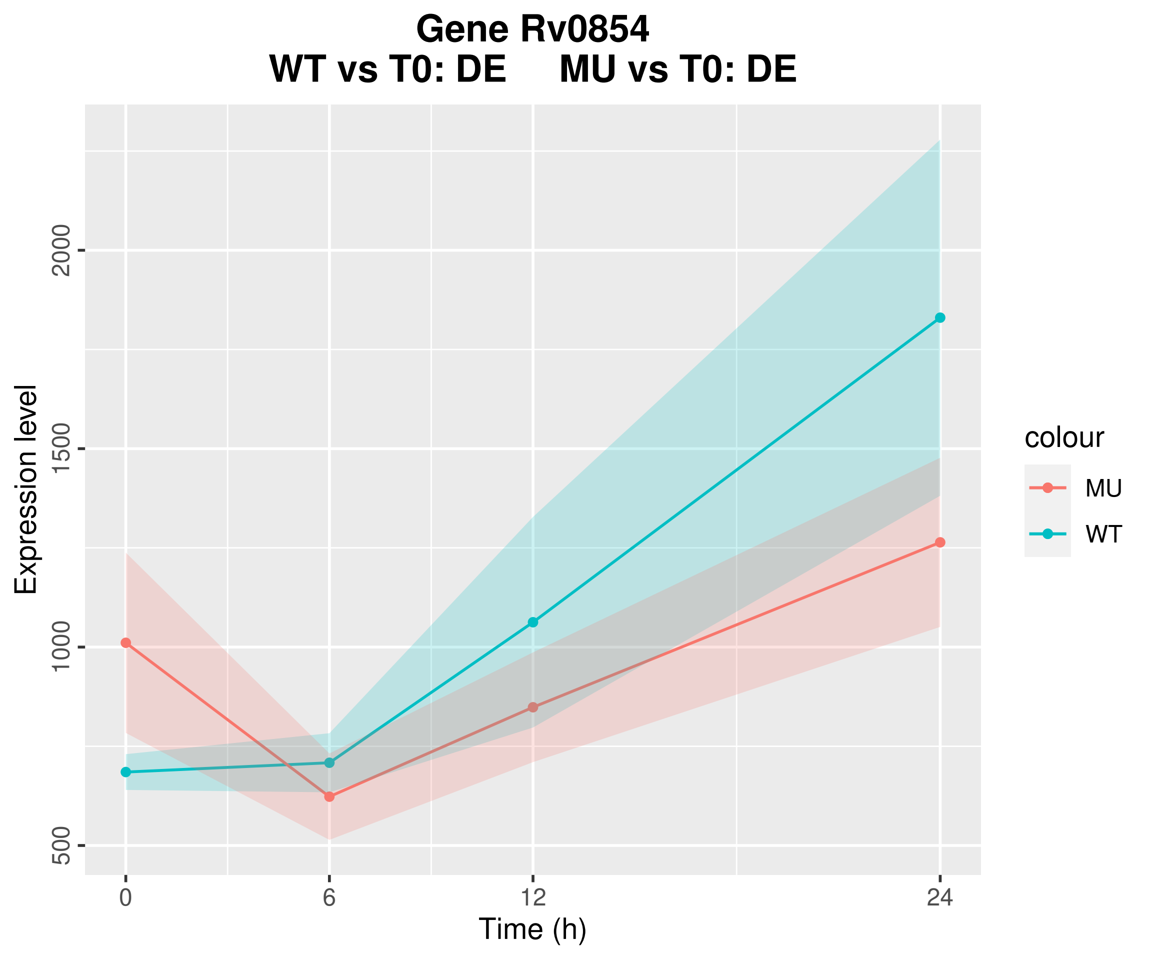
Gene Rv0854
WT vs T0: DE MU vs T0: DE
Expression level
2000
1500
1000
500
0
6
12
24
Time (h)
colour
MU
WT

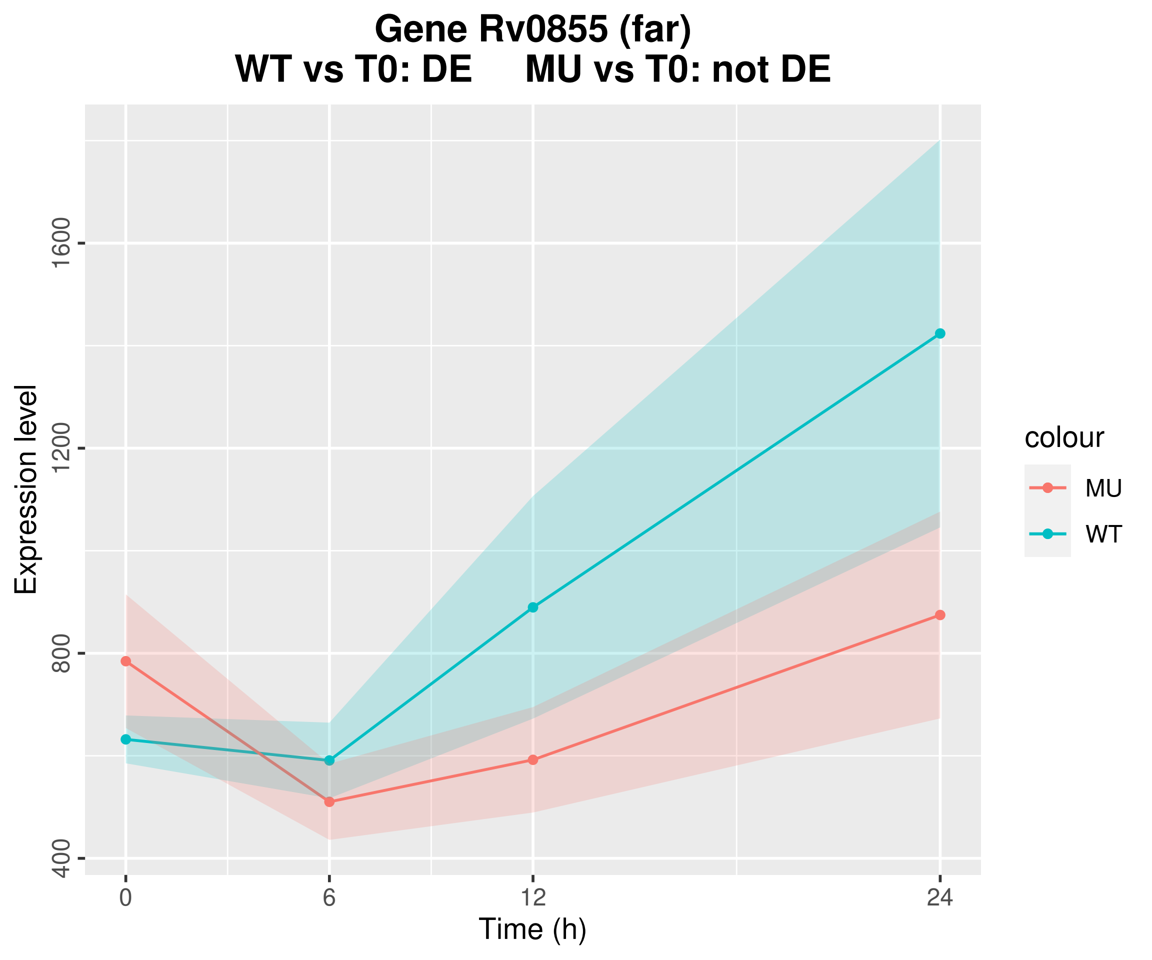

Gene Rv0855 (far)
WT vs T0: DE MU vs T0: not DE
Expression level
1600
1200
800
400
0
6
12
24
Time (h)
colour
MU
WT

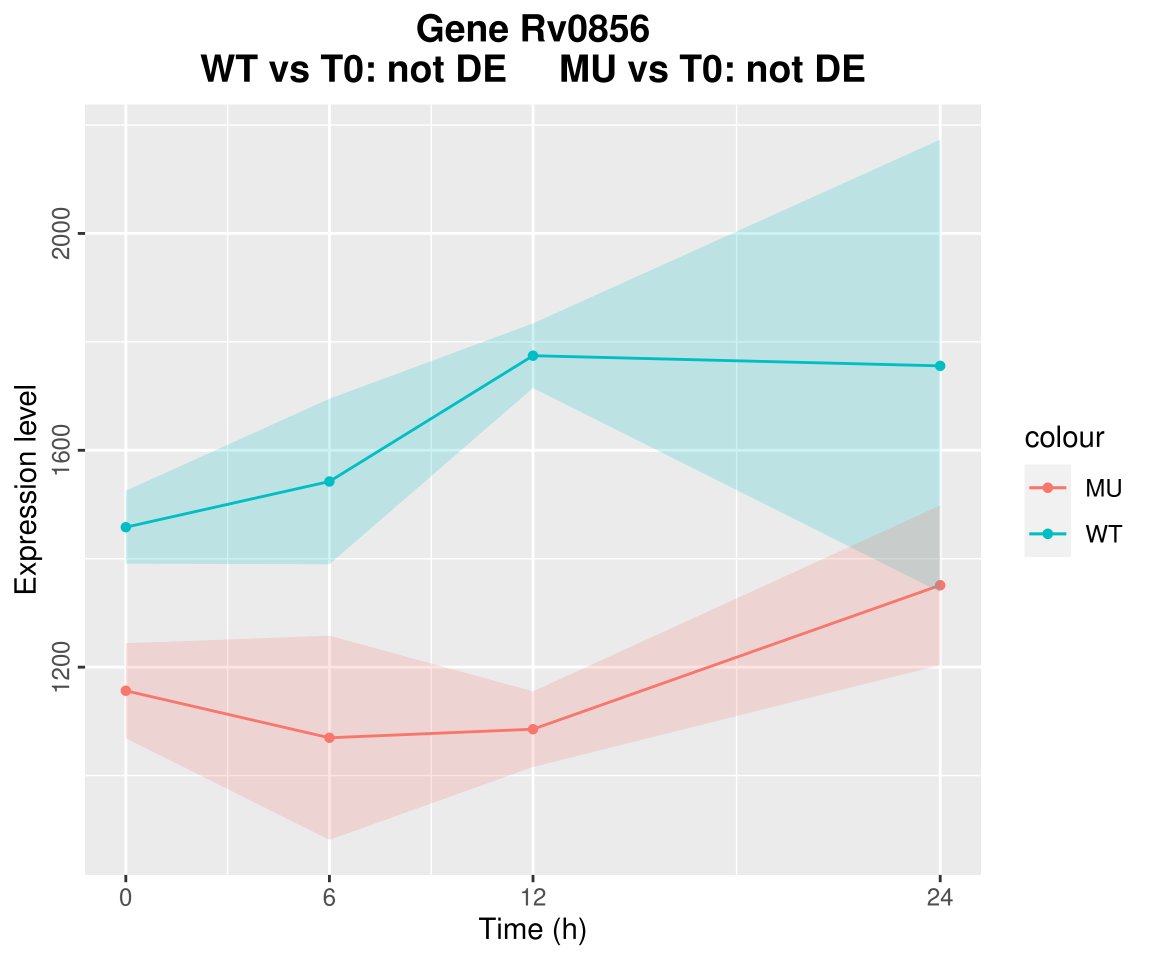

Gene Rv0856
WT vs T0: not DE MU vs T0: not DE
Expression level
2000
1600
1200
0
6
12
24
Time (h)
colour
MU
WT

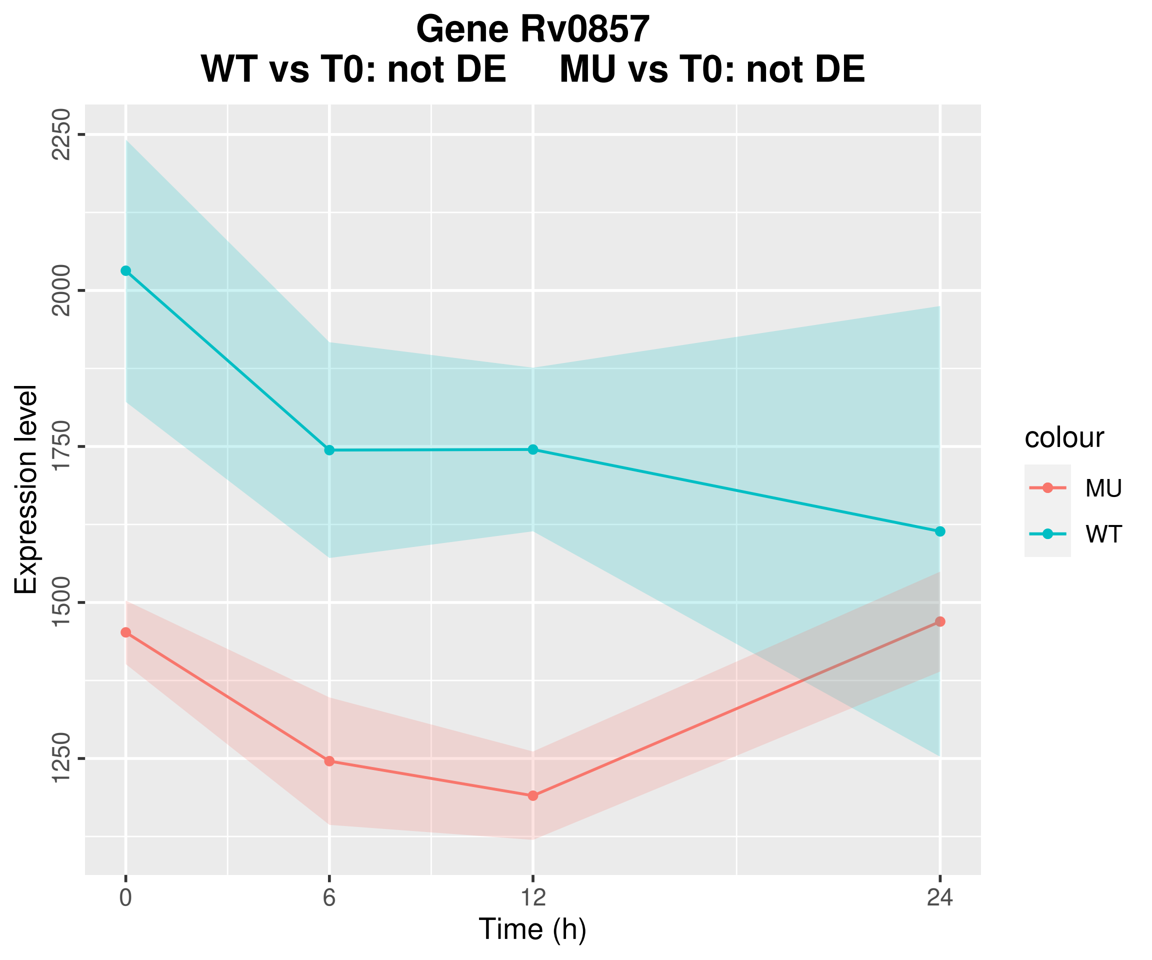
Gene Rv0857
WT vs T0: not DE MU vs T0: not DE
2250
2000
1750
1500
1250
Expression level
0
6
12
24
Time (h)
colour
MU
WT

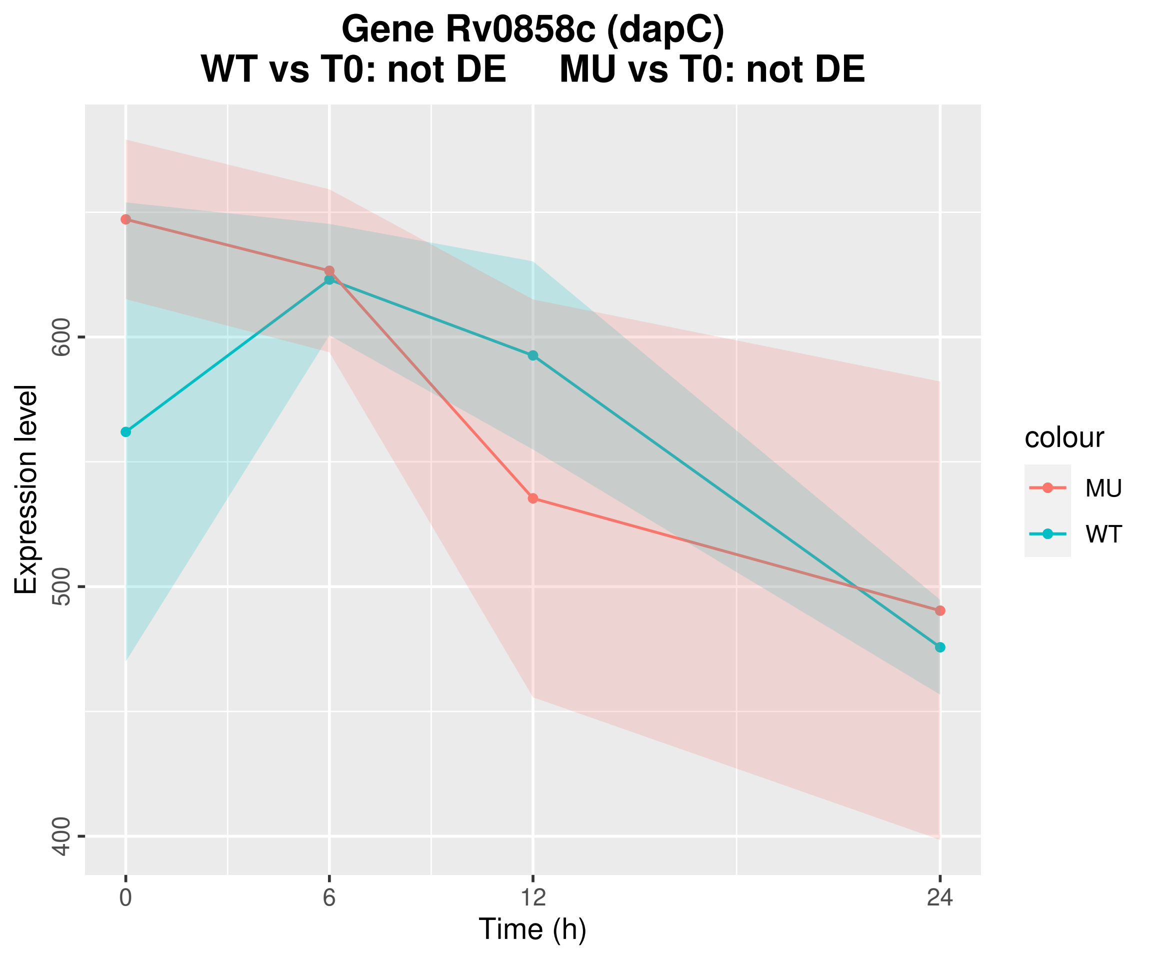

Gene Rv0858c (dapC)
WT vs T0: not DE   MU vs T0: not DE
Expression level
400
500
600
0
6
12
24
Time (h)
colour
MU
WT

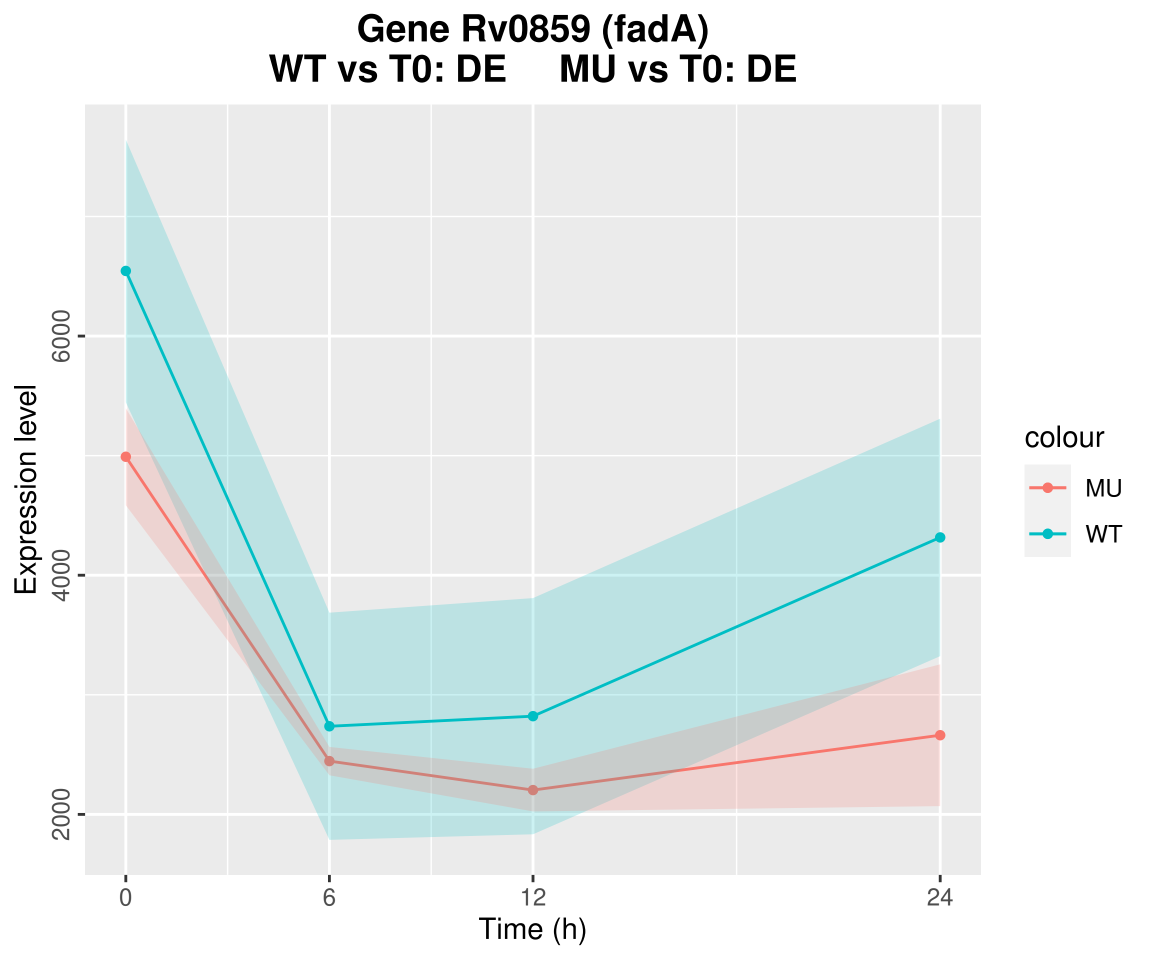

Gene Rv0859 (fadA)
WT vs T0: DE   MU vs T0: DE
Expression level
6000
4000
2000
0
6
12
24
Time (h)
colour
MU
WT

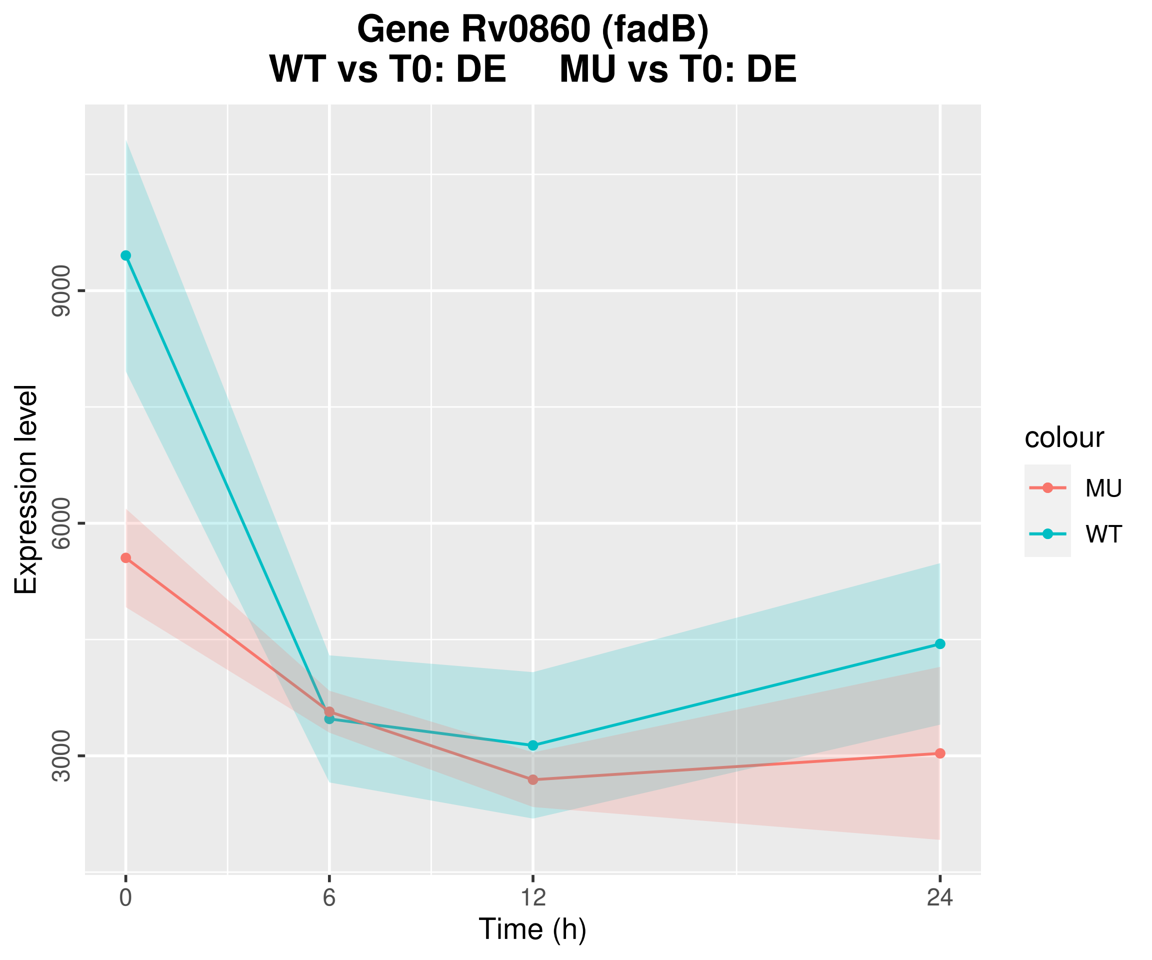

Gene Rv0860 (fadB)
WT vs T0: DE   MU vs T0: DE
Expression level
9000
6000
3000
0
6
12
24
Time (h)
colour
MU
WT

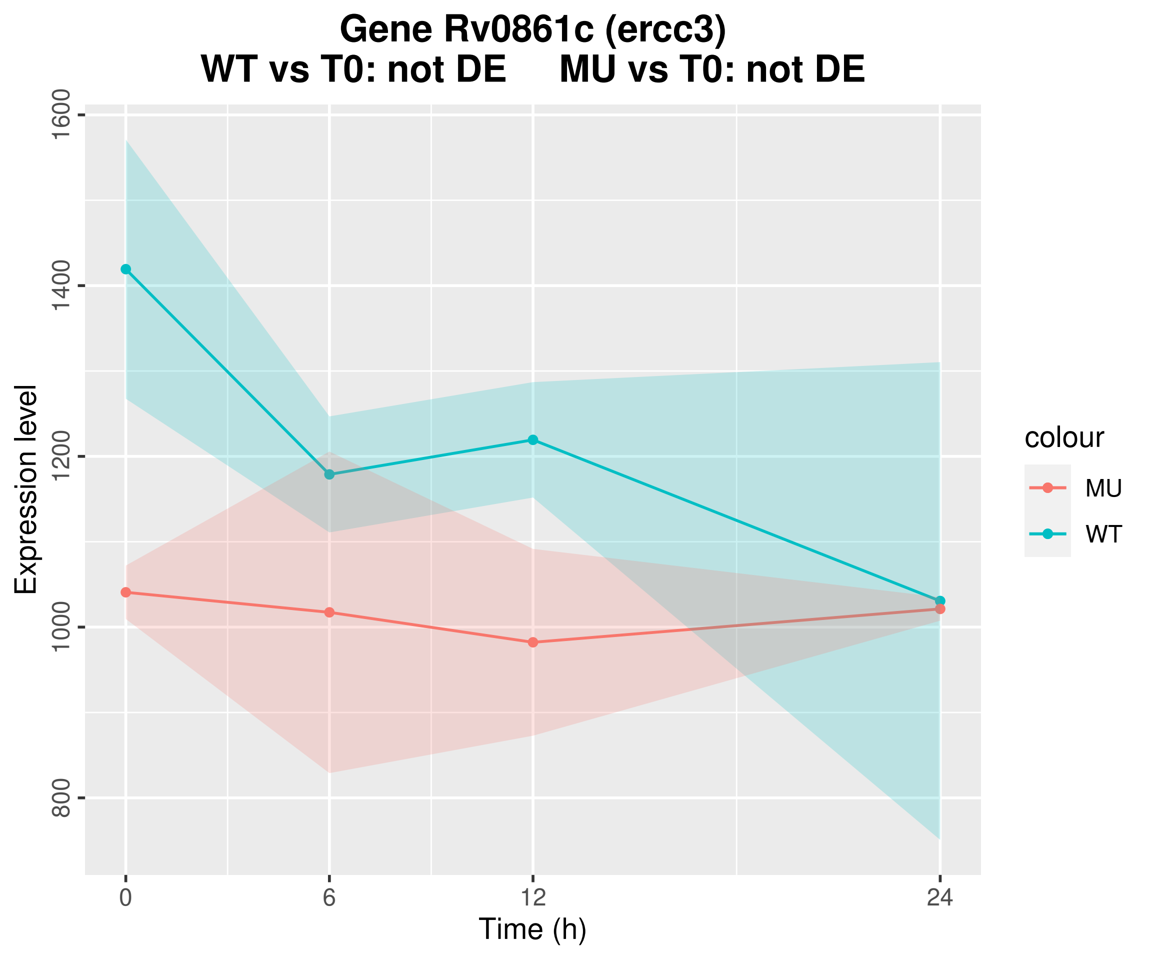
Gene Rv0861c (ercc3)
WT vs T0: not DE   MU vs T0: not DE
Expression level
1600
1400
1200
1000
800
0
6
12
24
Time (h)
colour
MU
WT

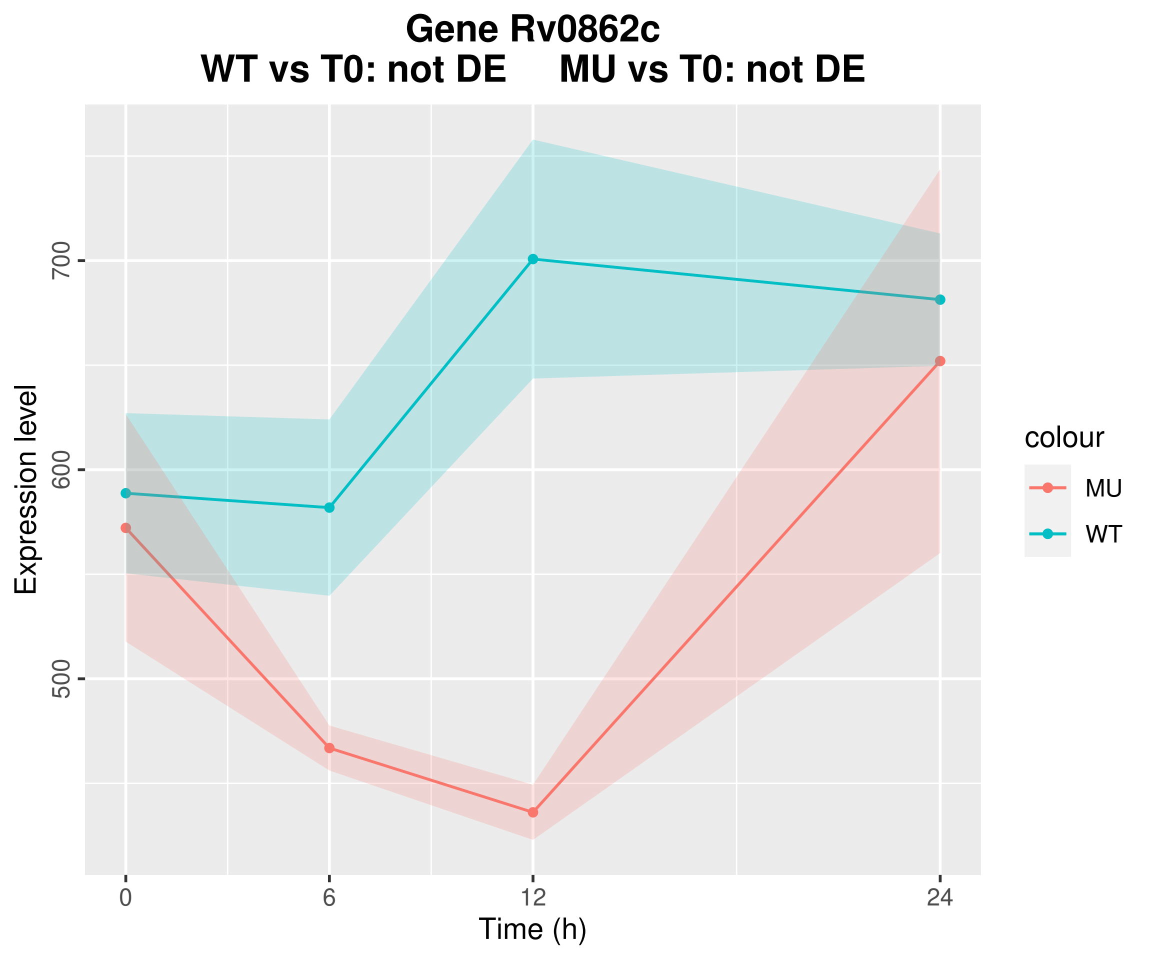

Gene Rv0862c
WT vs T0: not DE MU vs T0: not DE
Expression level
700
600
500
0
6
12
24
Time (h)
colour
MU
WT

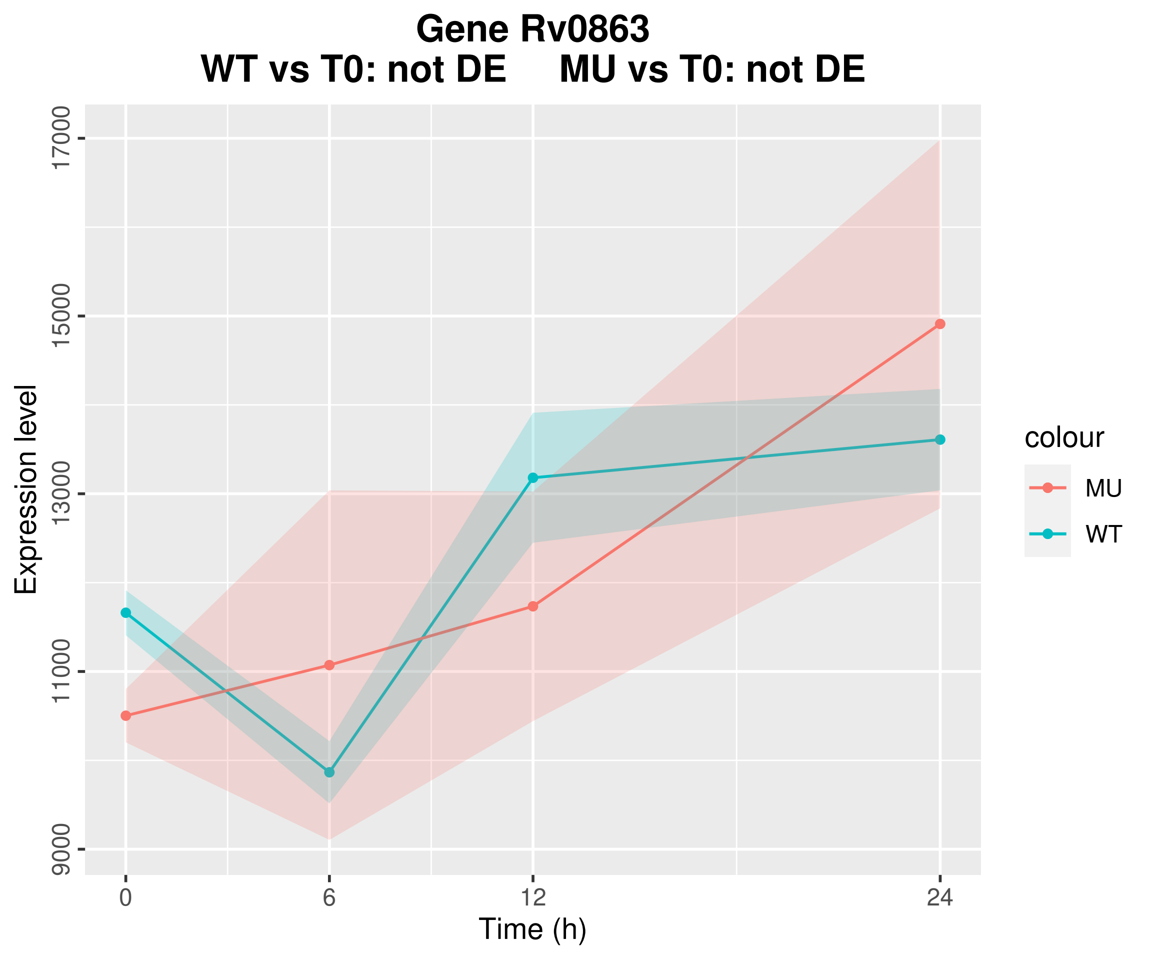
Gene Rv0863
WT vs T0: not DE   MU vs T0: not DE
Expression level
17000
15000
13000
11000
9000
0
6
12
24
Time (h)
colour
MU
WT

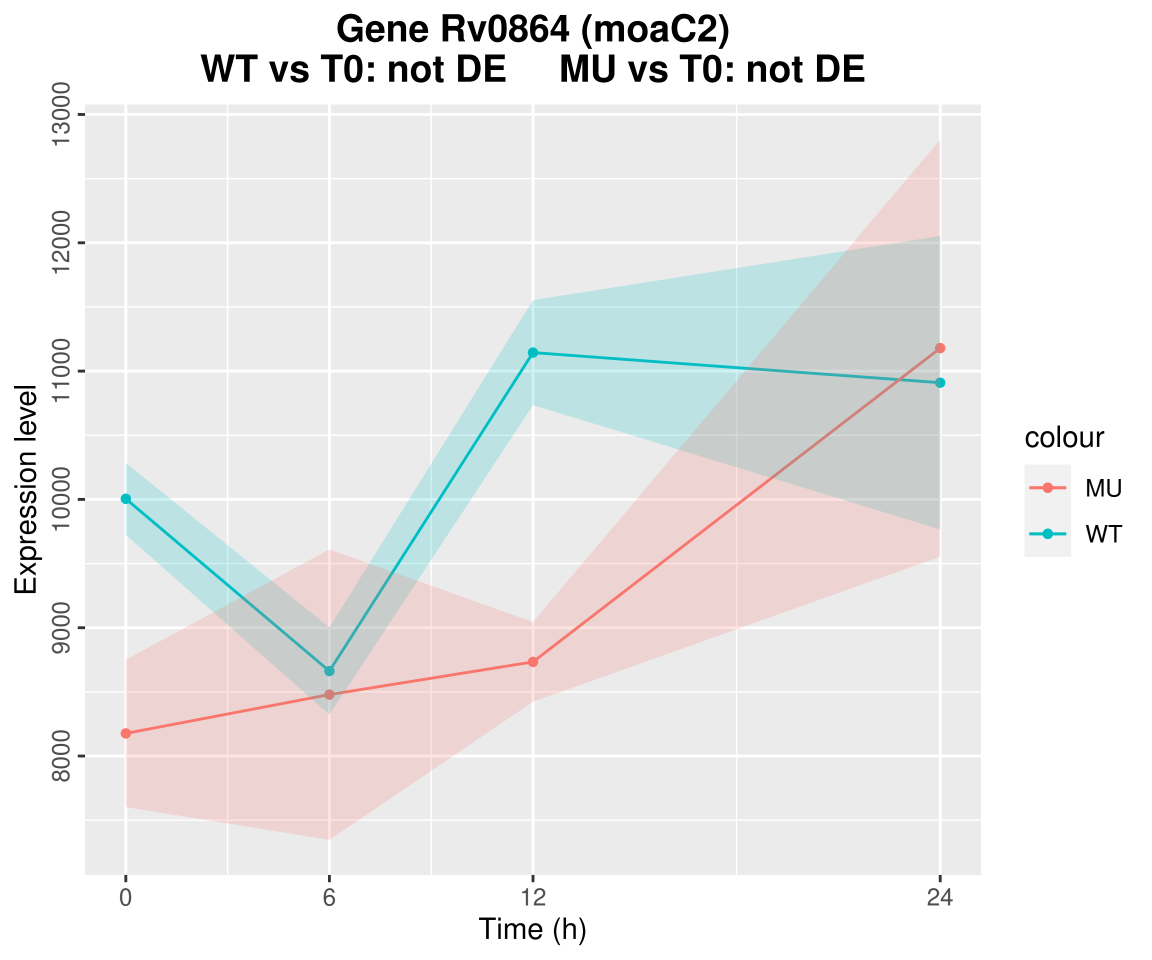
Gene Rv0864 (moaC2)
WT vs T0: not DE MU vs T0: not DE
Expression level
13000
12000
11000
10000
9000
8000
0
6
12
24
Time (h)
colour
MU
WT

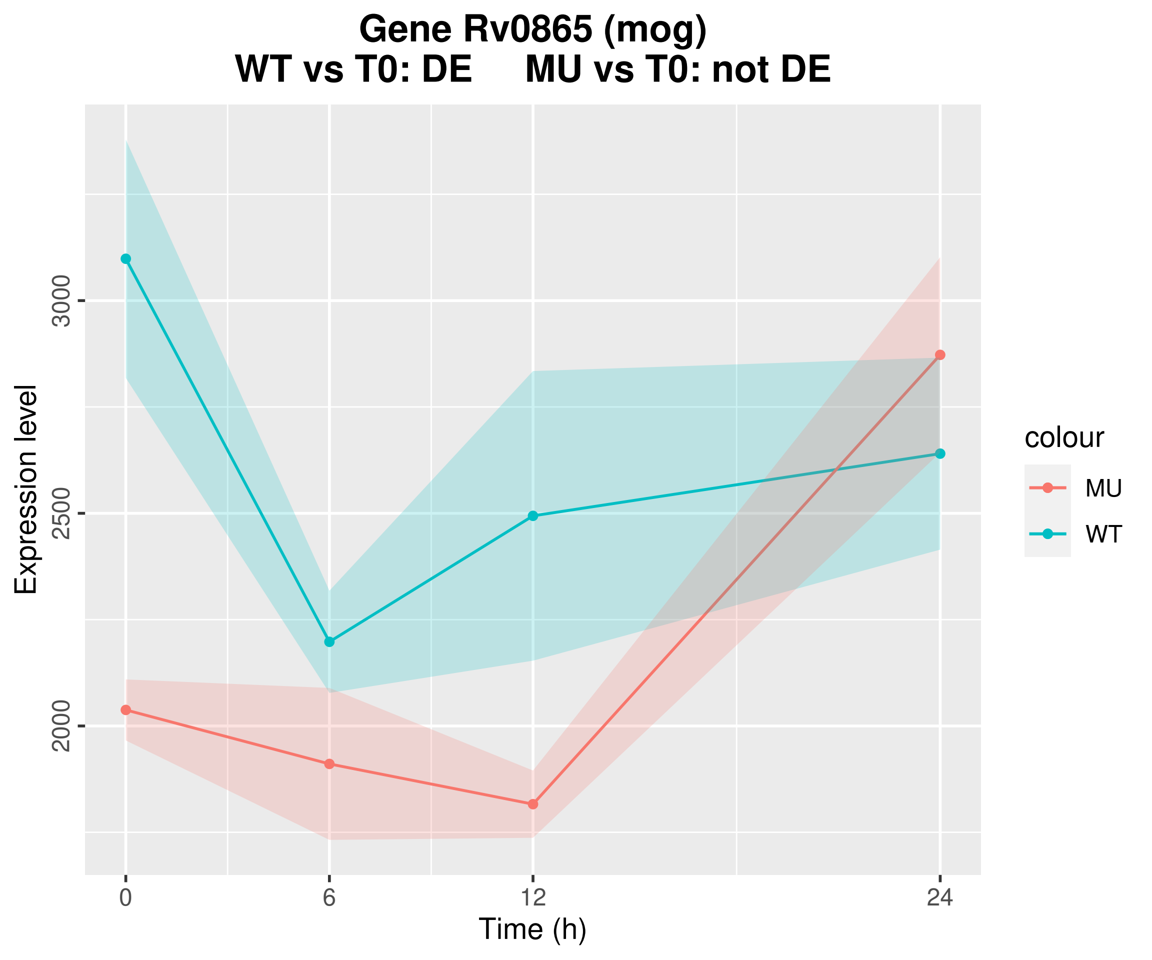
Gene Rv0865 (mog)
WT vs T0: DE   MU vs T0: not DE
Expression level
3000
2500
2000
0
6
12
24
Time (h)
colour
MU
WT

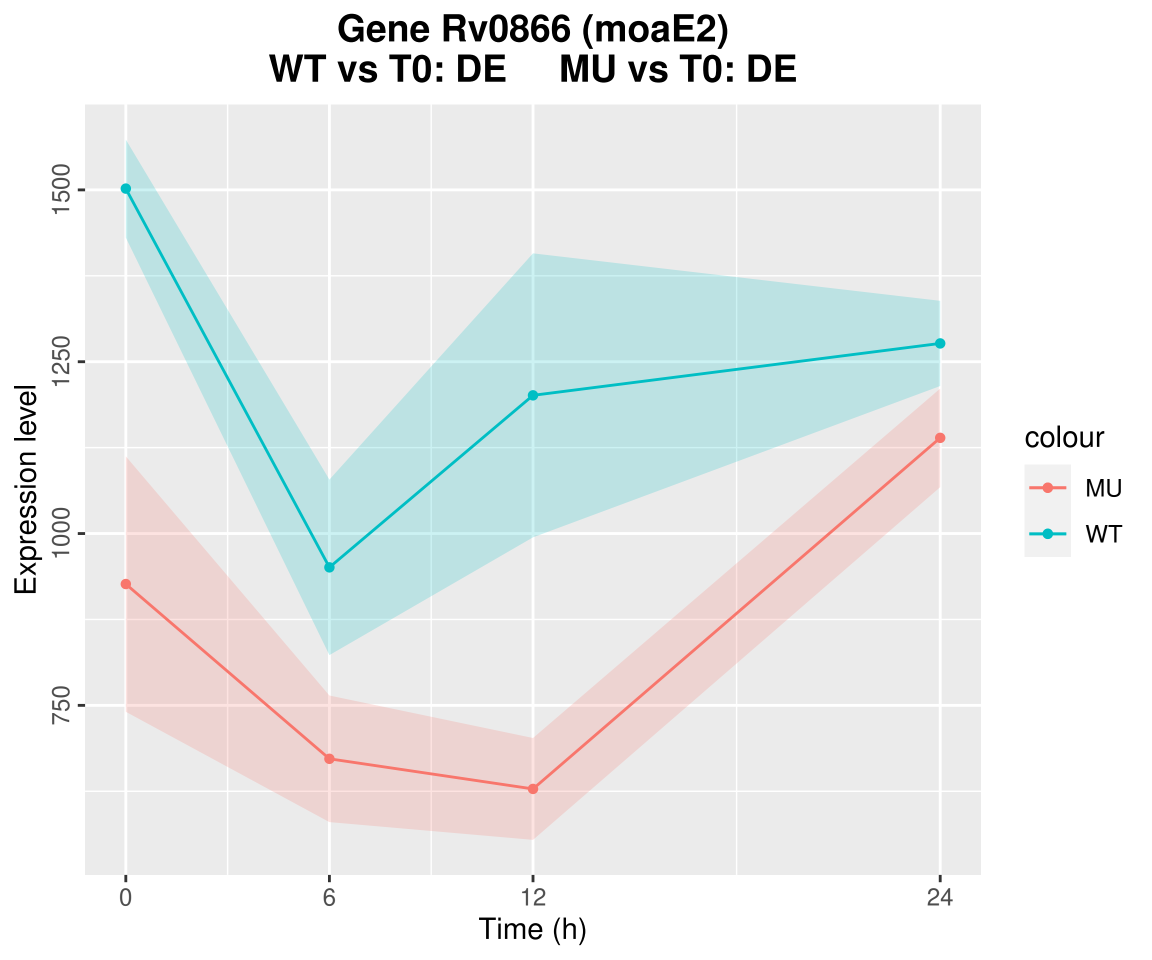

Gene Rv0866 (moaE2)
WT vs T0: DE   MU vs T0: DE
Expression level
1500
1250
1000
750
0
6
12
24
Time (h)
colour
MU
WT

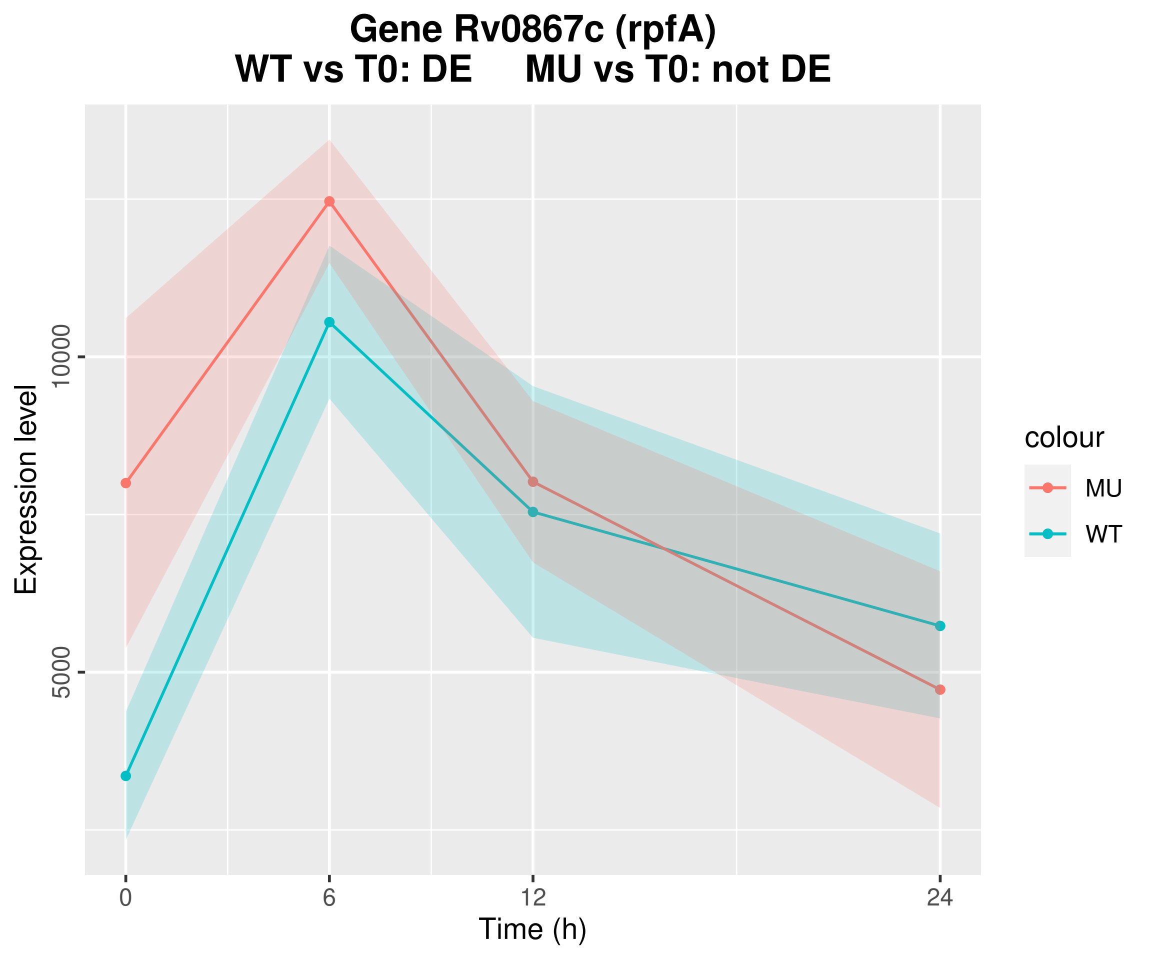

Gene Rv0867c (rpfA)
WT vs T0: DE MU vs T0: not DE
Expression level
10000
5000
0
6
12
24
Time (h)
colour
MU
WT

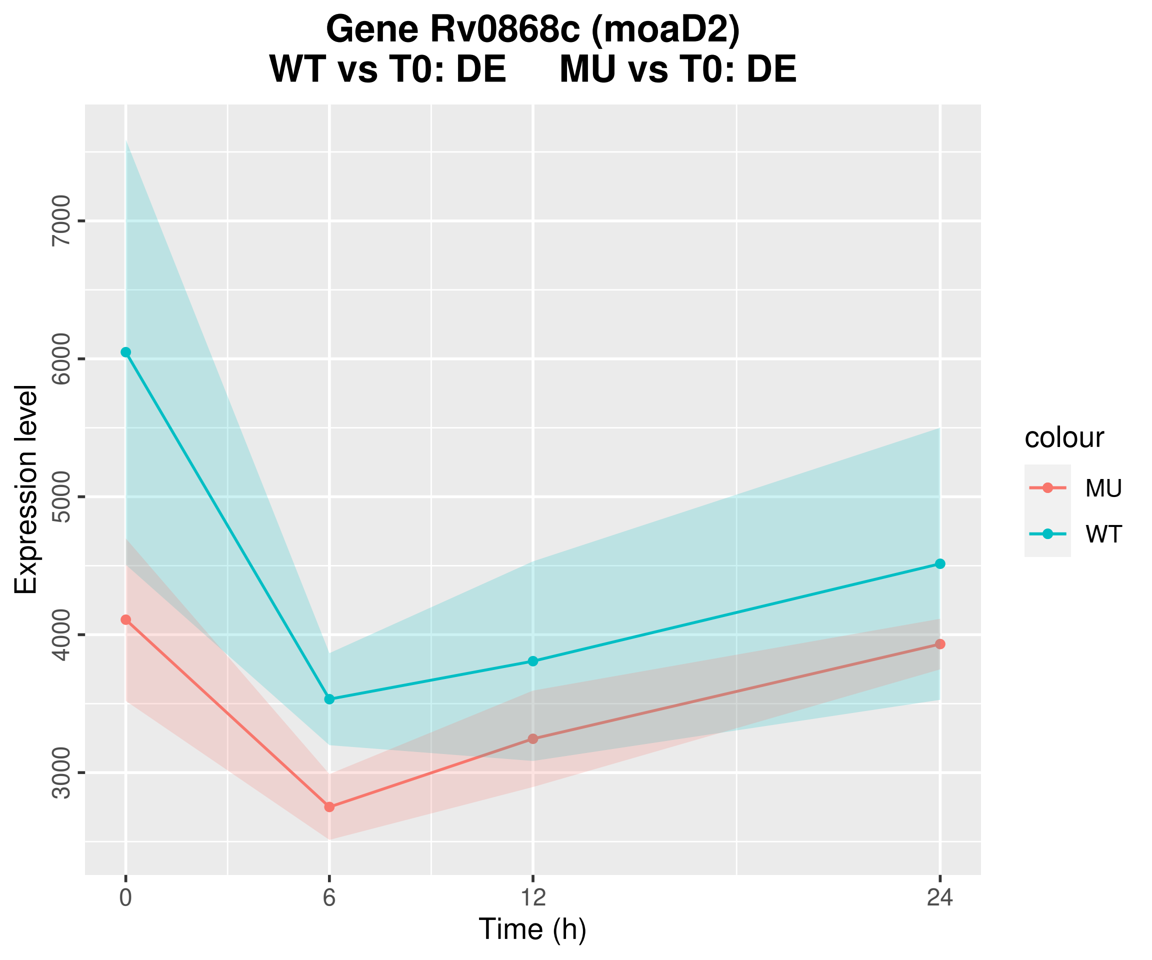

Gene Rv0868c (moaD2)
WT vs T0: DE MU vs T0: DE
Expression level
7000
6000
5000
4000
3000
0
6
12
24
Time (h)
colour
MU
WT

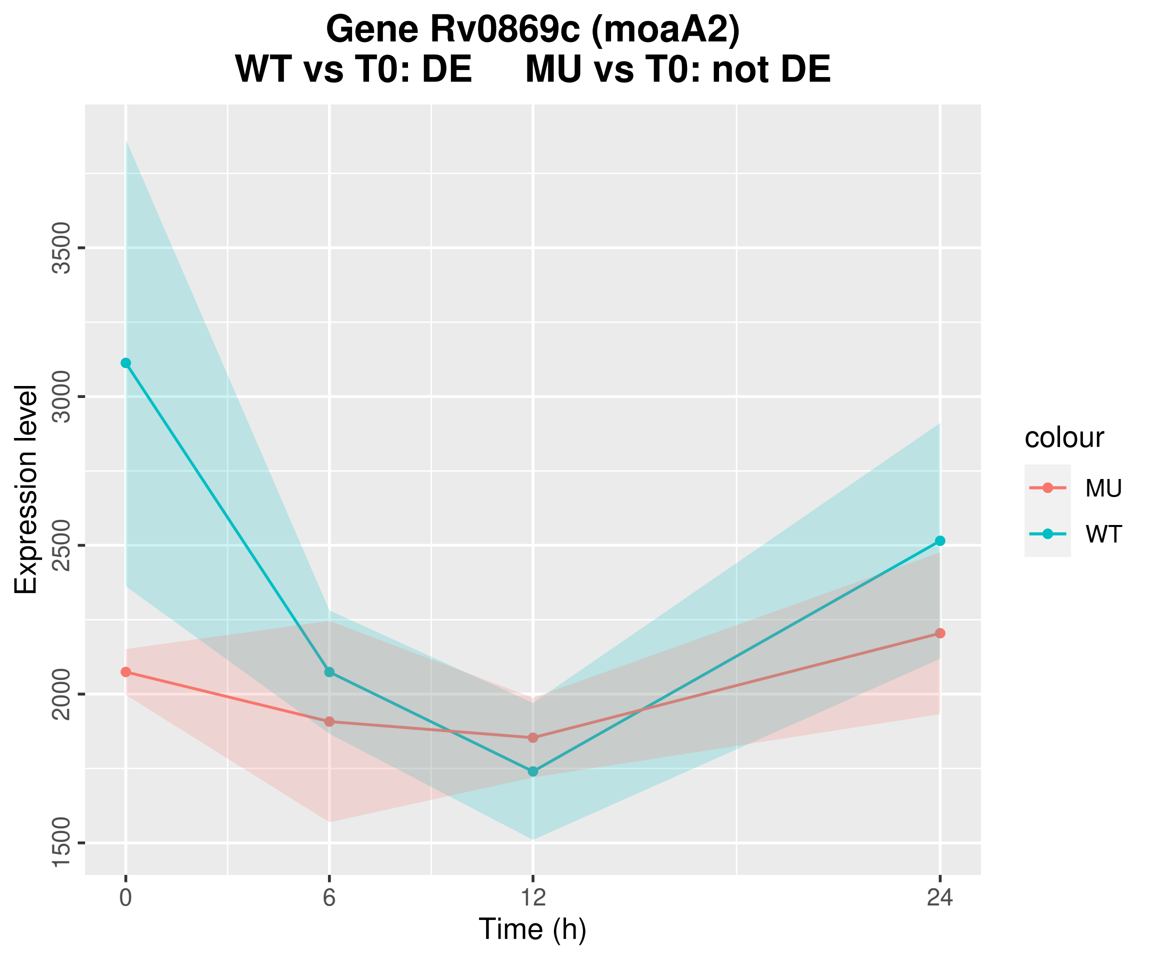

Gene Rv0869c (moaA2)
WT vs T0: DE   MU vs T0: not DE
Expression level
3500
3000
2500
2000
1500
0
6
12
24
Time (h)
colour
MU
WT

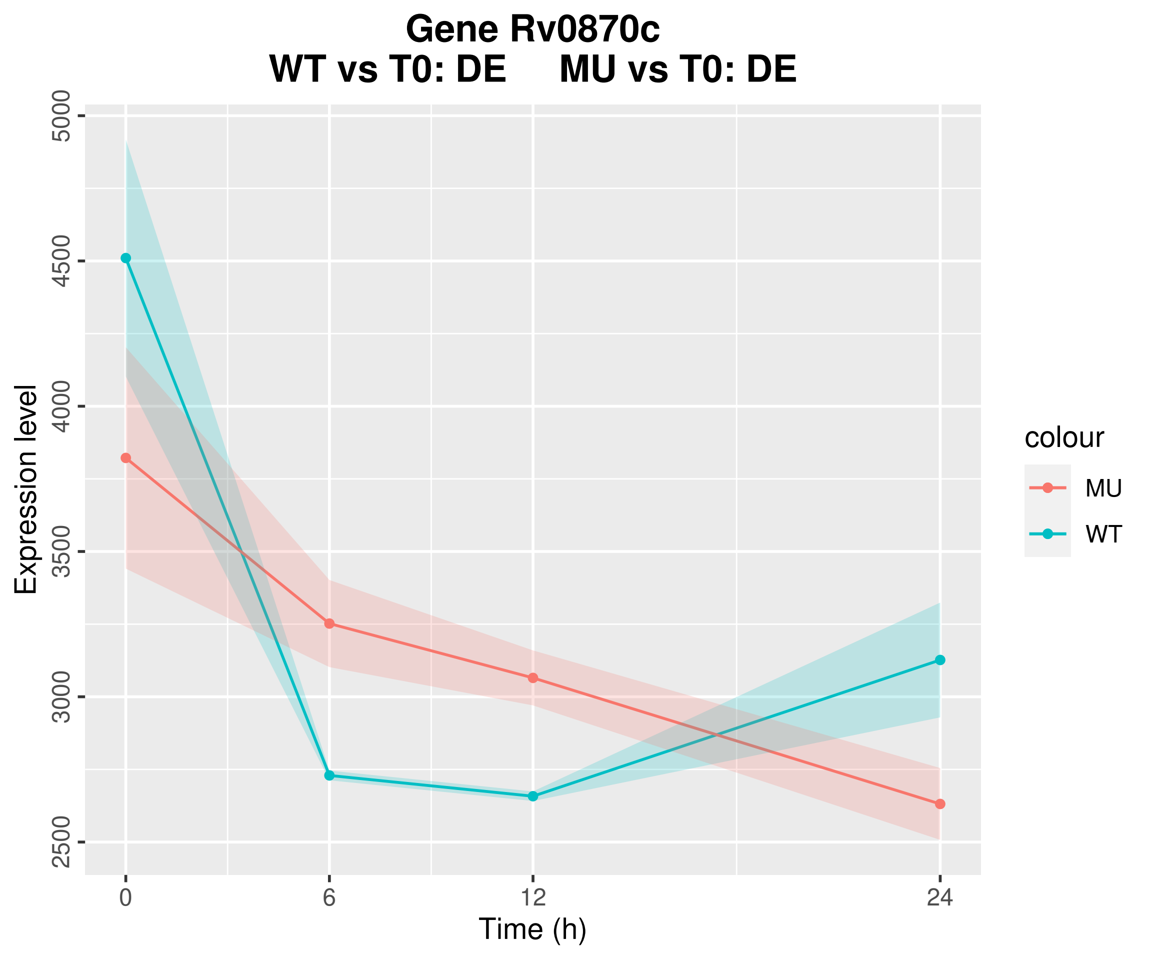

Gene Rv0870c
WT vs T0: DE   MU vs T0: DE
Expression level
5000
4500
4000
3500
3000
2500
0
6
12
24
Time (h)
colour
MU
WT

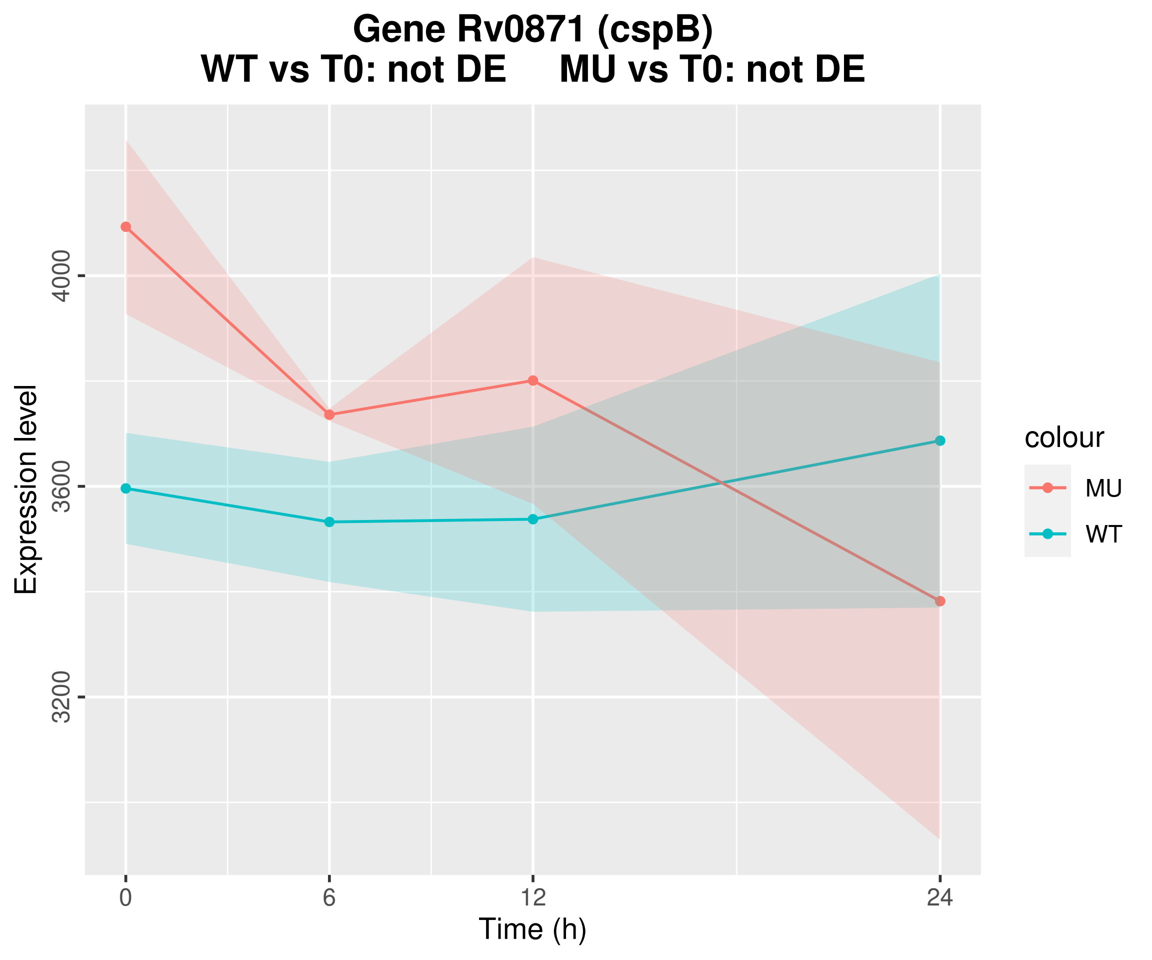

Gene Rv0871 (cspB)
WT vs T0: not DE MU vs T0: not DE
Expression level
4000
3600
3200
0
6
12
24
Time (h)
colour
MU
WT

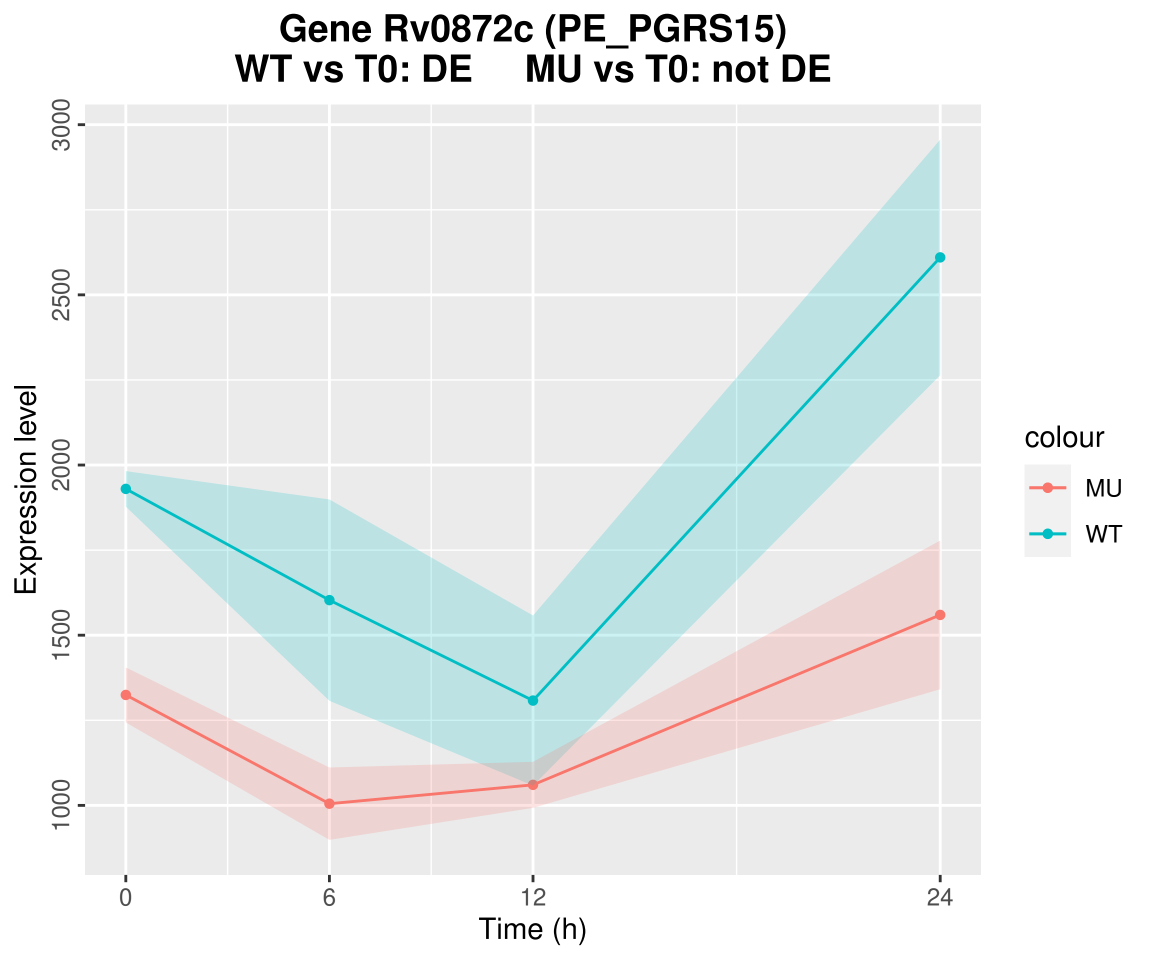

Gene Rv0872c (PE_PGRS15)
WT vs T0: DE MU vs T0: not DE
Expression level
3000
2500
2000
1500
1000
0
6
12
24
Time (h)
colour
MU
WT

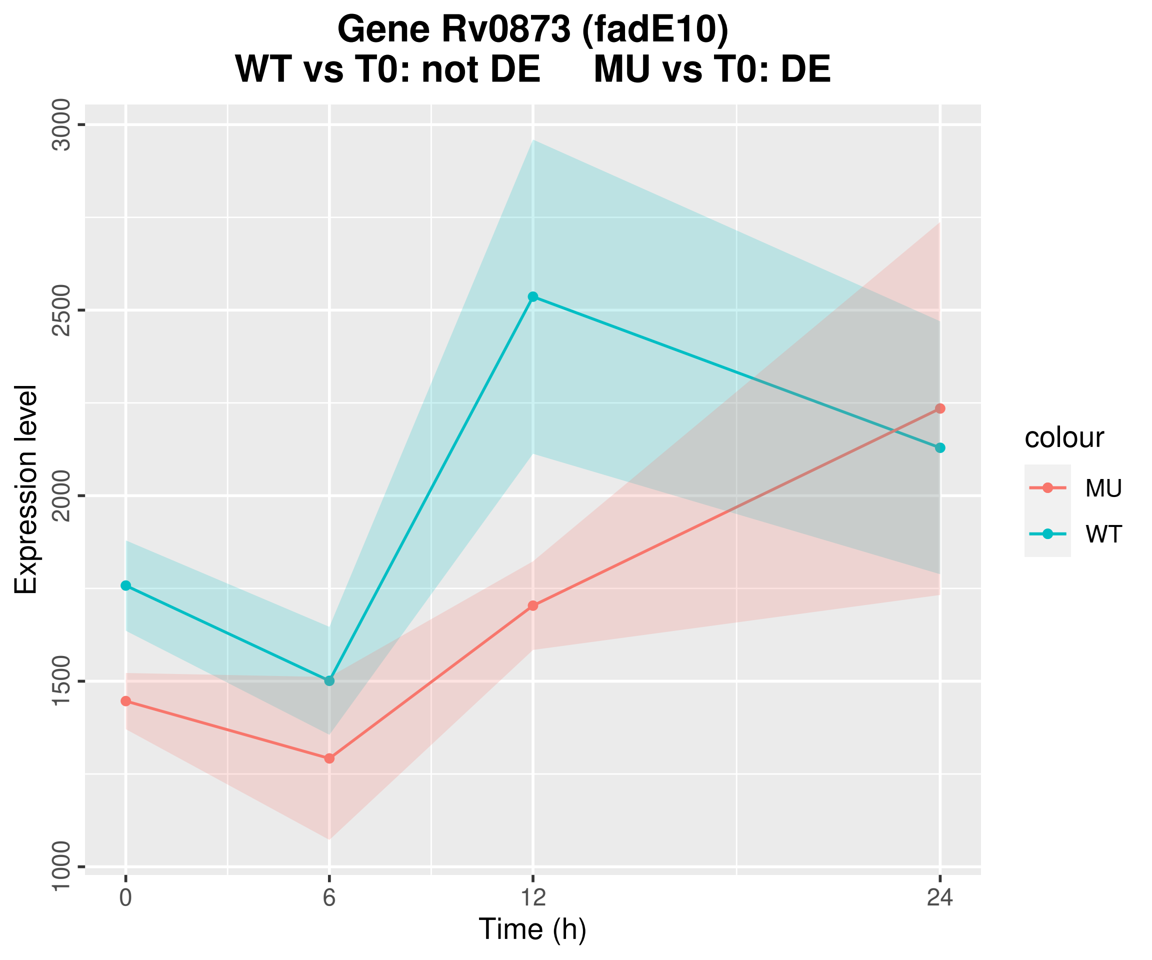

Gene Rv0873 (fadE10)
WT vs T0: not DE MU vs T0: DE
Expression level
3000
2500
2000
1500
1000
0
6
12
24
Time (h)
colour
MU
WT

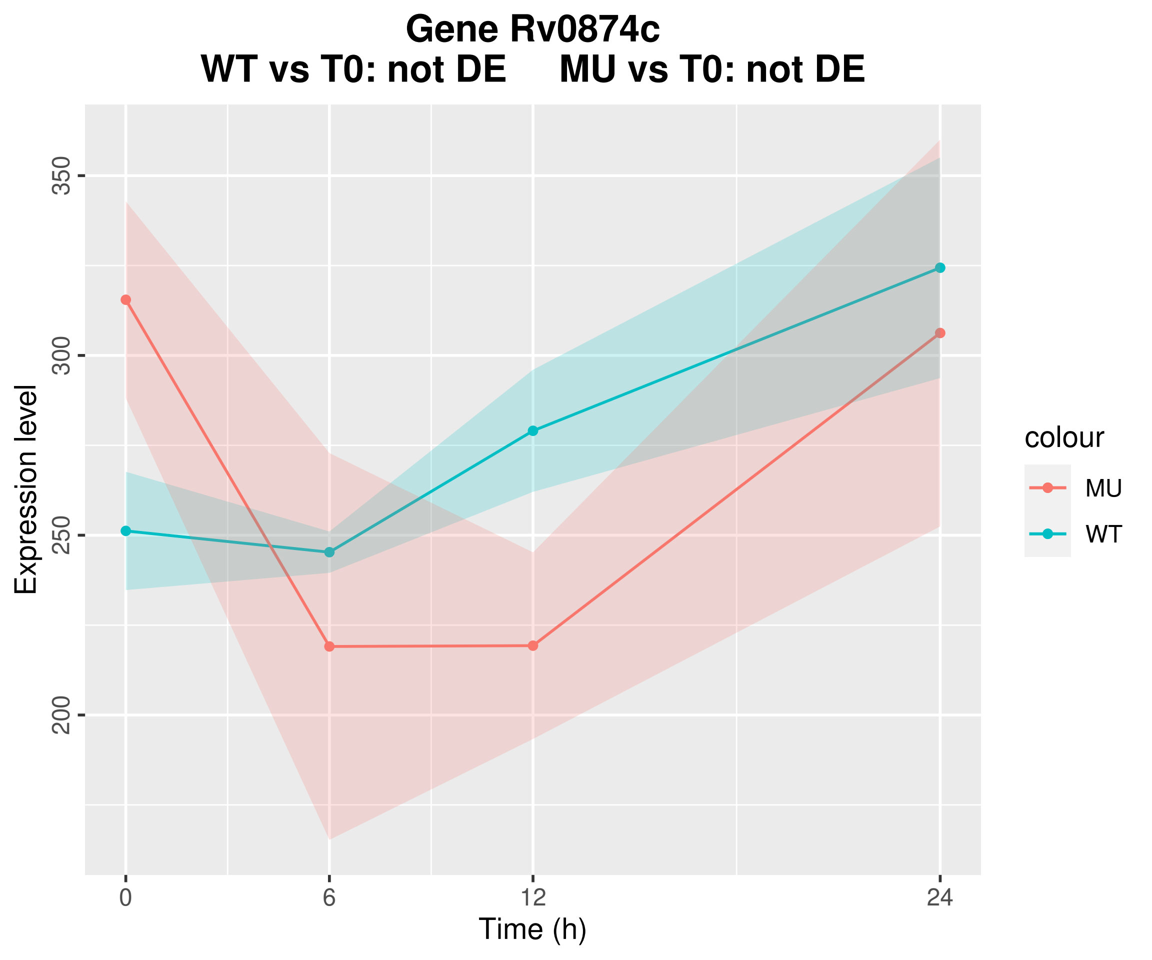
Gene Rv0874c
WT vs T0: not DE   MU vs T0: not DE
Expression level
350
300
250
200
0
6
12
24
Time (h)
colour
MU
WT

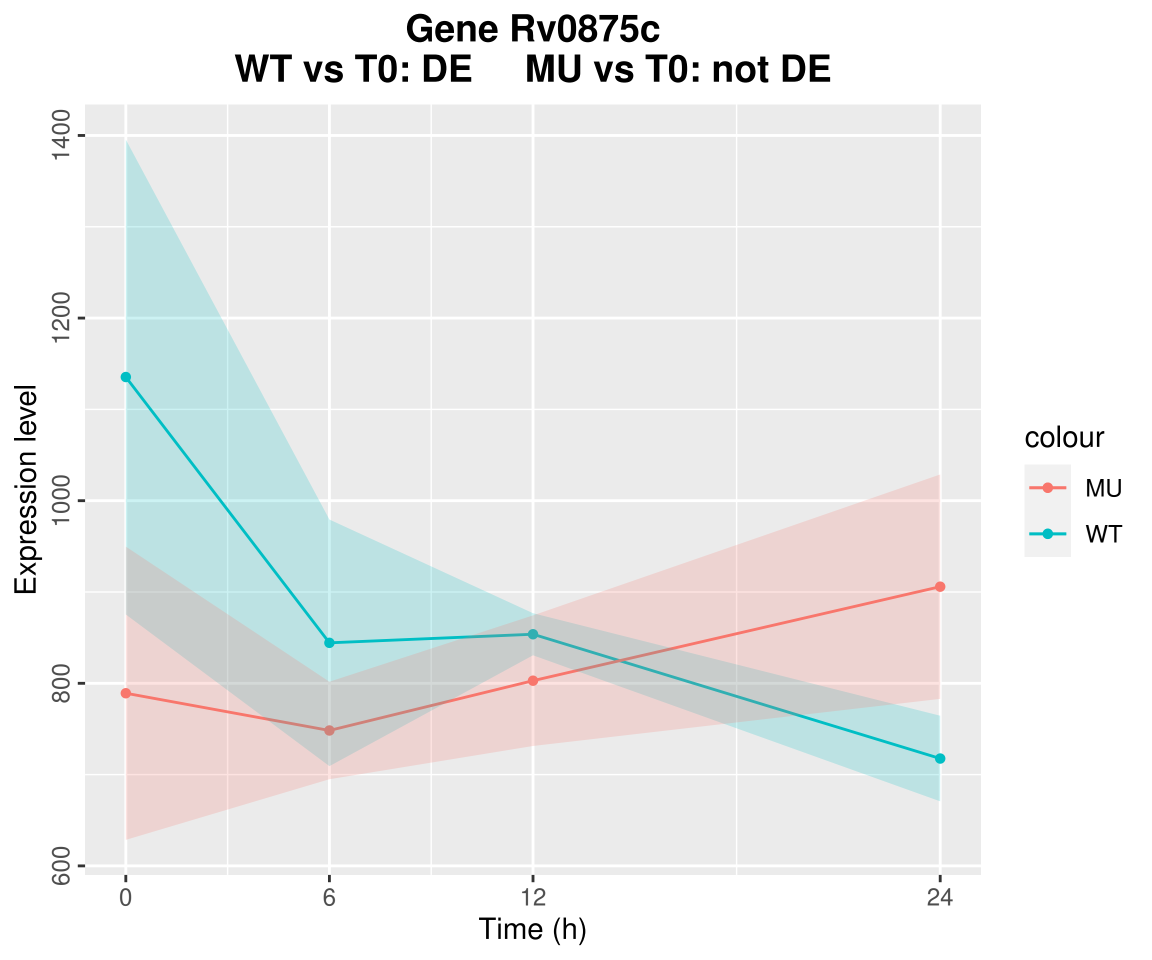

Gene Rv0875c
WT vs T0: DE   MU vs T0: not DE
Expression level
1400
1200
1000
800
600
0
6
12
24
Time (h)
colour
MU
WT

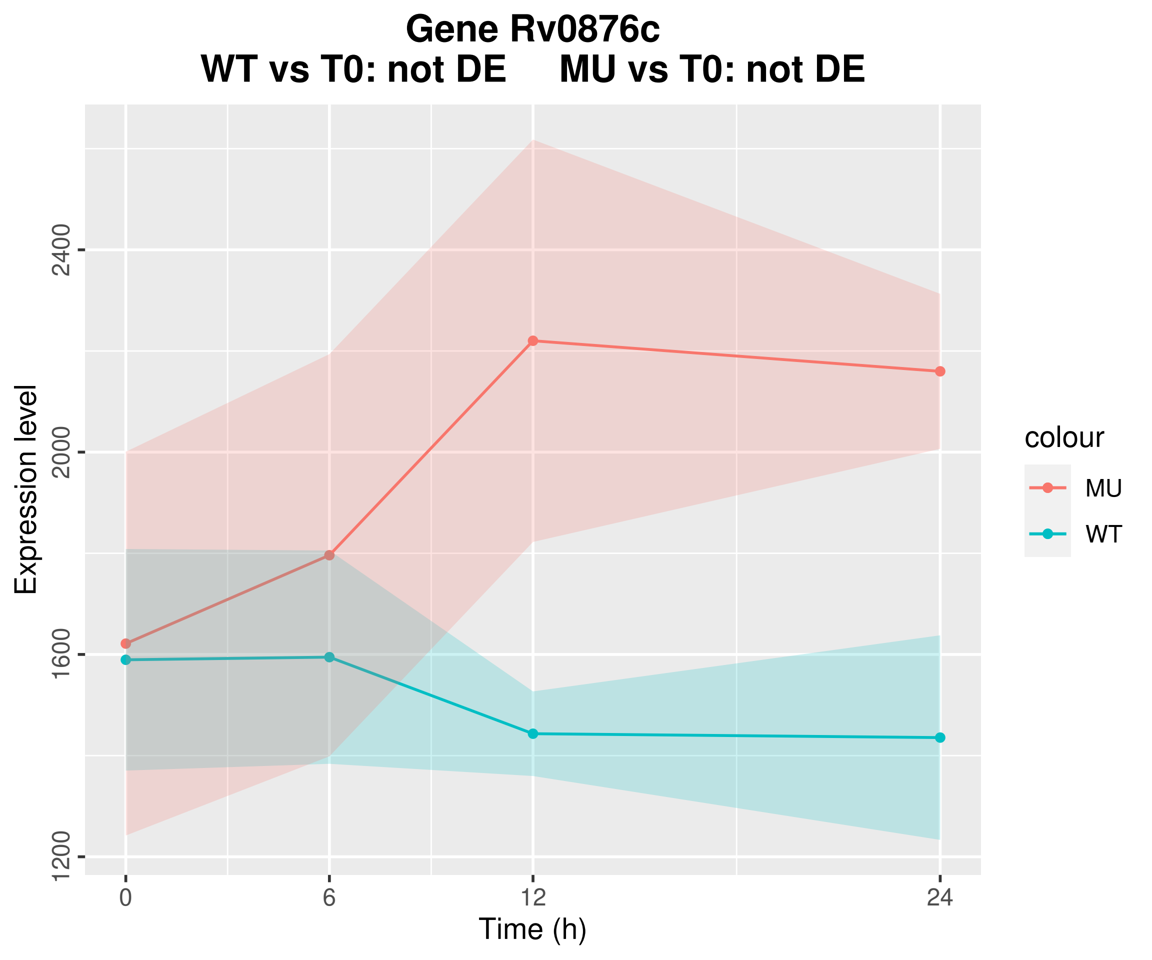

Gene Rv0876c
WT vs T0: not DE   MU vs T0: not DE
Expression level
2400
2000
1600
1200
0
6
12
24
Time (h)
colour
MU
WT

Gene Rv0877
WT vs T0: not DE   MU vs T0: DE

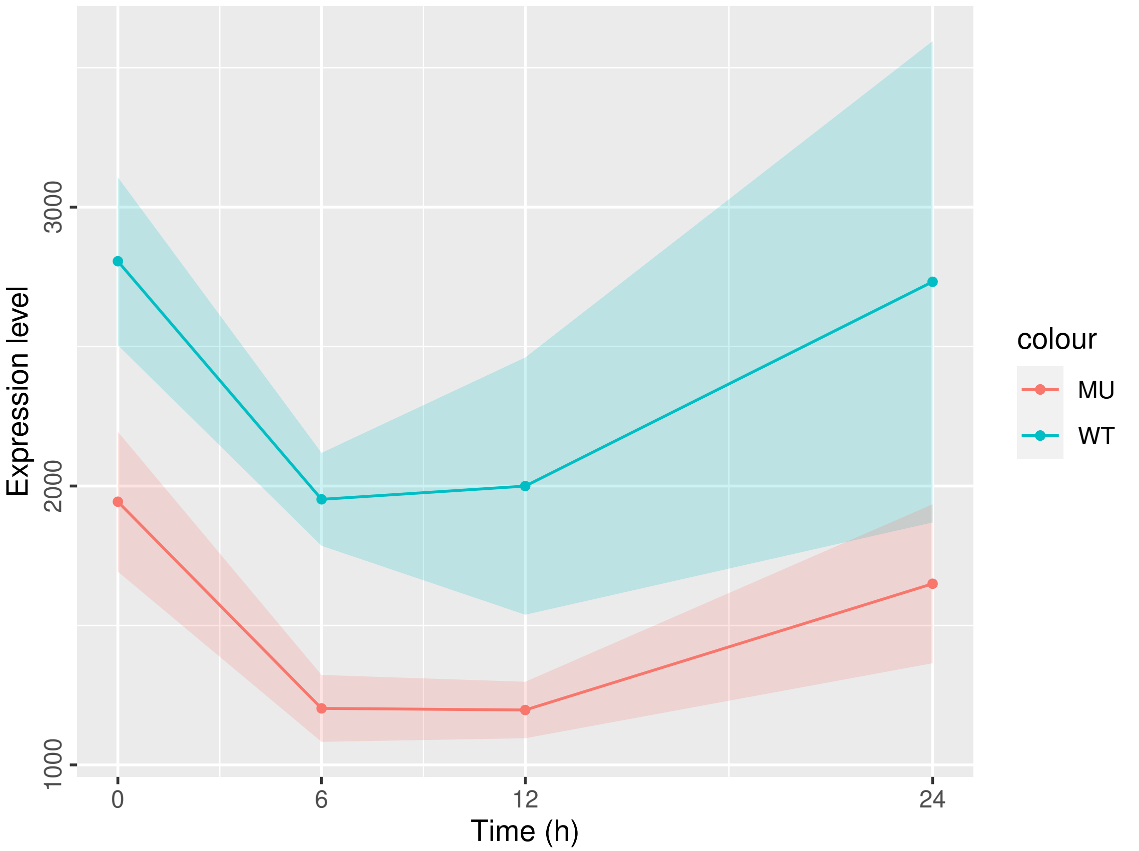

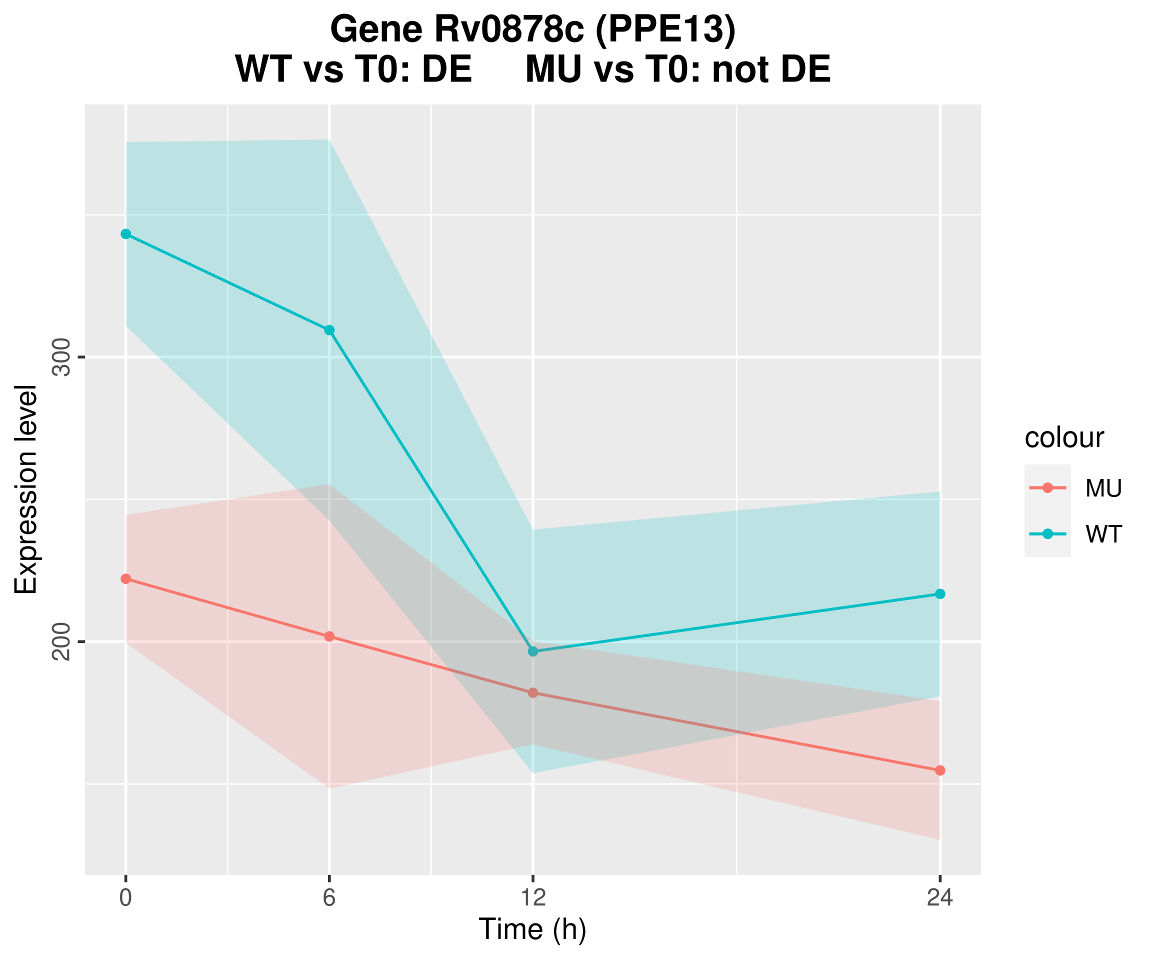
Gene Rv0878c (PPE13)
WT vs T0: DE   MU vs T0: not DE
Expression level
300
200
Time (h)
0
6
12
24
colour
MU
WT

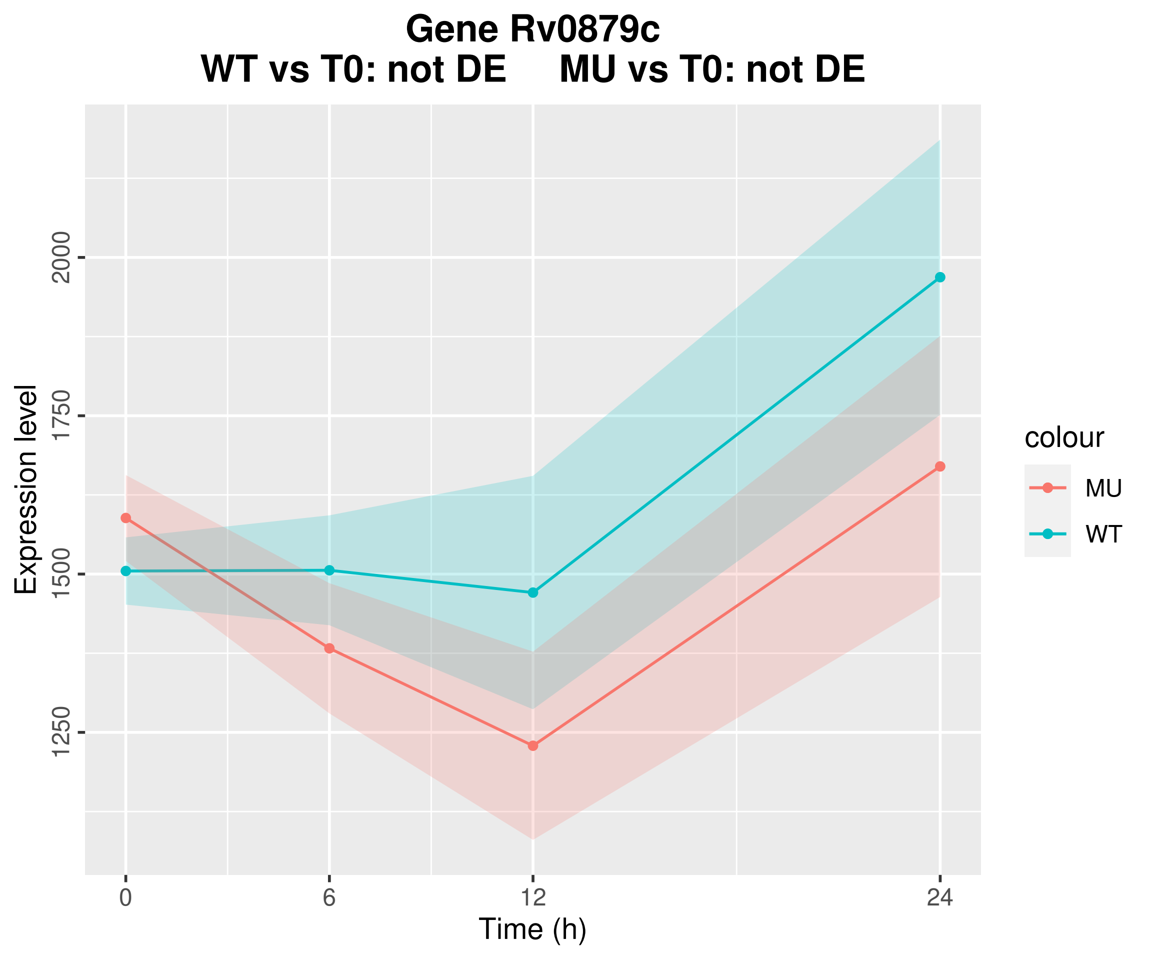
Gene Rv0879c
WT vs T0: not DE MU vs T0: not DE
Expression level
2000
1750
1500
1250
0
6
12
24
Time (h)
colour
MU
WT

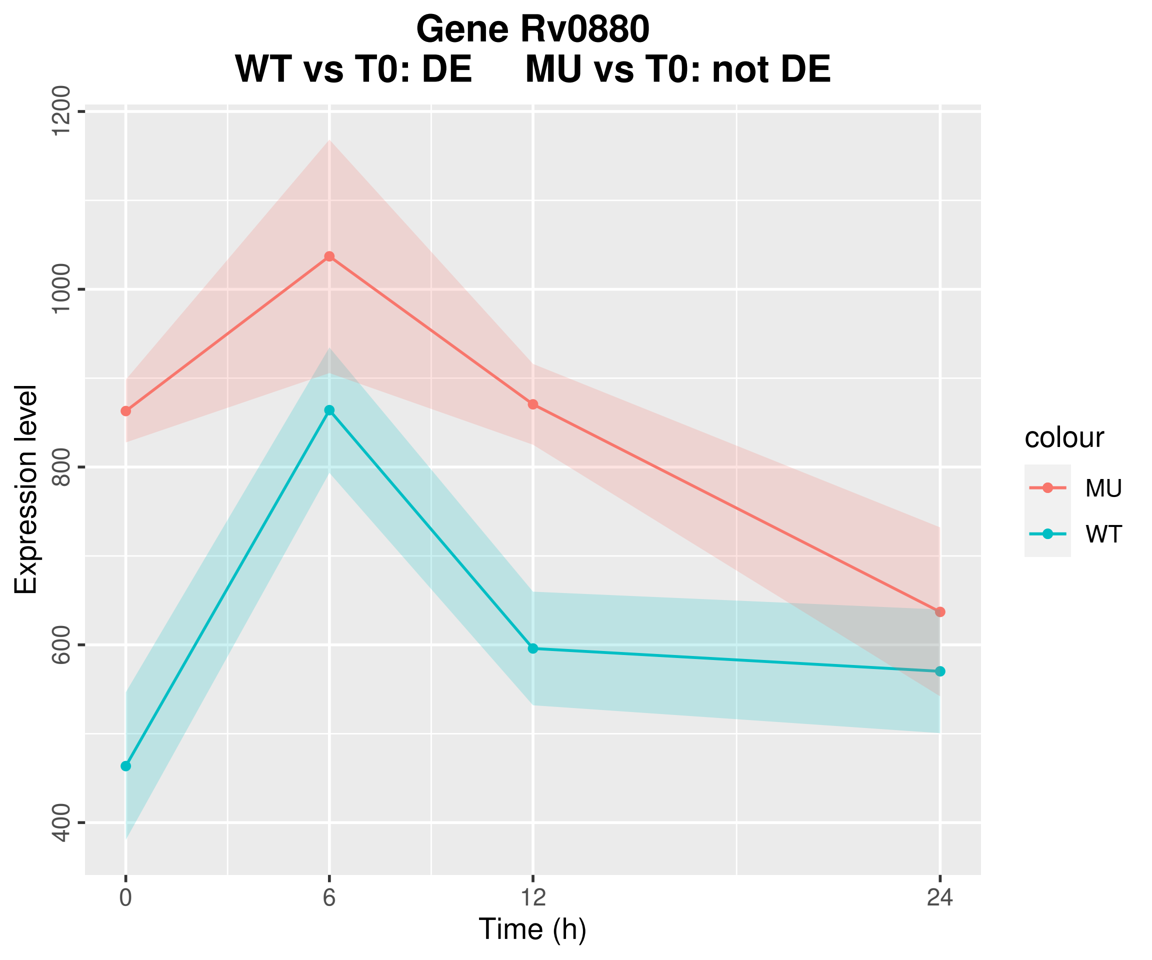
Gene Rv0880
WT vs T0: DE MU vs T0: not DE
Expression level
1200
1000
800
600
400
0
6
12
24
Time (h)
colour
MU
WT

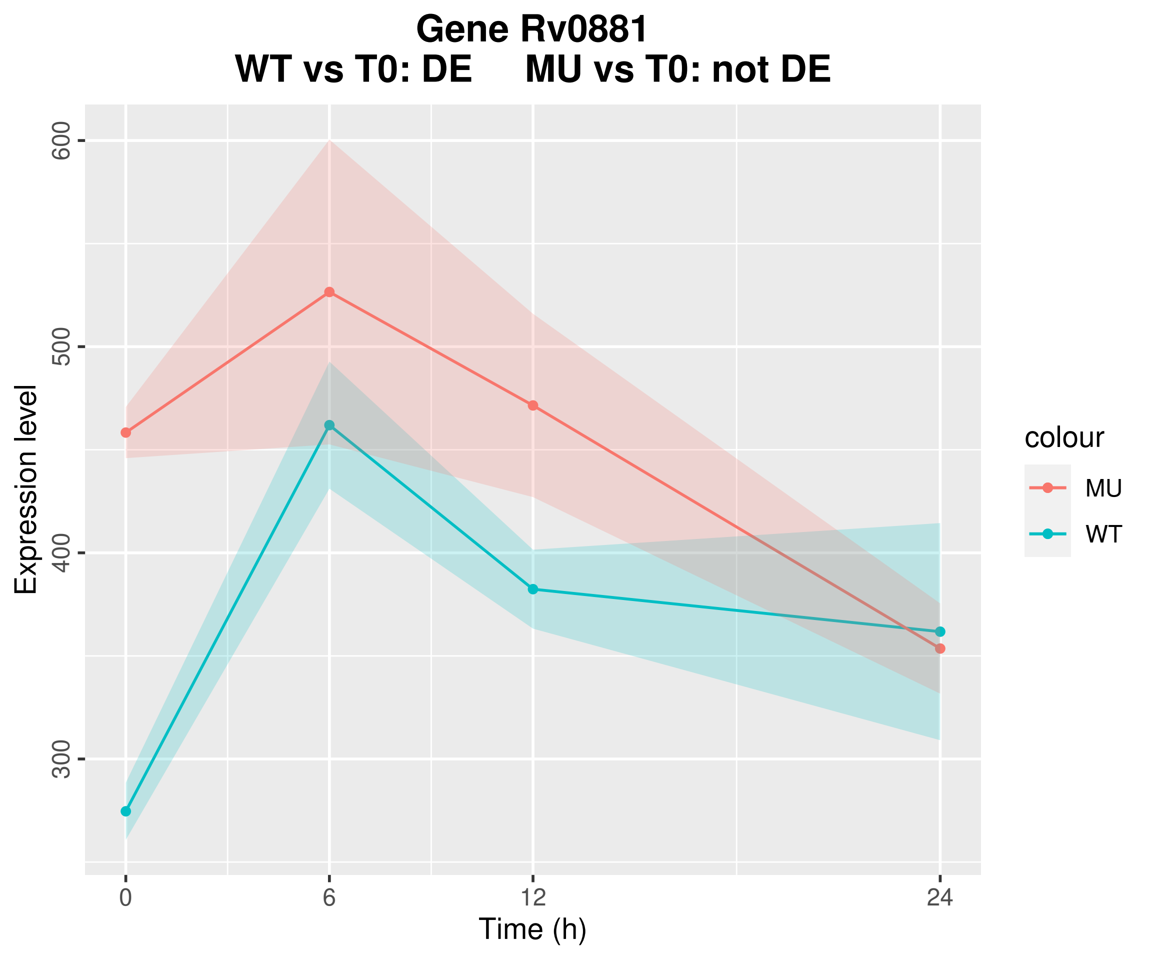

Gene Rv0881
WT vs T0: DE   MU vs T0: not DE
600
500
400
300
Expression level
0
6
12
24
Time (h)
colour
MU
WT

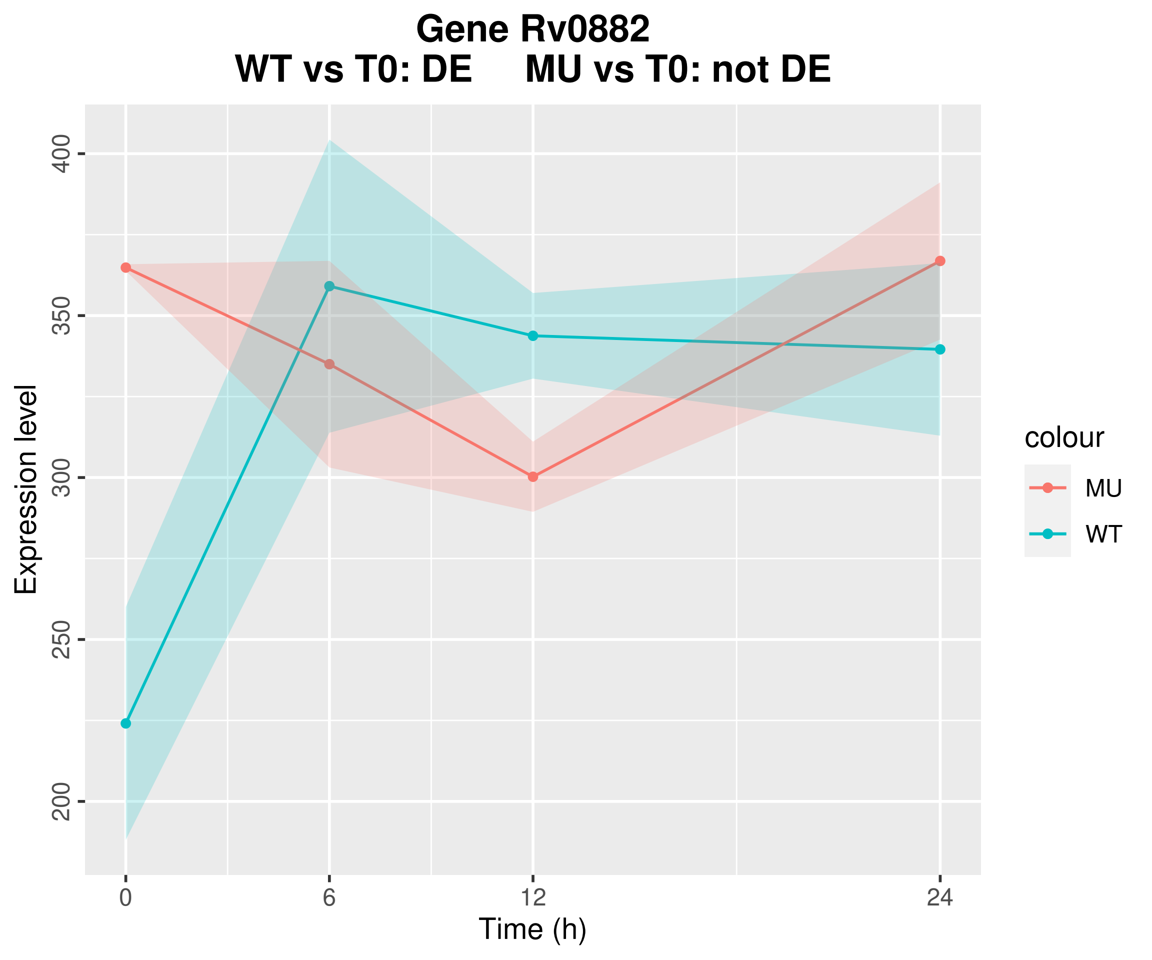
Gene Rv0882
WT vs T0: DE   MU vs T0: not DE
Expression level
400
350
300
250
200
0
6
12
24
Time (h)
colour
MU
WT

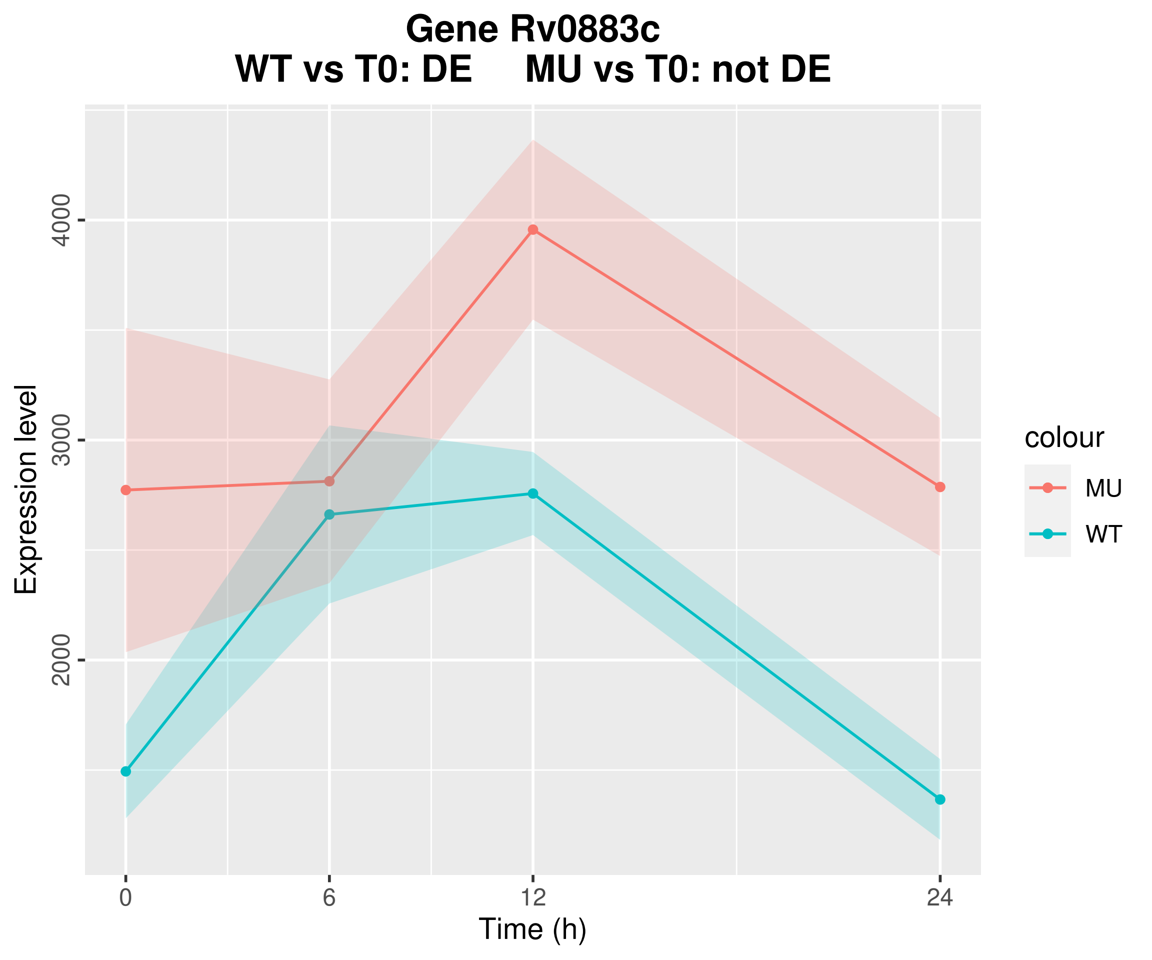
Gene Rv0883c
WT vs T0: DE   MU vs T0: not DE
Expression level
4000
3000
2000
0
6
12
24
Time (h)
colour
MU
WT

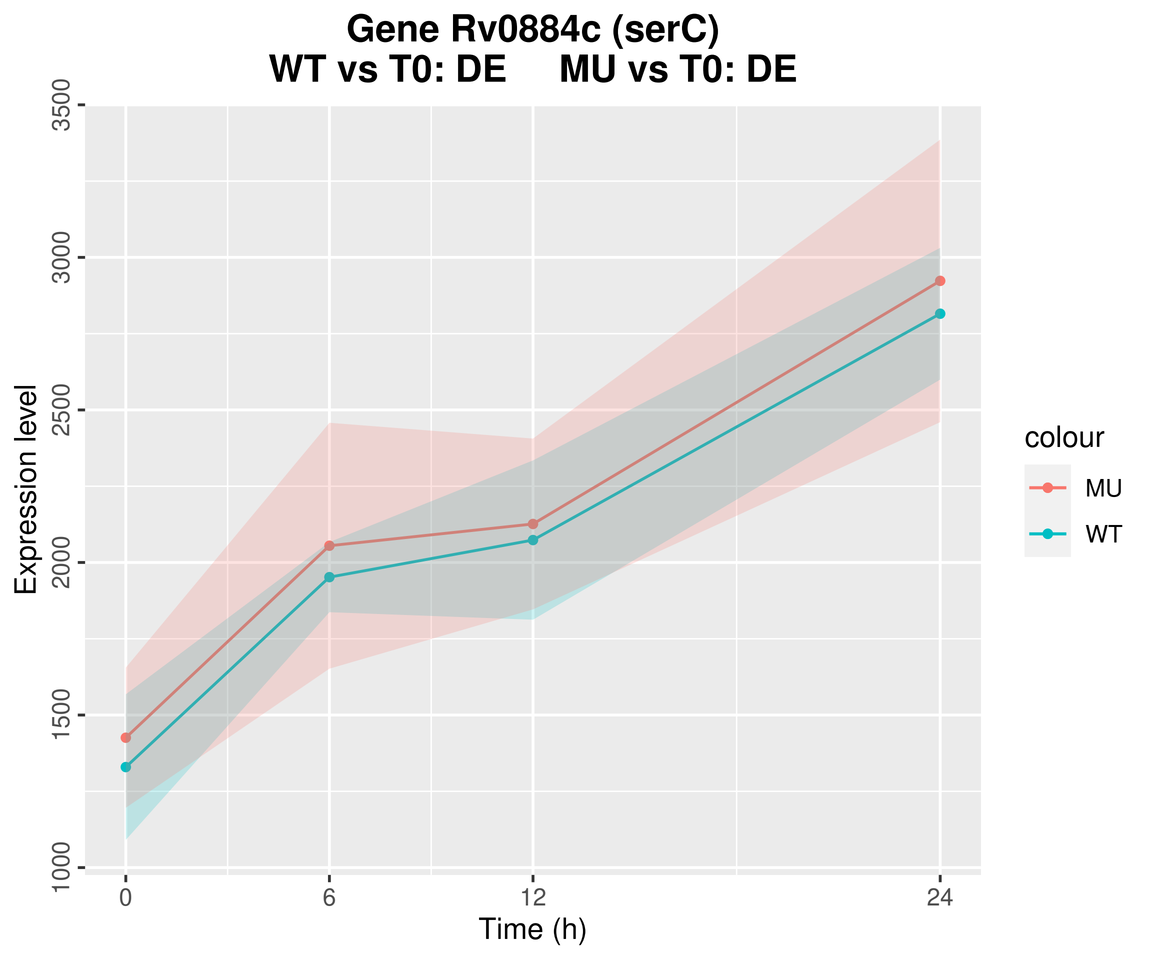

Gene Rv0884c (serC)
WT vs T0: DE MU vs T0: DE
Expression level
3500
3000
2500
2000
1500
1000
0
6
12
24
Time (h)
colour
MU
WT

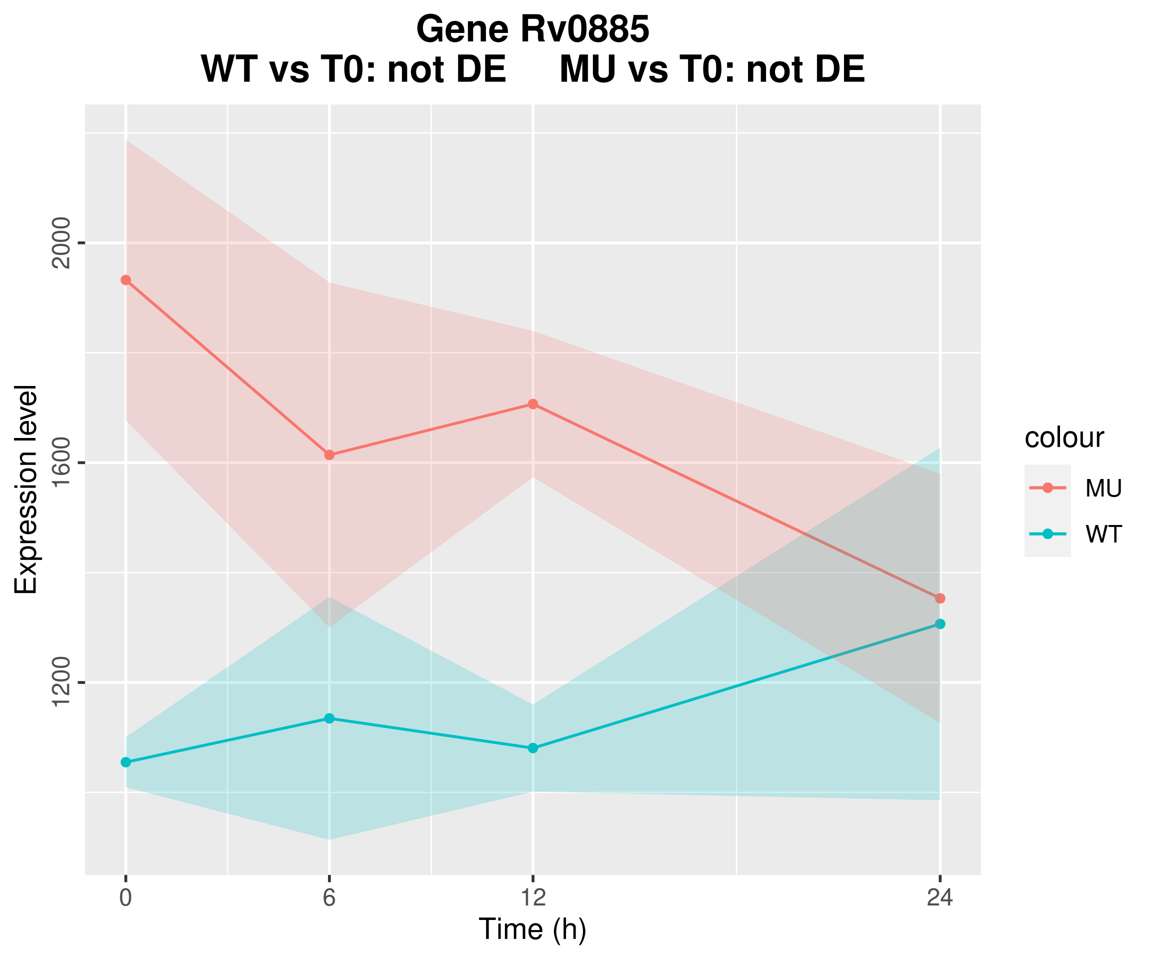
Gene Rv0885
WT vs T0: not DE MU vs T0: not DE
Expression level
2000
1600
1200
Time (h)
0
6
12
24
colour
MU
WT

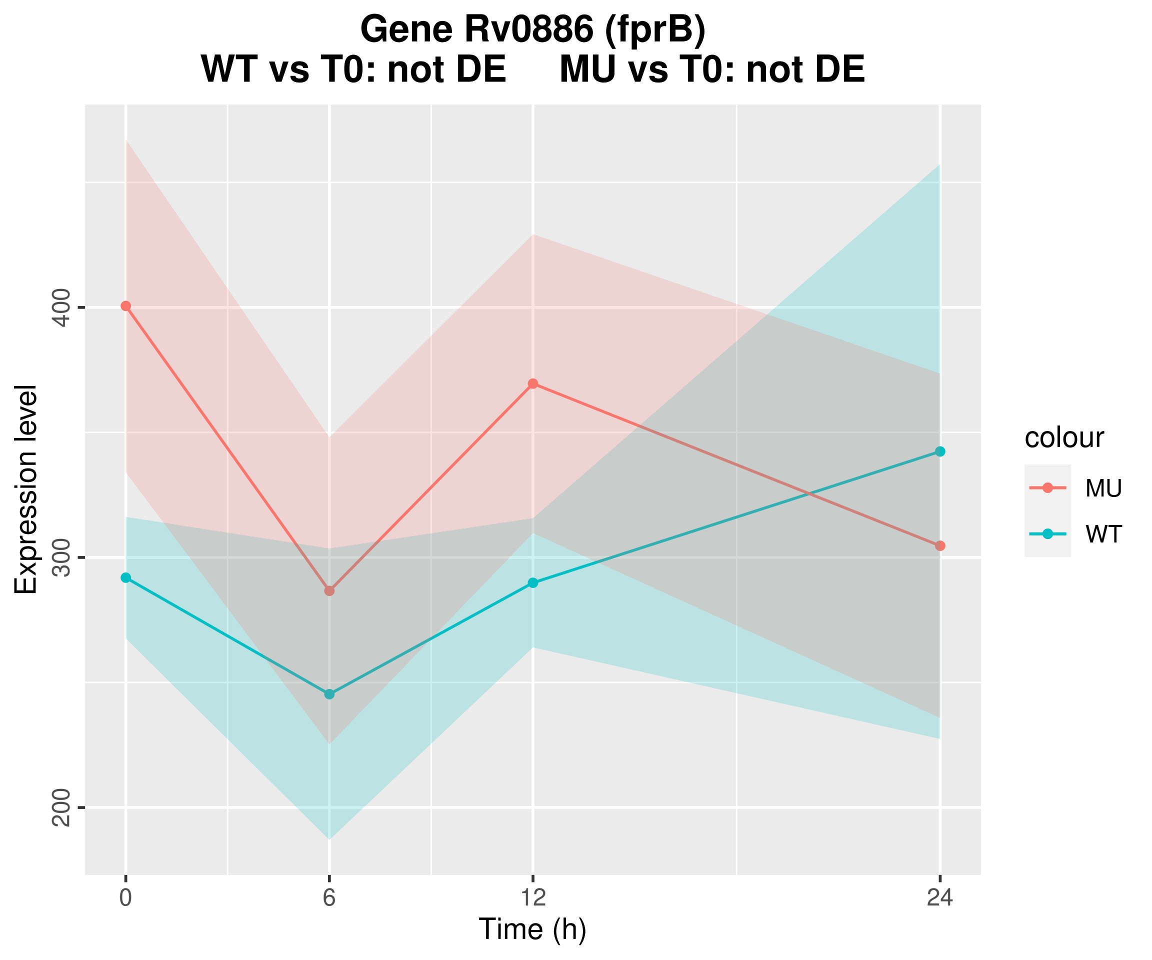
Gene Rv0886 (fprB)
WT vs T0: not DE   MU vs T0: not DE
Expression level
400
300
200
0
6
12
24
Time (h)
colour
MU
WT

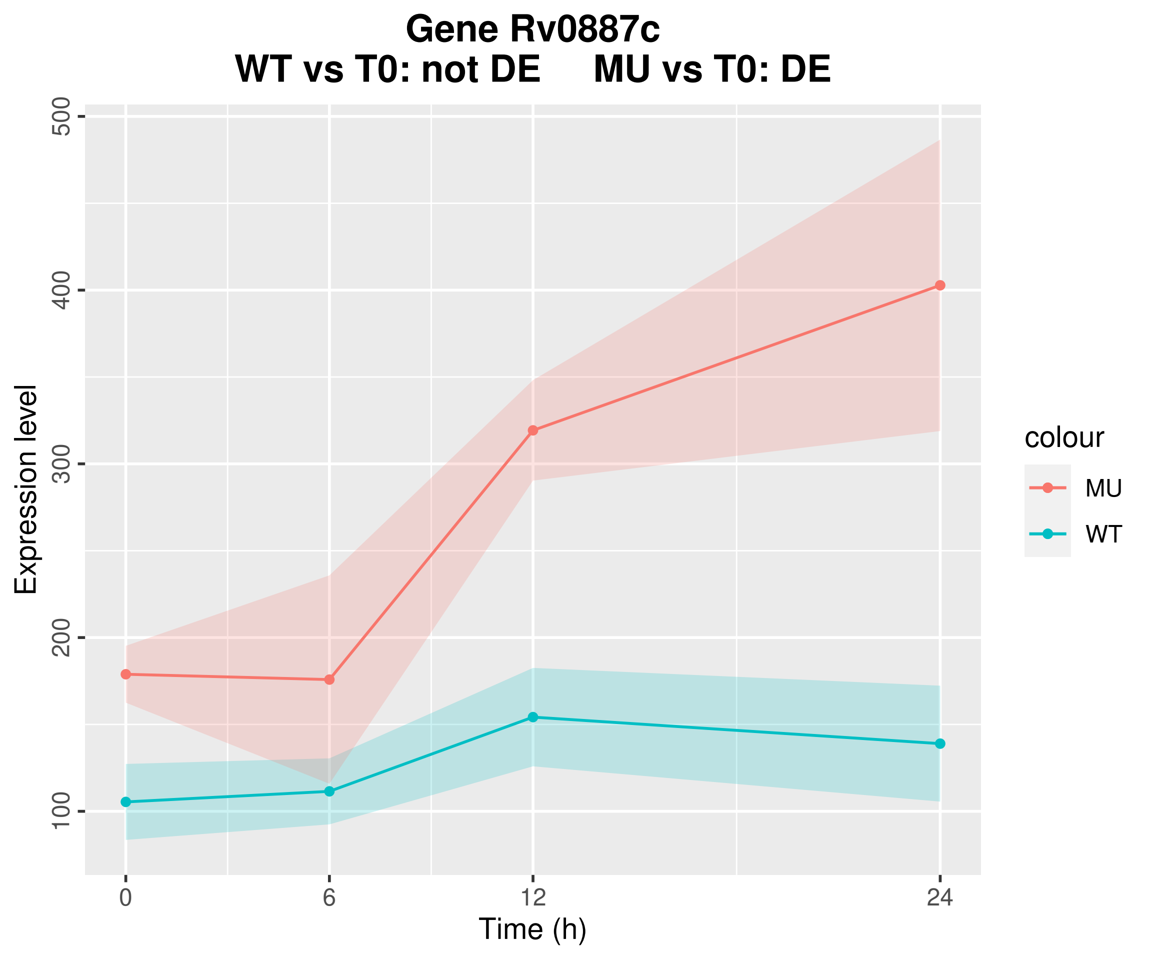
Gene Rv0887c
WT vs T0: not DE MU vs T0: DE
Expression level
100
200
300
400
500
0
6
12
24
Time (h)
colour
MU
WT

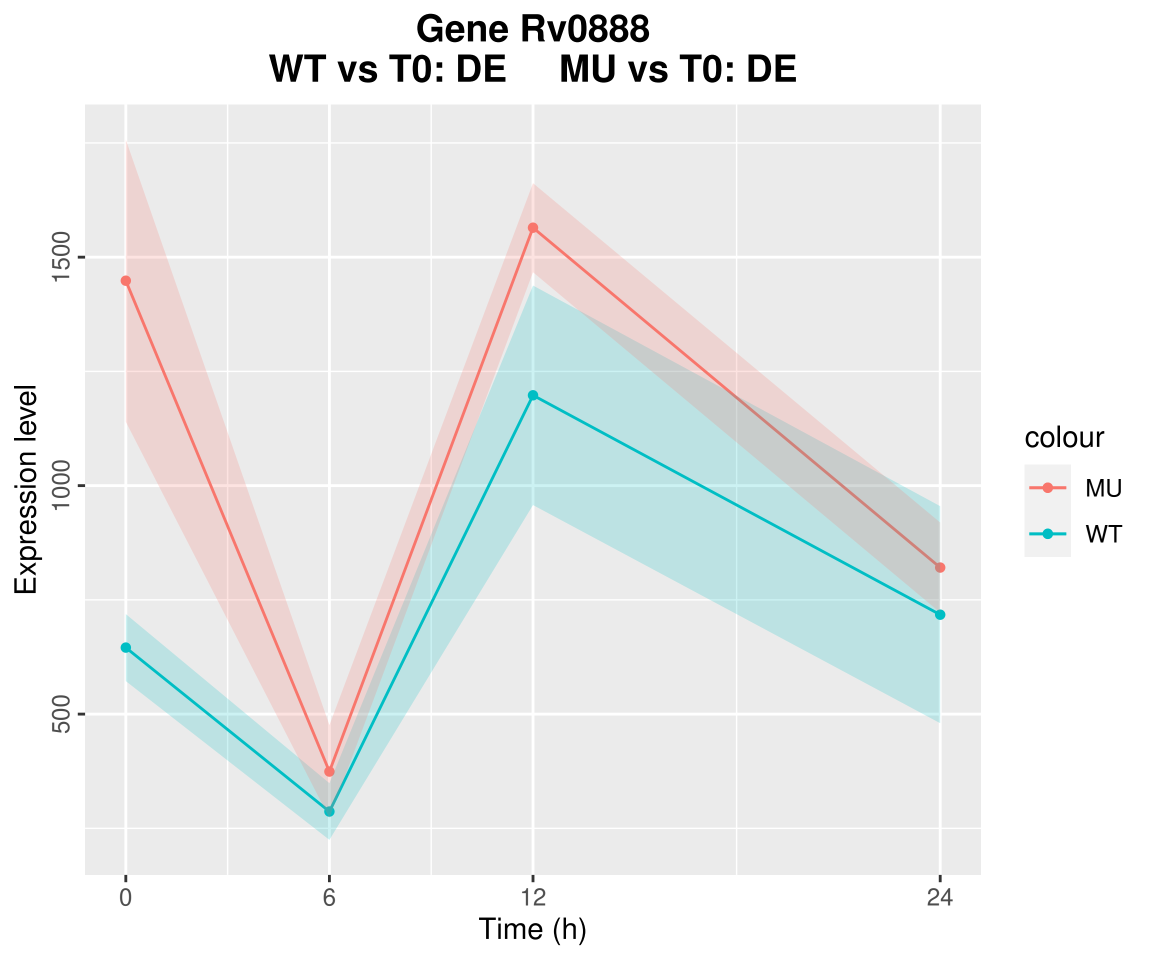

Gene Rv0888
WT vs T0: DE MU vs T0: DE
Expression level
1500
1000
500
0
6
12
24
Time (h)
colour
MU
WT

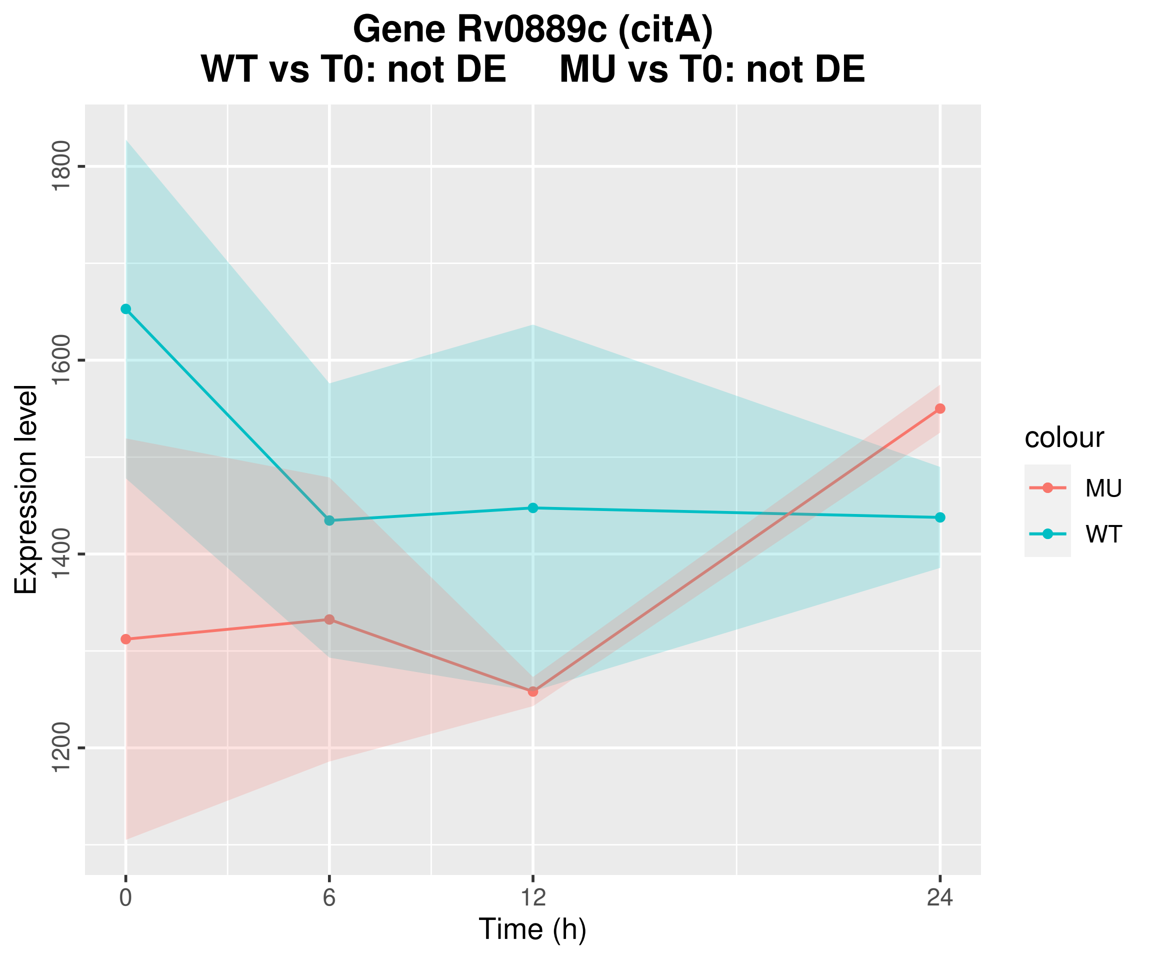
Gene Rv0889c (citA)
WT vs T0: not DE   MU vs T0: not DE
Expression level
1800
1600
1400
1200
0
6
12
24
Time (h)
colour
MU
WT

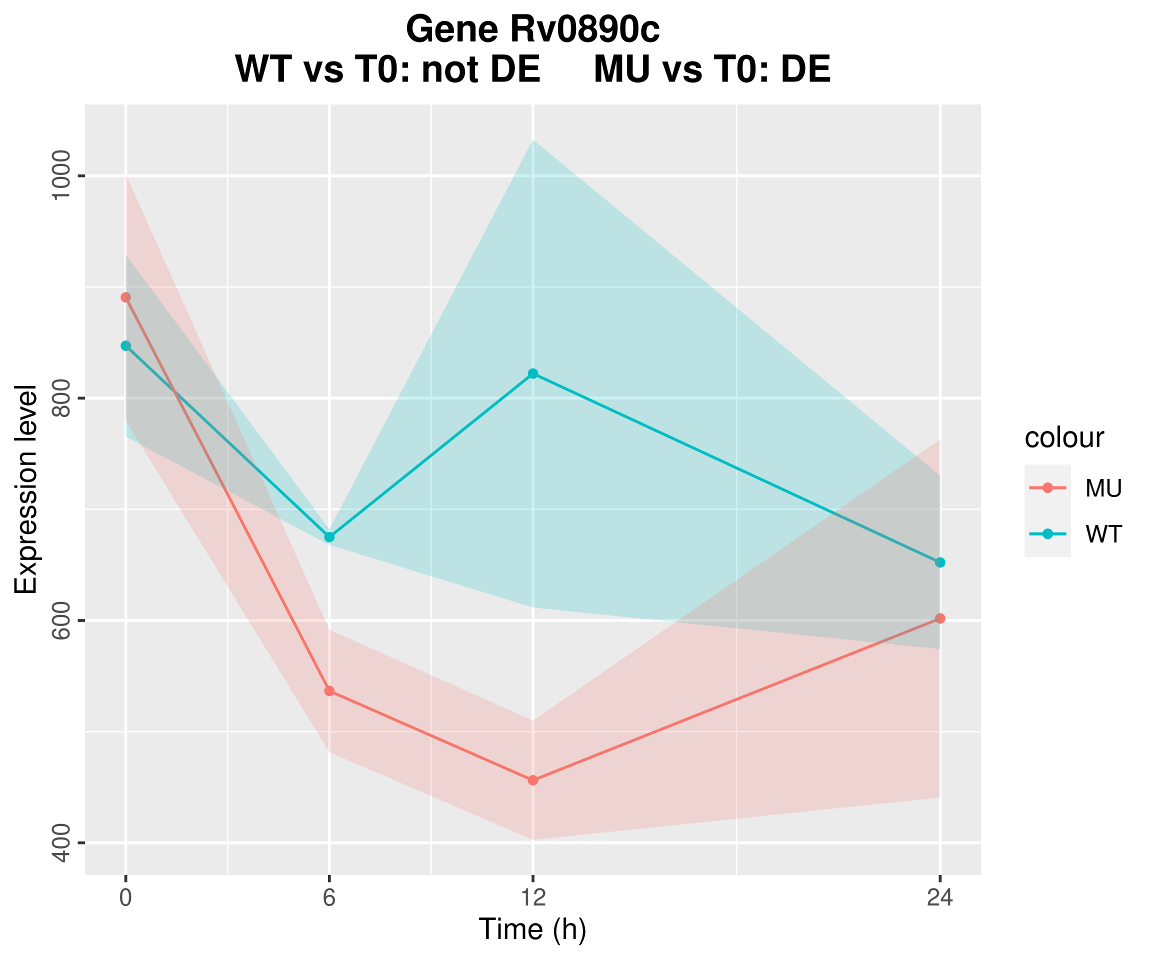

Gene Rv0890c
WT vs T0: not DE MU vs T0: DE
Expression level
1000
800
600
400
0
6
12
24
Time (h)
colour
MU
WT

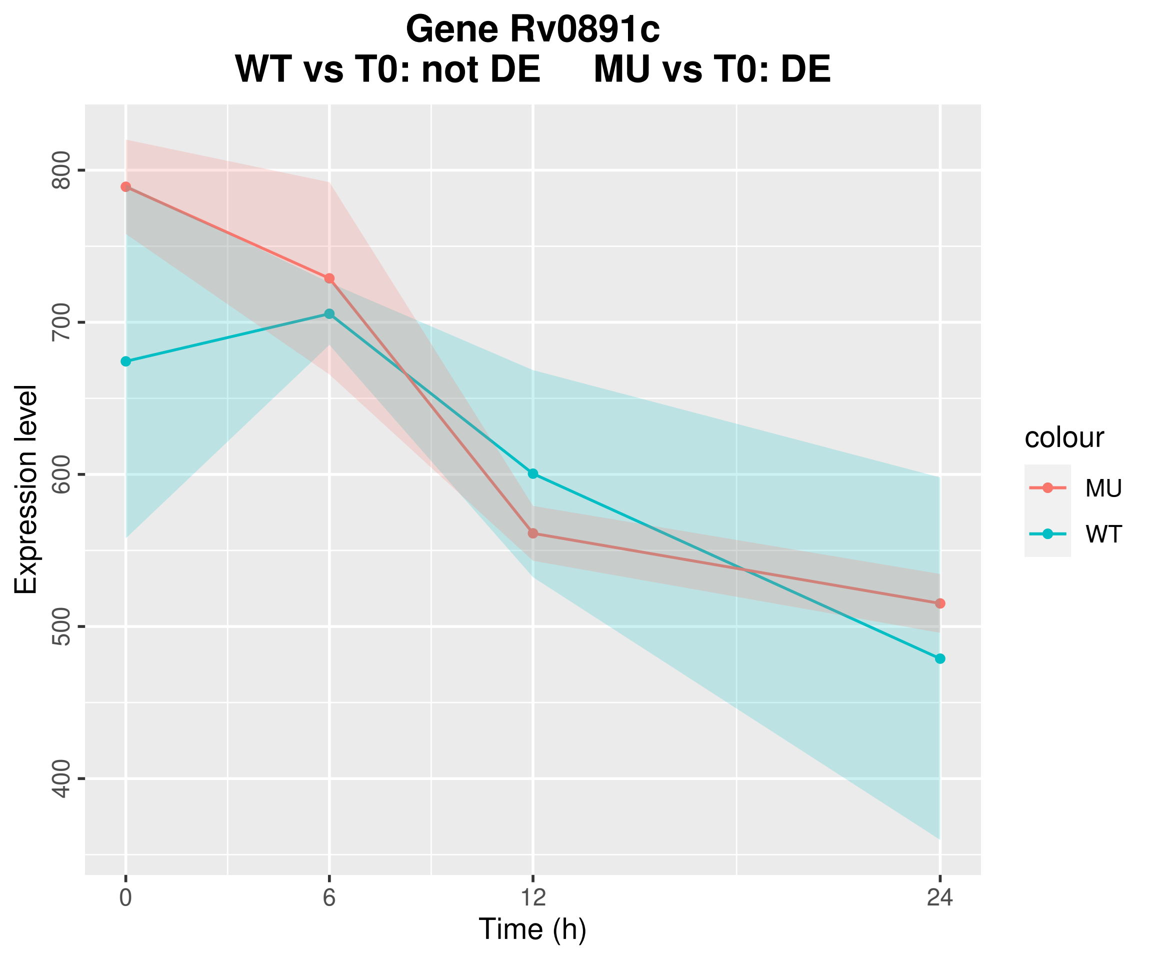
Gene Rv0891c
WT vs T0: not DE   MU vs T0: DE
Expression level
800
700
600
500
400
0
6
12
24
Time (h)
colour
MU
WT

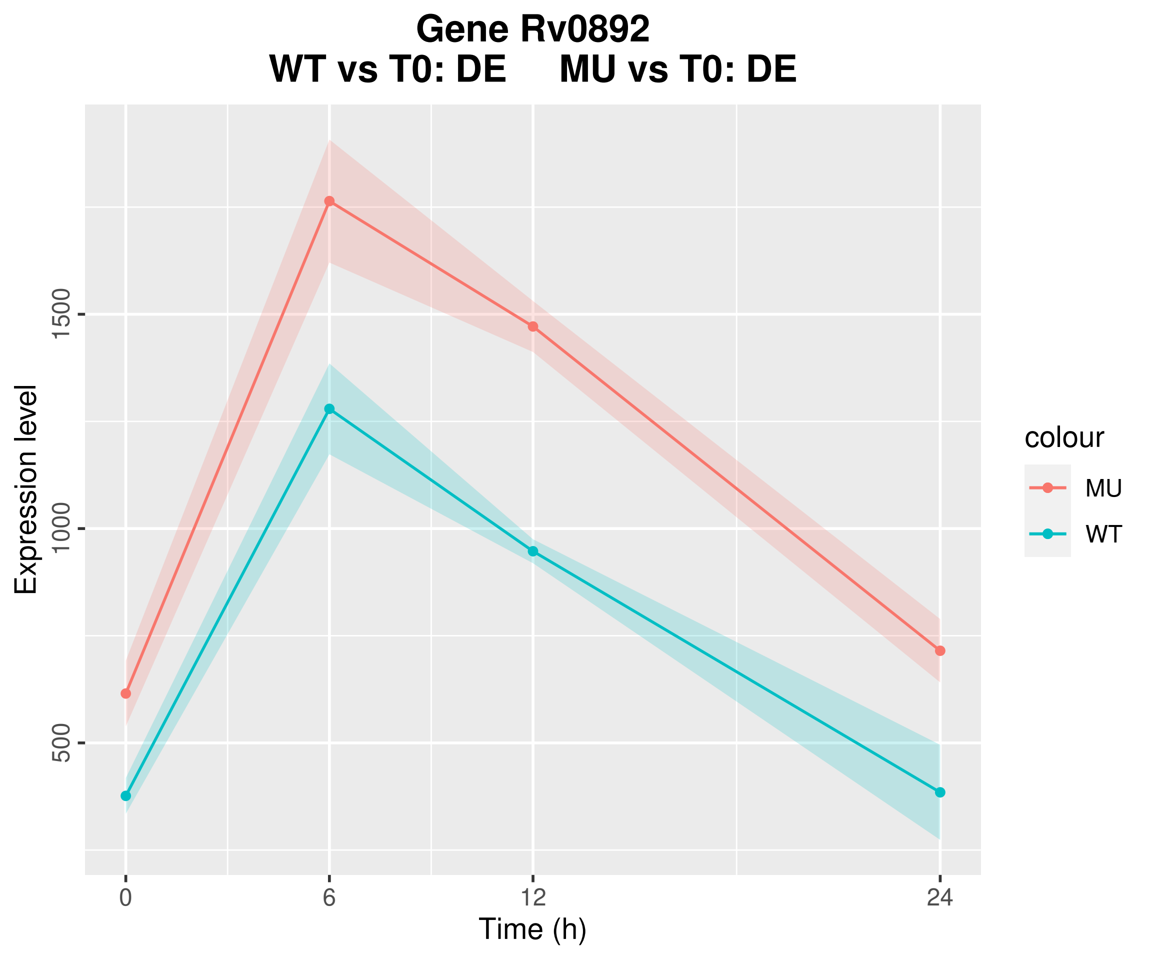

Gene Rv0892
WT vs T0: DE   MU vs T0: DE
Expression level
1500
1000
500
0
6
12
24
Time (h)
colour
MU
WT

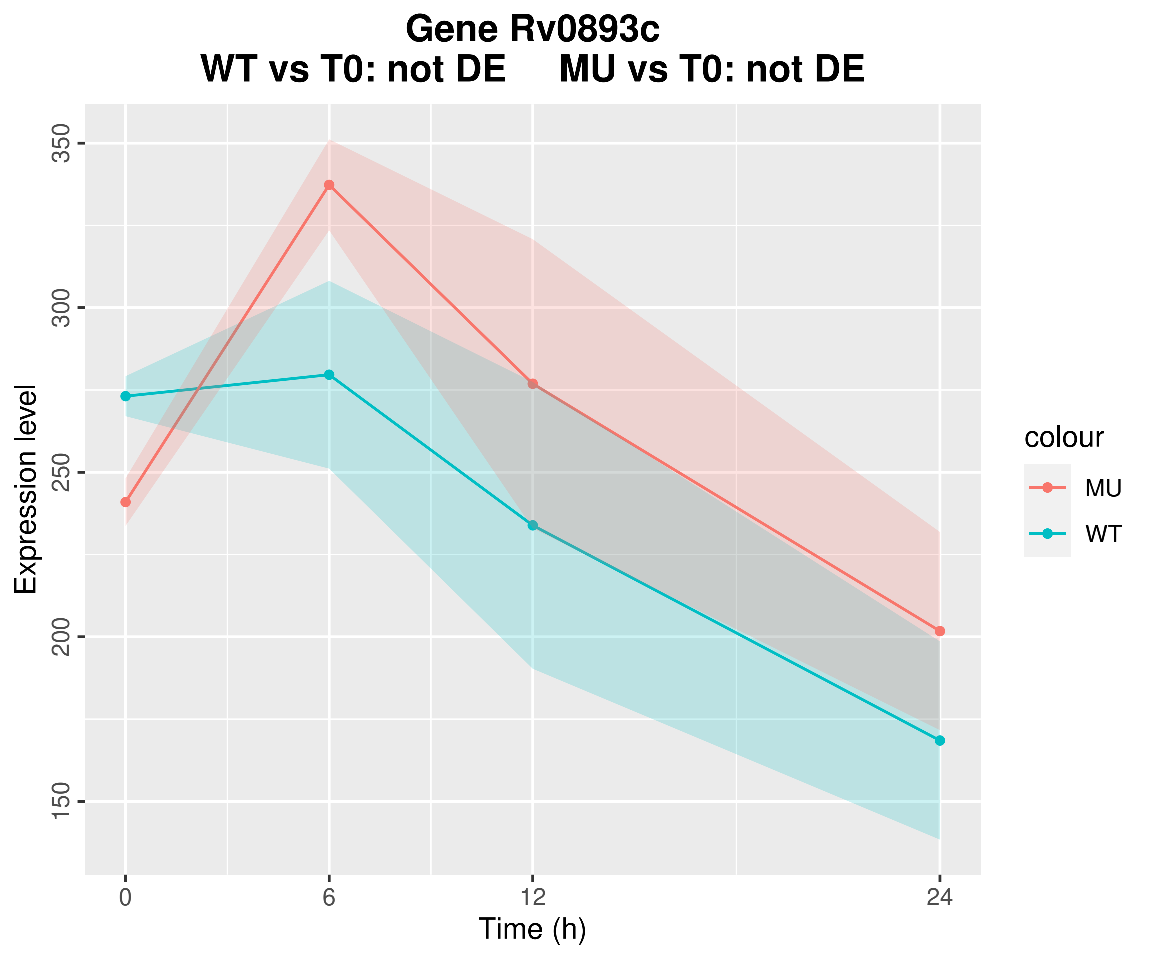

Gene Rv0893c
WT vs T0: not DE MU vs T0: not DE
Expression level
150
200
250
300
350
0
6
12
24
Time (h)
colour
MU
WT

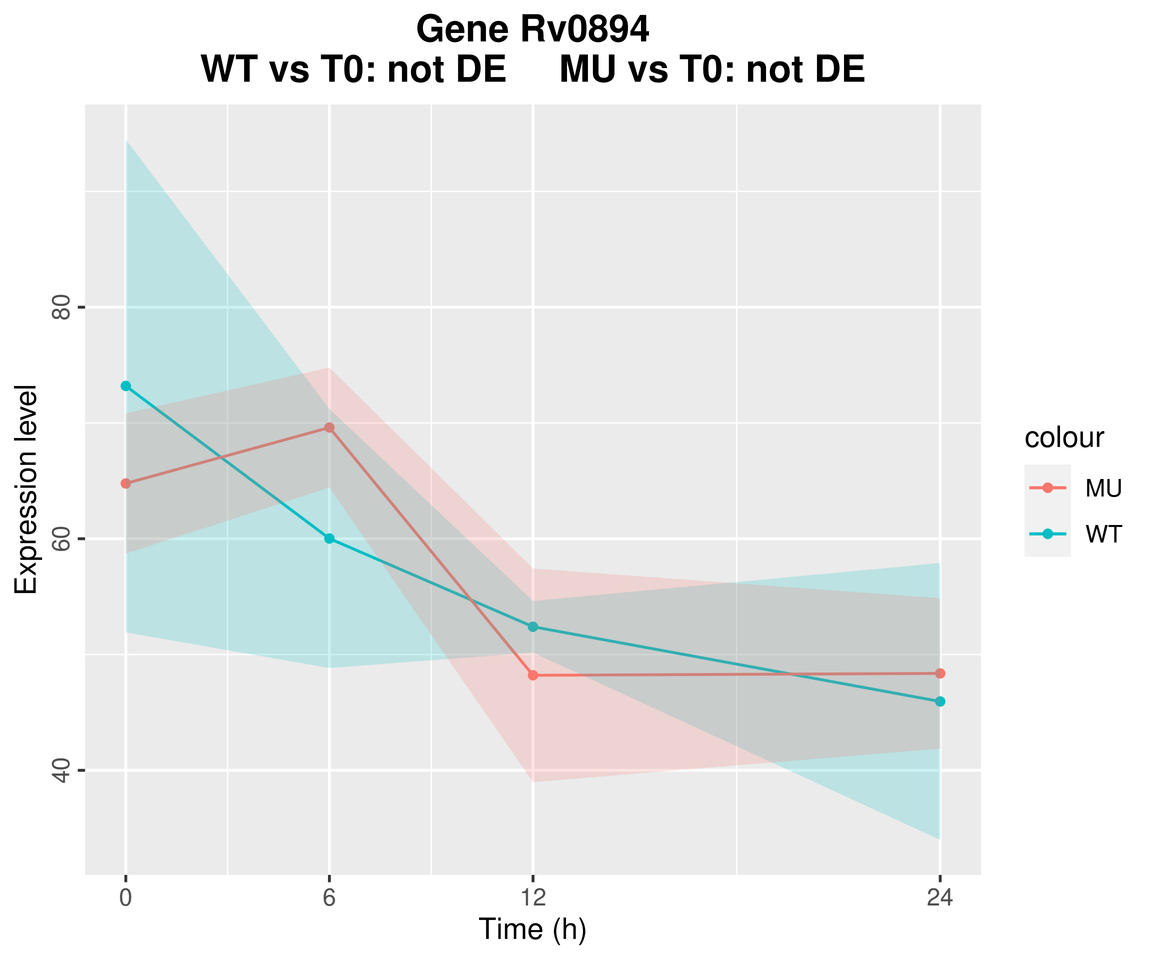
Gene Rv0894
WT vs T0: not DE MU vs T0: not DE
Expression level
80
60
40
Time (h)
0
6
12
24
colour
MU
WT

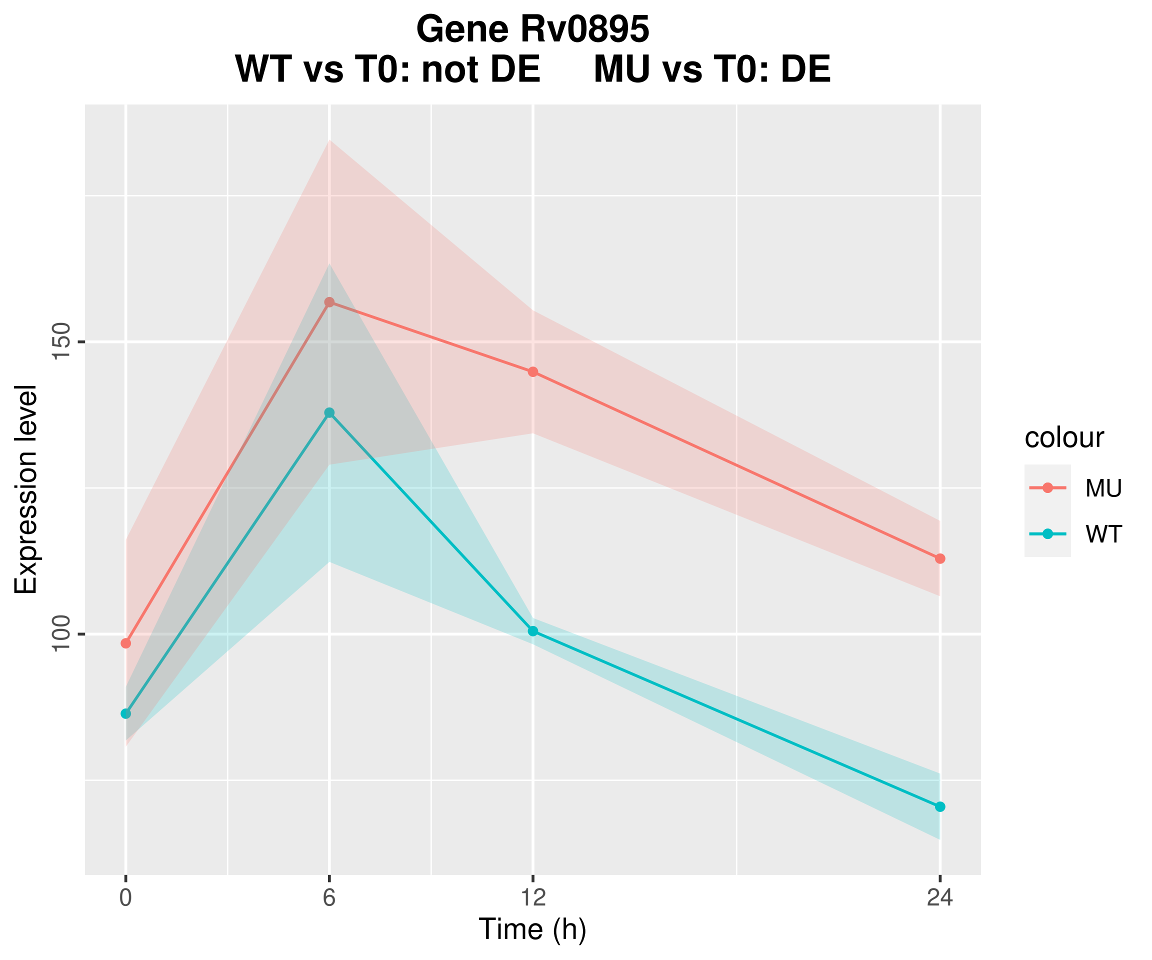

Gene Rv0895
WT vs T0: not DE   MU vs T0: DE
Expression level
150
100
Time (h)
0
6
12
24
colour
MU
WT

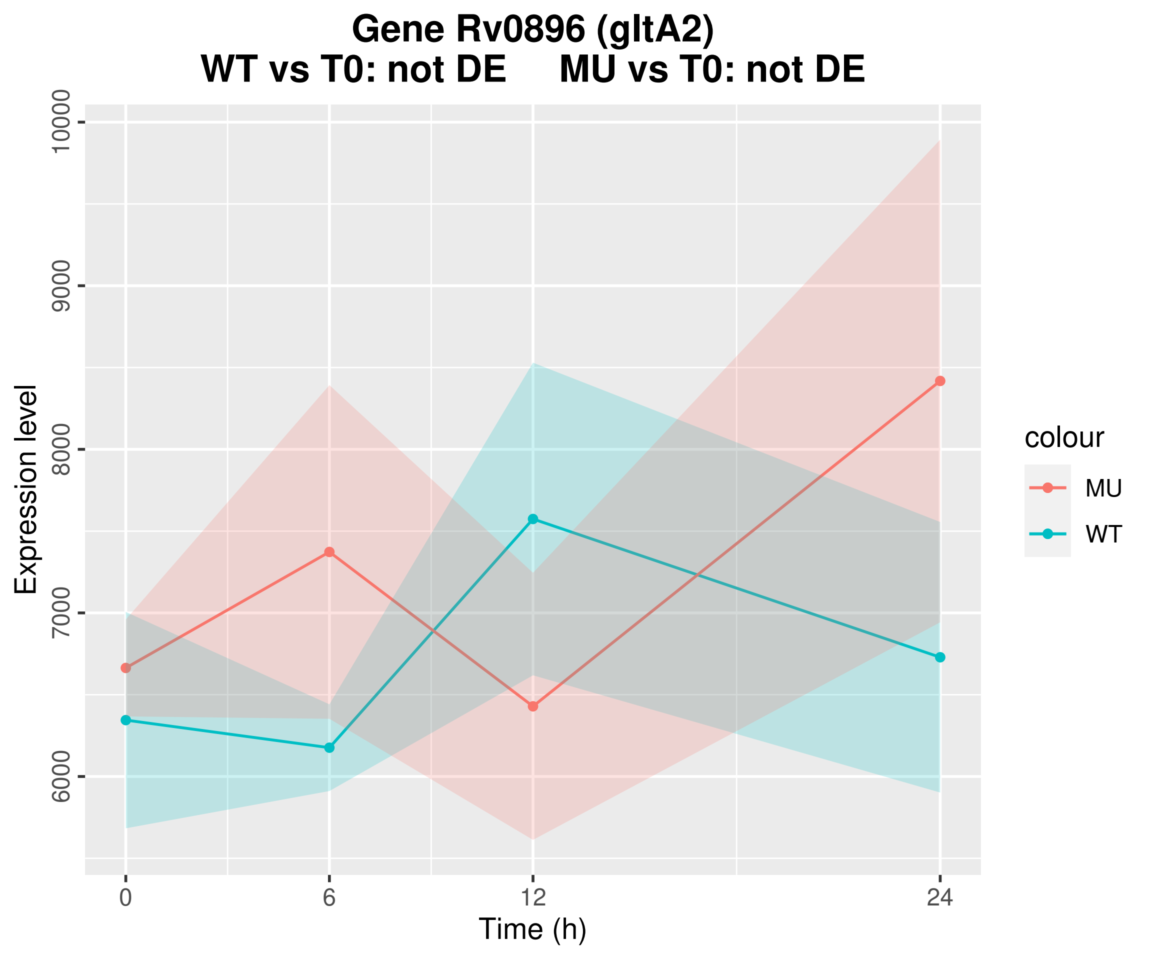

Gene Rv0896 (gltA2)
WT vs T0: not DE MU vs T0: not DE
Expression level
10000
9000
8000
7000
6000
0
6
12
24
Time (h)
colour
MU
WT

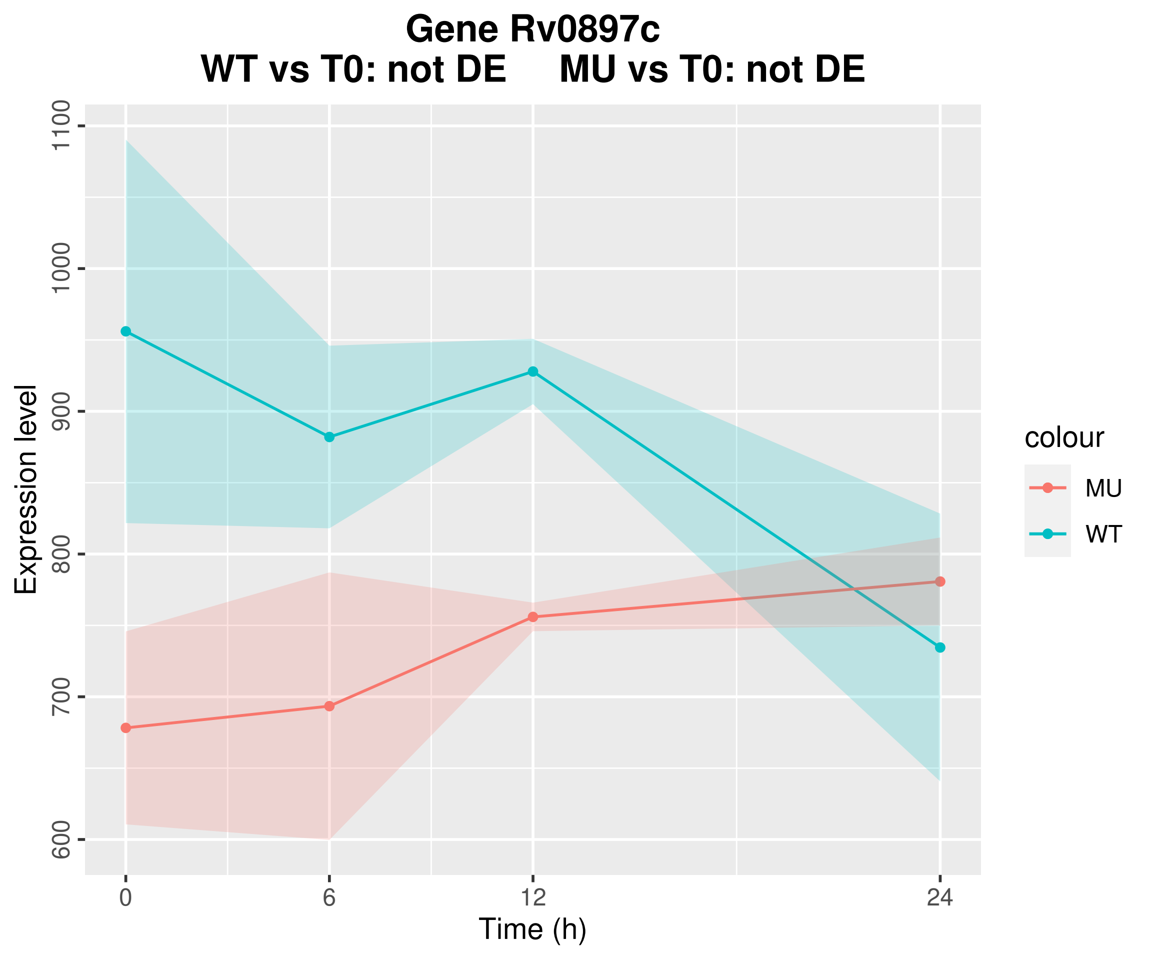

Gene Rv0897c
WT vs T0: not DE   MU vs T0: not DE
Expression level
1100
1000
900
800
700
600
0
6
12
24
Time (h)
colour
MU
WT

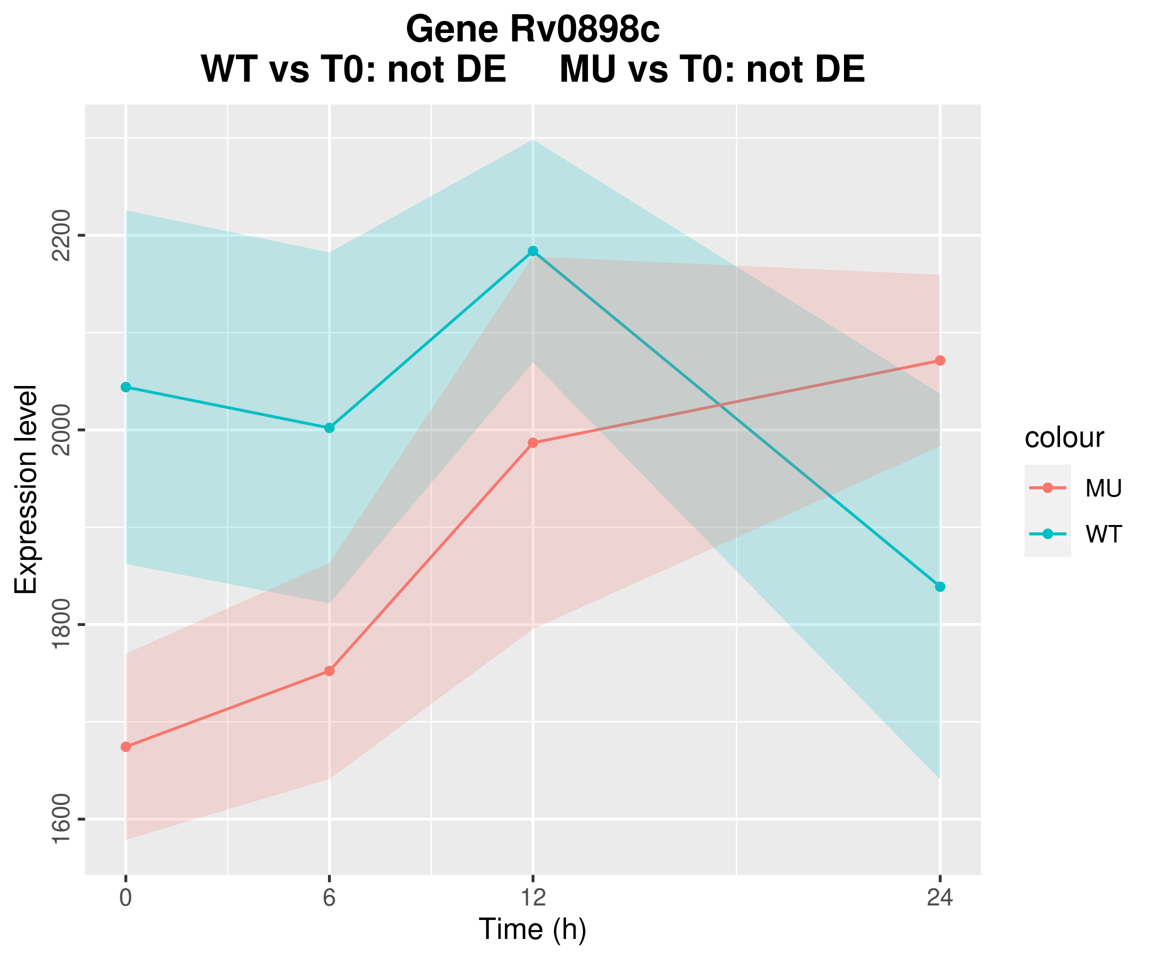
Gene Rv0898c
WT vs T0: not DE   MU vs T0: not DE
Expression level
1600
1800
2000
2200
0
6
12
24
Time (h)
colour
MU
WT

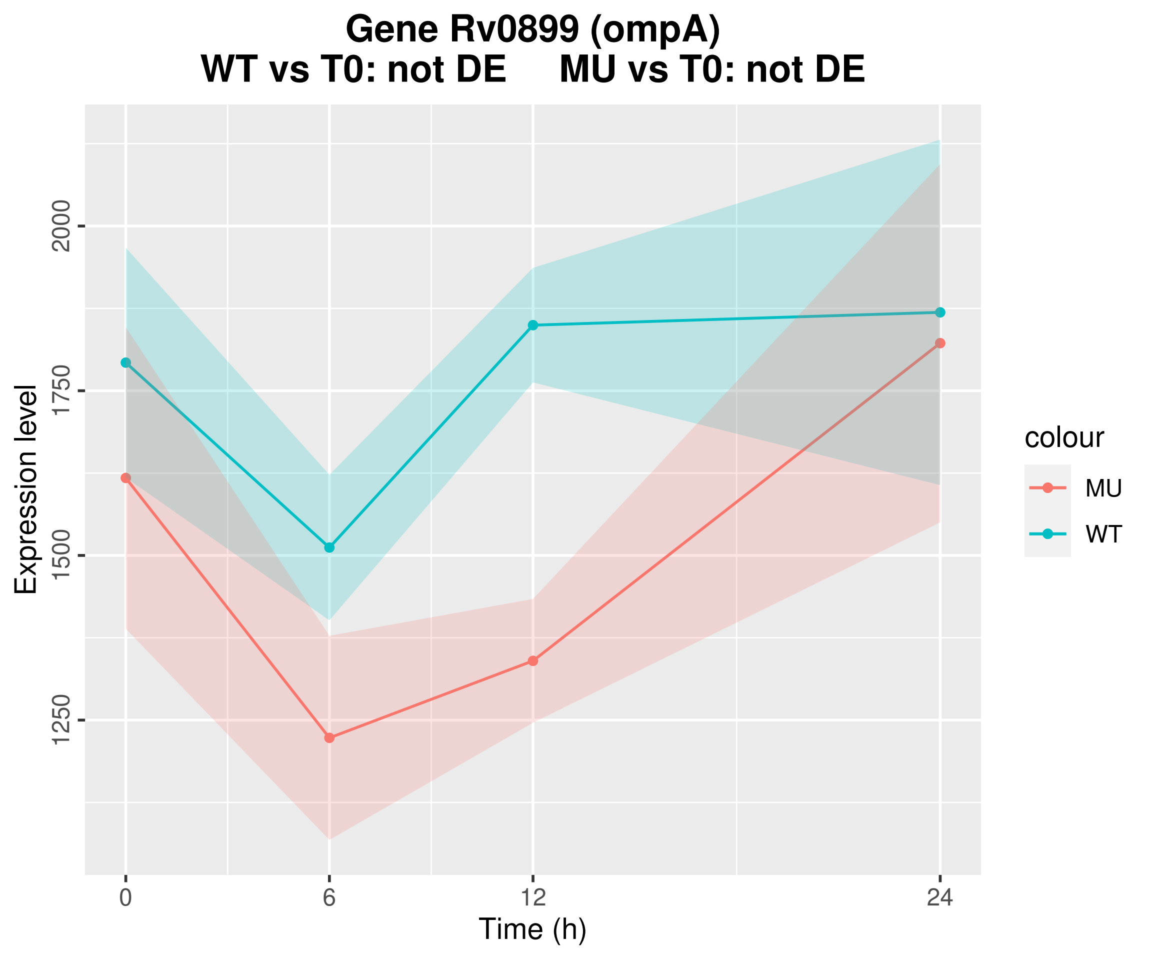
Gene Rv0899 (ompA)
WT vs T0: not DE   MU vs T0: not DE
Expression level
2000
1750
1500
1250
0
6
12
24
Time (h)
colour
MU
WT

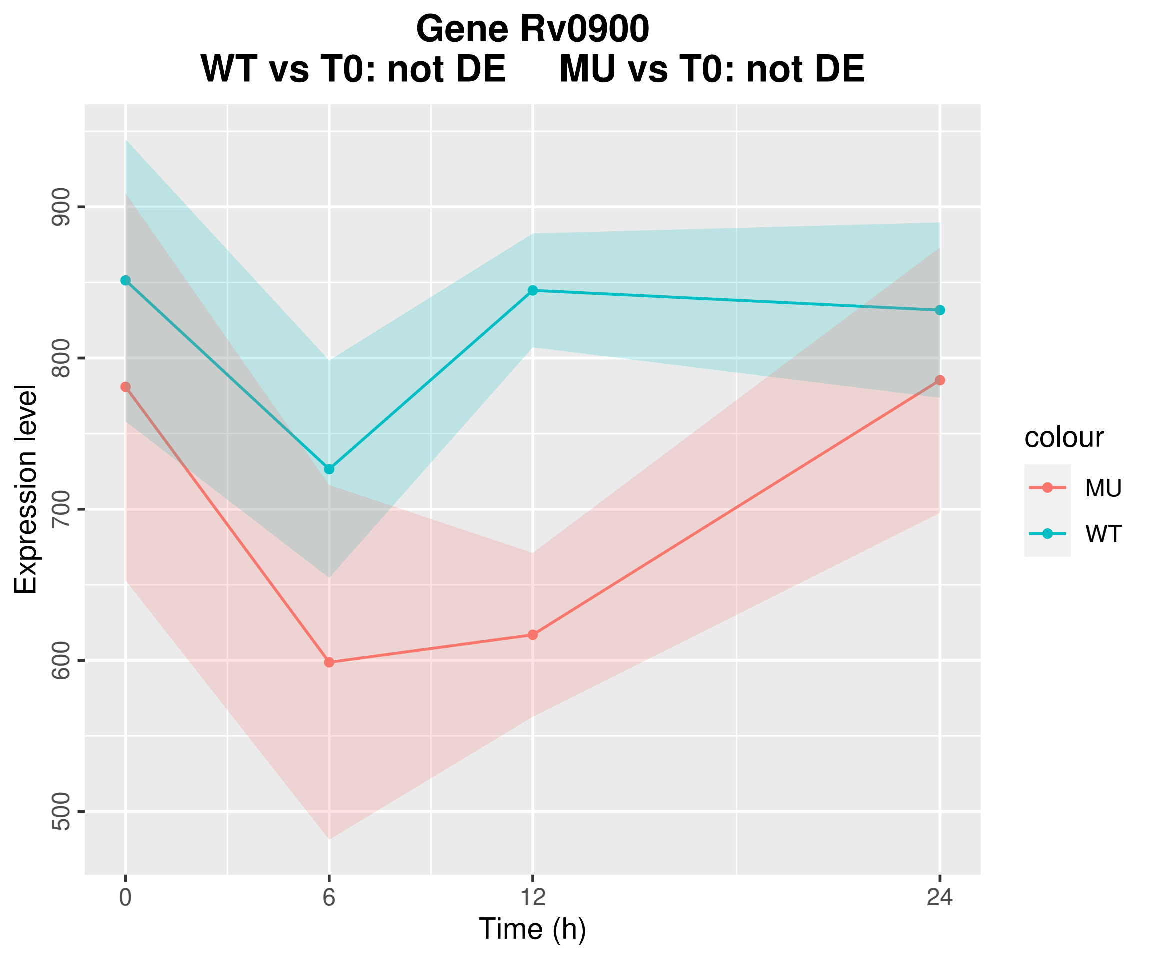
Gene Rv0900
WT vs T0: not DE   MU vs T0: not DE
Expression level
500
600
700
800
900
0
6
12
24
Time (h)
colour
MU
WT

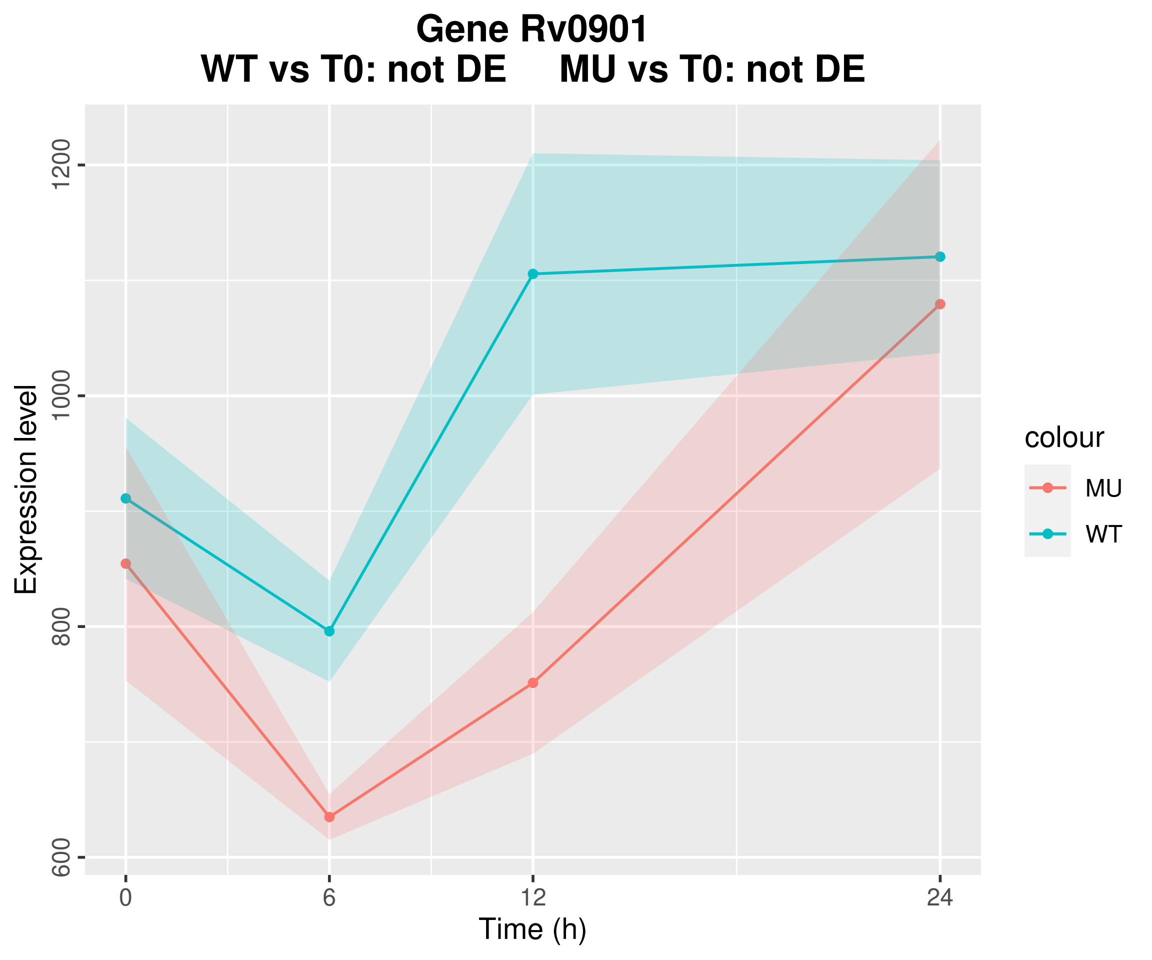
Gene Rv0901
WT vs T0: not DE   MU vs T0: not DE
Expression level
1200
1000
800
600
0
6
12
24
Time (h)
colour
MU
WT

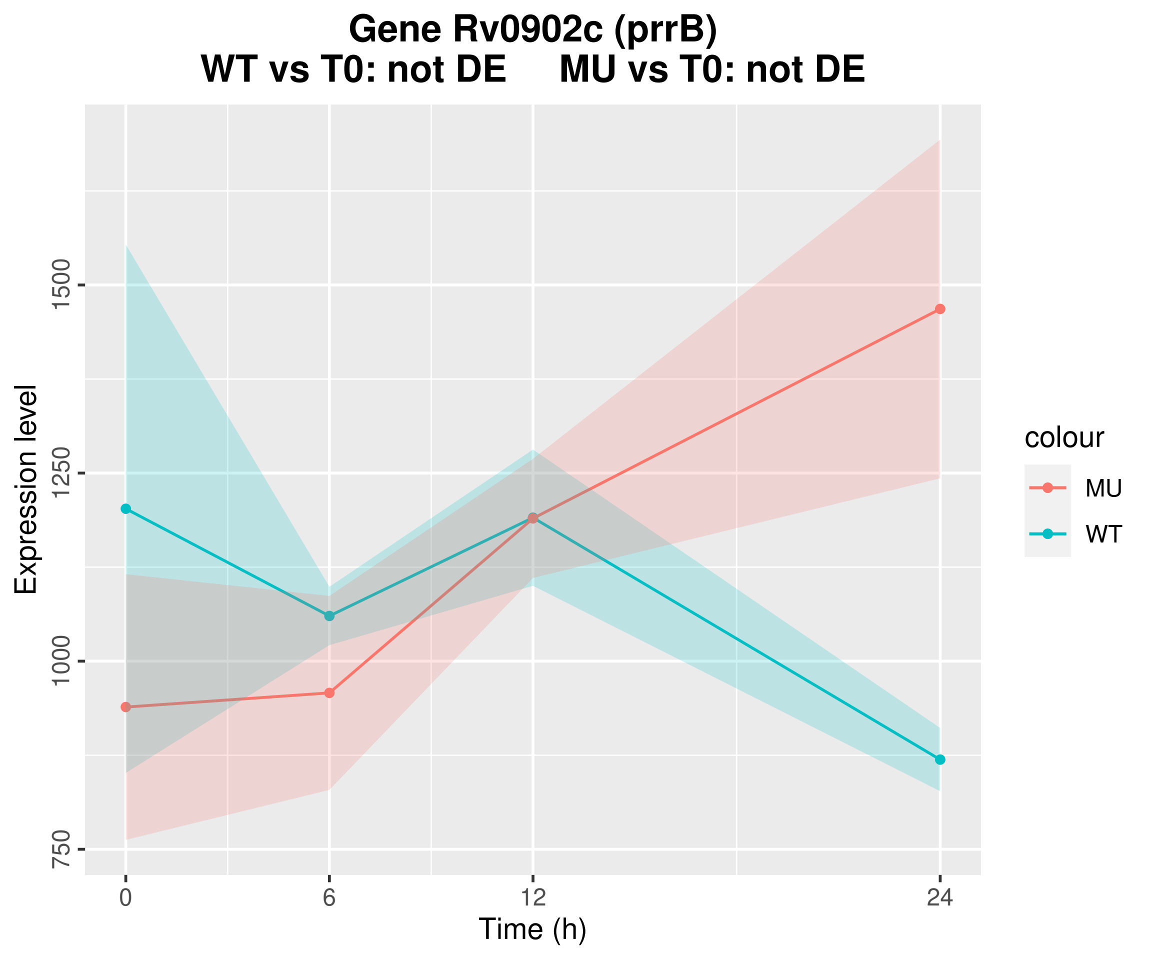
Gene Rv0902c (prrB)
WT vs T0: not DE   MU vs T0: not DE
Expression level
750
1000
1250
1500
0
6
12
24
Time (h)
colour
MU
WT

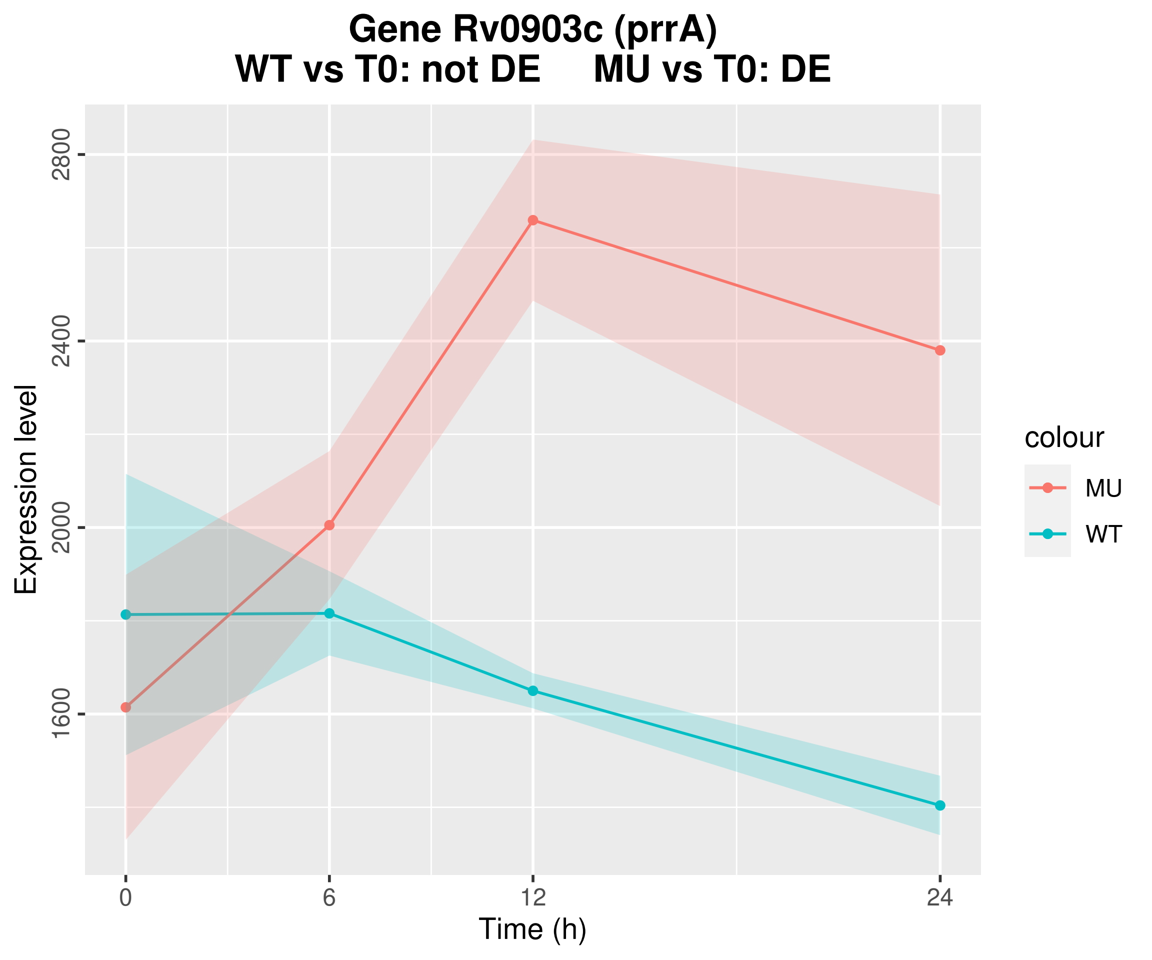

Gene Rv0903c (prrA)
WT vs T0: not DE MU vs T0: DE
Expression level
2800
2400
2000
1600
0
6
12
24
Time (h)
colour
MU
WT

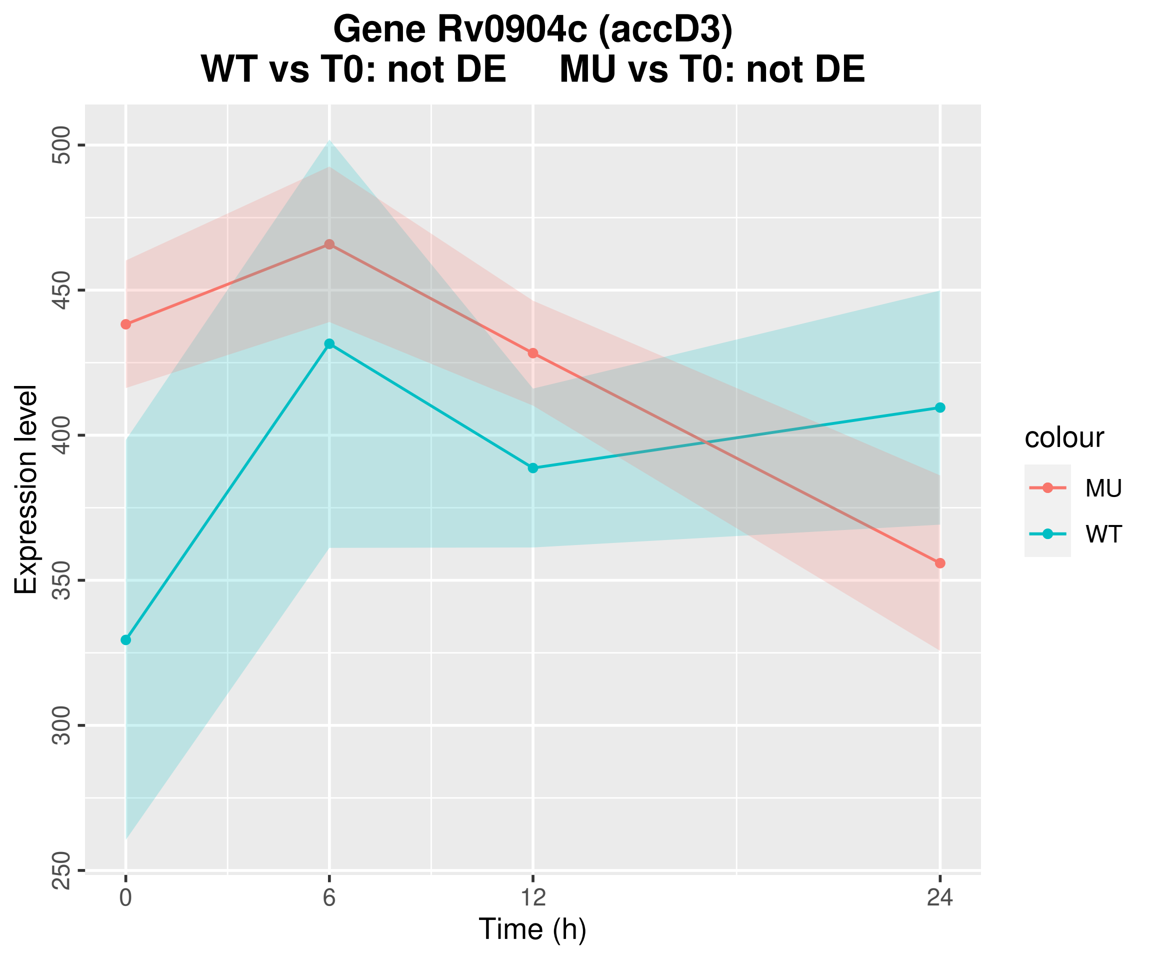

Gene Rv0904c (accD3)
WT vs T0: not DE   MU vs T0: not DE
Expression level
500
450
400
350
300
250
0
6
12
24
Time (h)
colour
MU
WT

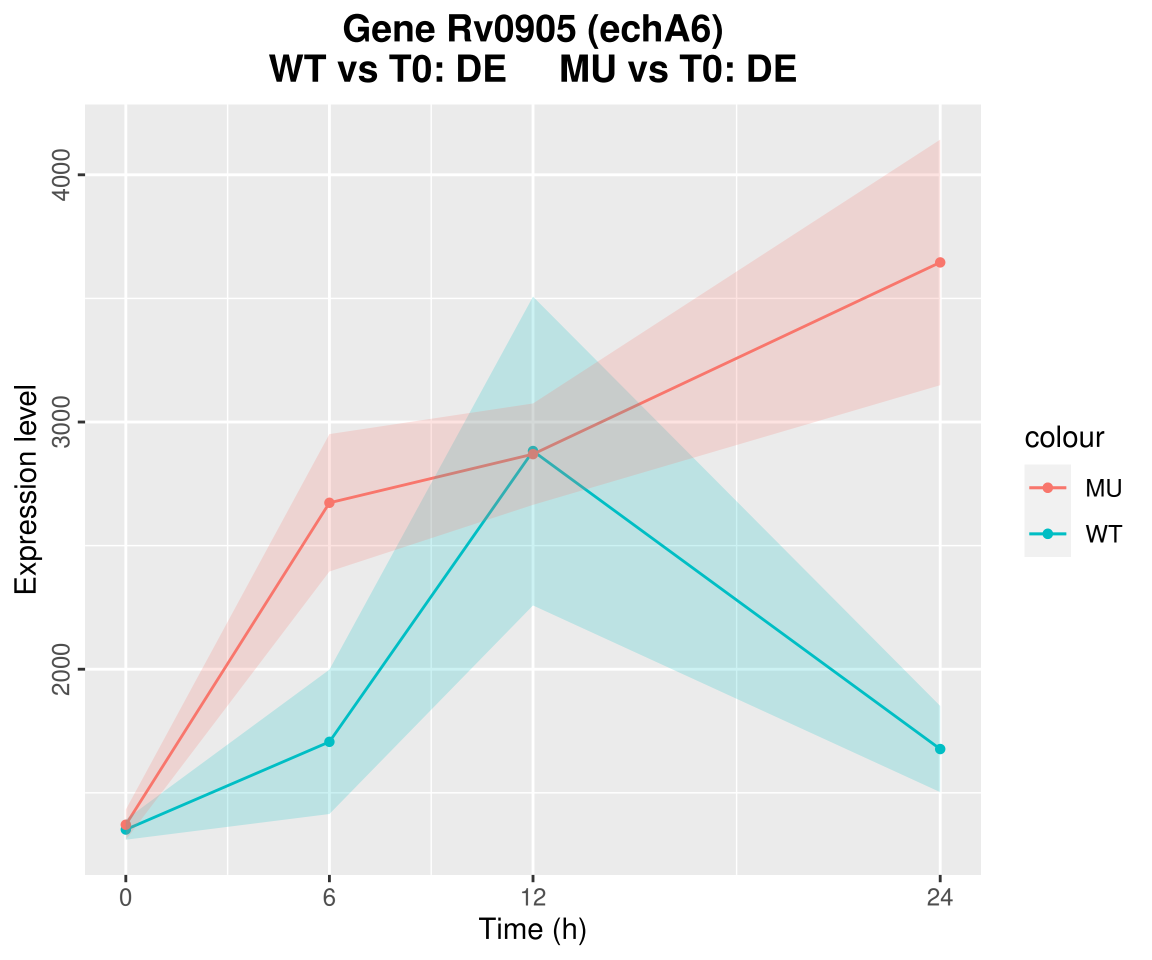

Gene Rv0905 (echA6)
WT vs T0: DE   MU vs T0: DE
4000
3000
2000
Expression level
0
6
12
24
Time (h)
colour
MU
WT

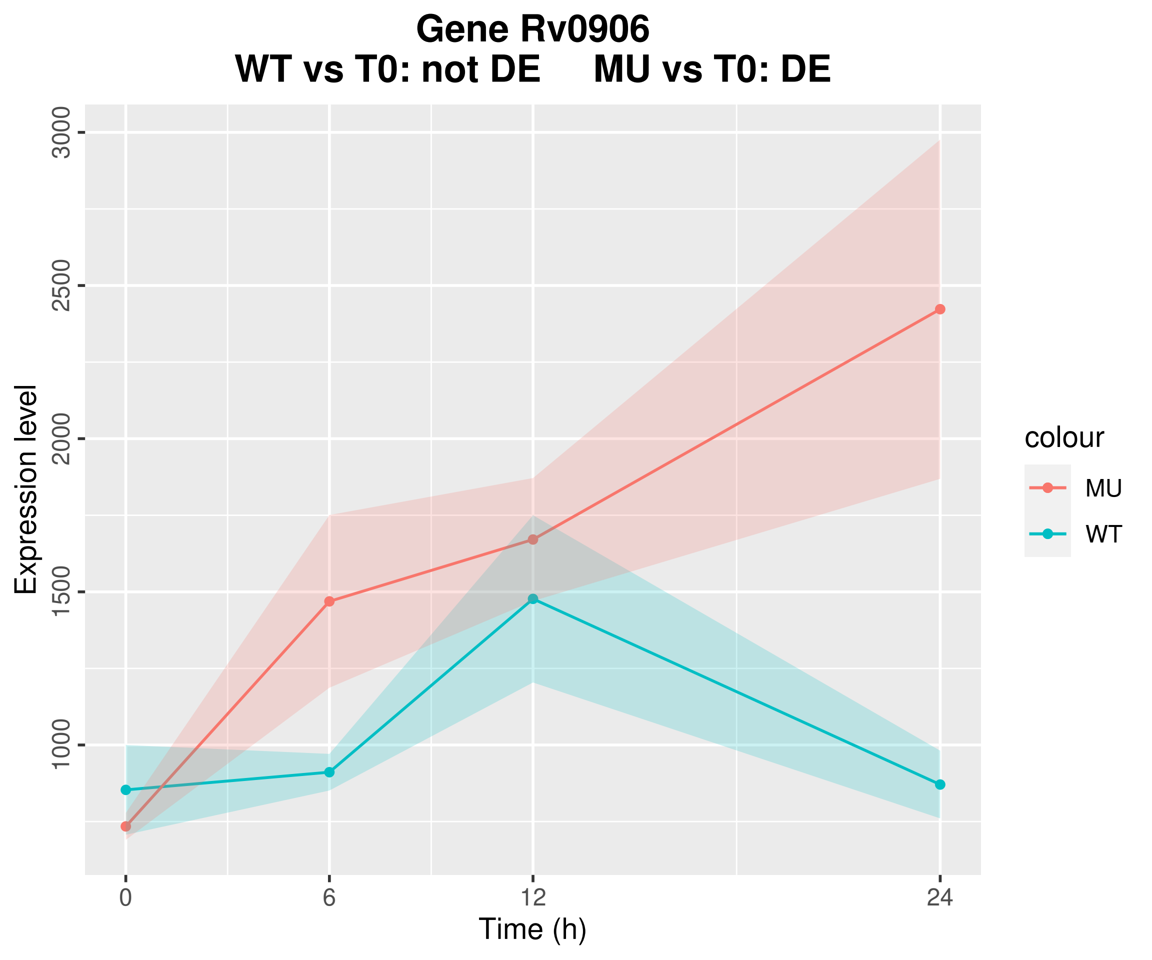

Gene Rv0906
WT vs T0: not DE   MU vs T0: DE
Expression level
3000
2500
2000
1500
1000
0
6
12
24
Time (h)
colour
MU
WT

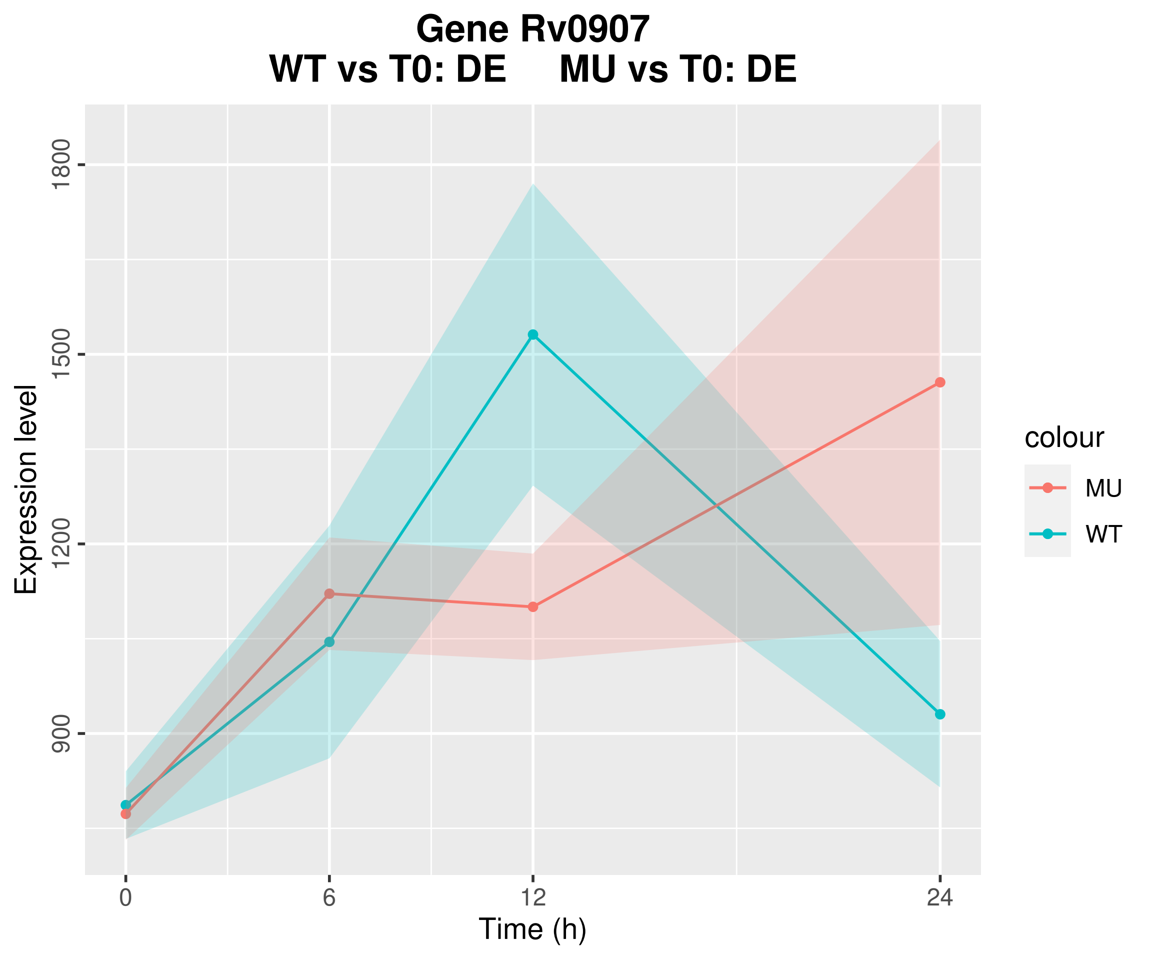
Gene Rv0907
WT vs T0: DE MU vs T0: DE
1800
1500
1200
900
Expression level
0
6
12
24
Time (h)
colour
MU
WT

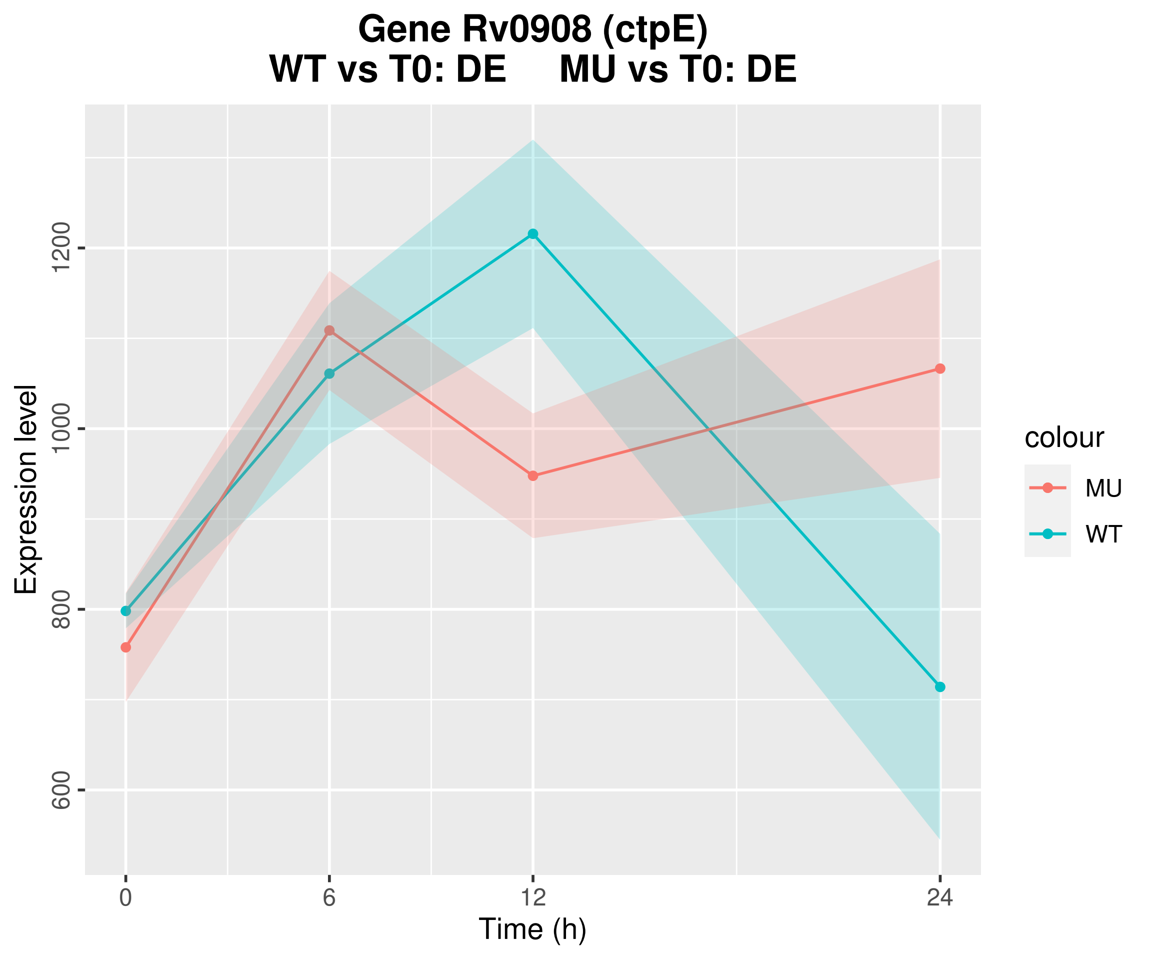

Gene Rv0908 (ctpE)
WT vs T0: DE   MU vs T0: DE
Expression level
1200
1000
800
600
0
6
12
24
Time (h)
colour
MU
WT

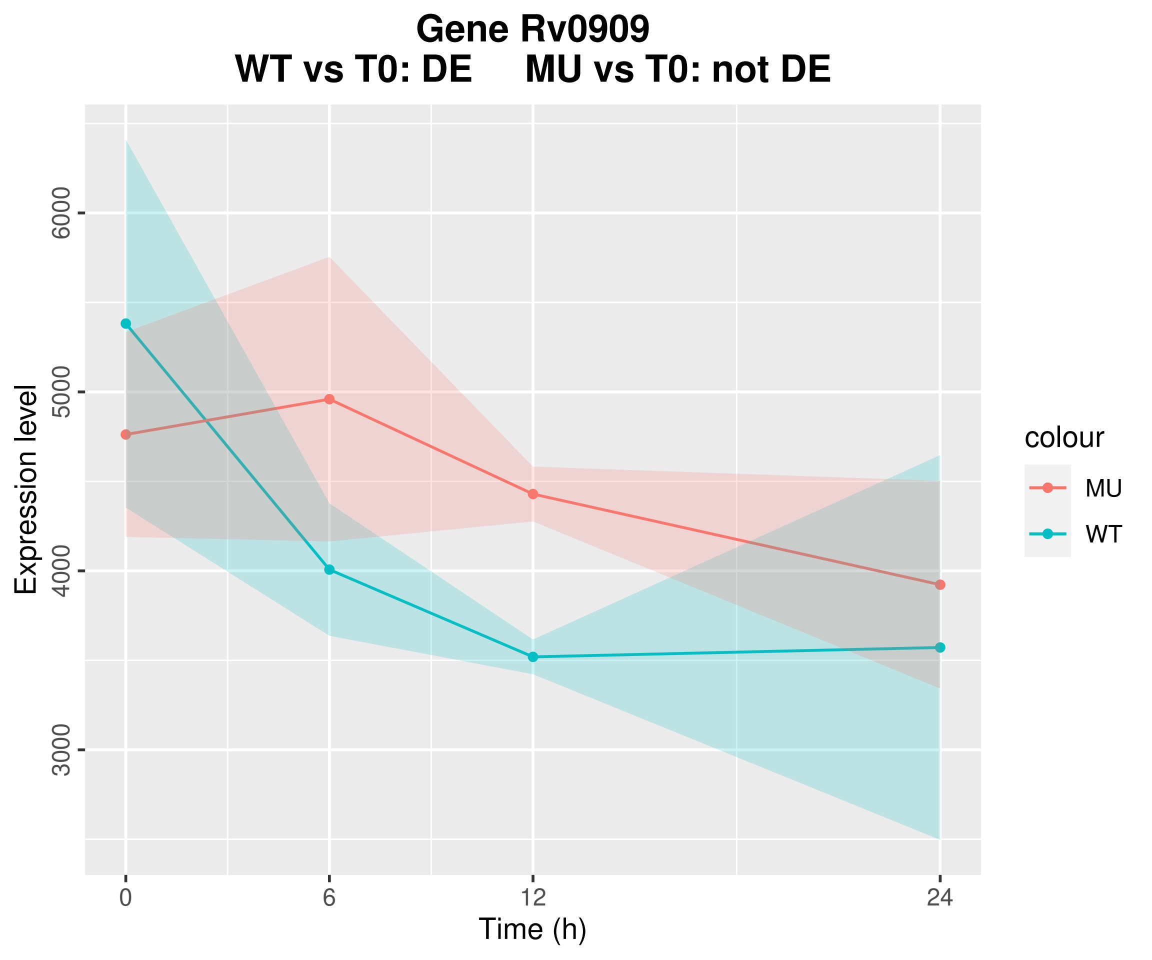

Gene Rv0909
WT vs T0: DE   MU vs T0: not DE
Expression level
6000
5000
4000
3000
0
6
12
24
Time (h)
colour
MU
WT

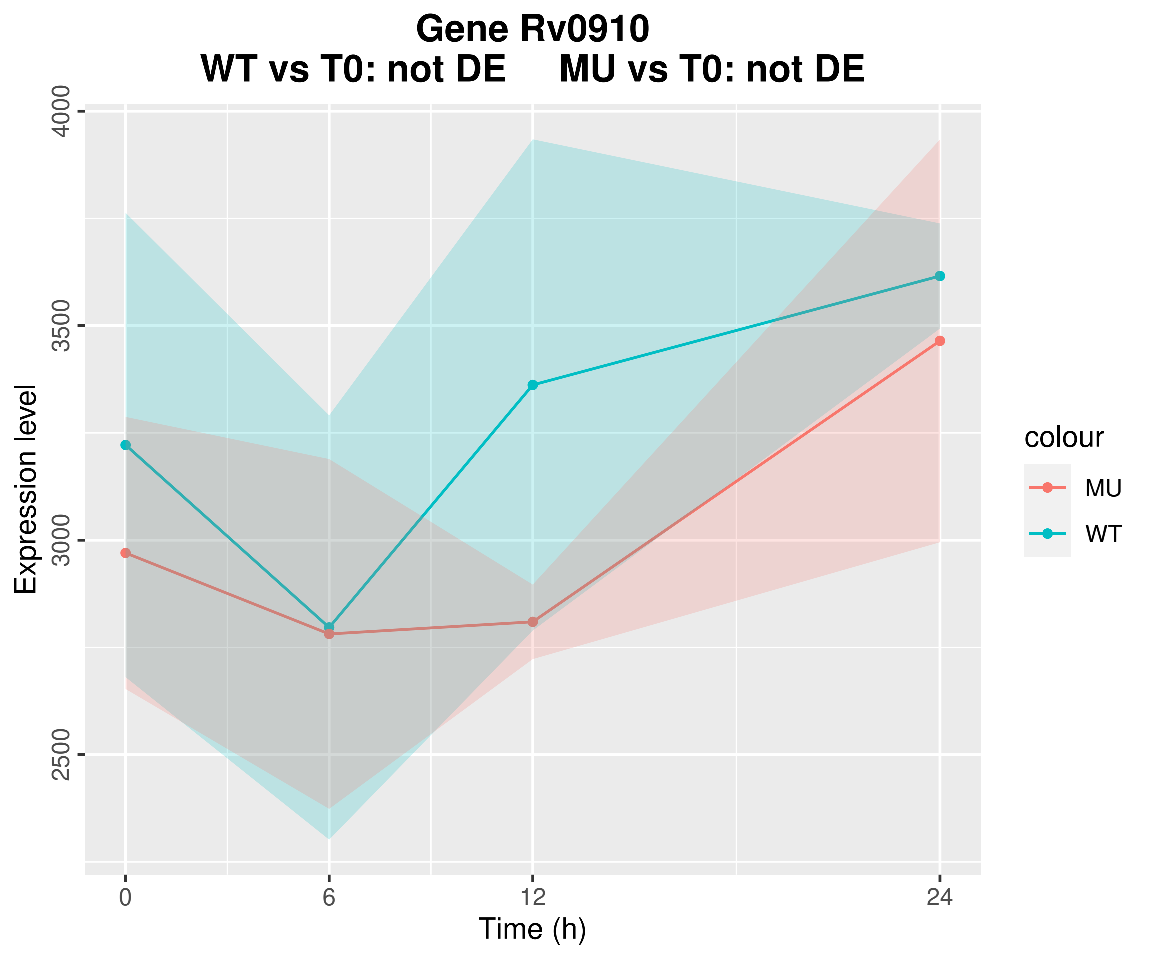

Gene Rv0910
WT vs T0: not DE MU vs T0: not DE
Expression level
4000
3500
3000
2500
0
6
12
24
Time (h)
colour
MU
WT

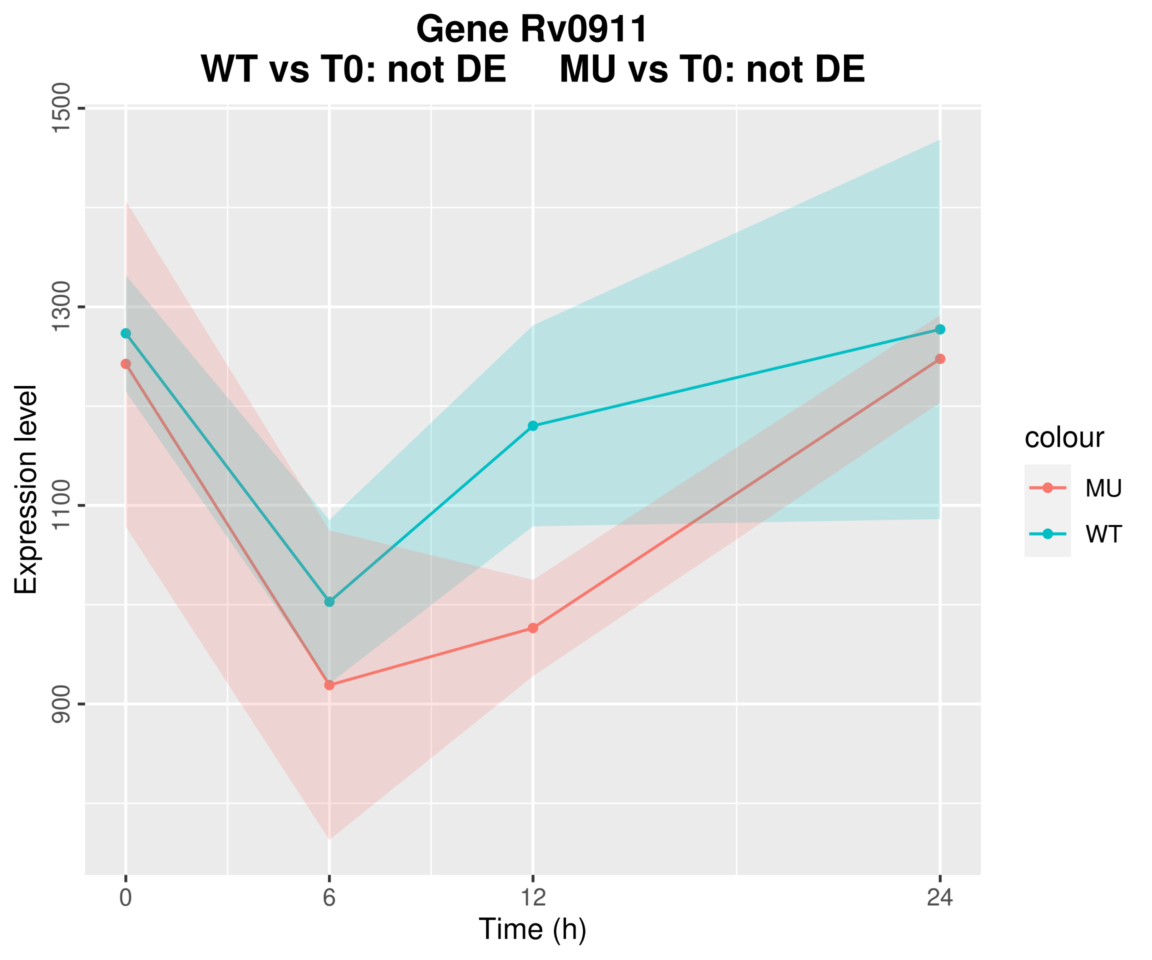

Gene Rv0911
WT vs T0: not DE MU vs T0: not DE
1500
1300
1100
900
Expression level
0
6
12
24
Time (h)
colour
MU
WT

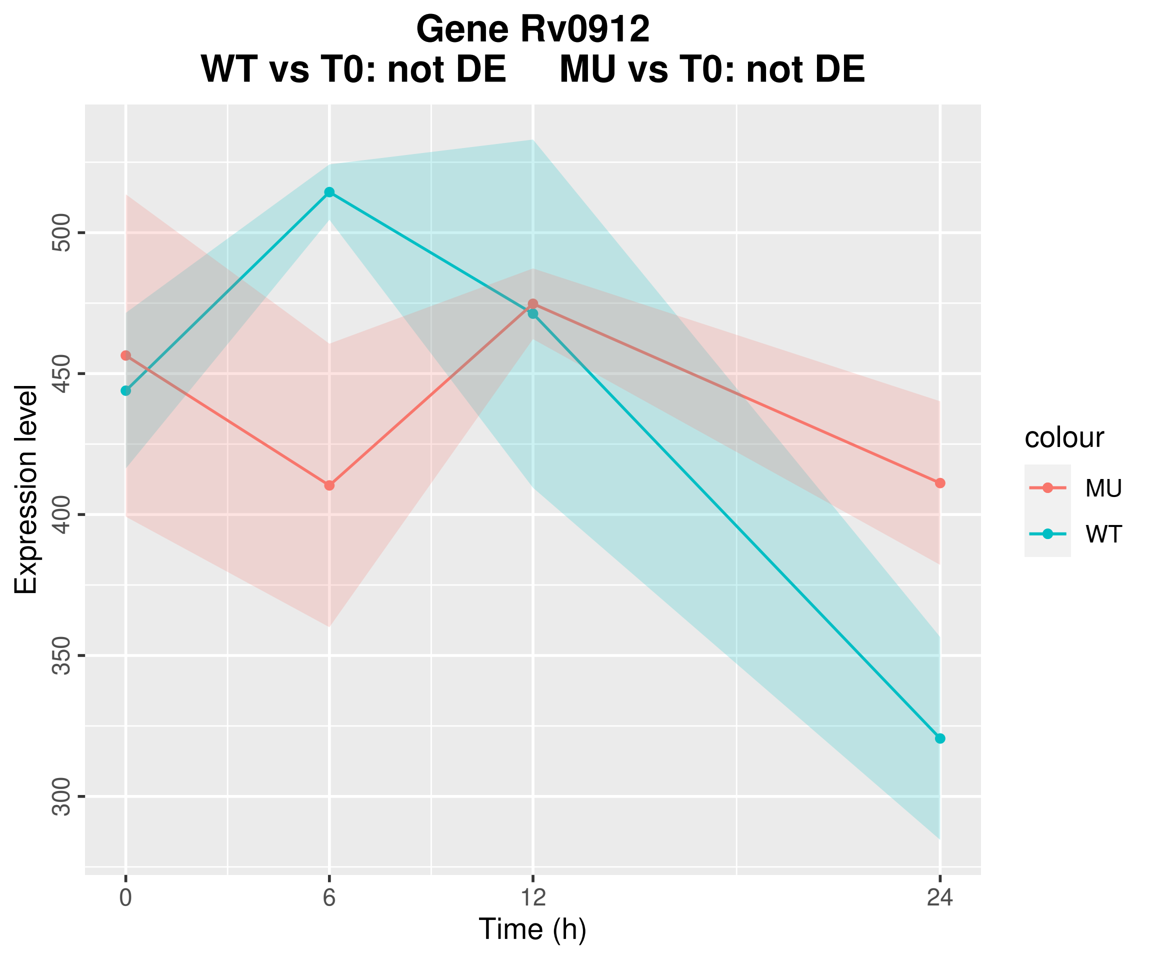

Gene Rv0912
WT vs T0: not DE MU vs T0: not DE
Expression level
500
450
400
350
300
0
6
12
24
Time (h)
colour
MU
WT

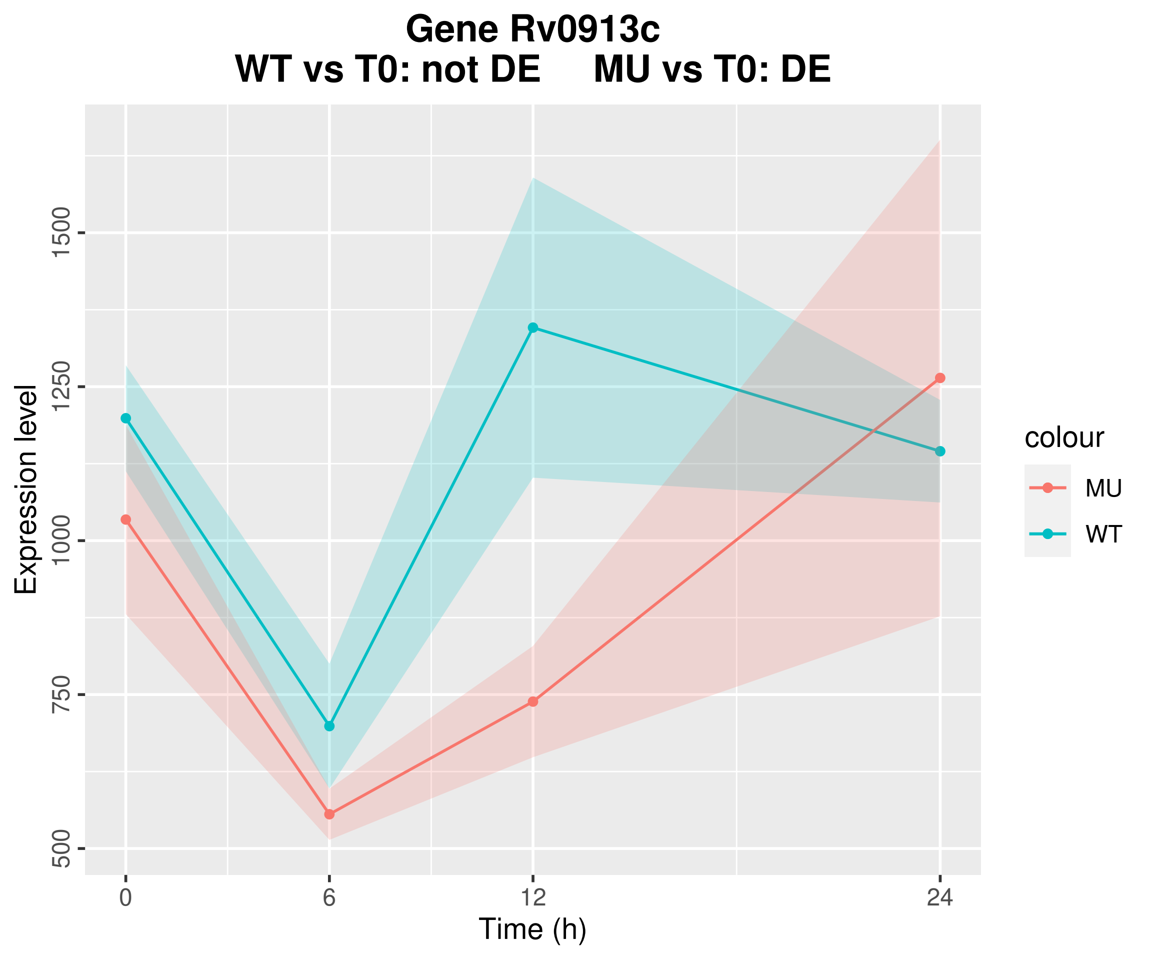

Gene Rv0913c
WT vs T0: not DE   MU vs T0: DE
Expression level
500
750
1000
1250
1500
0
6
12
24
Time (h)
colour
MU
WT

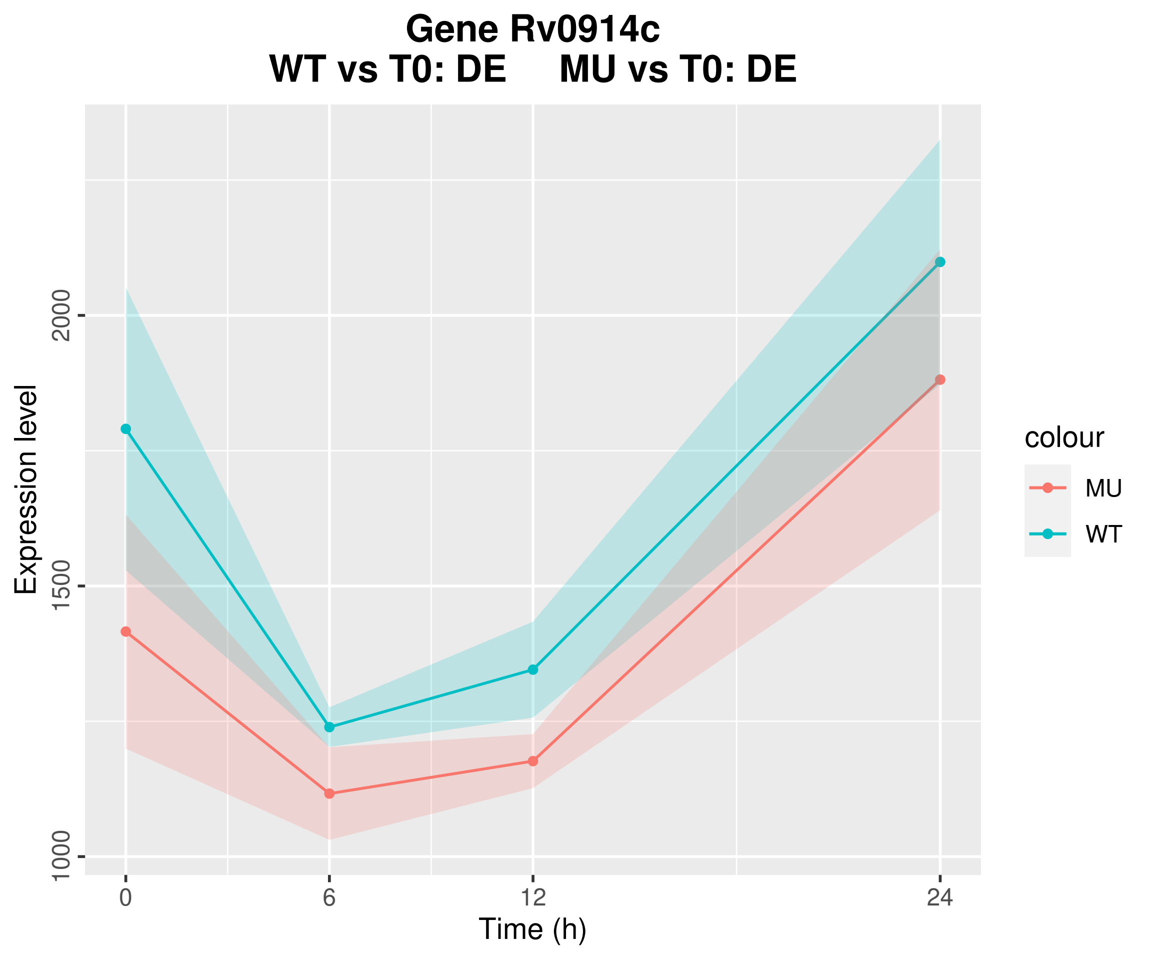
Gene Rv0914c
WT vs T0: DE   MU vs T0: DE
Expression level
2000
1500
1000
0
6
12
24
Time (h)
colour
MU
WT

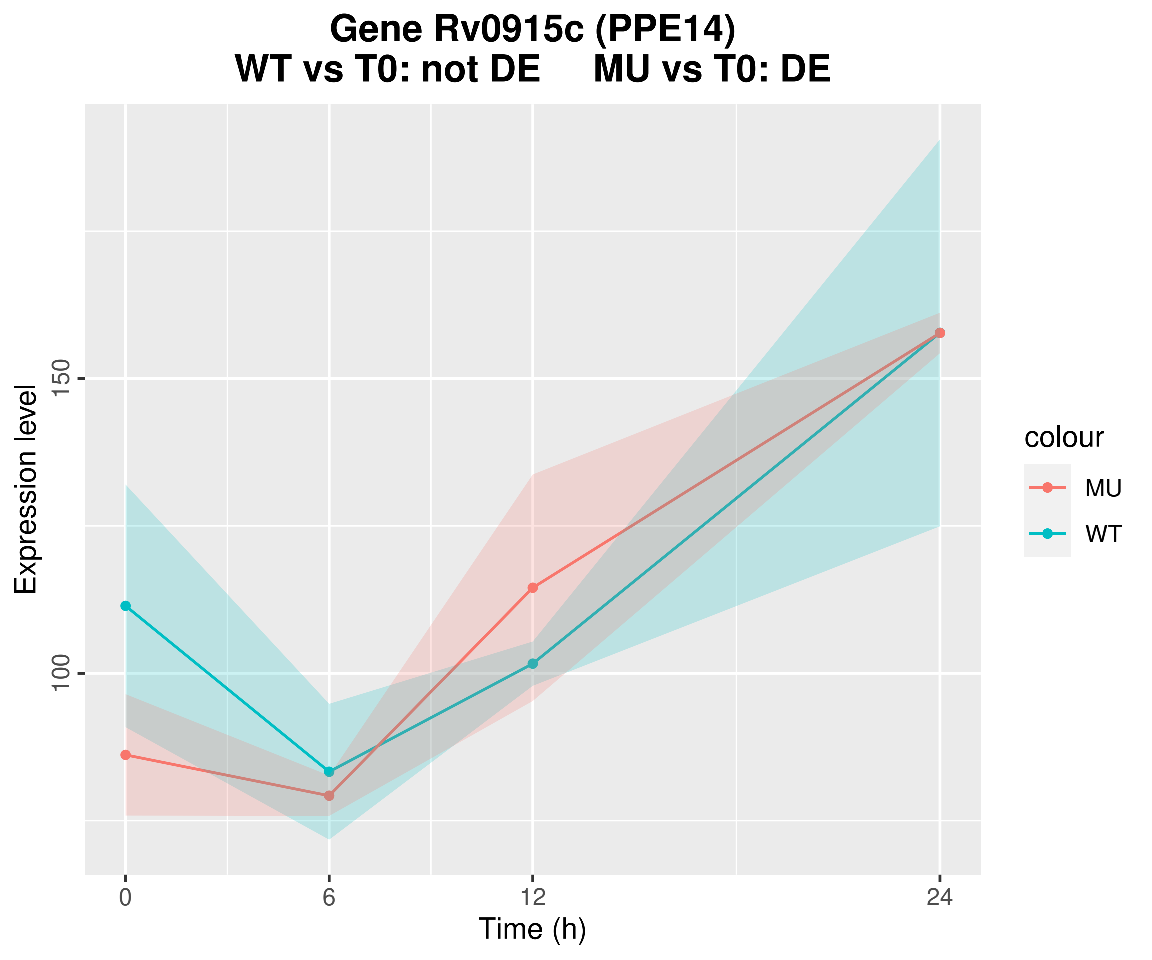
Gene Rv0915c (PPE14)
WT vs T0: not DE   MU vs T0: DE
Expression level
150
100
0
6
12
24
Time (h)
colour
MU
WT

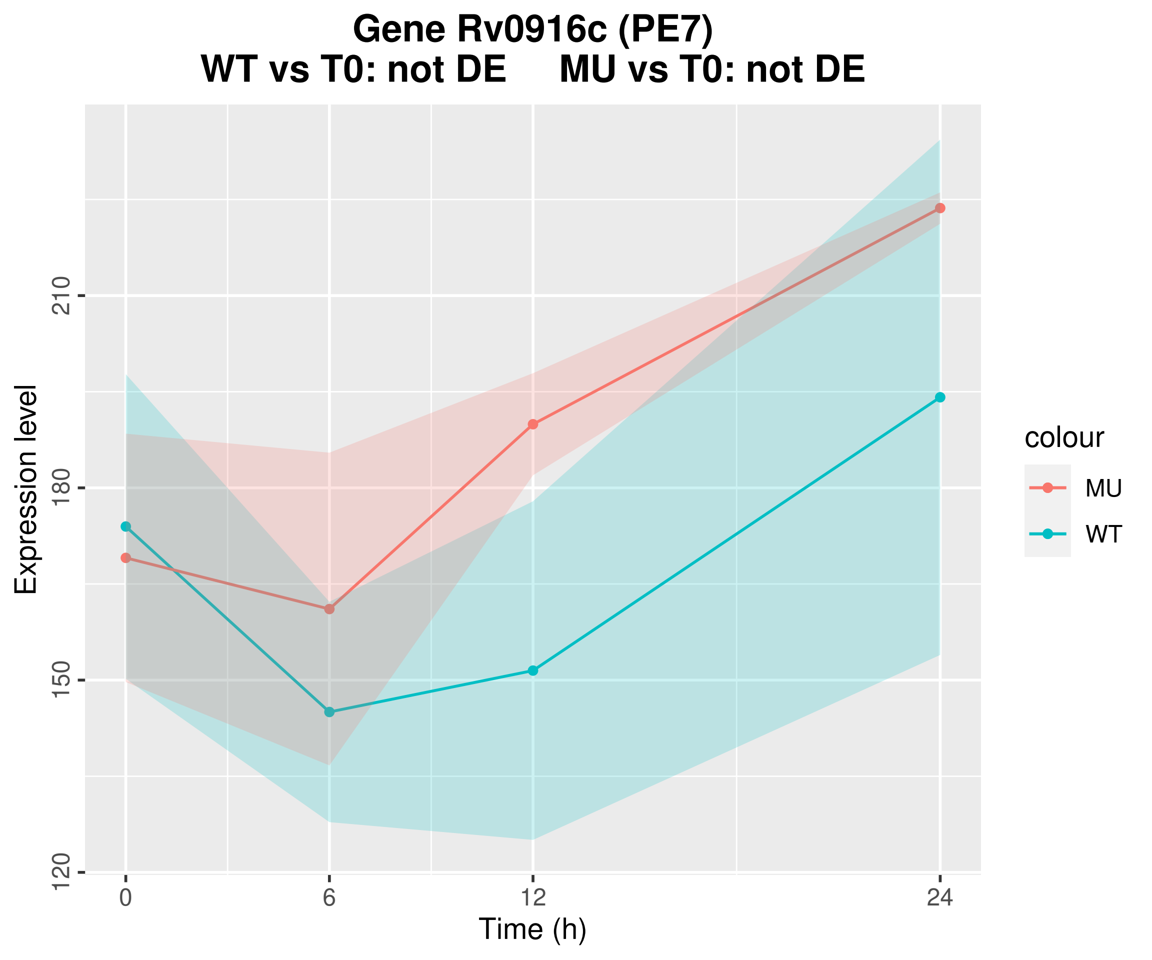
Gene Rv0916c (PE7)
WT vs T0: not DE MU vs T0: not DE
Expression level
210
180
150
120
0
6
12
24
Time (h)
colour
MU
WT

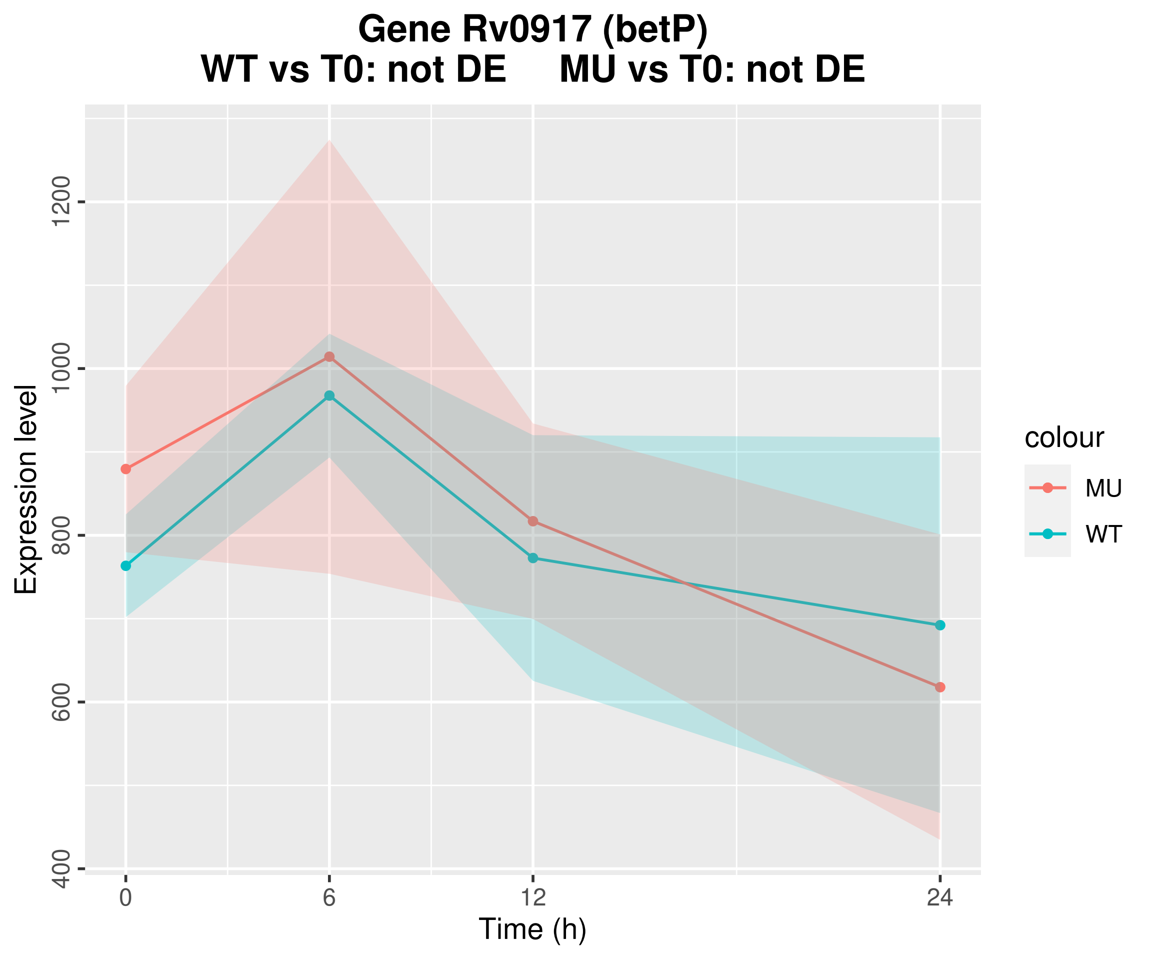

Gene Rv0917 (betP)
WT vs T0: not DE   MU vs T0: not DE
Expression level
1200
1000
800
600
400
0
6
12
24
Time (h)
colour
MU
WT

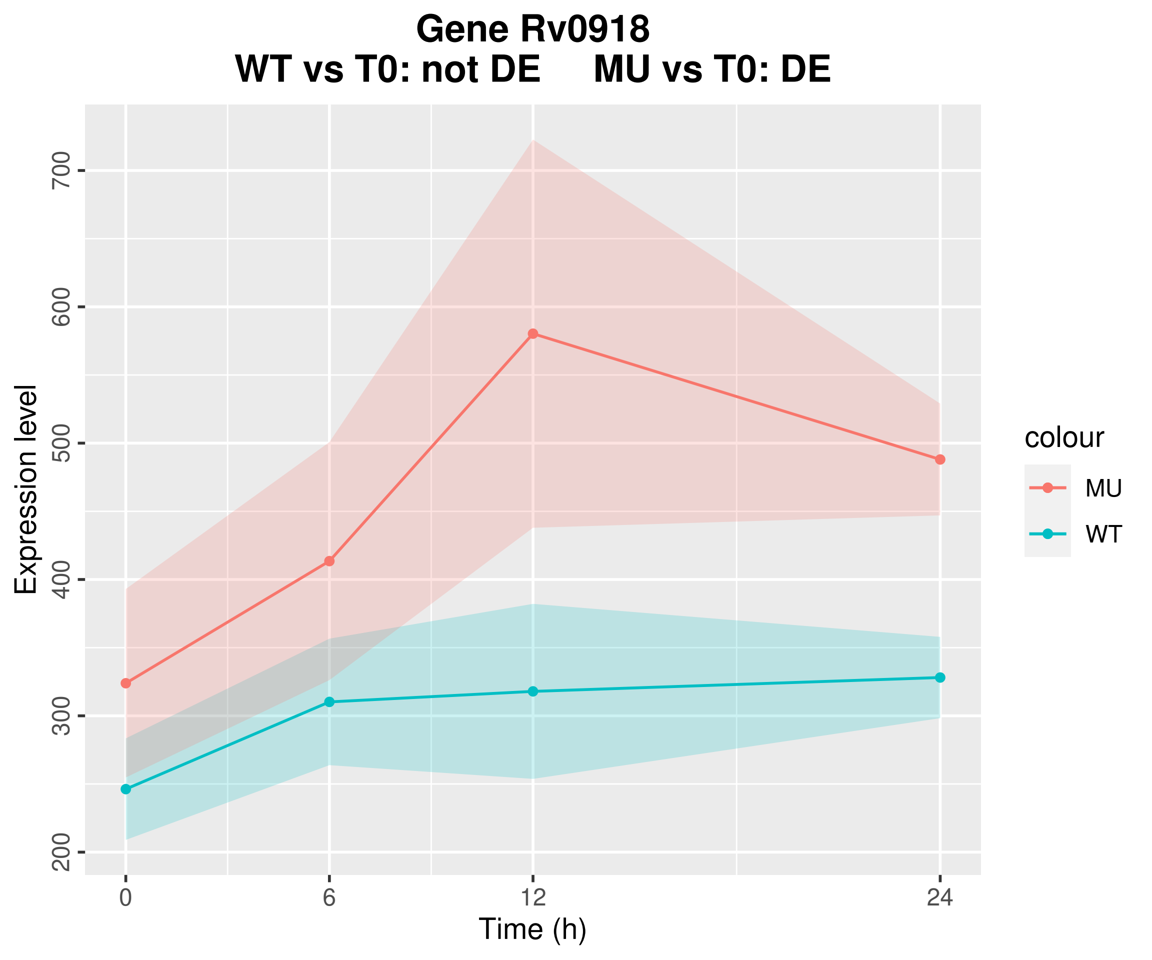
Gene Rv0918
WT vs T0: not DE MU vs T0: DE
Expression level
700
600
500
400
300
200
0
6
12
24
Time (h)
colour
MU
WT

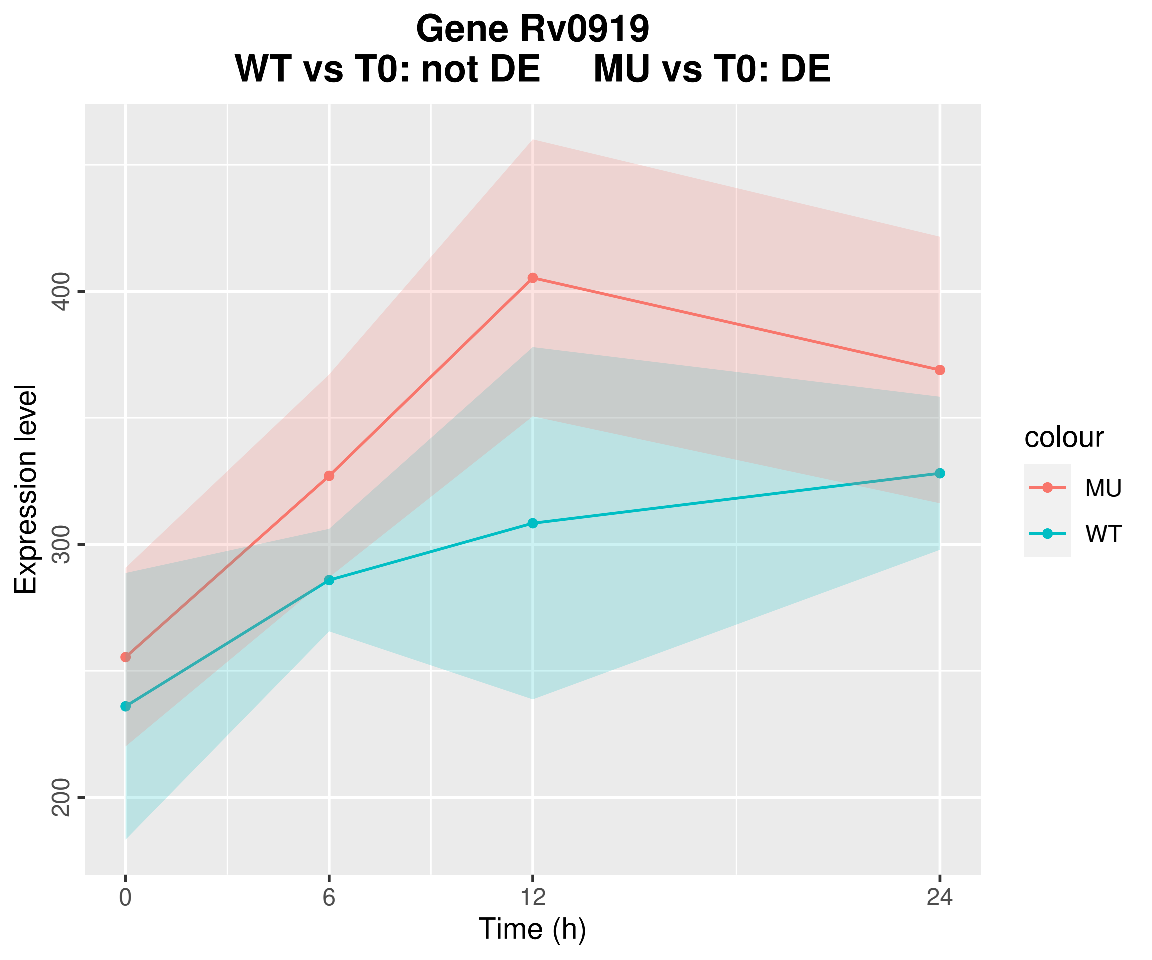

Gene Rv0919
WT vs T0: not DE   MU vs T0: DE
Expression level
200
300
400
0
6
12
24
Time (h)
colour
MU
WT

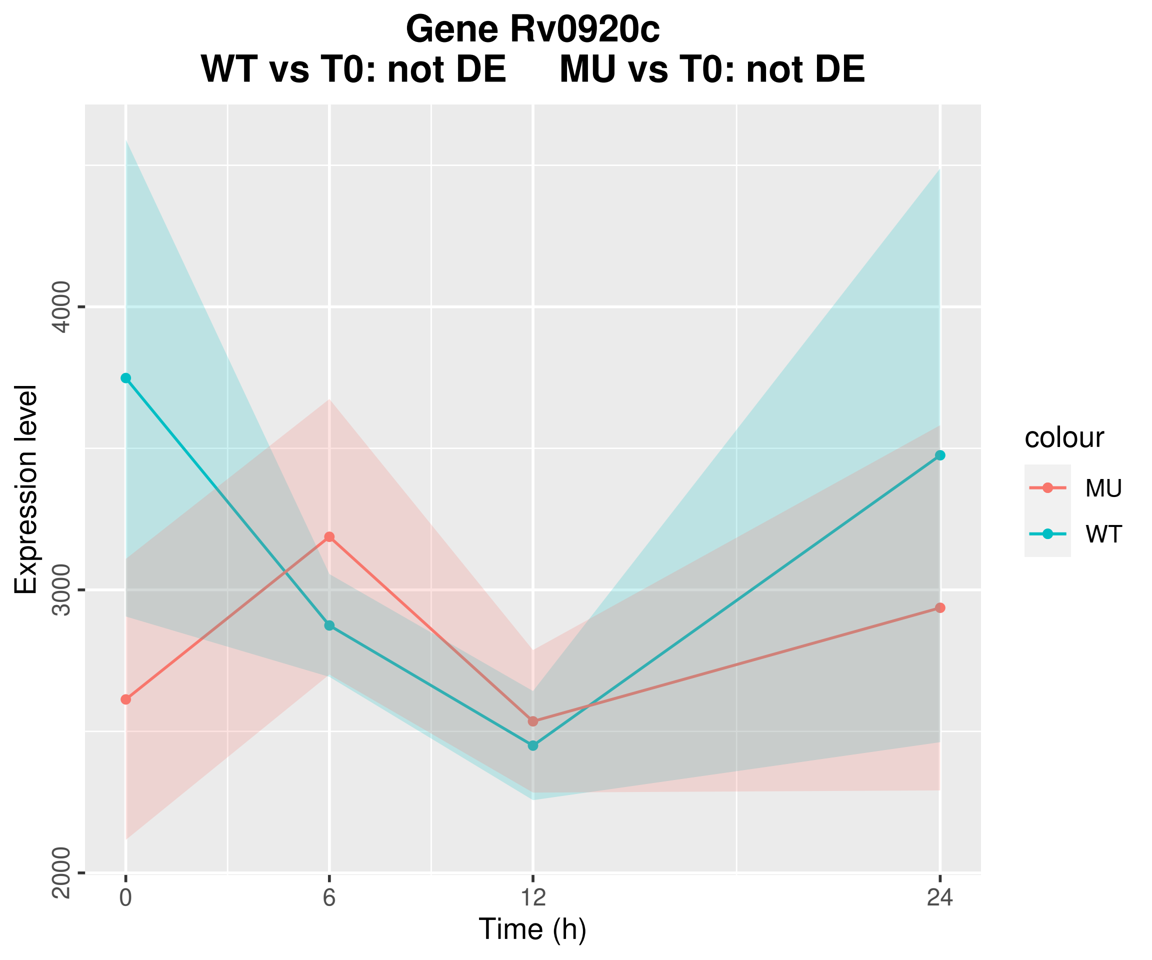
Gene Rv0920c
WT vs T0: not DE   MU vs T0: not DE
Expression level
4000
3000
2000
0
6
12
24
Time (h)
colour
MU
WT

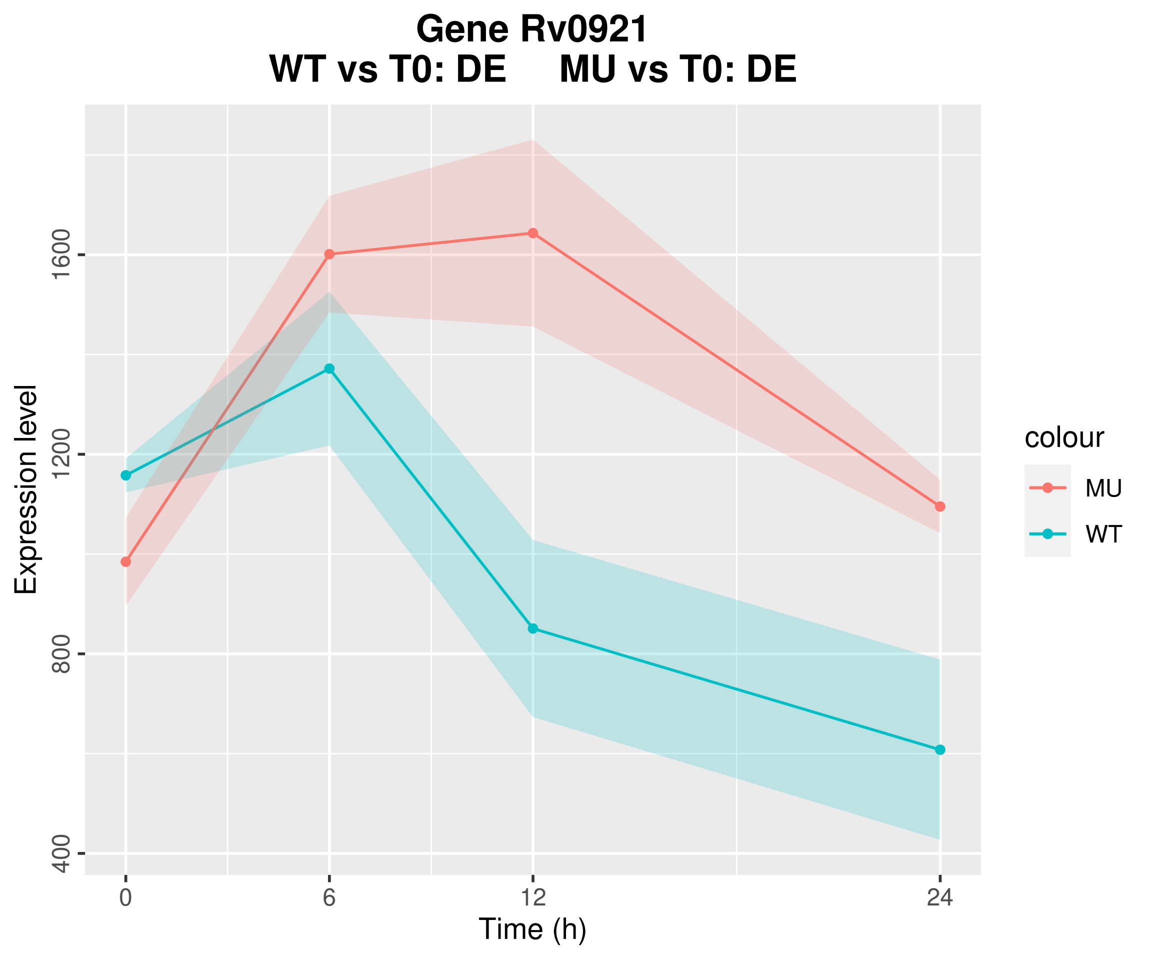
Gene Rv0921
WT vs T0: DE   MU vs T0: DE
Expression level
1600
1200
800
400
0
6
12
24
Time (h)
colour
MU
WT

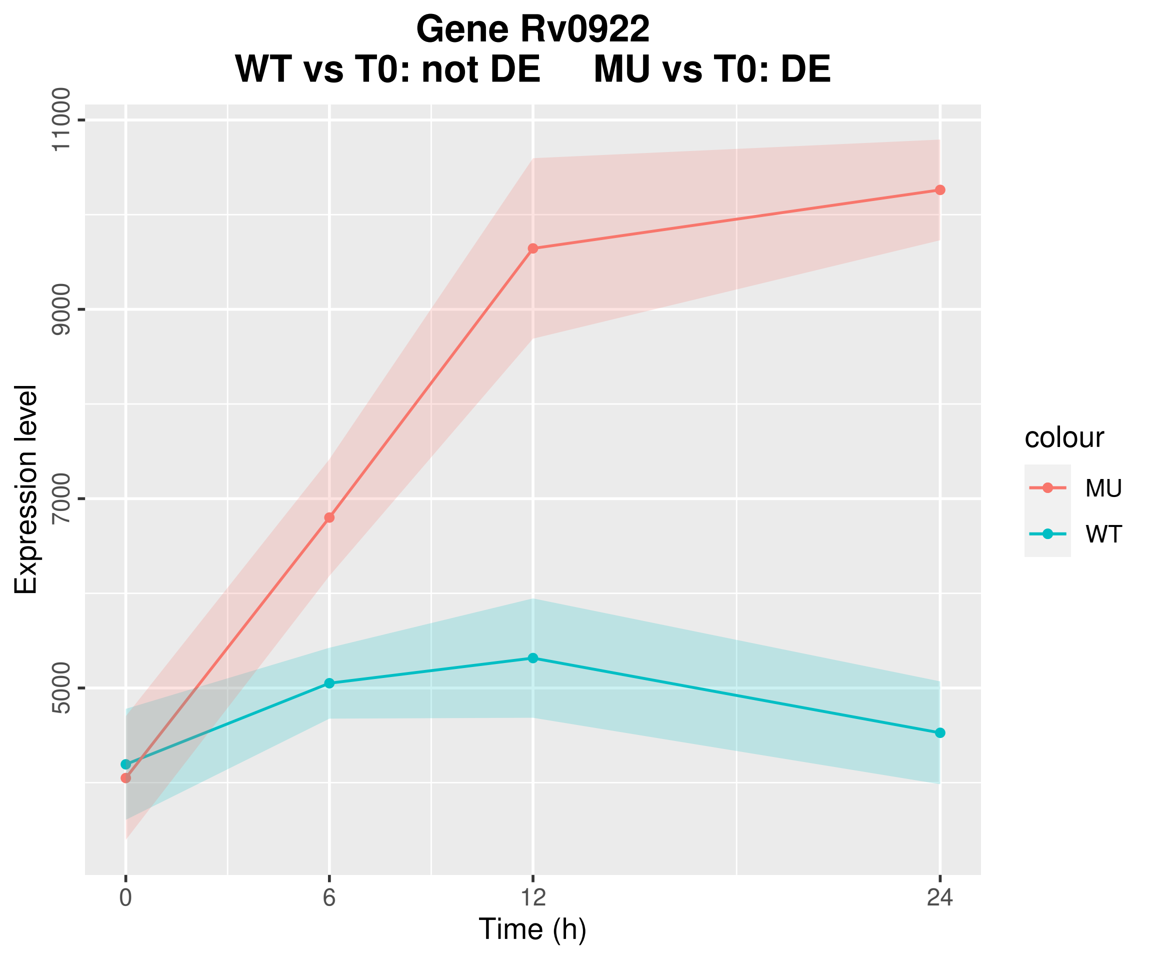

Gene Rv0922
WT vs T0: not DE   MU vs T0: DE
11000
9000
7000
5000
Expression level
0
6
12
24
Time (h)
colour
MU
WT

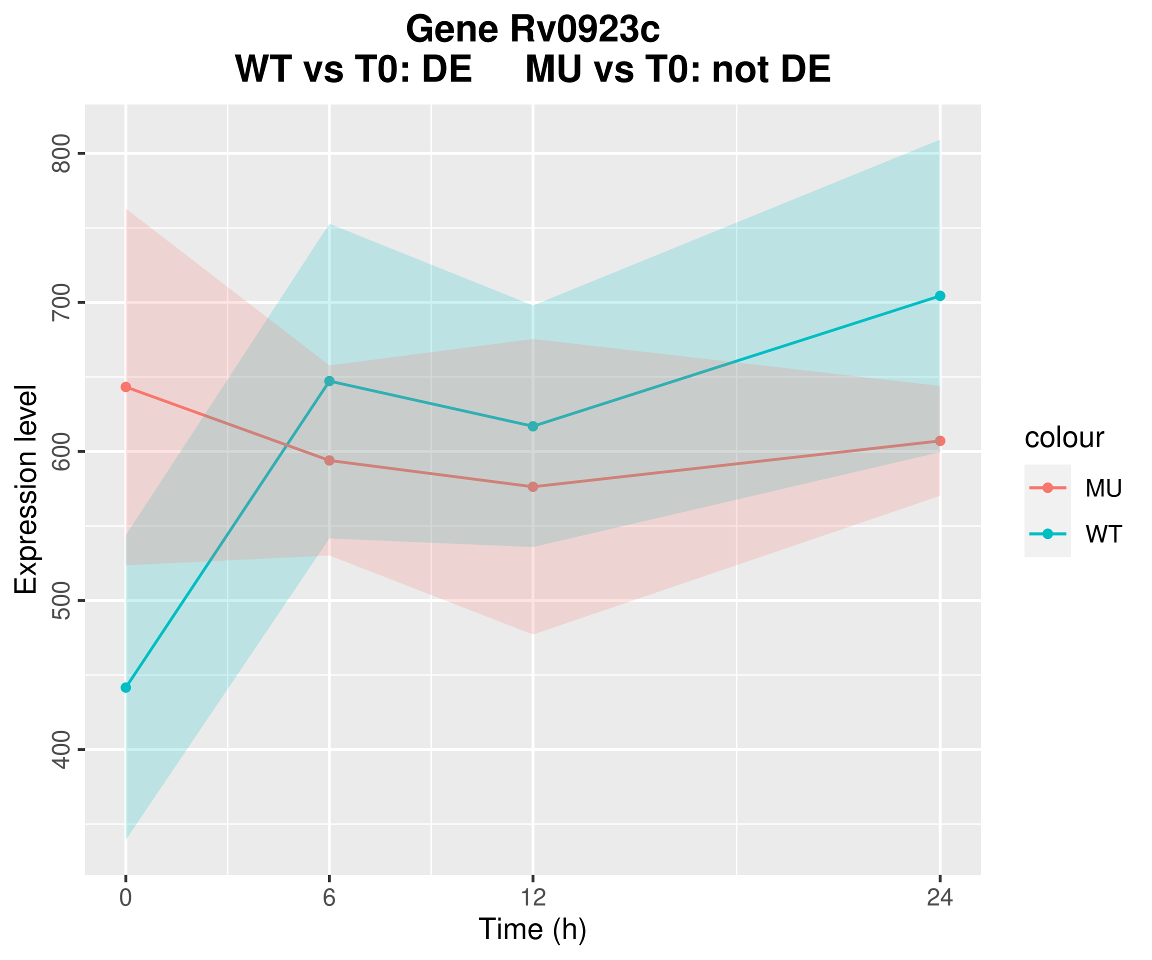

Gene Rv0923c
WT vs T0: DE MU vs T0: not DE
Expression level
800
700
600
500
400
0
6
12
24
Time (h)
colour
MU
WT

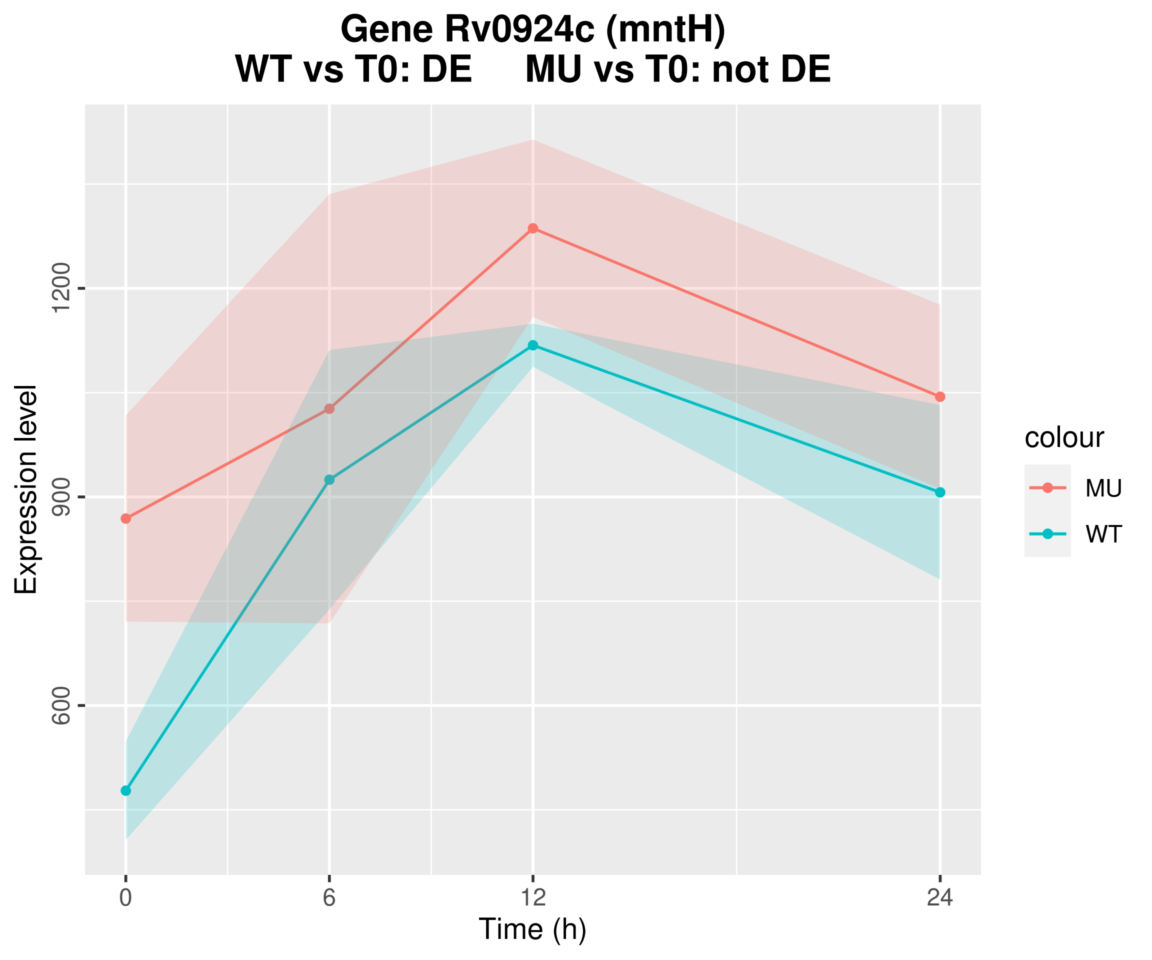
Gene Rv0924c (mntH)
WT vs T0: DE   MU vs T0: not DE
Expression level
1200
900
600
0
6
12
24
Time (h)
colour
MU
WT

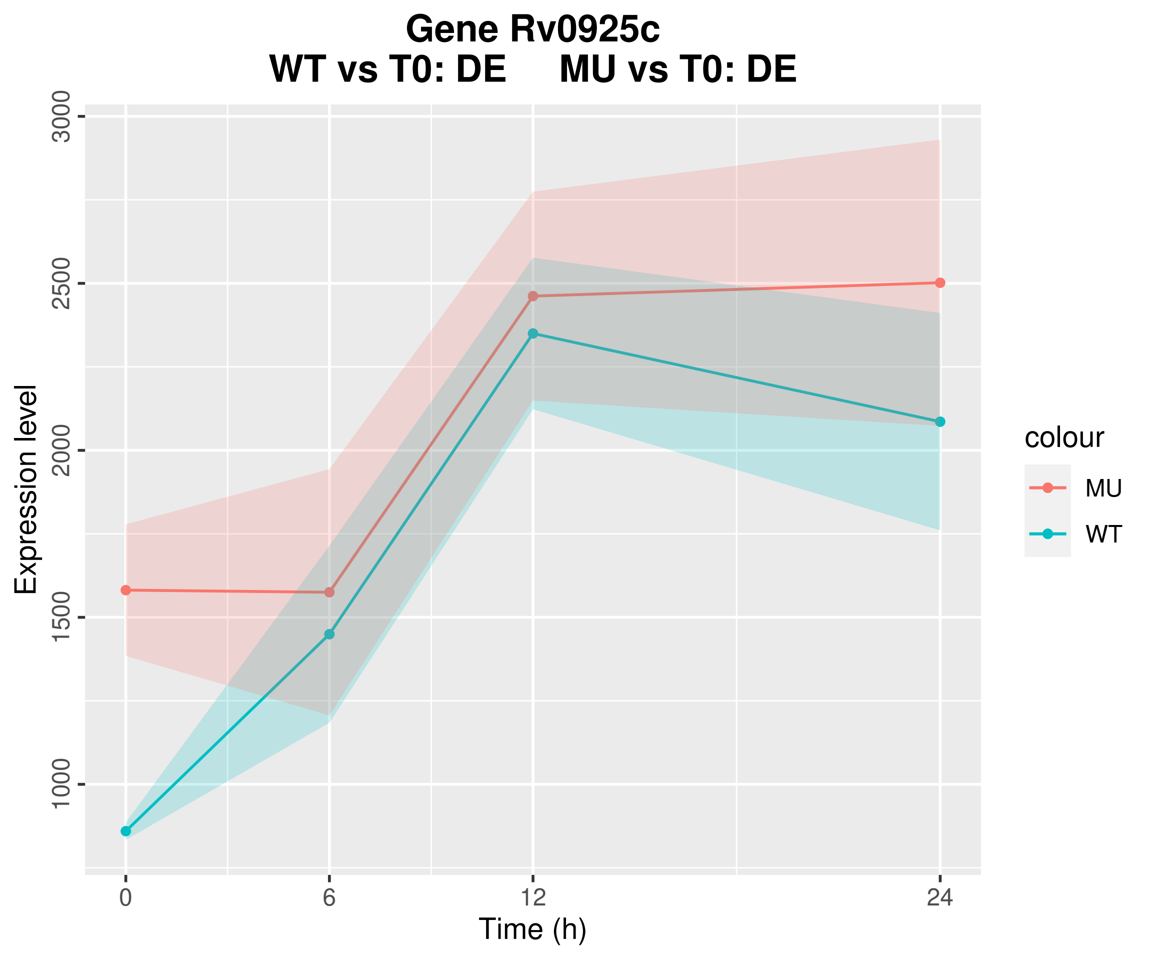
Gene Rv0925c
WT vs T0: DE   MU vs T0: DE
Expression level
3000
2500
2000
1500
1000
0
6
12
24
Time (h)
colour
MU
WT

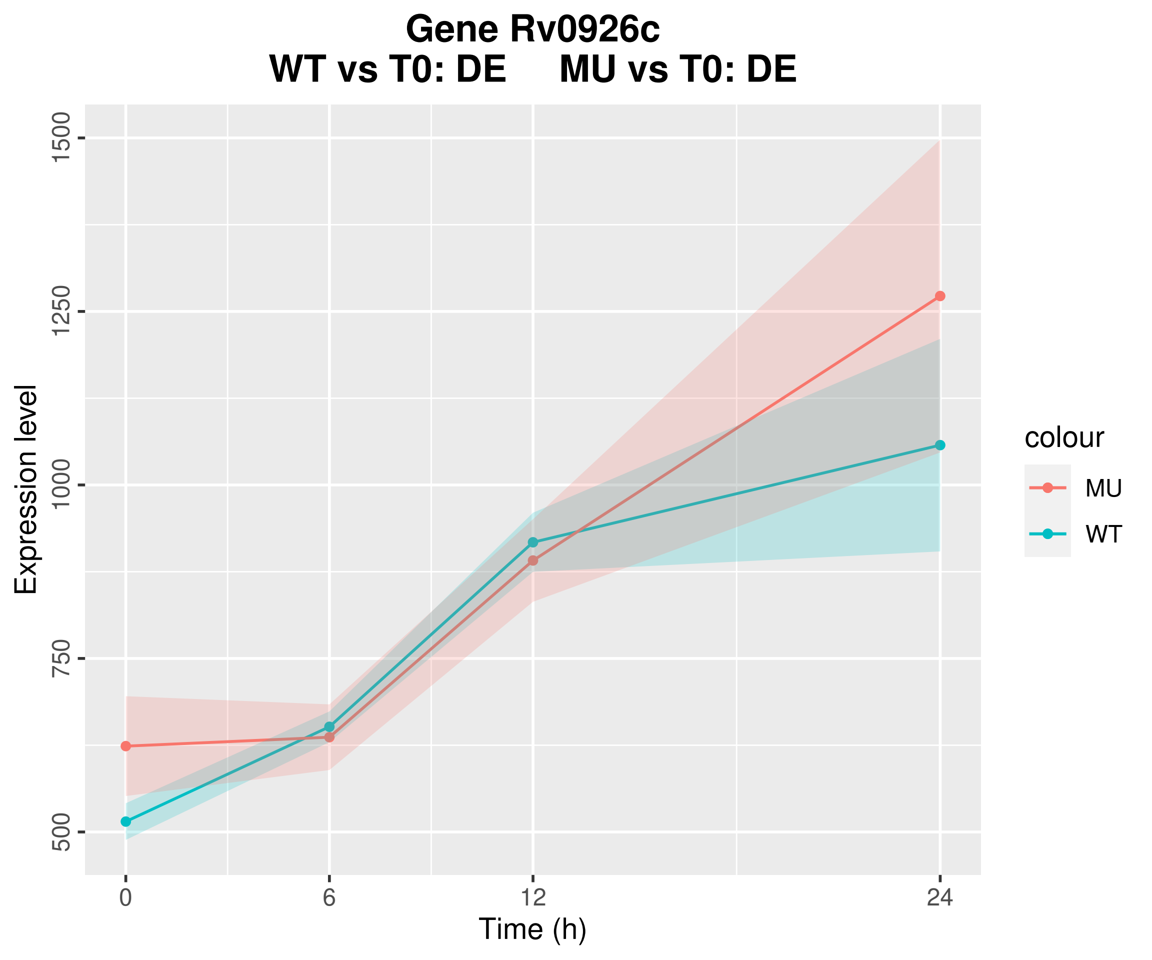

Gene Rv0926c
WT vs T0: DE   MU vs T0: DE
Expression level
1500
1250
1000
750
500
0
6
12
24
Time (h)
colour
MU
WT

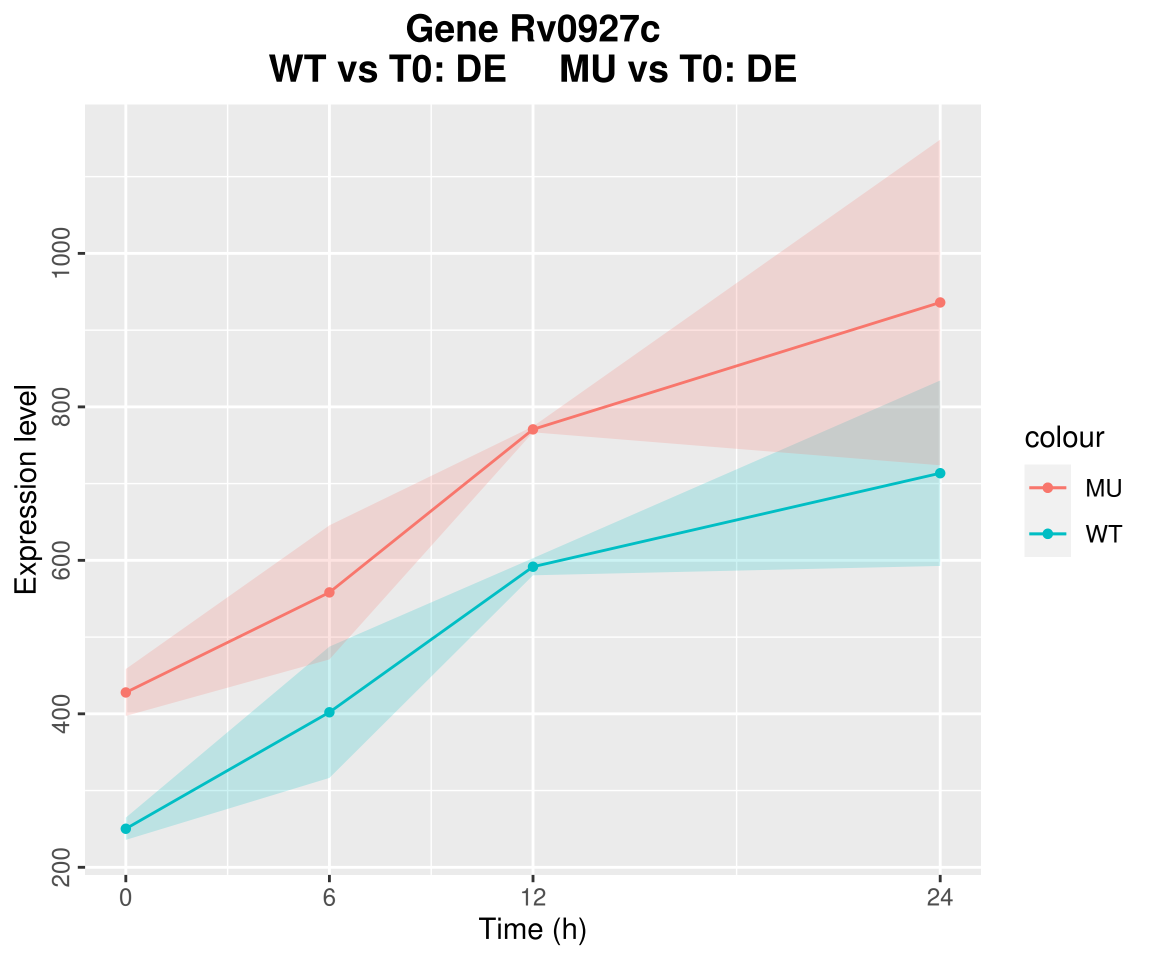
Gene Rv0927c
WT vs T0: DE MU vs T0: DE
Expression level
1000
800
600
400
200
0
6
12
24
Time (h)
colour
MU
WT

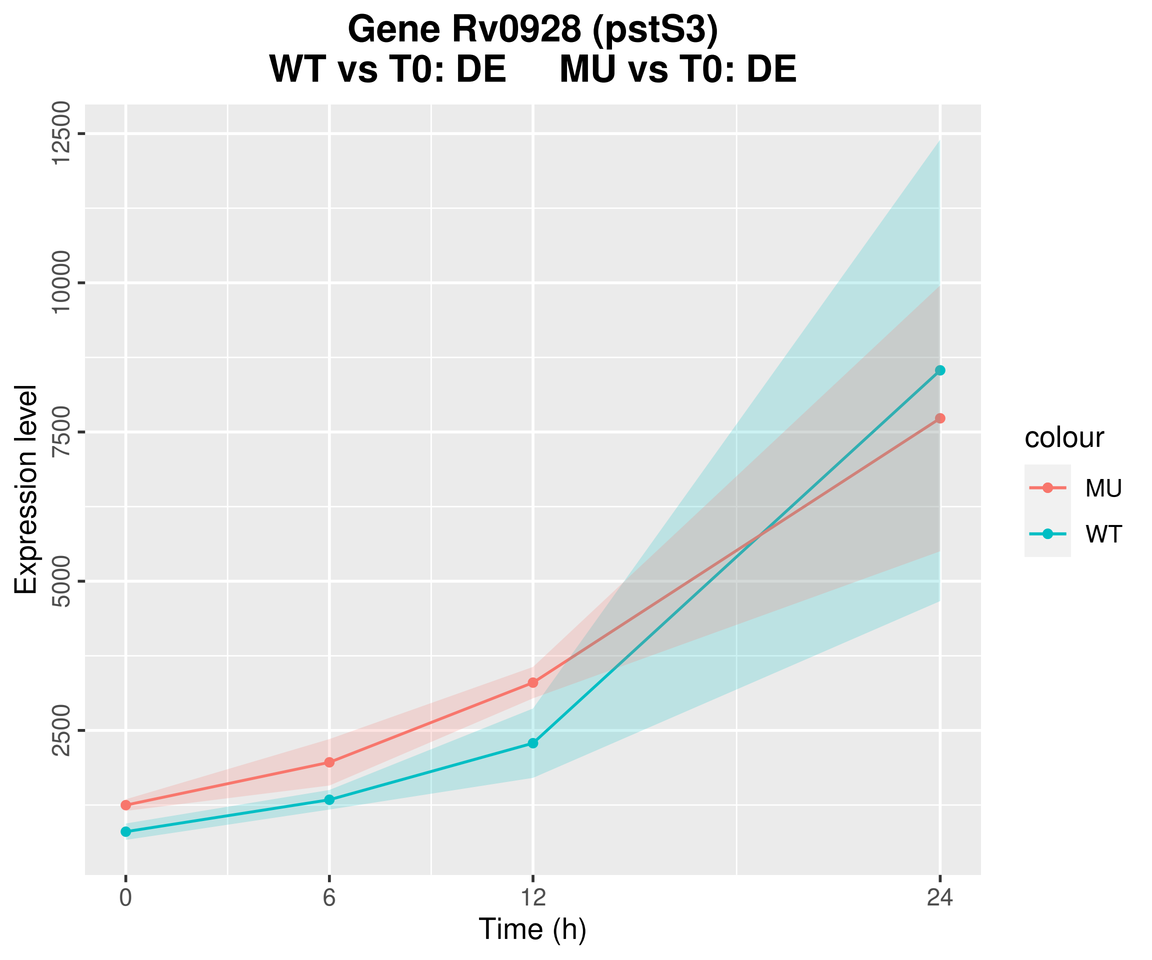

Gene Rv0928 (pstS3)
WT vs T0: DE MU vs T0: DE
Expression level
12500
10000
7500
5000
2500
0
6
12
24
Time (h)
colour
MU
WT

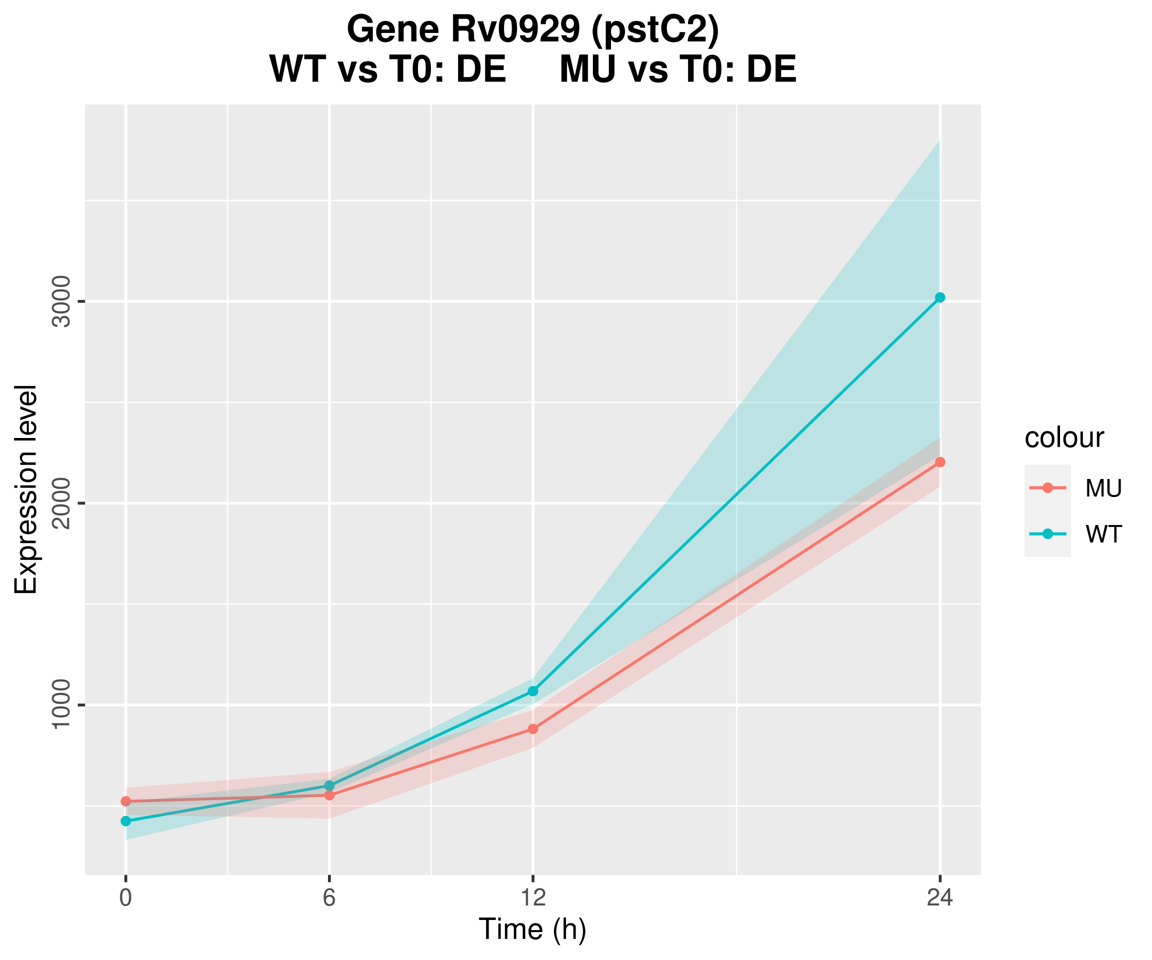
Gene Rv0929 (pstC2)
WT vs T0: DE   MU vs T0: DE
Expression level
3000
2000
1000
0
6
12
24
Time (h)
colour
MU
WT

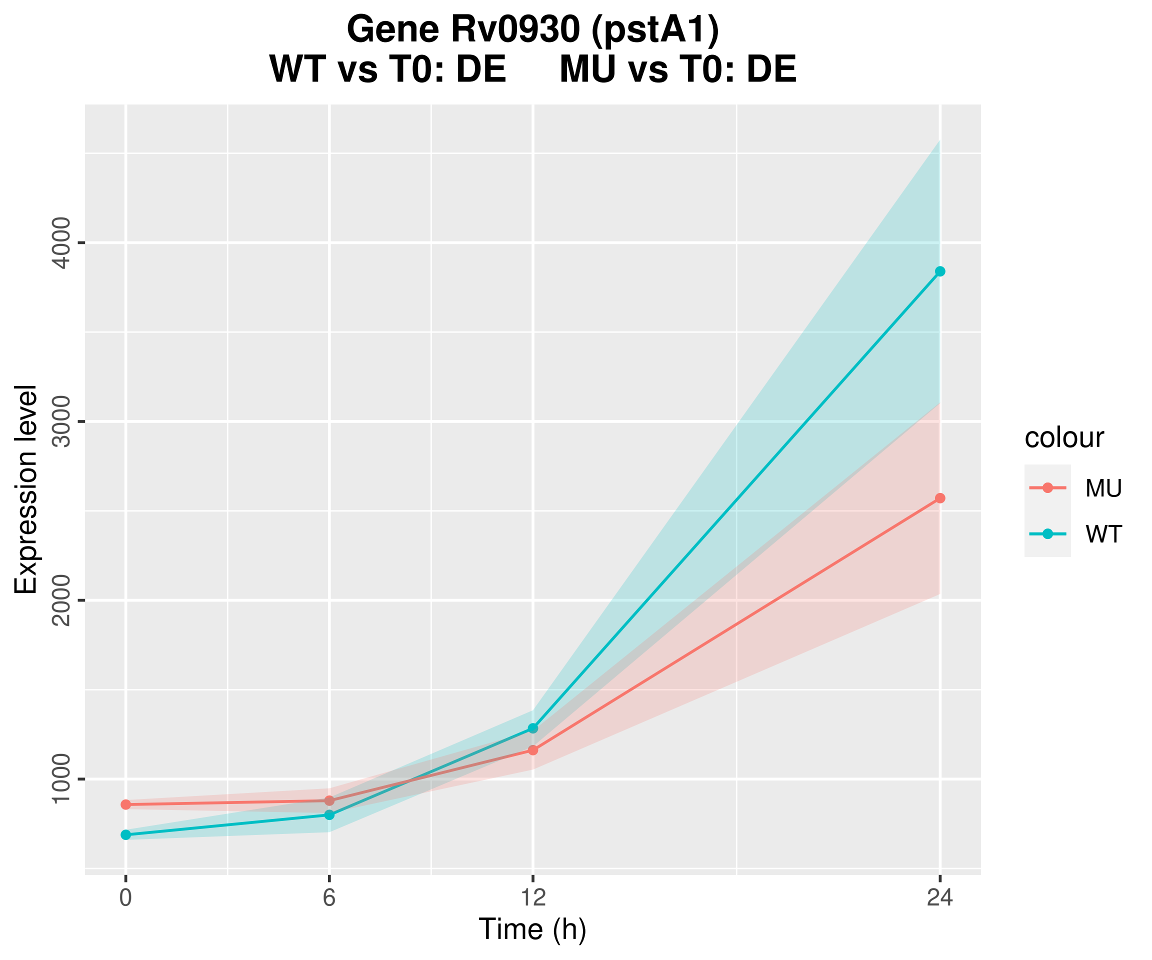

Gene Rv0930 (pstA1)
WT vs T0: DE MU vs T0: DE
Expression level
1000
2000
3000
4000
0
6
12
24
Time (h)
colour
MU
WT

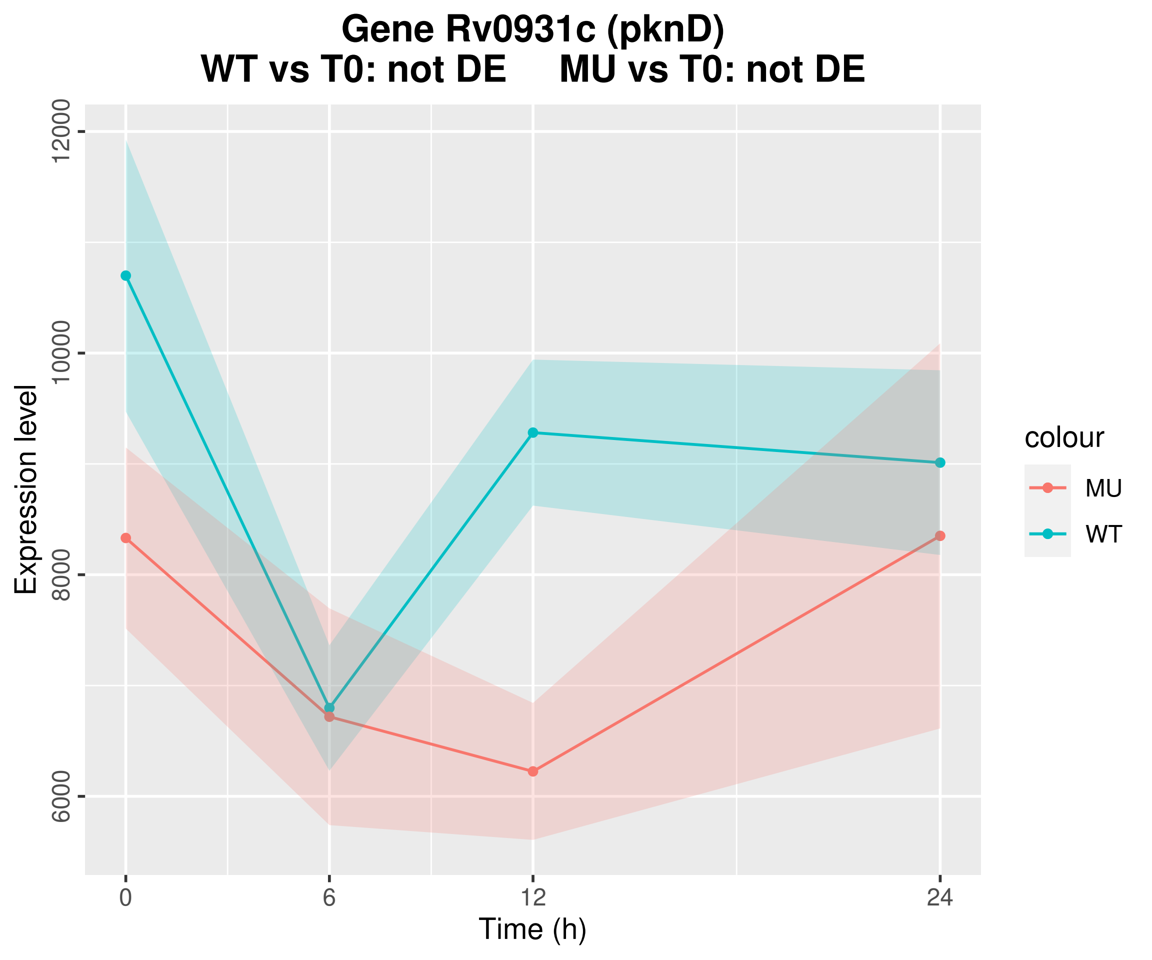

Gene Rv0931c (pknD)
WT vs T0: not DE MU vs T0: not DE
Expression level
12000
10000
8000
6000
0
6
12
24
Time (h)
colour
MU
WT

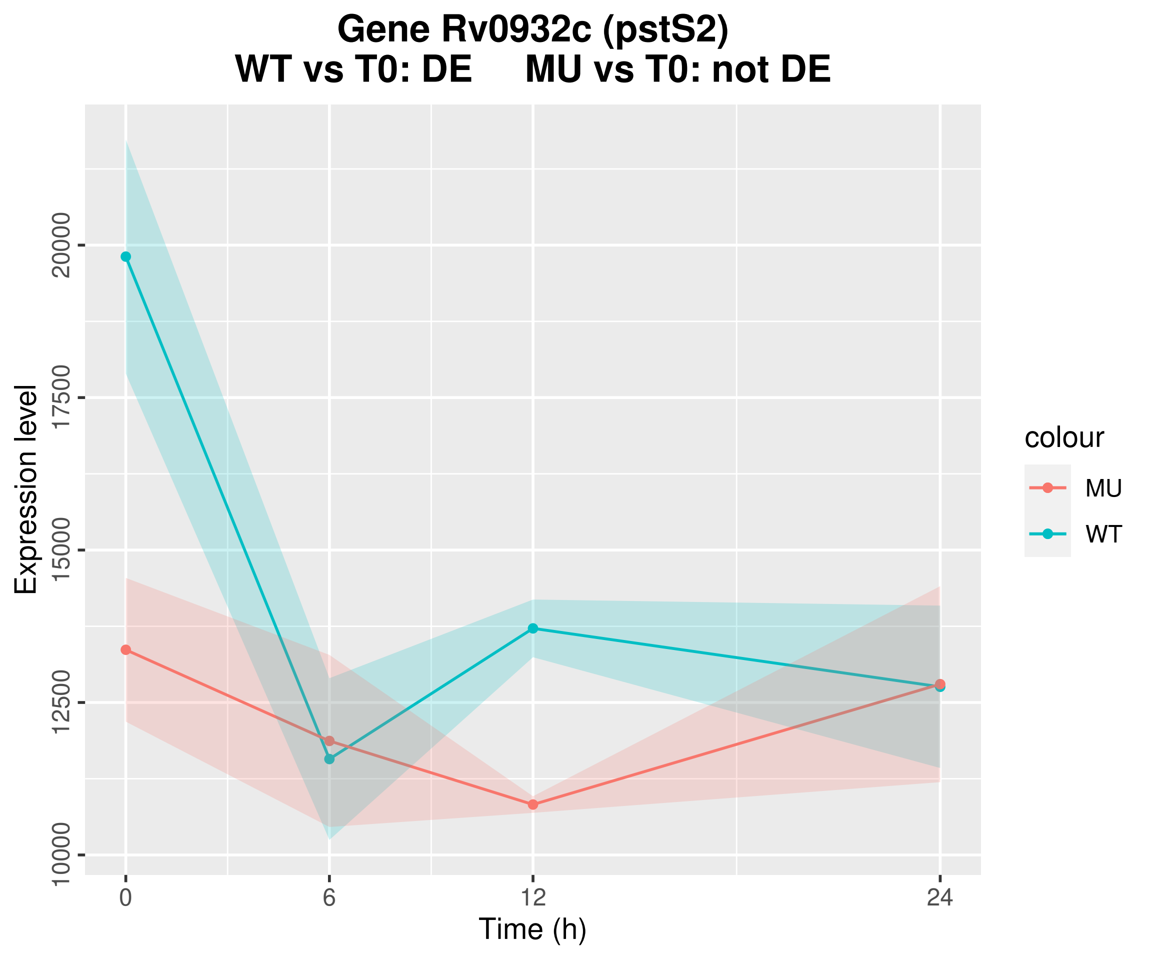

Gene Rv0932c (pstS2)
WT vs T0: DE MU vs T0: not DE
Expression level
20000
17500
15000
12500
10000
0
6
12
24
Time (h)
colour
MU
WT

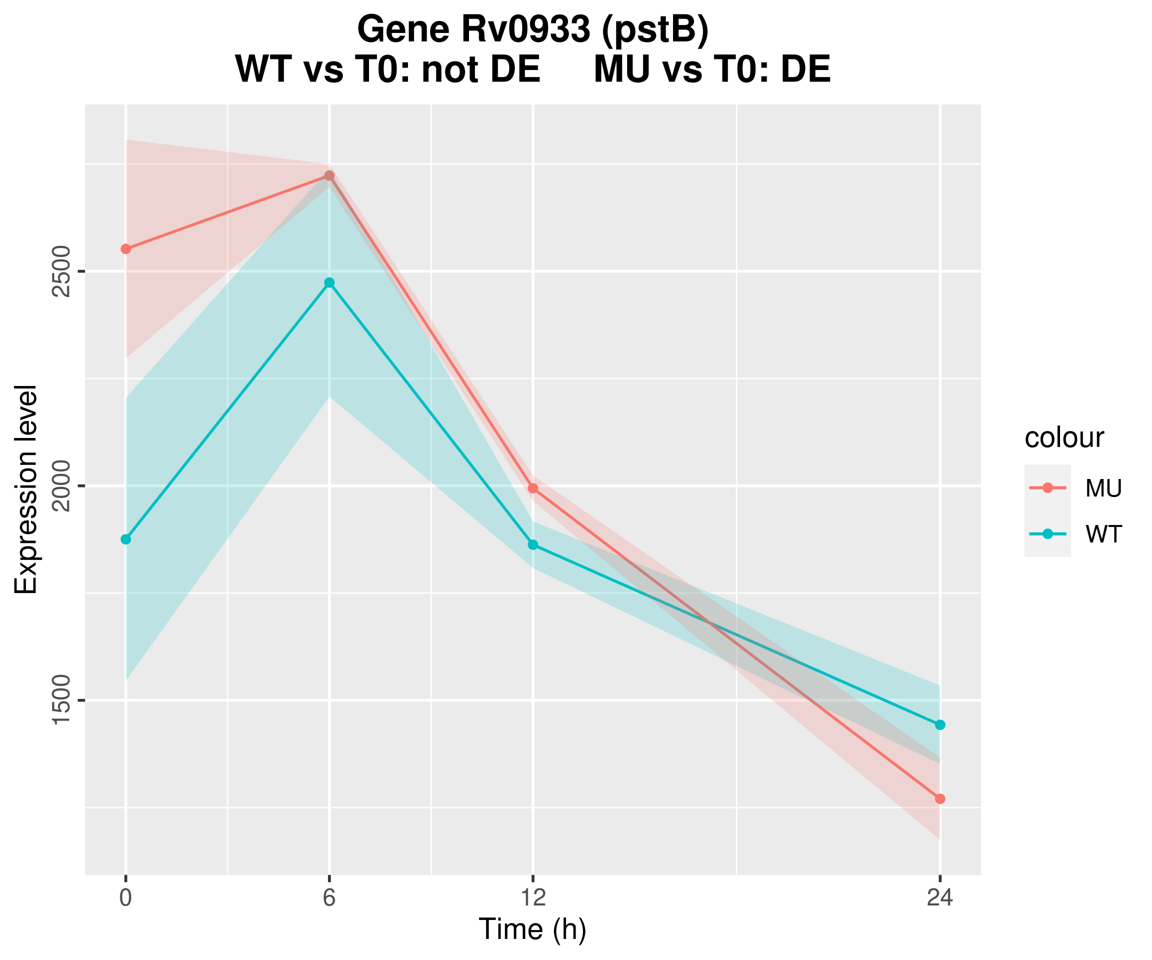
Gene Rv0933 (pstB)
WT vs T0: not DE   MU vs T0: DE
Expression level
2500
2000
1500
0
6
12
24
Time (h)
colour
MU
WT

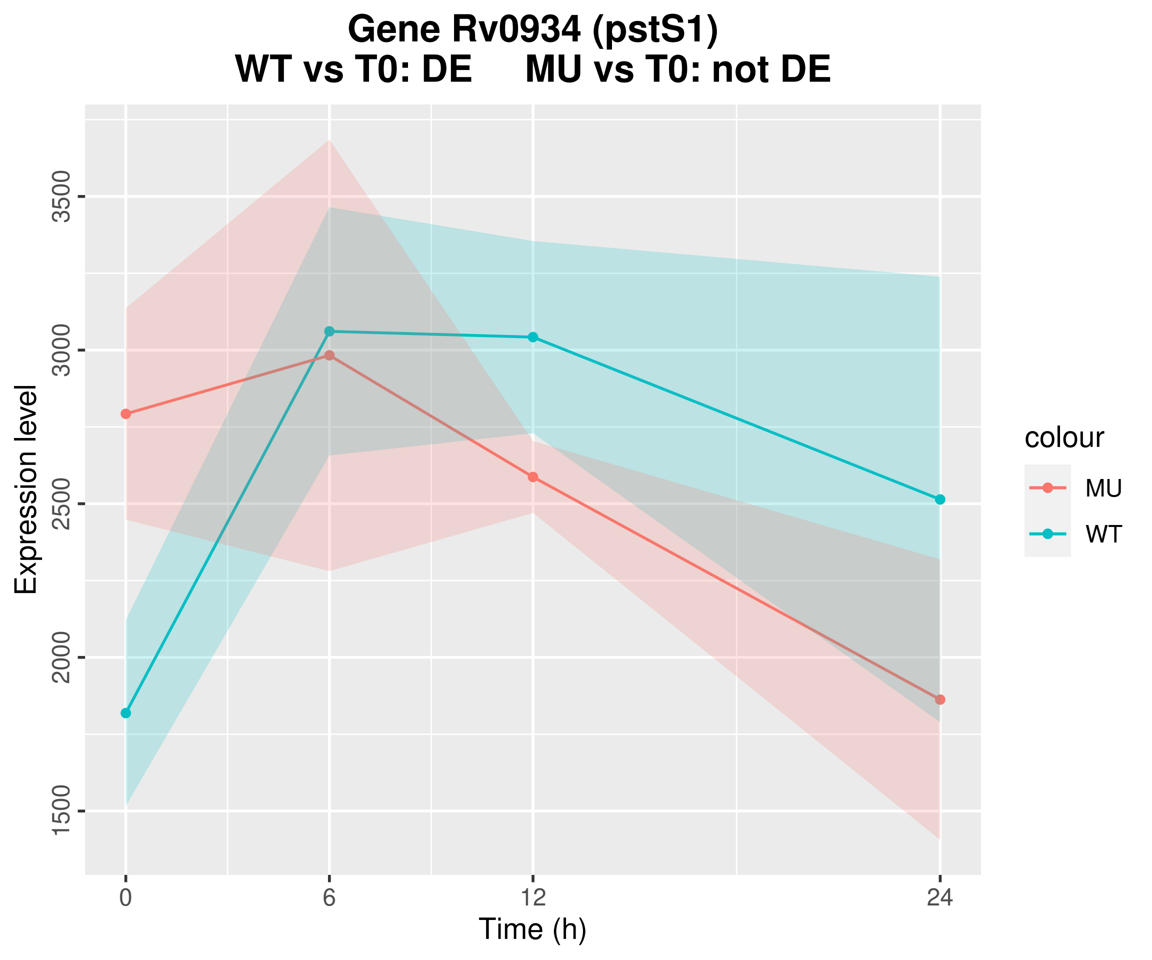
Gene Rv0934 (pstS1)
WT vs T0: DE   MU vs T0: not DE
Expression level
1500
2000
2500
3000
3500
0
6
12
24
Time (h)
colour
MU
WT

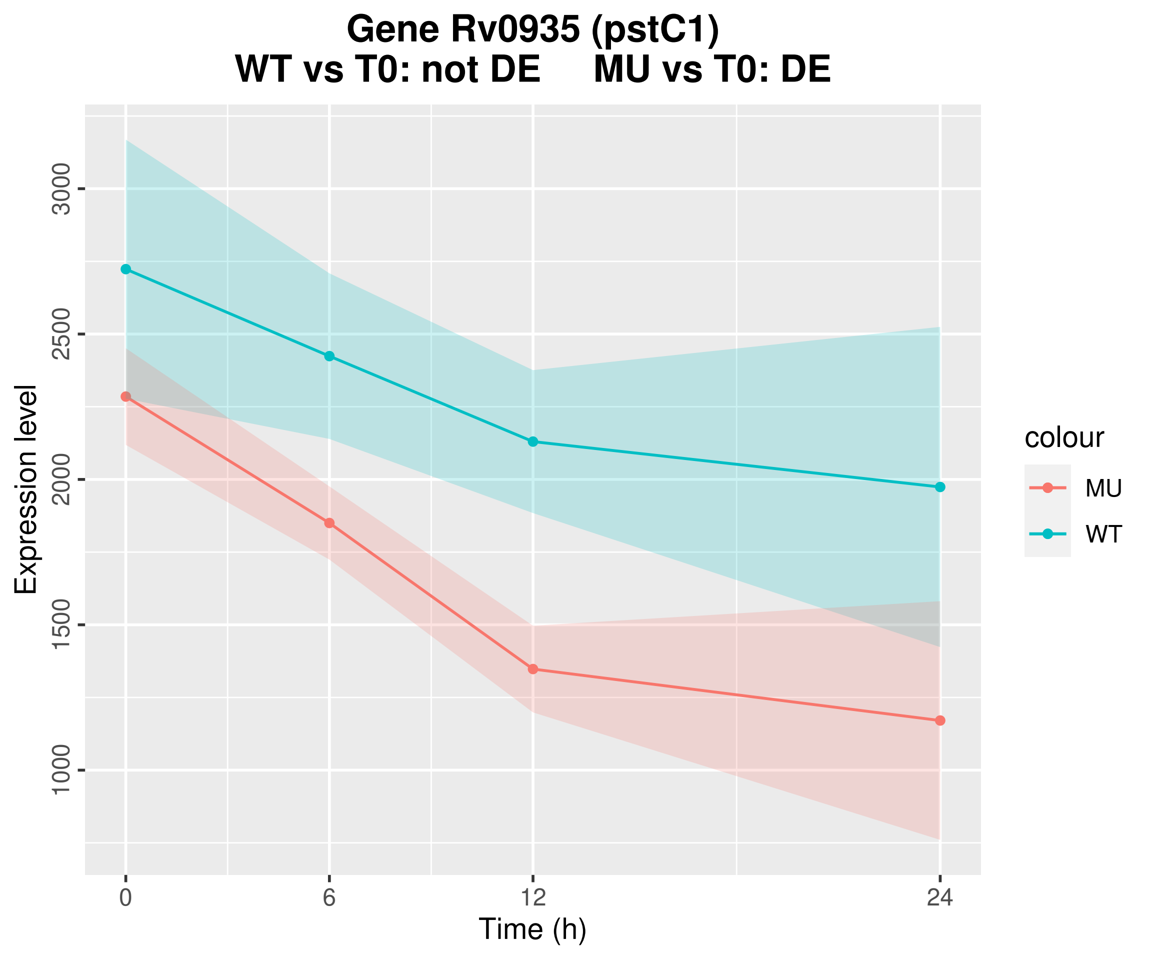
Gene Rv0935 (pstC1)
WT vs T0: not DE   MU vs T0: DE
Expression level
3000
2500
2000
1500
1000
Time (h)
0
6
12
24
colour
MU
WT

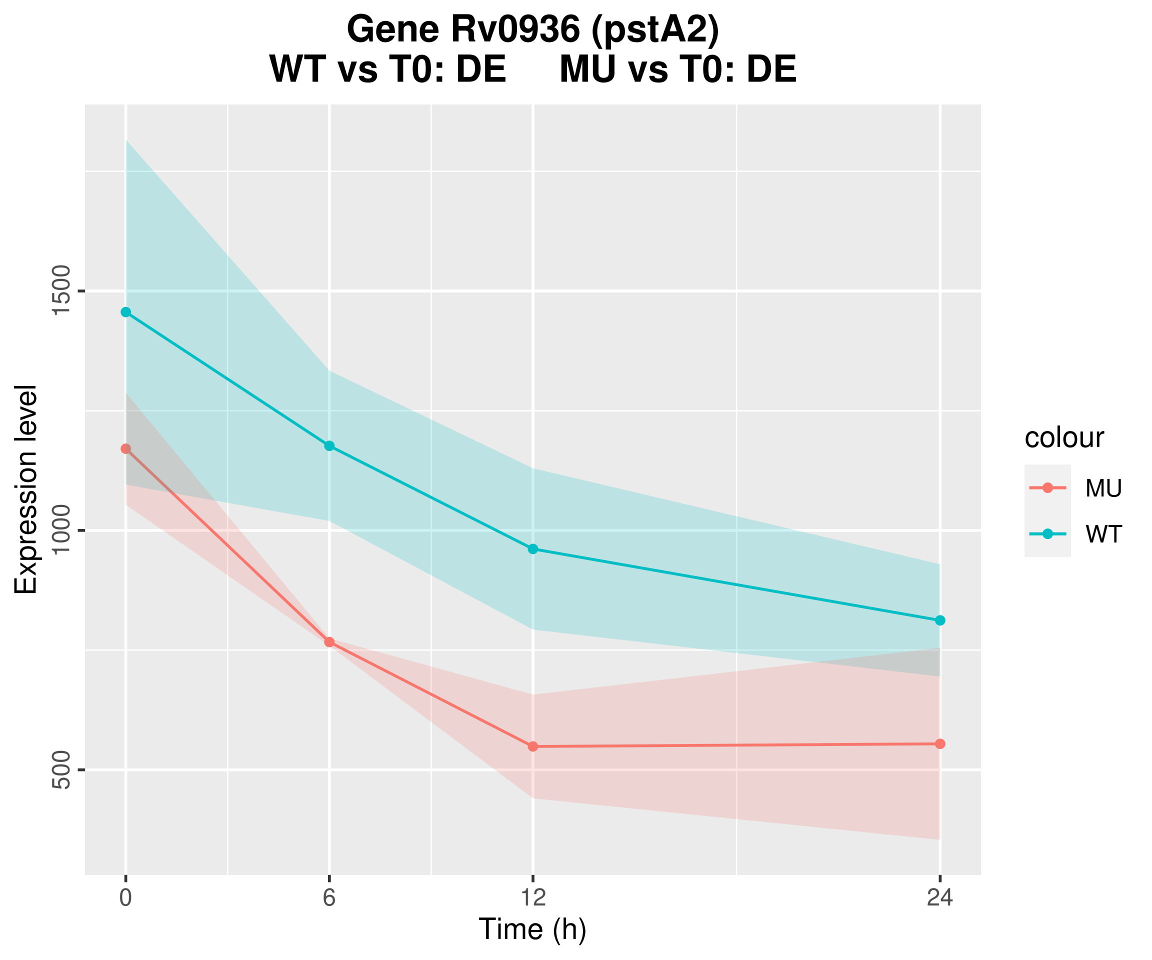
Gene Rv0936 (pstA2)
WT vs T0: DE MU vs T0: DE
Expression level
1500
1000
500
0
6
12
24
Time (h)
colour
MU
WT

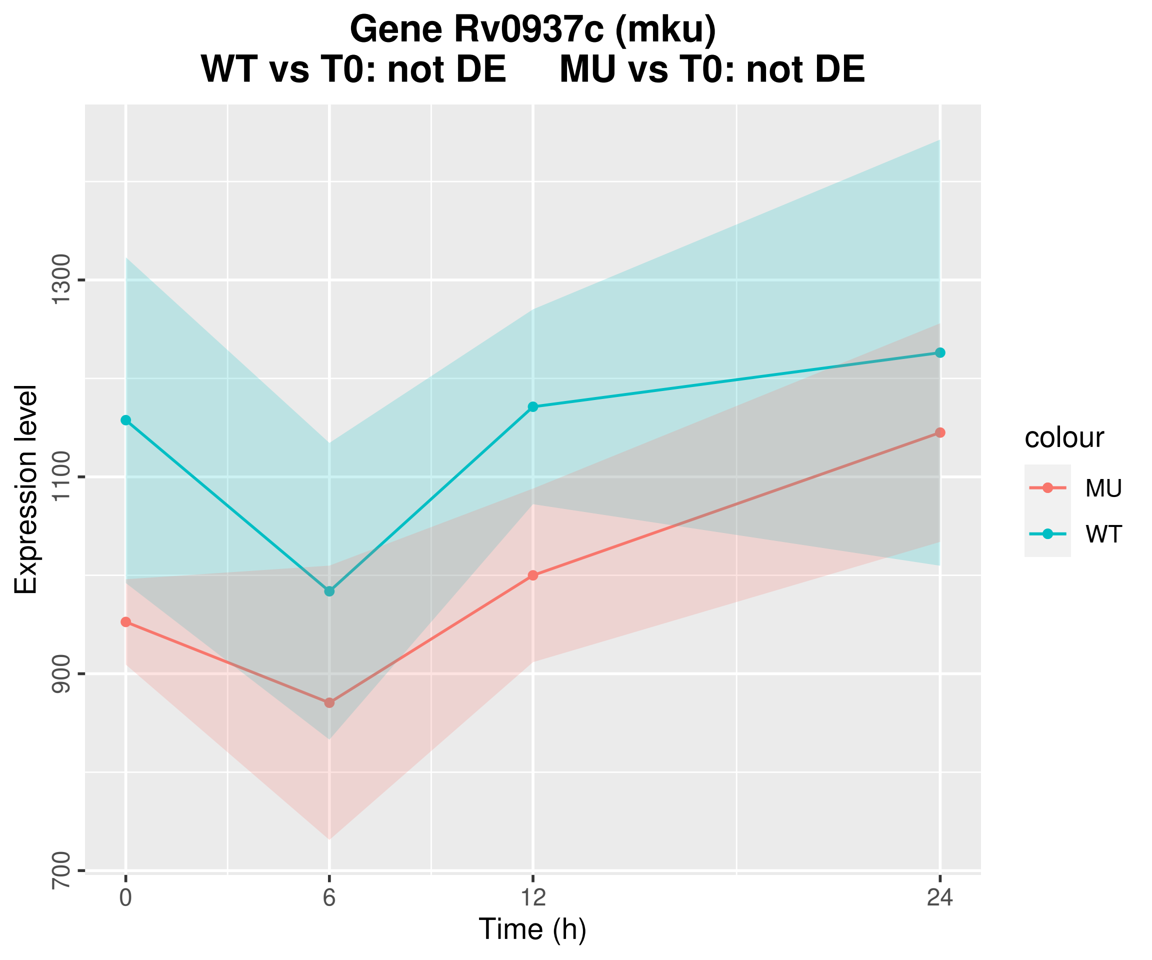
Gene Rv0937c (mku)
WT vs T0: not DE MU vs T0: not DE
Expression level
700
900
1100
1300
0
6
12
24
Time (h)
colour
MU
WT

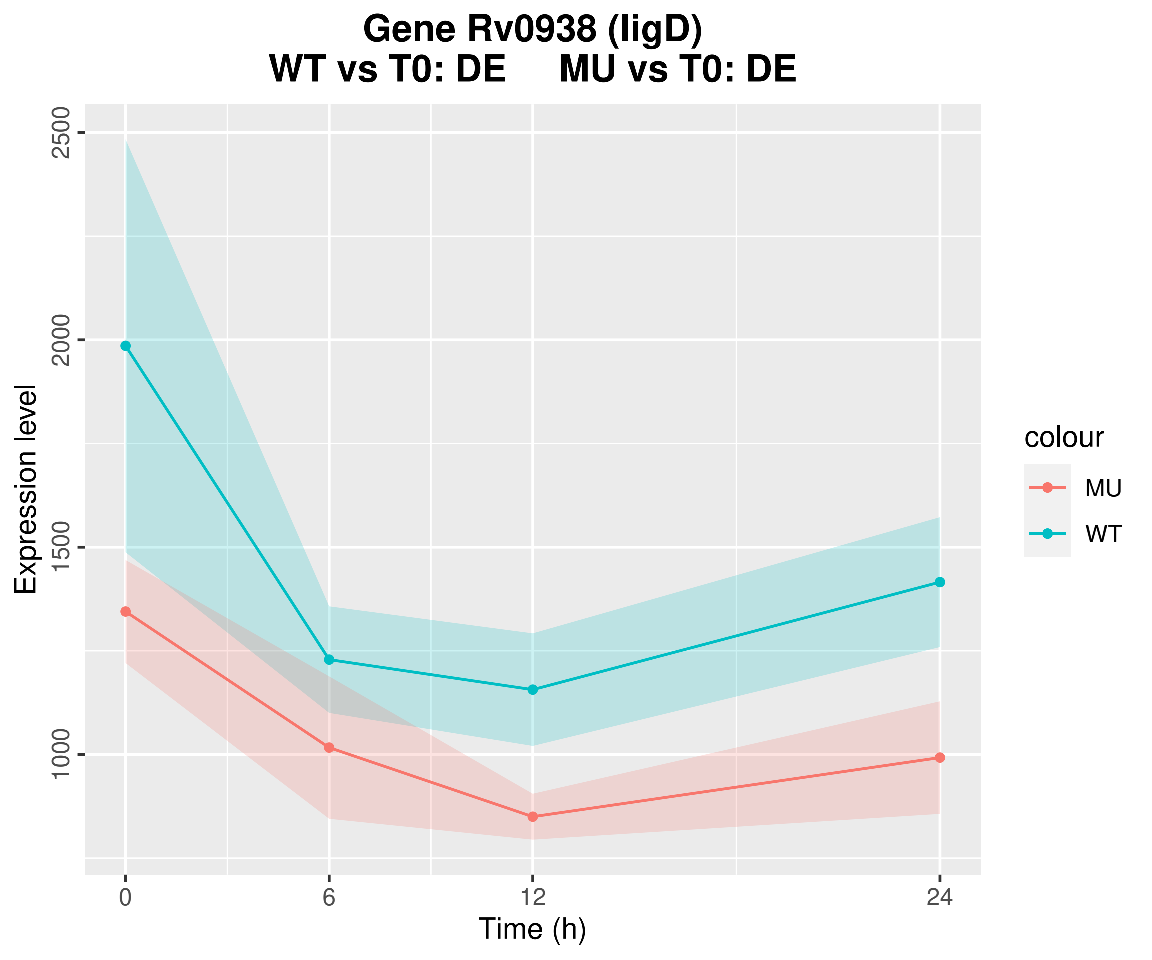
Gene Rv0938 (ligD)
WT vs T0: DE   MU vs T0: DE
2500
2000
1500
1000
Expression level
0
6
12
24
Time (h)
colour
MU
WT

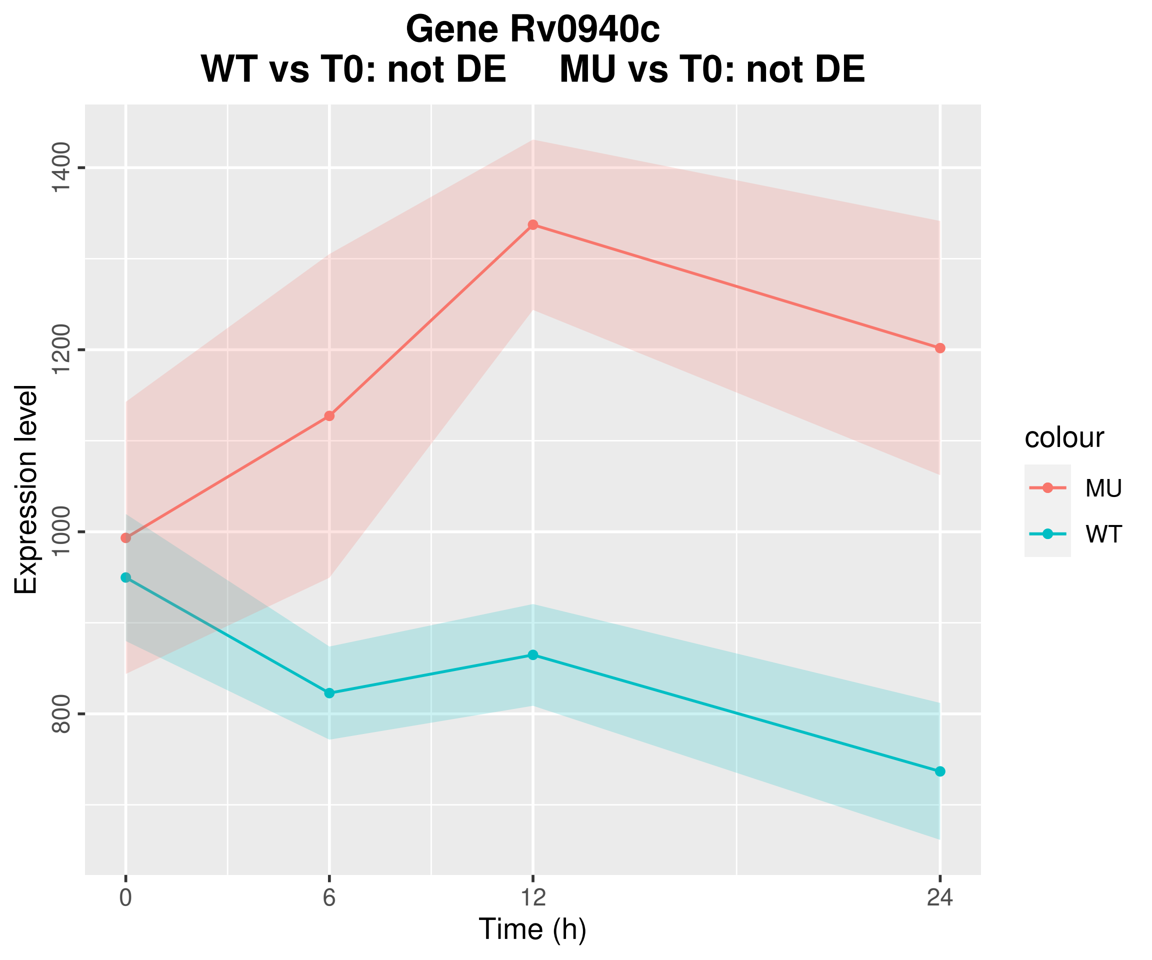

Gene Rv0940c
WT vs T0: not DE   MU vs T0: not DE
Expression level
1400
1200
1000
800
0
6
12
24
Time (h)
colour
MU
WT

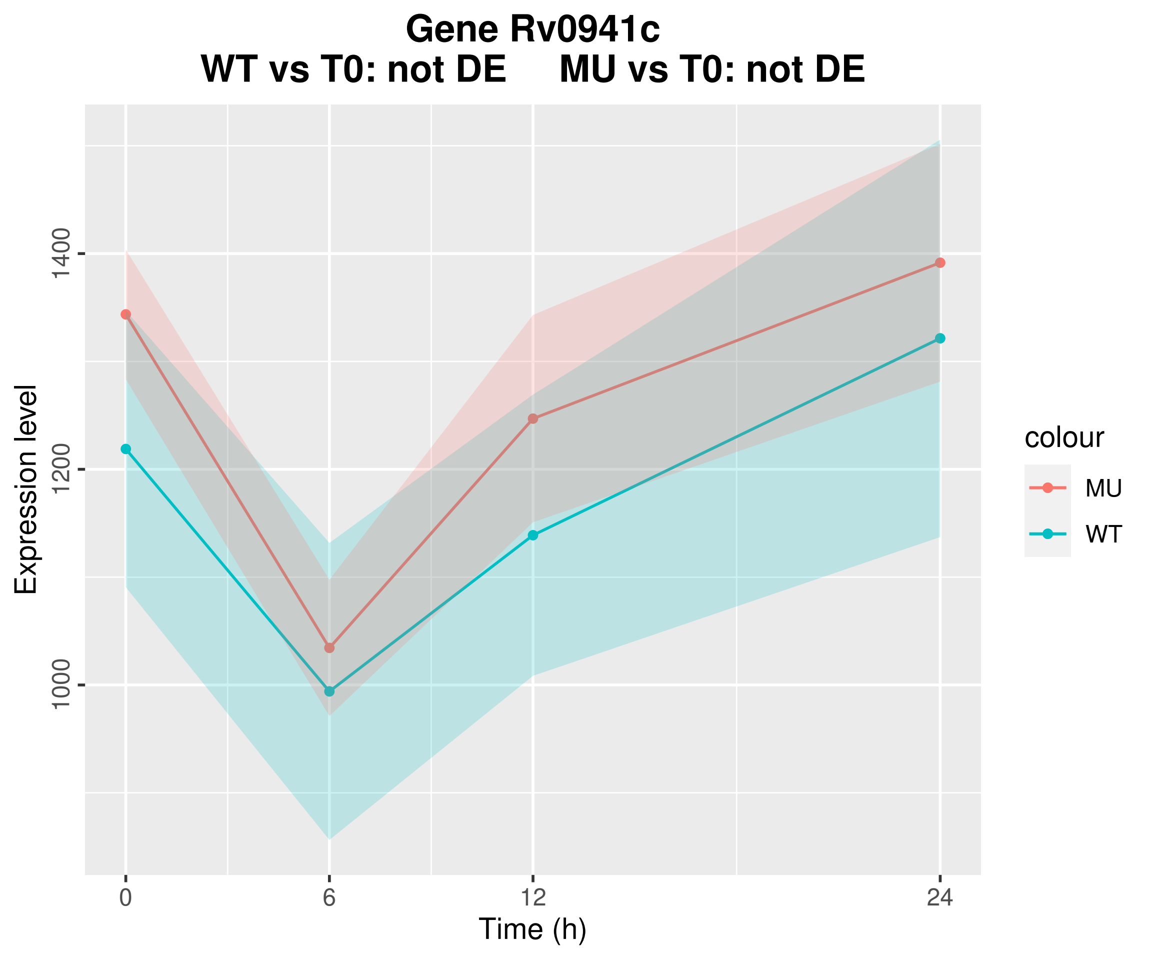
Gene Rv0941c
WT vs T0: not DE   MU vs T0: not DE
Expression level
1000
1200
1400
Time (h)
0
6
12
24
colour
MU
WT

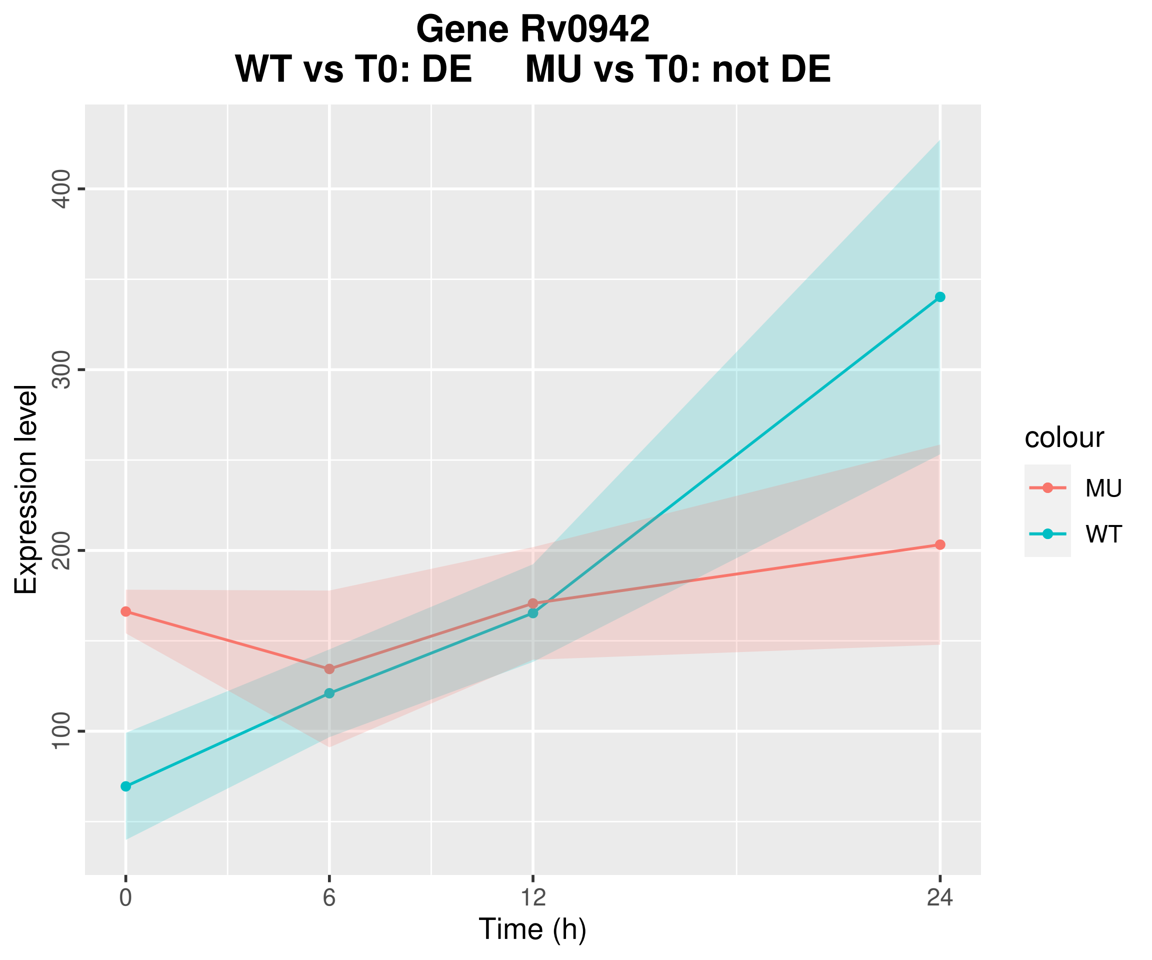

Gene Rv0942
WT vs T0: DE   MU vs T0: not DE
Expression level
100
200
300
400
0
6
12
24
Time (h)
colour
MU
WT

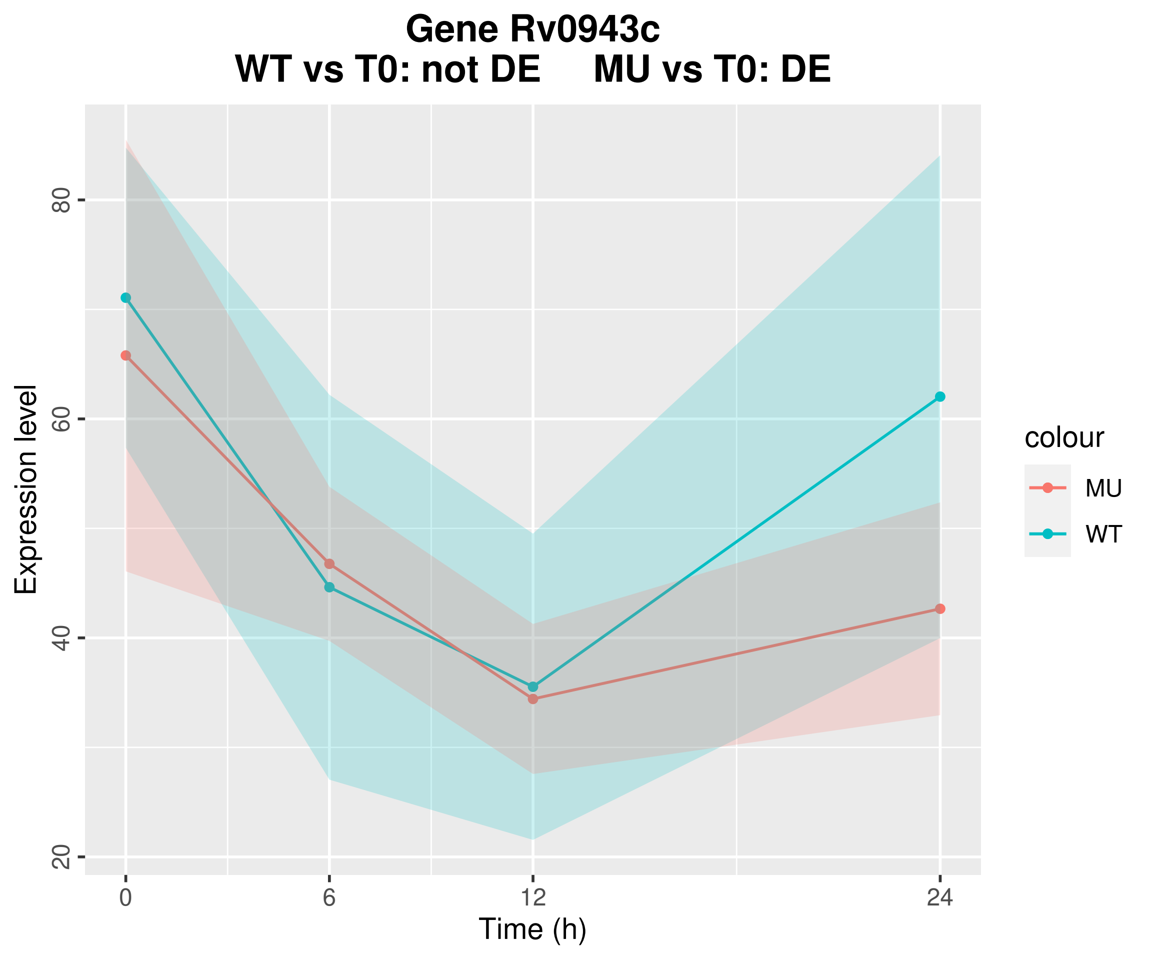

Gene Rv0943c
WT vs T0: not DE MU vs T0: DE
Expression level
80
60
40
20
0
6
12
24
Time (h)
colour
MU
WT

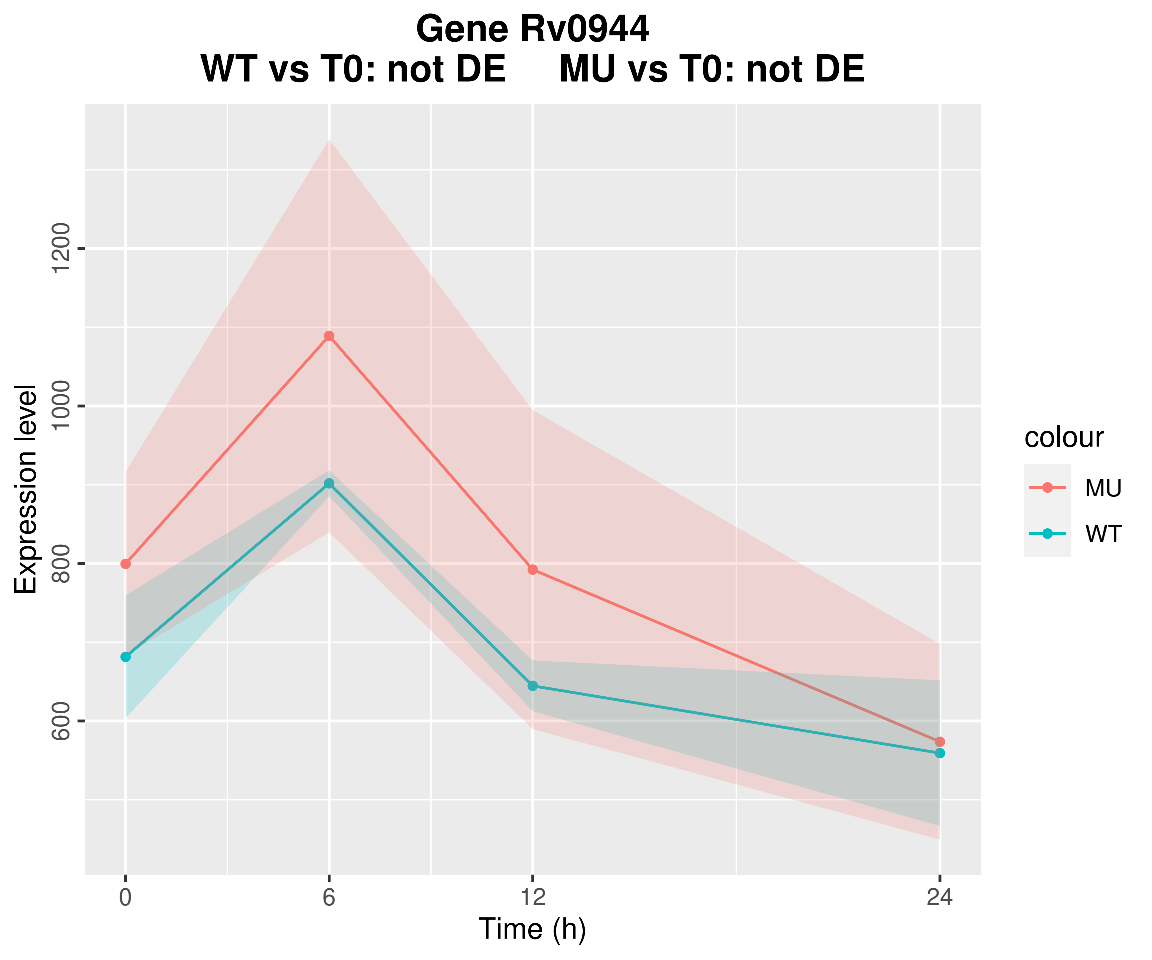
Gene Rv0944
WT vs T0: not DE   MU vs T0: not DE
Expression level
1200
1000
800
600
0
6
12
24
Time (h)
colour
MU
WT

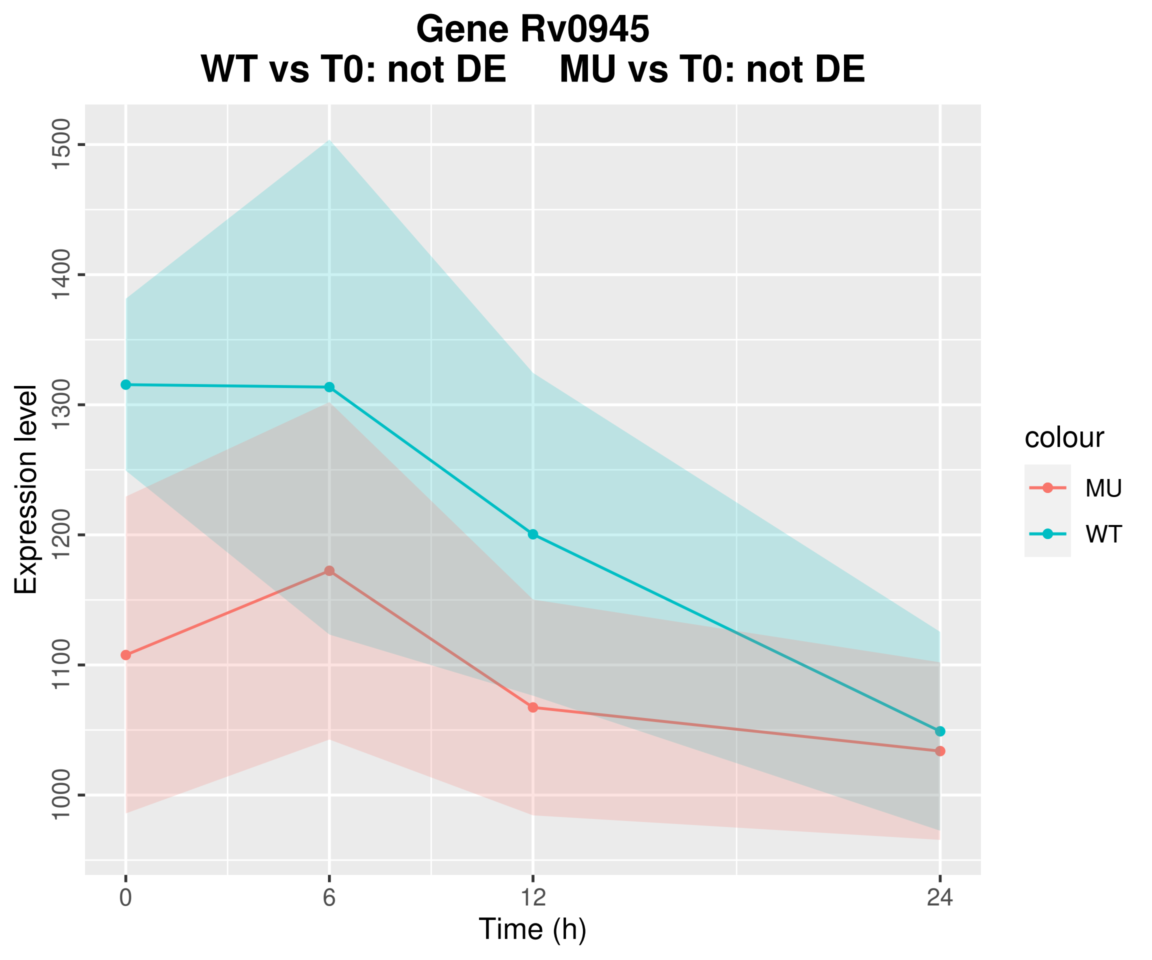

Gene Rv0945
WT vs T0: not DE MU vs T0: not DE
Expression level
1000
1100
1200
1300
1400
1500
0
6
12
24
Time (h)
colour
MU
WT

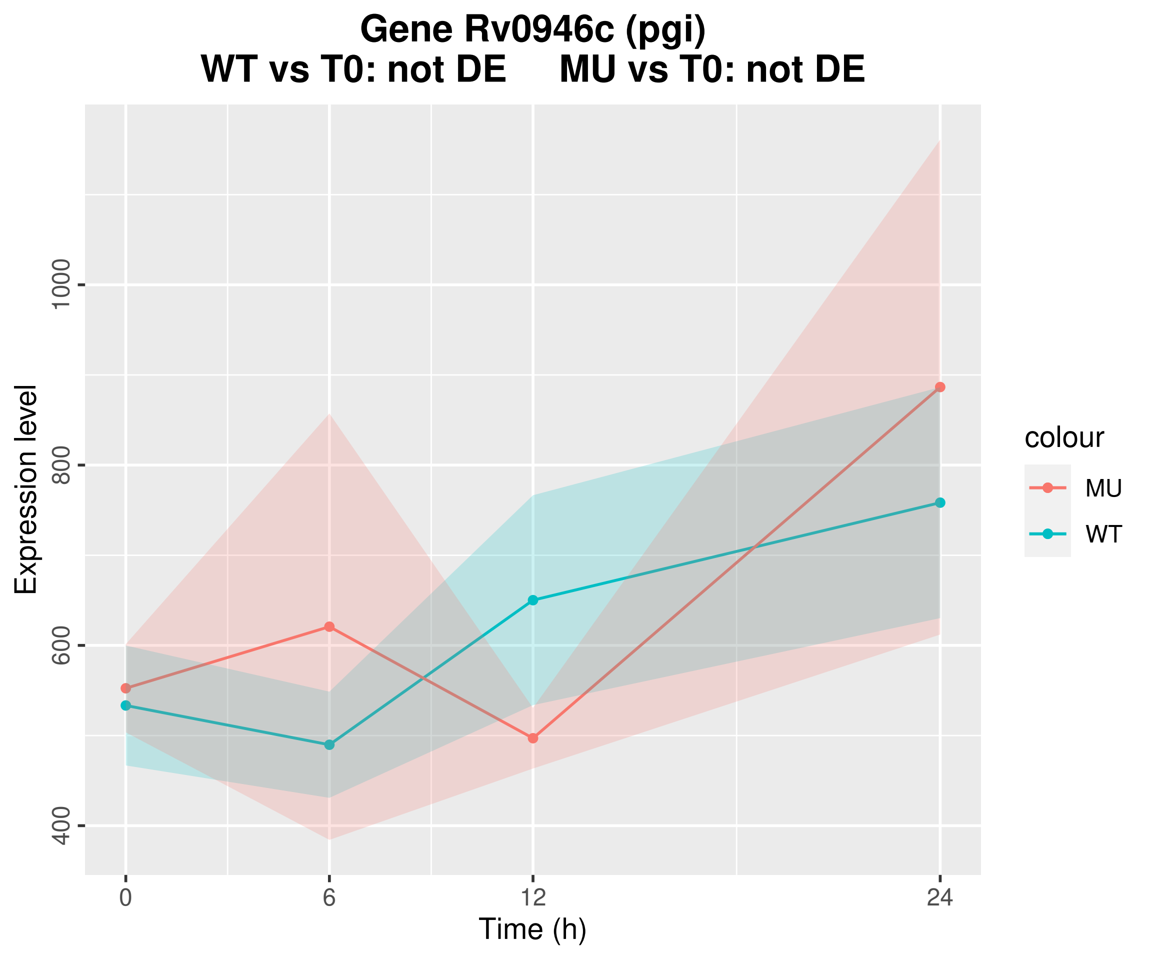
Gene Rv0946c (pgi)
WT vs T0: not DE   MU vs T0: not DE
Expression level
1000
800
600
400
0
6
12
24
Time (h)
colour
MU
WT

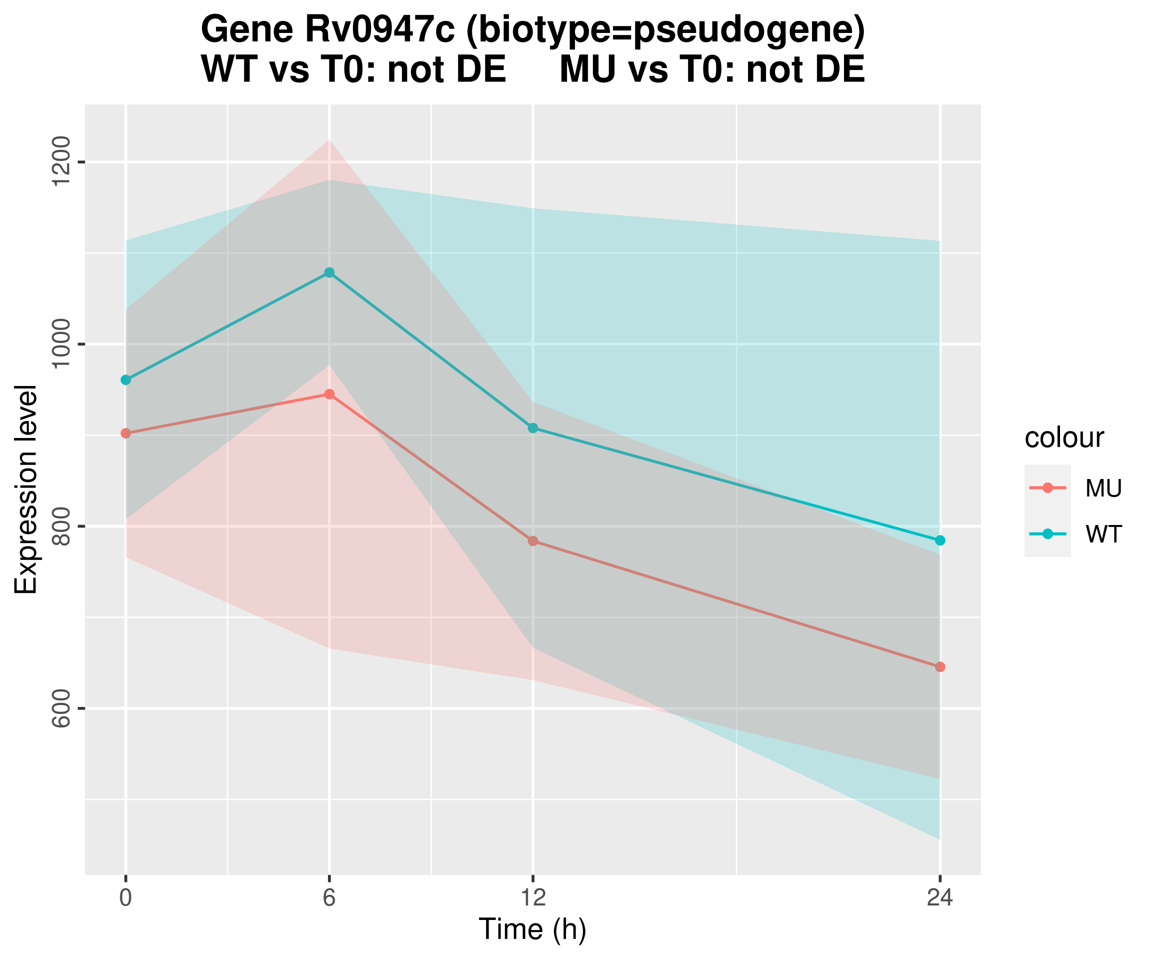

Gene Rv0947c (biotype=pseudogene)
WT vs T0: not DE MU vs T0: not DE
Expression level
1200
1000
800
600
0
6
12
24
Time (h)
colour
MU
WT

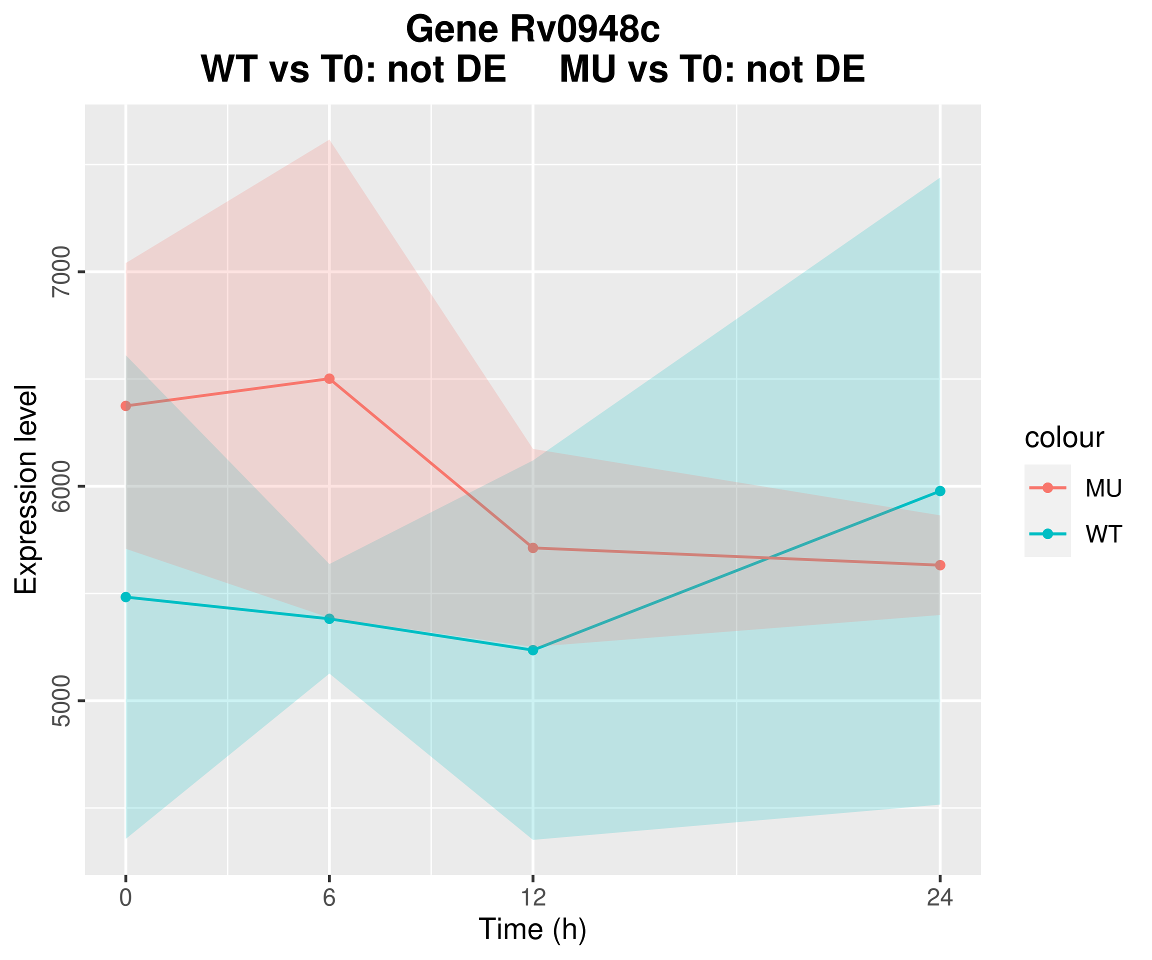

Gene Rv0948c
WT vs T0: not DE MU vs T0: not DE
Expression level
7000
6000
5000
0
6
12
24
Time (h)
colour
MU
WT

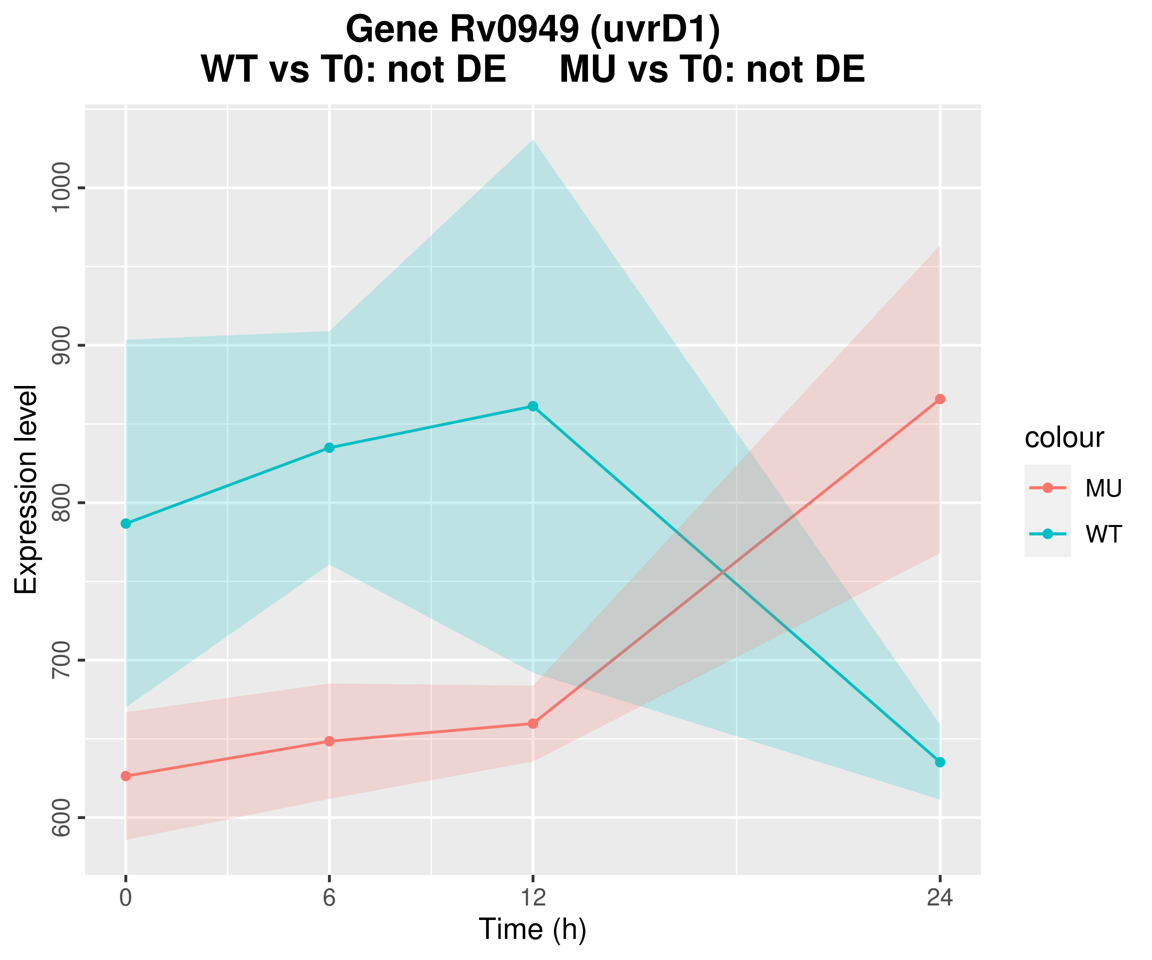
Gene Rv0949 (uvrD1)
WT vs T0: not DE   MU vs T0: not DE
Expression level
1000
900
800
700
600
0
6
12
24
Time (h)
colour
MU
WT

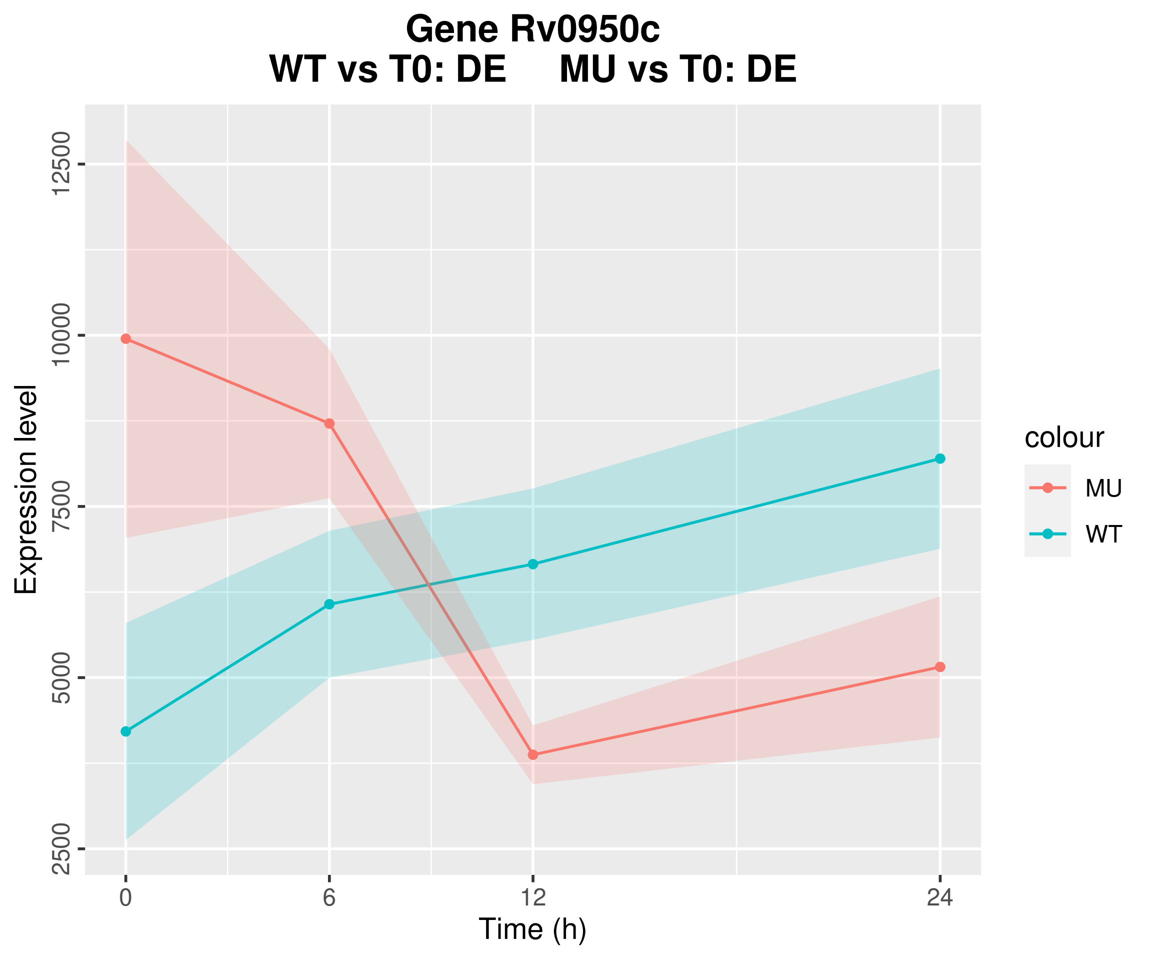

Gene Rv0950c
WT vs T0: DE   MU vs T0: DE
12500
10000
7500
5000
2500
Expression level
0
6
12
24
Time (h)
colour
MU
WT

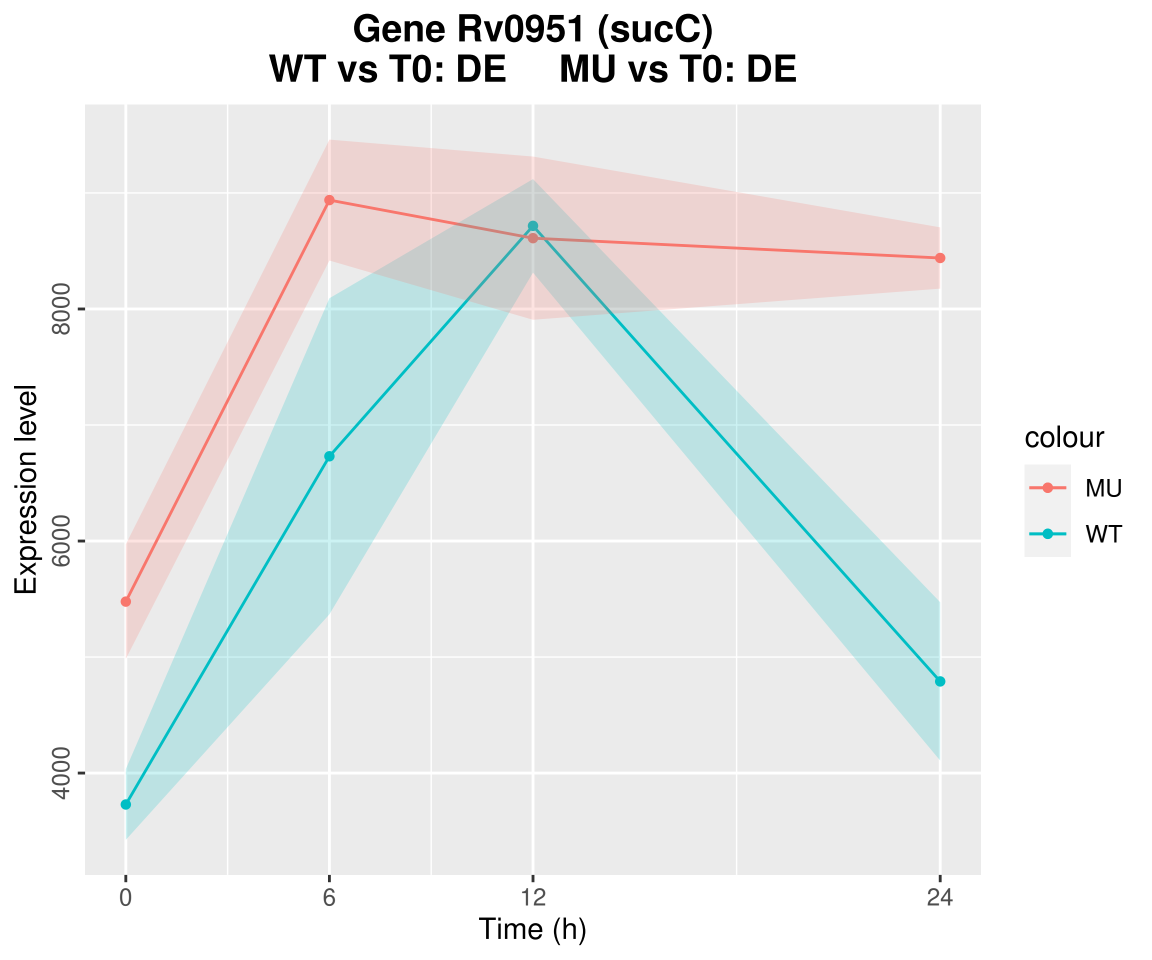

Gene Rv0951 (sucC)
WT vs T0: DE MU vs T0: DE
Expression level
4000
6000
8000
0
6
12
24
Time (h)
colour
MU
WT

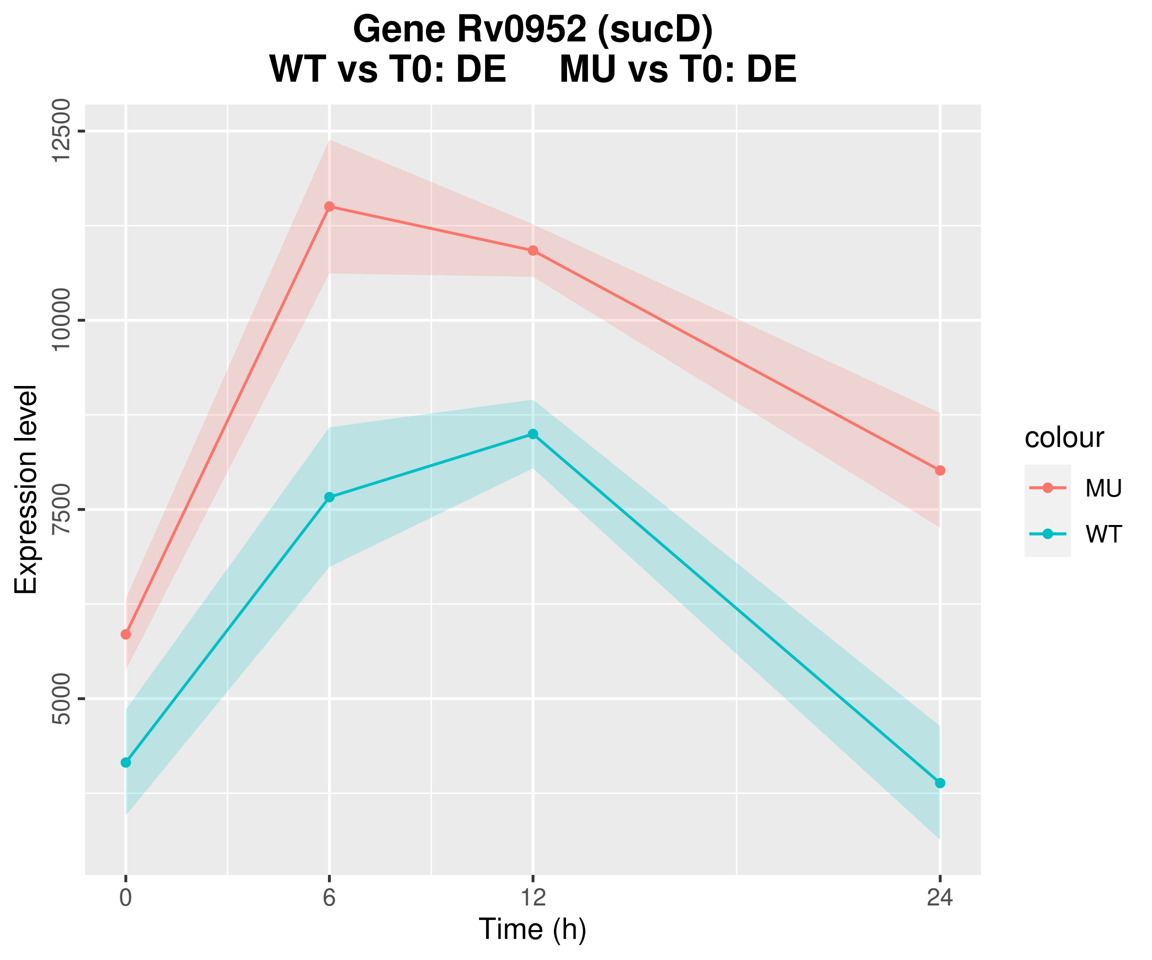

Gene Rv0952 (sucD)
WT vs T0: DE MU vs T0: DE
Expression level
12500
10000
7500
5000
0
6
12
24
Time (h)
colour
MU
WT

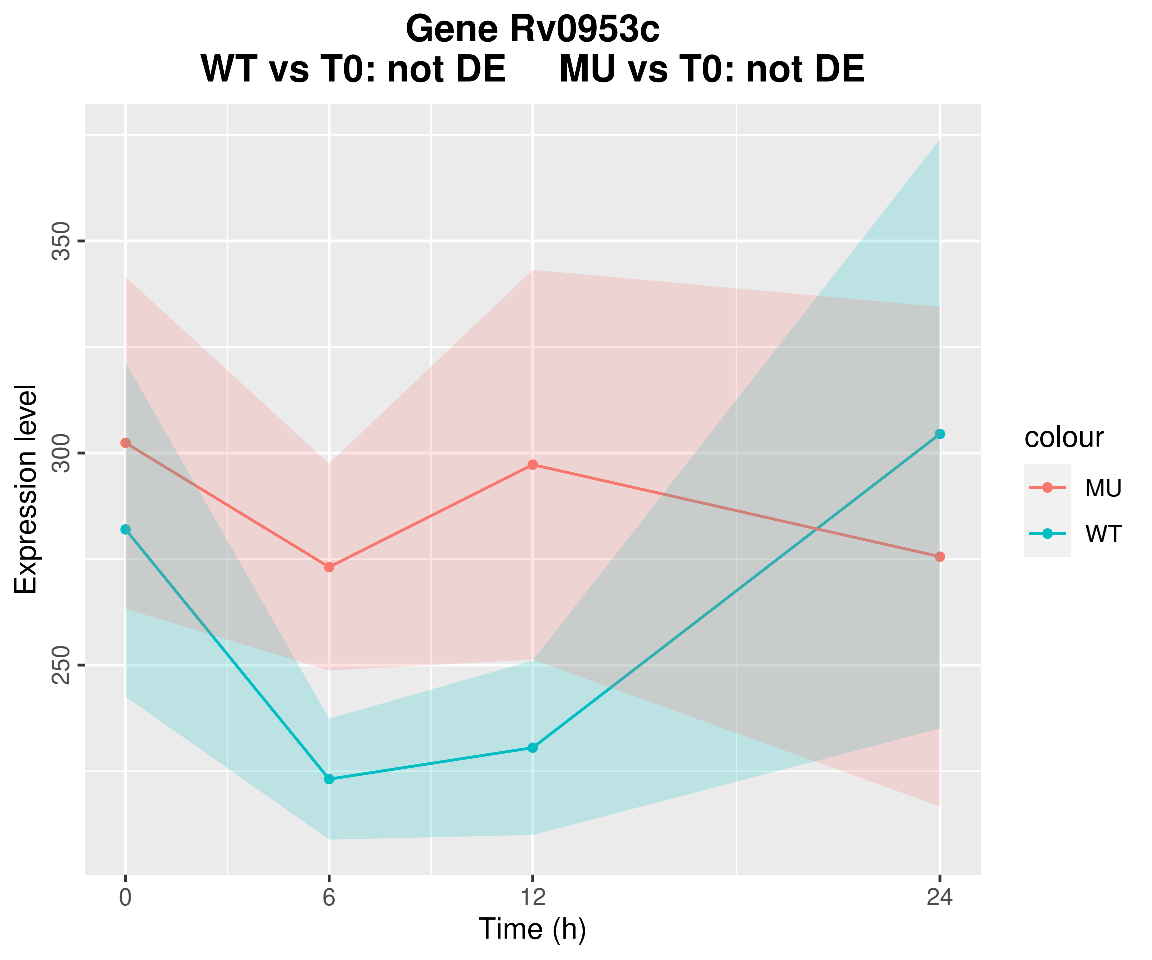

Gene Rv0953c
WT vs T0: not DE   MU vs T0: not DE
Expression level
350
300
250
0
6
12
24
Time (h)
colour
MU
WT

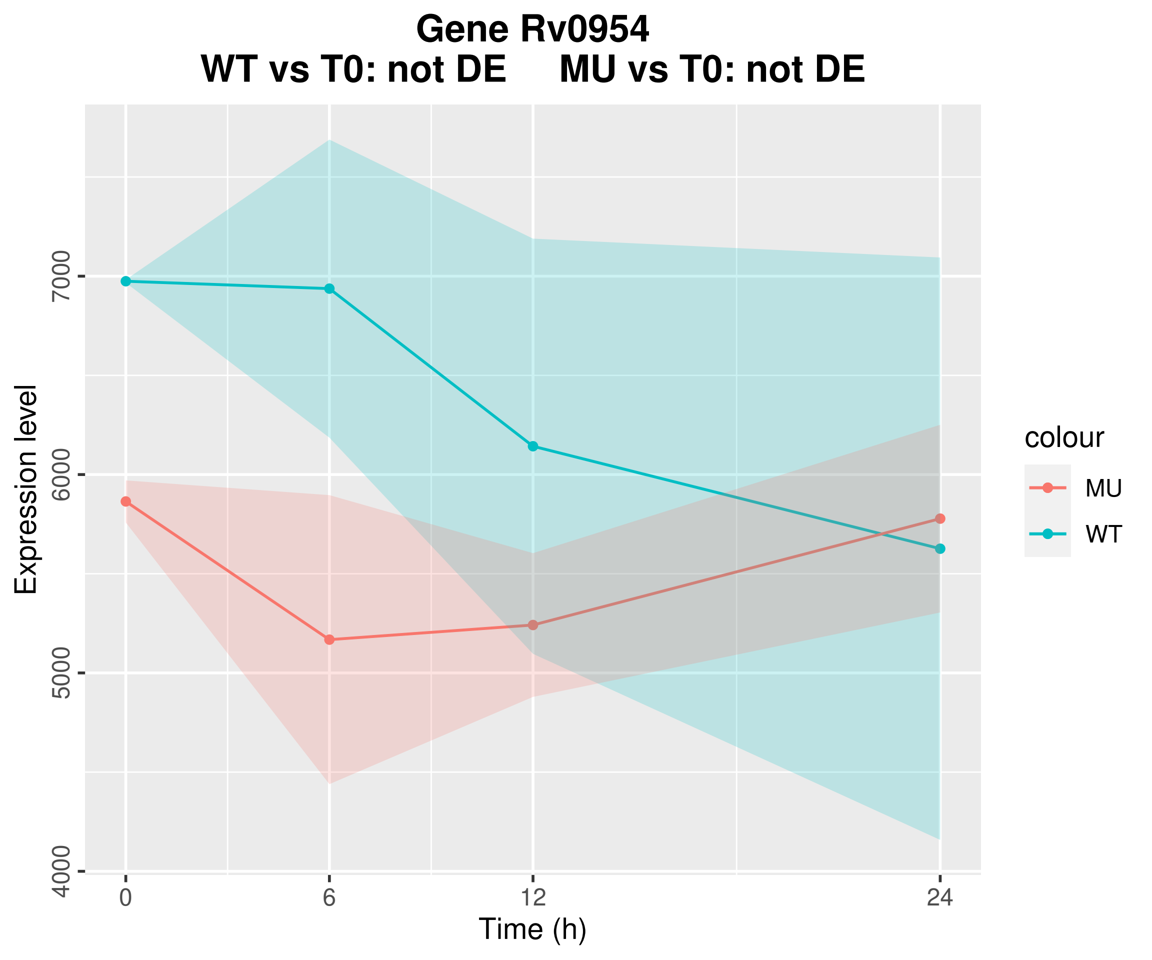

Gene Rv0954
WT vs T0: not DE   MU vs T0: not DE
Expression level
7000
6000
5000
4000
0
6
12
24
Time (h)
colour
MU
WT

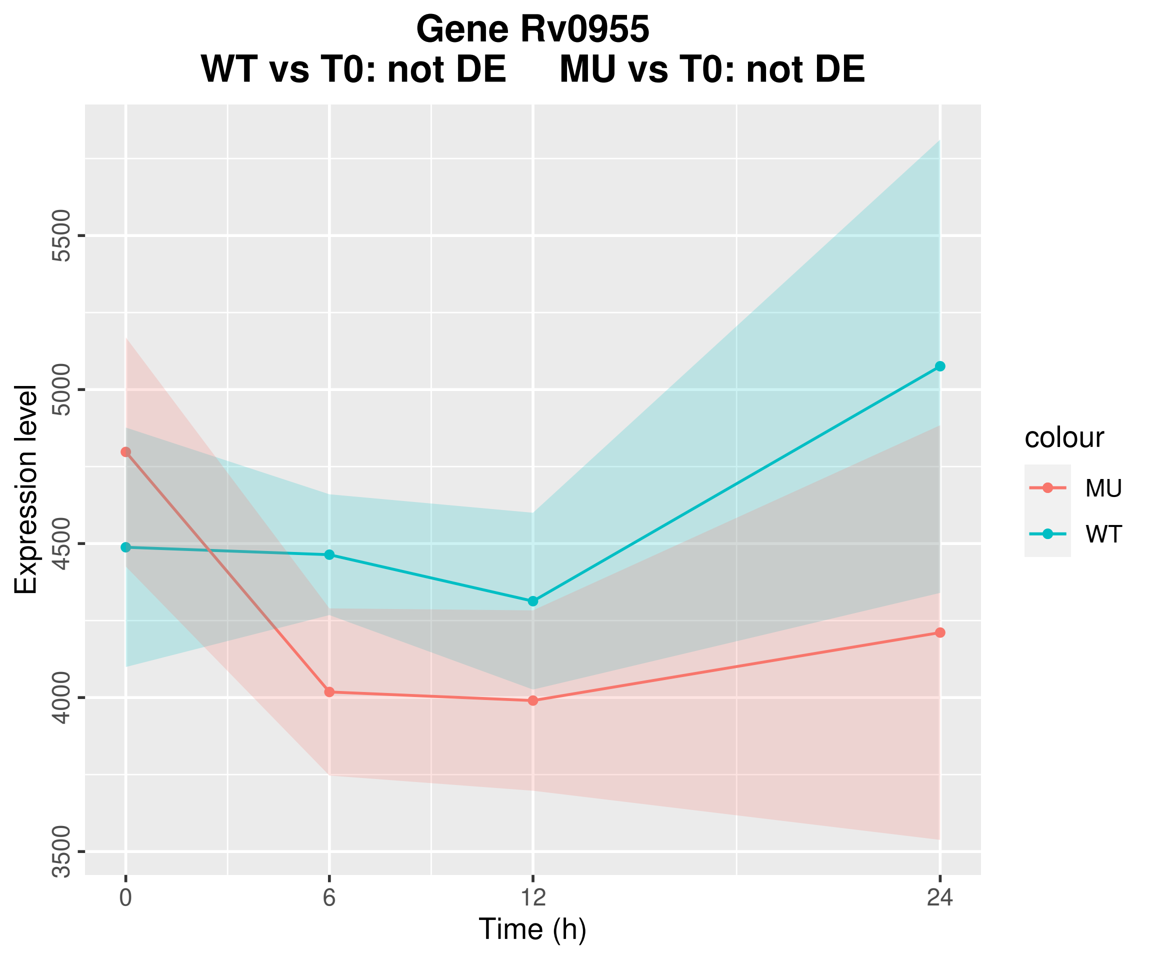
Gene Rv0955
WT vs T0: not DE   MU vs T0: not DE
Expression level
5500
5000
4500
4000
3500
0
6
12
24
Time (h)
colour
MU
WT

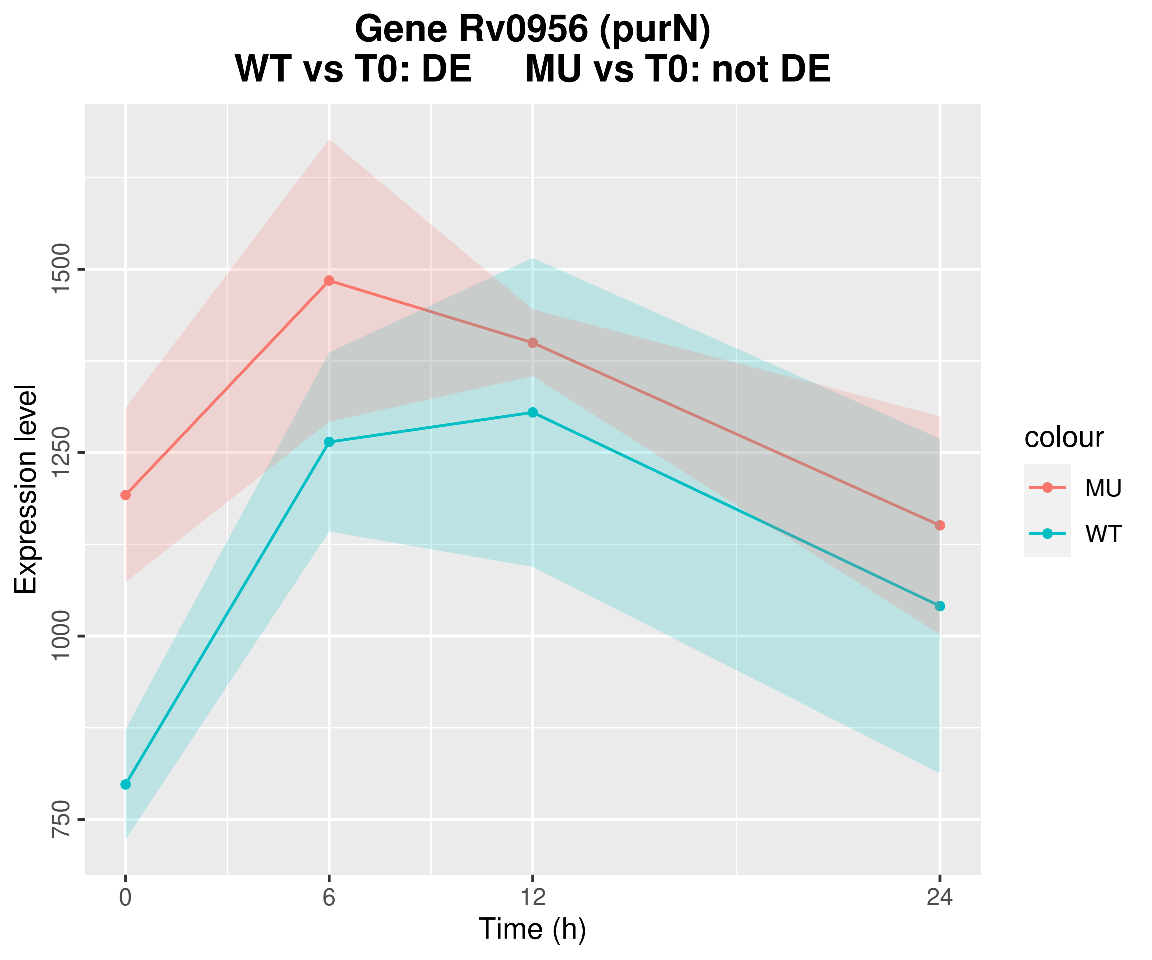

Gene Rv0956 (purN)
WT vs T0: DE   MU vs T0: not DE
Expression level
750
1000
1250
1500
0
6
12
24
Time (h)
colour
MU
WT

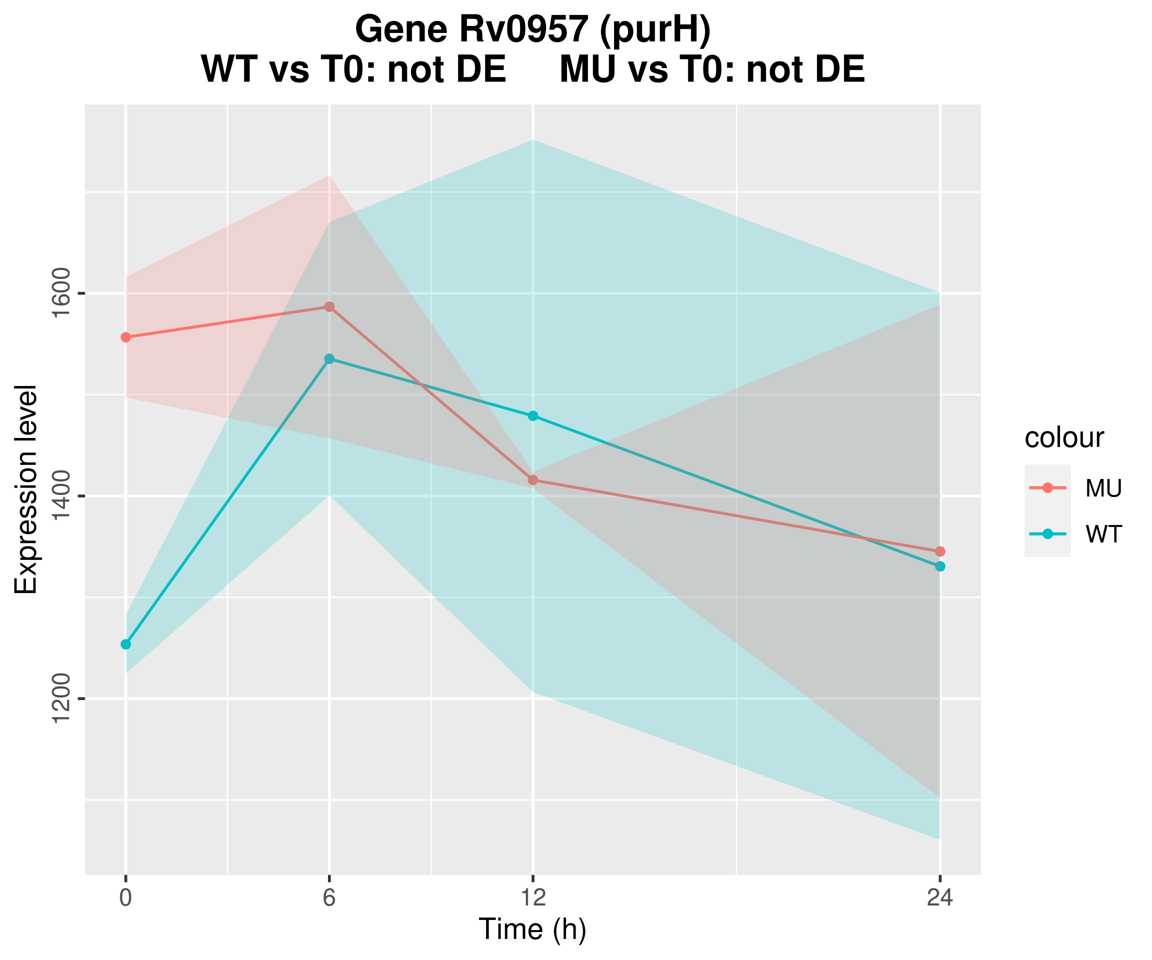
Gene Rv0957 (purH)
WT vs T0: not DE MU vs T0: not DE
Expression level
1200
1400
1600
0
6
12
24
Time (h)
colour
MU
WT

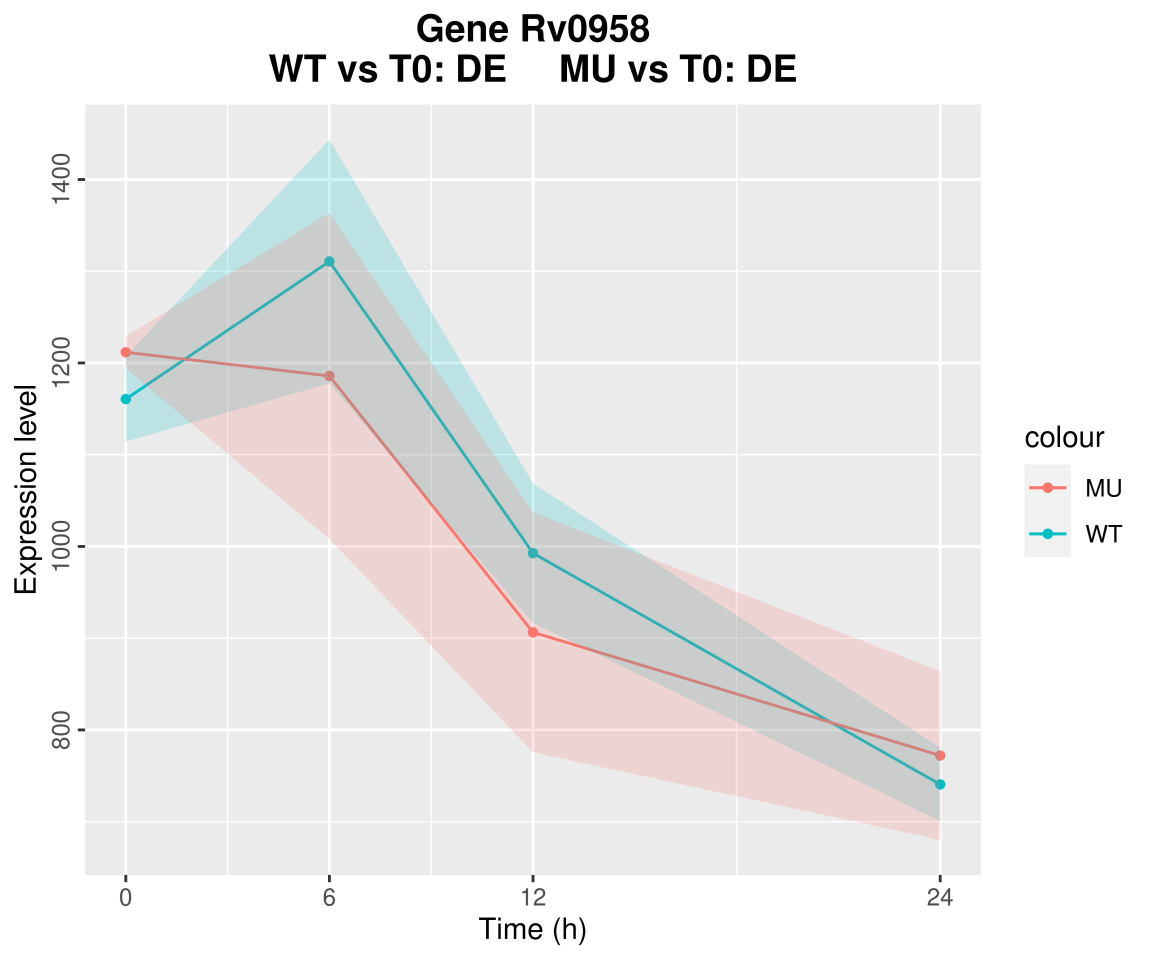

Gene Rv0958
WT vs T0: DE MU vs T0: DE
Expression level
1400
1200
1000
800
0
6
12
24
Time (h)
colour
MU
WT

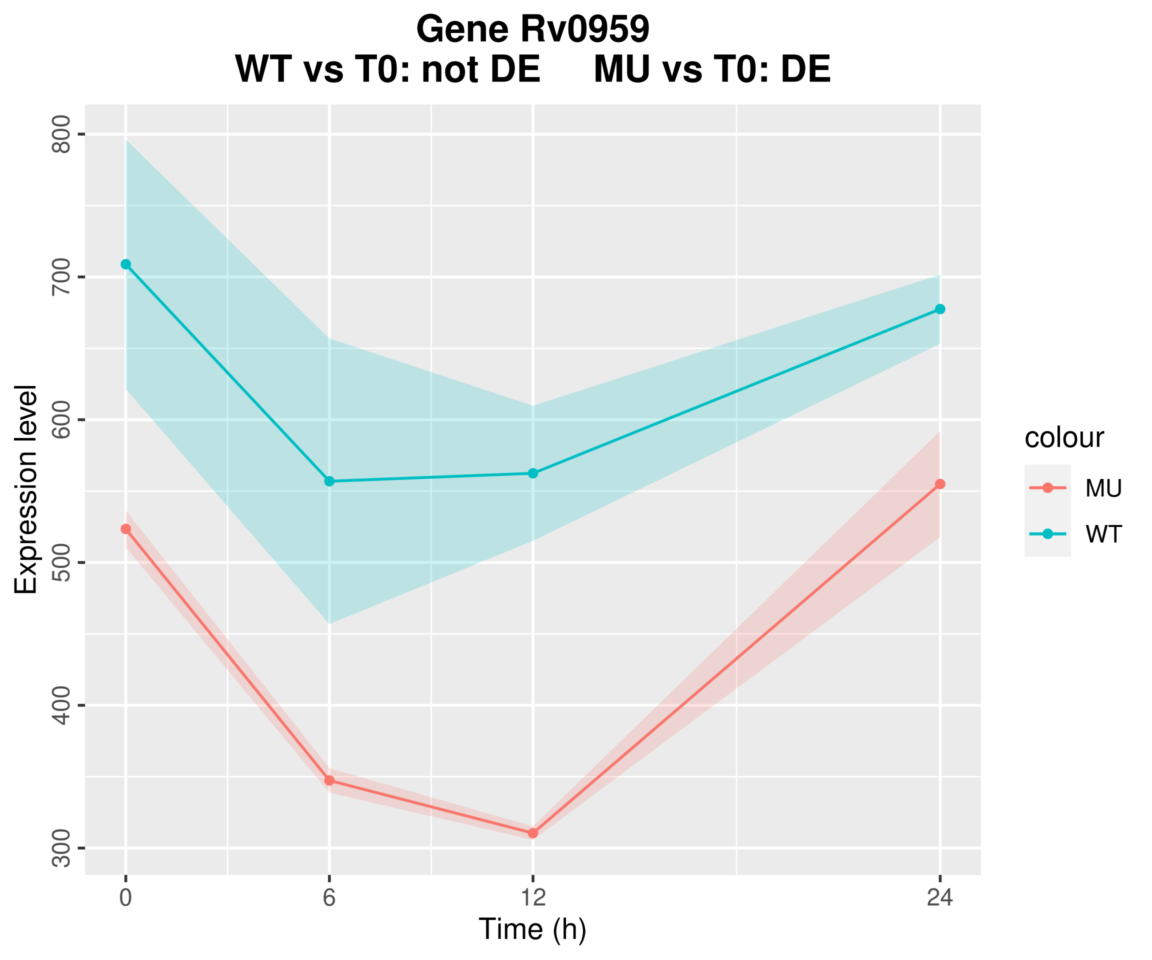

Gene Rv0959
WT vs T0: not DE MU vs T0: DE
Expression level
800
700
600
500
400
300
0
6
12
24
Time (h)
colour
MU
WT

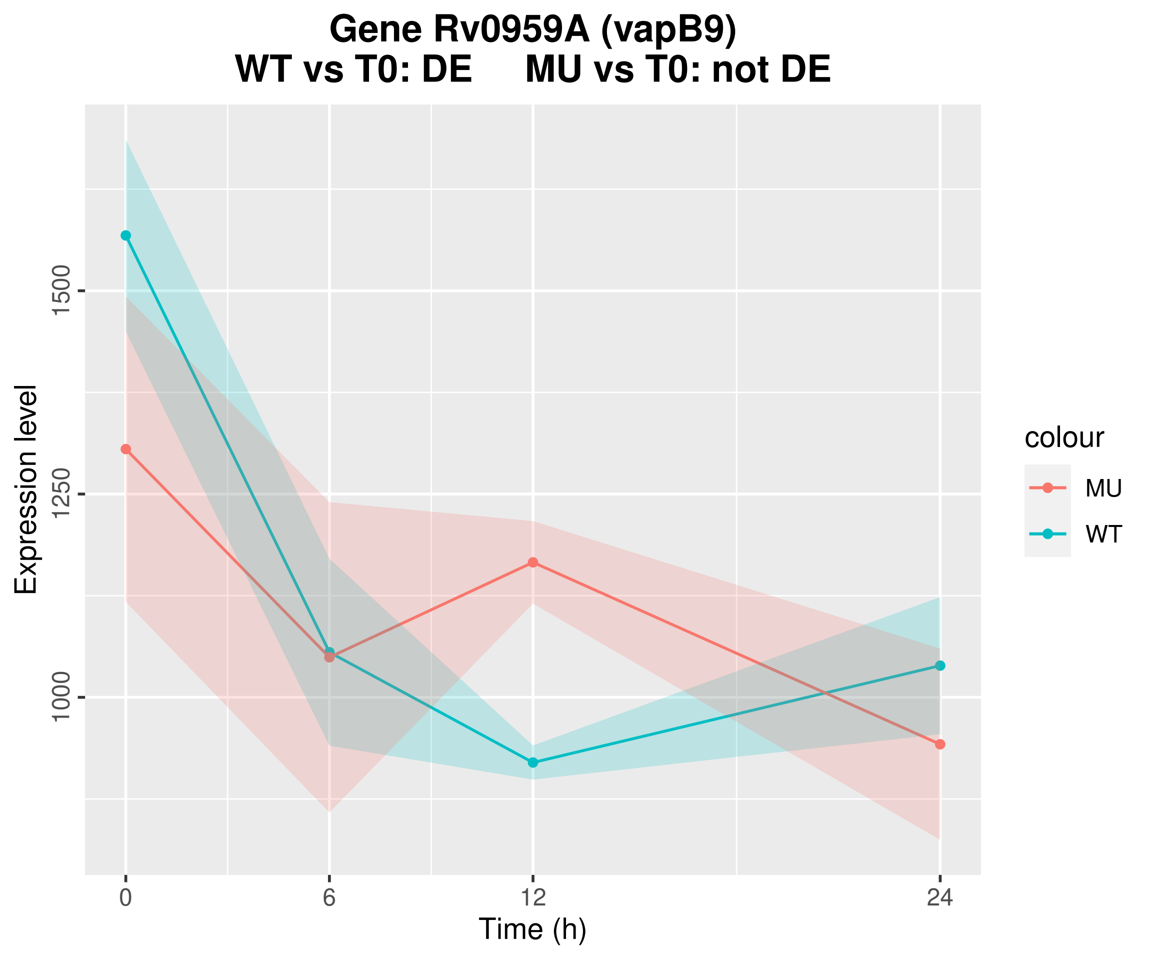
Gene Rv0959A (vapB9)
WT vs T0: DE   MU vs T0: not DE
Expression level
1500
1250
1000
0
6
12
24
Time (h)
colour
MU
WT

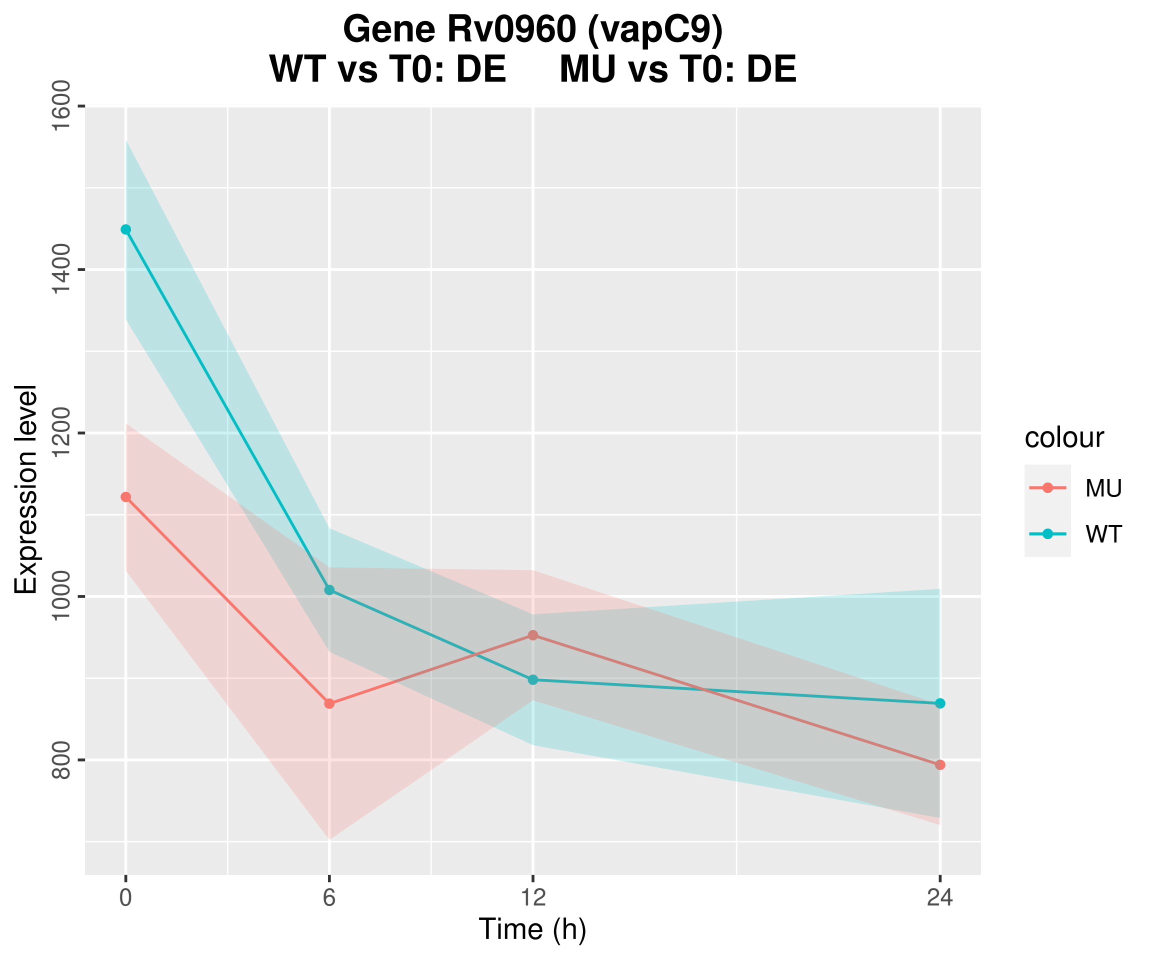

Gene Rv0960 (vapC9)
WT vs T0: DE MU vs T0: DE
Expression level
1600
1400
1200
1000
800
0
6
12
24
Time (h)
colour
MU
WT

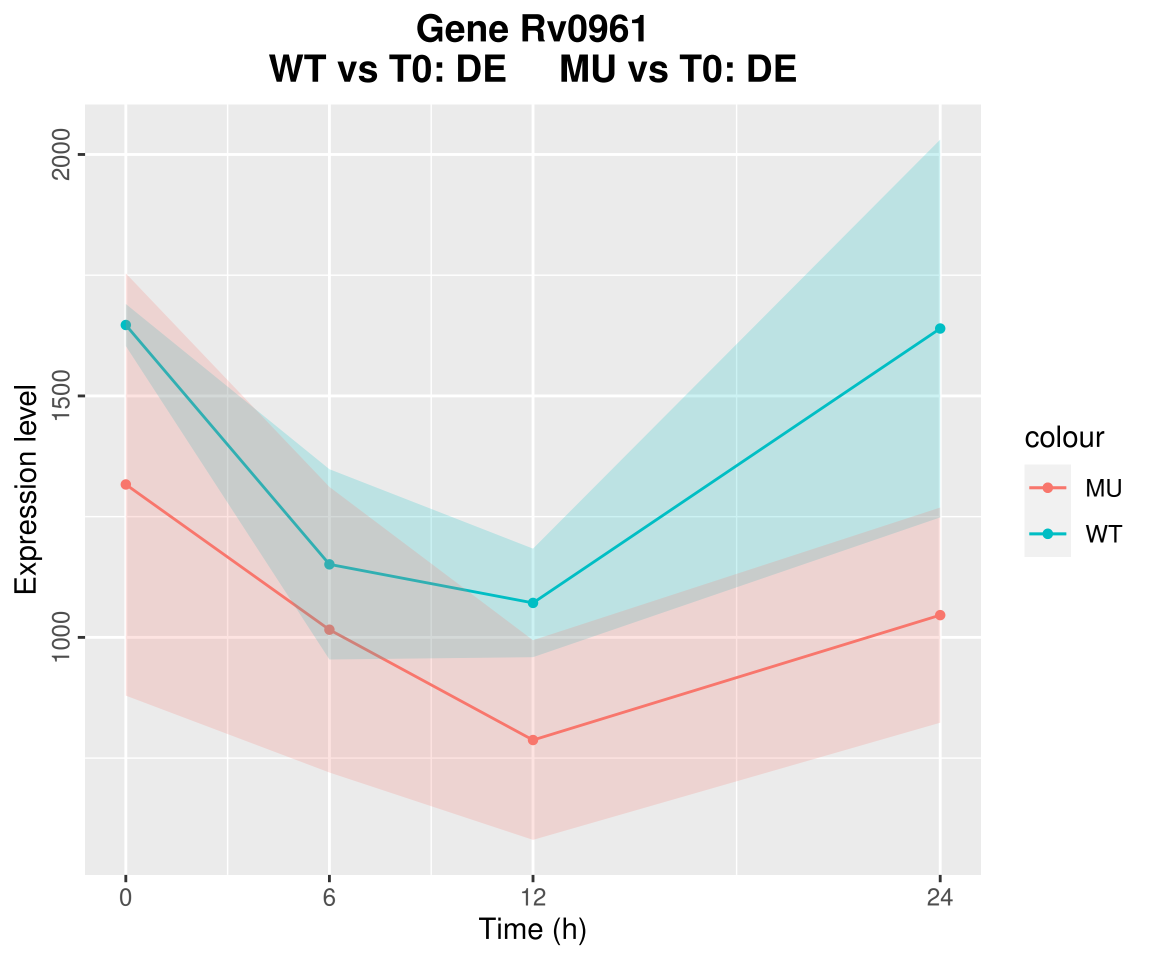

Gene Rv0961
WT vs T0: DE   MU vs T0: DE
Expression level
2000
1500
1000
0
6
12
24
Time (h)
colour
MU
WT

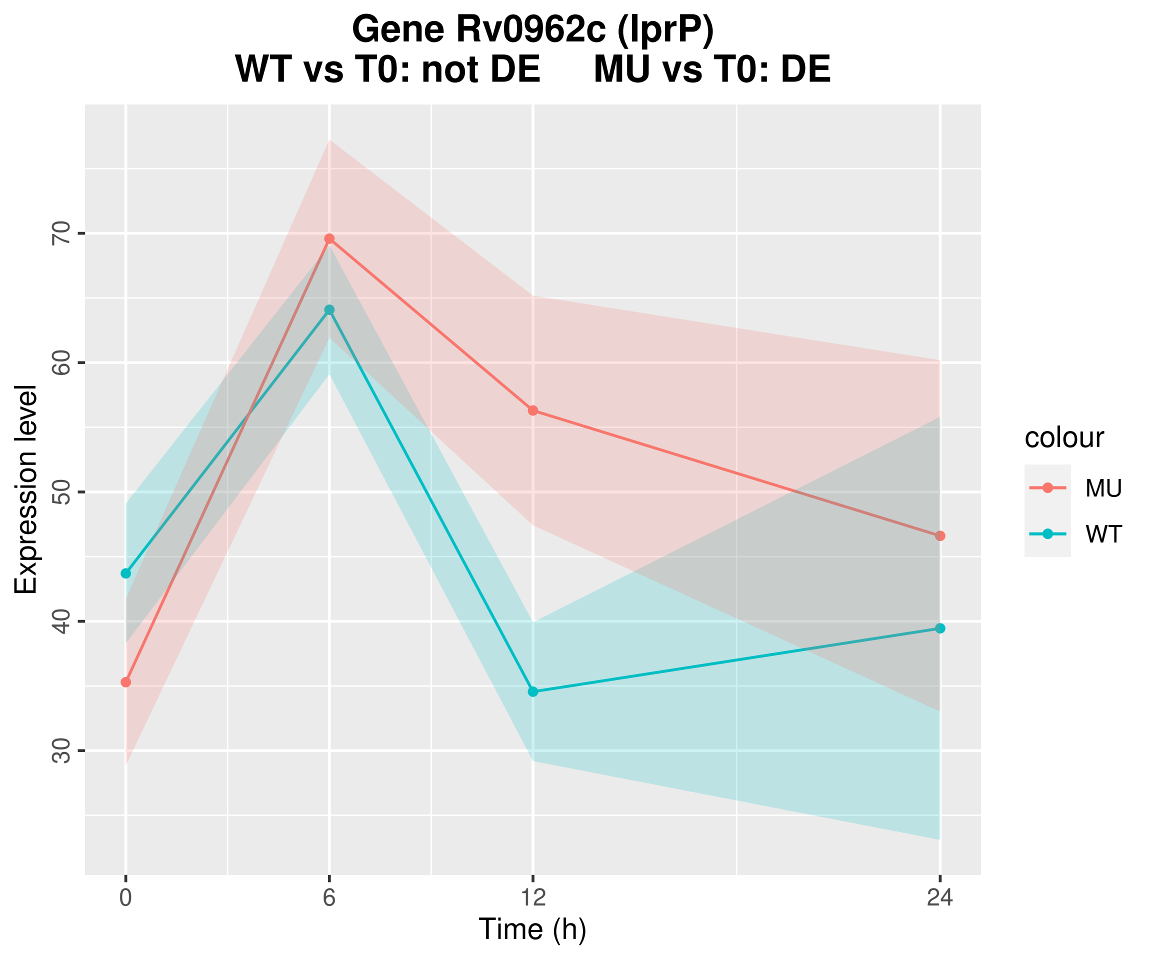

Gene Rv0962c (lprP)
WT vs T0: not DE MU vs T0: DE
Expression level
70
60
50
40
30
0
6
12
24
Time (h)
colour
MU
WT

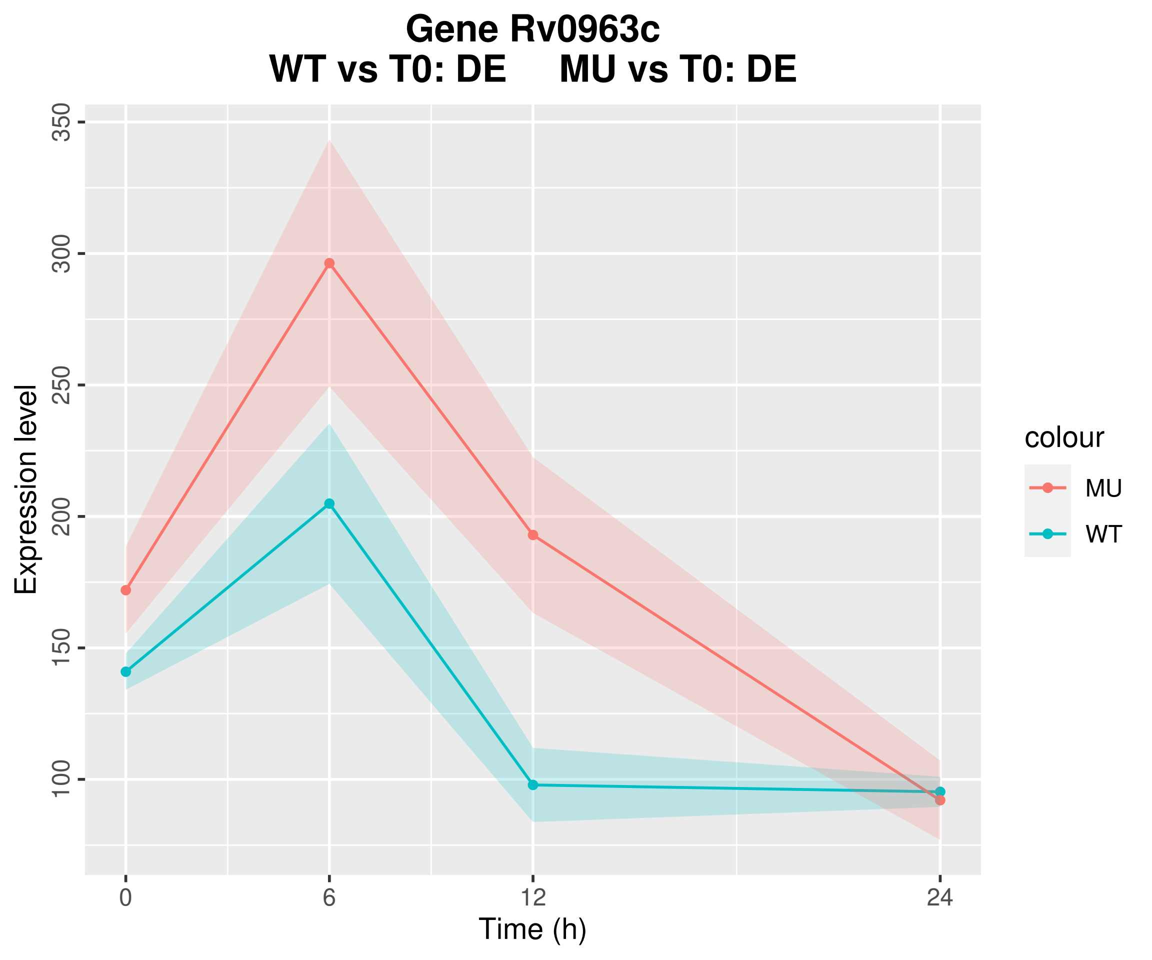
Gene Rv0963c
WT vs T0: DE   MU vs T0: DE
Expression level
350
300
250
200
150
100
0
6
12
24
Time (h)
colour
MU
WT

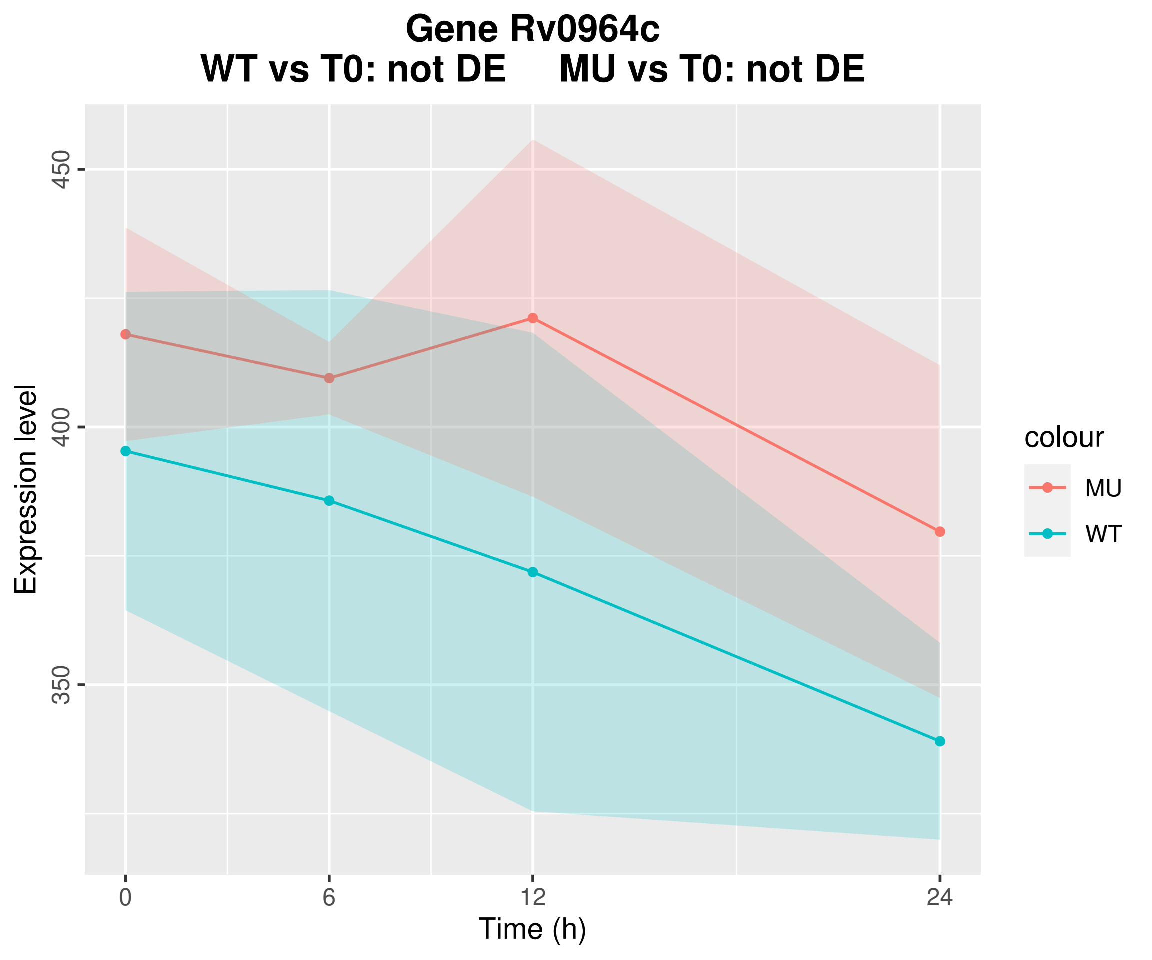

Gene Rv0964c
WT vs T0: not DE   MU vs T0: not DE
Expression level
450
400
350
0
6
12
24
Time (h)
colour
MU
WT

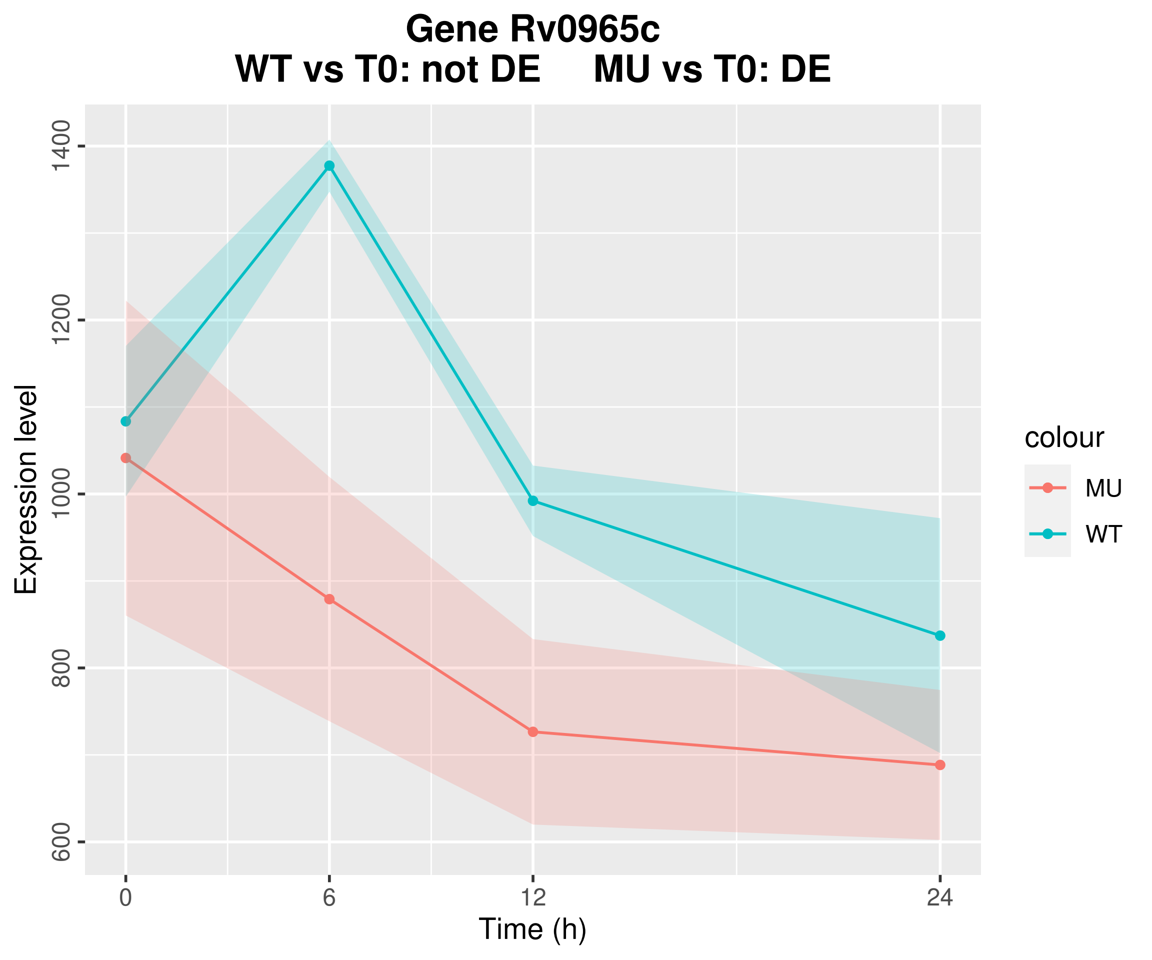
Gene Rv0965c
WT vs T0: not DE   MU vs T0: DE
Expression level
1400
1200
1000
800
600
0
6
12
24
Time (h)
colour
MU
WT

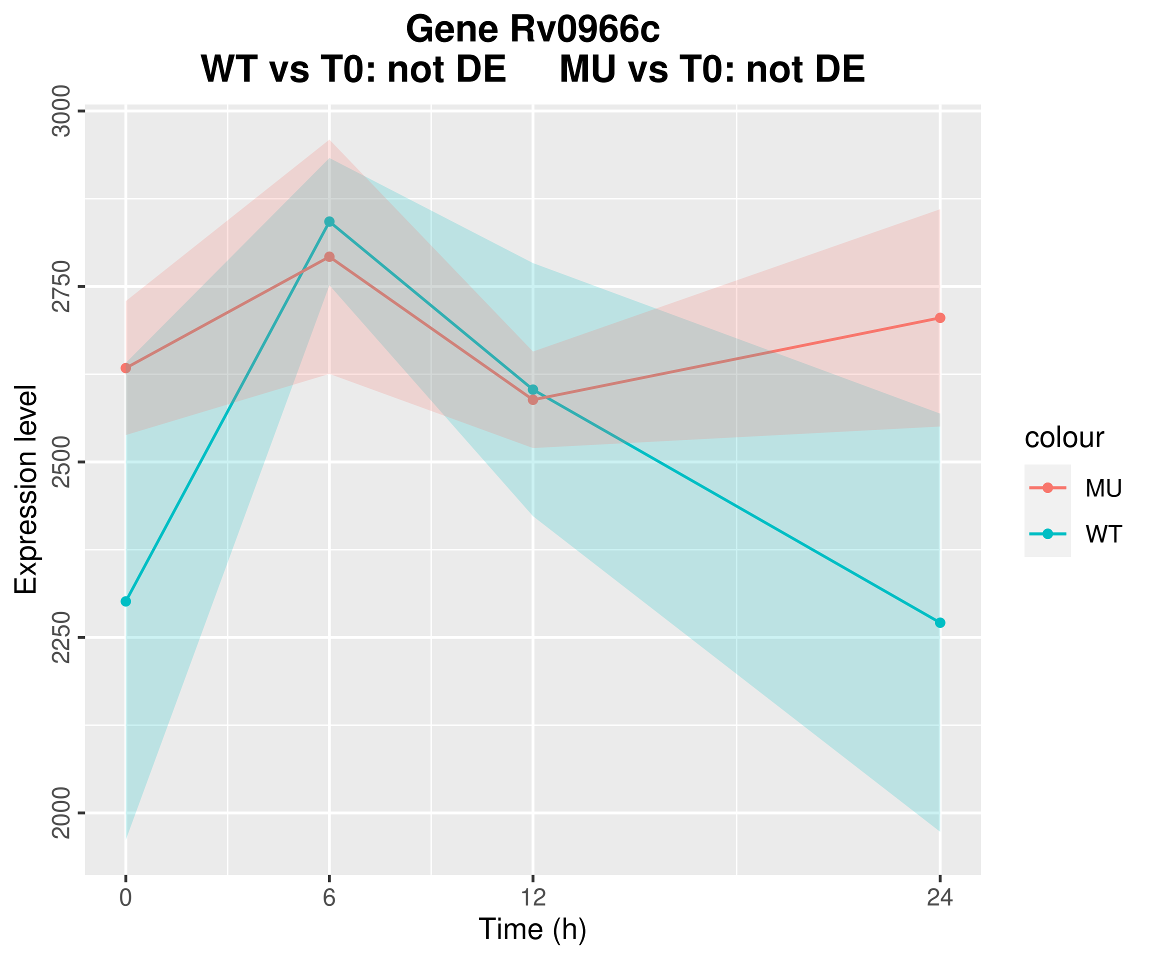

Gene Rv0966c
WT vs T0: not DE MU vs T0: not DE
Expression level
3000
2750
2500
2250
2000
0
6
12
24
Time (h)
colour
MU
WT

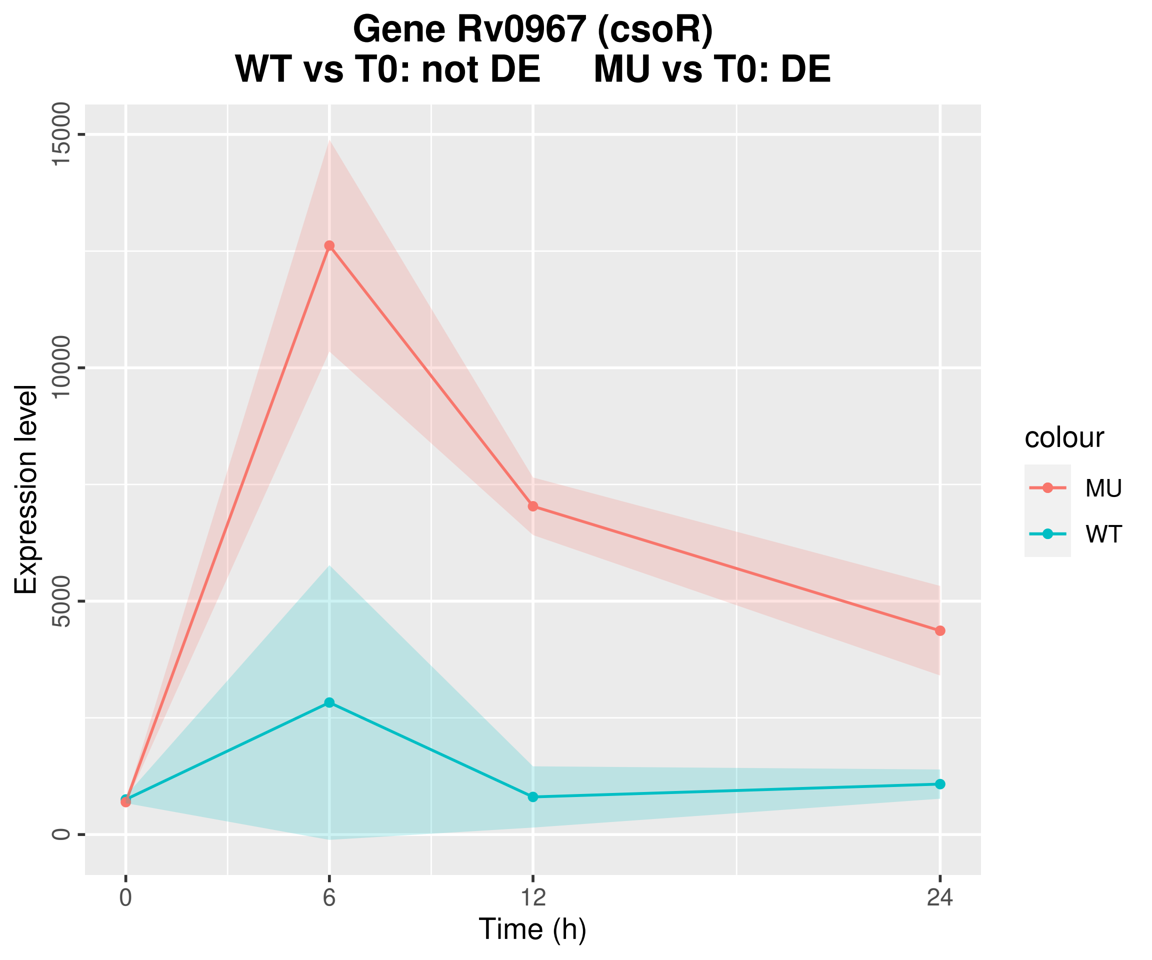
Gene Rv0967 (csoR)
WT vs T0: not DE MU vs T0: DE
Expression level
15000
10000
5000
0
0
6
12
24
Time (h)
colour
MU
WT

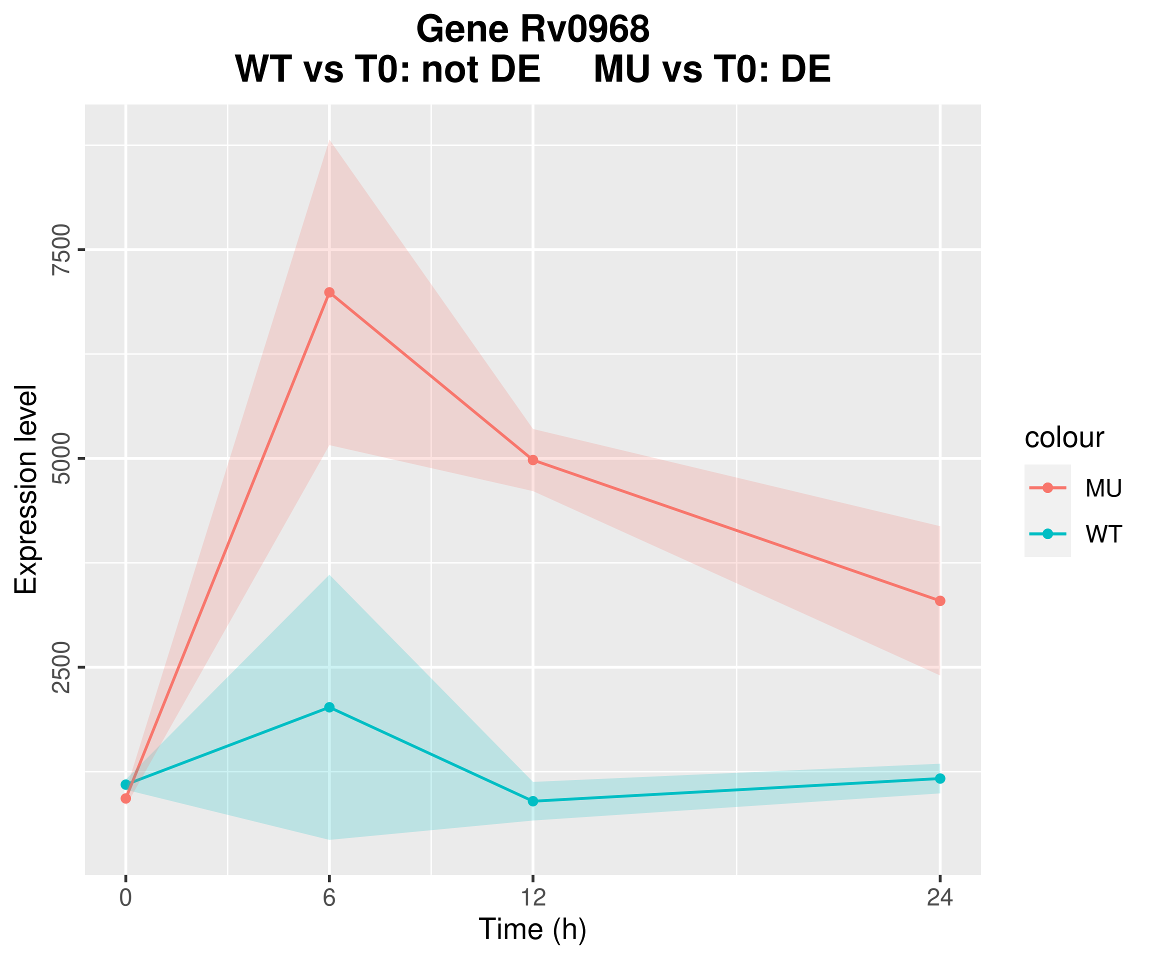

Gene Rv0968
WT vs T0: not DE MU vs T0: DE
Expression level
7500
5000
2500
0
6
12
24
Time (h)
colour
MU
WT

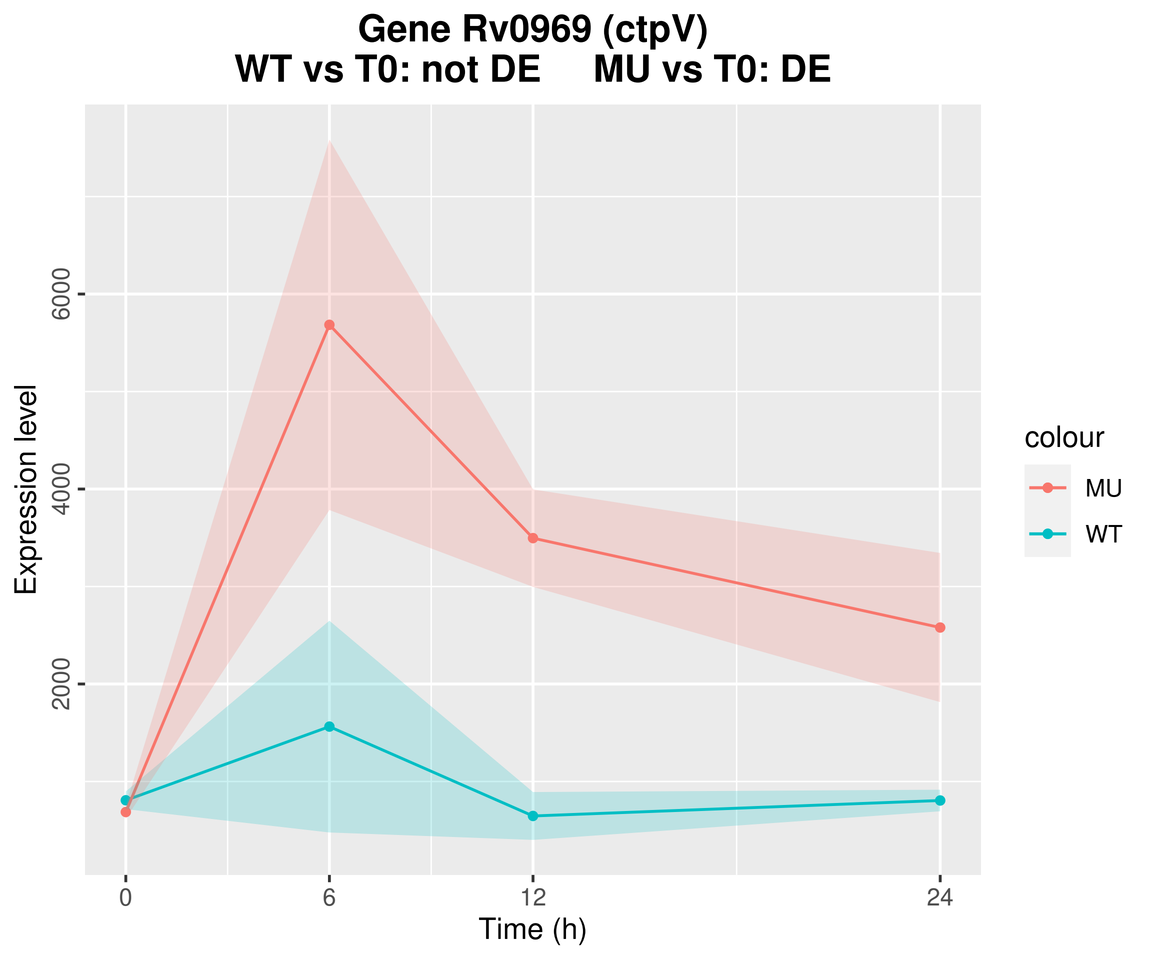
Gene Rv0969 (ctpV)
WT vs T0: not DE   MU vs T0: DE
Expression level
2000
4000
6000
0
6
12
24
Time (h)
colour
MU
WT

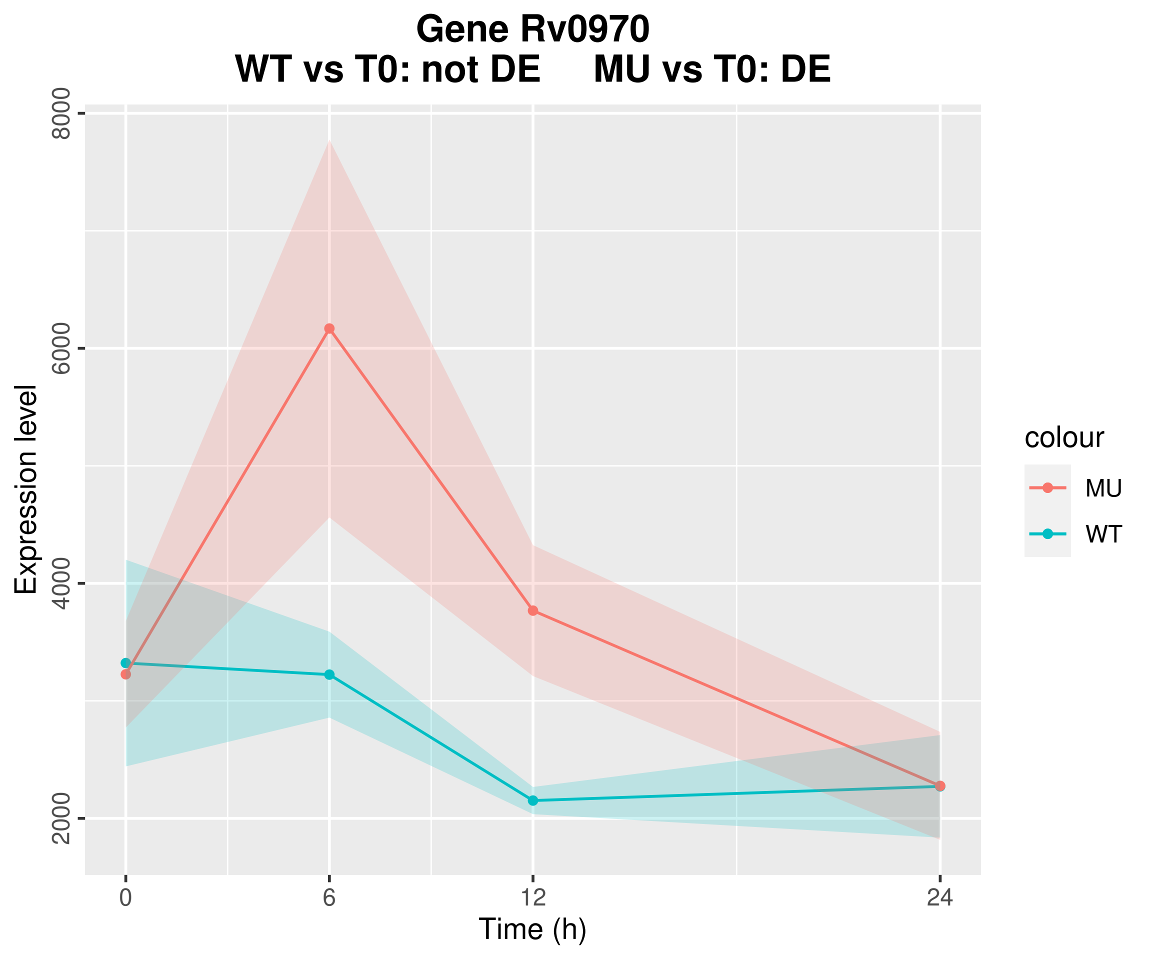
Gene Rv0970
WT vs T0: not DE MU vs T0: DE
Expression level
2000
4000
6000
8000
0
6
12
24
Time (h)
colour
MU
WT

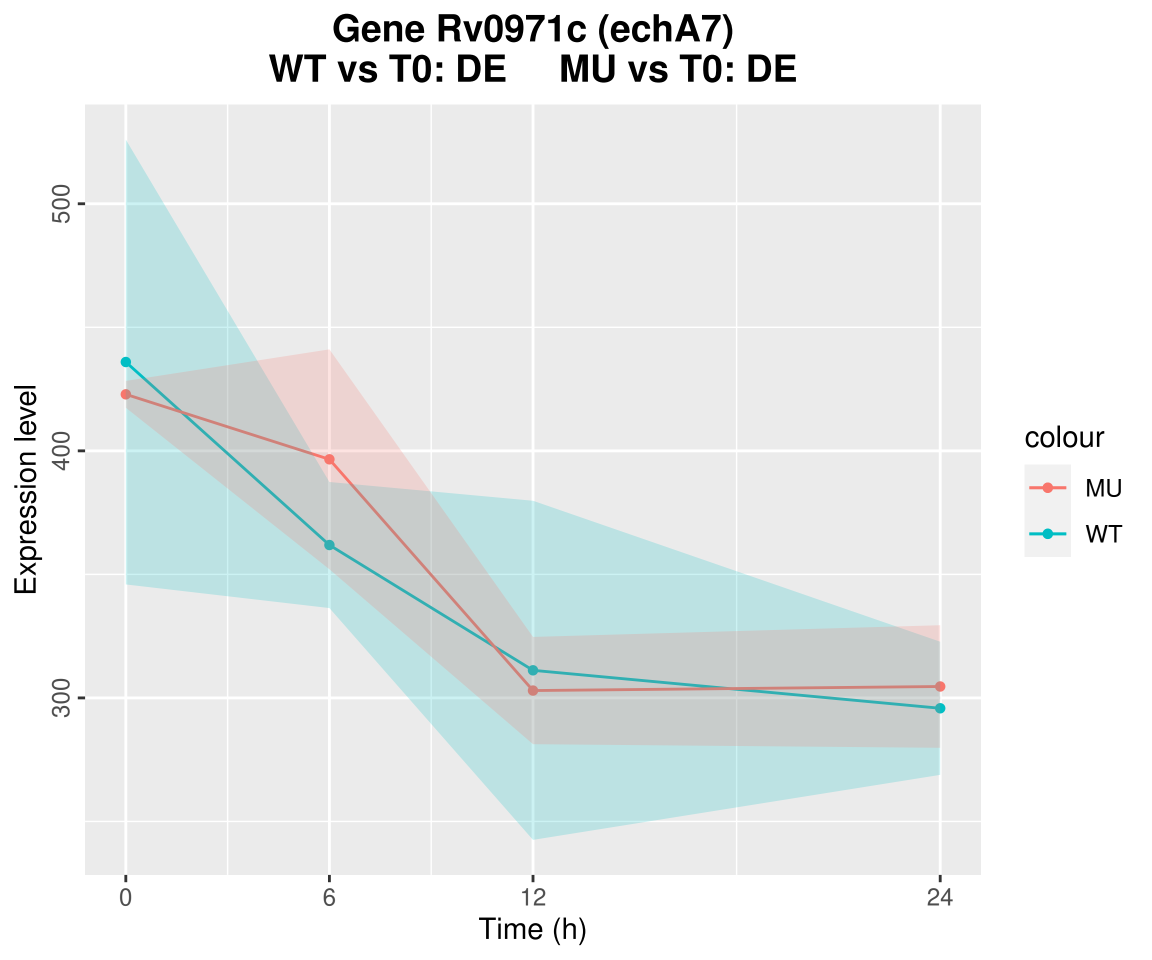

Gene Rv0971c (echA7)
WT vs T0: DE   MU vs T0: DE
Expression level
500
400
300
0
6
12
24
Time (h)
colour
MU
WT

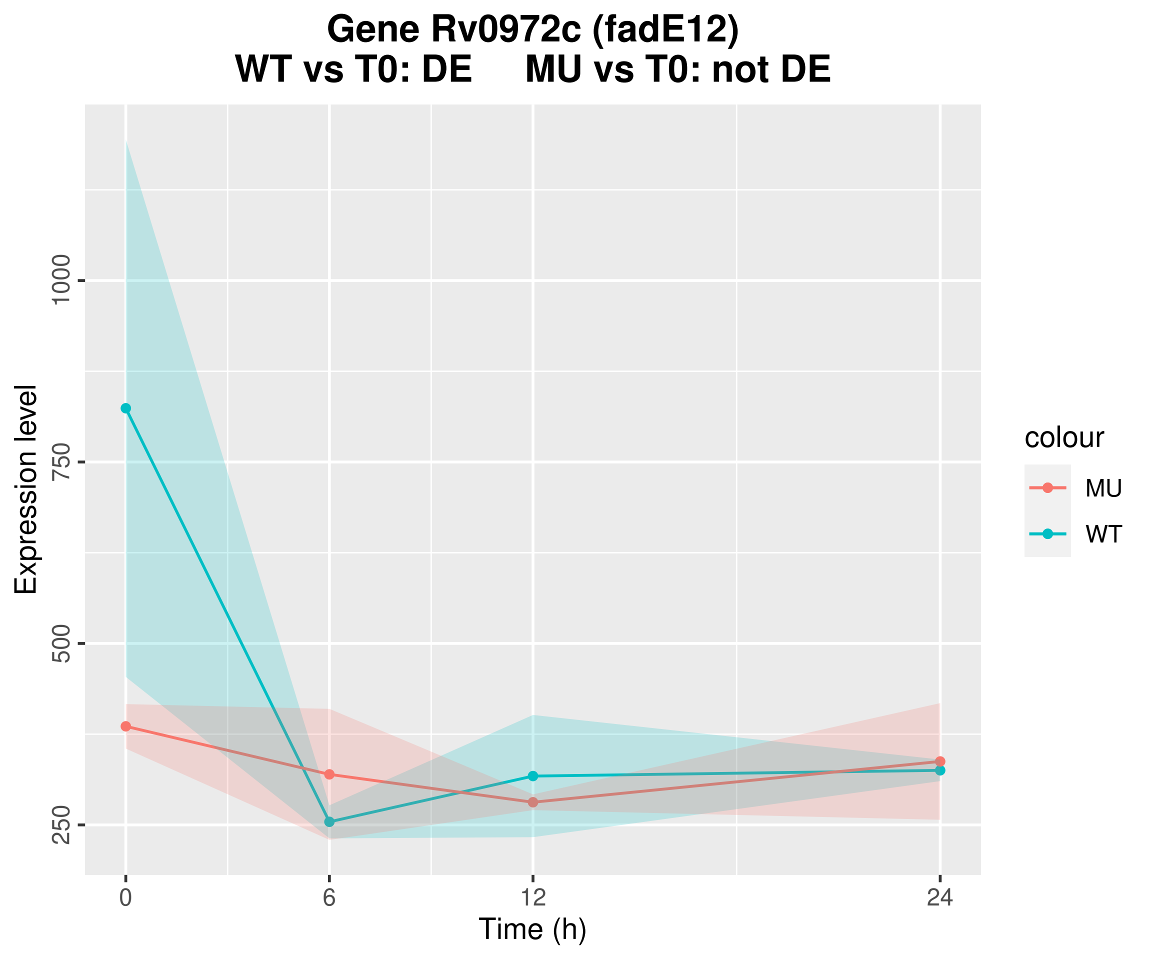

Gene Rv0972c (fadE12)
WT vs T0: DE MU vs T0: not DE
Expression level
1000
750
500
250
0
6
12
24
Time (h)
colour
MU
WT

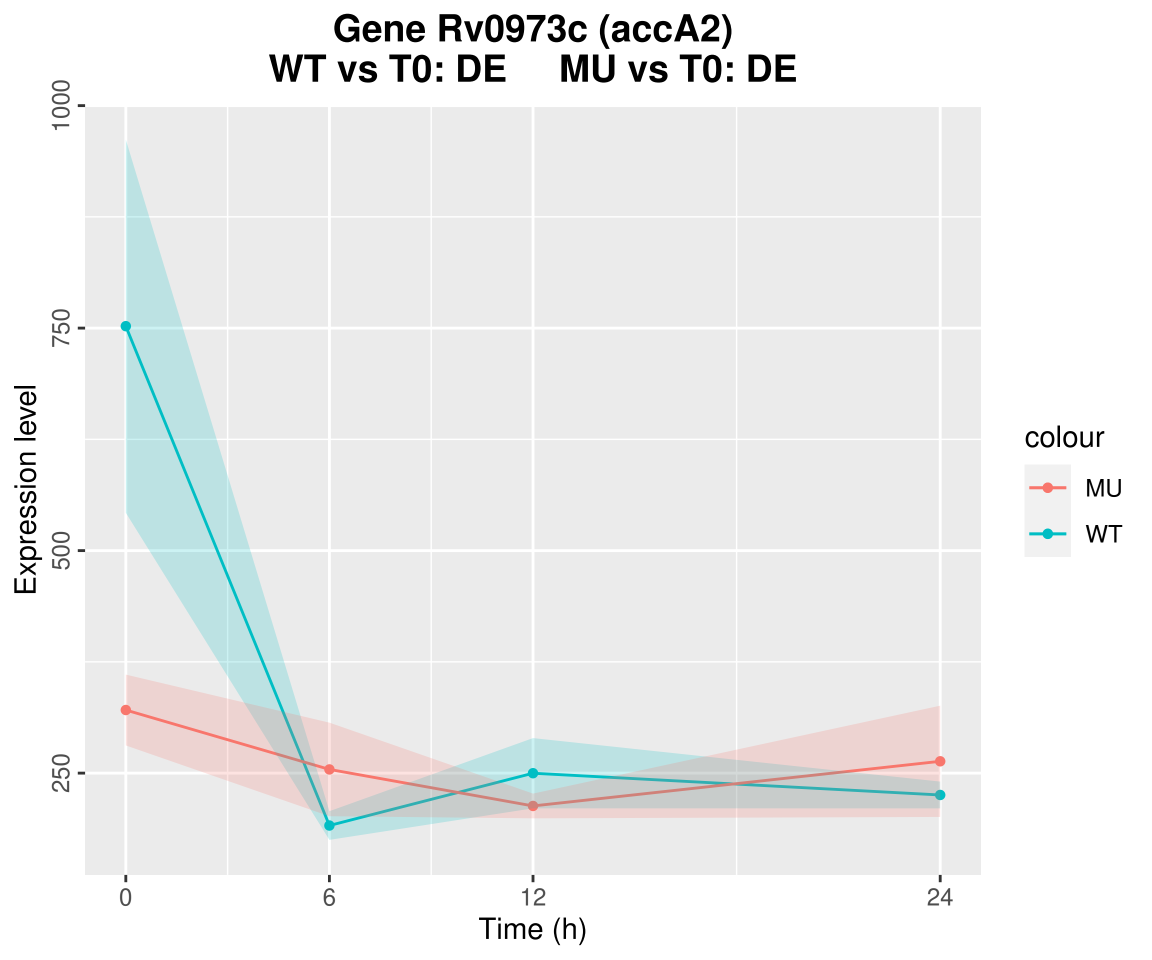
Gene Rv0973c (accA2)
WT vs T0: DE   MU vs T0: DE
Expression level
1000
750
500
250
0
6
12
24
Time (h)
colour
MU
WT

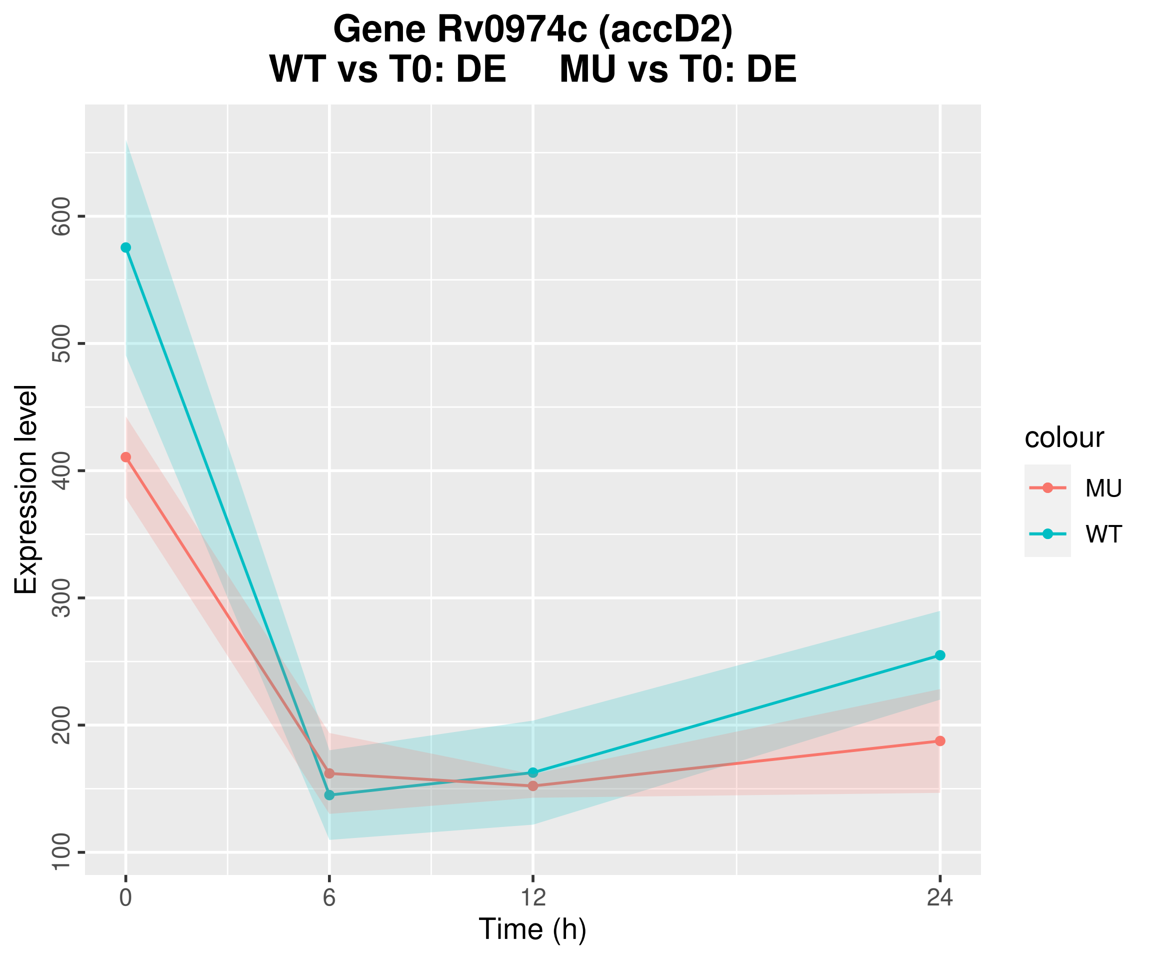

Gene Rv0974c (accD2)
WT vs T0: DE   MU vs T0: DE
Expression level
100
200
300
400
500
600
0
6
12
24
Time (h)
colour
MU
WT

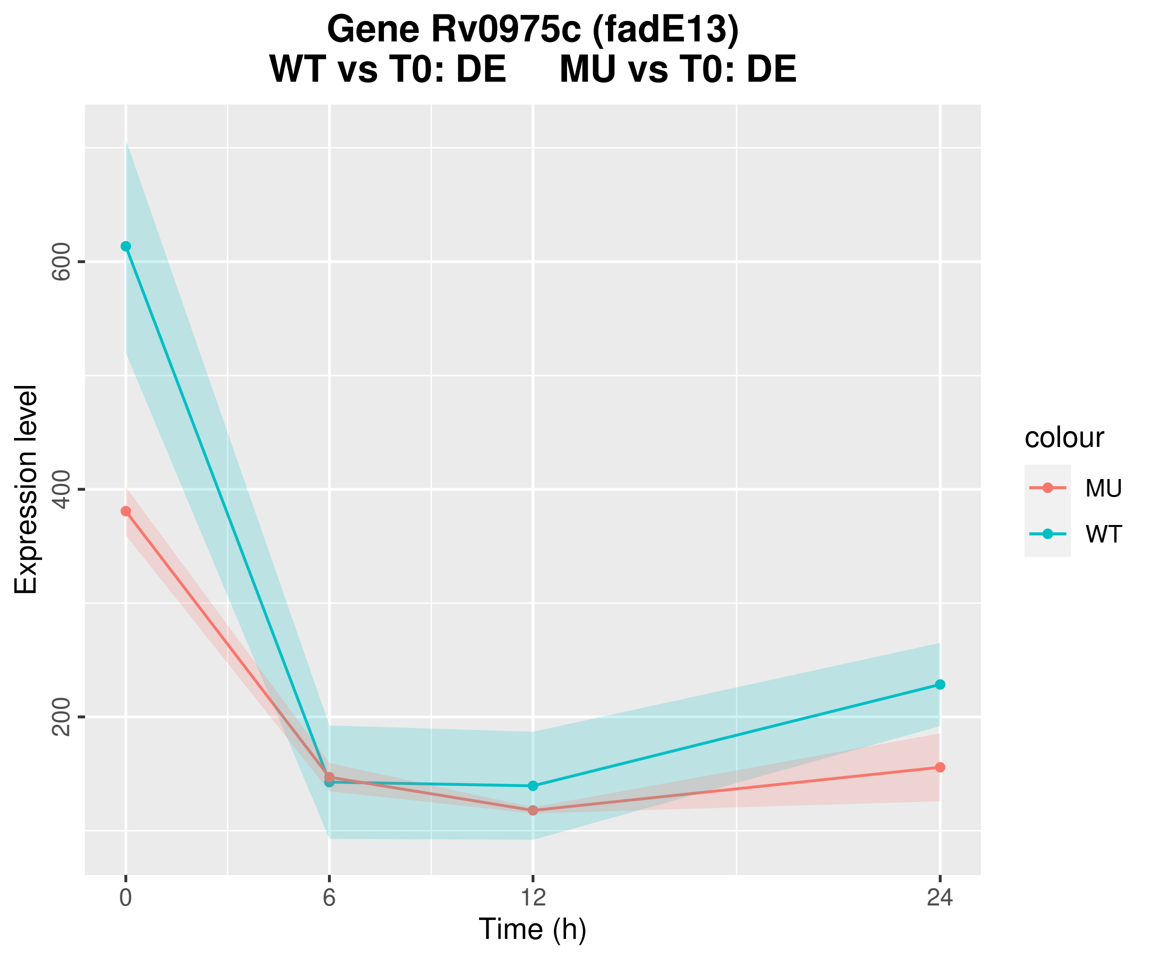

Gene Rv0975c (fadE13)
WT vs T0: DE   MU vs T0: DE
Expression level
600
400
200
0
6
12
24
Time (h)
colour
MU
WT

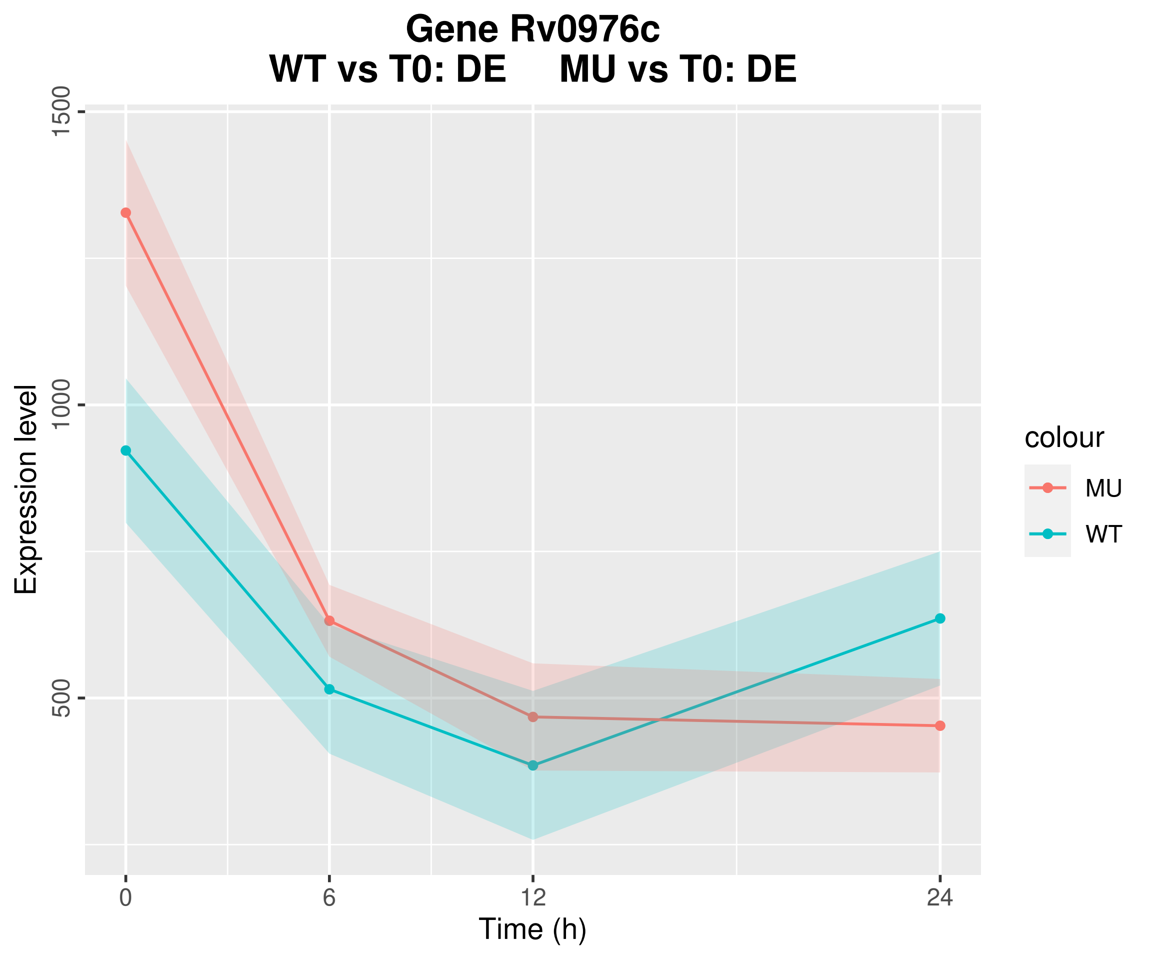

Gene Rv0976c
WT vs T0: DE   MU vs T0: DE
Expression level
1500
1000
500
0
6
12
24
Time (h)
colour
MU
WT

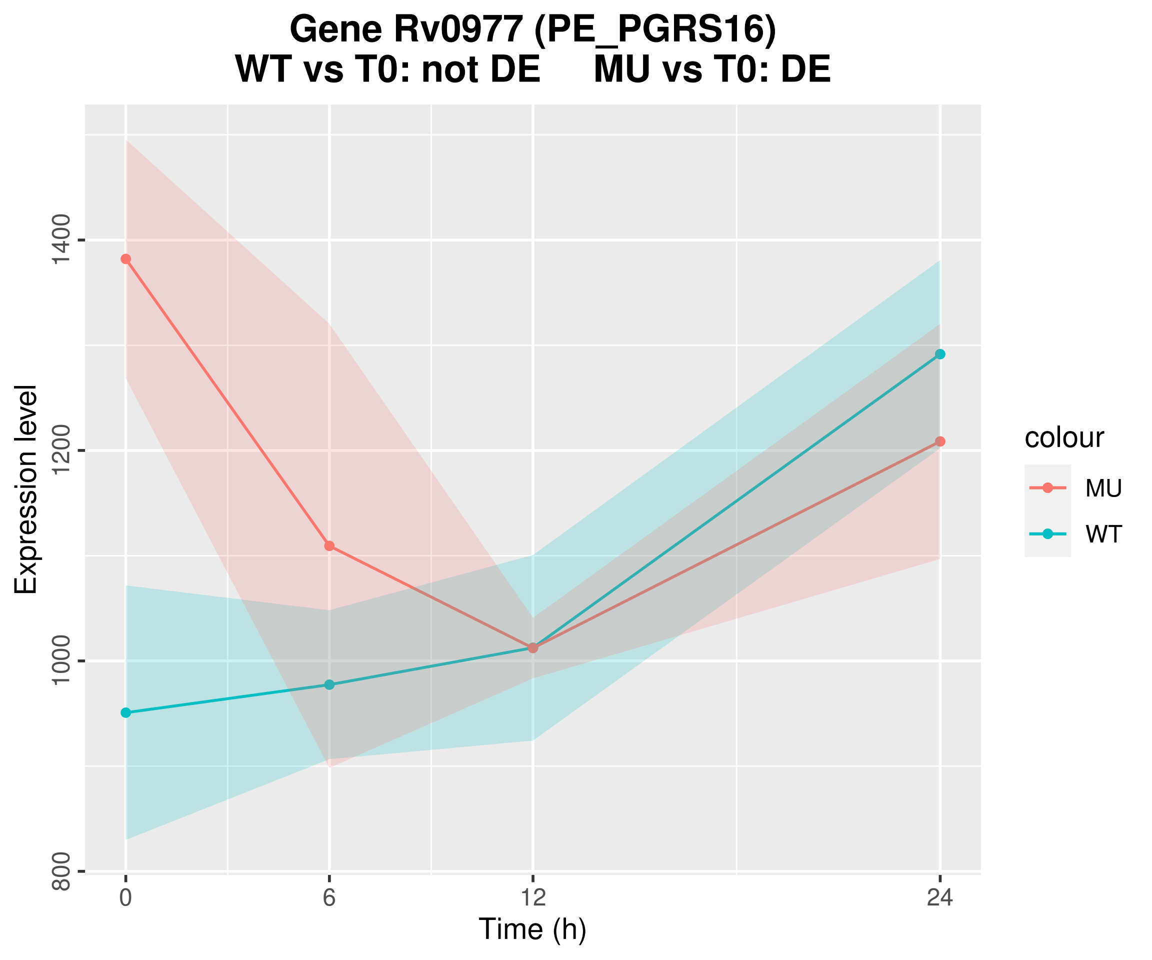

Gene Rv0977 (PE_PGRS16)
WT vs T0: not DE MU vs T0: DE
Expression level
1400
1200
1000
800
0
6
12
24
Time (h)
colour
MU
WT

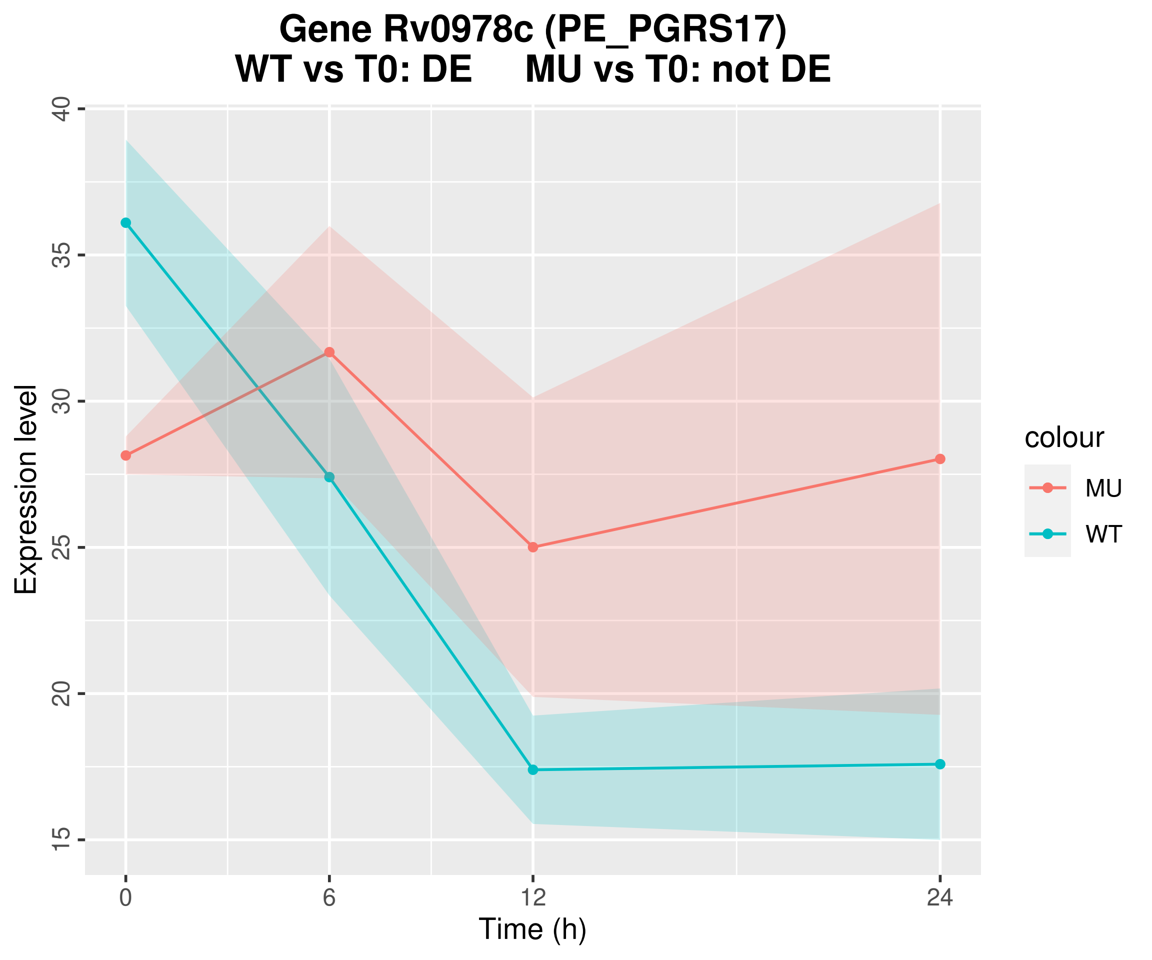

Gene Rv0978c (PE_PGRS17)
WT vs T0: DE   MU vs T0: not DE
Expression level
40
35
30
25
20
15
0
6
12
24
Time (h)
colour
MU
WT

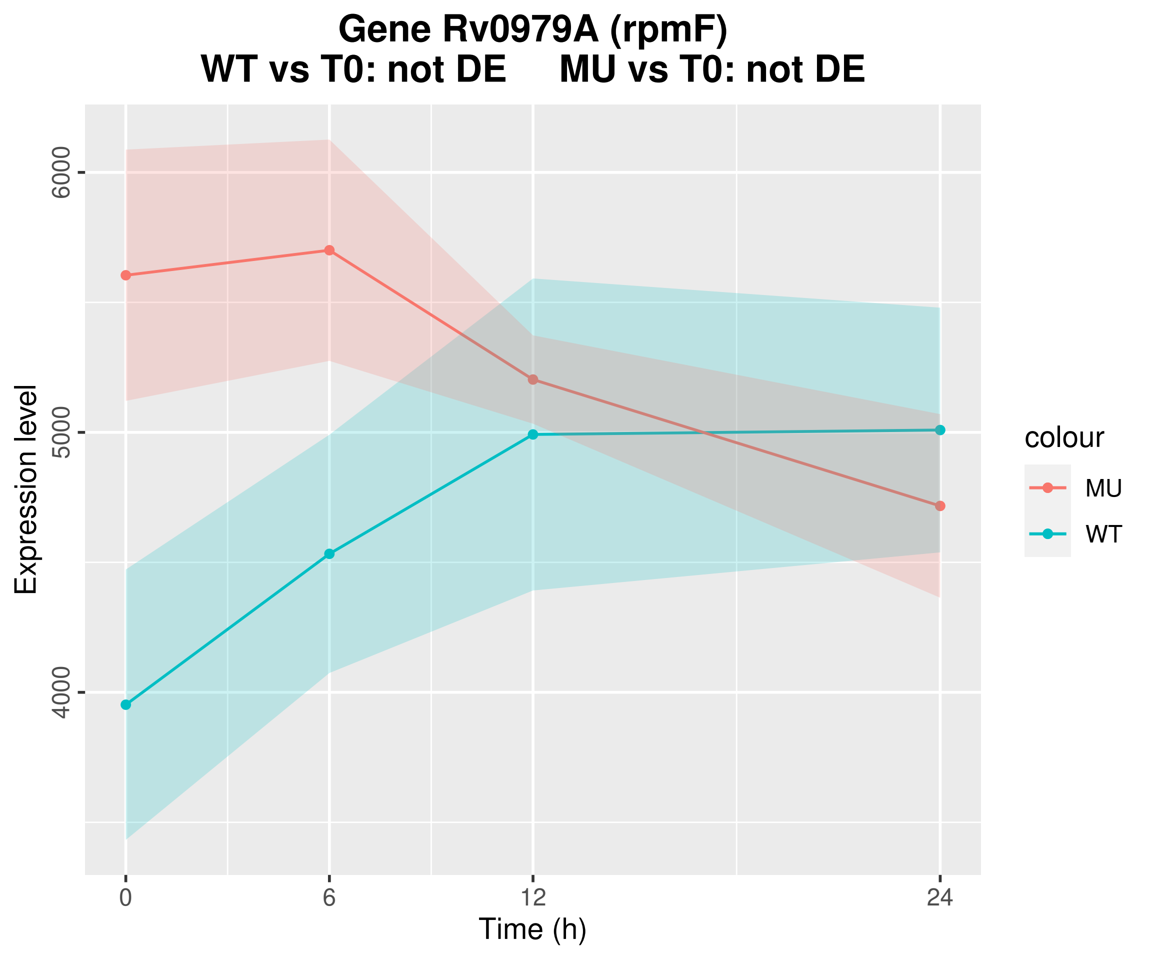
Gene Rv0979A (rpmF)
WT vs T0: not DE MU vs T0: not DE
Expression level
6000
5000
4000
Time (h)
0
6
12
24
colour
MU
WT

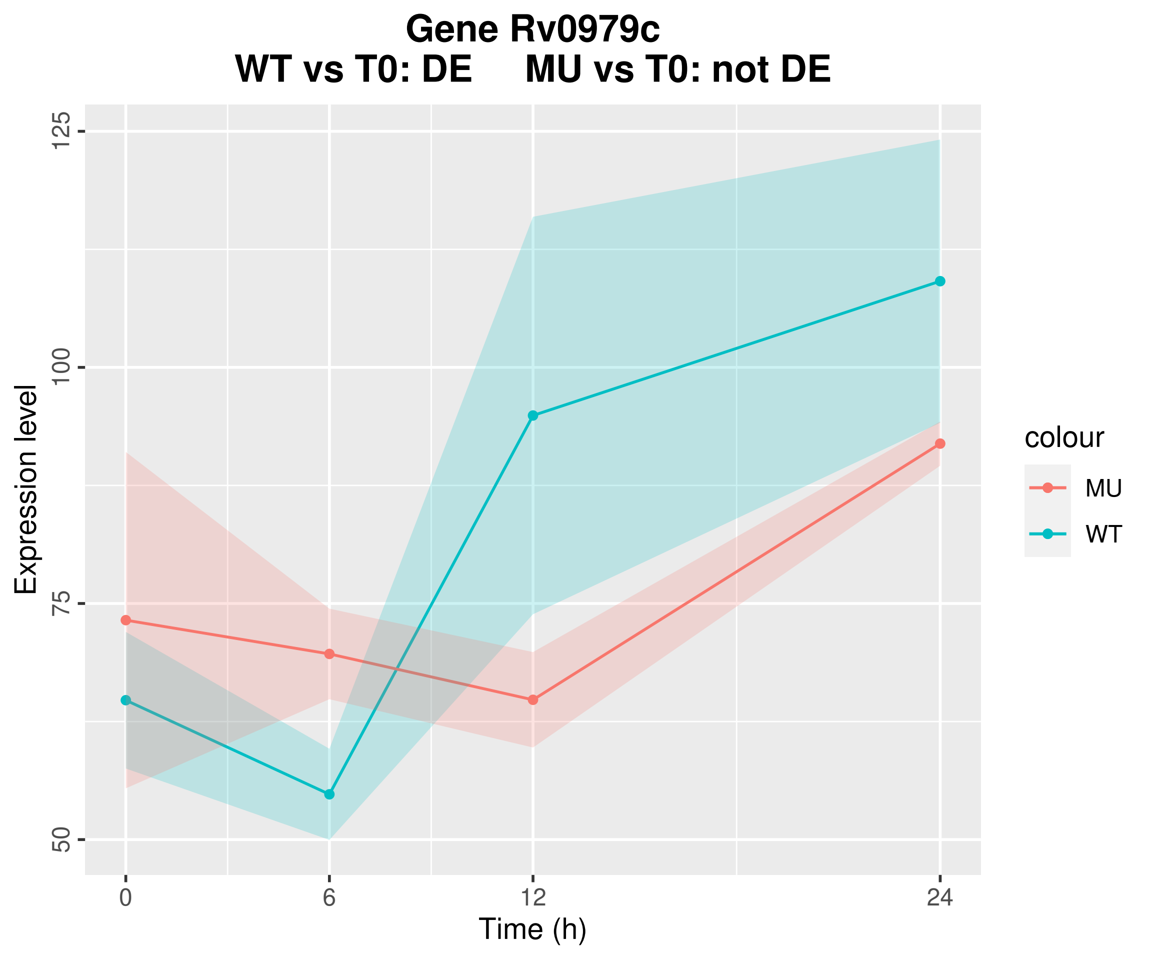

Gene Rv0979c
WT vs T0: DE   MU vs T0: not DE
Expression level
125
100
75
50
0
6
12
24
Time (h)
colour
MU
WT

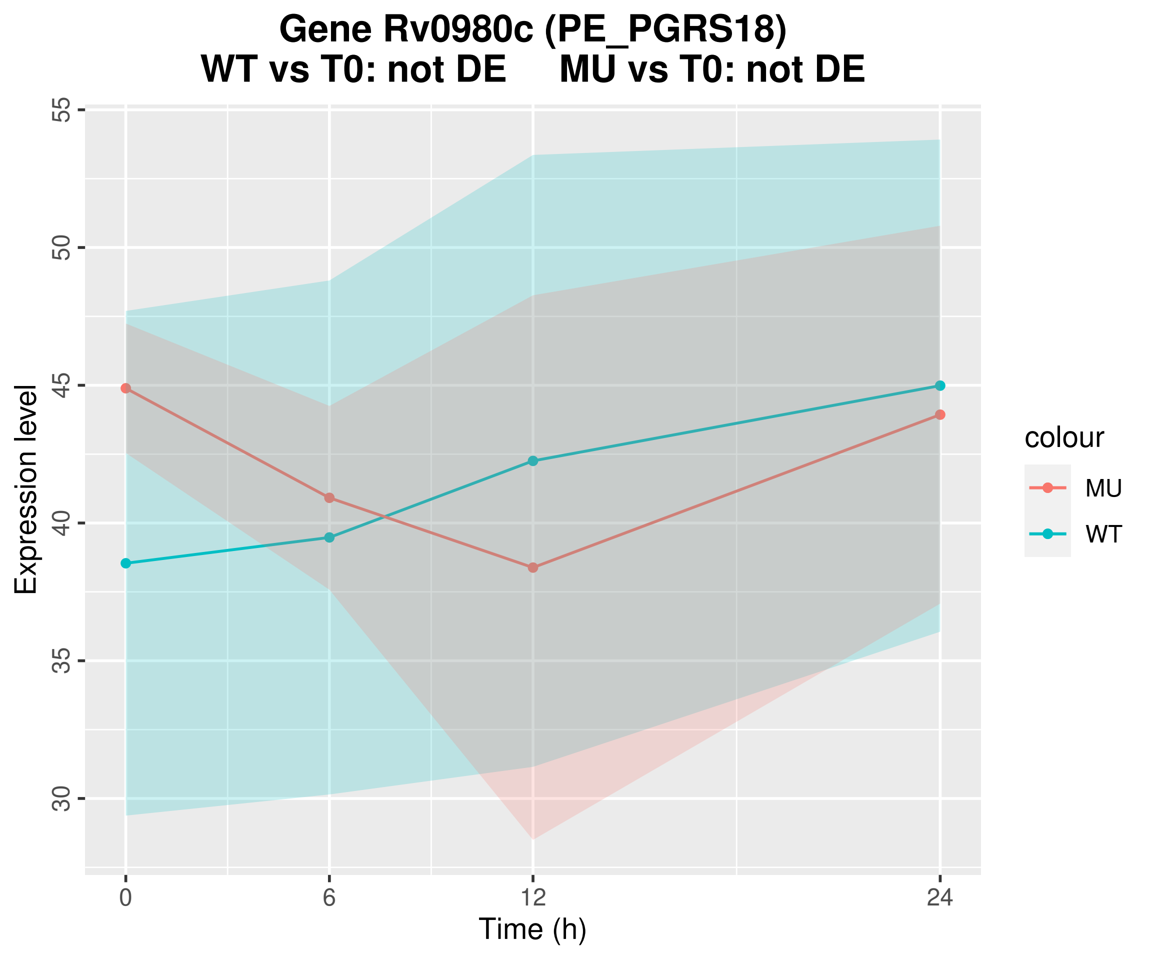
Gene Rv0980c (PE_PGRS18)
WT vs T0: not DE   MU vs T0: not DE
Expression level
55
50
45
40
35
30
0
6
12
24
Time (h)
colour
MU
WT

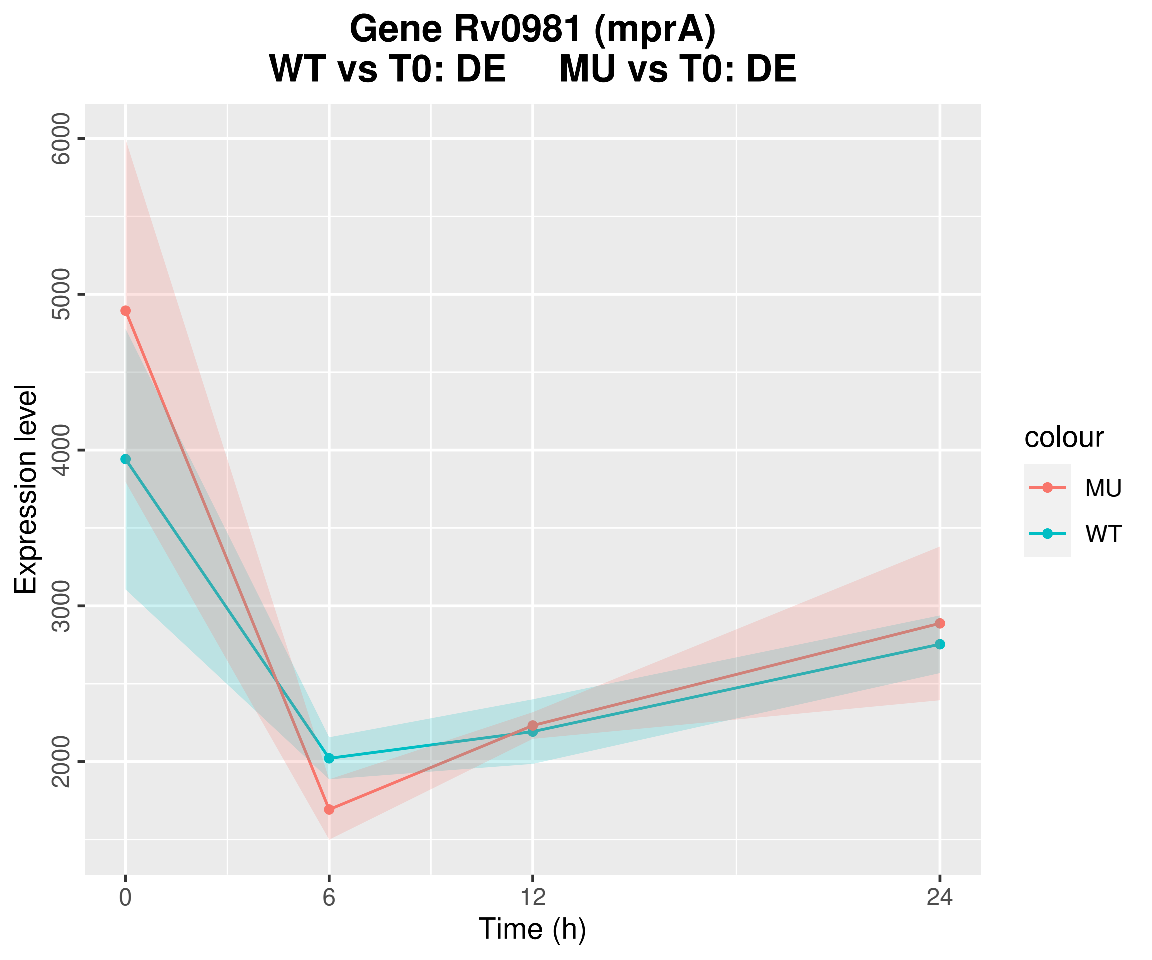

Gene Rv0981 (mprA)
WT vs T0: DE MU vs T0: DE
6000
5000
4000
3000
2000
Expression level
0
6
12
24
Time (h)
colour
MU
WT

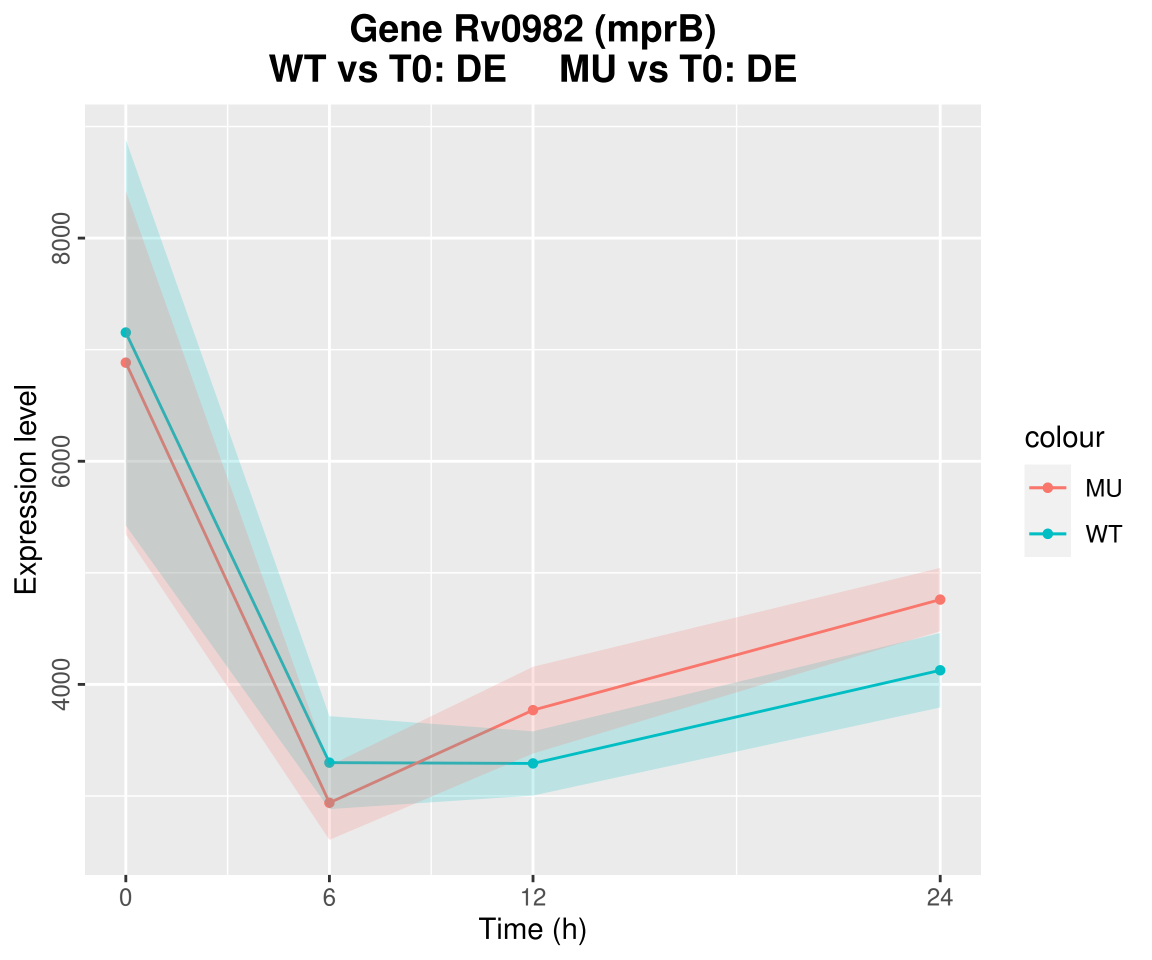
Gene Rv0982 (mprB)
WT vs T0: DE   MU vs T0: DE
Expression level
8000
6000
4000
0
6
12
24
Time (h)
colour
MU
WT

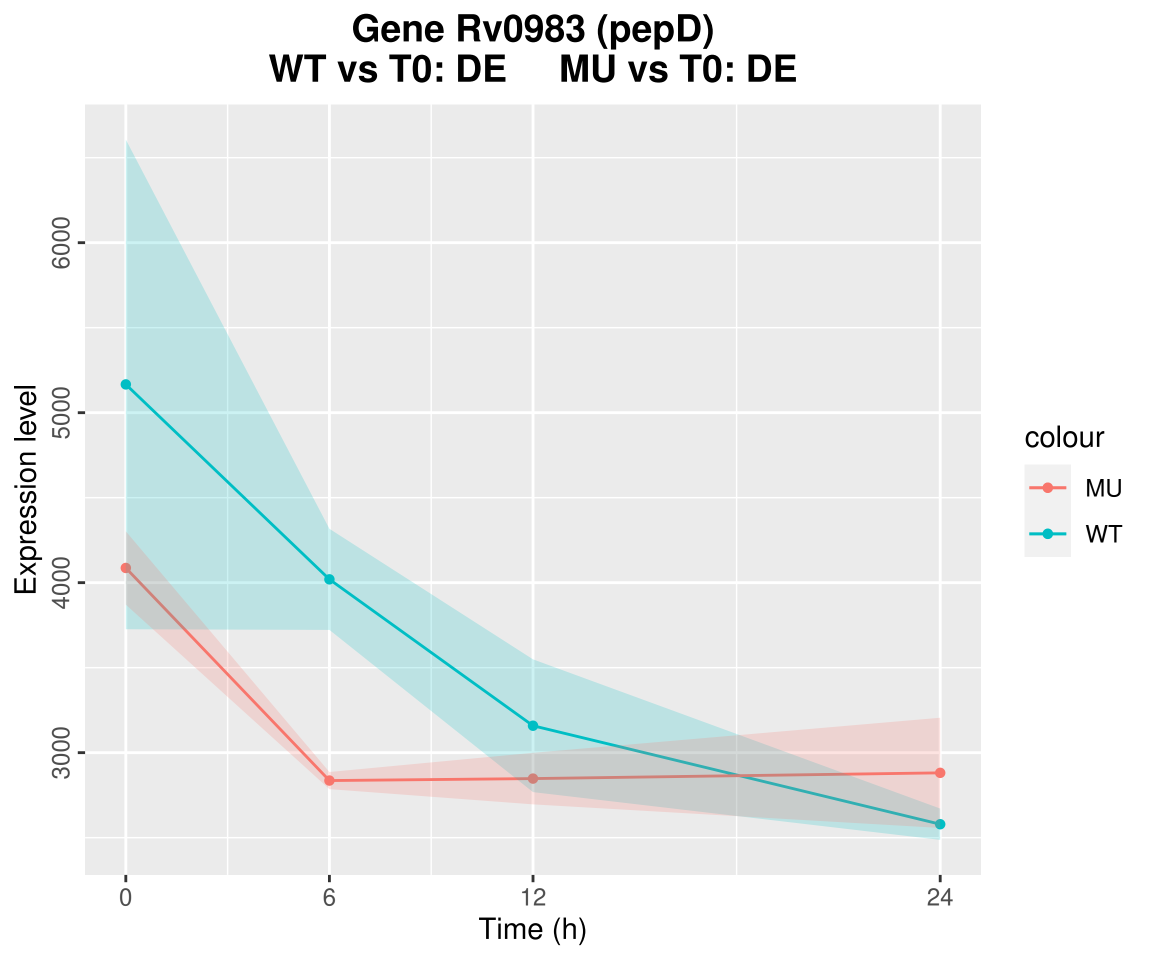

Gene Rv0983 (pepD)
WT vs T0: DE MU vs T0: DE
Expression level
6000
5000
4000
3000
0
6
12
24
Time (h)
colour
MU
WT

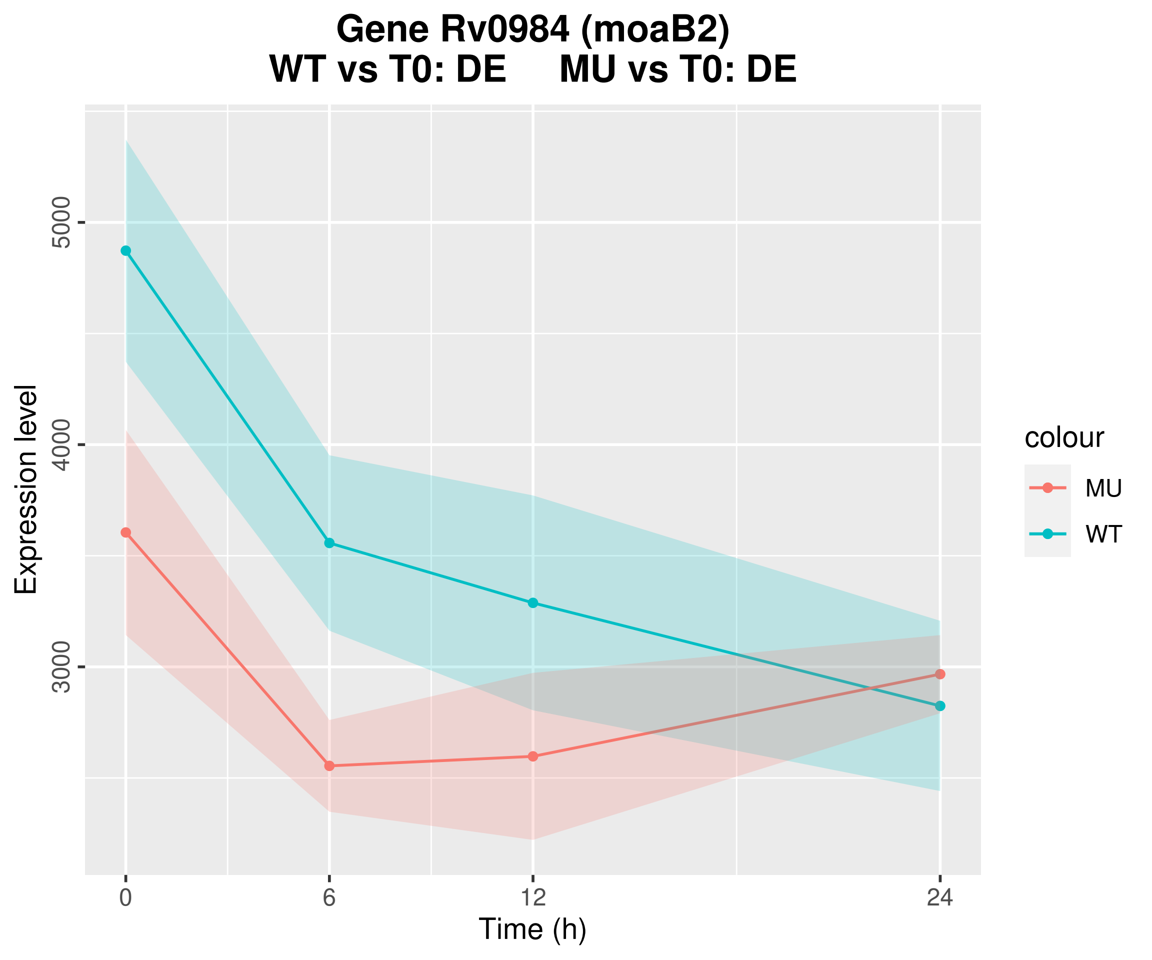

Gene Rv0984 (moaB2)
WT vs T0: DE MU vs T0: DE
5000
4000
3000
Expression level
0
6
12
24
Time (h)
colour
MU
WT

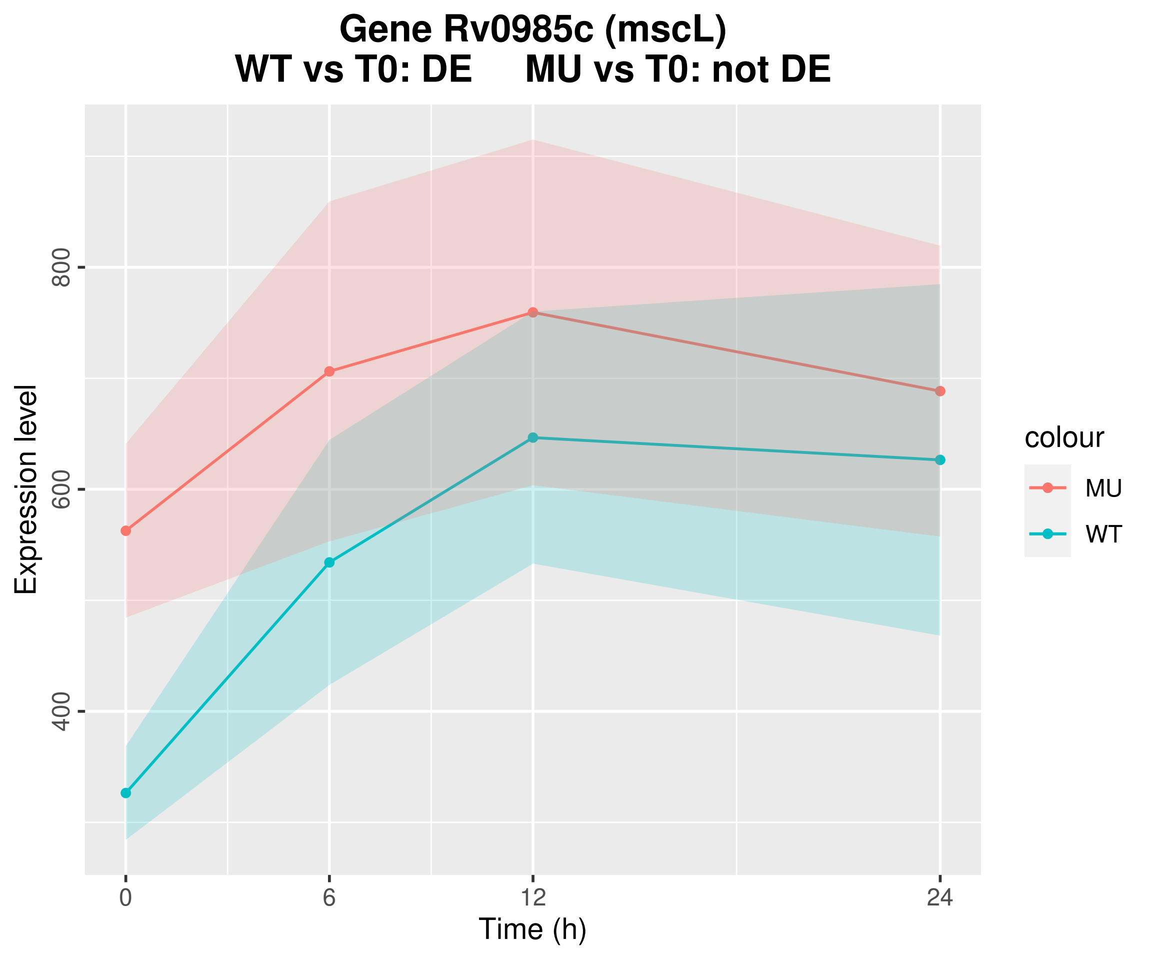
Gene Rv0985c (mscL)
WT vs T0: DE   MU vs T0: not DE
Expression level
800
600
400
0
6
12
24
Time (h)
colour
MU
WT

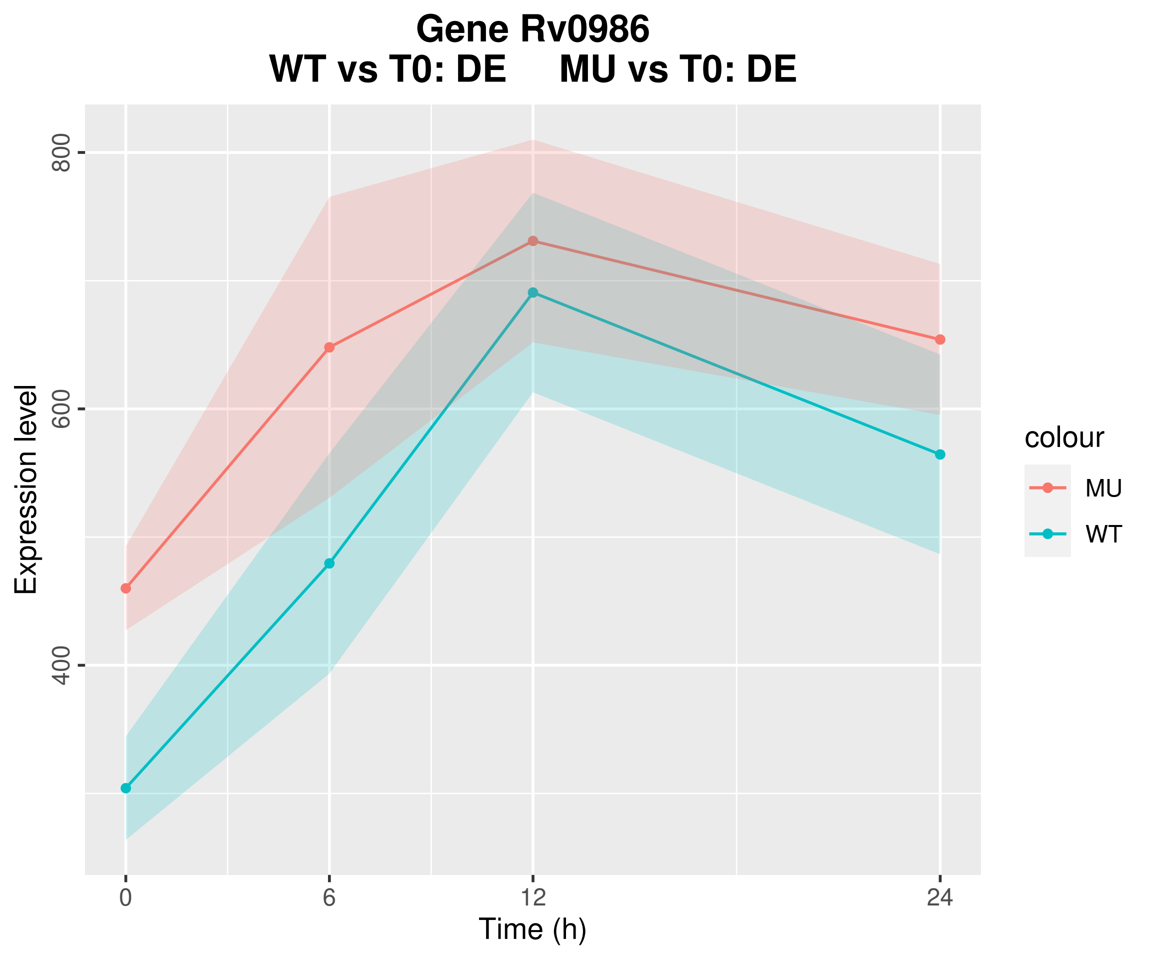

Gene Rv0986
WT vs T0: DE MU vs T0: DE
Expression level
800
600
400
0
6
12
24
Time (h)
colour
MU
WT

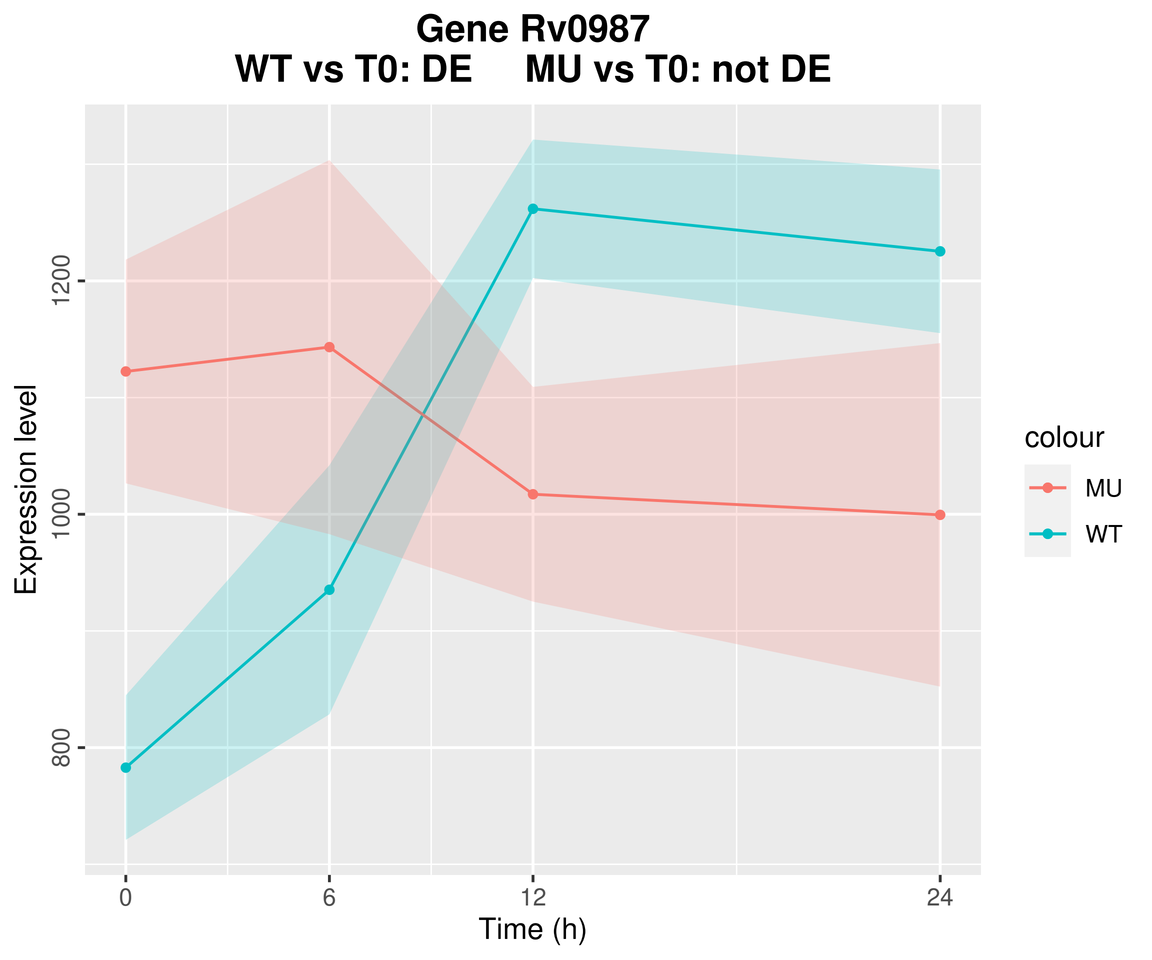

Gene Rv0987
WT vs T0: DE   MU vs T0: not DE
Expression level
1200
1000
800
0
6
12
24
Time (h)
colour
MU
WT

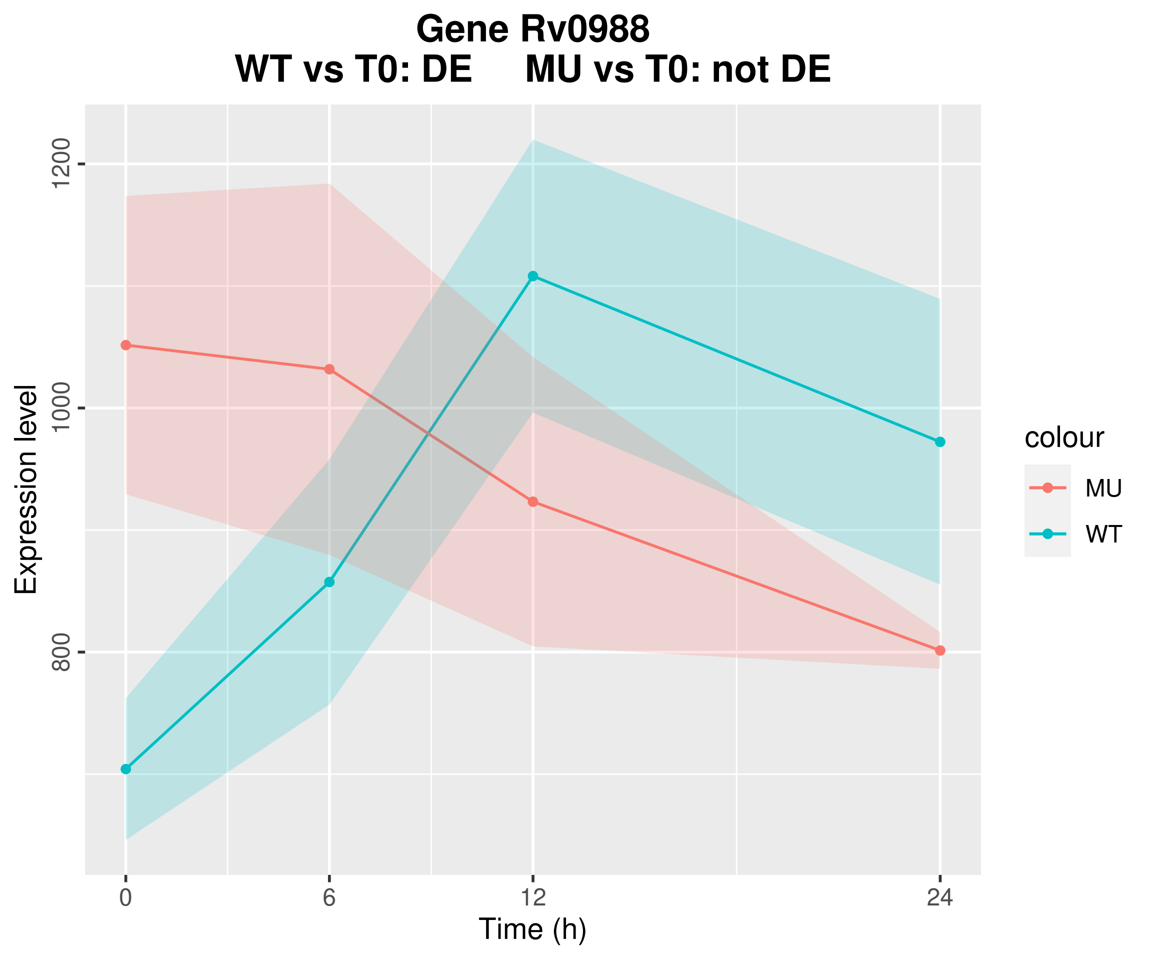
Gene Rv0988
WT vs T0: DE MU vs T0: not DE
Expression level
1200
1000
800
0
6
12
24
Time (h)
colour
MU
WT

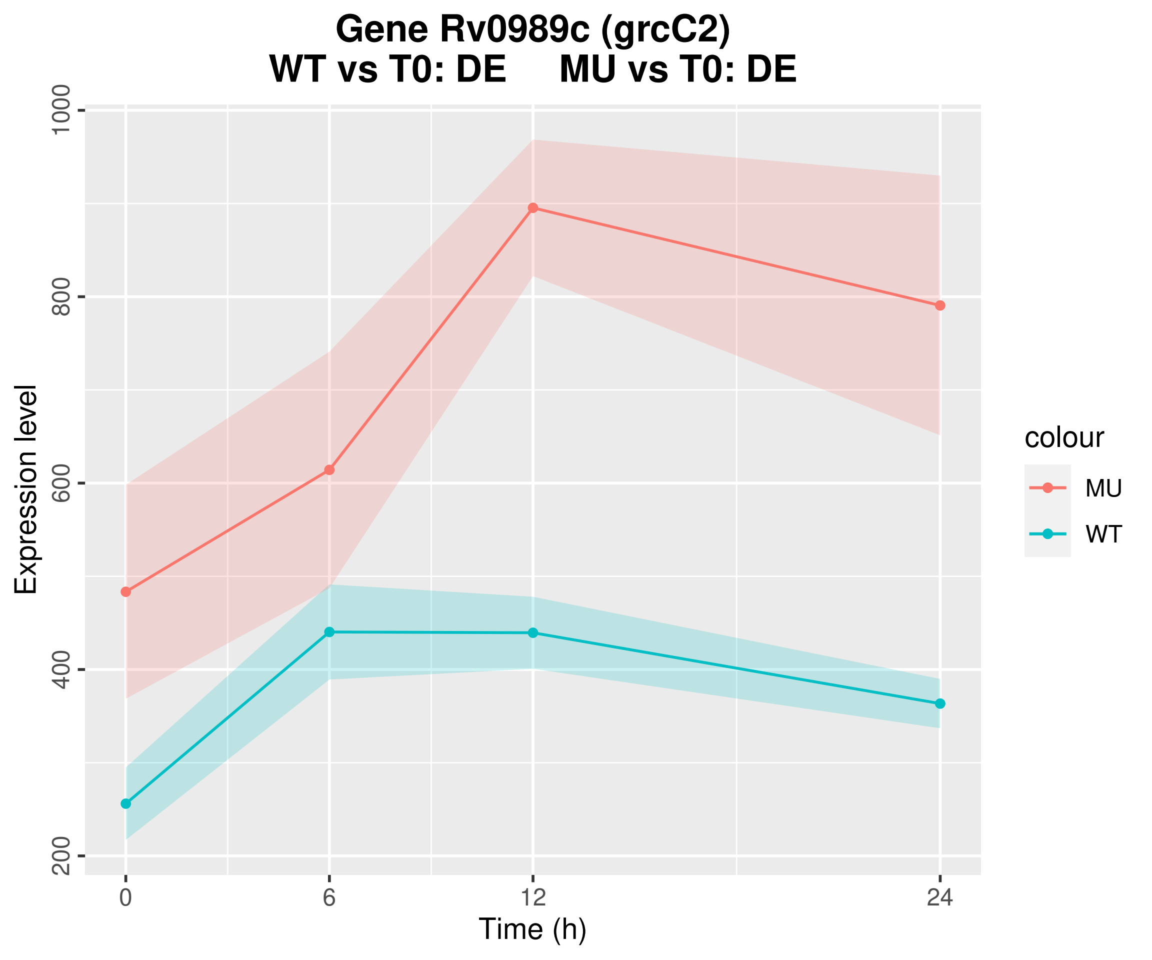
Gene Rv0989c (grcC2)
WT vs T0: DE   MU vs T0: DE
Expression level
1000
800
600
400
200
0
6
12
24
Time (h)
colour
MU
WT

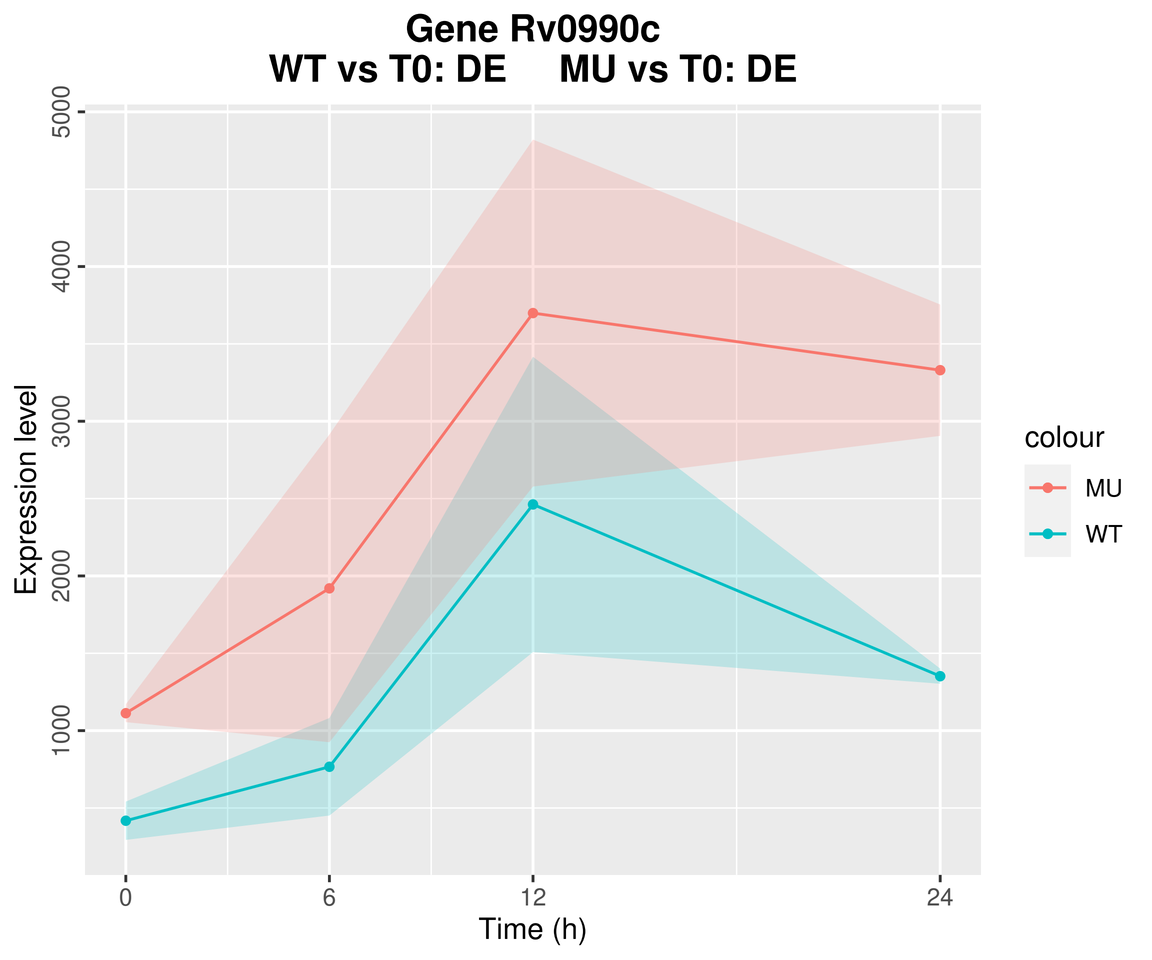
Gene Rv0990c
WT vs T0: DE   MU vs T0: DE
Expression level
5000
4000
3000
2000
1000
0
6
12
24
Time (h)
colour
MU
WT

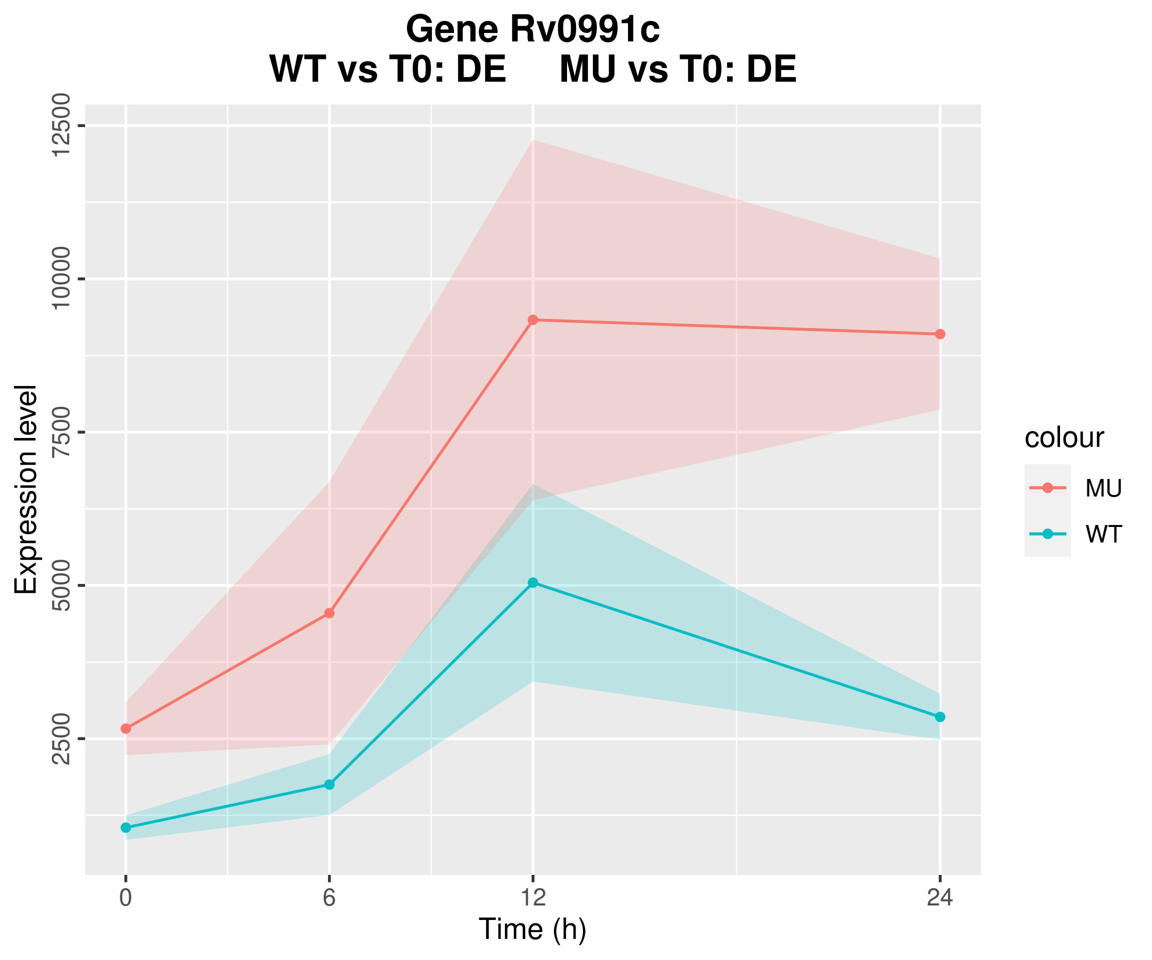

Gene Rv0991c
WT vs T0: DE   MU vs T0: DE
Expression level
12500
10000
7500
5000
2500
0
6
12
24
Time (h)
colour
MU
WT

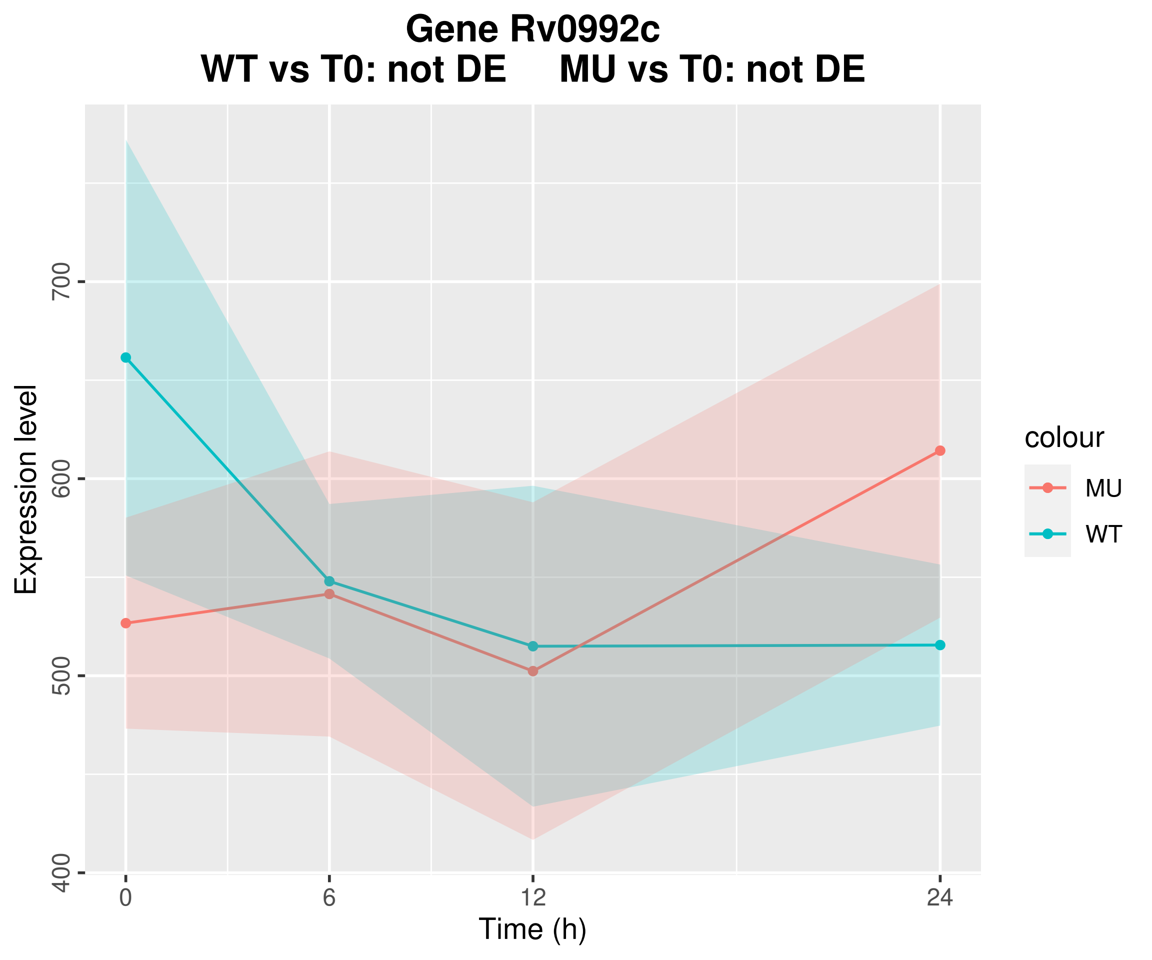

Gene Rv0992c
WT vs T0: not DE   MU vs T0: not DE
Expression level
700
600
500
400
0
6
12
24
Time (h)
colour
MU
WT

Gene Rv0993 (galU)
WT vs T0: DE   MU vs T0: DE

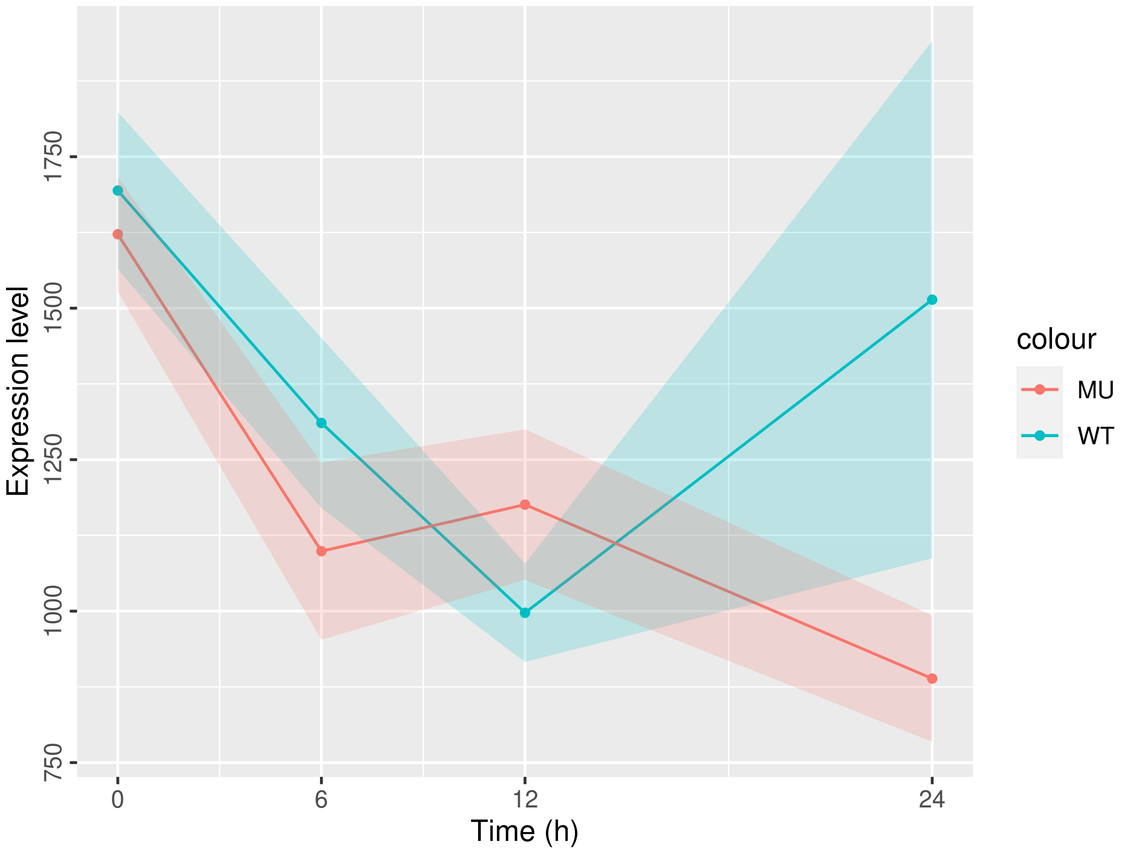

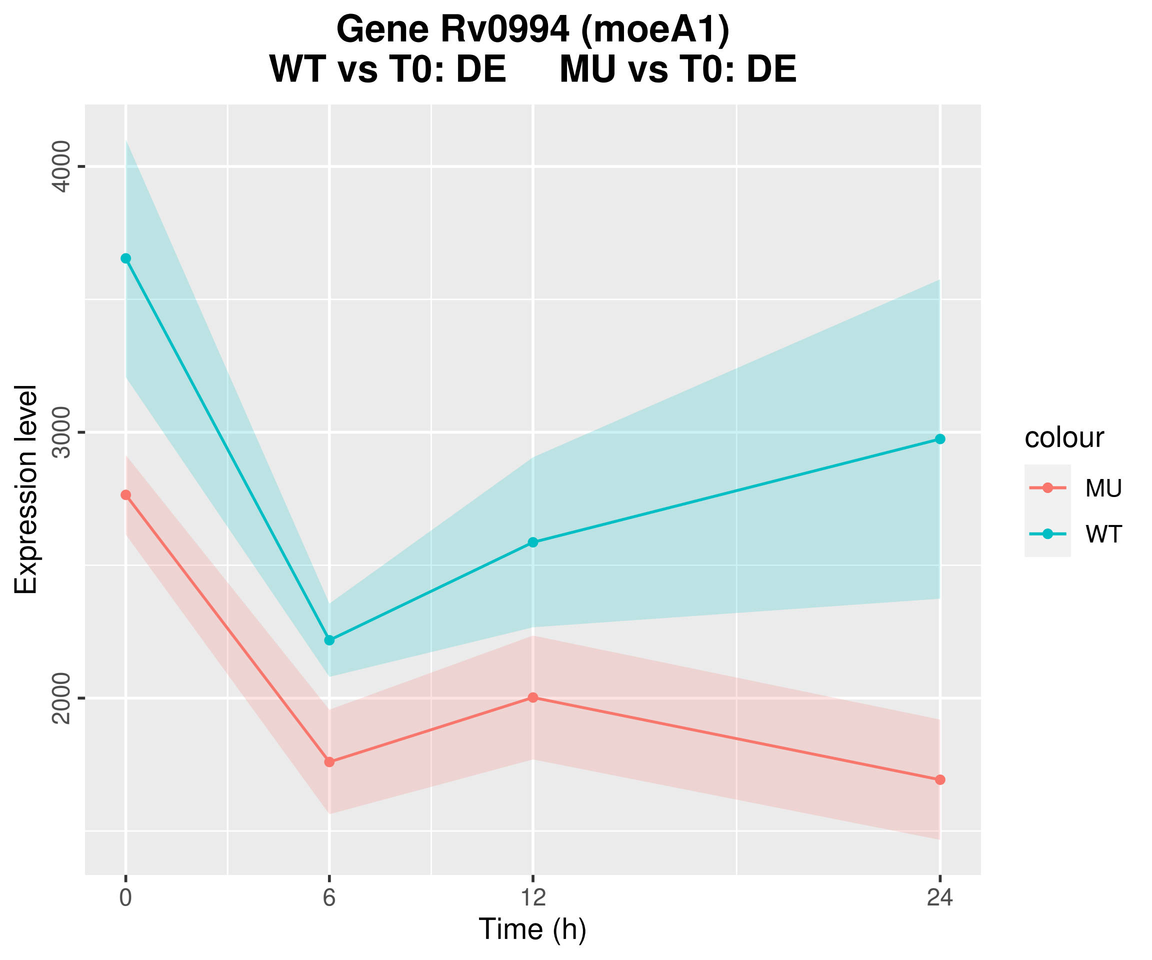
Gene Rv0994 (moeA1)
WT vs T0: DE   MU vs T0: DE
Expression level
4000
3000
2000
0
6
12
24
Time (h)
colour
MU
WT

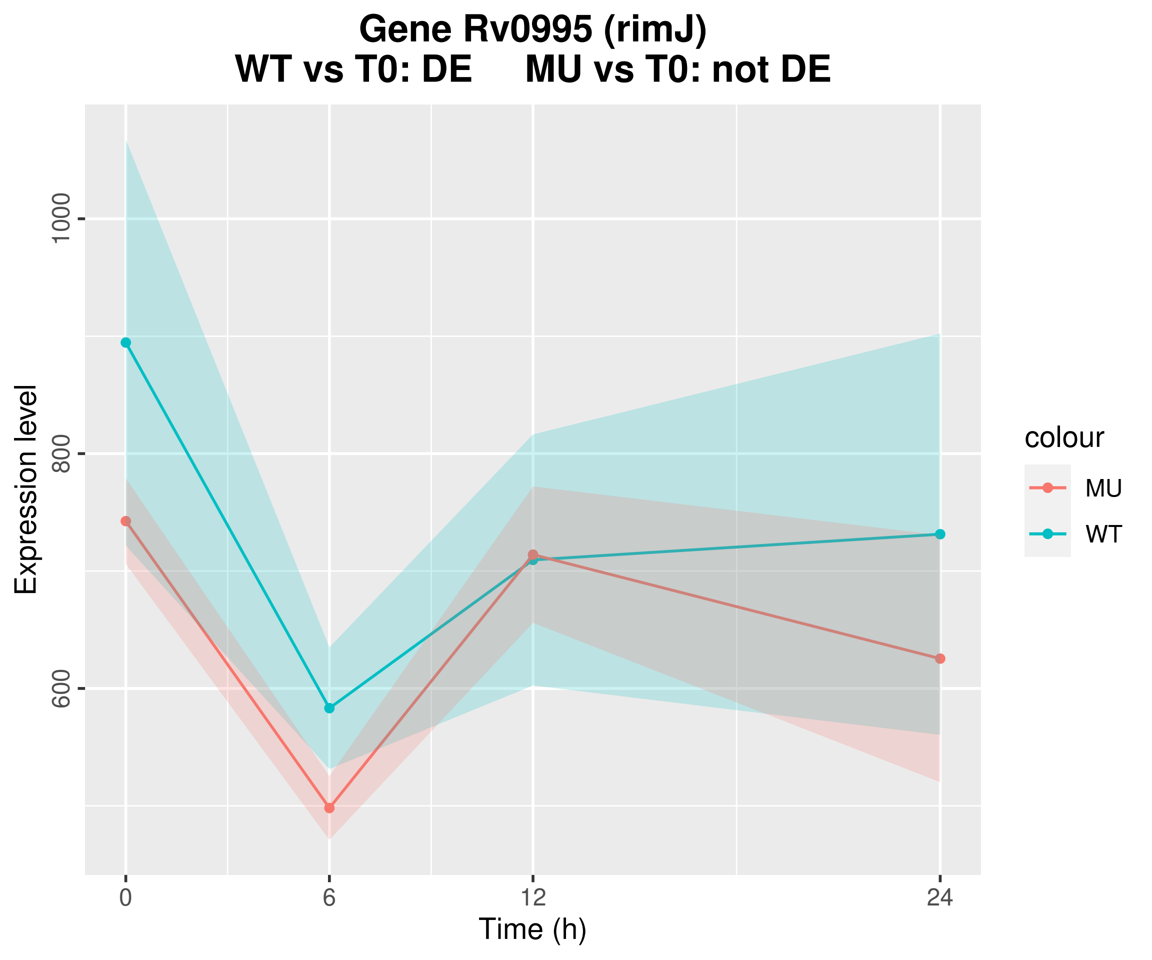

Gene Rv0995 (rimJ)
WT vs T0: DE MU vs T0: not DE
Expression level
1000
800
600
0
6
12
24
Time (h)
colour
MU
WT

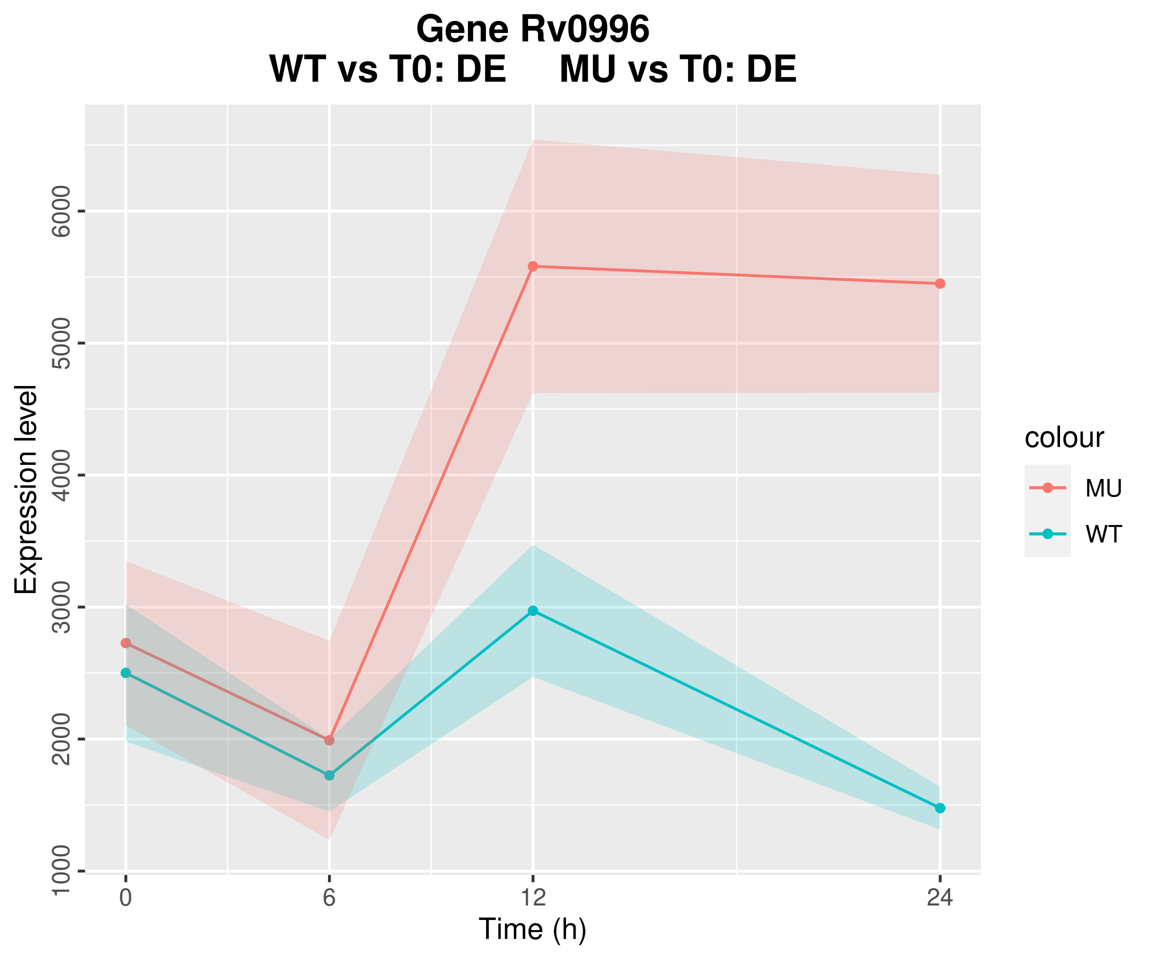

Gene Rv0996
WT vs T0: DE MU vs T0: DE
Expression level
1000
2000
3000
4000
5000
6000
0
6
12
24
Time (h)
colour
MU
WT

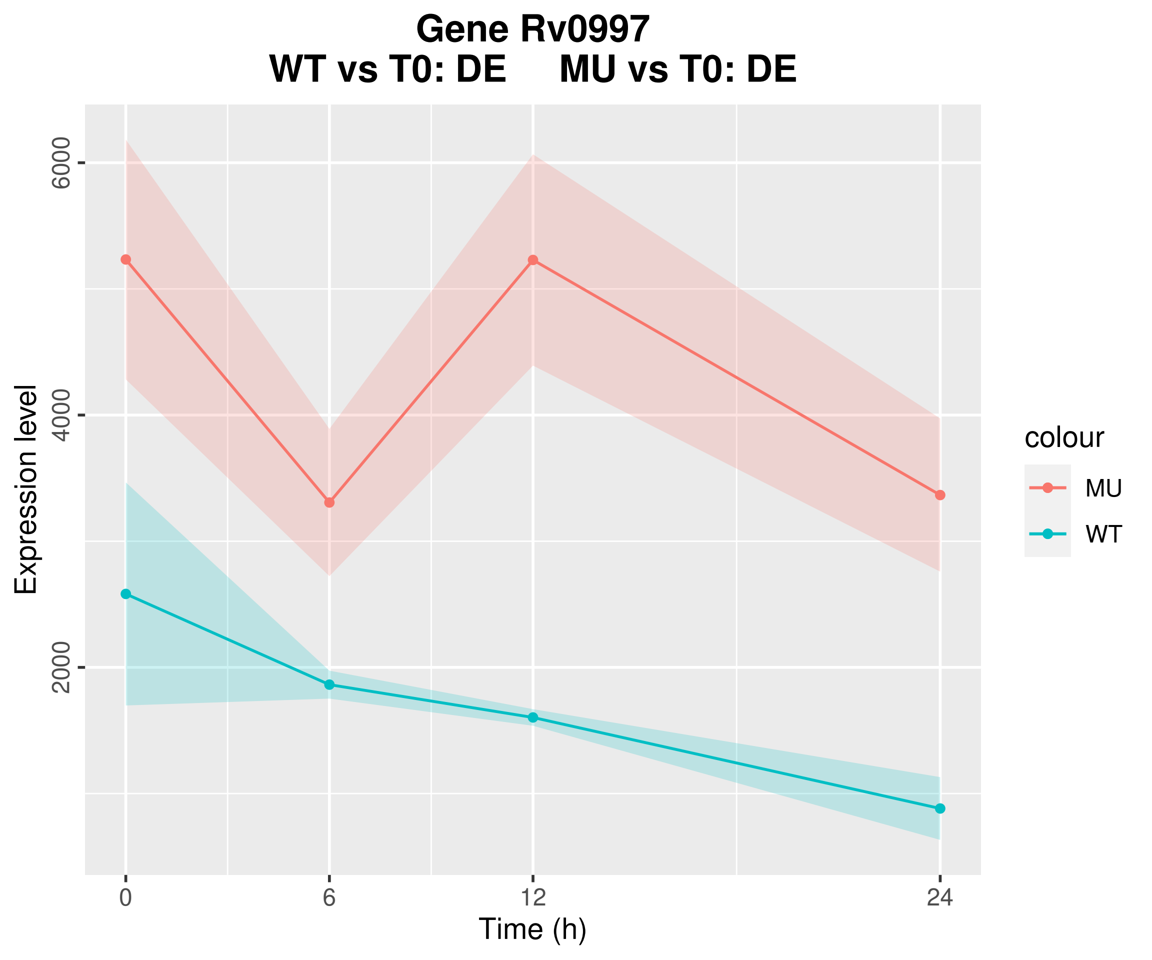

Gene Rv0997
WT vs T0: DE MU vs T0: DE
Expression level
6000
4000
2000
0
6
12
24
Time (h)
colour
MU
WT

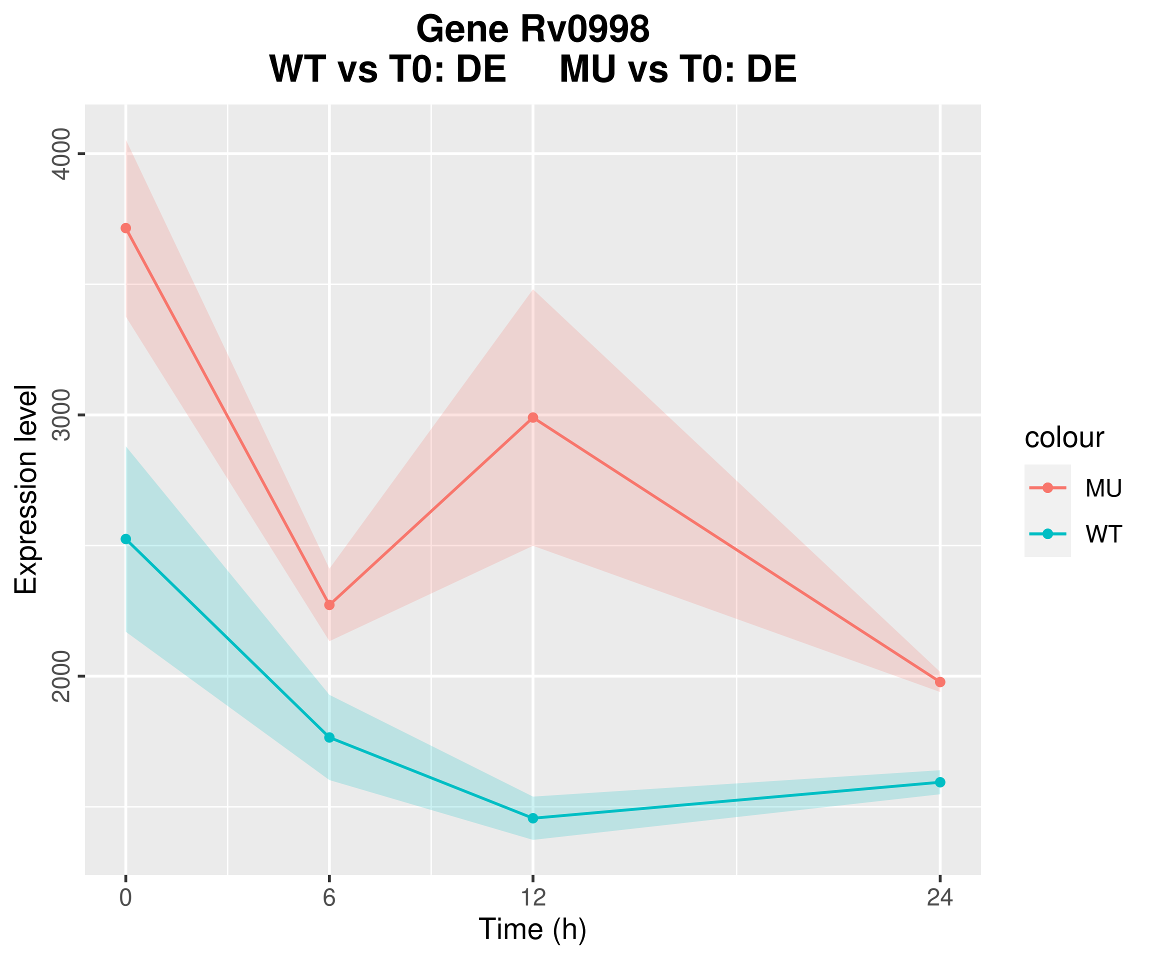
Gene Rv0998
WT vs T0: DE MU vs T0: DE
4000
3000
2000
0
6
12
24
Time (h)
Expression level
colour
MU
WT

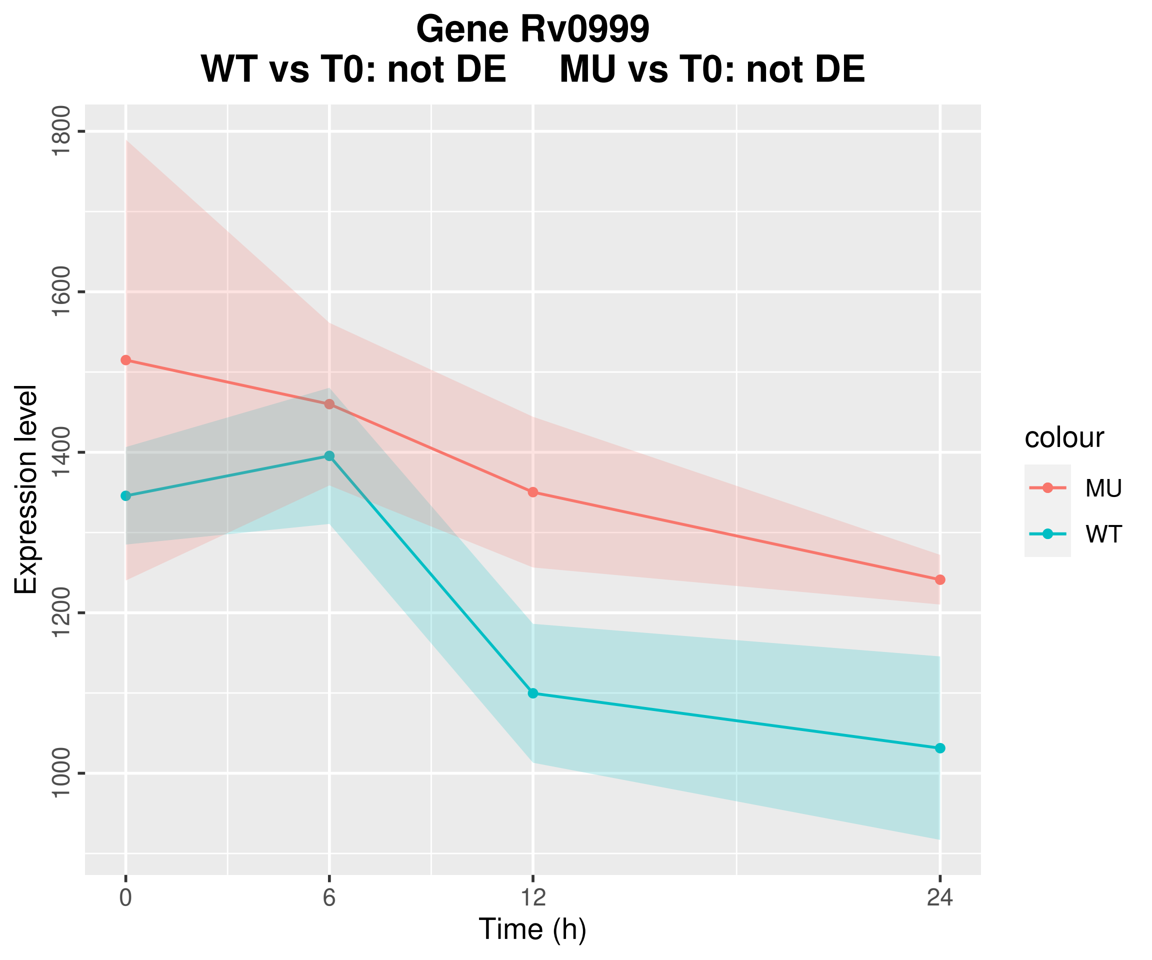
Gene Rv0999
WT vs T0: not DE   MU vs T0: not DE
Expression level
1800
1600
1400
1200
1000
0
6
12
24
Time (h)
colour
MU
WT

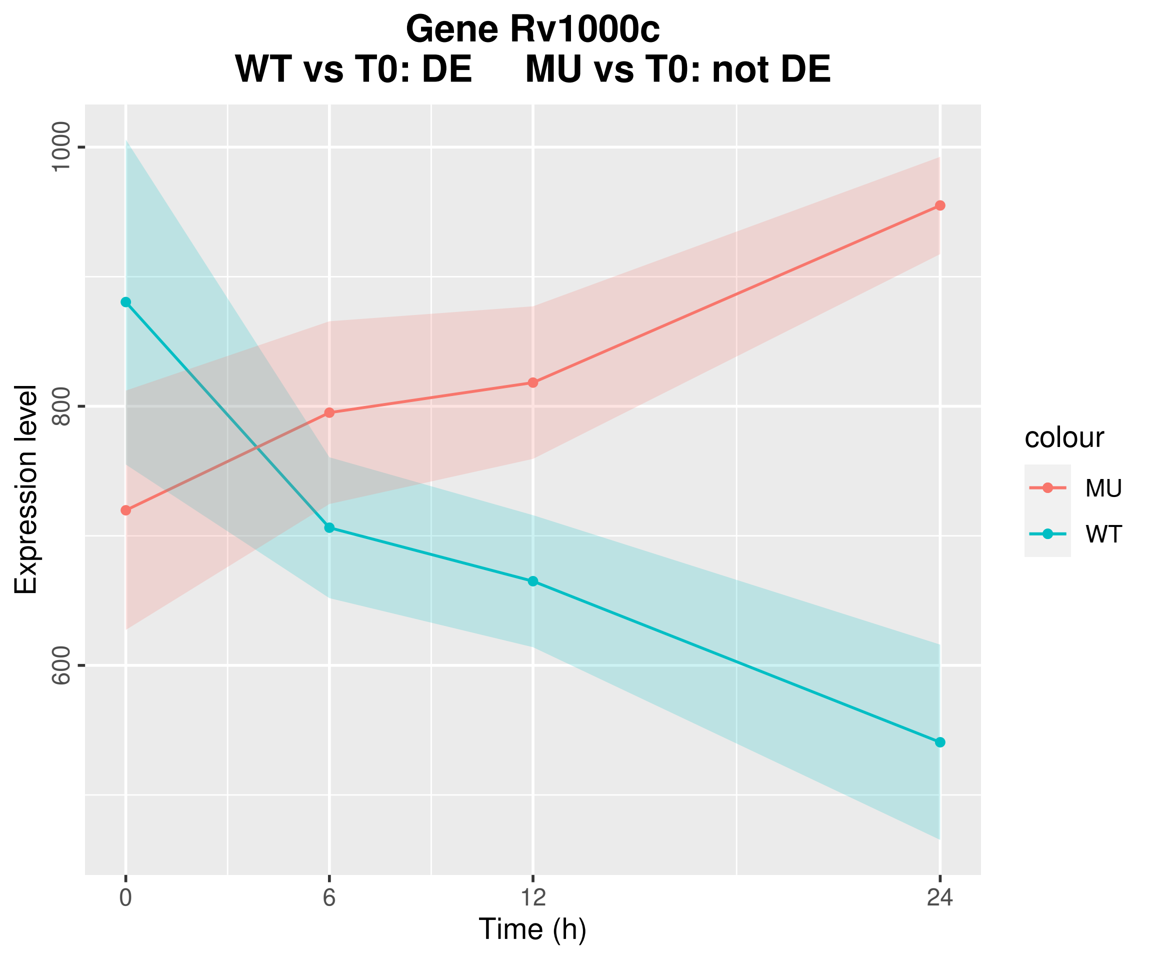

Gene Rv1000c
WT vs T0: DE MU vs T0: not DE
Expression level
1000
800
600
0
6
12
24
Time (h)
colour
MU
WT

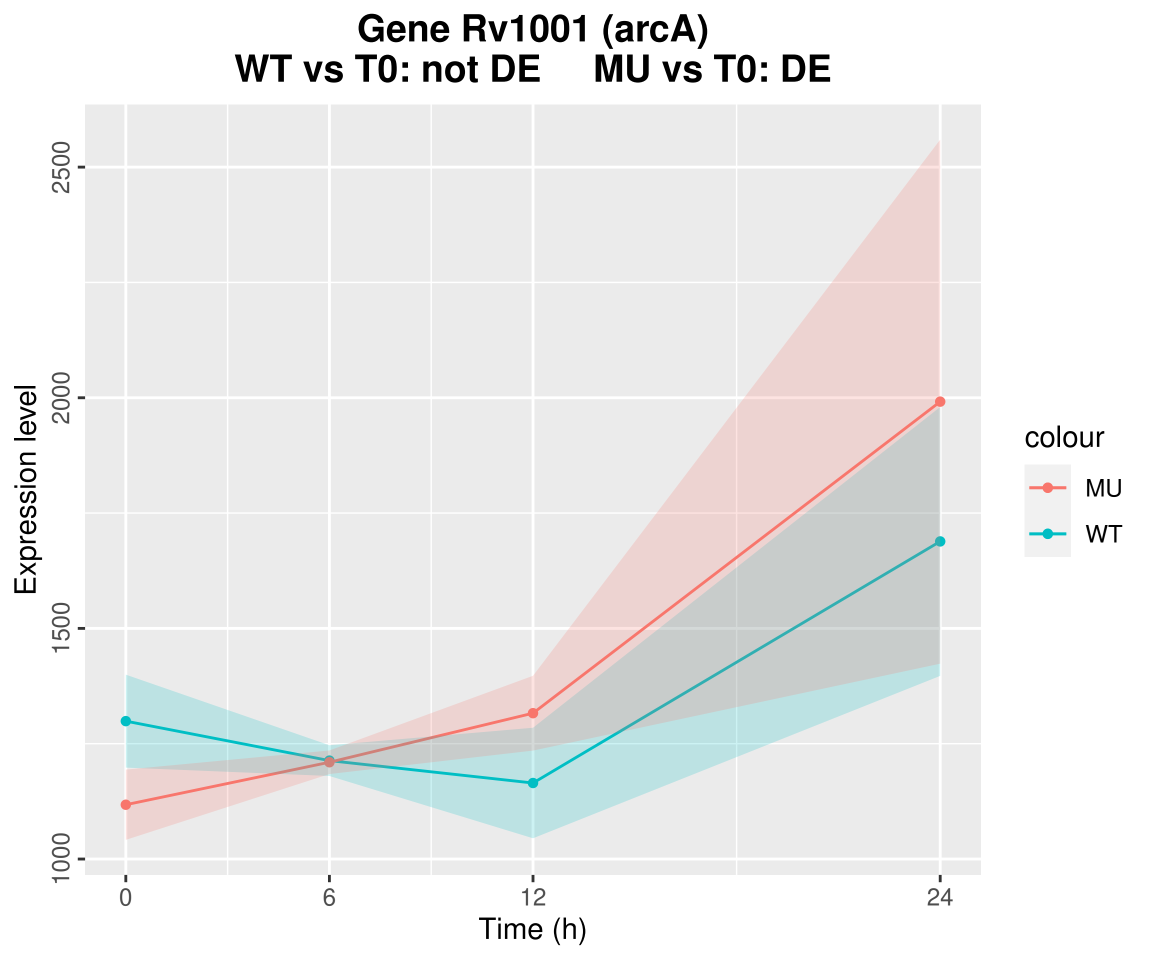
Gene Rv1001 (arcA)
WT vs T0: not DE MU vs T0: DE
Expression level
2500
2000
1500
1000
0
6
12
24
Time (h)
colour
MU
WT

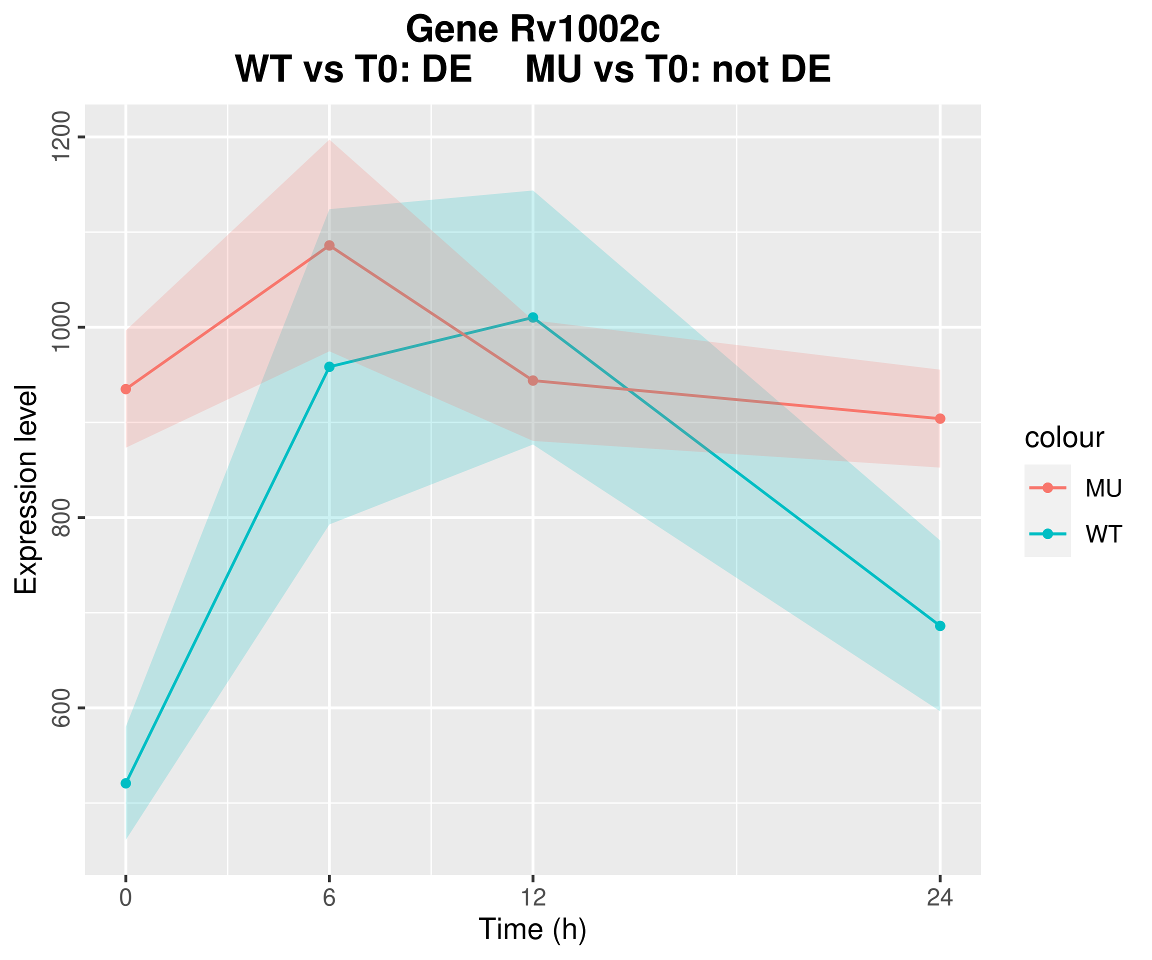
Gene Rv1002c
WT vs T0: DE   MU vs T0: not DE
Expression level
1200
1000
800
600
0
6
12
24
Time (h)
colour
MU
WT

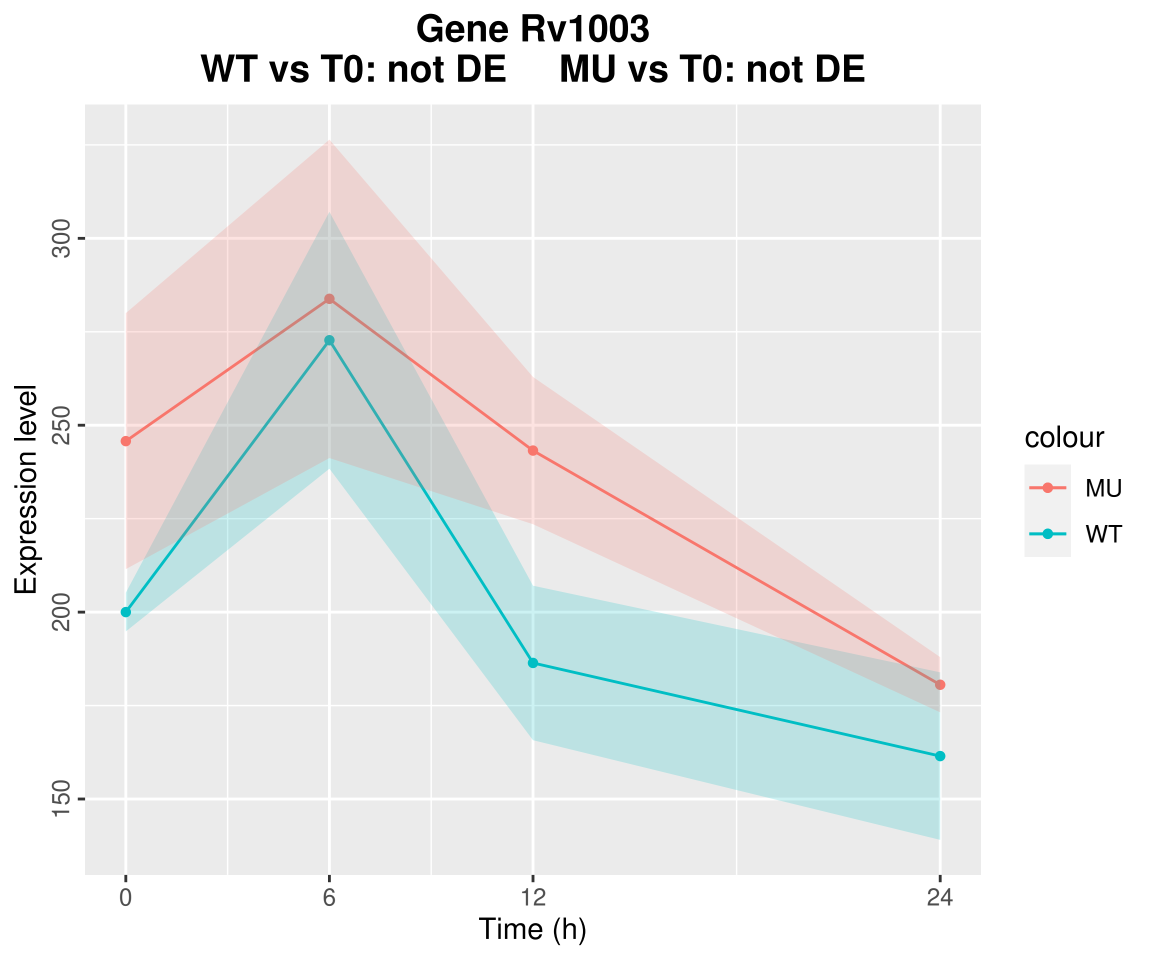

Gene Rv1003
WT vs T0: not DE MU vs T0: not DE
Expression level
300
250
200
150
0
6
12
24
Time (h)
colour
MU
WT

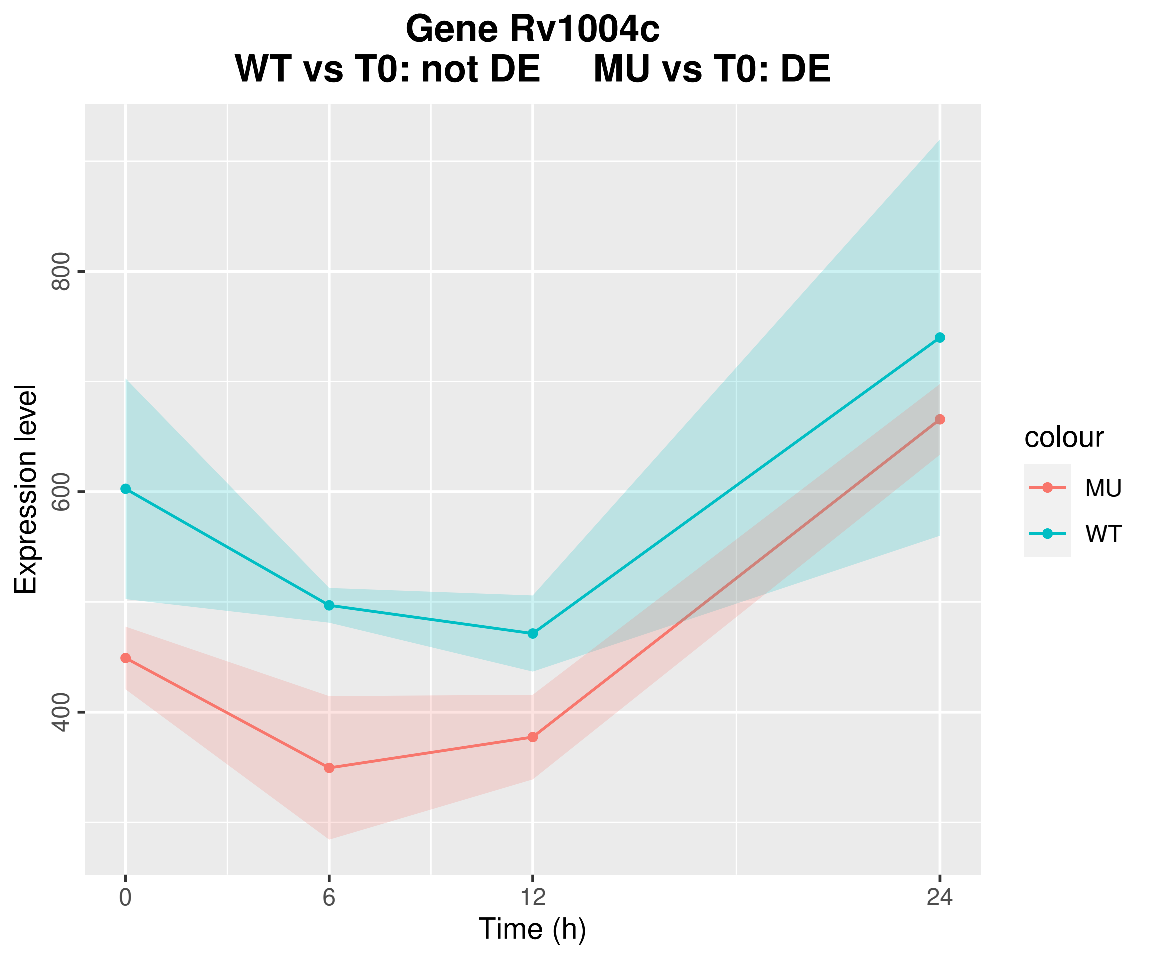
Gene Rv1004c
WT vs T0: not DE MU vs T0: DE
Expression level
800
600
400
0
6
12
24
Time (h)
colour
MU
WT

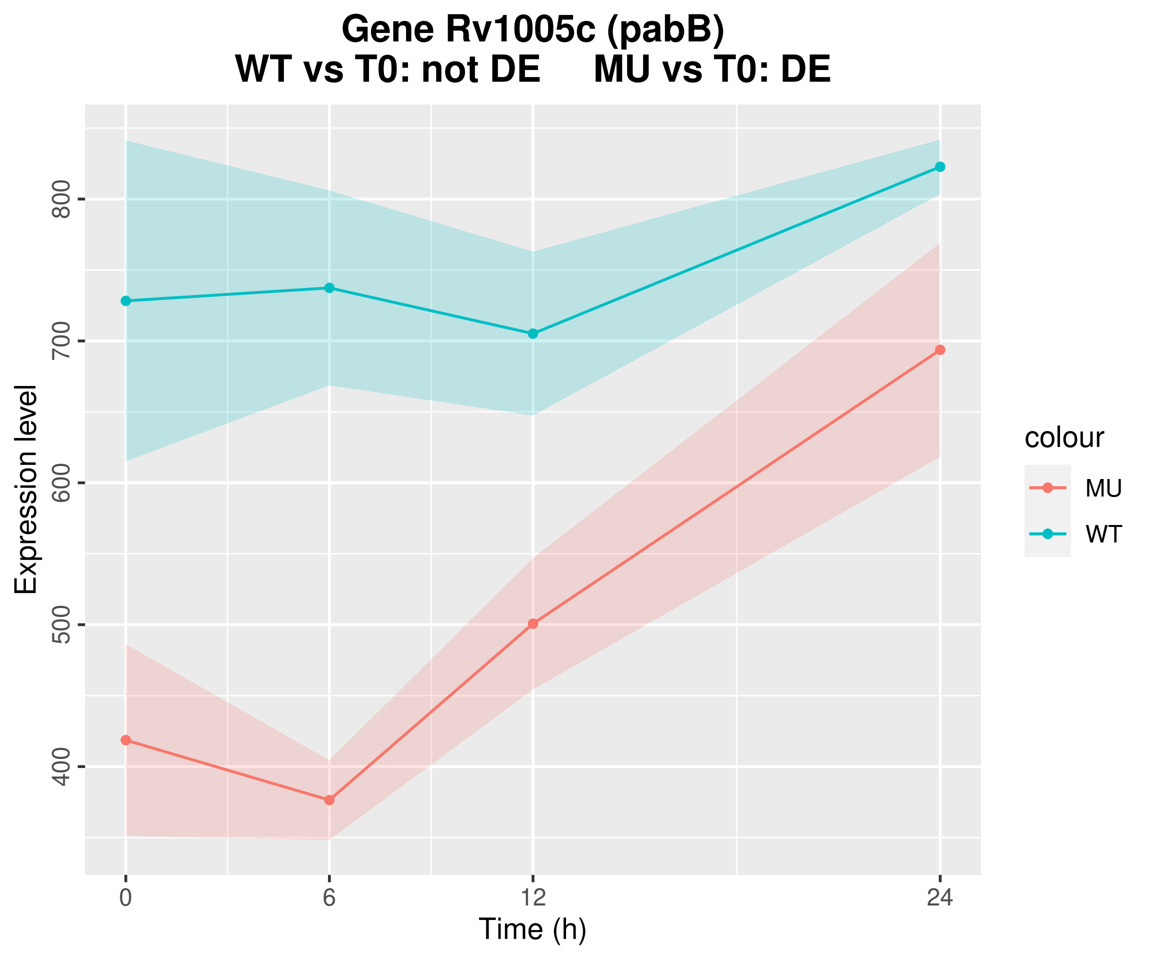
Gene Rv1005c (pabB)
WT vs T0: not DE   MU vs T0: DE
Expression level
800
700
600
500
400
0
6
12
24
Time (h)
colour
MU
WT

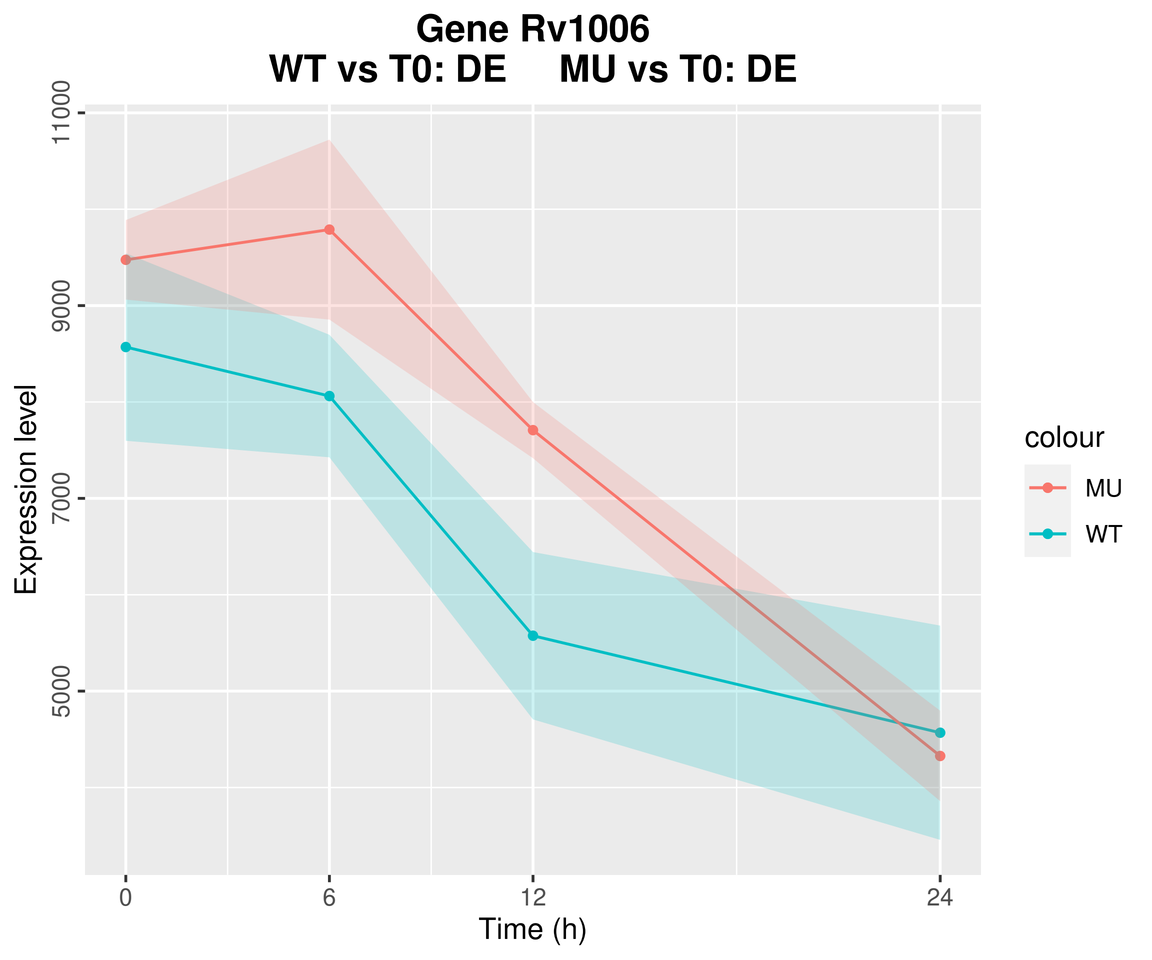

Gene Rv1006
WT vs T0: DE MU vs T0: DE
11000
9000
7000
5000
Expression level
0
6
12
24
Time (h)
colour
MU
WT

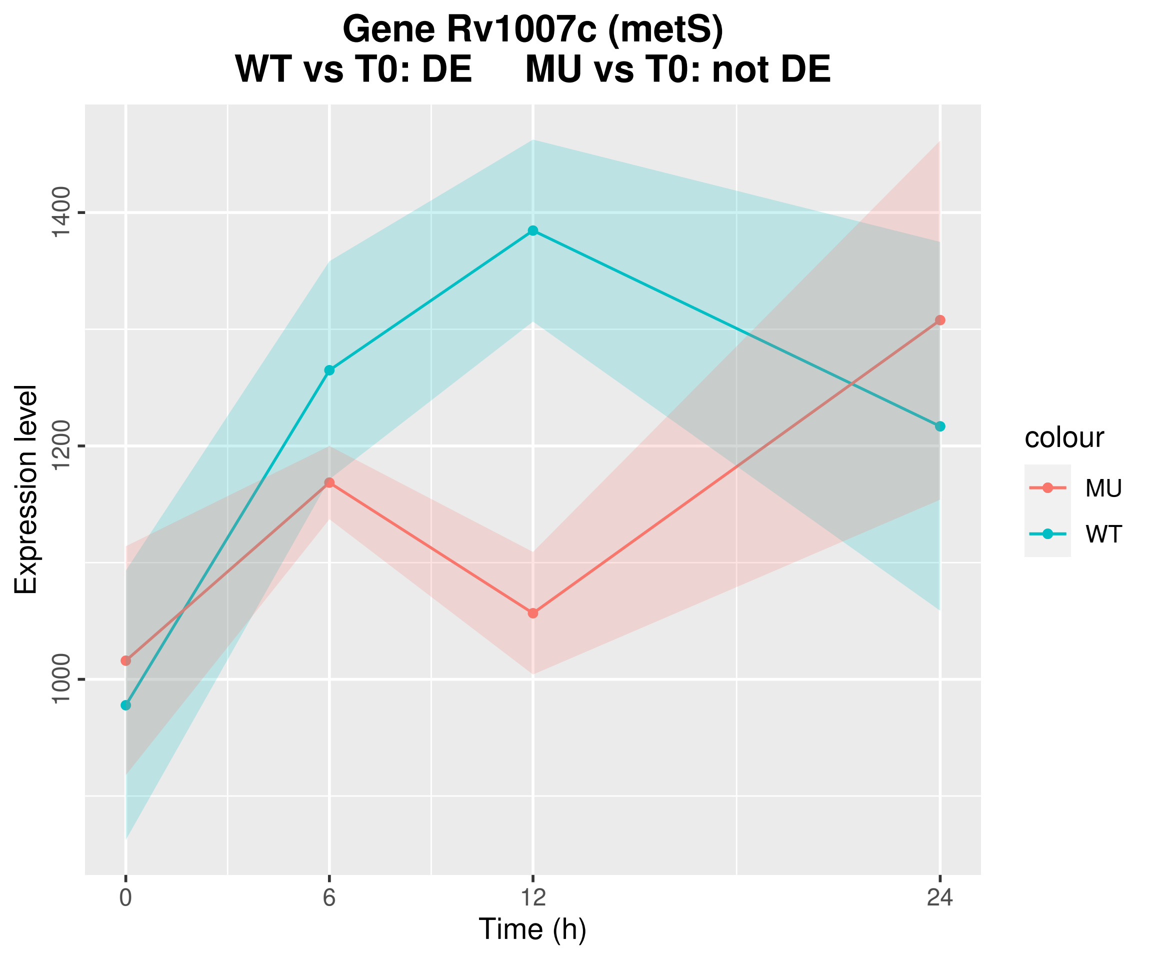

Gene Rv1007c (metS)
WT vs T0: DE   MU vs T0: not DE
Expression level
1400
1200
1000
0
6
12
24
Time (h)
colour
MU
WT

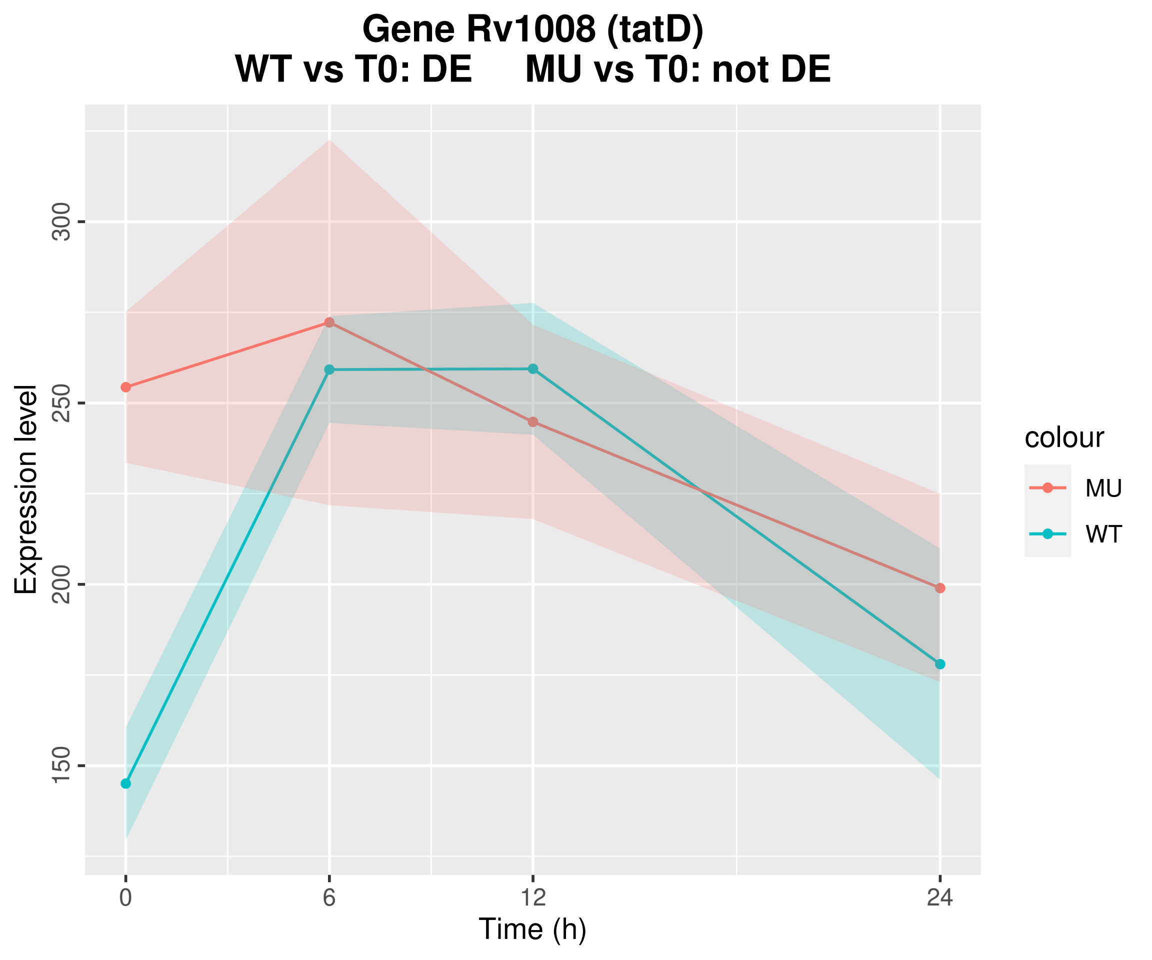

Gene Rv1008 (tatD)
WT vs T0: DE MU vs T0: not DE
Expression level
150
200
250
300
0
6
12
24
Time (h)
colour
MU
WT

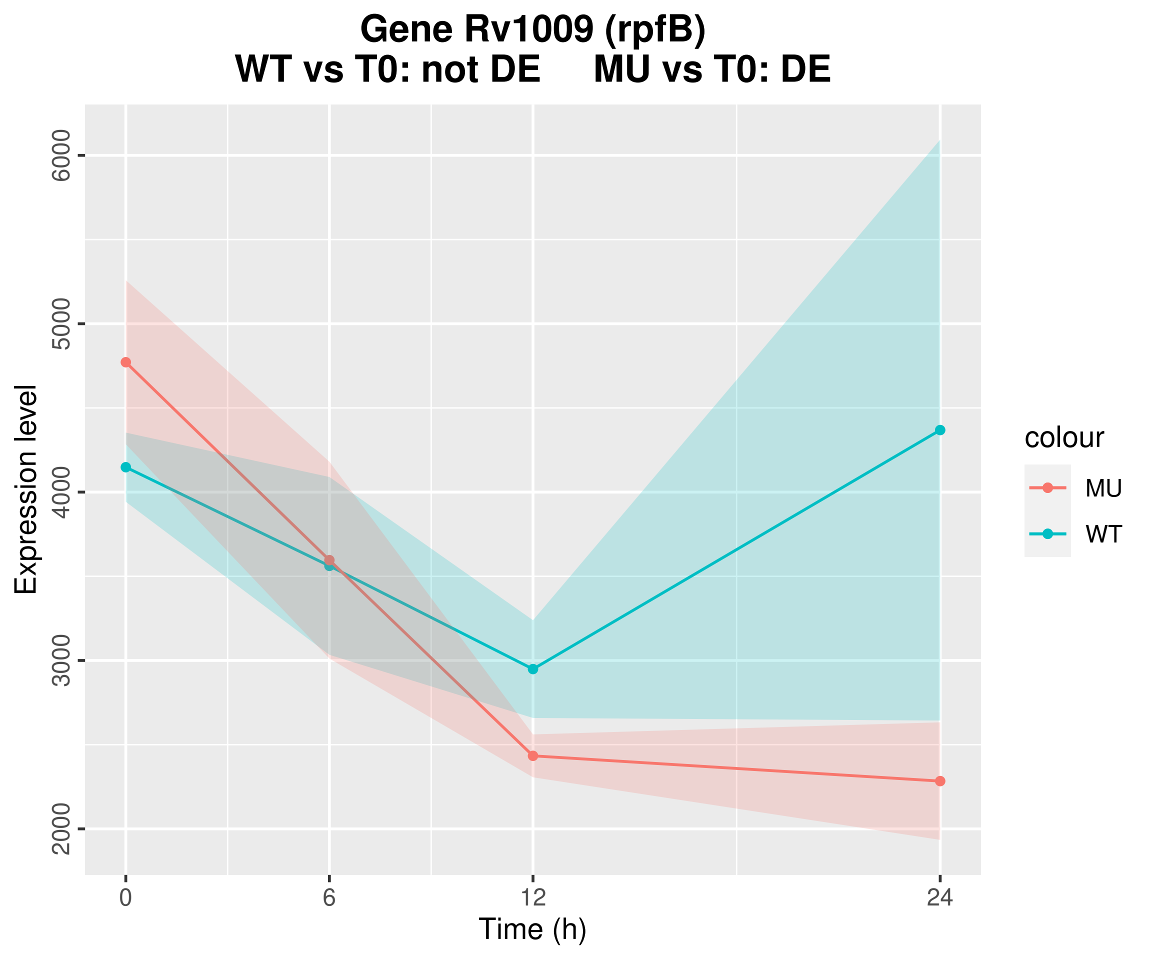

Gene Rv1009 (rpfB)
WT vs T0: not DE MU vs T0: DE
Expression level
6000
5000
4000
3000
2000
Time (h)
0
6
12
24
colour
MU
WT

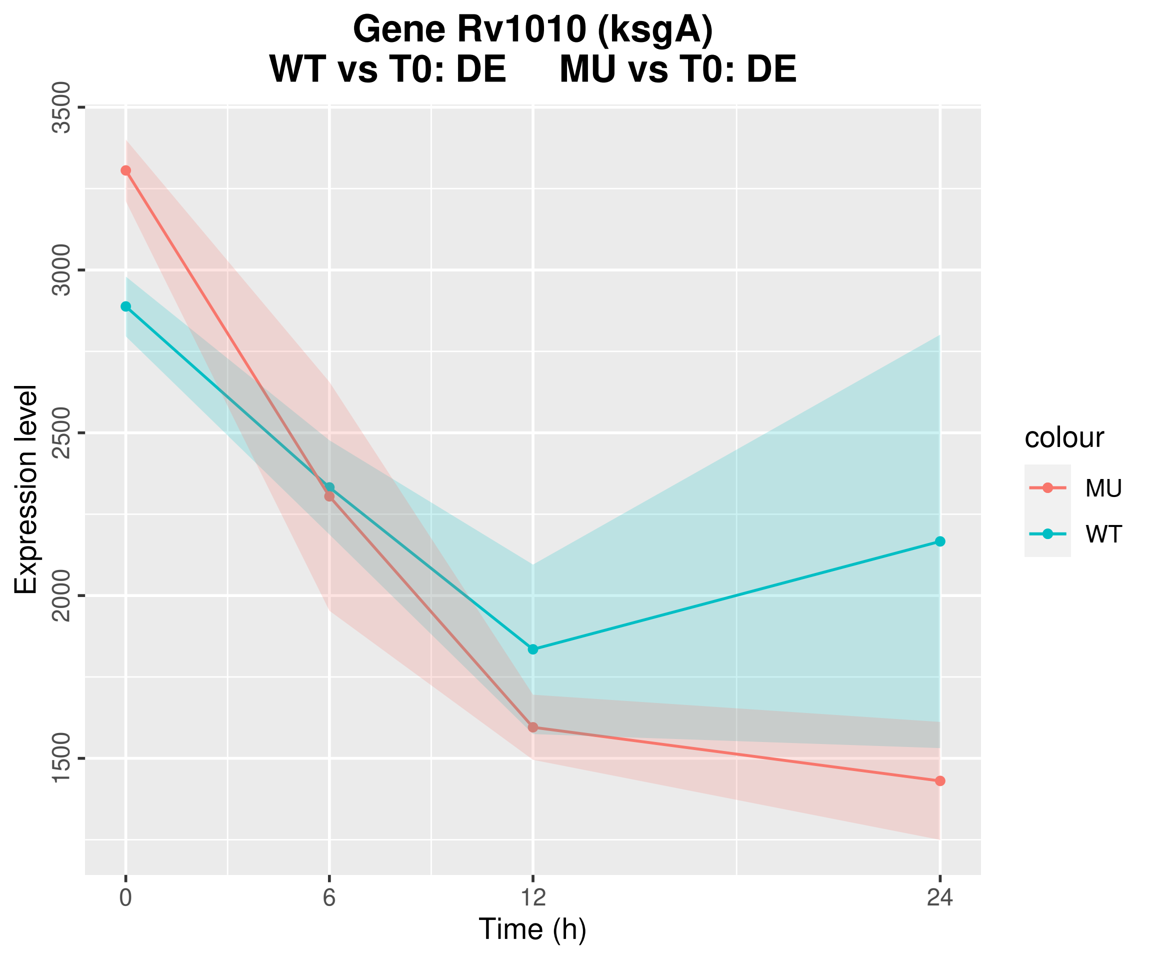
Gene Rv1010 (ksgA)
WT vs T0: DE   MU vs T0: DE
Expression level
3500
3000
2500
2000
1500
0
6
12
24
Time (h)
colour
MU
WT

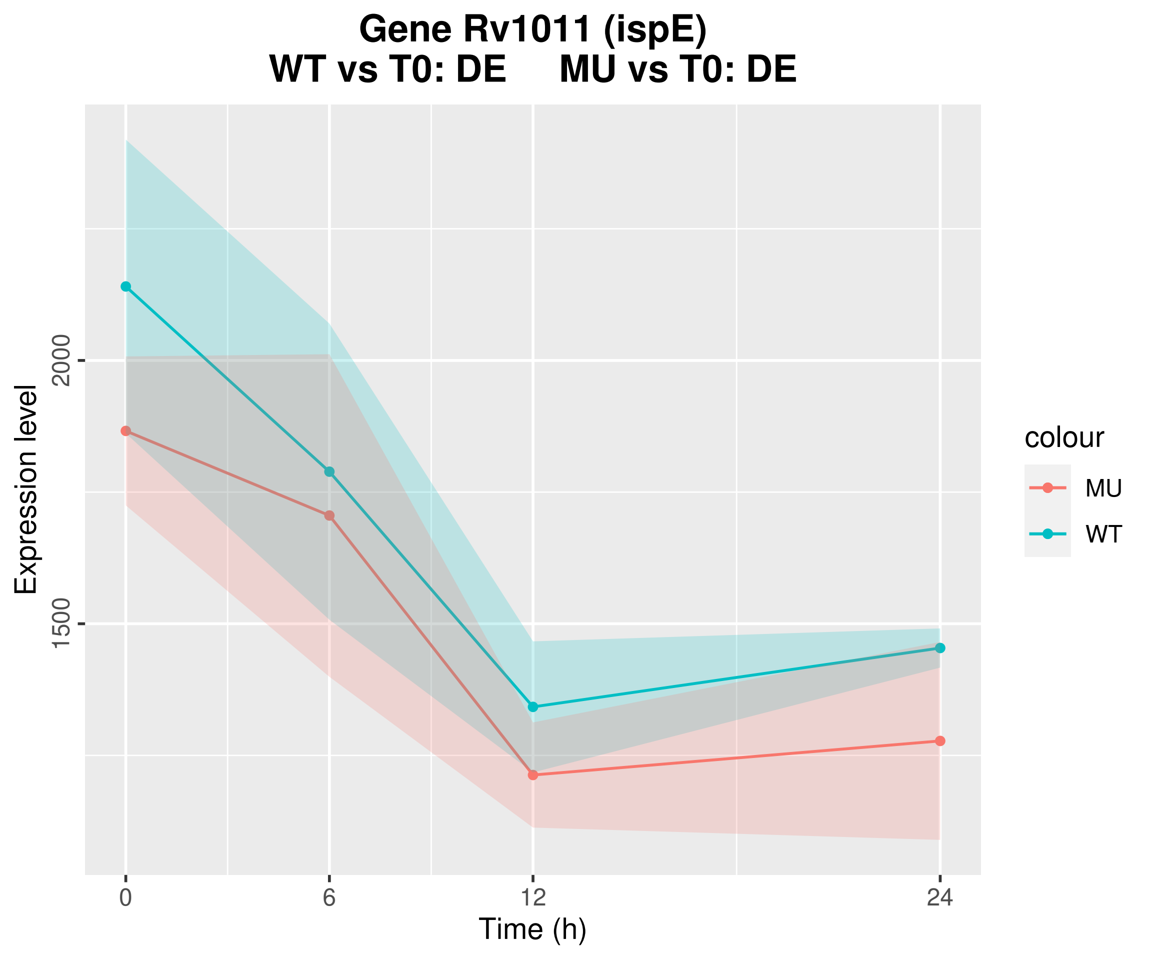
Gene Rv1011 (ispE)
WT vs T0: DE   MU vs T0: DE
Expression level
2000
1500
0
6
12
24
Time (h)
colour
MU
WT

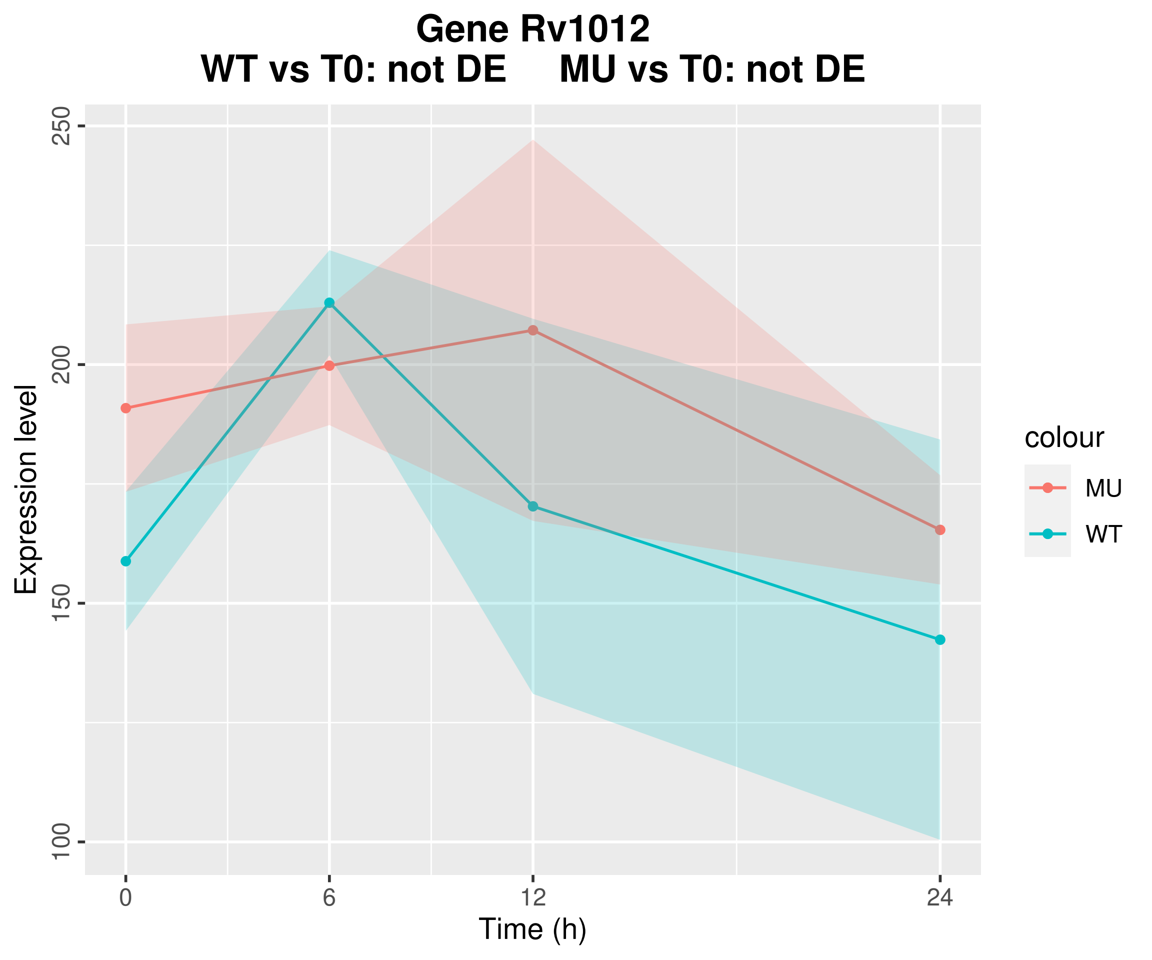

Gene Rv1012
WT vs T0: not DE MU vs T0: not DE
Expression level
250
200
150
100
0
6
12
24
Time (h)
colour
MU
WT

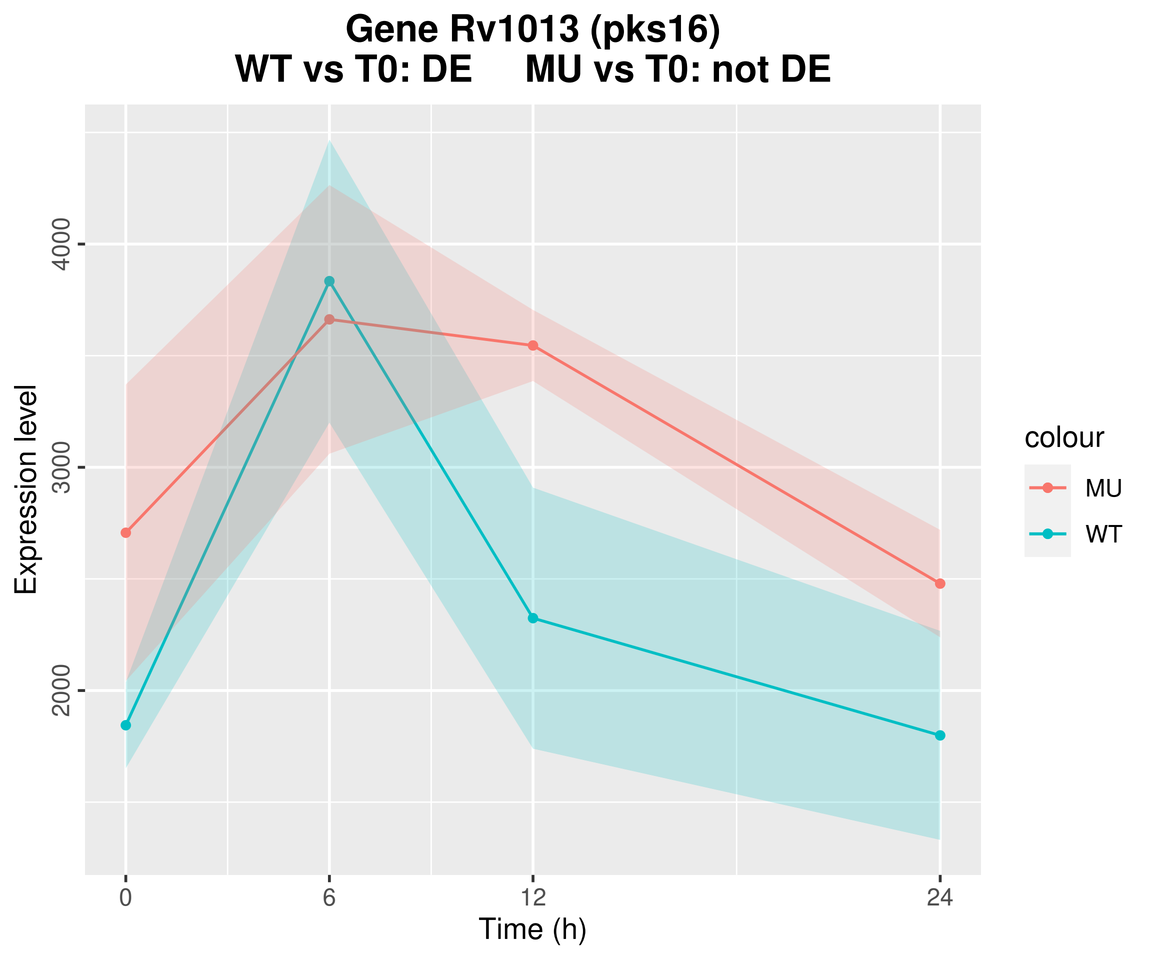
Gene Rv1013 (pks16)
WT vs T0: DE   MU vs T0: not DE
Expression level
4000
3000
2000
0
6
12
24
Time (h)
colour
MU
WT

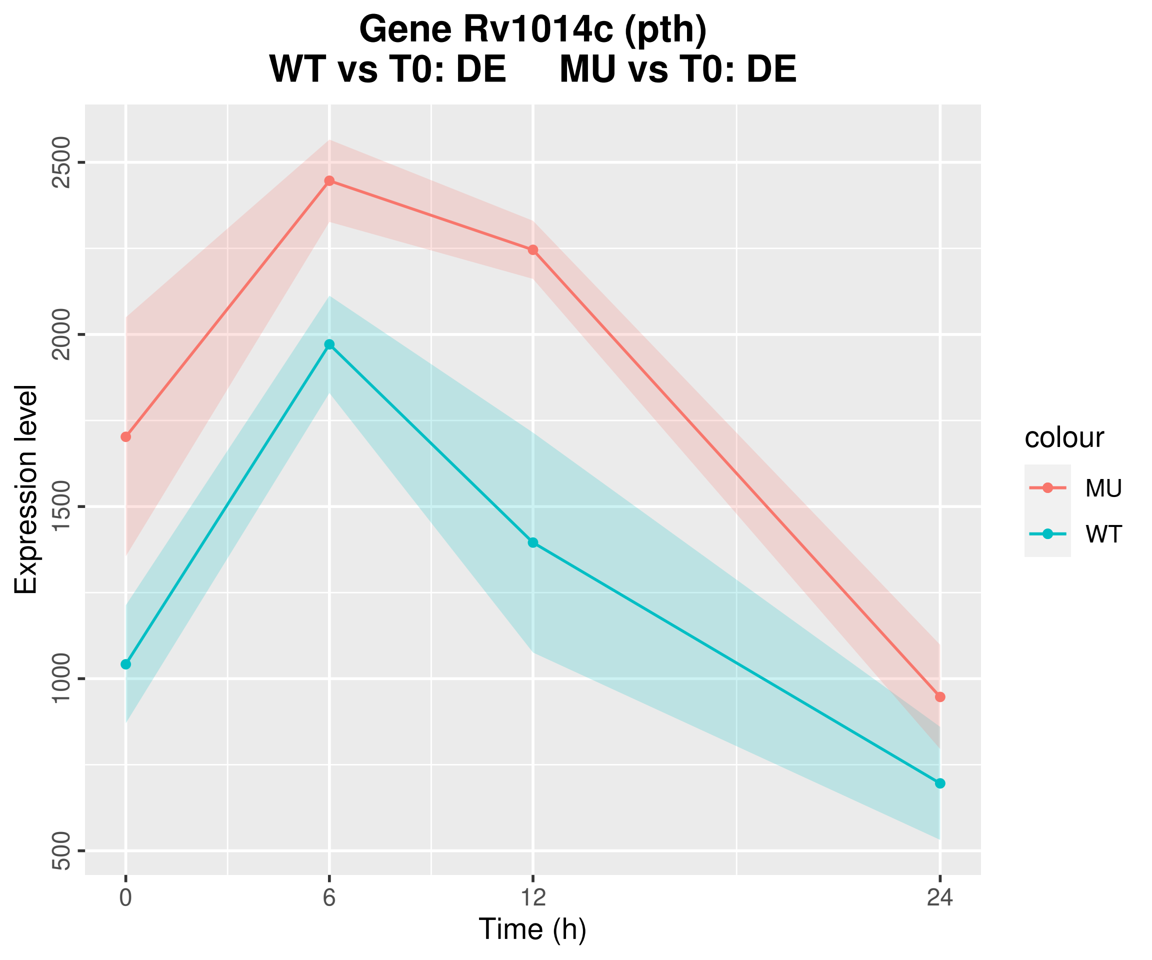
Gene Rv1014c (pth)
WT vs T0: DE   MU vs T0: DE
Expression level
2500
2000
1500
1000
500
0
6
12
24
Time (h)
colour
MU
WT

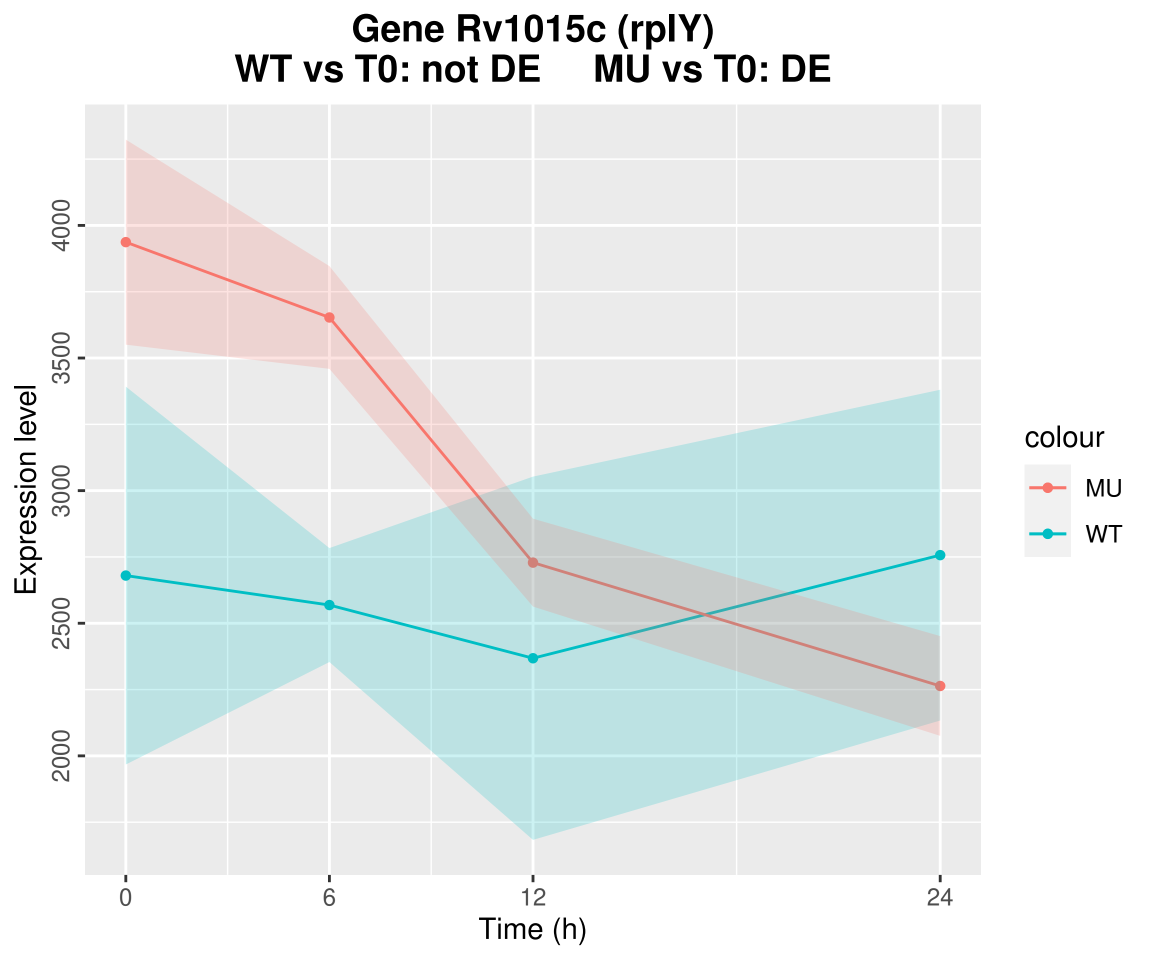

Gene Rv1015c (rplY)
WT vs T0: not DE   MU vs T0: DE
Expression level
4000
3500
3000
2500
2000
0
6
12
24
Time (h)
colour
MU
WT

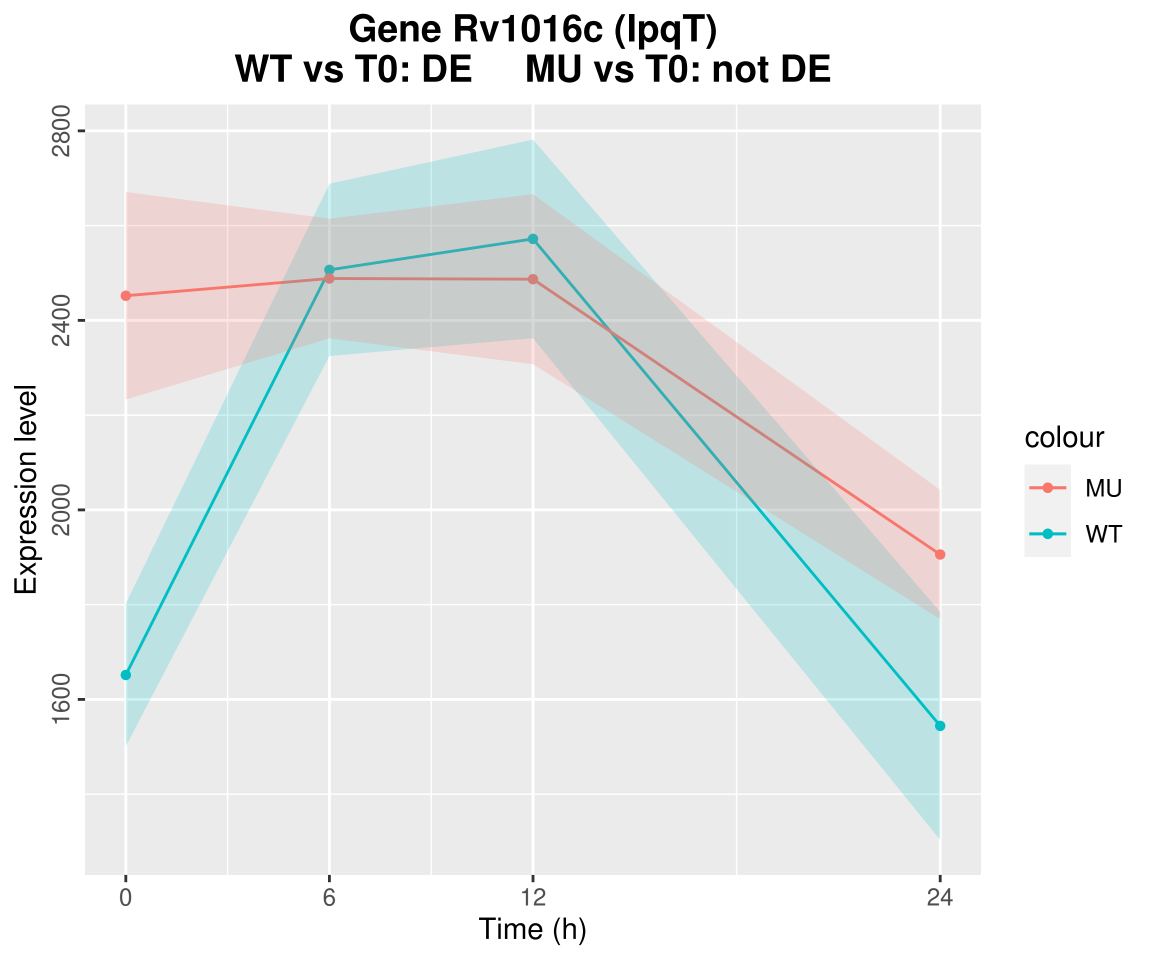

Gene Rv1016c (lpqT)
WT vs T0: DE   MU vs T0: not DE
2800
2400
2000
1600
Expression level
0
6
12
24
Time (h)
colour
MU
WT

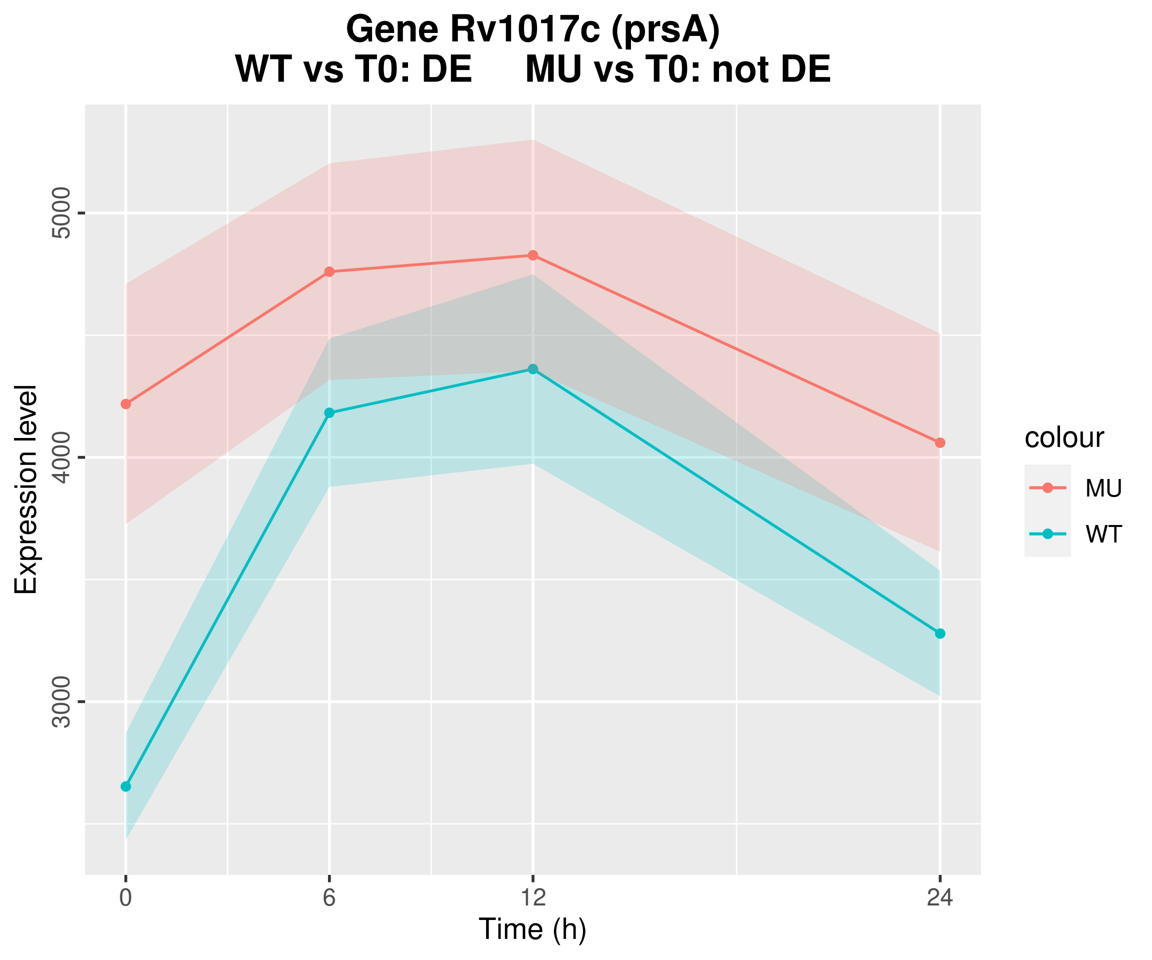
Gene Rv1017c (prsA)
WT vs T0: DE   MU vs T0: not DE
Expression level
5000
4000
3000
Time (h)
0
6
12
24
colour
MU
WT

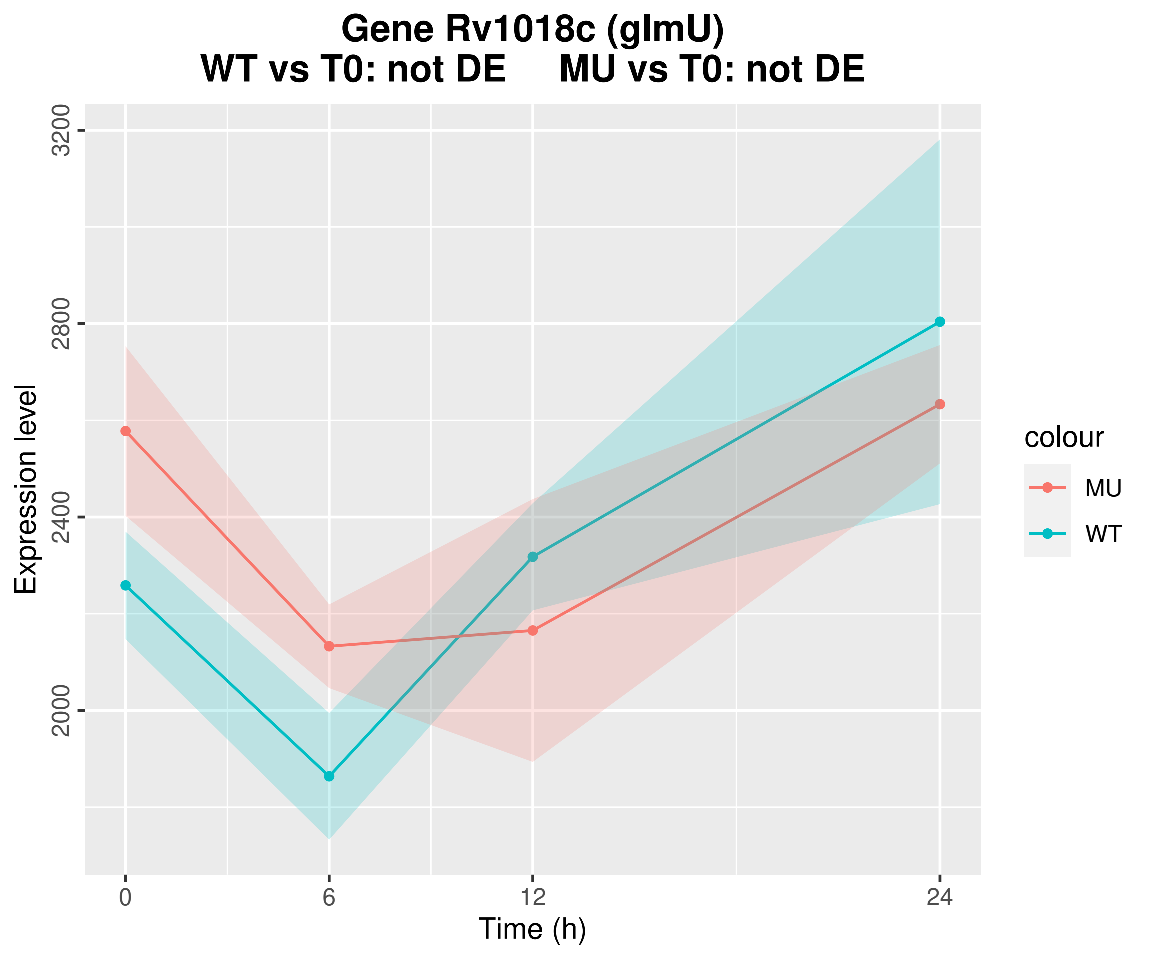

Gene Rv1018c (glmU)
WT vs T0: not DE MU vs T0: not DE
Expression level
3200
2800
2400
2000
0
6
12
24
Time (h)
colour
MU
WT

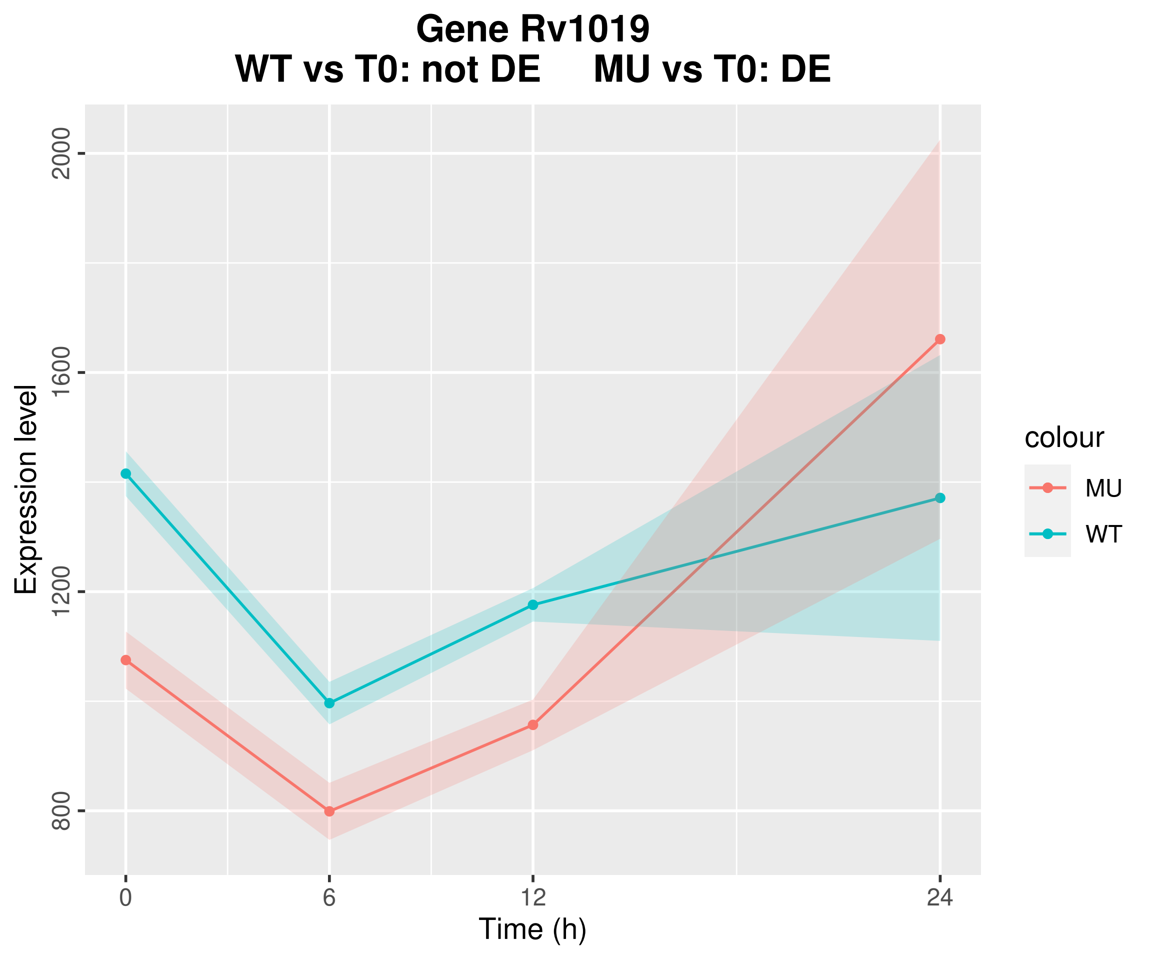

Gene Rv1019
WT vs T0: not DE MU vs T0: DE
Expression level
2000
1600
1200
800
0
6
12
24
Time (h)
colour
MU
WT

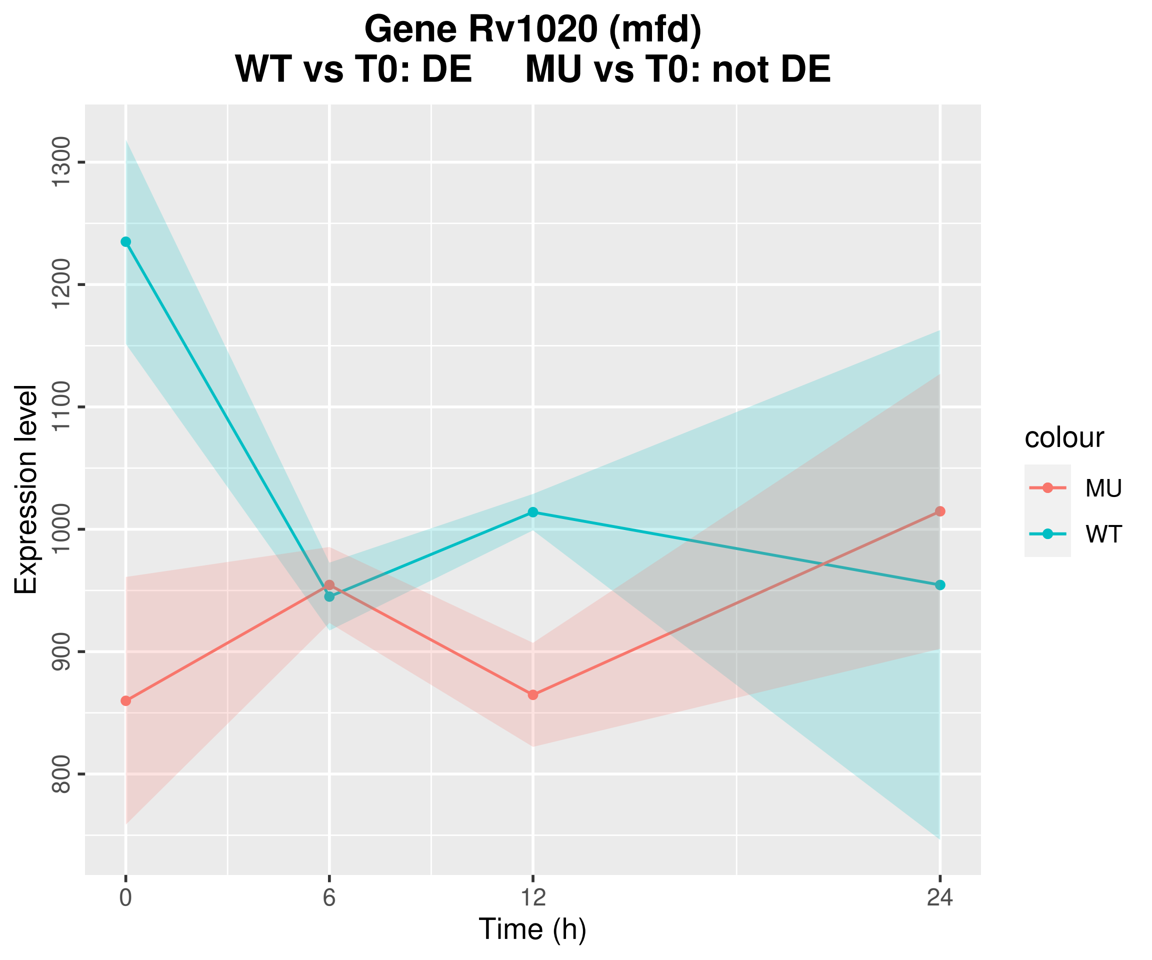

Gene Rv1020 (mfd)
WT vs T0: DE   MU vs T0: not DE
Expression level
1300
1200
1100
1000
900
800
0
6
12
24
Time (h)
colour
MU
WT

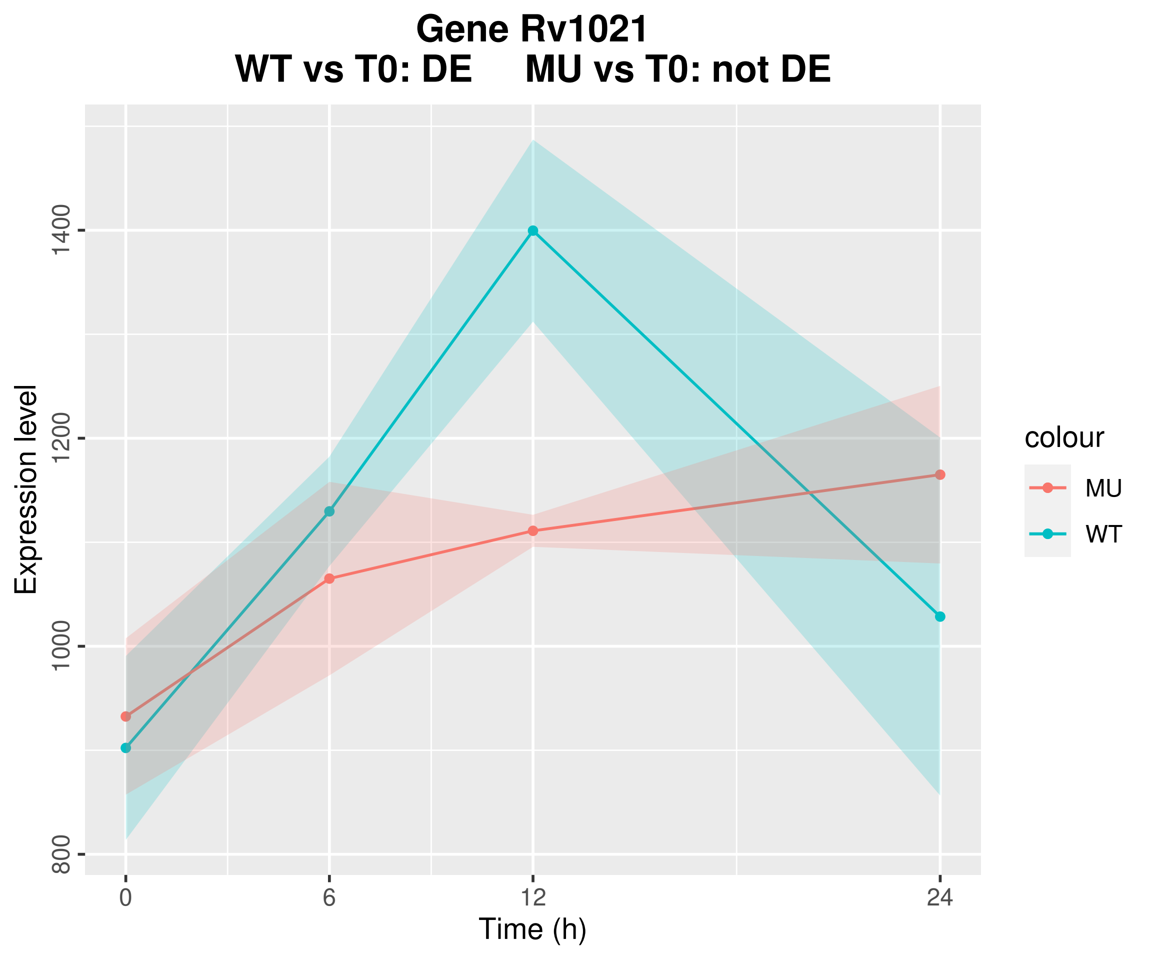

Gene Rv1021
WT vs T0: DE   MU vs T0: not DE
Expression level
800
1000
1200
1400
0
6
12
24
Time (h)
colour
MU
WT

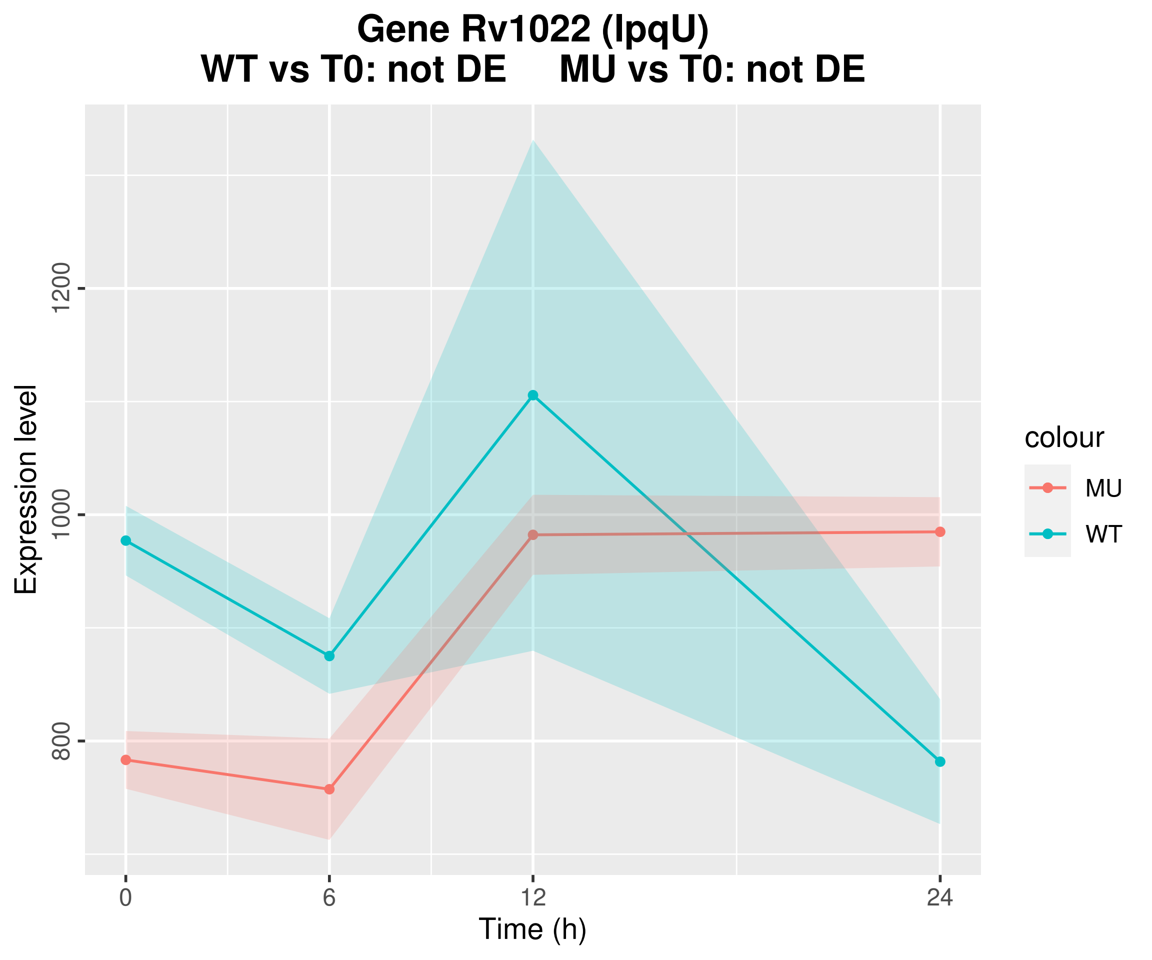

Gene Rv1022 (lpqU)
WT vs T0: not DE   MU vs T0: not DE
Expression level
1200
1000
800
0
6
12
24
Time (h)
colour
MU
WT

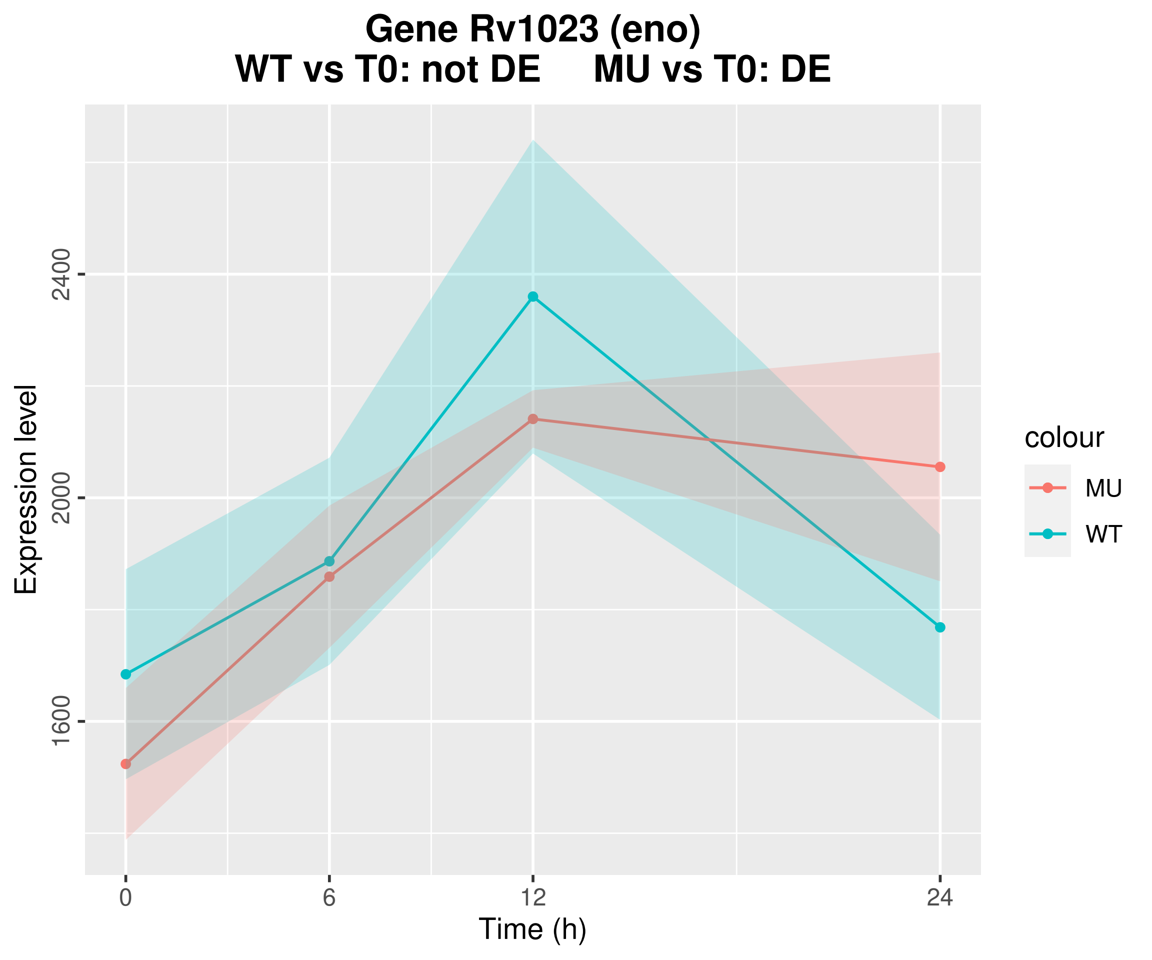
Gene Rv1023 (eno)
WT vs T0: not DE   MU vs T0: DE
Expression level
2400
2000
1600
Time (h)
0
6
12
24
colour
MU
WT

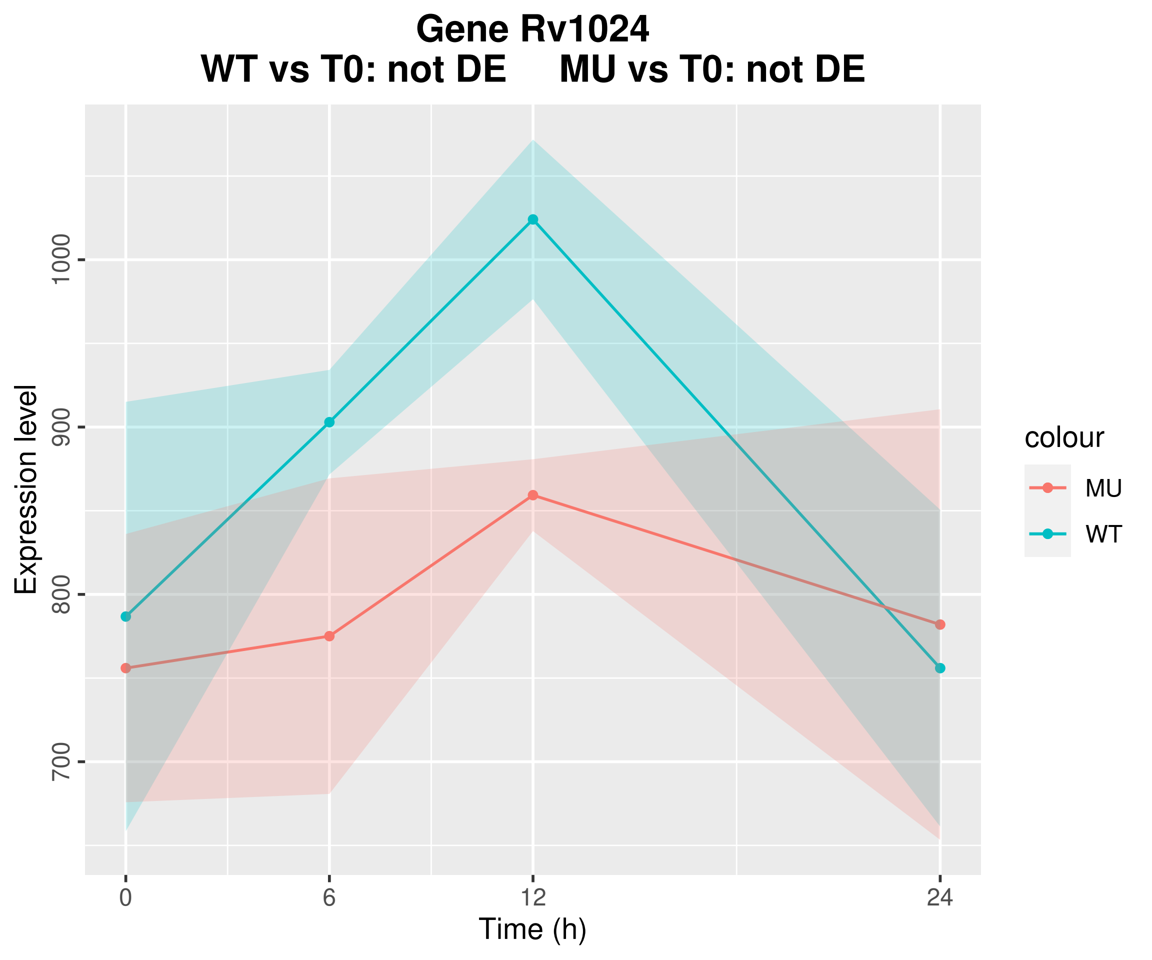

Gene Rv1024
WT vs T0: not DE MU vs T0: not DE
Expression level
700
800
900
1000
Time (h)
0
6
12
24
colour
MU
WT

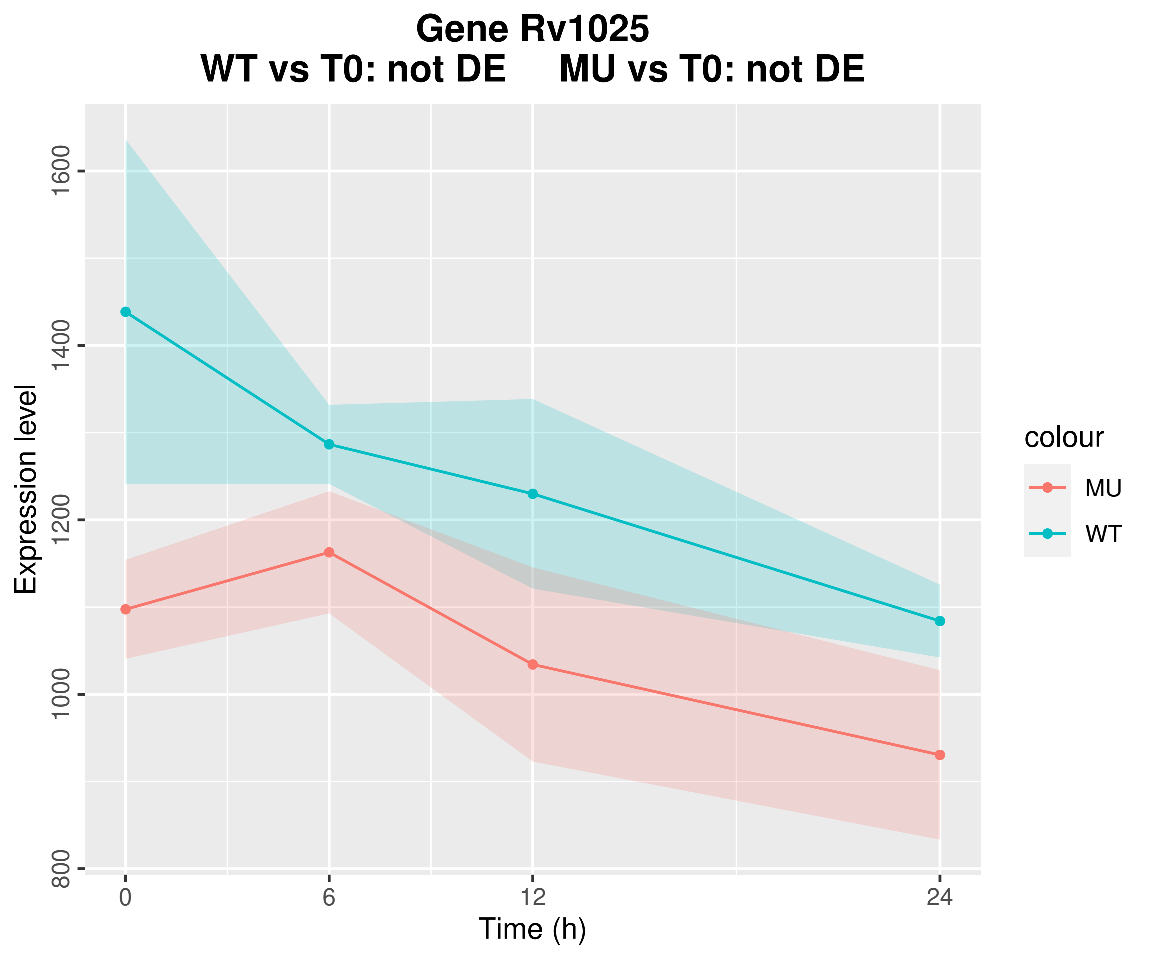
Gene Rv1025
WT vs T0: not DE   MU vs T0: not DE
Expression level
1600
1400
1200
1000
800
0
6
12
24
Time (h)
colour
MU
WT

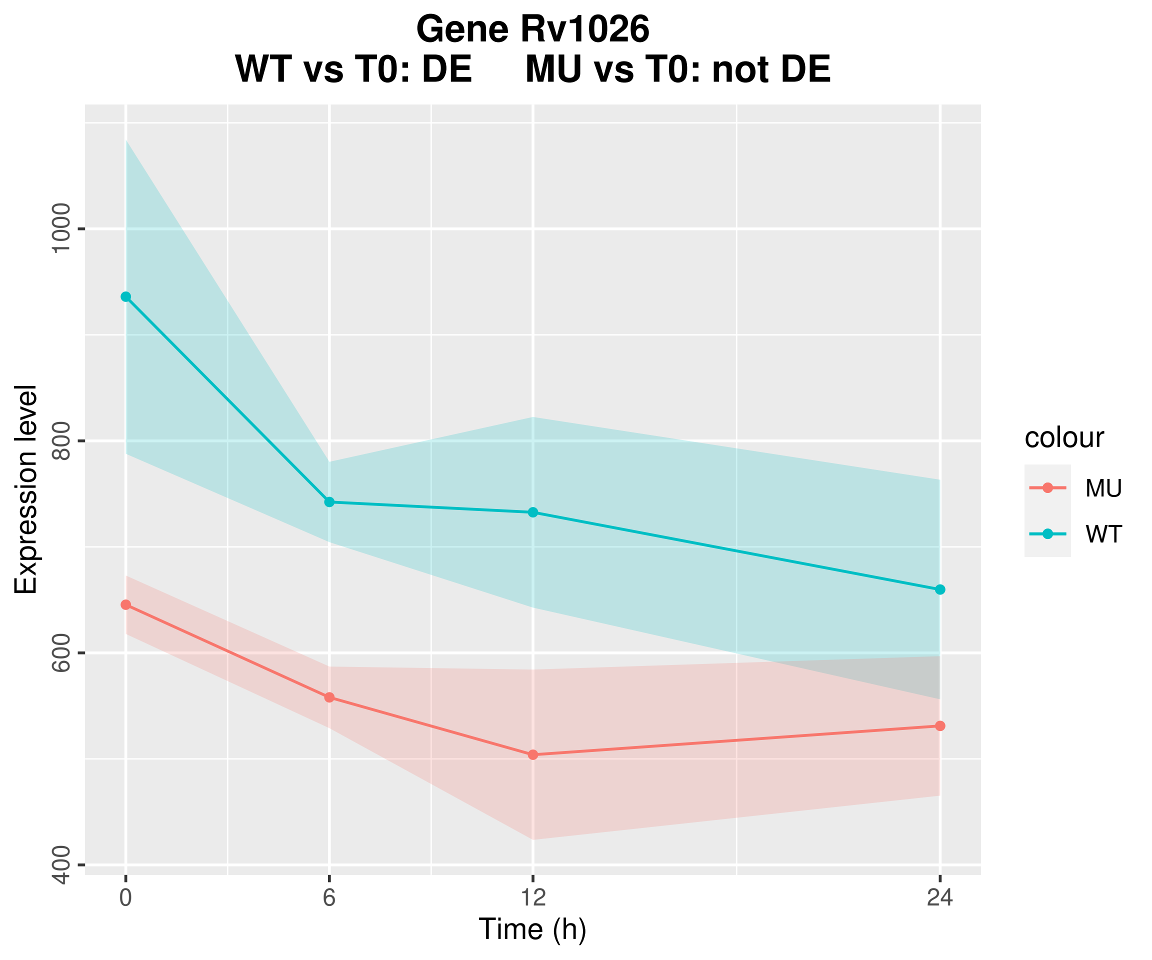
Gene Rv1026
WT vs T0: DE   MU vs T0: not DE
Expression level
1000
800
600
400
0
6
12
24
Time (h)
colour
MU
WT

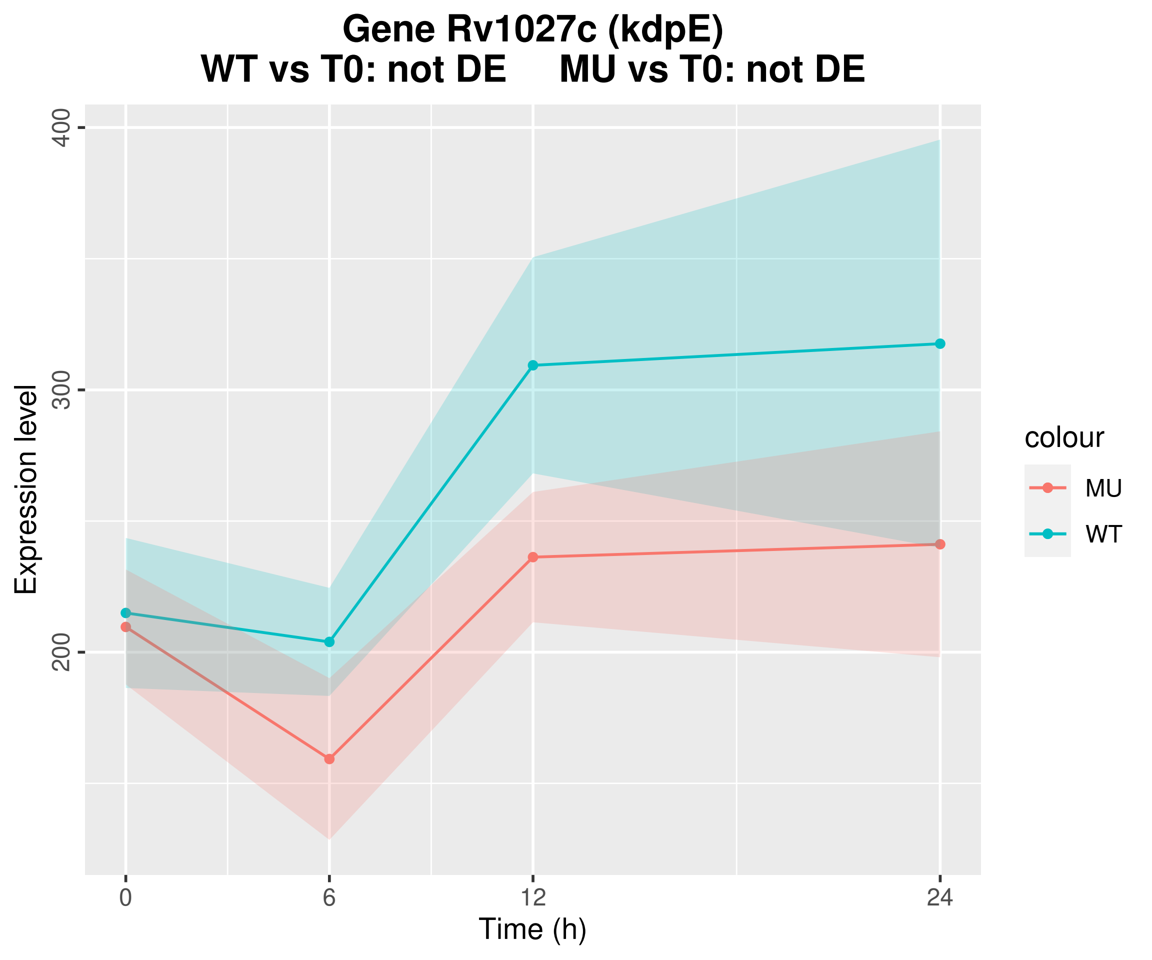
Gene Rv1027c (kdpE)
WT vs T0: not DE MU vs T0: not DE
Expression level
400
300
200
0
6
12
24
Time (h)
colour
MU
WT

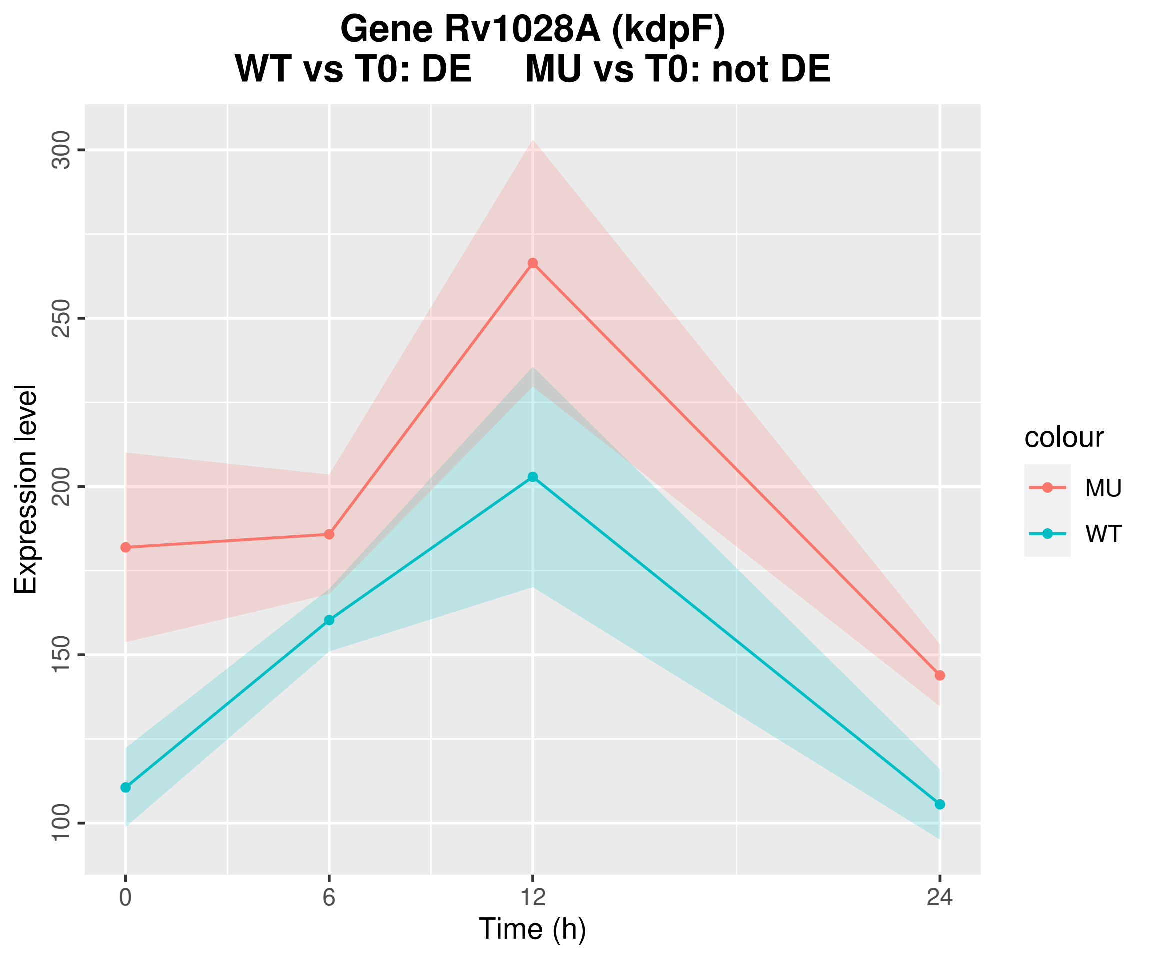

Gene Rv1028A (kdpF)
WT vs T0: DE   MU vs T0: not DE
Expression level
300
250
200
150
100
0
6
12
24
Time (h)
colour
MU
WT

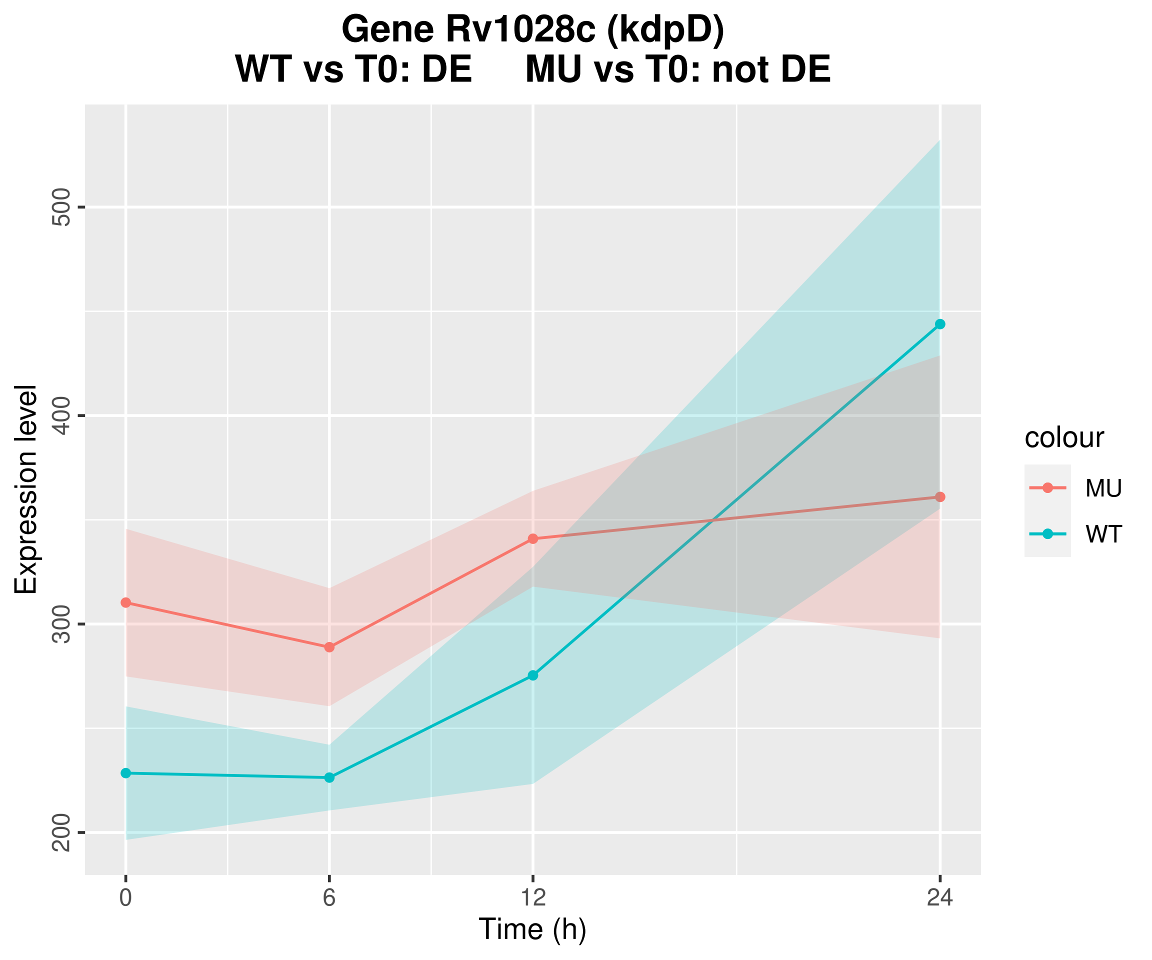
Gene Rv1028c (kdpD)
WT vs T0: DE   MU vs T0: not DE
Expression level
500
400
300
200
0
6
12
24
Time (h)
colour
MU
WT

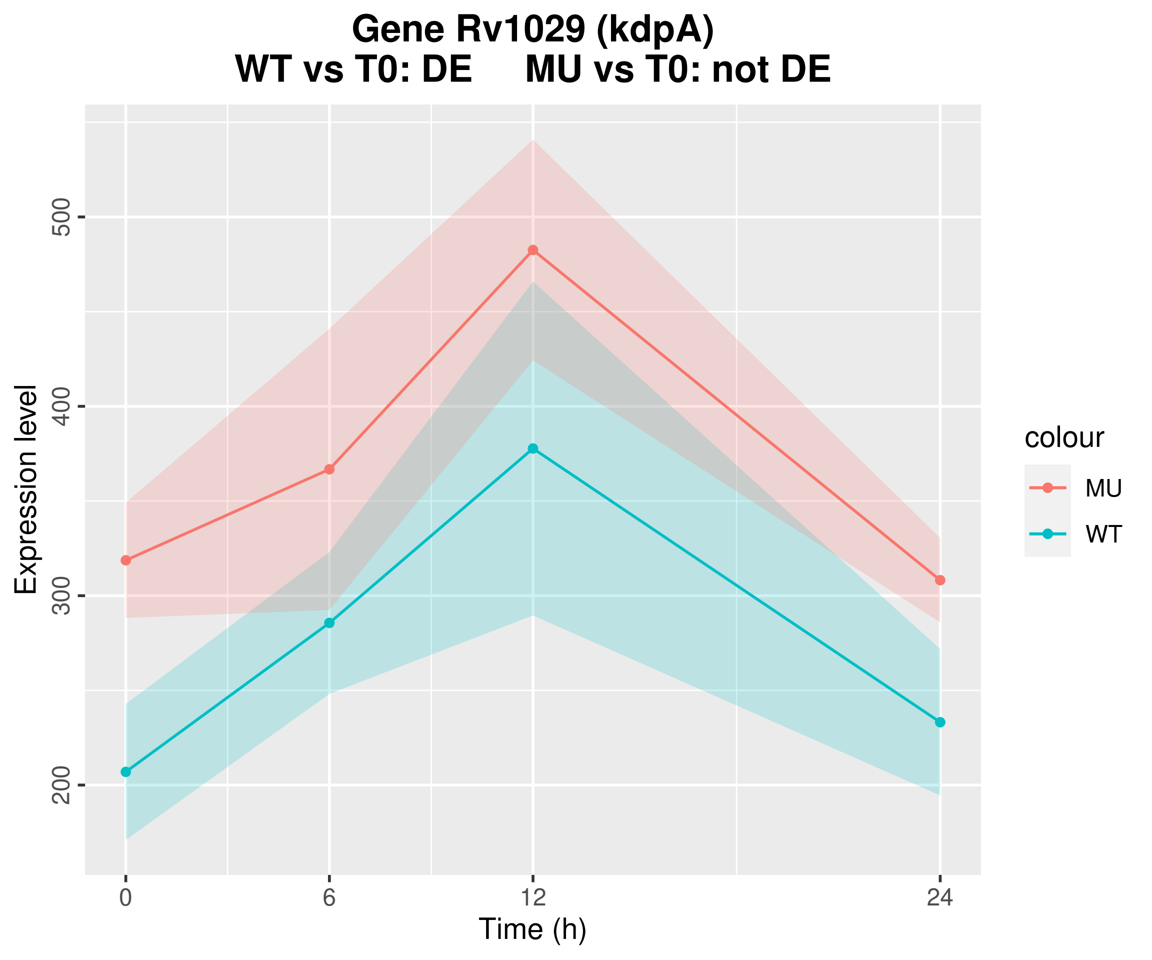
Gene Rv1029 (kdpA)
WT vs T0: DE   MU vs T0: not DE
Expression level
200
300
400
500
0
6
12
24
Time (h)
colour
MU
WT

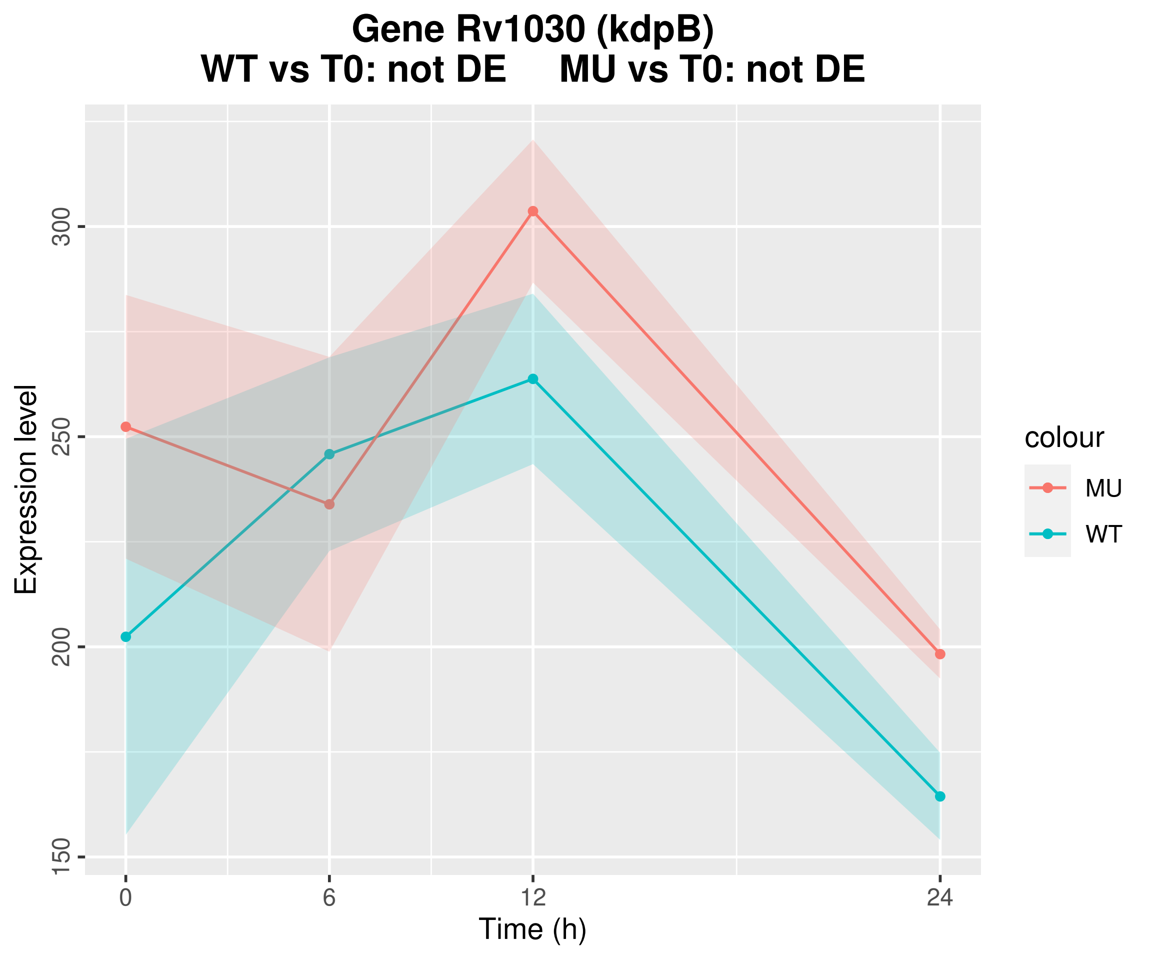

Gene Rv1030 (kdpB)
WT vs T0: not DE MU vs T0: not DE
Expression level
300
250
200
150
0
6
12
24
Time (h)
colour
MU
WT

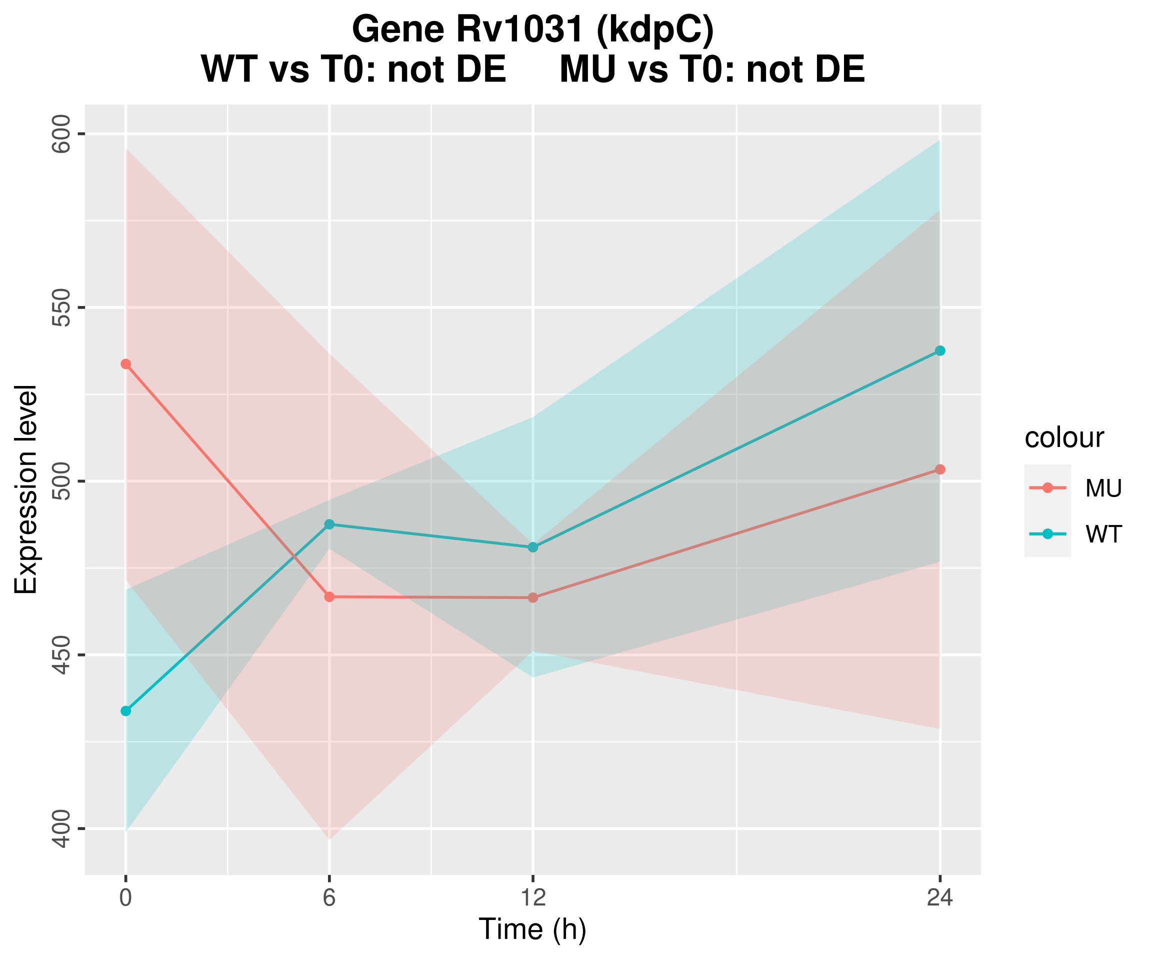
Gene Rv1031 (kdpC)
WT vs T0: not DE MU vs T0: not DE
600
550
500
450
400
Expression level
0
6
12
24
Time (h)
colour
MU
WT

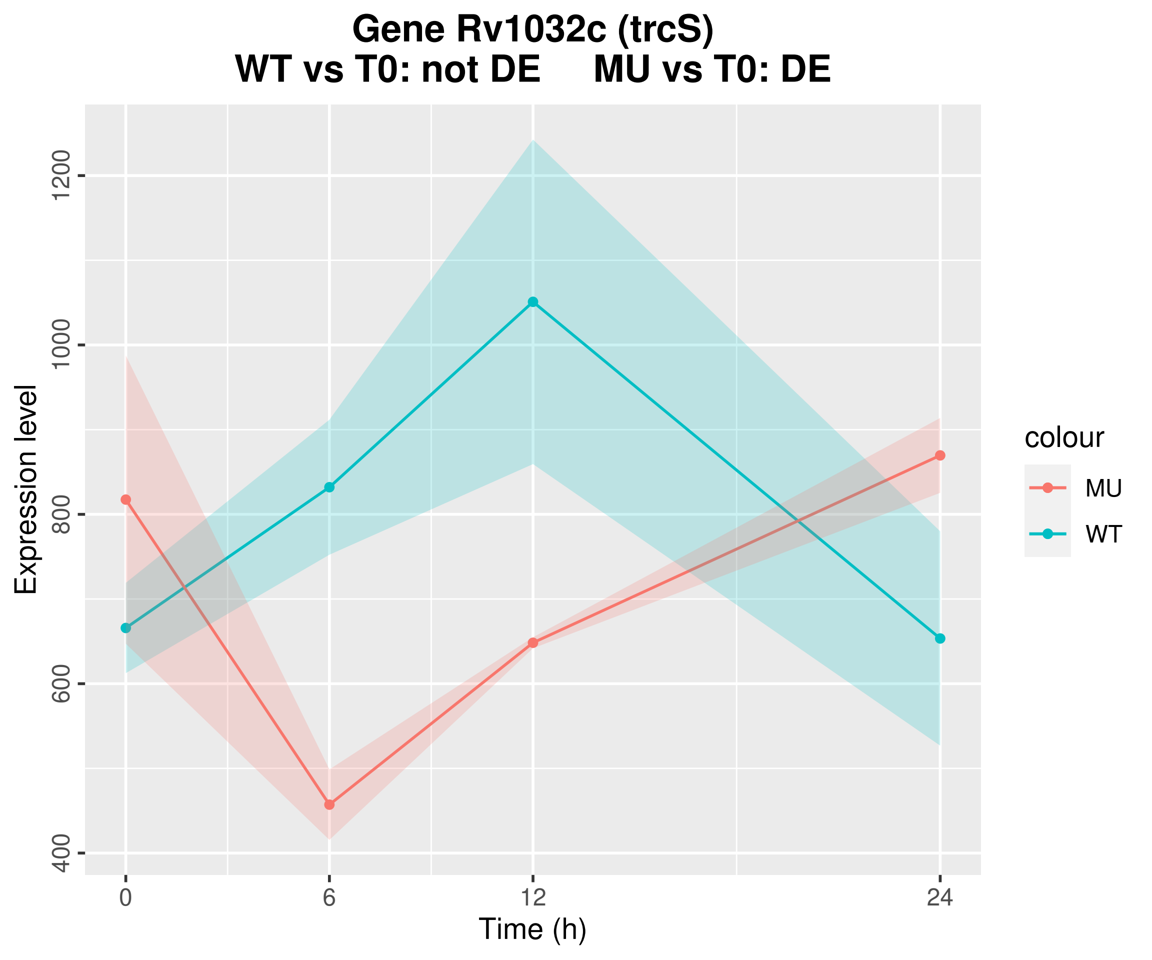
Gene Rv1032c (trcS)
WT vs T0: not DE MU vs T0: DE
Expression level
1200
1000
800
600
400
0
6
12
24
Time (h)
colour
MU
WT

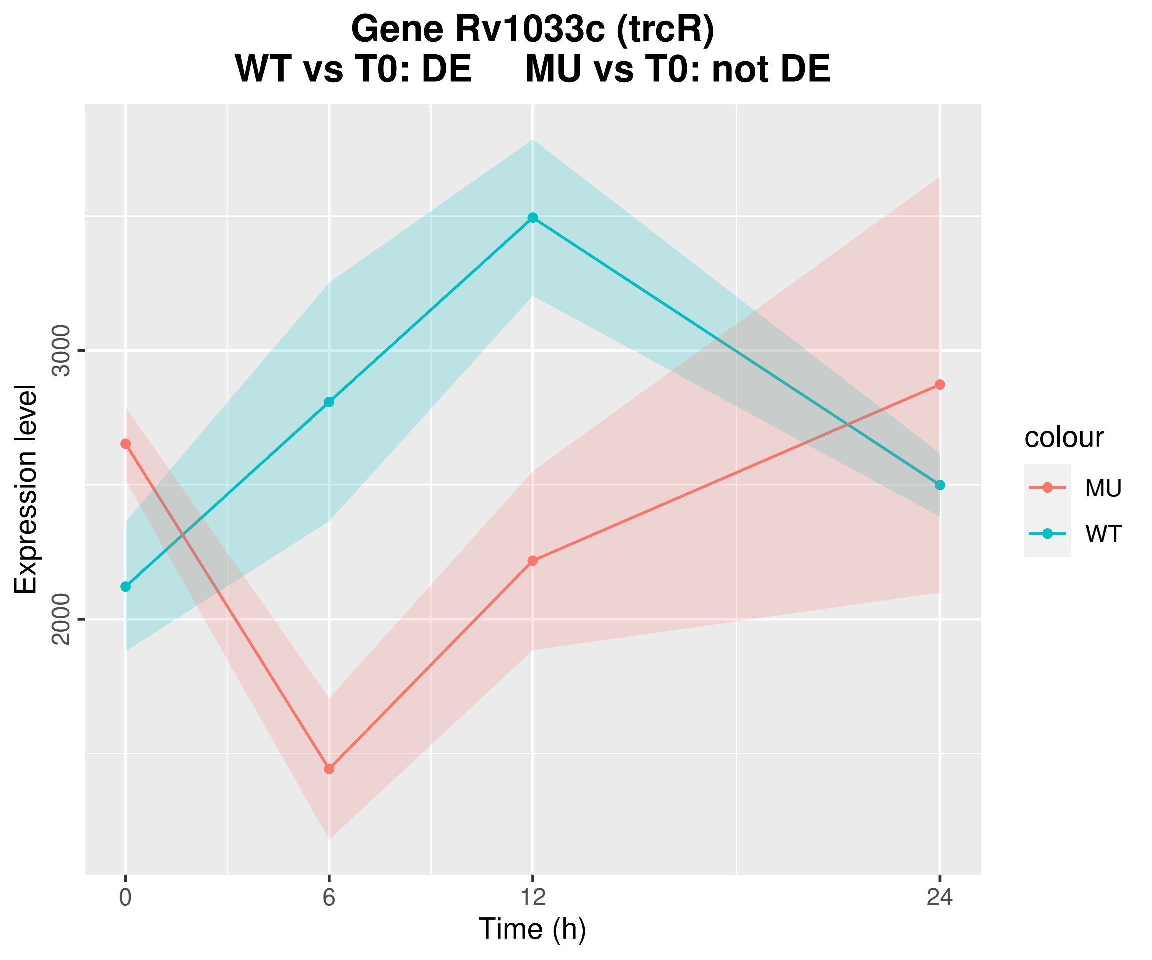
Gene Rv1033c (trcR)
WT vs T0: DE   MU vs T0: not DE
Expression level
3000
2000
Time (h)
0
6
12
24
colour
MU
WT

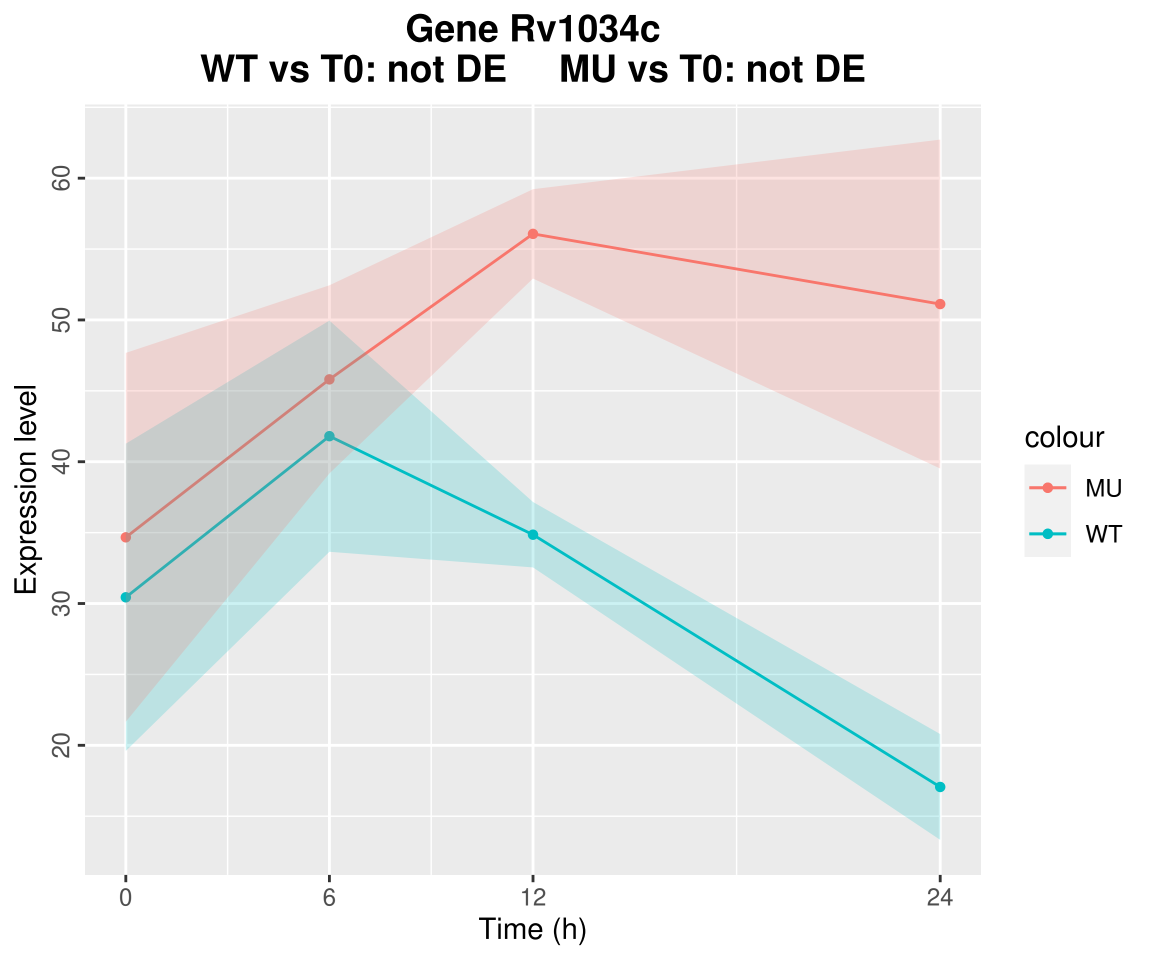
Gene Rv1034c
WT vs T0: not DE MU vs T0: not DE
Expression level
60
50
40
30
20
0
6
12
24
Time (h)
colour
MU
WT

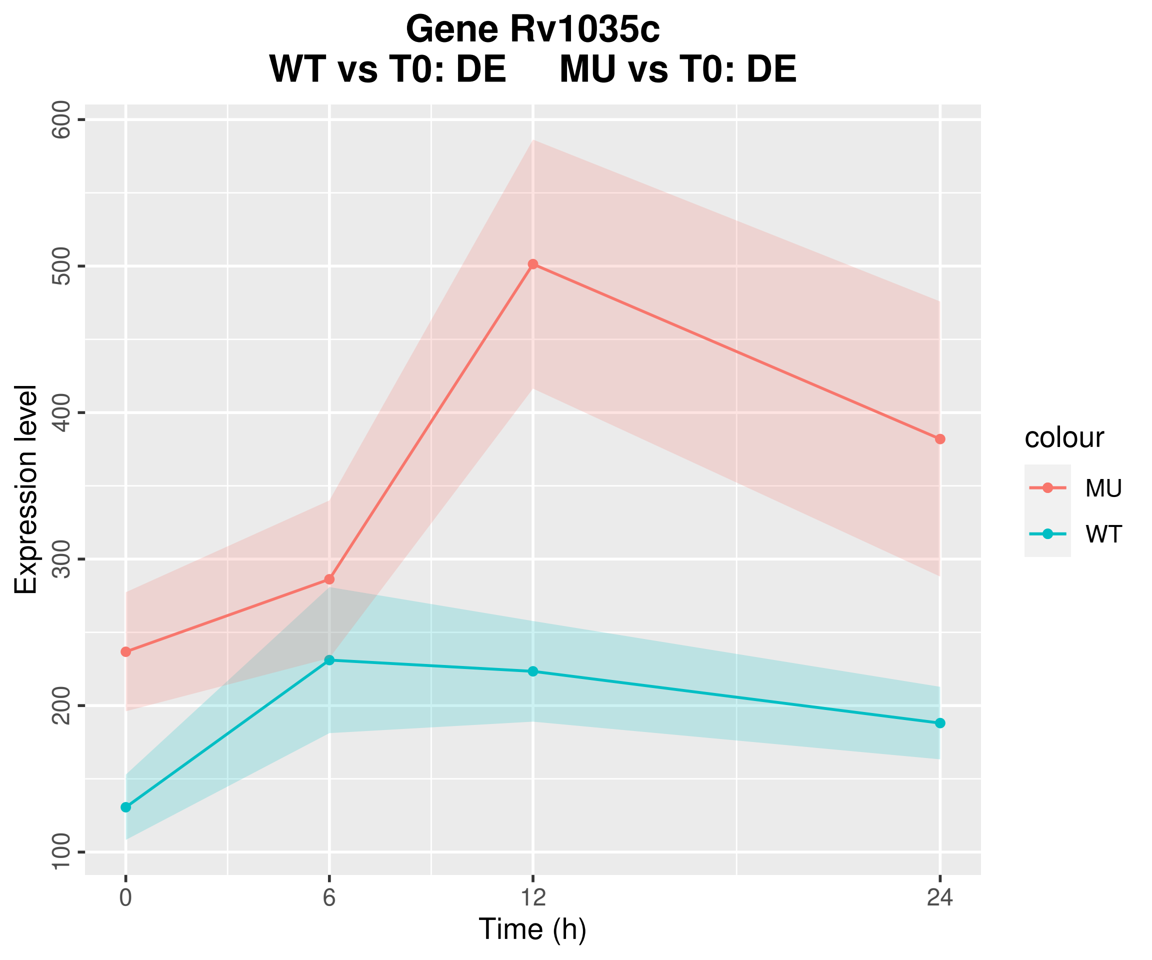

Gene Rv1035c
WT vs T0: DE MU vs T0: DE
Expression level
100
200
300
400
500
600
0
6
12
24
Time (h)
colour
MU
WT

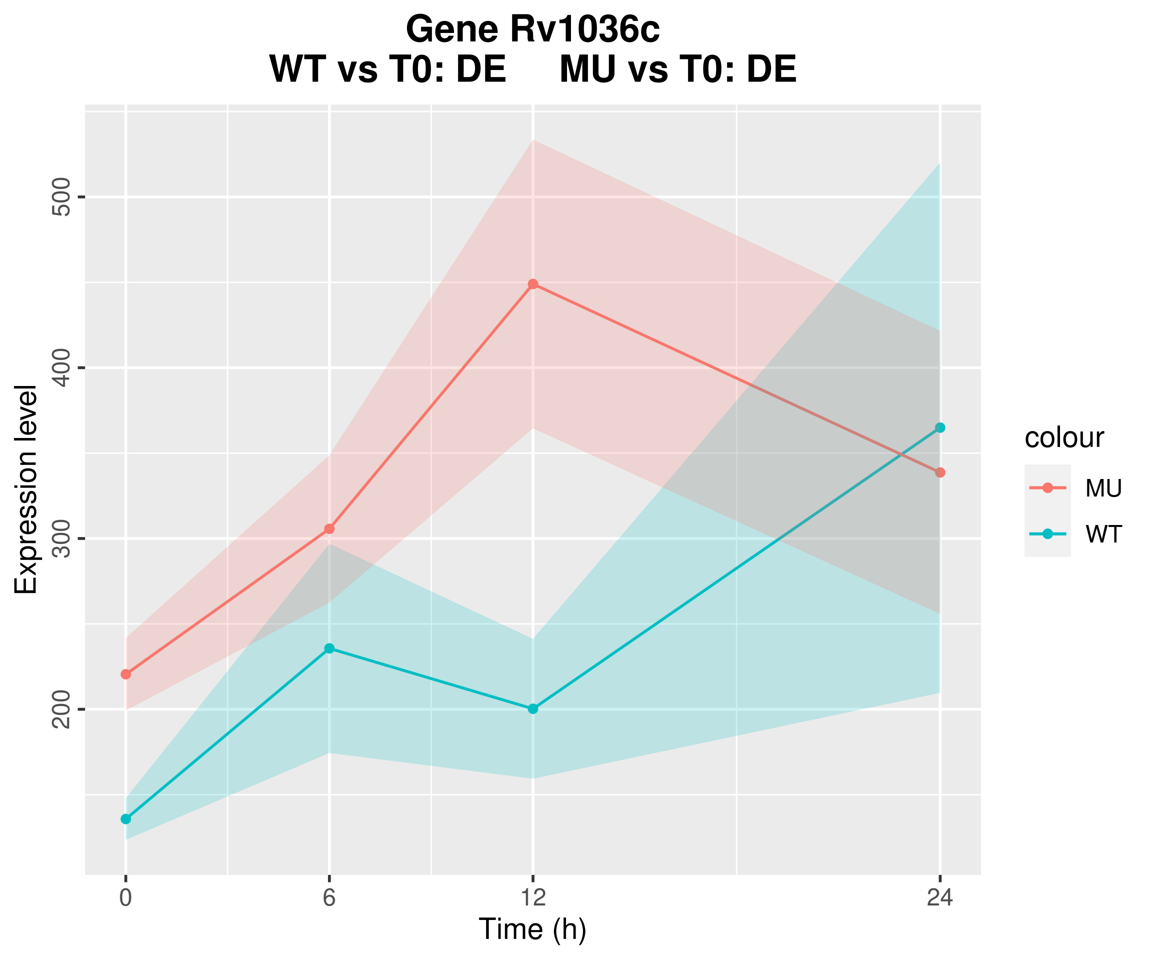

Gene Rv1036c
WT vs T0: DE   MU vs T0: DE
Expression level
500
400
300
200
0
6
12
24
Time (h)
colour
MU
WT

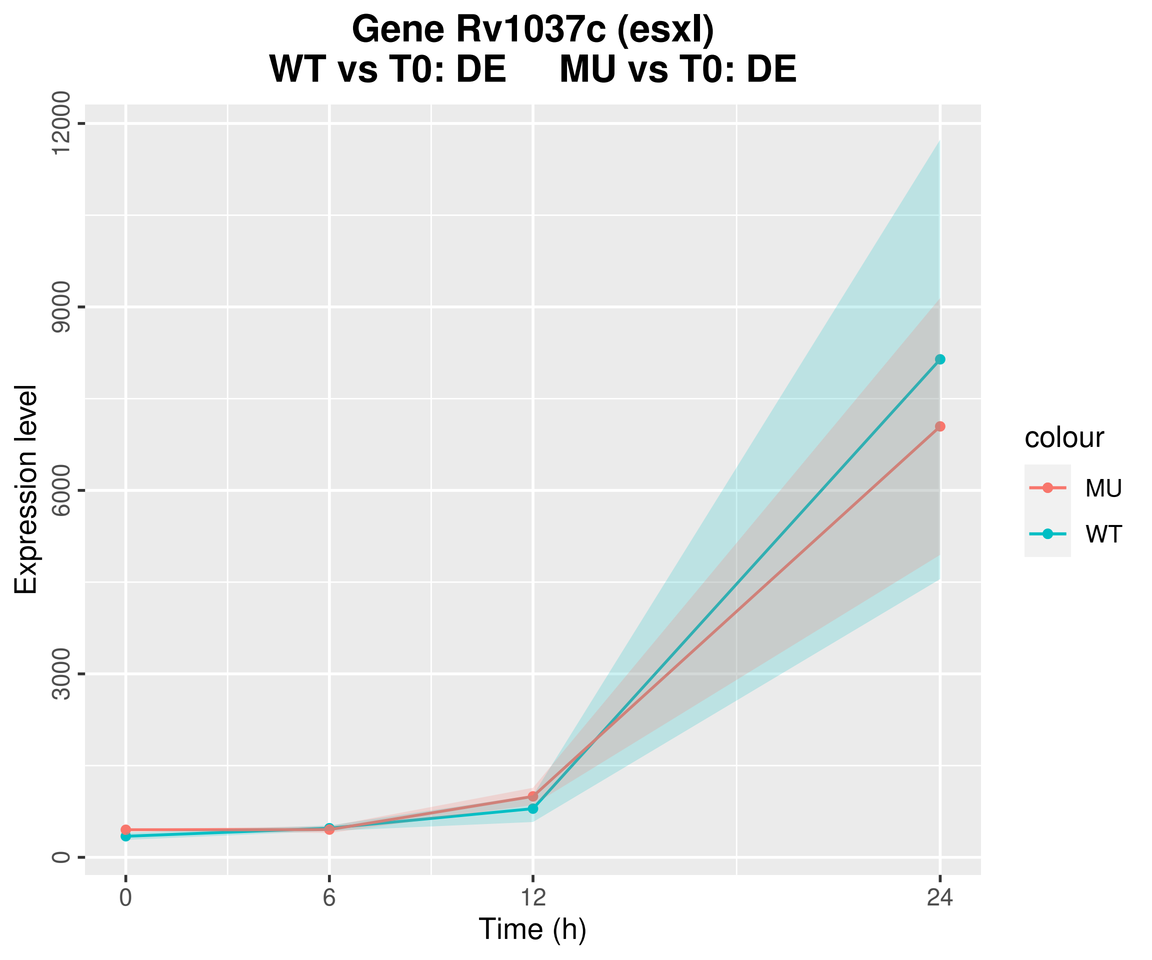

Gene Rv1037c (esxI)
WT vs T0: DE   MU vs T0: DE
Expression level
Time (h)
0
3000
6000
9000
12000
0
6
12
24
colour
MU
WT

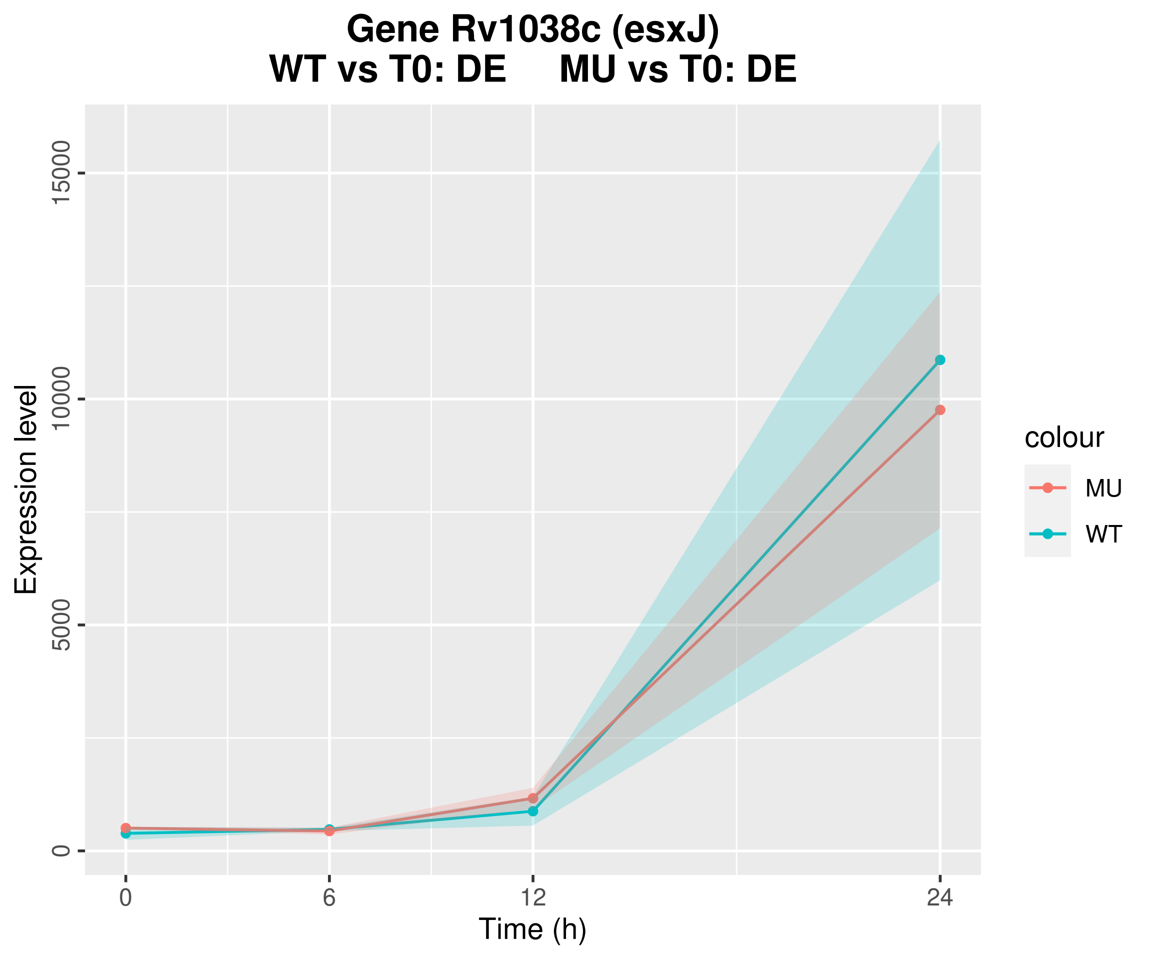
Gene Rv1038c (esxJ)
WT vs T0: DE MU vs T0: DE
Expression level
15000
10000
5000
0
0
6
12
24
Time (h)
colour
MU
WT

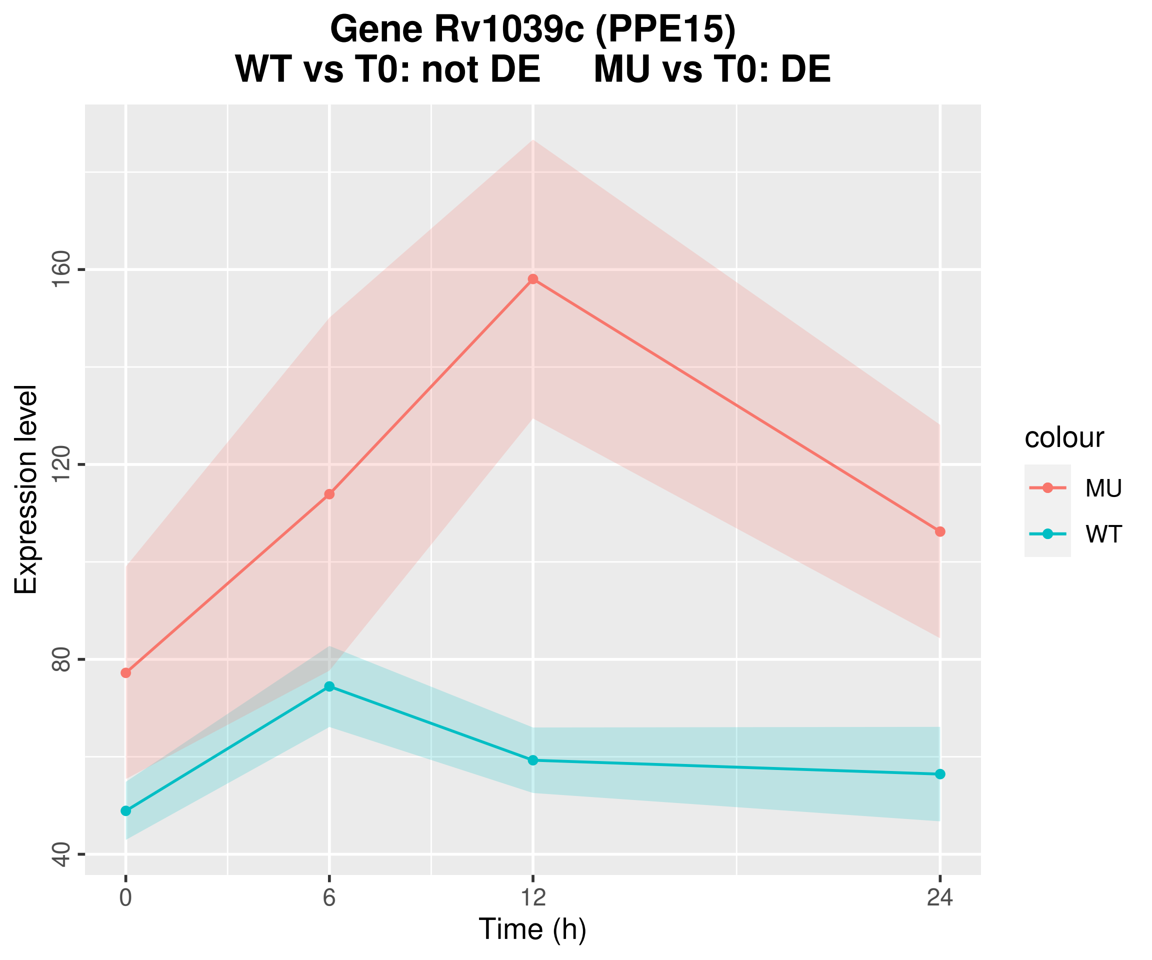

Gene Rv1039c (PPE15)
WT vs T0: not DE   MU vs T0: DE
Expression level
160
120
80
40
0
6
12
24
Time (h)
colour
MU
WT

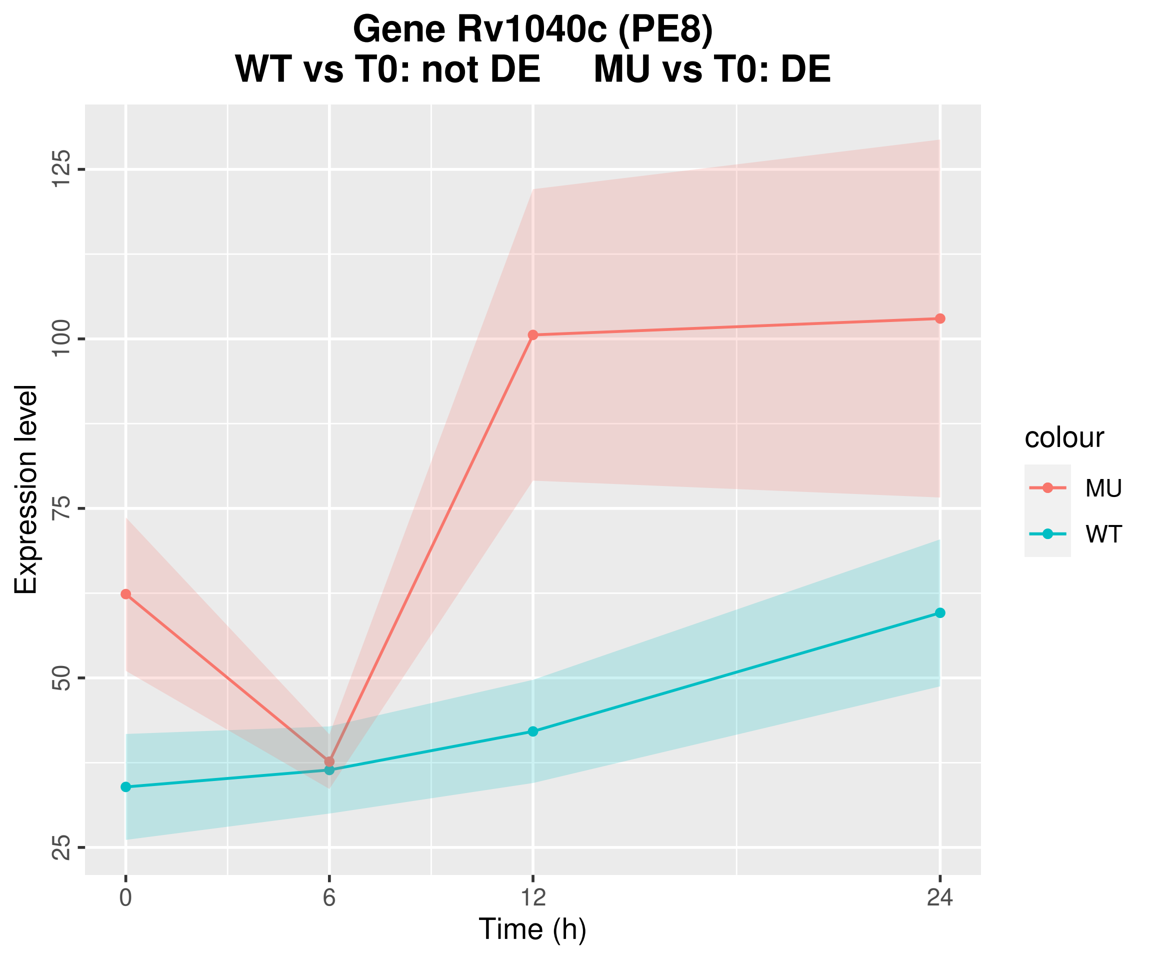

Gene Rv1040c (PE8)
WT vs T0: not DE MU vs T0: DE
Expression level
125
100
75
50
25
0
6
12
24
Time (h)
colour
MU
WT

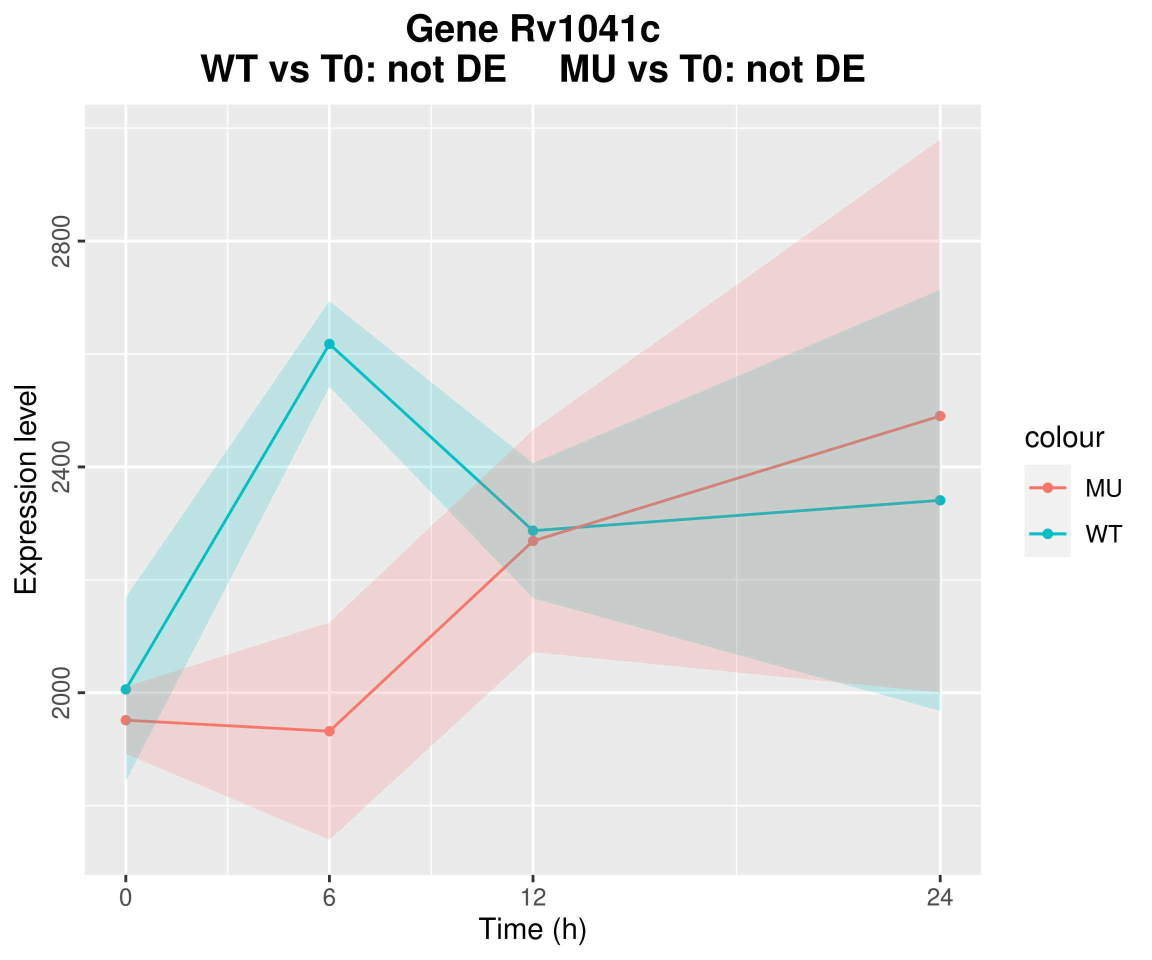

Gene Rv1041c
WT vs T0: not DE MU vs T0: not DE
Expression level
2000
2400
2800
0
6
12
24
Time (h)
colour
MU
WT

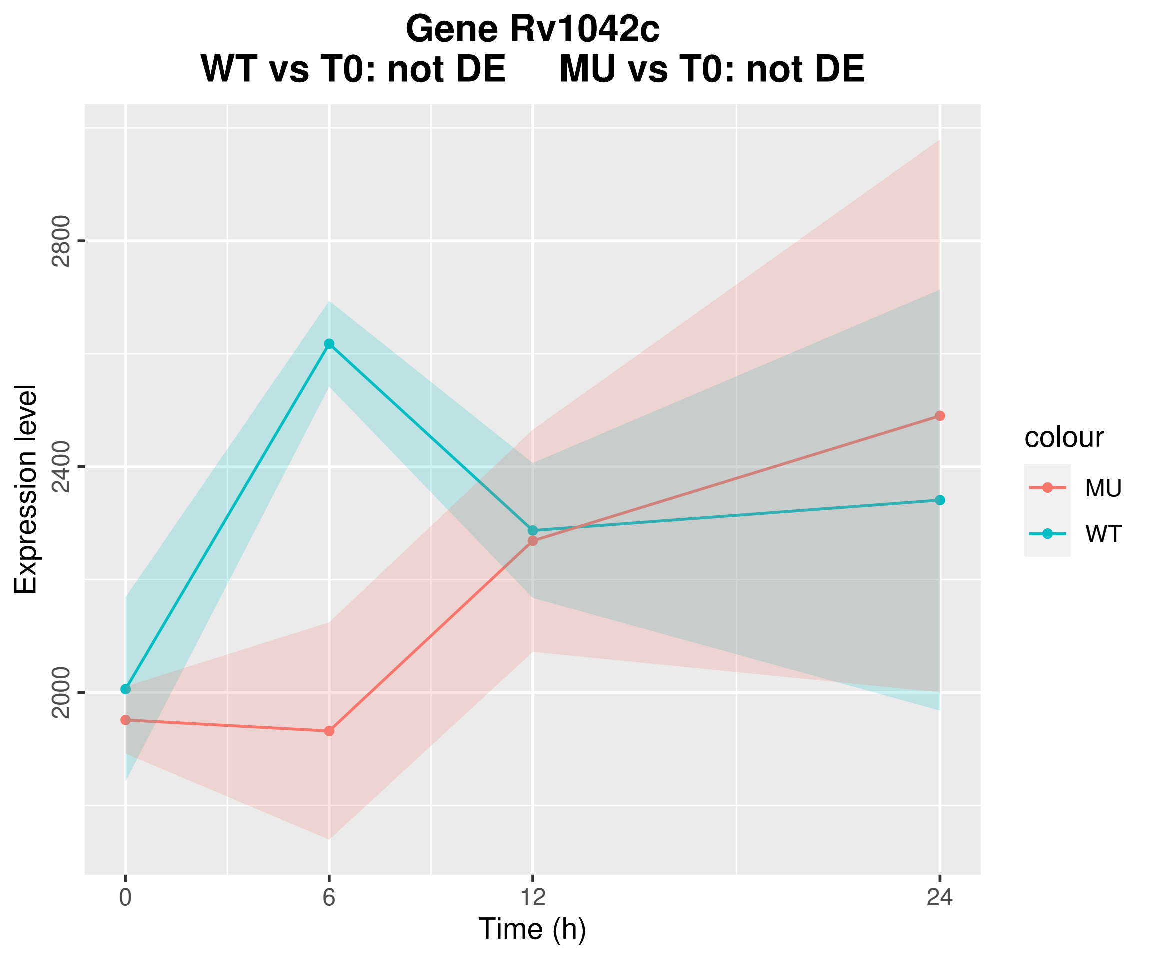

Gene Rv1042c
WT vs T0: not DE MU vs T0: not DE
Expression level
2000
2400
2800
Time (h)
0
6
12
24
colour
MU
WT

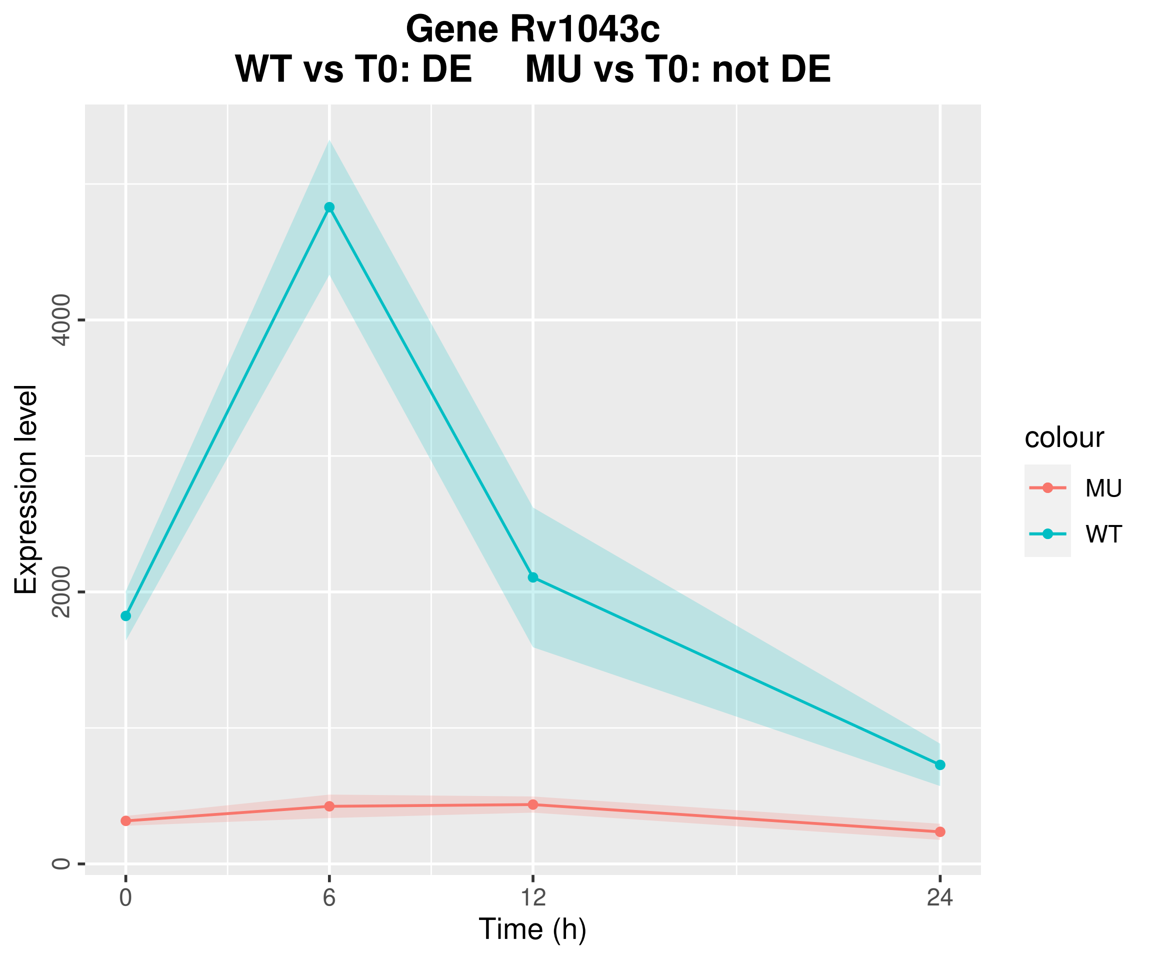
Gene Rv1043c
WT vs T0: DE MU vs T0: not DE
Expression level
0
2000
4000
0
6
12
24
Time (h)
colour
MU
WT

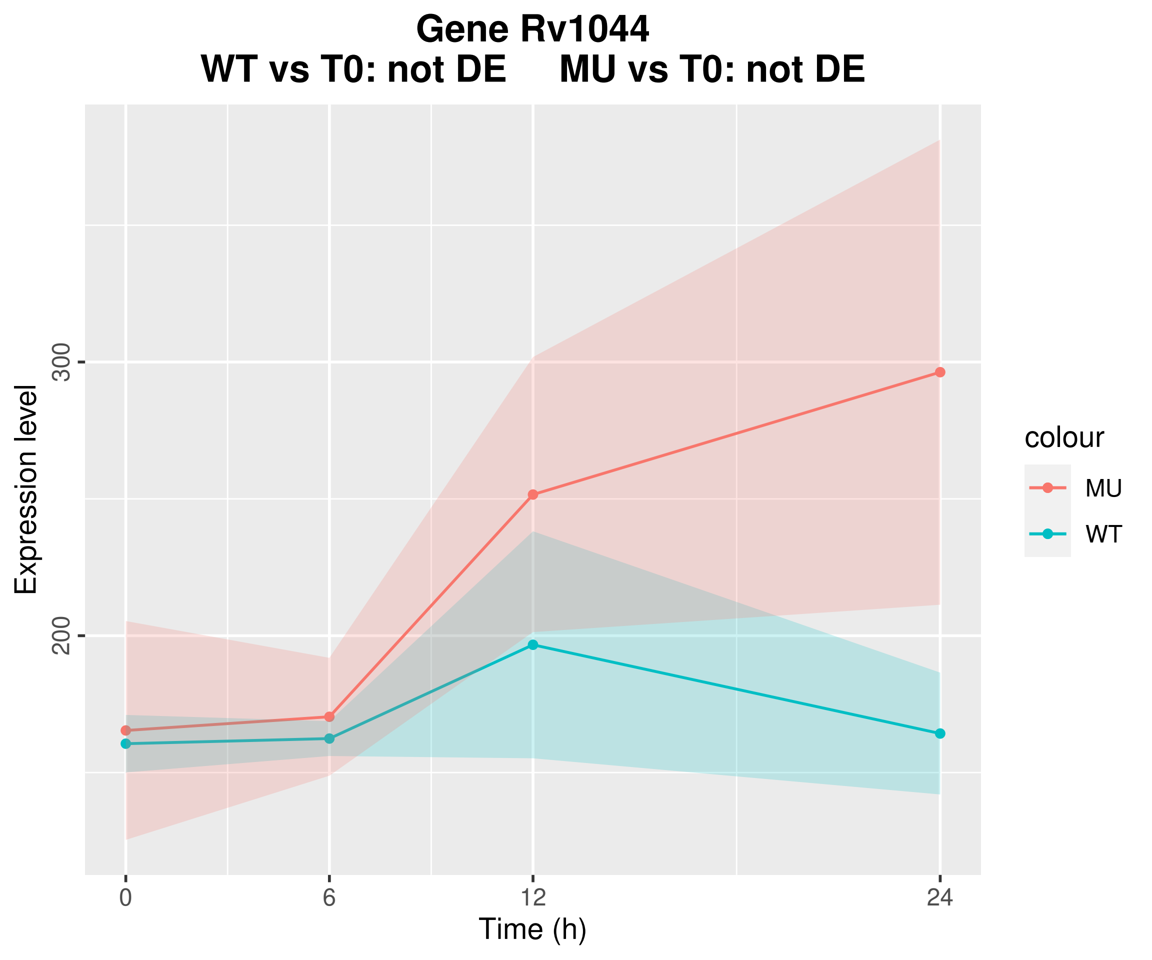
Gene Rv1044
WT vs T0: not DE   MU vs T0: not DE
Expression level
300
200
0
6
12
24
Time (h)
colour
MU
WT

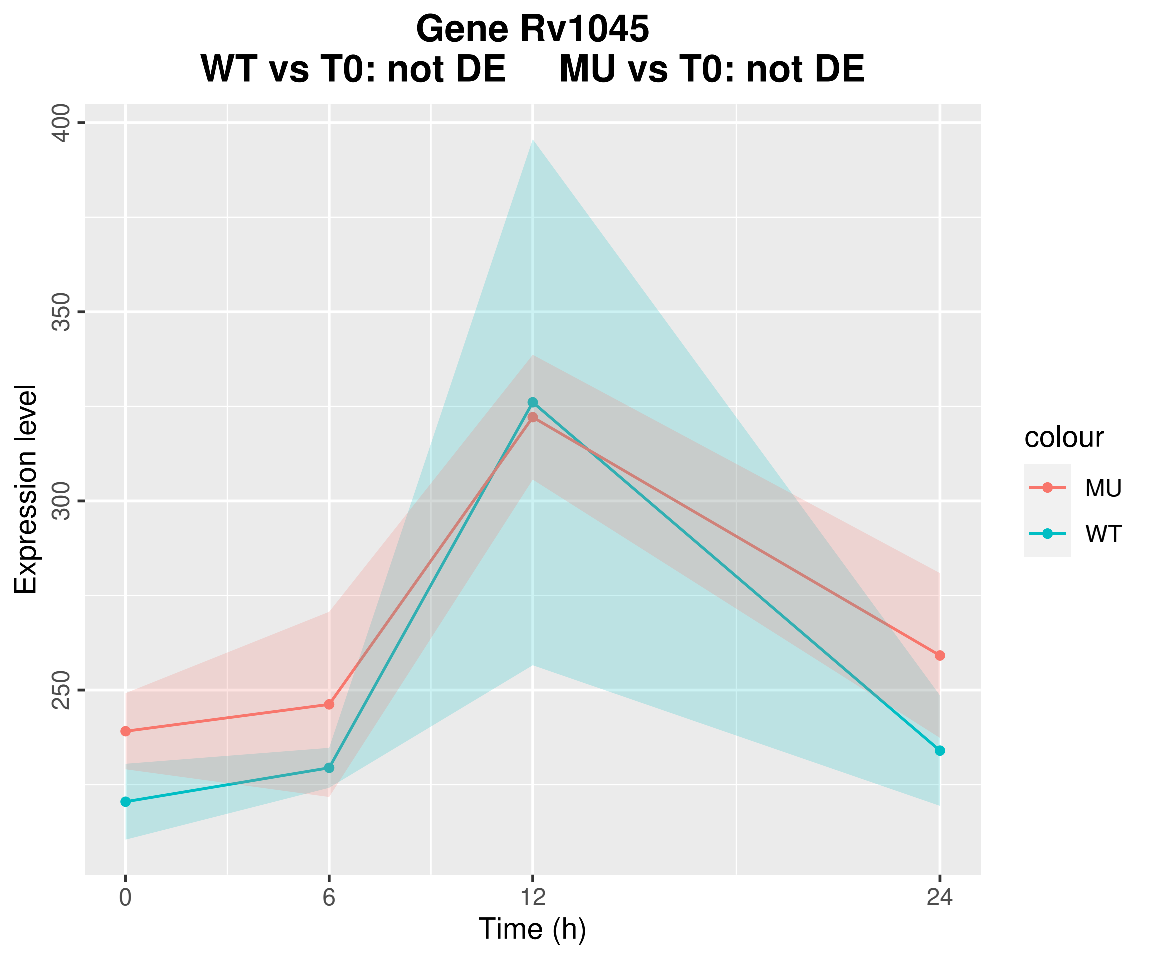

Gene Rv1045
WT vs T0: not DE   MU vs T0: not DE
Expression level
400
350
300
250
0
6
12
24
Time (h)
colour
MU
WT

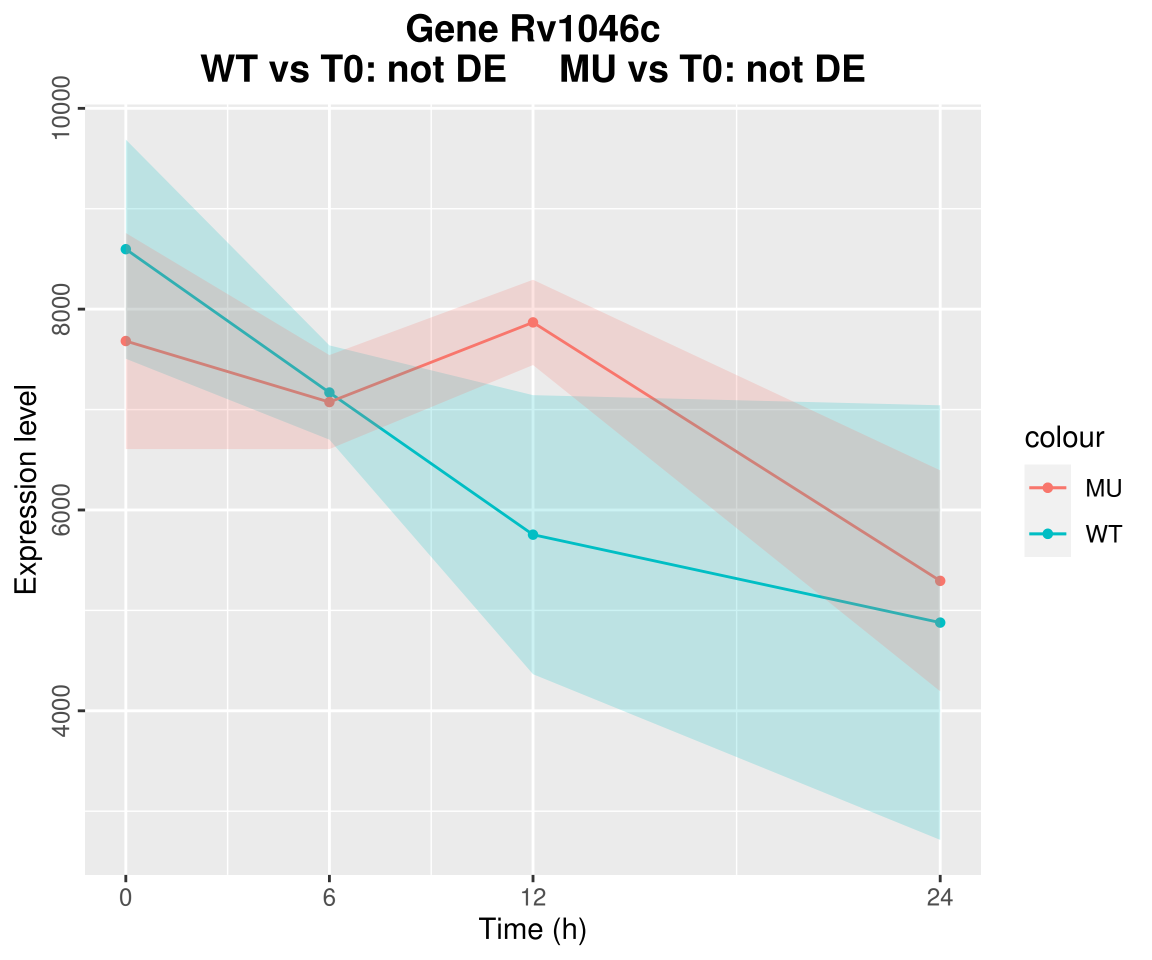

Gene Rv1046c
WT vs T0: not DE   MU vs T0: not DE
Expression level
10000
8000
6000
4000
0
6
12
24
Time (h)
colour
MU
WT

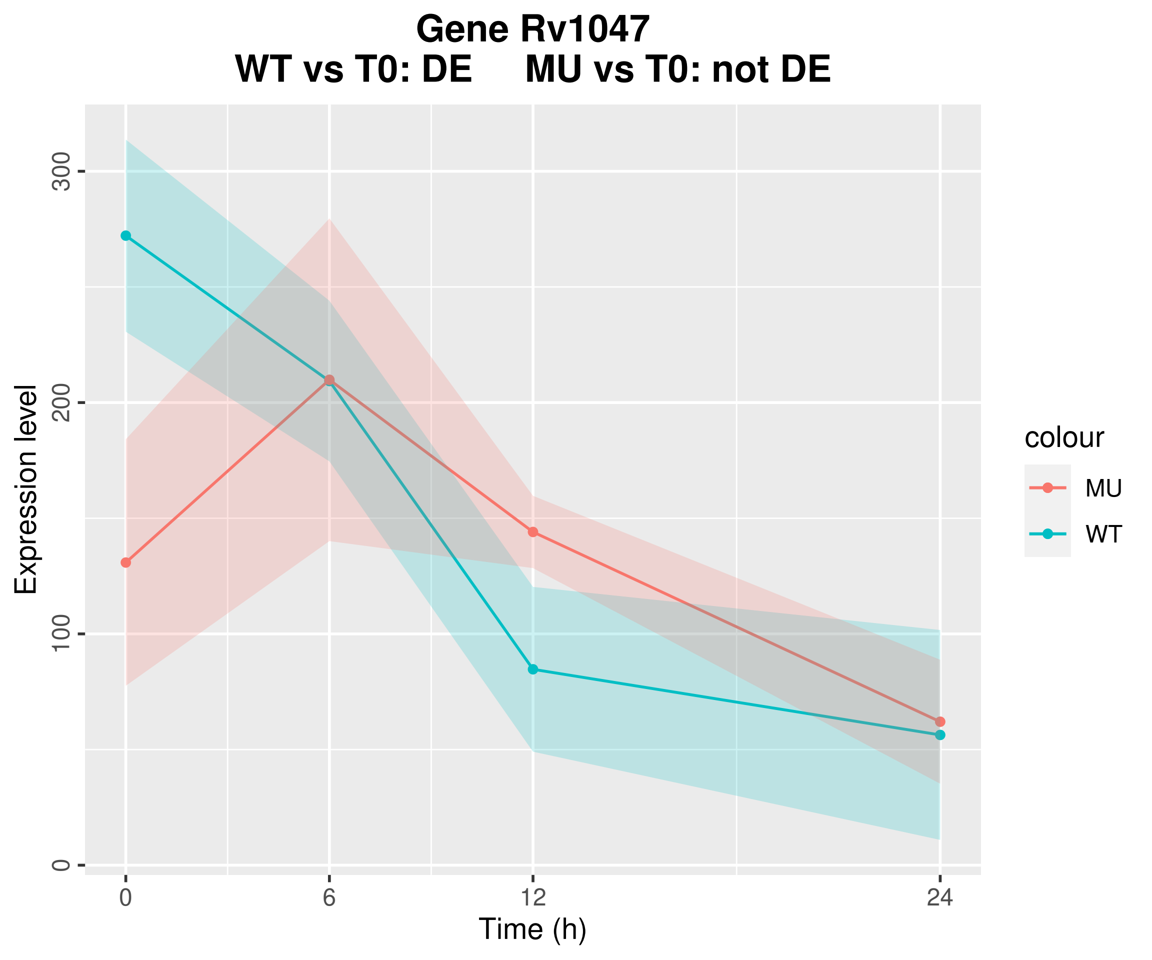
Gene Rv1047
WT vs T0: DE MU vs T0: not DE
Expression level
0
100
200
300
Time (h)
0
6
12
24
colour
MU
WT

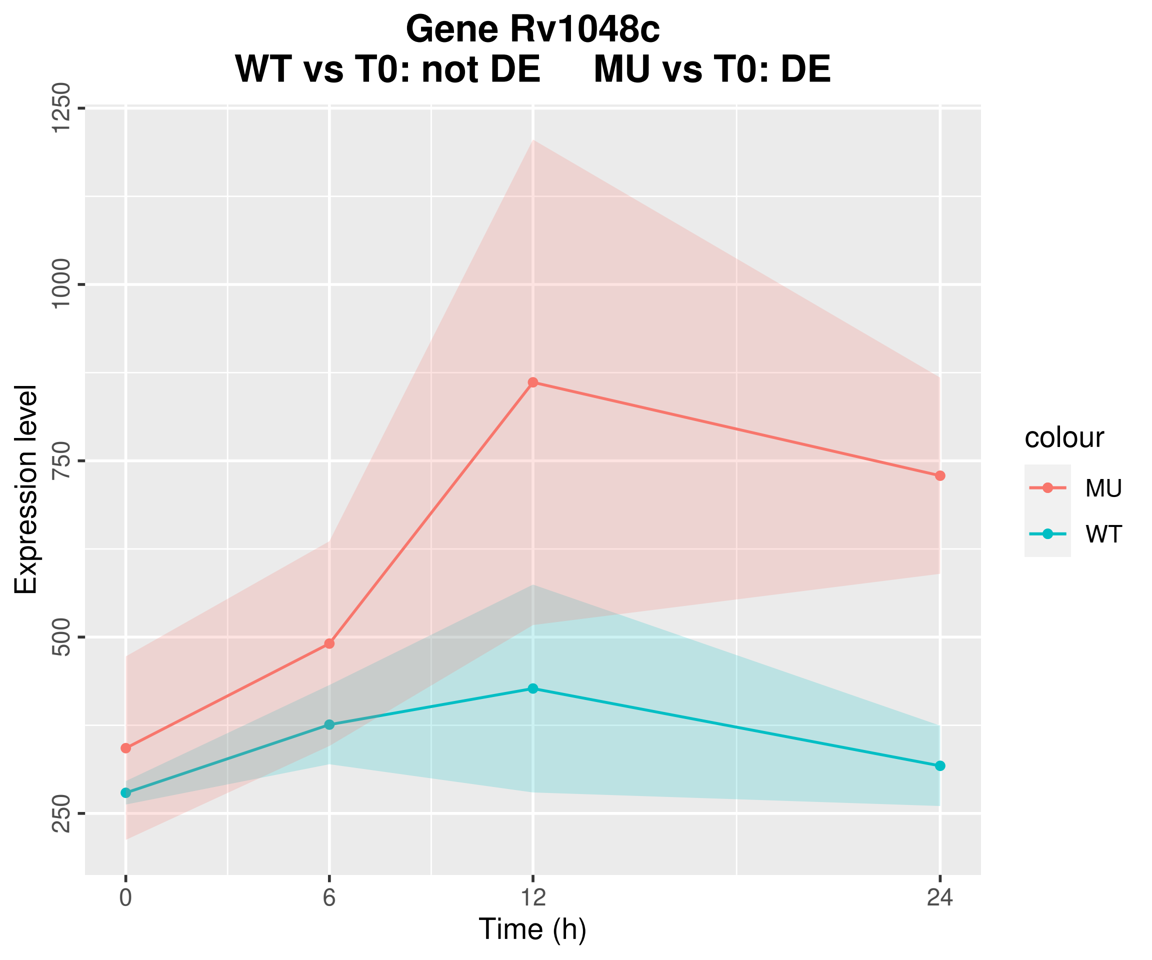
Gene Rv1048c
WT vs T0: not DE MU vs T0: DE
Expression level
1250
1000
750
500
250
0
6
12
24
Time (h)
colour
MU
WT

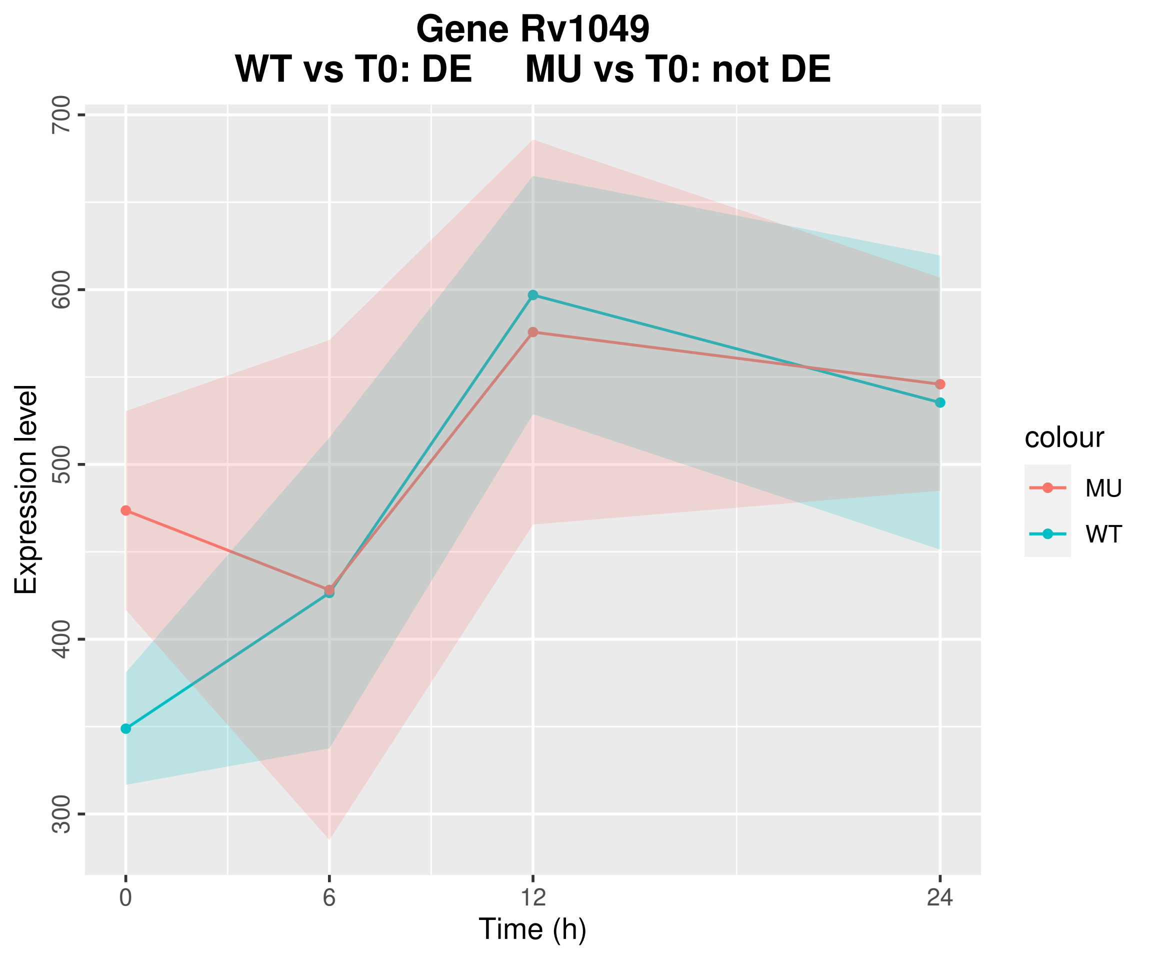

Gene Rv1049
WT vs T0: DE   MU vs T0: not DE
Expression level
700
600
500
400
300
0
6
12
24
Time (h)
colour
MU
WT

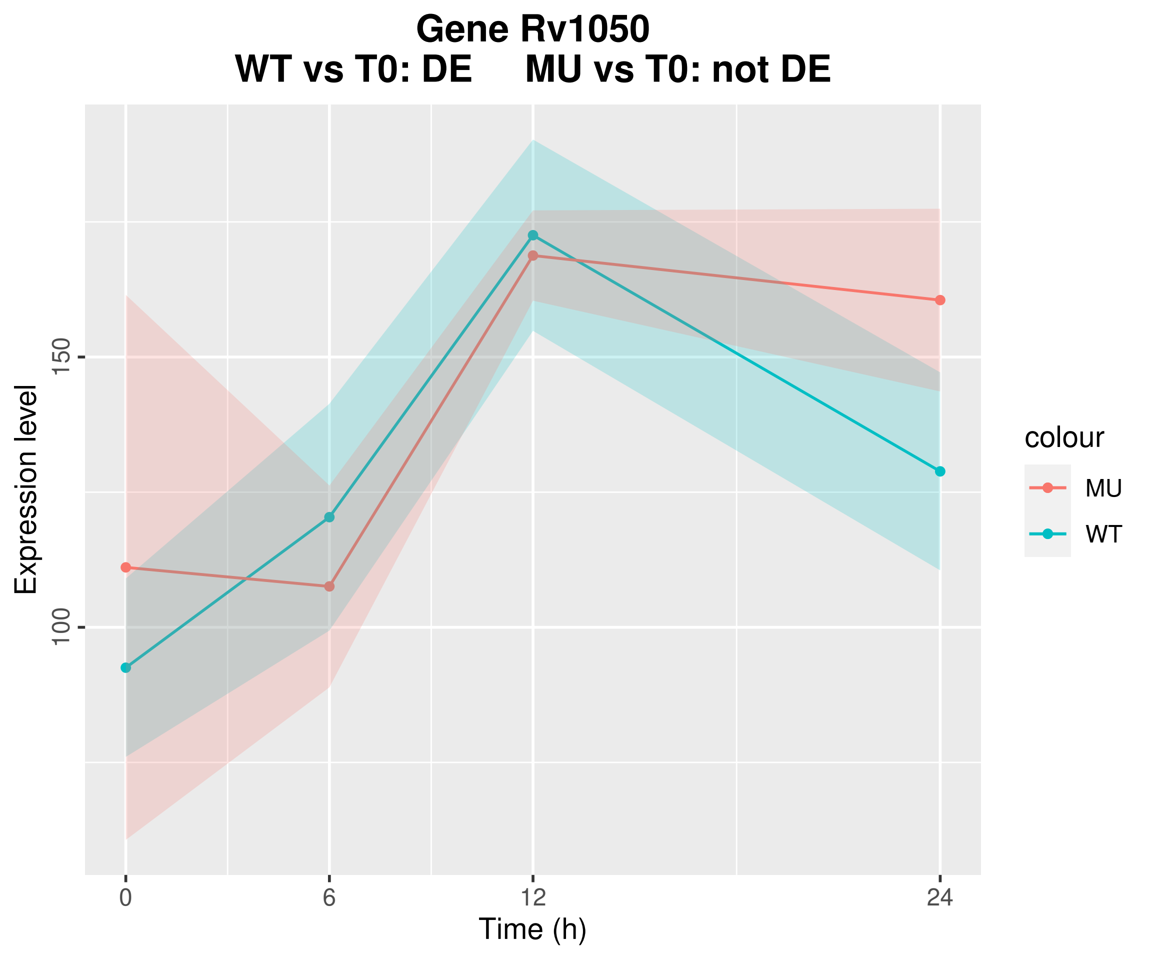

Gene Rv1050
WT vs T0: DE   MU vs T0: not DE
Expression level
150
100
0
6
12
24
Time (h)
colour
MU
WT

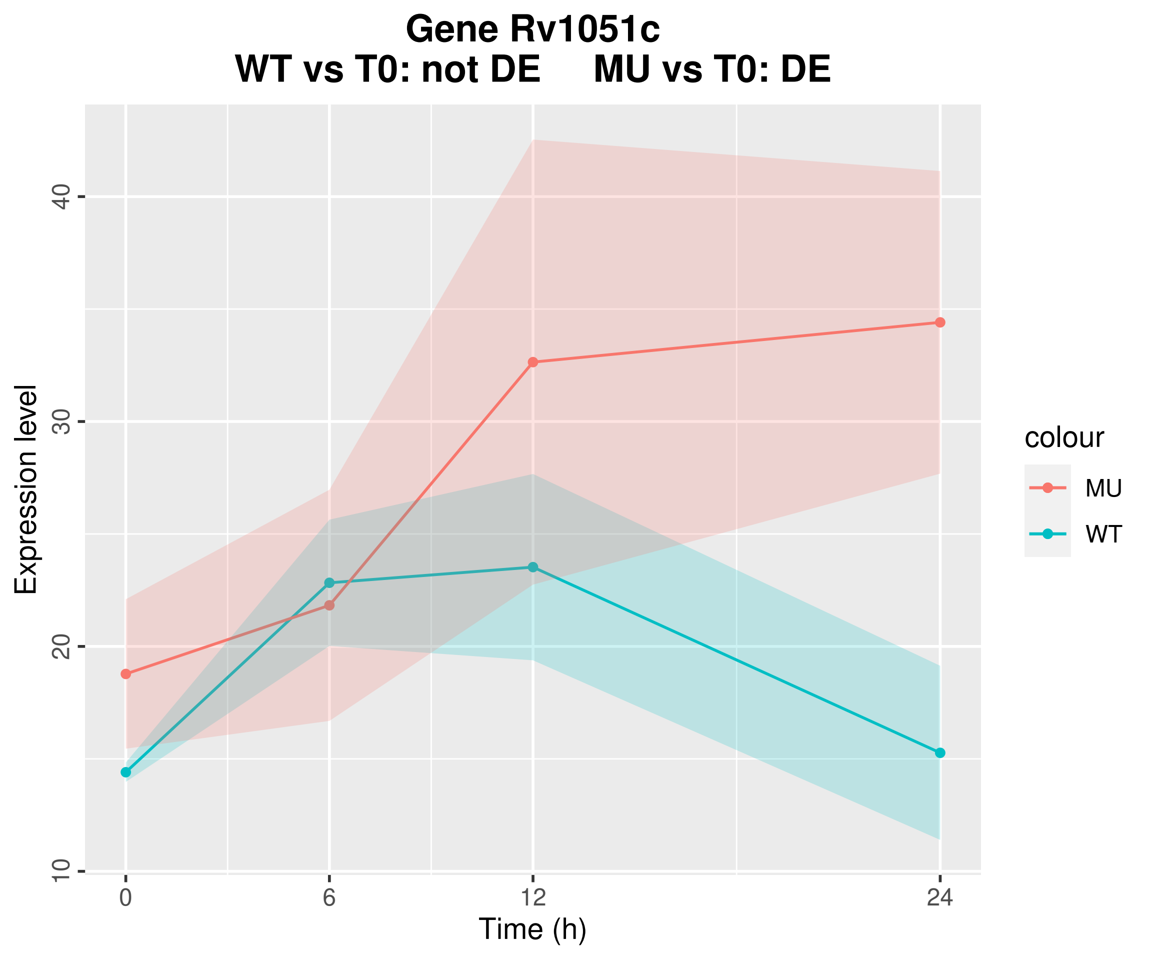

Gene Rv1051c
WT vs T0: not DE   MU vs T0: DE
Expression level
40
30
20
10
0
6
12
24
Time (h)
colour
MU
WT

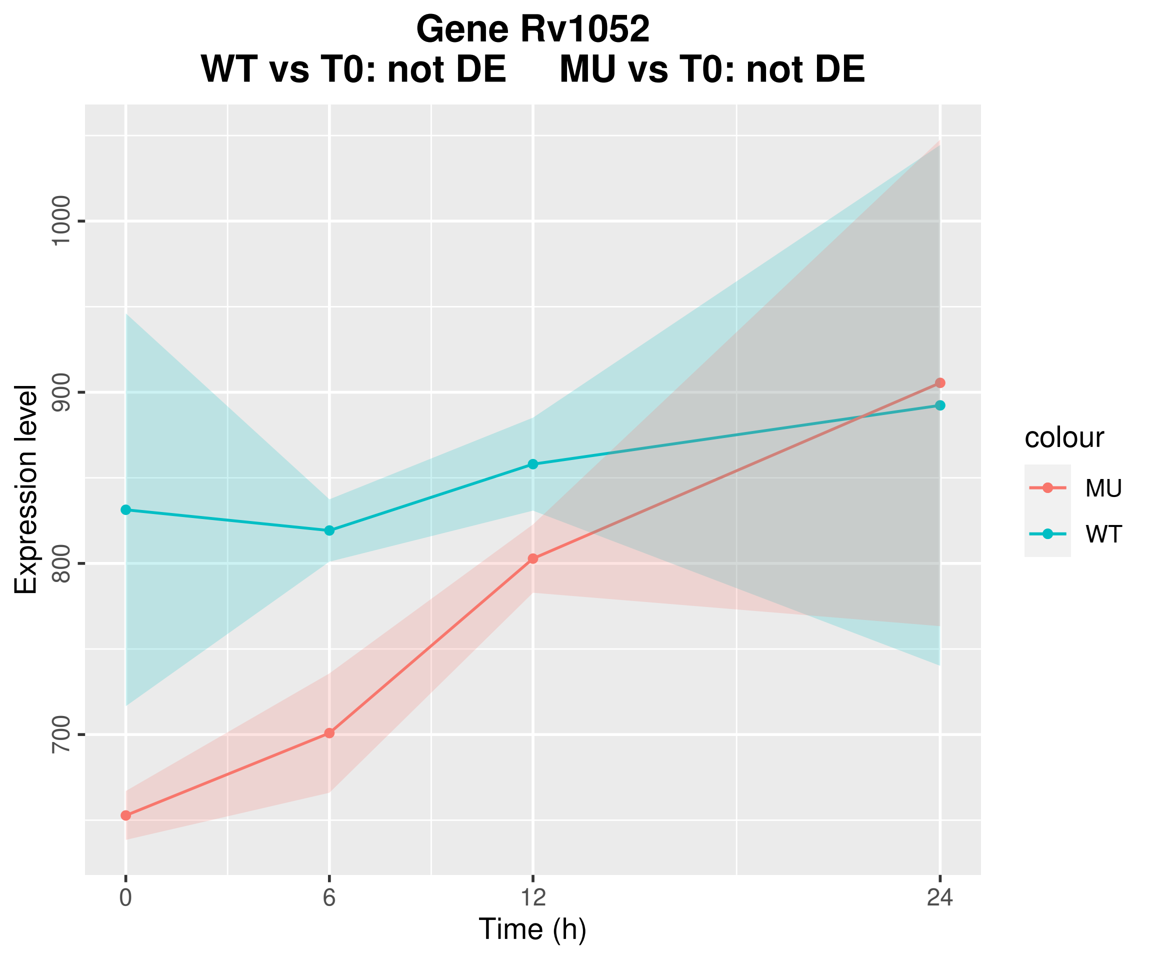

Gene Rv1052
WT vs T0: not DE   MU vs T0: not DE
Expression level
1000
900
800
700
0
6
12
24
Time (h)
colour
MU
WT

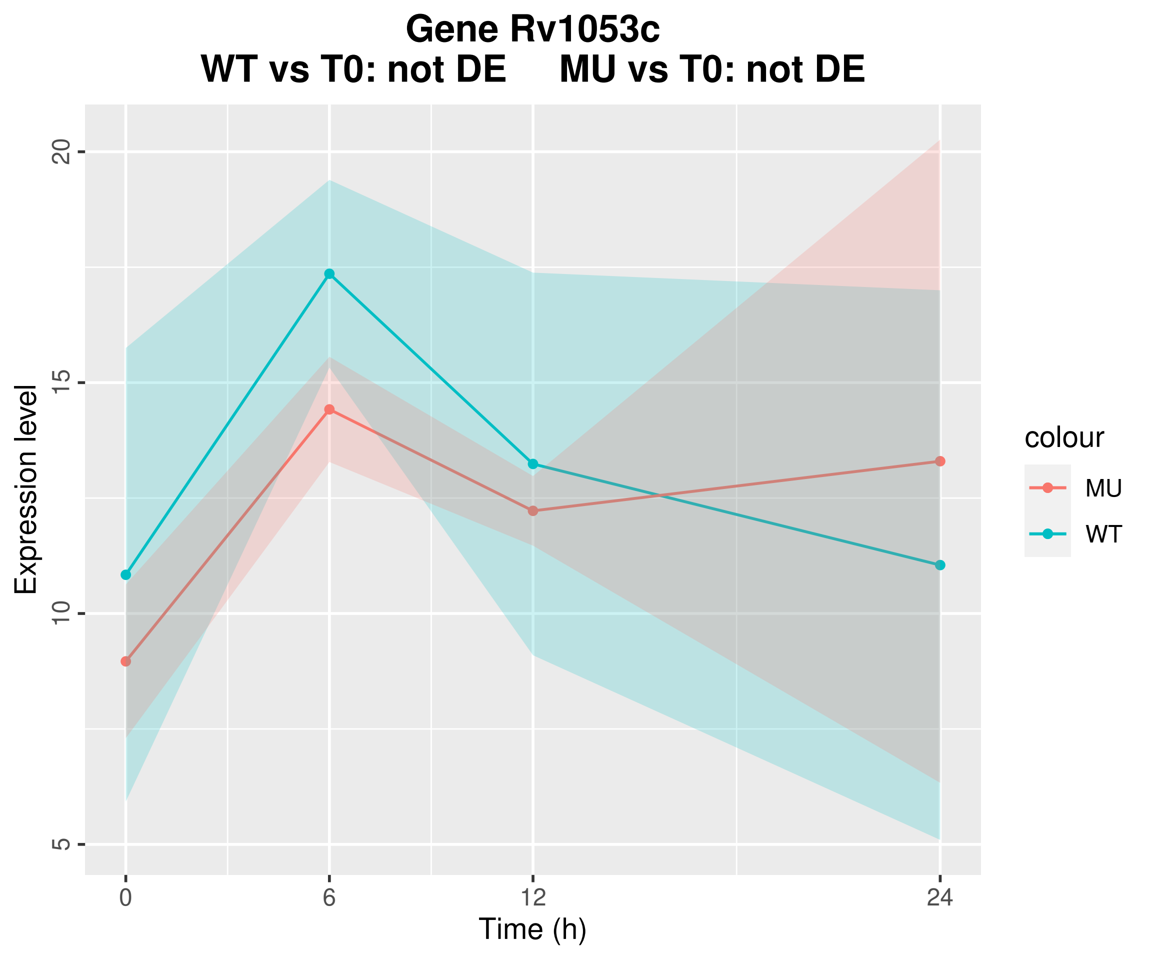
Gene Rv1053c
WT vs T0: not DE   MU vs T0: not DE
Expression level
20
15
10
5
0
6
12
24
Time (h)
colour
MU
WT

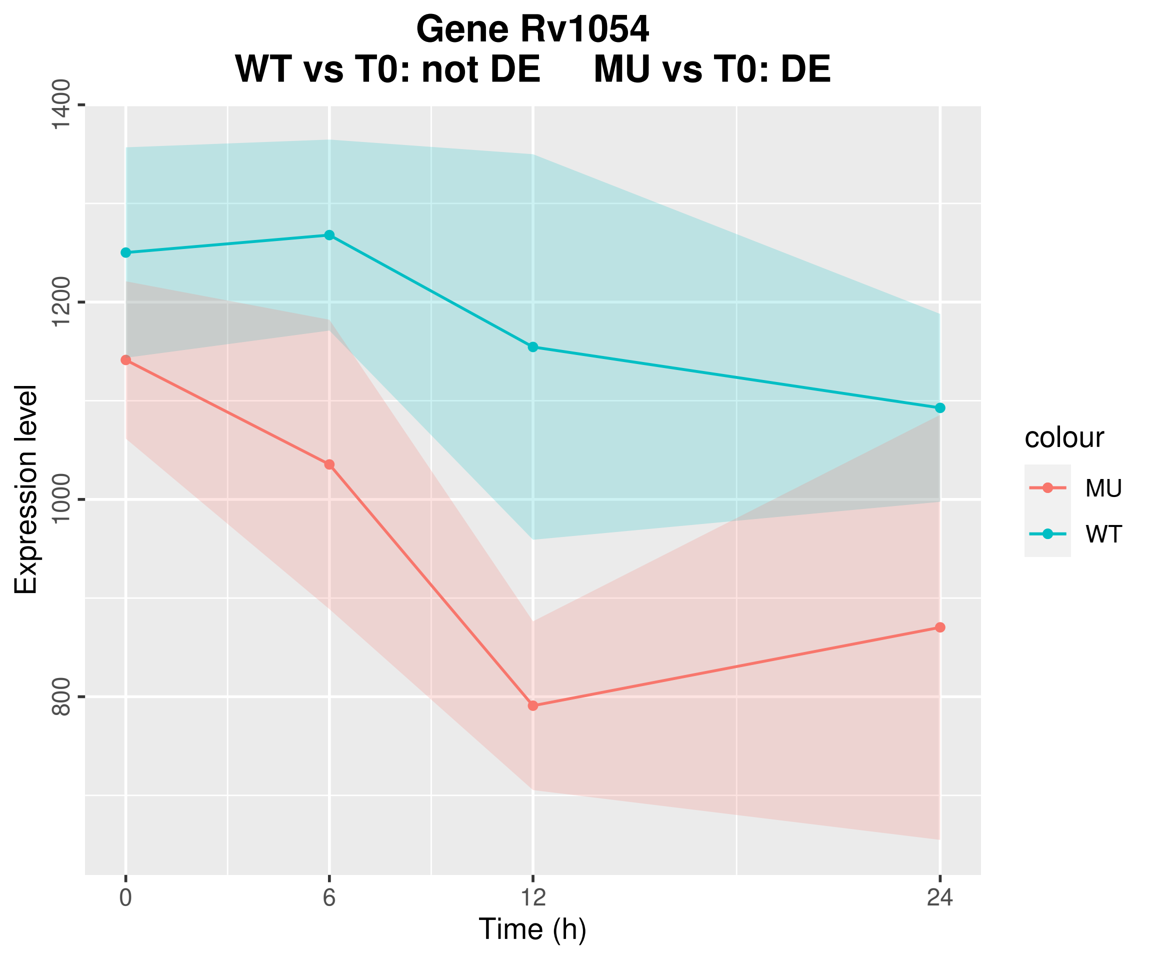
Gene Rv1054
WT vs T0: not DE   MU vs T0: DE
1400
1200
1000
800
Expression level
0
6
12
24
Time (h)
colour
MU
WT

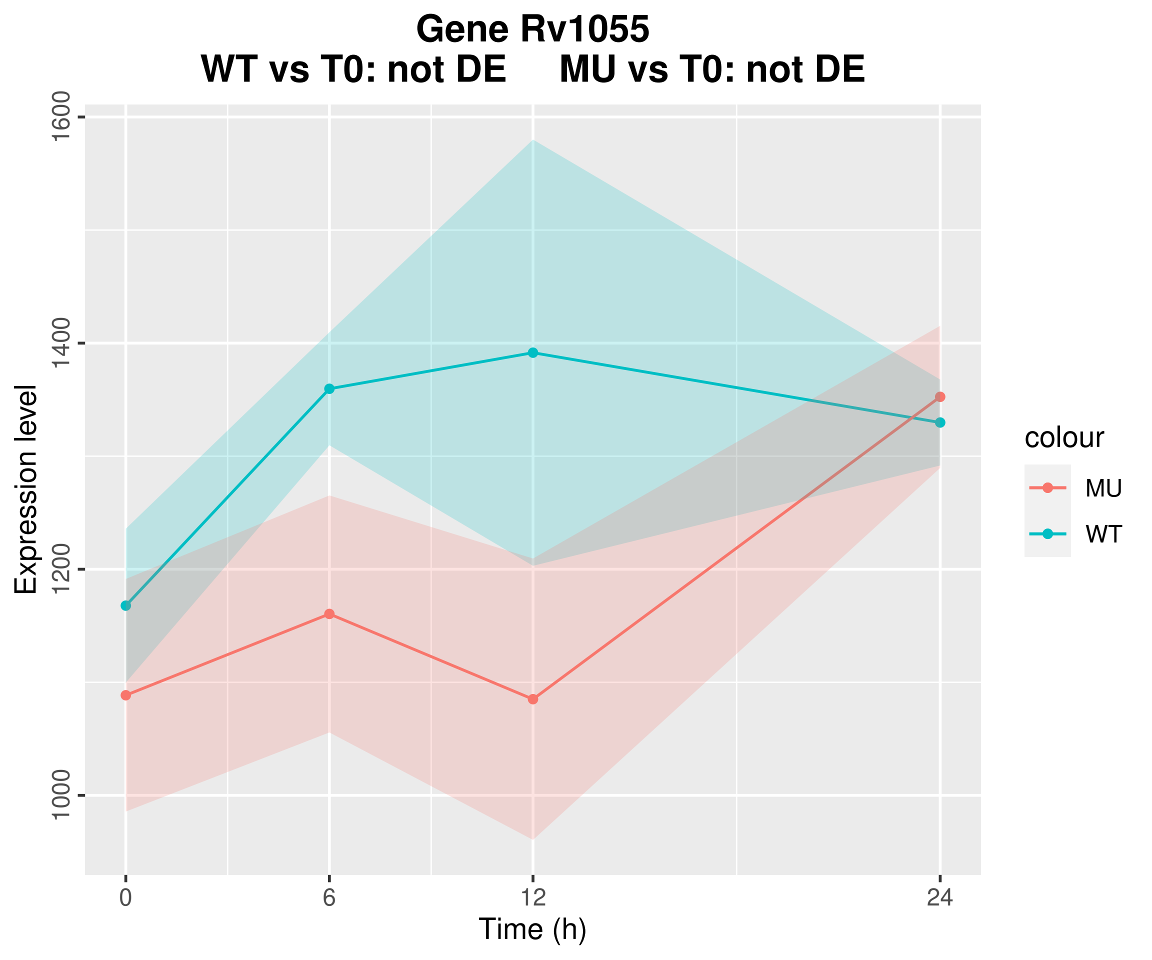
Gene Rv1055
WT vs T0: not DE   MU vs T0: not DE
1600
1400
1200
1000
Expression level
0
6
12
24
Time (h)
colour
MU
WT

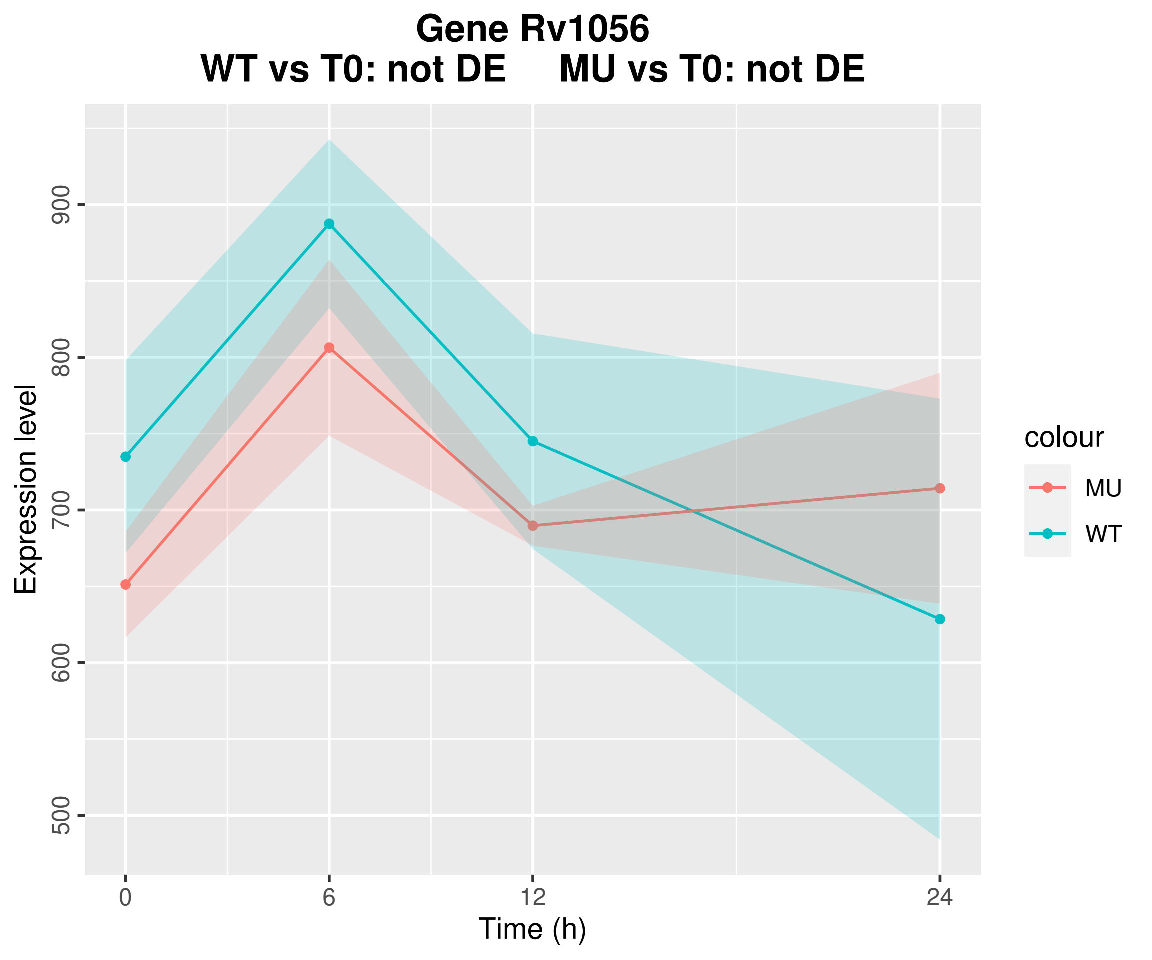

Gene Rv1056
WT vs T0: not DE   MU vs T0: not DE
Expression level
900
800
700
600
500
0
6
12
24
Time (h)
colour
MU
WT

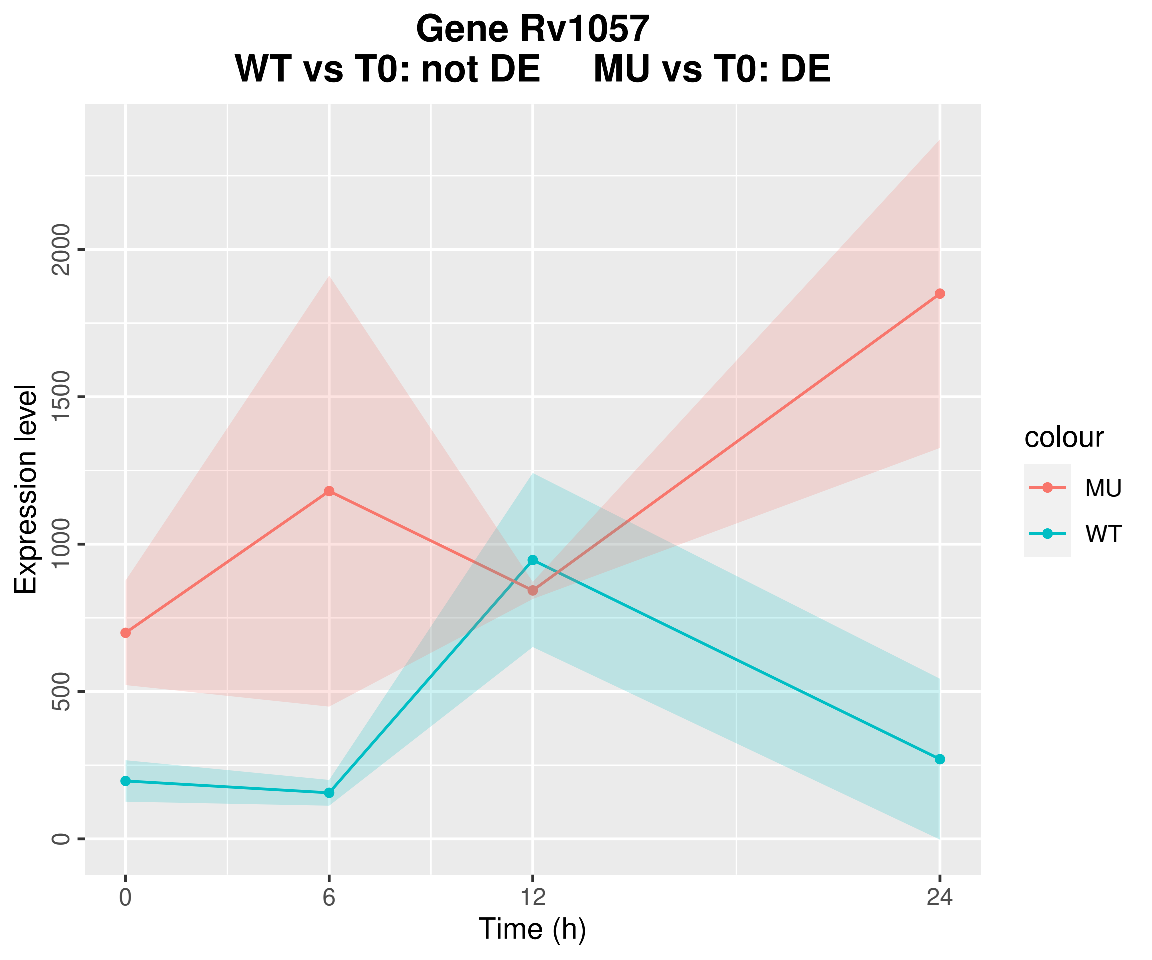
Gene Rv1057
WT vs T0: not DE   MU vs T0: DE
Expression level
2000
1500
1000
500
0
0
6
12
24
Time (h)
colour
MU
WT

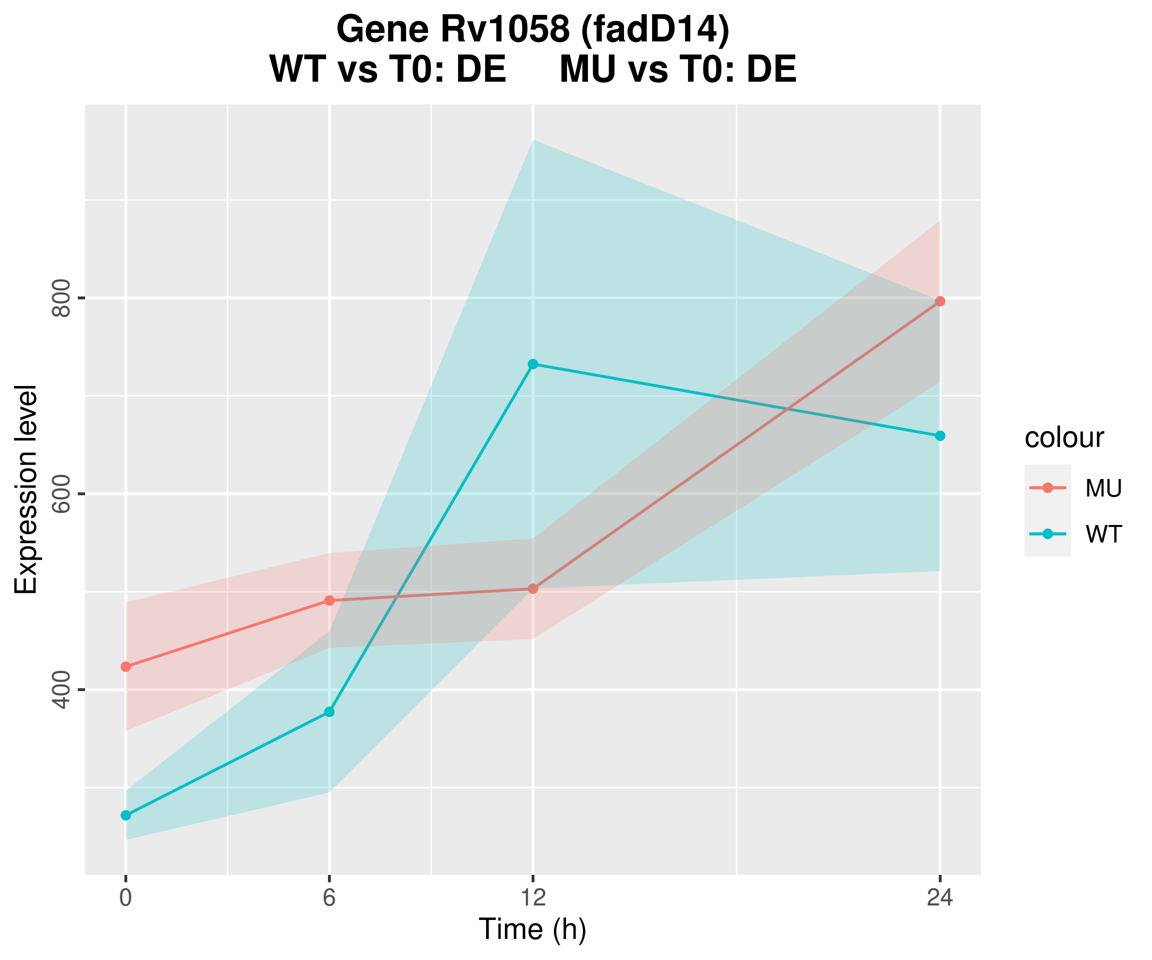

Gene Rv1058 (fadD14)
WT vs T0: DE MU vs T0: DE
Expression level
800
600
400
0
6
12
24
Time (h)
colour
MU
WT

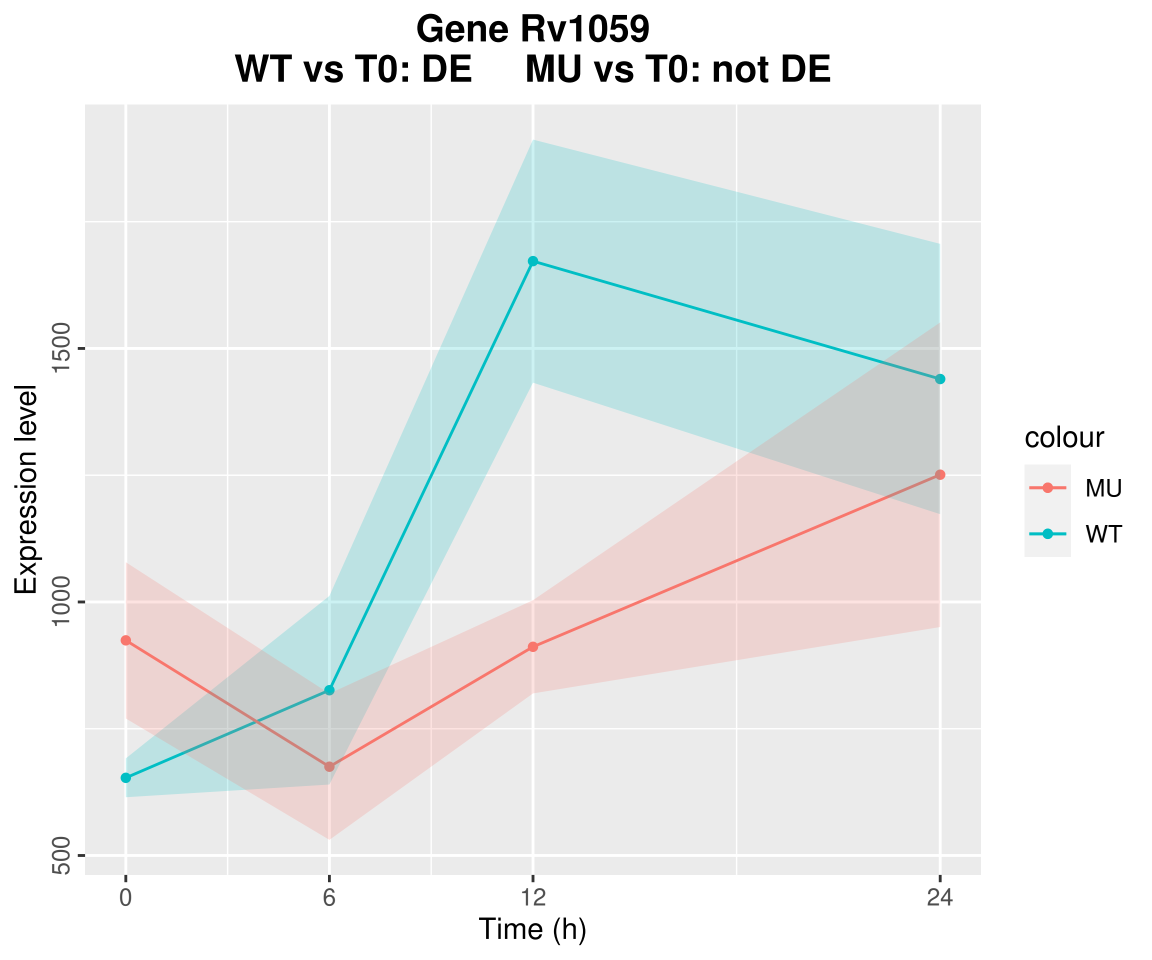
Gene Rv1059
WT vs T0: DE MU vs T0: not DE
Expression level
500
1000
1500
0
6
12
24
Time (h)
colour
MU
WT

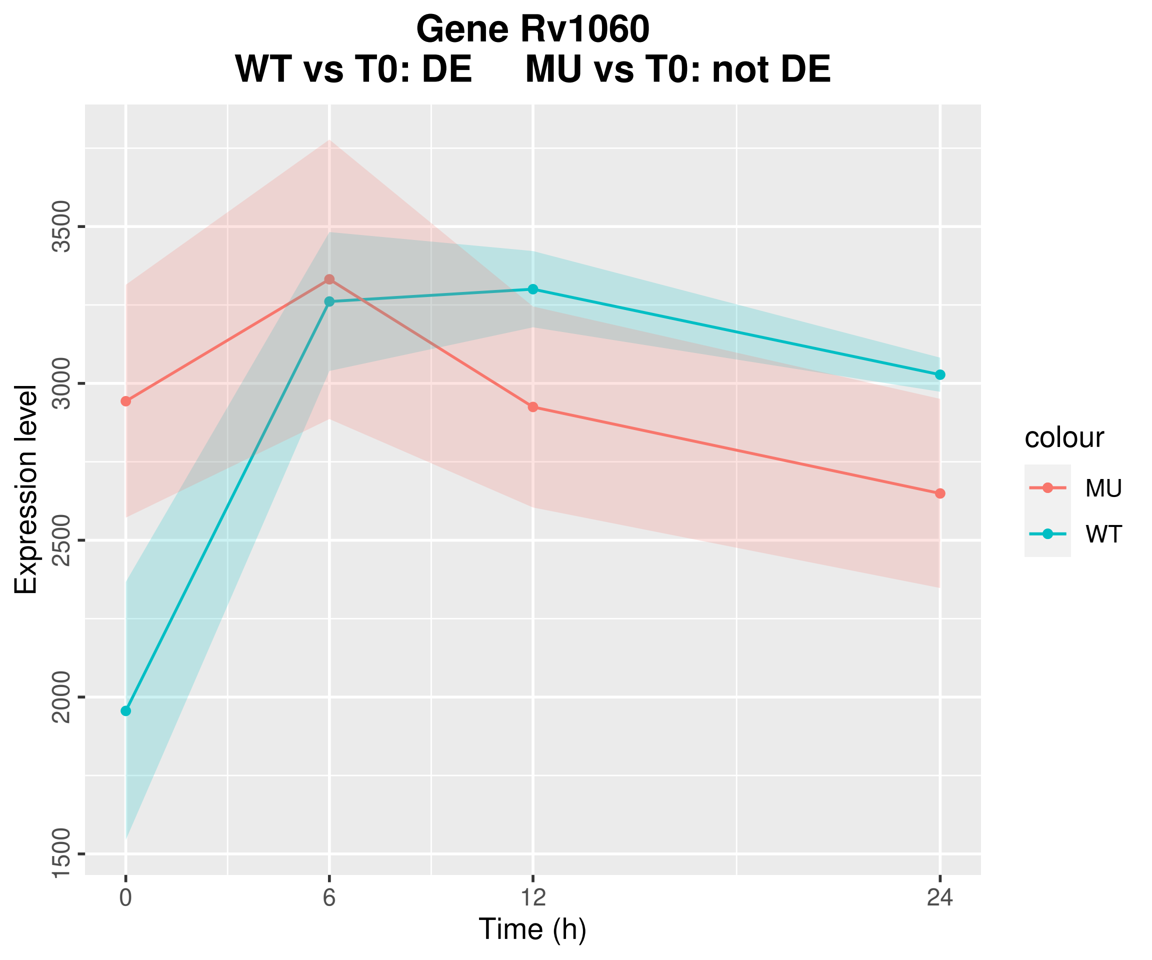
Gene Rv1060
WT vs T0: DE   MU vs T0: not DE
Expression level
1500
2000
2500
3000
3500
0
6
12
24
Time (h)
colour
MU
WT

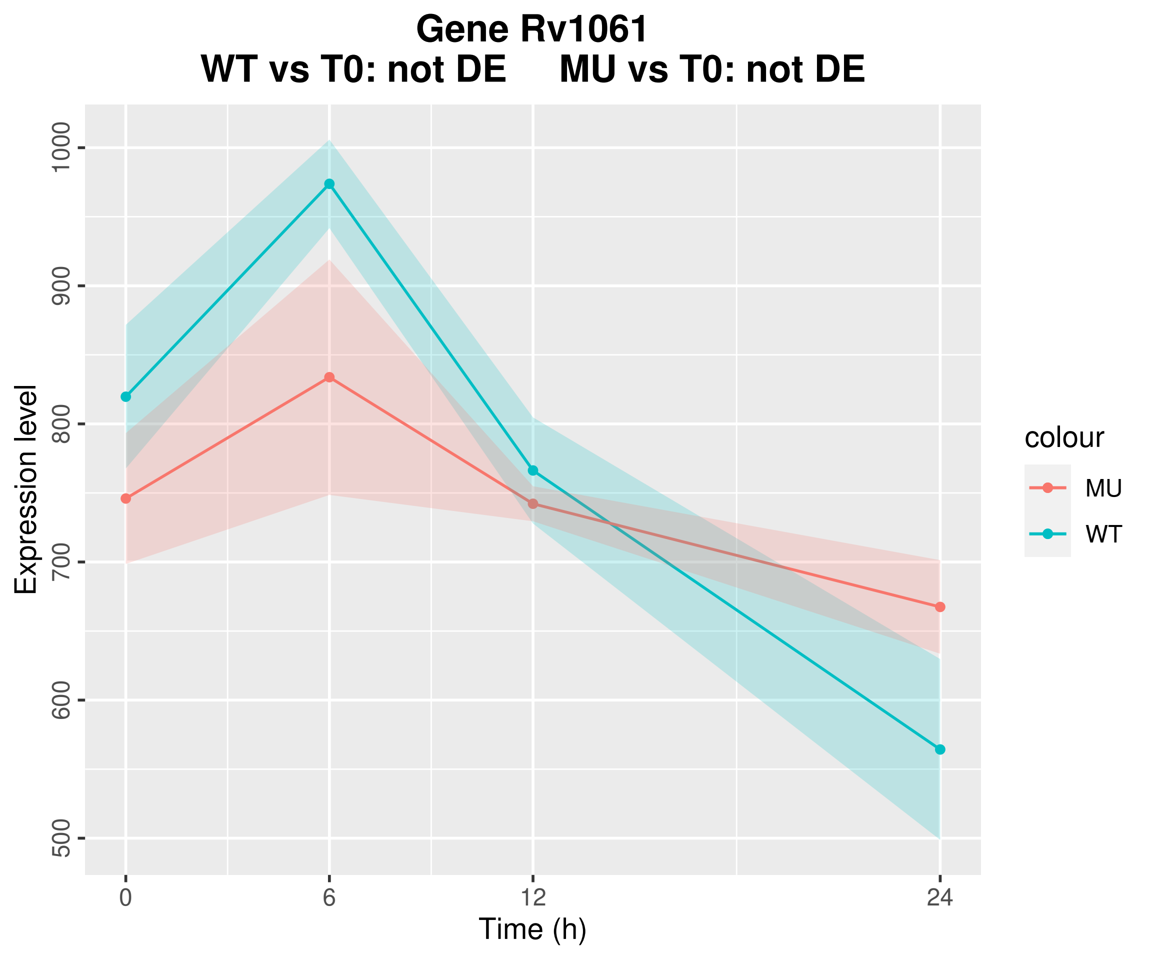
Gene Rv1061
WT vs T0: not DE MU vs T0: not DE
Expression level
1000
900
800
700
600
500
0
6
12
24
Time (h)
colour
MU
WT

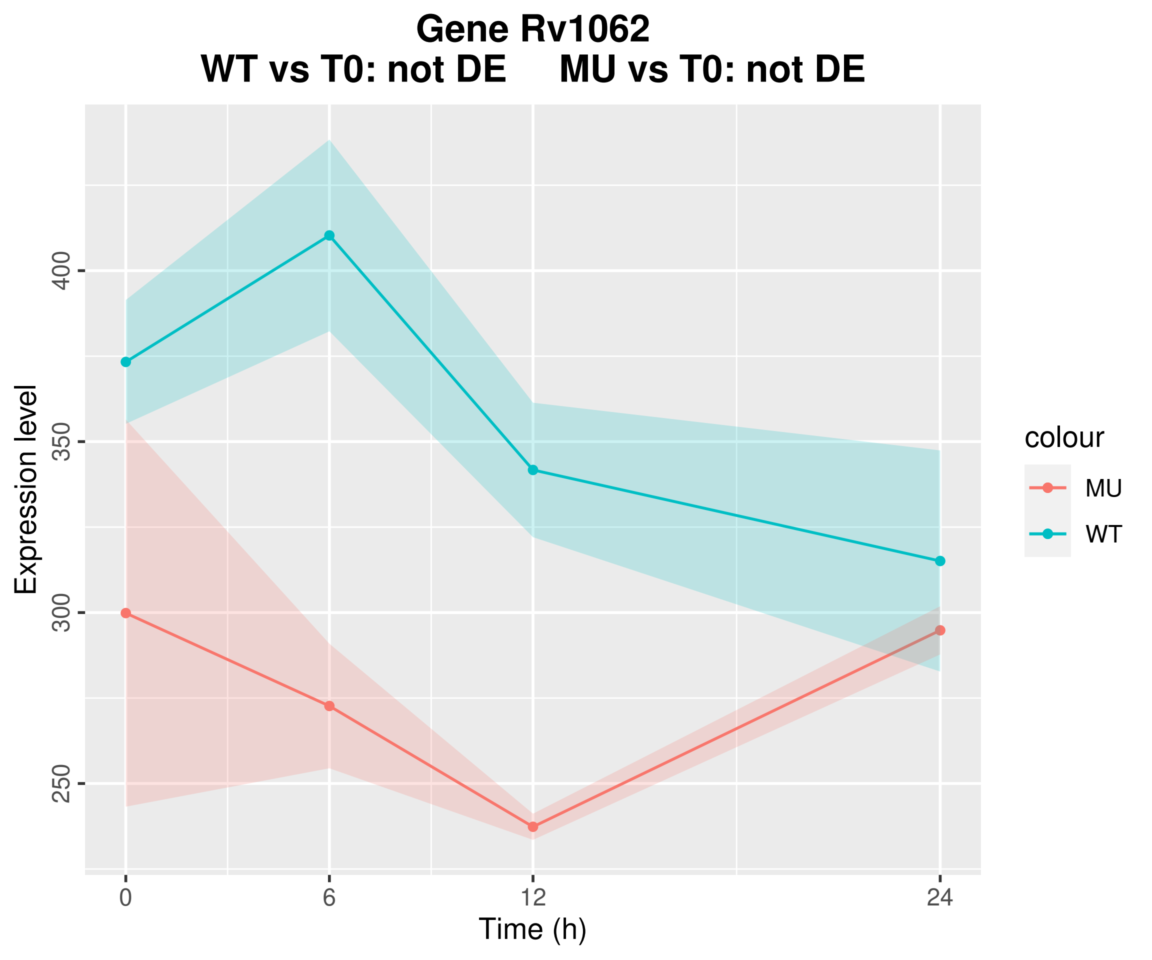

Gene Rv1062
WT vs T0: not DE MU vs T0: not DE
Expression level
400
350
300
250
0
6
12
24
Time (h)
colour
MU
WT

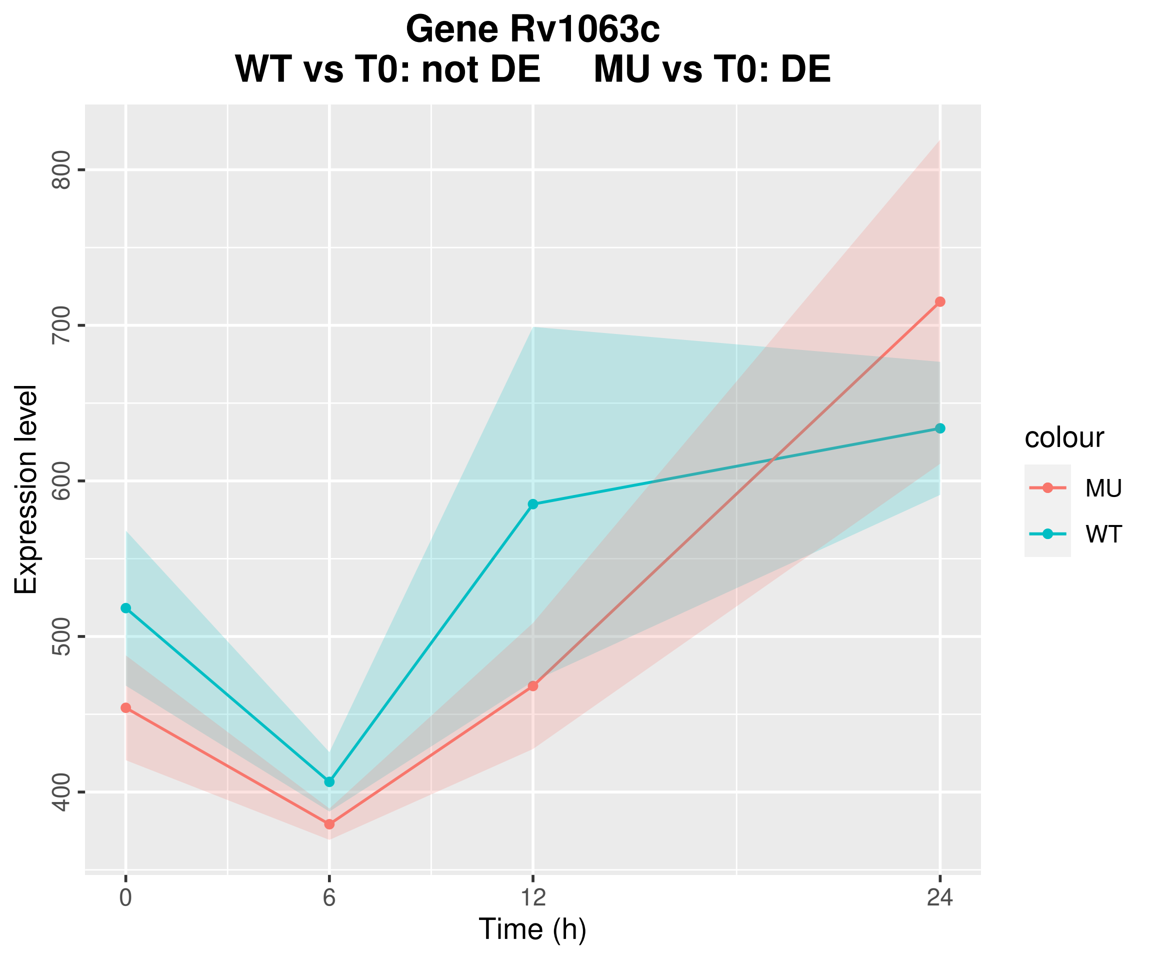
Gene Rv1063c
WT vs T0: not DE   MU vs T0: DE
Expression level
800
700
600
500
400
0
6
12
24
Time (h)
colour
MU
WT

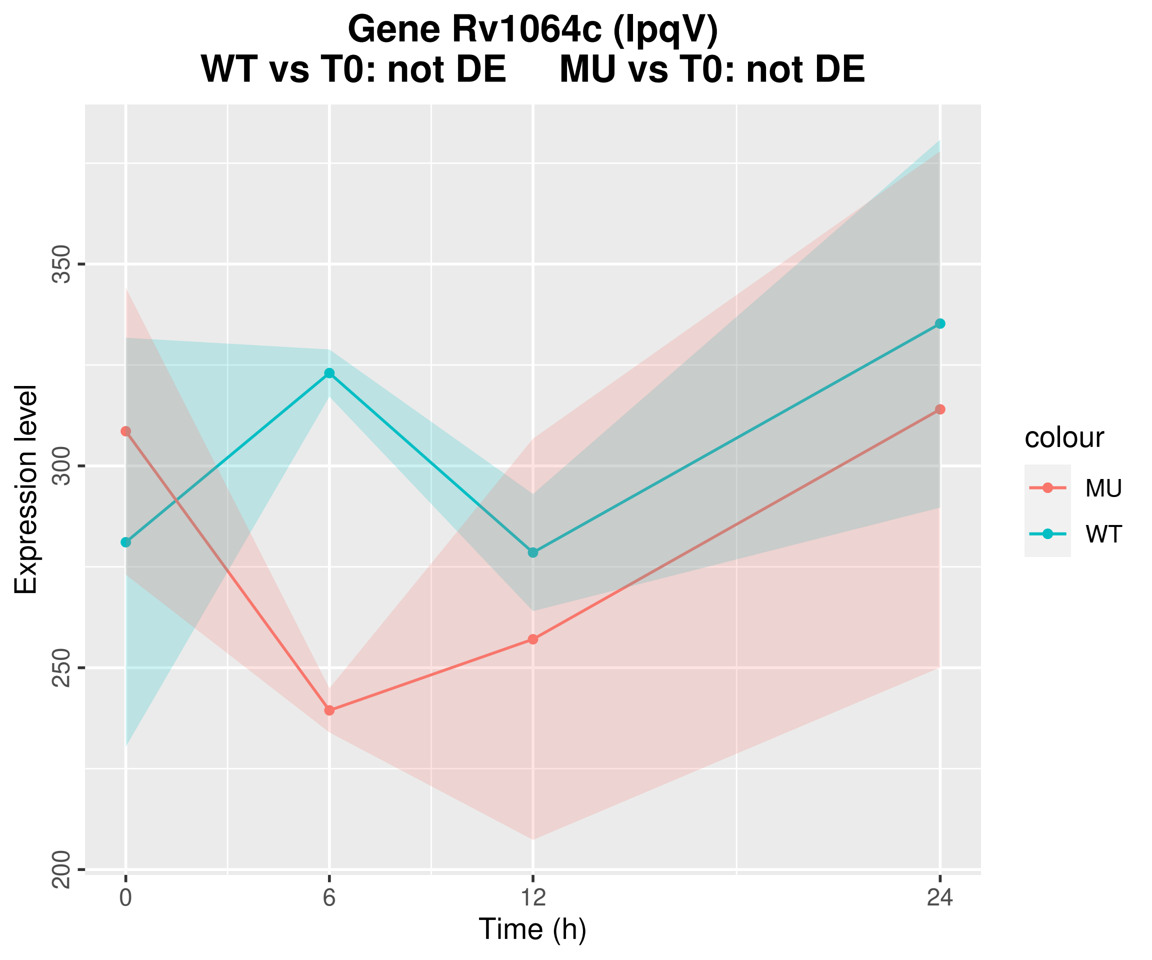

Gene Rv1064c (lpqV)
WT vs T0: not DE   MU vs T0: not DE
Expression level
350
300
250
200
0
6
12
24
Time (h)
colour
MU
WT

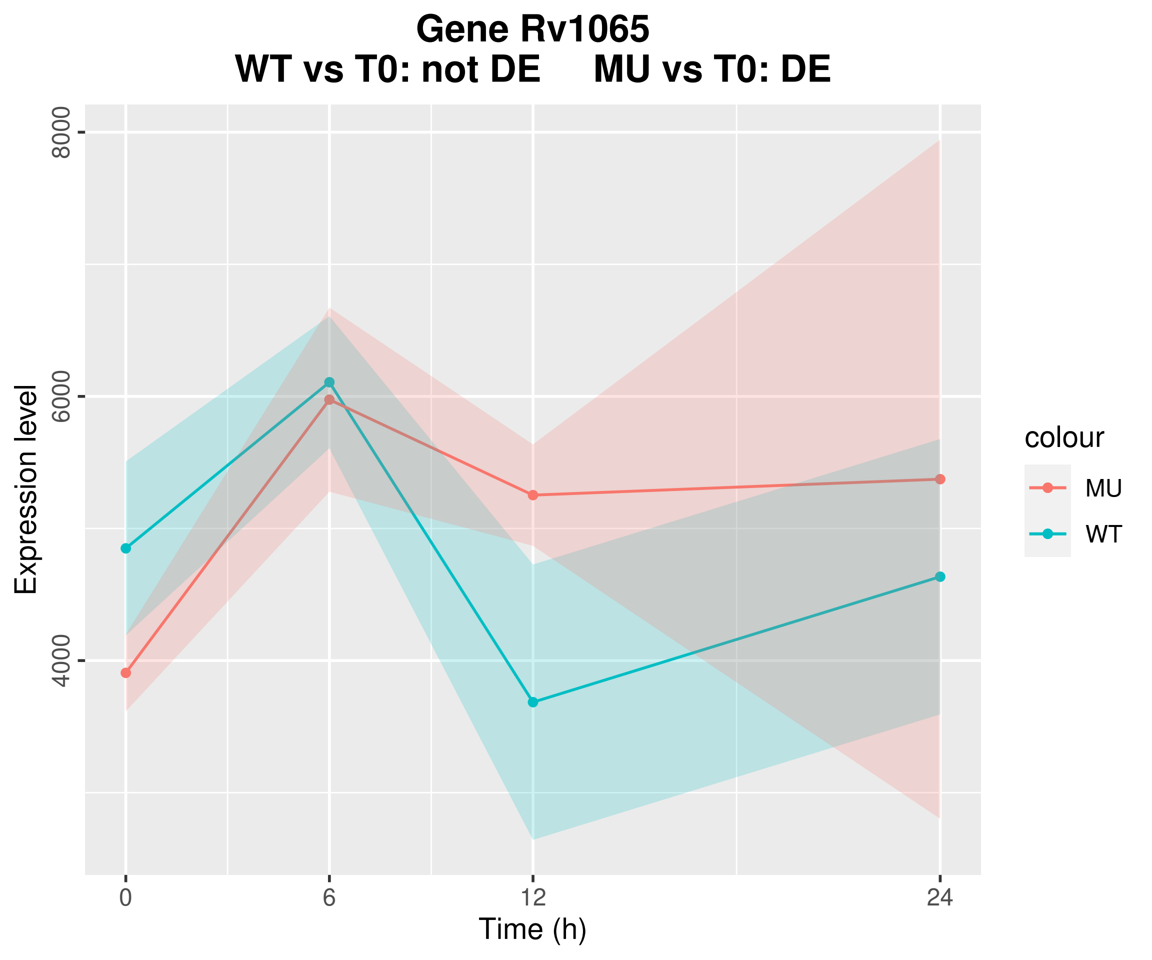

Gene Rv1065
WT vs T0: not DE   MU vs T0: DE
8000
6000
4000
Expression level
0
6
12
24
Time (h)
colour
MU
WT

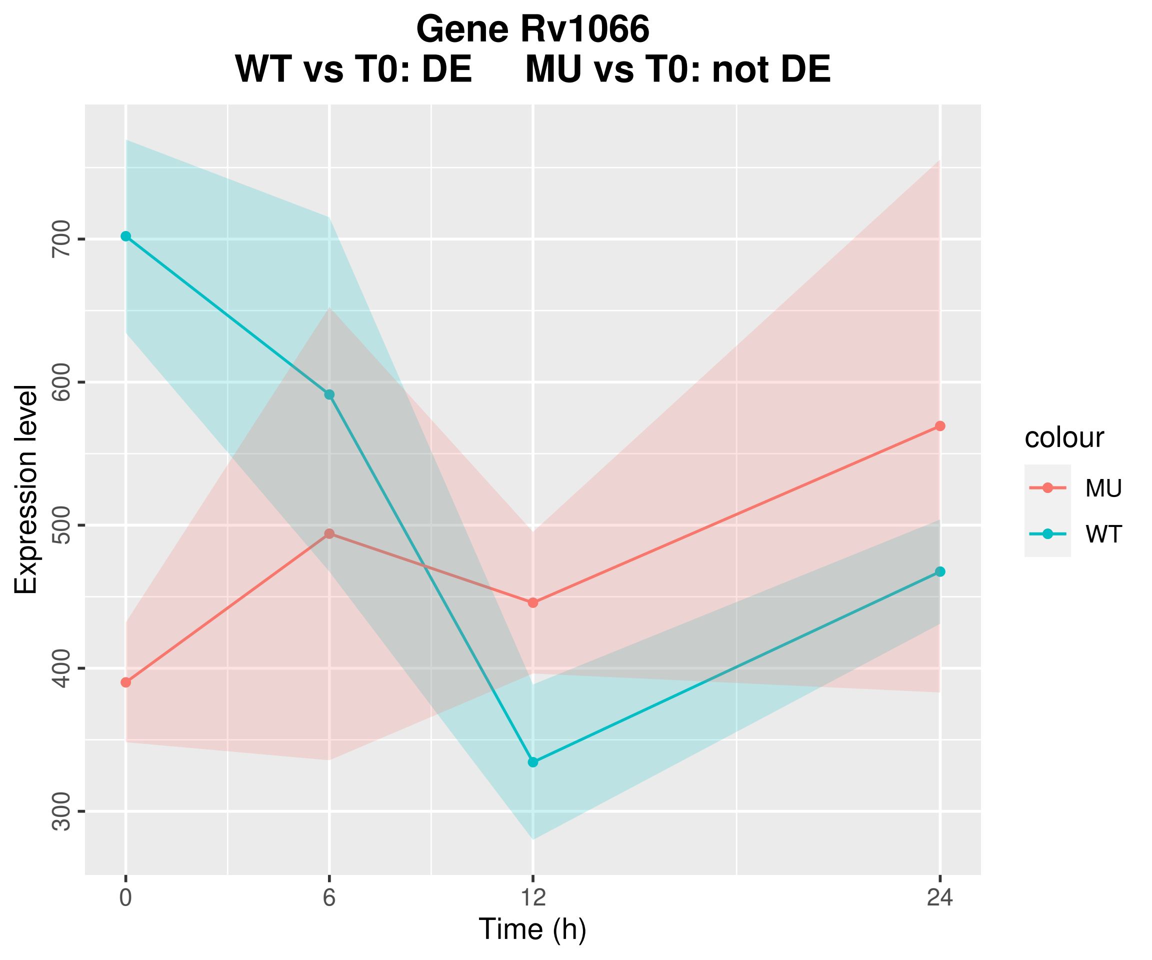
Gene Rv1066
WT vs T0: DE   MU vs T0: not DE
Expression level
700
600
500
400
300
0
6
12
24
Time (h)
colour
MU
WT

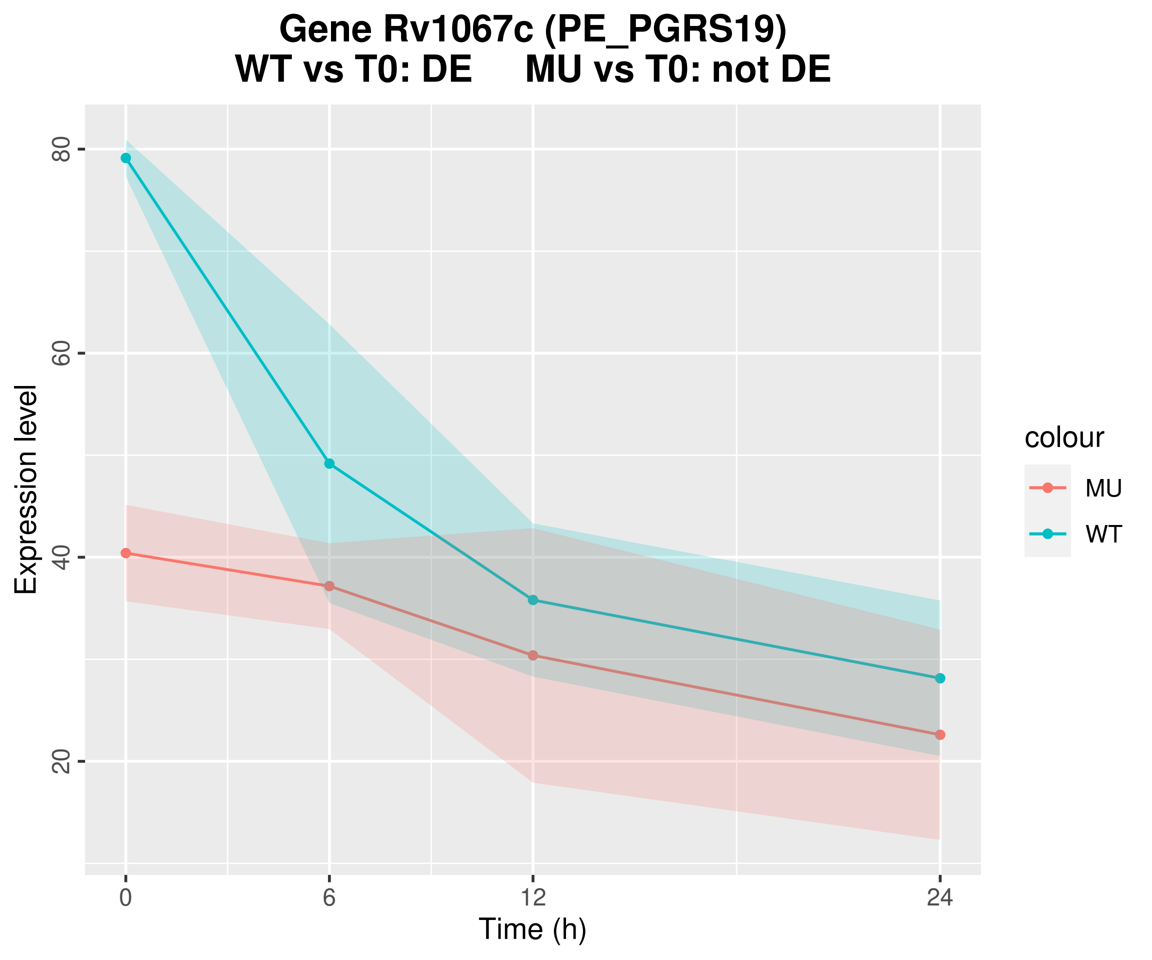
Gene Rv1067c (PE_PGRS19)
WT vs T0: DE   MU vs T0: not DE
Expression level
80
60
40
20
0
6
12
24
Time (h)
colour
MU
WT

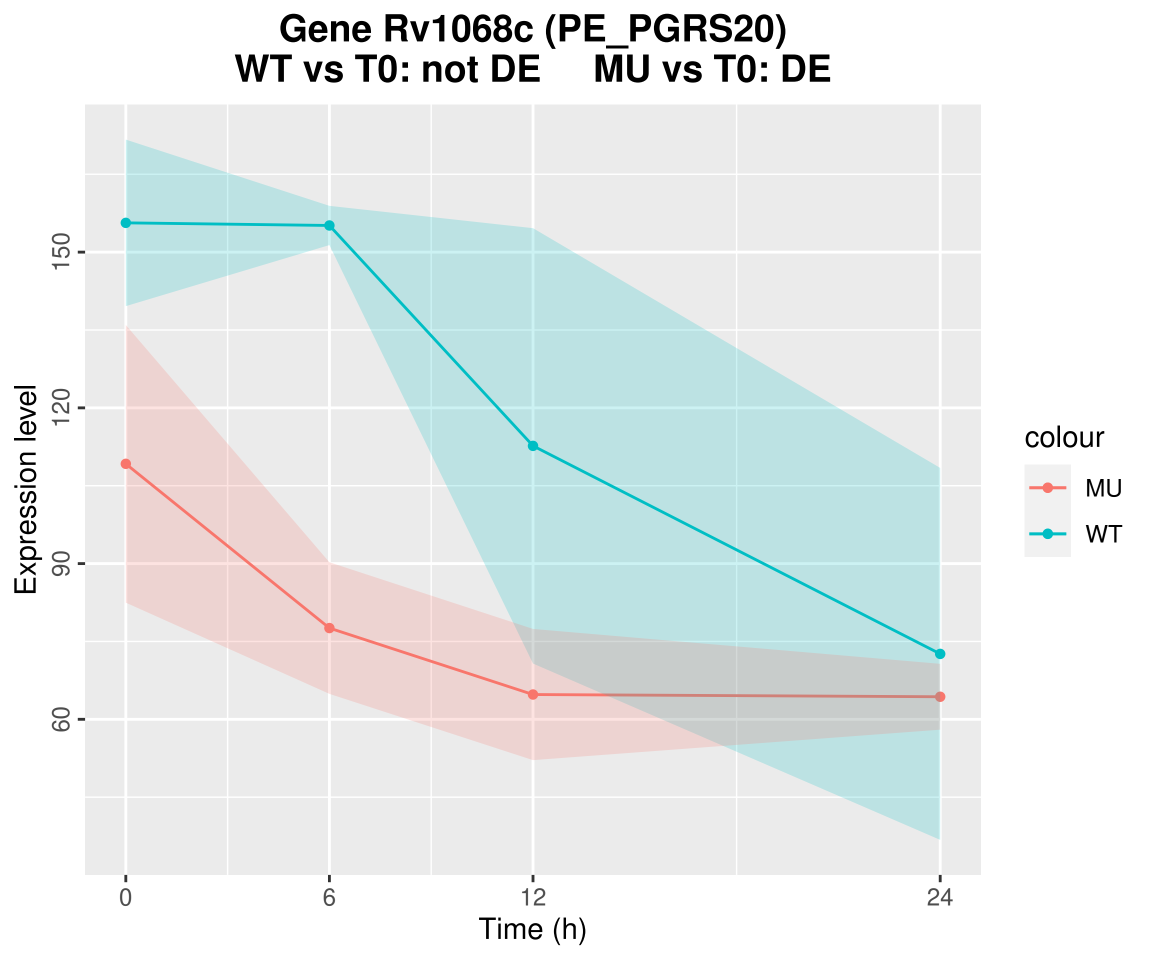

Gene Rv1068c (PE_PGRS20)
WT vs T0: not DE MU vs T0: DE
Expression level
150
120
90
60
0
6
12
24
Time (h)
colour
MU
WT

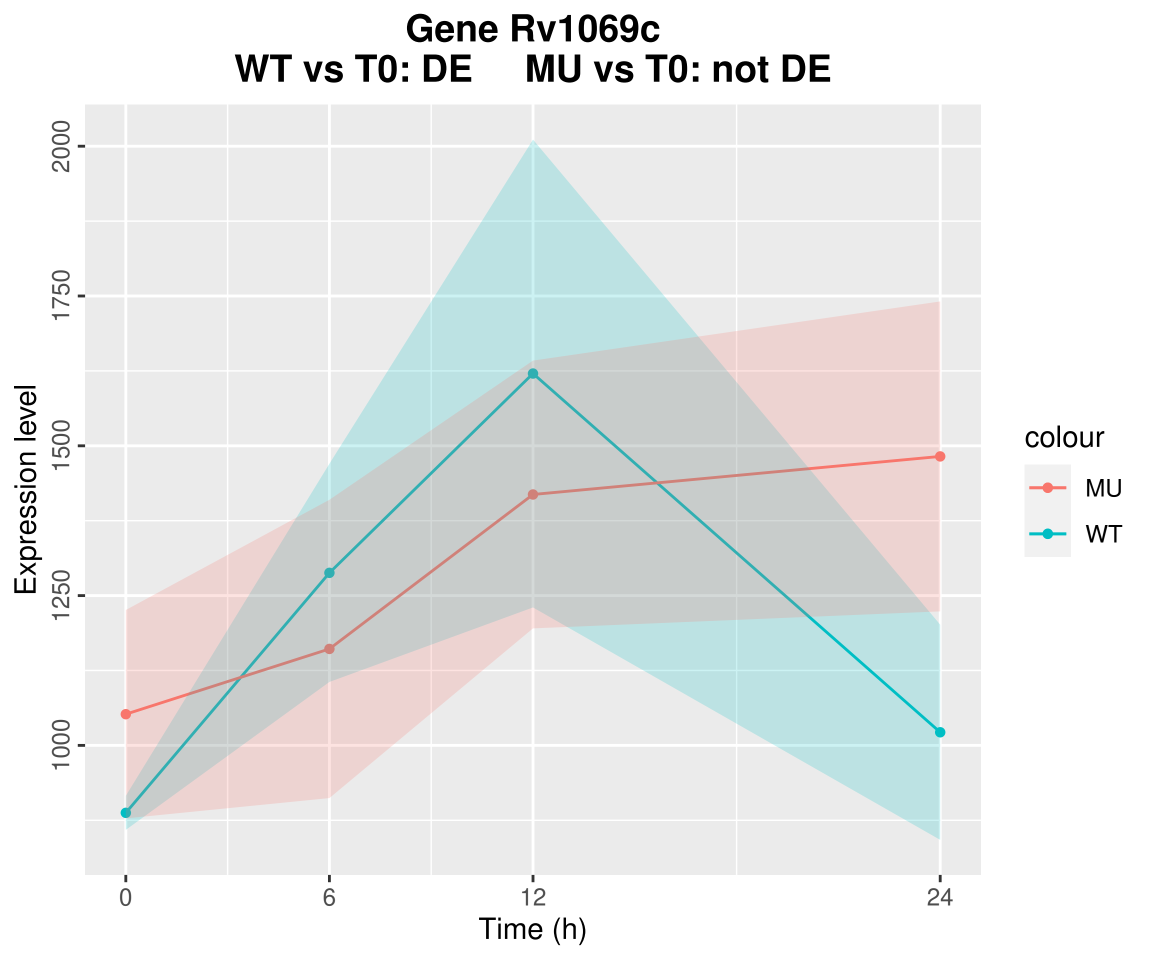

Gene Rv1069c
WT vs T0: DE MU vs T0: not DE
Expression level
2000
1750
1500
1250
1000
0
6
12
24
Time (h)
colour
MU
WT

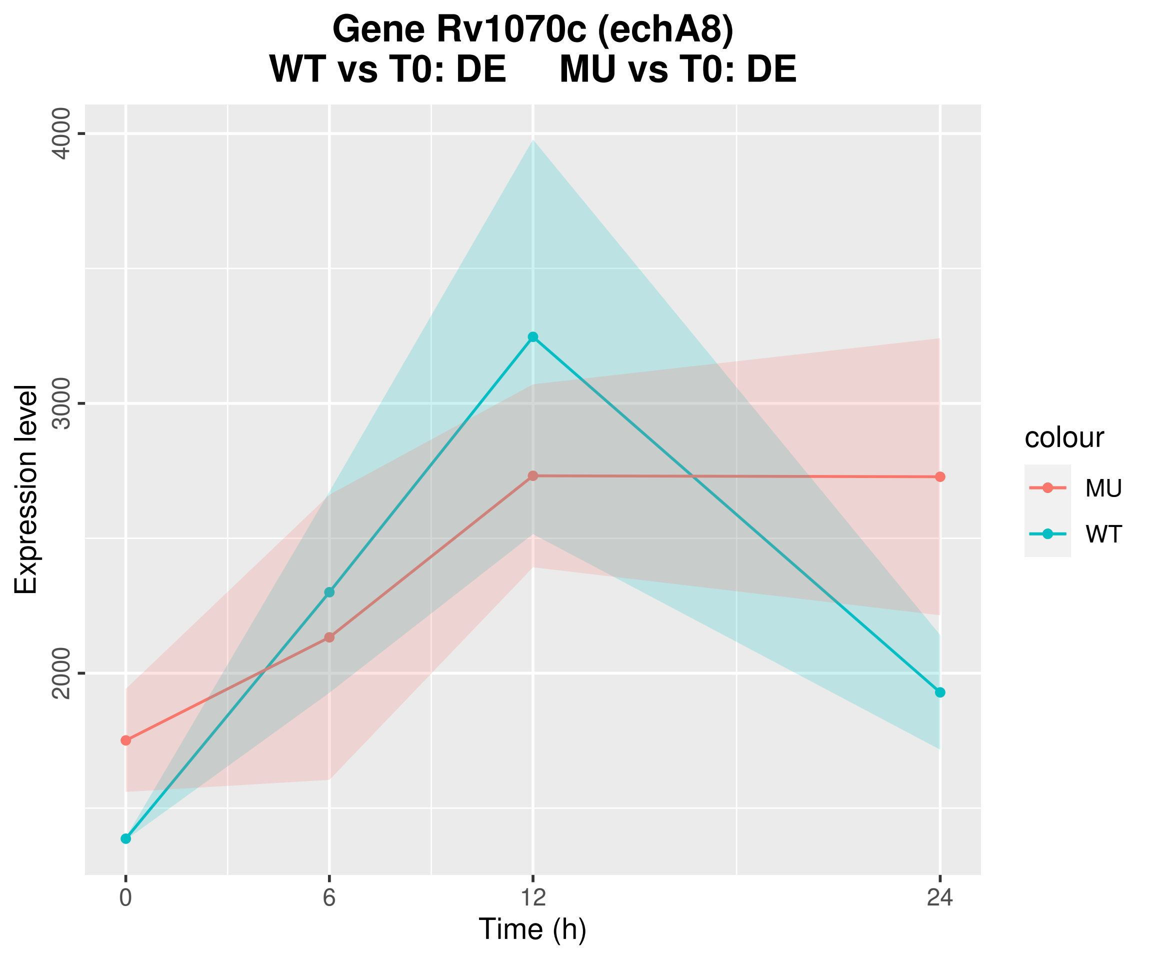

Gene Rv1070c (echA8)
WT vs T0: DE MU vs T0: DE
4000
3000
2000
Expression level
0
6
12
24
Time (h)
colour
MU
WT

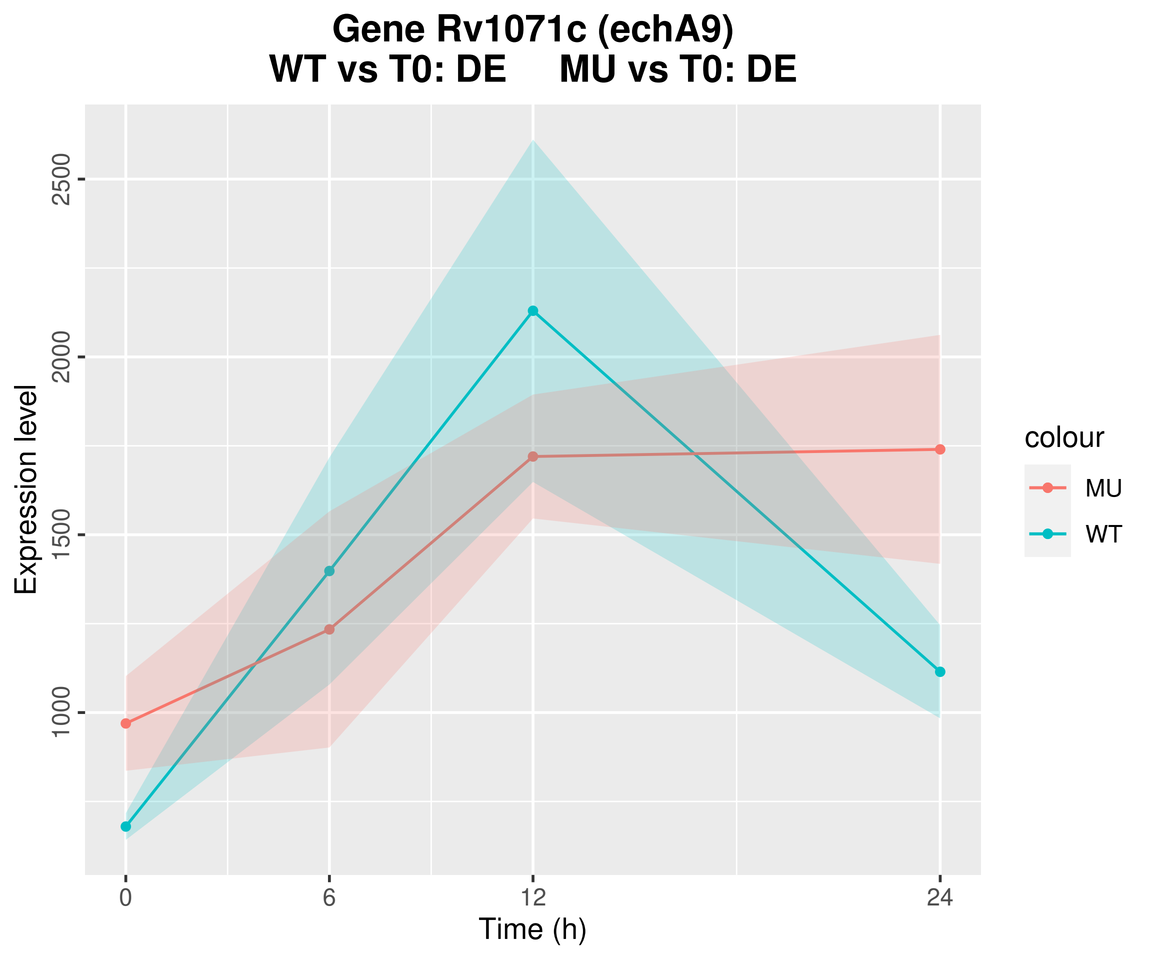
Gene Rv1071c (echA9)
WT vs T0: DE   MU vs T0: DE
Expression level
2500
2000
1500
1000
0
6
12
24
Time (h)
colour
MU
WT

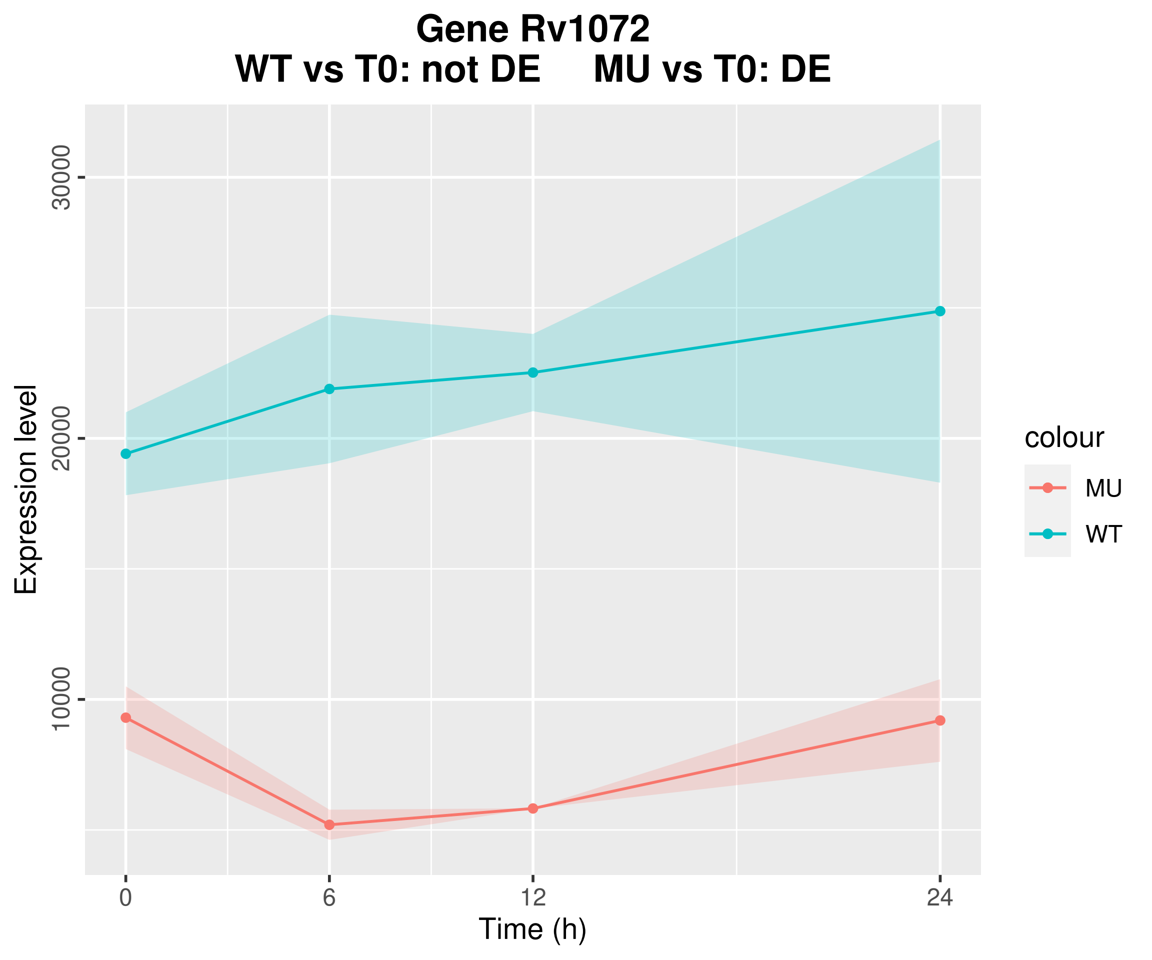

Gene Rv1072
WT vs T0: not DE   MU vs T0: DE
30000
20000
10000
Expression level
0
6
12
24
Time (h)
colour
MU
WT

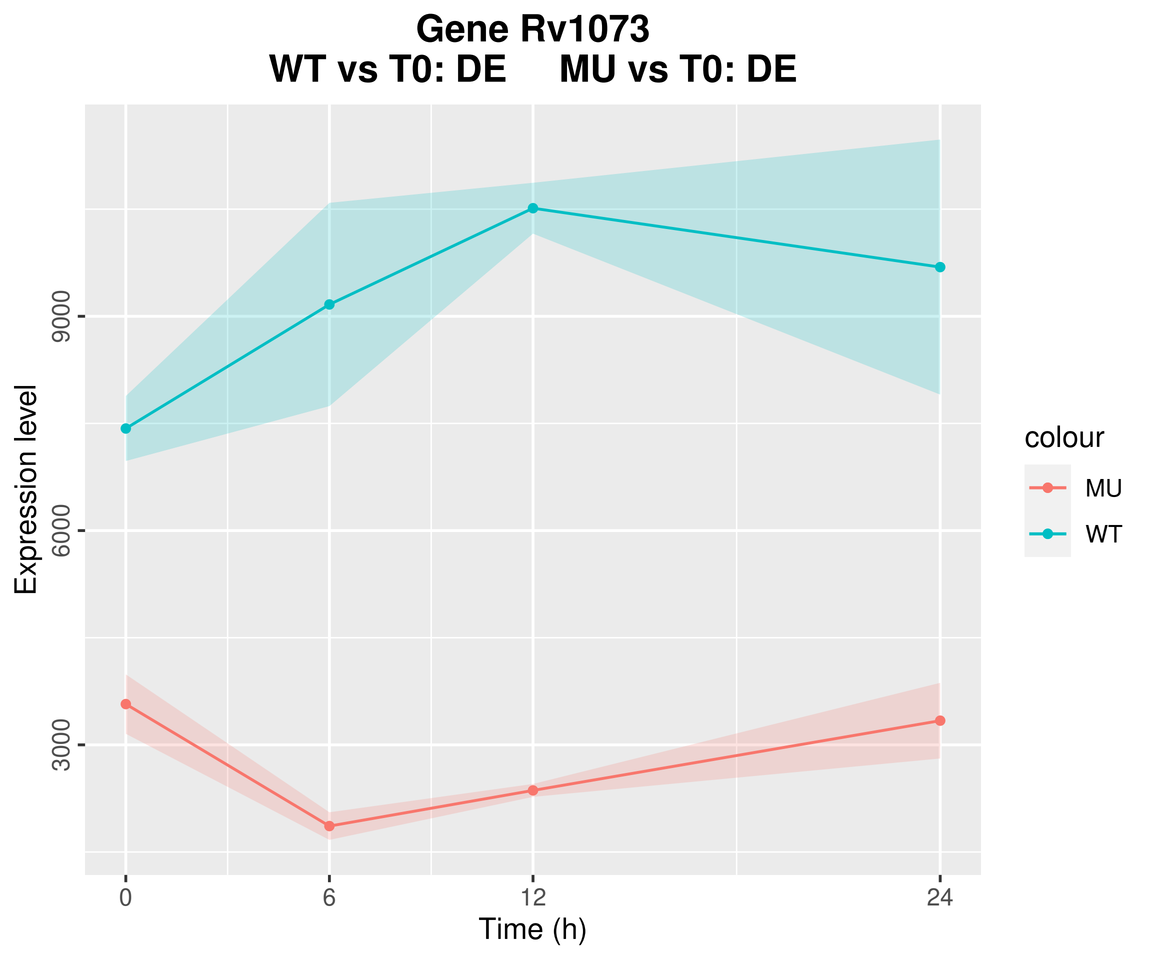

Gene Rv1073
WT vs T0: DE MU vs T0: DE
Expression level
9000
6000
3000
0
6
12
24
Time (h)
colour
MU
WT

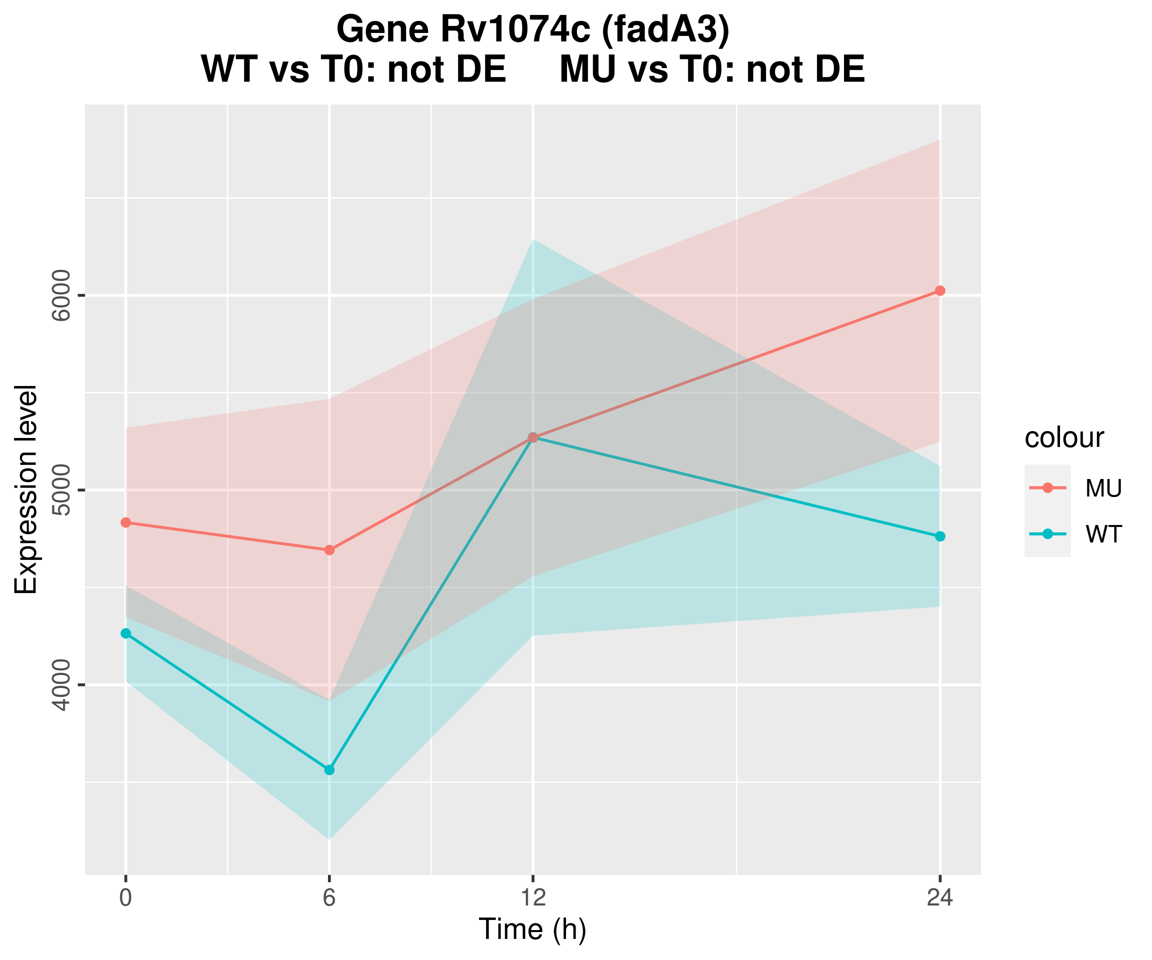

Gene Rv1074c (fadA3)
WT vs T0: not DE MU vs T0: not DE
Expression level
6000
5000
4000
0
6
12
24
Time (h)
colour
MU
WT

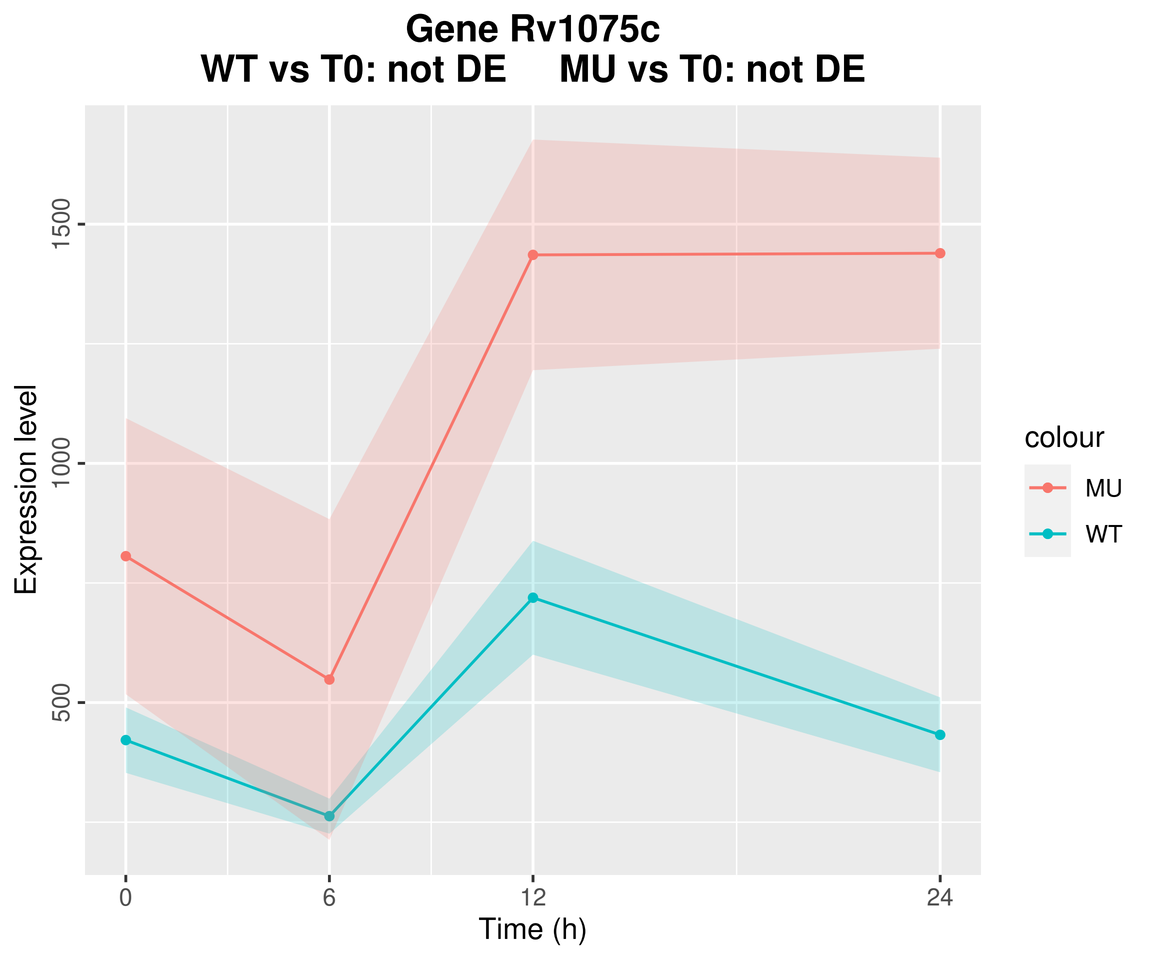
Gene Rv1075c
WT vs T0: not DE MU vs T0: not DE
Expression level
1500
1000
500
0
6
12
24
Time (h)
colour
MU
WT

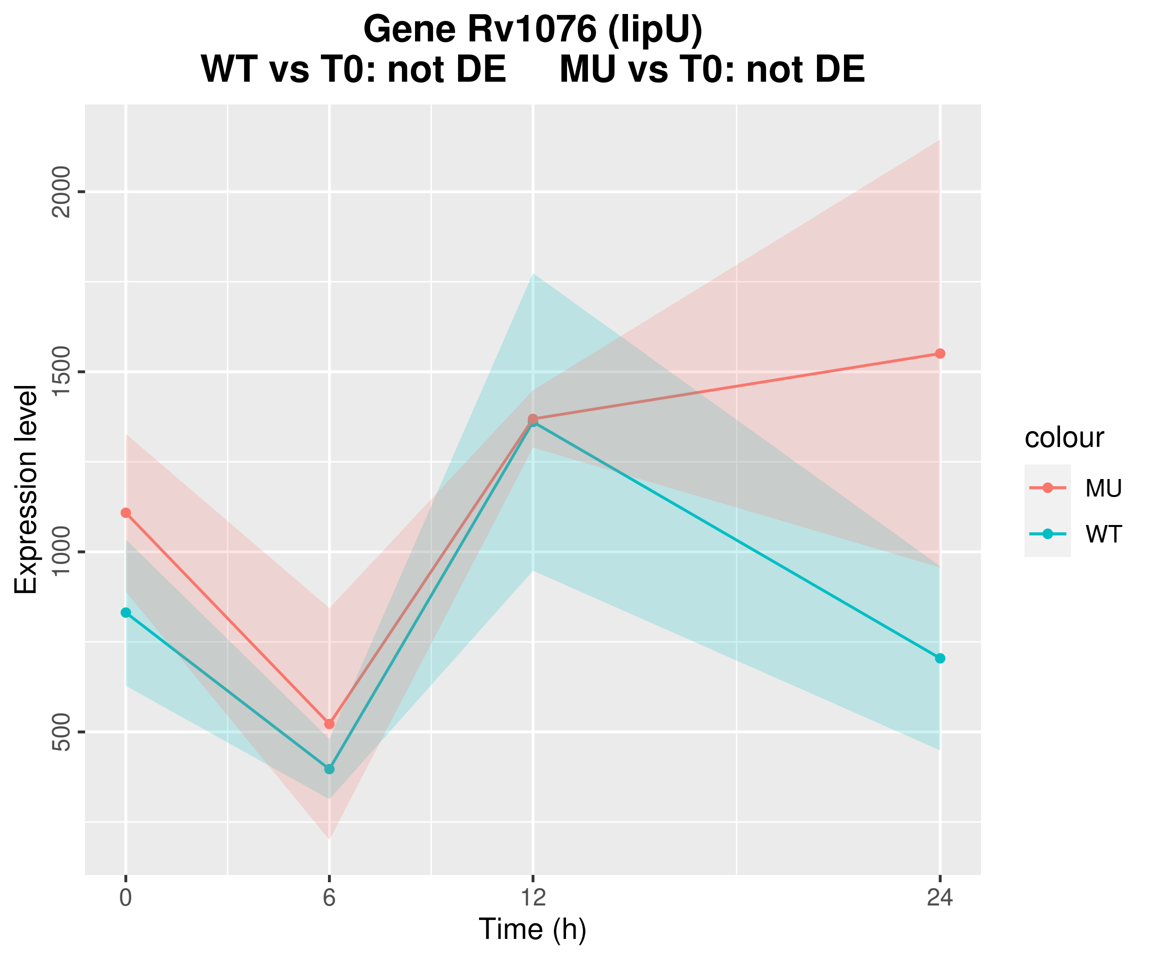

Gene Rv1076 (lipU)
WT vs T0: not DE   MU vs T0: not DE
Expression level
2000
1500
1000
500
0
6
12
24
Time (h)
colour
MU
WT

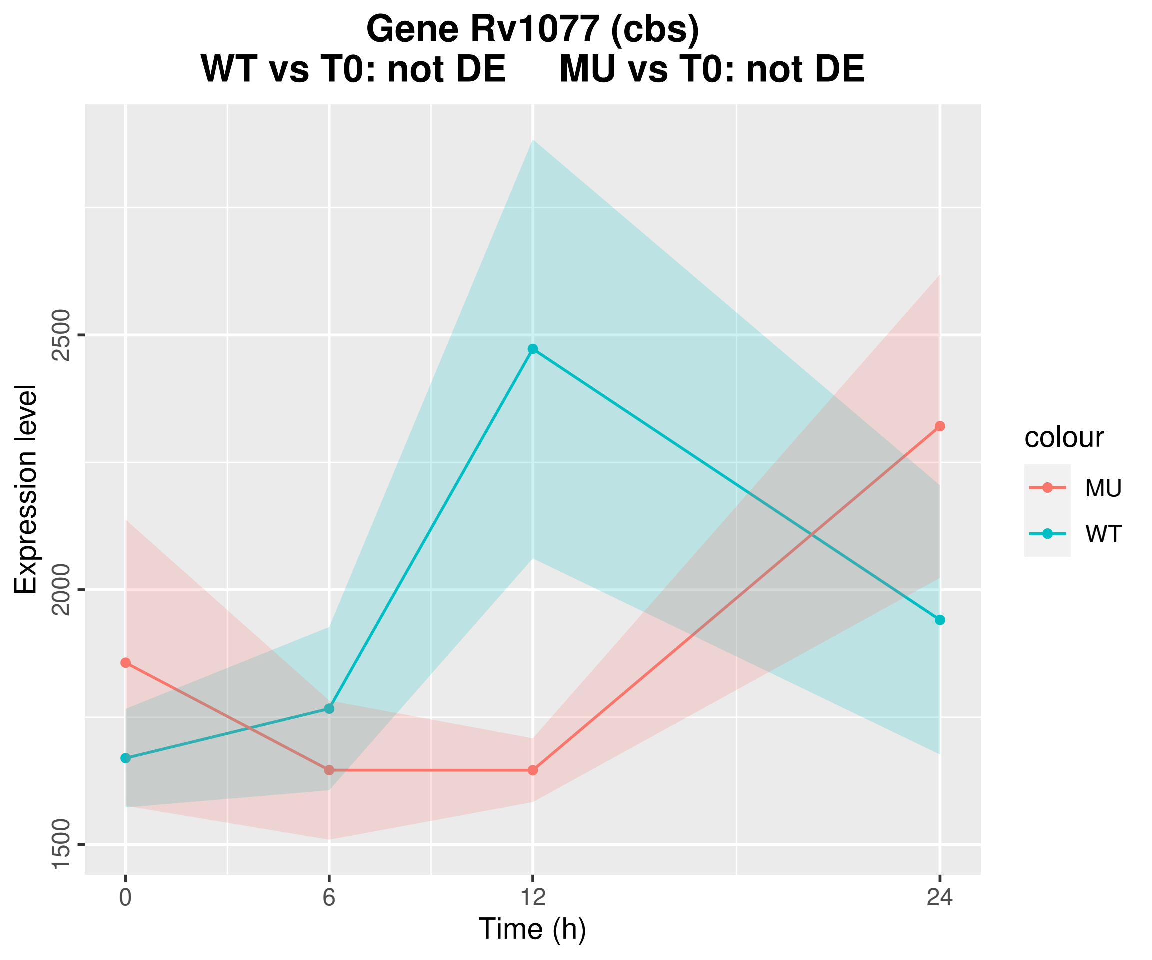

Gene Rv1077 (cbs)
WT vs T0: not DE   MU vs T0: not DE
Expression level
2500
2000
1500
0
6
12
24
Time (h)
colour
MU
WT

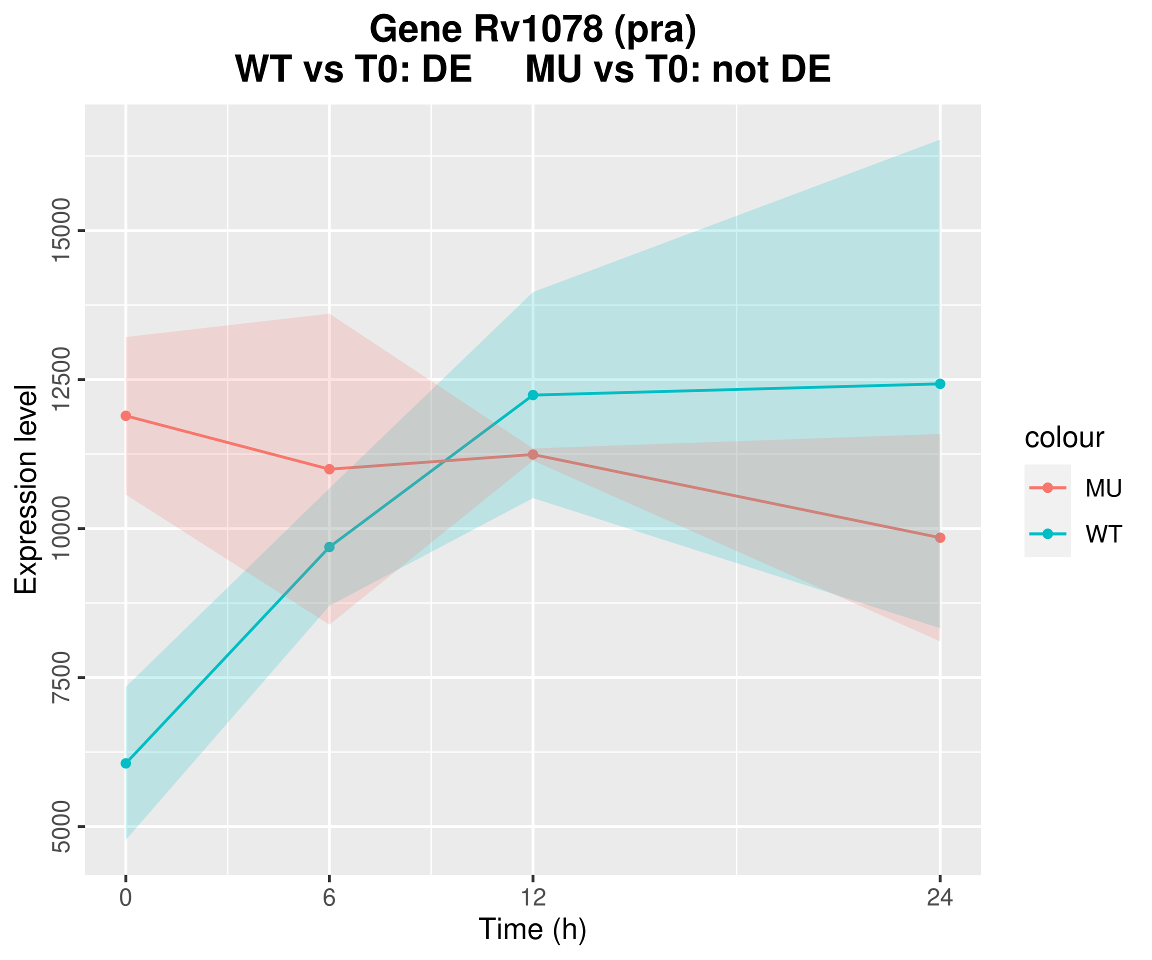

Gene Rv1078 (pra)
WT vs T0: DE MU vs T0: not DE
Expression level
15000
12500
10000
7500
5000
0
6
12
24
Time (h)
colour
MU
WT

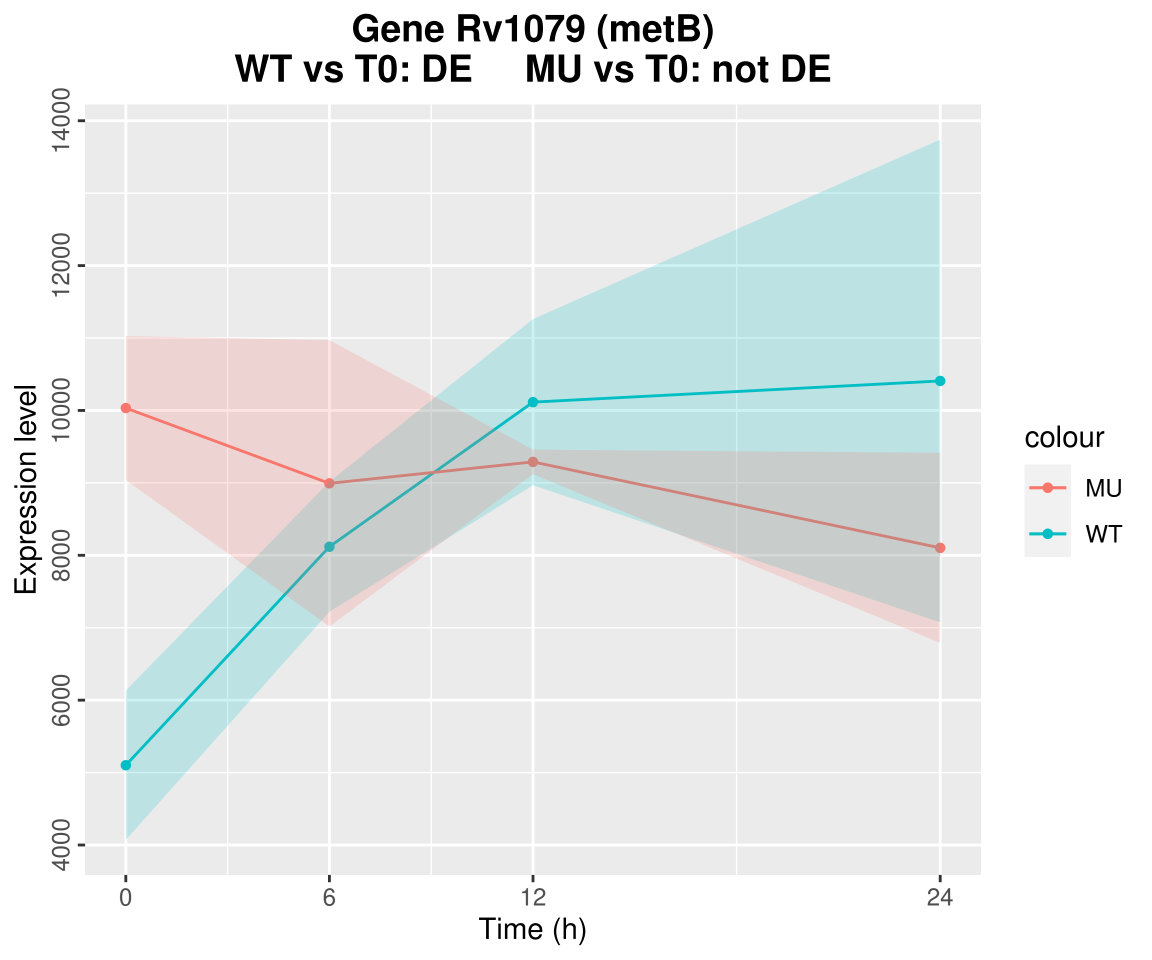

Gene Rv1079 (metB)
WT vs T0: DE MU vs T0: not DE
Expression level
4000
6000
8000
10000
12000
14000
0
6
12
24
Time (h)
colour
MU
WT

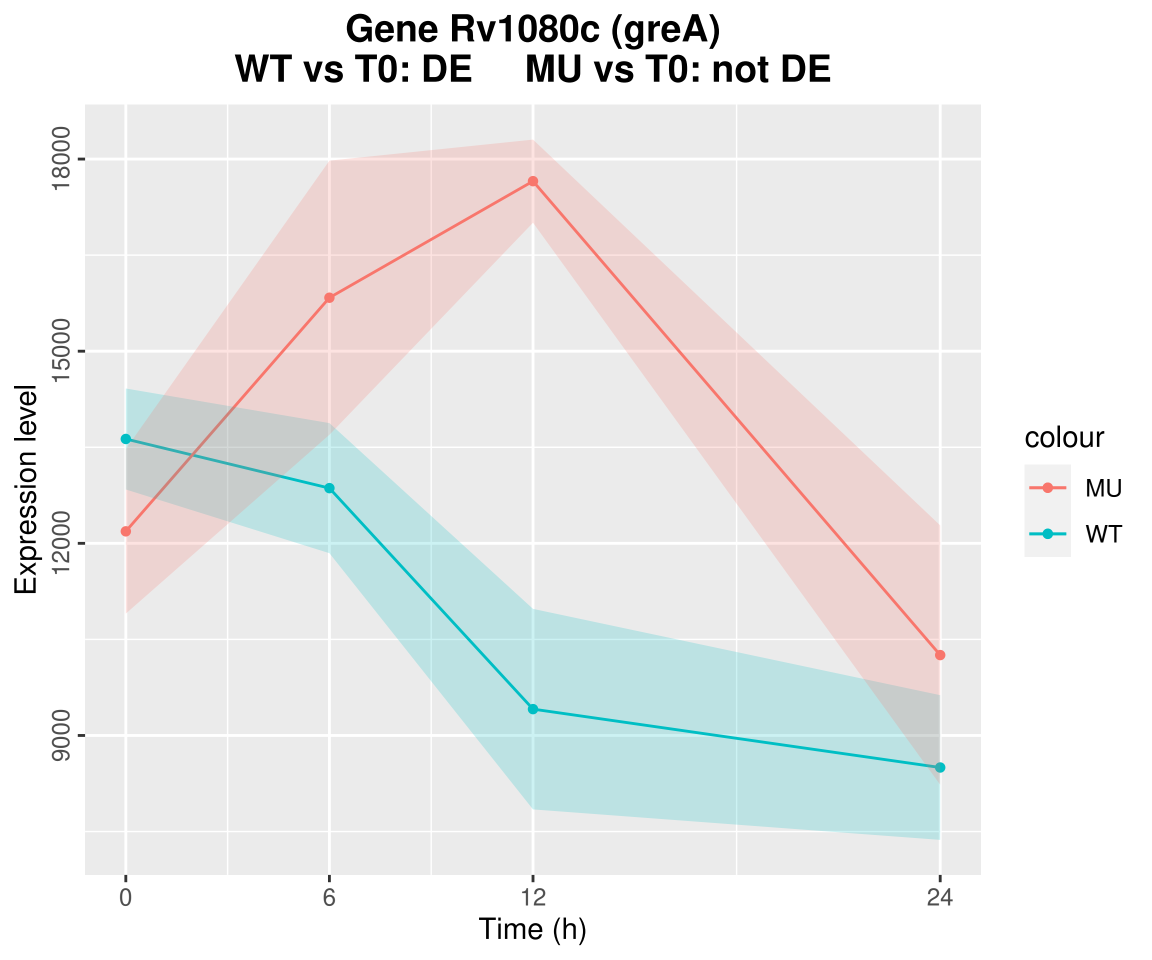

Gene Rv1080c (greA)
WT vs T0: DE   MU vs T0: not DE
Expression level
18000
15000
12000
9000
0
6
12
24
Time (h)
colour
MU
WT

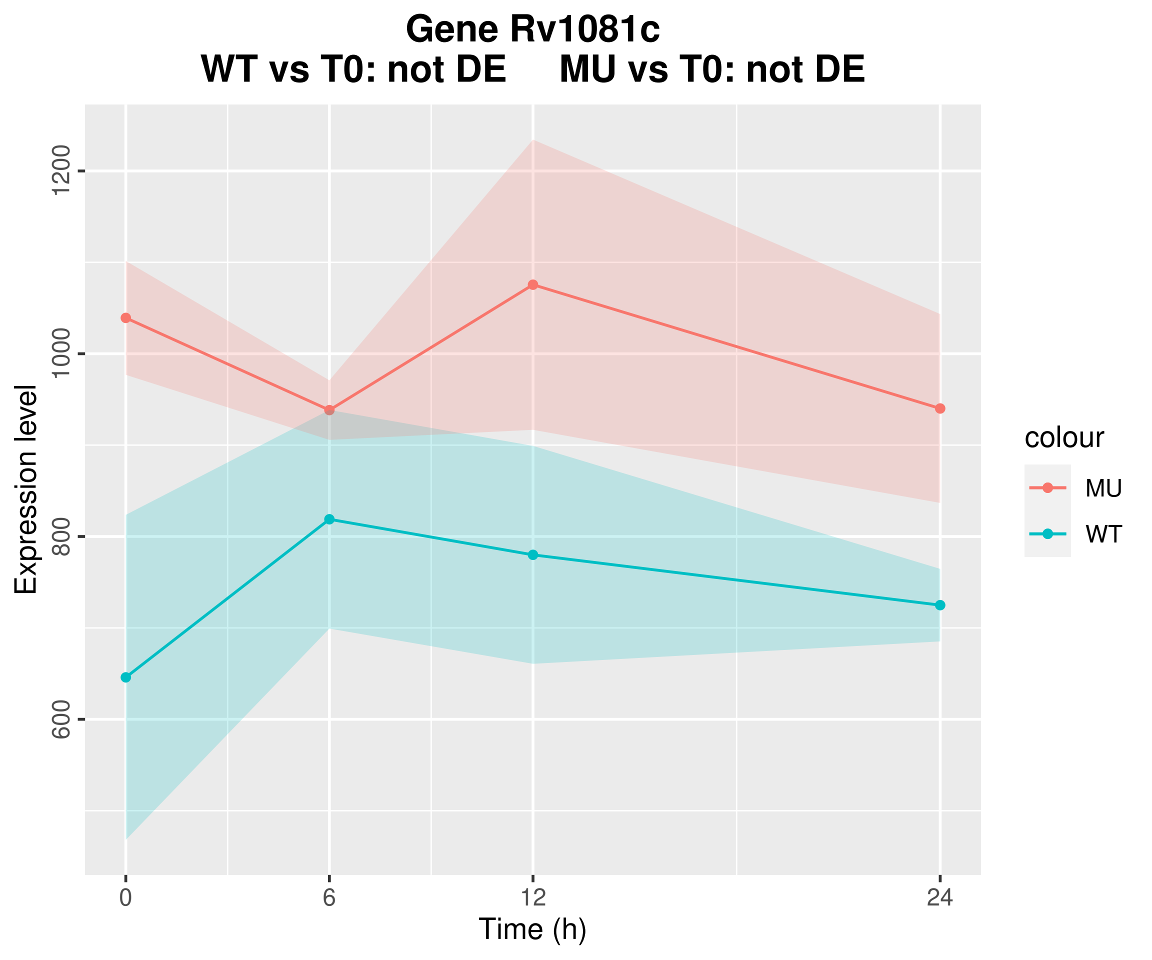
Gene Rv1081c
WT vs T0: not DE   MU vs T0: not DE
1200
1000
800
600
Expression level
0
6
12
24
Time (h)
colour
MU
WT

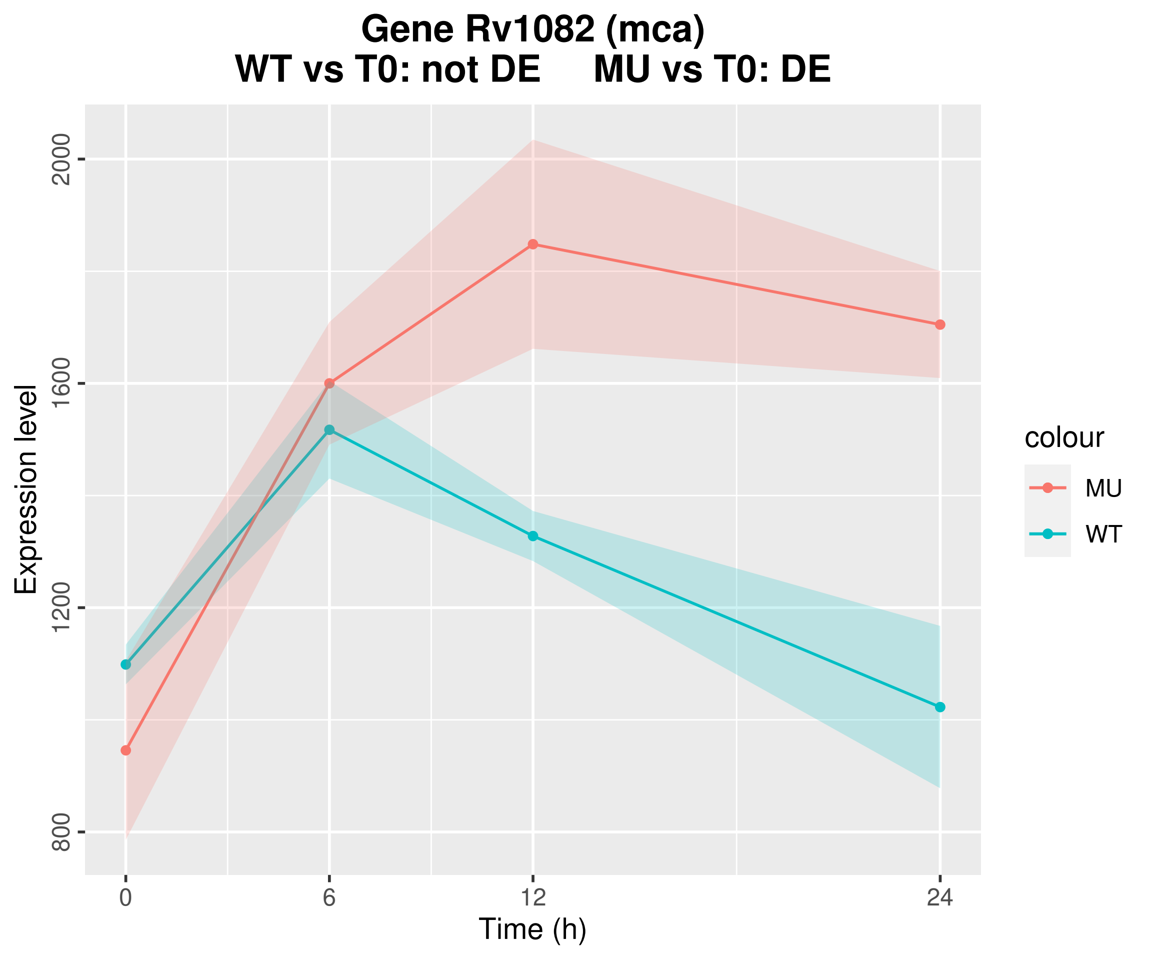
Gene Rv1082 (mca)
WT vs T0: not DE MU vs T0: DE
Expression level
2000
1600
1200
800
Time (h)
0
6
12
24
colour
MU
WT

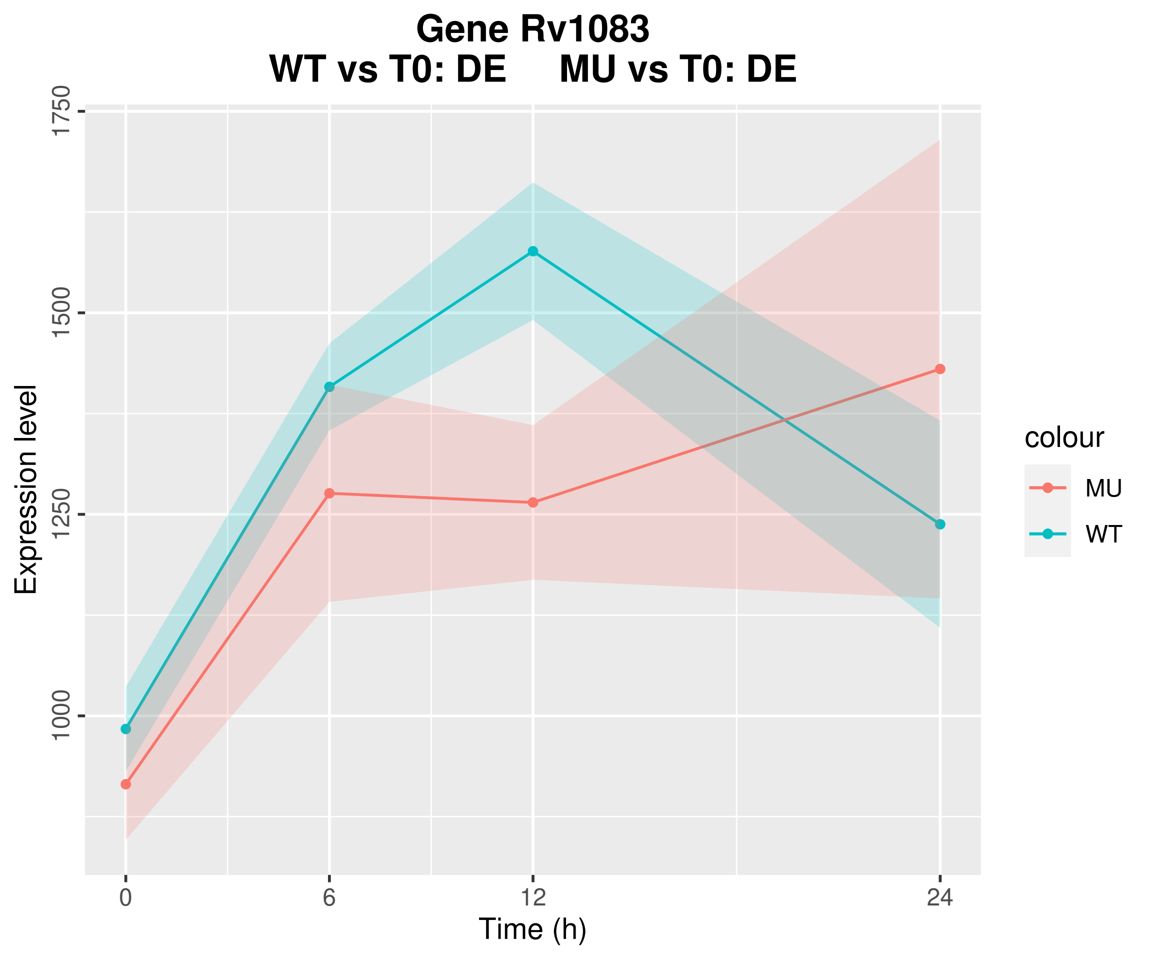

Gene Rv1083
WT vs T0: DE   MU vs T0: DE
Expression level
1750
1500
1250
1000
0
6
12
24
Time (h)
colour
MU
WT

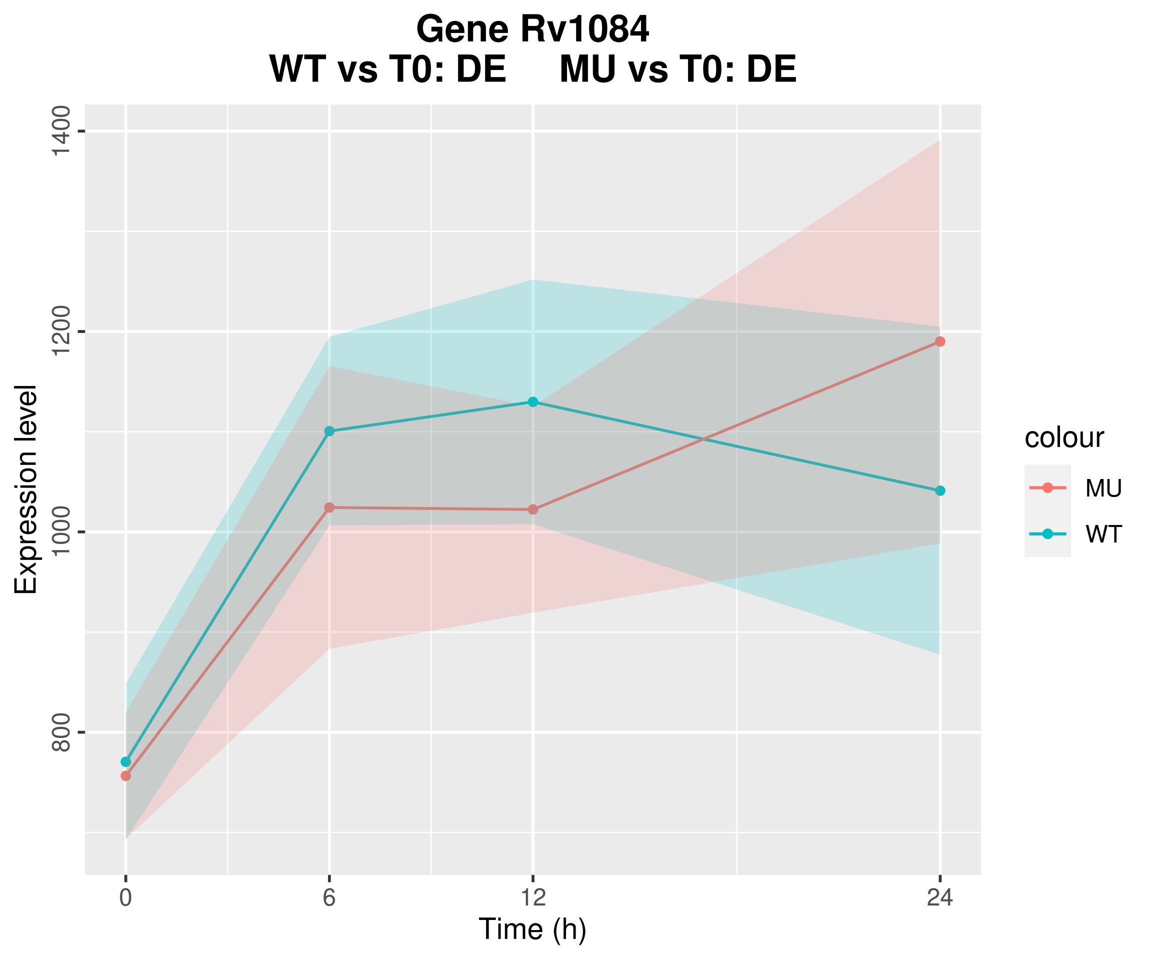
Gene Rv1084
WT vs T0: DE MU vs T0: DE
1400
1200
1000
800
Expression level
0
6
12
24
Time (h)
colour
MU
WT

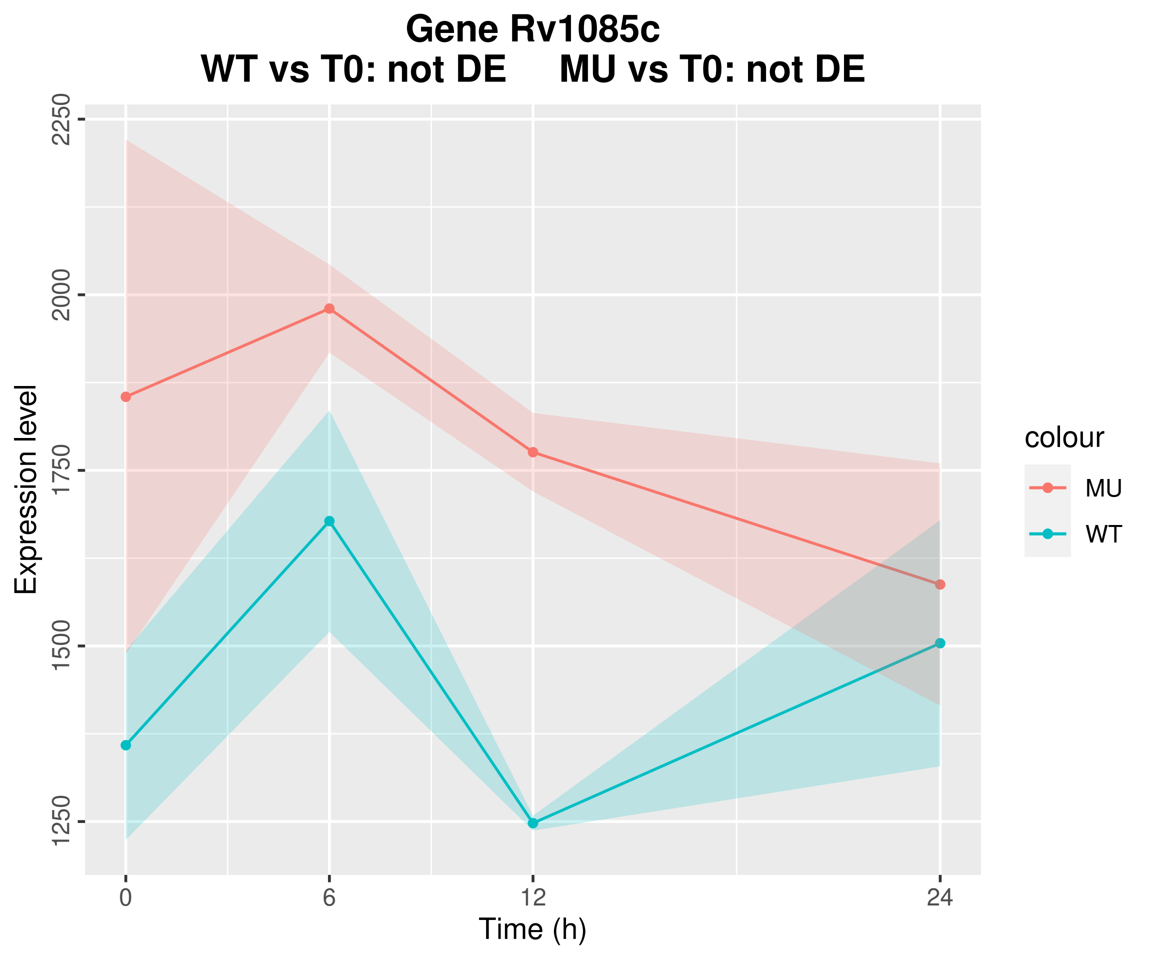
Gene Rv1085c
WT vs T0: not DE MU vs T0: not DE
Expression level
2250
2000
1750
1500
1250
0
6
12
24
Time (h)
colour
MU
WT

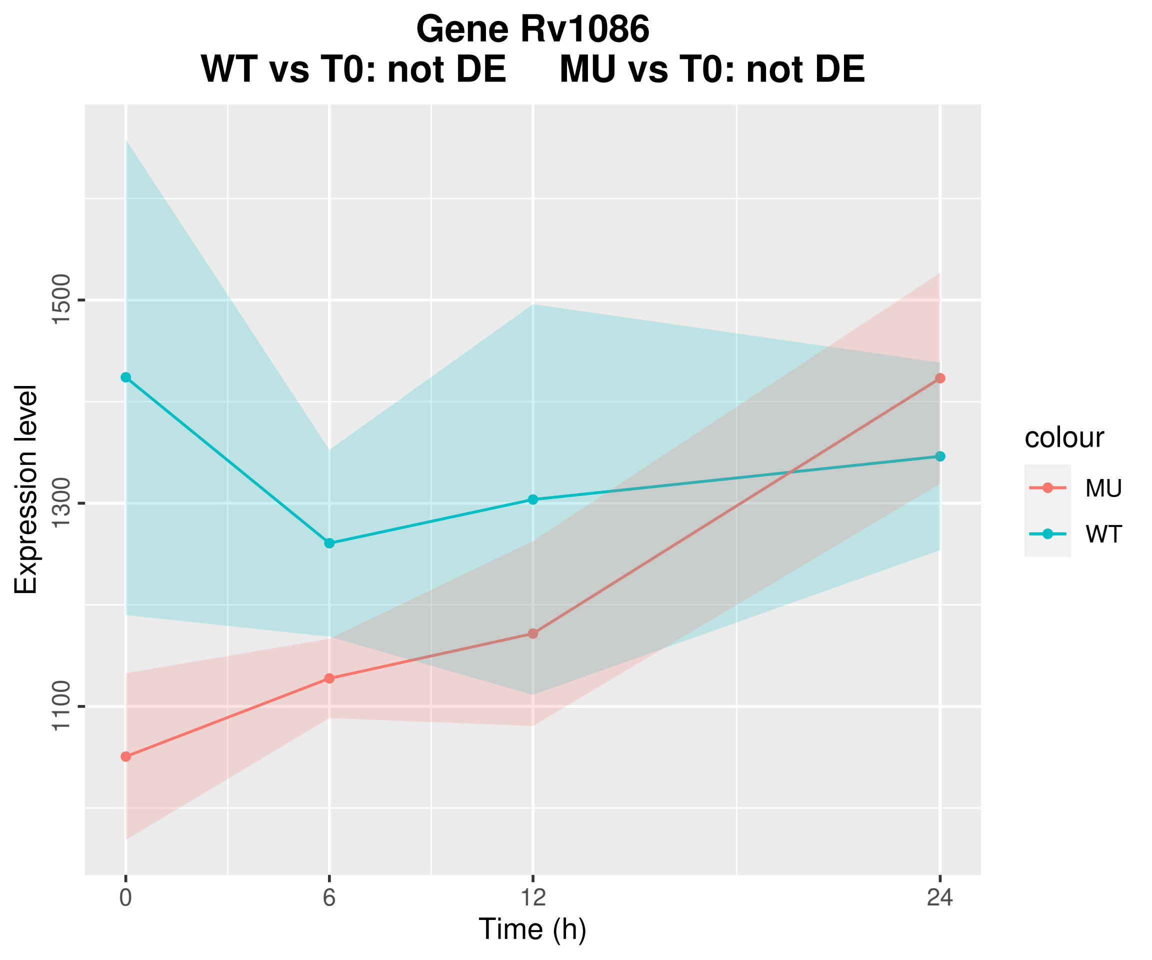

Gene Rv1086
WT vs T0: not DE   MU vs T0: not DE
Expression level
1500
1300
1100
0
6
12
24
Time (h)
colour
MU
WT

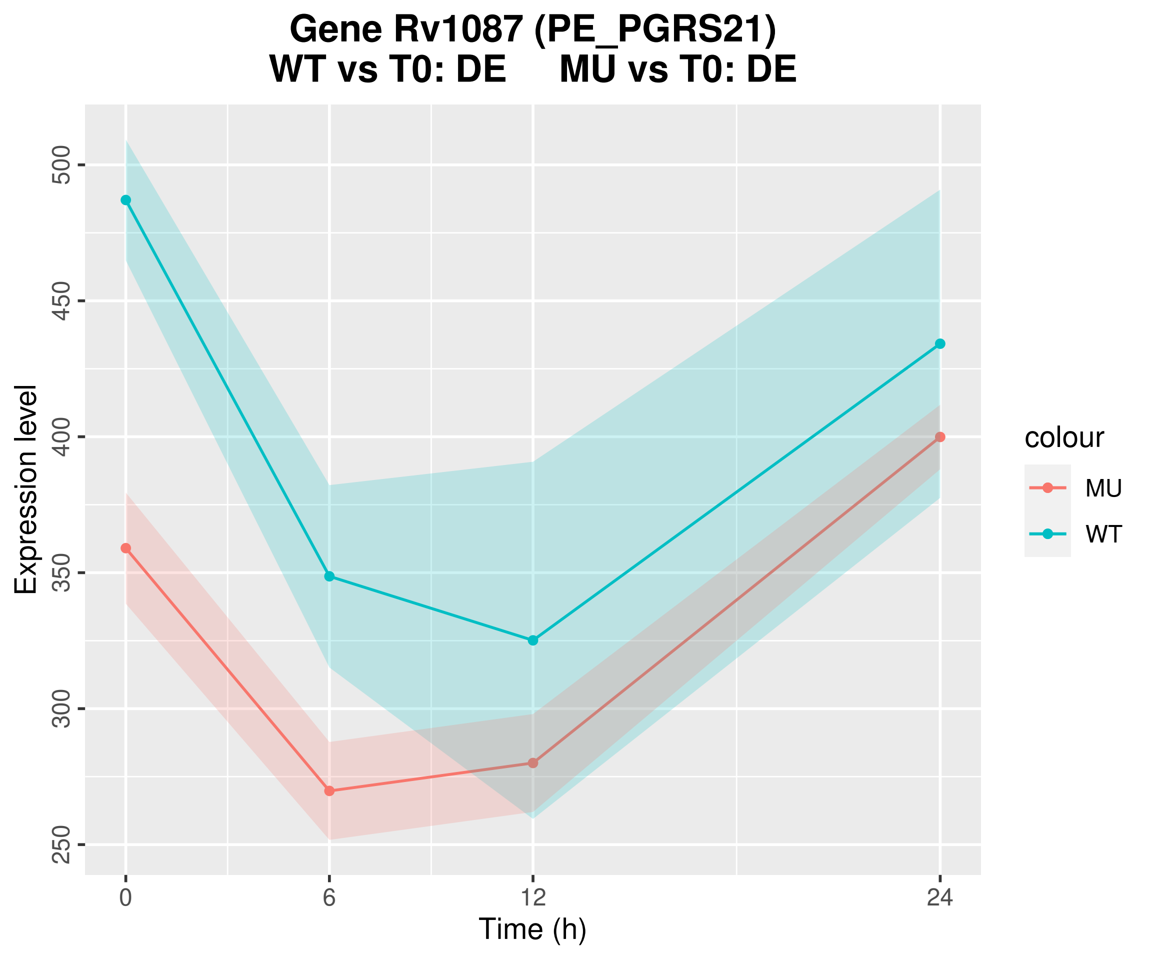

Gene Rv1087 (PE_PGRS21)
WT vs T0: DE   MU vs T0: DE
Expression level
500
450
400
350
300
250
0
6
12
24
Time (h)
colour
MU
WT

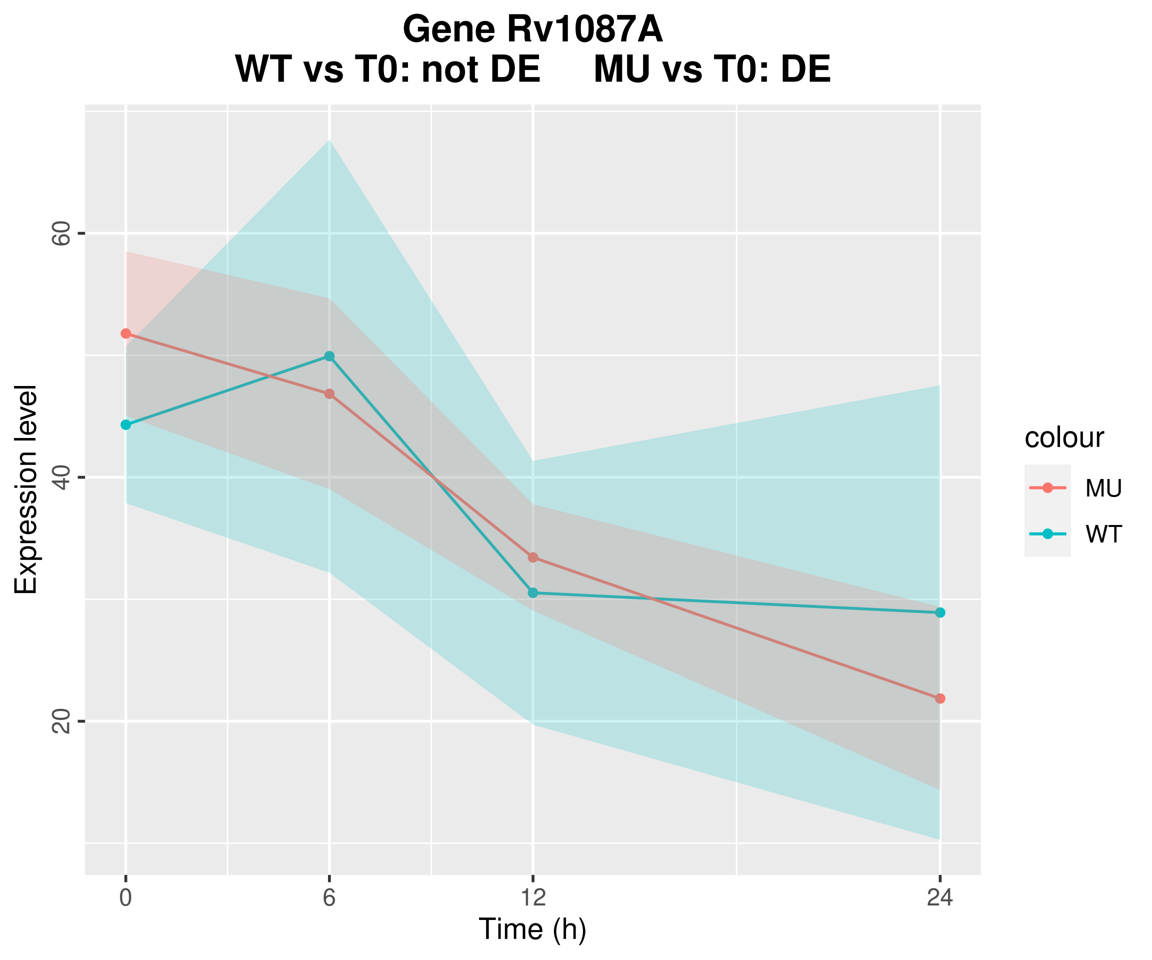
Gene Rv1087A
WT vs T0: not DE MU vs T0: DE
60
40
20
Expression level
0
6
12
24
Time (h)
colour
MU
WT

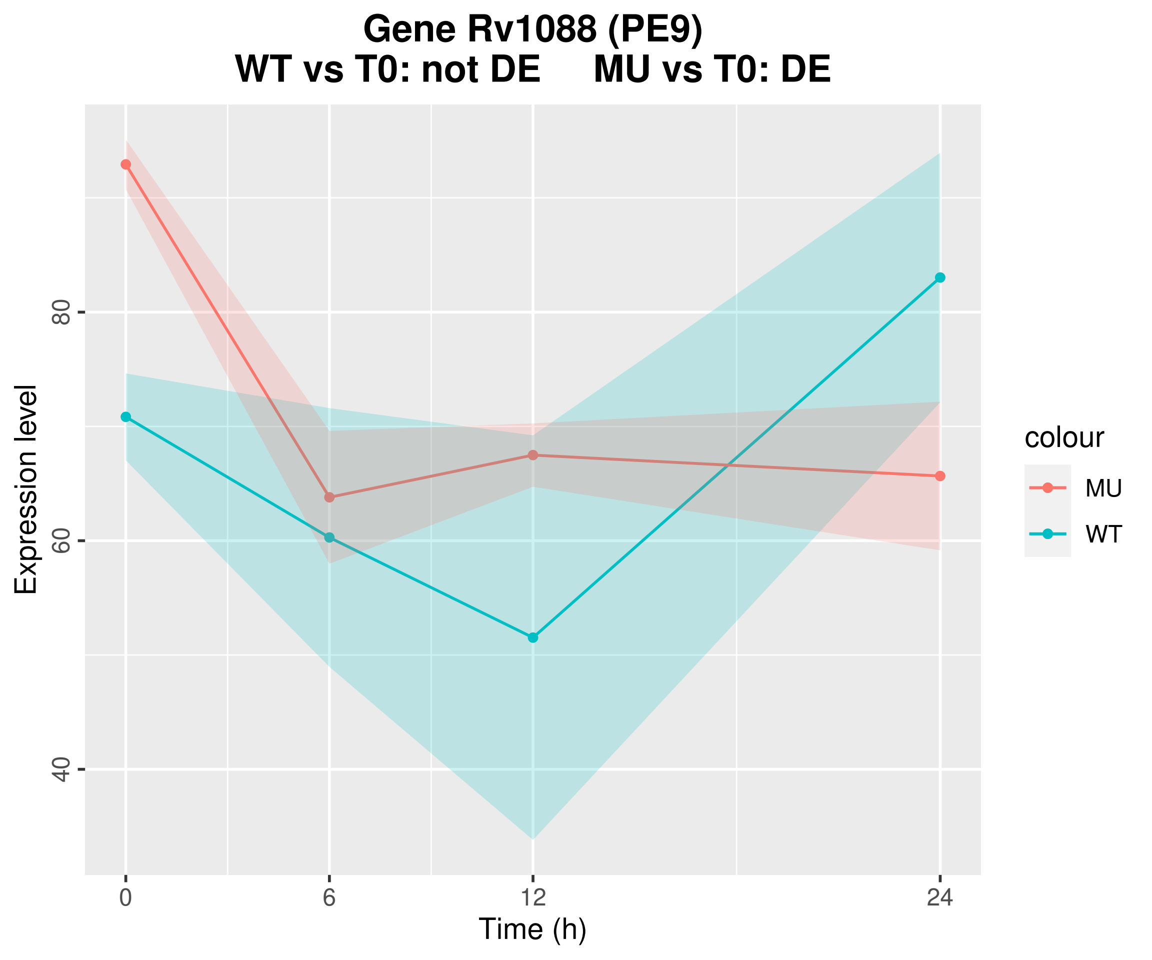

Gene Rv1088 (PE9)
WT vs T0: not DE MU vs T0: DE
Expression level
40
60
80
0
6
12
24
Time (h)
colour
MU
WT

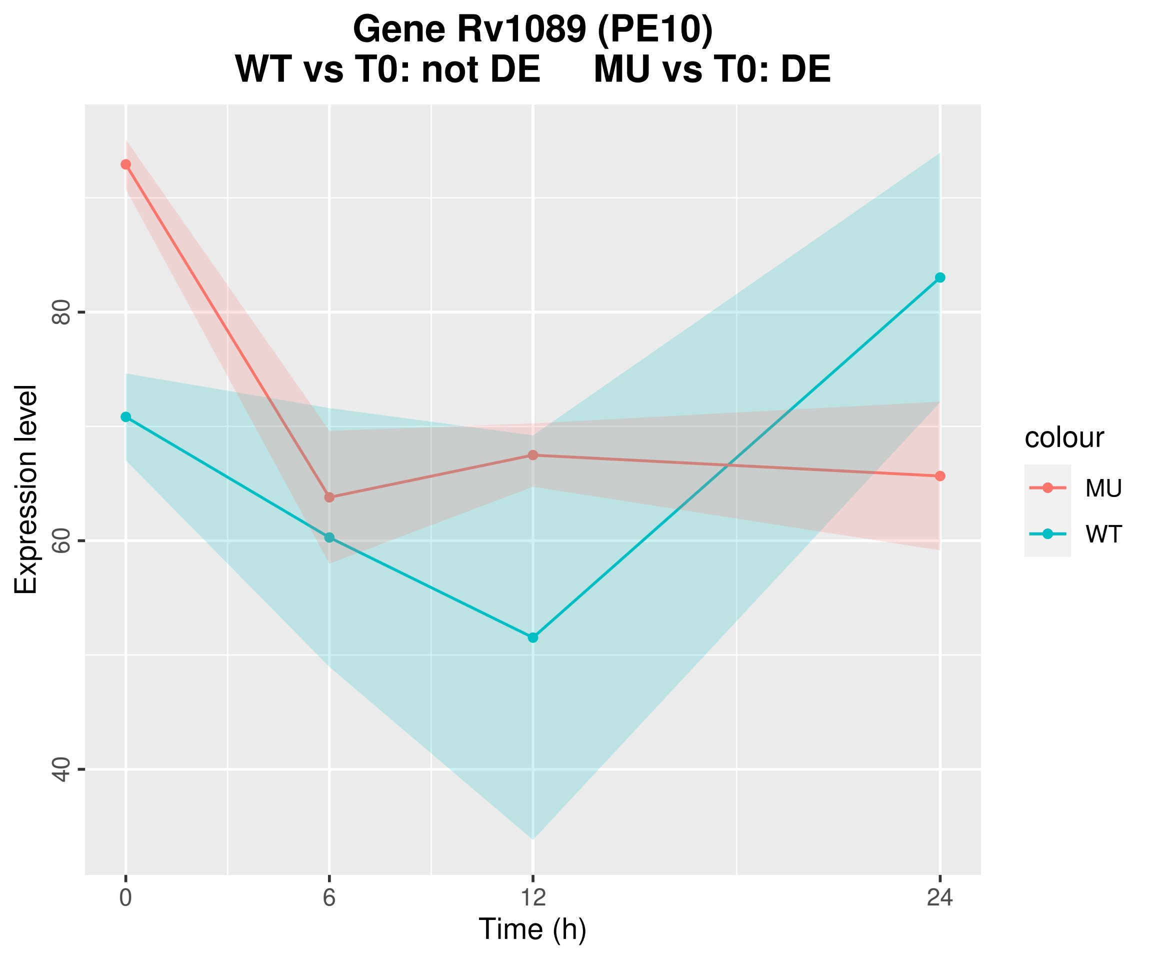

Gene Rv1089 (PE10)
WT vs T0: not DE   MU vs T0: DE
Expression level
80
60
40
0
6
12
24
Time (h)
colour
MU
WT

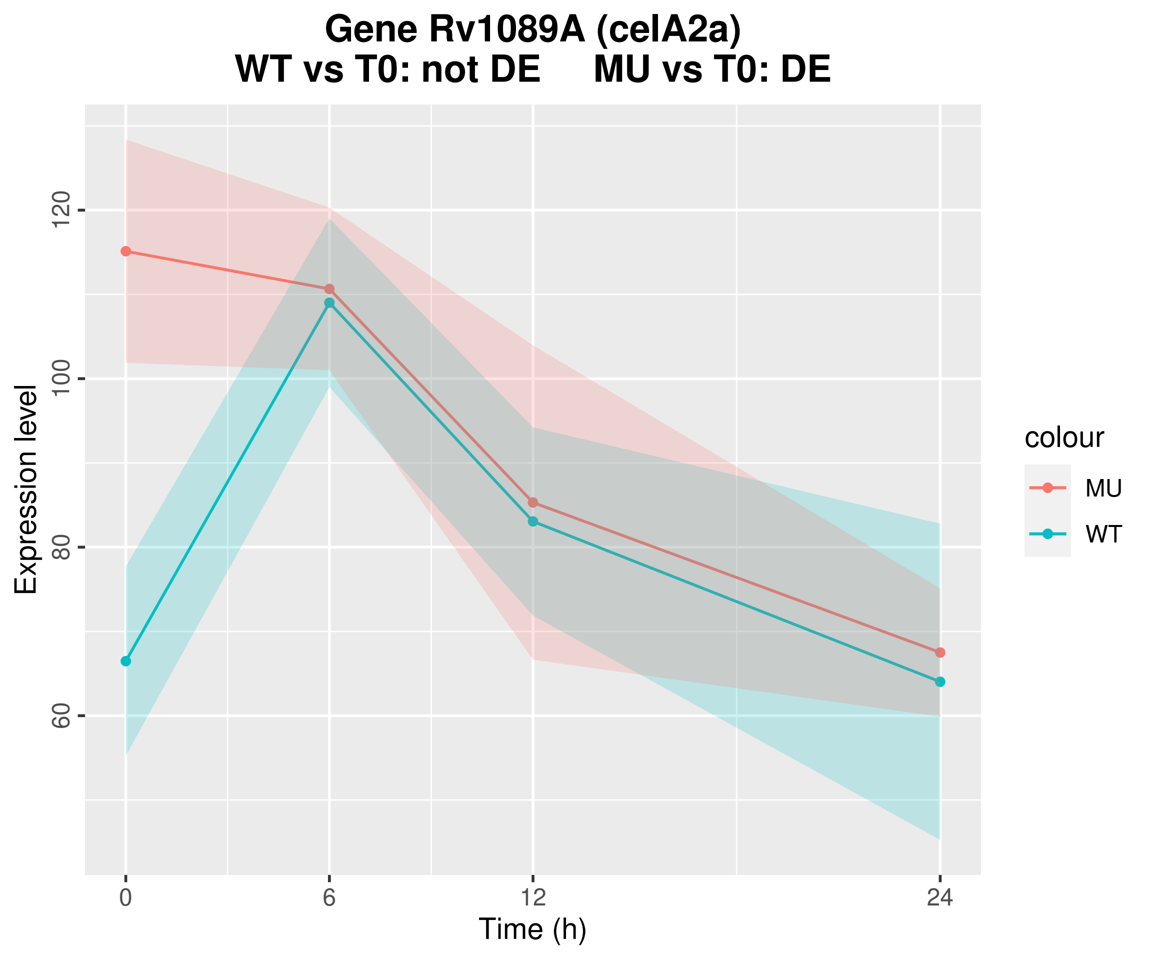

Gene Rv1089A (celA2a)
WT vs T0: not DE MU vs T0: DE
Expression level
120
100
80
60
0
6
12
24
Time (h)
colour
MU
WT

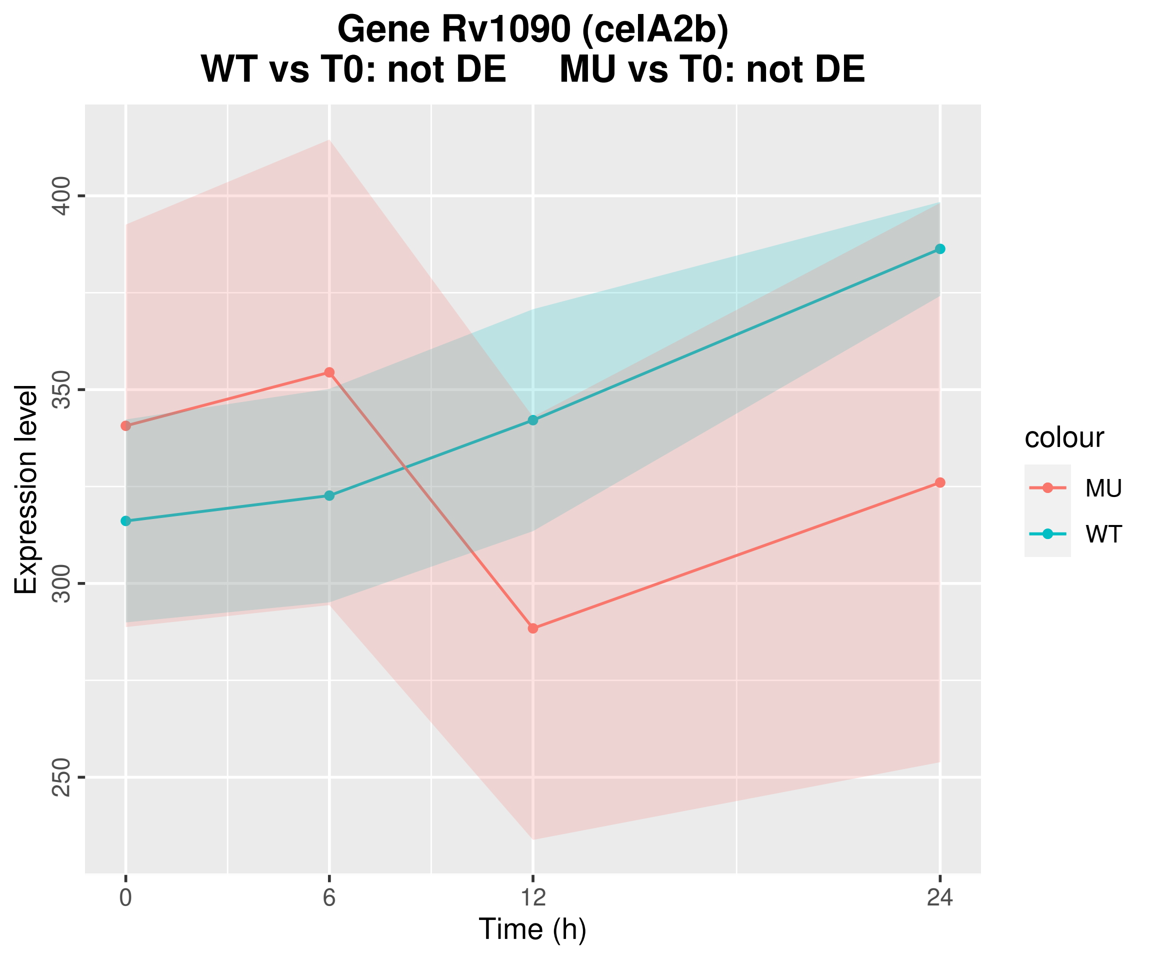

Gene Rv1090 (celA2b)
WT vs T0: not DE   MU vs T0: not DE
Expression level
400
350
300
250
0
6
12
24
Time (h)
colour
MU
WT

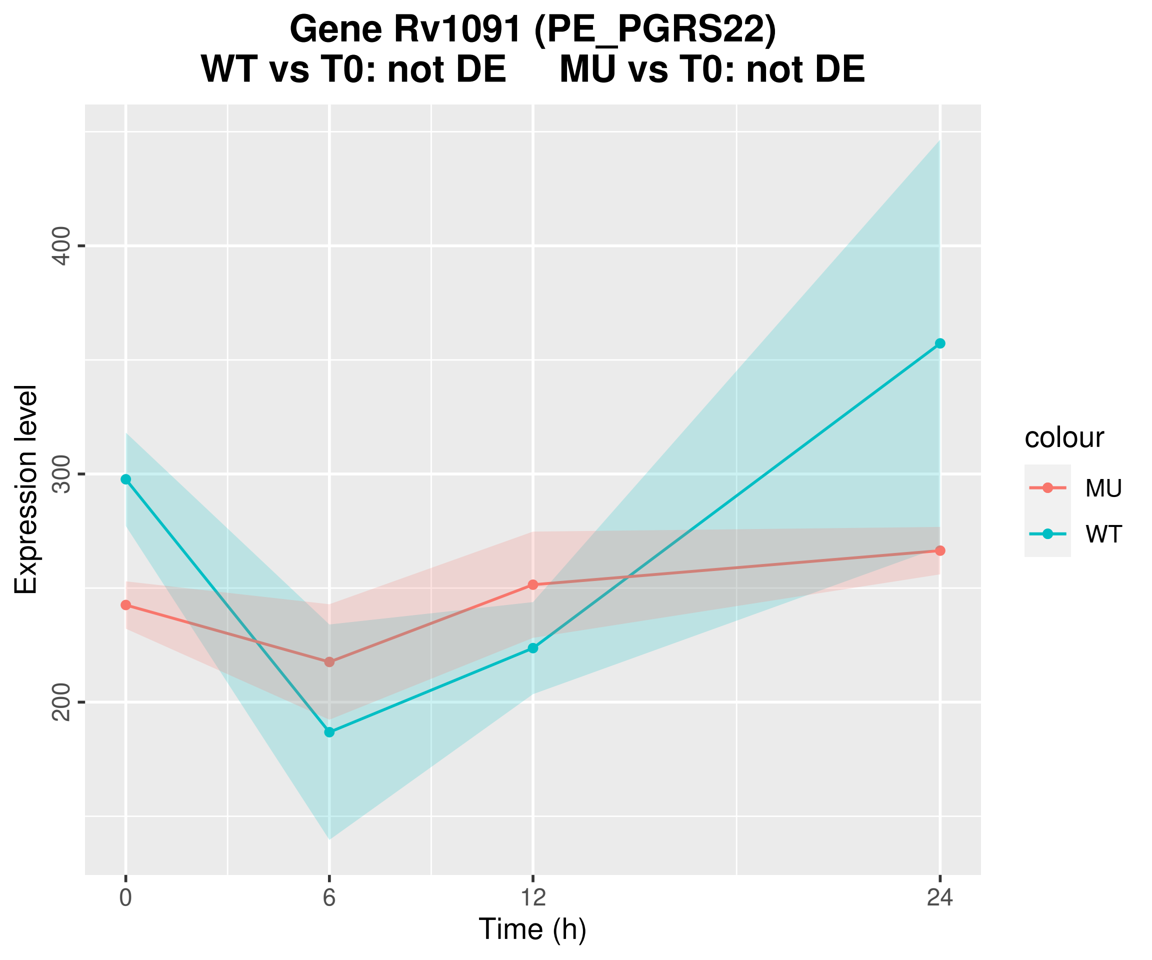
Gene Rv1091 (PE_PGRS22)
WT vs T0: not DE   MU vs T0: not DE
Expression level
400
300
200
0
6
12
24
Time (h)
colour
MU
WT

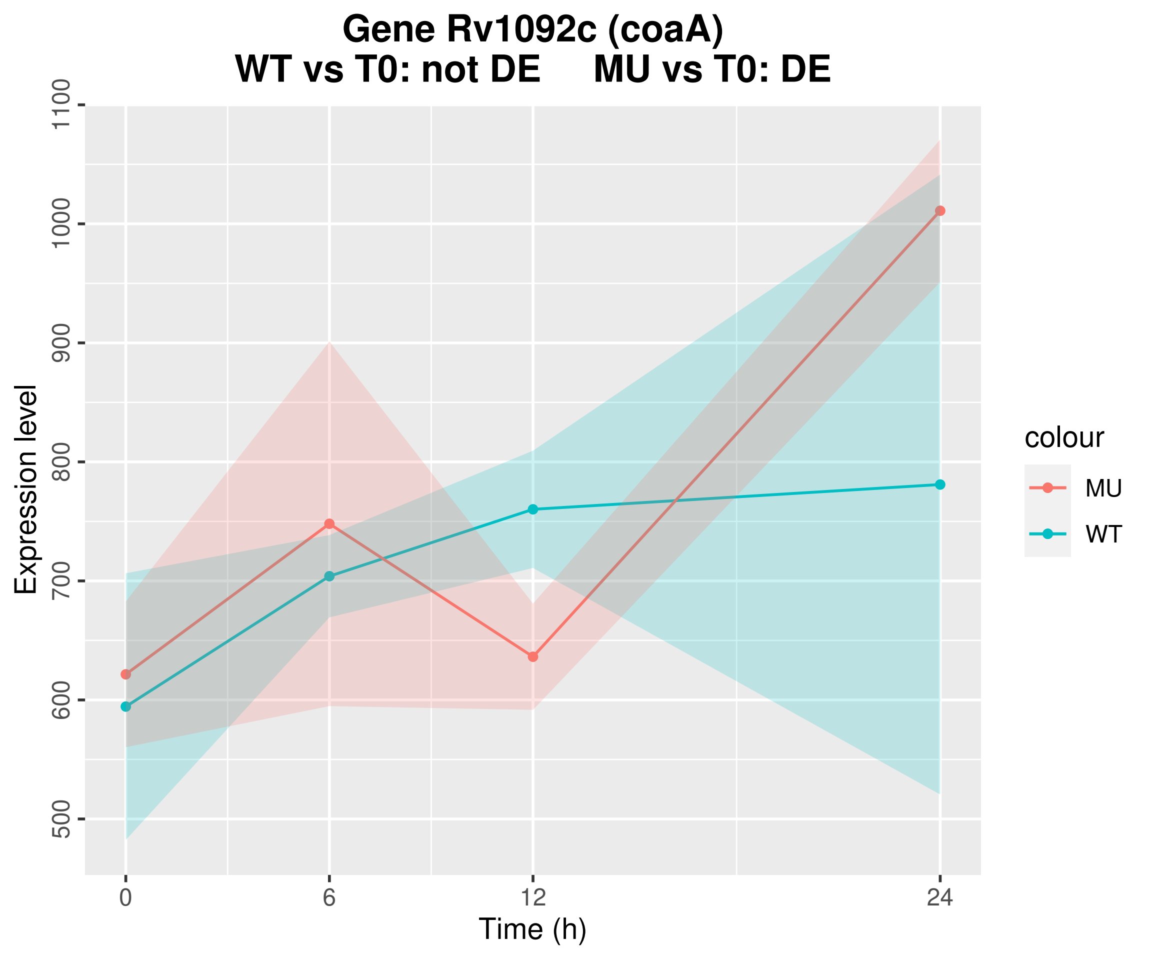

Gene Rv1092c (coaA)
WT vs T0: not DE MU vs T0: DE
Expression level
1100
1000
900
800
700
600
500
0
6
12
24
Time (h)
colour
MU
WT

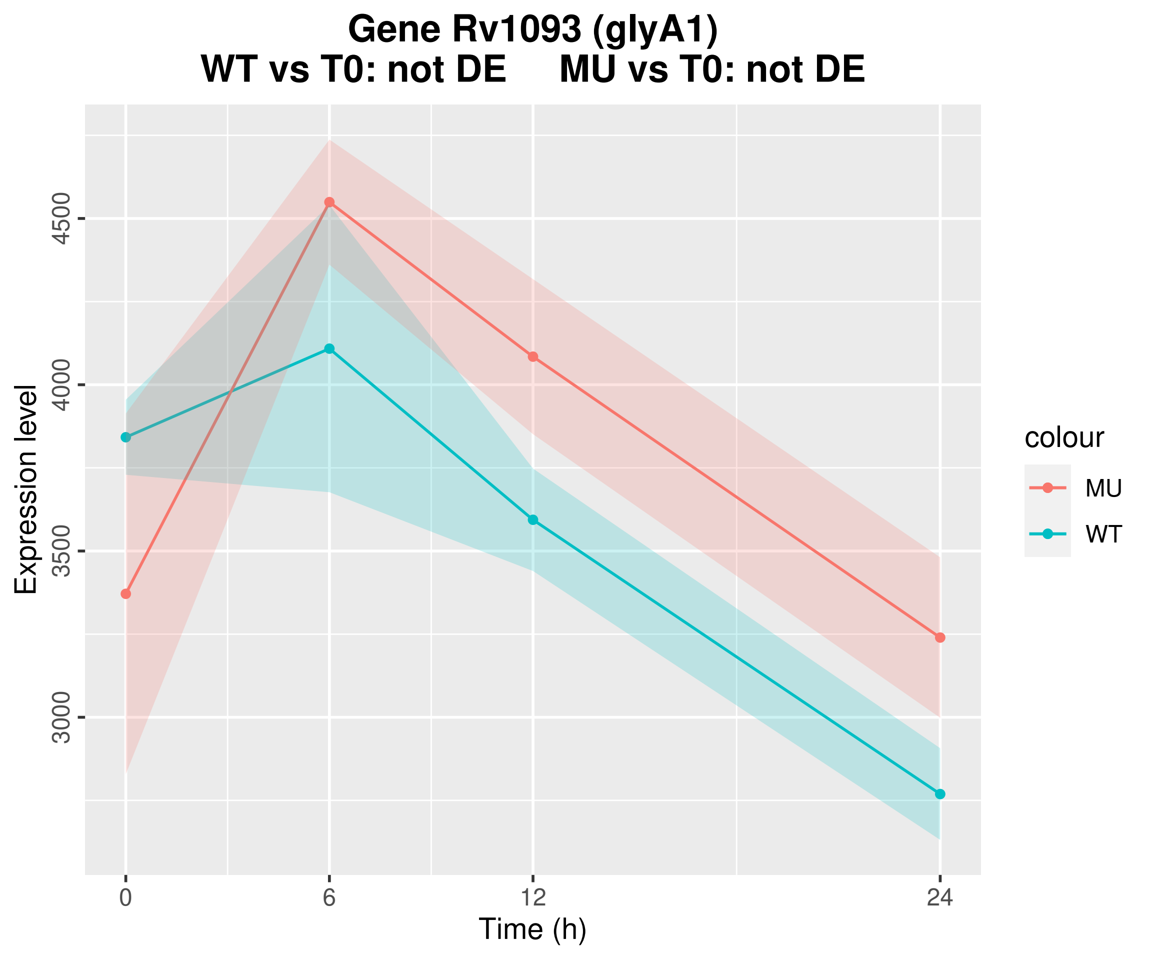
Gene Rv1093 (glyA1)
WT vs T0: not DE MU vs T0: not DE
Expression level
4500
4000
3500
3000
0
6
12
24
Time (h)
colour
MU
WT

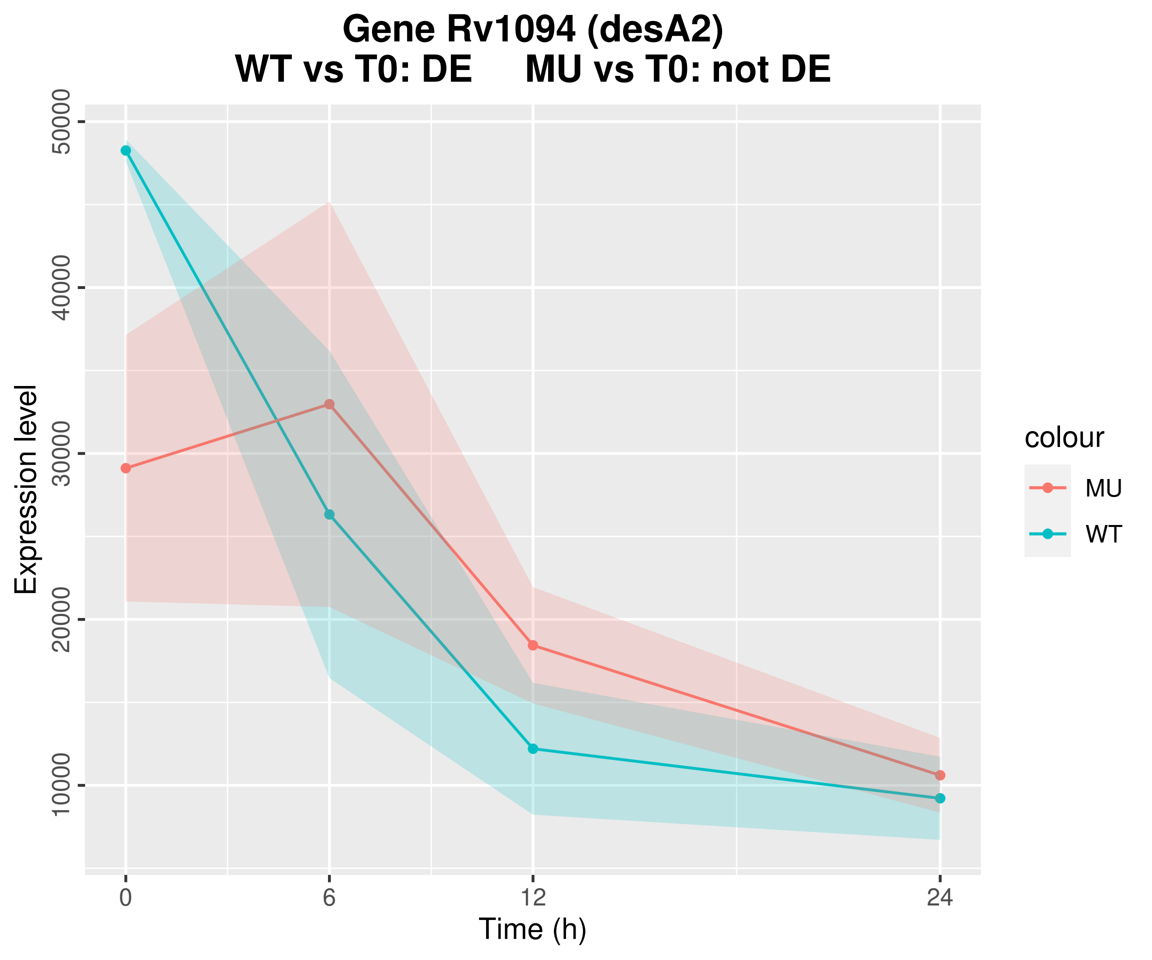
Gene Rv1094 (desA2)
WT vs T0: DE   MU vs T0: not DE
Expression level
50000
40000
30000
20000
10000
0
6
12
24
Time (h)
colour
MU
WT

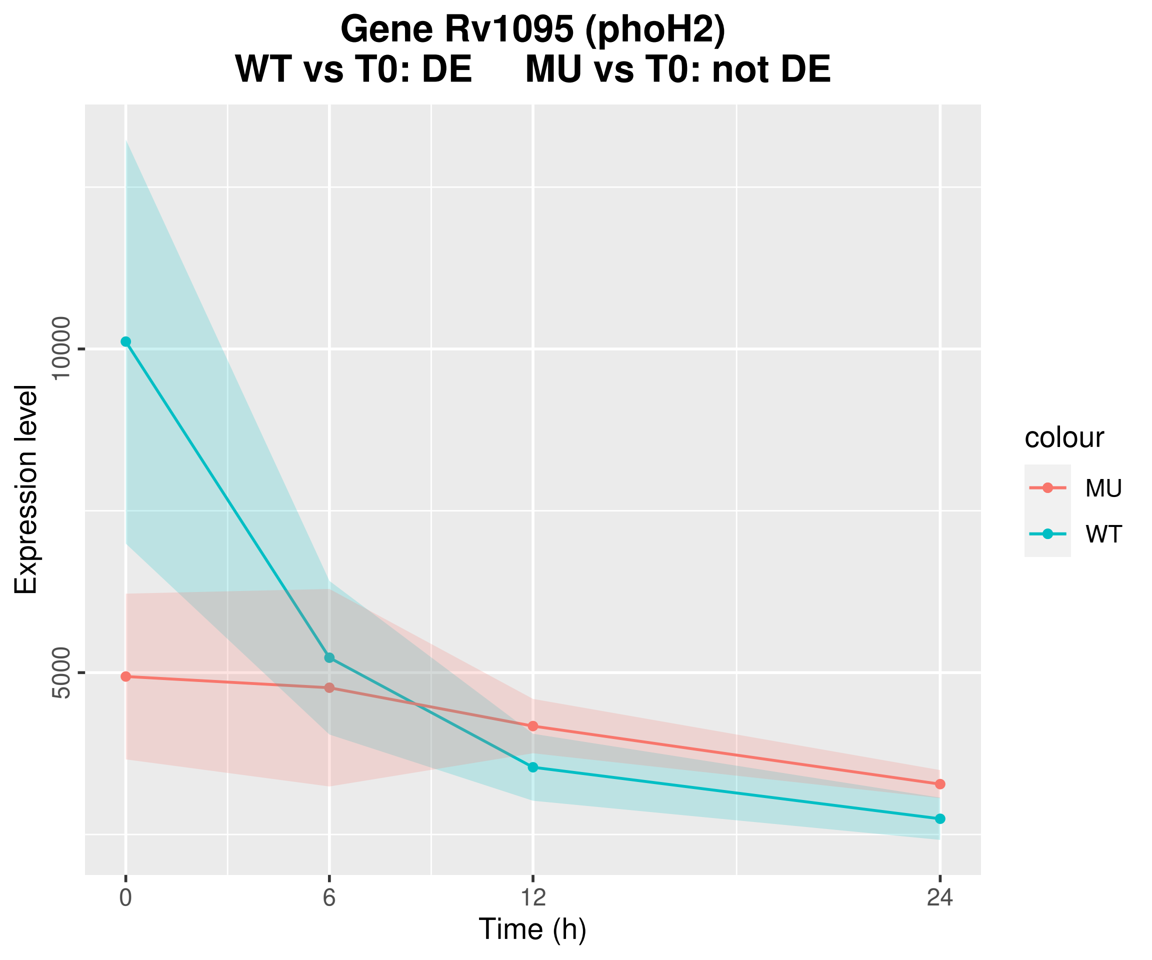

Gene Rv1095 (phoH2)
WT vs T0: DE MU vs T0: not DE
Expression level
10000
5000
0
6
12
24
Time (h)
colour
MU
WT

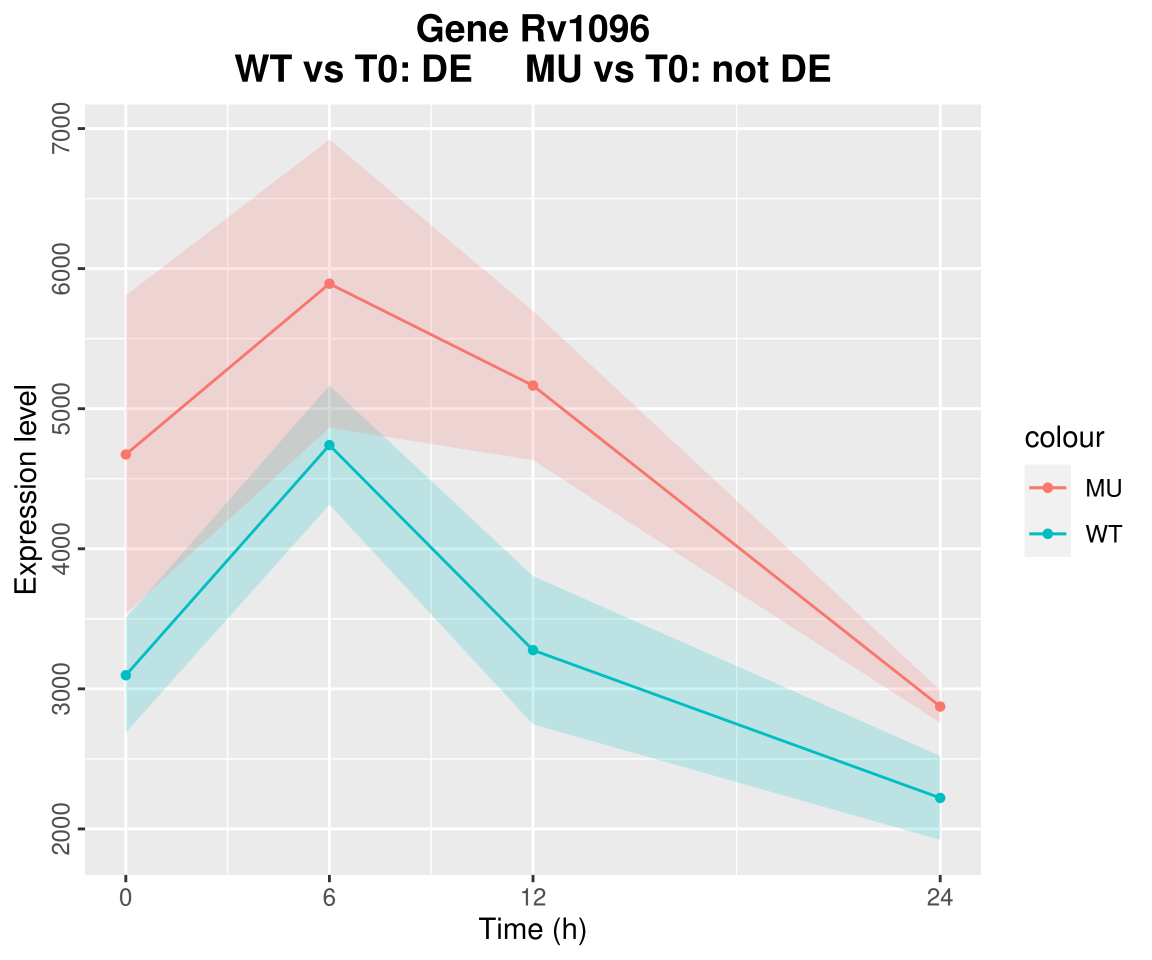

Gene Rv1096
WT vs T0: DE MU vs T0: not DE
Expression level
7000
6000
5000
4000
3000
2000
0
6
12
24
Time (h)
colour
MU
WT

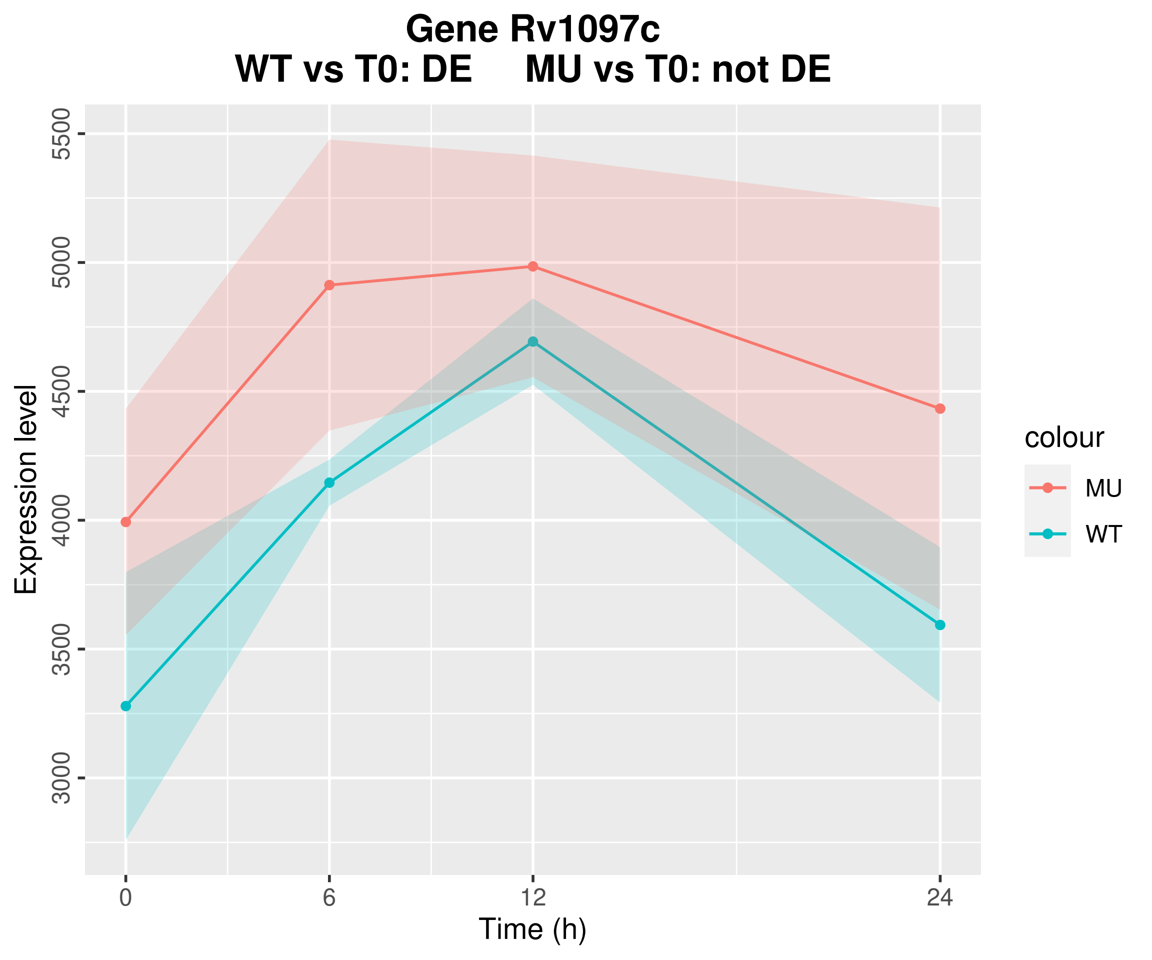
Gene Rv1097c
WT vs T0: DE   MU vs T0: not DE
Expression level
5500
5000
4500
4000
3500
3000
0
6
12
24
Time (h)
colour
MU
WT

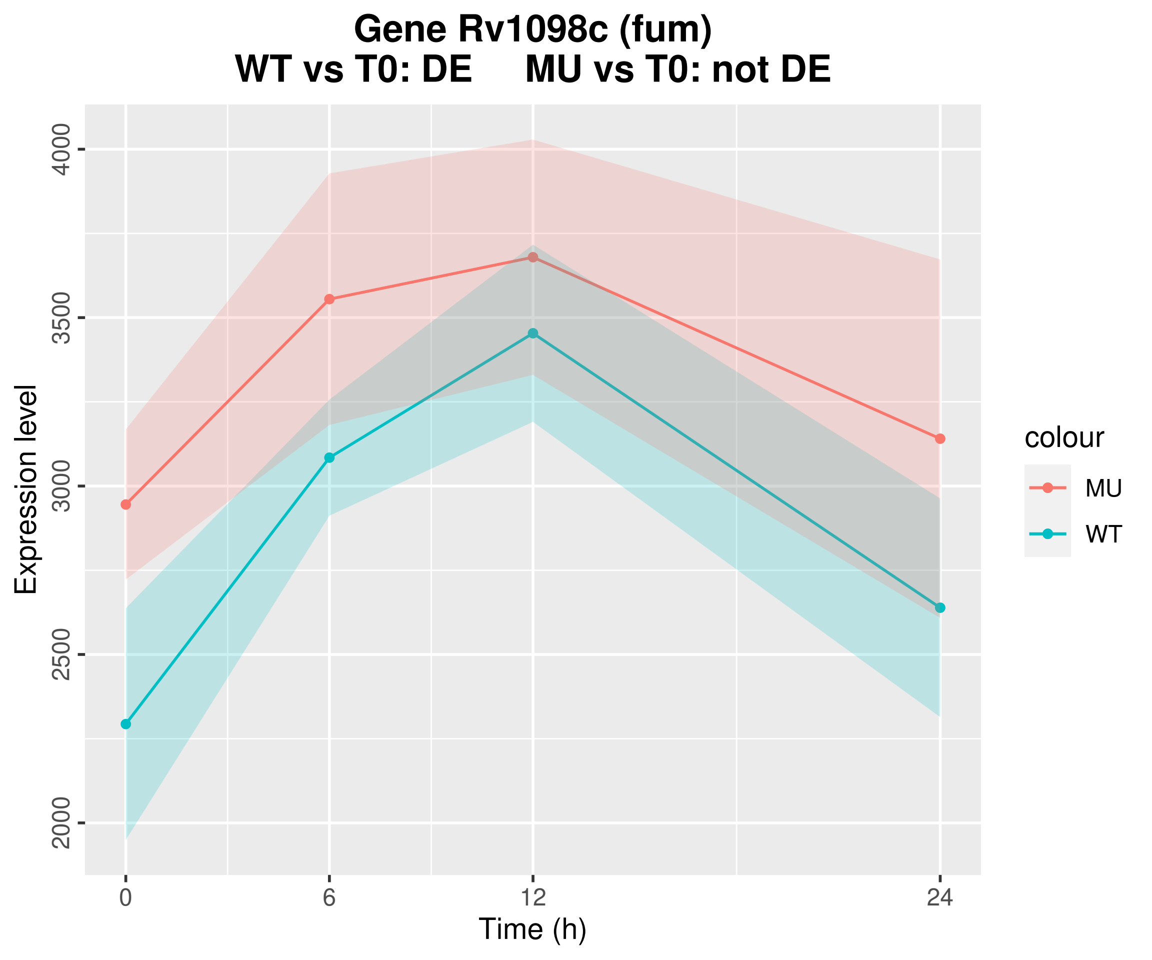
Gene Rv1098c (fum)
WT vs T0: DE   MU vs T0: not DE
Expression level
4000
3500
3000
2500
2000
0
6
12
24
Time (h)
colour
MU
WT

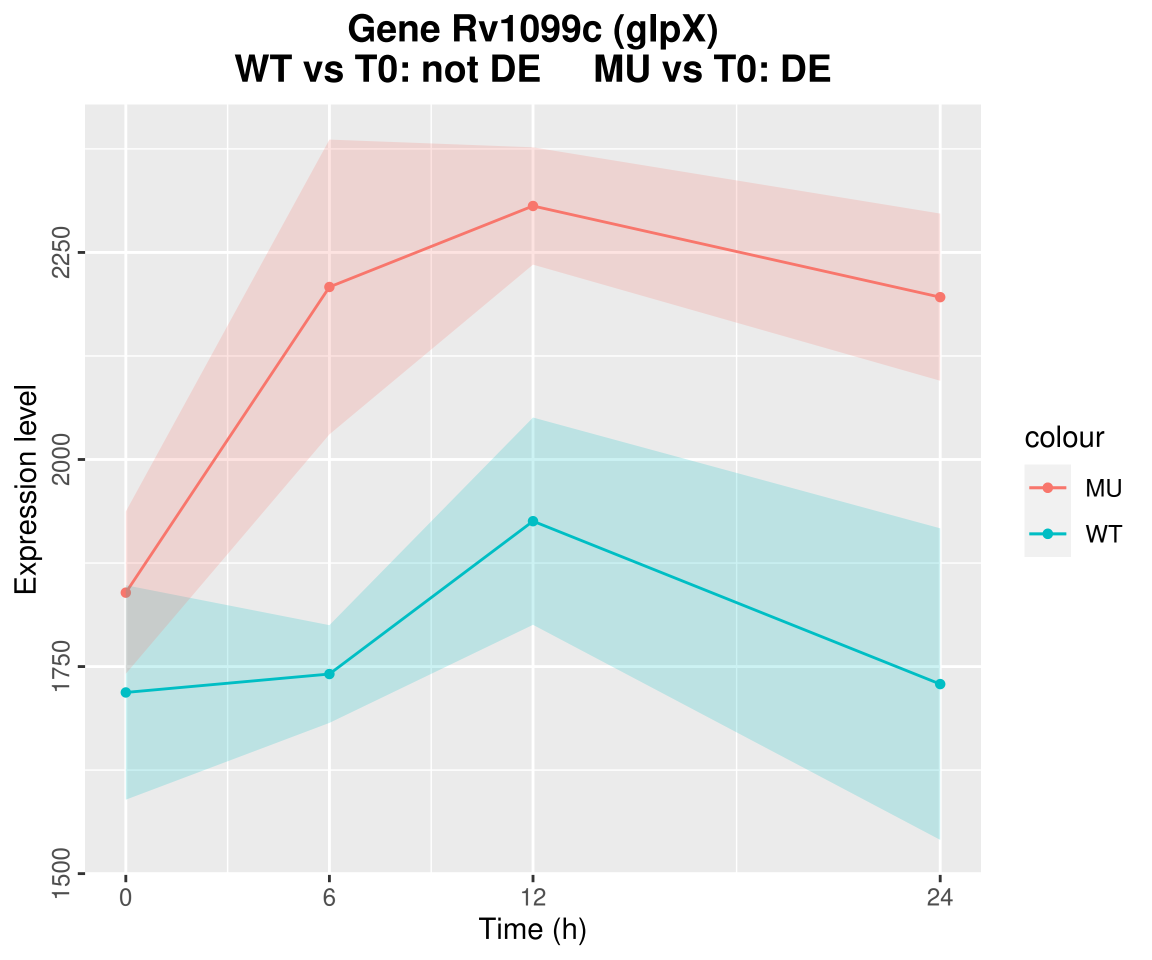

Gene Rv1099c (glpX)
WT vs T0: not DE   MU vs T0: DE
Expression level
2250
2000
1750
1500
0
6
12
24
Time (h)
colour
MU
WT

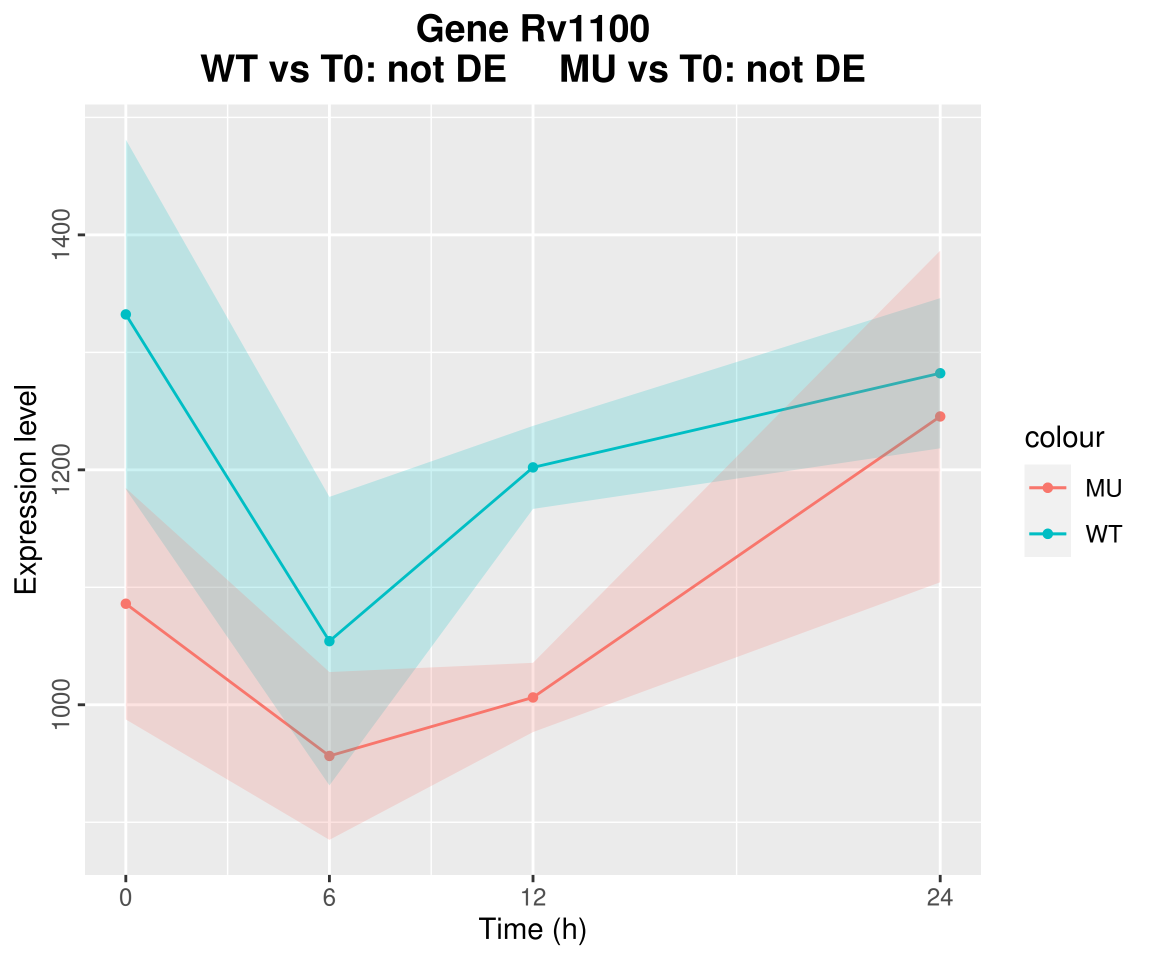

Gene Rv1100
WT vs T0: not DE   MU vs T0: not DE
Expression level
1000
1200
1400
0
6
12
24
Time (h)
colour
MU
WT

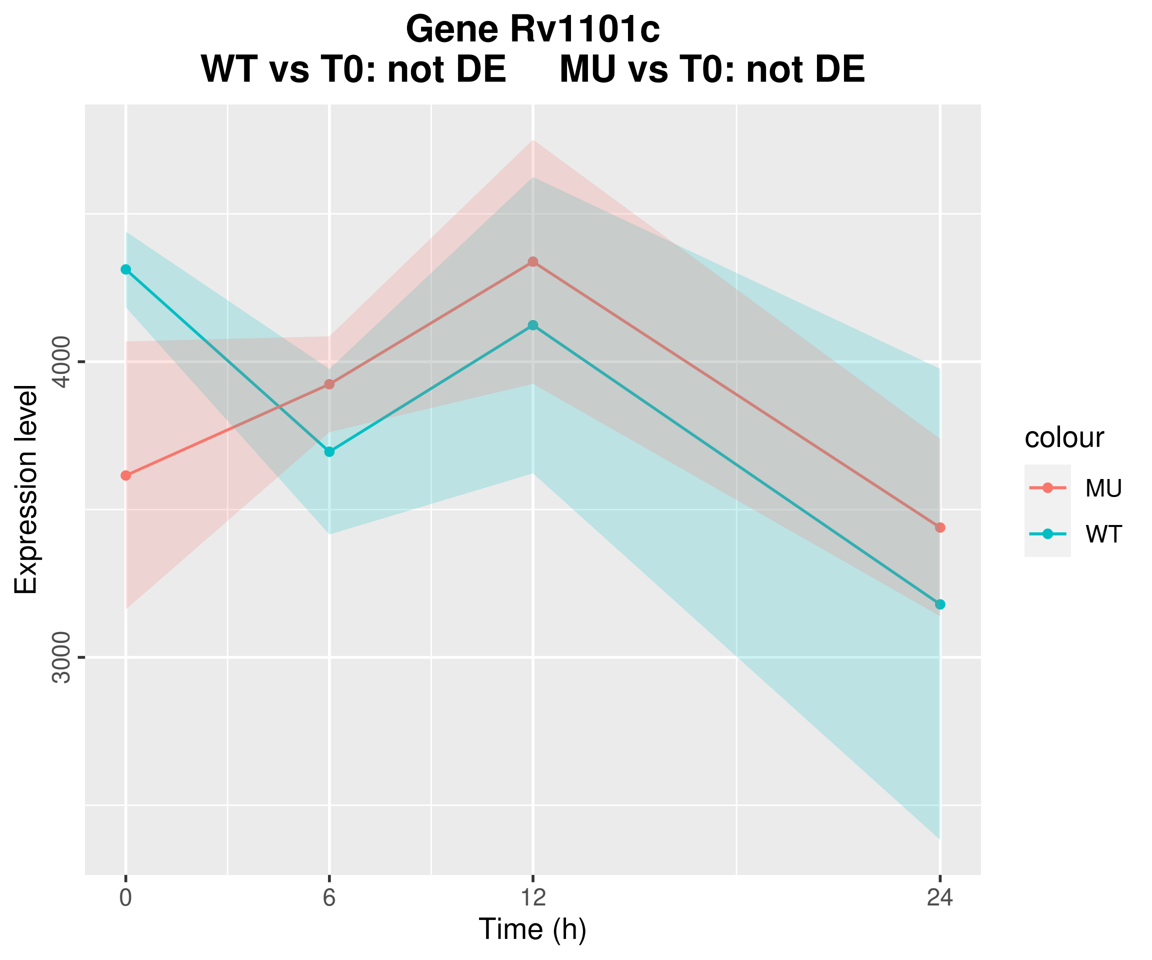

Gene Rv1101c
WT vs T0: not DE   MU vs T0: not DE
Expression level
4000
3000
0
6
12
24
Time (h)
colour
MU
WT

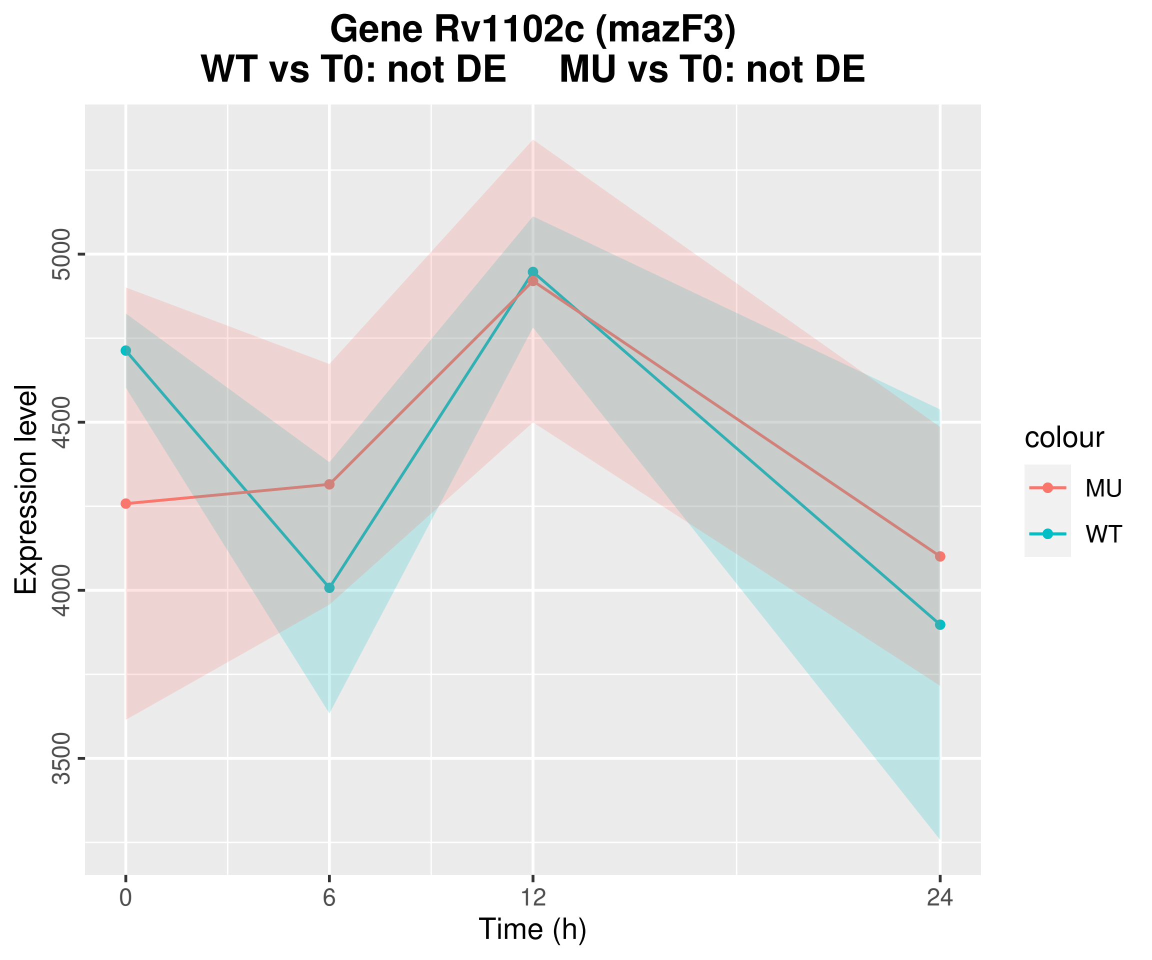
Gene Rv1102c (mazF3)
WT vs T0: not DE   MU vs T0: not DE
Expression level
5000
4500
4000
3500
0
6
12
24
Time (h)
colour
MU
WT

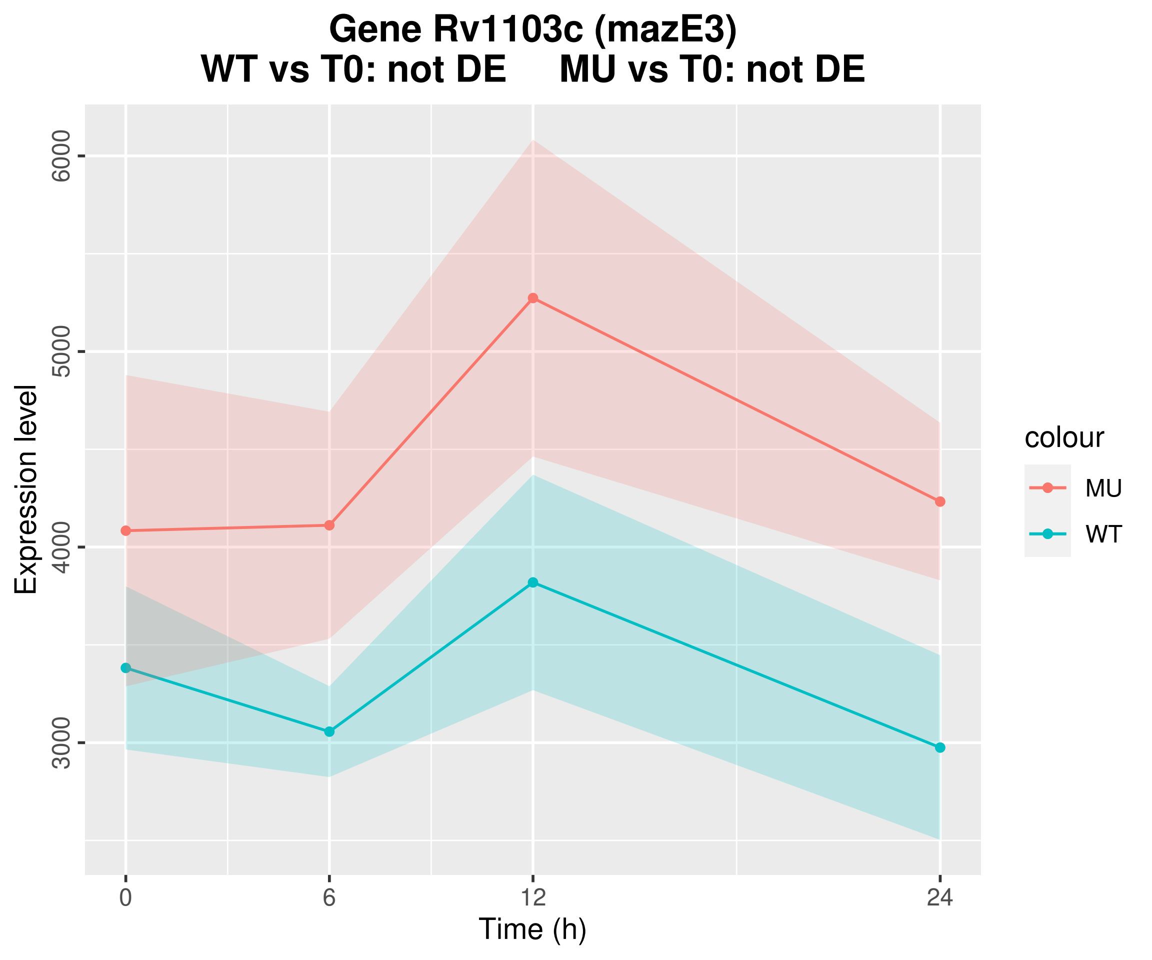

Gene Rv1103c (mazE3)
WT vs T0: not DE   MU vs T0: not DE
Expression level
6000
5000
4000
3000
0
6
12
24
Time (h)
colour
MU
WT

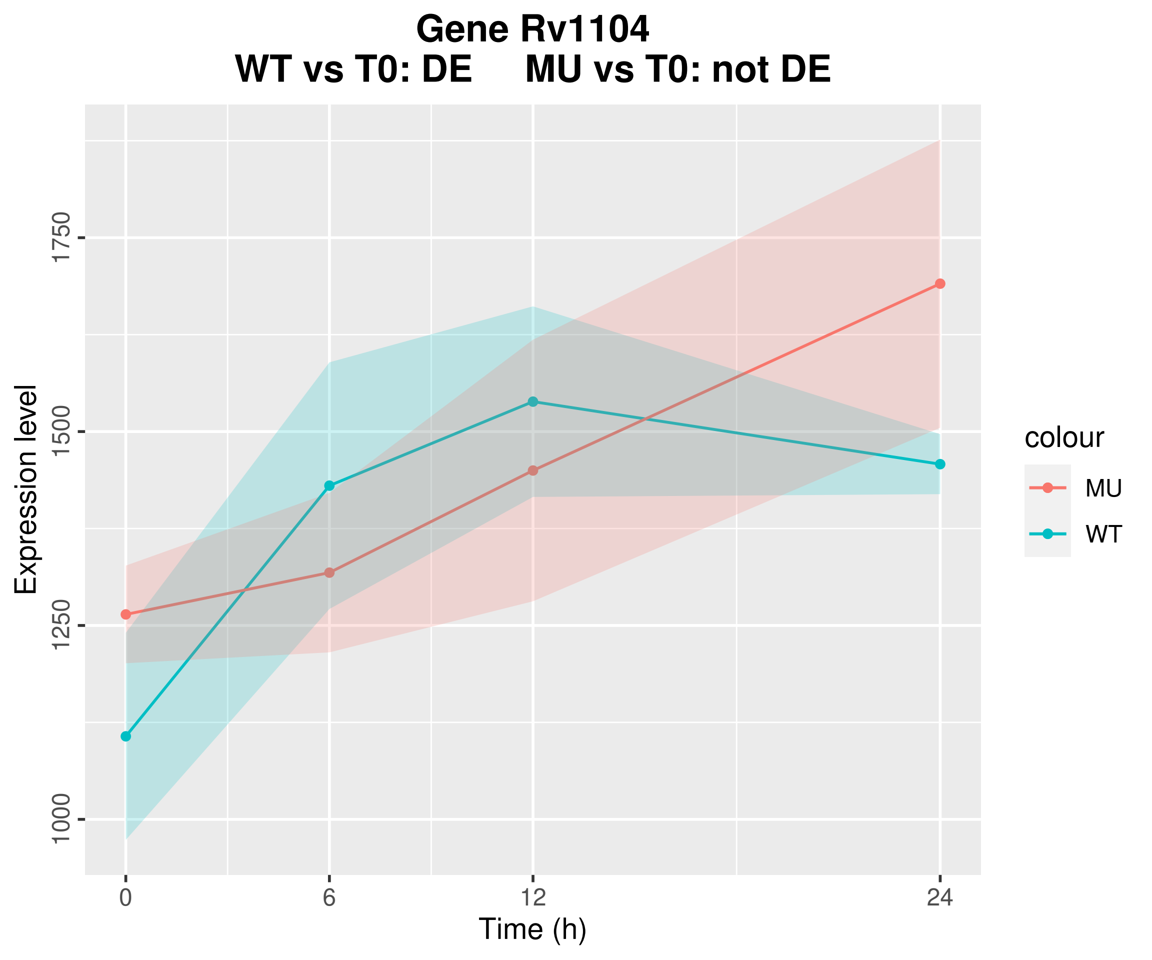
Gene Rv1104
WT vs T0: DE MU vs T0: not DE
Expression level
1000
1250
1500
1750
Time (h)
0
6
12
24
colour
MU
WT

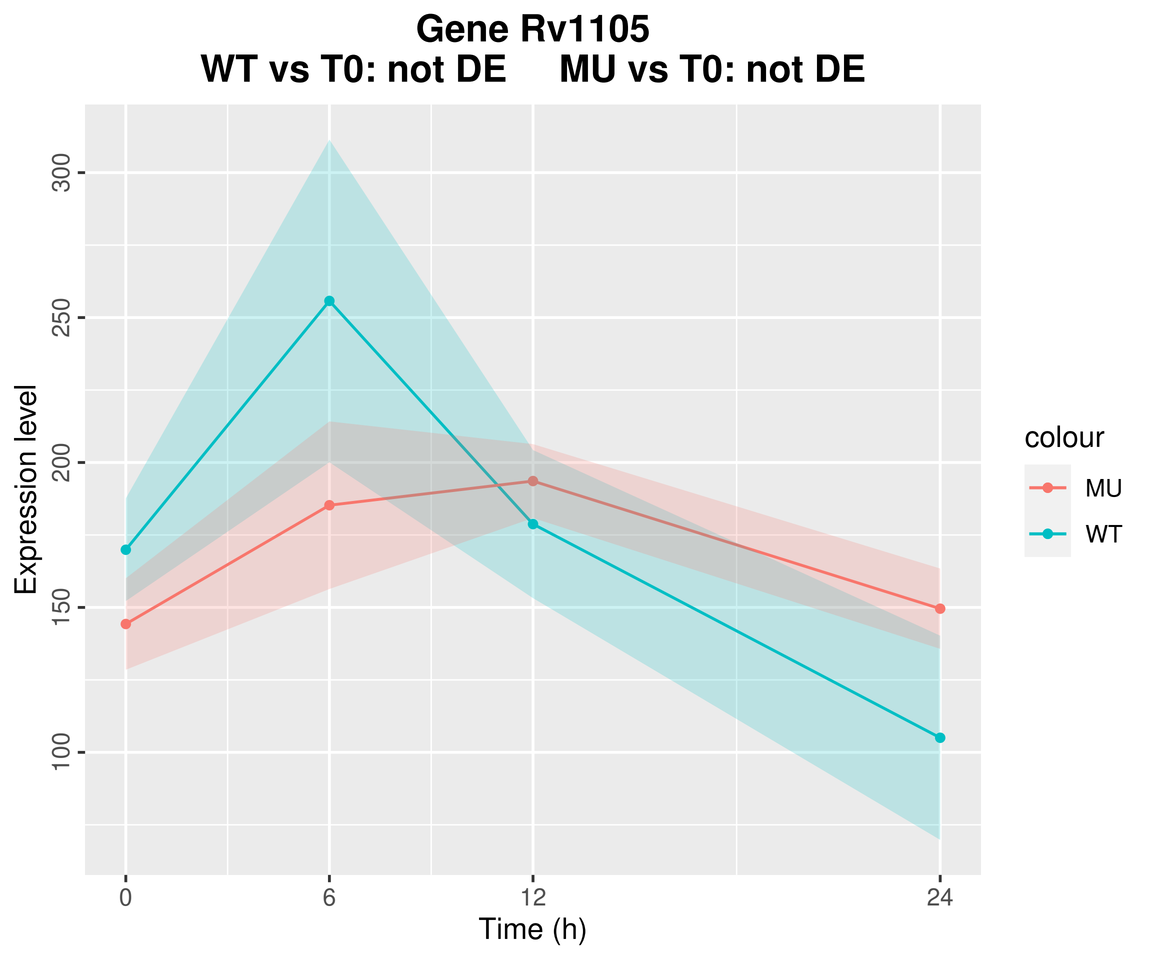

Gene Rv1105
WT vs T0: not DE   MU vs T0: not DE
Expression level
300
250
200
150
100
0
6
12
24
Time (h)
colour
MU
WT

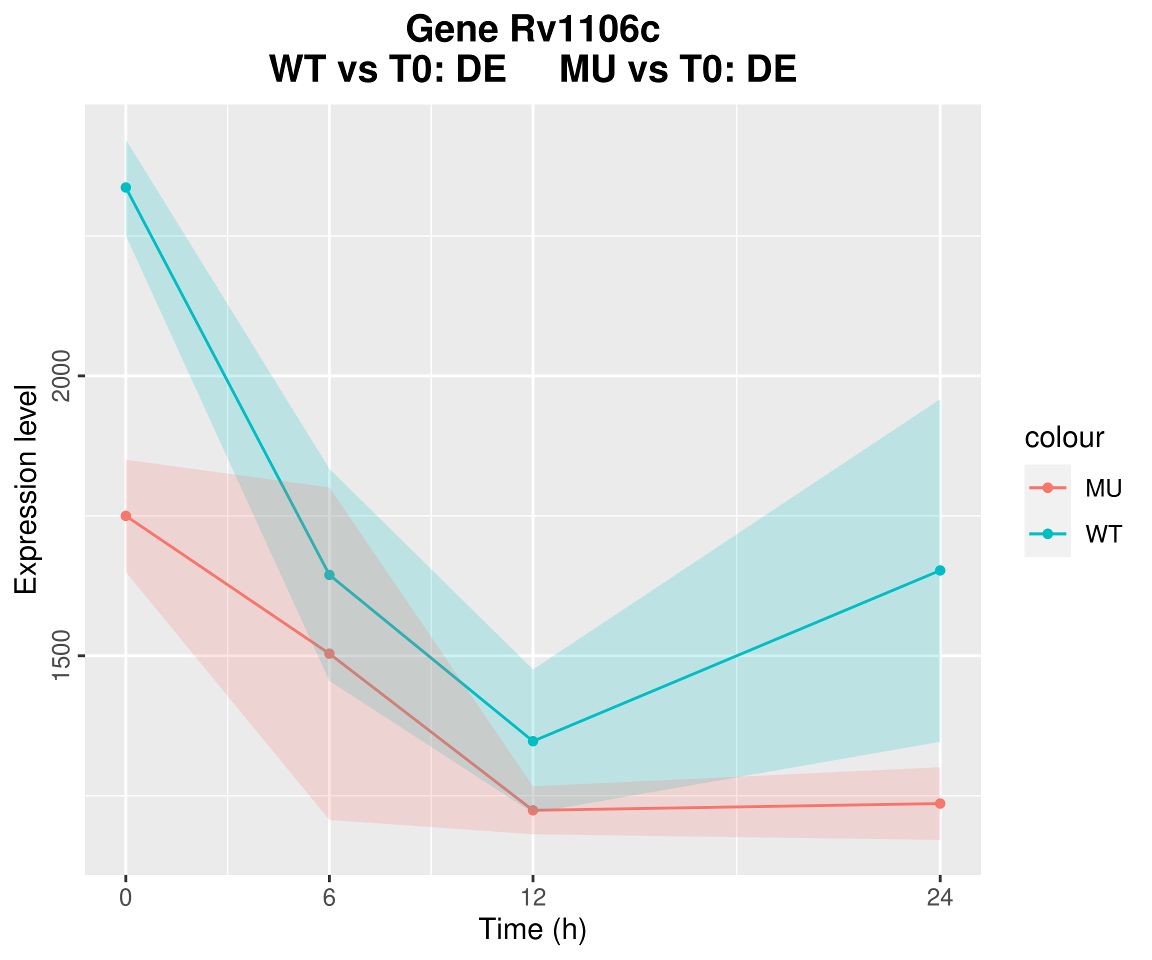
Gene Rv1106c
WT vs T0: DE   MU vs T0: DE
Expression level
2000
1500
0
6
12
24
Time (h)
colour
MU
WT

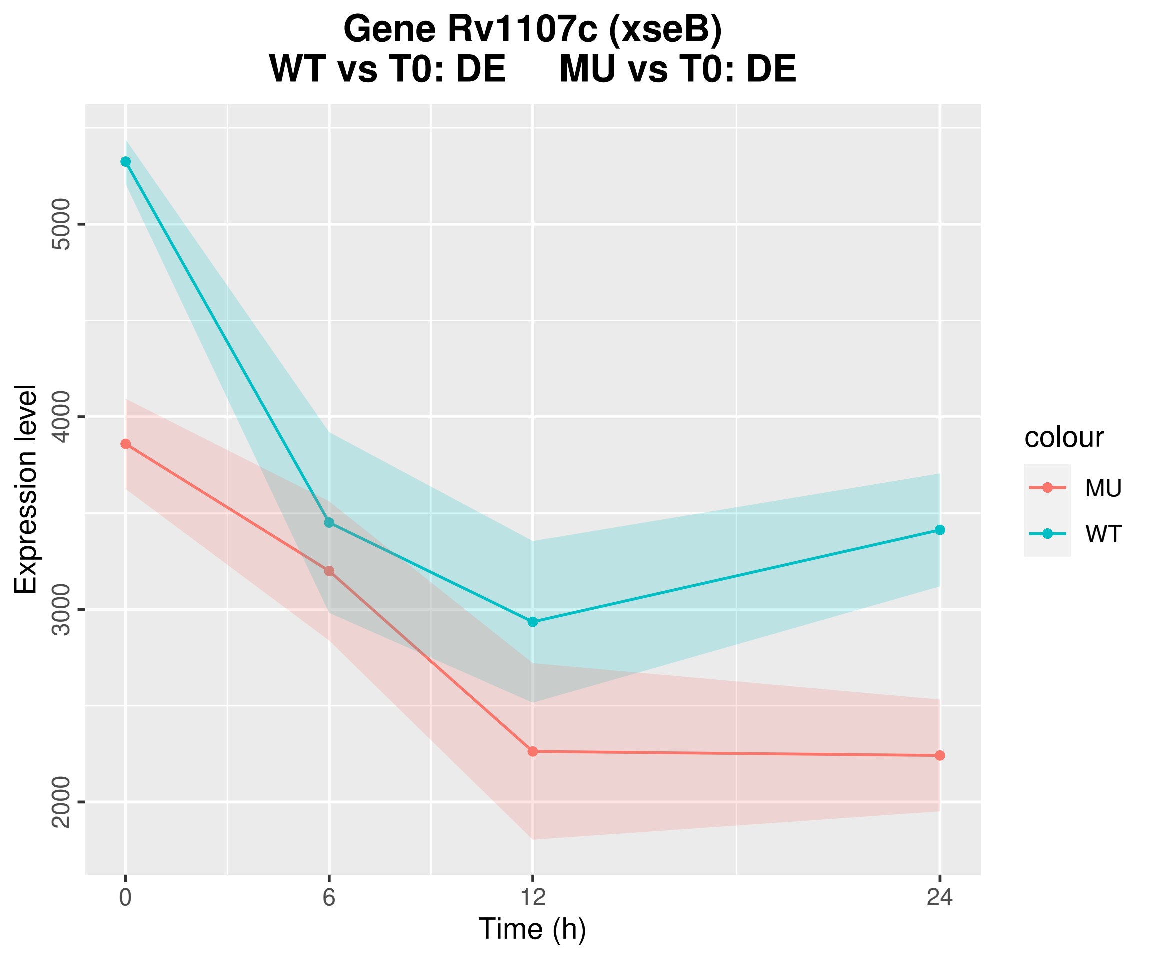

Gene Rv1107c (xseB)
WT vs T0: DE MU vs T0: DE
Expression level
5000
4000
3000
2000
0
6
12
24
Time (h)
colour
MU
WT

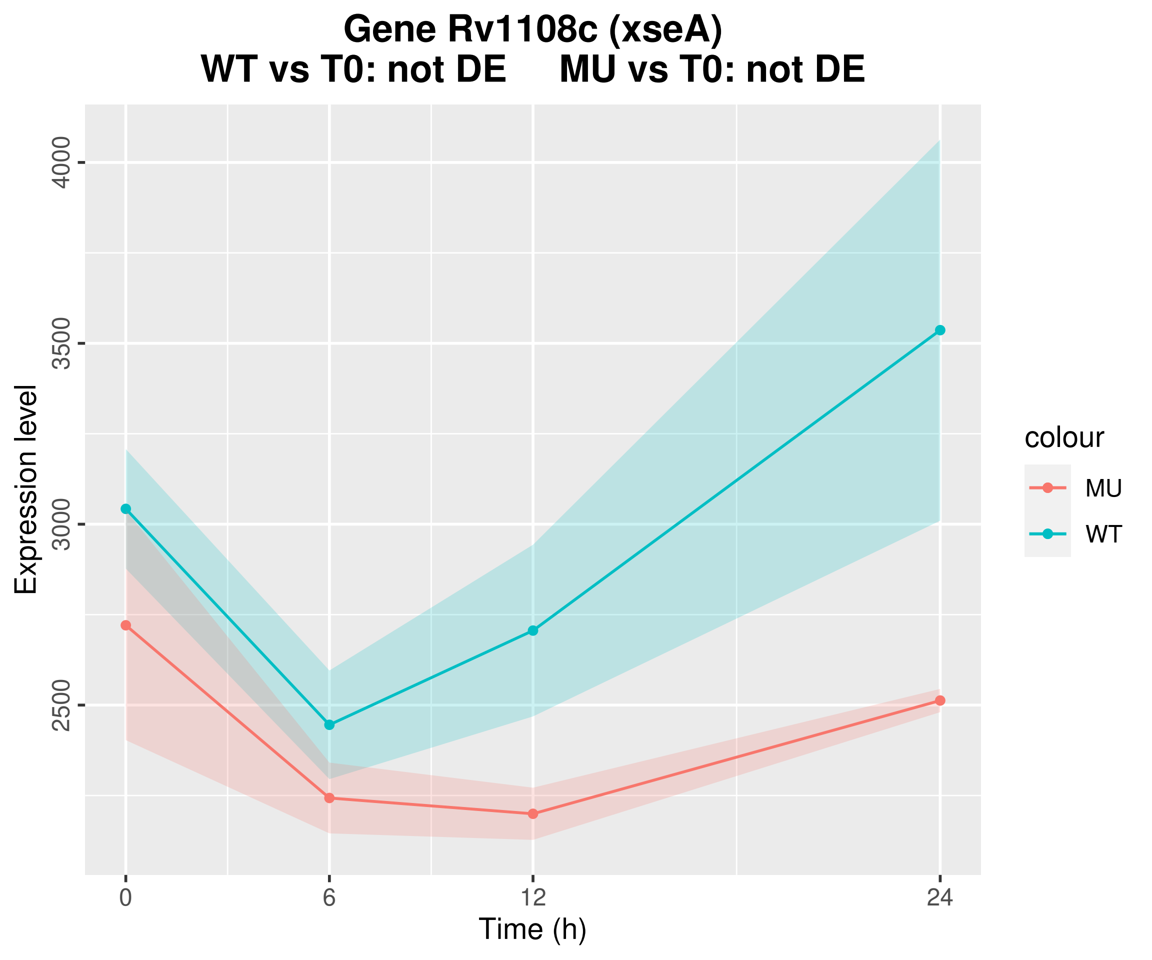
Gene Rv1108c (xseA)
WT vs T0: not DE MU vs T0: not DE
Expression level
4000
3500
3000
2500
0
6
12
24
Time (h)
colour
MU
WT

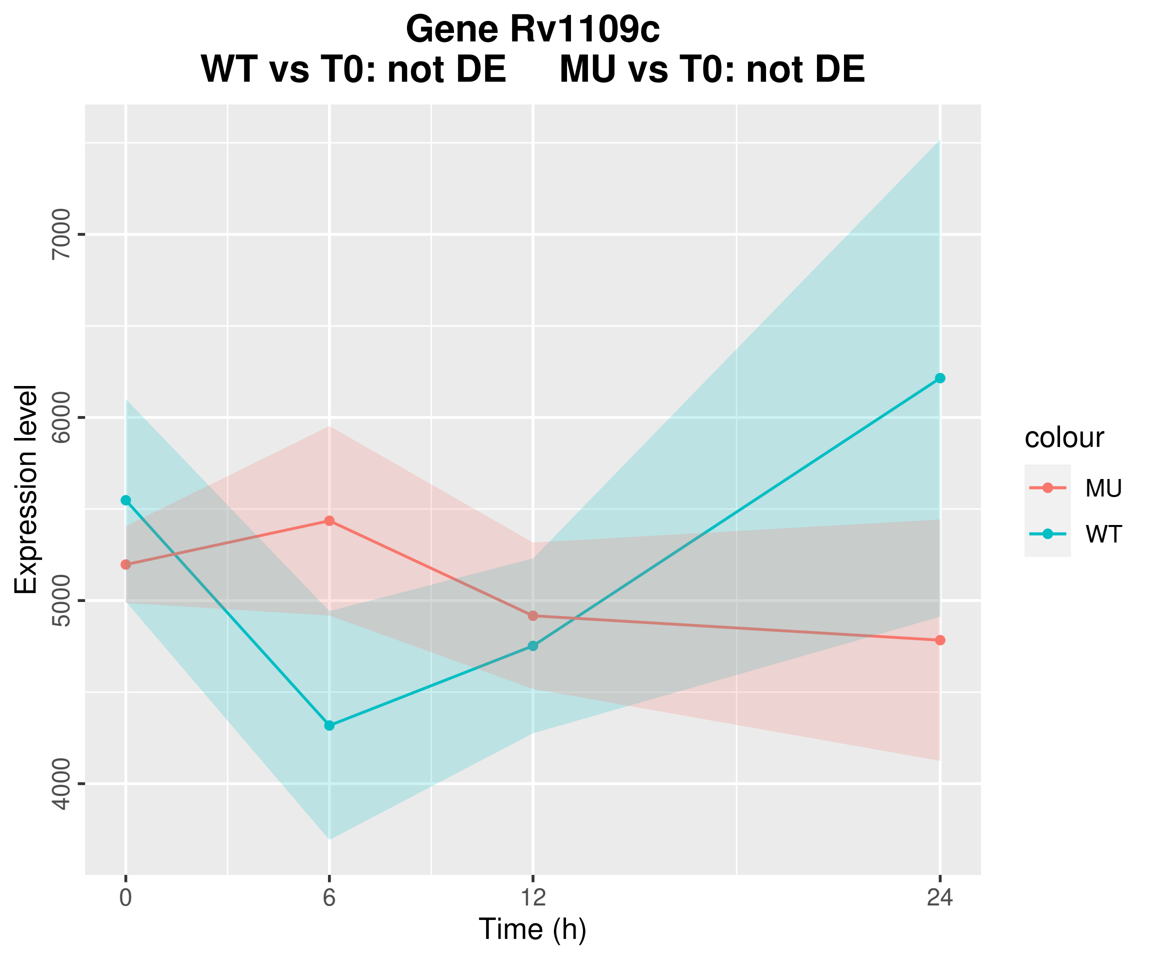

Gene Rv1109c
WT vs T0: not DE MU vs T0: not DE
Expression level
7000
6000
5000
4000
0
6
12
24
Time (h)
colour
MU
WT

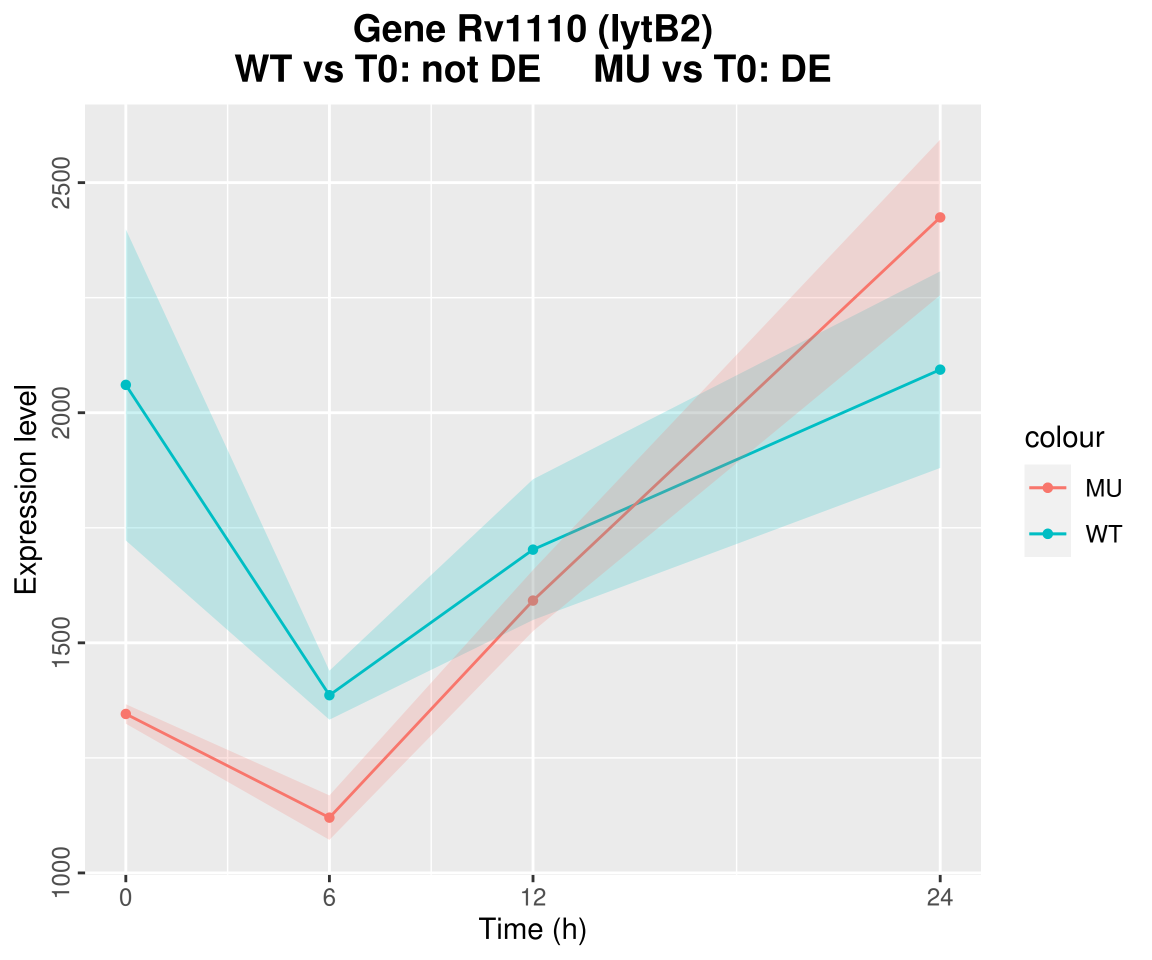

Gene Rv1110 (lytB2)
WT vs T0: not DE   MU vs T0: DE
Expression level
1000
1500
2000
2500
0
6
12
24
Time (h)
colour
MU
WT

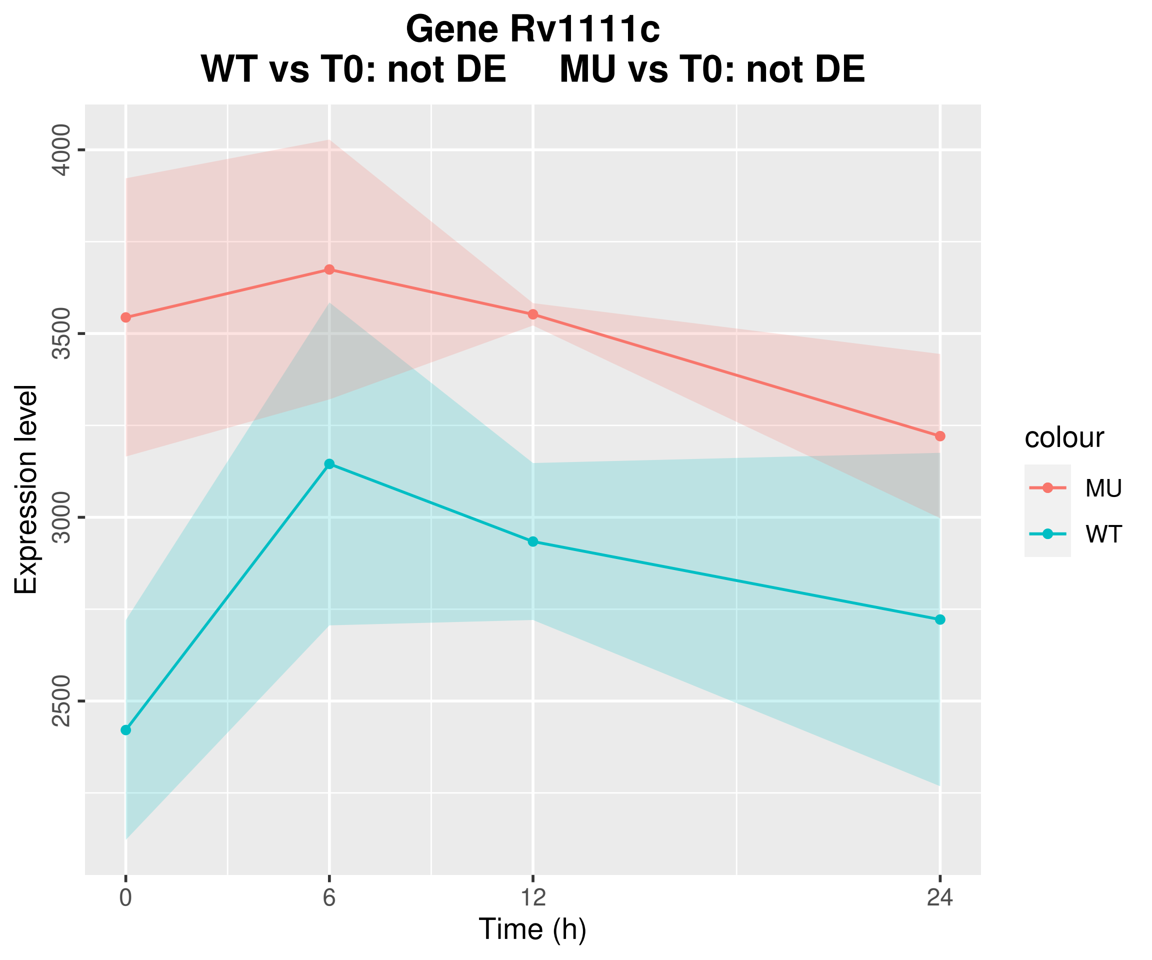

Gene Rv1111c
WT vs T0: not DE MU vs T0: not DE
Expression level
4000
3500
3000
2500
0
6
12
24
Time (h)
colour
MU
WT

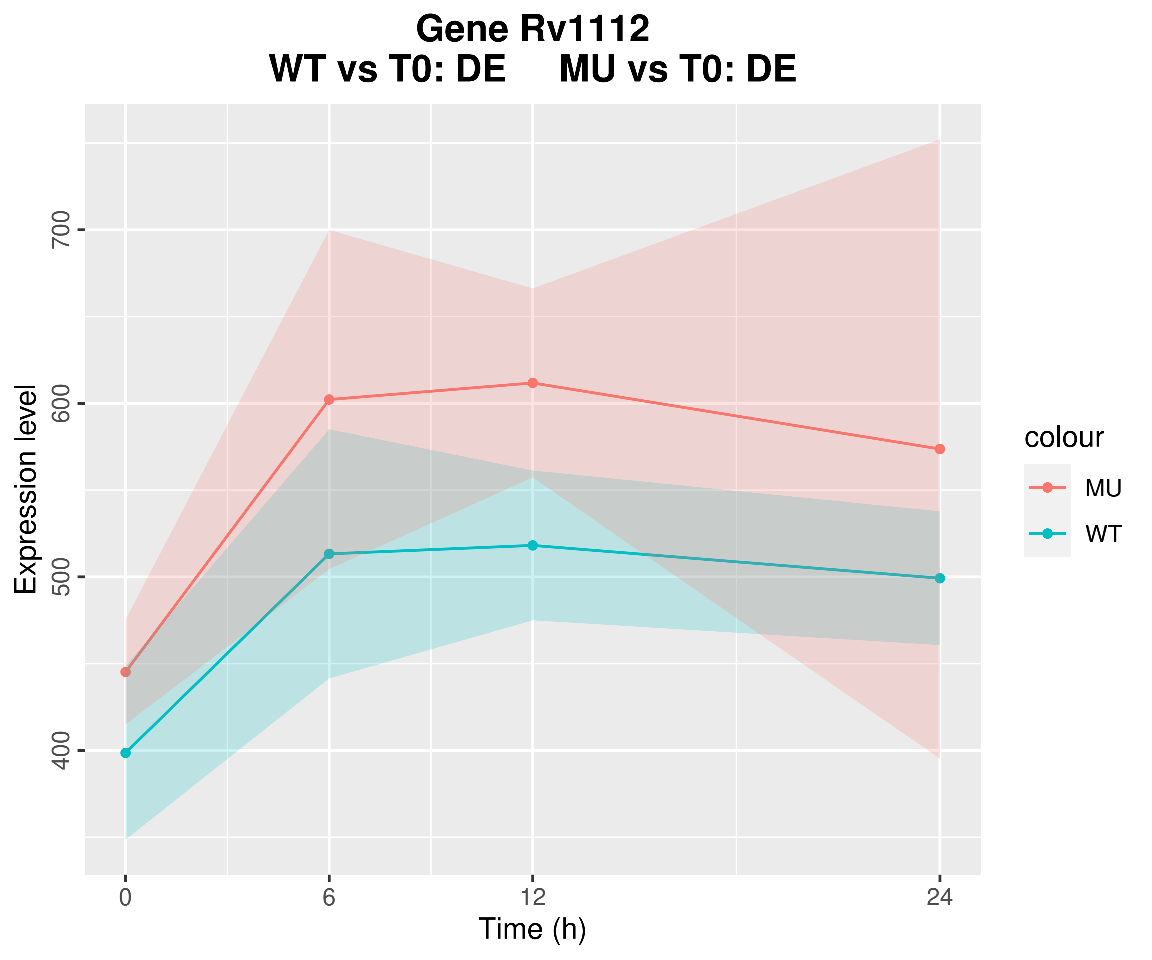
Gene Rv1112
WT vs T0: DE MU vs T0: DE
Expression level
700
600
500
400
0
6
12
24
Time (h)
colour
MU
WT

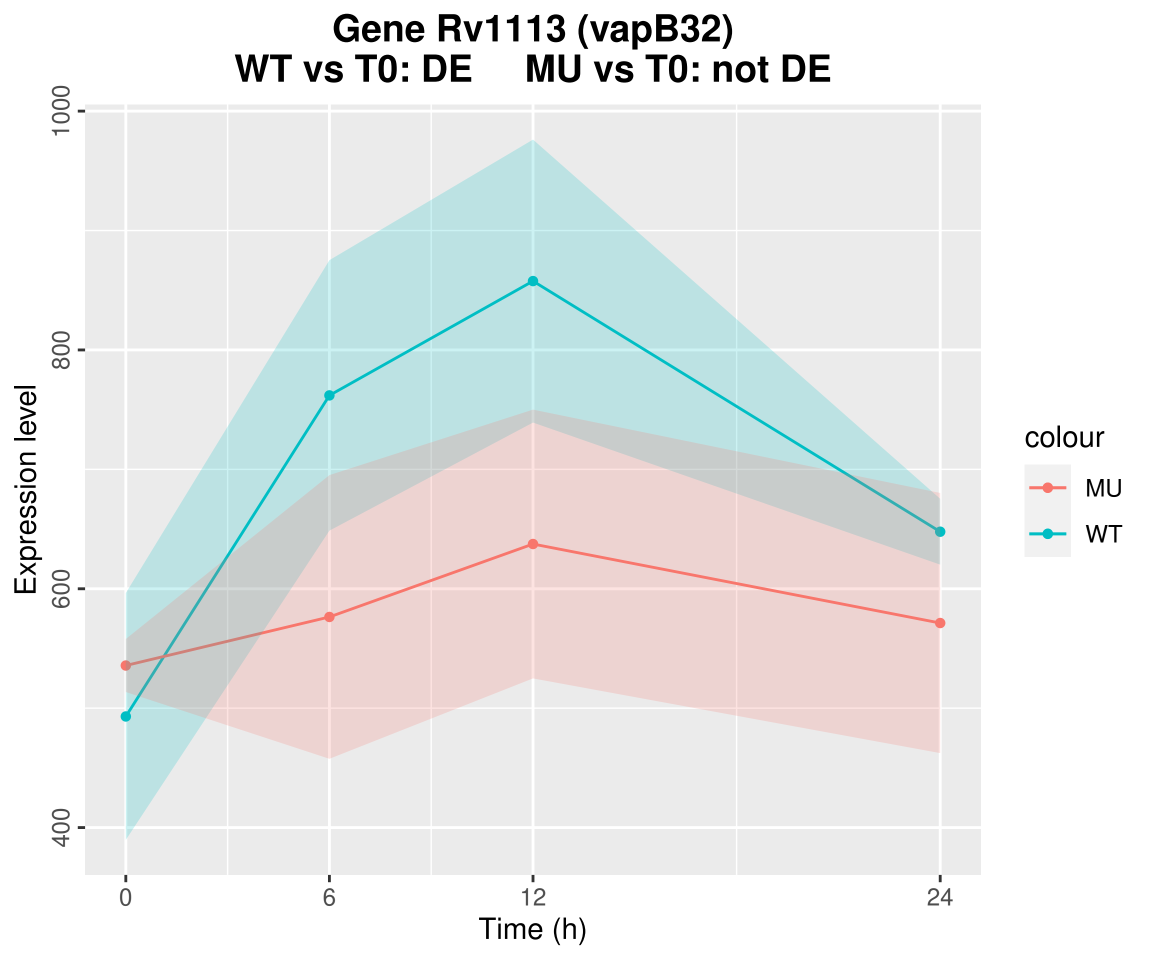
Gene Rv1113 (vapB32)
WT vs T0: DE   MU vs T0: not DE
Expression level
1000
800
600
400
0
6
12
24
Time (h)
colour
MU
WT

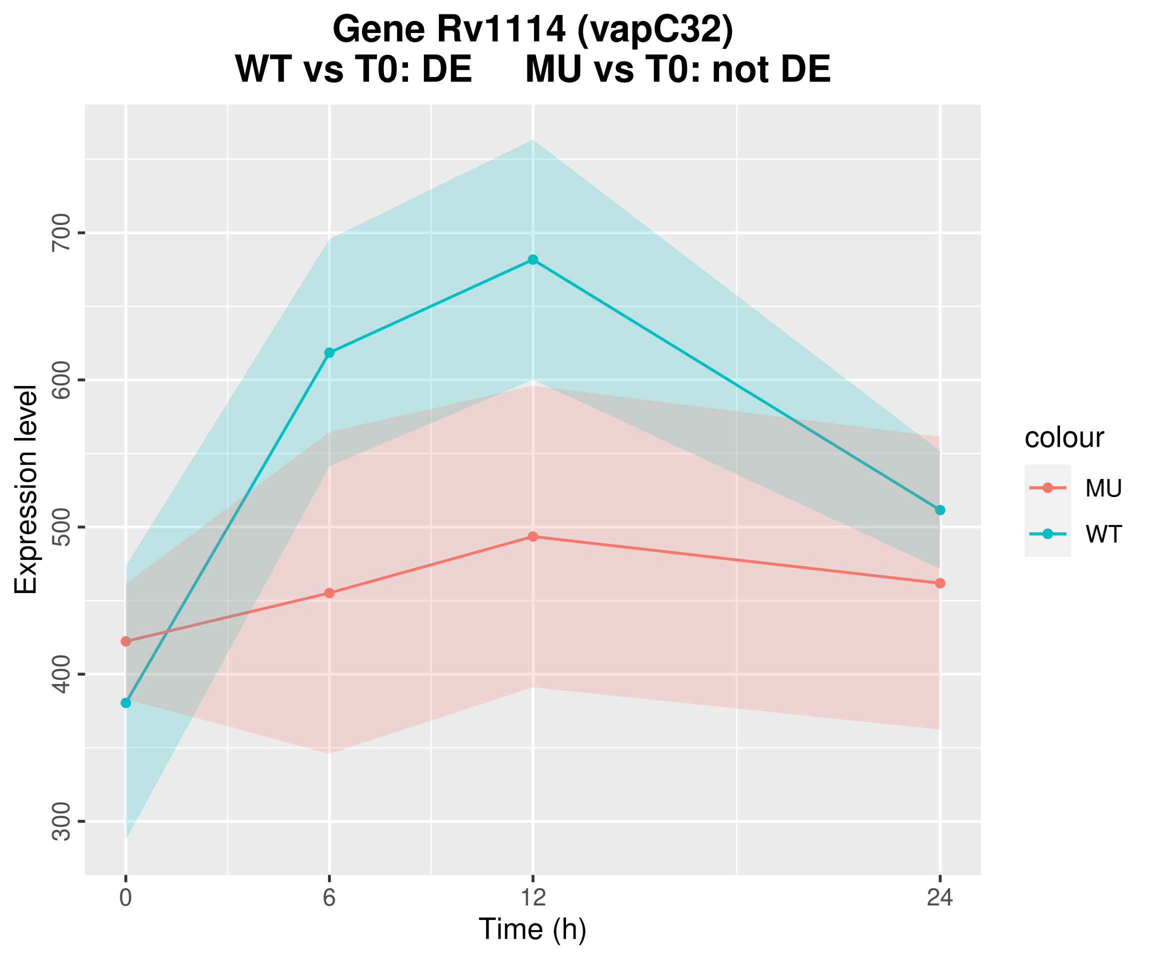
Gene Rv1114 (vapC32)
WT vs T0: DE   MU vs T0: not DE
Expression level
300
400
500
600
700
0
6
12
24
Time (h)
colour
MU
WT

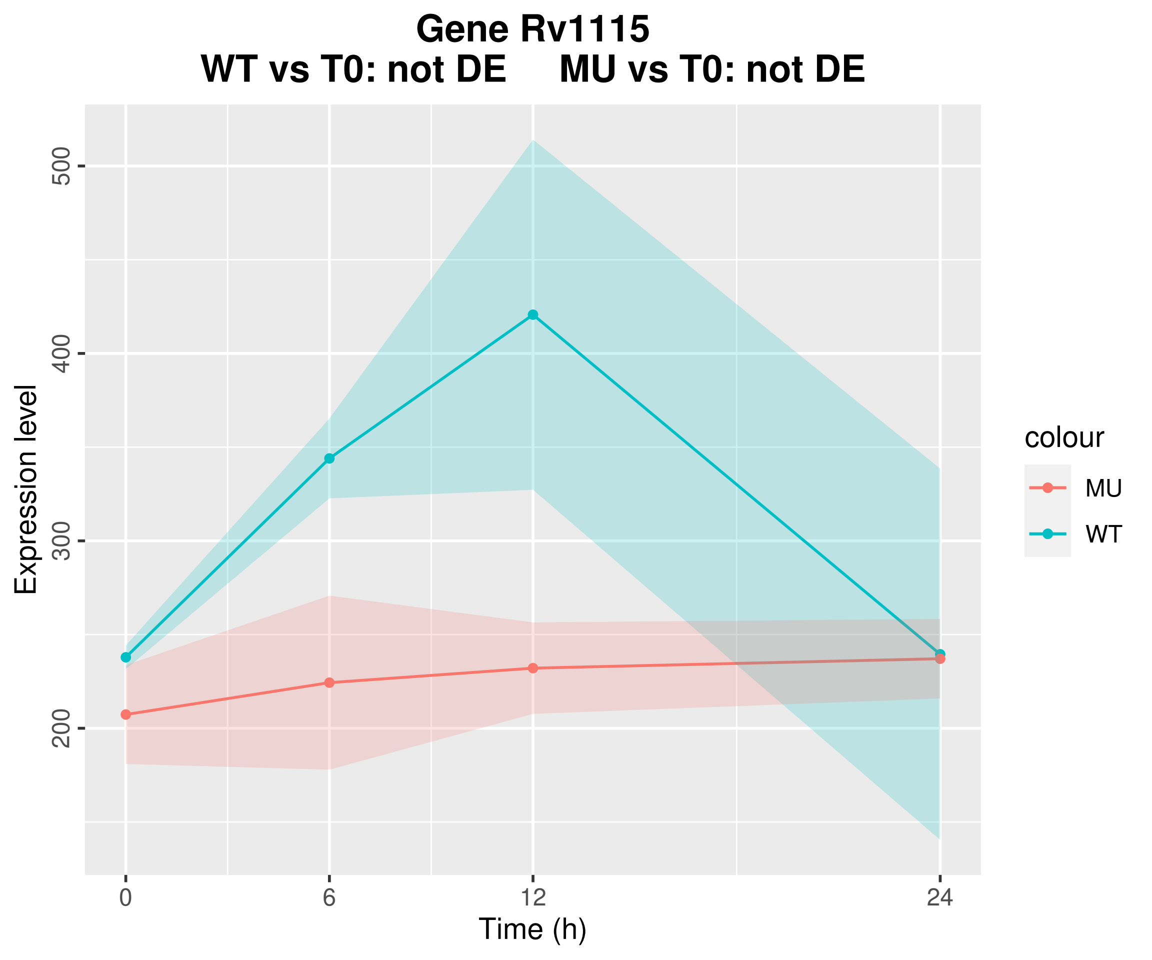
Gene Rv1115
WT vs T0: not DE   MU vs T0: not DE
Expression level
500
400
300
200
0
6
12
24
Time (h)
colour
MU
WT

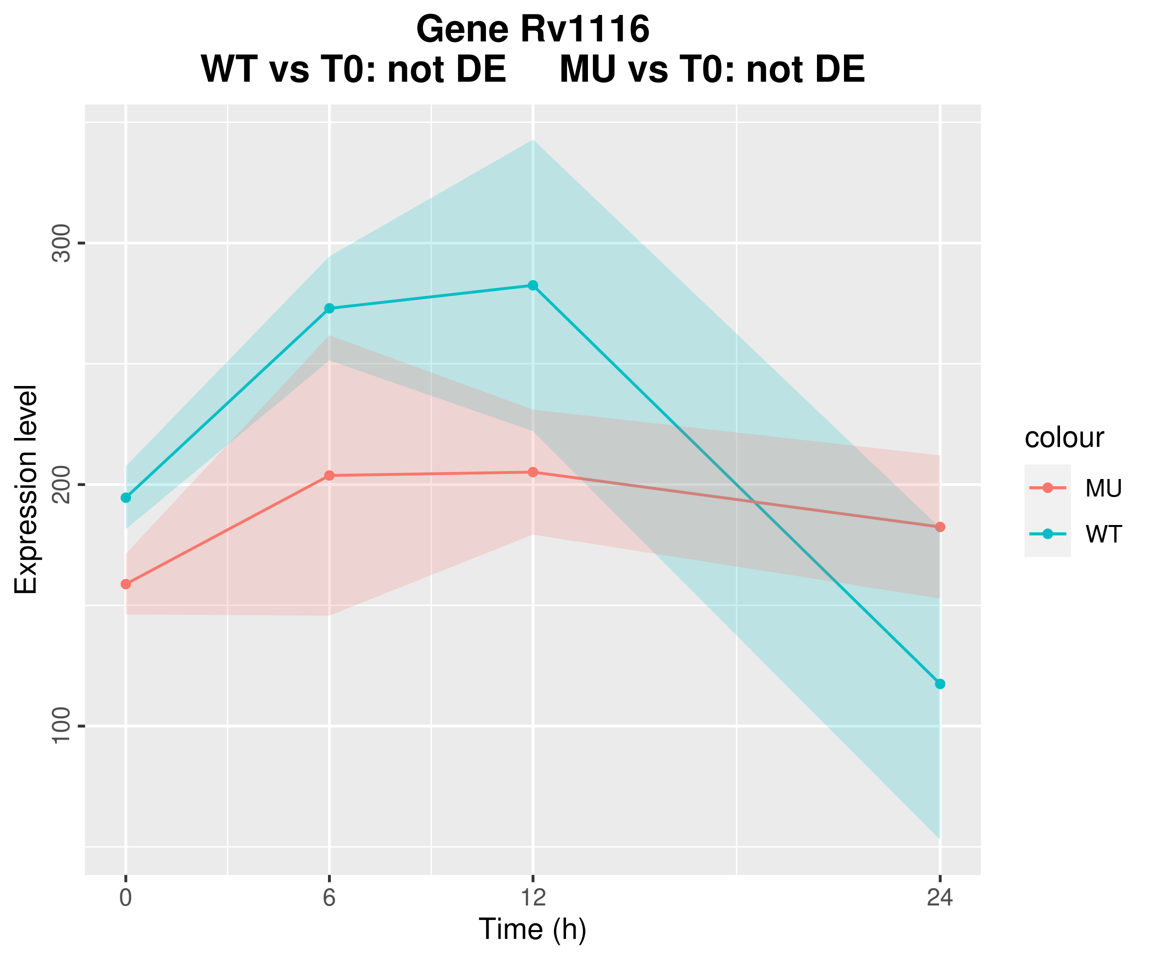

Gene Rv1116
WT vs T0: not DE MU vs T0: not DE
Expression level
300
200
100
0
6
12
24
Time (h)
colour
MU
WT

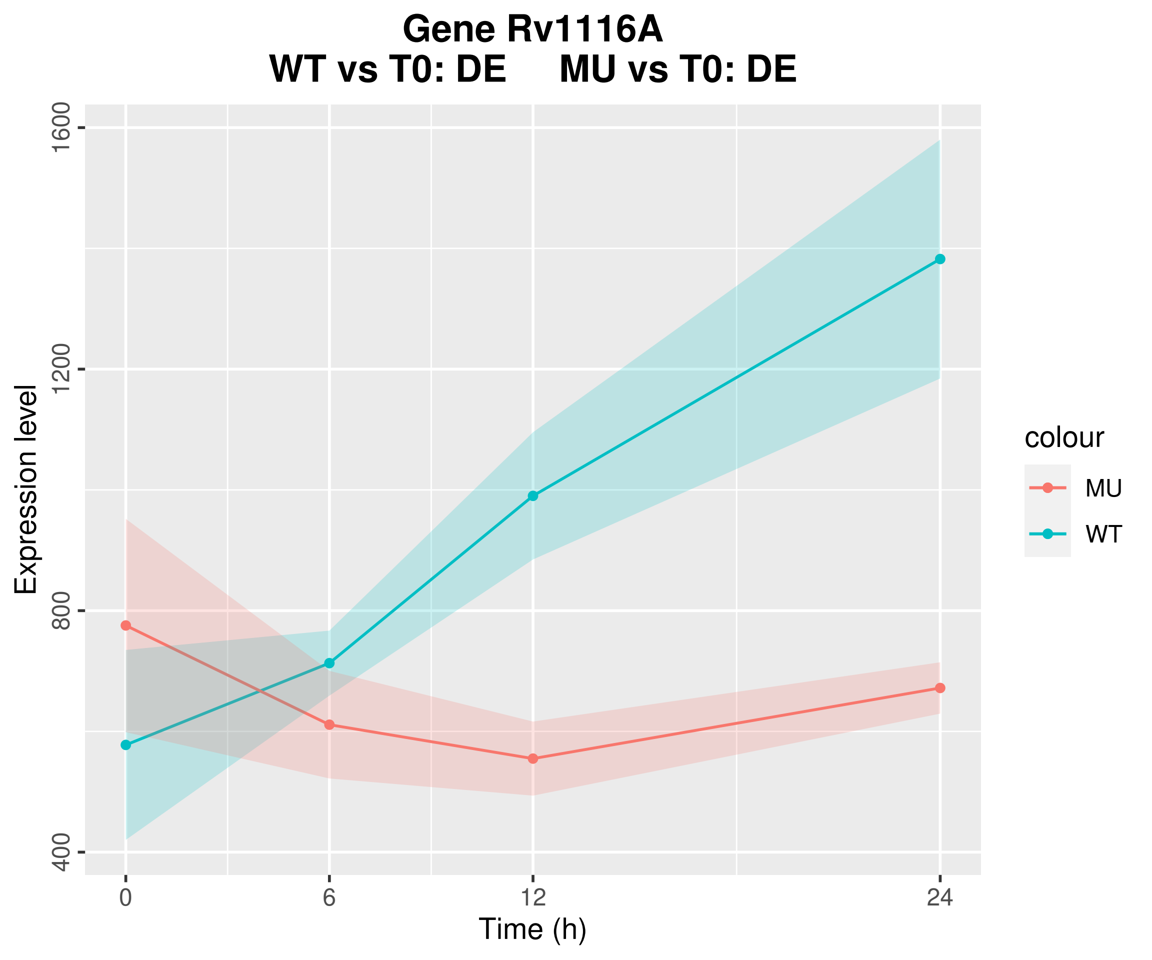

Gene Rv1116A
WT vs T0: DE MU vs T0: DE
Expression level
1600
1200
800
400
0
6
12
24
Time (h)
colour
MU
WT

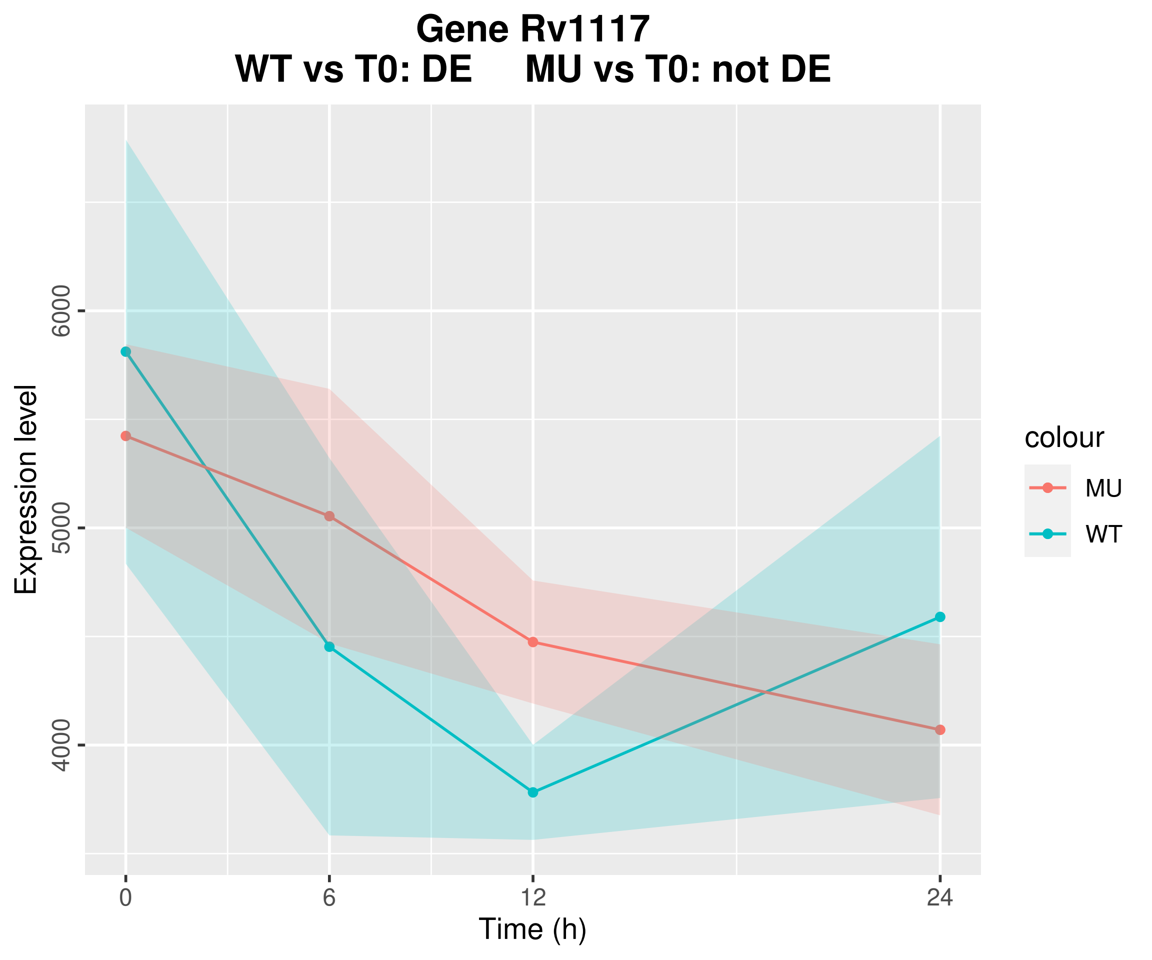

Gene Rv1117
WT vs T0: DE MU vs T0: not DE
Expression level
6000
5000
4000
0
6
12
24
Time (h)
colour
MU
WT

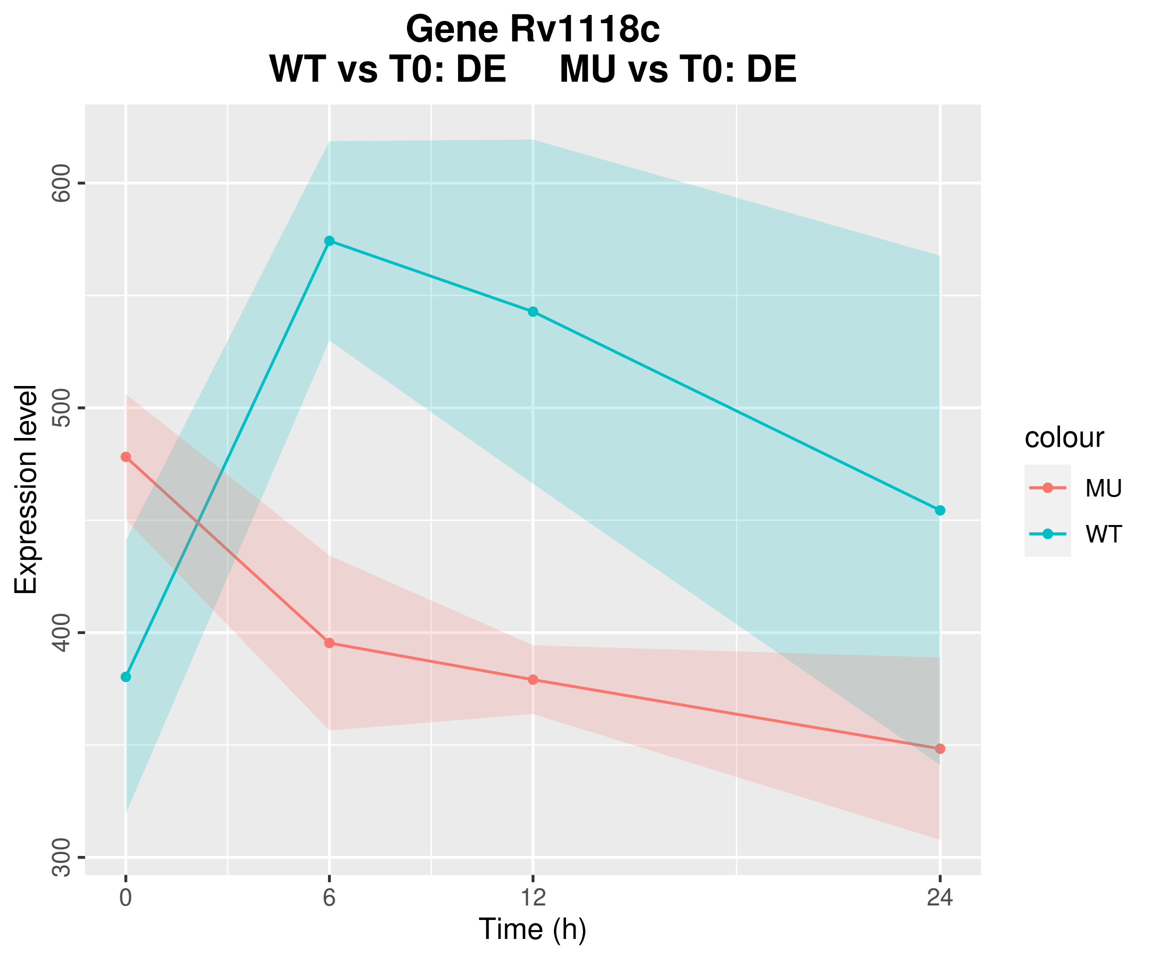
Gene Rv1118c
WT vs T0: DE MU vs T0: DE
Expression level
600
500
400
300
0
6
12
24
Time (h)
colour
MU
WT

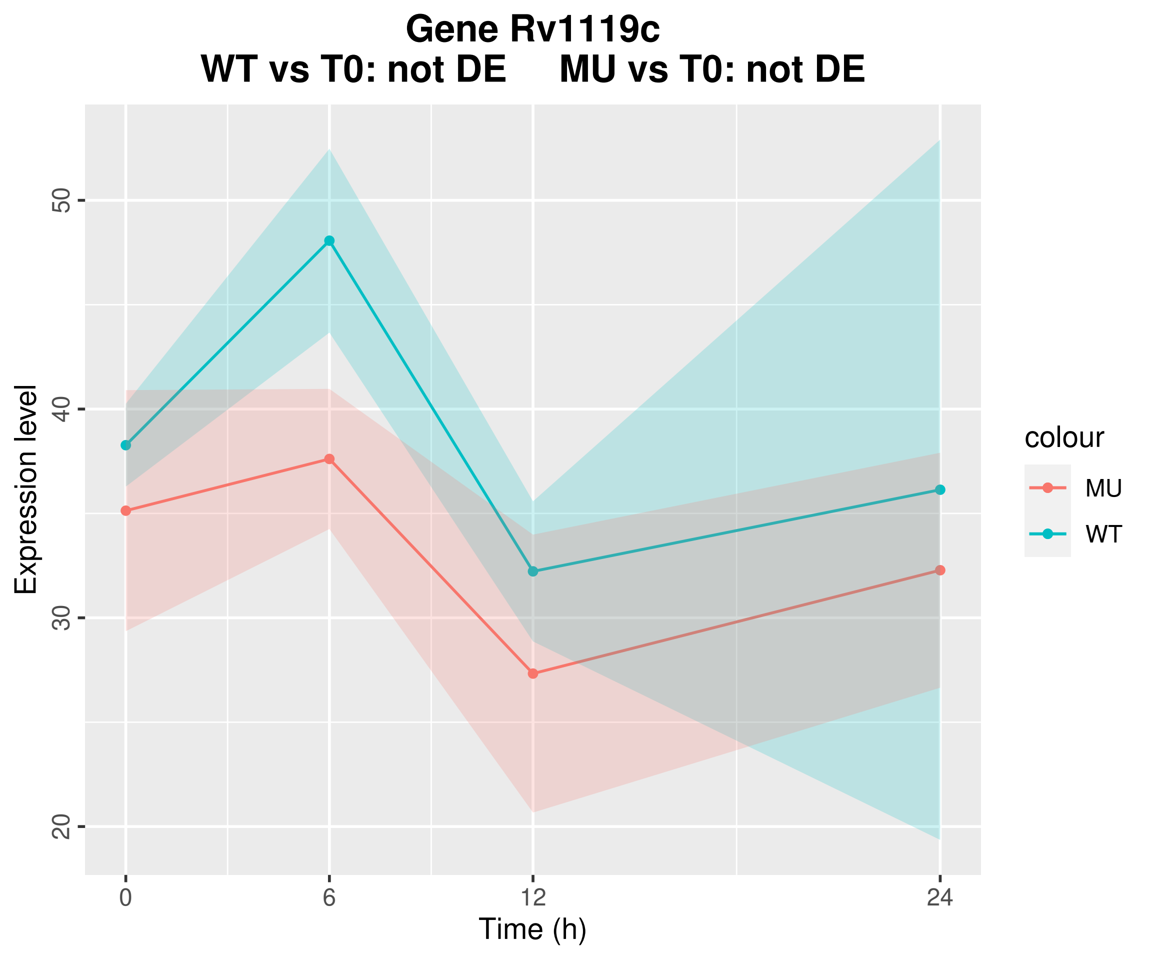

Gene Rv1119c
WT vs T0: not DE MU vs T0: not DE
Expression level
20
30
40
50
0
6
12
24
Time (h)
colour
MU
WT

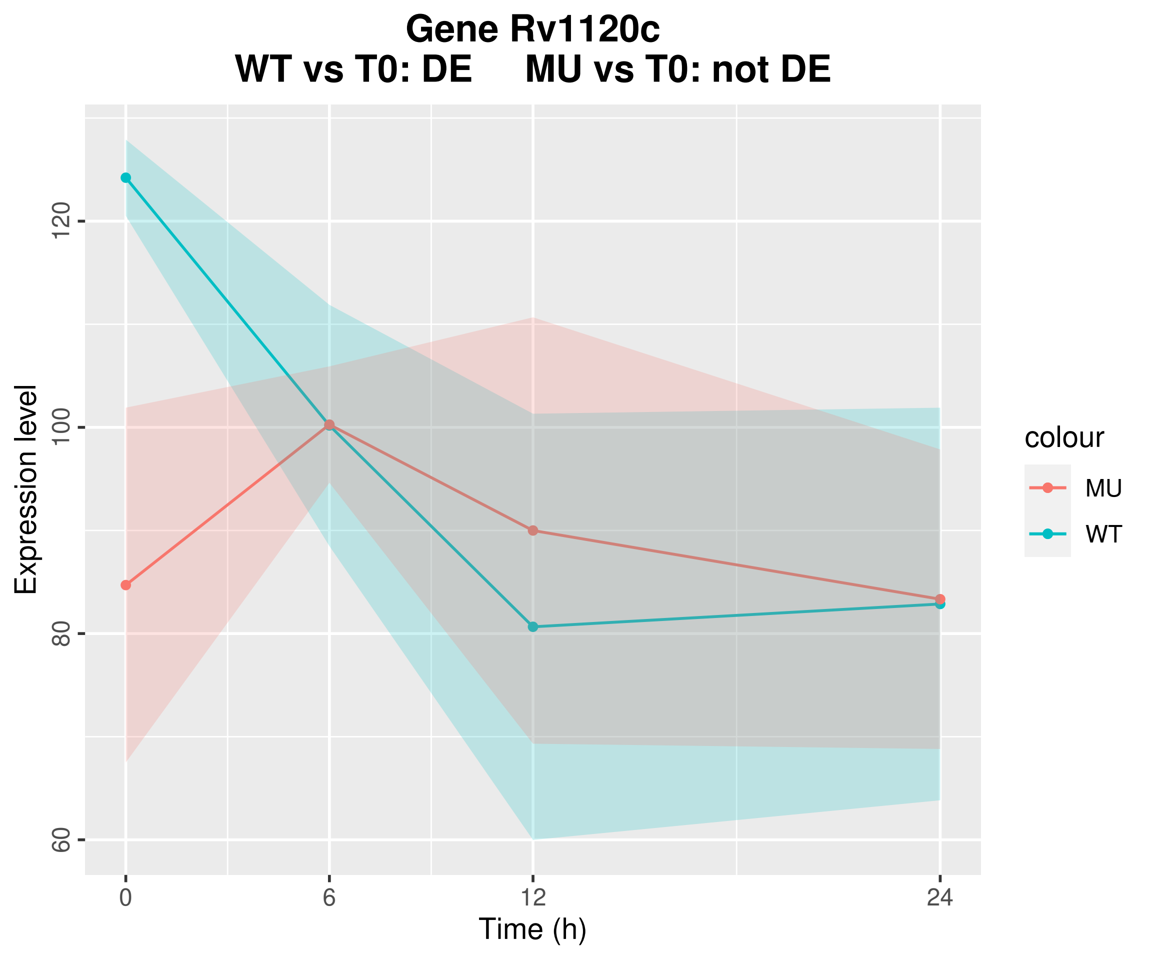

Gene Rv1120c
WT vs T0: DE MU vs T0: not DE
Expression level
120
100
80
60
0
6
12
24
Time (h)
colour
MU
WT

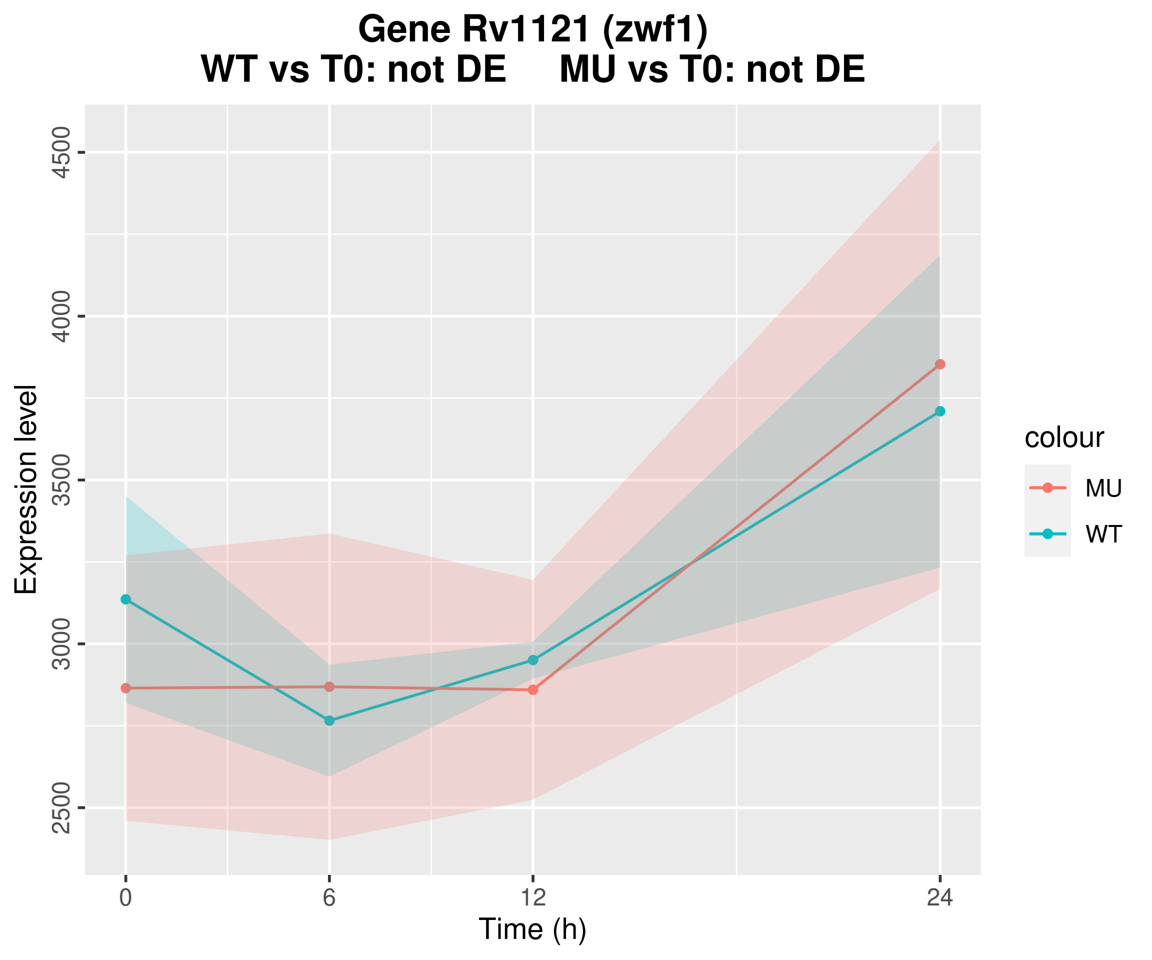

Gene Rv1121 (zwf1)
WT vs T0: not DE   MU vs T0: not DE
Expression level
4500
4000
3500
3000
2500
0
6
12
24
Time (h)
colour
MU
WT

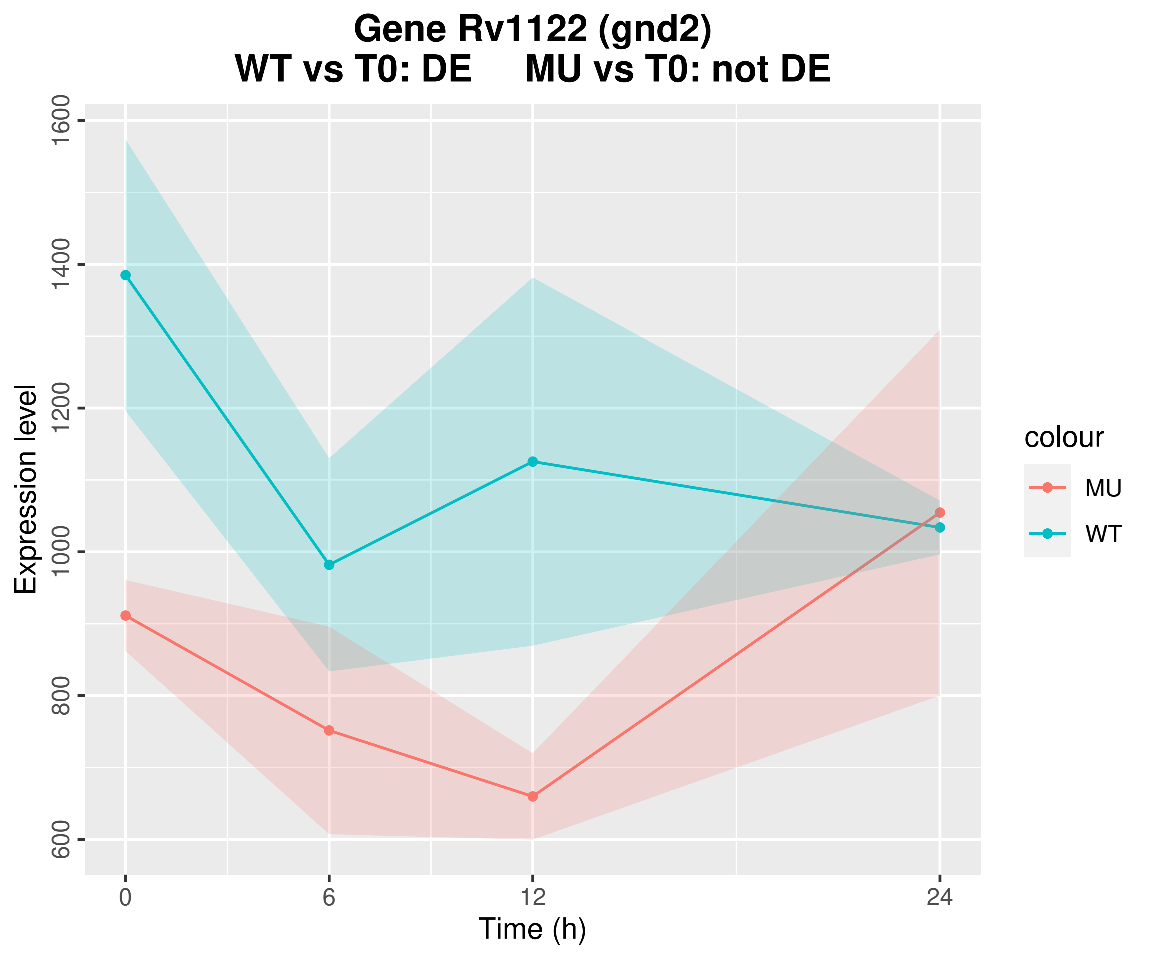

Gene Rv1122 (gnd2)
WT vs T0: DE MU vs T0: not DE
Expression level
1600
1400
1200
1000
800
600
0
6
12
24
Time (h)
colour
MU
WT

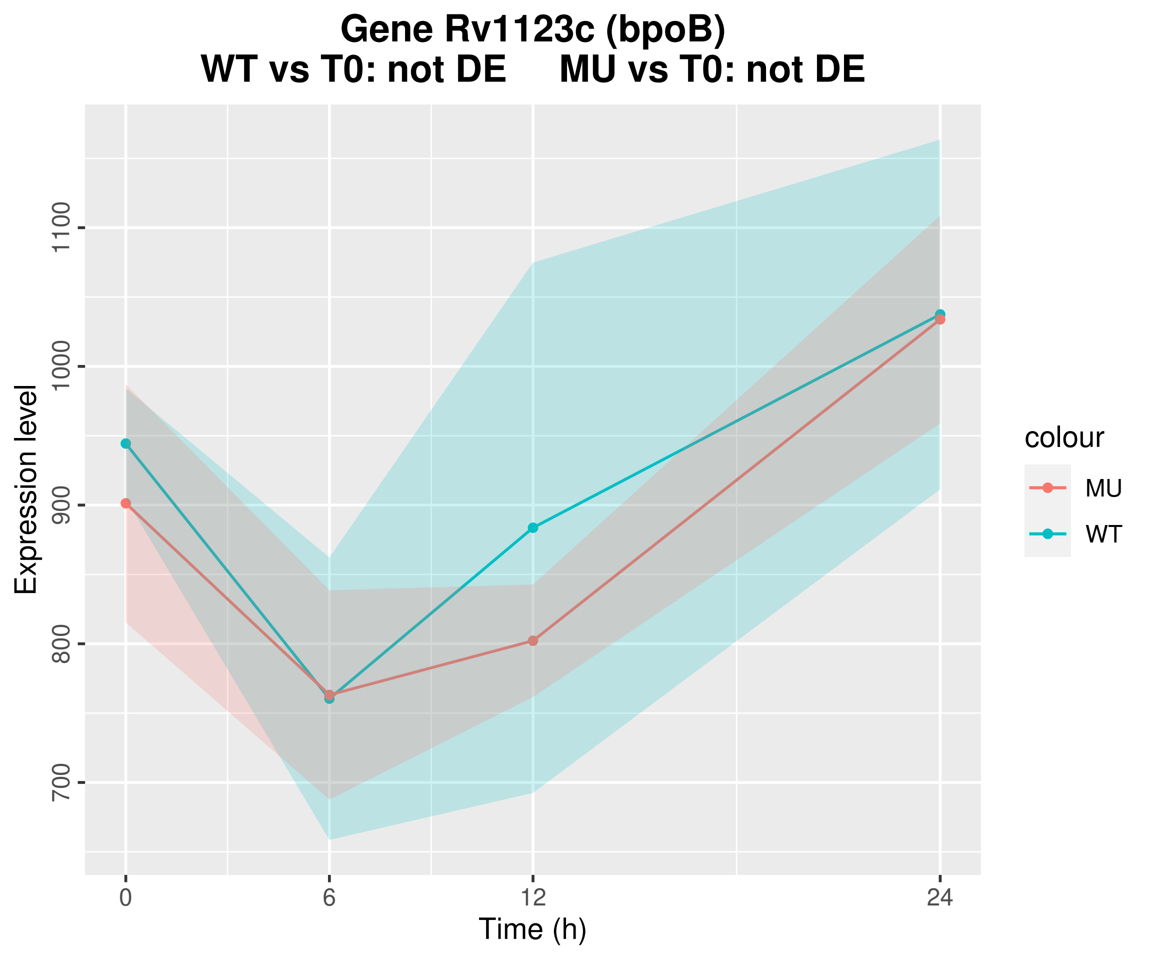
Gene Rv1123c (bpoB)
WT vs T0: not DE MU vs T0: not DE
Expression level
1100
1000
900
800
700
0
6
12
24
Time (h)
colour
MU
WT

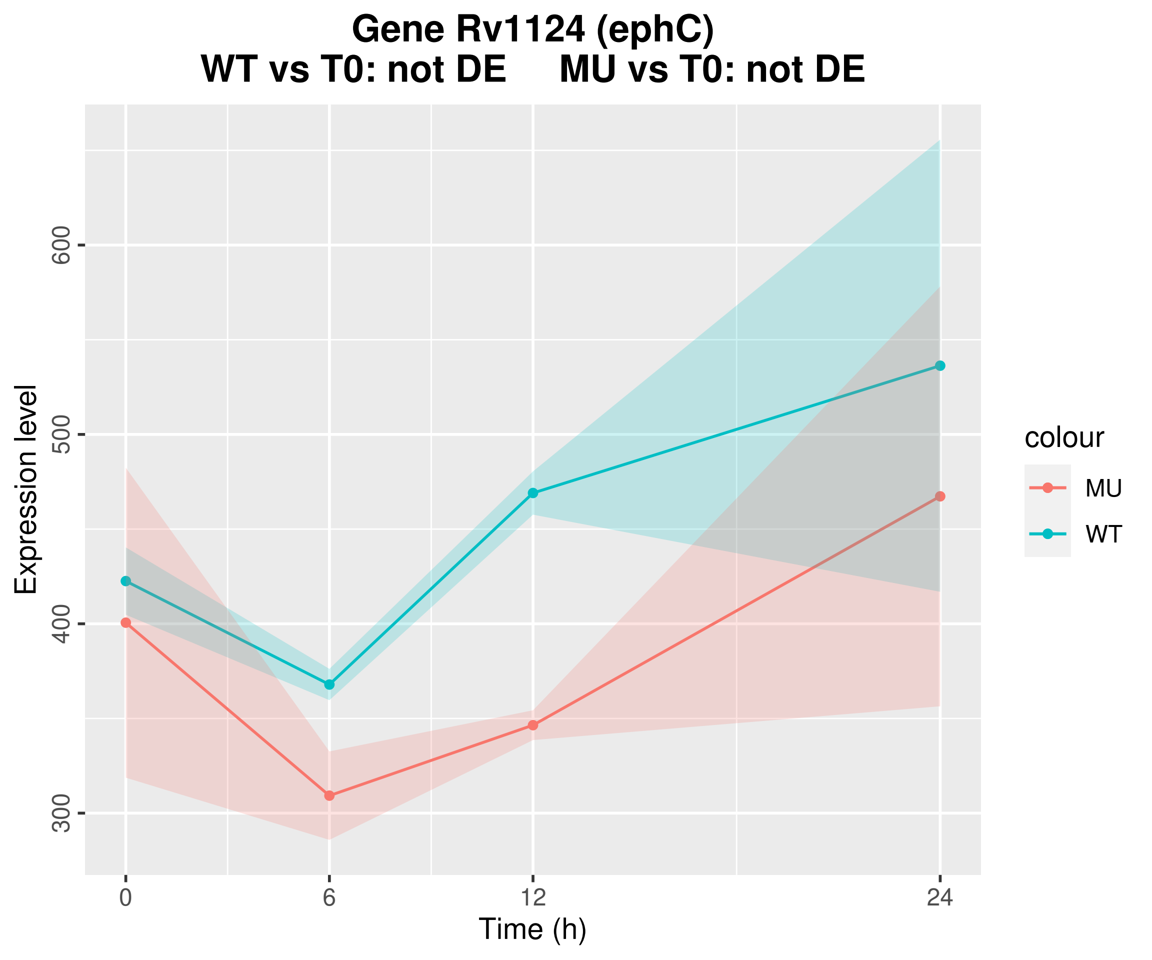

Gene Rv1124 (ephC)
WT vs T0: not DE   MU vs T0: not DE
Expression level
600
500
400
300
0
6
12
24
Time (h)
colour
MU
WT

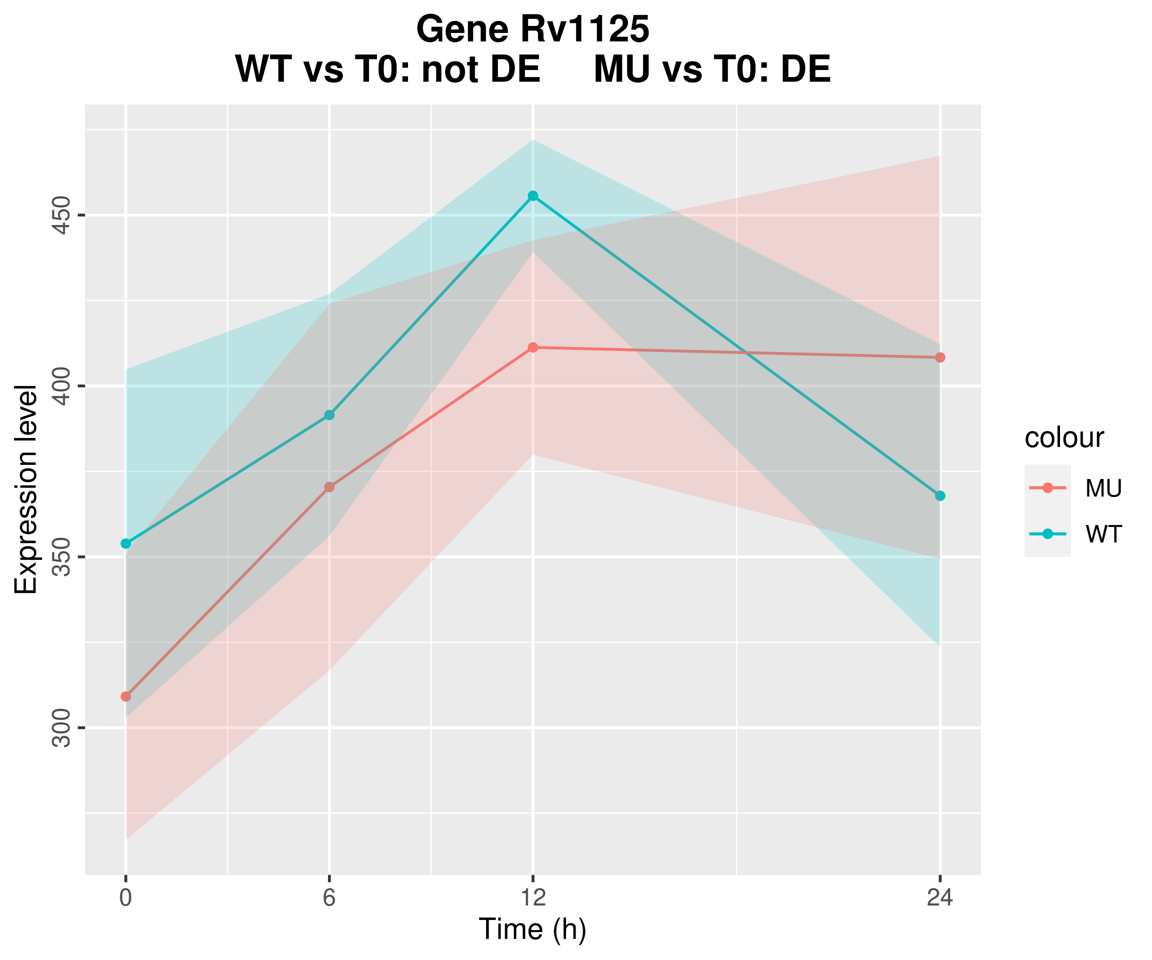
Gene Rv1125
WT vs T0: not DE   MU vs T0: DE
Expression level
300
350
400
450
0
6
12
24
Time (h)
colour
MU
WT

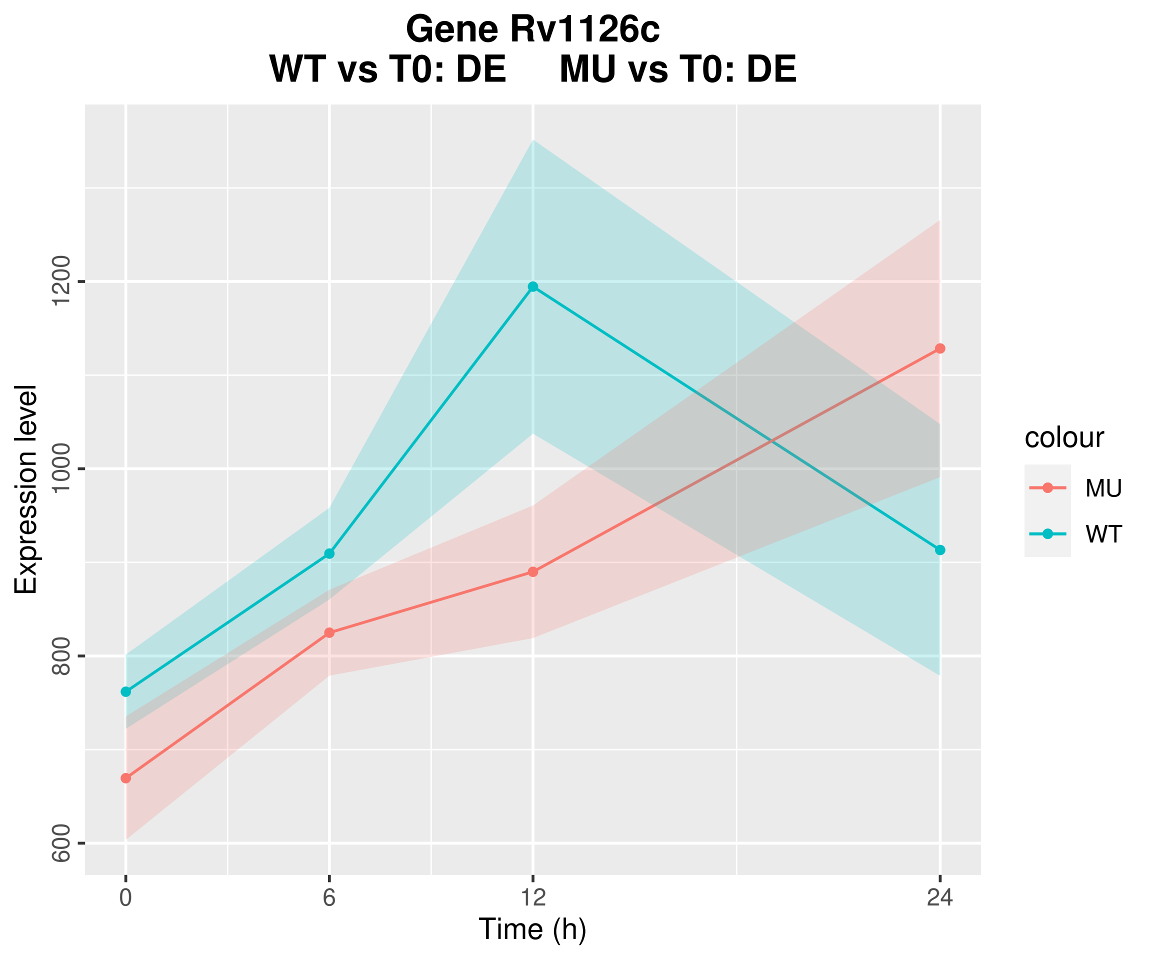

Gene Rv1126c
WT vs T0: DE MU vs T0: DE
Expression level
1200
1000
800
600
0
6
12
24
Time (h)
colour
MU
WT

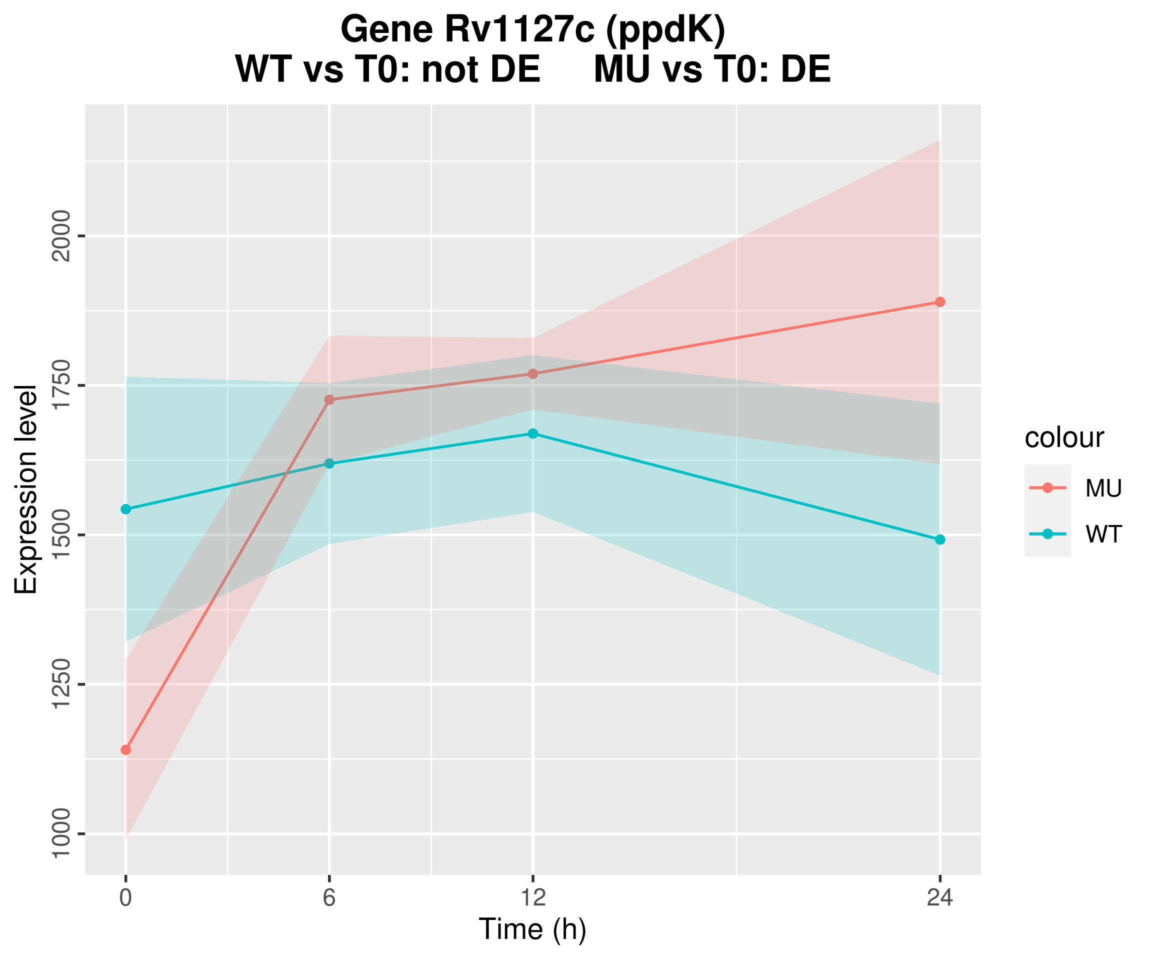
Gene Rv1127c (ppdK)
WT vs T0: not DE   MU vs T0: DE
Expression level
1000
1250
1500
1750
2000
0
6
12
24
Time (h)
colour
MU
WT

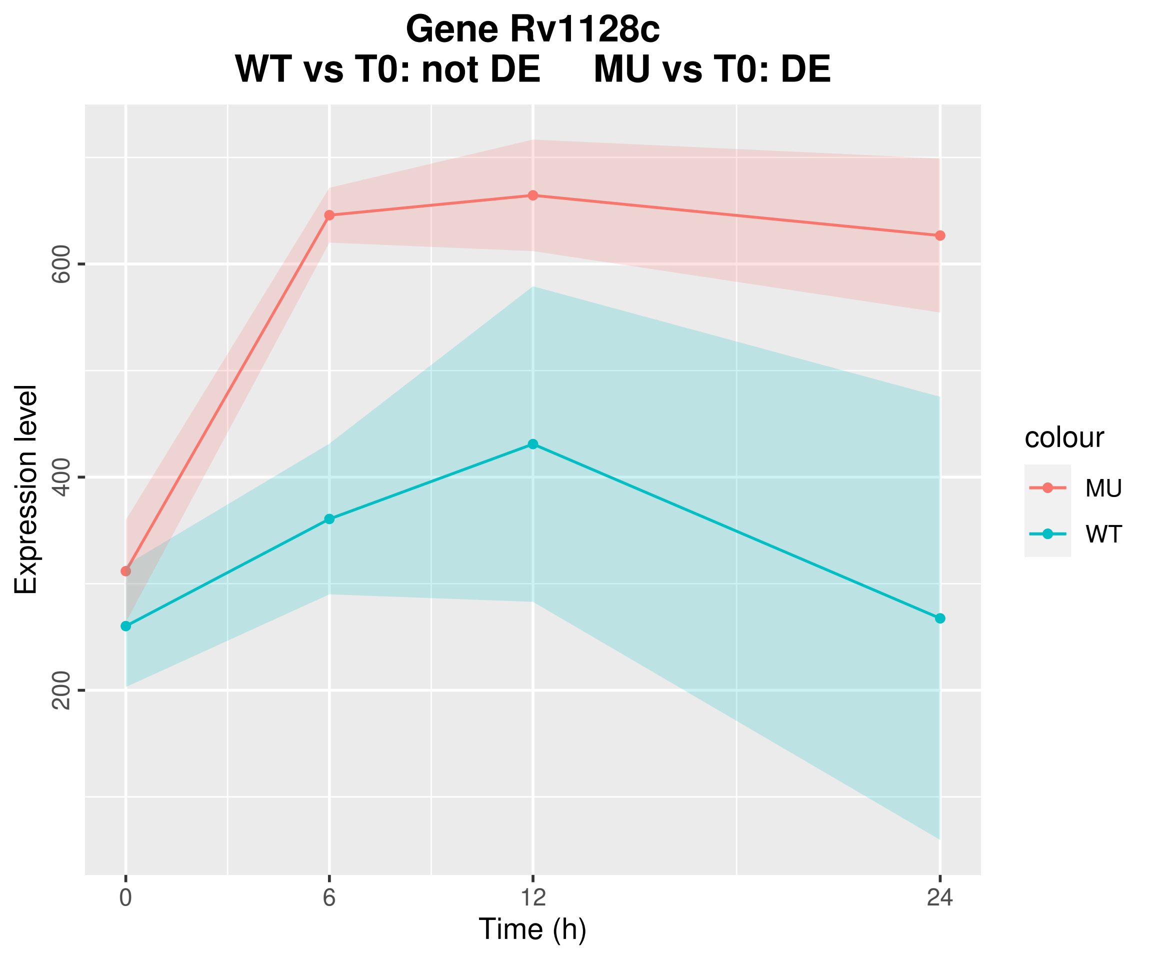
Gene Rv1128c
WT vs T0: not DE   MU vs T0: DE
Expression level
600
400
200
0
6
12
24
Time (h)
colour
MU
WT

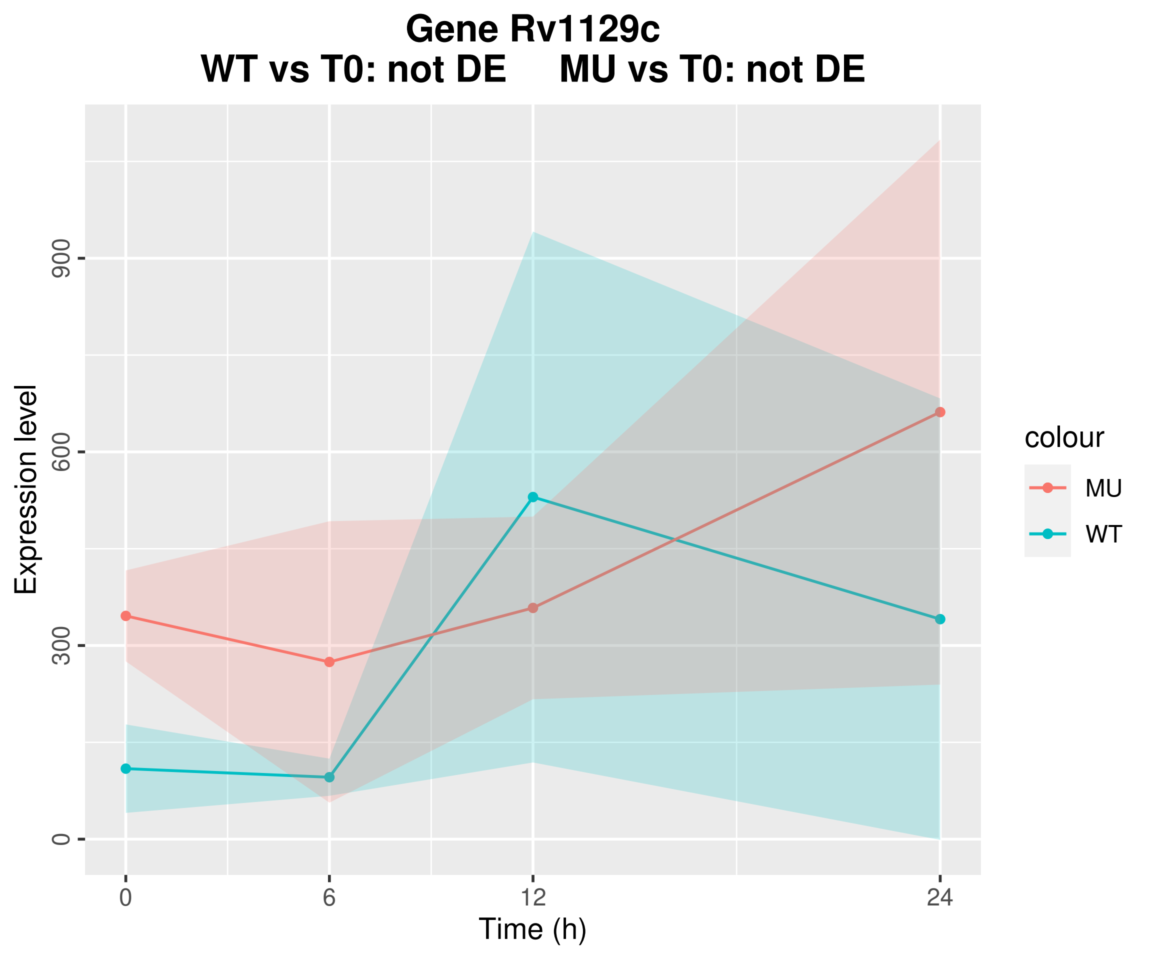
Gene Rv1129c
WT vs T0: not DE MU vs T0: not DE
Expression level
900
600
300
0
0
6
12
24
Time (h)
colour
MU
WT

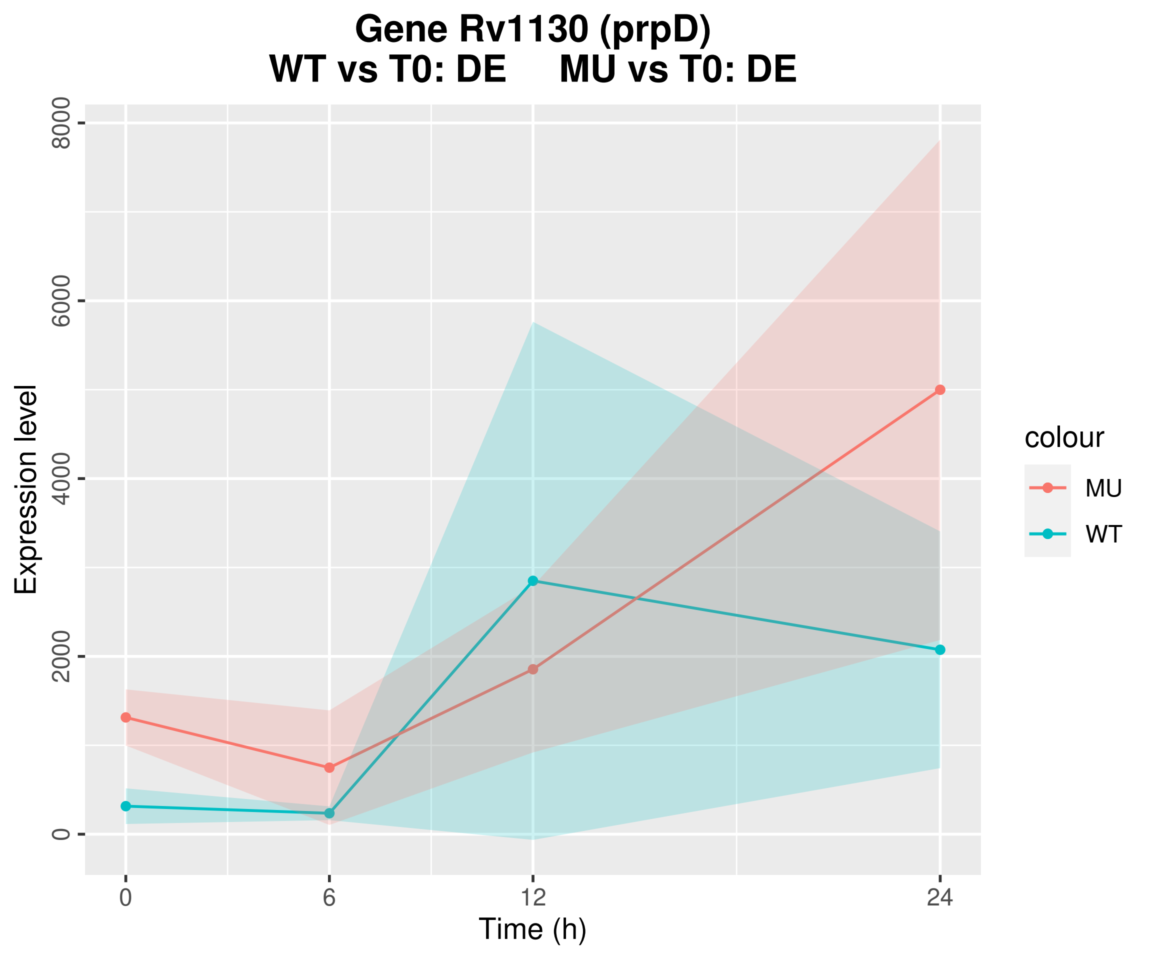
Gene Rv1130 (prpD)
WT vs T0: DE   MU vs T0: DE
Expression level
8000
6000
4000
2000
0
0
6
12
24
Time (h)
colour
MU
WT

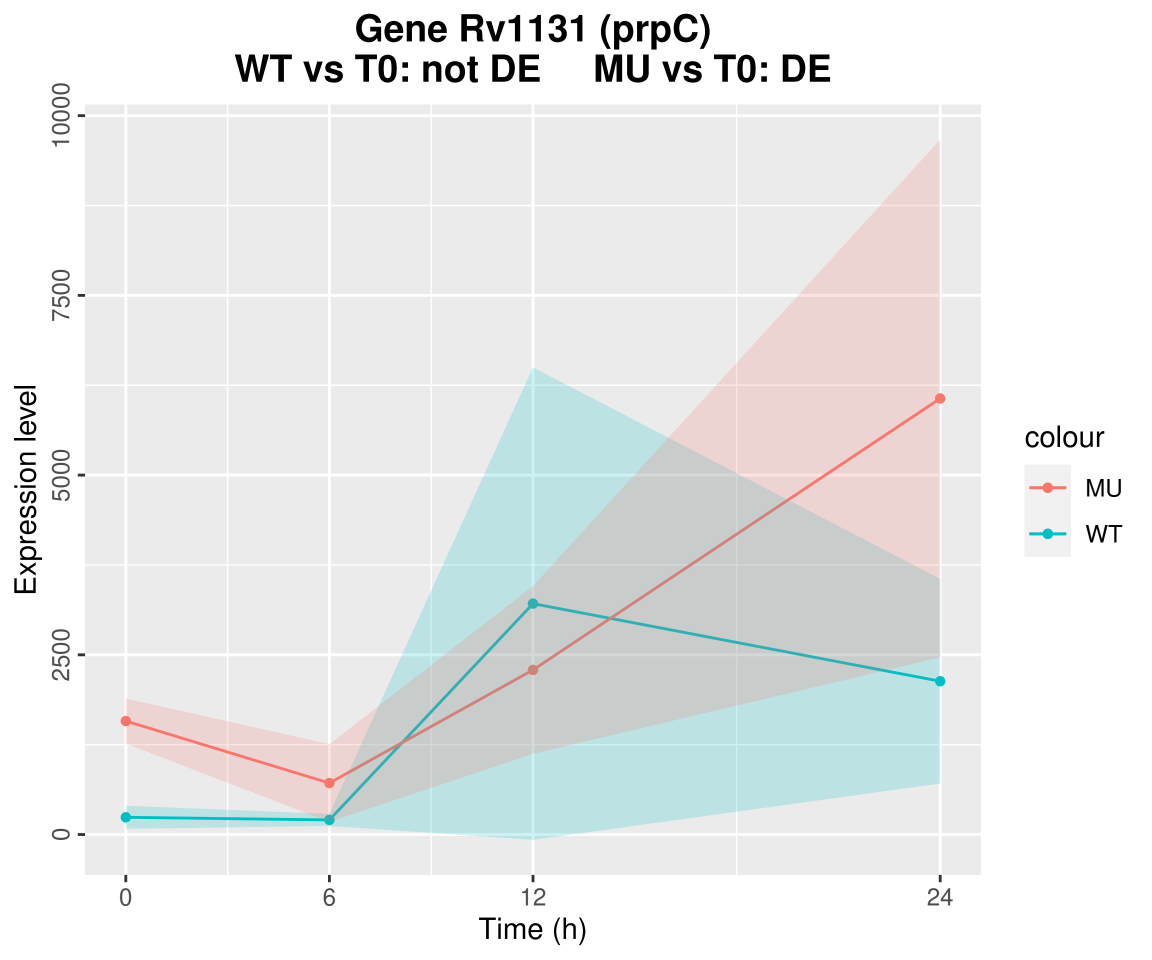

Gene Rv1131 (prpC)
WT vs T0: not DE MU vs T0: DE
Expression level
10000
7500
5000
2500
0
0
6
12
24
Time (h)
colour
MU
WT

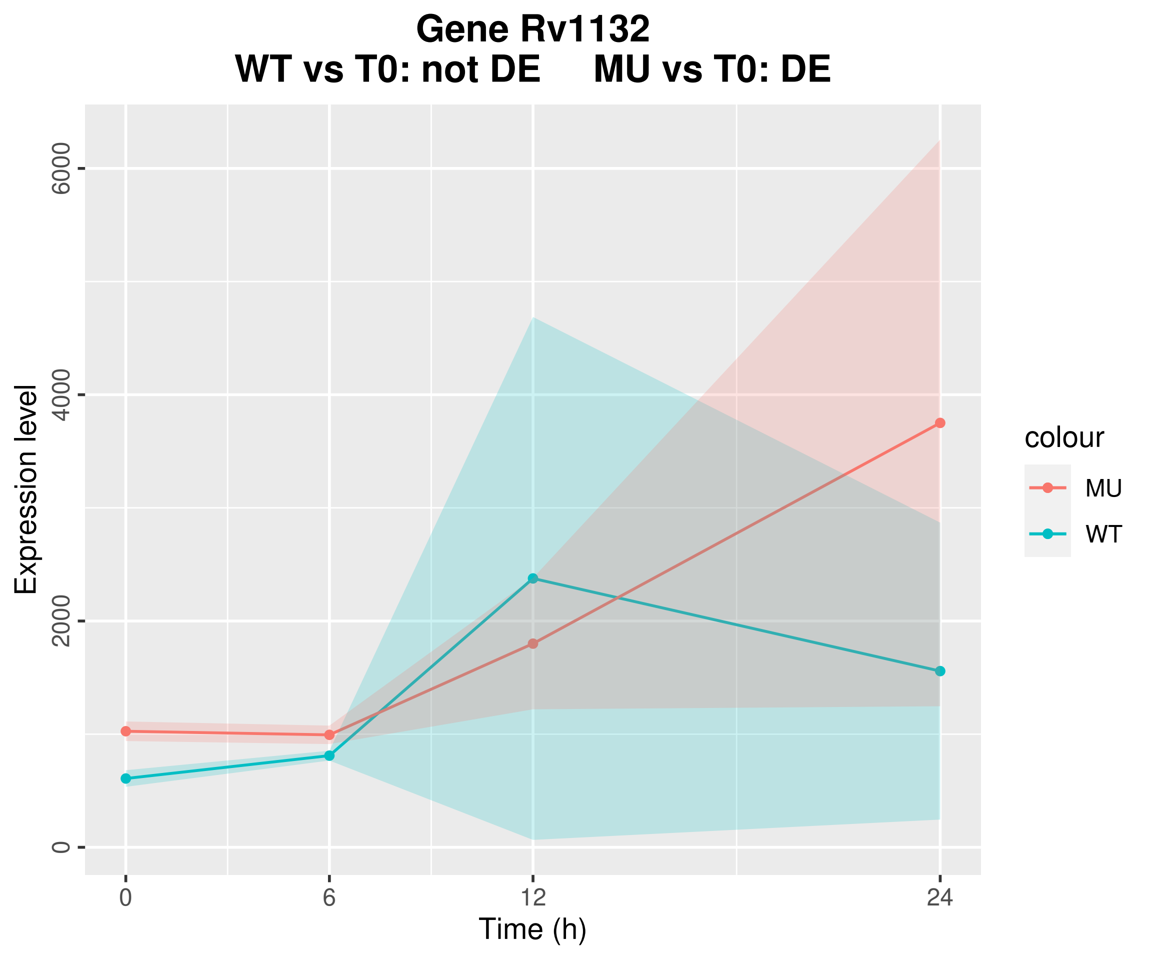
Gene Rv1132
WT vs T0: not DE   MU vs T0: DE
Expression level
6000
4000
2000
0
0
6
12
24
Time (h)
colour
MU
WT

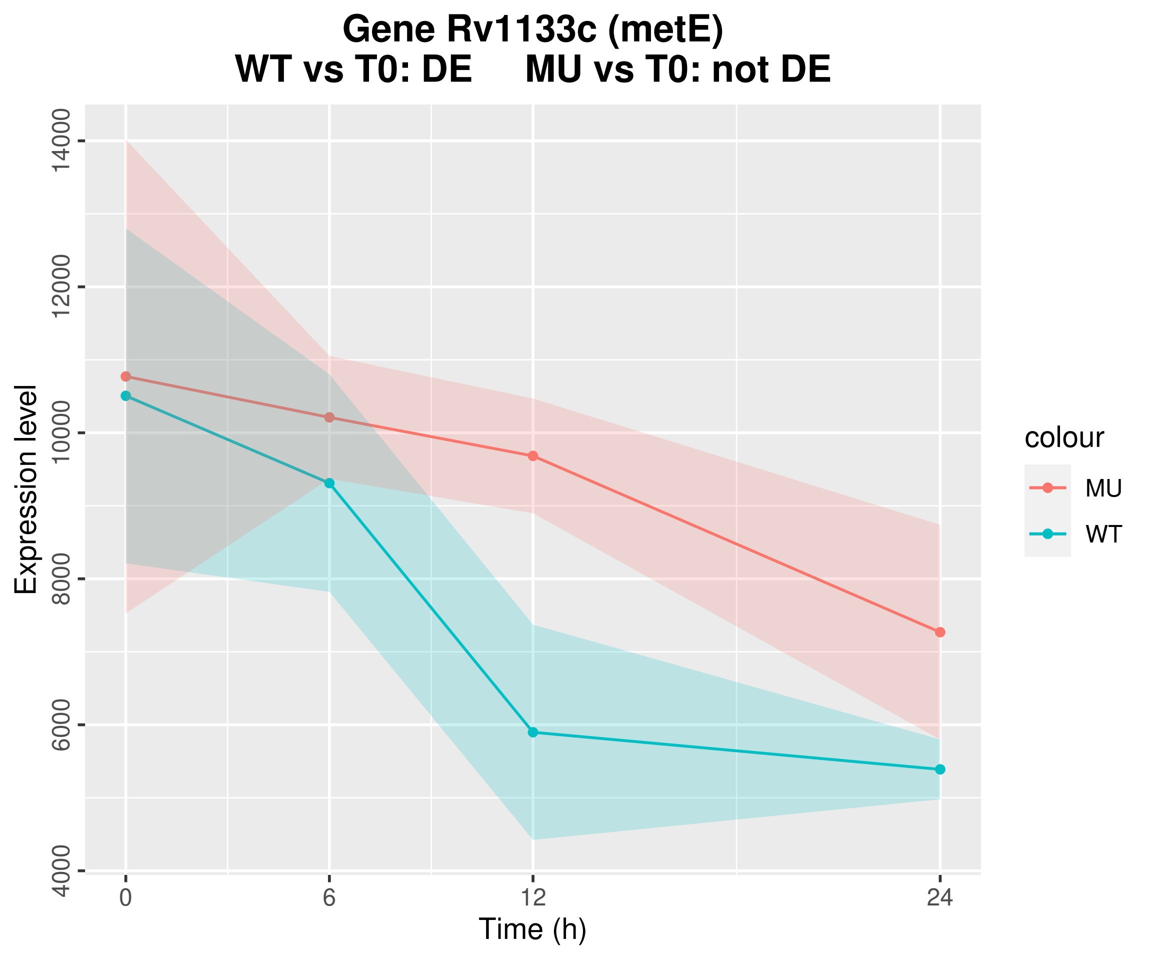

Gene Rv1133c (metE)
WT vs T0: DE   MU vs T0: not DE
Expression level
14000
12000
10000
8000
6000
4000
0
6
12
24
Time (h)
colour
MU
WT

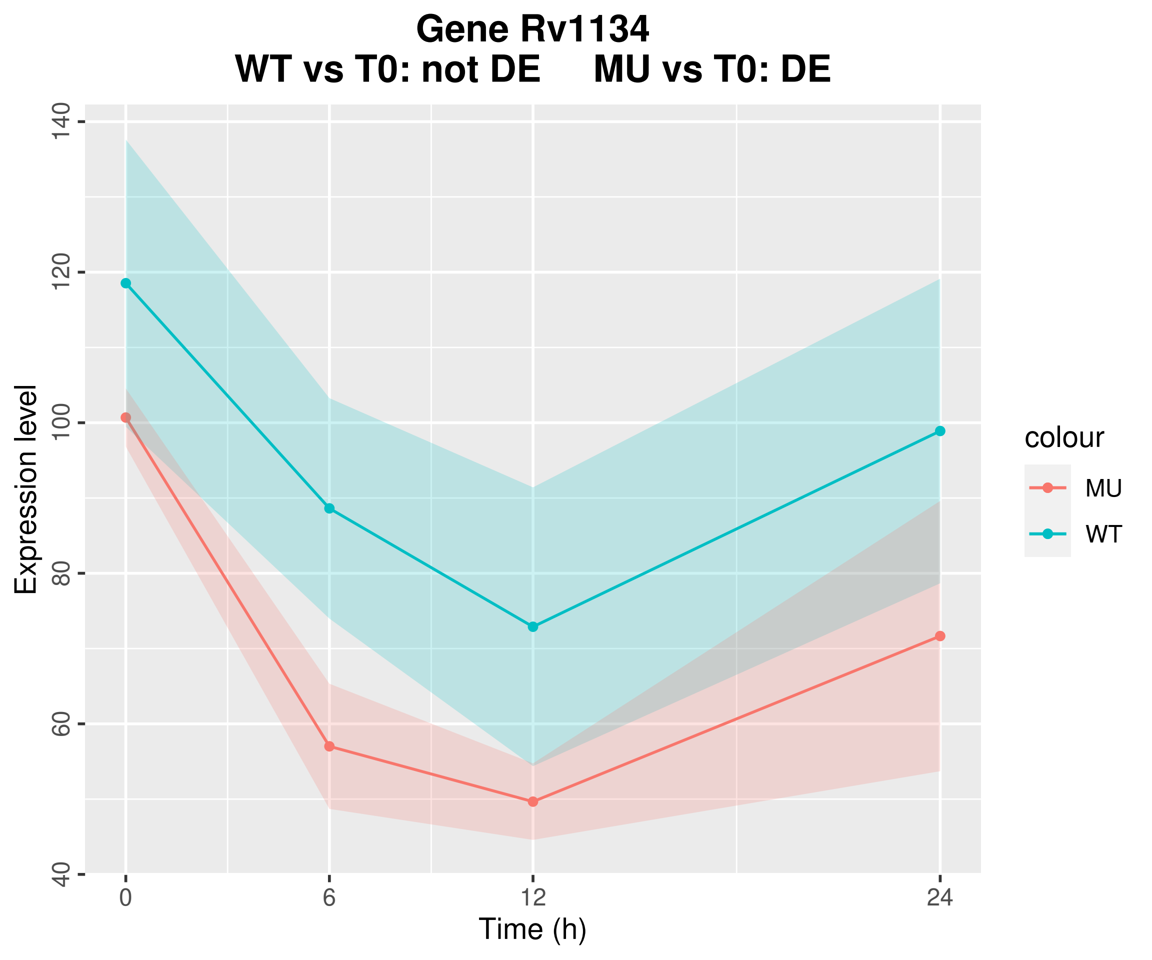
Gene Rv1134
WT vs T0: not DE MU vs T0: DE
Expression level
140
120
100
80
60
40
0
6
12
24
Time (h)
colour
MU
WT

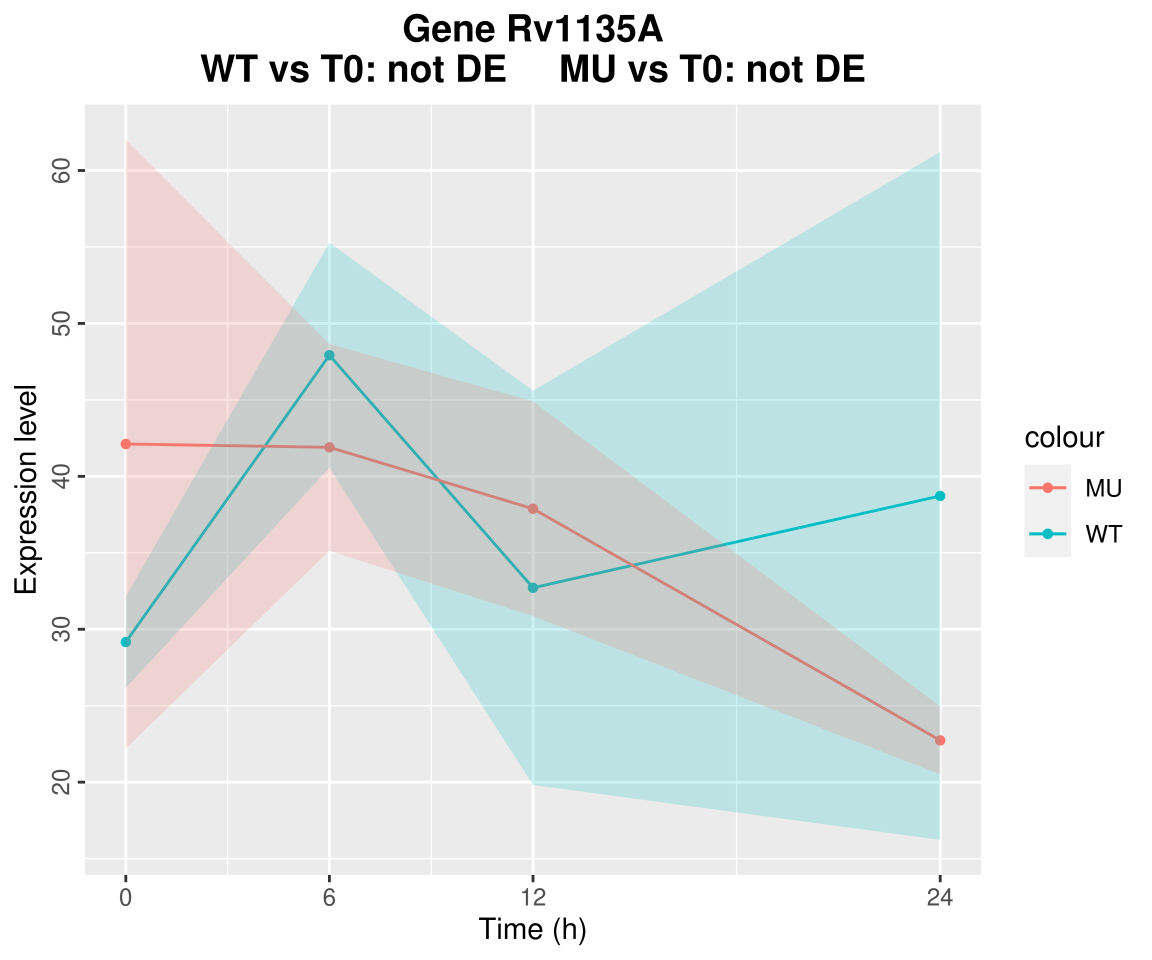
Gene Rv1135A
WT vs T0: not DE   MU vs T0: not DE
Expression level
20
30
40
50
60
0
6
12
24
Time (h)
colour
MU
WT

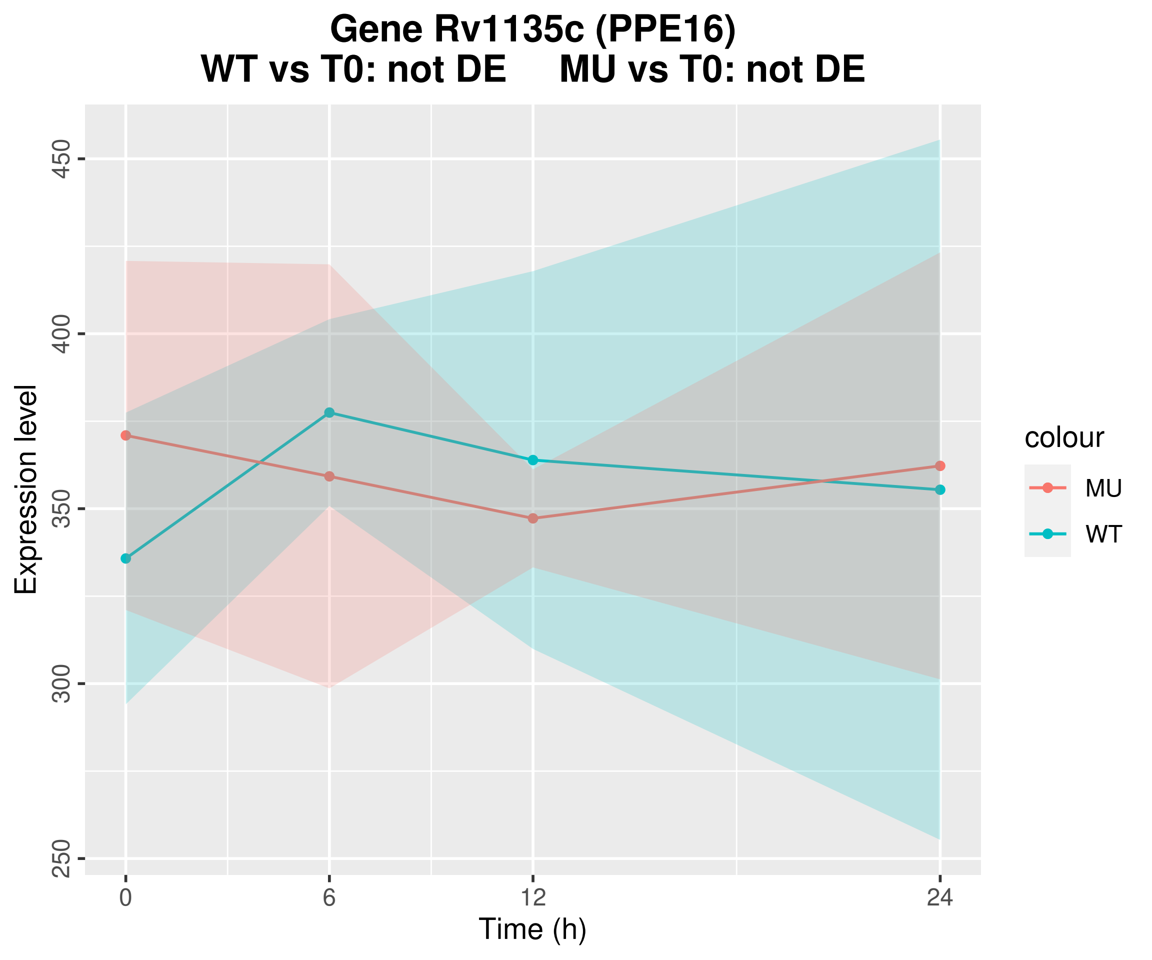

Gene Rv1135c (PPE16)
WT vs T0: not DE MU vs T0: not DE
Expression level
450
400
350
300
250
0
6
12
24
Time (h)
colour
MU
WT

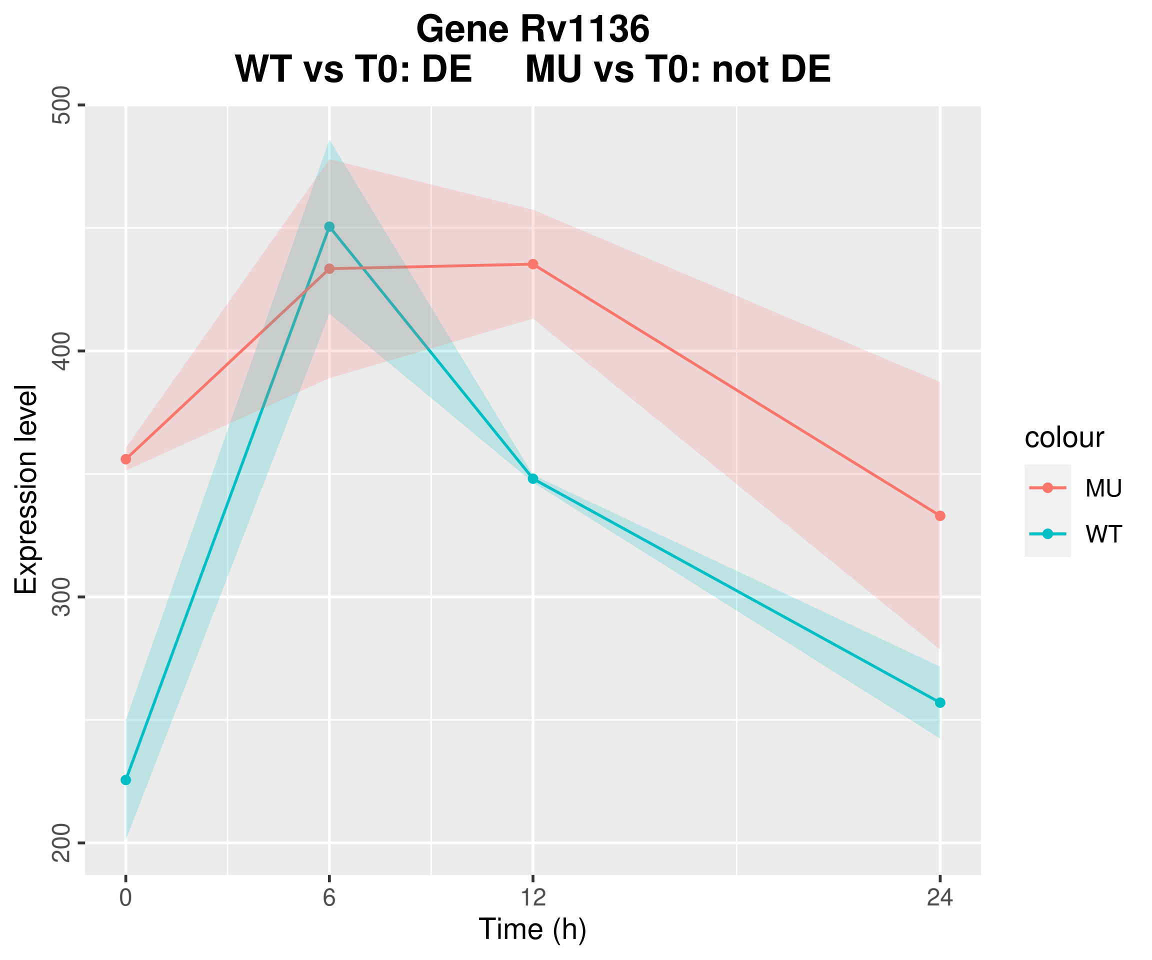

Gene Rv1136
WT vs T0: DE   MU vs T0: not DE
500
400
300
200
Expression level
0
6
12
24
Time (h)
colour
MU
WT

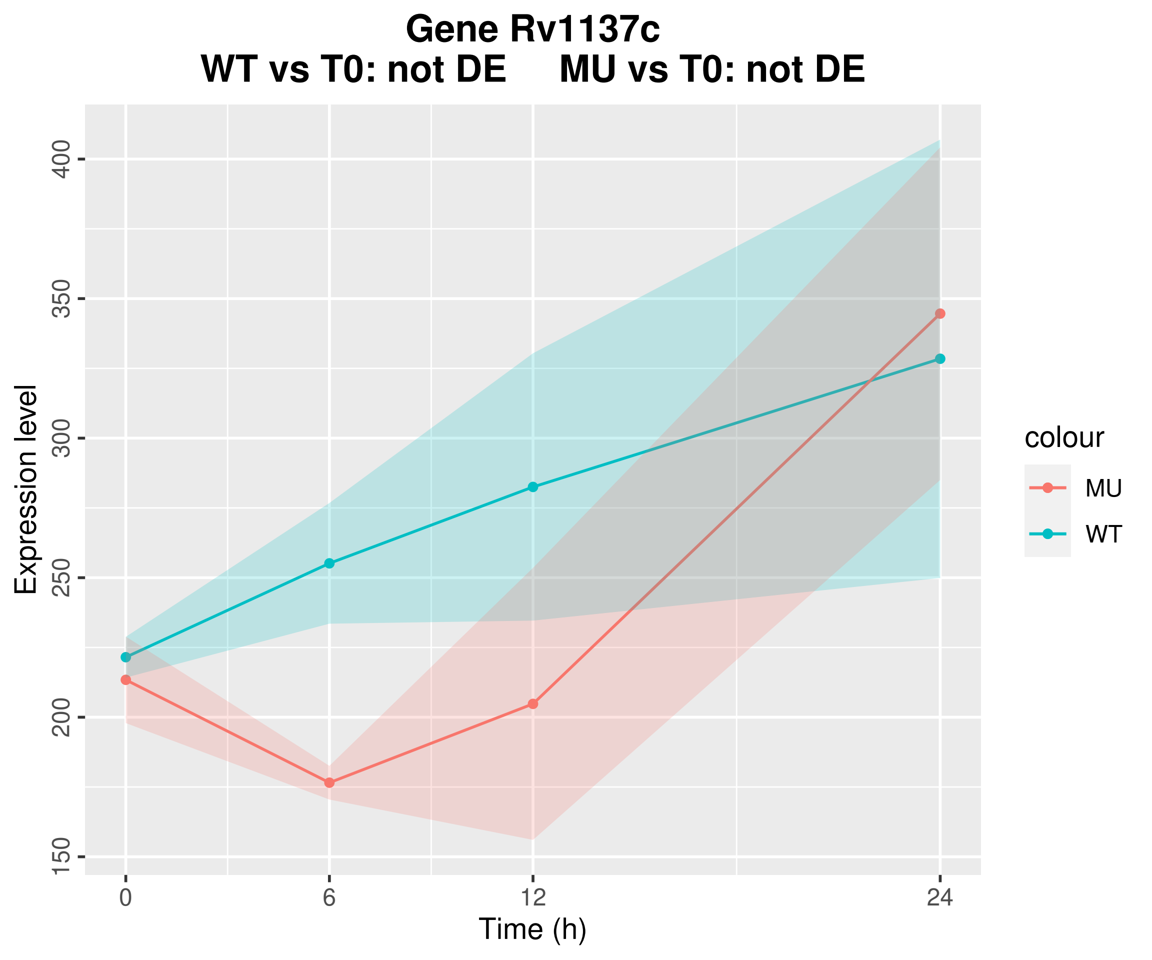
Gene Rv1137c
WT vs T0: not DE   MU vs T0: not DE
Expression level
400
350
300
250
200
150
0
6
12
24
Time (h)
colour
MU
WT

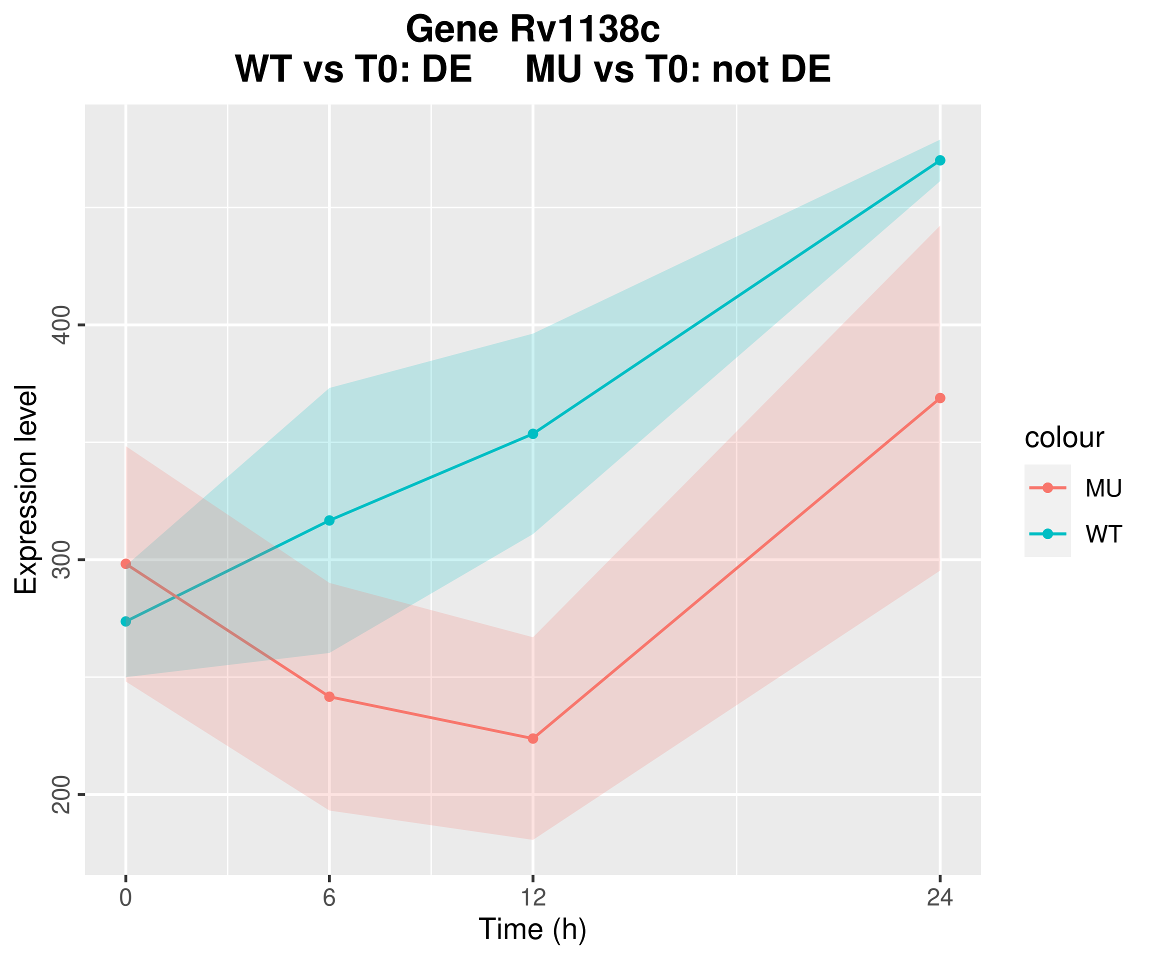

Gene Rv1138c
WT vs T0: DE MU vs T0: not DE
Expression level
400
300
200
0
6
12
24
Time (h)
colour
MU
WT

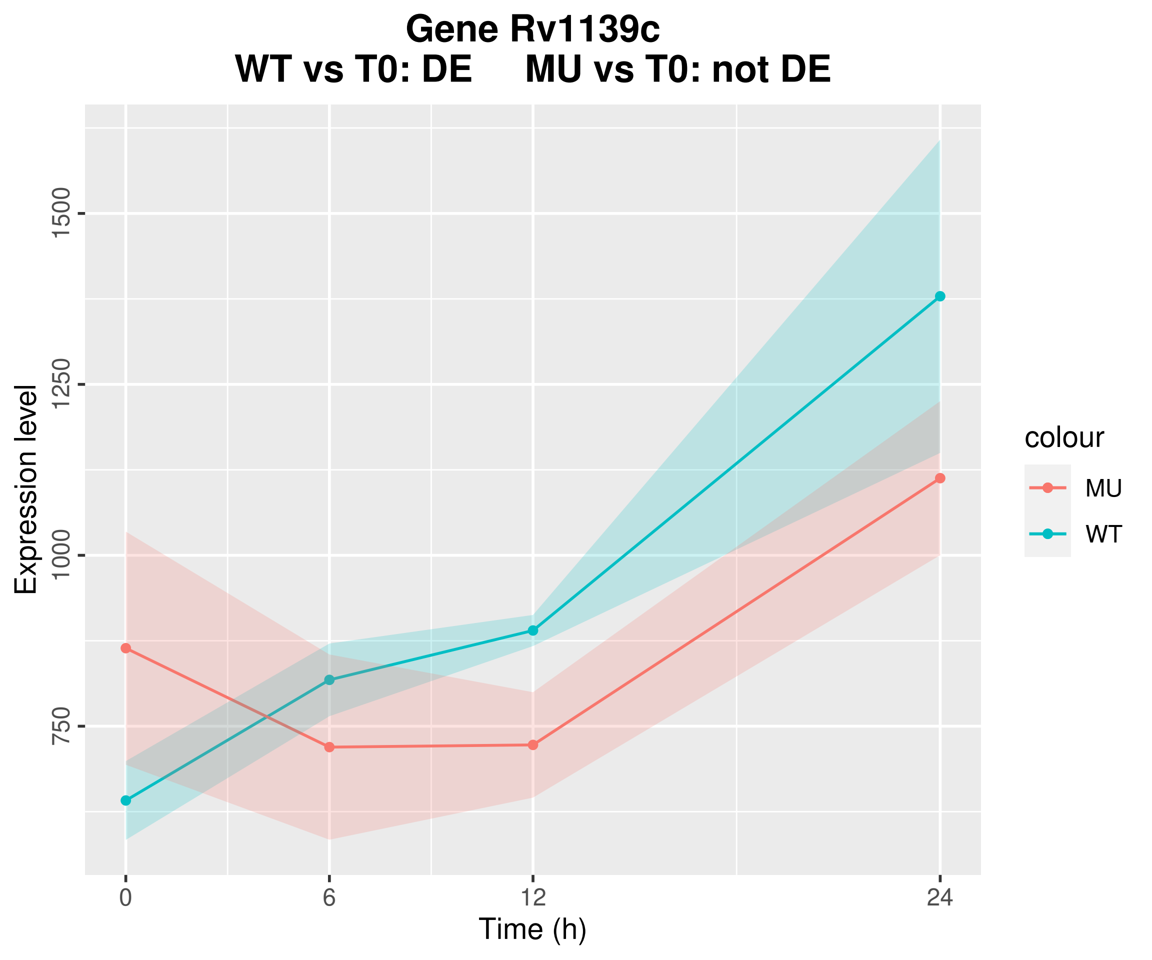
Gene Rv1139c
WT vs T0: DE   MU vs T0: not DE
Expression level
1500
1250
1000
750
0
6
12
24
Time (h)
colour
MU
WT

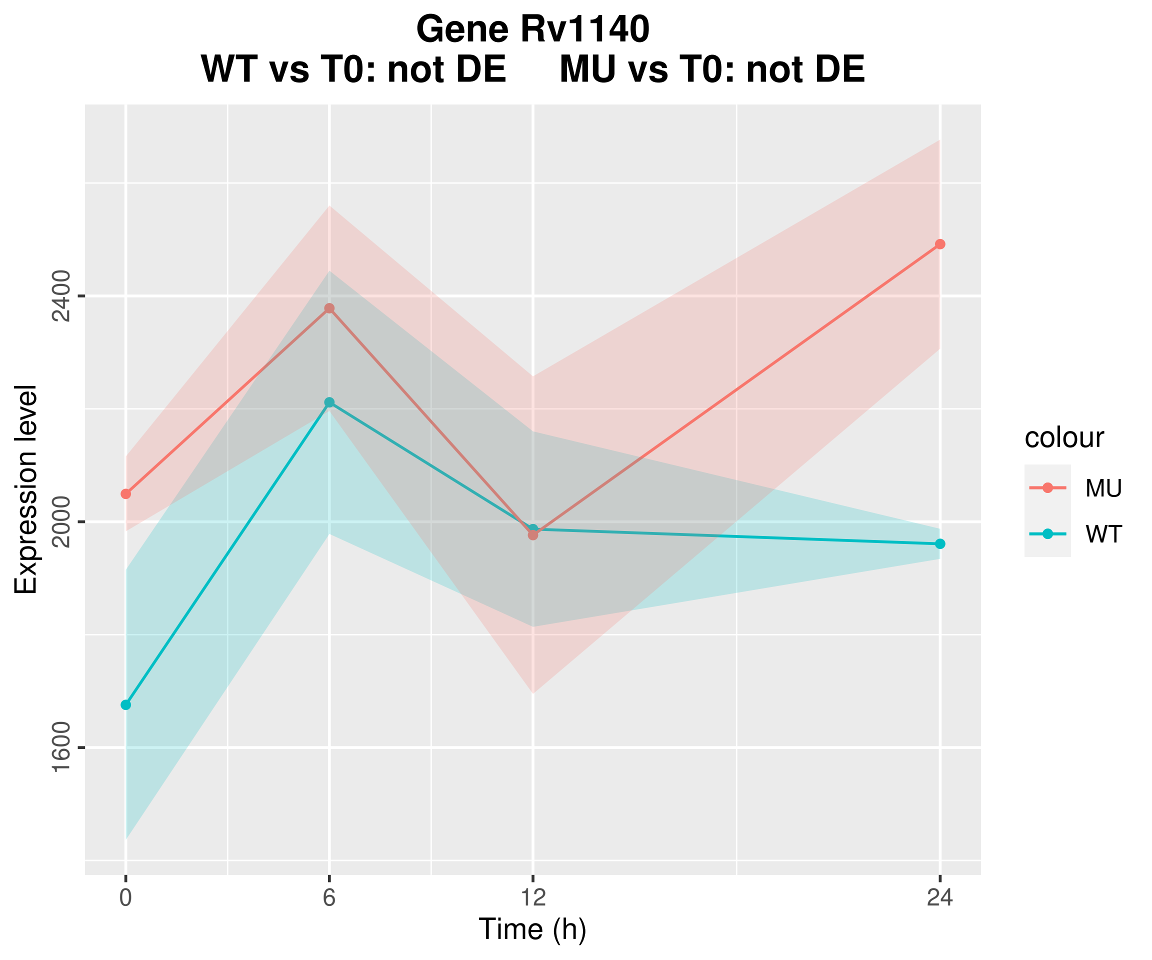
Gene Rv1140
WT vs T0: not DE MU vs T0: not DE
Expression level
2400
2000
1600
0
6
12
24
Time (h)
colour
MU
WT

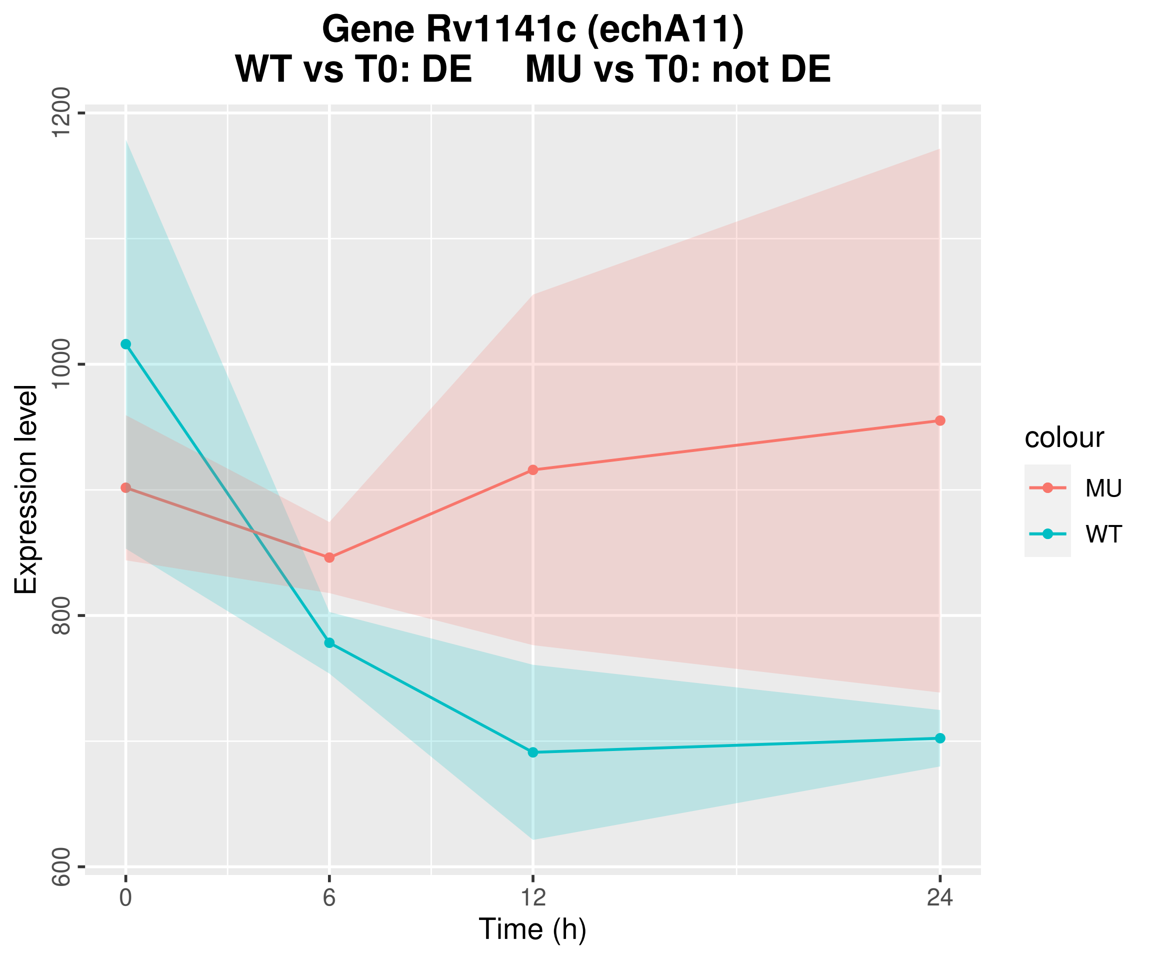

Gene Rv1141c (echA11)
WT vs T0: DE MU vs T0: not DE
1200
1000
800
600
Expression level
0
6
12
24
Time (h)
colour
MU
WT

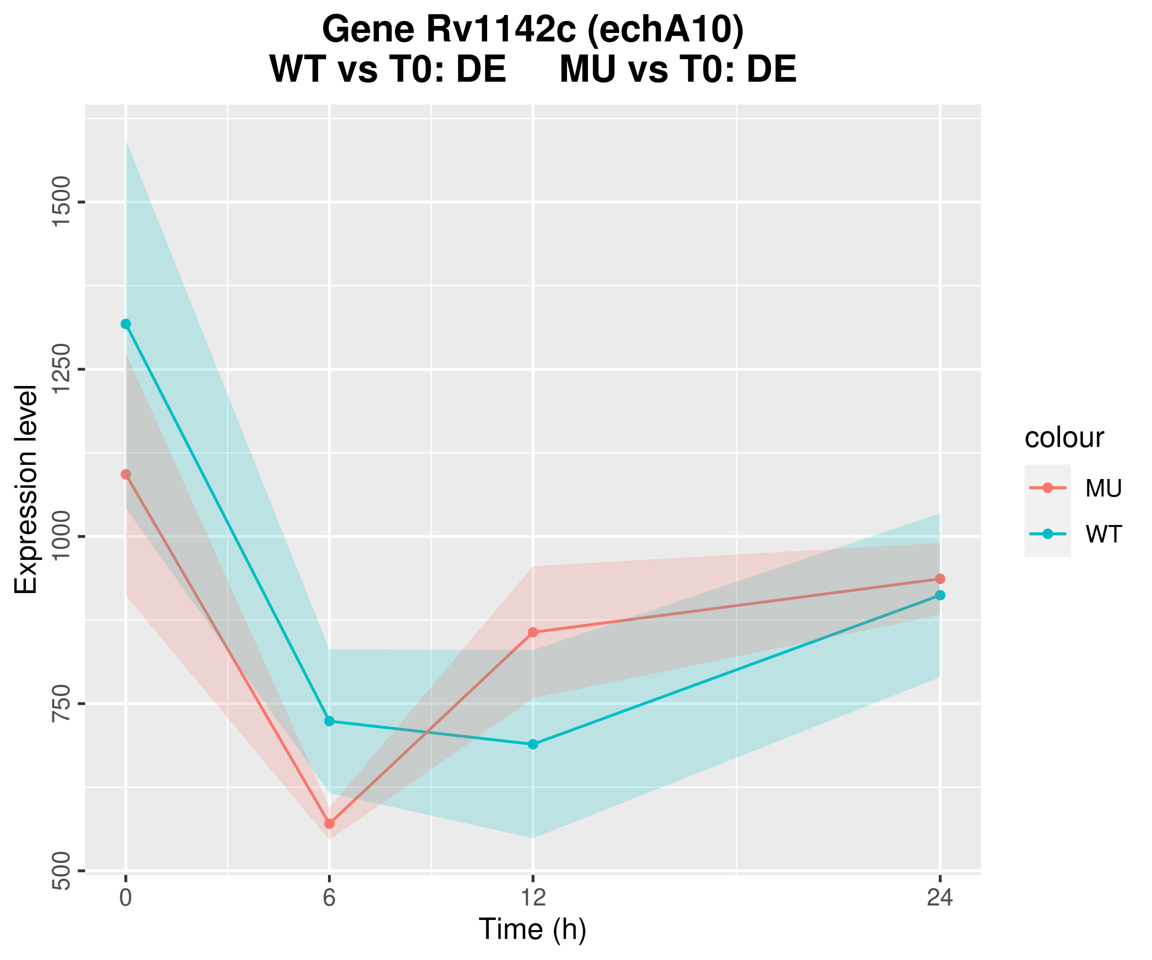
Gene Rv1142c (echA10)
WT vs T0: DE   MU vs T0: DE
Expression level
1500
1250
1000
750
500
0
6
12
24
Time (h)
colour
MU
WT

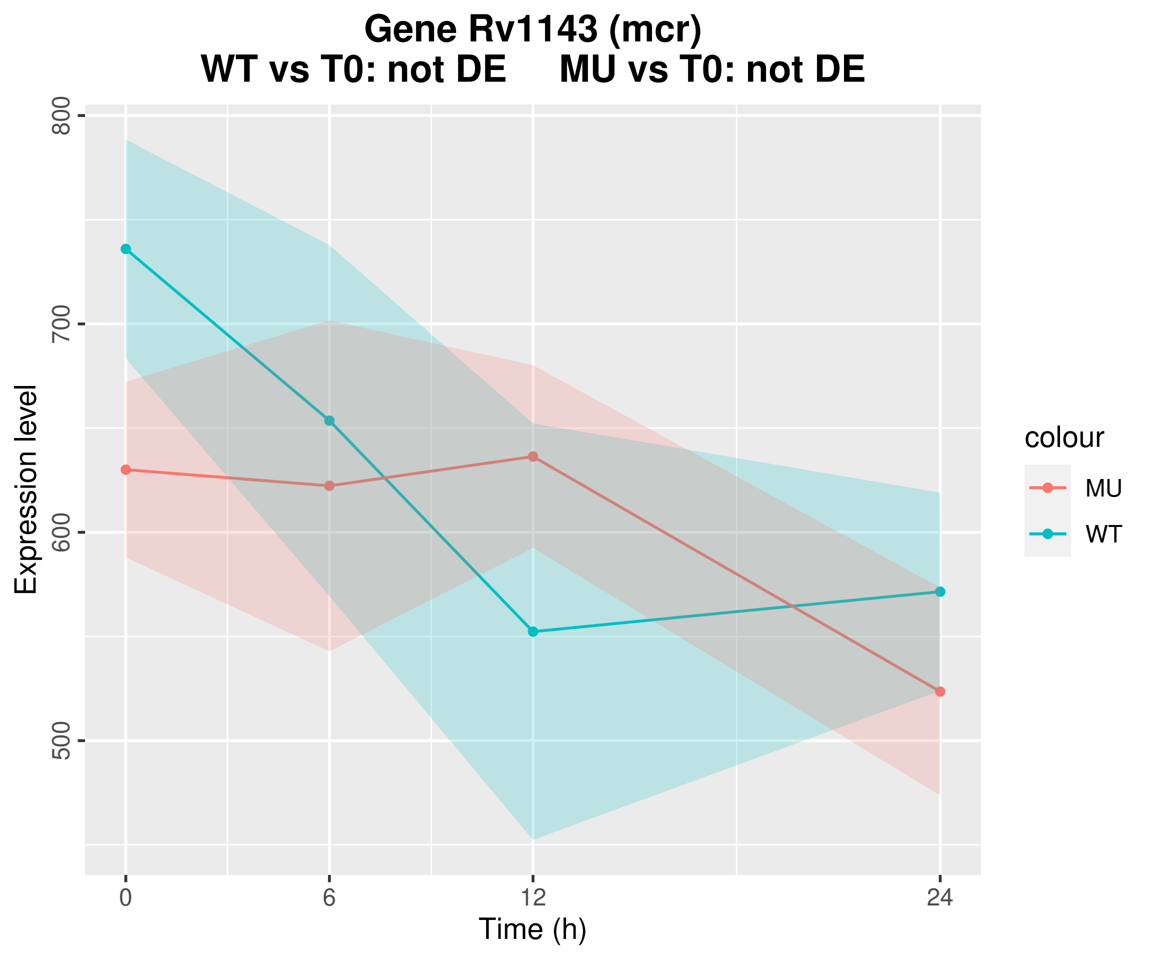
Gene Rv1143 (mcr)
WT vs T0: not DE MU vs T0: not DE
800
700
600
500
Expression level
0
6
12
24
Time (h)
colour
MU
WT

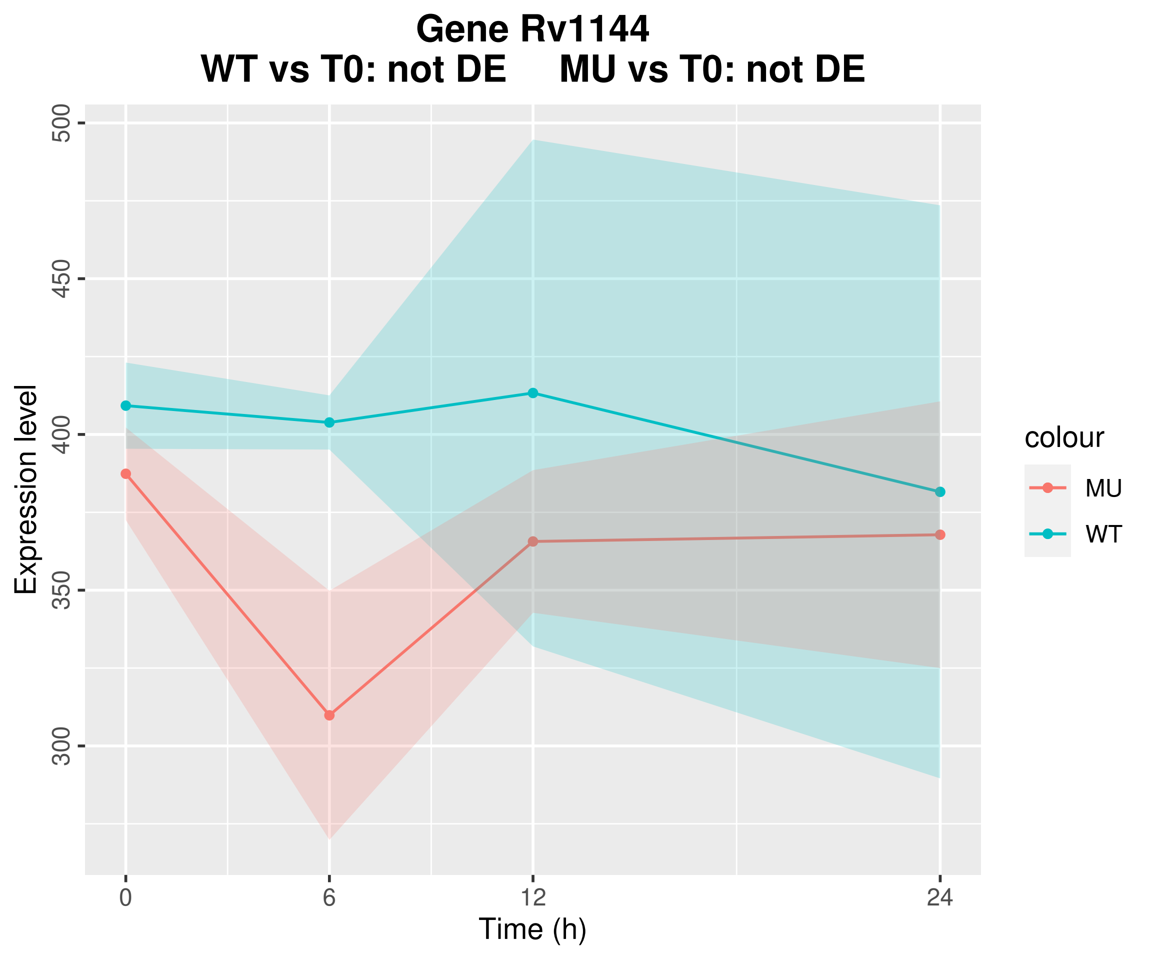

Gene Rv1144
WT vs T0: not DE   MU vs T0: not DE
Expression level
500
450
400
350
300
0
6
12
24
Time (h)
colour
MU
WT

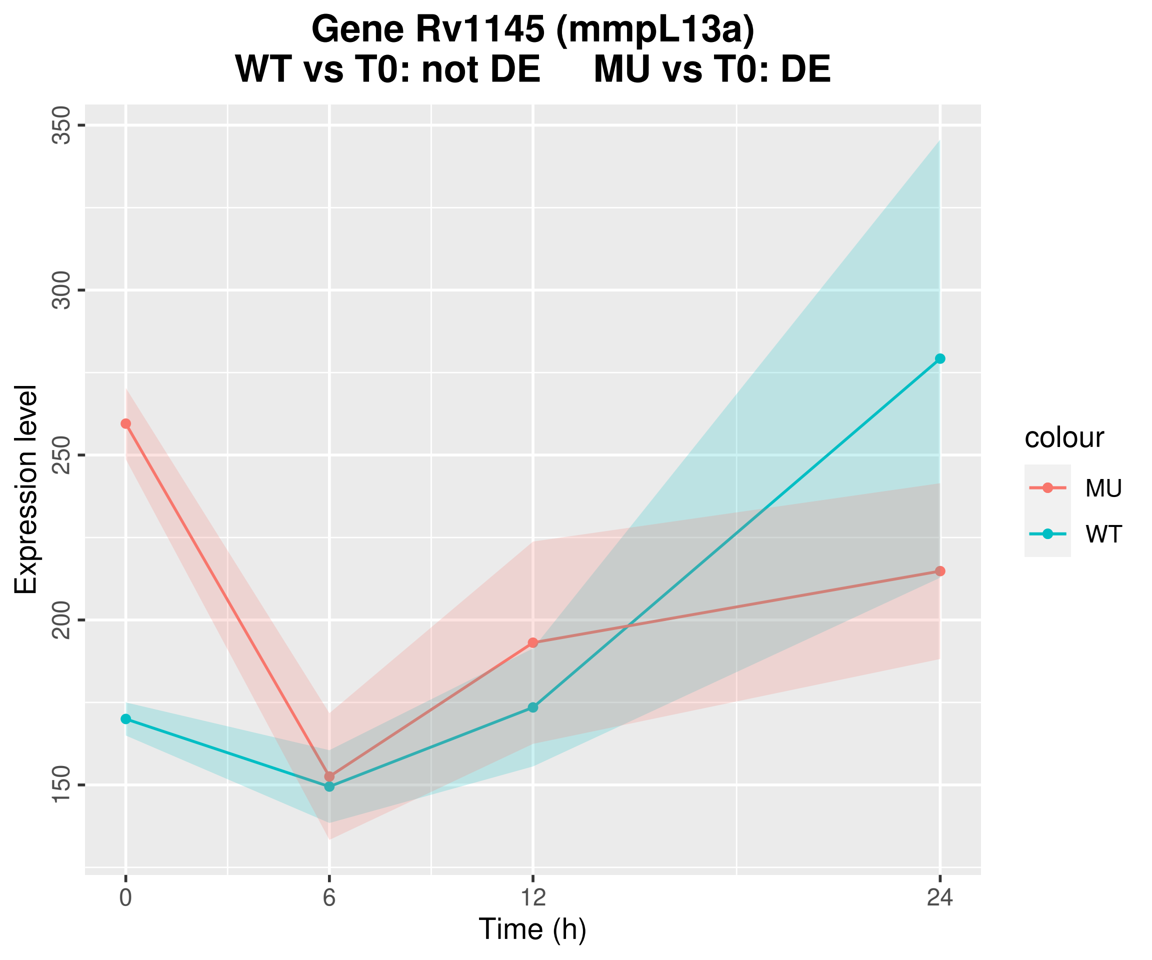
Gene Rv1145 (mmpL13a)
WT vs T0: not DE   MU vs T0: DE
Expression level
350
300
250
200
150
0
6
12
24
Time (h)
colour
MU
WT

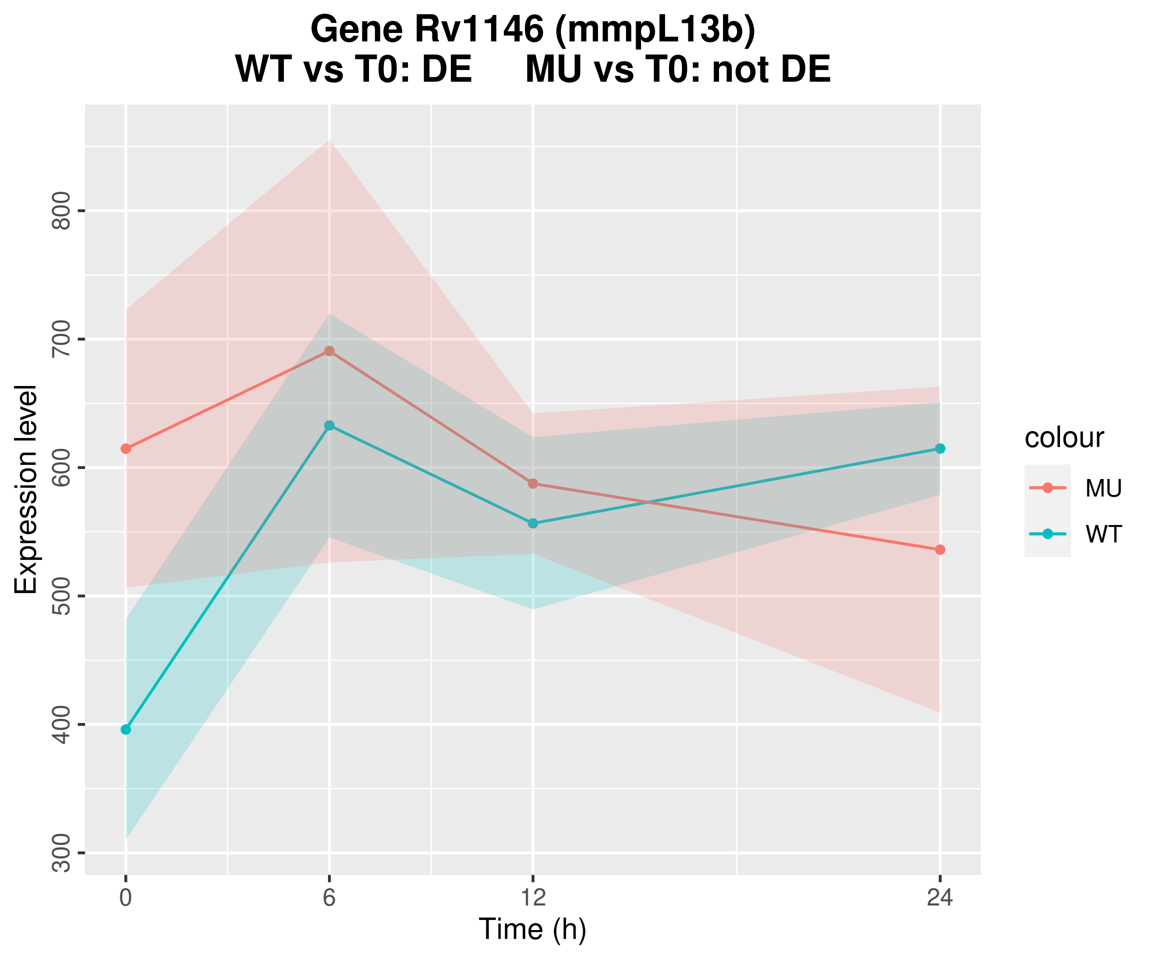
Gene Rv1146 (mmpL13b)
WT vs T0: DE MU vs T0: not DE
Expression level
300
400
500
600
700
800
0
6
12
24
Time (h)
colour
MU
WT

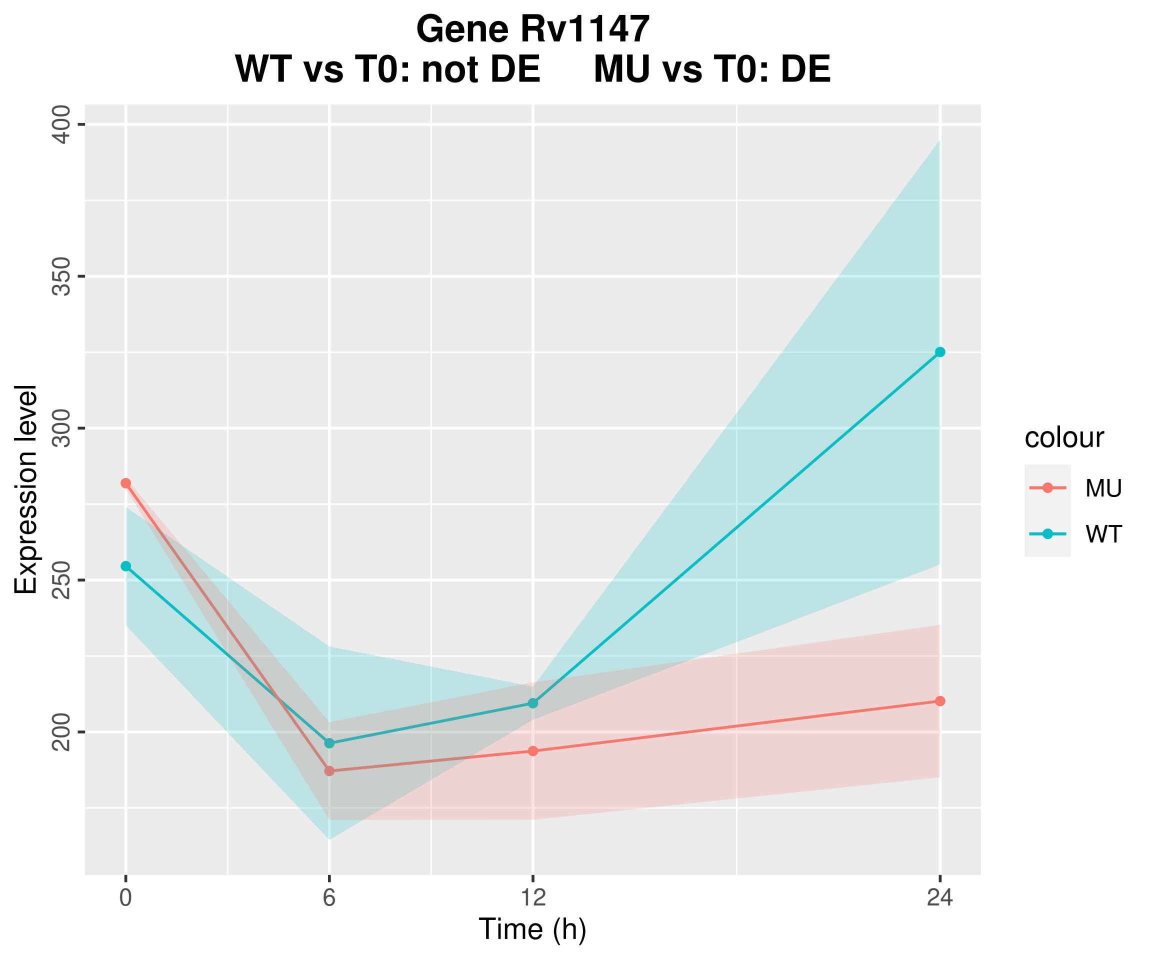

Gene Rv1147
WT vs T0: not DE MU vs T0: DE
Expression level
400
350
300
250
200
0
6
12
24
Time (h)
colour
MU
WT

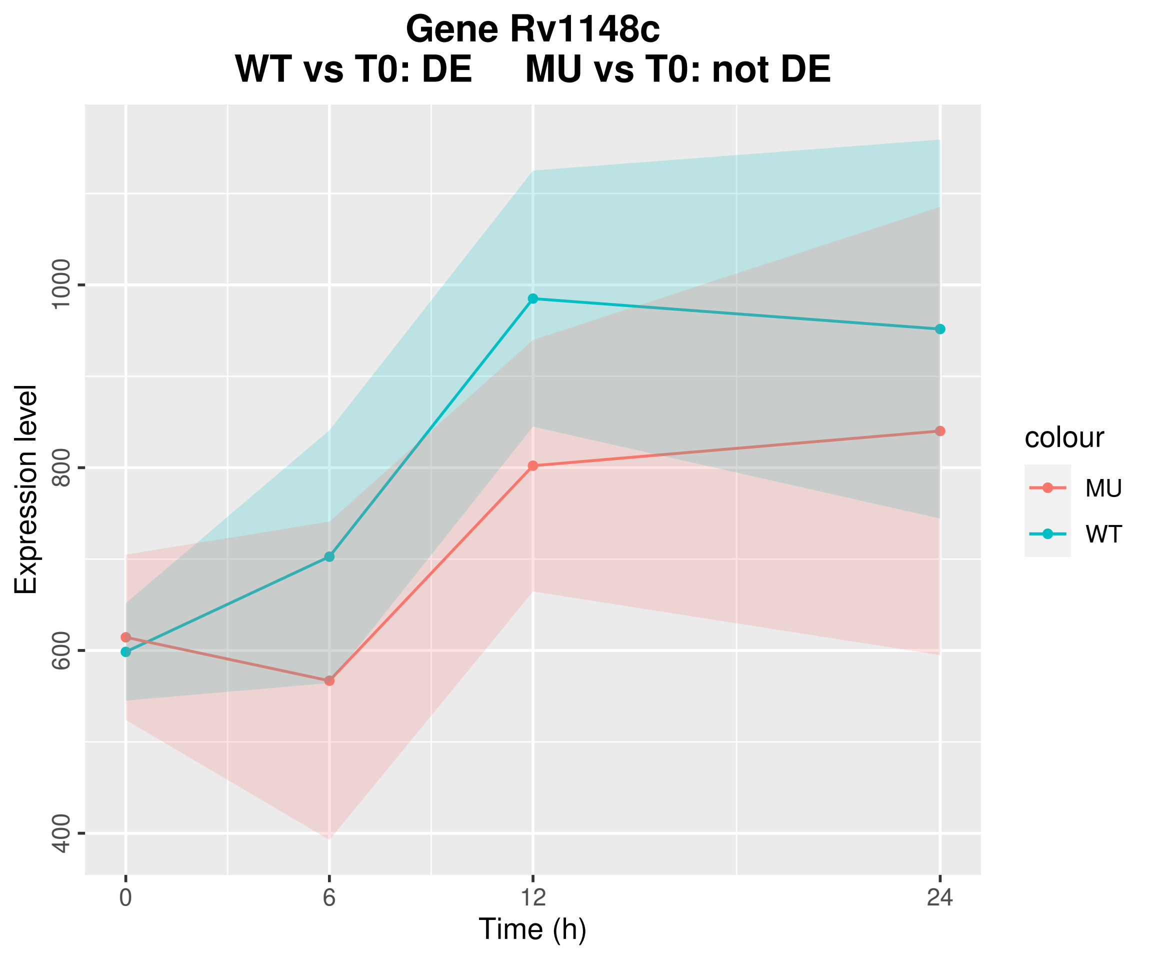
Gene Rv1148c
WT vs T0: DE   MU vs T0: not DE
Expression level
1000
800
600
400
0
6
12
24
Time (h)
colour
MU
WT

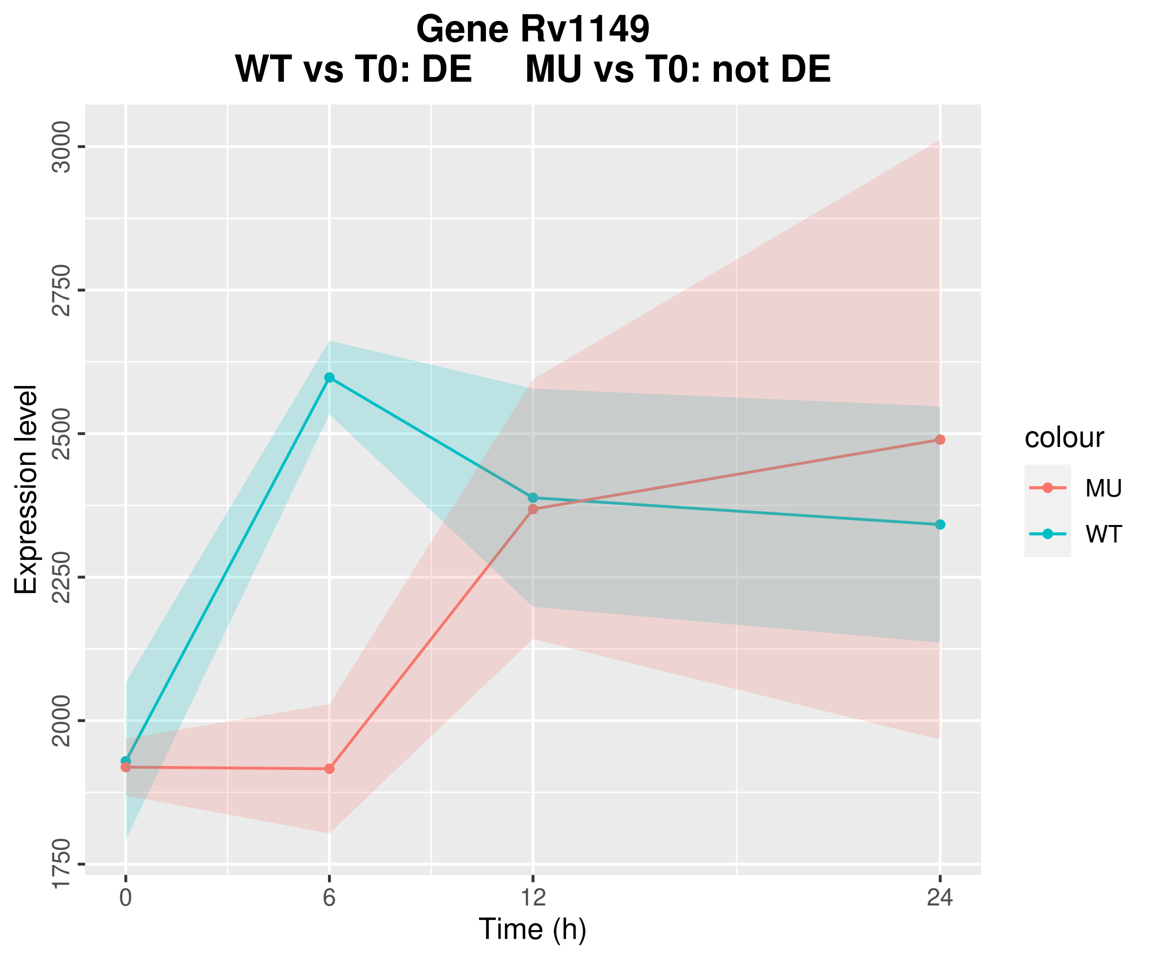

Gene Rv1149
WT vs T0: DE   MU vs T0: not DE
Expression level
3000
2750
2500
2250
2000
1750
0
6
12
24
Time (h)
colour
MU
WT

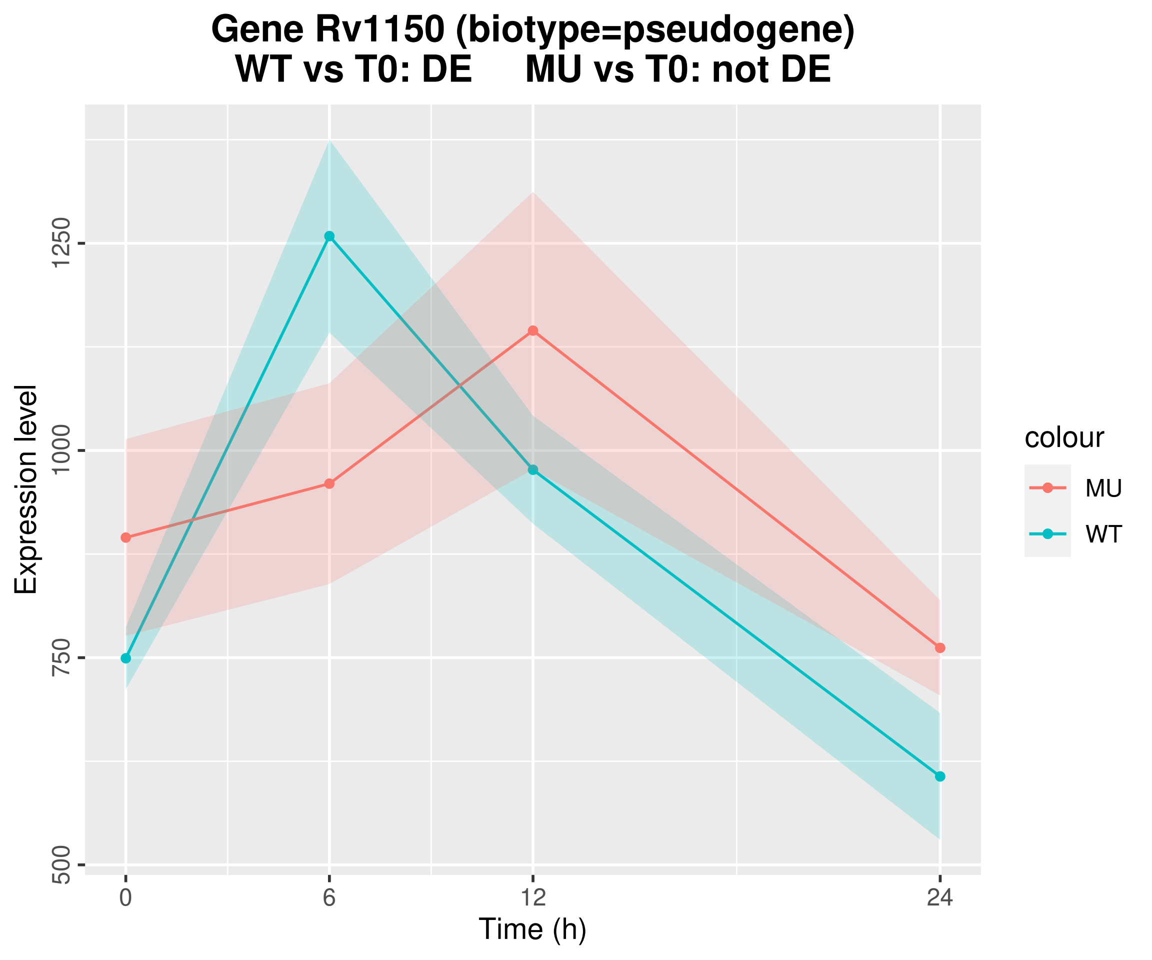

Gene Rv1150 (biotype=pseudogene)
WT vs T0: DE   MU vs T0: not DE
Expression level
500
750
1000
1250
0
6
12
24
Time (h)
colour
MU
WT

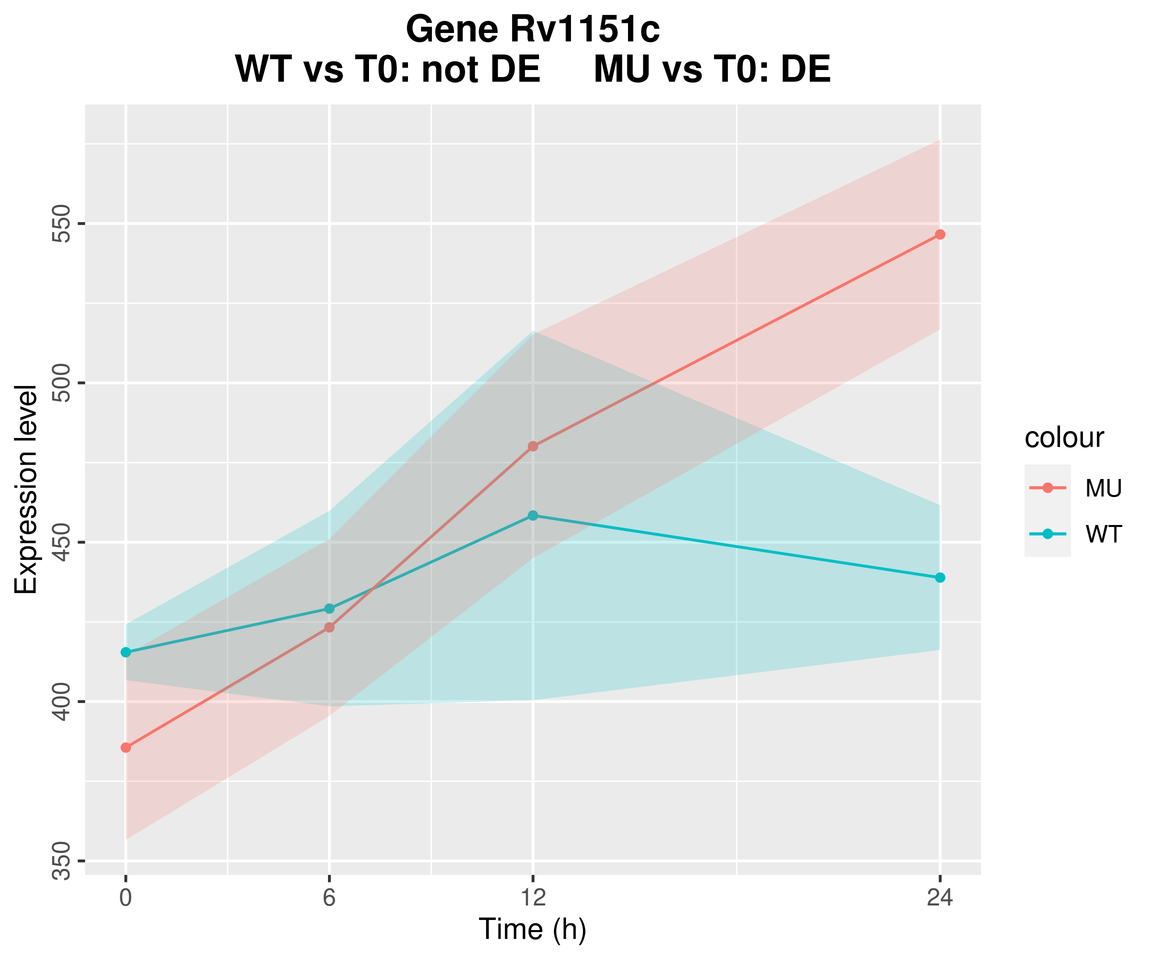

Gene Rv1151c
WT vs T0: not DE   MU vs T0: DE
Expression level
550
500
450
400
350
0
6
12
24
Time (h)
colour
MU
WT

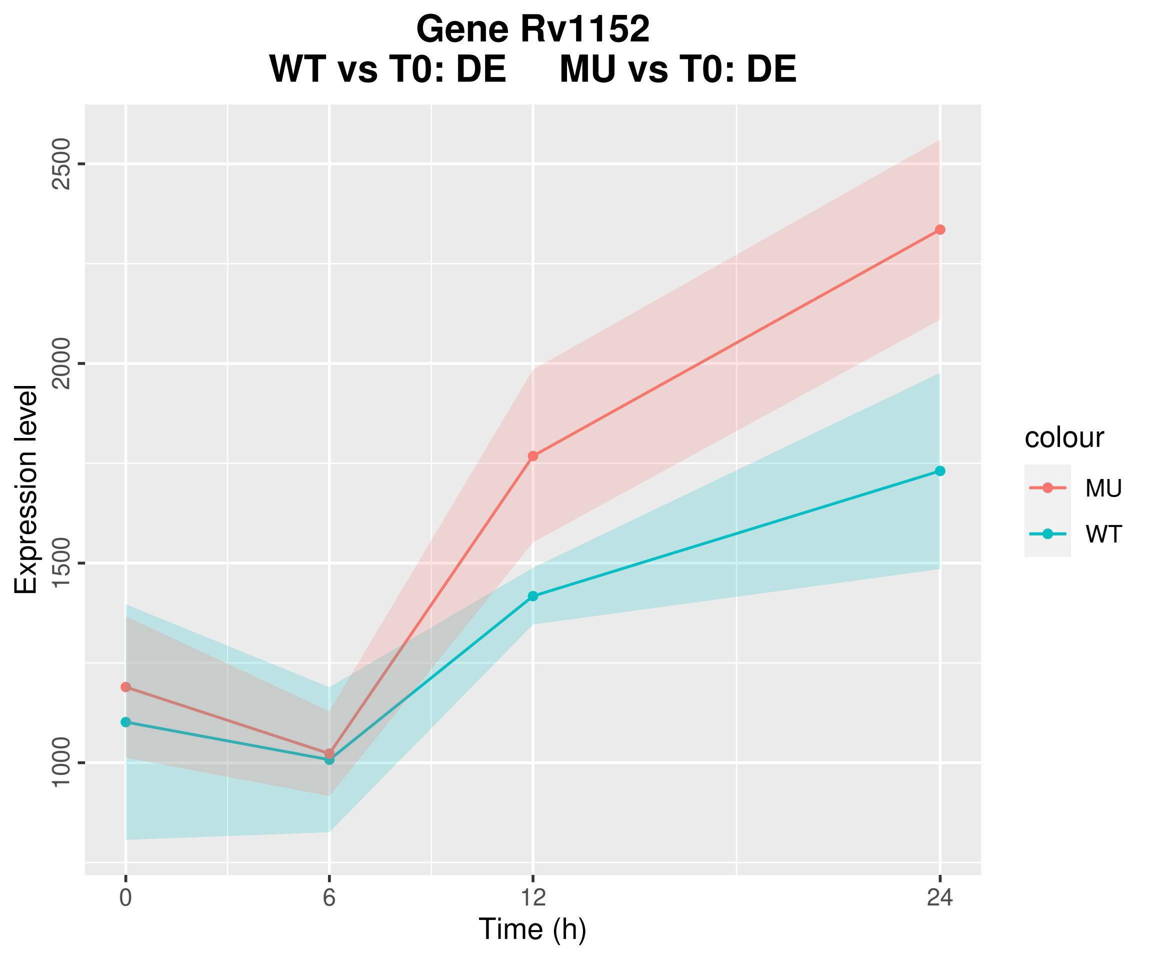
Gene Rv1152
WT vs T0: DE   MU vs T0: DE
Expression level
2500
2000
1500
1000
0
6
12
24
Time (h)
colour
MU
WT

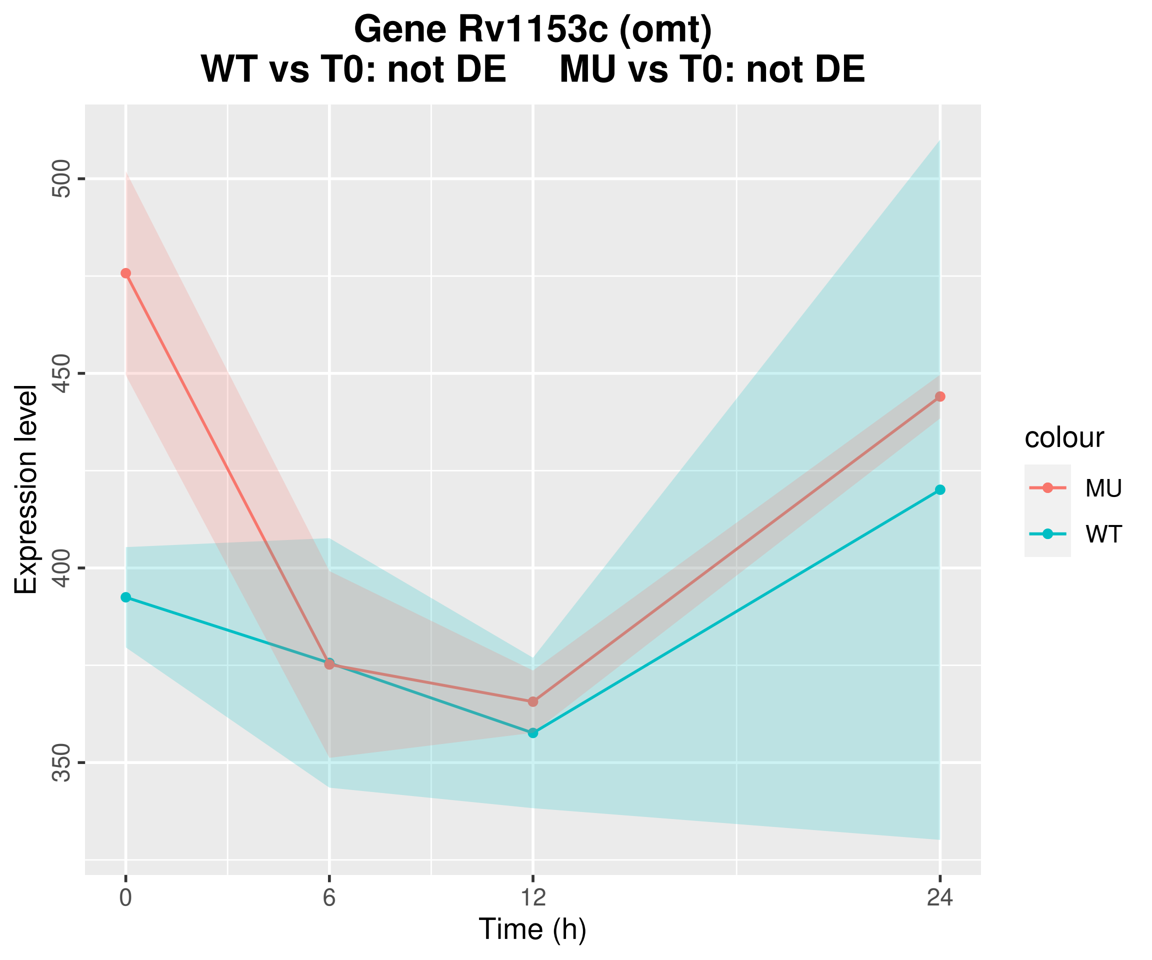

Gene Rv1153c (omt)
WT vs T0: not DE   MU vs T0: not DE
Expression level
500
450
400
350
0
6
12
24
Time (h)
colour
MU
WT

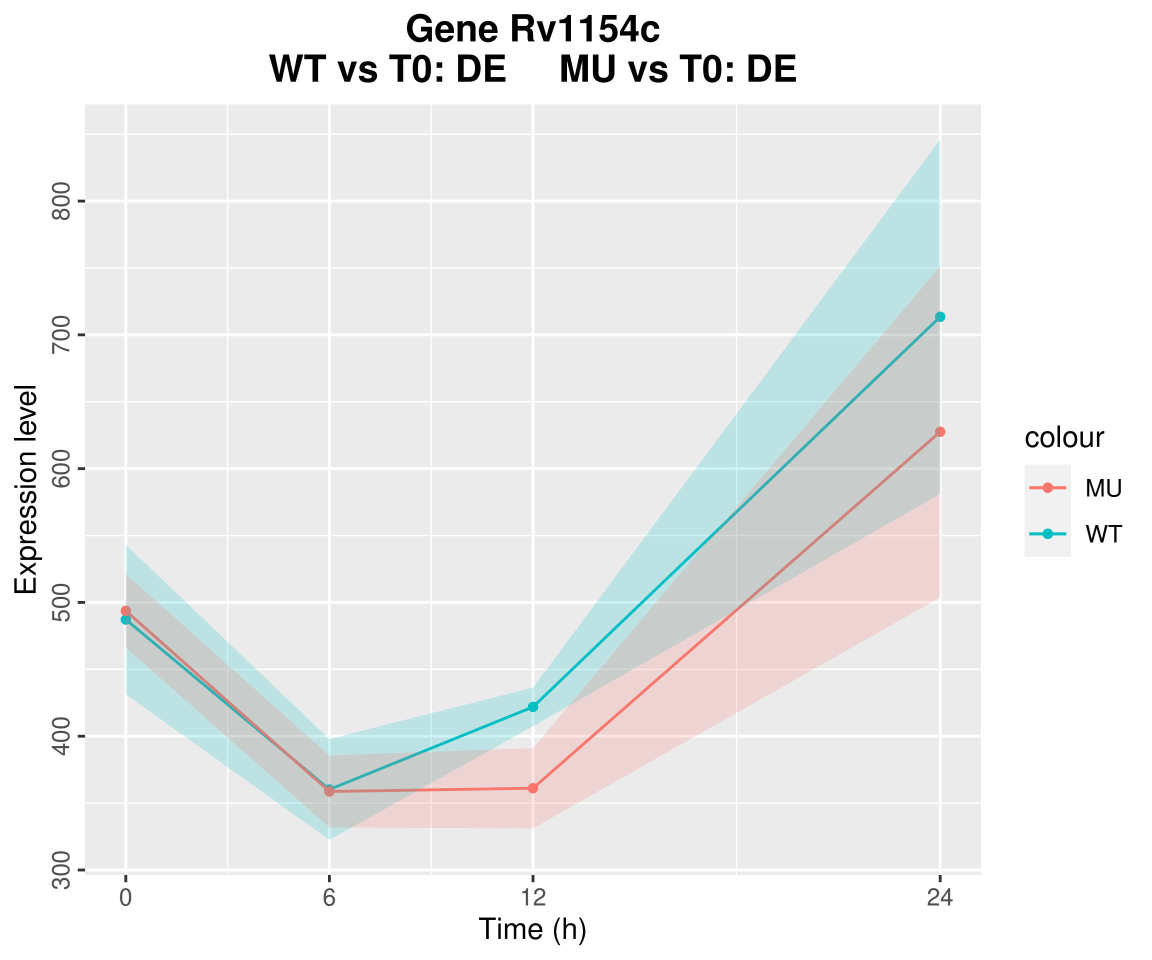
Gene Rv1154c
WT vs T0: DE MU vs T0: DE
Expression level
300
400
500
600
700
800
0
6
12
24
Time (h)
colour
MU
WT

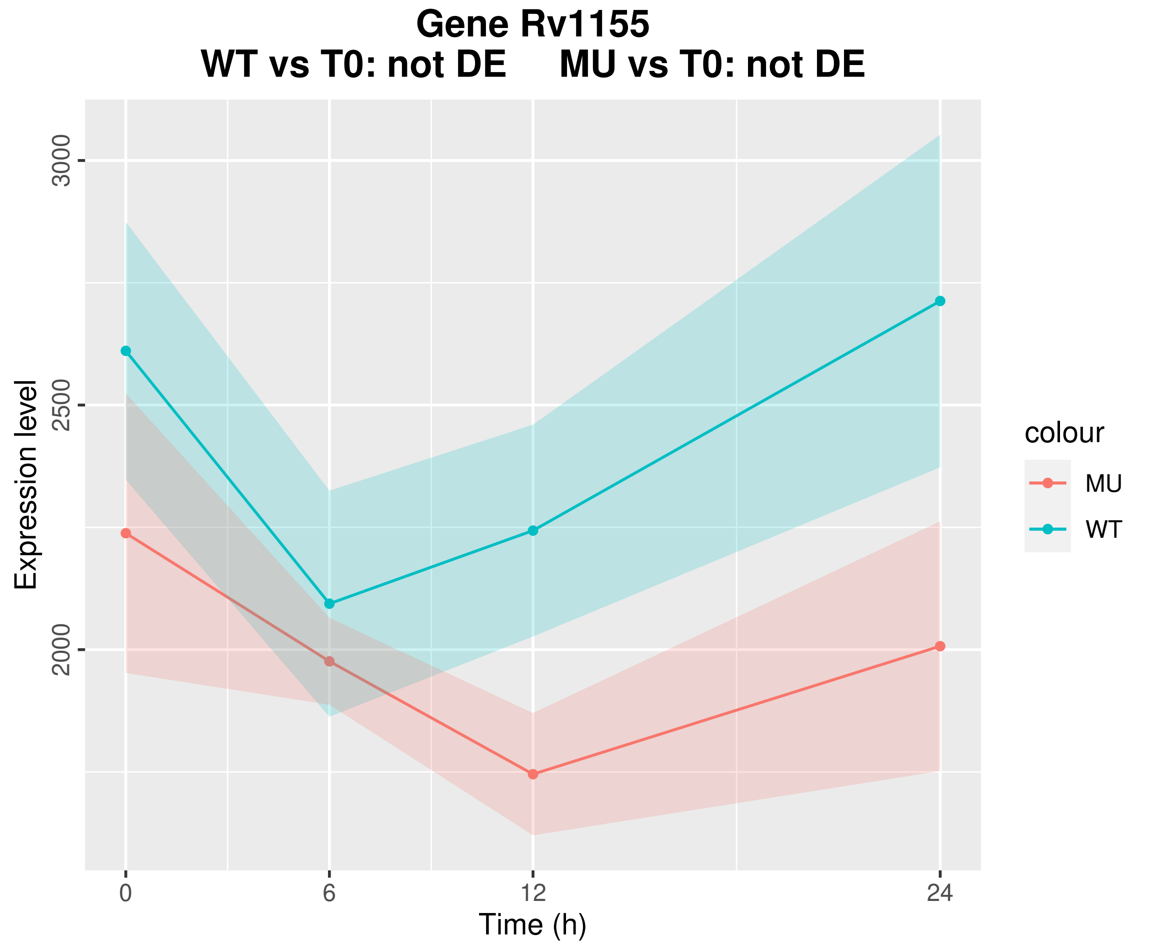

Gene Rv1155
WT vs T0: not DE   MU vs T0: not DE
Expression level
3000
2500
2000
0
6
12
24
Time (h)
colour
MU
WT

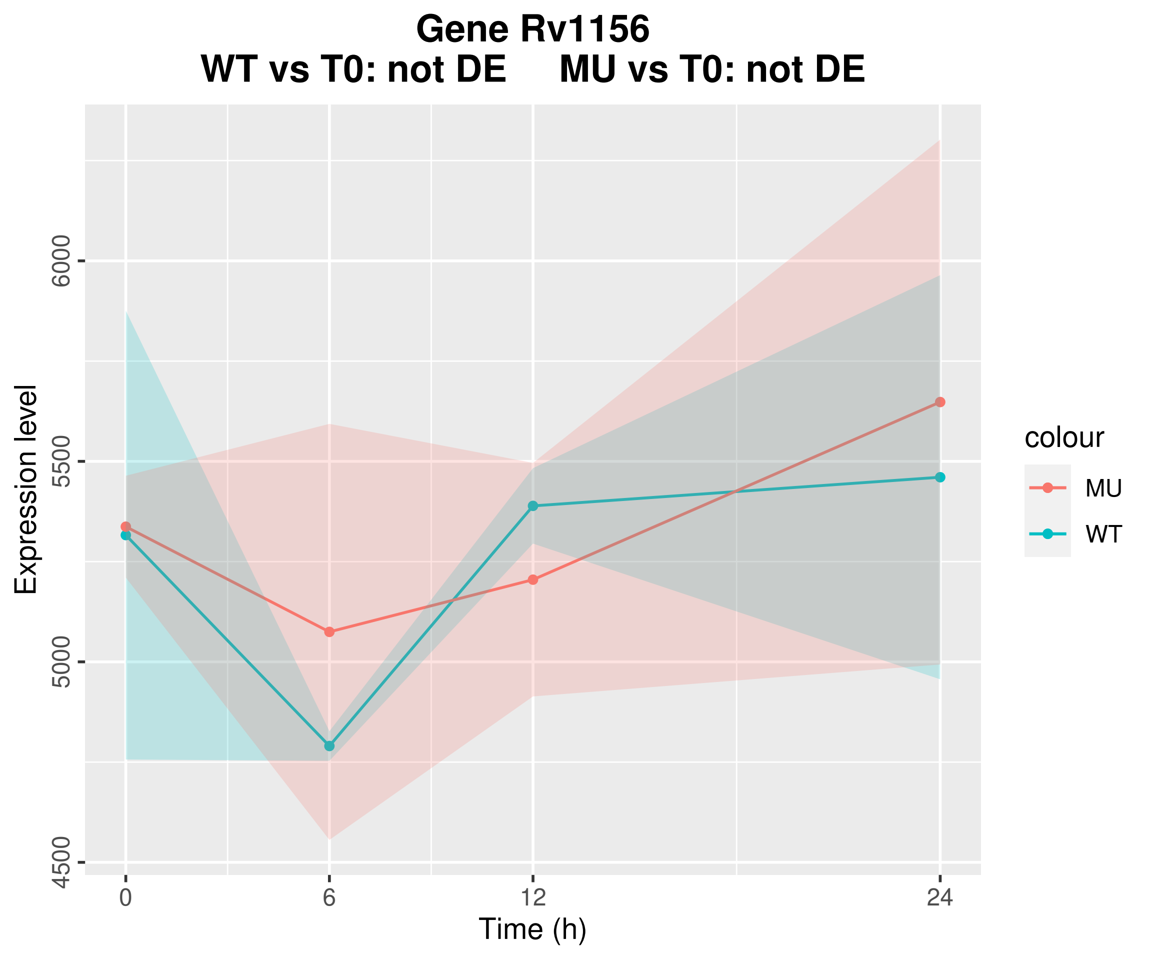
Gene Rv1156
WT vs T0: not DE MU vs T0: not DE
Expression level
6000
5500
5000
4500
0
6
12
24
Time (h)
colour
MU
WT

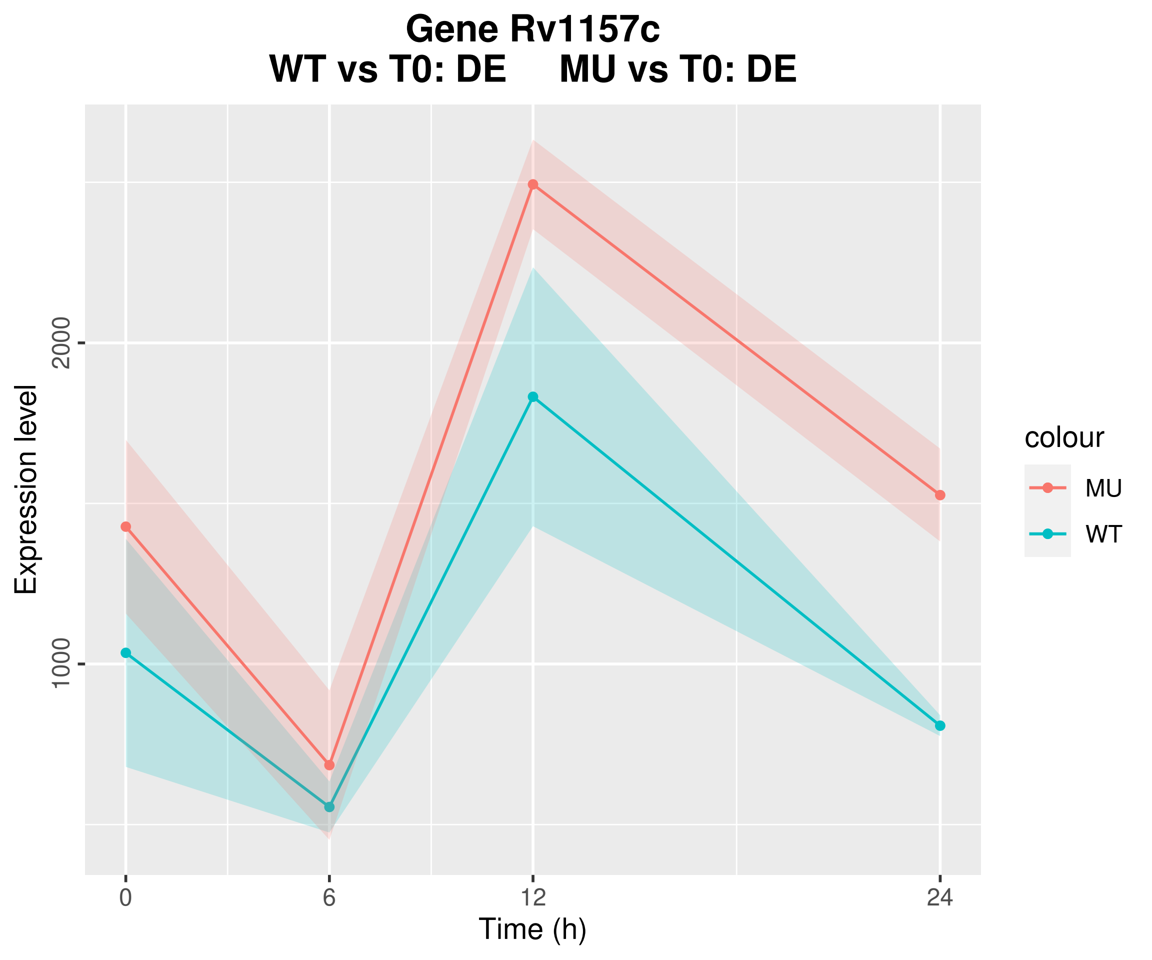

Gene Rv1157c
WT vs T0: DE   MU vs T0: DE
Expression level
2000
1000
0
6
12
24
Time (h)
colour
MU
WT

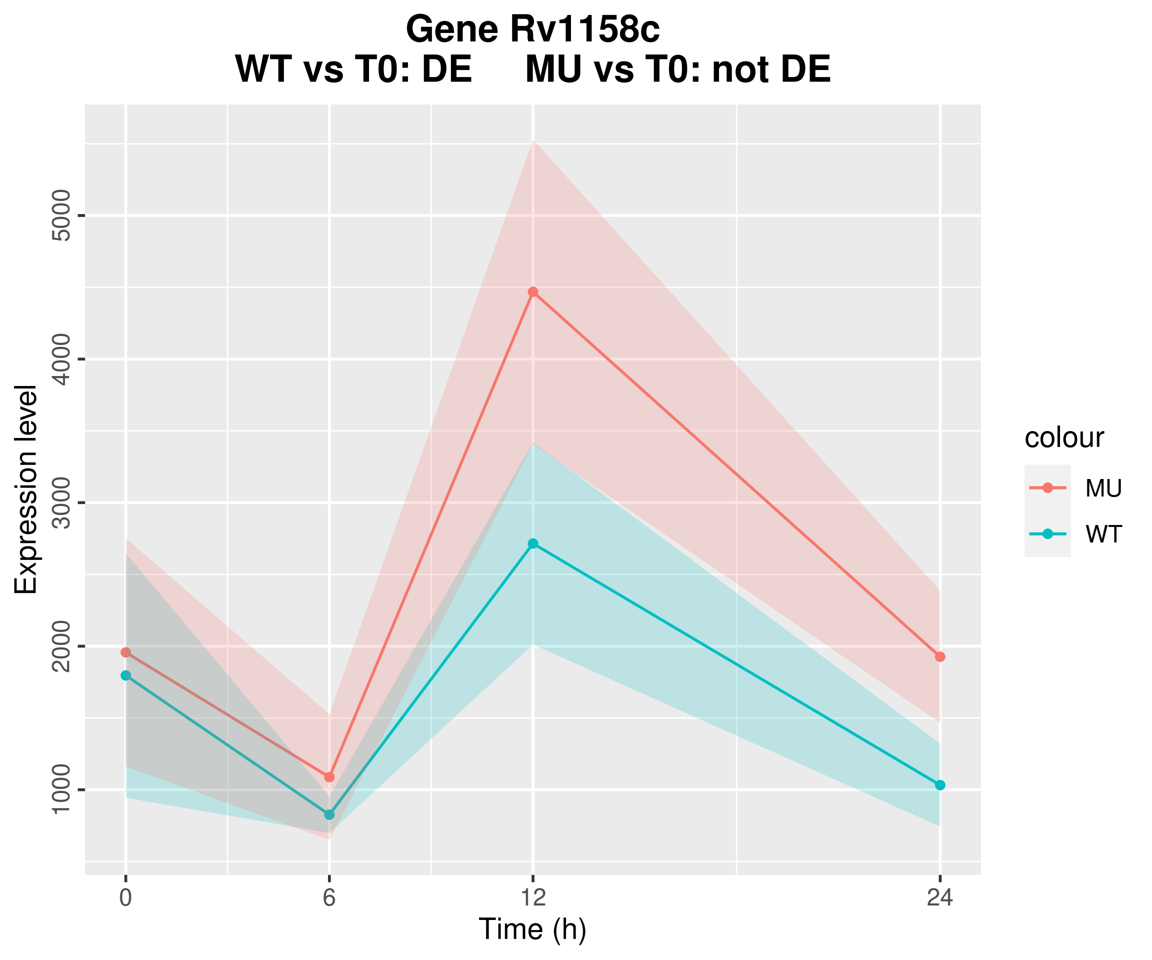
Gene Rv1158c
WT vs T0: DE MU vs T0: not DE
Expression level
5000
4000
3000
2000
1000
0
6
12
24
Time (h)
colour
MU
WT

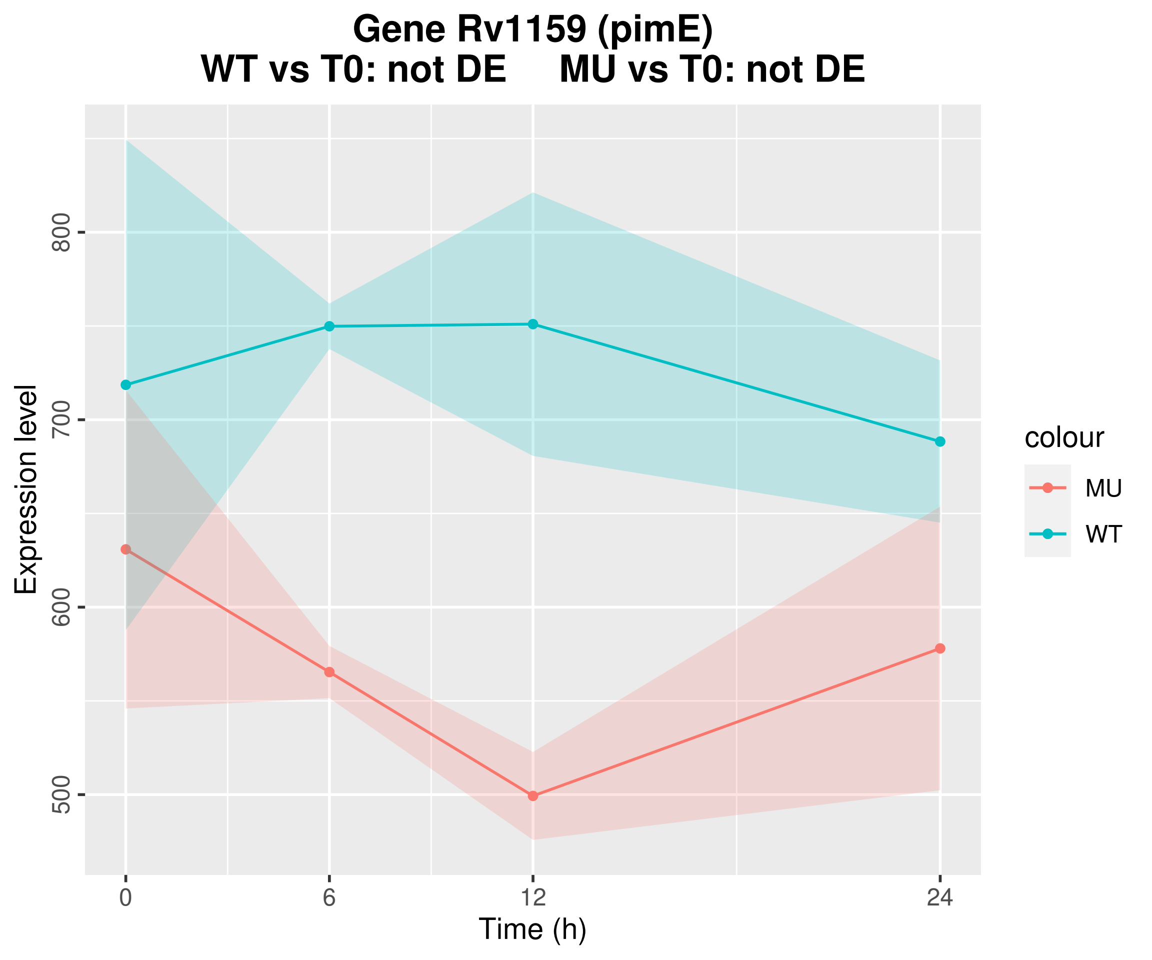
Gene Rv1159 (pimE)
WT vs T0: not DE   MU vs T0: not DE
Expression level
800
700
600
500
0
6
12
24
Time (h)
colour
MU
WT

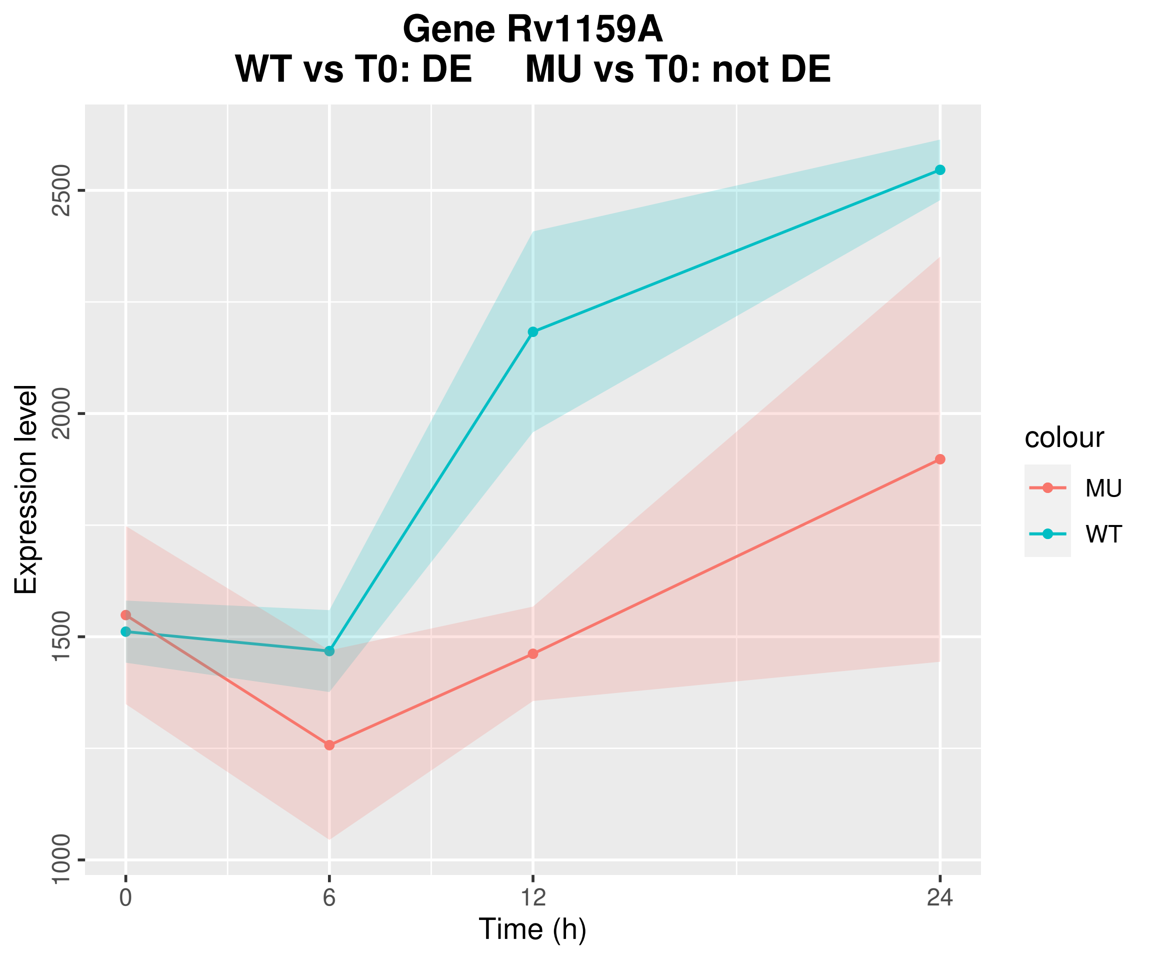

Gene Rv1159A
WT vs T0: DE MU vs T0: not DE
Expression level
2500
2000
1500
1000
0
6
12
24
Time (h)
colour
MU
WT

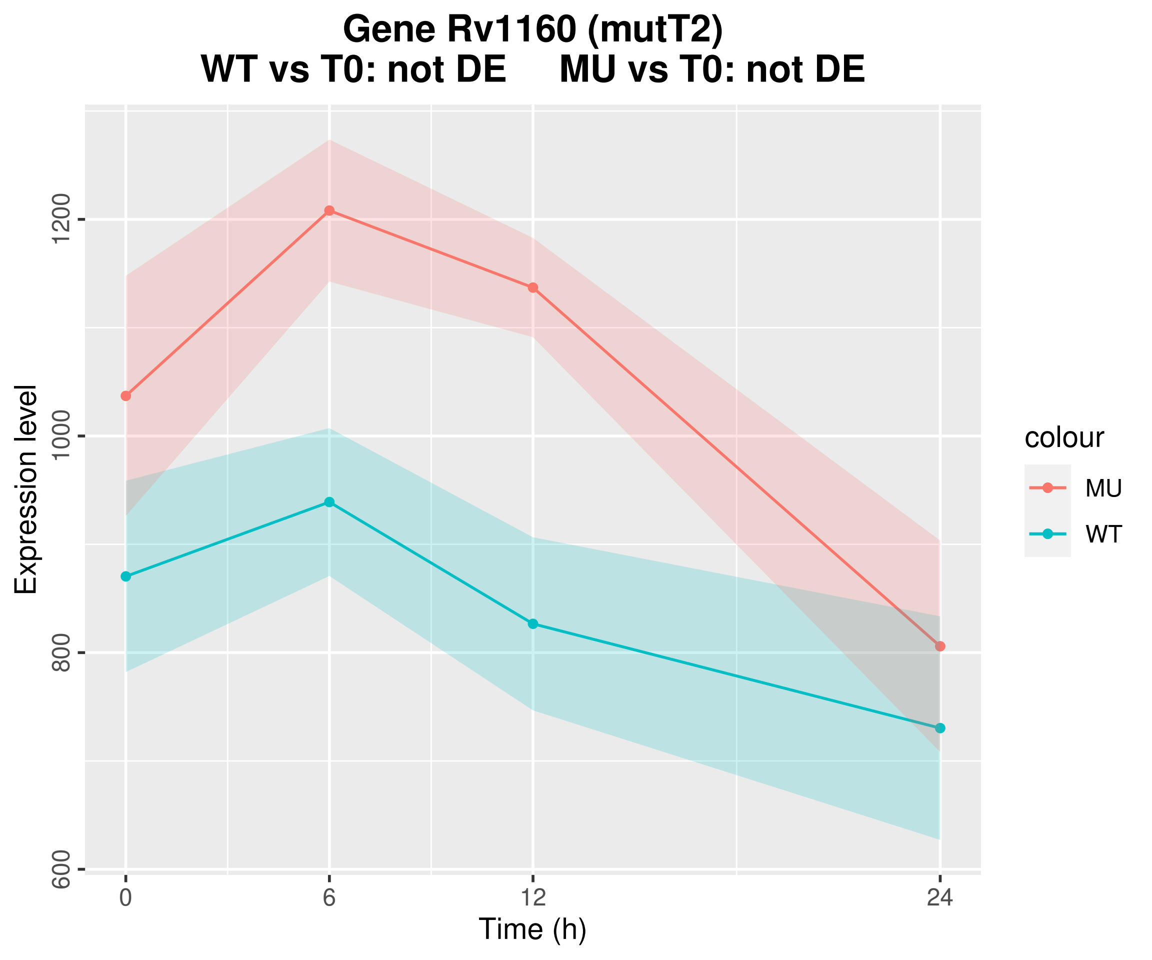

Gene Rv1160 (mutT2)
WT vs T0: not DE MU vs T0: not DE
Expression level
1200
1000
800
600
0
6
12
24
Time (h)
colour
MU
WT

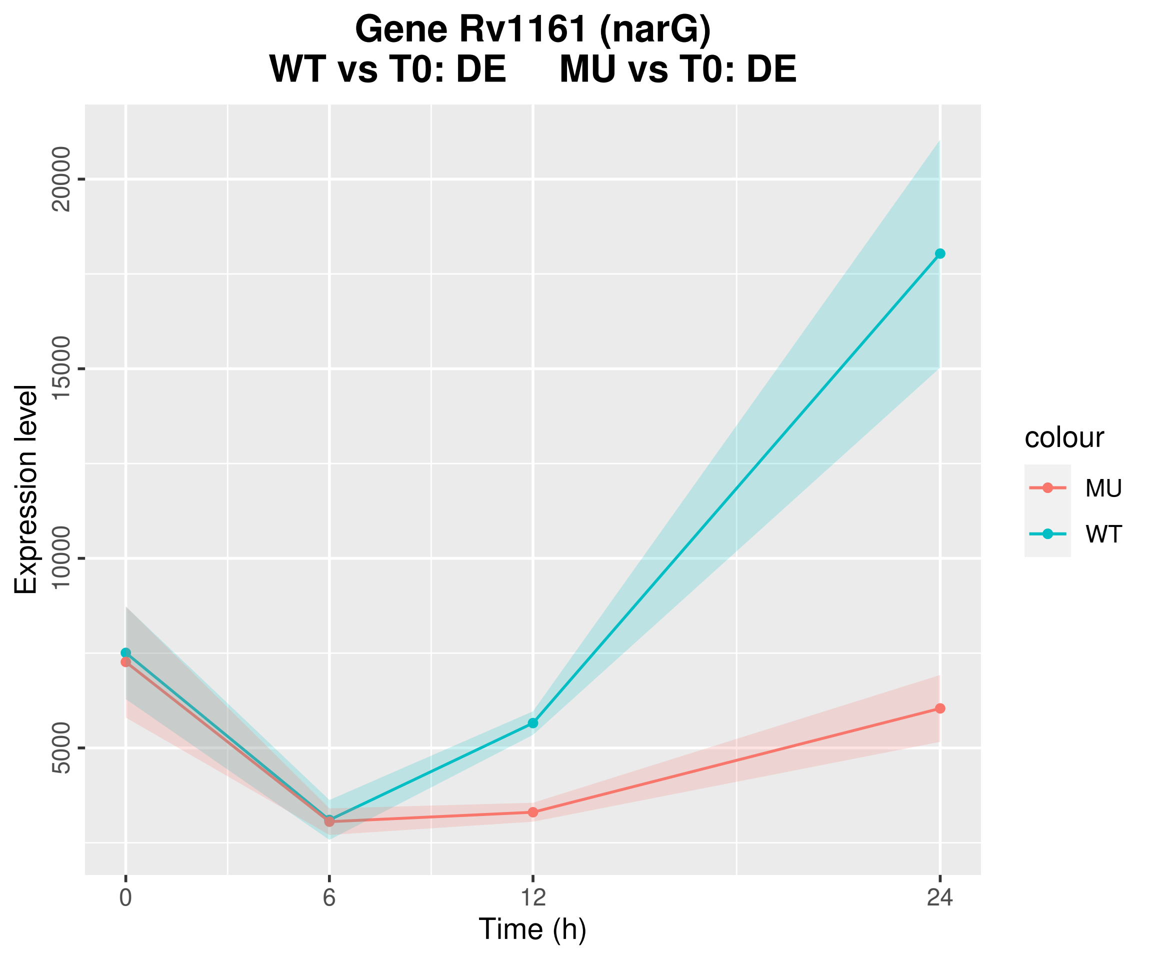

Gene Rv1161 (narG)
WT vs T0: DE MU vs T0: DE
Expression level
20000
15000
10000
5000
0
6
12
24
Time (h)
colour
MU
WT

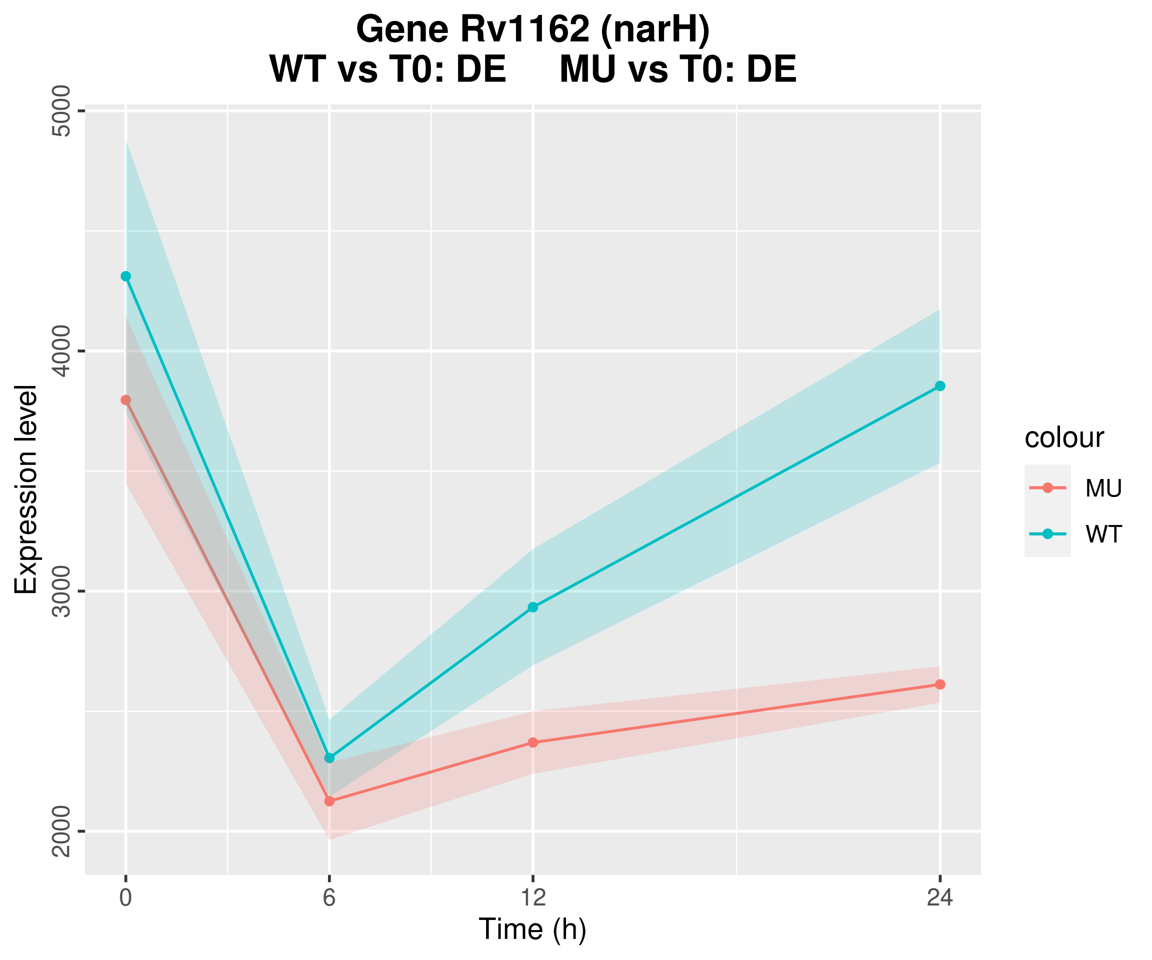

Gene Rv1162 (narH)
WT vs T0: DE MU vs T0: DE
Expression level
5000
4000
3000
2000
0
6
12
24
Time (h)
colour
MU
WT

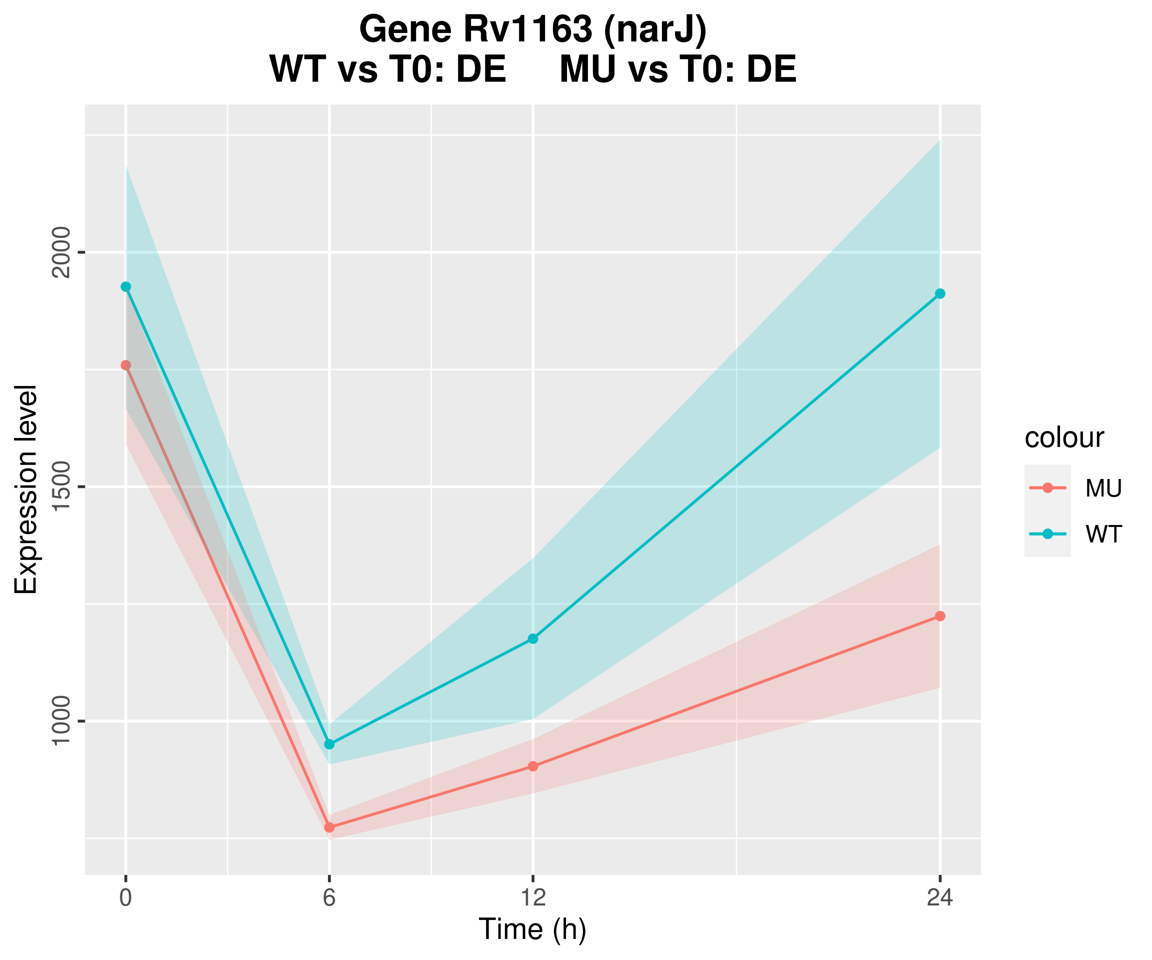

Gene Rv1163 (narJ)
WT vs T0: DE MU vs T0: DE
Expression level
2000
1500
1000
0
6
12
24
Time (h)
colour
MU
WT

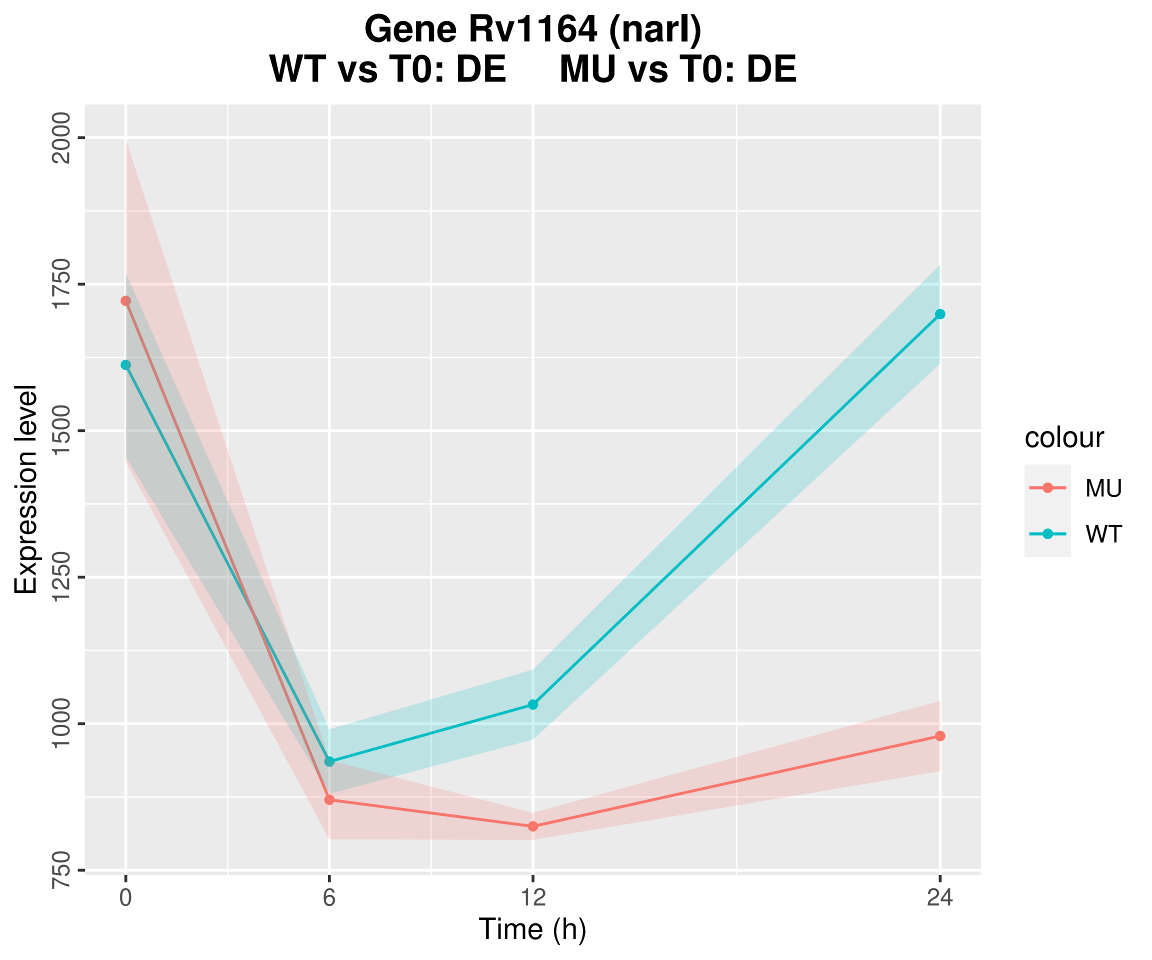
Gene Rv1164 (narI)
WT vs T0: DE MU vs T0: DE
Expression level
Time (h)
colour
MU
WT
2000
1750
1500
1250
1000
750
0
6
12
24

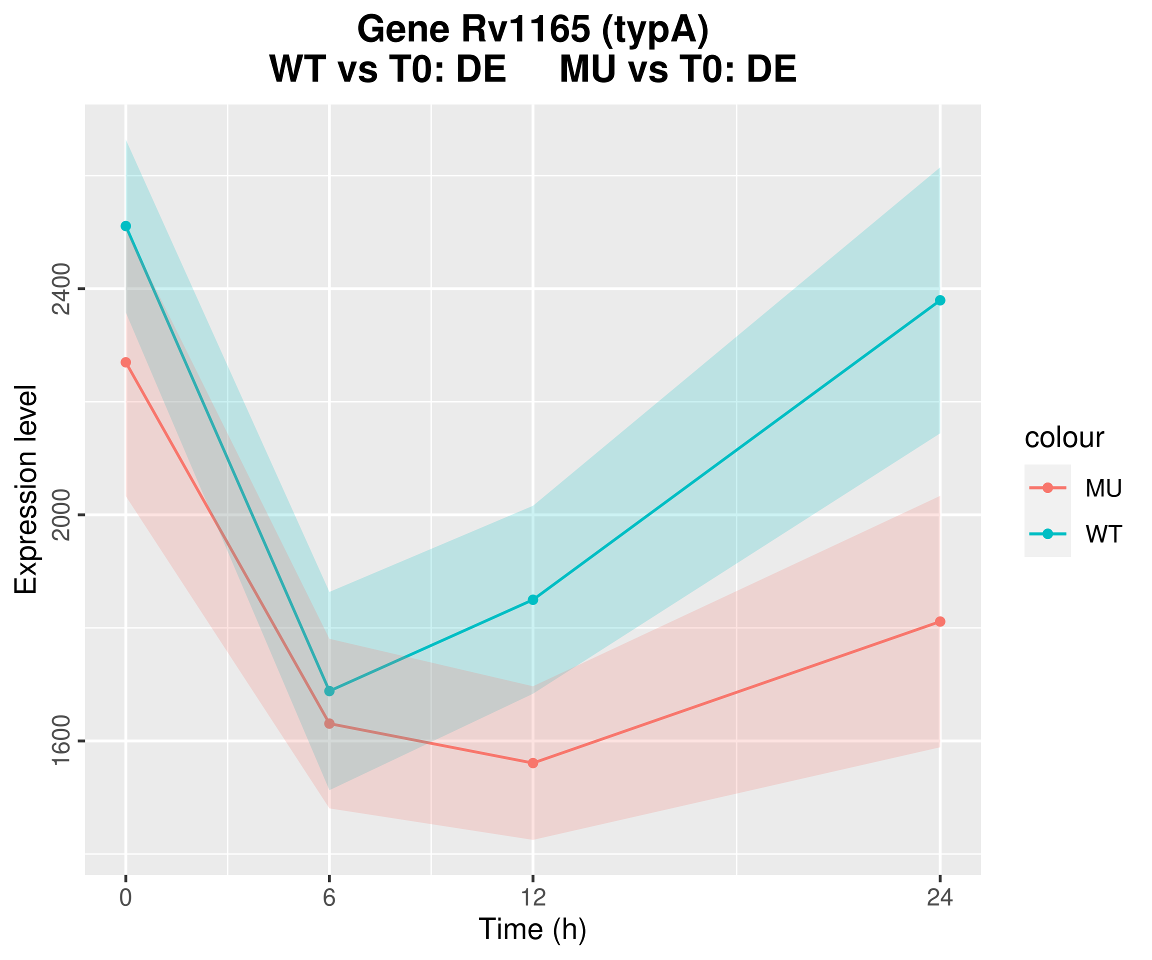

Gene Rv1165 (typA)
WT vs T0: DE   MU vs T0: DE
Expression level
2400
2000
1600
Time (h)
0
6
12
24
colour
MU
WT

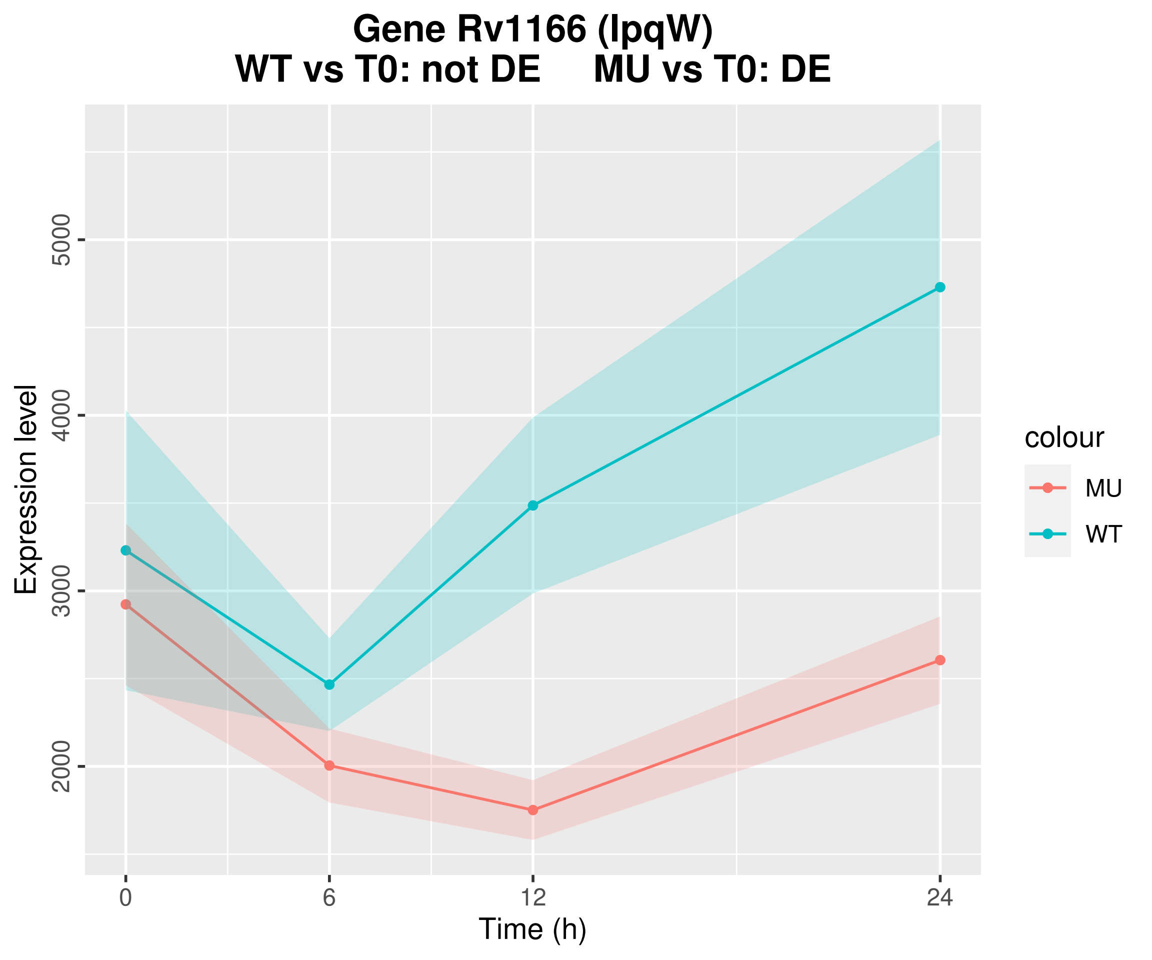

Gene Rv1166 (lpqW)
WT vs T0: not DE MU vs T0: DE
Expression level
5000
4000
3000
2000
0
6
12
24
Time (h)
colour
MU
WT

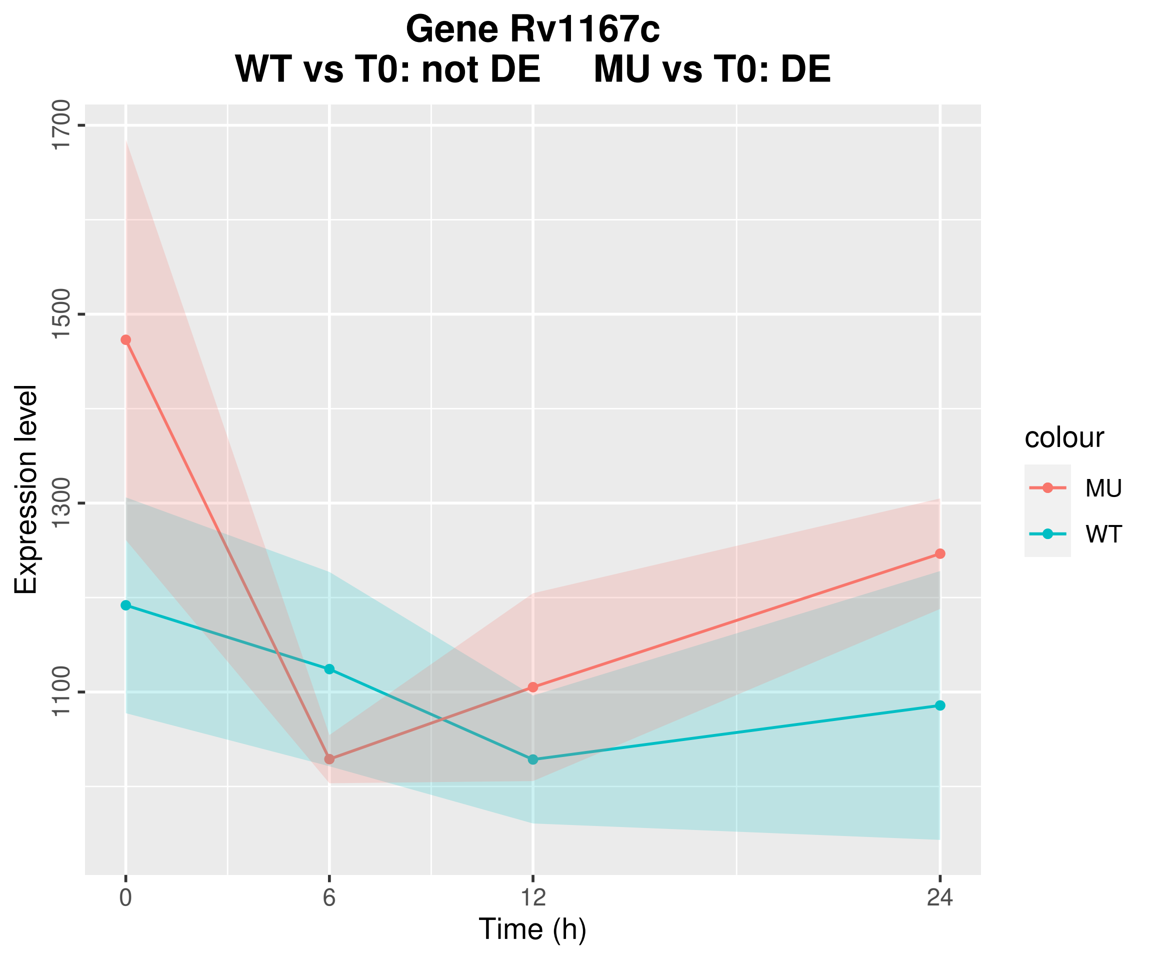
Gene Rv1167c
WT vs T0: not DE   MU vs T0: DE
Expression level
1700
1500
1300
1100
0
6
12
24
Time (h)
colour
MU
WT

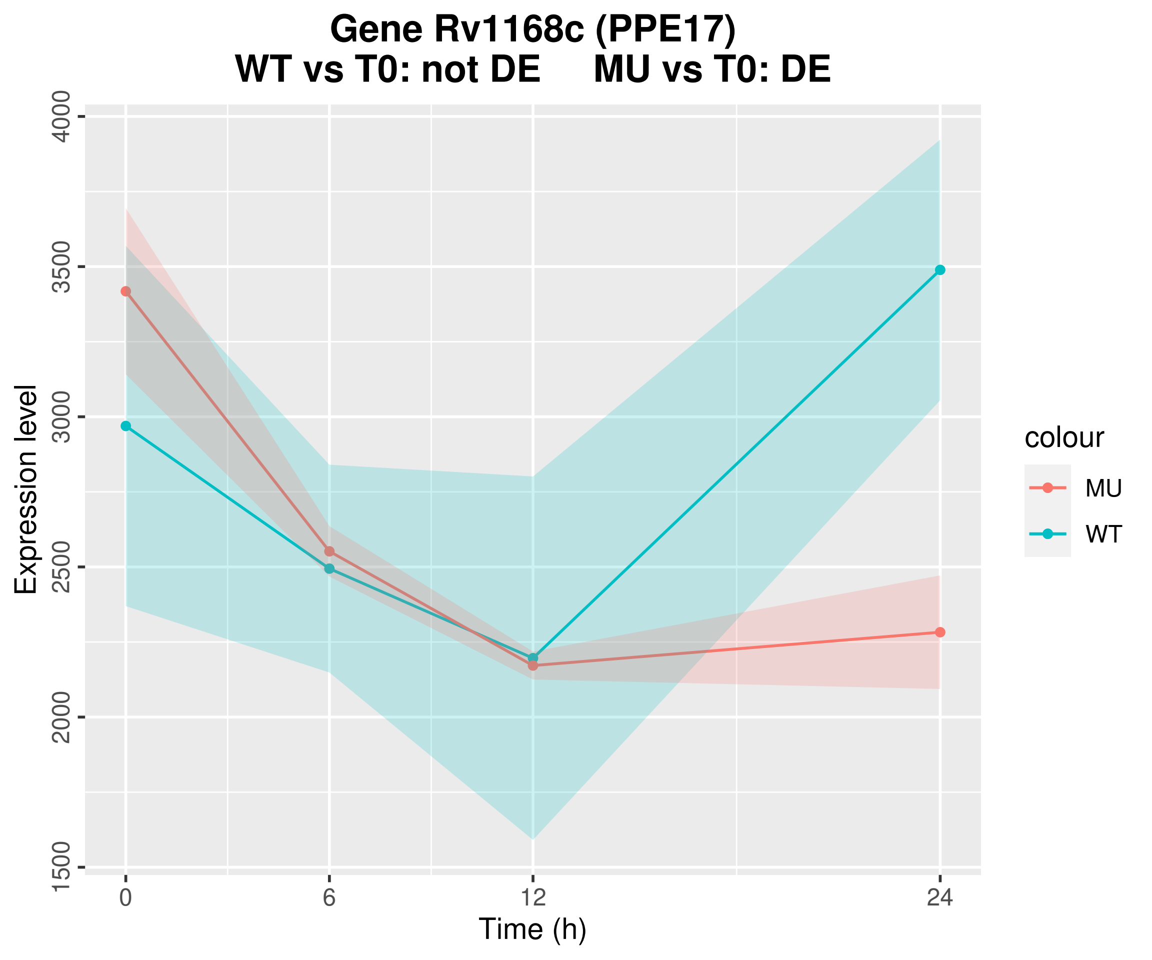
Gene Rv1168c (PPE17)
WT vs T0: not DE   MU vs T0: DE
Expression level
4000
3500
3000
2500
2000
1500
0
6
12
24
Time (h)
colour
MU
WT

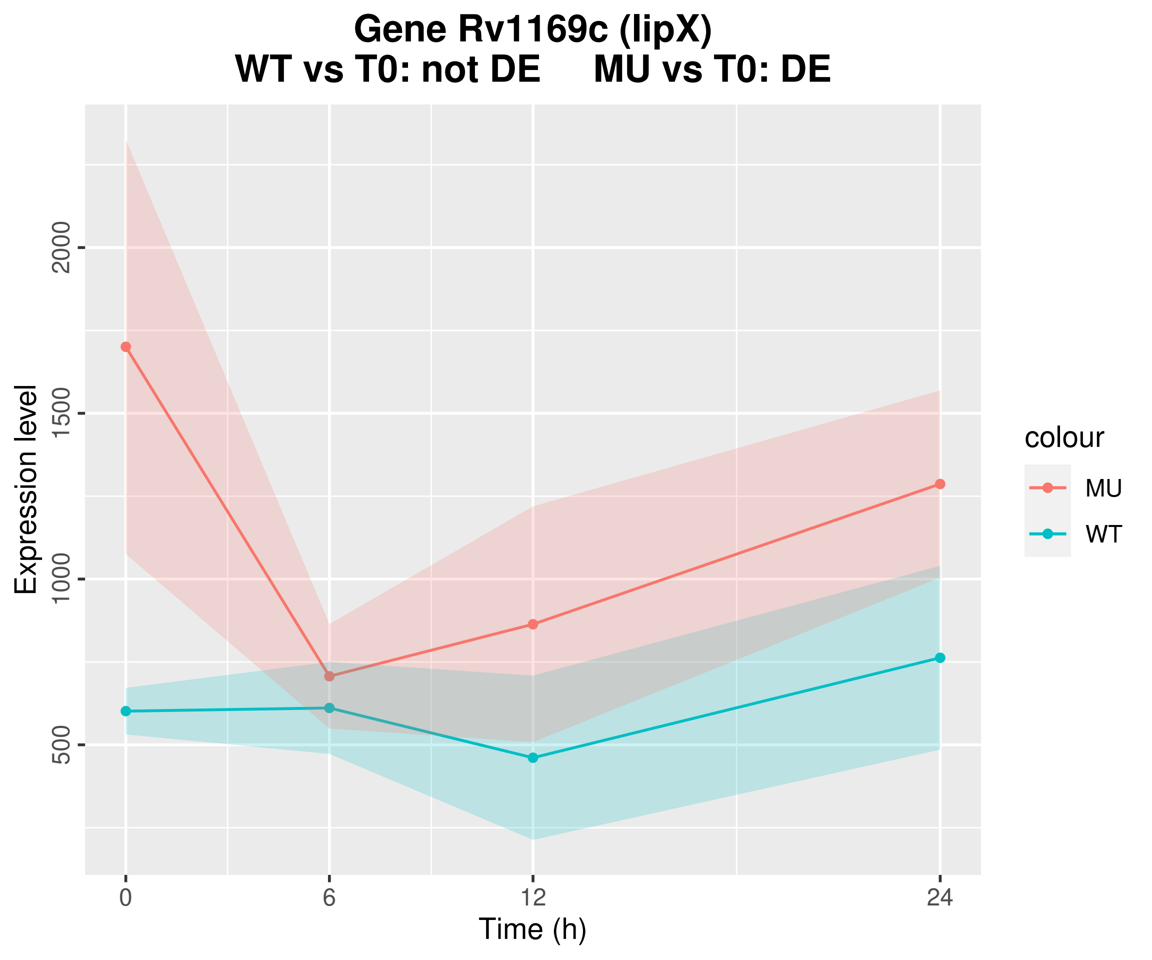

Gene Rv1169c (lipX)
WT vs T0: not DE MU vs T0: DE
Expression level
2000
1500
1000
500
0
6
12
24
Time (h)
colour
MU
WT

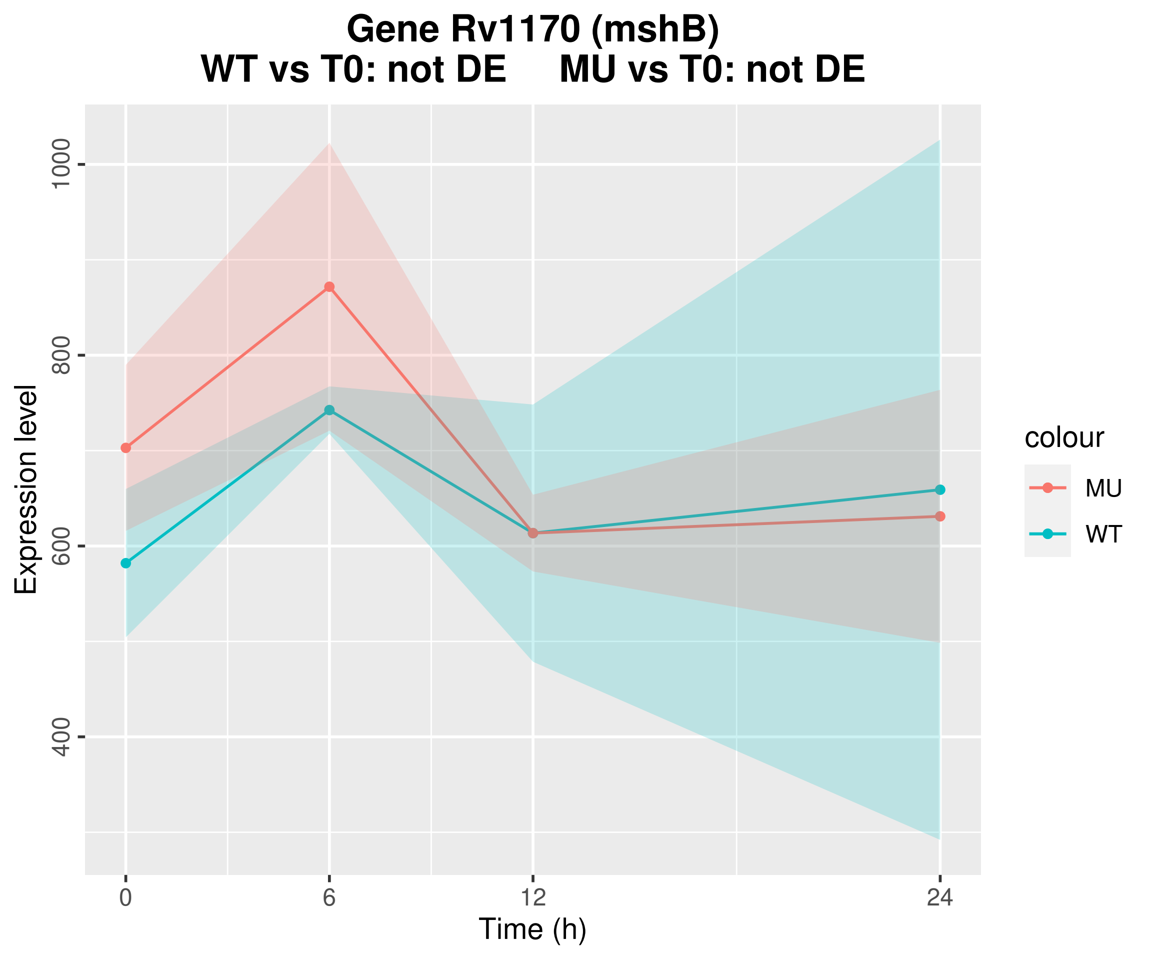

Gene Rv1170 (mshB)
WT vs T0: not DE   MU vs T0: not DE
Expression level
400
600
800
1000
0
6
12
24
Time (h)
colour
MU
WT

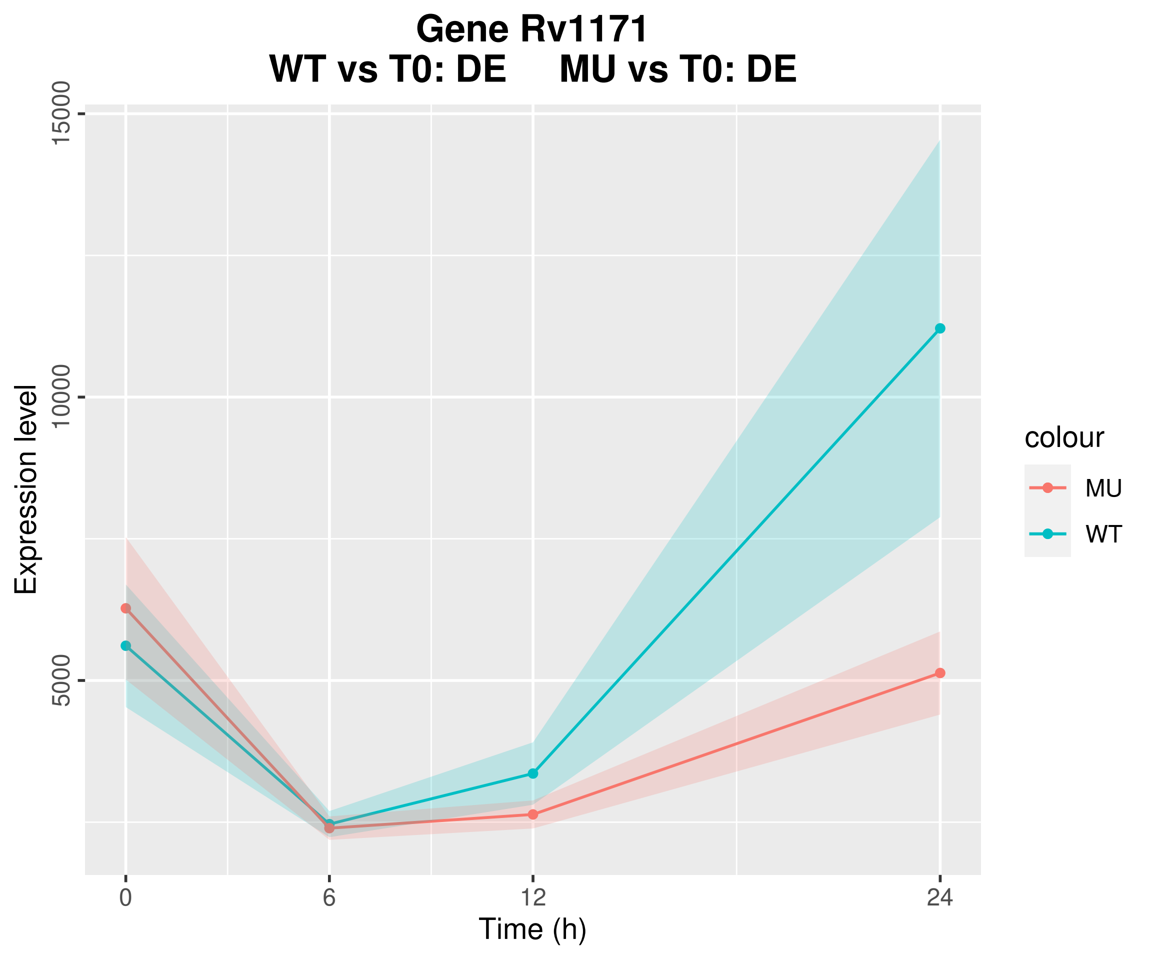

Gene Rv1171
WT vs T0: DE MU vs T0: DE
Expression level
15000
10000
5000
0
6
12
24
Time (h)
colour
MU
WT

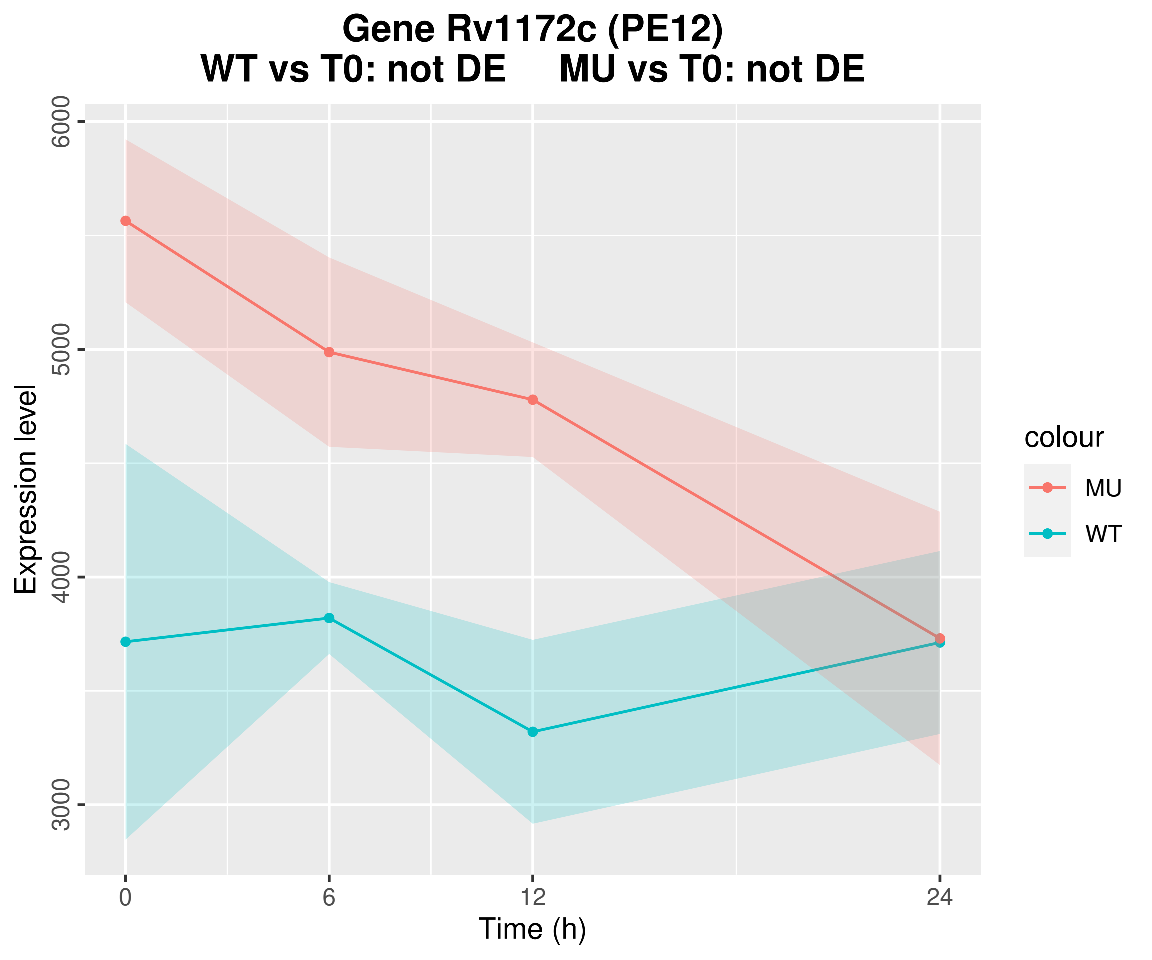

Gene Rv1172c (PE12)
WT vs T0: not DE   MU vs T0: not DE
Expression level
6000
5000
4000
3000
0
6
12
24
Time (h)
colour
MU
WT

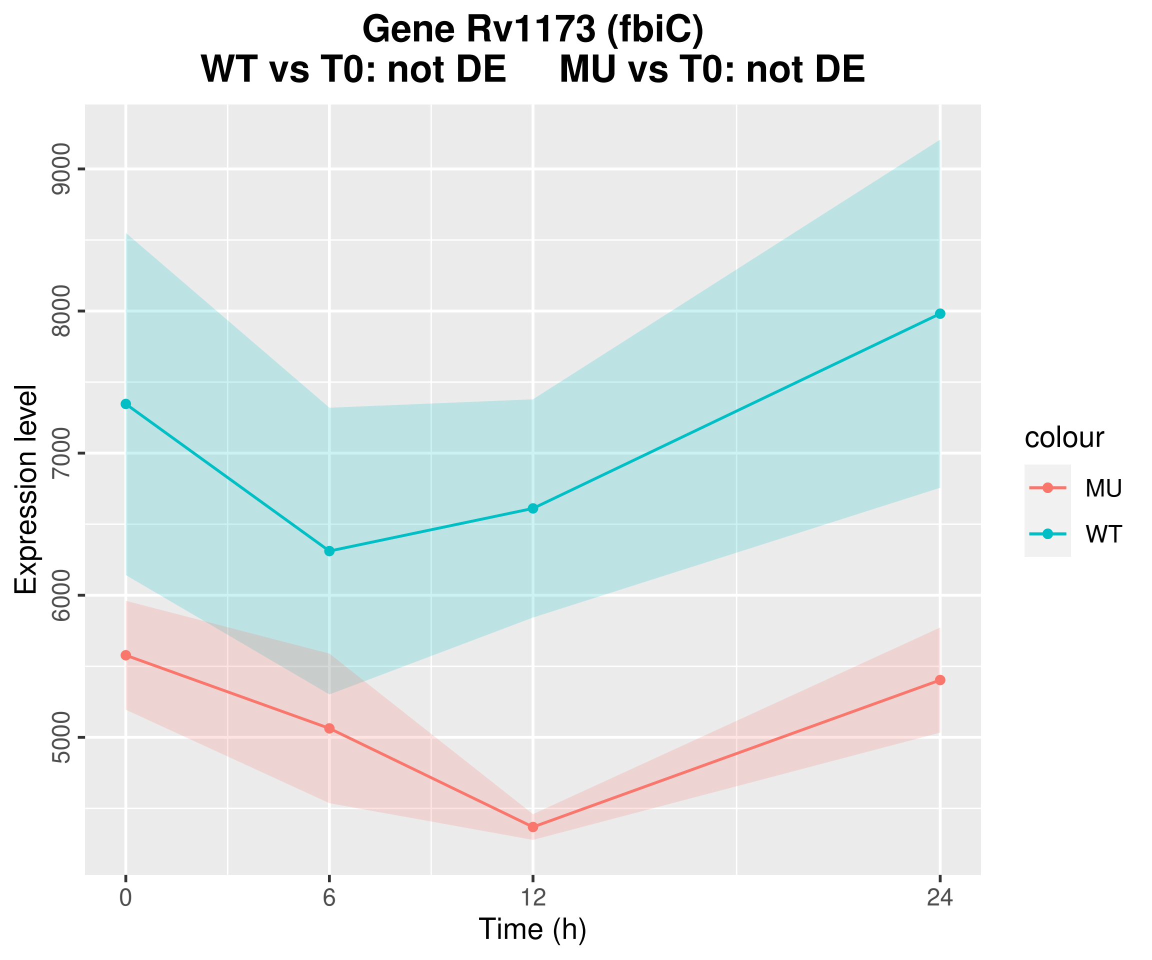

Gene Rv1173 (fbiC)
WT vs T0: not DE   MU vs T0: not DE
9000
8000
7000
6000
5000
Expression level
0
6
12
24
Time (h)
colour
MU
WT

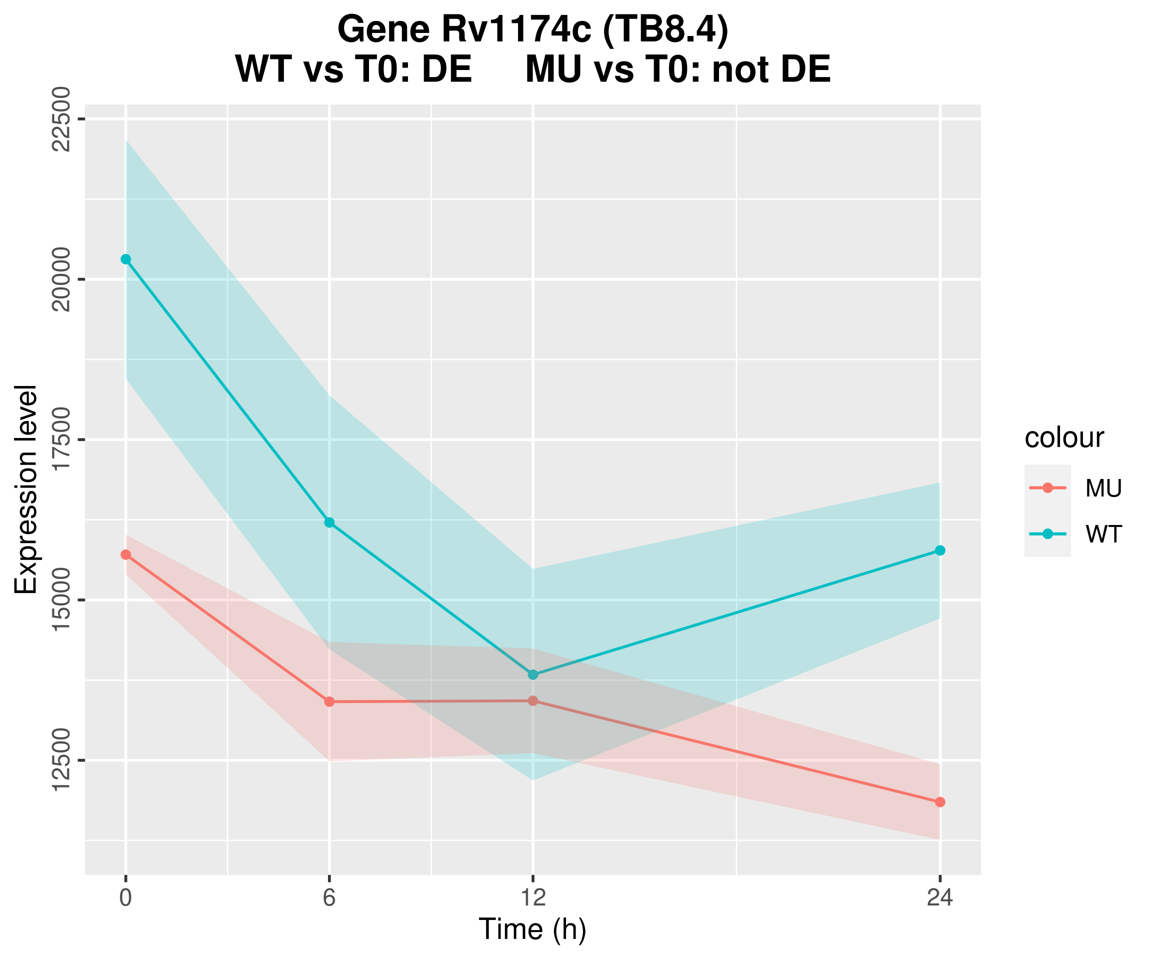

Gene Rv1174c (TB8.4)
WT vs T0: DE   MU vs T0: not DE
Expression level
22500
20000
17500
15000
12500
Time (h)
0
6
12
24
colour
MU
WT

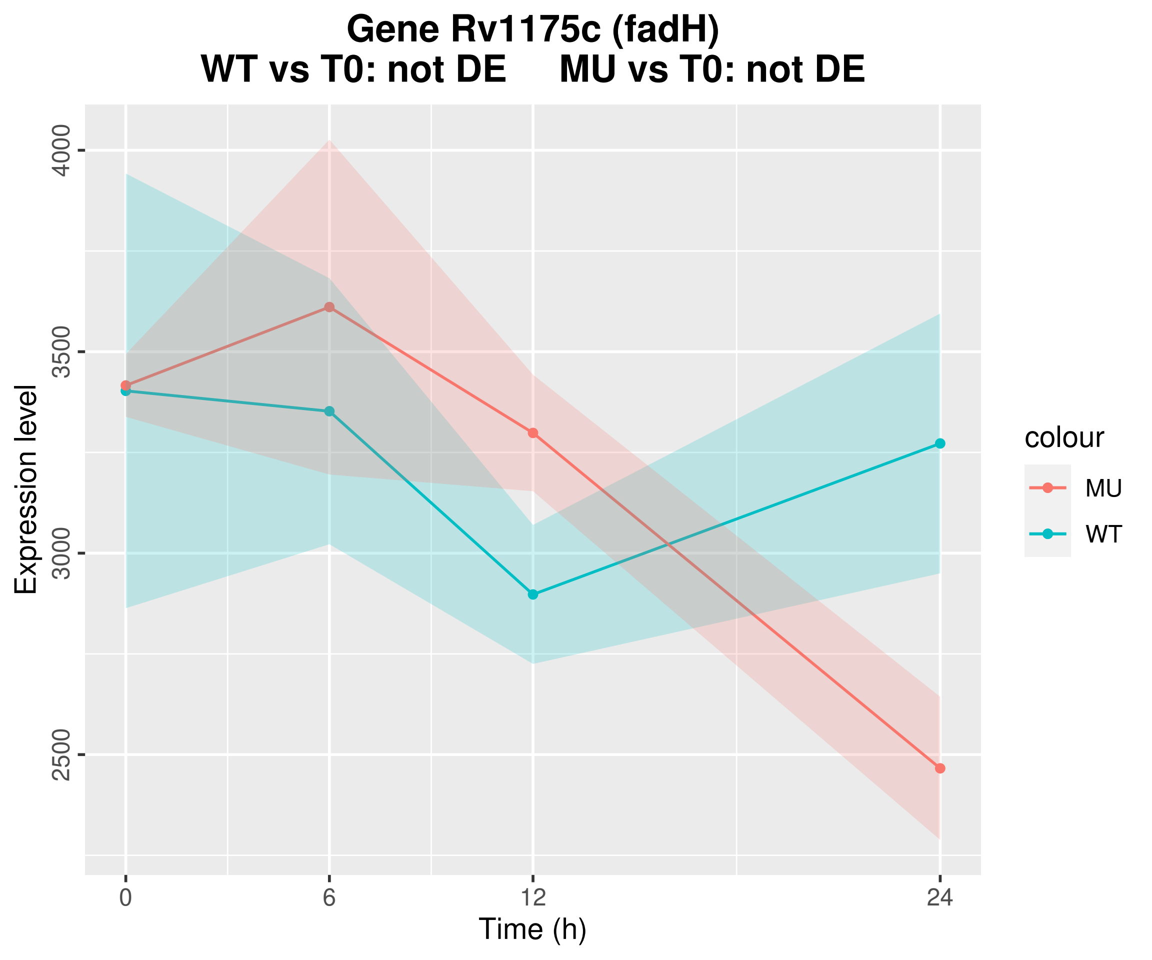
Gene Rv1175c (fadH)
WT vs T0: not DE MU vs T0: not DE
Expression level
4000
3500
3000
2500
0
6
12
24
Time (h)
colour
MU
WT

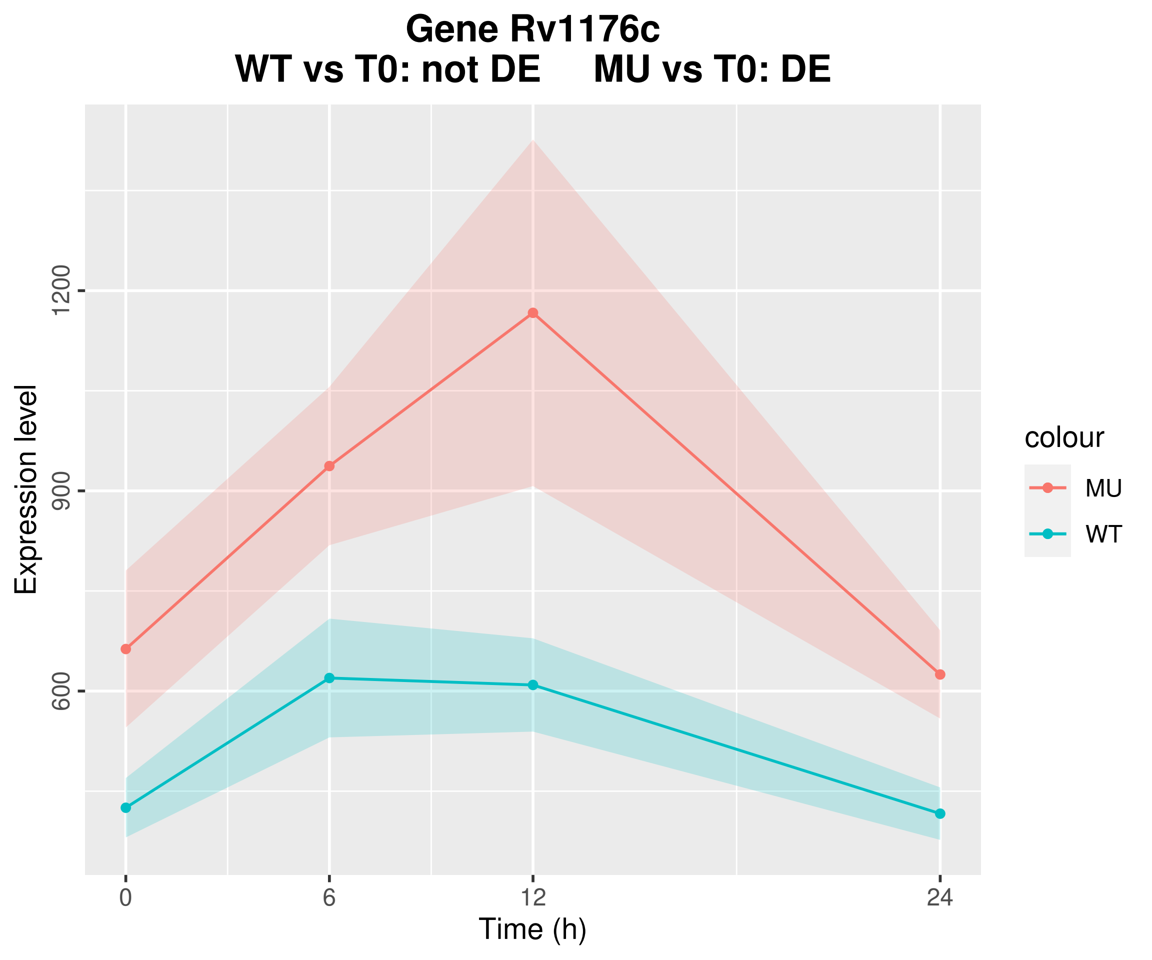

Gene Rv1176c
WT vs T0: not DE MU vs T0: DE
Expression level
1200
900
600
0
6
12
24
Time (h)
colour
MU
WT

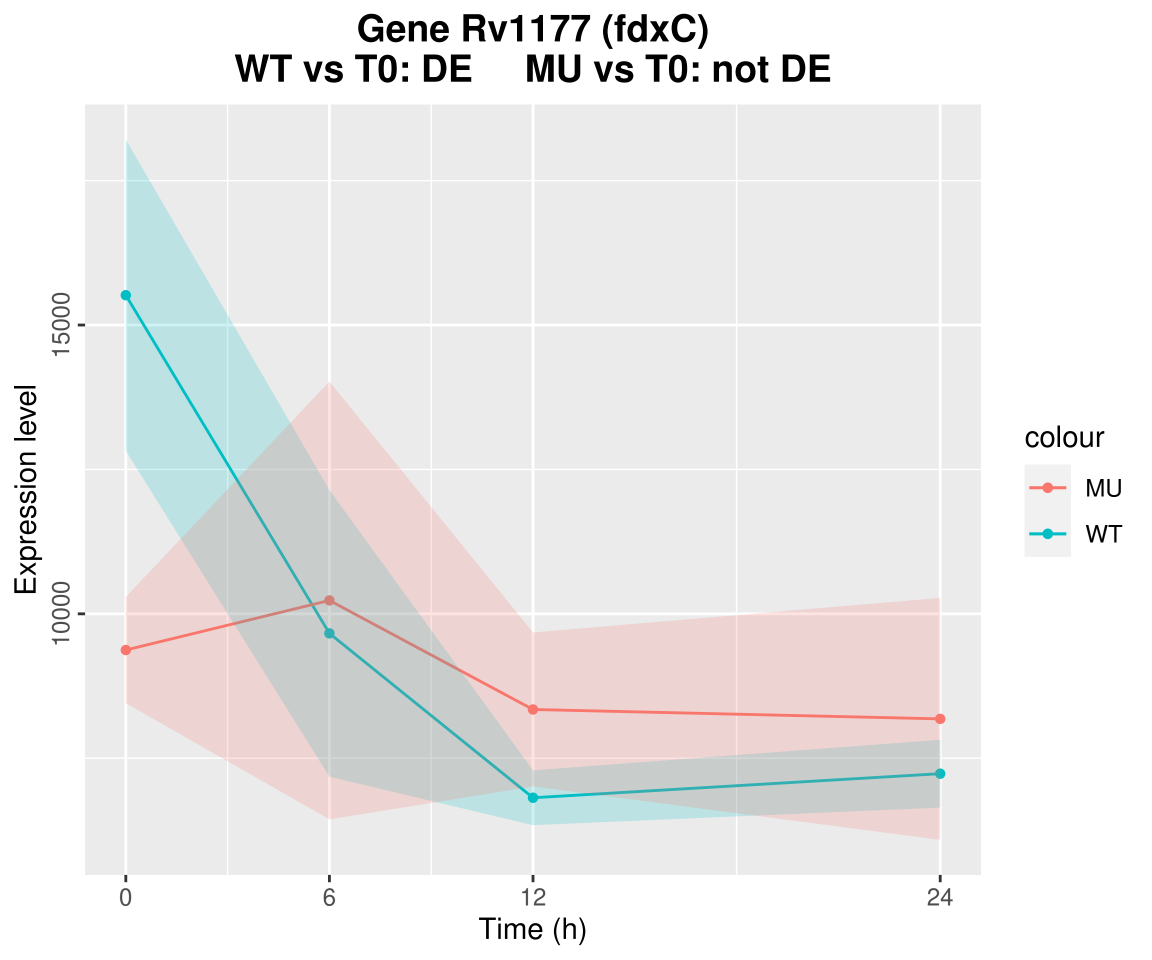

Gene Rv1177 (fdxC)
WT vs T0: DE   MU vs T0: not DE
Expression level
15000
10000
0
6
12
24
Time (h)
colour
MU
WT

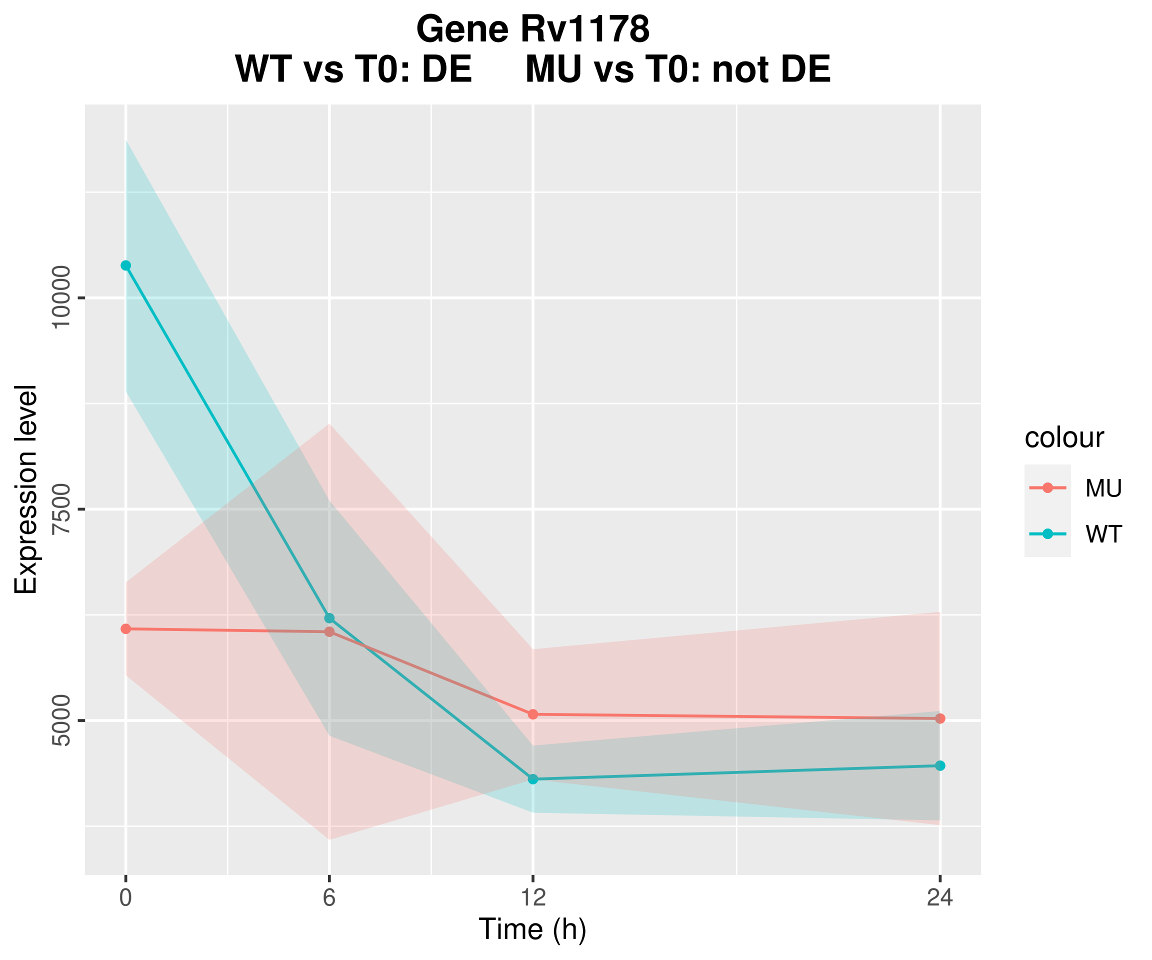

Gene Rv1178
WT vs T0: DE   MU vs T0: not DE
Expression level
10000
7500
5000
0
6
12
24
Time (h)
colour
MU
WT

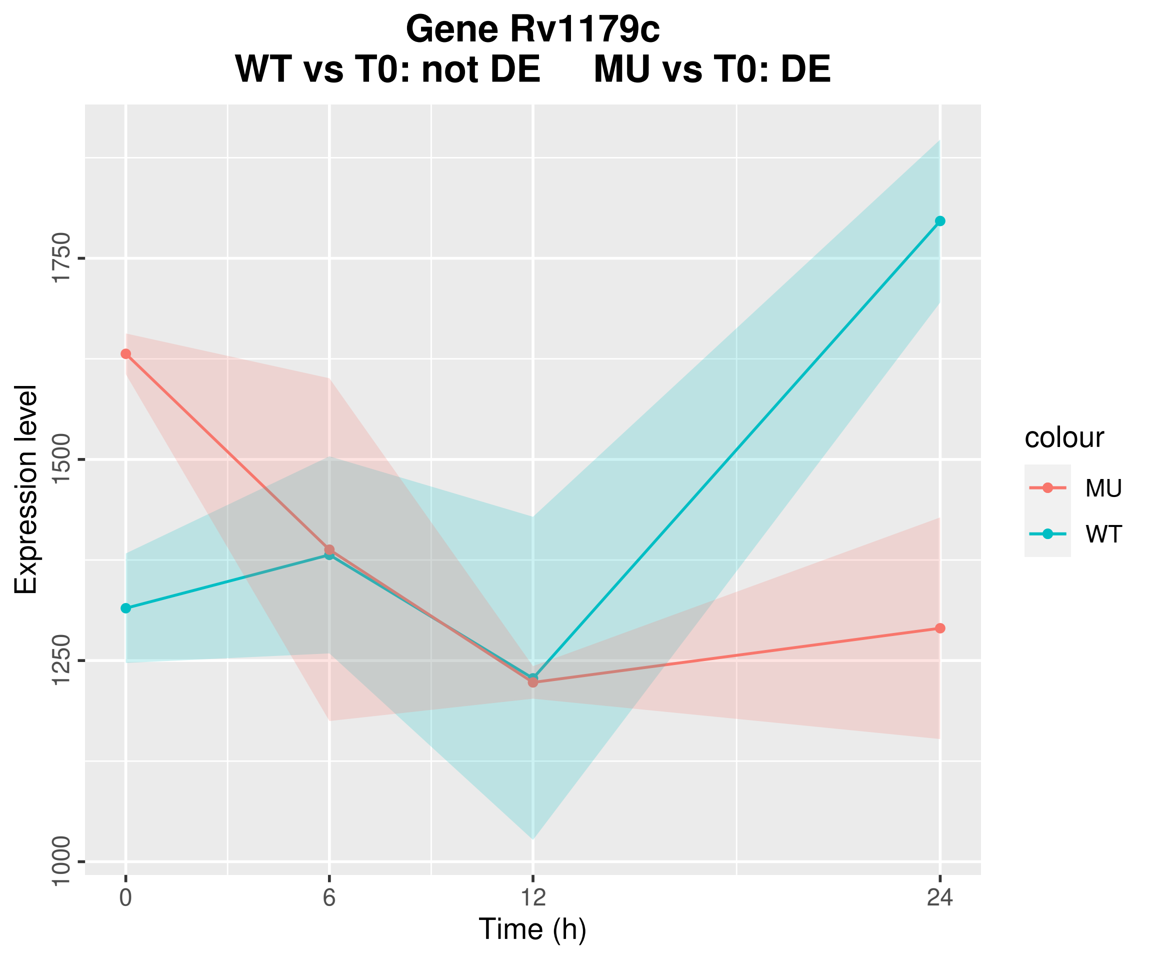
Gene Rv1179c
WT vs T0: not DE   MU vs T0: DE
Expression level
1000
1250
1500
1750
0
6
12
24
Time (h)
colour
MU
WT

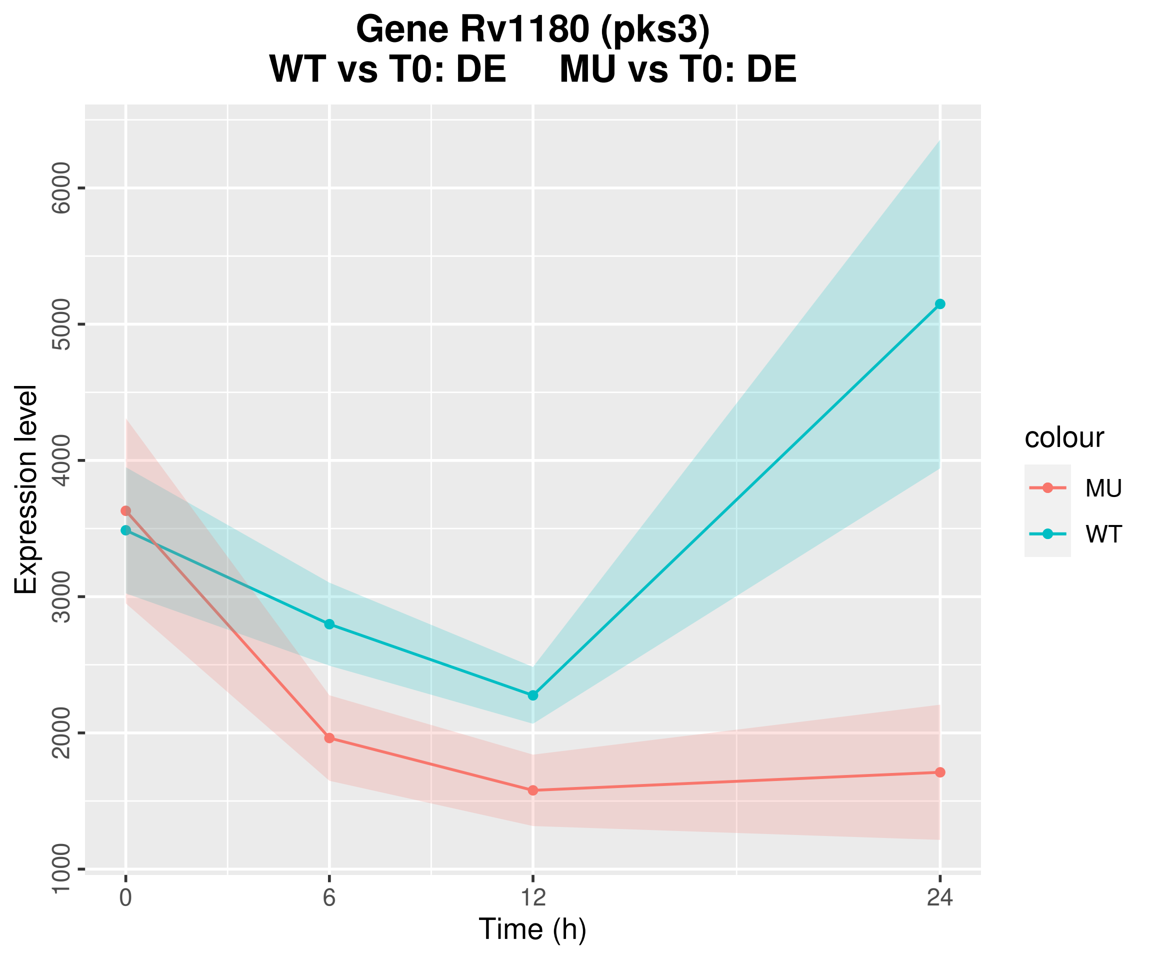

Gene Rv1180 (pks3)
WT vs T0: DE MU vs T0: DE
Expression level
6000
5000
4000
3000
2000
1000
0
6
12
24
Time (h)
colour
MU
WT

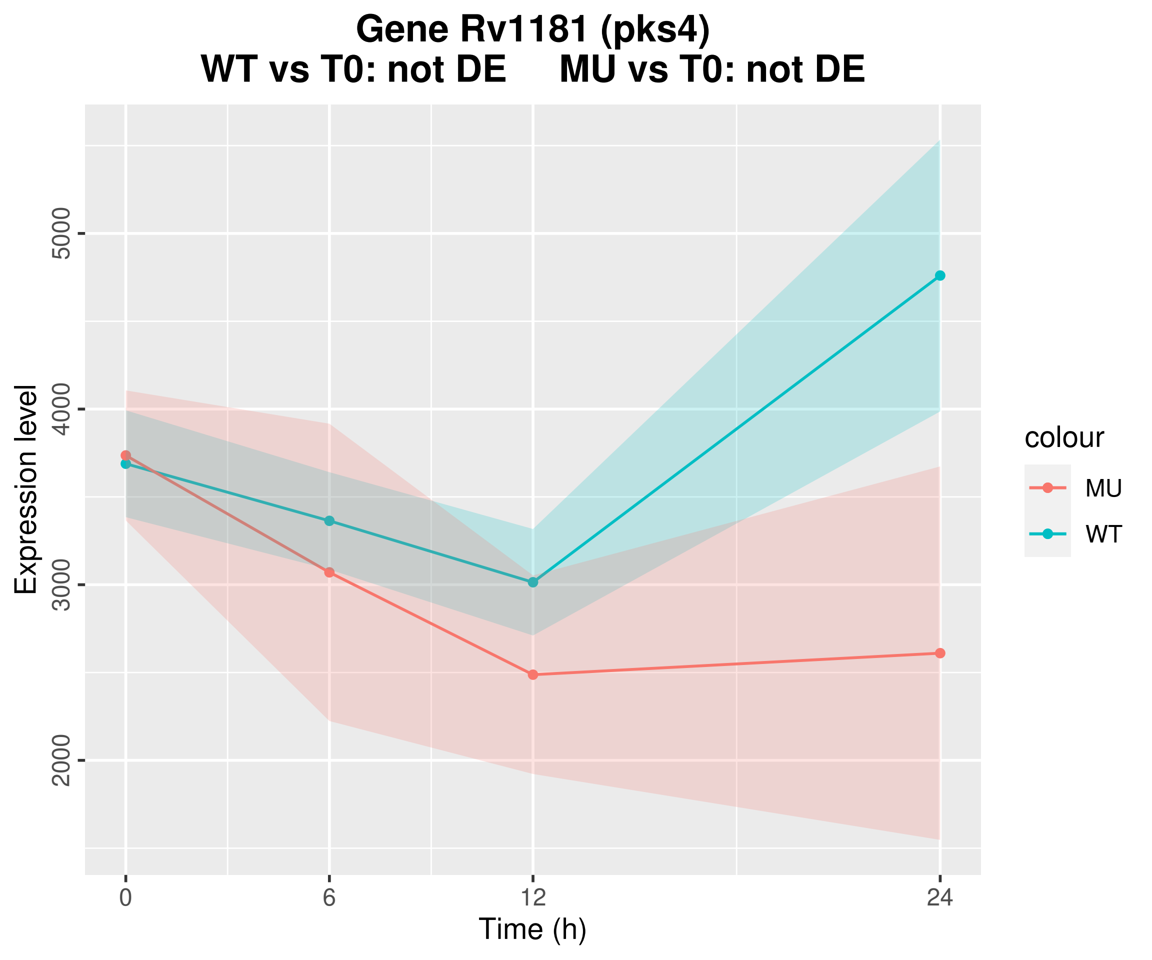
Gene Rv1181 (pks4)
WT vs T0: not DE   MU vs T0: not DE
Expression level
5000
4000
3000
2000
0
6
12
24
Time (h)
colour
MU
WT

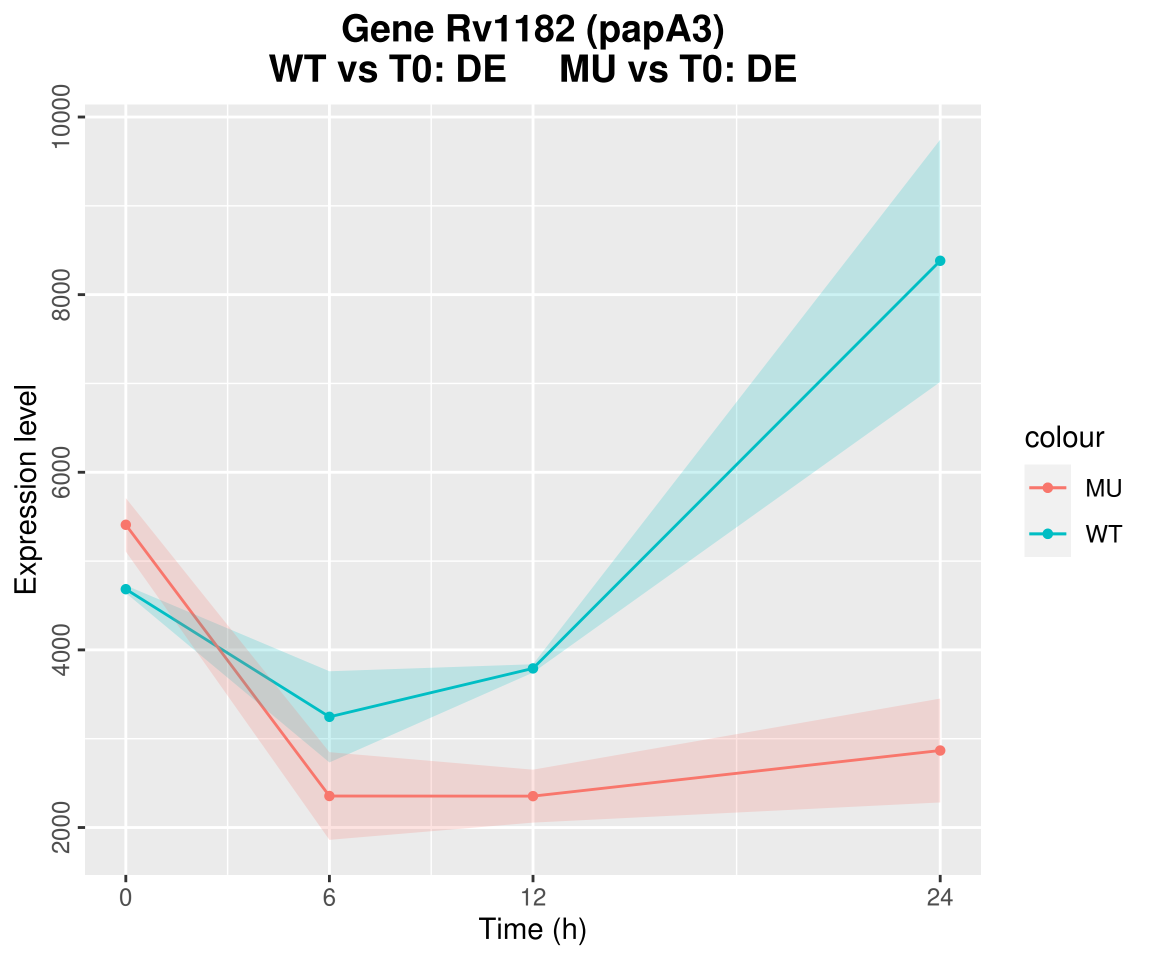
Gene Rv1182 (papA3)
WT vs T0: DE   MU vs T0: DE
Expression level
10000
8000
6000
4000
2000
0
6
12
24
Time (h)
colour
MU
WT

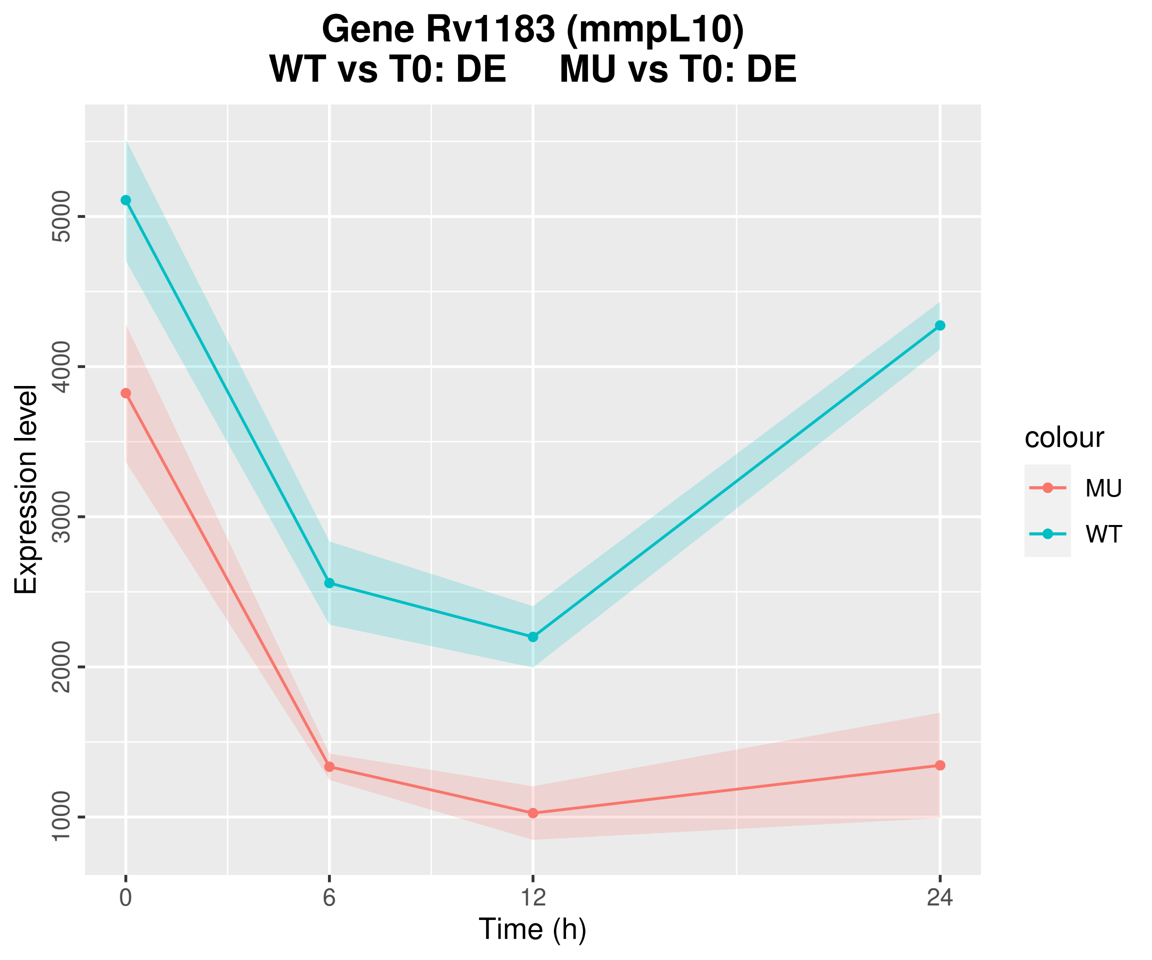
Gene Rv1183 (mmpL10)
WT vs T0: DE   MU vs T0: DE
Expression level
5000
4000
3000
2000
1000
0
6
12
24
Time (h)
colour
MU
WT

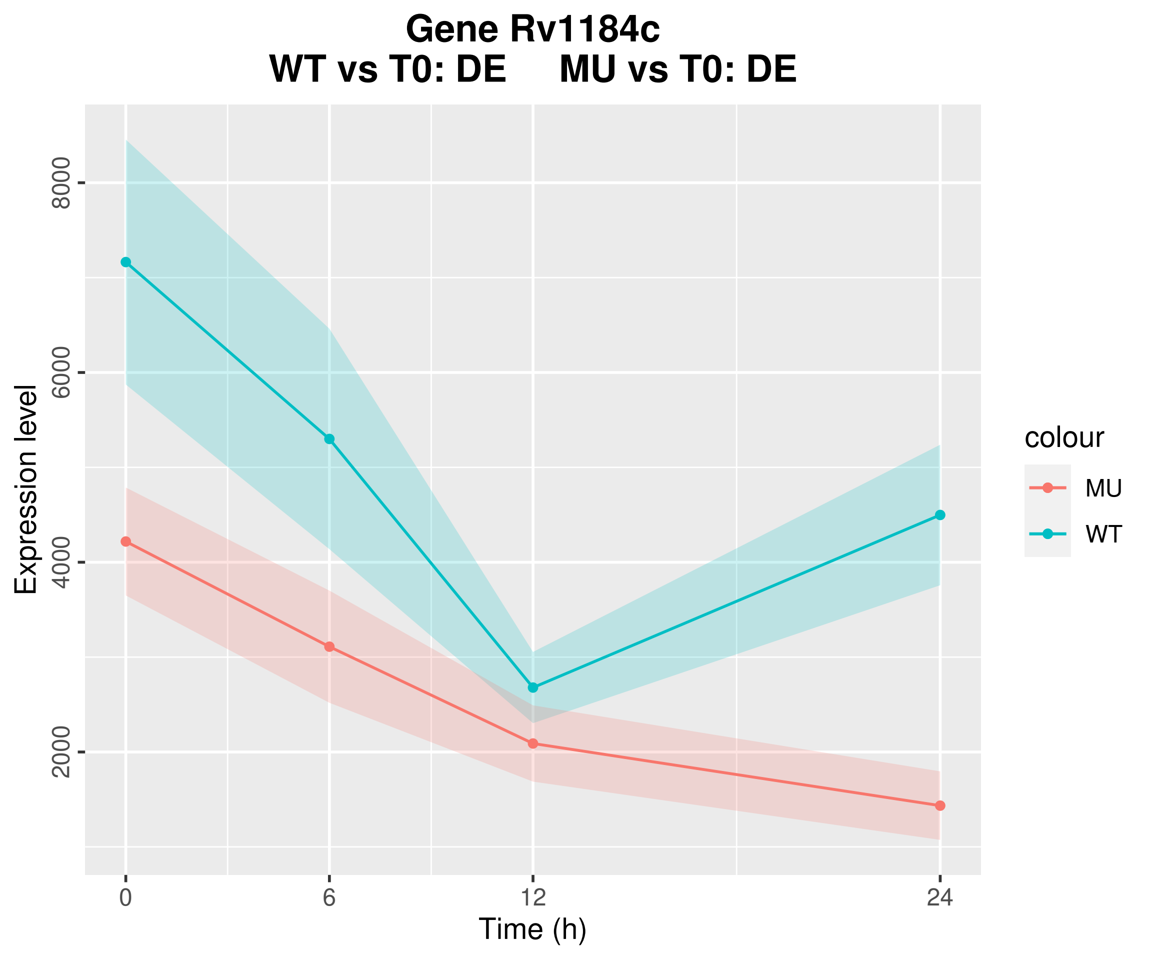
Gene Rv1184c
WT vs T0: DE MU vs T0: DE
Expression level
8000
6000
4000
2000
0
6
12
24
Time (h)
colour
MU
WT

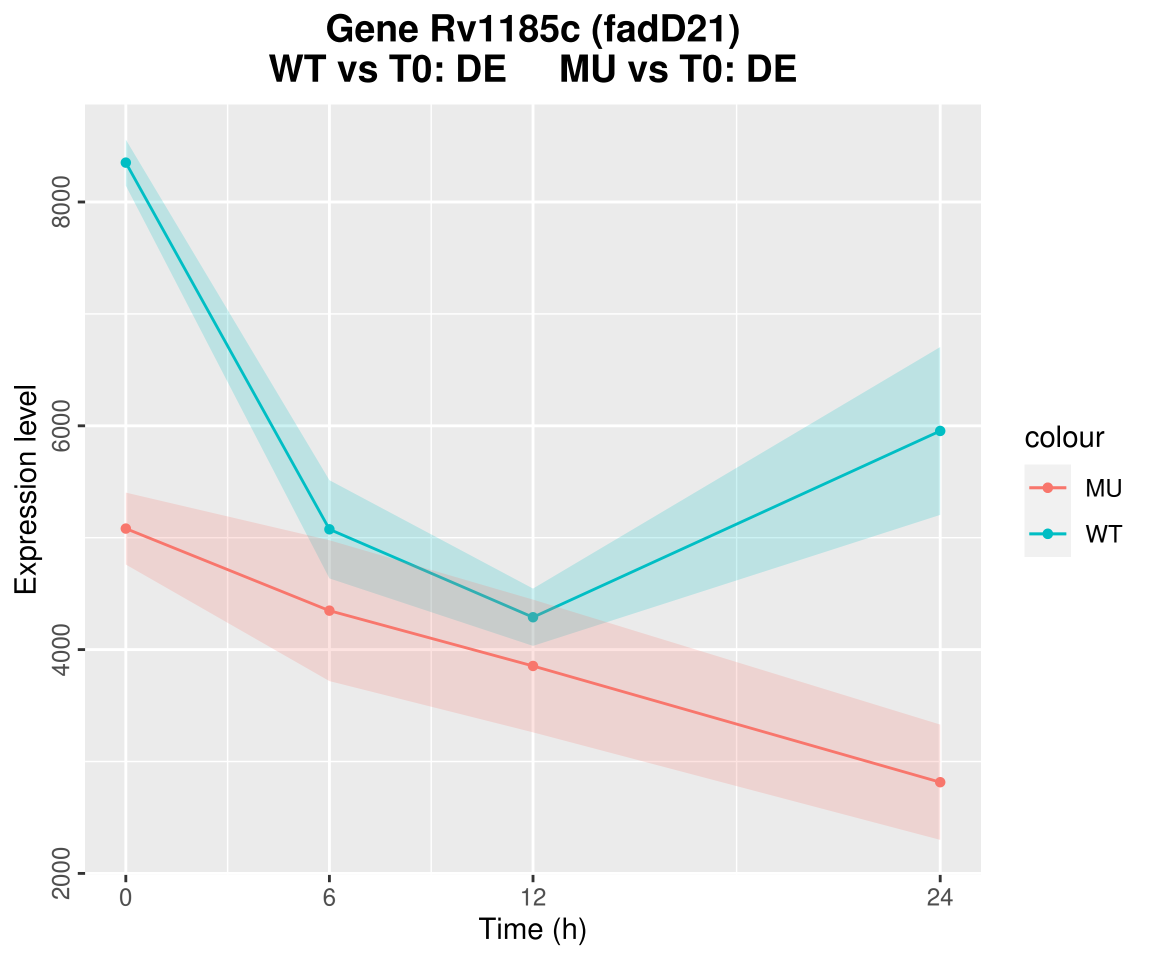

Gene Rv1185c (fadD21)
WT vs T0: DE MU vs T0: DE
Expression level
8000
6000
4000
2000
0
6
12
24
Time (h)
colour
MU
WT

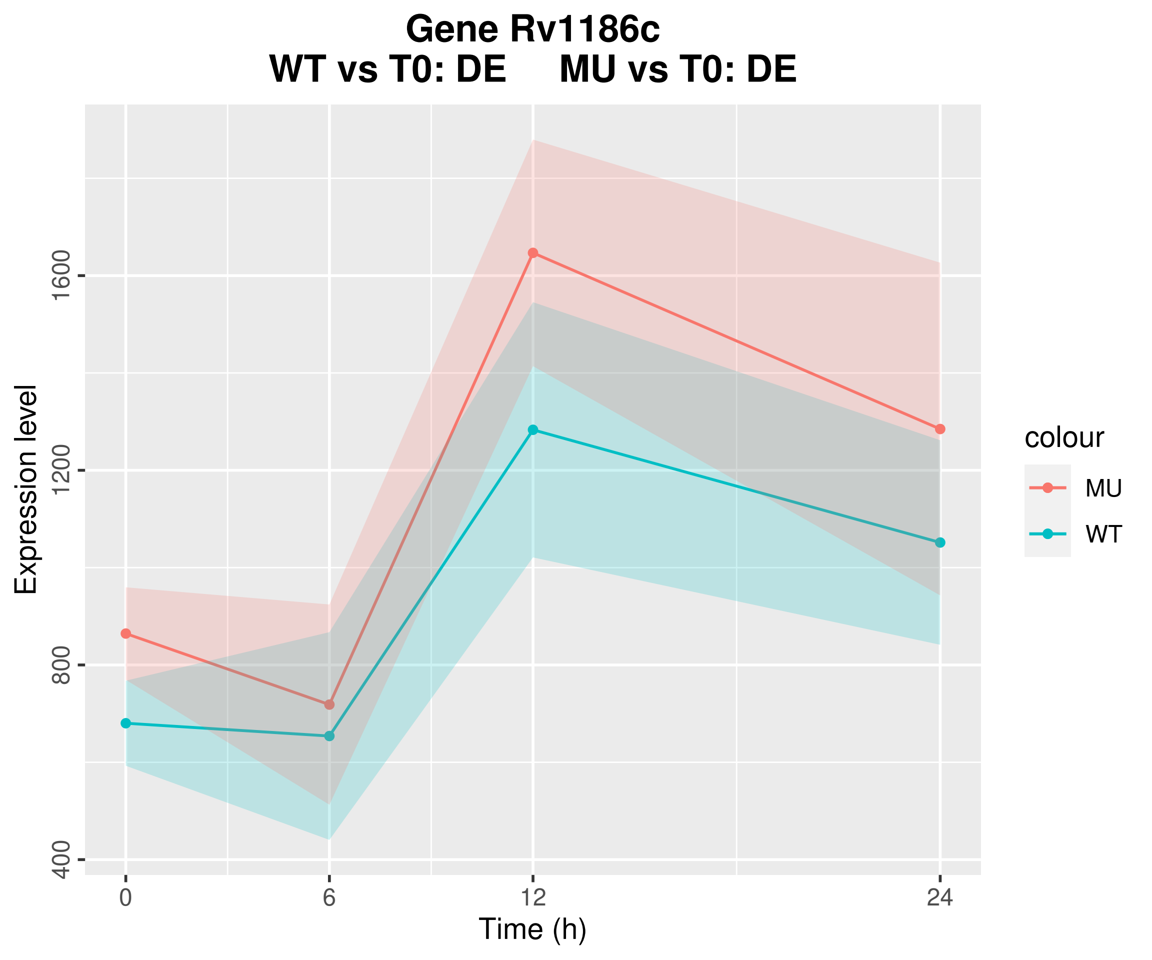
Gene Rv1186c
WT vs T0: DE MU vs T0: DE
Expression level
1600
1200
800
400
0
6
12
24
Time (h)
colour
MU
WT

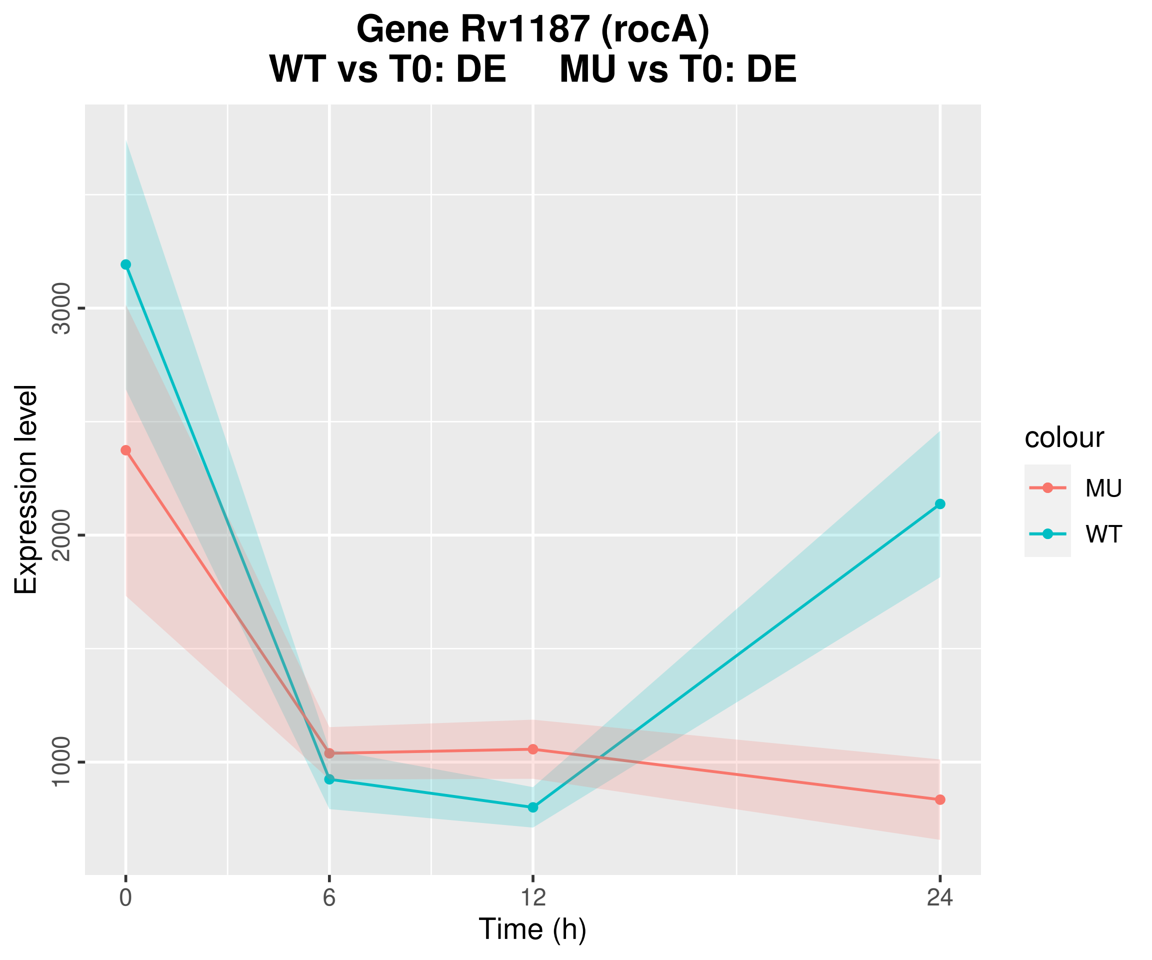

Gene Rv1187 (rocA)
WT vs T0: DE MU vs T0: DE
Expression level
3000
2000
1000
0
6
12
24
Time (h)
colour
MU
WT

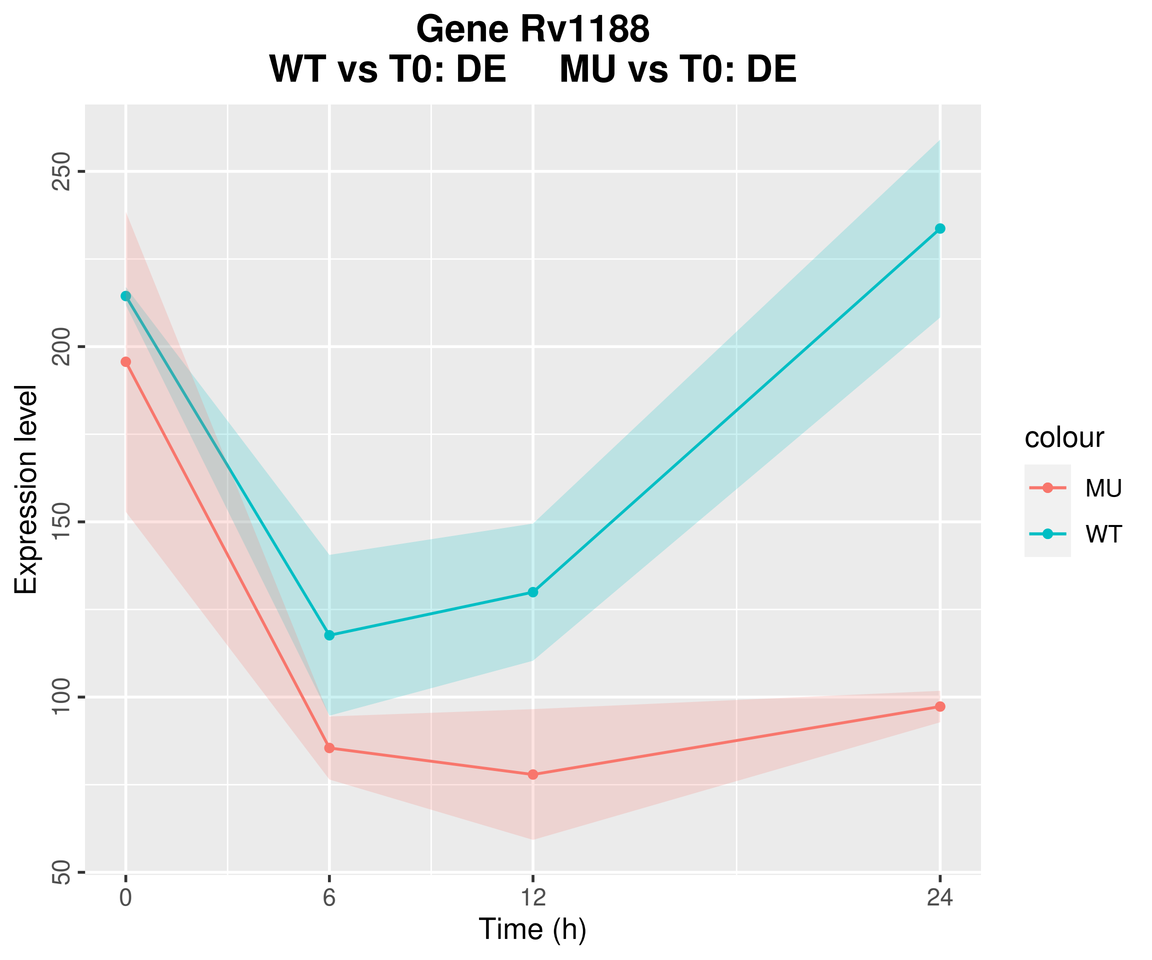
Gene Rv1188
WT vs T0: DE MU vs T0: DE
Expression level
250
200
150
100
50
0
6
12
24
Time (h)
colour
MU
WT

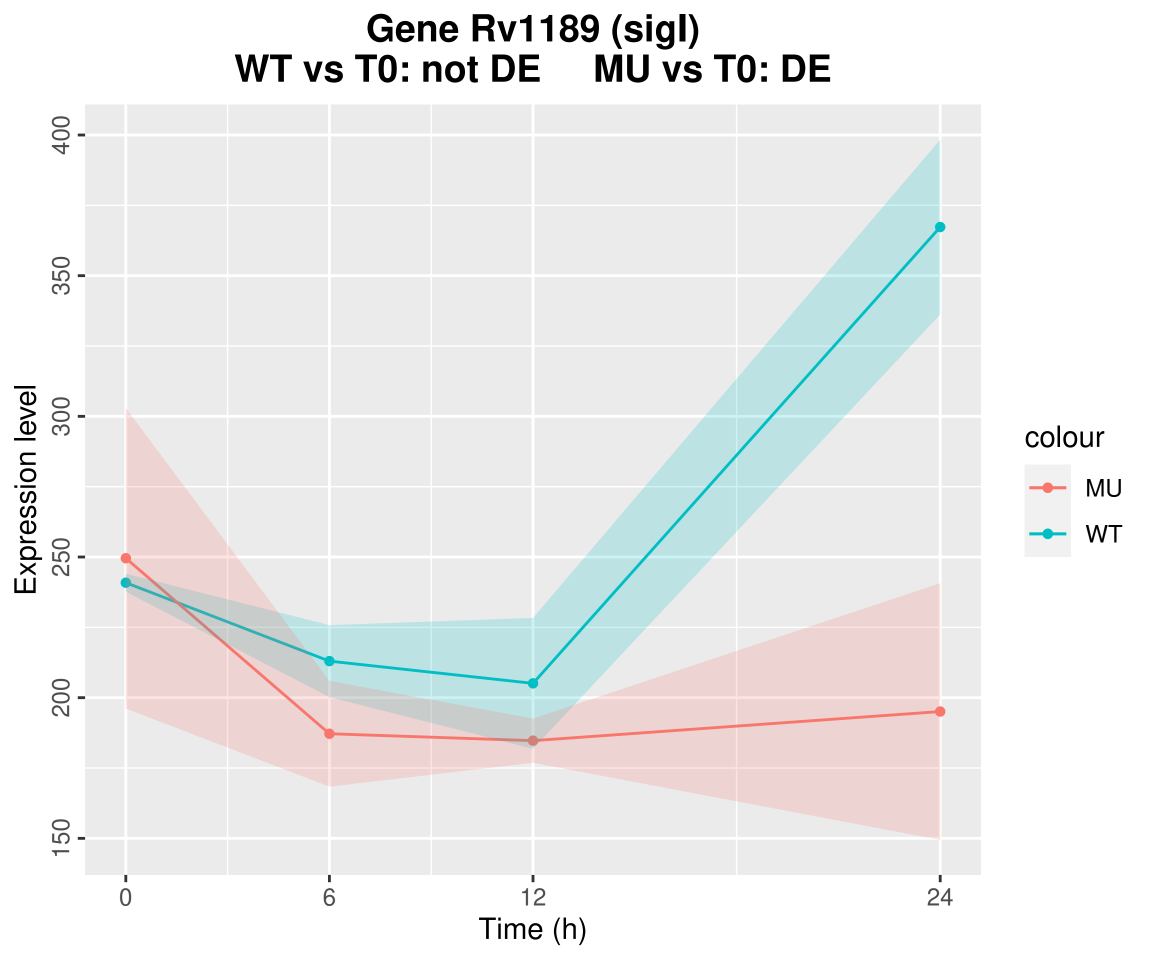
Gene Rv1189 (sigI)
WT vs T0: not DE MU vs T0: DE
Expression level
400
350
300
250
200
150
0
6
12
24
Time (h)
colour
MU
WT

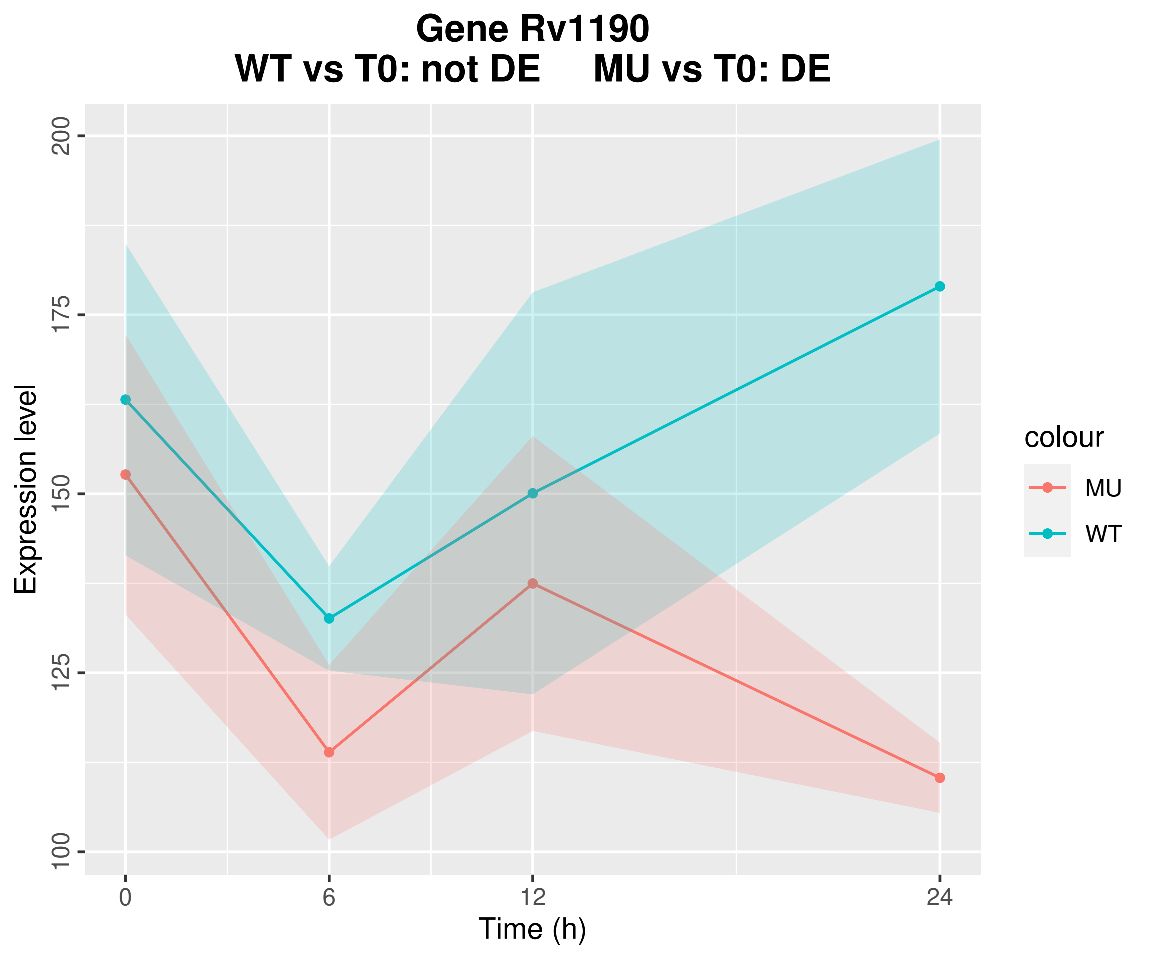
Gene Rv1190
WT vs T0: not DE MU vs T0: DE
Expression level
200
175
150
125
100
0
6
12
24
Time (h)
colour
MU
WT

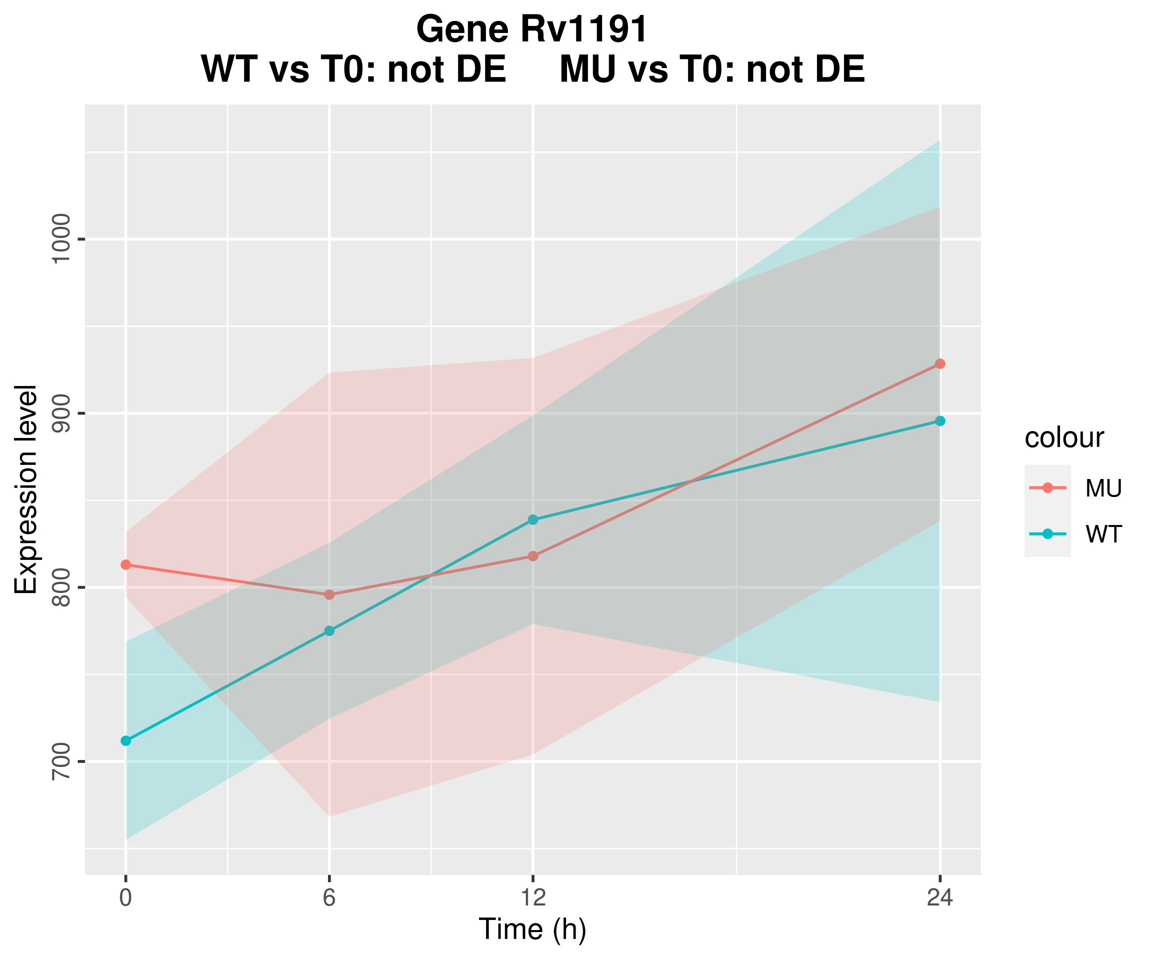

Gene Rv1191
WT vs T0: not DE MU vs T0: not DE
Expression level
1000
900
800
700
0
6
12
24
Time (h)
colour
MU
WT

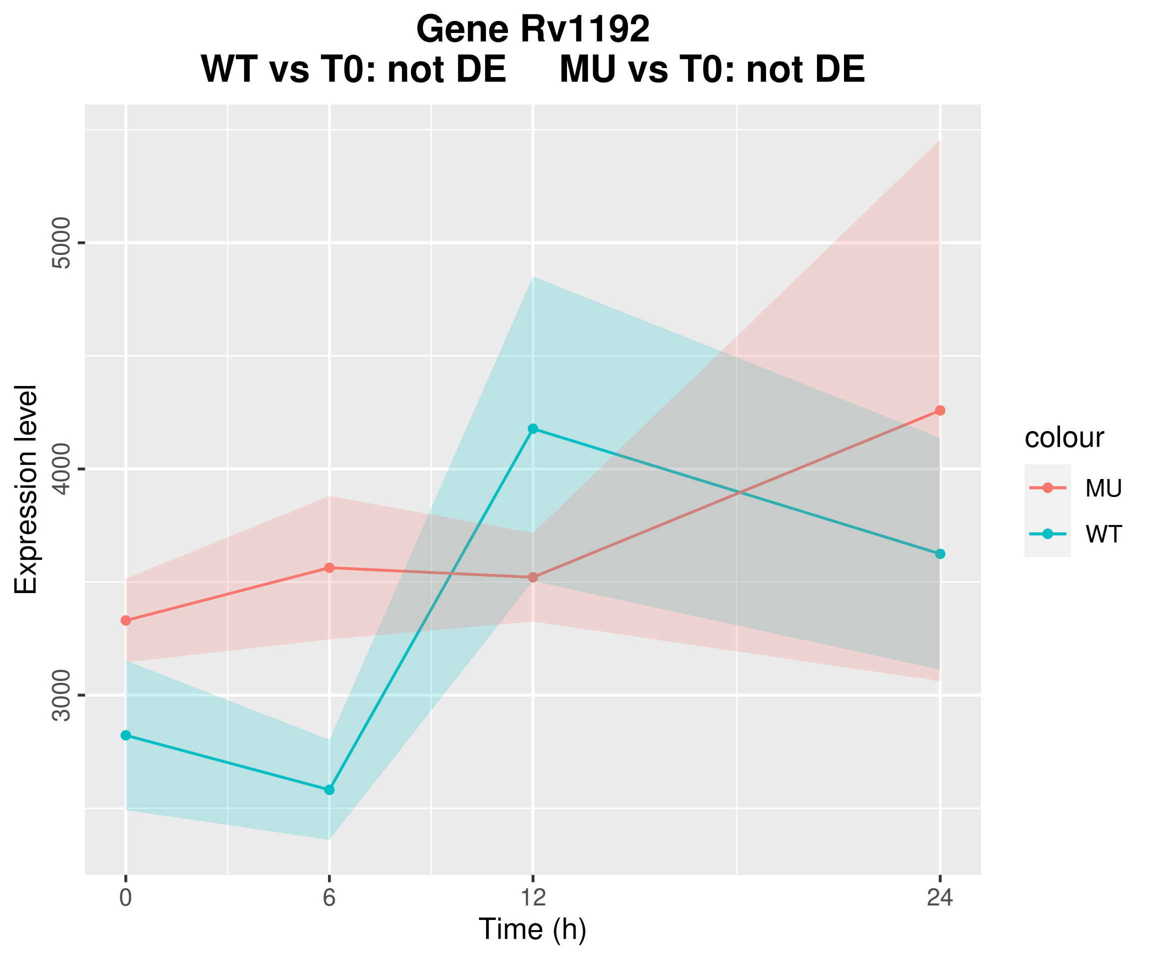

Gene Rv1192
WT vs T0: not DE MU vs T0: not DE
Expression level
5000
4000
3000
0
6
12
24
Time (h)
colour
MU
WT

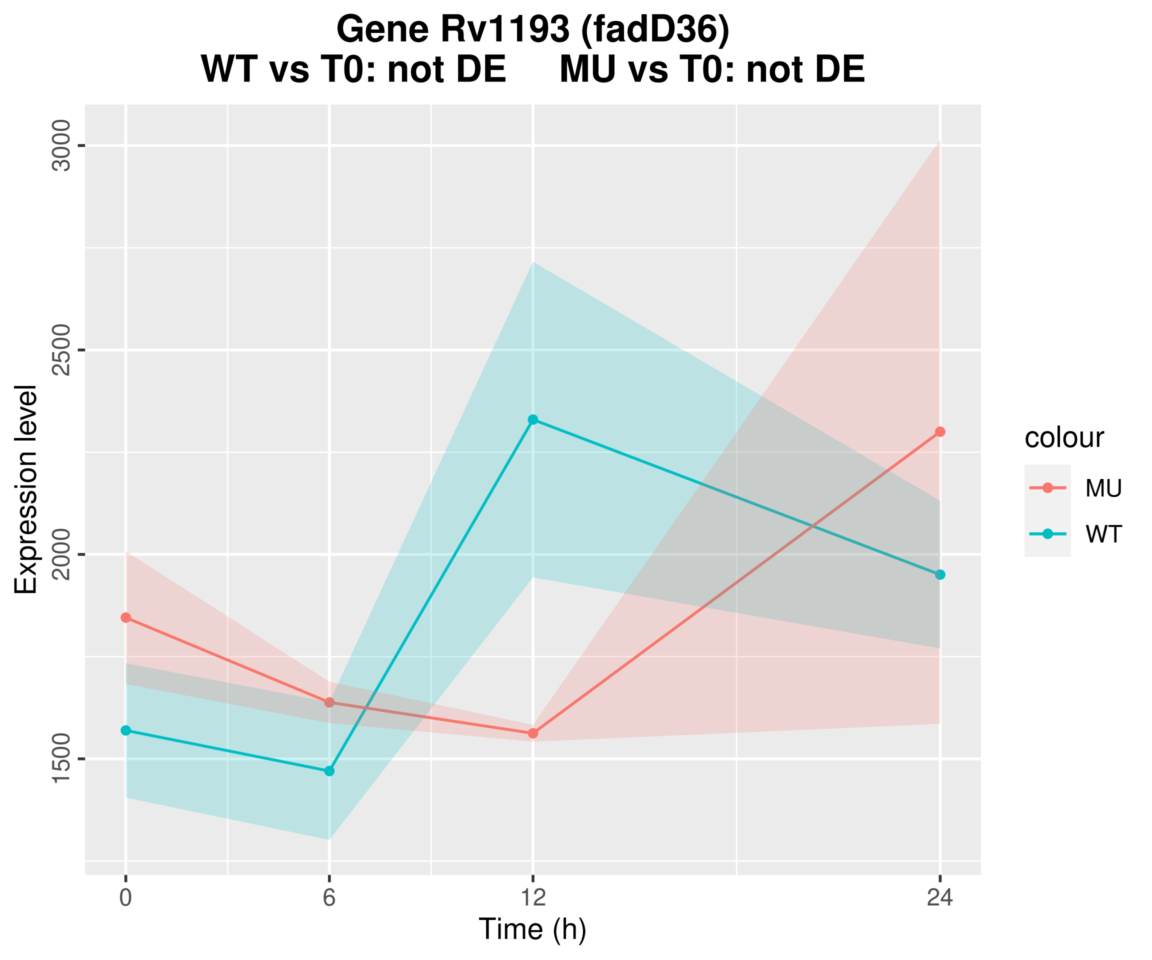

Gene Rv1193 (fadD36)
WT vs T0: not DE   MU vs T0: not DE
Expression level
3000
2500
2000
1500
0
6
12
24
Time (h)
colour
MU
WT

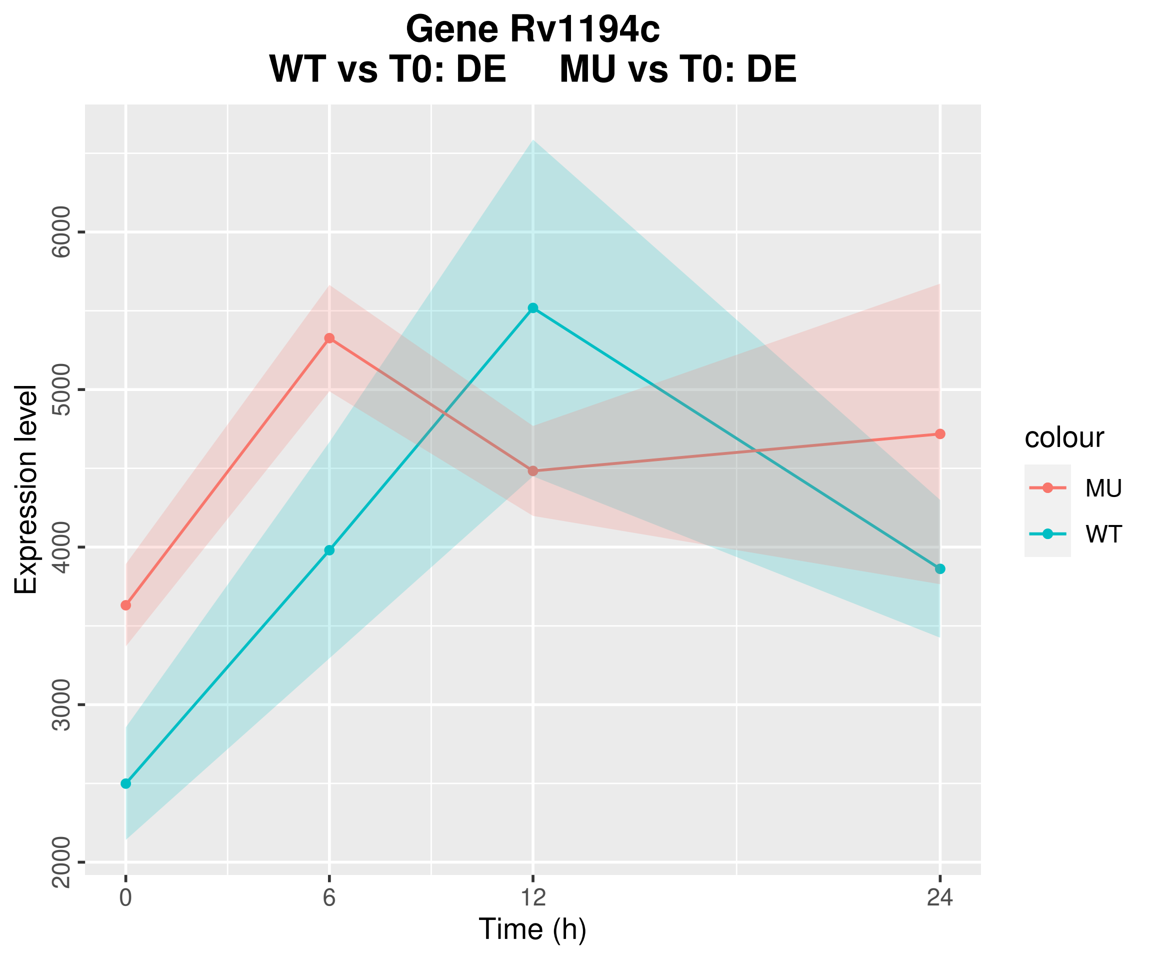

Gene Rv1194c
WT vs T0: DE   MU vs T0: DE
Expression level
6000
5000
4000
3000
2000
0
6
12
24
Time (h)
colour
MU
WT

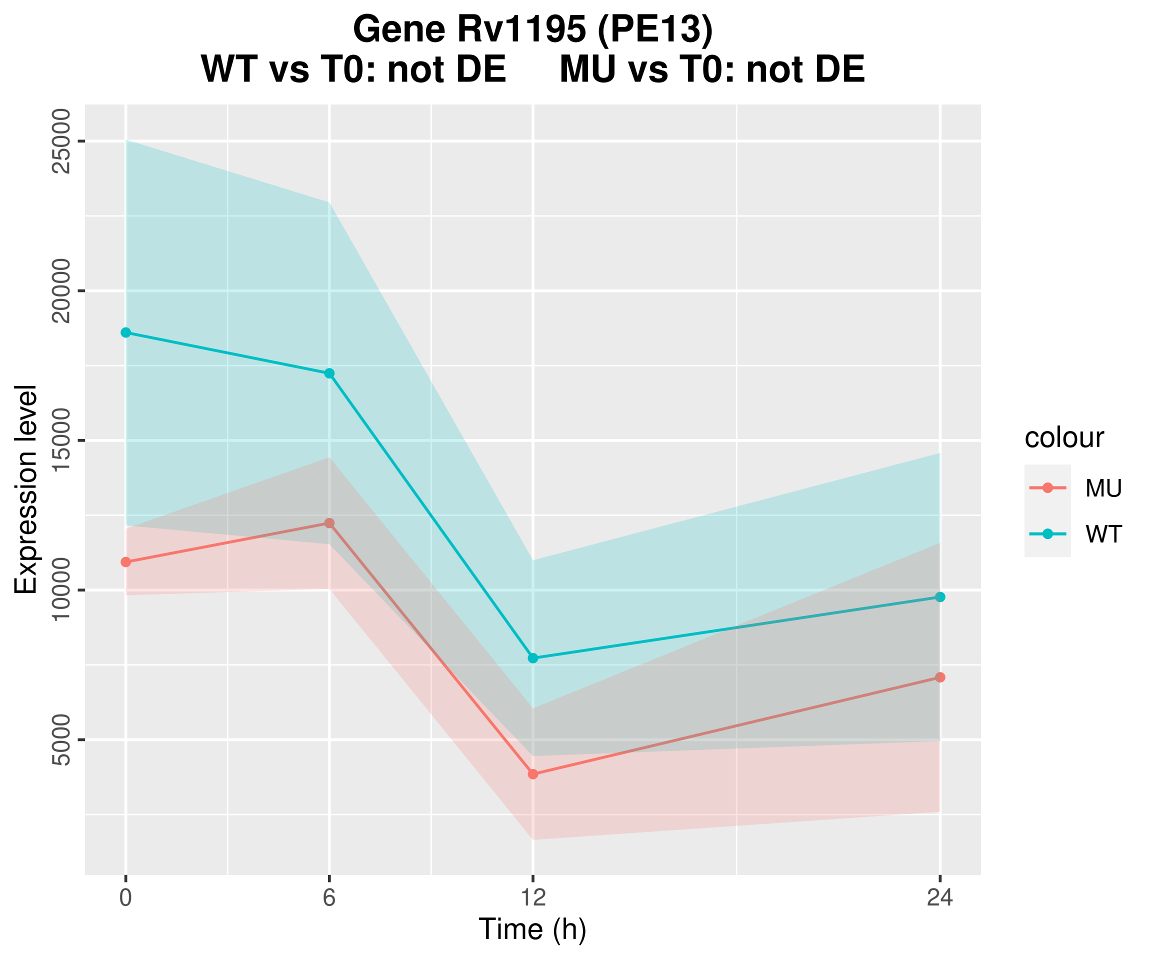
Gene Rv1195 (PE13)
WT vs T0: not DE MU vs T0: not DE
Expression level
25000
20000
15000
10000
5000
0
6
12
24
Time (h)
colour
MU
WT

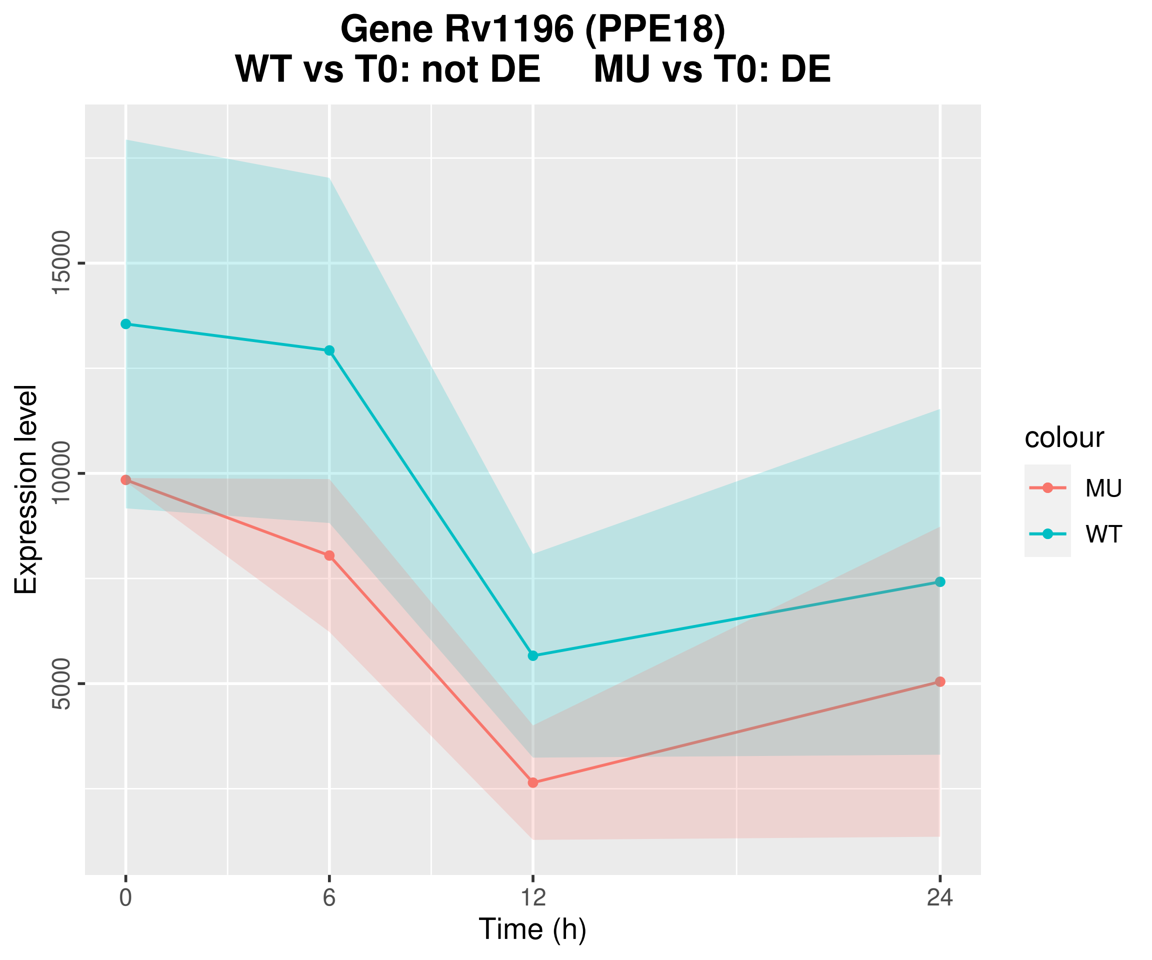

Gene Rv1196 (PPE18)
WT vs T0: not DE   MU vs T0: DE
Expression level
15000
10000
5000
0
6
12
24
Time (h)
colour
MU
WT

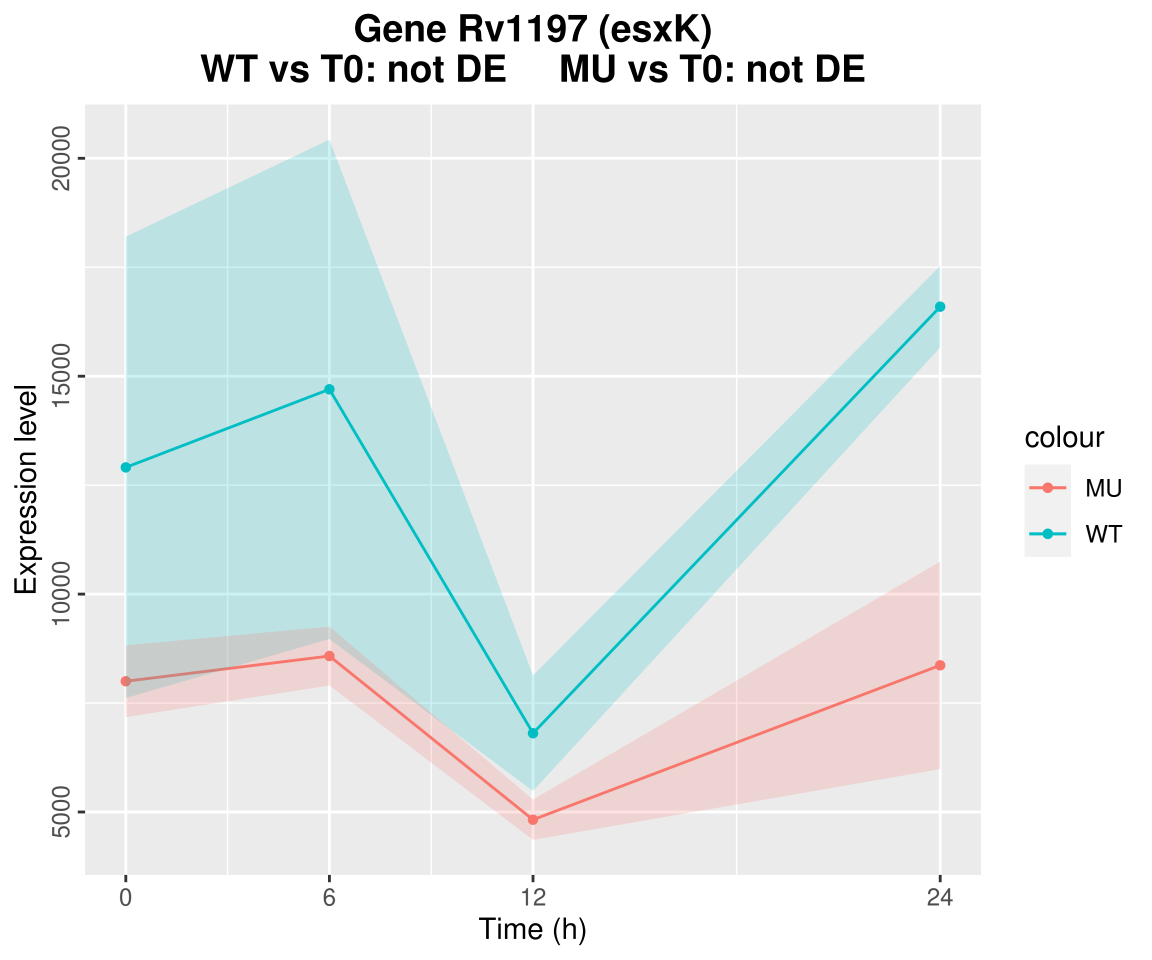
Gene Rv1197 (esxK)
WT vs T0: not DE MU vs T0: not DE
Expression level
20000
15000
10000
5000
0
6
12
24
Time (h)
colour
MU
WT

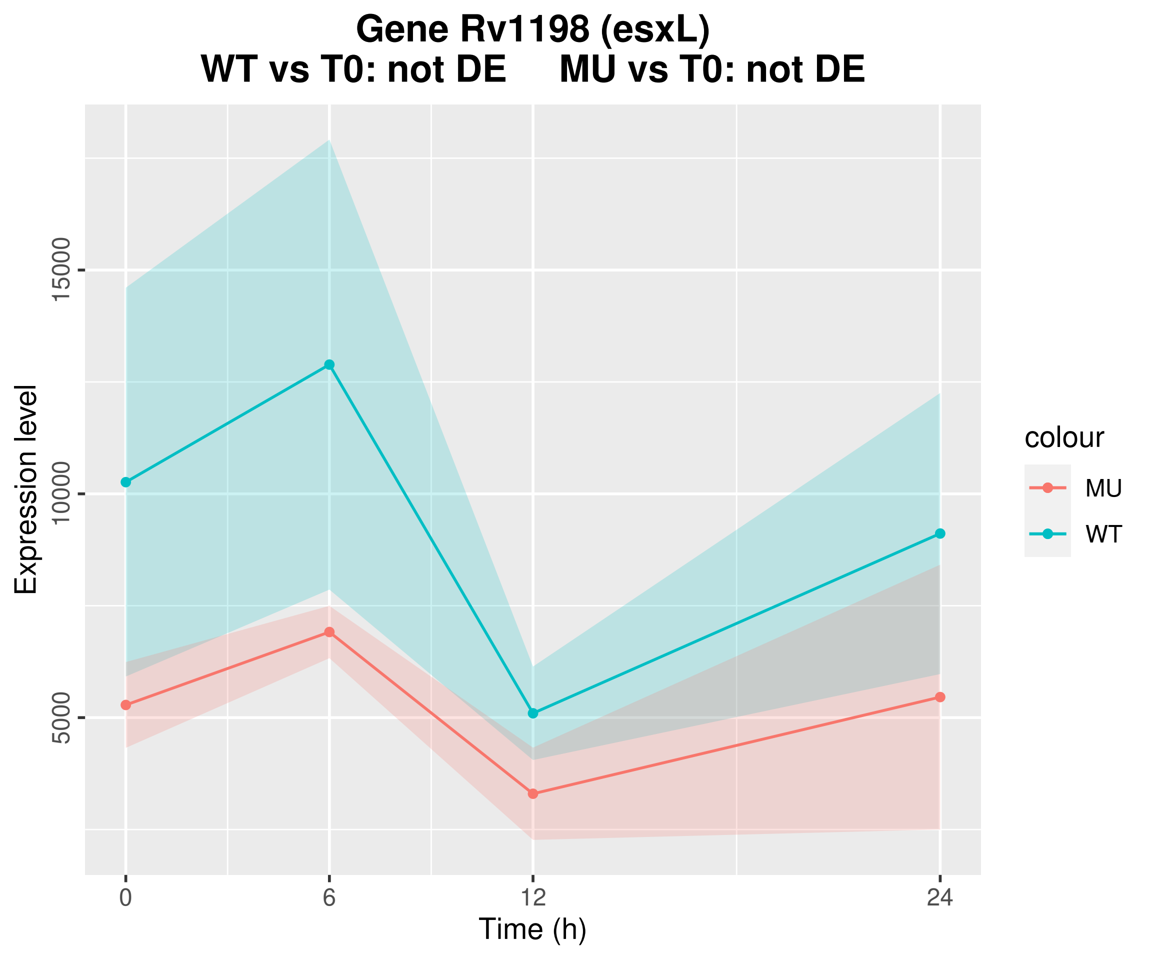
Gene Rv1198 (esxL)
WT vs T0: not DE   MU vs T0: not DE
Expression level
15000
10000
5000
0
6
12
24
Time (h)
colour
MU
WT

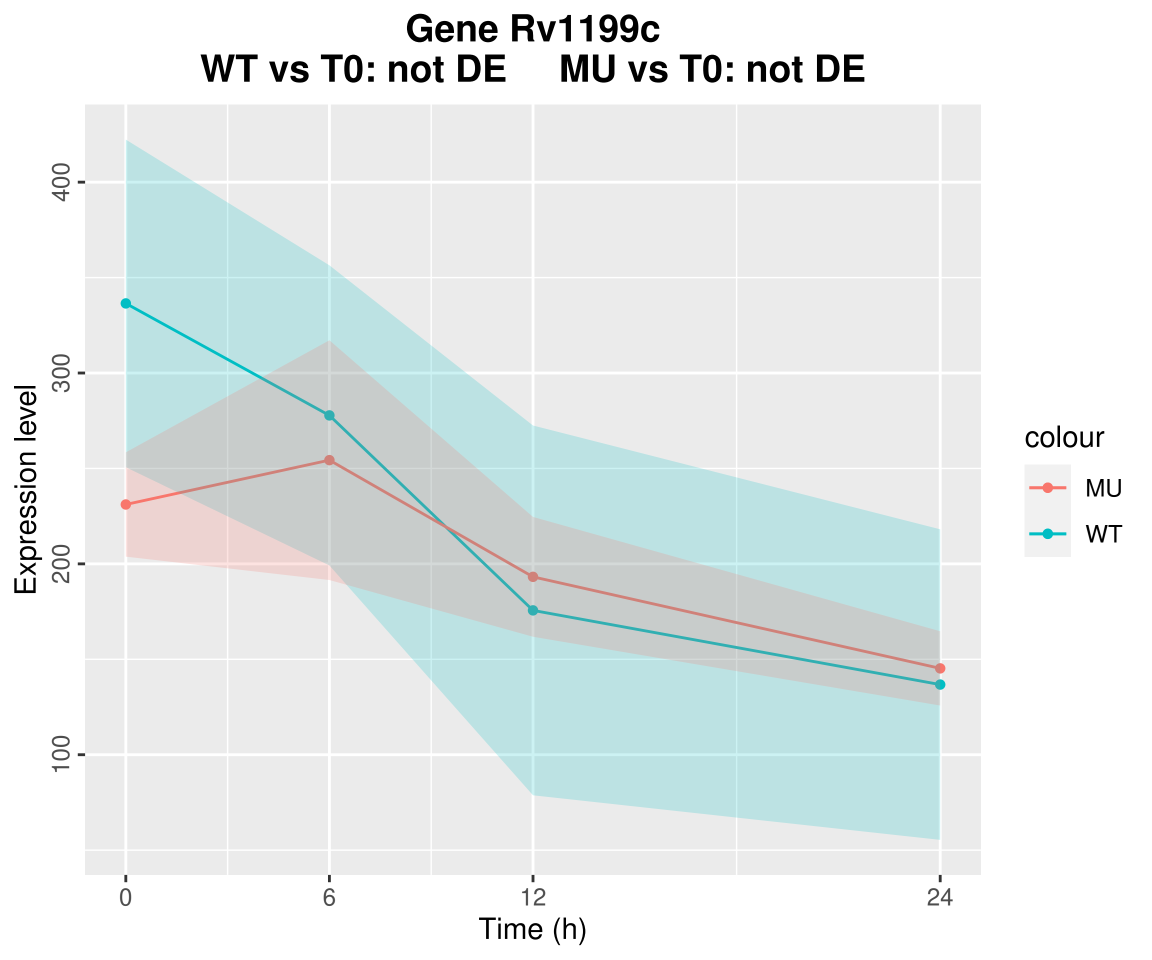

Gene Rv1199c
WT vs T0: not DE   MU vs T0: not DE
Expression level
400
300
200
100
0
6
12
24
Time (h)
colour
MU
WT

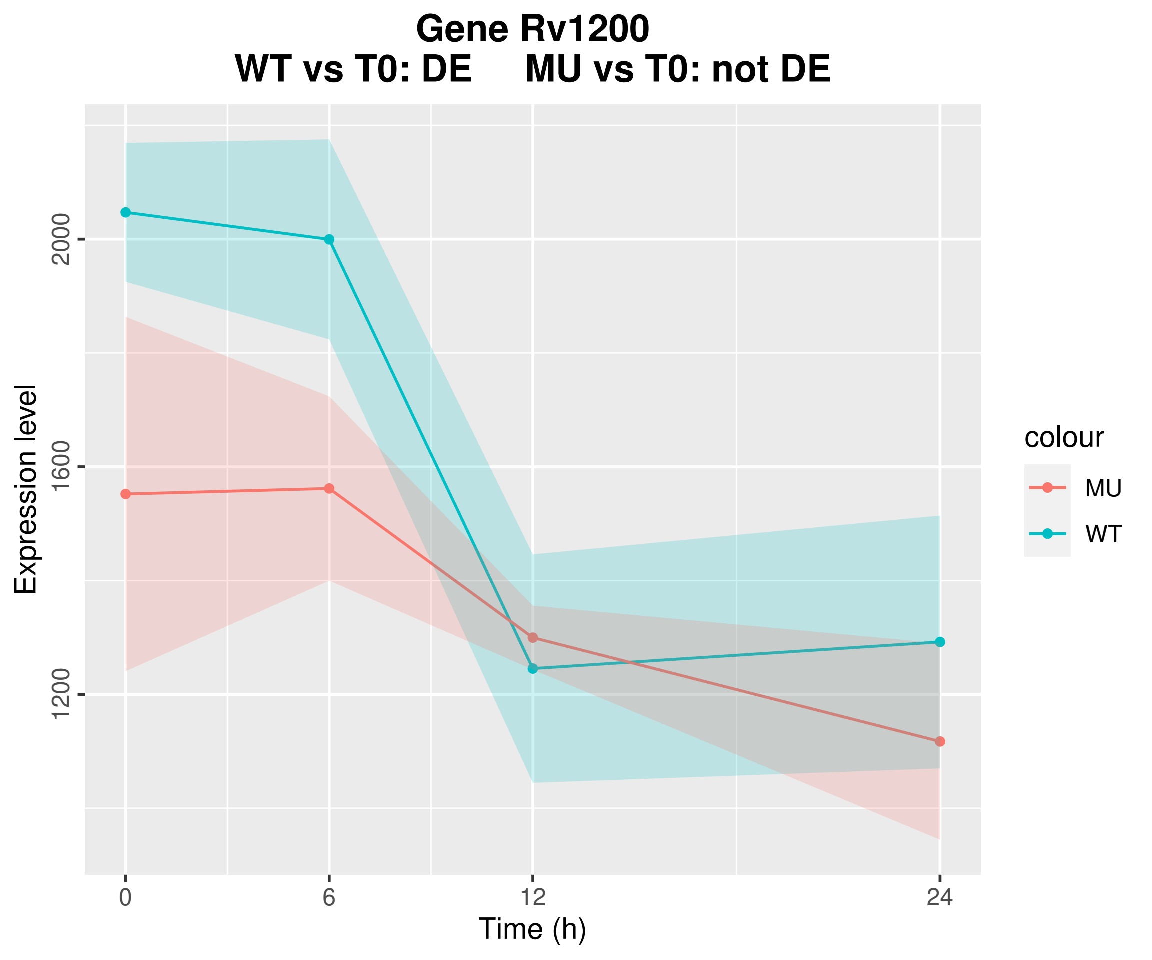
Gene Rv1200
WT vs T0: DE   MU vs T0: not DE
Expression level
2000
1600
1200
0
6
12
24
Time (h)
colour
MU
WT

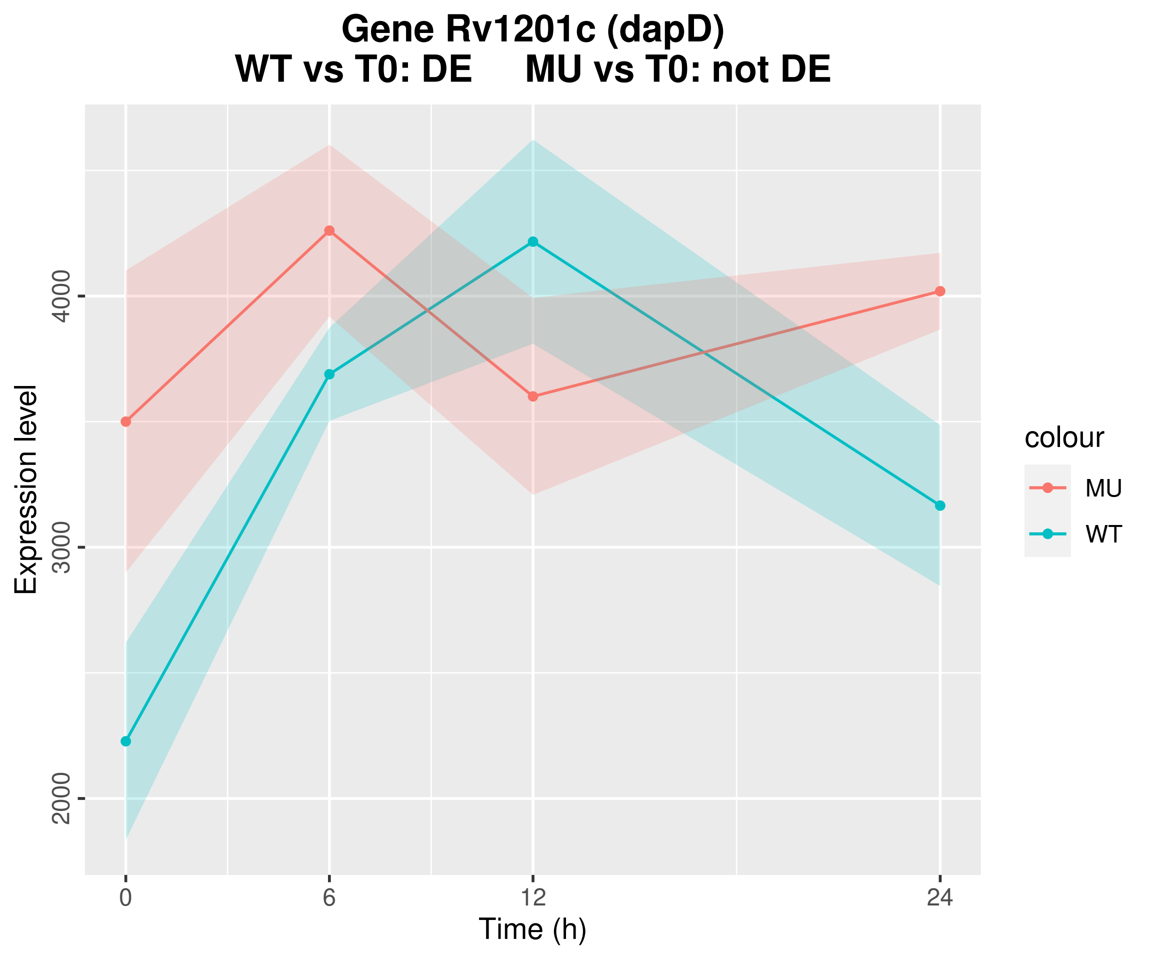

Gene Rv1201c (dapD)
WT vs T0: DE   MU vs T0: not DE
Expression level
2000
3000
4000
0
6
12
24
Time (h)
colour
MU
WT

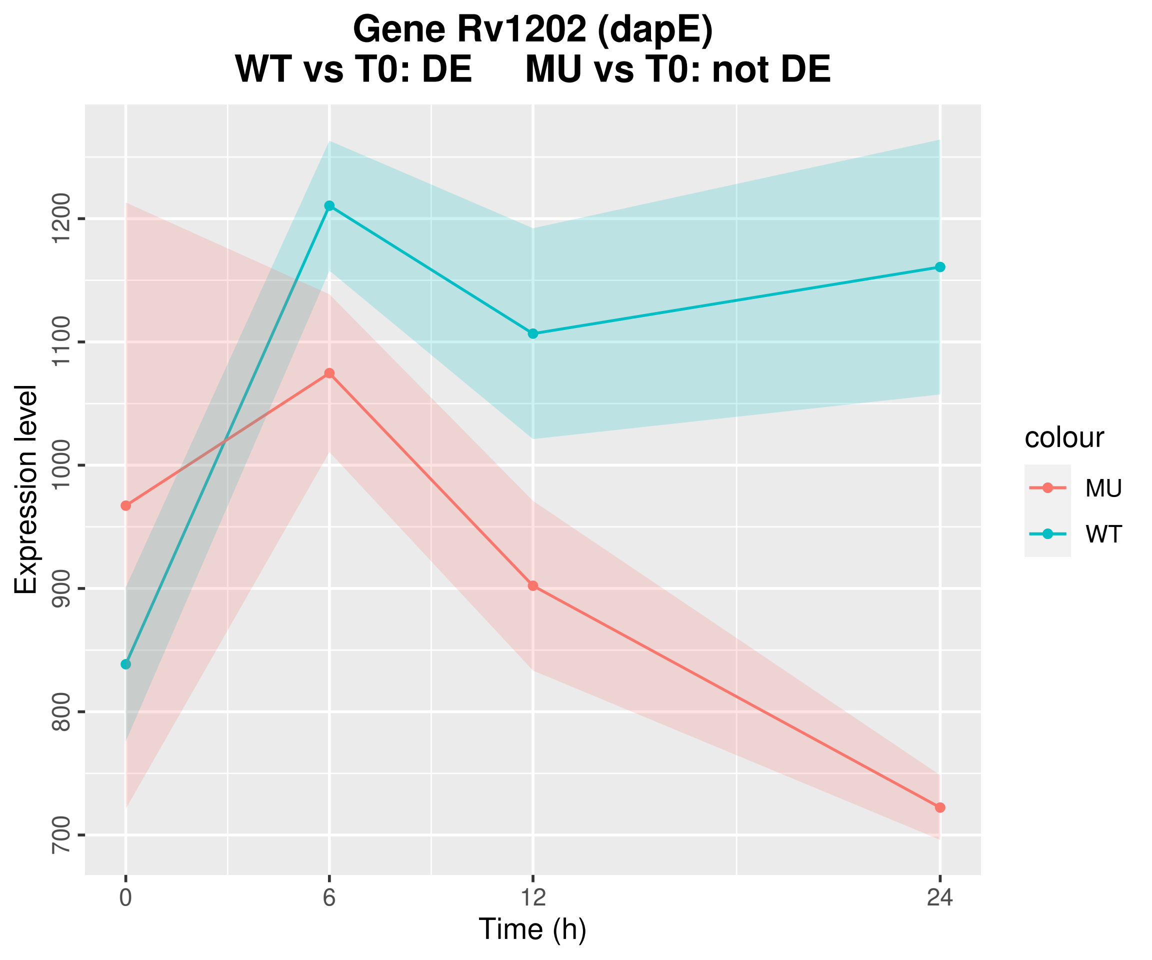
Gene Rv1202 (dapE)
WT vs T0: DE   MU vs T0: not DE
Expression level
700
800
900
1000
1100
1200
0
6
12
24
Time (h)
colour
MU
WT

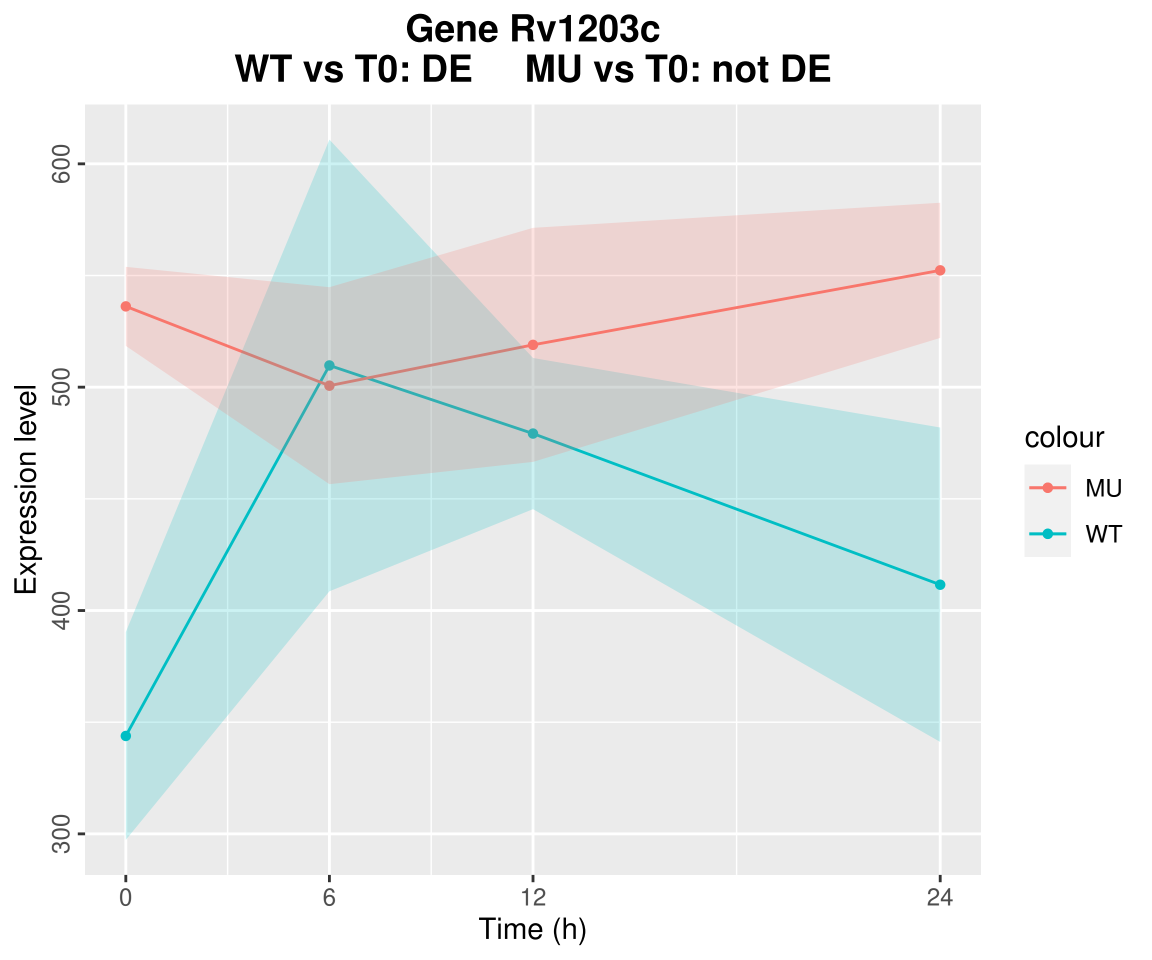

Gene Rv1203c
WT vs T0: DE MU vs T0: not DE
Expression level
600
500
400
300
0
6
12
24
Time (h)
colour
MU
WT

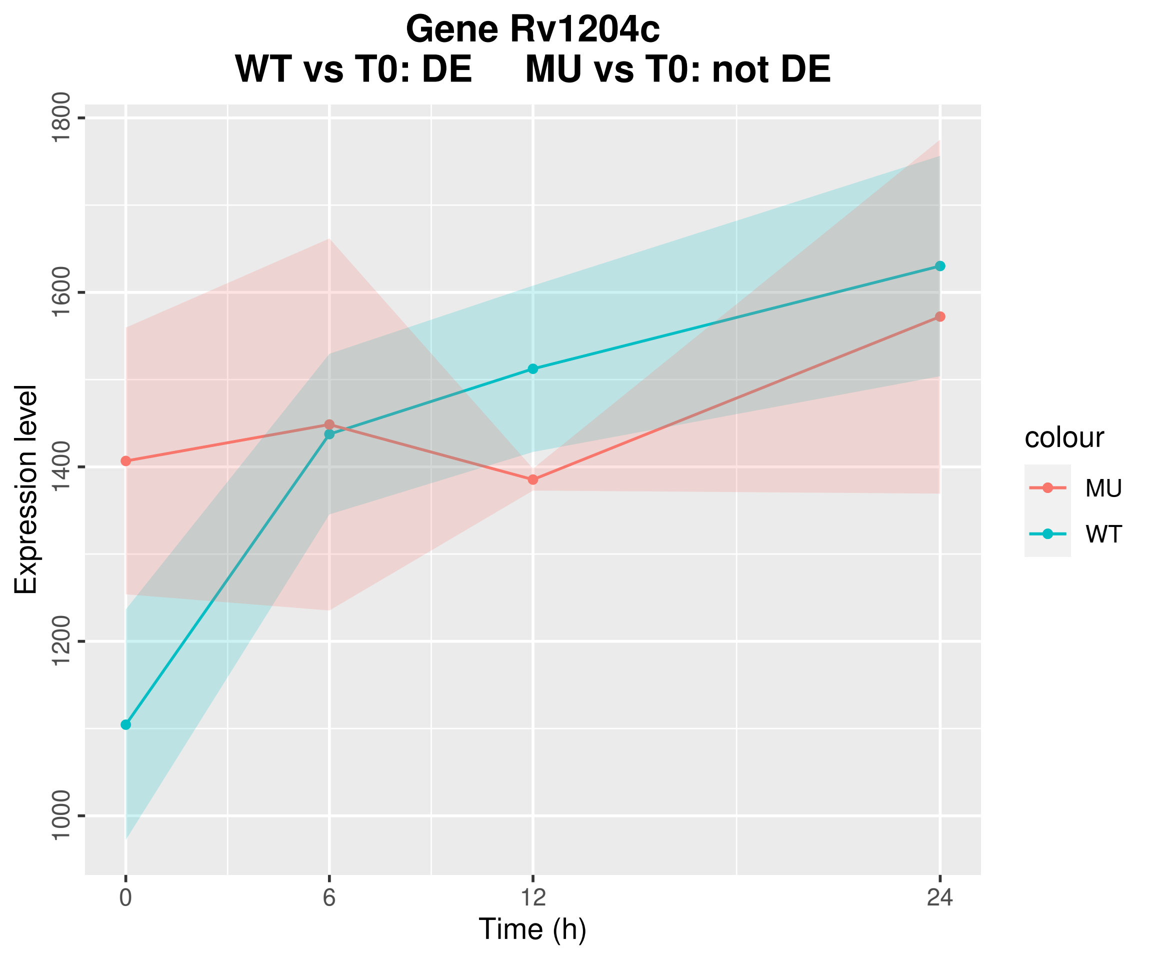

Gene Rv1204c
WT vs T0: DE   MU vs T0: not DE
Expression level
1000
1200
1400
1600
1800
0
6
12
24
Time (h)
colour
MU
WT

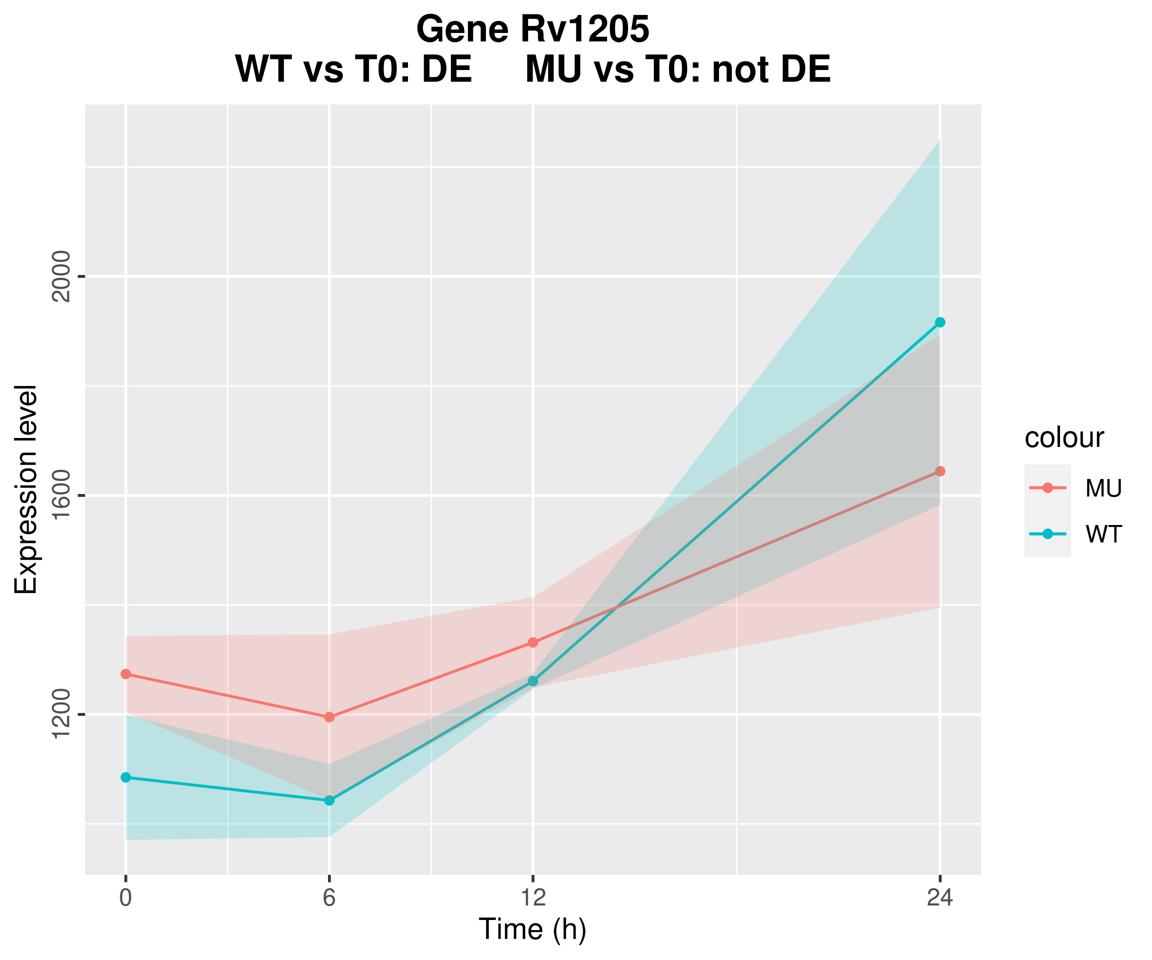

Gene Rv1205
WT vs T0: DE MU vs T0: not DE
Expression level
2000
1600
1200
0
6
12
24
Time (h)
colour
MU
WT

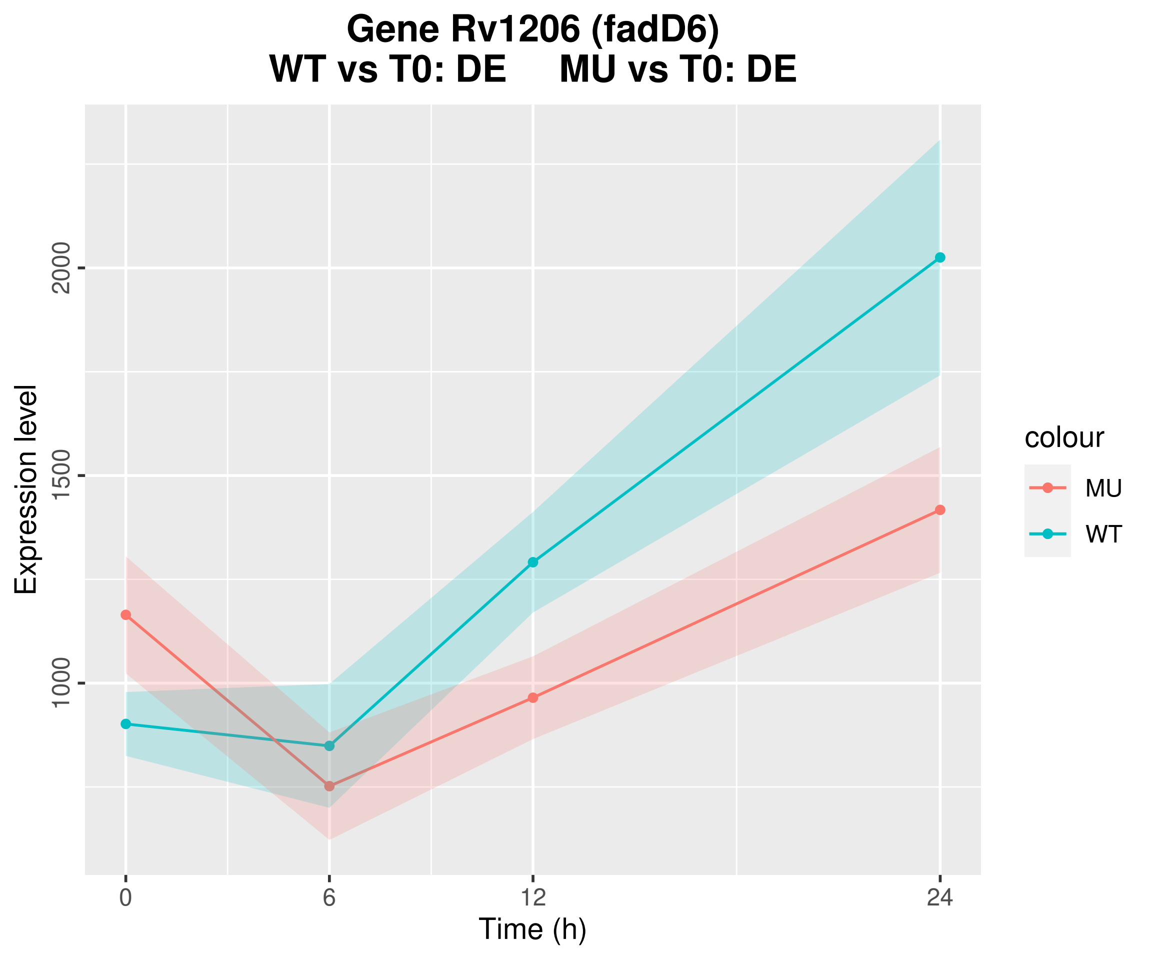
Gene Rv1206 (fadD6)
WT vs T0: DE   MU vs T0: DE
Expression level
2000
1500
1000
0
6
12
24
Time (h)
colour
MU
WT

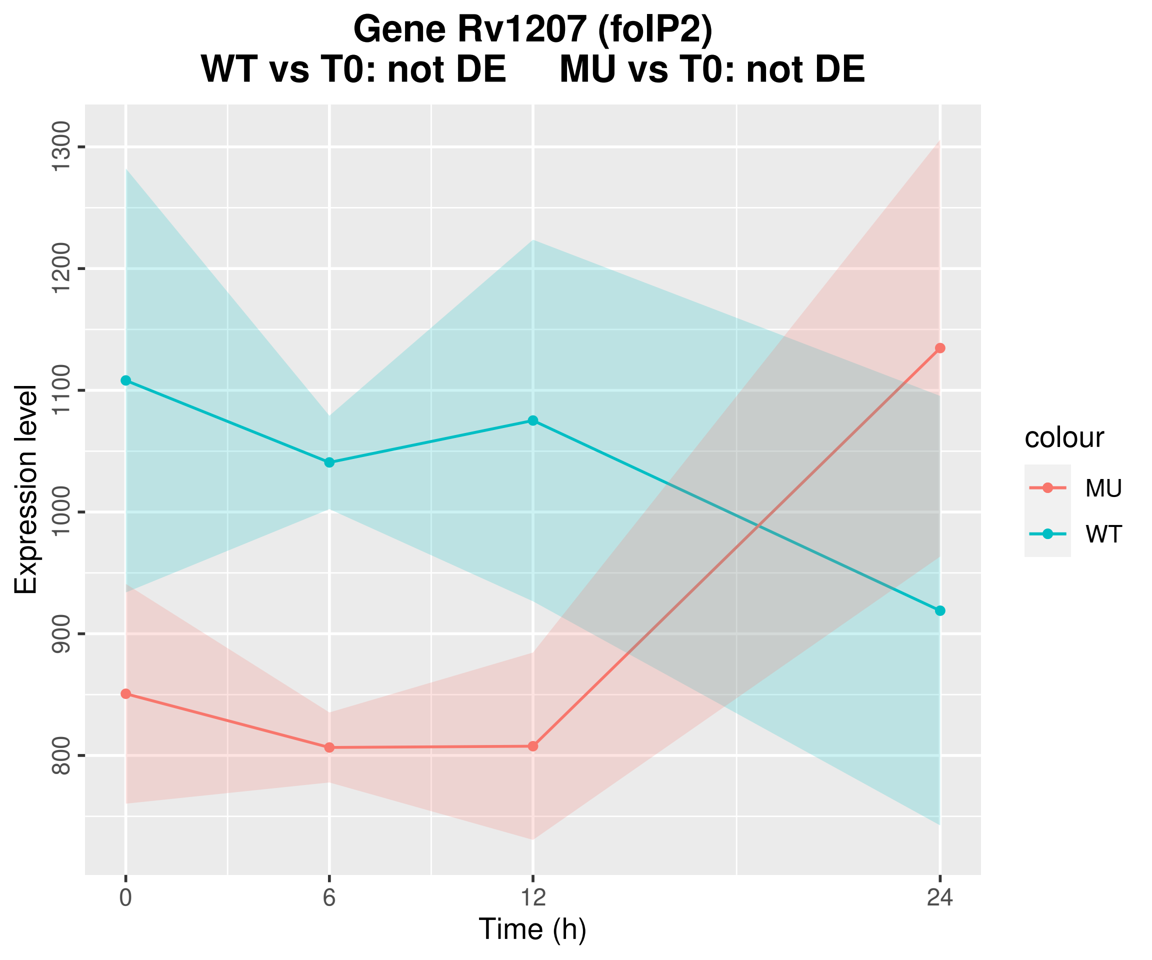
Gene Rv1207 (folP2)
WT vs T0: not DE   MU vs T0: not DE
Expression level
1300
1200
1100
1000
900
800
0
6
12
24
Time (h)
colour
MU
WT

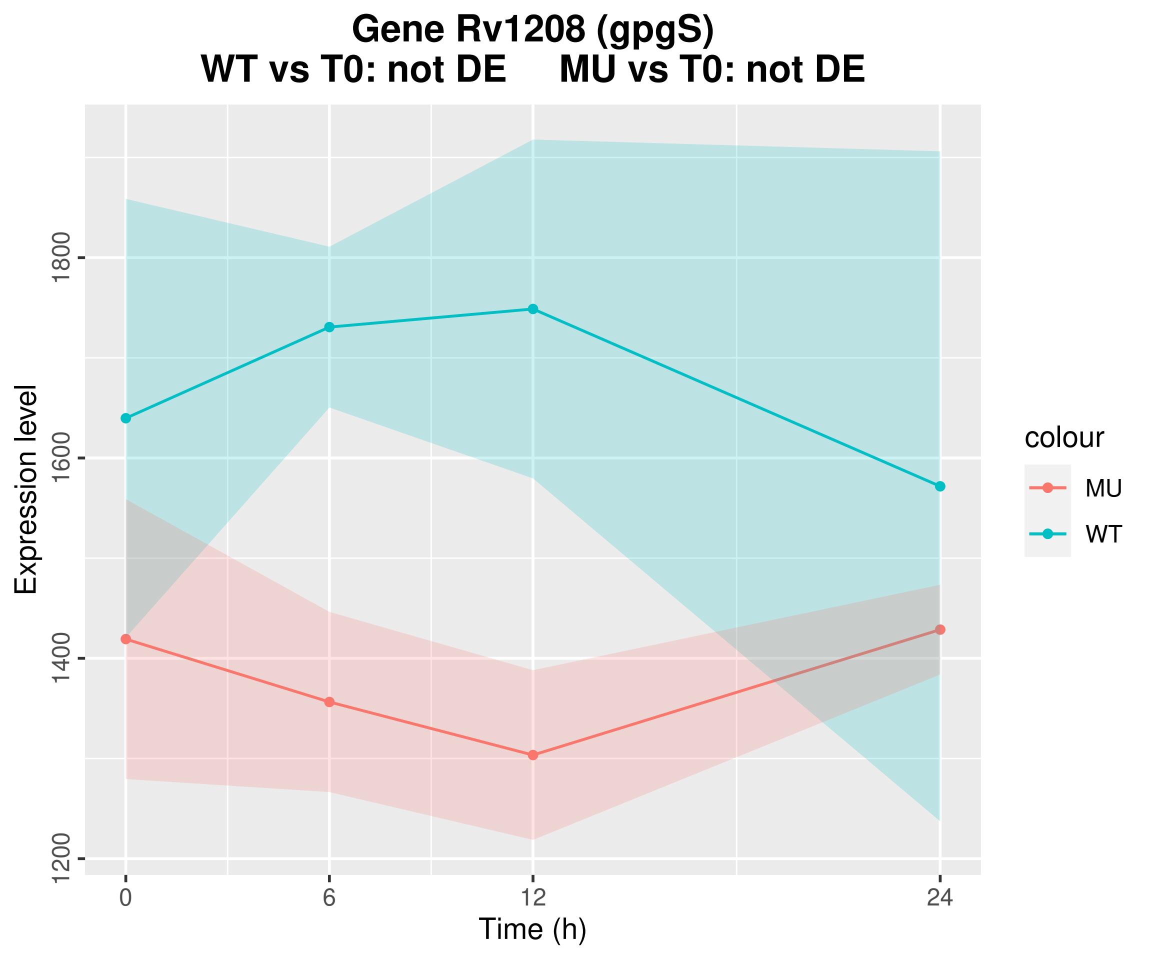
Gene Rv1208 (gpgS)
WT vs T0: not DE   MU vs T0: not DE
Expression level
1800
1600
1400
1200
0
6
12
24
Time (h)
colour
MU
WT

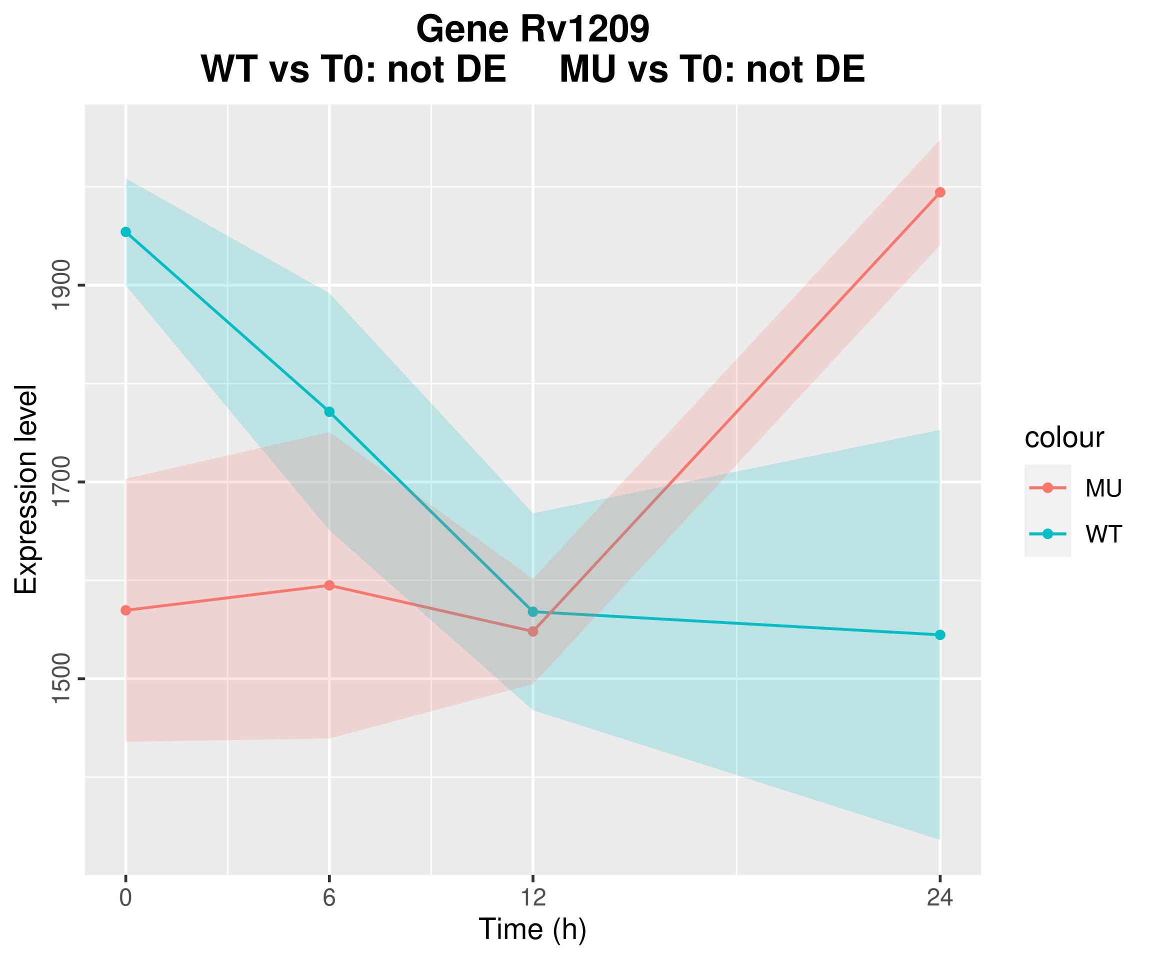

Gene Rv1209
WT vs T0: not DE MU vs T0: not DE
Expression level
1500
1700
1900
Time (h)
0
6
12
24
colour
MU
WT

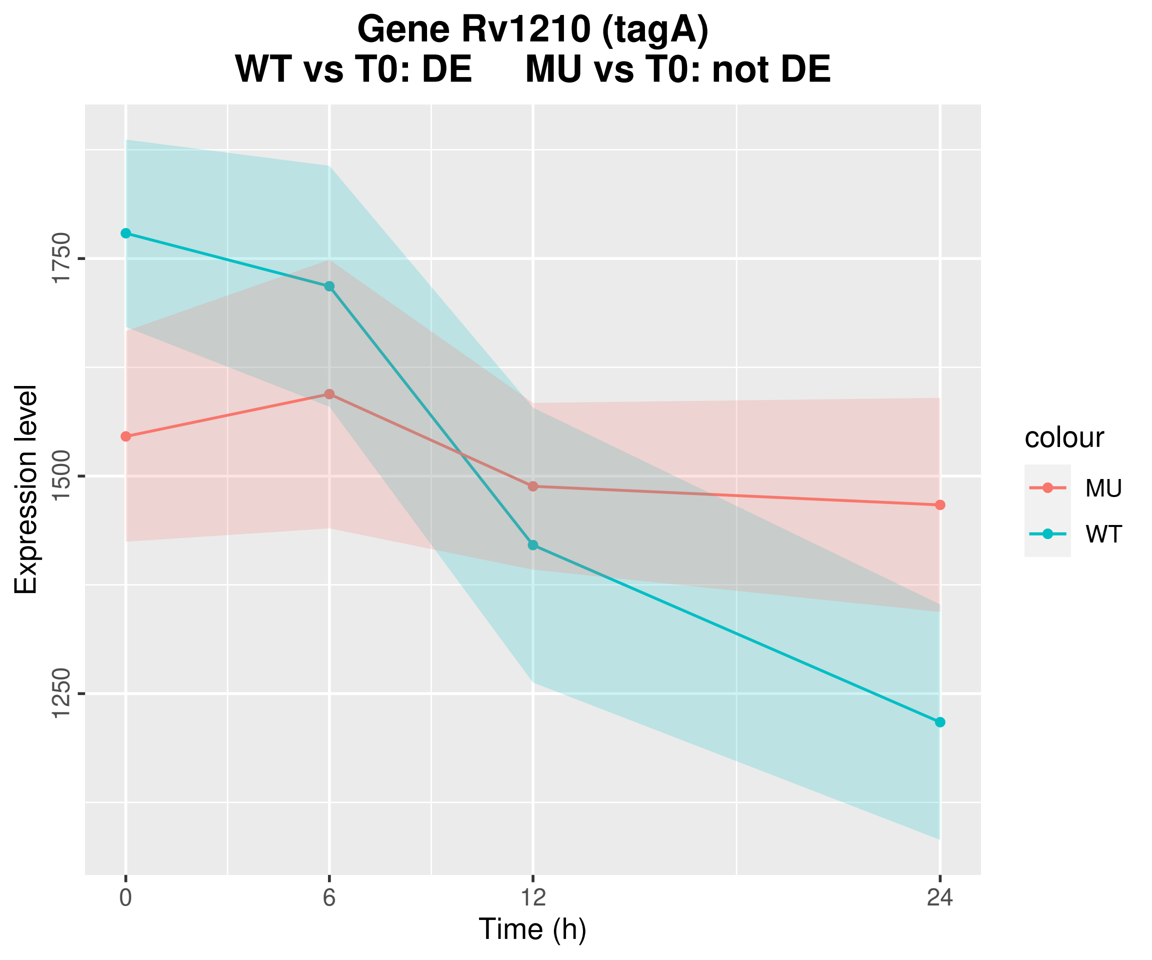

Gene Rv1210 (tagA)
WT vs T0: DE MU vs T0: not DE
Expression level
1750
1500
1250
0
6
12
24
Time (h)
colour
MU
WT

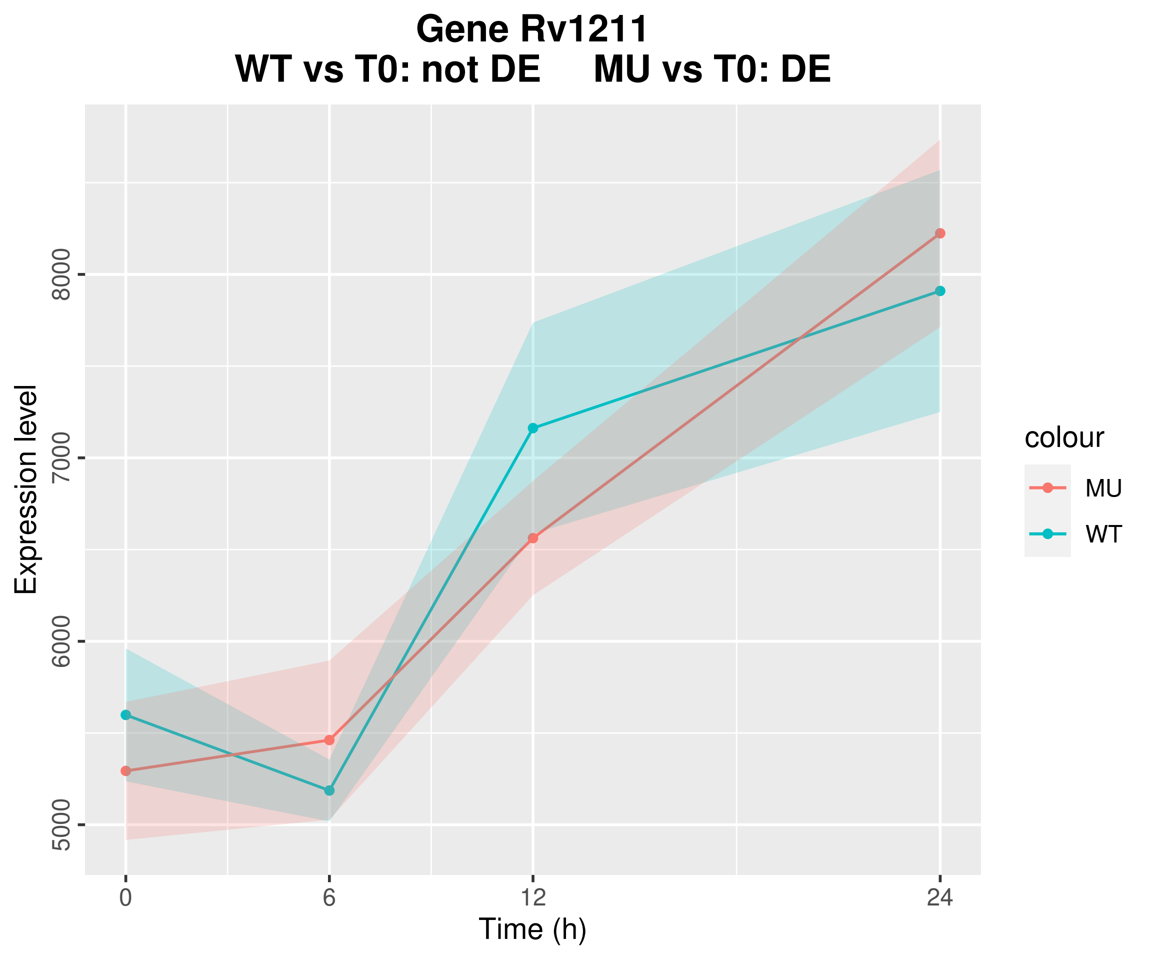
Gene Rv1211
WT vs T0: not DE   MU vs T0: DE
Expression level
8000
7000
6000
5000
0
6
12
24
Time (h)
colour
MU
WT

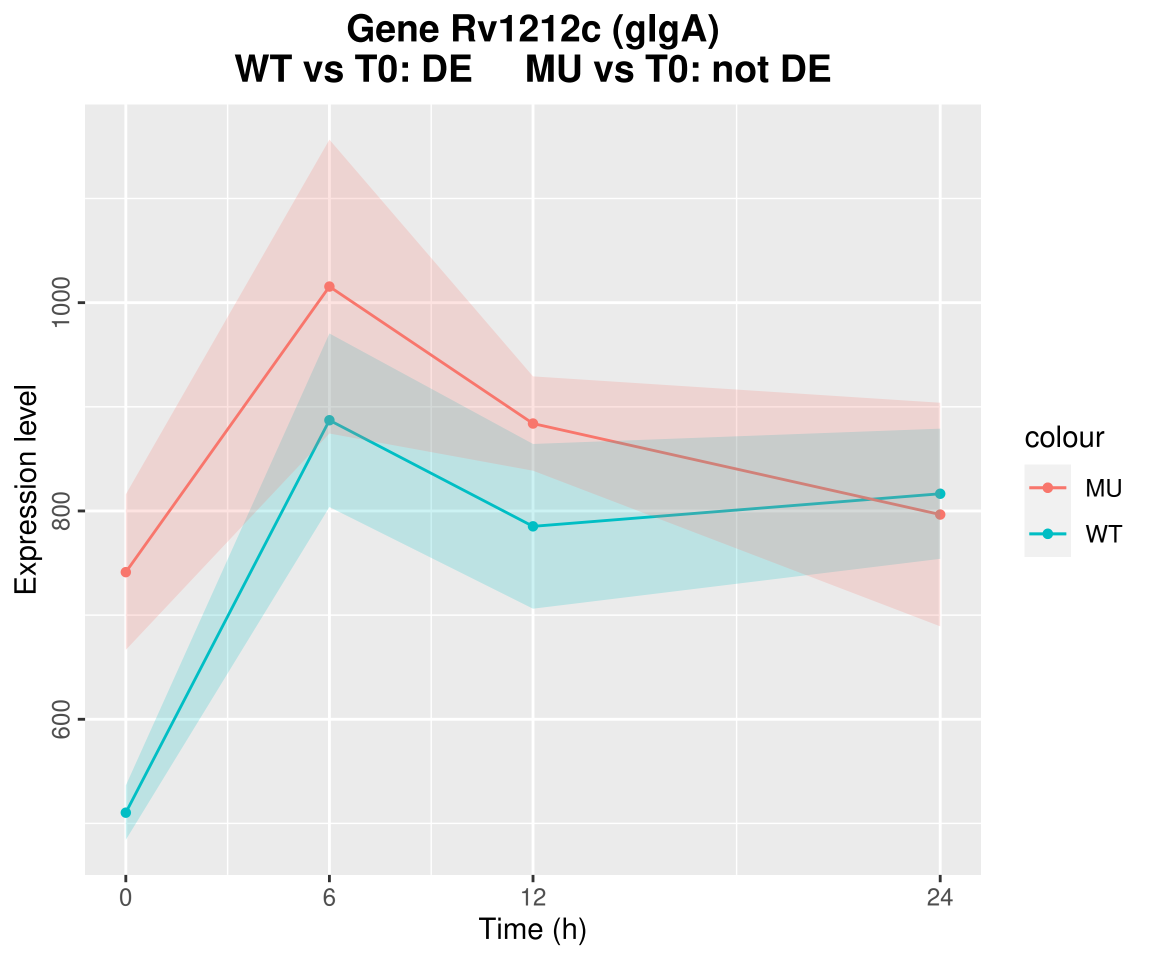
Gene Rv1212c (glgA)
WT vs T0: DE   MU vs T0: not DE
Expression level
1000
800
600
0
6
12
24
Time (h)
colour
MU
WT

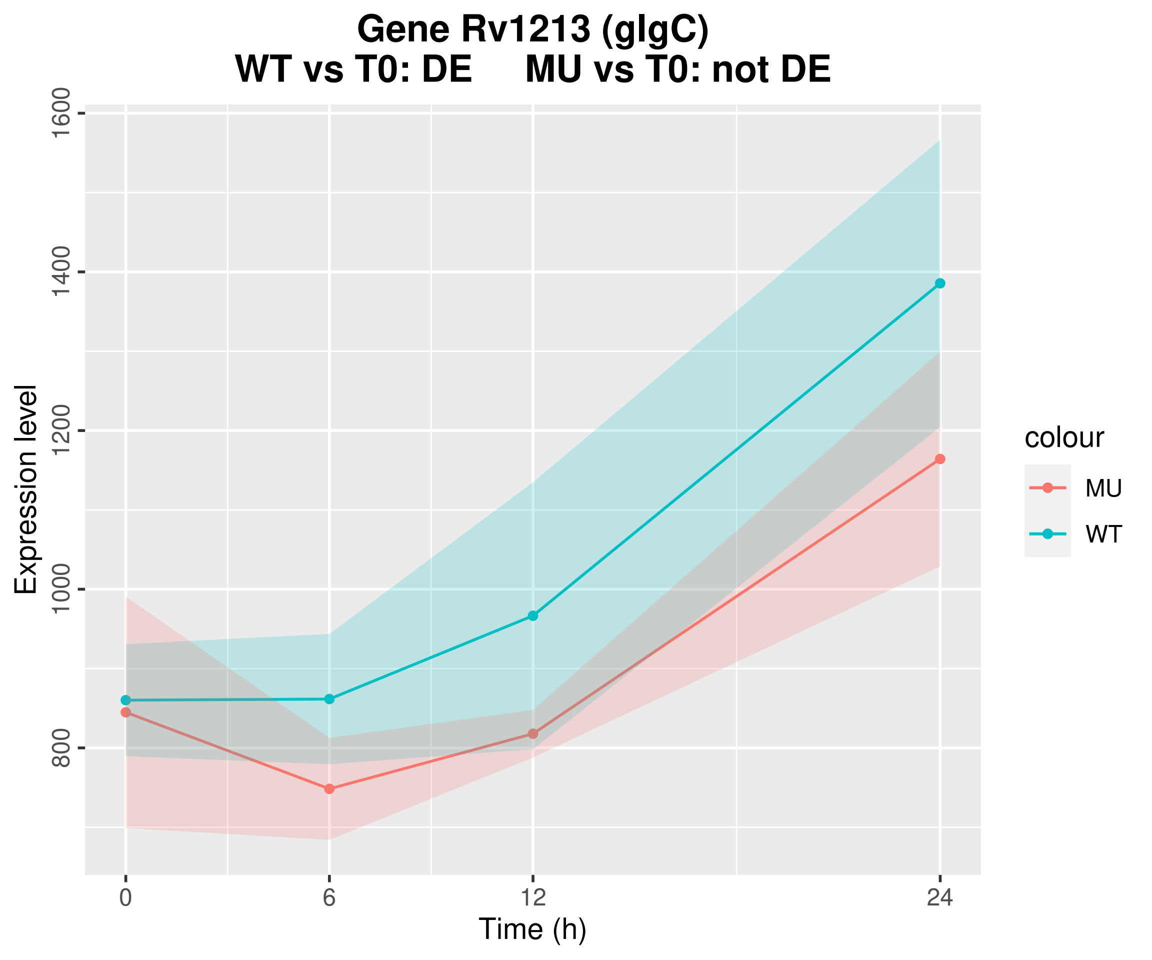

Gene Rv1213 (glgC)
WT vs T0: DE MU vs T0: not DE
Expression level
1600
1400
1200
1000
800
0
6
12
24
Time (h)
colour
MU
WT

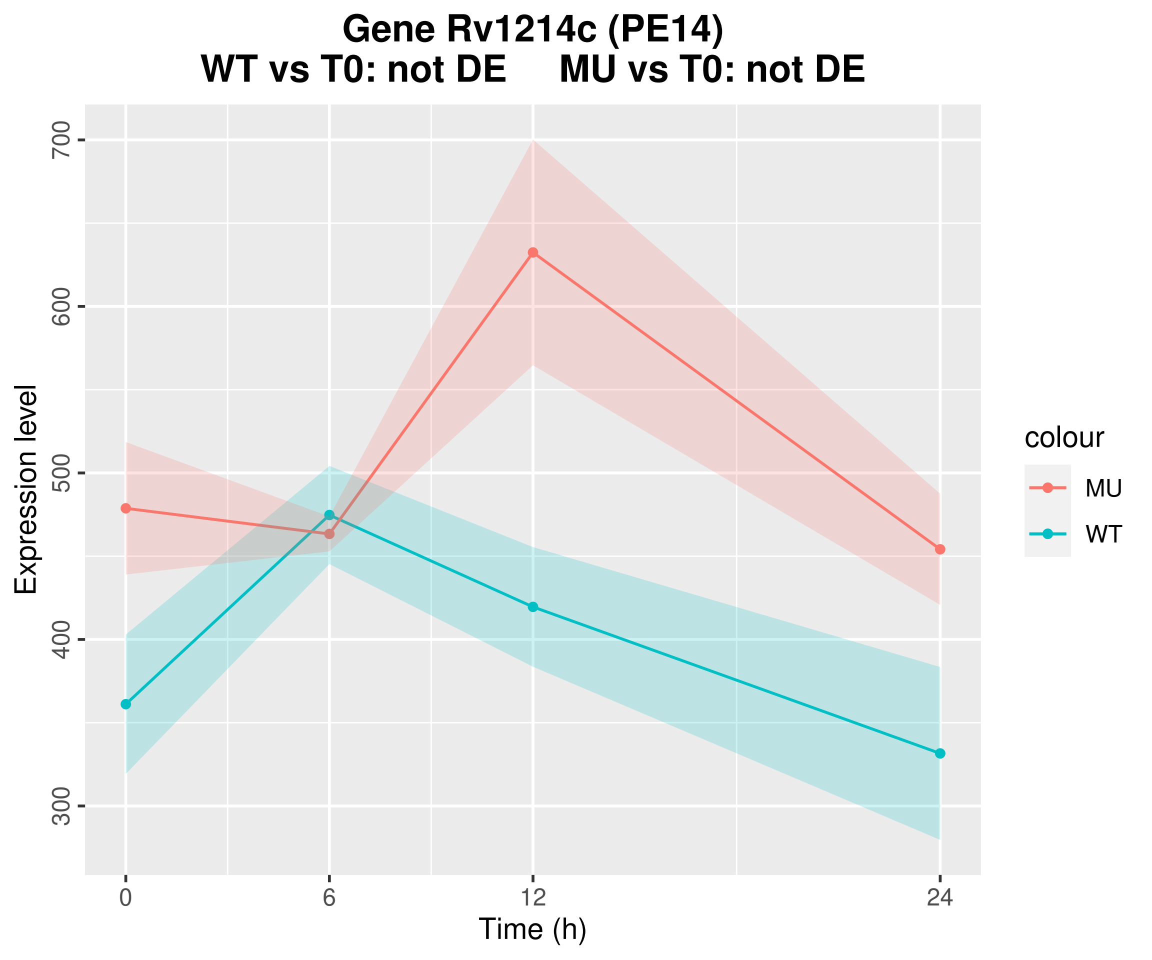

Gene Rv1214c (PE14)
WT vs T0: not DE   MU vs T0: not DE
Expression level
700
600
500
400
300
0
6
12
24
Time (h)
colour
MU
WT

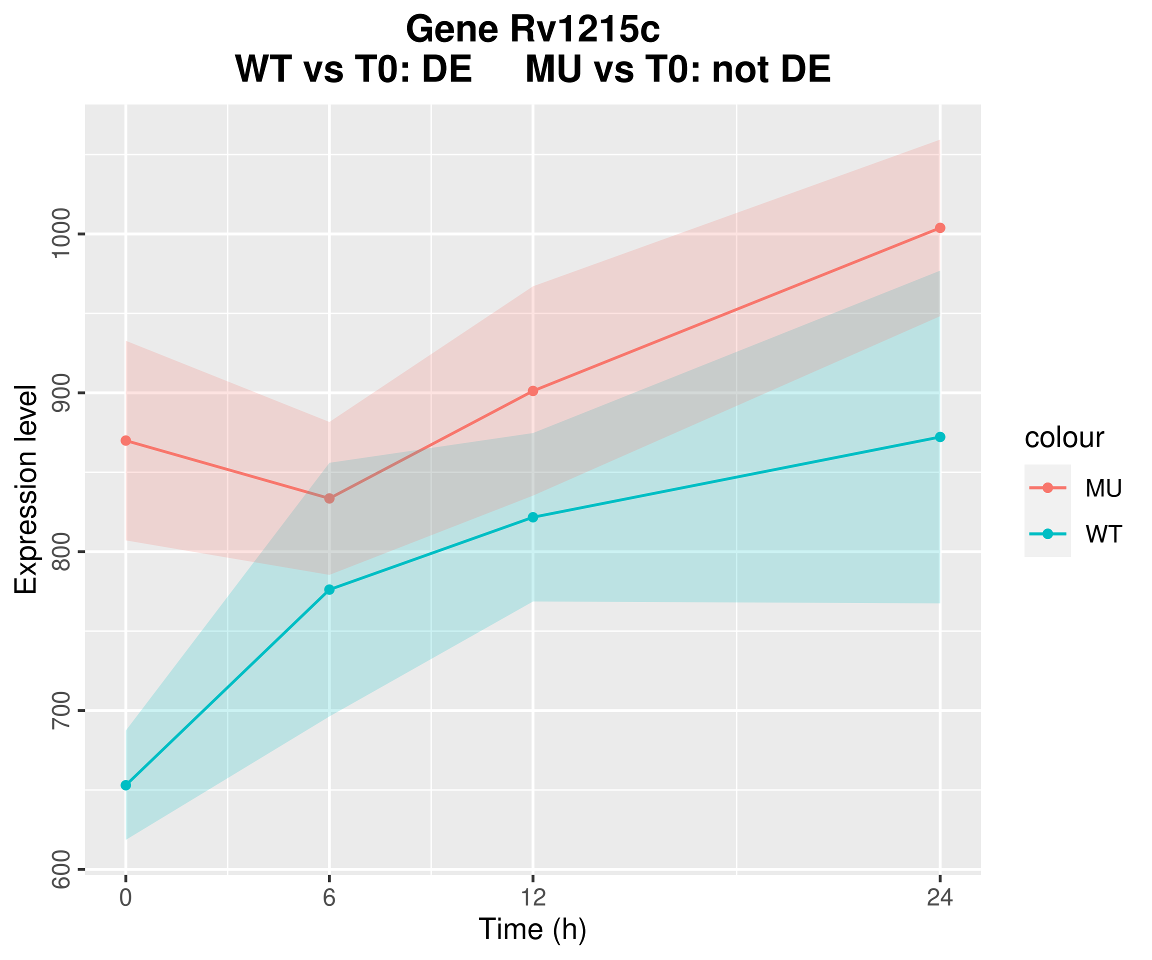
Gene Rv1215c
WT vs T0: DE   MU vs T0: not DE
Expression level
1000
900
800
700
600
0
6
12
24
Time (h)
colour
MU
WT

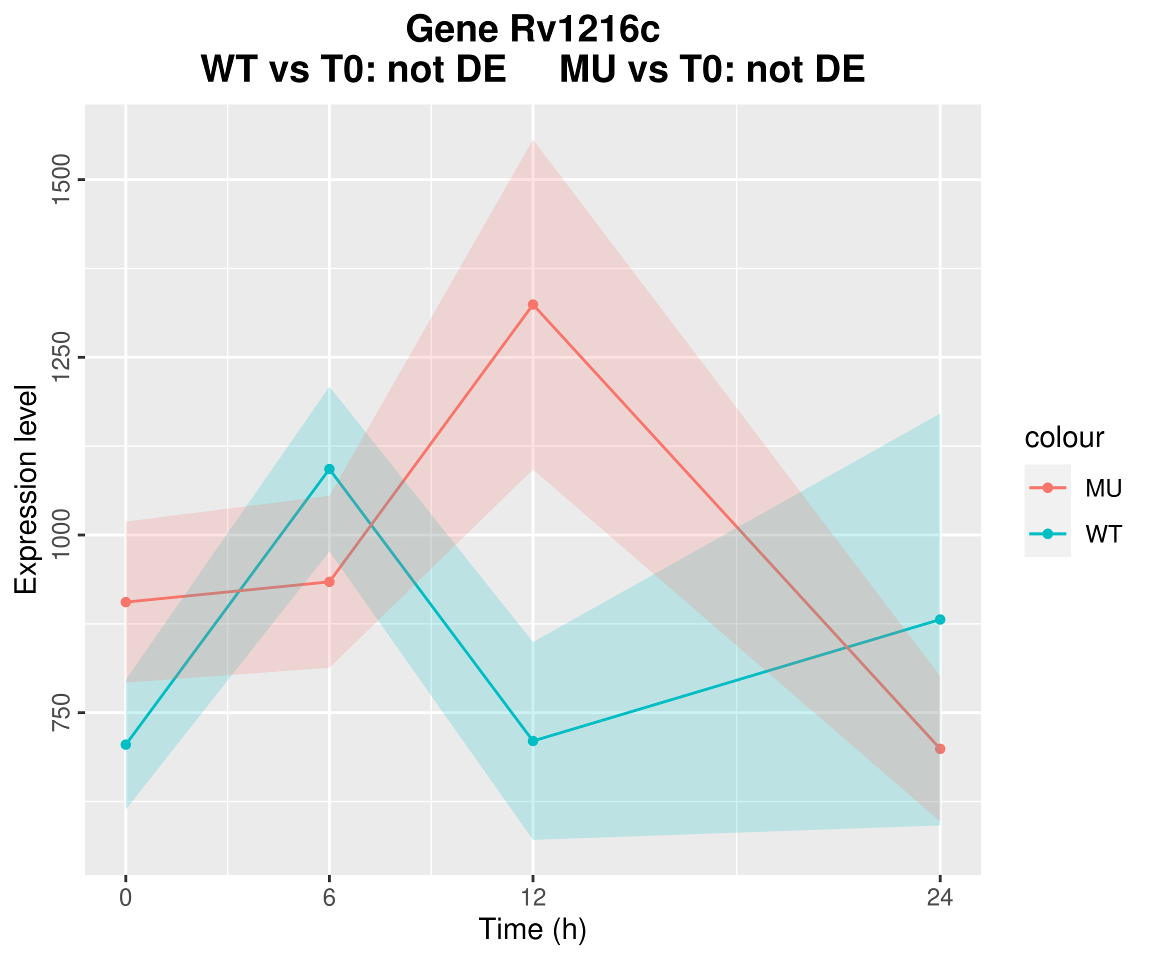

Gene Rv1216c
WT vs T0: not DE   MU vs T0: not DE
1500
1250
1000
750
Expression level
0
6
12
24
Time (h)
colour
MU
WT

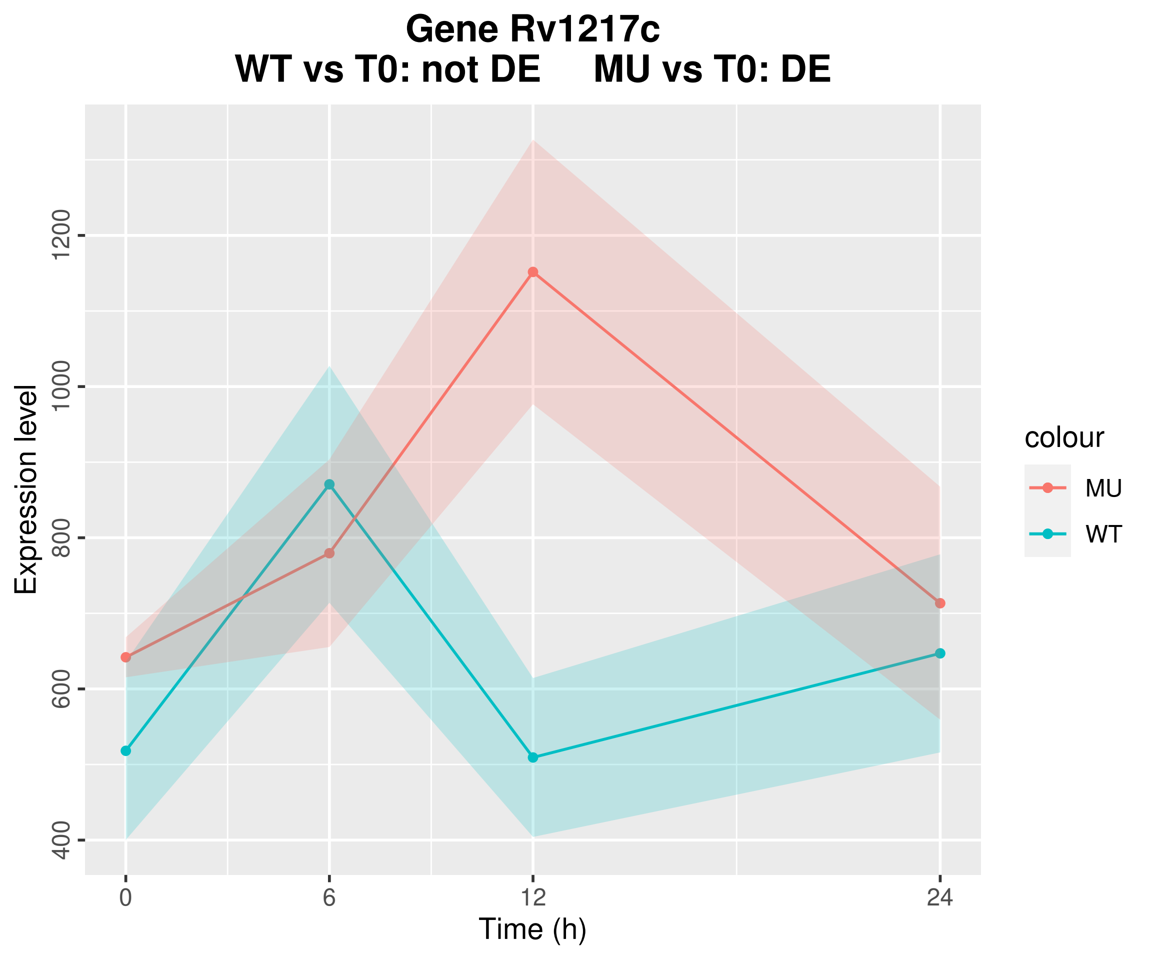
Gene Rv1217c
WT vs T0: not DE MU vs T0: DE
Expression level
1200
1000
800
600
400
0
6
12
24
Time (h)
colour
MU
WT

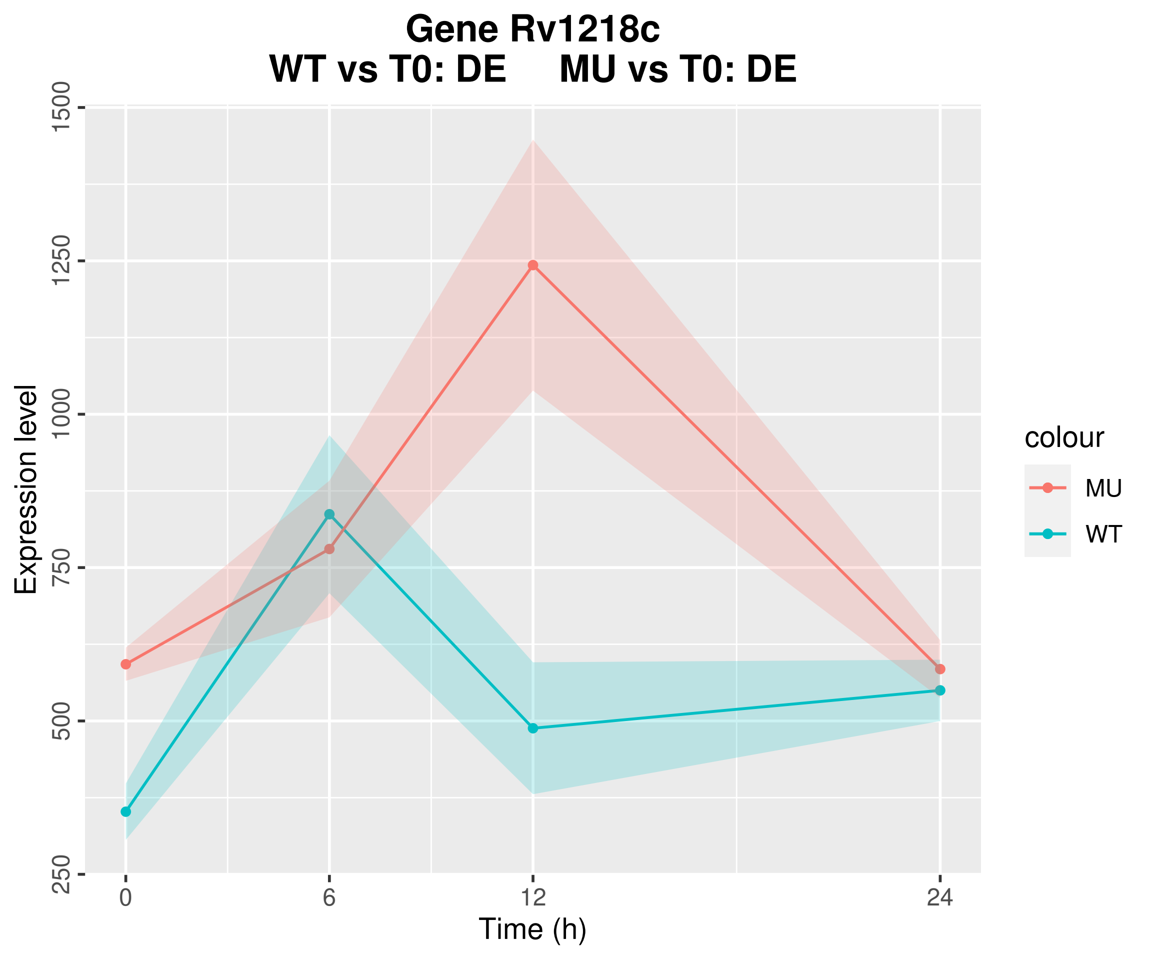
Gene Rv1218c
WT vs T0: DE MU vs T0: DE
Expression level
1500
1250
1000
750
500
250
0
6
12
24
Time (h)
colour
MU
WT

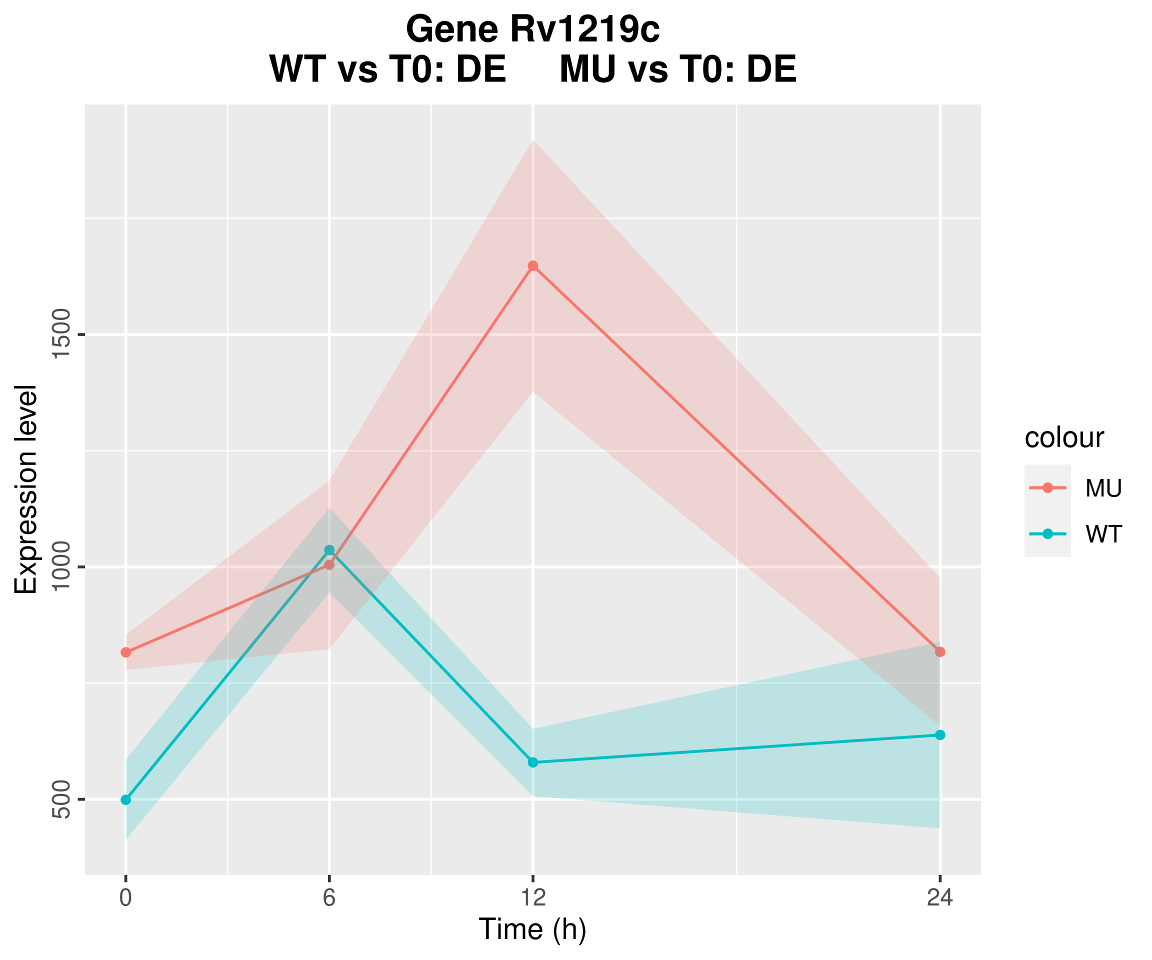
Gene Rv1219c
WT vs T0: DE   MU vs T0: DE
Expression level
500
1000
1500
0
6
12
24
Time (h)
colour
MU
WT

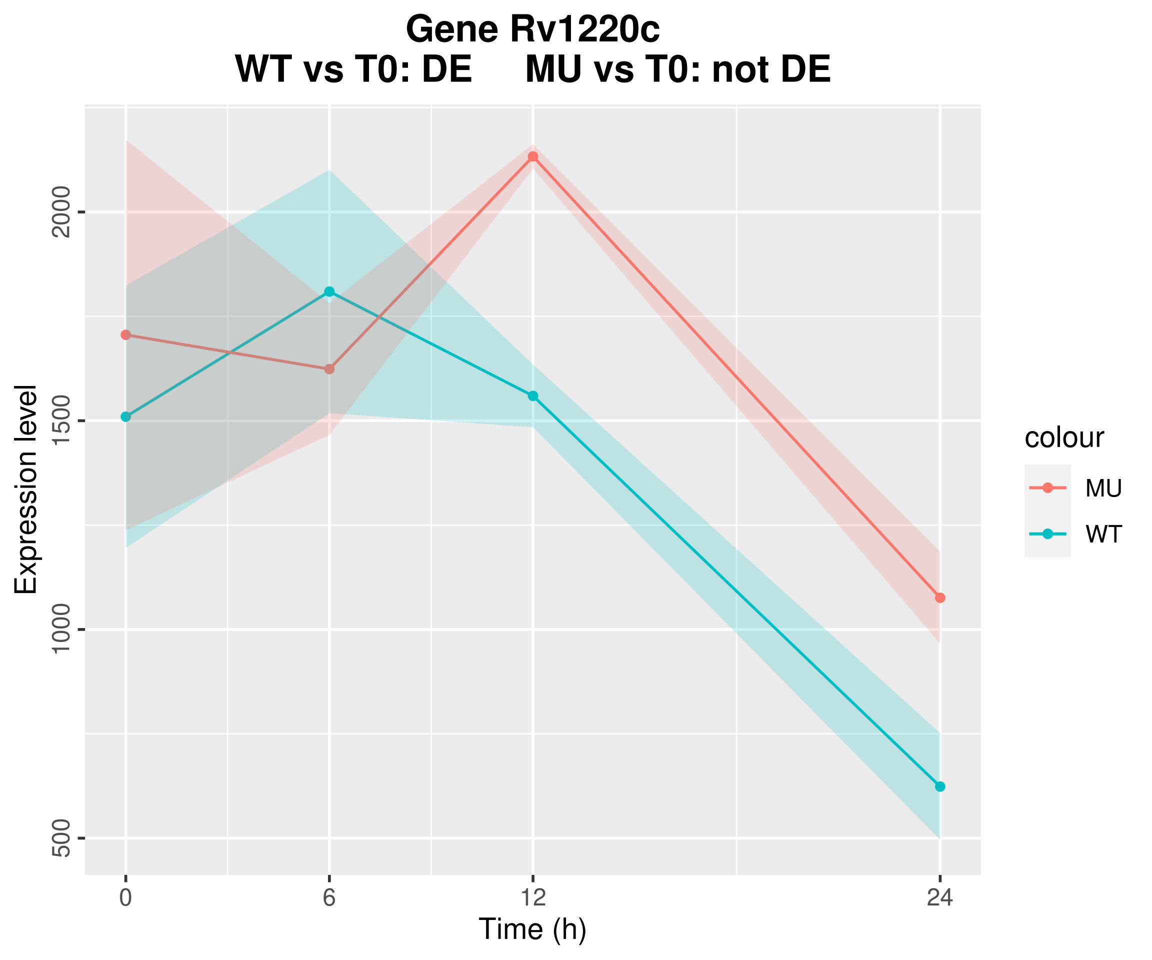
Gene Rv1220c
WT vs T0: DE MU vs T0: not DE
Expression level
500
1000
1500
2000
0
6
12
24
Time (h)
colour
MU
WT

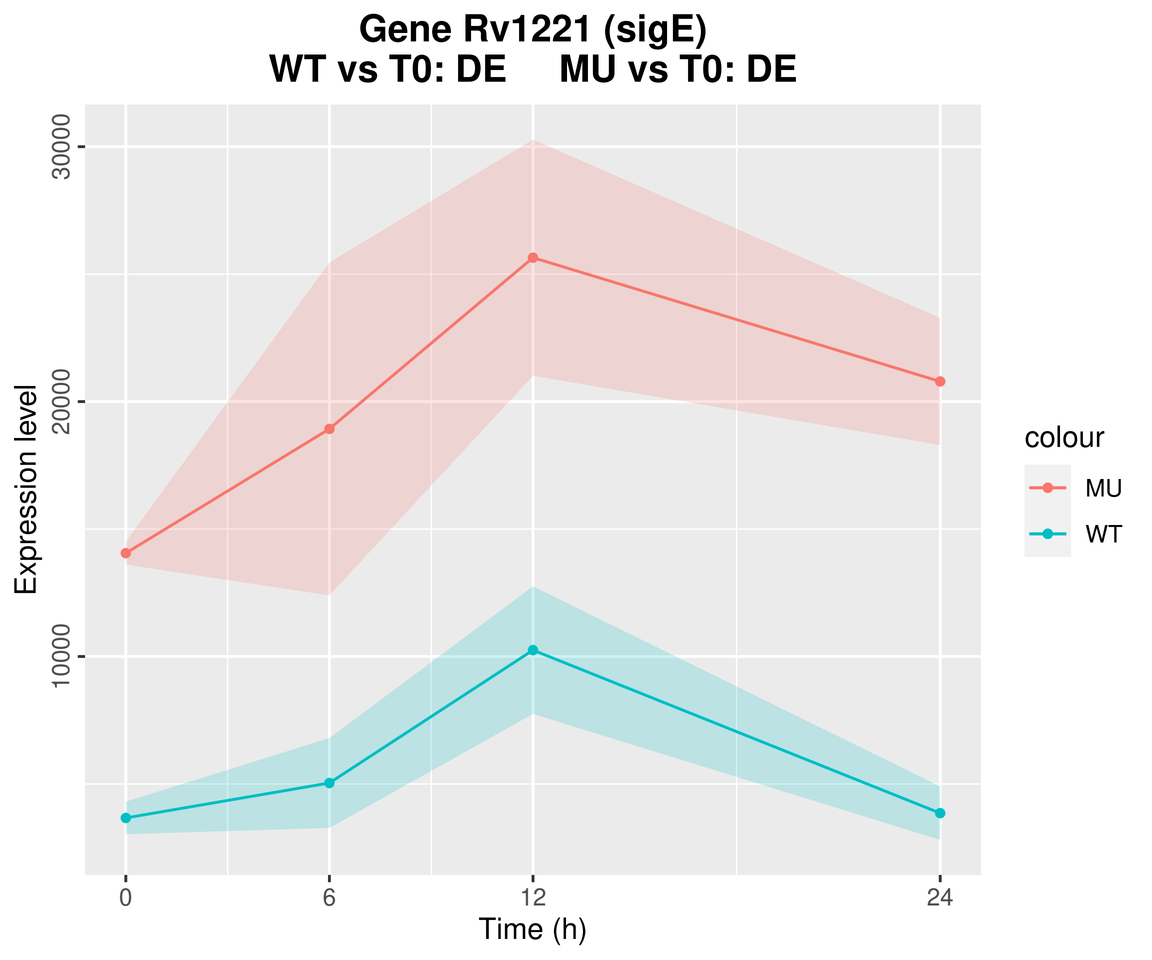

Gene Rv1221 (sigE)
WT vs T0: DE   MU vs T0: DE
30000
20000
10000
Expression level
0
6
12
24
Time (h)
colour
MU
WT

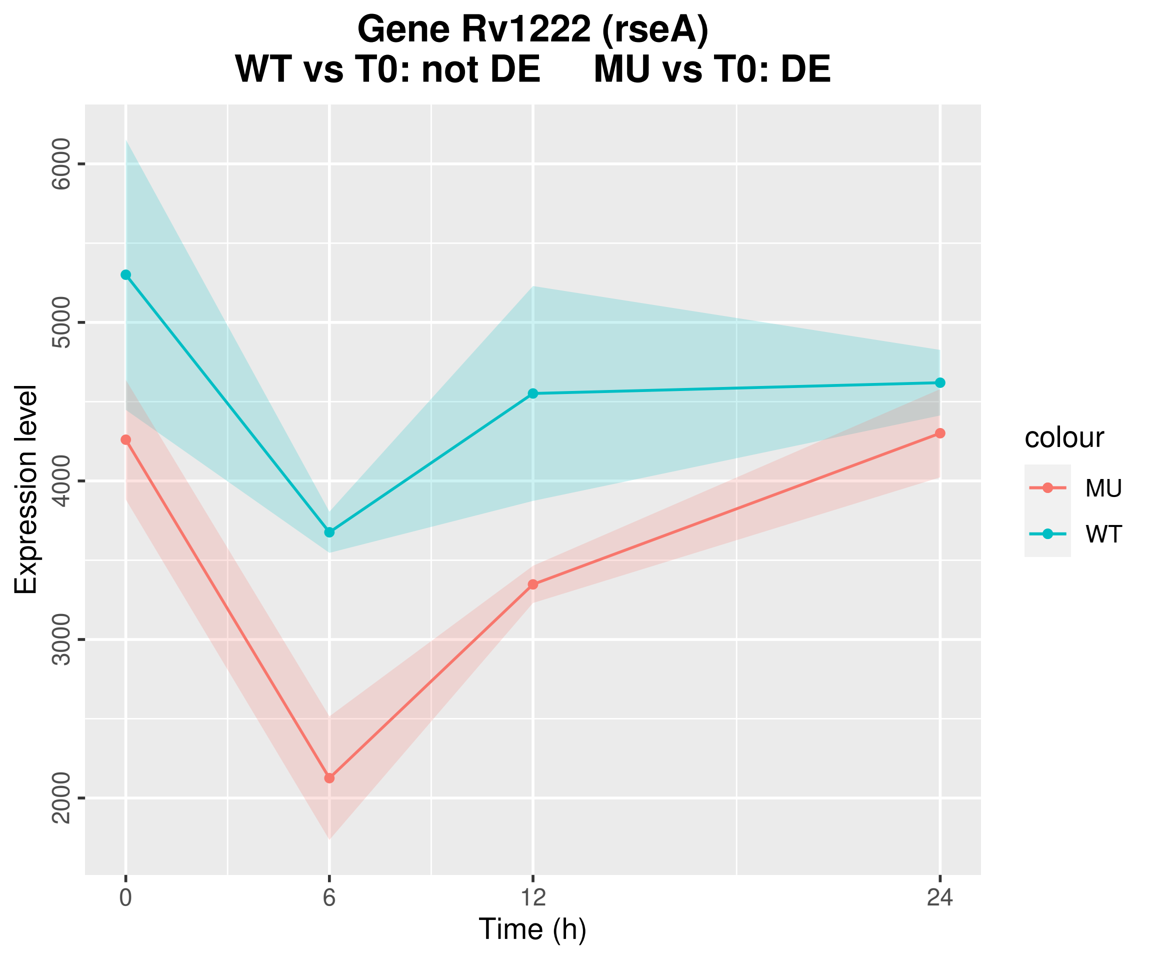
Gene Rv1222 (rseA)
WT vs T0: not DE   MU vs T0: DE
6000
5000
4000
3000
2000
Expression level
0
6
12
24
Time (h)
colour
MU
WT

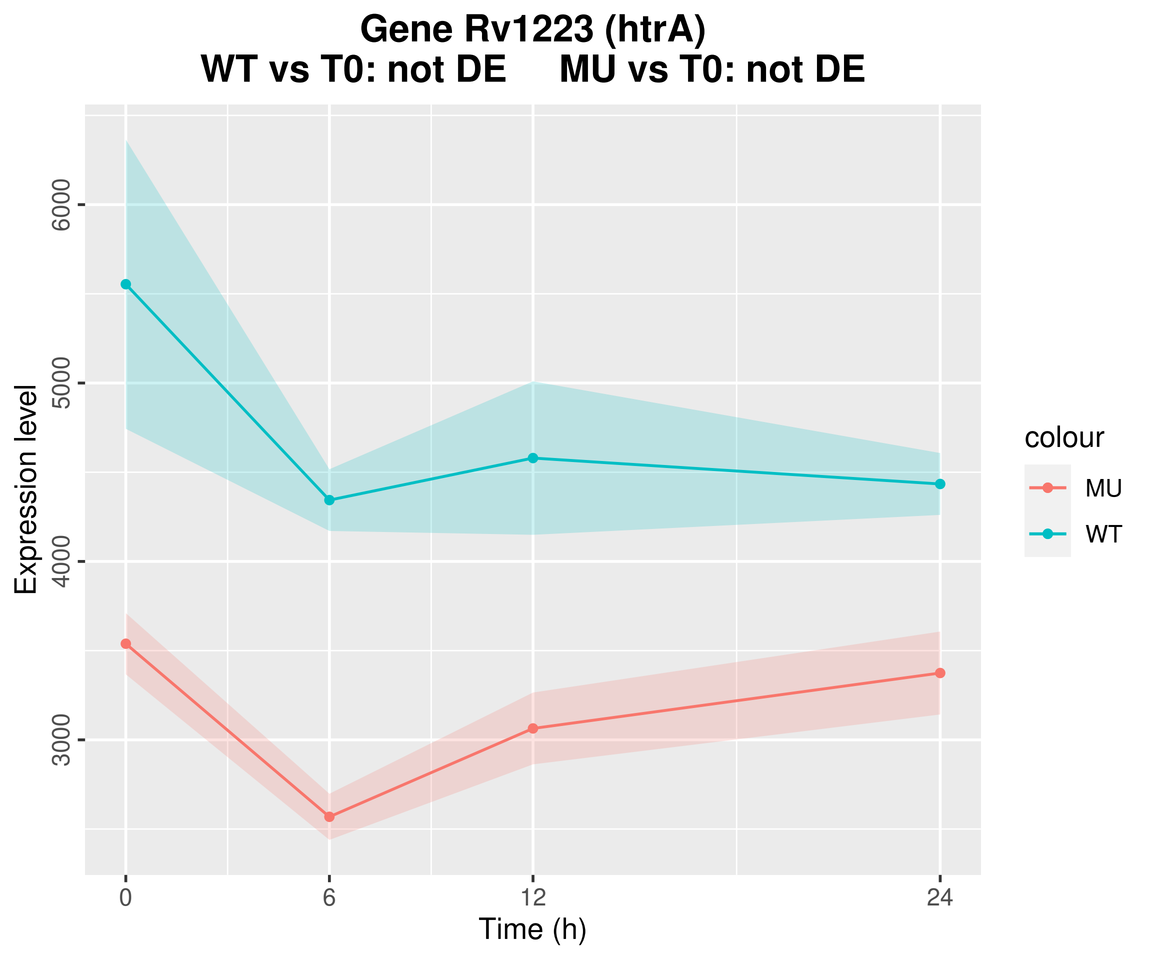
Gene Rv1223 (htrA)
WT vs T0: not DE   MU vs T0: not DE
Expression level
6000
5000
4000
3000
0
6
12
24
Time (h)
colour
MU
WT

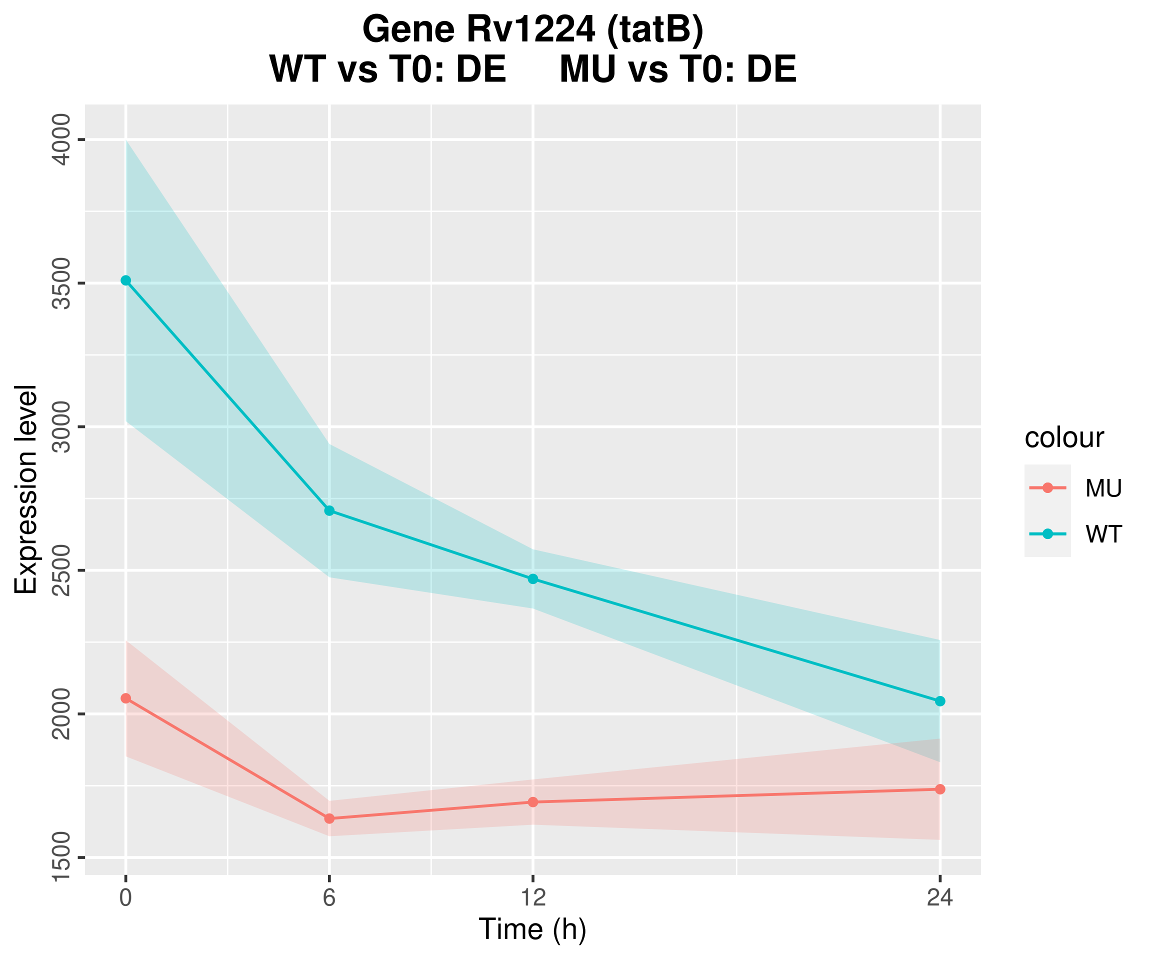
Gene Rv1224 (tatB)
WT vs T0: DE MU vs T0: DE
Expression level
4000
3500
3000
2500
2000
1500
0
6
12
24
Time (h)
colour
MU
WT

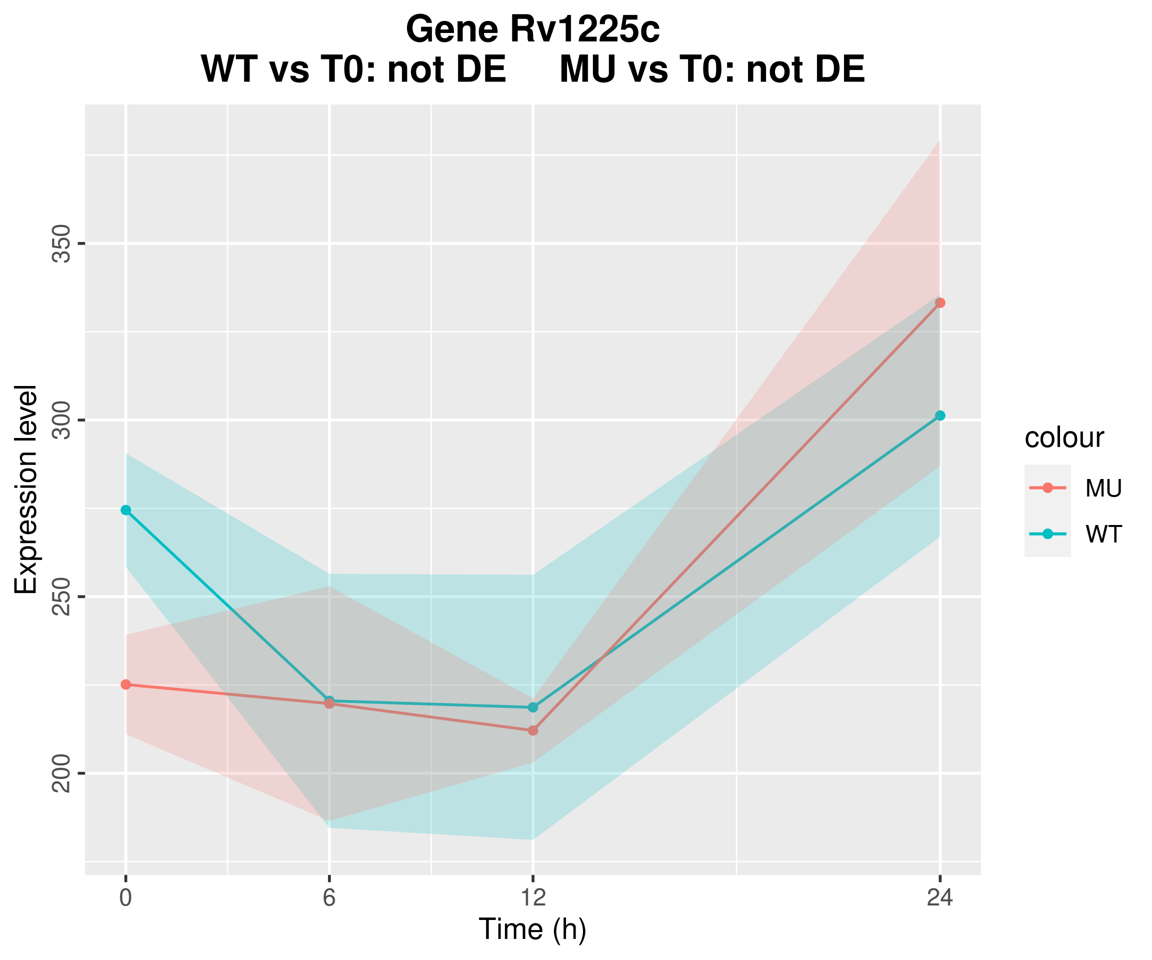

Gene Rv1225c
WT vs T0: not DE   MU vs T0: not DE
Expression level
350
300
250
200
0
6
12
24
Time (h)
colour
MU
WT

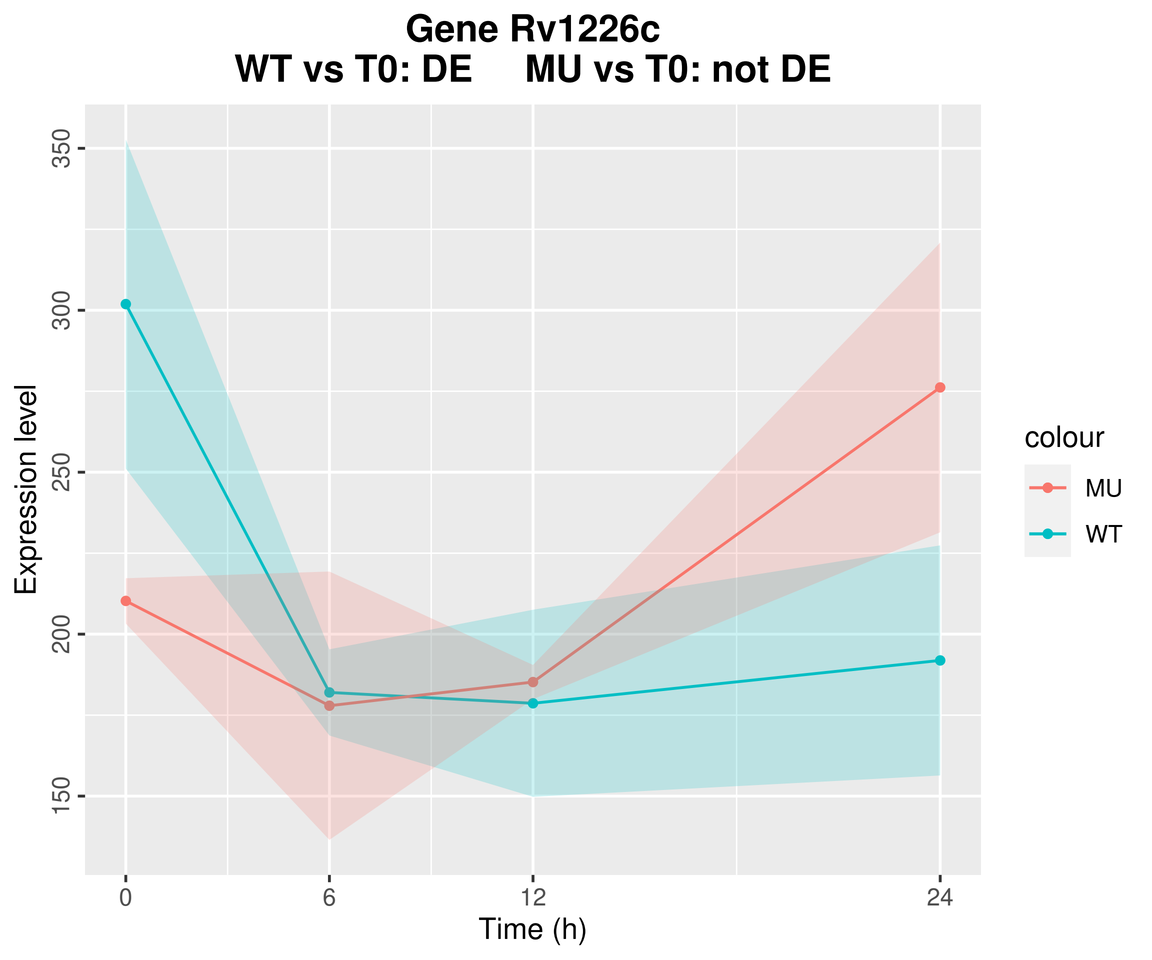

Gene Rv1226c
WT vs T0: DE MU vs T0: not DE
Expression level
350
300
250
200
150
0
6
12
24
Time (h)
colour
MU
WT

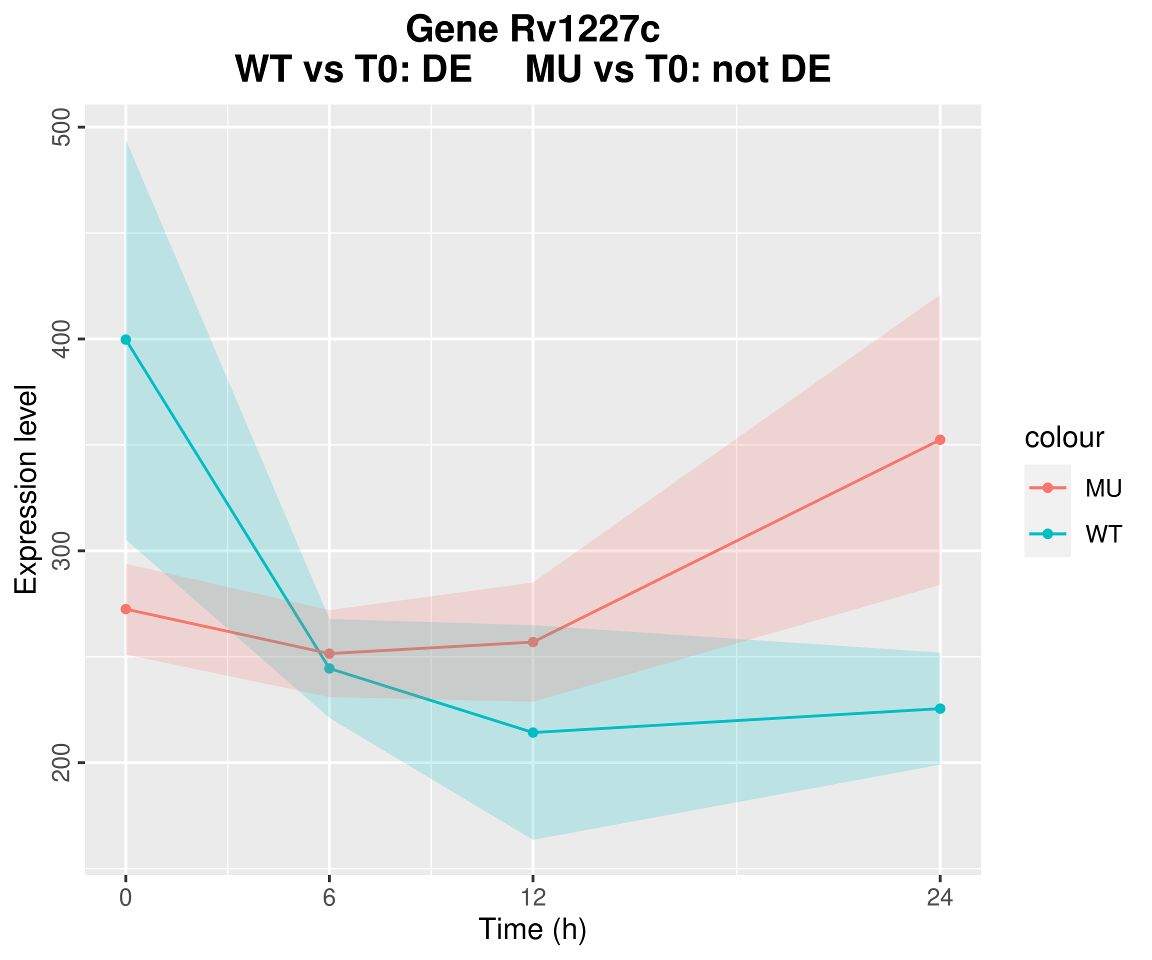
Gene Rv1227c
WT vs T0: DE   MU vs T0: not DE
500
400
300
200
Expression level
0
6
12
24
Time (h)
colour
MU
WT

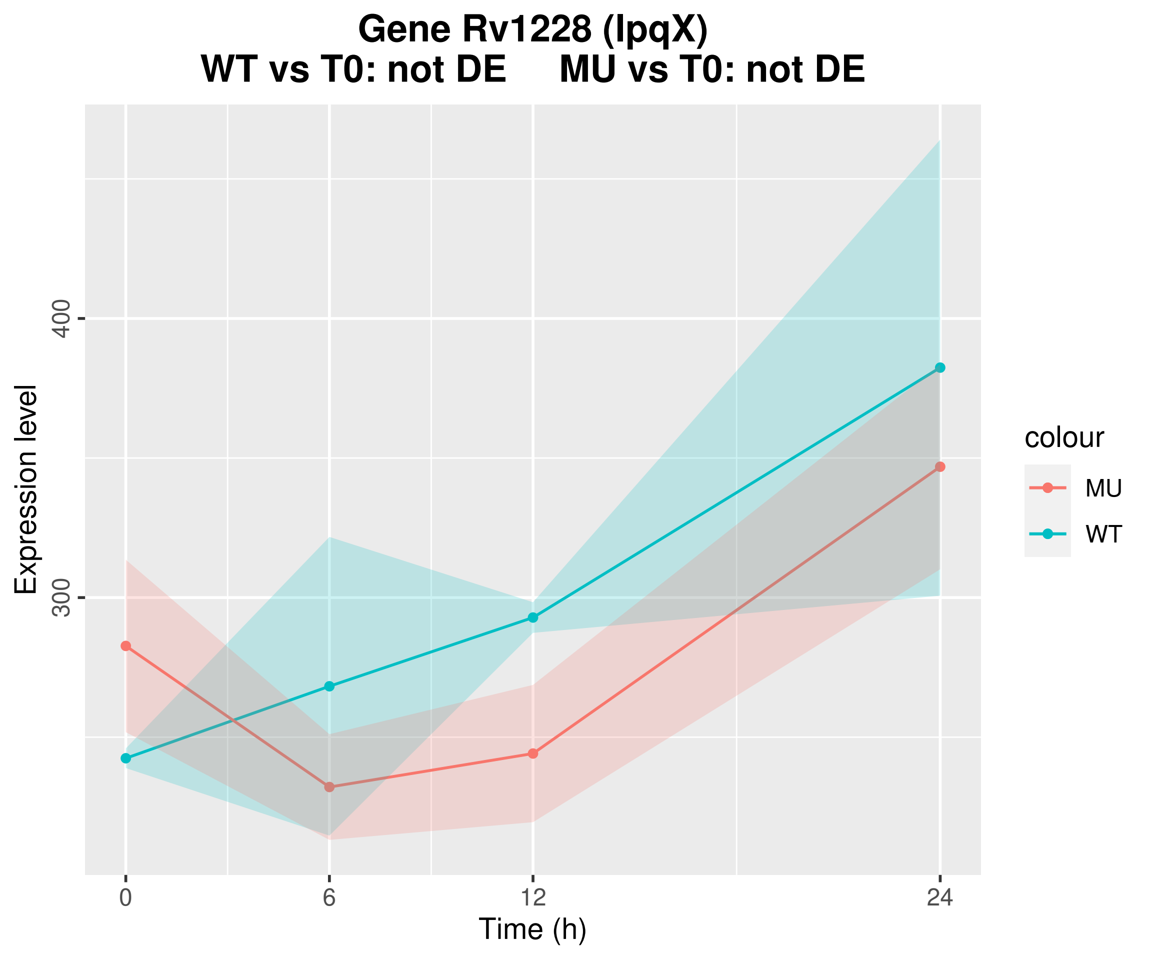

Gene Rv1228 (lpqX)
WT vs T0: not DE   MU vs T0: not DE
400
300
Expression level
0
6
12
24
Time (h)
colour
MU
WT

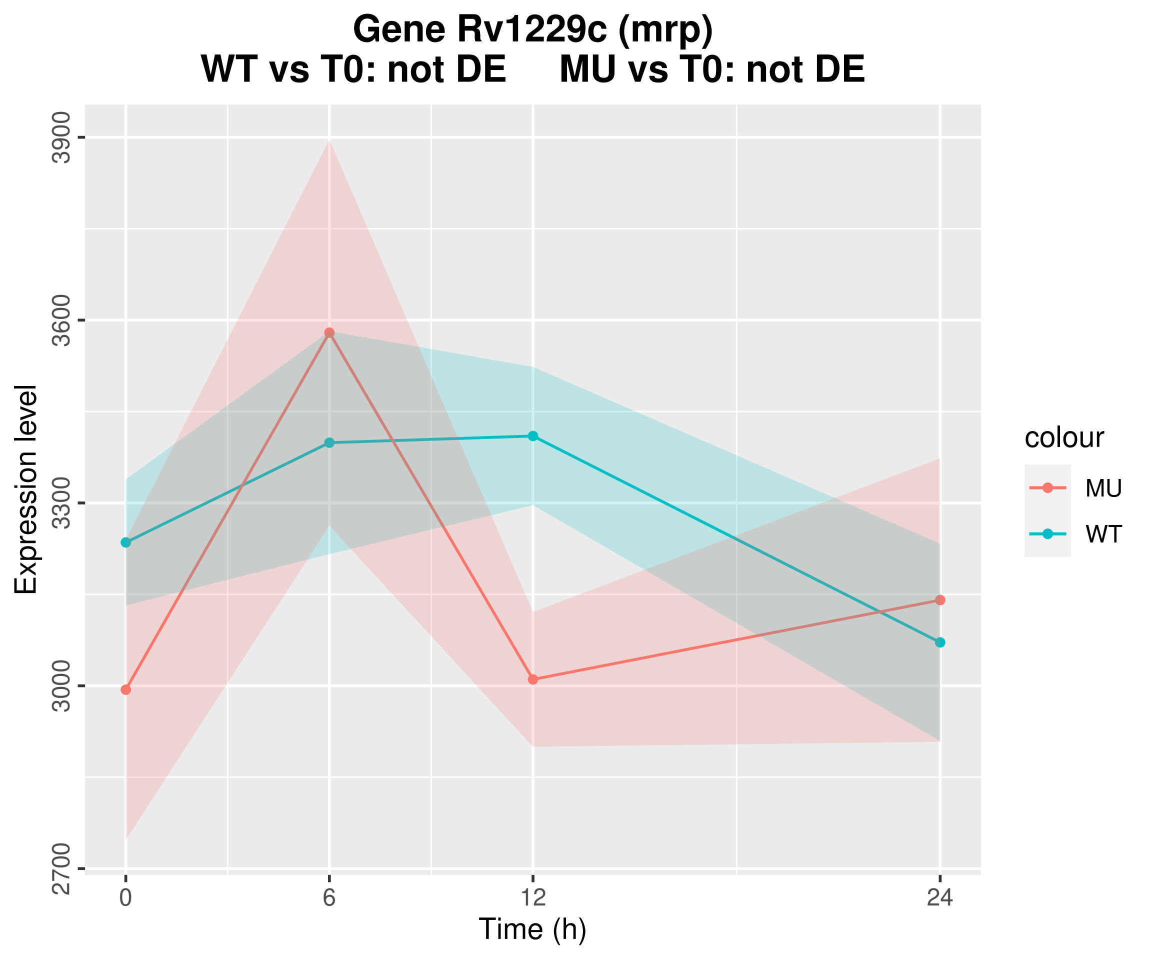

Gene Rv1229c (mrp)
WT vs T0: not DE   MU vs T0: not DE
Expression level
3900
3600
3300
3000
2700
0
6
12
24
Time (h)
colour
MU
WT

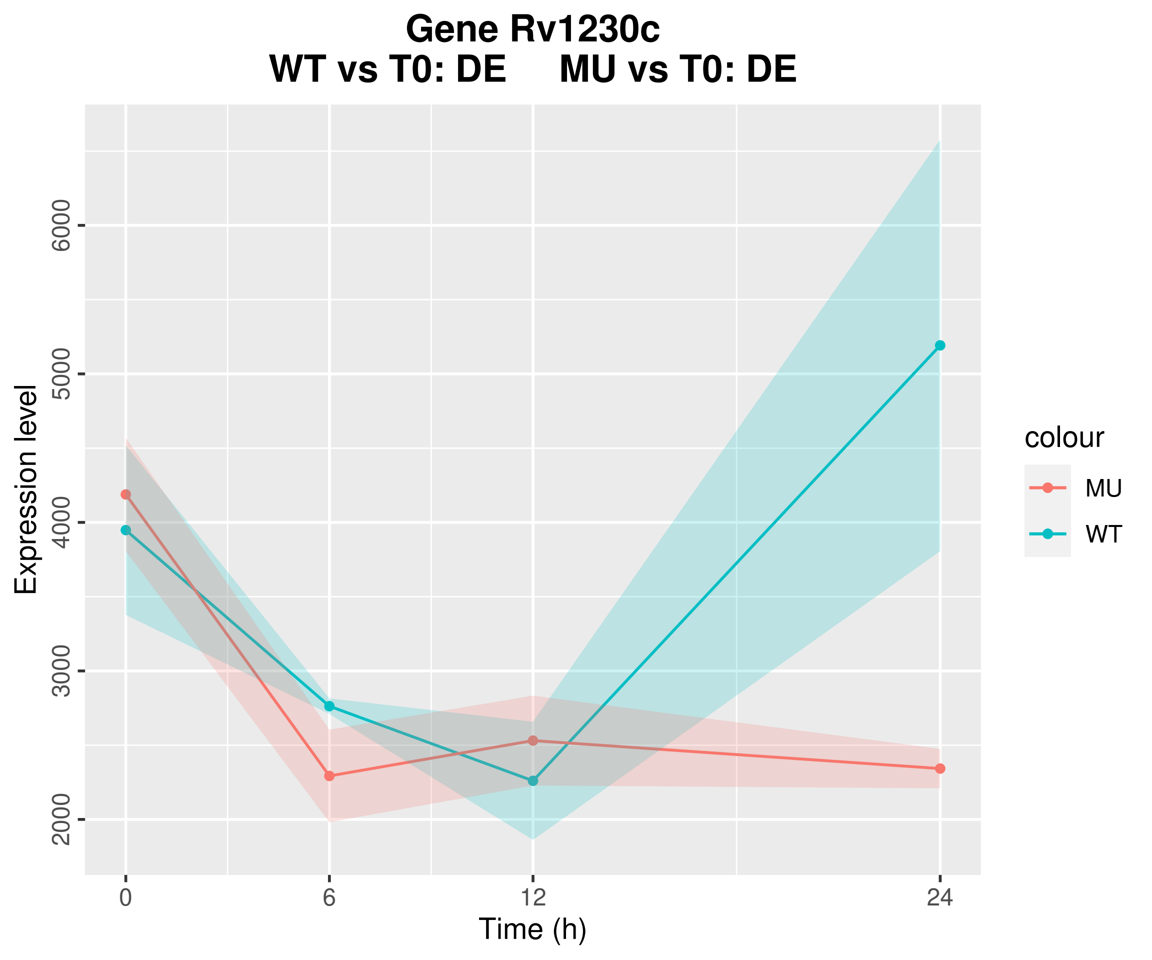

Gene Rv1230c
WT vs T0: DE MU vs T0: DE
Expression level
6000
5000
4000
3000
2000
0
6
12
24
Time (h)
colour
MU
WT

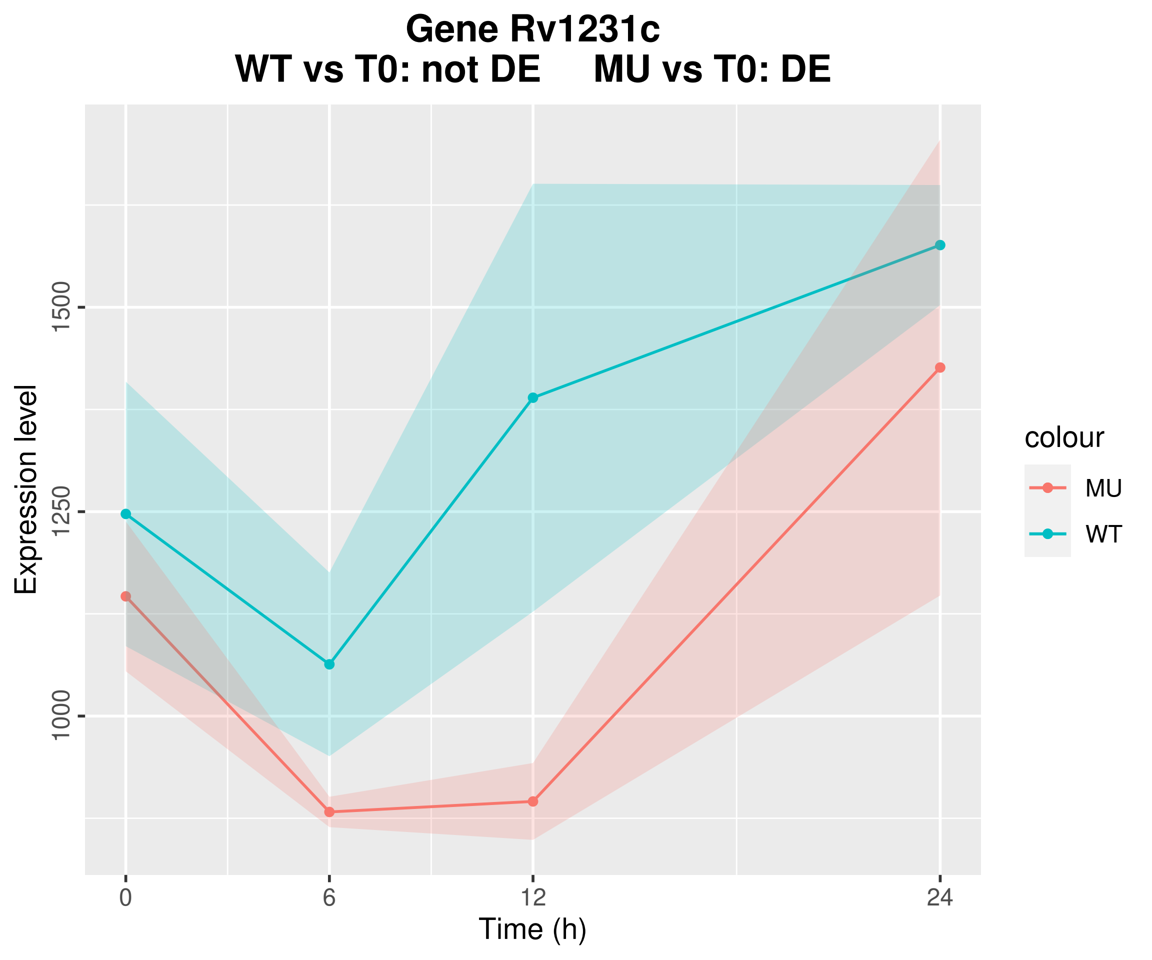
Gene Rv1231c
WT vs T0: not DE MU vs T0: DE
Expression level
1000
1250
1500
0
6
12
24
Time (h)
colour
MU
WT

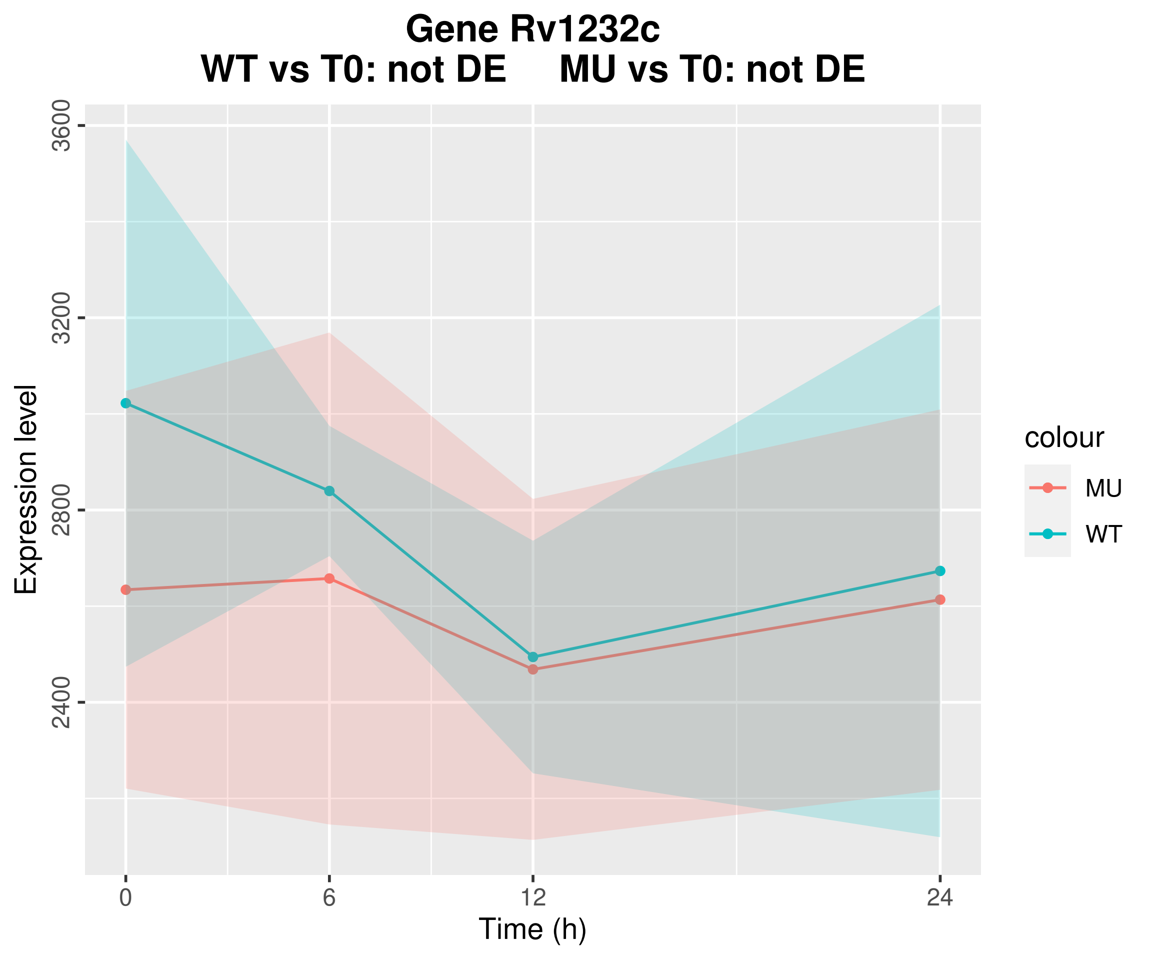
Gene Rv1232c
WT vs T0: not DE   MU vs T0: not DE
Expression level
3600
3200
2800
2400
0
6
12
24
Time (h)
colour
MU
WT

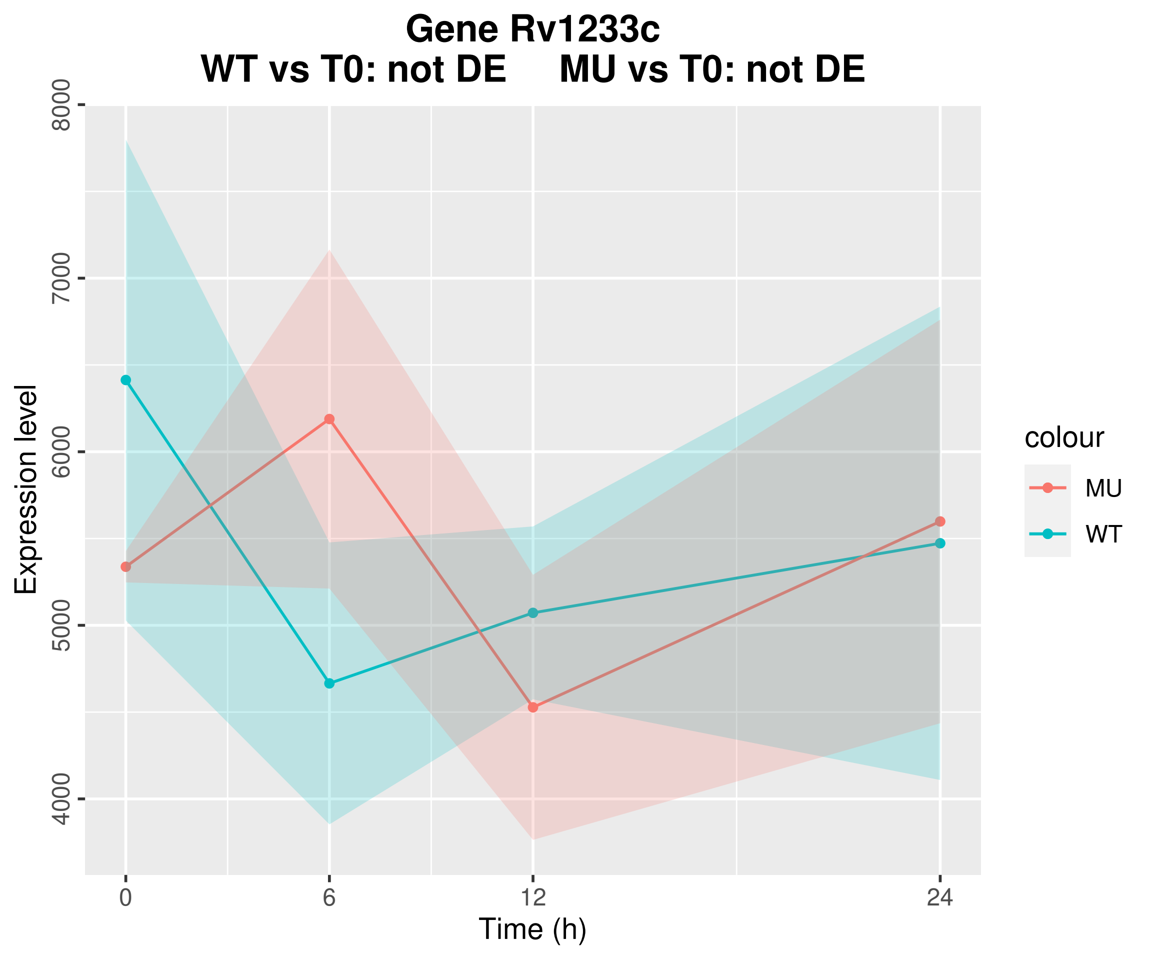
Gene Rv1233c
WT vs T0: not DE MU vs T0: not DE
Expression level
8000
7000
6000
5000
4000
0
6
12
24
Time (h)
colour
MU
WT

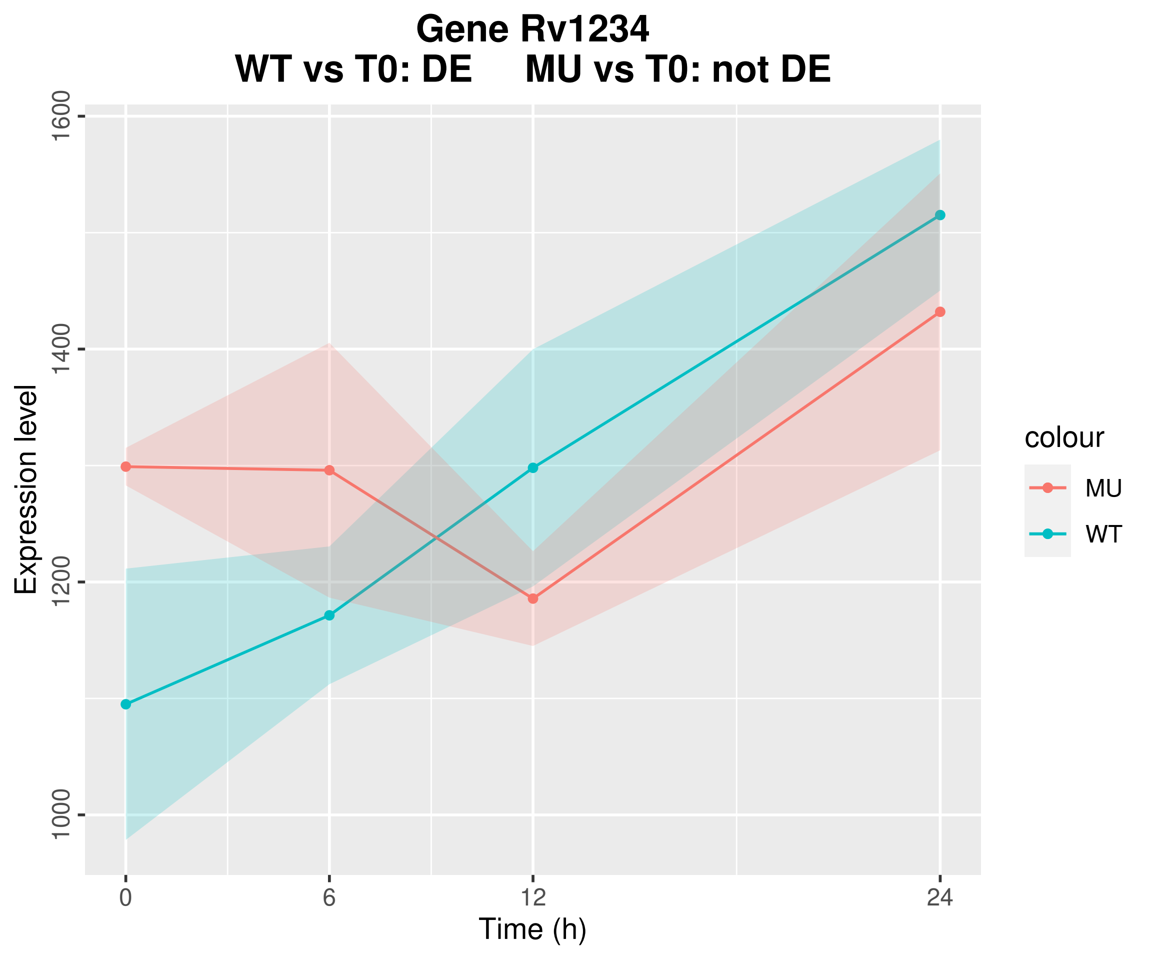

Gene Rv1234
WT vs T0: DE   MU vs T0: not DE
Expression level
1000
1200
1400
1600
0
6
12
24
Time (h)
colour
MU
WT

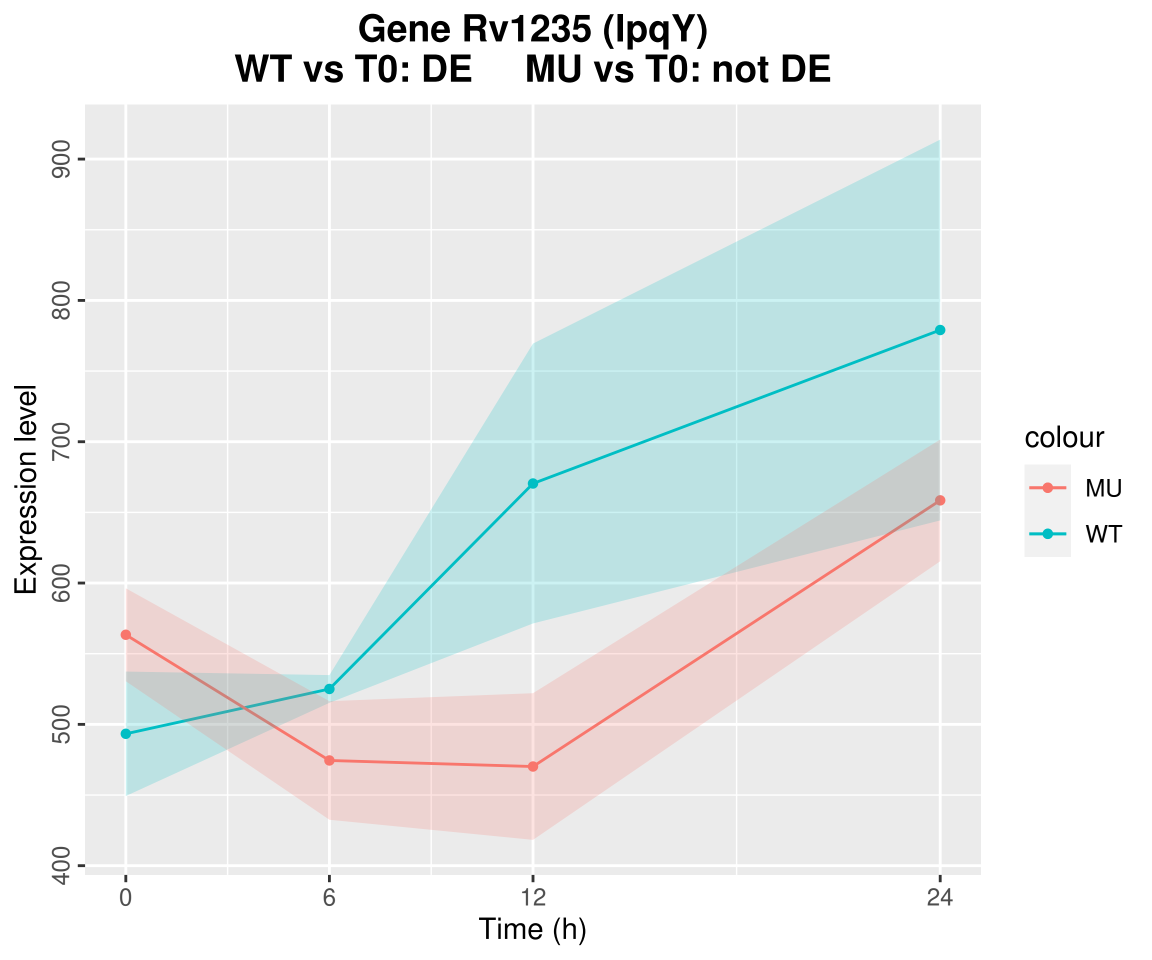
Gene Rv1235 (lpqY)
WT vs T0: DE   MU vs T0: not DE
Expression level
900
800
700
600
500
400
0
6
12
24
Time (h)
colour
MU
WT

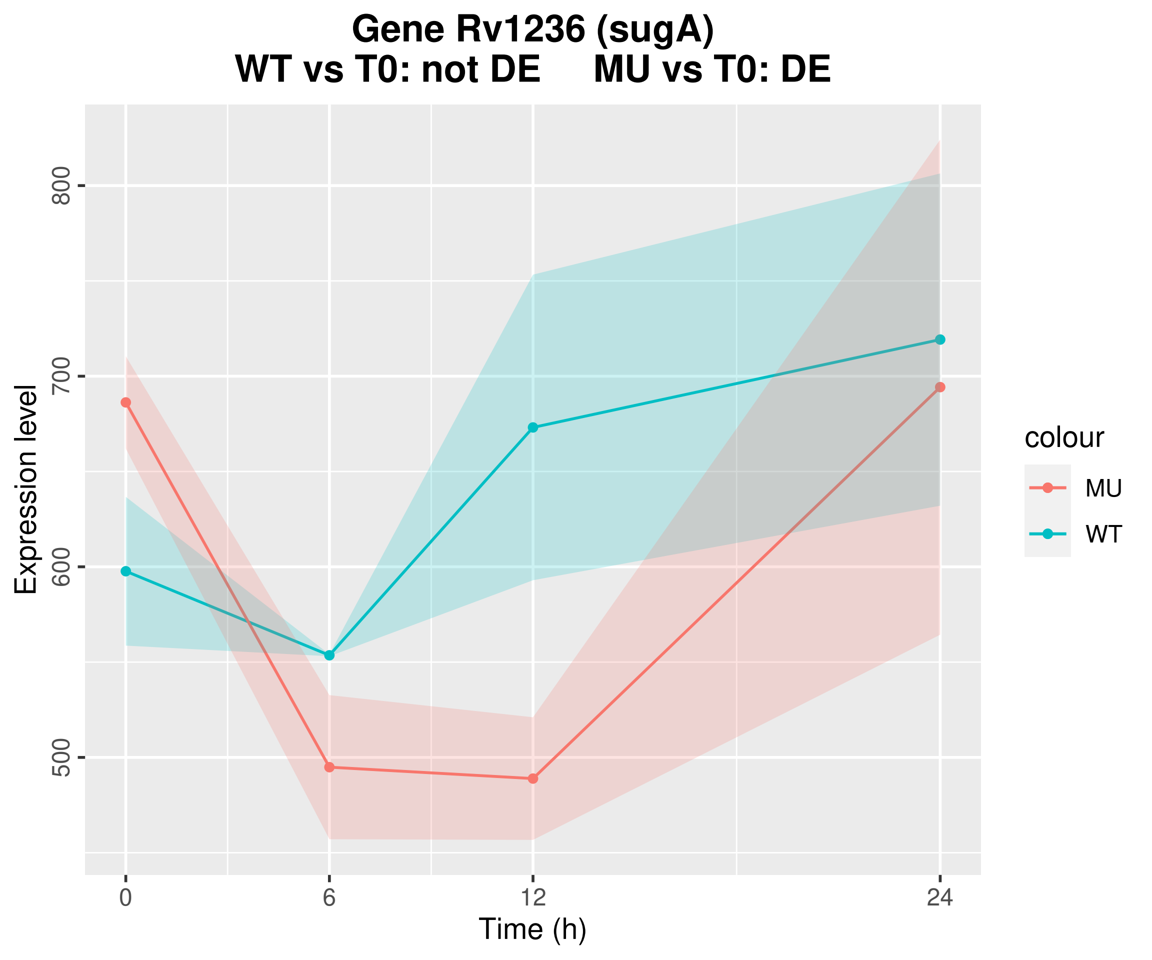
Gene Rv1236 (sugA)
WT vs T0: not DE MU vs T0: DE
Expression level
800
700
600
500
0
6
12
24
Time (h)
colour
MU
WT

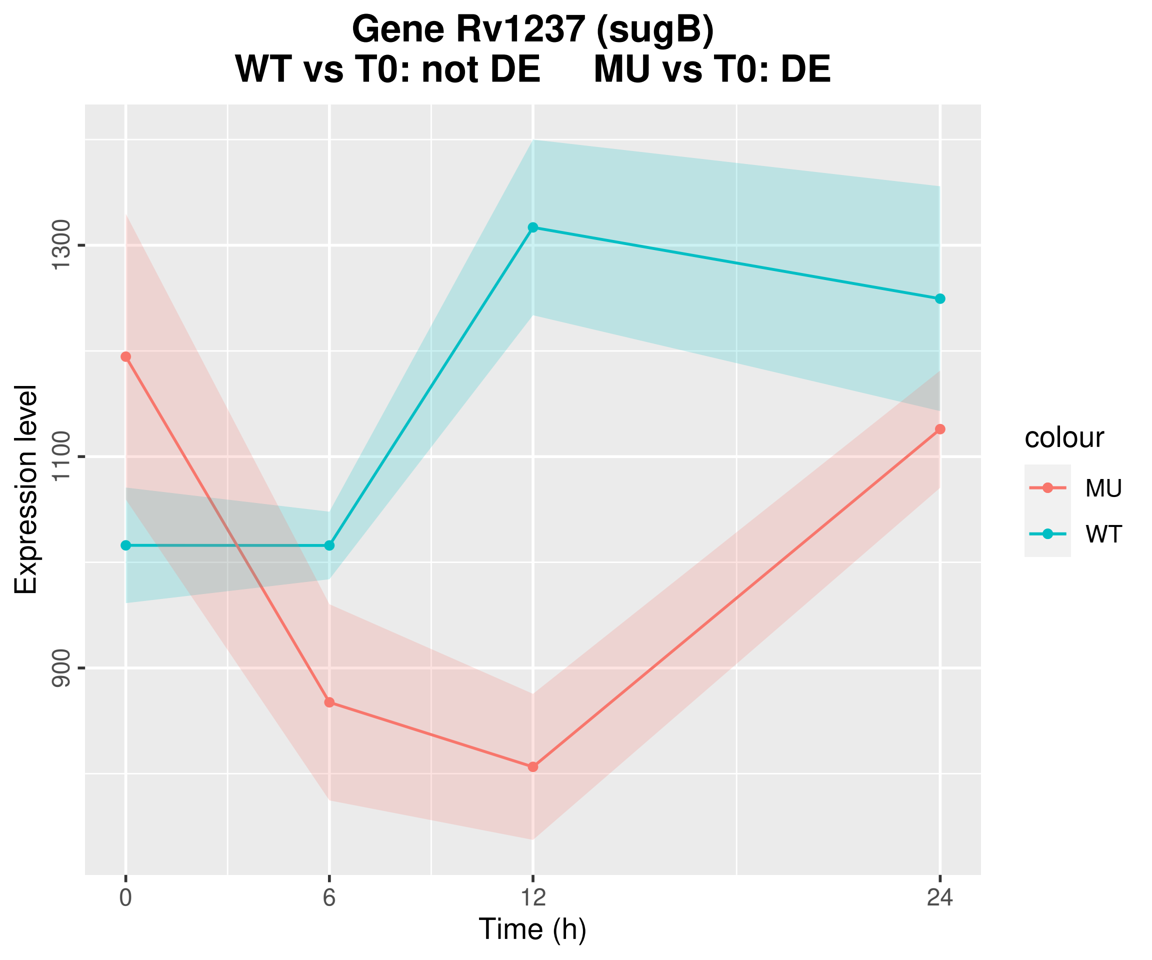
Gene Rv1237 (sugB)
WT vs T0: not DE   MU vs T0: DE
Expression level
1300
1100
900
0
6
12
24
Time (h)
colour
MU
WT

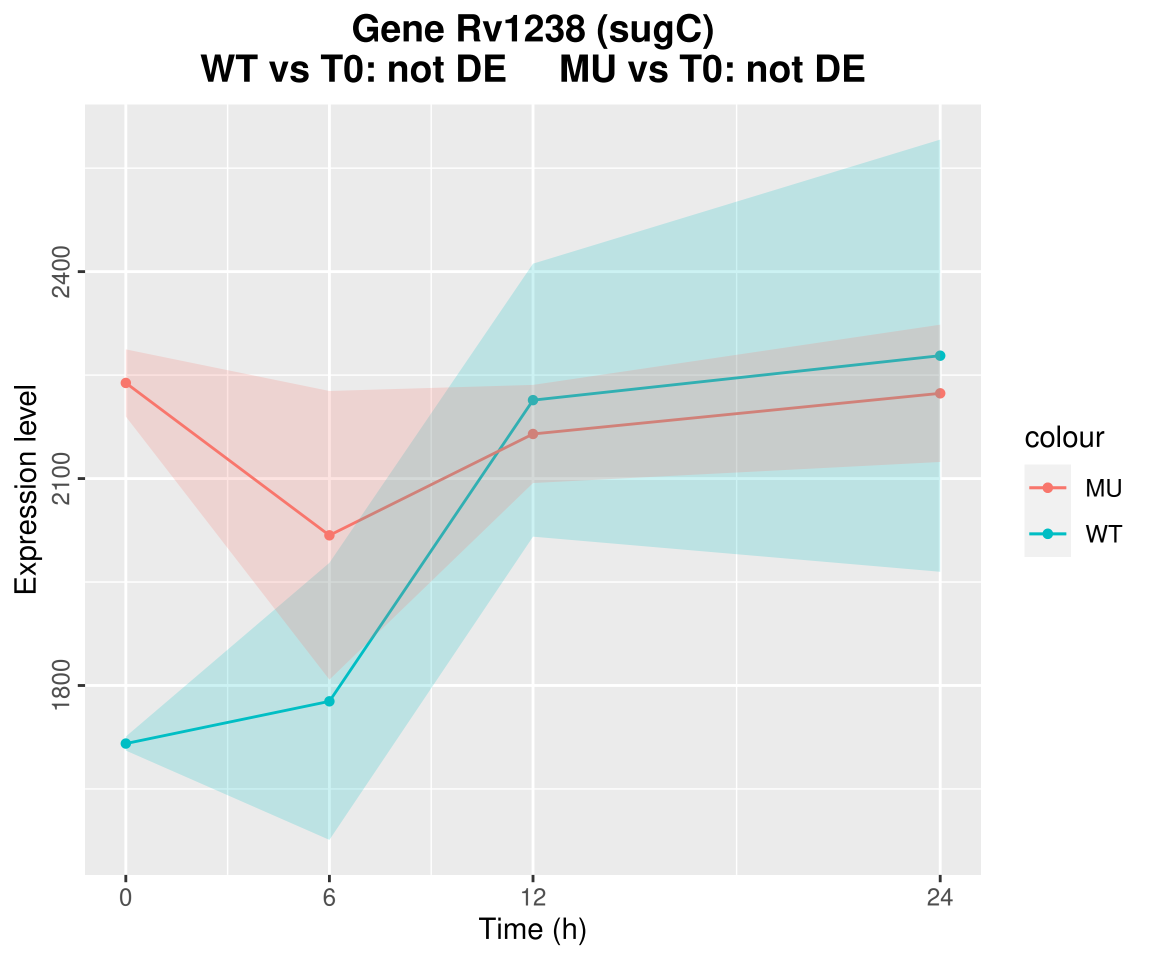
Gene Rv1238 (sugC)
WT vs T0: not DE MU vs T0: not DE
Expression level
2400
2100
1800
0
6
12
24
Time (h)
colour
MU
WT

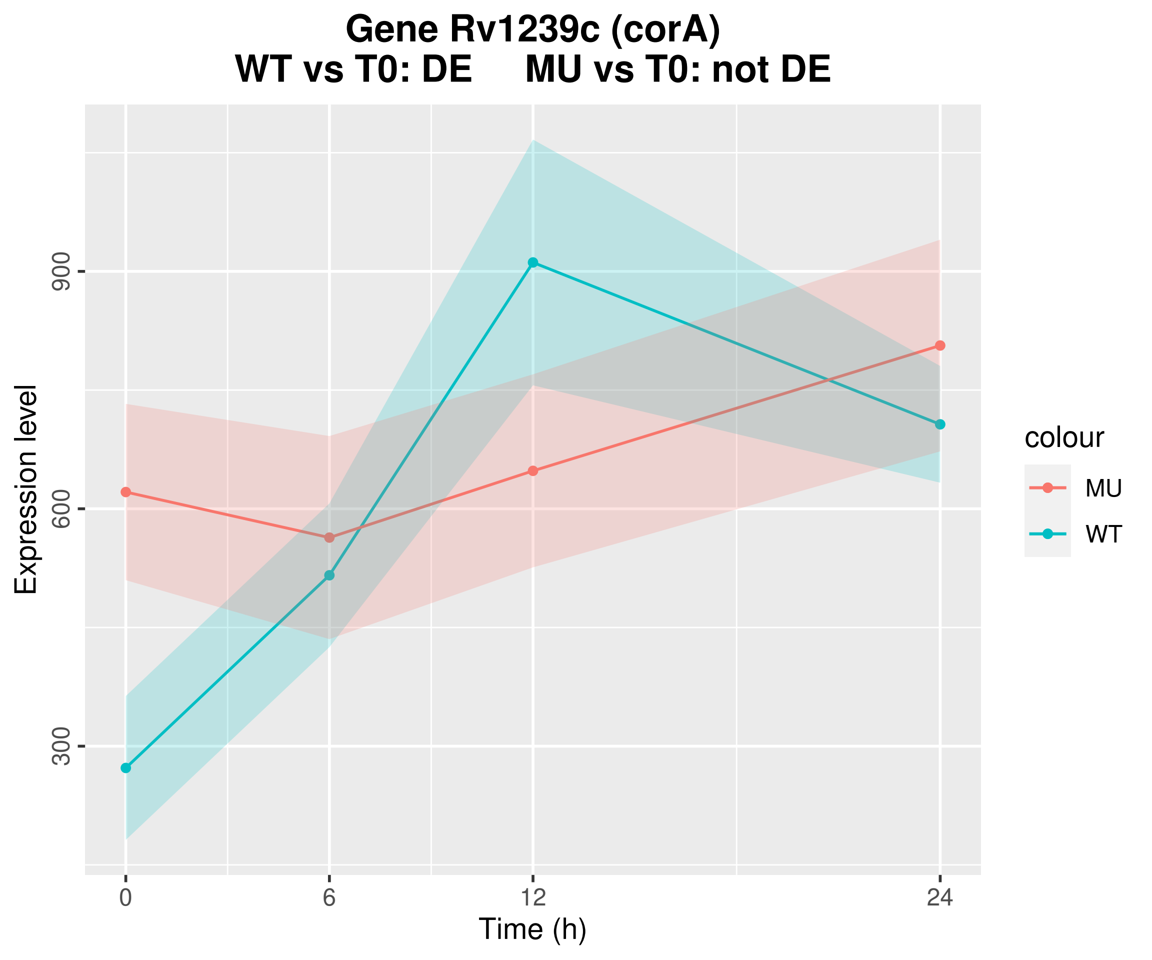
Gene Rv1239c (corA)
WT vs T0: DE   MU vs T0: not DE
Expression level
900
600
300
0
6
12
24
Time (h)
colour
MU
WT

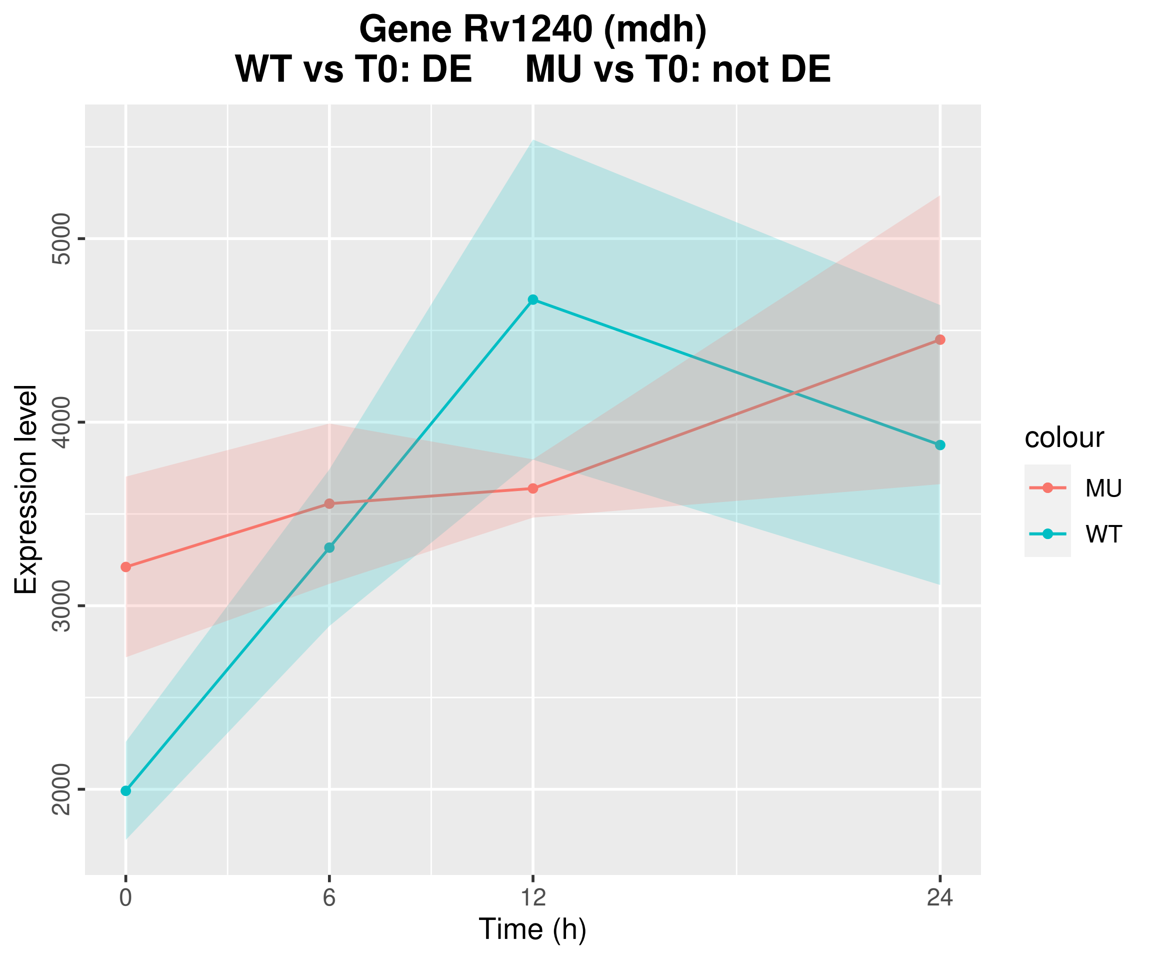

Gene Rv1240 (mdh)
WT vs T0: DE   MU vs T0: not DE
Expression level
5000
4000
3000
2000
0
6
12
24
Time (h)
colour
MU
WT

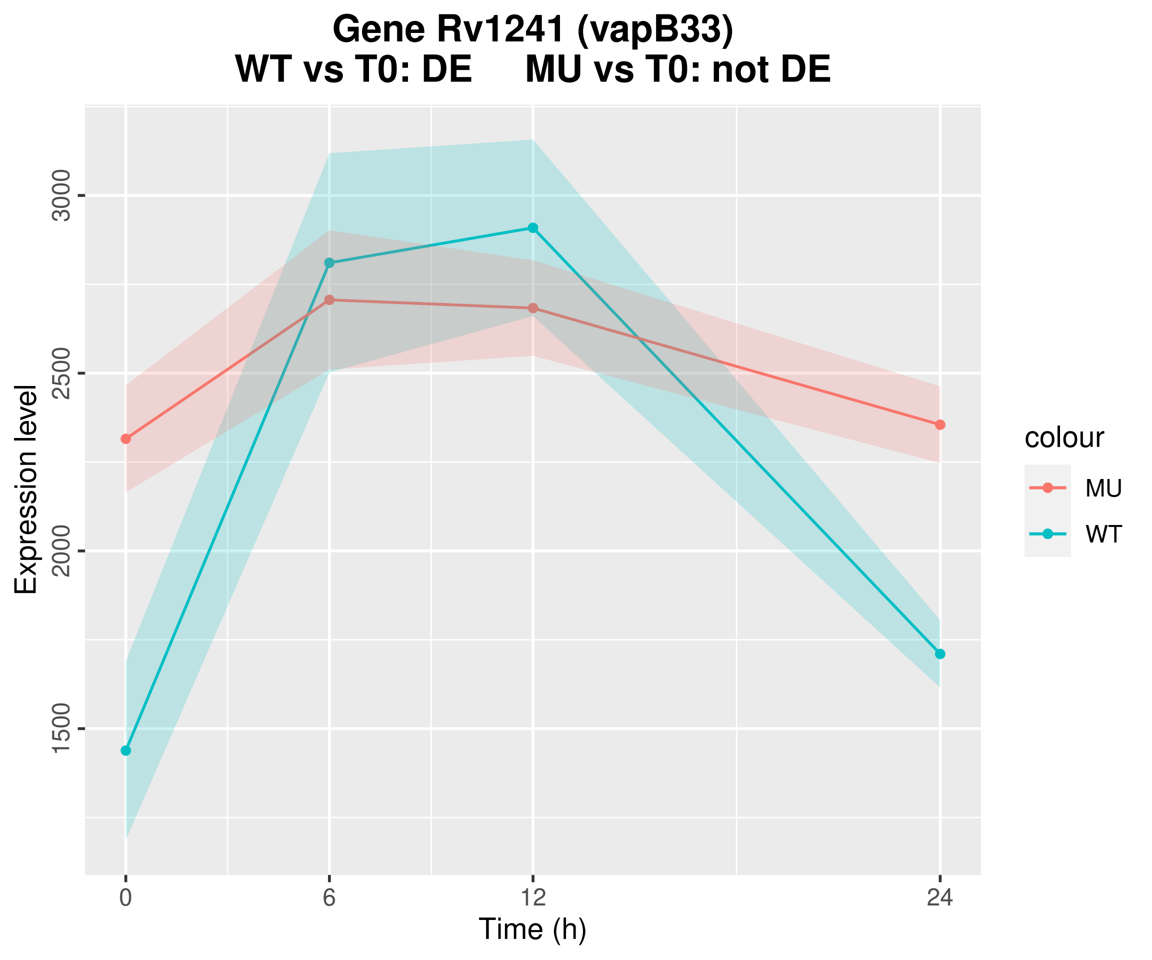
Gene Rv1241 (vapB33)
WT vs T0: DE   MU vs T0: not DE
Expression level
3000
2500
2000
1500
0
6
12
24
Time (h)
colour
MU
WT

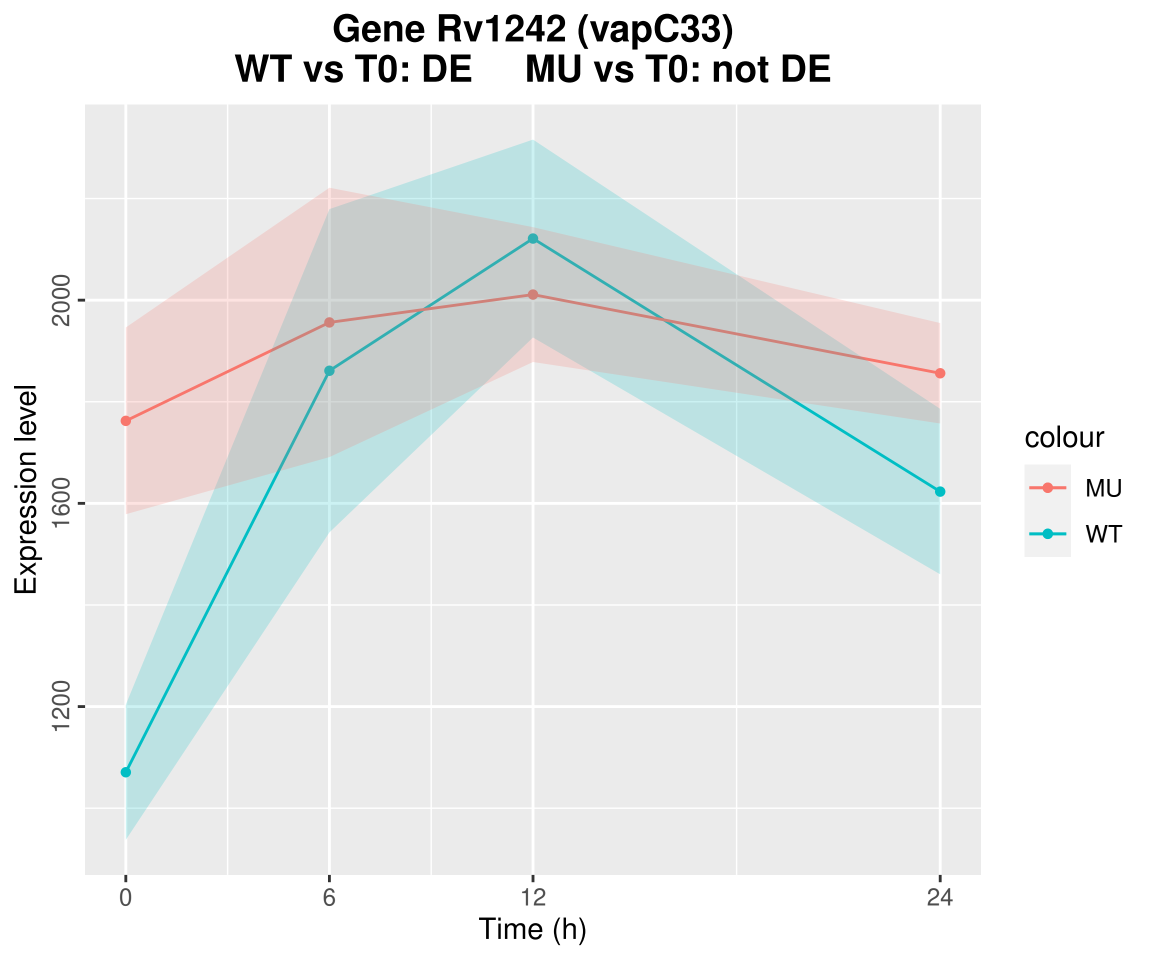

Gene Rv1242 (vapC33)
WT vs T0: DE   MU vs T0: not DE
Expression level
2000
1600
1200
0
6
12
24
Time (h)
colour
MU
WT

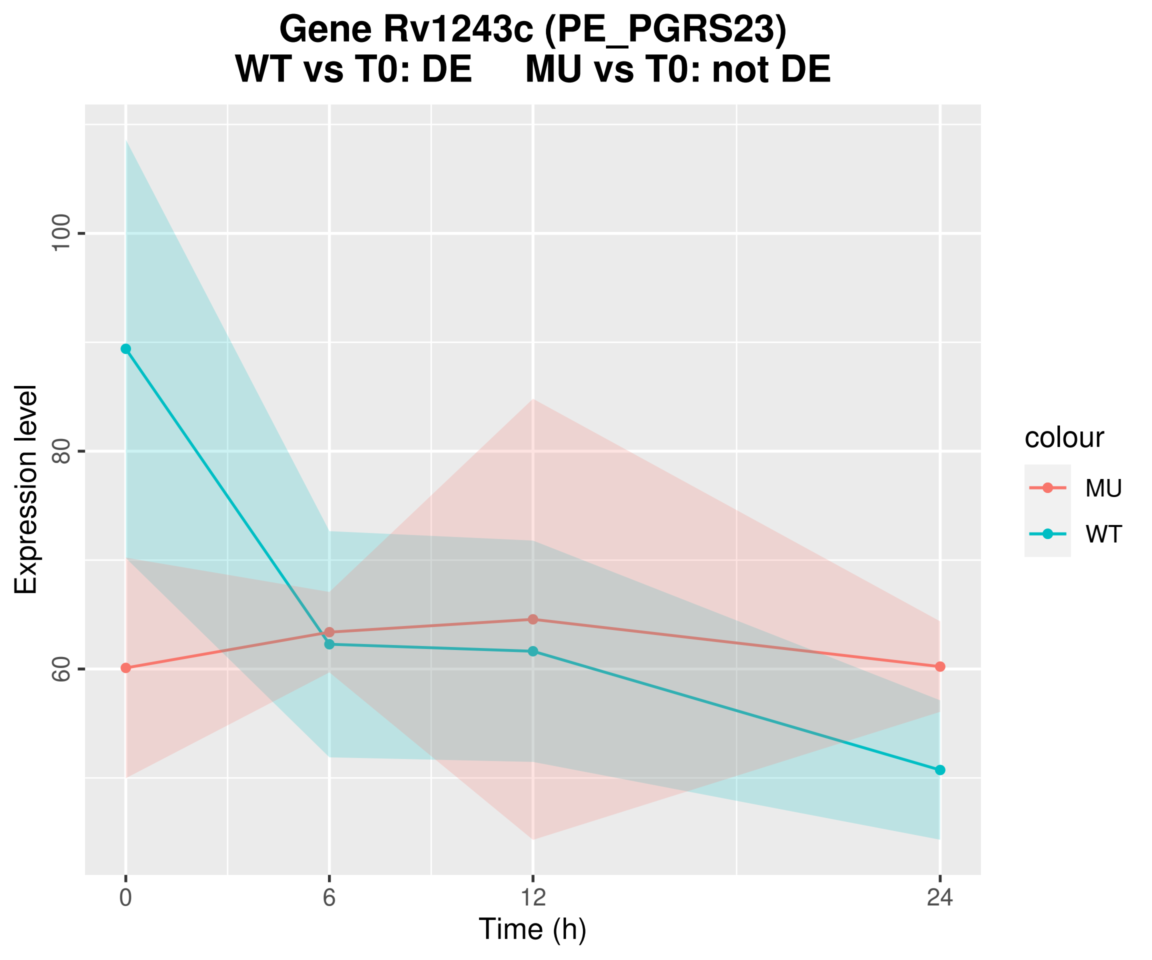
Gene Rv1243c (PE_PGRS23)
WT vs T0: DE   MU vs T0: not DE
Expression level
100
80
60
0
6
12
24
Time (h)
colour
MU
WT

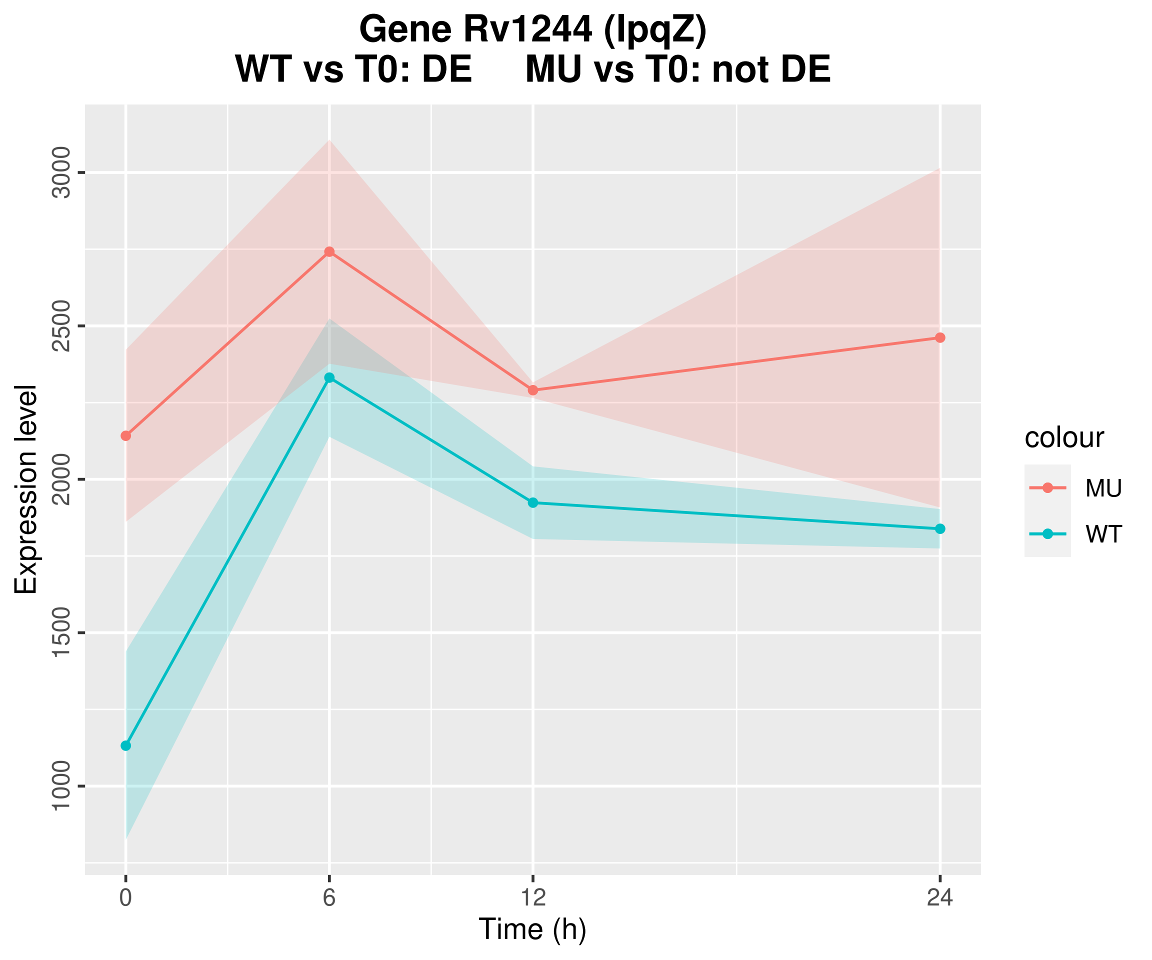
Gene Rv1244 (lpqZ)
WT vs T0: DE   MU vs T0: not DE
Expression level
3000
2500
2000
1500
1000
0
6
12
24
Time (h)
colour
MU
WT

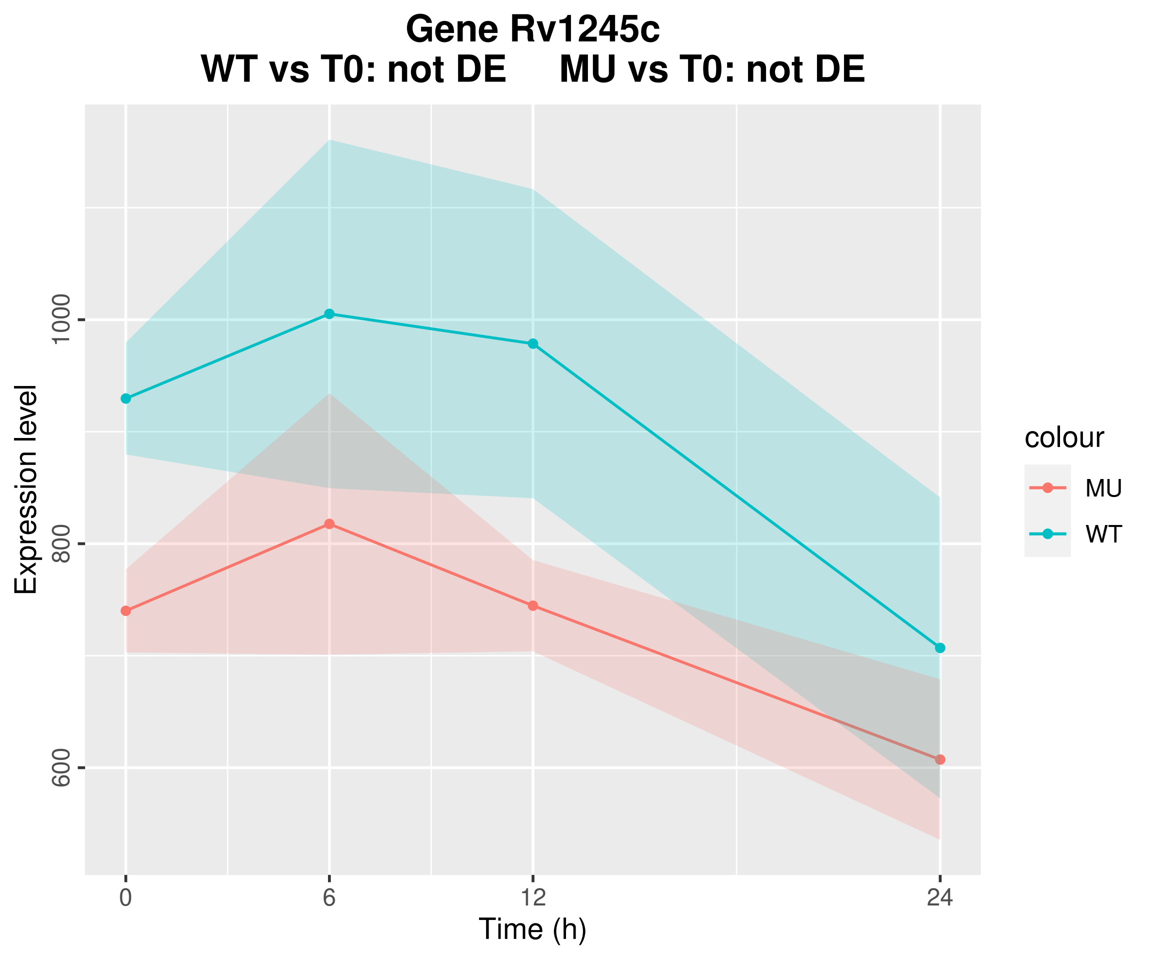
Gene Rv1245c
WT vs T0: not DE MU vs T0: not DE
Expression level
600
800
1000
0
6
12
24
Time (h)
colour
MU
WT

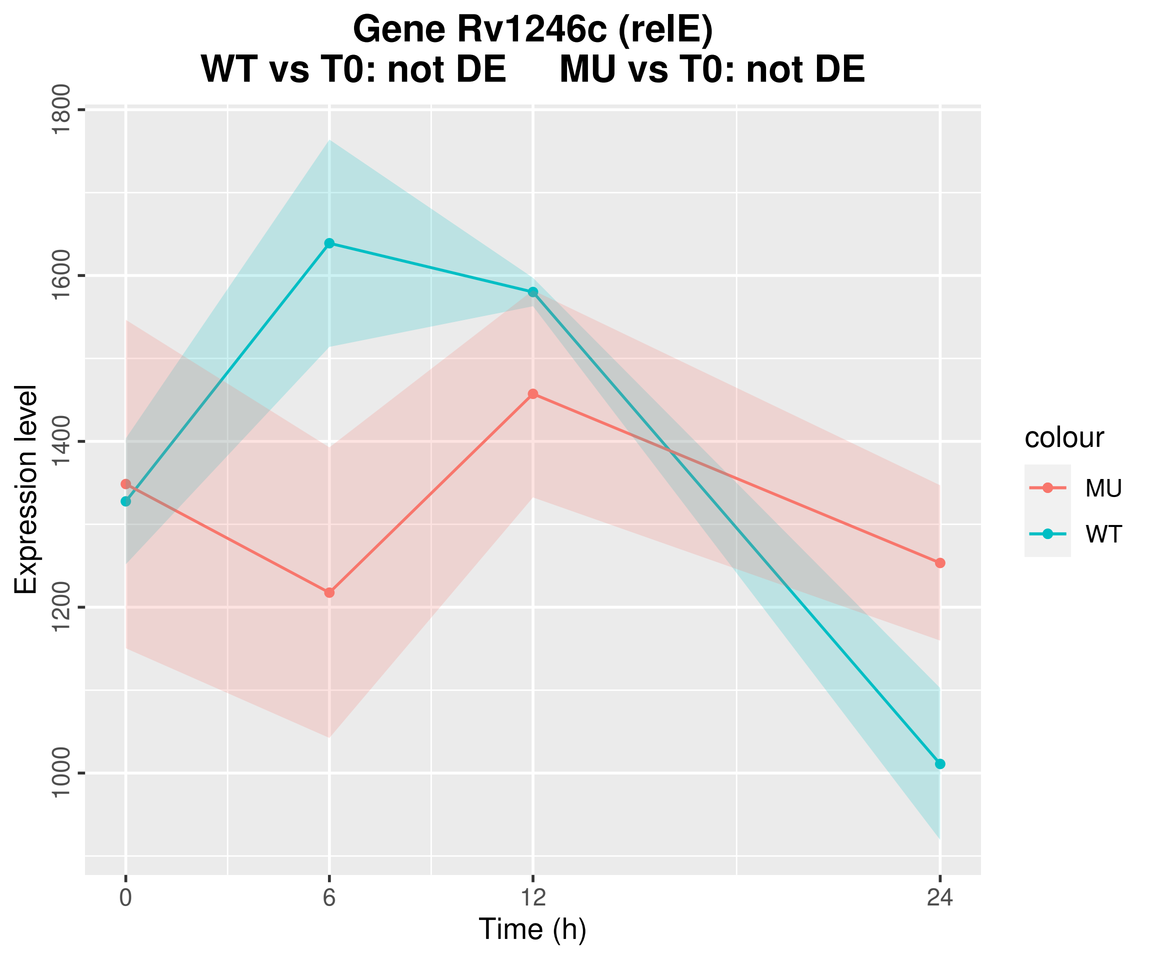

Gene Rv1246c (relE)
WT vs T0: not DE   MU vs T0: not DE
Expression level
1800
1600
1400
1200
1000
0
6
12
24
Time (h)
colour
MU
WT

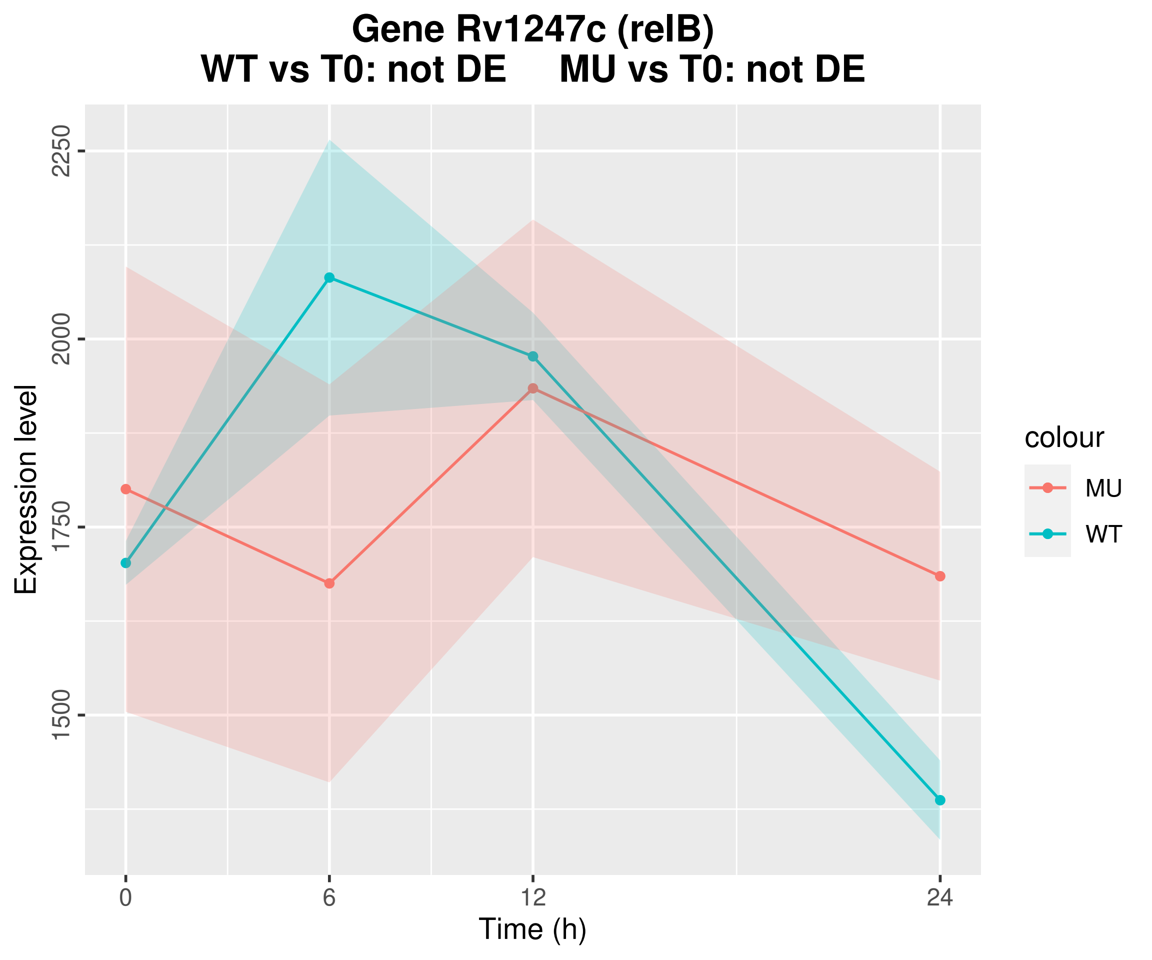
Gene Rv1247c (relB)
WT vs T0: not DE   MU vs T0: not DE
Expression level
2250
2000
1750
1500
0
6
12
24
Time (h)
colour
MU
WT

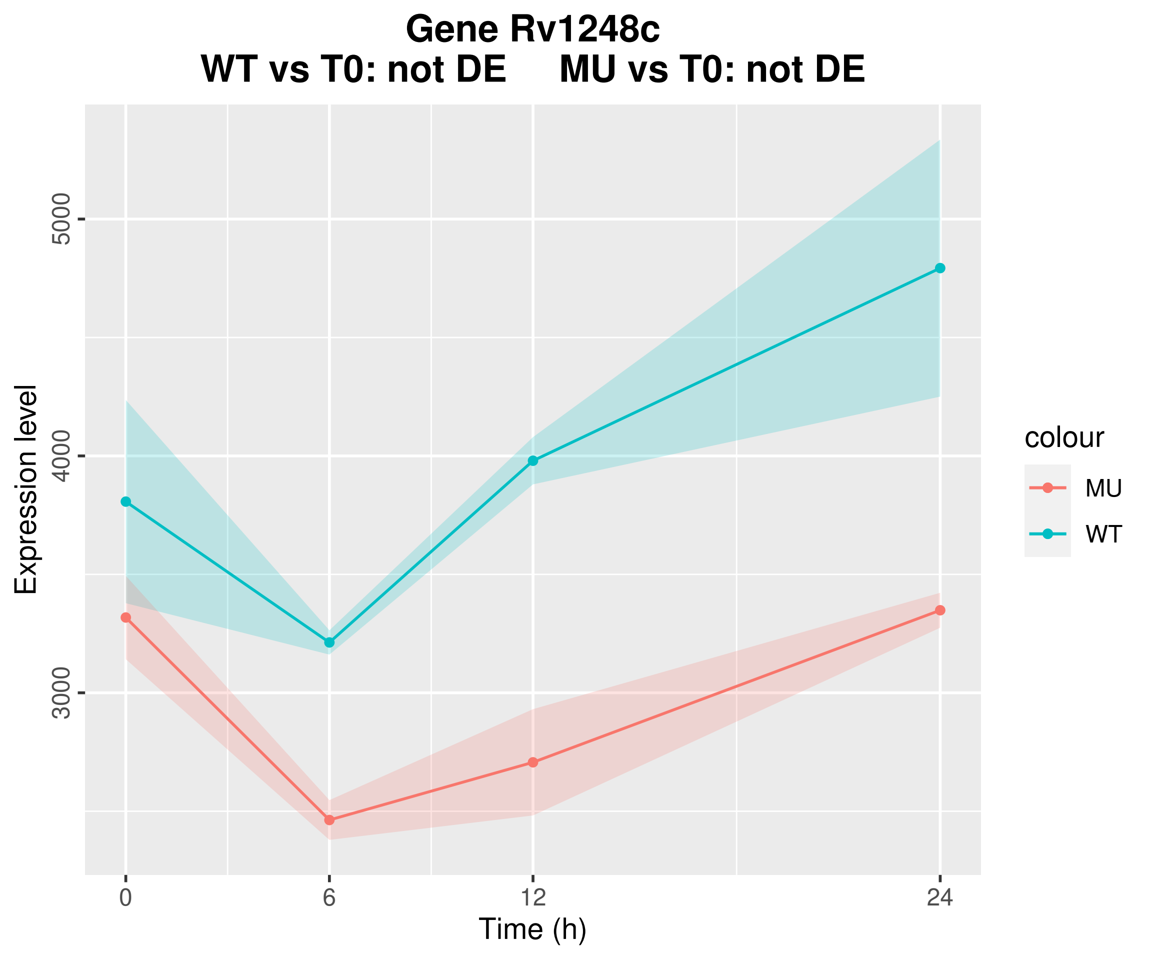
Gene Rv1248c
WT vs T0: not DE MU vs T0: not DE
Expression level
5000
4000
3000
Time (h)
0
6
12
24
colour
MU
WT

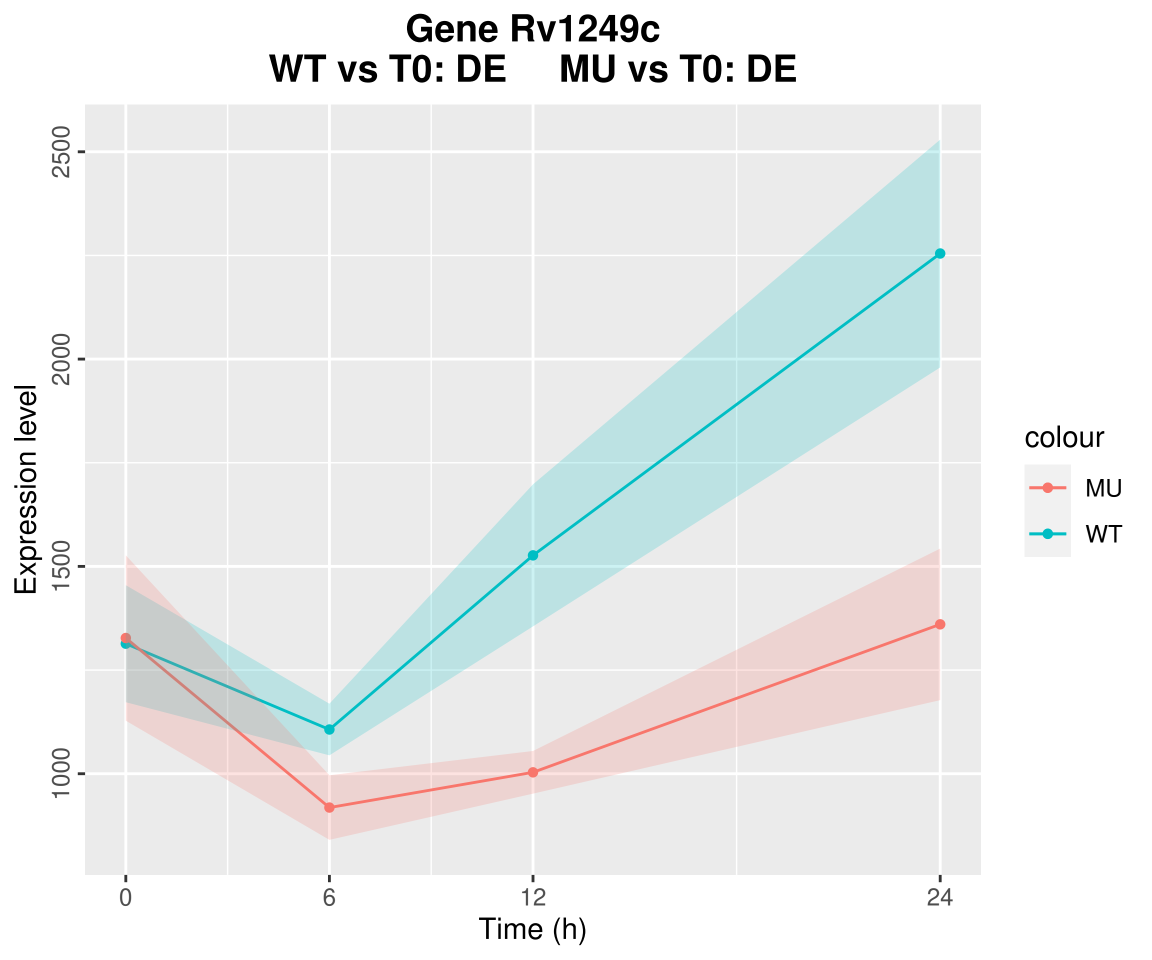
Gene Rv1249c
WT vs T0: DE   MU vs T0: DE
Expression level
2500
2000
1500
1000
0
6
12
24
Time (h)
colour
MU
WT

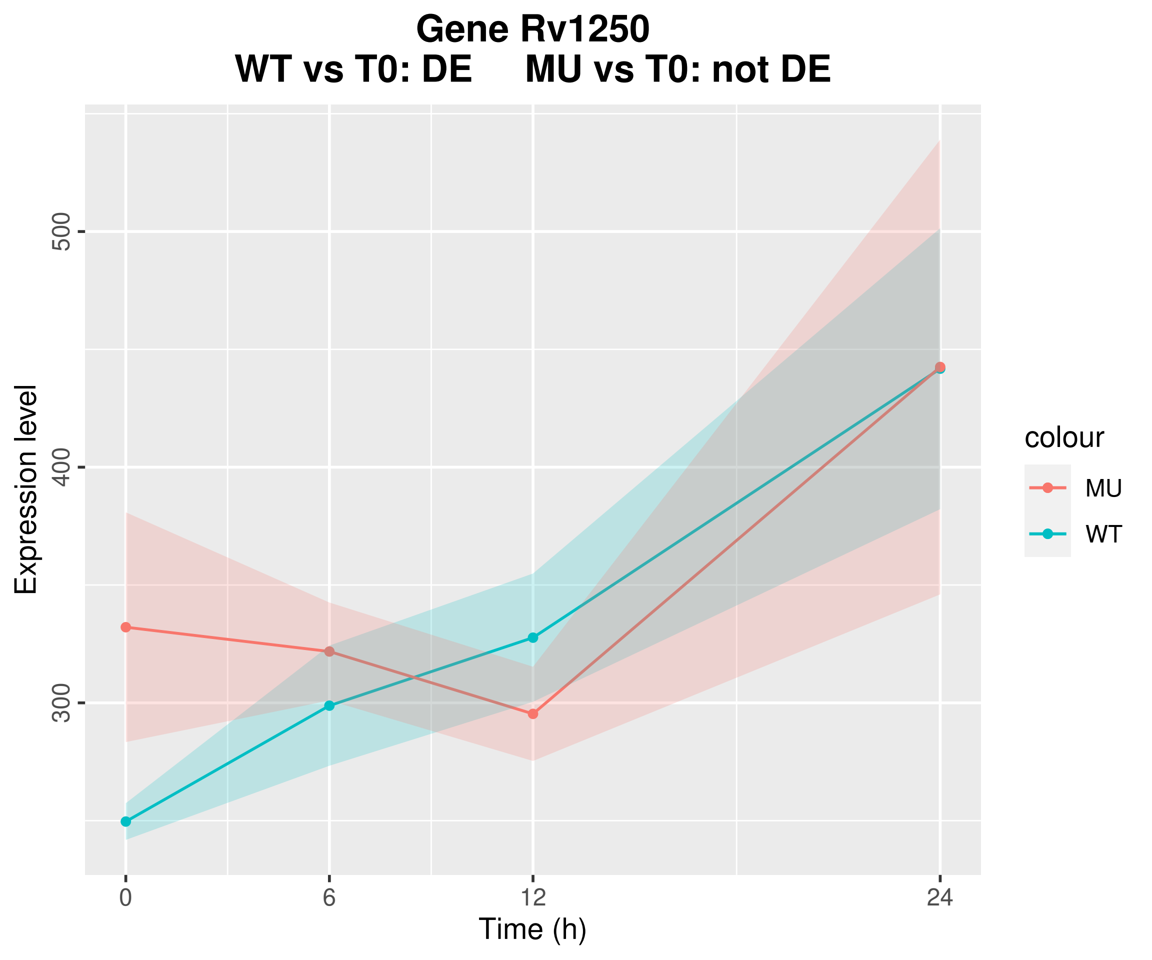
Gene Rv1250
WT vs T0: DE   MU vs T0: not DE
Expression level
500
400
300
0
6
12
24
Time (h)
colour
MU
WT

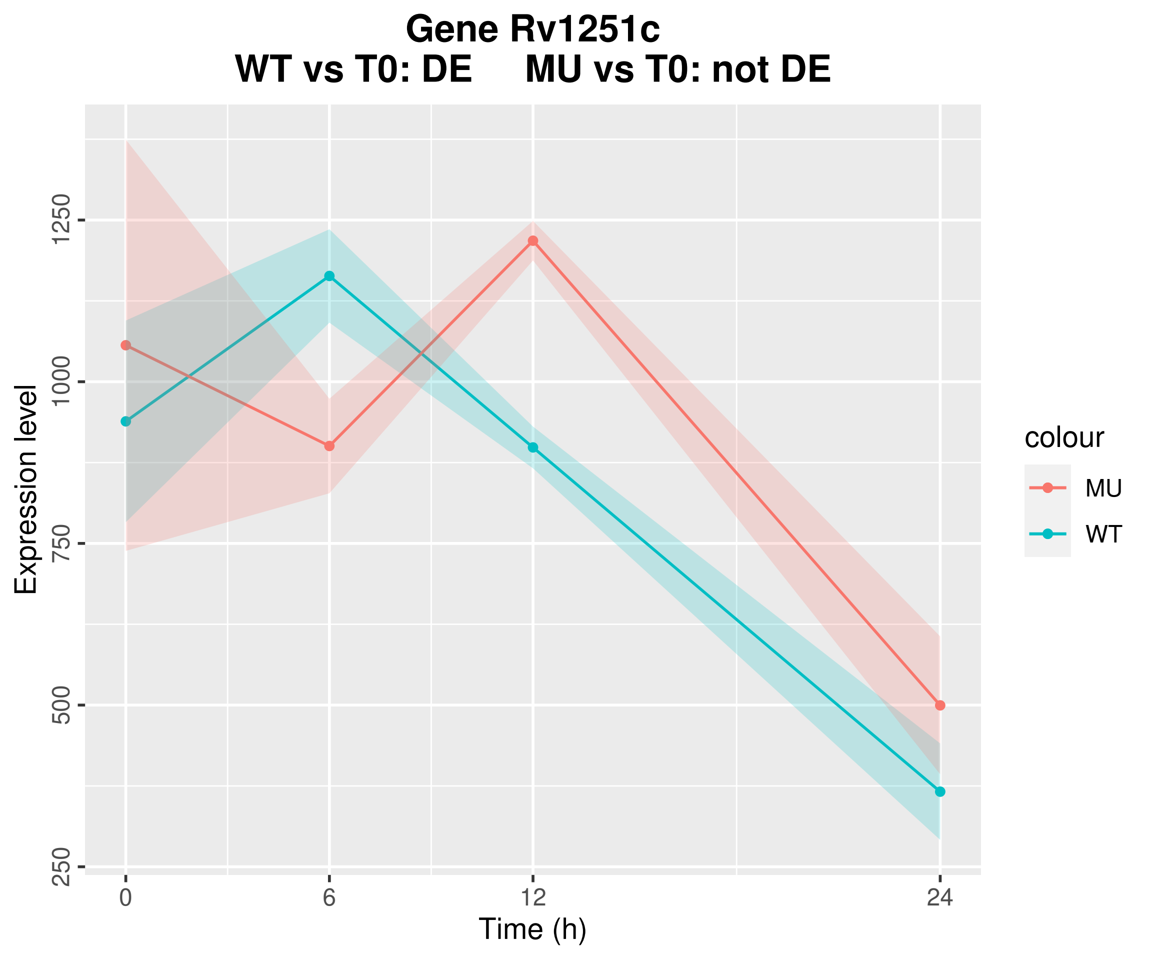
Gene Rv1251c
WT vs T0: DE MU vs T0: not DE
Expression level
1250
1000
750
500
250
0
6
12
24
Time (h)
colour
MU
WT

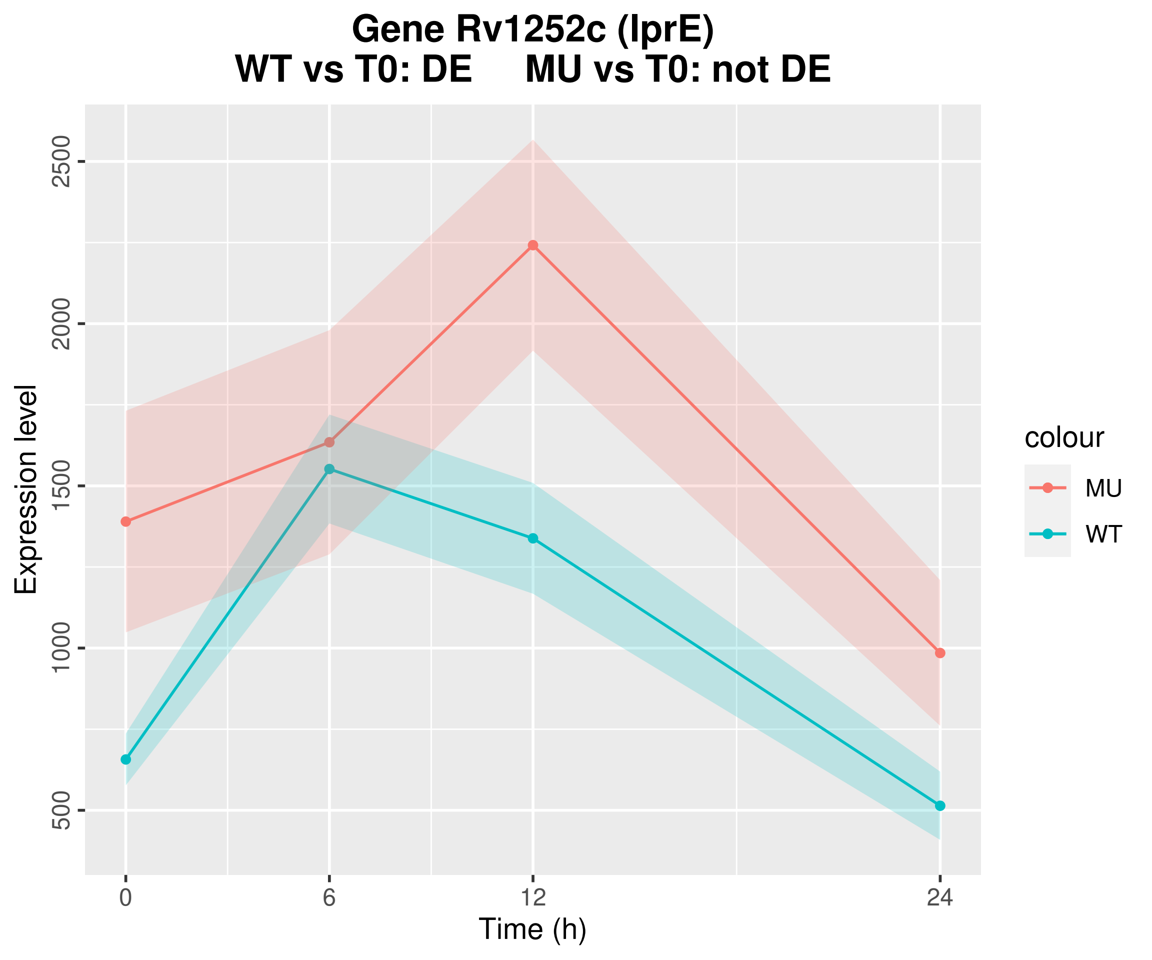
Gene Rv1252c (lprE)
WT vs T0: DE   MU vs T0: not DE
Expression level
2500
2000
1500
1000
500
0
6
12
24
Time (h)
colour
MU
WT

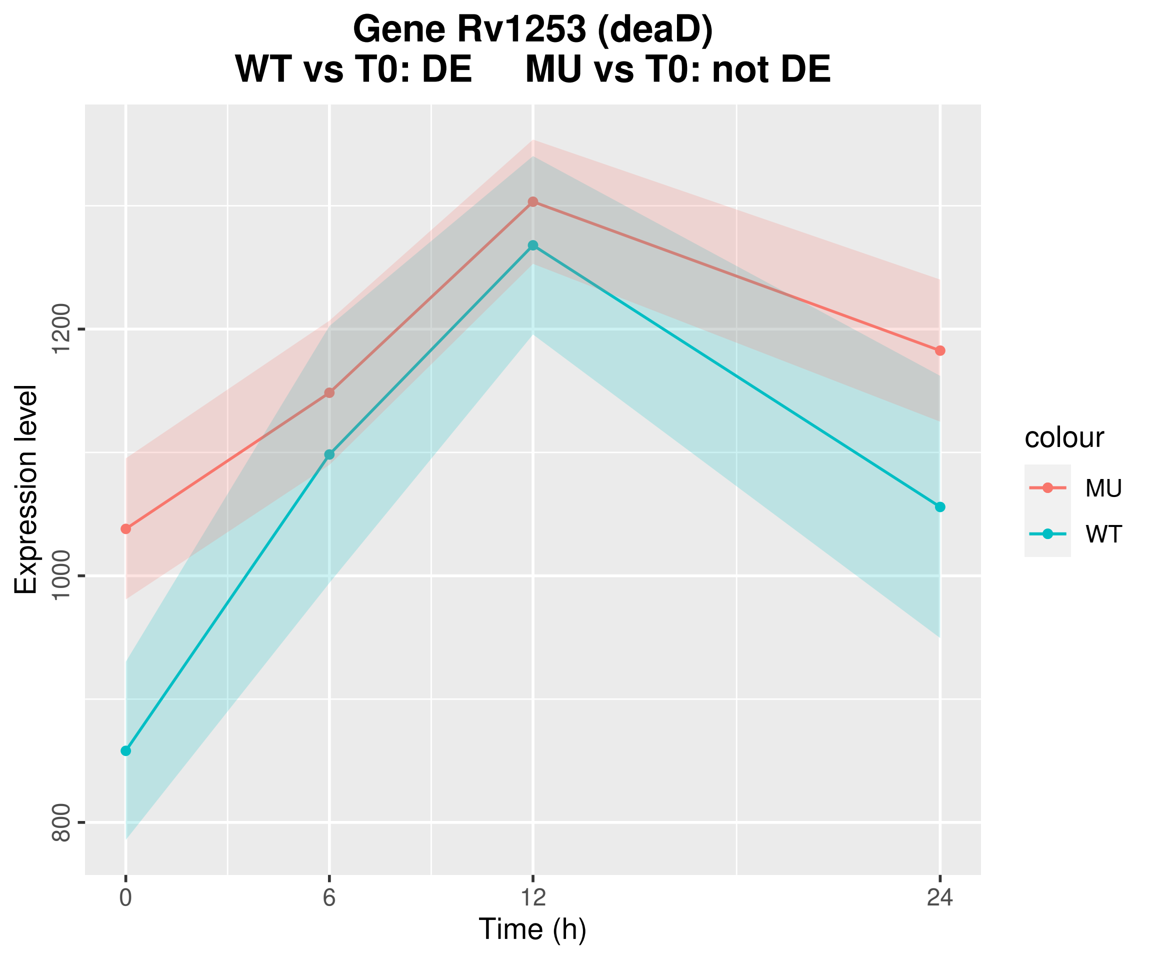
Gene Rv1253 (deaD)
WT vs T0: DE MU vs T0: not DE
Expression level
1200
1000
800
0
6
12
24
Time (h)
colour
MU
WT

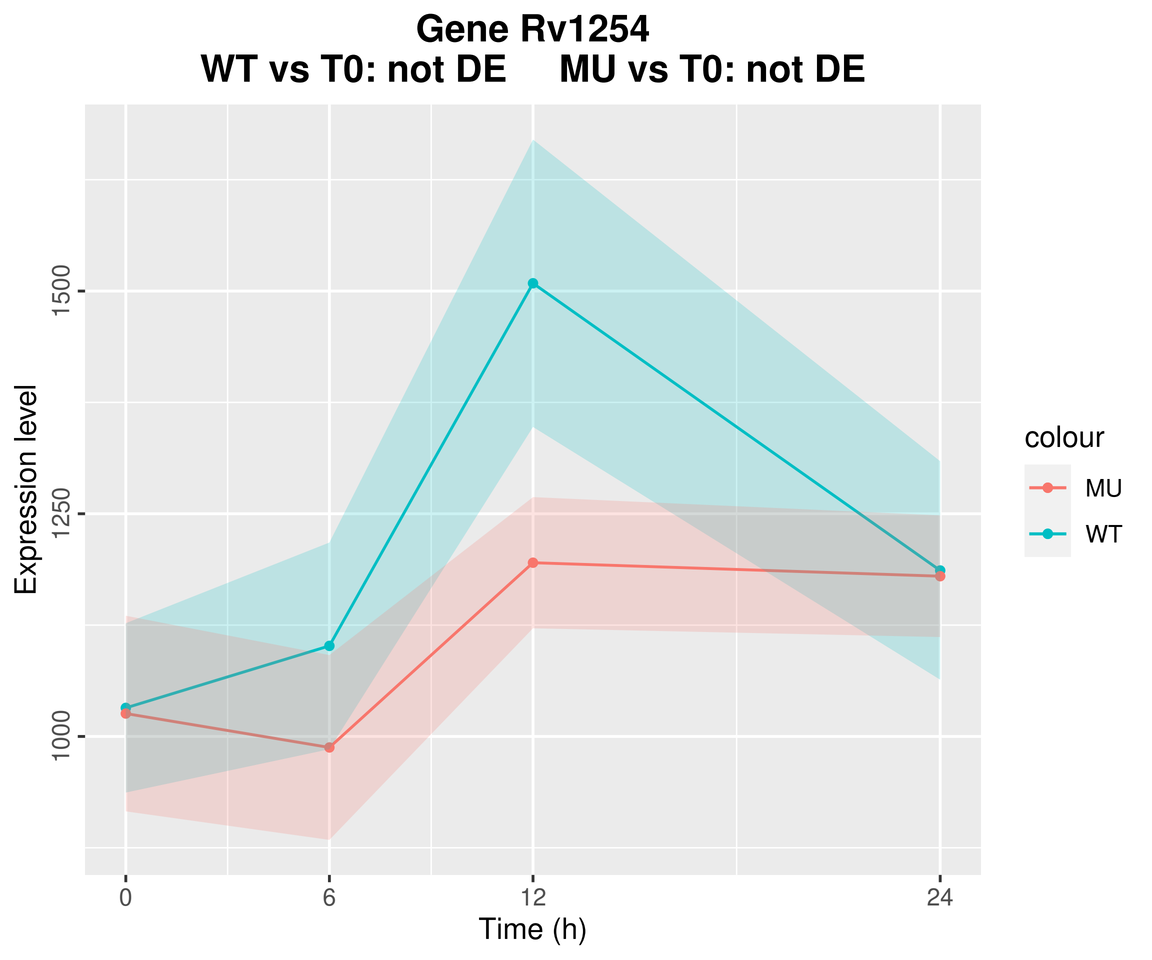
Gene Rv1254
WT vs T0: not DE   MU vs T0: not DE
Expression level
1000
1250
1500
0
6
12
24
Time (h)
colour
MU
WT

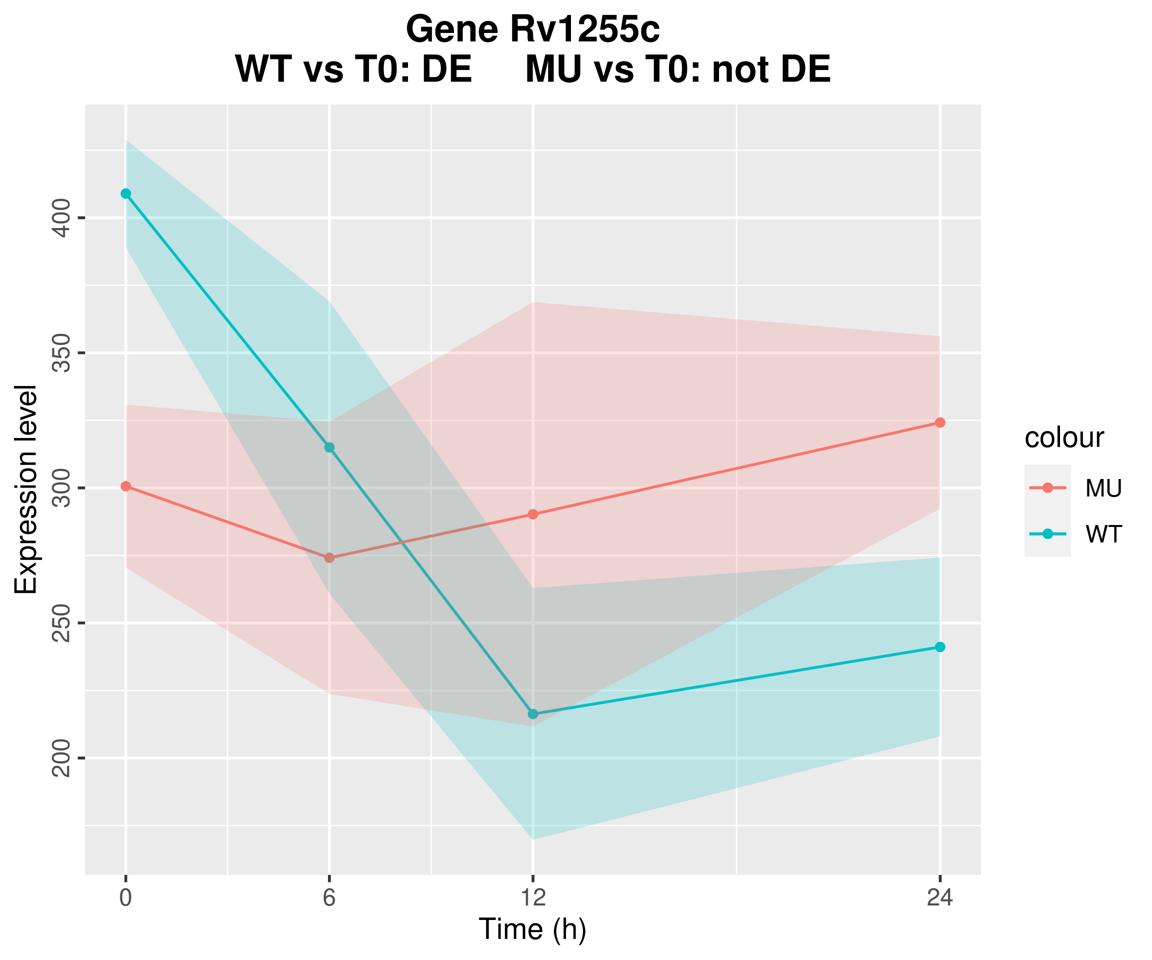

Gene Rv1255c
WT vs T0: DE   MU vs T0: not DE
Expression level
200
250
300
350
400
0
6
12
24
Time (h)
colour
MU
WT

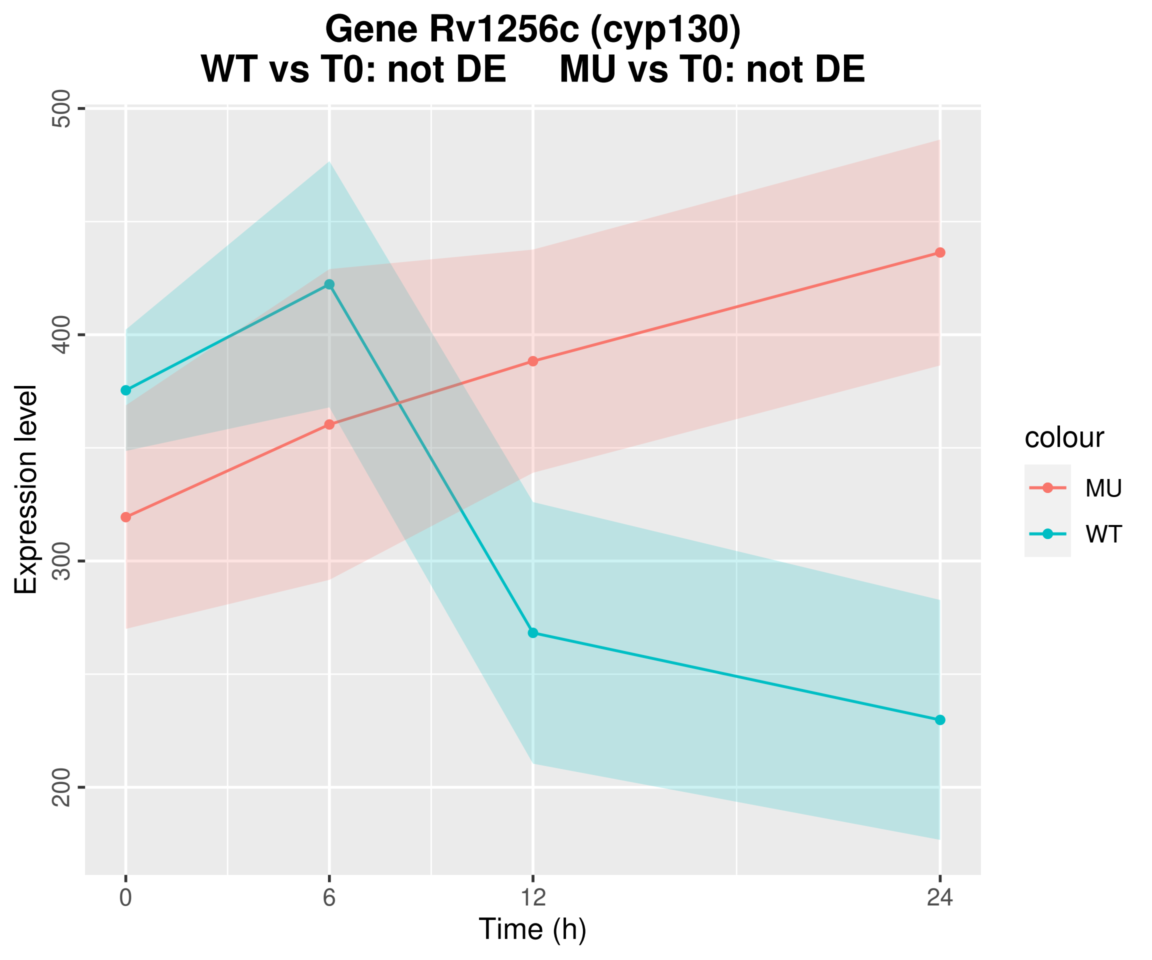
Gene Rv1256c (cyp130)
WT vs T0: not DE   MU vs T0: not DE
500
400
300
200
Expression level
0
6
12
24
Time (h)
colour
MU
WT

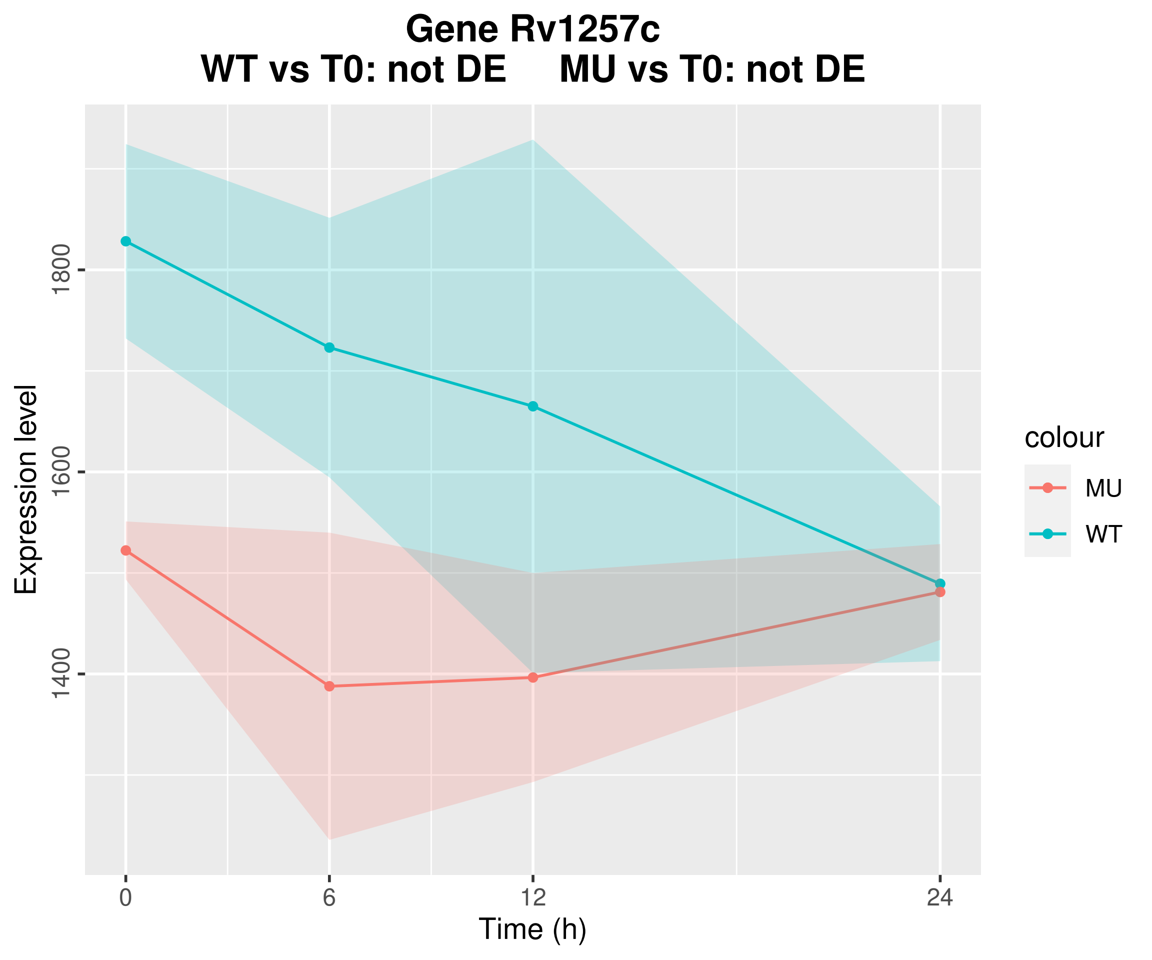

Gene Rv1257c
WT vs T0: not DE MU vs T0: not DE
Expression level
1400
1600
1800
0
6
12
24
Time (h)
colour
MU
WT

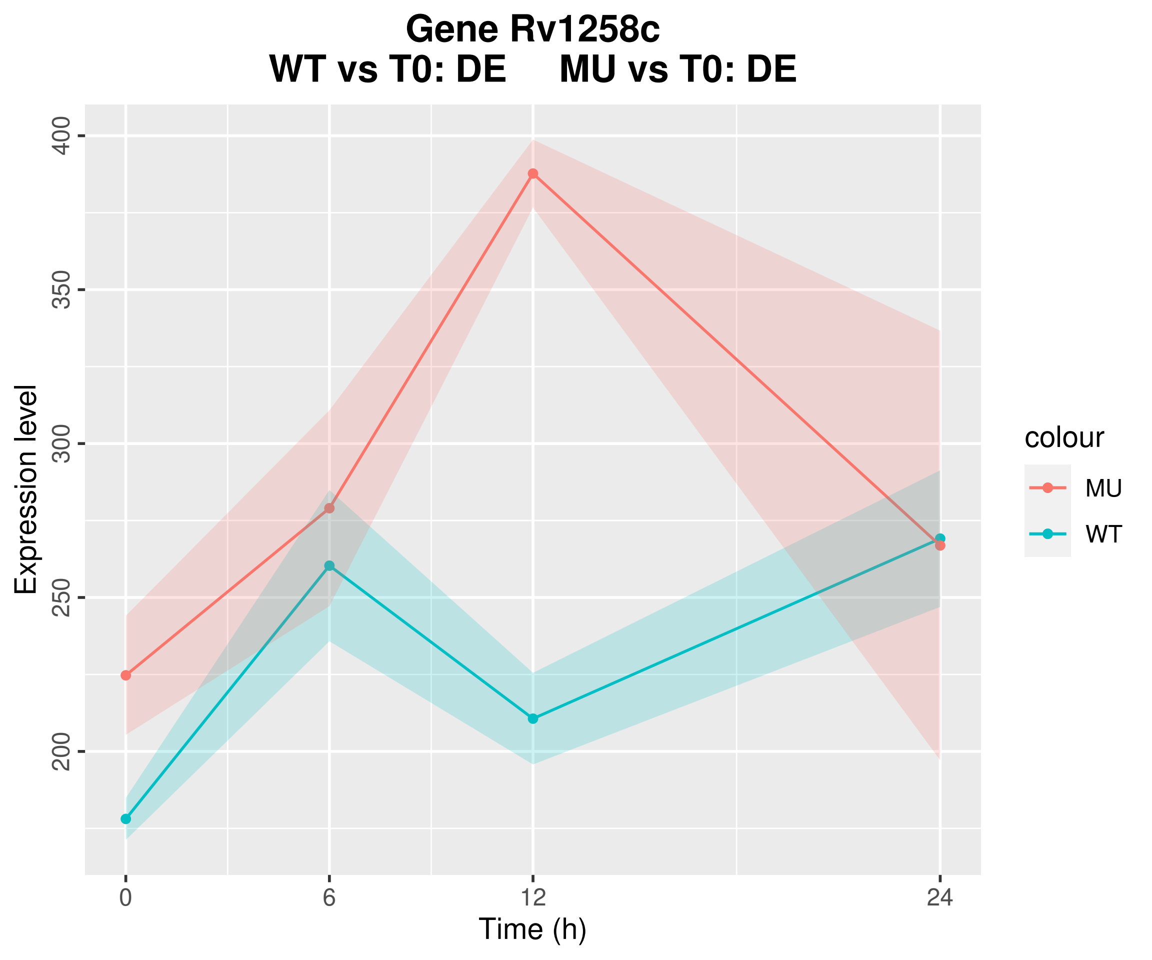

Gene Rv1258c
WT vs T0: DE   MU vs T0: DE
Expression level
400
350
300
250
200
0
6
12
24
Time (h)
colour
MU
WT

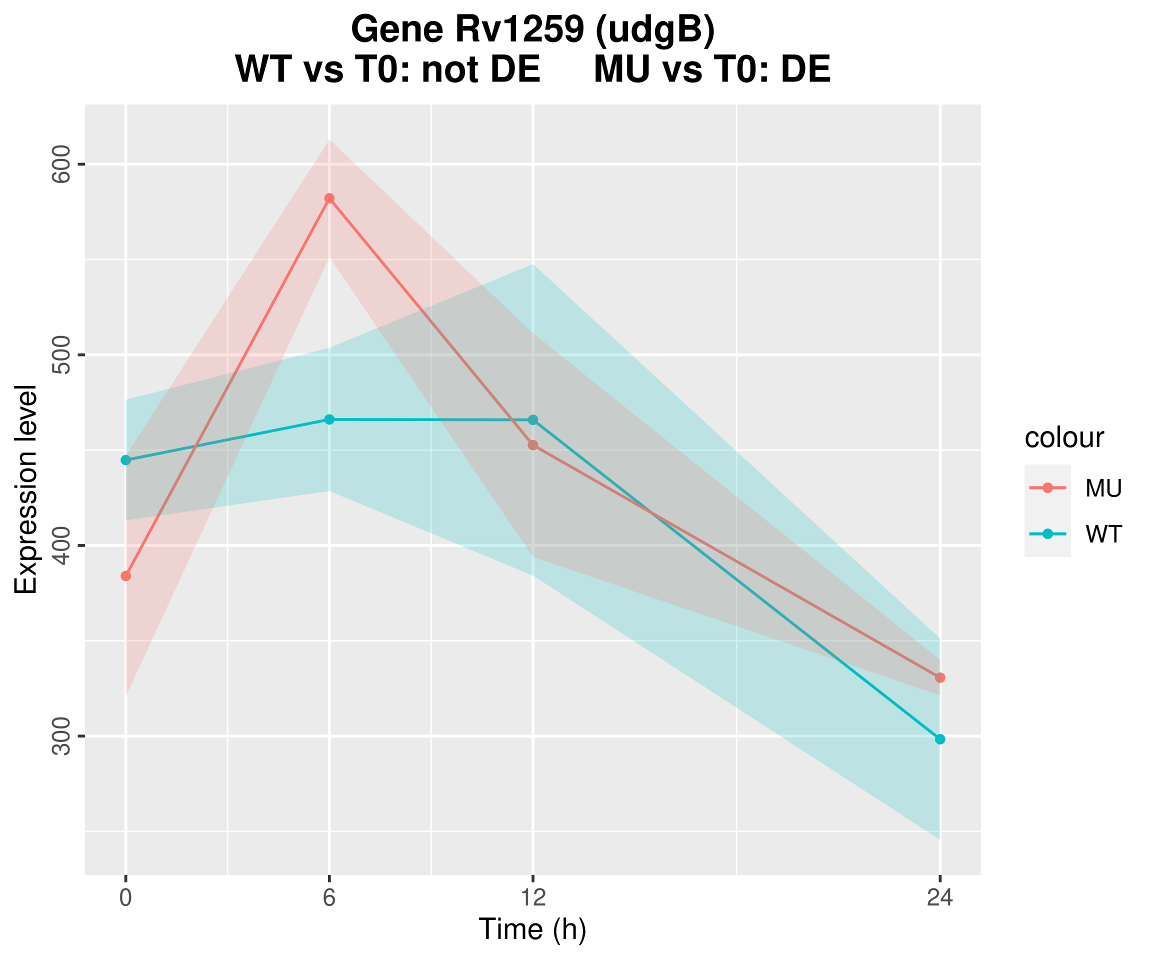

Gene Rv1259 (udgB)
WT vs T0: not DE MU vs T0: DE
600
500
400
300
Expression level
0
6
12
24
Time (h)
colour
MU
WT

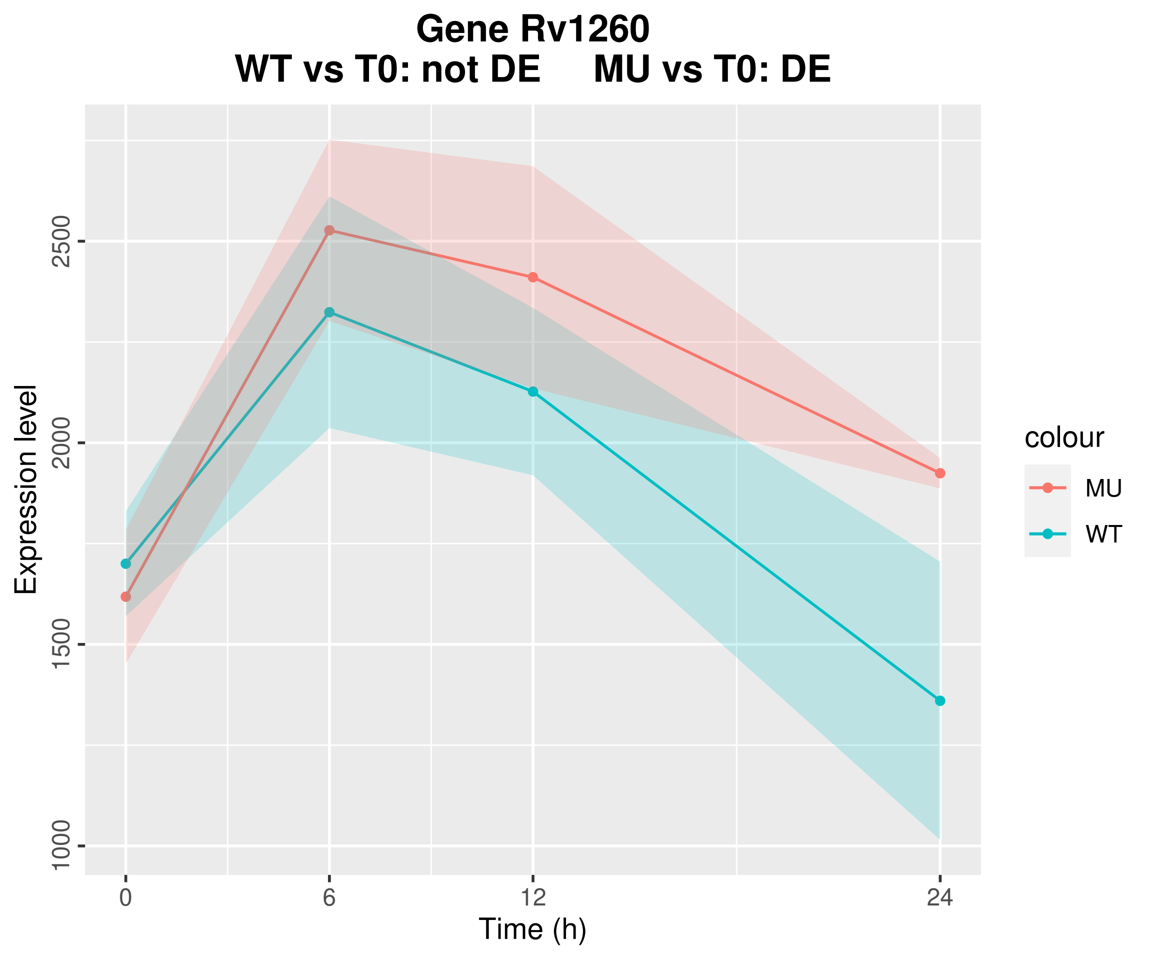

Gene Rv1260
WT vs T0: not DE MU vs T0: DE
Expression level
2500
2000
1500
1000
0
6
12
24
Time (h)
colour
MU
WT

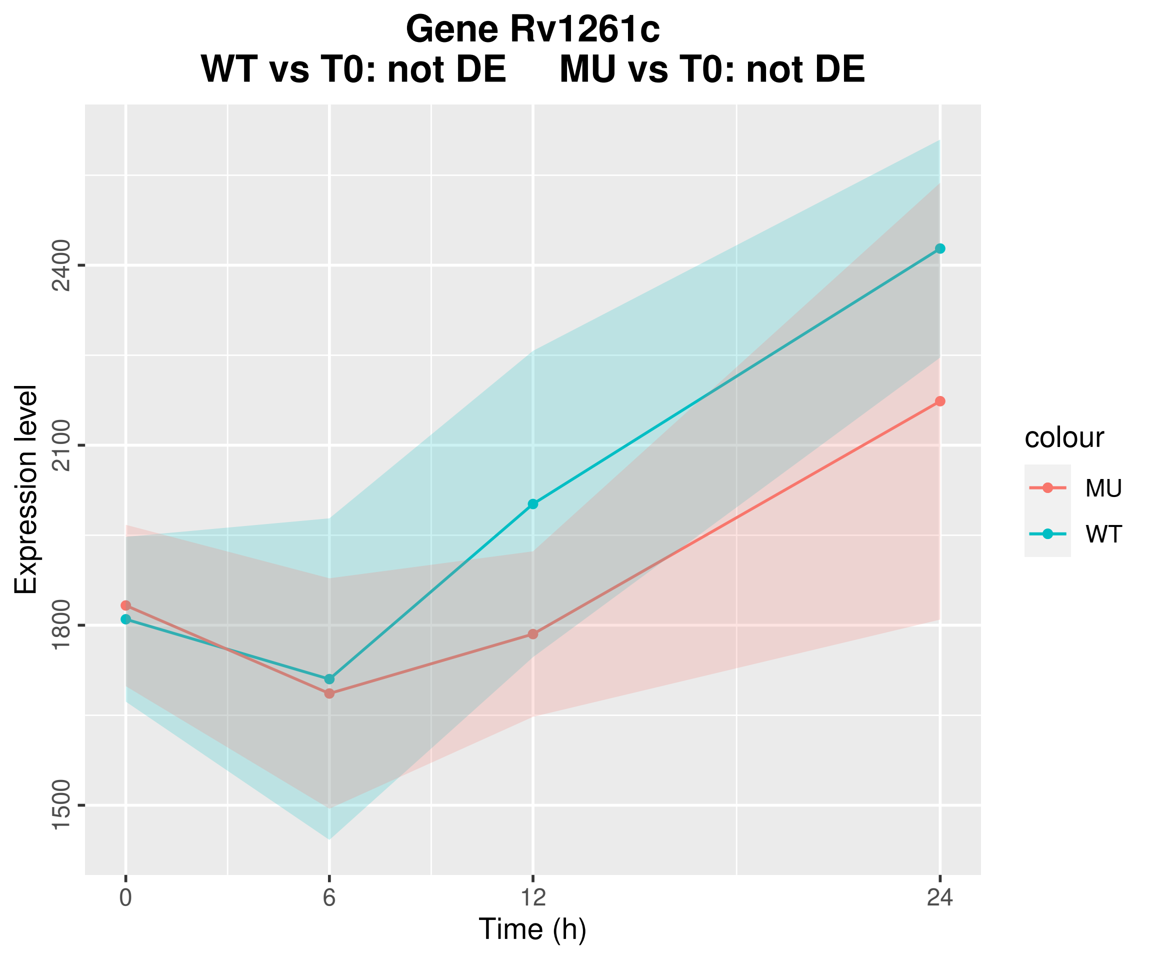

Gene Rv1261c
WT vs T0: not DE MU vs T0: not DE
Expression level
2400
2100
1800
1500
0
6
12
24
Time (h)
colour
MU
WT

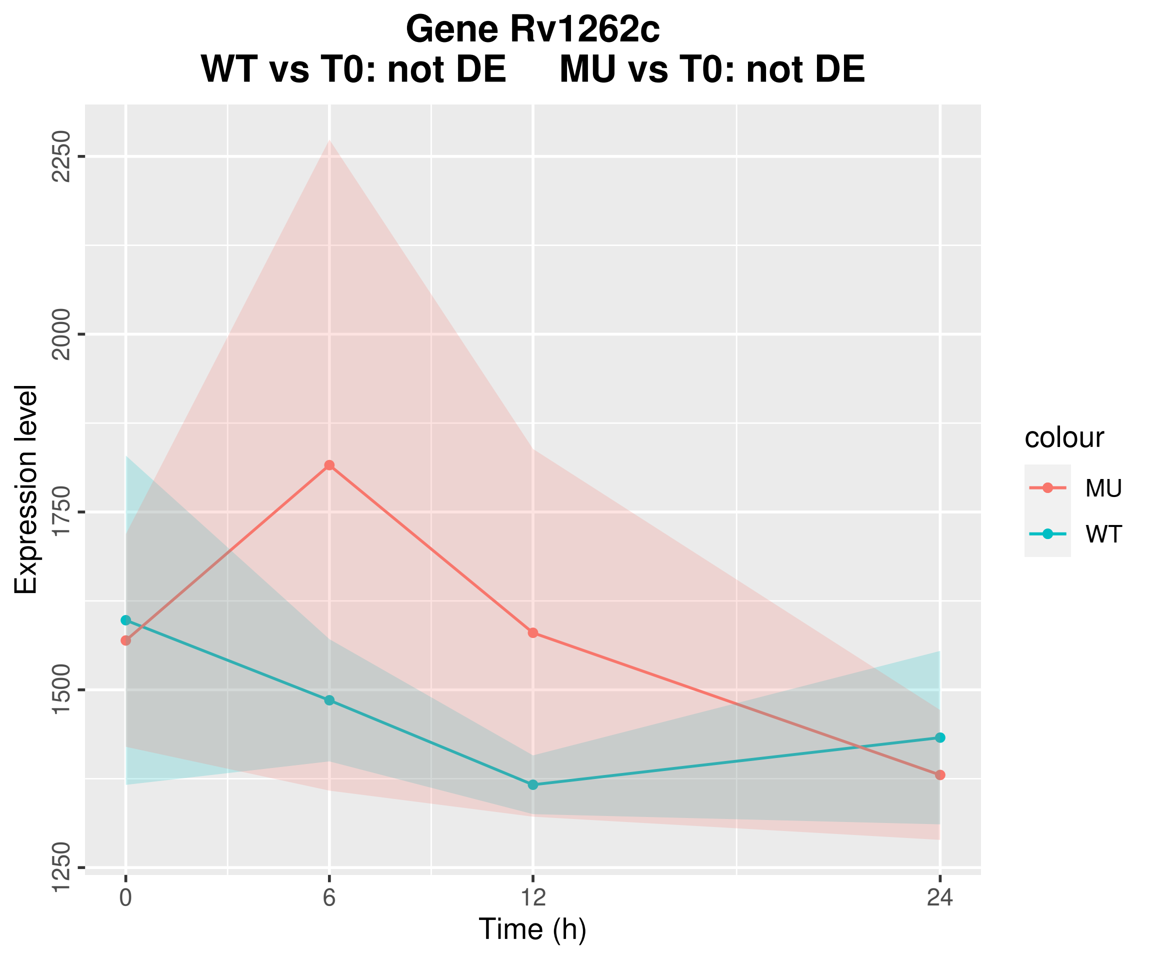
Gene Rv1262c
WT vs T0: not DE   MU vs T0: not DE
Expression level
2250
2000
1750
1500
1250
Time (h)
0
6
12
24
colour
MU
WT

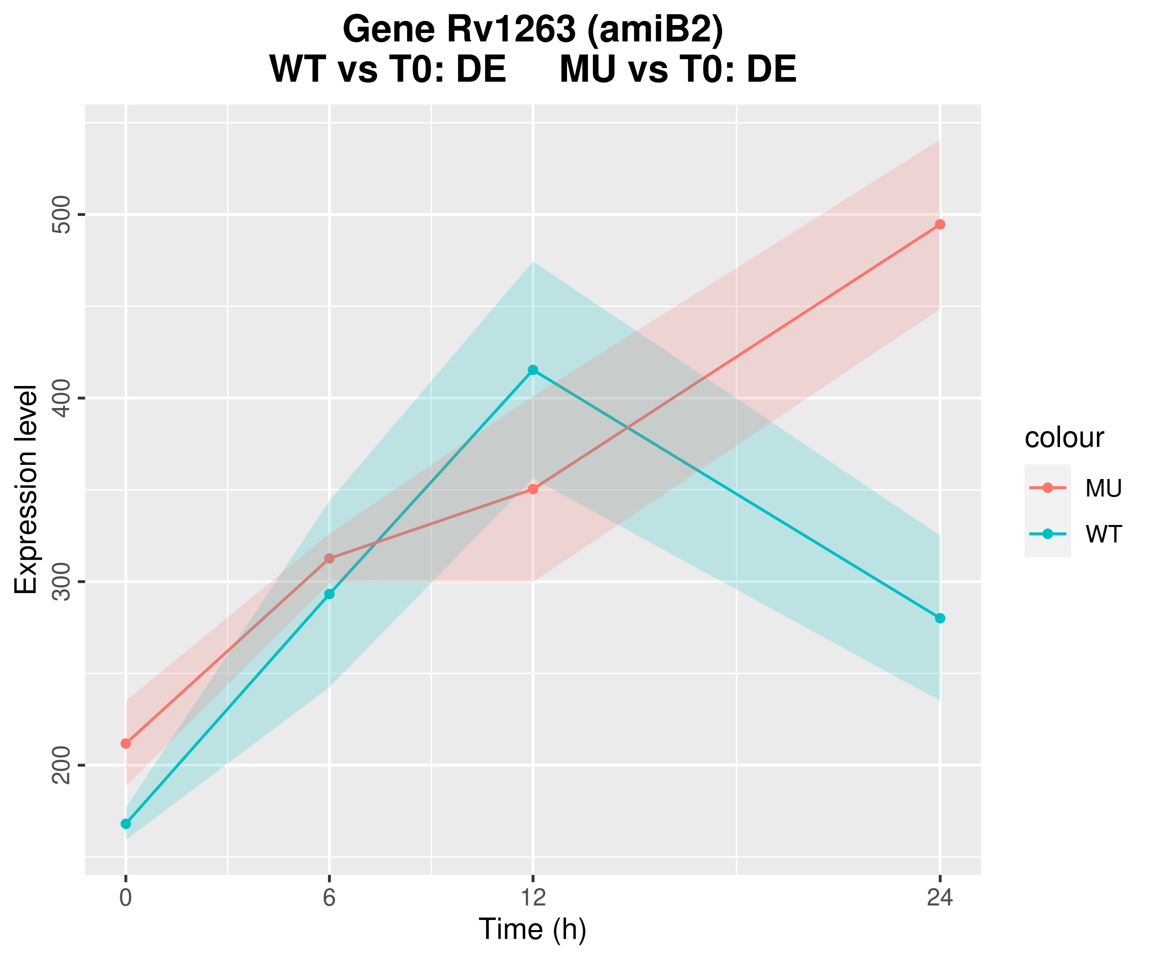

Gene Rv1263 (amiB2)
WT vs T0: DE MU vs T0: DE
Expression level
500
400
300
200
0
6
12
24
Time (h)
colour
MU
WT

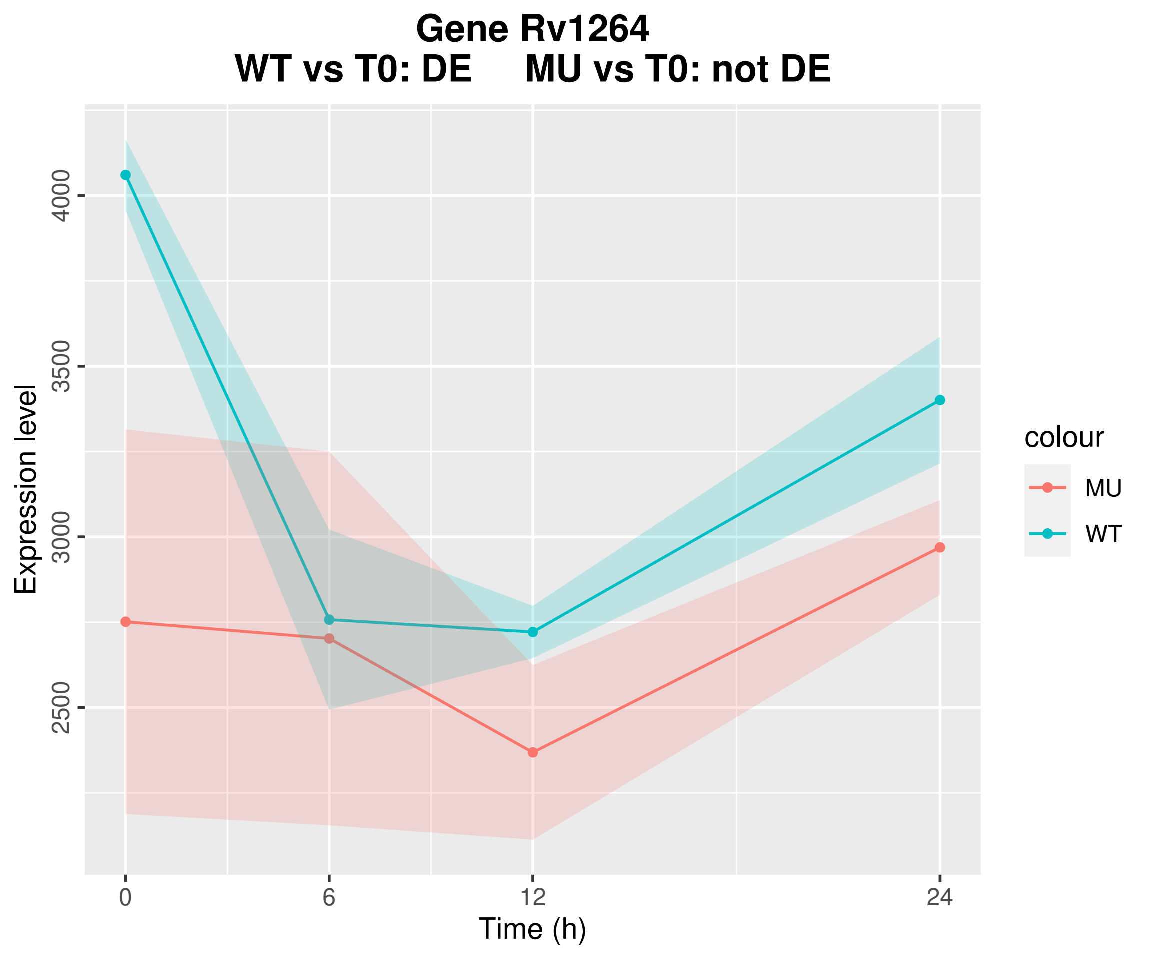

Gene Rv1264
WT vs T0: DE   MU vs T0: not DE
Expression level
4000
3500
3000
2500
0
6
12
24
Time (h)
colour
MU
WT

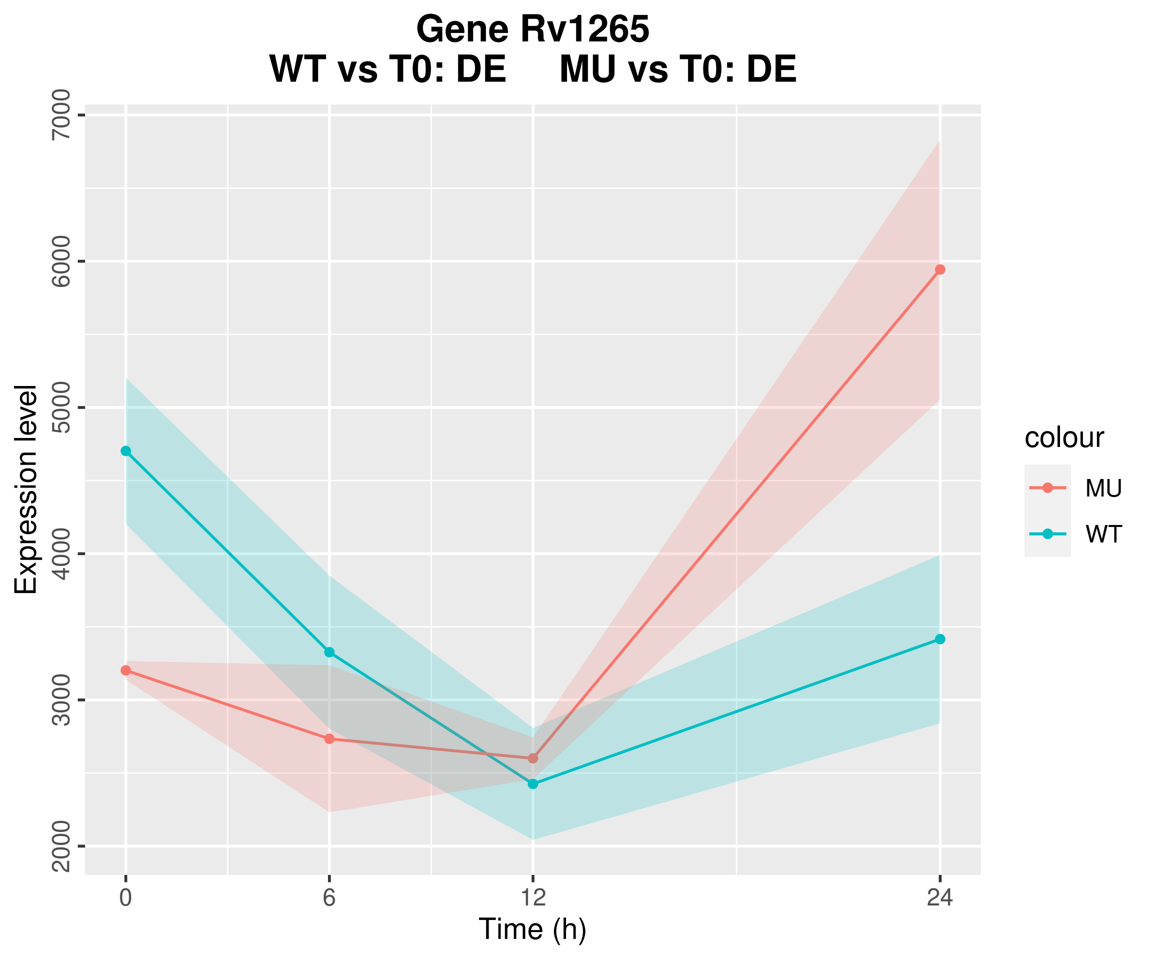

Gene Rv1265
WT vs T0: DE MU vs T0: DE
Expression level
7000
6000
5000
4000
3000
2000
0
6
12
24
Time (h)
colour
MU
WT

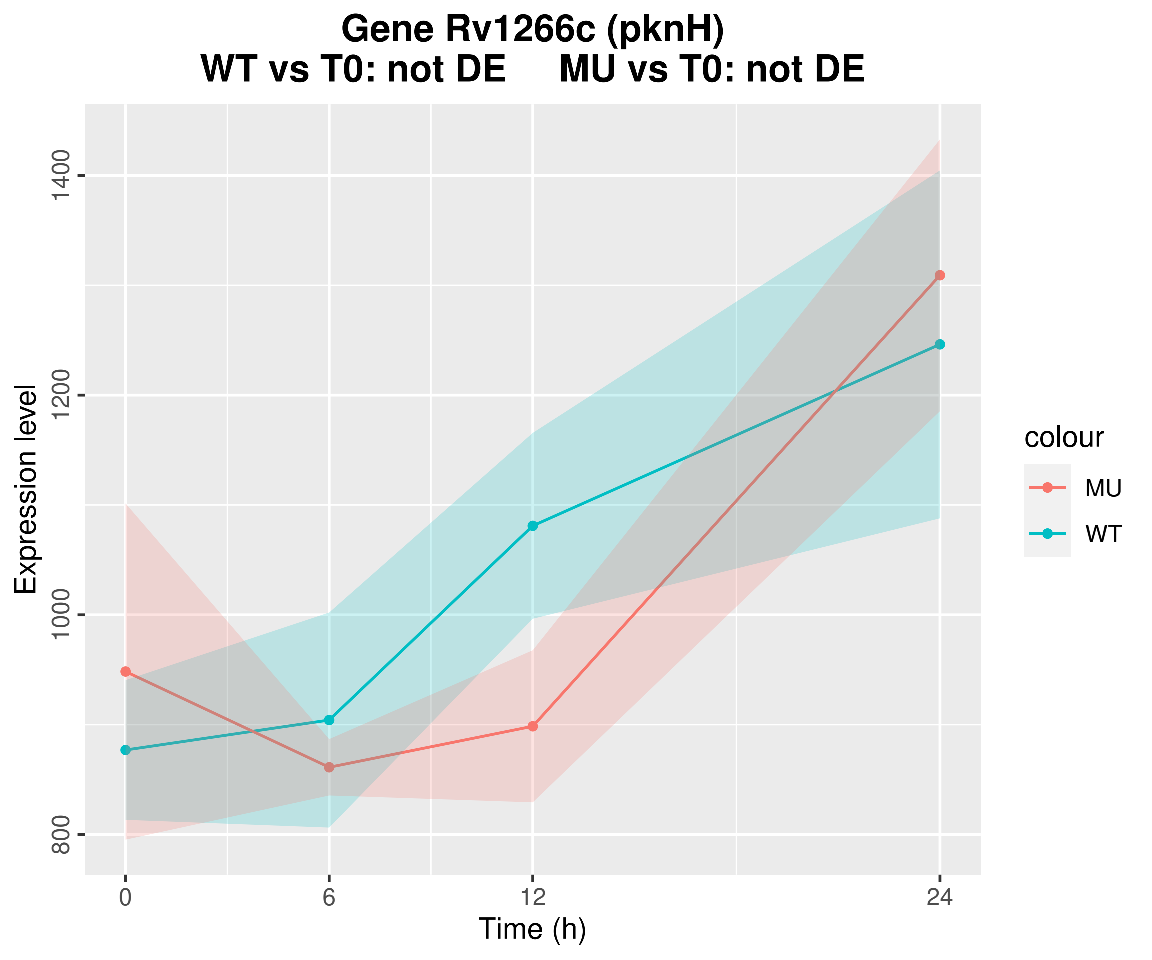

Gene Rv1266c (pknH)
WT vs T0: not DE   MU vs T0: not DE
Expression level
800
1000
1200
1400
0
6
12
24
Time (h)
colour
MU
WT

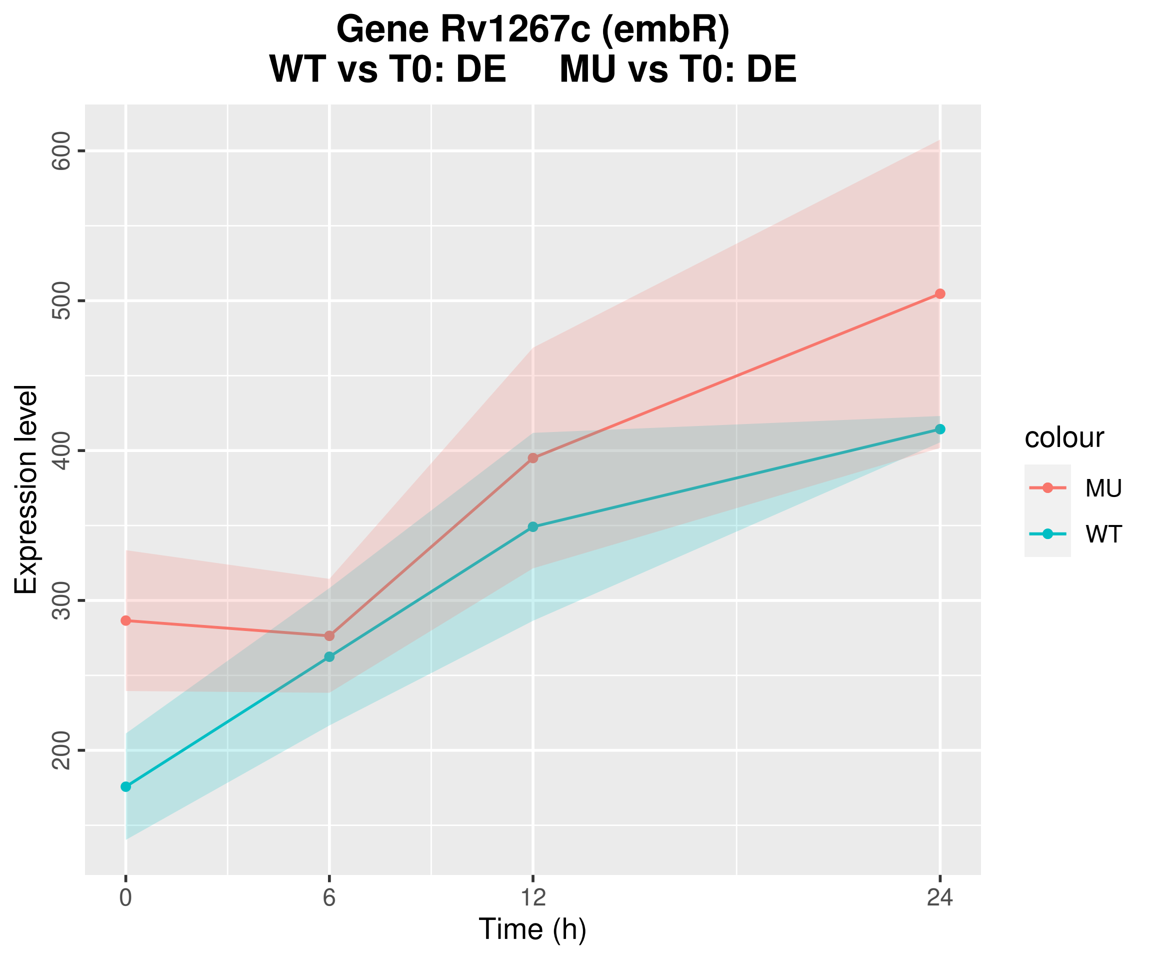
Gene Rv1267c (embR)
WT vs T0: DE MU vs T0: DE
Expression level
600
500
400
300
200
Time (h)
0
6
12
24
colour
MU
WT

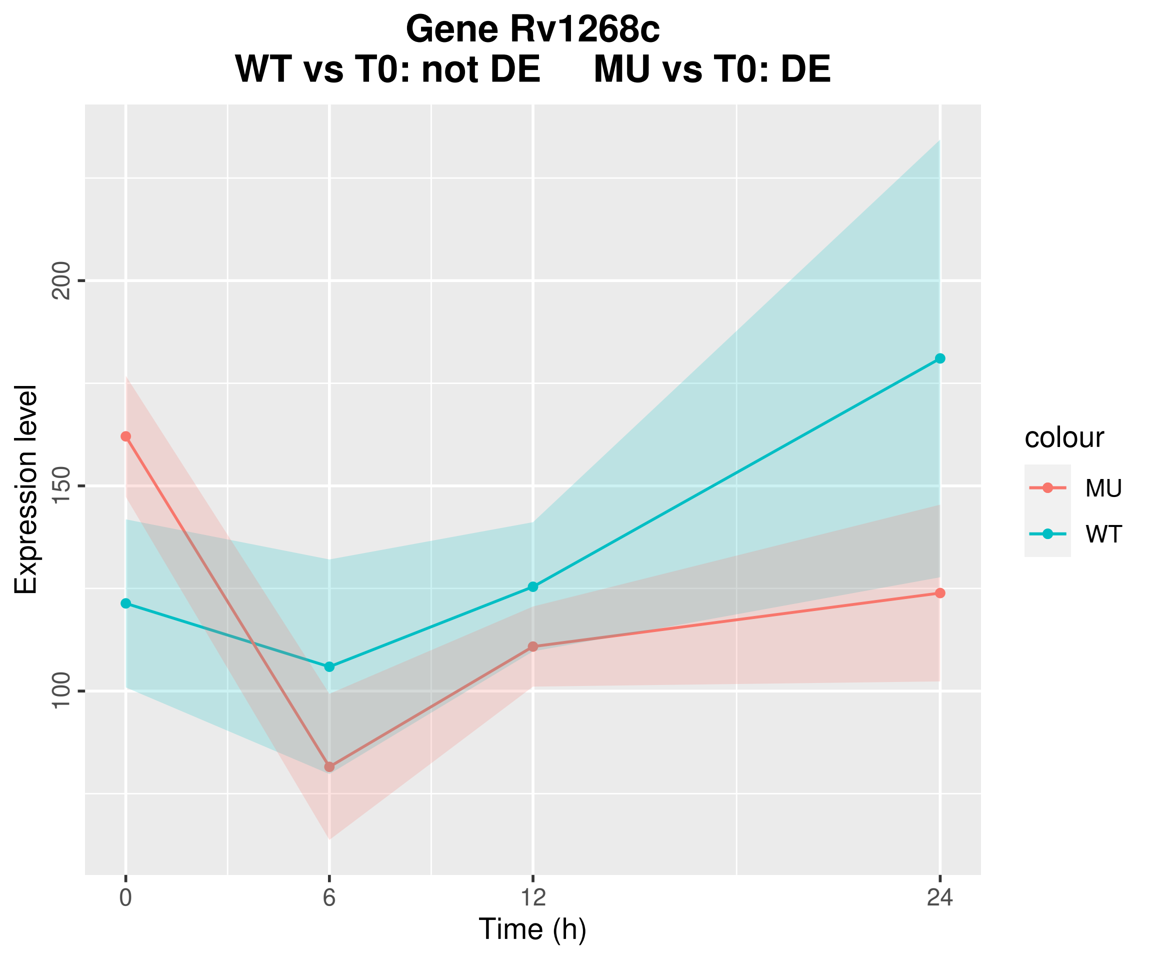
Gene Rv1268c
WT vs T0: not DE   MU vs T0: DE
Expression level
200
150
100
0
6
12
24
Time (h)
colour
MU
WT

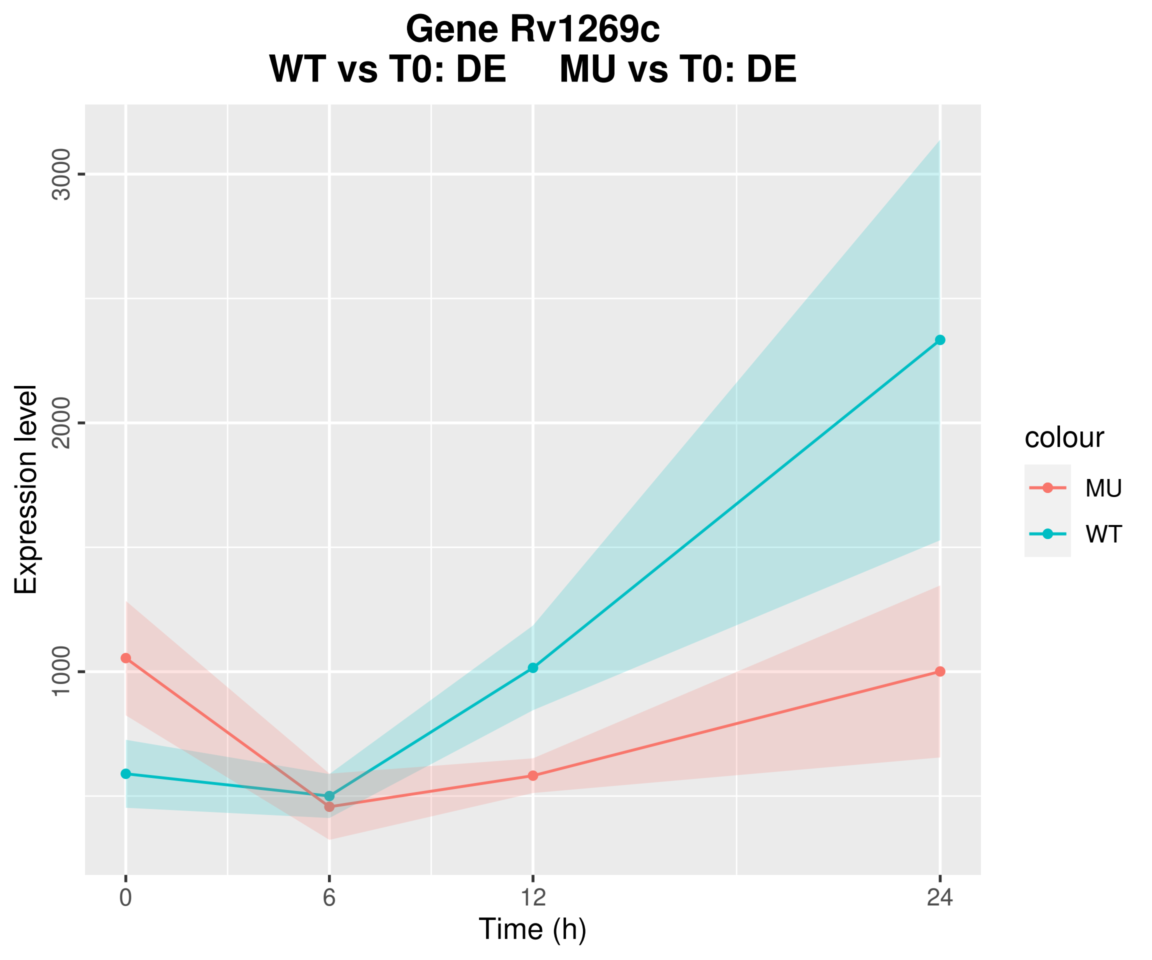

Gene Rv1269c
WT vs T0: DE MU vs T0: DE
3000
2000
1000
Expression level
0
6
12
24
Time (h)
colour
MU
WT

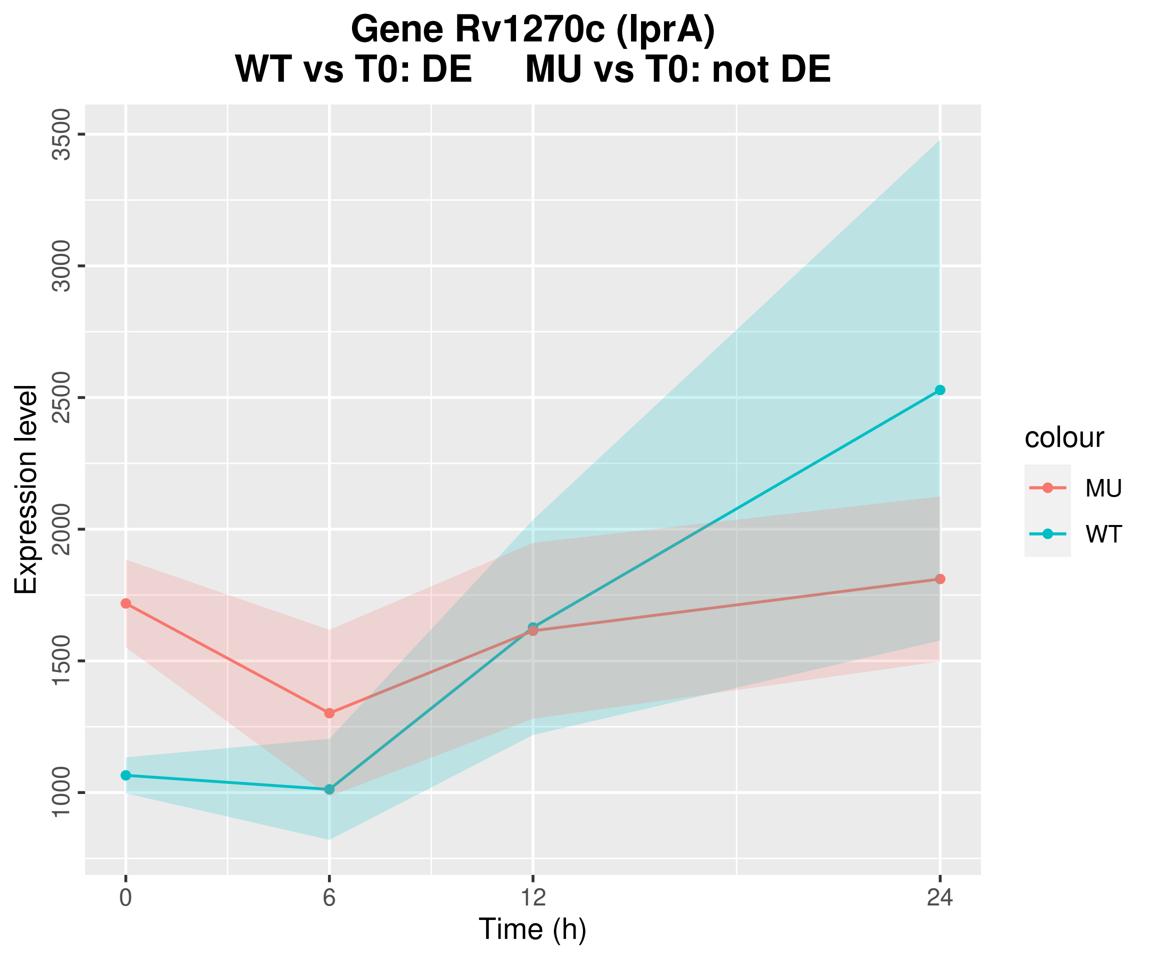
Gene Rv1270c (lprA)
WT vs T0: DE   MU vs T0: not DE
Expression level
1000
1500
2000
2500
3000
3500
0
6
12
24
Time (h)
colour
MU
WT

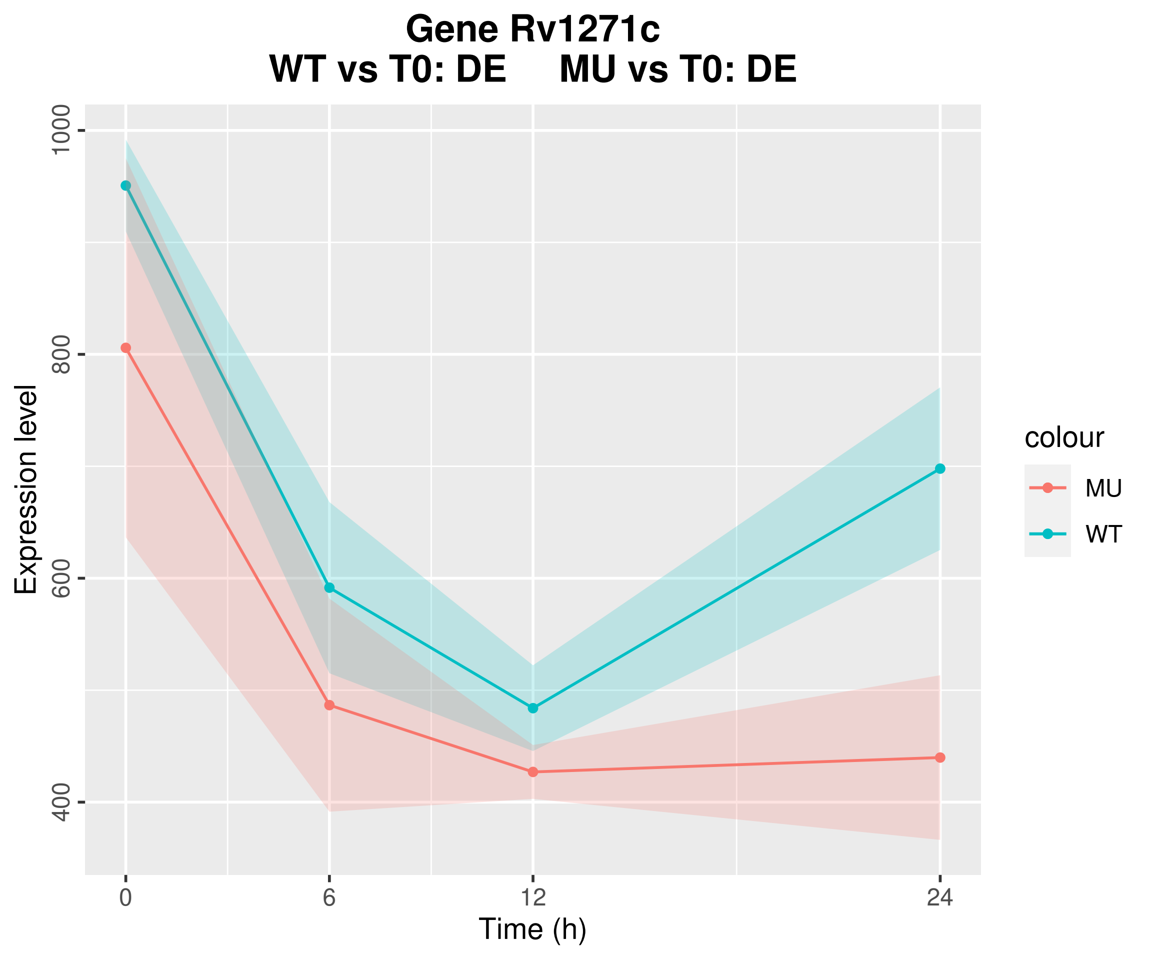

Gene Rv1271c
WT vs T0: DE   MU vs T0: DE
Expression level
1000
800
600
400
0
6
12
24
Time (h)
colour
MU
WT

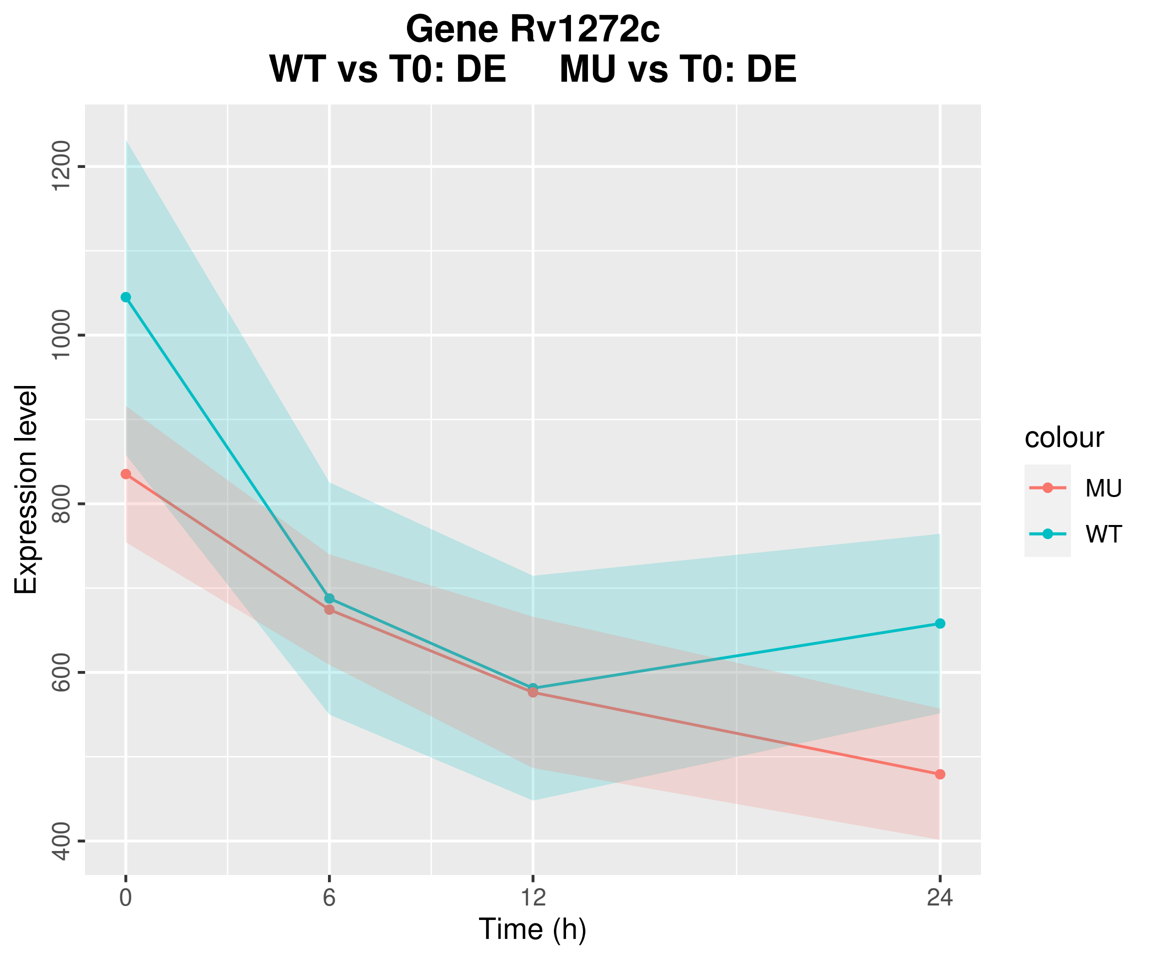
Gene Rv1272c
WT vs T0: DE MU vs T0: DE
Expression level
1200
1000
800
600
400
0
6
12
24
Time (h)
colour
MU
WT

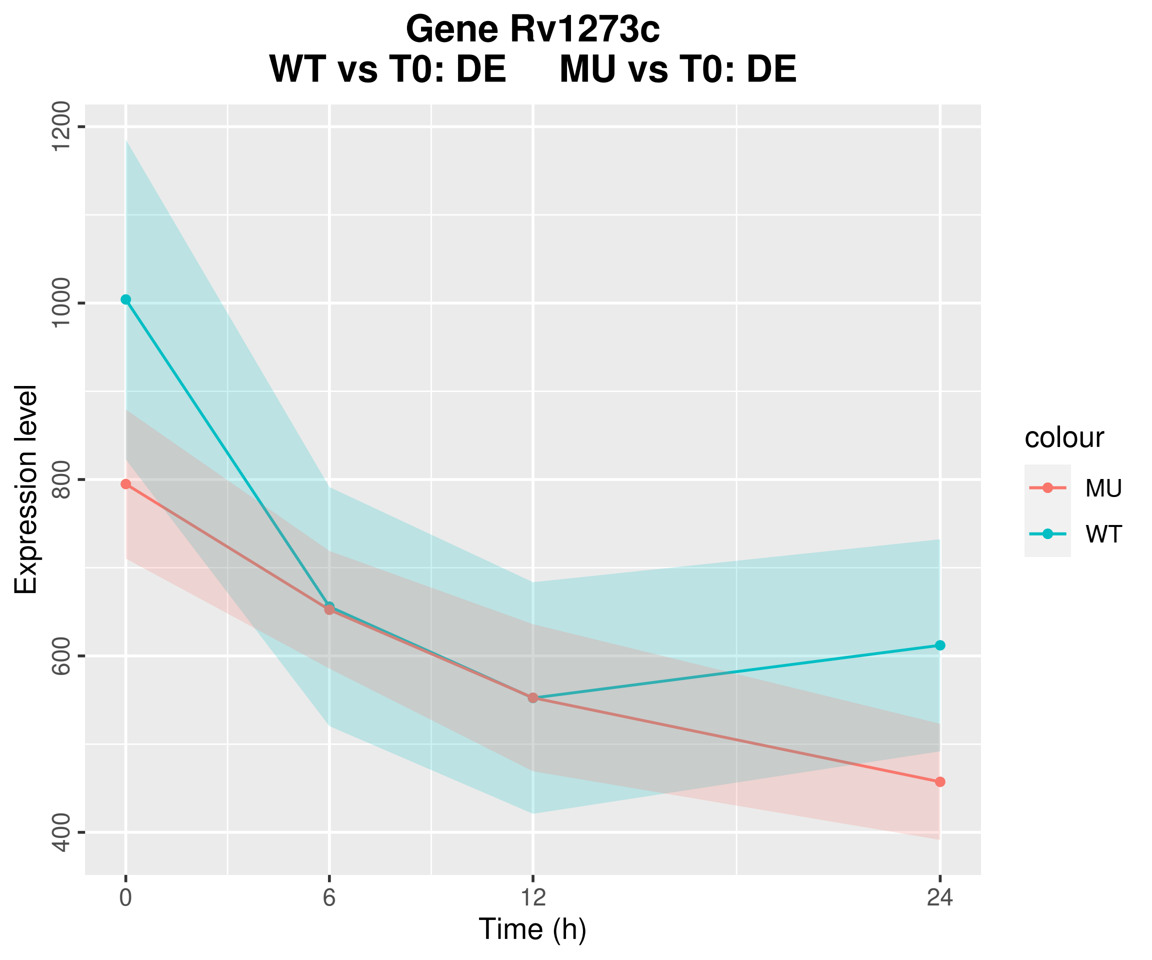
Gene Rv1273c
WT vs T0: DE   MU vs T0: DE
Expression level
1200
1000
800
600
400
0
6
12
24
Time (h)
colour
MU
WT

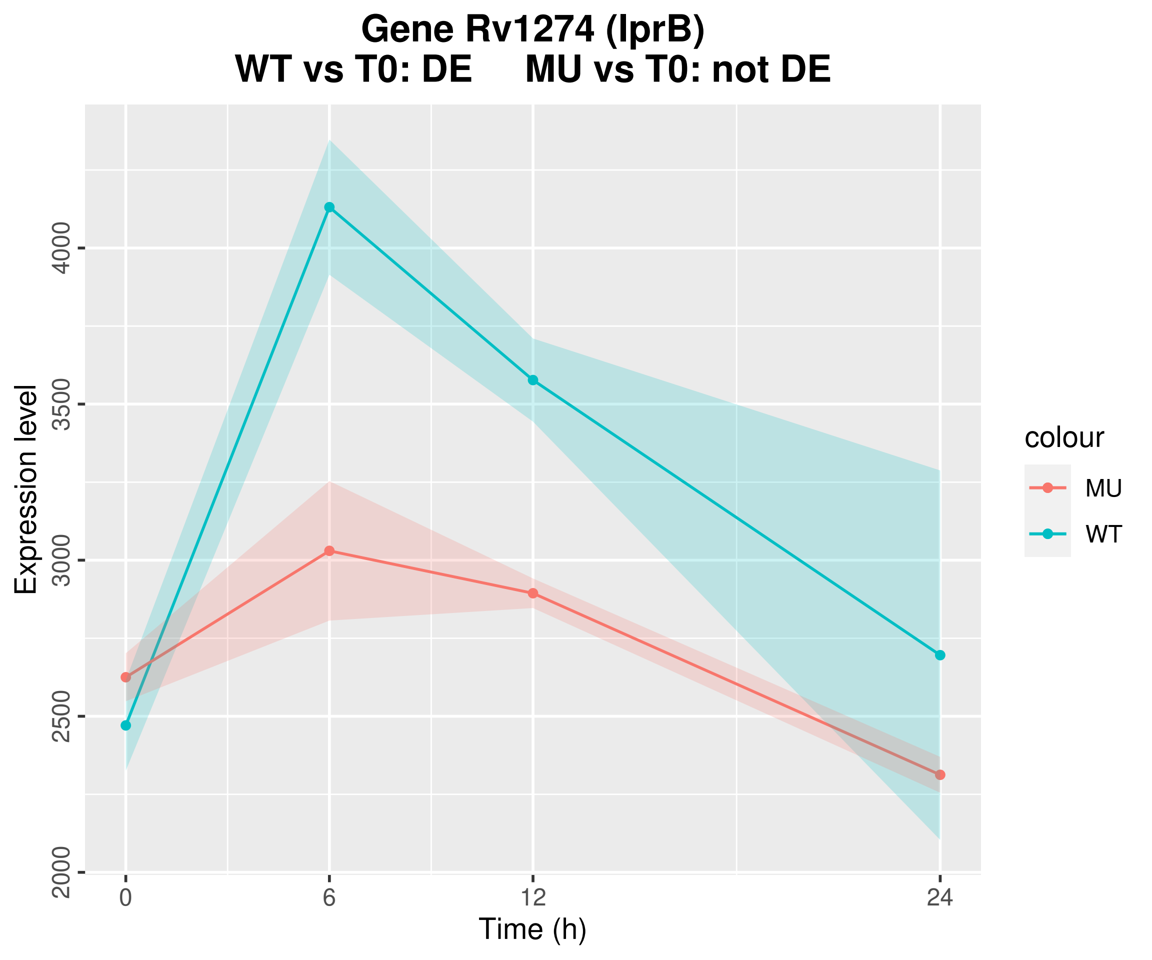

Gene Rv1274 (lprB)
WT vs T0: DE MU vs T0: not DE
Expression level
2000
2500
3000
3500
4000
0
6
12
24
Time (h)
colour
MU
WT

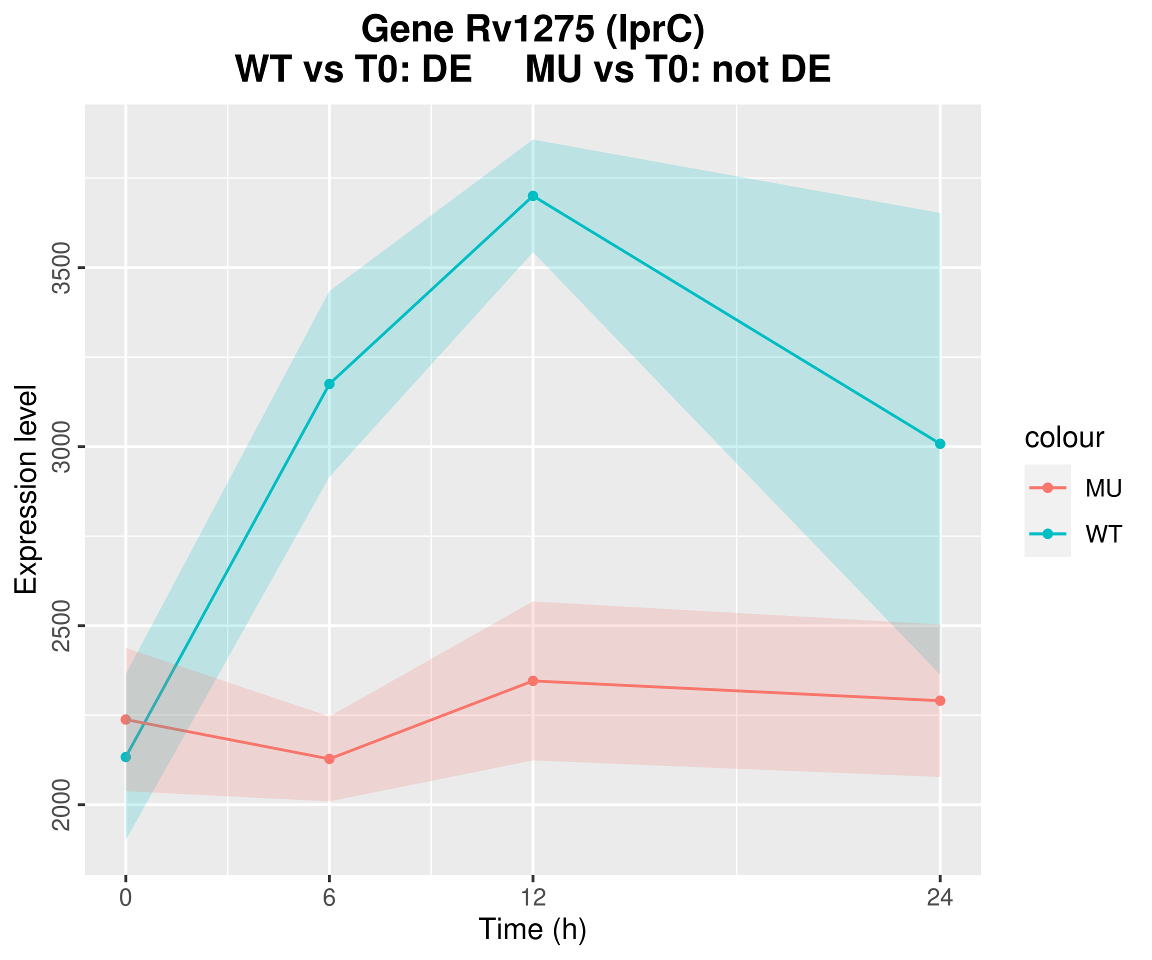
Gene Rv1275 (lprC)
WT vs T0: DE   MU vs T0: not DE
Expression level
3500
3000
2500
2000
0
6
12
24
Time (h)
colour
MU
WT

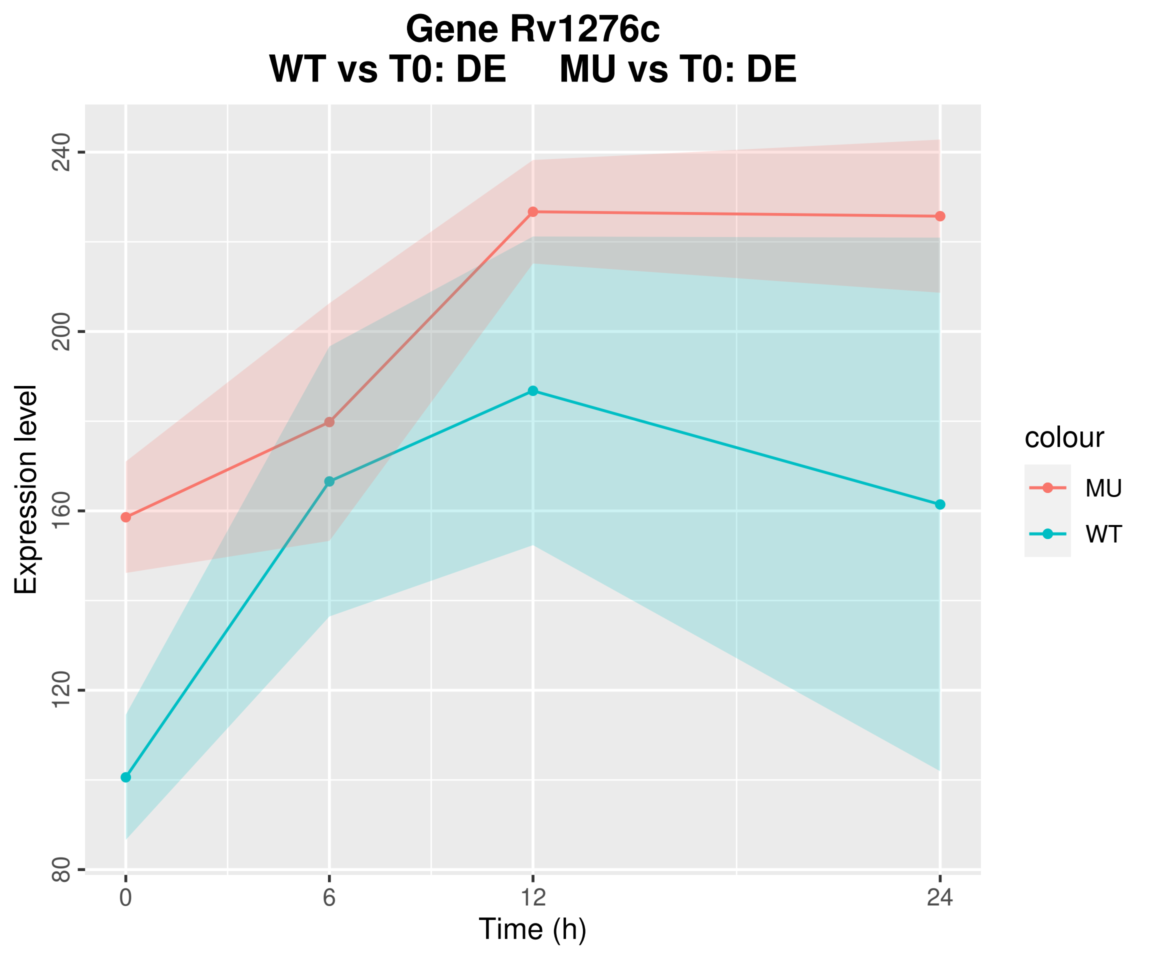
Gene Rv1276c
WT vs T0: DE MU vs T0: DE
Expression level
240
200
160
120
80
0
6
12
24
Time (h)
colour
MU
WT

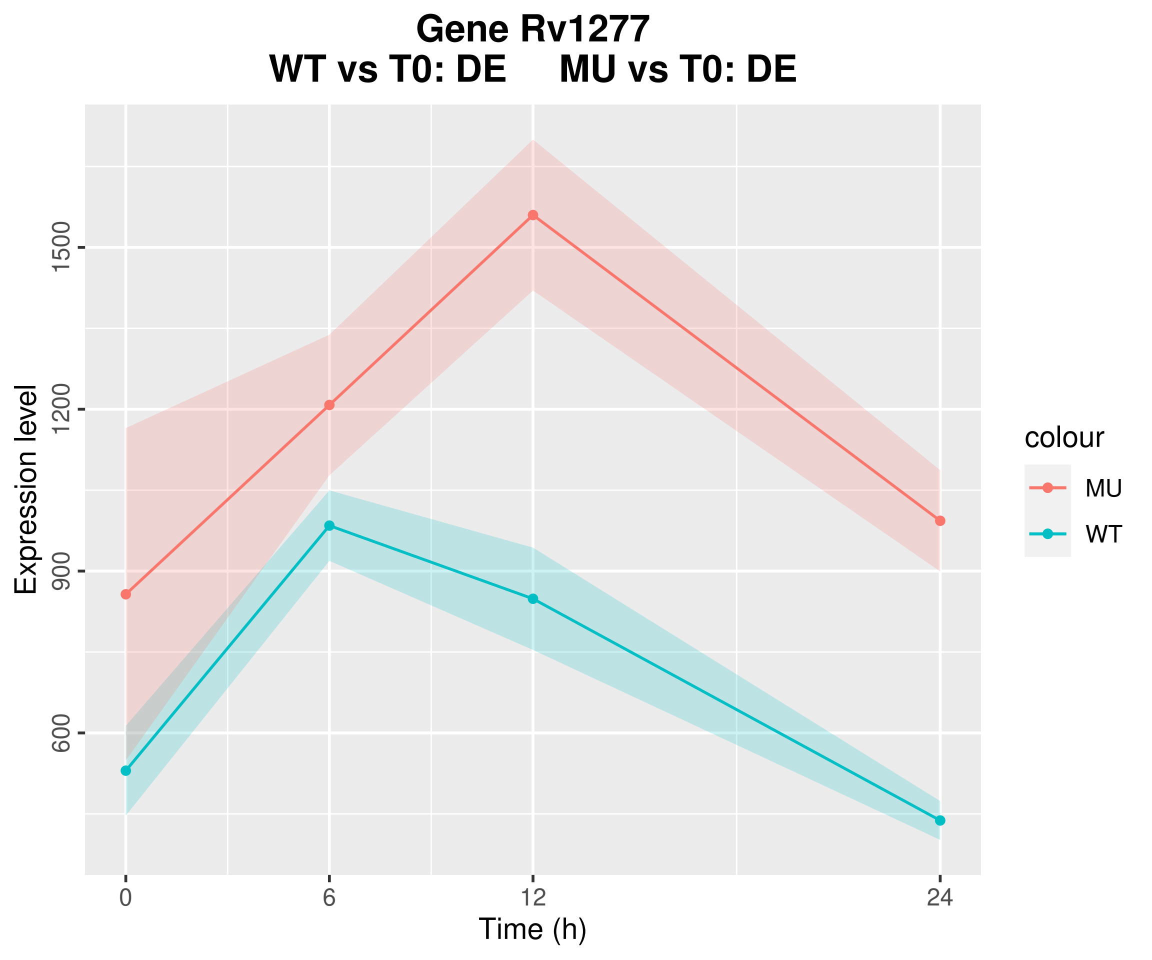

Gene Rv1277
WT vs T0: DE   MU vs T0: DE
Expression level
1500
1200
900
600
0
6
12
24
Time (h)
colour
MU
WT

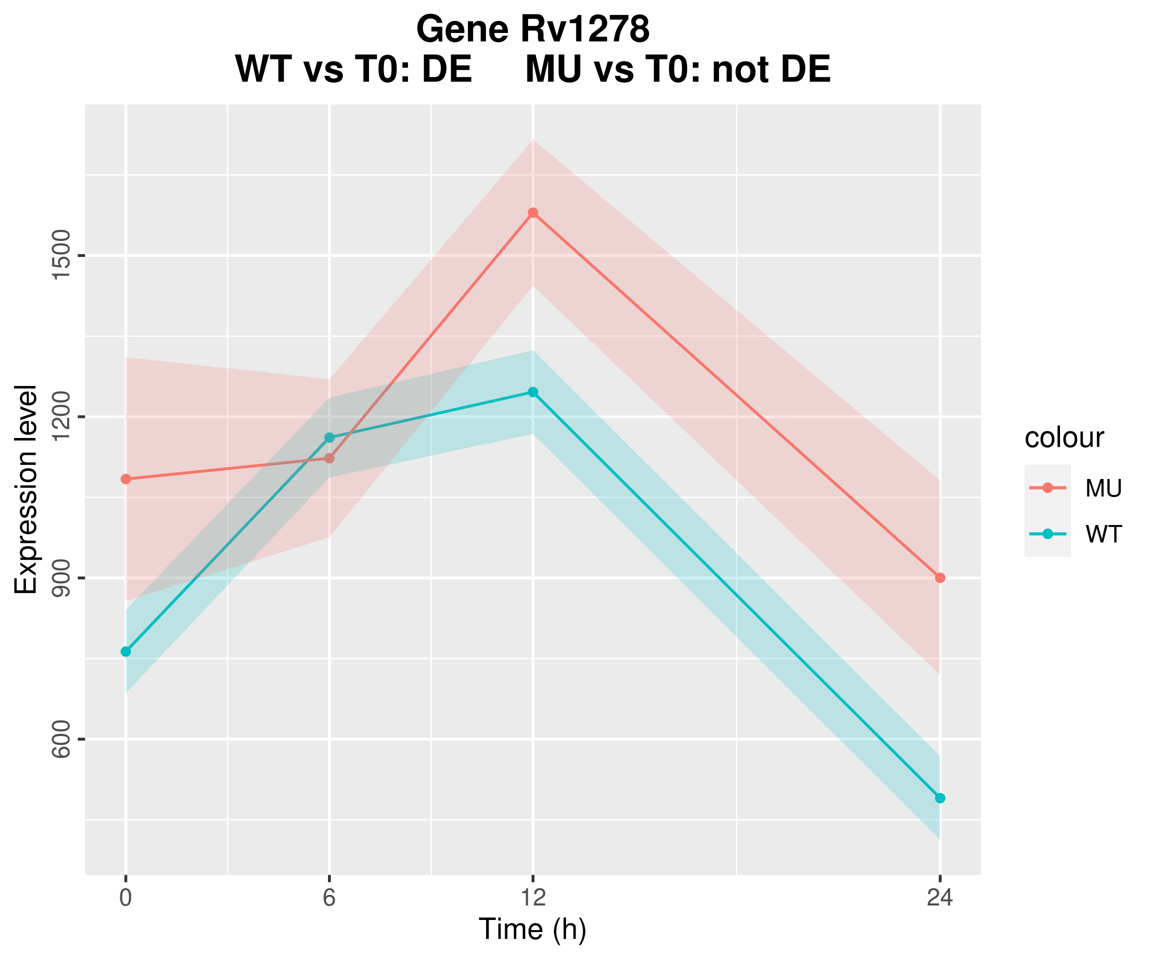
Gene Rv1278
WT vs T0: DE   MU vs T0: not DE
Expression level
1500
1200
900
600
0
6
12
24
Time (h)
colour
MU
WT

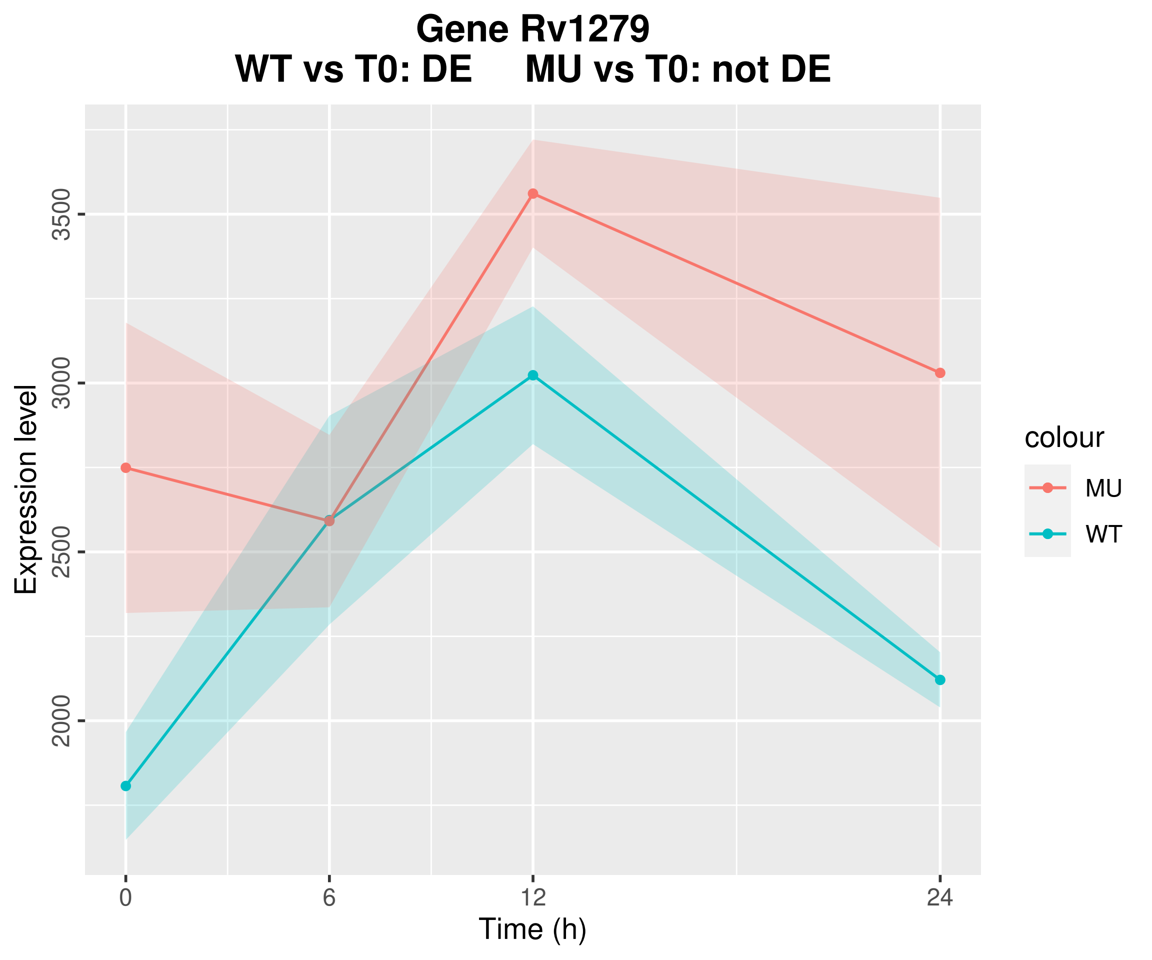

Gene Rv1279
WT vs T0: DE   MU vs T0: not DE
Expression level
2000
2500
3000
3500
0
6
12
24
Time (h)
colour
MU
WT

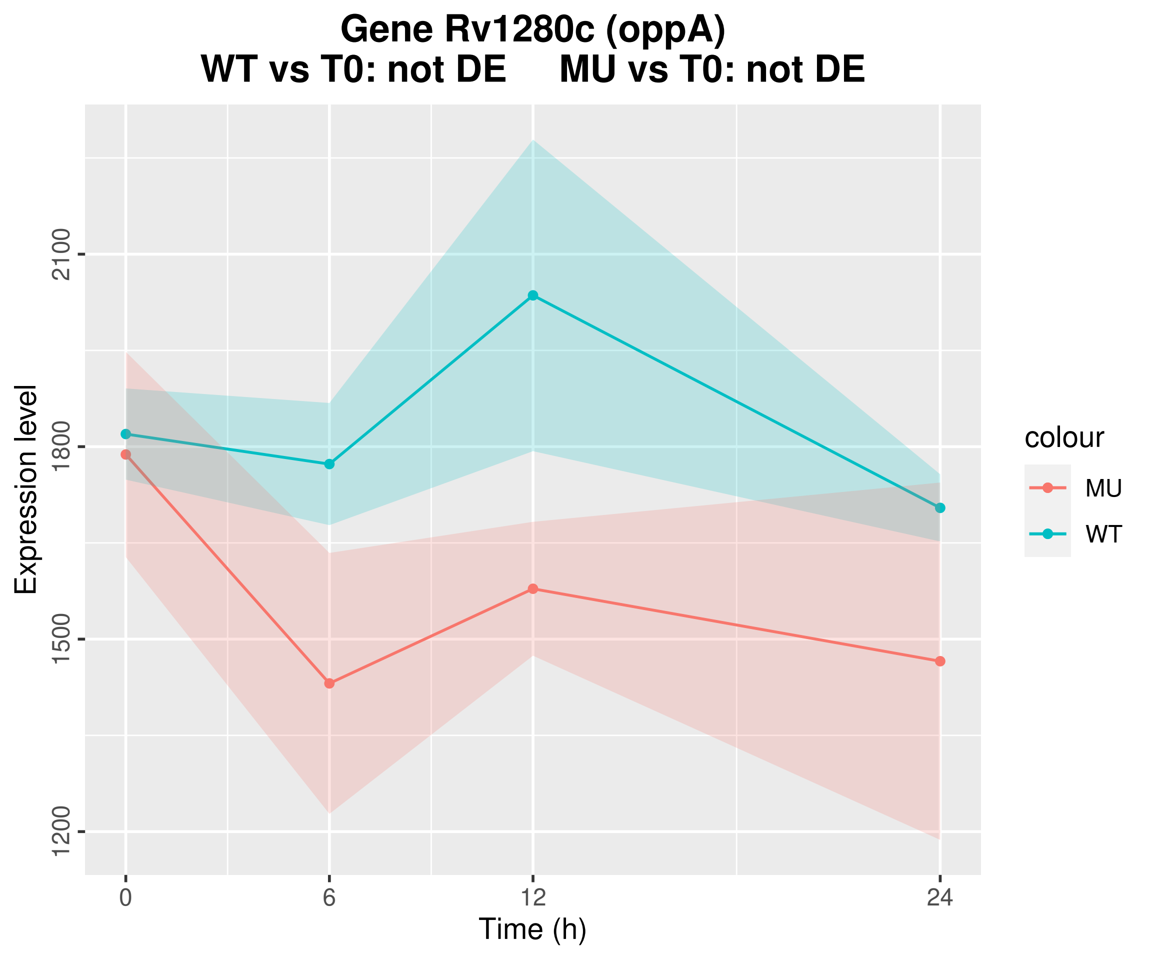

Gene Rv1280c (oppA)
WT vs T0: not DE   MU vs T0: not DE
Expression level
2100
1800
1500
1200
0
6
12
24
Time (h)
colour
MU
WT

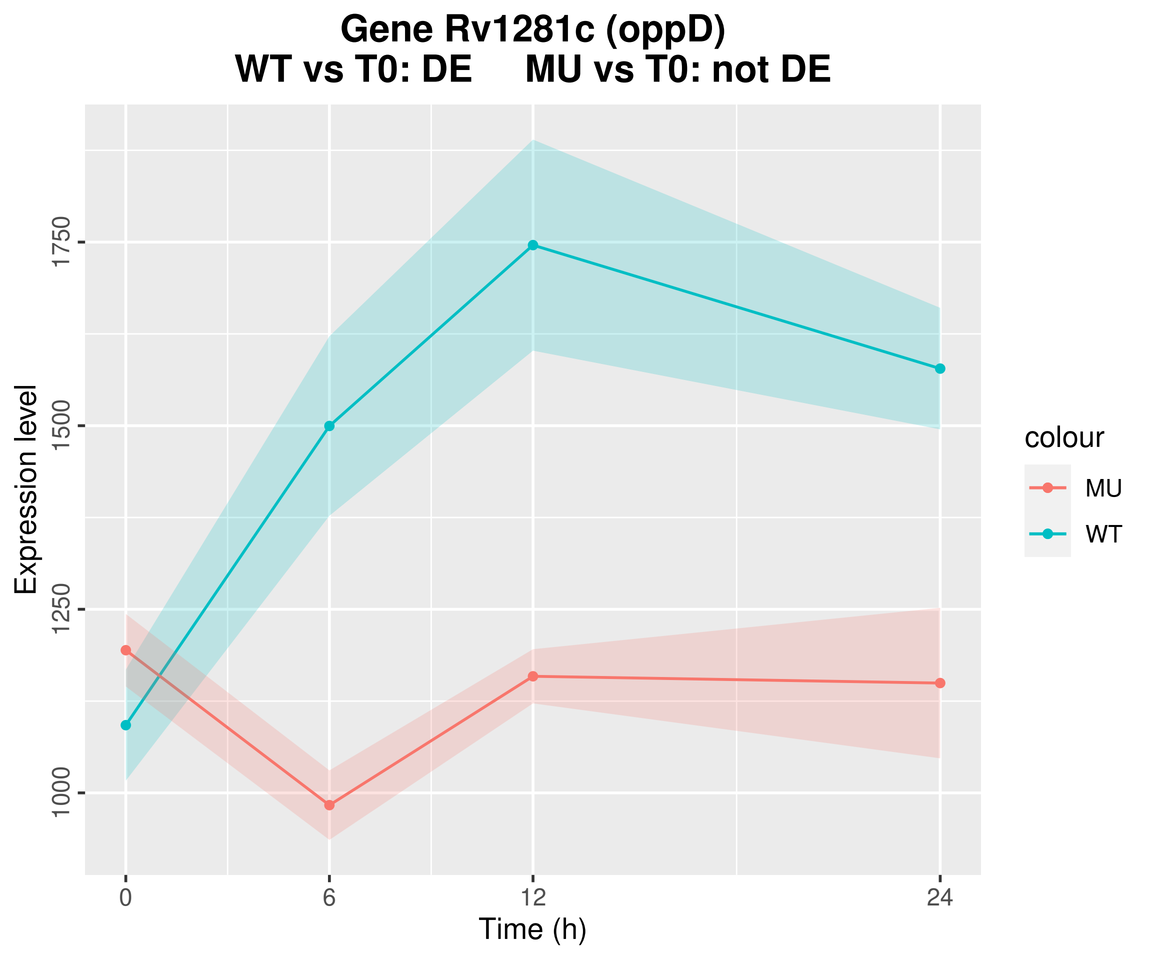

Gene Rv1281c (oppD)
WT vs T0: DE   MU vs T0: not DE
Expression level
1750
1500
1250
1000
0
6
12
24
Time (h)
colour
MU
WT

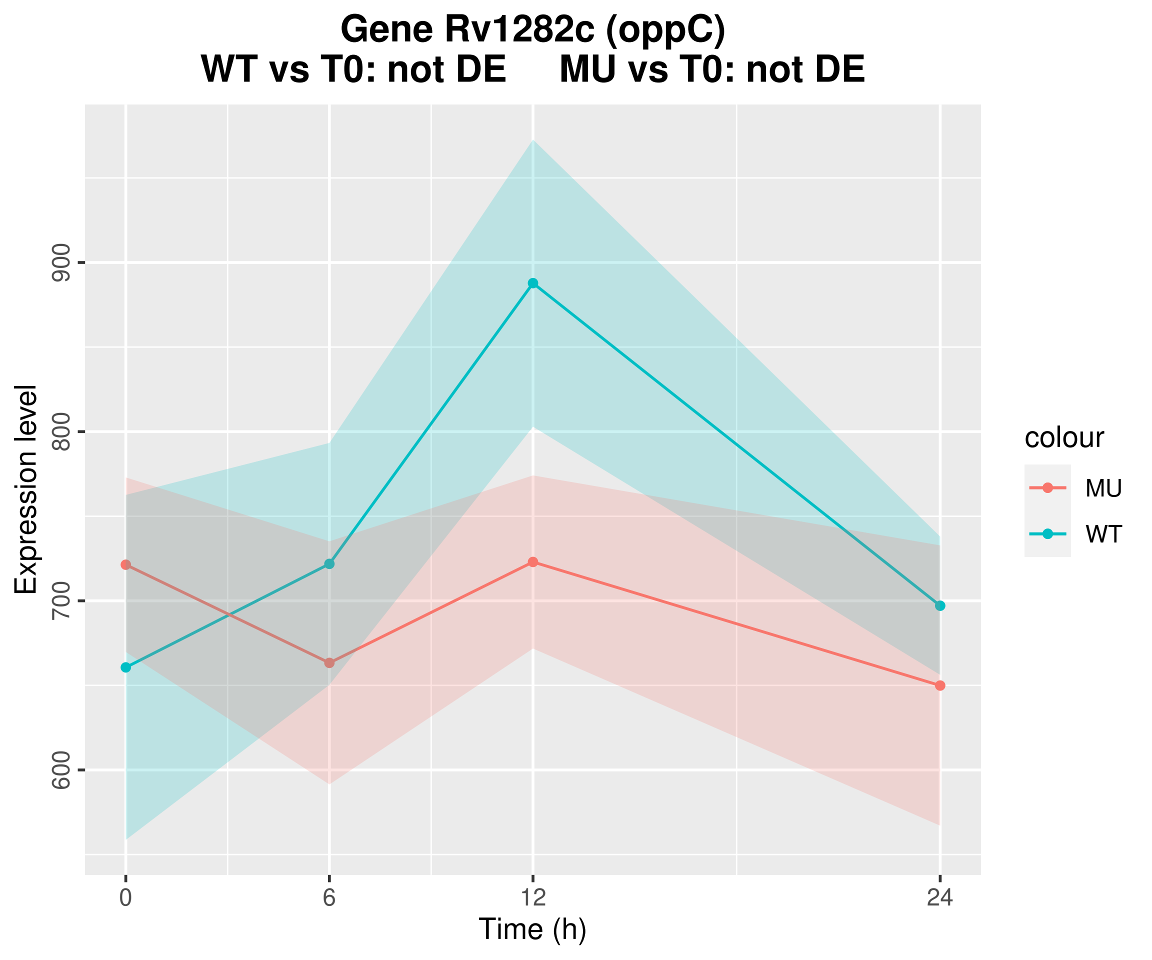
Gene Rv1282c (oppC)
WT vs T0: not DE   MU vs T0: not DE
Expression level
900
800
700
600
0
6
12
24
Time (h)
colour
MU
WT

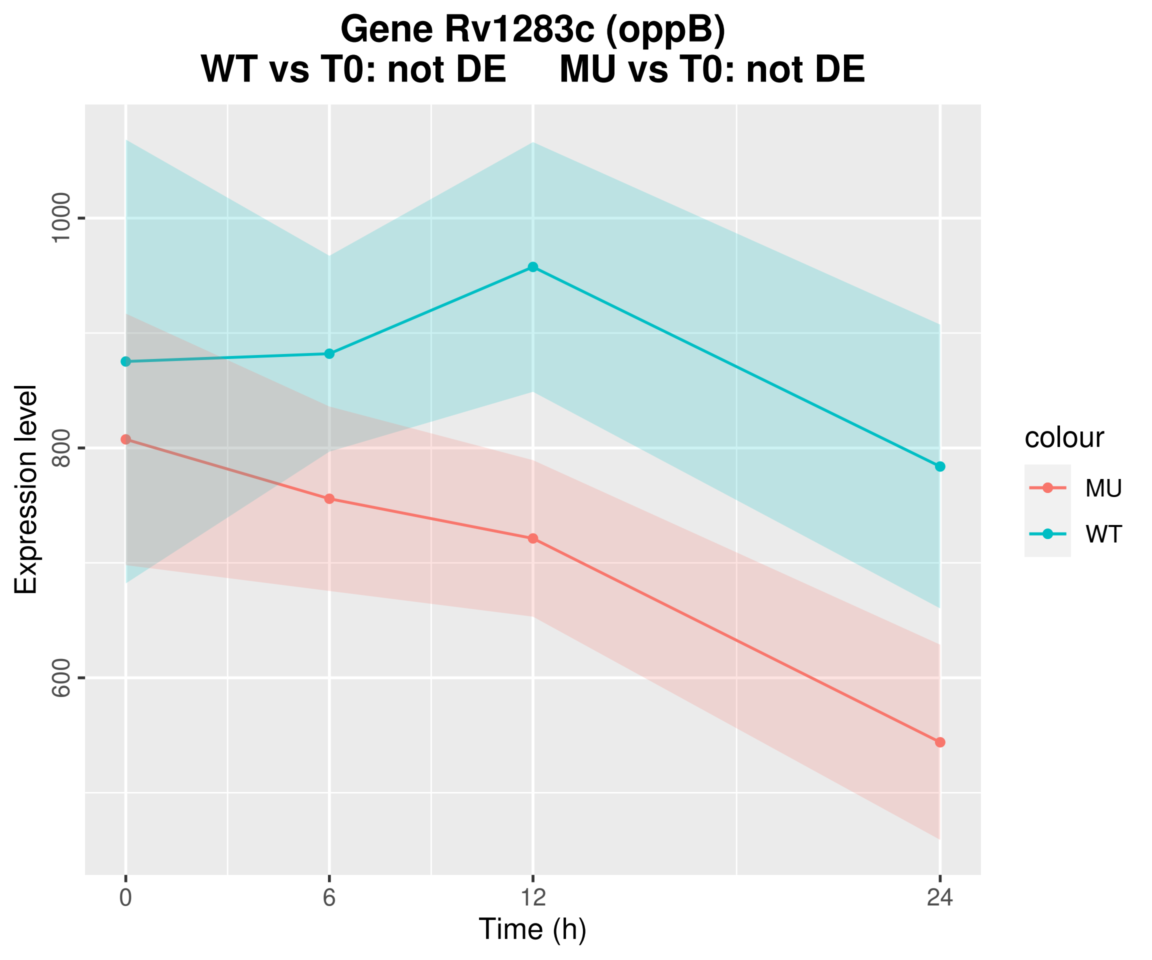

Gene Rv1283c (oppB)
WT vs T0: not DE   MU vs T0: not DE
1000
800
600
Expression level
0
6
12
24
Time (h)
colour
MU
WT

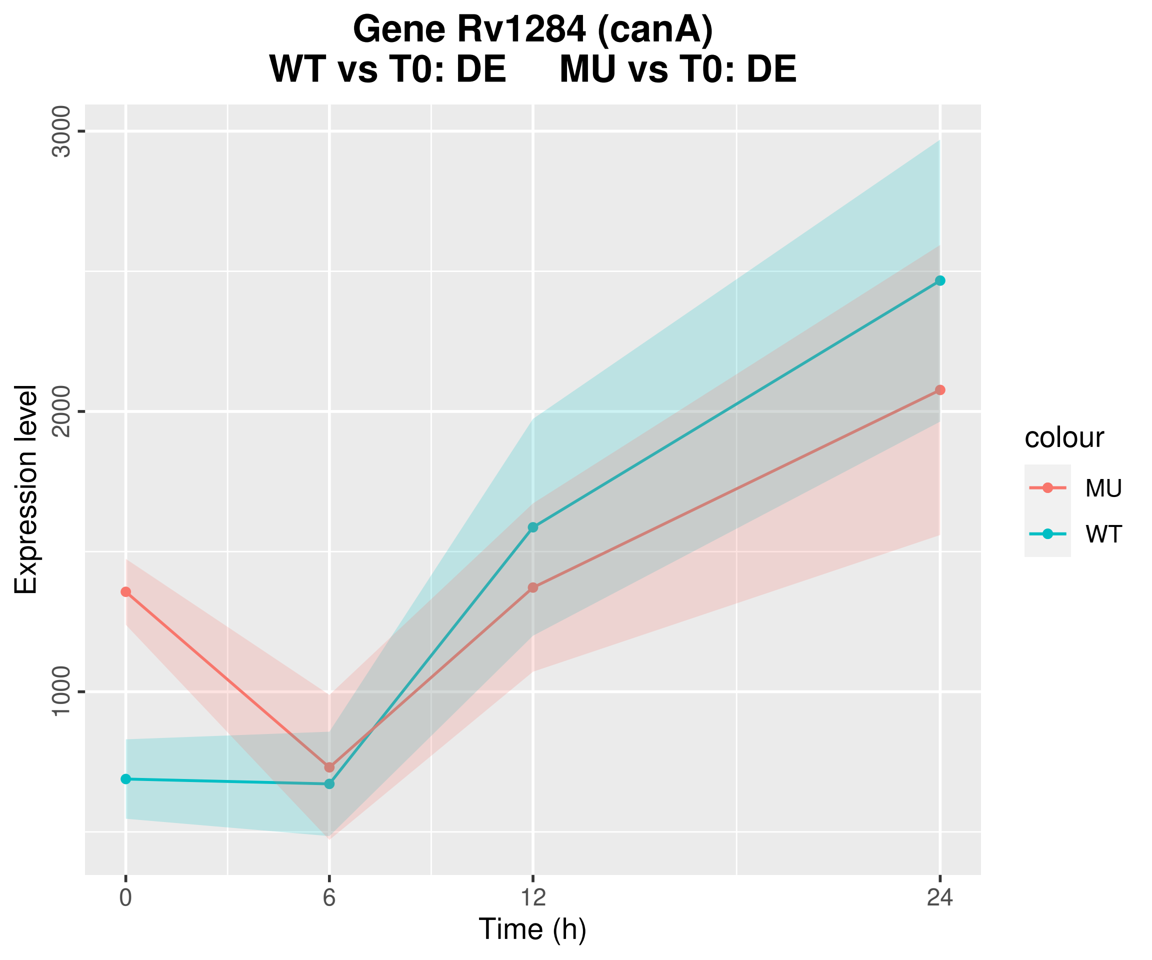
Gene Rv1284 (canA)
WT vs T0: DE MU vs T0: DE
Expression level
3000
2000
1000
0
6
12
24
Time (h)
colour
MU
WT

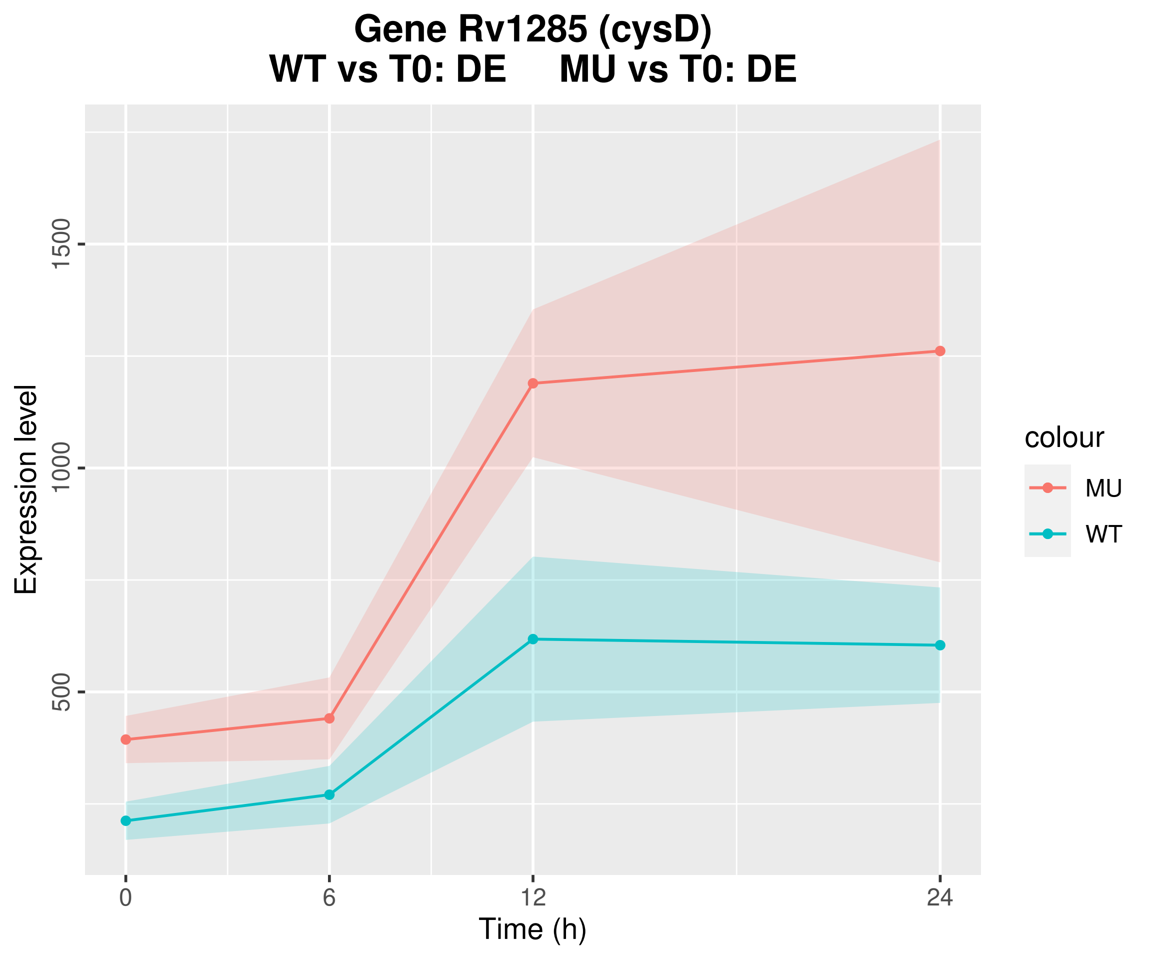
Gene Rv1285 (cysD)
WT vs T0: DE   MU vs T0: DE
Expression level
1500
1000
500
0
6
12
24
Time (h)
colour
MU
WT

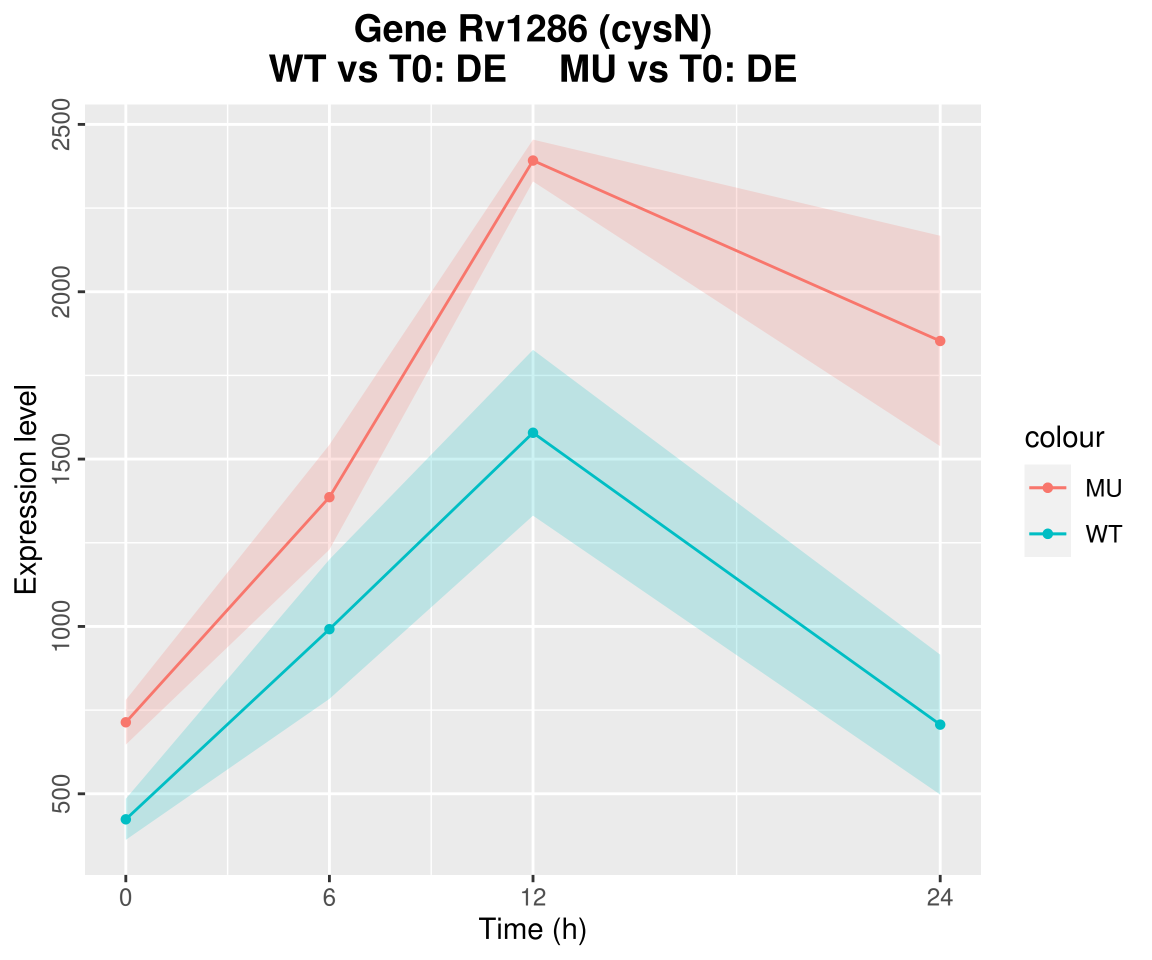

Gene Rv1286 (cysN)
WT vs T0: DE MU vs T0: DE
Expression level
2500
2000
1500
1000
500
0
6
12
24
Time (h)
colour
MU
WT

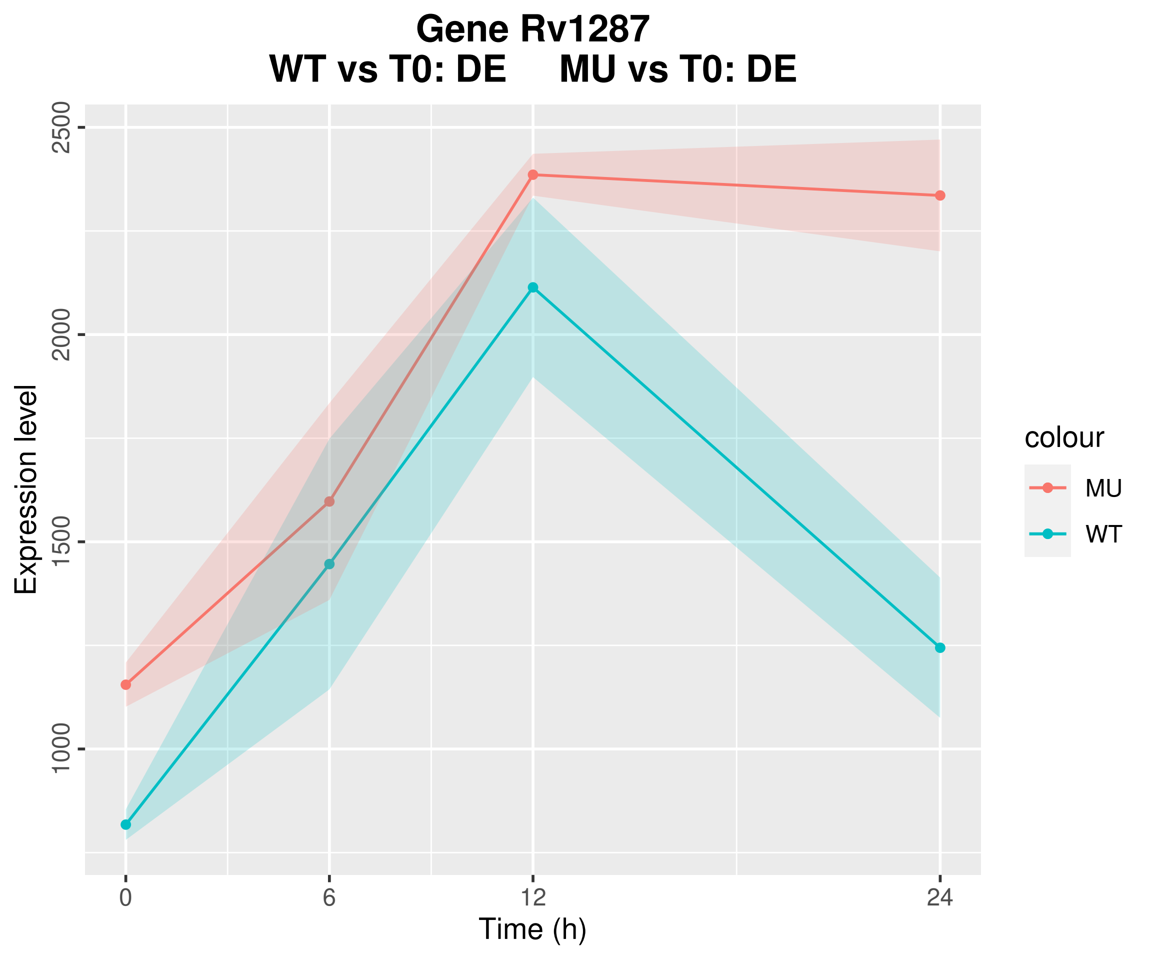

Gene Rv1287
WT vs T0: DE MU vs T0: DE
Expression level
2500
2000
1500
1000
0
6
12
24
Time (h)
colour
MU
WT

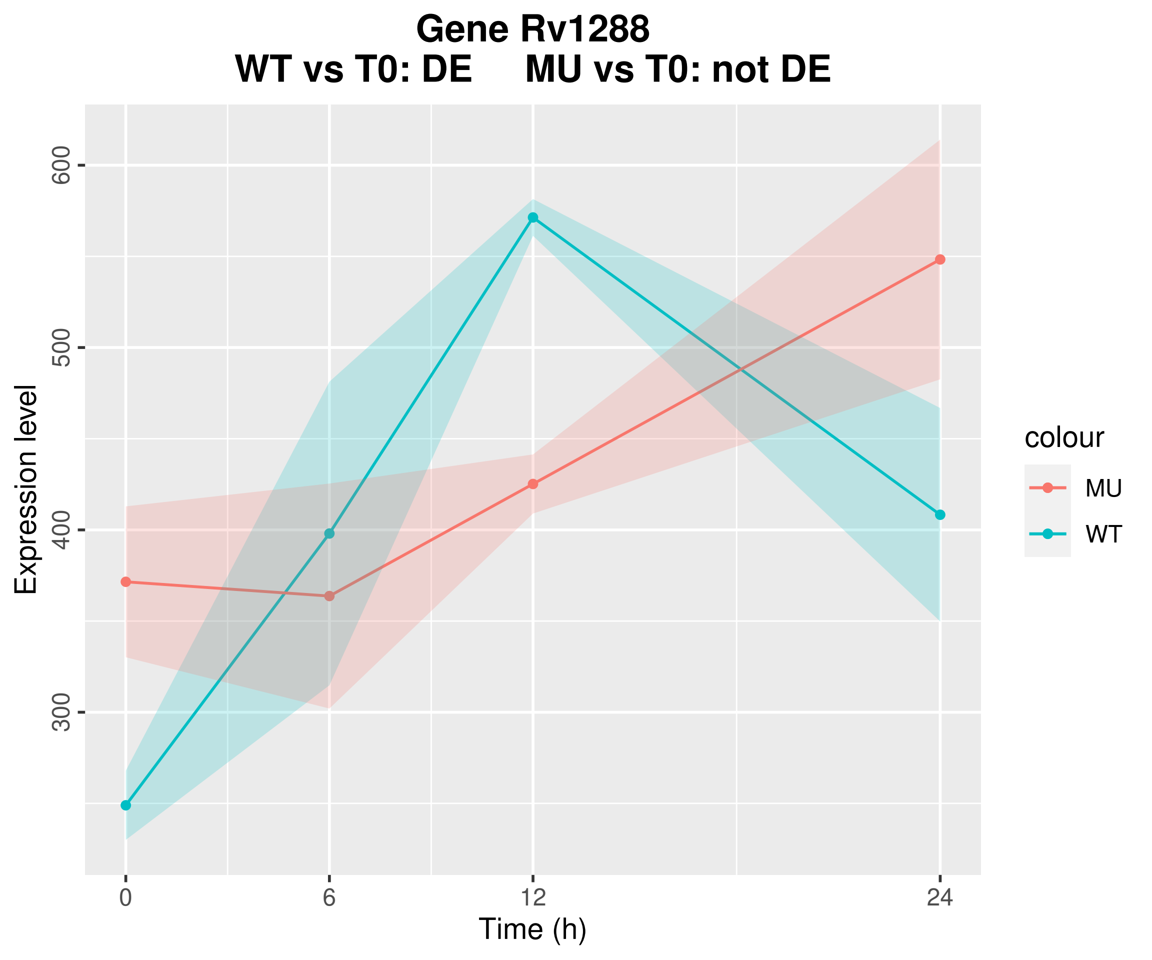
Gene Rv1288
WT vs T0: DE   MU vs T0: not DE
Expression level
600
500
400
300
0
6
12
24
Time (h)
colour
MU
WT

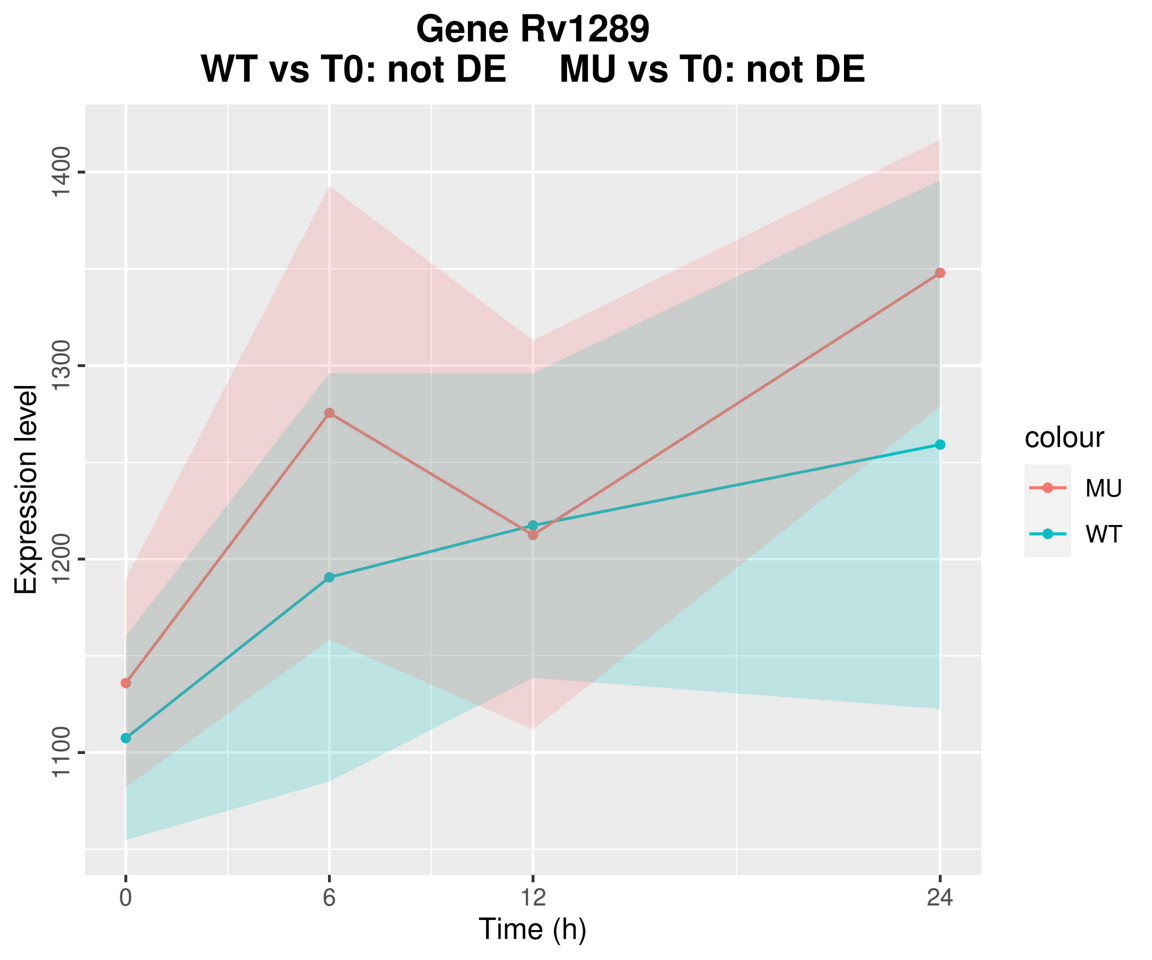

Gene Rv1289
WT vs T0: not DE   MU vs T0: not DE
1100
1200
1300
1400
Expression level
0
6
12
24
Time (h)
colour
MU
WT

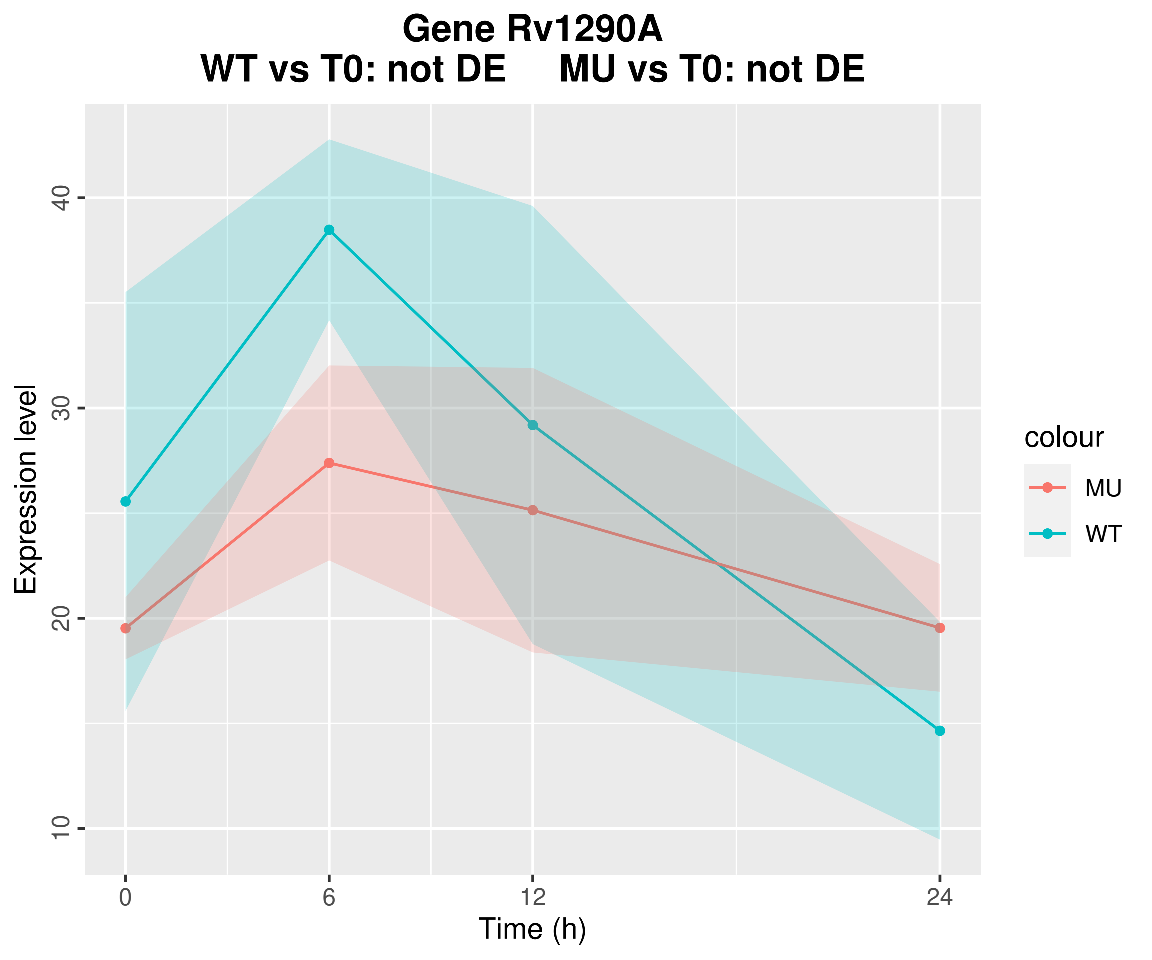

Gene Rv1290A
WT vs T0: not DE   MU vs T0: not DE
Expression level
40
30
20
10
0
6
12
24
Time (h)
colour
MU
WT

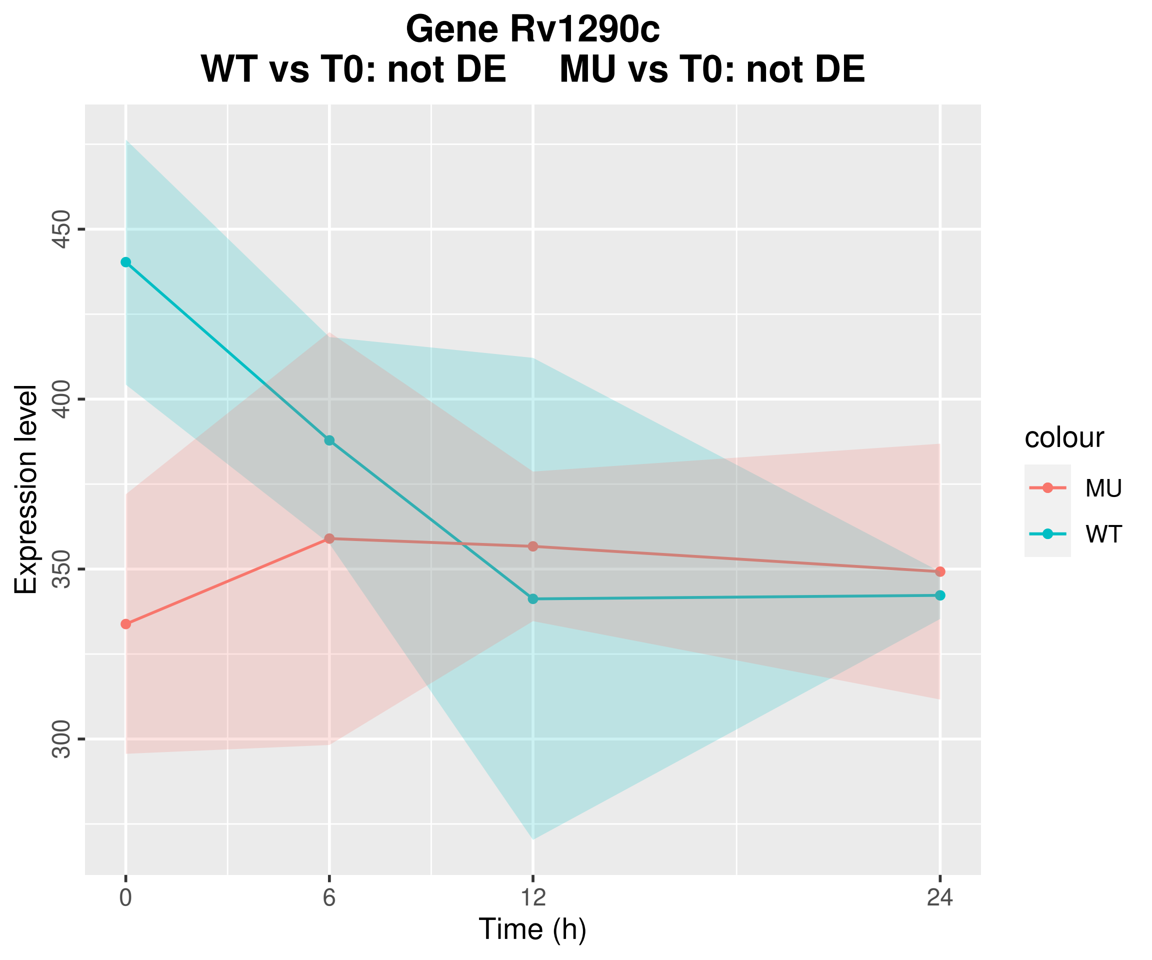
Gene Rv1290c
WT vs T0: not DE   MU vs T0: not DE
Expression level
450
400
350
300
0
6
12
24
Time (h)
colour
MU
WT

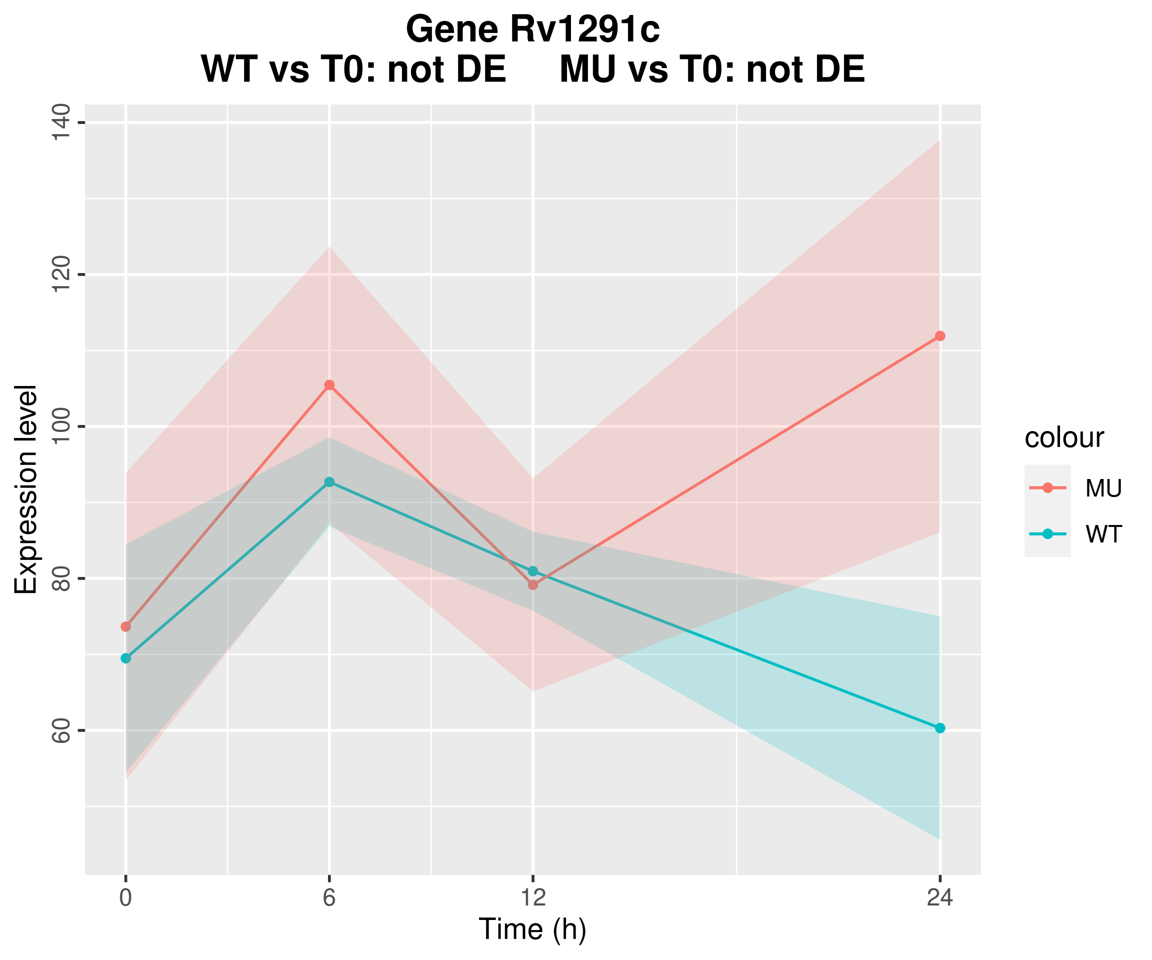
Gene Rv1291c
WT vs T0: not DE MU vs T0: not DE
Expression level
140
120
100
80
60
0
6
12
24
Time (h)
colour
MU
WT

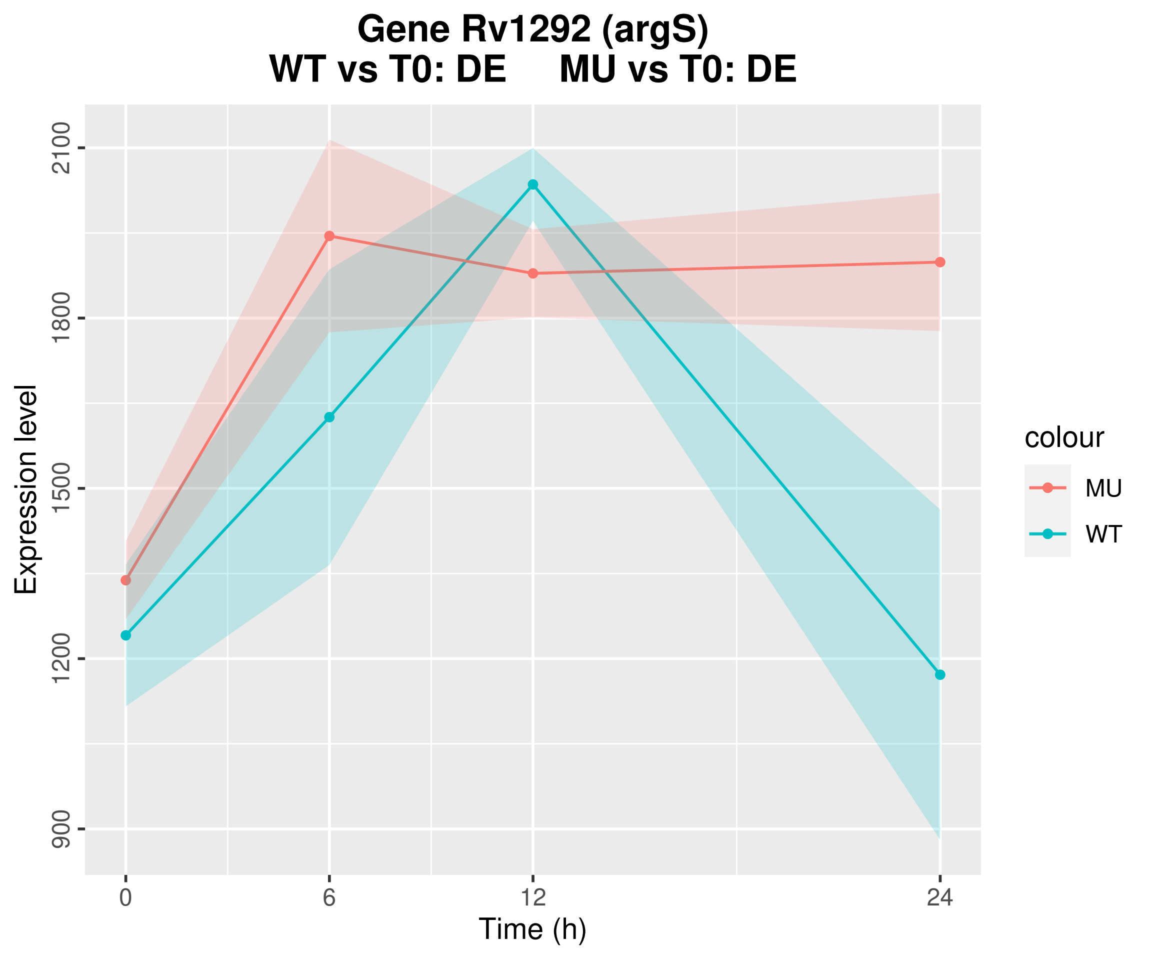

Gene Rv1292 (argS)
WT vs T0: DE MU vs T0: DE
Expression level
2100
1800
1500
1200
900
0
6
12
24
Time (h)
colour
MU
WT

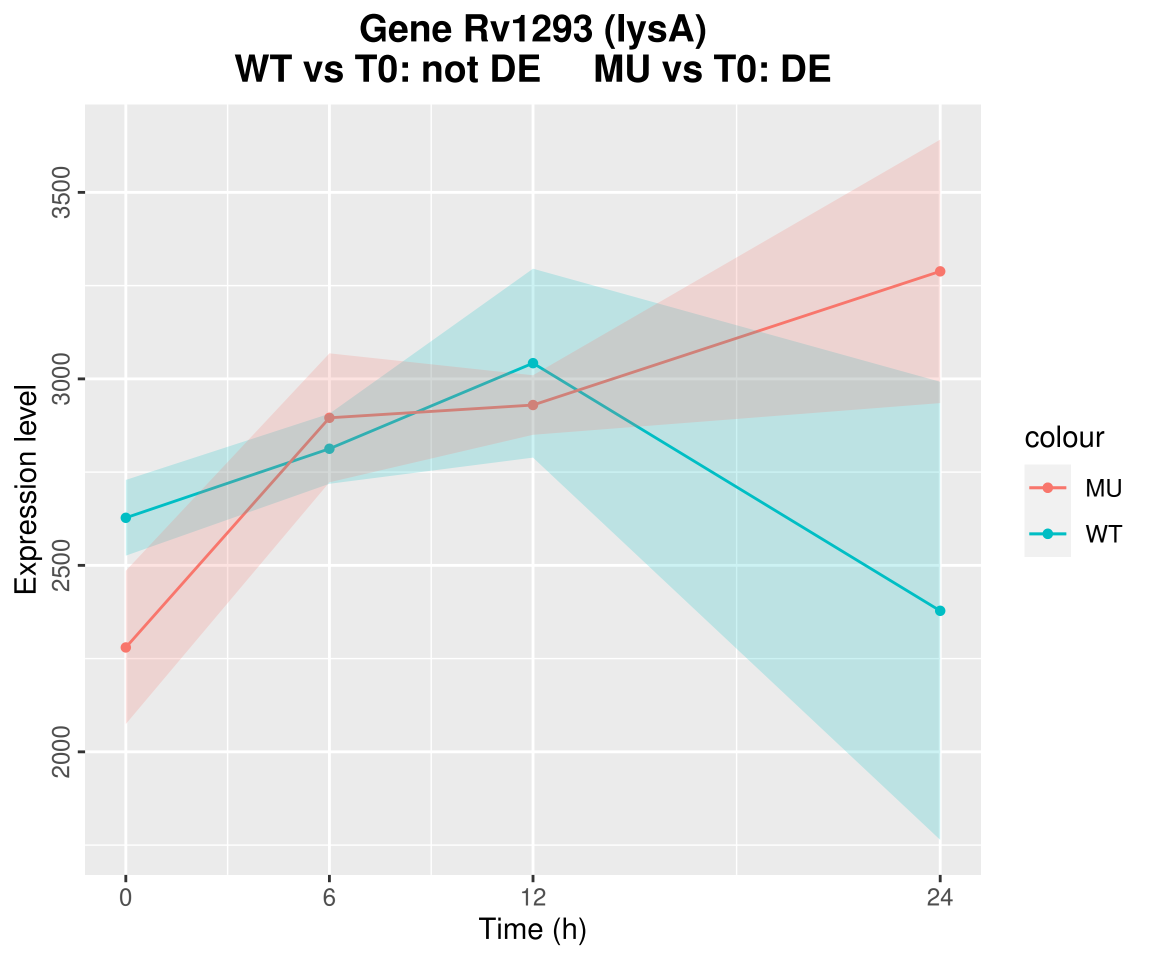

Gene Rv1293 (lysA)
WT vs T0: not DE   MU vs T0: DE
Expression level
3500
3000
2500
2000
0
6
12
24
Time (h)
colour
MU
WT

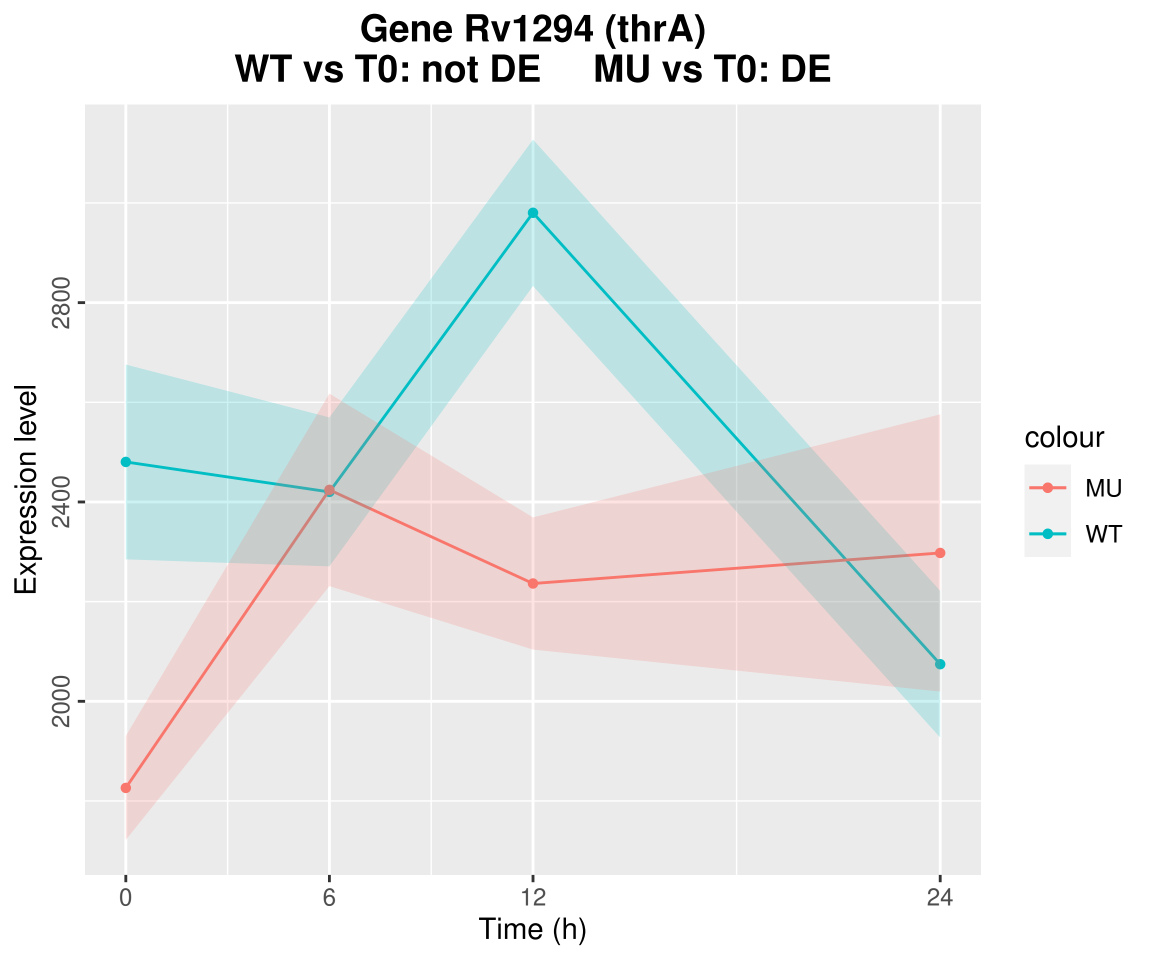

Gene Rv1294 (thrA)
WT vs T0: not DE   MU vs T0: DE
Expression level
2000
2400
2800
0
6
12
24
Time (h)
colour
MU
WT

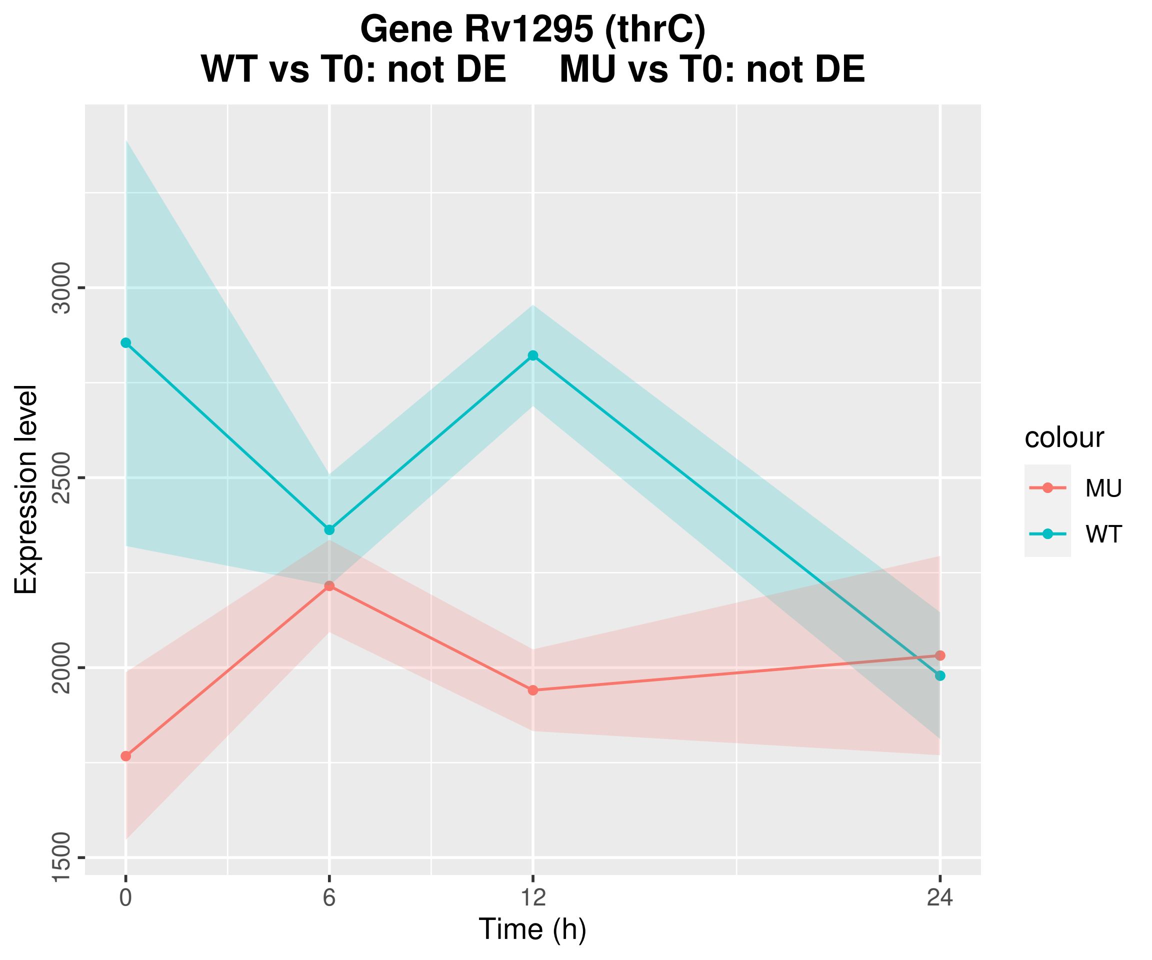

Gene Rv1295 (thrC)
WT vs T0: not DE MU vs T0: not DE
Expression level
1500
2000
2500
3000
0
6
12
24
Time (h)
colour
MU
WT

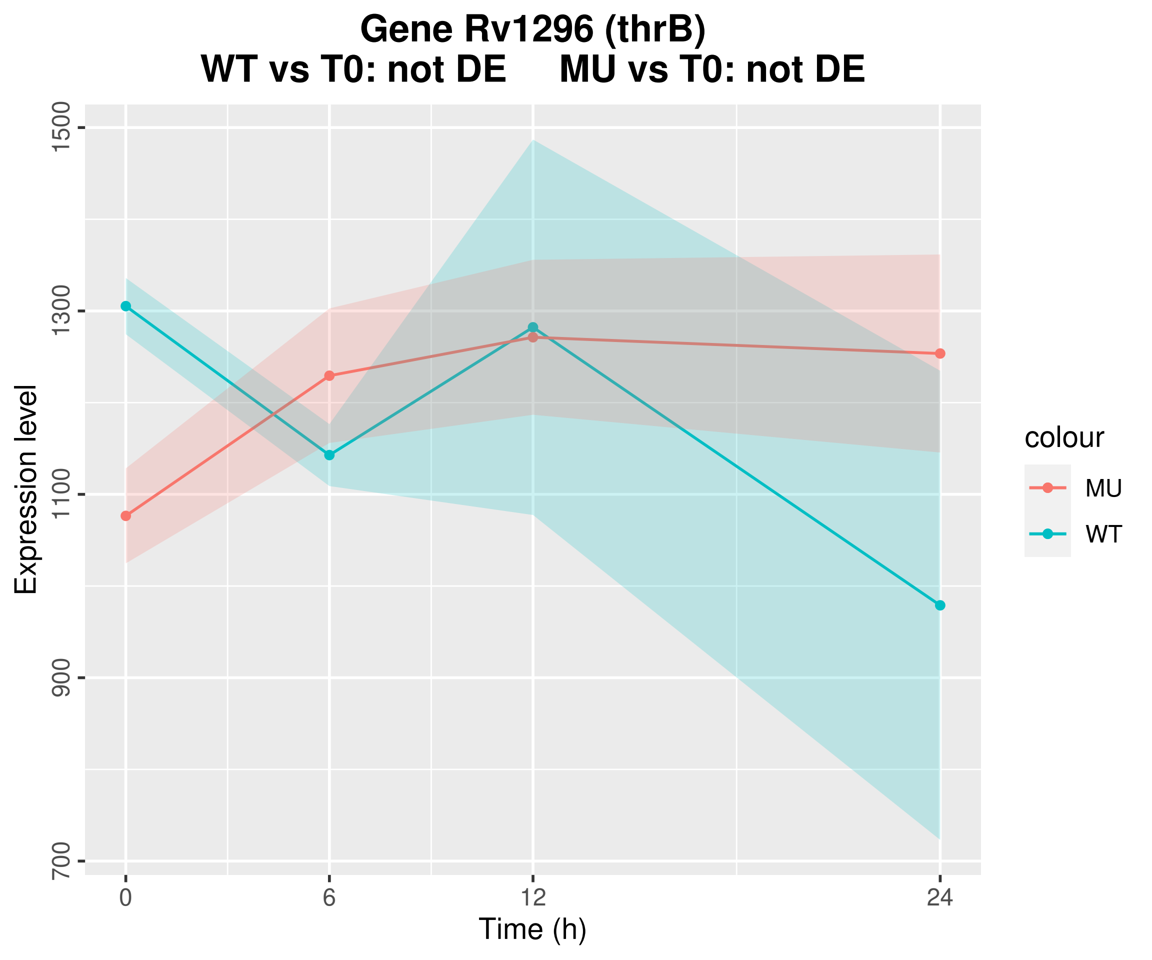

Gene Rv1296 (thrB)
WT vs T0: not DE MU vs T0: not DE
Expression level
1500
1300
1100
900
700
0
6
12
24
Time (h)
colour
MU
WT

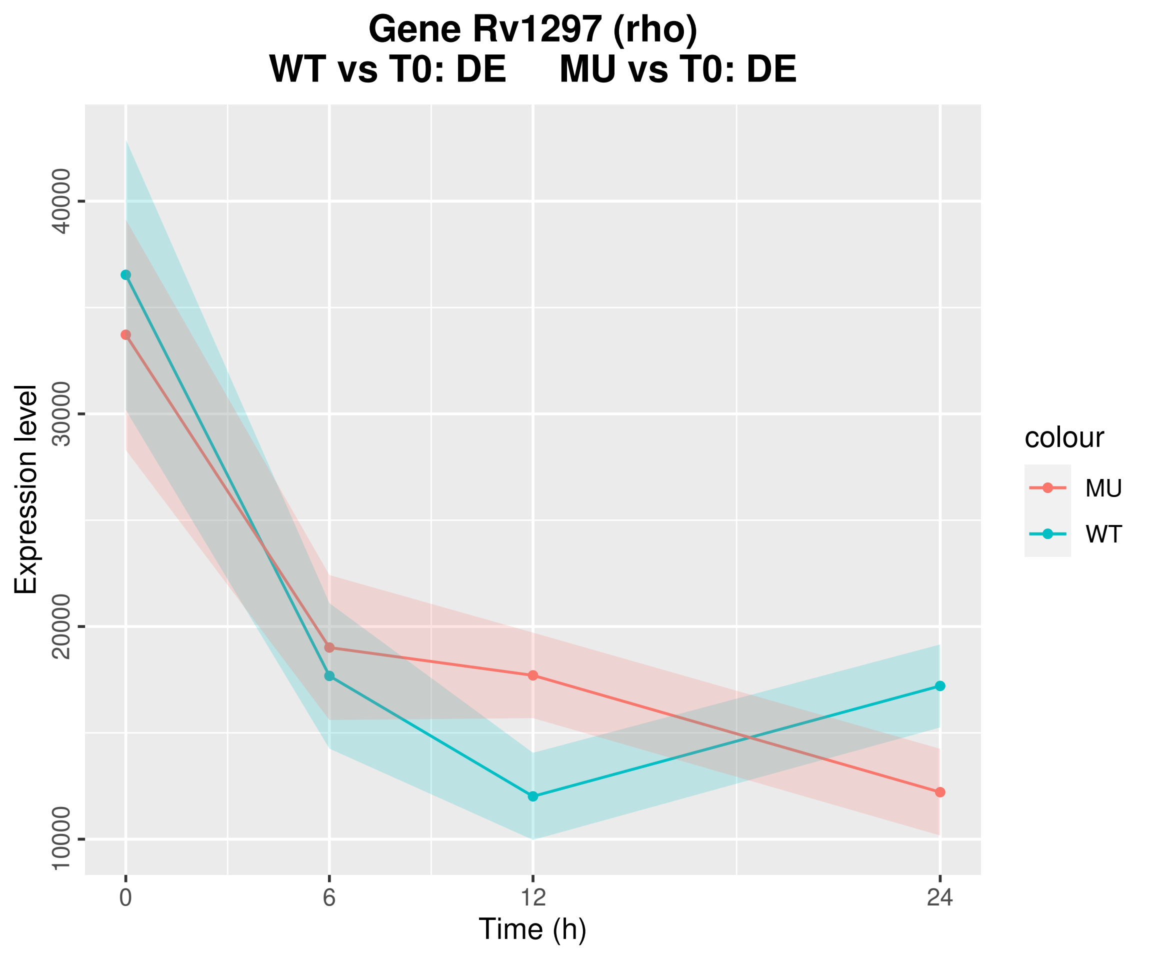

Gene Rv1297 (rho)
WT vs T0: DE   MU vs T0: DE
Expression level
40000
30000
20000
10000
0
6
12
24
Time (h)
colour
MU
WT

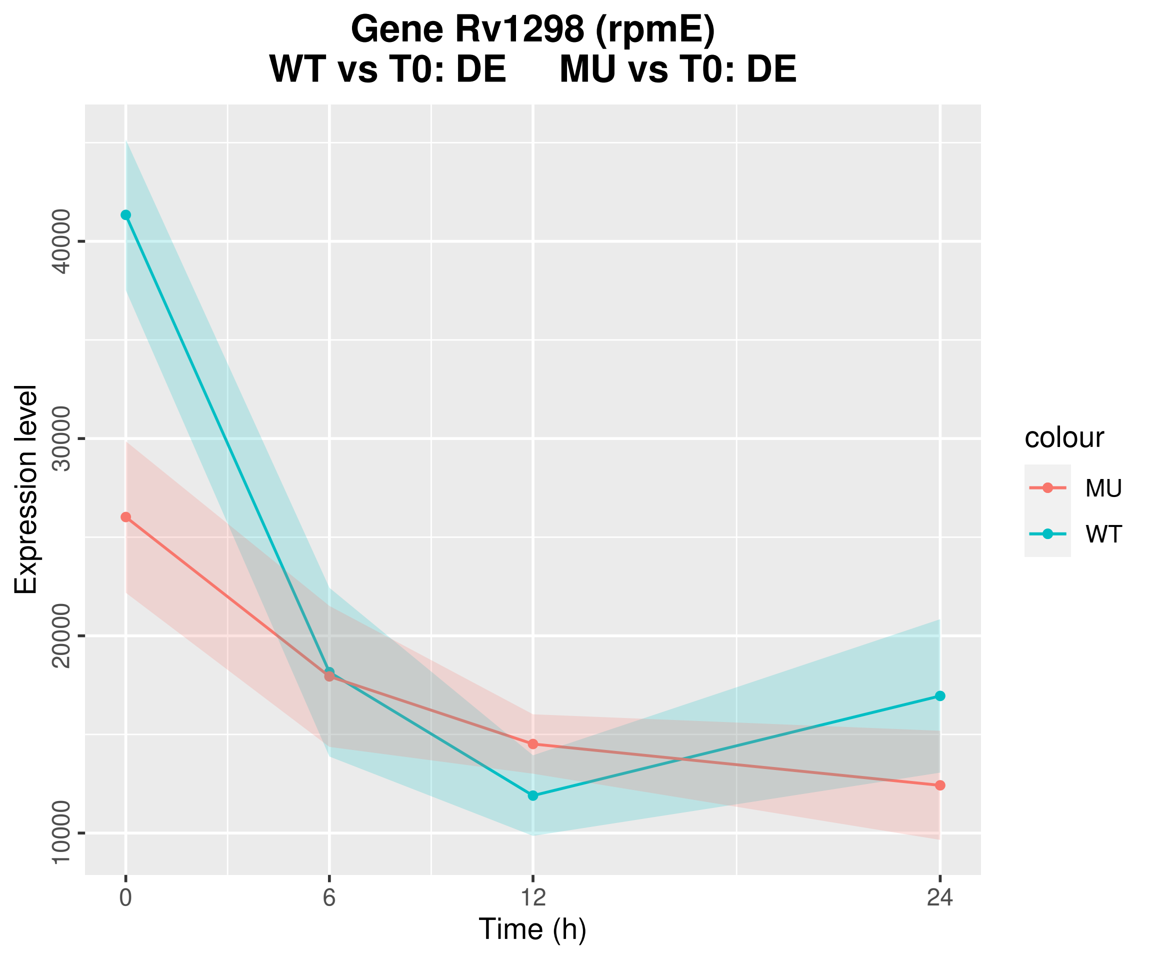
Gene Rv1298 (rpmE)
WT vs T0: DE   MU vs T0: DE
Expression level
40000
30000
20000
10000
0
6
12
24
Time (h)
colour
MU
WT

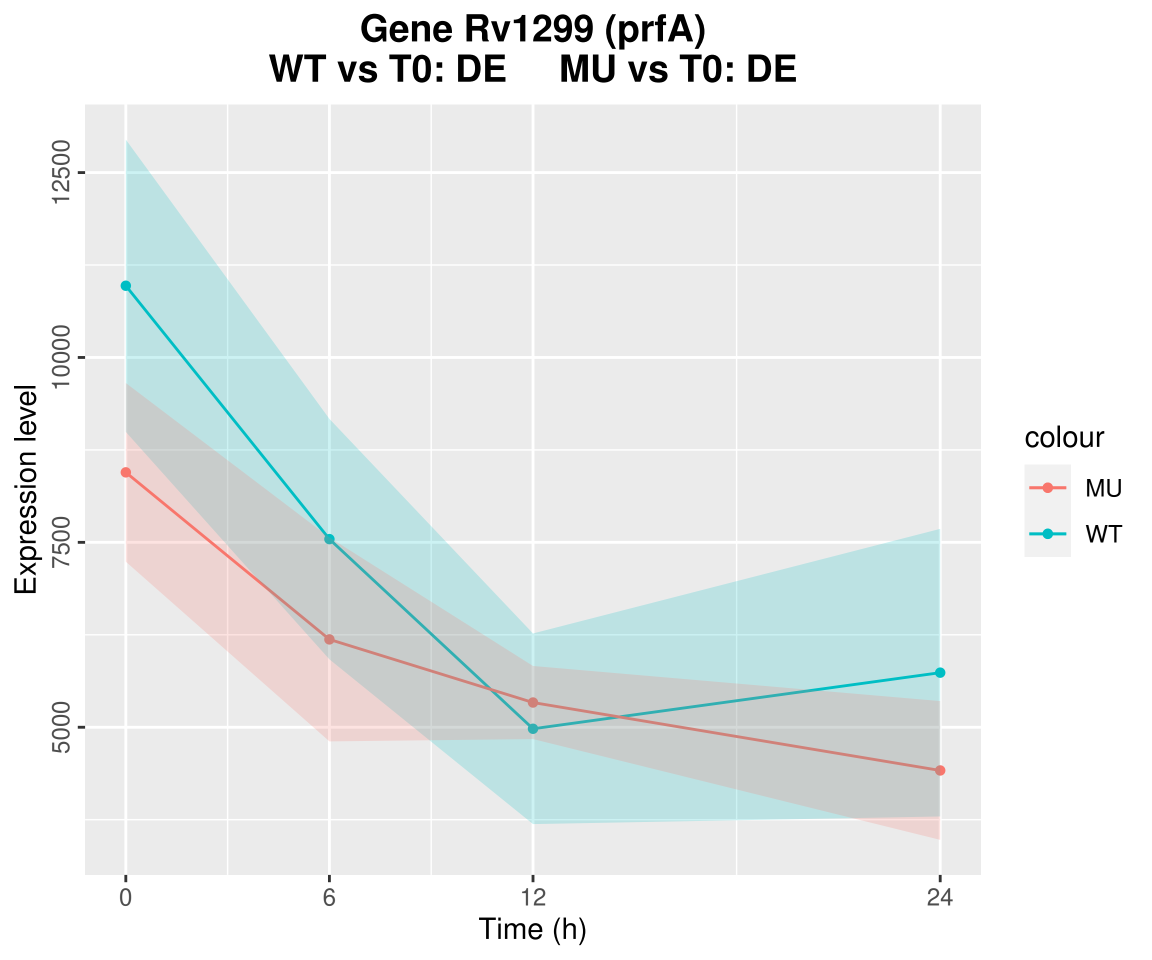

Gene Rv1299 (prfA)
WT vs T0: DE   MU vs T0: DE
12500
10000
7500
5000
Expression level
0
6
12
24
Time (h)
colour
MU
WT

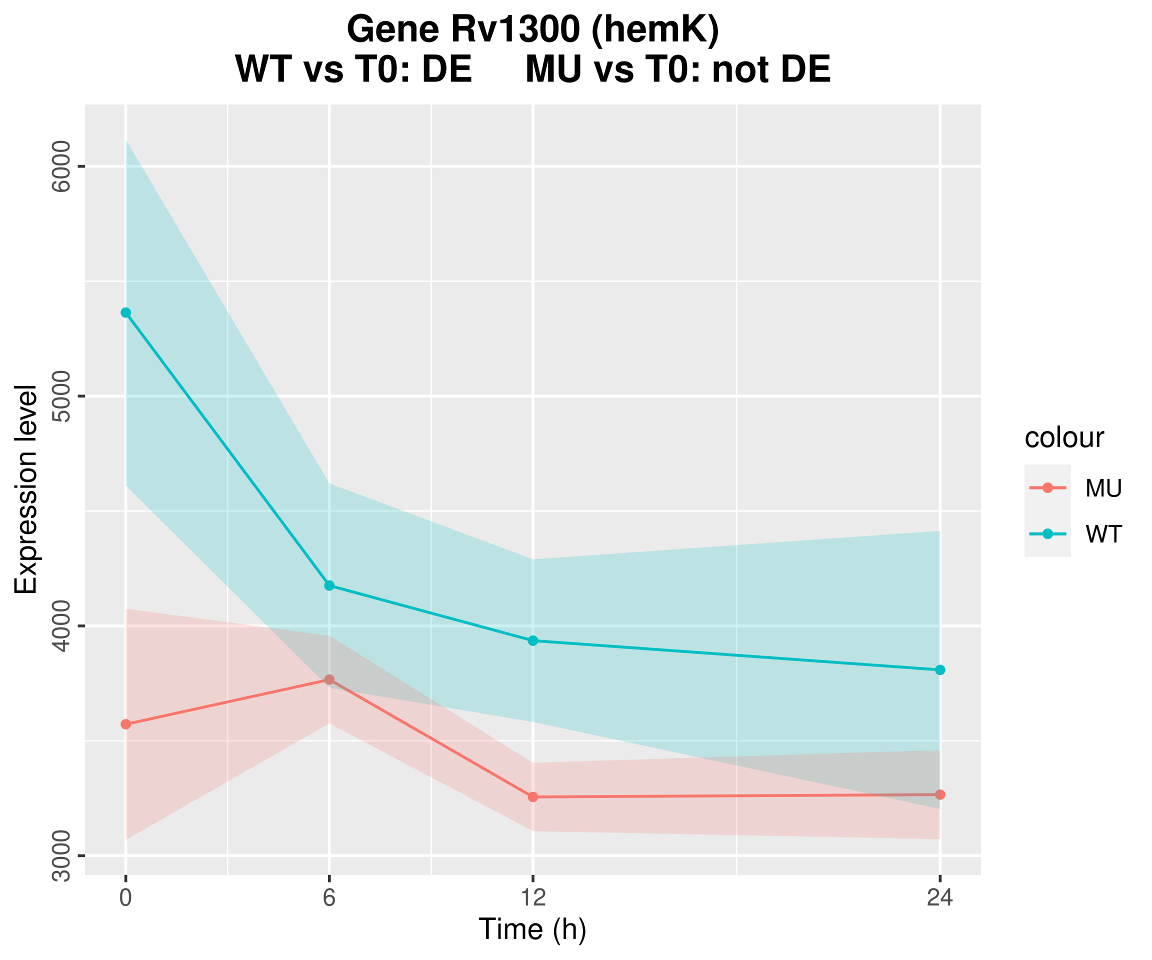

Gene Rv1300 (hemK)
WT vs T0: DE MU vs T0: not DE
Expression level
6000
5000
4000
3000
0
6
12
24
Time (h)
colour
MU
WT

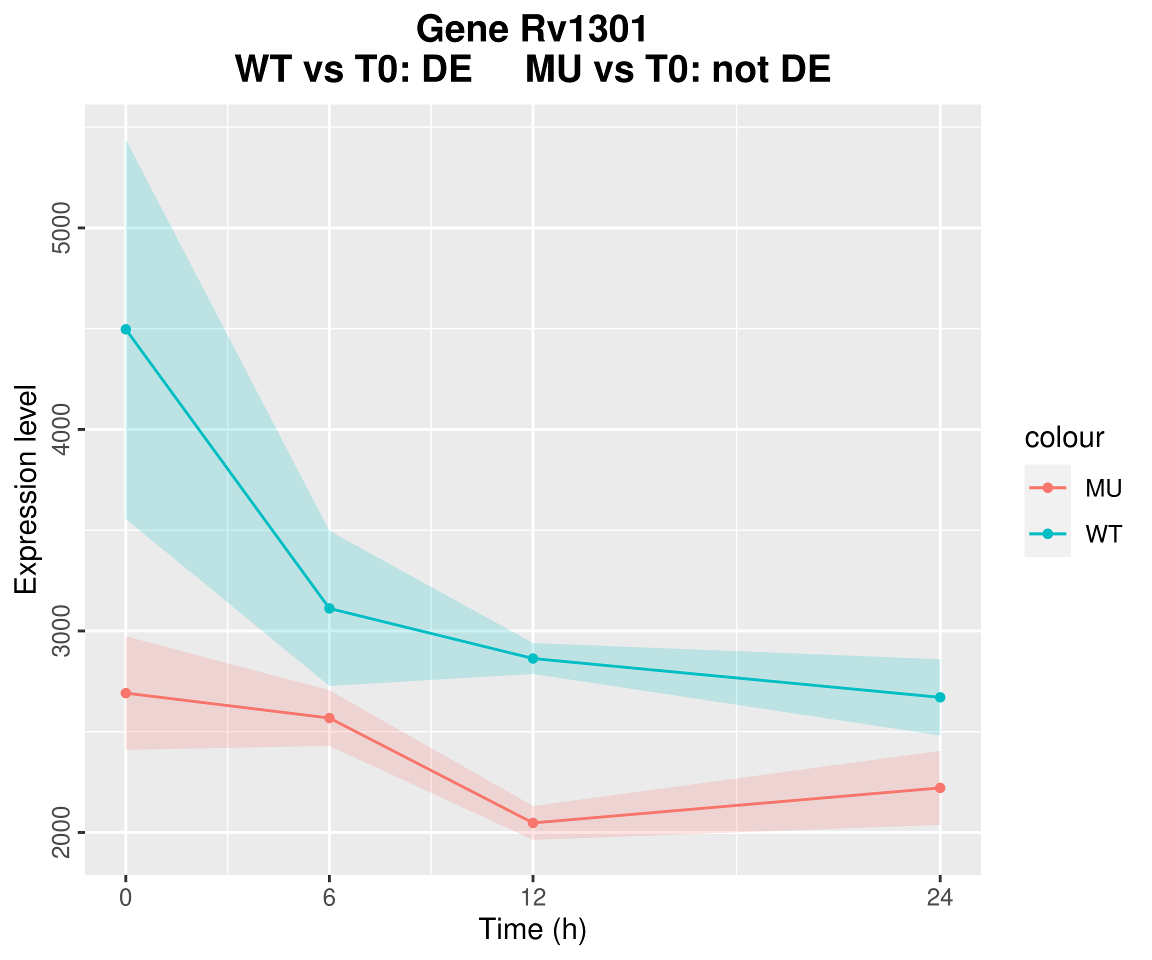

Gene Rv1301
WT vs T0: DE MU vs T0: not DE
Expression level
5000
4000
3000
2000
0
6
12
24
Time (h)
colour
MU
WT

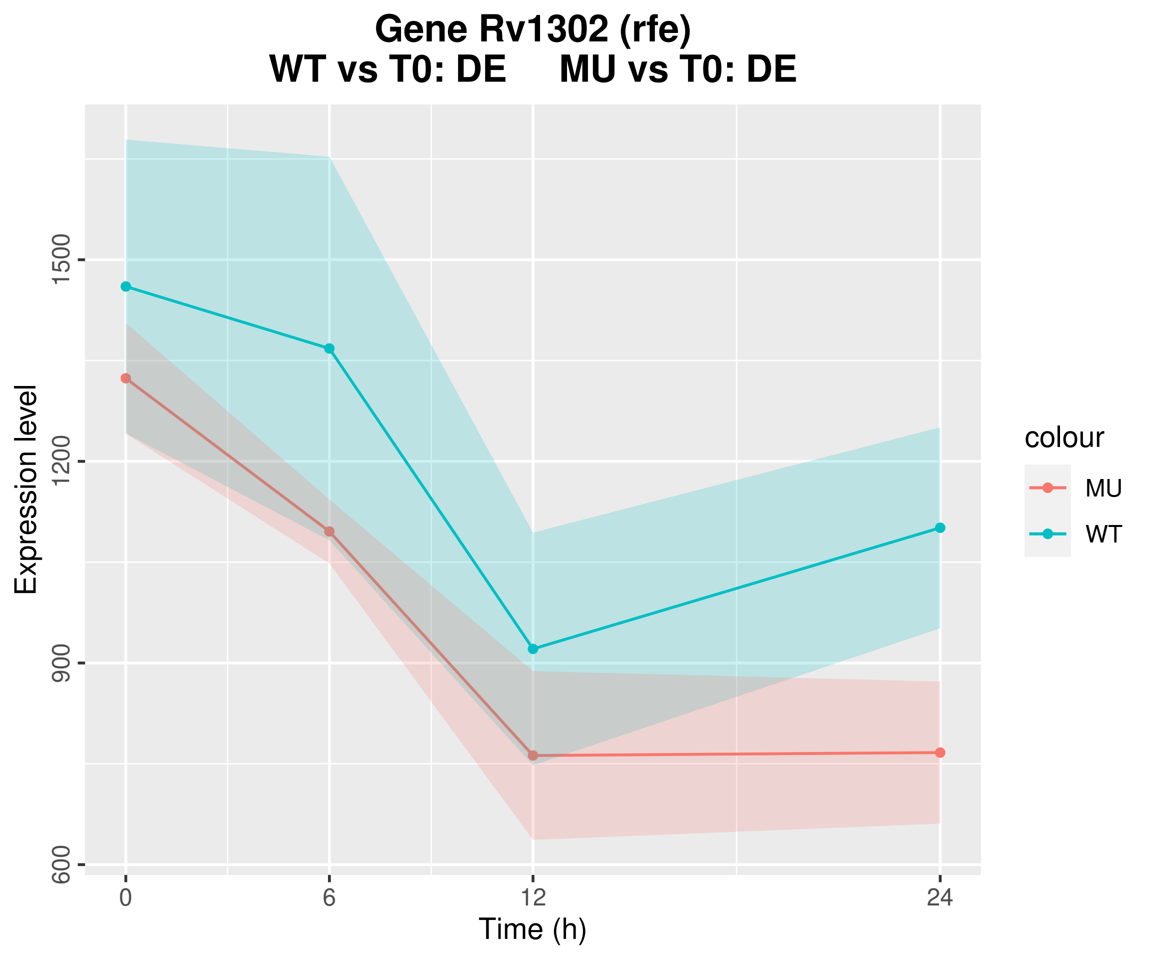

Gene Rv1302 (rfe)
WT vs T0: DE MU vs T0: DE
Expression level
1500
1200
900
600
0
6
12
24
Time (h)
colour
MU
WT

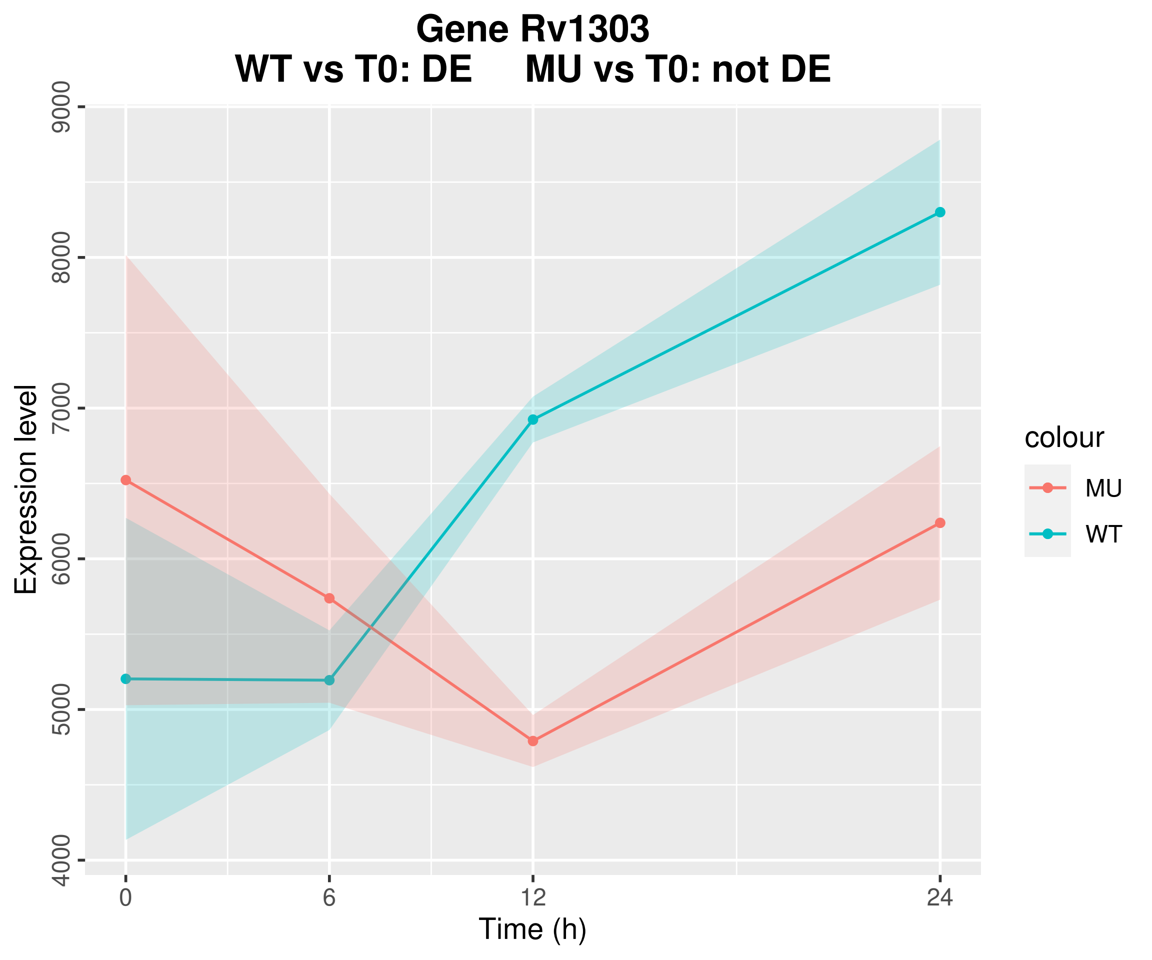

Gene Rv1303
WT vs T0: DE   MU vs T0: not DE
Expression level
9000
8000
7000
6000
5000
4000
0
6
12
24
Time (h)
colour
MU
WT

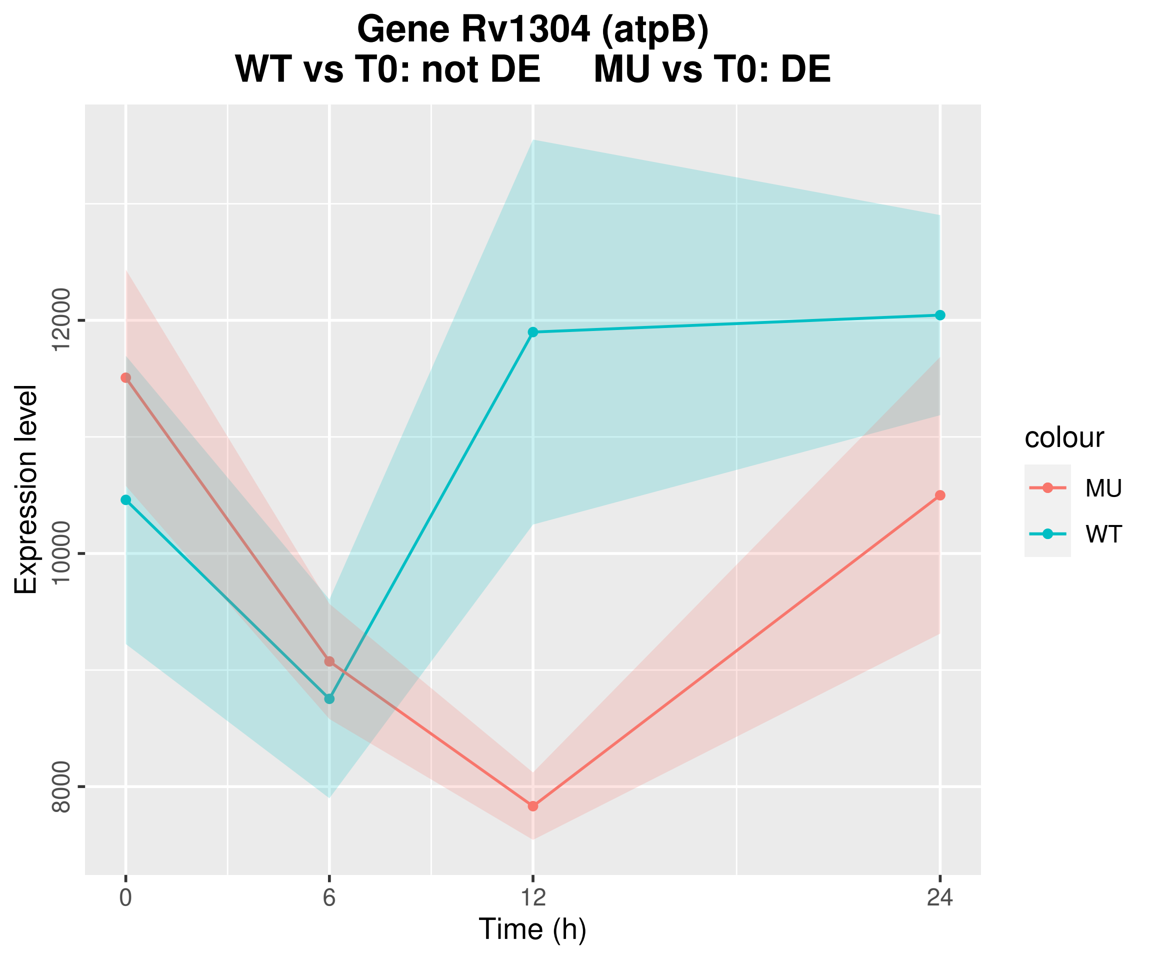
Gene Rv1304 (atpB)
WT vs T0: not DE MU vs T0: DE
Expression level
12000
10000
8000
0
6
12
24
Time (h)
colour
MU
WT

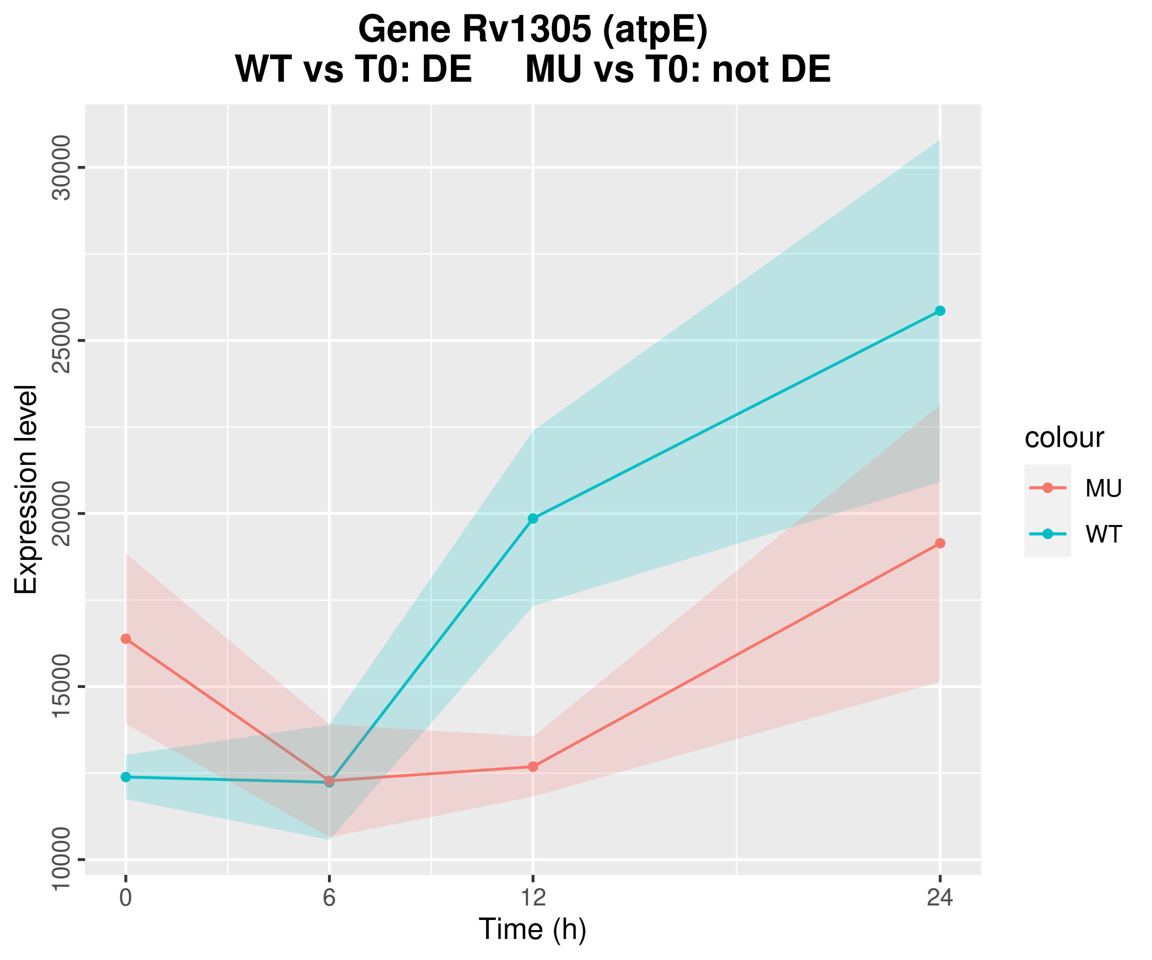
Gene Rv1305 (atpE)
WT vs T0: DE MU vs T0: not DE
Expression level
30000
25000
20000
15000
10000
0
6
12
24
Time (h)
colour
MU
WT

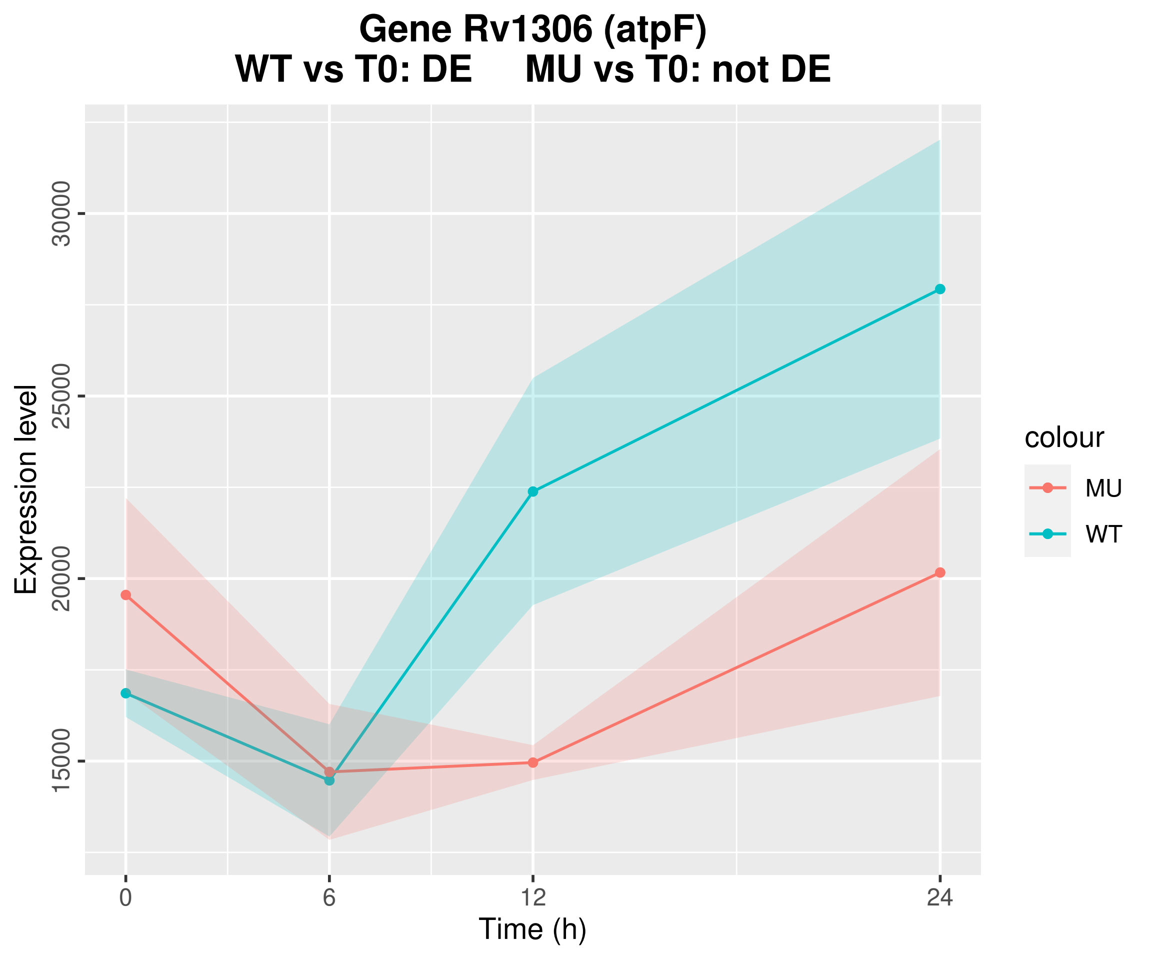
Gene Rv1306 (atpF)
WT vs T0: DE   MU vs T0: not DE
Expression level
30000
25000
20000
15000
0
6
12
24
Time (h)
colour
MU
WT

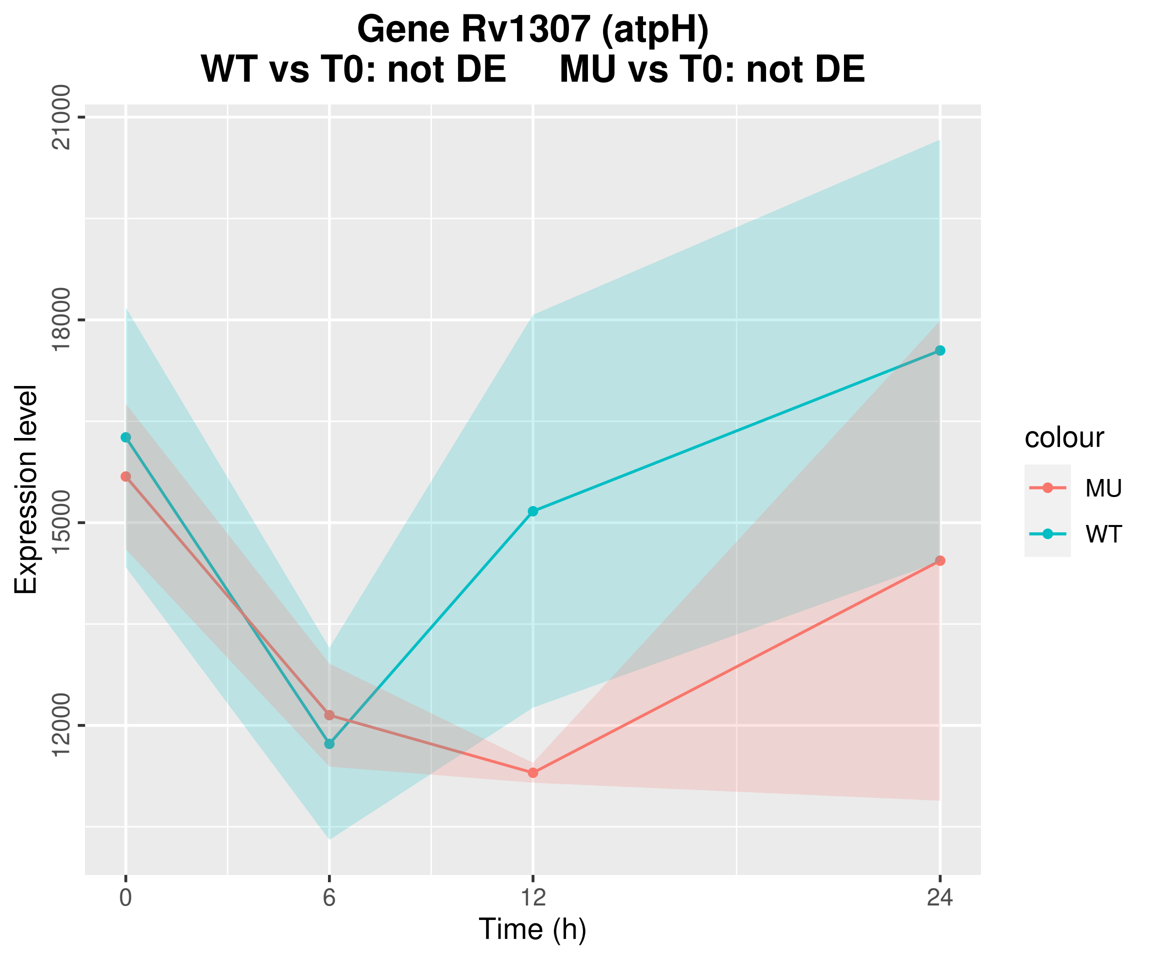

Gene Rv1307 (atpH)
WT vs T0: not DE   MU vs T0: not DE
Expression level
21000
18000
15000
12000
0
6
12
24
Time (h)
colour
MU
WT

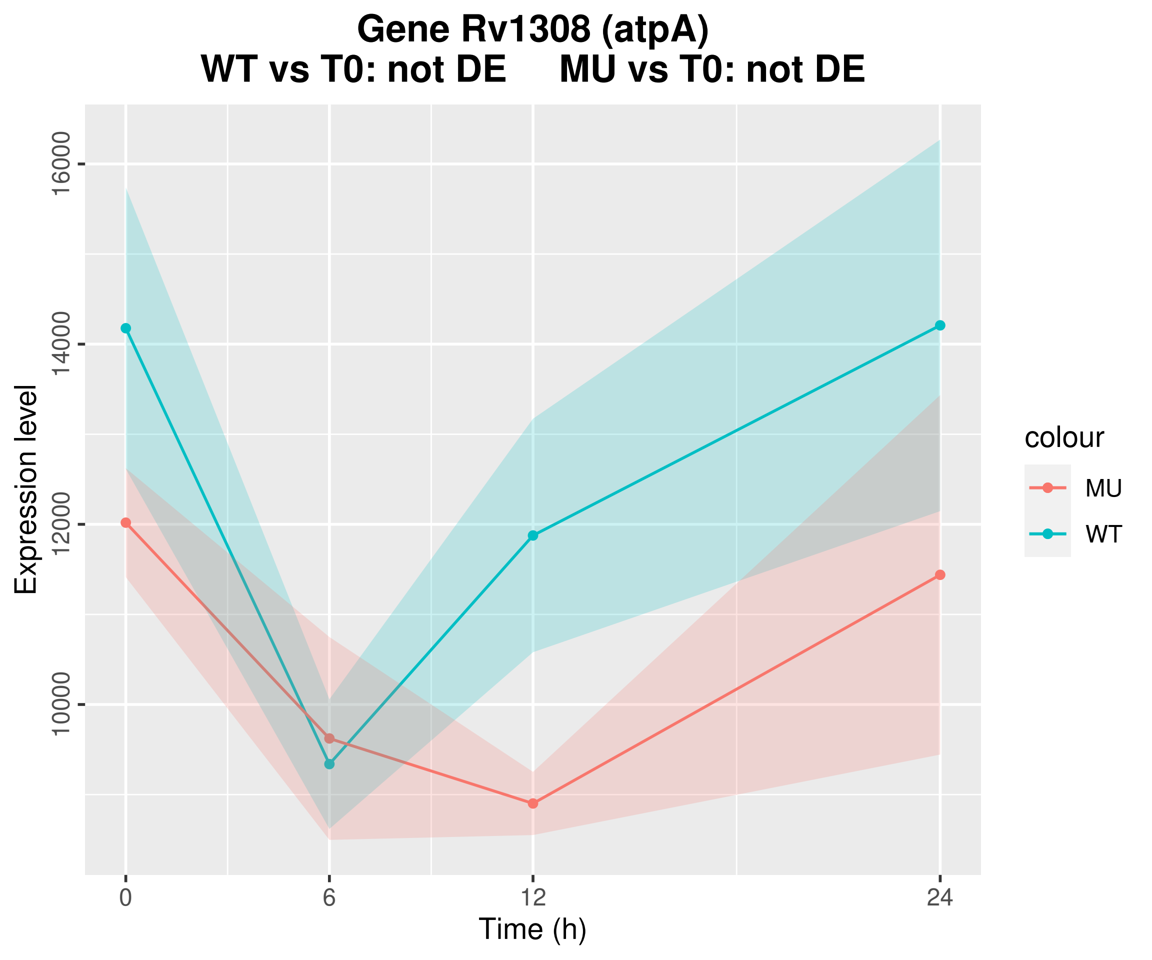

Gene Rv1308 (atpA)
WT vs T0: not DE   MU vs T0: not DE
Expression level
16000
14000
12000
10000
0
6
12
24
Time (h)
colour
MU
WT

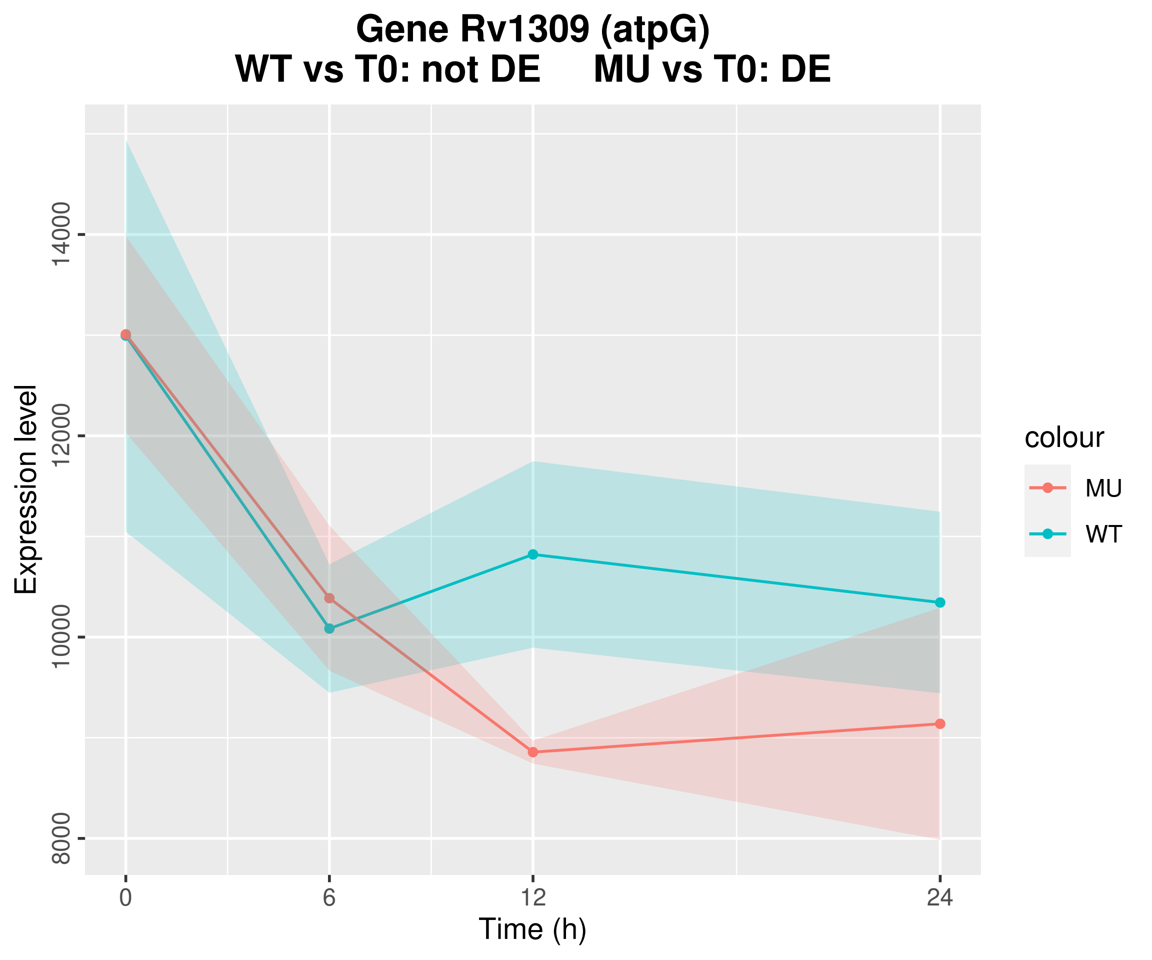
Gene Rv1309 (atpG)
WT vs T0: not DE MU vs T0: DE
Expression level
14000
12000
10000
8000
0
6
12
24
Time (h)
colour
MU
WT

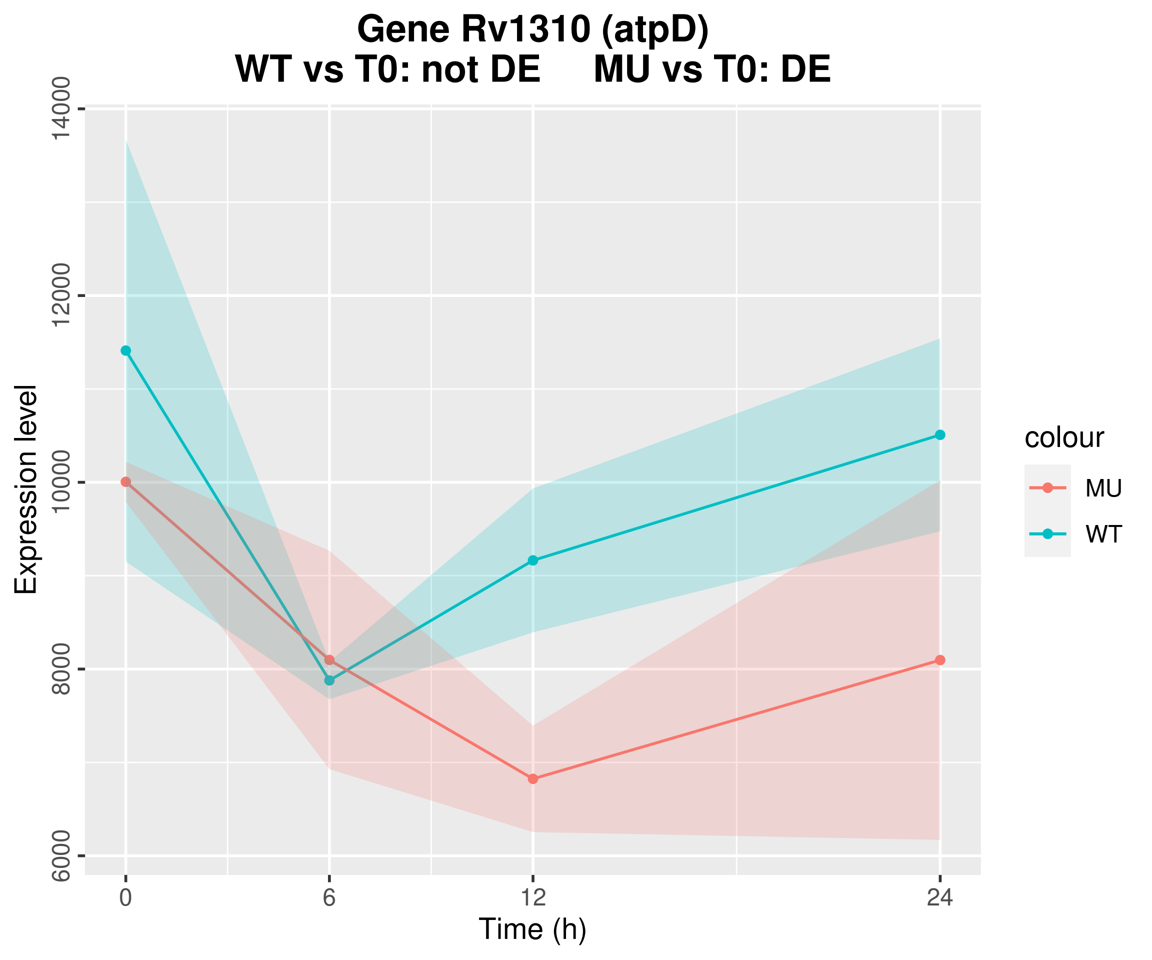

Gene Rv1310 (atpD)
WT vs T0: not DE MU vs T0: DE
Expression level
14000
12000
10000
8000
6000
0
6
12
24
Time (h)
colour
MU
WT

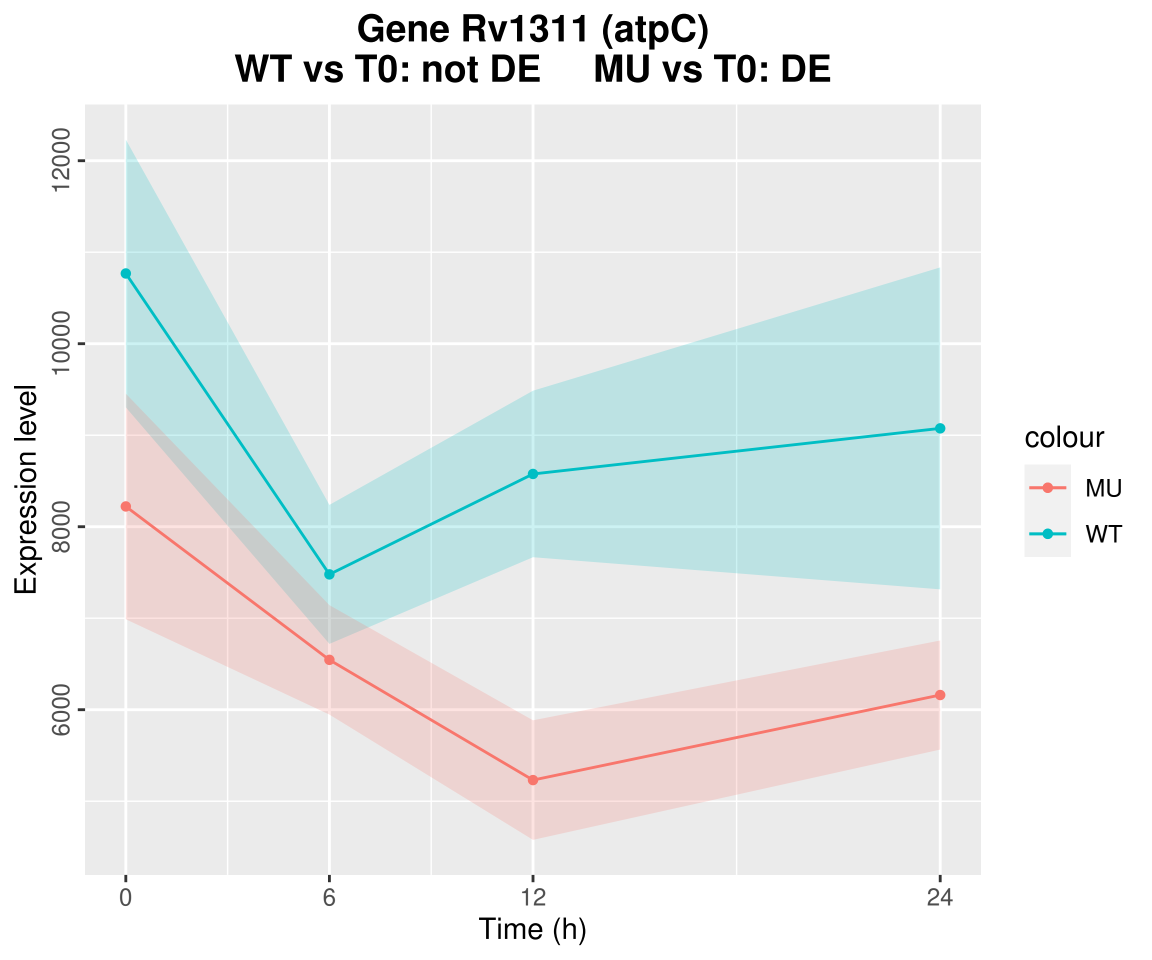

Gene Rv1311 (atpC)
WT vs T0: not DE   MU vs T0: DE
Expression level
12000
10000
8000
6000
0
6
12
24
Time (h)
colour
MU
WT

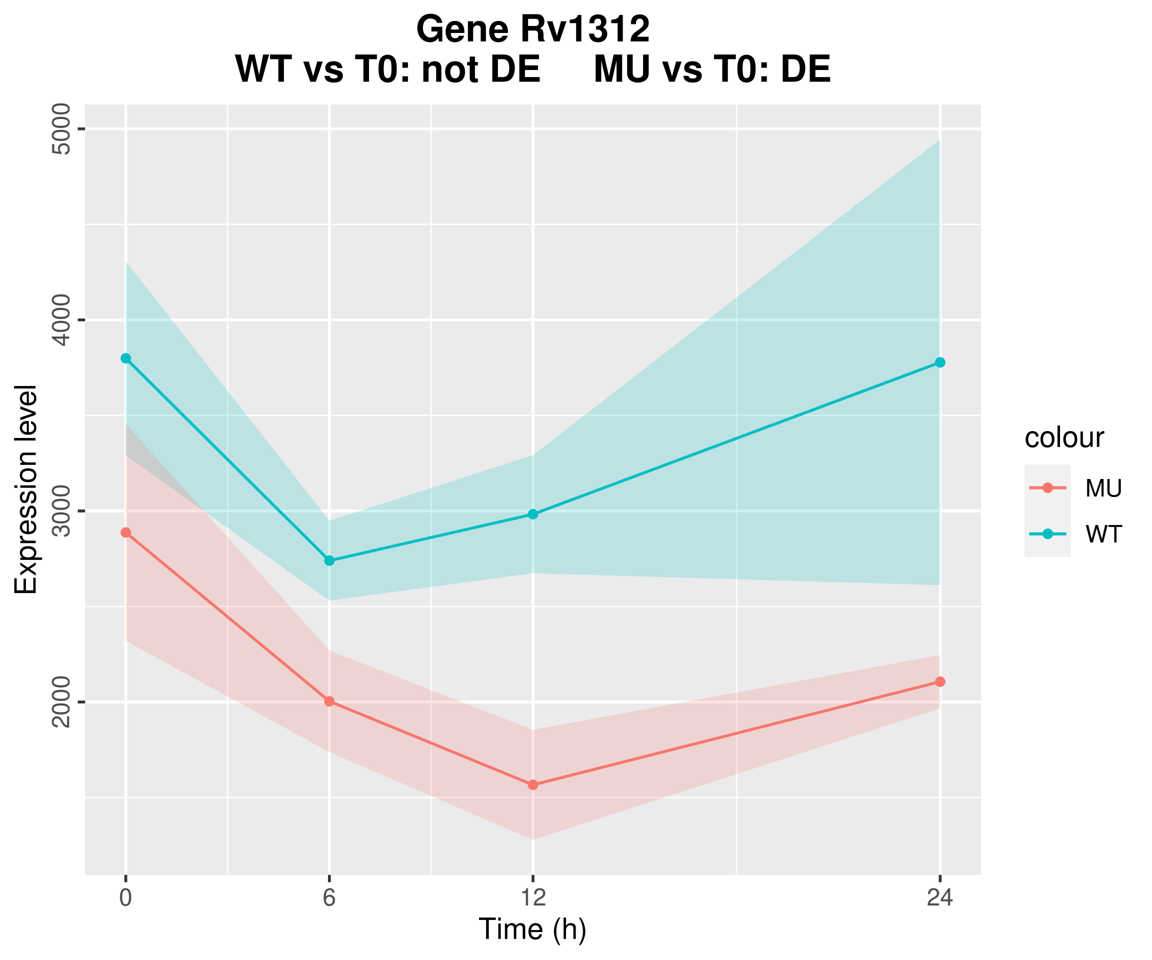

Gene Rv1312
WT vs T0: not DE   MU vs T0: DE
5000
4000
3000
2000
Expression level
0
6
12
24
Time (h)
colour
MU
WT

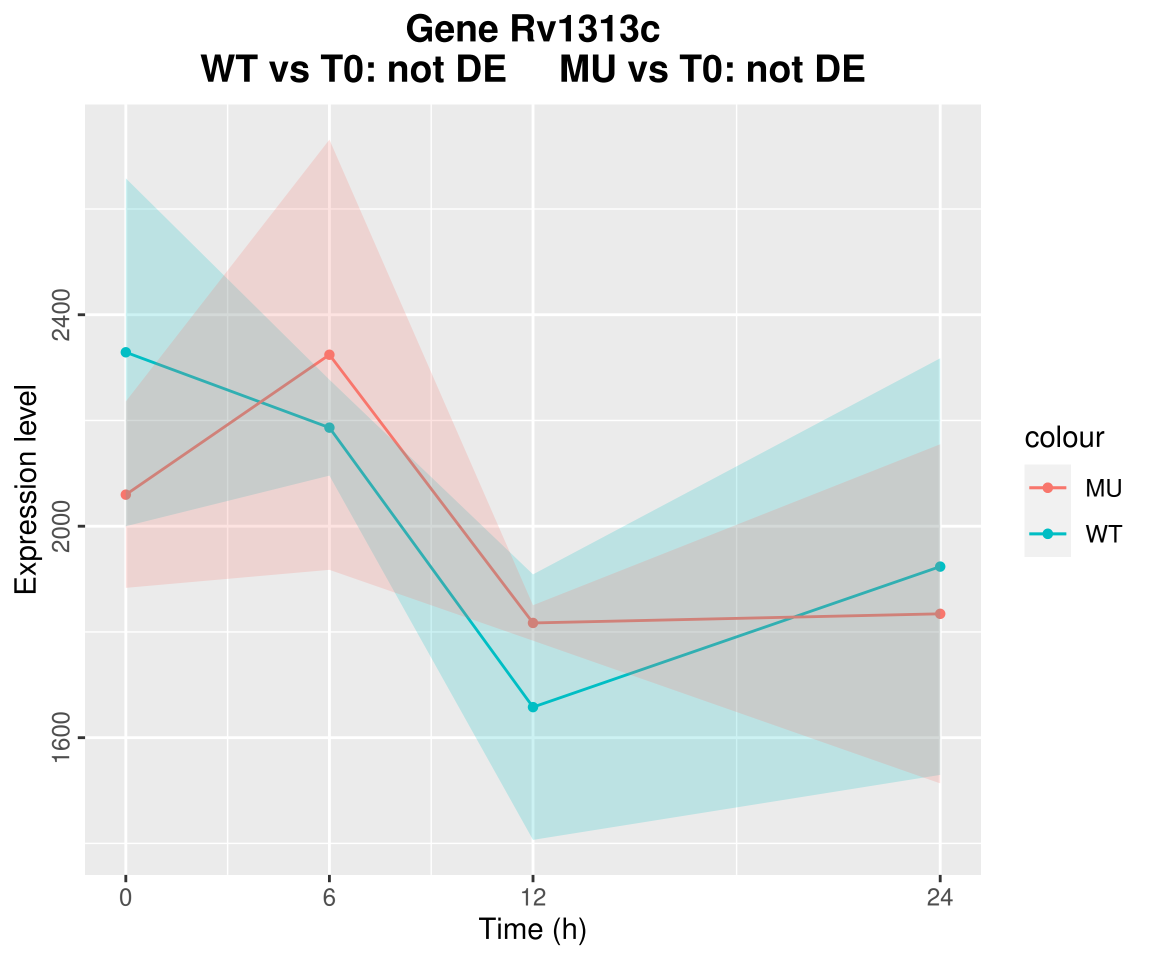
Gene Rv1313c
WT vs T0: not DE MU vs T0: not DE
Expression level
2400
2000
1600
0
6
12
24
Time (h)
colour
MU
WT

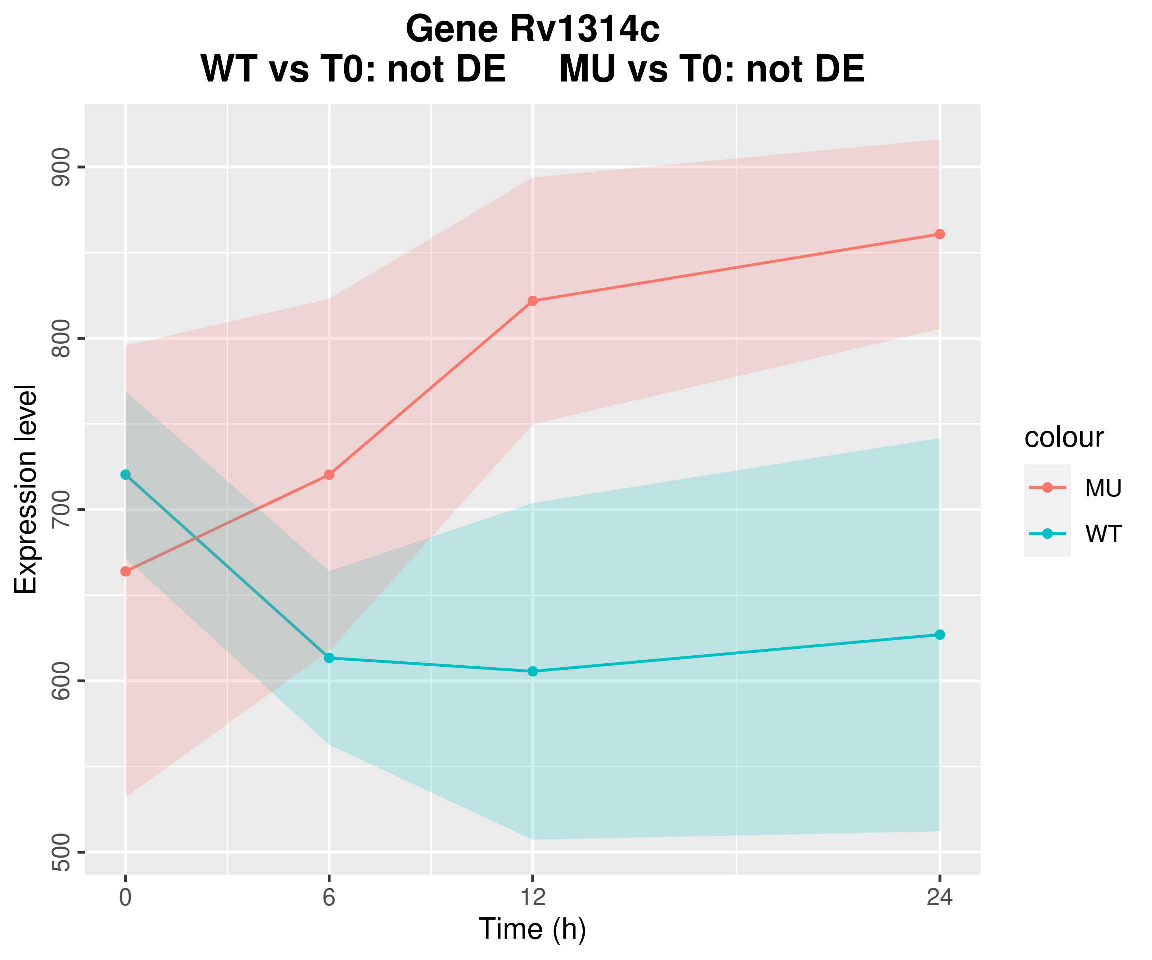

Gene Rv1314c
WT vs T0: not DE MU vs T0: not DE
Expression level
900
800
700
600
500
0
6
12
24
Time (h)
colour
MU
WT

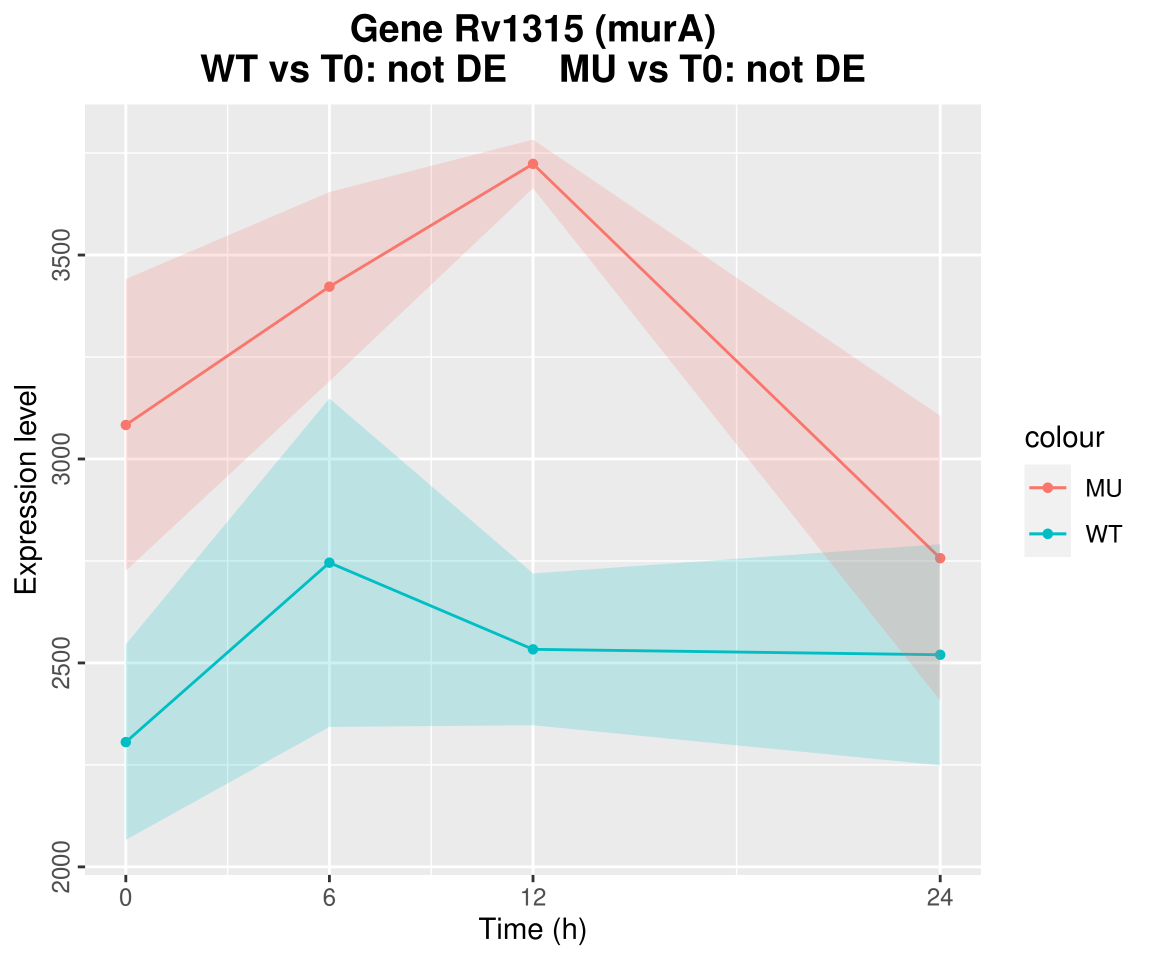

Gene Rv1315 (murA)
WT vs T0: not DE   MU vs T0: not DE
Expression level
3500
3000
2500
2000
0
6
12
24
Time (h)
colour
MU
WT

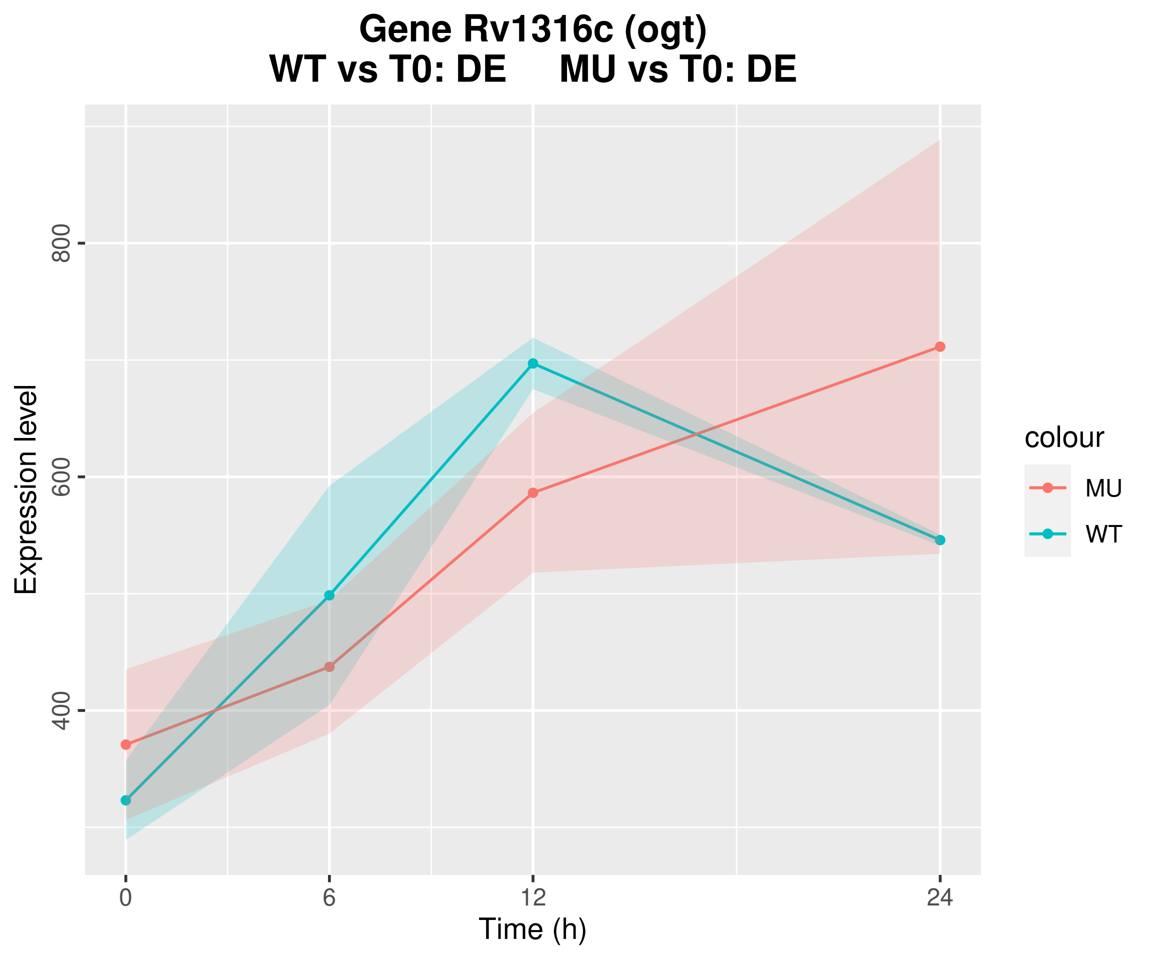
Gene Rv1316c (ogt)
WT vs T0: DE MU vs T0: DE
Expression level
800
600
400
0
6
12
24
Time (h)
colour
MU
WT

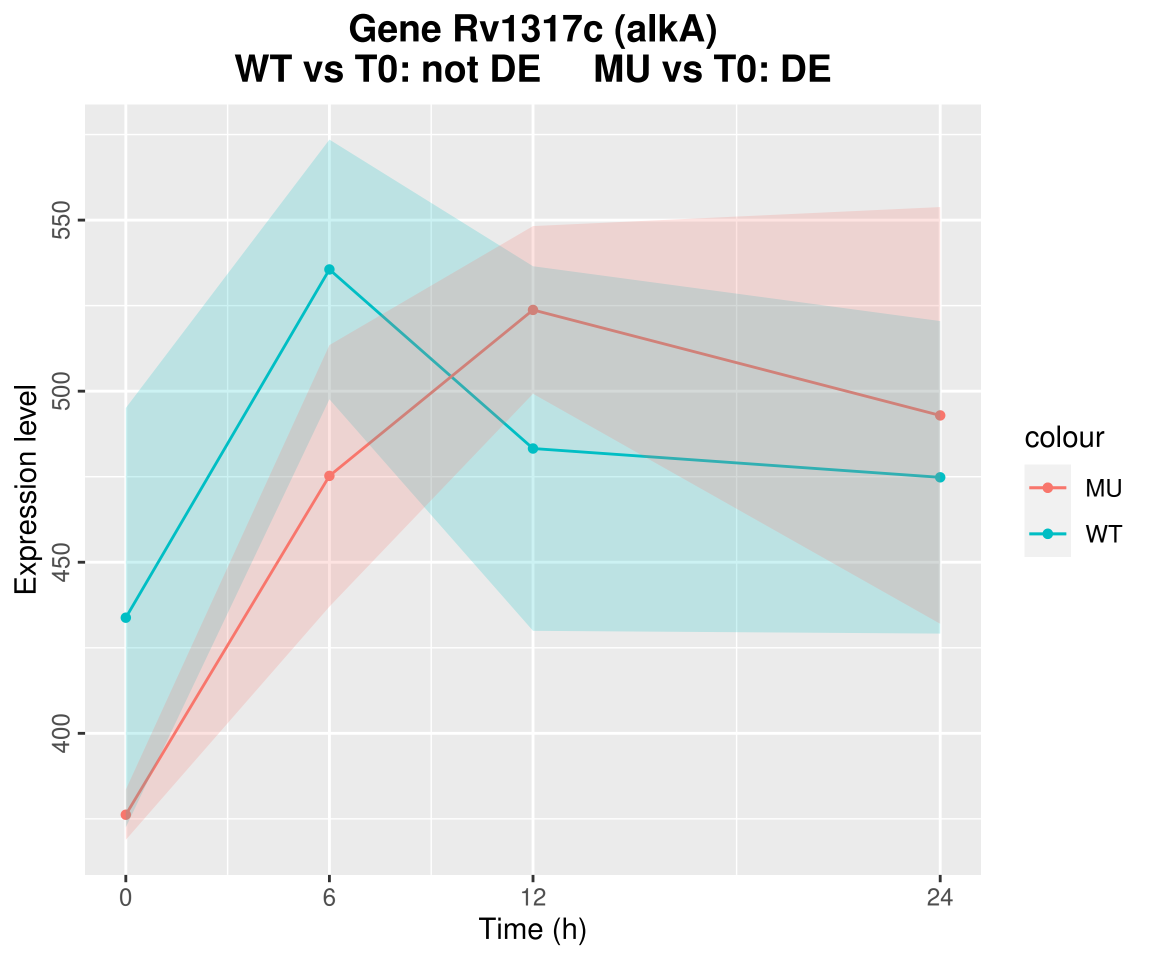

Gene Rv1317c (alkA)
WT vs T0: not DE MU vs T0: DE
Expression level
550
500
450
400
0
6
12
24
Time (h)
colour
MU
WT

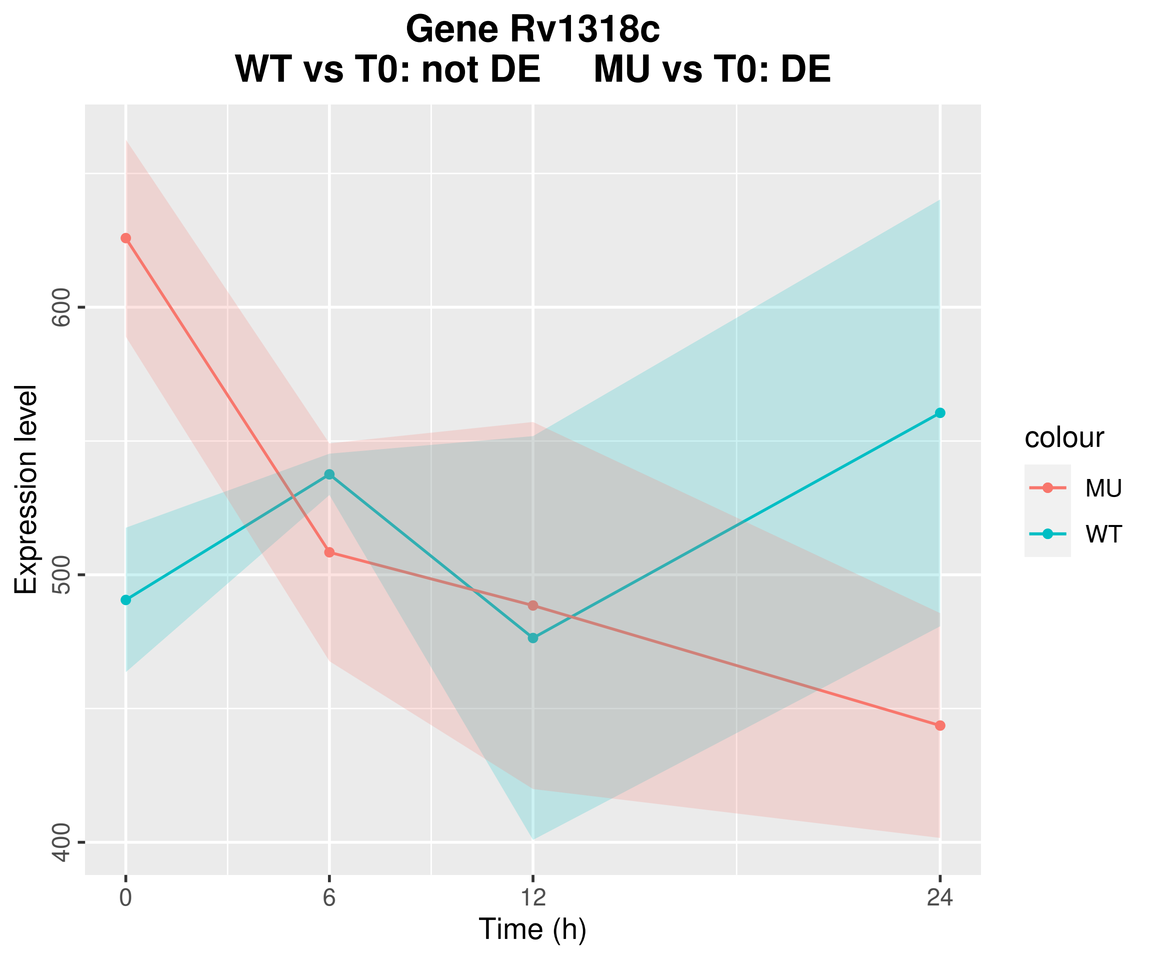

Gene Rv1318c
WT vs T0: not DE   MU vs T0: DE
Expression level
600
500
400
0
6
12
24
Time (h)
colour
MU
WT

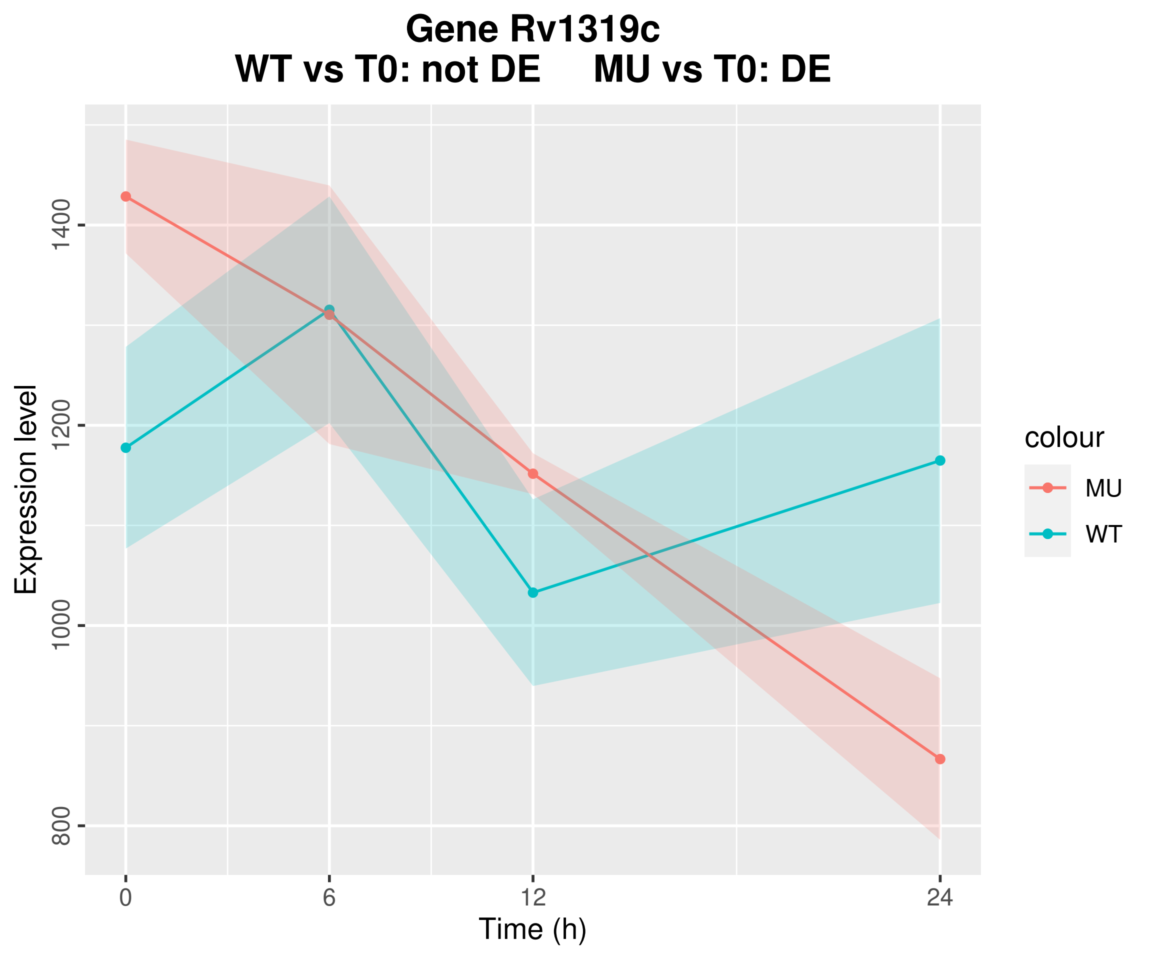
Gene Rv1319c
WT vs T0: not DE MU vs T0: DE
Expression level
1400
1200
1000
800
0
6
12
24
Time (h)
colour
MU
WT

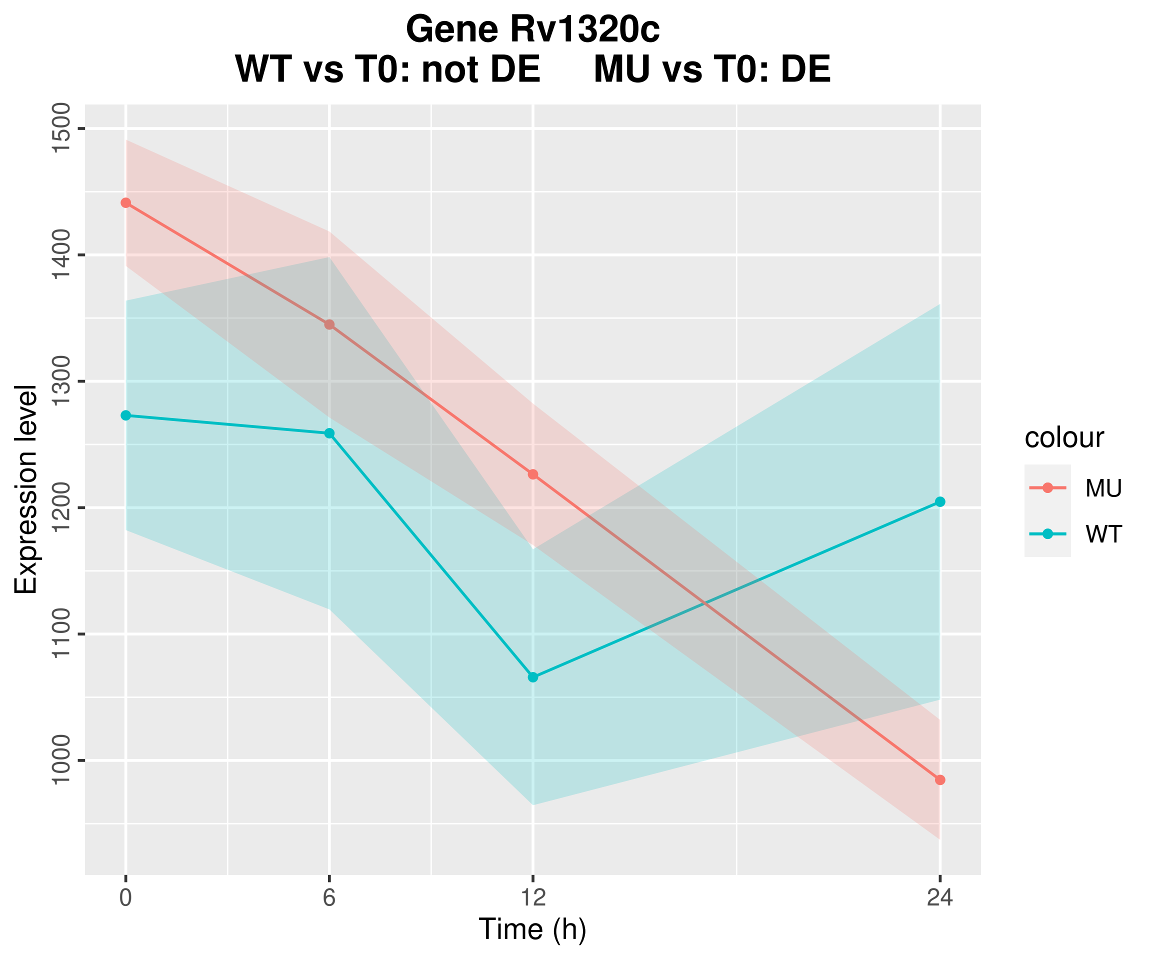
Gene Rv1320c
WT vs T0: not DE MU vs T0: DE
Expression level
1000
1100
1200
1300
1400
1500
0
6
12
24
Time (h)
colour
MU
WT

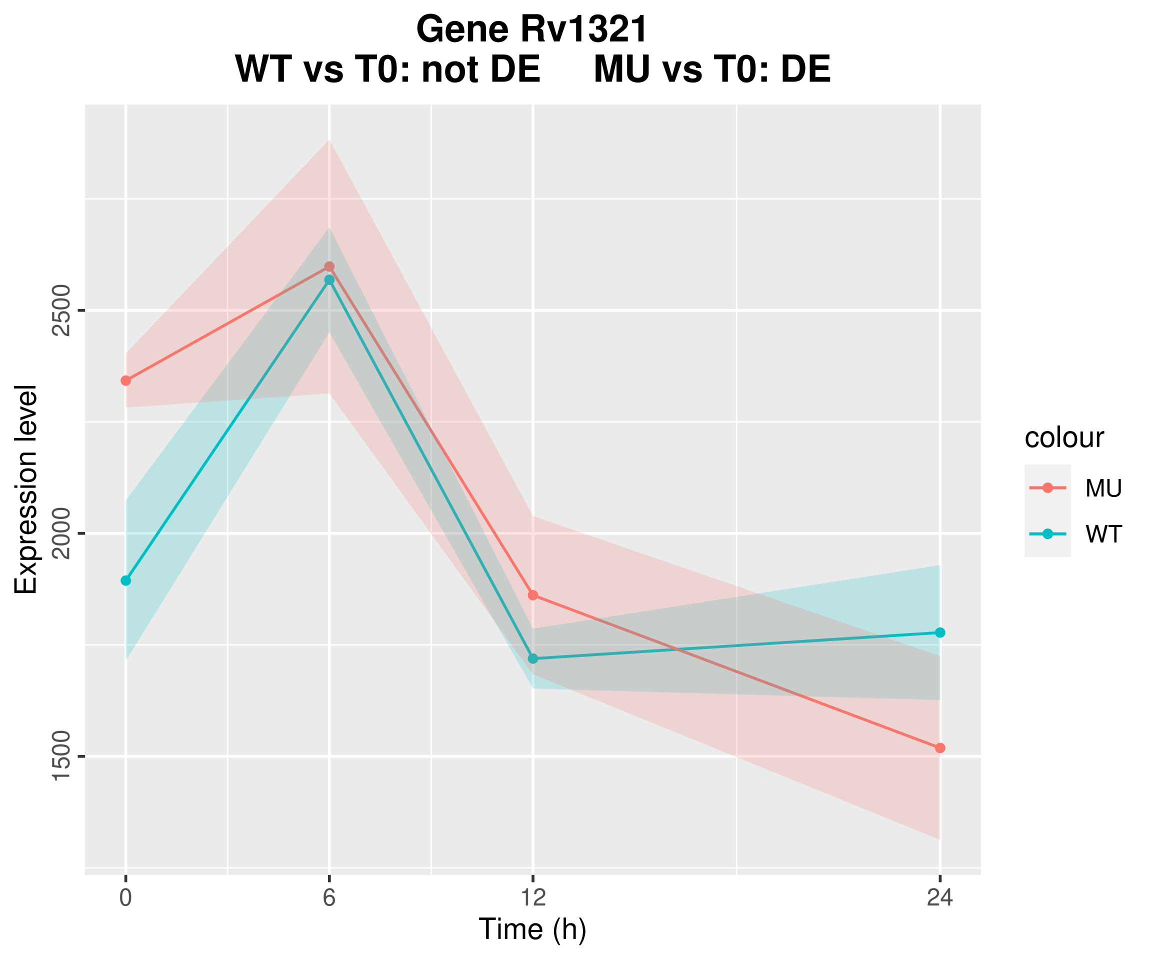

Gene Rv1321
WT vs T0: not DE   MU vs T0: DE
Expression level
2500
2000
1500
0
6
12
24
Time (h)
colour
MU
WT

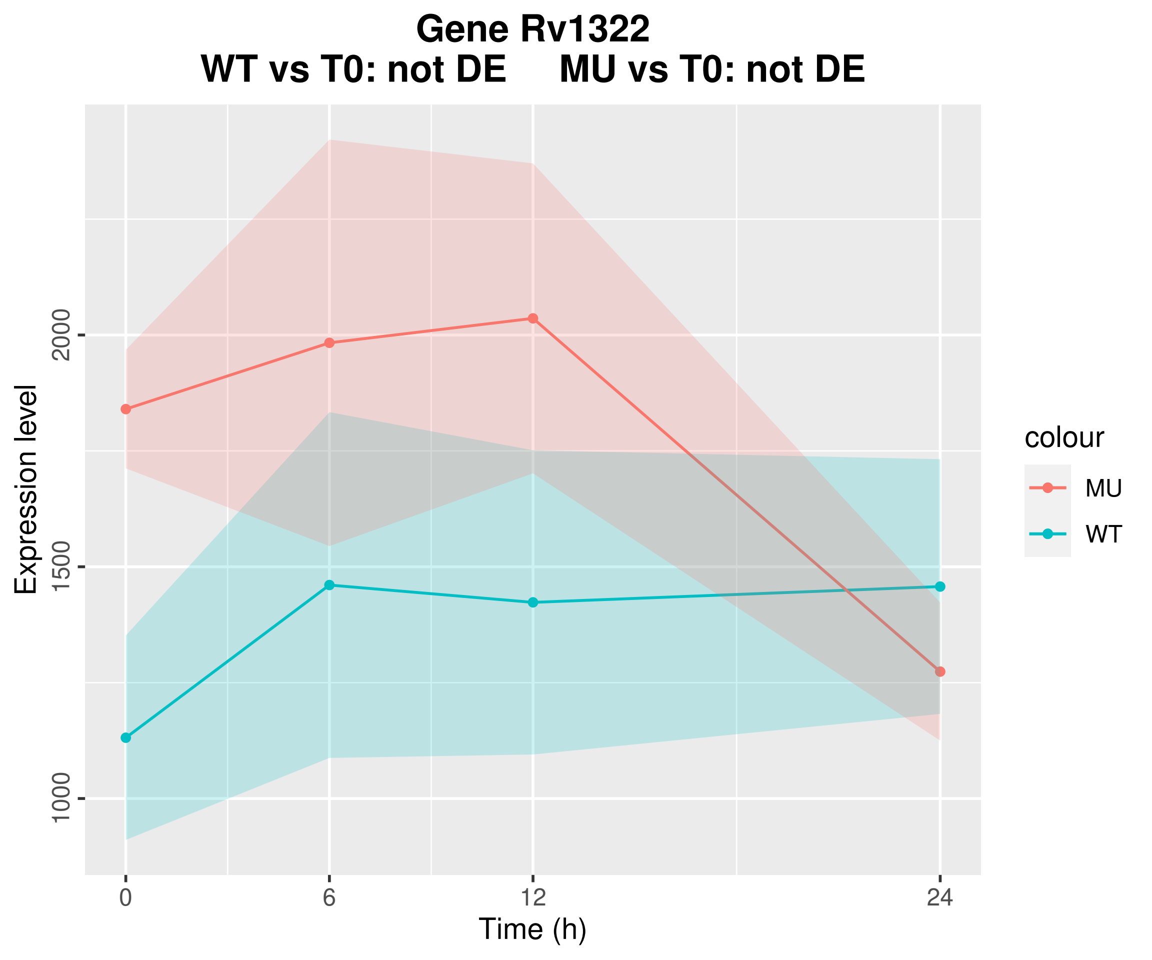

Gene Rv1322
WT vs T0: not DE   MU vs T0: not DE
Expression level
2000
1500
1000
0
6
12
24
Time (h)
colour
MU
WT

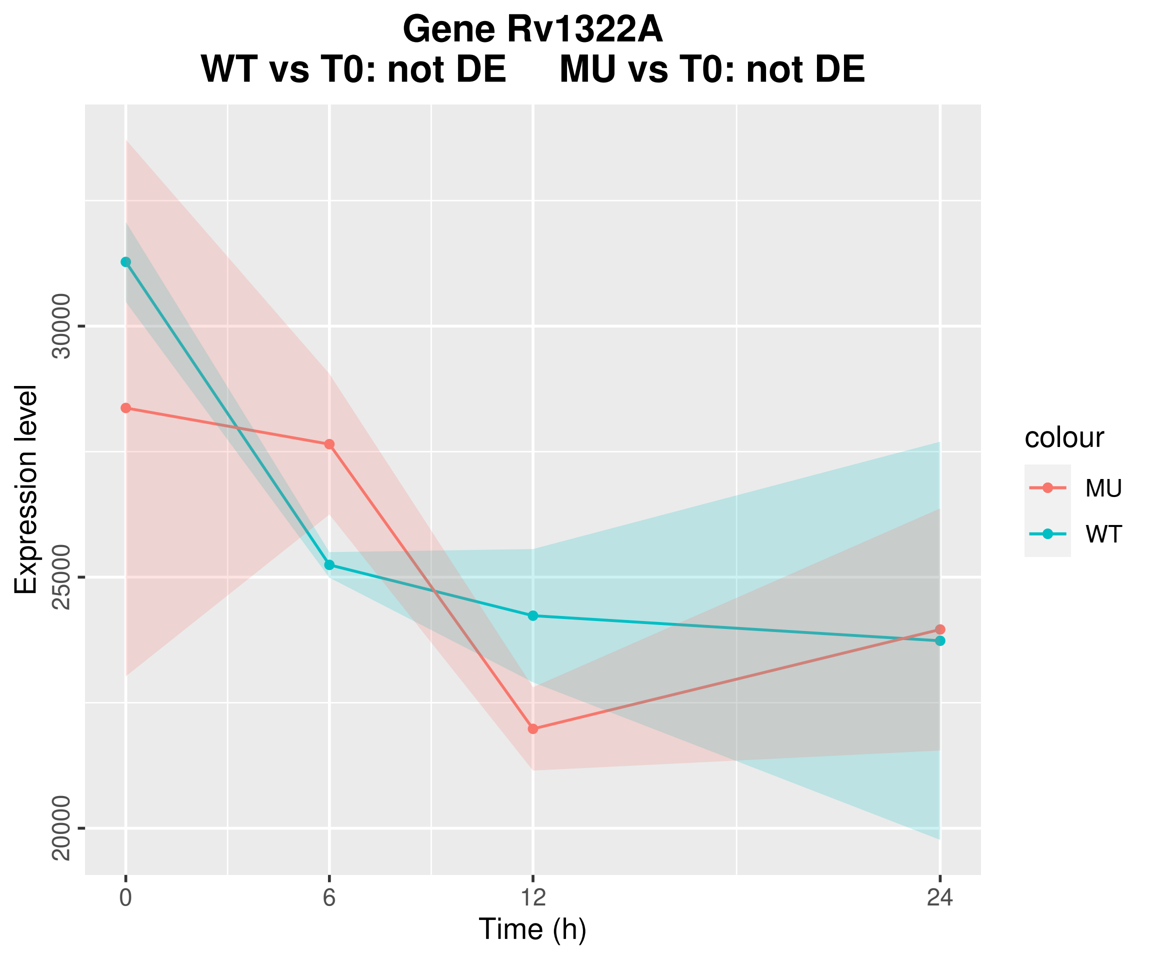

Gene Rv1322A
WT vs T0: not DE   MU vs T0: not DE
Expression level
30000
25000
20000
0
6
12
24
Time (h)
colour
MU
WT

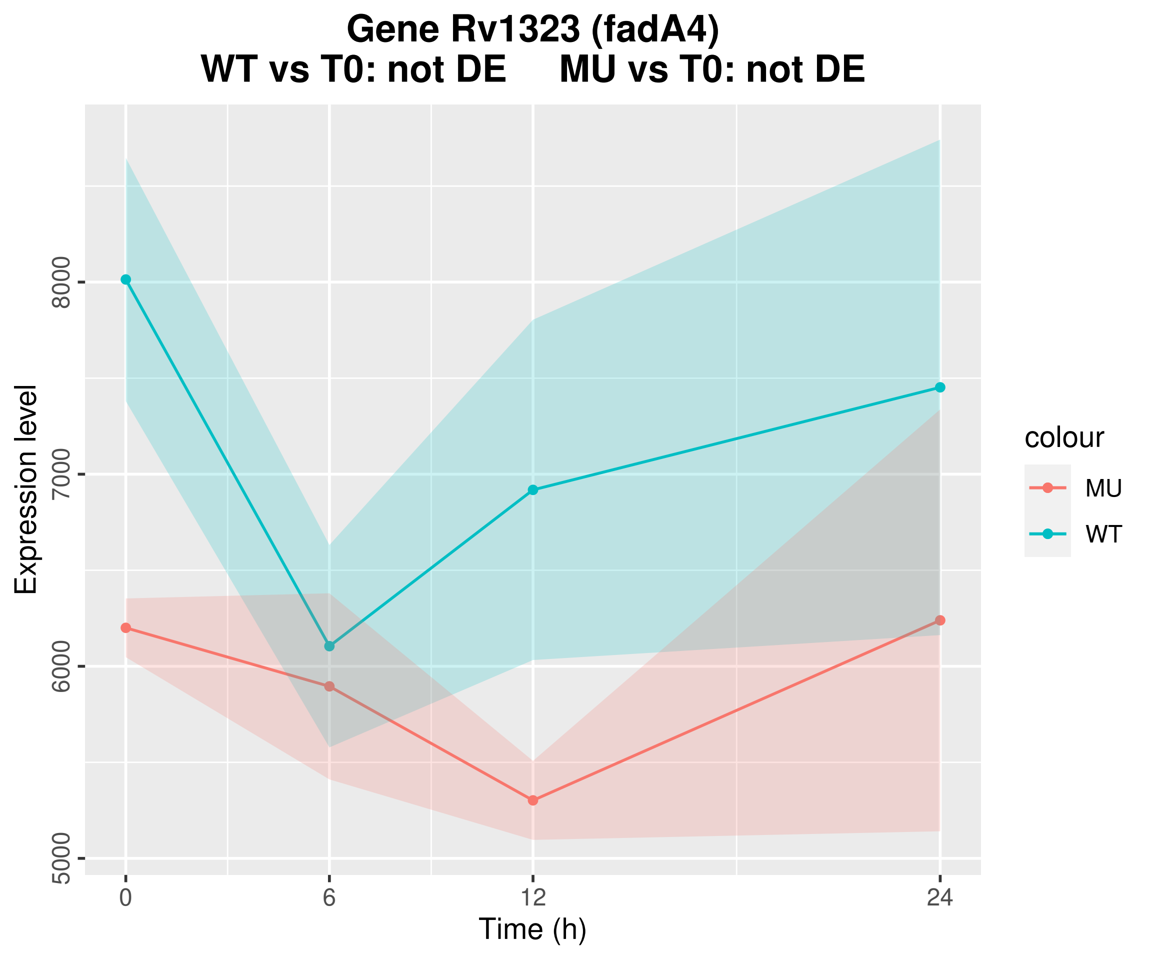
Gene Rv1323 (fadA4)
WT vs T0: not DE   MU vs T0: not DE
Expression level
8000
7000
6000
5000
0
6
12
24
Time (h)
colour
MU
WT

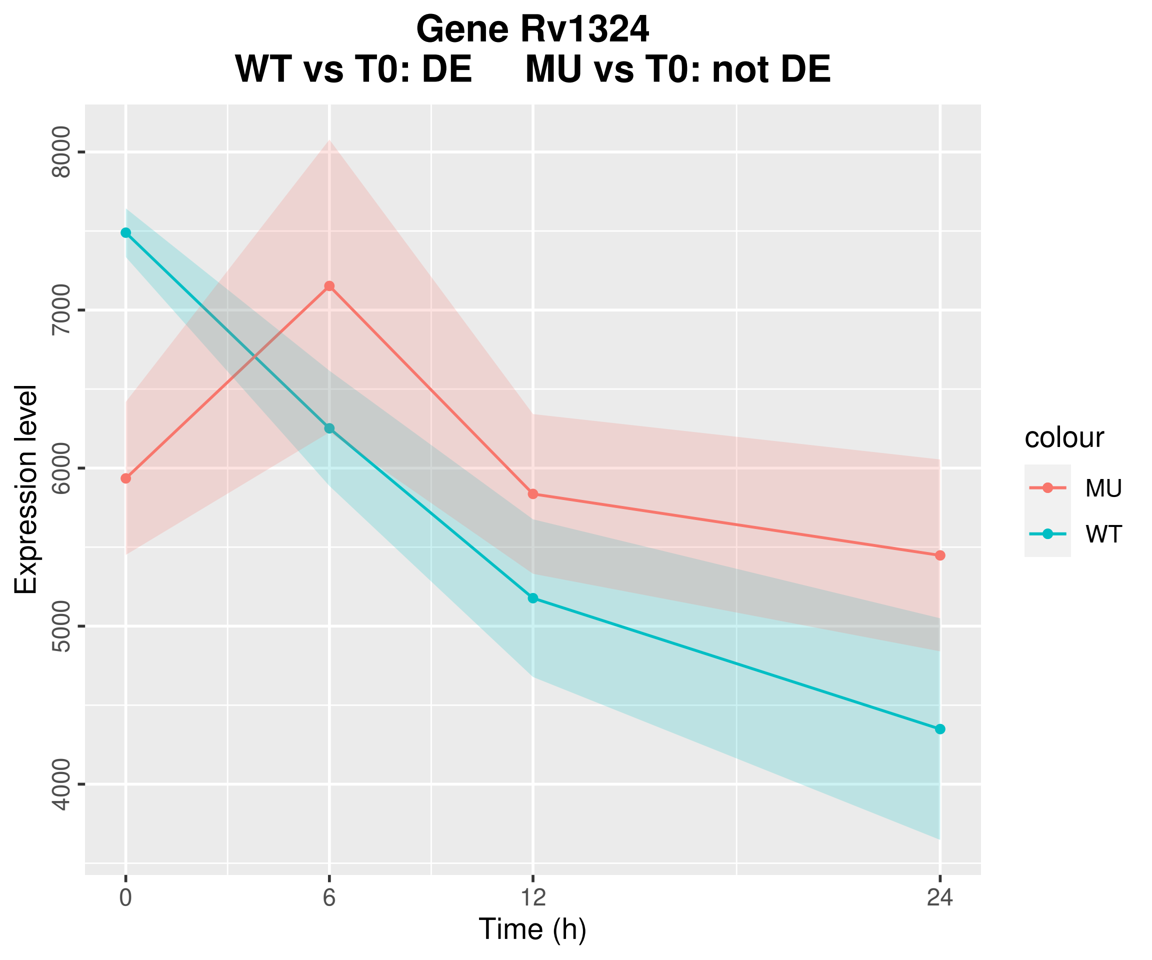

Gene Rv1324
WT vs T0: DE MU vs T0: not DE
Expression level
8000
7000
6000
5000
4000
0
6
12
24
Time (h)
colour
MU
WT

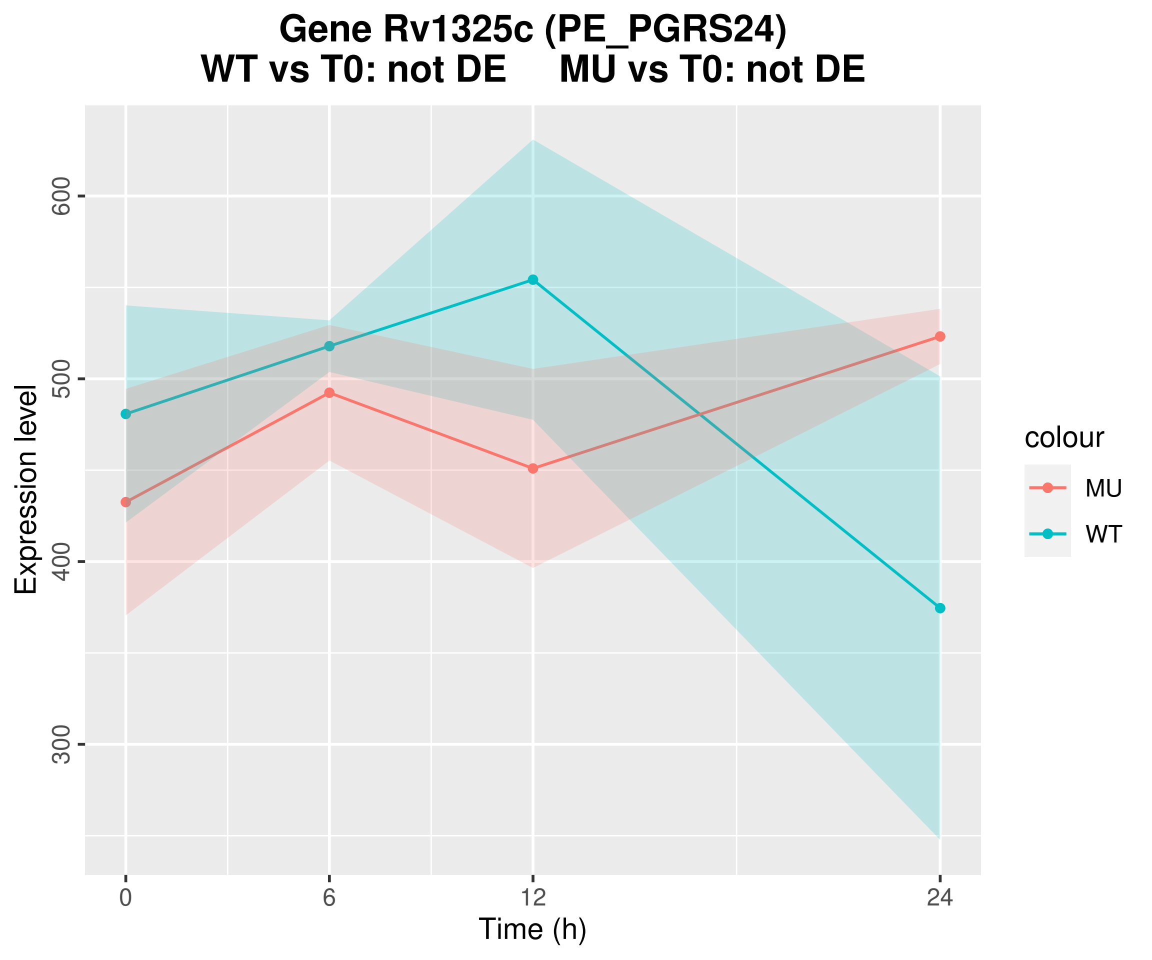

Gene Rv1325c (PE_PGRS24)
WT vs T0: not DE MU vs T0: not DE
Expression level
600
500
400
300
0
6
12
24
Time (h)
colour
MU
WT

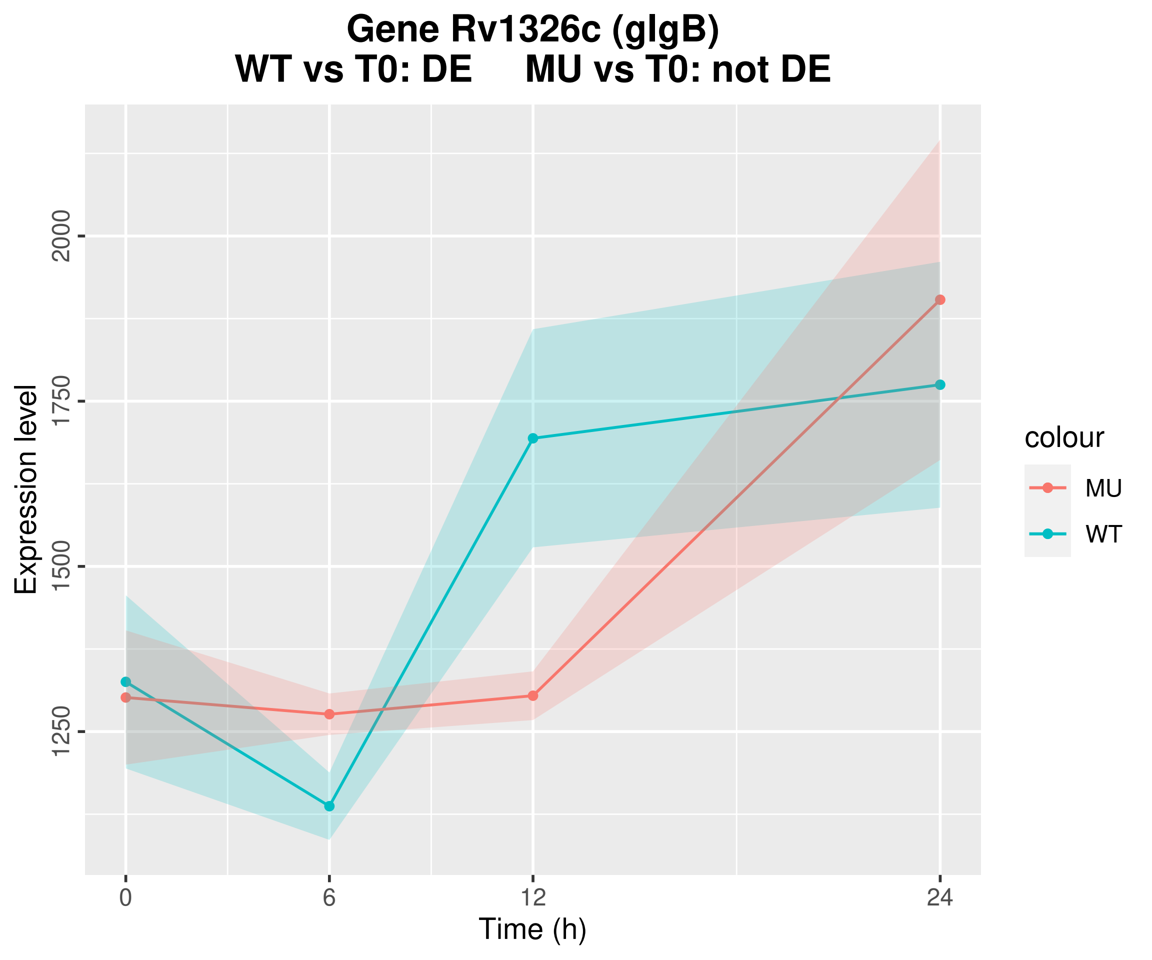

Gene Rv1326c (glgB)
WT vs T0: DE   MU vs T0: not DE
Expression level
2000
1750
1500
1250
0
6
12
24
Time (h)
colour
MU
WT

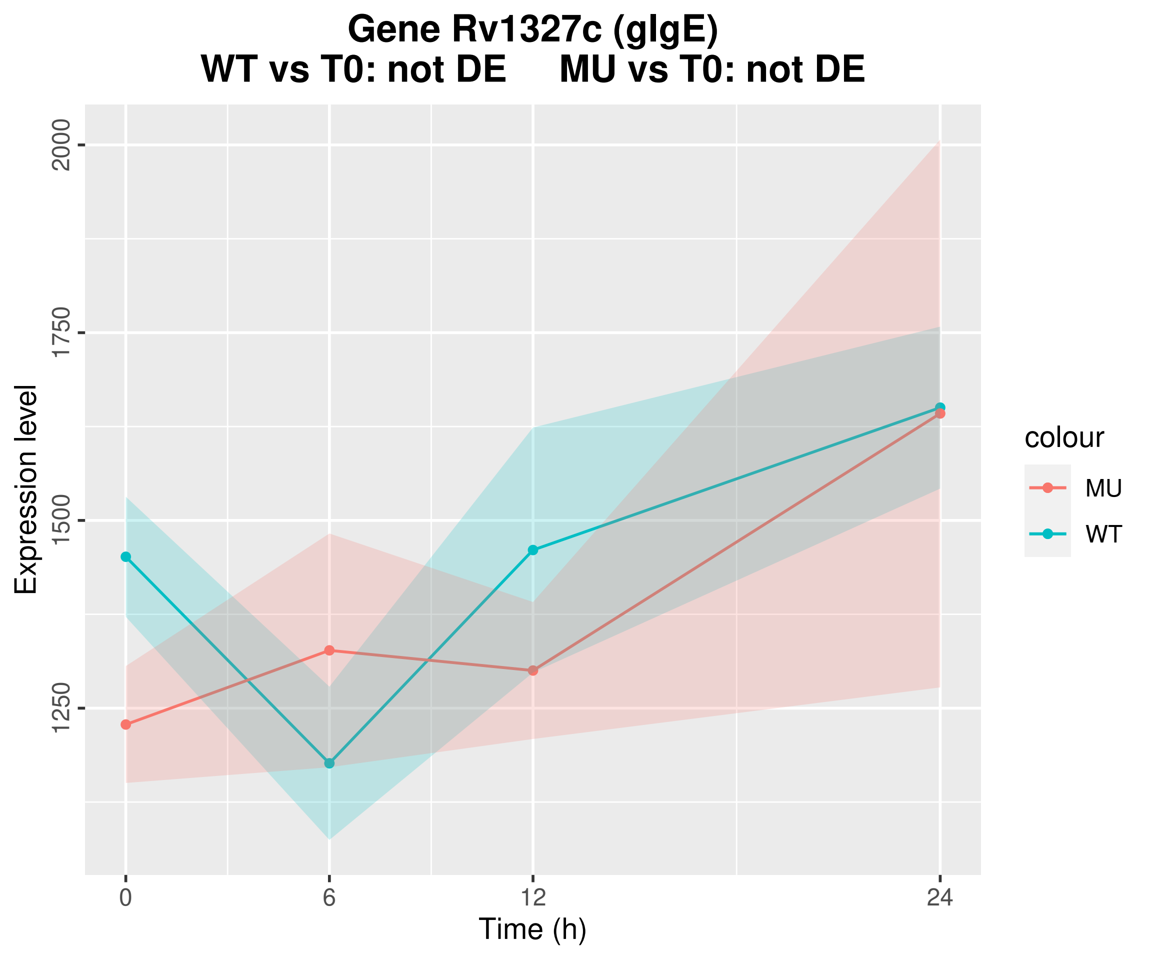
Gene Rv1327c (glgE)
WT vs T0: not DE MU vs T0: not DE
Expression level
2000
1750
1500
1250
0
6
12
24
Time (h)
colour
MU
WT

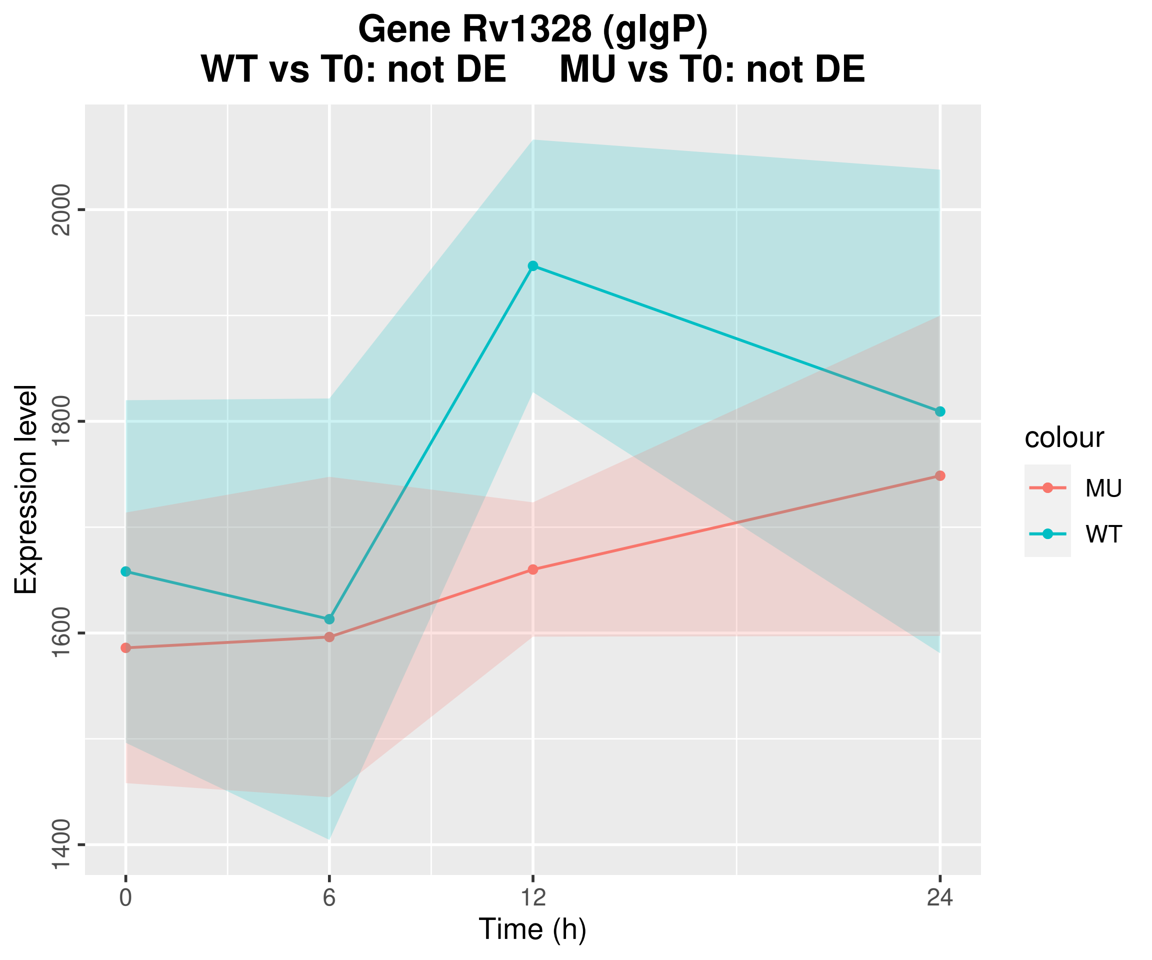
Gene Rv1328 (glgP)
WT vs T0: not DE   MU vs T0: not DE
Expression level
2000
1800
1600
1400
0
6
12
24
Time (h)
colour
MU
WT

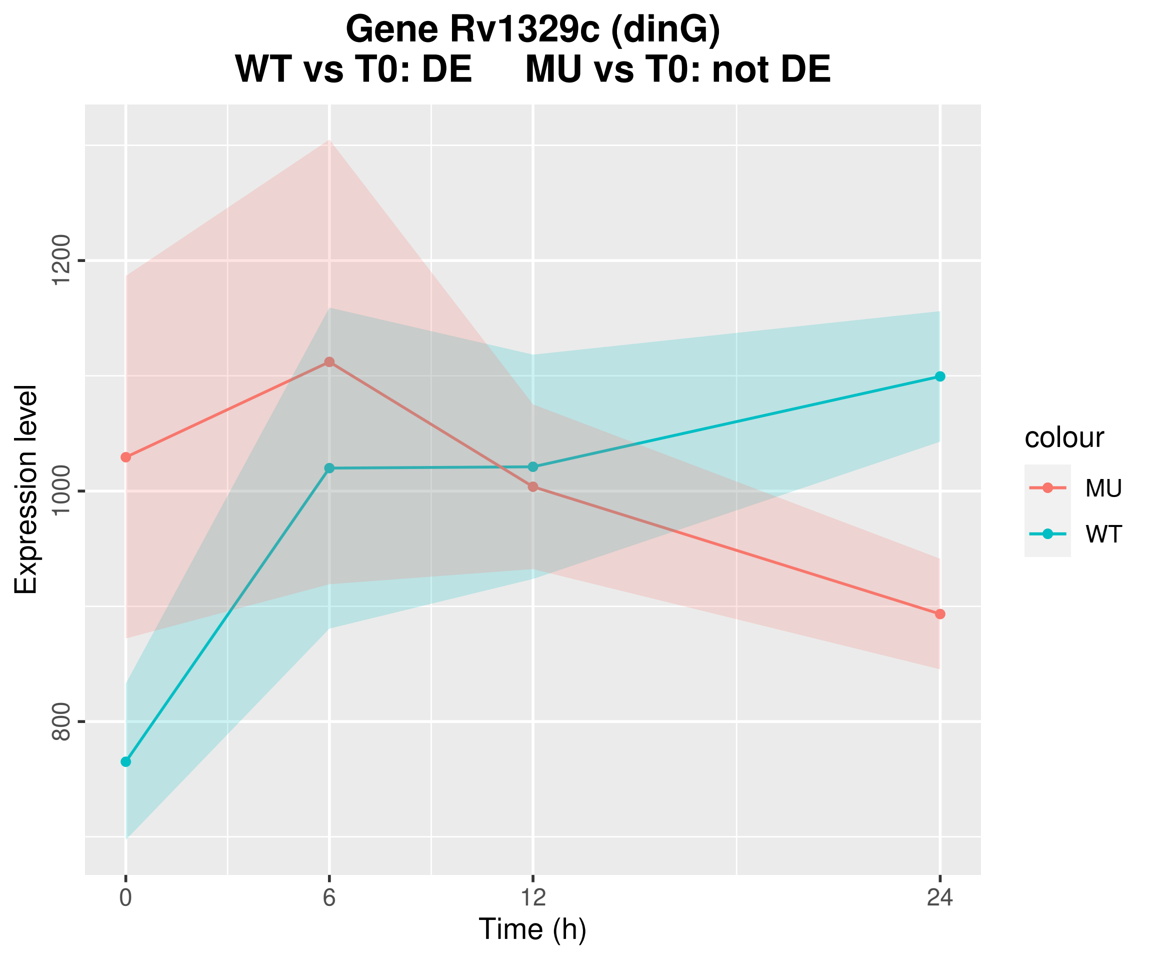
Gene Rv1329c (dinG)
WT vs T0: DE   MU vs T0: not DE
Expression level
1200
1000
800
0
6
12
24
Time (h)
colour
MU
WT

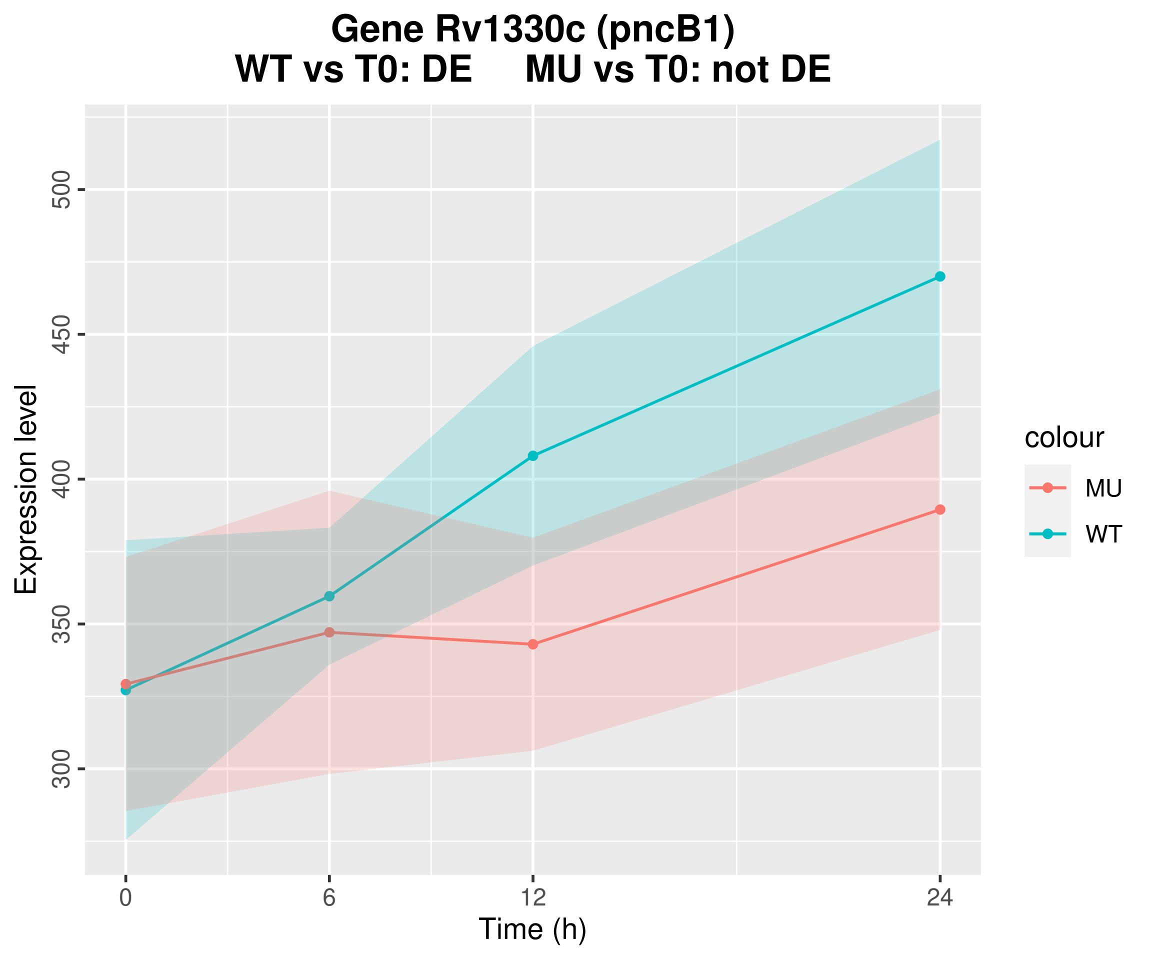
Gene Rv1330c (pncB1)
WT vs T0: DE MU vs T0: not DE
Expression level
300
350
400
450
500
0
6
12
24
Time (h)
colour
MU
WT

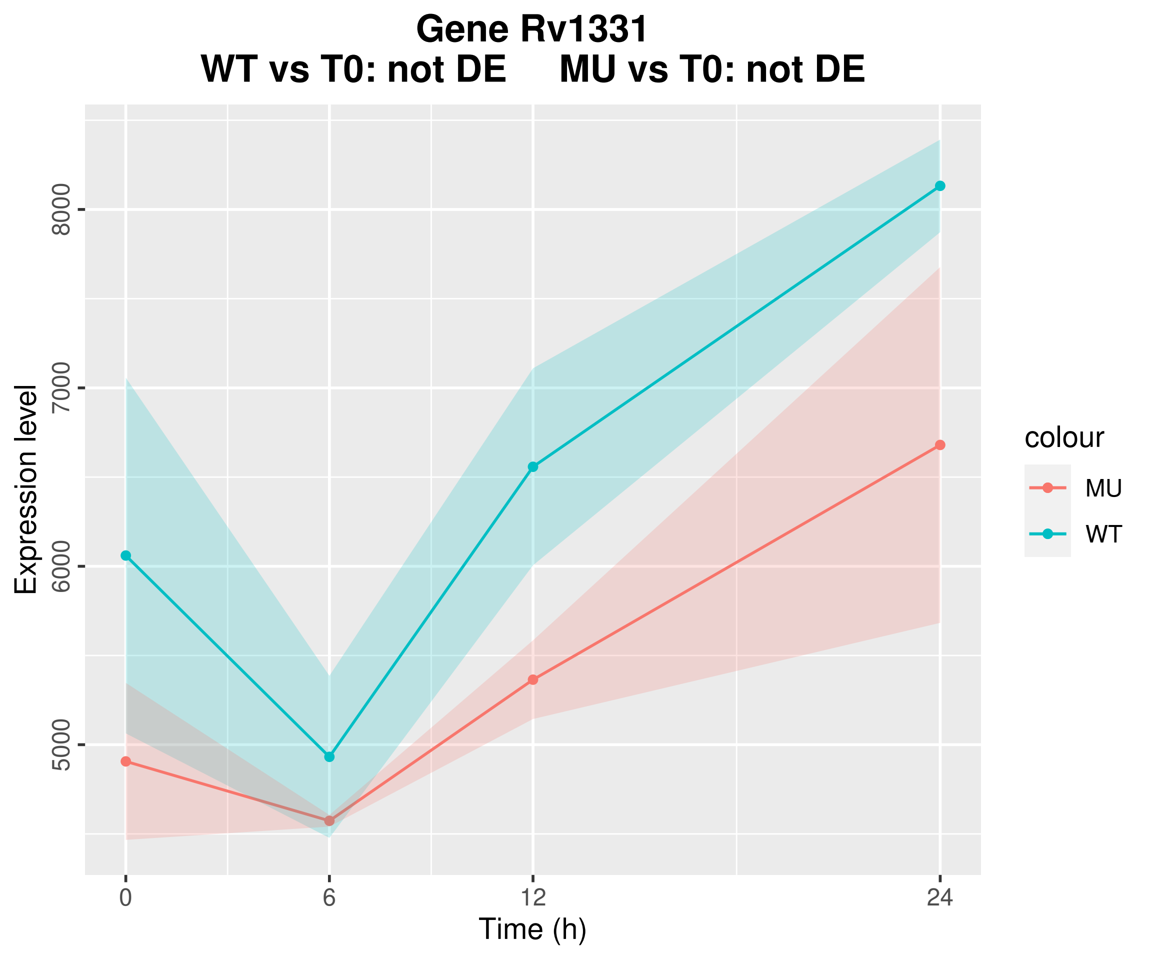

Gene Rv1331
WT vs T0: not DE MU vs T0: not DE
Expression level
8000
7000
6000
5000
0
6
12
24
Time (h)
colour
MU
WT

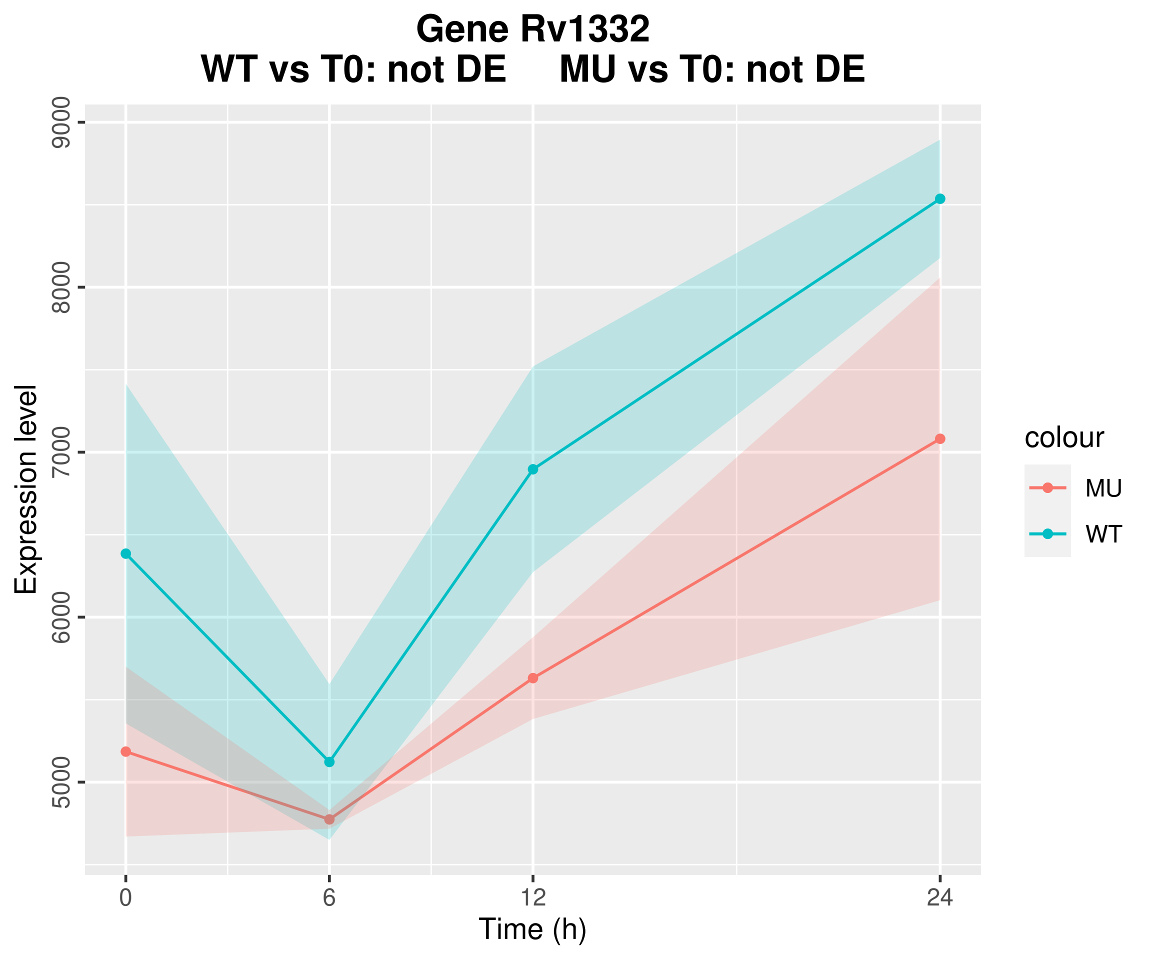
Gene Rv1332
WT vs T0: not DE   MU vs T0: not DE
Expression level
9000
8000
7000
6000
5000
0
6
12
24
Time (h)
colour
MU
WT

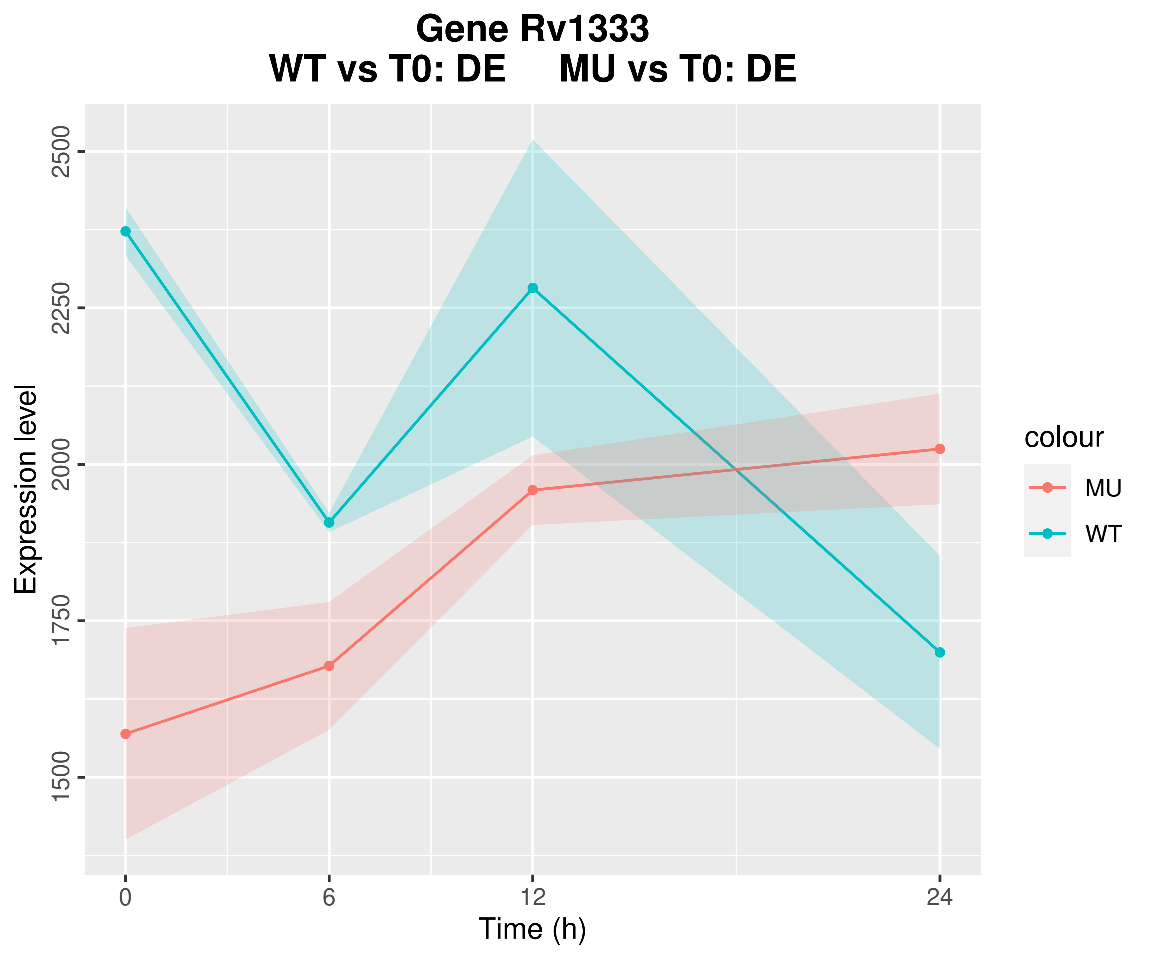

Gene Rv1333
WT vs T0: DE MU vs T0: DE
Expression level
2500
2250
2000
1750
1500
0
6
12
24
Time (h)
colour
MU
WT

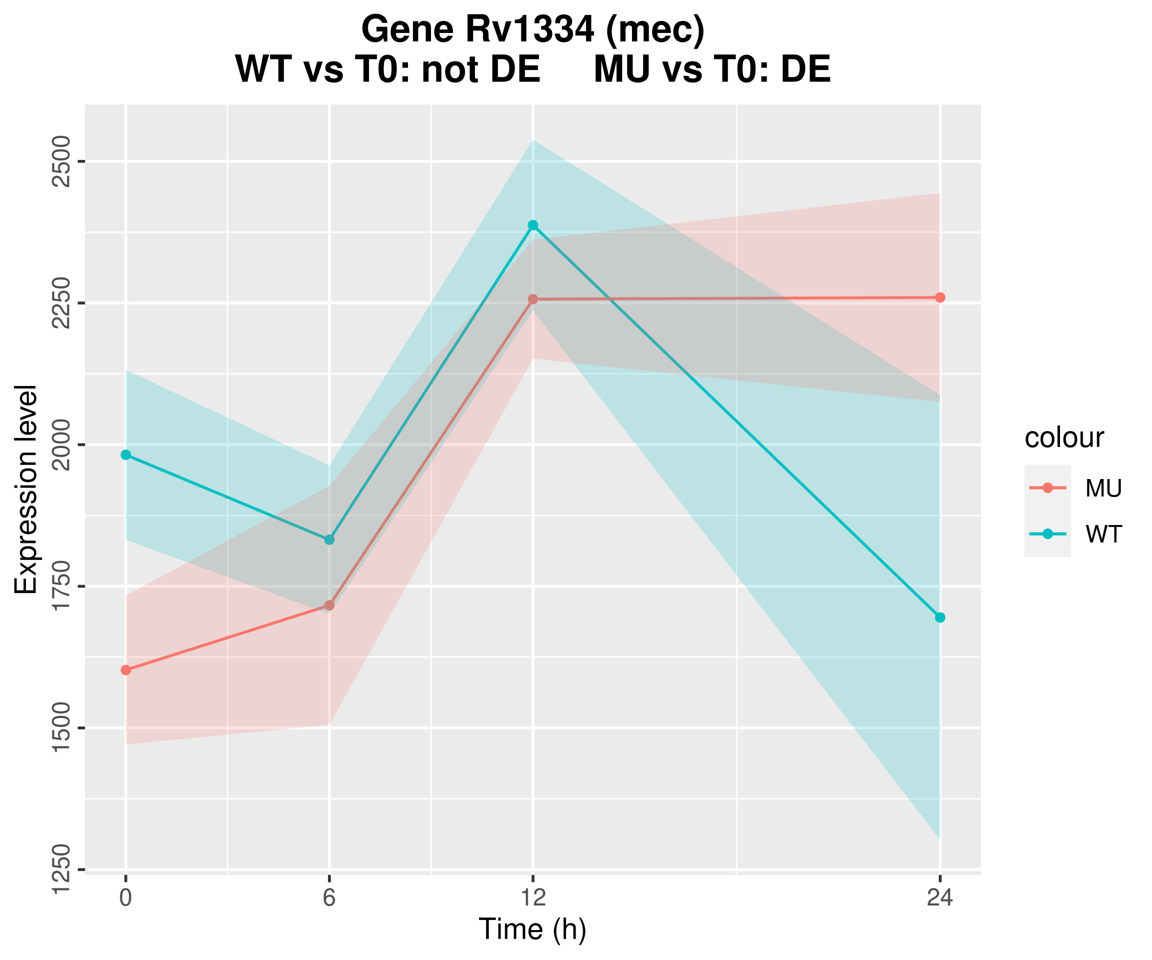
Gene Rv1334 (mec)
WT vs T0: not DE MU vs T0: DE
Expression level
2500
2250
2000
1750
1500
1250
0
6
12
24
Time (h)
colour
MU
WT

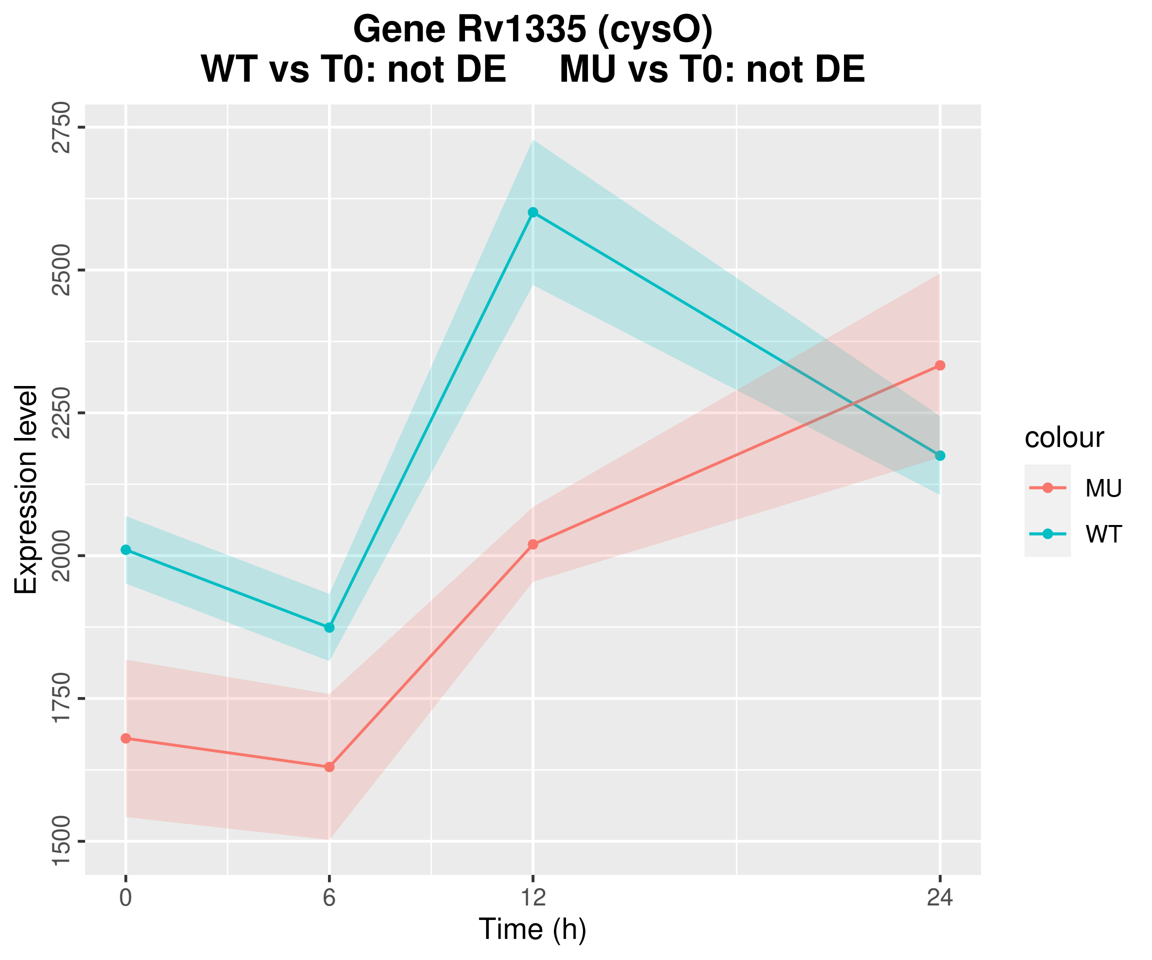

Gene Rv1335 (cysO)
WT vs T0: not DE MU vs T0: not DE
2750
2500
2250
2000
1750
1500
Expression level
0
6
12
24
Time (h)
colour
MU
WT

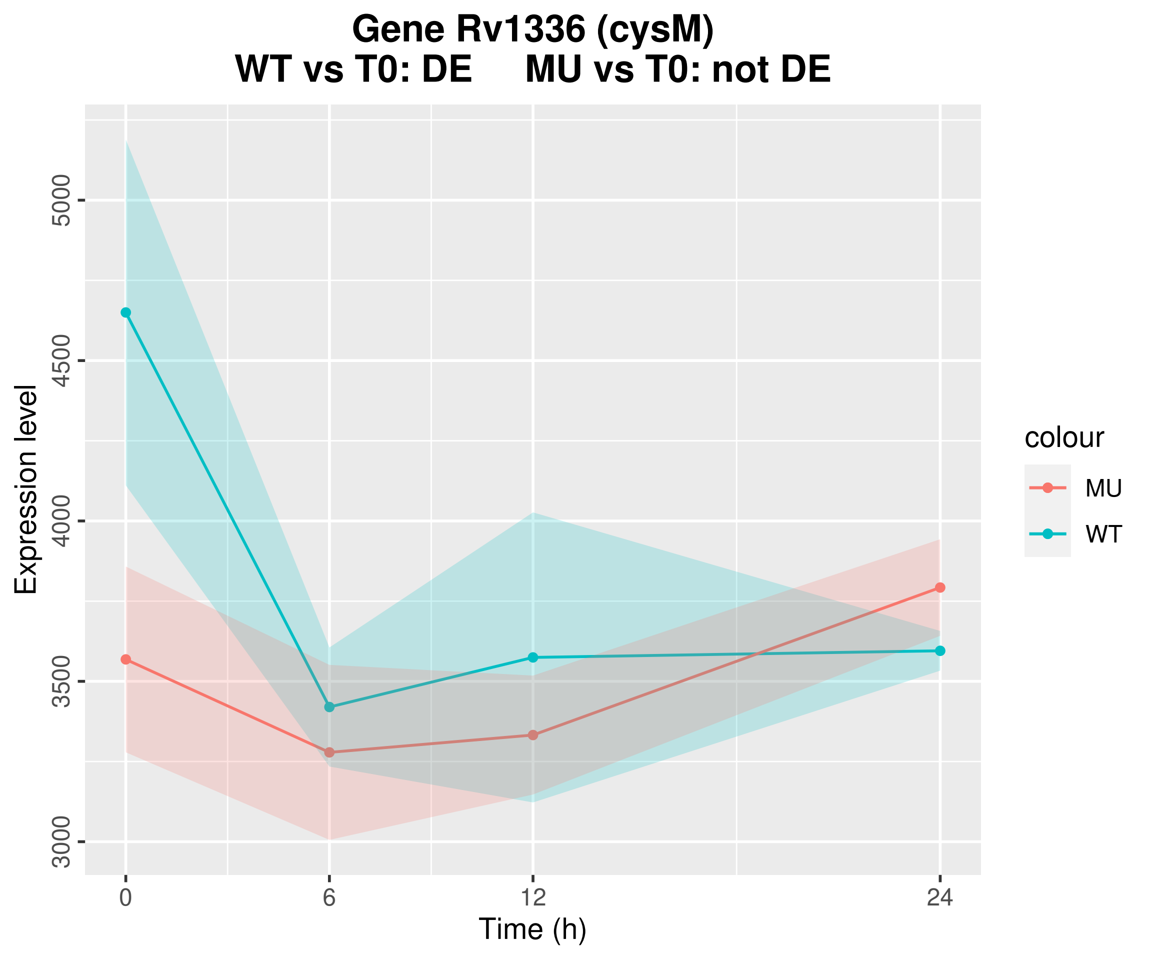
Gene Rv1336 (cysM)
WT vs T0: DE   MU vs T0: not DE
Expression level
5000
4500
4000
3500
3000
0
6
12
24
Time (h)
colour
MU
WT

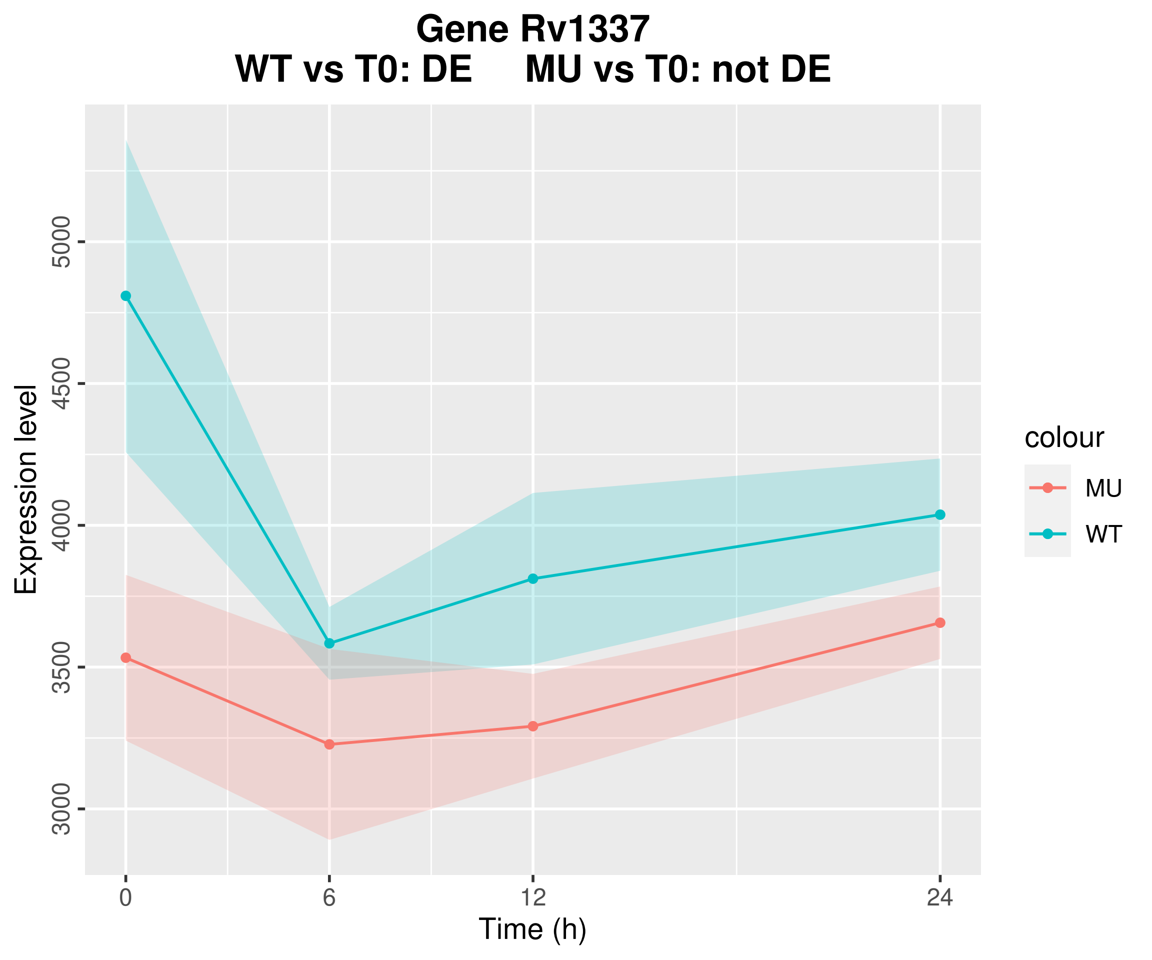
Gene Rv1337
WT vs T0: DE   MU vs T0: not DE
Expression level
5000
4500
4000
3500
3000
0
6
12
24
Time (h)
colour
MU
WT

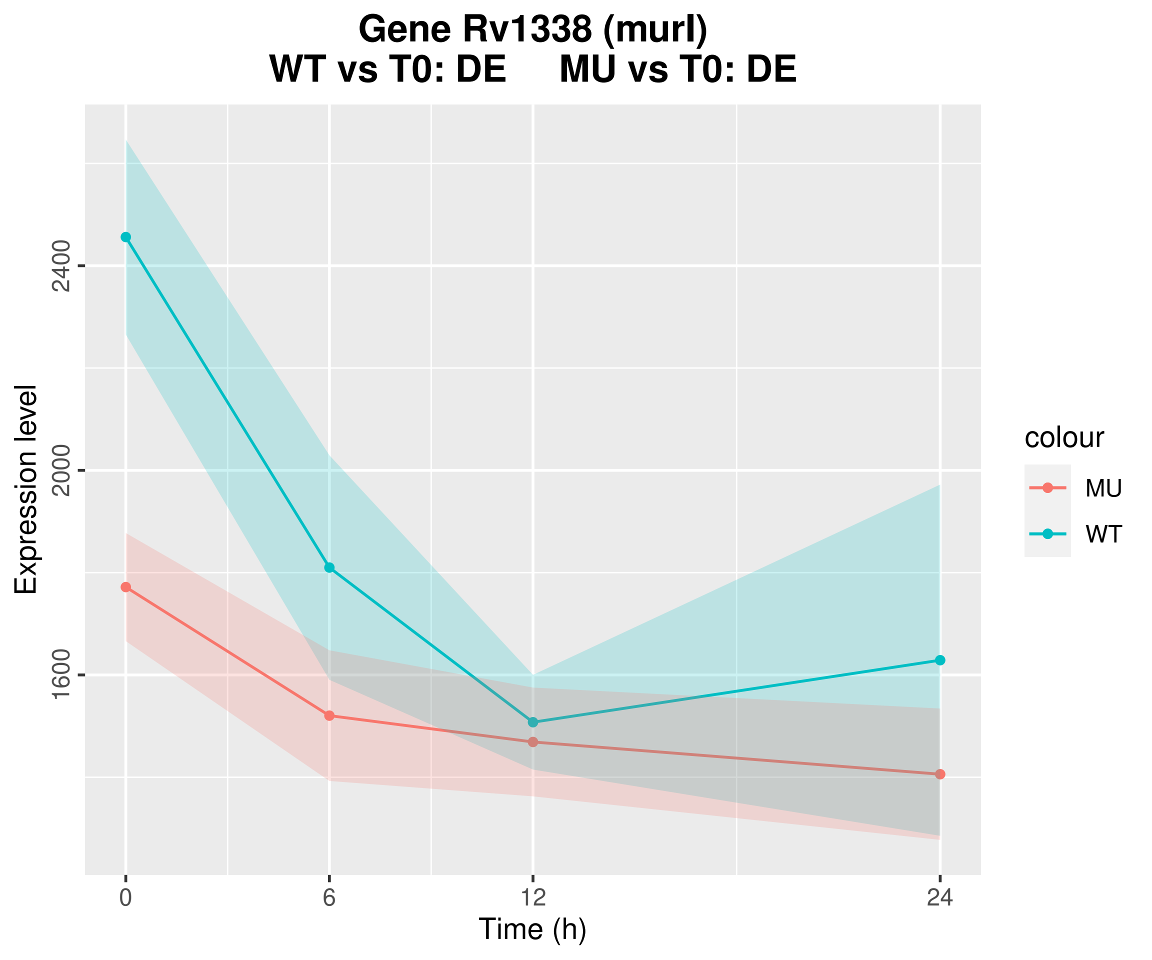
Gene Rv1338 (murI)
WT vs T0: DE MU vs T0: DE
Expression level
2400
2000
1600
0
6
12
24
Time (h)
colour
MU
WT

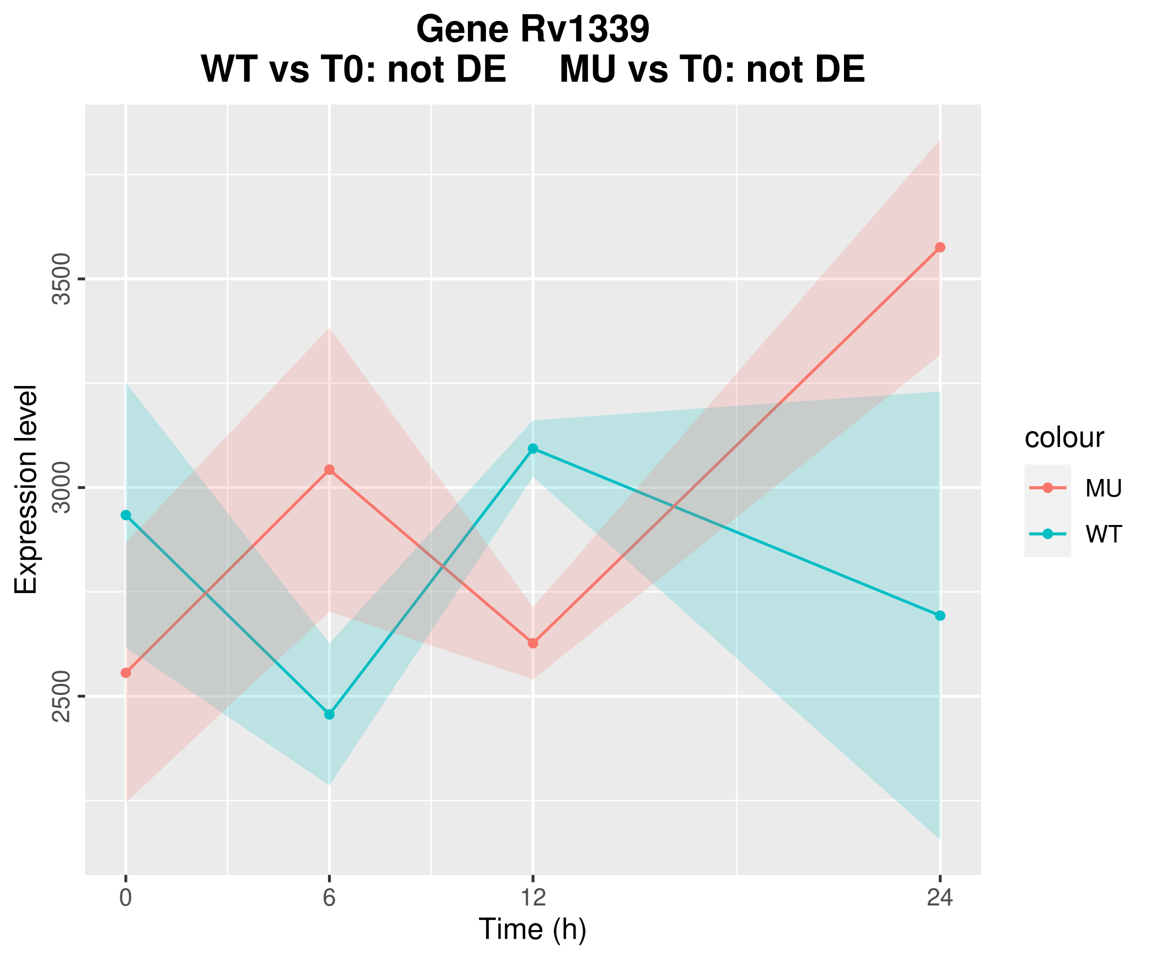
Gene Rv1339
WT vs T0: not DE MU vs T0: not DE
Expression level
3500
3000
2500
0
6
12
24
Time (h)
colour
MU
WT

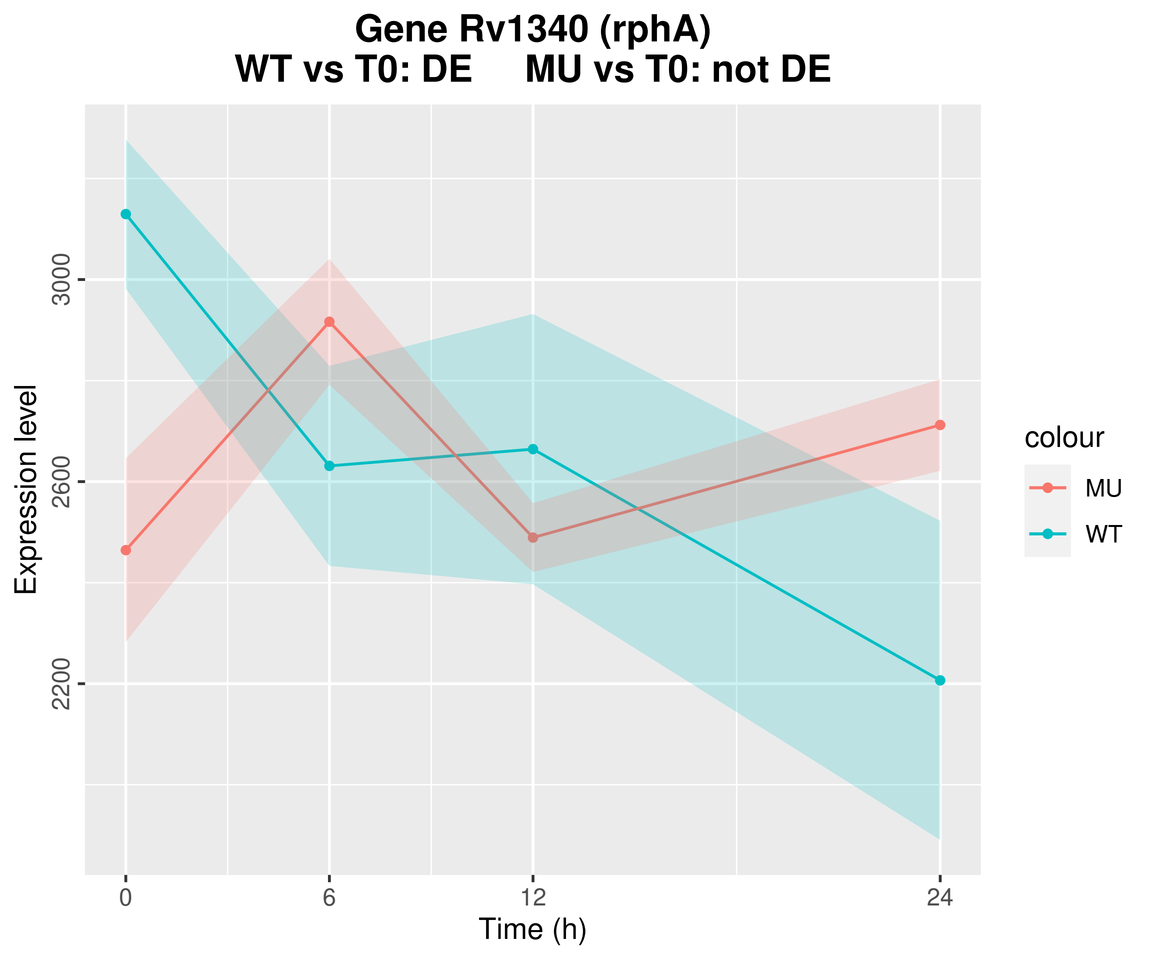
Gene Rv1340 (rphA)
WT vs T0: DE   MU vs T0: not DE
Expression level
3000
2600
2200
0
6
12
24
Time (h)
colour
MU
WT

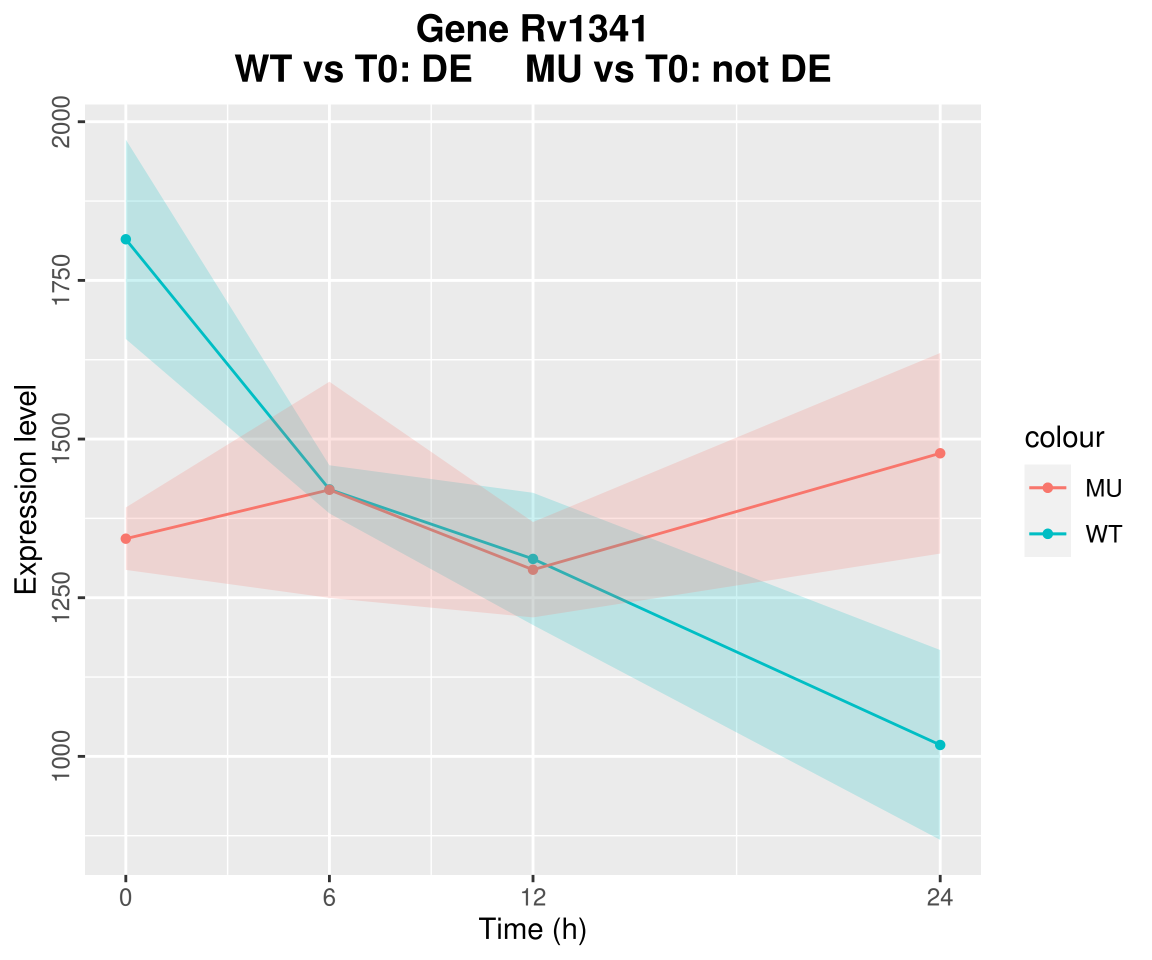
Gene Rv1341
WT vs T0: DE MU vs T0: not DE
Expression level
2000
1750
1500
1250
1000
0
6
12
24
Time (h)
colour
MU
WT

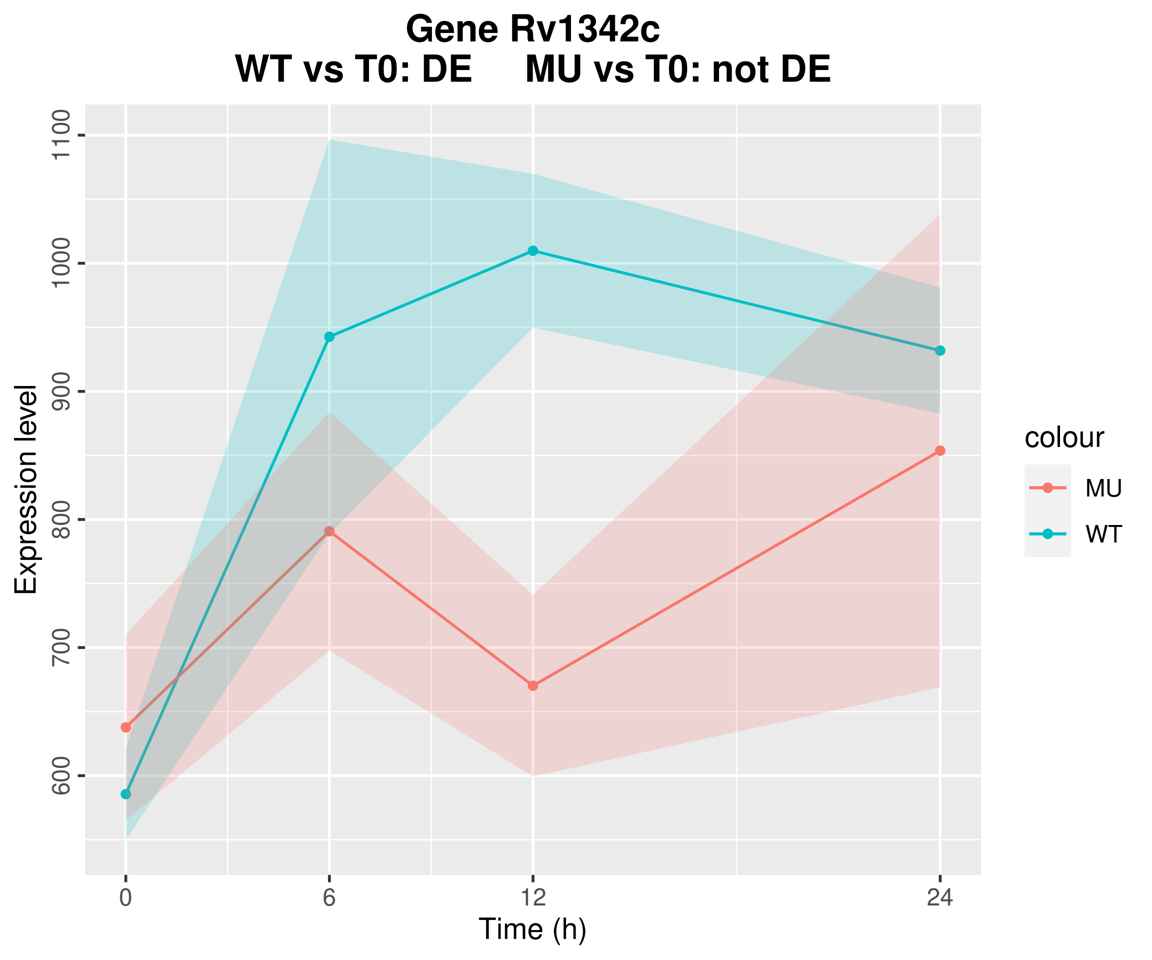
Gene Rv1342c
WT vs T0: DE   MU vs T0: not DE
Expression level
1100
1000
900
800
700
600
0
6
12
24
Time (h)
colour
MU
WT

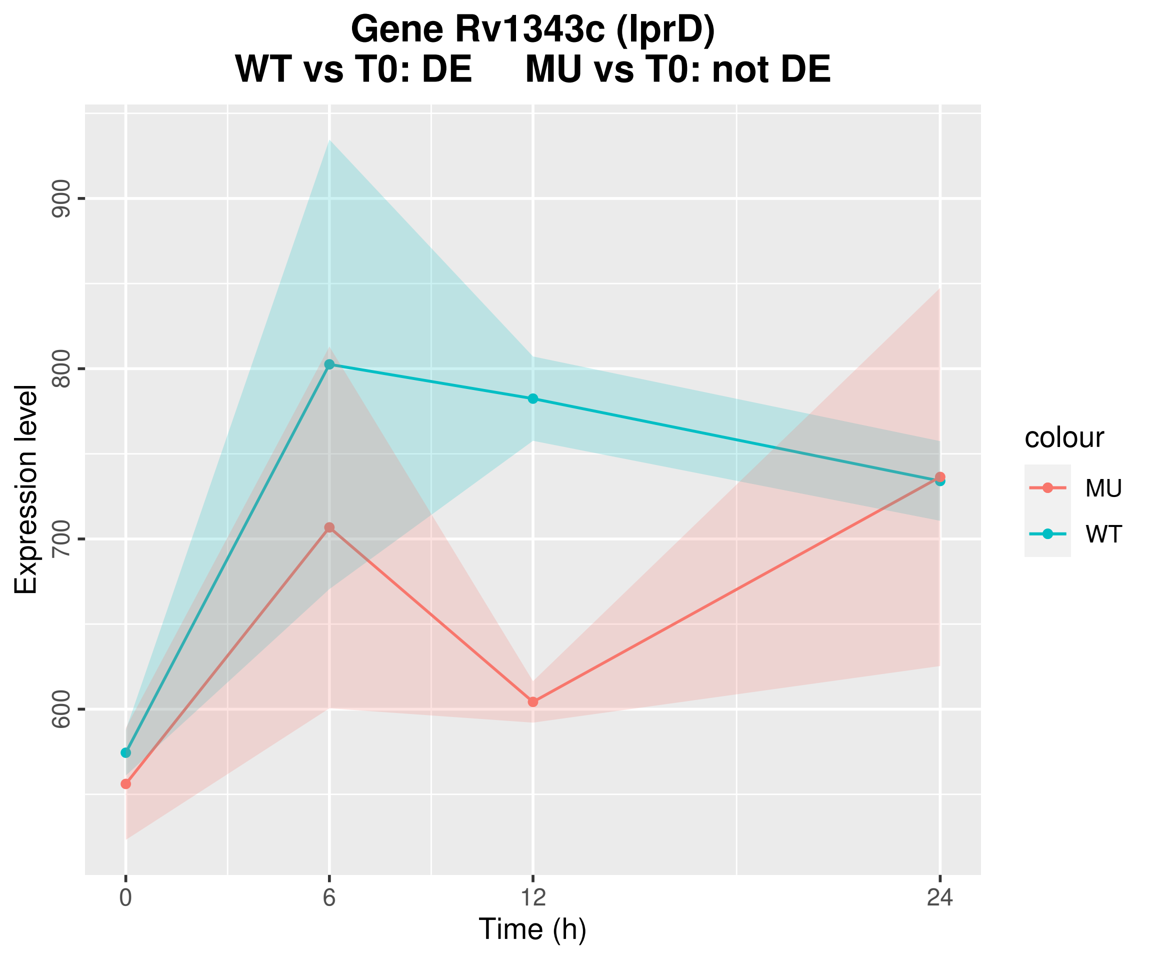
Gene Rv1343c (lprD)
WT vs T0: DE   MU vs T0: not DE
Expression level
900
800
700
600
0
6
12
24
Time (h)
colour
MU
WT

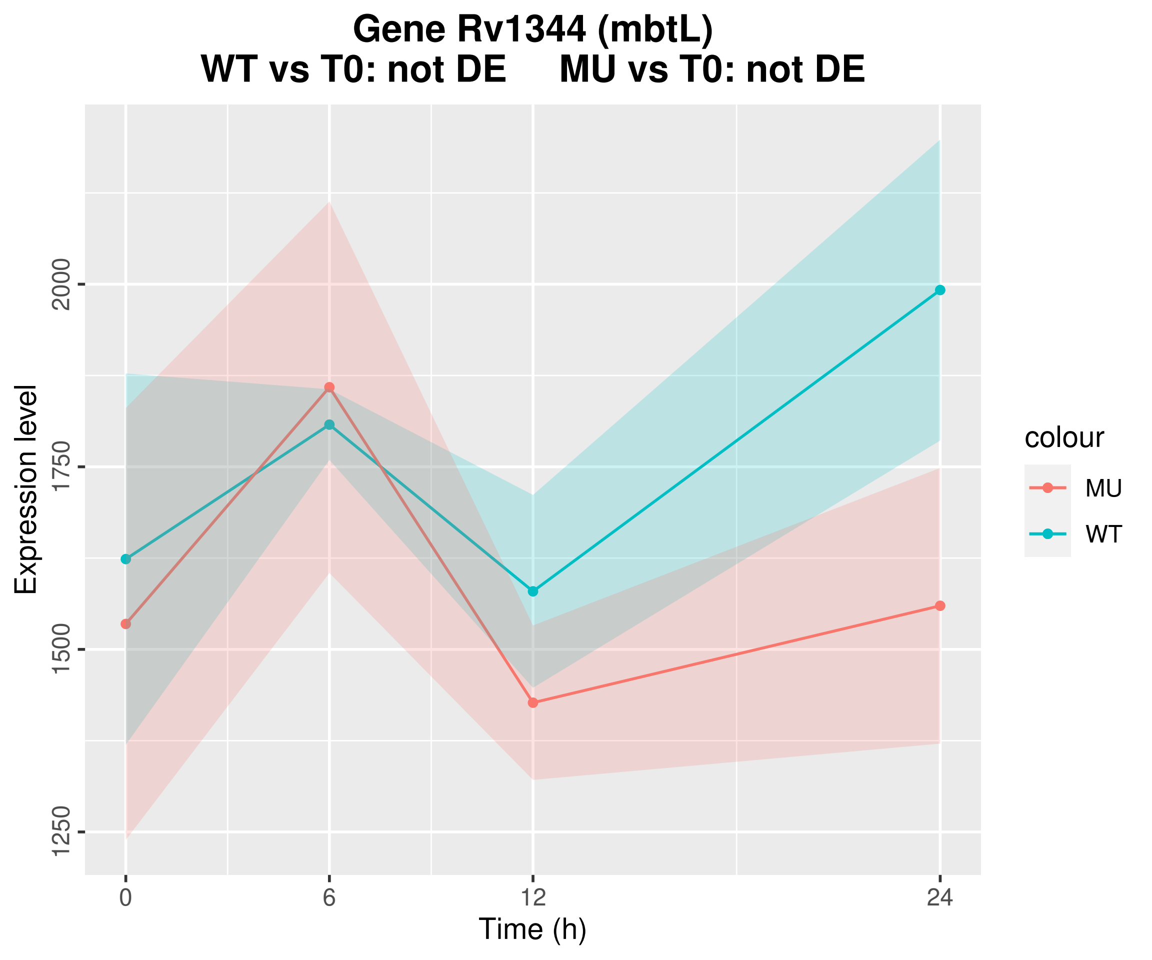

Gene Rv1344 (mbtL)
WT vs T0: not DE   MU vs T0: not DE
Expression level
1250
1500
1750
2000
Time (h)
0
6
12
24
colour
MU
WT

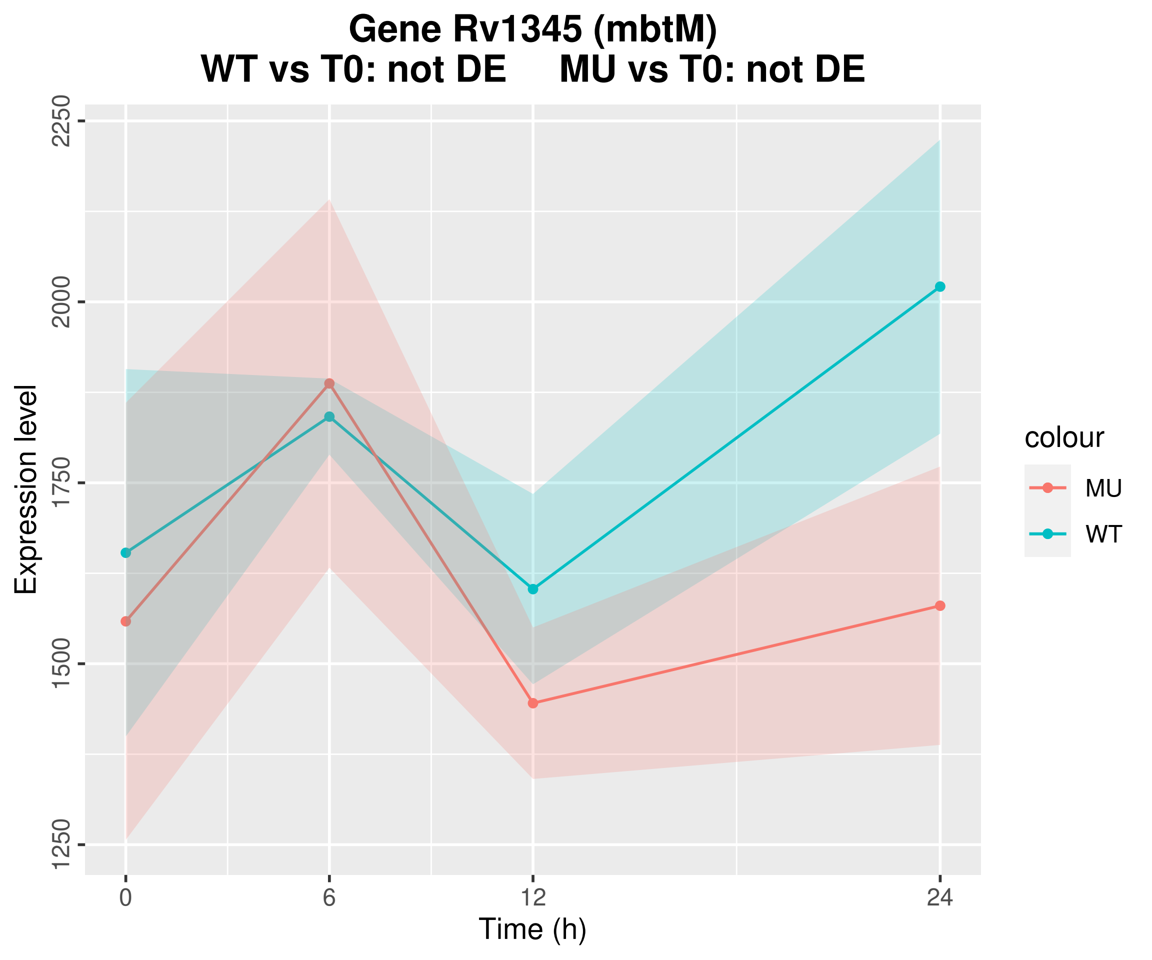
Gene Rv1345 (mbtM)
WT vs T0: not DE   MU vs T0: not DE
Expression level
2250
2000
1750
1500
1250
Time (h)
0
6
12
24
colour
MU
WT

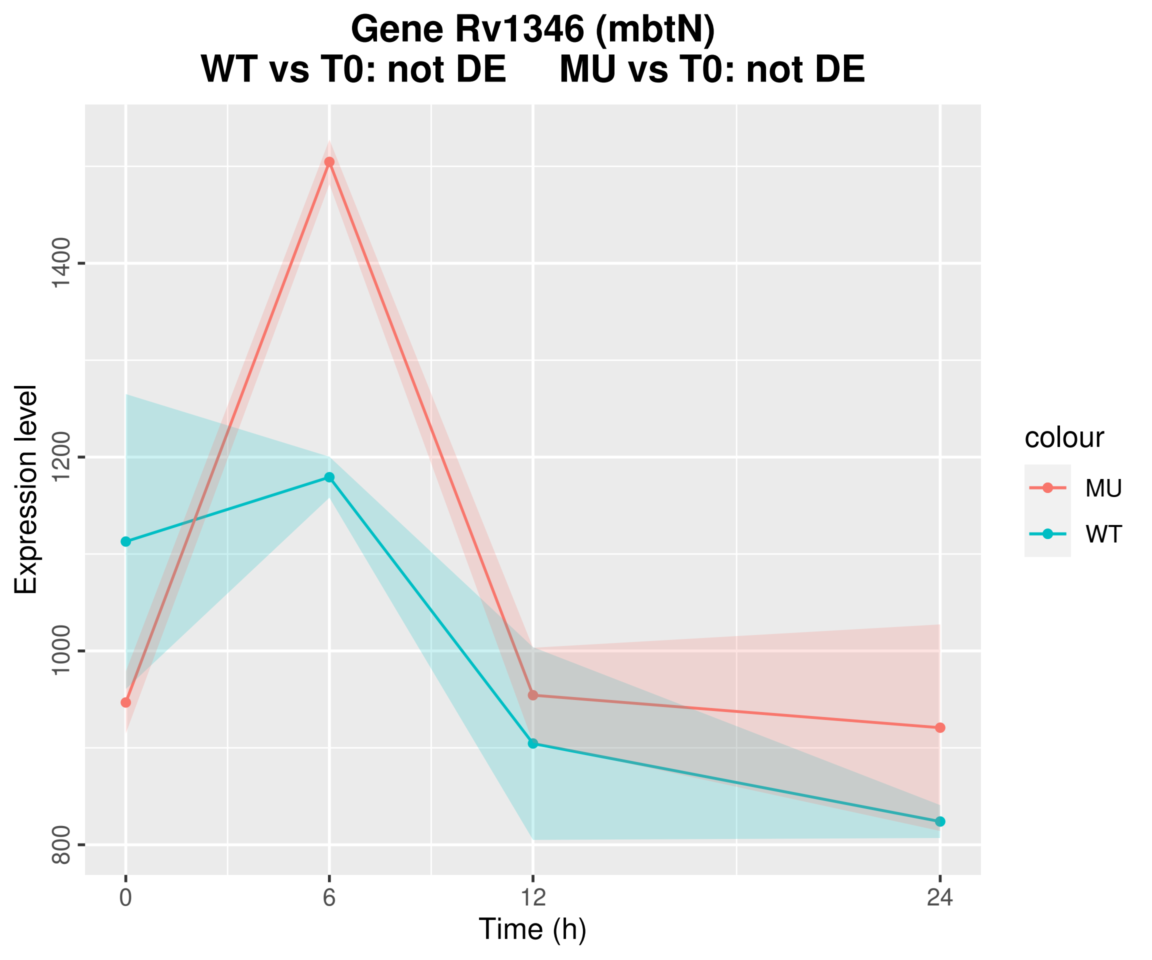
Gene Rv1346 (mbtN)
WT vs T0: not DE   MU vs T0: not DE
Expression level
1400
1200
1000
800
0
6
12
24
Time (h)
colour
MU
WT

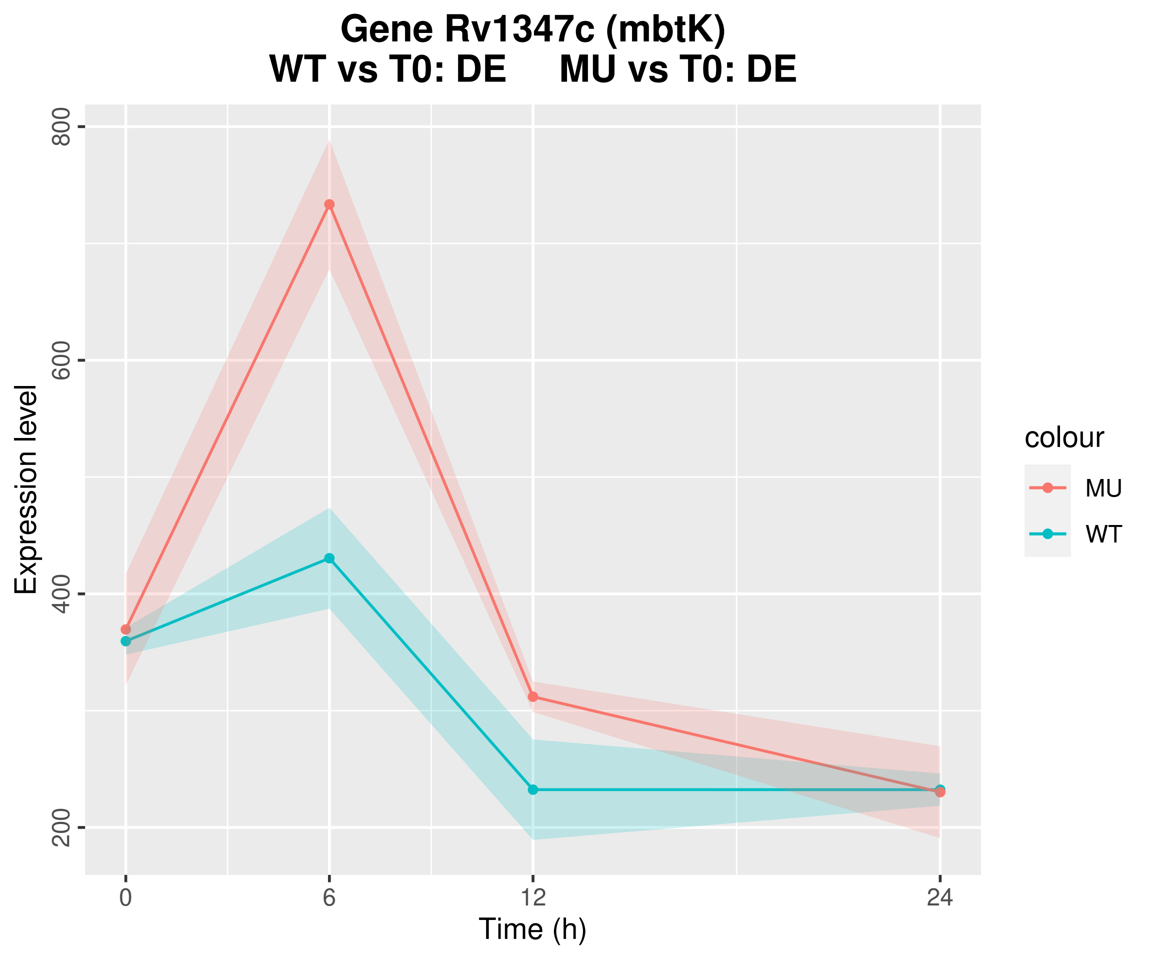
Gene Rv1347c (mbtK)
WT vs T0: DE   MU vs T0: DE
800
600
400
200
Expression level
0
6
12
24
Time (h)
colour
MU
WT

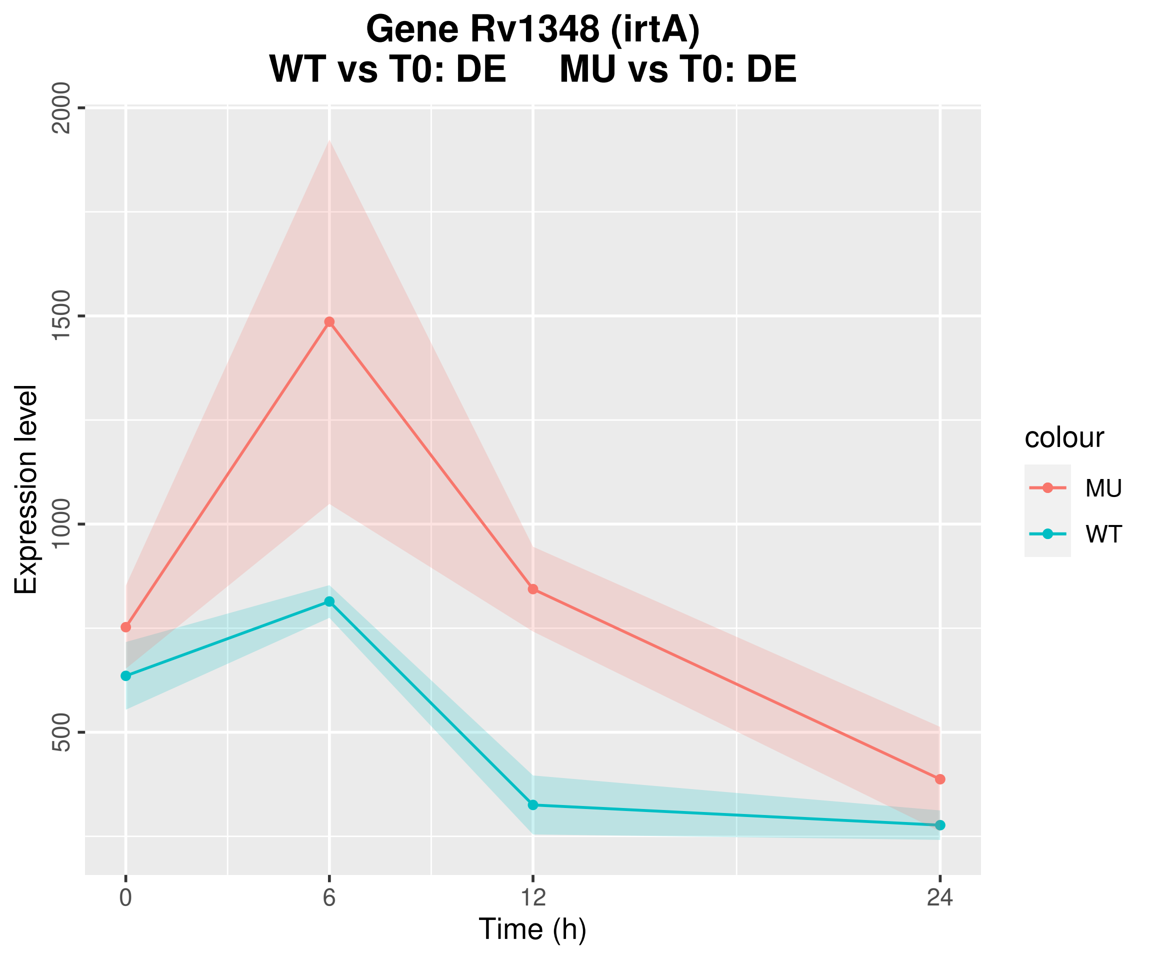

Gene Rv1348 (irtA)
WT vs T0: DE   MU vs T0: DE
Expression level
2000
1500
1000
500
0
6
12
24
Time (h)
colour
MU
WT

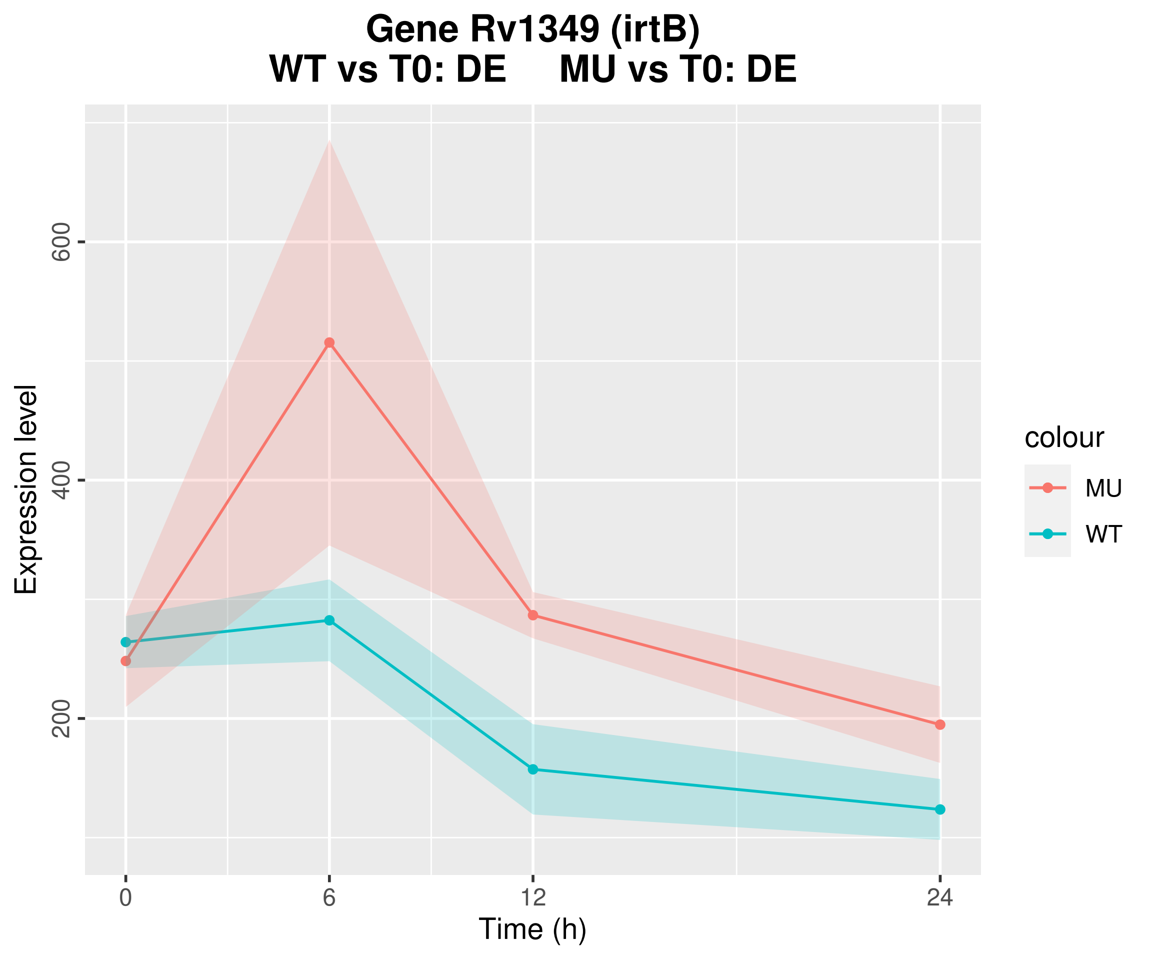
Gene Rv1349 (irtB)
WT vs T0: DE MU vs T0: DE
Expression level
600
400
200
0
6
12
24
Time (h)
colour
MU
WT

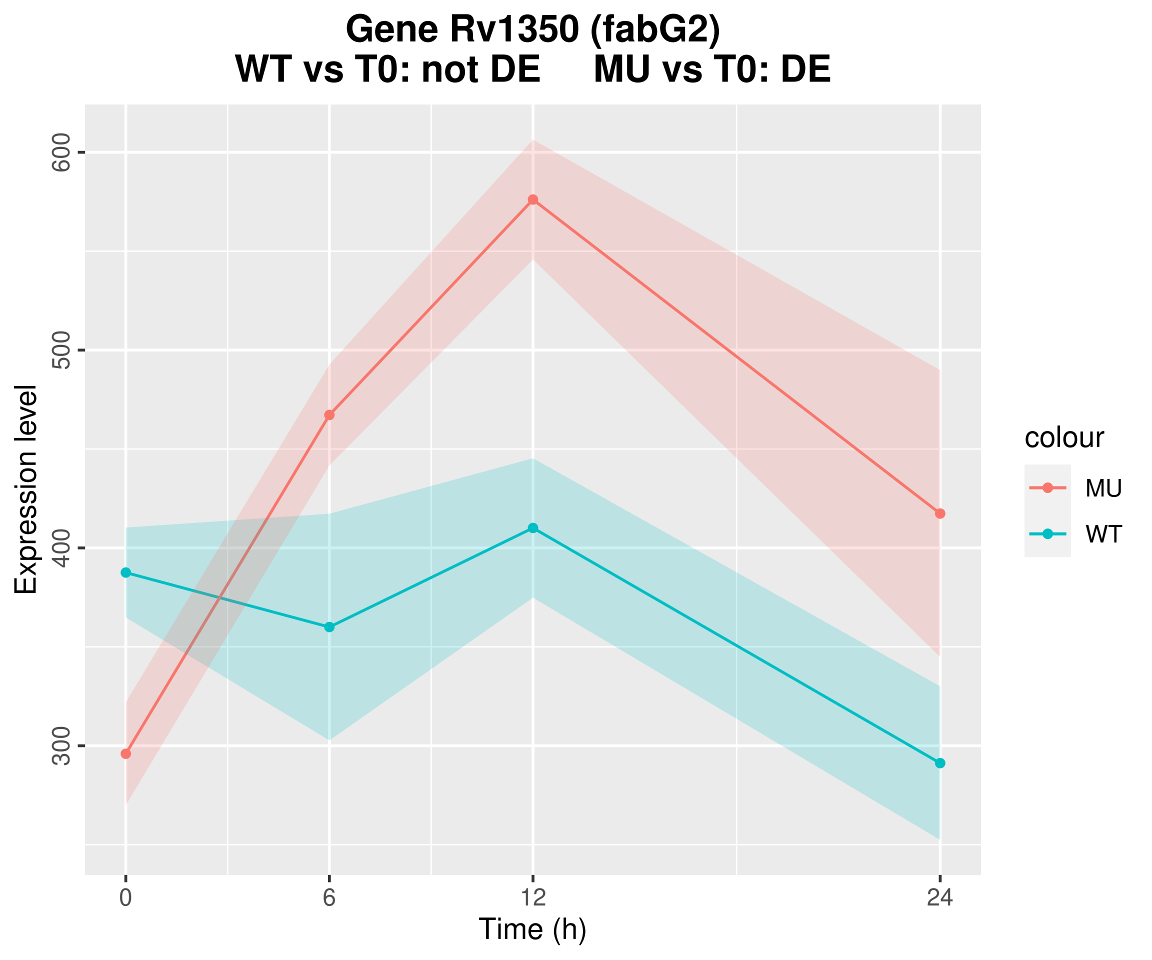

Gene Rv1350 (fabG2)
WT vs T0: not DE   MU vs T0: DE
600
500
400
300
Expression level
0
6
12
24
Time (h)
colour
MU
WT

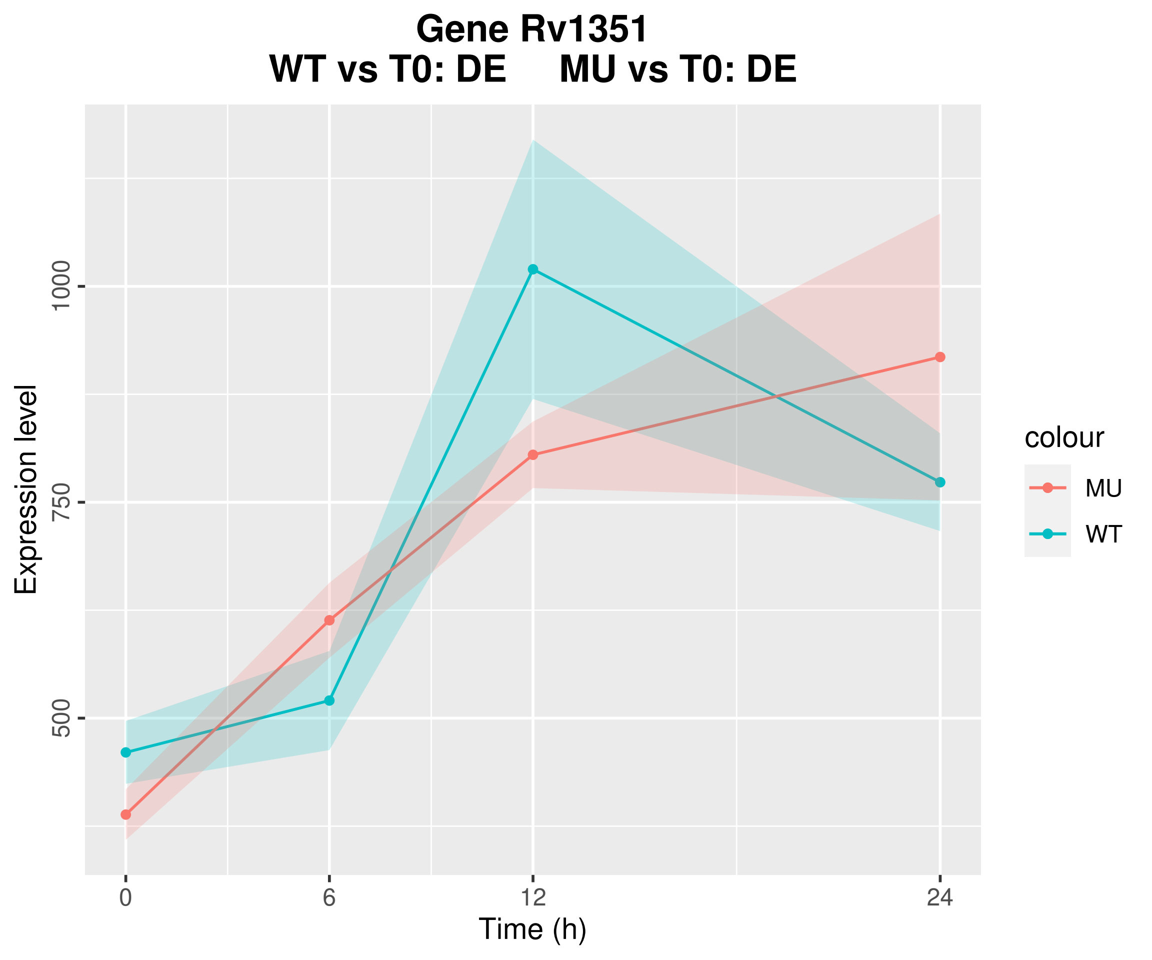

Gene Rv1351
WT vs T0: DE MU vs T0: DE
Expression level
1000
750
500
0
6
12
24
Time (h)
colour
MU
WT

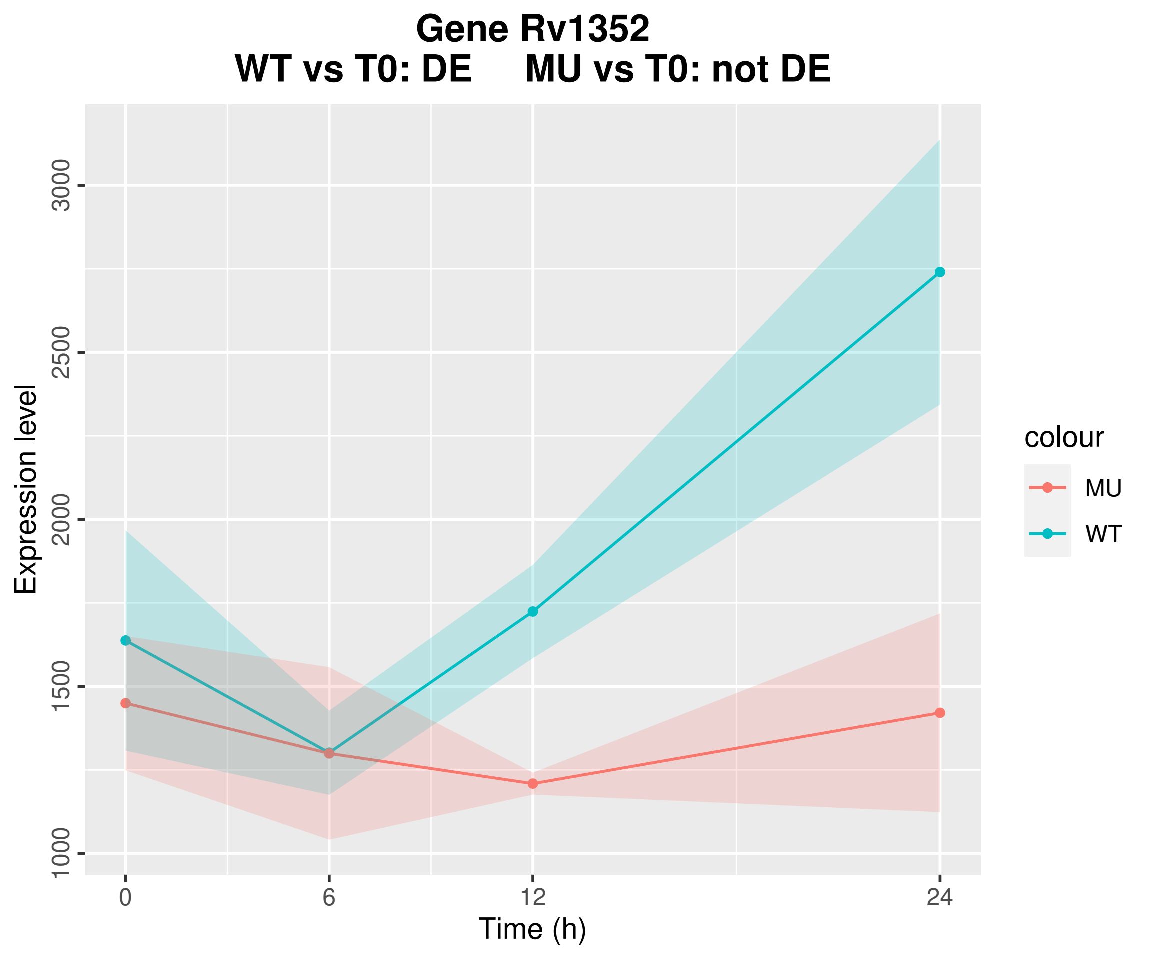
Gene Rv1352
WT vs T0: DE   MU vs T0: not DE
Expression level
3000
2500
2000
1500
1000
0
6
12
24
Time (h)
colour
MU
WT

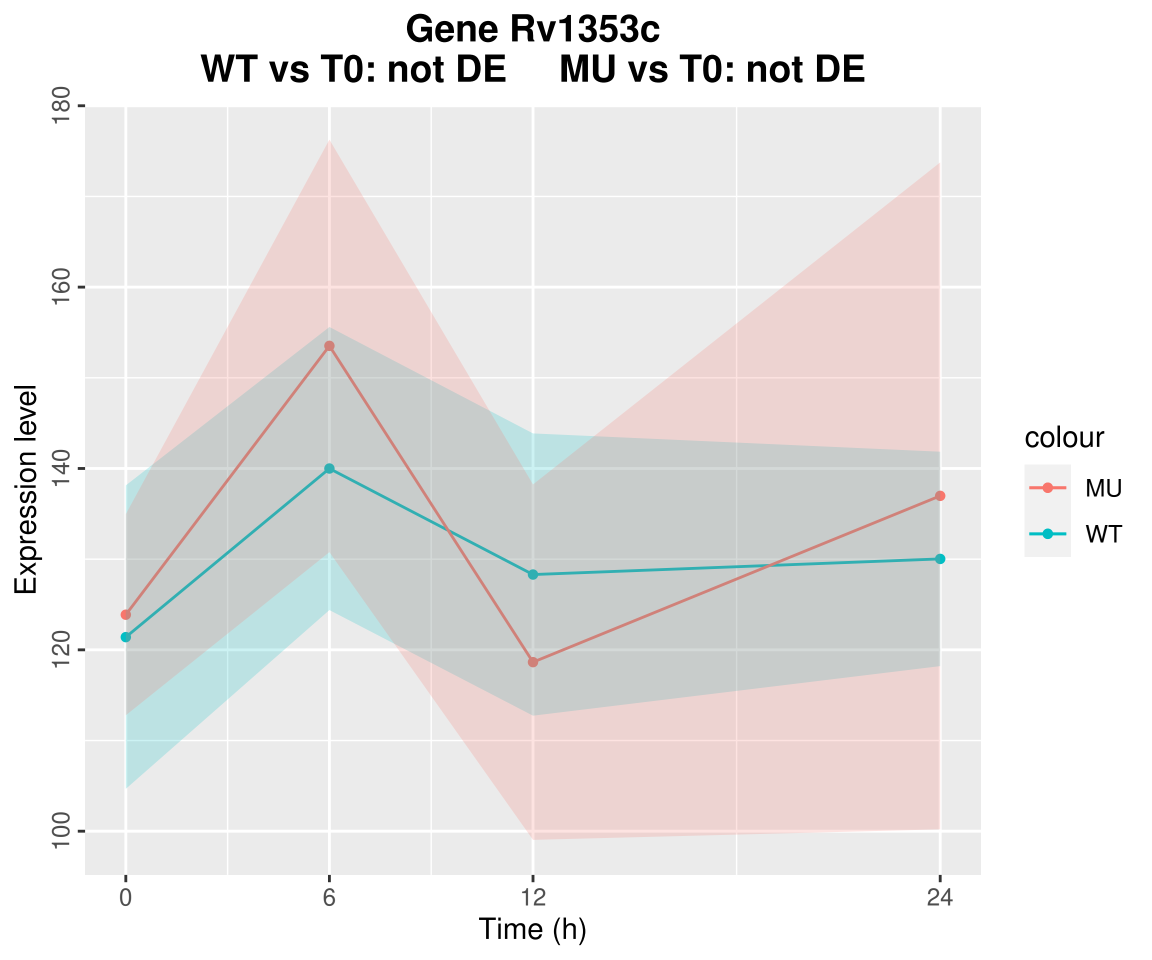

Gene Rv1353c
WT vs T0: not DE   MU vs T0: not DE
180
160
140
120
100
Expression level
0
6
12
24
Time (h)
colour
MU
WT

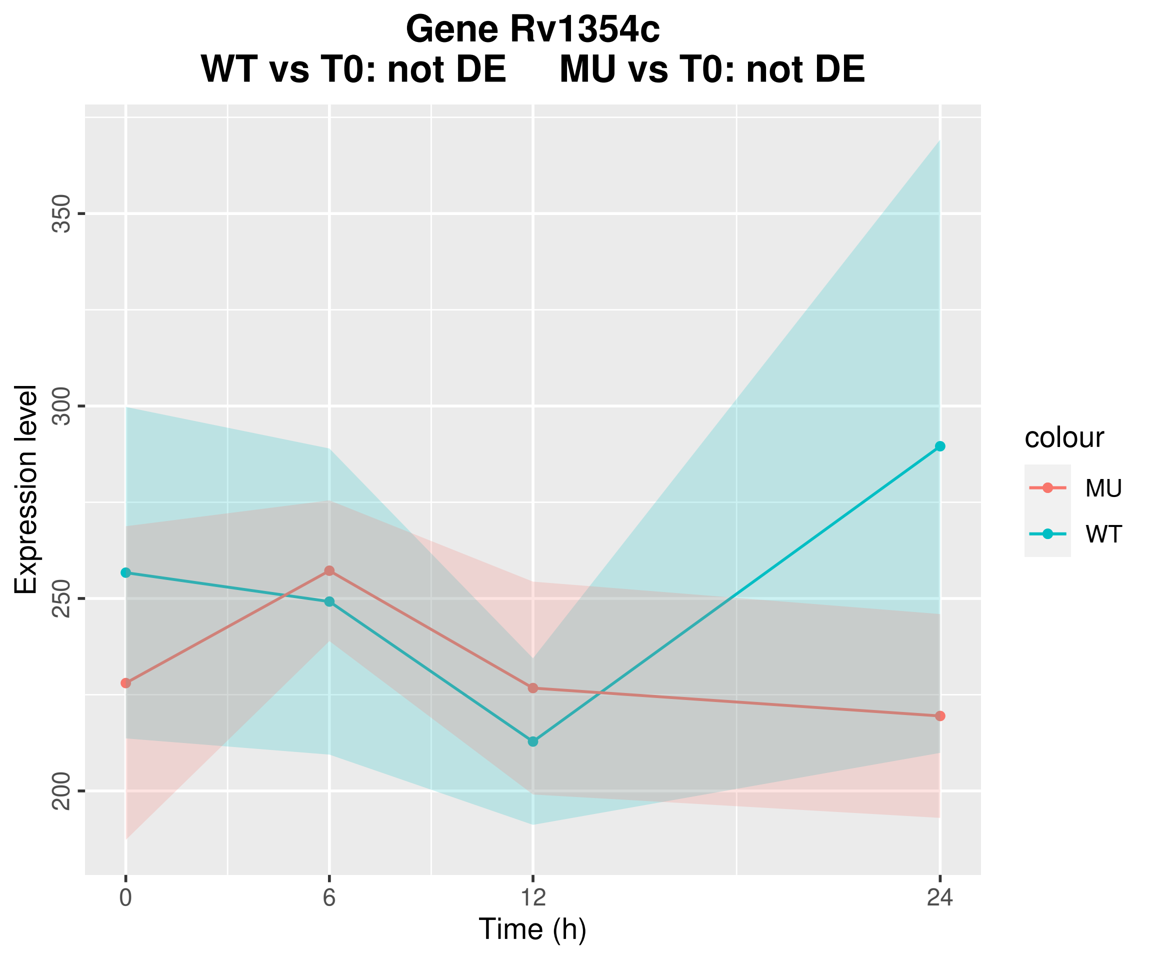

Gene Rv1354c
WT vs T0: not DE   MU vs T0: not DE
Expression level
350
300
250
200
0
6
12
24
Time (h)
colour
MU
WT

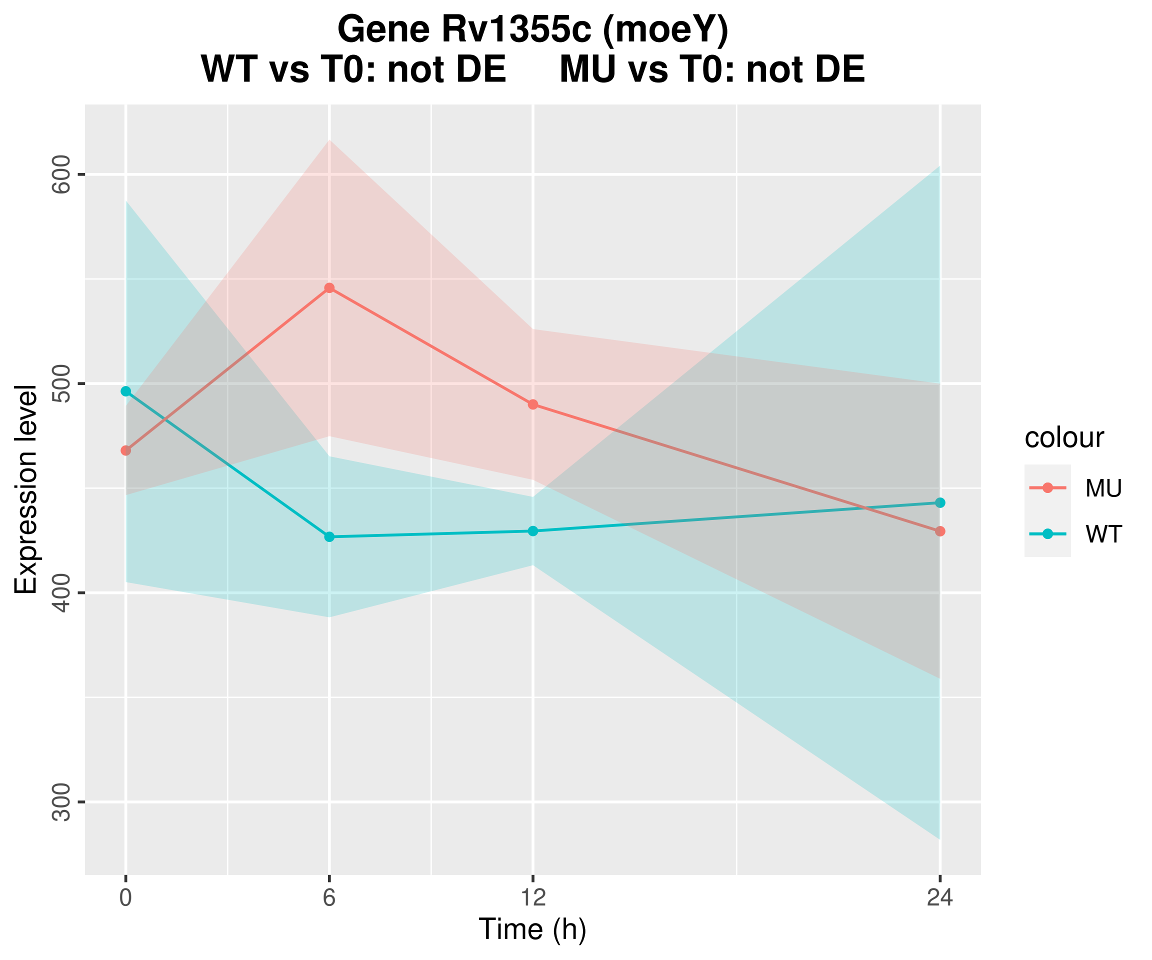

Gene Rv1355c (moeY)
WT vs T0: not DE MU vs T0: not DE
600
500
400
300
Expression level
0
6
12
24
Time (h)
colour
MU
WT

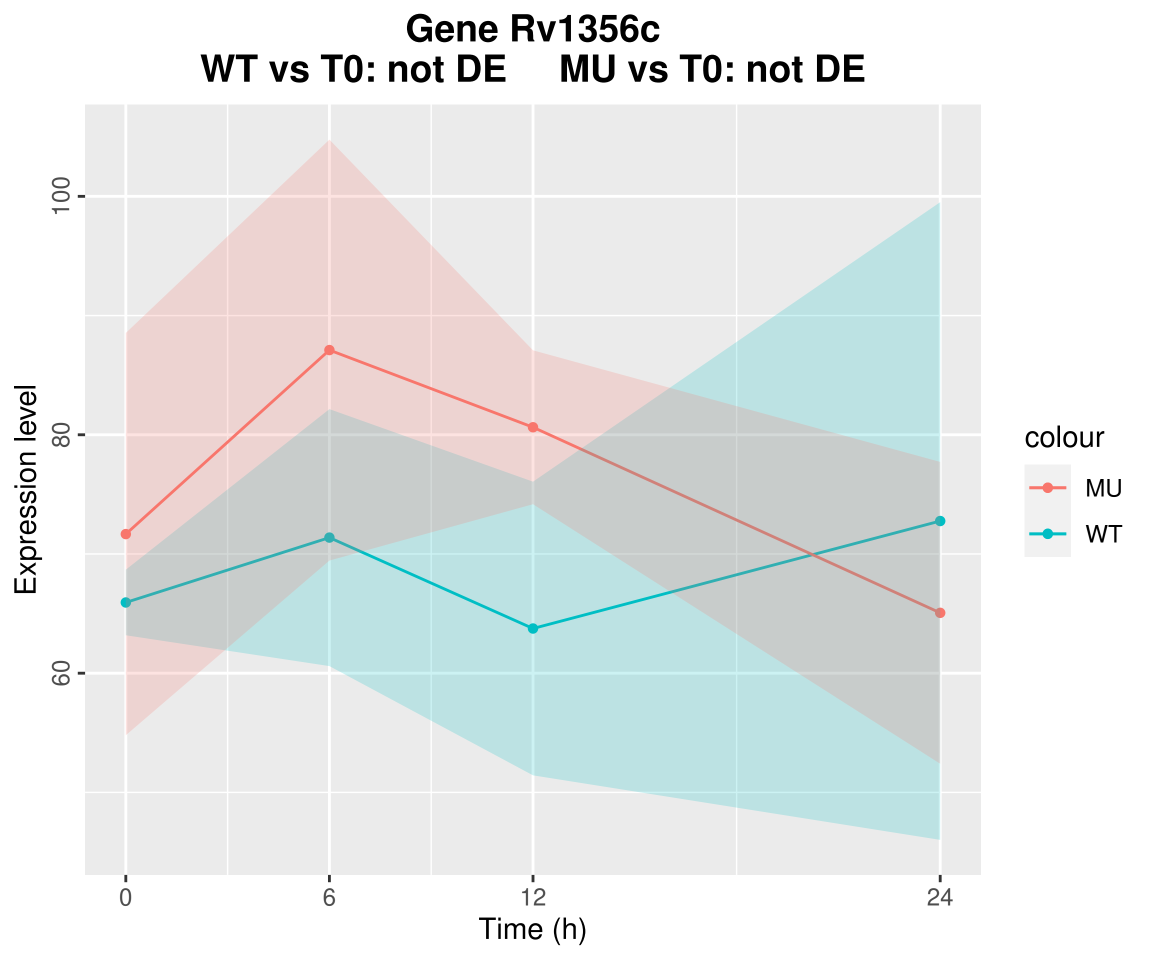

Gene Rv1356c
WT vs T0: not DE   MU vs T0: not DE
Expression level
100
80
60
0
6
12
24
Time (h)
colour
MU
WT

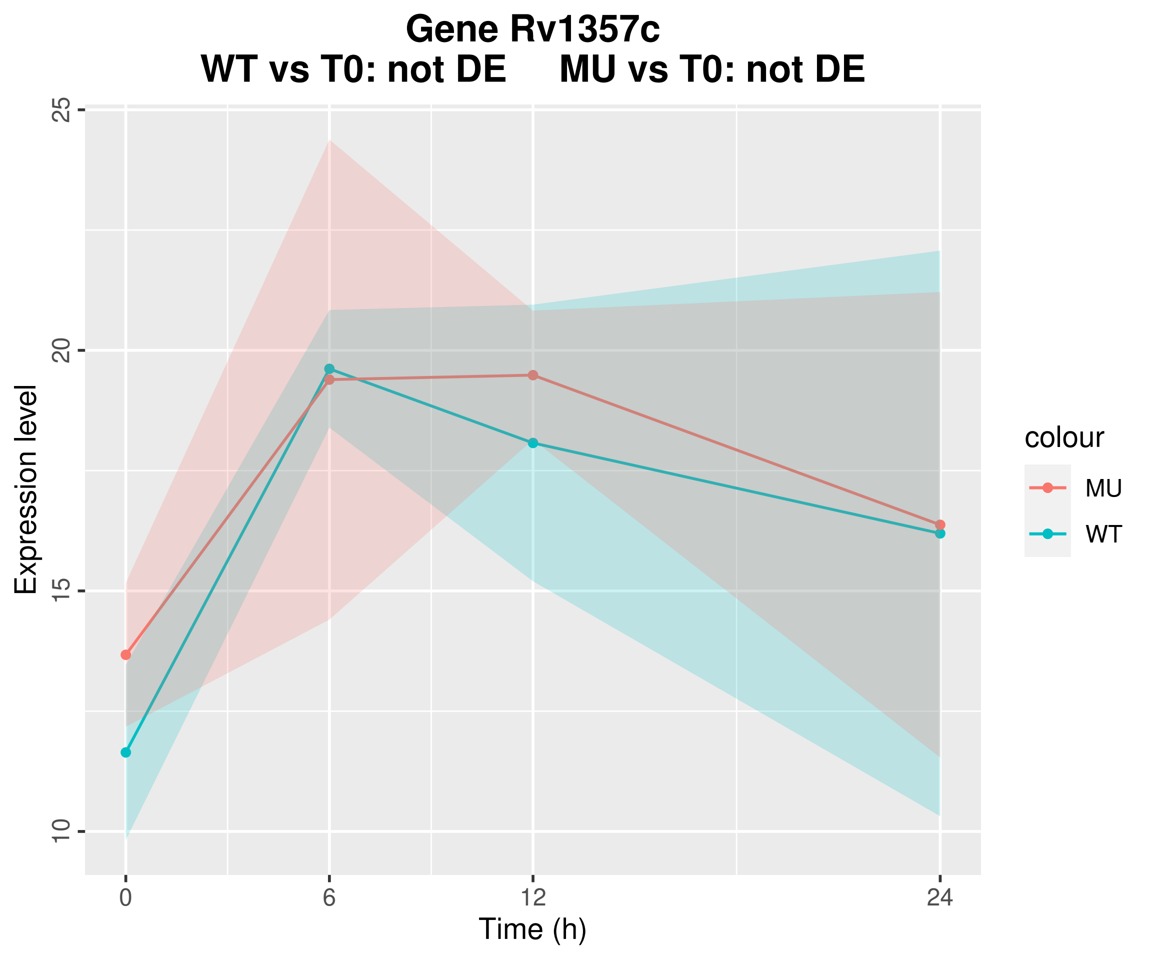

Gene Rv1357c
WT vs T0: not DE MU vs T0: not DE
25
20
15
10
Expression level
0
6
12
24
Time (h)
colour
MU
WT

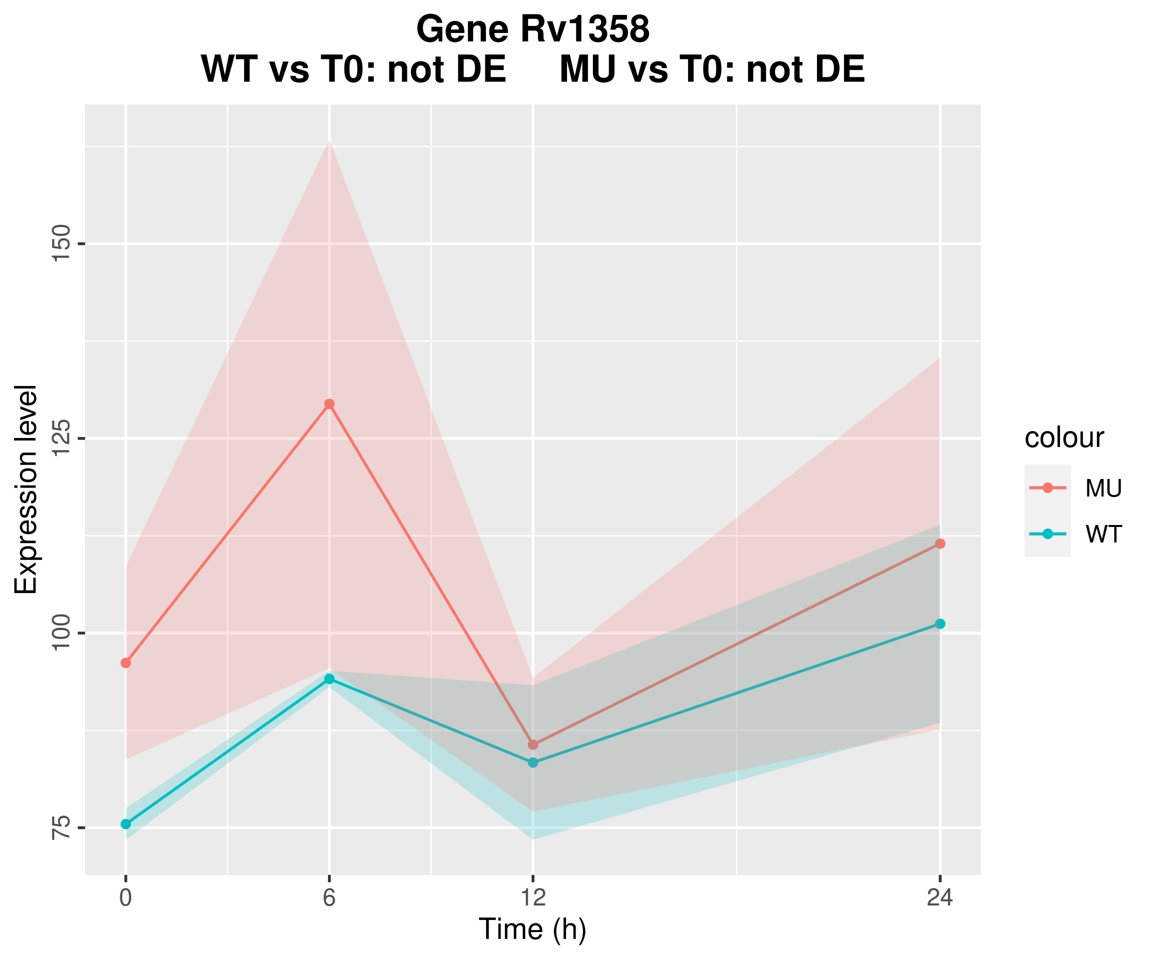
Gene Rv1358
WT vs T0: not DE   MU vs T0: not DE
Expression level
150
125
100
75
0
6
12
24
Time (h)
colour
MU
WT

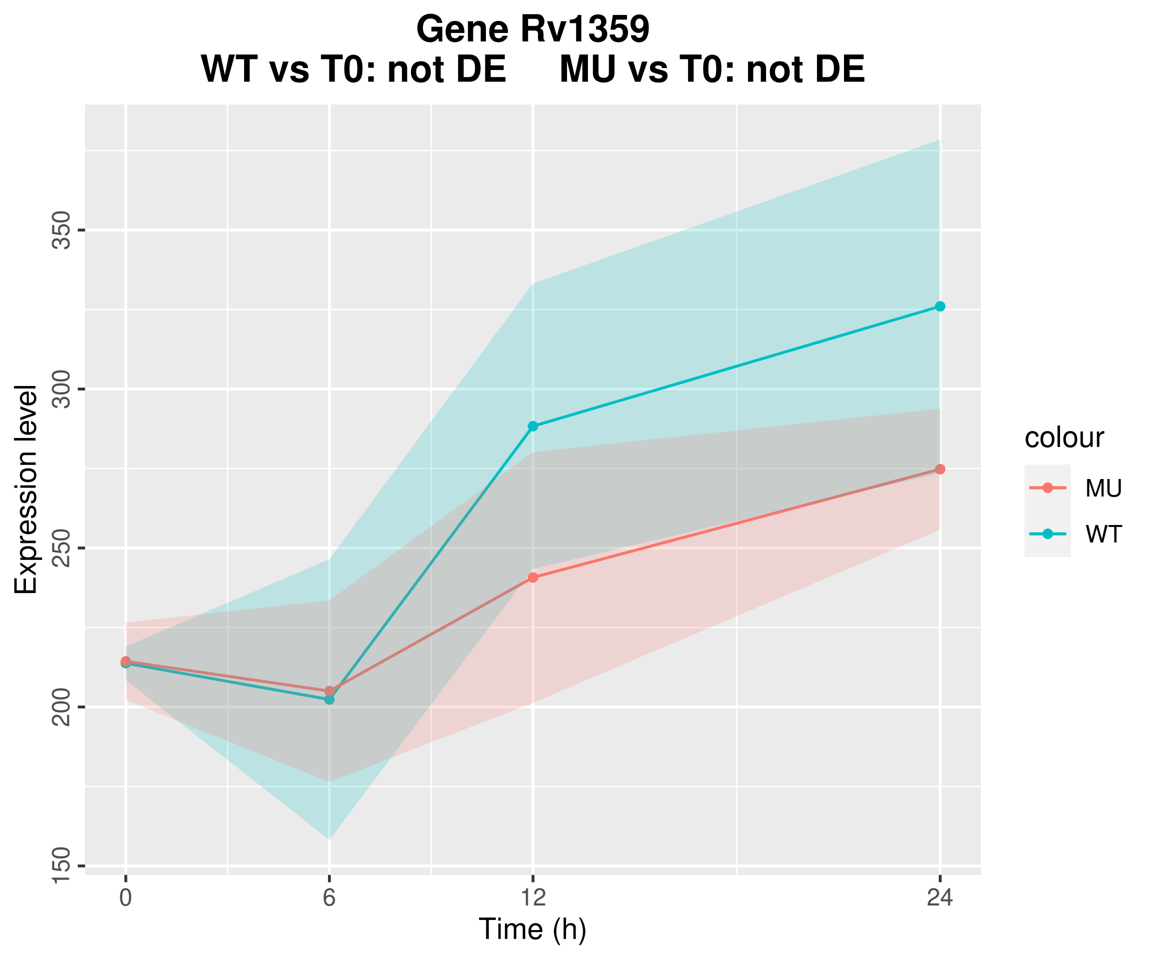
Gene Rv1359
WT vs T0: not DE MU vs T0: not DE
Expression level
150
200
250
300
350
0
6
12
24
Time (h)
colour
MU
WT

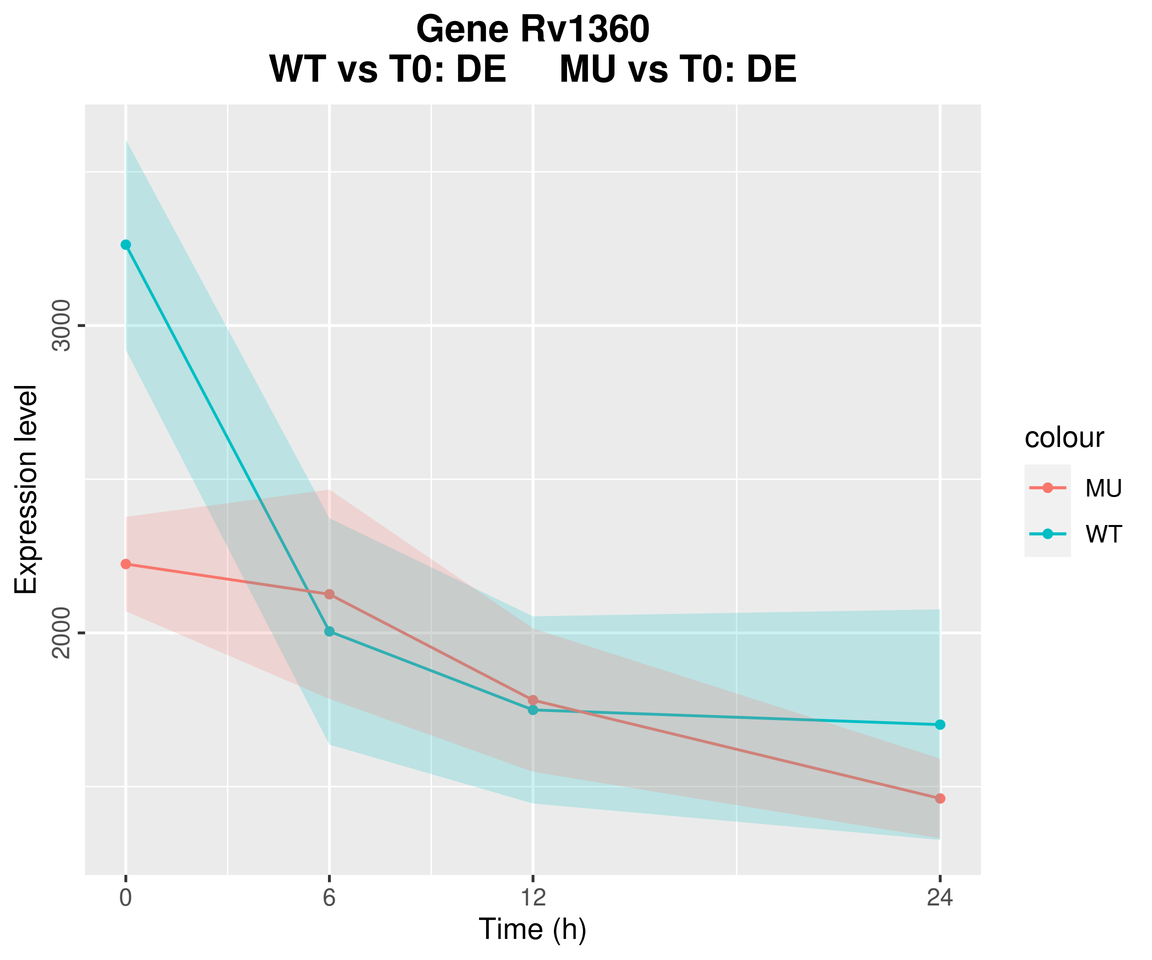

Gene Rv1360
WT vs T0: DE   MU vs T0: DE
Expression level
3000
2000
0
6
12
24
Time (h)
colour
MU
WT

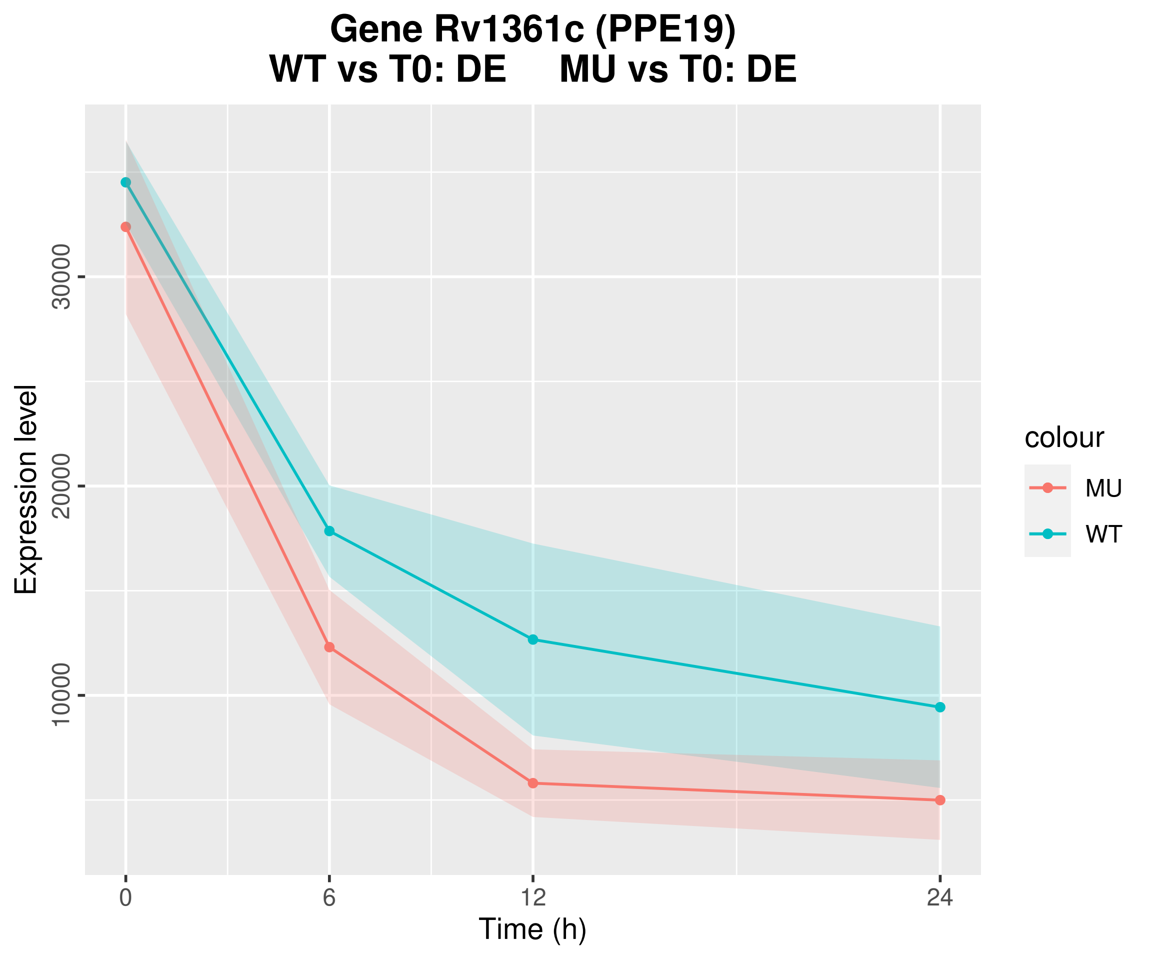

Gene Rv1361c (PPE19)
WT vs T0: DE MU vs T0: DE
Expression level
30000
20000
10000
0
6
12
24
Time (h)
colour
MU
WT

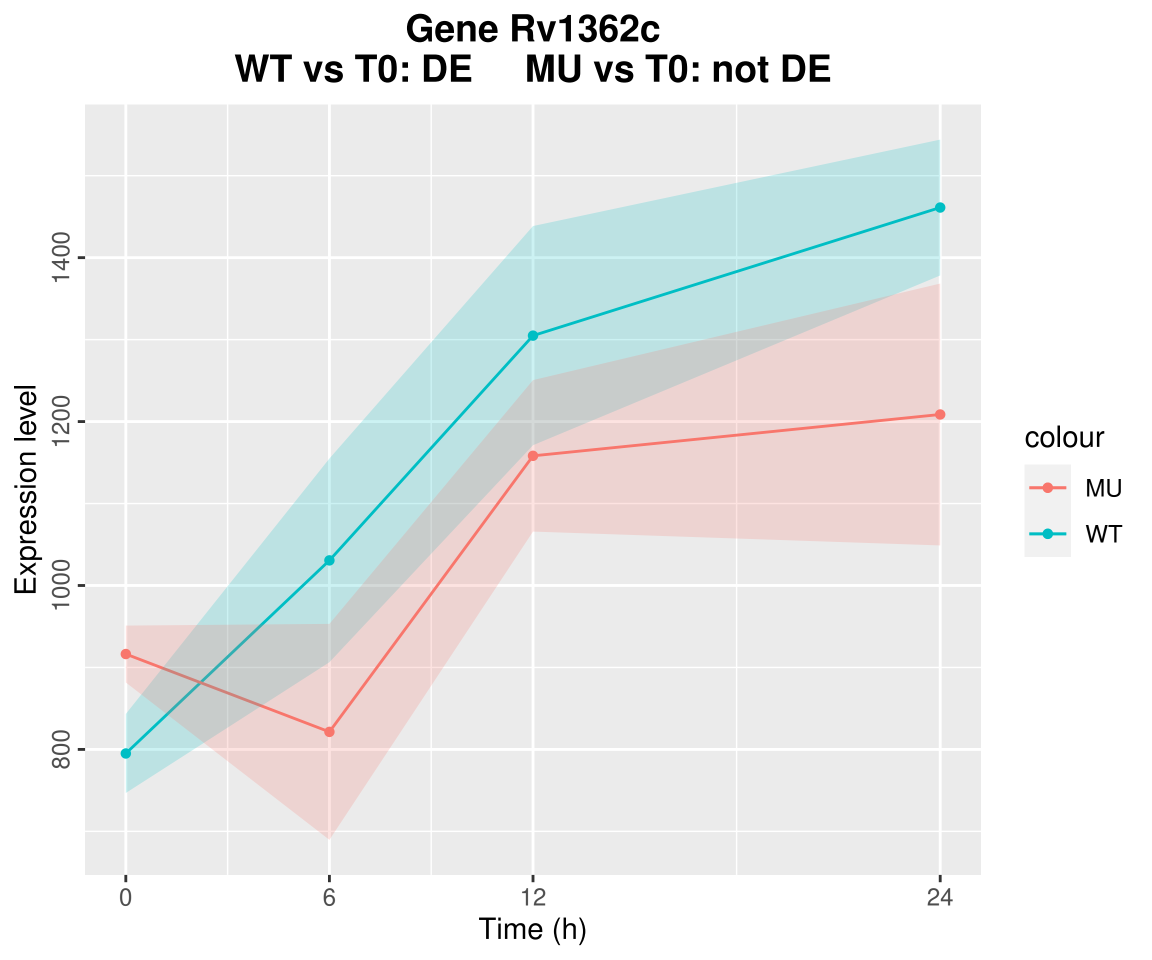
Gene Rv1362c
WT vs T0: DE   MU vs T0: not DE
Expression level
1400
1200
1000
800
0
6
12
24
Time (h)
colour
MU
WT

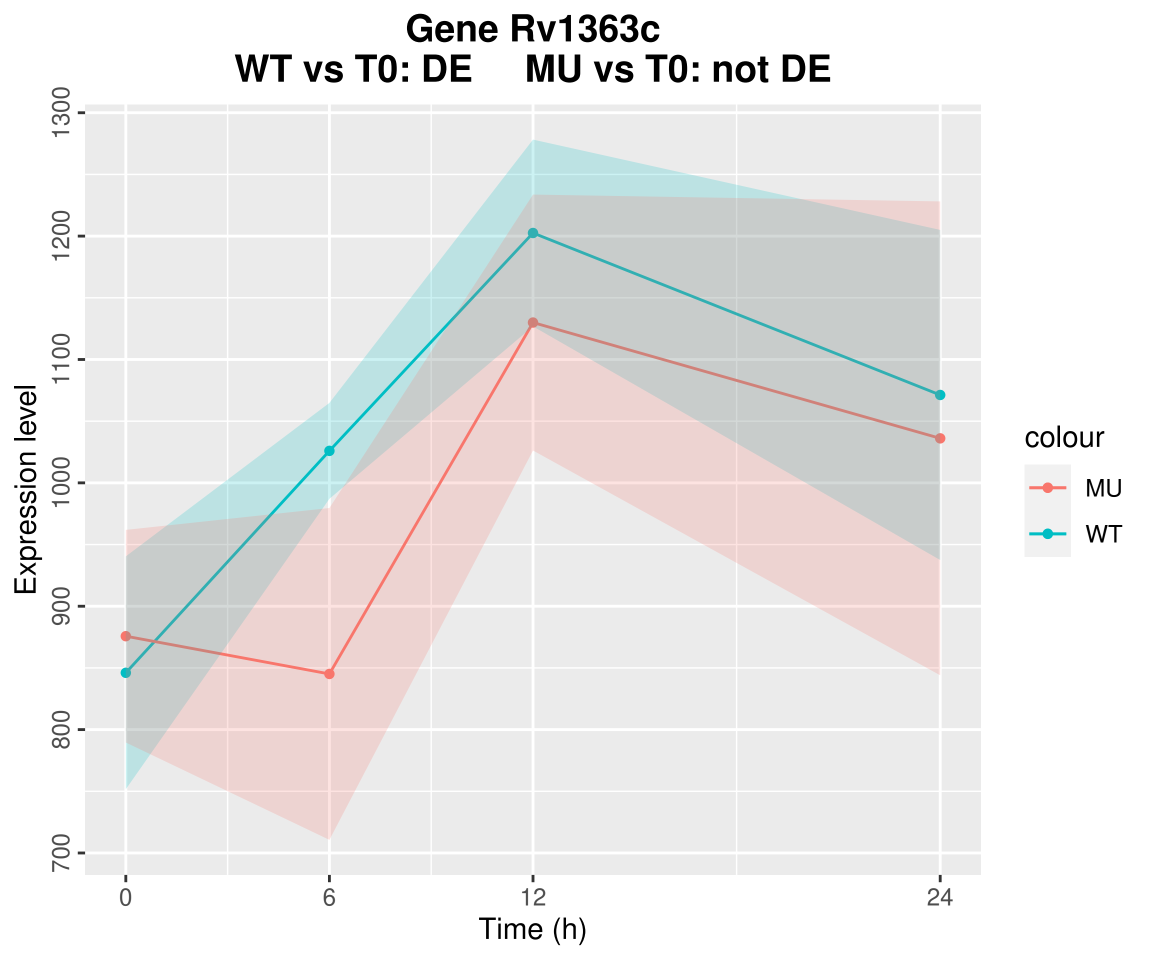

Gene Rv1363c
WT vs T0: DE   MU vs T0: not DE
Expression level
1300
1200
1100
1000
900
800
700
0
6
12
24
Time (h)
colour
MU
WT

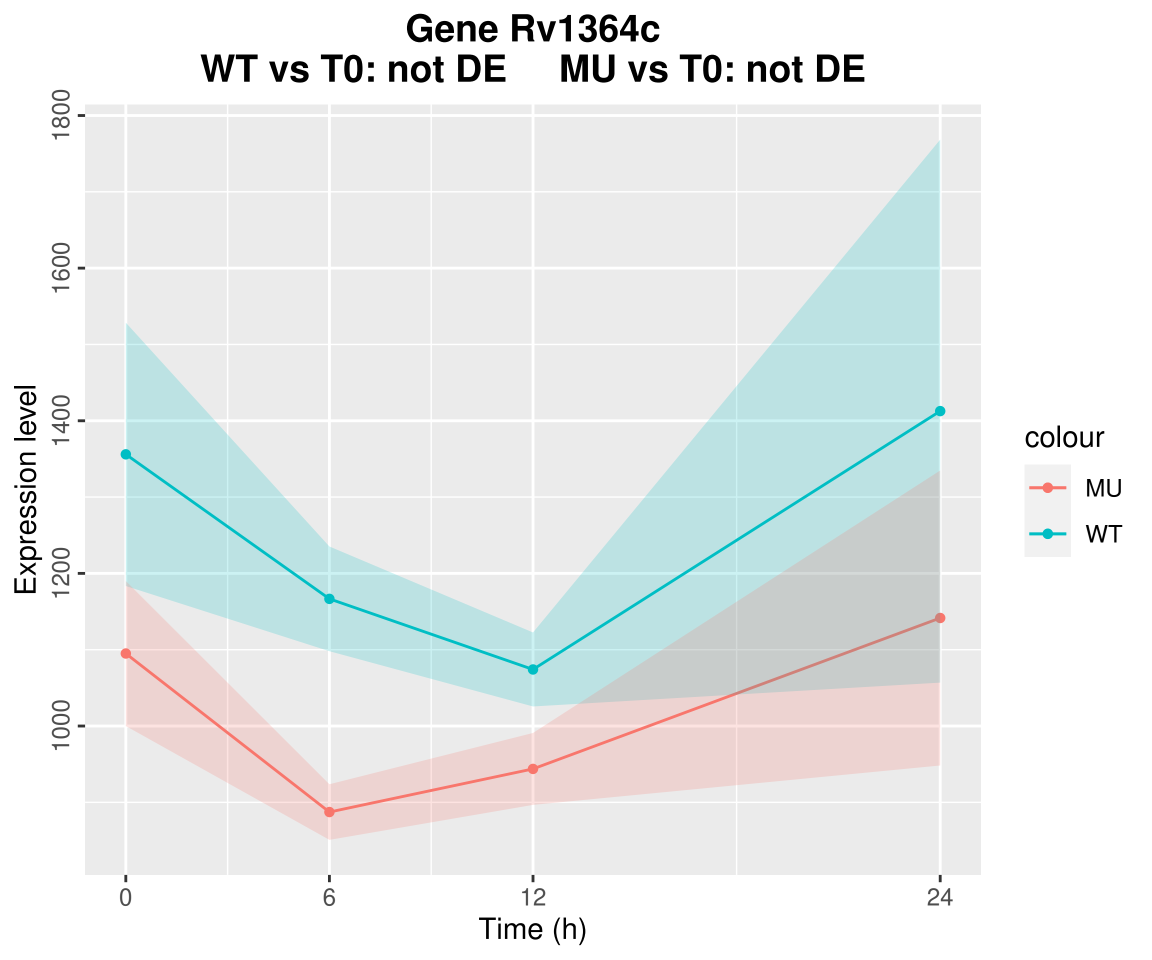

Gene Rv1364c
WT vs T0: not DE   MU vs T0: not DE
1800
1600
1400
1200
1000
Expression level
0
6
12
24
Time (h)
colour
MU
WT

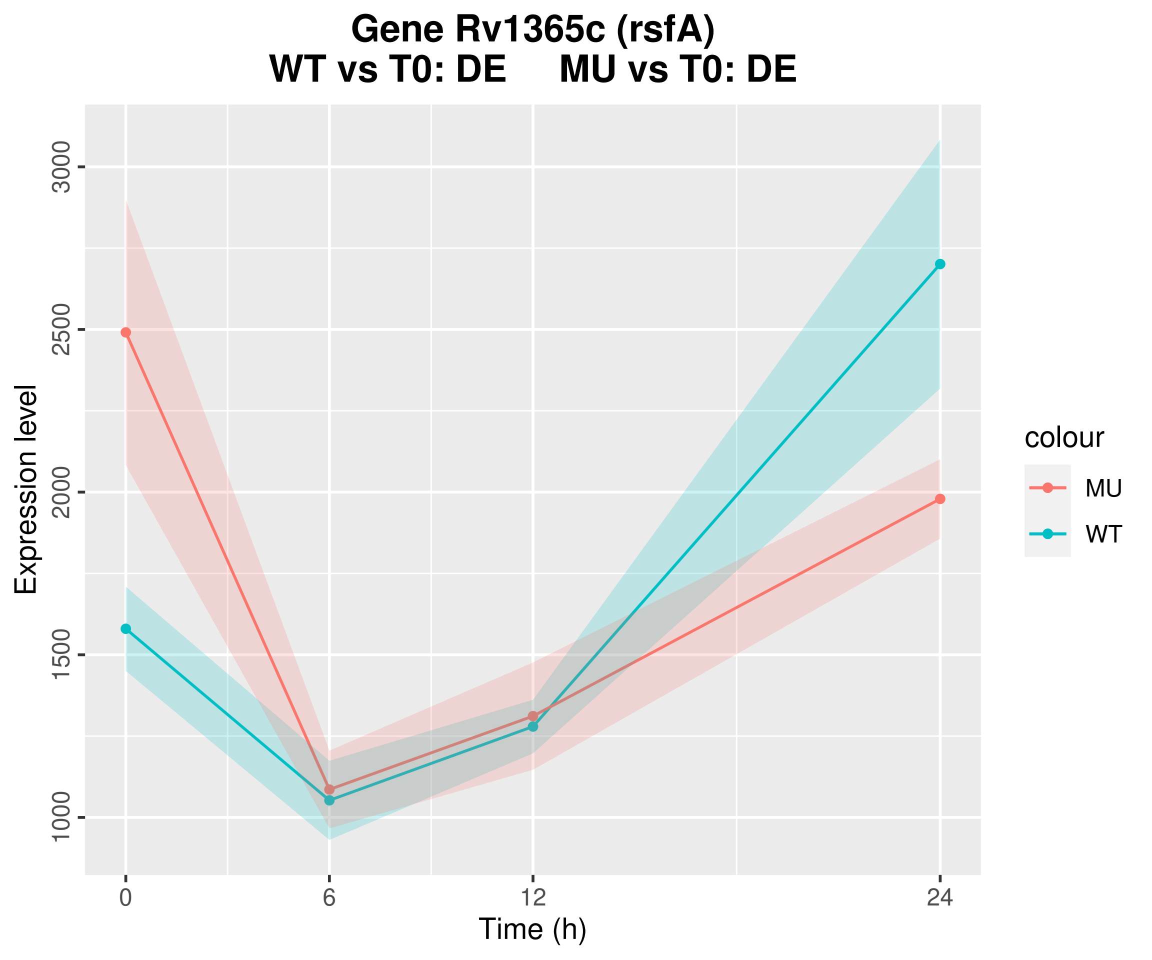

Gene Rv1365c (rsfA)
WT vs T0: DE MU vs T0: DE
Expression level
3000
2500
2000
1500
1000
0
6
12
24
Time (h)
colour
MU
WT

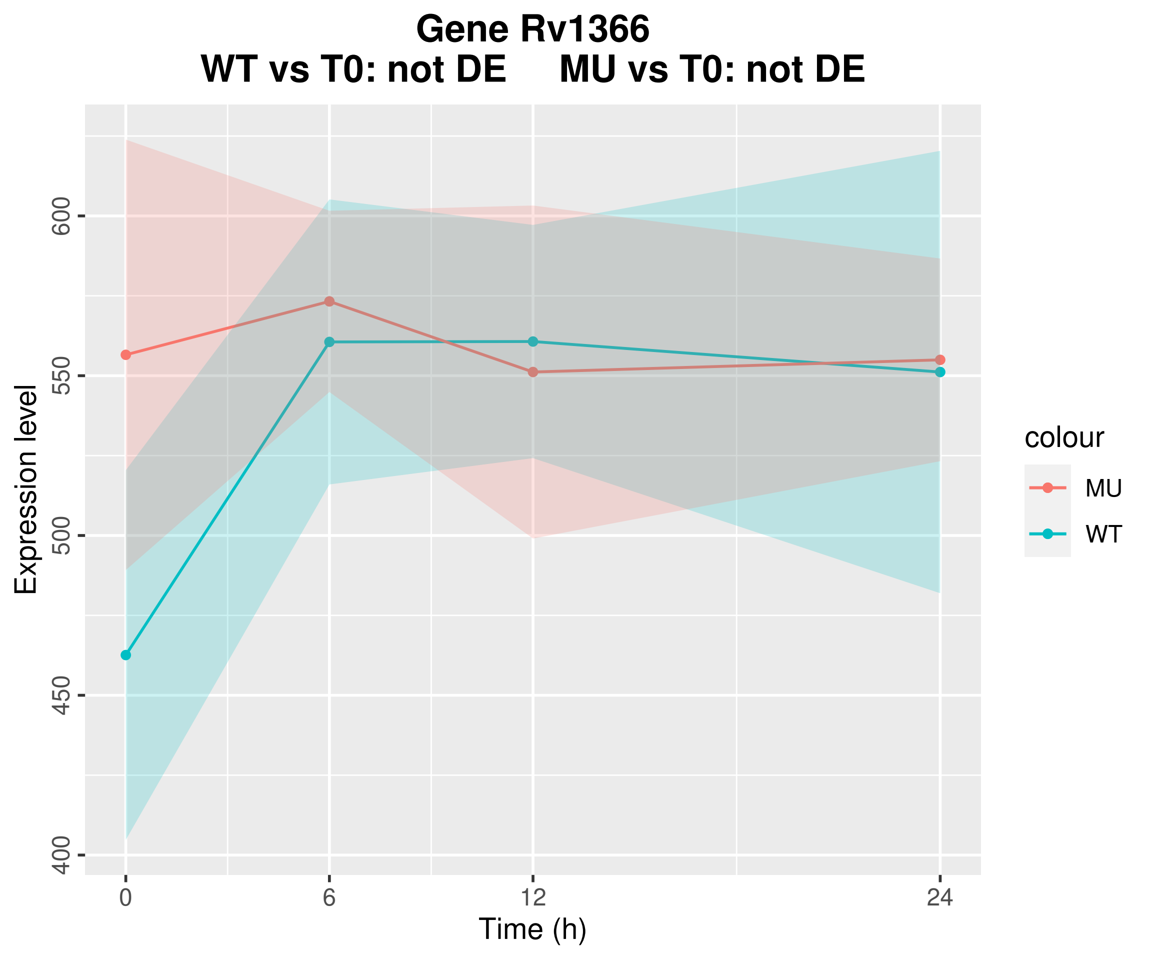

Gene Rv1366
WT vs T0: not DE   MU vs T0: not DE
Expression level
600
550
500
450
400
0
6
12
24
Time (h)
colour
MU
WT

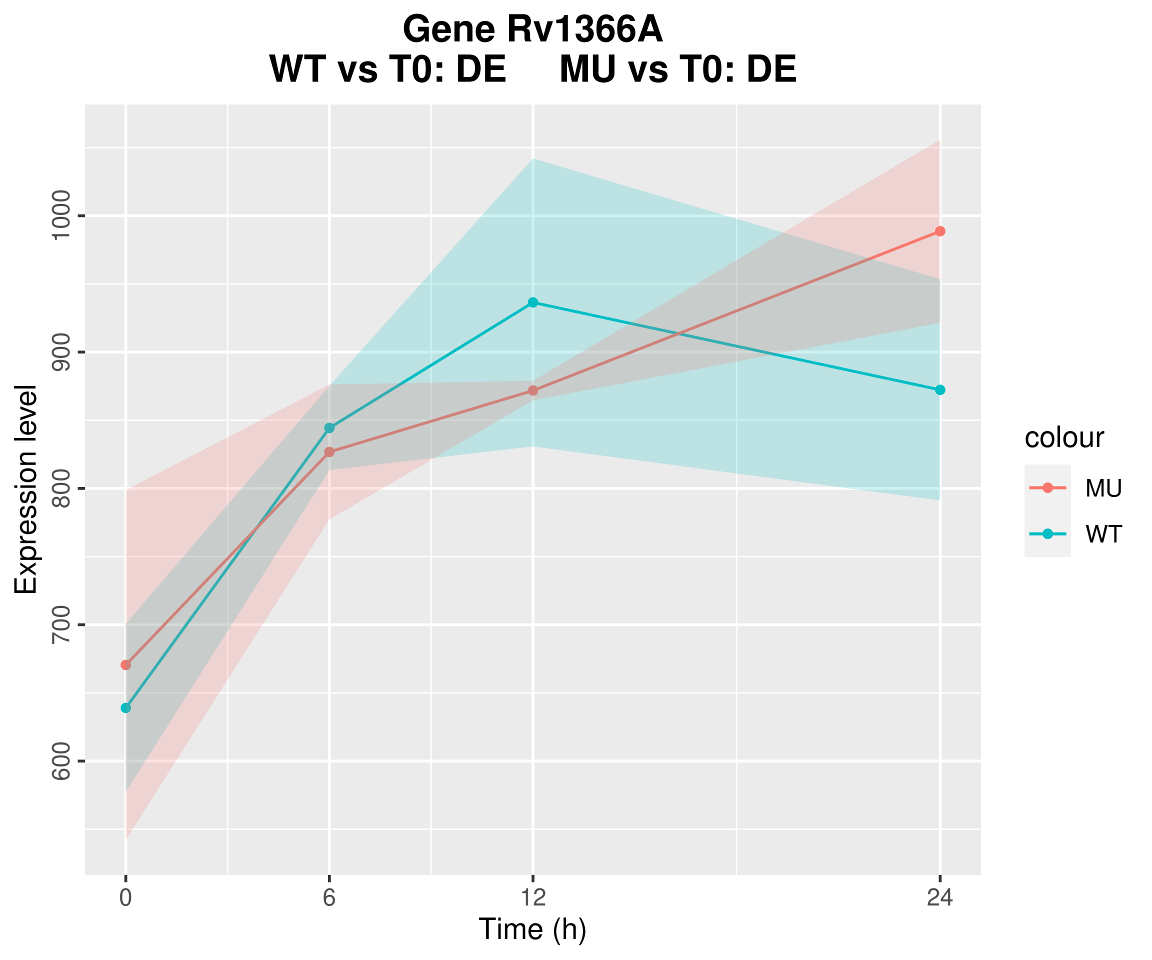

Gene Rv1366A
WT vs T0: DE MU vs T0: DE
Expression level
1000
900
800
700
600
0
6
12
24
Time (h)
colour
MU
WT

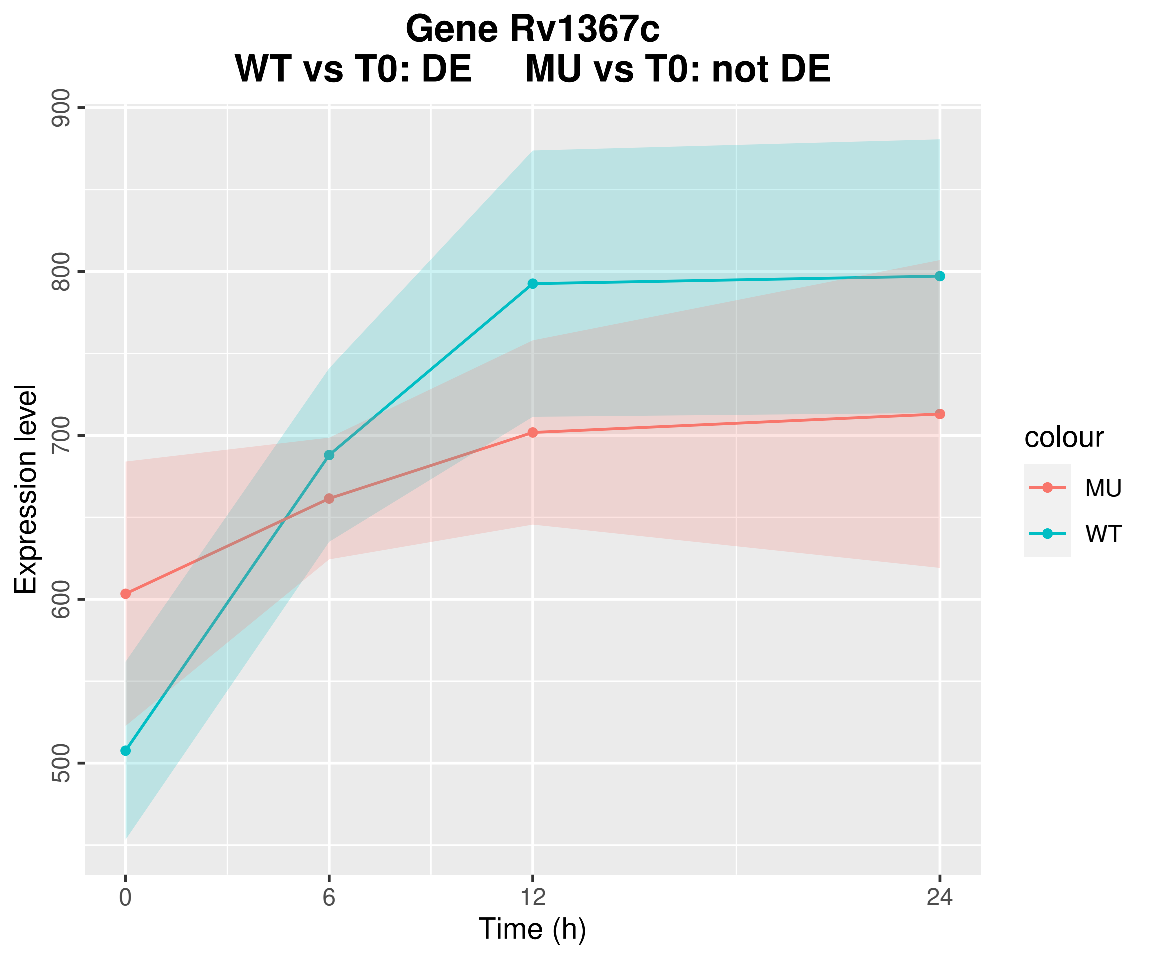

Gene Rv1367c
WT vs T0: DE MU vs T0: not DE
900
800
700
600
500
Expression level
0
6
12
24
Time (h)
colour
MU
WT

Gene Rv1368 (lprF)
WT vs T0: not DE MU vs T0: DE

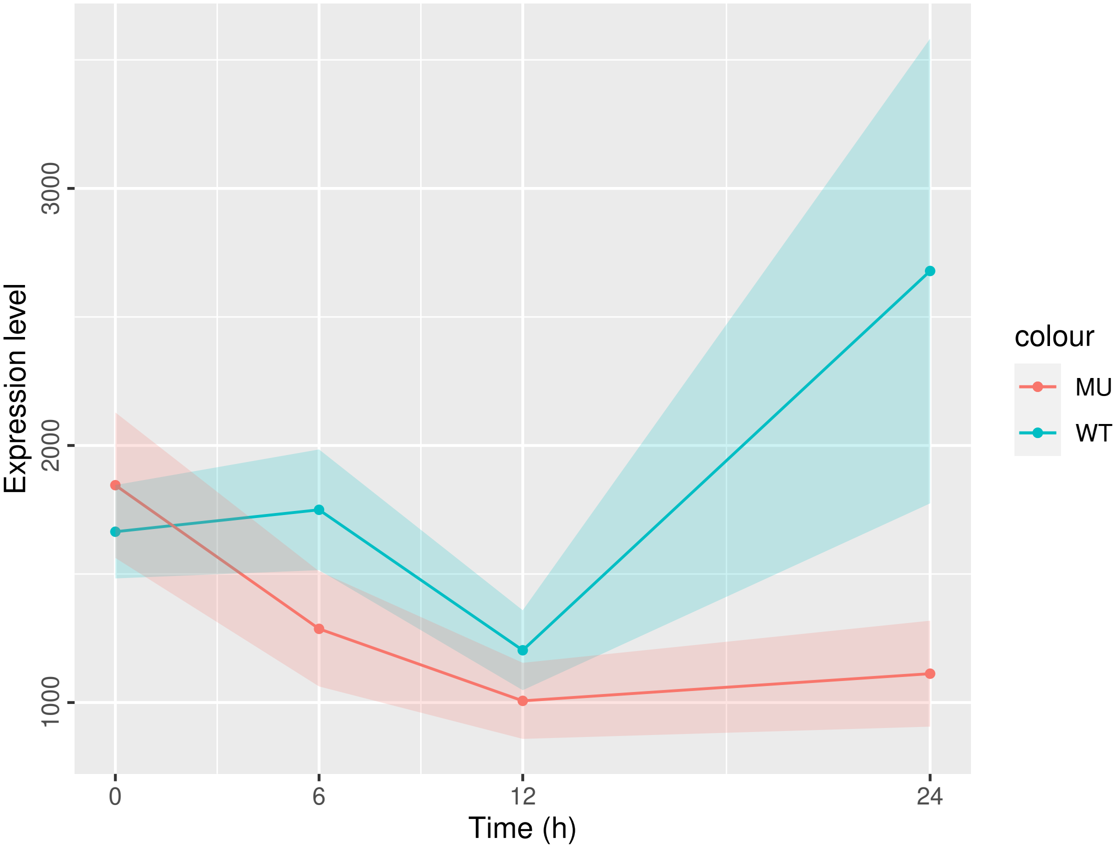

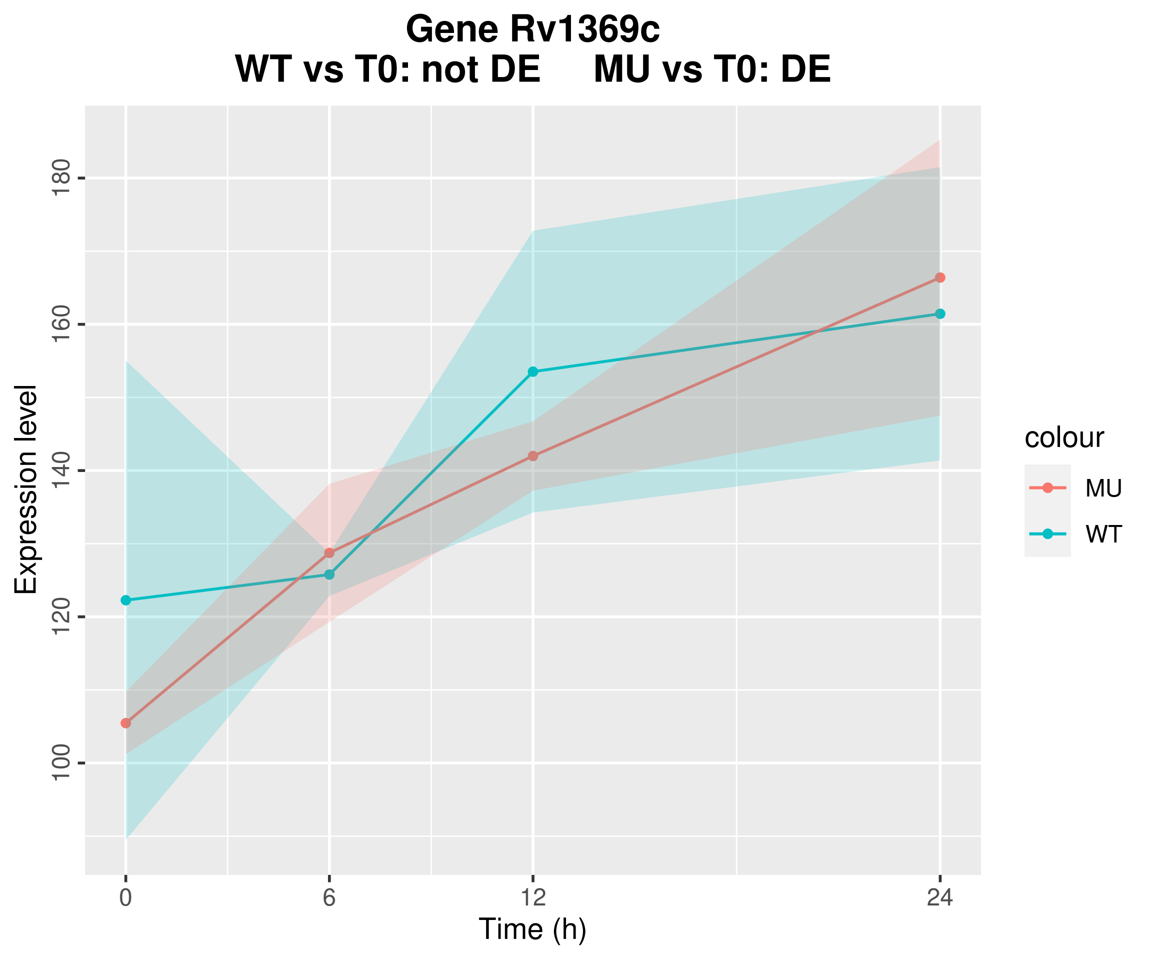

Gene Rv1369c
WT vs T0: not DE MU vs T0: DE
Expression level
100
120
140
160
180
0
6
12
24
Time (h)
colour
MU
WT

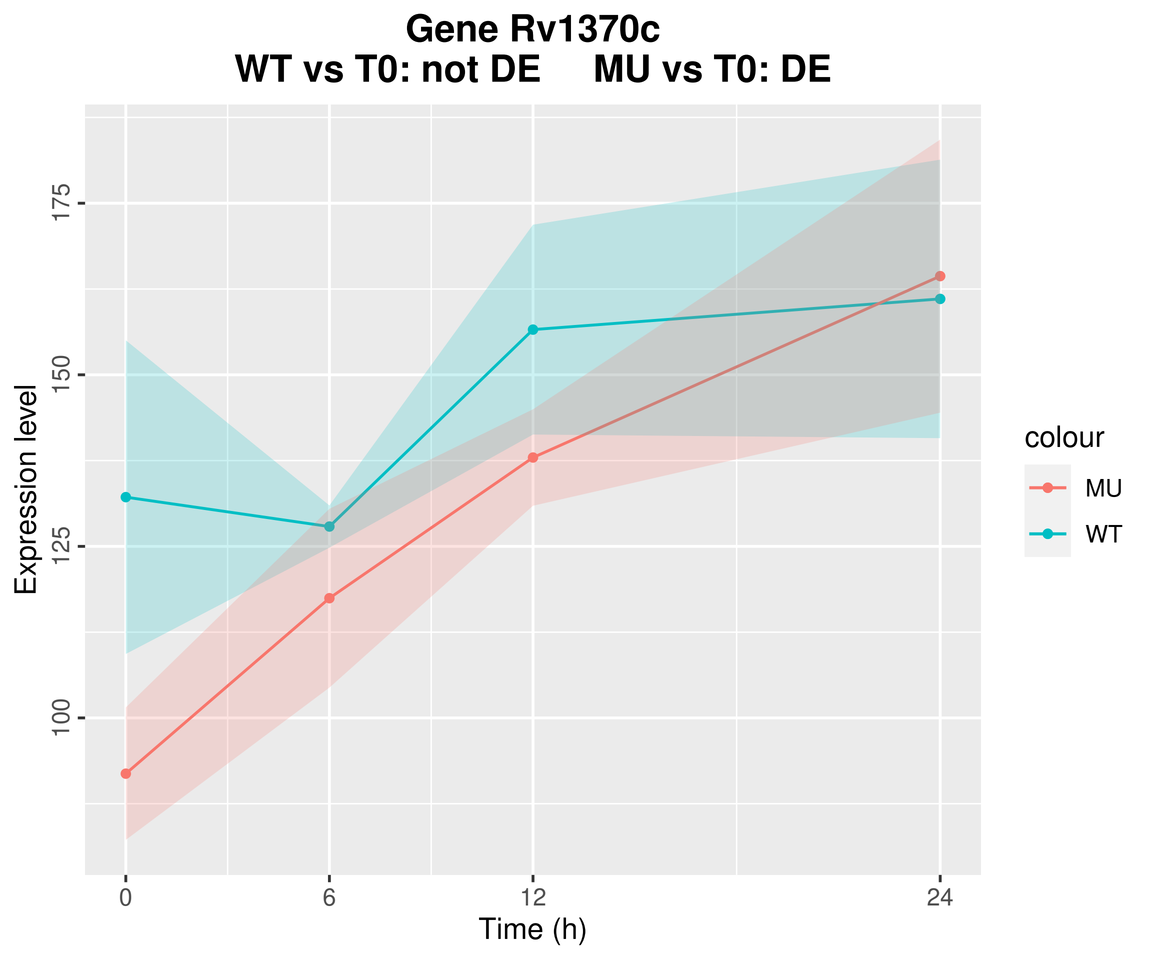

Gene Rv1370c
WT vs T0: not DE MU vs T0: DE
Expression level
175
150
125
100
0
6
12
24
Time (h)
colour
MU
WT

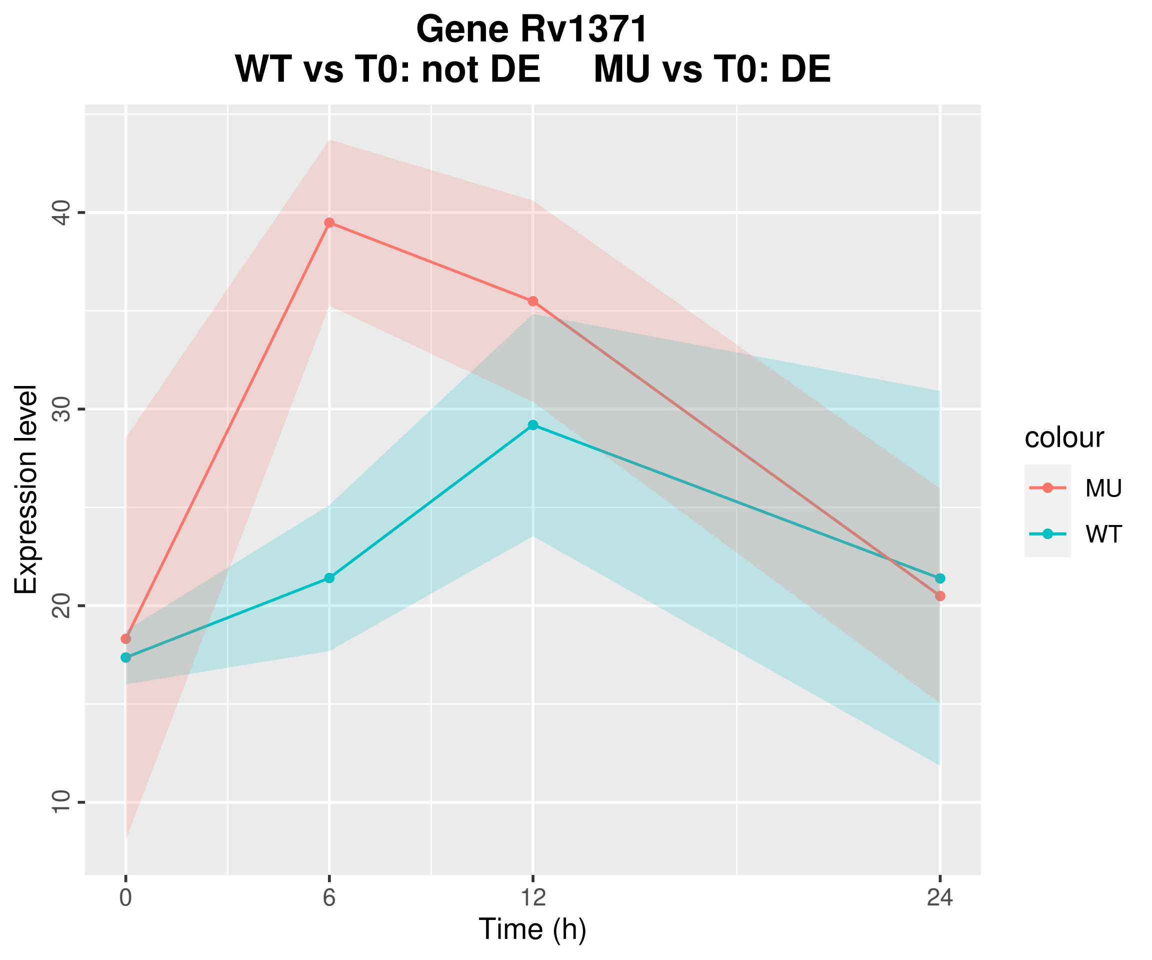

Gene Rv1371
WT vs T0: not DE   MU vs T0: DE
Expression level
10
20
30
40
0
6
12
24
Time (h)
colour
MU
WT

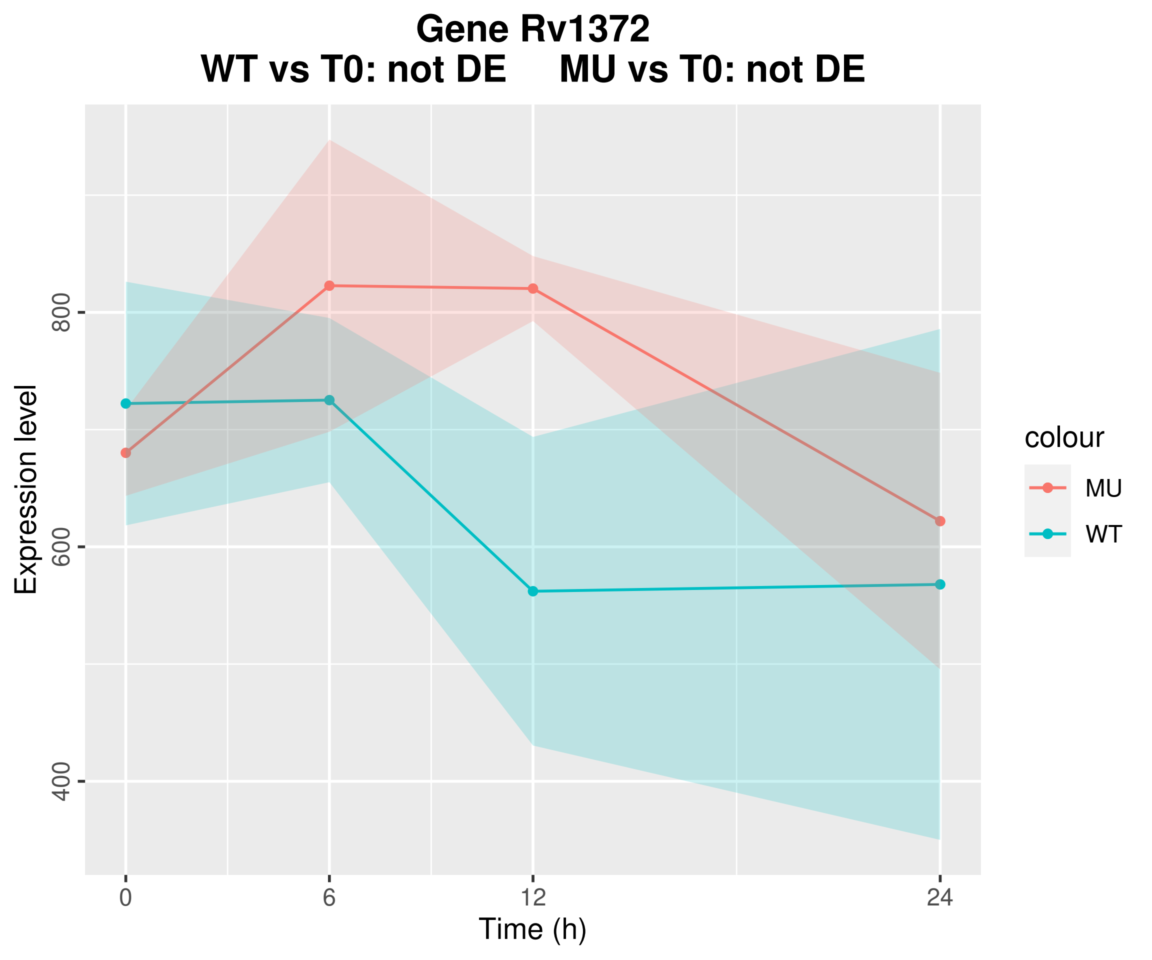

Gene Rv1372
WT vs T0: not DE MU vs T0: not DE
Expression level
400
600
800
0
6
12
24
Time (h)
colour
MU
WT

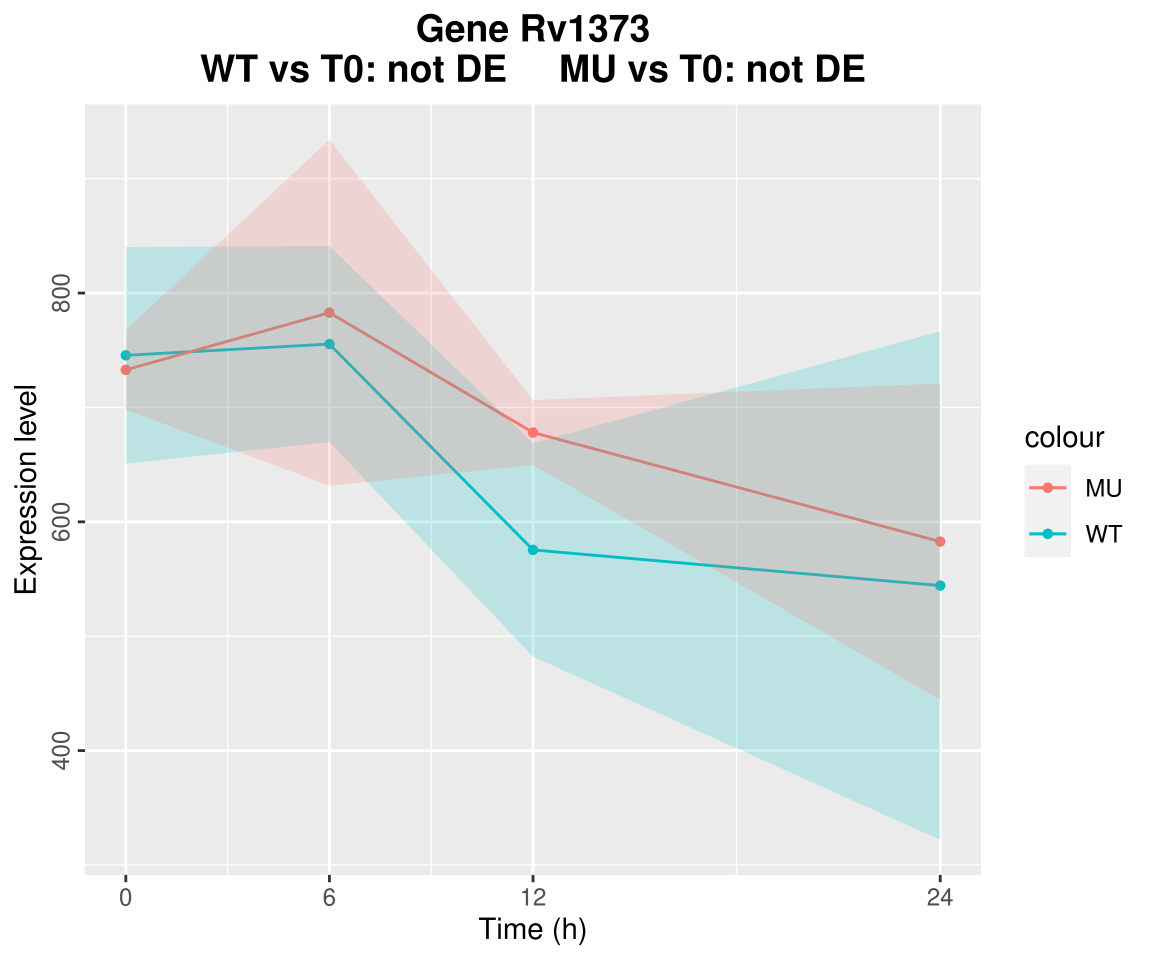
Gene Rv1373
WT vs T0: not DE MU vs T0: not DE
800
600
400
Expression level
0
6
12
24
Time (h)
colour
MU
WT

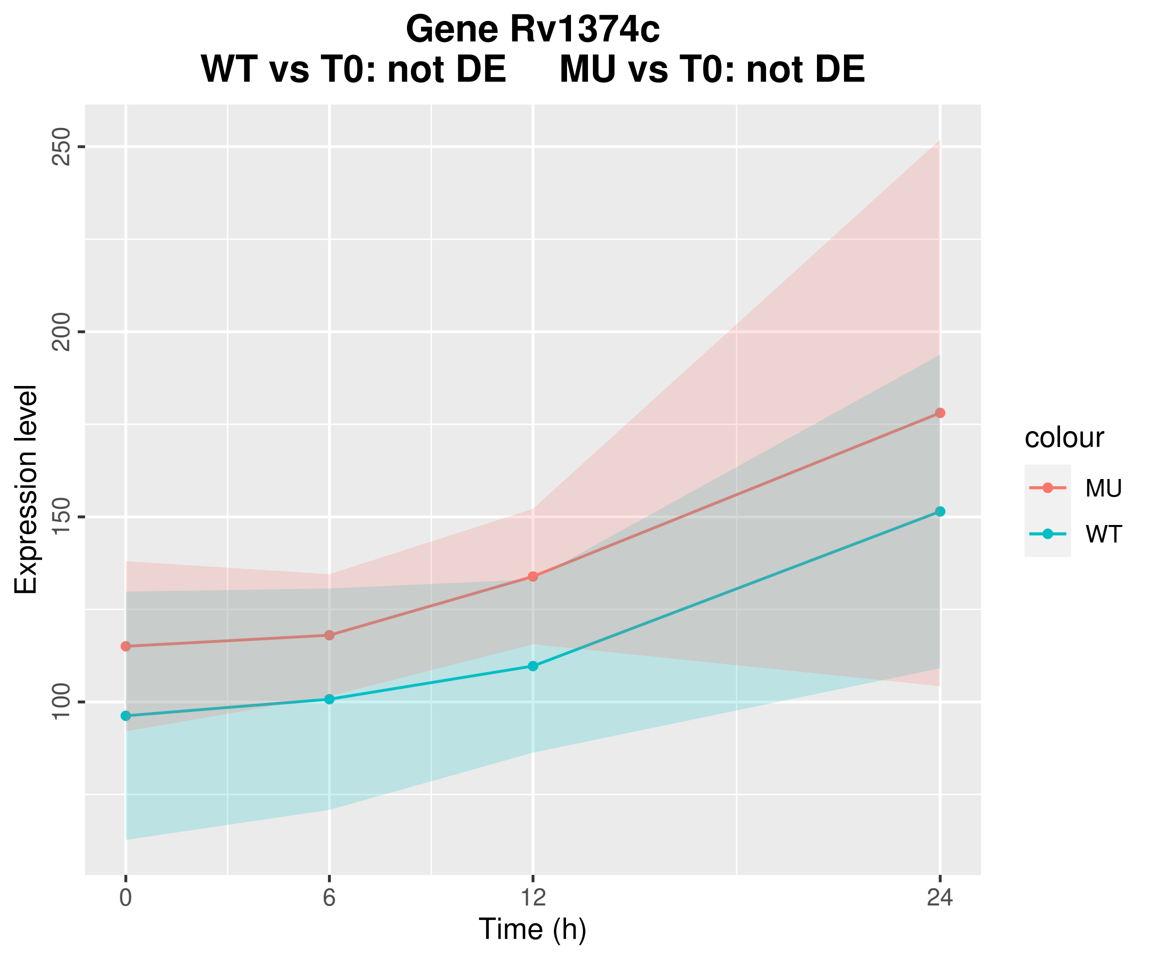
Gene Rv1374c
WT vs T0: not DE   MU vs T0: not DE
Expression level
250
200
150
100
0
6
12
24
Time (h)
colour
MU
WT

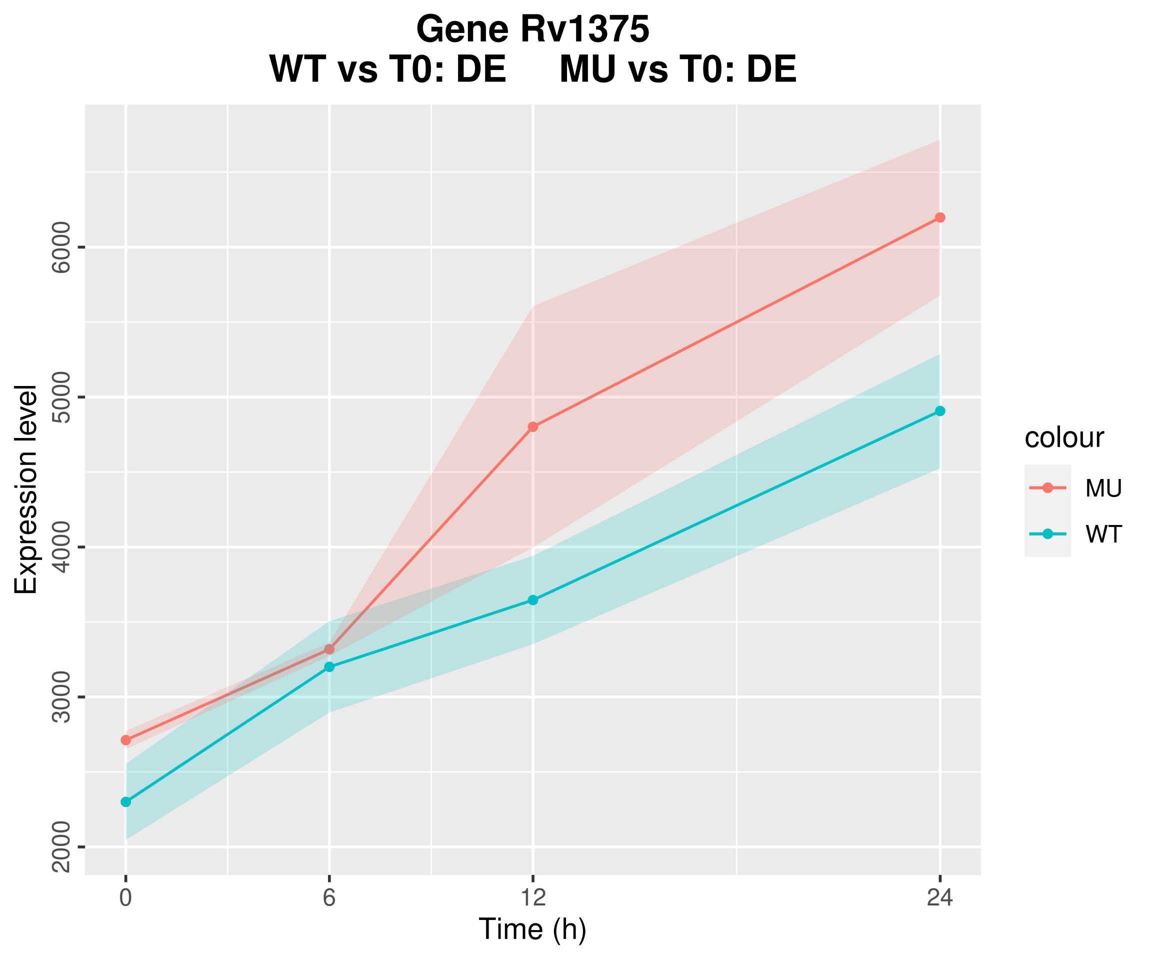
Gene Rv1375
WT vs T0: DE   MU vs T0: DE
Expression level
2000
3000
4000
5000
6000
0
6
12
24
Time (h)
colour
MU
WT

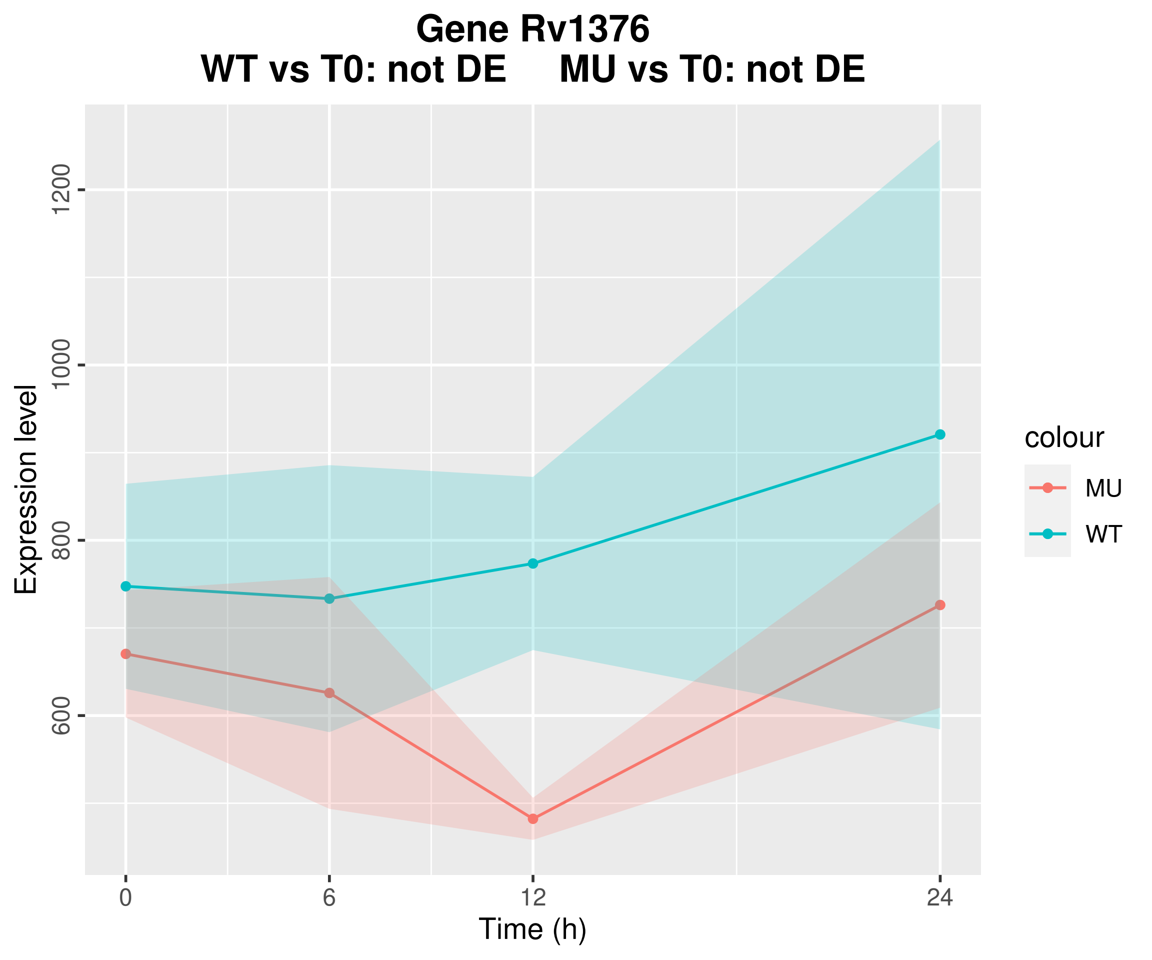
Gene Rv1376
WT vs T0: not DE MU vs T0: not DE
Expression level
1200
1000
800
600
0
6
12
24
Time (h)
colour
MU
WT

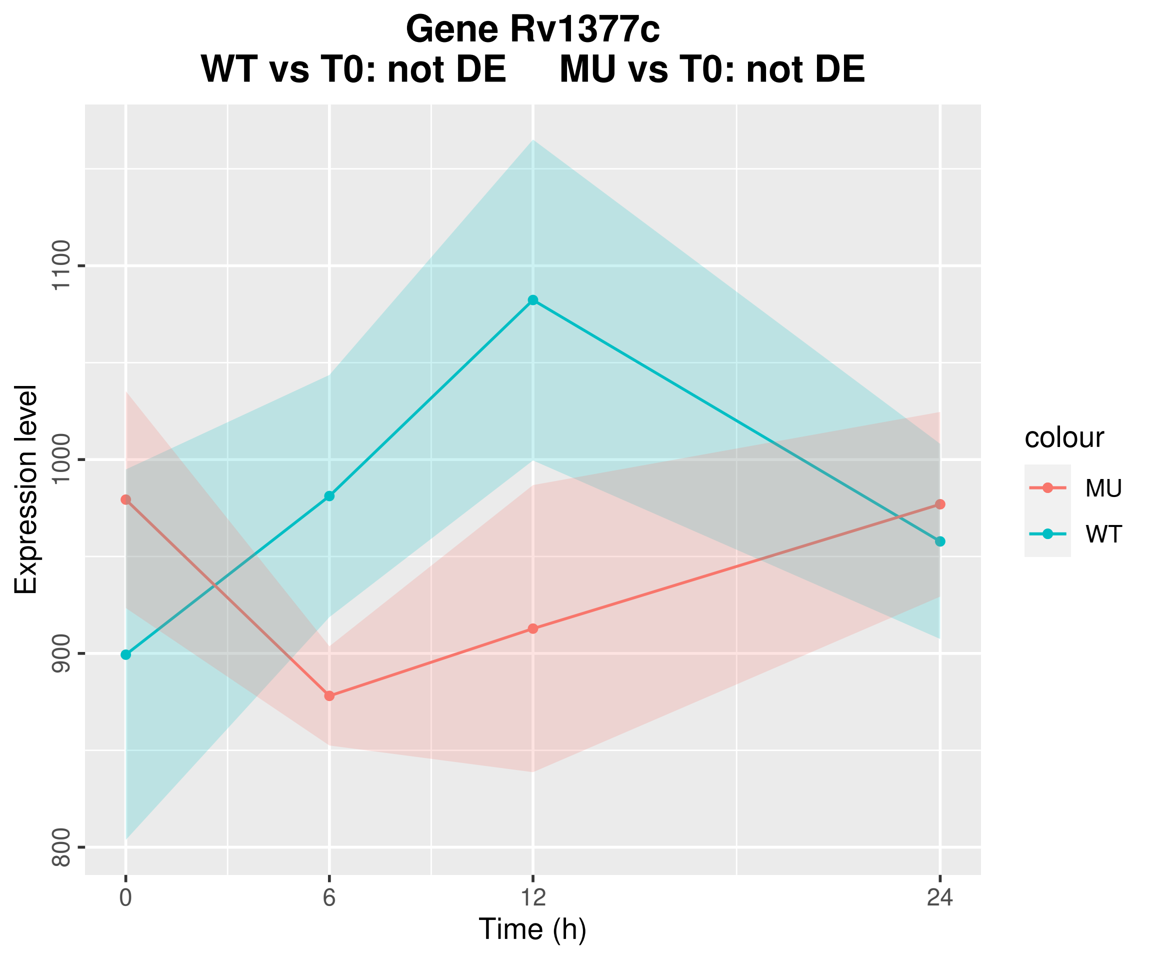
Gene Rv1377c
WT vs T0: not DE   MU vs T0: not DE
Expression level
800
900
1000
1100
0
6
12
24
Time (h)
colour
MU
WT

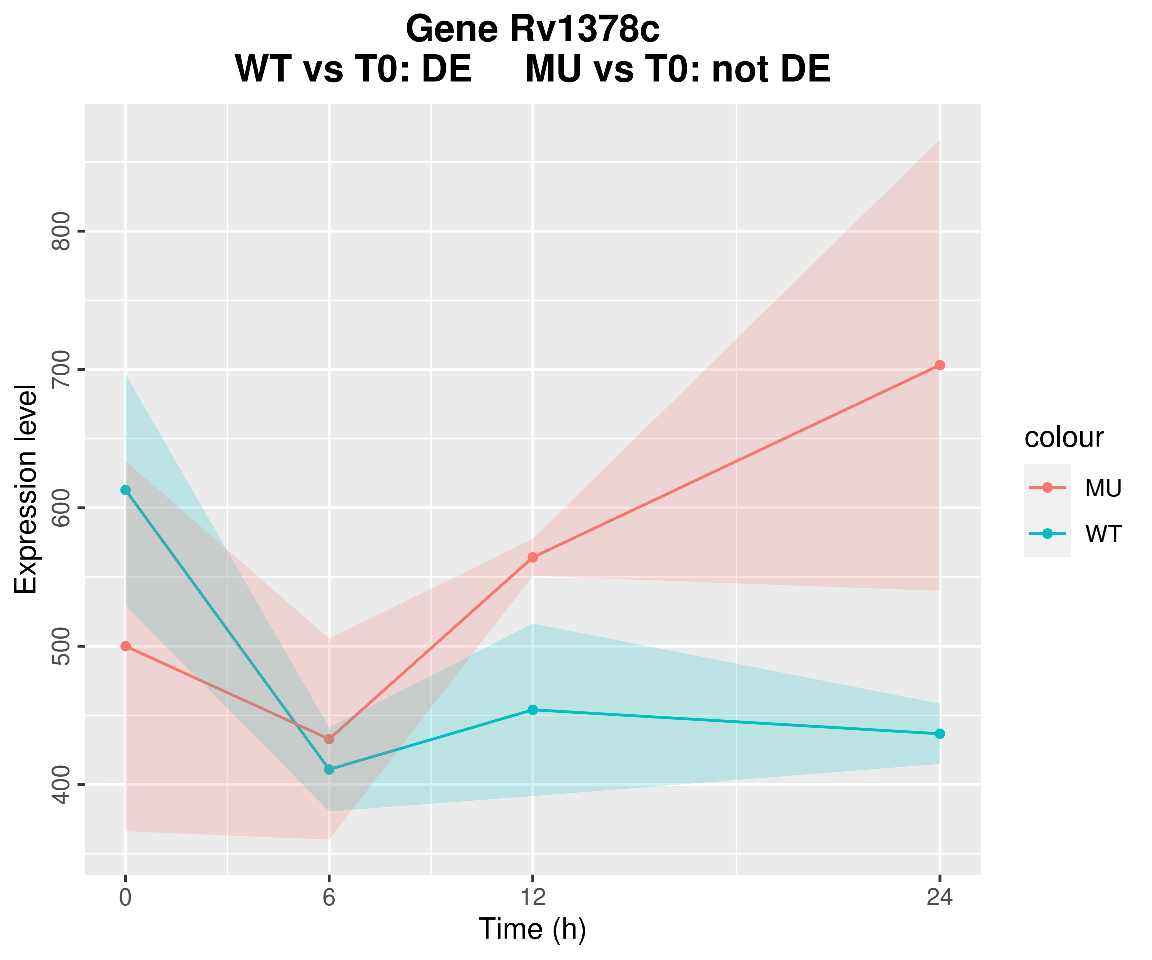

Gene Rv1378c
WT vs T0: DE MU vs T0: not DE
Expression level
800
700
600
500
400
0
6
12
24
Time (h)
colour
MU
WT

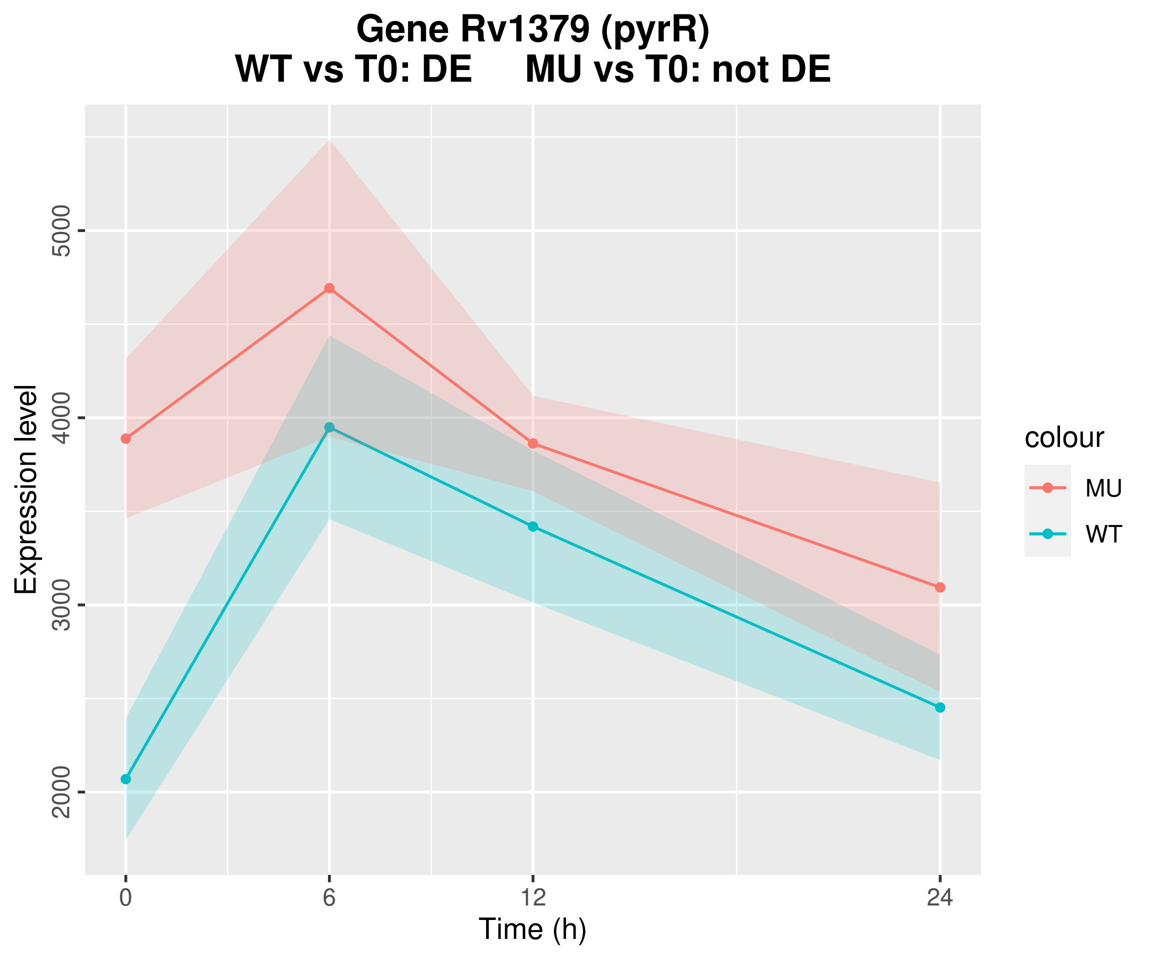
Gene Rv1379 (pyrR)
WT vs T0: DE   MU vs T0: not DE
Expression level
5000
4000
3000
2000
0
6
12
24
Time (h)
colour
MU
WT

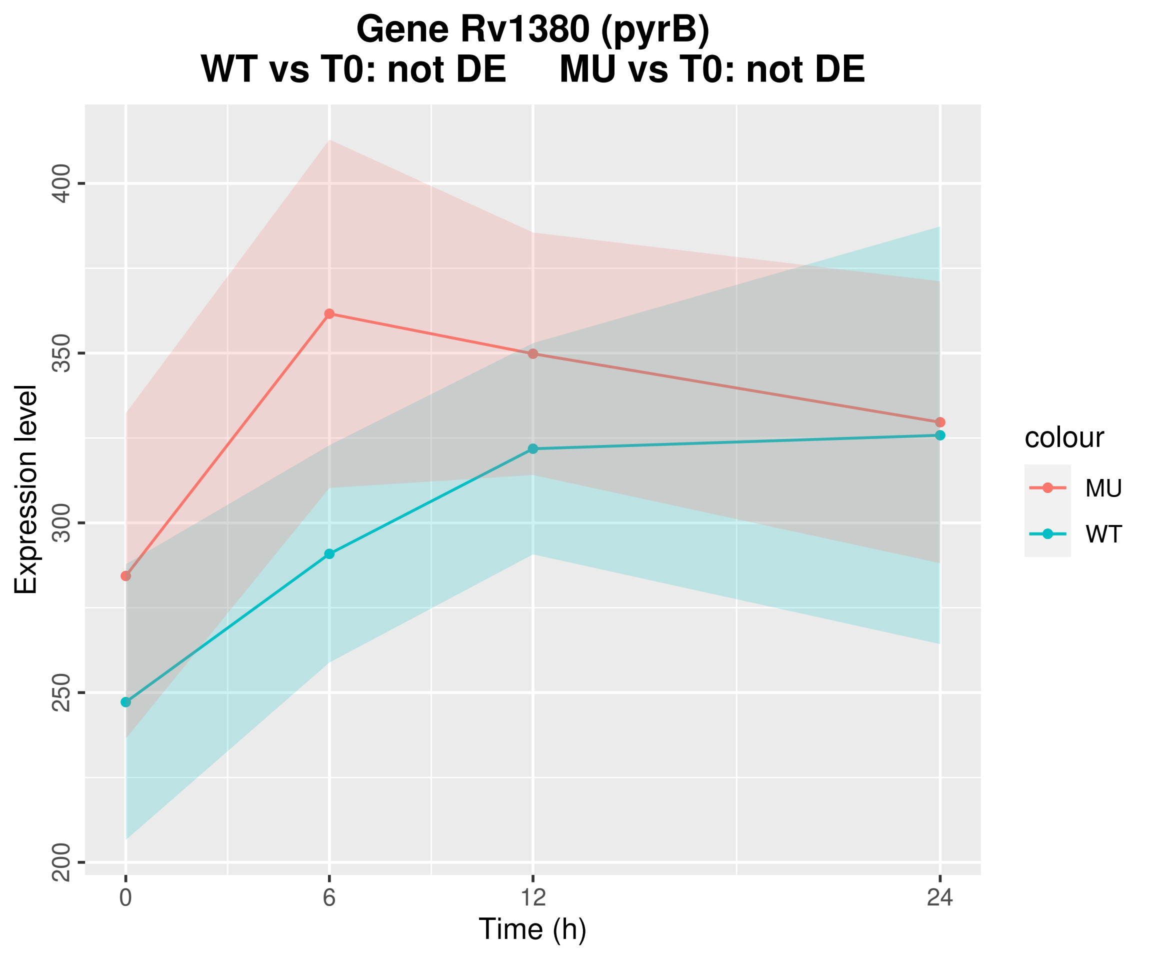

Gene Rv1380 (pyrB)
WT vs T0: not DE   MU vs T0: not DE
Expression level
400
350
300
250
200
0
6
12
24
Time (h)
colour
MU
WT

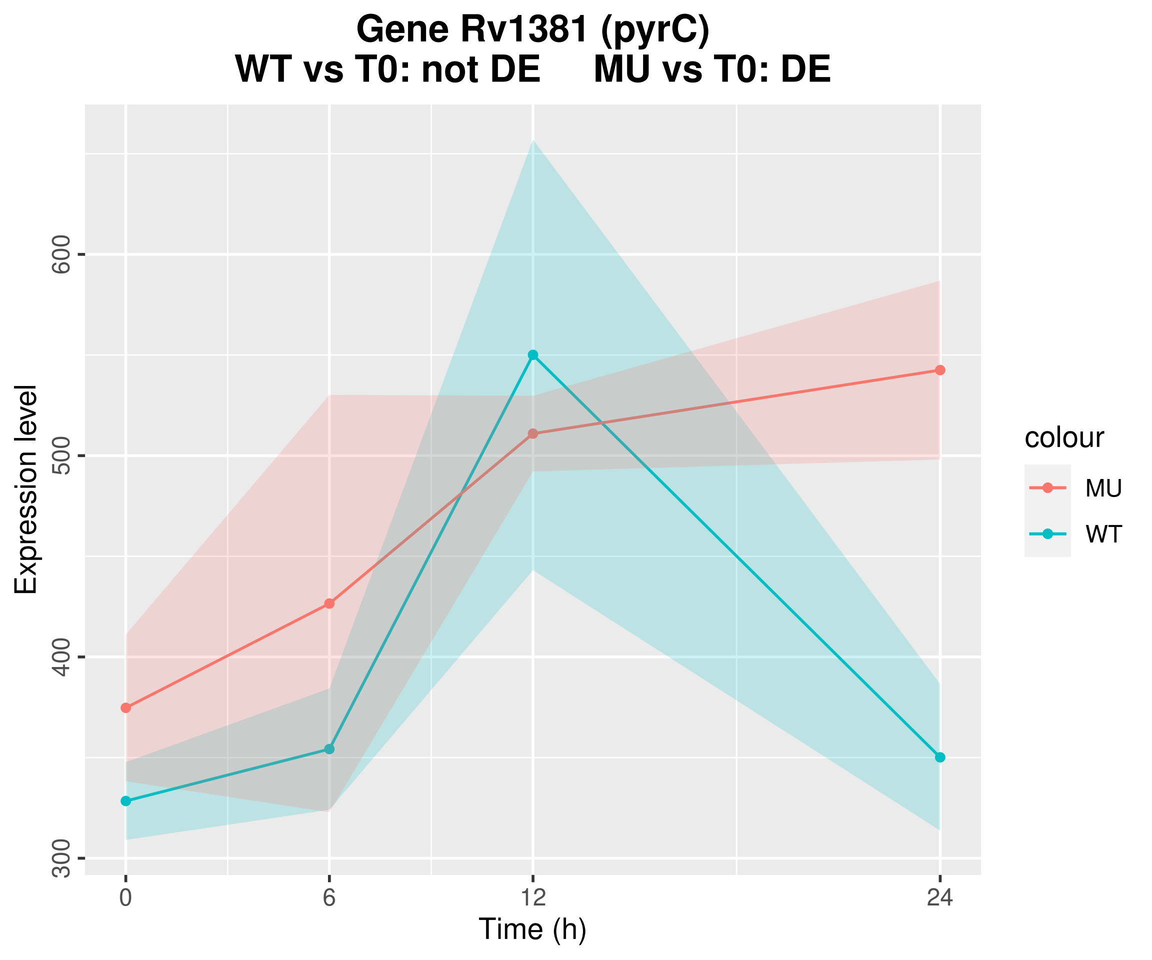
Gene Rv1381 (pyrC)
WT vs T0: not DE   MU vs T0: DE
Expression level
600
500
400
300
0
6
12
24
Time (h)
colour
MU
WT

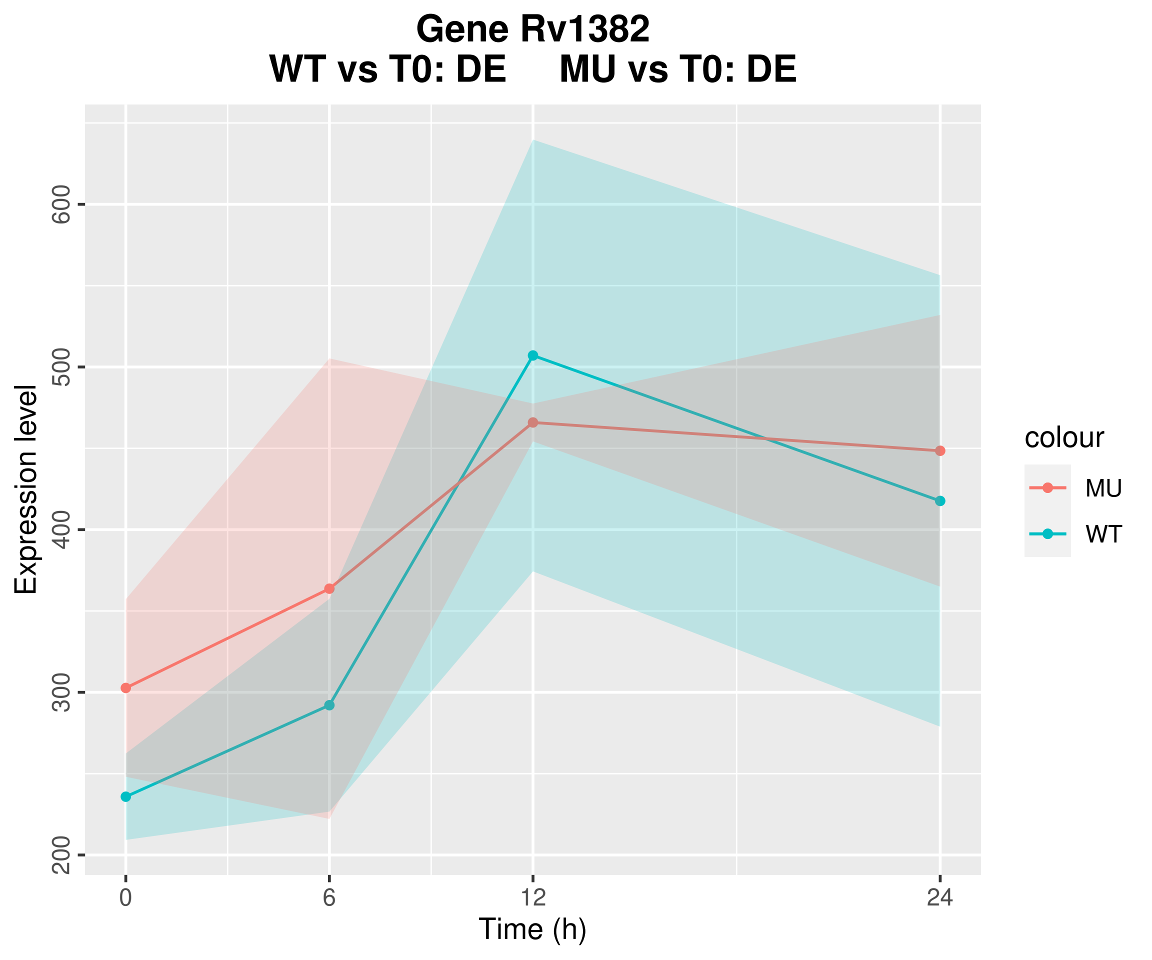

Gene Rv1382
WT vs T0: DE   MU vs T0: DE
Expression level
200
300
400
500
600
0
6
12
24
Time (h)
colour
MU
WT

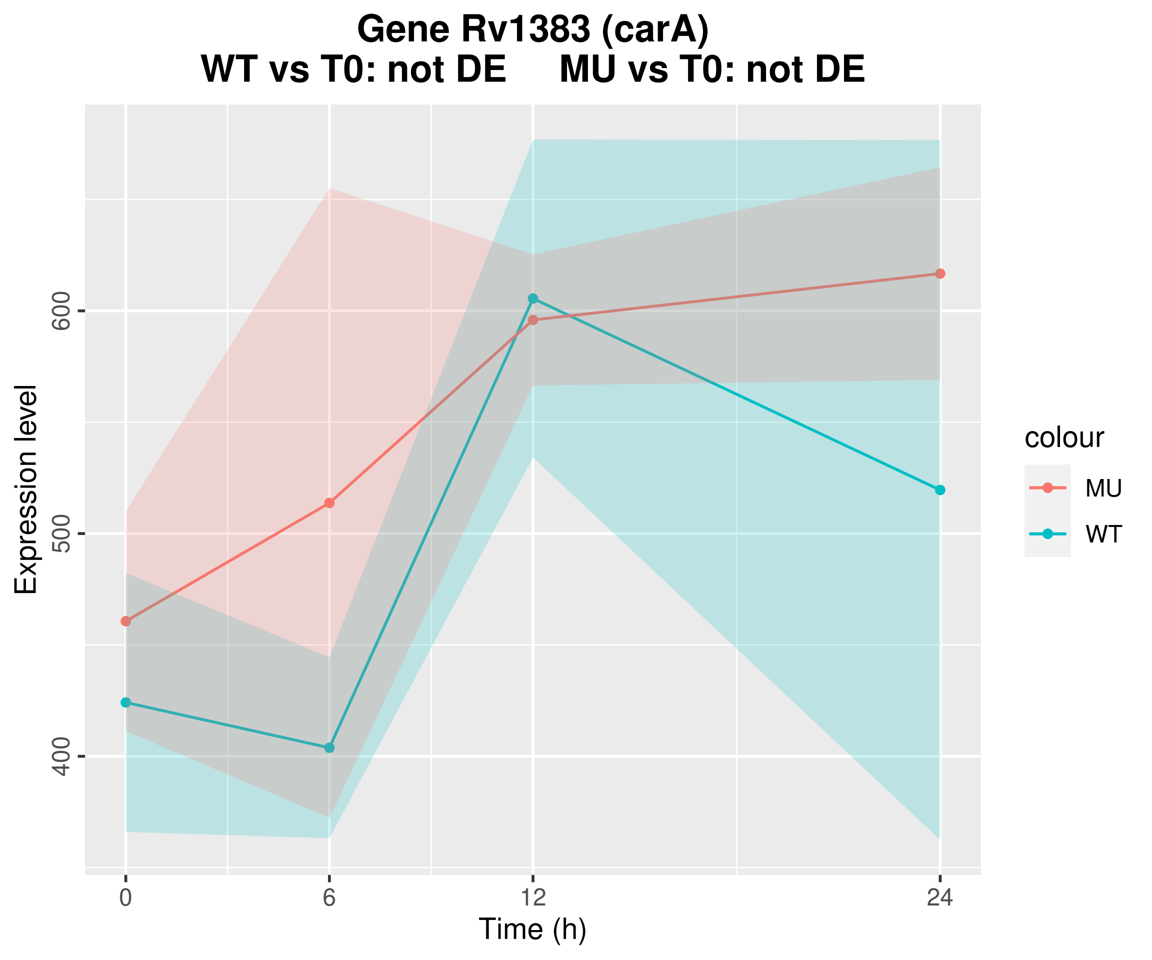

Gene Rv1383 (carA)
WT vs T0: not DE MU vs T0: not DE
Expression level
400
500
600
0
6
12
24
Time (h)
colour
MU
WT

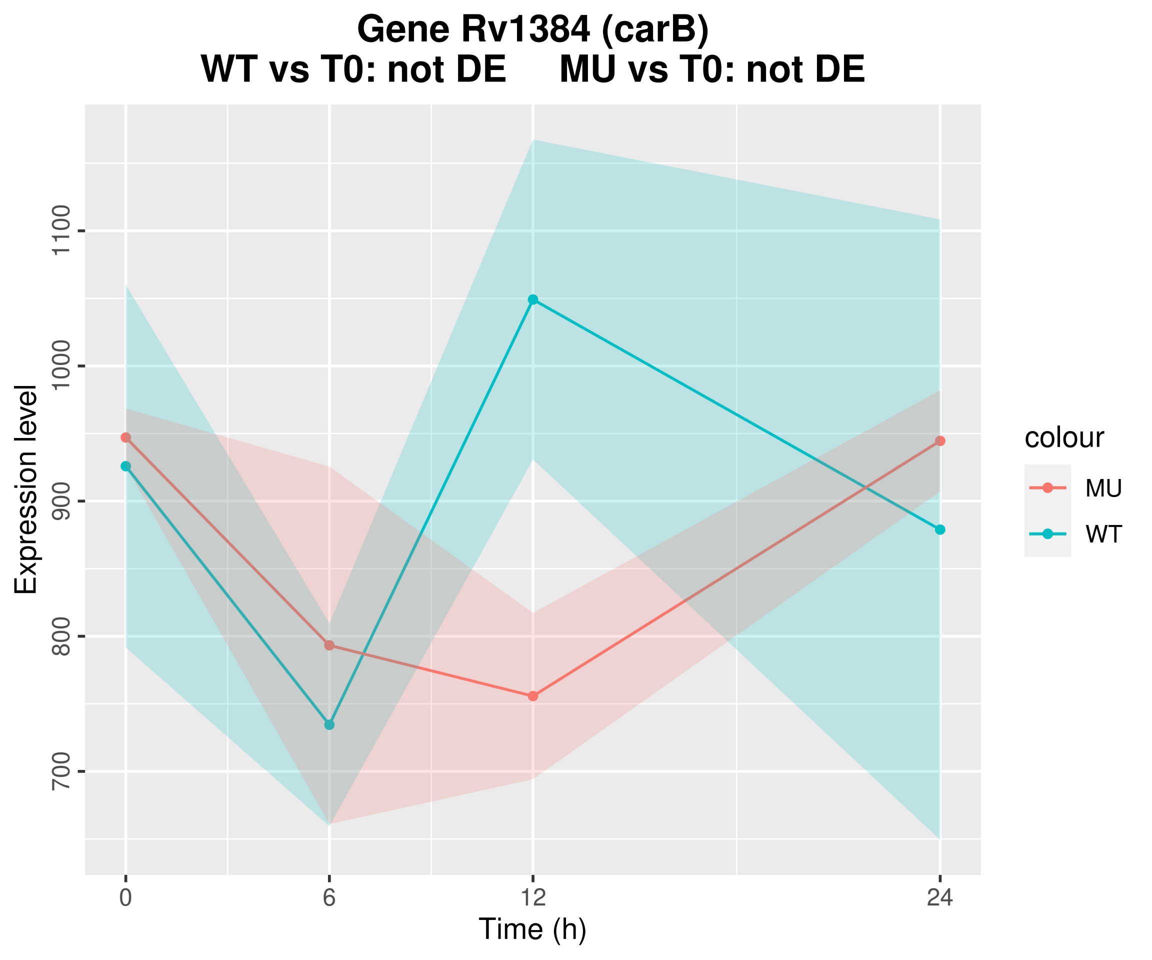

Gene Rv1384 (carB)
WT vs T0: not DE MU vs T0: not DE
Expression level
1100
1000
900
800
700
0
6
12
24
Time (h)
colour
MU
WT

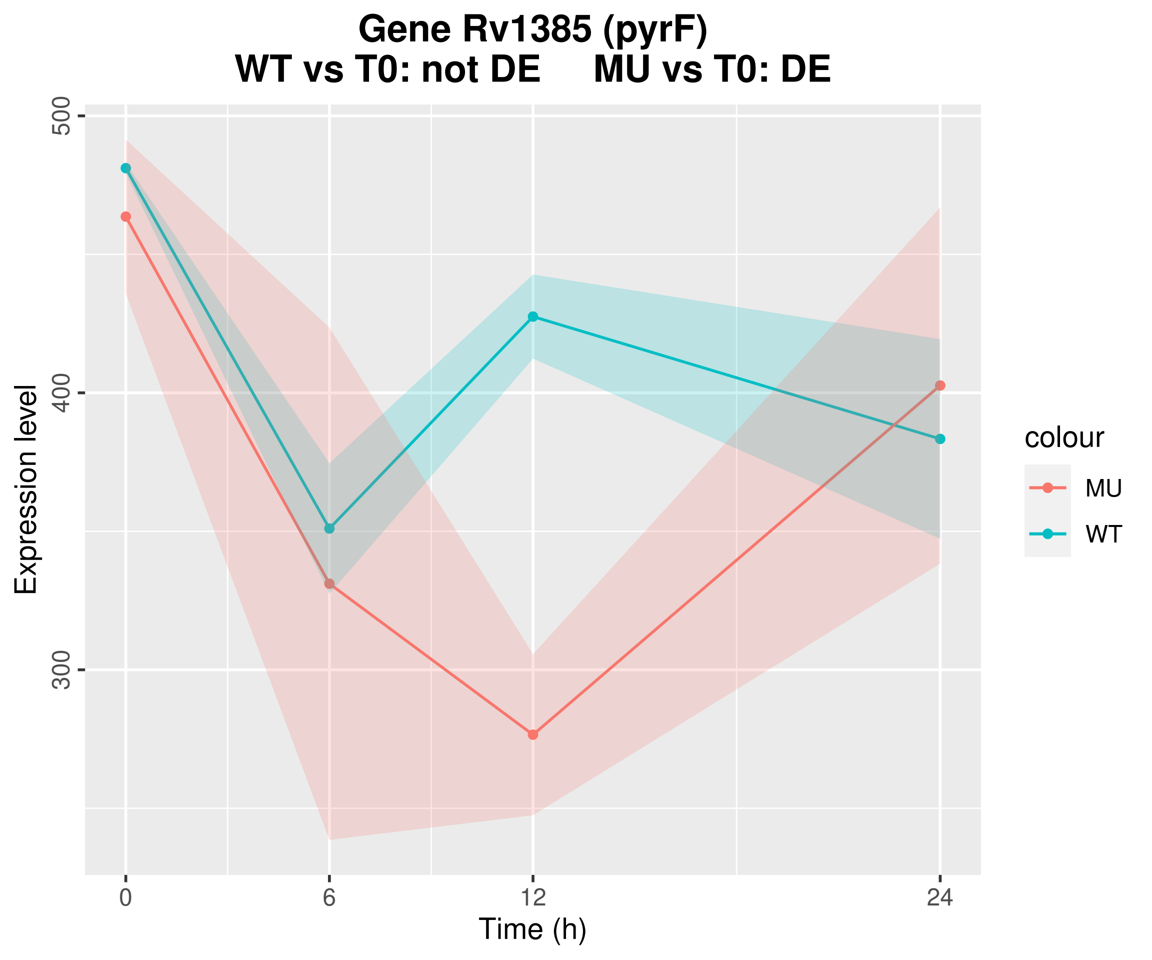
Gene Rv1385 (pyrF)
WT vs T0: not DE   MU vs T0: DE
500
400
300
Expression level
0
6
12
24
Time (h)
colour
MU
WT

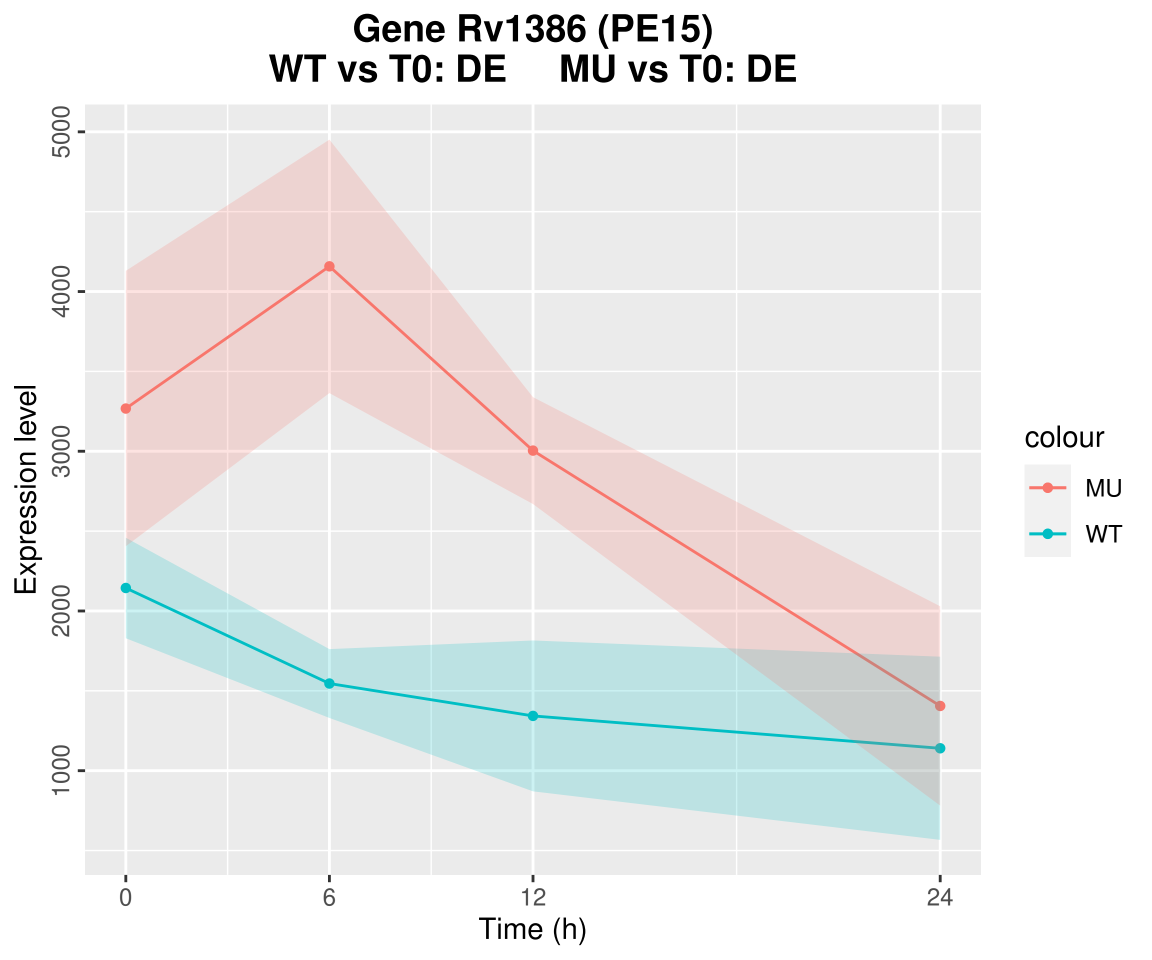
Gene Rv1386 (PE15)
WT vs T0: DE MU vs T0: DE
Expression level
5000
4000
3000
2000
1000
0
6
12
24
Time (h)
colour
MU
WT

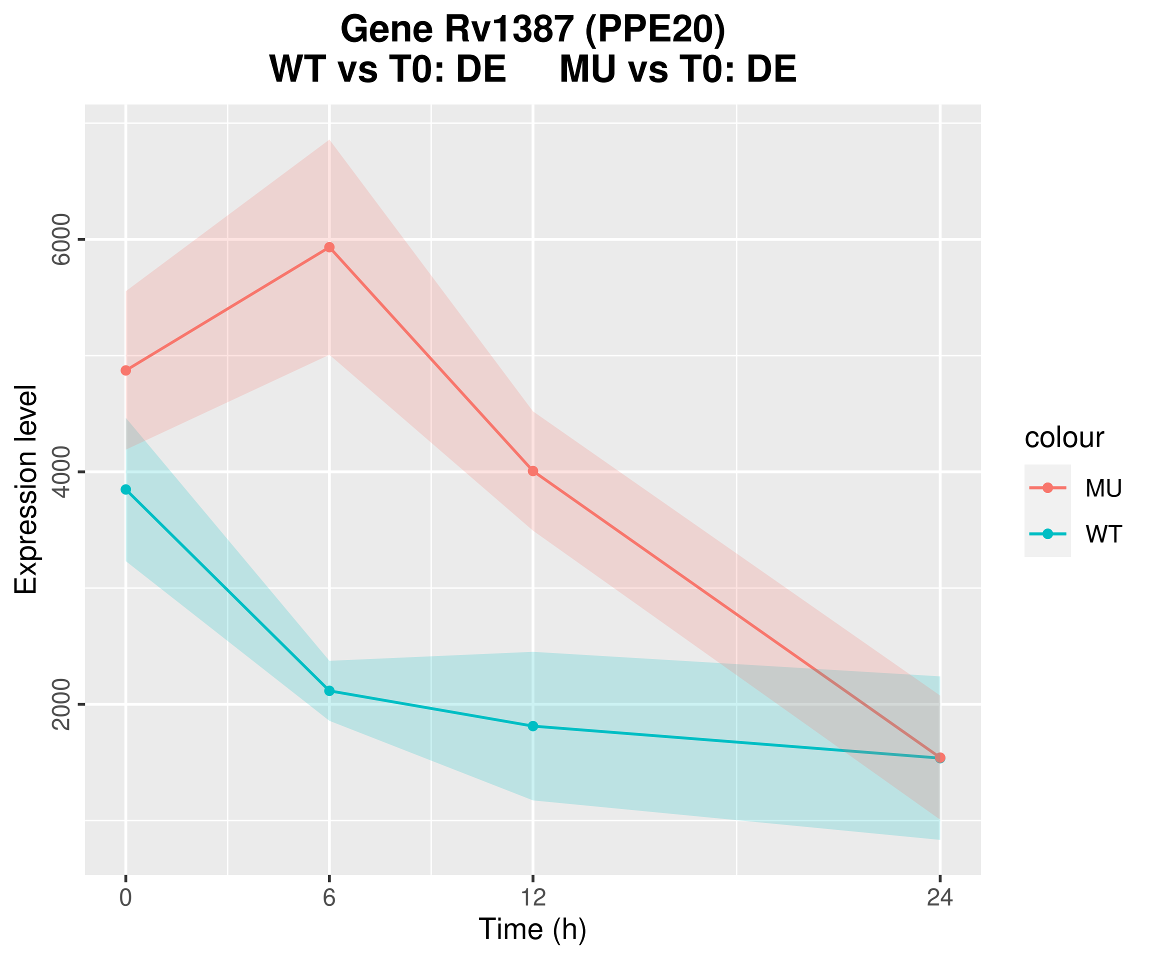

Gene Rv1387 (PPE20)
WT vs T0: DE MU vs T0: DE
Expression level
6000
4000
2000
0
6
12
24
Time (h)
colour
MU
WT

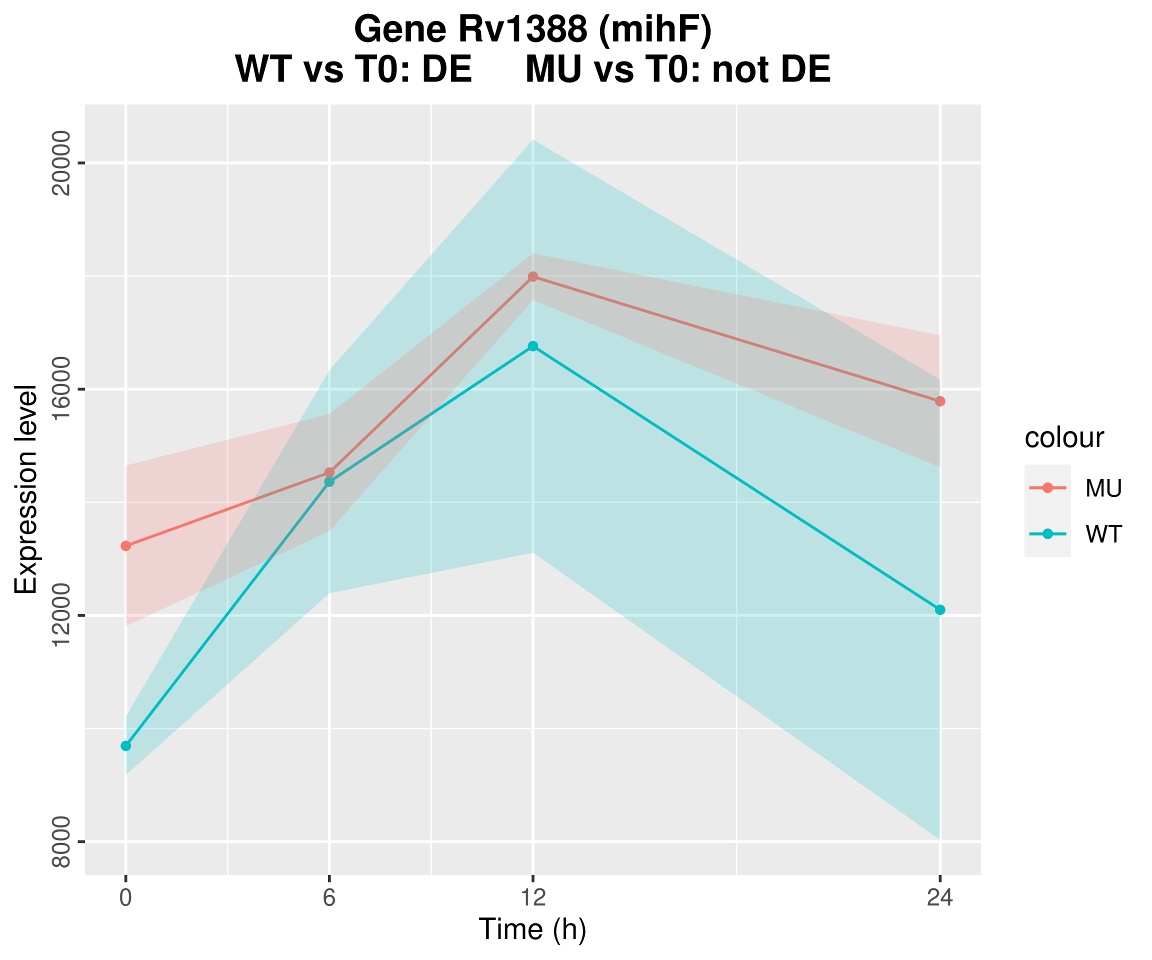

Gene Rv1388 (mihF)
WT vs T0: DE   MU vs T0: not DE
Expression level
20000
16000
12000
8000
0
6
12
24
Time (h)
colour
MU
WT

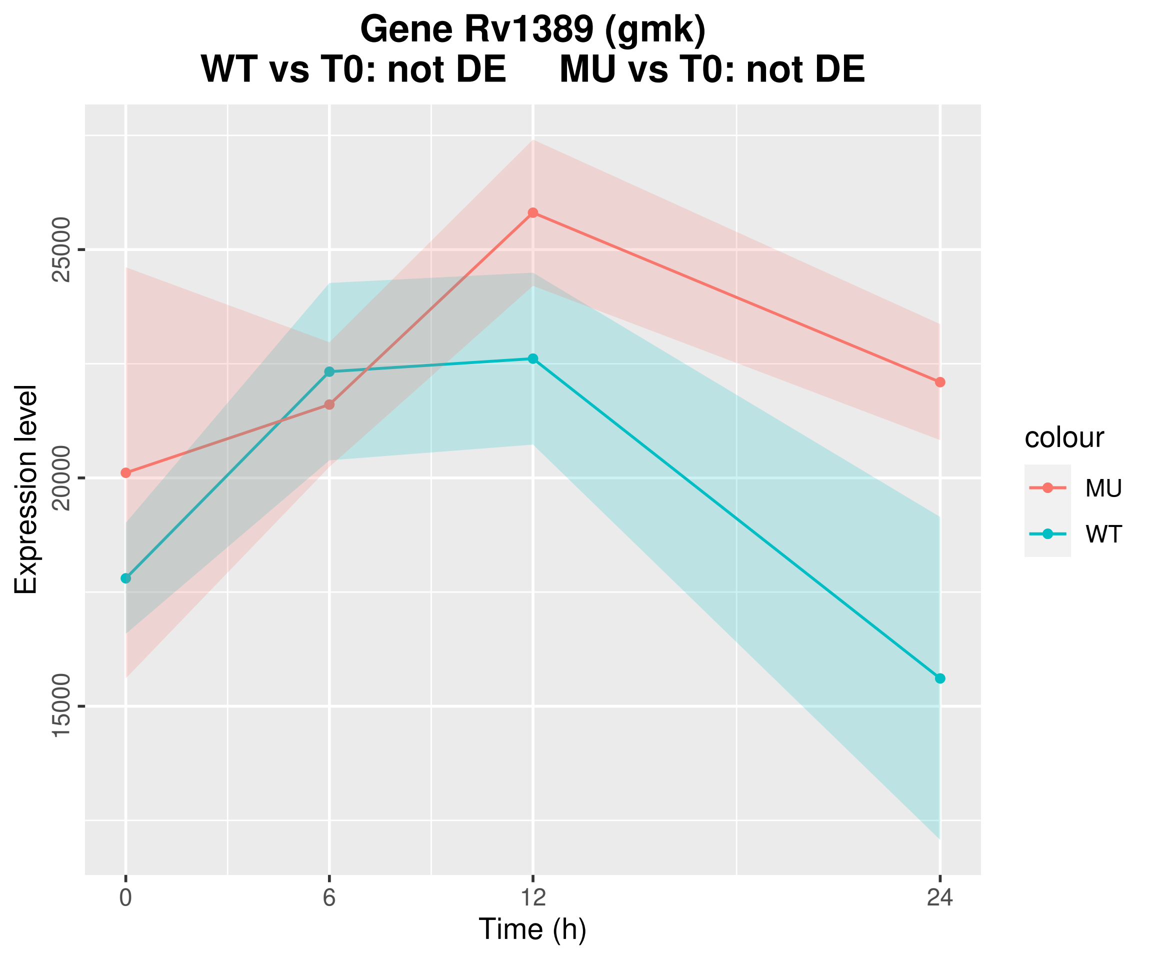

Gene Rv1389 (gmk)
WT vs T0: not DE   MU vs T0: not DE
Expression level
25000
20000
15000
0
6
12
24
Time (h)
colour
MU
WT

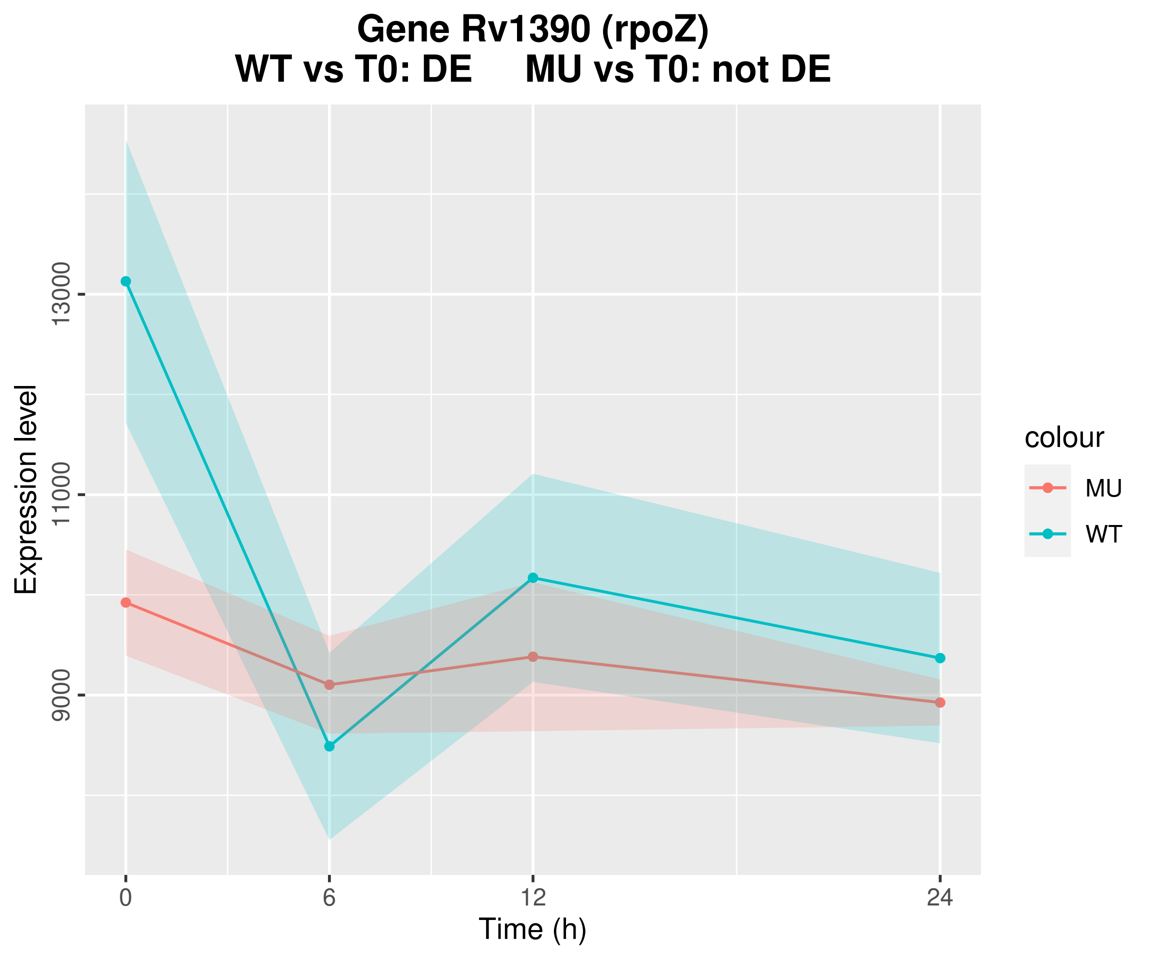
Gene Rv1390 (rpoZ)
WT vs T0: DE   MU vs T0: not DE
Expression level
13000
11000
9000
0
6
12
24
Time (h)
colour
MU
WT

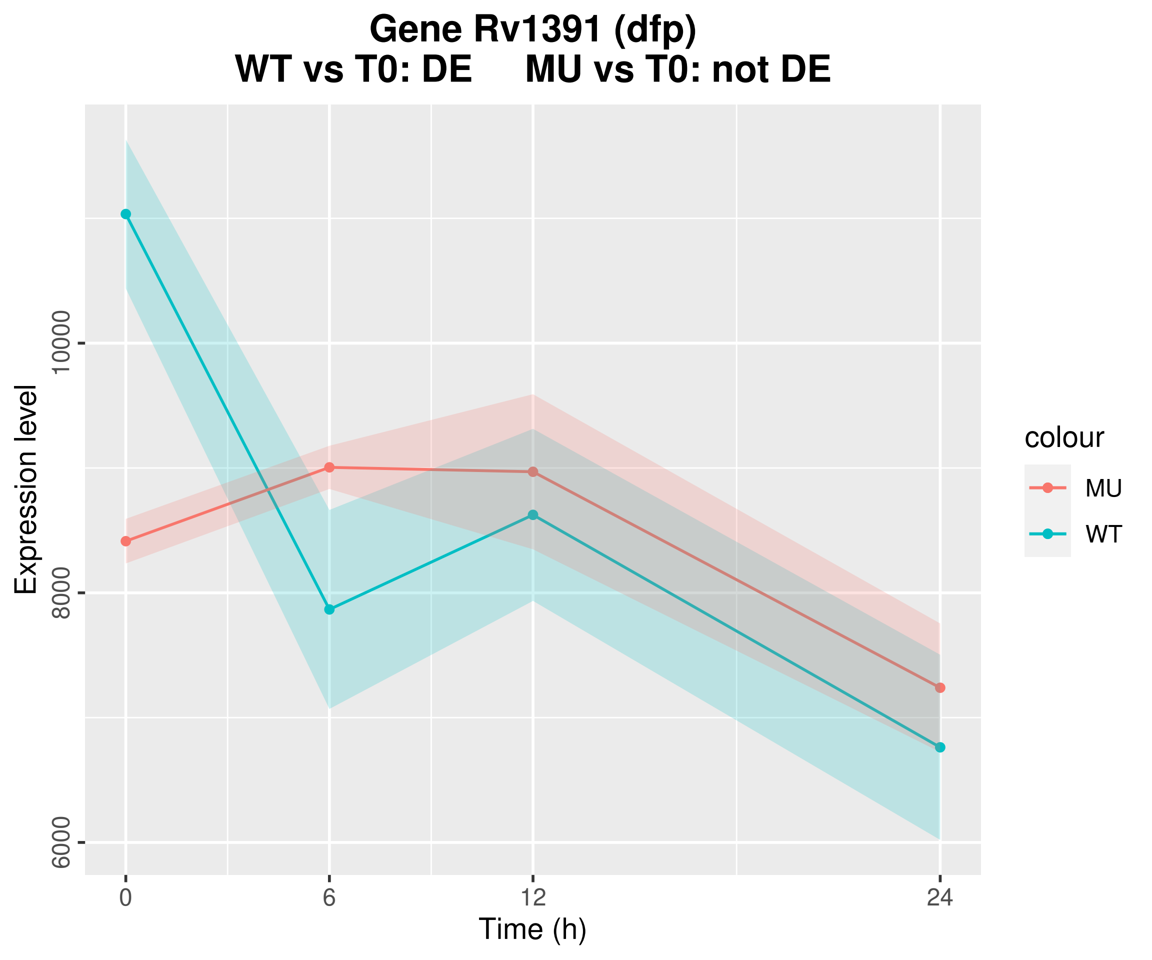

Gene Rv1391 (dfp)
WT vs T0: DE   MU vs T0: not DE
Expression level
10000
8000
6000
0
6
12
24
Time (h)
colour
MU
WT

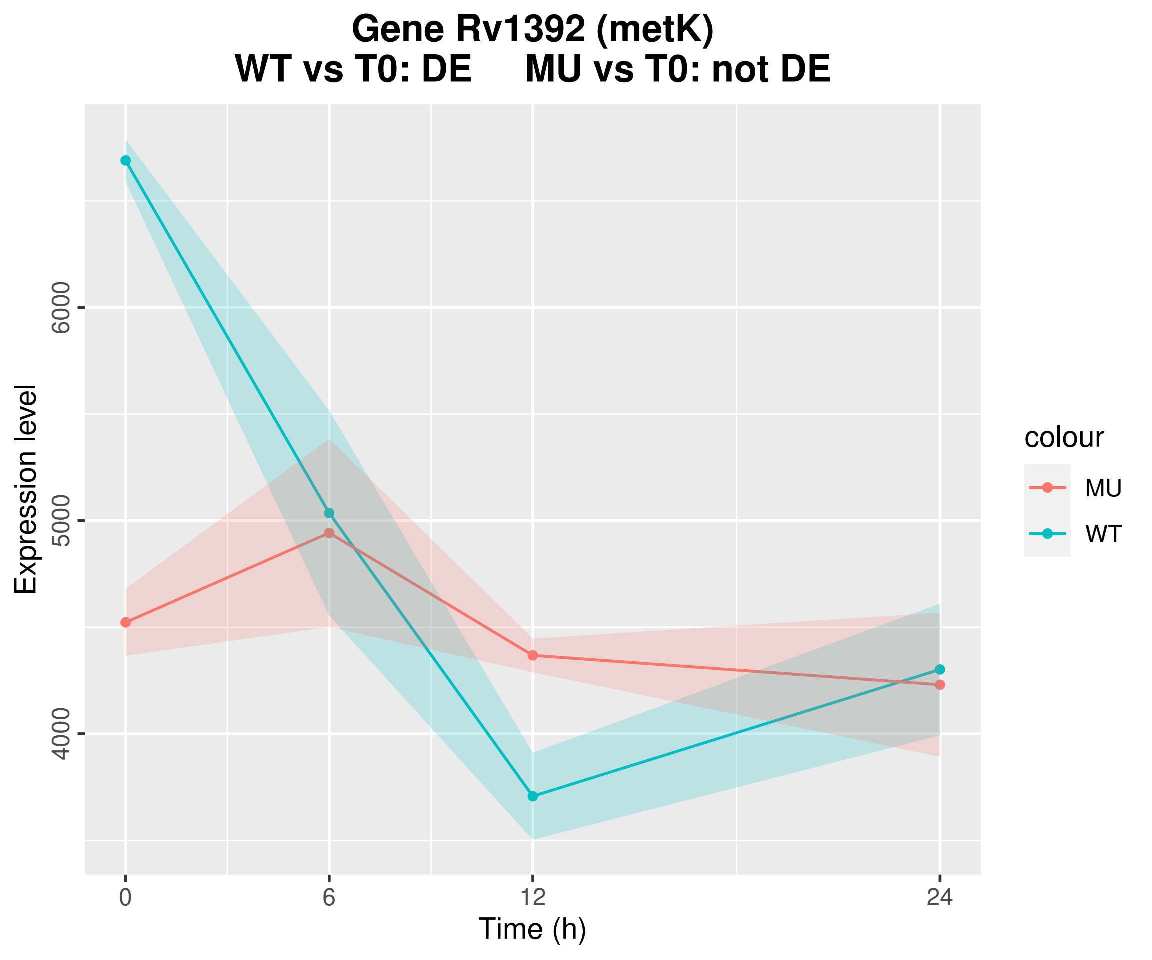
Gene Rv1392 (metK)
WT vs T0: DE   MU vs T0: not DE
Expression level
6000
5000
4000
0
6
12
24
Time (h)
colour
MU
WT

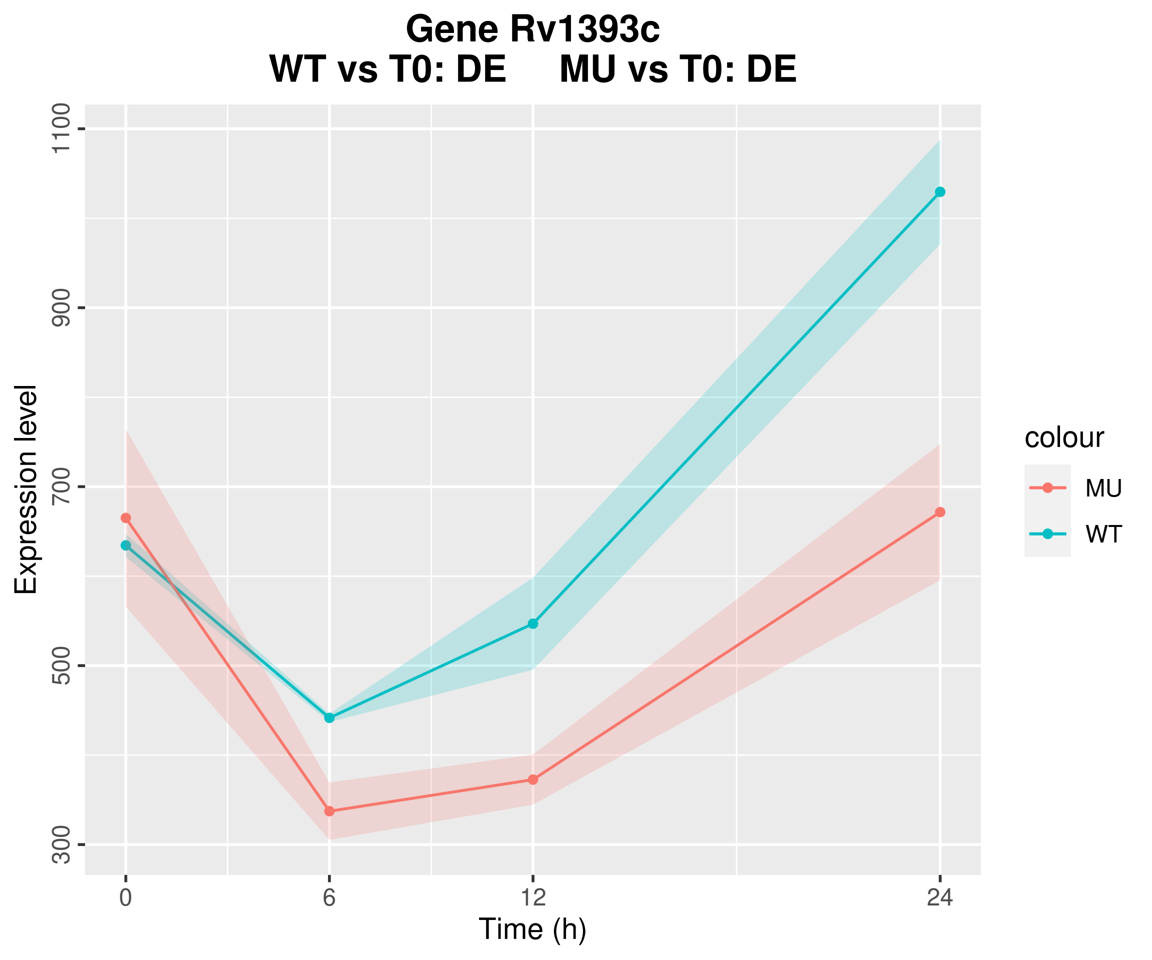

Gene Rv1393c
WT vs T0: DE   MU vs T0: DE
Expression level
Time (h)
1100
900
700
500
300
0
6
12
24
colour
MU
WT

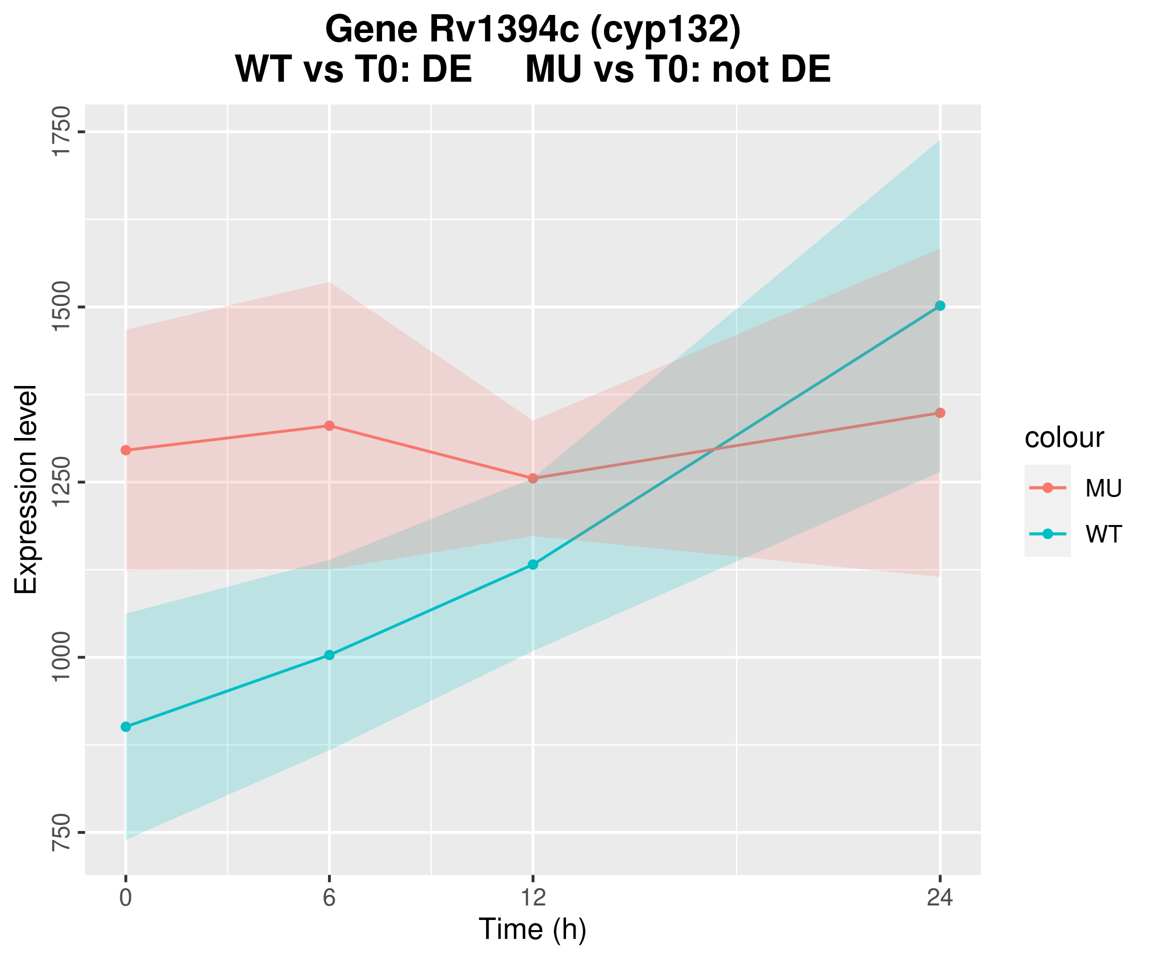
Gene Rv1394c (cyp132)
WT vs T0: DE   MU vs T0: not DE
Expression level
Time (h)
750
1000
1250
1500
1750
0
6
12
24
colour
MU
WT

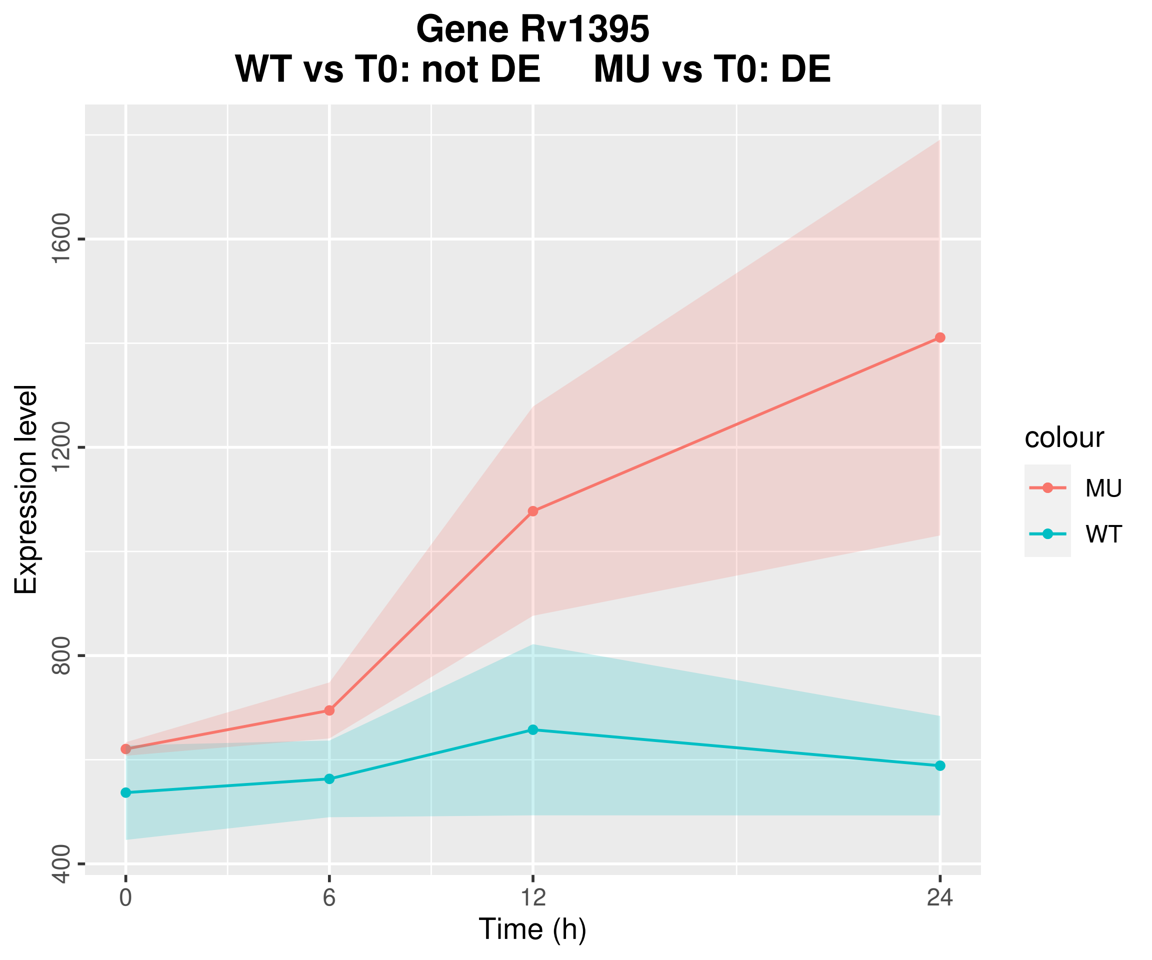
Gene Rv1395
WT vs T0: not DE MU vs T0: DE
Expression level
1600
1200
800
400
0
6
12
24
Time (h)
colour
MU
WT

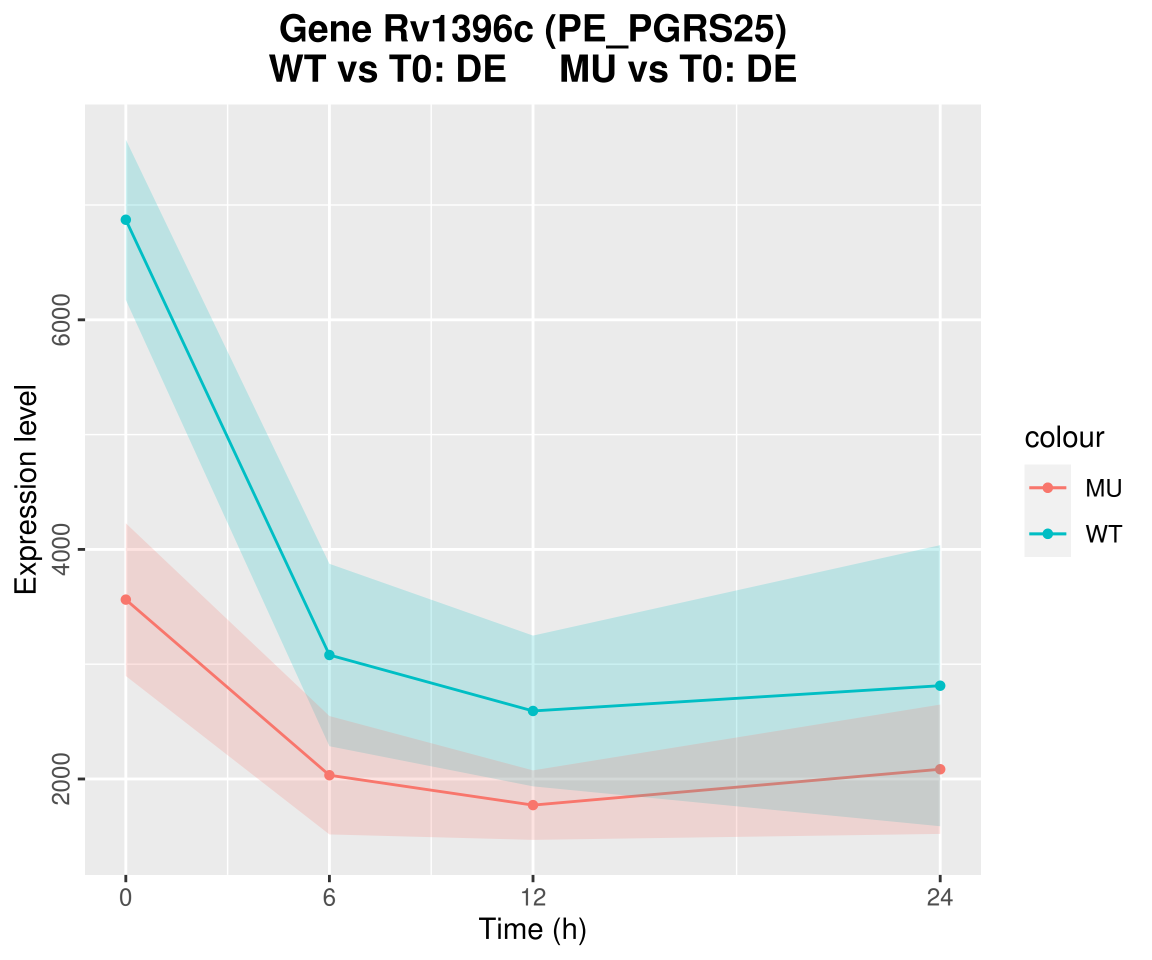

Gene Rv1396c (PE_PGRS25)
WT vs T0: DE MU vs T0: DE
Expression level
6000
4000
2000
0
6
12
24
Time (h)
colour
MU
WT

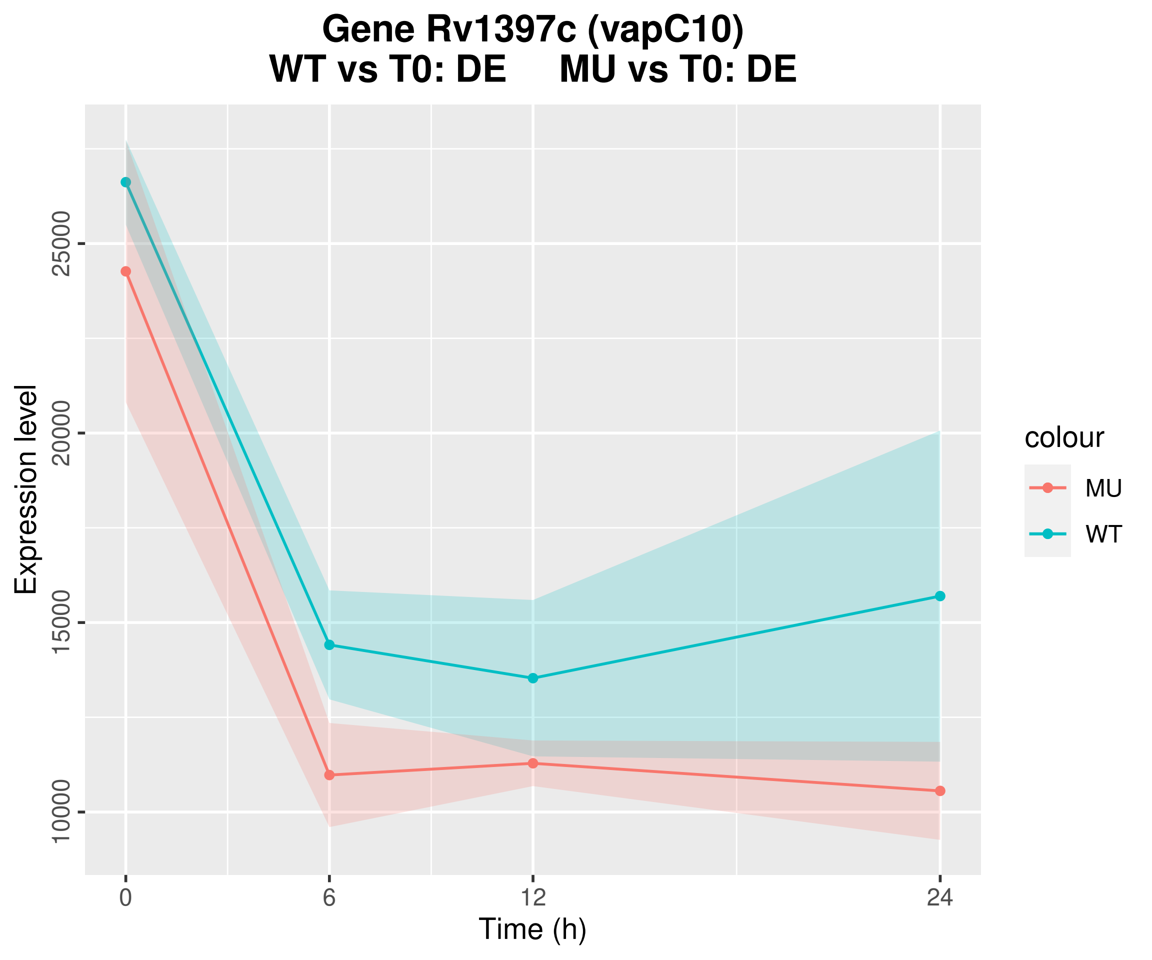
Gene Rv1397c (vapC10)
WT vs T0: DE   MU vs T0: DE
Expression level
25000
20000
15000
10000
0
6
12
24
Time (h)
colour
MU
WT

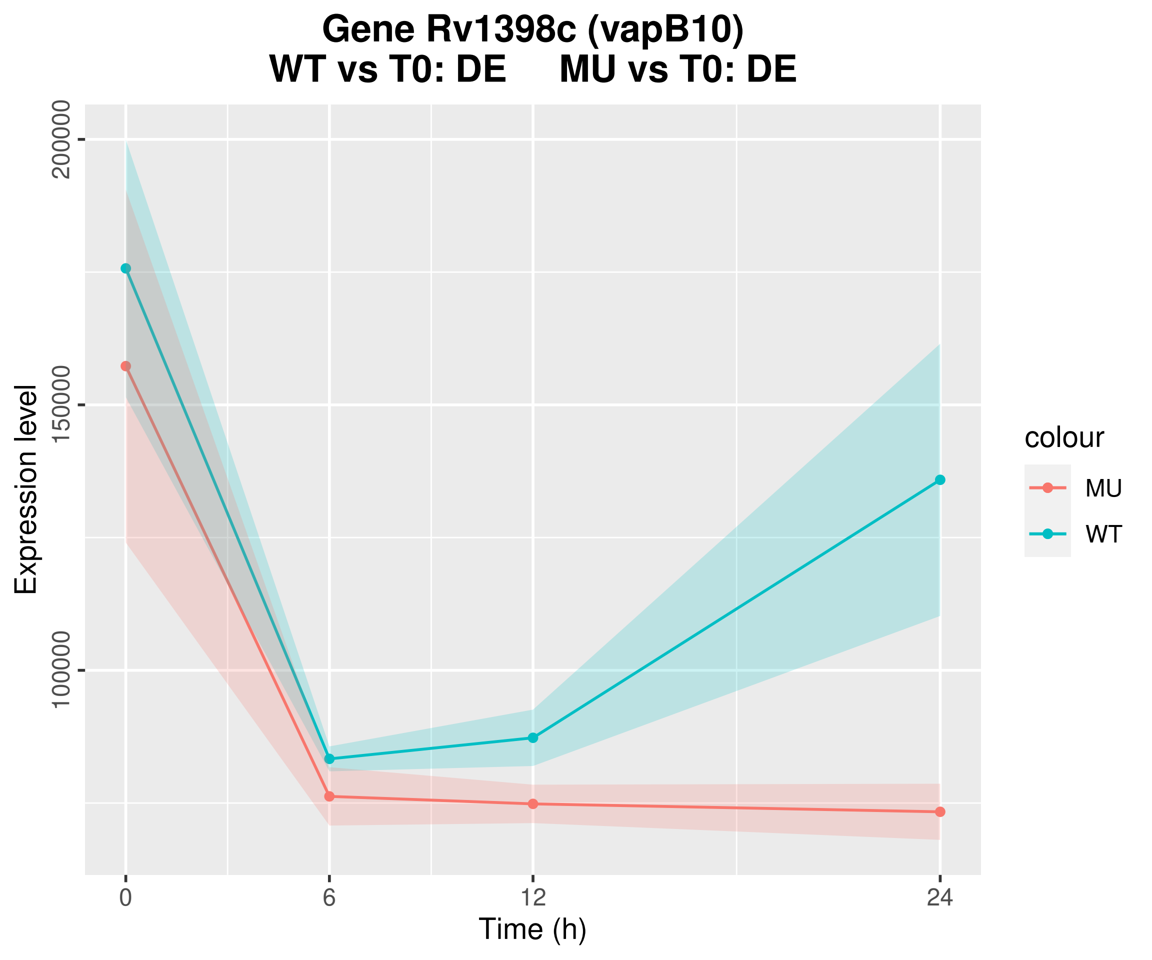
Gene Rv1398c (vapB10)
WT vs T0: DE   MU vs T0: DE
Expression level
200000
150000
100000
0
6
12
24
Time (h)
colour
MU
WT

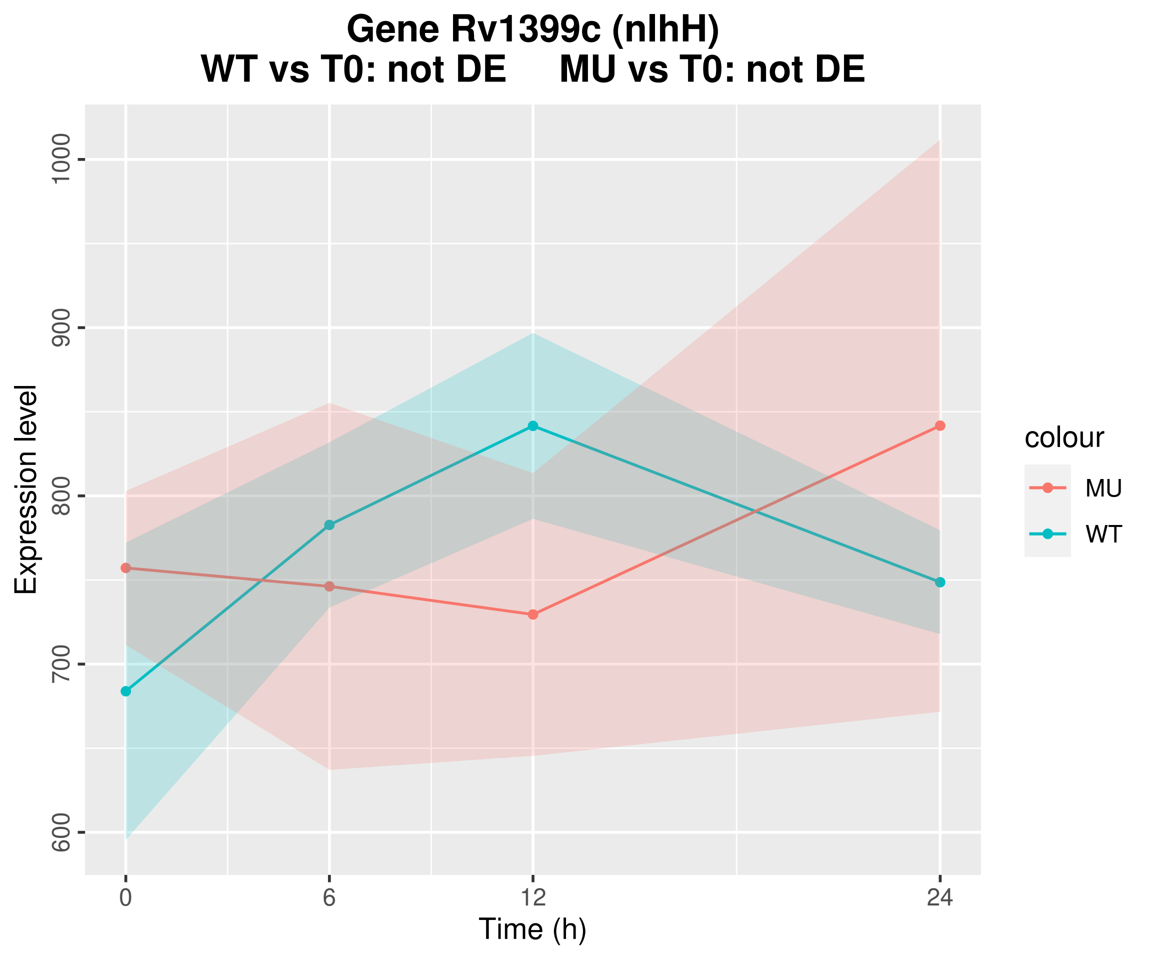
Gene Rv1399c (nlhH)
WT vs T0: not DE   MU vs T0: not DE
Expression level
1000
900
800
700
600
0
6
12
24
Time (h)
colour
MU
WT

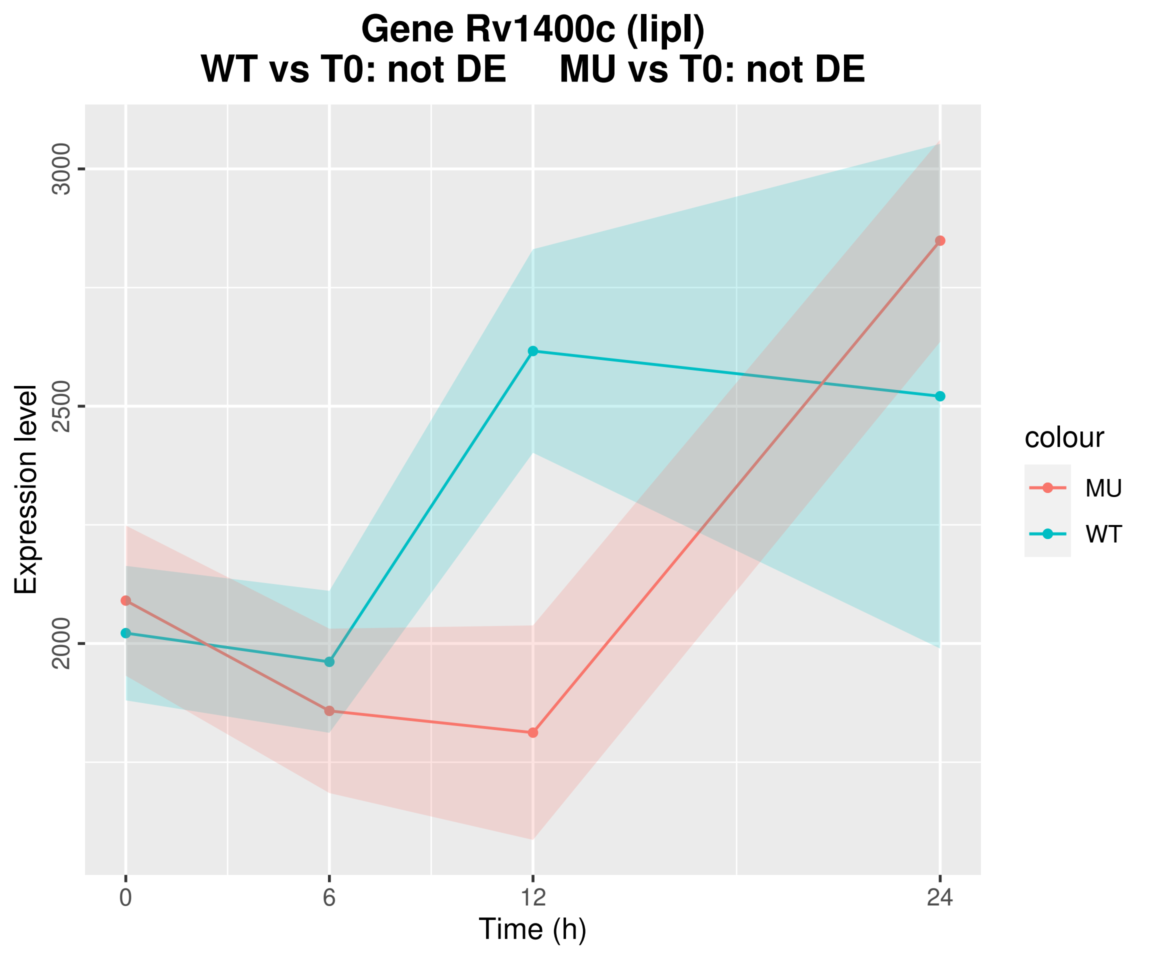

Gene Rv1400c (lipI)
WT vs T0: not DE   MU vs T0: not DE
3000
2500
2000
Expression level
0
6
12
24
Time (h)
colour
MU
WT

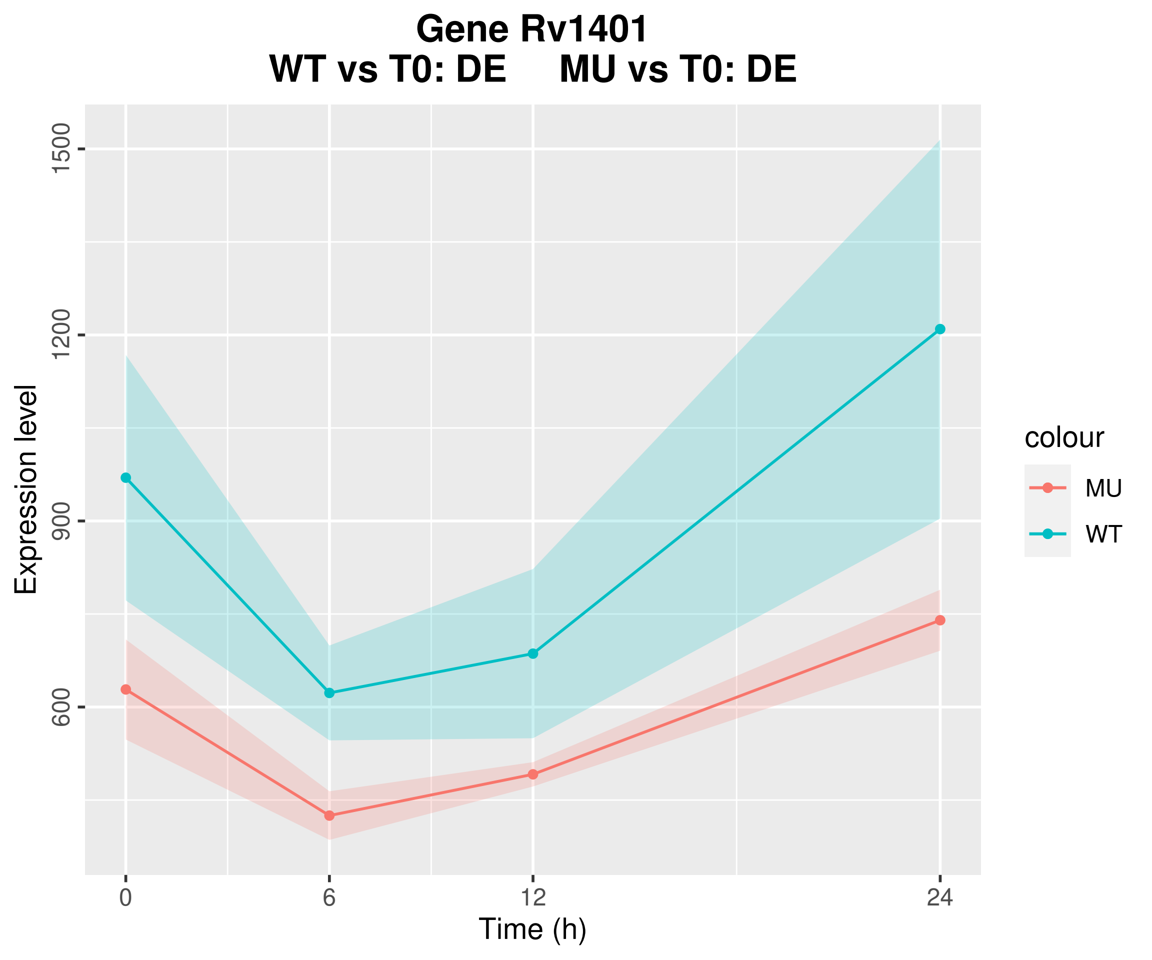

Gene Rv1401
WT vs T0: DE   MU vs T0: DE
Expression level
1500
1200
900
600
0
6
12
24
Time (h)
colour
MU
WT

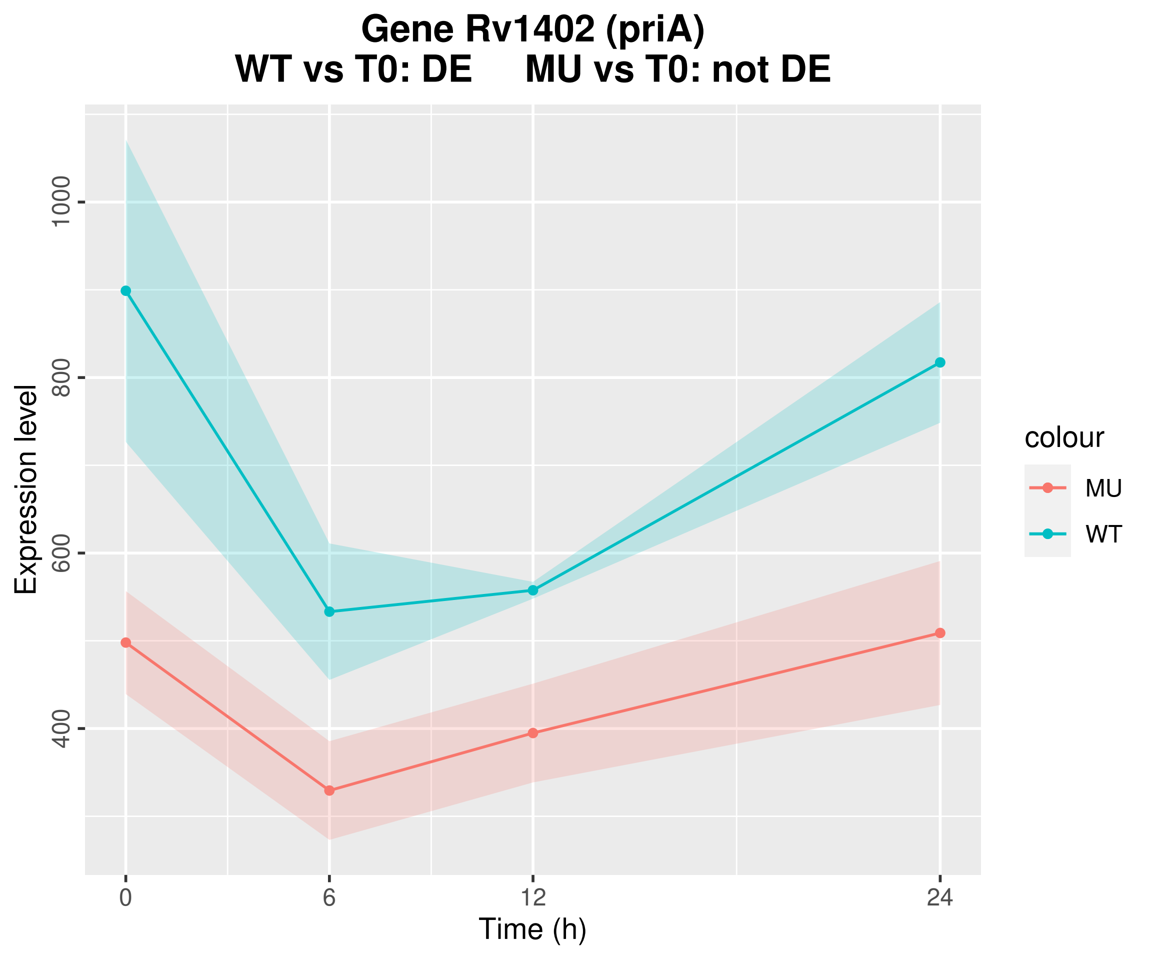
Gene Rv1402 (priA)
WT vs T0: DE   MU vs T0: not DE
Expression level
1000
800
600
400
0
6
12
24
Time (h)
colour
MU
WT

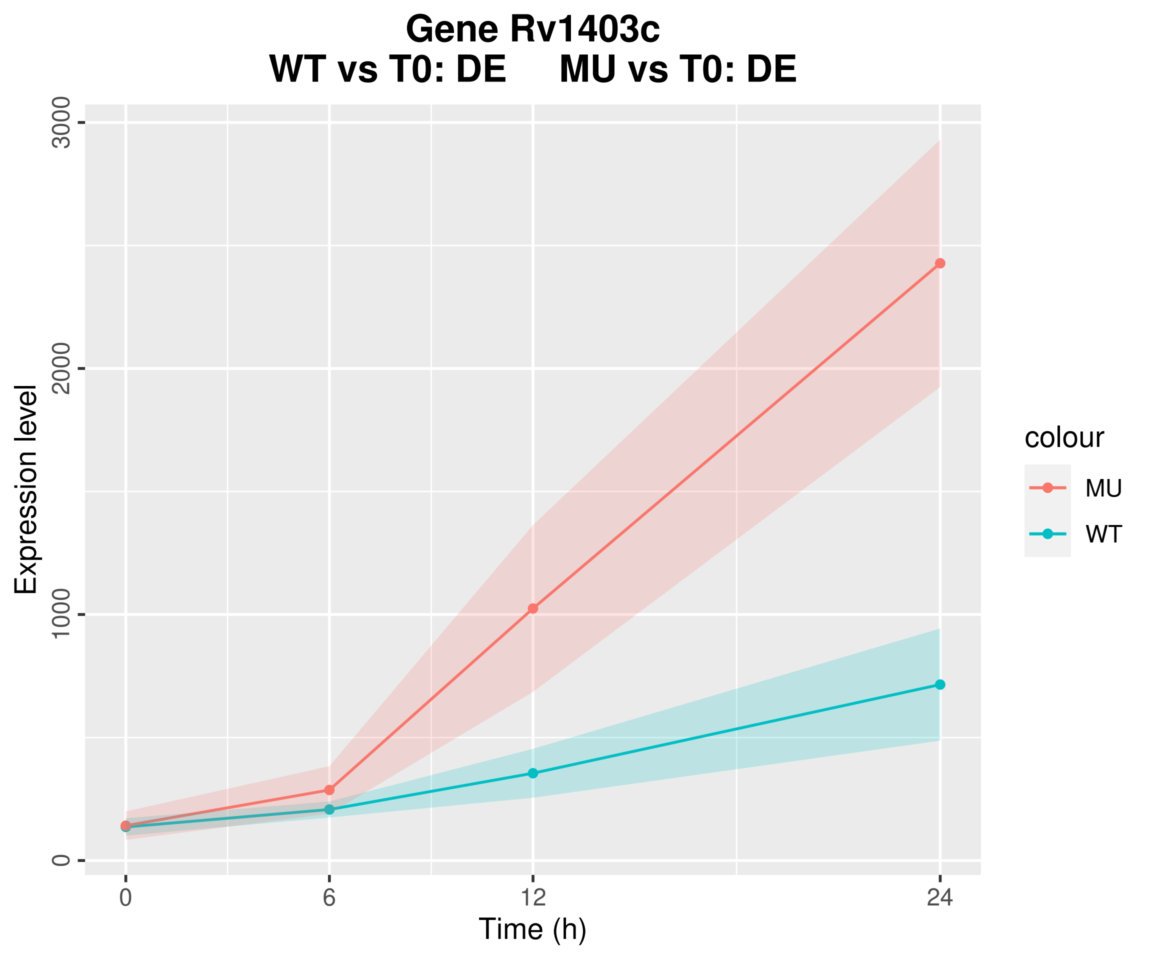
Gene Rv1403c
WT vs T0: DE MU vs T0: DE
Expression level
3000
2000
1000
0
0
6
12
24
Time (h)
colour
MU
WT

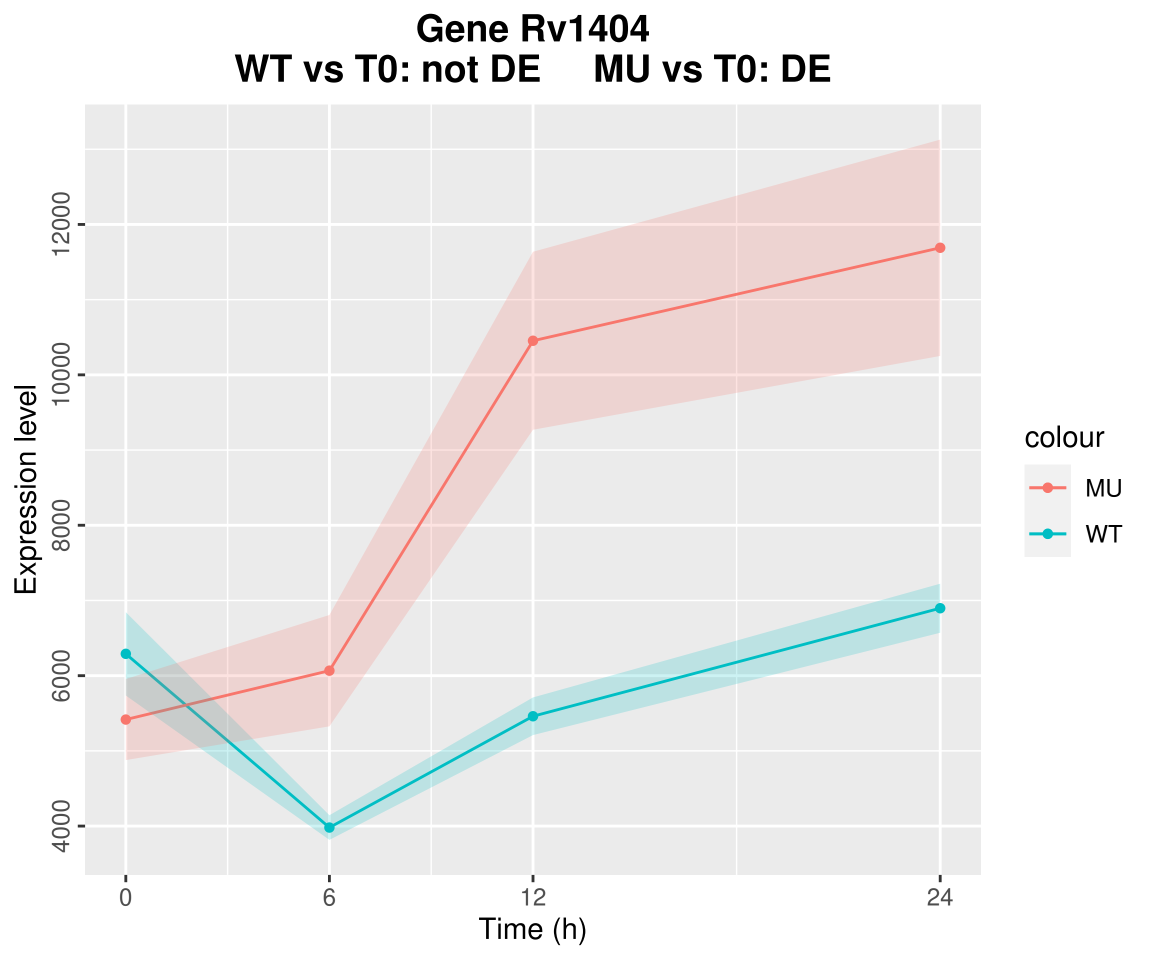
Gene Rv1404
WT vs T0: not DE   MU vs T0: DE
Expression level
12000
10000
8000
6000
4000
0
6
12
24
Time (h)
colour
MU
WT

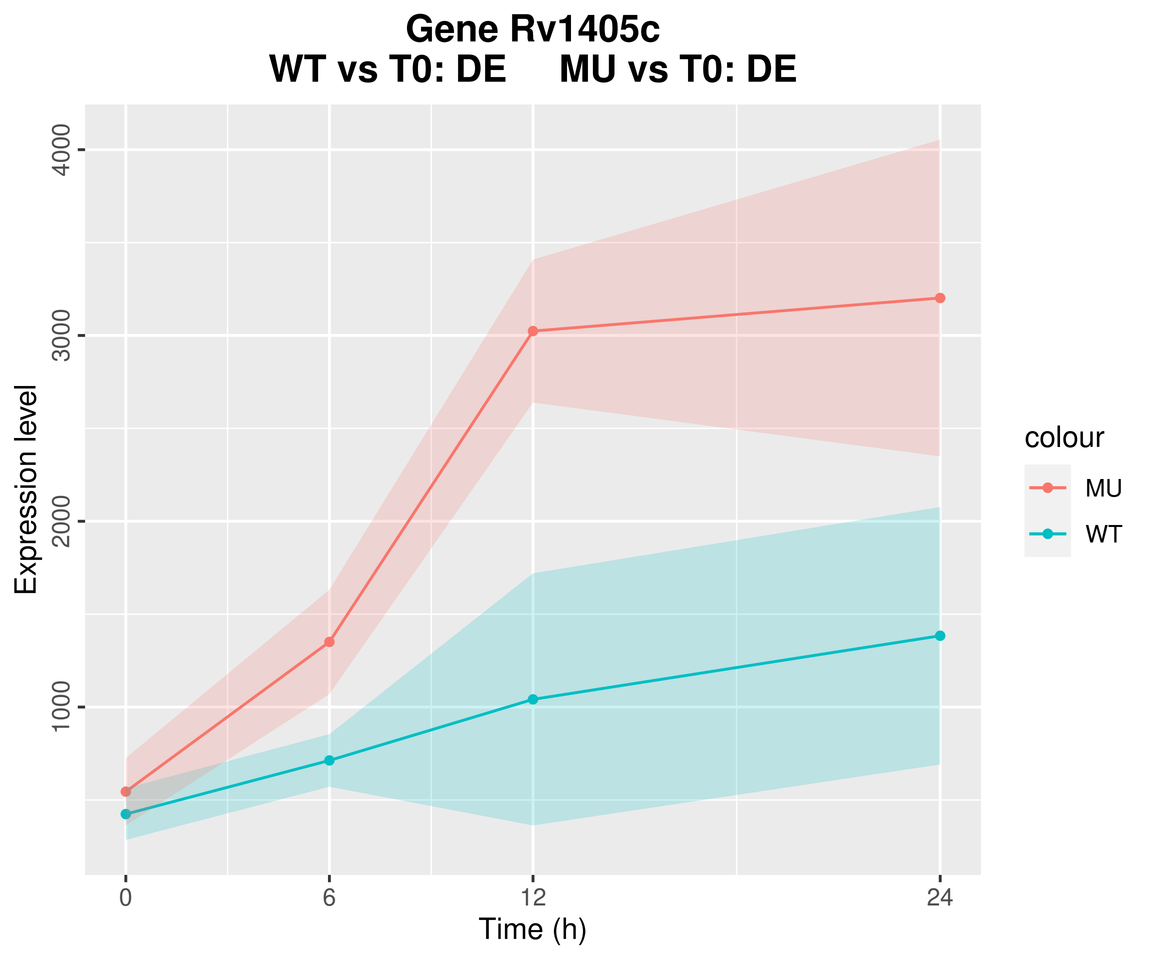
Gene Rv1405c
WT vs T0: DE   MU vs T0: DE
Expression level
4000
3000
2000
1000
0
6
12
24
Time (h)
colour
MU
WT

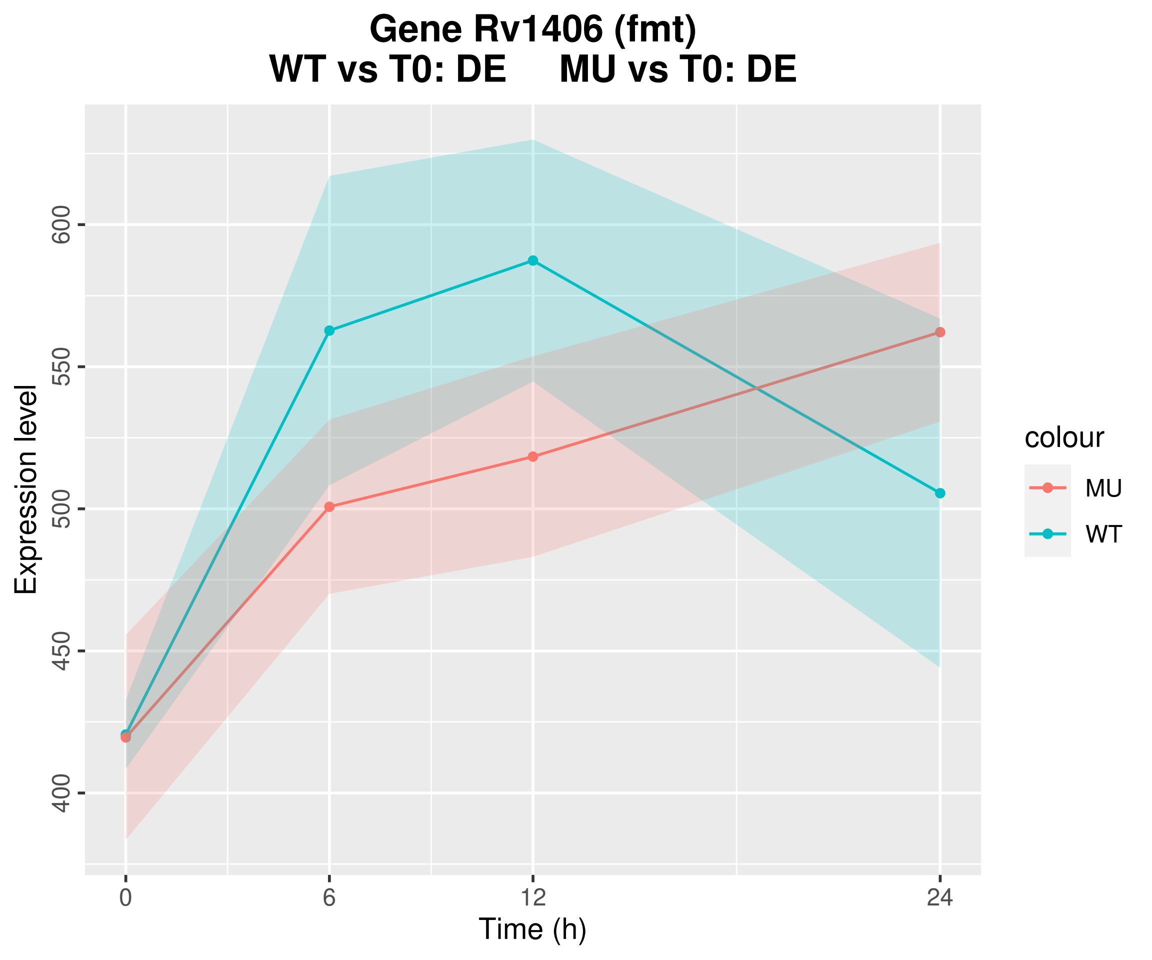
Gene Rv1406 (fmt)
WT vs T0: DE   MU vs T0: DE
Expression level
600
550
500
450
400
0
6
12
24
Time (h)
colour
MU
WT

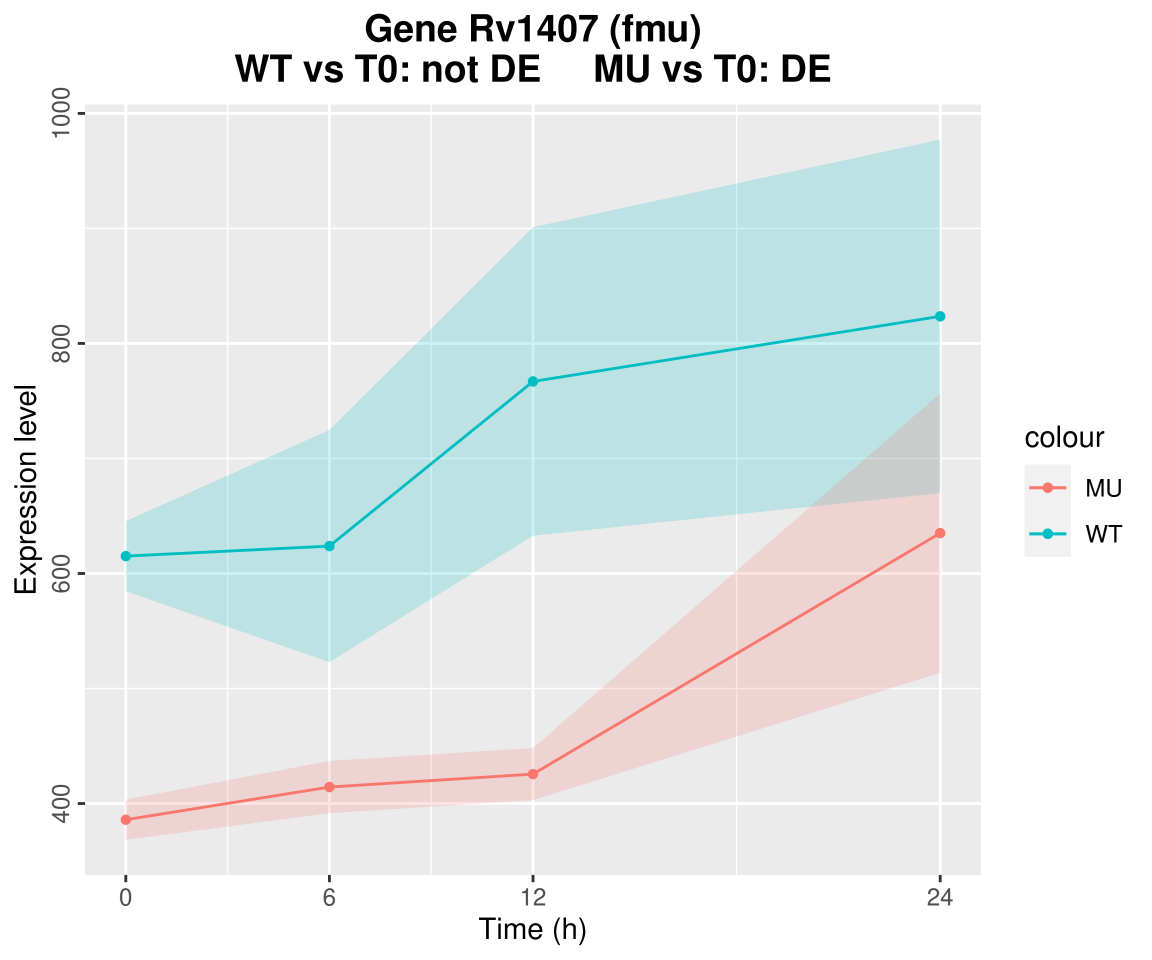

Gene Rv1407 (fmu)
WT vs T0: not DE   MU vs T0: DE
1000
800
600
400
Expression level
0
6
12
24
Time (h)
colour
MU
WT

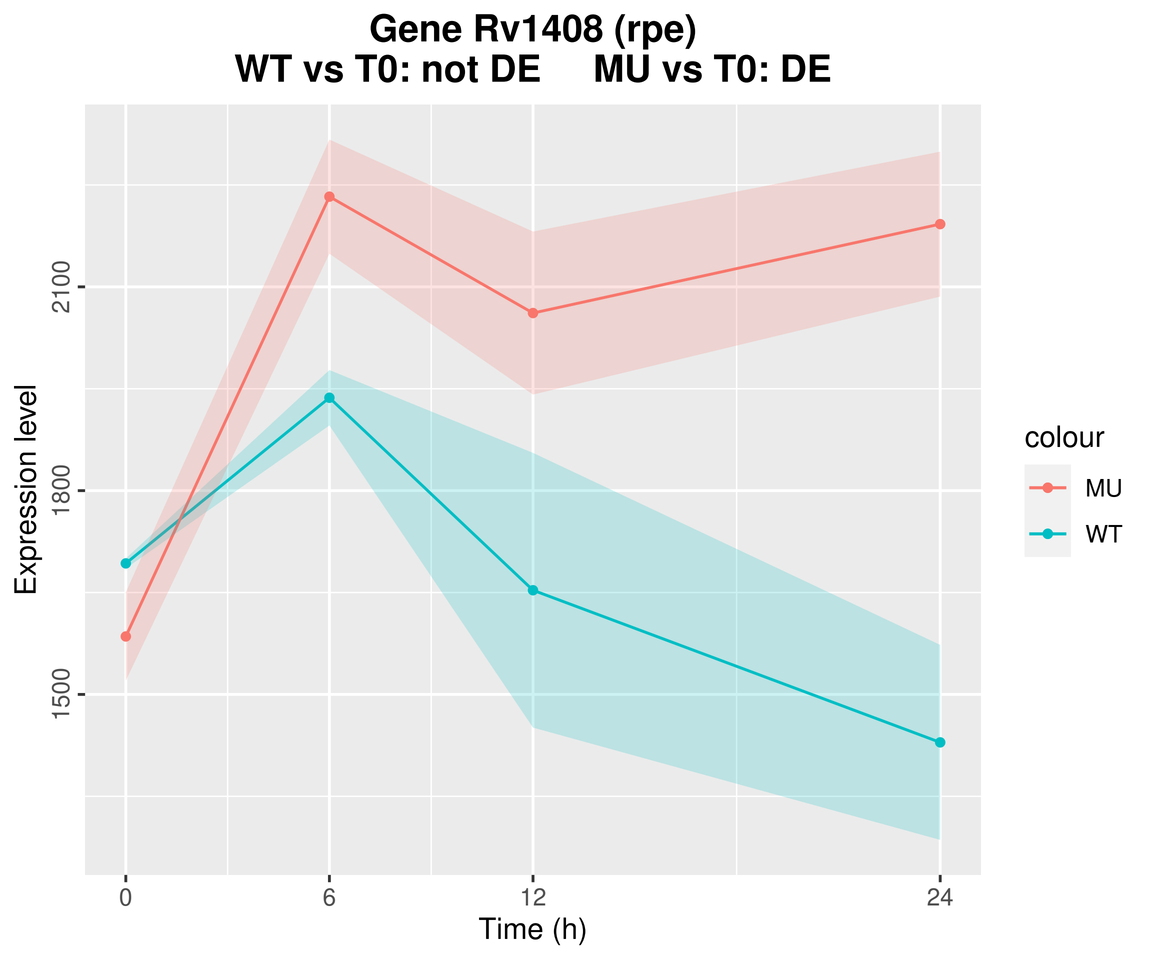
Gene Rv1408 (rpe)
WT vs T0: not DE   MU vs T0: DE
Expression level
2100
1800
1500
0
6
12
24
Time (h)
colour
MU
WT

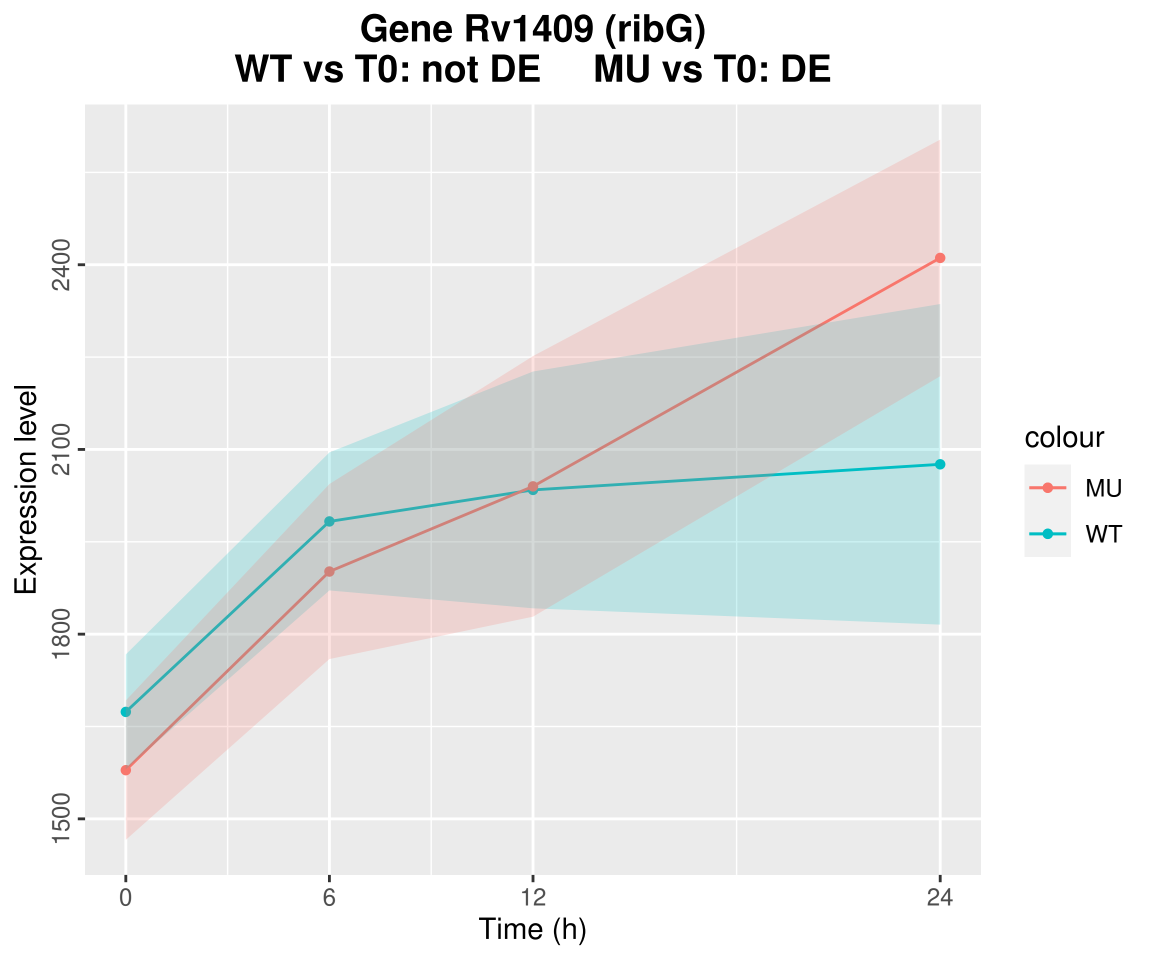

Gene Rv1409 (ribG)
WT vs T0: not DE   MU vs T0: DE
Expression level
1500
1800
2100
2400
Time (h)
0
6
12
24
colour
MU
WT

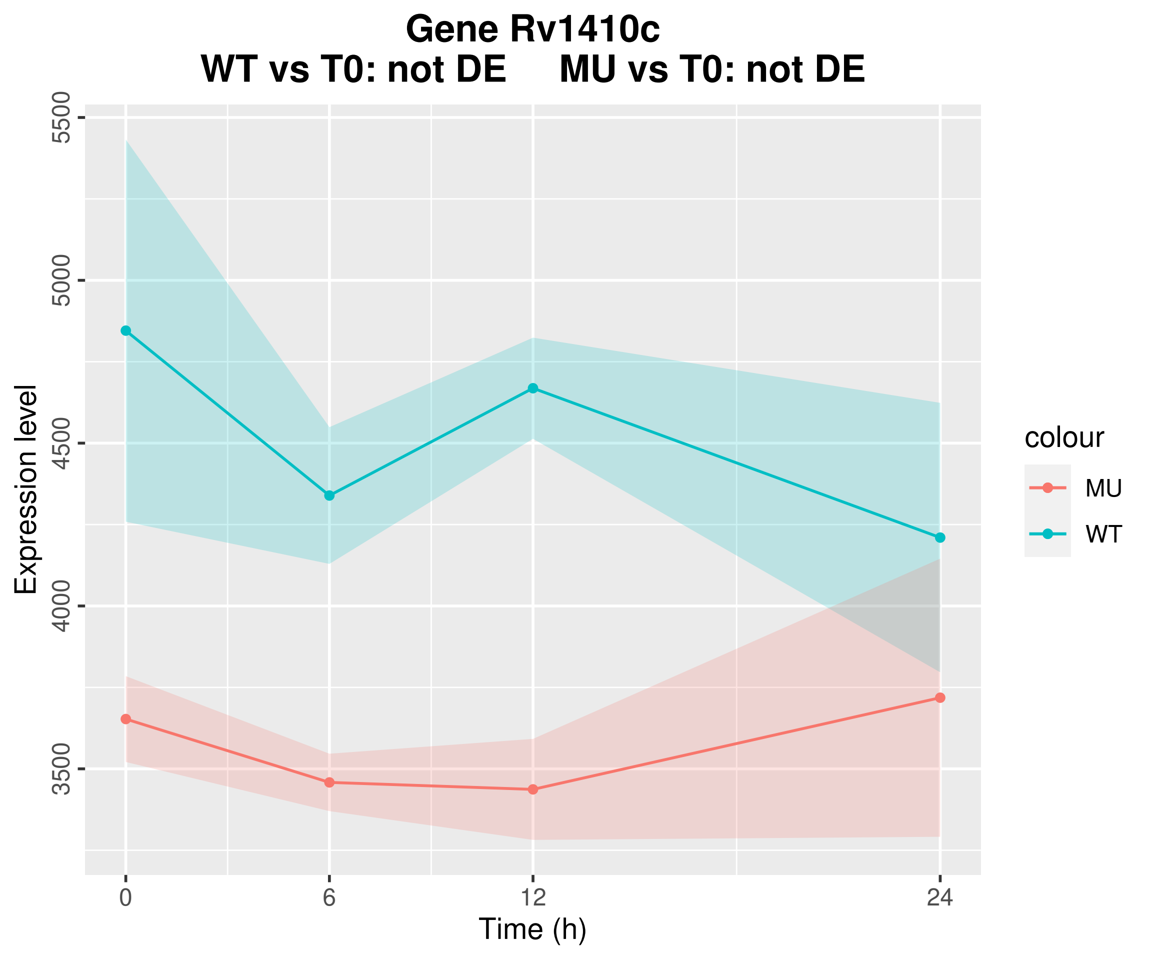
Gene Rv1410c
WT vs T0: not DE   MU vs T0: not DE
Expression level
5500
5000
4500
4000
3500
0
6
12
24
Time (h)
colour
MU
WT

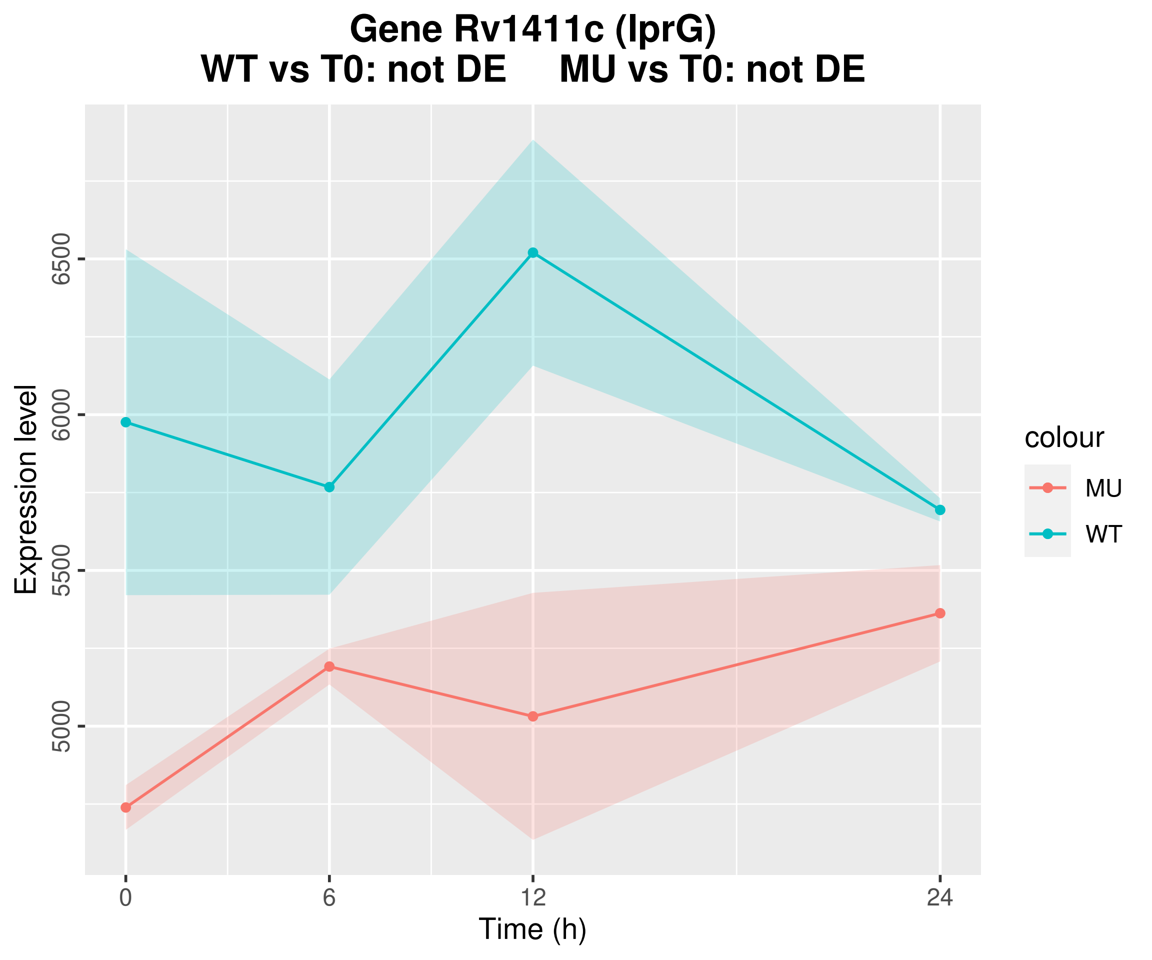
Gene Rv1411c (lprG)
WT vs T0: not DE   MU vs T0: not DE
Expression level
6500
6000
5500
5000
0
6
12
24
Time (h)
colour
MU
WT

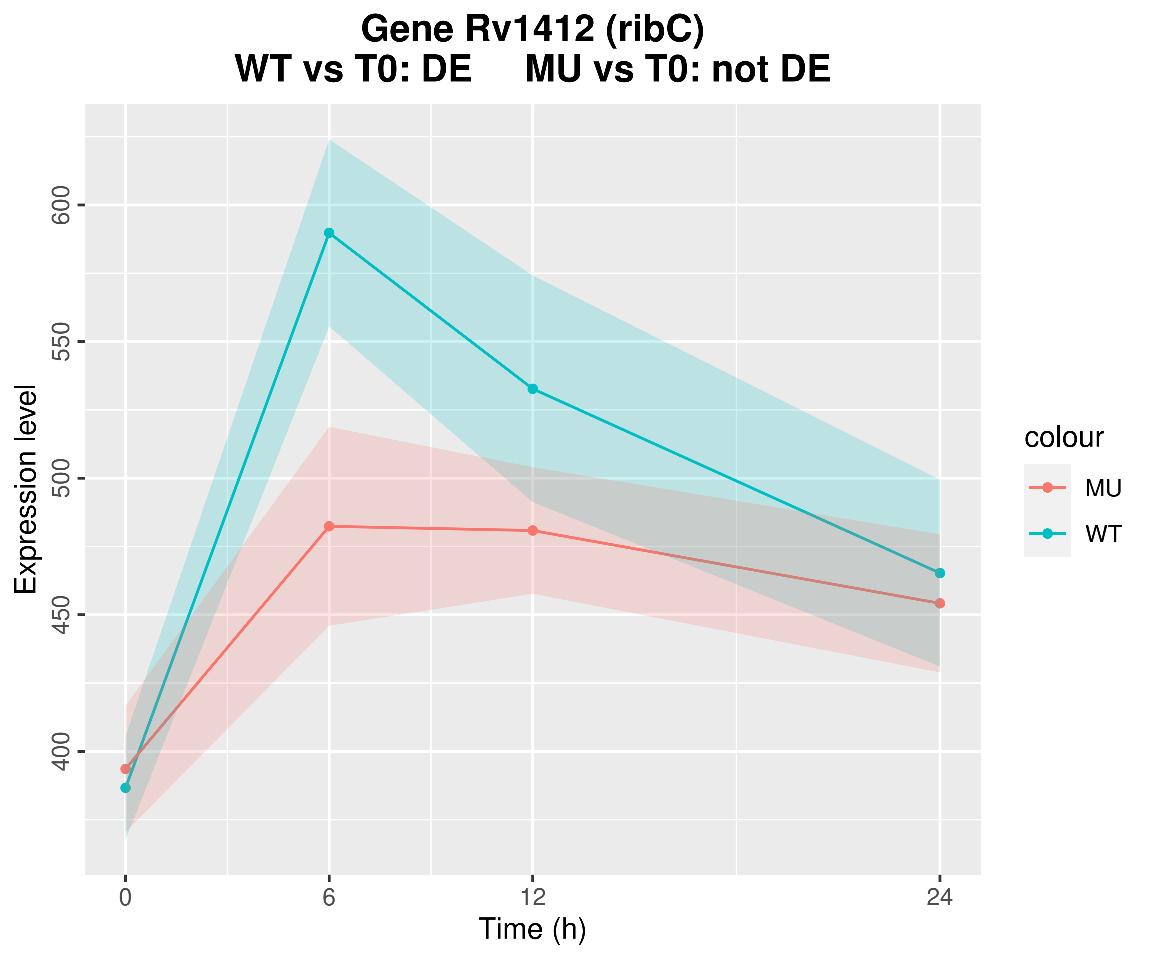
Gene Rv1412 (ribC)
WT vs T0: DE   MU vs T0: not DE
Expression level
600
550
500
450
400
0
6
12
24
Time (h)
colour
MU
WT

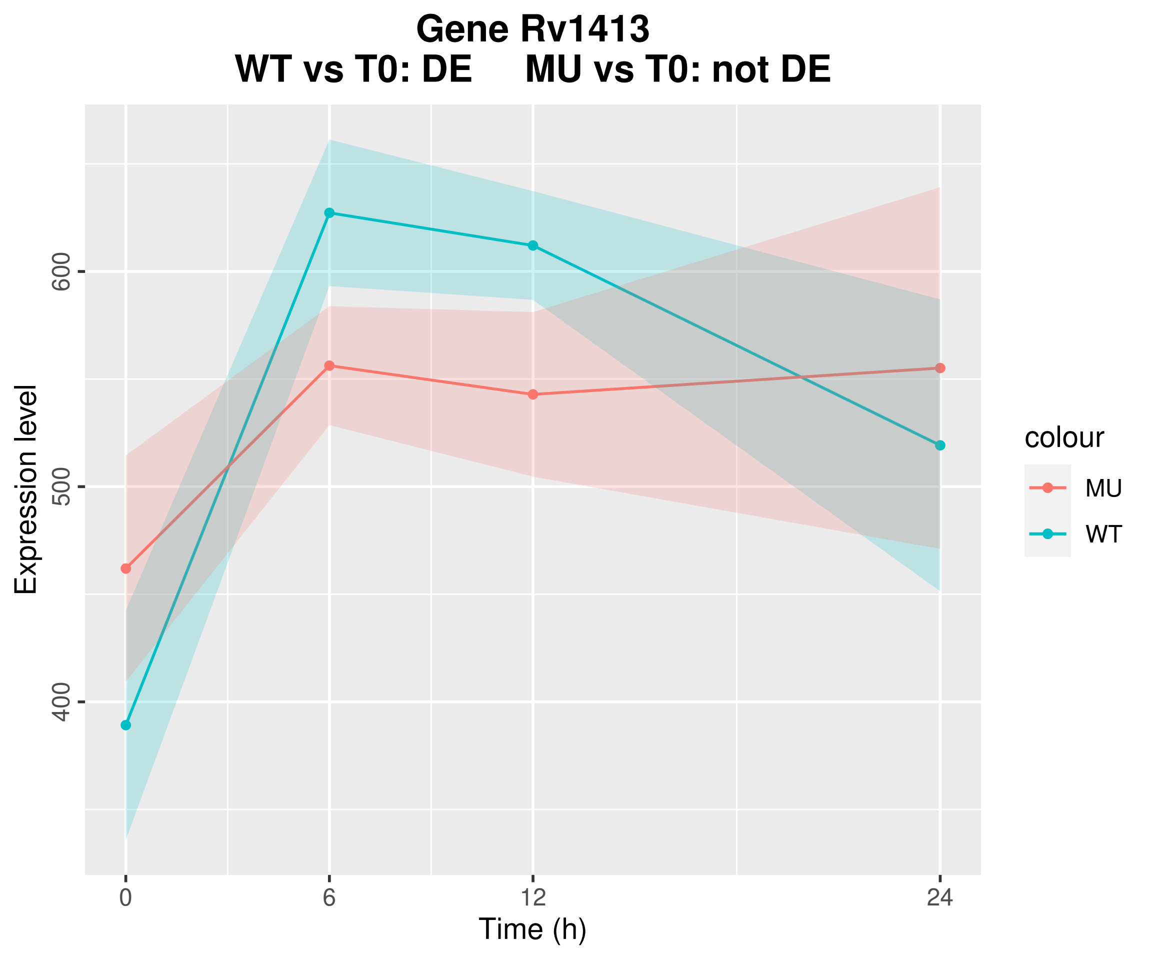
Gene Rv1413
WT vs T0: DE   MU vs T0: not DE
600
500
400
Expression level
0
6
12
24
Time (h)
colour
MU
WT

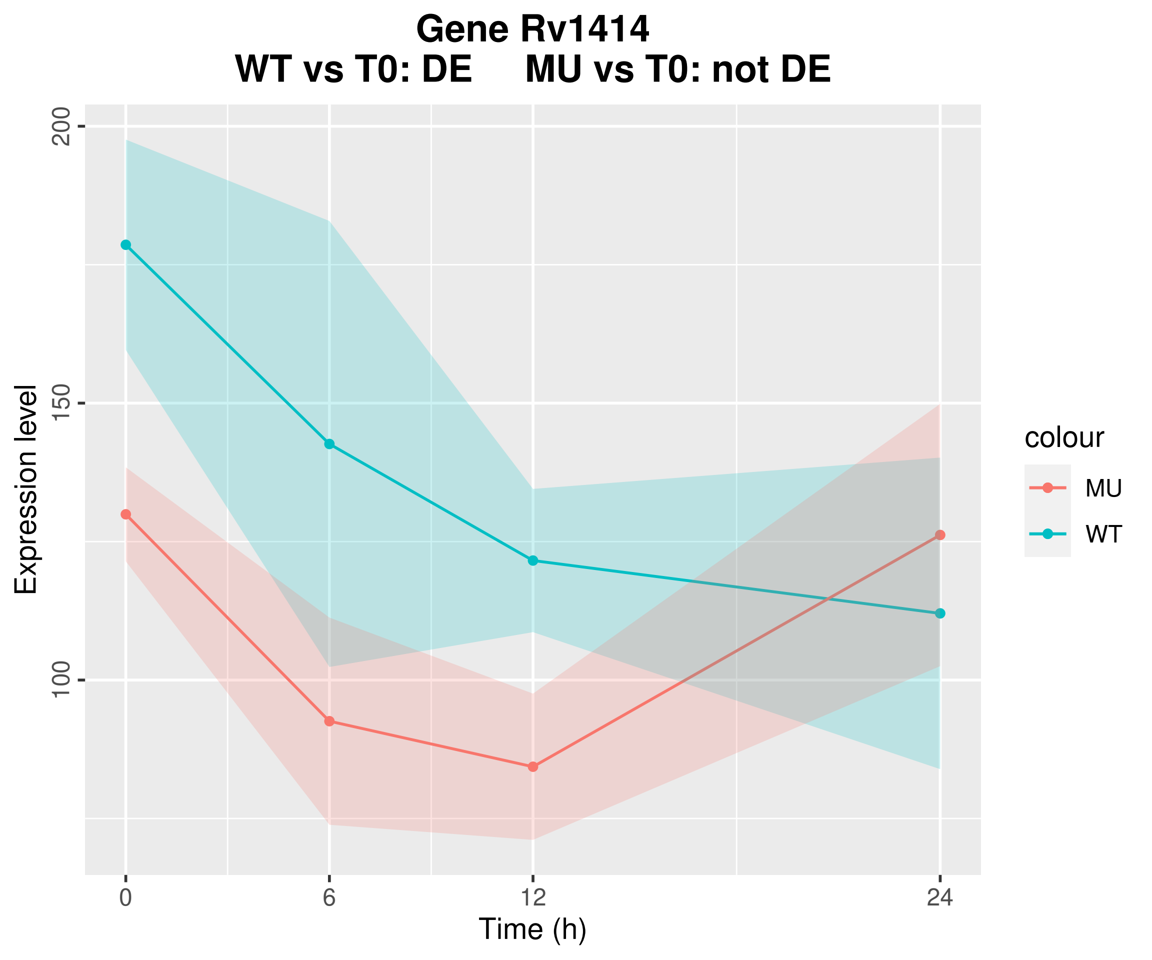
Gene Rv1414
WT vs T0: DE MU vs T0: not DE
200
150
100
Expression level
0
6
12
24
Time (h)
colour
MU
WT

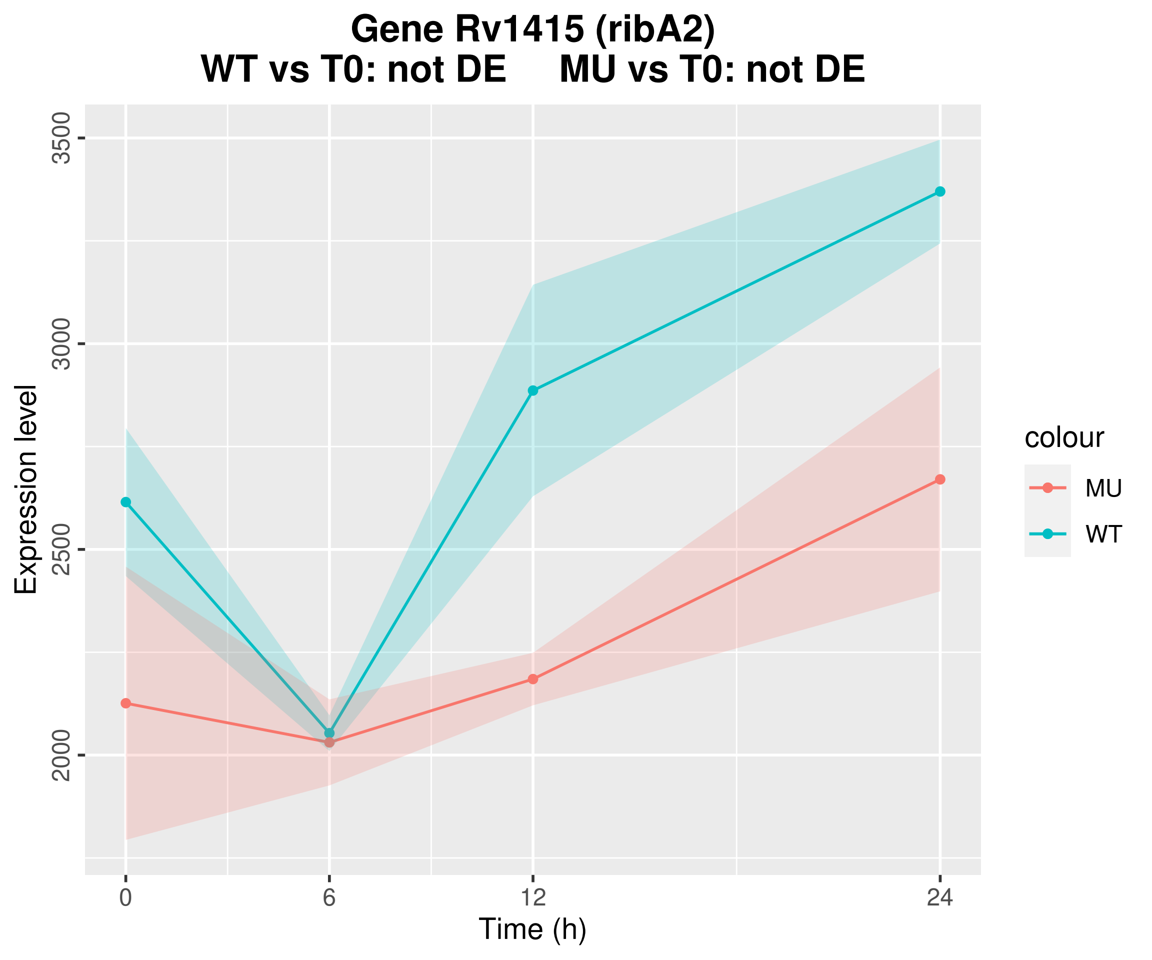
Gene Rv1415 (ribA2)
WT vs T0: not DE   MU vs T0: not DE
Expression level
3500
3000
2500
2000
Time (h)
0
6
12
24
colour
MU
WT

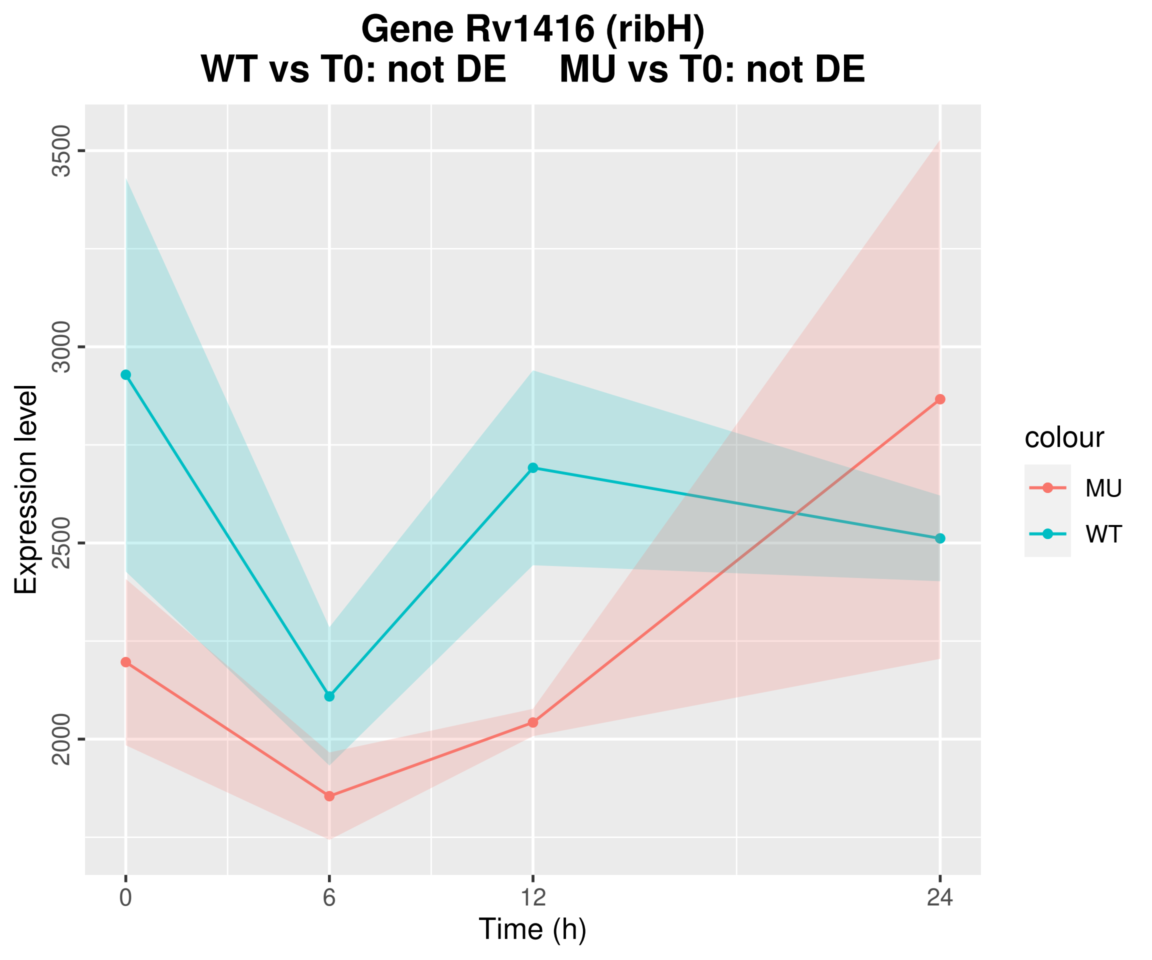
Gene Rv1416 (ribH)
WT vs T0: not DE   MU vs T0: not DE
Expression level
3500
3000
2500
2000
0
6
12
24
Time (h)
colour
MU
WT

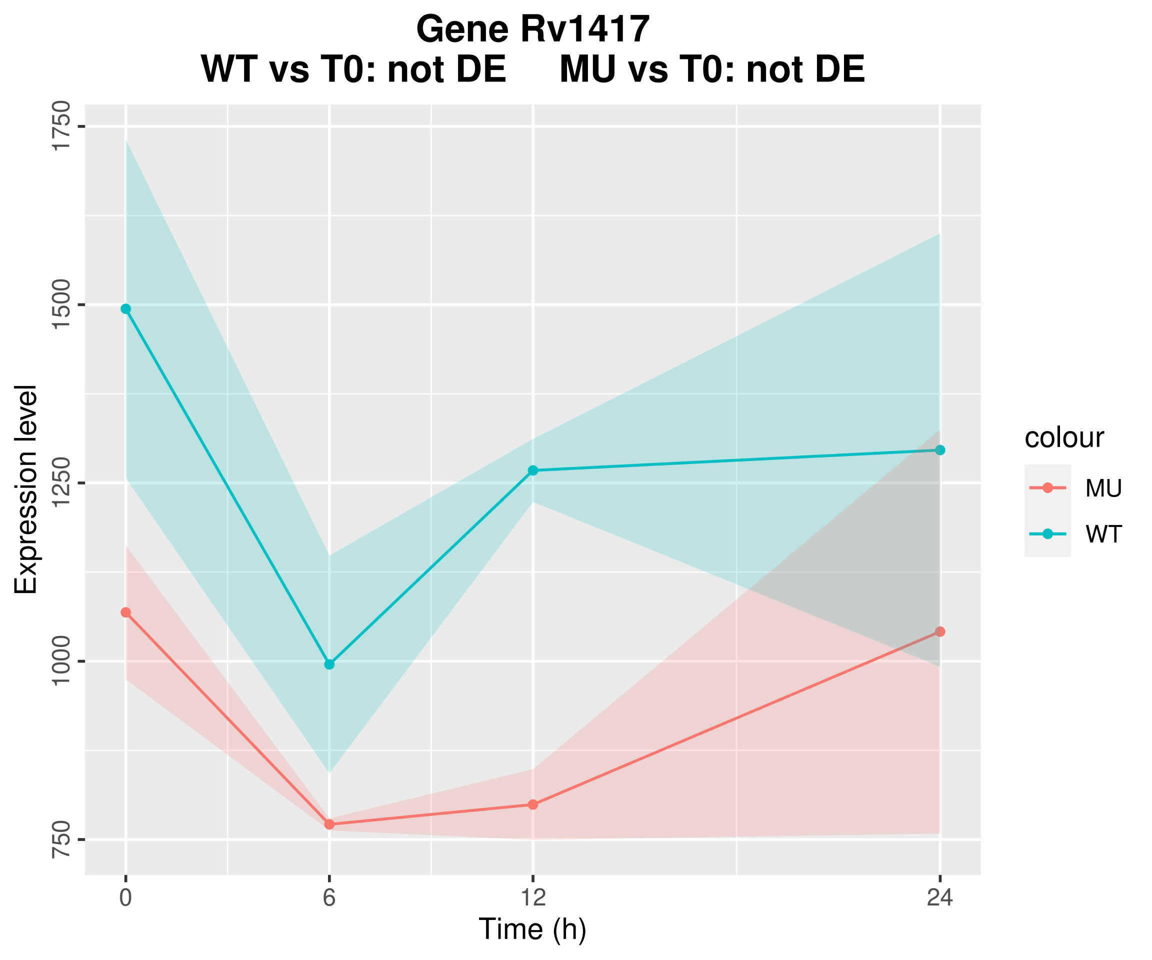
Gene Rv1417
WT vs T0: not DE   MU vs T0: not DE
Expression level
1750
1500
1250
1000
750
0
6
12
24
Time (h)
colour
MU
WT

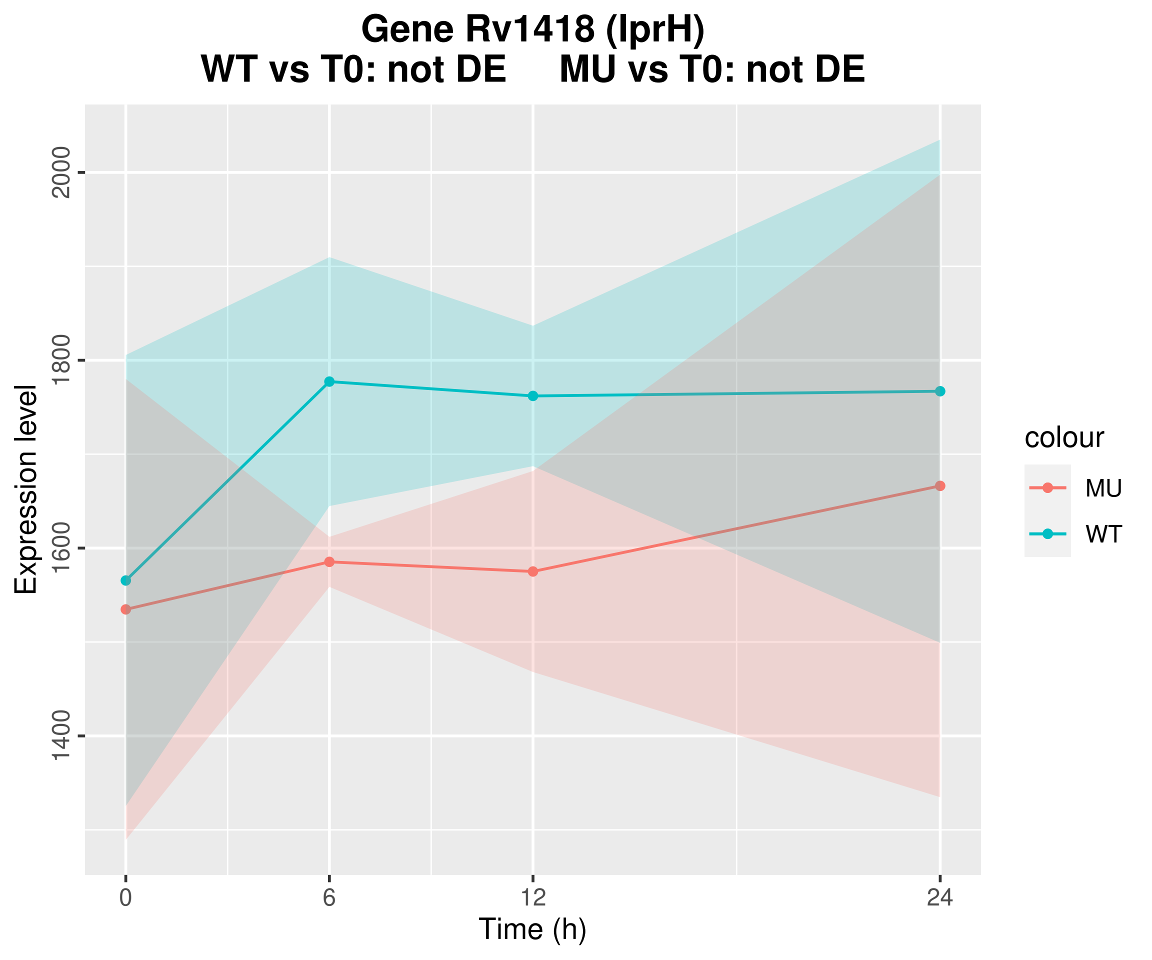

Gene Rv1418 (lprH)
WT vs T0: not DE MU vs T0: not DE
2000
1800
1600
1400
Expression level
0
6
12
24
Time (h)
colour
MU
WT

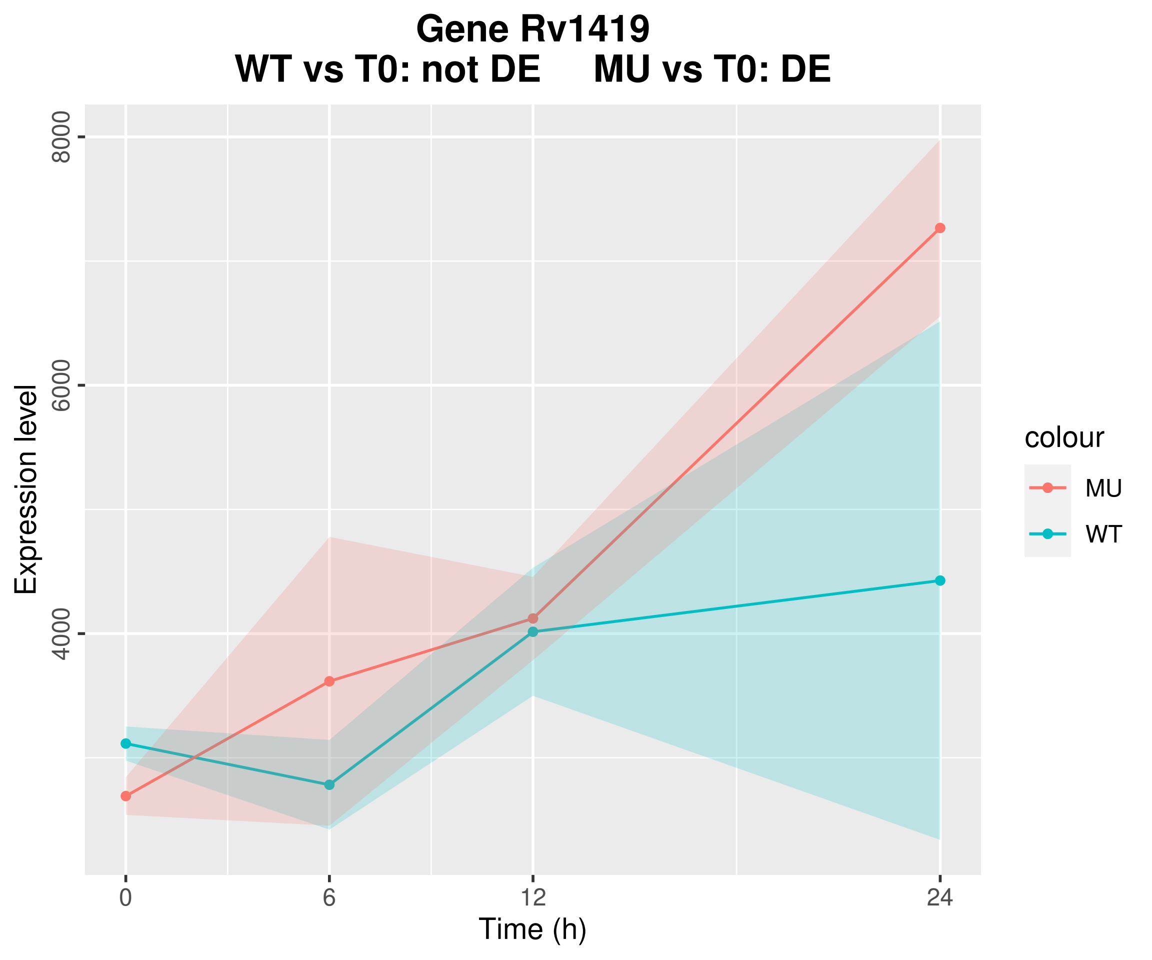
Gene Rv1419
WT vs T0: not DE   MU vs T0: DE
Expression level
8000
6000
4000
0
6
12
24
Time (h)
colour
MU
WT

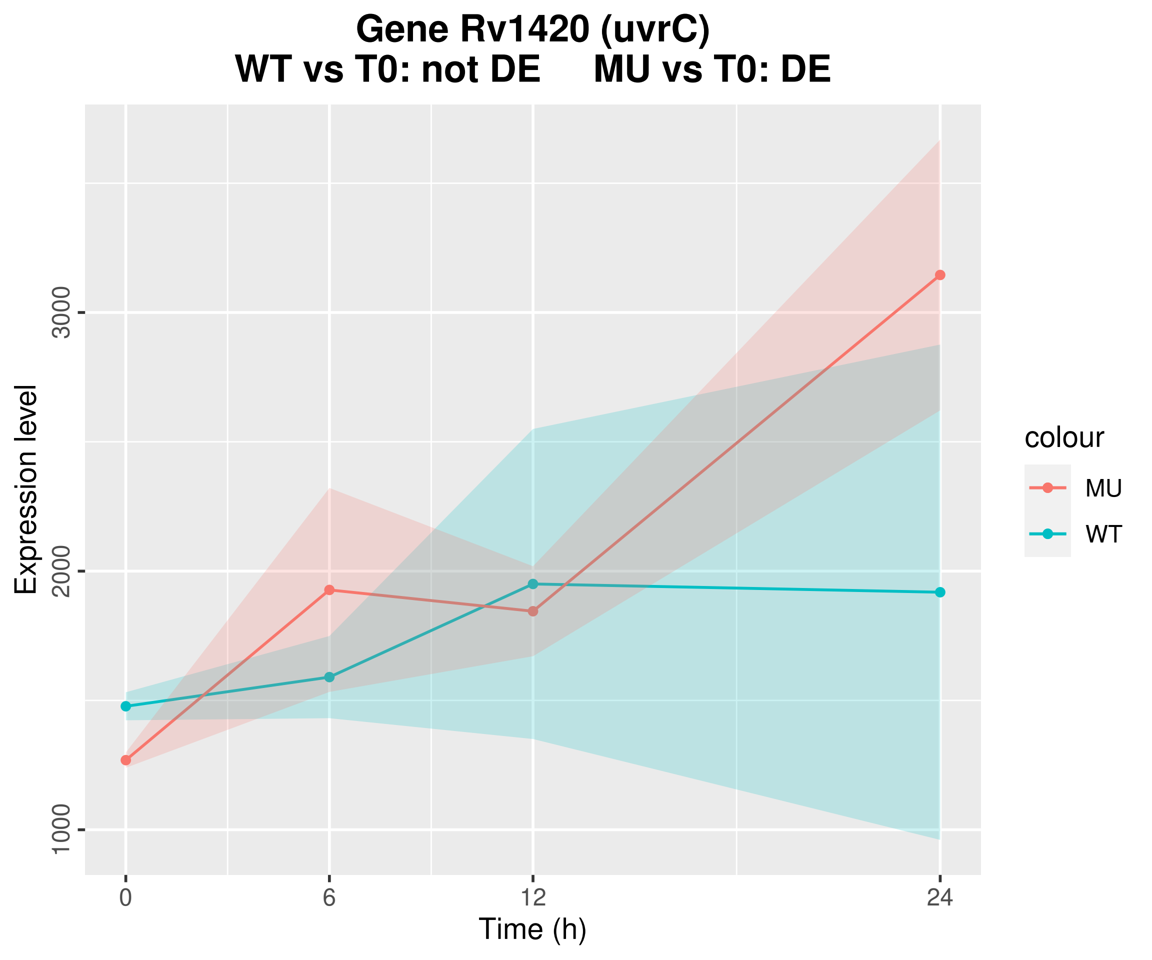

Gene Rv1420 (uvrC)
WT vs T0: not DE   MU vs T0: DE
Expression level
1000
2000
3000
0
6
12
24
Time (h)
colour
MU
WT

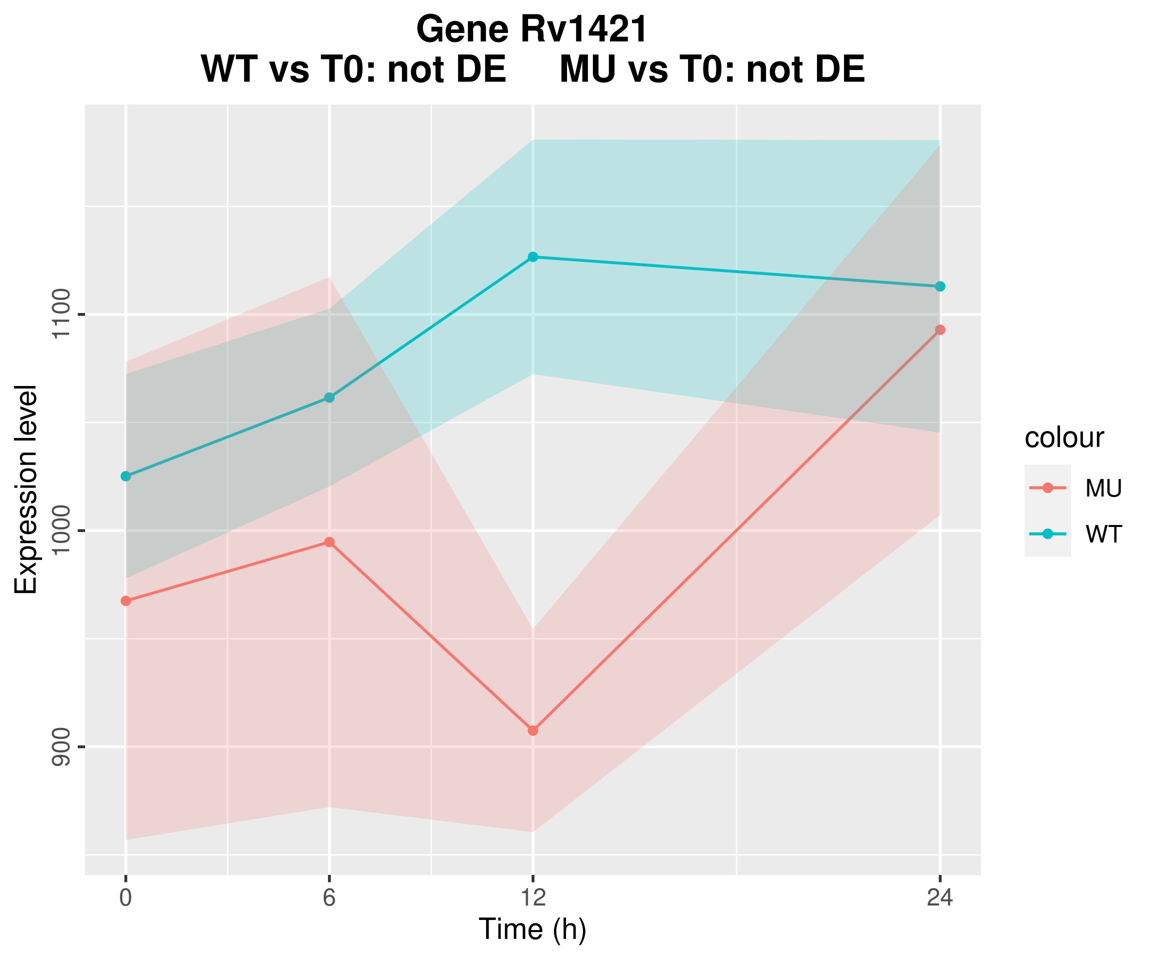
Gene Rv1421
WT vs T0: not DE MU vs T0: not DE
Expression level
1100
1000
900
0
6
12
24
Time (h)
colour
MU
WT

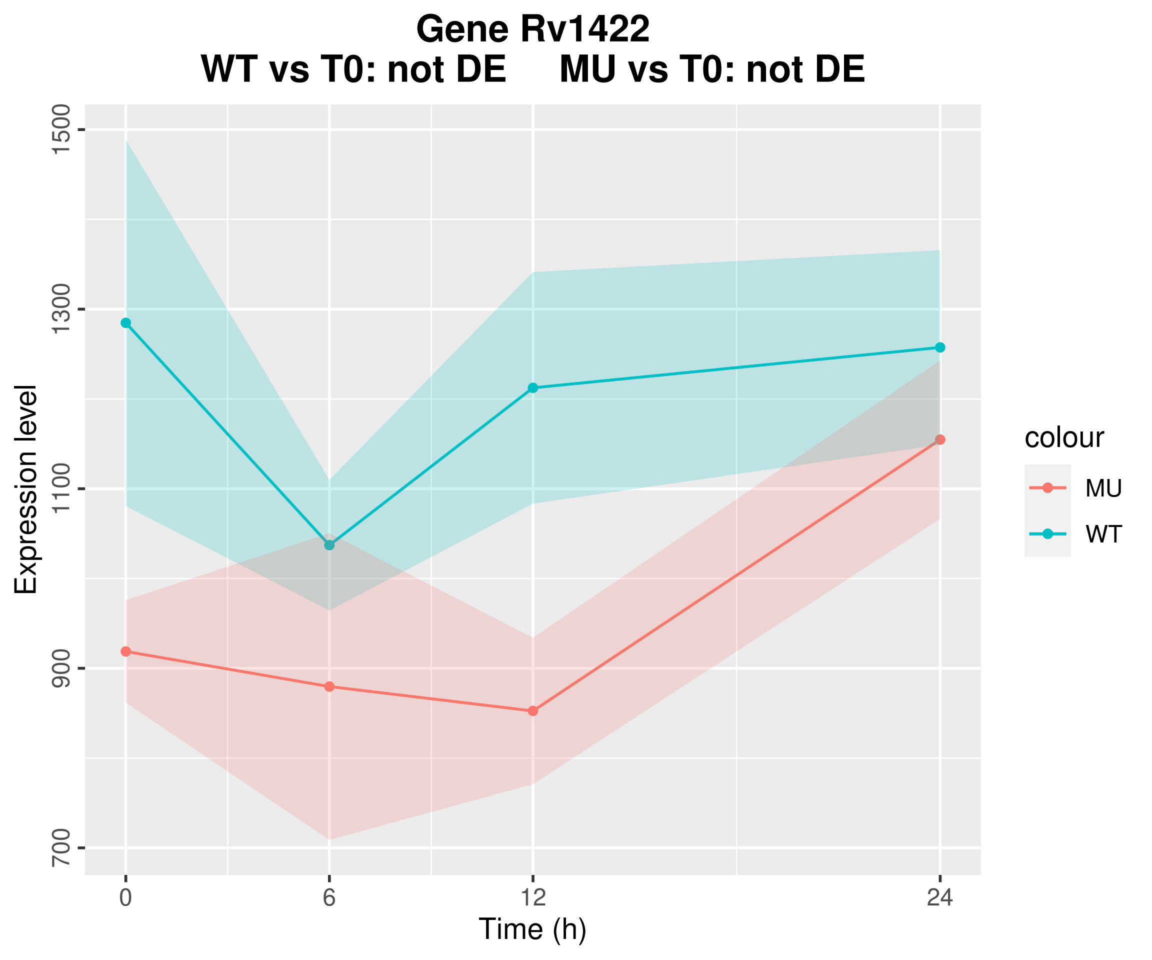
Gene Rv1422
WT vs T0: not DE   MU vs T0: not DE
Expression level
1500
1300
1100
900
700
Time (h)
0
6
12
24
colour
MU
WT

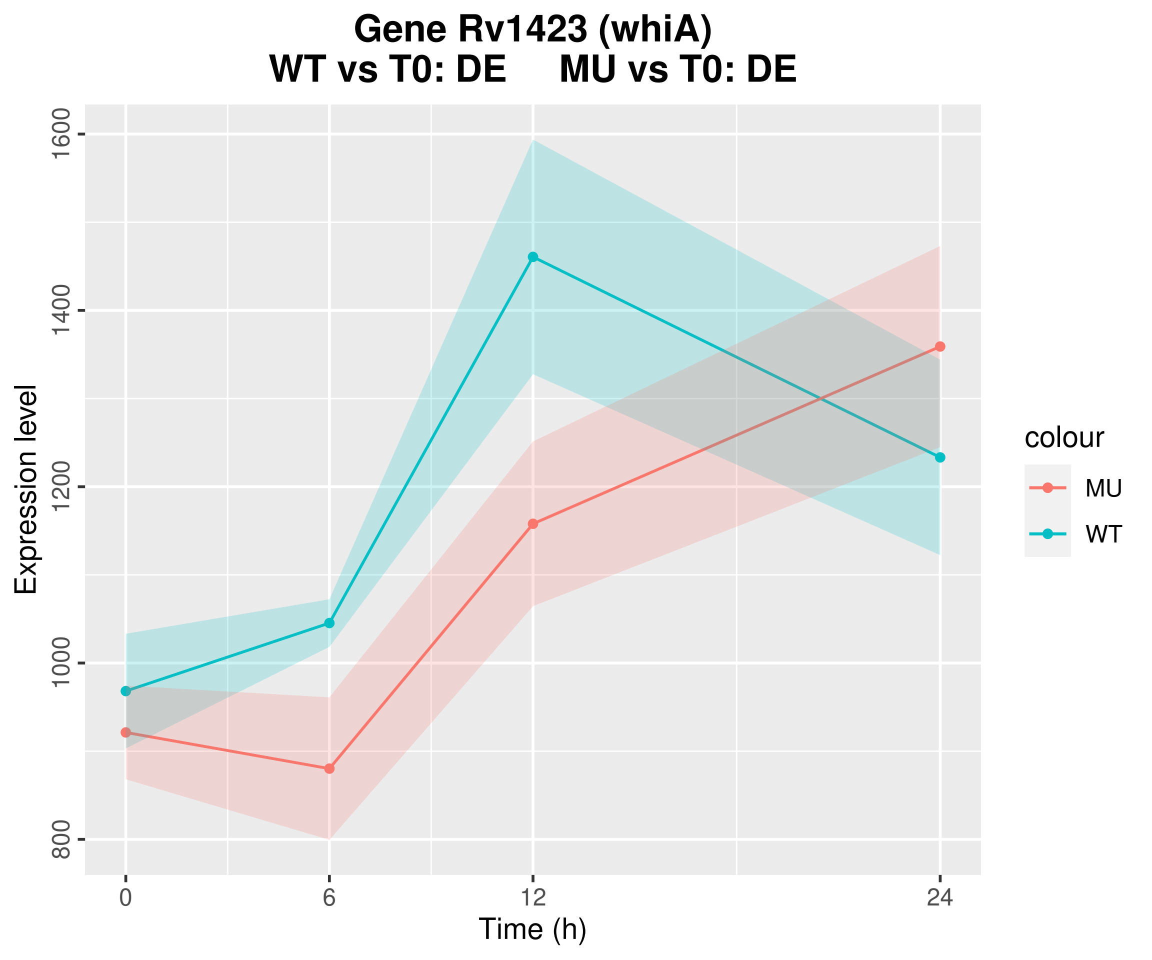

Gene Rv1423 (whiA)
WT vs T0: DE   MU vs T0: DE
Expression level
1600
1400
1200
1000
800
0
6
12
24
Time (h)
colour
MU
WT

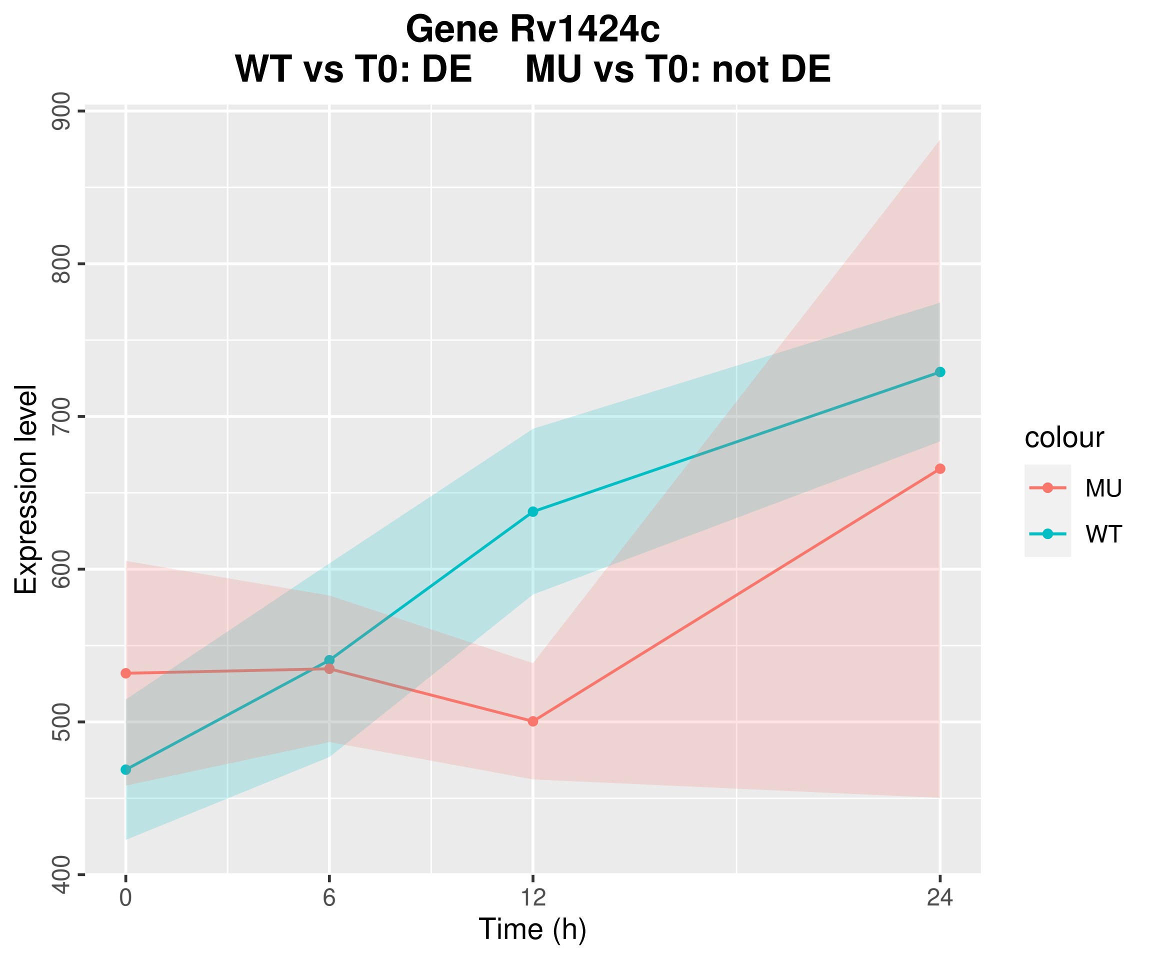

Gene Rv1424c
WT vs T0: DE   MU vs T0: not DE
Expression level
900
800
700
600
500
400
0
6
12
24
Time (h)
colour
MU
WT

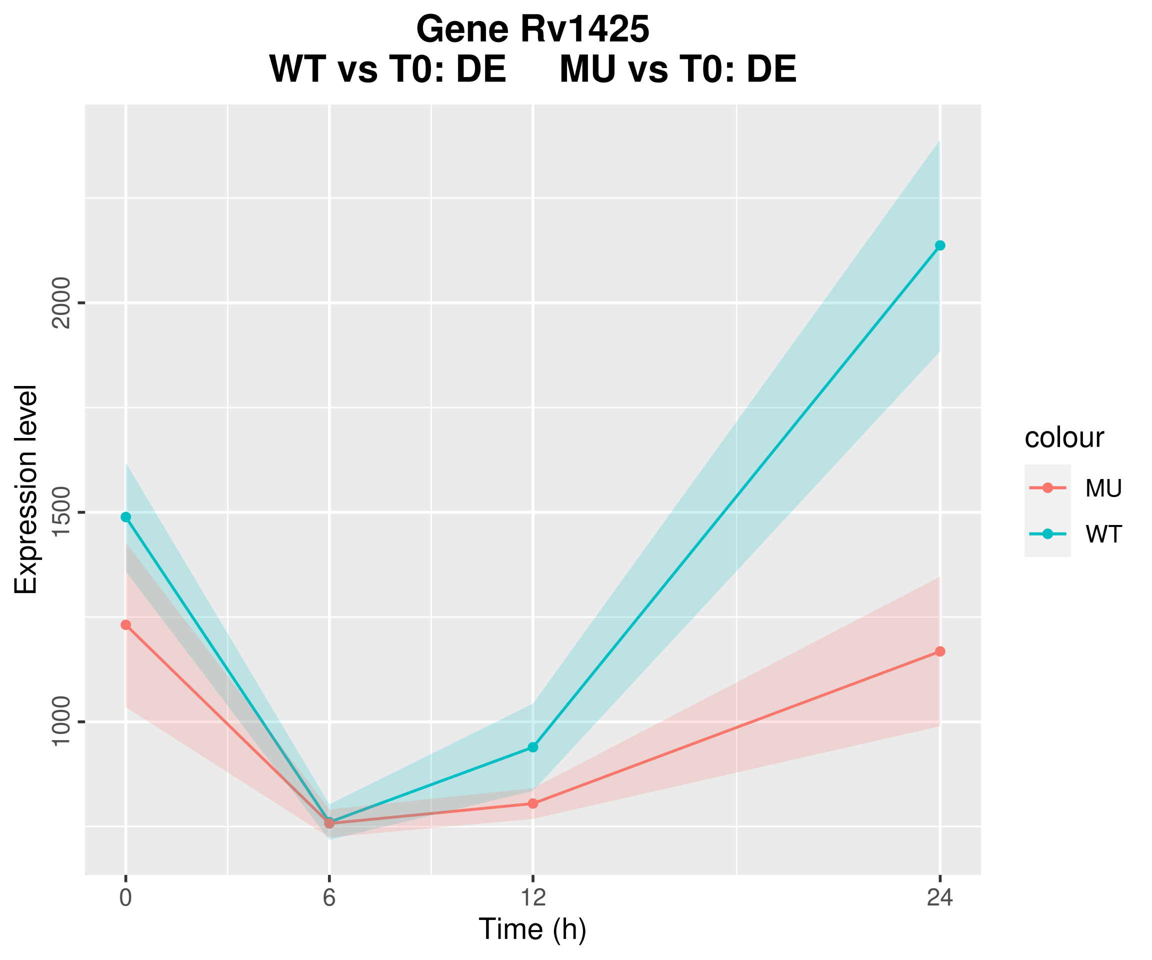
Gene Rv1425
WT vs T0: DE   MU vs T0: DE
Expression level
2000
1500
1000
0
6
12
24
Time (h)
colour
MU
WT

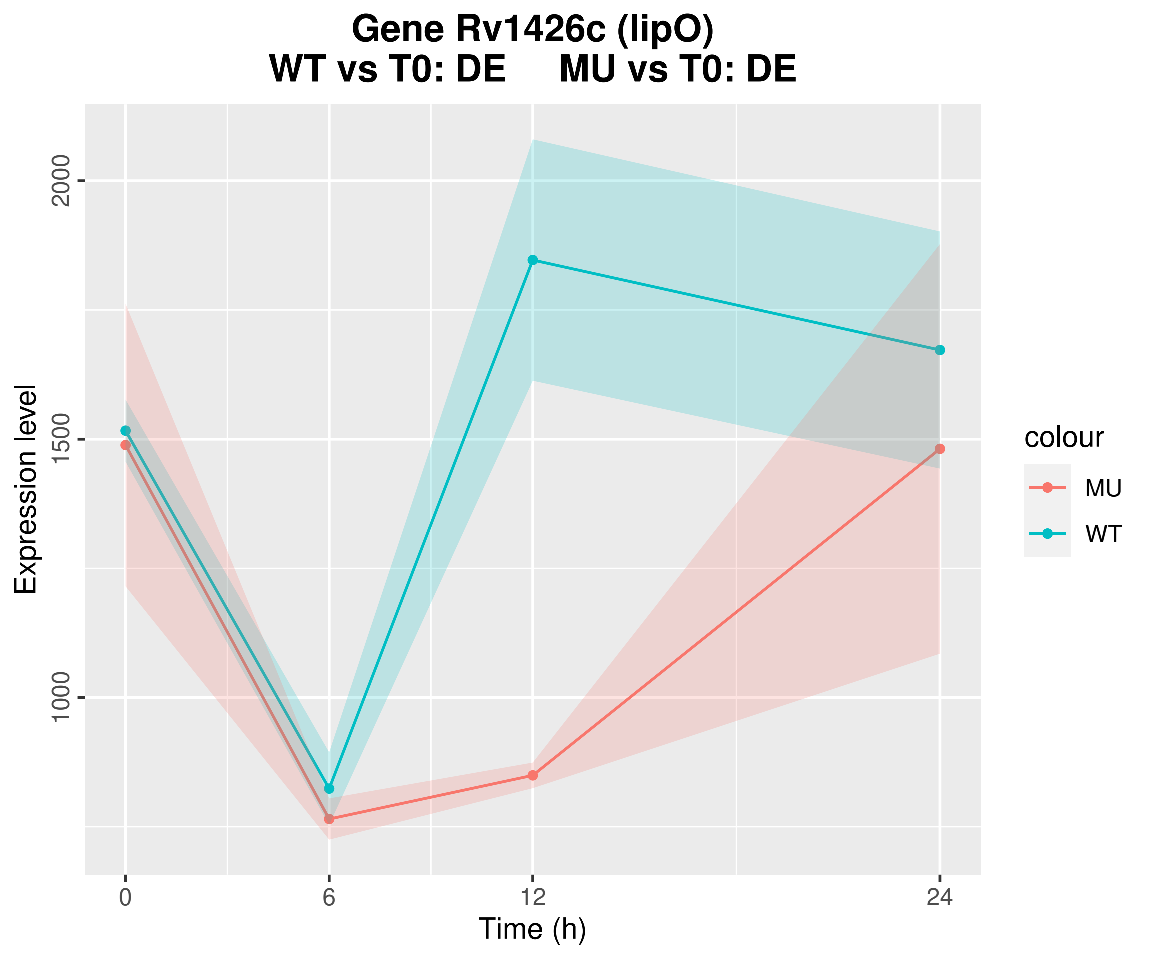
Gene Rv1426c (lipO)
WT vs T0: DE   MU vs T0: DE
Expression level
2000
1500
1000
0
6
12
24
Time (h)
colour
MU
WT

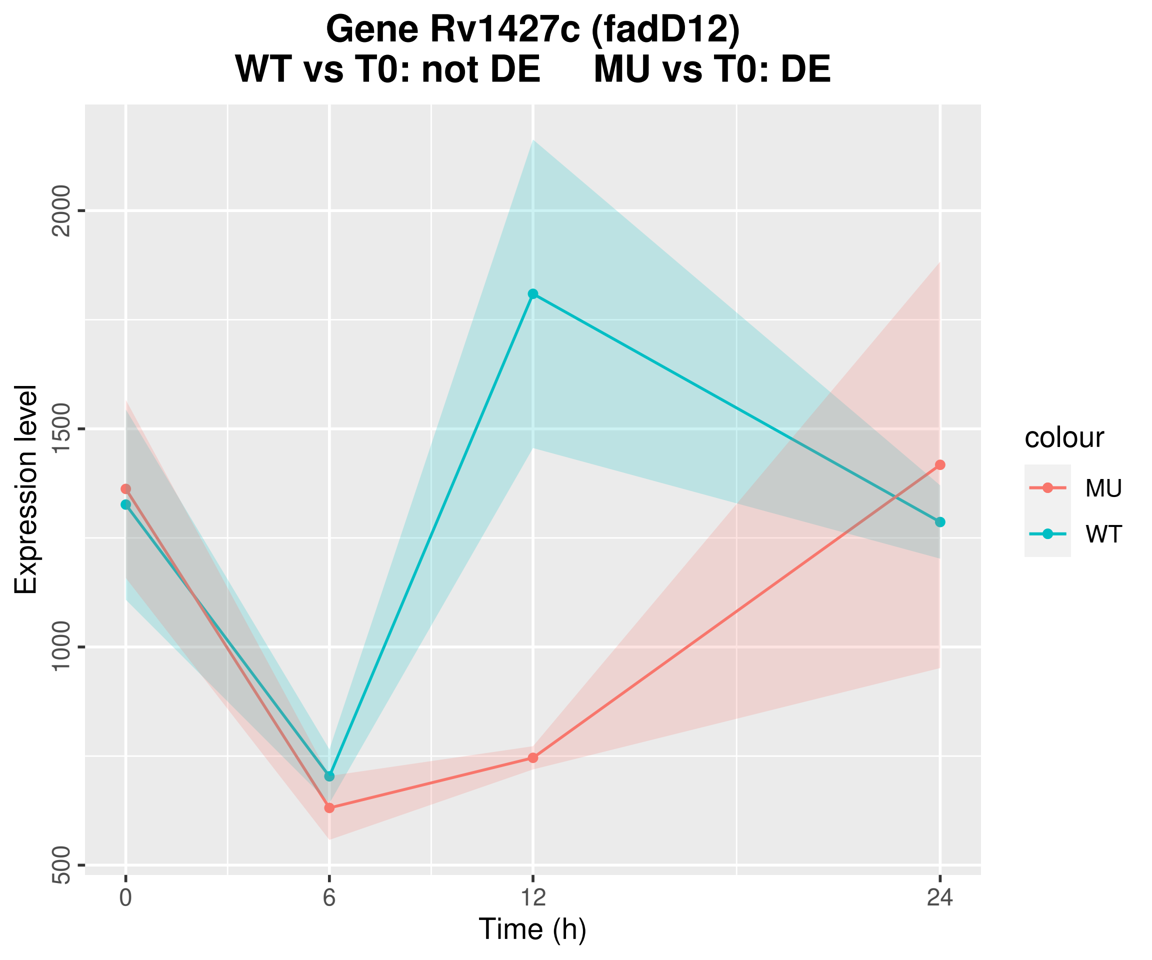

Gene Rv1427c (fadD12)
WT vs T0: not DE MU vs T0: DE
Expression level
2000
1500
1000
500
0
6
12
24
Time (h)
colour
MU
WT

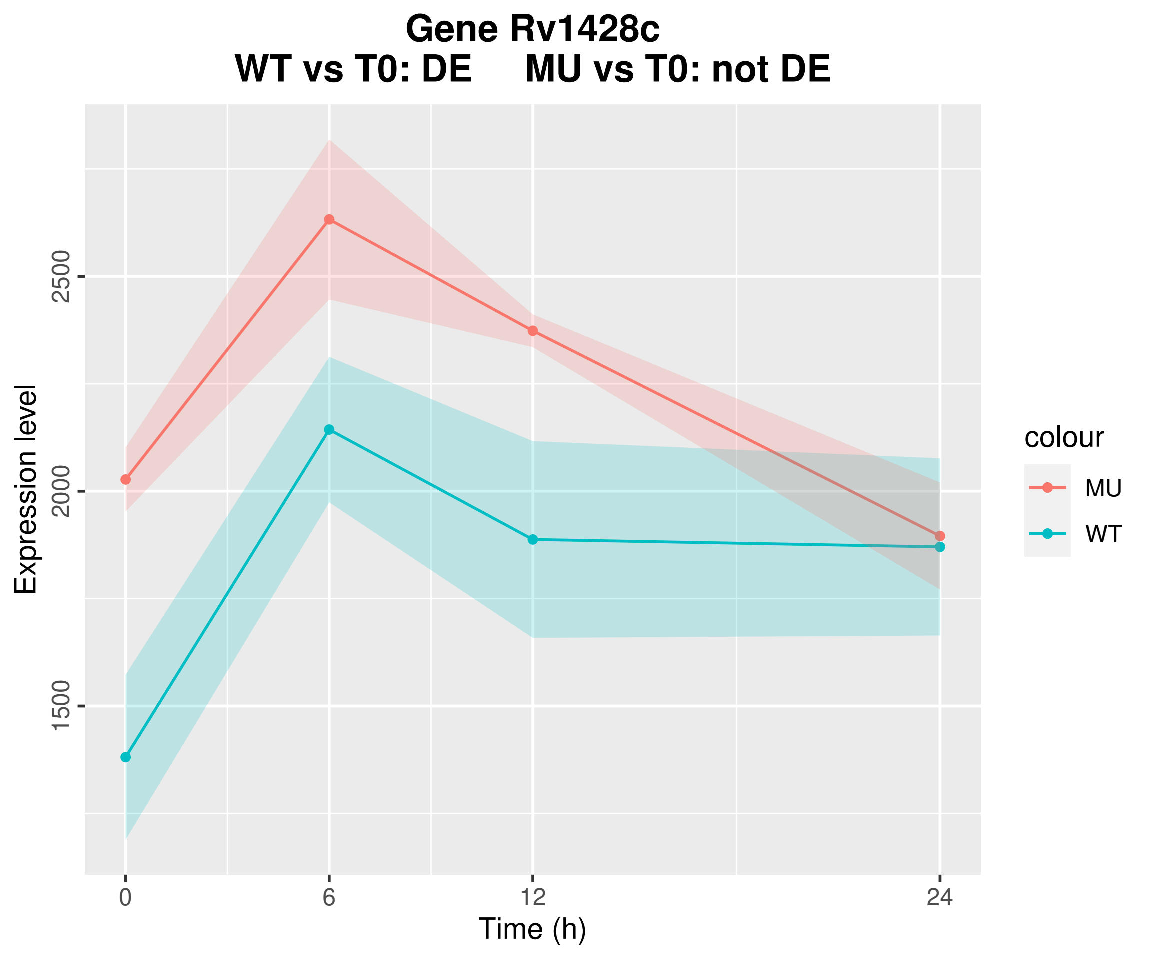
Gene Rv1428c
WT vs T0: DE   MU vs T0: not DE
Expression level
2500
2000
1500
Time (h)
0
6
12
24
colour
MU
WT

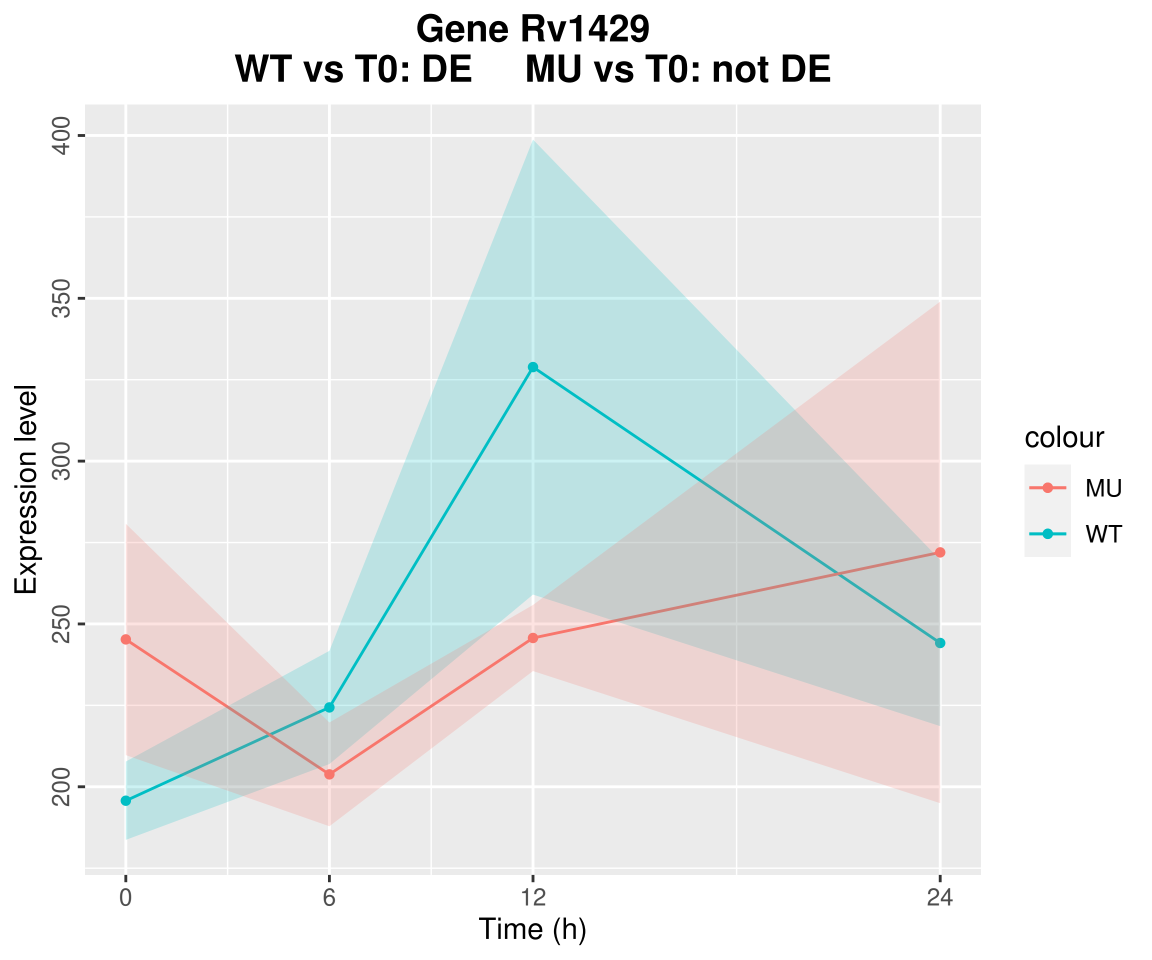
Gene Rv1429
WT vs T0: DE MU vs T0: not DE
Expression level
400
350
300
250
200
0
6
12
24
Time (h)
colour
MU
WT

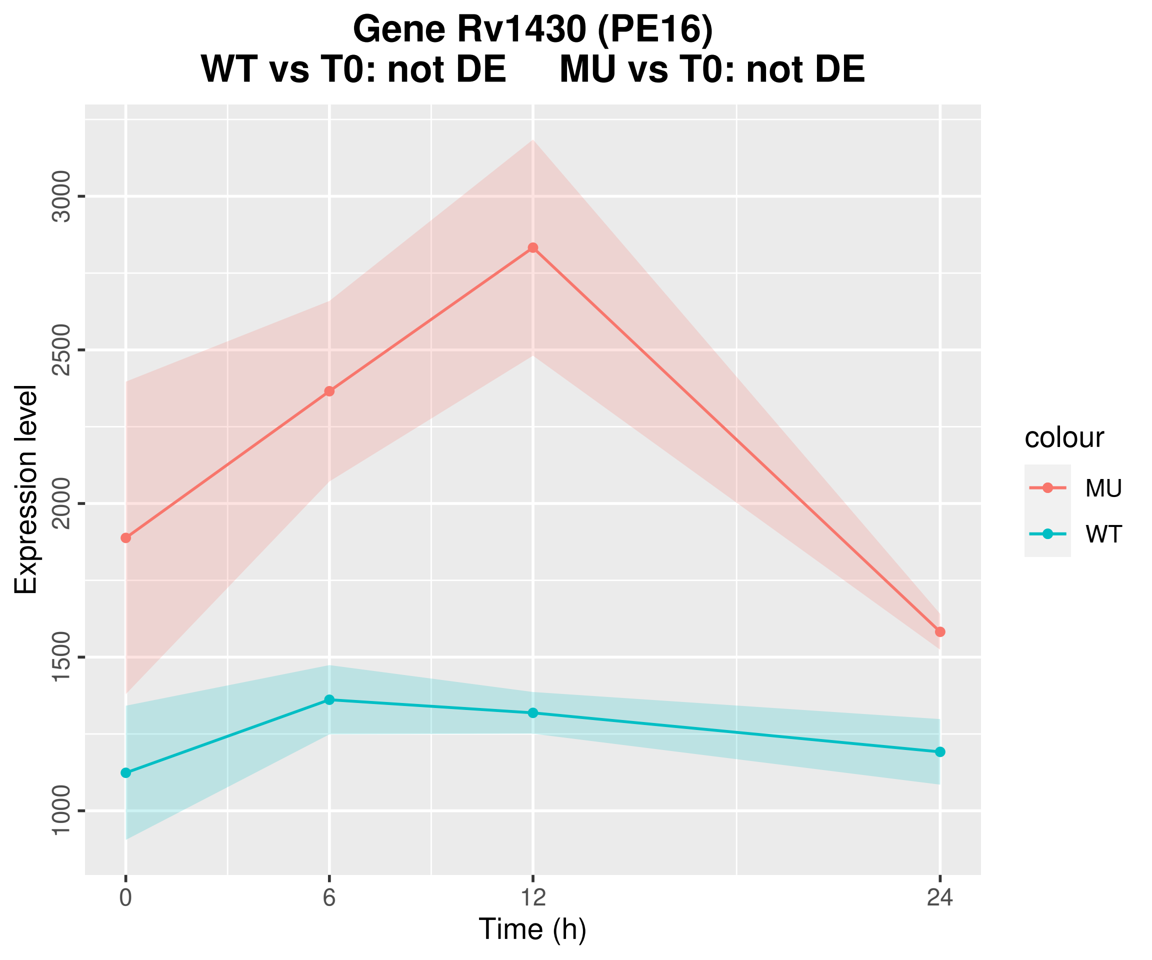
Gene Rv1430 (PE16)
WT vs T0: not DE   MU vs T0: not DE
Expression level
3000
2500
2000
1500
1000
0
6
12
24
Time (h)
colour
MU
WT

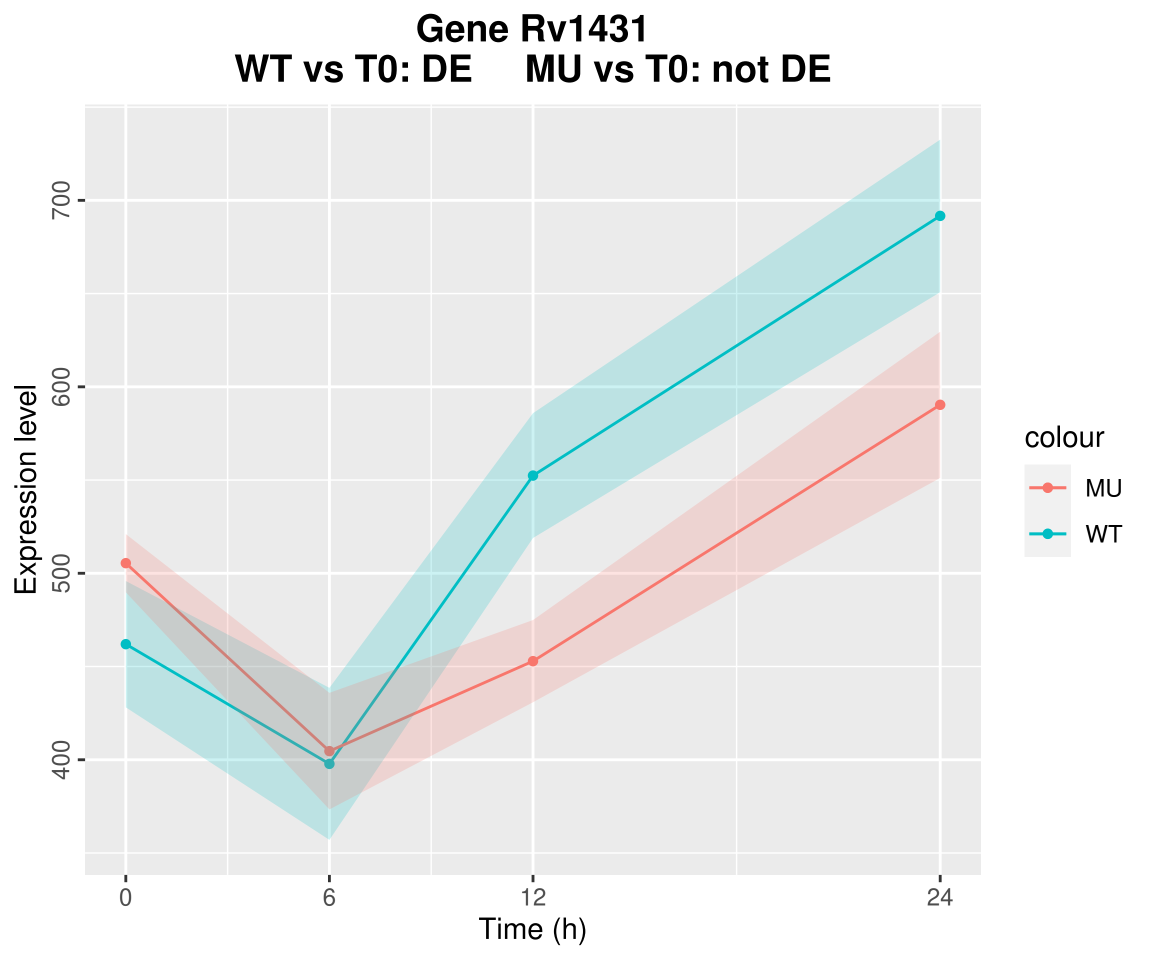

Gene Rv1431
WT vs T0: DE MU vs T0: not DE
Expression level
400
500
600
700
0
6
12
24
Time (h)
colour
MU
WT

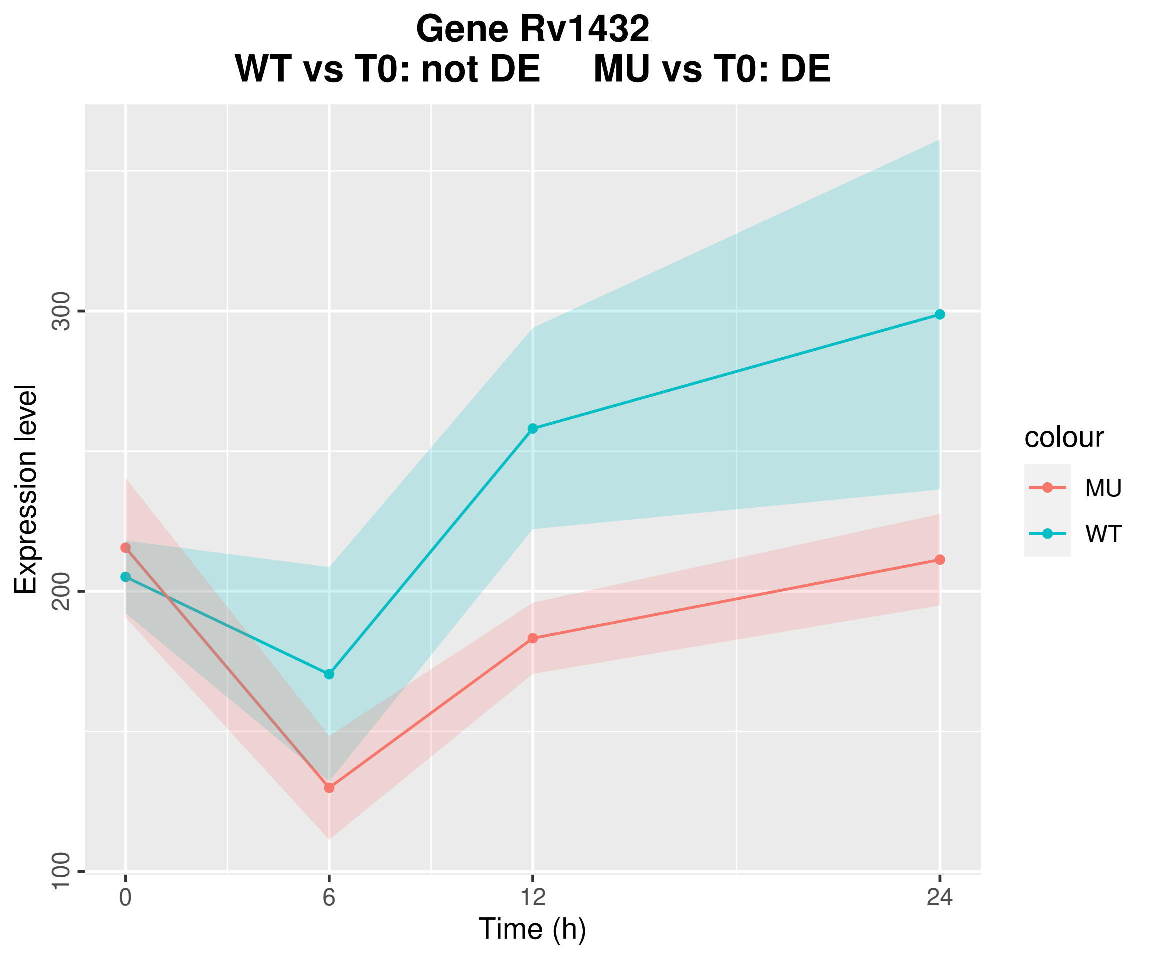

Gene Rv1432
WT vs T0: not DE MU vs T0: DE
Expression level
300
200
100
0
6
12
24
Time (h)
colour
MU
WT

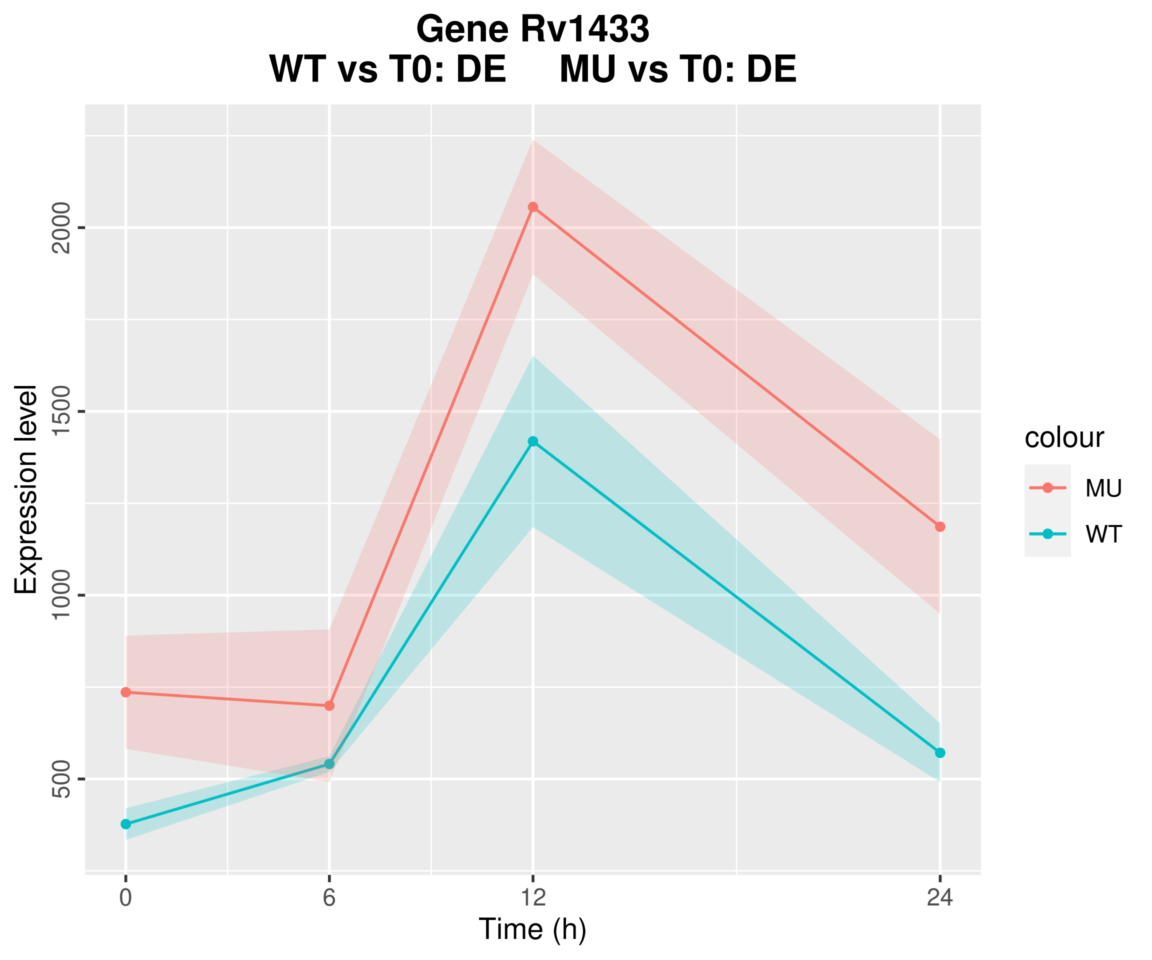
Gene Rv1433
WT vs T0: DE MU vs T0: DE
Expression level
2000
1500
1000
500
0
6
12
24
Time (h)
colour
MU
WT

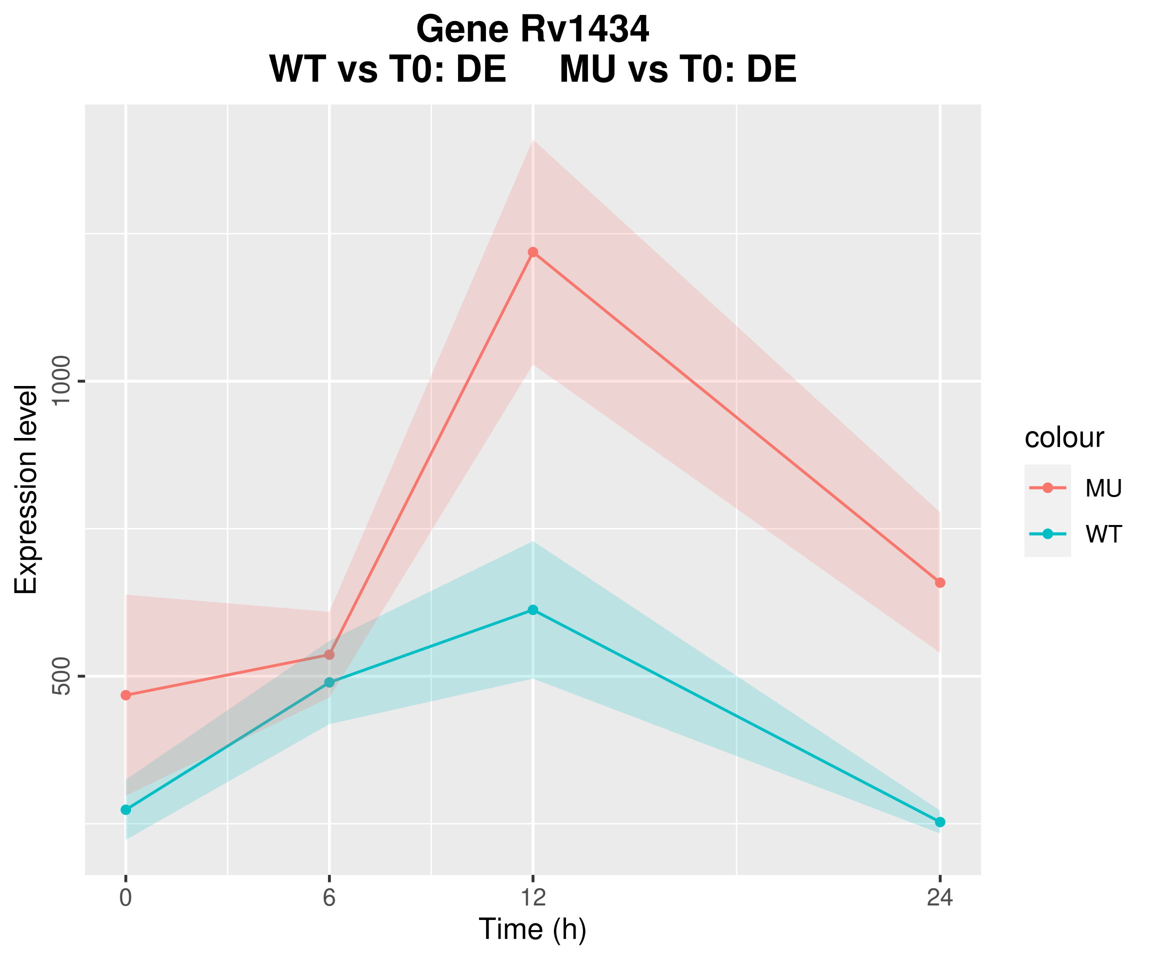

Gene Rv1434
WT vs T0: DE   MU vs T0: DE
Expression level
1000
500
0
6
12
24
Time (h)
colour
MU
WT

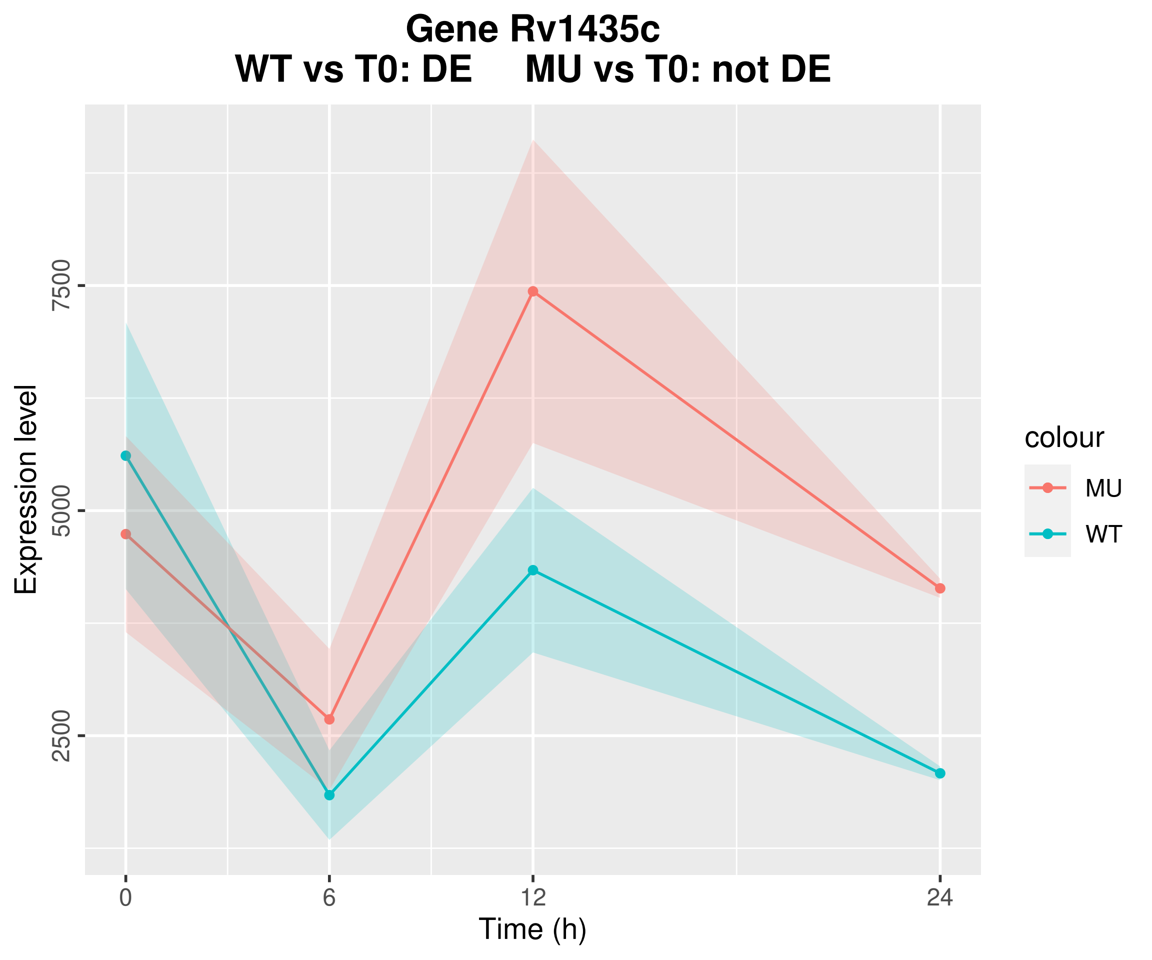
Gene Rv1435c
WT vs T0: DE MU vs T0: not DE
Expression level
7500
5000
2500
0
6
12
24
Time (h)
colour
MU
WT

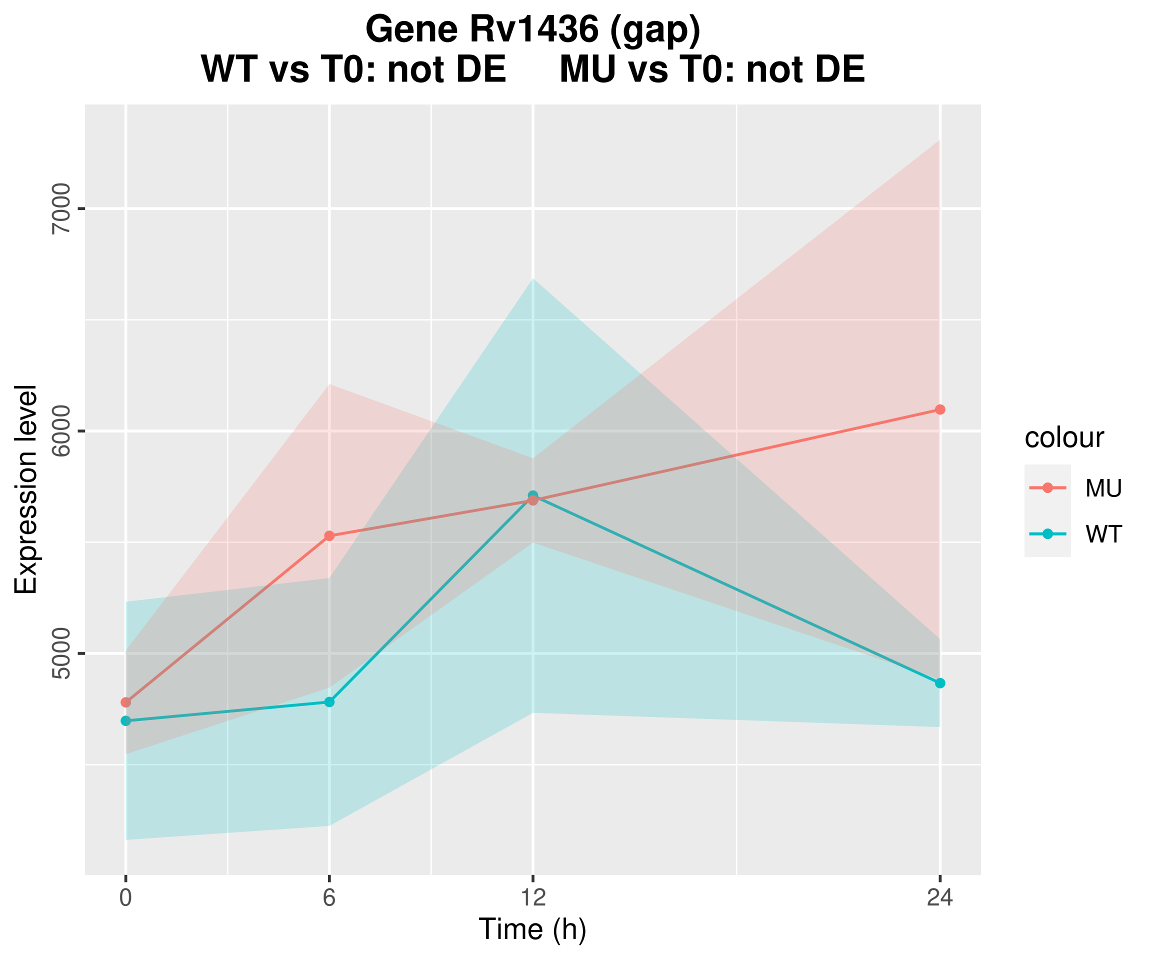

Gene Rv1436 (gap)
WT vs T0: not DE MU vs T0: not DE
7000
6000
5000
Expression level
0
6
12
24
Time (h)
colour
MU
WT

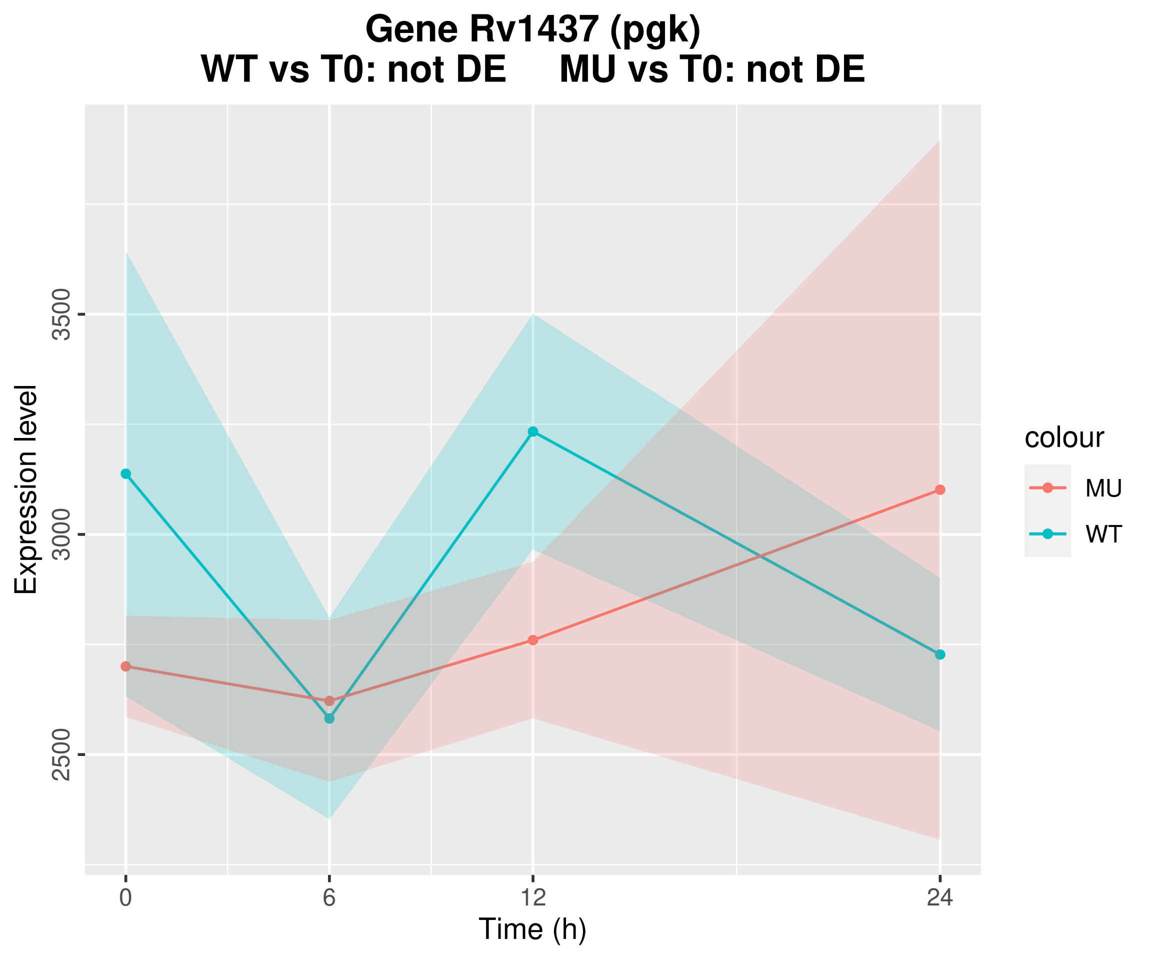

Gene Rv1437 (pgk)
WT vs T0: not DE   MU vs T0: not DE
Expression level
2500
3000
3500
0
6
12
24
Time (h)
colour
MU
WT

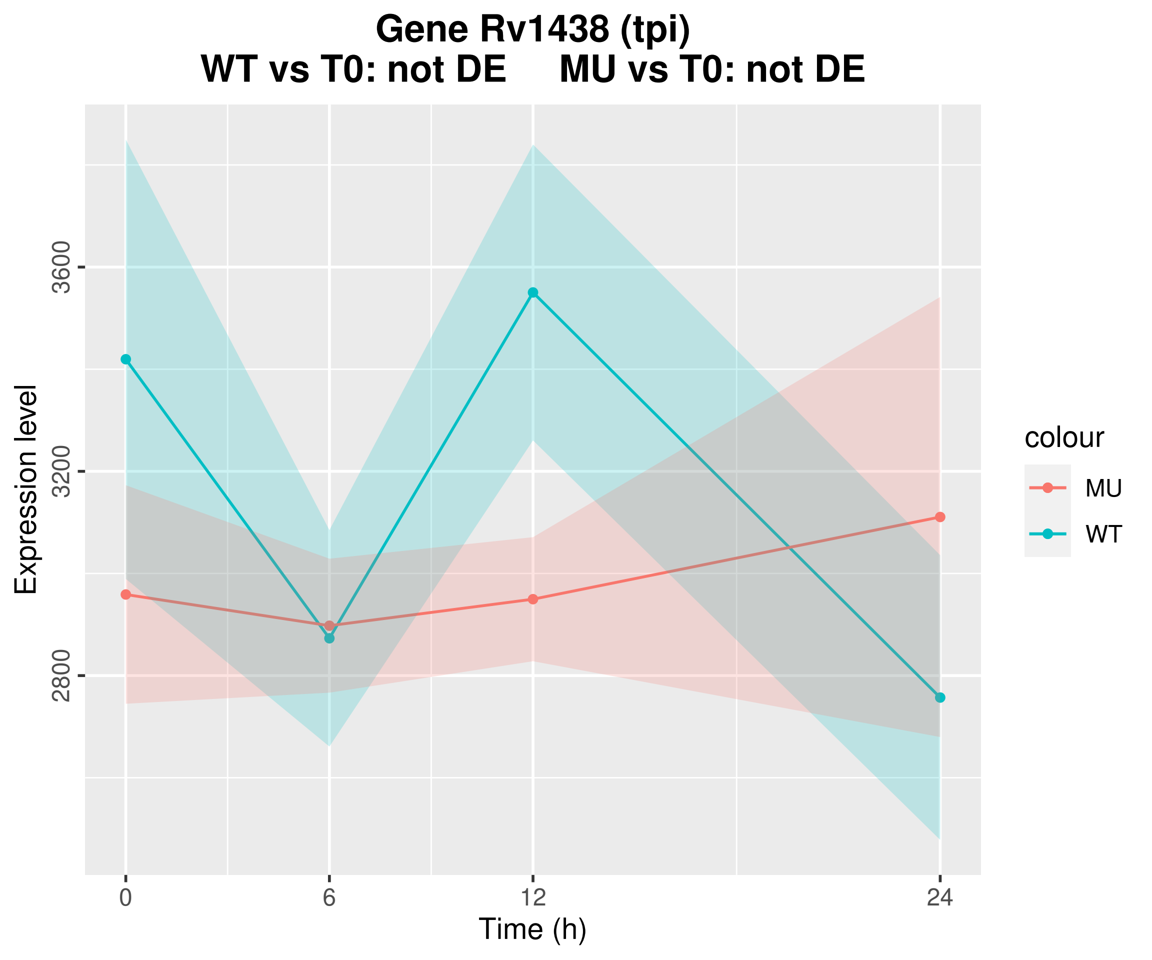

Gene Rv1438 (tpi)
WT vs T0: not DE   MU vs T0: not DE
Expression level
3600
3200
2800
0
6
12
24
Time (h)
colour
MU
WT

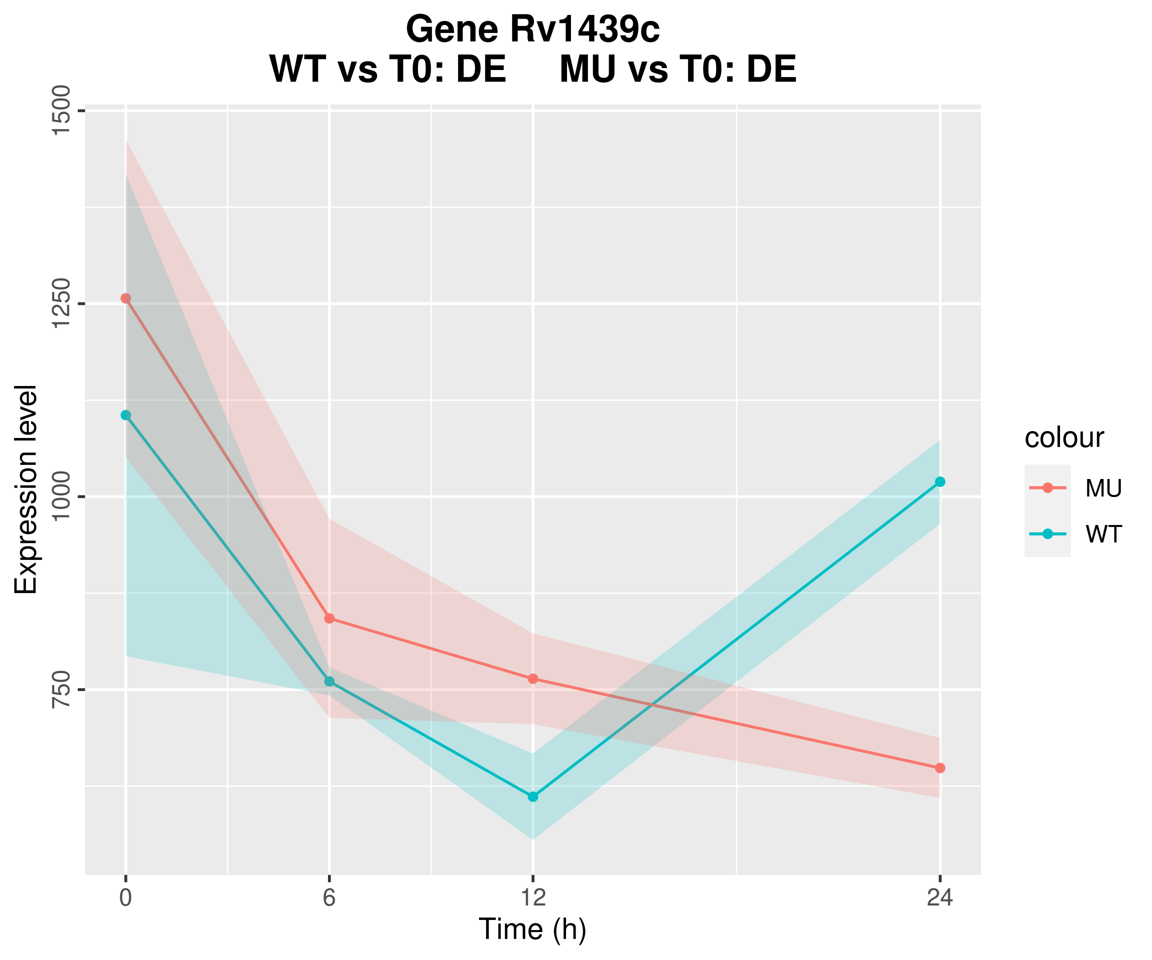

Gene Rv1439c
WT vs T0: DE   MU vs T0: DE
1500
1250
1000
750
Expression level
0
6
12
24
Time (h)
colour
MU
WT

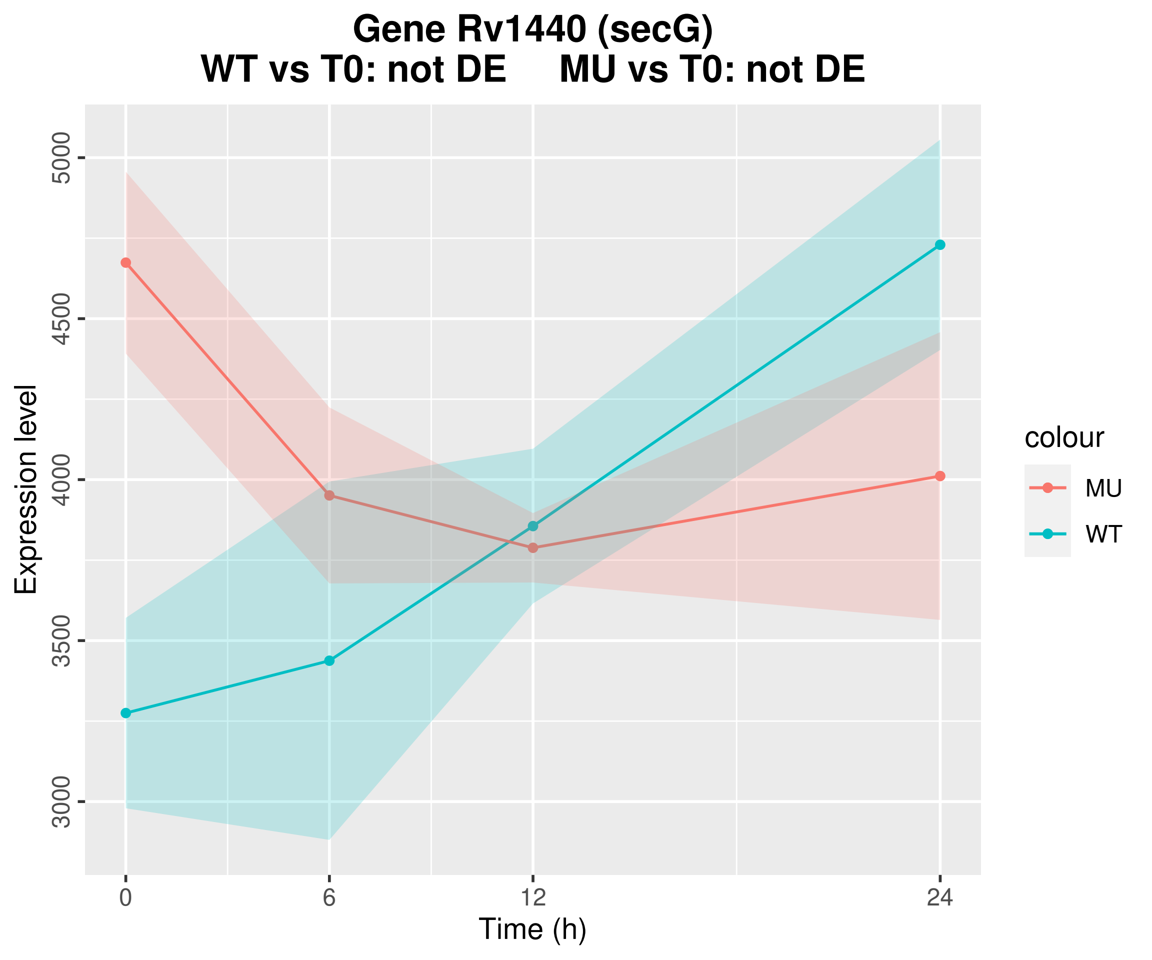

Gene Rv1440 (secG)
WT vs T0: not DE   MU vs T0: not DE
Expression level
5000
4500
4000
3500
3000
0
6
12
24
Time (h)
colour
MU
WT

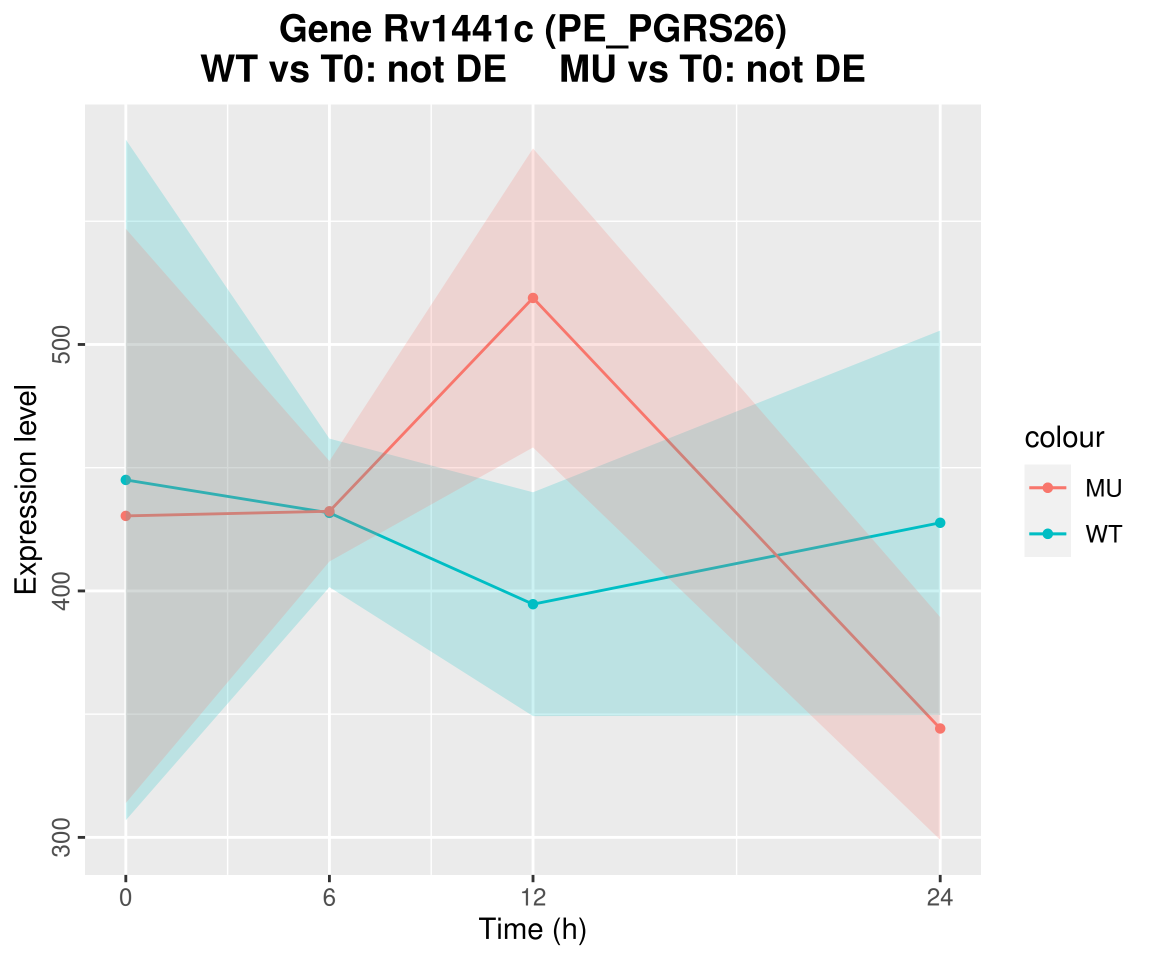

Gene Rv1441c (PE_PGRS26)
WT vs T0: not DE   MU vs T0: not DE
Expression level
500
400
300
0
6
12
24
Time (h)
colour
MU
WT

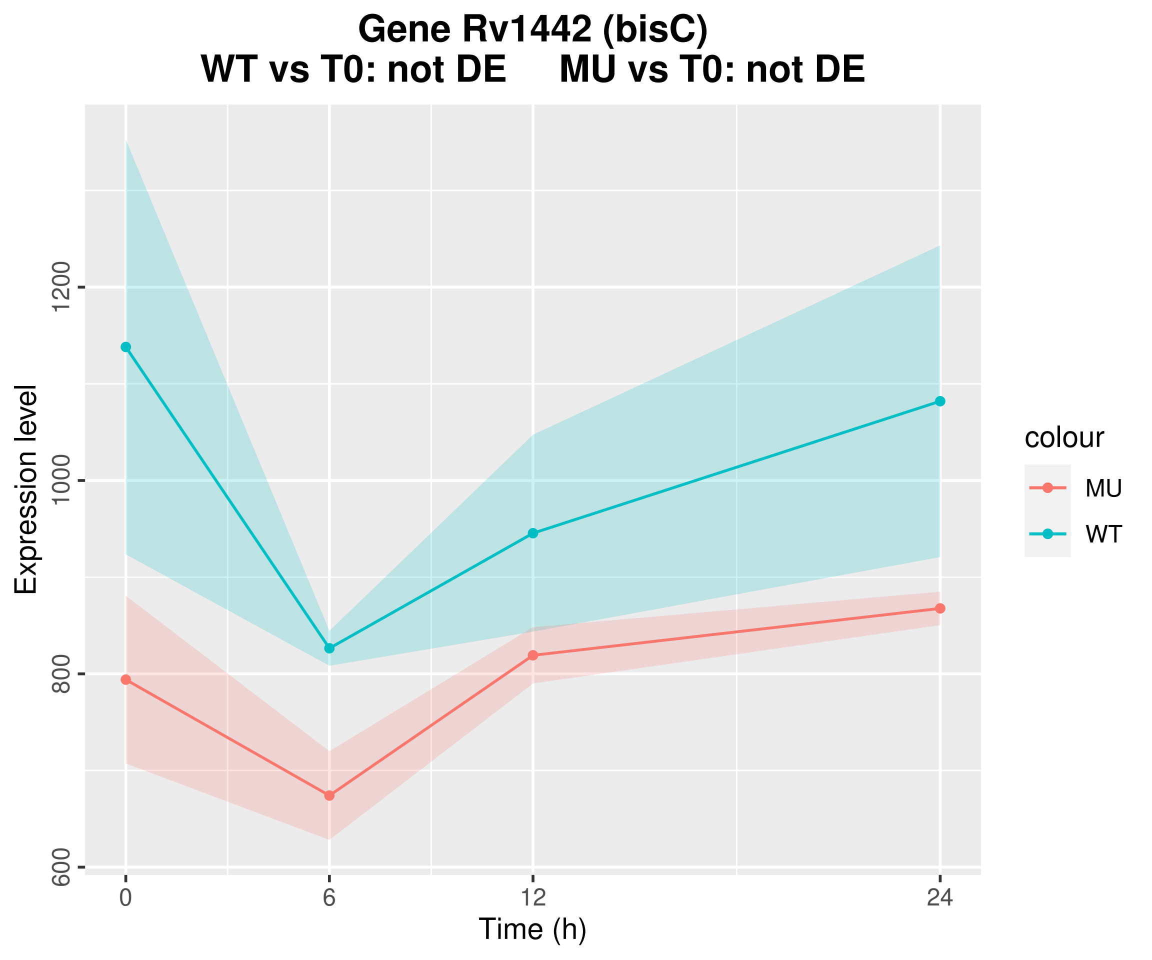

Gene Rv1442 (bisC)
WT vs T0: not DE MU vs T0: not DE
Expression level
1200
1000
800
600
0
6
12
24
Time (h)
colour
MU
WT

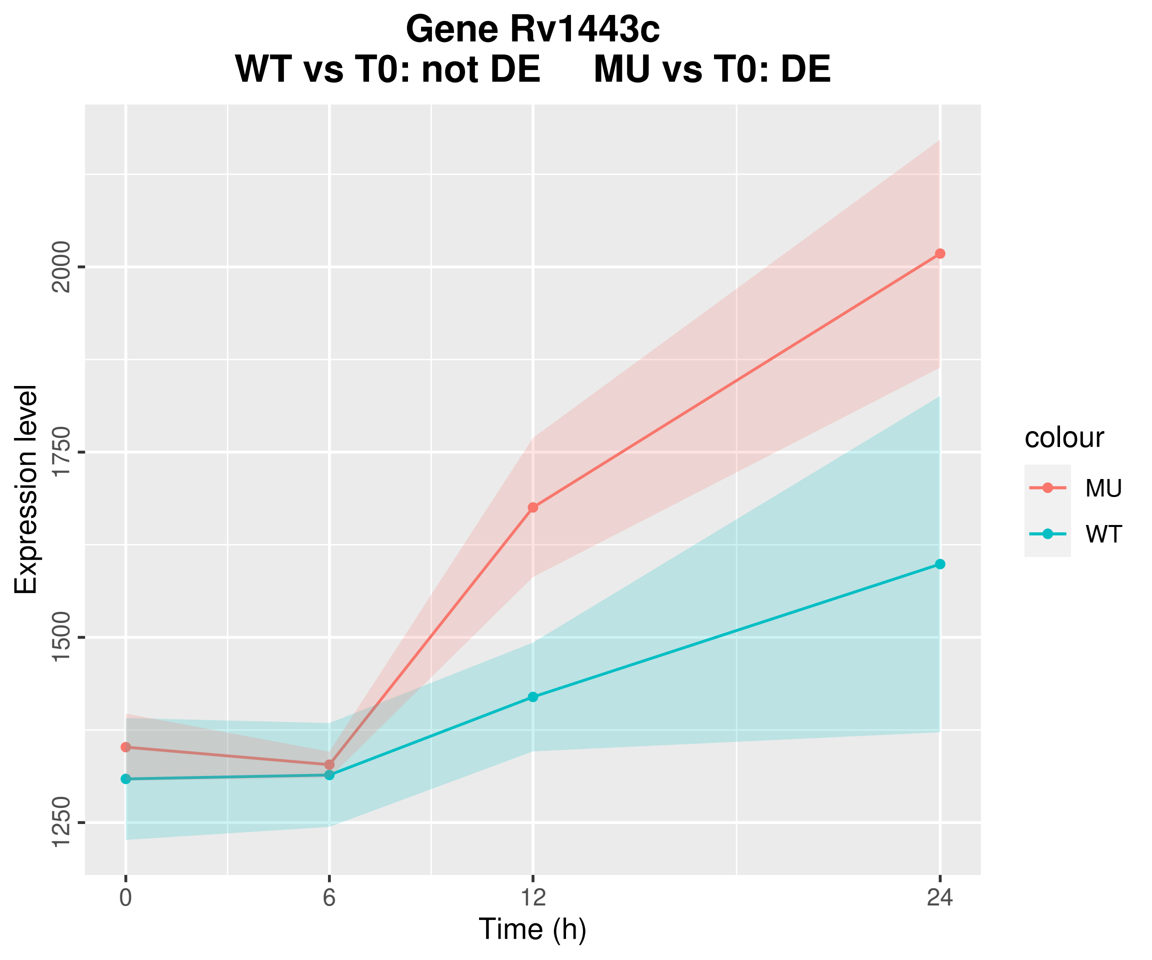

Gene Rv1443c
WT vs T0: not DE MU vs T0: DE
Expression level
Time (h)
1250
1500
1750
2000
0
6
12
24
colour
MU
WT

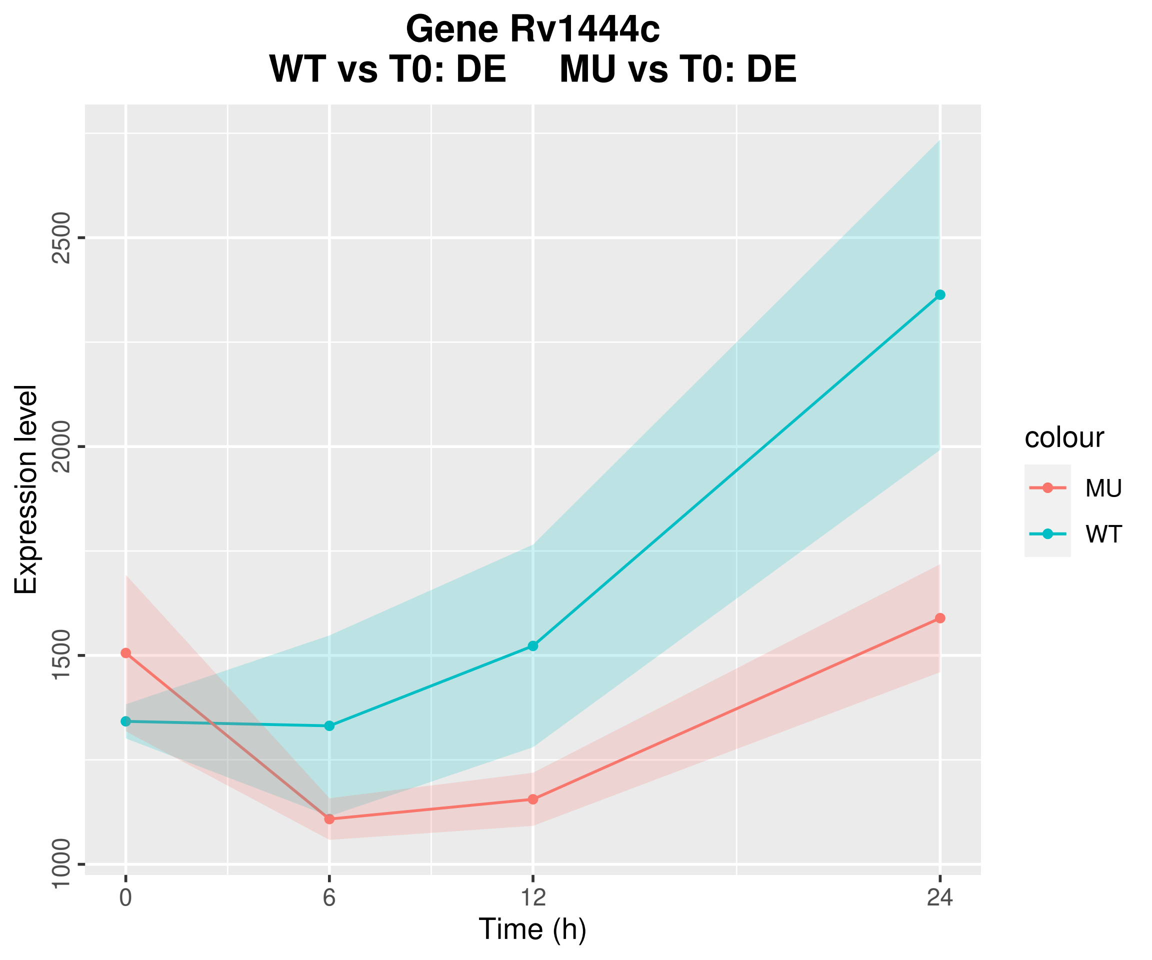
Gene Rv1444c
WT vs T0: DE MU vs T0: DE
Expression level
2500
2000
1500
1000
0
6
12
24
Time (h)
colour
MU
WT

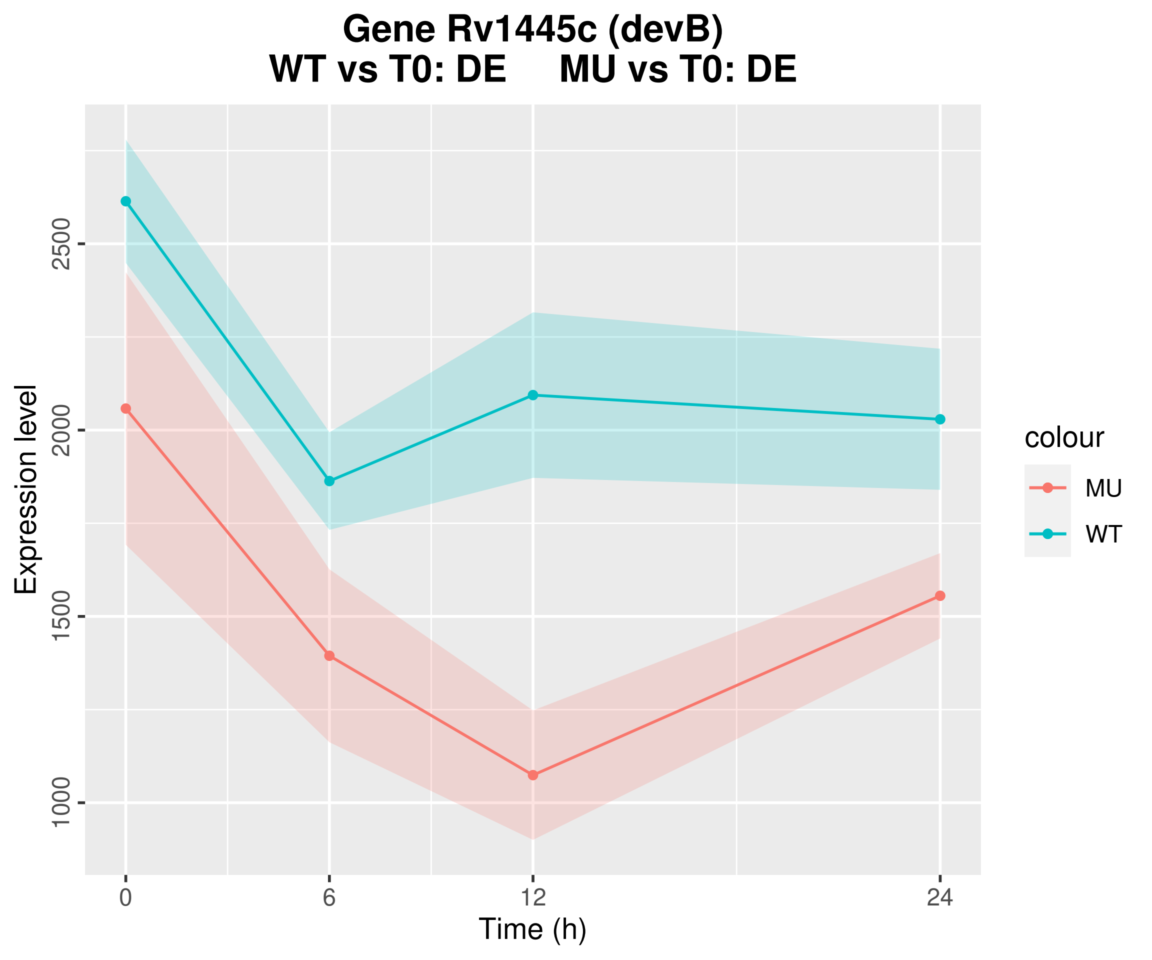
Gene Rv1445c (devB)
WT vs T0: DE MU vs T0: DE
Expression level
2500
2000
1500
1000
0
6
12
24
Time (h)
colour
MU
WT

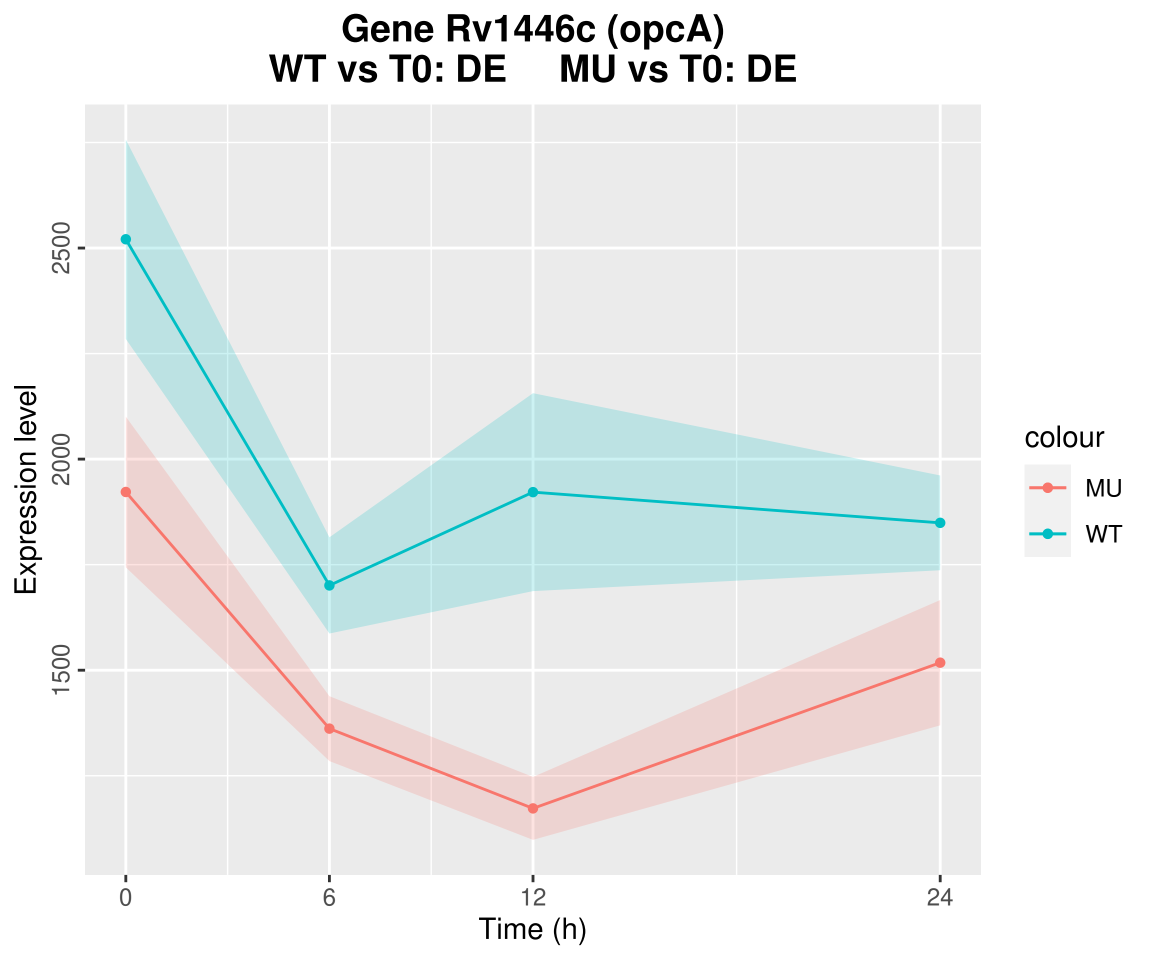
Gene Rv1446c (opcA)
WT vs T0: DE   MU vs T0: DE
Expression level
2500
2000
1500
0
6
12
24
Time (h)
colour
MU
WT

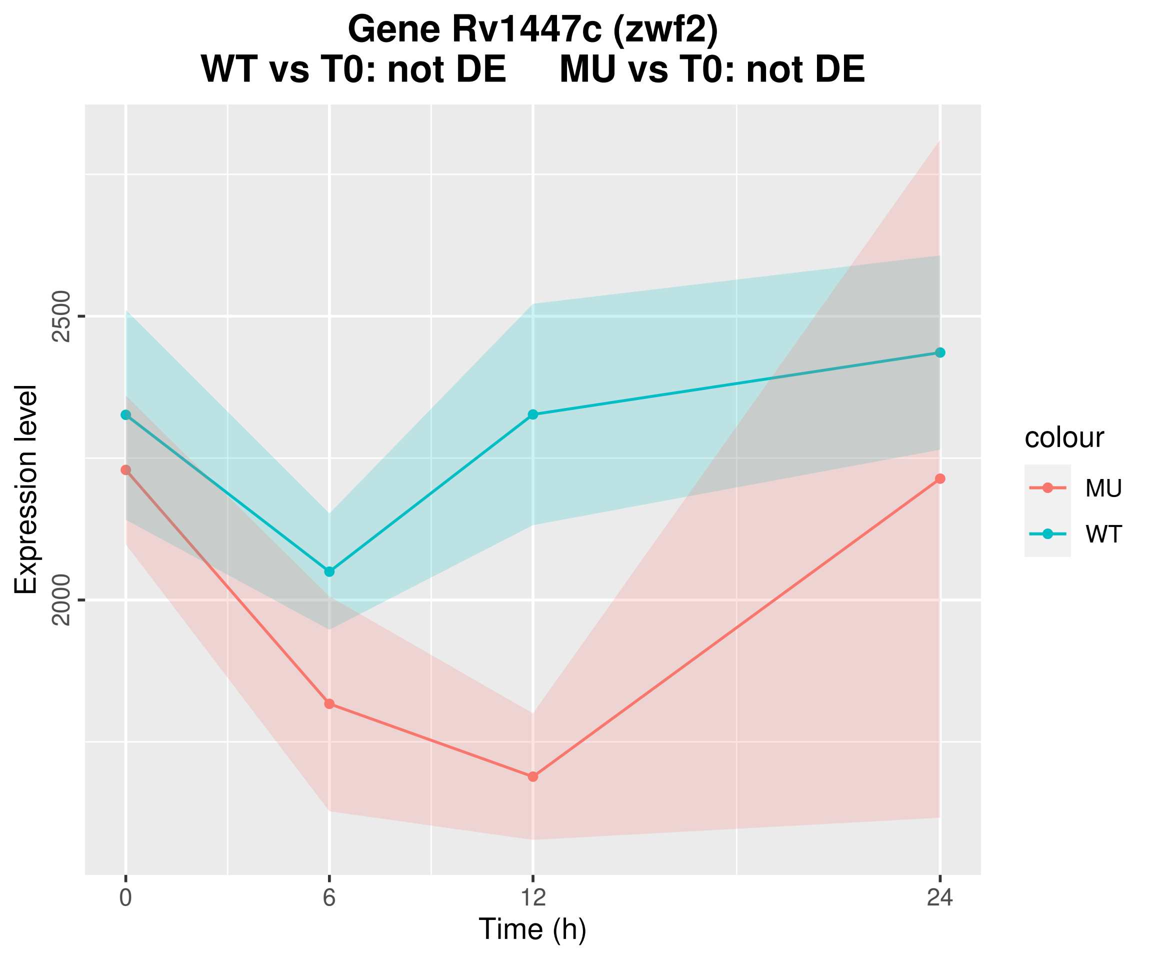

Gene Rv1447c (zwf2)
WT vs T0: not DE MU vs T0: not DE
Expression level
2500
2000
0
6
12
24
Time (h)
colour
MU
WT

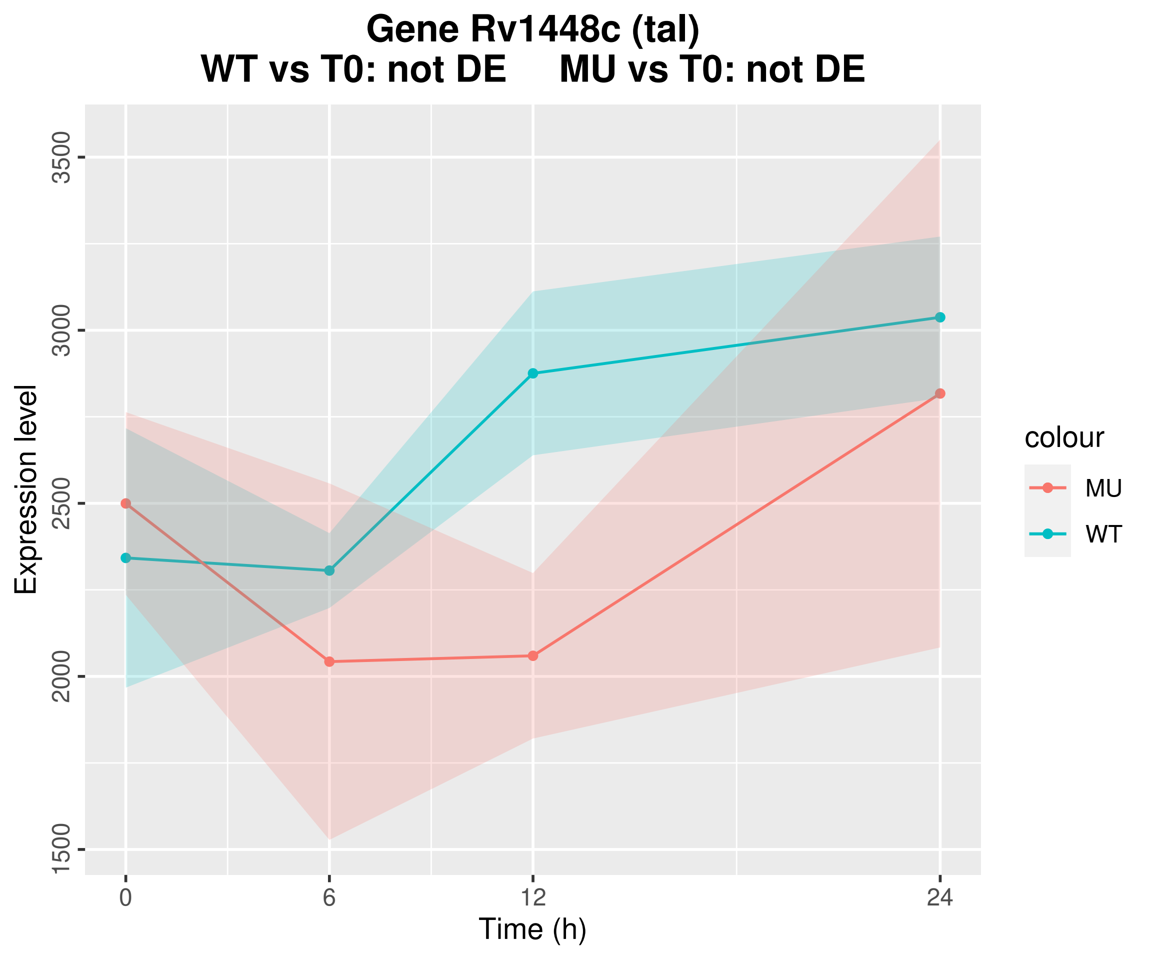

Gene Rv1448c (tal)
WT vs T0: not DE MU vs T0: not DE
Expression level
3500
3000
2500
2000
1500
0
6
12
24
Time (h)
colour
MU
WT

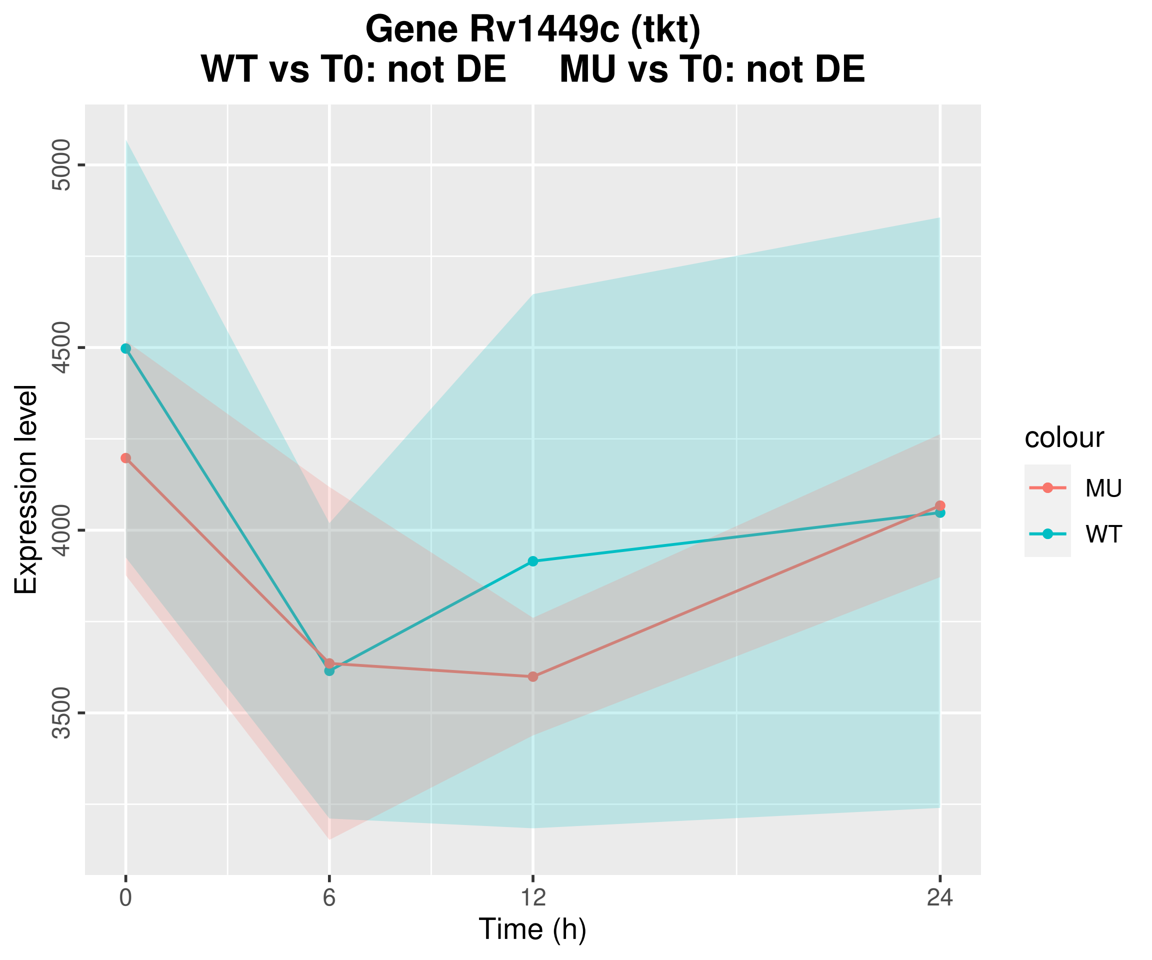

Gene Rv1449c (tkt)
WT vs T0: not DE   MU vs T0: not DE
Expression level
5000
4500
4000
3500
0
6
12
24
Time (h)
colour
MU
WT

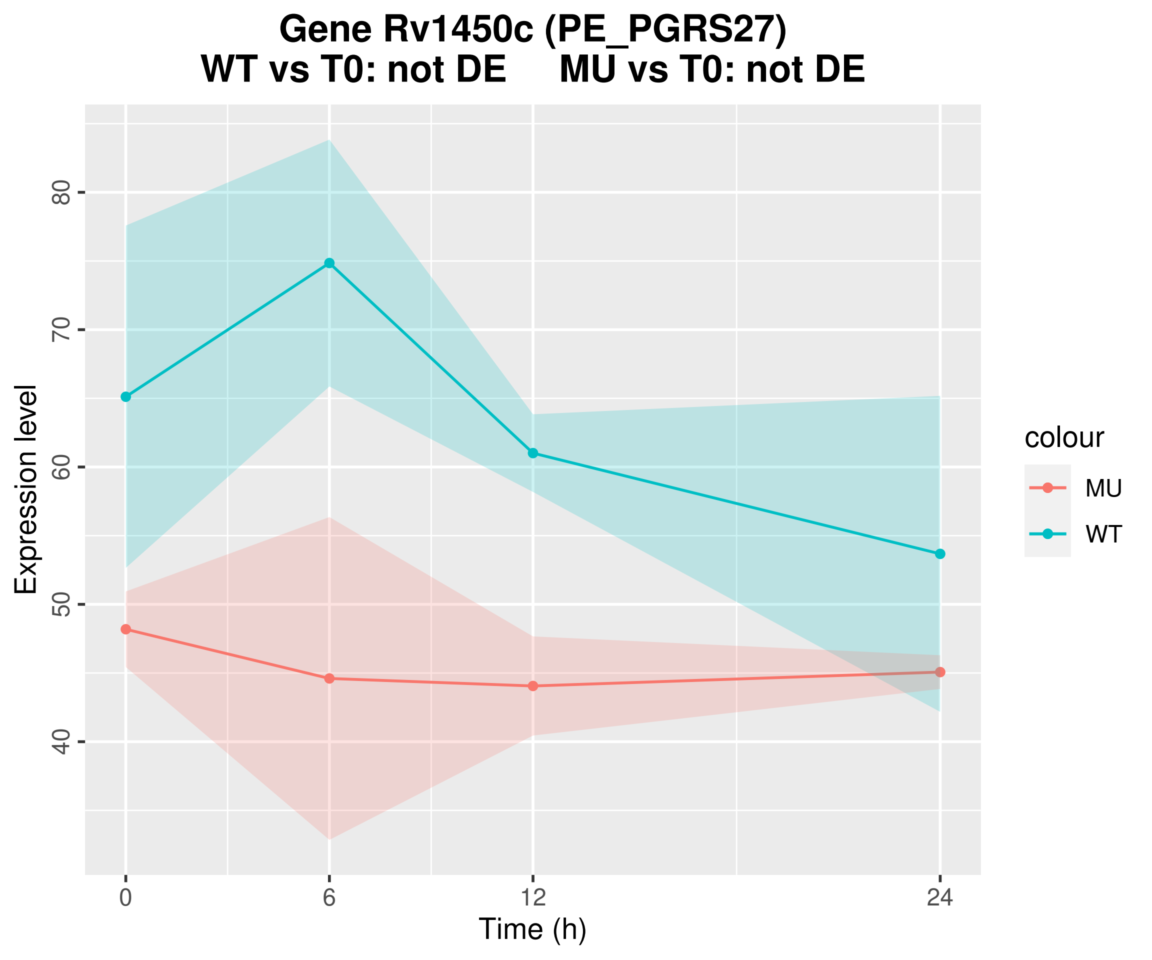
Gene Rv1450c (PE_PGRS27)
WT vs T0: not DE   MU vs T0: not DE
Expression level
80
70
60
50
40
0
6
12
24
Time (h)
colour
MU
WT

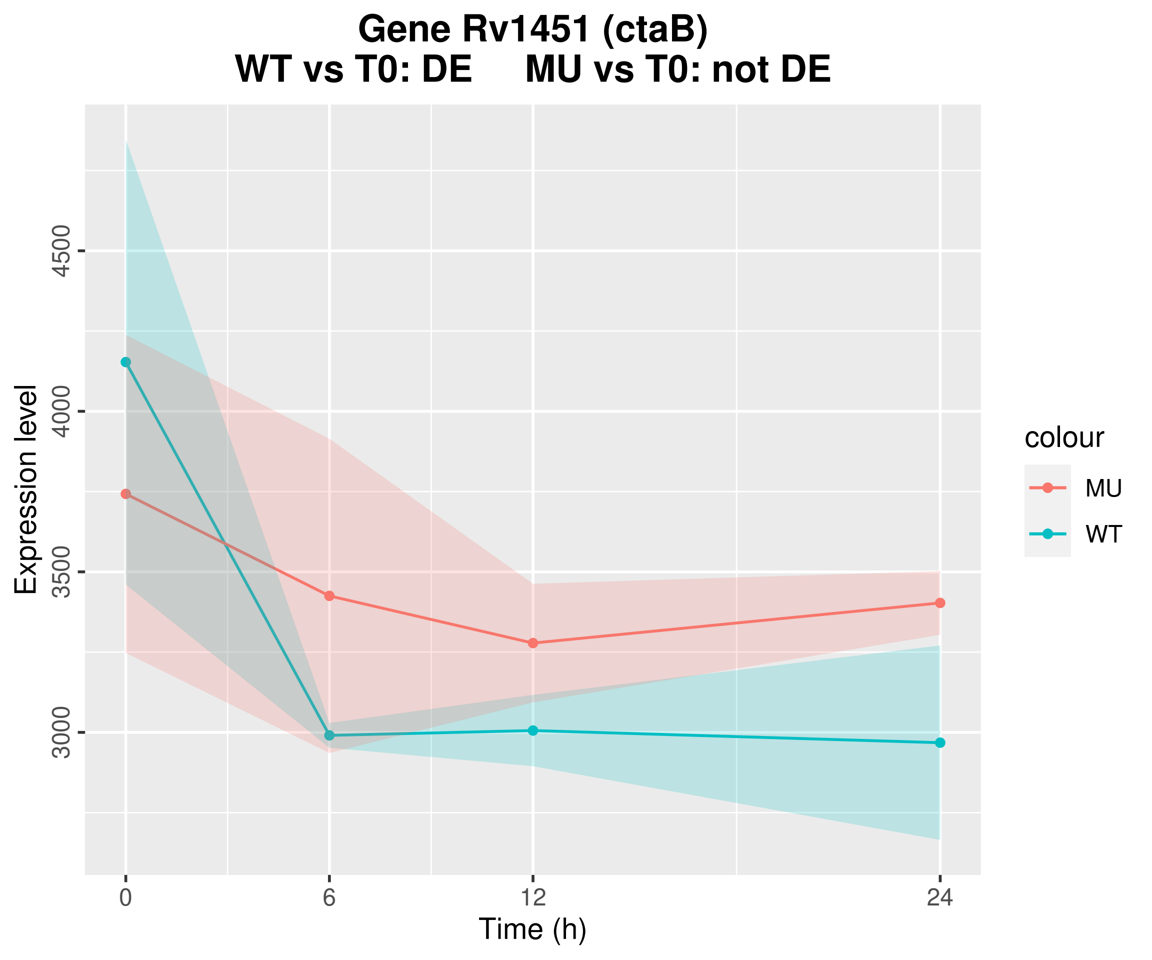
Gene Rv1451 (ctaB)
WT vs T0: DE   MU vs T0: not DE
Expression level
4500
4000
3500
3000
0
6
12
24
Time (h)
colour
MU
WT

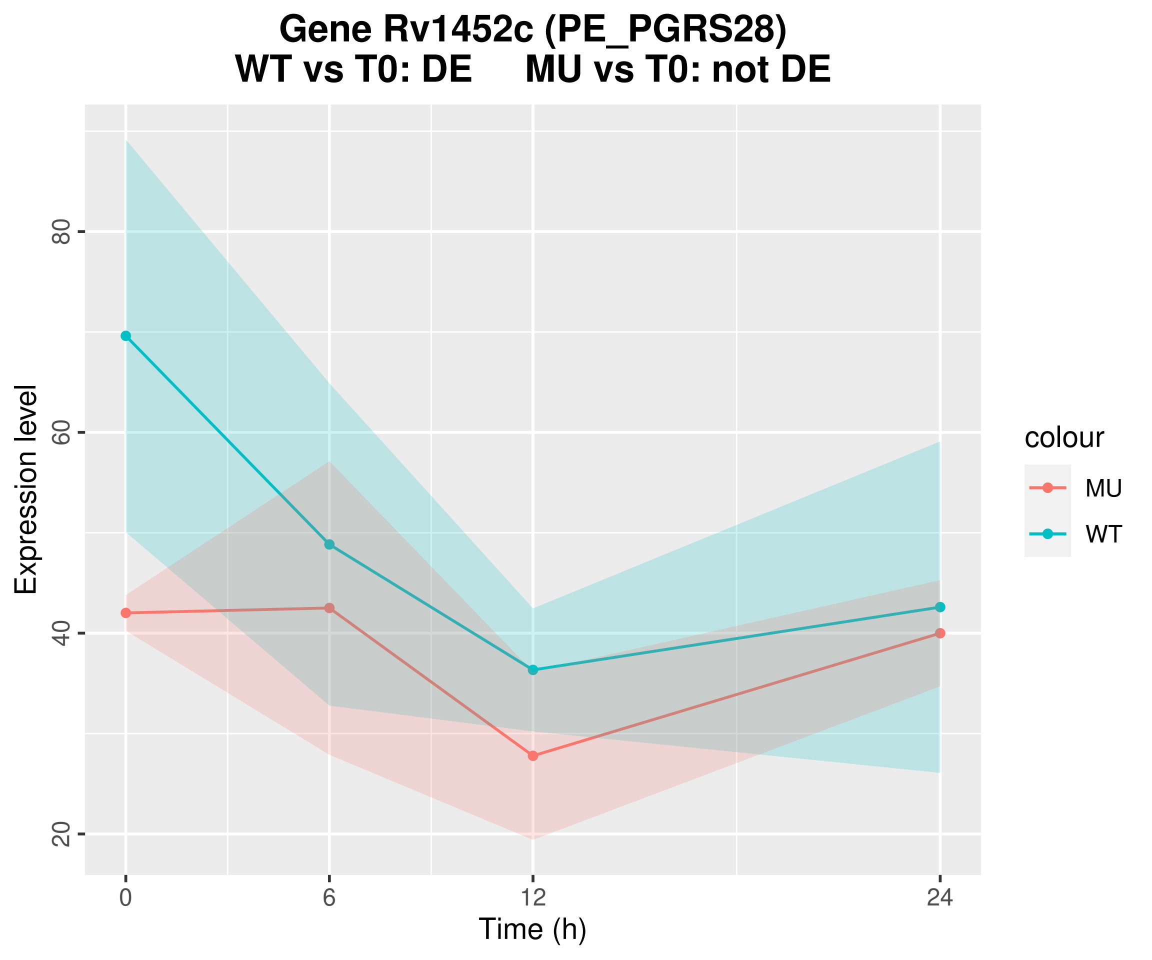

Gene Rv1452c (PE_PGRS28)
WT vs T0: DE MU vs T0: not DE
Expression level
Time (h)
20
40
60
80
0
6
12
24
colour
MU
WT

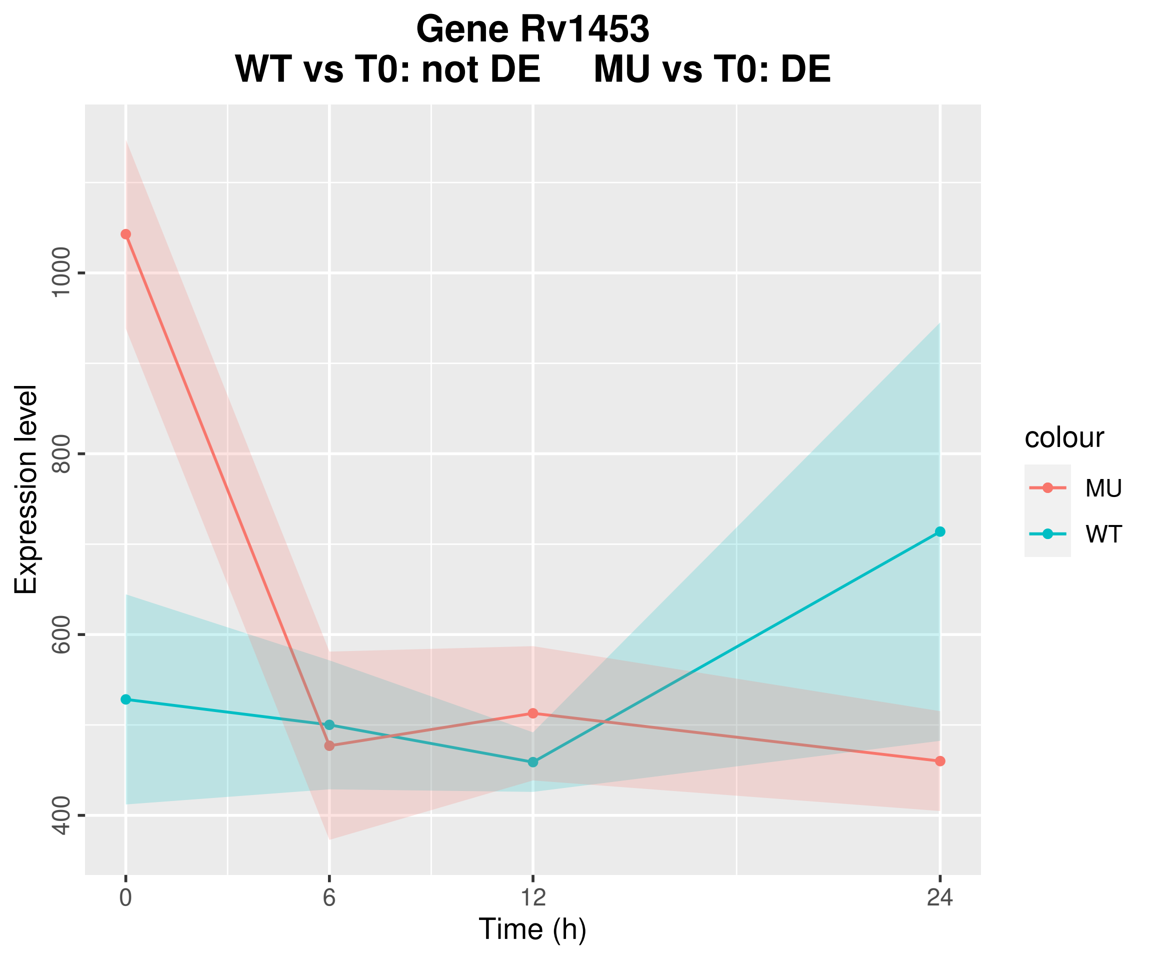

Gene Rv1453
WT vs T0: not DE   MU vs T0: DE
Expression level
400
600
800
1000
0
6
12
24
Time (h)
colour
MU
WT

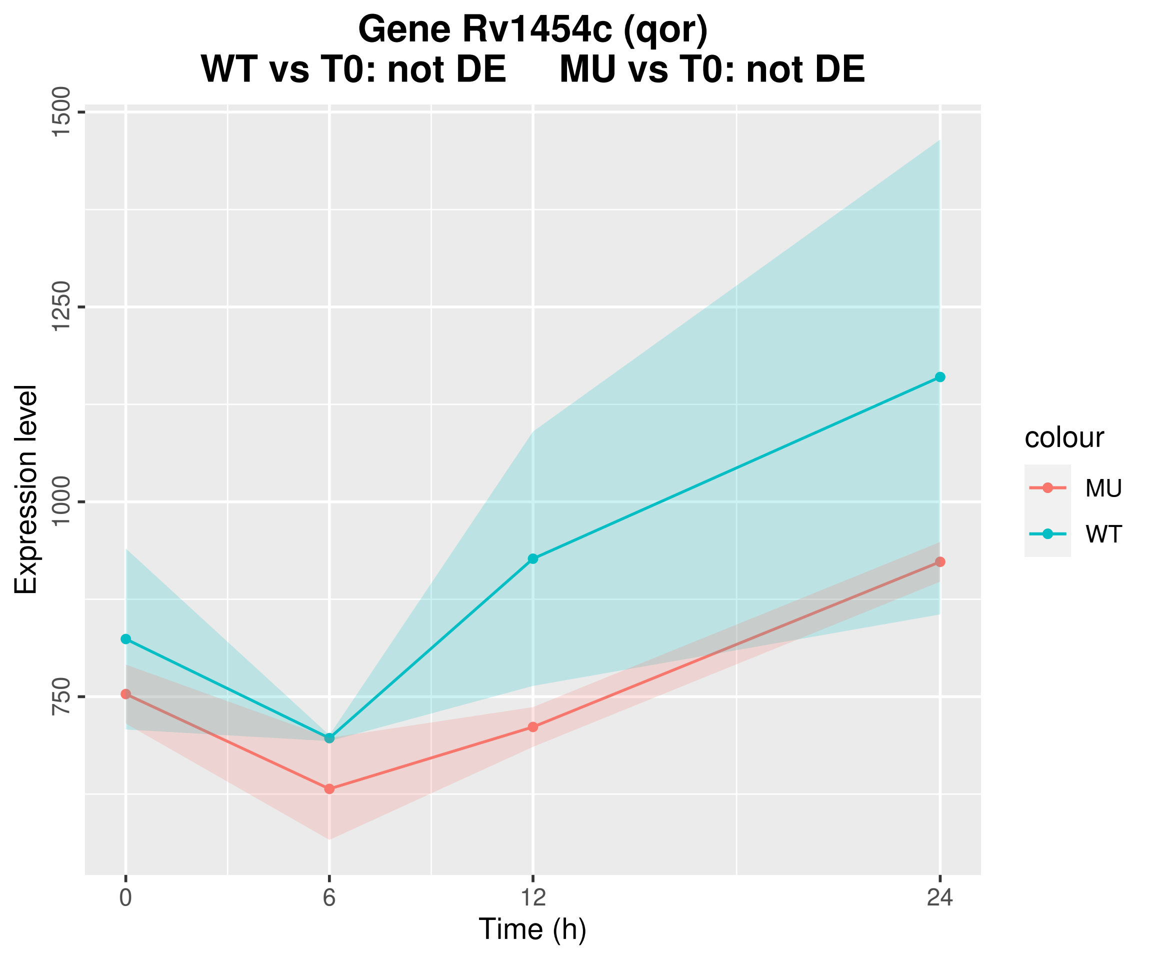

Gene Rv1454c (qor)
WT vs T0: not DE   MU vs T0: not DE
Expression level
1500
1250
1000
750
0
6
12
24
Time (h)
colour
MU
WT

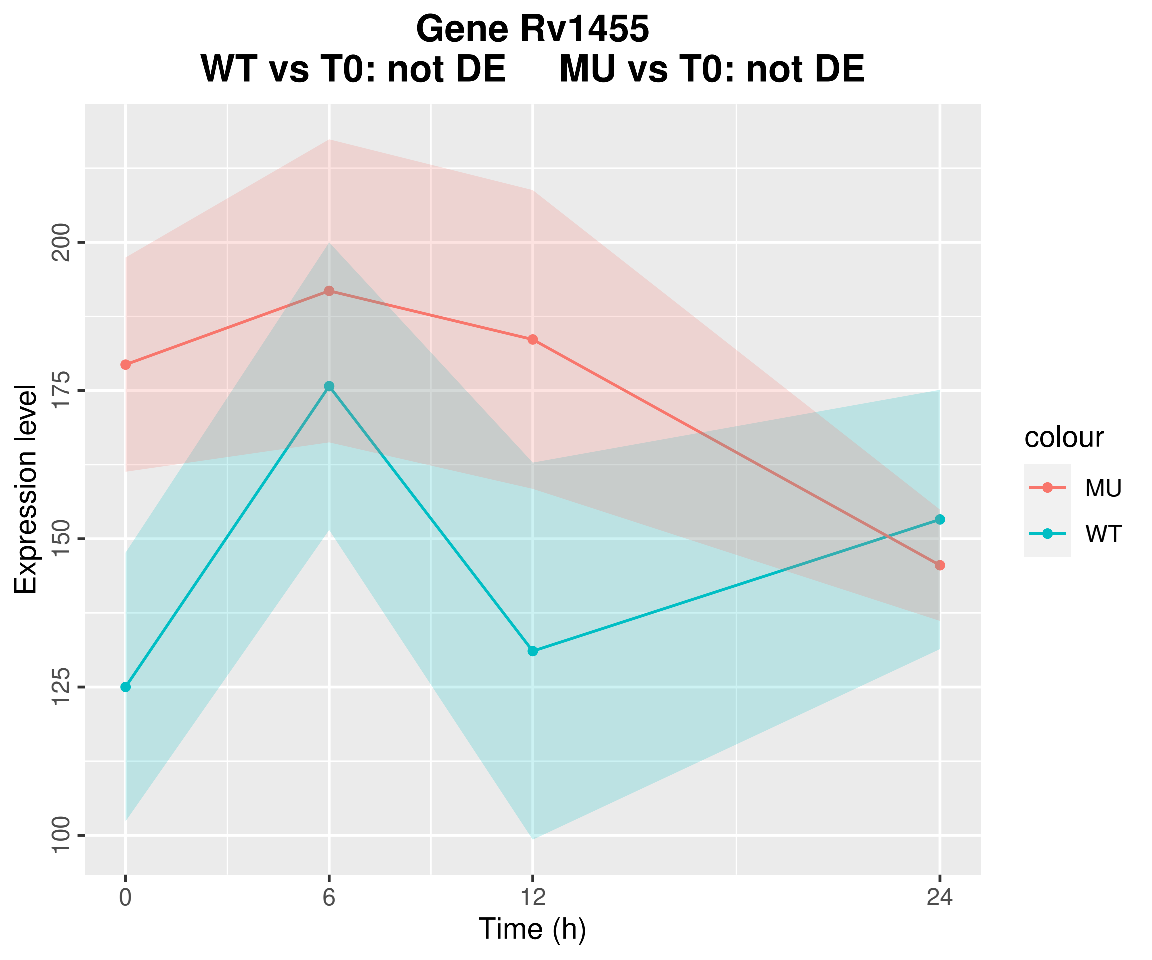
Gene Rv1455
WT vs T0: not DE   MU vs T0: not DE
Expression level
100
125
150
175
200
0
6
12
24
Time (h)
colour
MU
WT

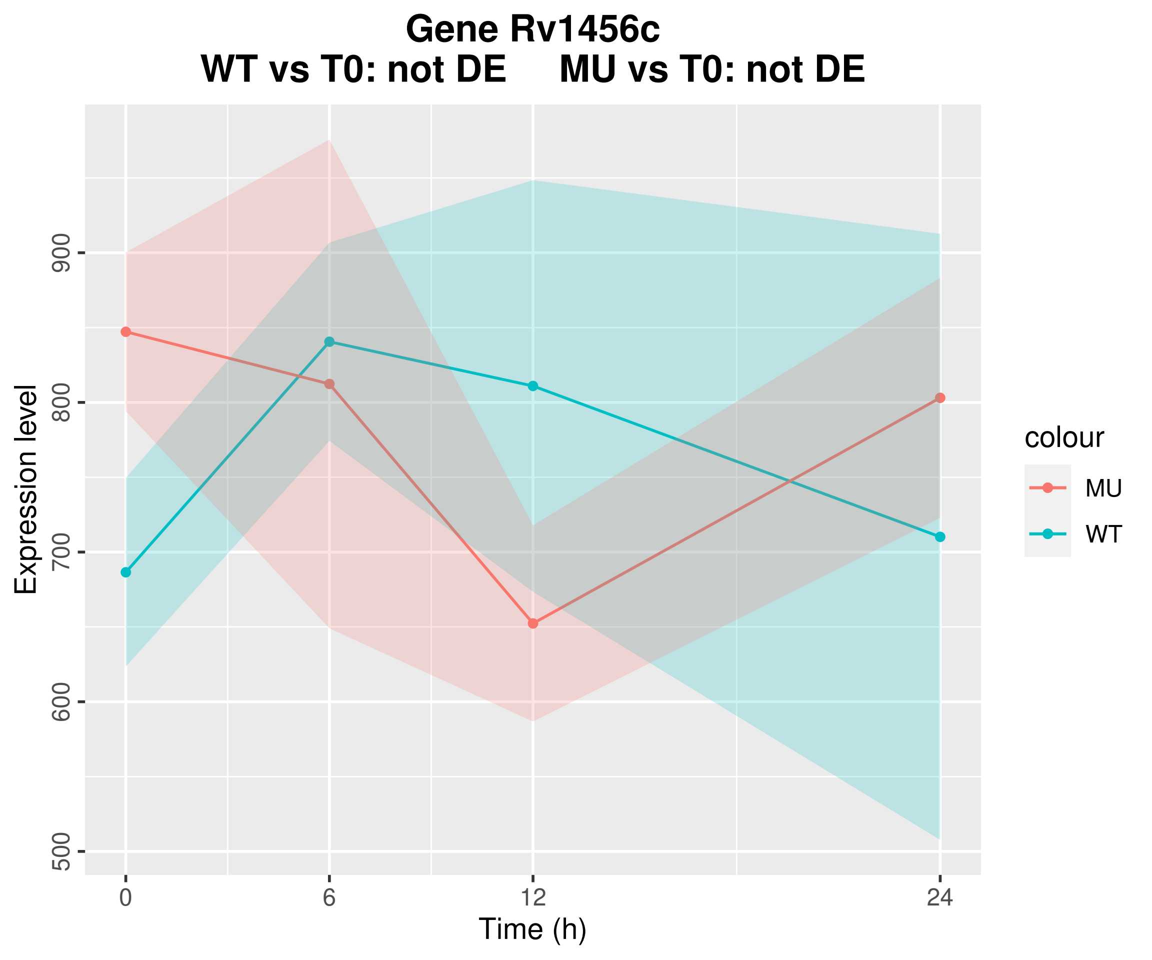
Gene Rv1456c
WT vs T0: not DE
MU vs T0: not DE
Expression level
900
800
700
600
500
0
6
12
24
Time (h)
colour
MU
WT

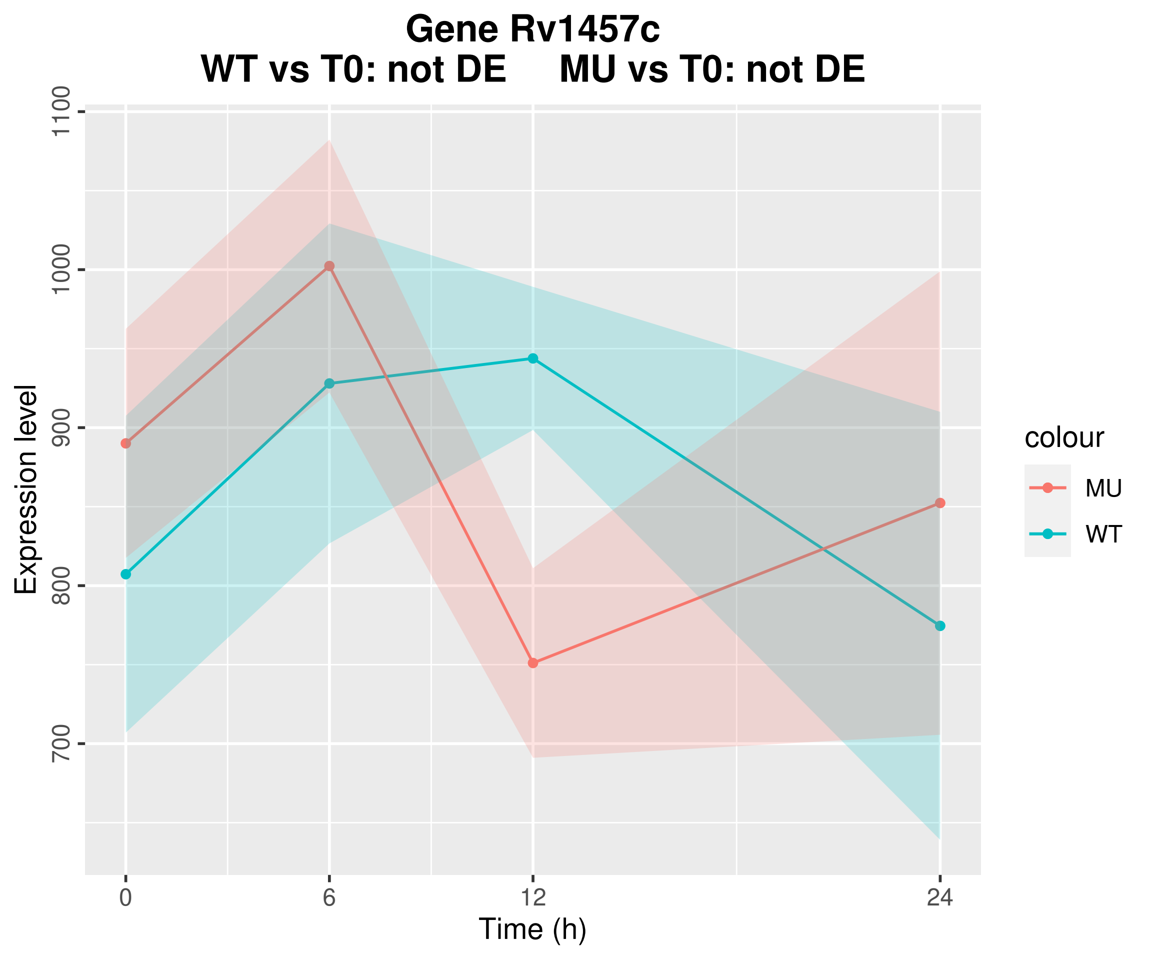

Gene Rv1457c
WT vs T0: not DE MU vs T0: not DE
Expression level
1100
1000
900
800
700
0
6
12
24
Time (h)
colour
MU
WT

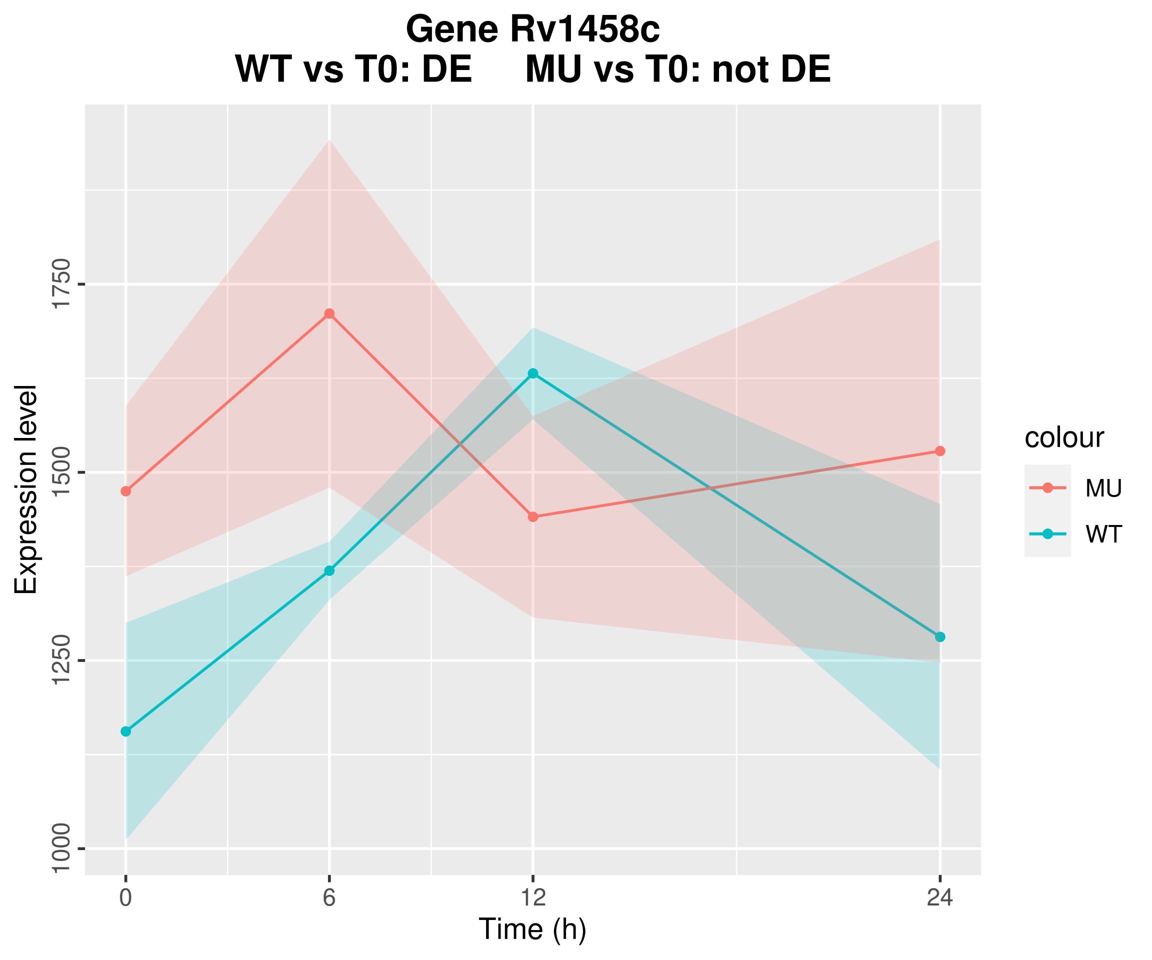
Gene Rv1458c
WT vs T0: DE MU vs T0: not DE
Expression level
1000
1250
1500
1750
0
6
12
24
Time (h)
colour
MU
WT

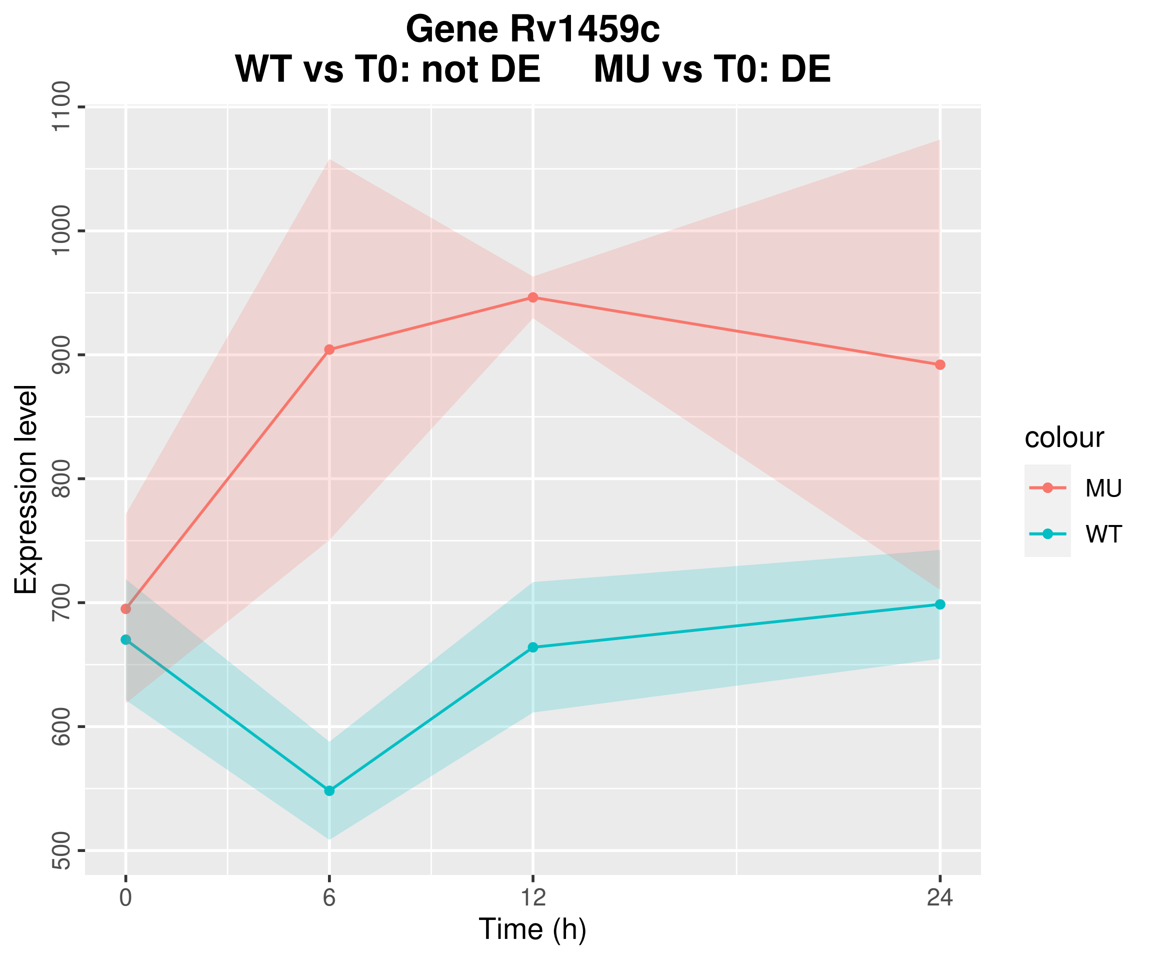
Gene Rv1459c
WT vs T0: not DE MU vs T0: DE
Expression level
1100
1000
900
800
700
600
500
0
6
12
24
Time (h)
colour
MU
WT

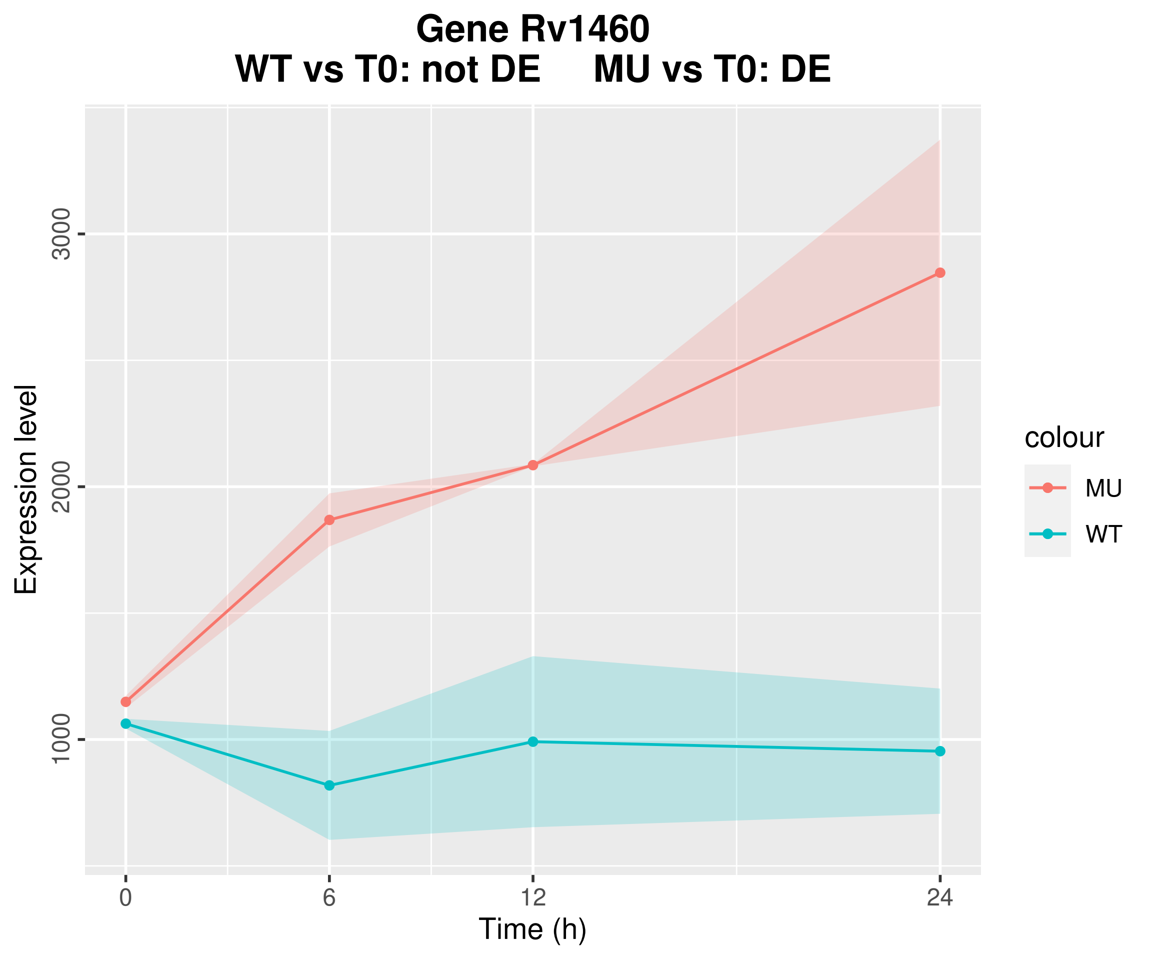

Gene Rv1460
WT vs T0: not DE MU vs T0: DE
Expression level
3000
2000
1000
0
6
12
24
Time (h)
colour
MU
WT

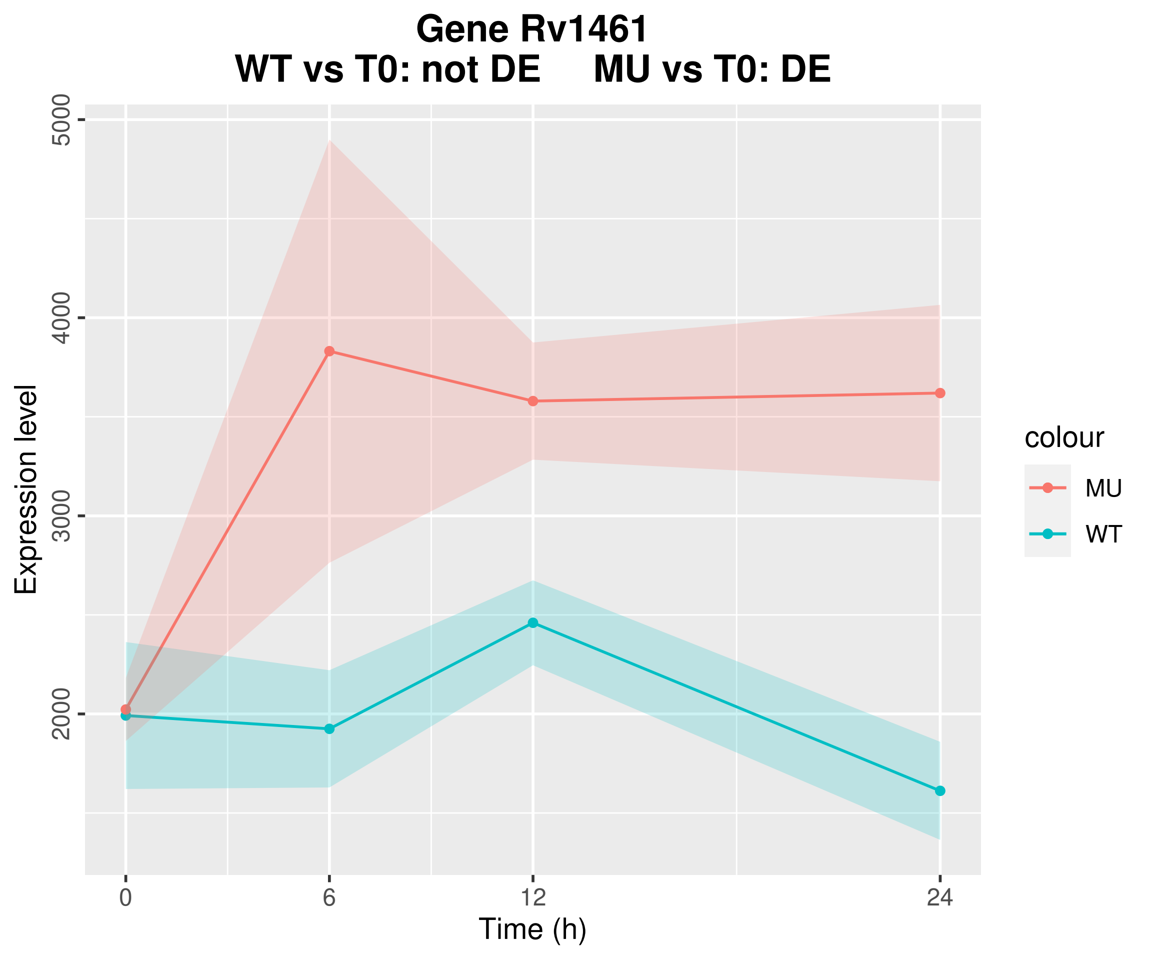

Gene Rv1461
WT vs T0: not DE MU vs T0: DE
Expression level
5000
4000
3000
2000
0
6
12
24
Time (h)
colour
MU
WT

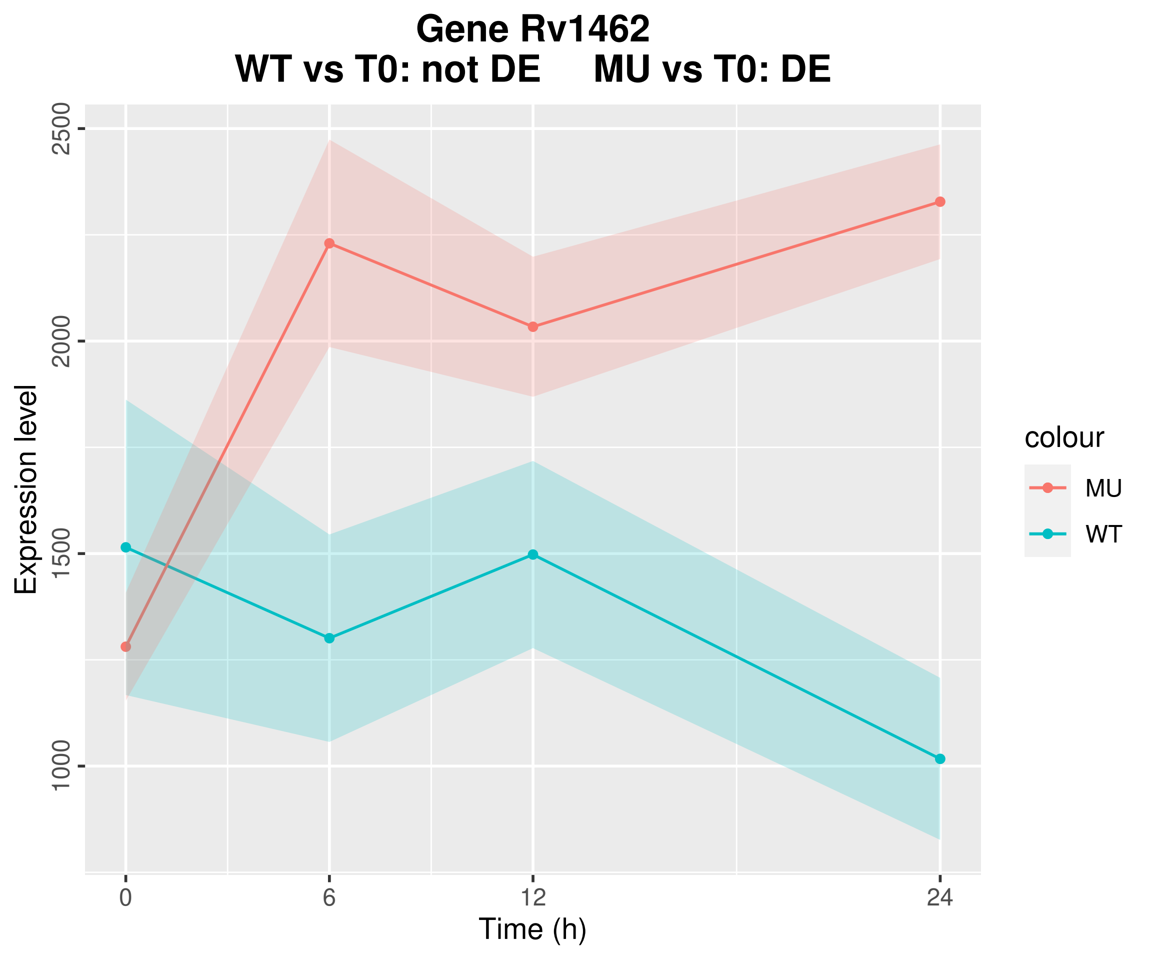

Gene Rv1462
WT vs T0: not DE MU vs T0: DE
Expression level
2500
2000
1500
1000
0
6
12
24
Time (h)
colour
MU
WT

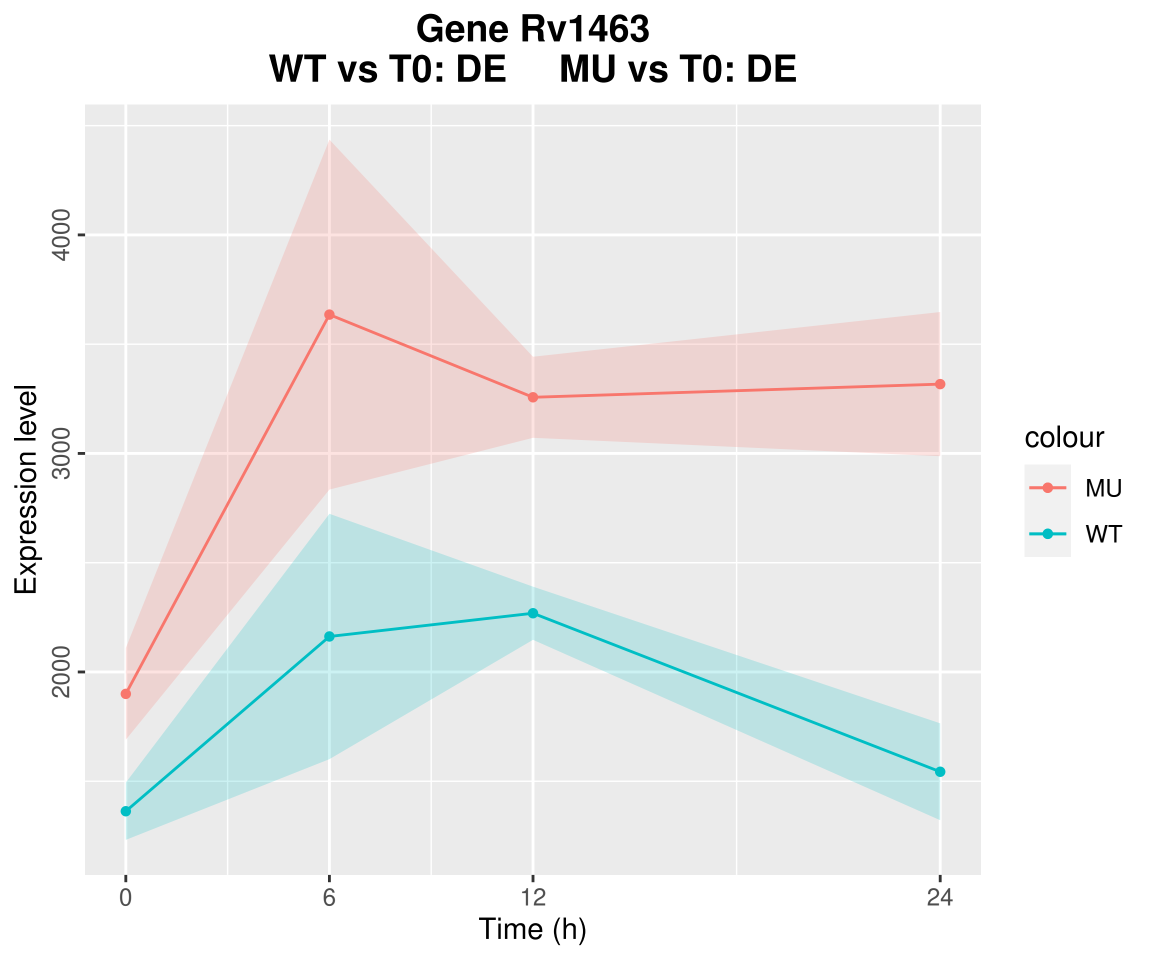

Gene Rv1463
WT vs T0: DE MU vs T0: DE
Expression level
4000
3000
2000
0
6
12
24
Time (h)
colour
MU
WT

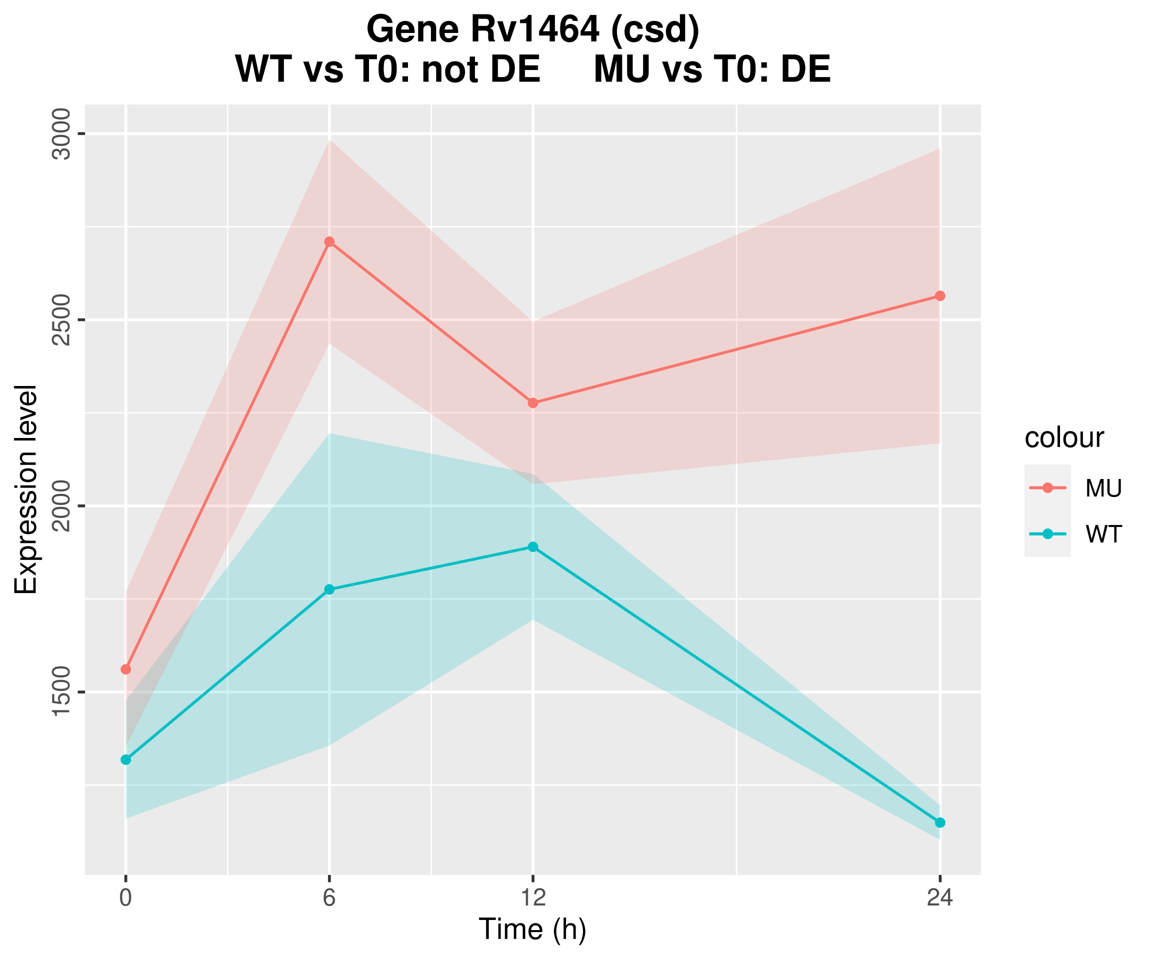
Gene Rv1464 (csd)
WT vs T0: not DE MU vs T0: DE
Expression level
3000
2500
2000
1500
0
6
12
24
Time (h)
colour
MU
WT

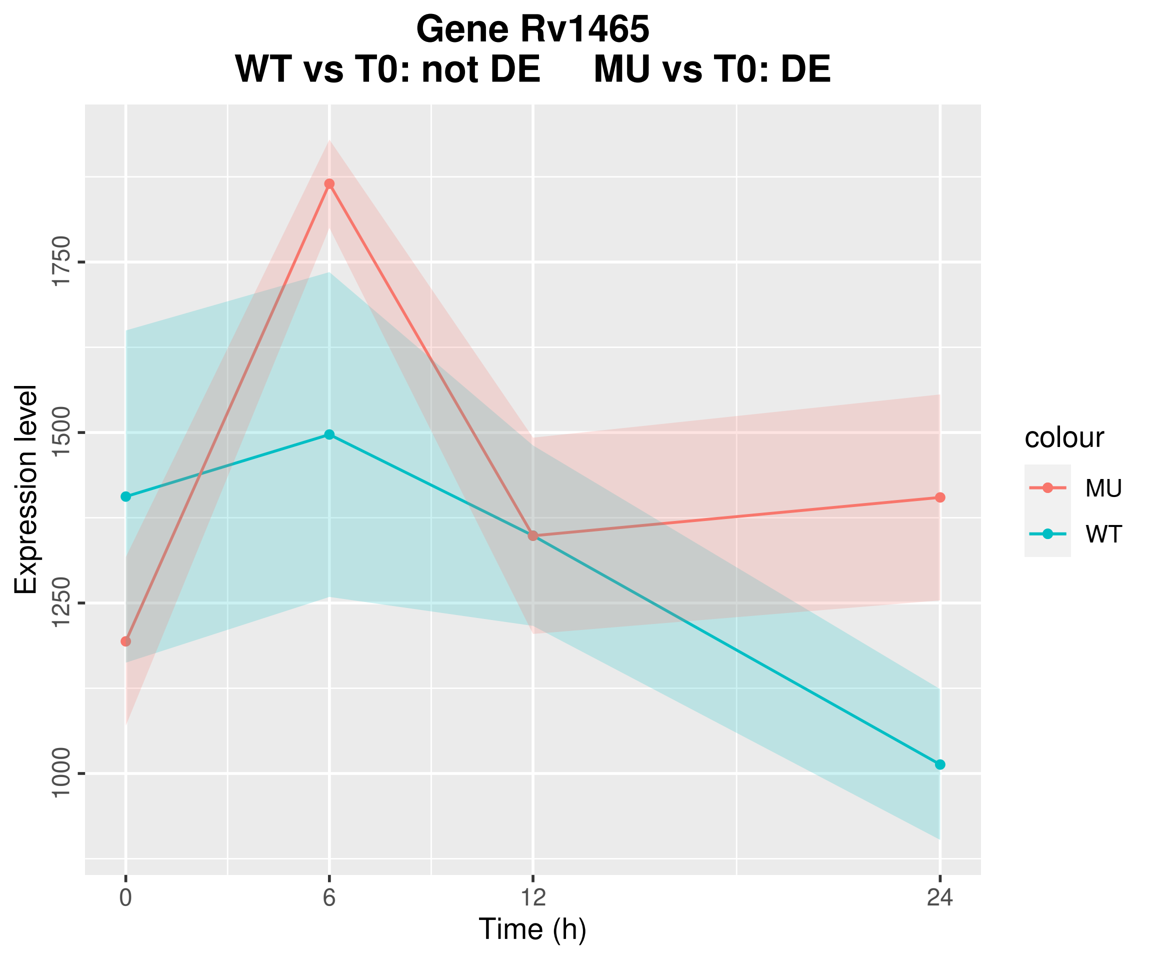
Gene Rv1465
WT vs T0: not DE MU vs T0: DE
Expression level
1000
1250
1500
1750
0
6
12
24
Time (h)
colour
MU
WT

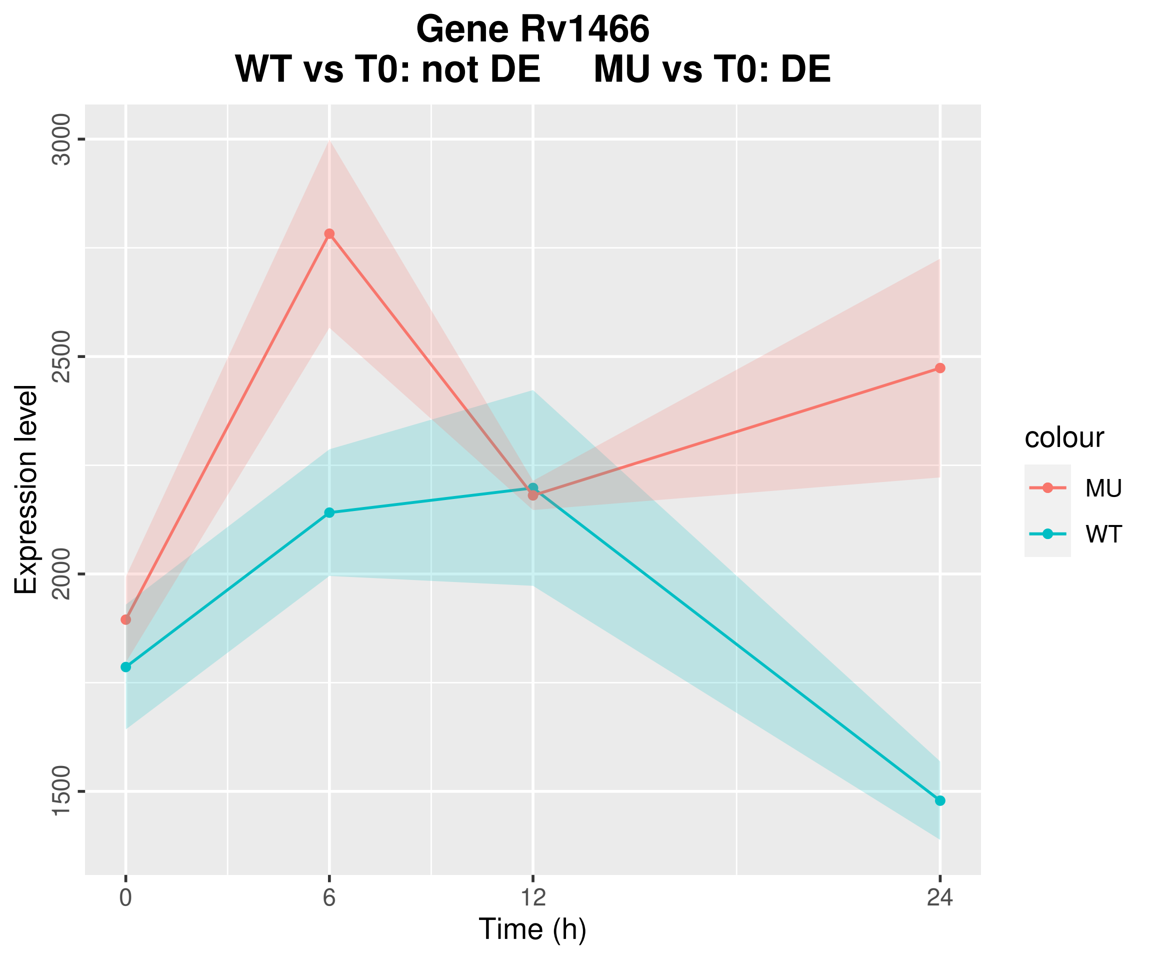

Gene Rv1466
WT vs T0: not DE MU vs T0: DE
Expression level
3000
2500
2000
1500
0
6
12
24
Time (h)
colour
MU
WT

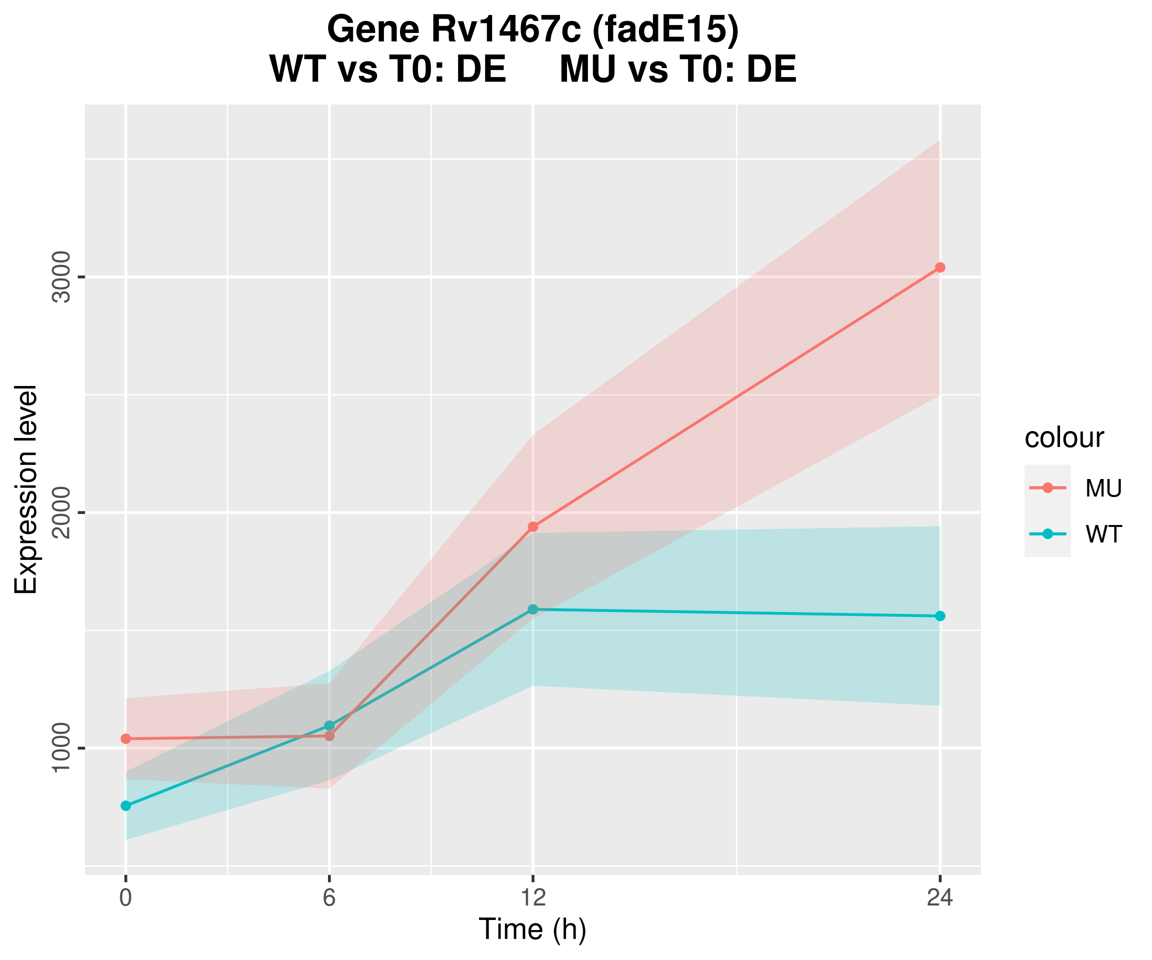

Gene Rv1467c (fadE15)
WT vs T0: DE MU vs T0: DE
3000
2000
1000
0
6
12
24
Time (h)
Expression level
colour
MU
WT

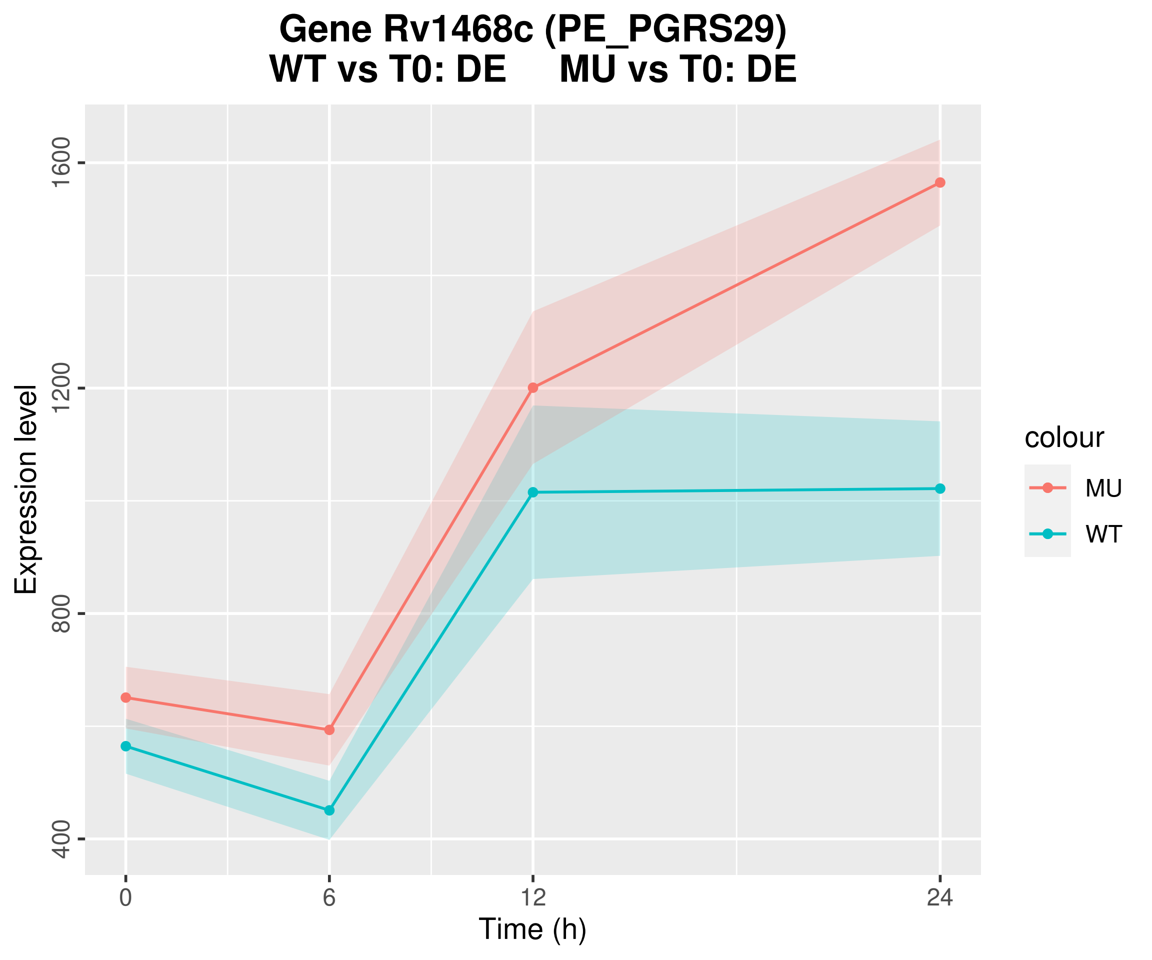

Gene Rv1468c (PE_PGRS29)
WT vs T0: DE MU vs T0: DE
1600
1200
800
400
Expression level
0
6
12
24
Time (h)
colour
MU
WT

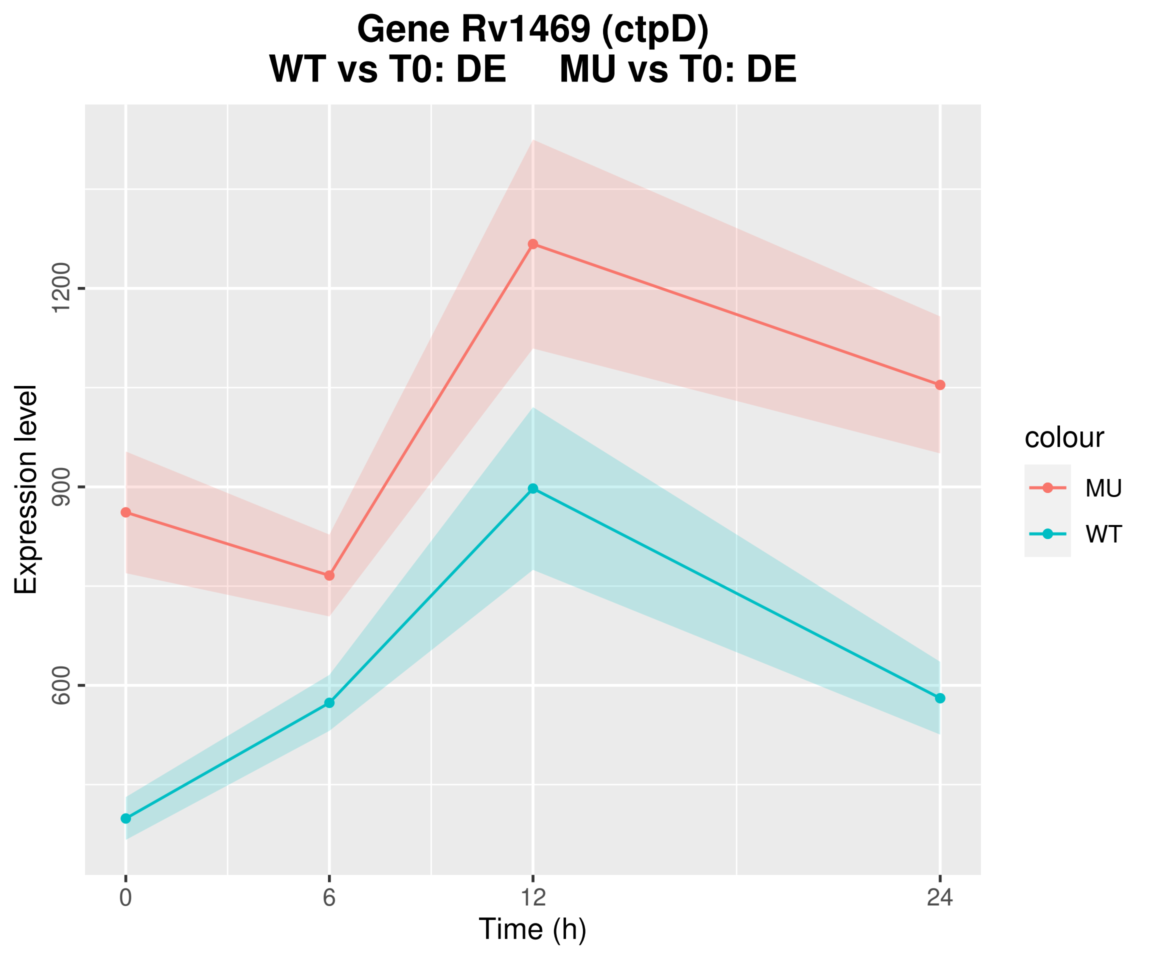
Gene Rv1469 (ctpD)
WT vs T0: DE   MU vs T0: DE
Expression level
1200
900
600
0
6
12
24
Time (h)
colour
MU
WT

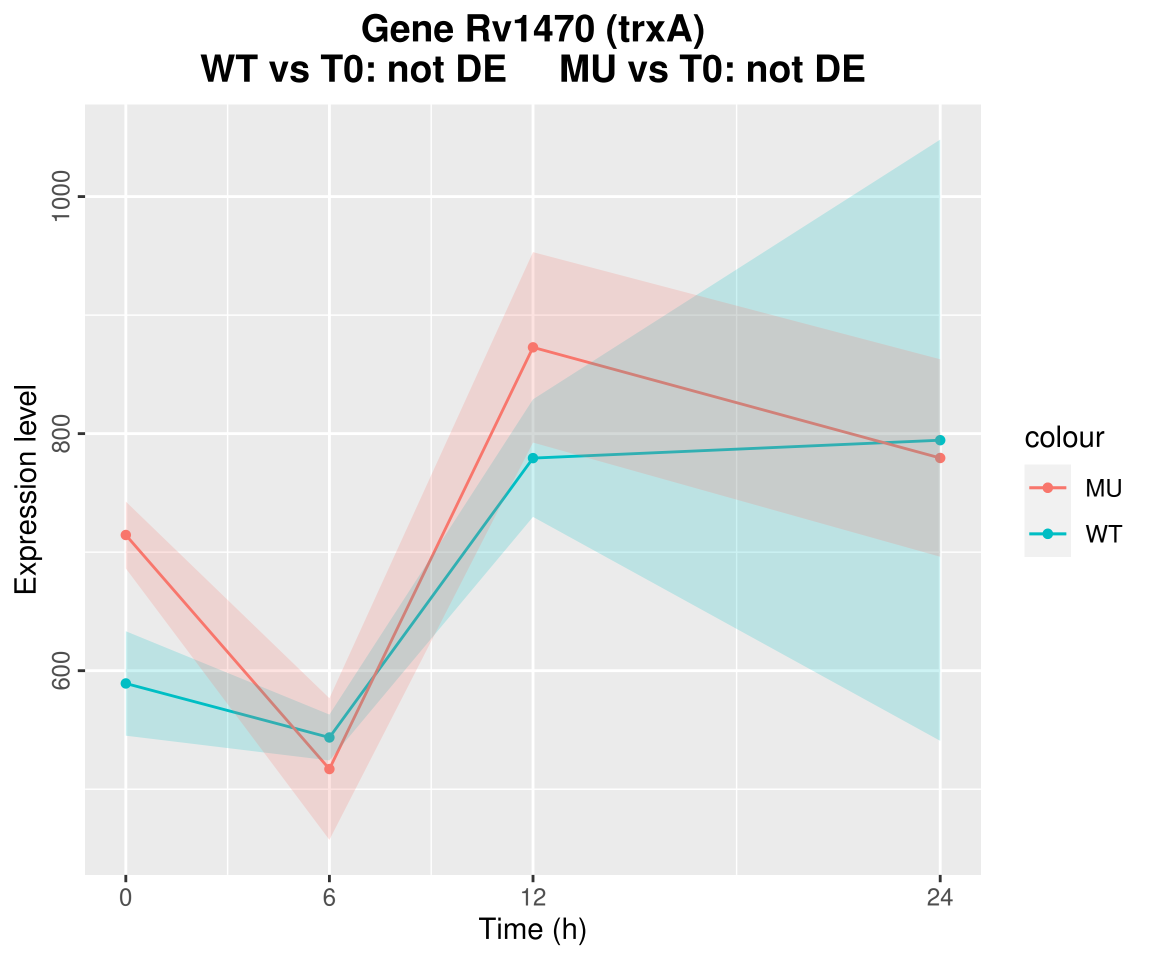
Gene Rv1470 (trxA)
WT vs T0: not DE   MU vs T0: not DE
1000
800
600
Expression level
0
6
12
24
Time (h)
colour
MU
WT

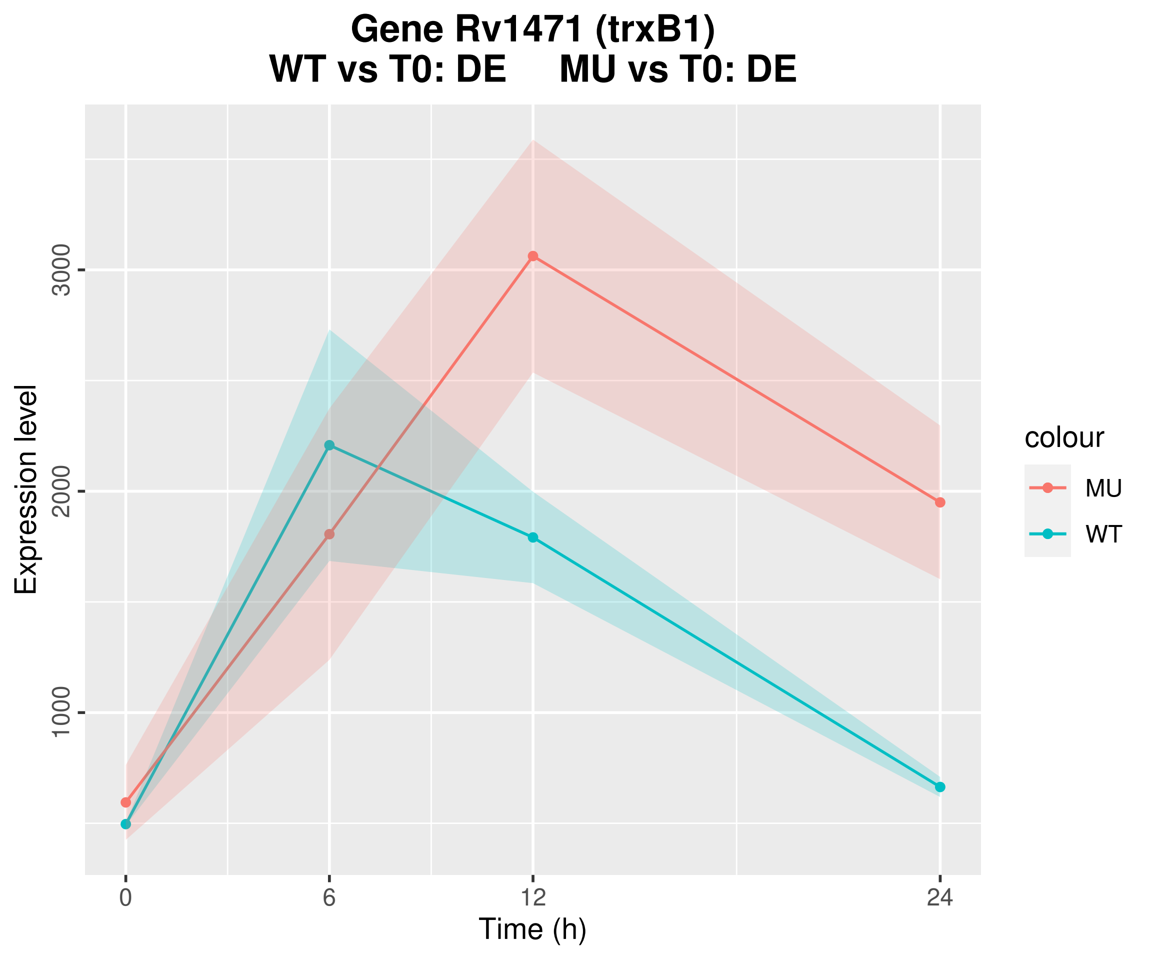
Gene Rv1471 (trxB1)
WT vs T0: DE MU vs T0: DE
Expression level
3000
2000
1000
0
6
12
24
Time (h)
colour
MU
WT

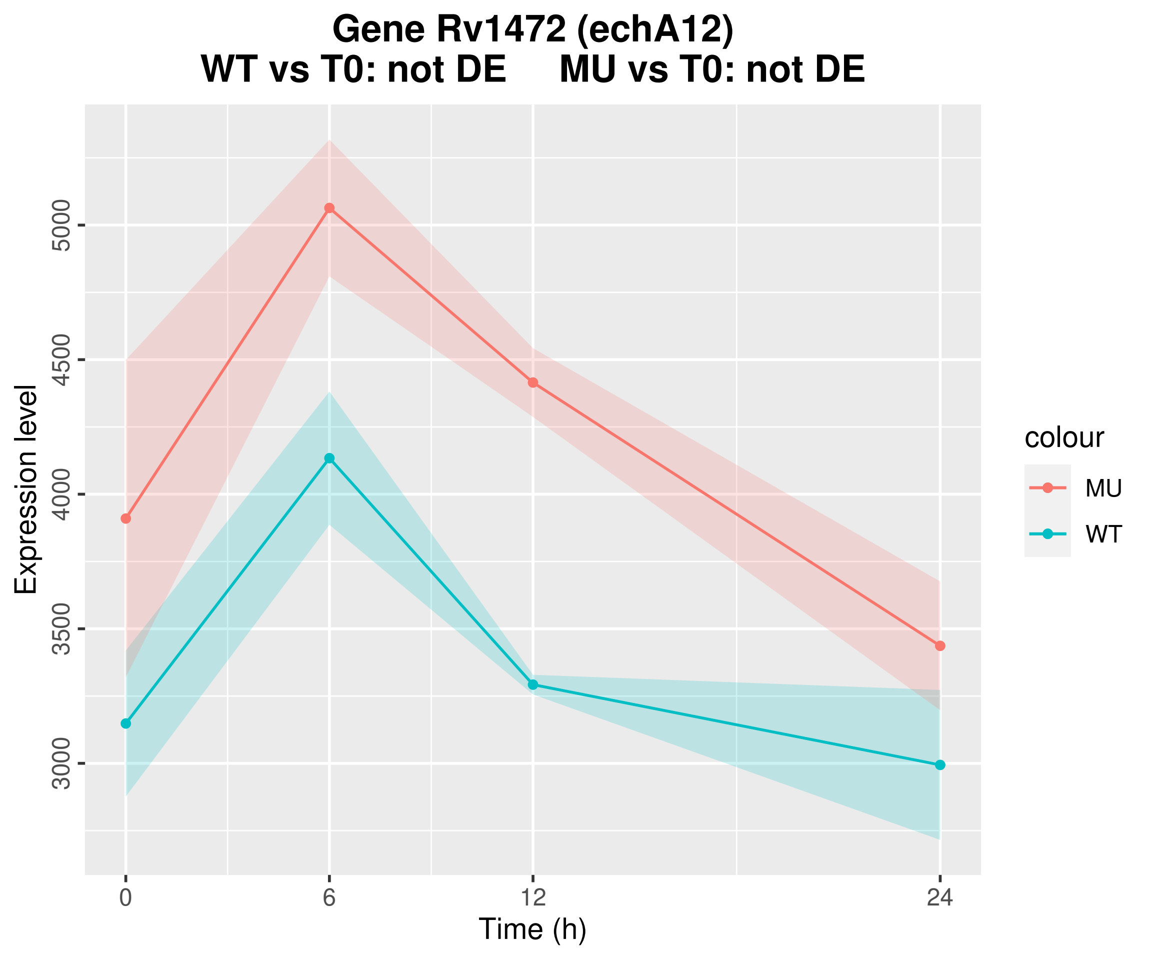
Gene Rv1472 (echA12)
WT vs T0: not DE MU vs T0: not DE
Expression level
5000
4500
4000
3500
3000
0
6
12
24
Time (h)
colour
MU
WT

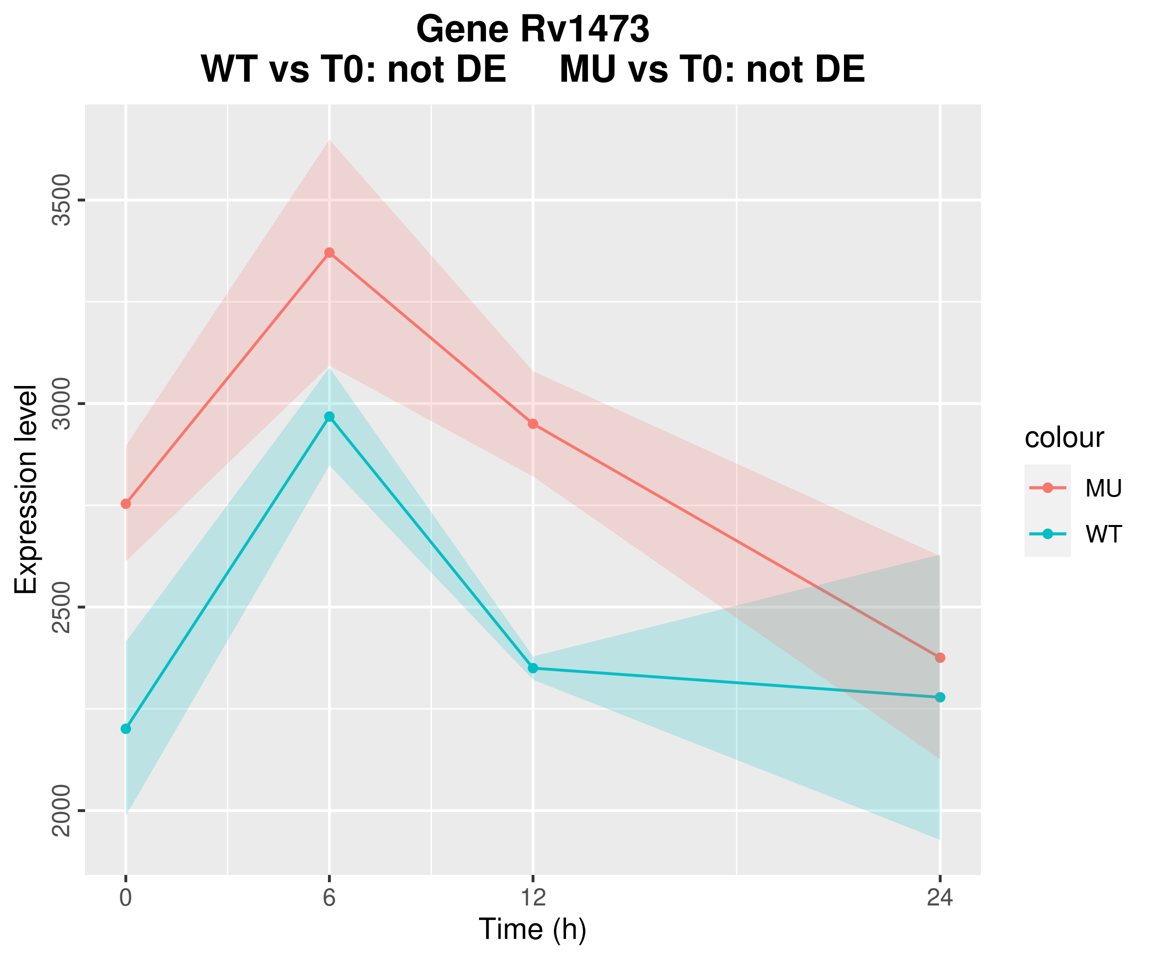

Gene Rv1473
WT vs T0: not DE   MU vs T0: not DE
Expression level
3500
3000
2500
2000
0
6
12
24
Time (h)
colour
MU
WT

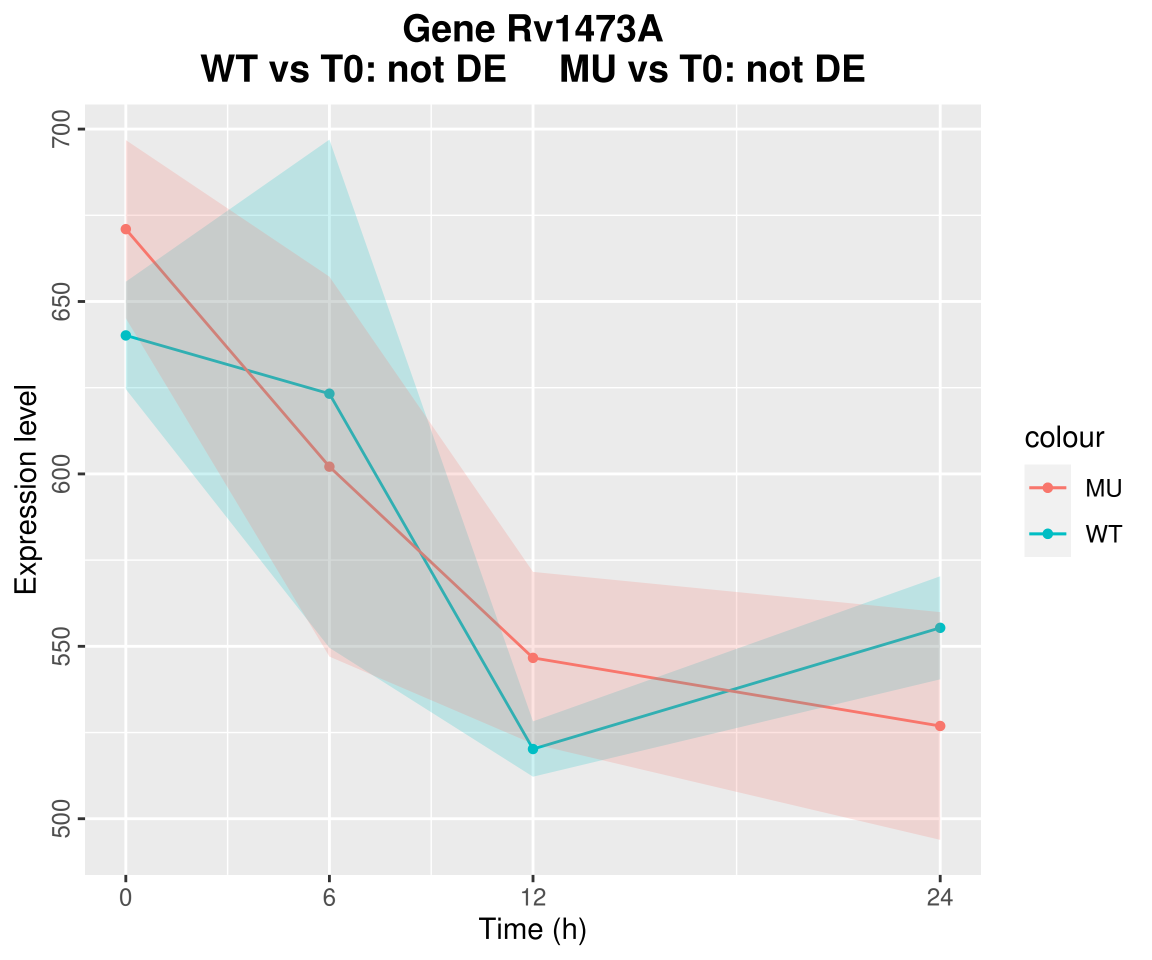
Gene Rv1473A
WT vs T0: not DE   MU vs T0: not DE
Expression level
700
650
600
550
500
0
6
12
24
Time (h)
colour
MU
WT

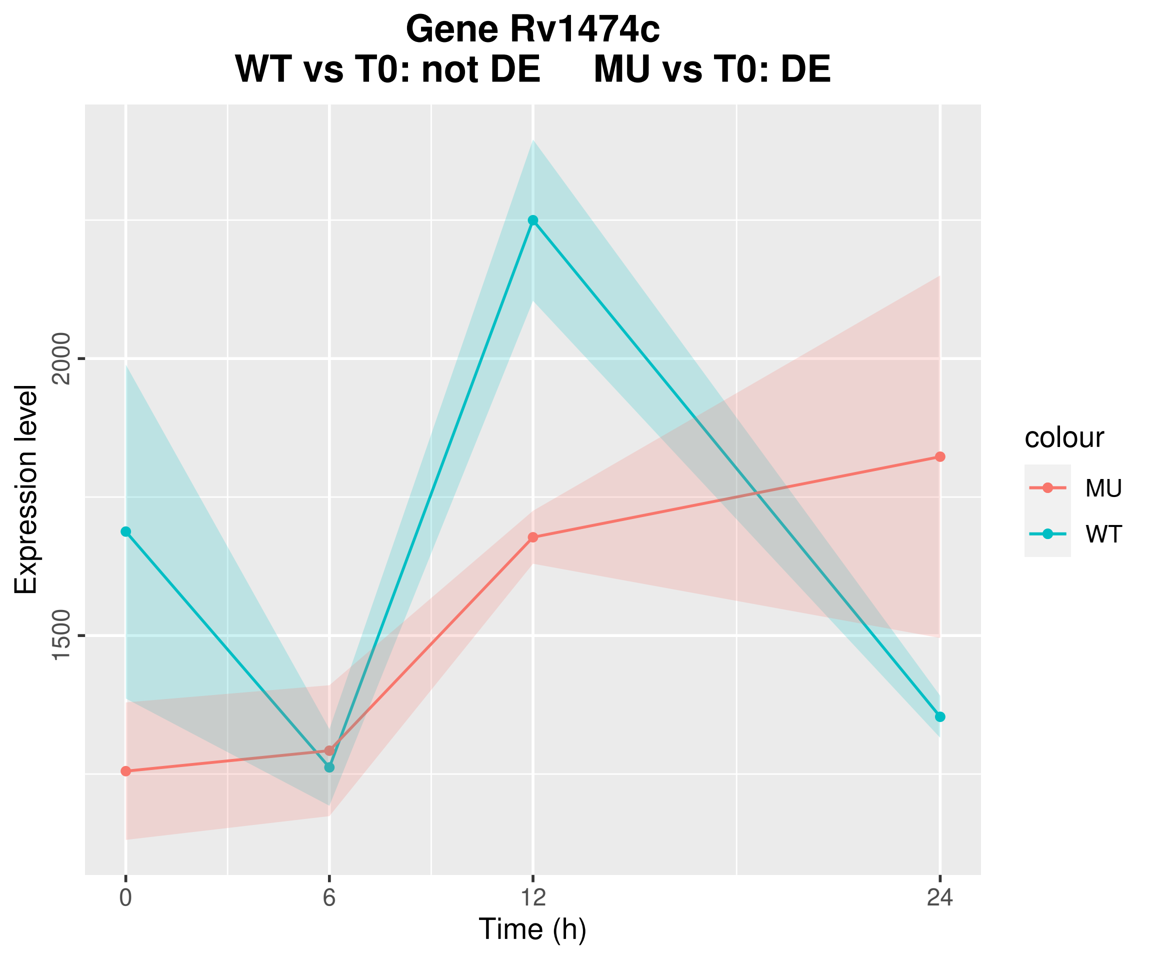

Gene Rv1474c
WT vs T0: not DE MU vs T0: DE
Expression level
1500
2000
0
6
12
24
Time (h)
colour
MU
WT

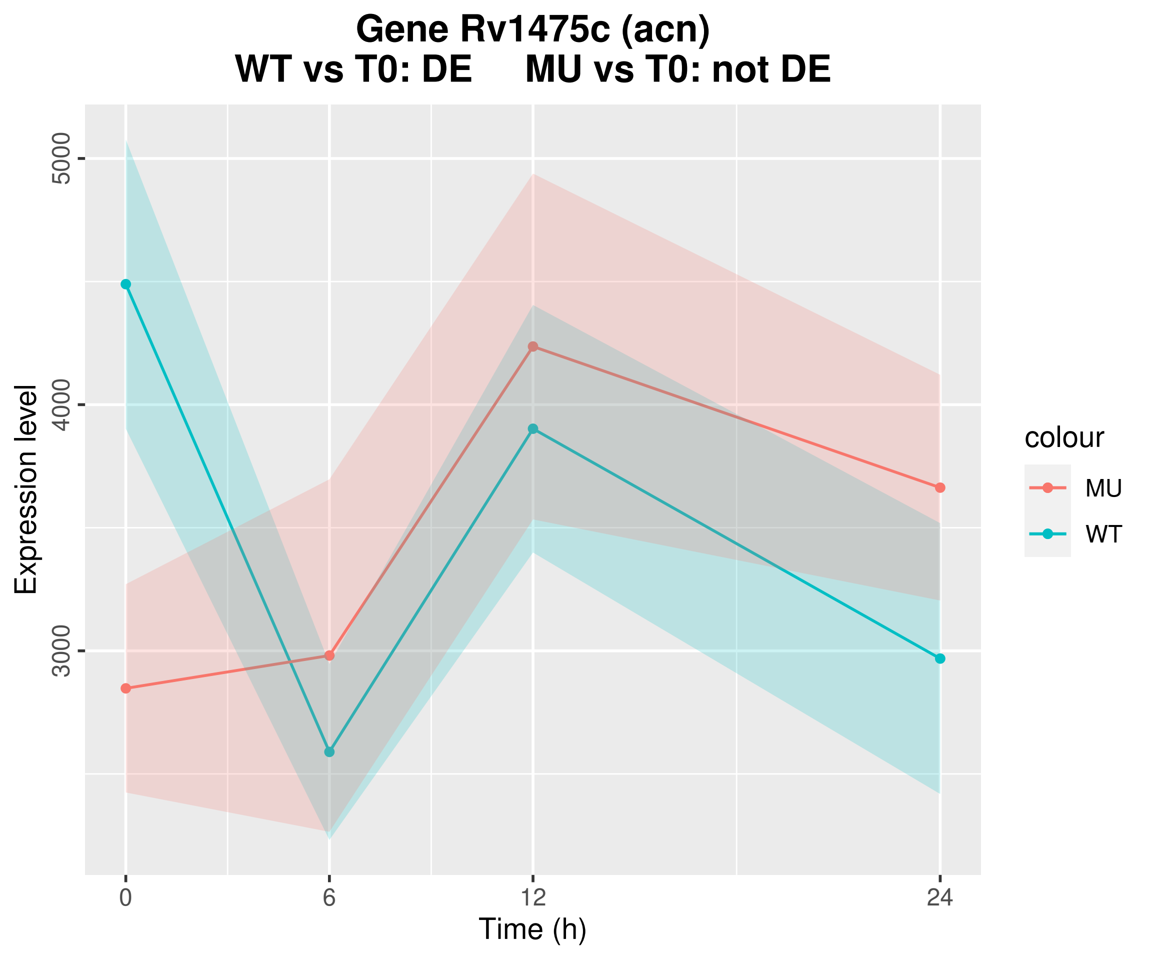
Gene Rv1475c (acn)
WT vs T0: DE   MU vs T0: not DE
Expression level
5000
4000
3000
Time (h)
0
6
12
24
colour
MU
WT

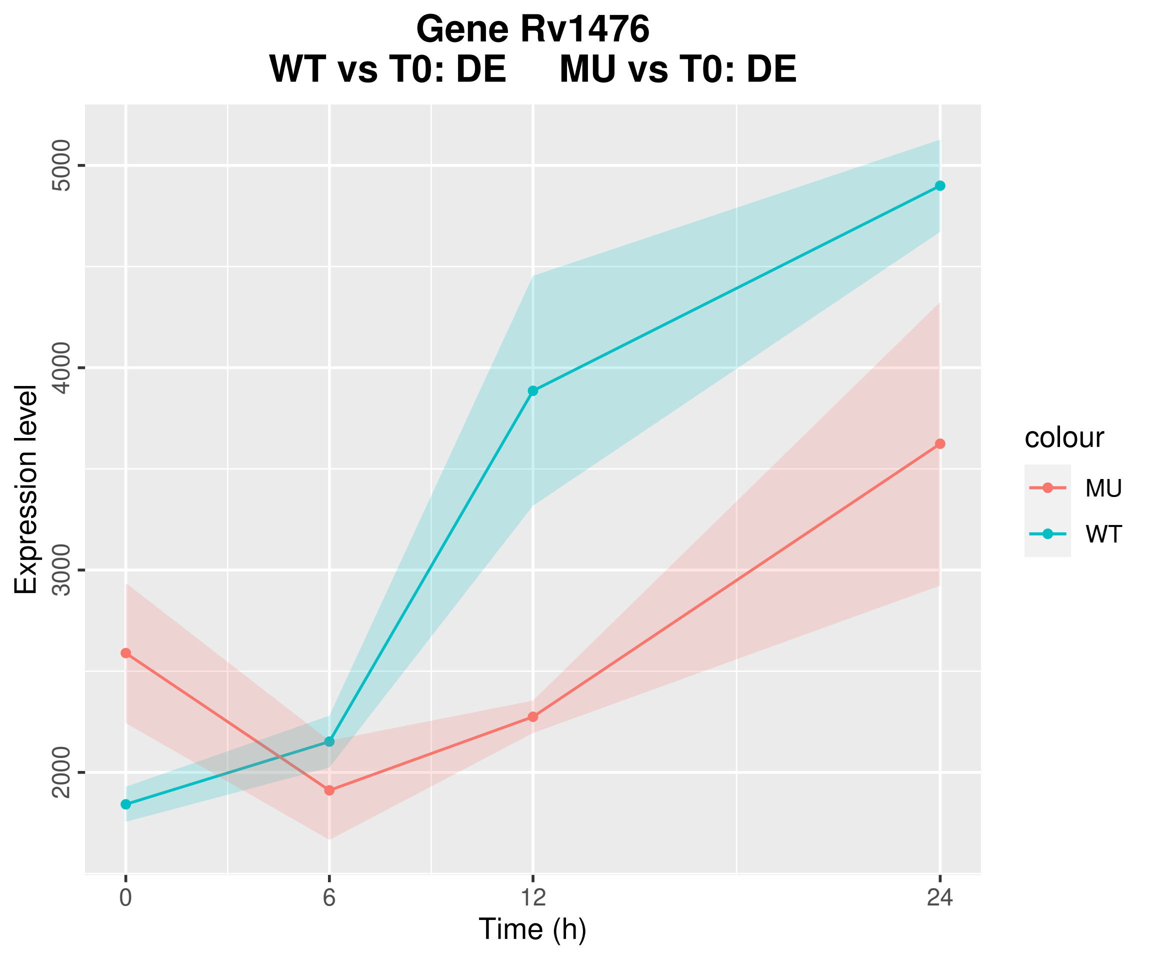

Gene Rv1476
WT vs T0: DE   MU vs T0: DE
Expression level
5000
4000
3000
2000
0
6
12
24
Time (h)
colour
MU
WT

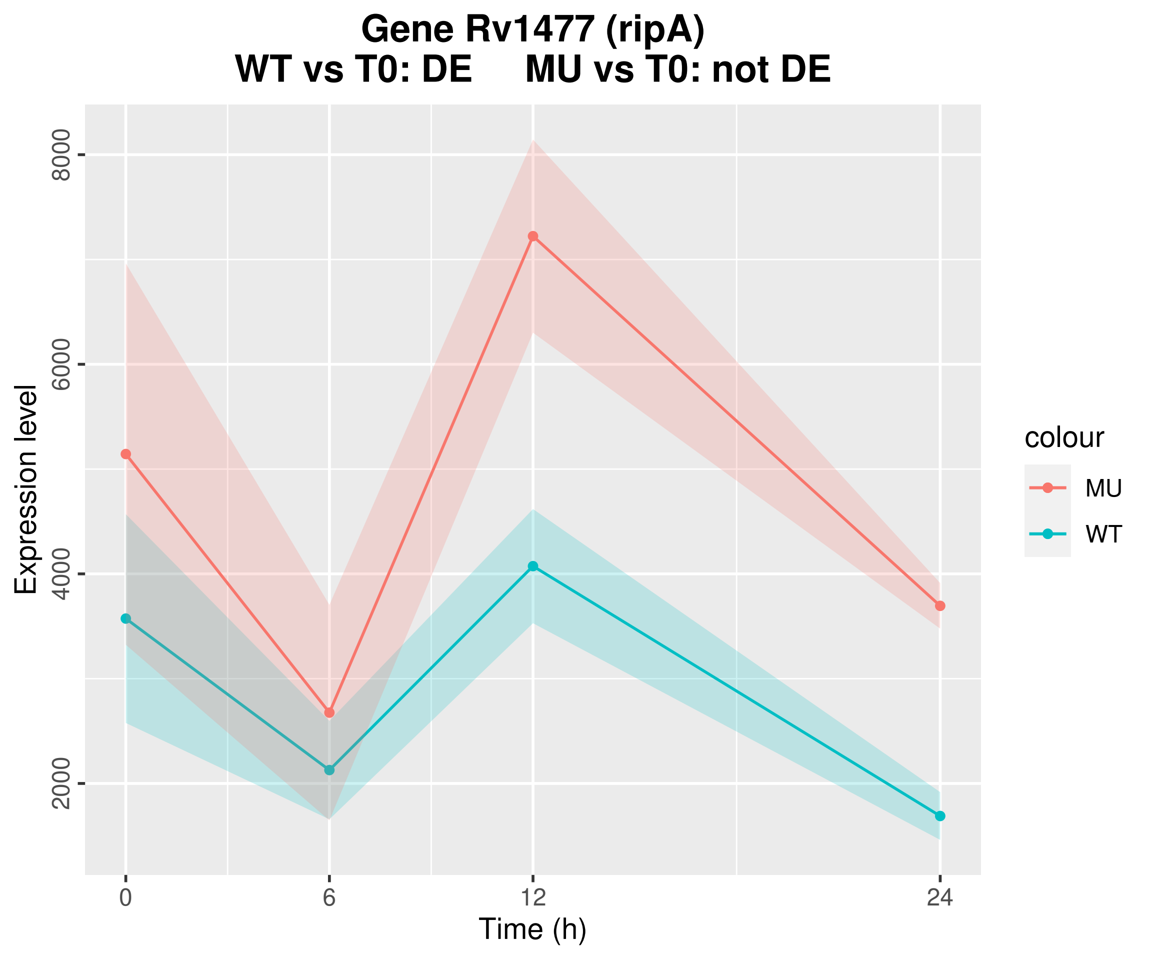

Gene Rv1477 (ripA)
WT vs T0: DE   MU vs T0: not DE
Expression level
8000
6000
4000
2000
0
6
12
24
Time (h)
colour
MU
WT

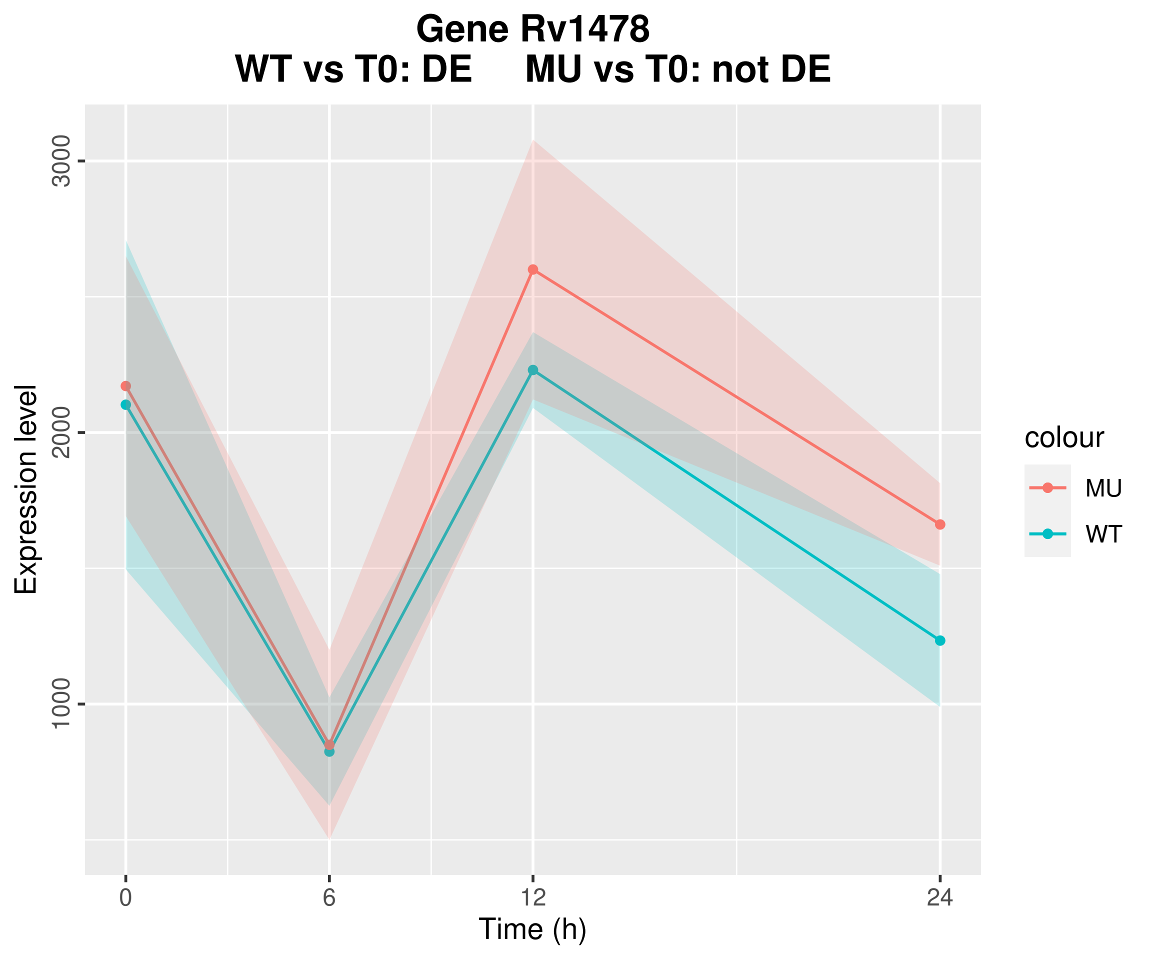
Gene Rv1478
WT vs T0: DE MU vs T0: not DE
Expression level
3000
2000
1000
0
6
12
24
Time (h)
colour
MU
WT

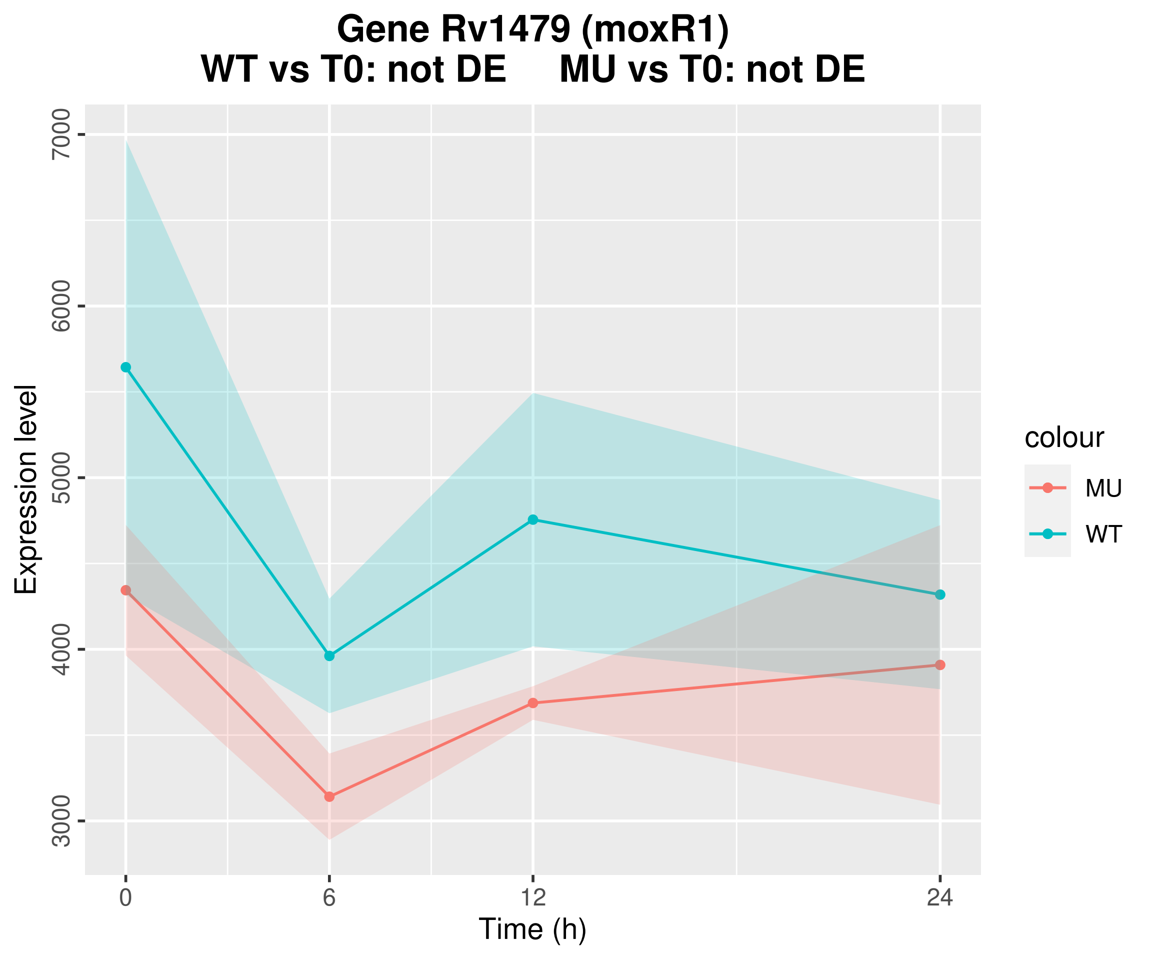
Gene Rv1479 (moxR1)
WT vs T0: not DE   MU vs T0: not DE
Expression level
7000
6000
5000
4000
3000
0
6
12
24
Time (h)
colour
MU
WT

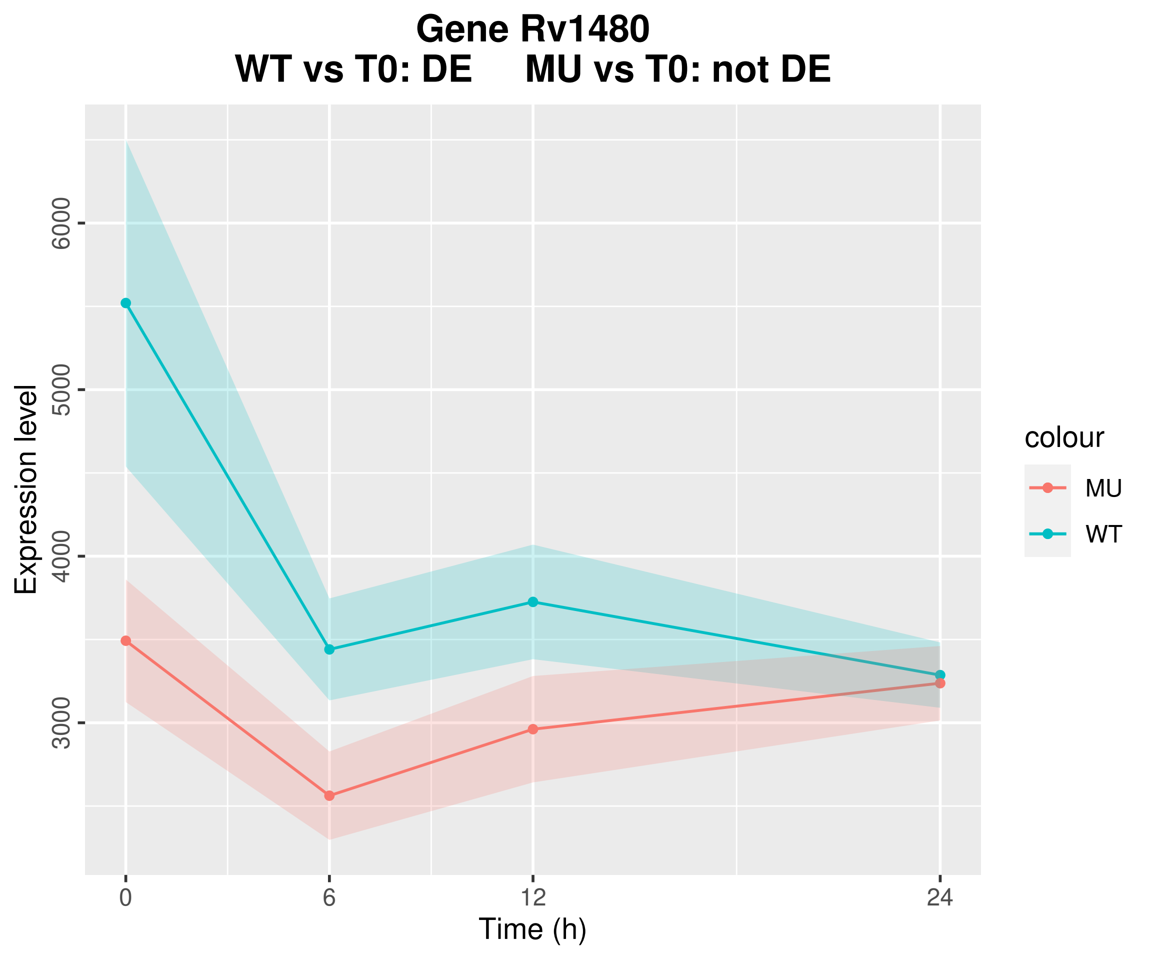

Gene Rv1480
WT vs T0: DE MU vs T0: not DE
Expression level
6000
5000
4000
3000
0
6
12
24
Time (h)
colour
MU
WT

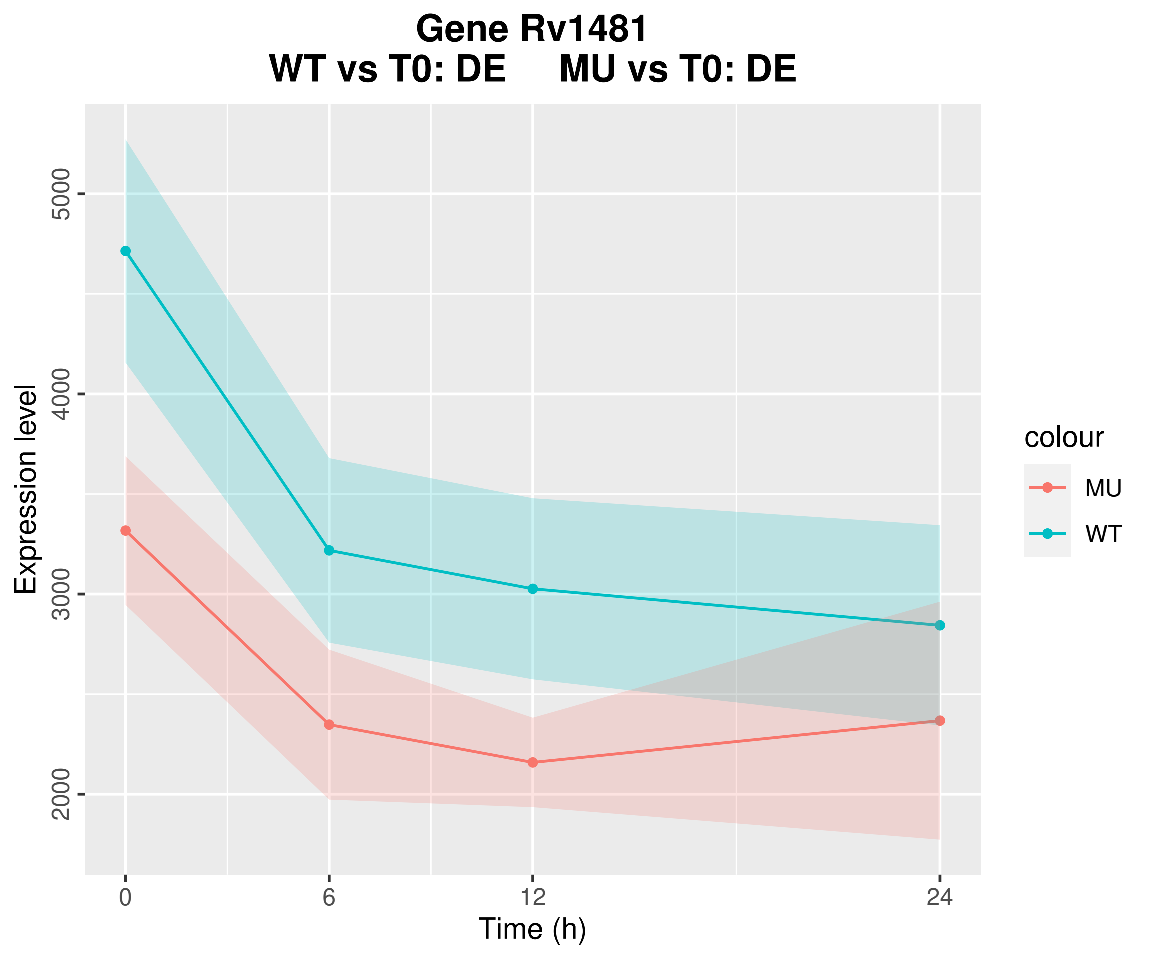

Gene Rv1481
WT vs T0: DE MU vs T0: DE
Expression level
5000
4000
3000
2000
0
6
12
24
Time (h)
colour
MU
WT

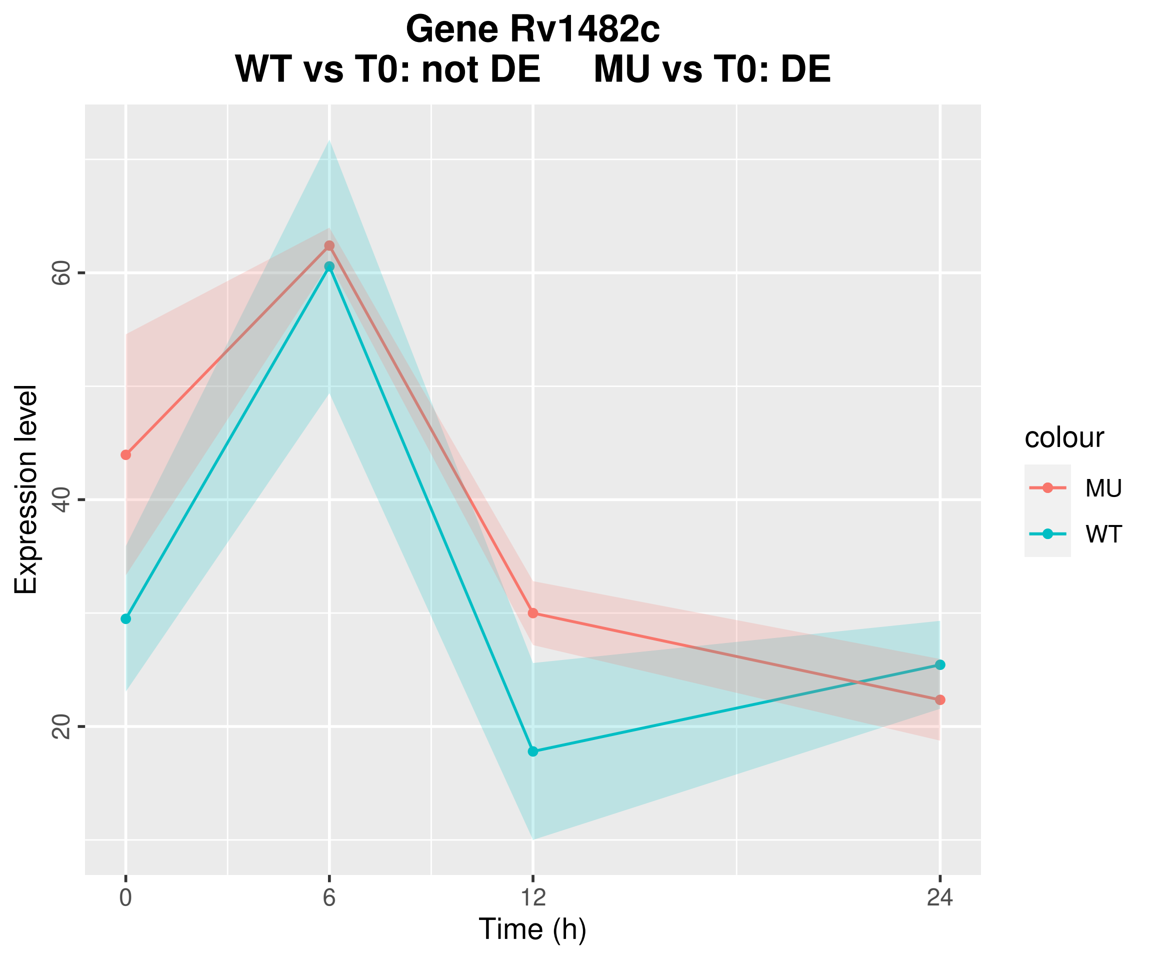

Gene Rv1482c
WT vs T0: not DE MU vs T0: DE
Expression level
60
40
20
0
6
12
24
Time (h)
colour
MU
WT

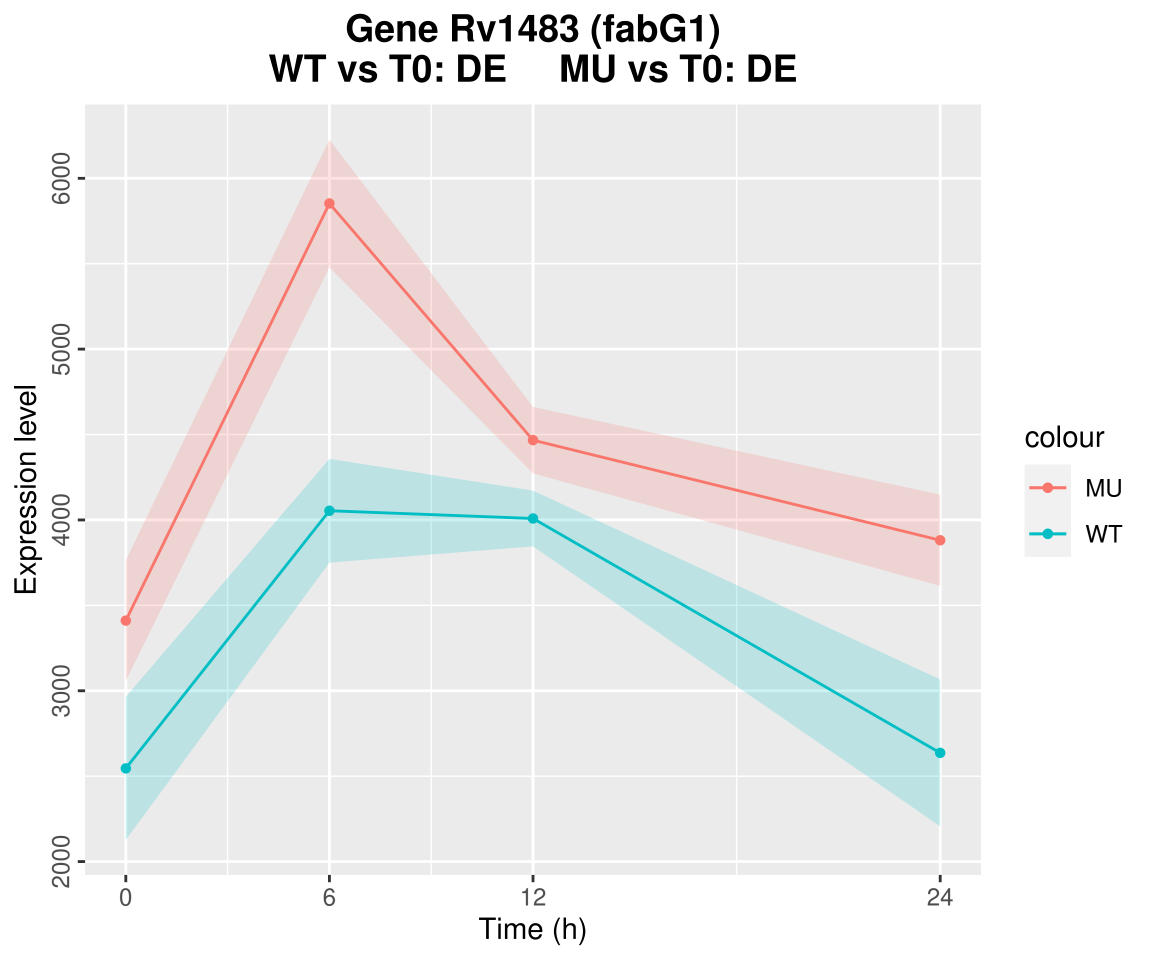
Gene Rv1483 (fabG1)
WT vs T0: DE   MU vs T0: DE
Expression level
6000
5000
4000
3000
2000
0
6
12
24
Time (h)
colour
MU
WT

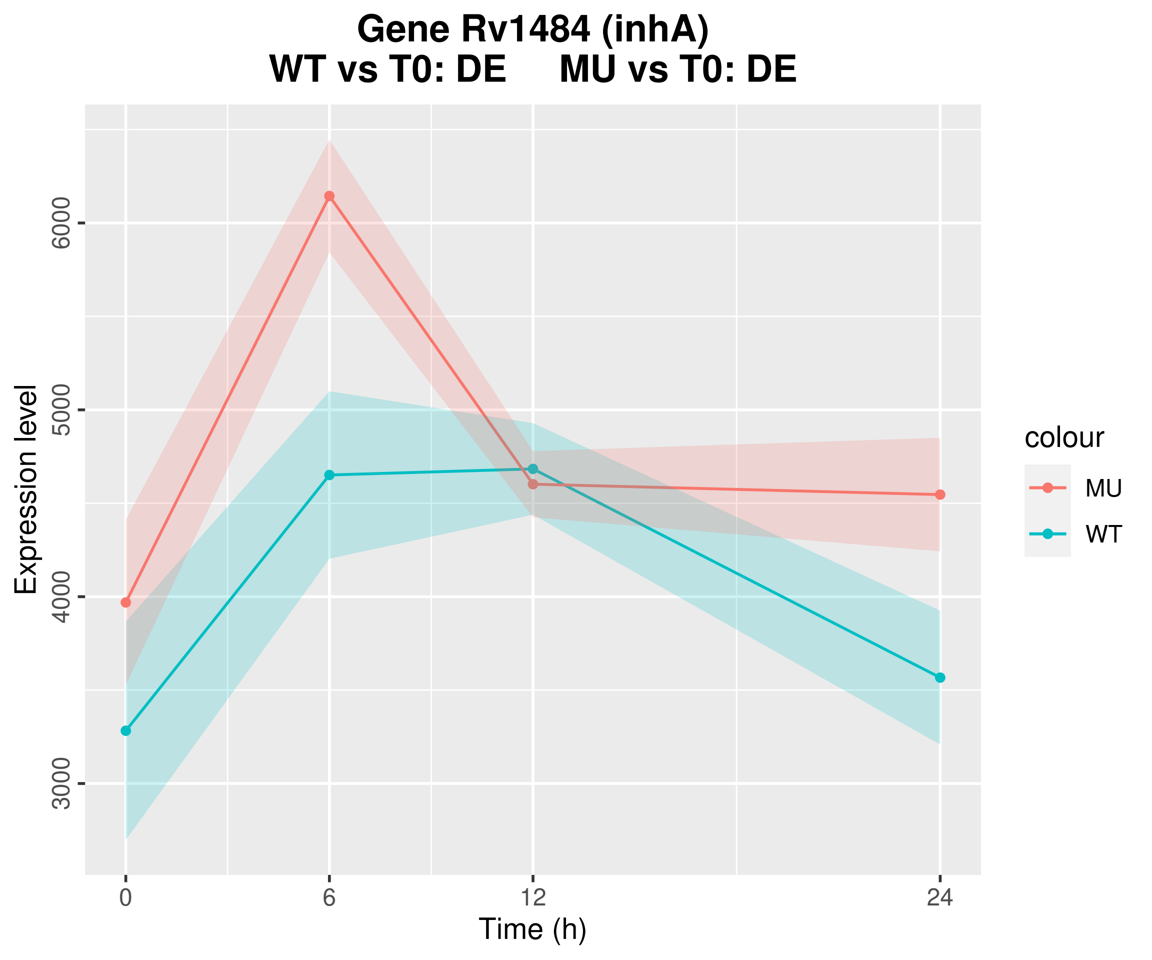

Gene Rv1484 (inhA)
WT vs T0: DE   MU vs T0: DE
Expression level
6000
5000
4000
3000
0
6
12
24
Time (h)
colour
MU
WT

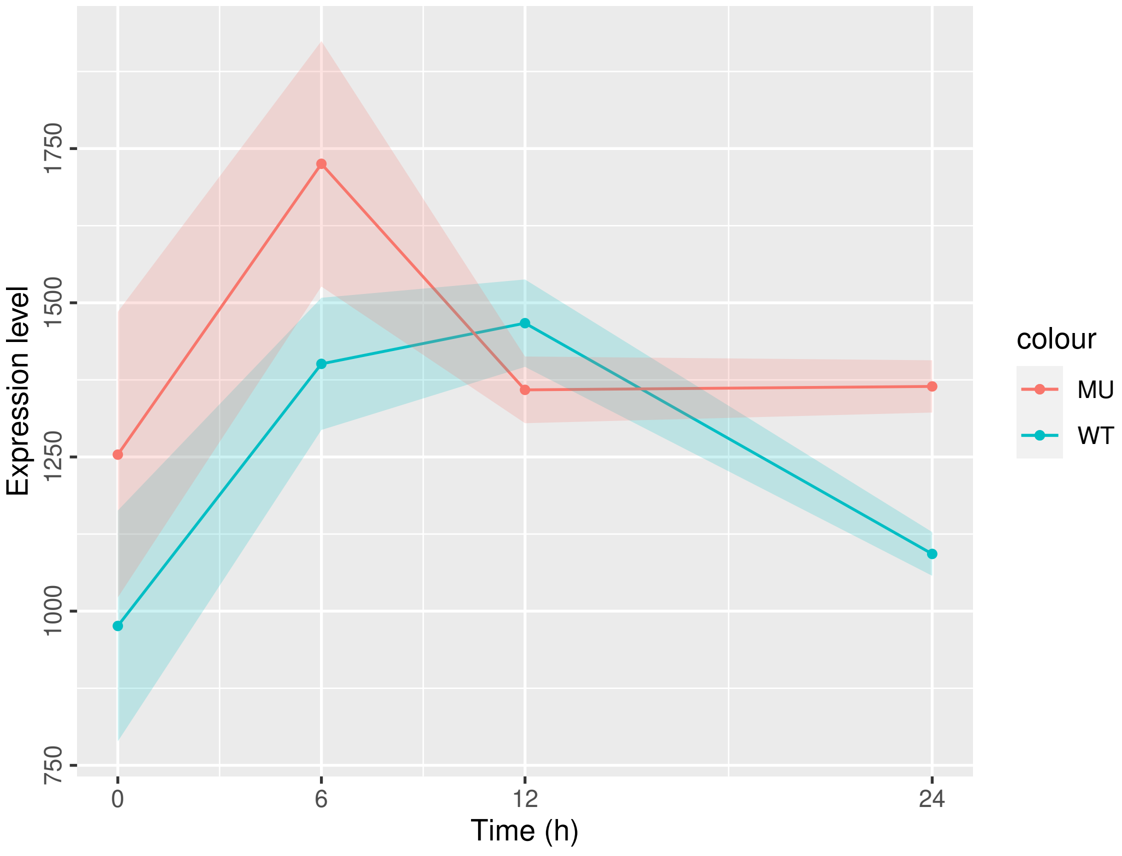

Gene Rv1485 (hemZ)
WT vs T0: DE   MU vs T0: not DE

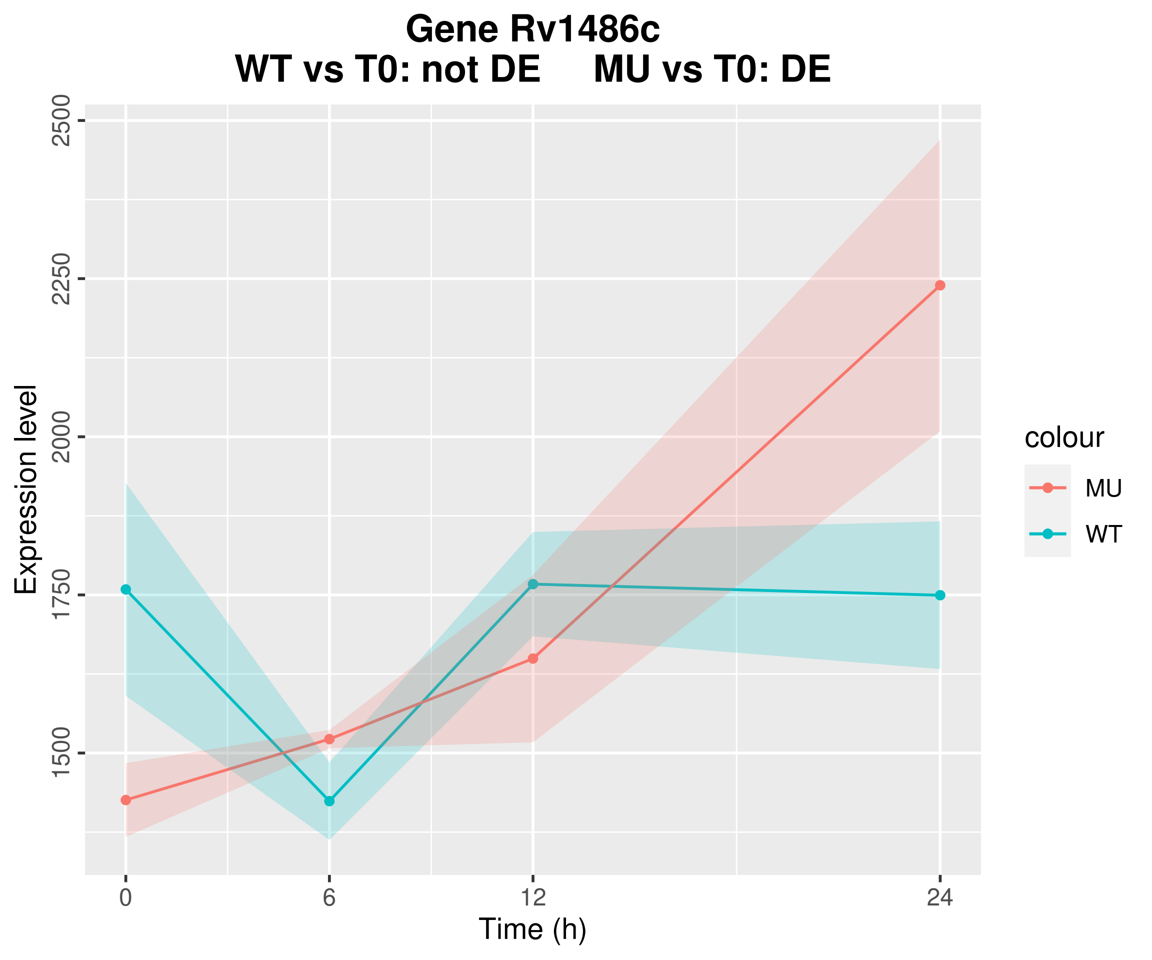

Gene Rv1486c
WT vs T0: not DE MU vs T0: DE
Expression level
2500
2250
2000
1750
1500
0
6
12
24
Time (h)
colour
MU
WT

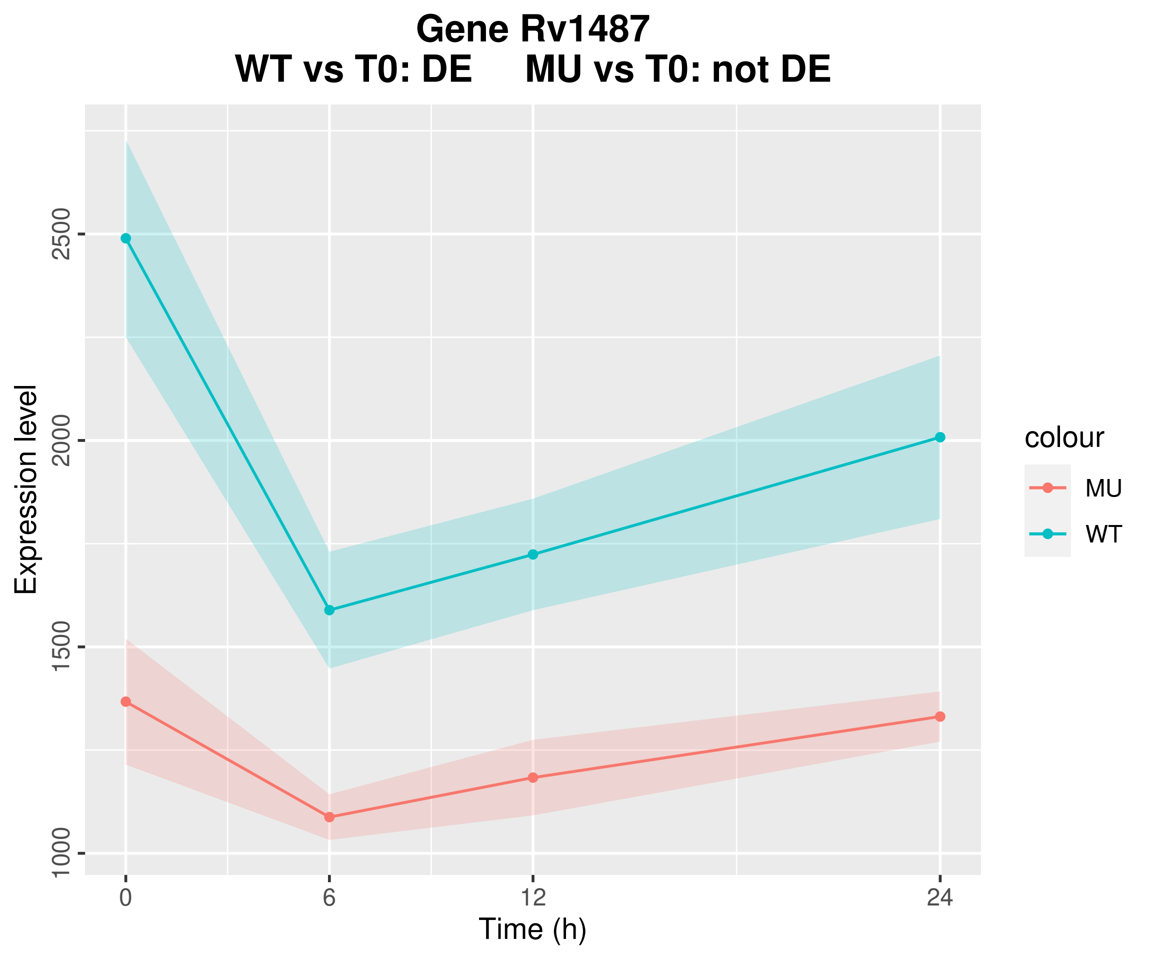
Gene Rv1487
WT vs T0: DE   MU vs T0: not DE
Expression level
2500
2000
1500
1000
0
6
12
24
Time (h)
colour
MU
WT

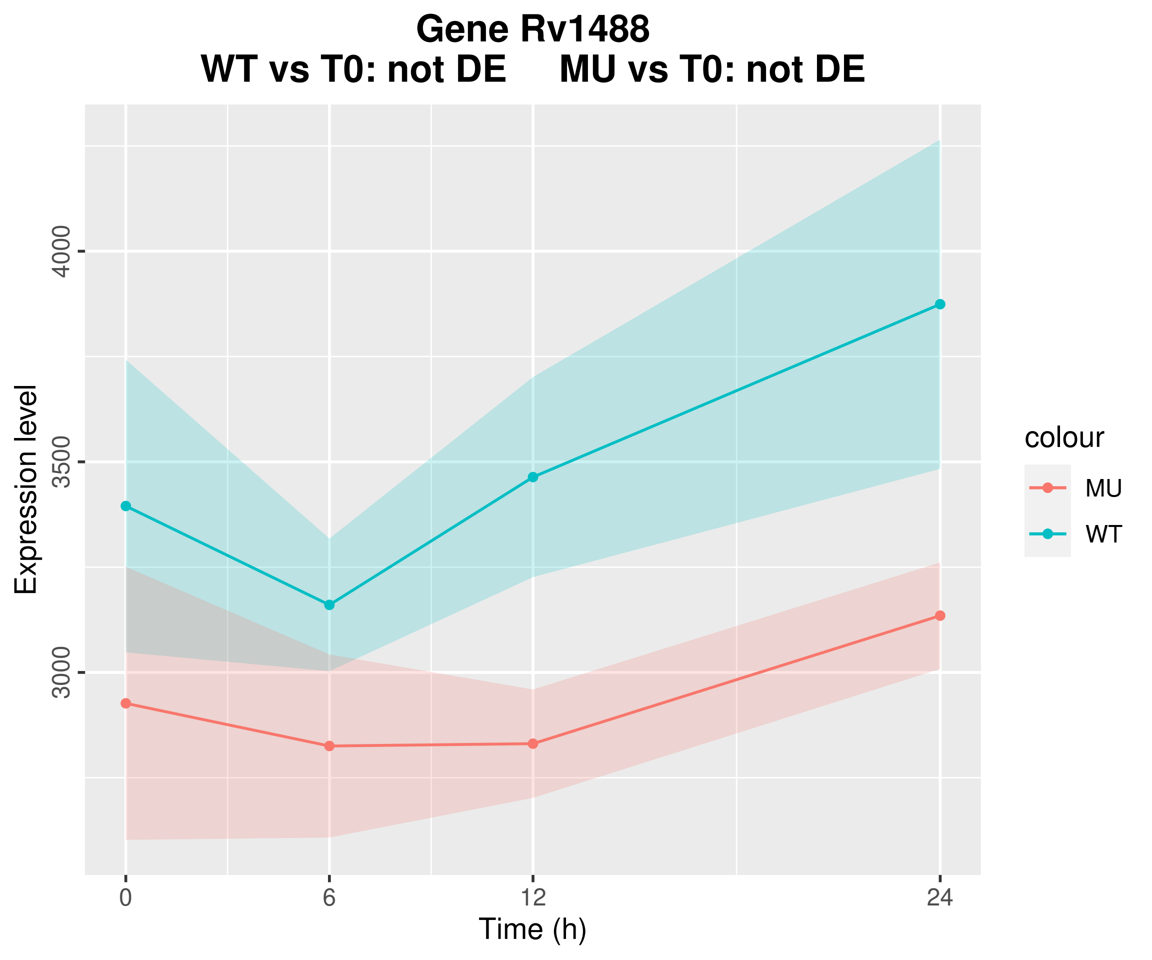

Gene Rv1488
WT vs T0: not DE   MU vs T0: not DE
Expression level
4000
3500
3000
0
6
12
24
Time (h)
colour
MU
WT

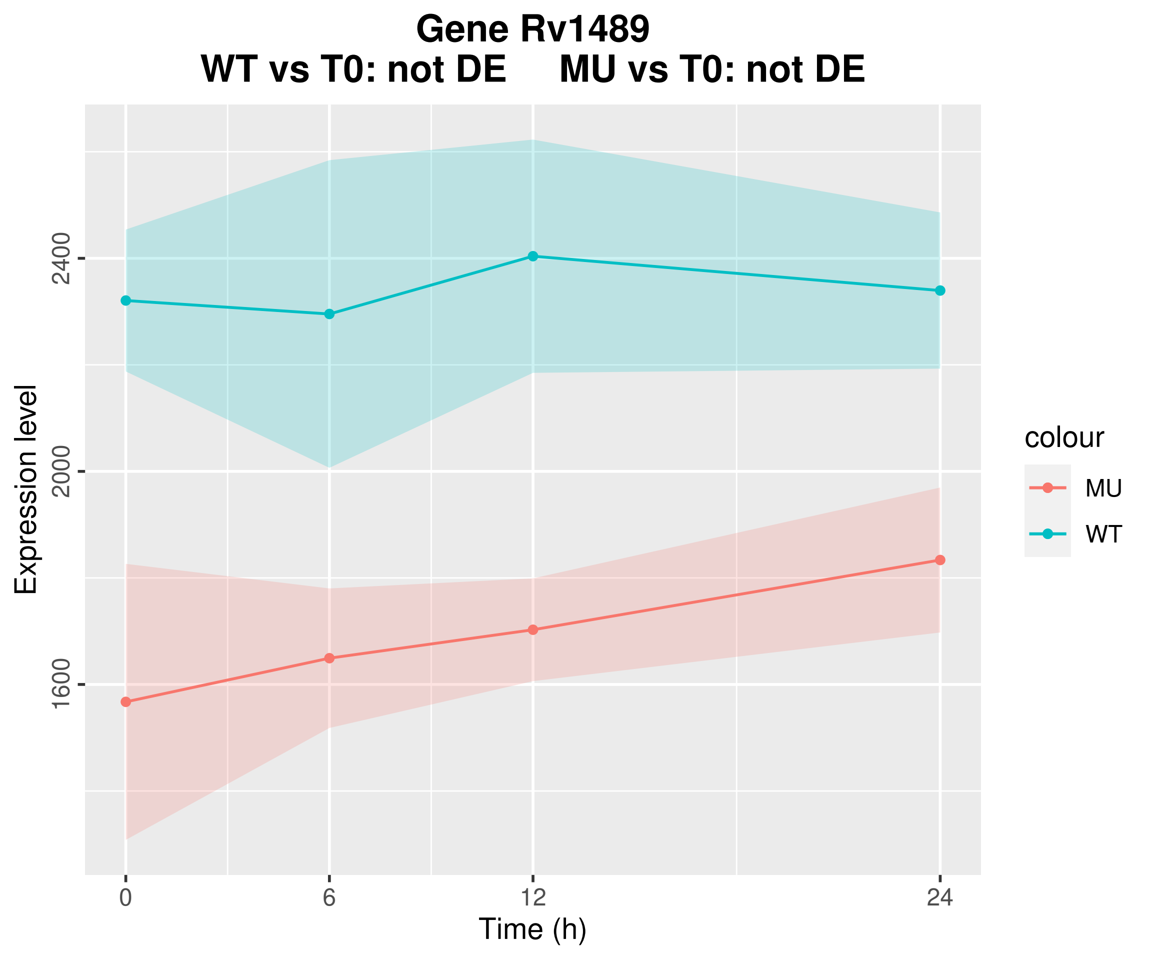

Gene Rv1489
WT vs T0: not DE   MU vs T0: not DE
Expression level
2400
2000
1600
Time (h)
0
6
12
24
colour
MU
WT

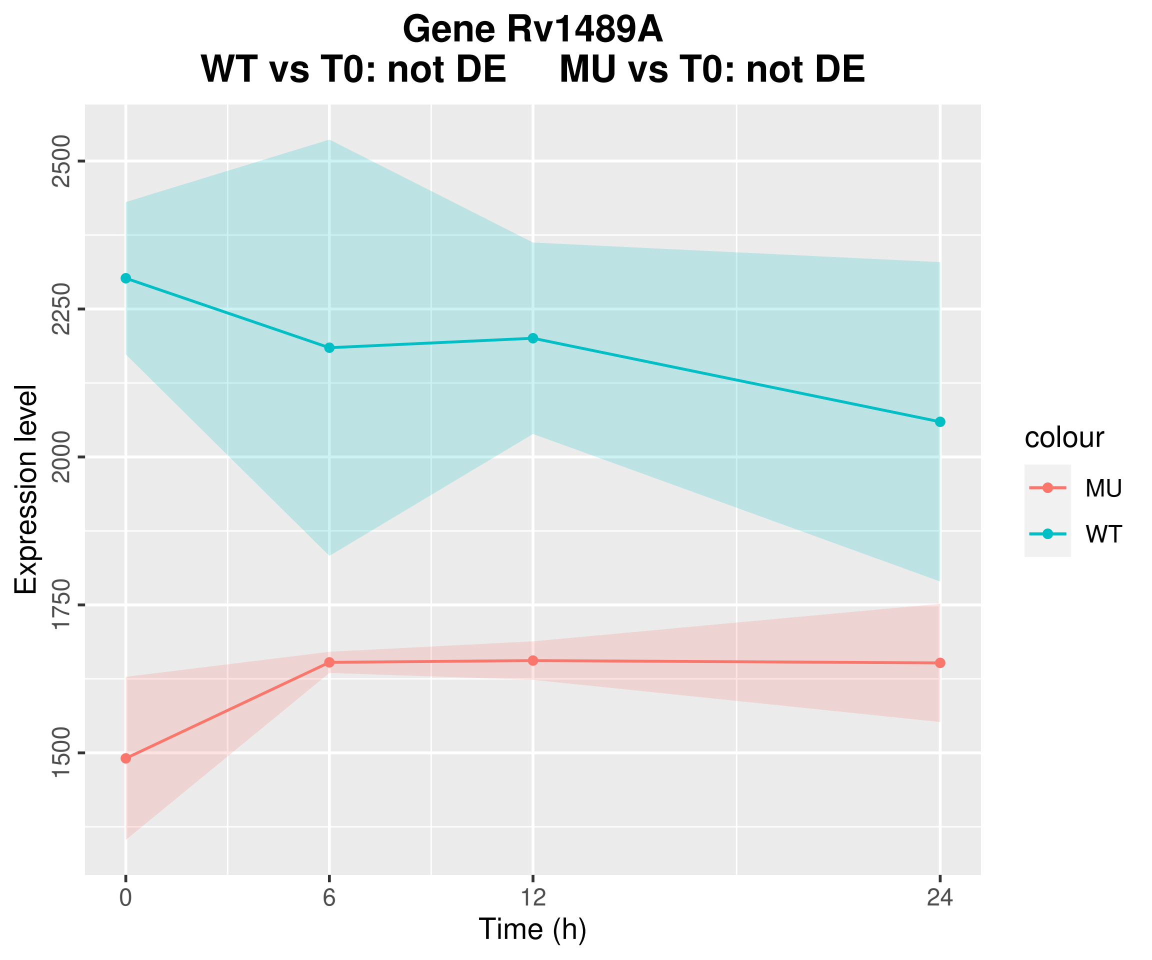

Gene Rv1489A
WT vs T0: not DE   MU vs T0: not DE
Expression level
2500
2250
2000
1750
1500
0
6
12
24
Time (h)
colour
MU
WT

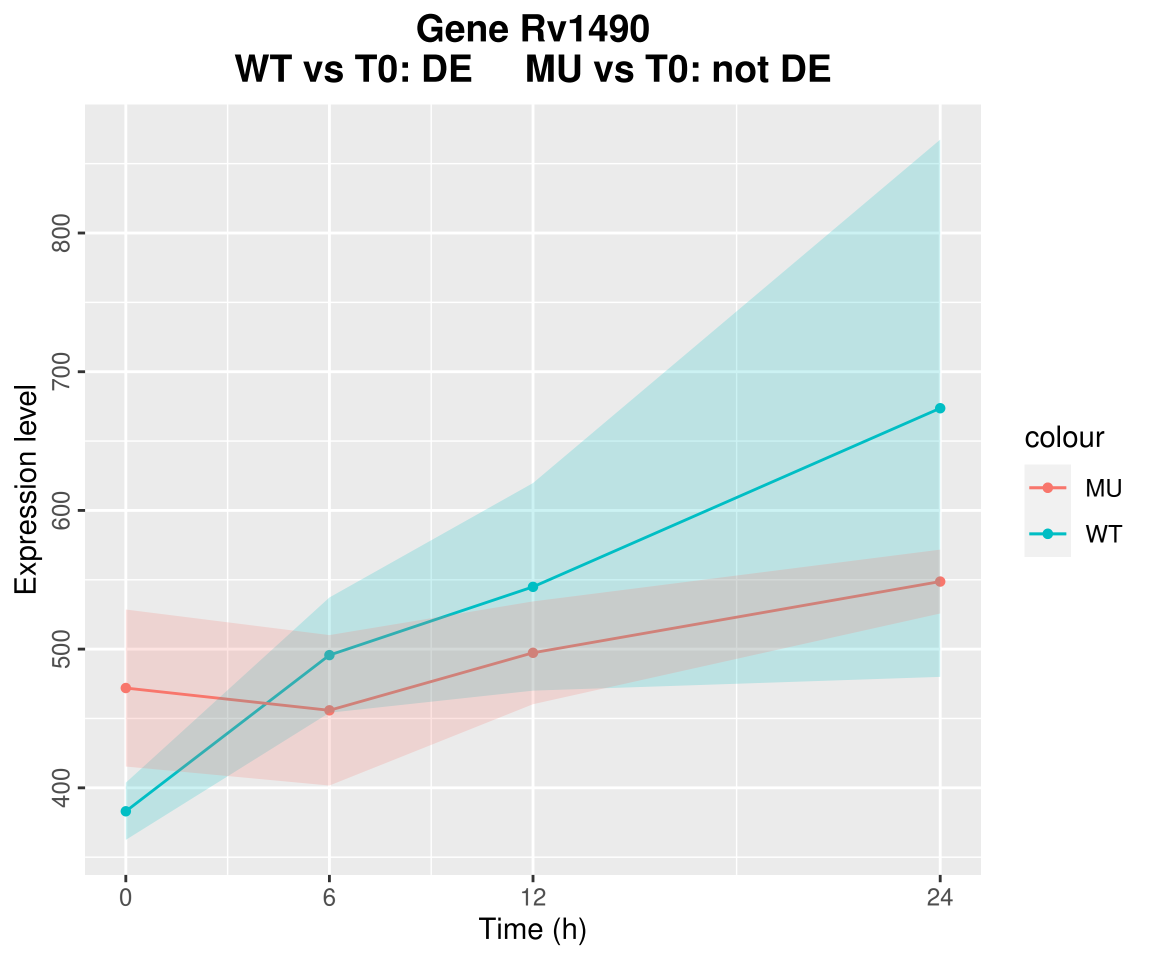

Gene Rv1490
WT vs T0: DE MU vs T0: not DE
Expression level
800
700
600
500
400
0
6
12
24
Time (h)
colour
MU
WT

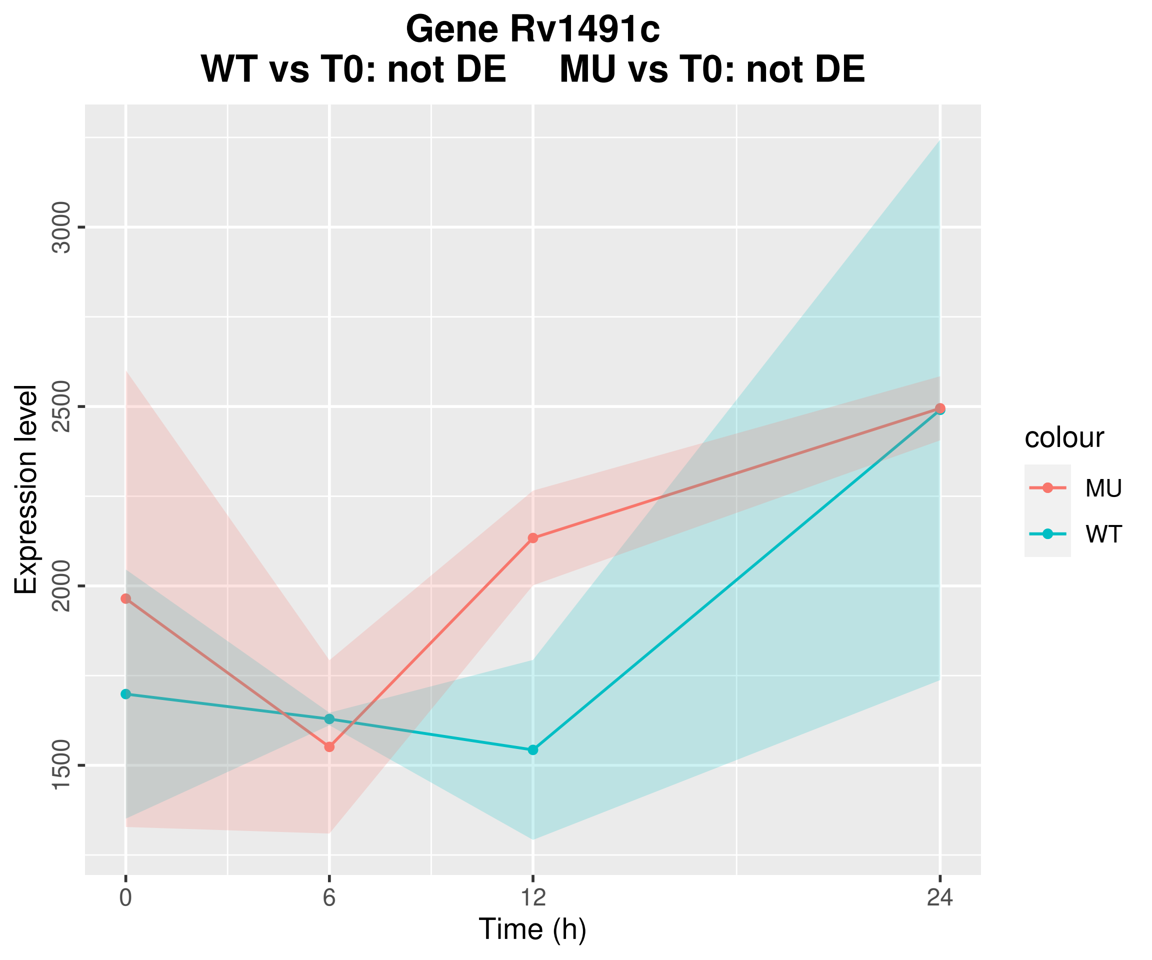
Gene Rv1491c
WT vs T0: not DE   MU vs T0: not DE
Expression level
3000
2500
2000
1500
0
6
12
24
Time (h)
colour
MU
WT

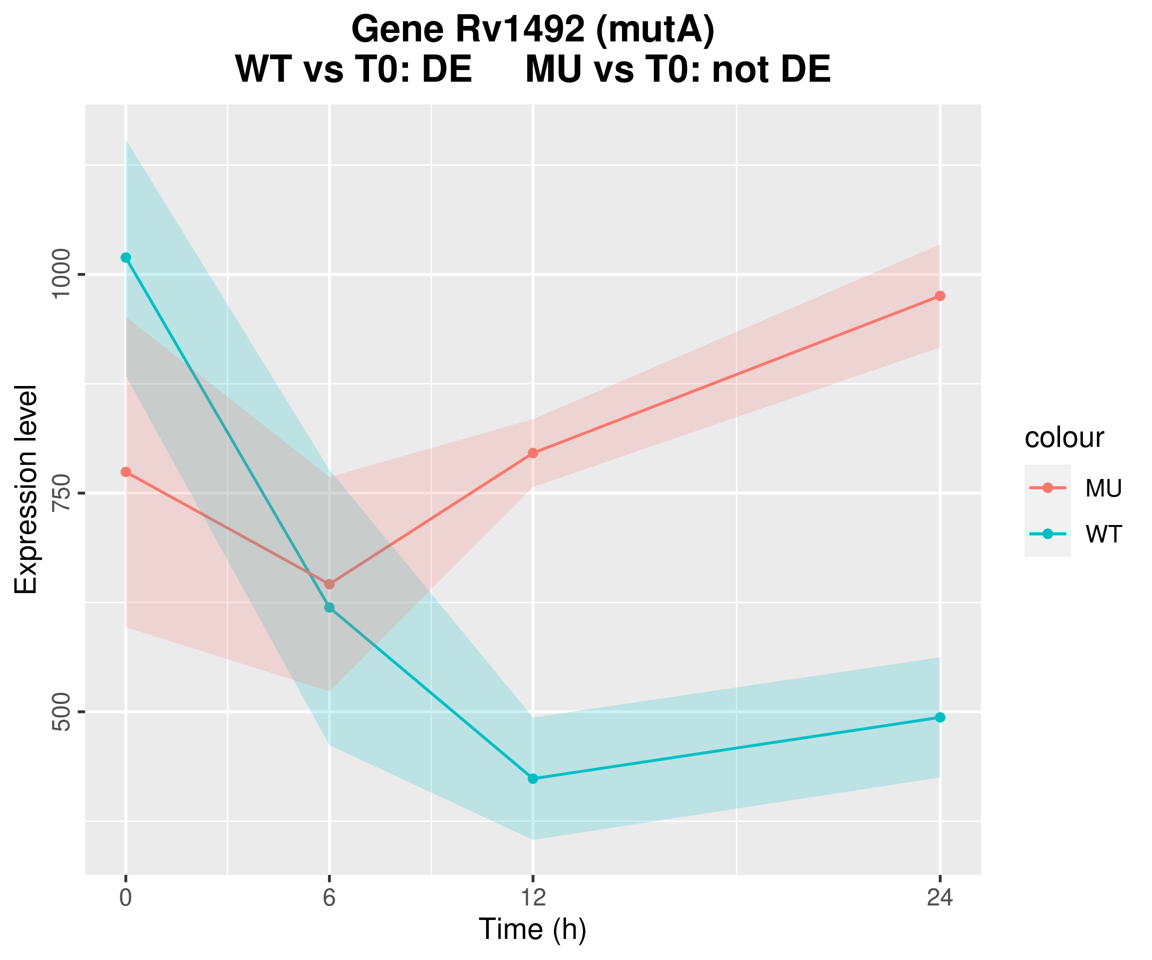
Gene Rv1492 (mutA)
WT vs T0: DE   MU vs T0: not DE
Expression level
1000
750
500
0
6
12
24
Time (h)
colour
MU
WT

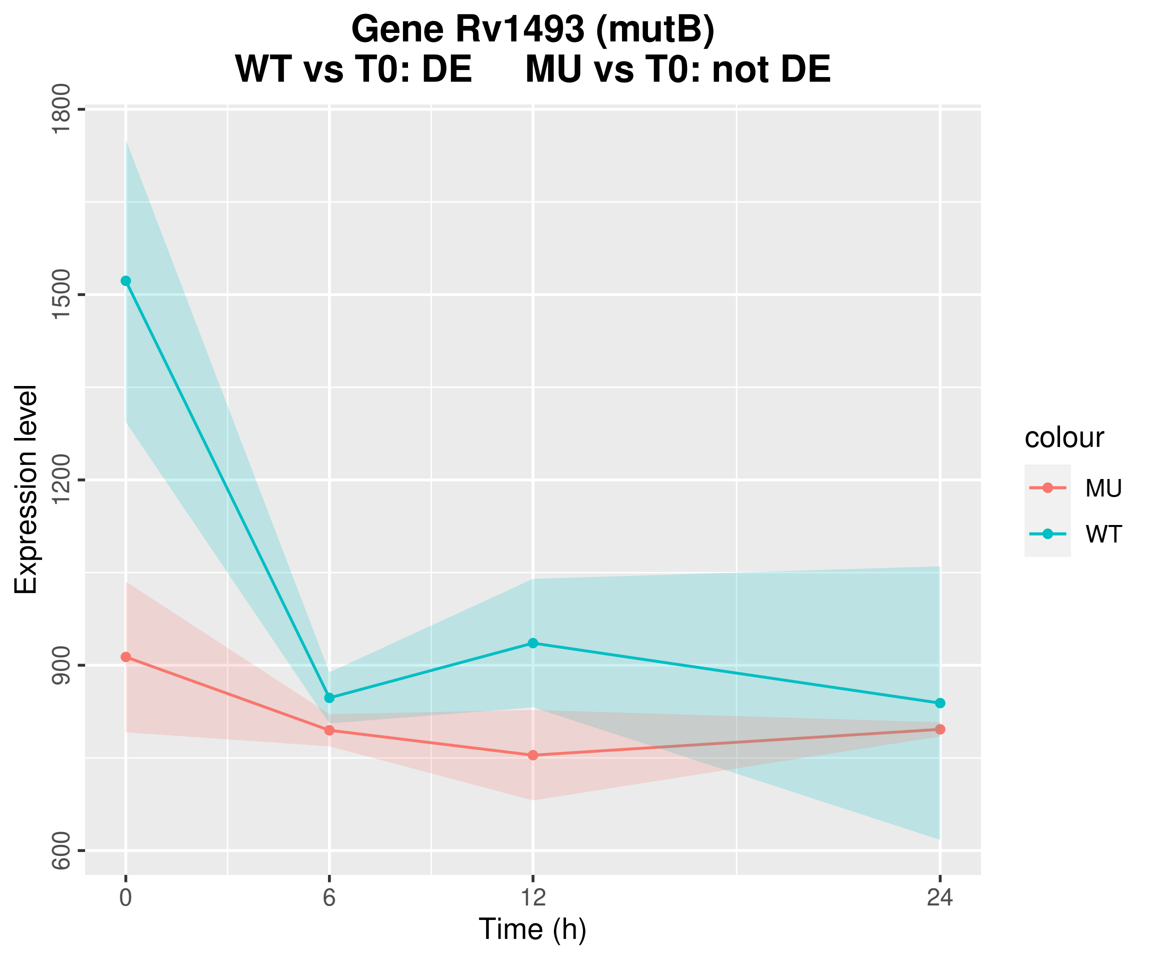

Gene Rv1493 (mutB)
WT vs T0: DE   MU vs T0: not DE
Expression level
1800
1500
1200
900
600
0
6
12
24
Time (h)
colour
MU
WT

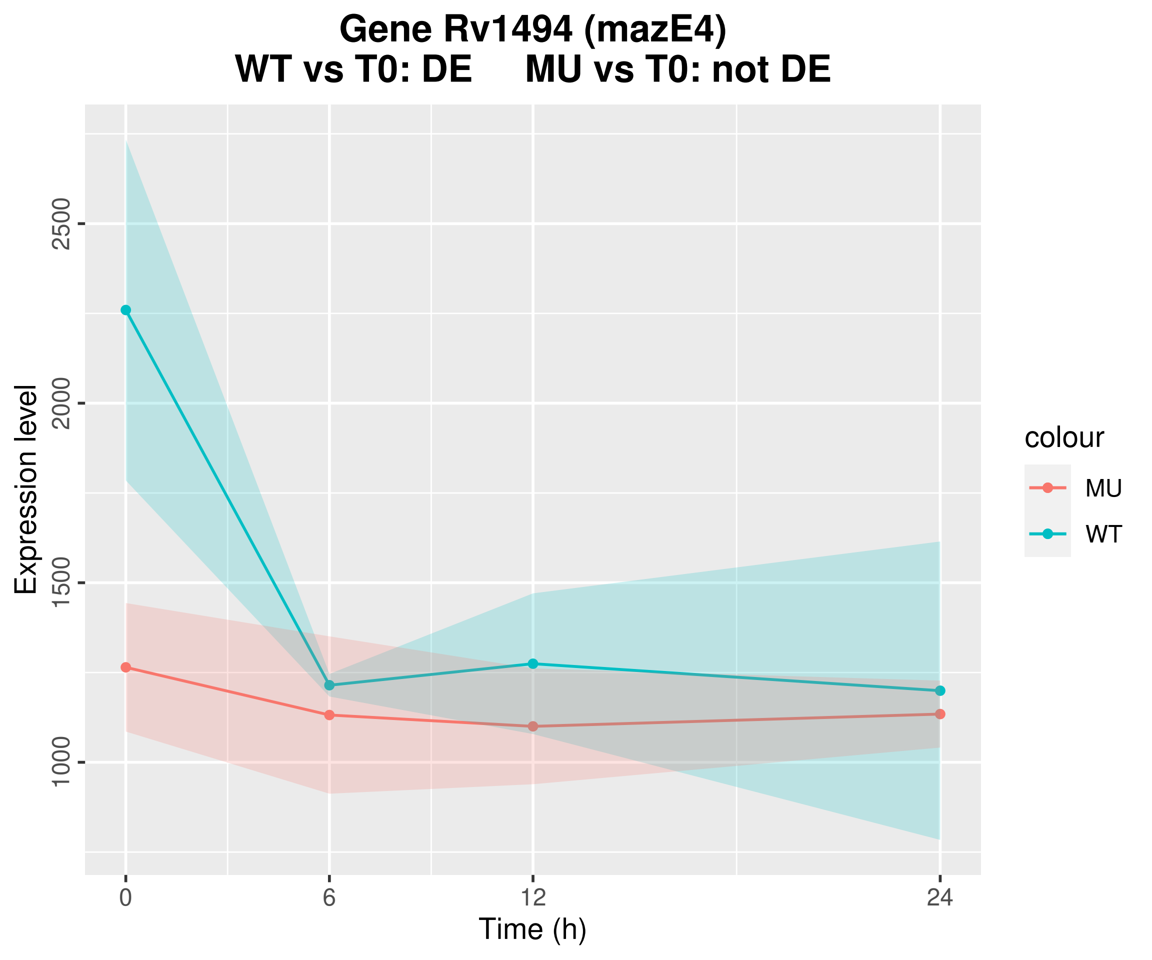

Gene Rv1494 (mazE4)
WT vs T0: DE   MU vs T0: not DE
Expression level
2500
2000
1500
1000
0
6
12
24
Time (h)
colour
MU
WT

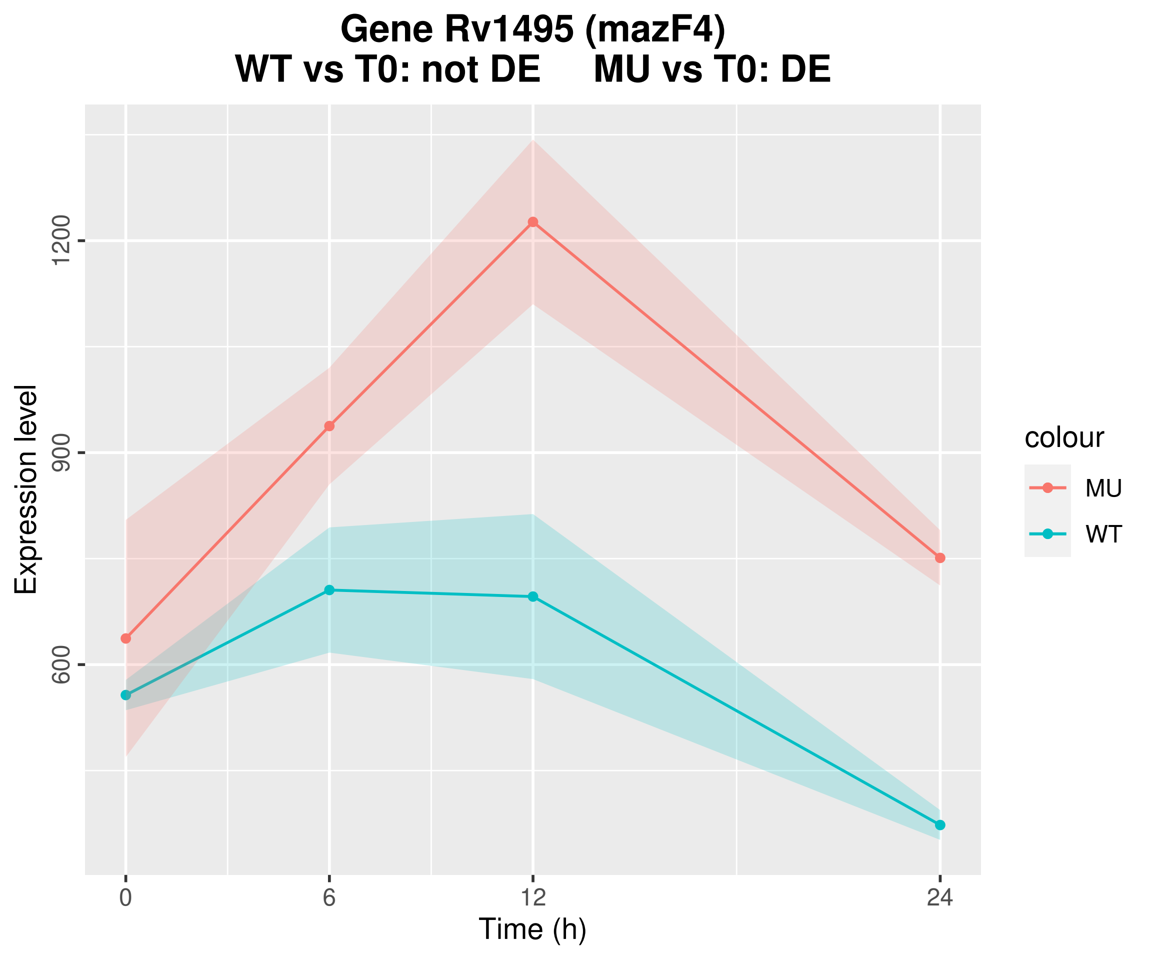
Gene Rv1495 (mazF4)
WT vs T0: not DE MU vs T0: DE
Expression level
1200
900
600
0
6
12
24
Time (h)
colour
MU
WT

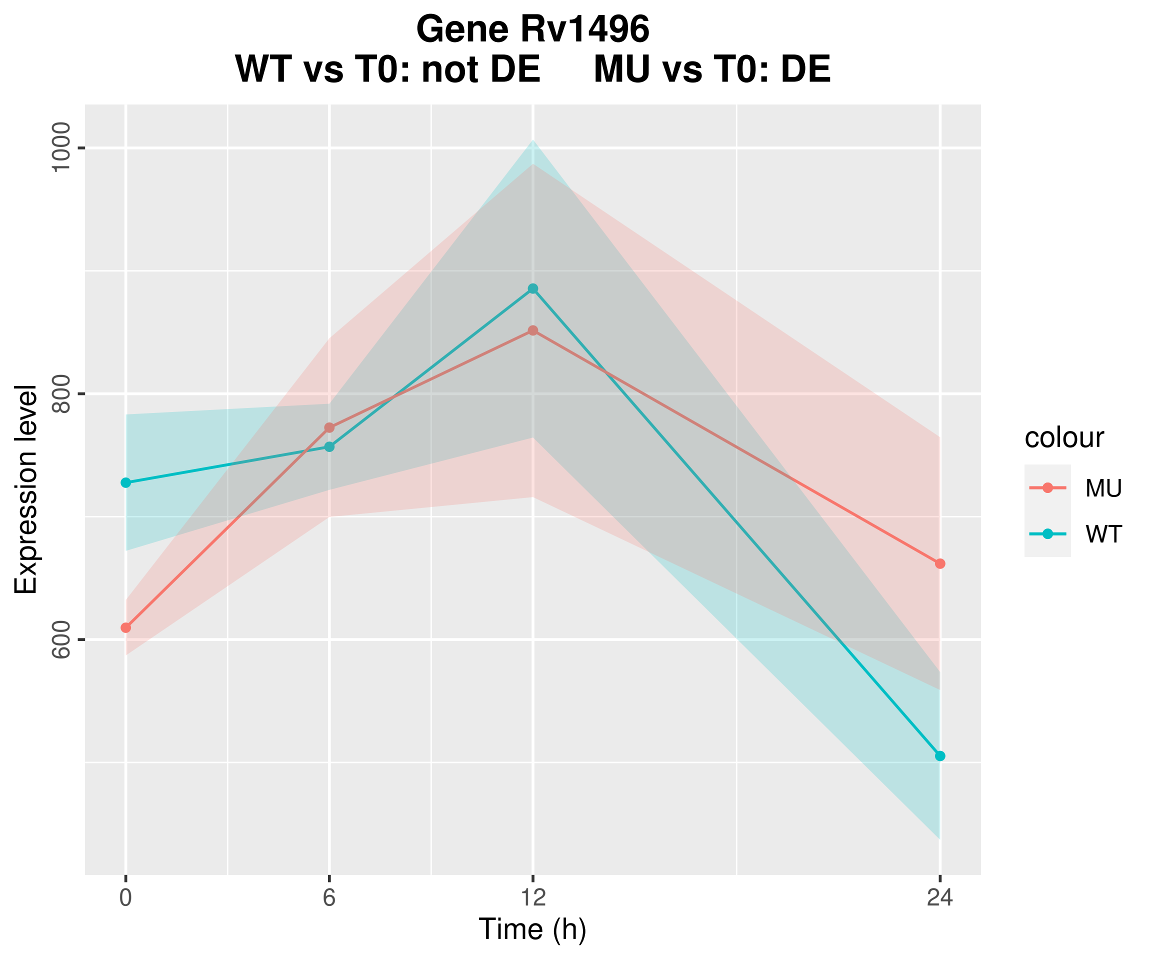

Gene Rv1496
WT vs T0: not DE MU vs T0: DE
1000
800
600
Expression level
0
6
12
24
Time (h)
colour
MU
WT

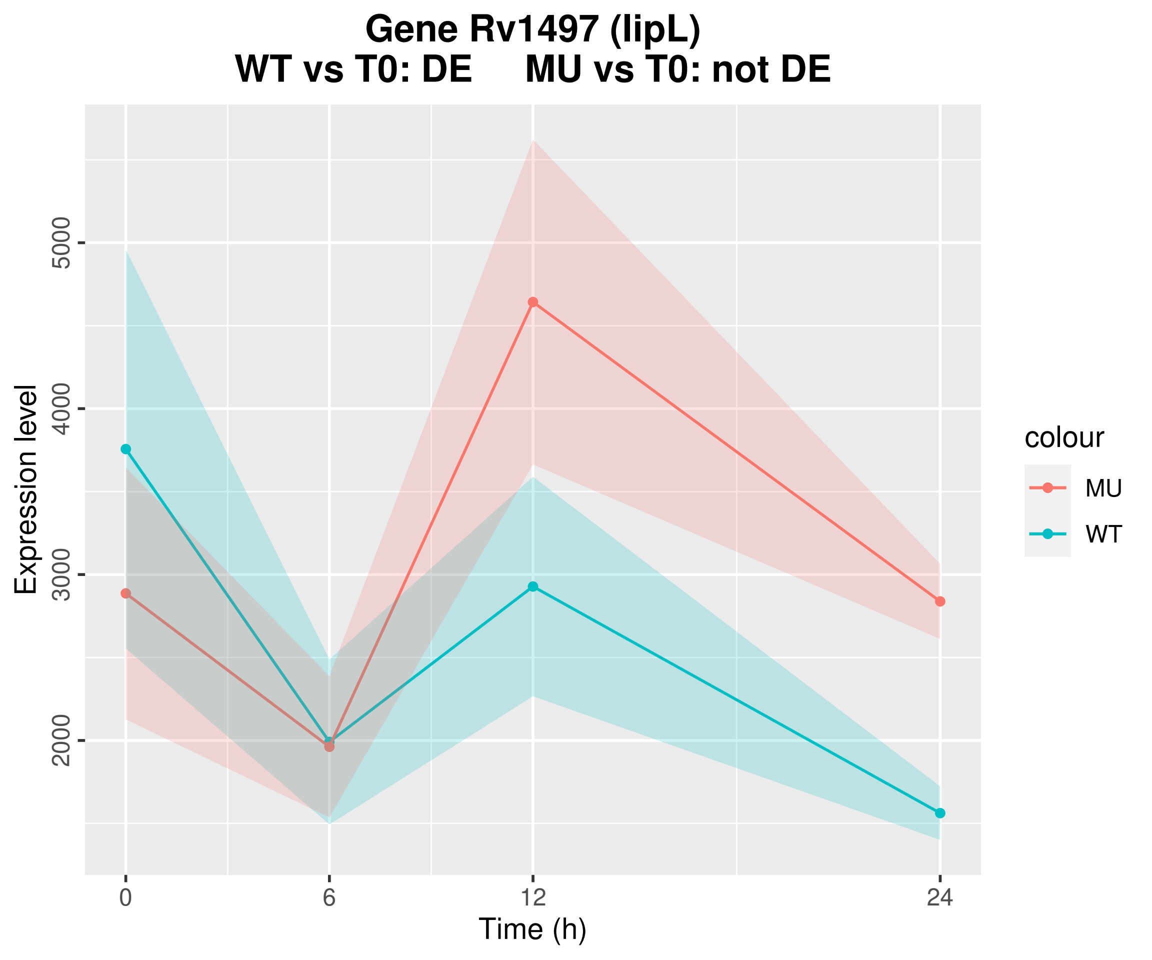

Gene Rv1497 (lipL)
WT vs T0: DE MU vs T0: not DE
Expression level
5000
4000
3000
2000
0
6
12
24
Time (h)
colour
MU
WT

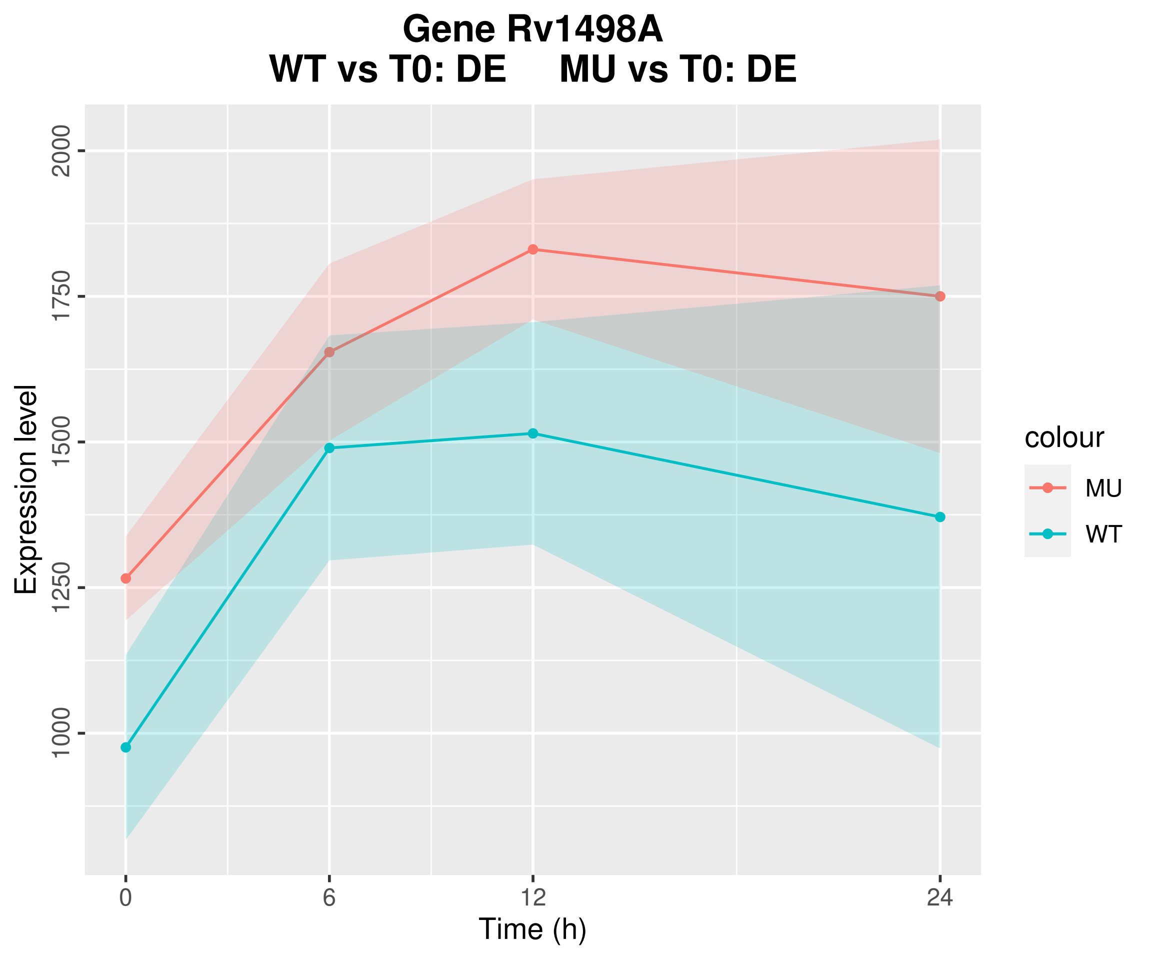
Gene Rv1498A
WT vs T0: DE   MU vs T0: DE
Expression level
2000
1750
1500
1250
1000
0
6
12
24
Time (h)
colour
MU
WT

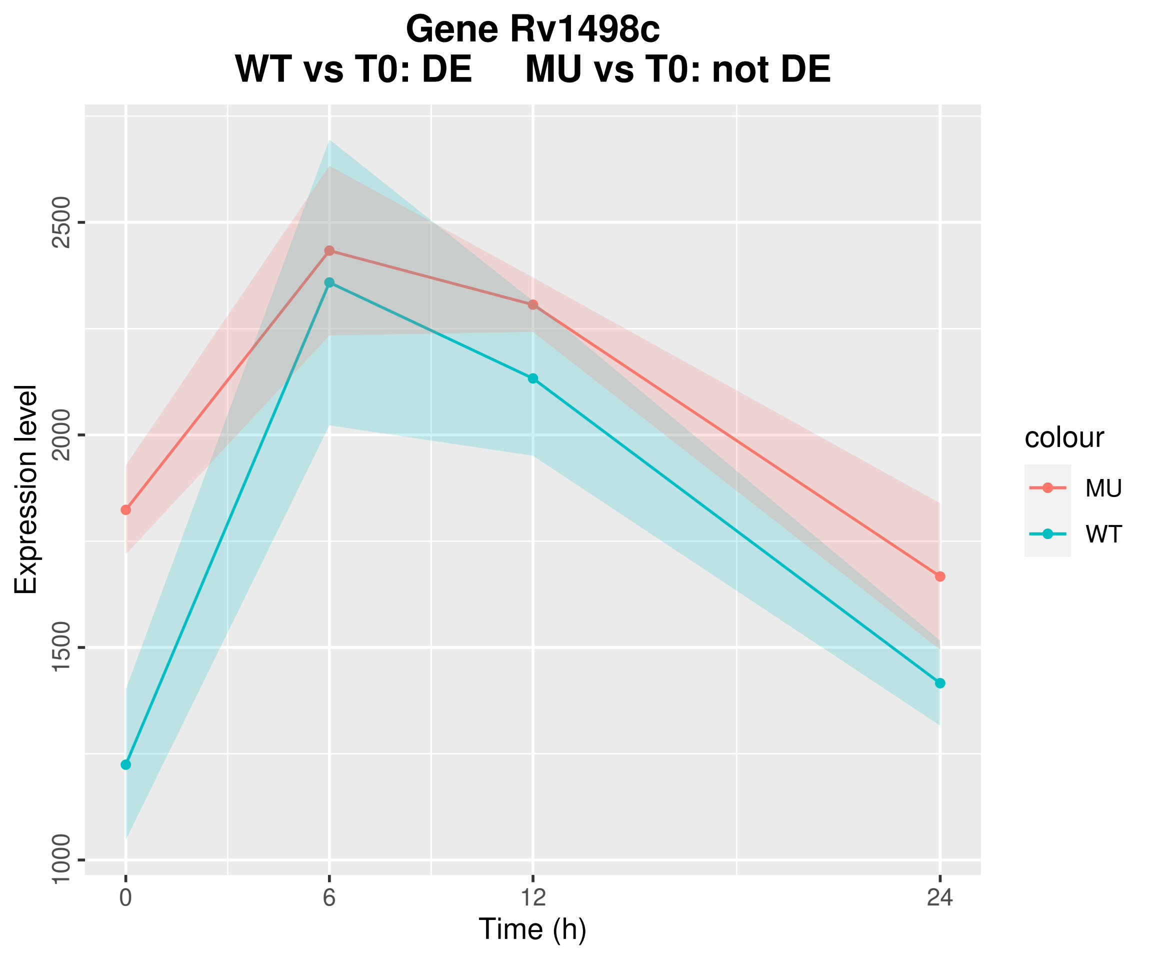

Gene Rv1498c
WT vs T0: DE   MU vs T0: not DE
Expression level
2500
2000
1500
1000
0
6
12
24
Time (h)
colour
MU
WT

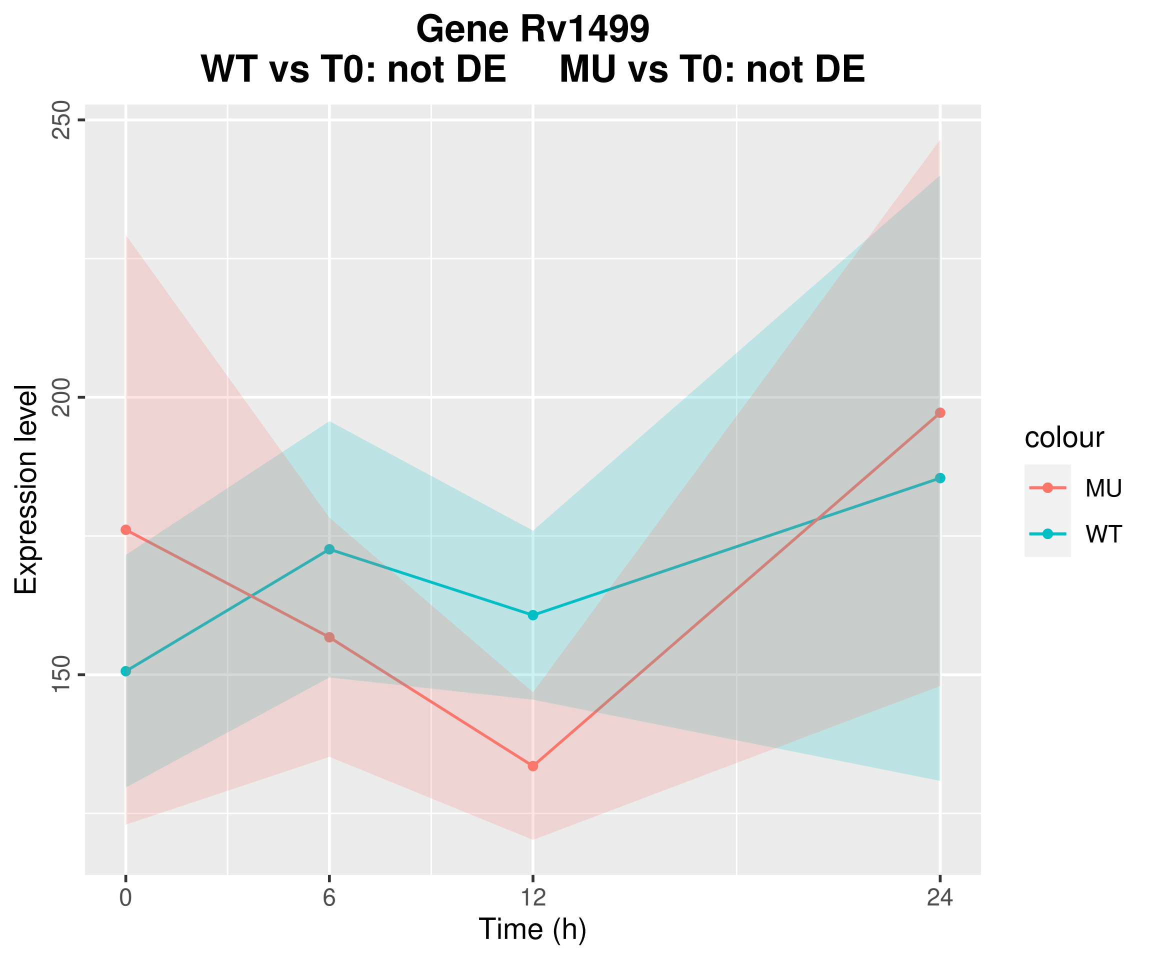

Gene Rv1499
WT vs T0: not DE   MU vs T0: not DE
Expression level
250
200
150
Time (h)
0
6
12
24
colour
MU
WT

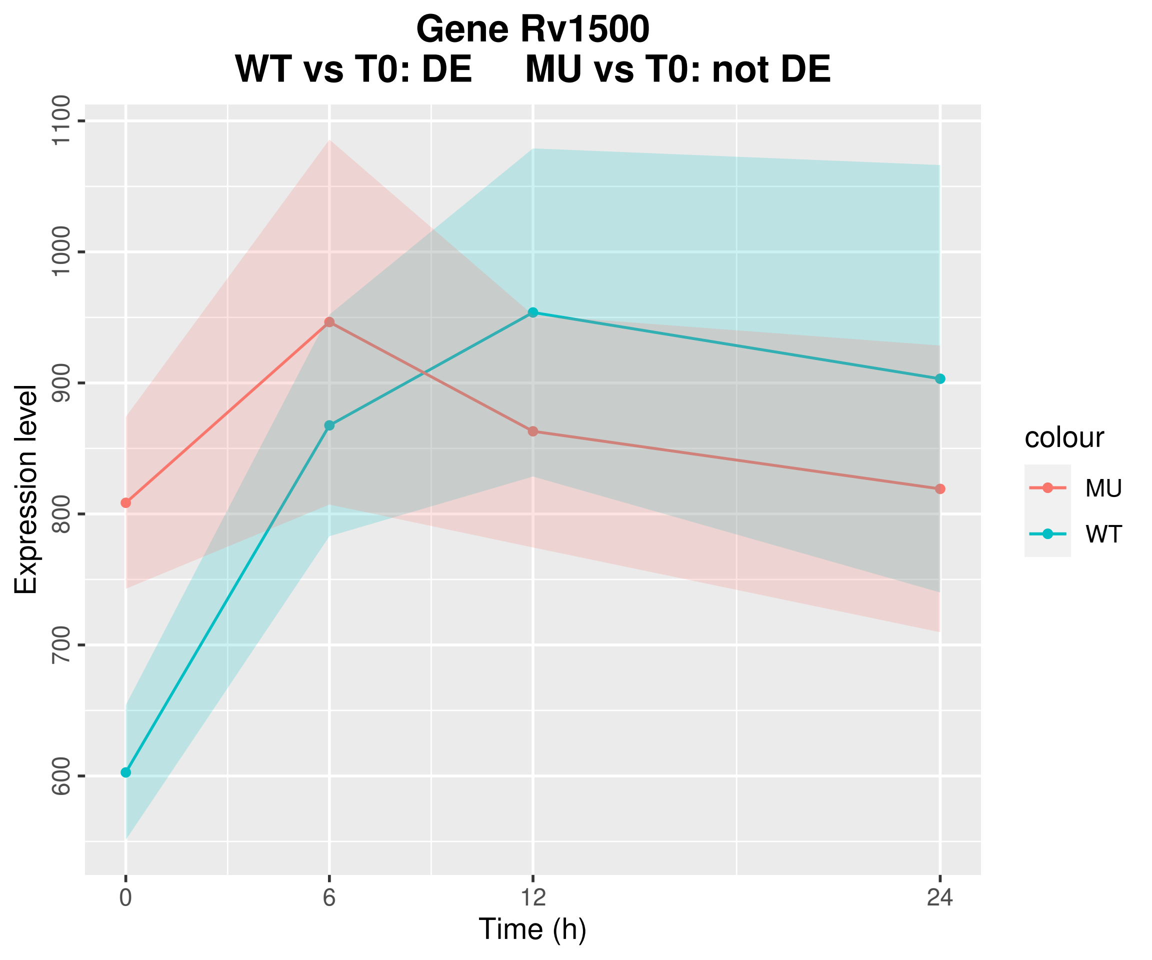

Gene Rv1500
WT vs T0: DE   MU vs T0: not DE
Expression level
1100
1000
900
800
700
600
0
6
12
24
Time (h)
colour
MU
WT

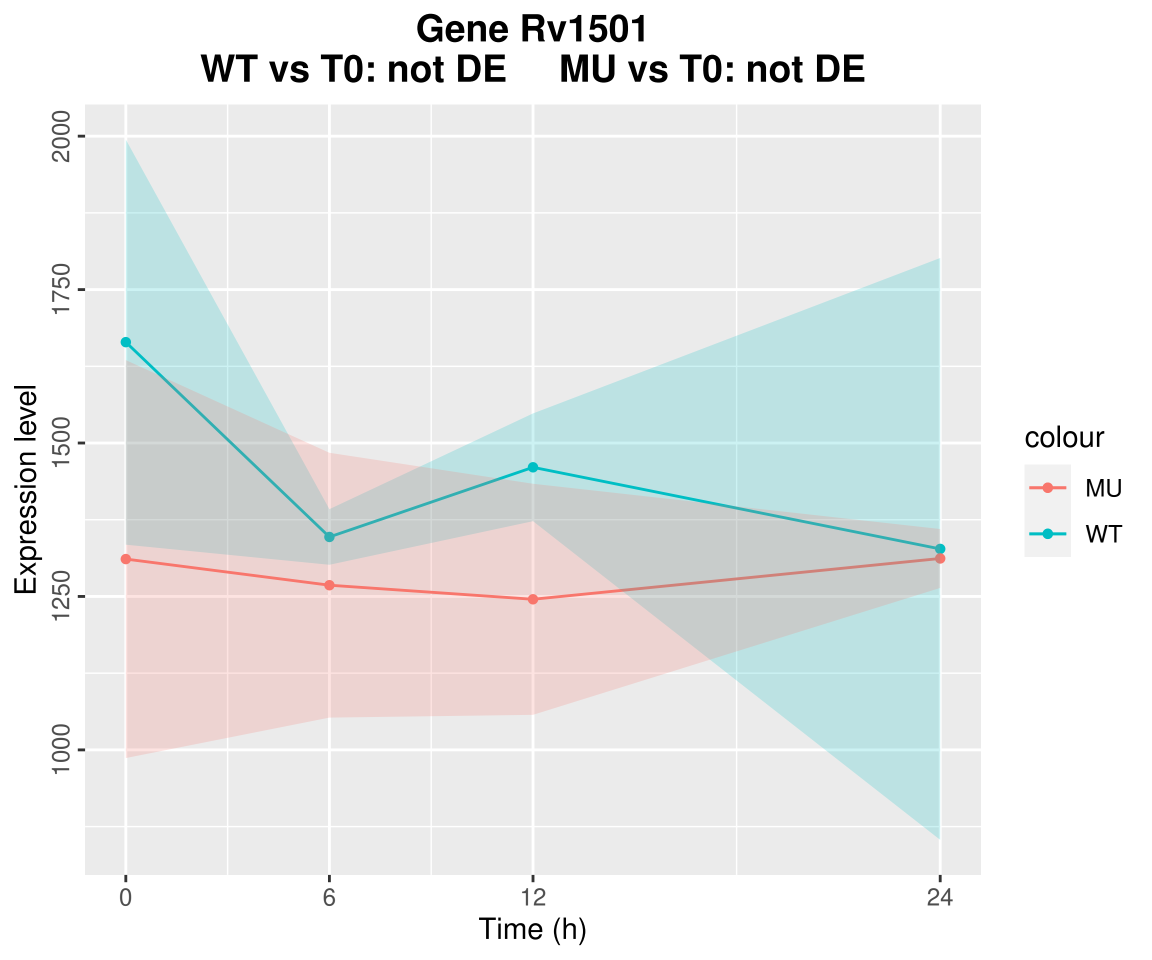
Gene Rv1501
WT vs T0: not DE   MU vs T0: not DE
Expression level
2000
1750
1500
1250
1000
0
6
12
24
Time (h)
colour
MU
WT

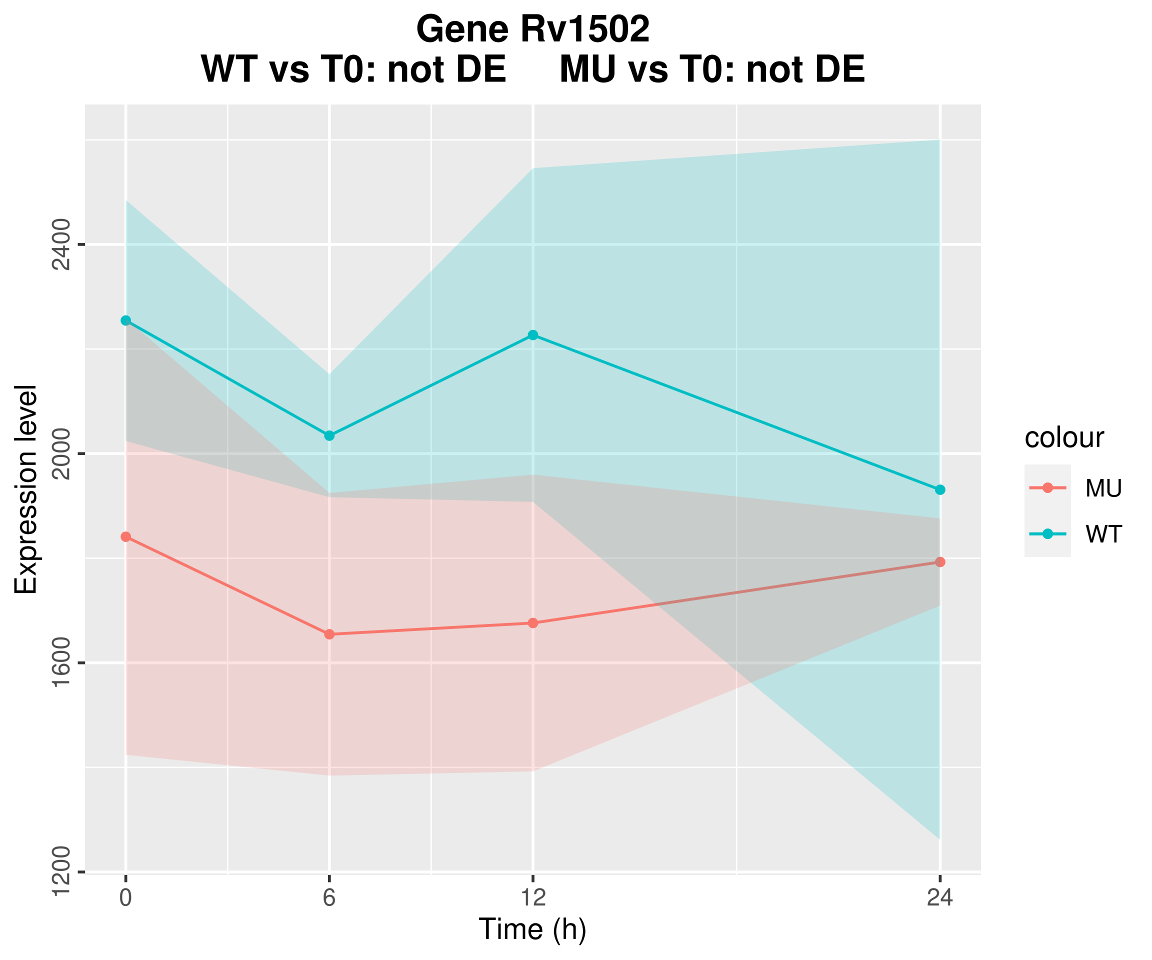
Gene Rv1502
WT vs T0: not DE   MU vs T0: not DE
Expression level
2400
2000
1600
1200
0
6
12
24
Time (h)
colour
MU
WT

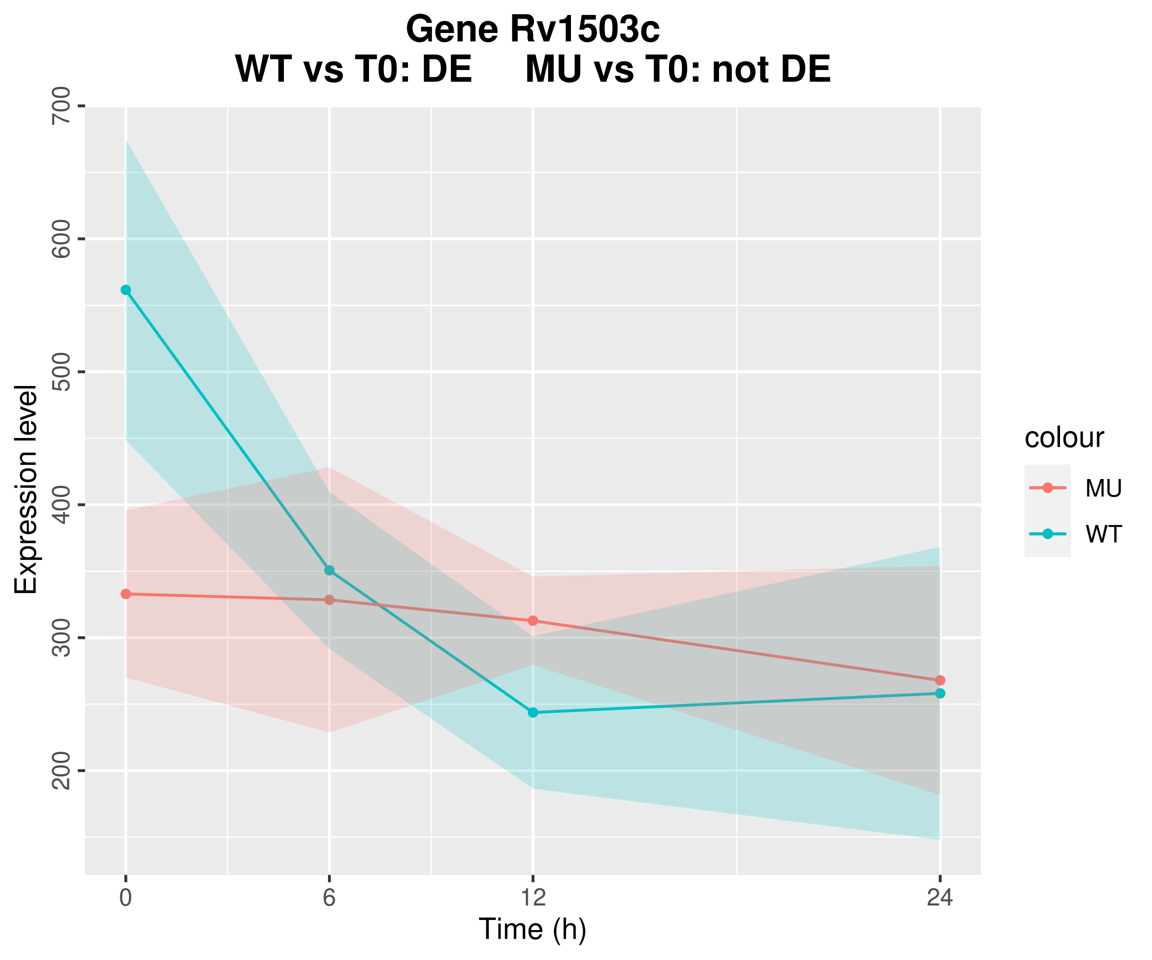
Gene Rv1503c
WT vs T0: DE MU vs T0: not DE
Expression level
Time (h)
700
600
500
400
300
200
0
6
12
24
colour
MU
WT

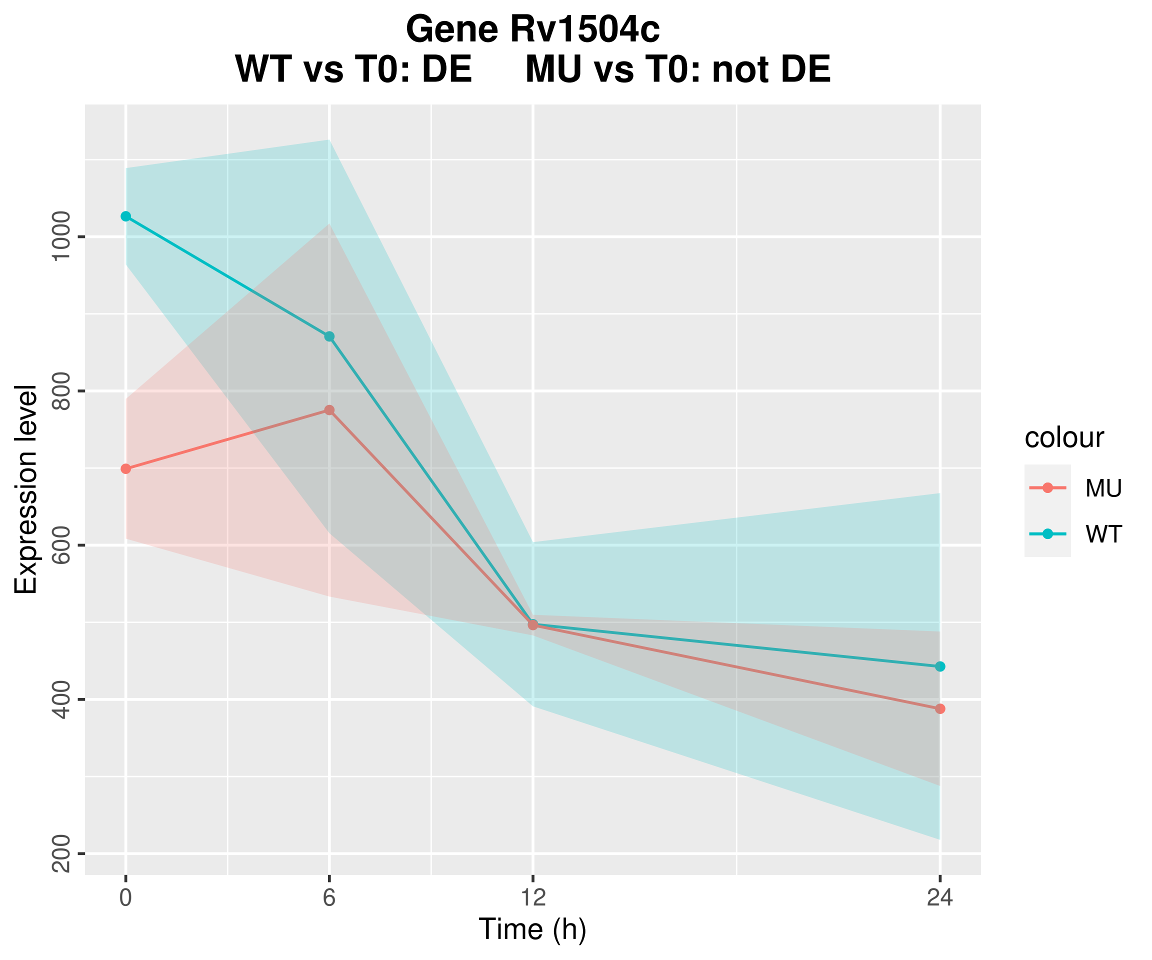

Gene Rv1504c
WT vs T0: DE MU vs T0: not DE
Expression level
1000
800
600
400
200
0
6
12
24
Time (h)
colour
MU
WT

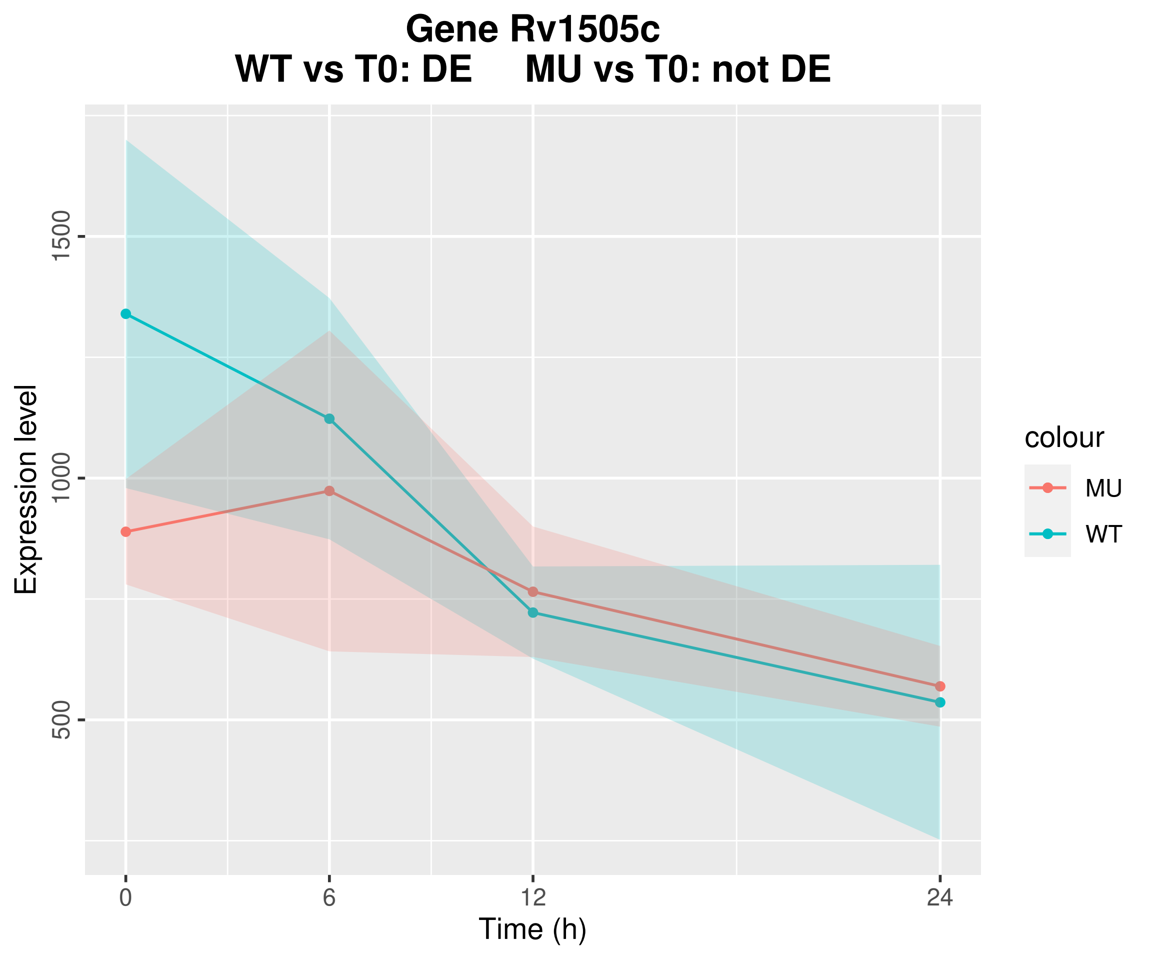
Gene Rv1505c
WT vs T0: DE   MU vs T0: not DE
Expression level
1500
1000
500
0
6
12
24
Time (h)
colour
MU
WT

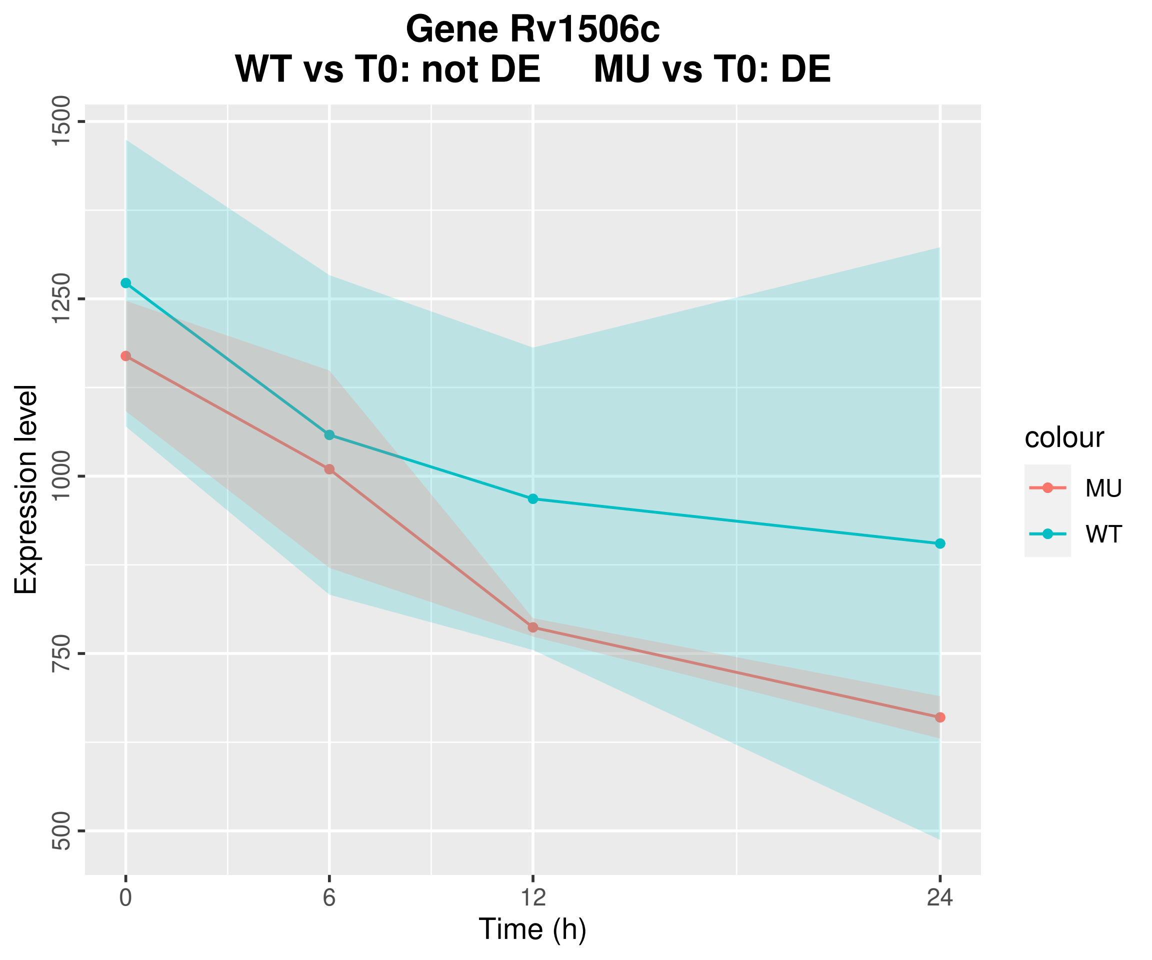

Gene Rv1506c
WT vs T0: not DE MU vs T0: DE
Expression level
1500
1250
1000
750
500
0
6
12
24
Time (h)
colour
MU
WT

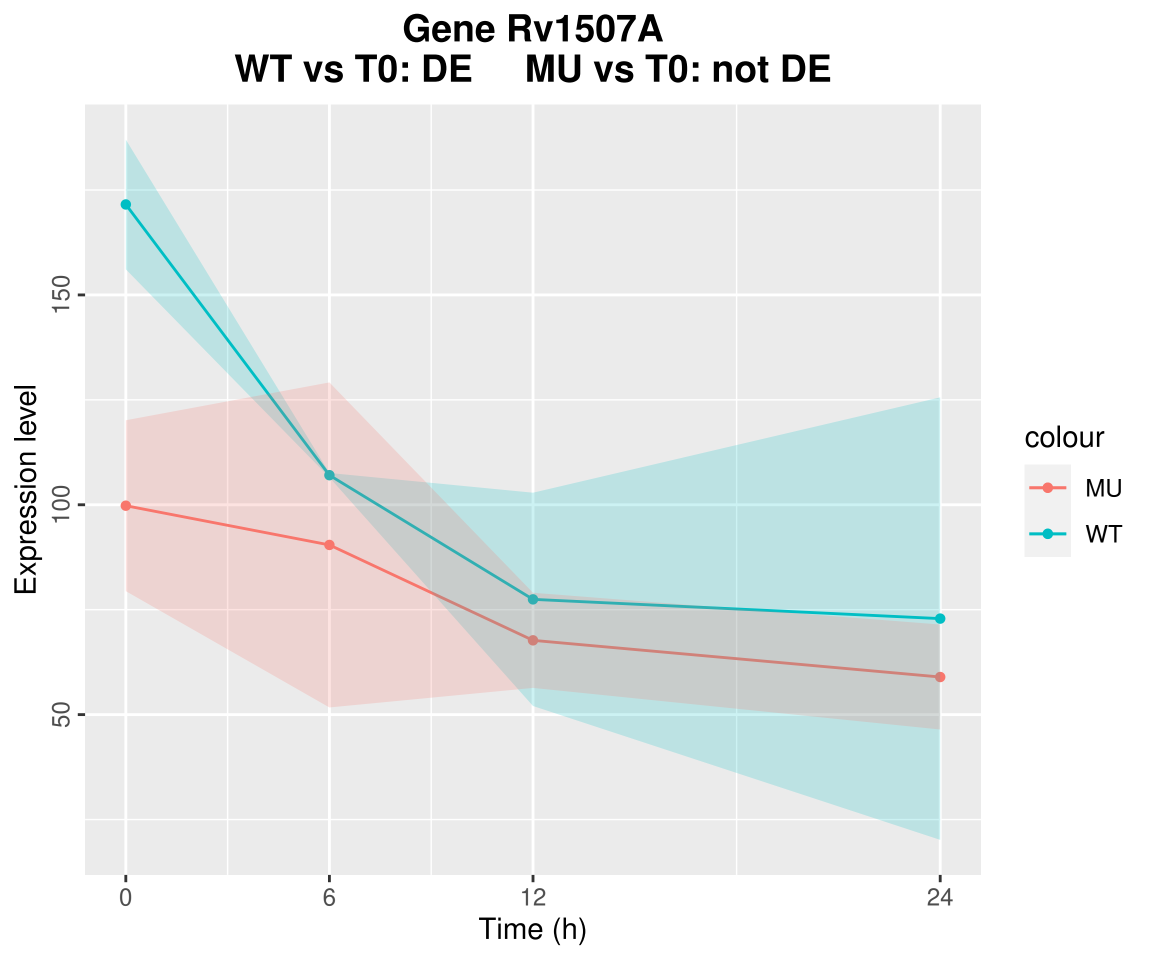
Gene Rv1507A
WT vs T0: DE   MU vs T0: not DE
Expression level
150
100
50
0
6
12
24
Time (h)
colour
MU
WT

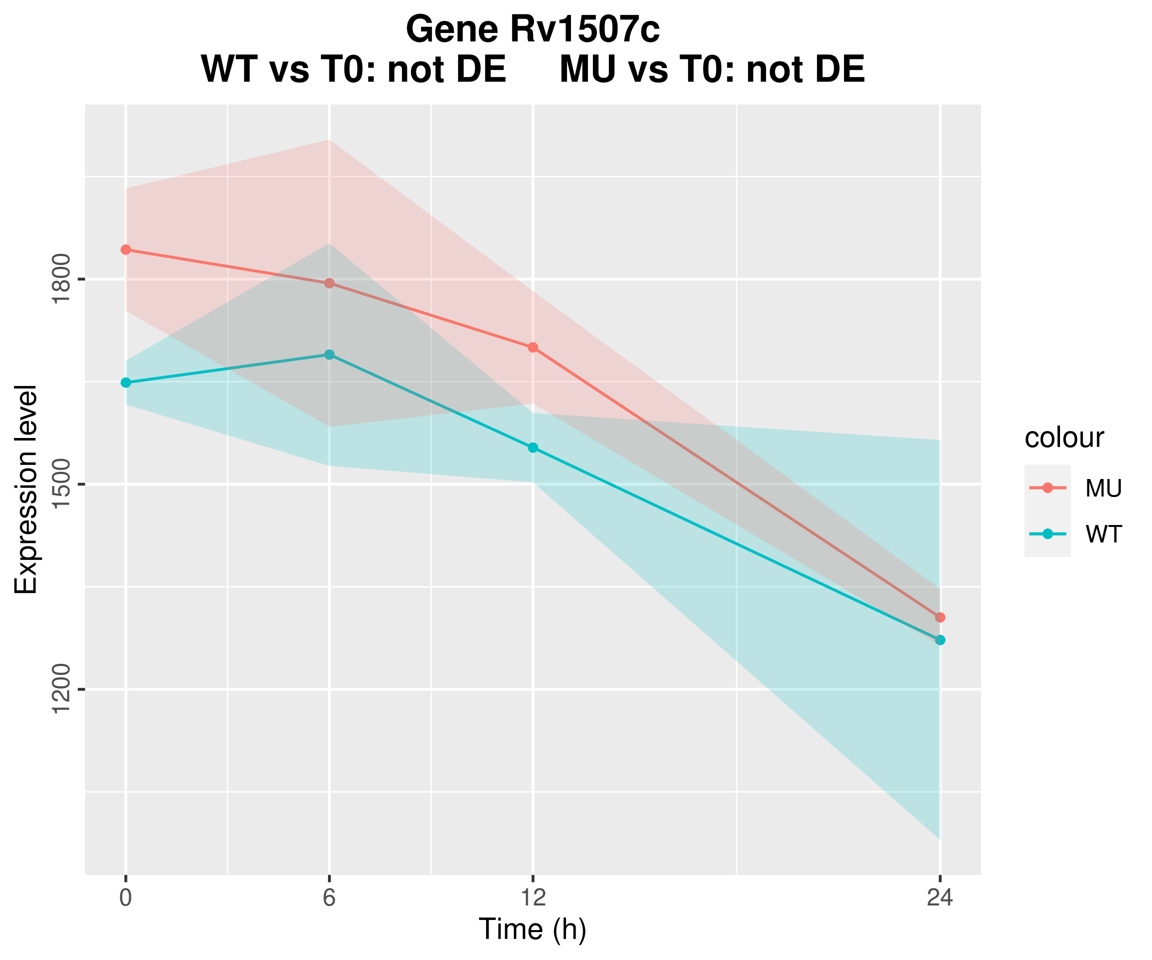
Gene Rv1507c
WT vs T0: not DE   MU vs T0: not DE
Expression level
1200
1500
1800
Time (h)
0
6
12
24
colour
MU
WT

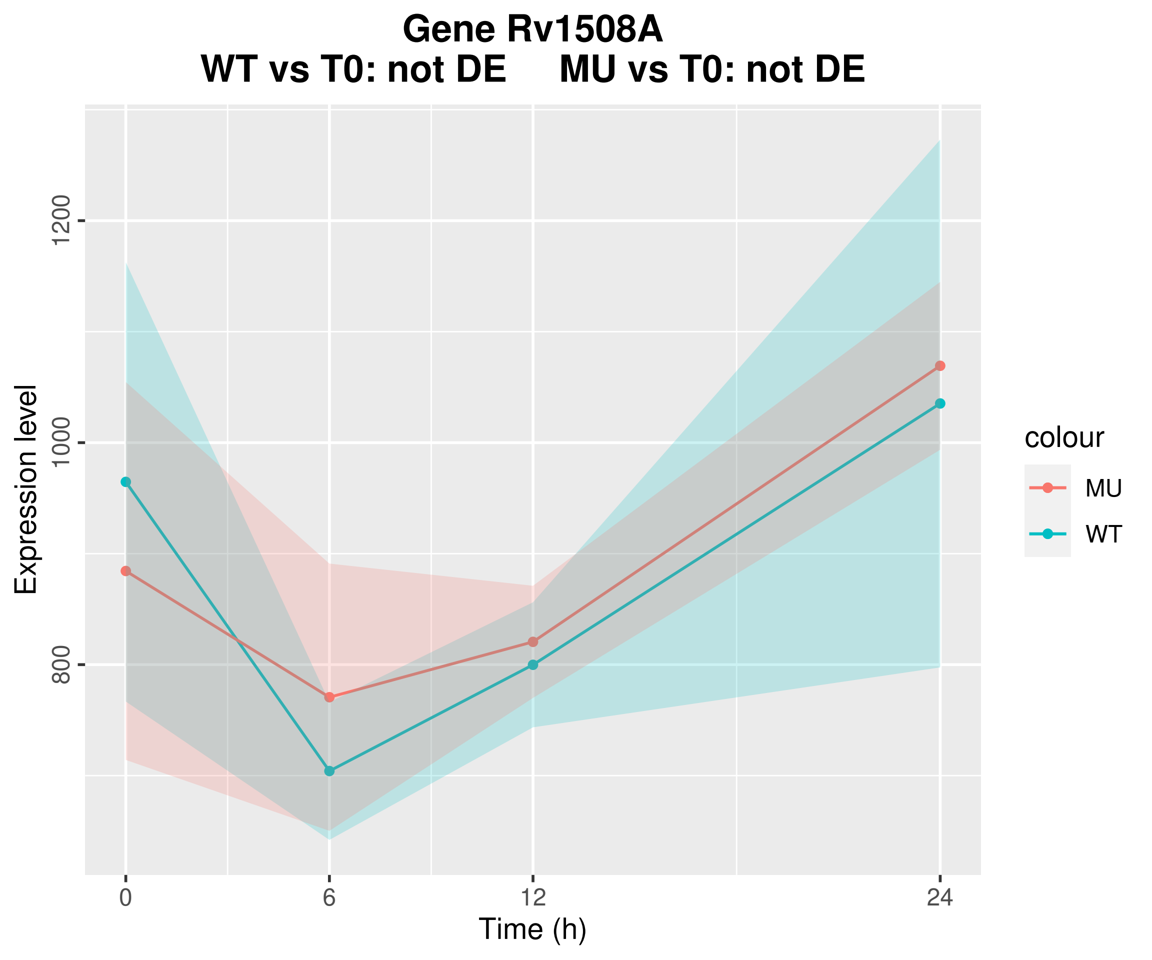
Gene Rv1508A
WT vs T0: not DE   MU vs T0: not DE
Expression level
1200
1000
800
0
6
12
24
Time (h)
colour
MU
WT

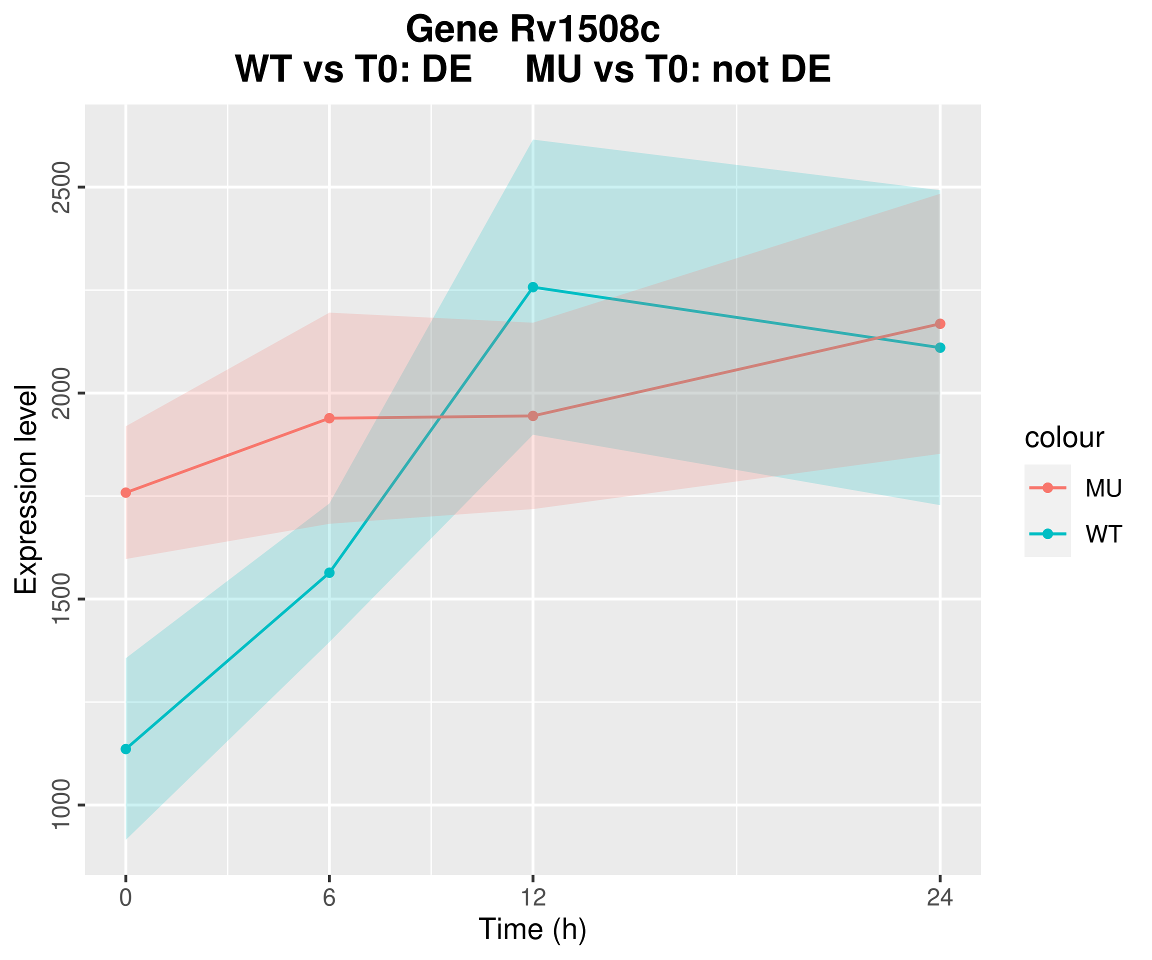
Gene Rv1508c
WT vs T0: DE   MU vs T0: not DE
Expression level
2500
2000
1500
1000
0
6
12
24
Time (h)
colour
MU
WT

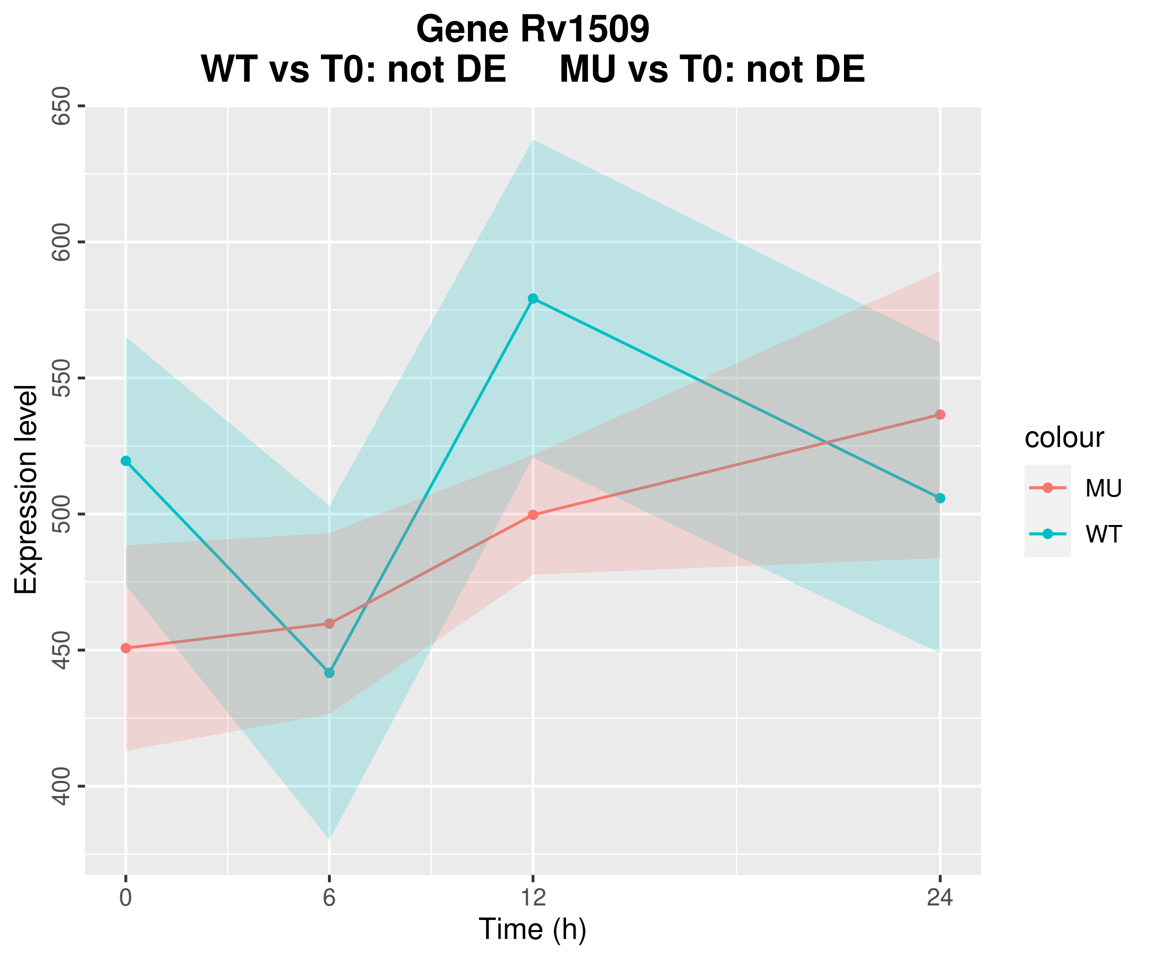

Gene Rv1509
WT vs T0: not DE MU vs T0: not DE
Expression level
650
600
550
500
450
400
0
6
12
24
Time (h)
colour
MU
WT

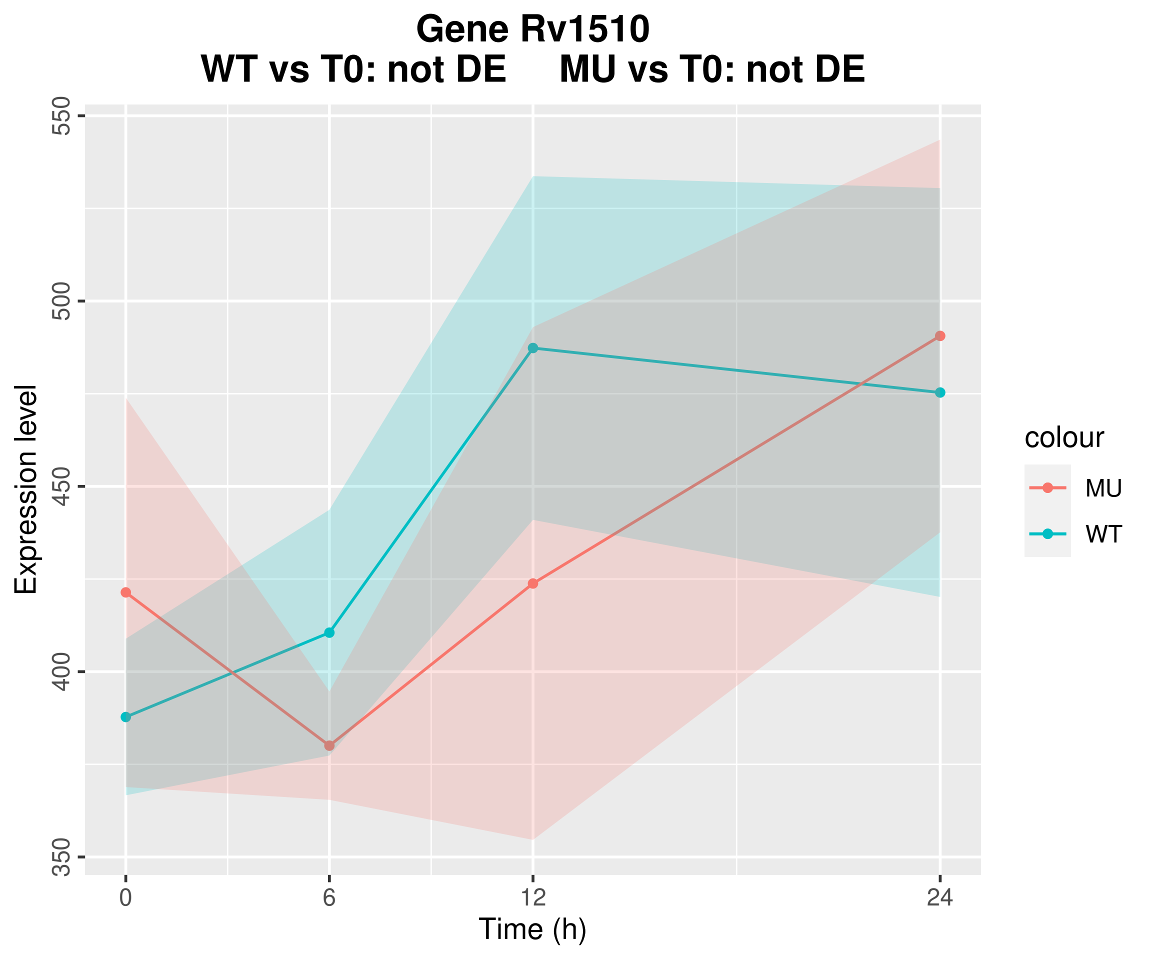

Gene Rv1510
WT vs T0: not DE MU vs T0: not DE
Expression level
550
500
450
400
350
0
6
12
24
Time (h)
colour
MU
WT

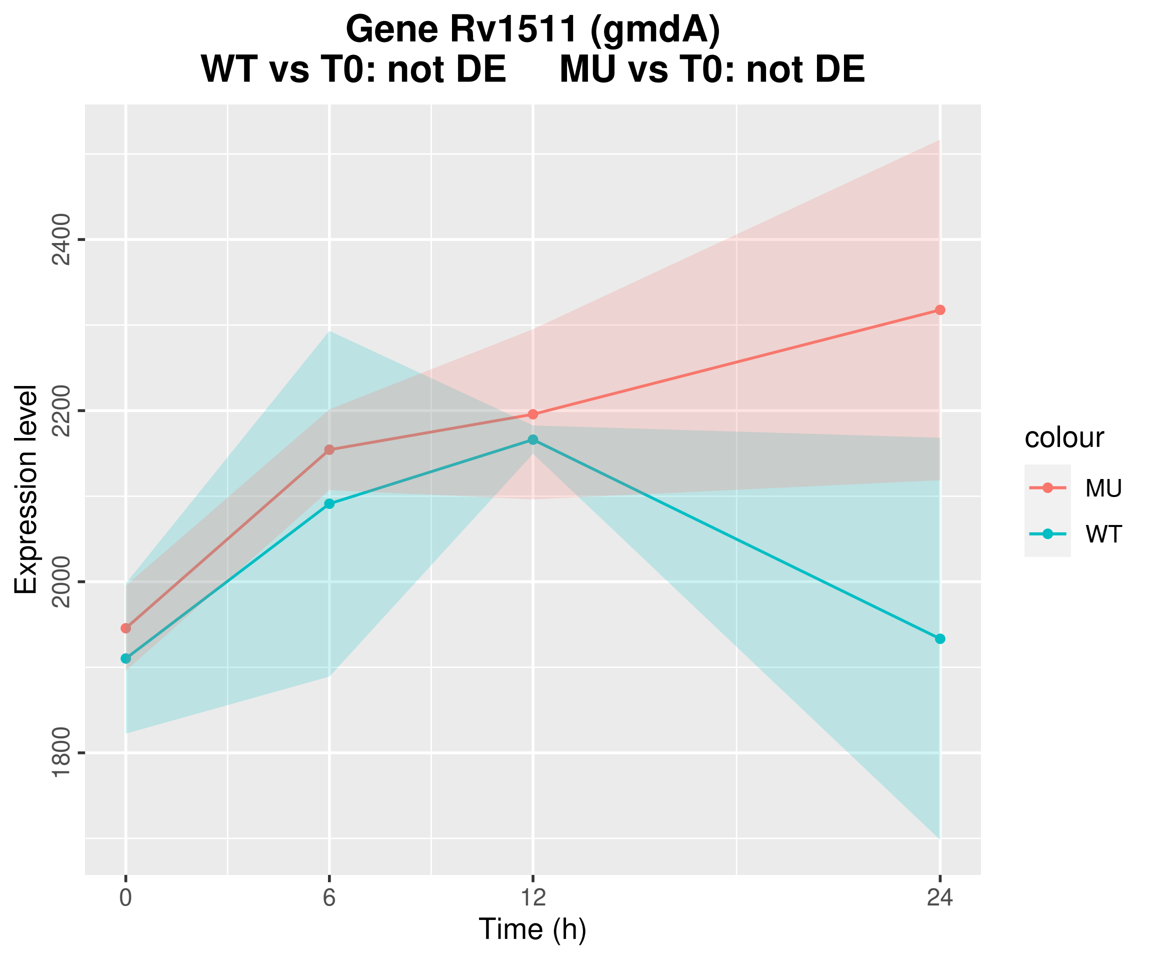

Gene Rv1511 (gmdA)
WT vs T0: not DE MU vs T0: not DE
Expression level
1800
2000
2200
2400
0
6
12
24
Time (h)
colour
MU
WT

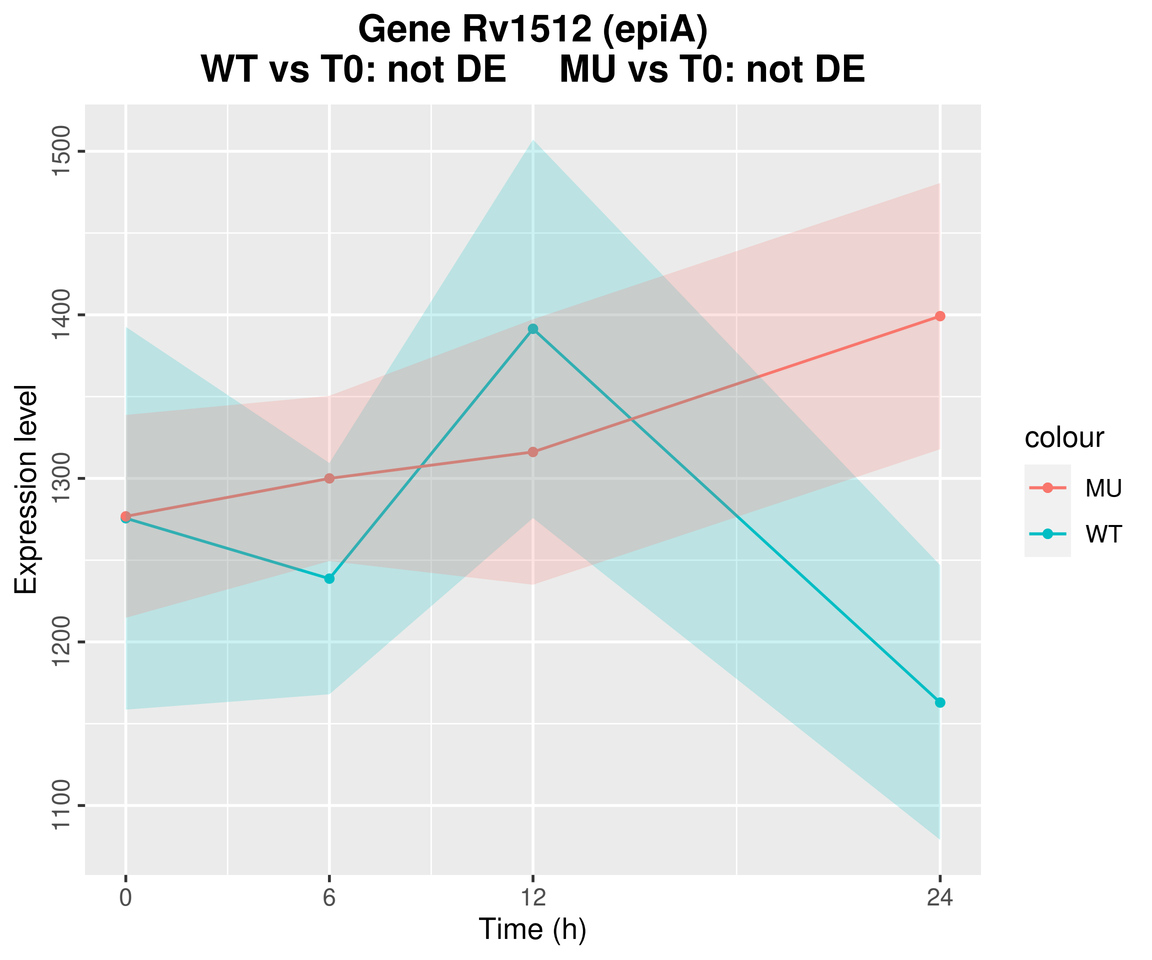

Gene Rv1512 (epiA)
WT vs T0: not DE   MU vs T0: not DE
Expression level
1100
1200
1300
1400
1500
Time (h)
0
6
12
24
colour
MU
WT

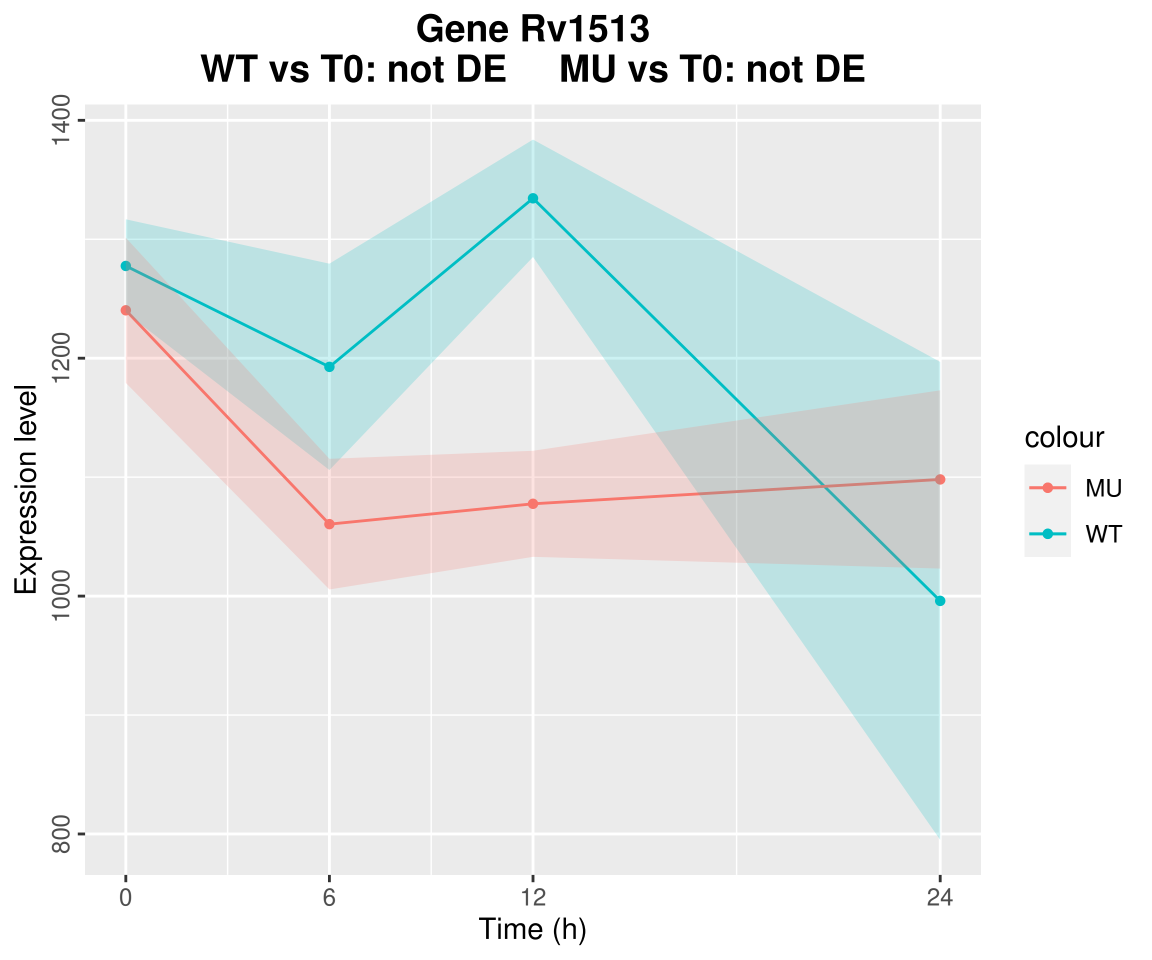

Gene Rv1513
WT vs T0: not DE   MU vs T0: not DE
Expression level
1400
1200
1000
800
0
6
12
24
Time (h)
colour
MU
WT

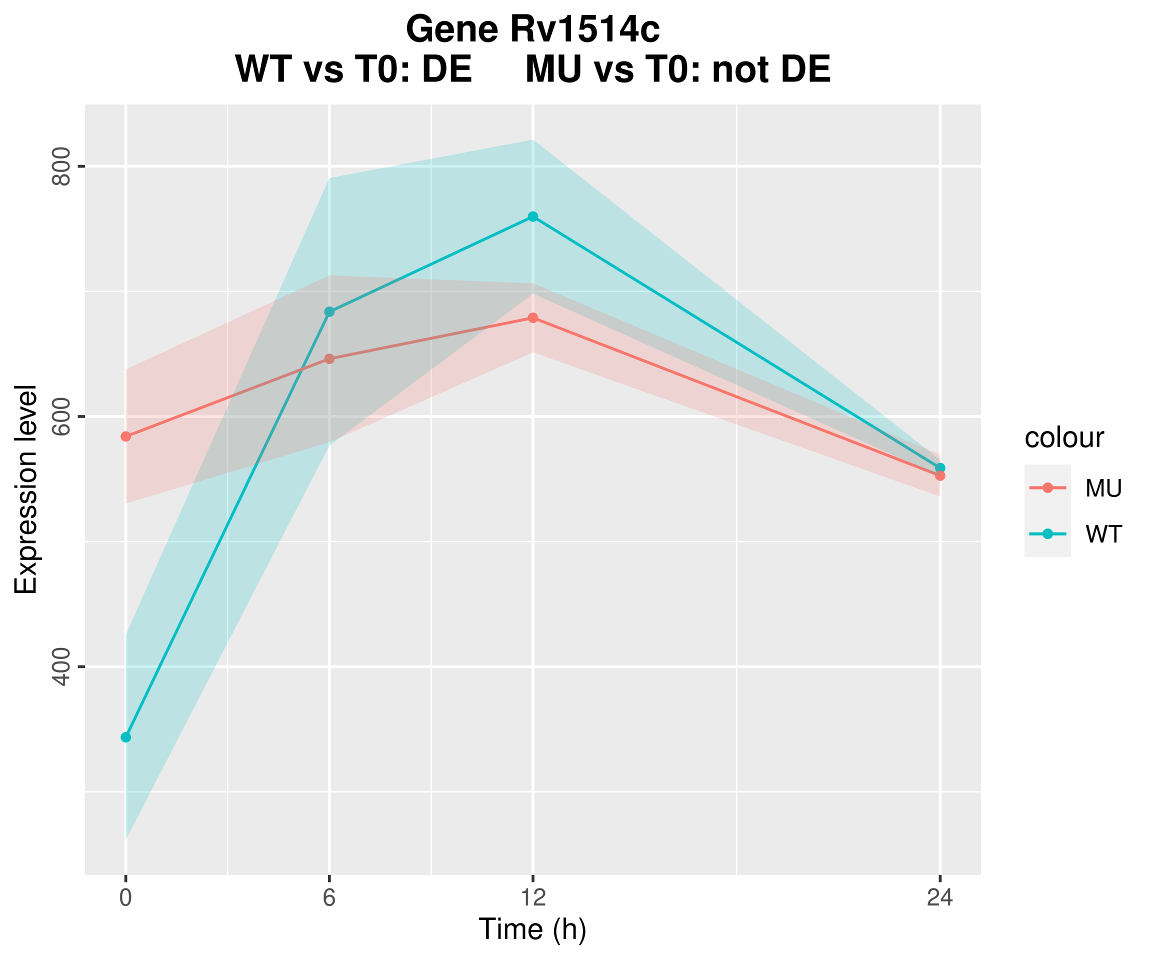

Gene Rv1514c
WT vs T0: DE   MU vs T0: not DE
Expression level
800
600
400
0
6
12
24
Time (h)
colour
MU
WT

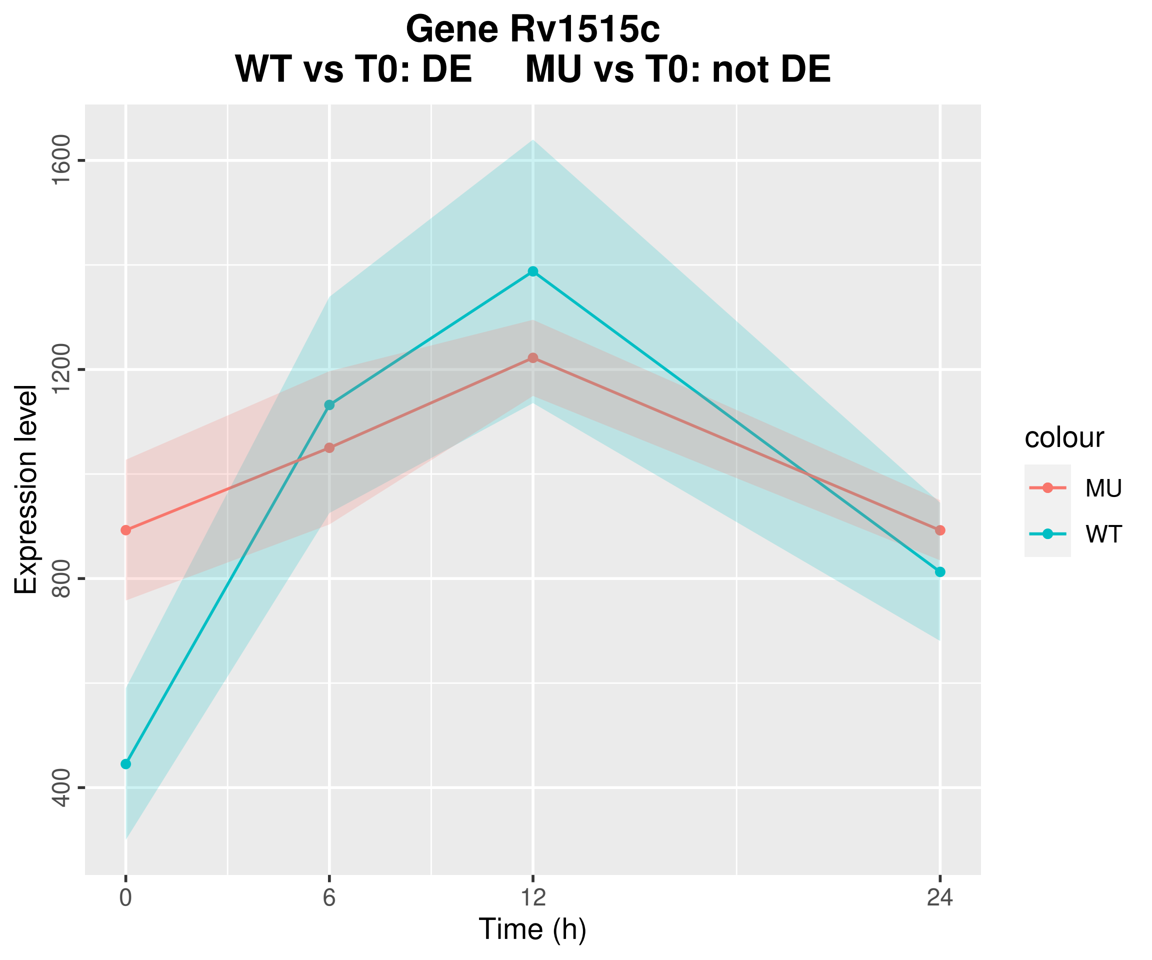
Gene Rv1515c
WT vs T0: DE MU vs T0: not DE
Expression level
1600
1200
800
400
0
6
12
24
Time (h)
colour
MU
WT

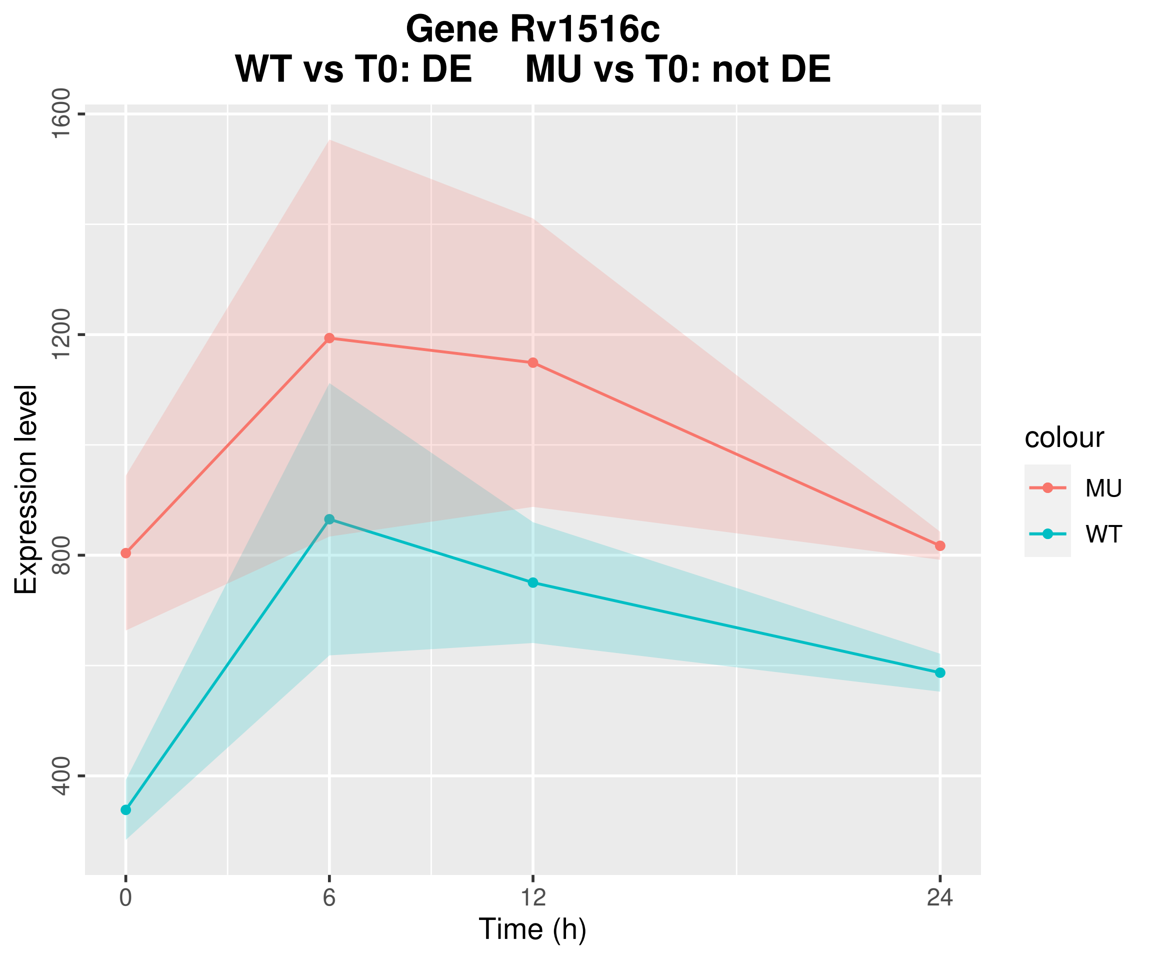
Gene Rv1516c
WT vs T0: DE MU vs T0: not DE
Expression level
1600
1200
800
400
0
6
12
24
Time (h)
colour
MU
WT

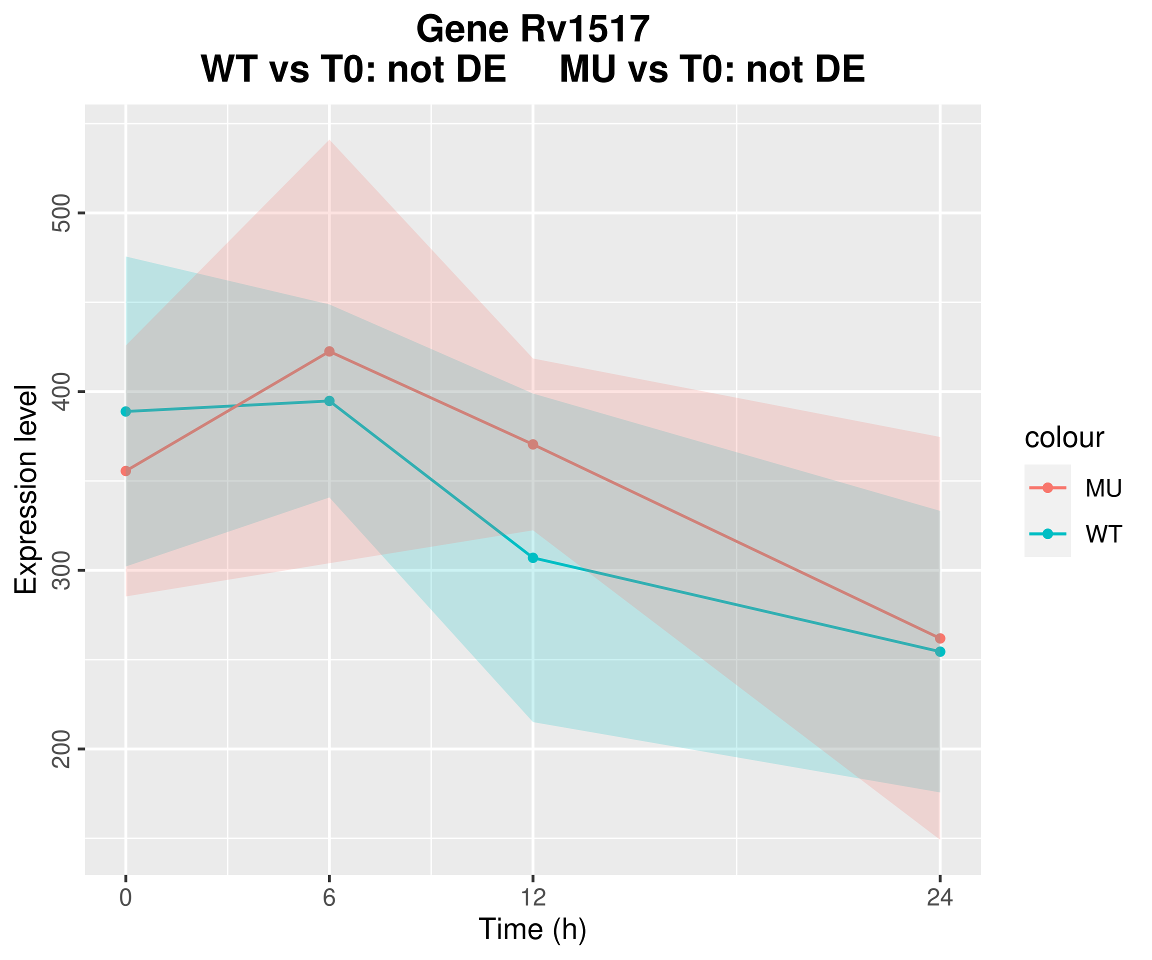

Gene Rv1517
WT vs T0: not DE   MU vs T0: not DE
Expression level
500
400
300
200
Time (h)
0
6
12
24
colour
MU
WT

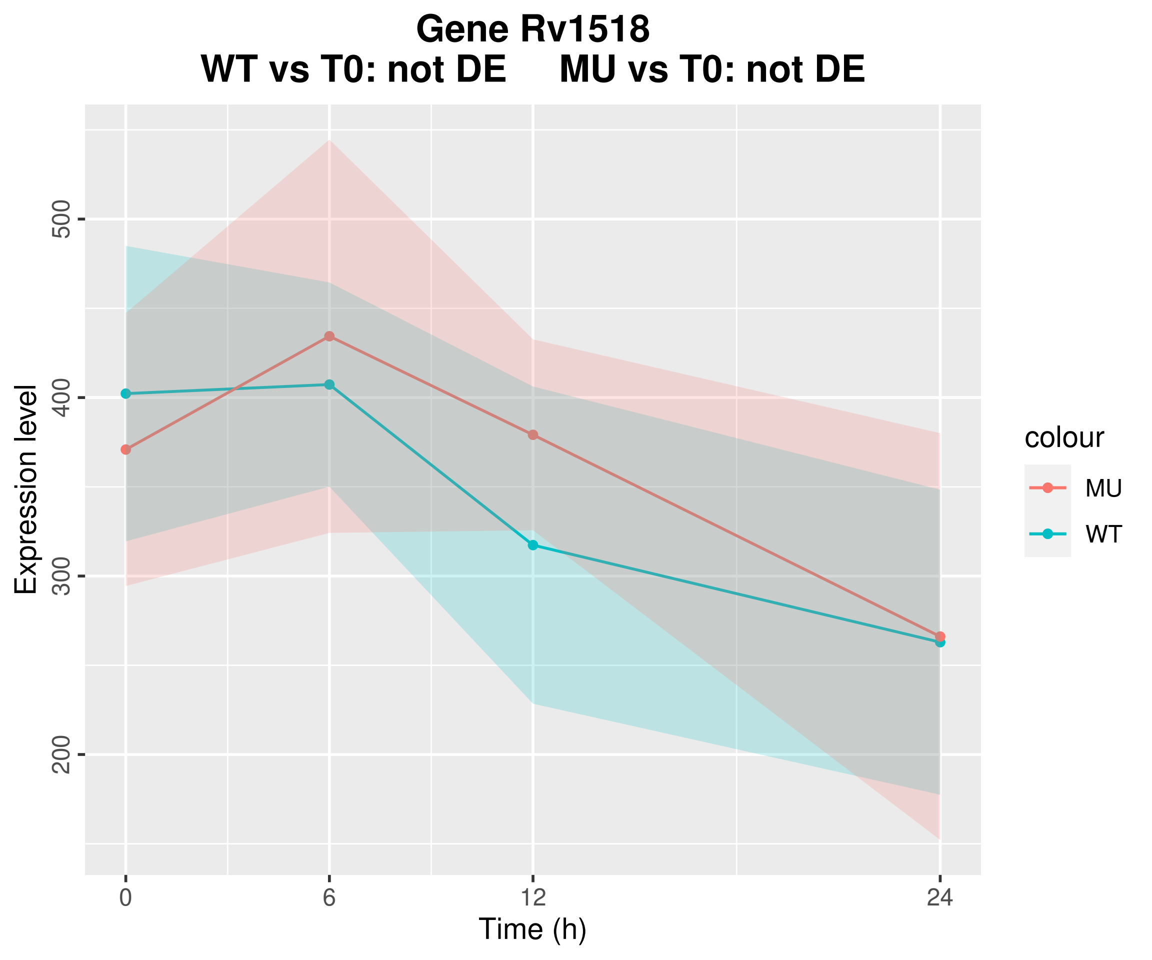

Gene Rv1518
WT vs T0: not DE MU vs T0: not DE
Expression level
500
400
300
200
0
6
12
24
Time (h)
colour
MU
WT

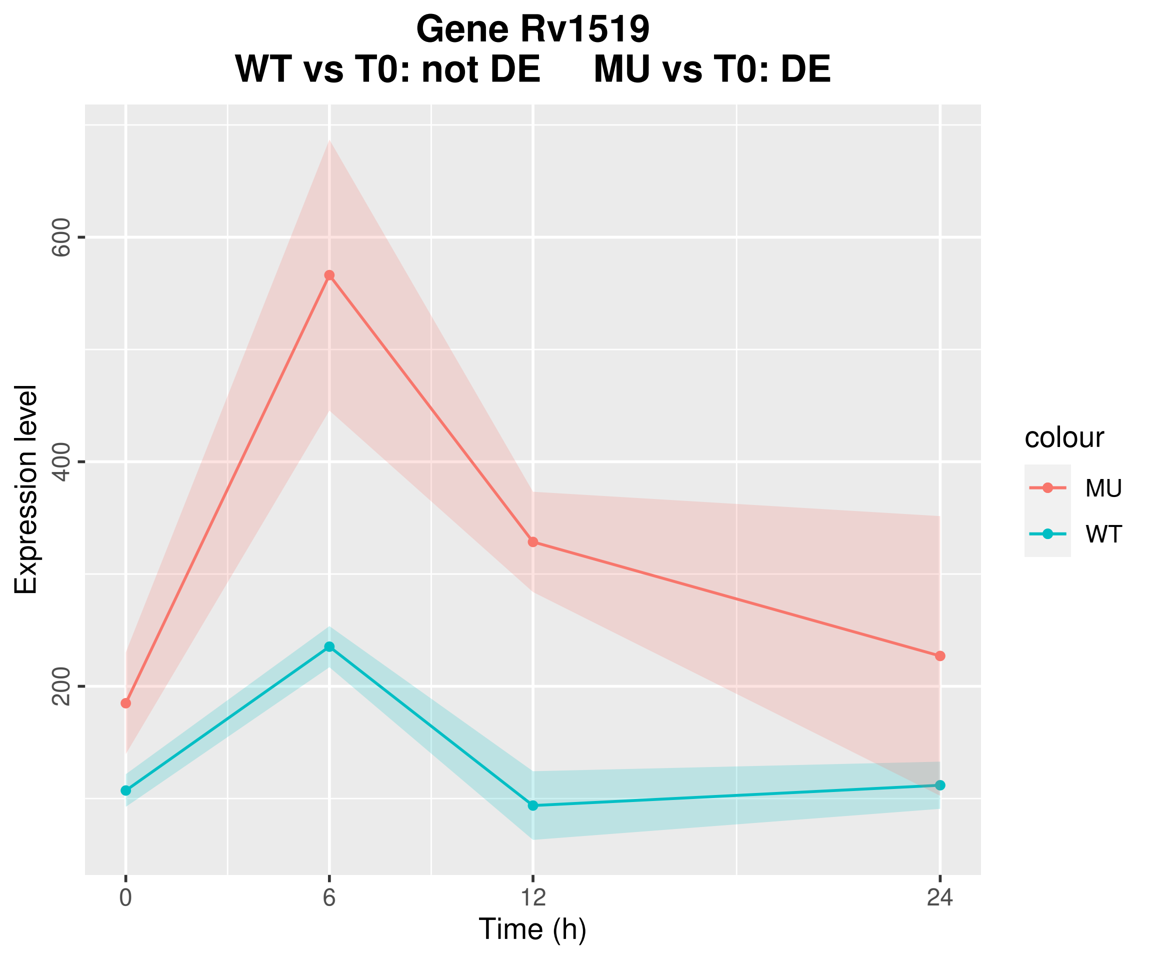
Gene Rv1519
WT vs T0: not DE MU vs T0: DE
Expression level
600
400
200
0
6
12
24
Time (h)
colour
MU
WT

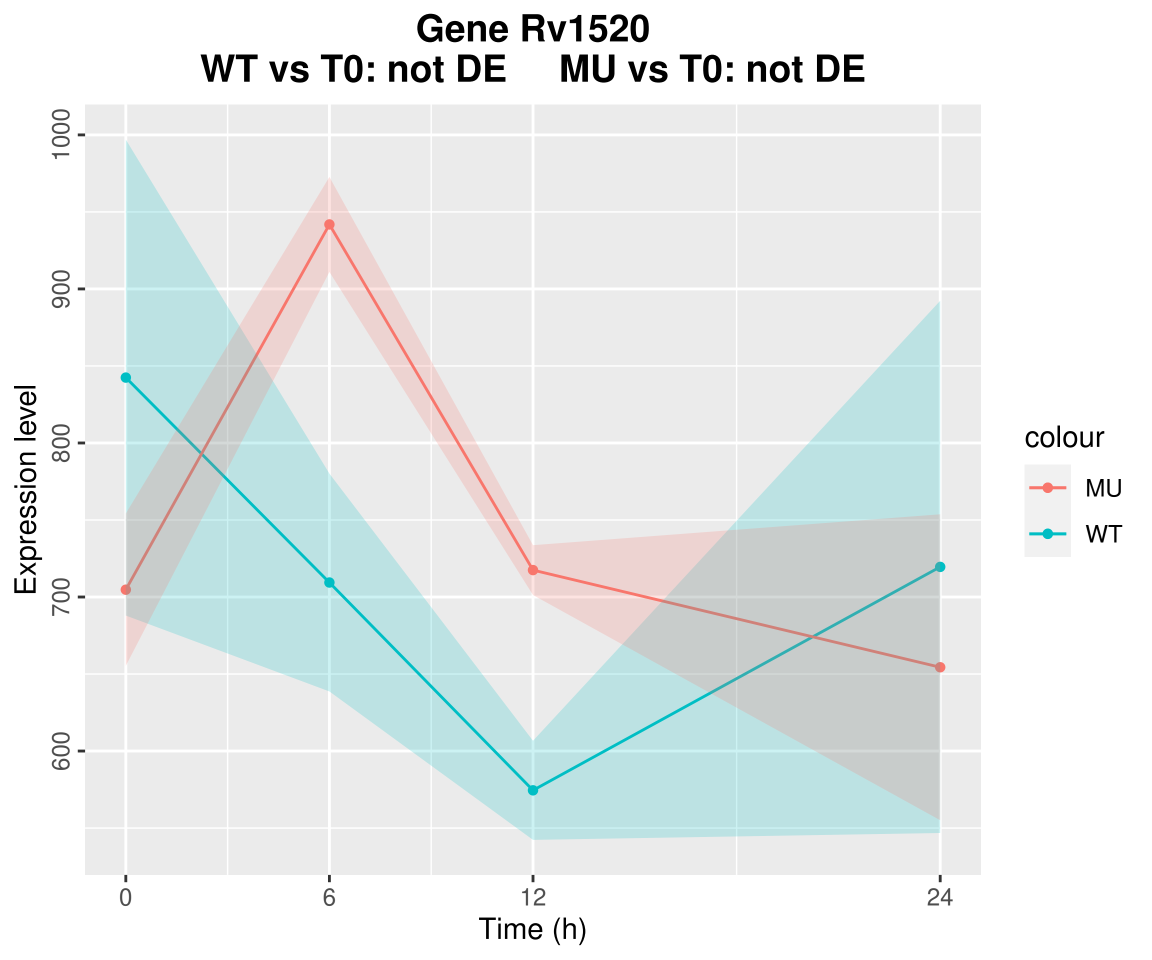

Gene Rv1520
WT vs T0: not DE MU vs T0: not DE
Expression level
1000
900
800
700
600
0
6
12
24
Time (h)
colour
MU
WT

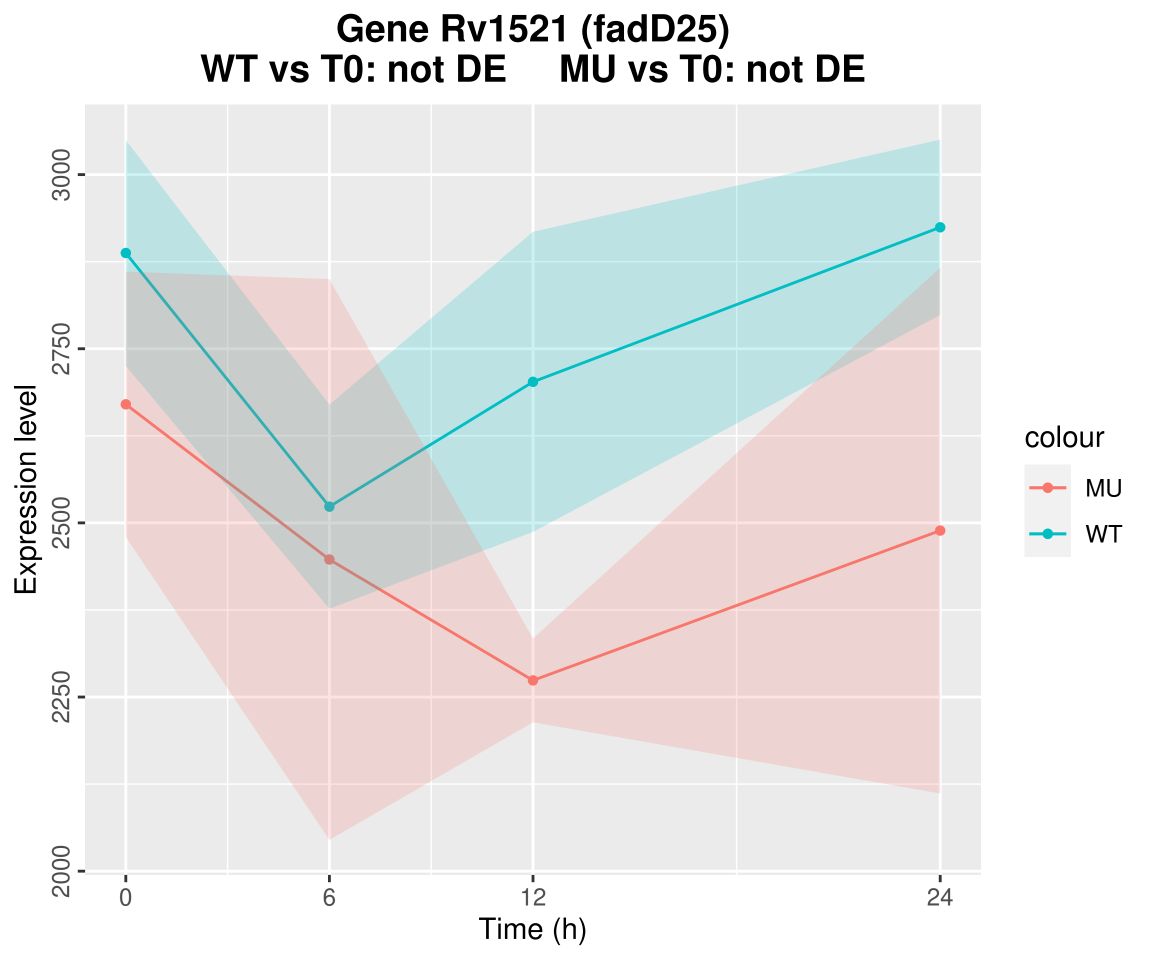
Gene Rv1521 (fadD25)
WT vs T0: not DE MU vs T0: not DE
Expression level
3000
2750
2500
2250
2000
0
6
12
24
Time (h)
colour
MU
WT

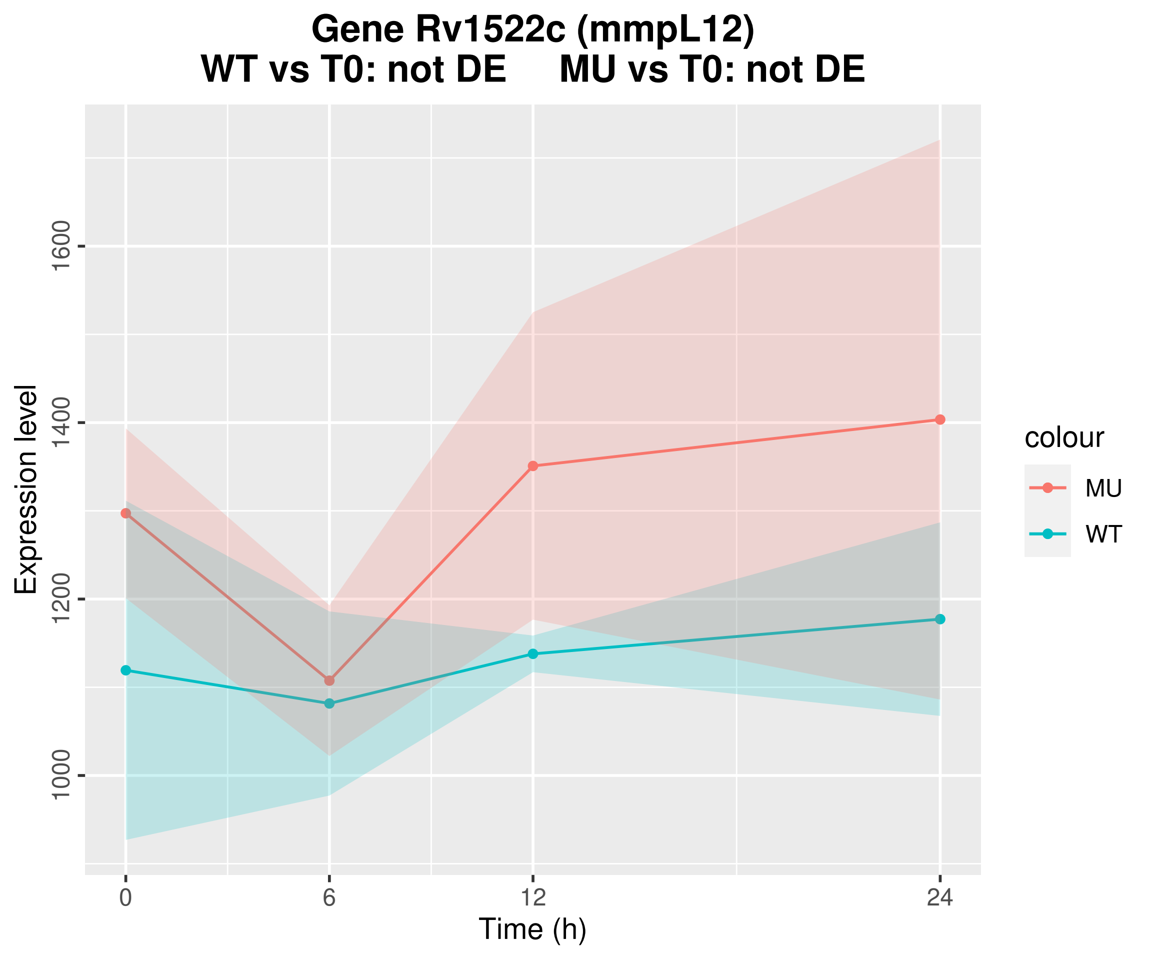
Gene Rv1522c (mmpL12)
WT vs T0: not DE   MU vs T0: not DE
Expression level
1000
1200
1400
1600
Time (h)
0
6
12
24
colour
MU
WT

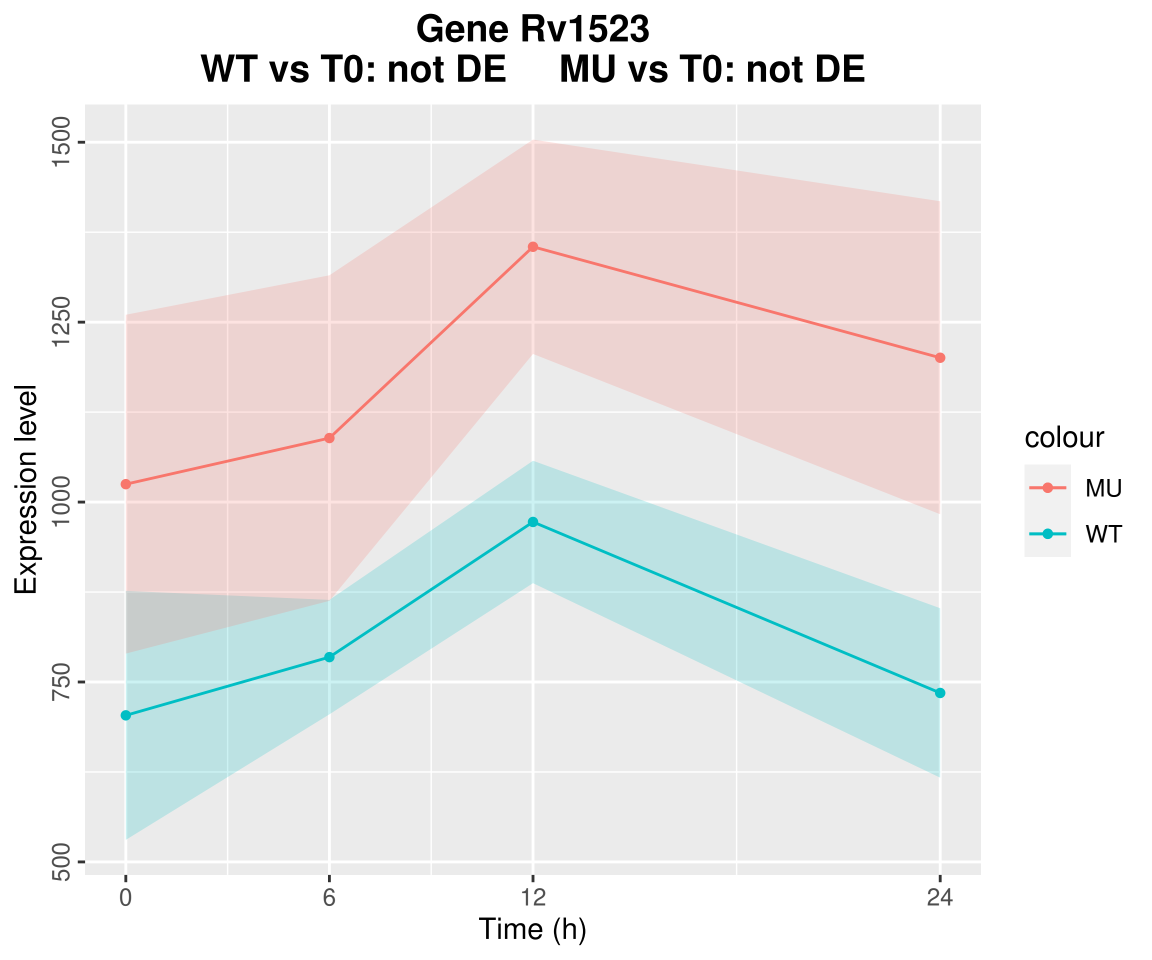
Gene Rv1523
WT vs T0: not DE   MU vs T0: not DE
Expression level
1500
1250
1000
750
500
0
6
12
24
Time (h)
colour
MU
WT

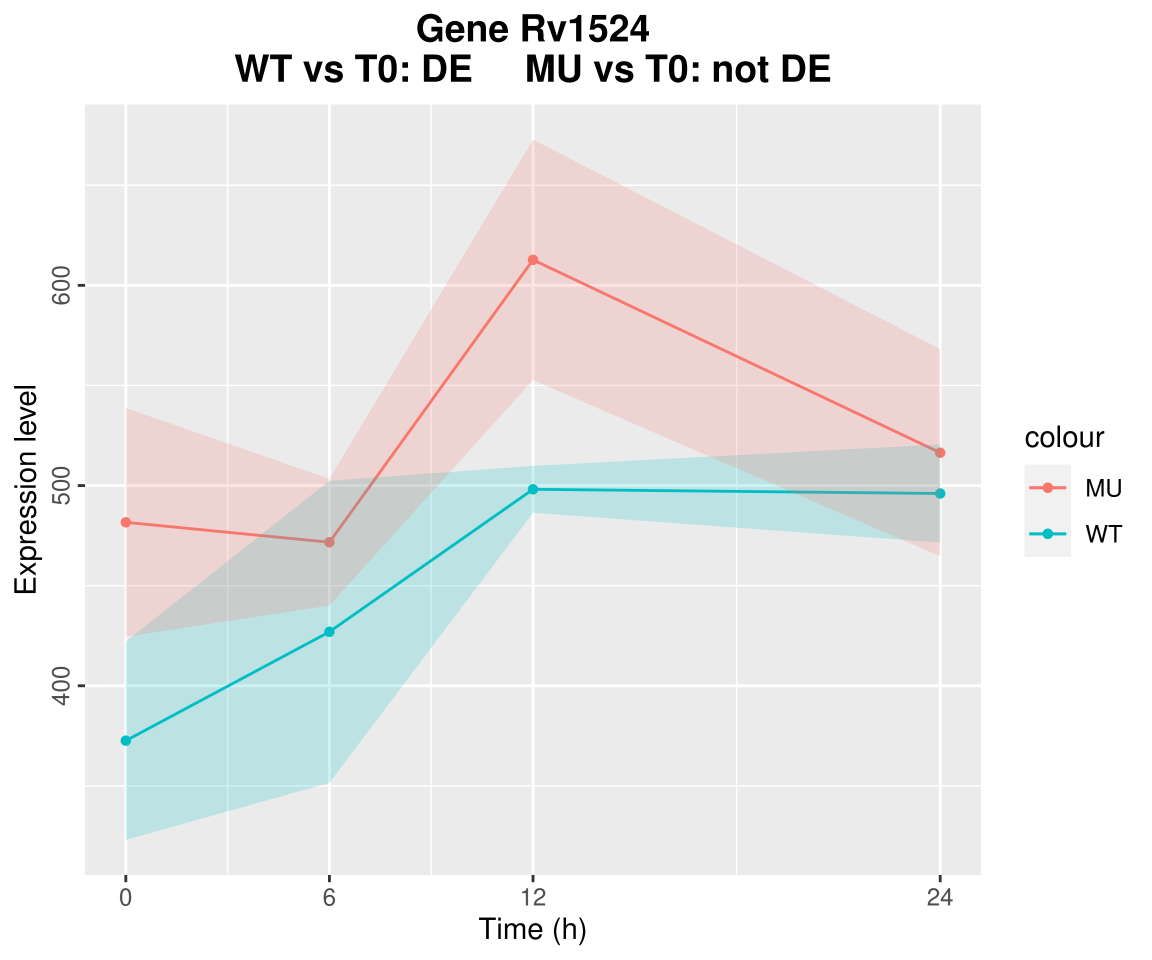

Gene Rv1524
WT vs T0: DE MU vs T0: not DE
Expression level
600
500
400
0
6
12
24
Time (h)
colour
MU
WT

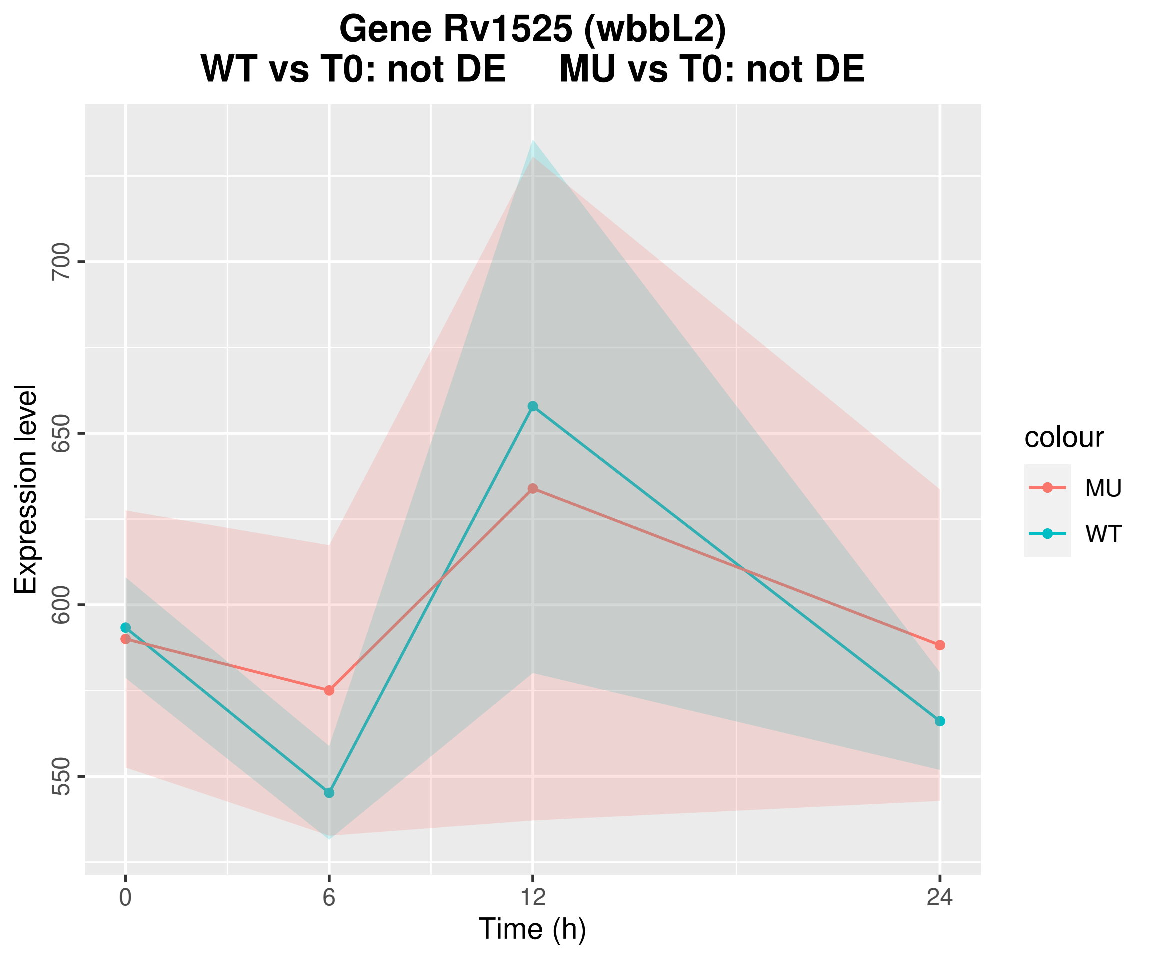
Gene Rv1525 (wbbL2)
WT vs T0: not DE   MU vs T0: not DE
Expression level
700
650
600
550
0
6
12
24
Time (h)
colour
MU
WT

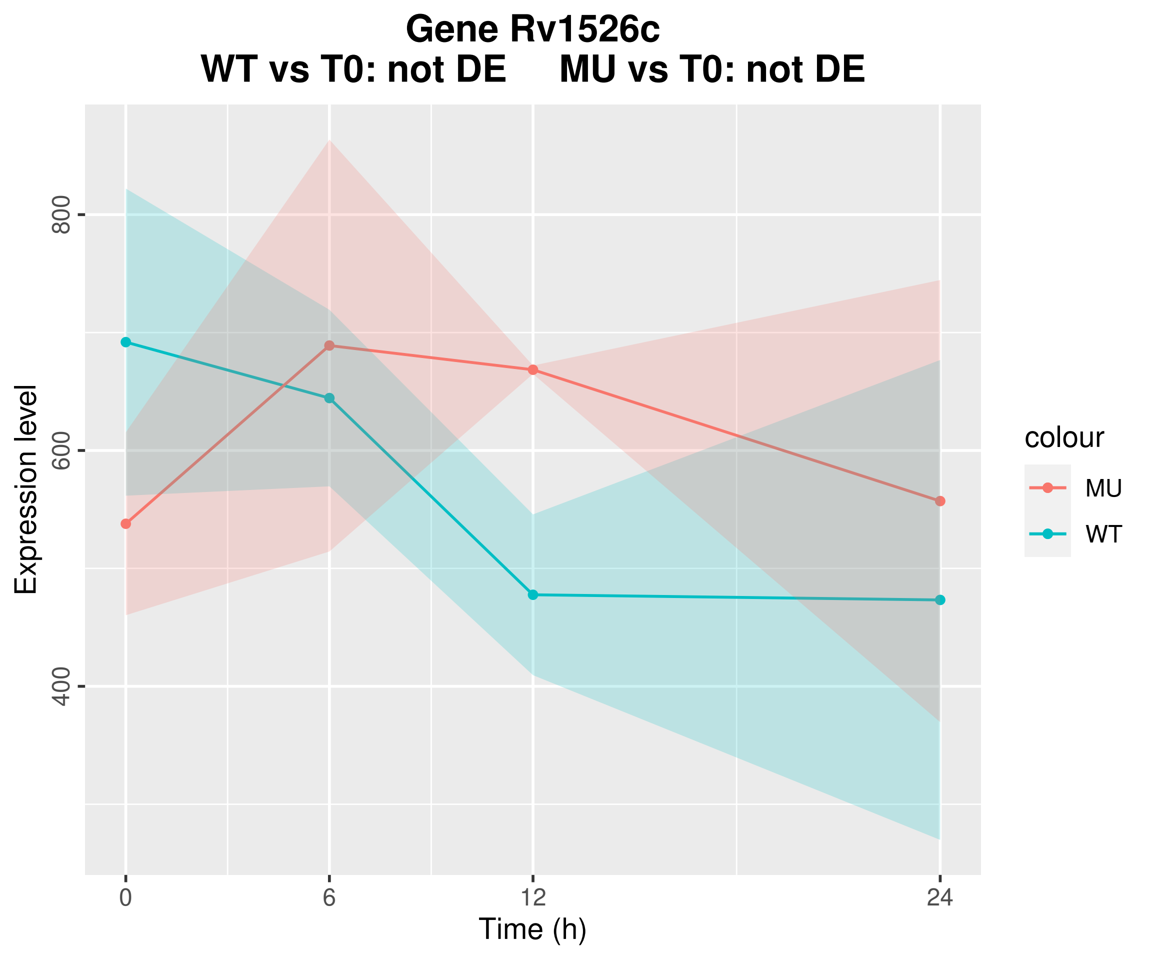
Gene Rv1526c
WT vs T0: not DE   MU vs T0: not DE
800
600
400
Expression level
0
6
12
24
Time (h)
colour
MU
WT

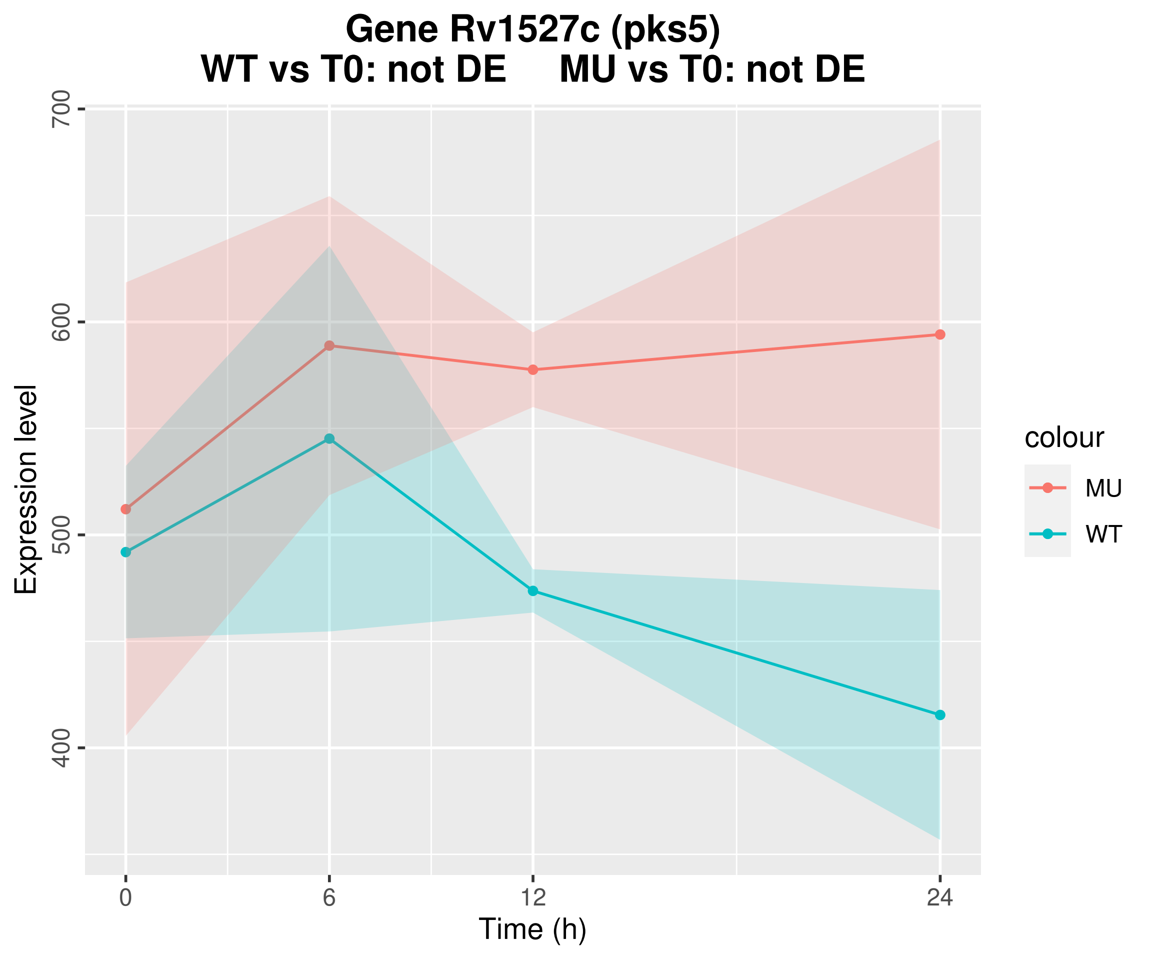

Gene Rv1527c (pks5)
WT vs T0: not DE MU vs T0: not DE
700
600
500
400
Expression level
0
6
12
24
Time (h)
colour
MU
WT

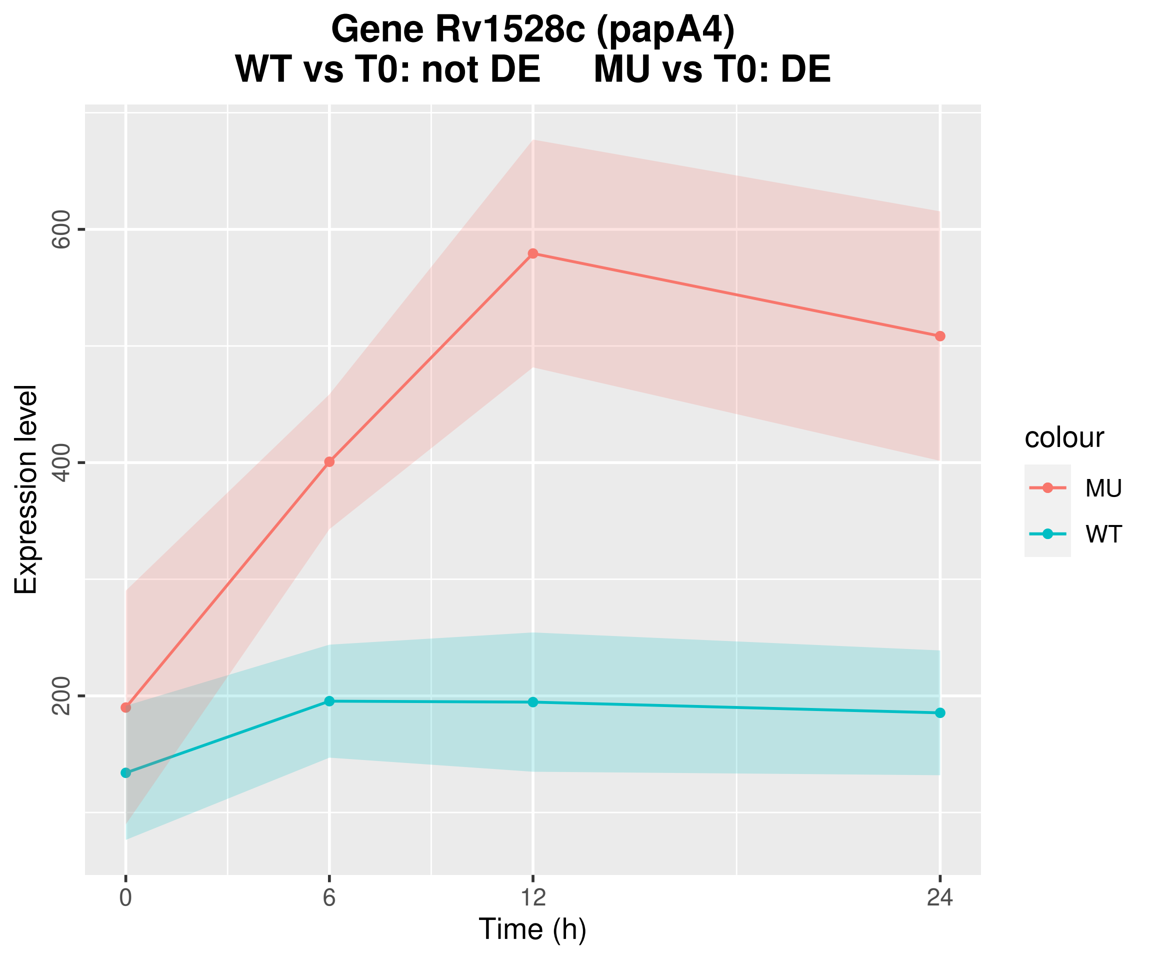

Gene Rv1528c (papA4)
WT vs T0: not DE   MU vs T0: DE
Expression level
600
400
200
0
6
12
24
Time (h)
colour
MU
WT

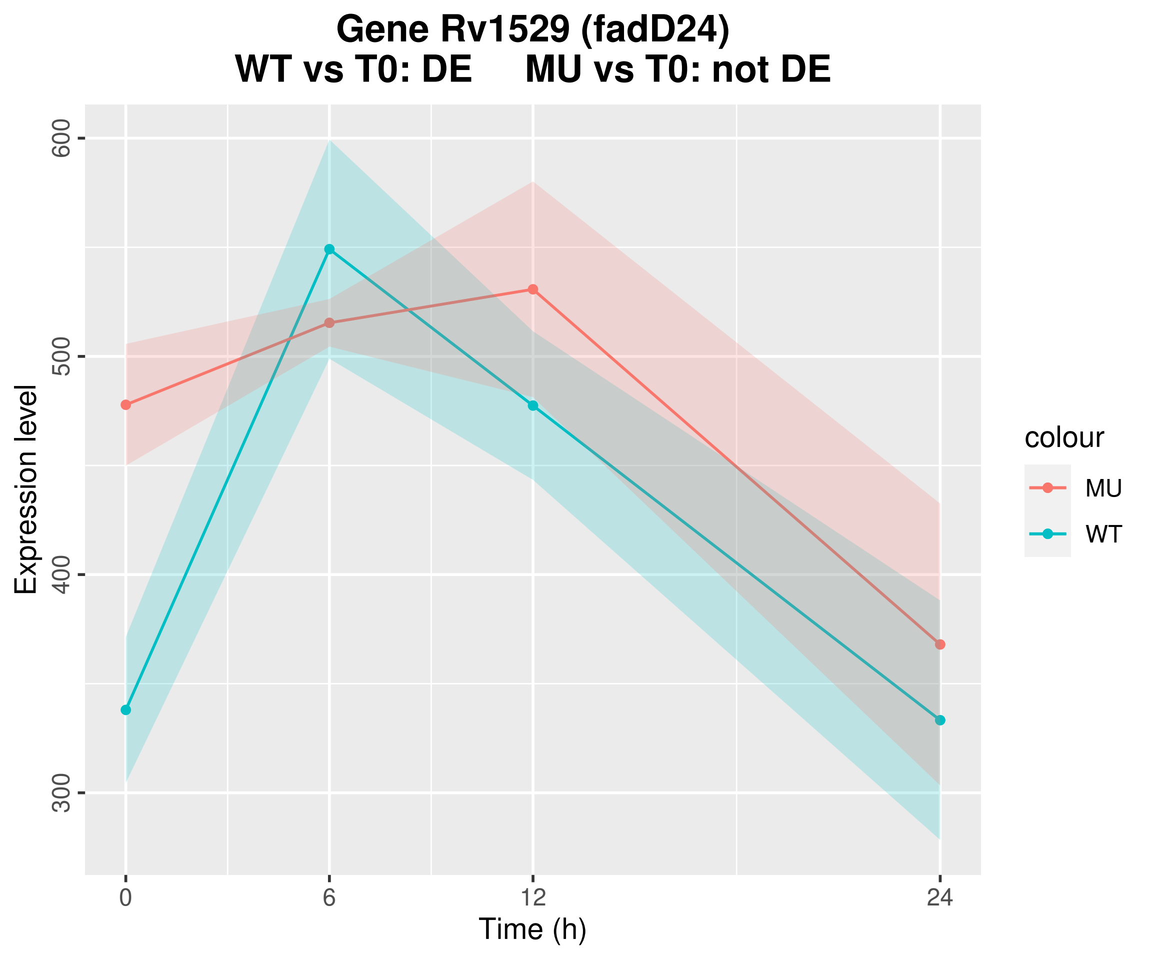

Gene Rv1529 (fadD24)
WT vs T0: DE MU vs T0: not DE
Expression level
600
500
400
300
0
6
12
24
Time (h)
colour
MU
WT

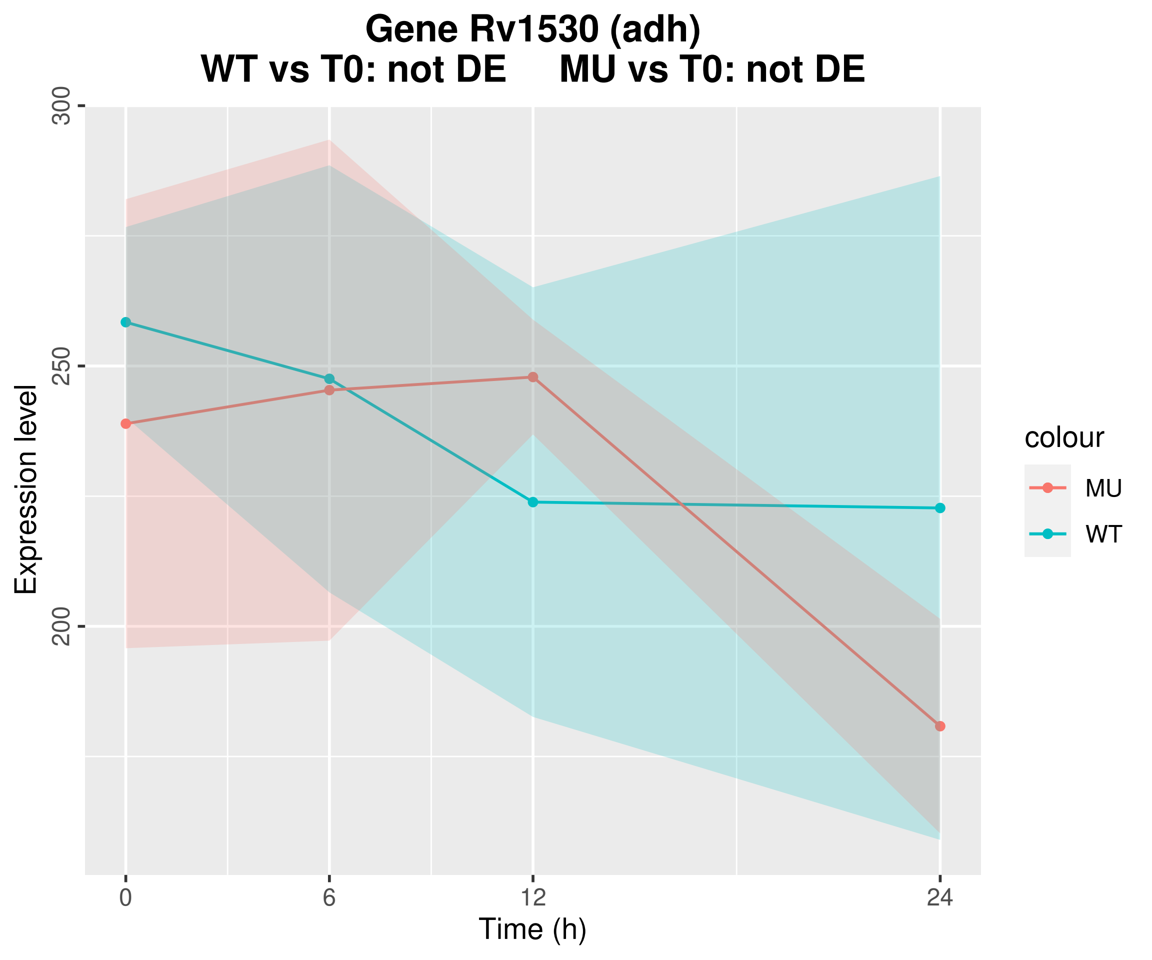

Gene Rv1530 (adh)
WT vs T0: not DE   MU vs T0: not DE
300
250
200
Expression level
0
6
12
24
Time (h)
colour
MU
WT

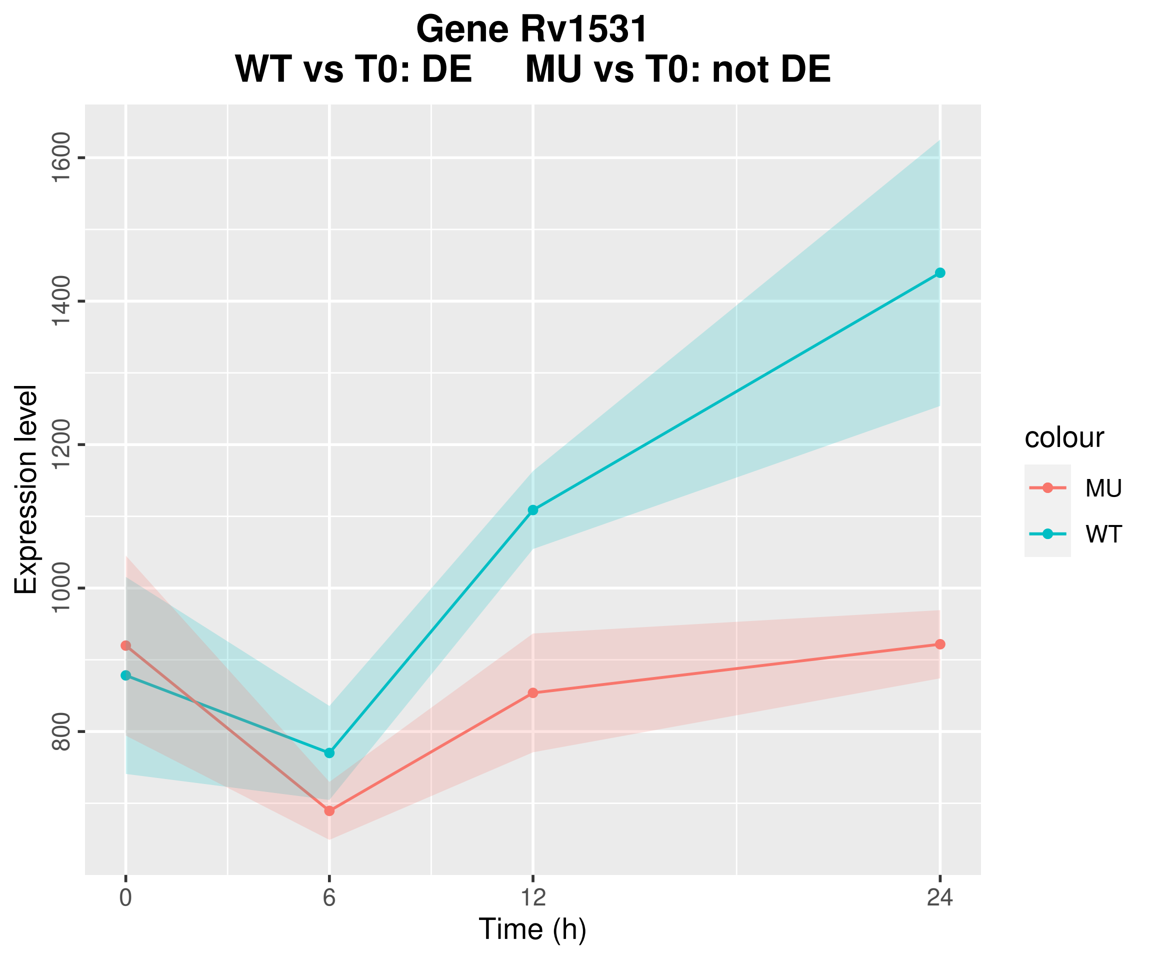

Gene Rv1531
WT vs T0: DE   MU vs T0: not DE
1600
1400
1200
1000
800
Expression level
0
6
12
24
Time (h)
colour
MU
WT

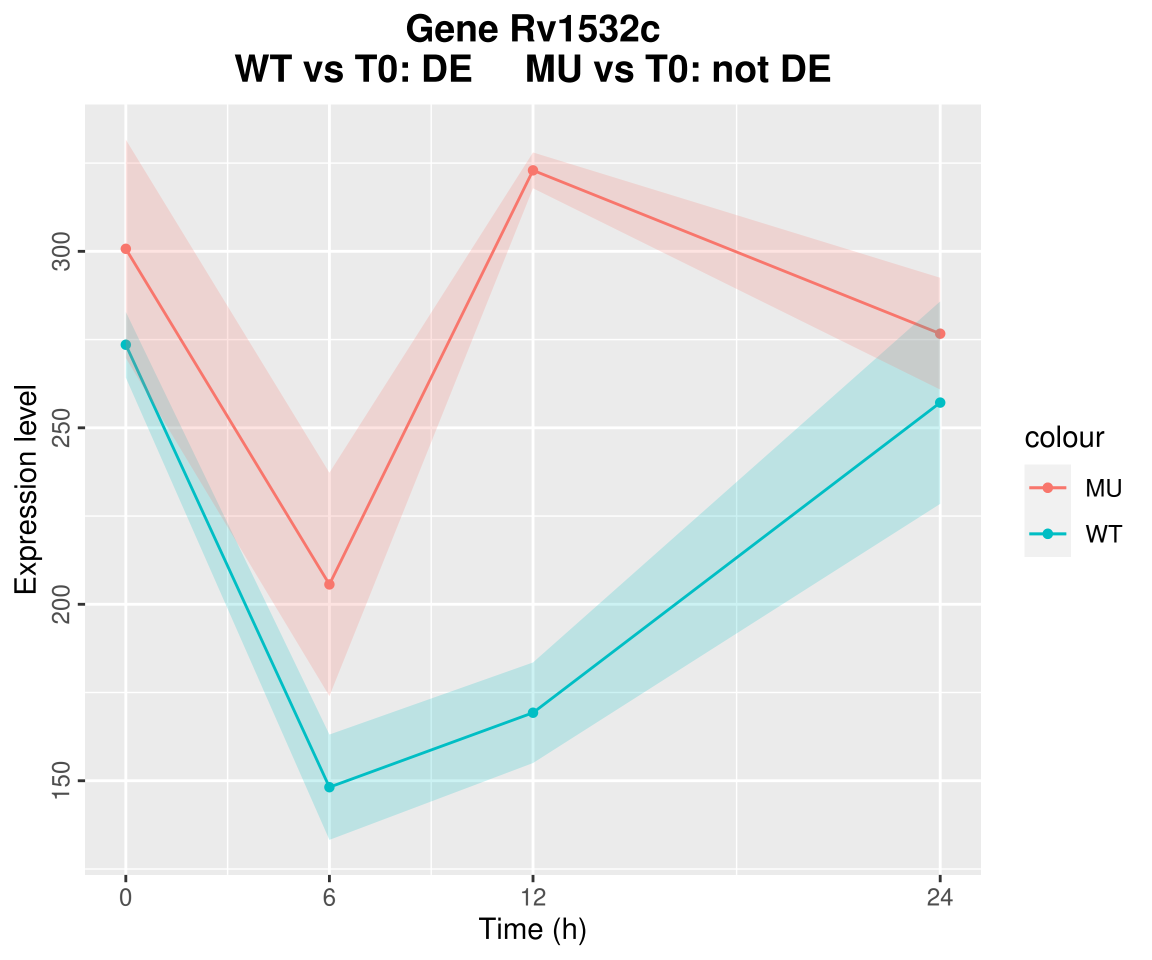

Gene Rv1532c
WT vs T0: DE MU vs T0: not DE
Expression level
300
250
200
150
0
6
12
24
Time (h)
colour
MU
WT

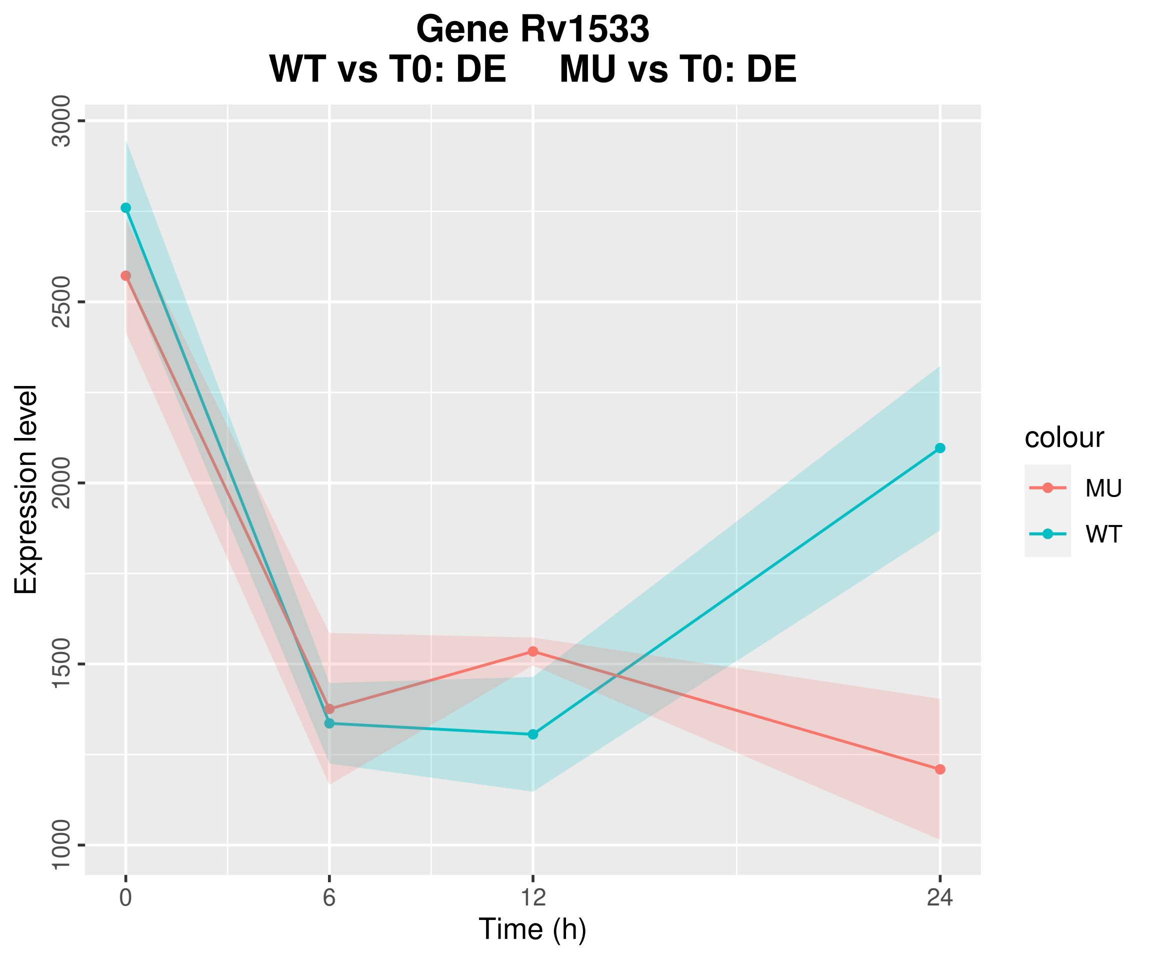

Gene Rv1533
WT vs T0: DE   MU vs T0: DE
Expression level
3000
2500
2000
1500
1000
0
6
12
24
Time (h)
colour
MU
WT

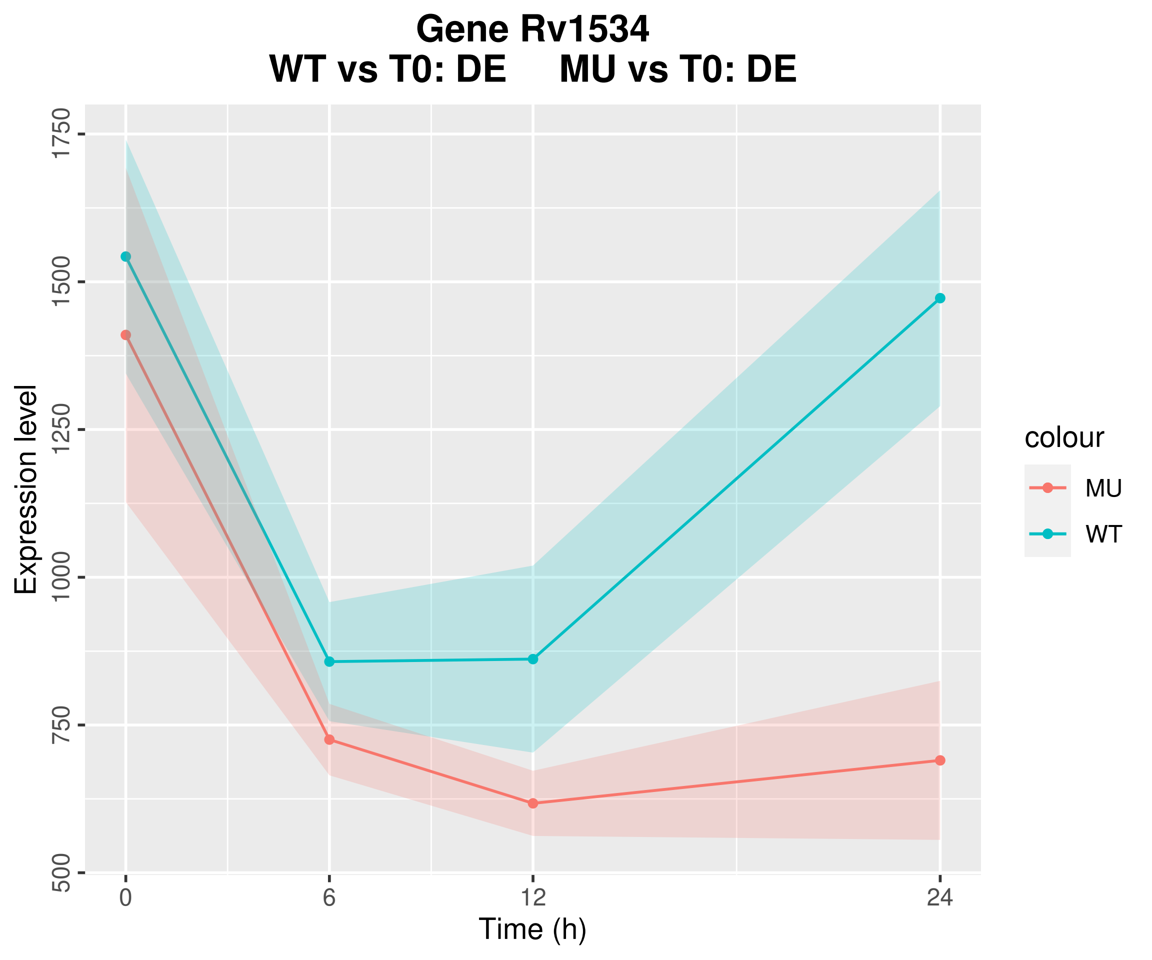

Gene Rv1534
WT vs T0: DE MU vs T0: DE
Expression level
1750
1500
1250
1000
750
500
0
6
12
24
Time (h)
colour
MU
WT

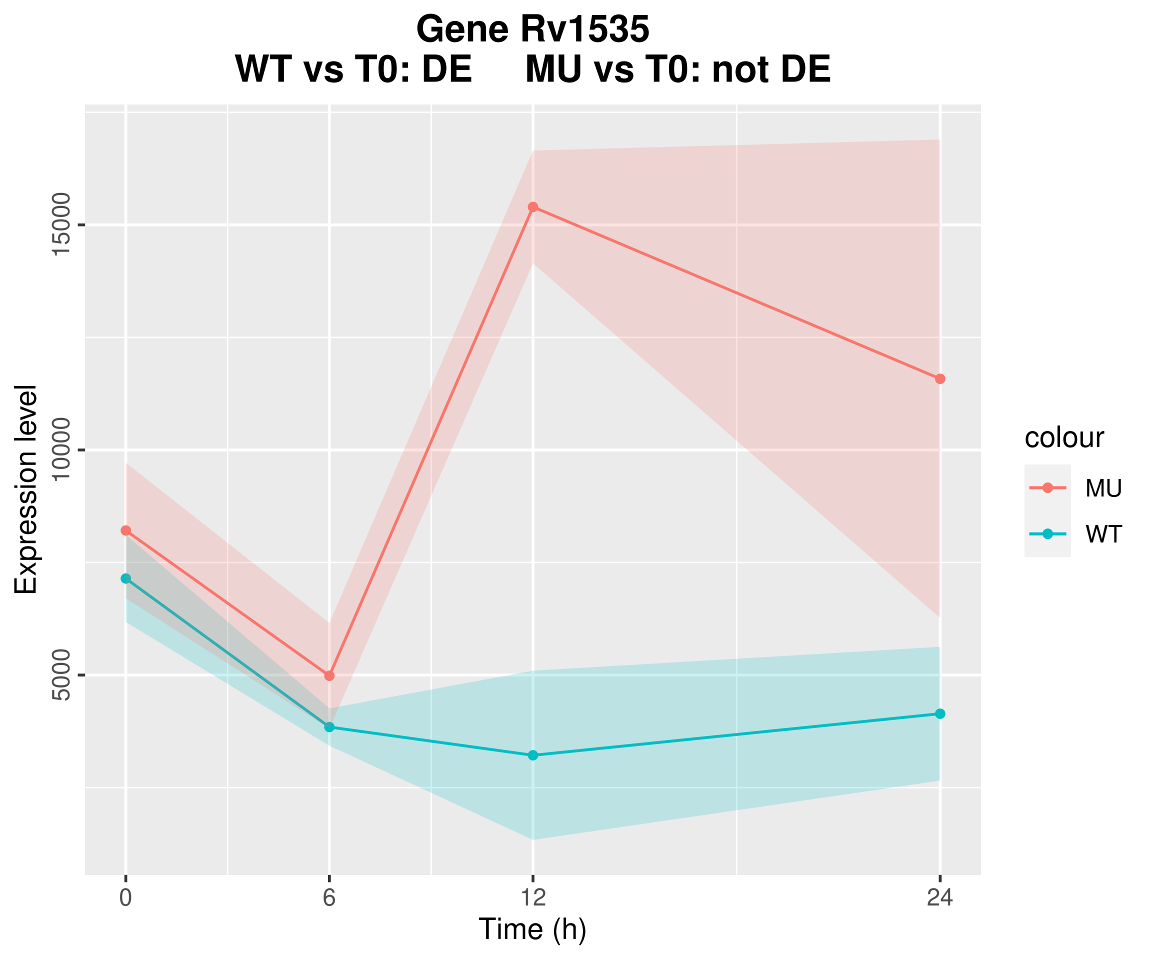
Gene Rv1535
WT vs T0: DE   MU vs T0: not DE
Expression level
15000
10000
5000
0
6
12
24
Time (h)
colour
MU
WT

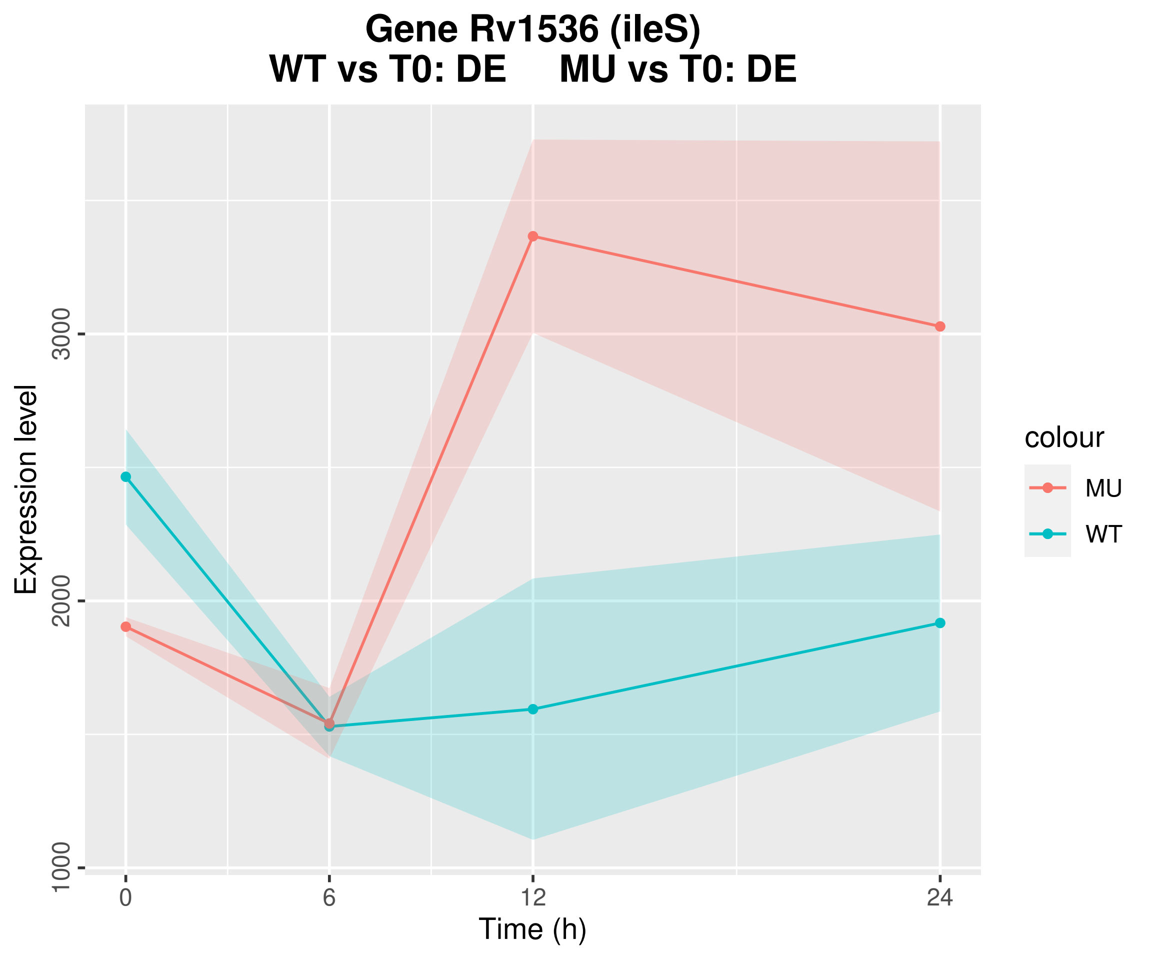

Gene Rv1536 (ileS)
WT vs T0: DE MU vs T0: DE
Expression level
3000
2000
1000
0
6
12
24
Time (h)
colour
MU
WT

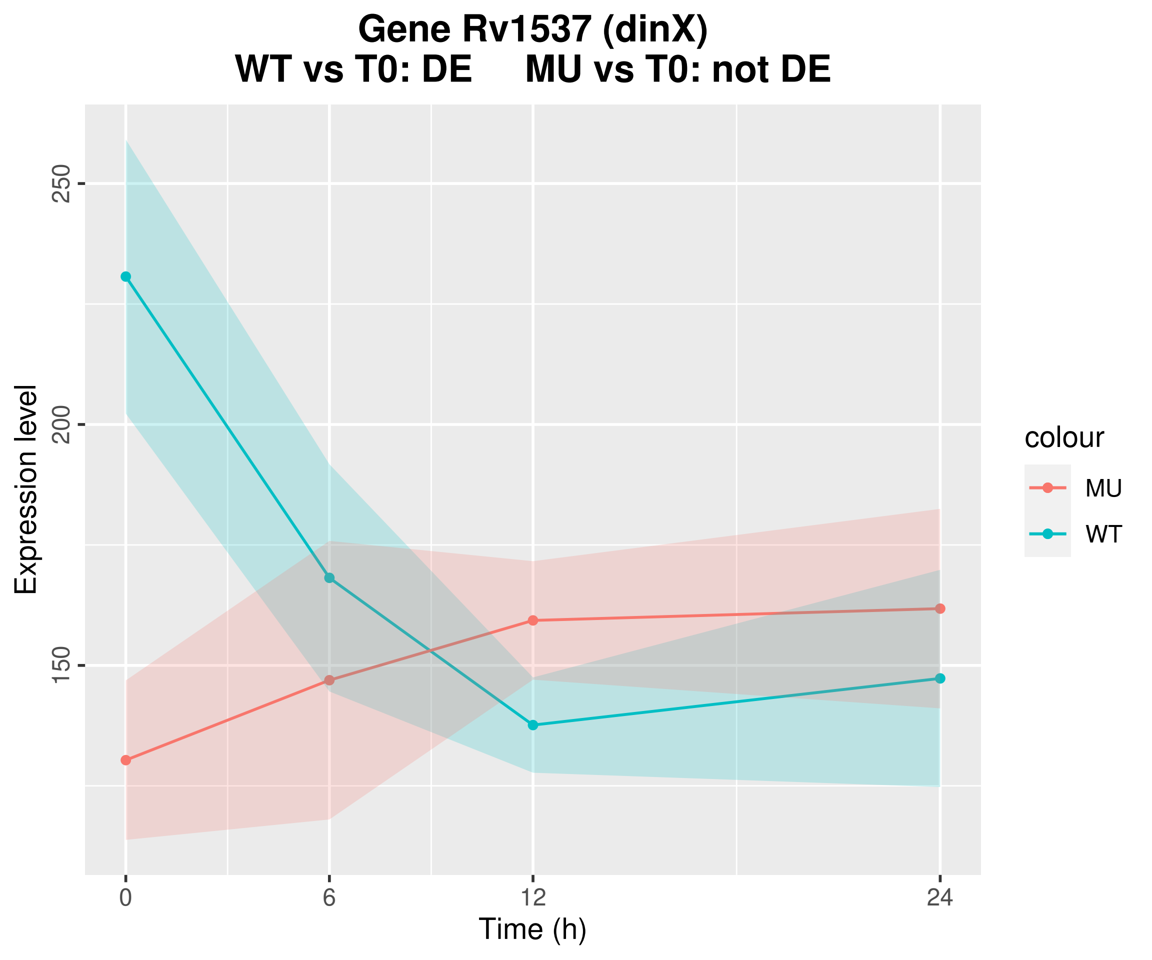

Gene Rv1537 (dinX)
WT vs T0: DE   MU vs T0: not DE
Expression level
250
200
150
0
6
12
24
Time (h)
colour
MU
WT

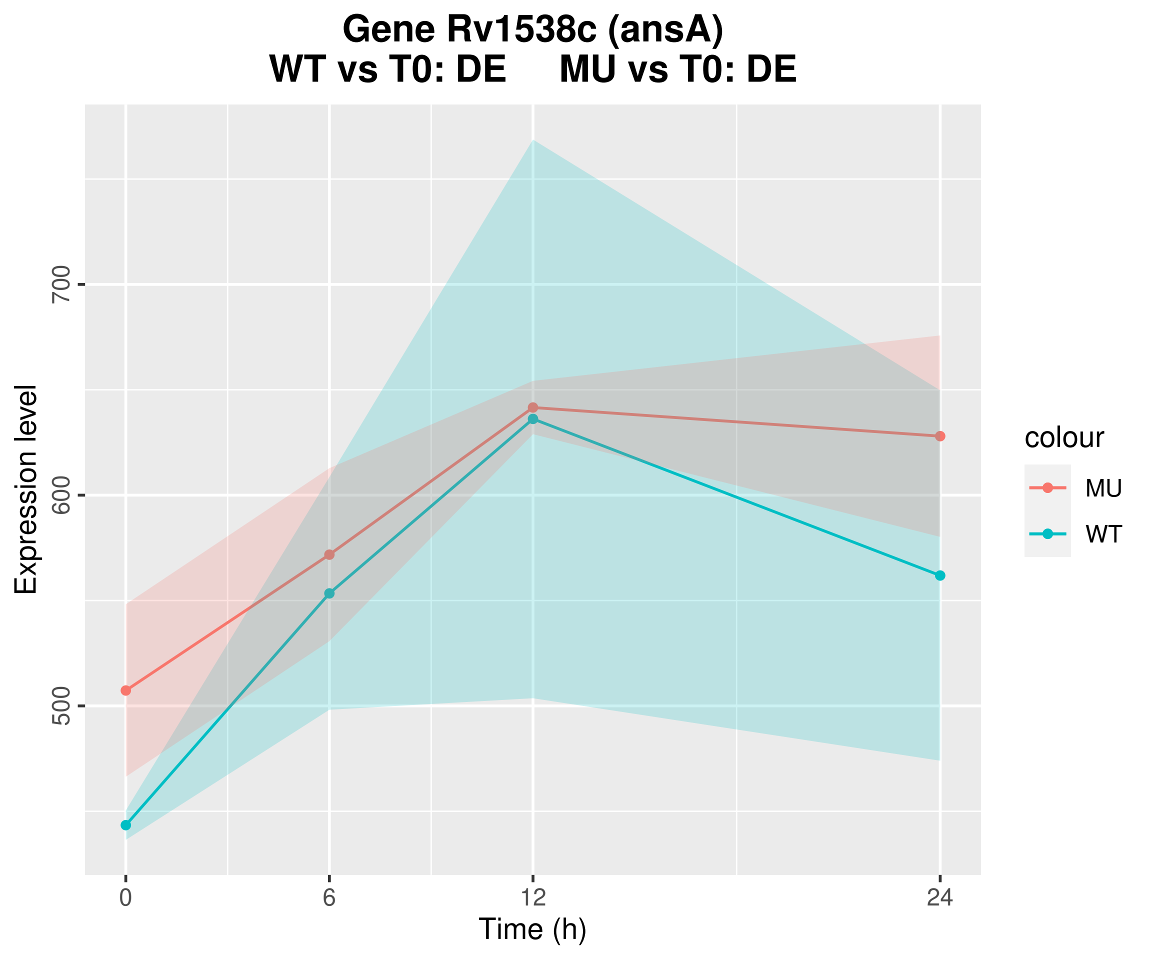
Gene Rv1538c (ansA)
WT vs T0: DE MU vs T0: DE
Expression level
700
600
500
0
6
12
24
Time (h)
colour
MU
WT

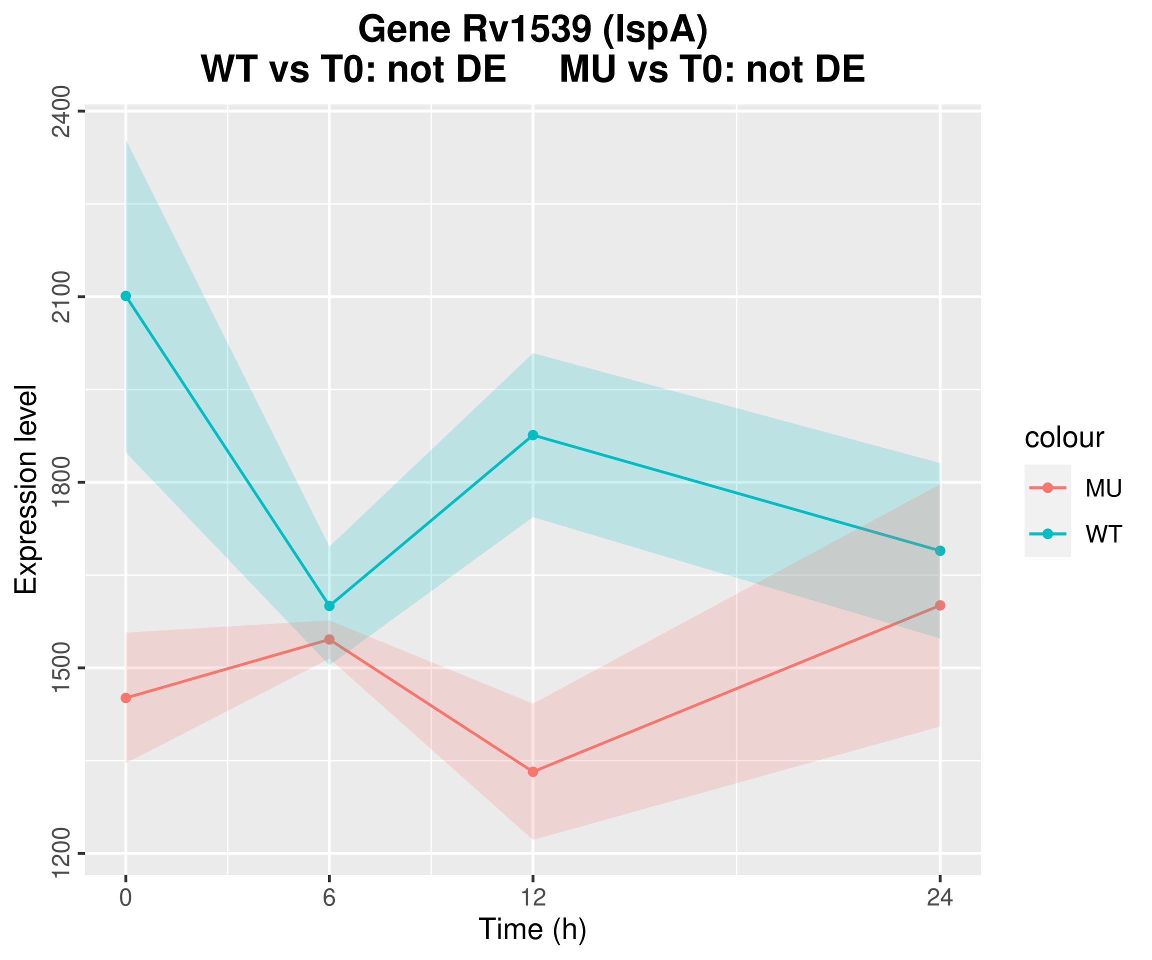
Gene Rv1539 (lspA)
WT vs T0: not DE   MU vs T0: not DE
Expression level
2400
2100
1800
1500
1200
0
6
12
24
Time (h)
colour
MU
WT

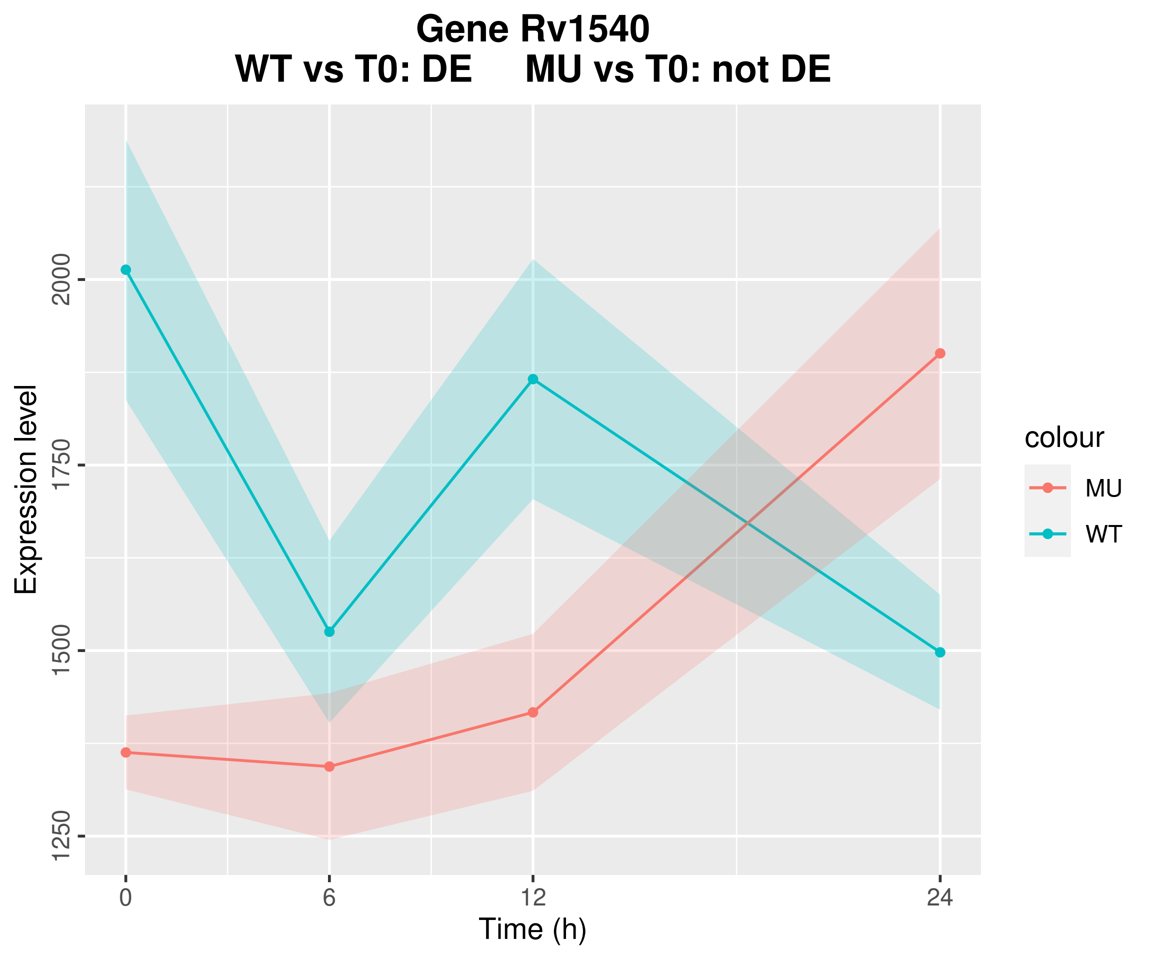
Gene Rv1540
WT vs T0: DE MU vs T0: not DE
Expression level
2000
1750
1500
1250
0
6
12
24
Time (h)
colour
MU
WT

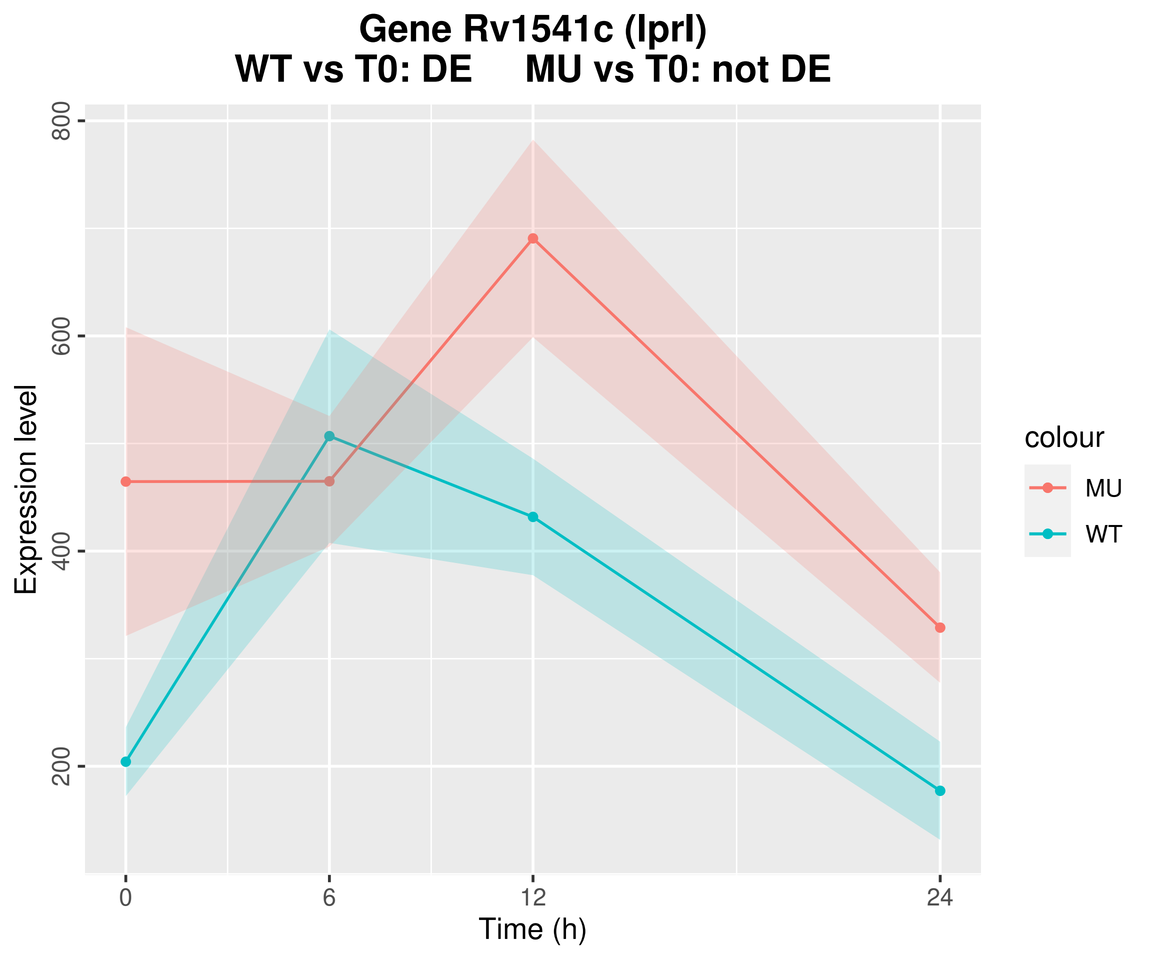

Gene Rv1541c (lprI)
WT vs T0: DE MU vs T0: not DE
800
600
400
200
Expression level
0
6
12
24
Time (h)
colour
MU
WT

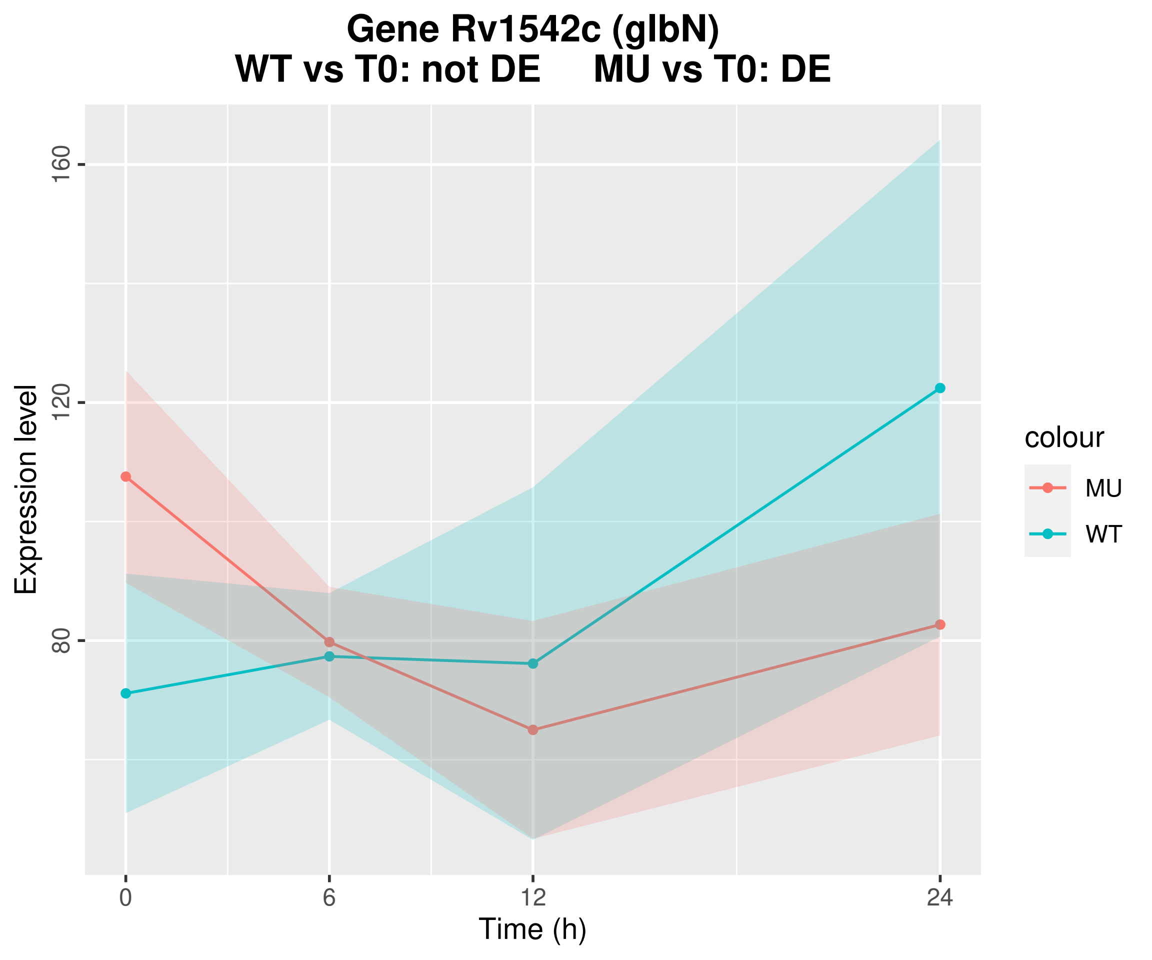
Gene Rv1542c (glbN)
WT vs T0: not DE   MU vs T0: DE
Expression level
160
120
80
0
6
12
24
Time (h)
colour
MU
WT

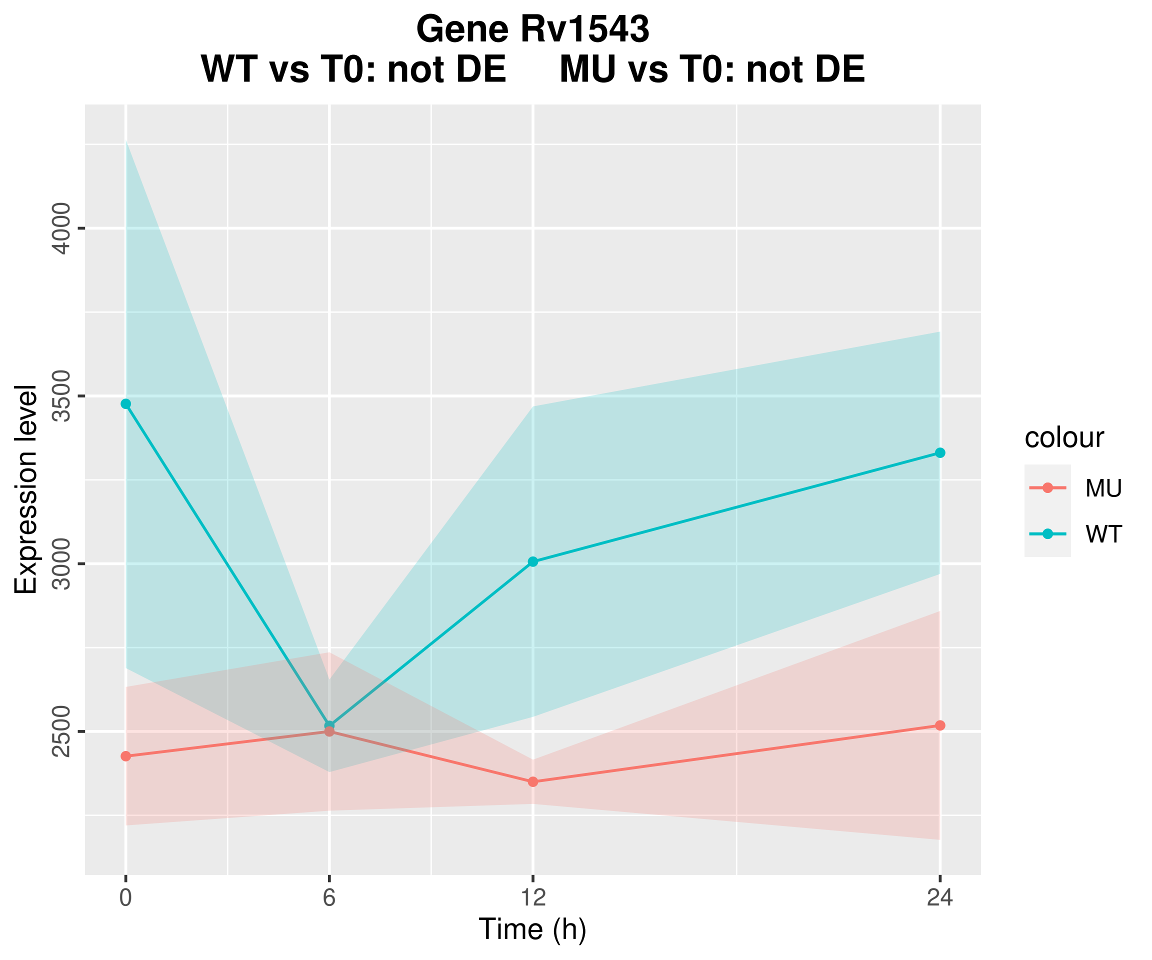
Gene Rv1543
WT vs T0: not DE MU vs T0: not DE
Expression level
4000
3500
3000
2500
0
6
12
24
Time (h)
colour
MU
WT

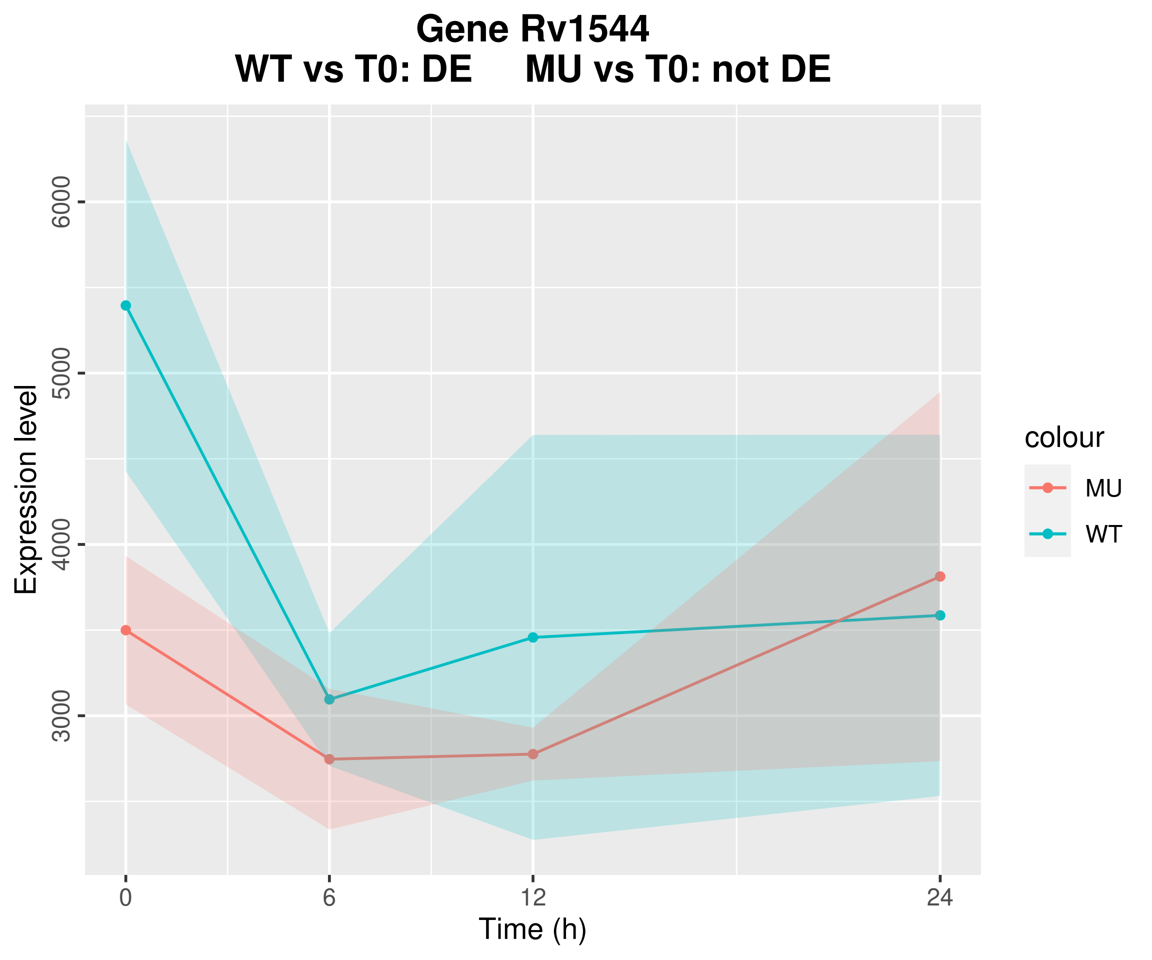

Gene Rv1544
WT vs T0: DE MU vs T0: not DE
6000
5000
4000
3000
Expression level
0
6
12
24
Time (h)
colour
MU
WT

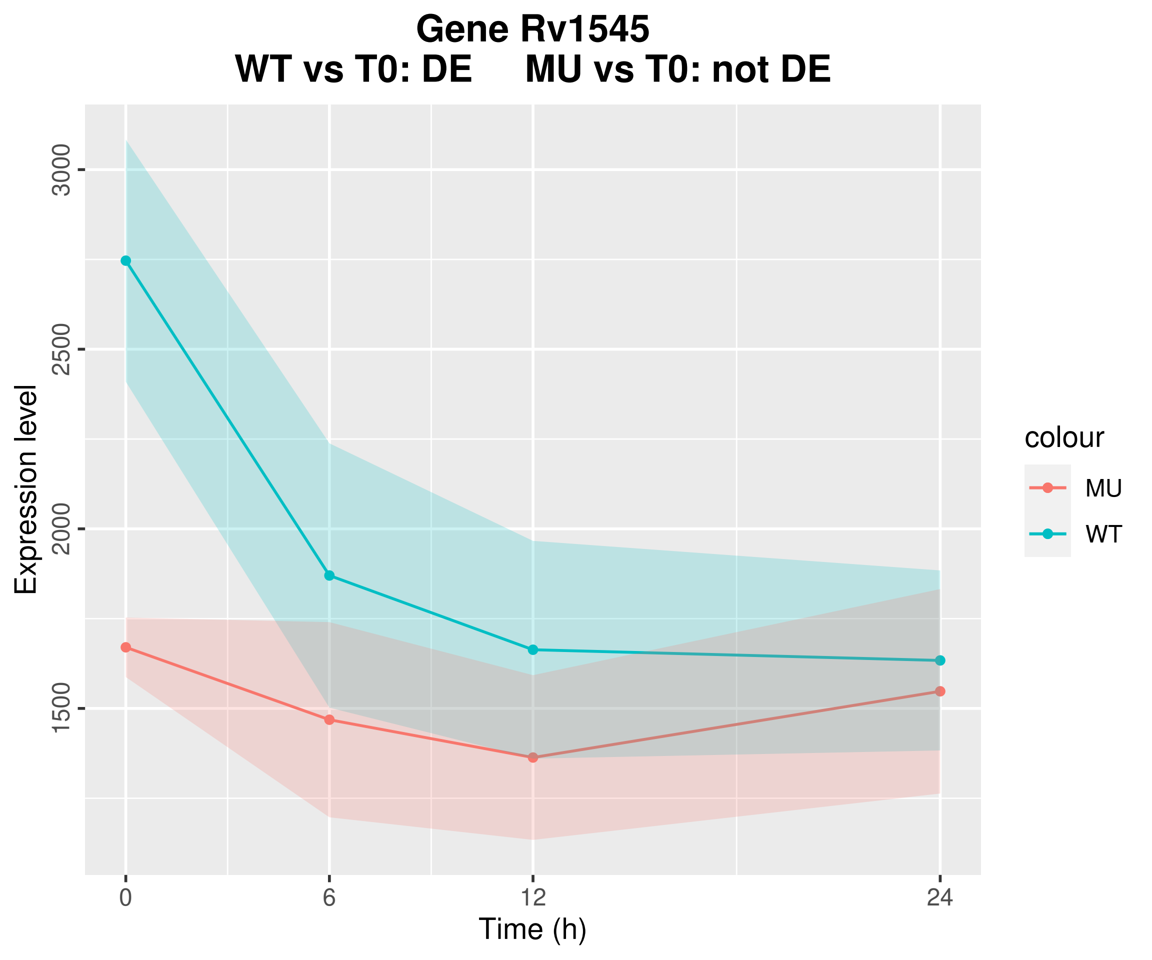
Gene Rv1545
WT vs T0: DE   MU vs T0: not DE
Expression level
3000
2500
2000
1500
0
6
12
24
Time (h)
colour
MU
WT

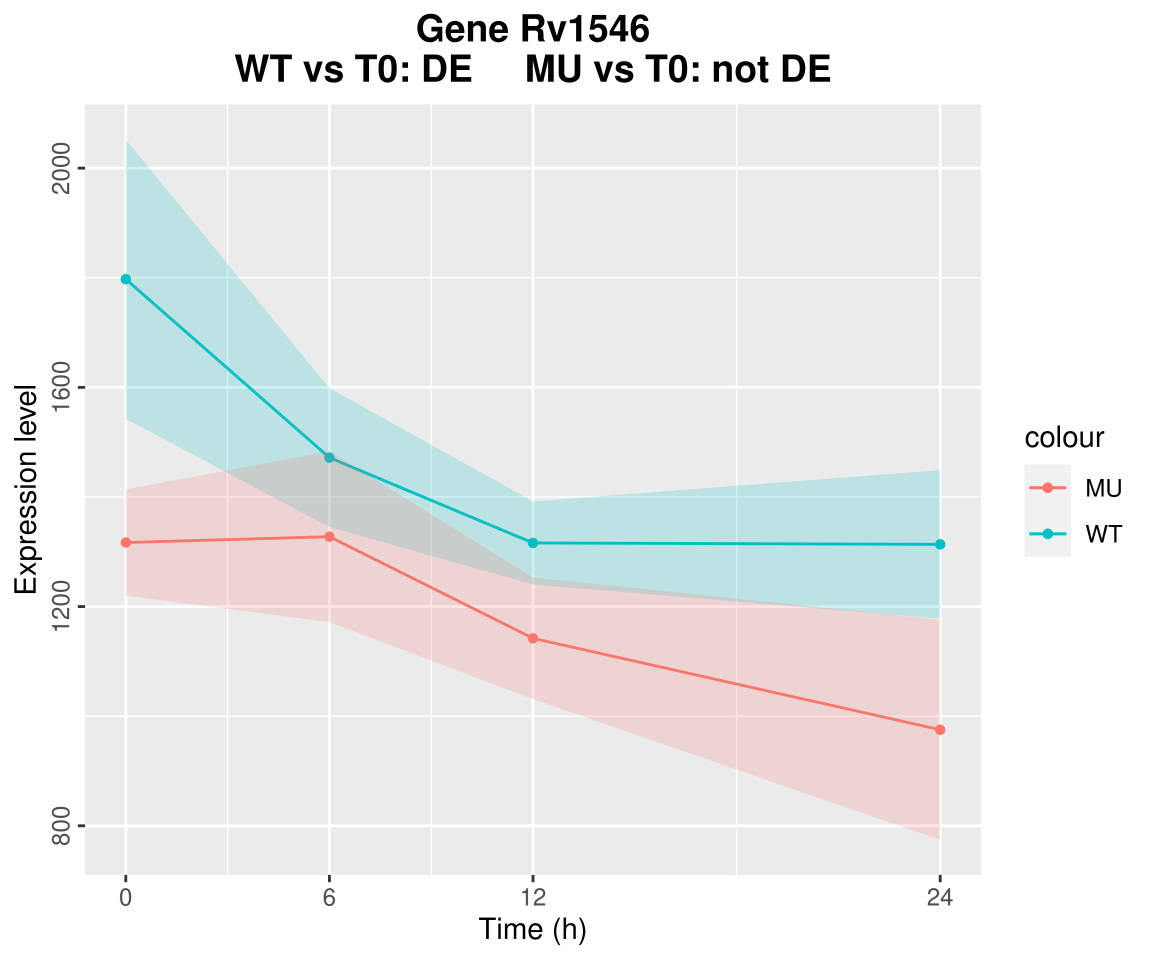

Gene Rv1546
WT vs T0: DE MU vs T0: not DE
Expression level
2000
1600
1200
800
0
6
12
24
Time (h)
colour
MU
WT

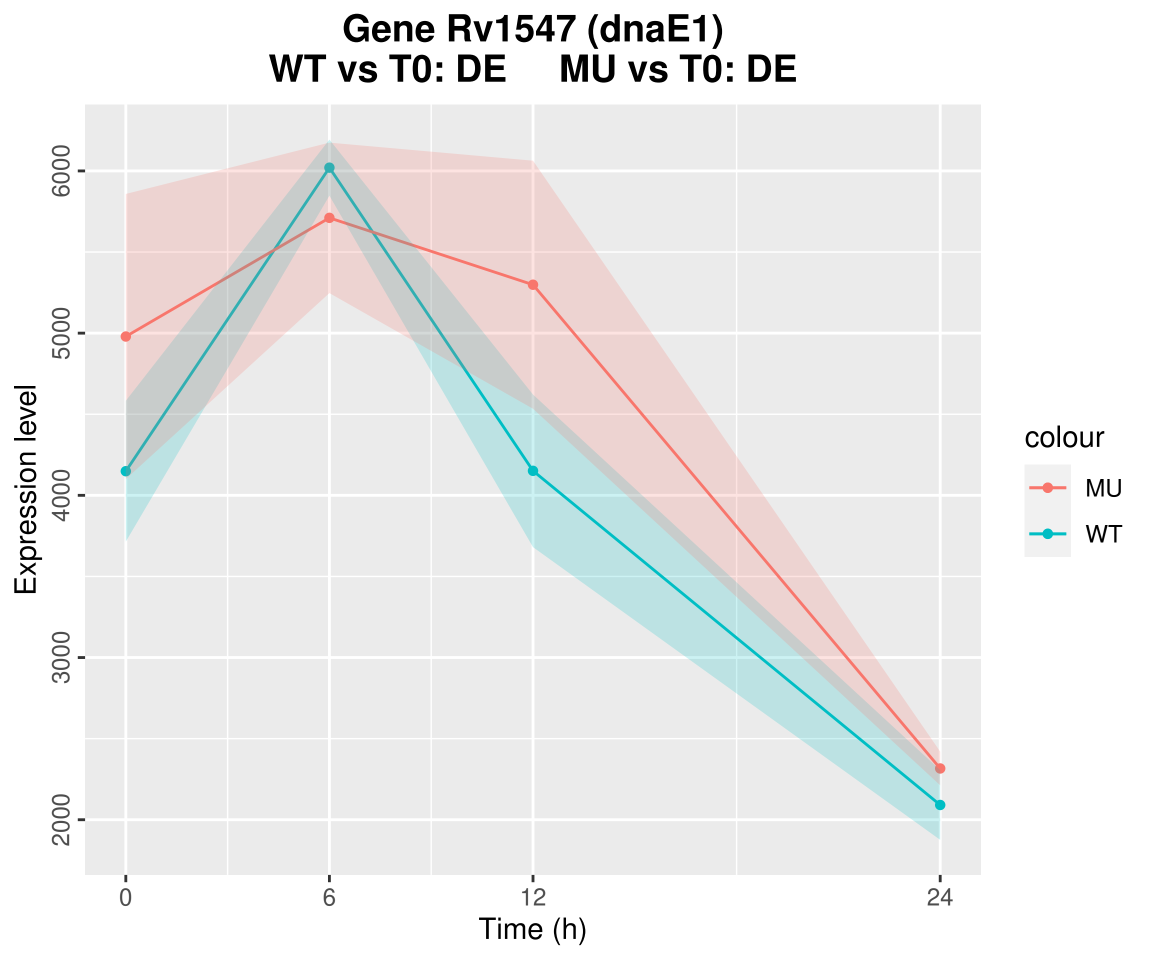
Gene Rv1547 (dnaE1)
WT vs T0: DE   MU vs T0: DE
Expression level
6000
5000
4000
3000
2000
0
6
12
24
Time (h)
colour
MU
WT

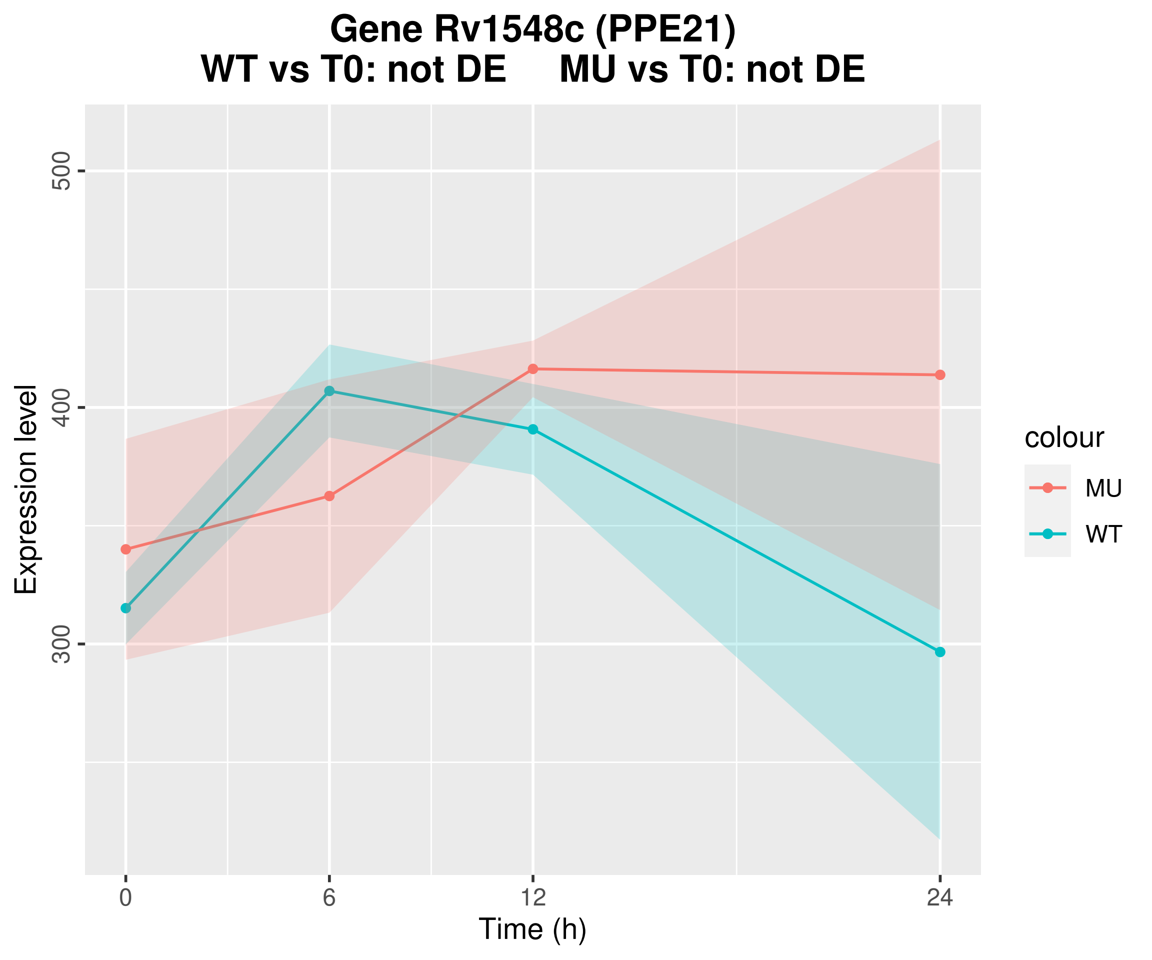

Gene Rv1548c (PPE21)
WT vs T0: not DE MU vs T0: not DE
500
400
300
Expression level
0
6
12
24
Time (h)
colour
MU
WT

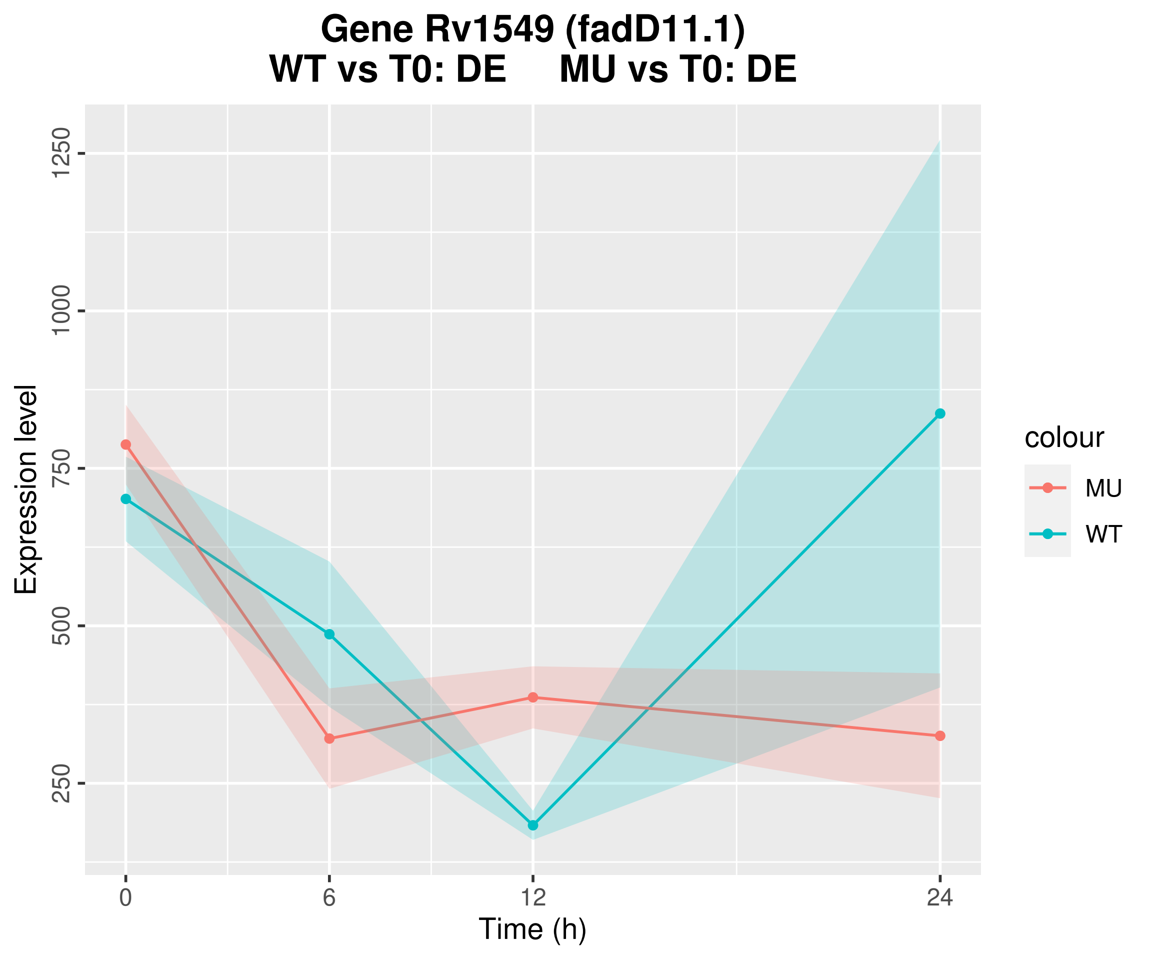

Gene Rv1549 (fadD11.1)
WT vs T0: DE   MU vs T0: DE
Expression level
1250
1000
750
500
250
0
6
12
24
Time (h)
colour
MU
WT

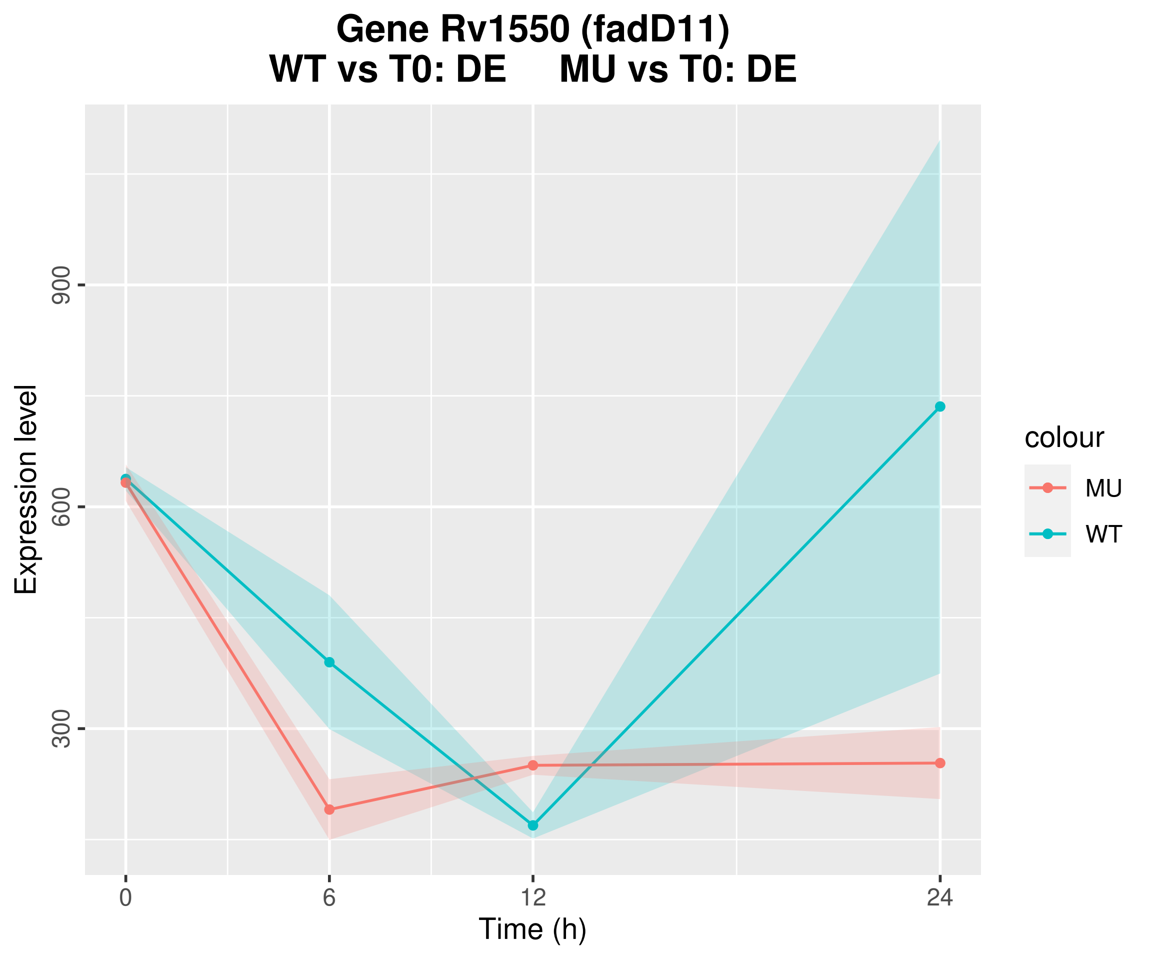

Gene Rv1550 (fadD11)
WT vs T0: DE MU vs T0: DE
Expression level
900
600
300
0
6
12
24
Time (h)
colour
MU
WT

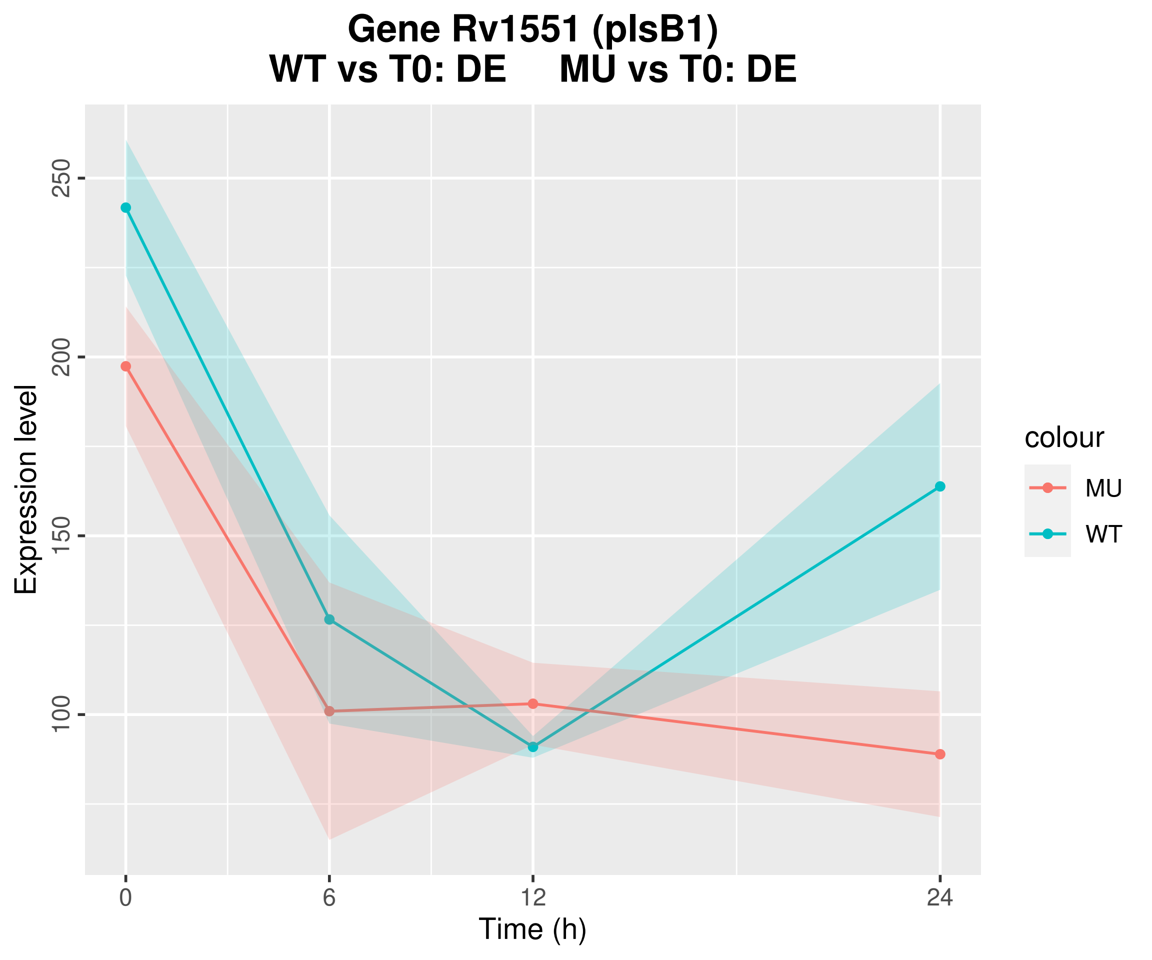
Gene Rv1551 (plsB1)
WT vs T0: DE   MU vs T0: DE
Expression level
250
200
150
100
0
6
12
24
Time (h)
colour
MU
WT

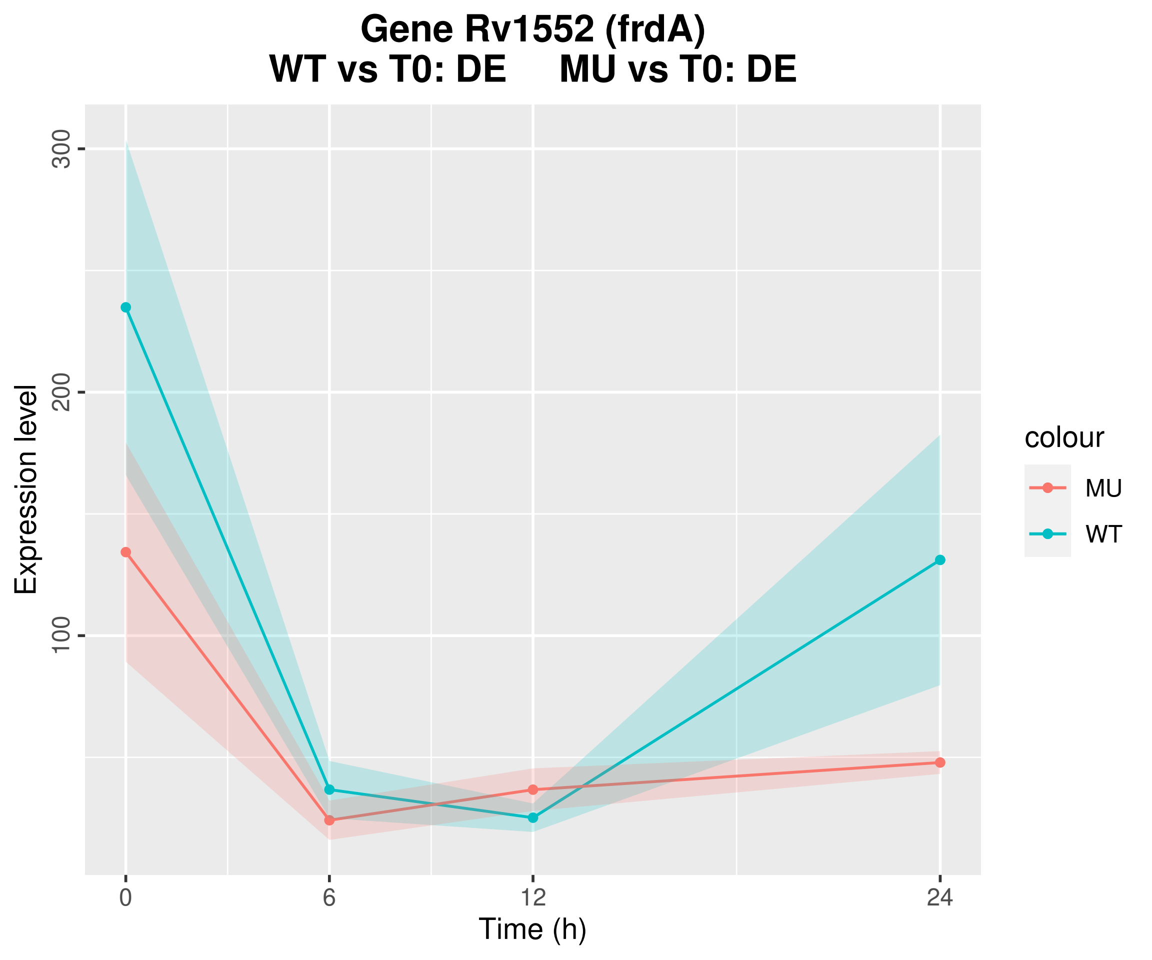

Gene Rv1552 (frdA)
WT vs T0: DE   MU vs T0: DE
300
200
100
Expression level
0
6
12
24
Time (h)
colour
MU
WT

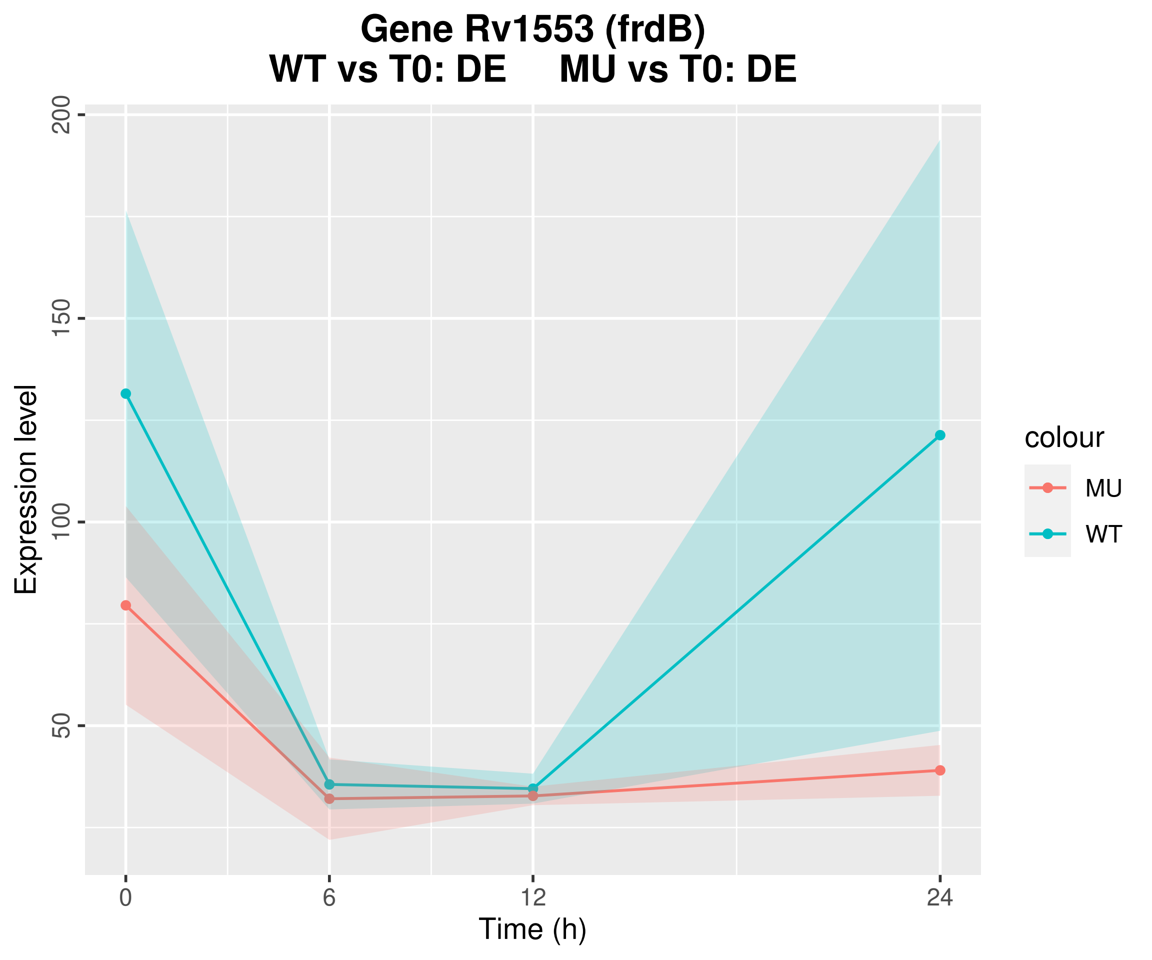

Gene Rv1553 (frdB)
WT vs T0: DE   MU vs T0: DE
200
150
100
50
Expression level
0
6
12
24
Time (h)
colour
MU
WT

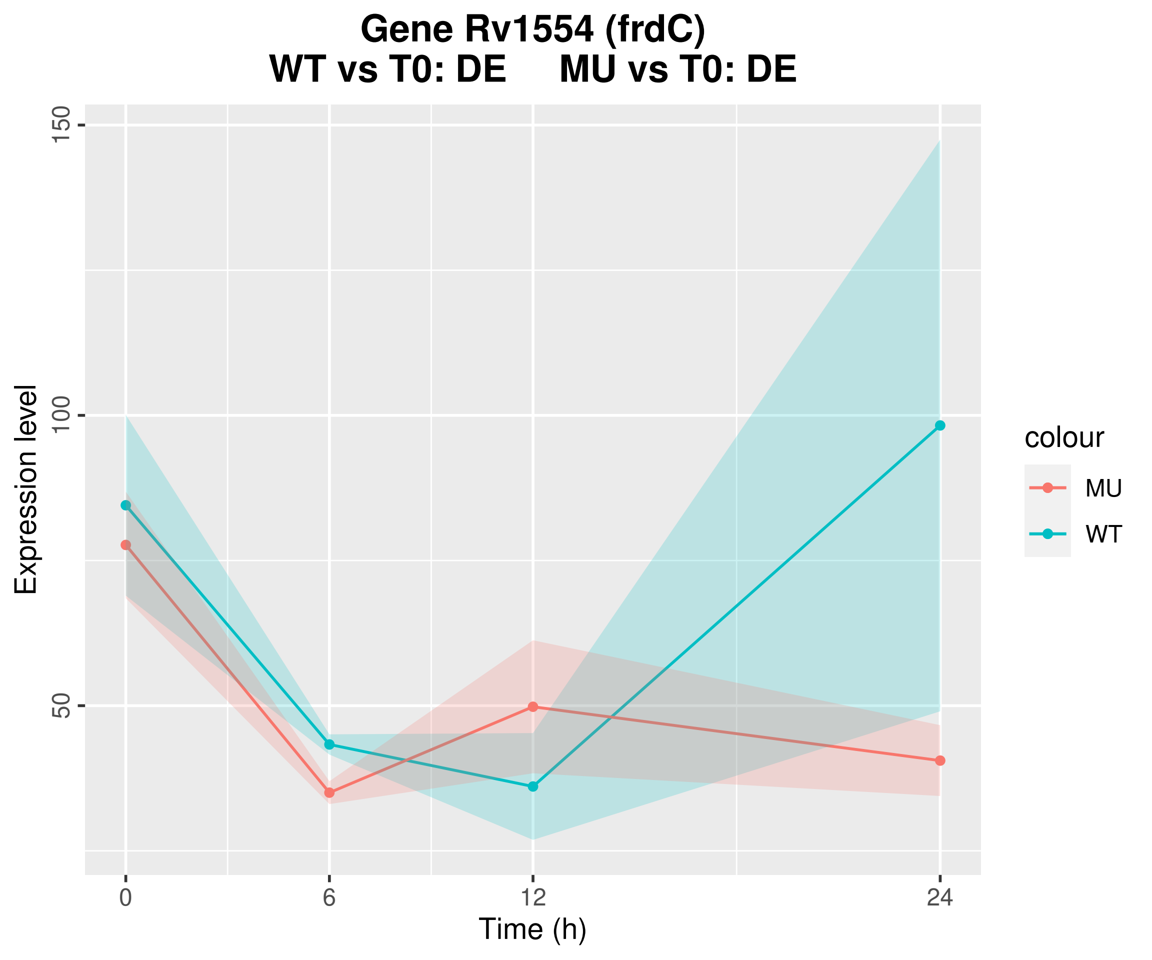
Gene Rv1554 (frdC)
WT vs T0: DE MU vs T0: DE
150
100
50
Expression level
0
6
12
24
Time (h)
colour
MU
WT

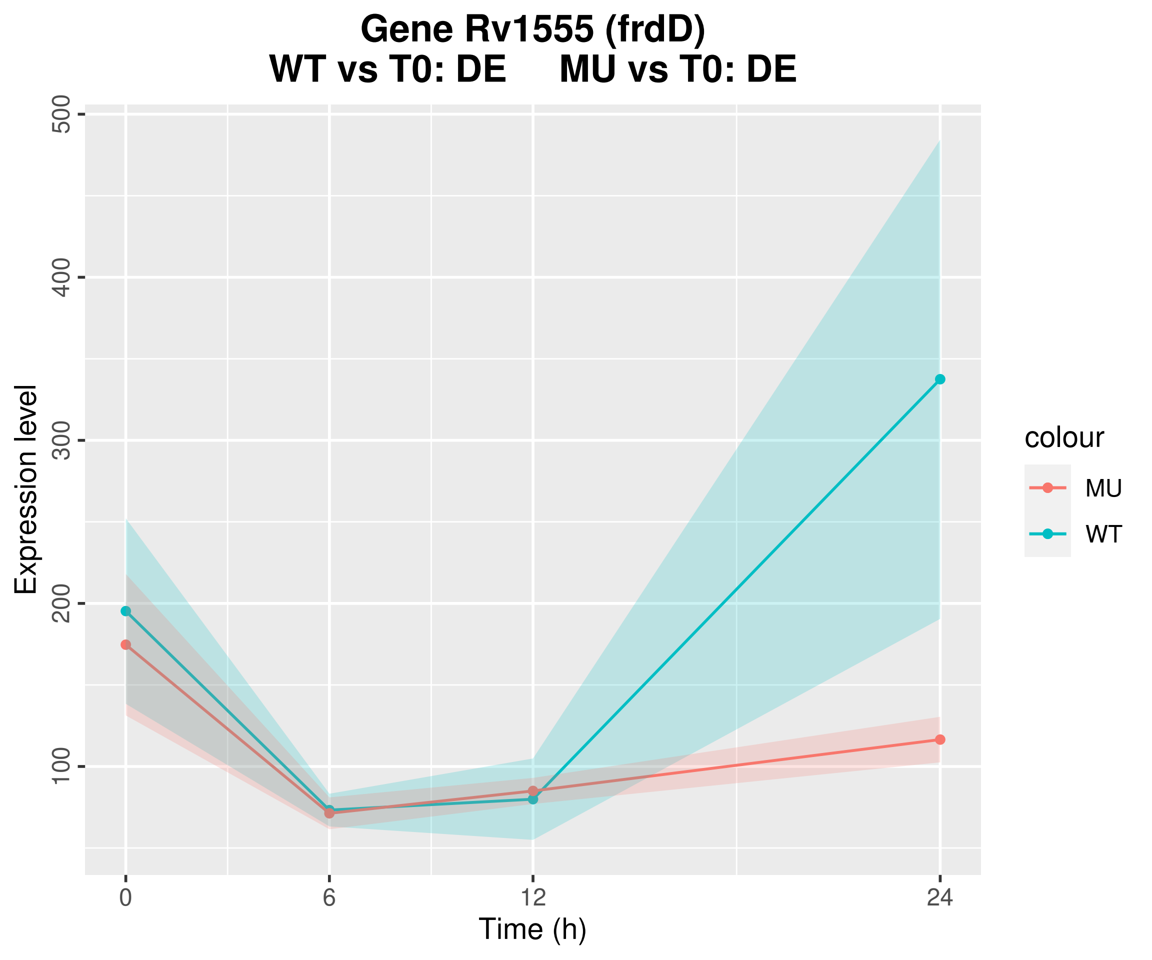
Gene Rv1555 (frdD)
WT vs T0: DE MU vs T0: DE
Expression level
500
400
300
200
100
0
6
12
24
Time (h)
colour
MU
WT

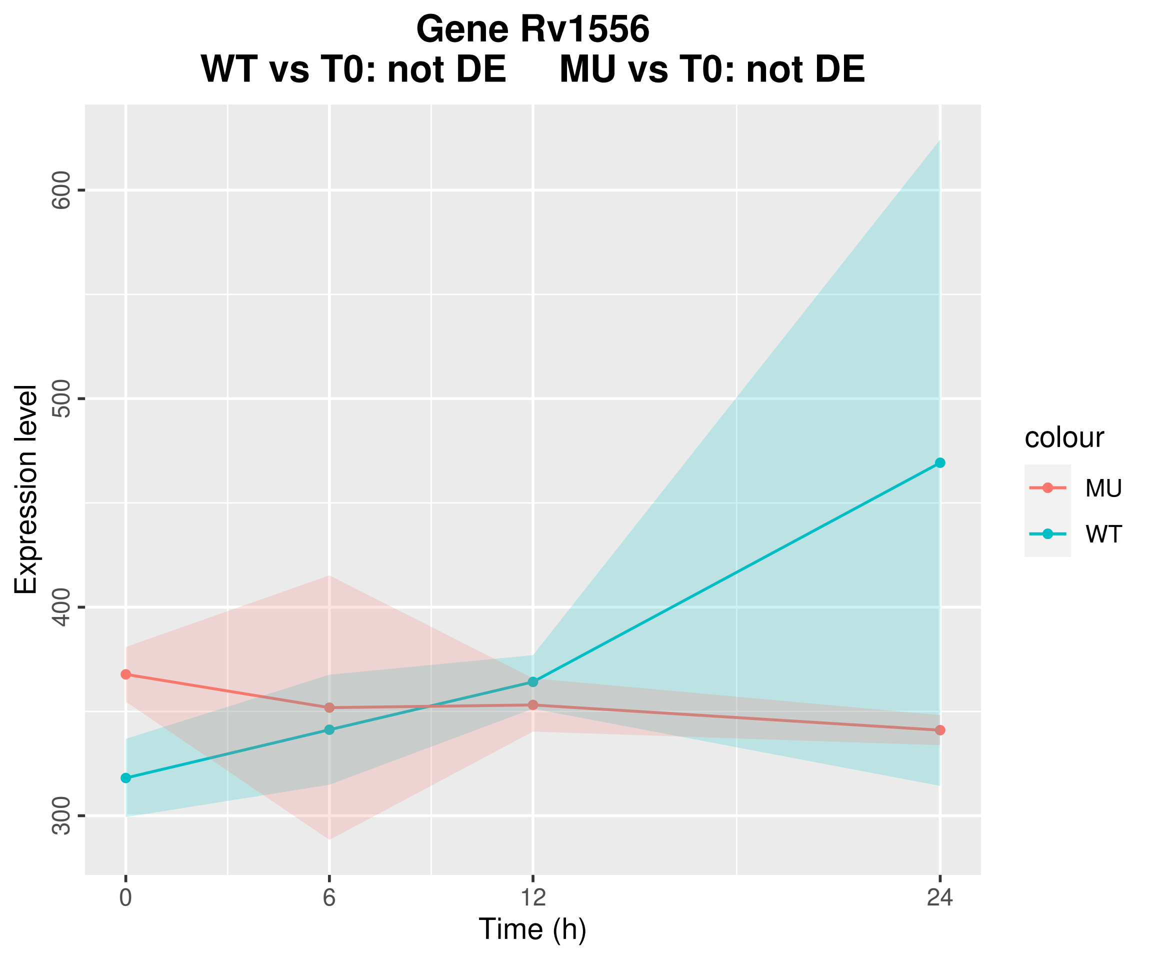

Gene Rv1556
WT vs T0: not DE MU vs T0: not DE
Expression level
600
500
400
300
0
6
12
24
Time (h)
colour
MU
WT

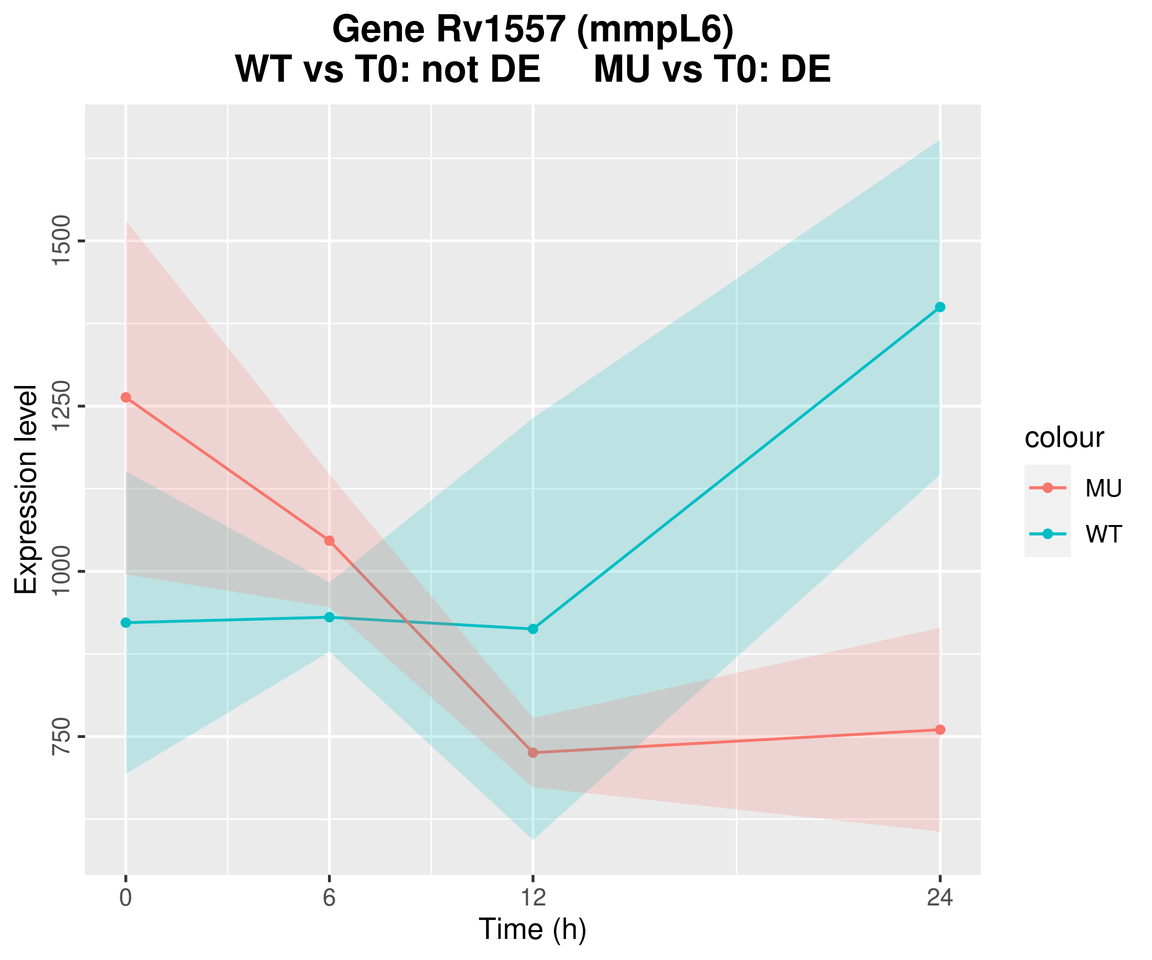
Gene Rv1557 (mmpL6)
WT vs T0: not DE MU vs T0: DE
Expression level
1500
1250
1000
750
0
6
12
24
Time (h)
colour
MU
WT

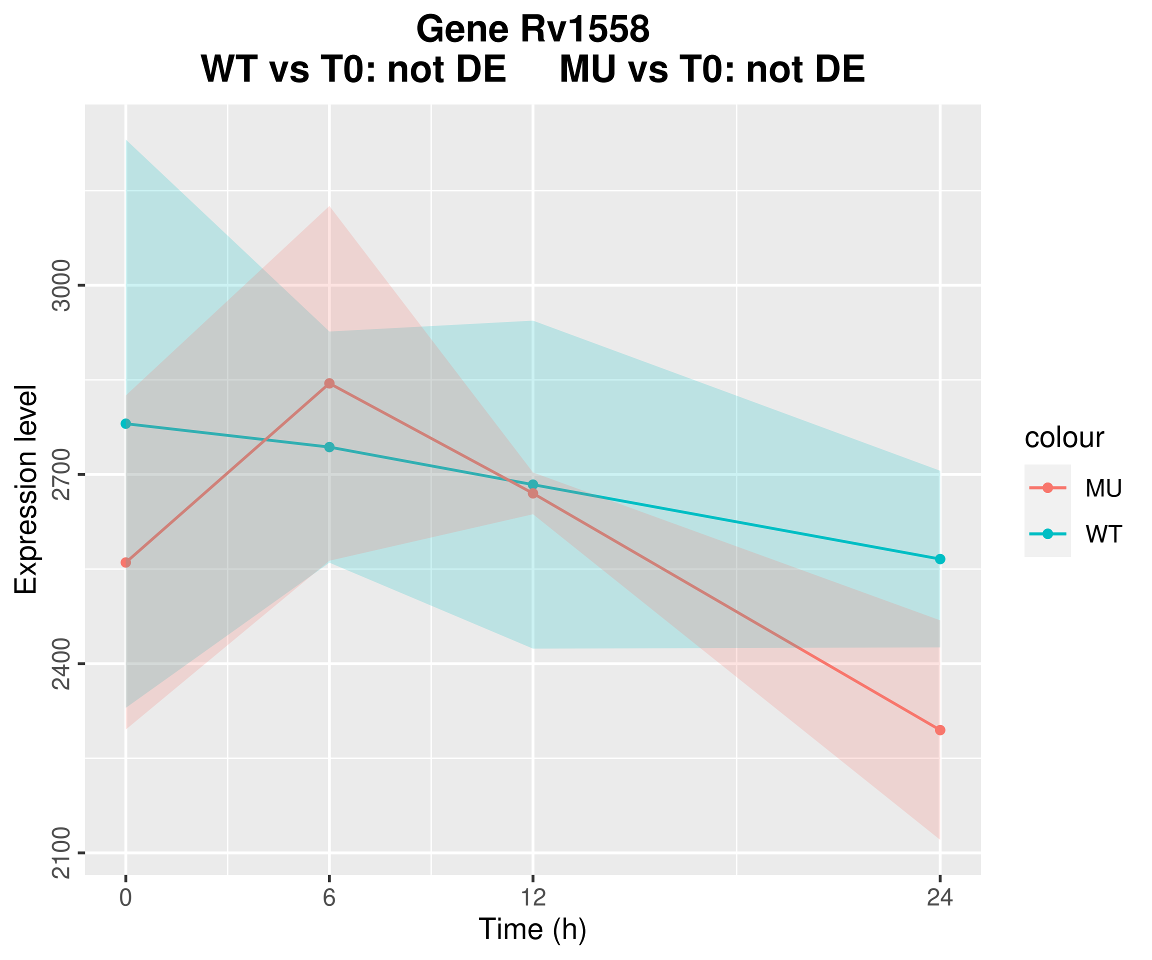
Gene Rv1558
WT vs T0: not DE MU vs T0: not DE
Expression level
3000
2700
2400
2100
0
6
12
24
Time (h)
colour
MU
WT

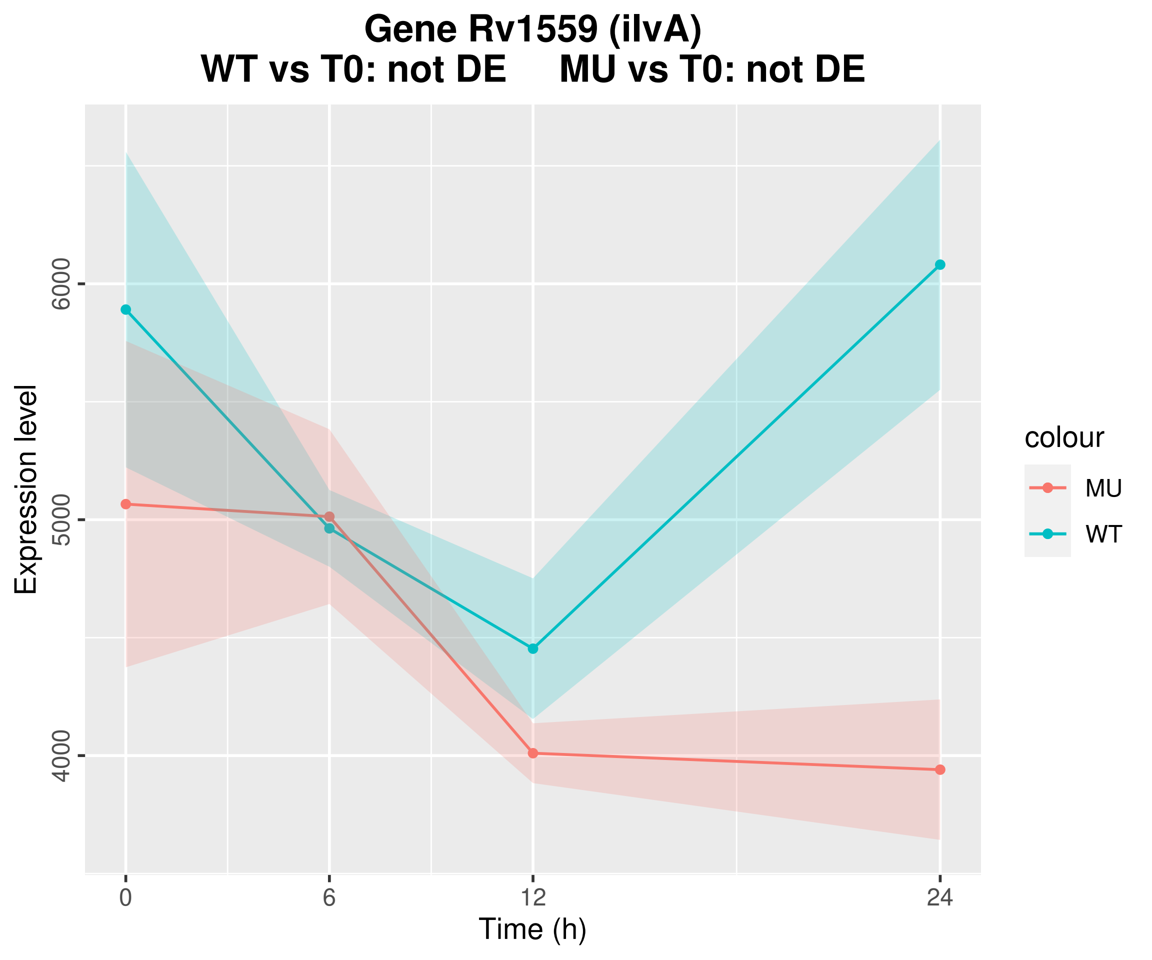
Gene Rv1559 (ilvA)
WT vs T0: not DE   MU vs T0: not DE
Expression level
6000
5000
4000
0
6
12
24
Time (h)
colour
MU
WT

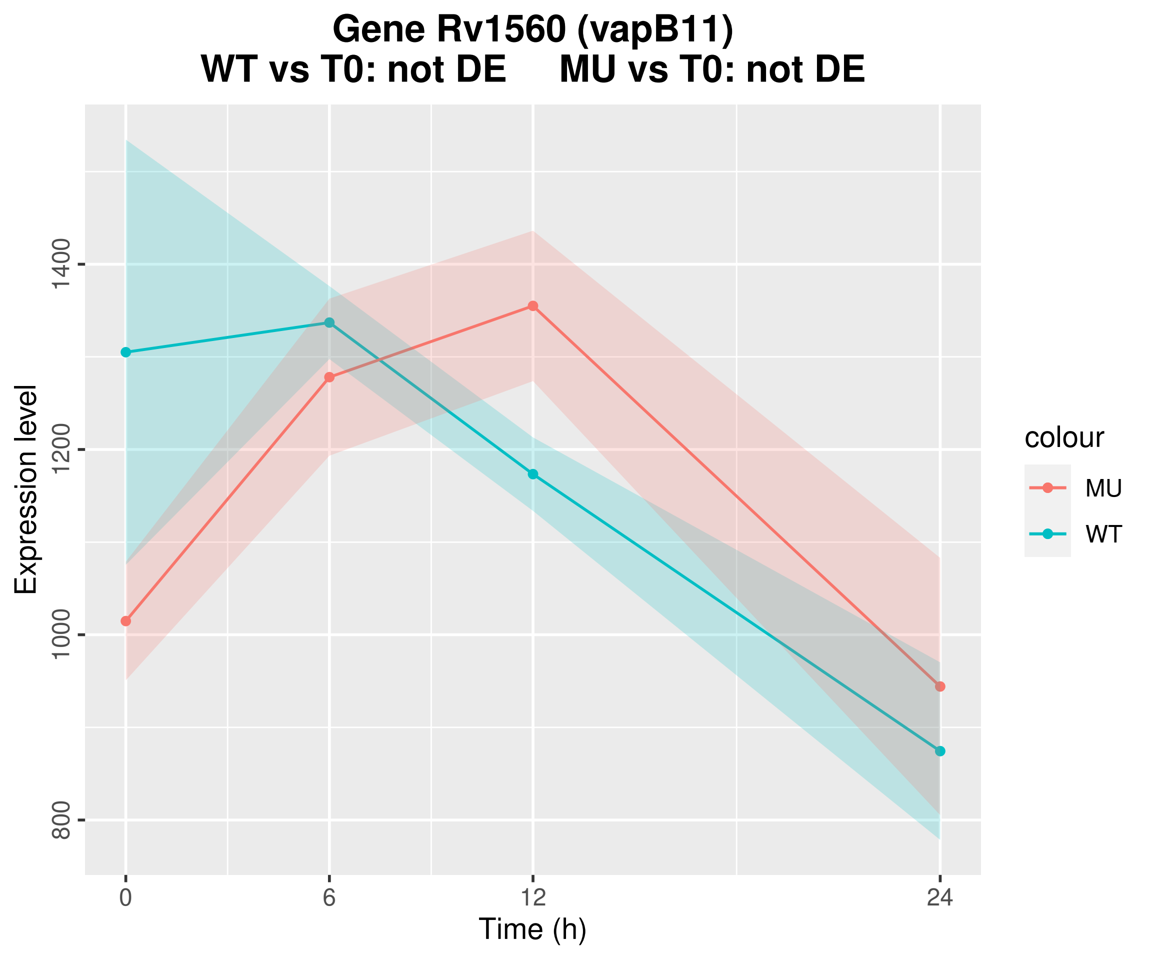
Gene Rv1560 (vapB11)
WT vs T0: not DE
MU vs T0: not DE
Expression level
1400
1200
1000
800
0
6
12
24
Time (h)
colour
MU
WT

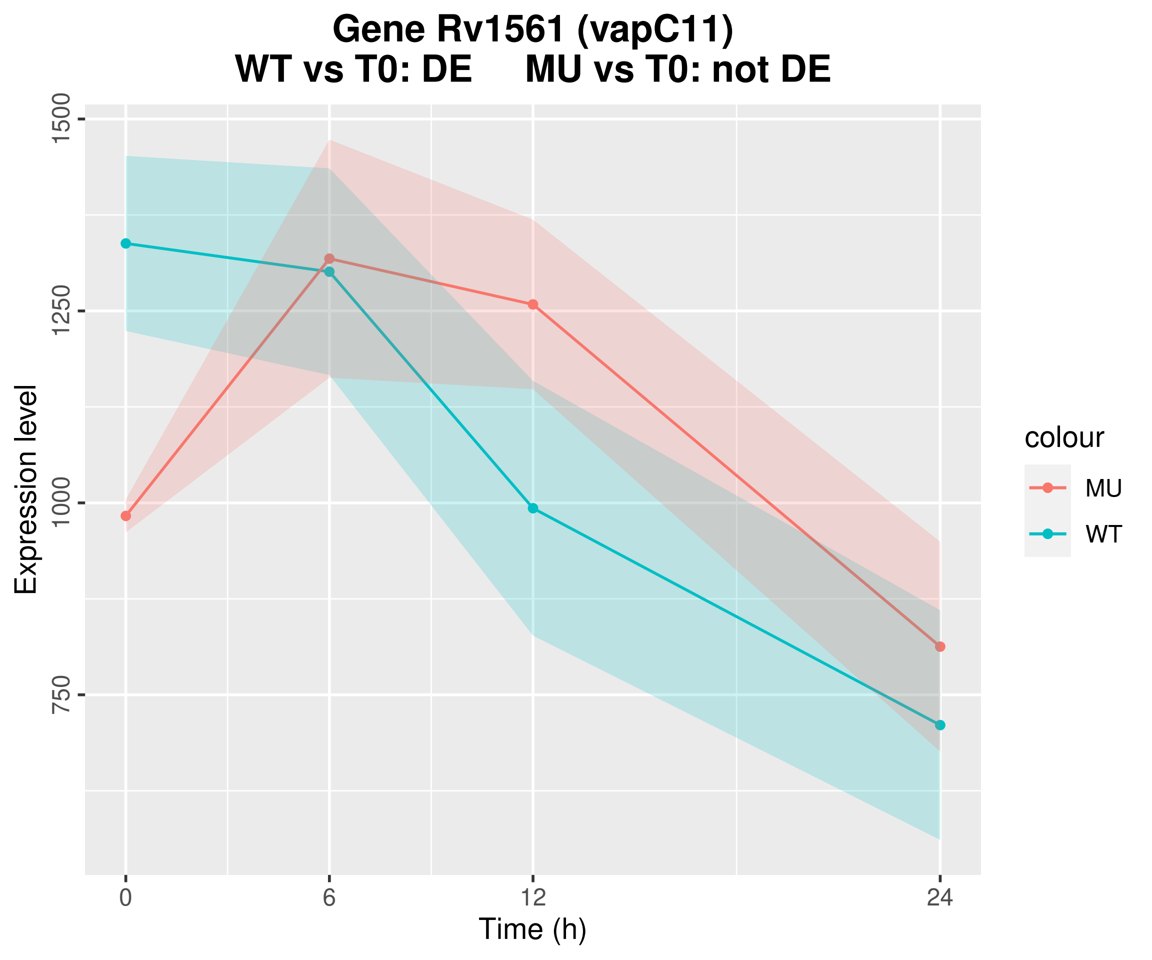
Gene Rv1561 (vapC11)
WT vs T0: DE   MU vs T0: not DE
Expression level
1500
1250
1000
750
0
6
12
24
Time (h)
colour
MU
WT

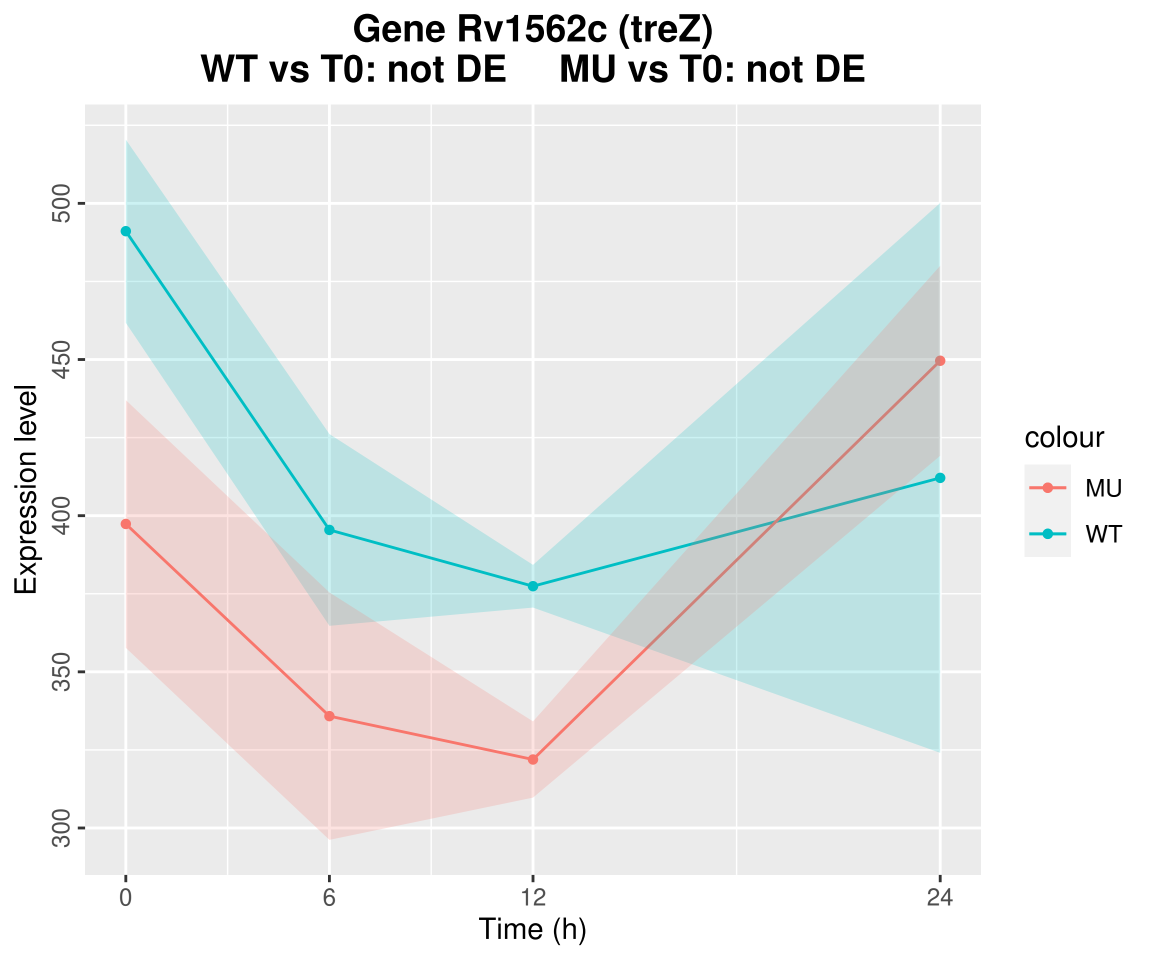

Gene Rv1562c (treZ)
WT vs T0: not DE   MU vs T0: not DE
Expression level
500
450
400
350
300
0
6
12
24
Time (h)
colour
MU
WT

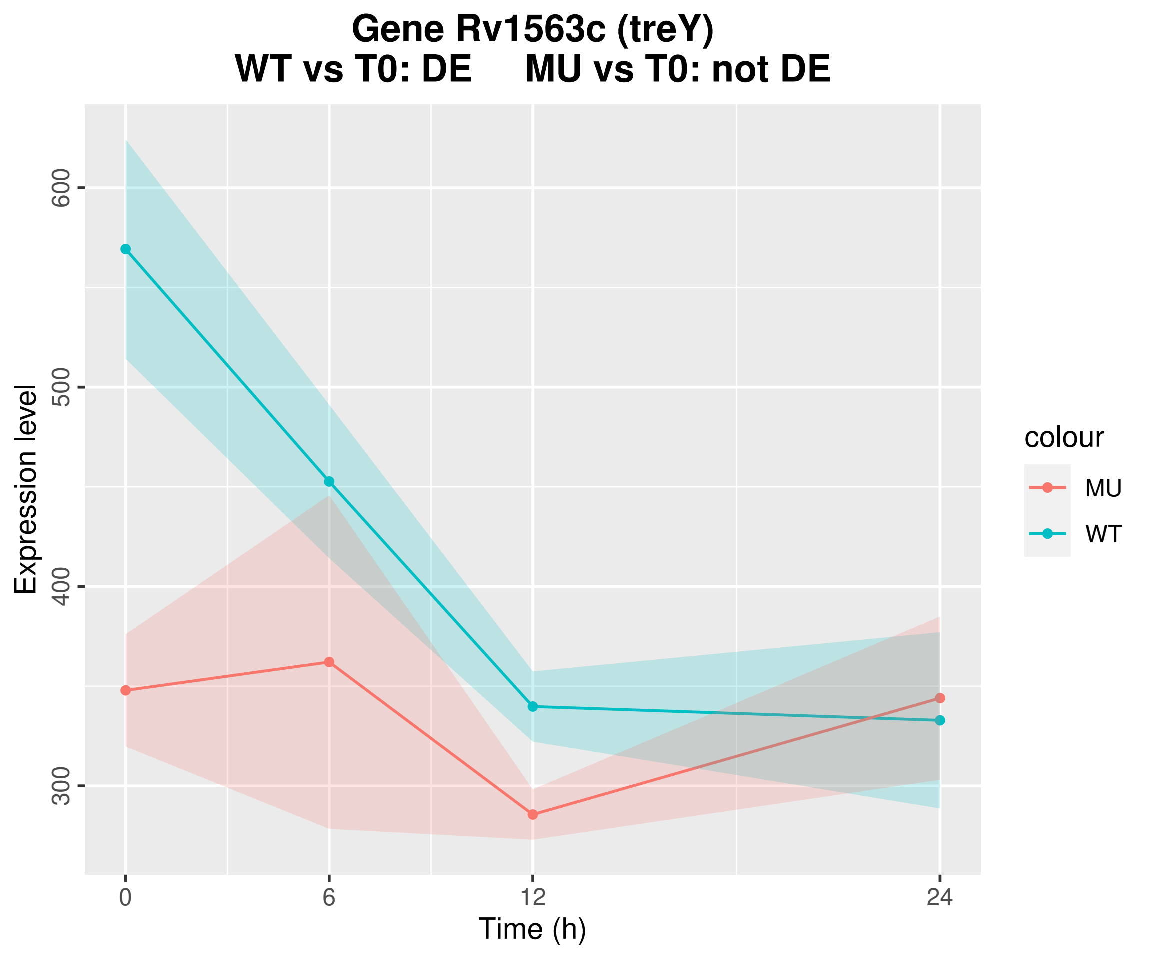

Gene Rv1563c (treY)
WT vs T0: DE   MU vs T0: not DE
Expression level
600
500
400
300
0
6
12
24
Time (h)
colour
MU
WT

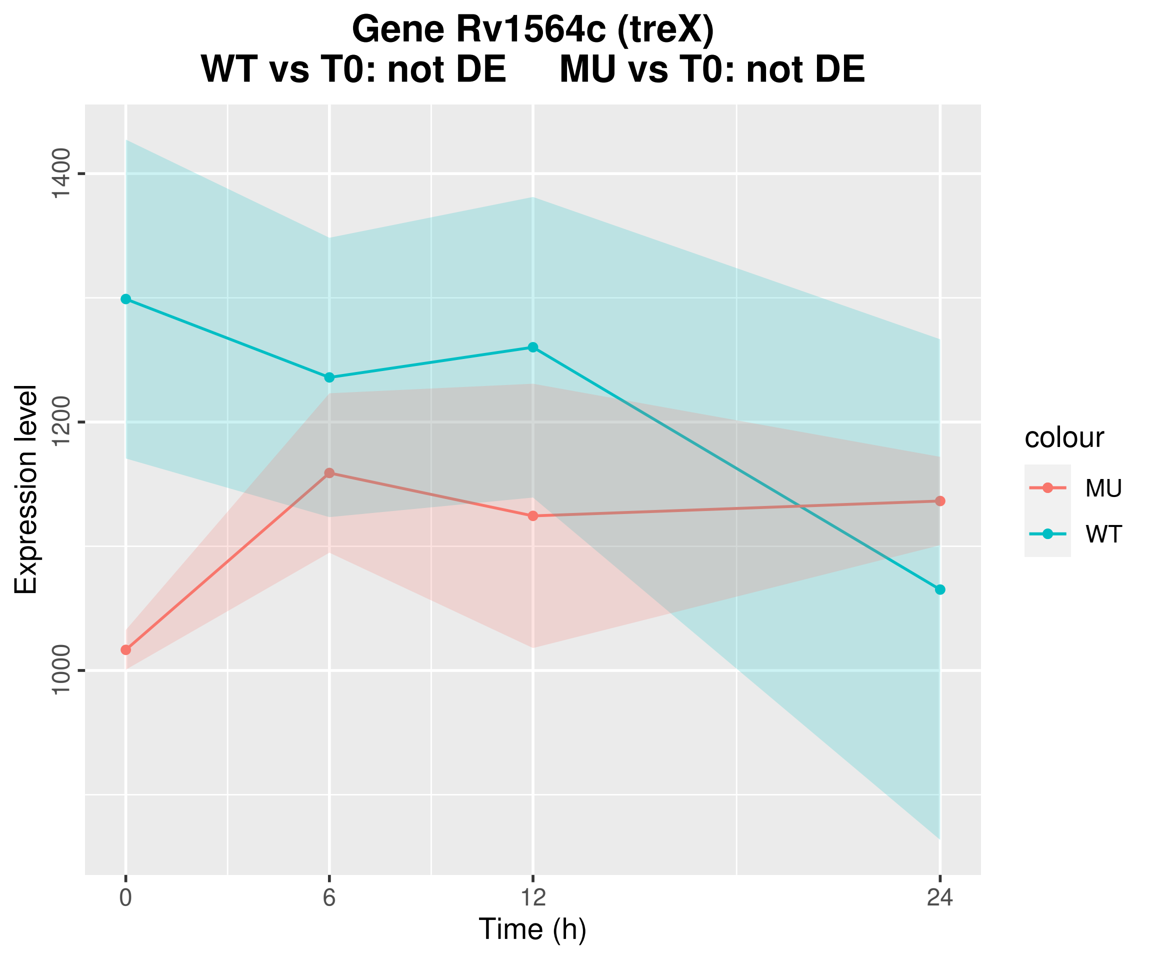

Gene Rv1564c (treX)
WT vs T0: not DE   MU vs T0: not DE
Expression level
1400
1200
1000
0
6
12
24
Time (h)
colour
MU
WT

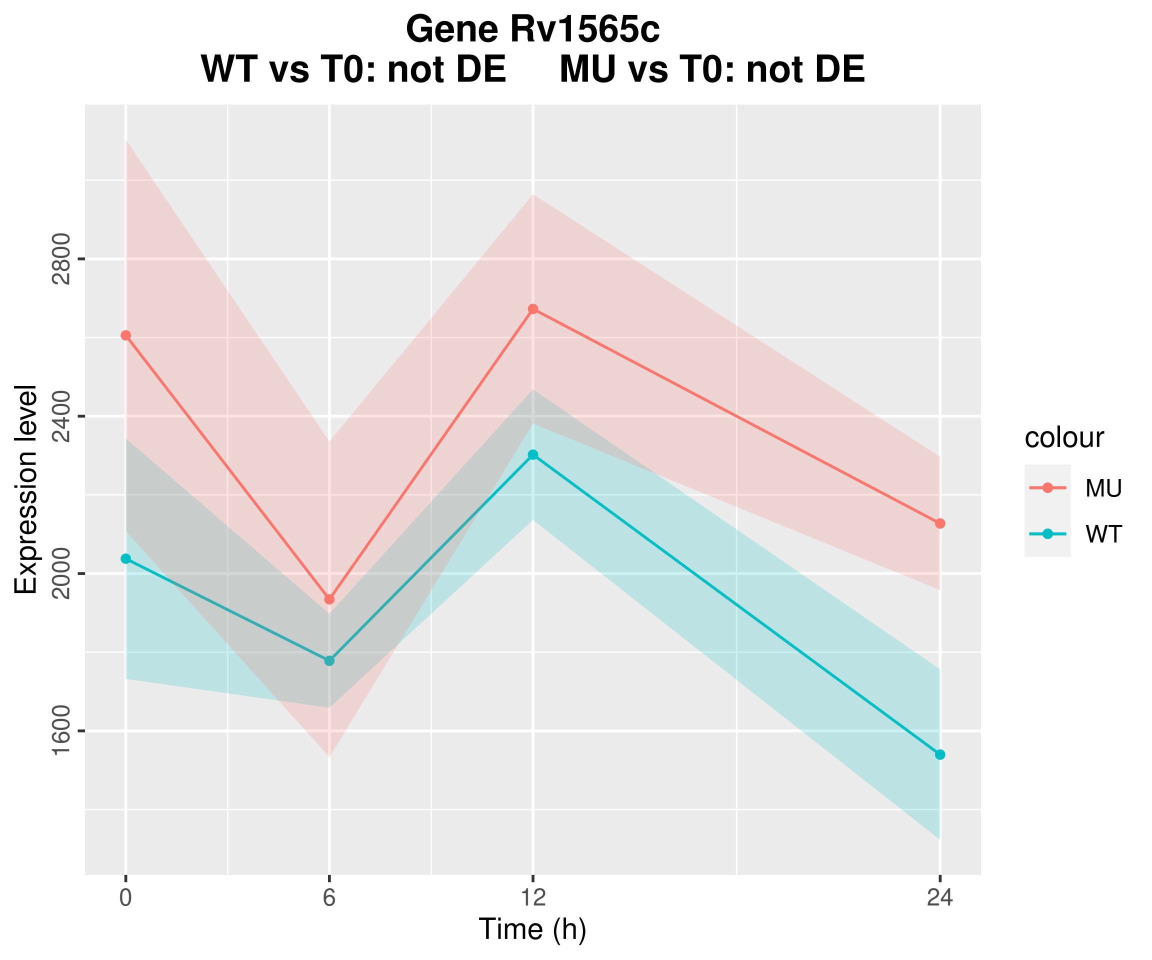
Gene Rv1565c
WT vs T0: not DE MU vs T0: not DE
Expression level
2800
2400
2000
1600
0
6
12
24
Time (h)
colour
MU
WT

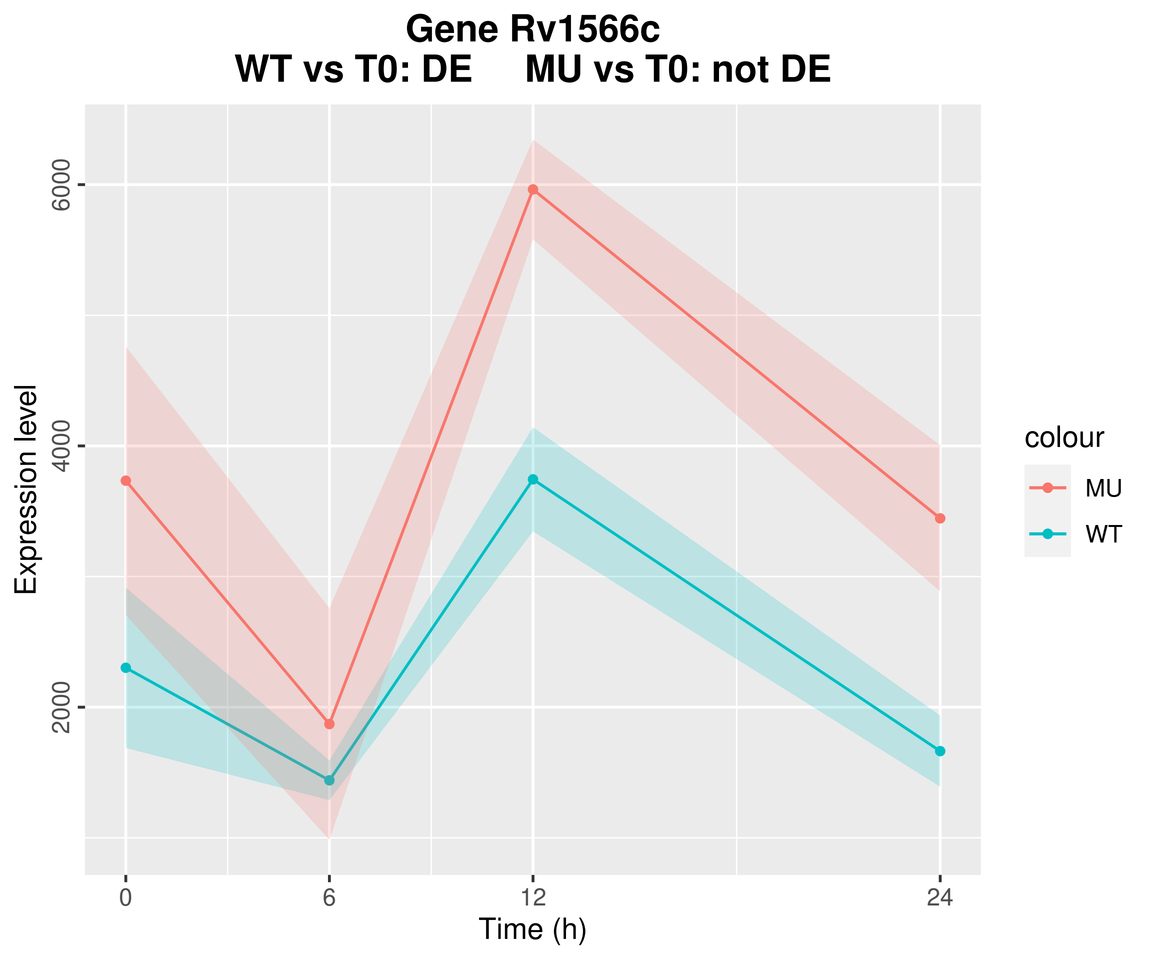

Gene Rv1566c
WT vs T0: DE MU vs T0: not DE
Expression level
6000
4000
2000
0
6
12
24
Time (h)
colour
MU
WT

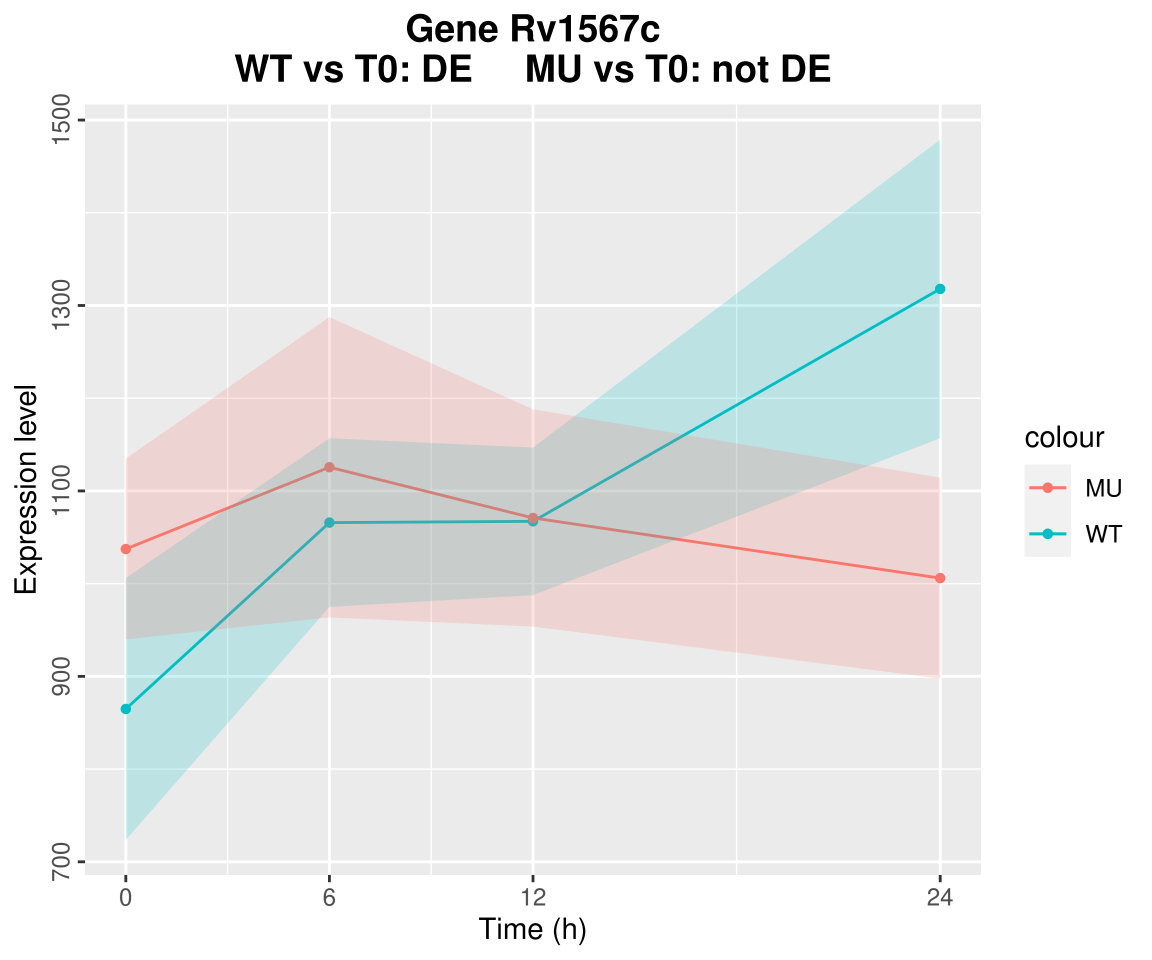

Gene Rv1567c
WT vs T0: DE   MU vs T0: not DE
1500
1300
1100
900
700
Expression level
0
6
12
24
Time (h)
colour
MU
WT

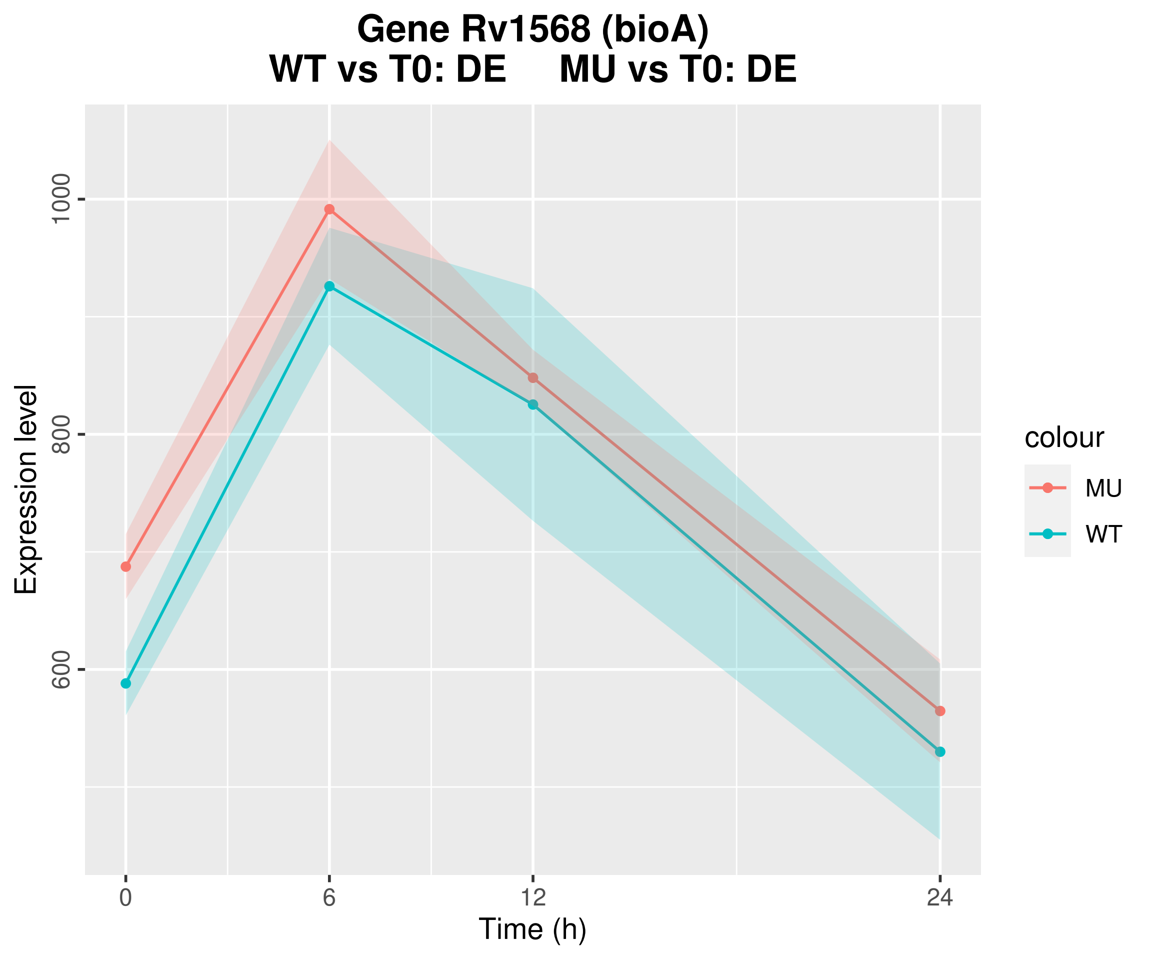
Gene Rv1568 (bioA)
WT vs T0: DE MU vs T0: DE
Expression level
1000
800
600
0
6
12
24
Time (h)
colour
MU
WT

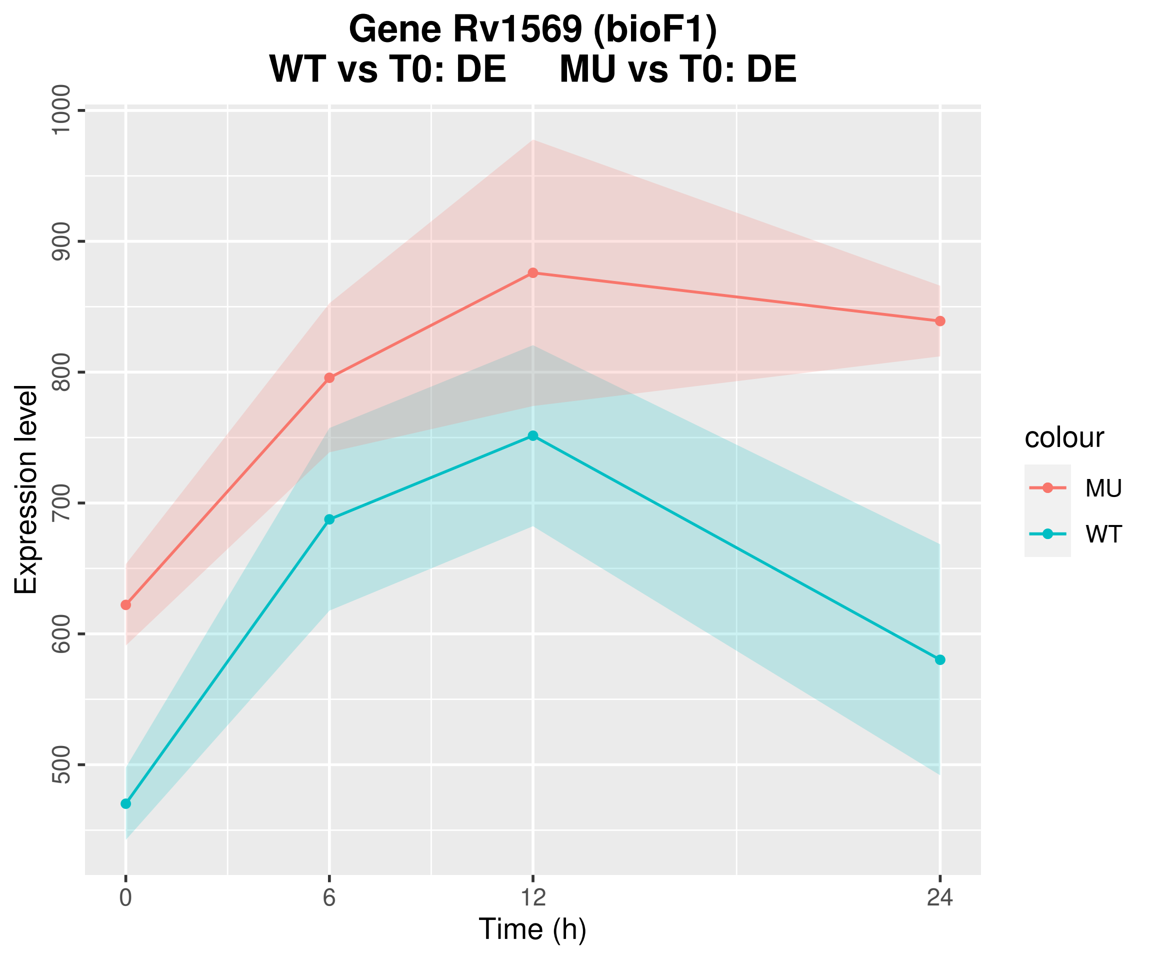

Gene Rv1569 (bioF1)
WT vs T0: DE MU vs T0: DE
Expression level
1000
900
800
700
600
500
0
6
12
24
Time (h)
colour
MU
WT

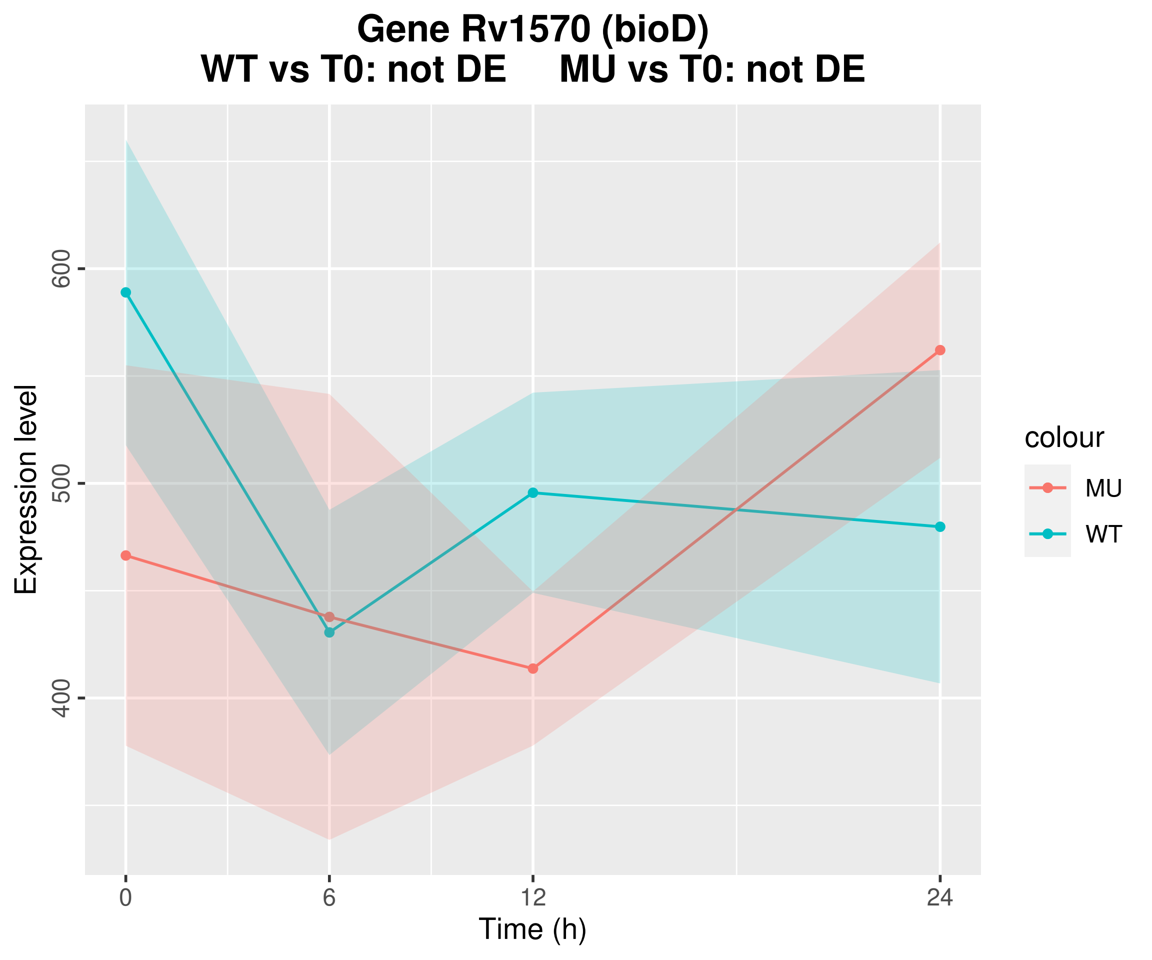

Gene Rv1570 (bioD)
WT vs T0: not DE MU vs T0: not DE
Expression level
600
500
400
0
6
12
24
Time (h)
colour
MU
WT

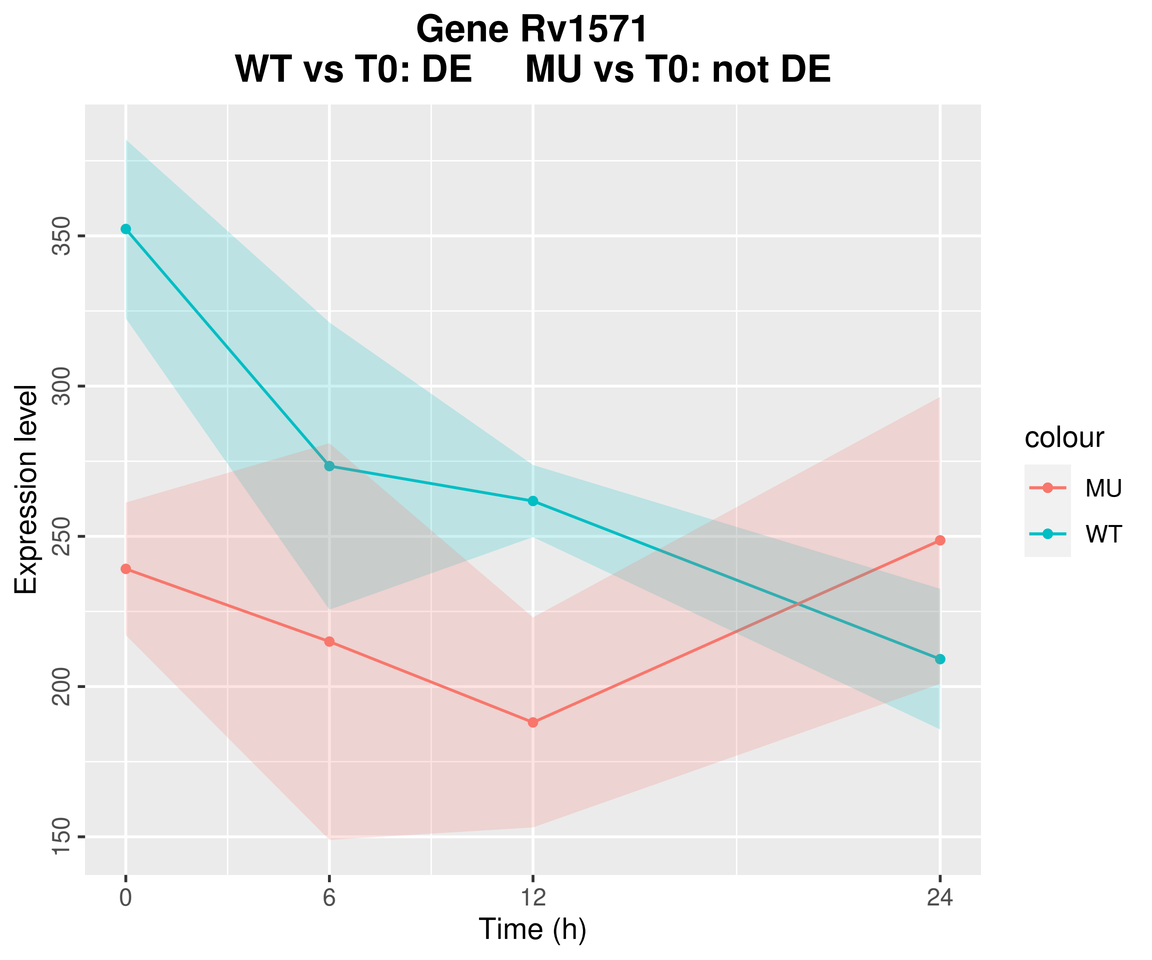
Gene Rv1571
WT vs T0: DE   MU vs T0: not DE
Expression level
350
300
250
200
150
0
6
12
24
Time (h)
colour
MU
WT

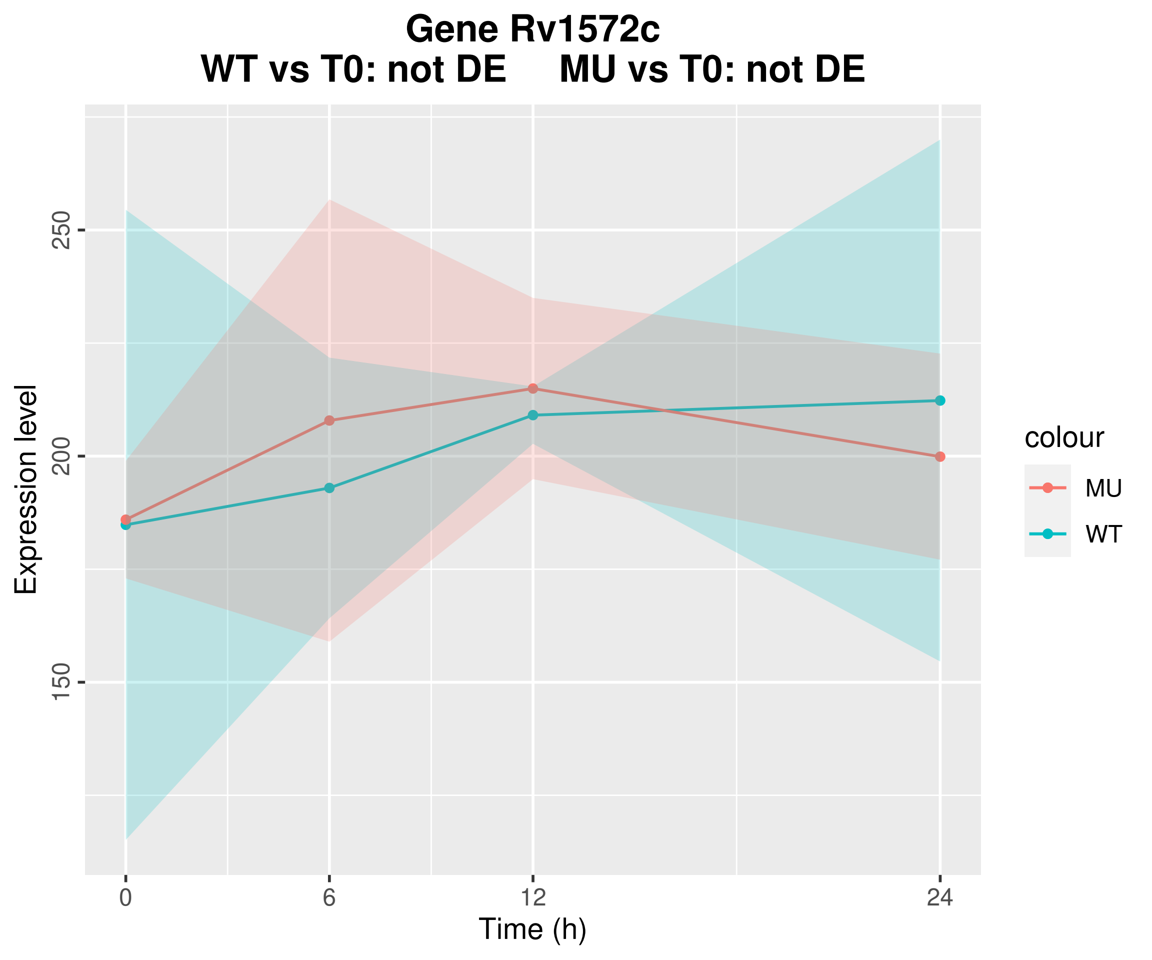
Gene Rv1572c
WT vs T0: not DE MU vs T0: not DE
Expression level
250
200
150
0
6
12
24
Time (h)
colour
MU
WT

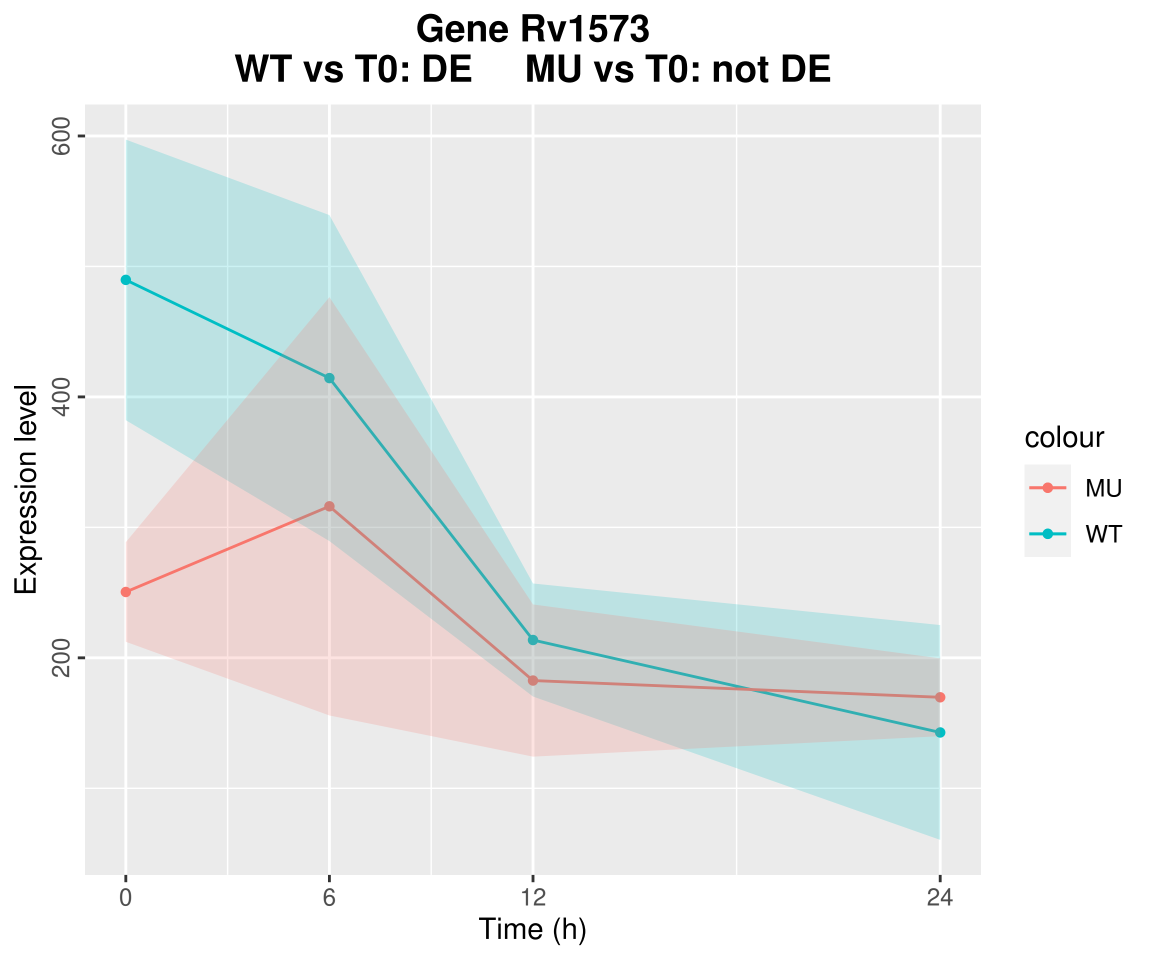
Gene Rv1573
WT vs T0: DE   MU vs T0: not DE
Expression level
600
400
200
0
6
12
24
Time (h)
colour
MU
WT

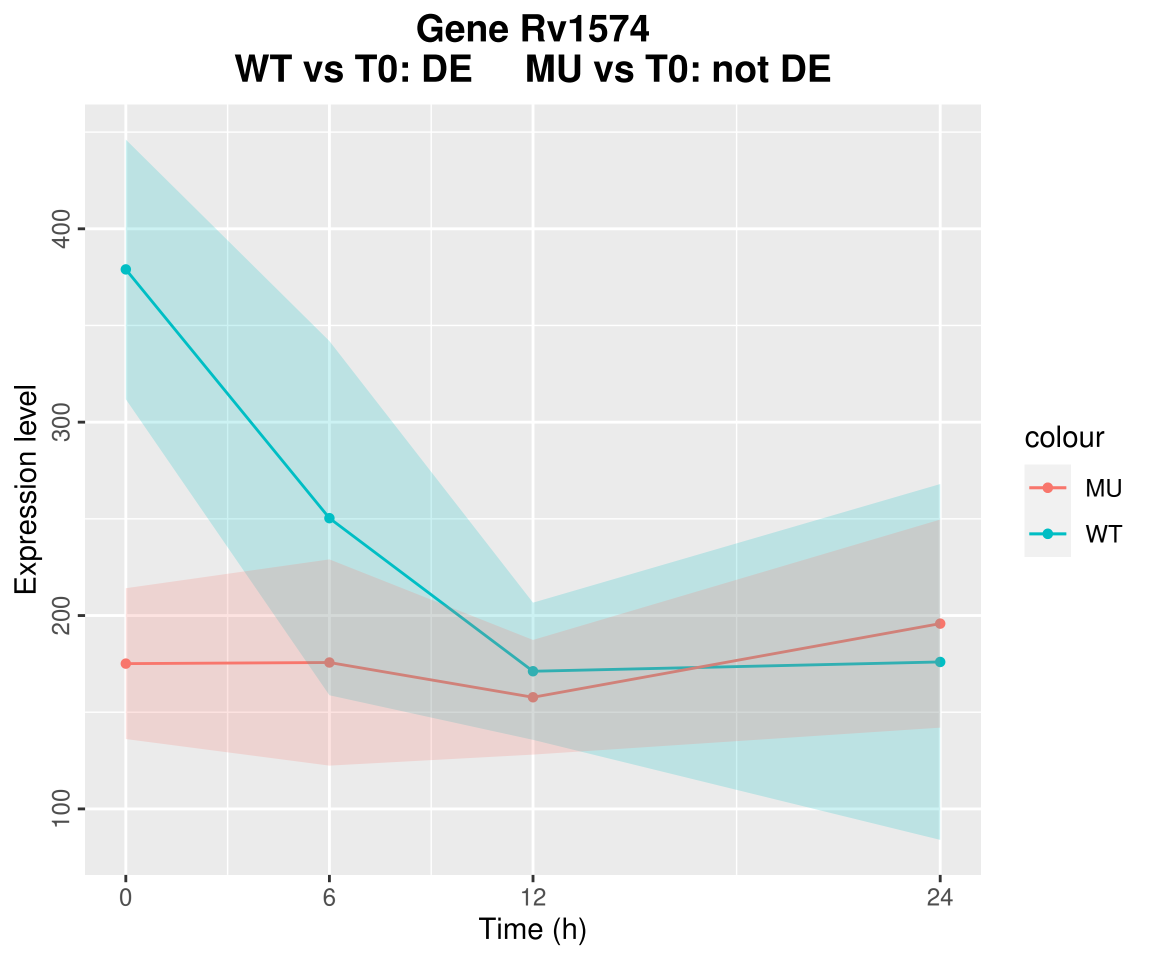
Gene Rv1574
WT vs T0: DE   MU vs T0: not DE
Expression level
100
200
300
400
0
6
12
24
Time (h)
colour
MU
WT

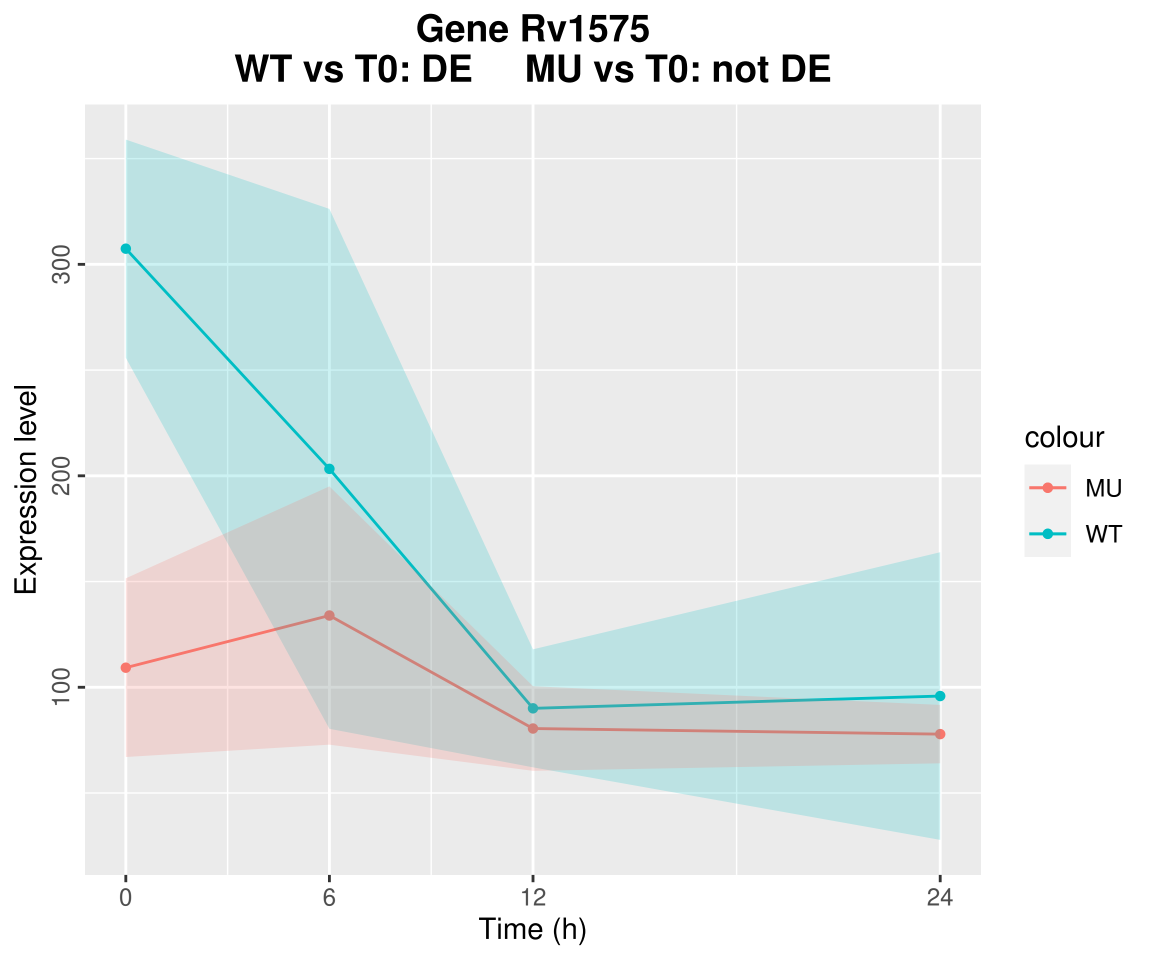
Gene Rv1575
WT vs T0: DE   MU vs T0: not DE
Expression level
300
200
100
0
6
12
24
Time (h)
colour
MU
WT

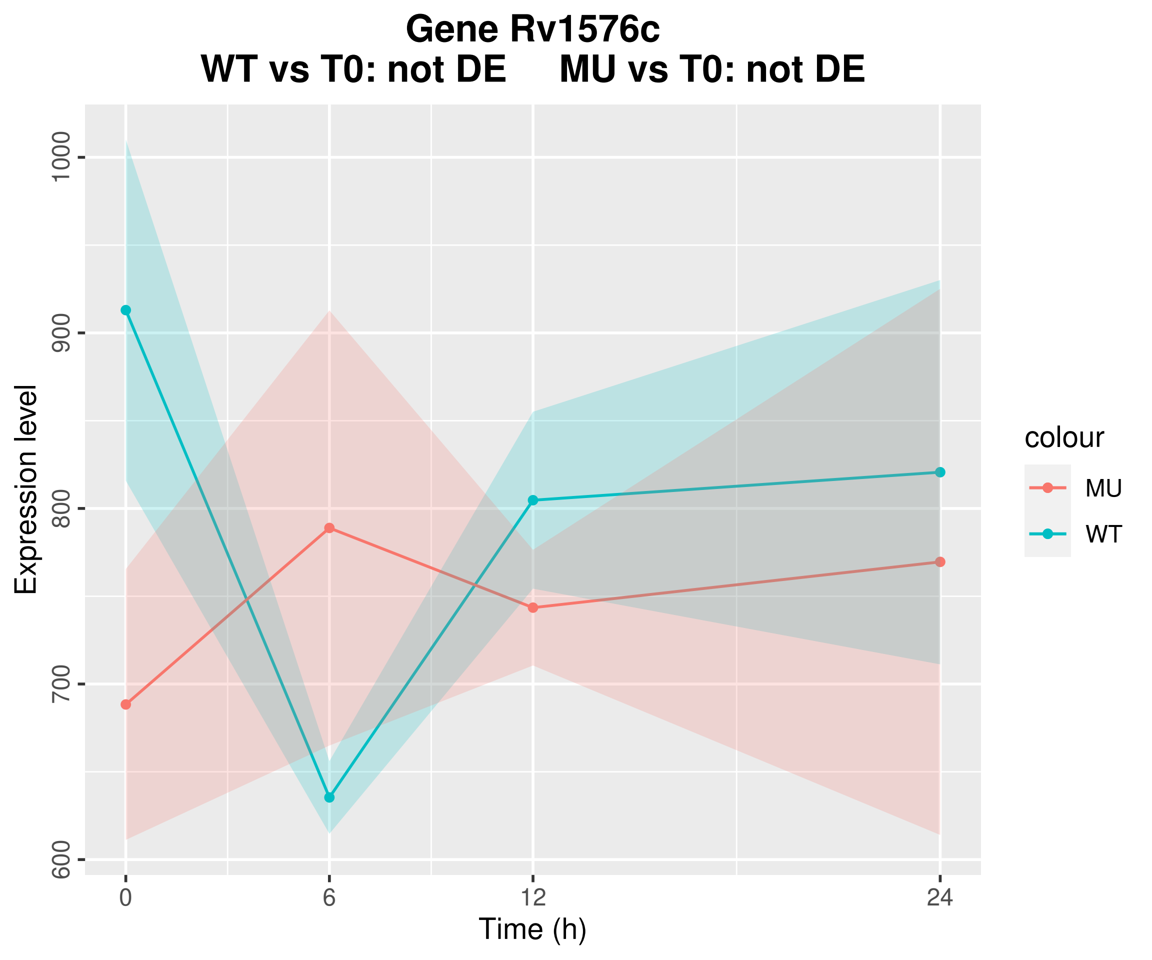
Gene Rv1576c
WT vs T0: not DE   MU vs T0: not DE
Expression level
1000
900
800
700
600
0
6
12
24
Time (h)
colour
MU
WT

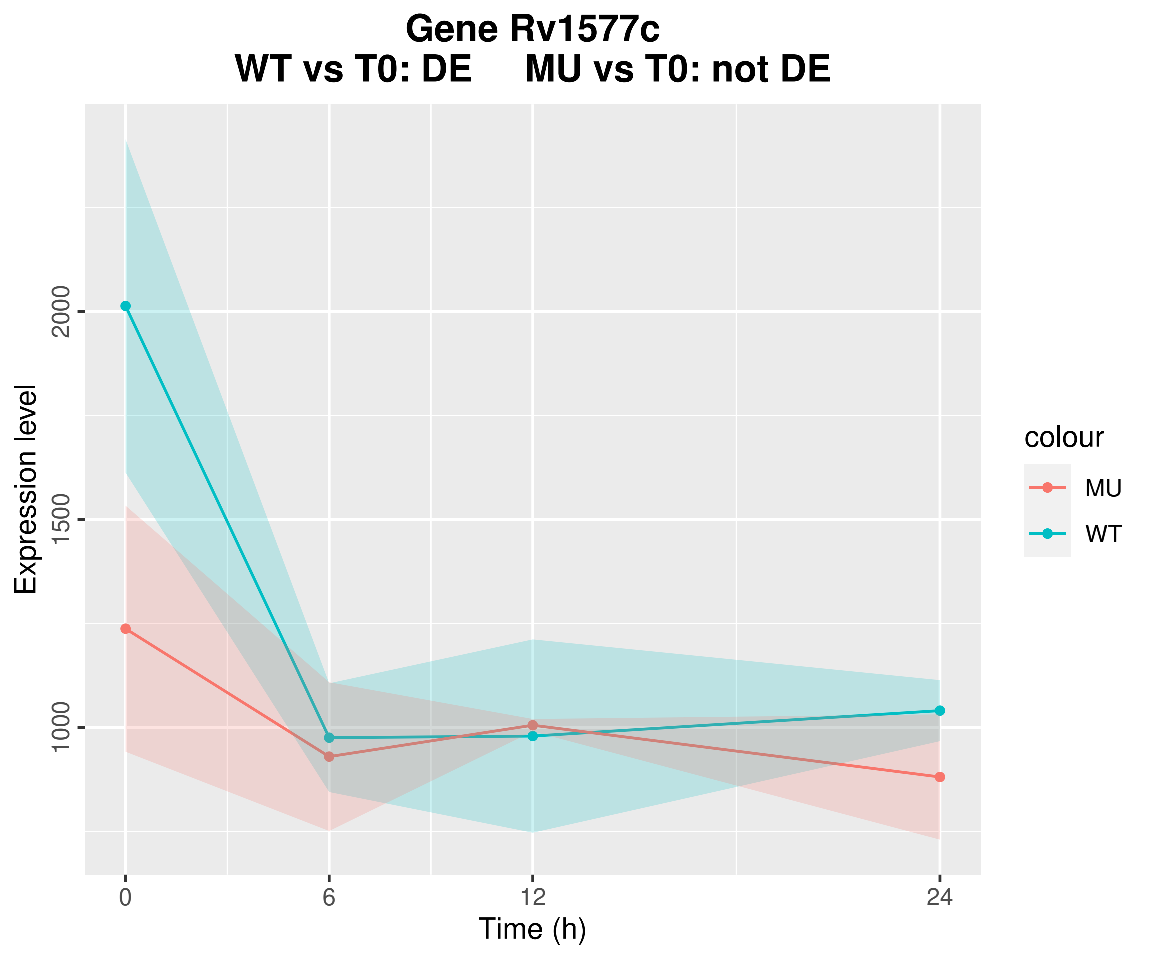
Gene Rv1577c
WT vs T0: DE   MU vs T0: not DE
Expression level
2000
1500
1000
0
6
12
24
Time (h)
colour
MU
WT

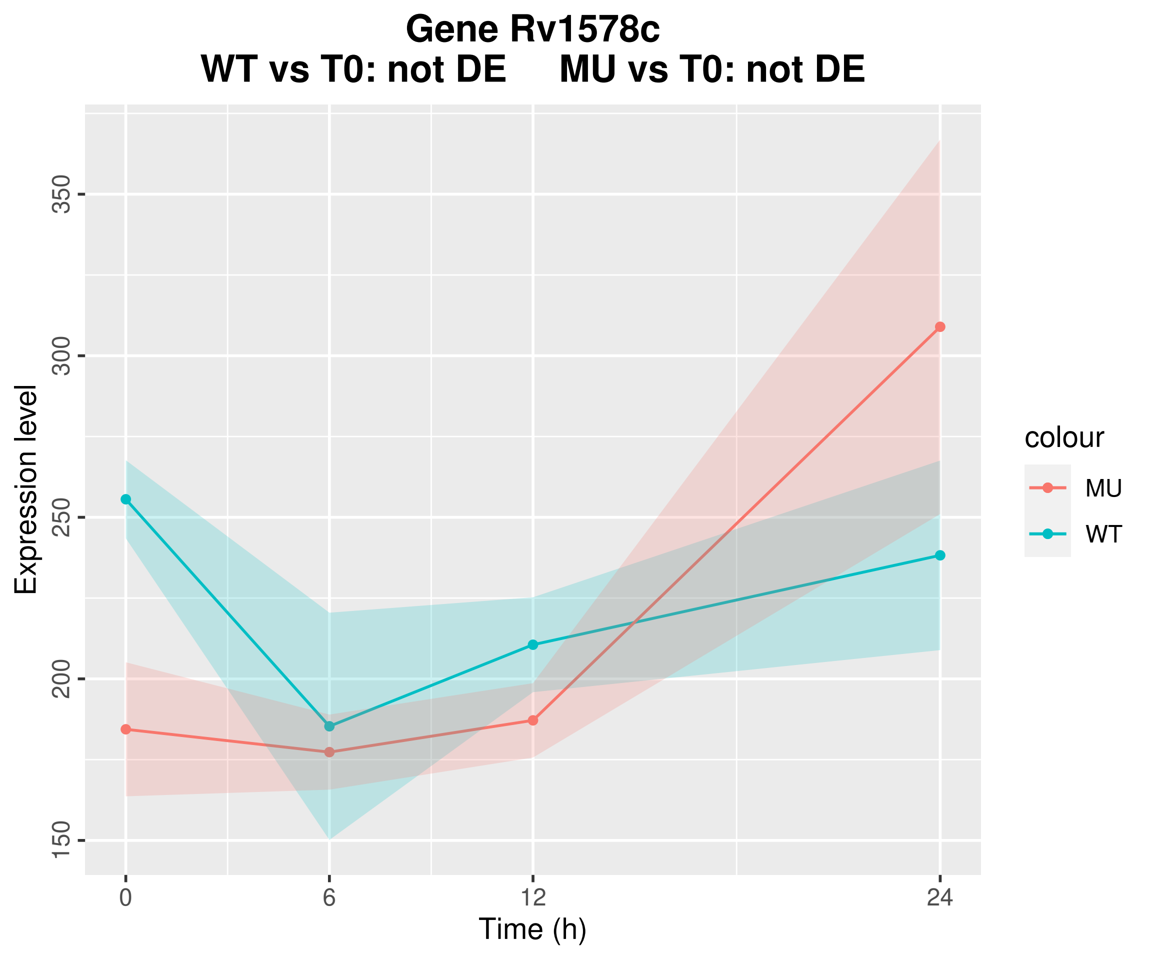
Gene Rv1578c
WT vs T0: not DE   MU vs T0: not DE
Expression level
350
300
250
200
150
0
6
12
24
Time (h)
colour
MU
WT

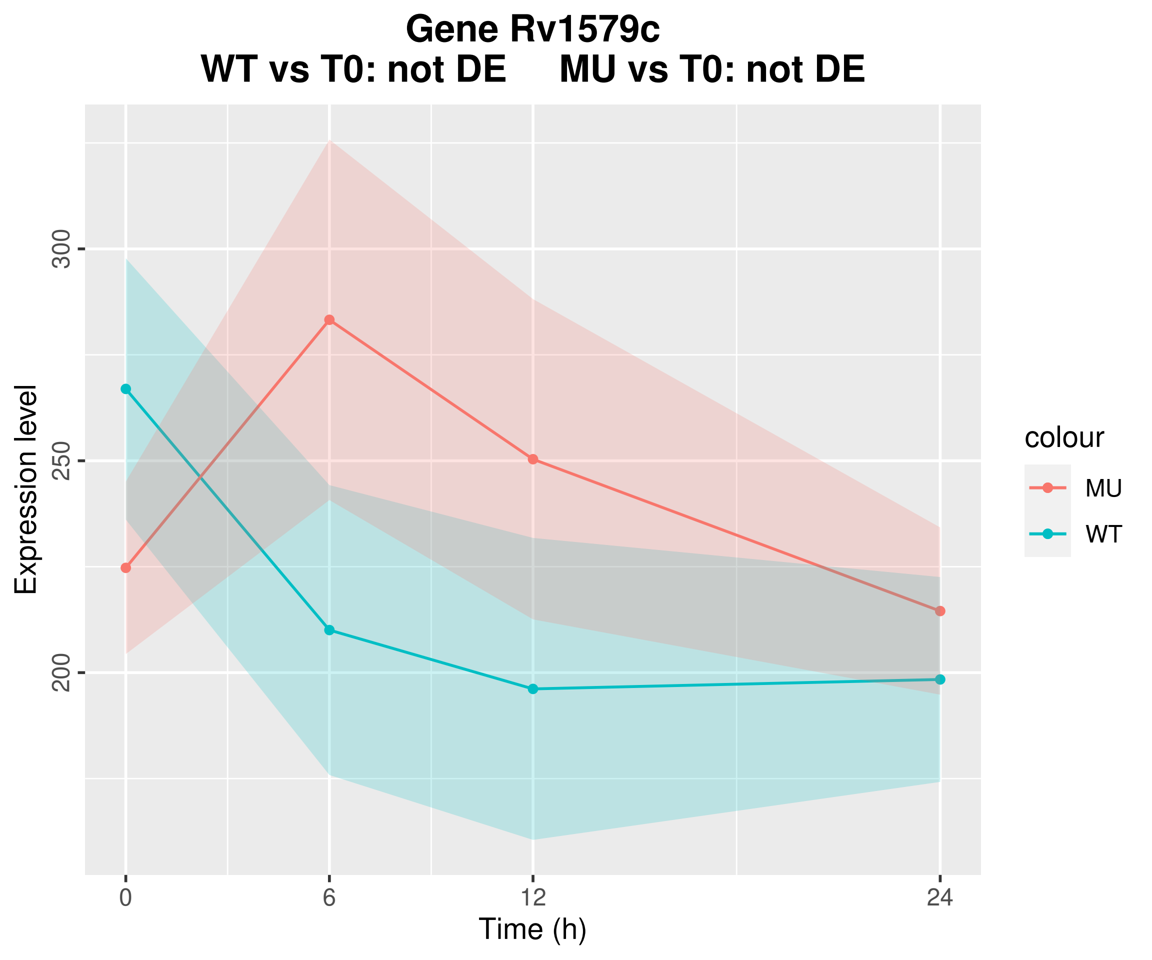

Gene Rv1579c
WT vs T0: not DE   MU vs T0: not DE
Expression level
300
250
200
0
6
12
24
Time (h)
colour
MU
WT

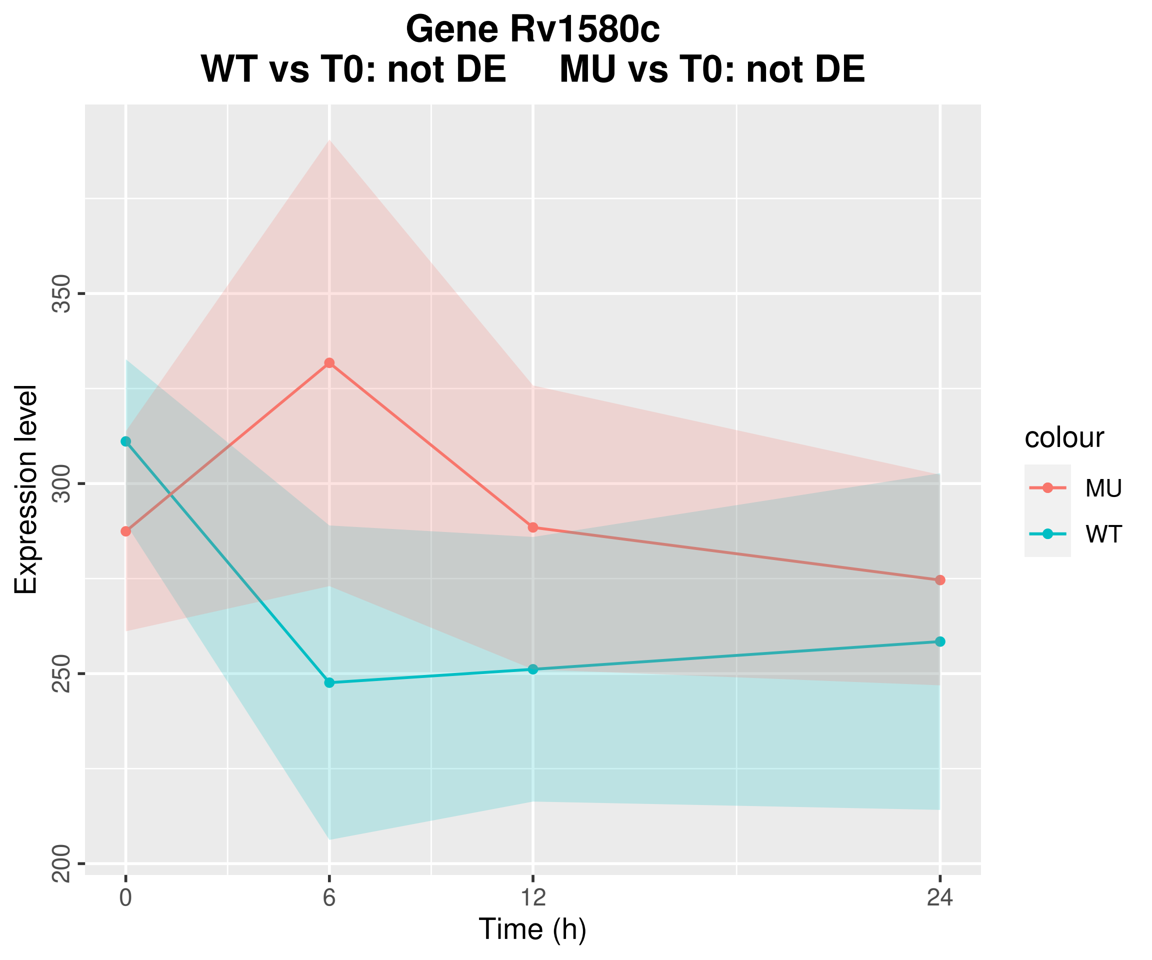
Gene Rv1580c
WT vs T0: not DE   MU vs T0: not DE
Expression level
350
300
250
200
0
6
12
24
Time (h)
colour
MU
WT

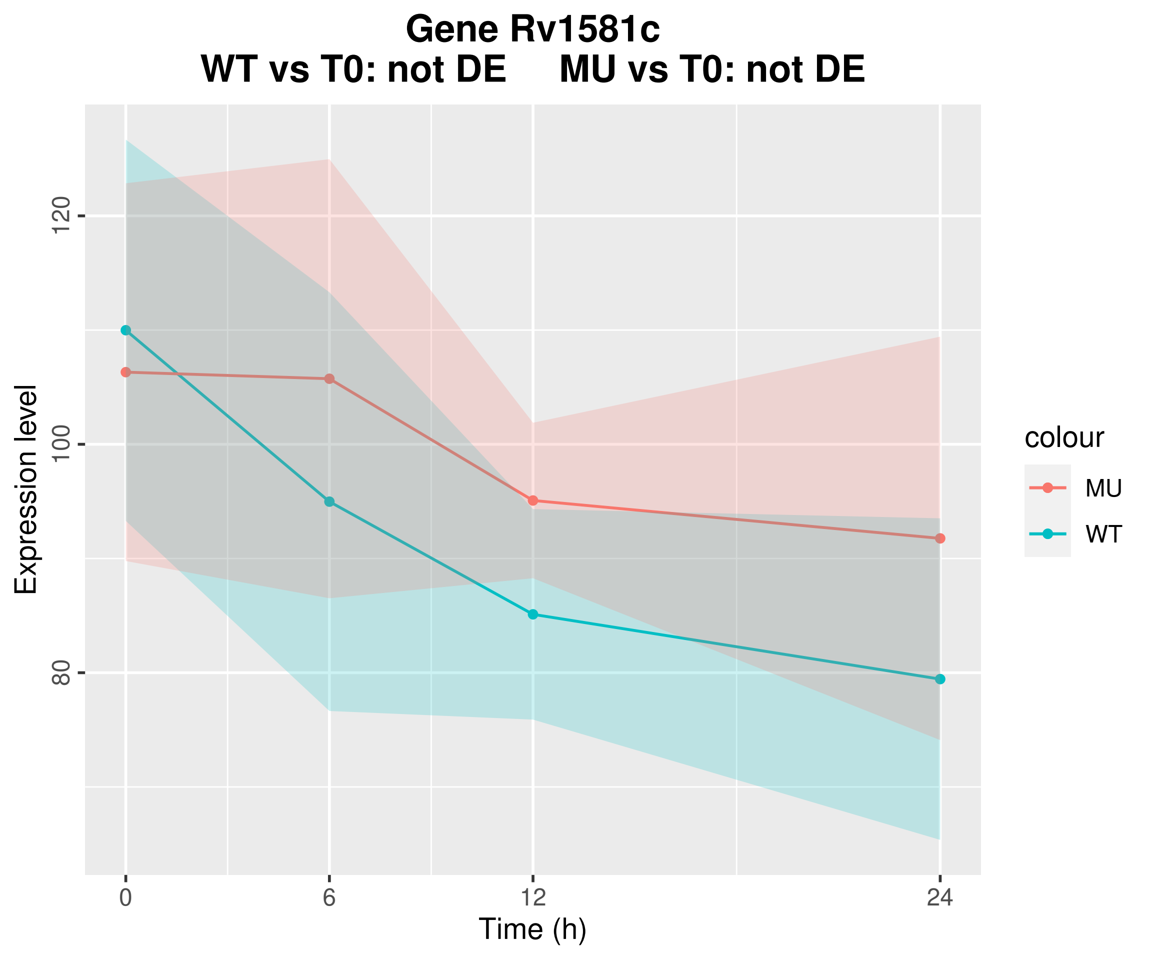
Gene Rv1581c
WT vs T0: not DE MU vs T0: not DE
Expression level
120
100
80
0
6
12
24
Time (h)
colour
MU
WT

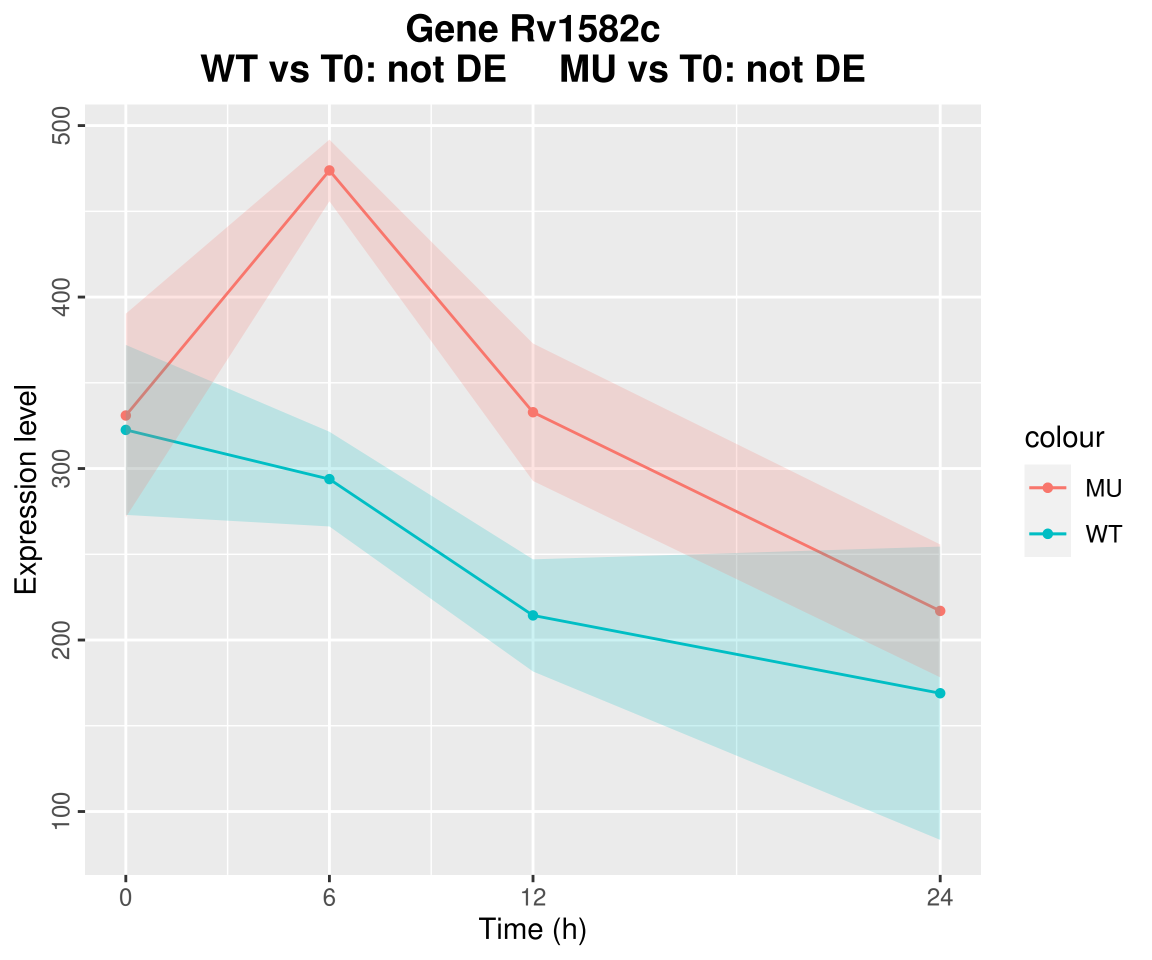
Gene Rv1582c
WT vs T0: not DE   MU vs T0: not DE
Expression level
100
200
300
400
500
Time (h)
0
6
12
24
colour
MU
WT

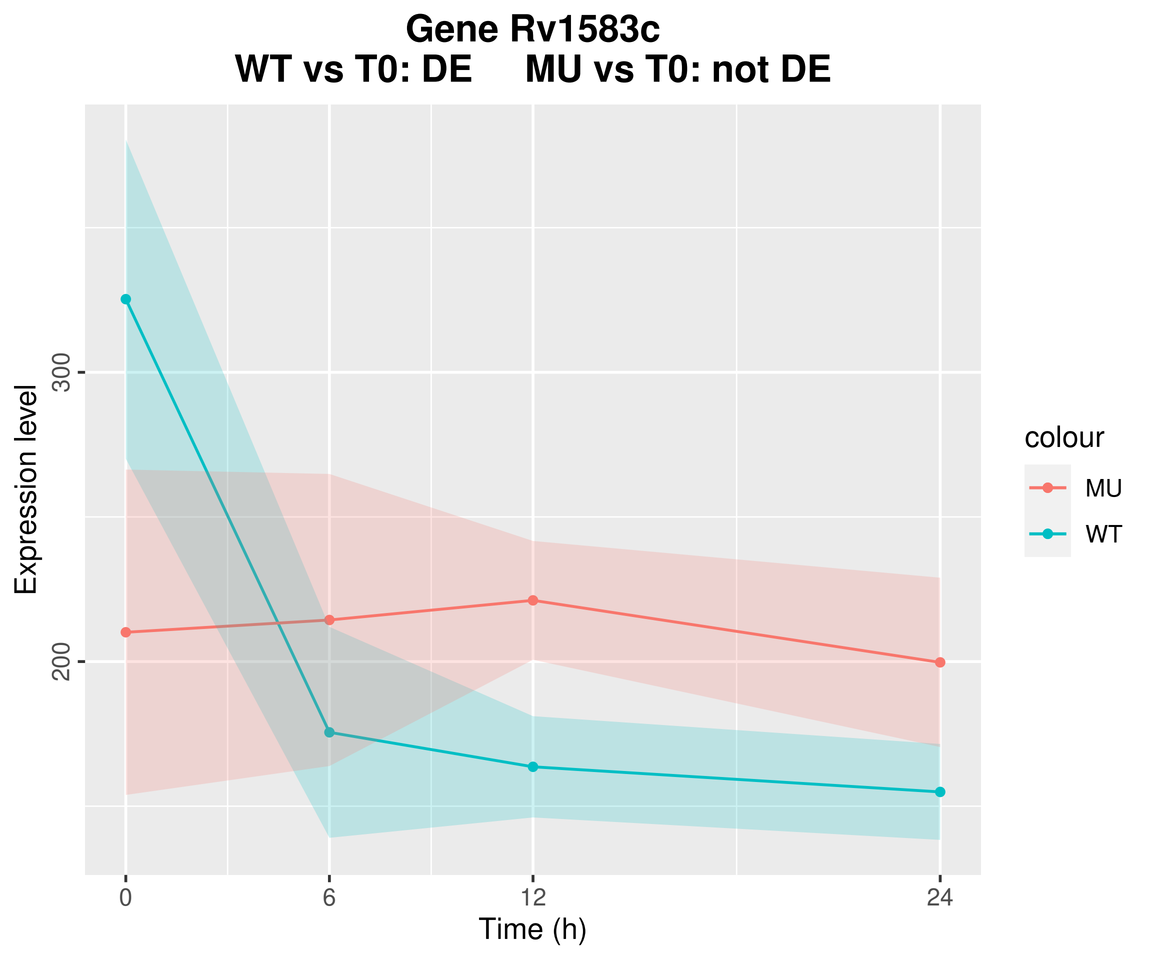
Gene Rv1583c
WT vs T0: DE MU vs T0: not DE
Expression level
300
200
0
6
12
24
Time (h)
colour
MU
WT

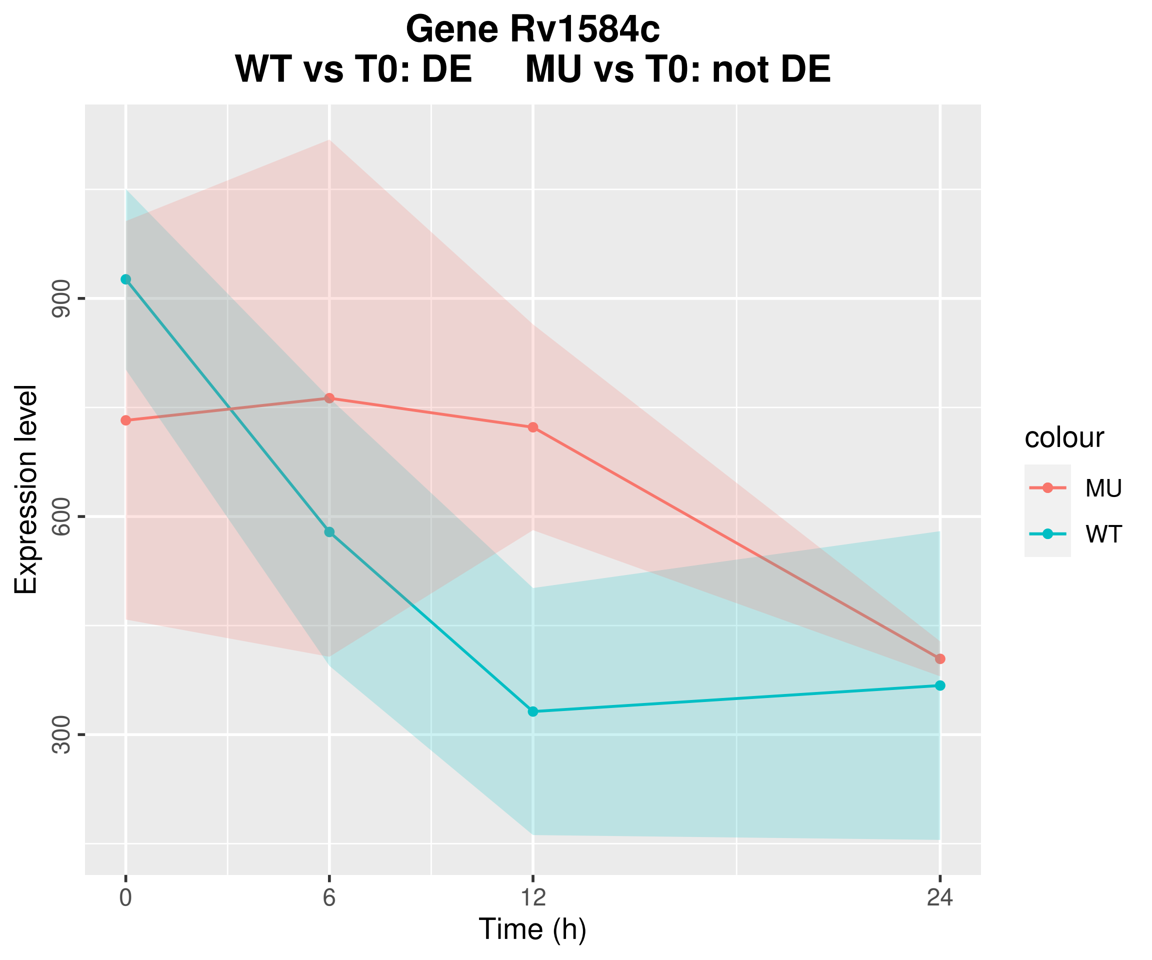
Gene Rv1584c
WT vs T0: DE MU vs T0: not DE
Expression level
900
600
300
0
6
12
24
Time (h)
colour
MU
WT

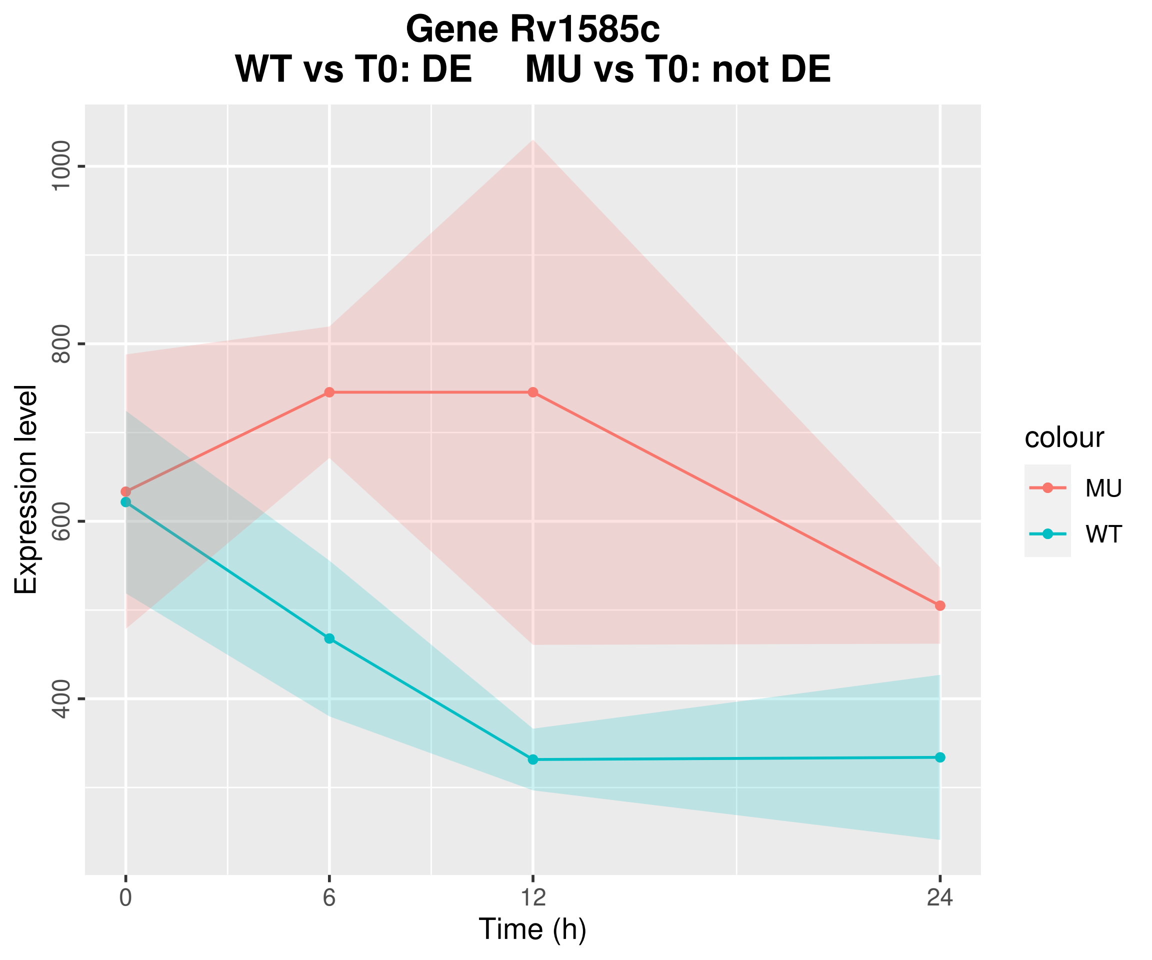

Gene Rv1585c
WT vs T0: DE   MU vs T0: not DE
Expression level
1000
800
600
400
0
6
12
24
Time (h)
colour
MU
WT

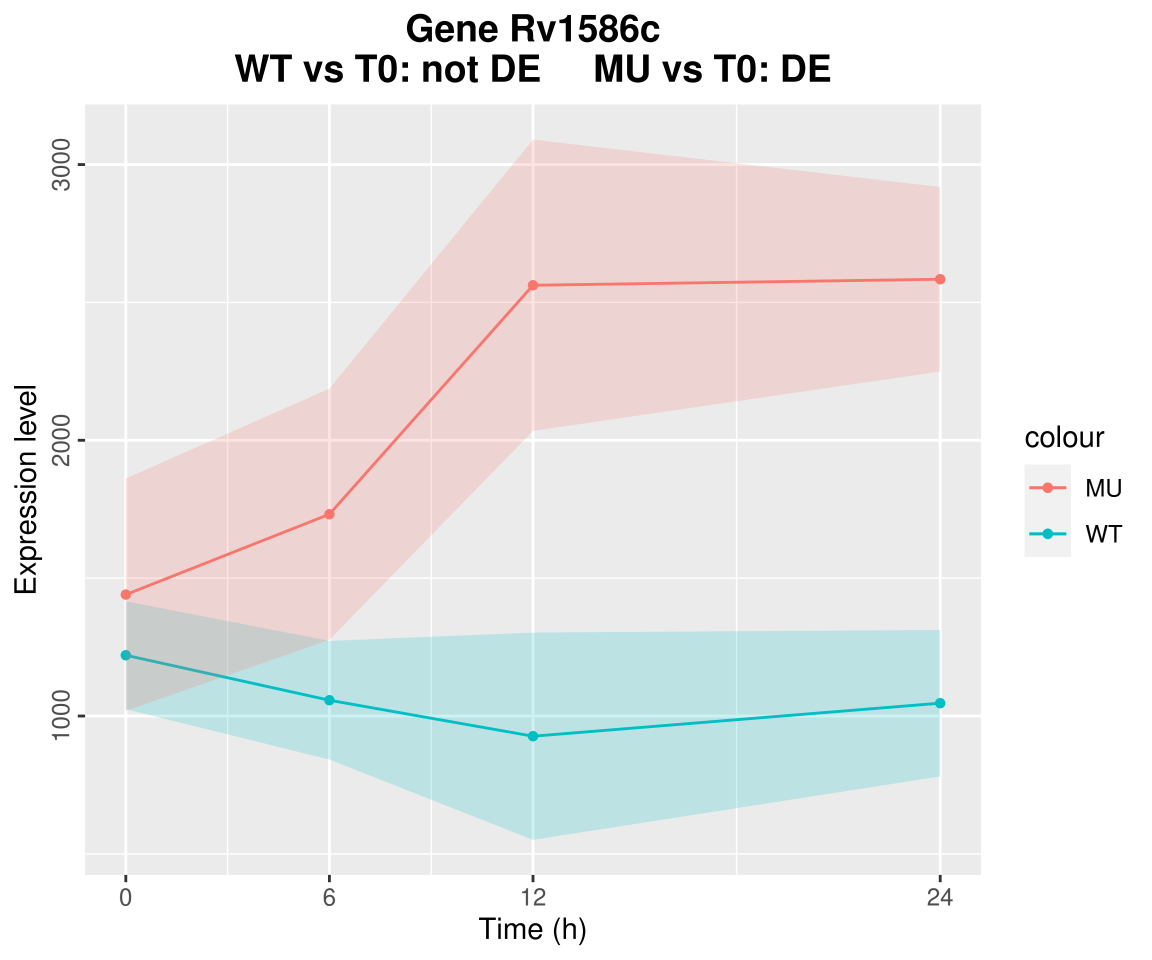
Gene Rv1586c
WT vs T0: not DE MU vs T0: DE
Expression level
3000
2000
1000
0
6
12
24
Time (h)
colour
MU
WT

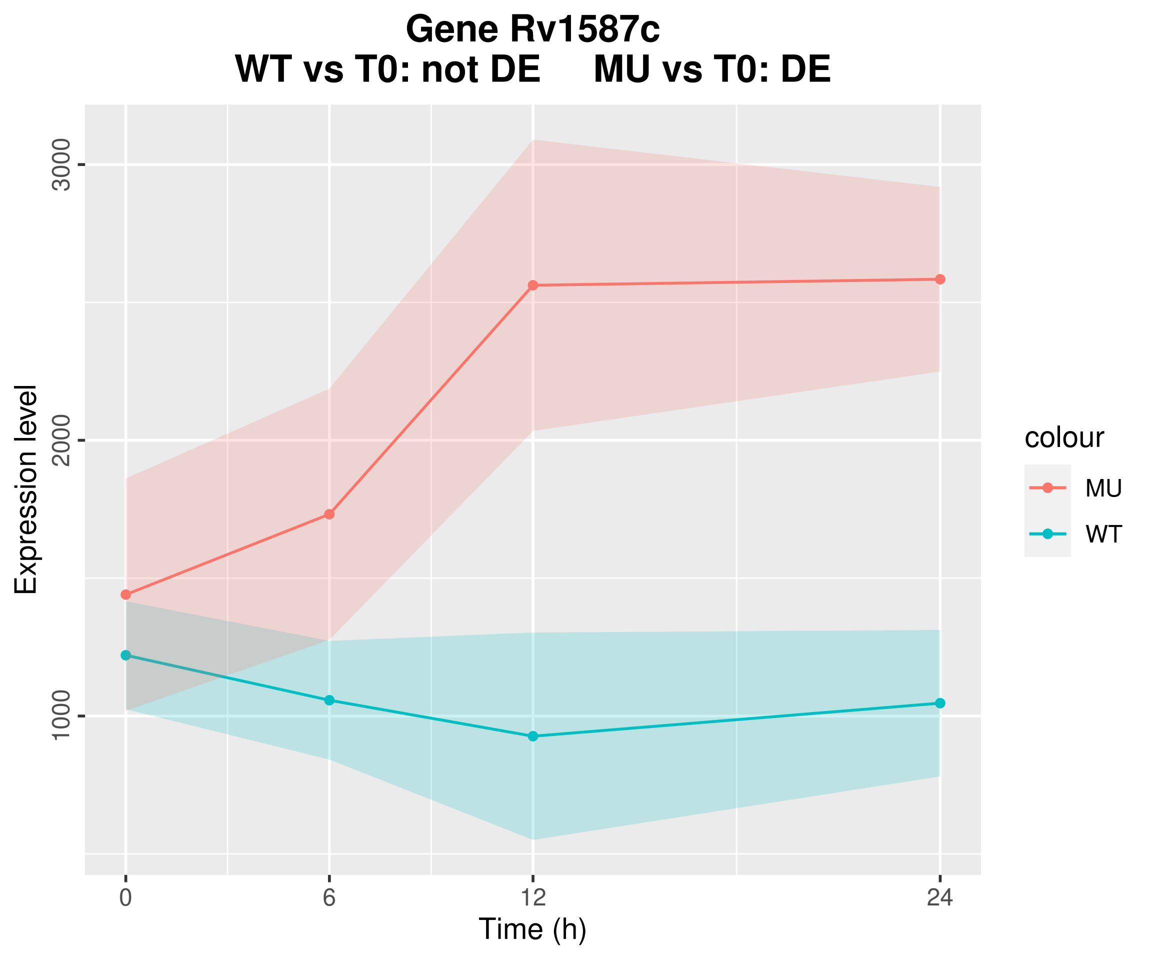

Gene Rv1587c
WT vs T0: not DE   MU vs T0: DE
Expression level
3000
2000
1000
0
6
12
24
Time (h)
colour
MU
WT

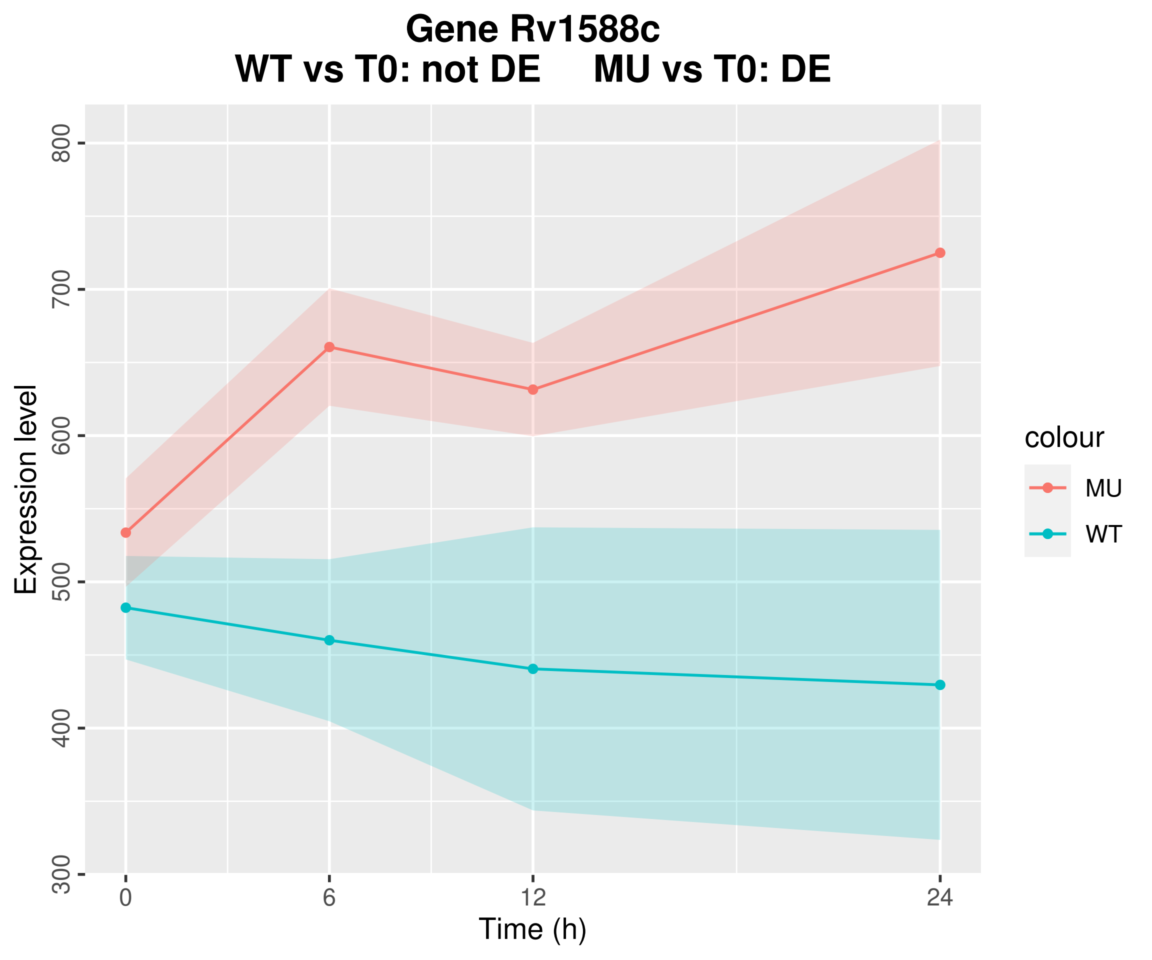
Gene Rv1588c
WT vs T0: not DE MU vs T0: DE
Expression level
800
700
600
500
400
300
0
6
12
24
Time (h)
colour
MU
WT

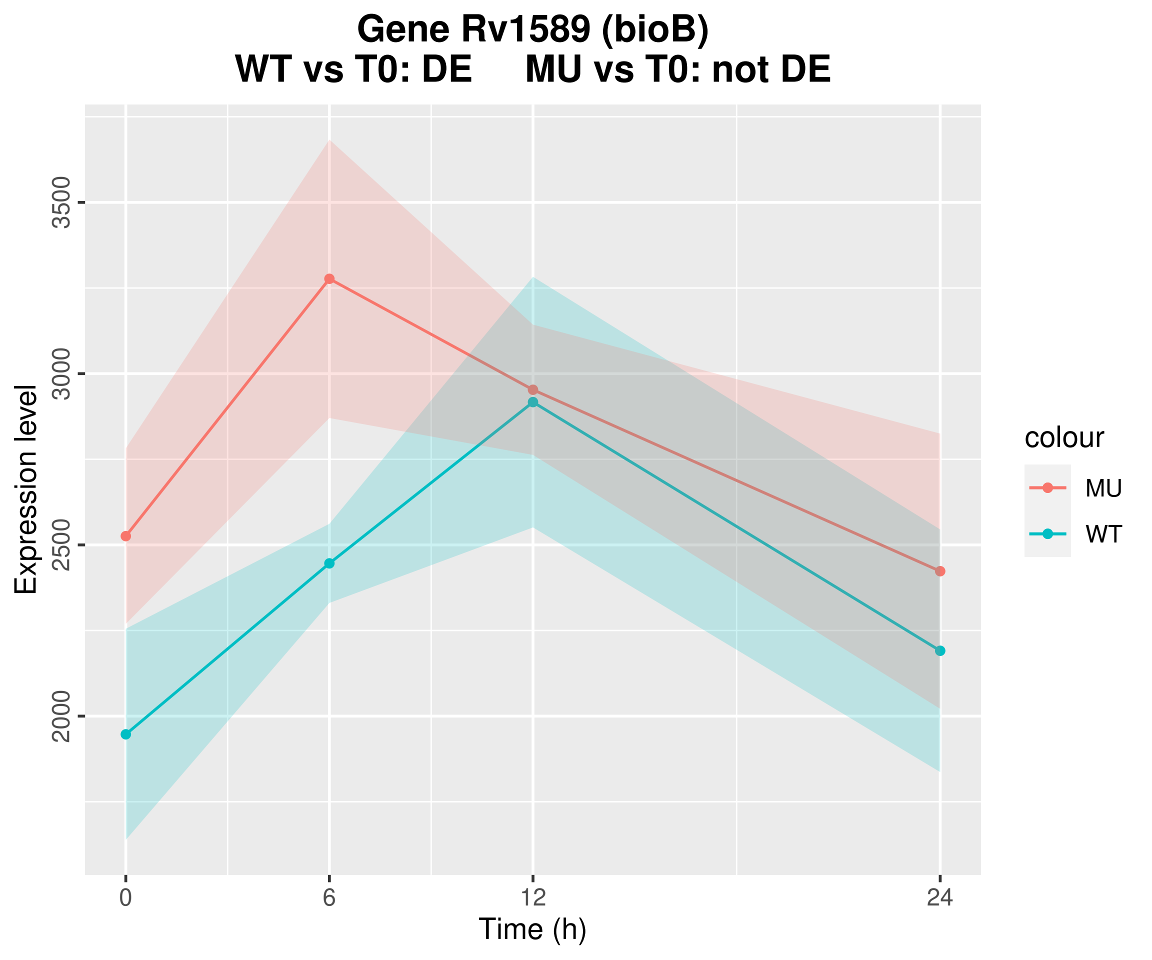
Gene Rv1589 (bioB)
WT vs T0: DE MU vs T0: not DE
Expression level
3500
3000
2500
2000
0
6
12
24
Time (h)
colour
MU
WT

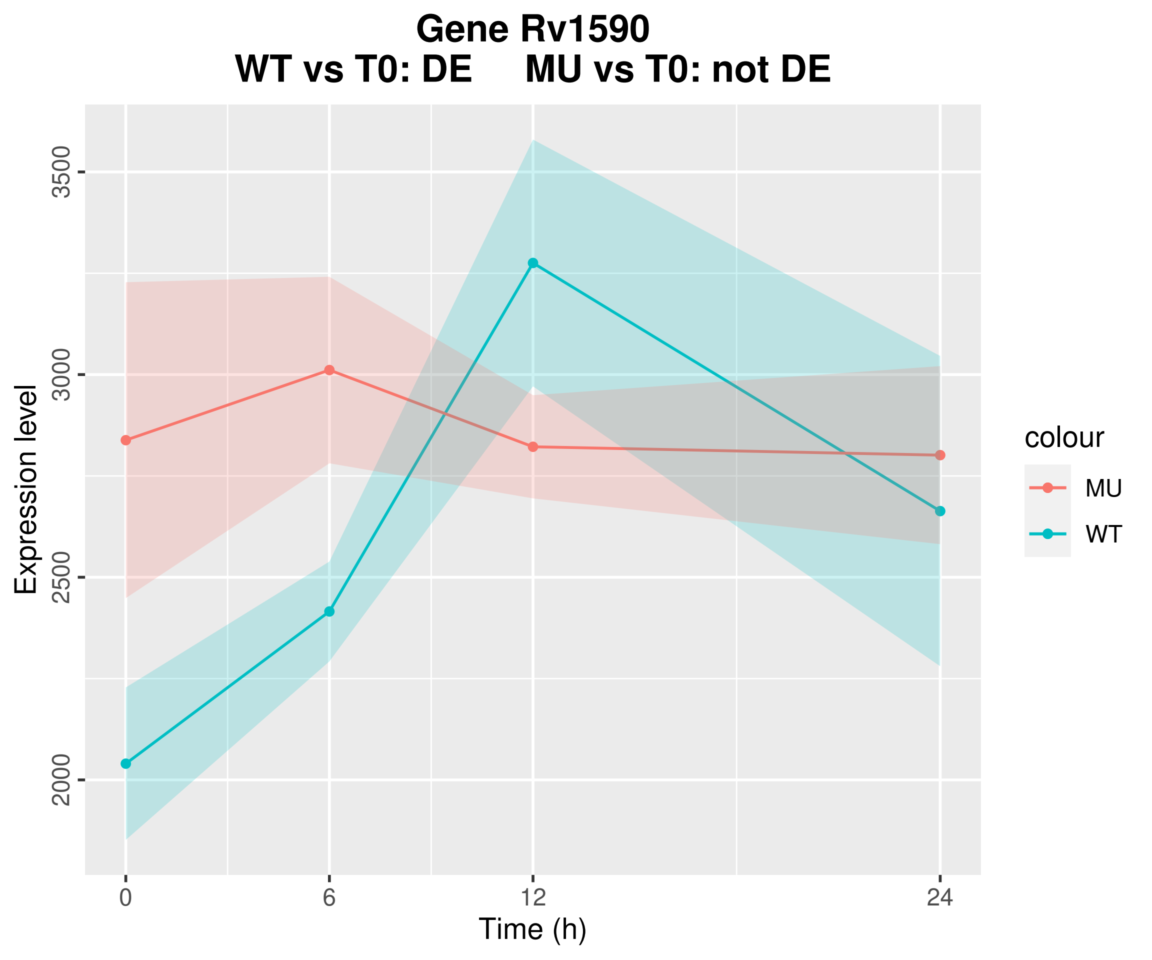

Gene Rv1590
WT vs T0: DE MU vs T0: not DE
Expression level
3500
3000
2500
2000
0
6
12
24
Time (h)
colour
MU
WT

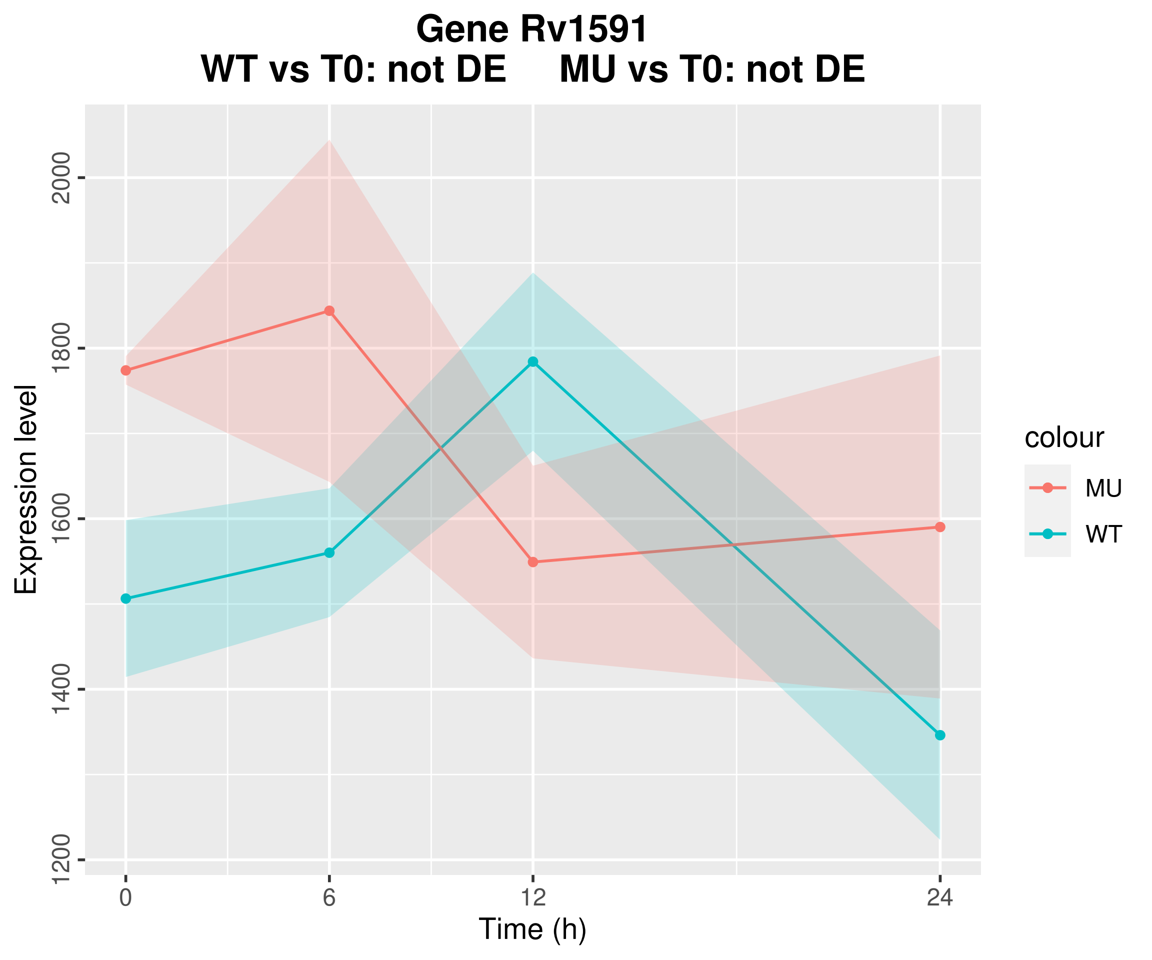

Gene Rv1591
WT vs T0: not DE MU vs T0: not DE
Expression level
2000
1800
1600
1400
1200
0
6
12
24
Time (h)
colour
MU
WT

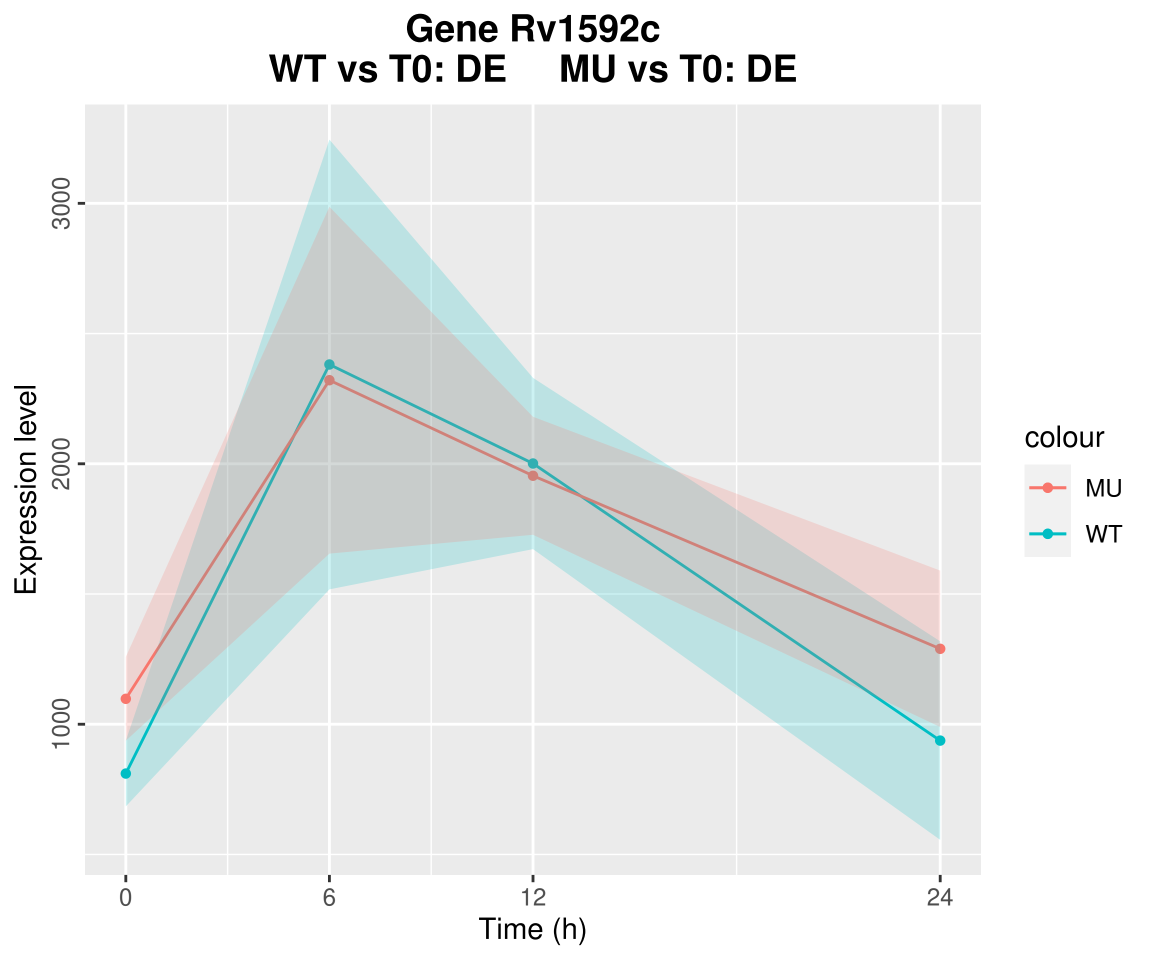
Gene Rv1592c
WT vs T0: DE MU vs T0: DE
Expression level
3000
2000
1000
0
6
12
24
Time (h)
colour
MU
WT

Gene Rv1593c
WT vs T0: not DE   MU vs T0: DE

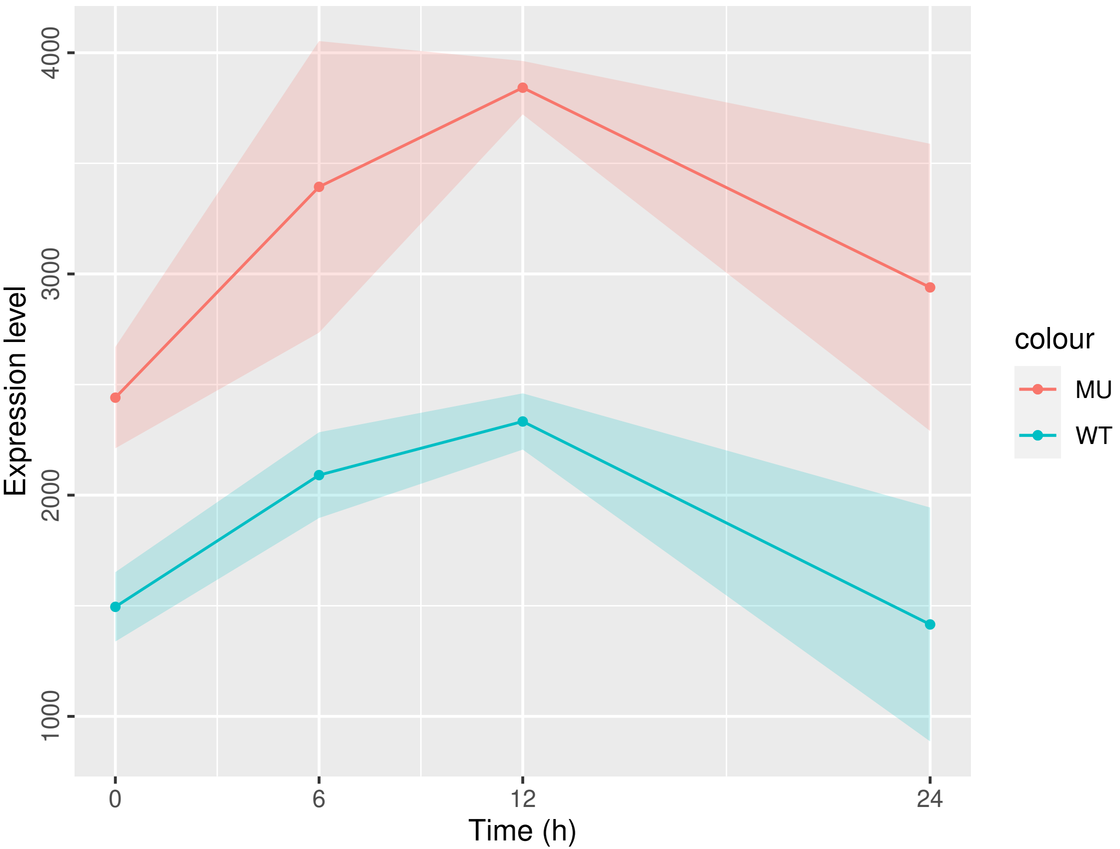

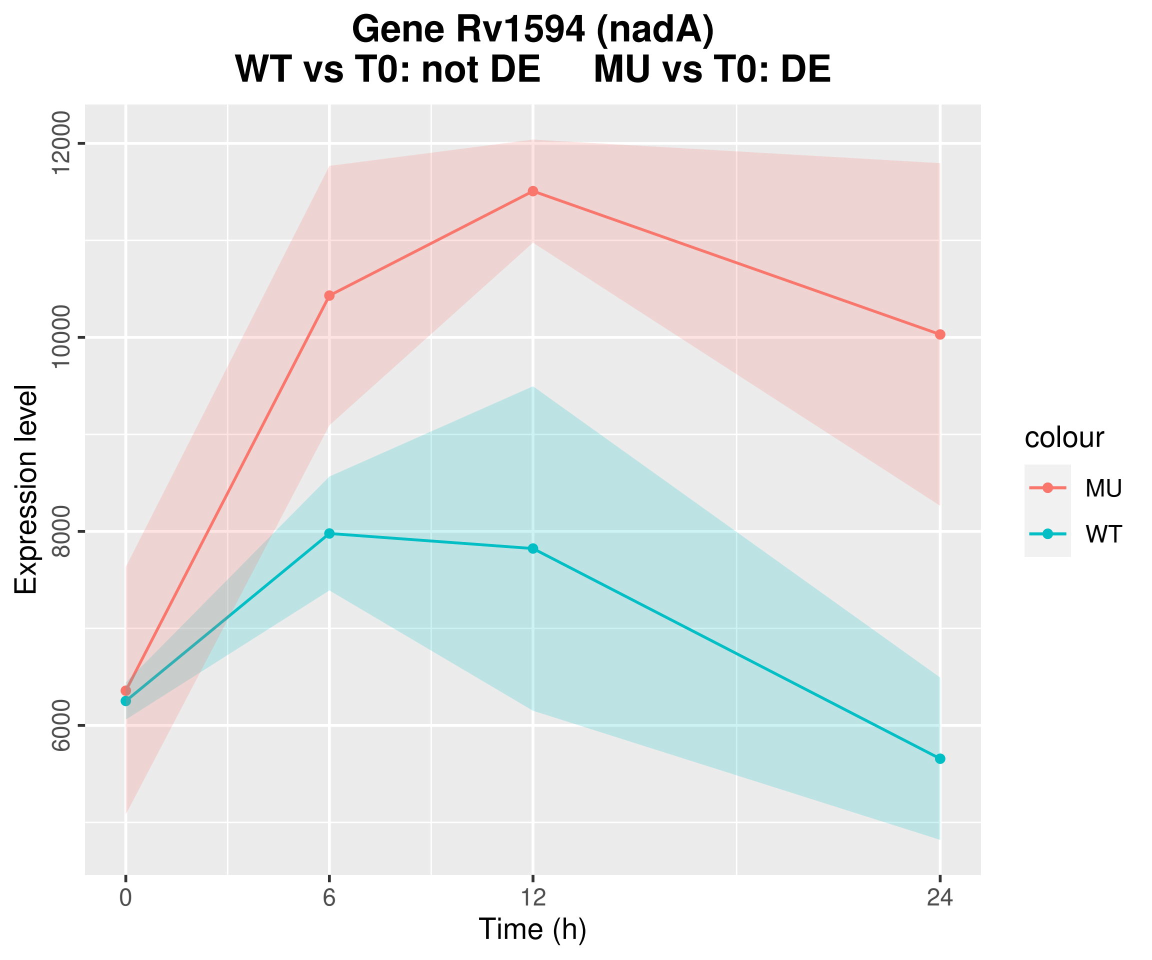

Gene Rv1594 (nadA)
WT vs T0: not DE MU vs T0: DE
Expression level
12000
10000
8000
6000
0
6
12
24
Time (h)
colour
MU
WT

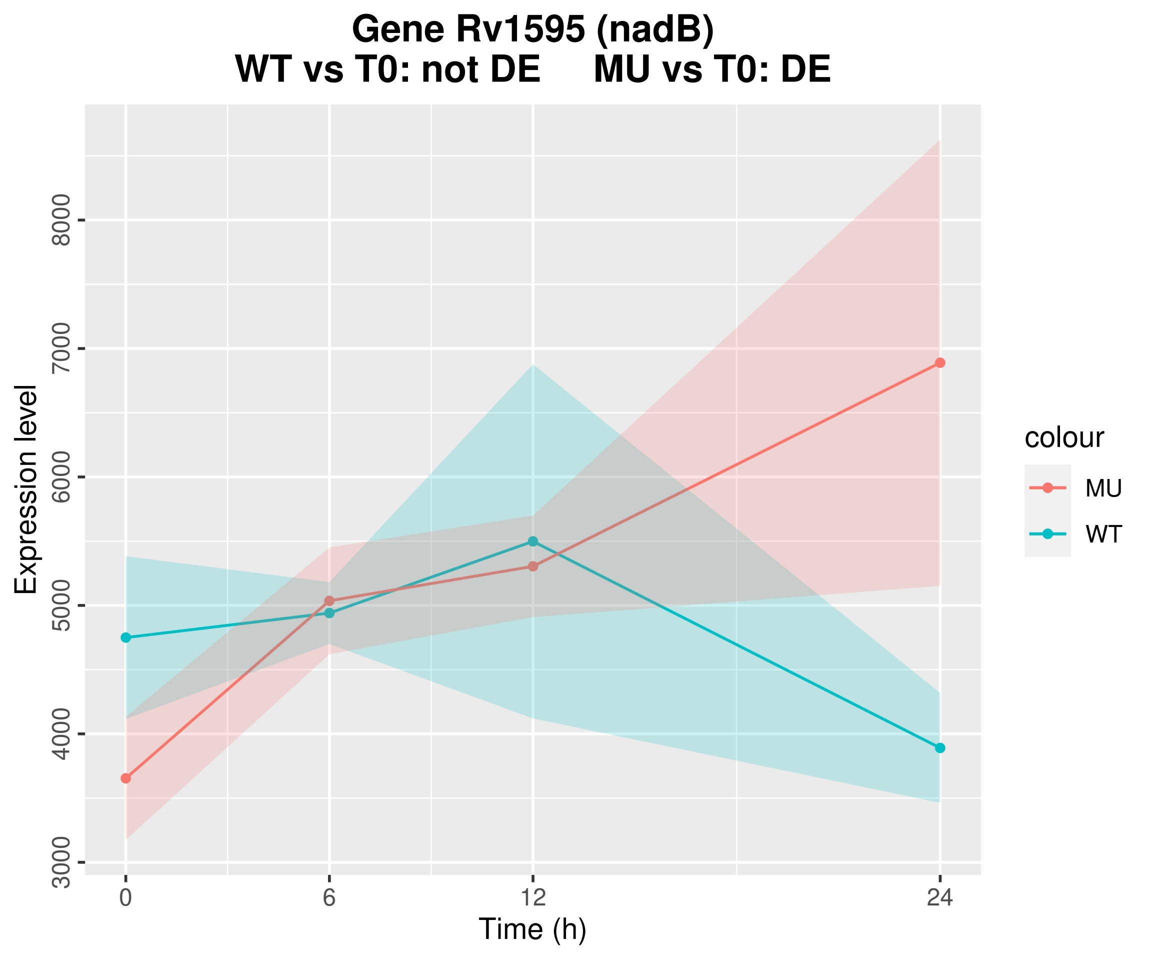
Gene Rv1595 (nadB)
WT vs T0: not DE   MU vs T0: DE
Expression level
8000
7000
6000
5000
4000
3000
0
6
12
24
Time (h)
colour
MU
WT

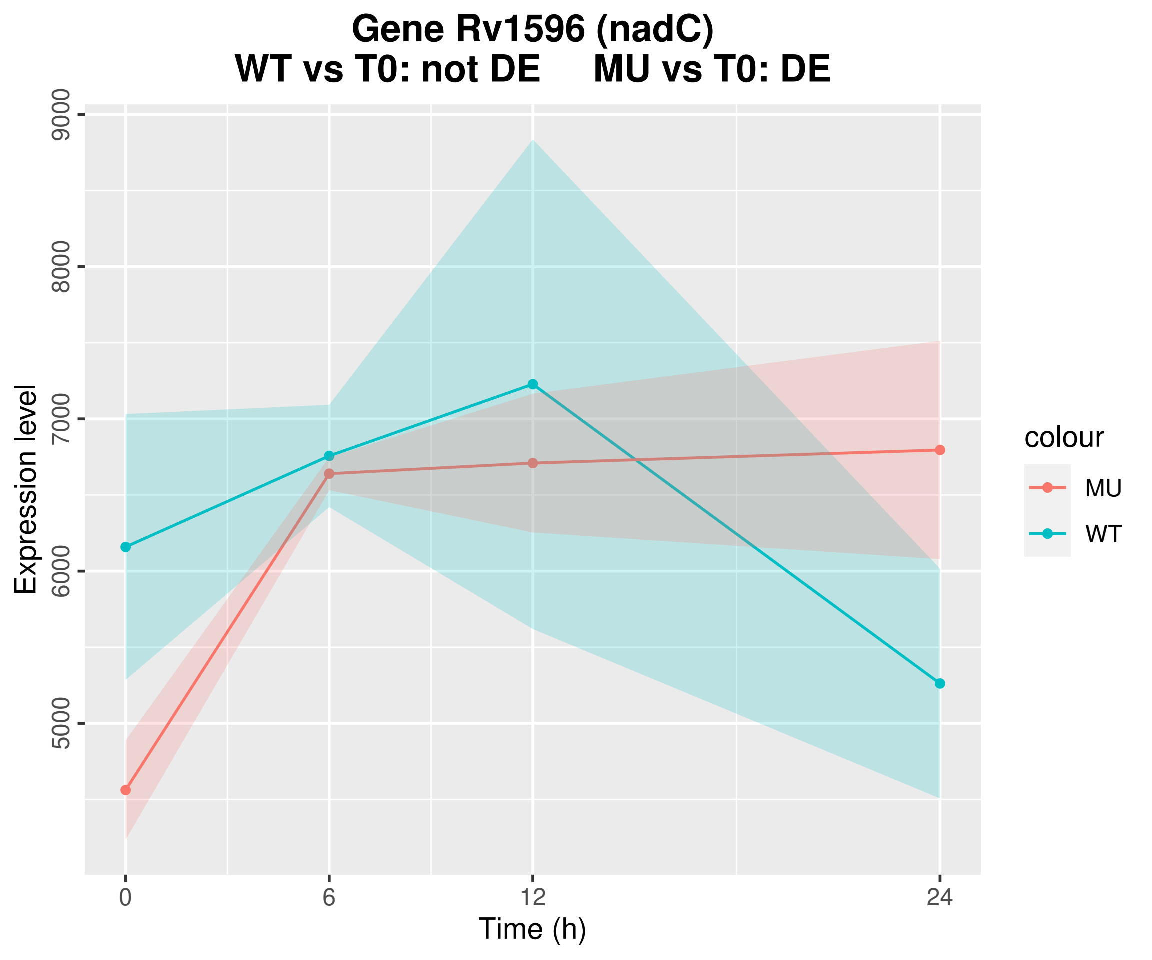
Gene Rv1596 (nadC)
WT vs T0: not DE   MU vs T0: DE
Expression level
9000
8000
7000
6000
5000
0
6
12
24
Time (h)
colour
MU
WT

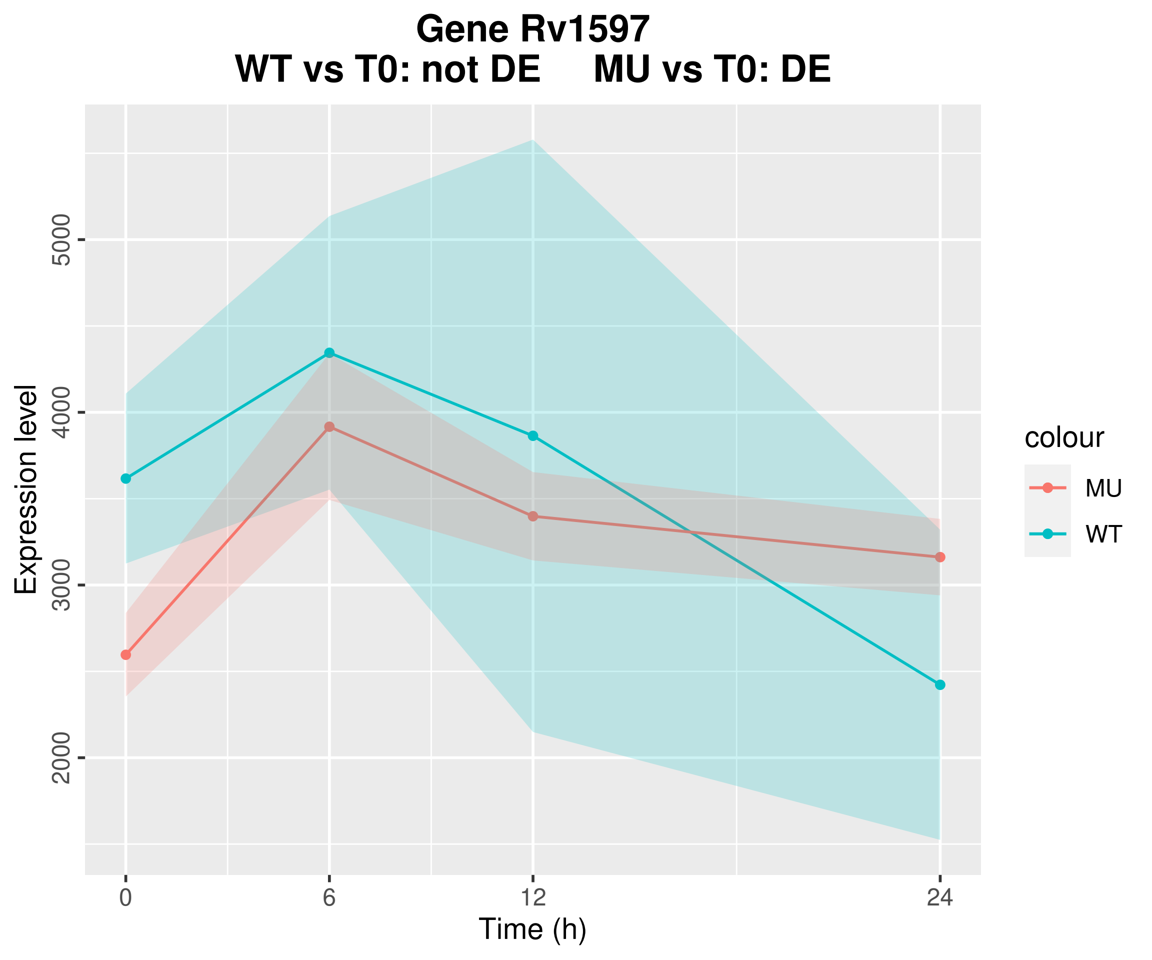
Gene Rv1597
WT vs T0: not DE MU vs T0: DE
Expression level
5000
4000
3000
2000
0
6
12
24
Time (h)
colour
MU
WT

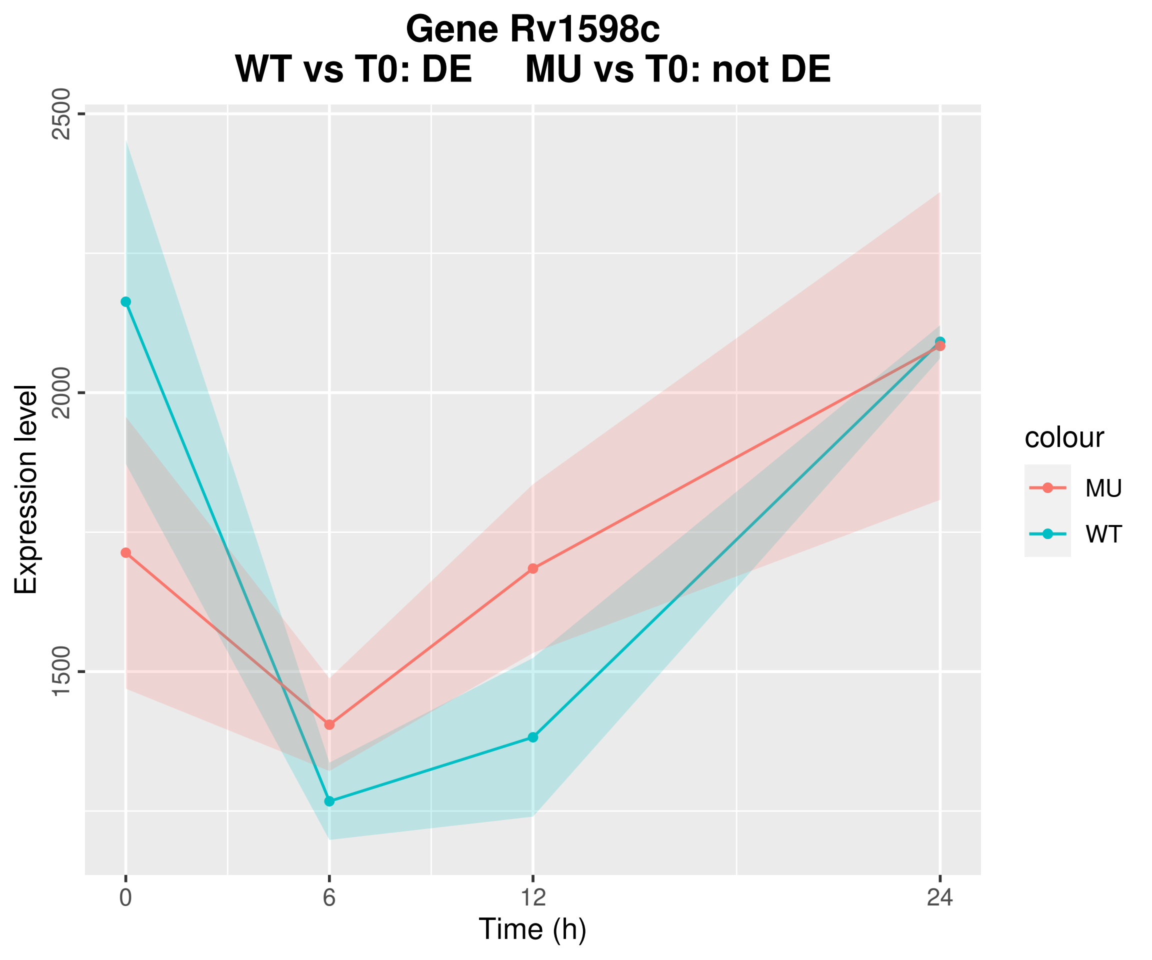

Gene Rv1598c
WT vs T0: DE MU vs T0: not DE
Expression level
2500
2000
1500
Time (h)
0
6
12
24
colour
MU
WT

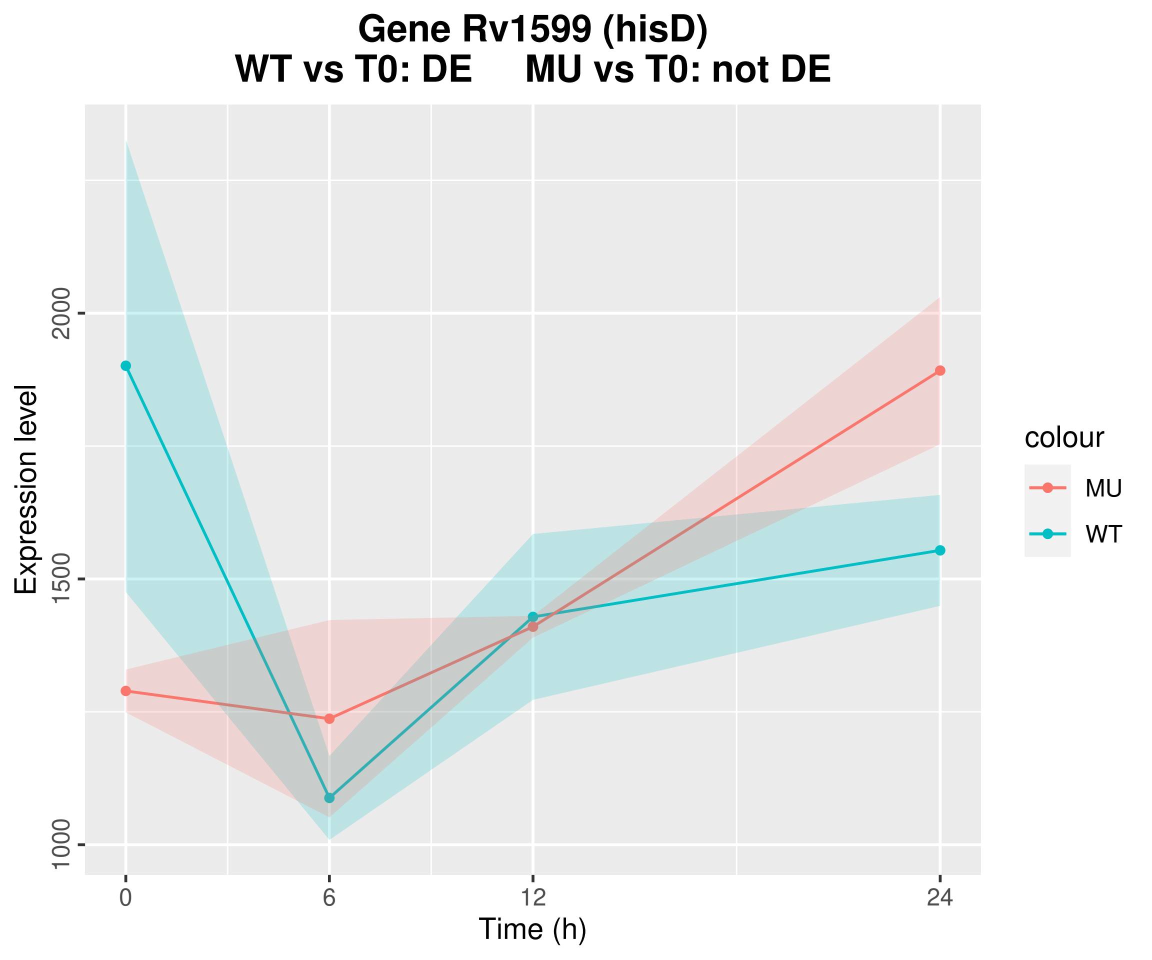
Gene Rv1599 (hisD)
WT vs T0: DE   MU vs T0: not DE
Expression level
2000
1500
1000
0
6
12
24
Time (h)
colour
MU
WT

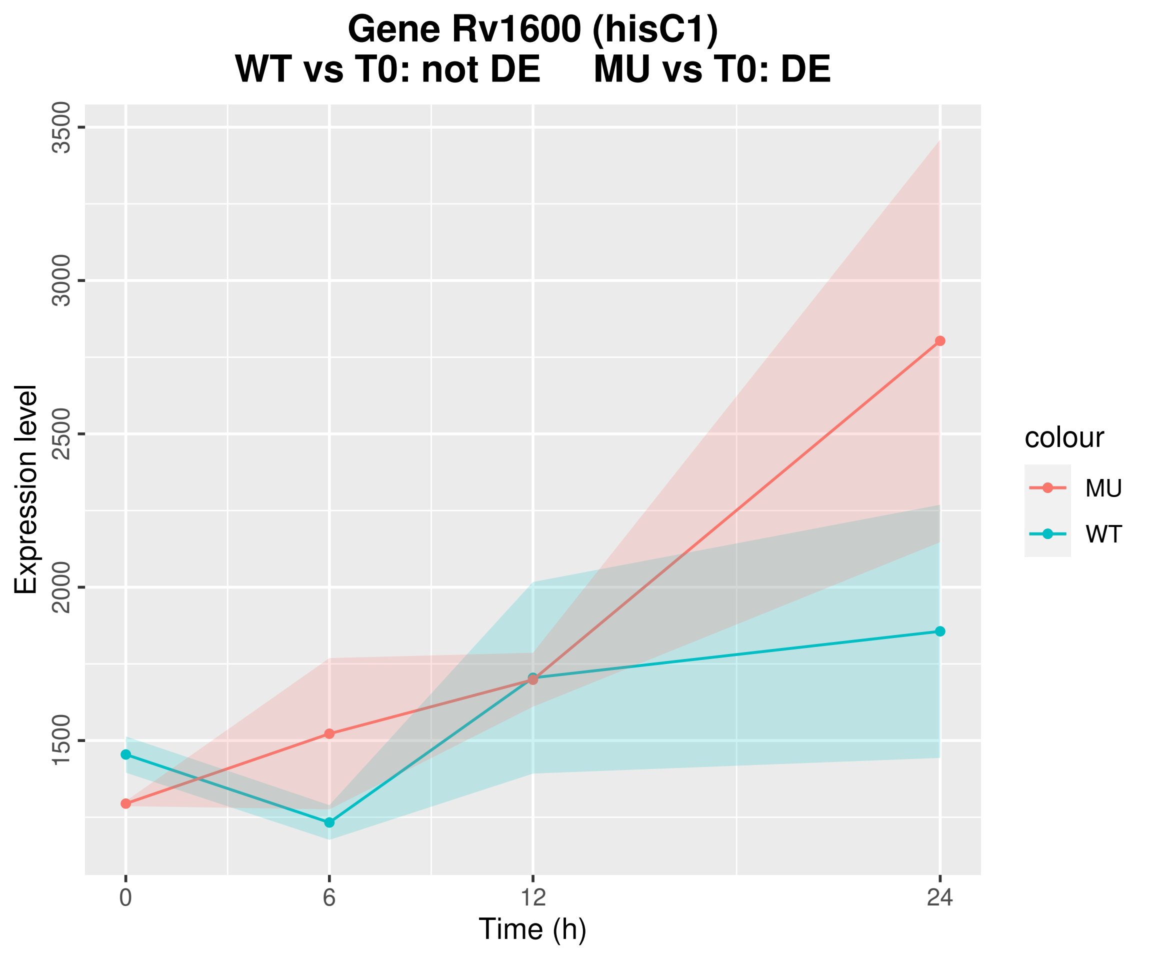

Gene Rv1600 (hisC1)
WT vs T0: not DE   MU vs T0: DE
3500
3000
2500
2000
1500
Expression level
0
6
12
24
Time (h)
colour
MU
WT

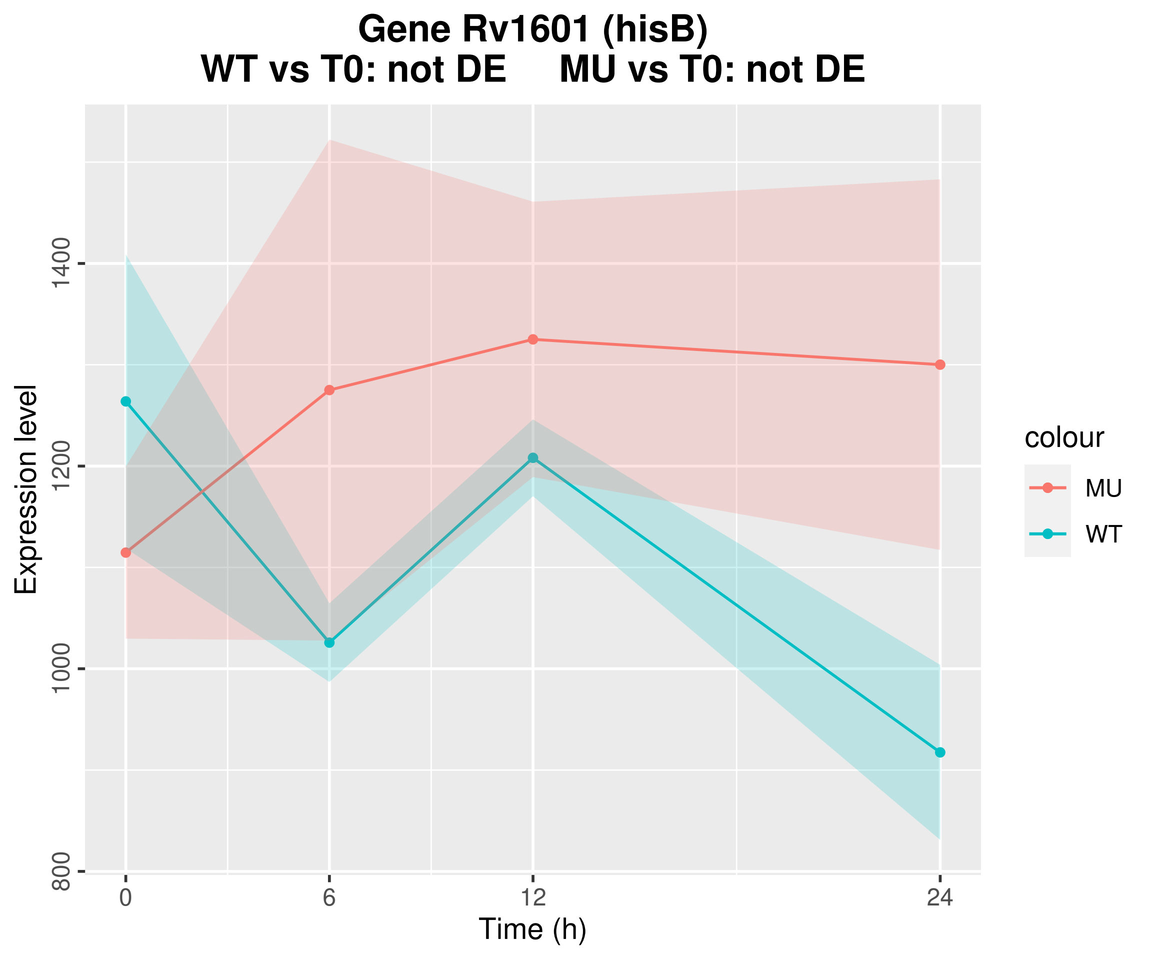
Gene Rv1601 (hisB)
WT vs T0: not DE   MU vs T0: not DE
Expression level
1400
1200
1000
800
0
6
12
24
Time (h)
colour
MU
WT

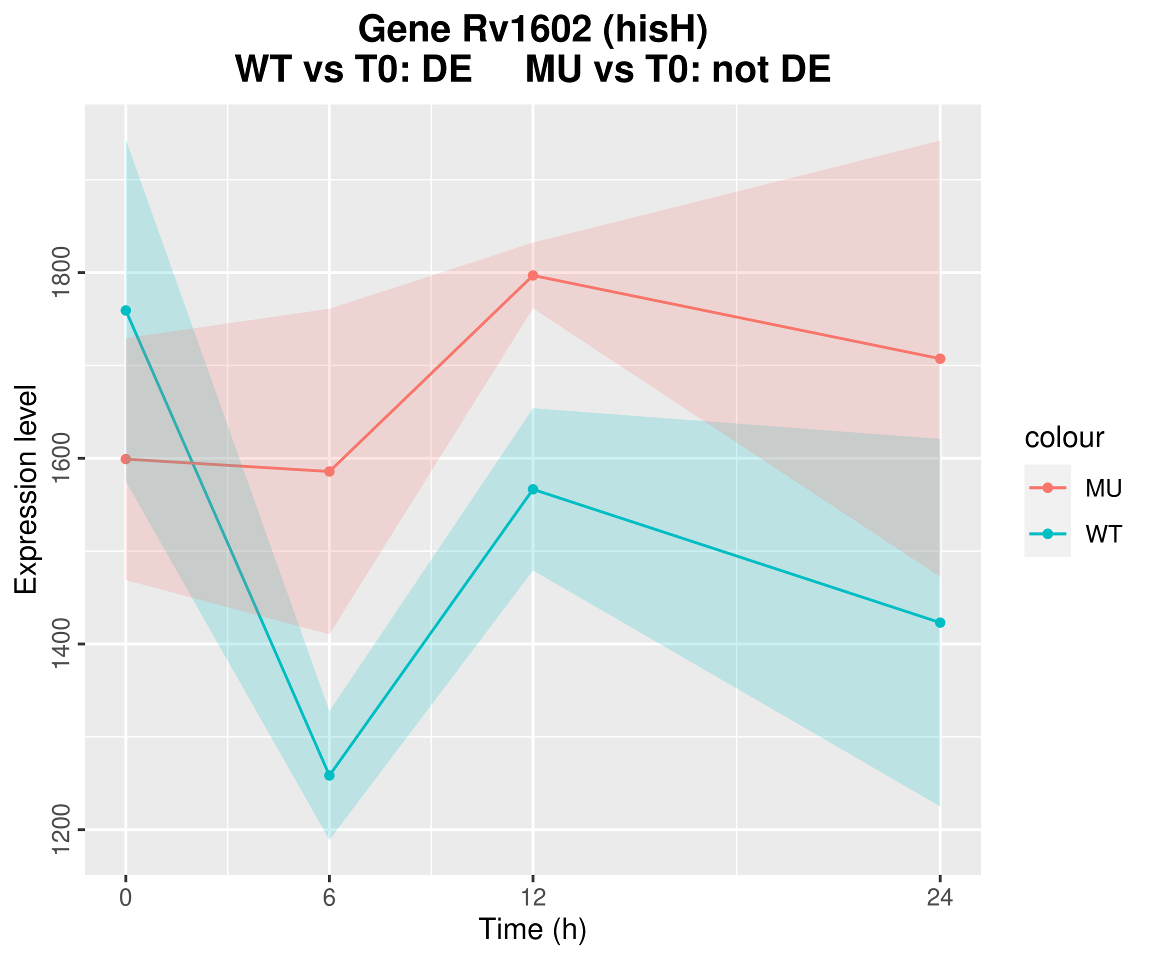
Gene Rv1602 (hisH)
WT vs T0: DE   MU vs T0: not DE
Expression level
1200
1400
1600
1800
0
6
12
24
Time (h)
colour
MU
WT

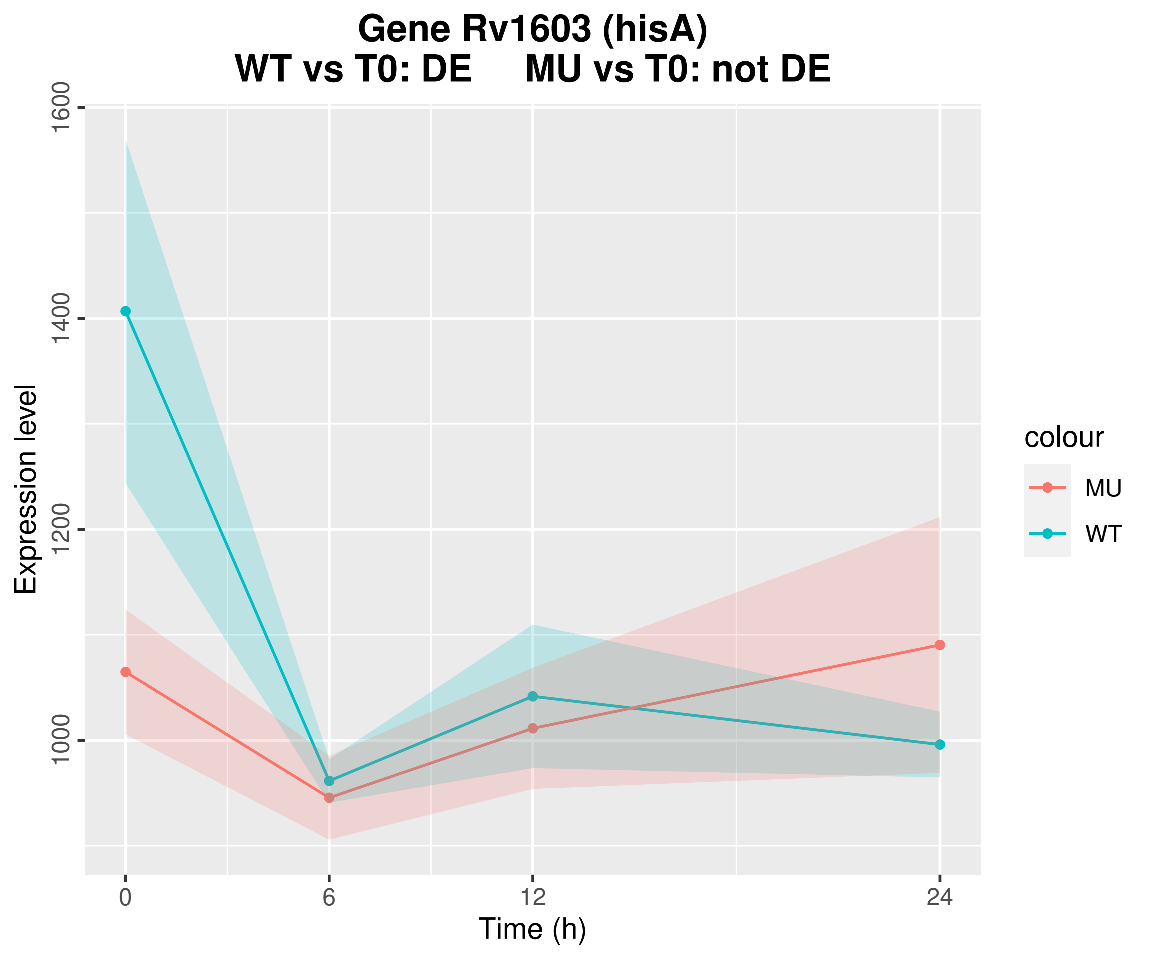
Gene Rv1603 (hisA)
WT vs T0: DE   MU vs T0: not DE
Expression level
1600
1400
1200
1000
0
6
12
24
Time (h)
colour
MU
WT

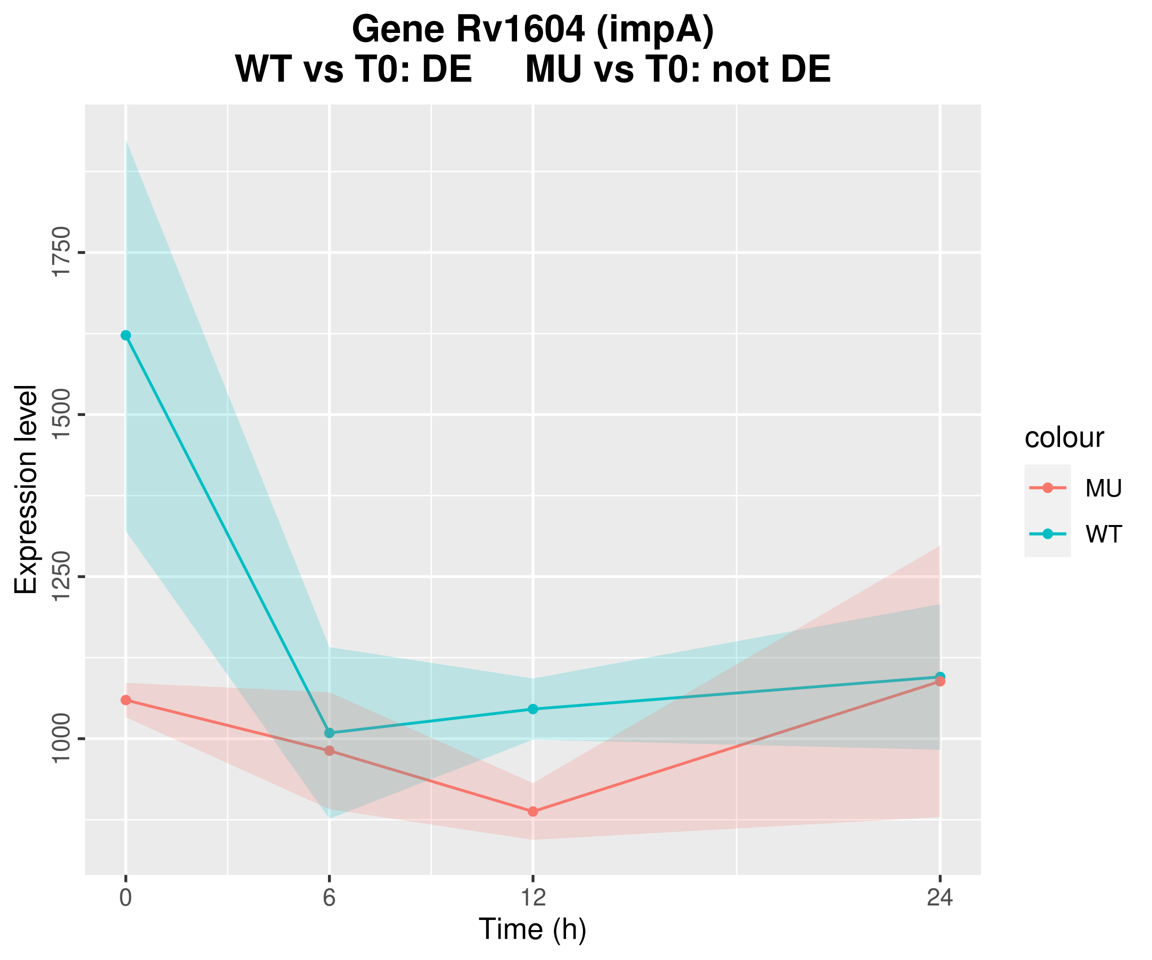

Gene Rv1604 (impA)
WT vs T0: DE MU vs T0: not DE
Expression level
1750
1500
1250
1000
0
6
12
24
Time (h)
colour
MU
WT

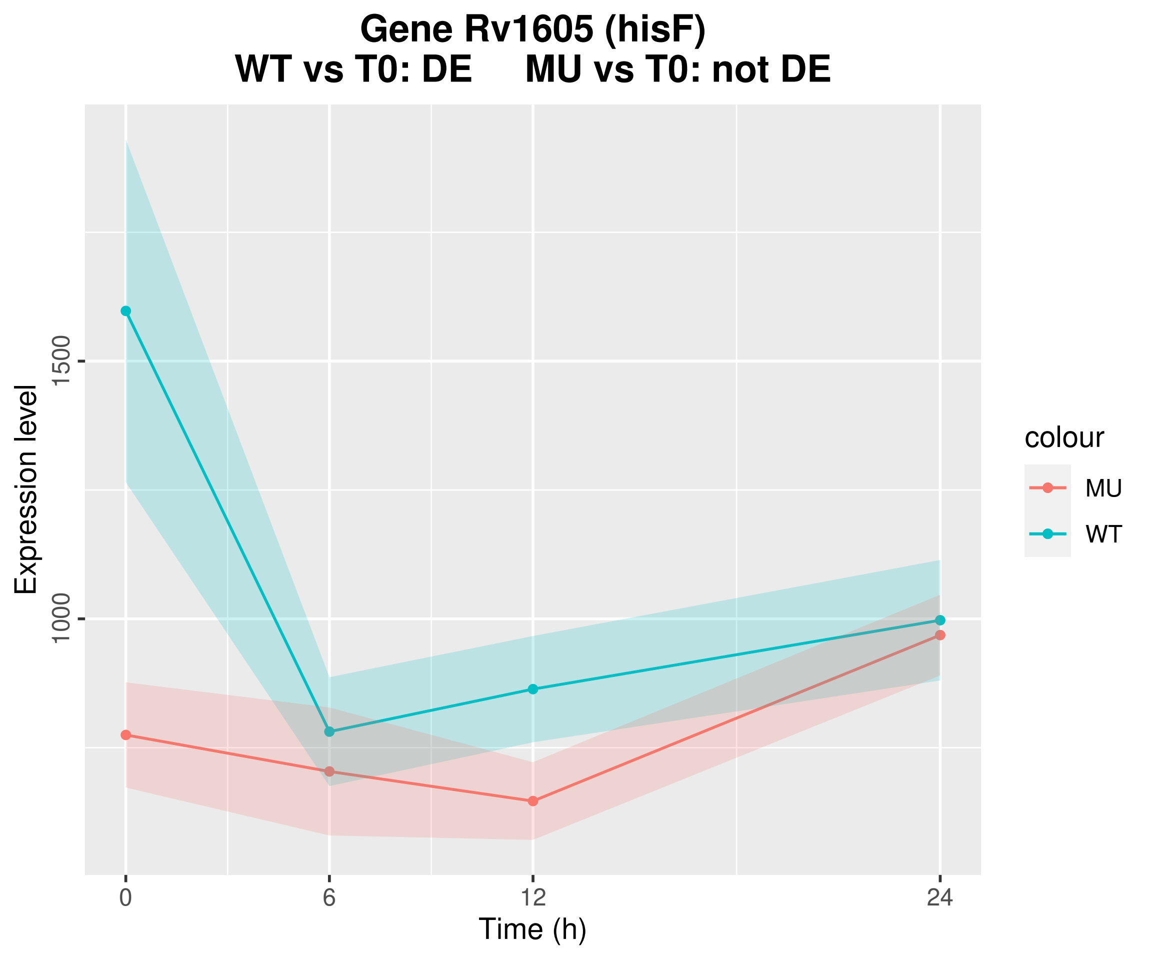
Gene Rv1605 (hisF)
WT vs T0: DE   MU vs T0: not DE
Expression level
1500
1000
0
6
12
24
Time (h)
colour
MU
WT

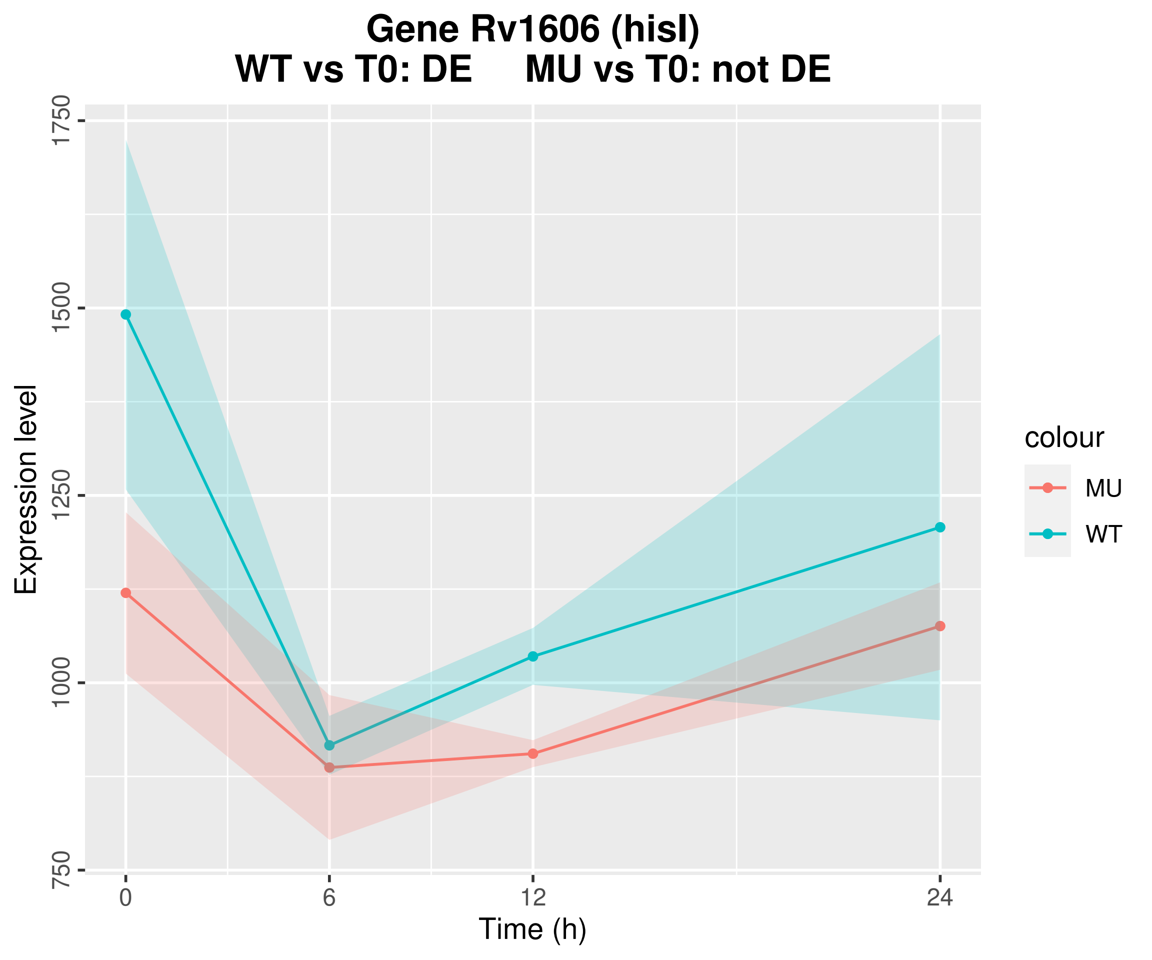
Gene Rv1606 (hisI)
WT vs T0: DE MU vs T0: not DE
Expression level
1750
1500
1250
1000
750
0
6
12
24
Time (h)
colour
MU
WT

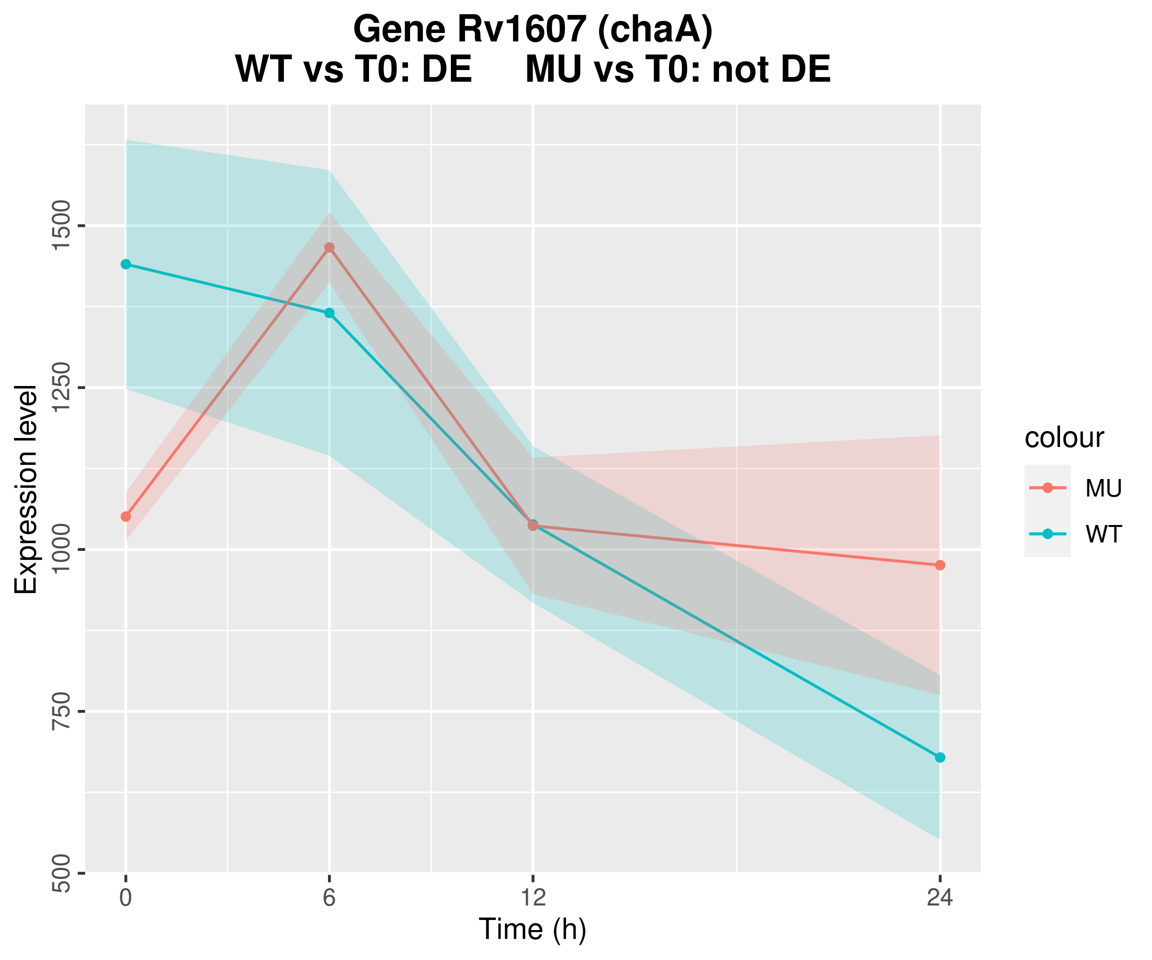
Gene Rv1607 (chaA)
WT vs T0: DE   MU vs T0: not DE
Expression level
1500
1250
1000
750
500
0
6
12
24
Time (h)
colour
MU
WT

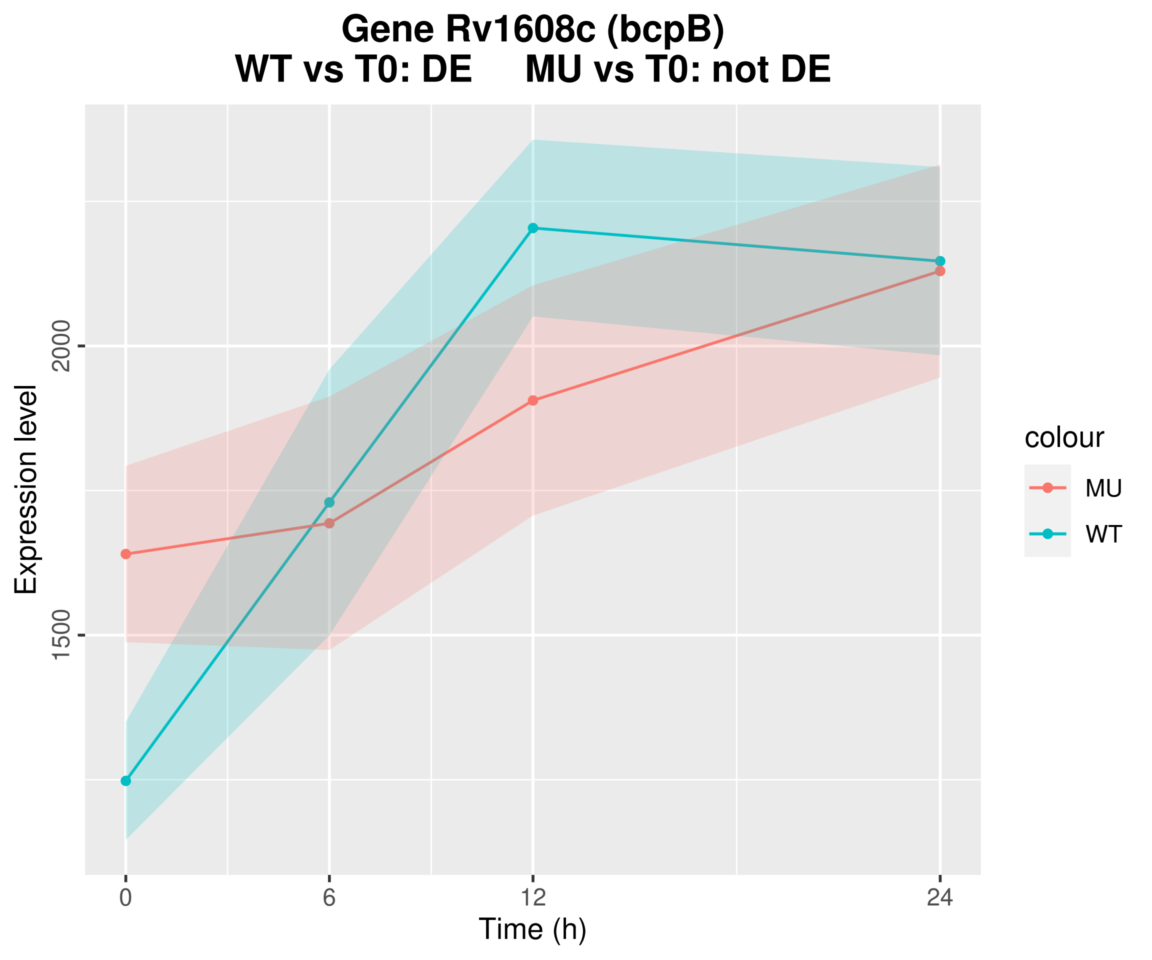

Gene Rv1608c (bcpB)
WT vs T0: DE   MU vs T0: not DE
Expression level
2000
1500
0
6
12
24
Time (h)
colour
MU
WT

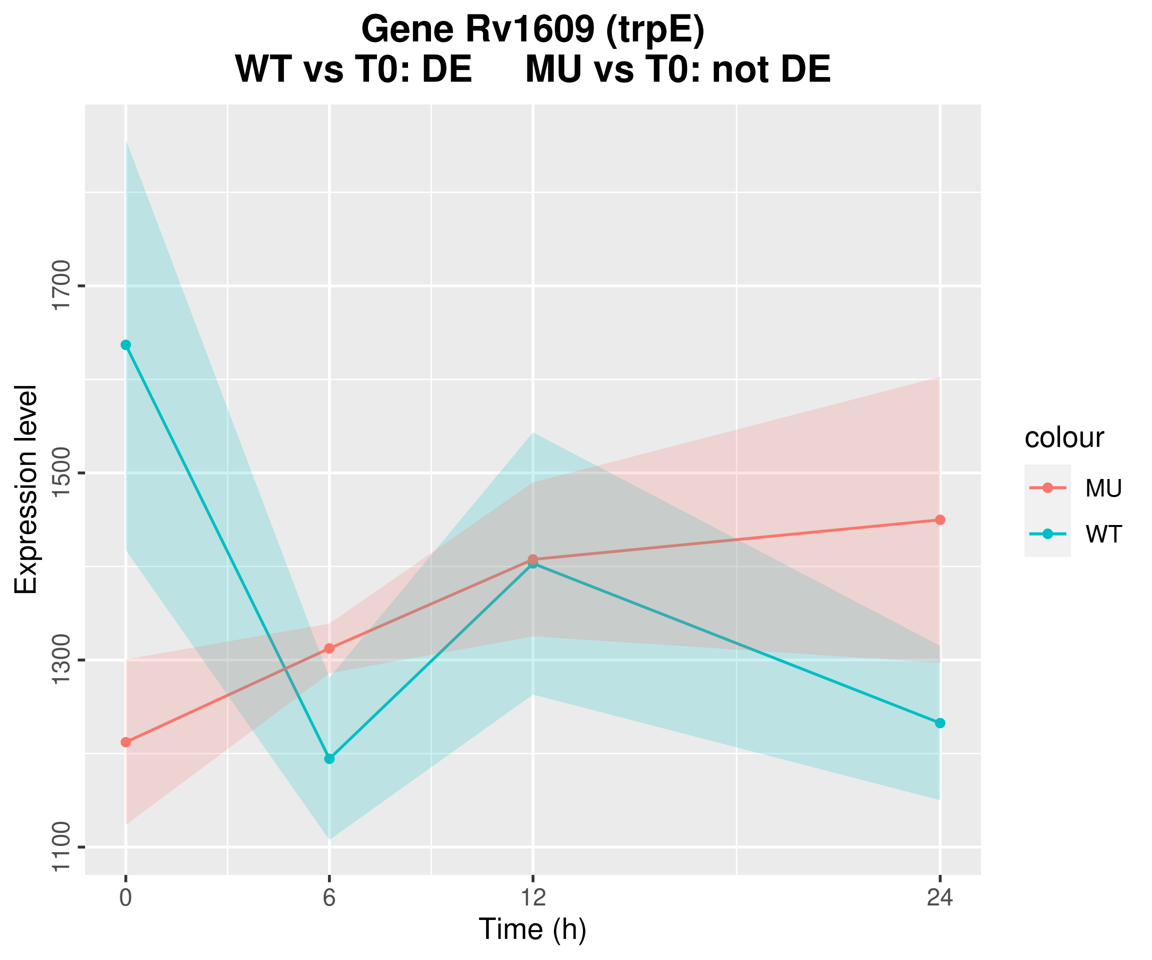

Gene Rv1609 (trpE)
WT vs T0: DE MU vs T0: not DE
Expression level
1100
1300
1500
1700
Time (h)
0
6
12
24
colour
MU
WT

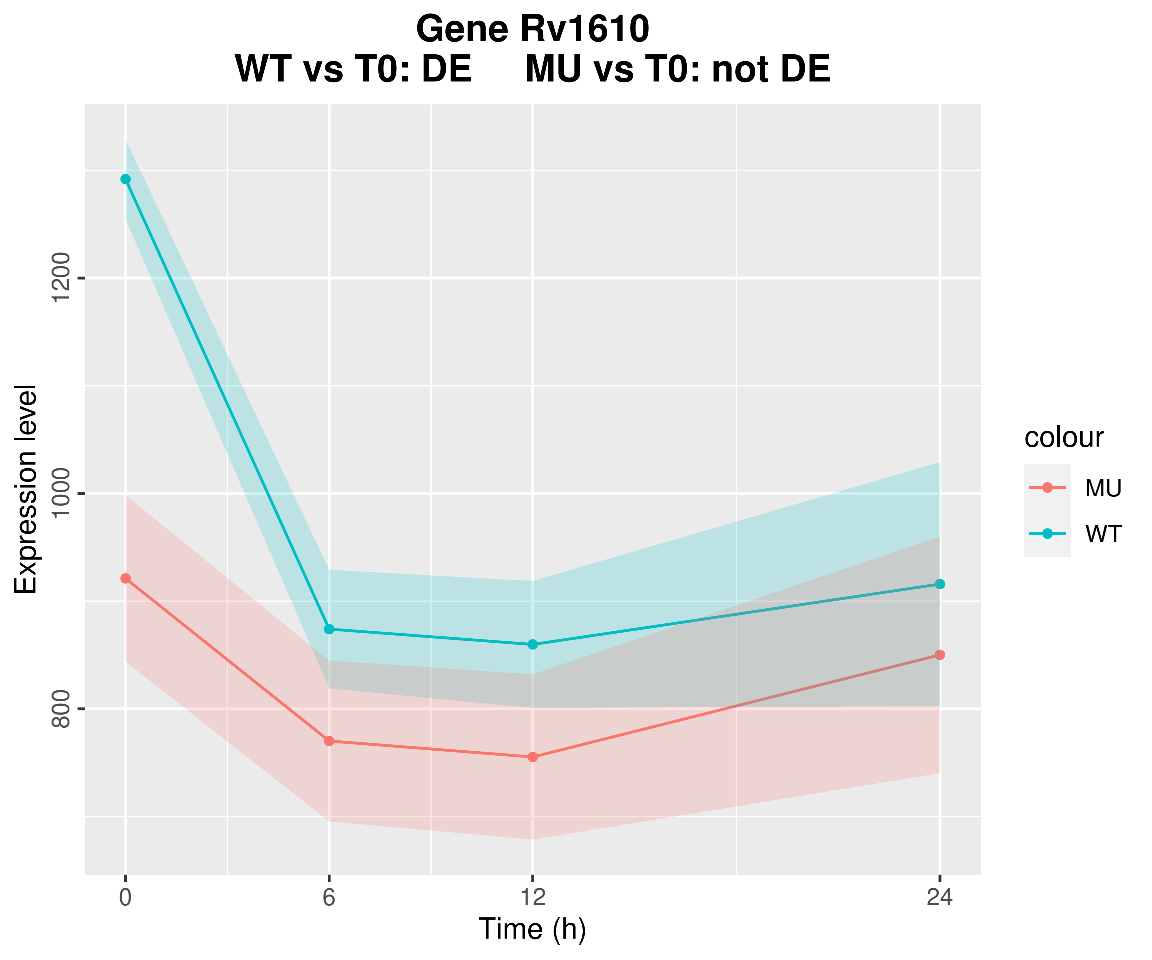
Gene Rv1610
WT vs T0: DE MU vs T0: not DE
Expression level
1200
1000
800
0
6
12
24
Time (h)
colour
MU
WT

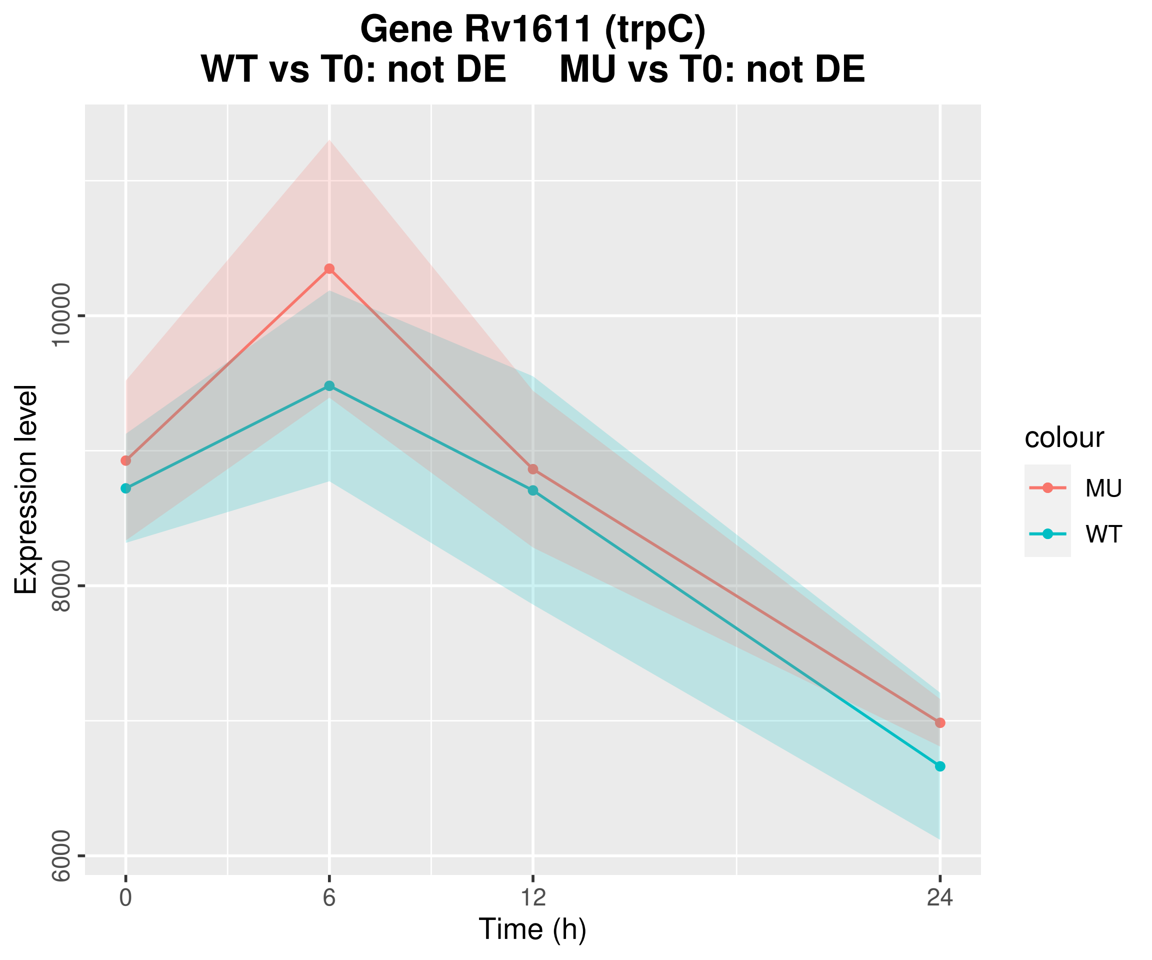

Gene Rv1611 (trpC)
WT vs T0: not DE MU vs T0: not DE
Expression level
10000
8000
6000
0
6
12
24
Time (h)
colour
MU
WT

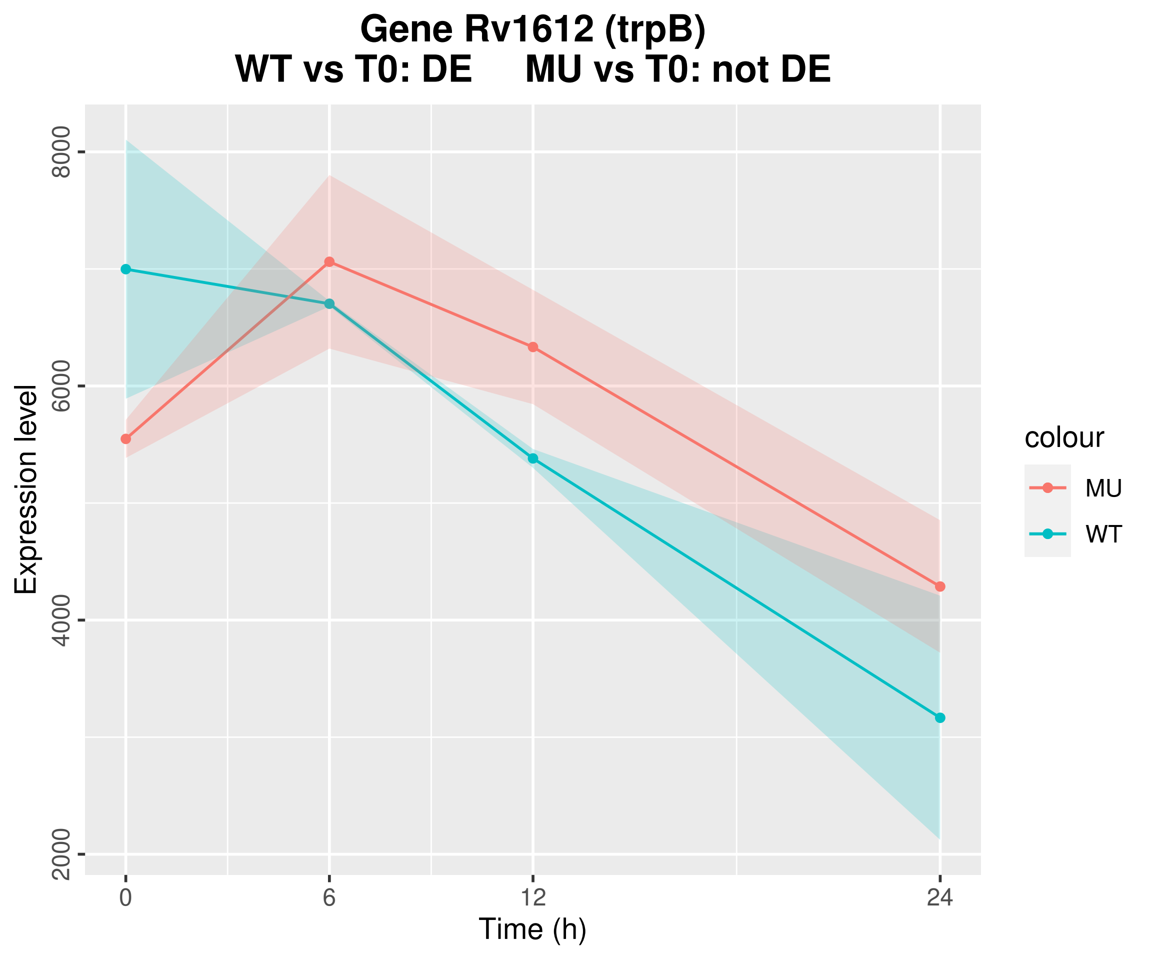

Gene Rv1612 (trpB)
WT vs T0: DE MU vs T0: not DE
8000
6000
4000
2000
Expression level
0
6
12
24
Time (h)
colour
MU
WT

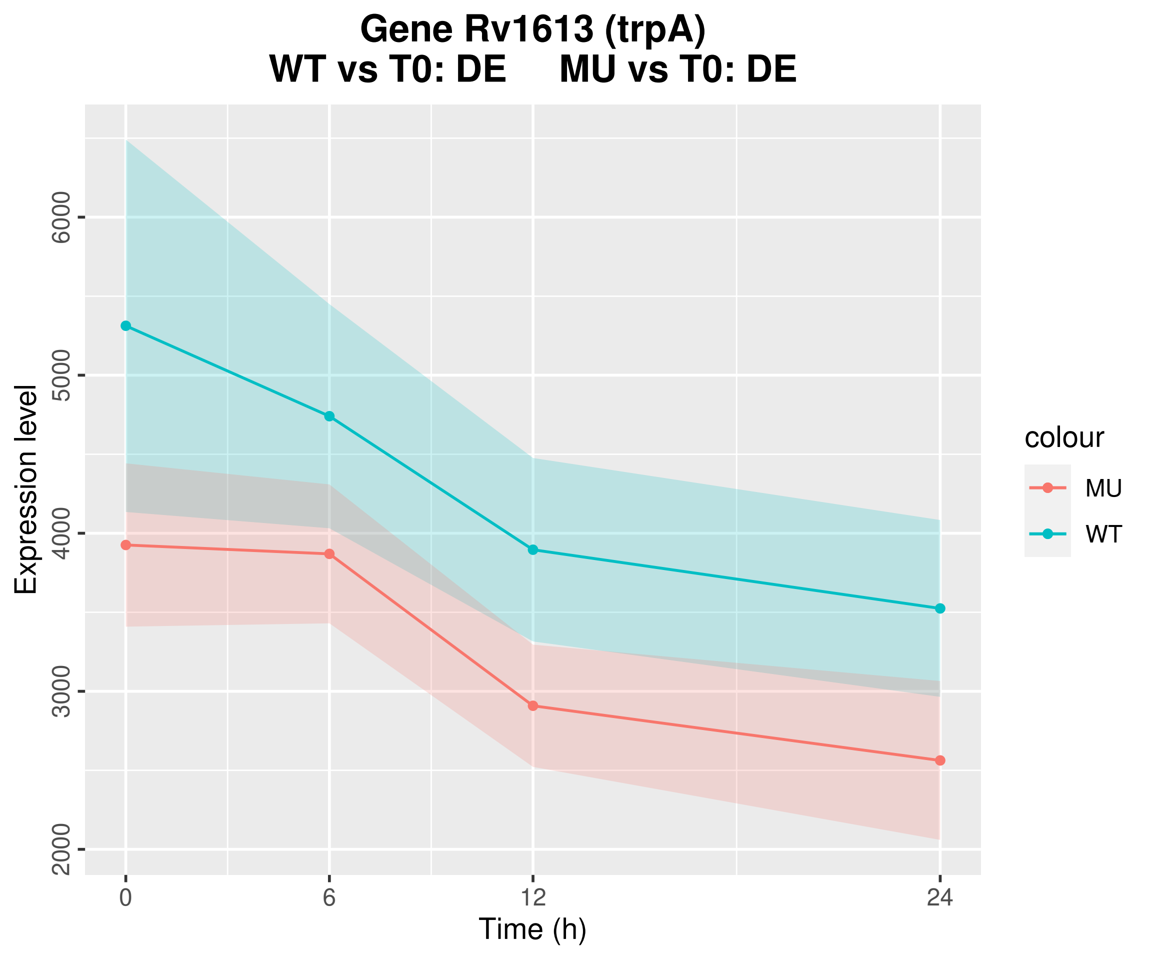

Gene Rv1613 (trpA)
WT vs T0: DE   MU vs T0: DE
Expression level
2000
3000
4000
5000
6000
0
6
12
24
Time (h)
colour
MU
WT

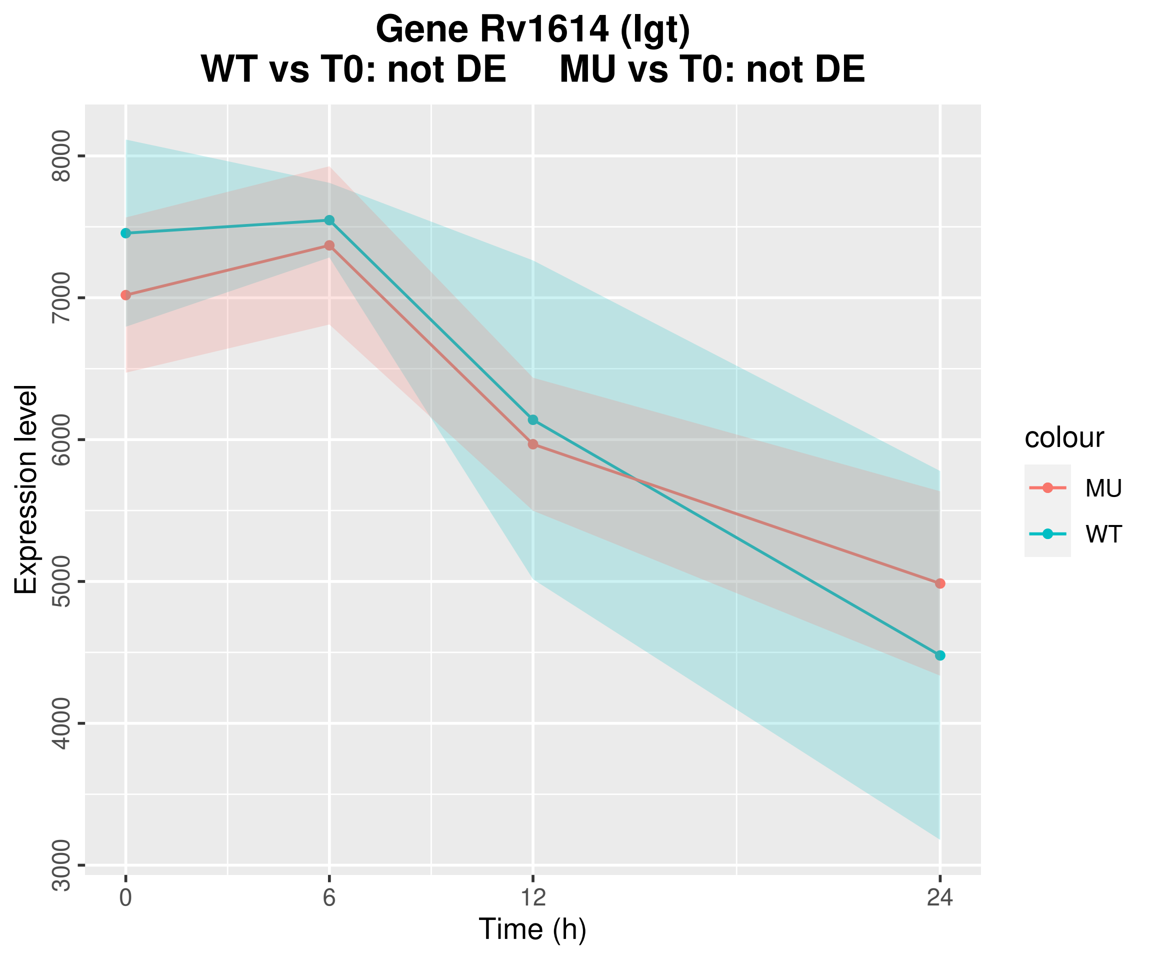

Gene Rv1614 (lgt)
WT vs T0: not DE MU vs T0: not DE
Expression level
8000
7000
6000
5000
4000
3000
0
6
12
24
Time (h)
colour
MU
WT

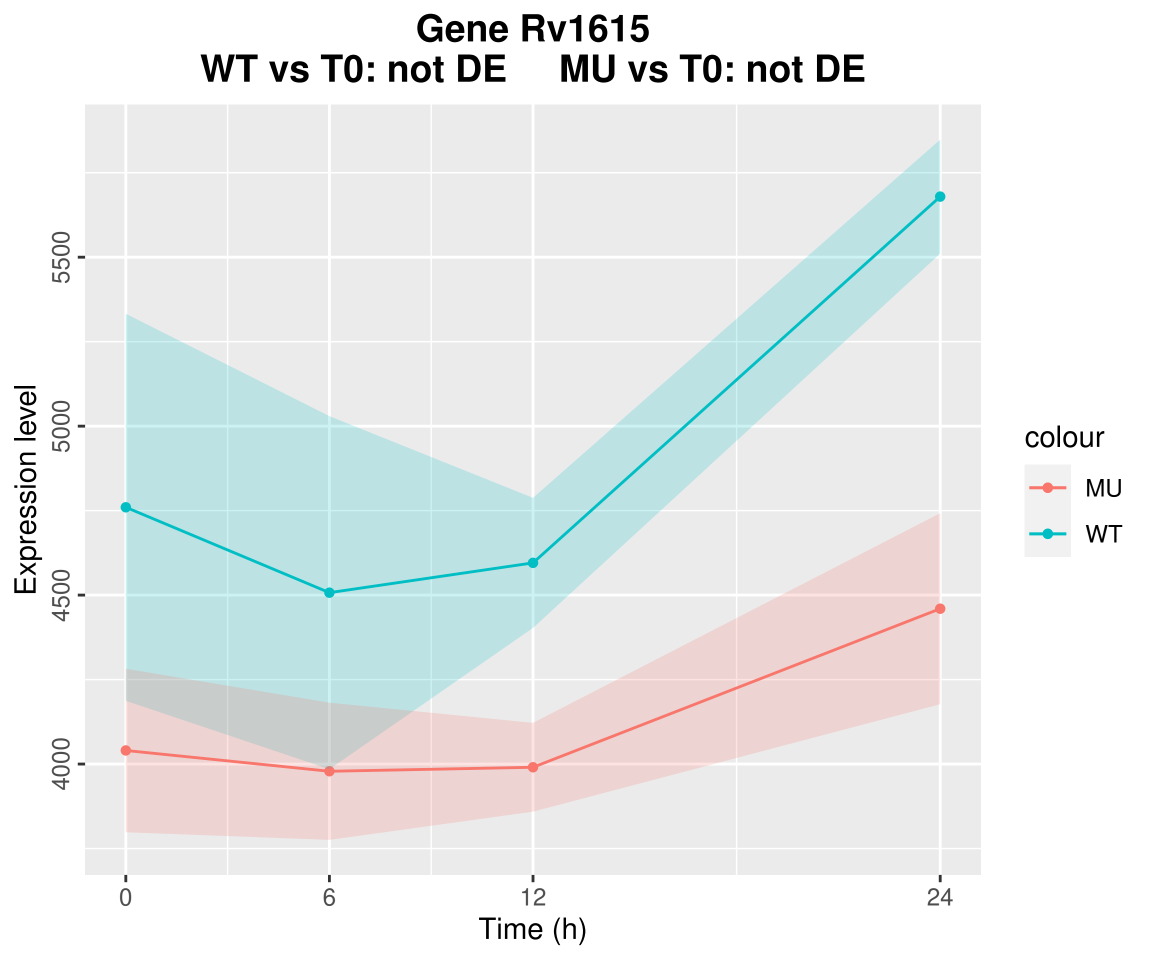

Gene Rv1615
WT vs T0: not DE MU vs T0: not DE
Expression level
5500
5000
4500
4000
0
6
12
24
Time (h)
colour
MU
WT

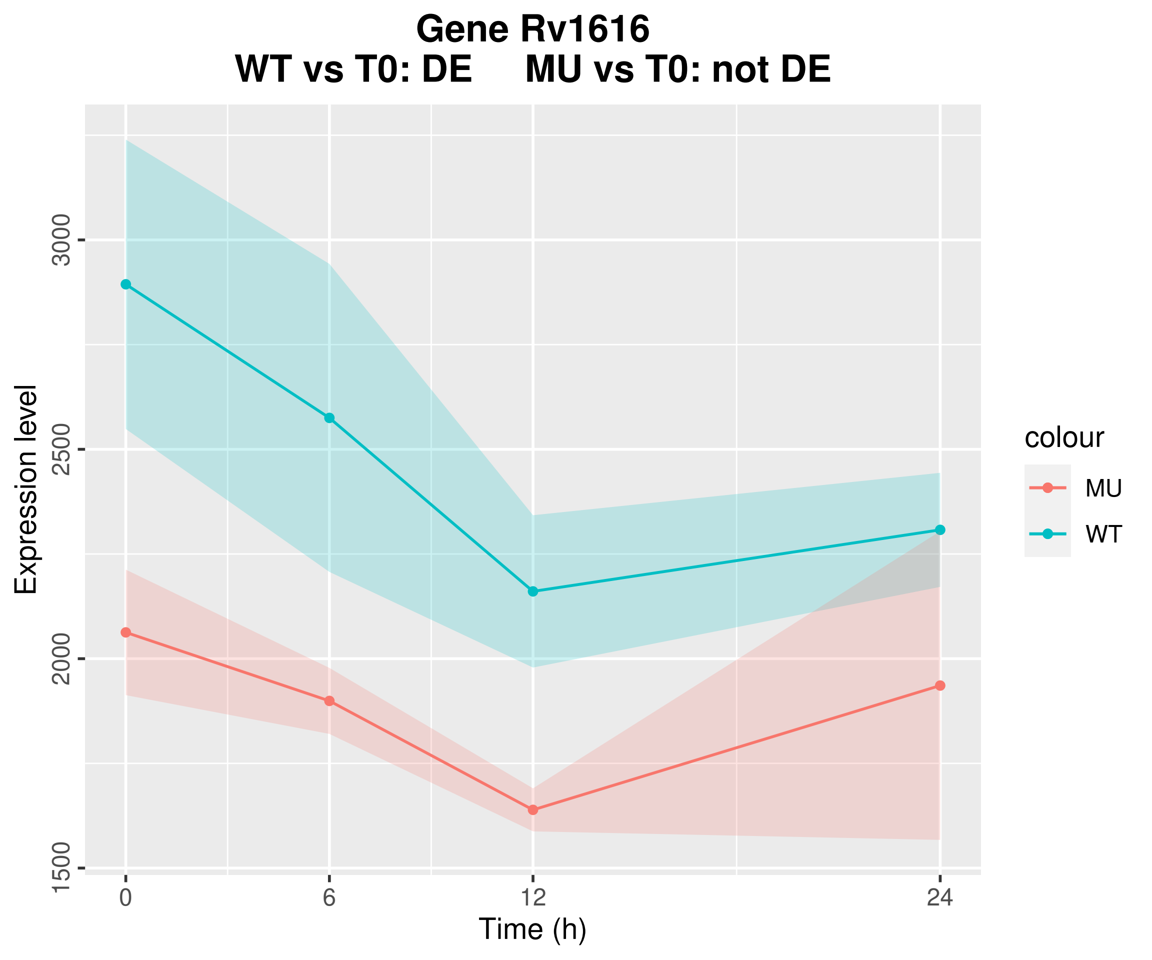
Gene Rv1616
WT vs T0: DE   MU vs T0: not DE
Expression level
3000
2500
2000
1500
0
6
12
24
Time (h)
colour
MU
WT

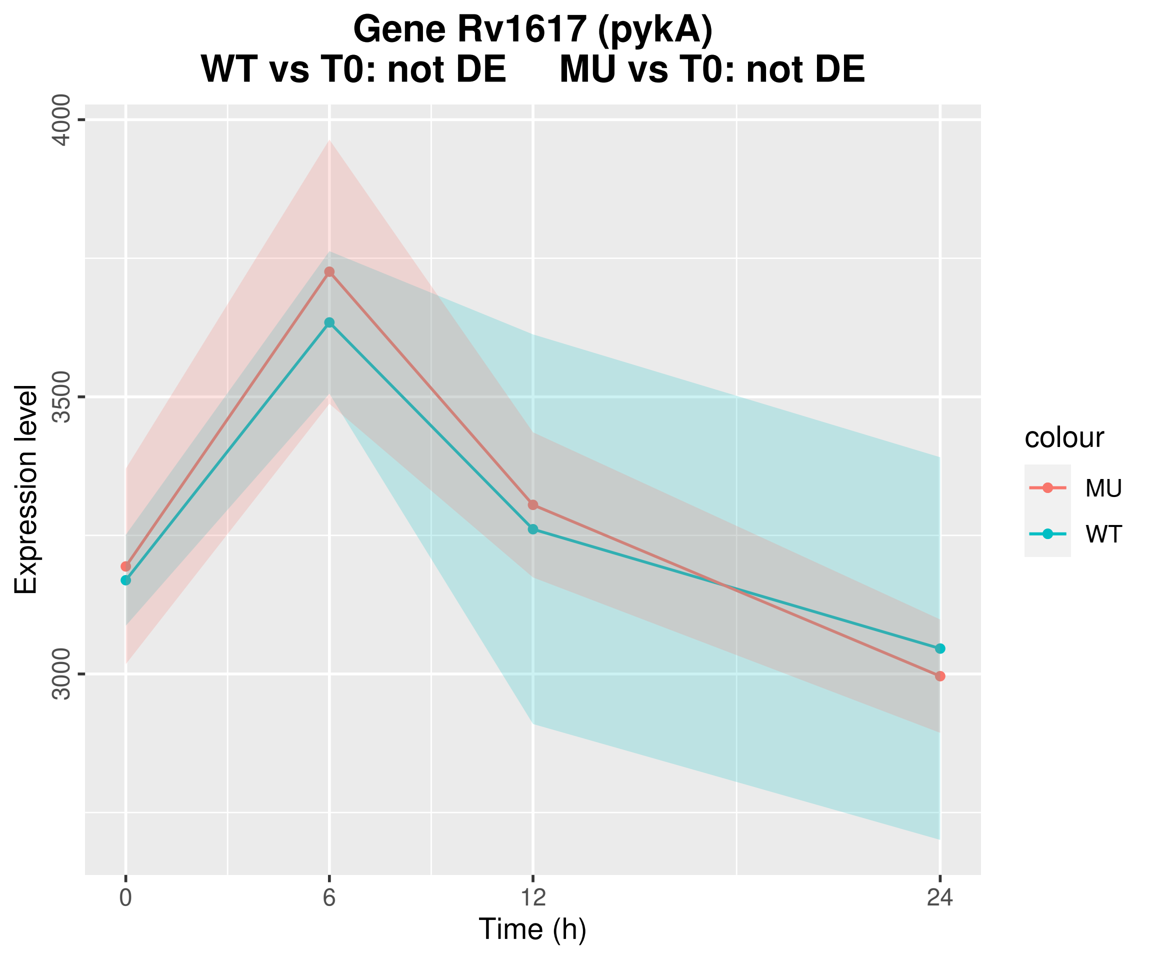

Gene Rv1617 (pykA)
WT vs T0: not DE   MU vs T0: not DE
4000
3500
3000
Expression level
0
6
12
24
Time (h)
colour
MU
WT

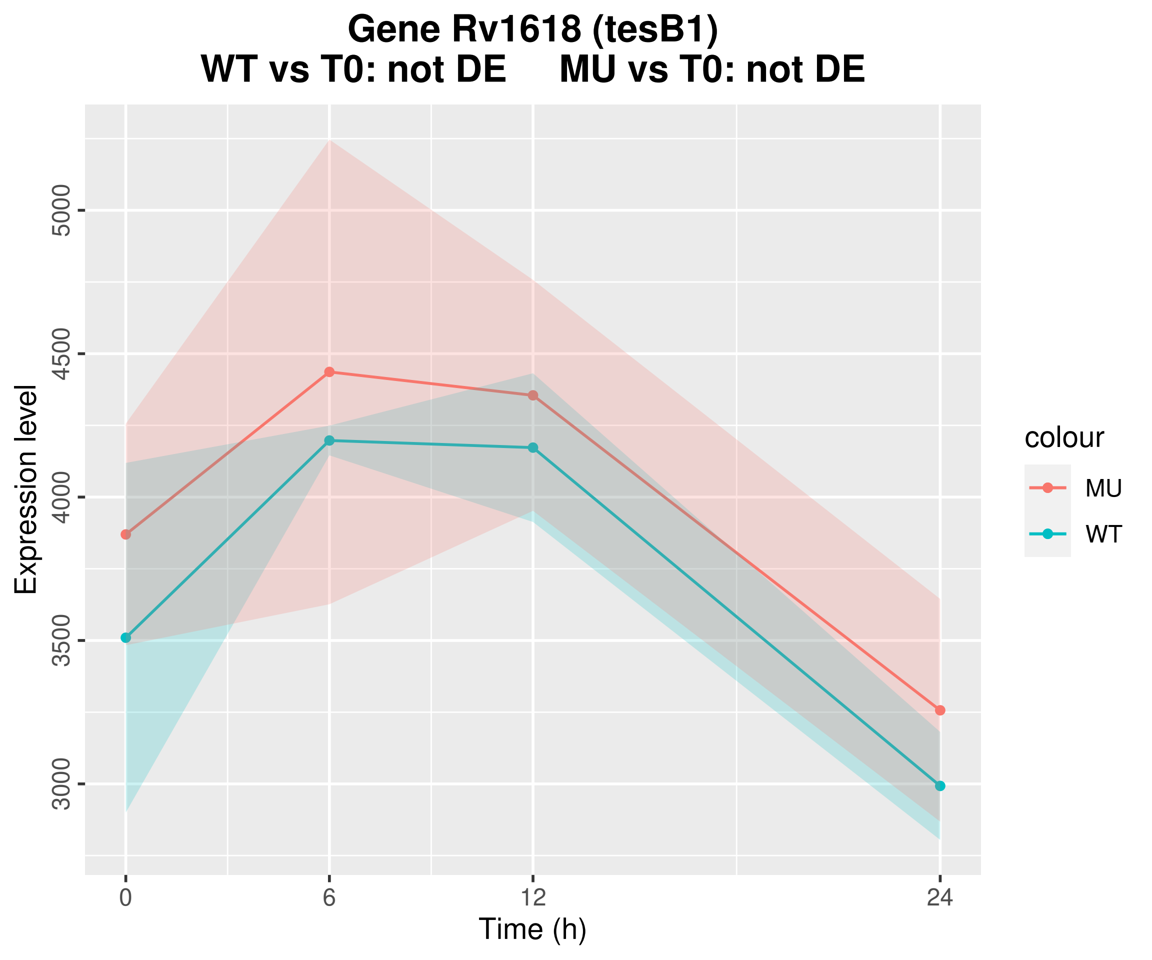

Gene Rv1618 (tesB1)
WT vs T0: not DE MU vs T0: not DE
Expression level
5000
4500
4000
3500
3000
0
6
12
24
Time (h)
colour
MU
WT

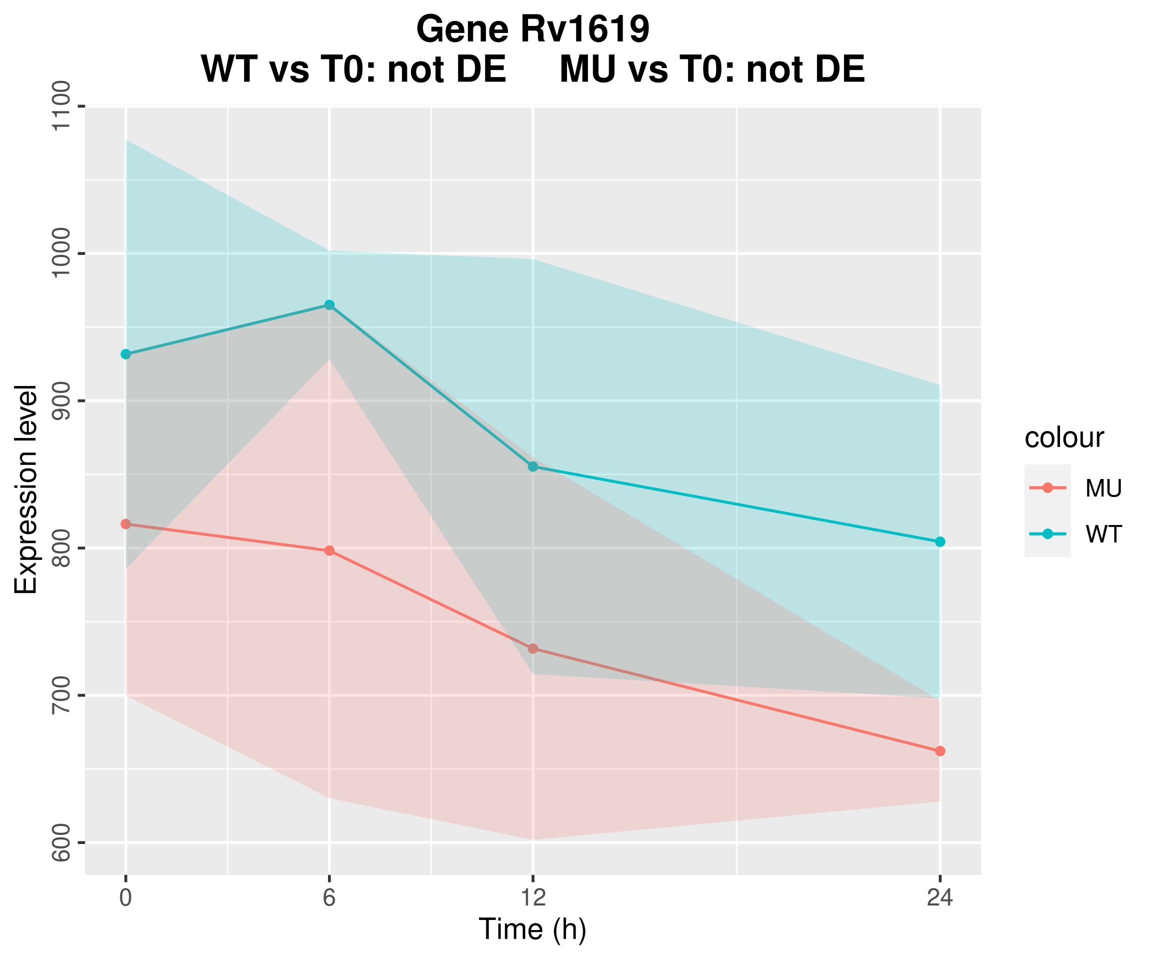

Gene Rv1619
WT vs T0: not DE MU vs T0: not DE
Expression level
1100
1000
900
800
700
600
0
6
12
24
Time (h)
colour
MU
WT

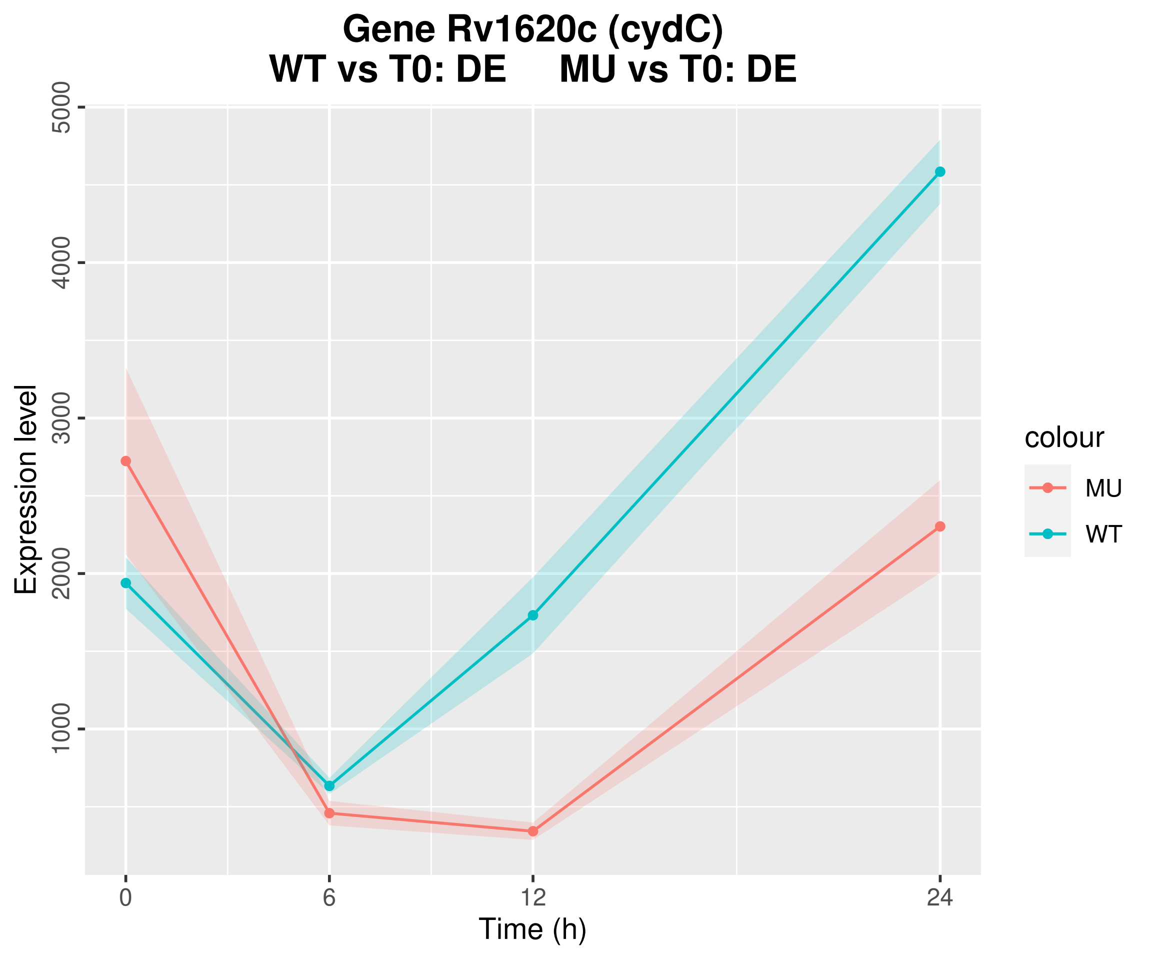

Gene Rv1620c (cydC)
WT vs T0: DE MU vs T0: DE
Expression level
5000
4000
3000
2000
1000
0
6
12
24
Time (h)
colour
MU
WT

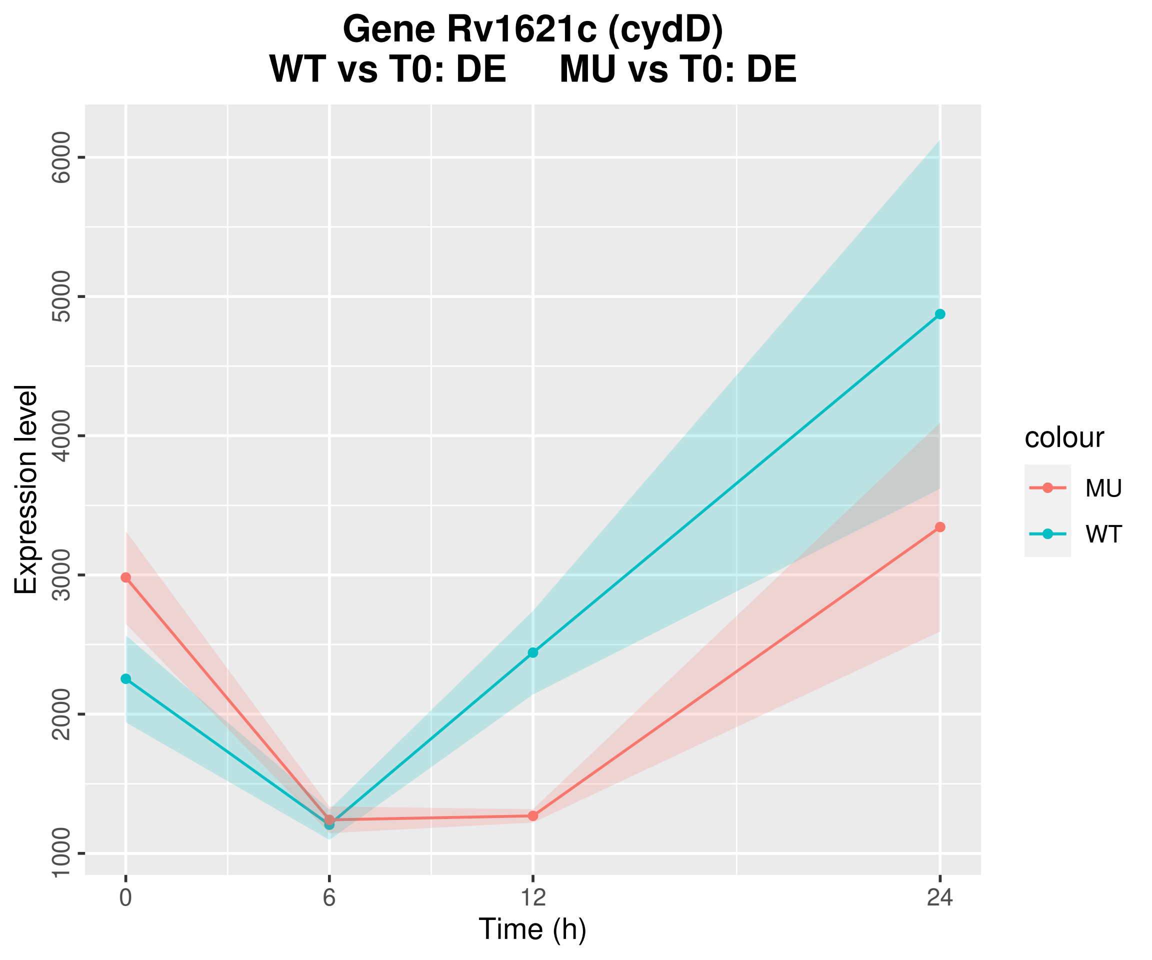
Gene Rv1621c (cydD)
WT vs T0: DE   MU vs T0: DE
Expression level
6000
5000
4000
3000
2000
1000
0
6
12
24
Time (h)
colour
MU
WT

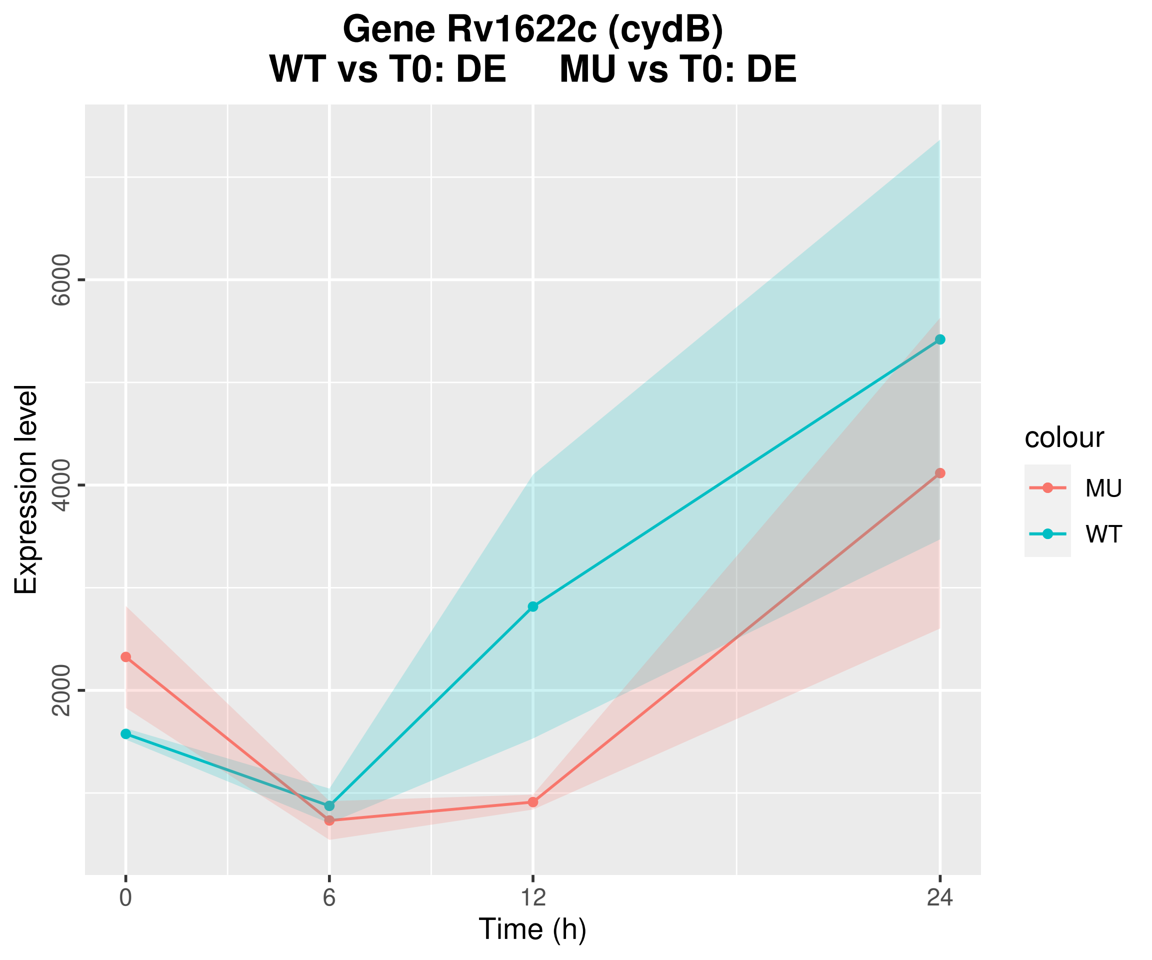
Gene Rv1622c (cydB)
WT vs T0: DE   MU vs T0: DE
Expression level
6000
4000
2000
0
6
12
24
Time (h)
colour
MU
WT

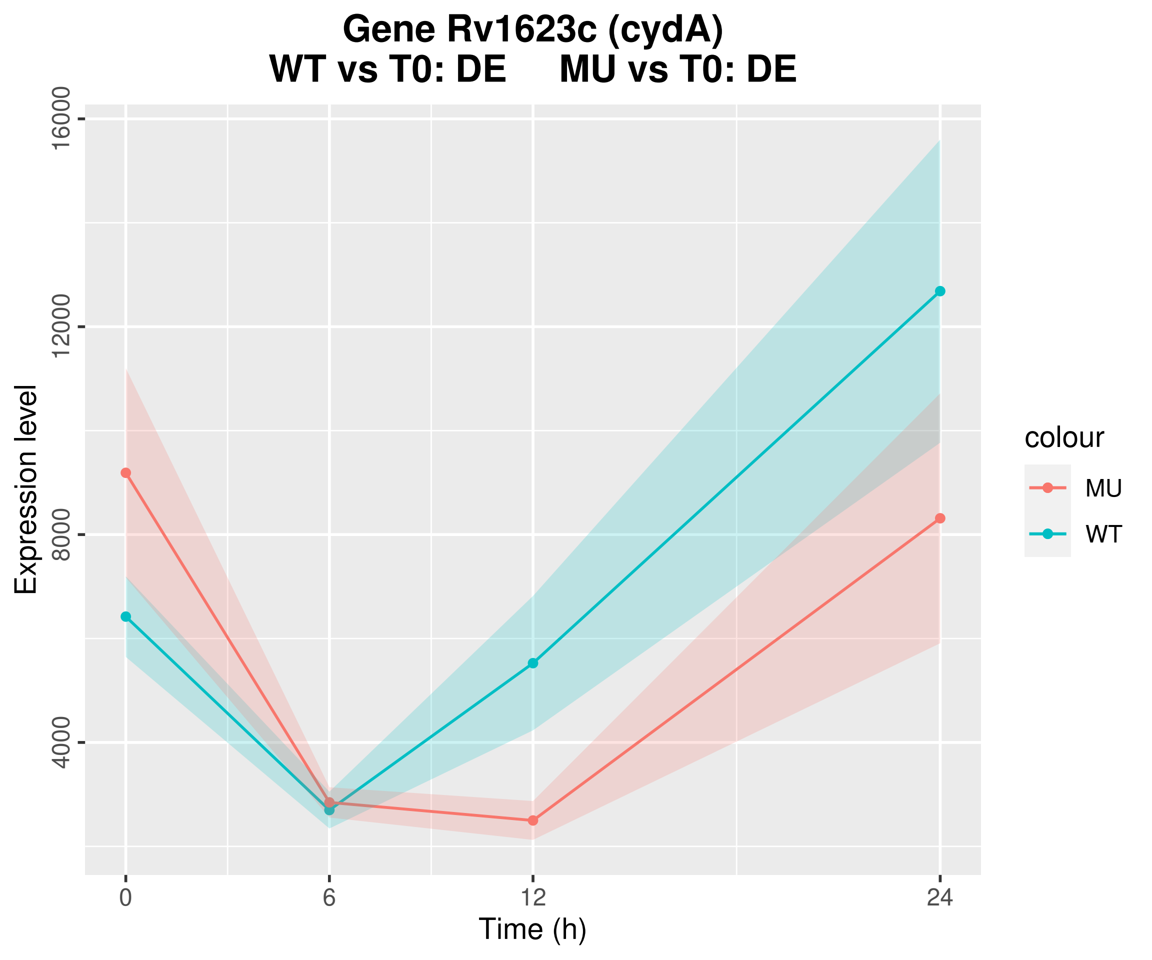
Gene Rv1623c (cydA)
WT vs T0: DE   MU vs T0: DE
Expression level
16000
12000
8000
4000
0
6
12
24
Time (h)
colour
MU
WT

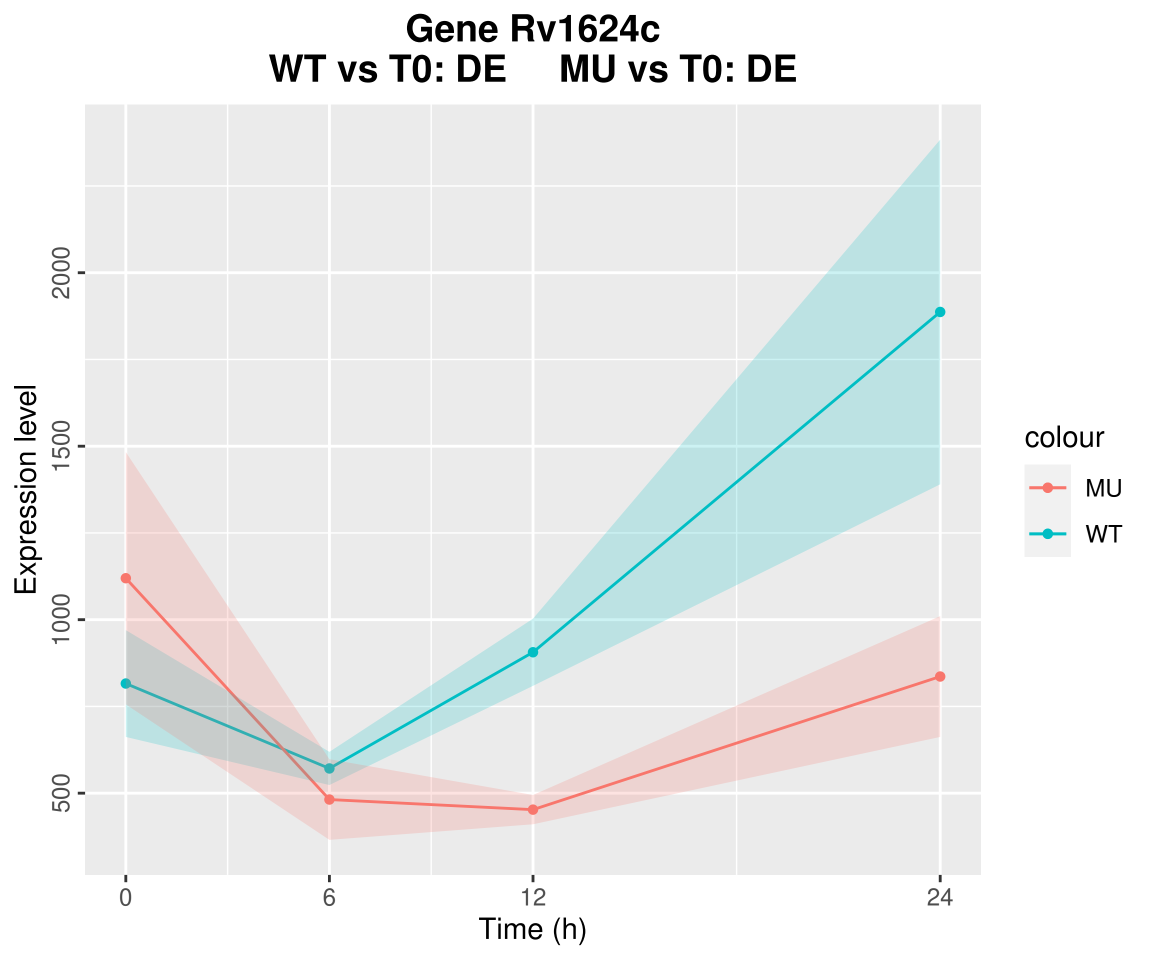
Gene Rv1624c
WT vs T0: DE MU vs T0: DE
Expression level
2000
1500
1000
500
0
6
12
24
Time (h)
colour
MU
WT

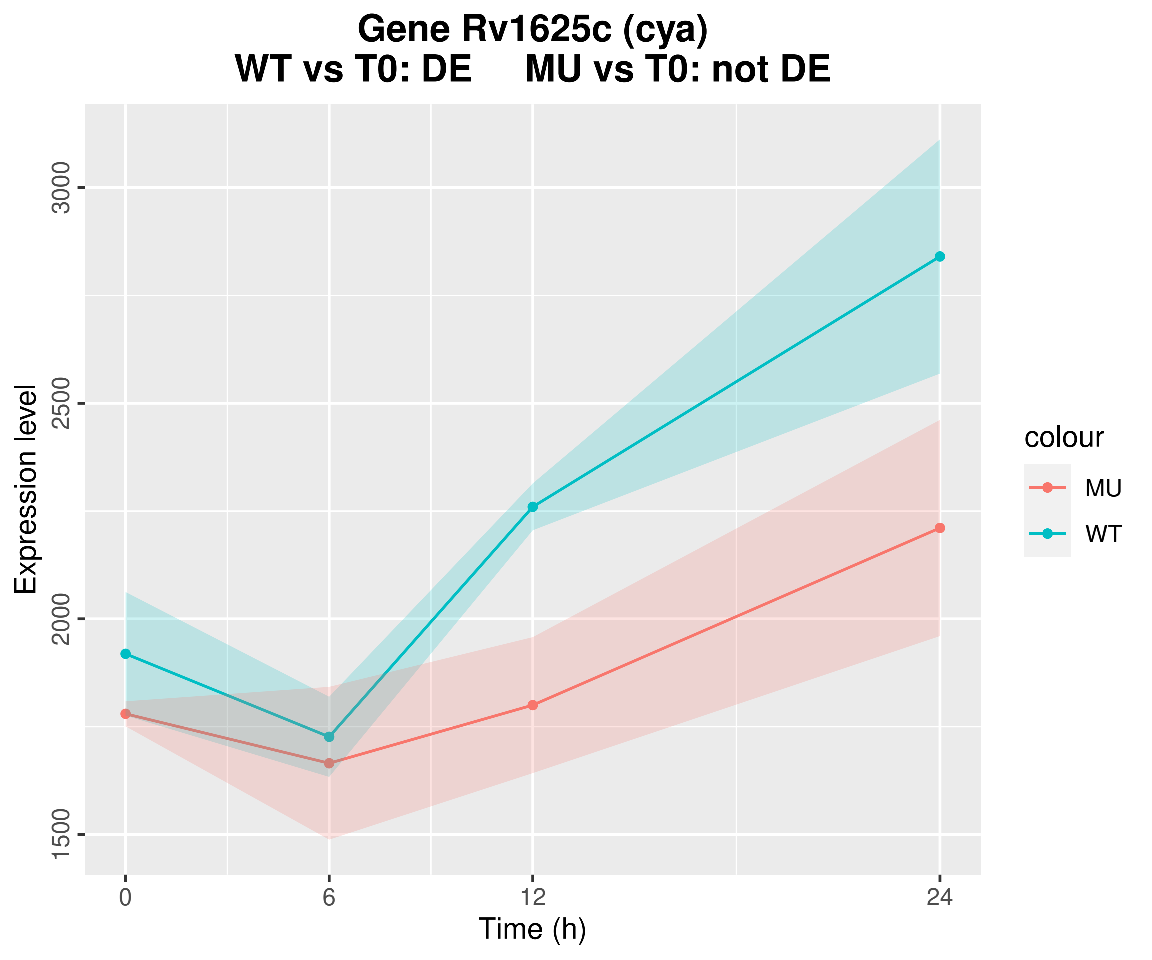
Gene Rv1625c (cya)
WT vs T0: DE MU vs T0: not DE
Expression level
3000
2500
2000
1500
0
6
12
24
Time (h)
colour
MU
WT

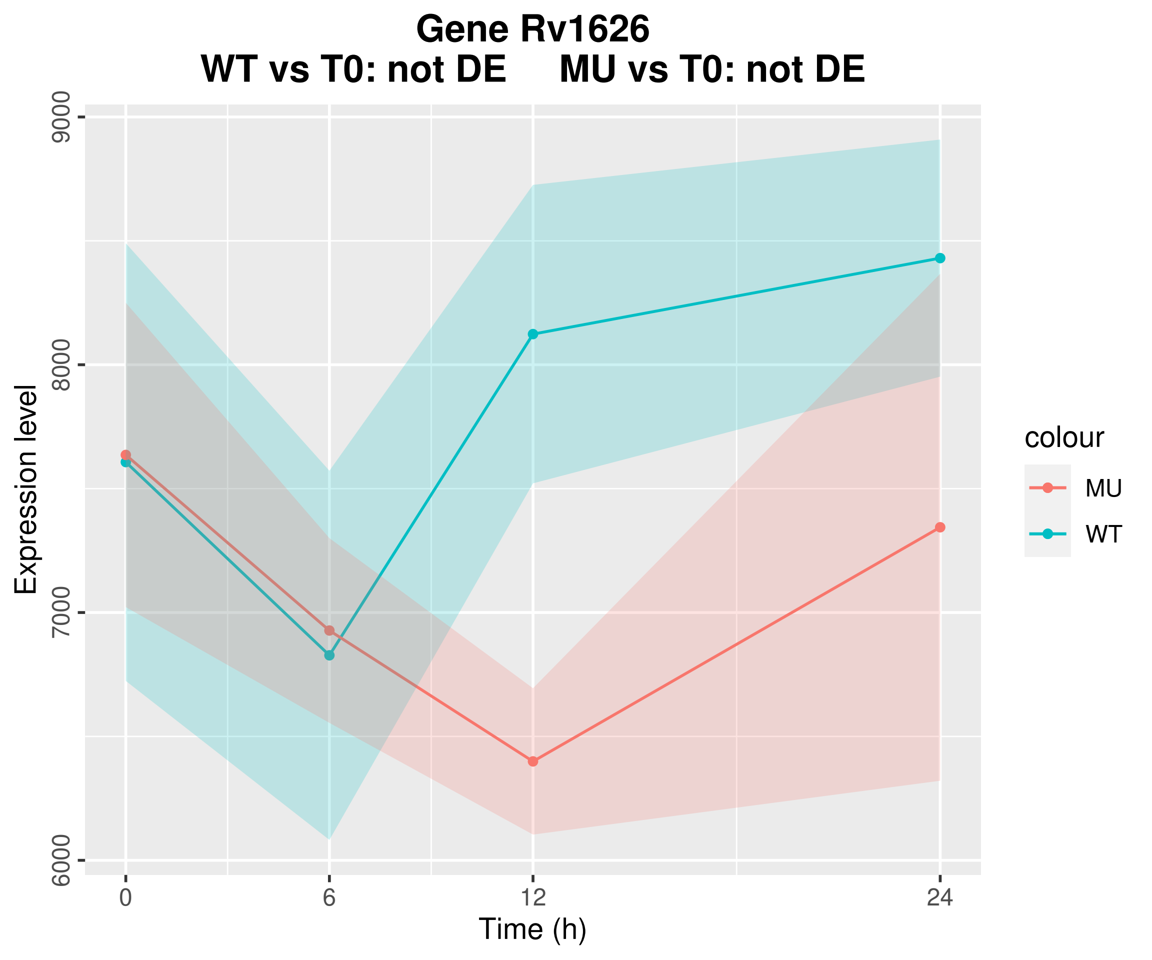
Gene Rv1626
WT vs T0: not DE MU vs T0: not DE
9000
8000
7000
6000
Expression level
0
6
12
24
Time (h)
colour
MU
WT

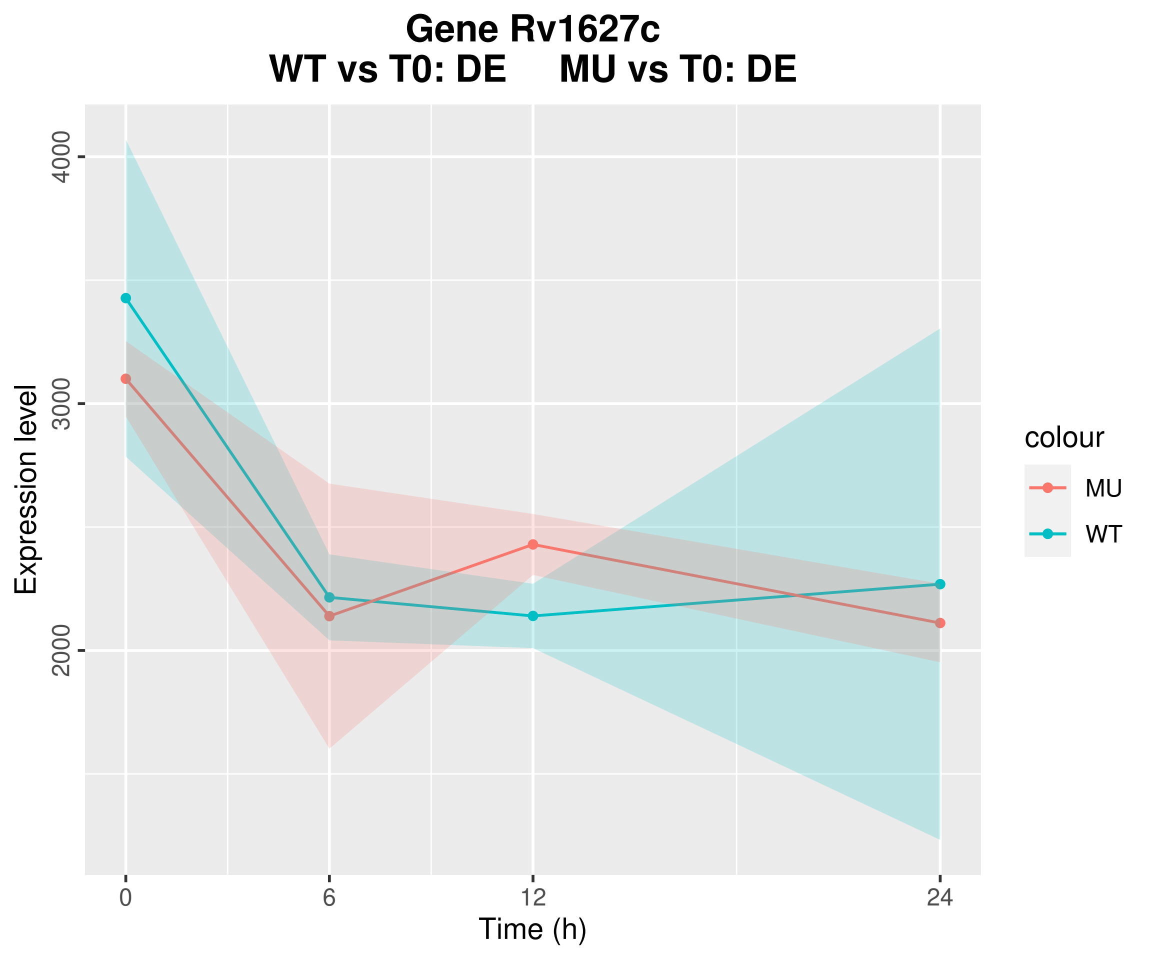
Gene Rv1627c
WT vs T0: DE MU vs T0: DE
Expression level
4000
3000
2000
Time (h)
0
6
12
24
colour
MU
WT

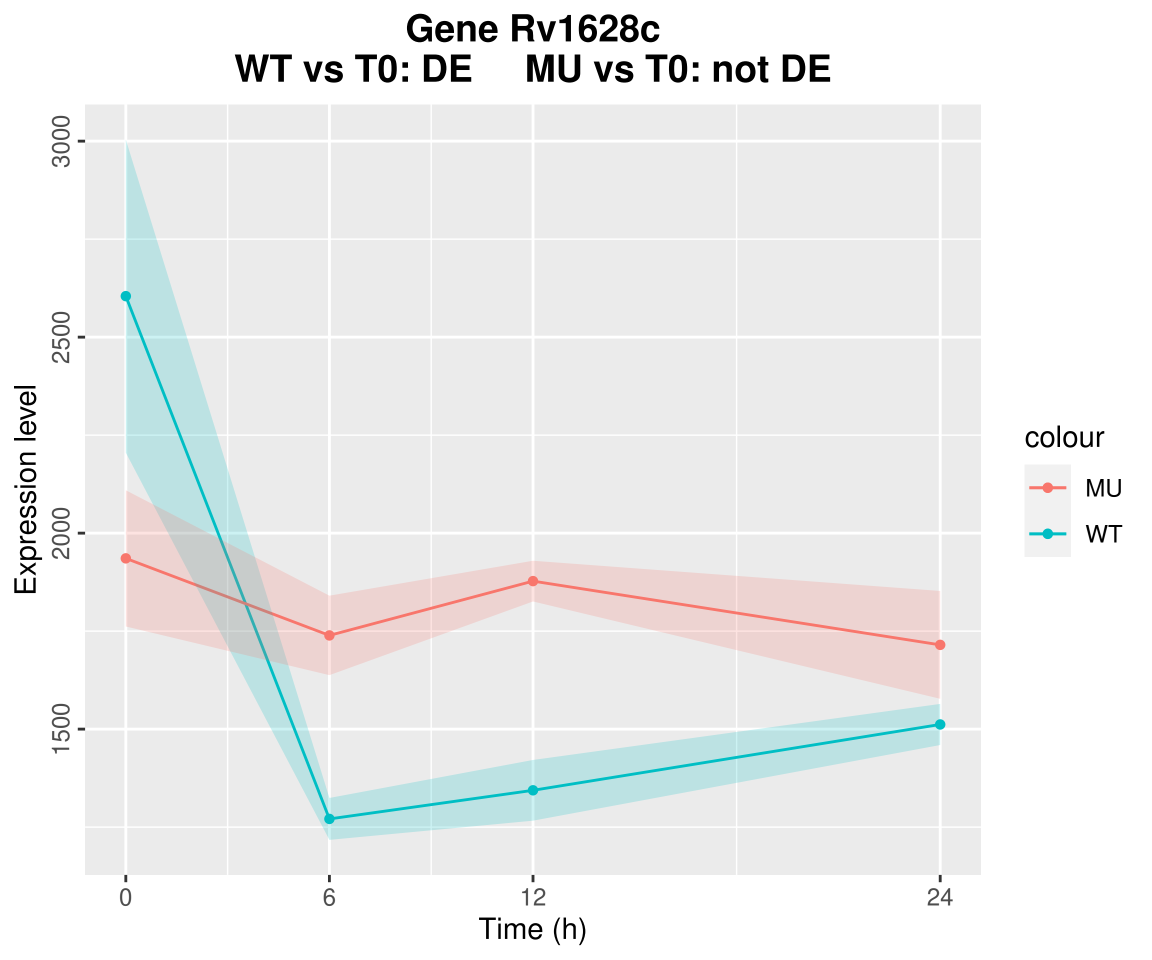
Gene Rv1628c
WT vs T0: DE   MU vs T0: not DE
Expression level
3000
2500
2000
1500
0
6
12
24
Time (h)
colour
MU
WT

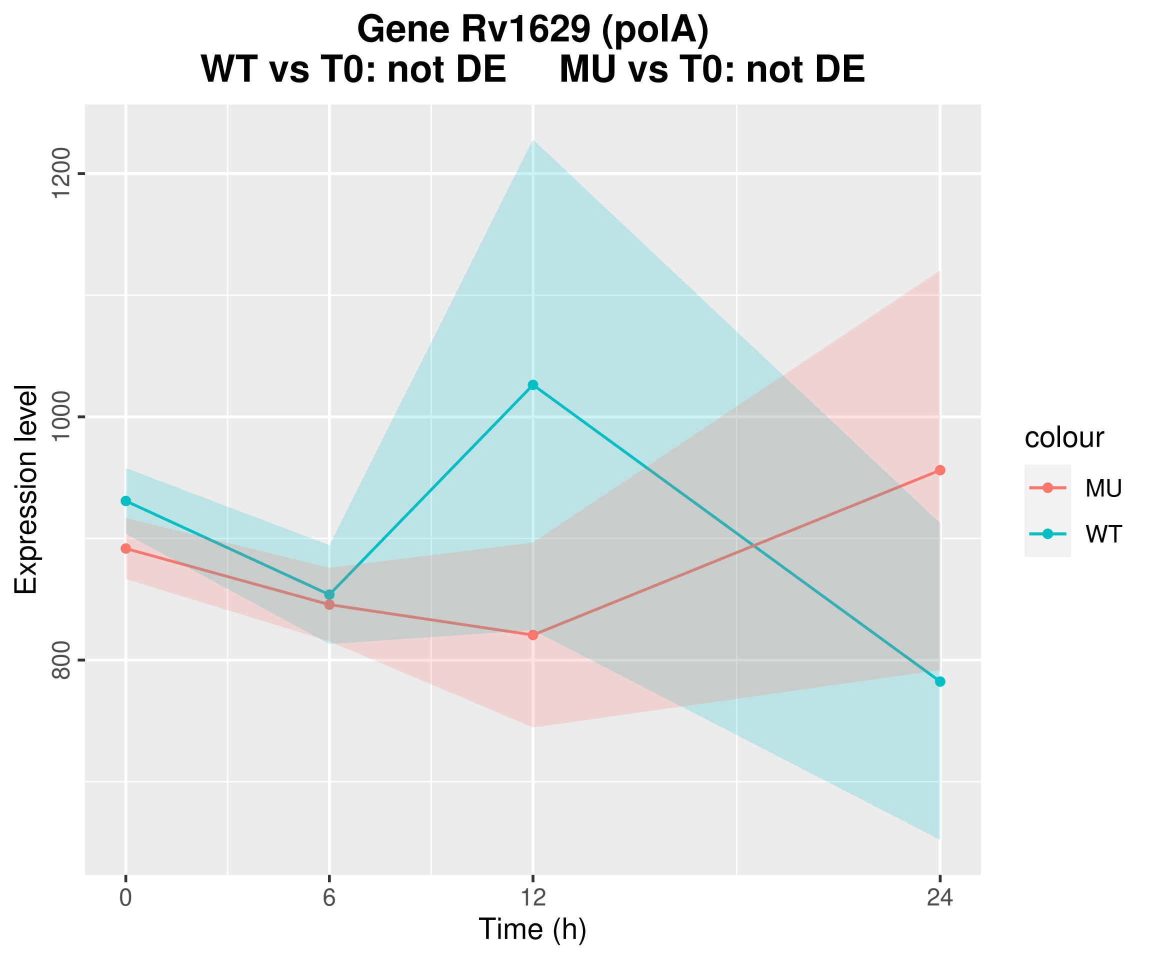
Gene Rv1629 (polA)
WT vs T0: not DE MU vs T0: not DE
1200
1000
800
Expression level
0
6
12
24
Time (h)
colour
MU
WT

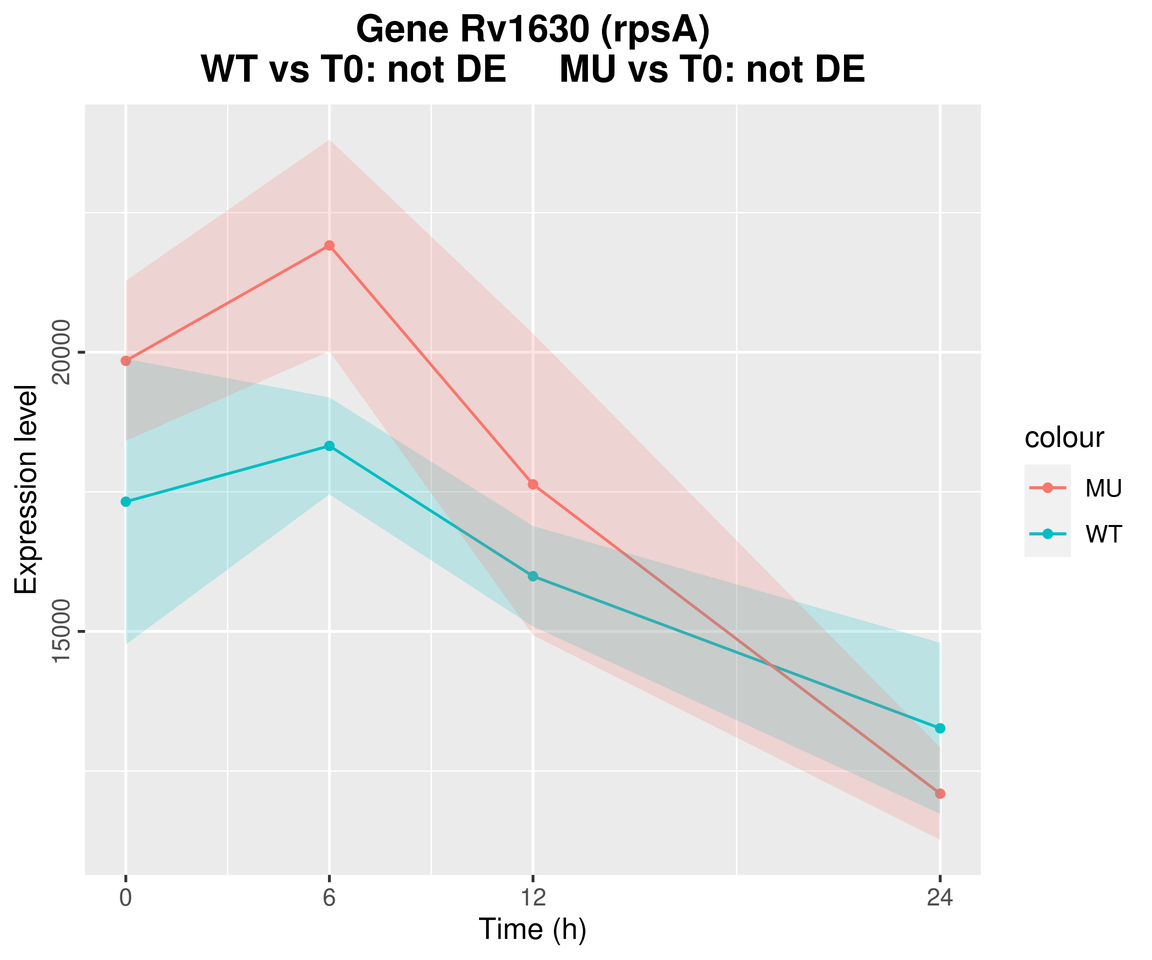

Gene Rv1630 (rpsA)
WT vs T0: not DE   MU vs T0: not DE
Expression level
20000
15000
0
6
12
24
Time (h)
colour
MU
WT

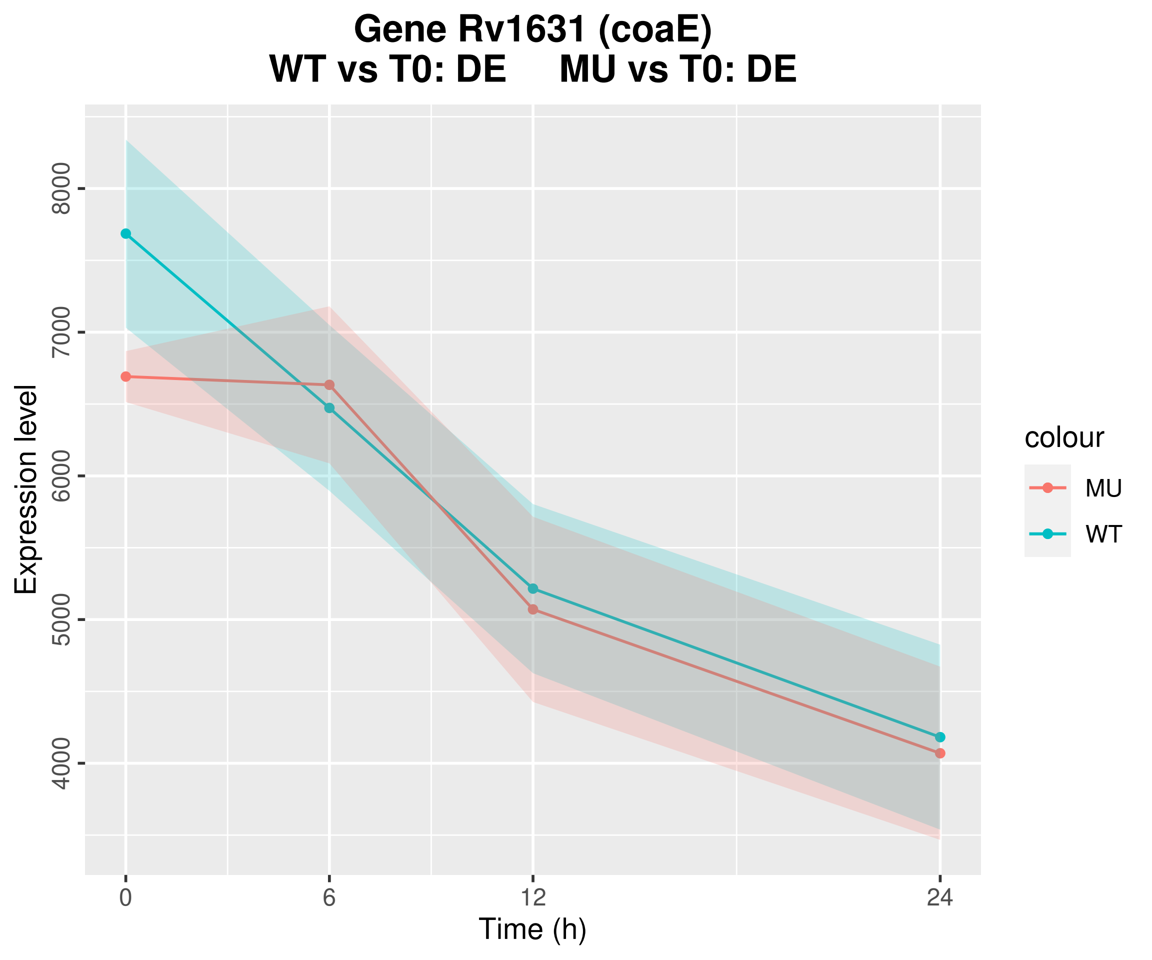
Gene Rv1631 (coaE)
WT vs T0: DE   MU vs T0: DE
Expression level
8000
7000
6000
5000
4000
Time (h)
0
6
12
24
colour
MU
WT

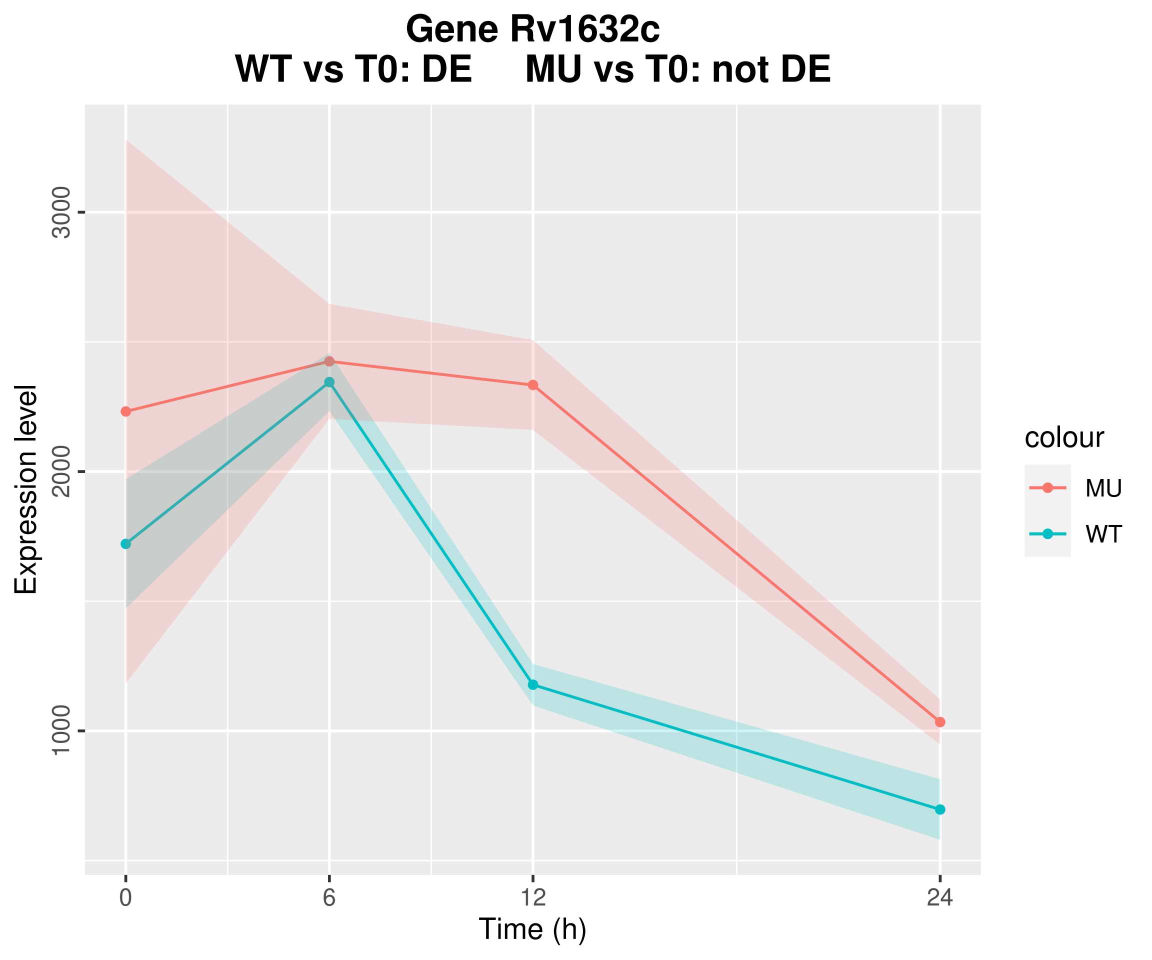
Gene Rv1632c
WT vs T0: DE   MU vs T0: not DE
3000
2000
1000
Expression level
0
6
12
24
Time (h)
colour
MU
WT

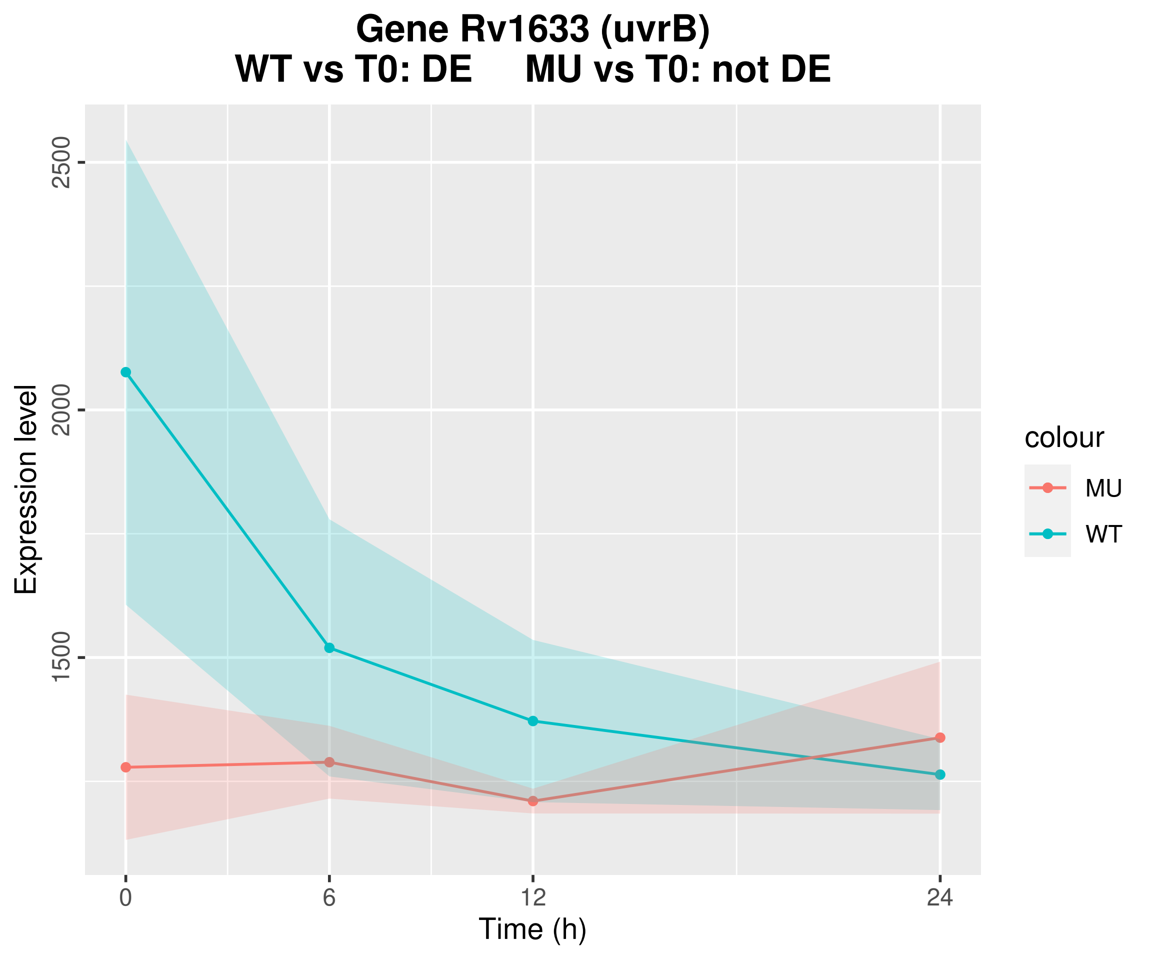

Gene Rv1633 (uvrB)
WT vs T0: DE MU vs T0: not DE
Expression level
2500
2000
1500
Time (h)
0
6
12
24
colour
MU
WT

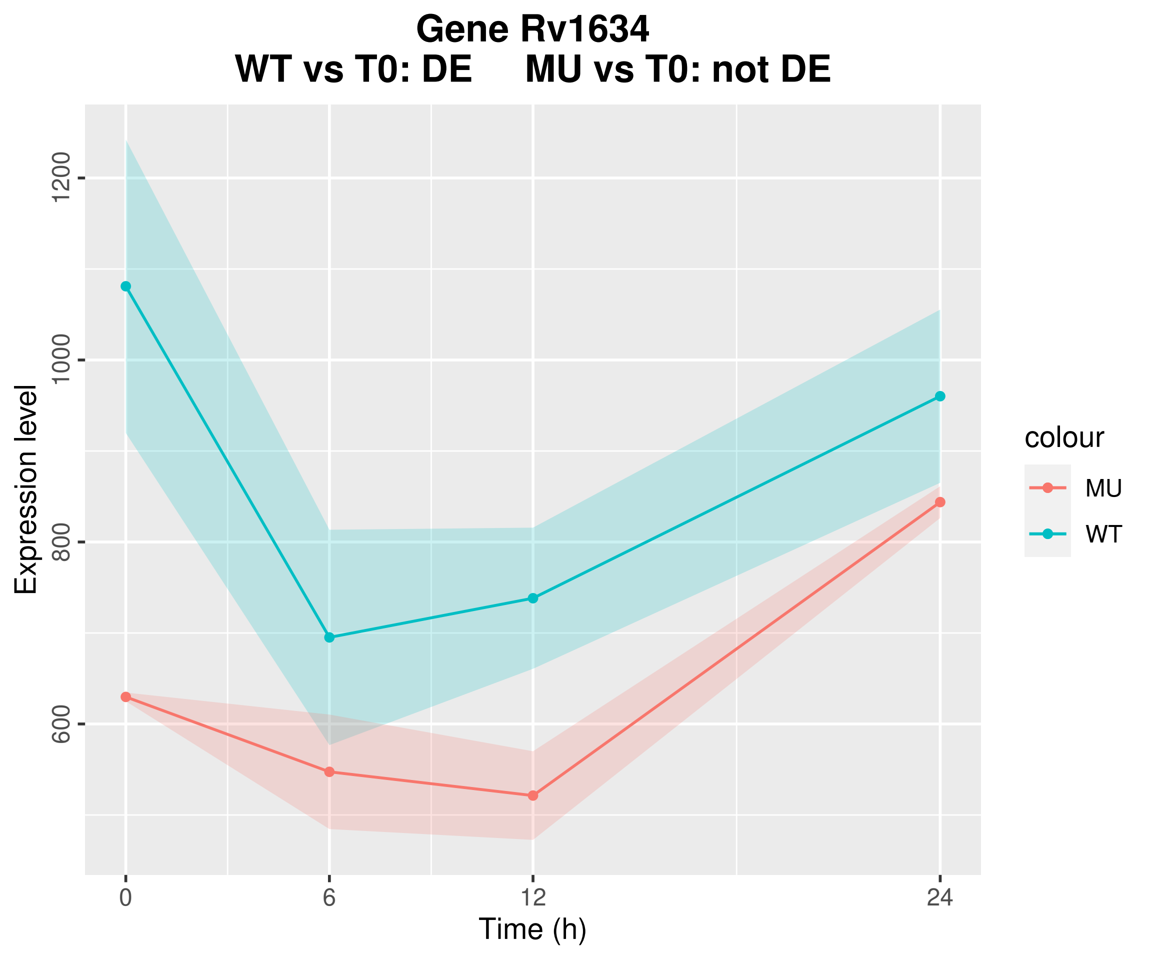

Gene Rv1634
WT vs T0: DE   MU vs T0: not DE
1200
1000
800
600
Expression level
0
6
12
24
Time (h)
colour
MU
WT

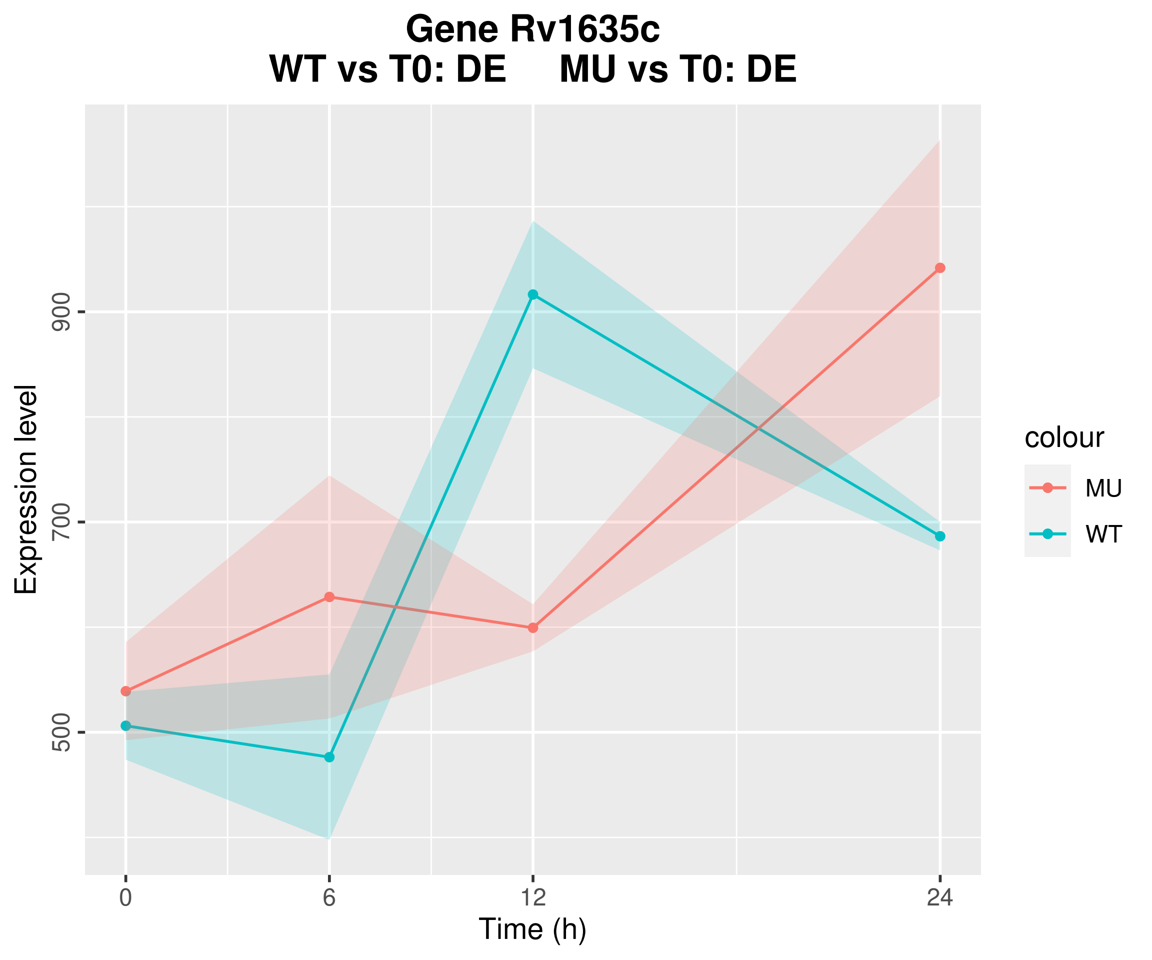
Gene Rv1635c
WT vs T0: DE MU vs T0: DE
Expression level
900
700
500
0
6
12
24
Time (h)
colour
MU
WT

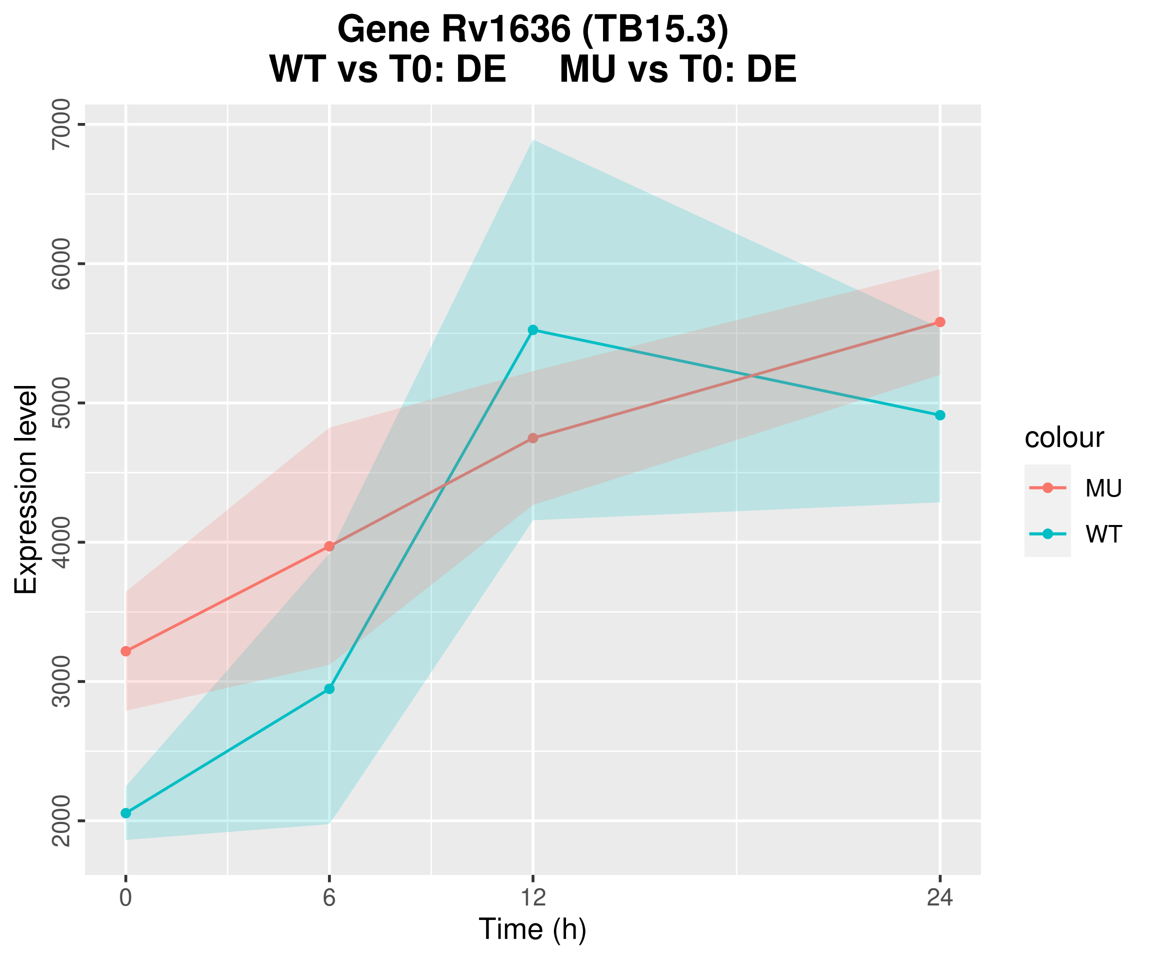
Gene Rv1636 (TB15.3)
WT vs T0: DE MU vs T0: DE
Expression level
7000
6000
5000
4000
3000
2000
0
6
12
24
Time (h)
colour
MU
WT

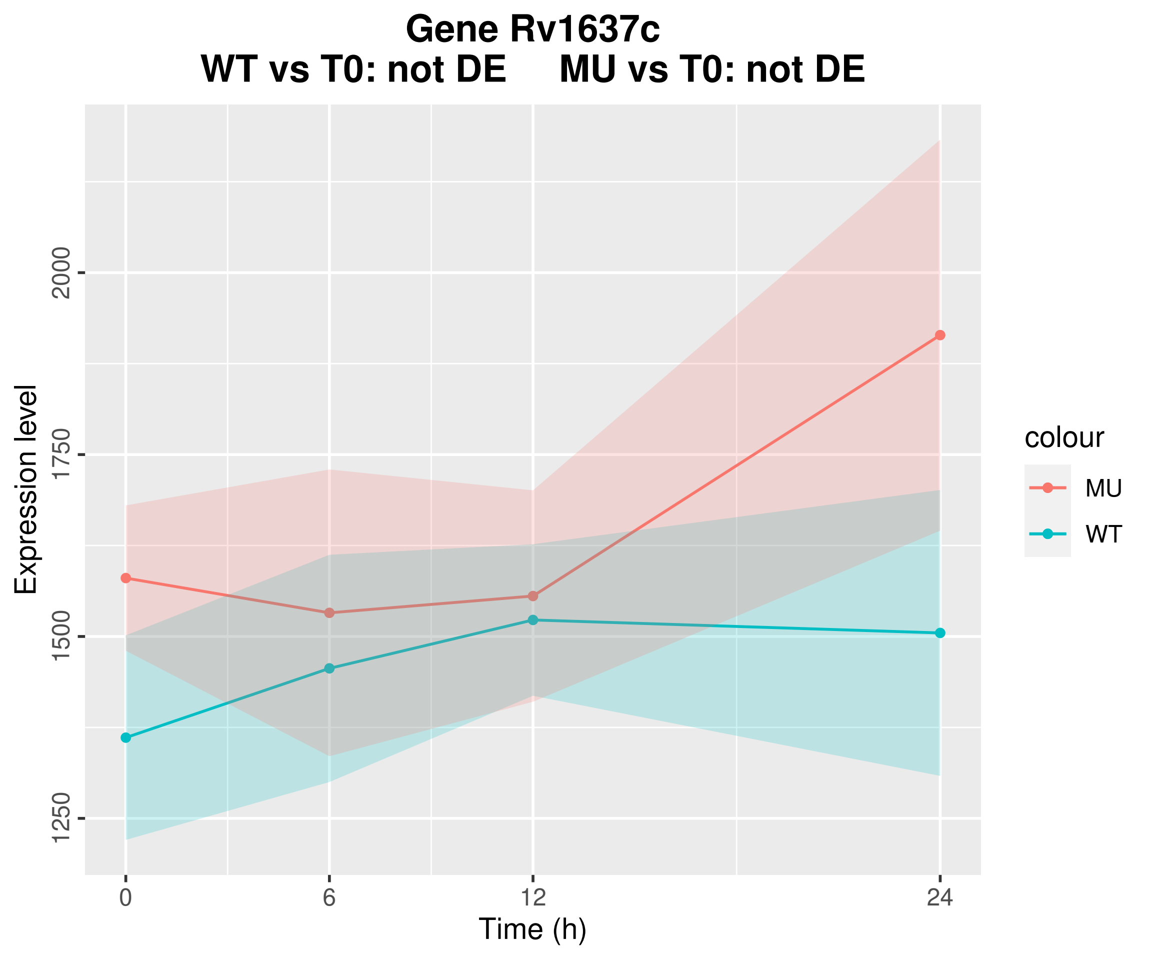

Gene Rv1637c
WT vs T0: not DE MU vs T0: not DE
Expression level
2000
1750
1500
1250
0
6
12
24
Time (h)
colour
MU
WT

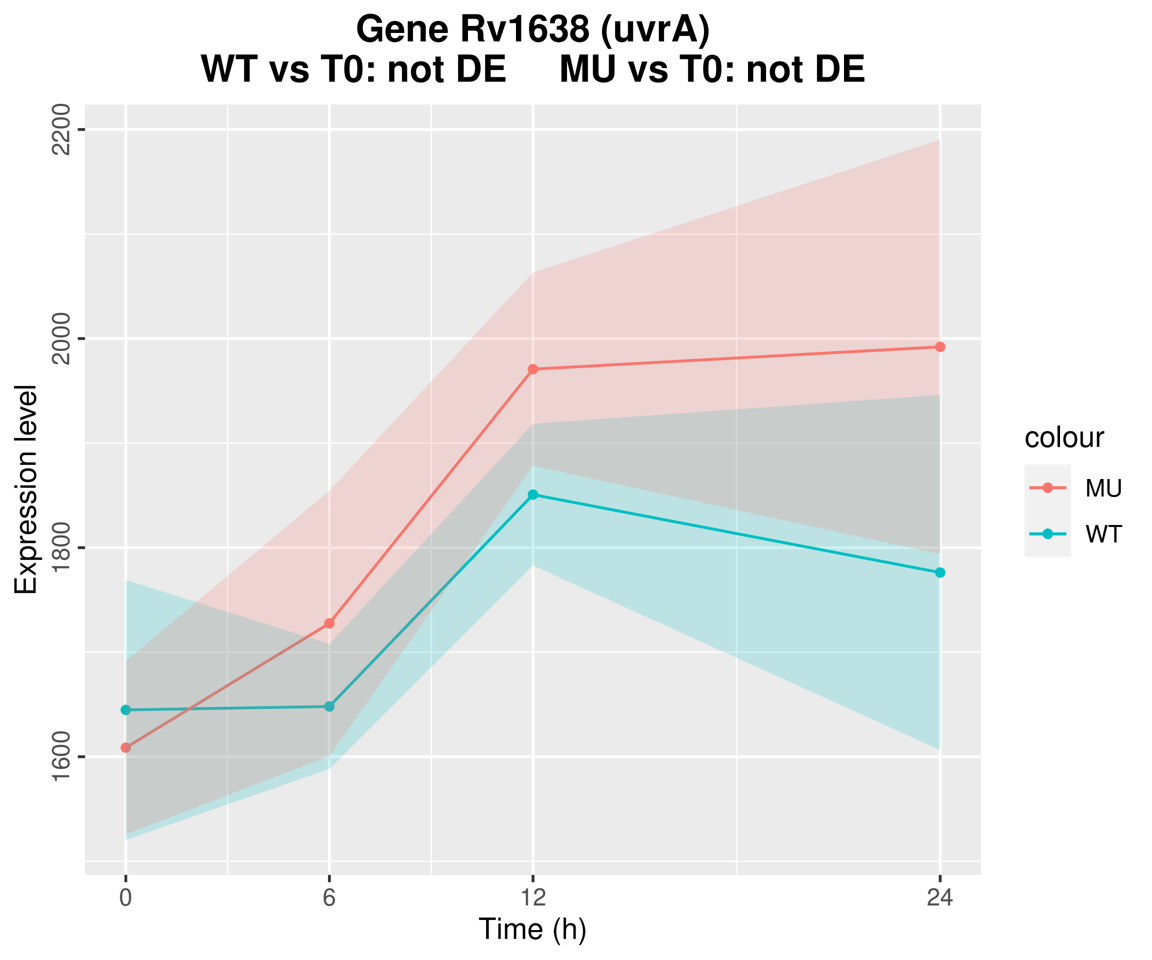
Gene Rv1638 (uvrA)
WT vs T0: not DE   MU vs T0: not DE
Expression level
2200
2000
1800
1600
0
6
12
24
Time (h)
colour
MU
WT

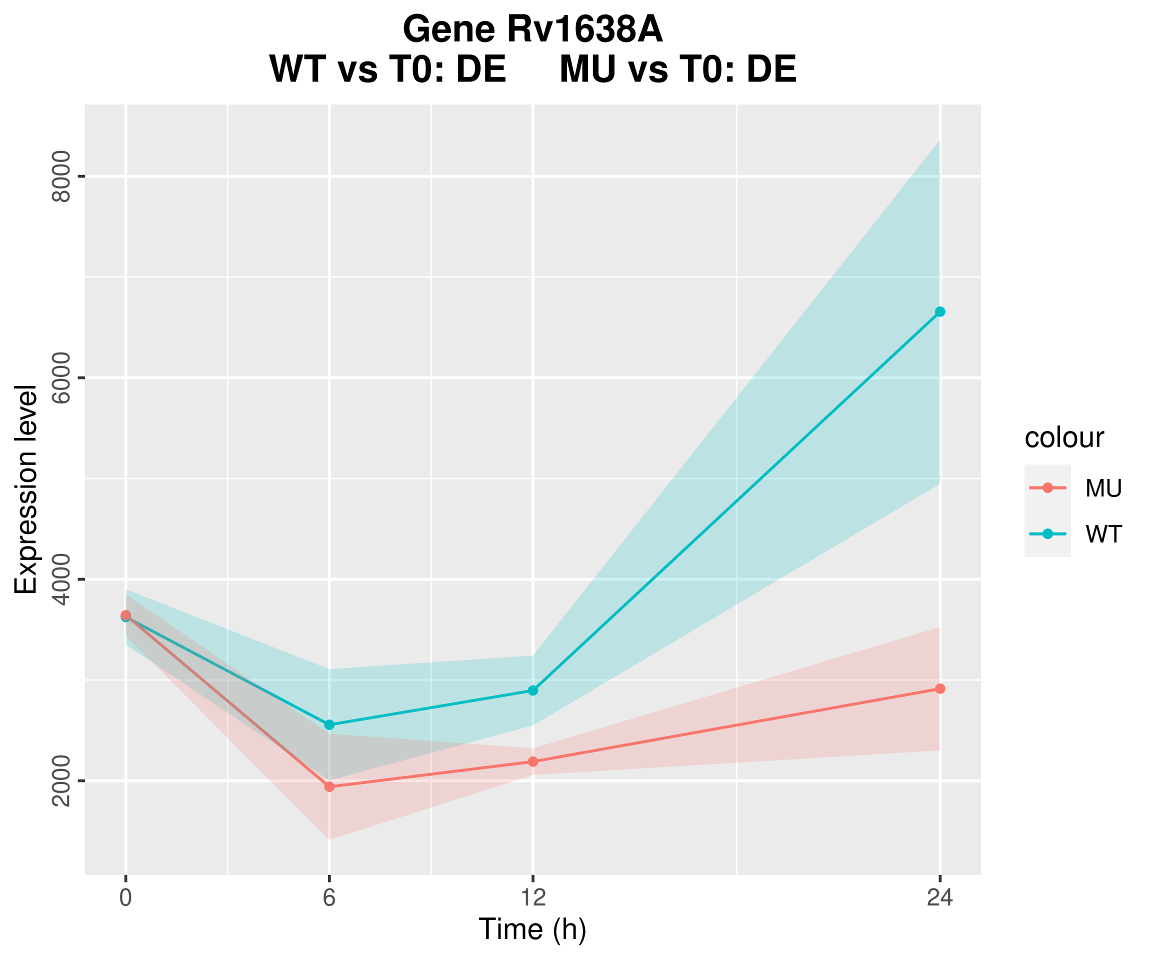
Gene Rv1638A
WT vs T0: DE   MU vs T0: DE
Expression level
8000
6000
4000
2000
0
6
12
24
Time (h)
colour
MU
WT

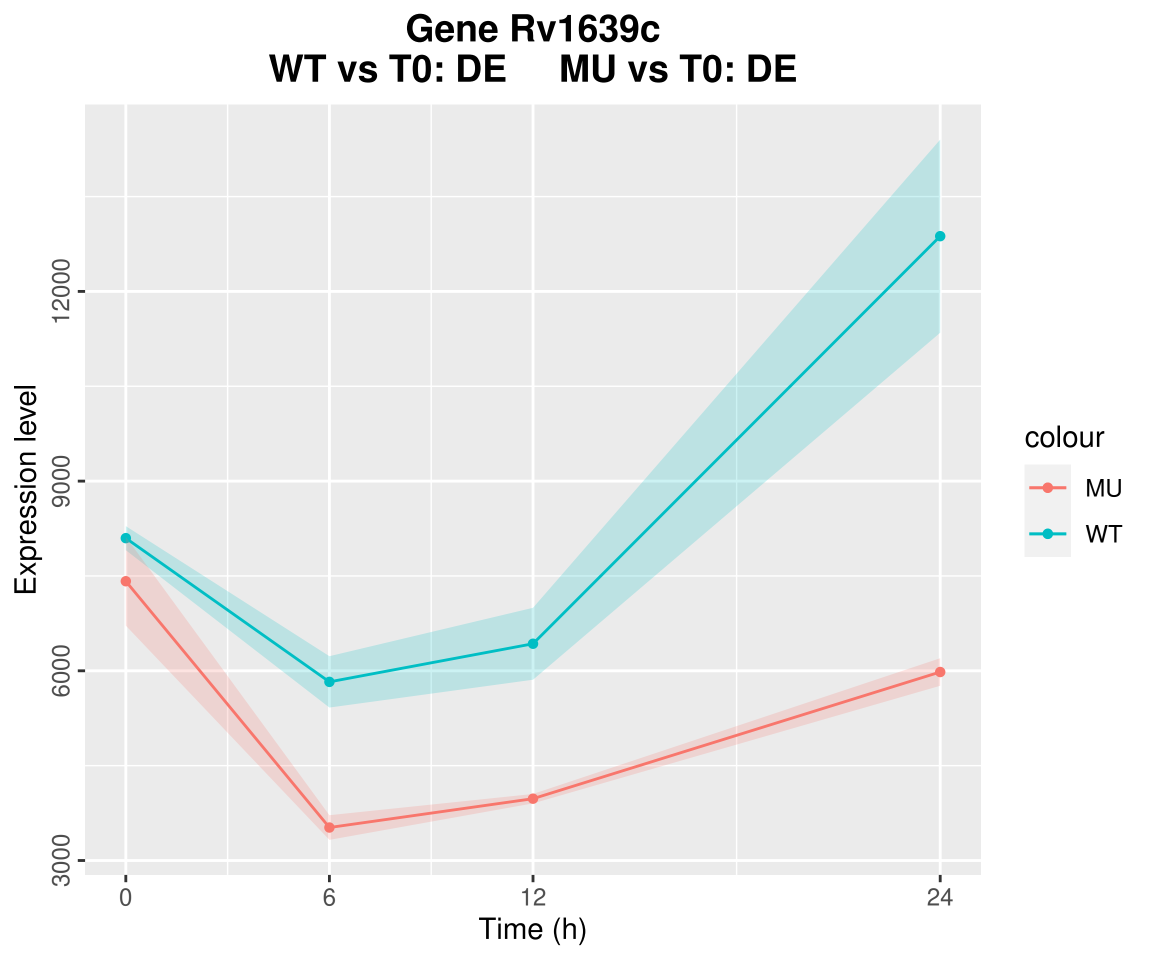
Gene Rv1639c
WT vs T0: DE MU vs T0: DE
Expression level
3000
6000
9000
12000
0
6
12
24
Time (h)
colour
MU
WT

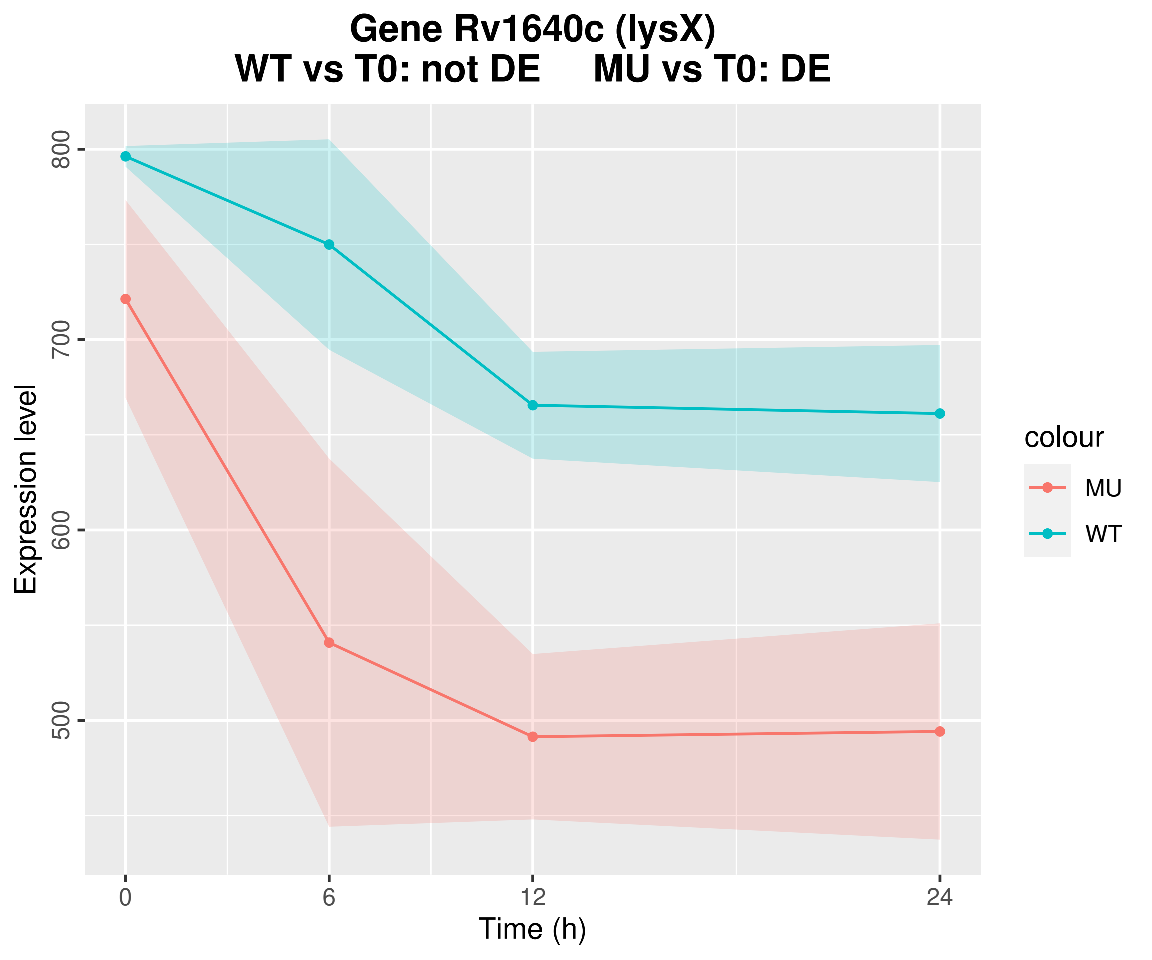

Gene Rv1640c (lysX)
WT vs T0: not DE MU vs T0: DE
Expression level
800
700
600
500
0
6
12
24
Time (h)
colour
MU
WT

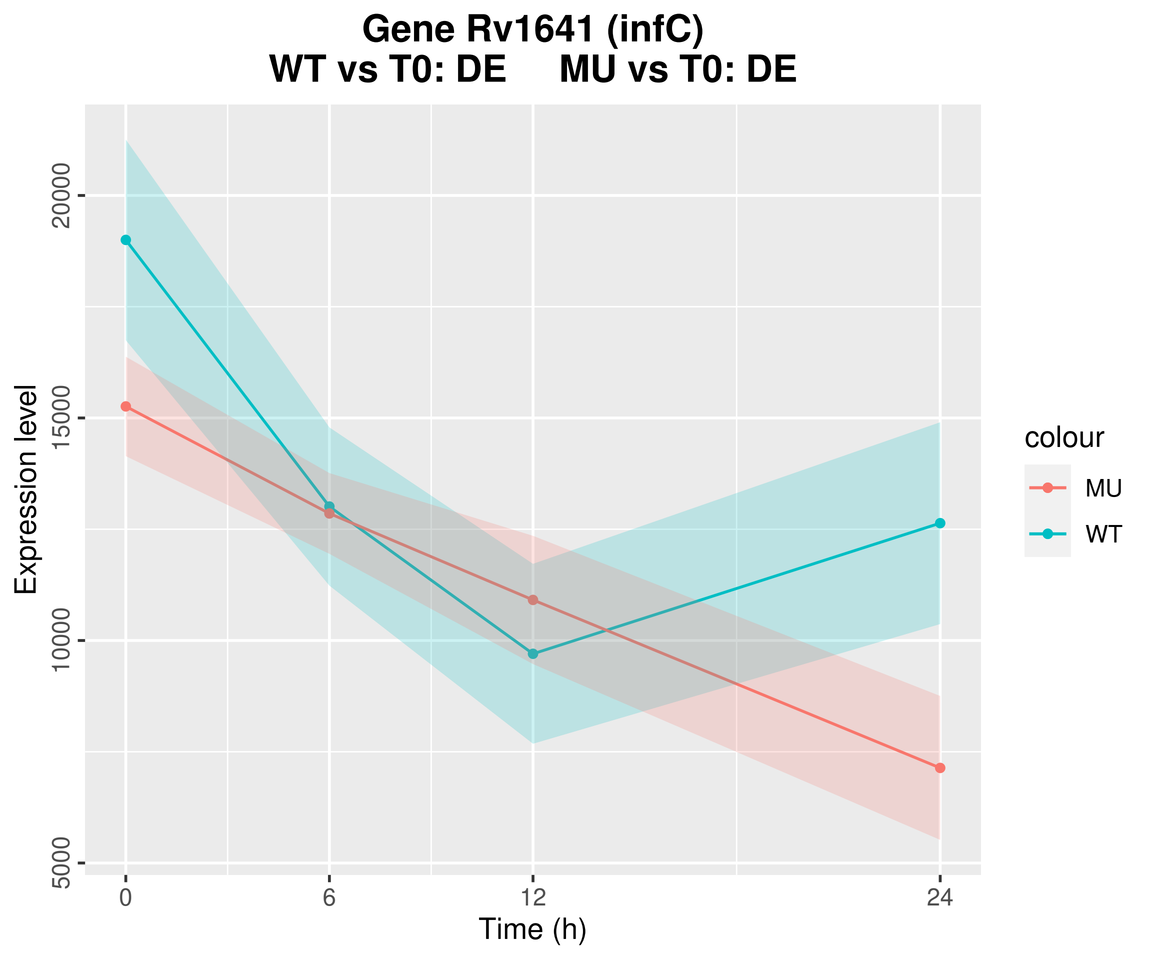
Gene Rv1641 (infC)
WT vs T0: DE MU vs T0: DE
Expression level
20000
15000
10000
5000
0
6
12
24
Time (h)
colour
MU
WT

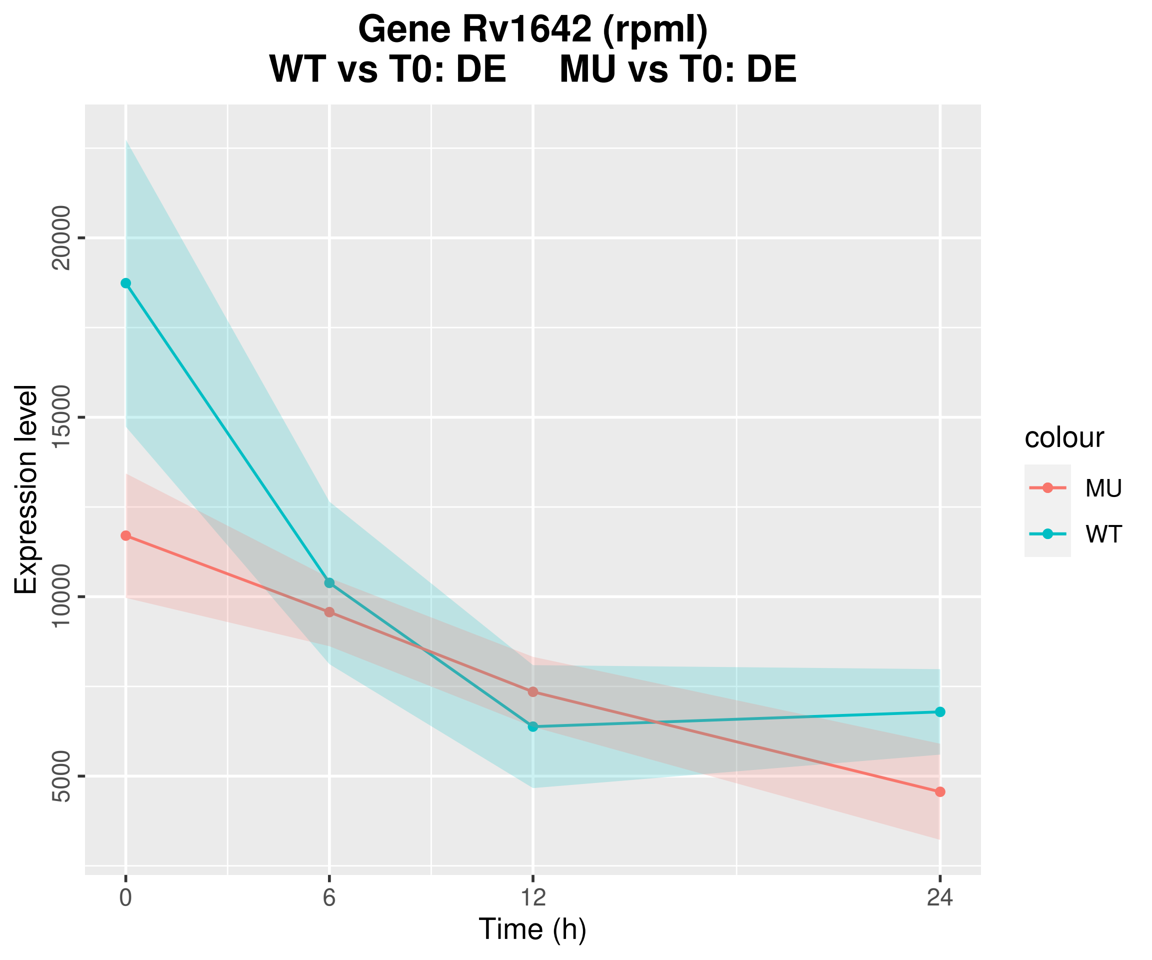
Gene Rv1642 (rpml)
WT vs T0: DE MU vs T0: DE
Expression level
20000
15000
10000
5000
0
6
12
24
Time (h)
colour
MU
WT

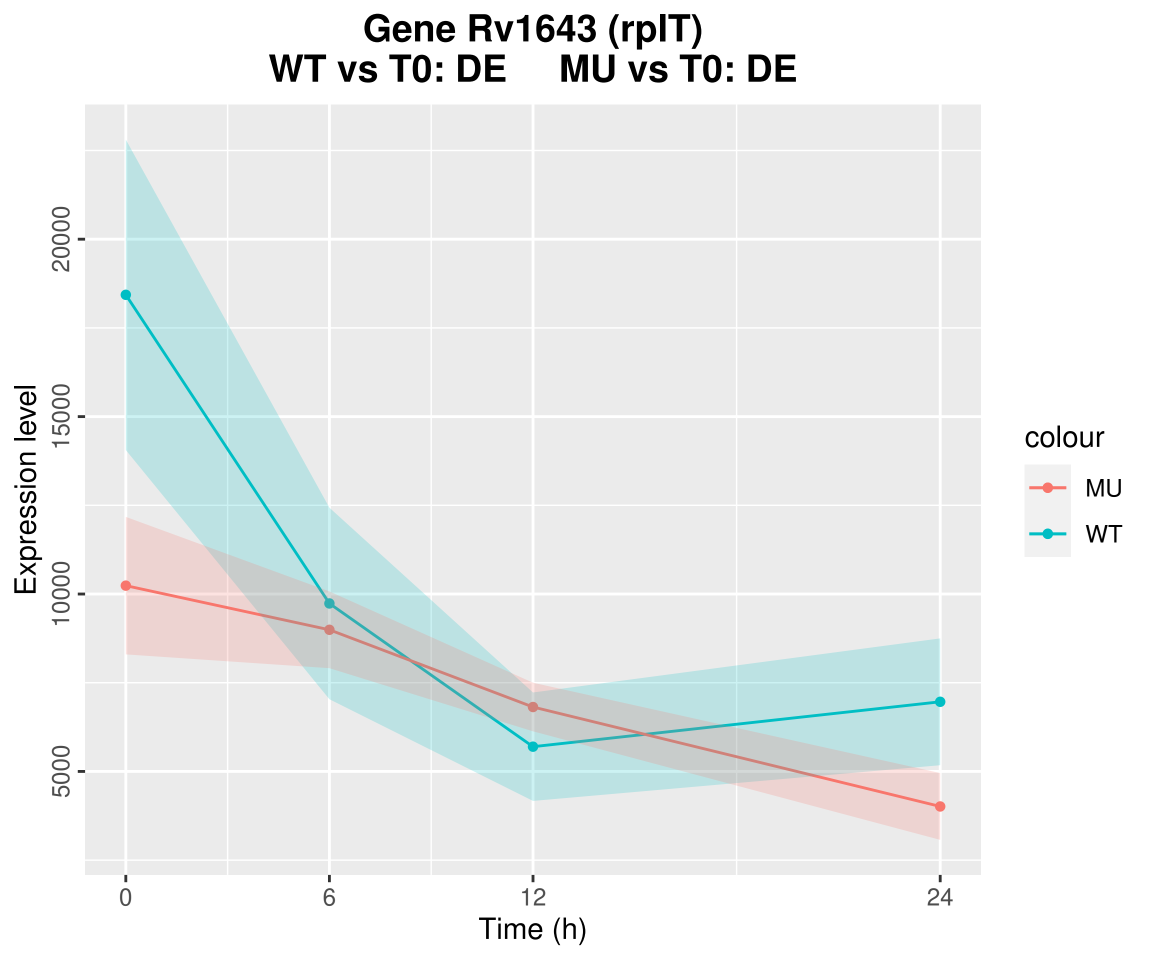
Gene Rv1643 (rplT)
WT vs T0: DE MU vs T0: DE
Expression level
20000
15000
10000
5000
0
6
12
24
Time (h)
colour
MU
WT

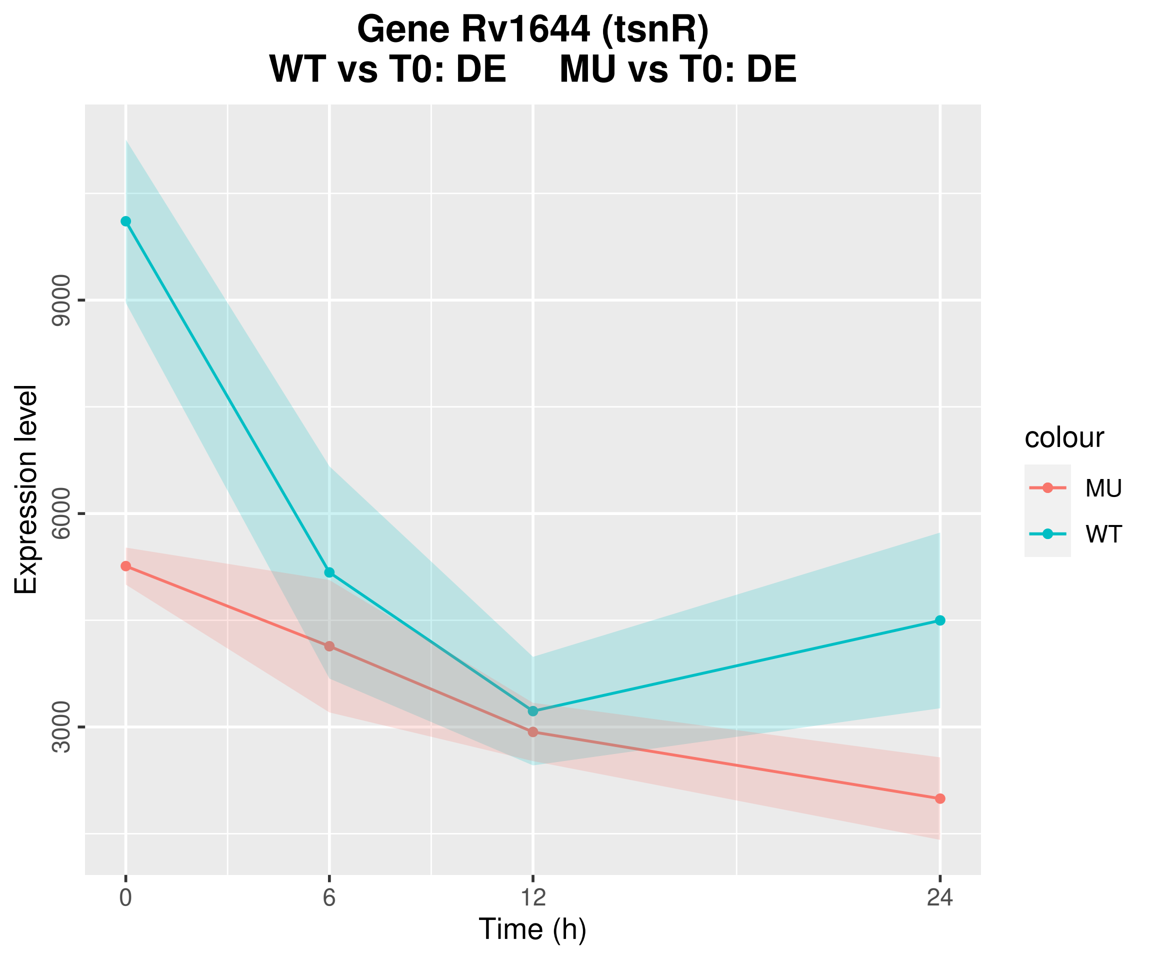
Gene Rv1644 (tsnR)
WT vs T0: DE MU vs T0: DE
Expression level
9000
6000
3000
0
6
12
24
Time (h)
colour
MU
WT

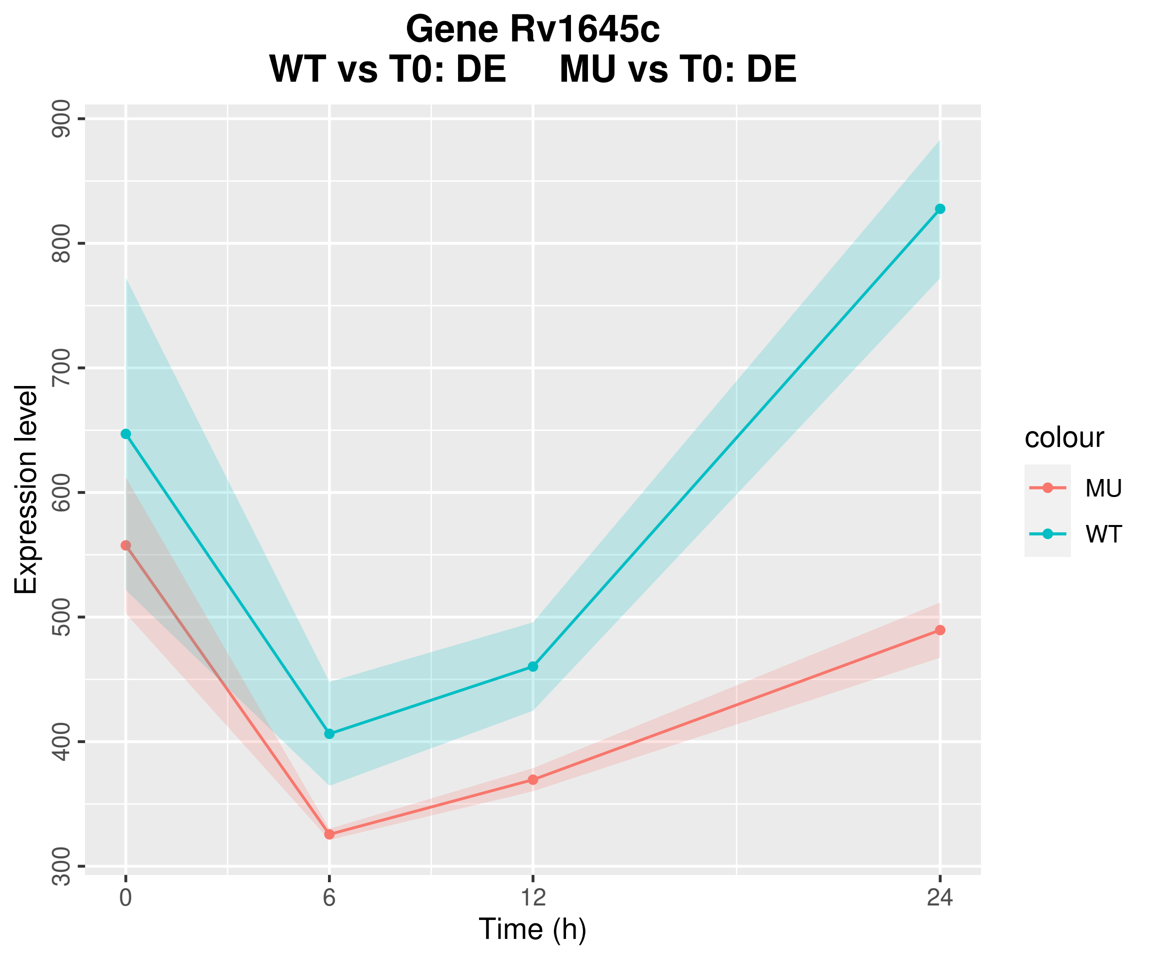
Gene Rv1645c
WT vs T0: DE MU vs T0: DE
Expression level
900
800
700
600
500
400
300
0
6
12
24
Time (h)
colour
MU
WT

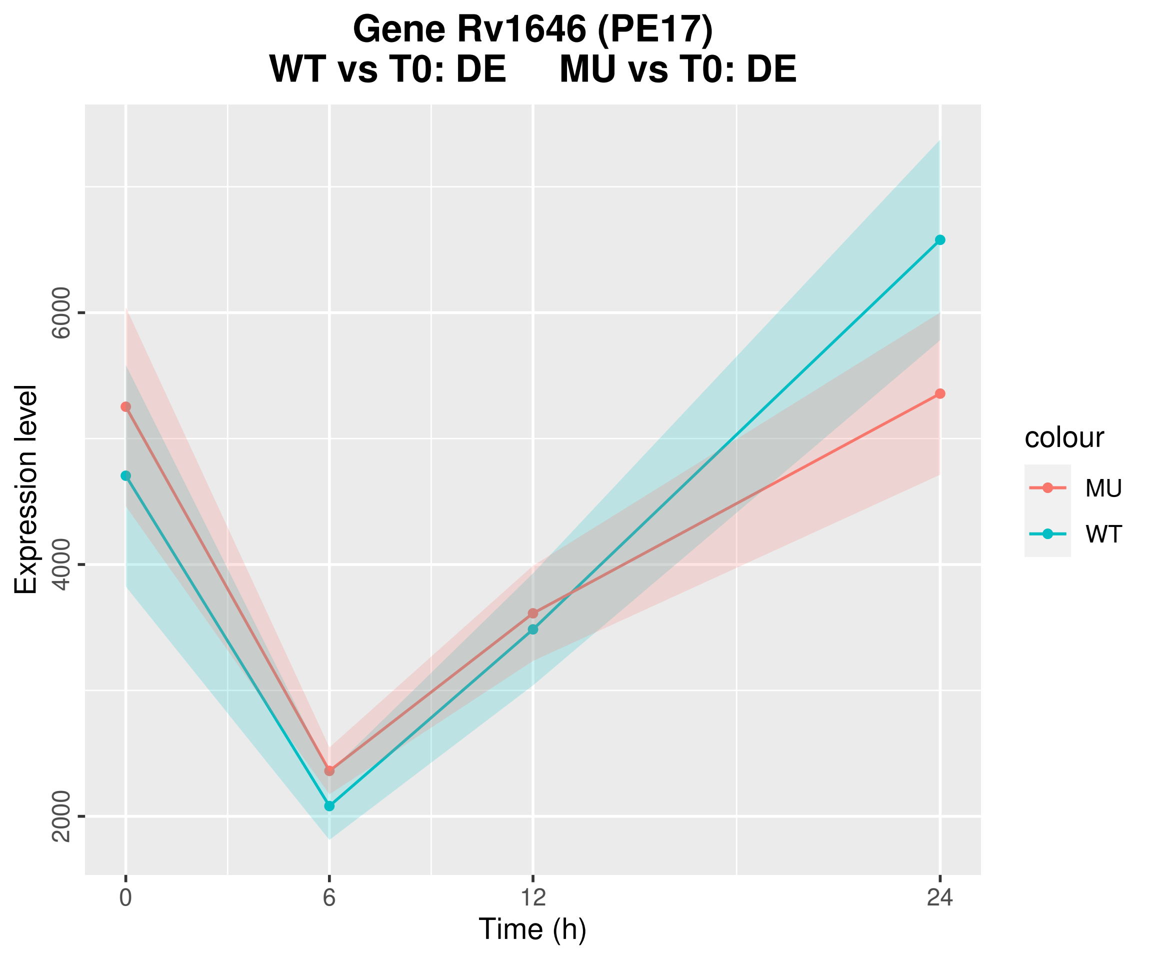

Gene Rv1646 (PE17)
WT vs T0: DE MU vs T0: DE
Expression level
6000
4000
2000
0
6
12
24
Time (h)
colour
MU
WT

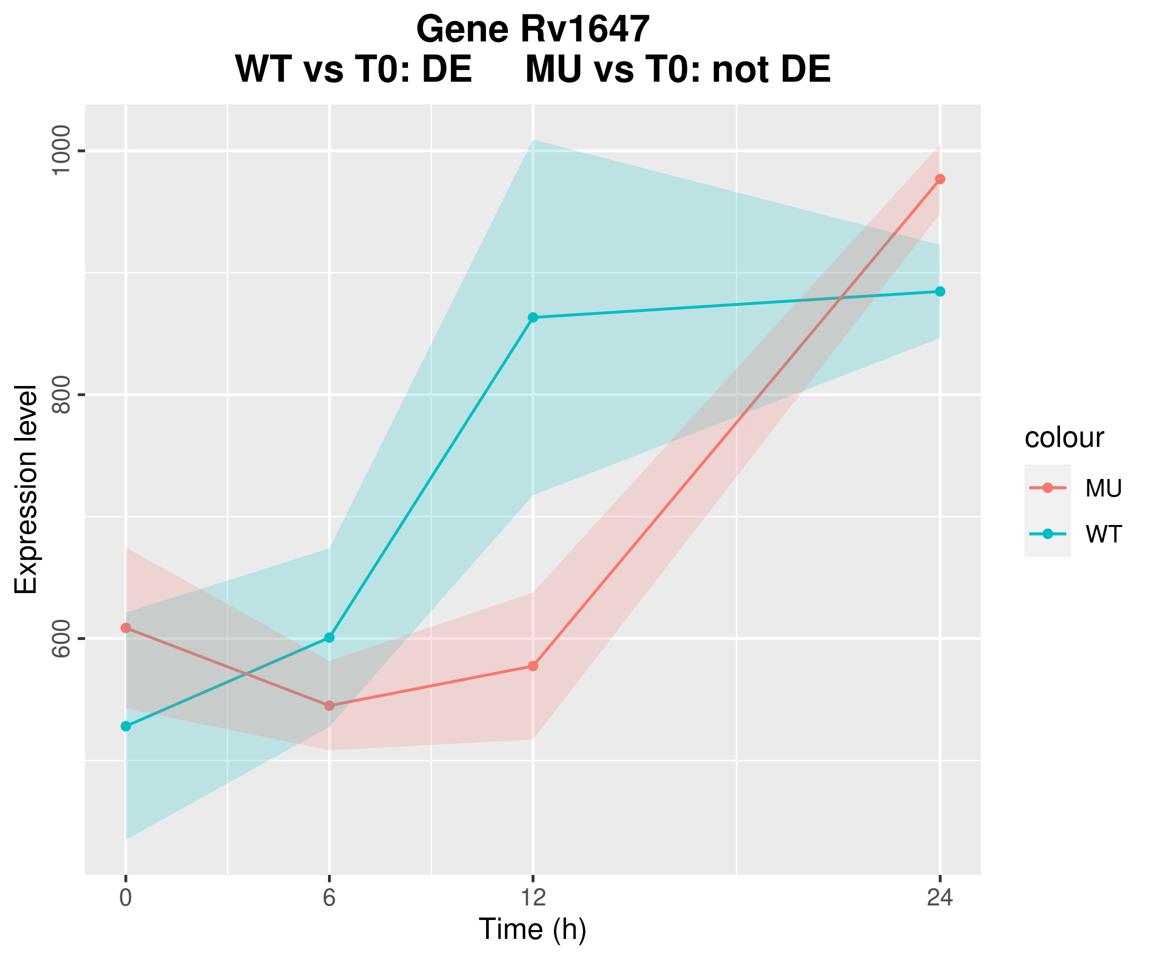

Gene Rv1647
WT vs T0: DE MU vs T0: not DE
Expression level
1000
800
600
0
6
12
24
Time (h)
colour
MU
WT

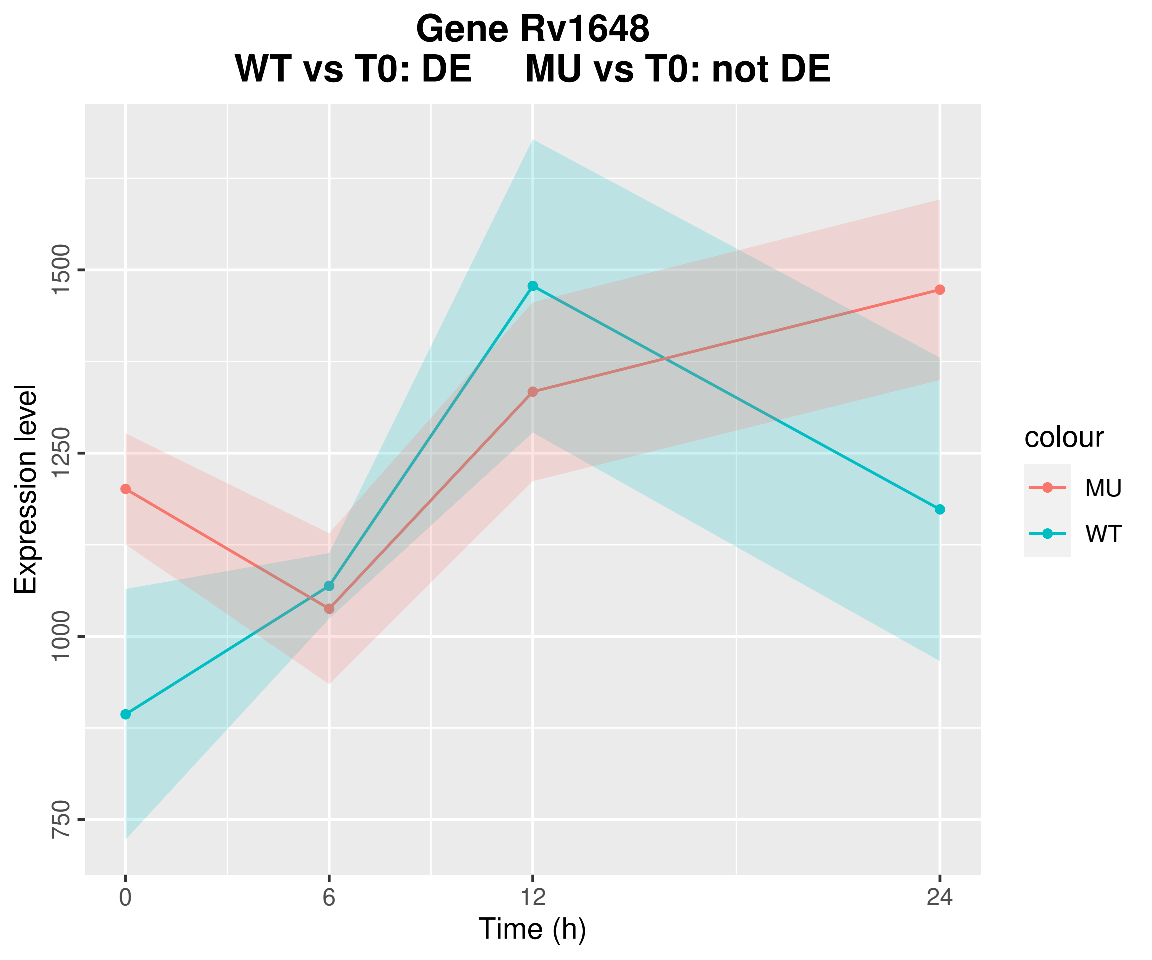
Gene Rv1648
WT vs T0: DE MU vs T0: not DE
Expression level
750
1000
1250
1500
0
6
12
24
Time (h)
colour
MU
WT

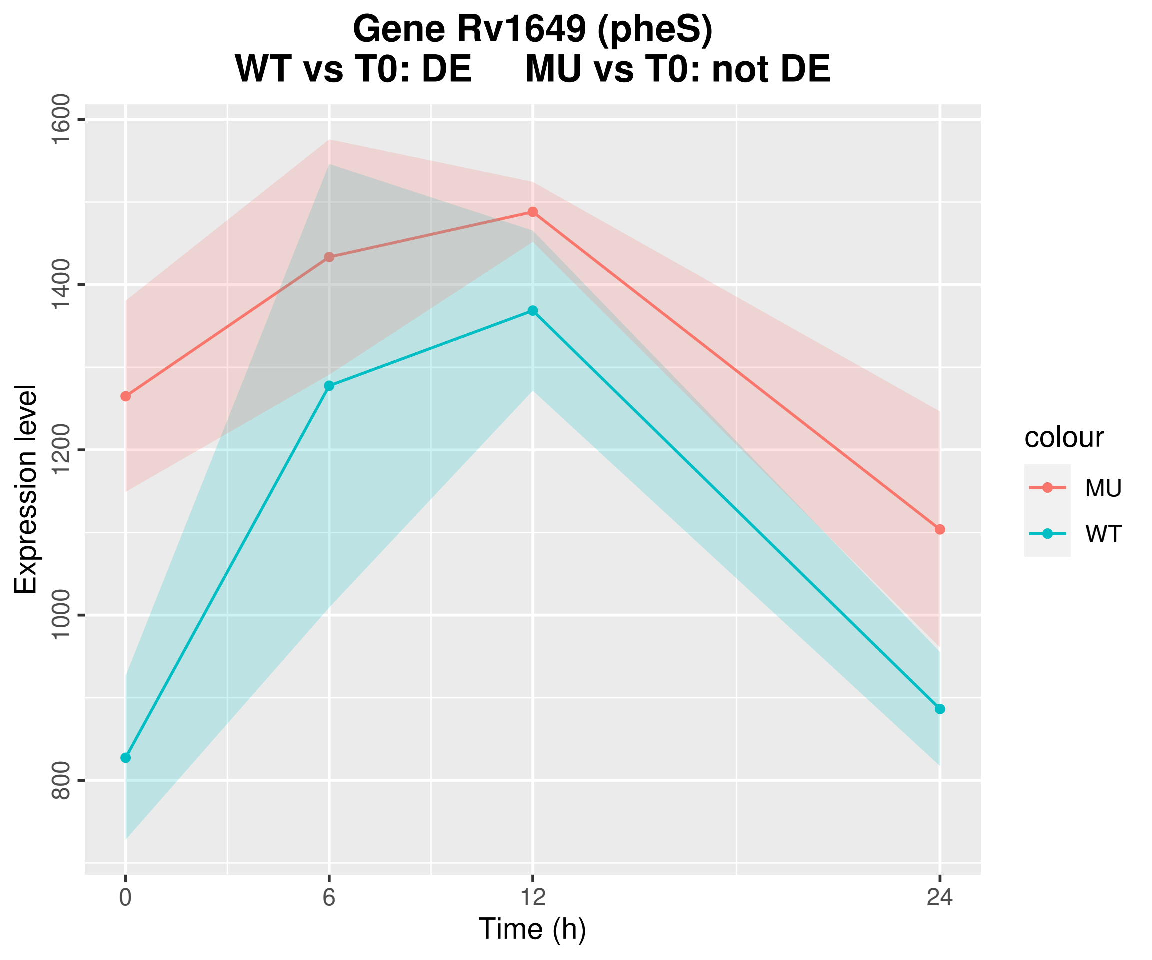
Gene Rv1649 (pheS)
WT vs T0: DE   MU vs T0: not DE
Expression level
1600
1400
1200
1000
800
0
6
12
24
Time (h)
colour
MU
WT

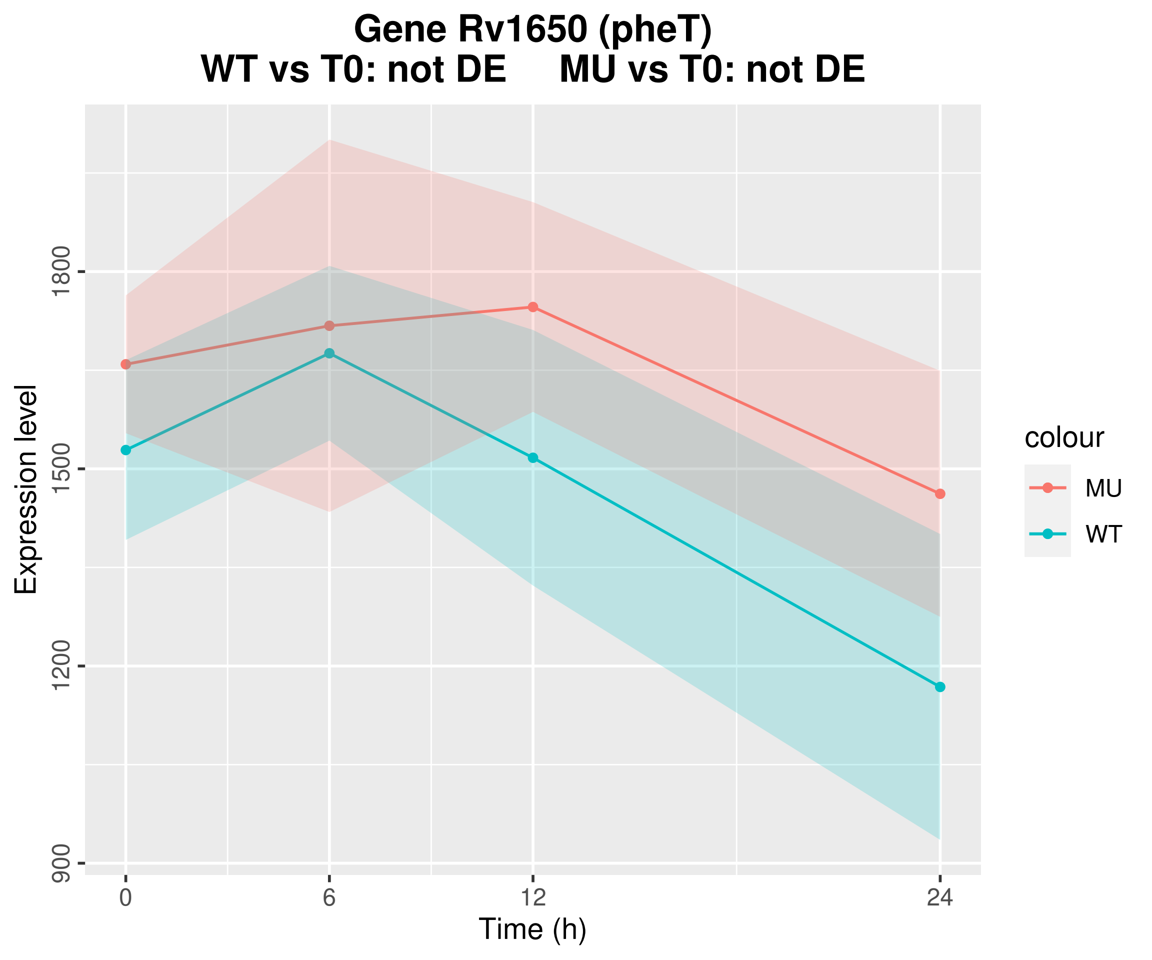

Gene Rv1650 (pheT)
WT vs T0: not DE   MU vs T0: not DE
1800
1500
1200
900
Expression level
0
6
12
24
Time (h)
colour
MU
WT

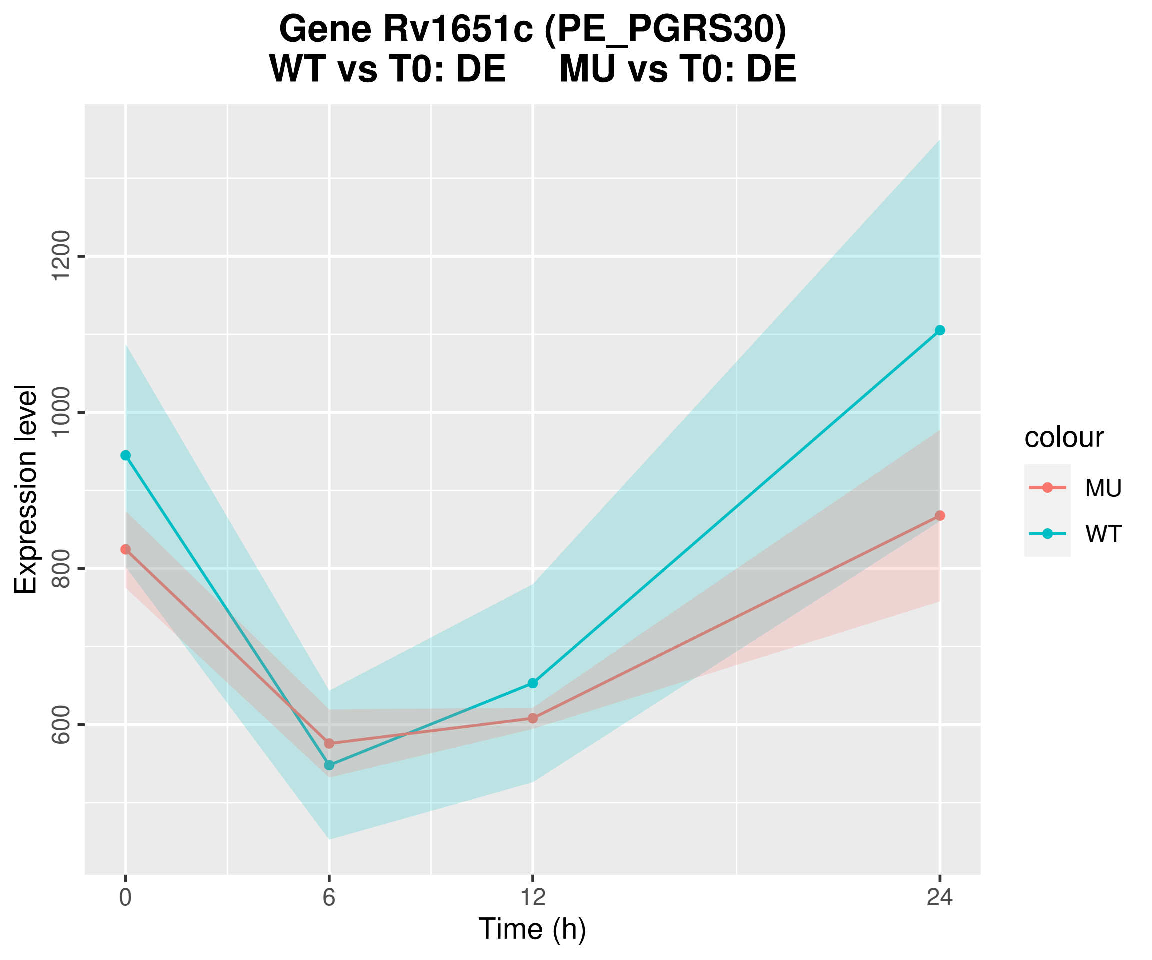

Gene Rv1651c (PE_PGRS30)
WT vs T0: DE MU vs T0: DE
Expression level
1200
1000
800
600
0
6
12
24
Time (h)
colour
MU
WT

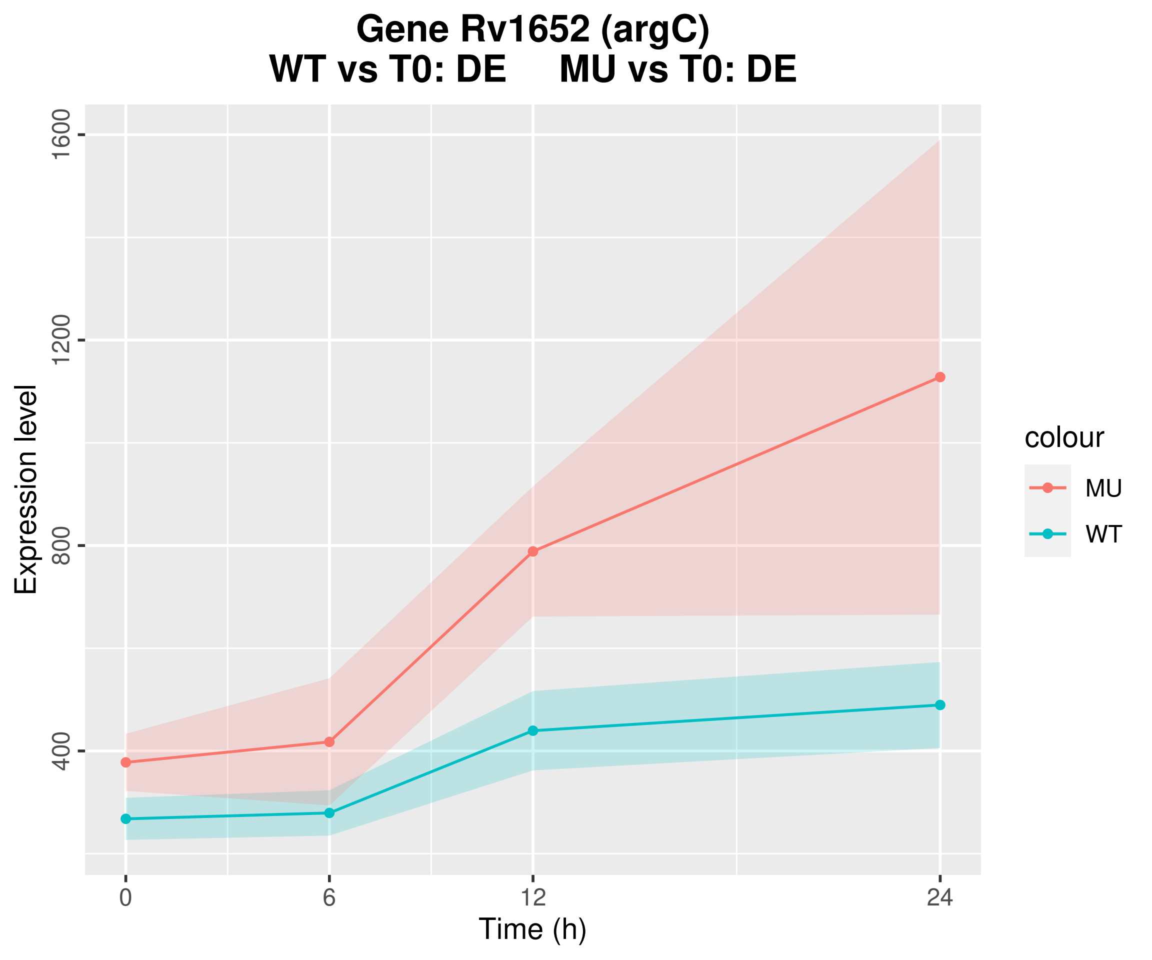

Gene Rv1652 (argC)
WT vs T0: DE   MU vs T0: DE
Expression level
1600
1200
800
400
0
6
12
24
Time (h)
colour
MU
WT

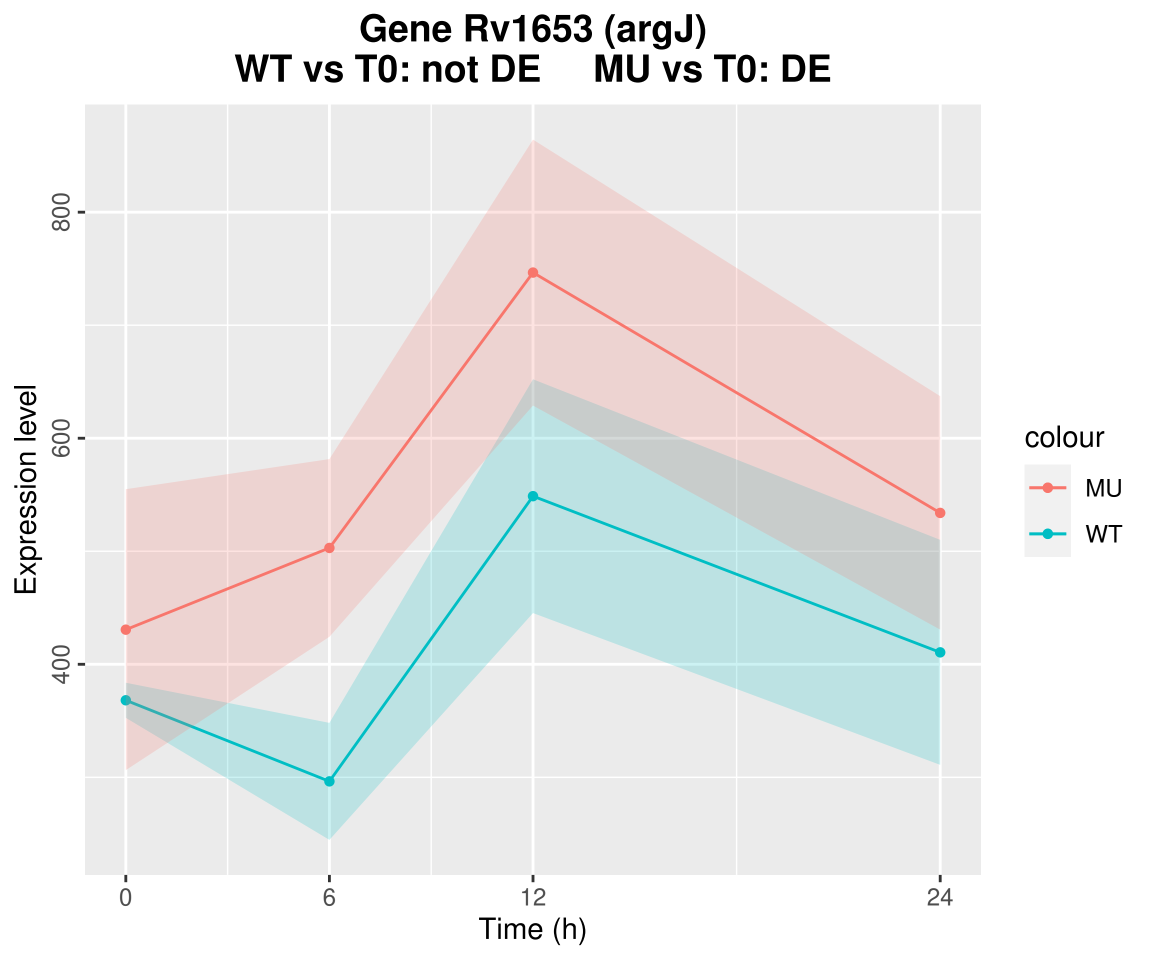

Gene Rv1653 (argJ)
WT vs T0: not DE MU vs T0: DE
Expression level
800
600
400
0
6
12
24
Time (h)
colour
MU
WT

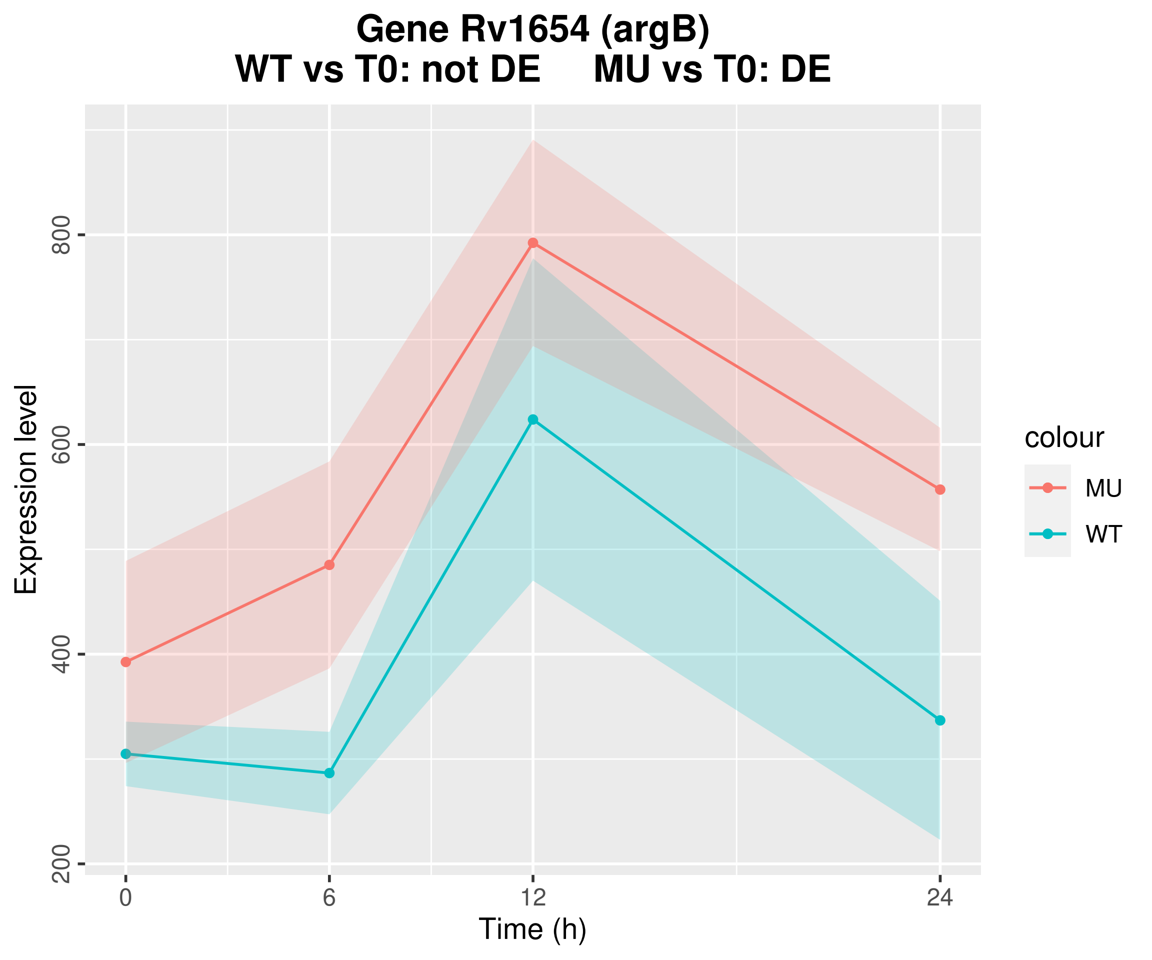

Gene Rv1654 (argB)
WT vs T0: not DE MU vs T0: DE
Expression level
200
400
600
800
0
6
12
24
Time (h)
colour
MU
WT

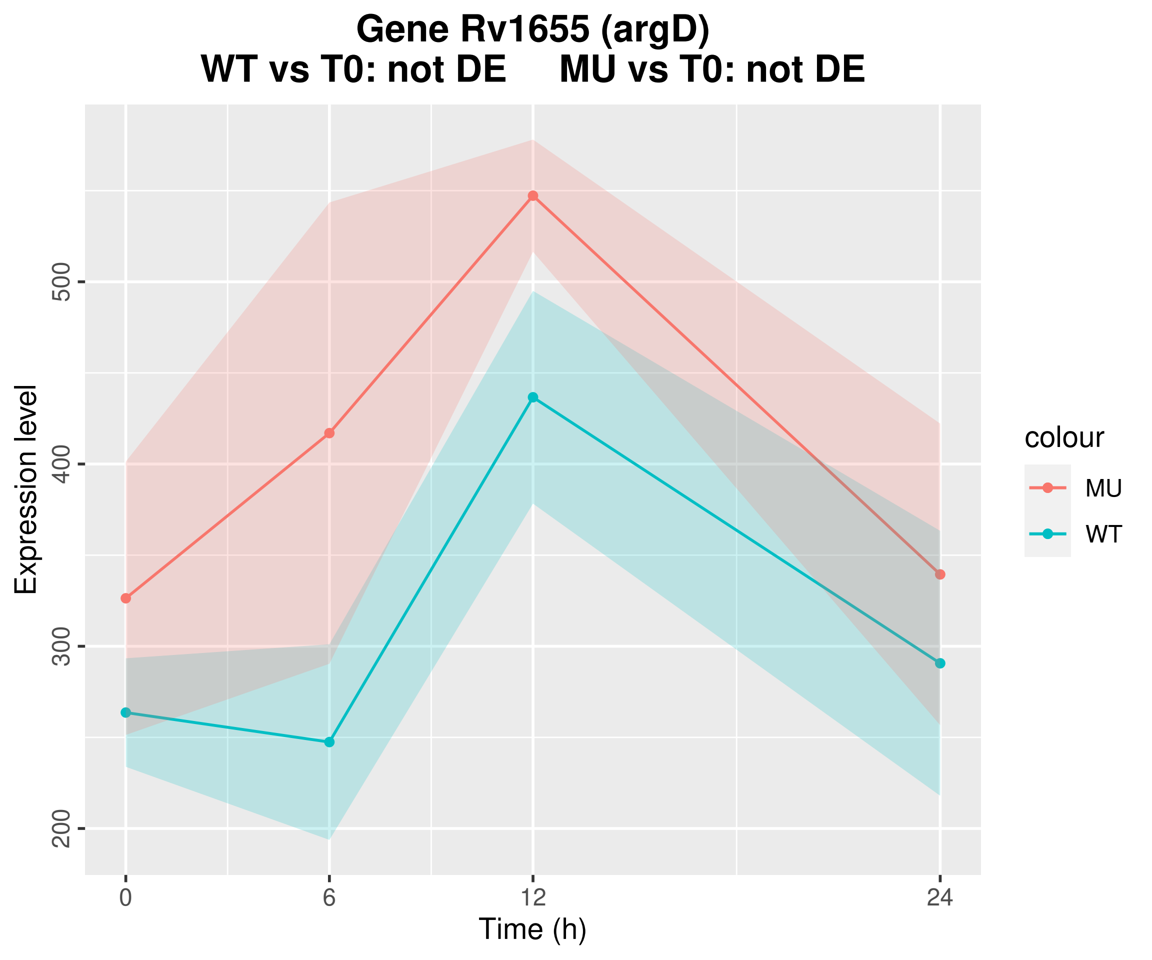
Gene Rv1655 (argD)
WT vs T0: not DE   MU vs T0: not DE
Expression level
200
300
400
500
0
6
12
24
Time (h)
colour
MU
WT

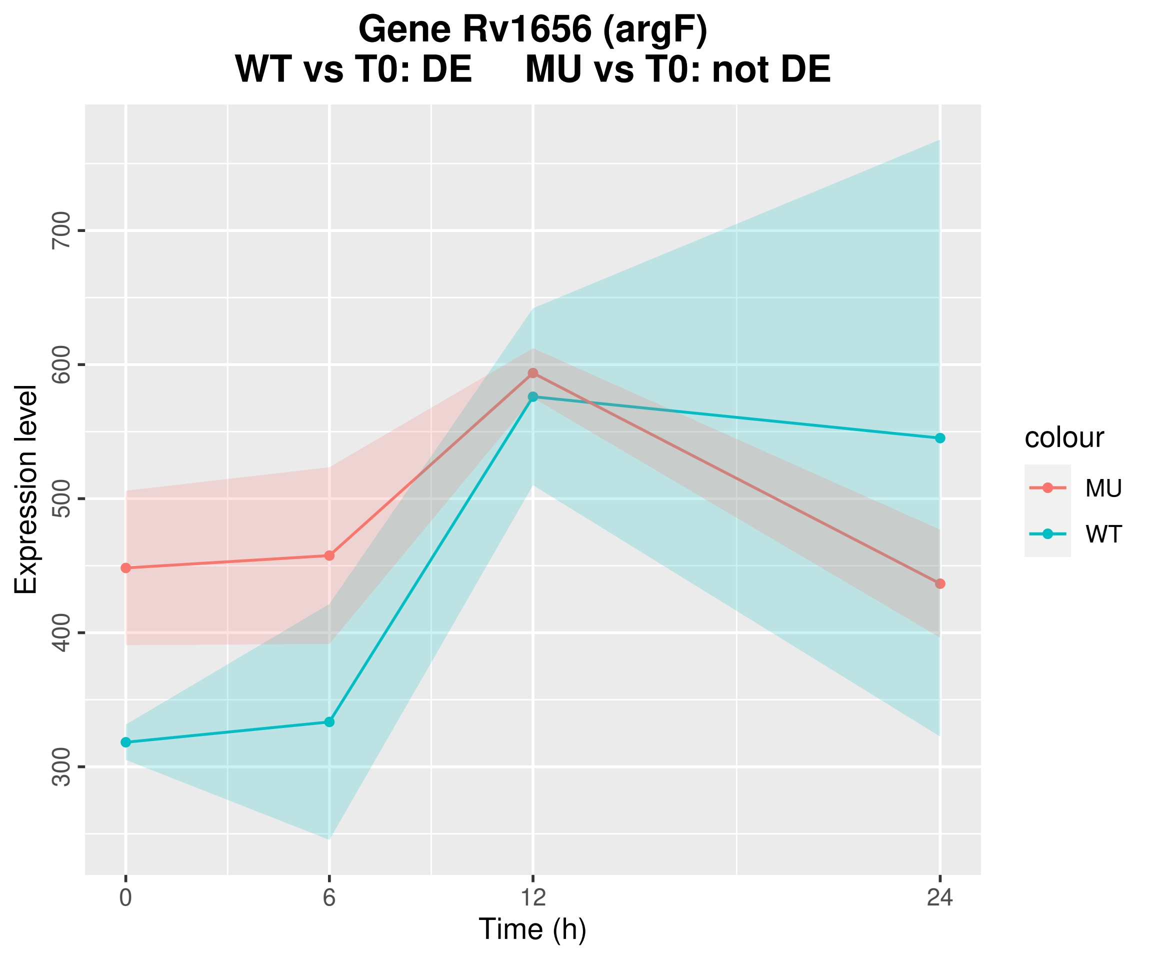
Gene Rv1656 (argF)
WT vs T0: DE   MU vs T0: not DE
Expression level
700
600
500
400
300
0
6
12
24
Time (h)
colour
MU
WT

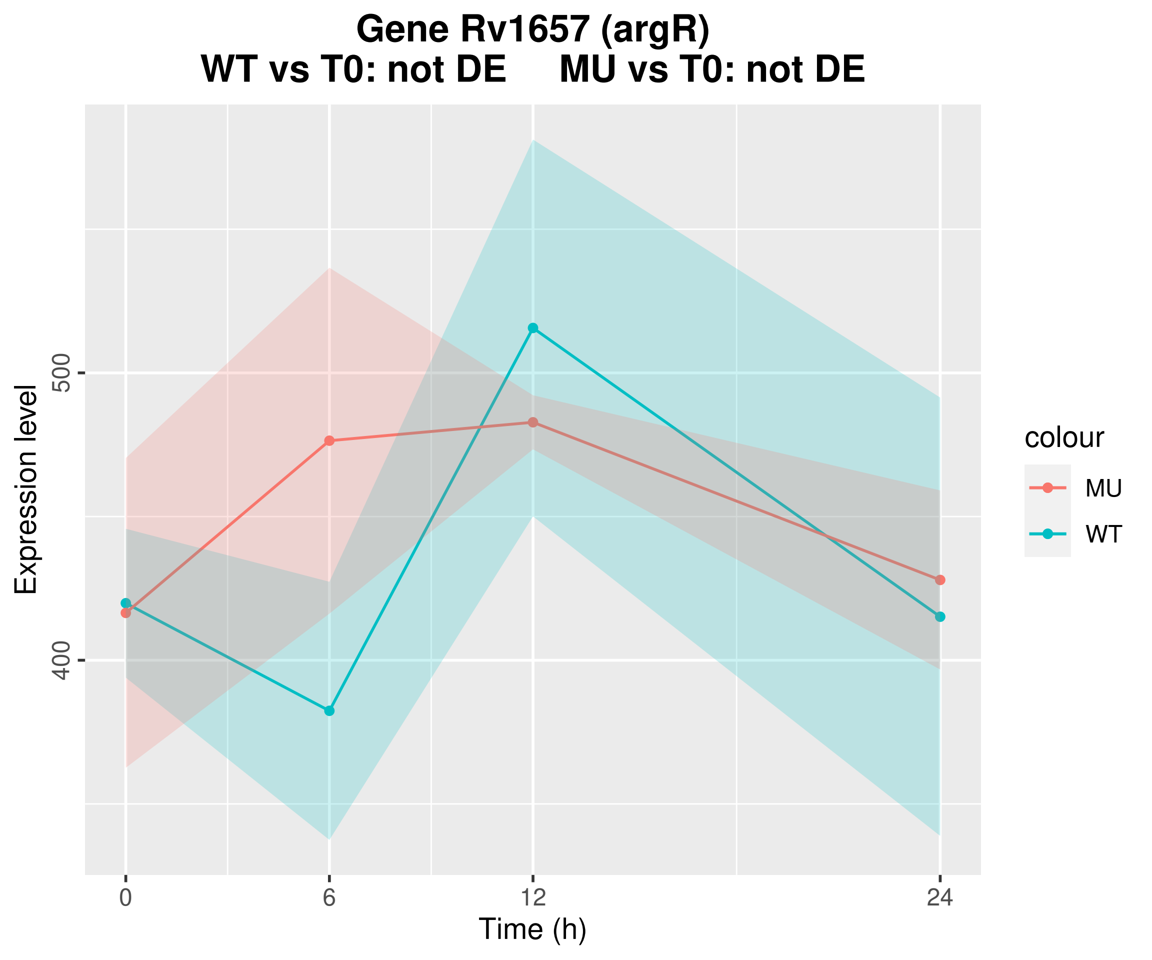

Gene Rv1657 (argR)
WT vs T0: not DE MU vs T0: not DE
Expression level
500
400
0
6
12
24
Time (h)
colour
MU
WT

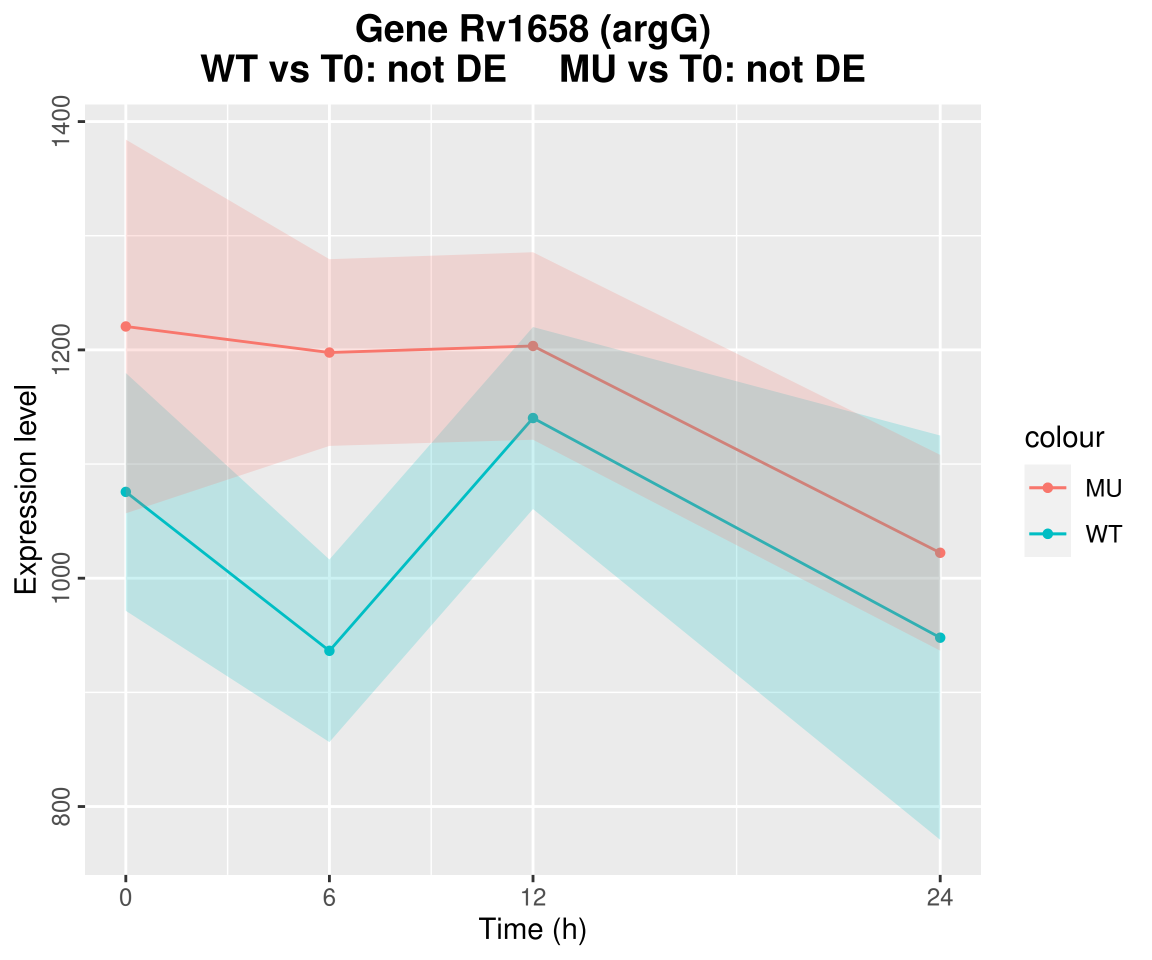

Gene Rv1658 (argG)
WT vs T0: not DE MU vs T0: not DE
Expression level
1400
1200
1000
800
0
6
12
24
Time (h)
colour
MU
WT

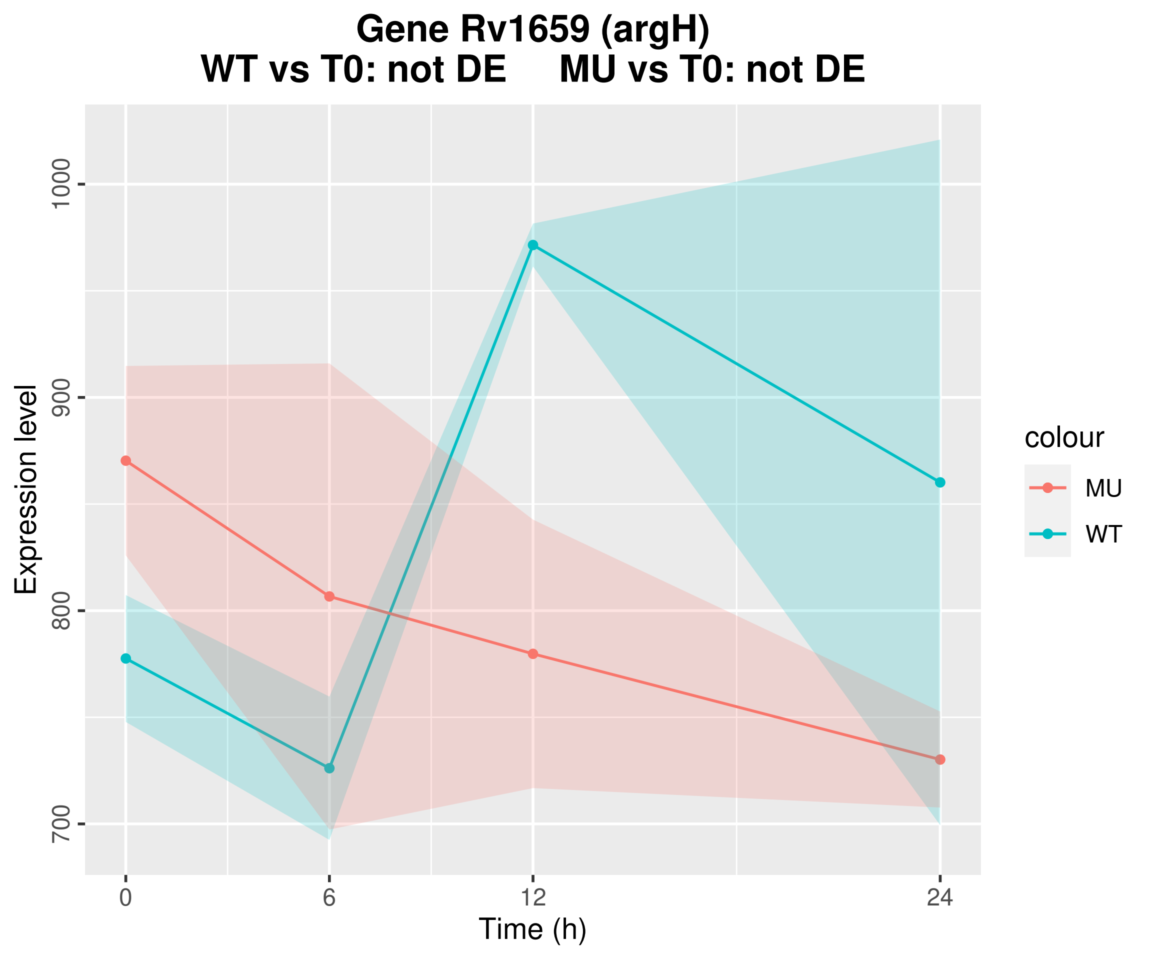
Gene Rv1659 (argH)
WT vs T0: not DE   MU vs T0: not DE
Expression level
1000
900
800
700
0
6
12
24
Time (h)
colour
MU
WT

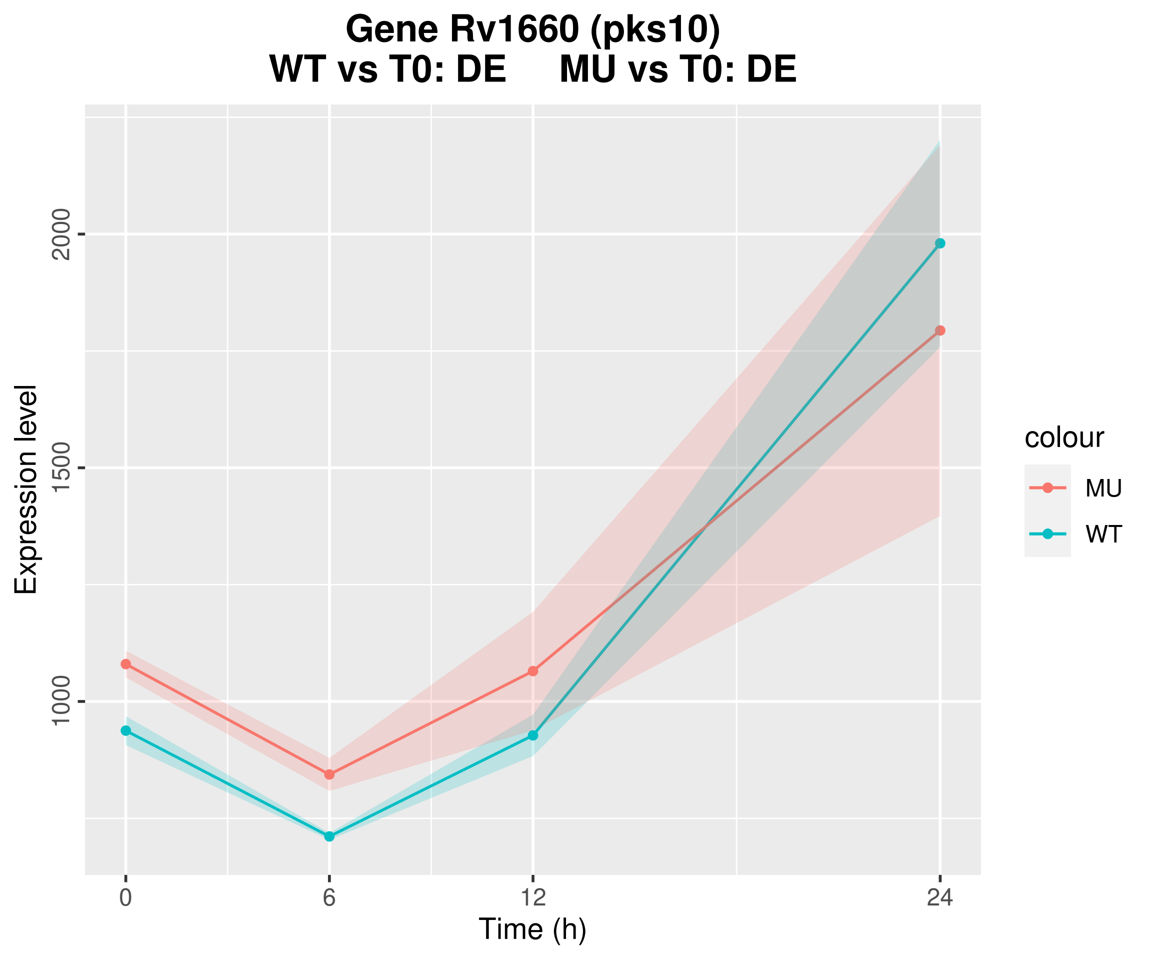
Gene Rv1660 (pks10)
WT vs T0: DE   MU vs T0: DE
Expression level
2000
1500
1000
0
6
12
24
Time (h)
colour
MU
WT

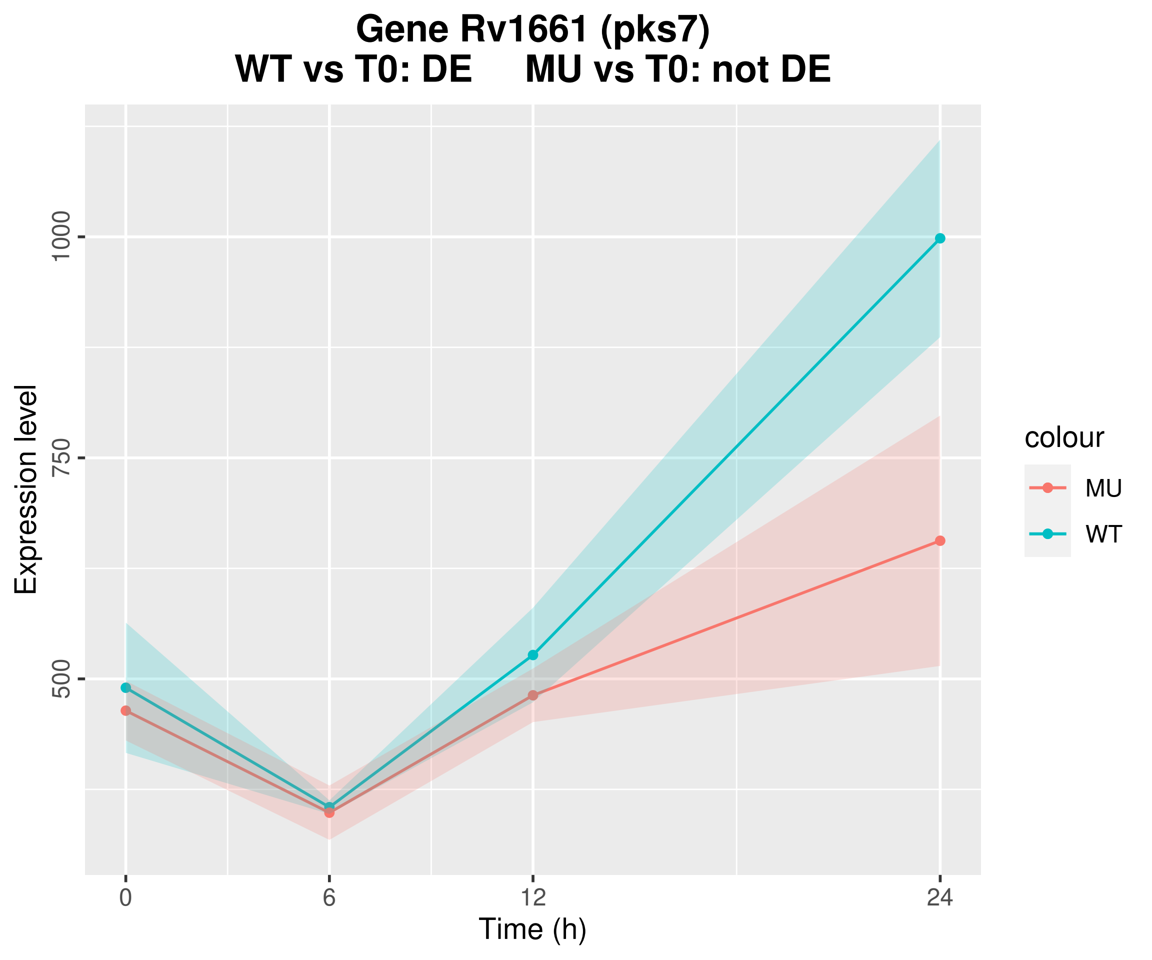
Gene Rv1661 (pks7)
WT vs T0: DE   MU vs T0: not DE
Expression level
1000
750
500
0
6
12
24
Time (h)
colour
MU
WT

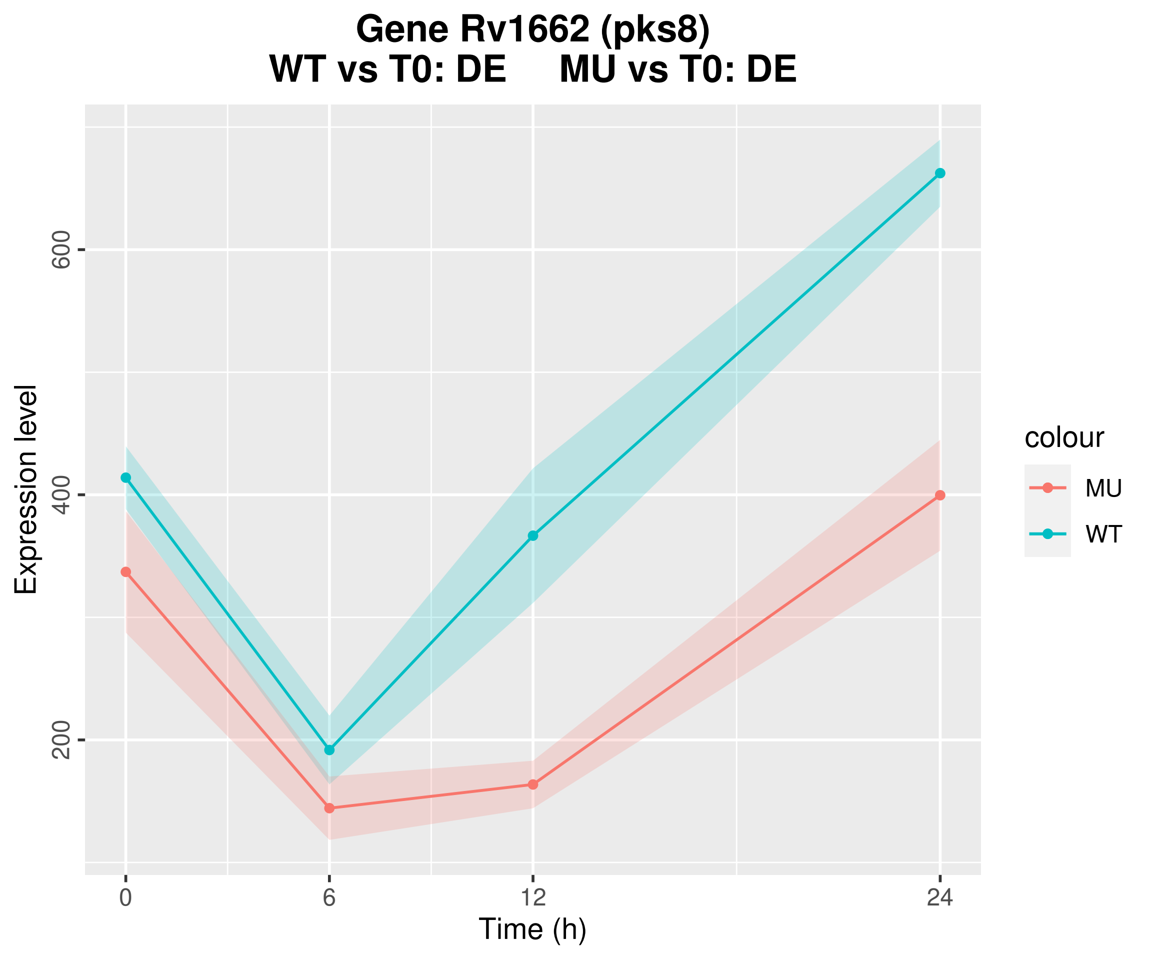
Gene Rv1662 (pks8)
WT vs T0: DE
MU vs T0: DE
Expression level
600
400
200
0
6
12
24
Time (h)
colour
MU
WT

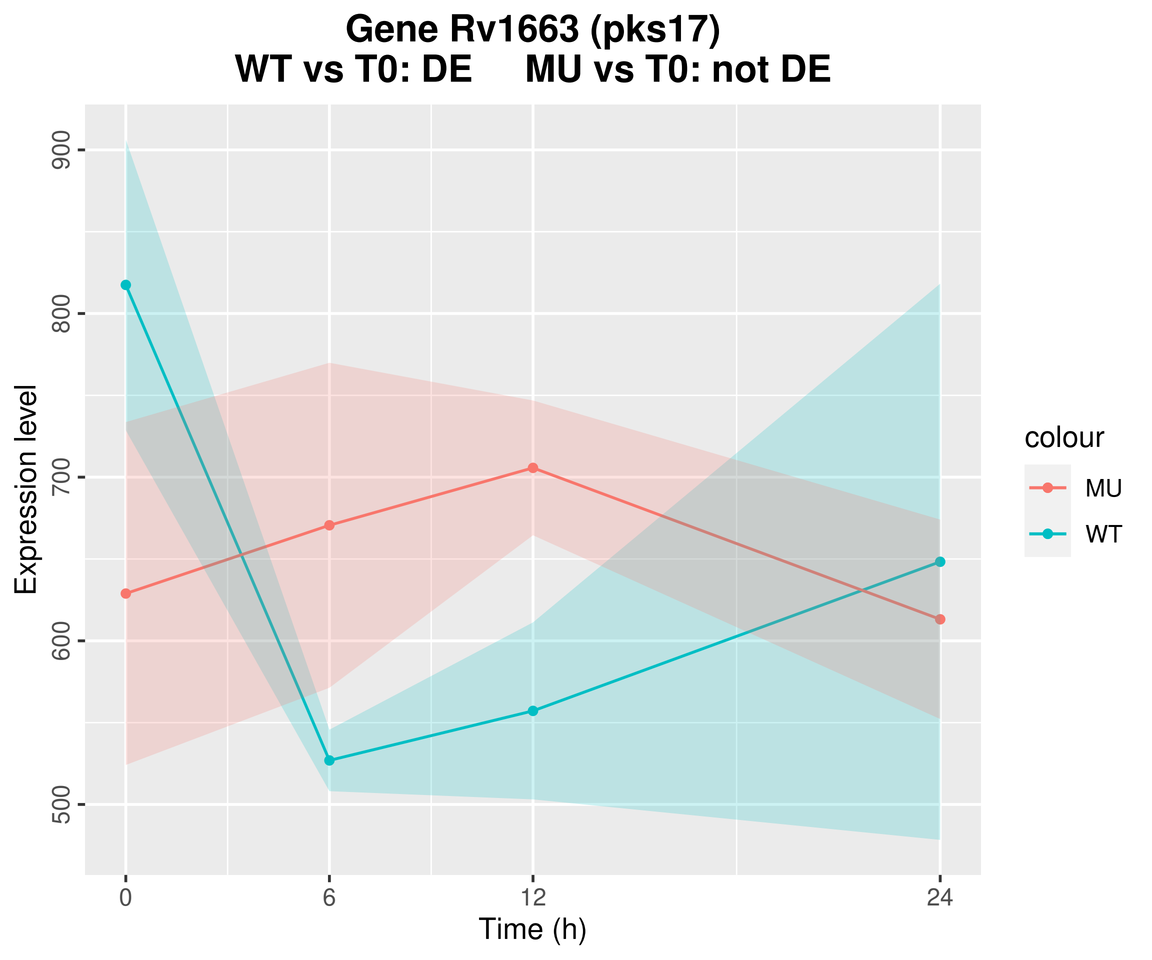
Gene Rv1663 (pks17)
WT vs T0: DE   MU vs T0: not DE
Expression level
900
800
700
600
500
0
6
12
24
Time (h)
colour
MU
WT

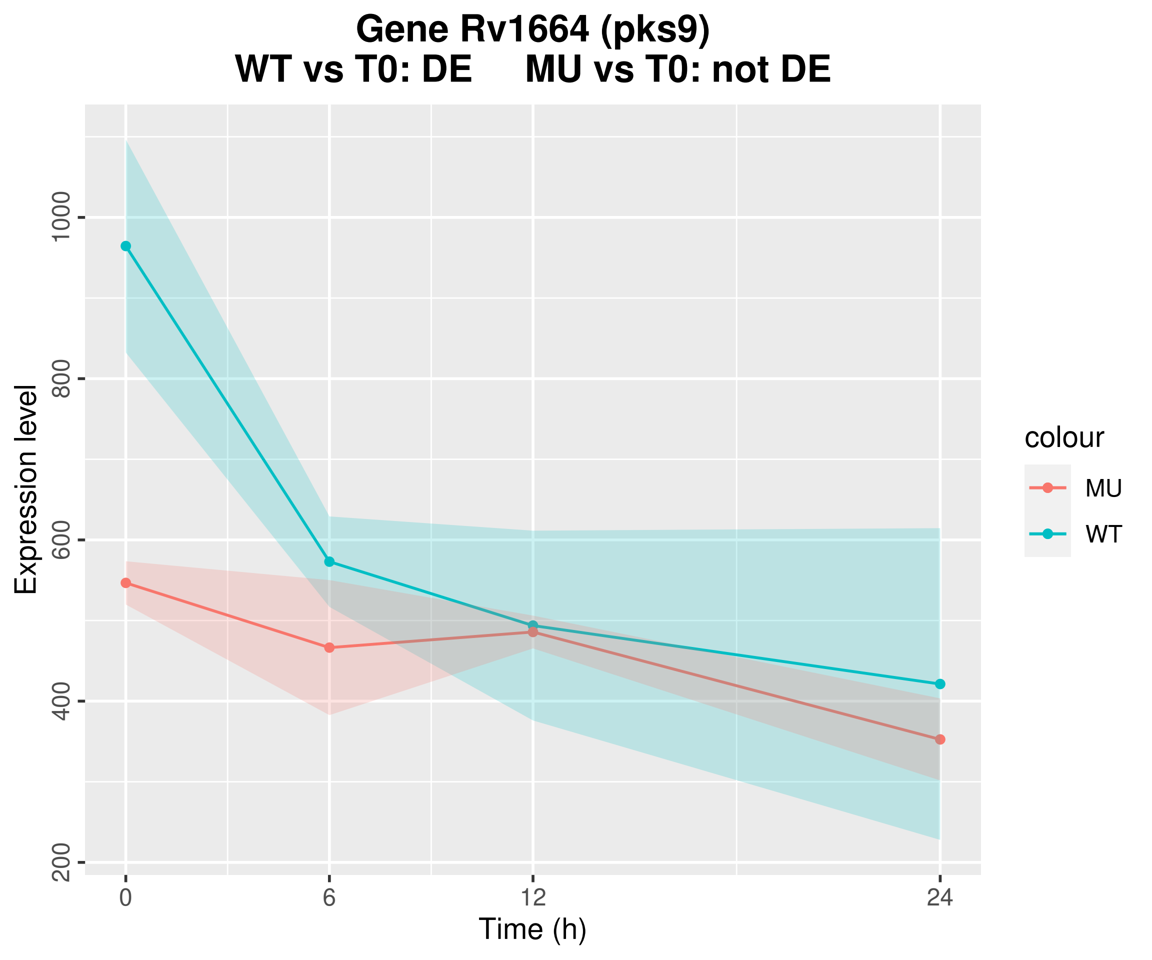

Gene Rv1664 (pks9)
WT vs T0: DE MU vs T0: not DE
Expression level
1000
800
600
400
200
0
6
12
24
Time (h)
colour
MU
WT

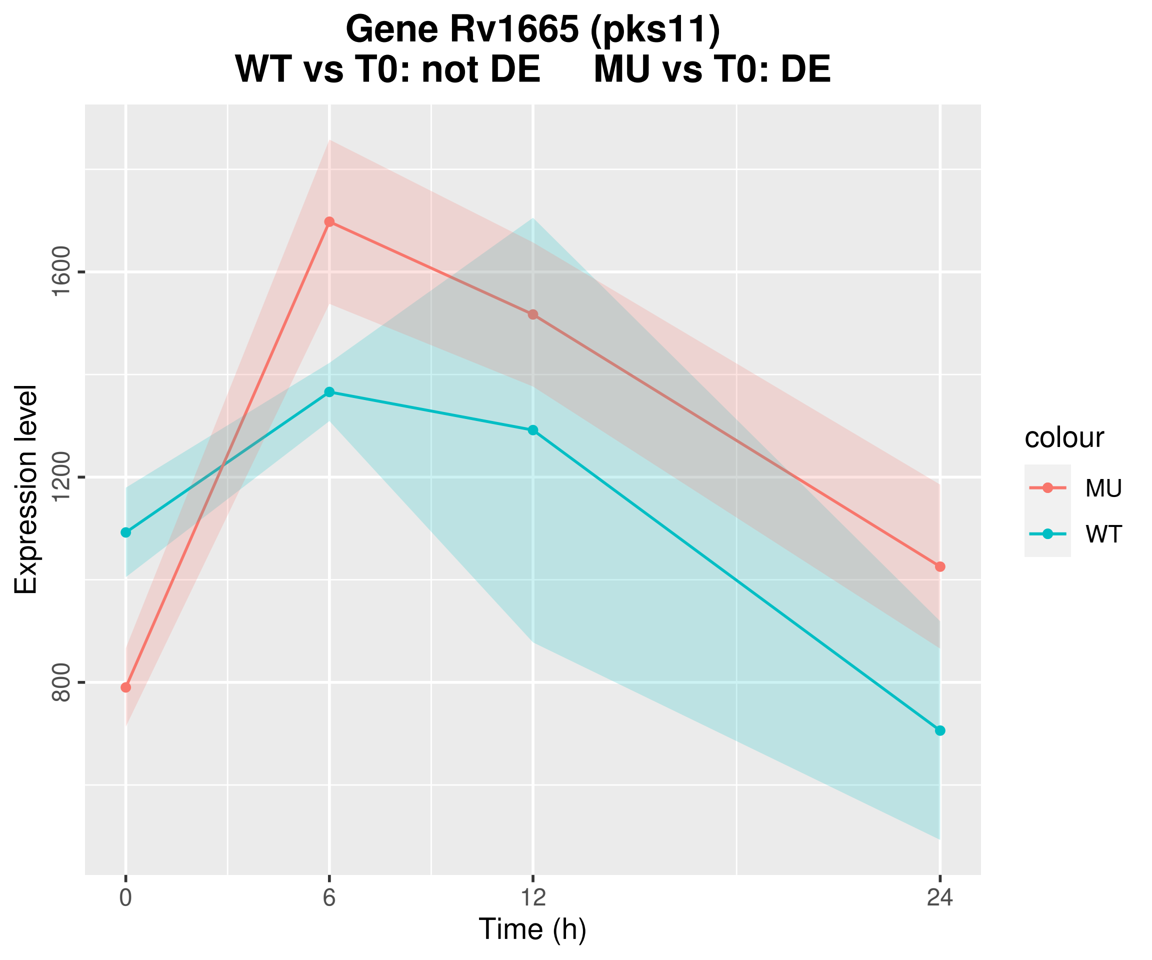

Gene Rv1665 (pks11)
WT vs T0: not DE   MU vs T0: DE
Expression level
1600
1200
800
0
6
12
24
Time (h)
colour
MU
WT

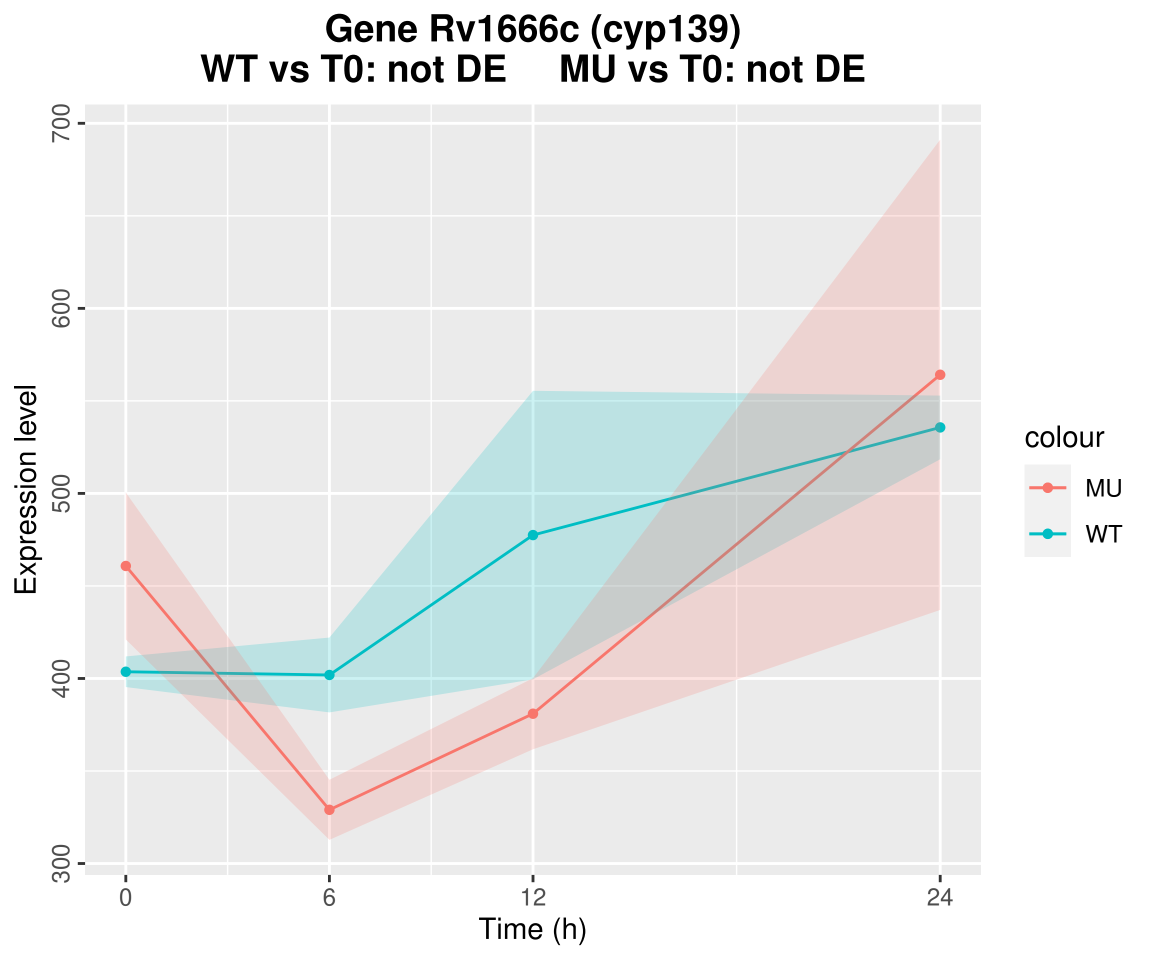

Gene Rv1666c (cyp139)
WT vs T0: not DE MU vs T0: not DE
Expression level
700
600
500
400
300
0
6
12
24
Time (h)
colour
MU
WT

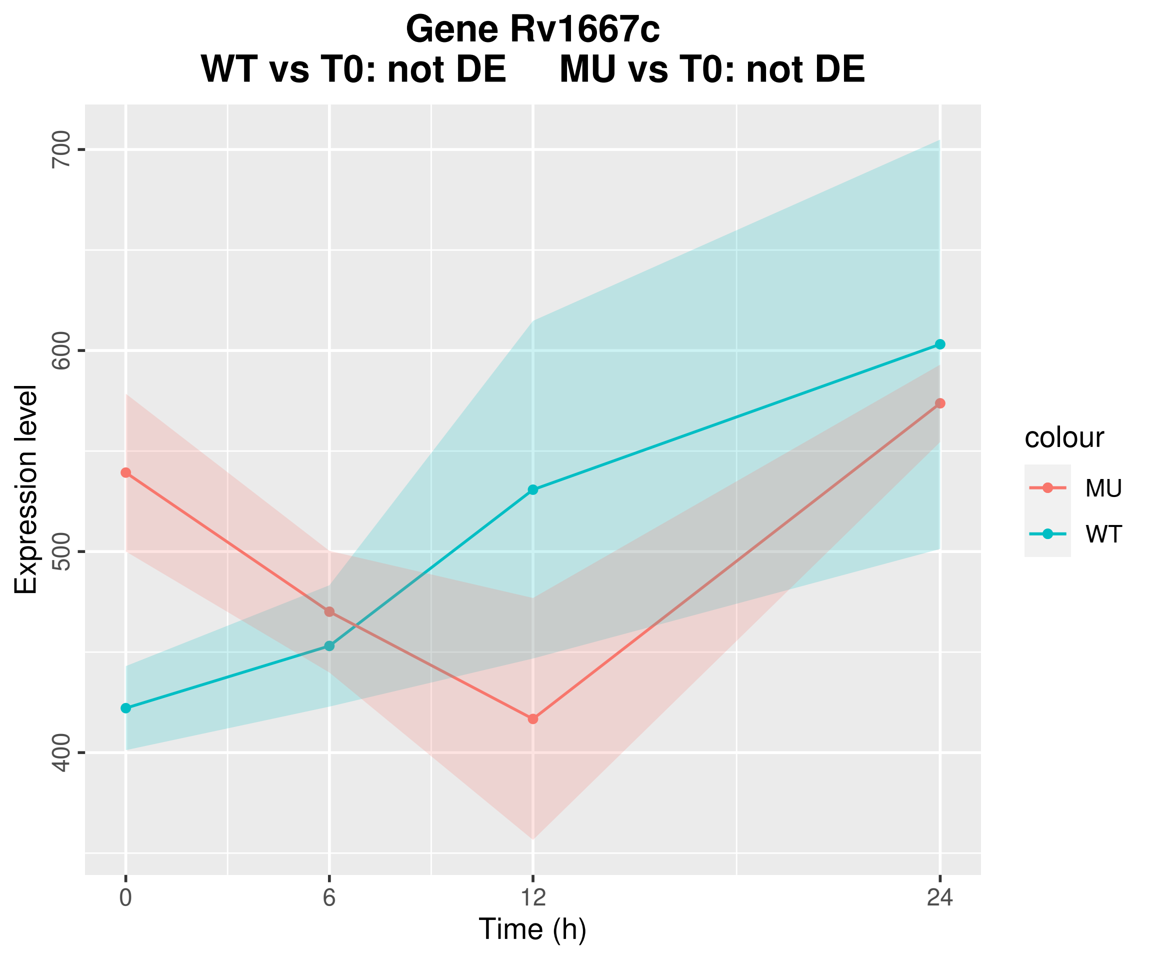

Gene Rv1667c
WT vs T0: not DE MU vs T0: not DE
Expression level
700
600
500
400
0
6
12
24
Time (h)
colour
MU
WT

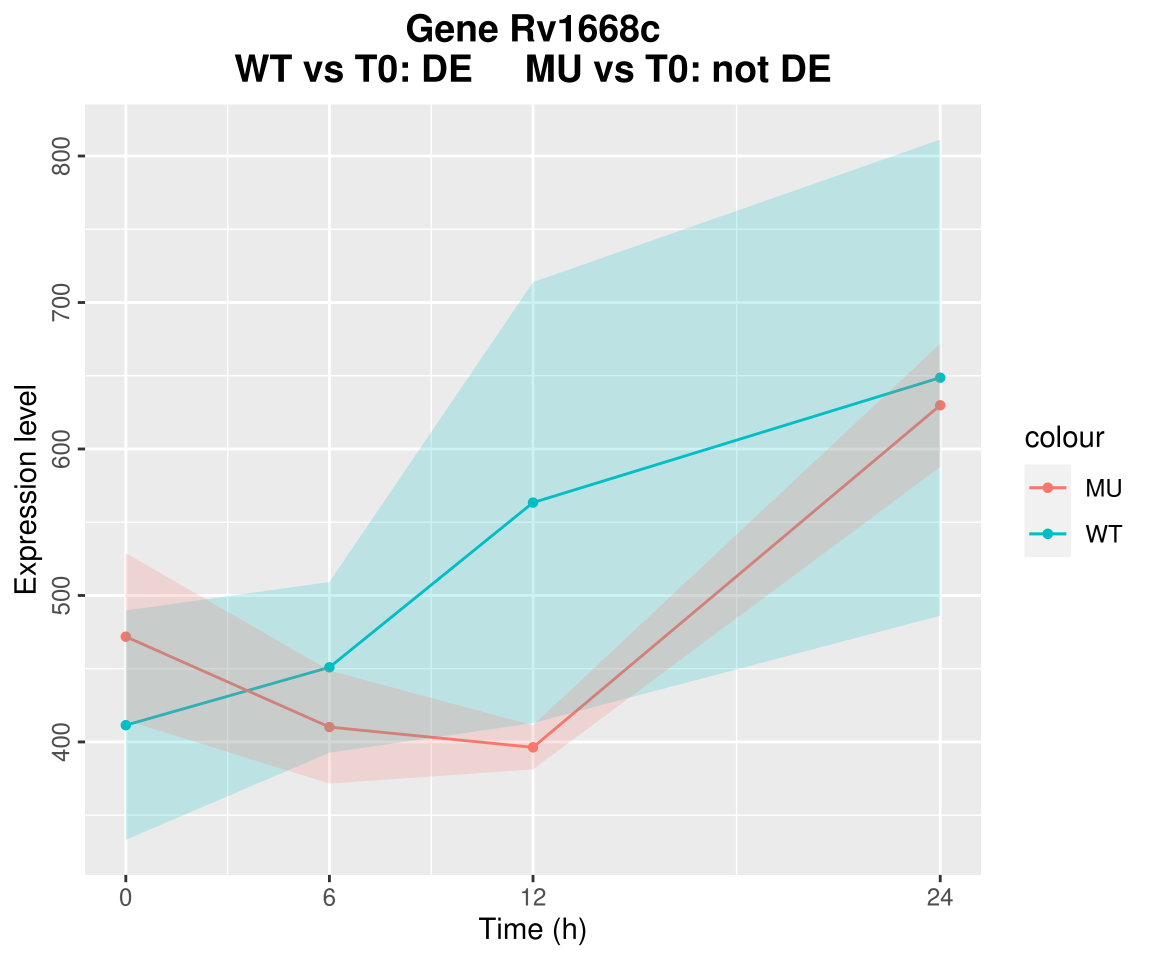
Gene Rv1668c
WT vs T0: DE   MU vs T0: not DE
Expression level
800
700
600
500
400
0
6
12
24
Time (h)
colour
MU
WT

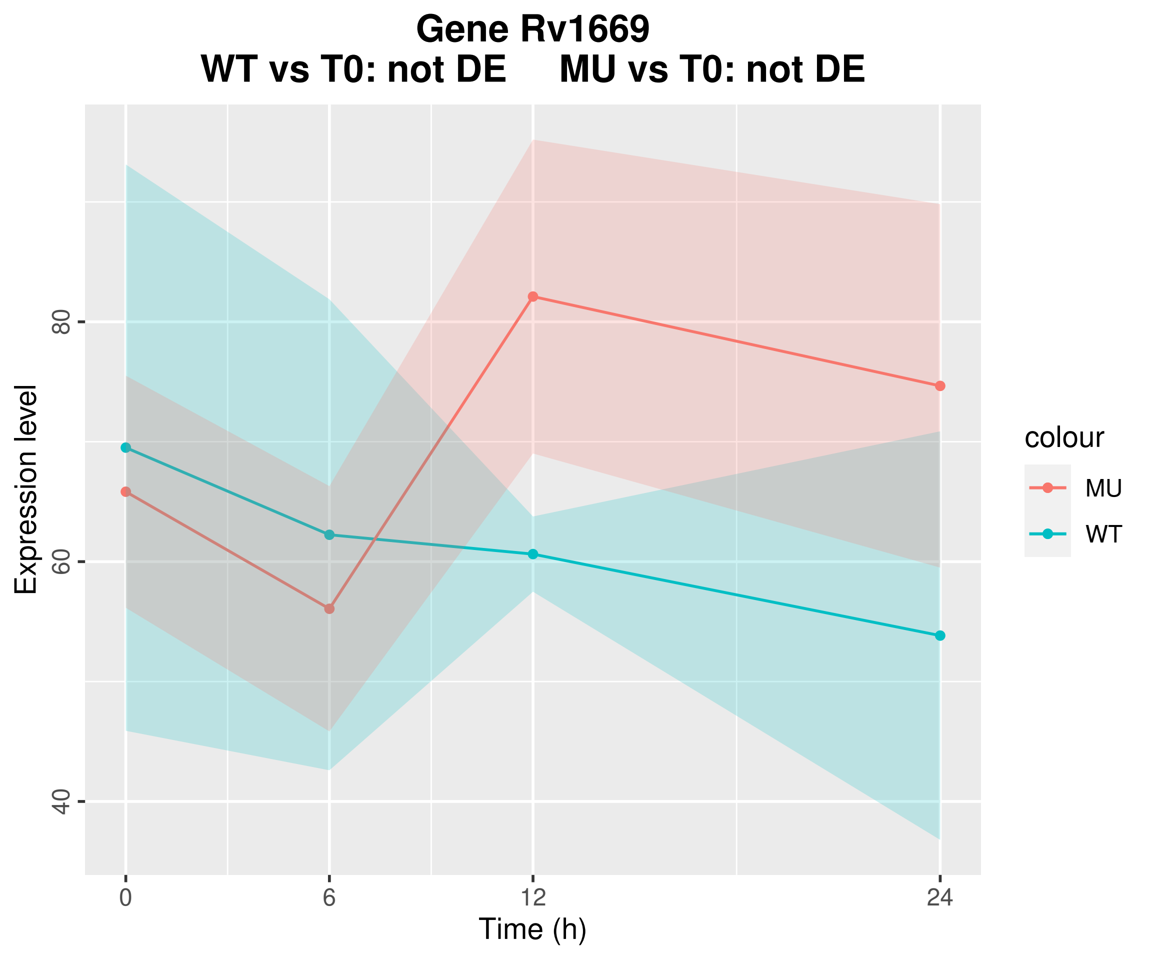
Gene Rv1669
WT vs T0: not DE   MU vs T0: not DE
Expression level
80
60
40
0
6
12
24
Time (h)
colour
MU
WT

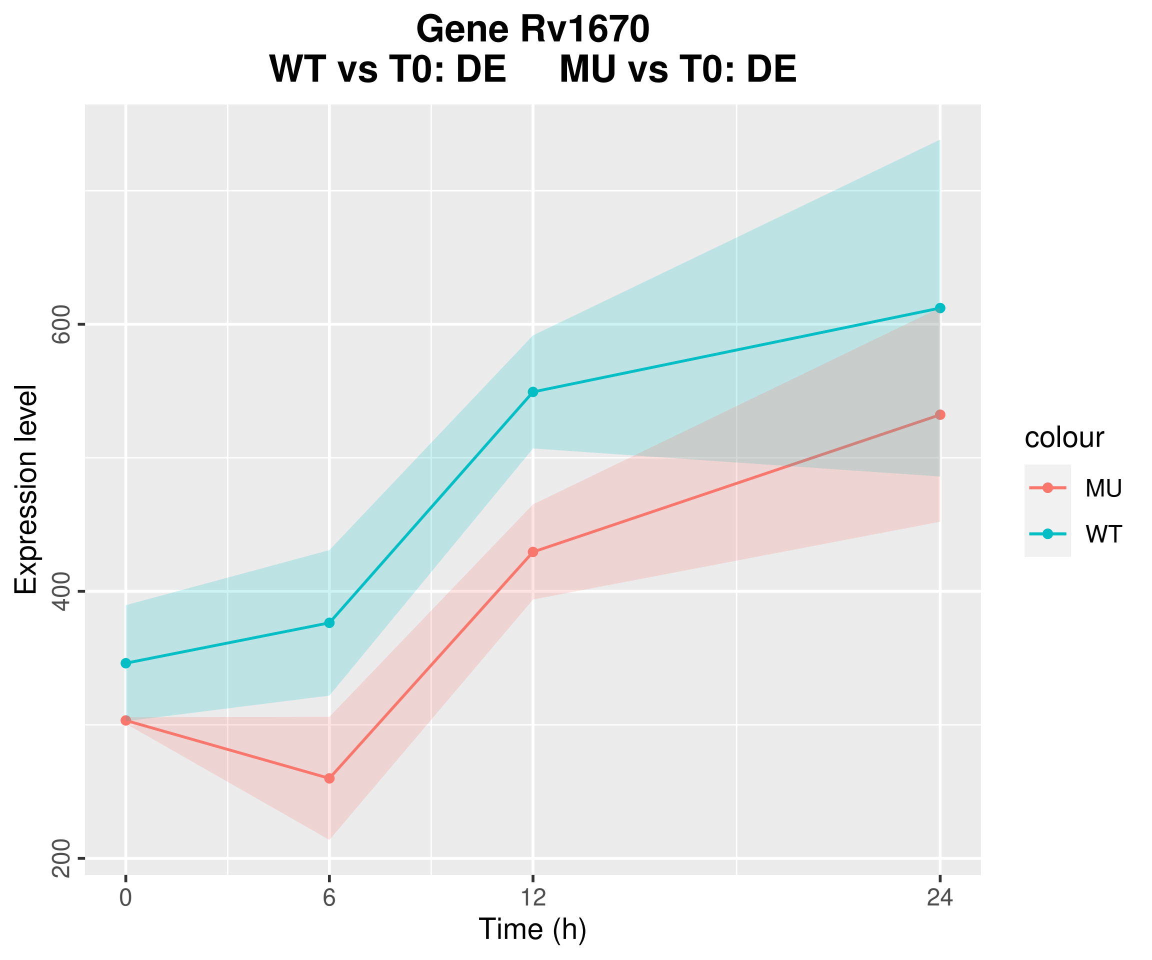
Gene Rv1670
WT vs T0: DE MU vs T0: DE
Expression level
600
400
200
0
6
12
24
Time (h)
colour
MU
WT

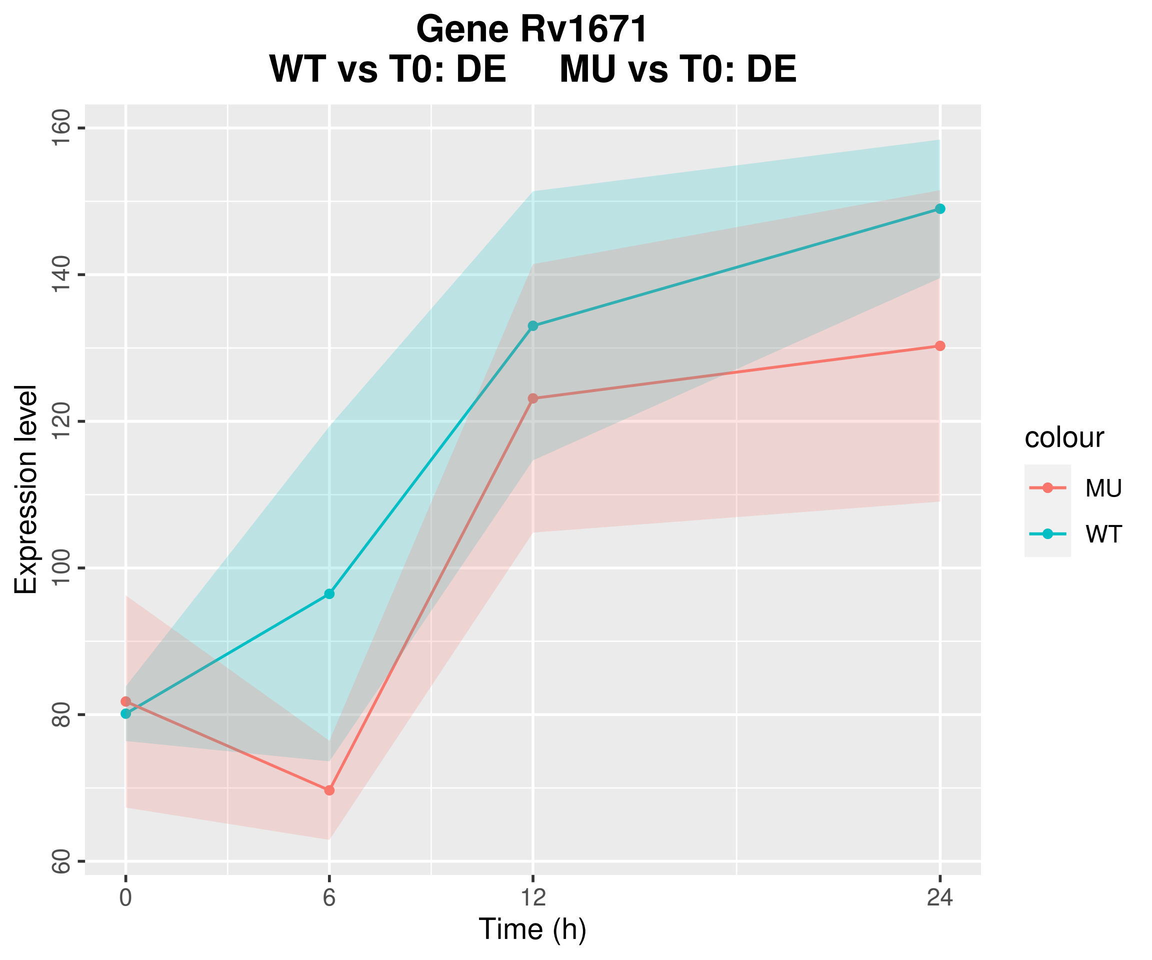
Gene Rv1671
WT vs T0: DE   MU vs T0: DE
Expression level
160
140
120
100
80
60
0
6
12
24
Time (h)
colour
MU
WT

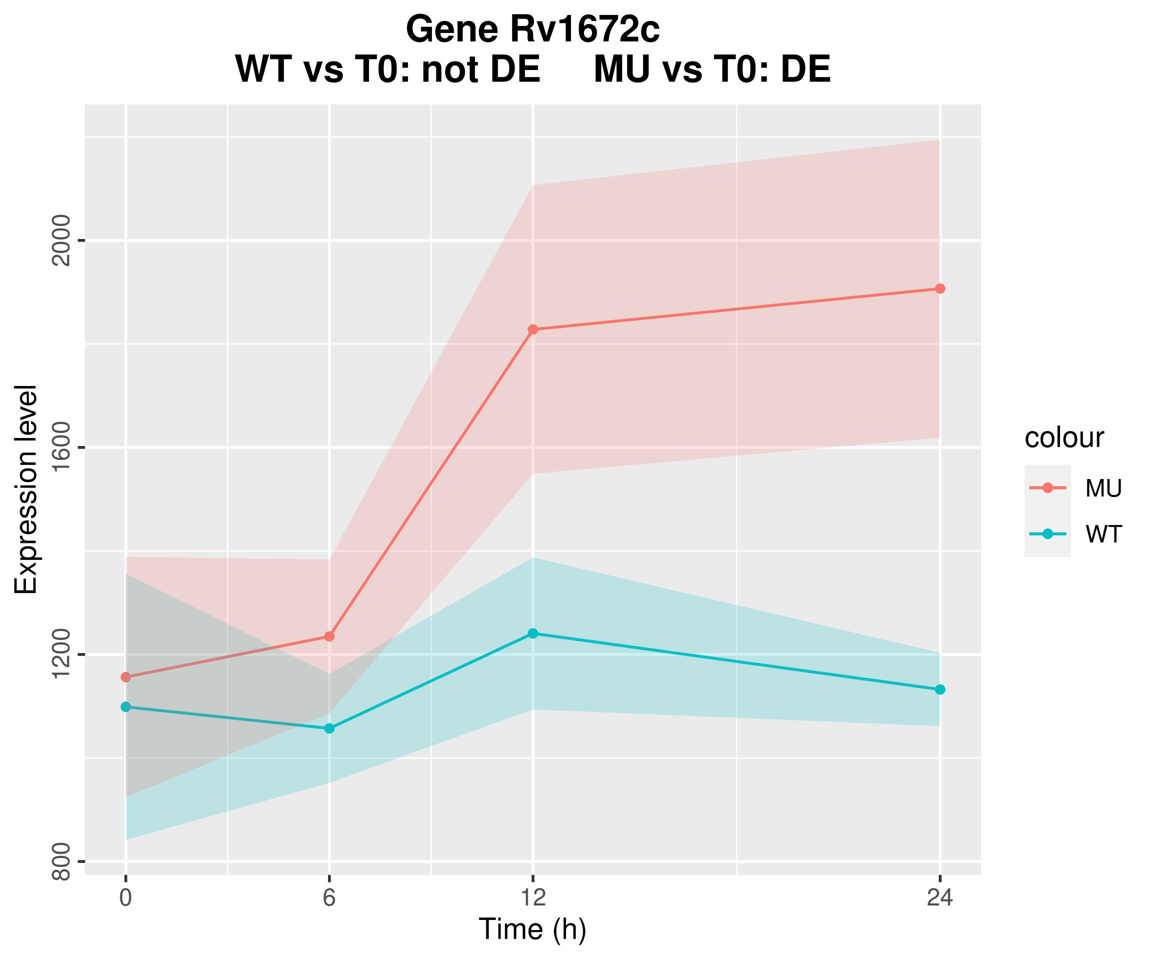

Gene Rv1672c
WT vs T0: not DE   MU vs T0: DE
Expression level
2000
1600
1200
800
0
6
12
24
Time (h)
colour
MU
WT

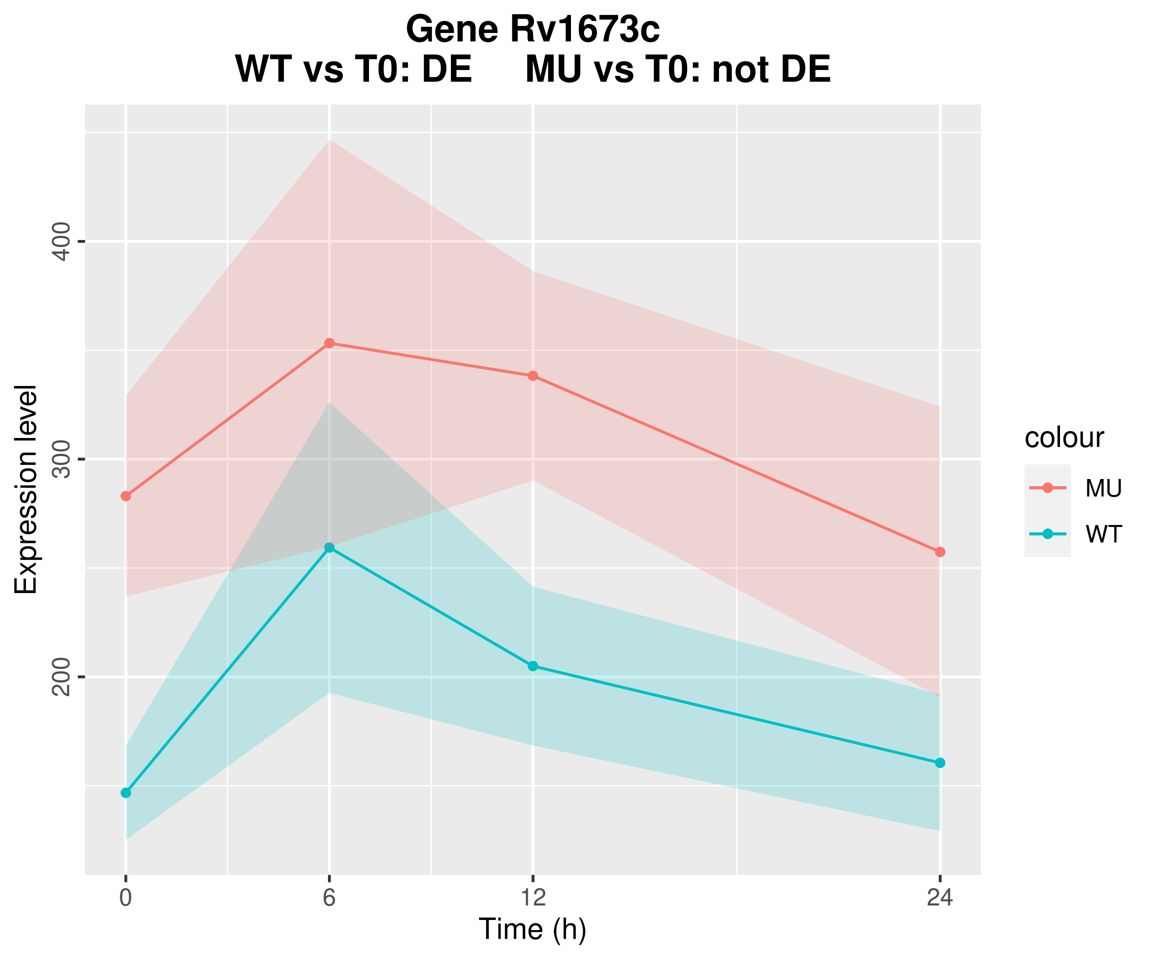

Gene Rv1673c
WT vs T0: DE   MU vs T0: not DE
Expression level
400
300
200
0
6
12
24
Time (h)
colour
MU
WT

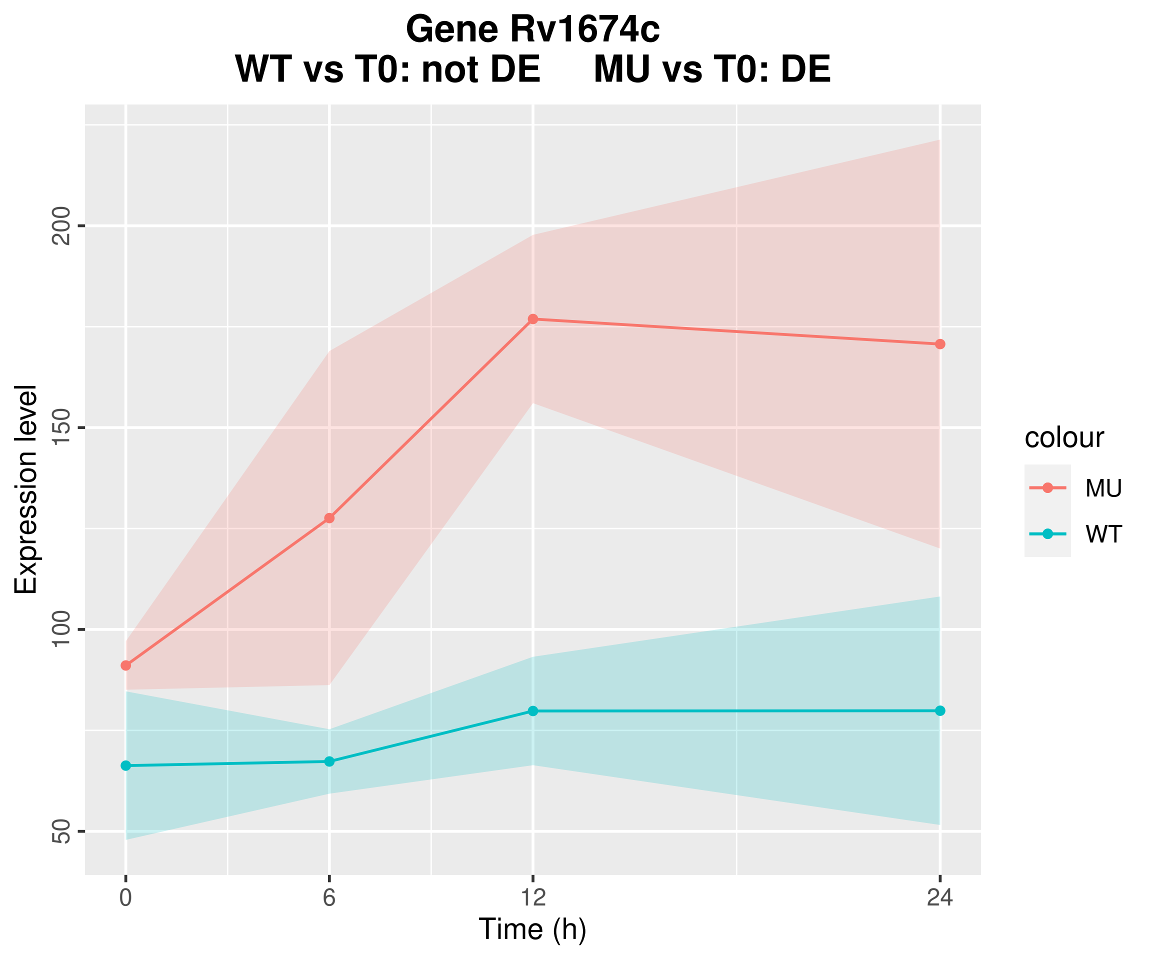

Gene Rv1674c
WT vs T0: not DE   MU vs T0: DE
Expression level
200
150
100
50
0
6
12
24
Time (h)
colour
MU
WT

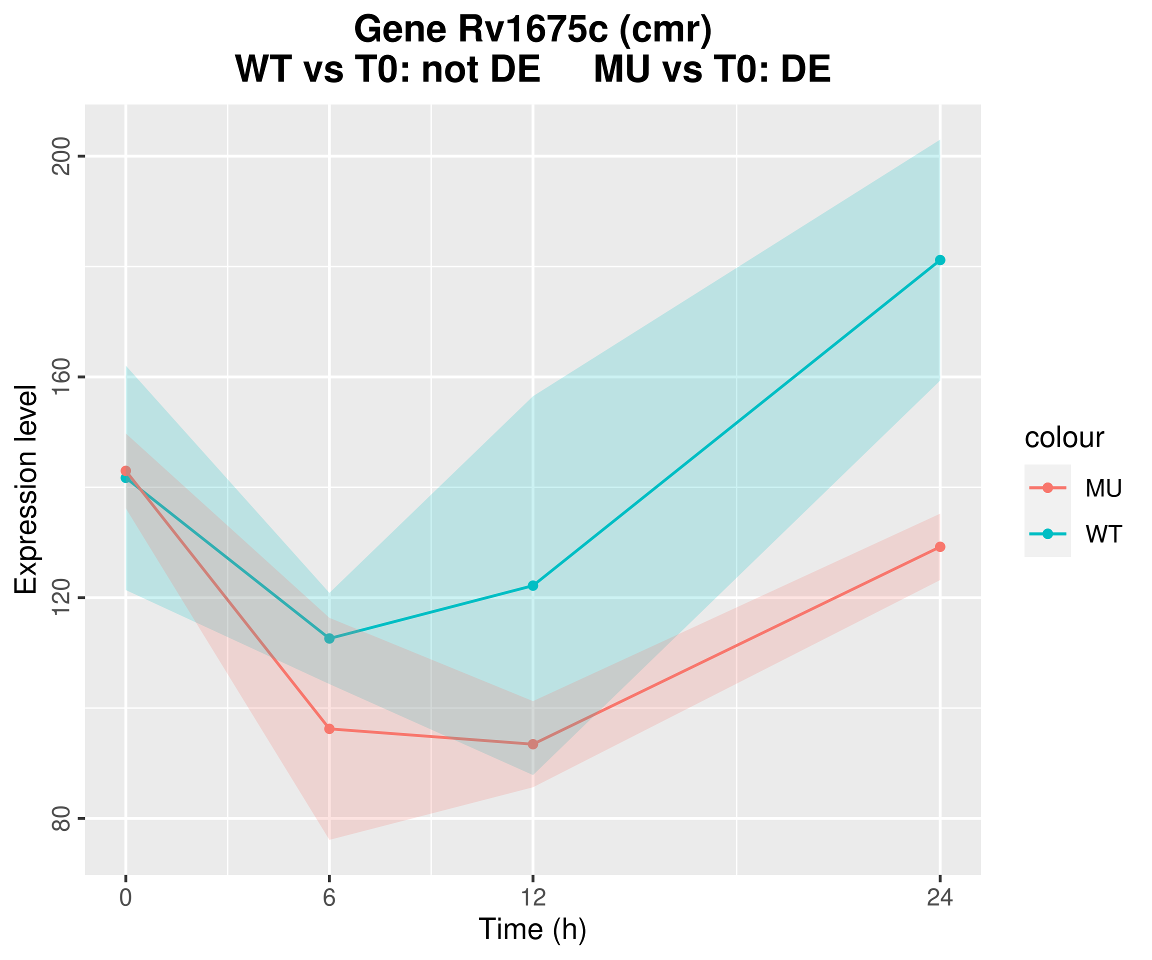

Gene Rv1675c (cmr)
WT vs T0: not DE MU vs T0: DE
200
160
120
80
Expression level
0
6
12
24
Time (h)
colour
MU
WT

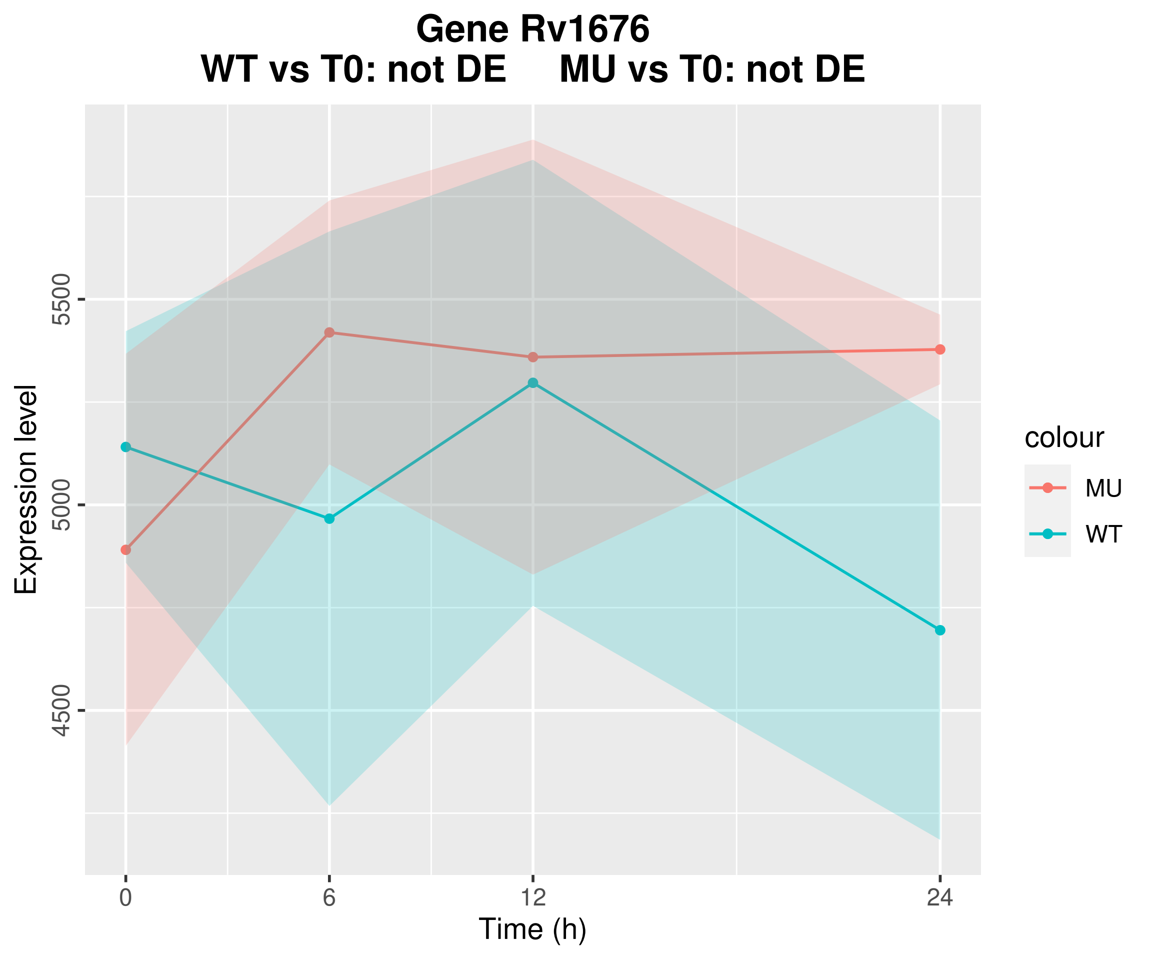

Gene Rv1676
WT vs T0: not DE MU vs T0: not DE
Expression level
5500
5000
4500
0
6
12
24
Time (h)
colour
MU
WT

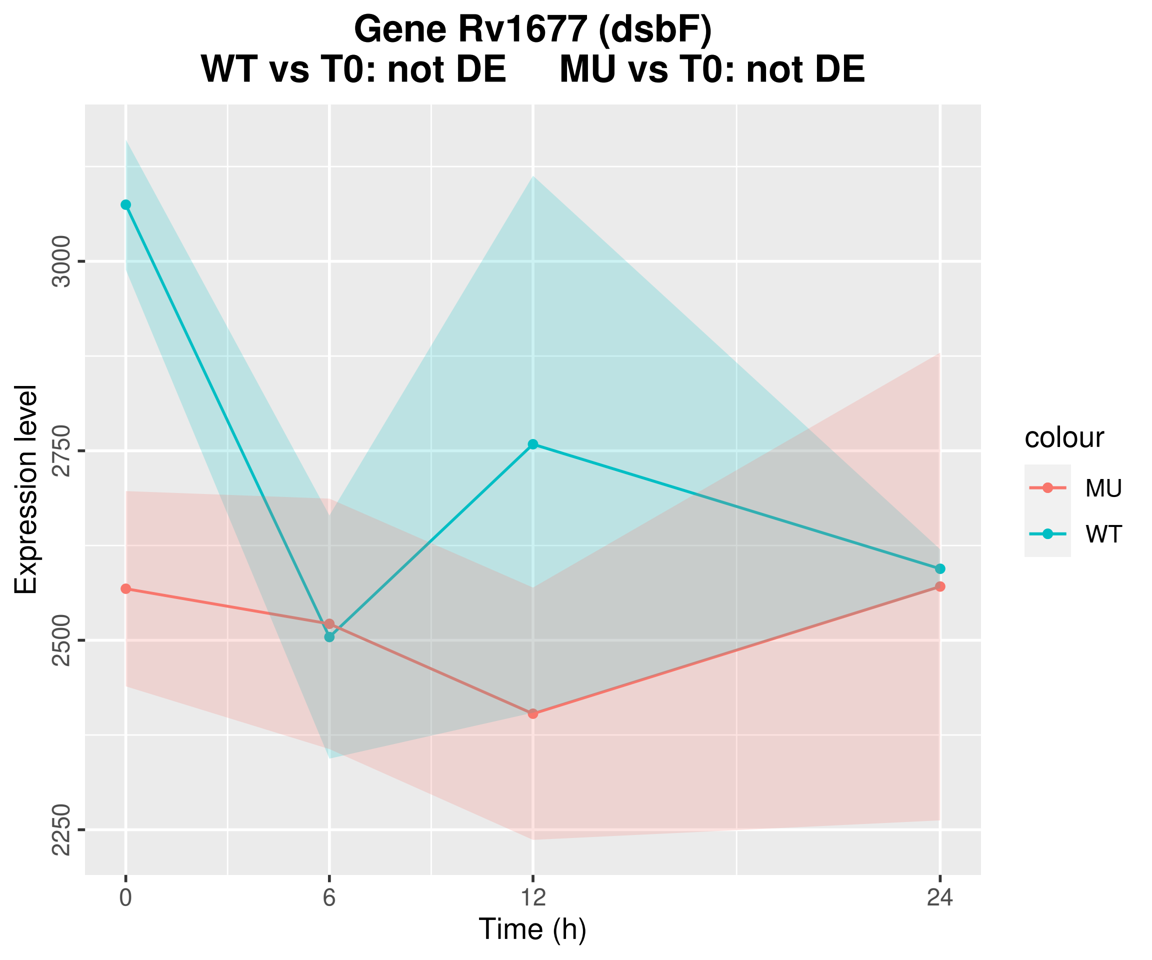
Gene Rv1677 (dsbF)
WT vs T0: not DE   MU vs T0: not DE
Expression level
3000
2750
2500
2250
0
6
12
24
Time (h)
colour
MU
WT

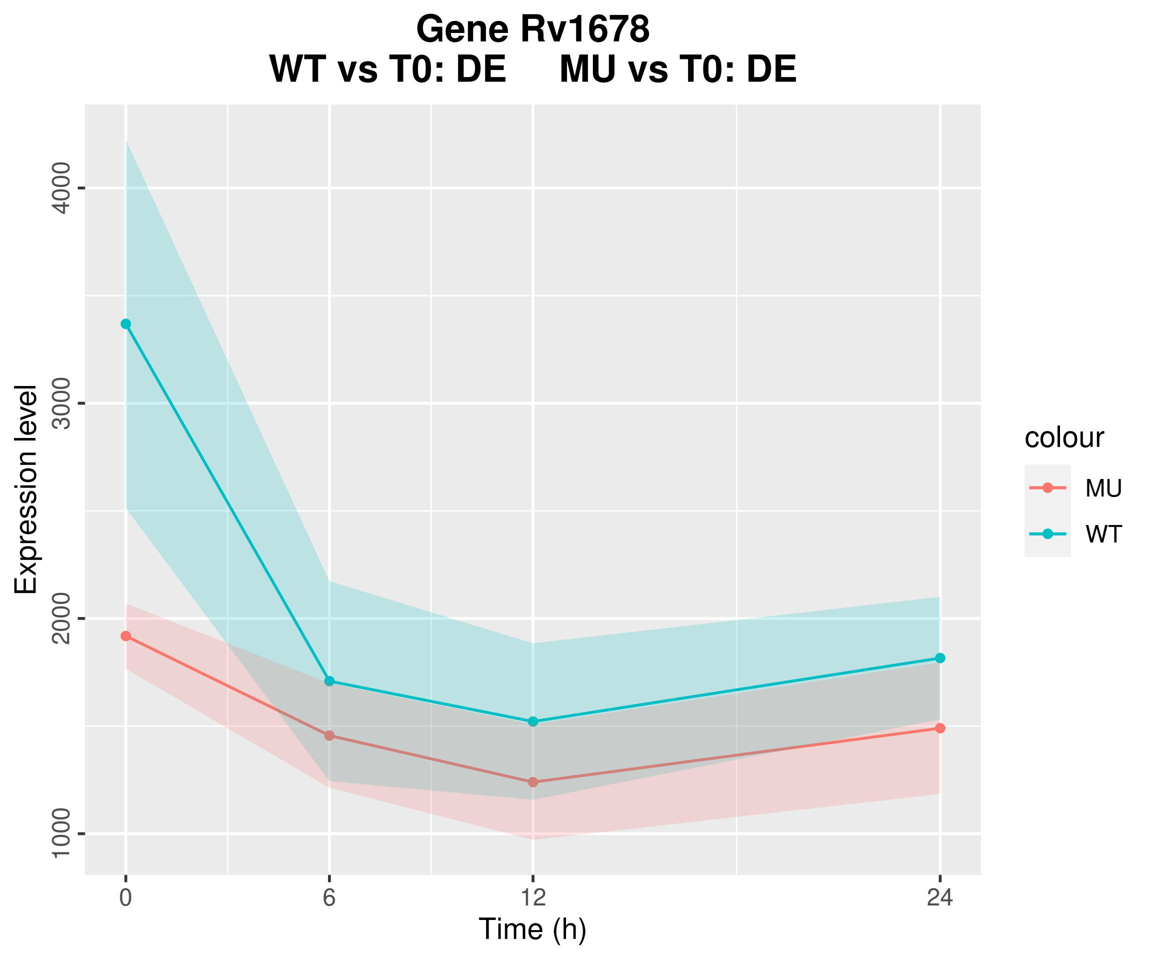

Gene Rv1678
WT vs T0: DE   MU vs T0: DE
Expression level
4000
3000
2000
1000
0
6
12
24
Time (h)
colour
MU
WT

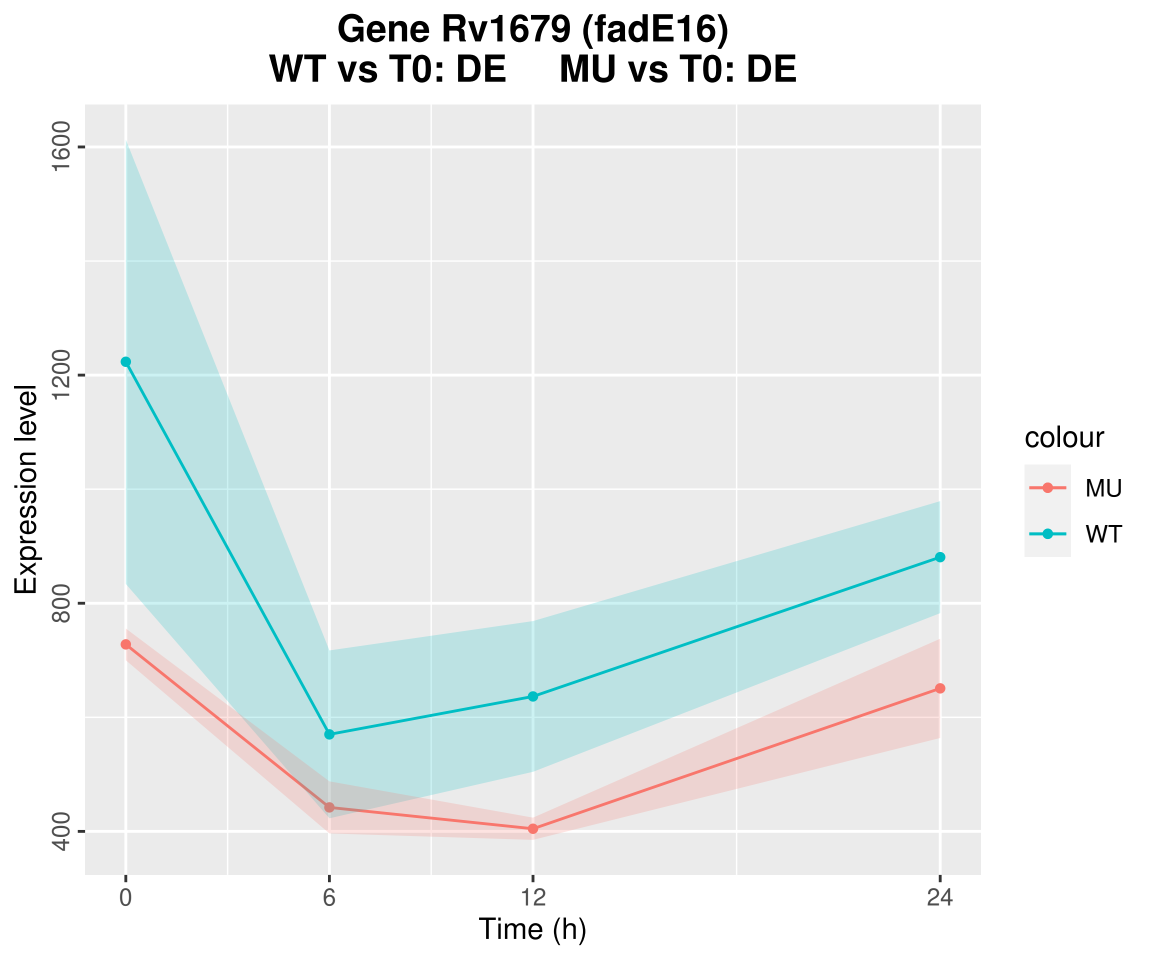
Gene Rv1679 (fadE16)
WT vs T0: DE MU vs T0: DE
Expression level
1600
1200
800
400
0
6
12
24
Time (h)
colour
MU
WT

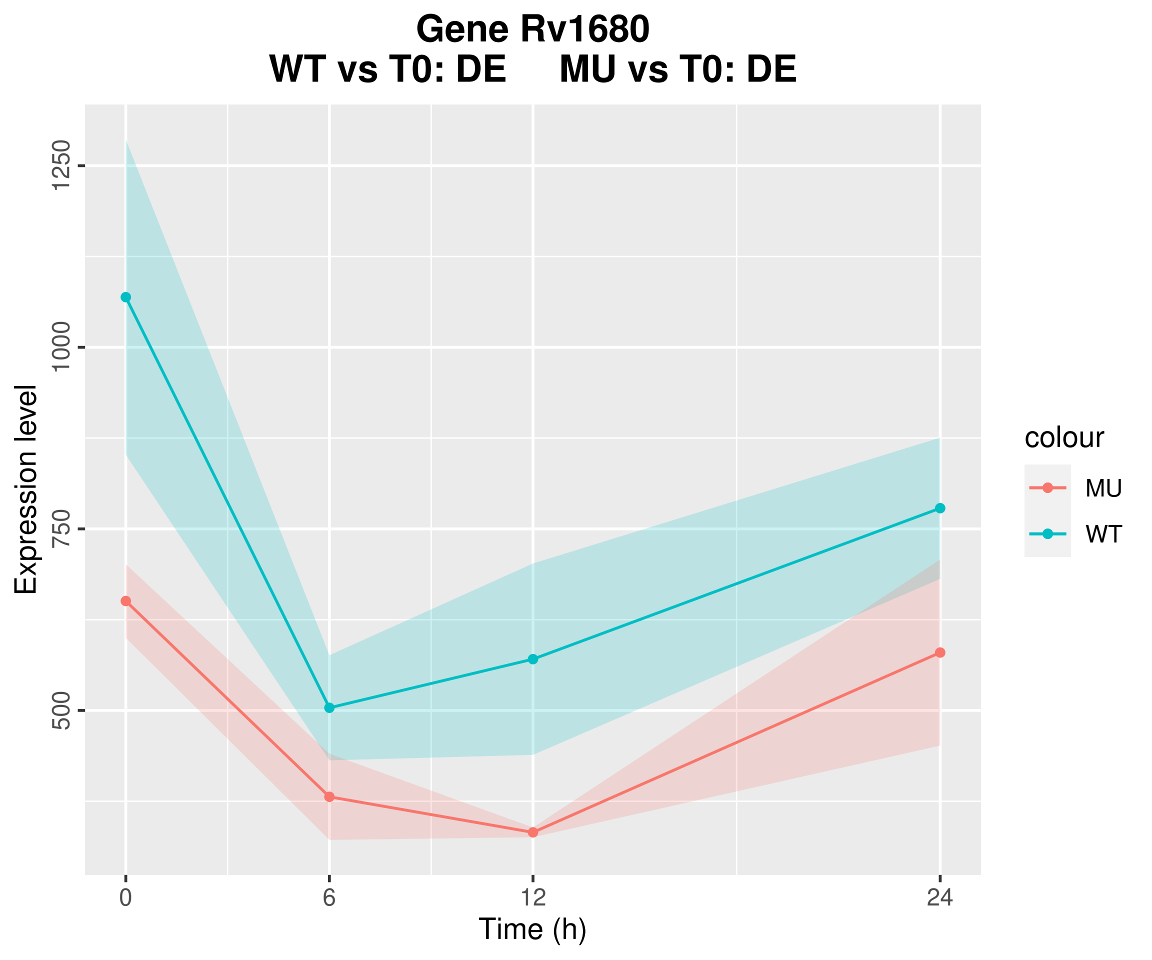
Gene Rv1680
WT vs T0: DE   MU vs T0: DE
Expression level
1250
1000
750
500
0
6
12
24
Time (h)
colour
MU
WT

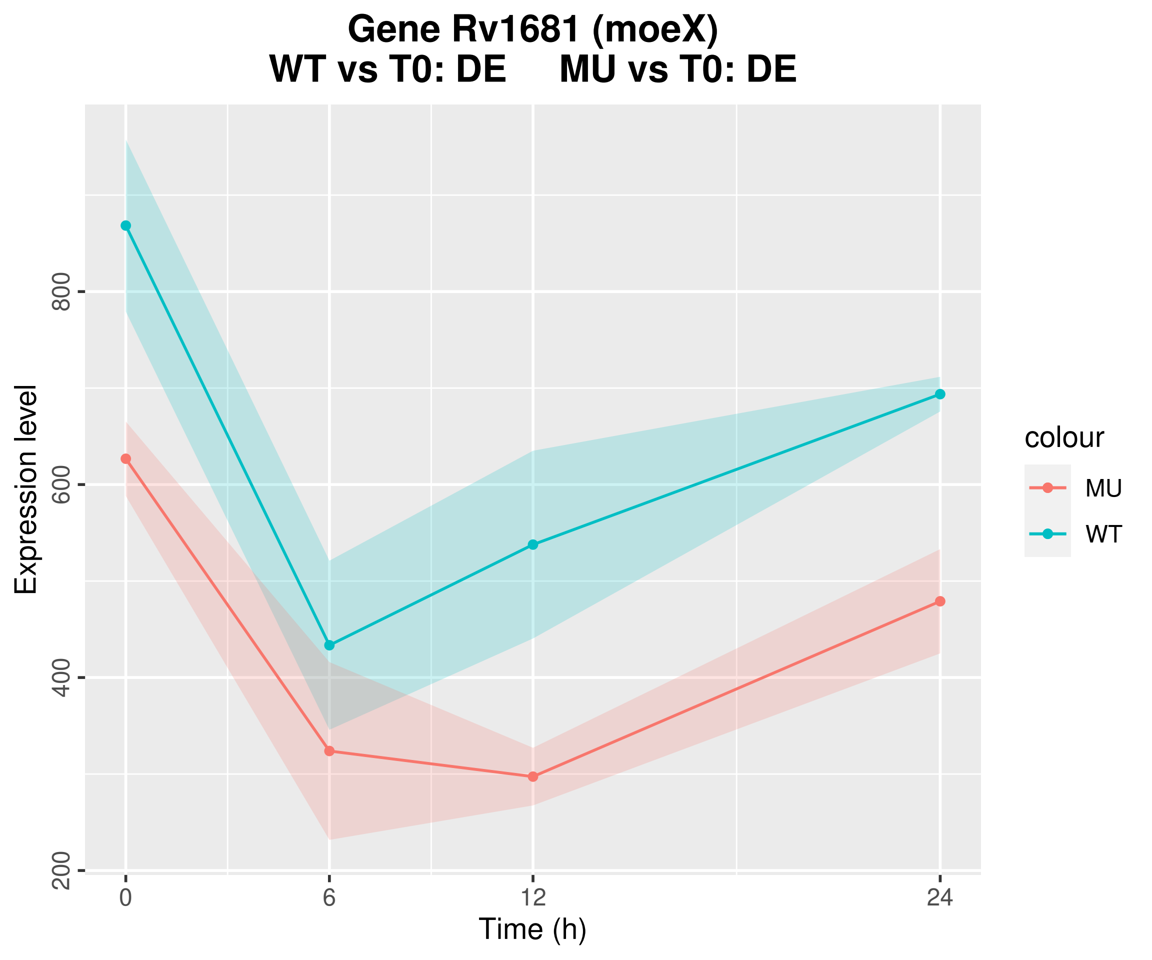
Gene Rv1681 (moeX)
WT vs T0: DE   MU vs T0: DE
Expression level
800
600
400
200
0
6
12
24
Time (h)
colour
MU
WT

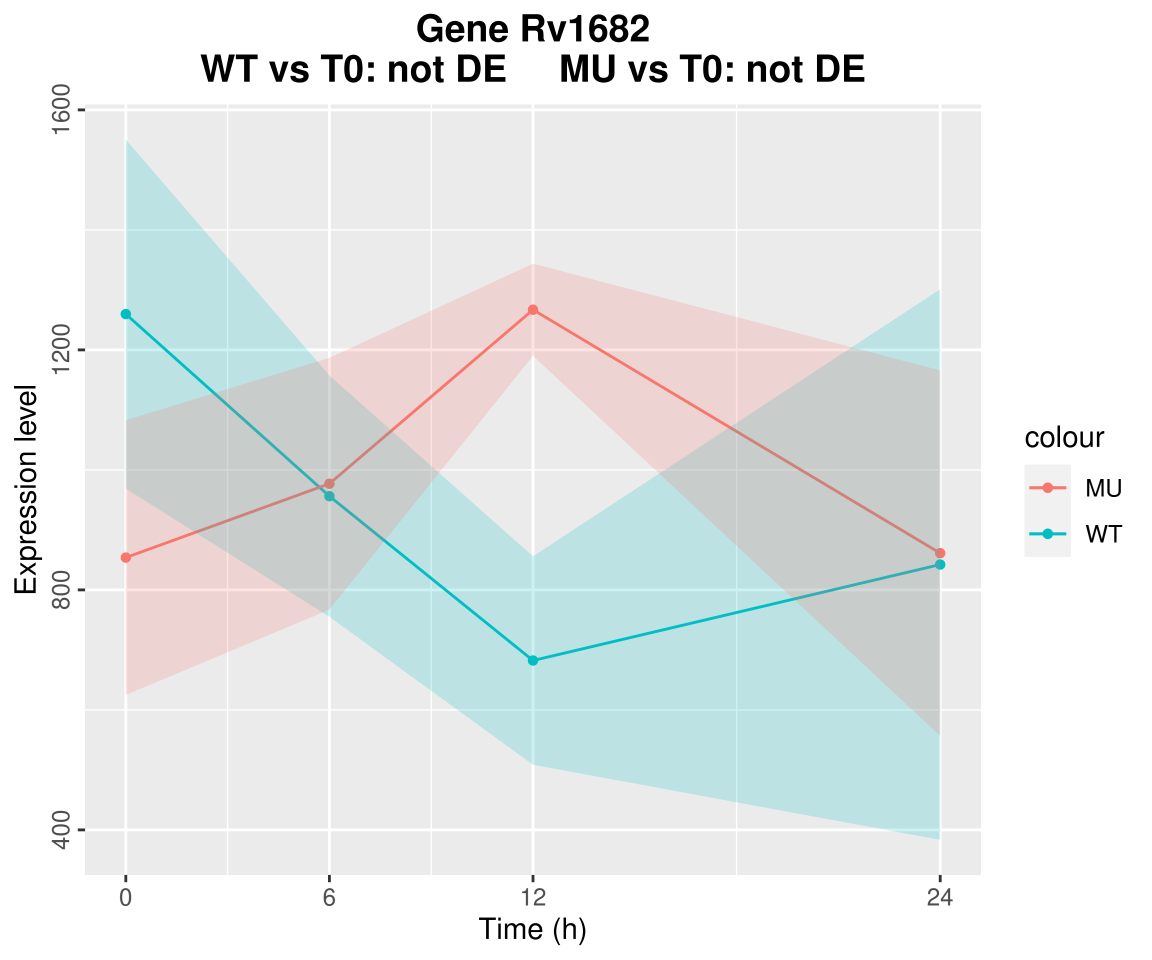

Gene Rv1682
WT vs T0: not DE MU vs T0: not DE
1600
1200
800
400
Expression level
0
6
12
24
Time (h)
colour
MU
WT

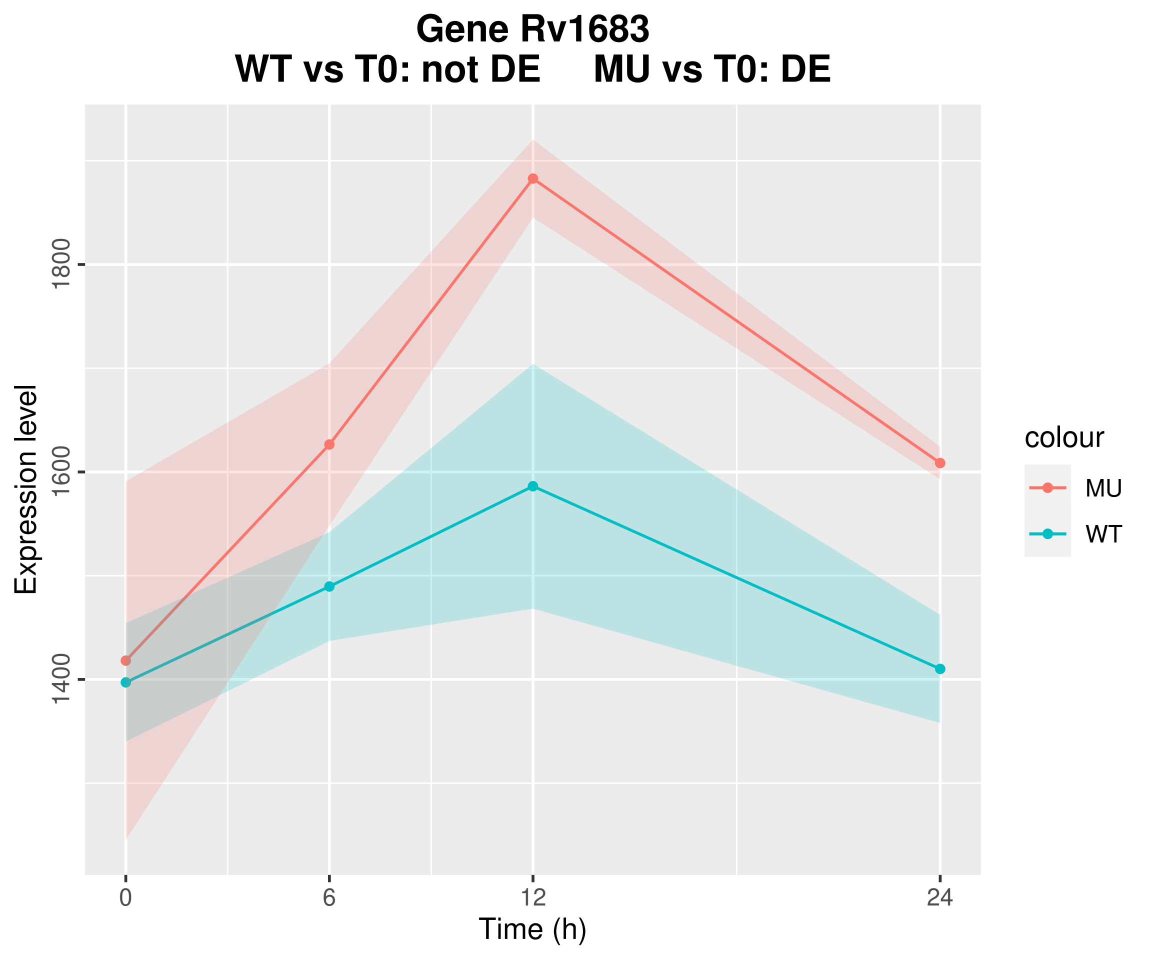
Gene Rv1683
WT vs T0: not DE MU vs T0: DE
Expression level
1400
1600
1800
Time (h)
0
6
12
24
colour
MU
WT

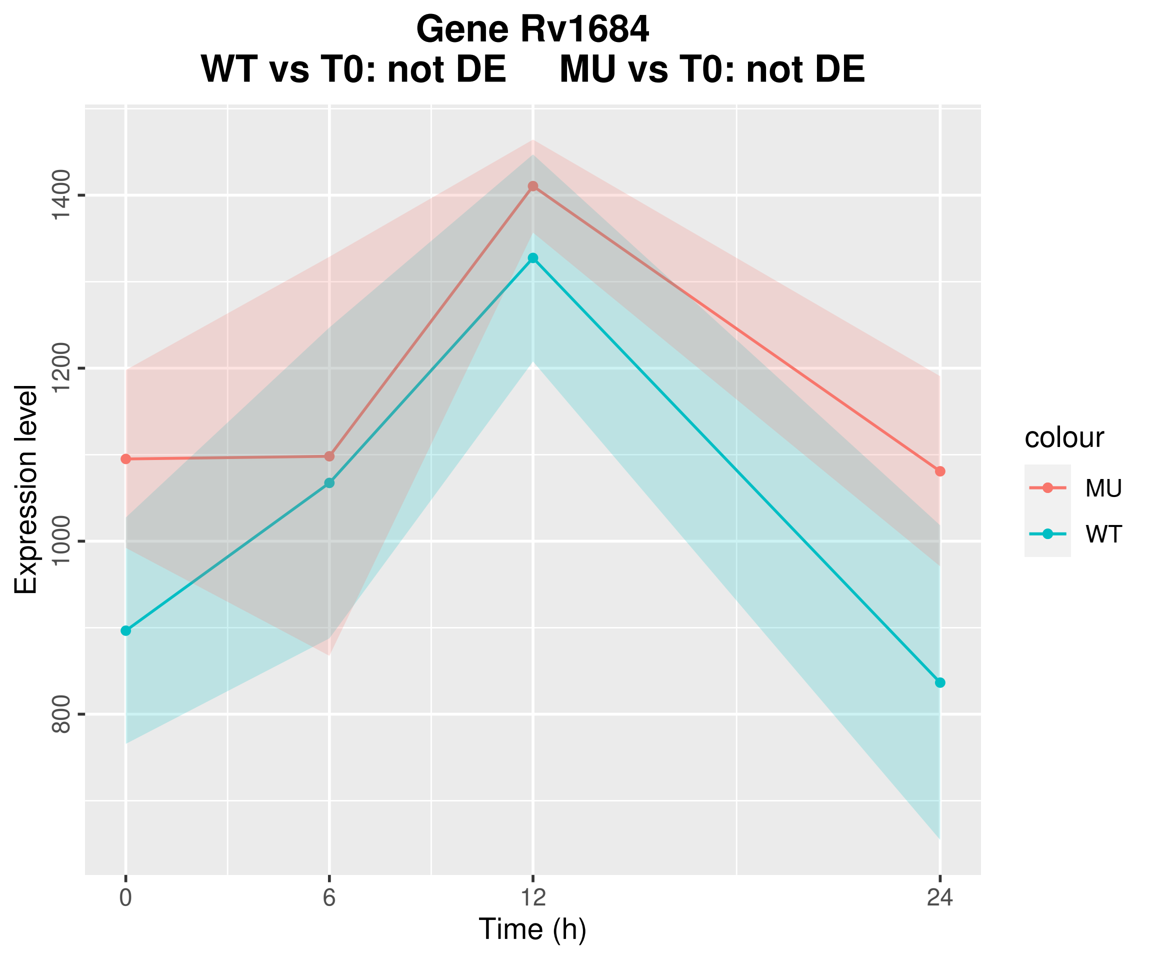

Gene Rv1684
WT vs T0: not DE   MU vs T0: not DE
Expression level
1400
1200
1000
800
0
6
12
24
Time (h)
colour
MU
WT

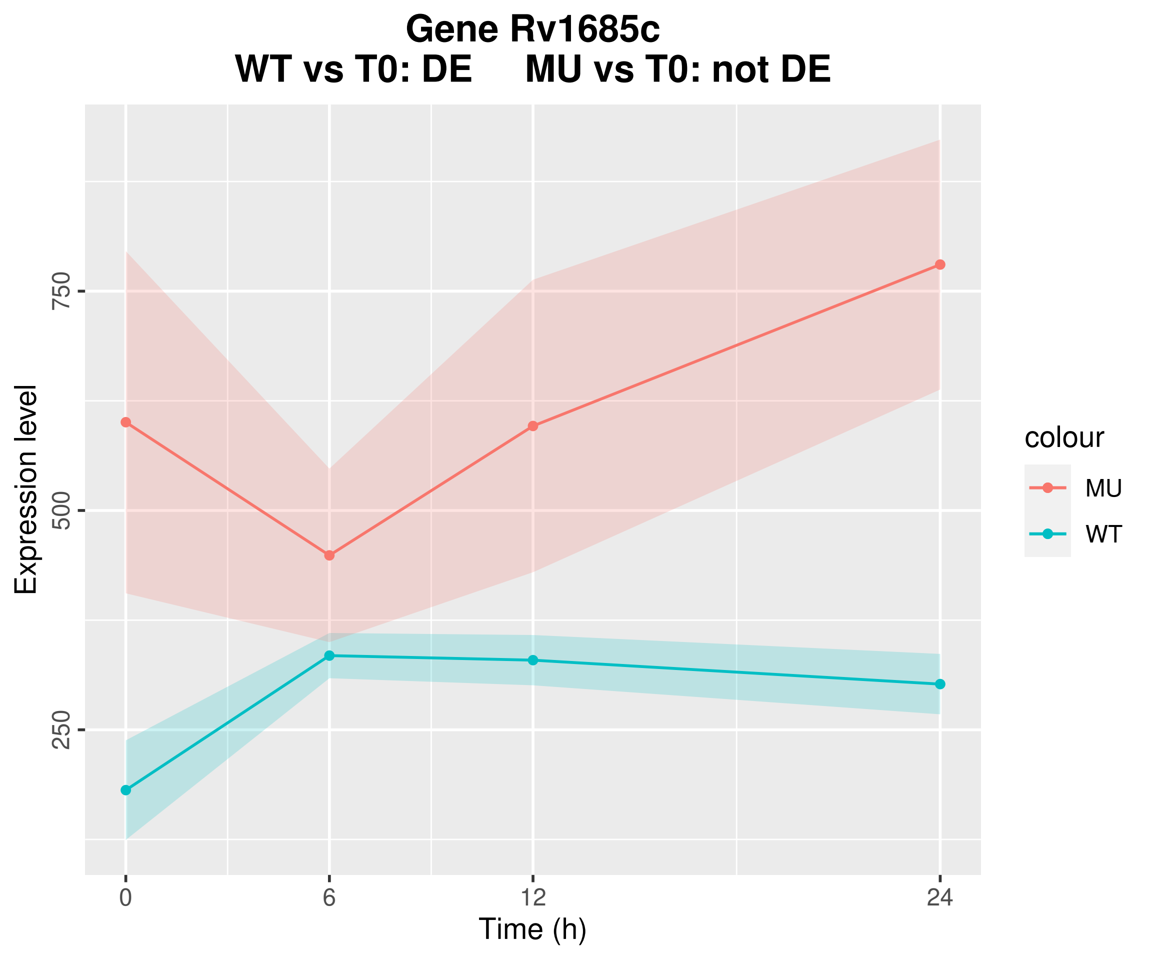

Gene Rv1685c
WT vs T0: DE MU vs T0: not DE
Expression level
750
500
250
0
6
12
24
Time (h)
colour
MU
WT

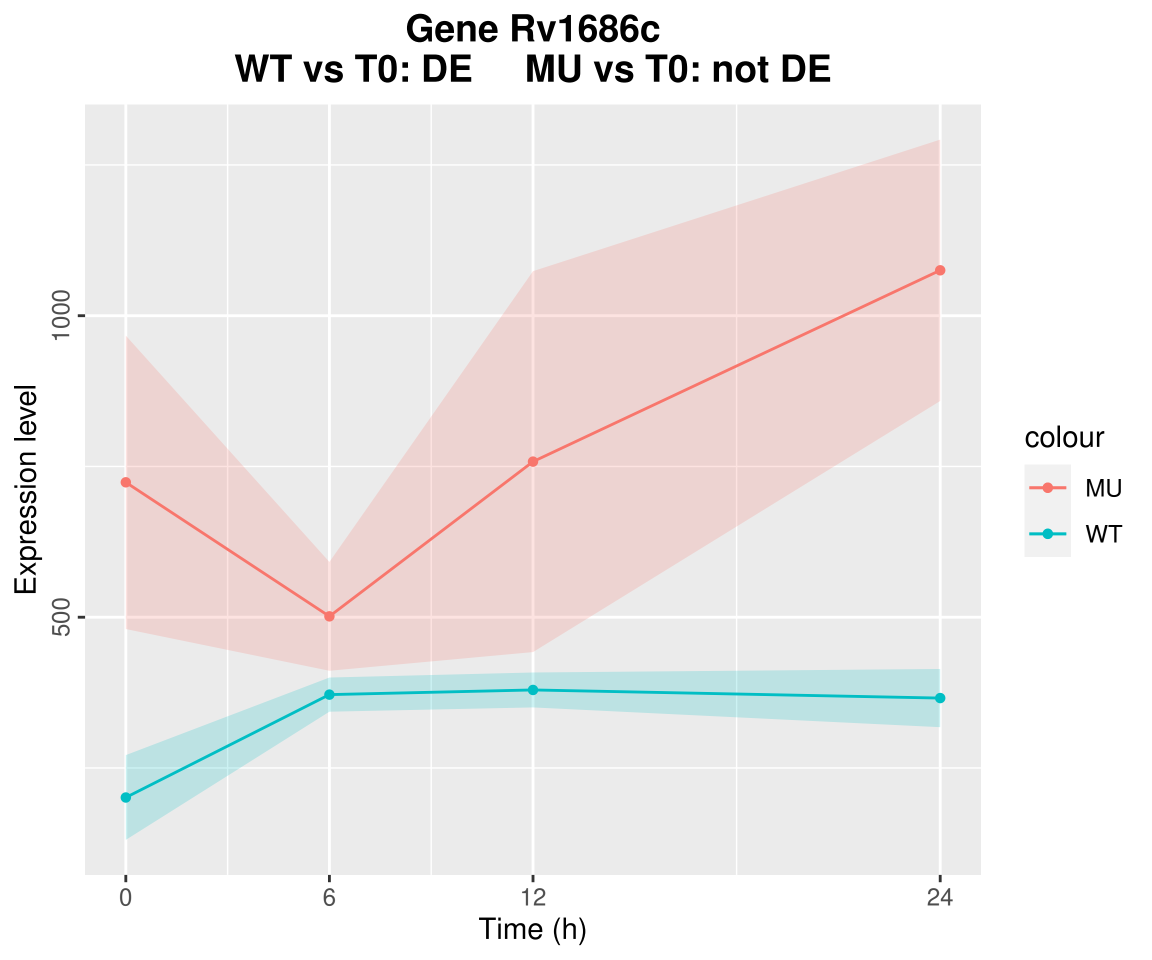

Gene Rv1686c
WT vs T0: DE MU vs T0: not DE
Expression level
1000
500
0
6
12
24
Time (h)
colour
MU
WT

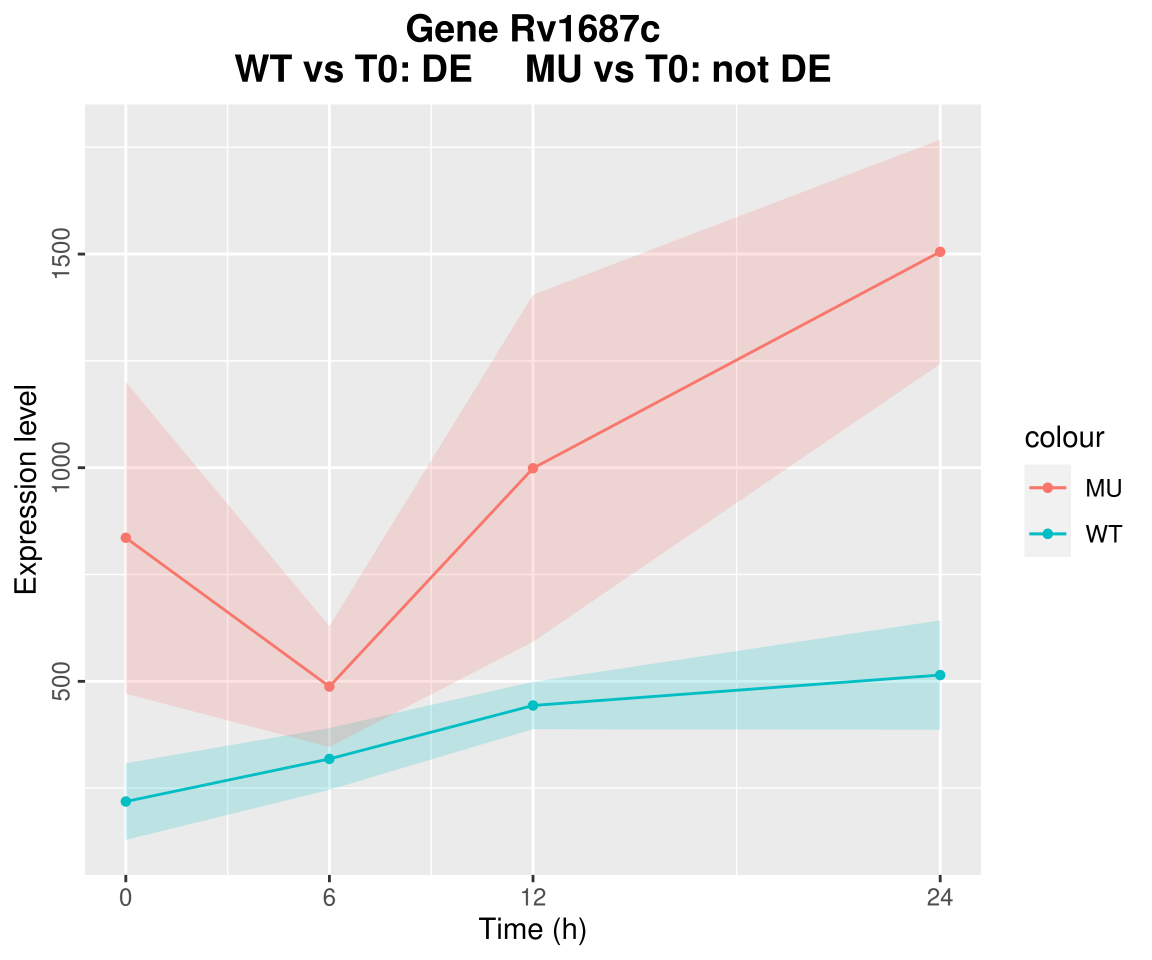

Gene Rv1687c
WT vs T0: DE   MU vs T0: not DE
Expression level
1500
1000
500
0
6
12
24
Time (h)
colour
MU
WT

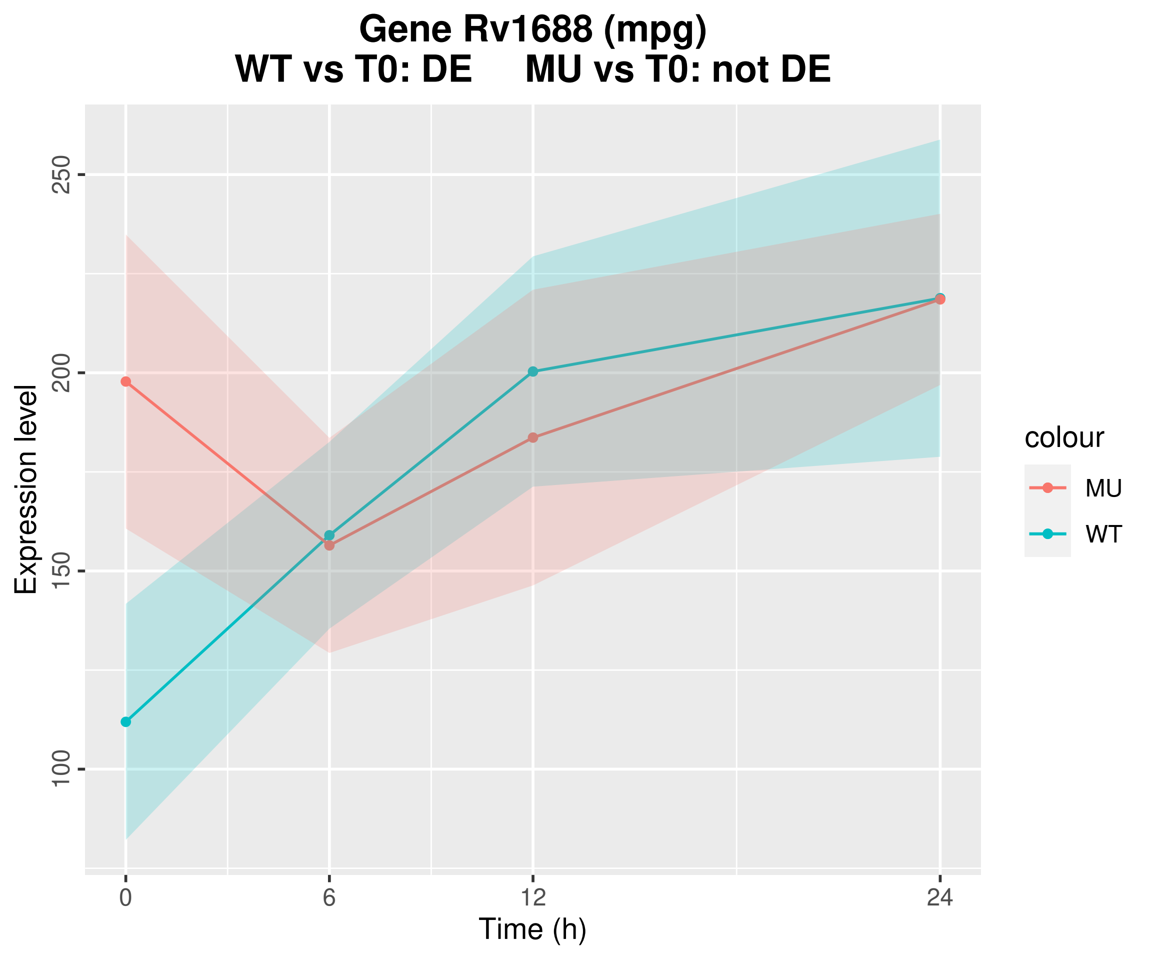

Gene Rv1688 (mpg)
WT vs T0: DE MU vs T0: not DE
Expression level
250
200
150
100
0
6
12
24
Time (h)
colour
MU
WT

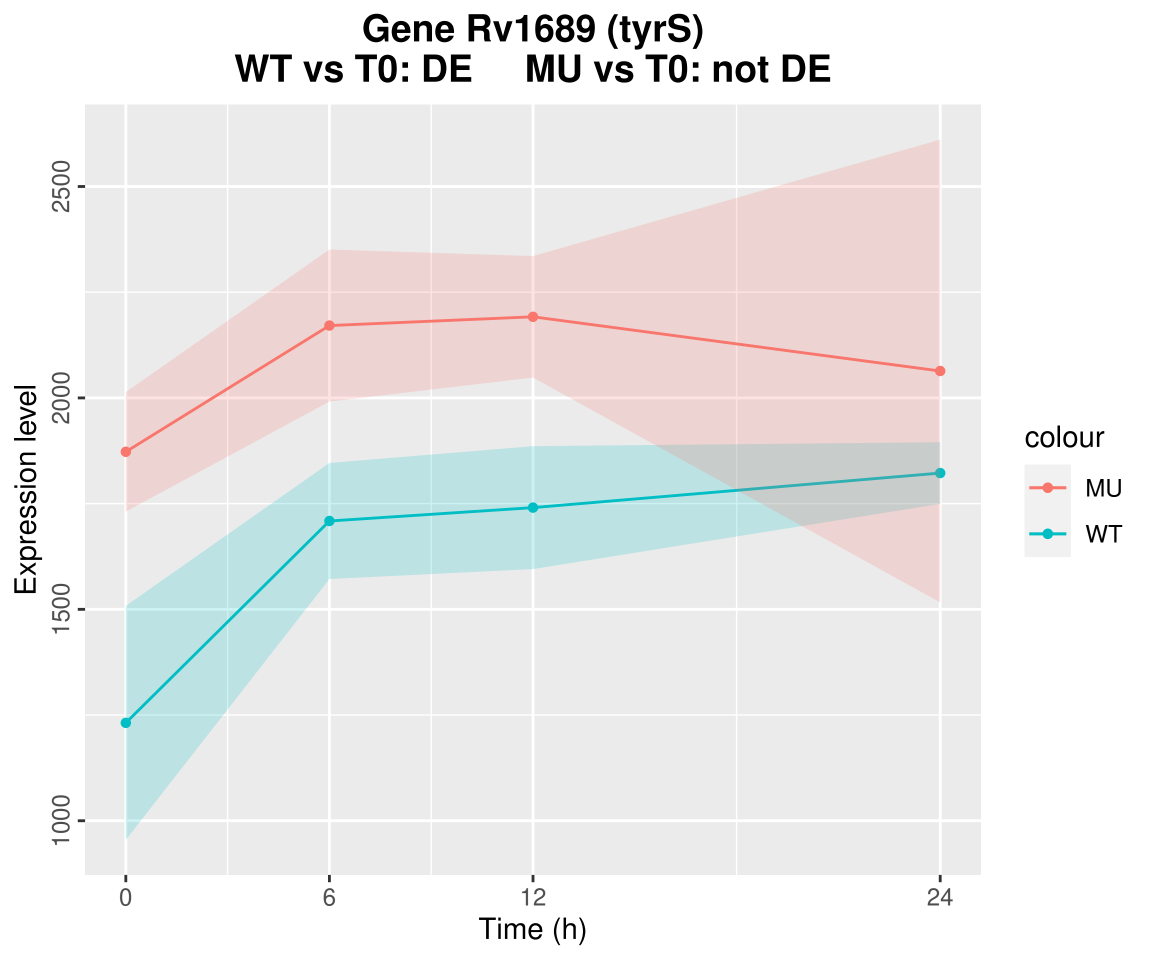

Gene Rv1689 (tyrS)
WT vs T0: DE   MU vs T0: not DE
Expression level
2500
2000
1500
1000
0
6
12
24
Time (h)
colour
MU
WT

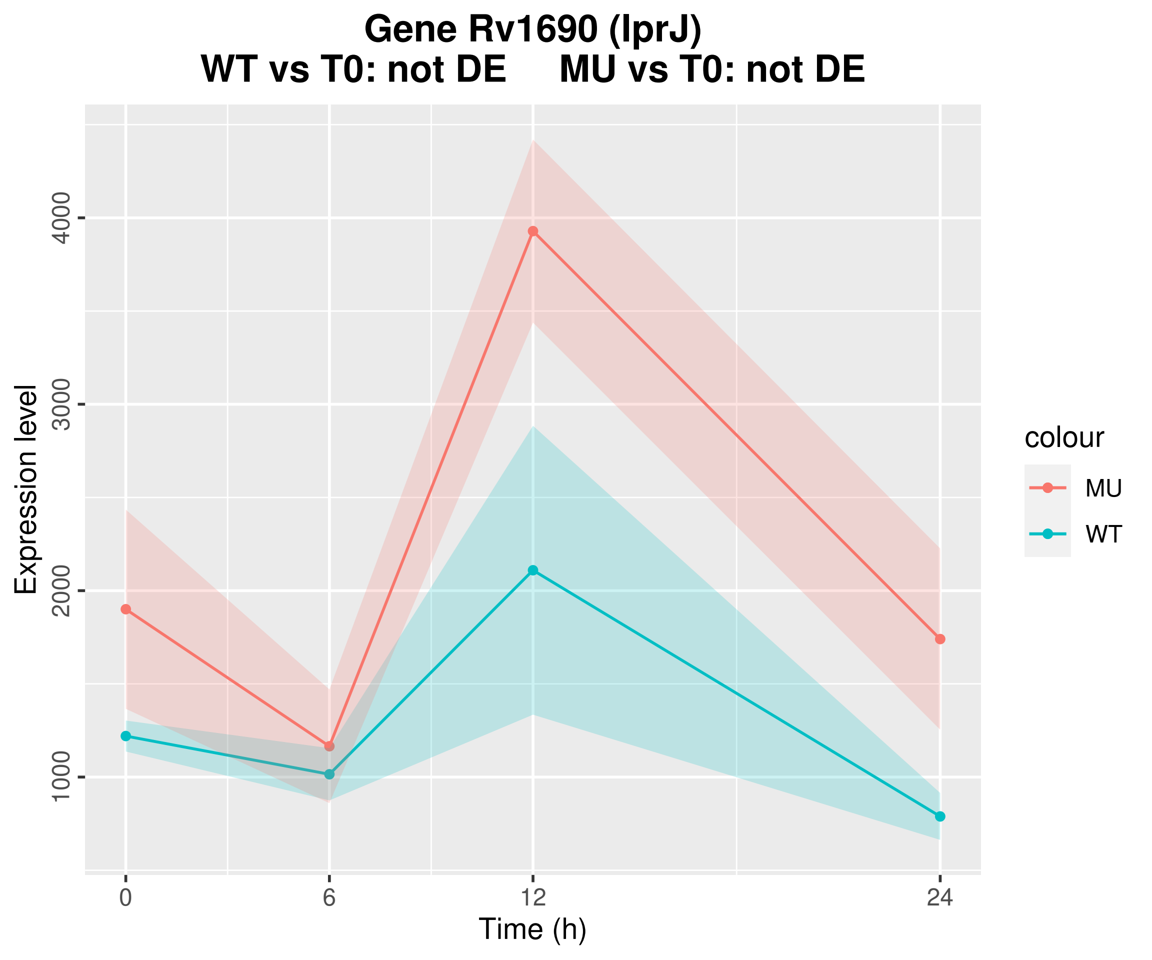
Gene Rv1690 (lprJ)
WT vs T0: not DE   MU vs T0: not DE
Expression level
4000
3000
2000
1000
0
6
12
24
Time (h)
colour
MU
WT

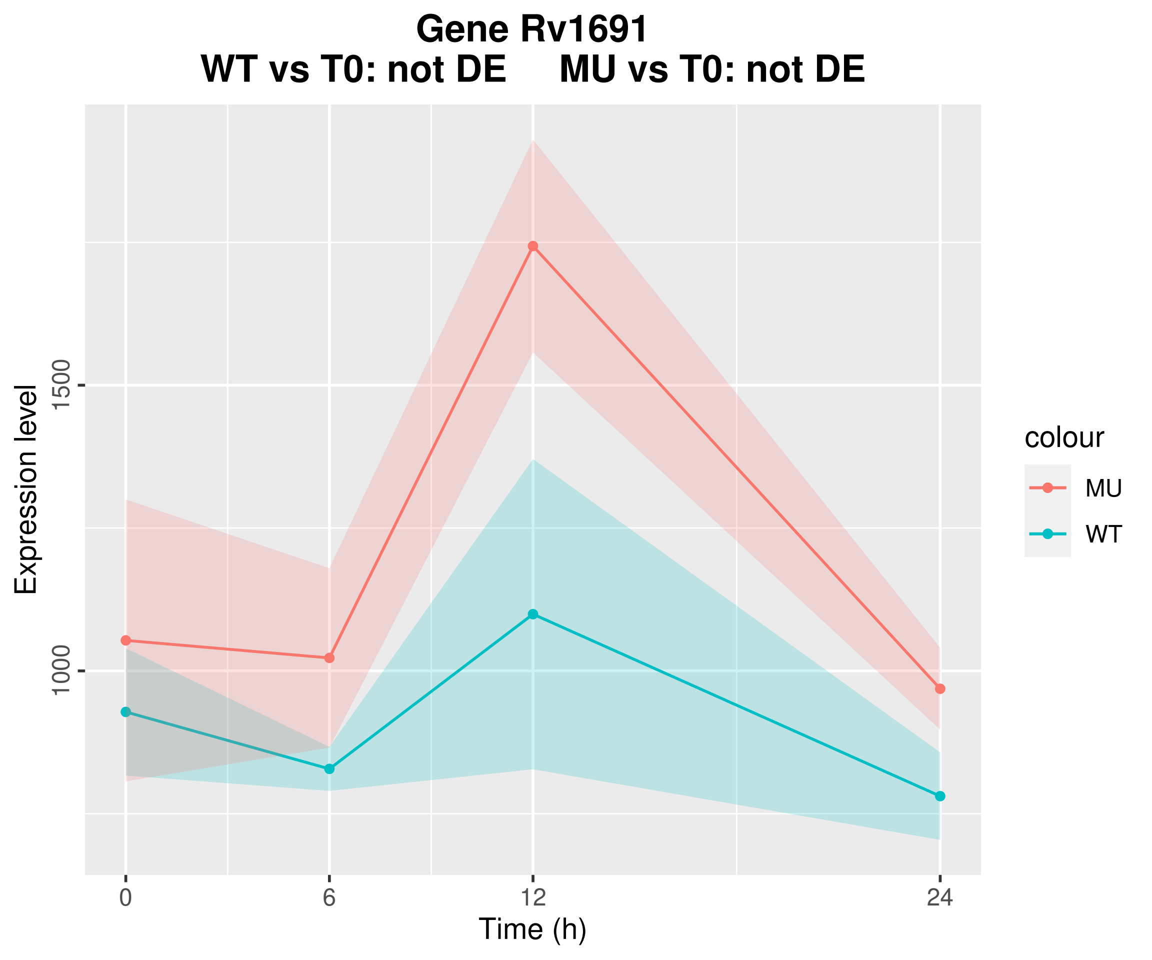

Gene Rv1691
WT vs T0: not DE   MU vs T0: not DE
Expression level
1500
1000
0
6
12
24
Time (h)
colour
MU
WT

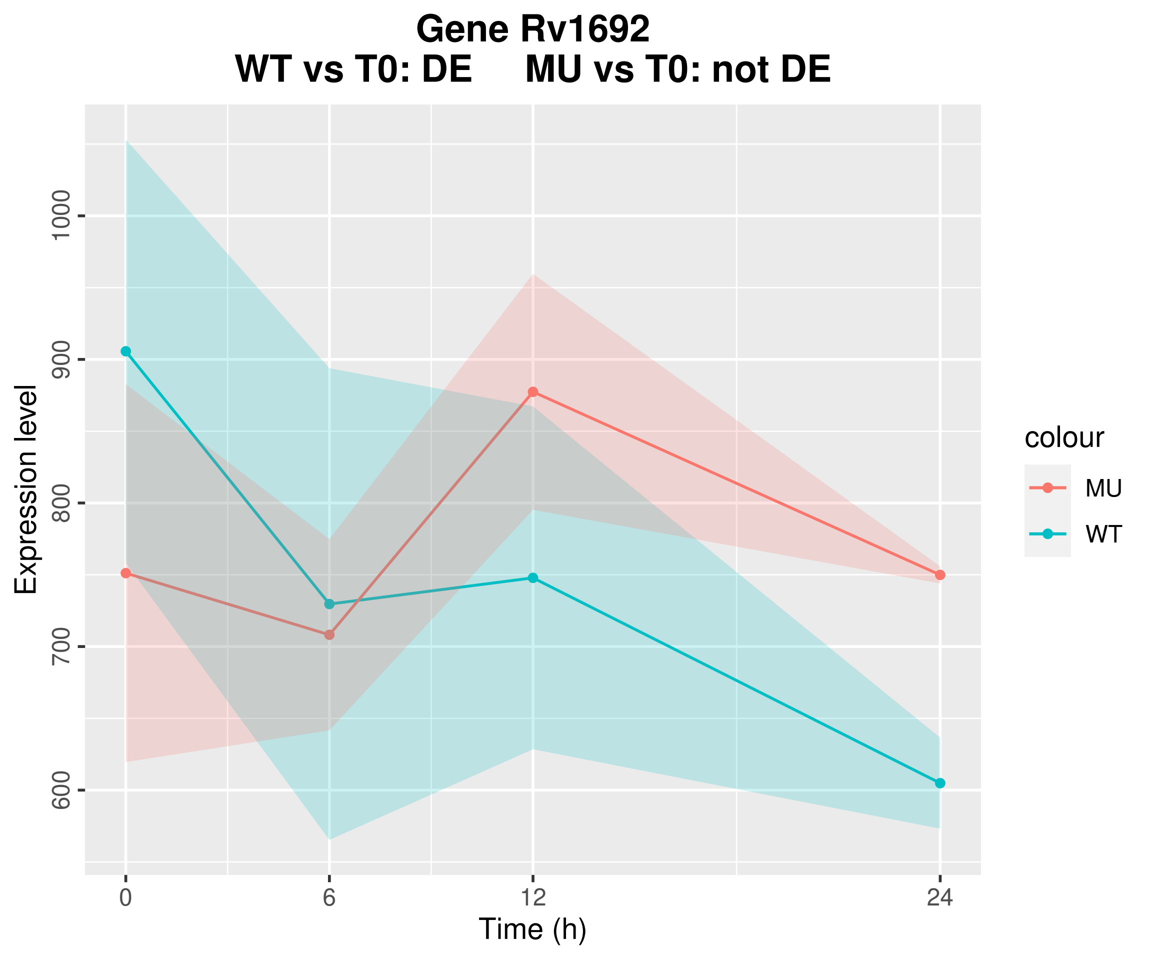

Gene Rv1692
WT vs T0: DE   MU vs T0: not DE
Expression level
1000
900
800
700
600
0
6
12
24
Time (h)
colour
MU
WT

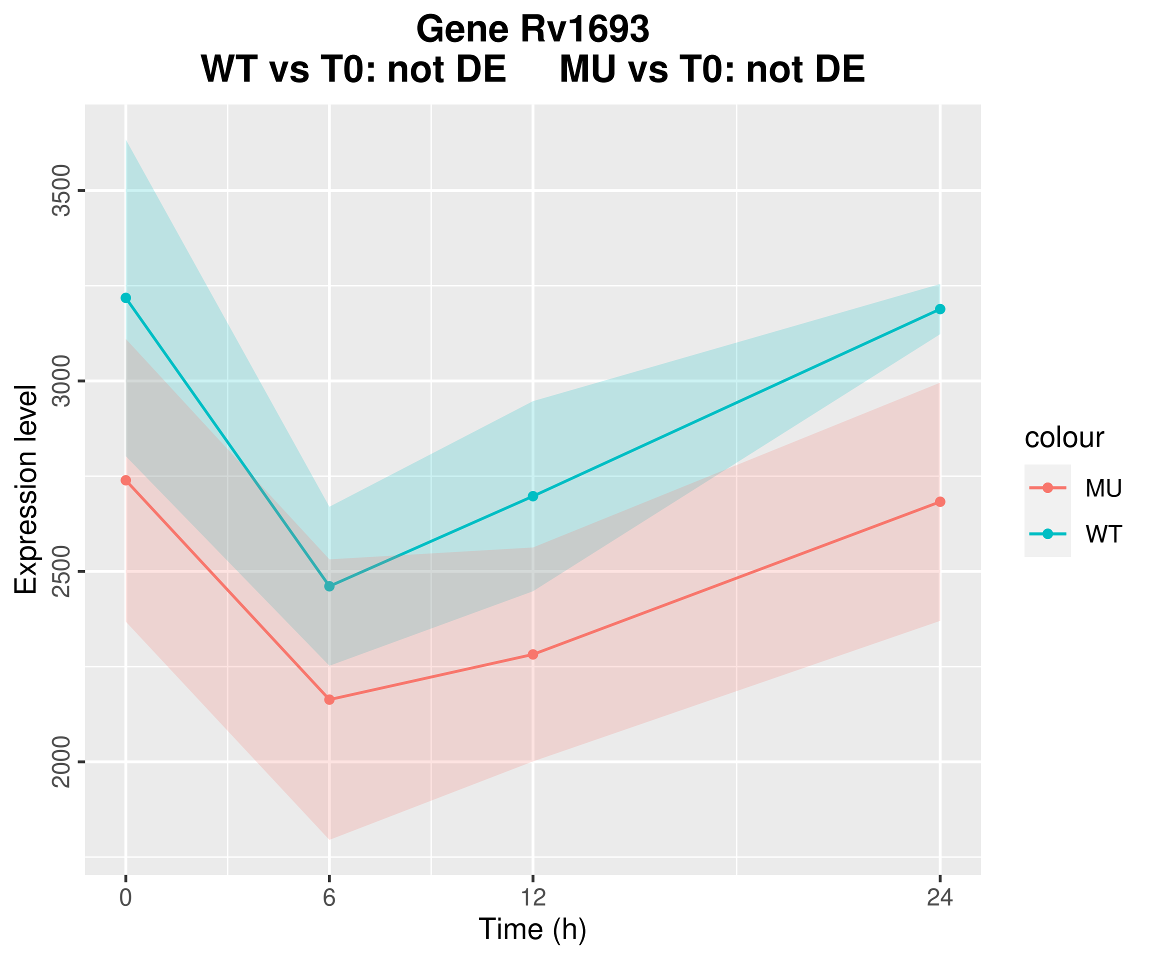

Gene Rv1693
WT vs T0: not DE   MU vs T0: not DE
Expression level
3500
3000
2500
2000
0
6
12
24
Time (h)
colour
MU
WT

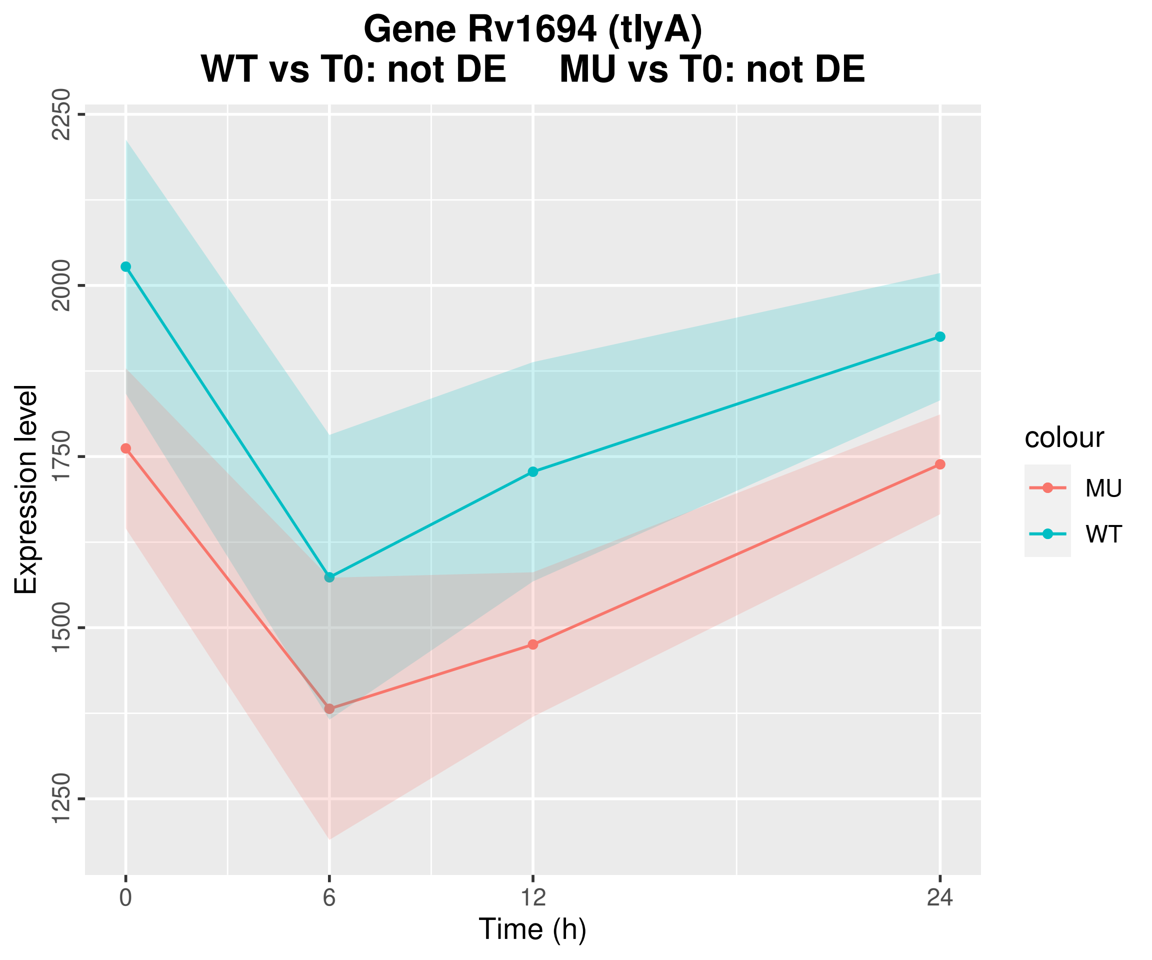
Gene Rv1694 (tlyA)
WT vs T0: not DE MU vs T0: not DE
Expression level
2250
2000
1750
1500
1250
0
6
12
24
Time (h)
colour
MU
WT

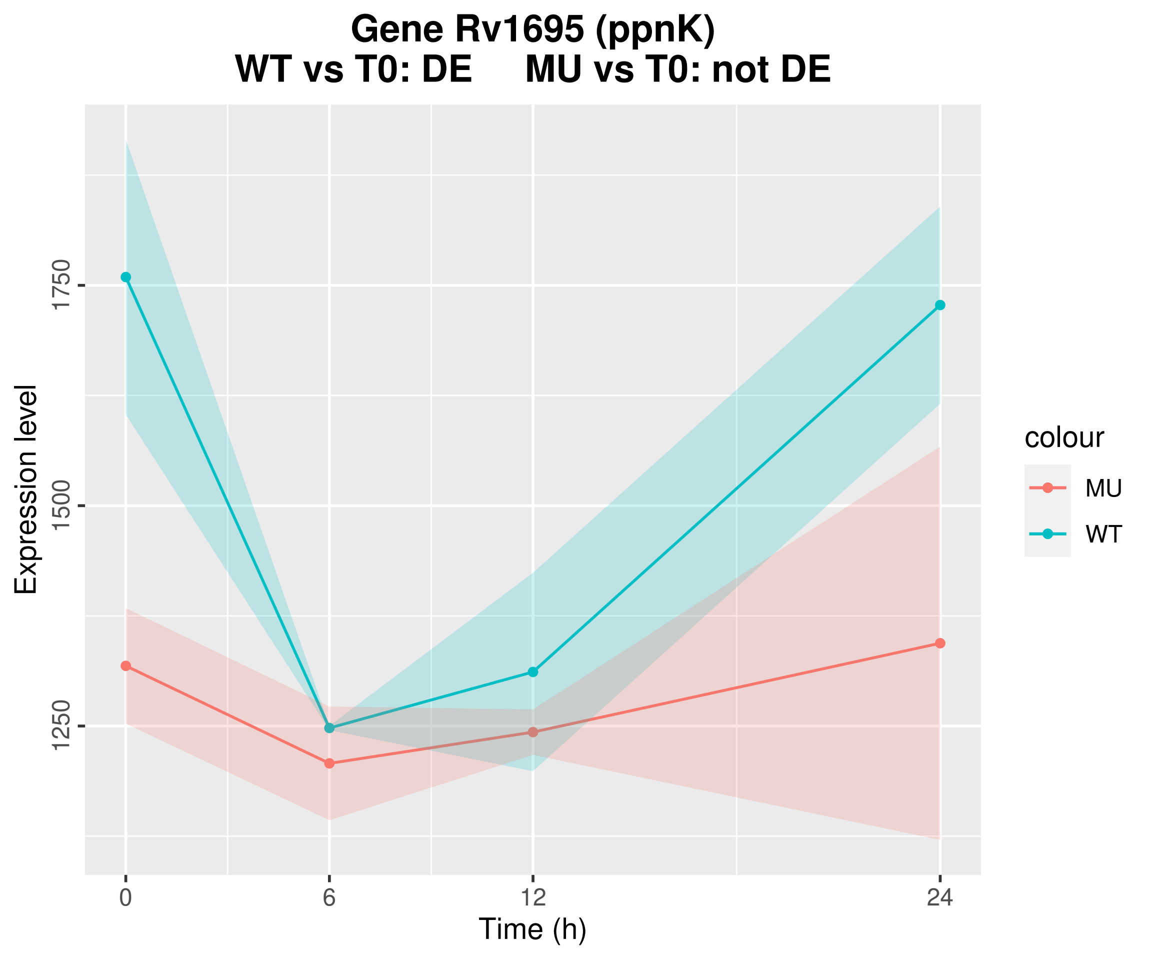

Gene Rv1695 (ppnK)
WT vs T0: DE   MU vs T0: not DE
Expression level
1250
1500
1750
0
6
12
24
Time (h)
colour
MU
WT

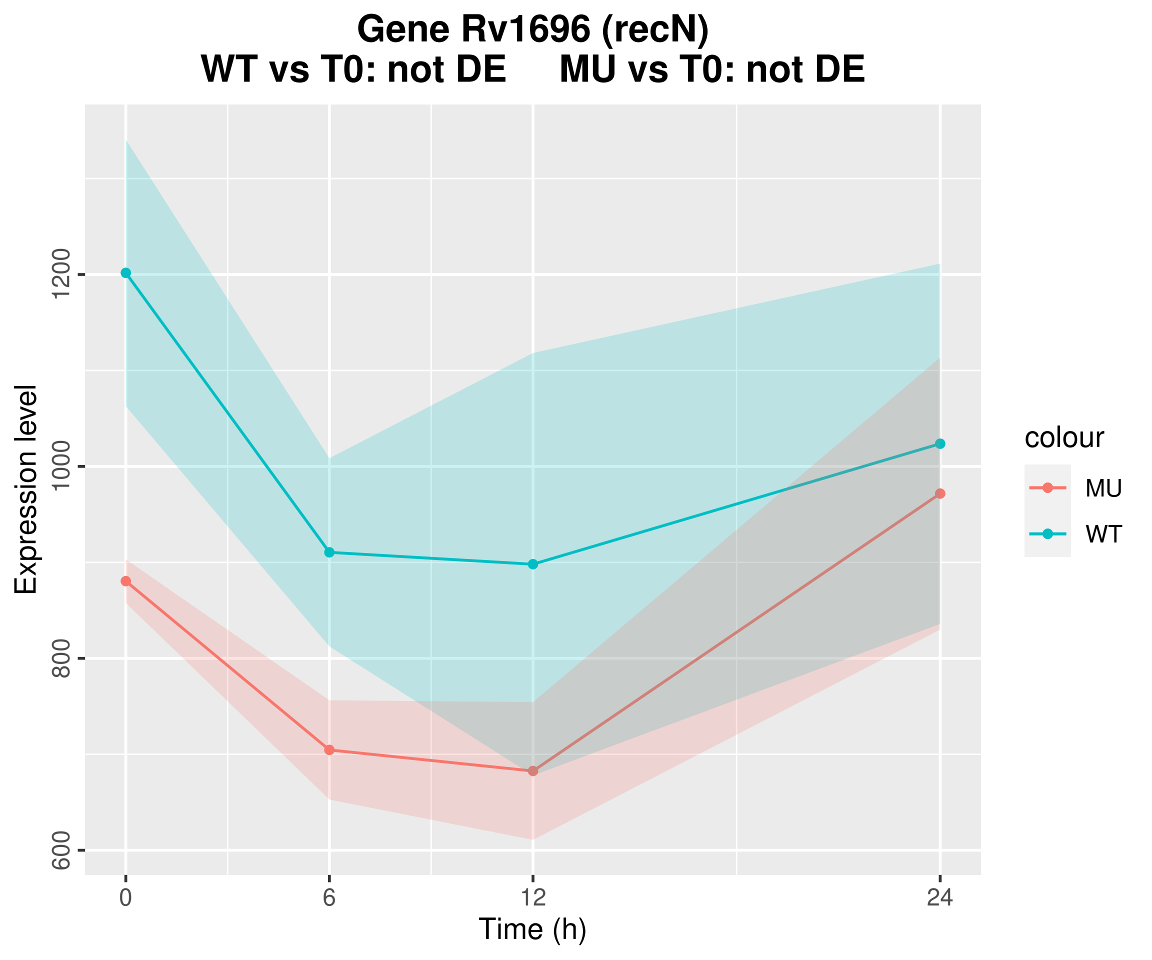
Gene Rv1696 (recN)
WT vs T0: not DE   MU vs T0: not DE
Expression level
1200
1000
800
600
0
6
12
24
Time (h)
colour
MU
WT

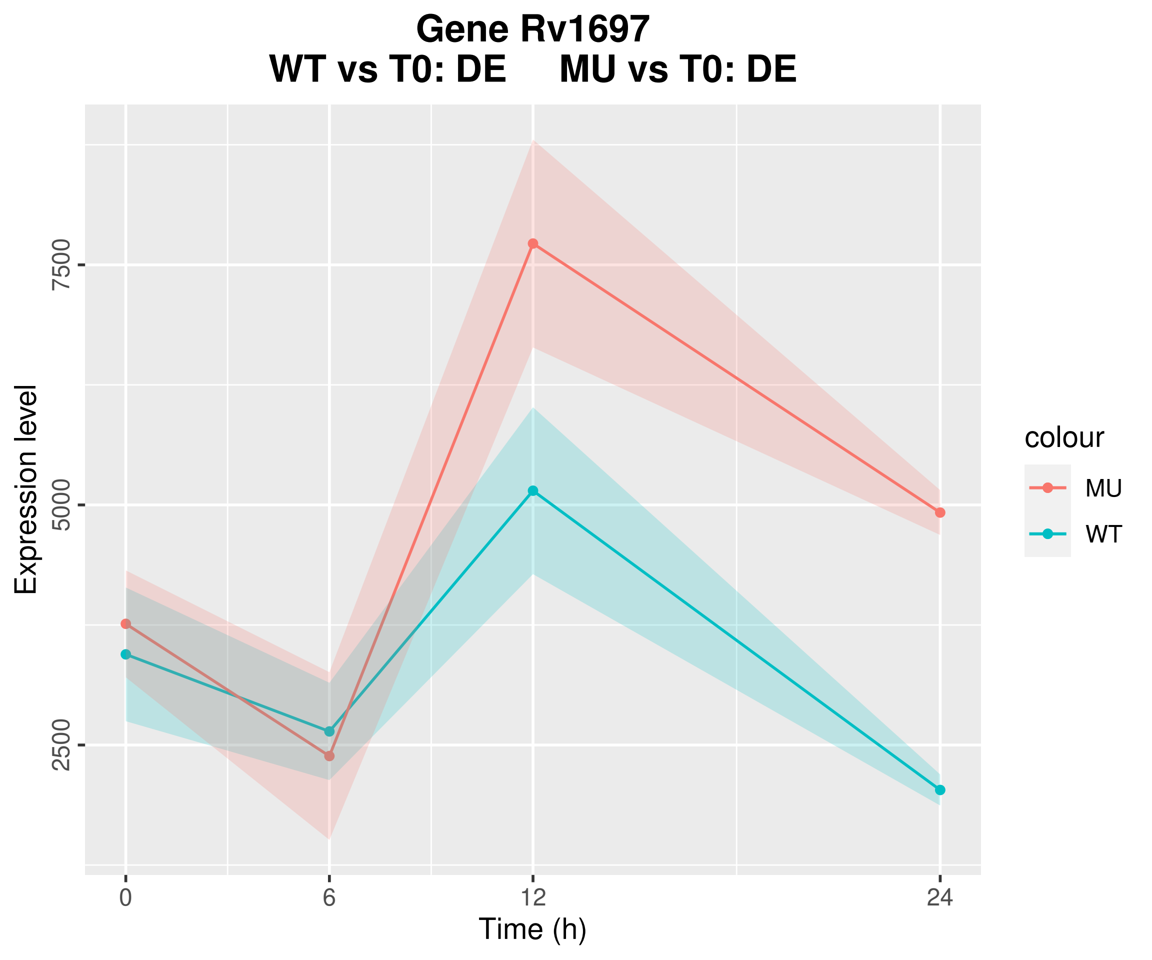
Gene Rv1697
WT vs T0: DE   MU vs T0: DE
Expression level
2500
5000
7500
0
6
12
24
Time (h)
colour
MU
WT

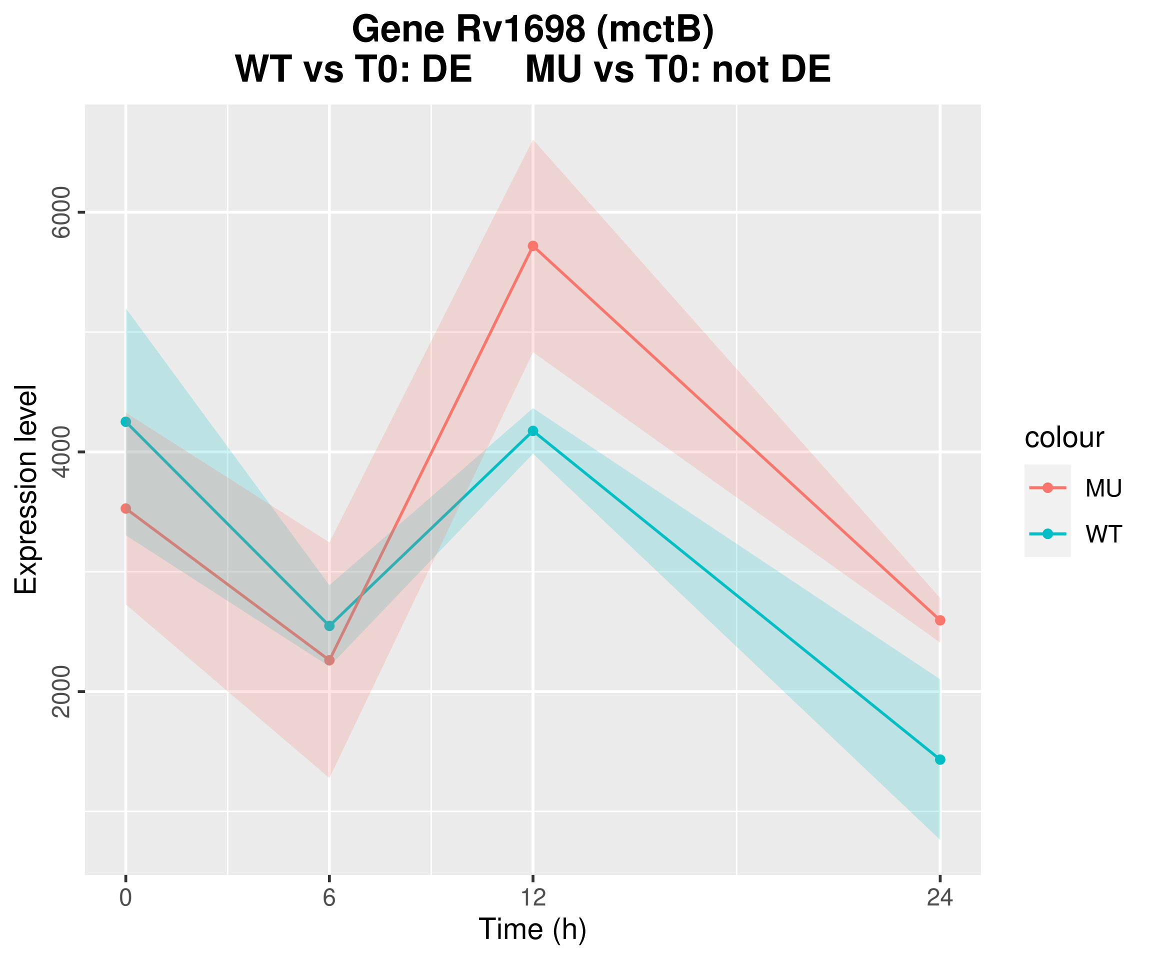
Gene Rv1698 (mctB)
WT vs T0: DE   MU vs T0: not DE
Expression level
6000
4000
2000
0
6
12
24
Time (h)
colour
MU
WT

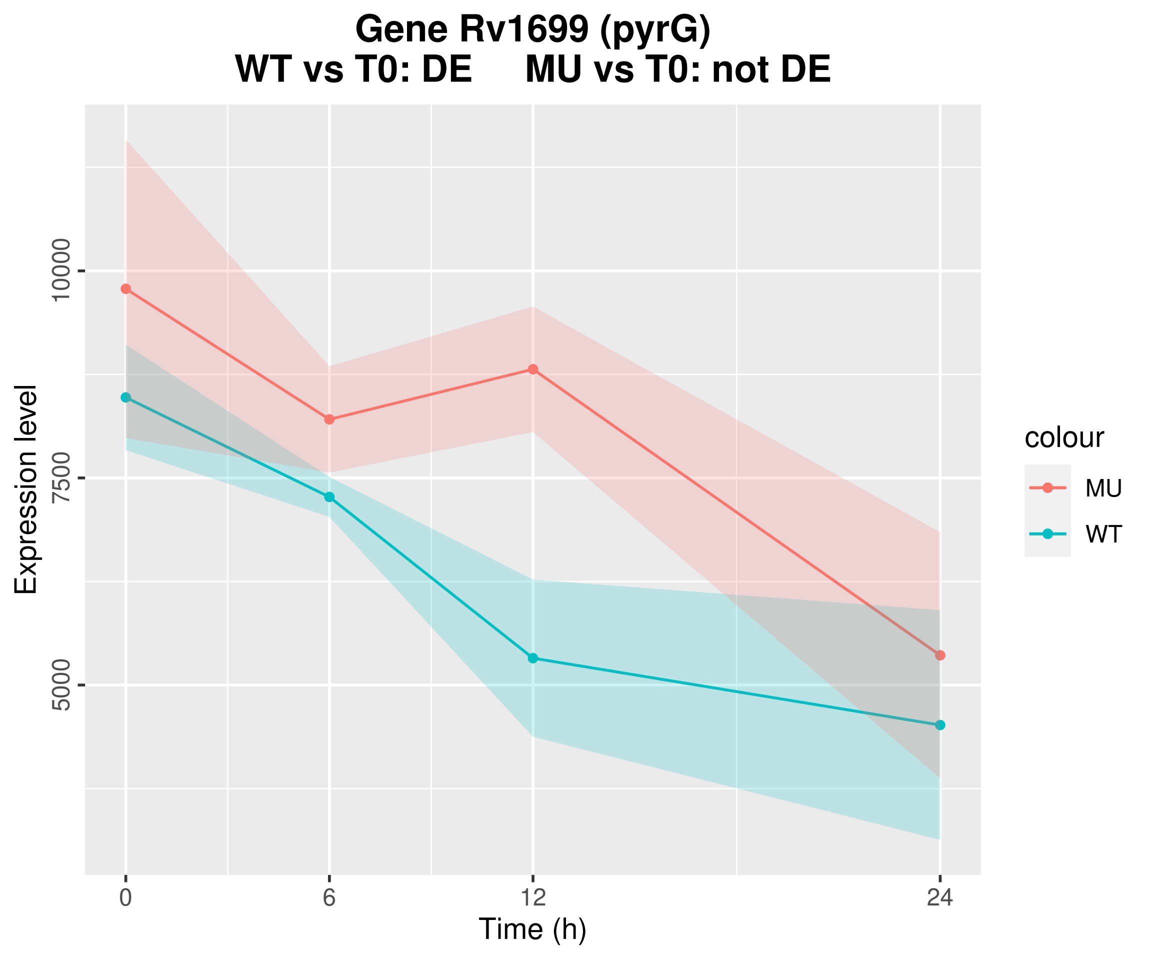
Gene Rv1699 (pyrG)
WT vs T0: DE   MU vs T0: not DE
Expression level
10000
7500
5000
0
6
12
24
Time (h)
colour
MU
WT

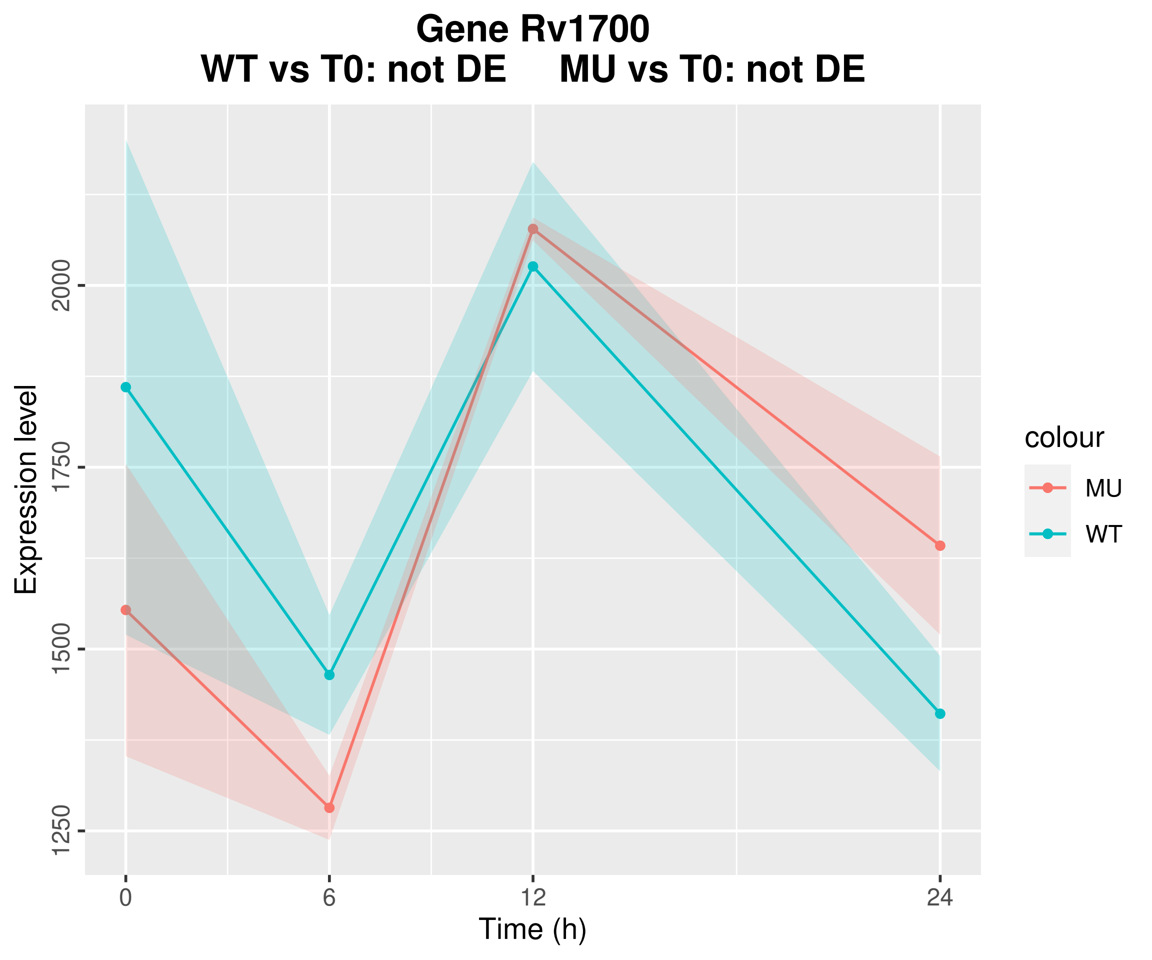

Gene Rv1700
WT vs T0: not DE MU vs T0: not DE
Expression level
2000
1750
1500
1250
0
6
12
24
Time (h)
colour
MU
WT

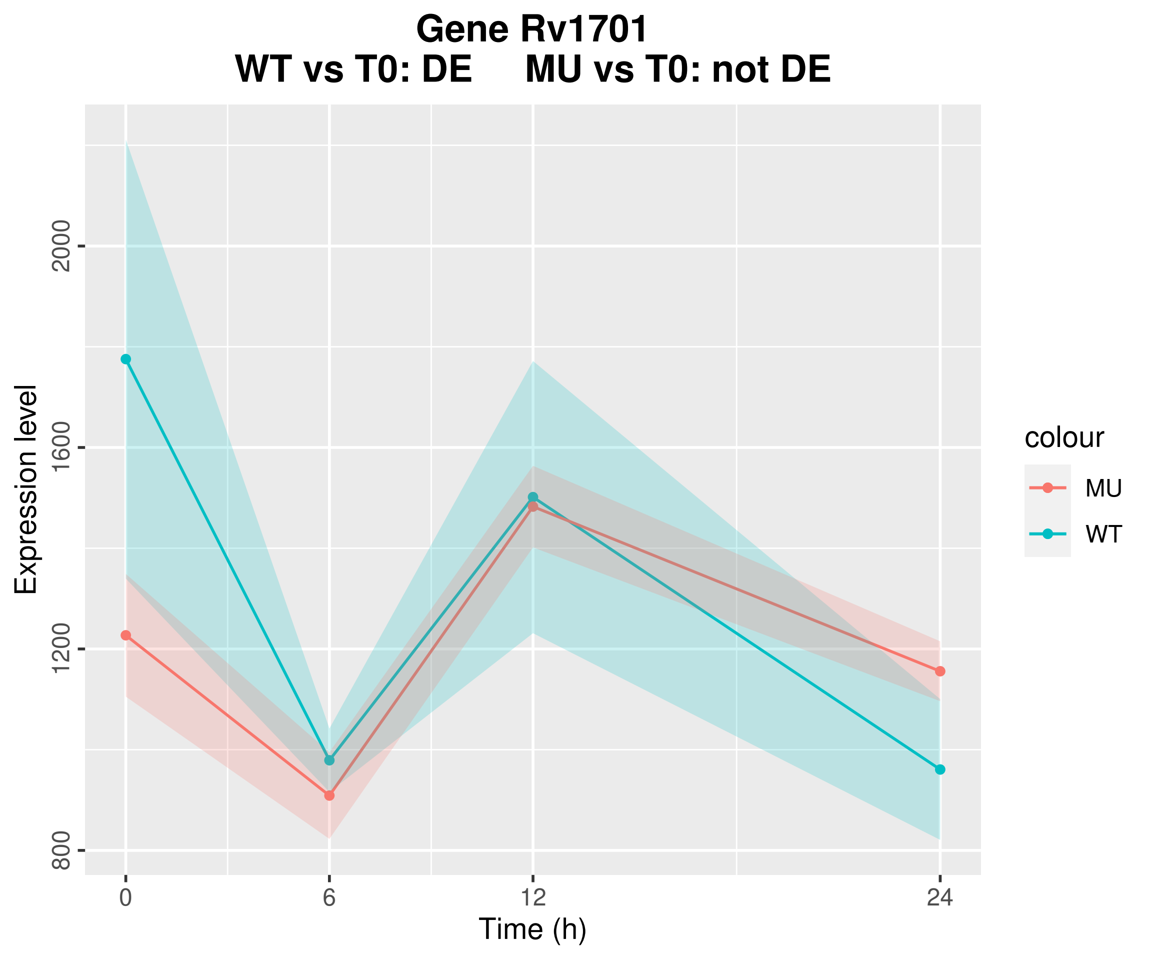

Gene Rv1701
WT vs T0: DE MU vs T0: not DE
Expression level
2000
1600
1200
800
0
6
12
24
Time (h)
colour
MU
WT

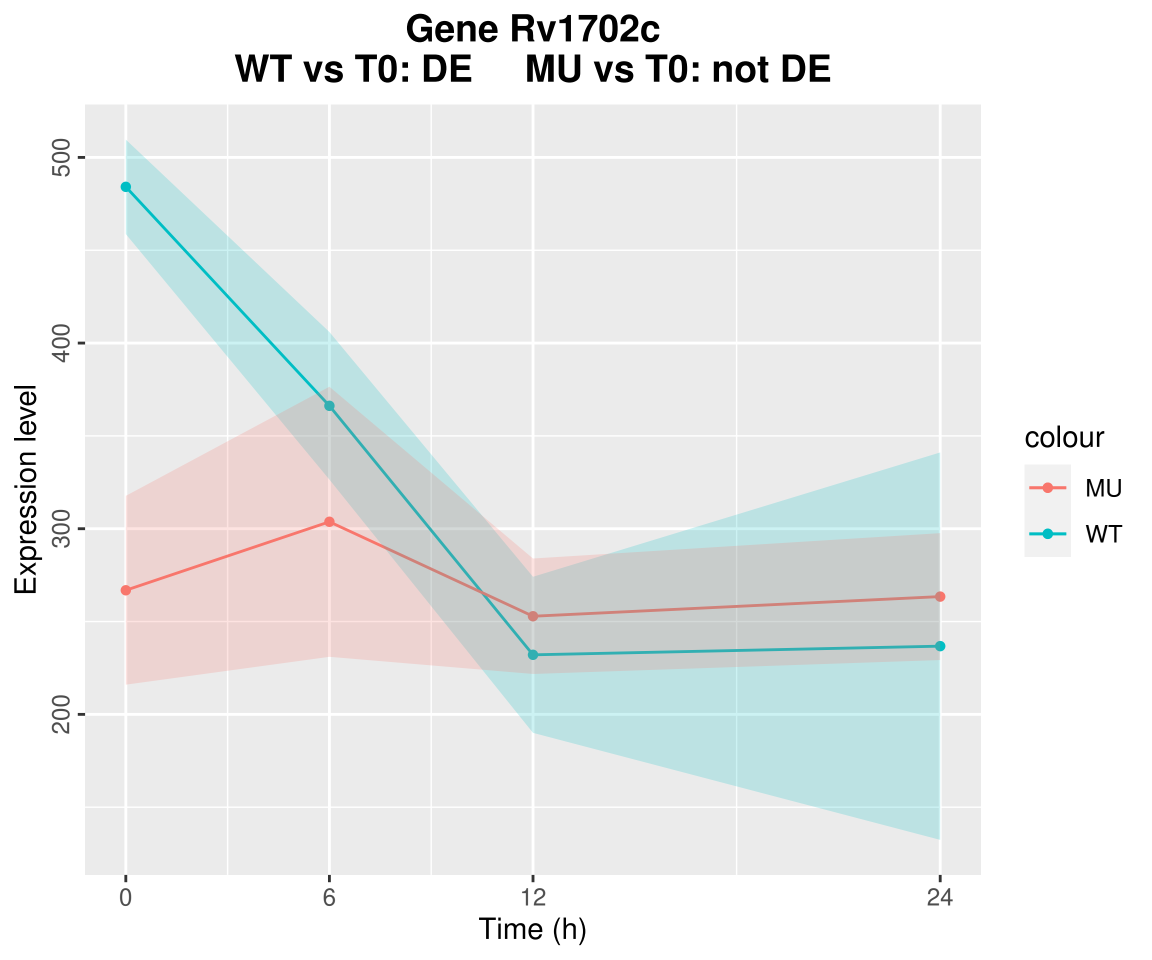
Gene Rv1702c
WT vs T0: DE   MU vs T0: not DE
Expression level
500
400
300
200
0
6
12
24
Time (h)
colour
MU
WT

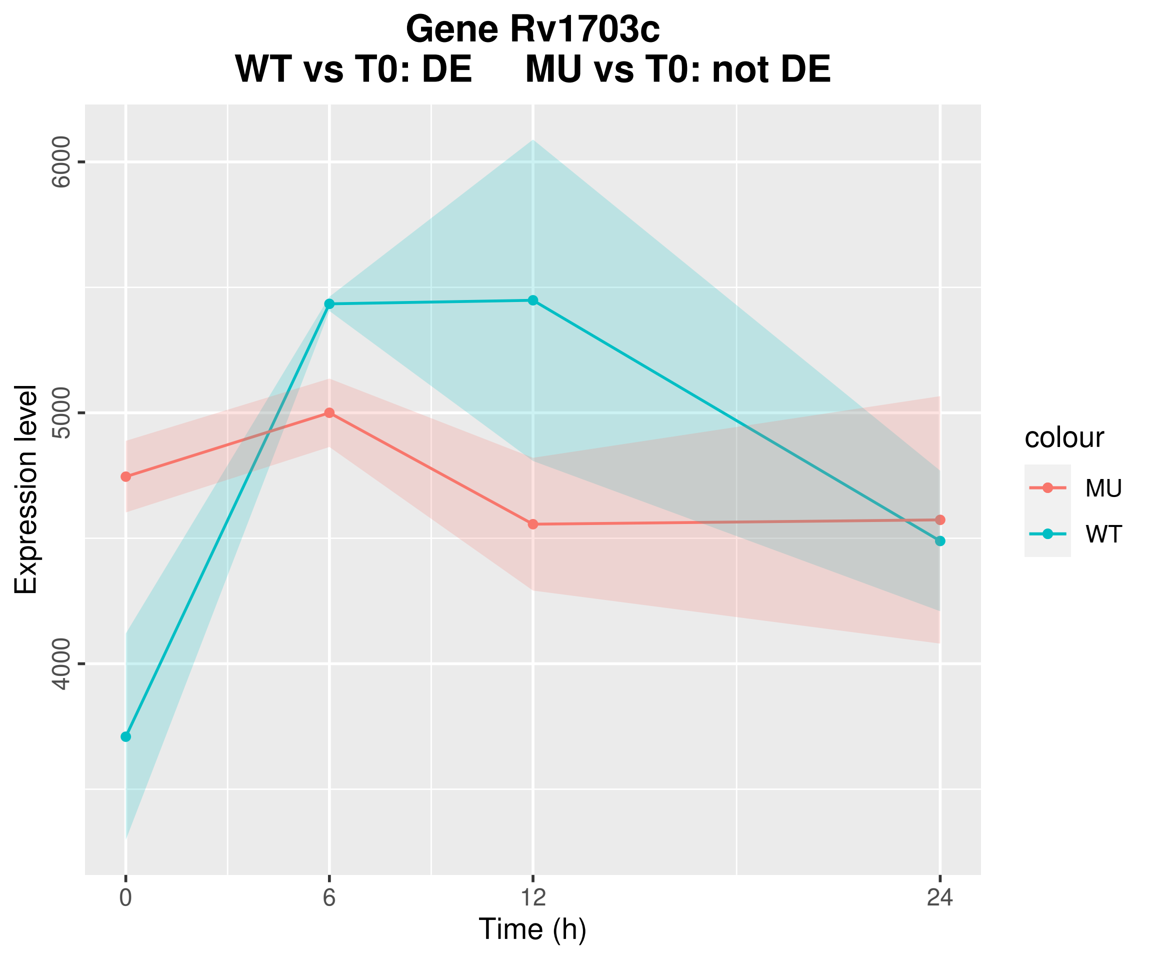
Gene Rv1703c
WT vs T0: DE MU vs T0: not DE
Expression level
6000
5000
4000
0
6
12
24
Time (h)
colour
MU
WT

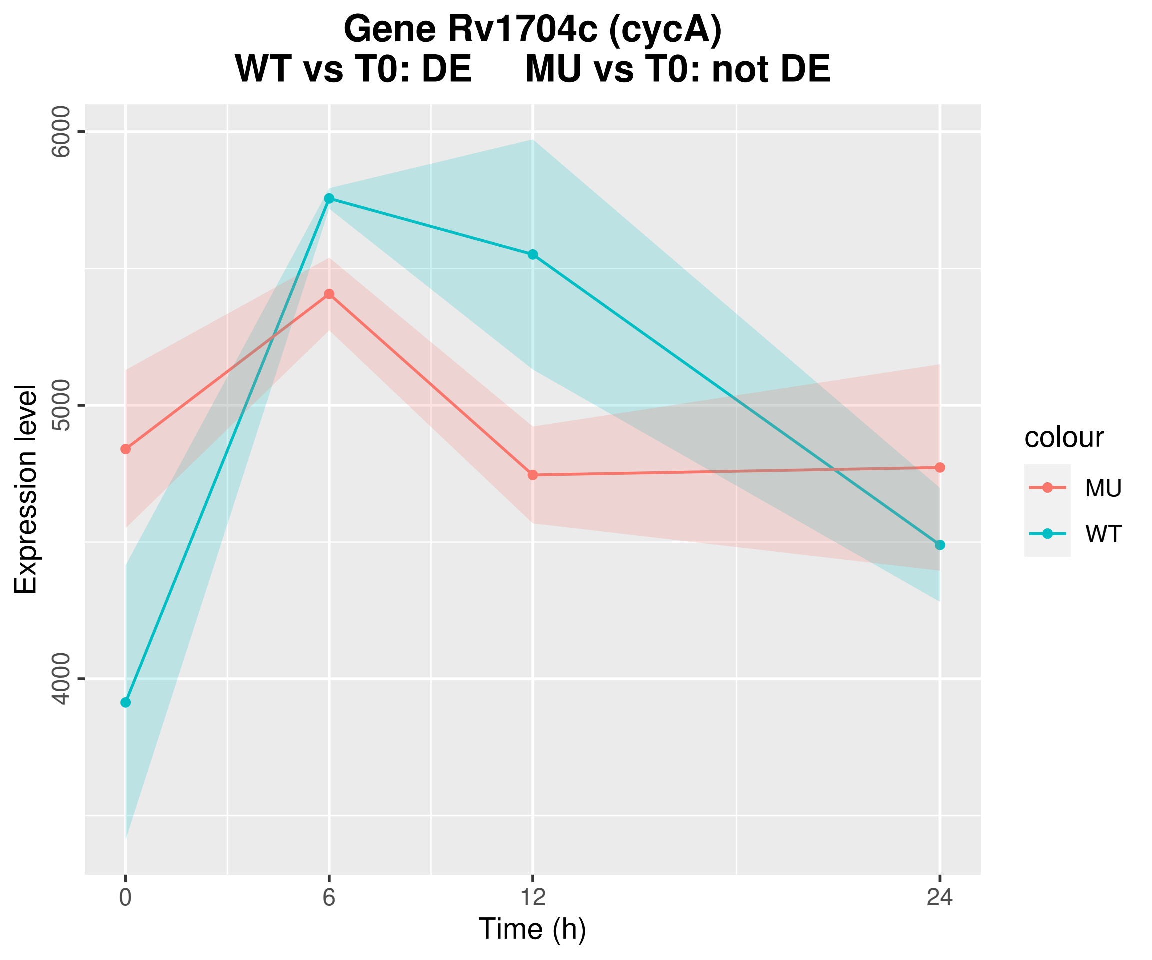

Gene Rv1704c (cycA)
WT vs T0: DE   MU vs T0: not DE
Expression level
6000
5000
4000
0
6
12
24
Time (h)
colour
MU
WT

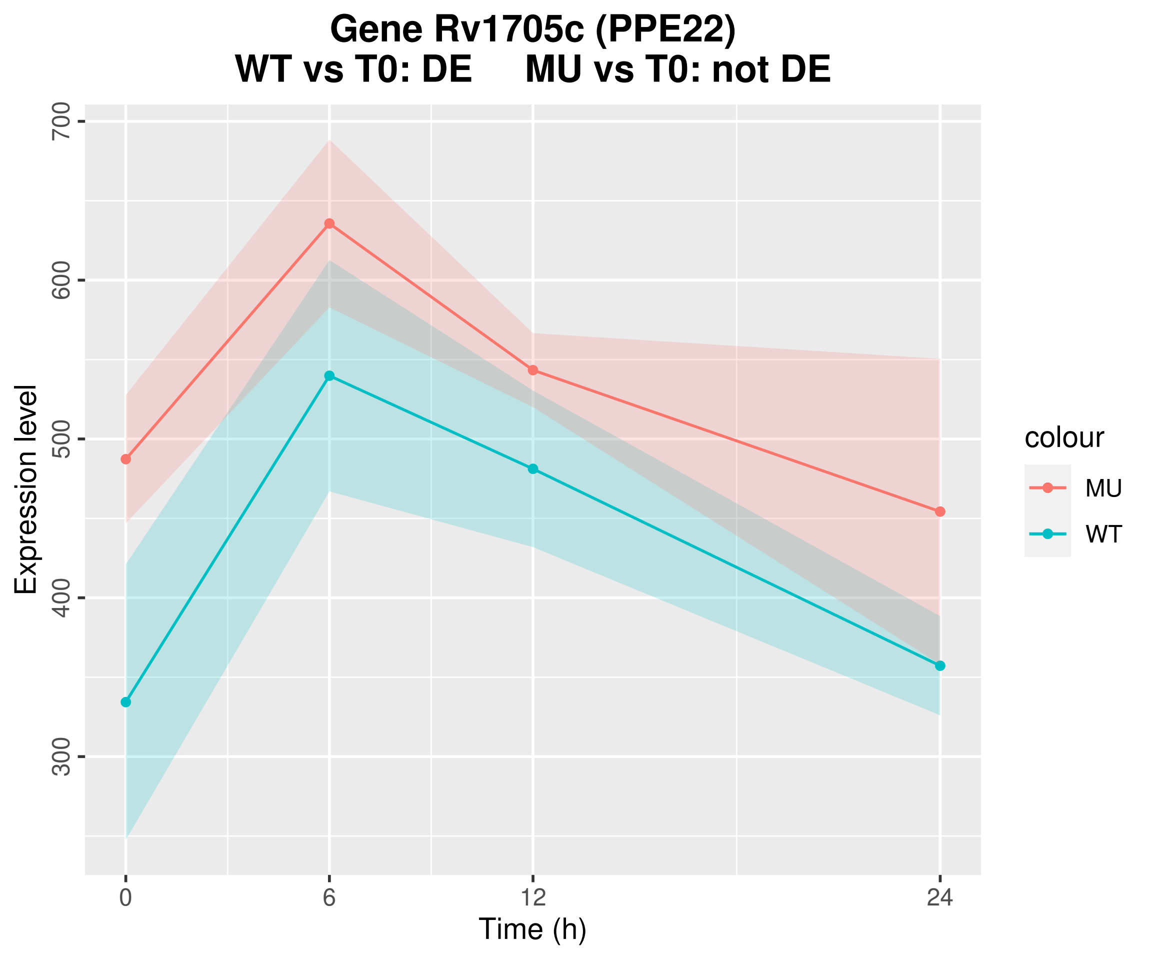
Gene Rv1705c (PPE22)
WT vs T0: DE   MU vs T0: not DE
Expression level
700
600
500
400
300
0
6
12
24
Time (h)
colour
MU
WT

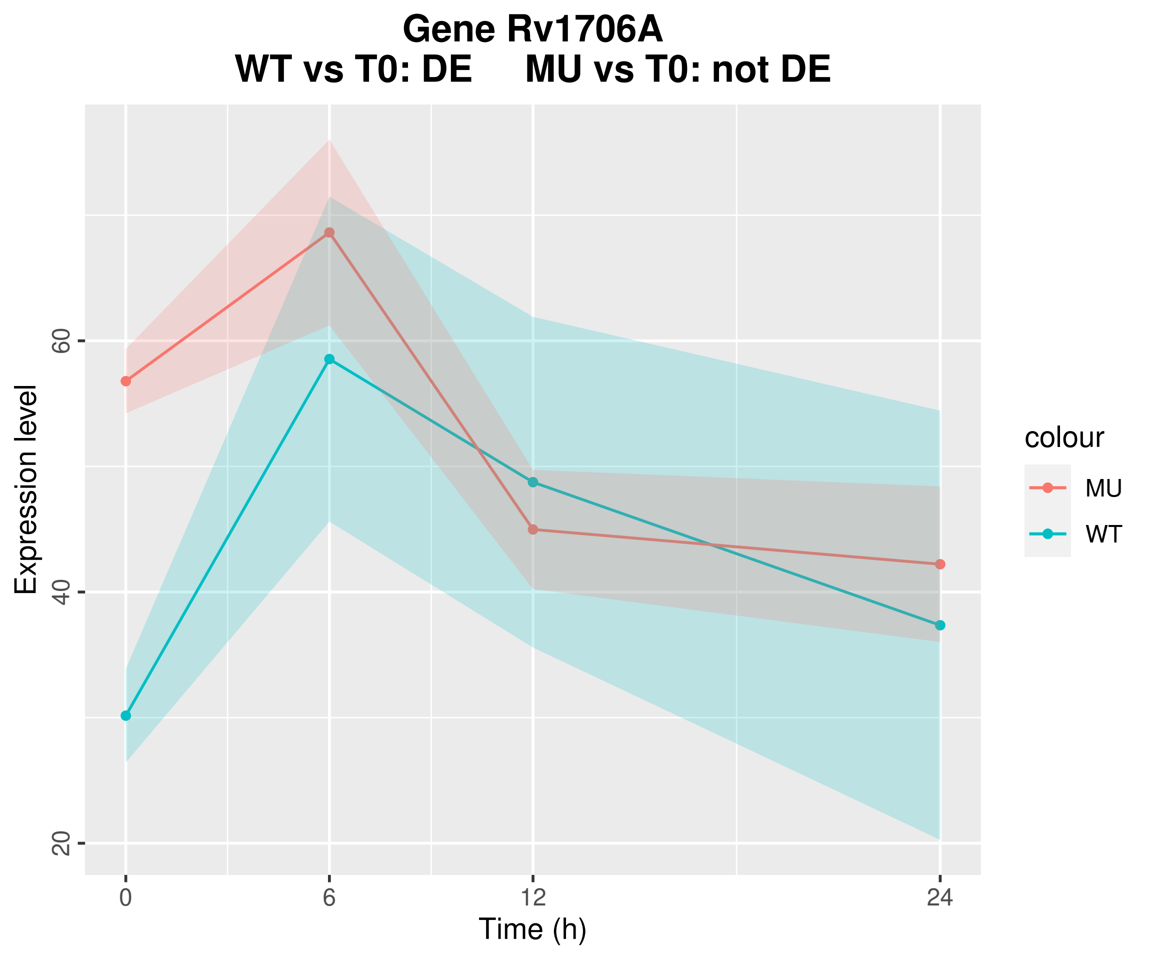
Gene Rv1706A
WT vs T0: DE MU vs T0: not DE
Expression level
20
40
60
0
6
12
24
Time (h)
colour
MU
WT

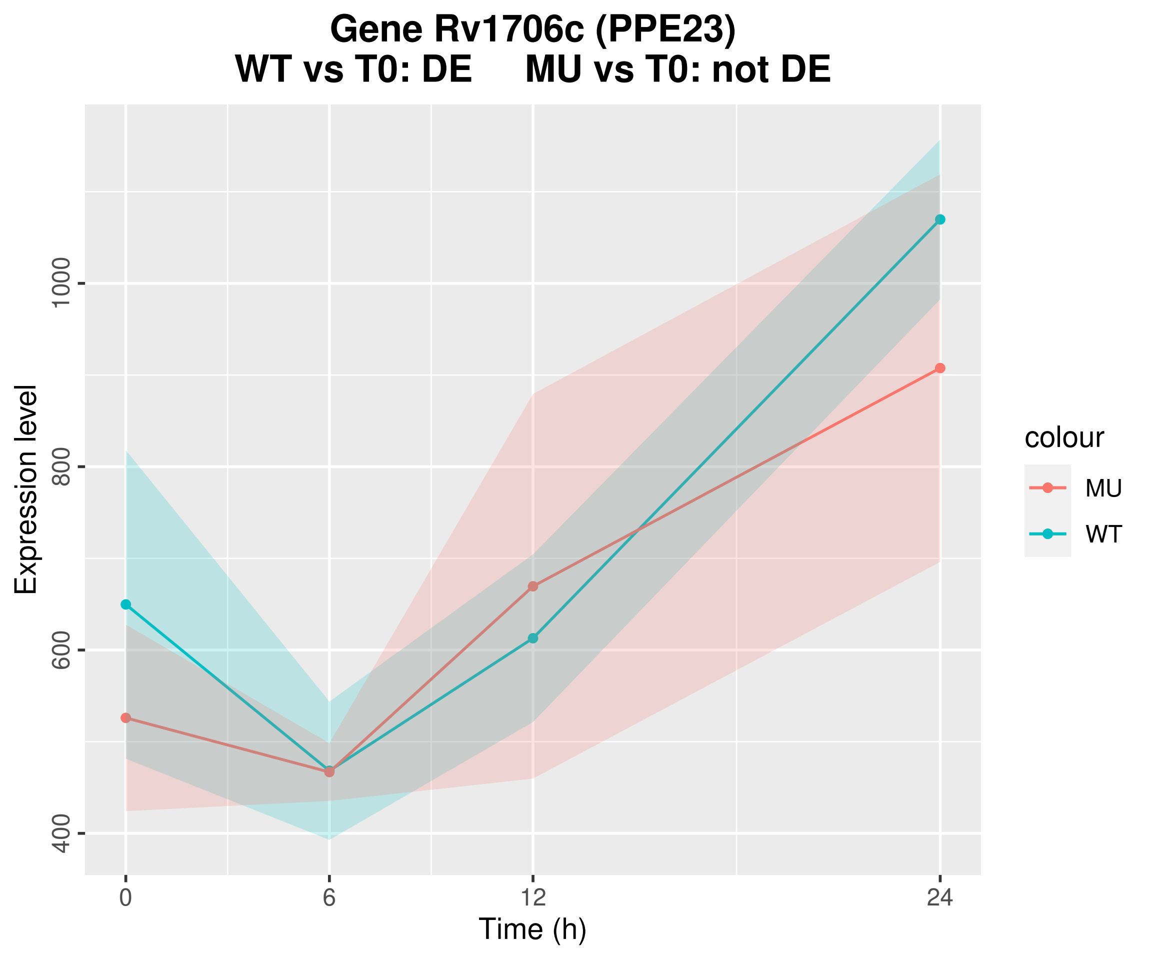

Gene Rv1706c (PPE23)
WT vs T0: DE MU vs T0: not DE
Expression level
1000
800
600
400
0
6
12
24
Time (h)
colour
MU
WT

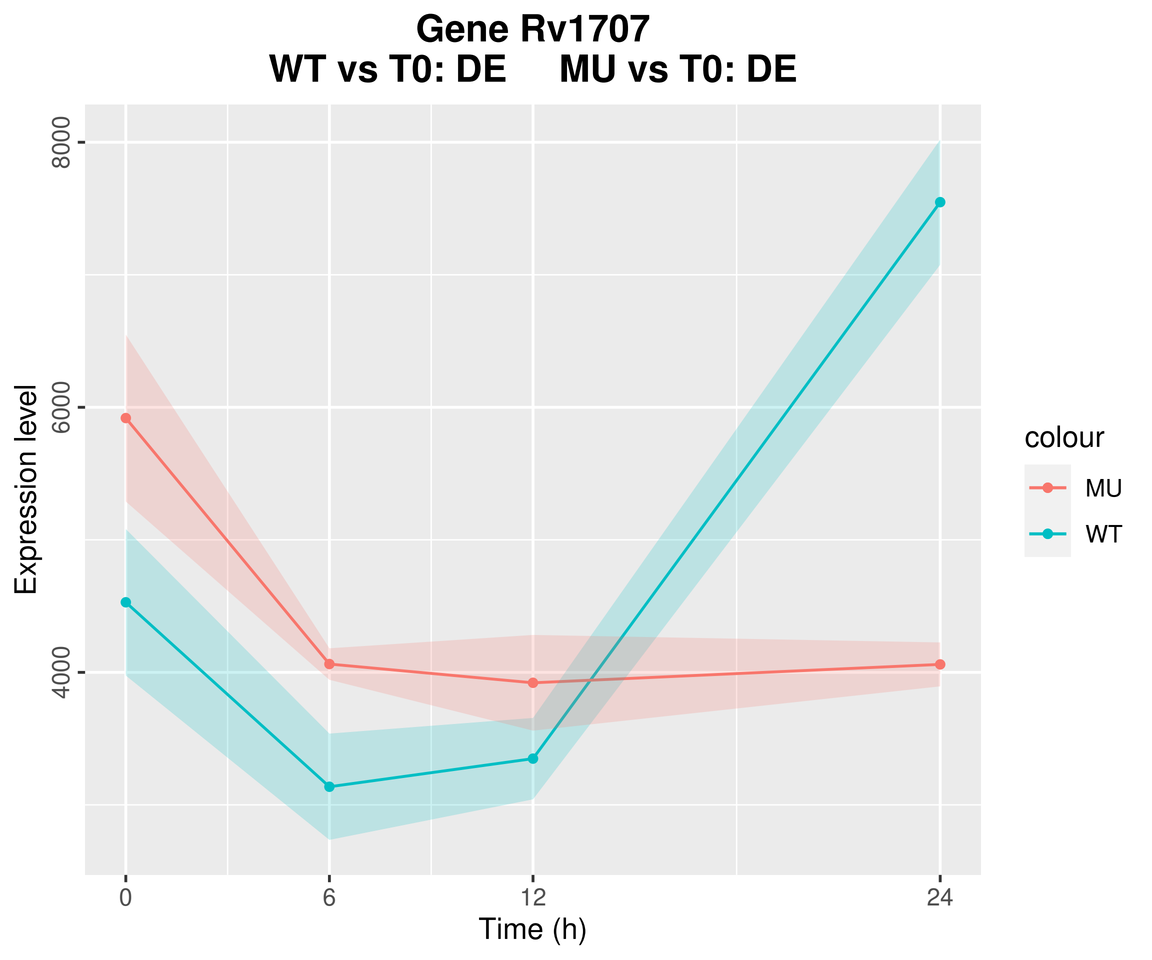

Gene Rv1707
WT vs T0: DE   MU vs T0: DE
8000
6000
4000
Expression level
0
6
12
24
Time (h)
colour
MU
WT

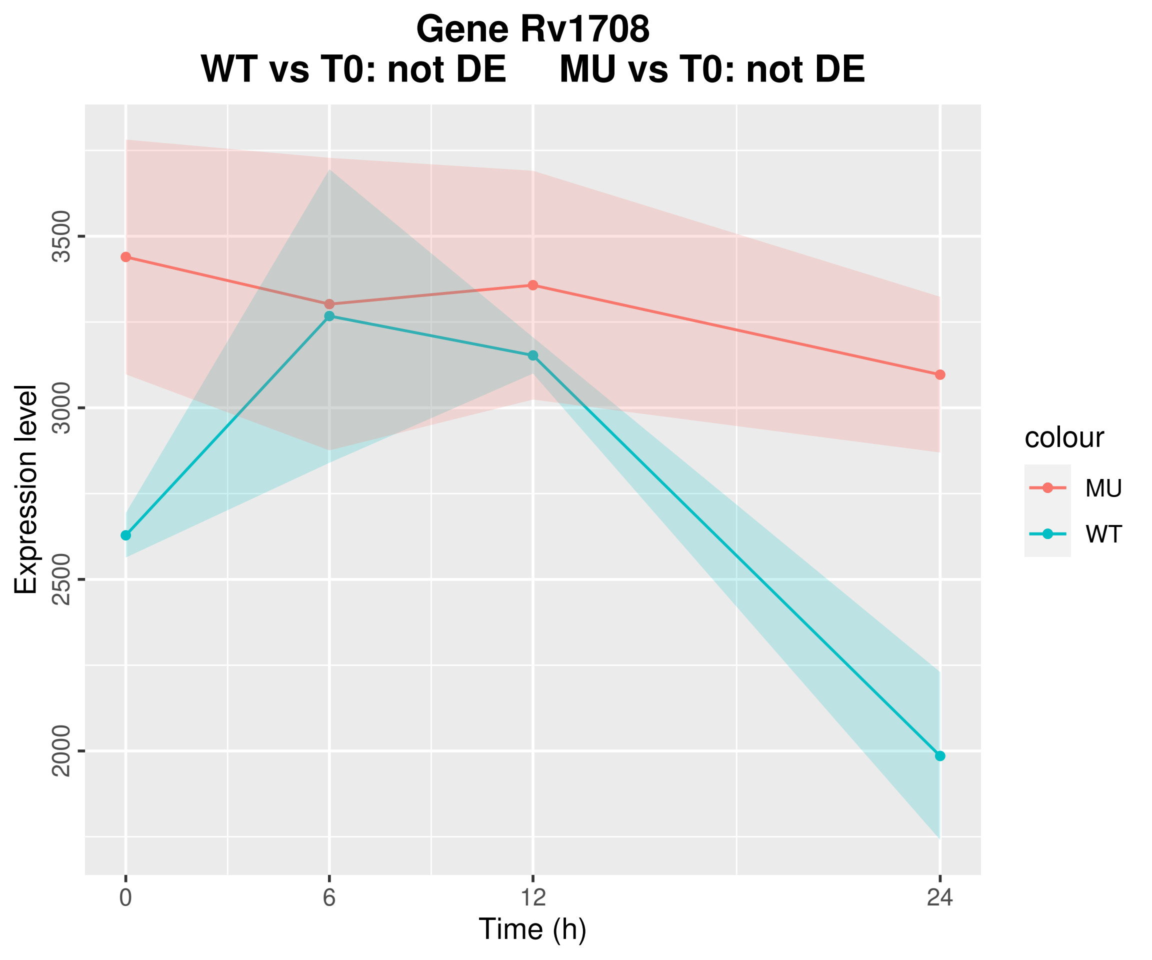

Gene Rv1708
WT vs T0: not DE MU vs T0: not DE
Expression level
3500
3000
2500
2000
0
6
12
24
Time (h)
colour
MU
WT

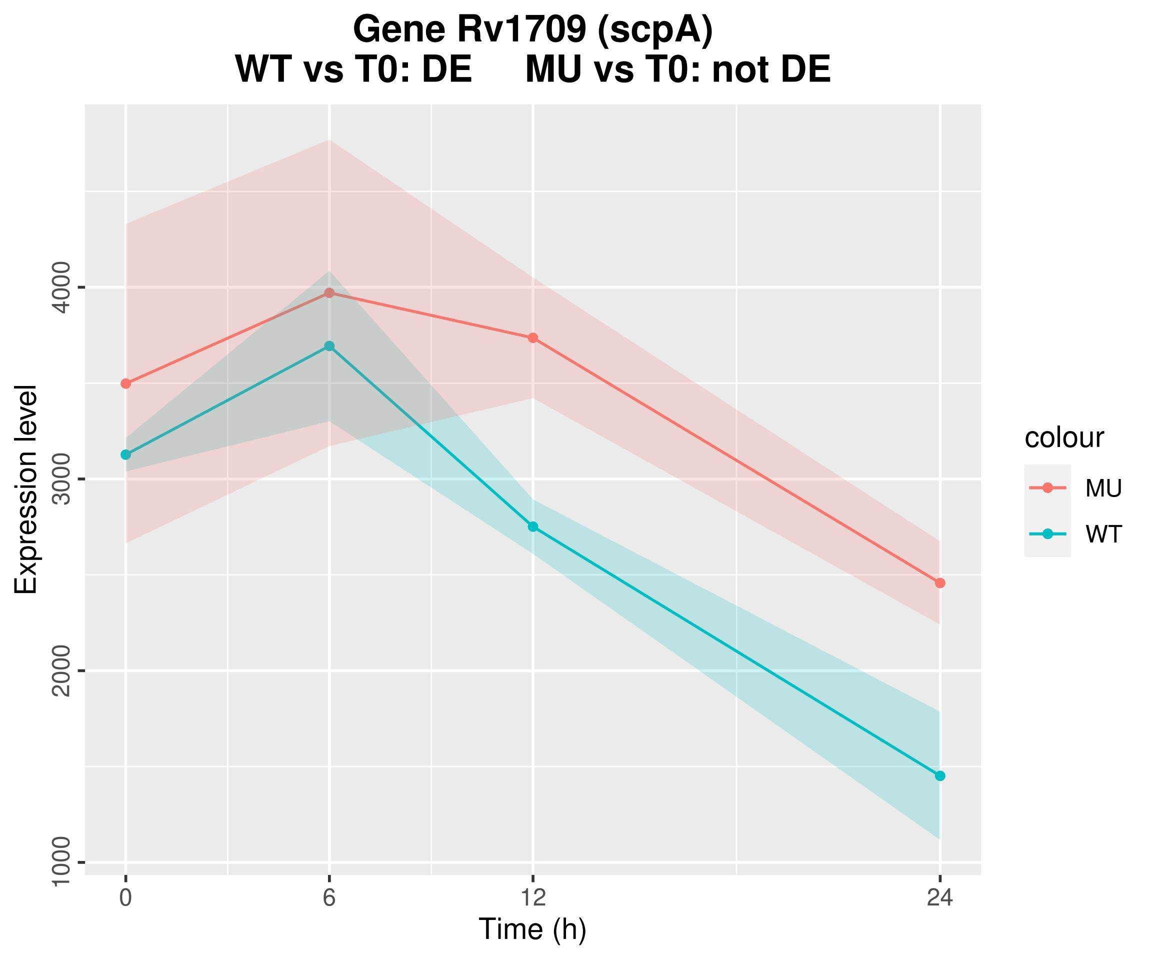
Gene Rv1709 (scpA)
WT vs T0: DE   MU vs T0: not DE
Expression level
1000
2000
3000
4000
0
6
12
24
Time (h)
colour
MU
WT

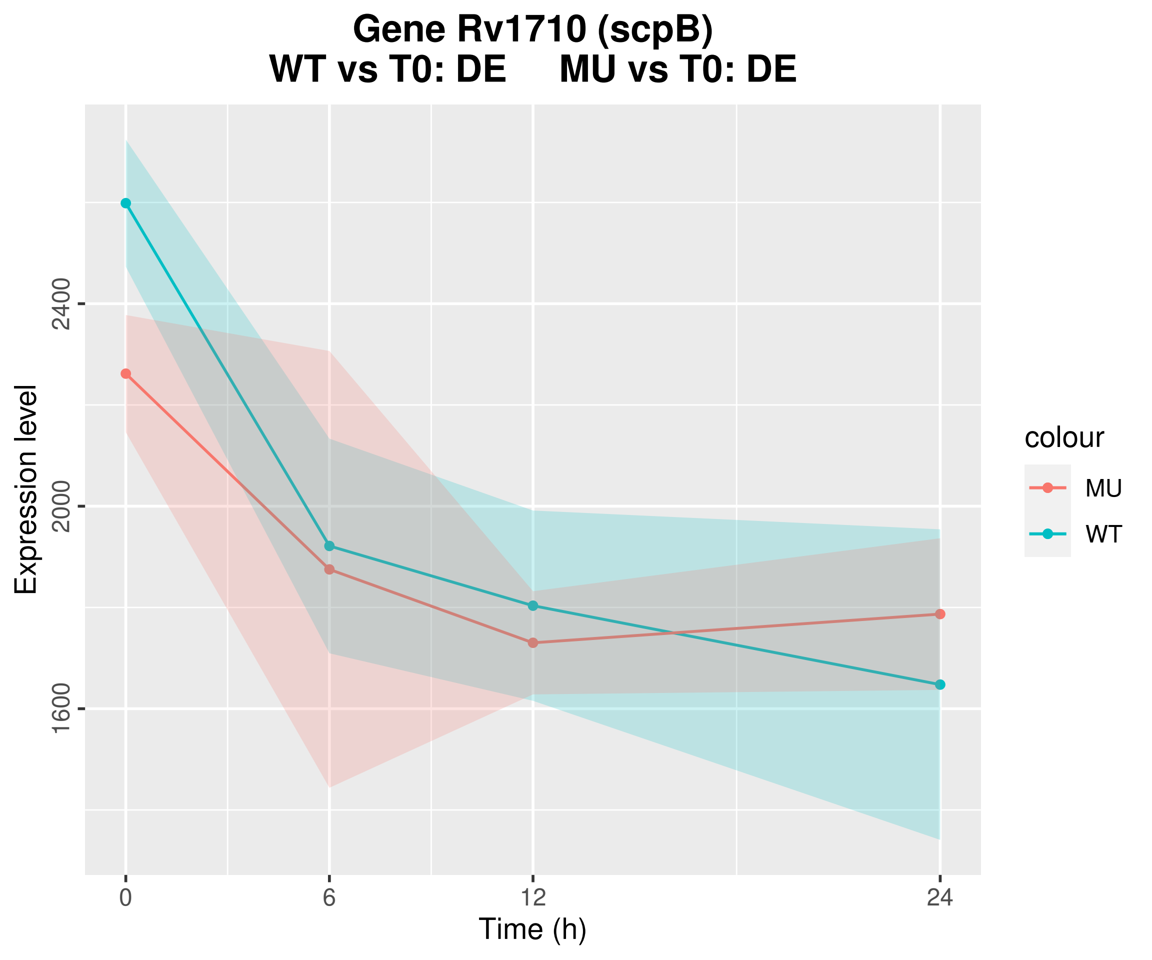

Gene Rv1710 (scpB)
WT vs T0: DE   MU vs T0: DE
Expression level
2400
2000
1600
0
6
12
24
Time (h)
colour
MU
WT

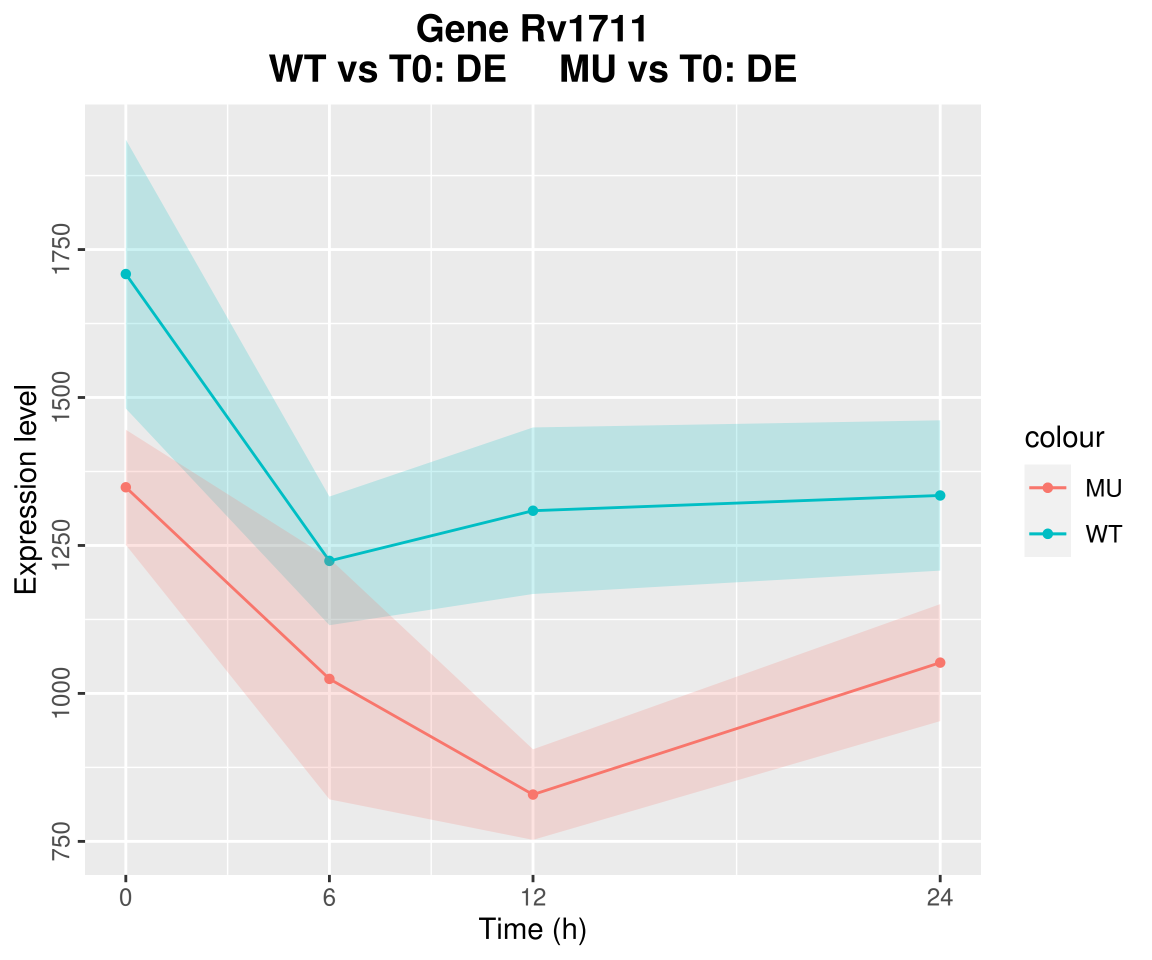
Gene Rv1711
WT vs T0: DE   MU vs T0: DE
Expression level
1750
1500
1250
1000
750
0
6
12
24
Time (h)
colour
MU
WT

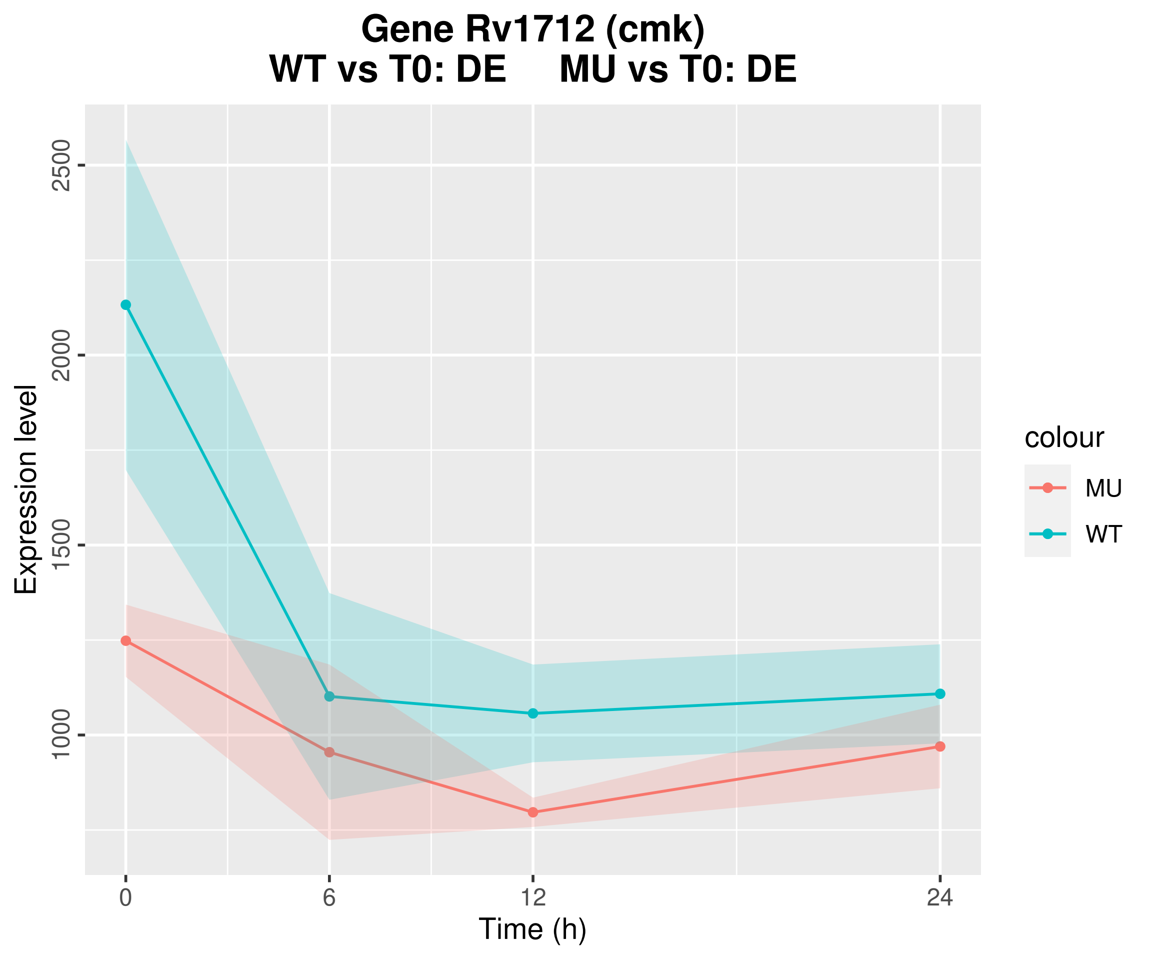
Gene Rv1712 (cmk)
WT vs T0: DE   MU vs T0: DE
Expression level
2500
2000
1500
1000
0
6
12
24
Time (h)
colour
MU
WT

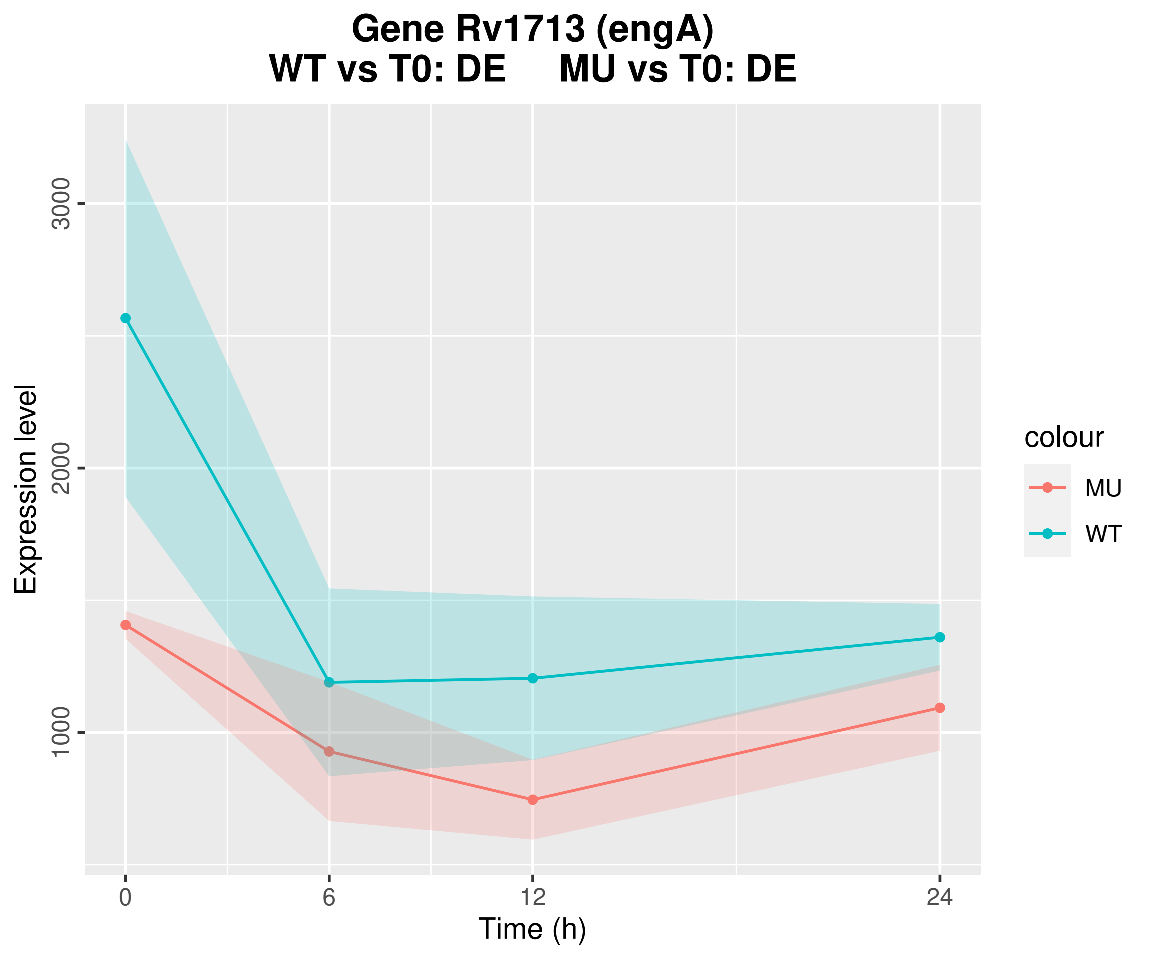
Gene Rv1713 (engA)
WT vs T0: DE MU vs T0: DE
Expression level
3000
2000
1000
0
6
12
24
Time (h)
colour
MU
WT

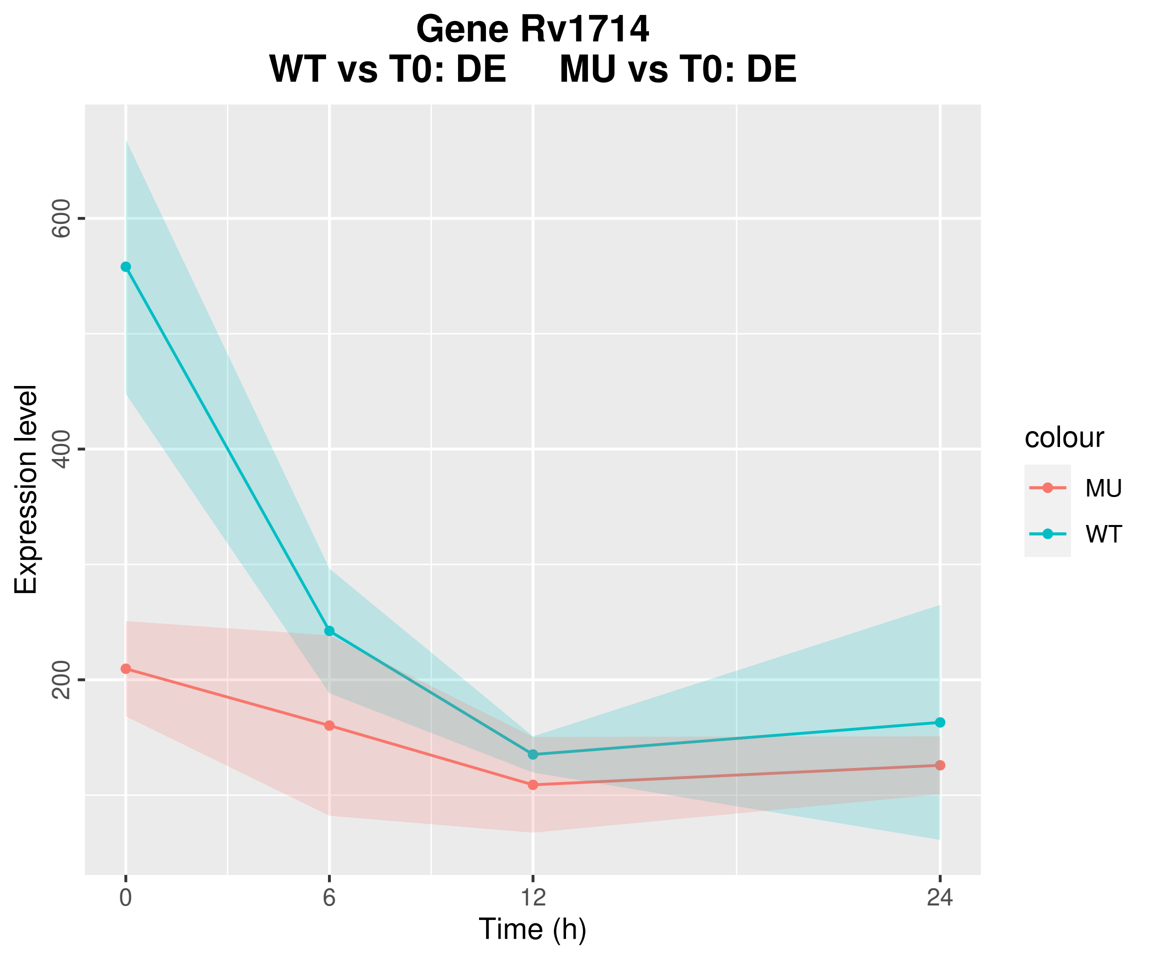

Gene Rv1714
WT vs T0: DE   MU vs T0: DE
Expression level
600
400
200
0
6
12
24
Time (h)
colour
MU
WT

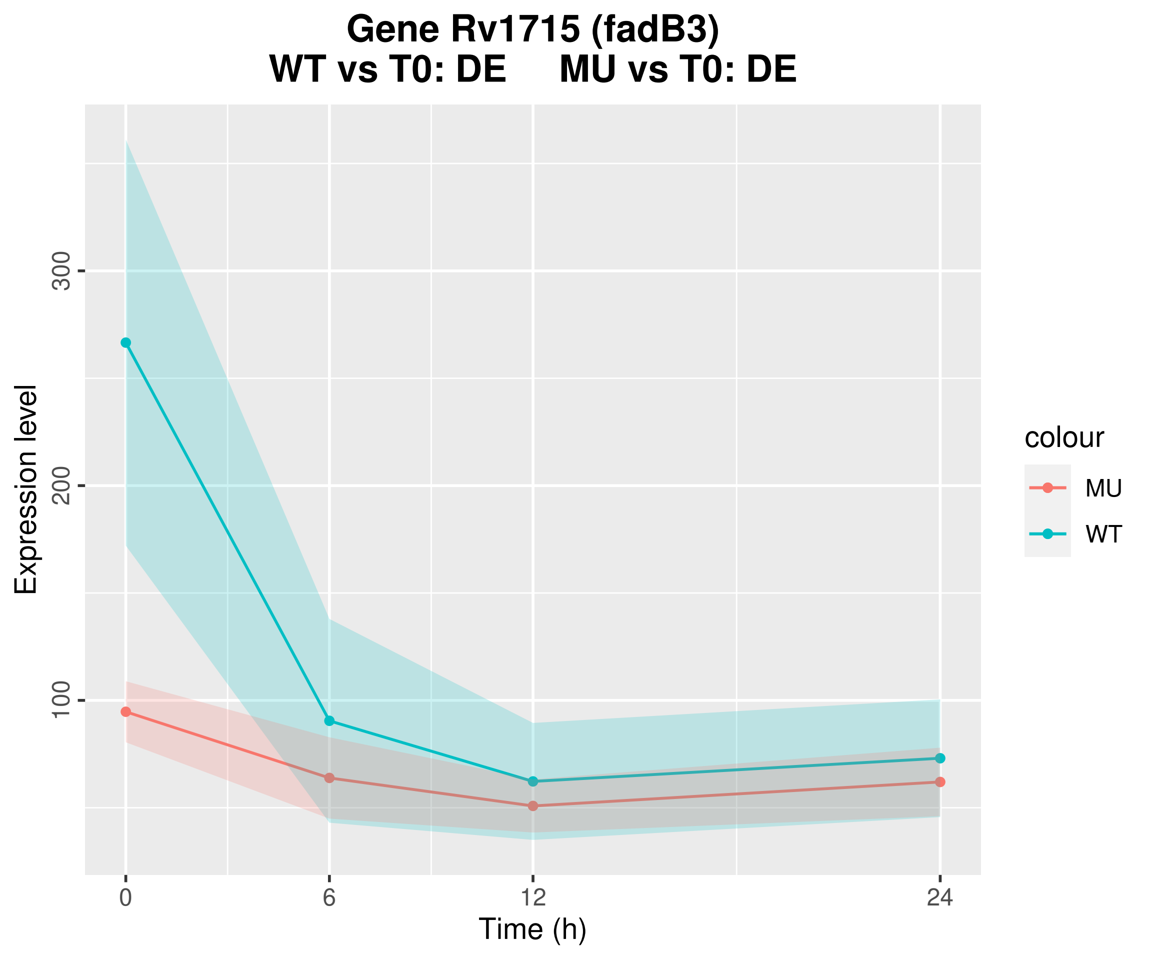
Gene Rv1715 (fadB3)
WT vs T0: DE   MU vs T0: DE
Expression level
300
200
100
0
6
12
24
Time (h)
colour
MU
WT

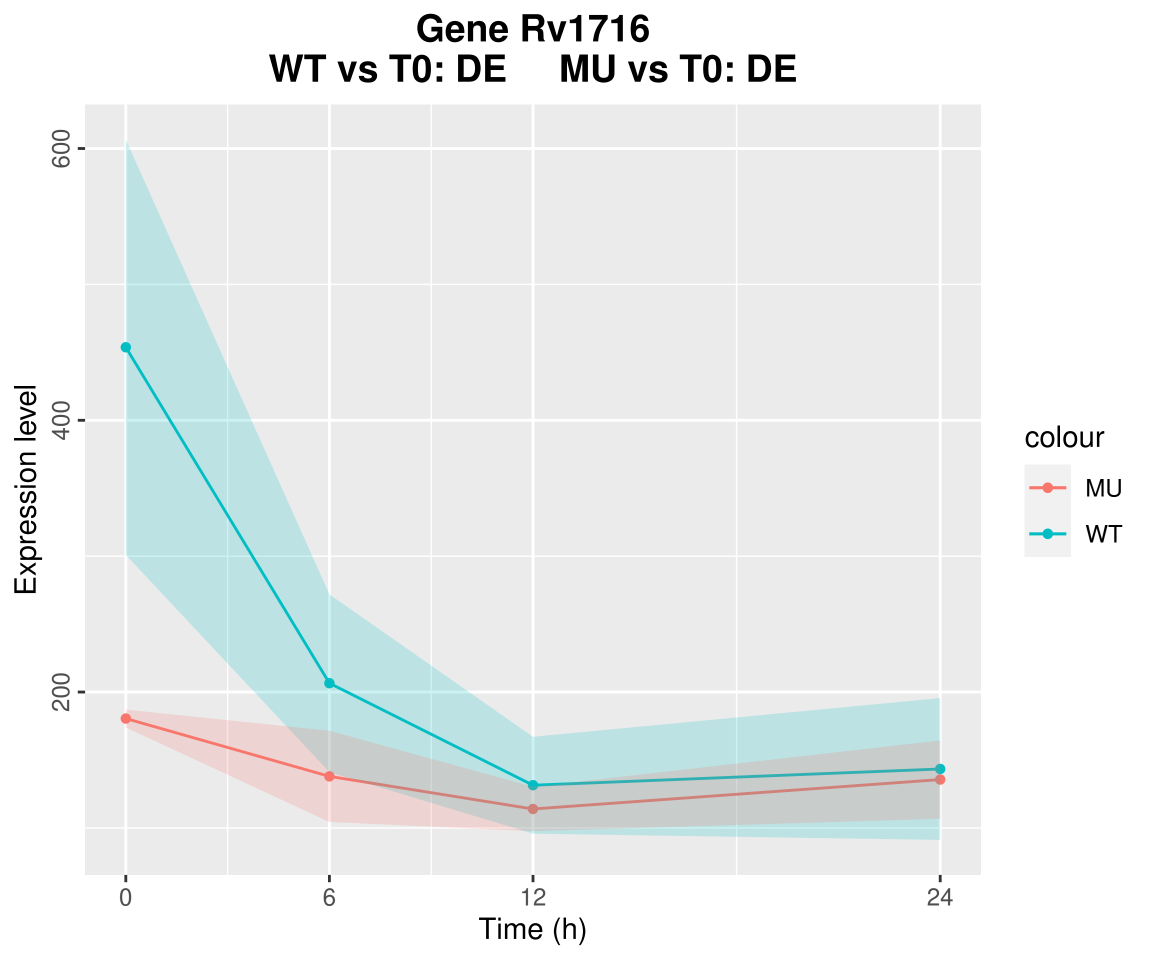

Gene Rv1716
WT vs T0: DE MU vs T0: DE
Expression level
600
400
200
0
6
12
24
Time (h)
colour
MU
WT

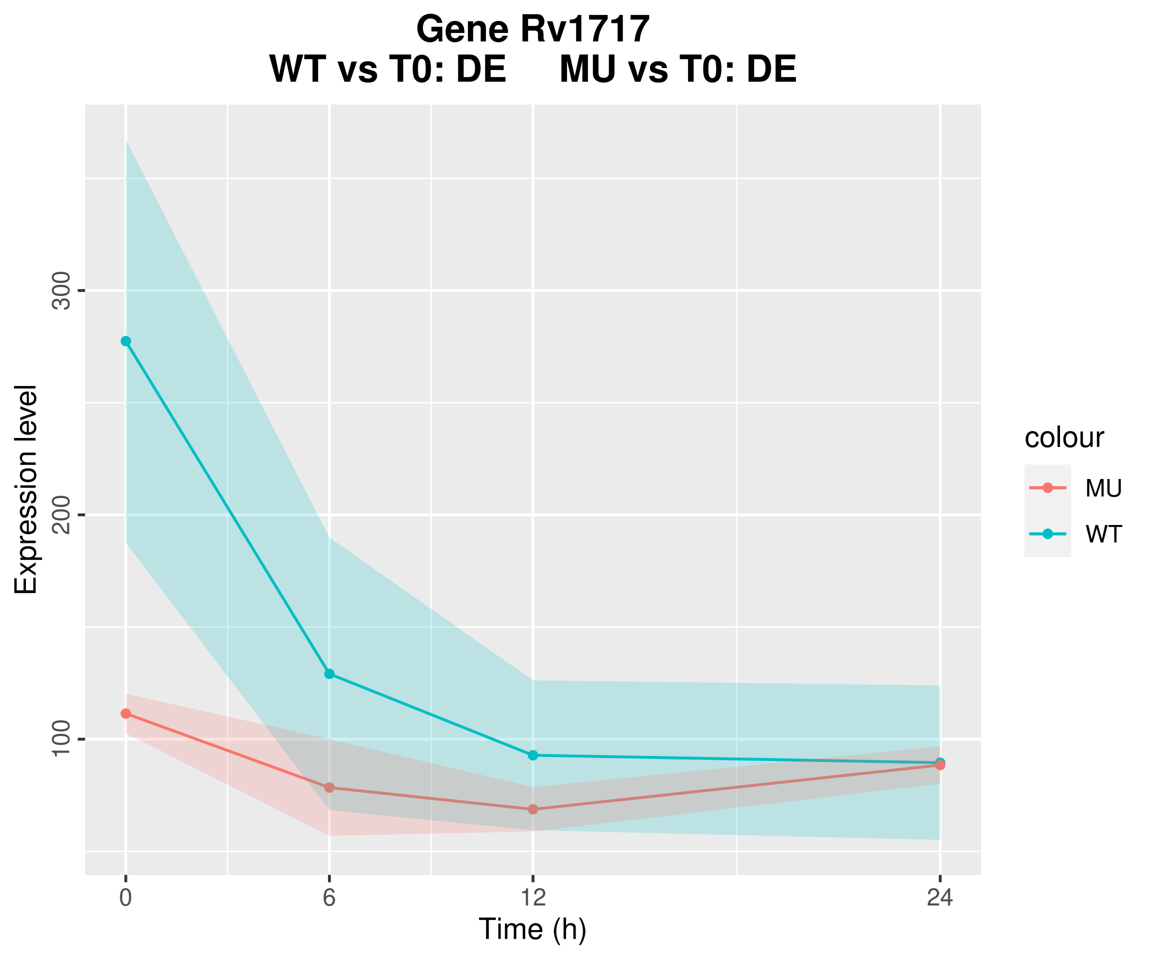

Gene Rv1717
WT vs T0: DE MU vs T0: DE
Expression level
300
200
100
0
6
12
24
Time (h)
colour
MU
WT

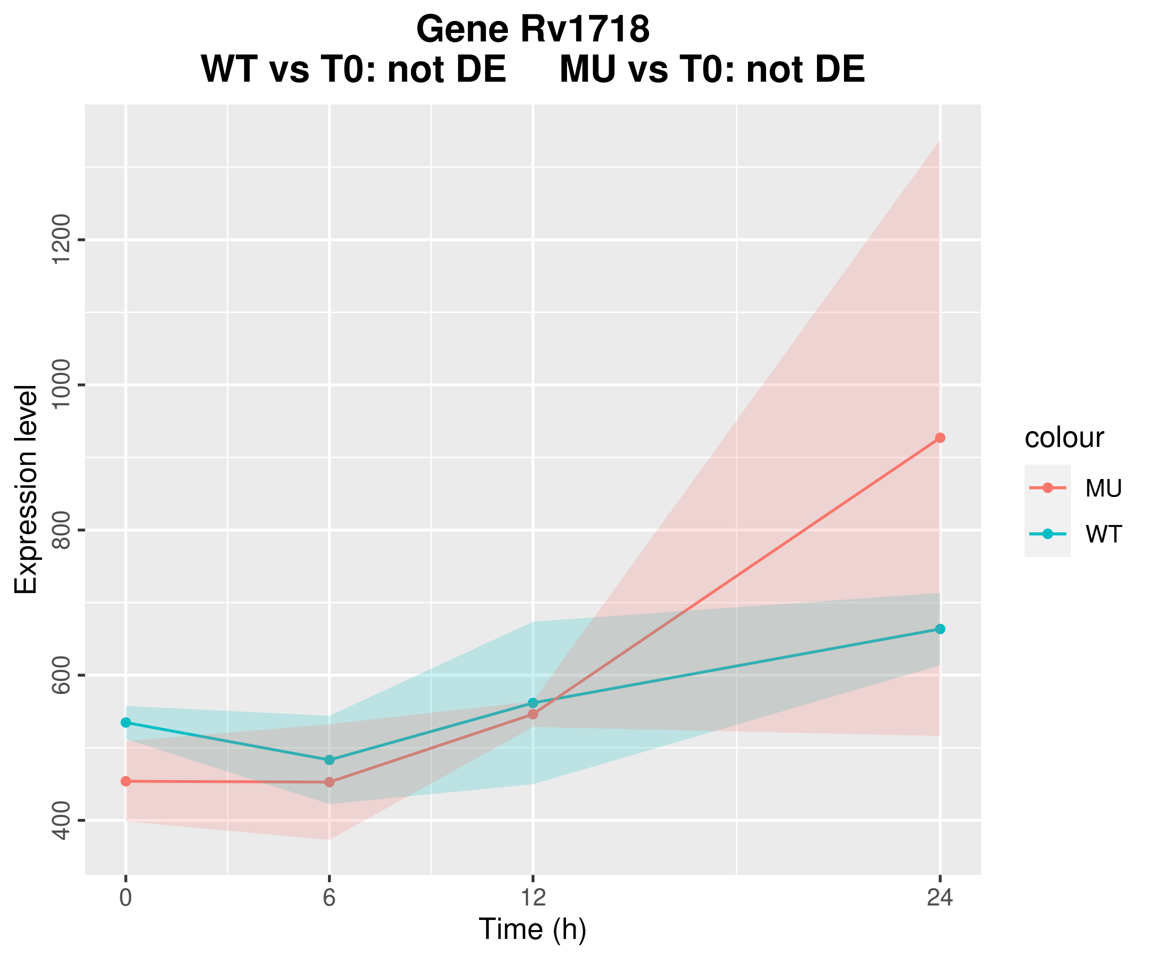
Gene Rv1718
WT vs T0: not DE   MU vs T0: not DE
Expression level
1200
1000
800
600
400
0
6
12
24
Time (h)
colour
MU
WT

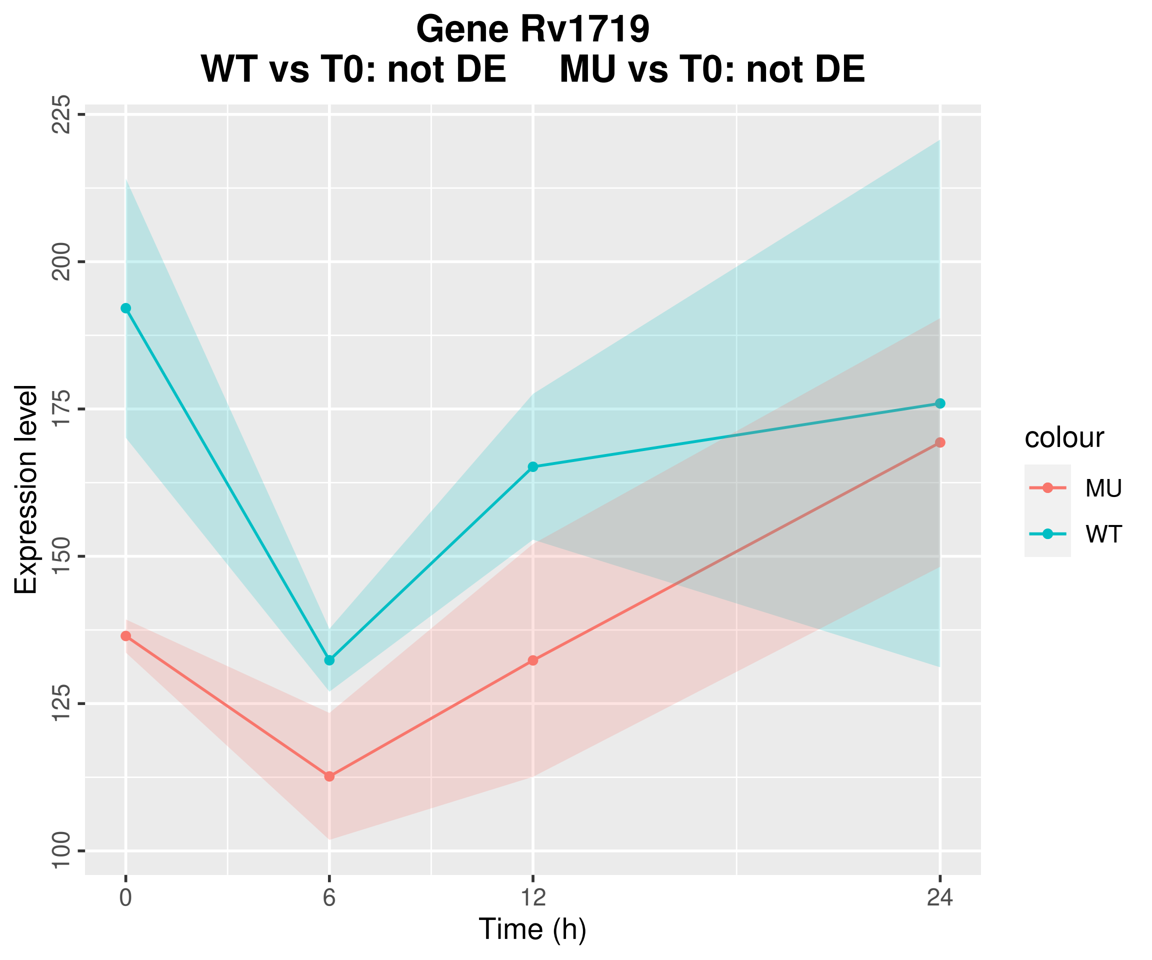
Gene Rv1719
WT vs T0: not DE
MU vs T0: not DE
Expression level
225
200
175
150
125
100
0
6
12
24
Time (h)
colour
MU
WT

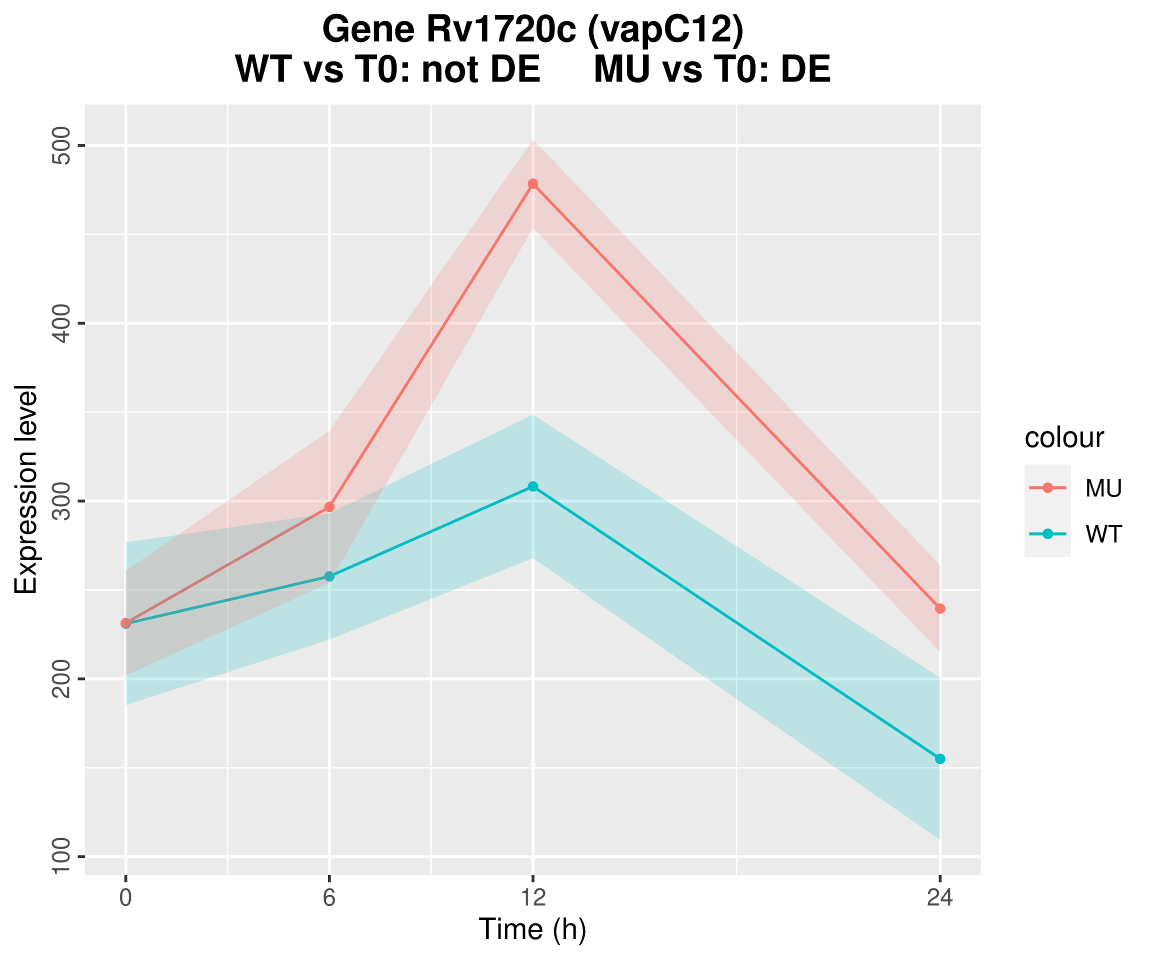

Gene Rv1720c (vapC12)
WT vs T0: not DE   MU vs T0: DE
500
400
300
200
100
Expression level
0
6
12
24
Time (h)
colour
MU
WT

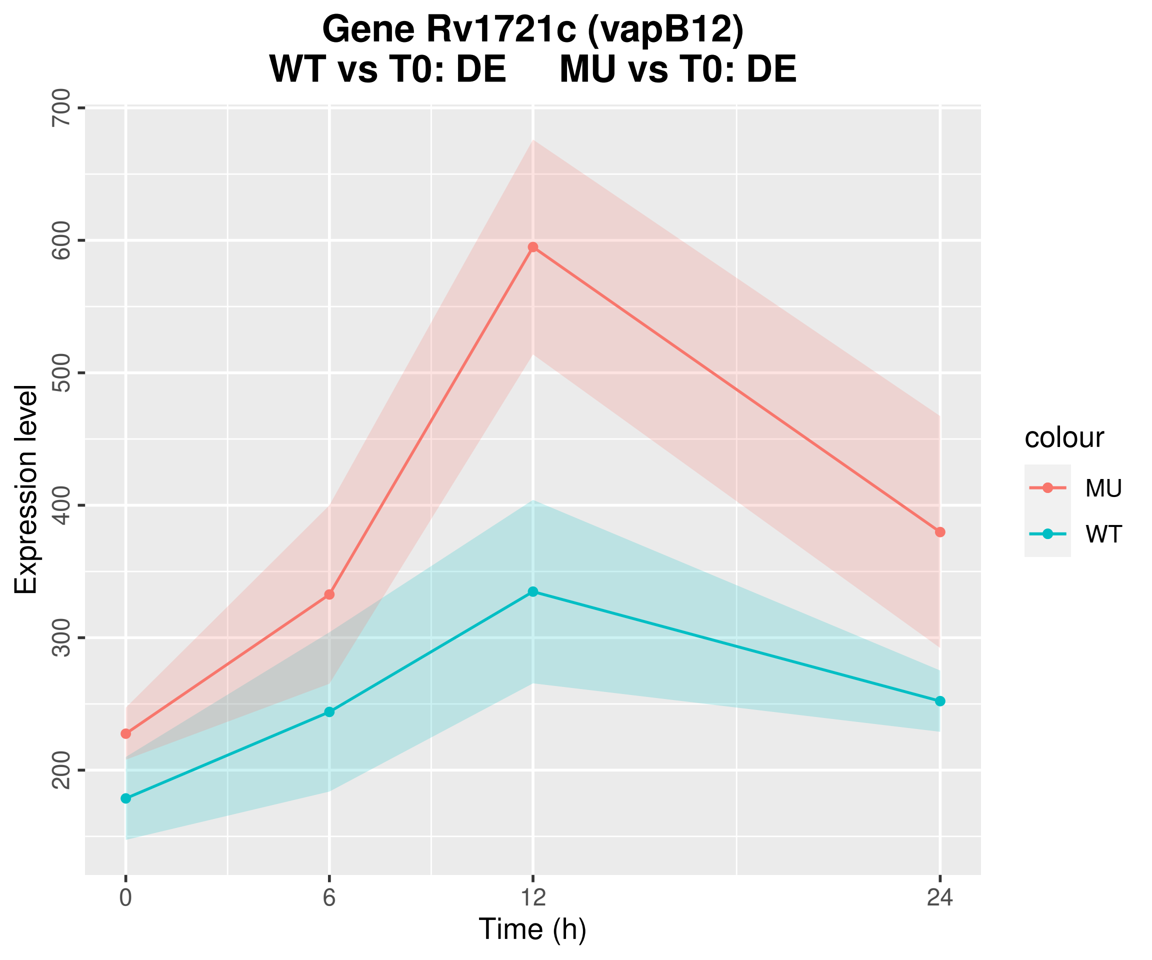

Gene Rv1721c (vapB12)
WT vs T0: DE   MU vs T0: DE
Expression level
700
600
500
400
300
200
0
6
12
24
Time (h)
colour
MU
WT

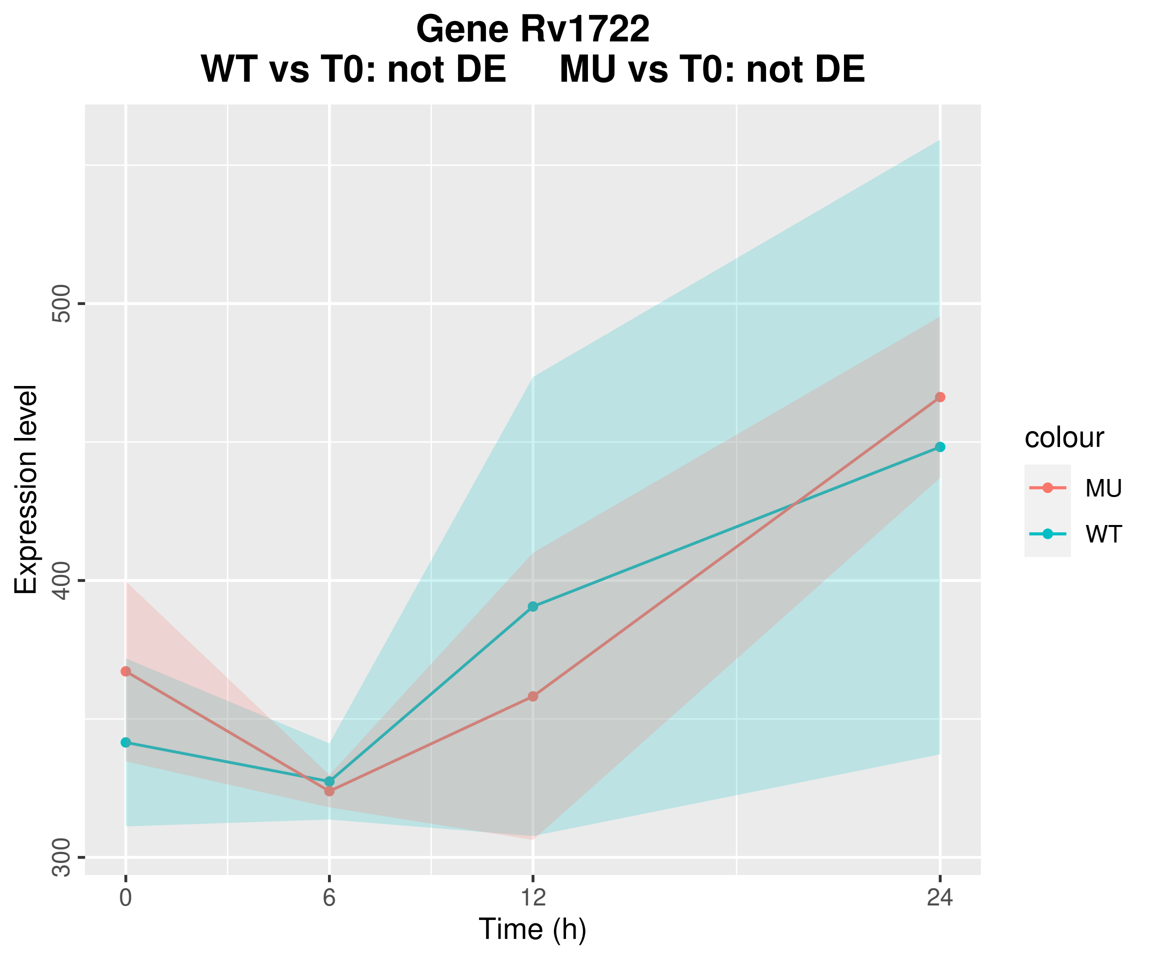

Gene Rv1722
WT vs T0: not DE MU vs T0: not DE
Expression level
500
400
300
0
6
12
24
Time (h)
colour
MU
WT

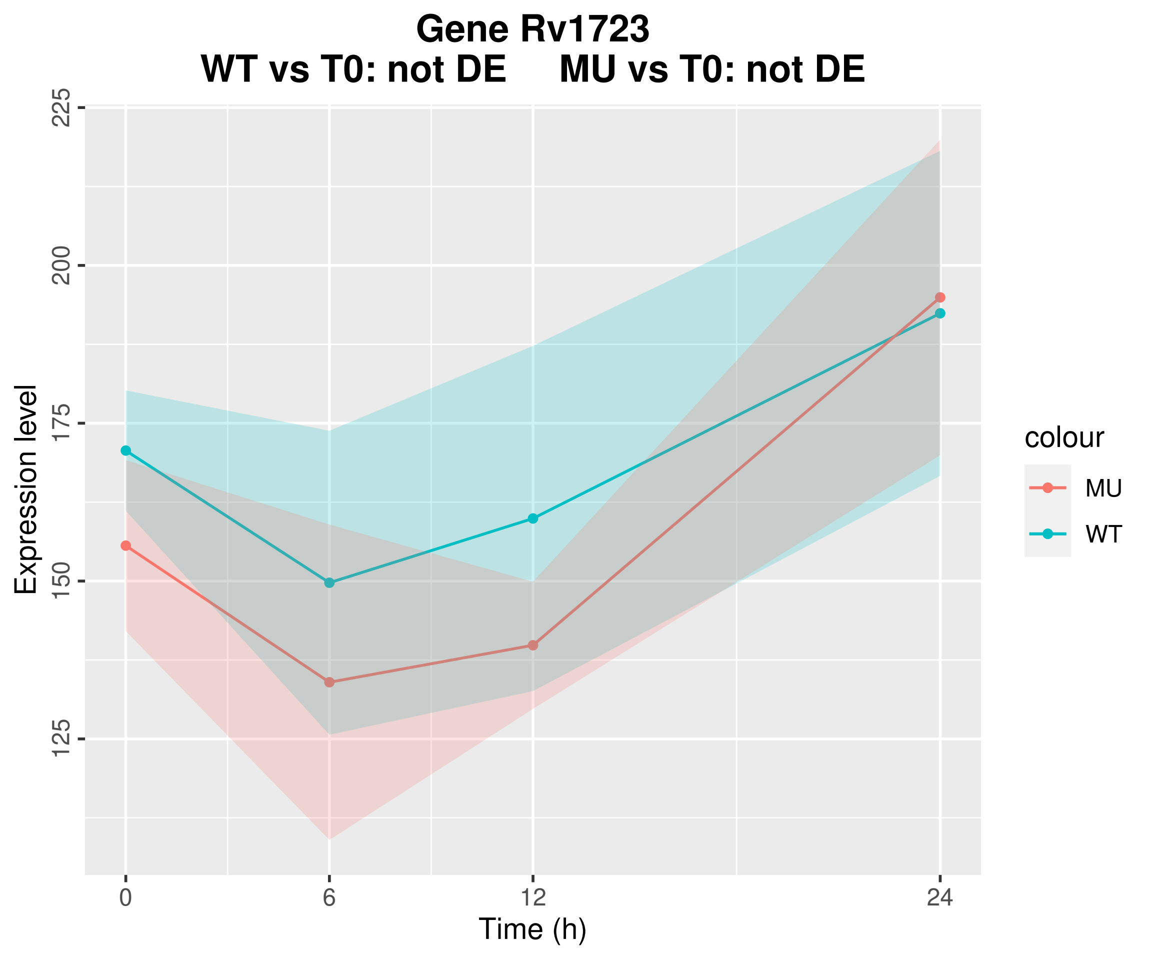
Gene Rv1723
WT vs T0: not DE MU vs T0: not DE
Expression level
225
200
175
150
125
0
6
12
24
Time (h)
colour
MU
WT

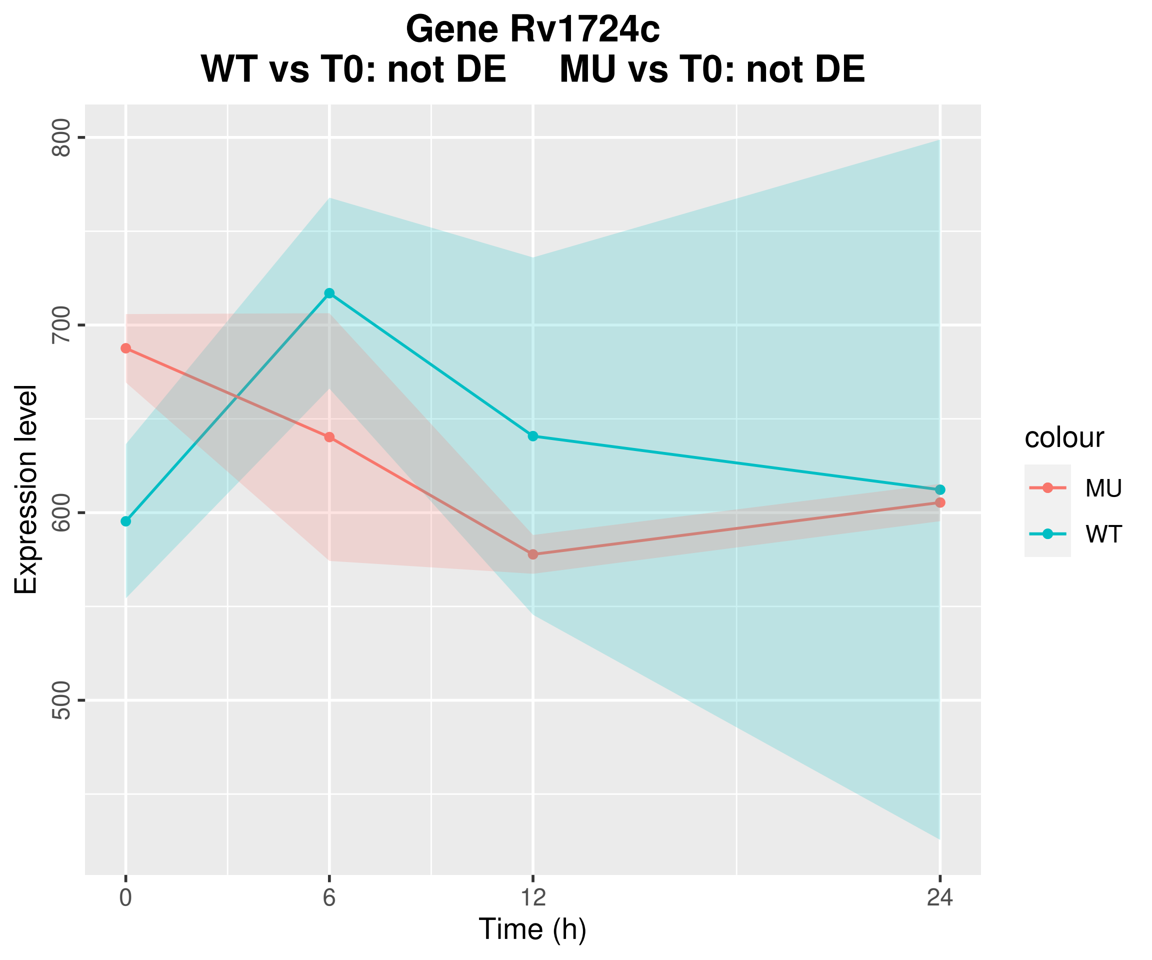

Gene Rv1724c
WT vs T0: not DE   MU vs T0: not DE
800
700
600
500
Expression level
0
6
12
24
Time (h)
colour
MU
WT

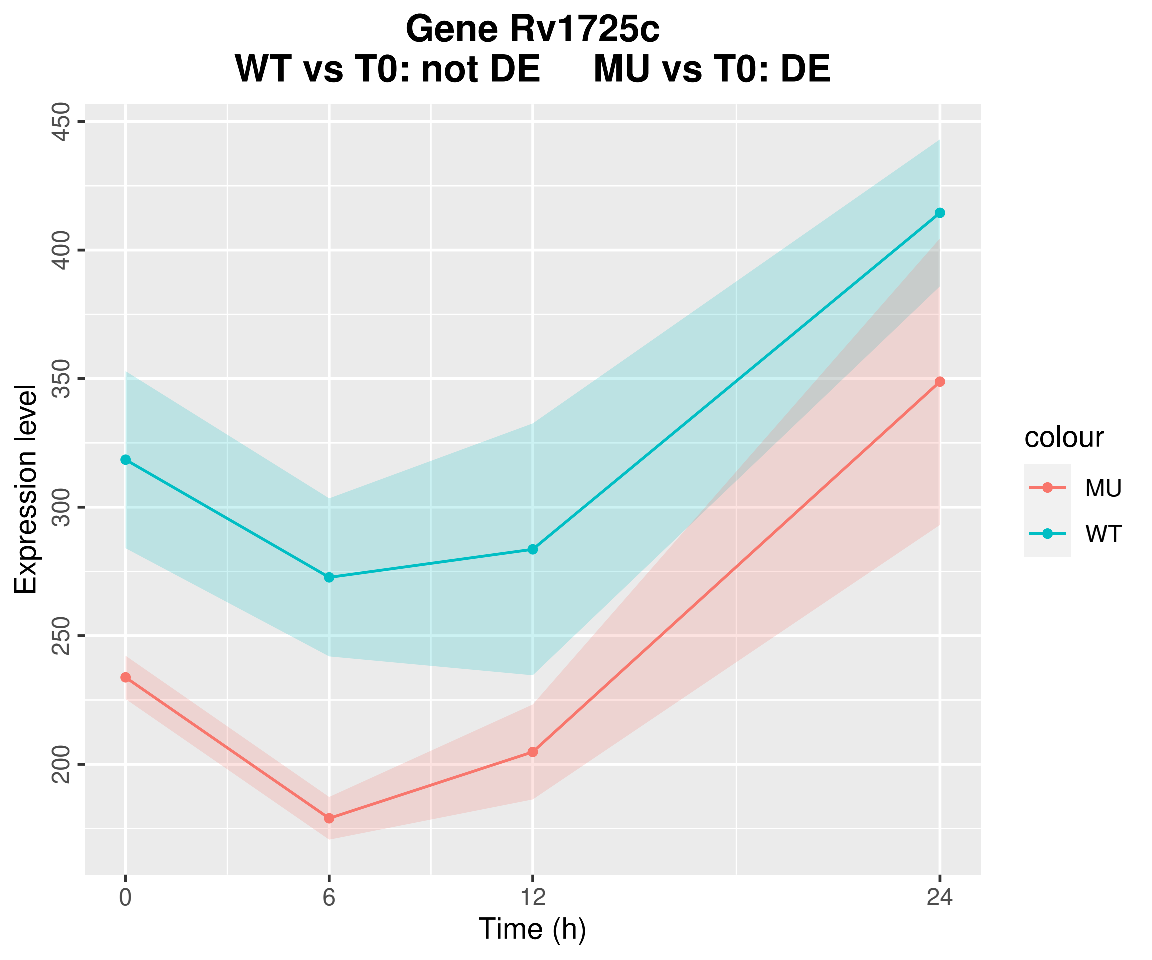

Gene Rv1725c
WT vs T0: not DE MU vs T0: DE
Expression level
450
400
350
300
250
200
0
6
12
24
Time (h)
colour
MU
WT

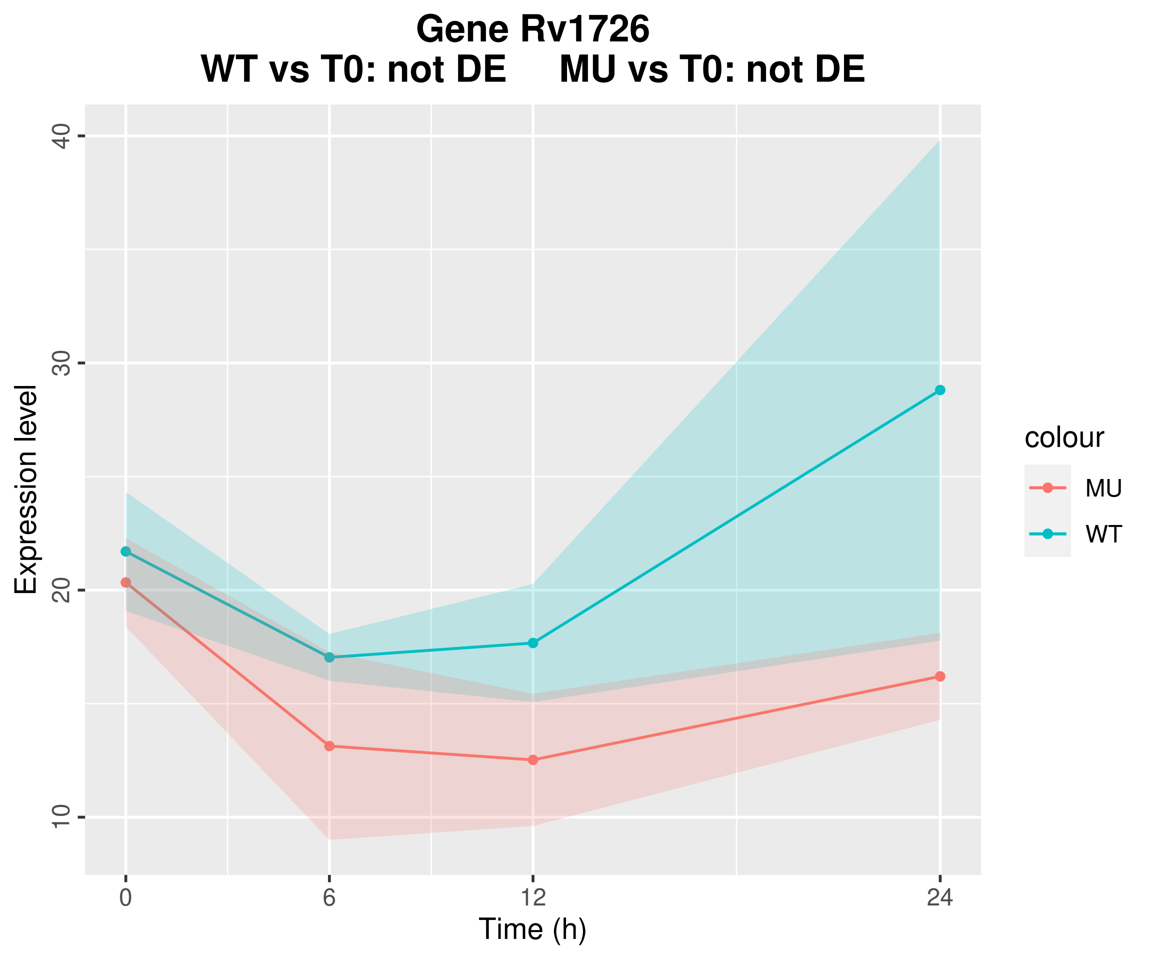
Gene Rv1726
WT vs T0: not DE MU vs T0: not DE
Expression level
40
30
20
10
0
6
12
24
Time (h)
colour
MU
WT

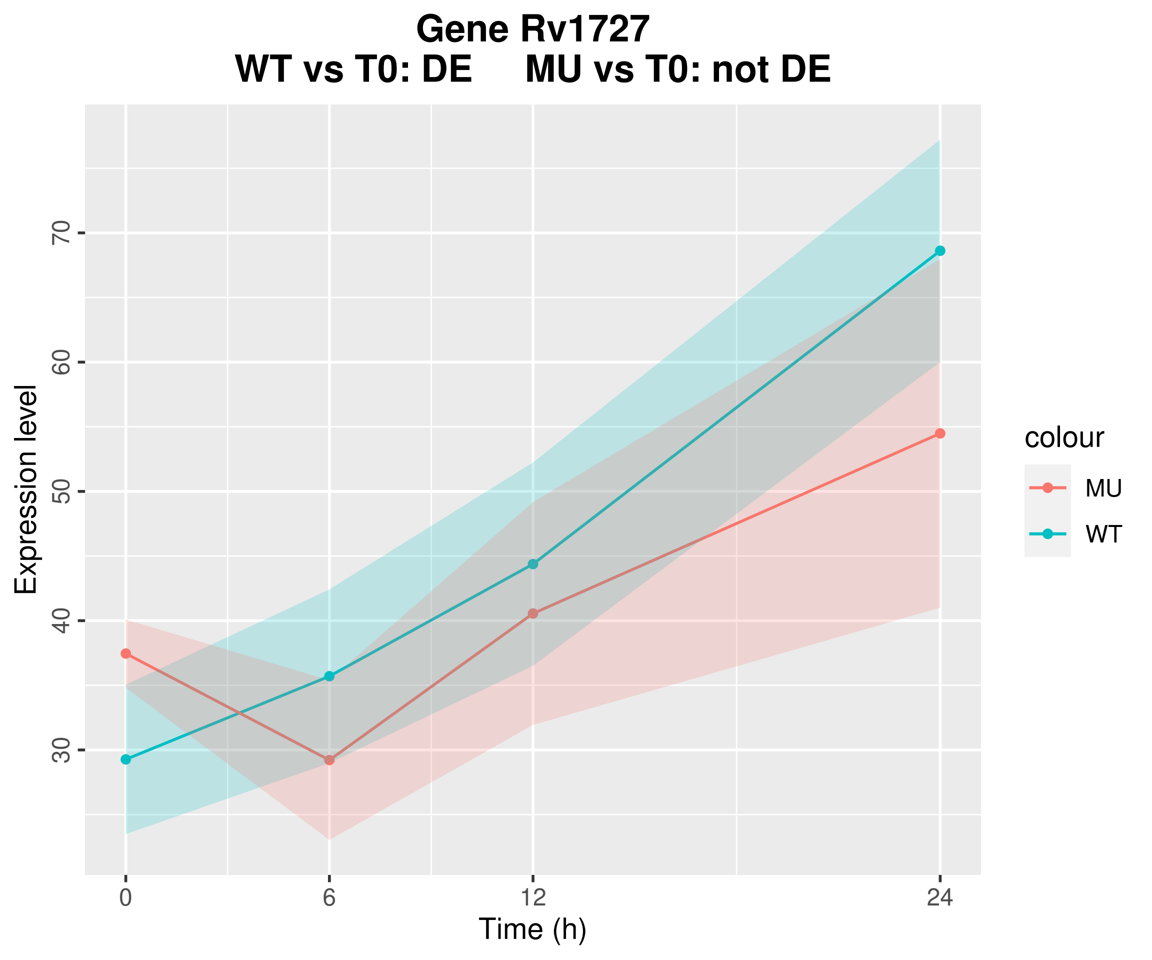
Gene Rv1727
WT vs T0: DE MU vs T0: not DE
Expression level
70
60
50
40
30
0
6
12
24
Time (h)
colour
MU
WT

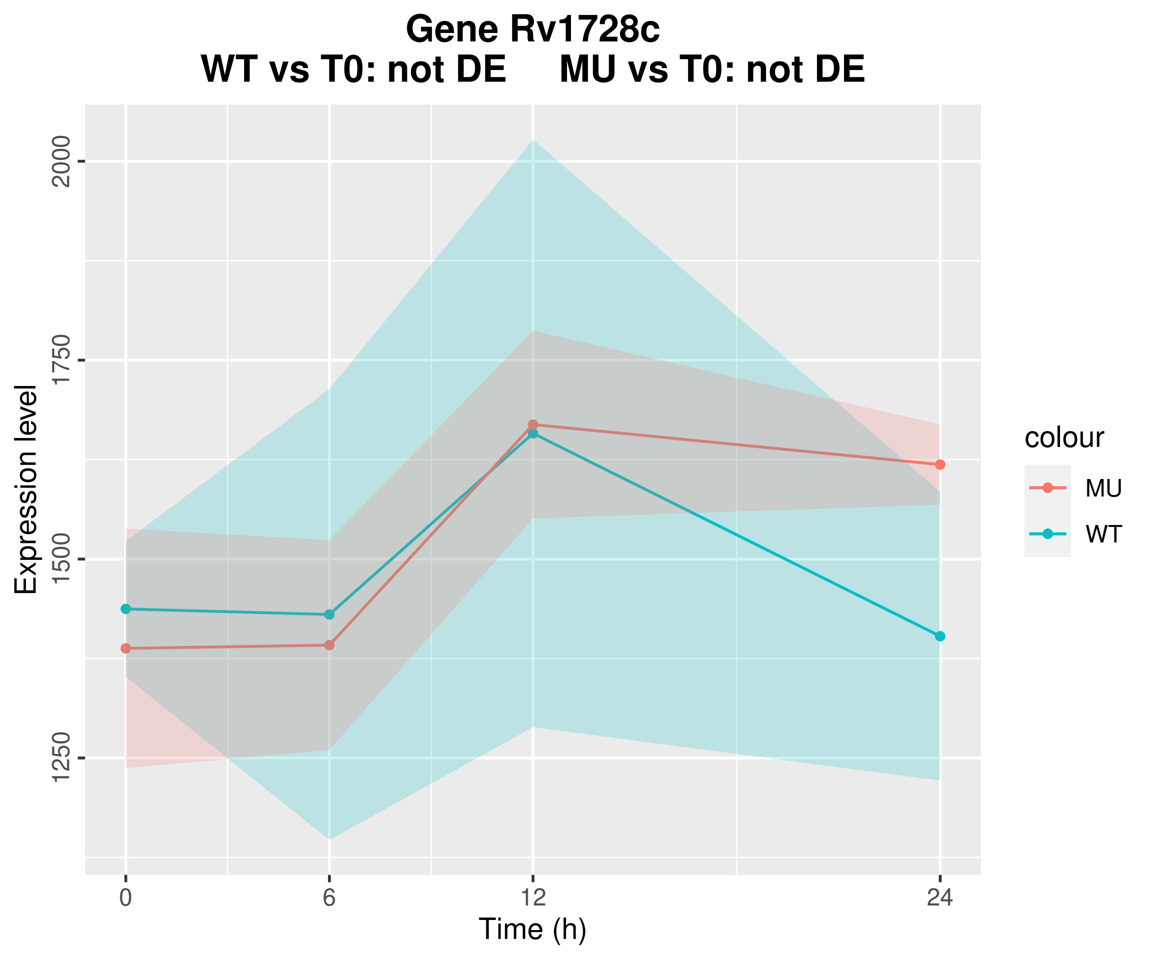
Gene Rv1728c
WT vs T0: not DE   MU vs T0: not DE
Expression level
2000
1750
1500
1250
0
6
12
24
Time (h)
colour
MU
WT

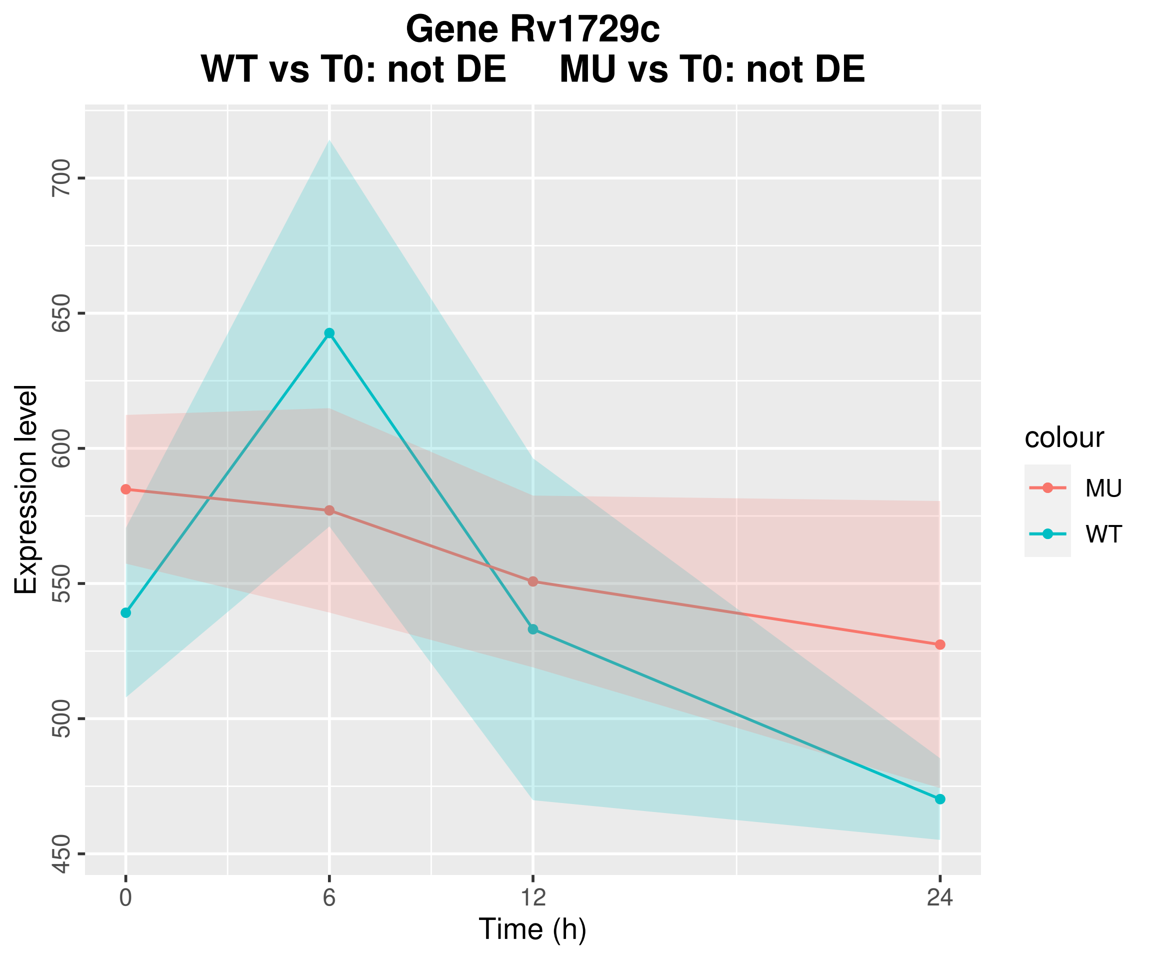

Gene Rv1729c
WT vs T0: not DE   MU vs T0: not DE
Expression level
700
650
600
550
500
450
0
6
12
24
Time (h)
colour
MU
WT

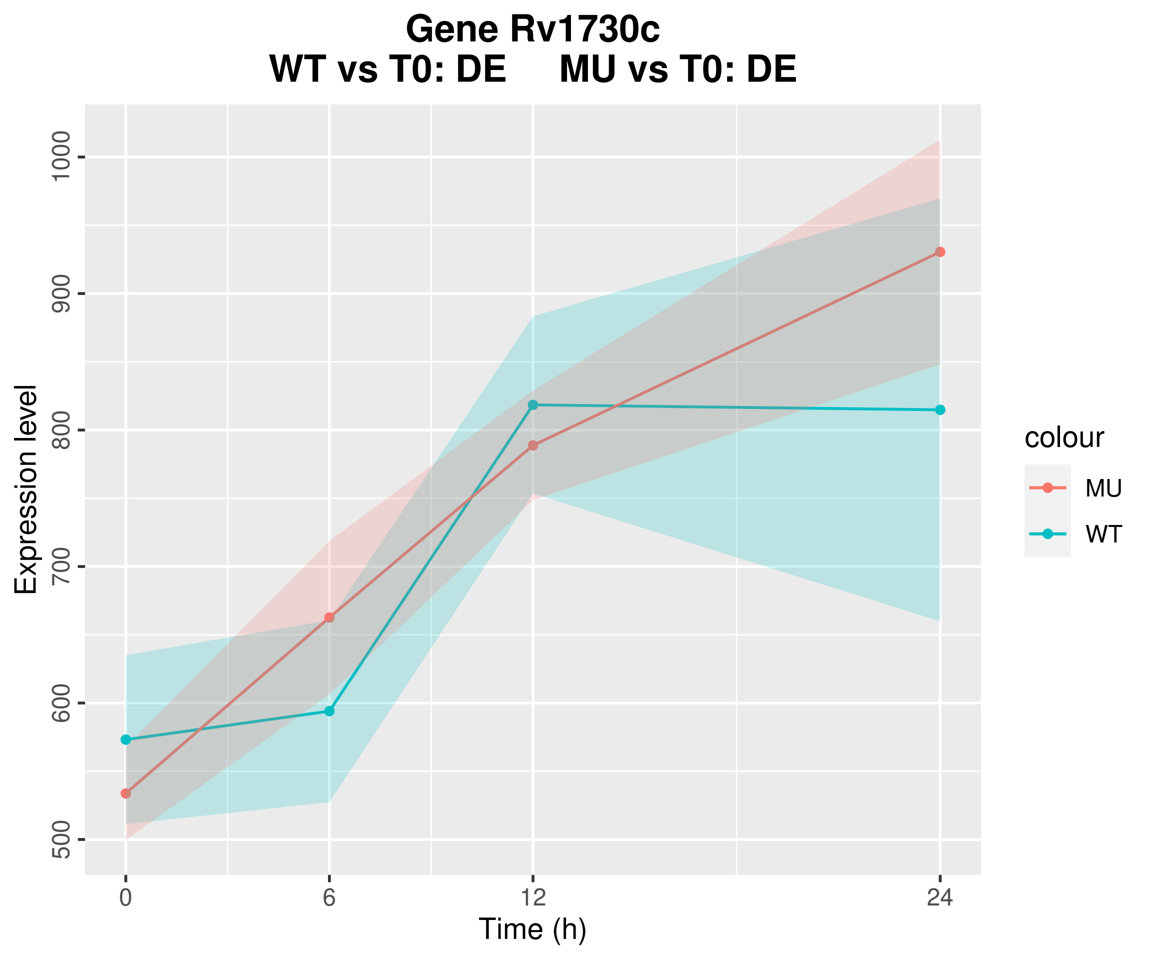
Gene Rv1730c
WT vs T0: DE MU vs T0: DE
Expression level
500
600
700
800
900
1000
0
6
12
24
Time (h)
colour
MU
WT

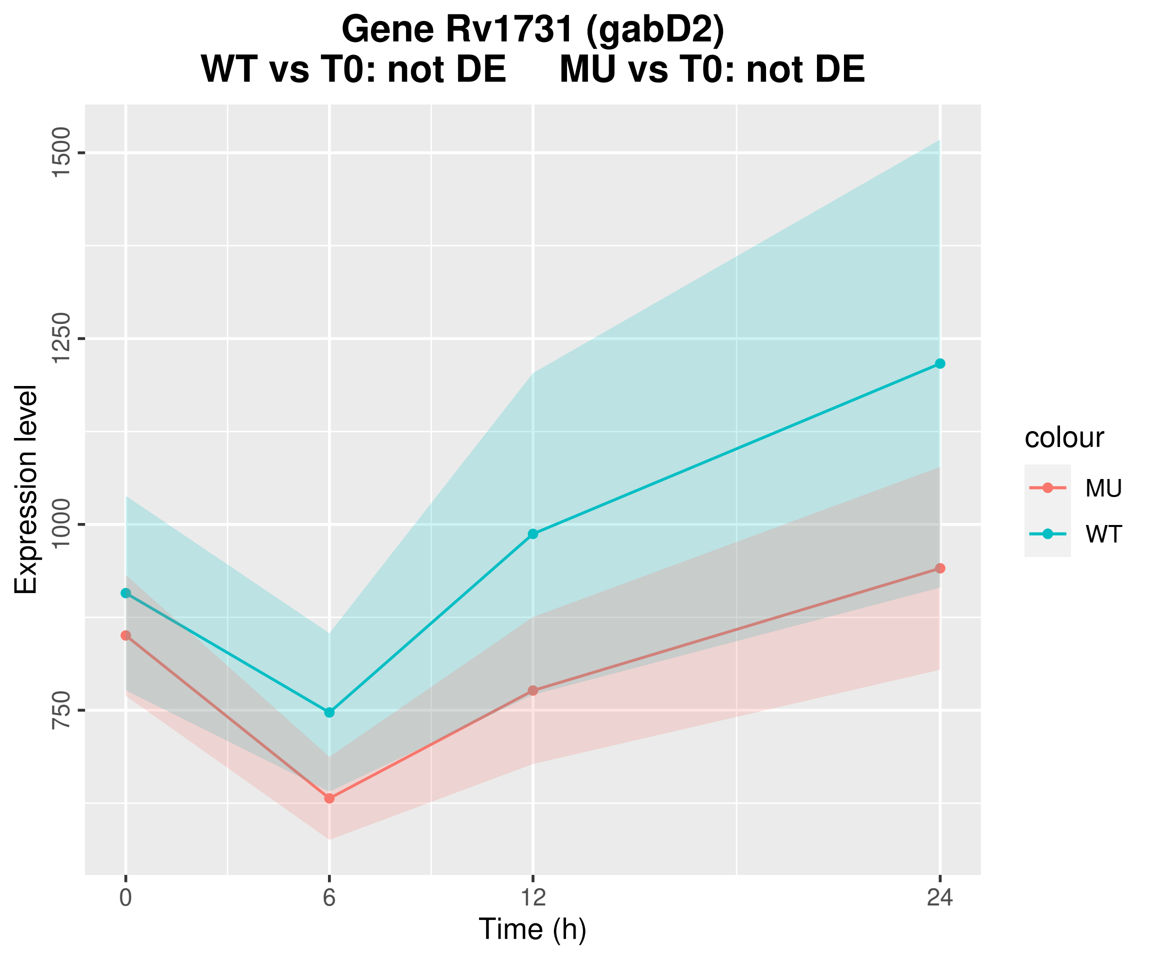

Gene Rv1731 (gabD2)
WT vs T0: not DE   MU vs T0: not DE
Expression level
1500
1250
1000
750
0
6
12
24
Time (h)
colour
MU
WT

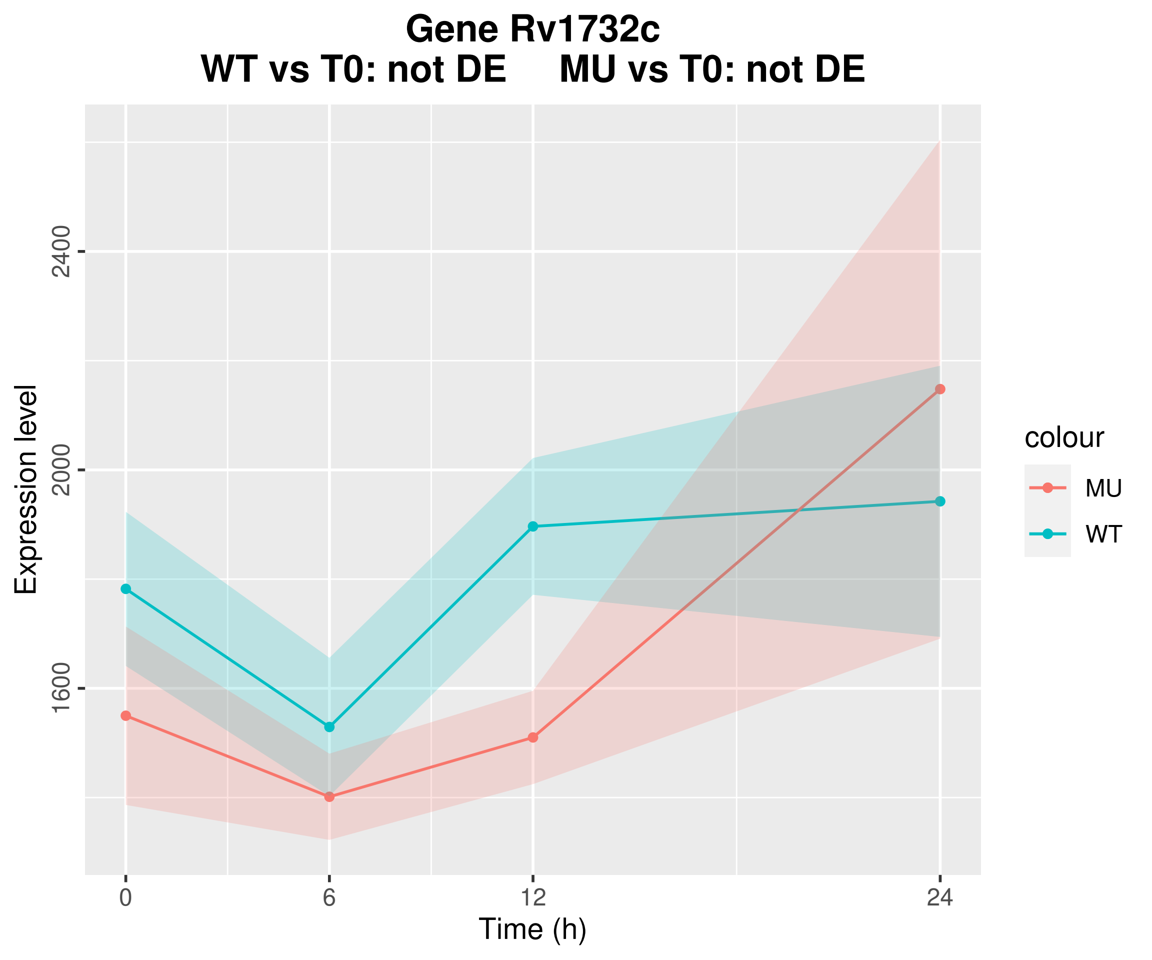

Gene Rv1732c
WT vs T0: not DE MU vs T0: not DE
Expression level
2400
2000
1600
0
6
12
24
Time (h)
colour
MU
WT

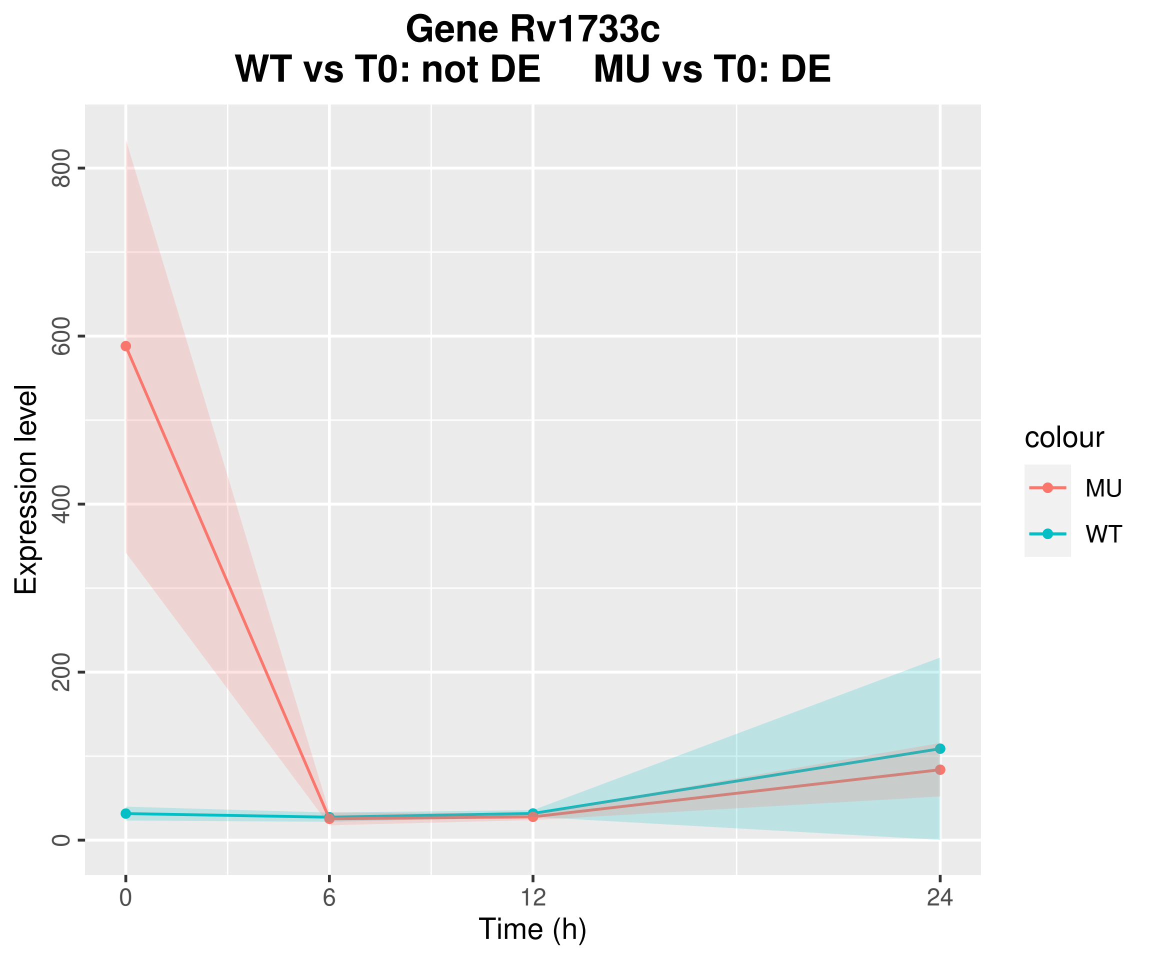

Gene Rv1733c
WT vs T0: not DE MU vs T0: DE
Expression level
800
600
400
200
0
0
6
12
24
Time (h)
colour
MU
WT

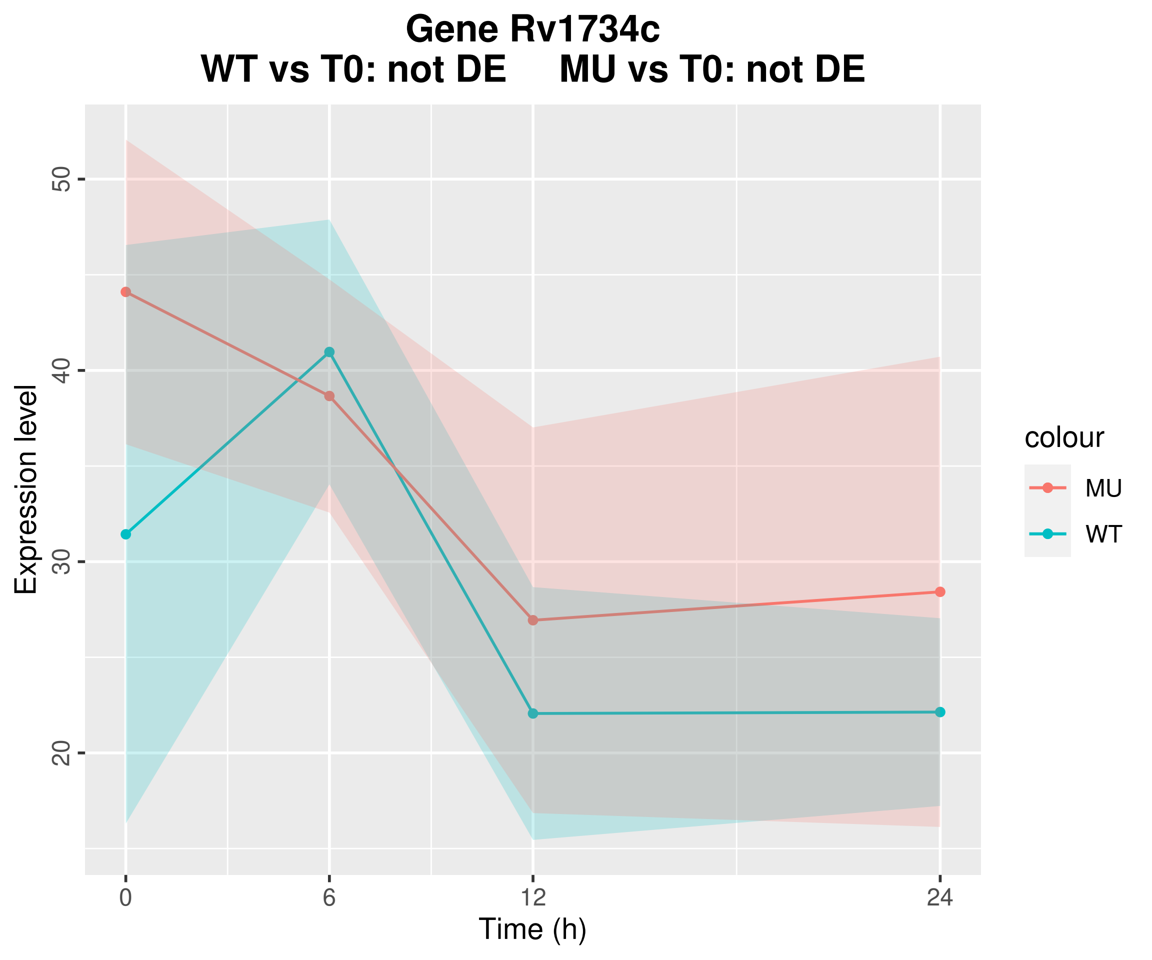
Gene Rv1734c
WT vs T0: not DE MU vs T0: not DE
Expression level
50
40
30
20
0
6
12
24
Time (h)
colour
MU
WT

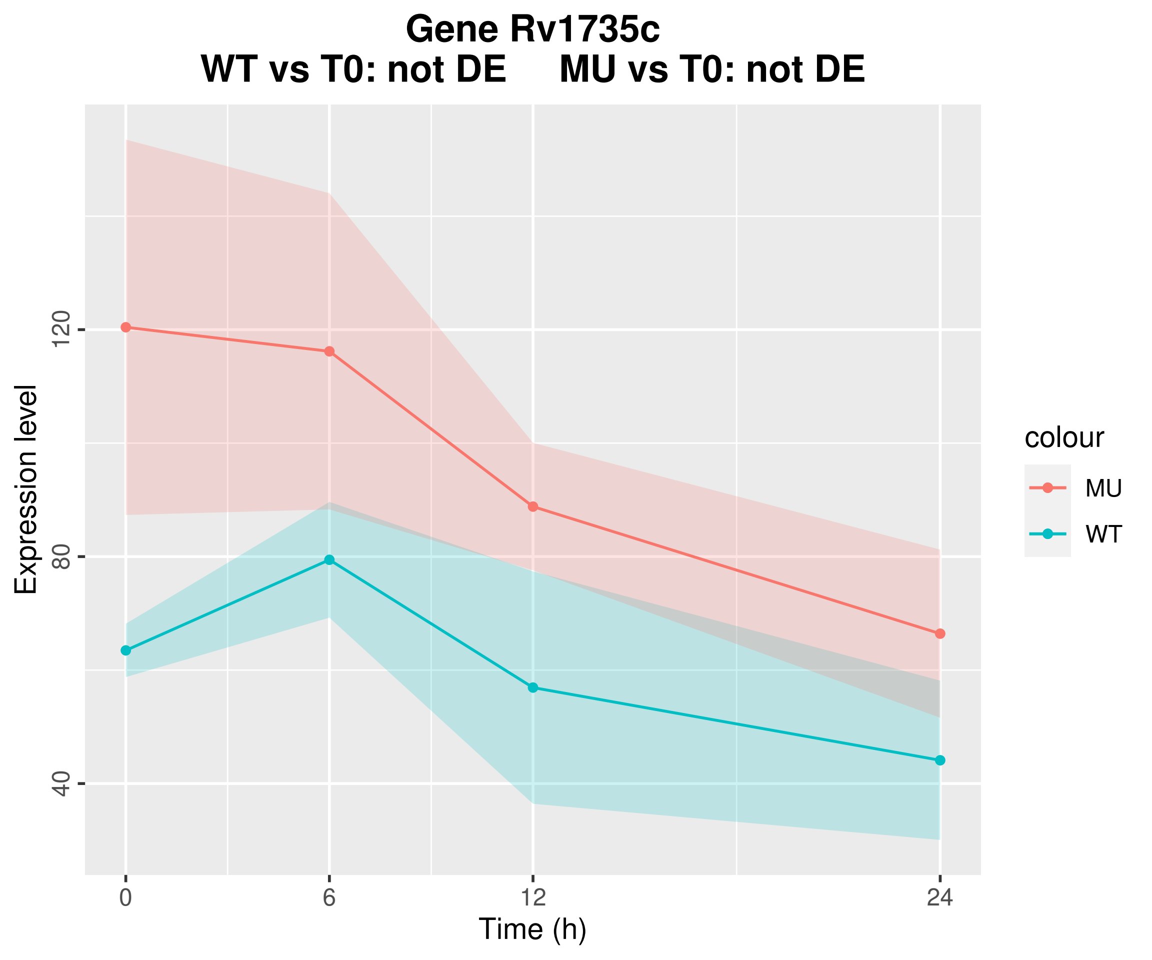

Gene Rv1735c
WT vs T0: not DE   MU vs T0: not DE
Expression level
120
80
40
0
6
12
24
Time (h)
colour
MU
WT

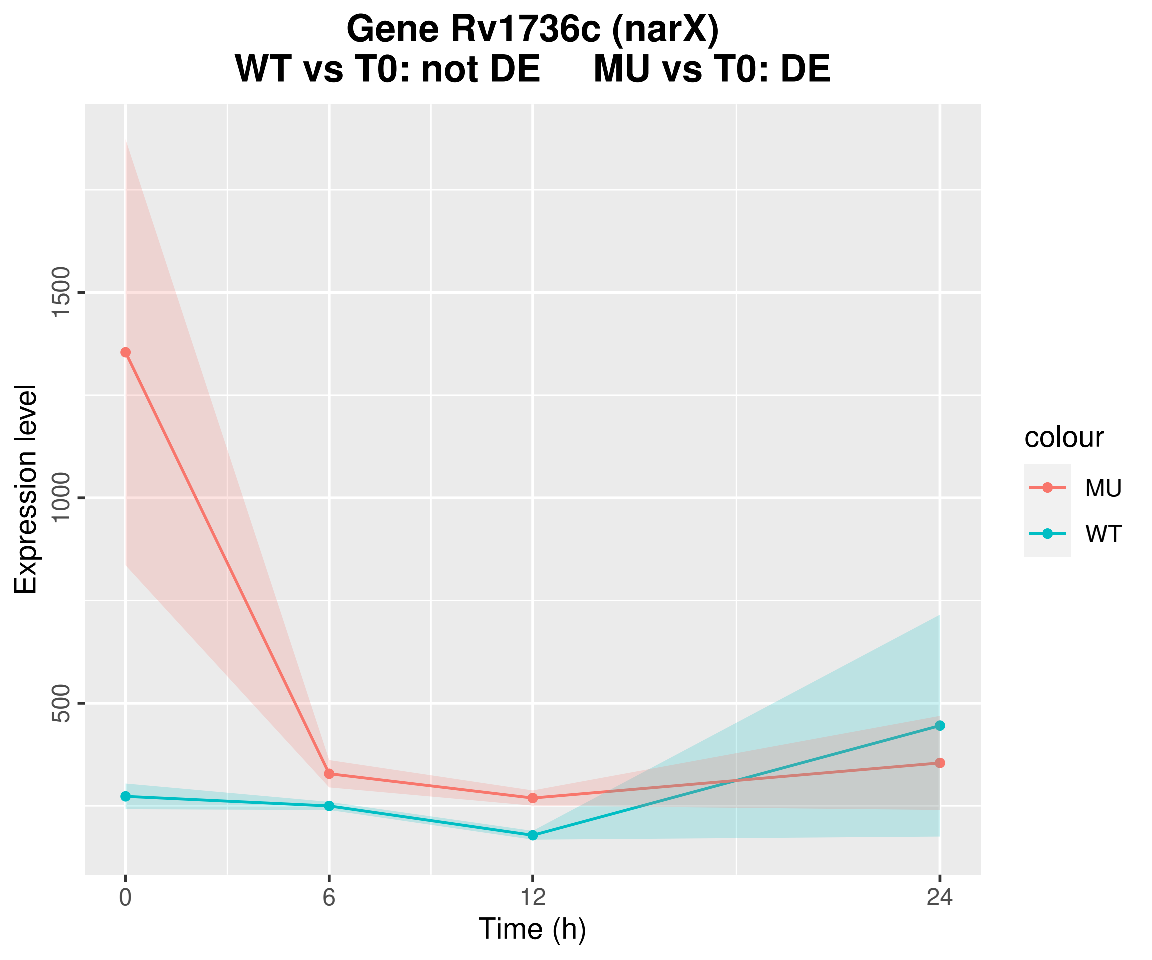
Gene Rv1736c (narX)
WT vs T0: not DE MU vs T0: DE
Expression level
1500
1000
500
0
6
12
24
Time (h)
colour
MU
WT

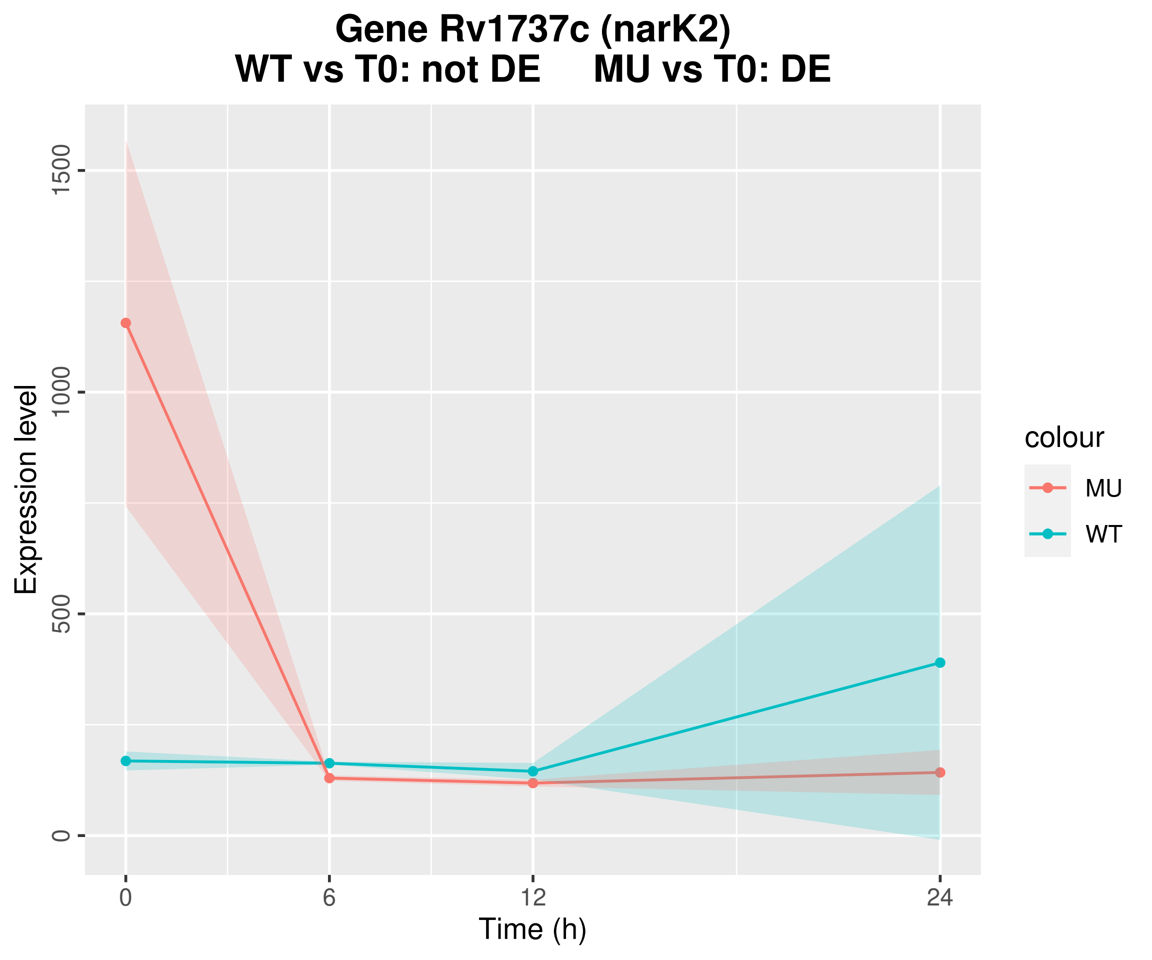
Gene Rv1737c (narK2)
WT vs T0: not DE MU vs T0: DE
Expression level
1500
1000
500
0
0
6
12
24
Time (h)
colour
MU
WT

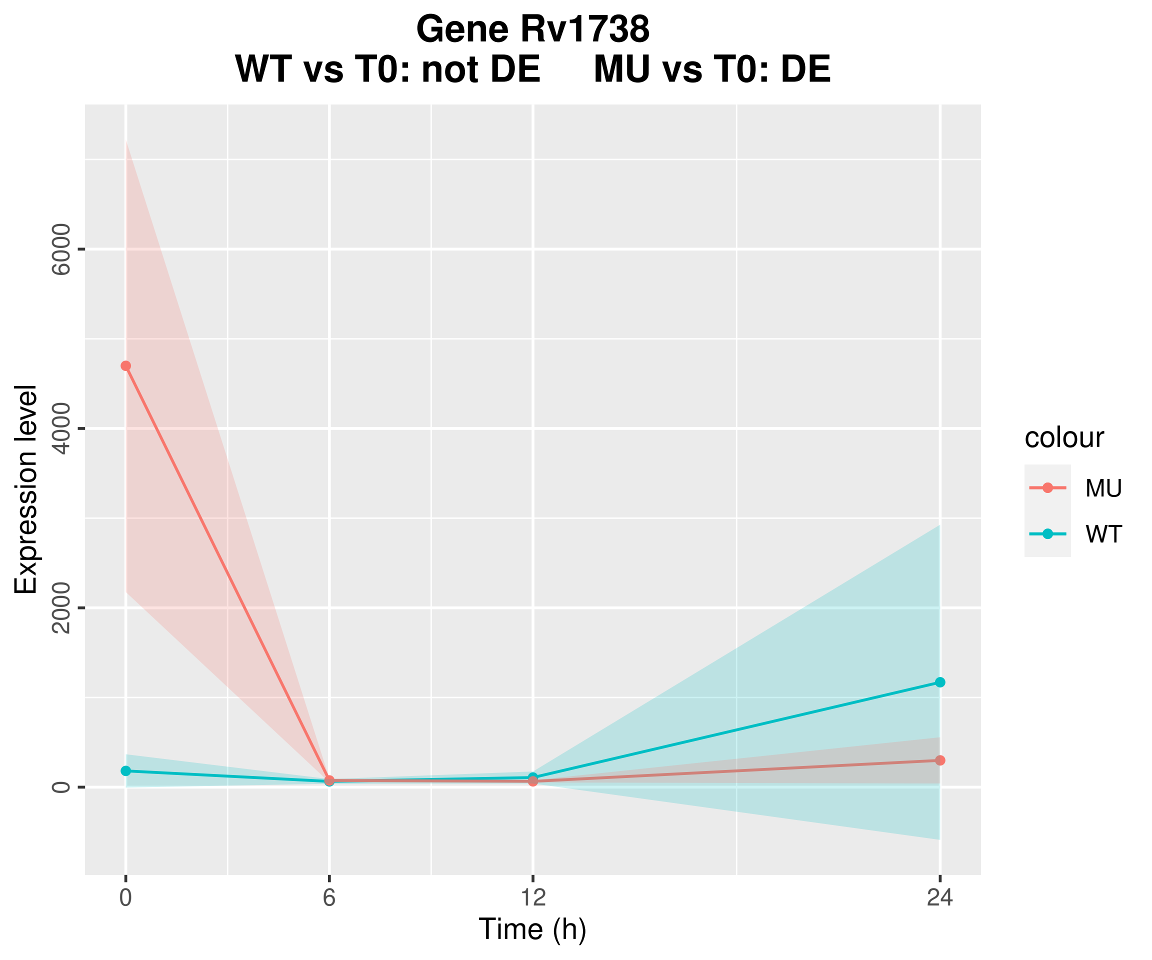

Gene Rv1738
WT vs T0: not DE MU vs T0: DE
Expression level
6000
4000
2000
0
0
6
12
24
Time (h)
colour
MU
WT

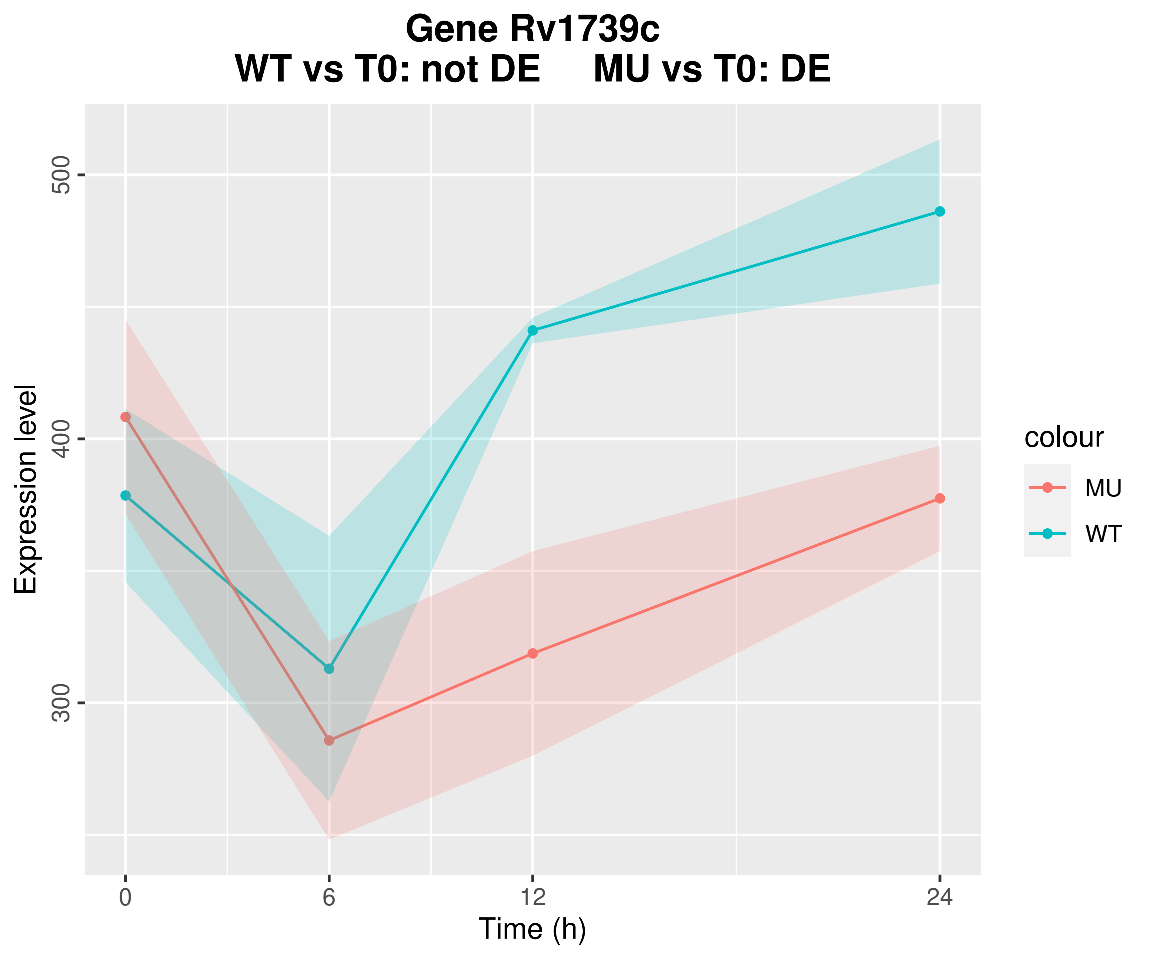
Gene Rv1739c
WT vs T0: not DE   MU vs T0: DE
Expression level
500
400
300
0
6
12
24
Time (h)
colour
MU
WT

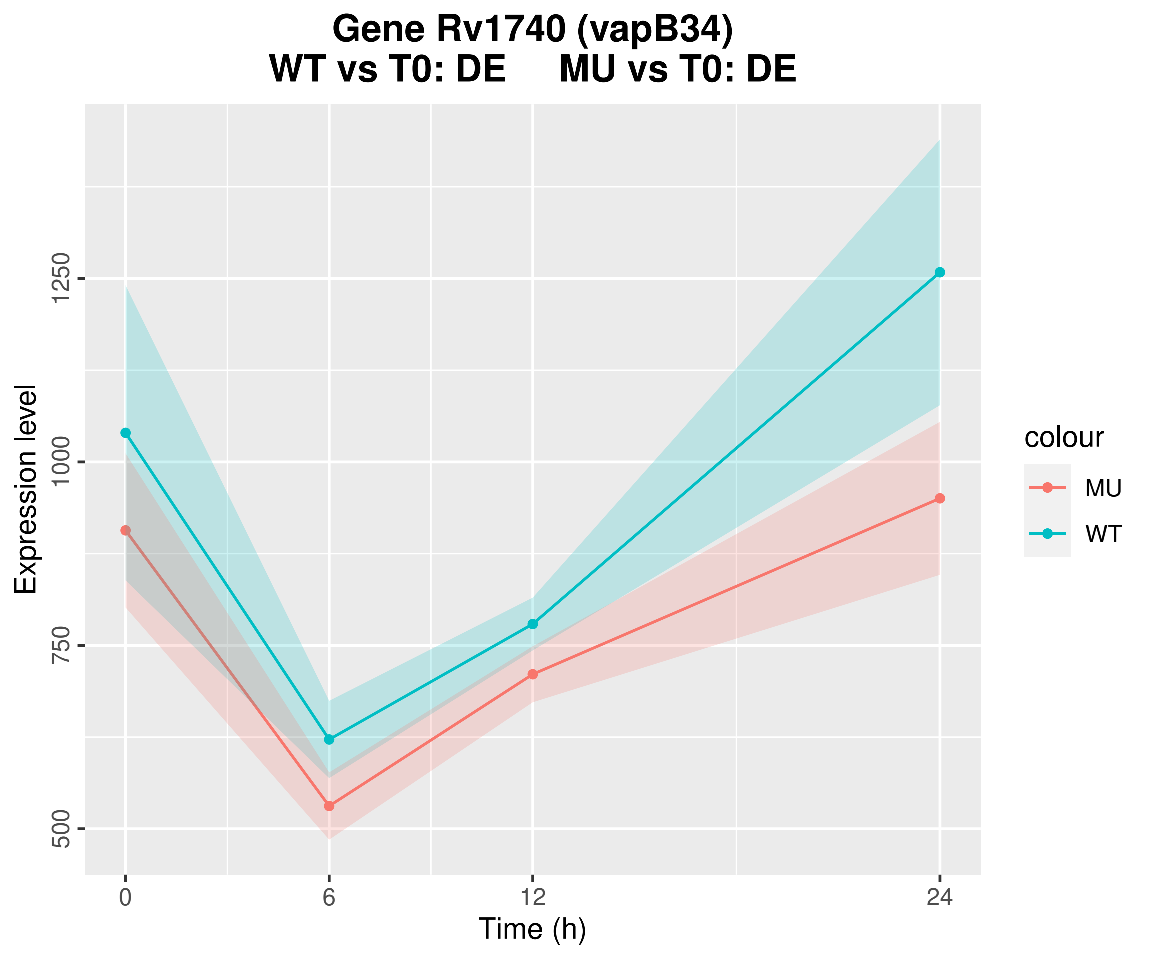

Gene Rv1740 (vapB34)
WT vs T0: DE MU vs T0: DE
Expression level
1250
1000
750
500
0
6
12
24
Time (h)
colour
MU
WT

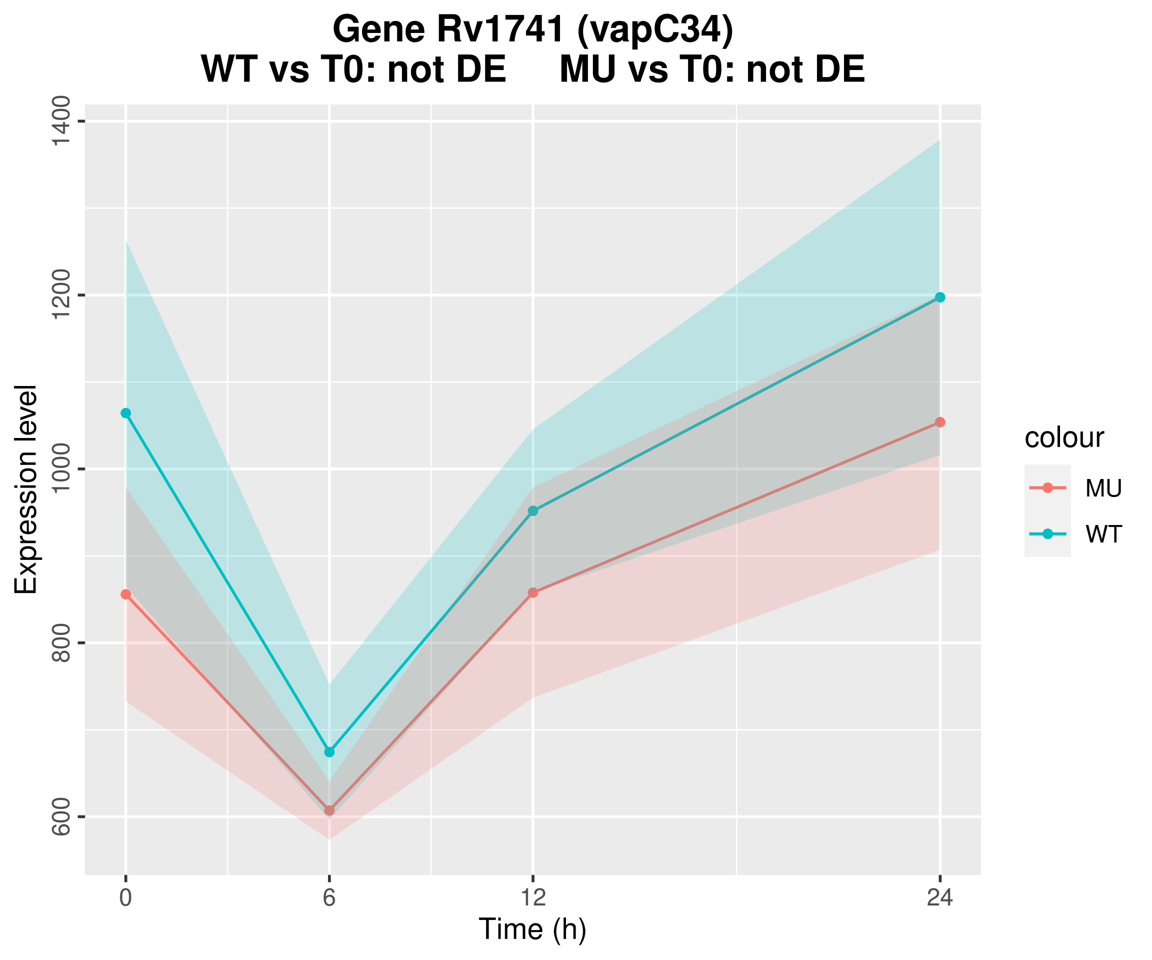
Gene Rv1741 (vapC34)
WT vs T0: not DE   MU vs T0: not DE
1400
1200
1000
800
600
Expression level
0
6
12
24
Time (h)
colour
MU
WT

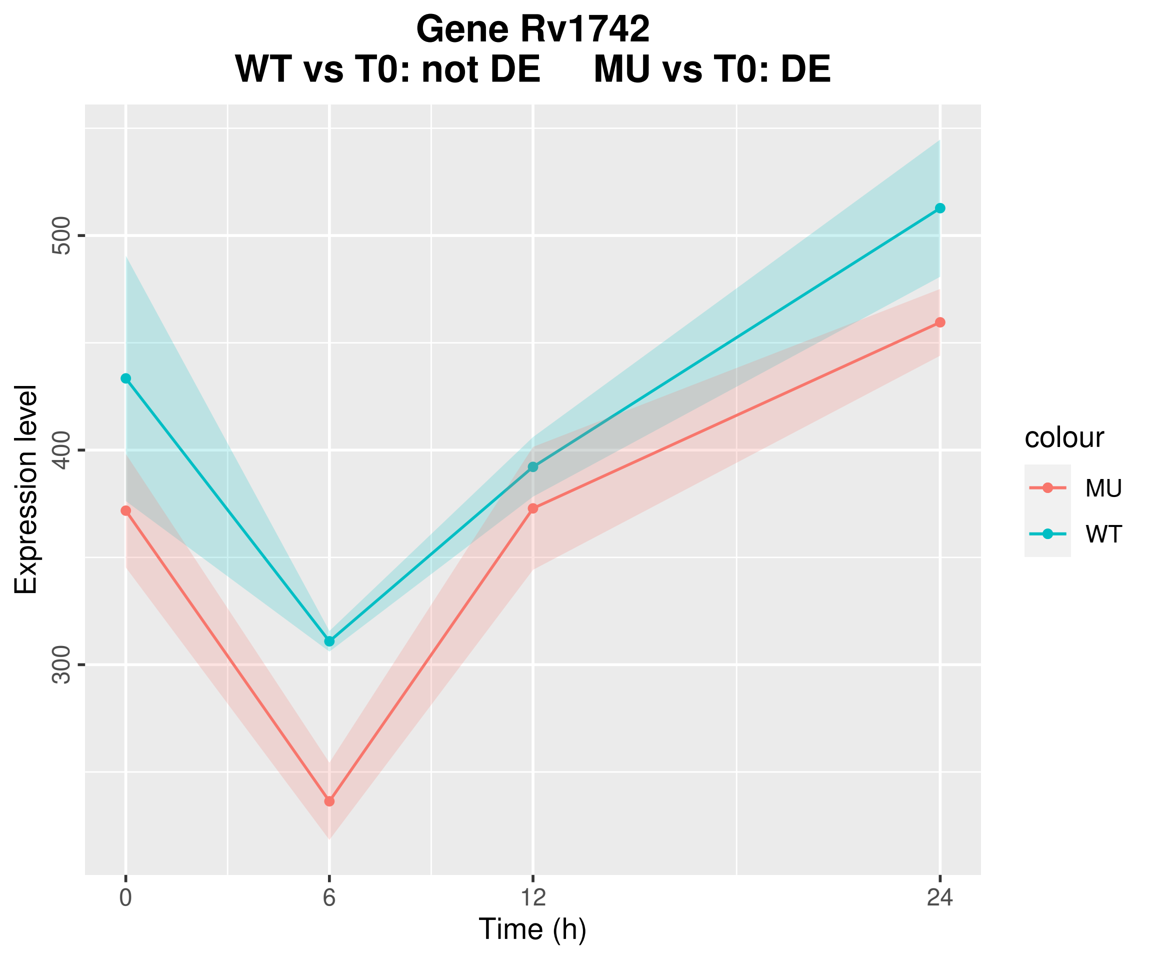

Gene Rv1742
WT vs T0: not DE   MU vs T0: DE
Expression level
500
400
300
0
6
12
24
Time (h)
colour
MU
WT

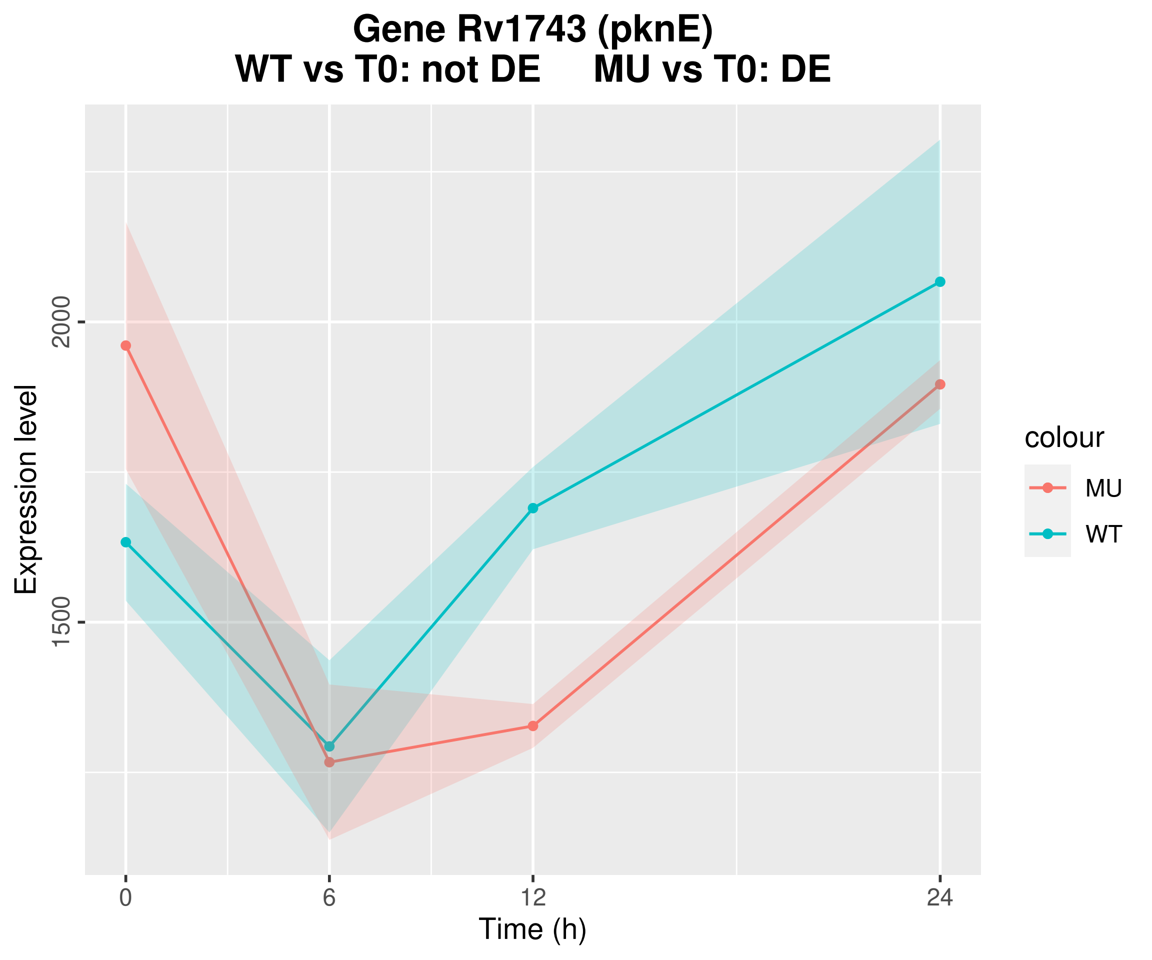

Gene Rv1743 (pknE)
WT vs T0: not DE MU vs T0: DE
Expression level
2000
1500
0
6
12
24
Time (h)
colour
MU
WT

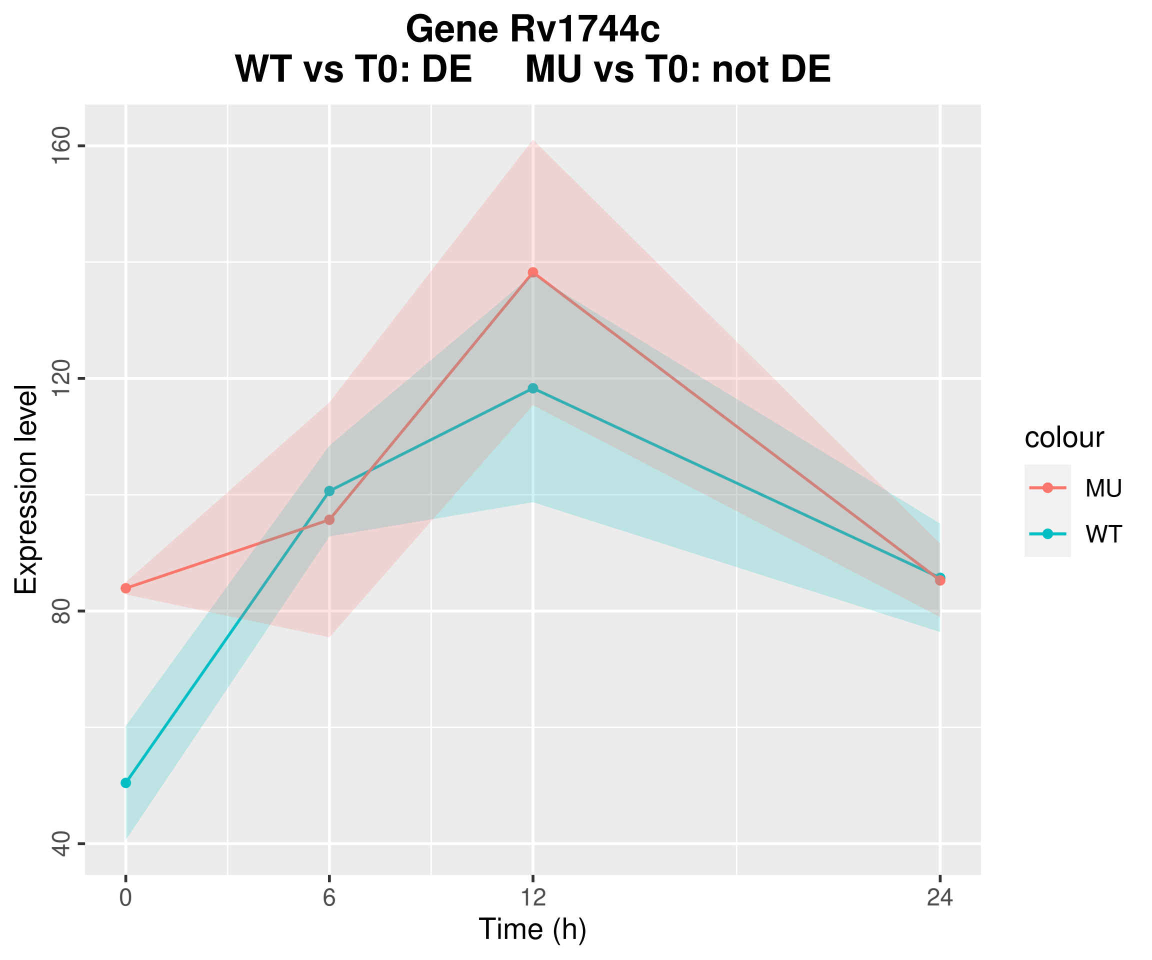
Gene Rv1744c
WT vs T0: DE MU vs T0: not DE
Expression level
160
120
80
40
0
6
12
24
Time (h)
colour
MU
WT

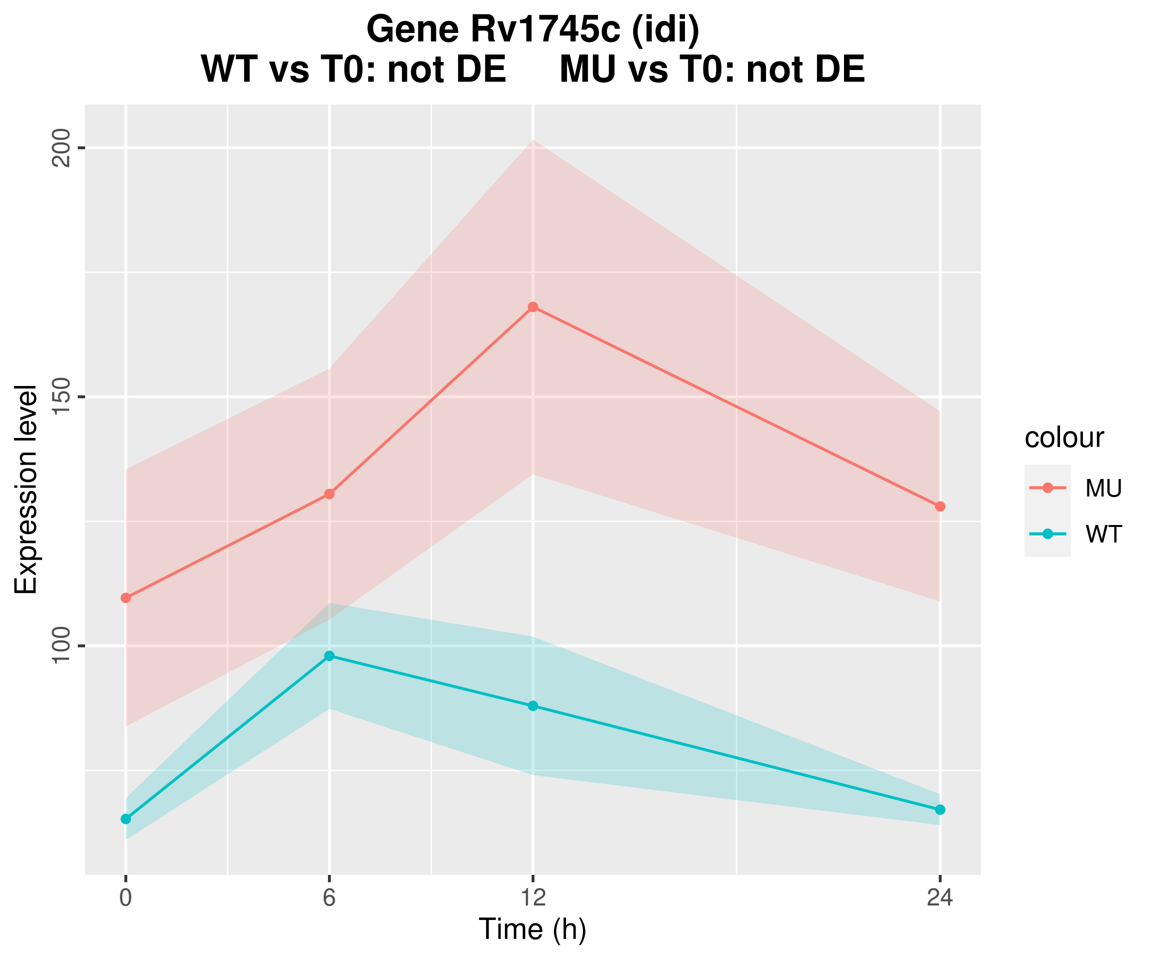
Gene Rv1745c (idi)
WT vs T0: not DE   MU vs T0: not DE
200
150
100
Expression level
0
6
12
24
Time (h)
colour
MU
WT

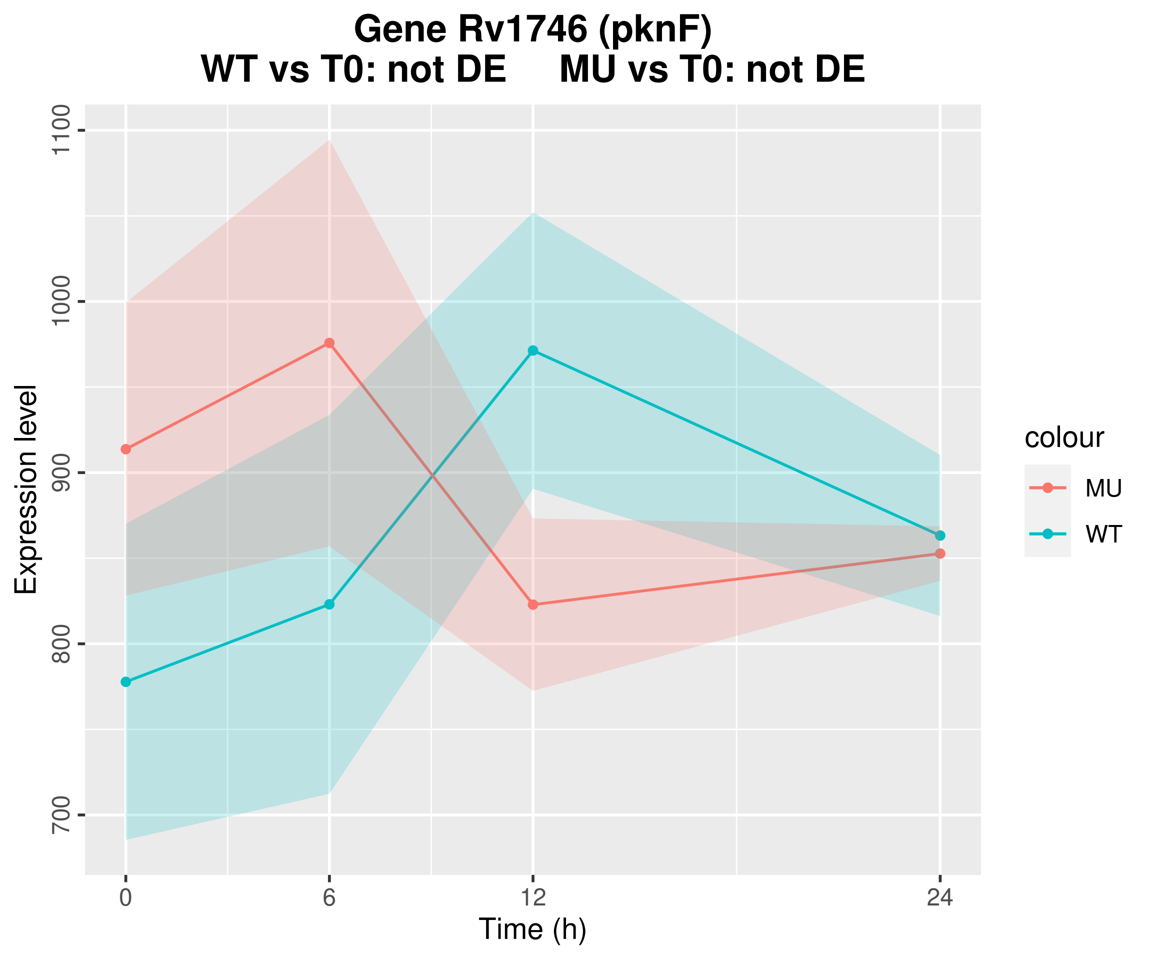
Gene Rv1746 (pknF)
WT vs T0: not DE MU vs T0: not DE
Expression level
1100
1000
900
800
700
0
6
12
24
Time (h)
colour
MU
WT

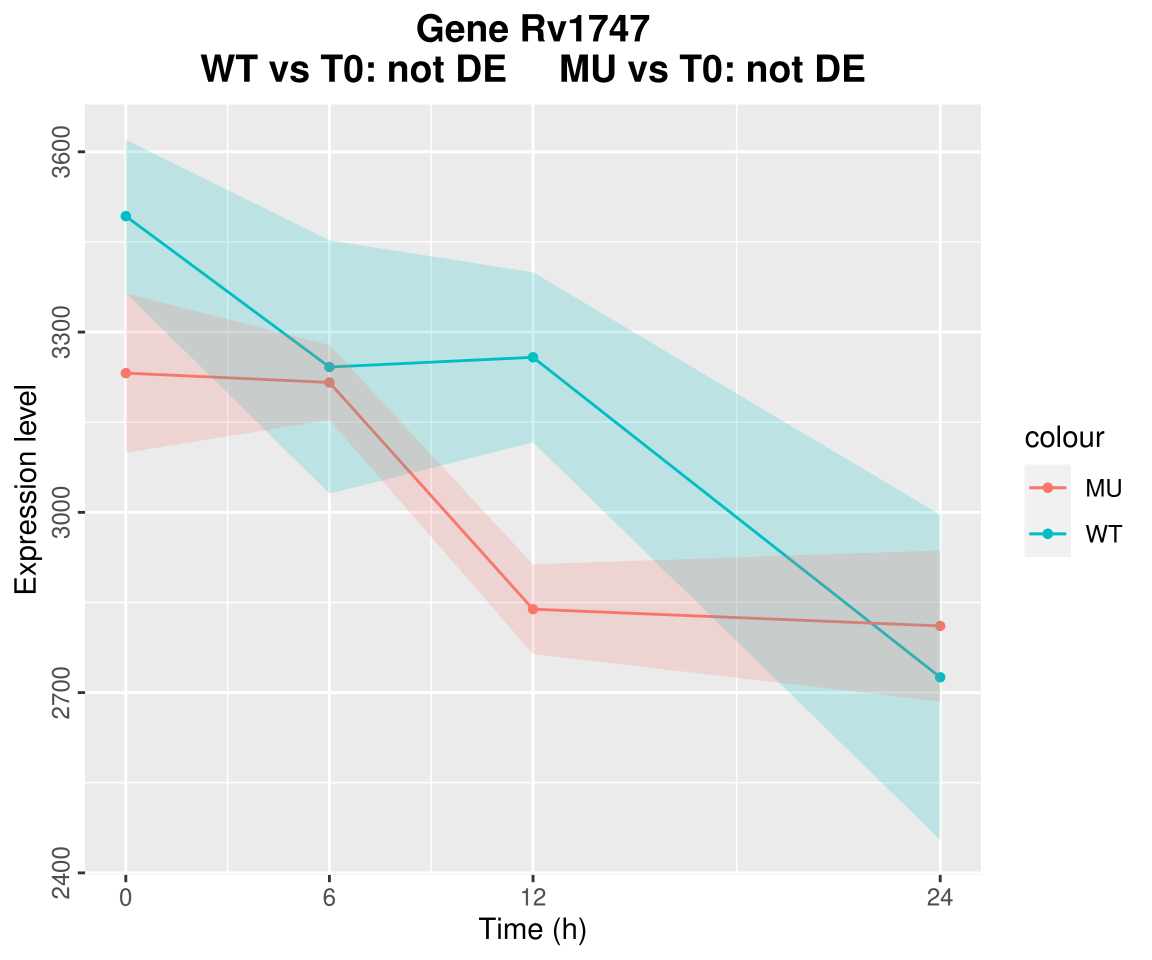
Gene Rv1747
WT vs T0: not DE   MU vs T0: not DE
Expression level
3600
3300
3000
2700
2400
0
6
12
24
Time (h)
colour
MU
WT

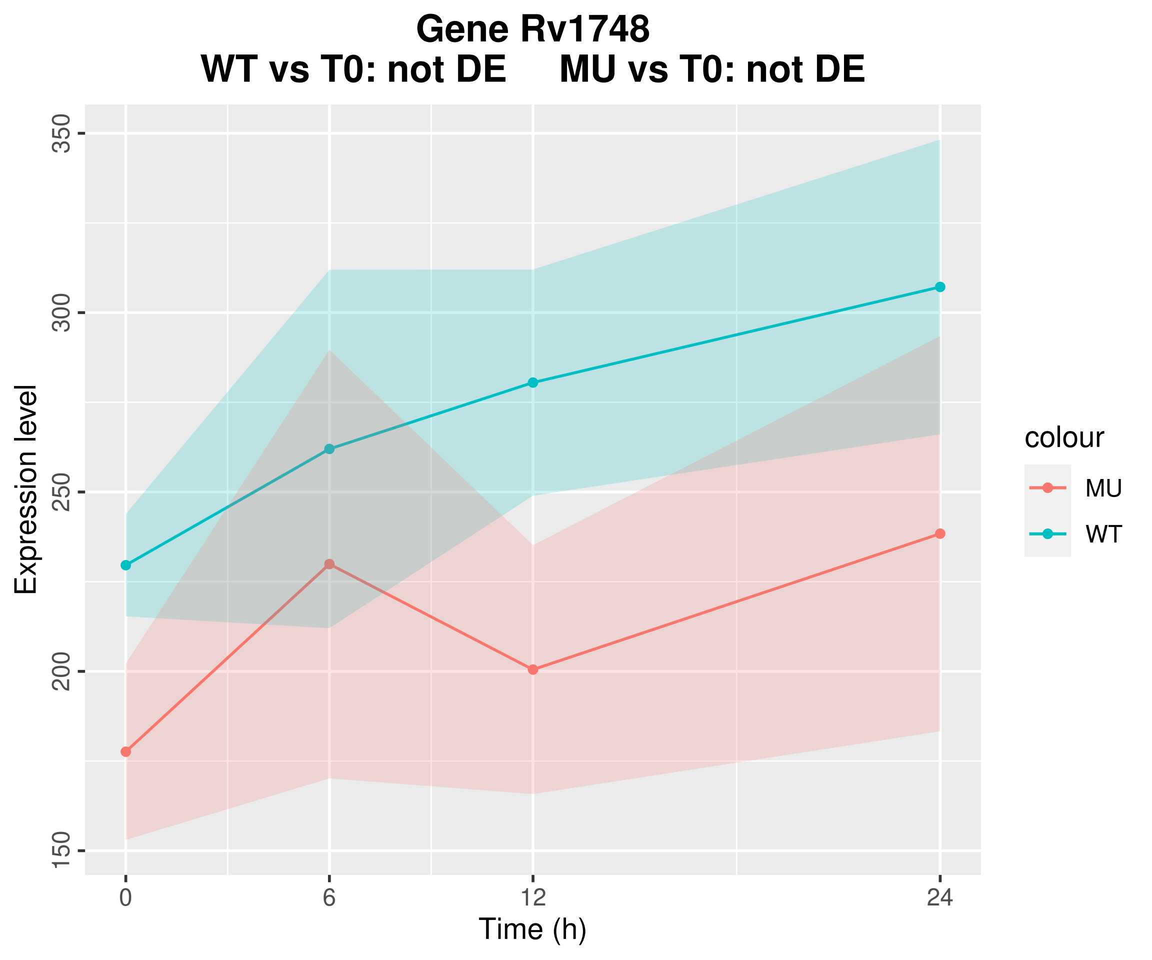
Gene Rv1748
WT vs T0: not DE   MU vs T0: not DE
Expression level
350
300
250
200
150
0
6
12
24
Time (h)
colour
MU
WT

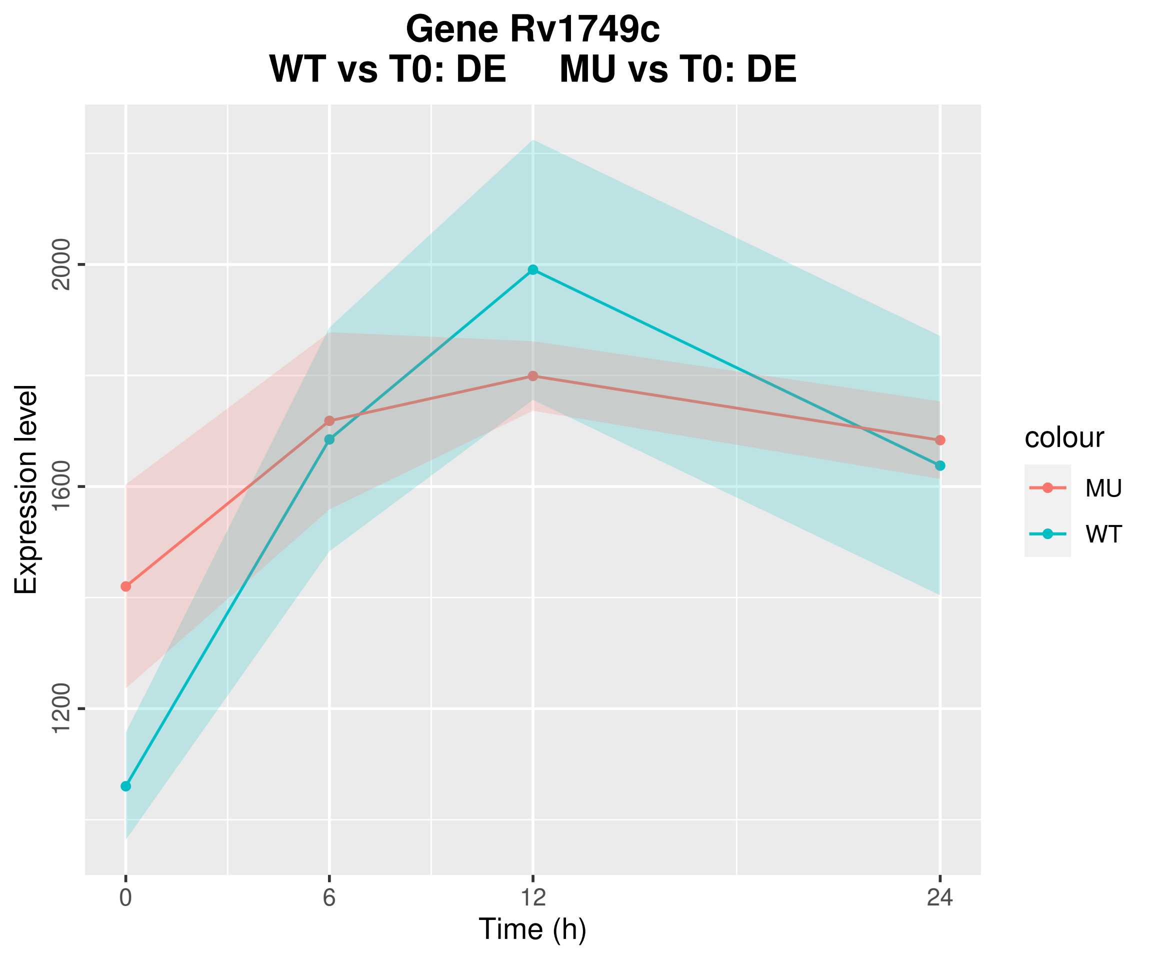

Gene Rv1749c
WT vs T0: DE MU vs T0: DE
Expression level
2000
1600
1200
0
6
12
24
Time (h)
colour
MU
WT

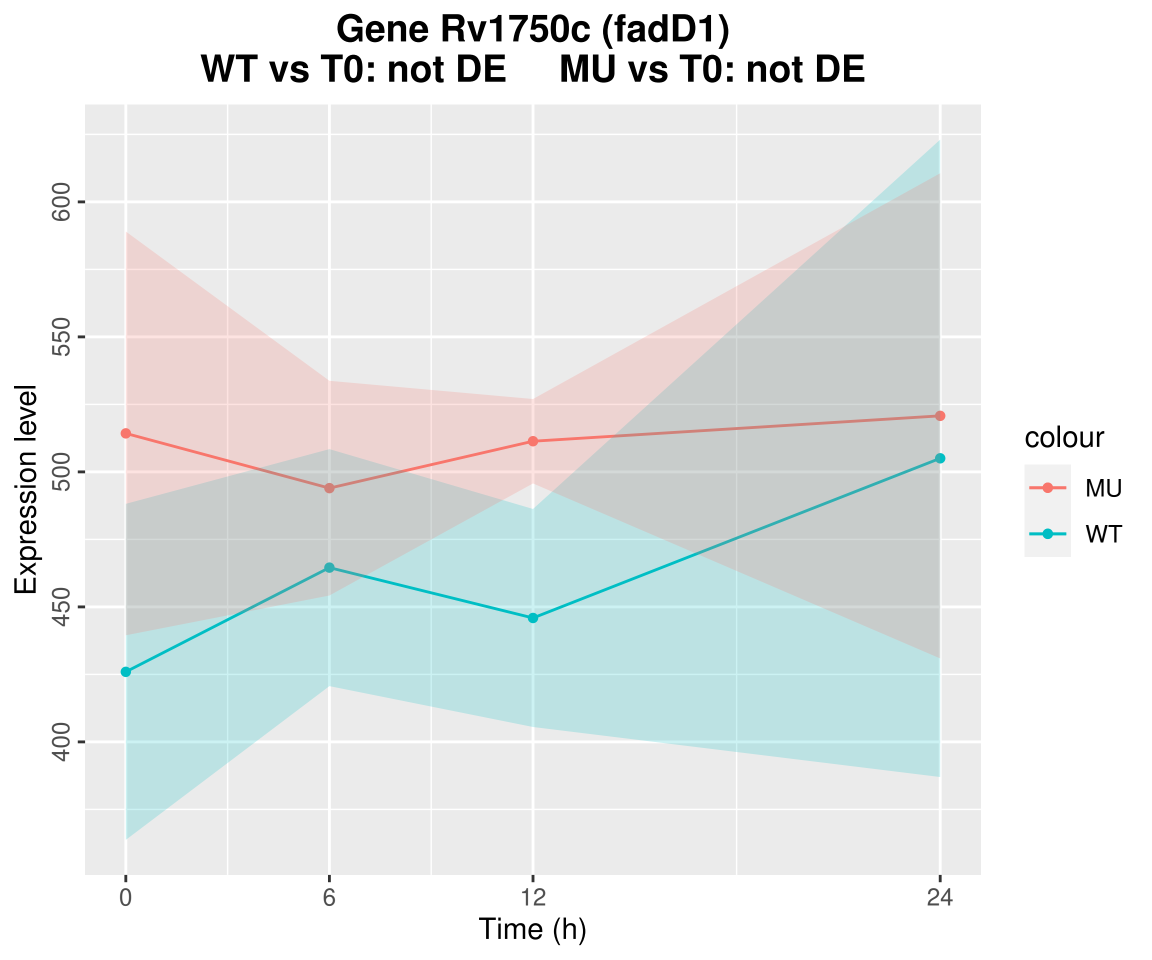
Gene Rv1750c (fadD1)
WT vs T0: not DE   MU vs T0: not DE
Expression level
600
550
500
450
400
0
6
12
24
Time (h)
colour
MU
WT

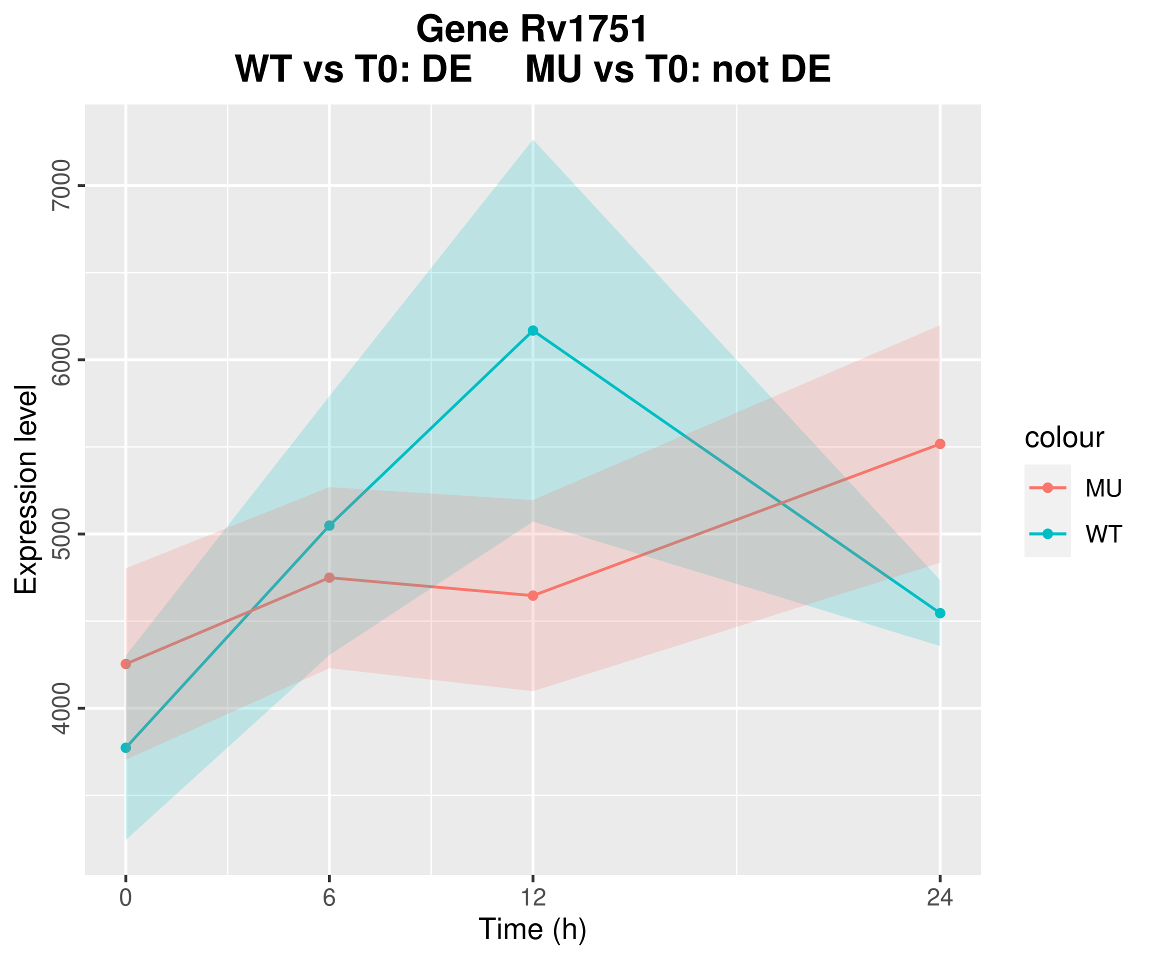
Gene Rv1751
WT vs T0: DE   MU vs T0: not DE
Expression level
7000
6000
5000
4000
0
6
12
24
Time (h)
colour
MU
WT

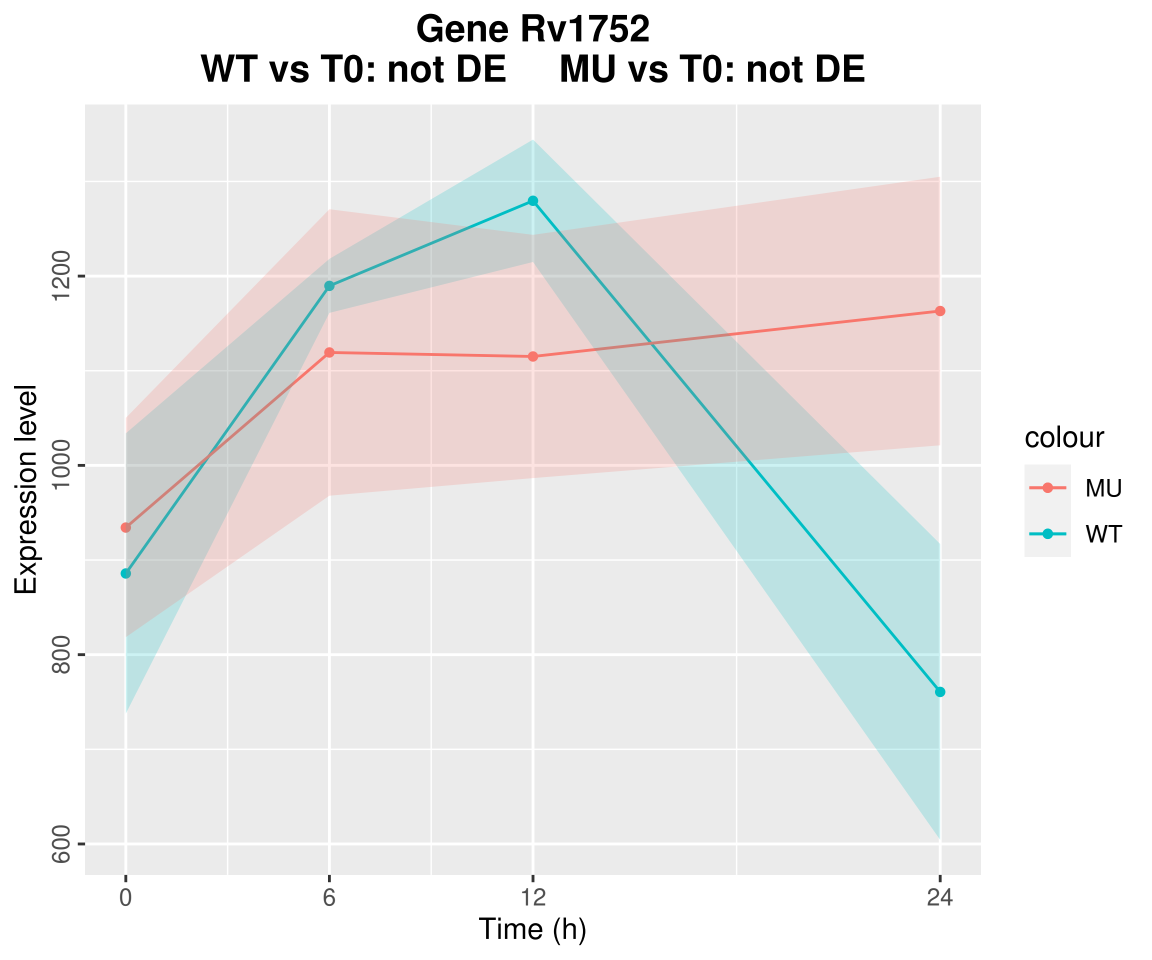

Gene Rv1752
WT vs T0: not DE   MU vs T0: not DE
Expression level
1200
1000
800
600
0
6
12
24
Time (h)
colour
MU
WT

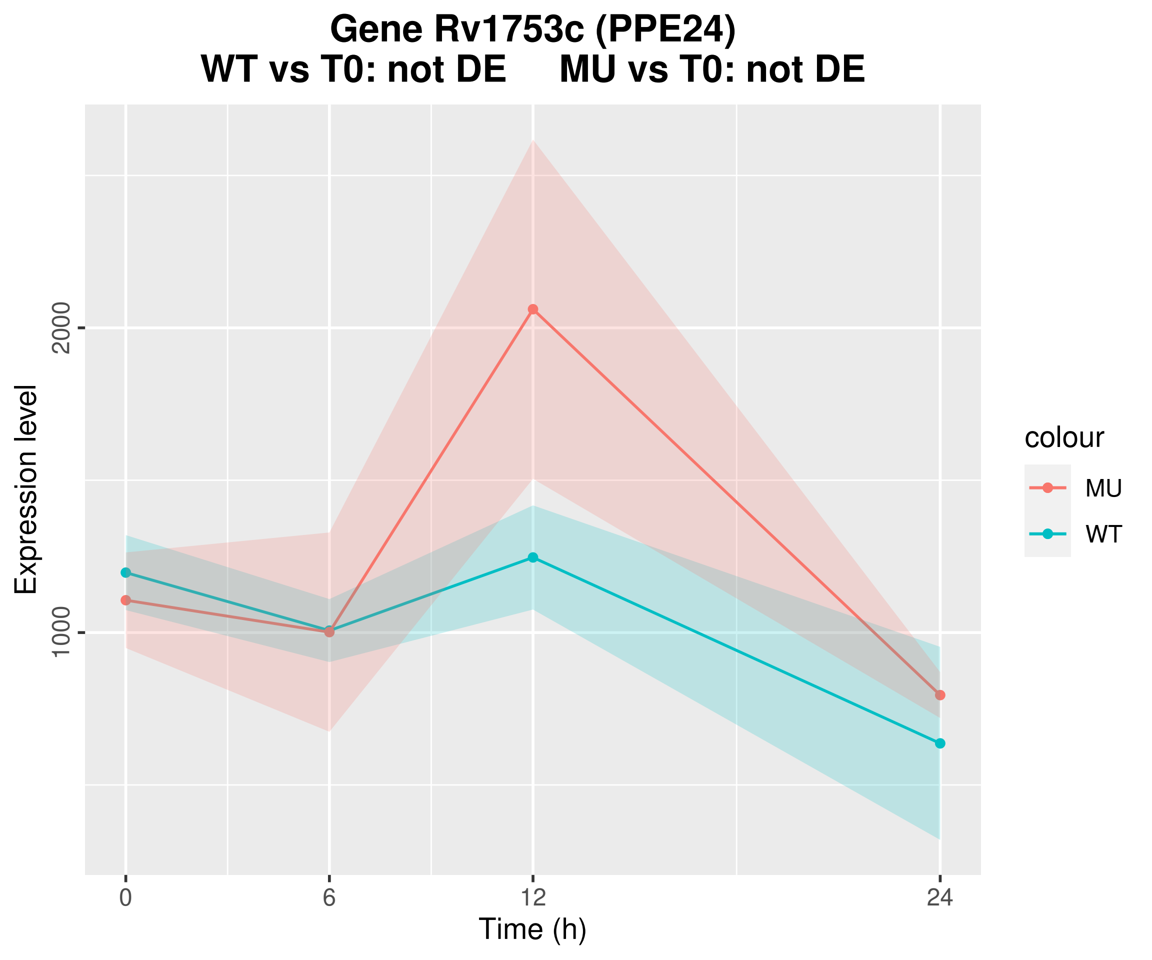

Gene Rv1753c (PPE24)
WT vs T0: not DE   MU vs T0: not DE
Expression level
2000
1000
0
6
12
24
Time (h)
colour
MU
WT

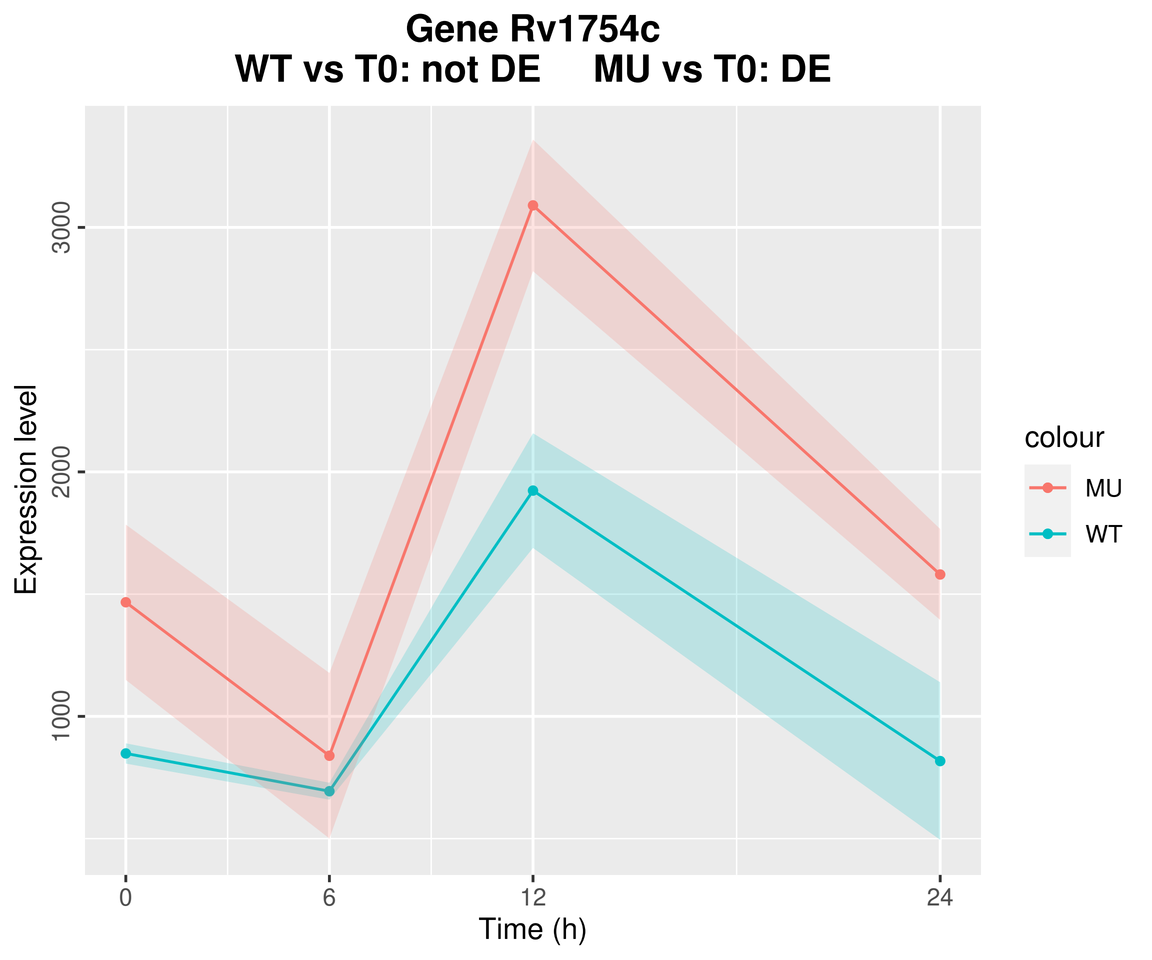
Gene Rv1754c
WT vs T0: not DE MU vs T0: DE
Expression level
3000
2000
1000
0
6
12
24
Time (h)
colour
MU
WT

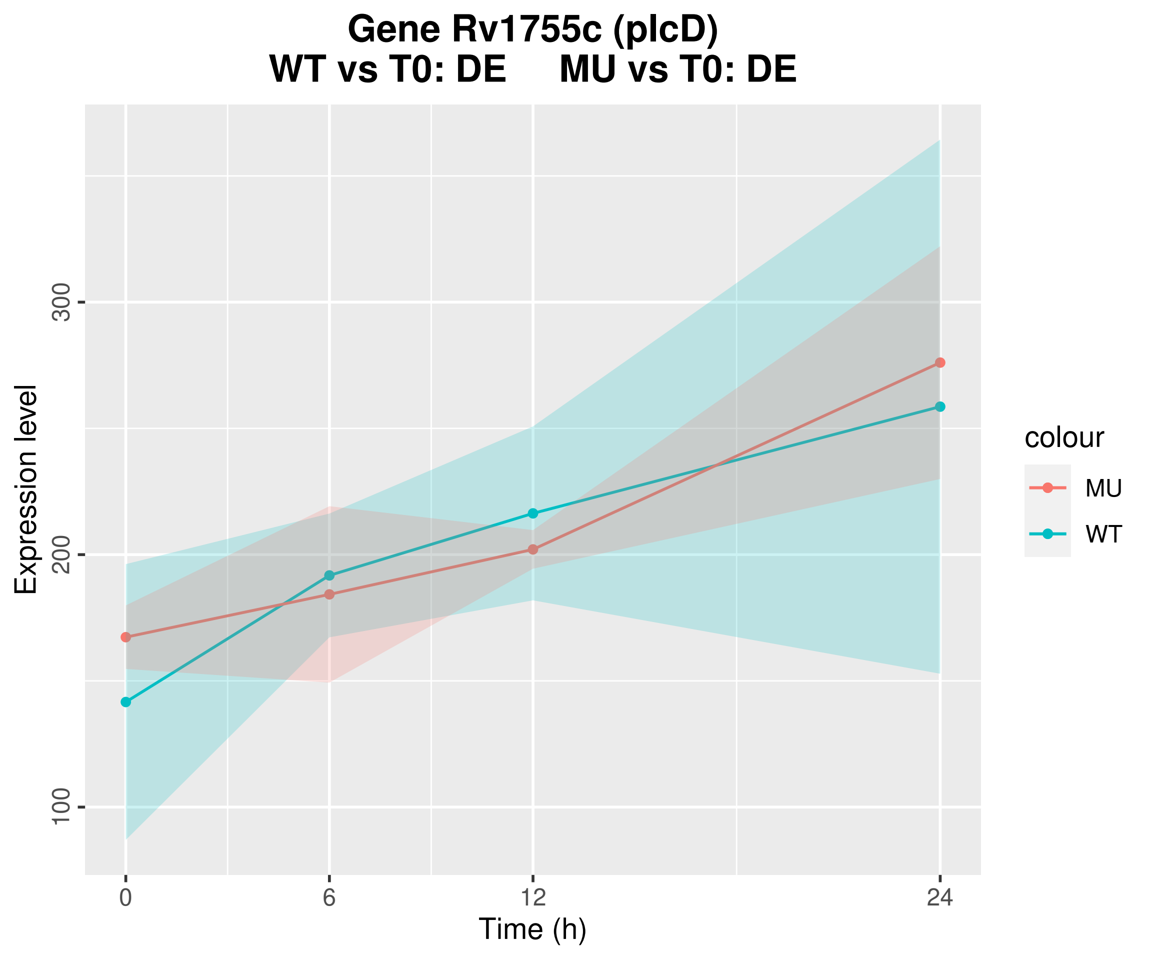
Gene Rv1755c (plcD)
WT vs T0: DE MU vs T0: DE
Expression level
300
200
100
0
6
12
24
Time (h)
colour
MU
WT

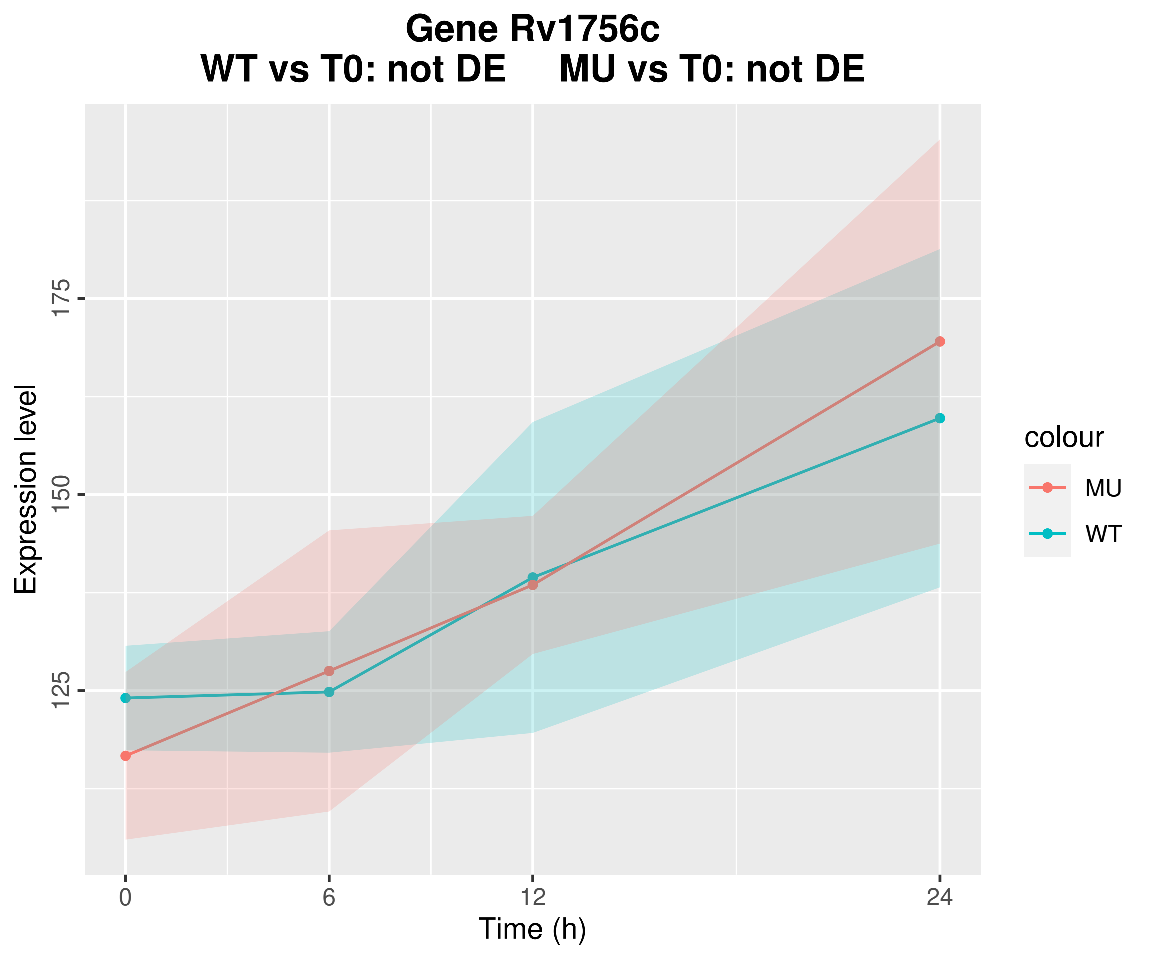

Gene Rv1756c
WT vs T0: not DE MU vs T0: not DE
Expression level
125
150
175
0
6
12
24
Time (h)
colour
MU
WT

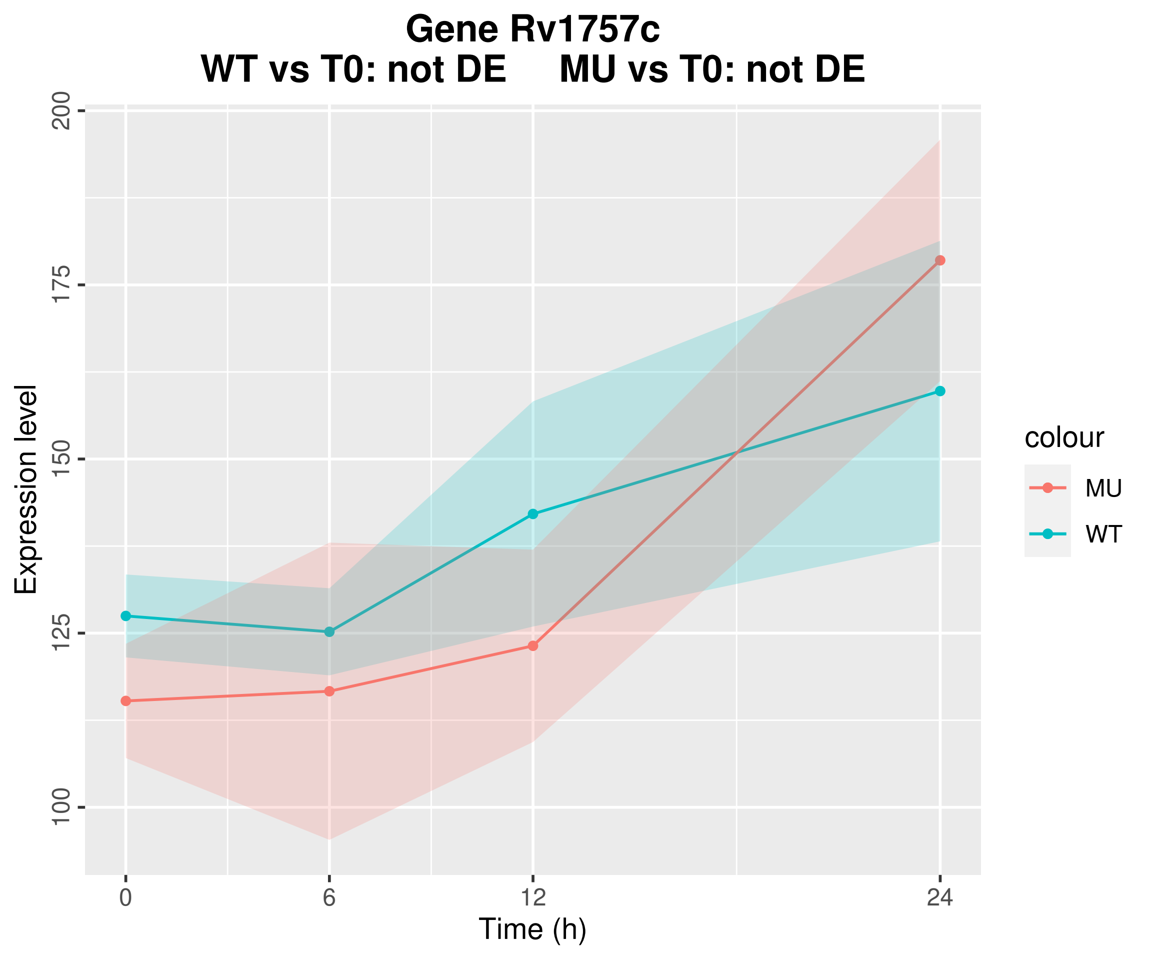

Gene Rv1757c
WT vs T0: not DE   MU vs T0: not DE
Expression level
200
175
150
125
100
0
6
12
24
Time (h)
colour
MU
WT

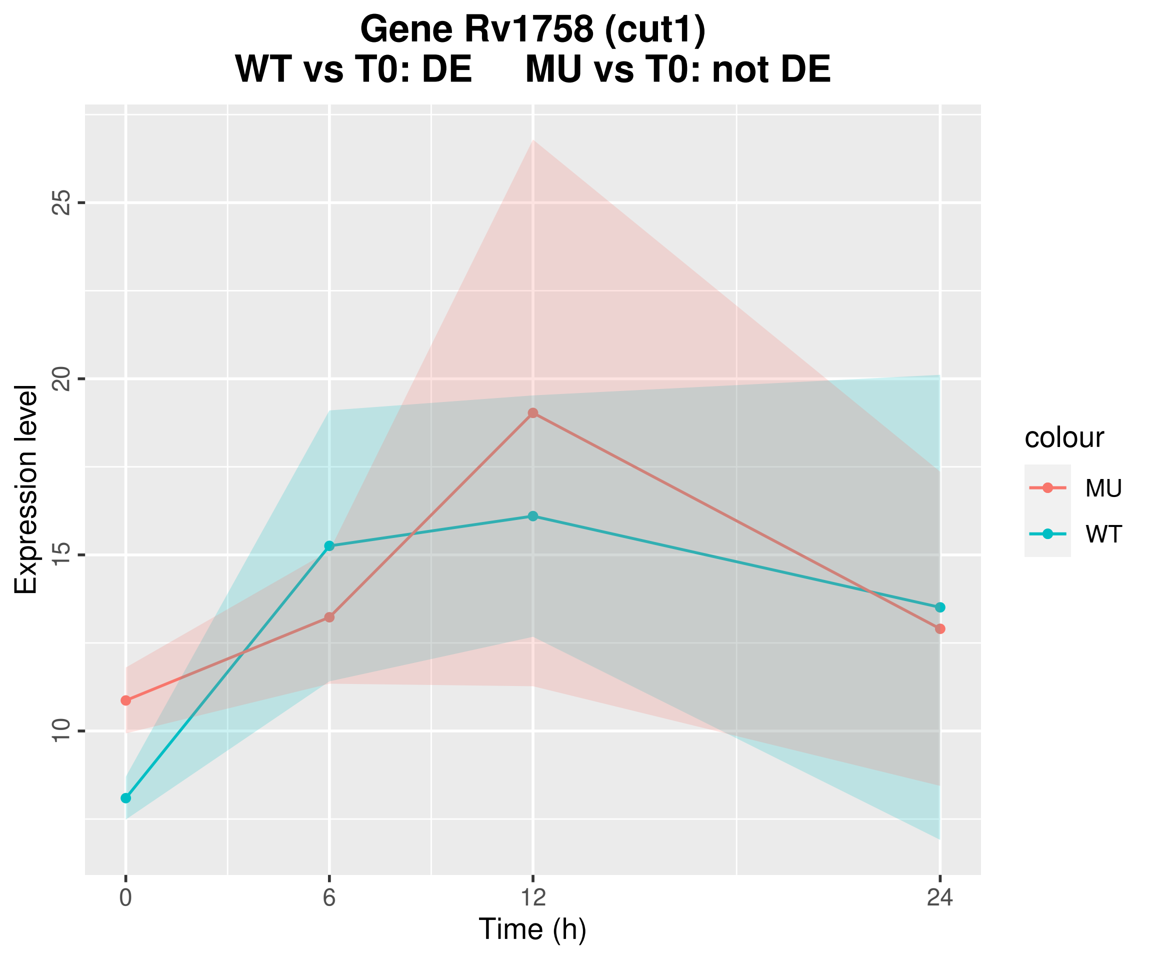
Gene Rv1758 (cut1)
WT vs T0: DE   MU vs T0: not DE
Expression level
25
20
15
10
Time (h)
0
6
12
24
colour
MU
WT

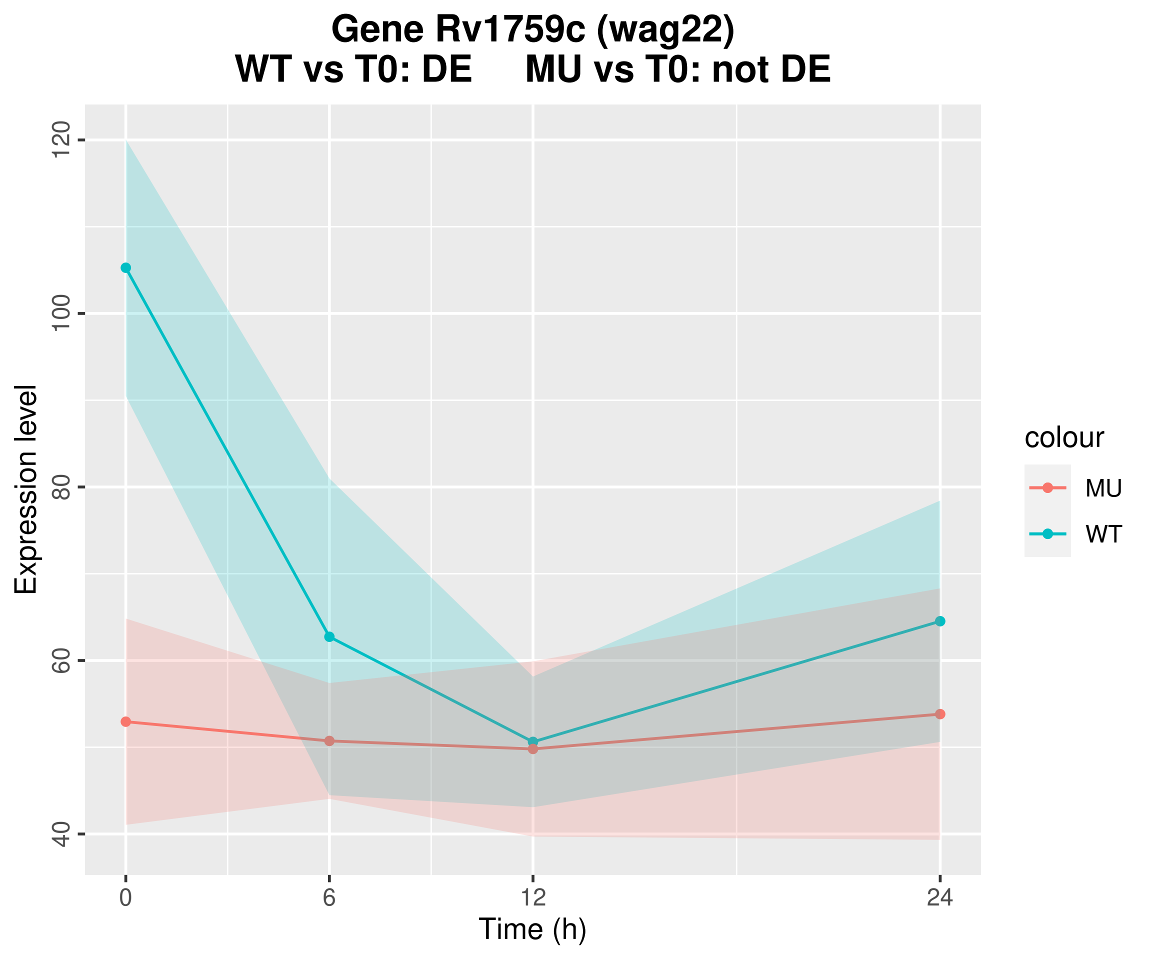
Gene Rv1759c (wag22)
WT vs T0: DE   MU vs T0: not DE
Expression level
120
100
80
60
40
0
6
12
24
Time (h)
colour
MU
WT

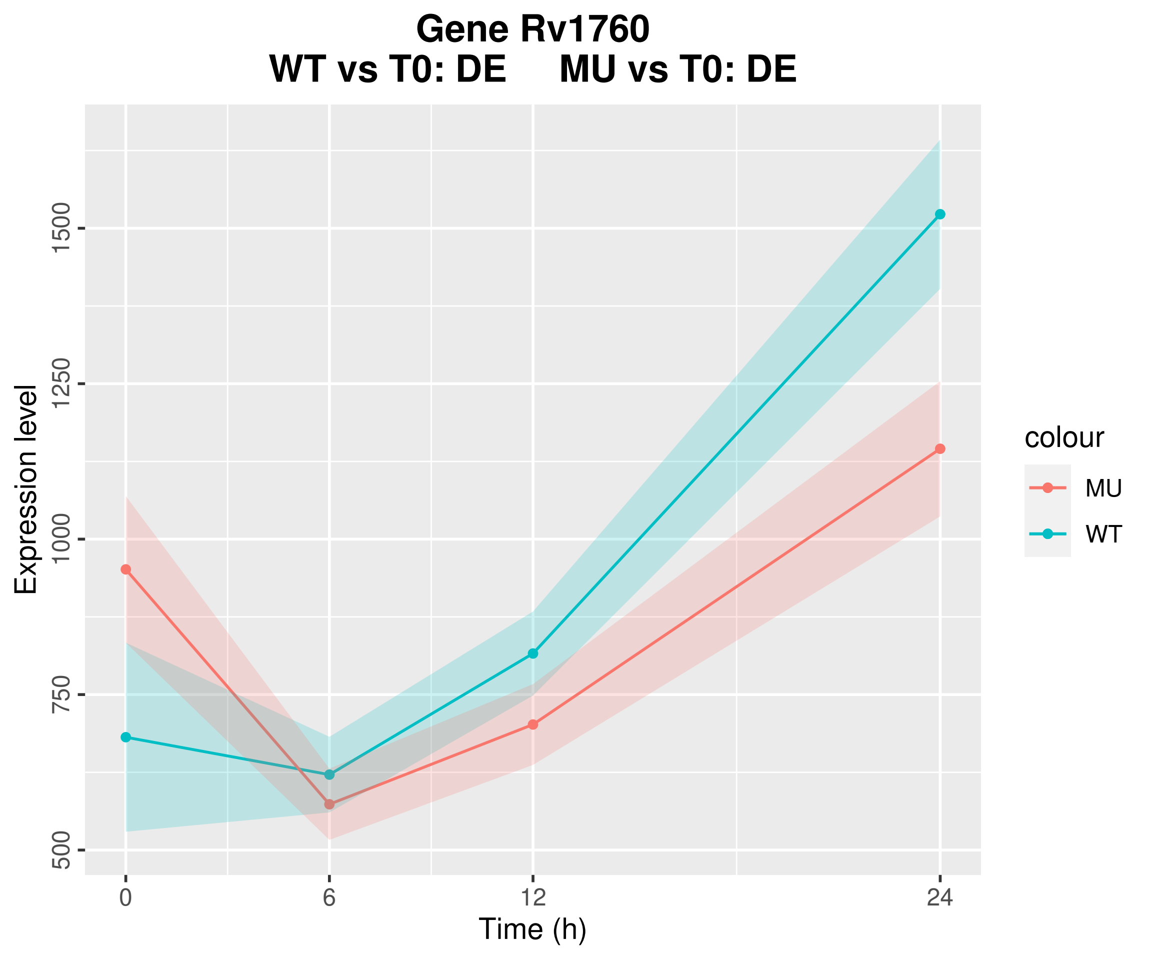

Gene Rv1760
WT vs T0: DE MU vs T0: DE
Expression level
500
750
1000
1250
1500
0
6
12
24
Time (h)
colour
MU
WT

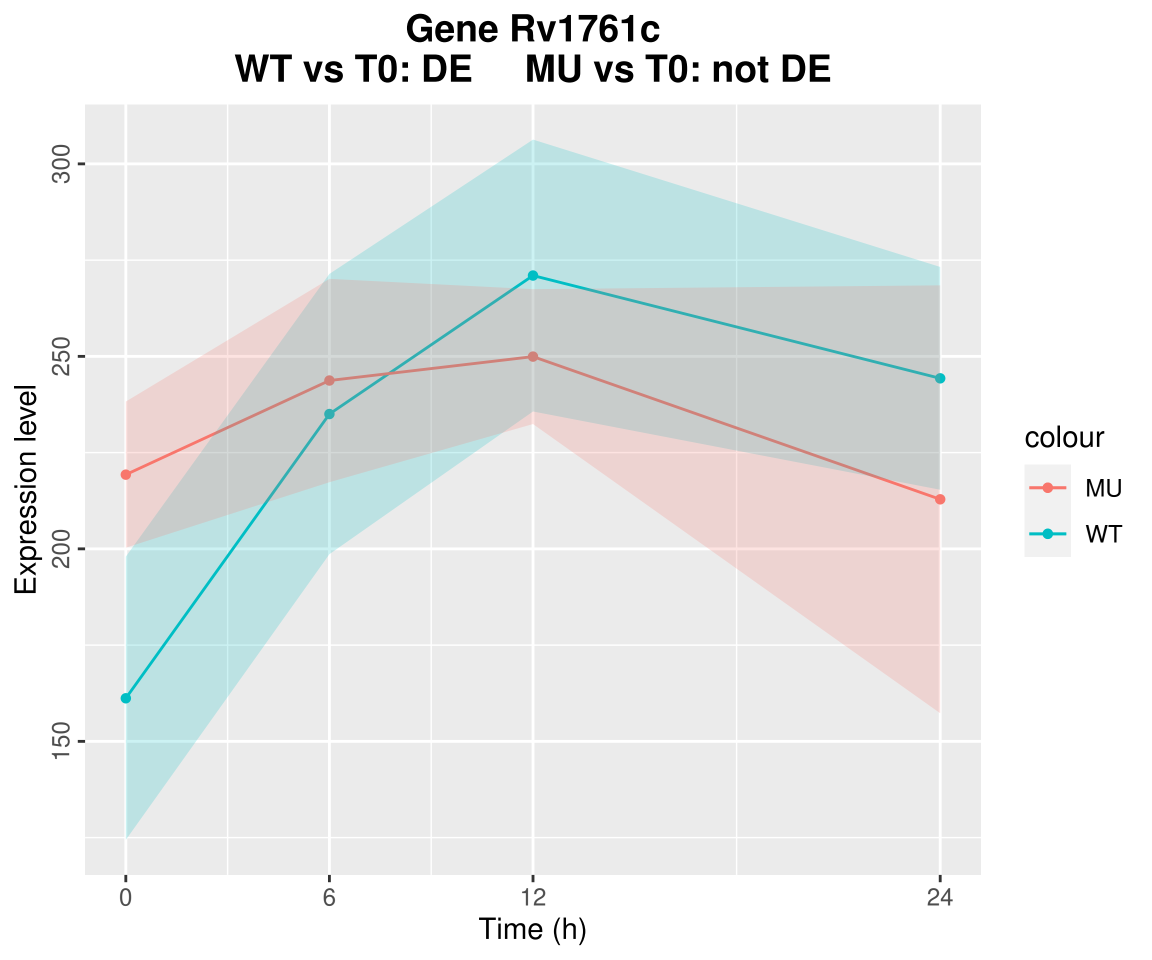

Gene Rv1761c
WT vs T0: DE MU vs T0: not DE
Expression level
300
250
200
150
0
6
12
24
Time (h)
colour
MU
WT

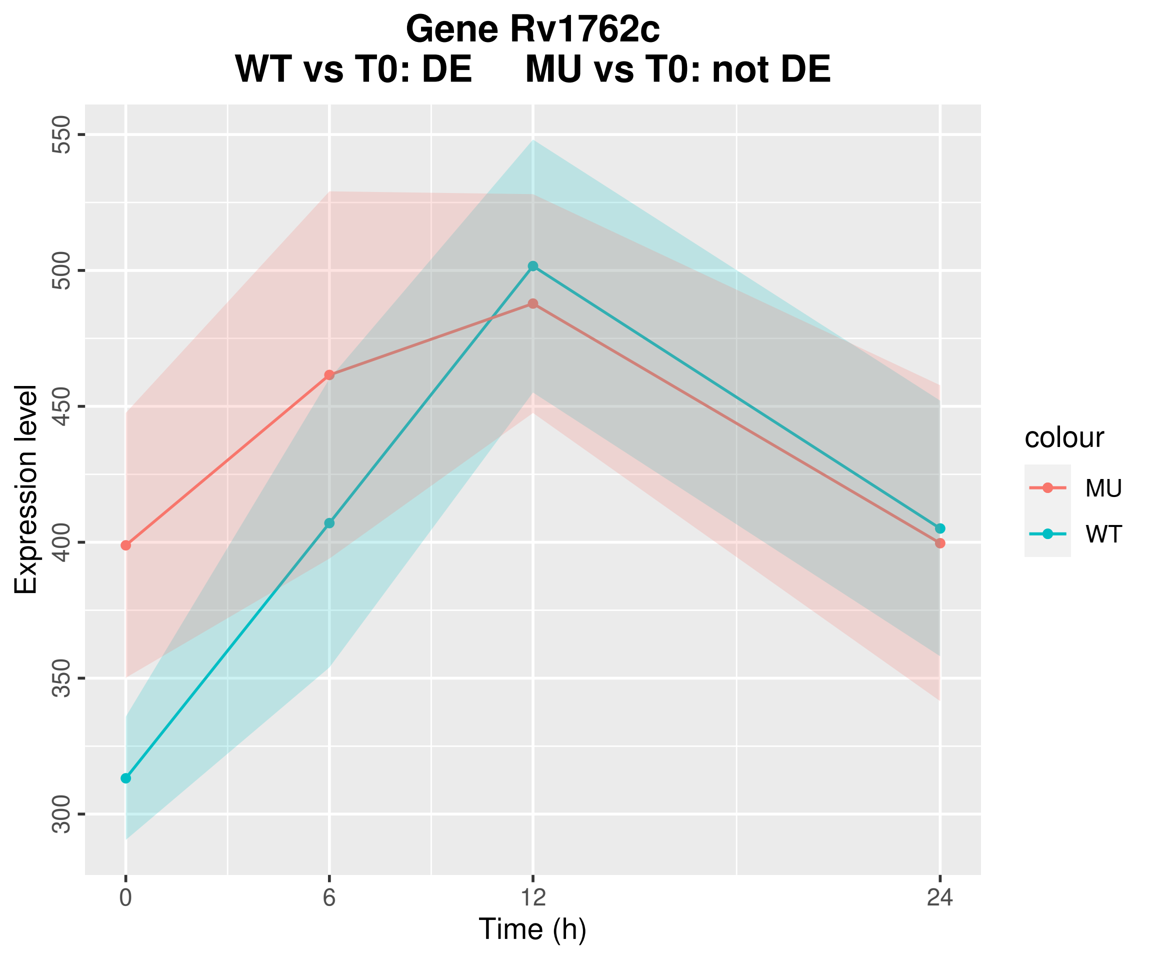
Gene Rv1762c
WT vs T0: DE   MU vs T0: not DE
Expression level
550
500
450
400
350
300
0
6
12
24
Time (h)
colour
MU
WT

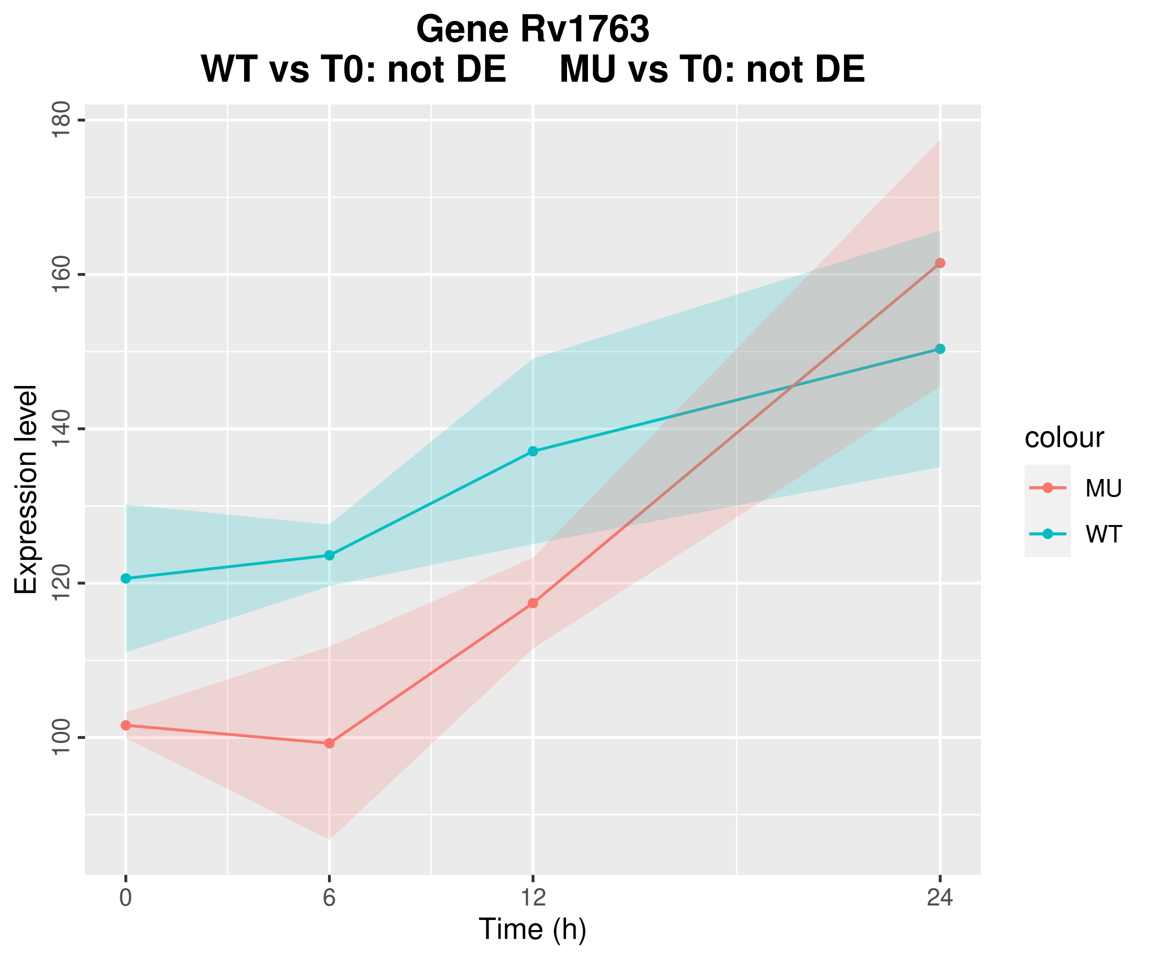

Gene Rv1763
WT vs T0: not DE MU vs T0: not DE
Expression level
Time (h)
colour
MU
WT
180
160
140
120
100
0
6
12
24

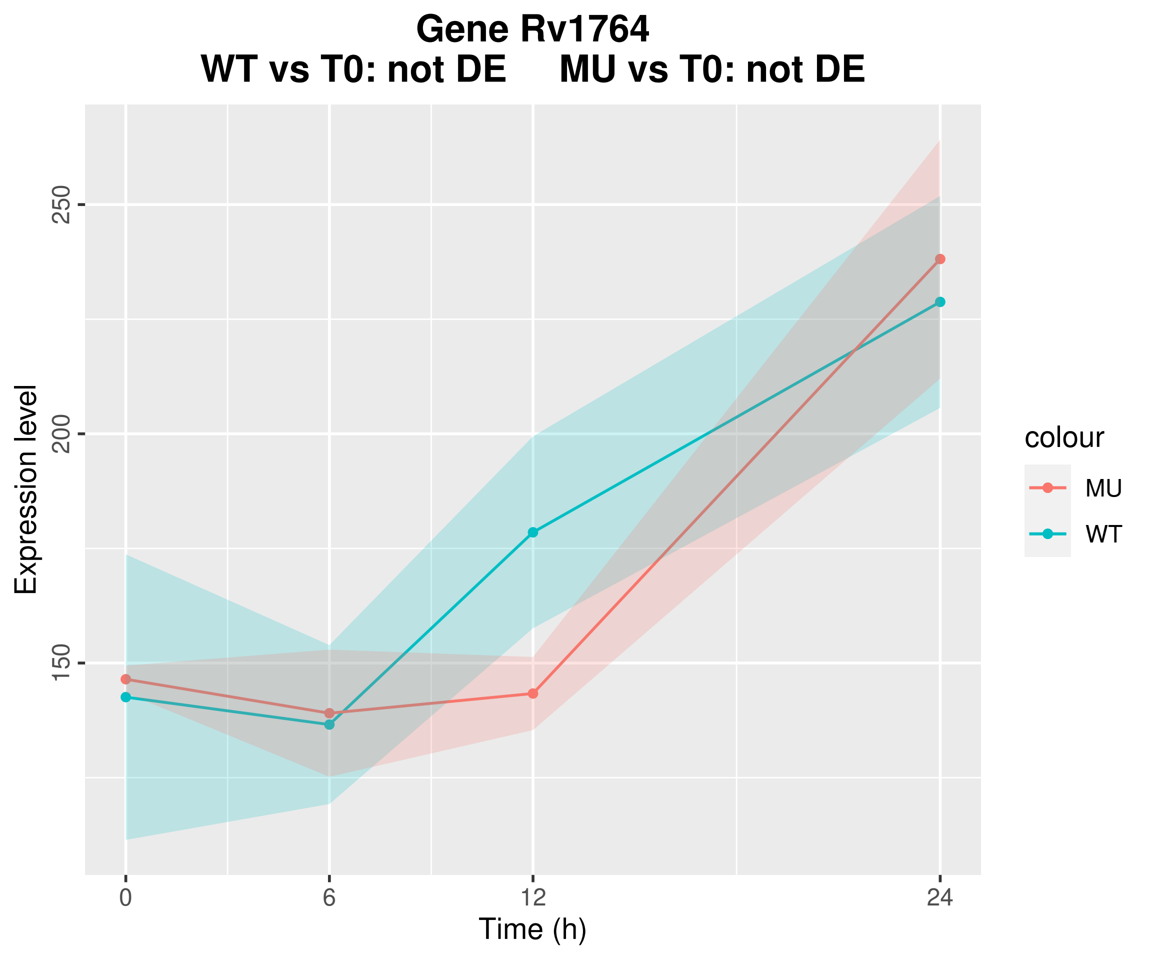

Gene Rv1764
WT vs T0: not DE   MU vs T0: not DE
Expression level
250
200
150
0
6
12
24
Time (h)
colour
MU
WT

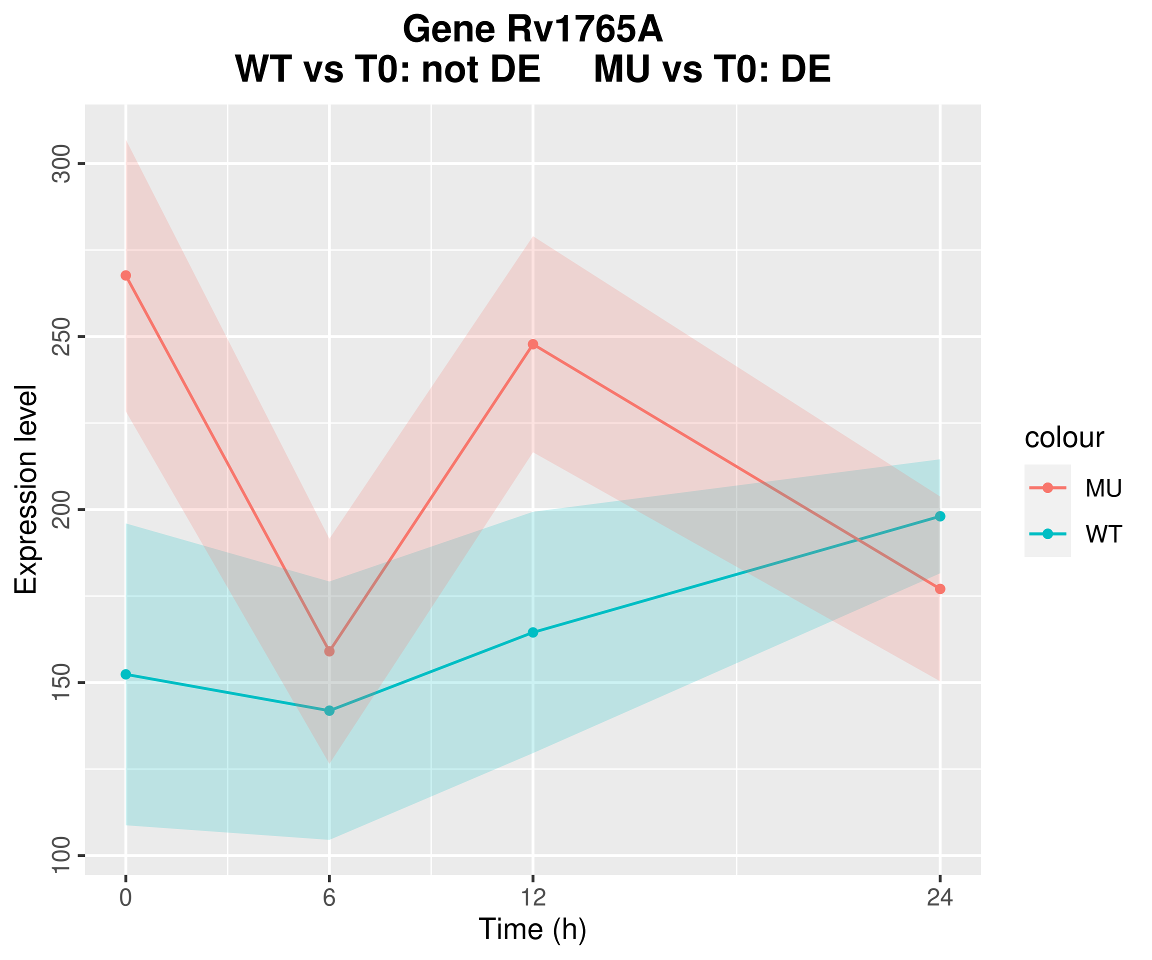
Gene Rv1765A
WT vs T0: not DE   MU vs T0: DE
Expression level
300
250
200
150
100
0
6
12
24
Time (h)
colour
MU
WT

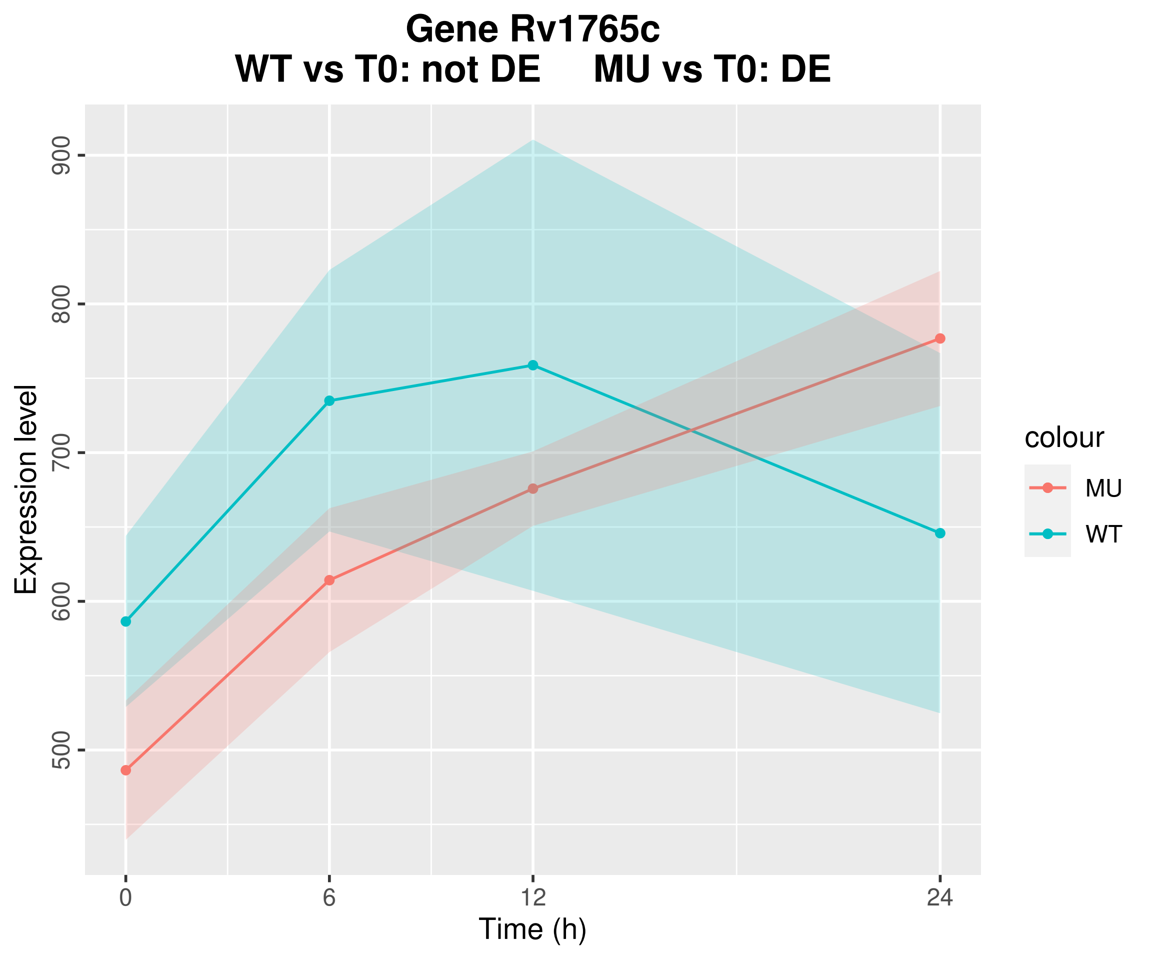

Gene Rv1765c
WT vs T0: not DE   MU vs T0: DE
Expression level
900
800
700
600
500
0
6
12
24
Time (h)
colour
MU
WT

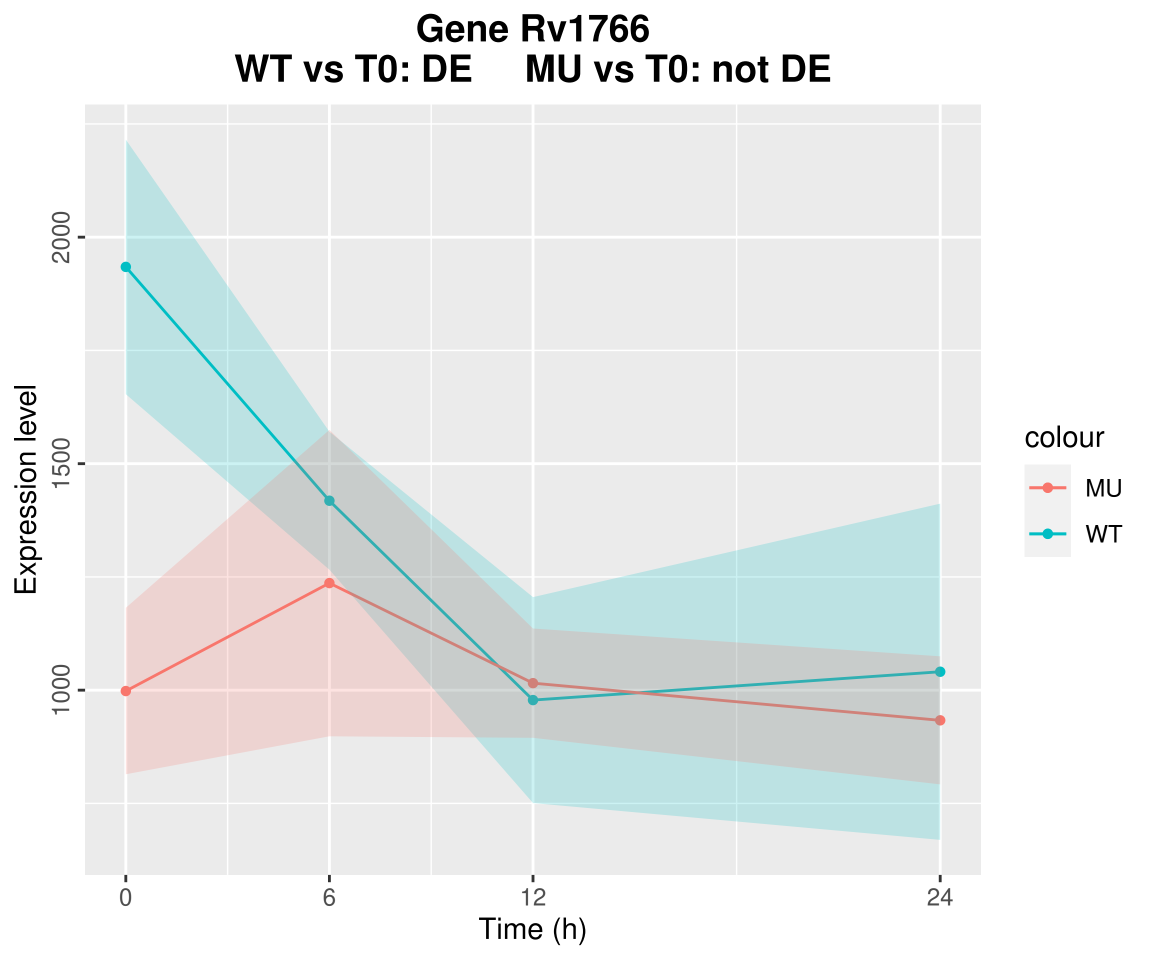
Gene Rv1766
WT vs T0: DE MU vs T0: not DE
Expression level
2000
1500
1000
0
6
12
24
Time (h)
colour
MU
WT

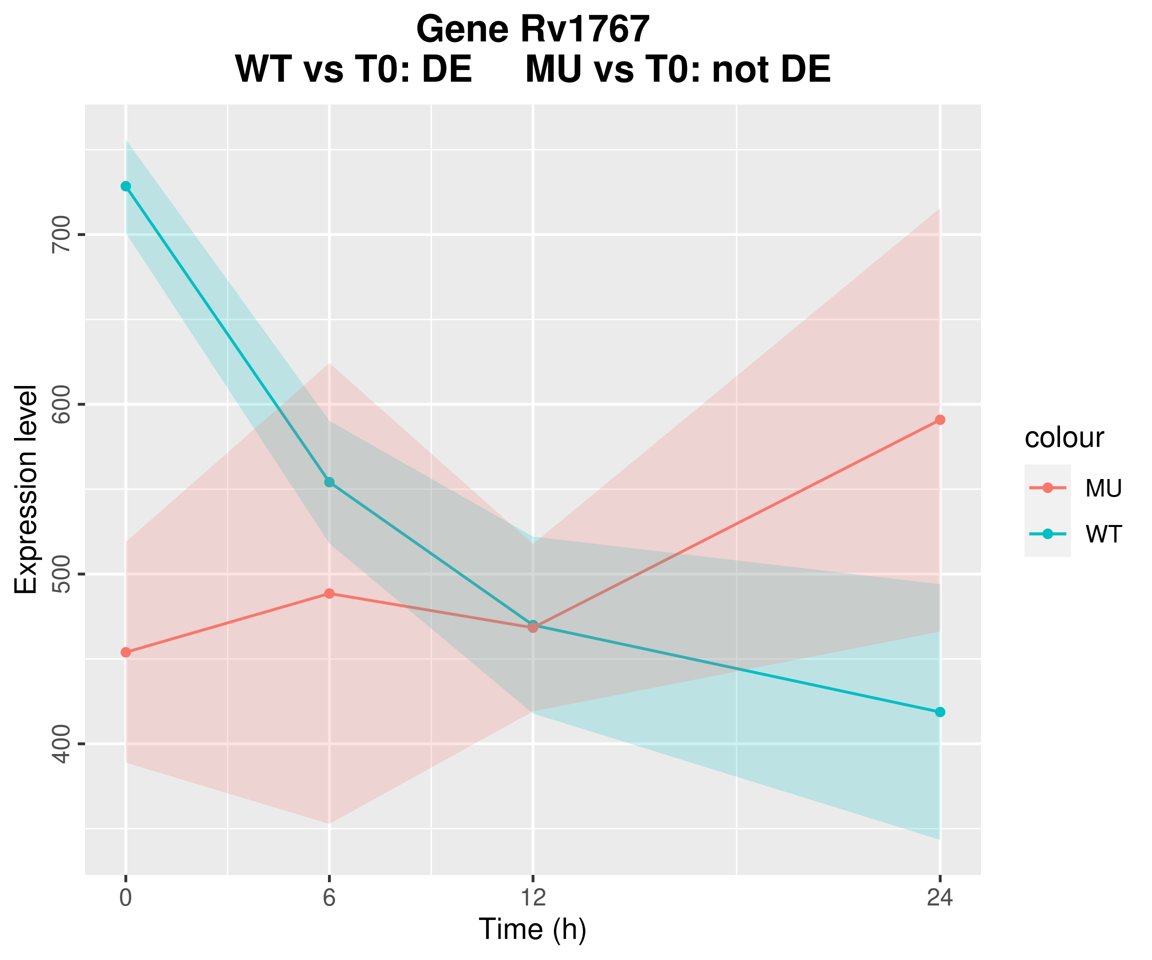
Gene Rv1767
WT vs T0: DE   MU vs T0: not DE
Expression level
700
600
500
400
0
6
12
24
Time (h)
colour
MU
WT

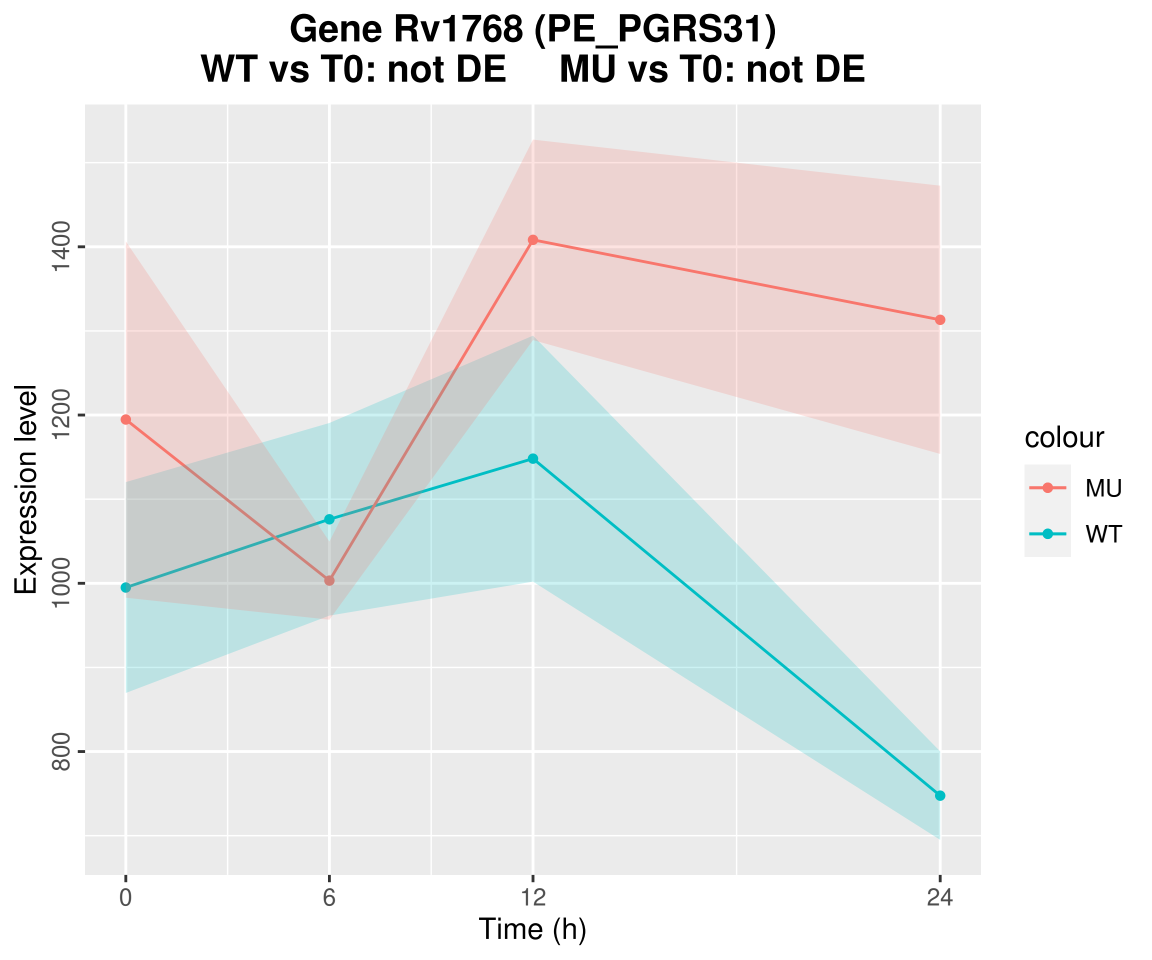
Gene Rv1768 (PE_PGRS31)
WT vs T0: not DE   MU vs T0: not DE
Expression level
1400
1200
1000
800
0
6
12
24
Time (h)
colour
MU
WT

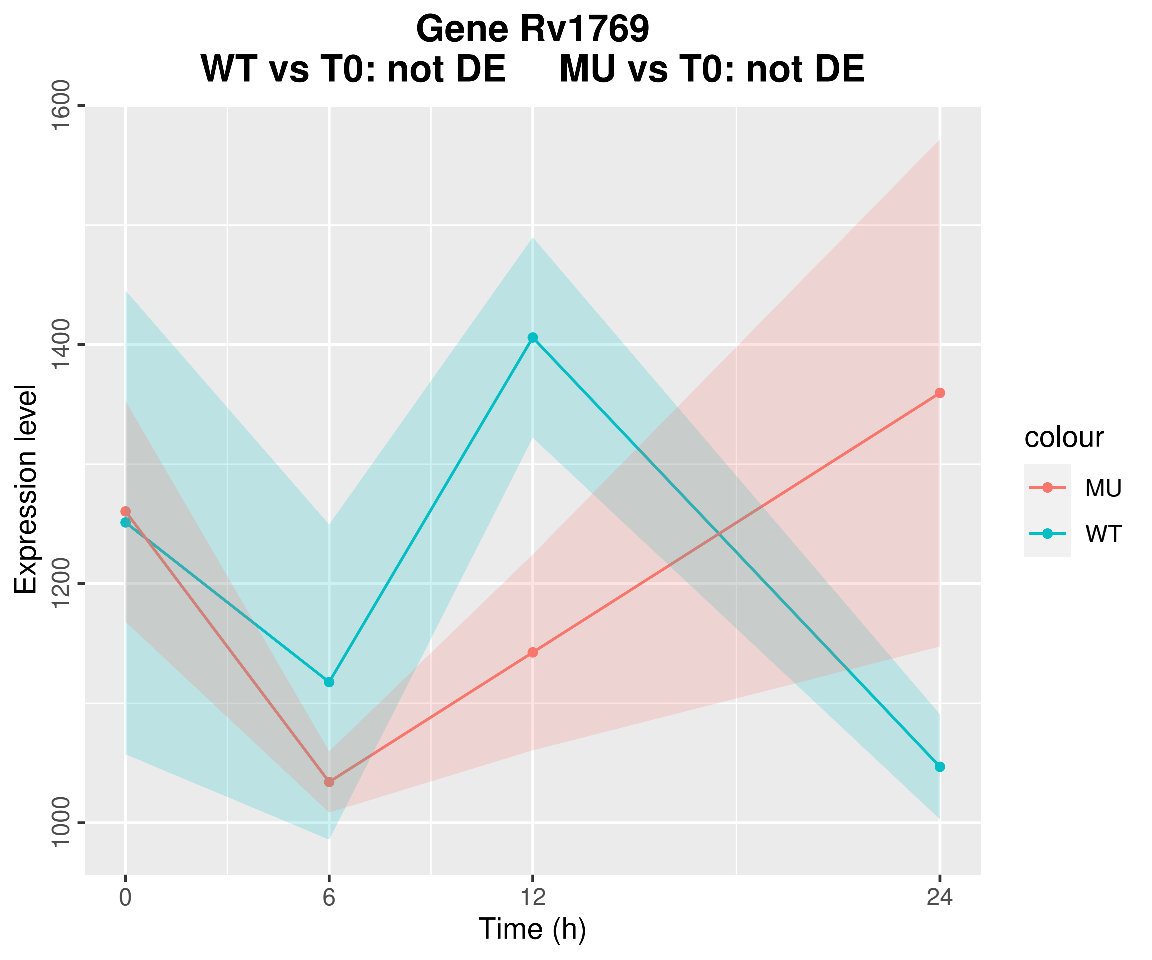
Gene Rv1769
WT vs T0: not DE MU vs T0: not DE
Expression level
1600
1400
1200
1000
0
6
12
24
Time (h)
colour
MU
WT

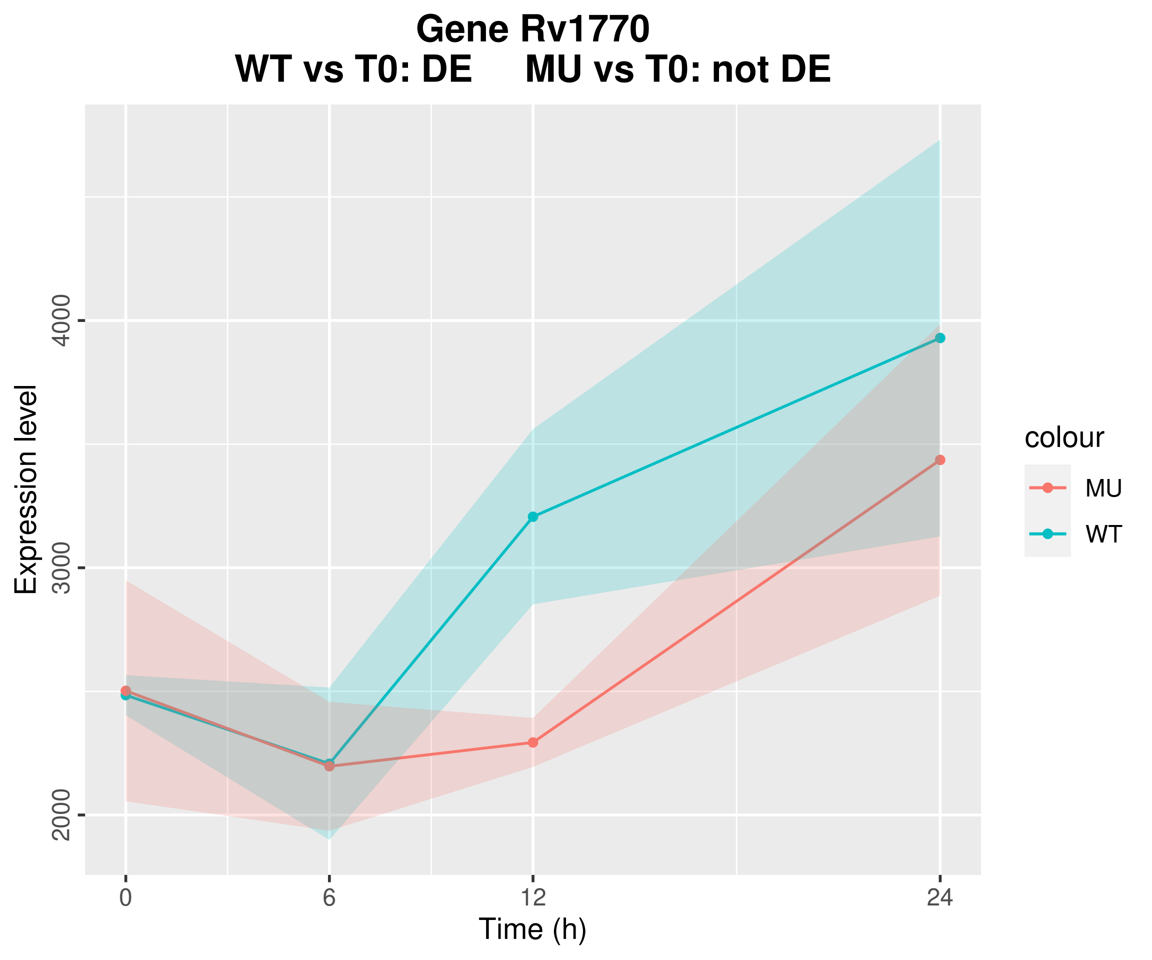

Gene Rv1770
WT vs T0: DE MU vs T0: not DE
Expression level
2000
3000
4000
0
6
12
24
Time (h)
colour
MU
WT

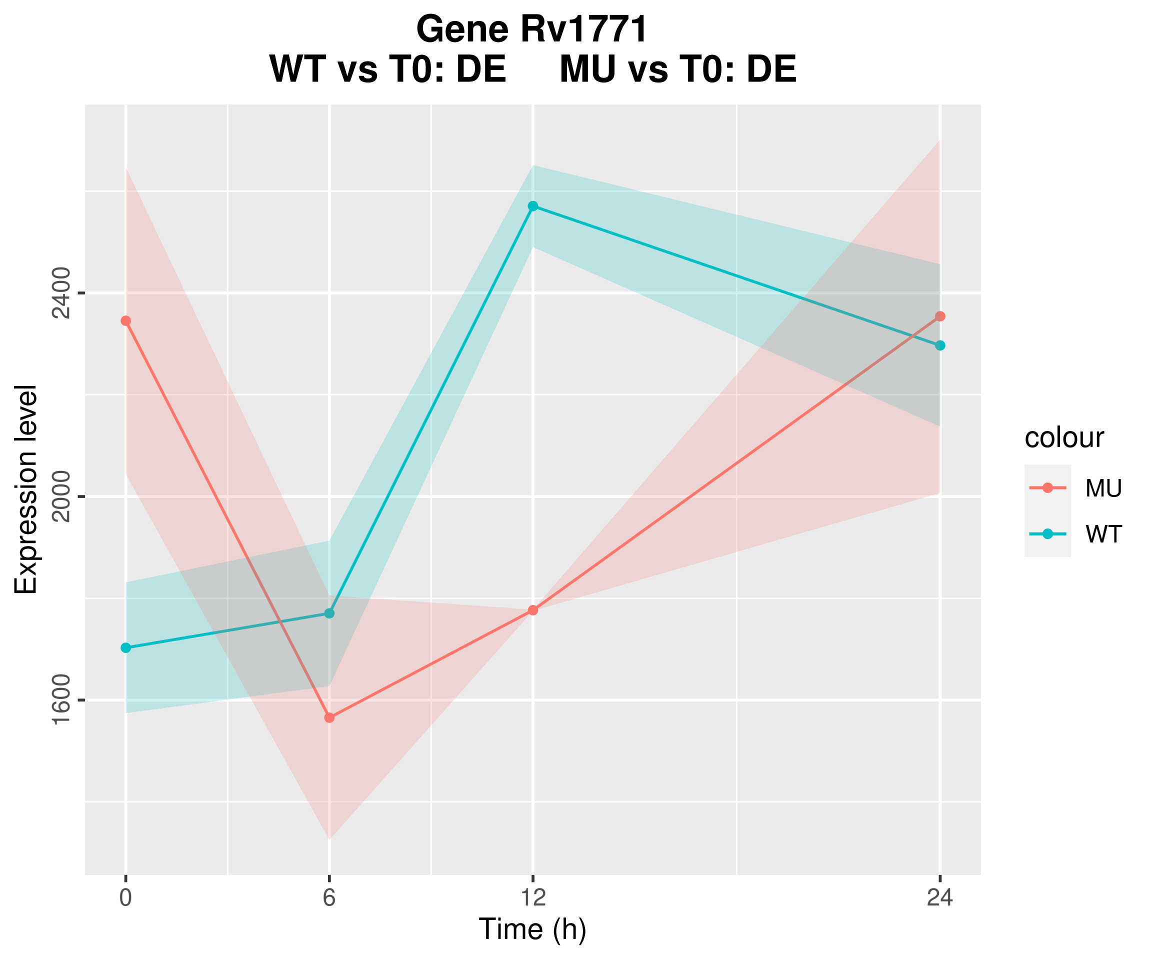
Gene Rv1771
WT vs T0: DE MU vs T0: DE
Expression level
2400
2000
1600
Time (h)
0
6
12
24
colour
MU
WT

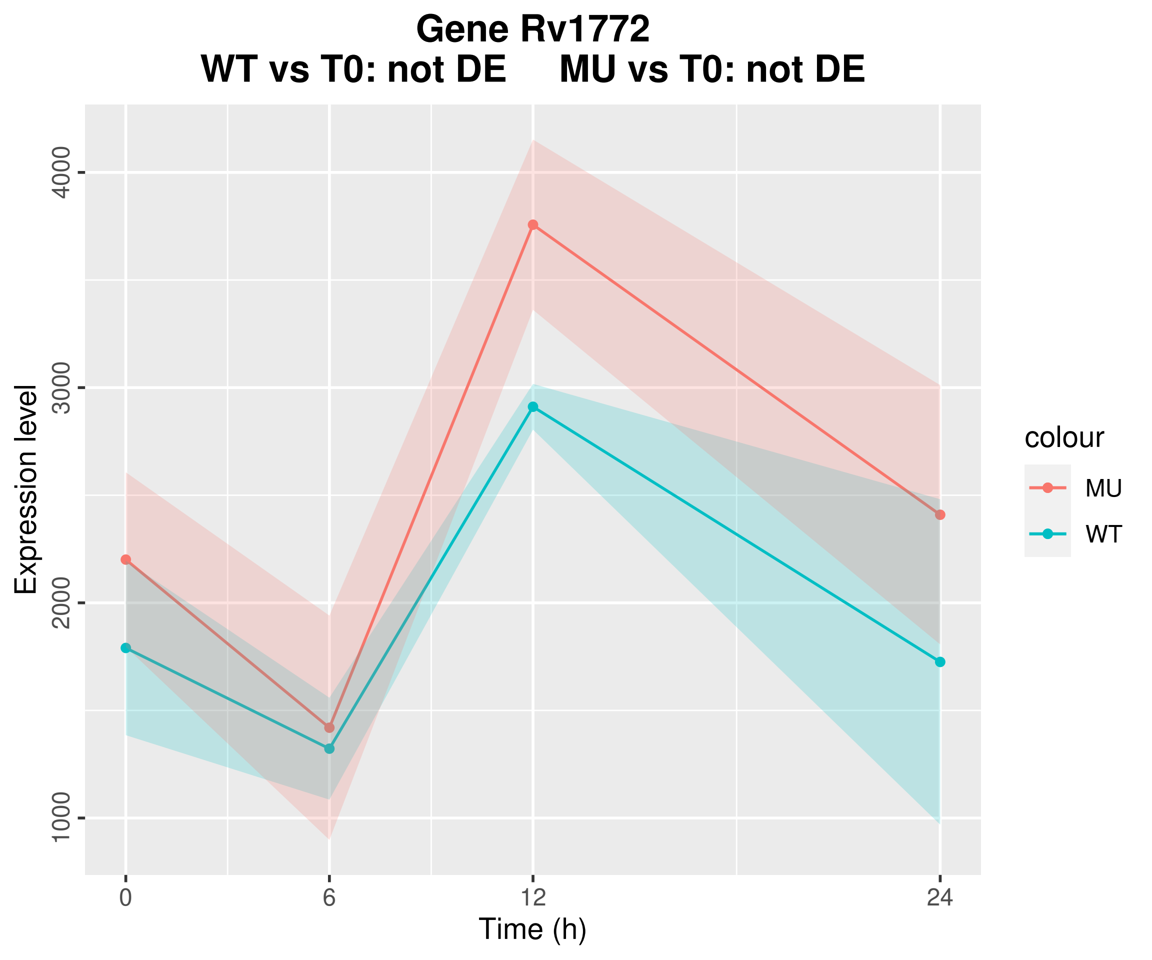

Gene Rv1772
WT vs T0: not DE   MU vs T0: not DE
Expression level
4000
3000
2000
1000
0
6
12
24
Time (h)
colour
MU
WT

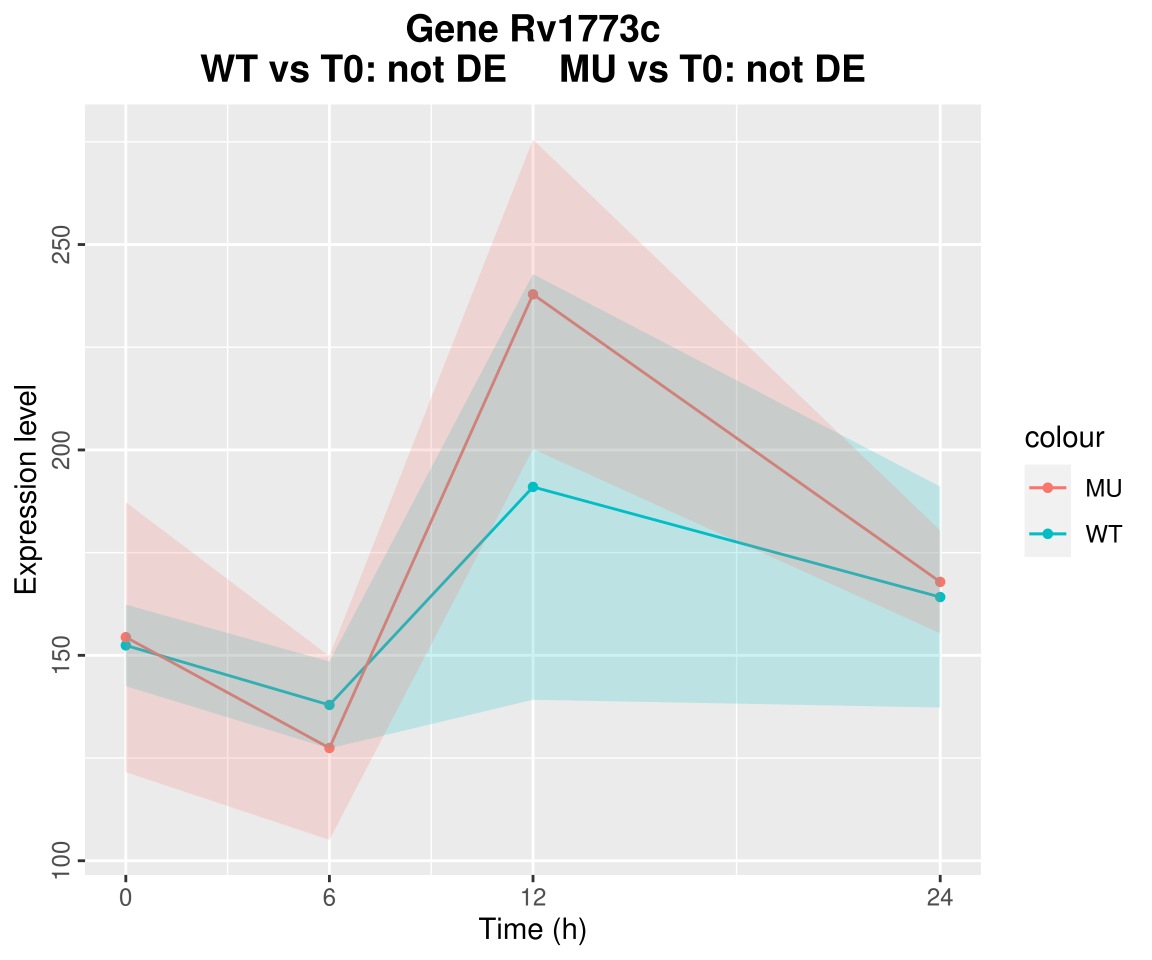

Gene Rv1773c
WT vs T0: not DE MU vs T0: not DE
Expression level
250
200
150
100
0
6
12
24
Time (h)
colour
MU
WT

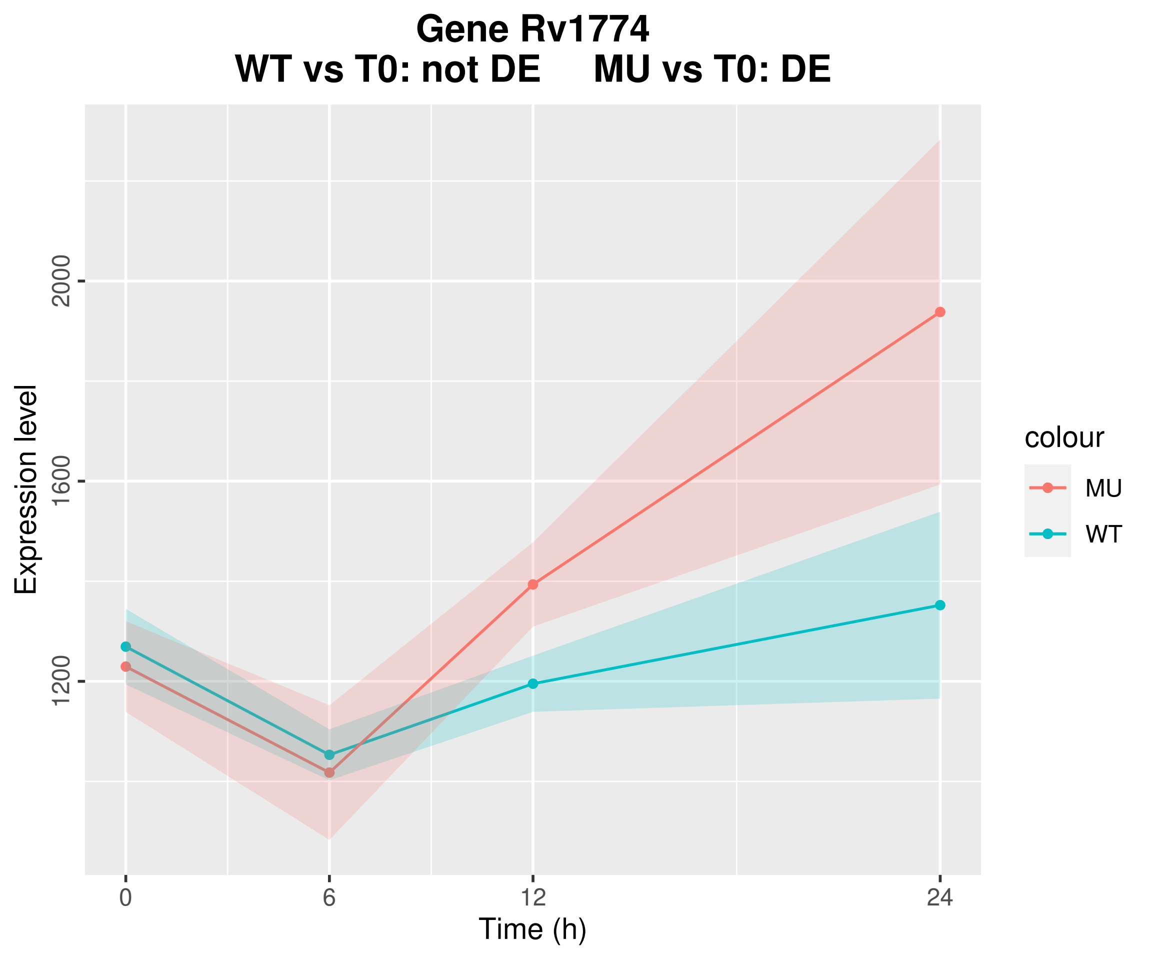

Gene Rv1774
WT vs T0: not DE MU vs T0: DE
Expression level
2000
1600
1200
0
6
12
24
Time (h)
colour
MU
WT

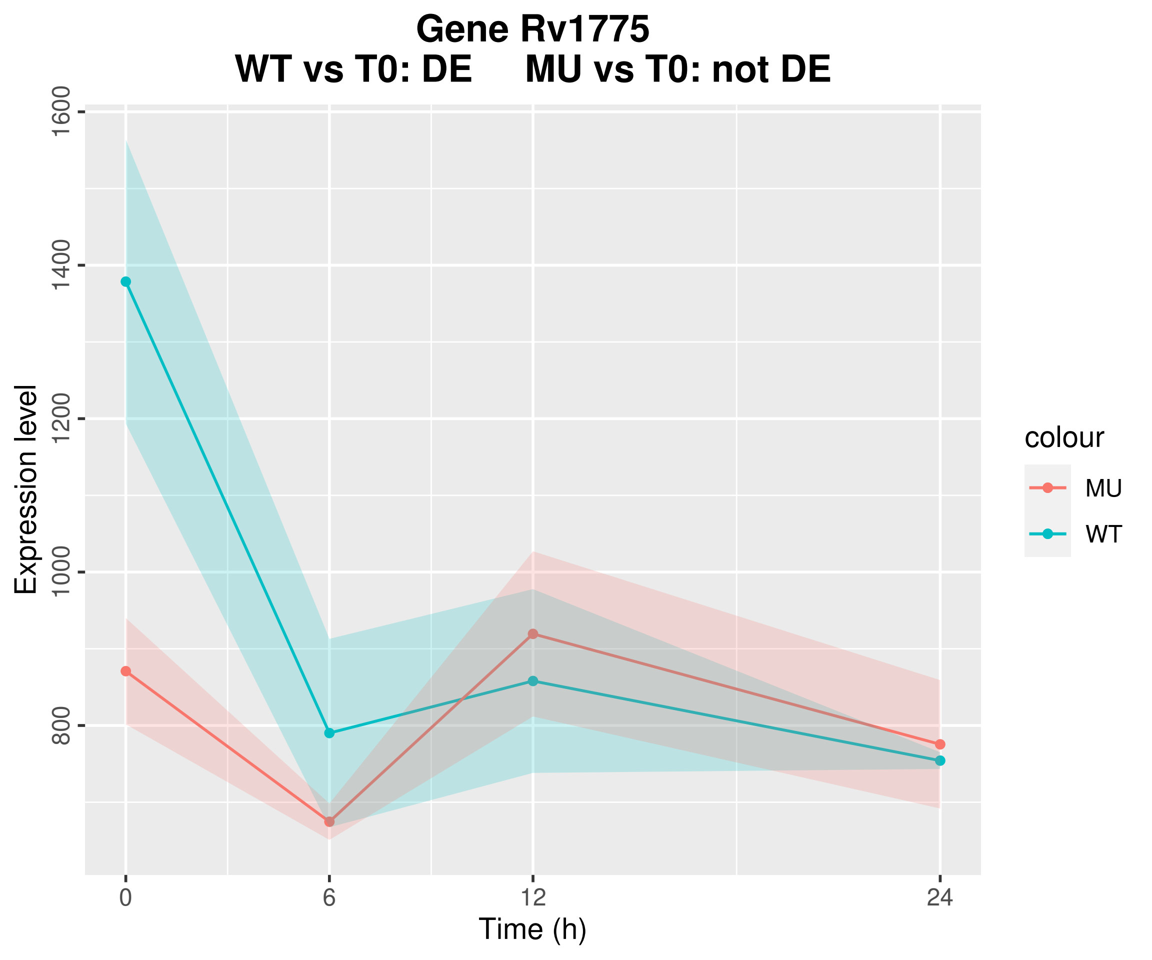

Gene Rv1775
WT vs T0: DE MU vs T0: not DE
Expression level
1600
1400
1200
1000
800
0
6
12
24
Time (h)
colour
MU
WT

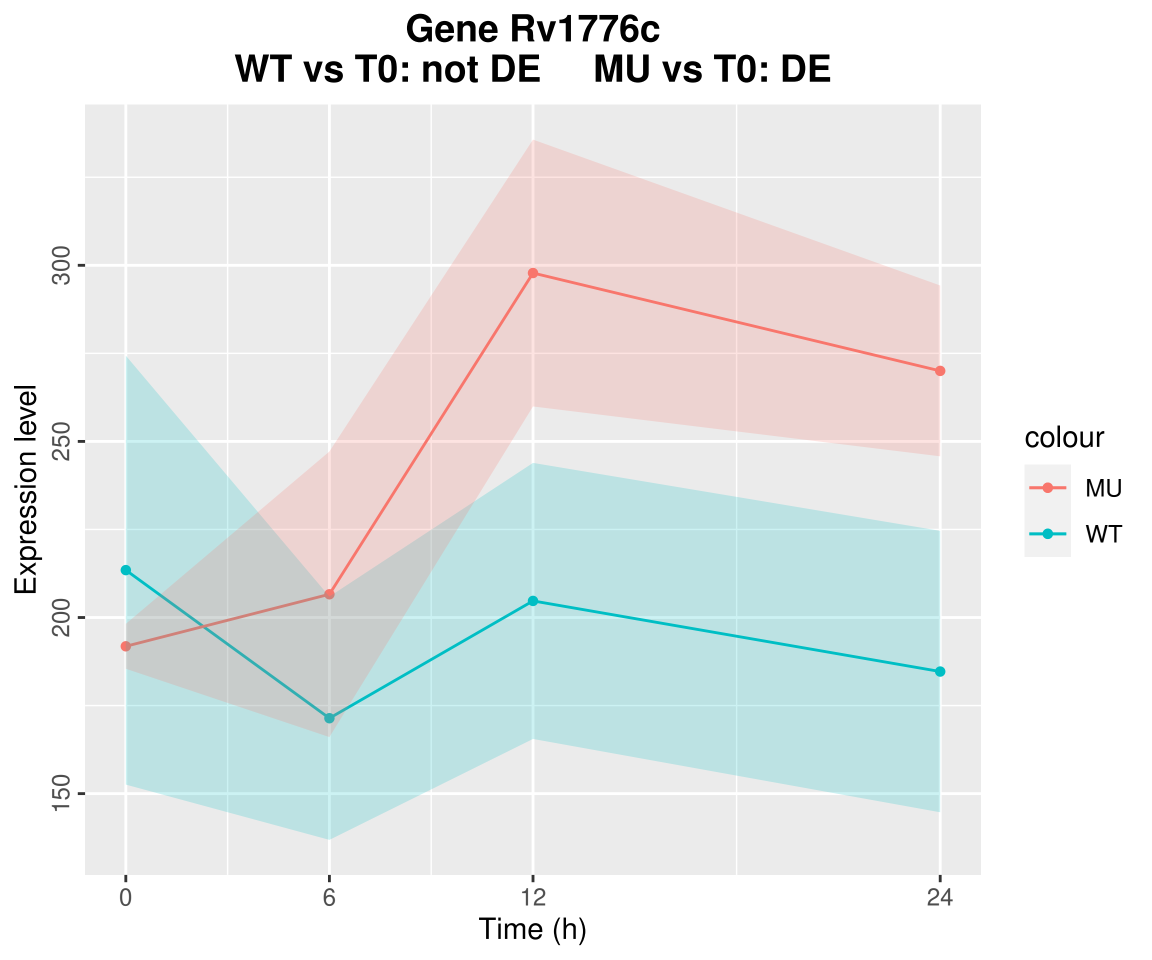

Gene Rv1776c
WT vs T0: not DE   MU vs T0: DE
Expression level
300
250
200
150
0
6
12
24
Time (h)
colour
MU
WT

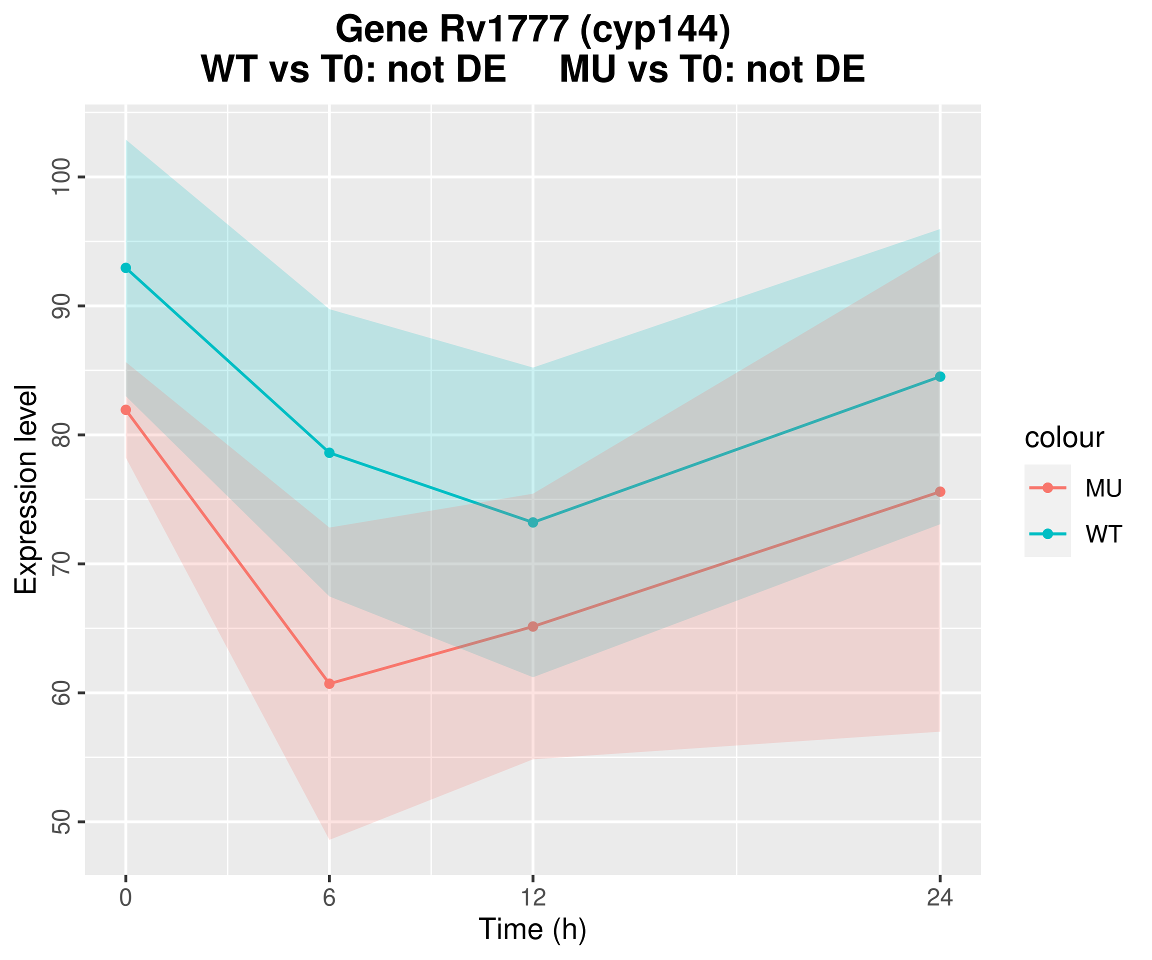

Gene Rv1777 (cyp144)
WT vs T0: not DE MU vs T0: not DE
Expression level
100
90
80
70
60
50
0
6
12
24
Time (h)
colour
MU
WT

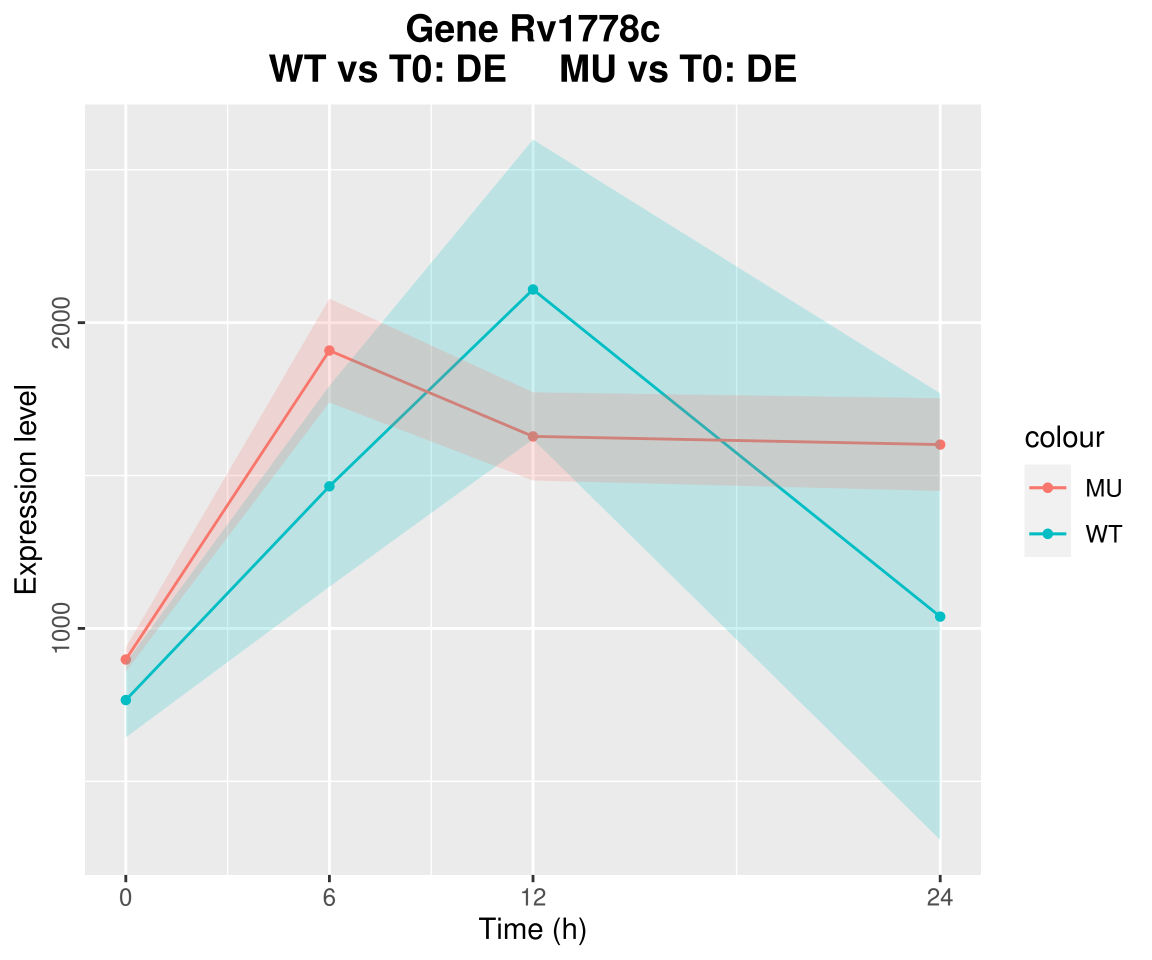

Gene Rv1778c
WT vs T0: DE MU vs T0: DE
Expression level
2000
1000
0
6
12
24
Time (h)
colour
MU
WT

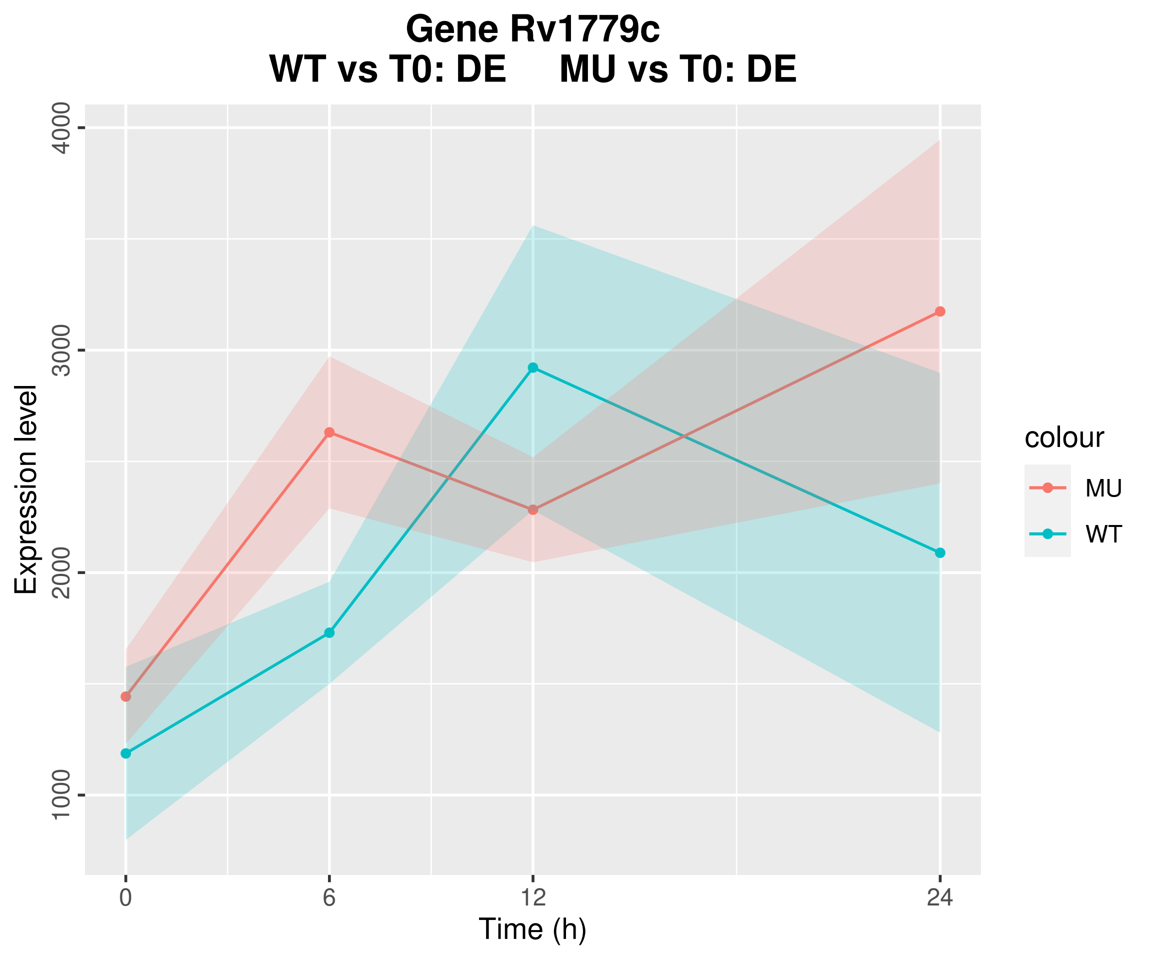
Gene Rv1779c
WT vs T0: DE MU vs T0: DE
Expression level
4000
3000
2000
1000
0
6
12
24
Time (h)
colour
MU
WT

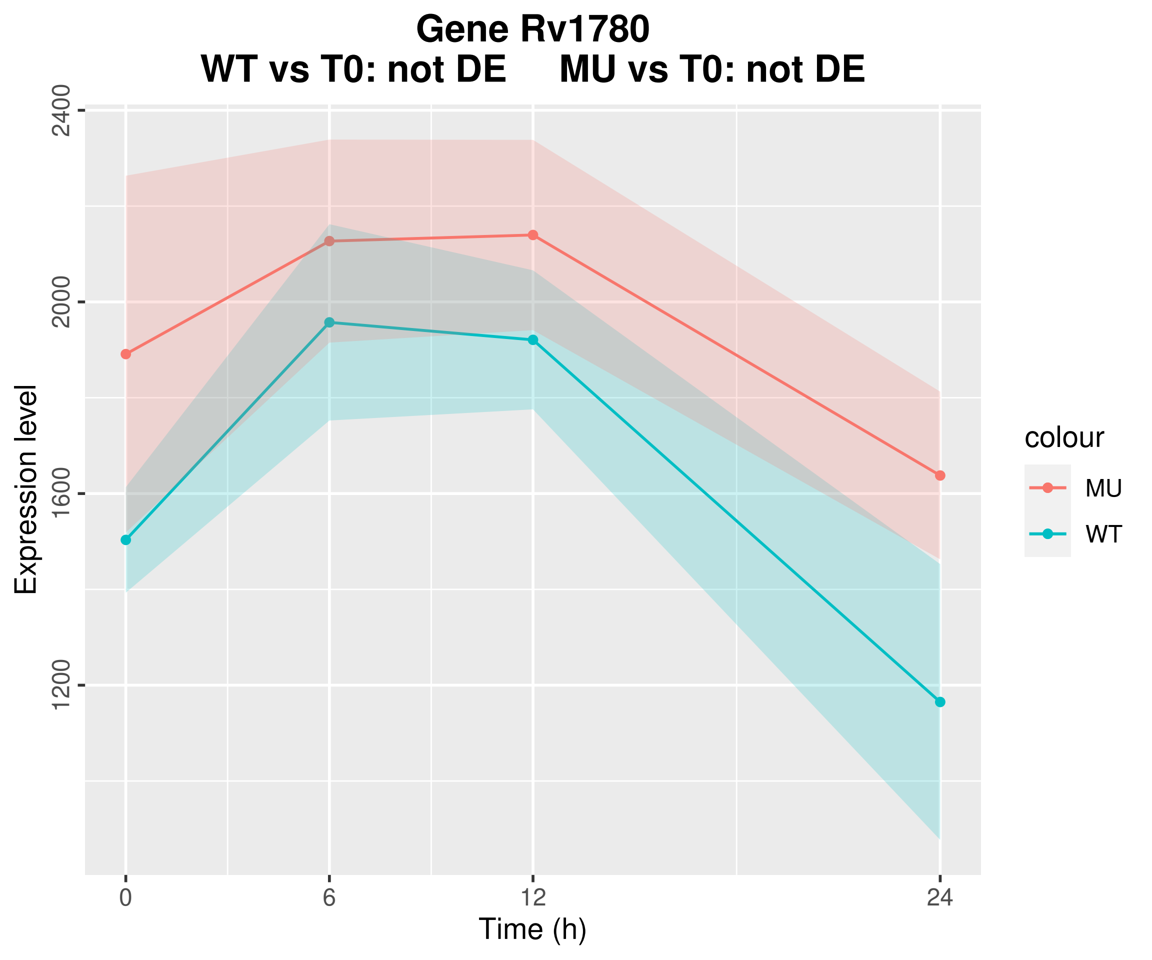

Gene Rv1780
WT vs T0: not DE MU vs T0: not DE
Expression level
2400
2000
1600
1200
0
6
12
24
Time (h)
colour
MU
WT

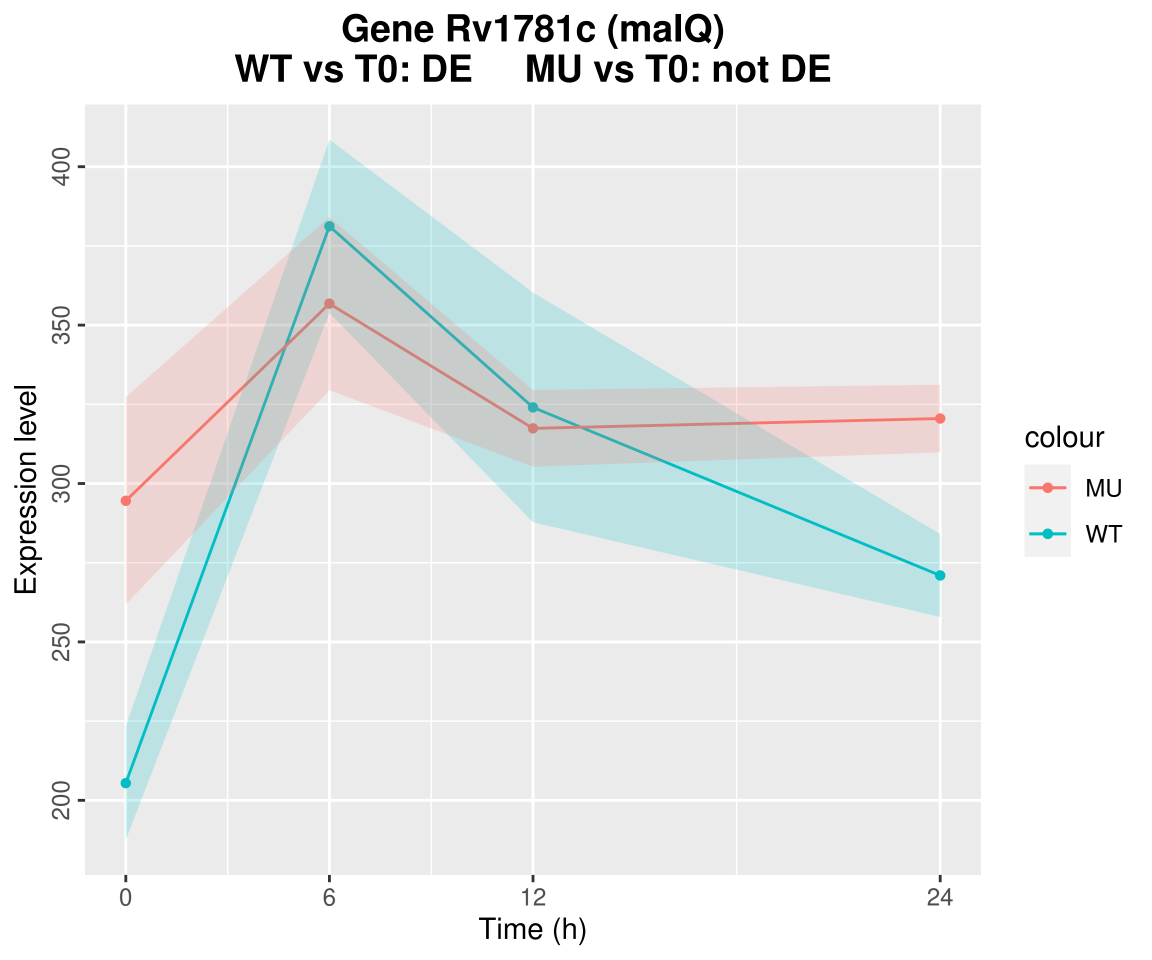
Gene Rv1781c (malQ)
WT vs T0: DE MU vs T0: not DE
Expression level
400
350
300
250
200
0
6
12
24
Time (h)
colour
MU
WT

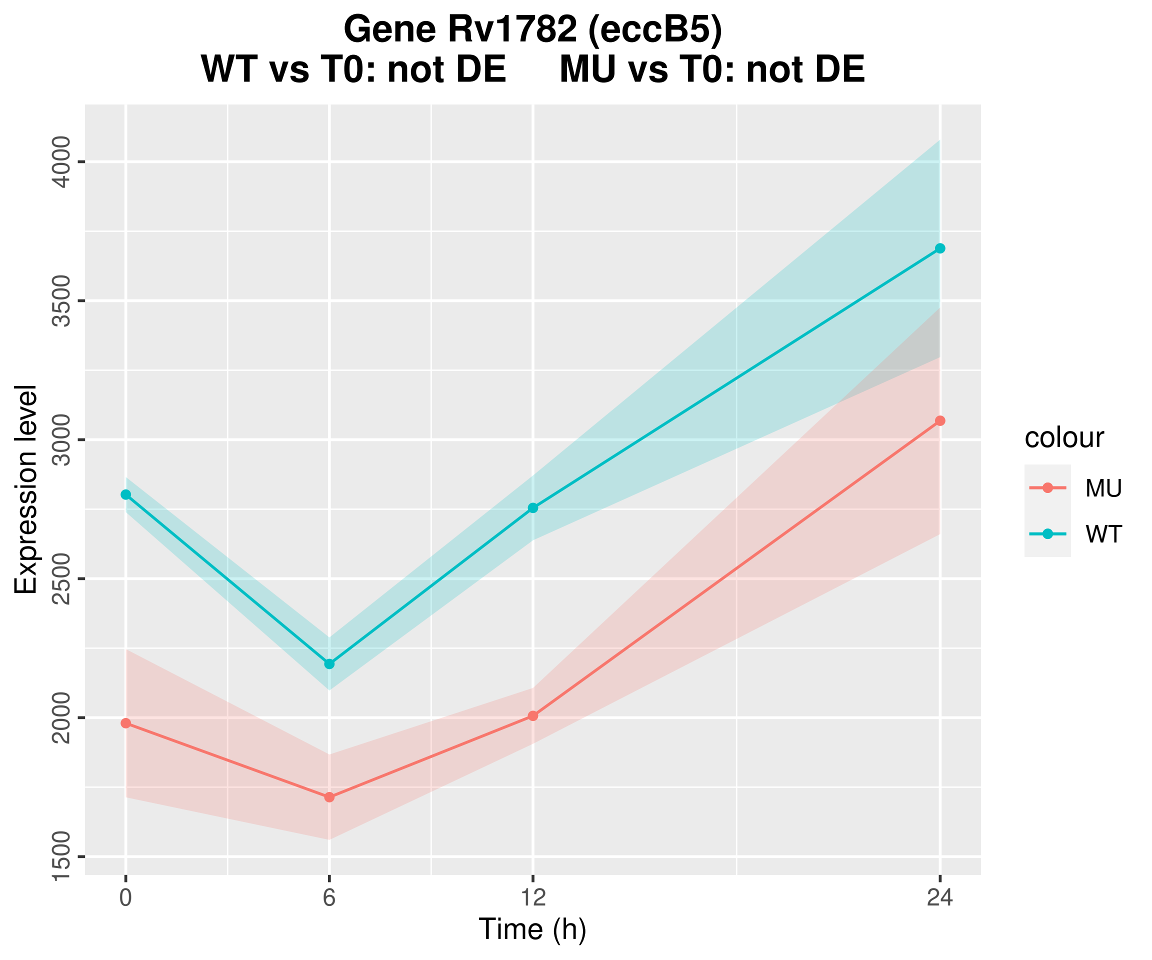

Gene Rv1782 (eccB5)
WT vs T0: not DE   MU vs T0: not DE
Expression level
4000
3500
3000
2500
2000
1500
0
6
12
24
Time (h)
colour
MU
WT

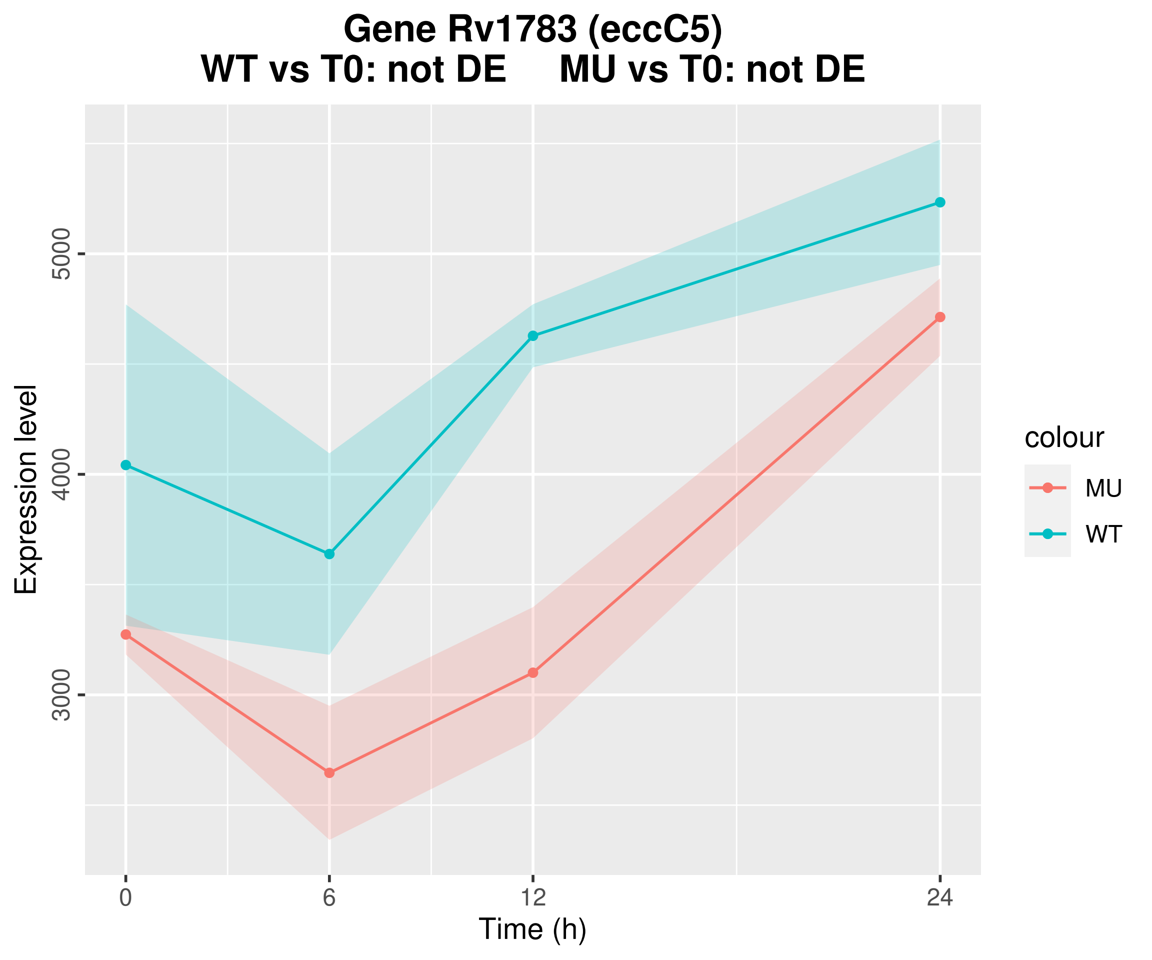

Gene Rv1783 (eccC5)
WT vs T0: not DE   MU vs T0: not DE
Expression level
5000
4000
3000
0
6
12
24
Time (h)
colour
MU
WT

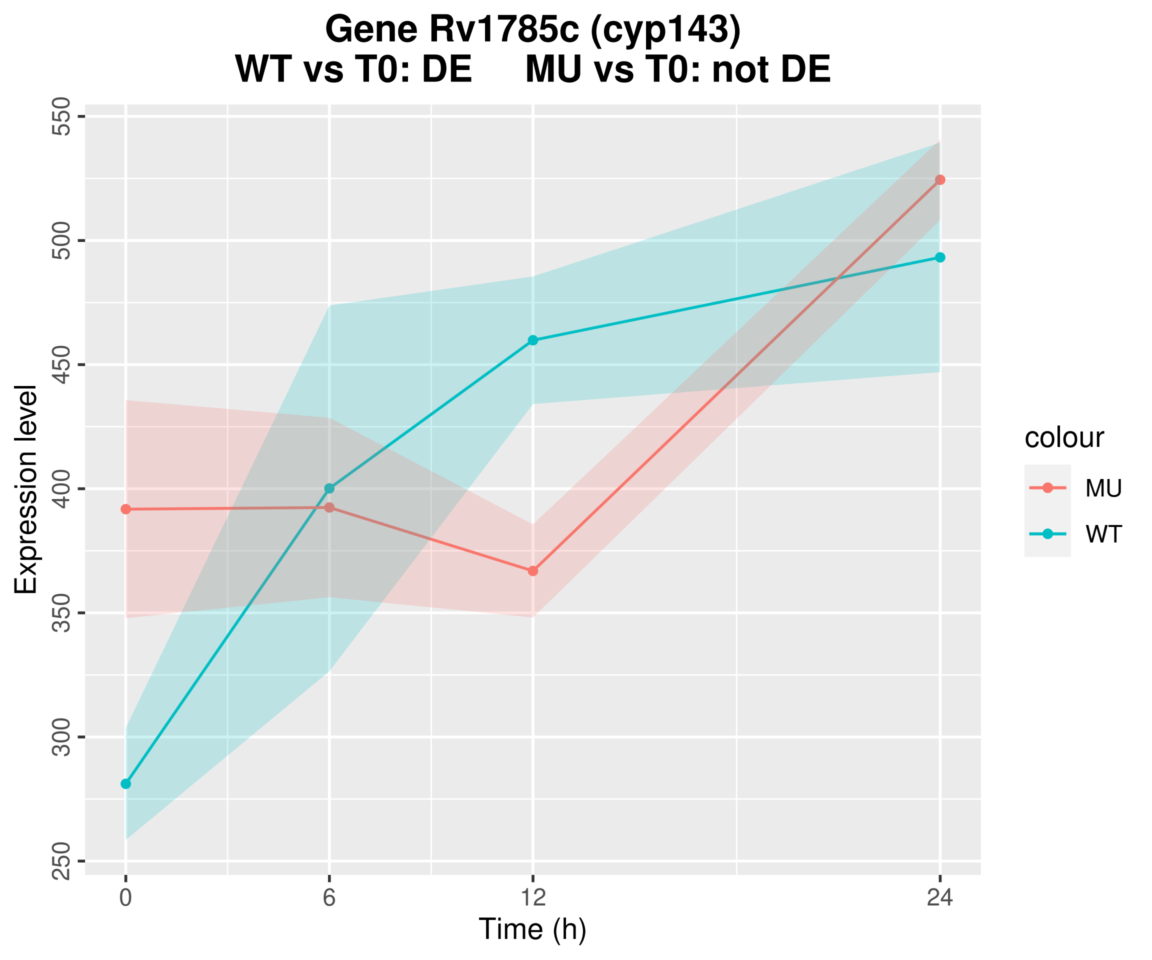
Gene Rv1785c (cyp143)
WT vs T0: DE MU vs T0: not DE
Expression level
550
500
450
400
350
300
250
0
6
12
24
Time (h)
colour
MU
WT

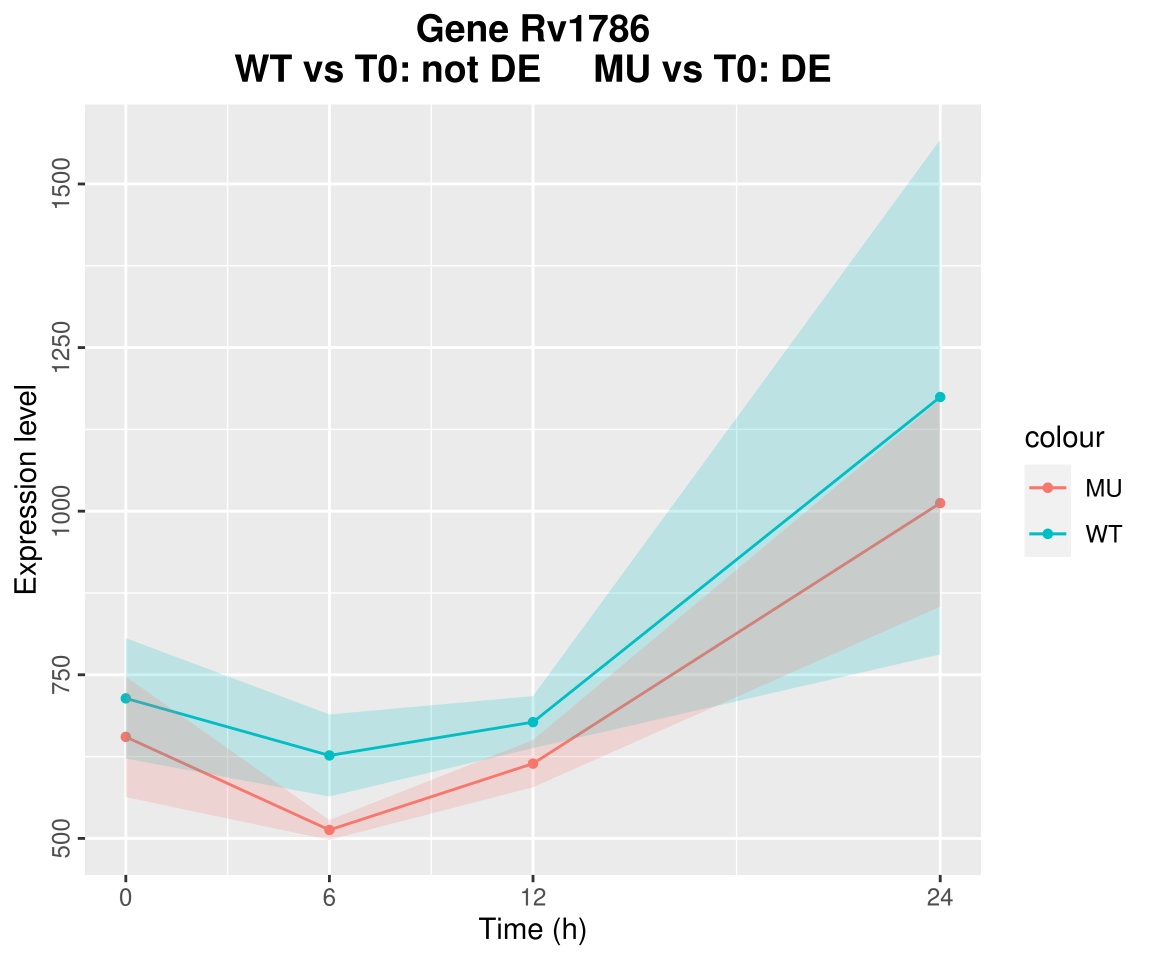
Gene Rv1786
WT vs T0: not DE MU vs T0: DE
Expression level
1500
1250
1000
750
500
0
6
12
24
Time (h)
colour
MU
WT

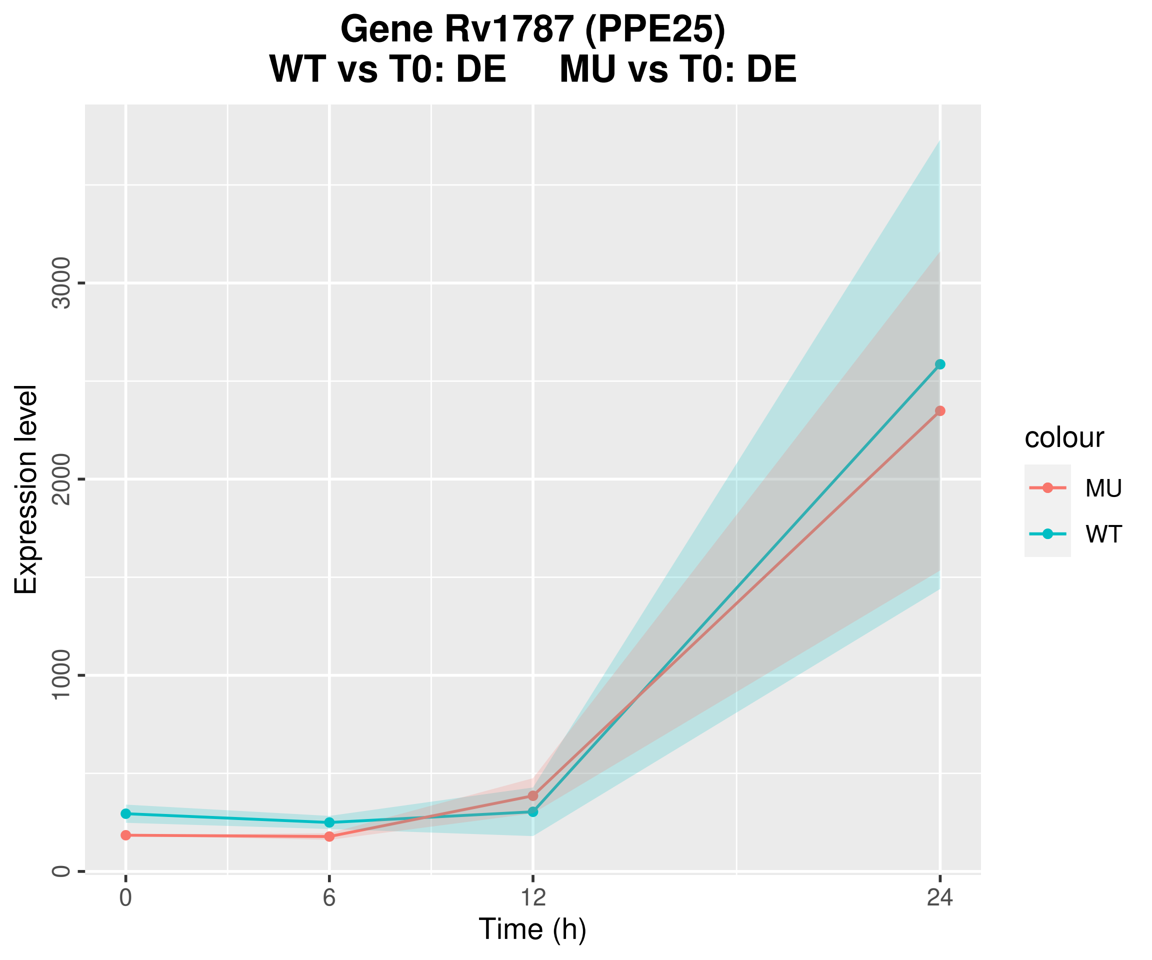

Gene Rv1787 (PPE25)
WT vs T0: DE   MU vs T0: DE
Expression level
0
1000
2000
3000
0
6
12
24
Time (h)
colour
MU
WT

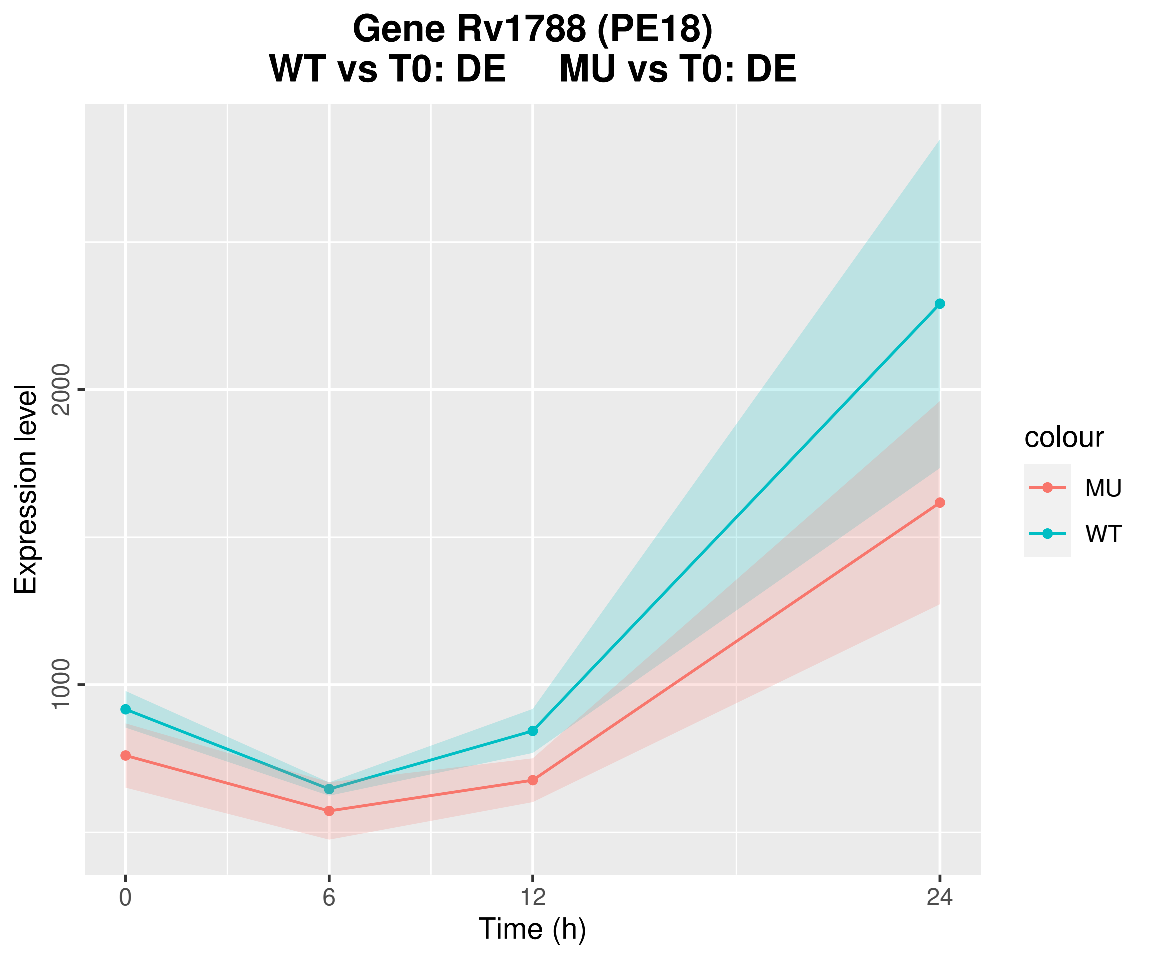
Gene Rv1788 (PE18)
WT vs T0: DE MU vs T0: DE
Expression level
2000
1000
0
6
12
24
Time (h)
colour
MU
WT

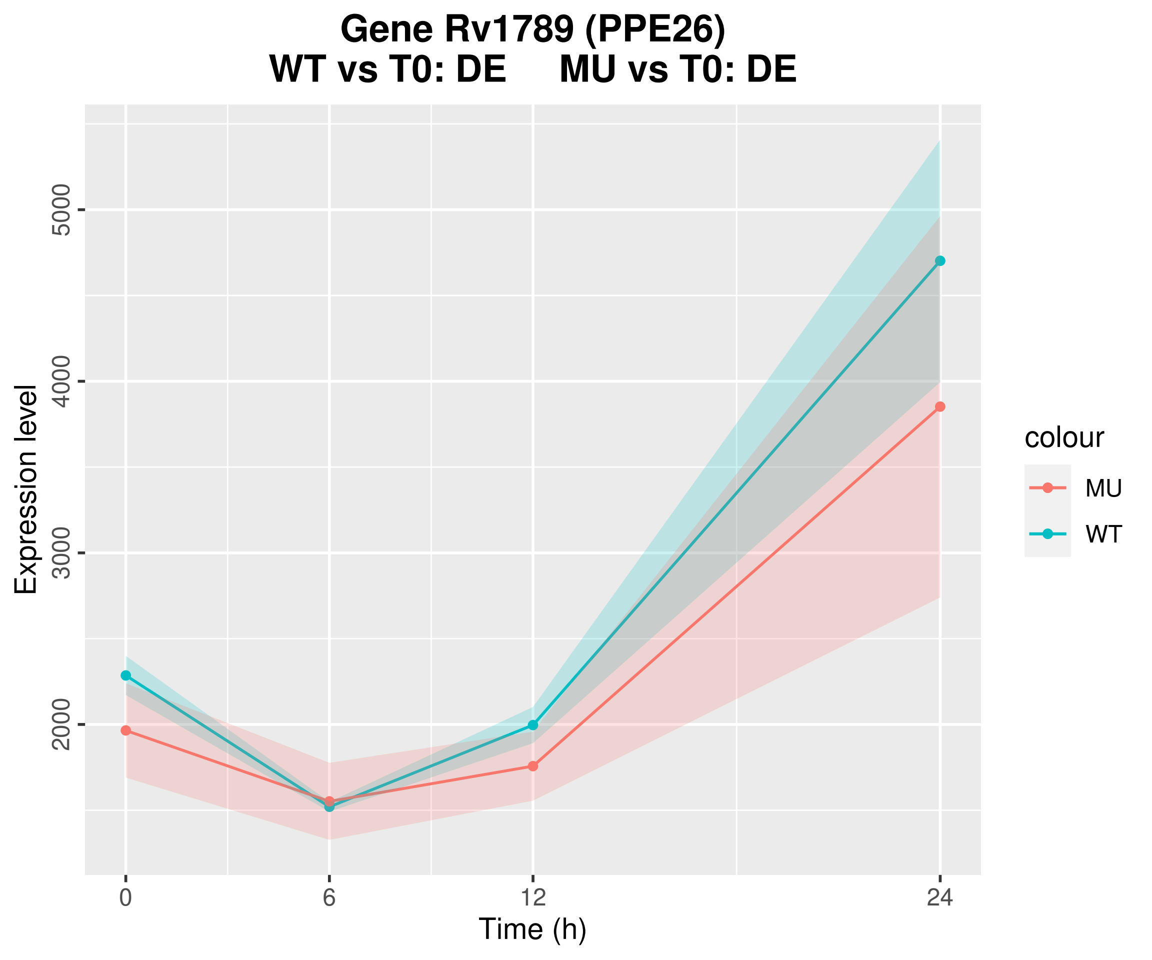

Gene Rv1789 (PPE26)
WT vs T0: DE MU vs T0: DE
Expression level
5000
4000
3000
2000
0
6
12
24
Time (h)
colour
MU
WT

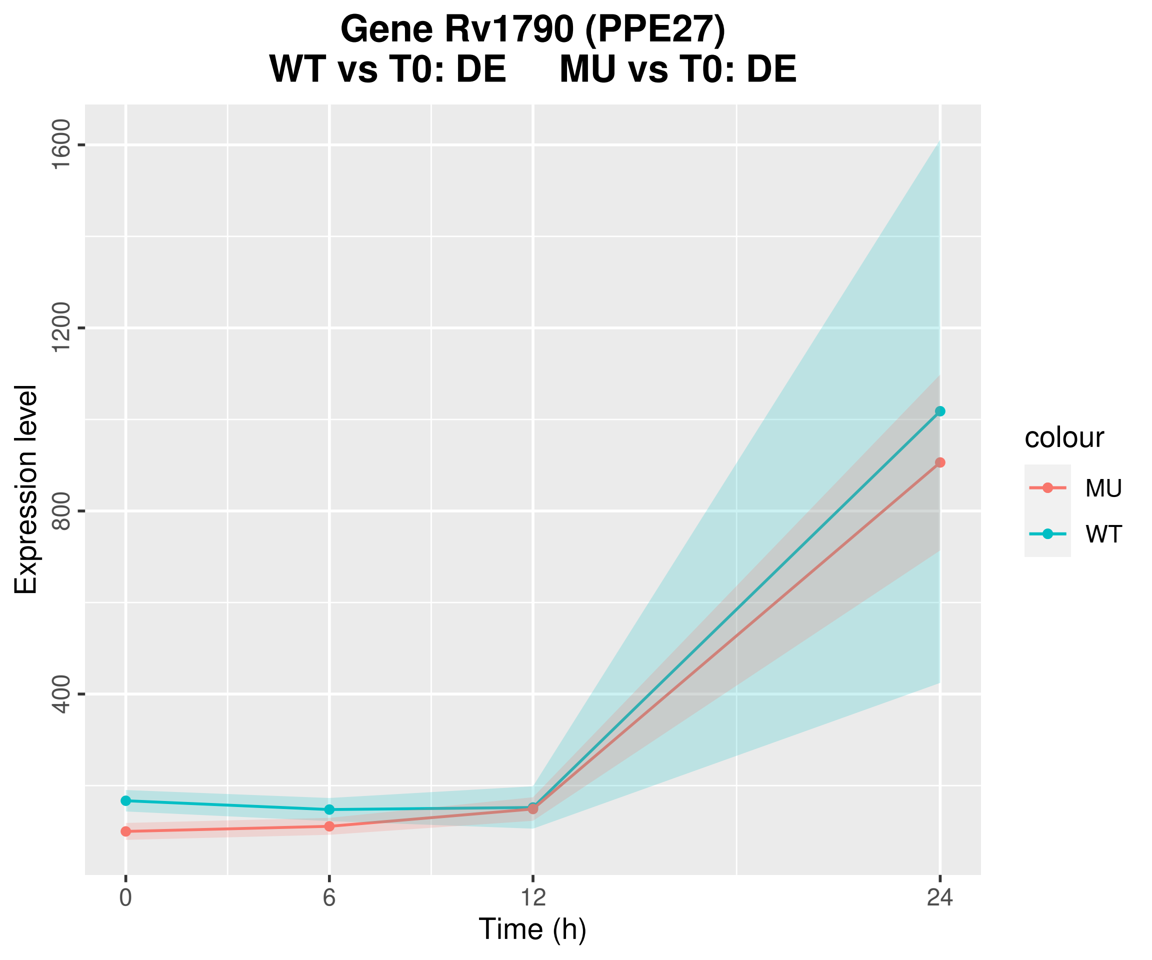
Gene Rv1790 (PPE27)
WT vs T0: DE MU vs T0: DE
Expression level
1600
1200
800
400
0
6
12
24
Time (h)
colour
MU
WT

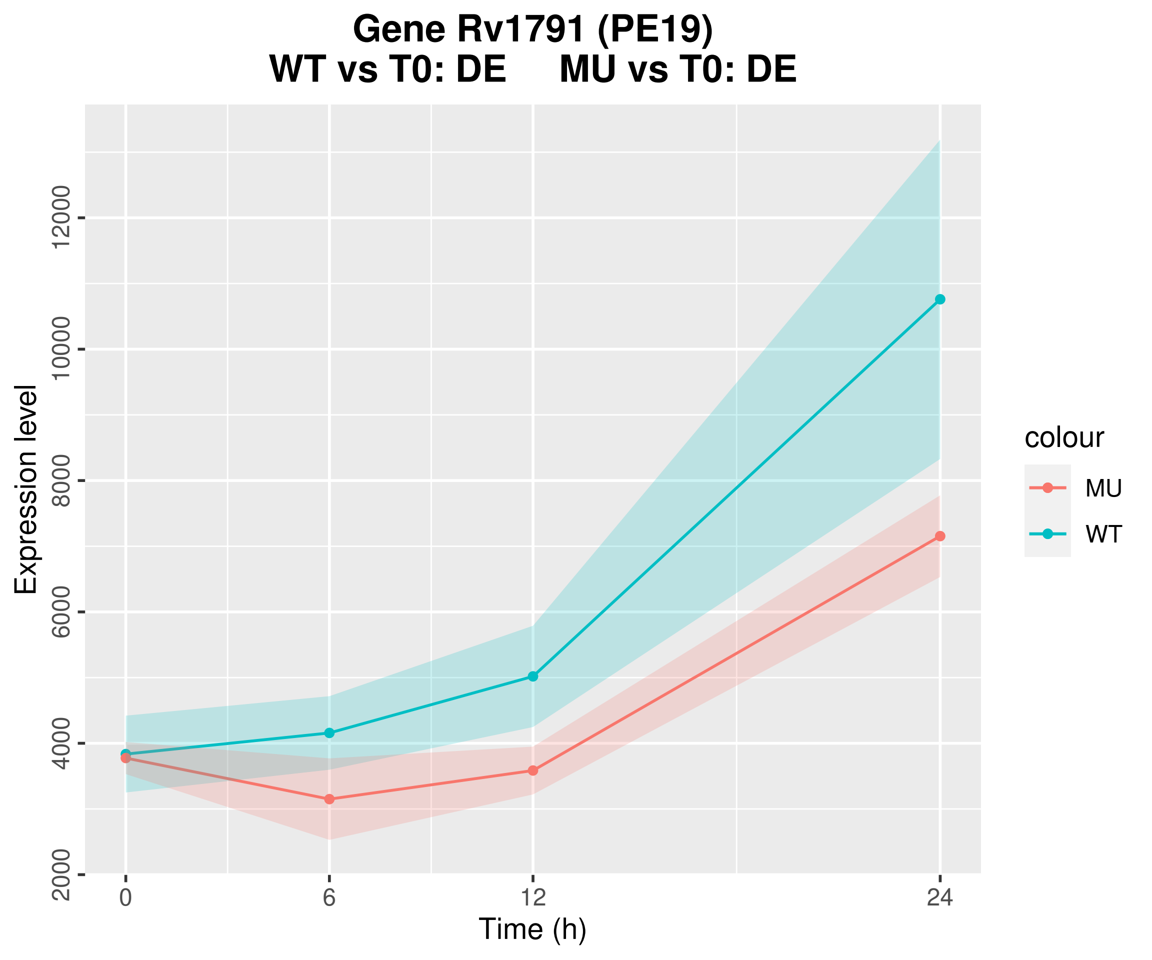

Gene Rv1791 (PE19)
WT vs T0: DE MU vs T0: DE
Expression level
2000
4000
6000
8000
10000
12000
0
6
12
24
Time (h)
colour
MU
WT

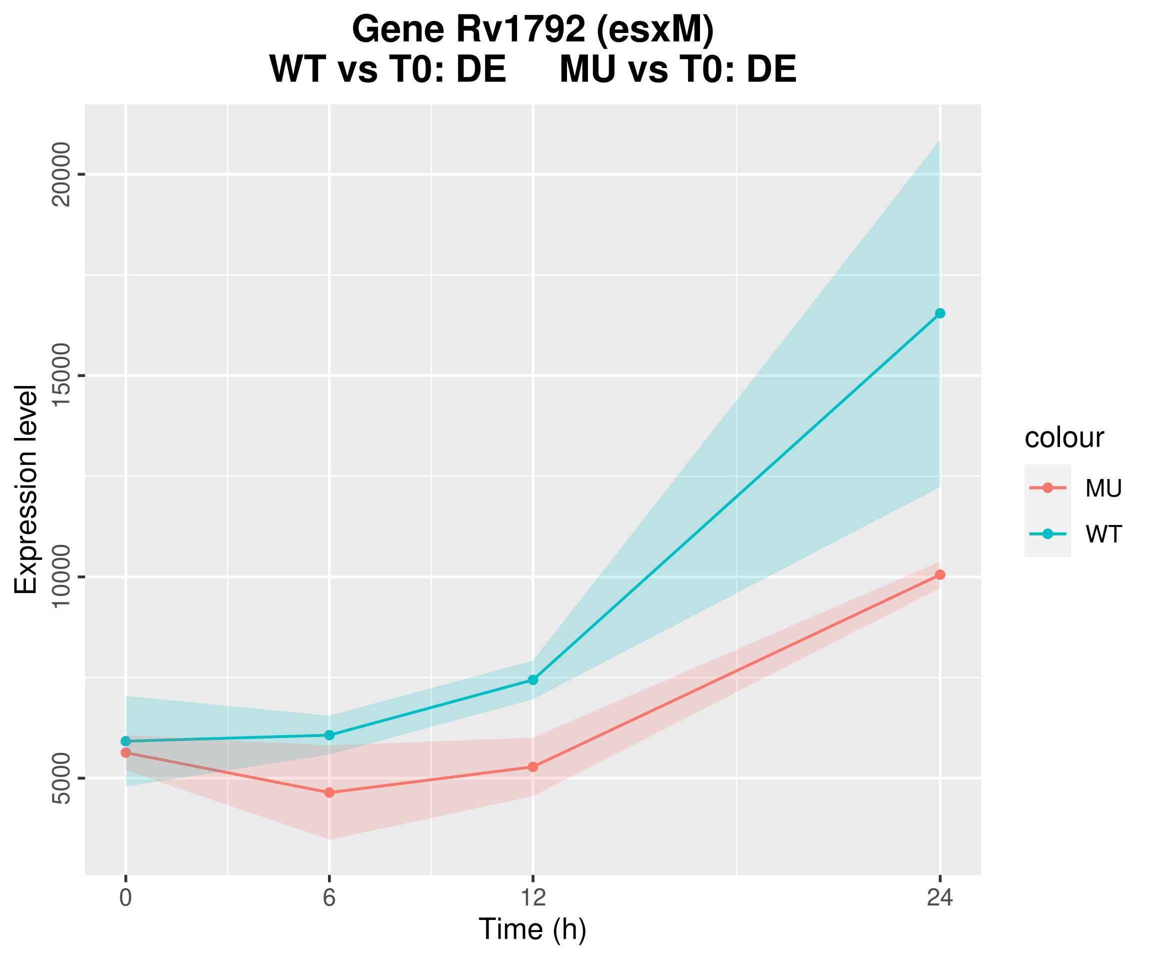

Gene Rv1792 (esxM)
WT vs T0: DE   MU vs T0: DE
Expression level
20000
15000
10000
5000
0
6
12
24
Time (h)
colour
MU
WT

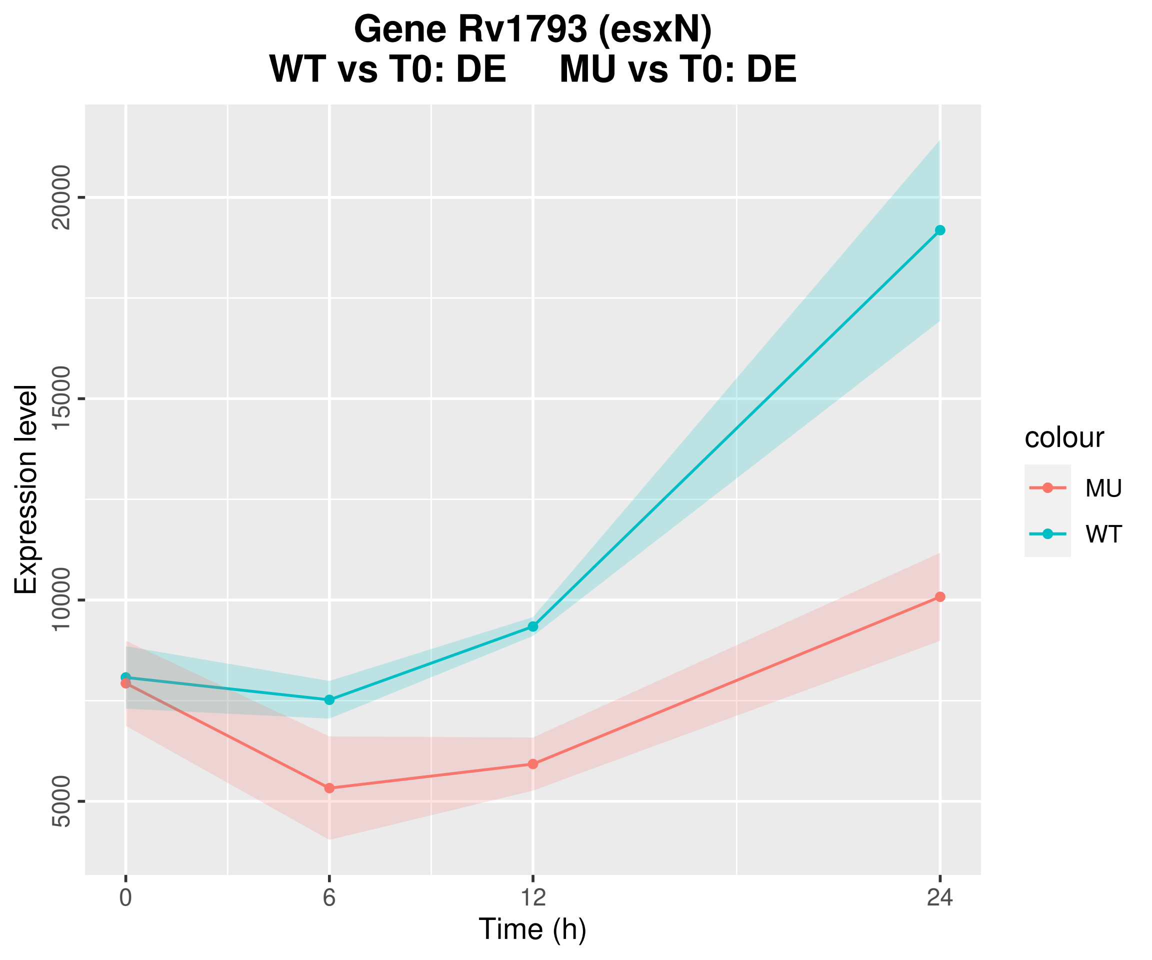

Gene Rv1793 (esxN)
WT vs T0: DE MU vs T0: DE
Expression level
20000
15000
10000
5000
0
6
12
24
Time (h)
colour
MU
WT

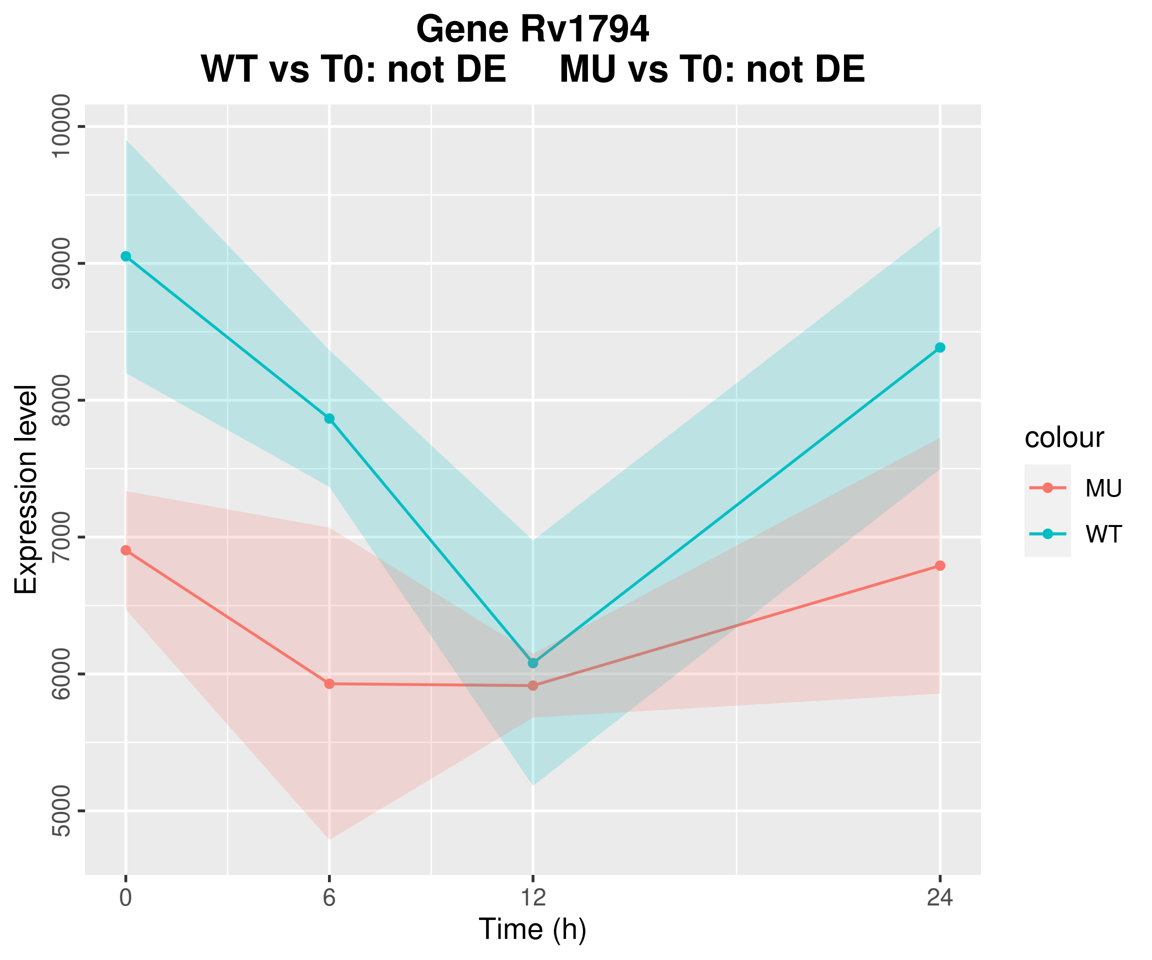

Gene Rv1794
WT vs T0: not DE MU vs T0: not DE
Expression level
10000
9000
8000
7000
6000
5000
0
6
12
24
Time (h)
colour
MU
WT

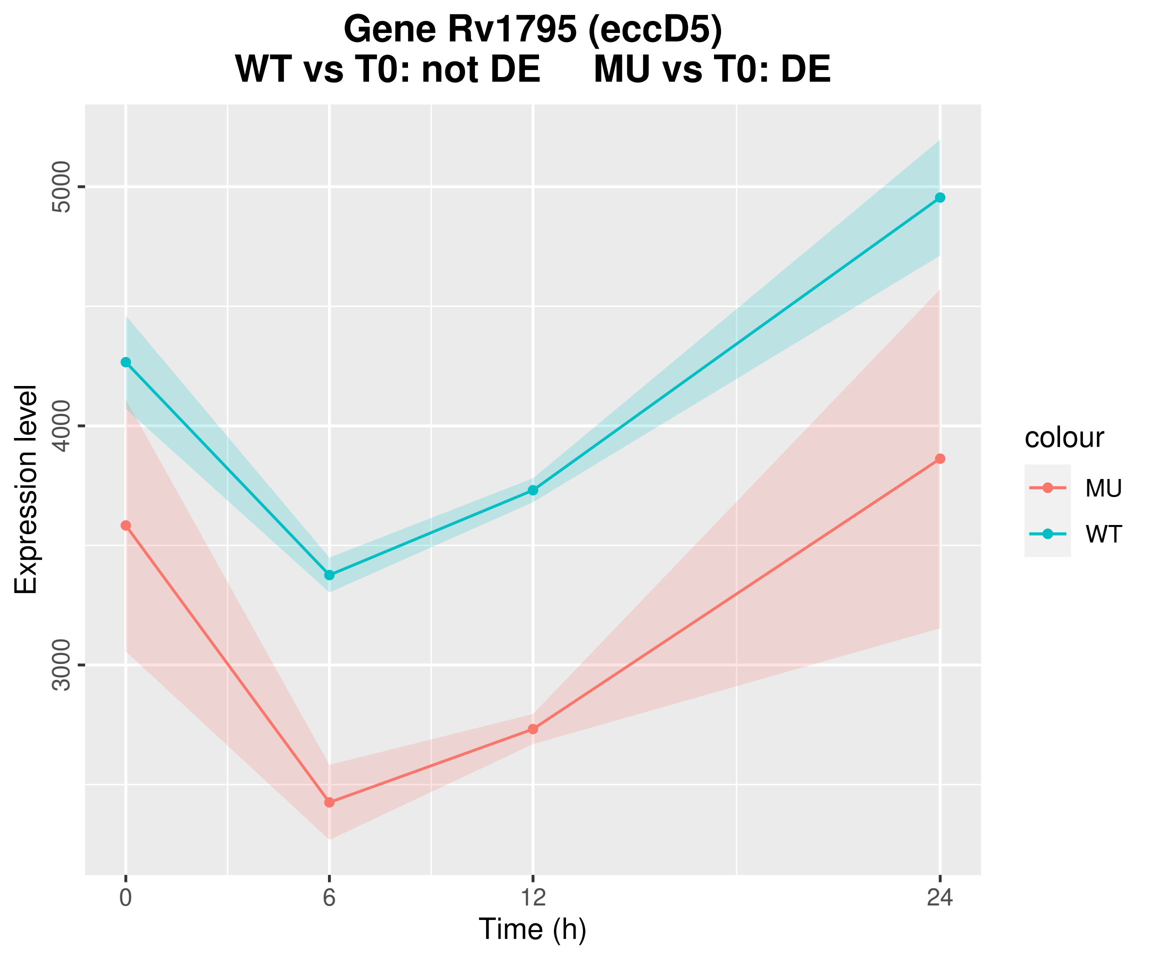
Gene Rv1795 (eccD5)
WT vs T0: not DE MU vs T0: DE
Expression level
5000
4000
3000
0
6
12
24
Time (h)
colour
MU
WT

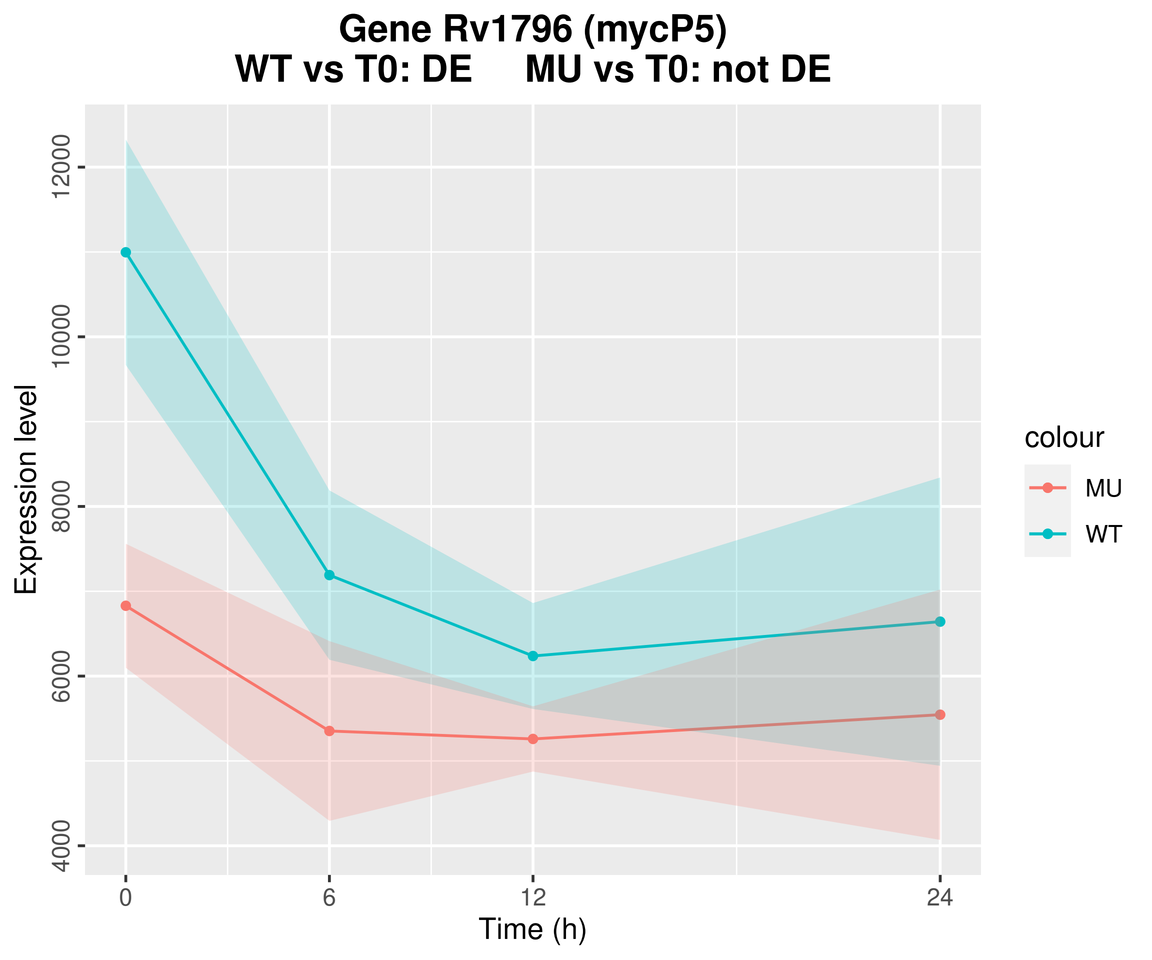
Gene Rv1796 (mycP5)
WT vs T0: DE MU vs T0: not DE
Expression level
12000
10000
8000
6000
4000
0
6
12
24
Time (h)
colour
MU
WT

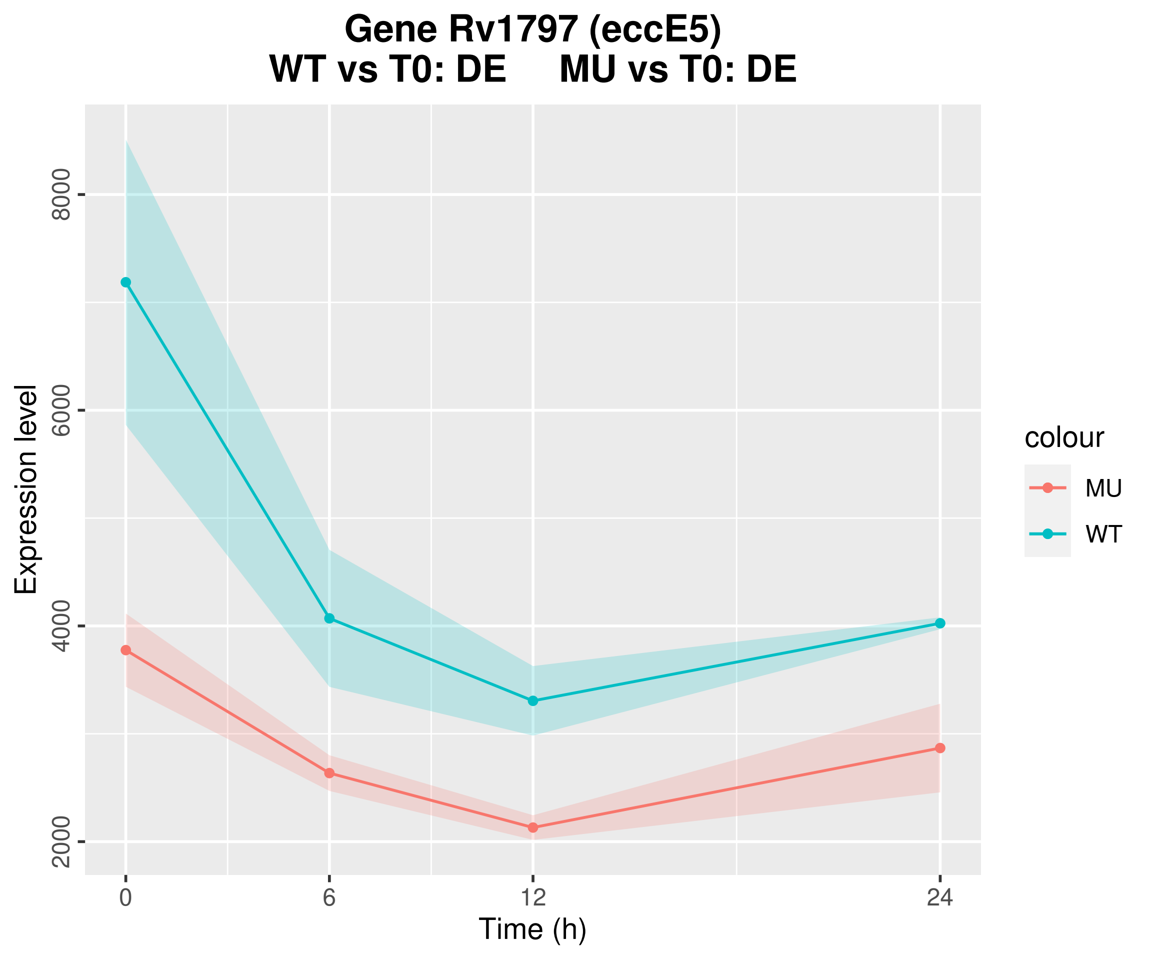

Gene Rv1797 (eccE5)
WT vs T0: DE MU vs T0: DE
Expression level
8000
6000
4000
2000
0
6
12
24
Time (h)
colour
MU
WT

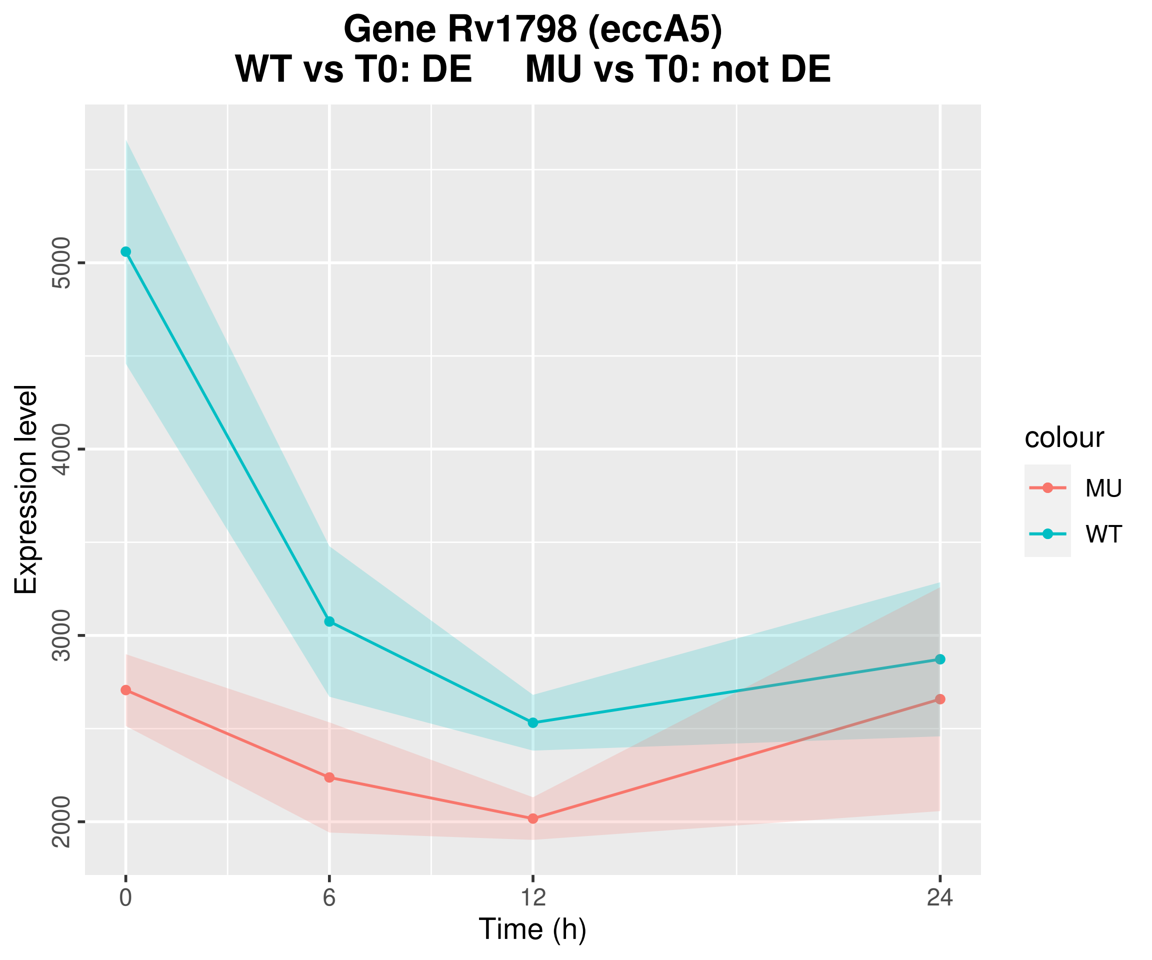
Gene Rv1798 (eccA5)
WT vs T0: DE   MU vs T0: not DE
Expression level
5000
4000
3000
2000
0
6
12
24
Time (h)
colour
MU
WT

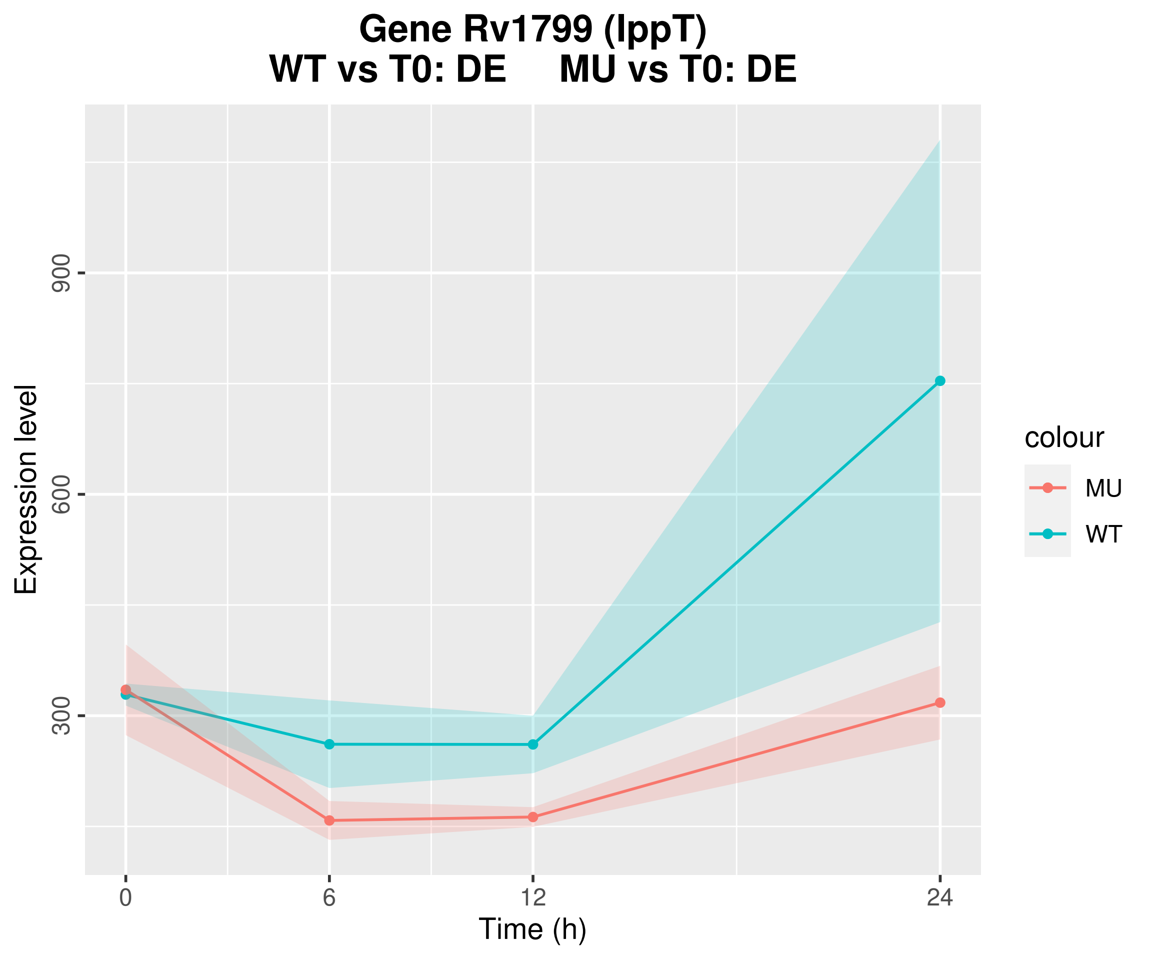

Gene Rv1799 (lppT)
WT vs T0: DE MU vs T0: DE
Expression level
900
600
300
0
6
12
24
Time (h)
colour
MU
WT

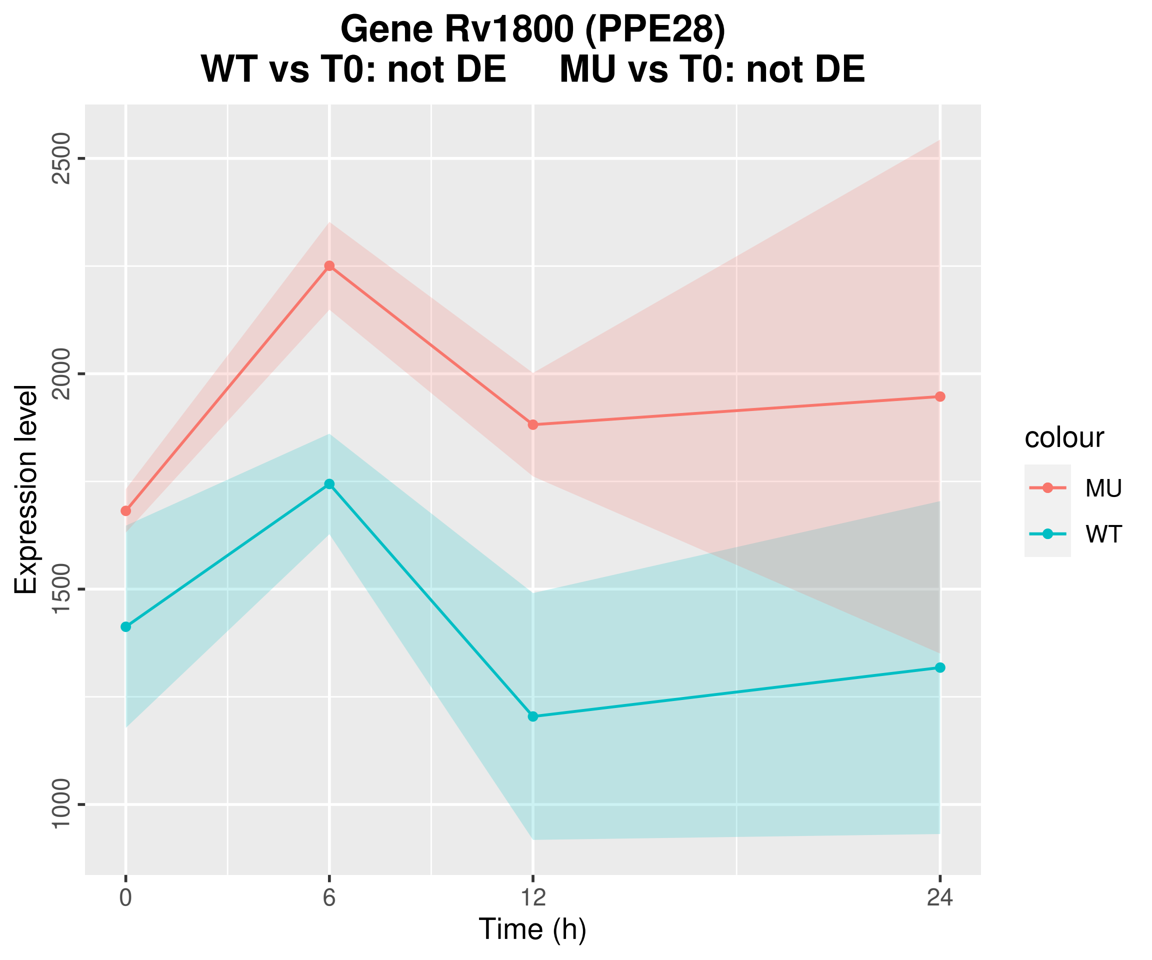

Gene Rv1800 (PPE28)
WT vs T0: not DE   MU vs T0: not DE
Expression level
2500
2000
1500
1000
0
6
12
24
Time (h)
colour
MU
WT

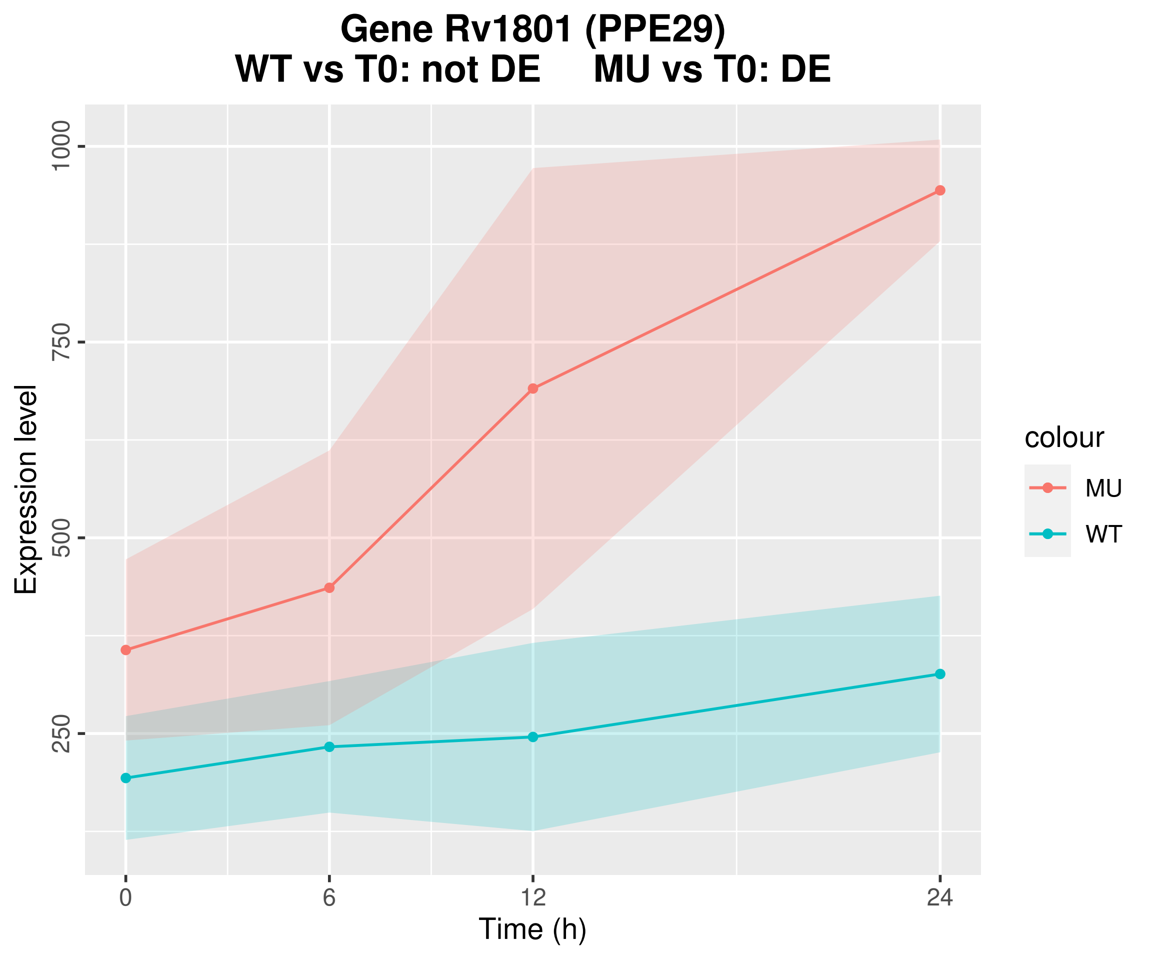
Gene Rv1801 (PPE29)
WT vs T0: not DE MU vs T0: DE
Expression level
1000
750
500
250
0
6
12
24
Time (h)
colour
MU
WT

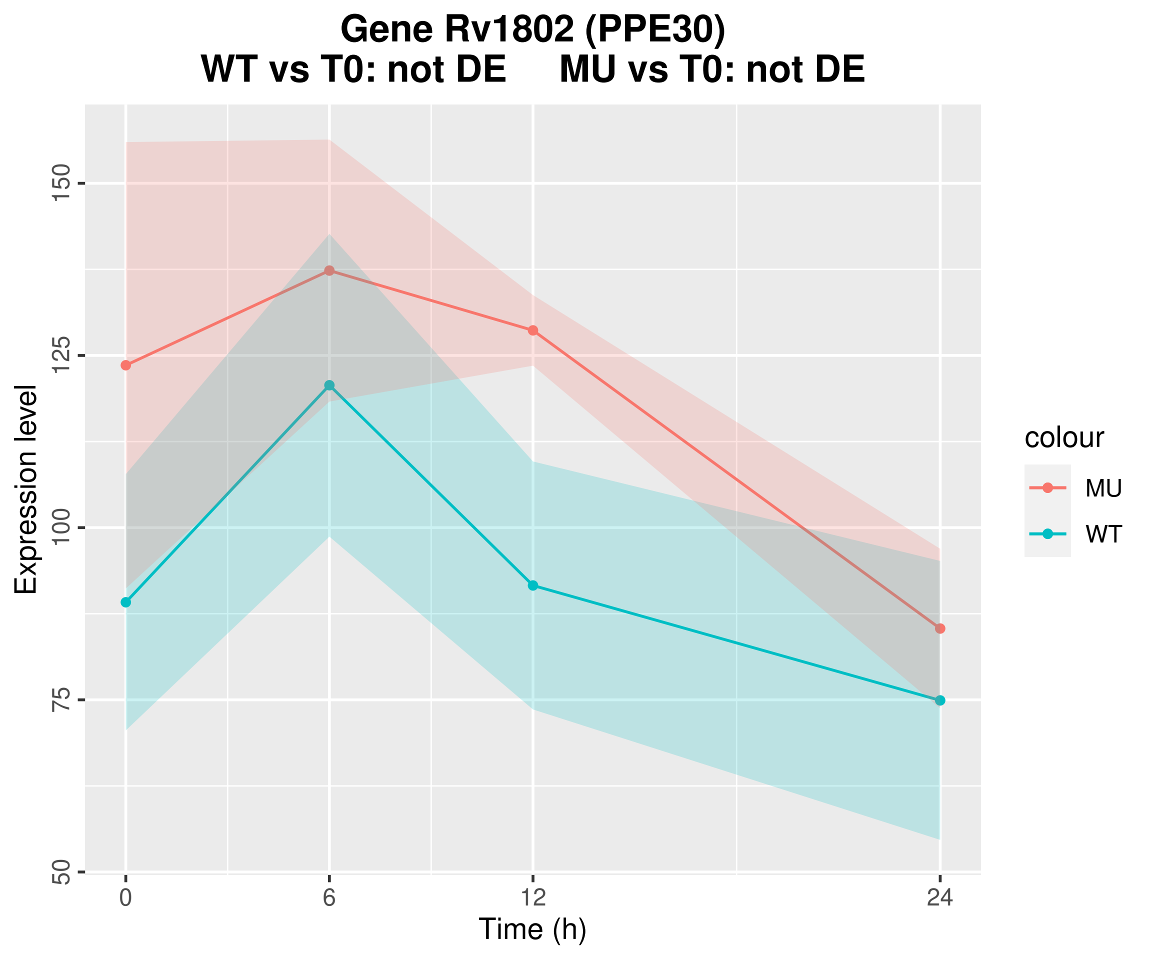

Gene Rv1802 (PPE30)
WT vs T0: not DE   MU vs T0: not DE
Expression level
150
125
100
75
50
0
6
12
24
Time (h)
colour
MU
WT

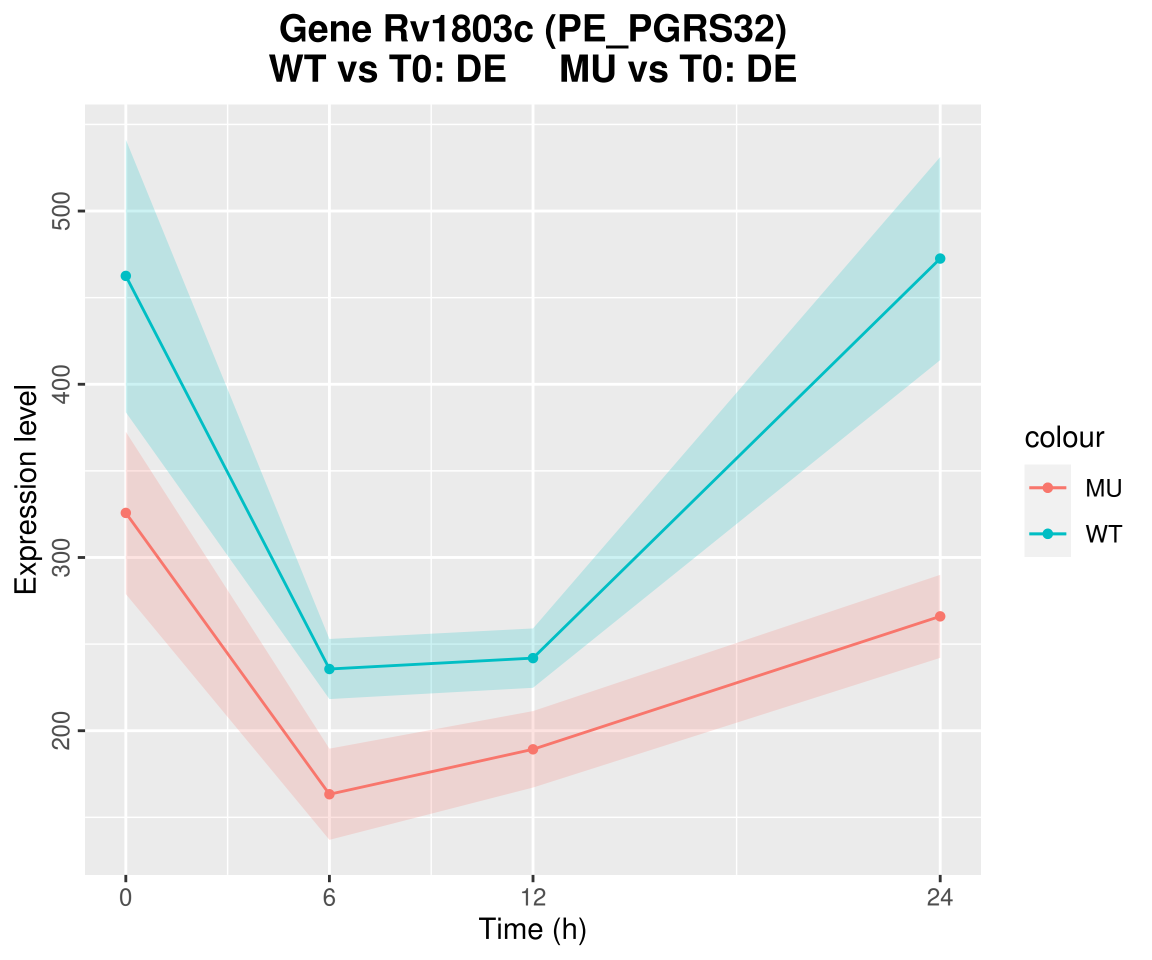

Gene Rv1803c (PE_PGRS32)
WT vs T0: DE MU vs T0: DE
Expression level
500
400
300
200
0
6
12
24
Time (h)
colour
MU
WT

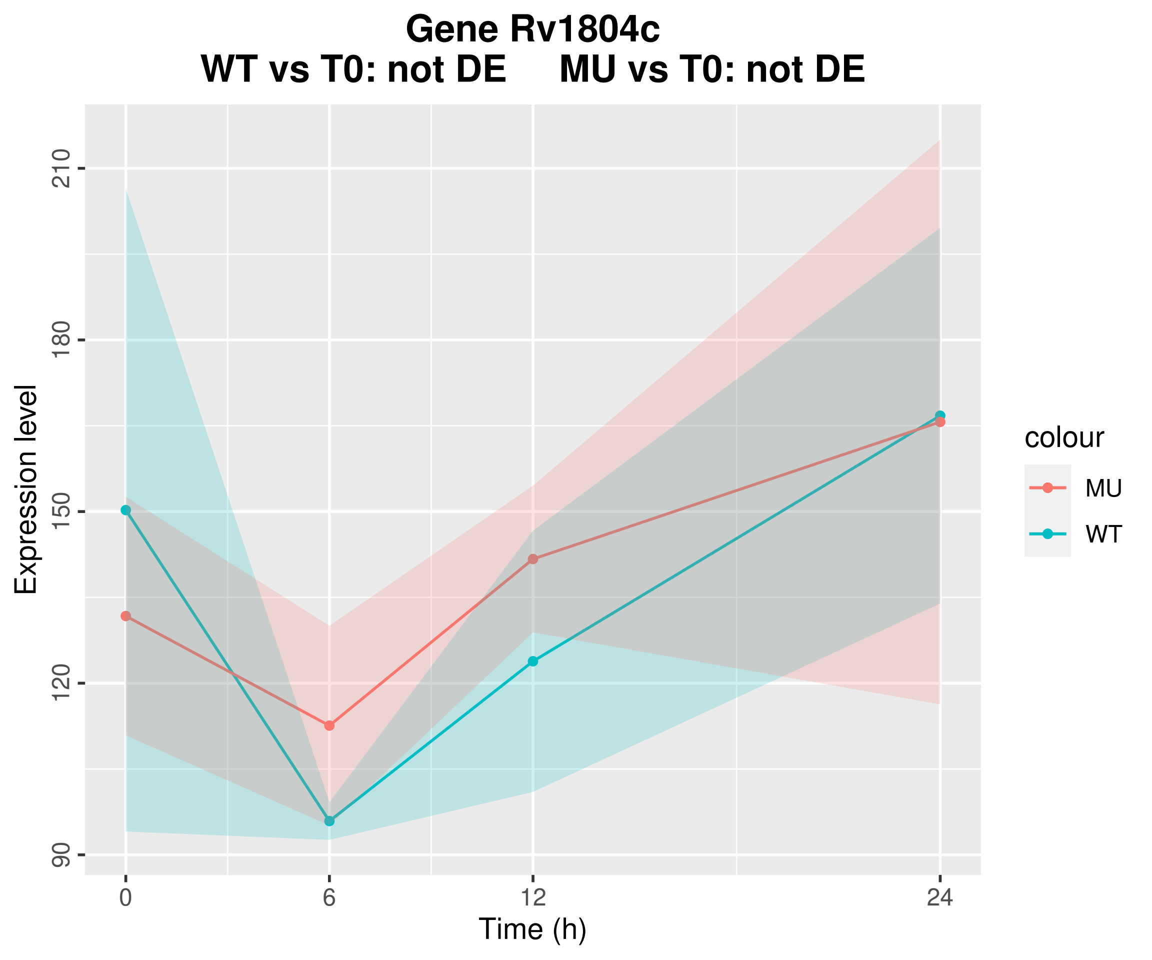

Gene Rv1804c
WT vs T0: not DE   MU vs T0: not DE
Expression level
210
180
150
120
90
0
6
12
24
Time (h)
colour
MU
WT

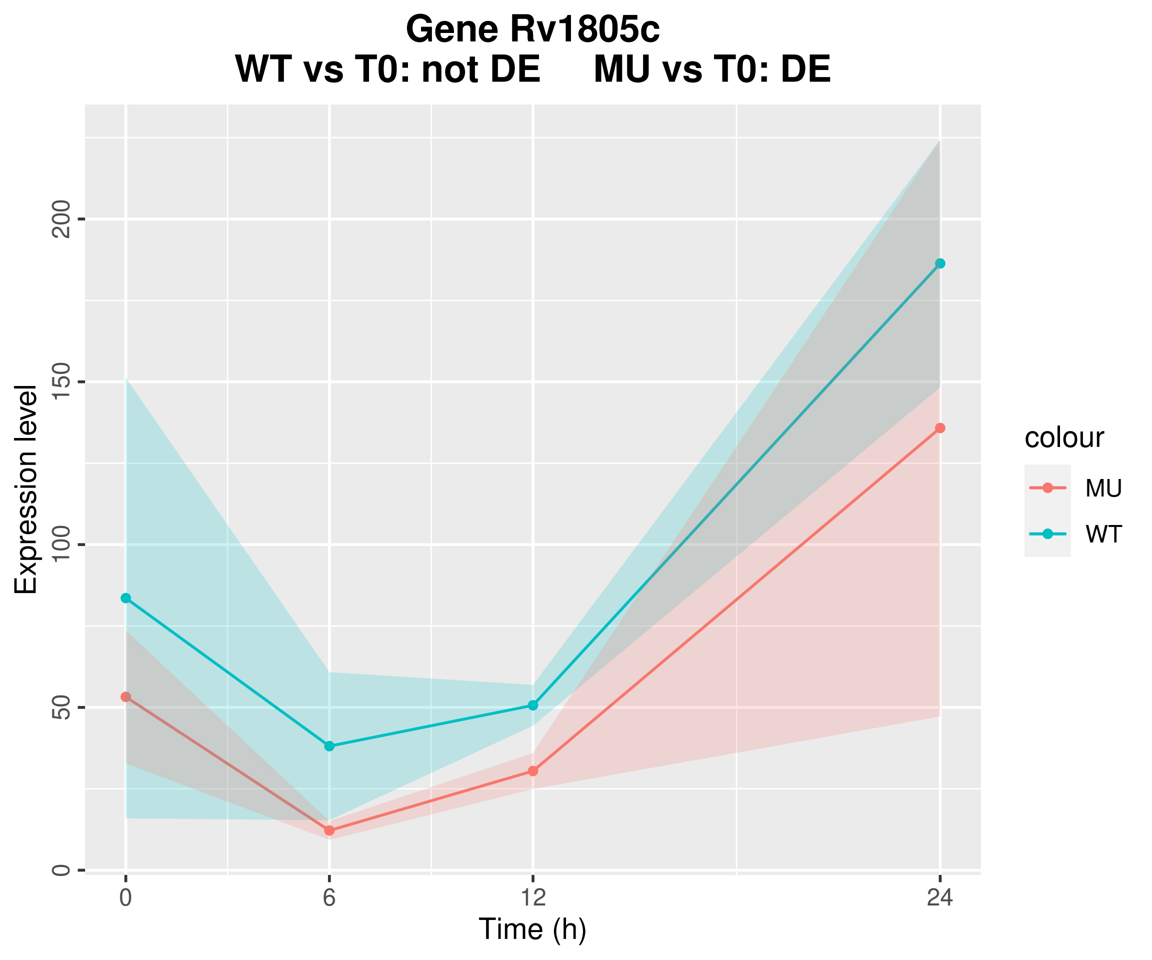
Gene Rv1805c
WT vs T0: not DE MU vs T0: DE
Expression level
0
50
100
150
200
0
6
12
24
Time (h)
colour
MU
WT

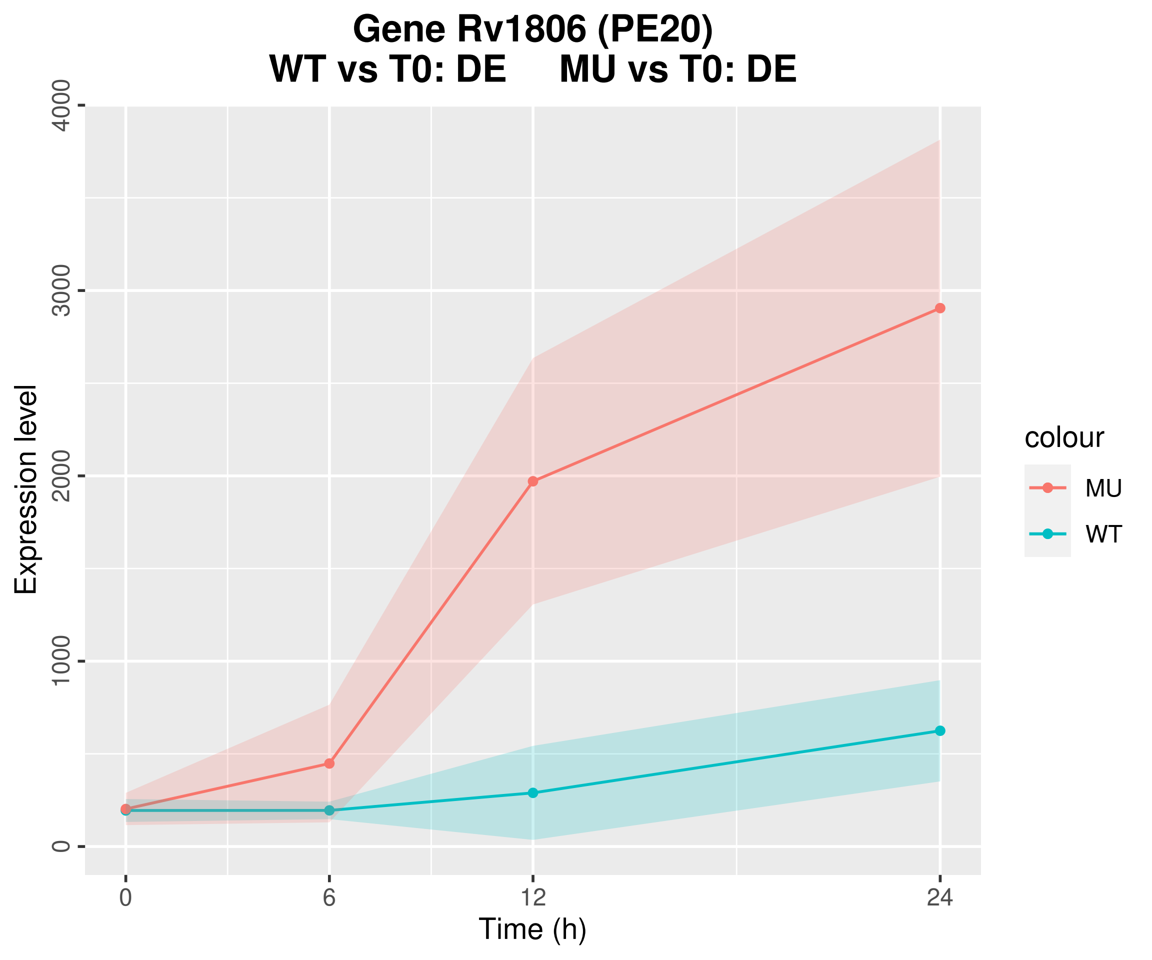

Gene Rv1806 (PE20)
WT vs T0: DE   MU vs T0: DE
Expression level
Time (h)
0
1000
2000
3000
4000
0
6
12
24
colour
MU
WT

Gene Rv1807 (PPE31)
WT vs T0: not DE   MU vs T0: DE

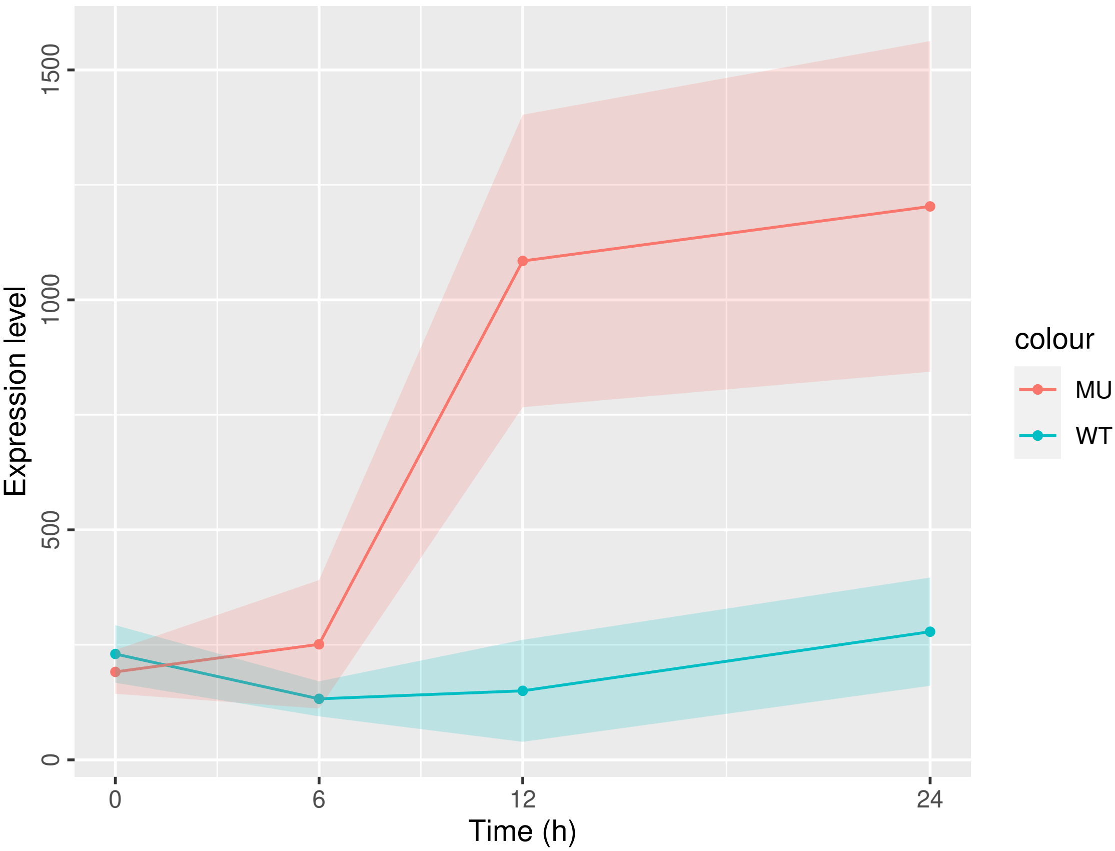

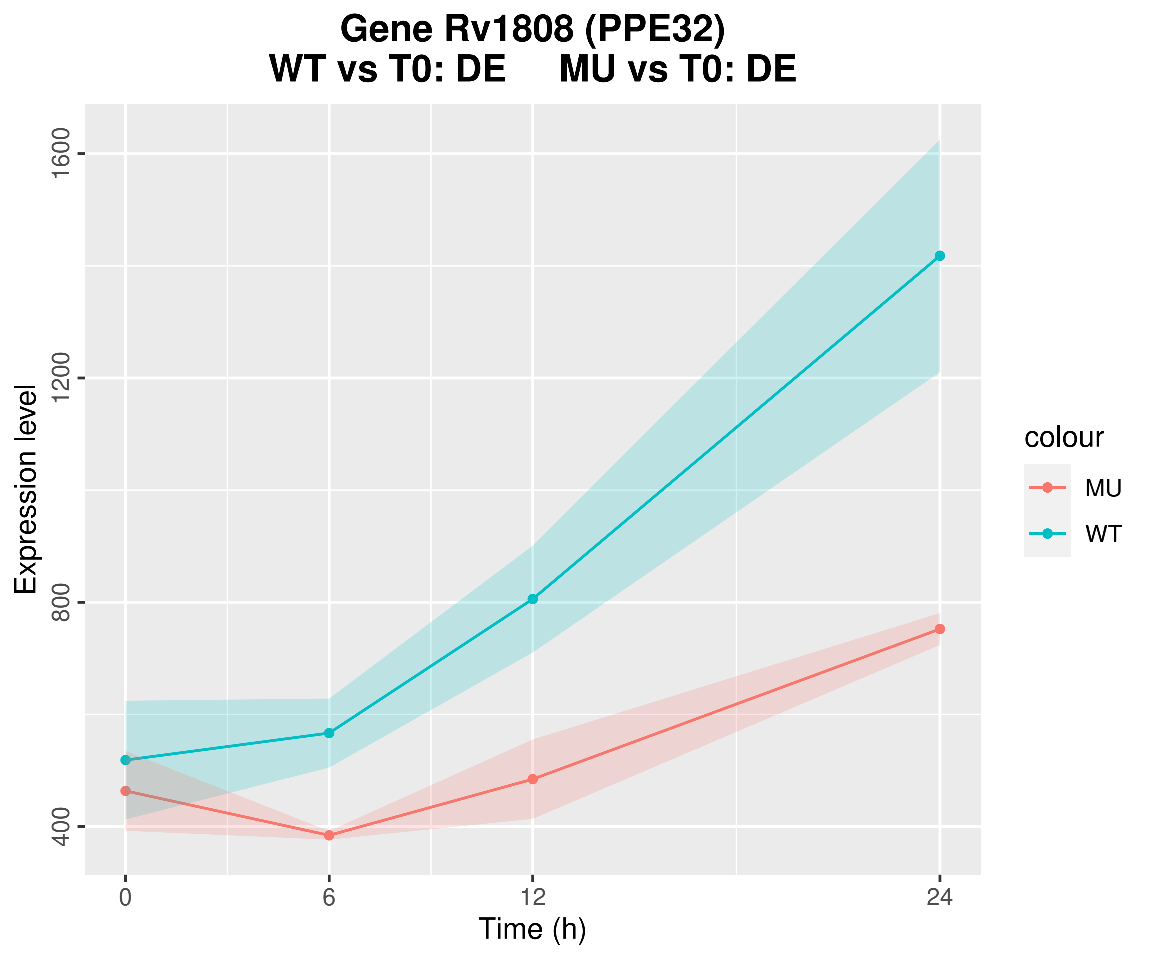

Gene Rv1808 (PPE32)
WT vs T0: DE MU vs T0: DE
Expression level
1600
1200
800
400
0
6
12
24
Time (h)
colour
MU
WT

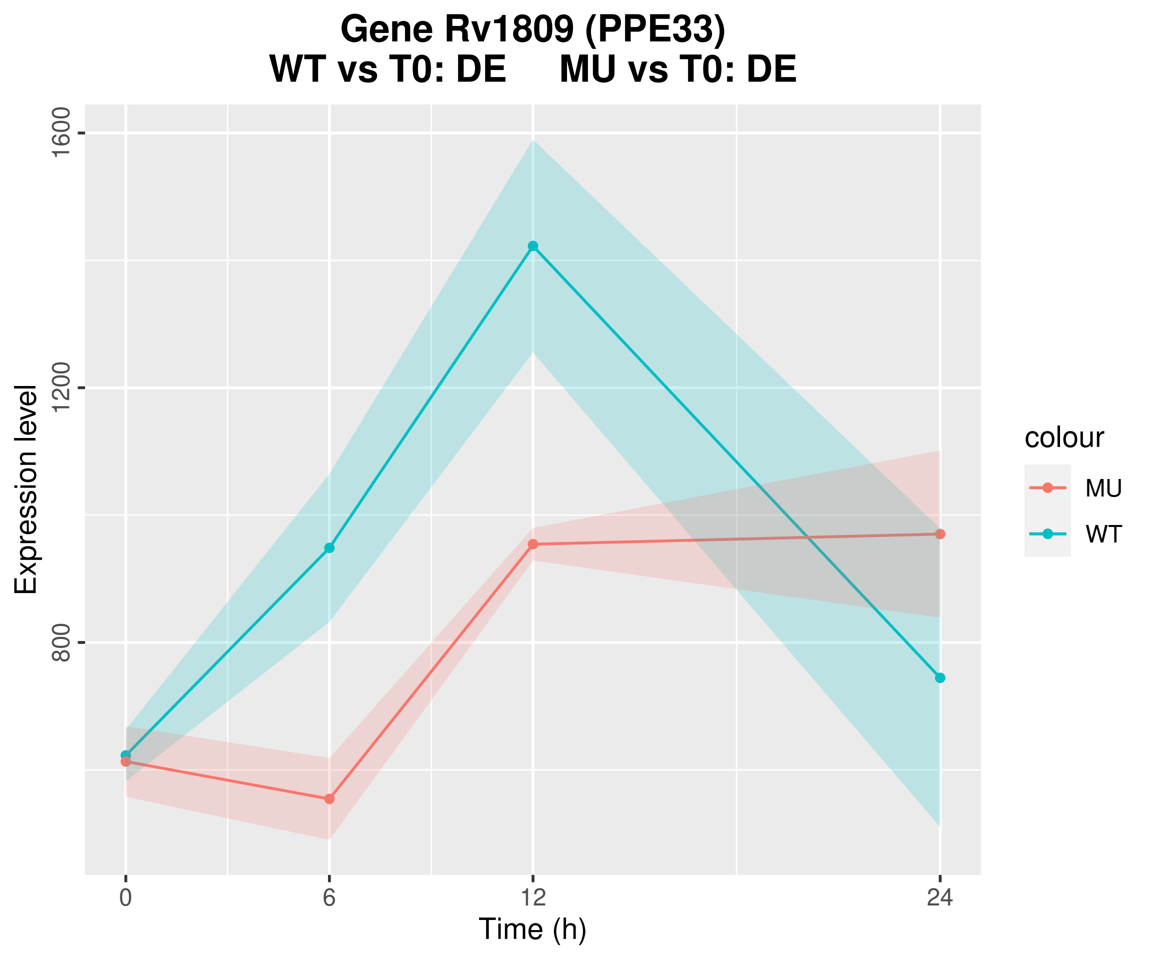
Gene Rv1809 (PPE33)
WT vs T0: DE   MU vs T0: DE
Expression level
1600
1200
800
0
6
12
24
Time (h)
colour
MU
WT

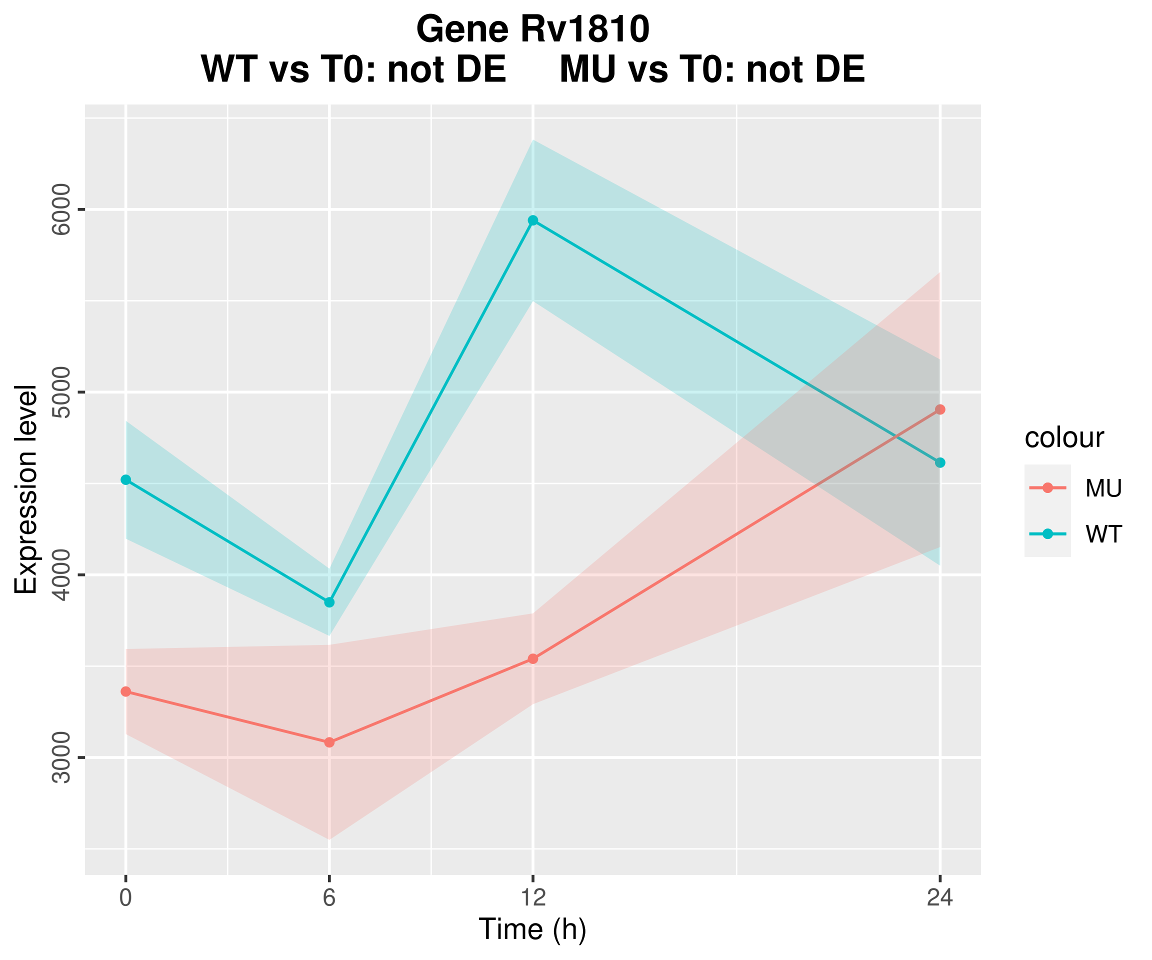
Gene Rv1810
WT vs T0: not DE MU vs T0: not DE
Expression level
6000
5000
4000
3000
0
6
12
24
Time (h)
colour
MU
WT

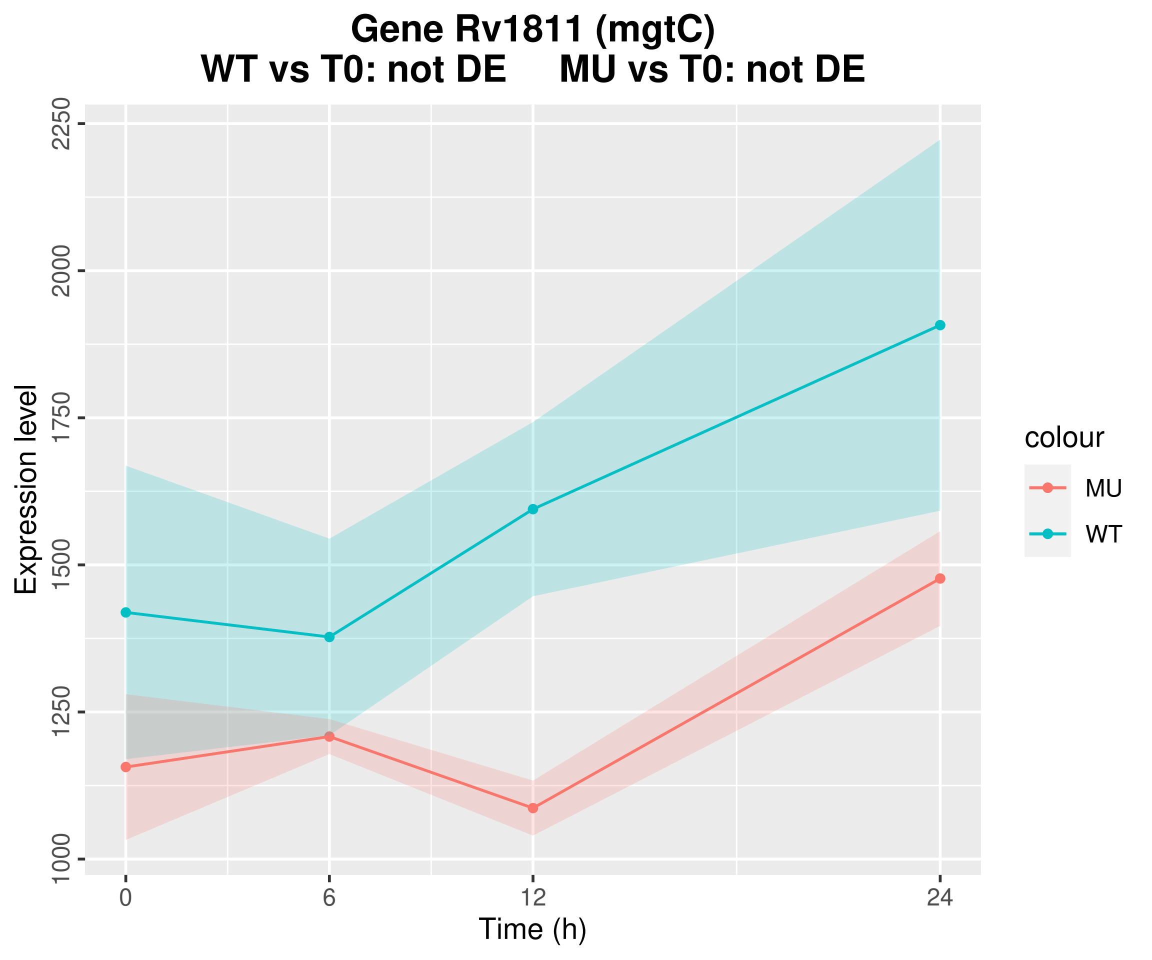
Gene Rv1811 (mgtC)
WT vs T0: not DE MU vs T0: not DE
Expression level
1000
1250
1500
1750
2000
2250
0
6
12
24
Time (h)
colour
MU
WT

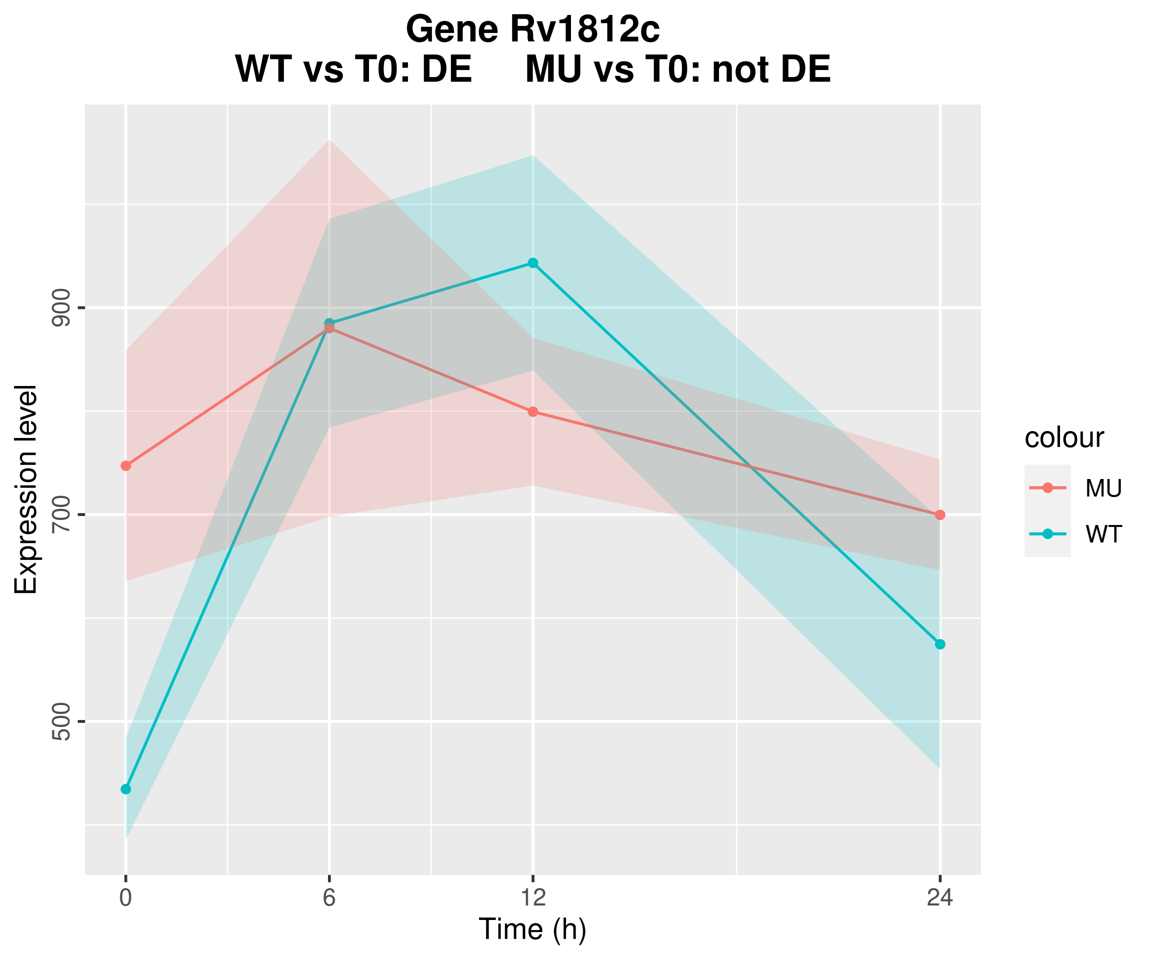

Gene Rv1812c
WT vs T0: DE   MU vs T0: not DE
Expression level
900
700
500
0
6
12
24
Time (h)
colour
MU
WT

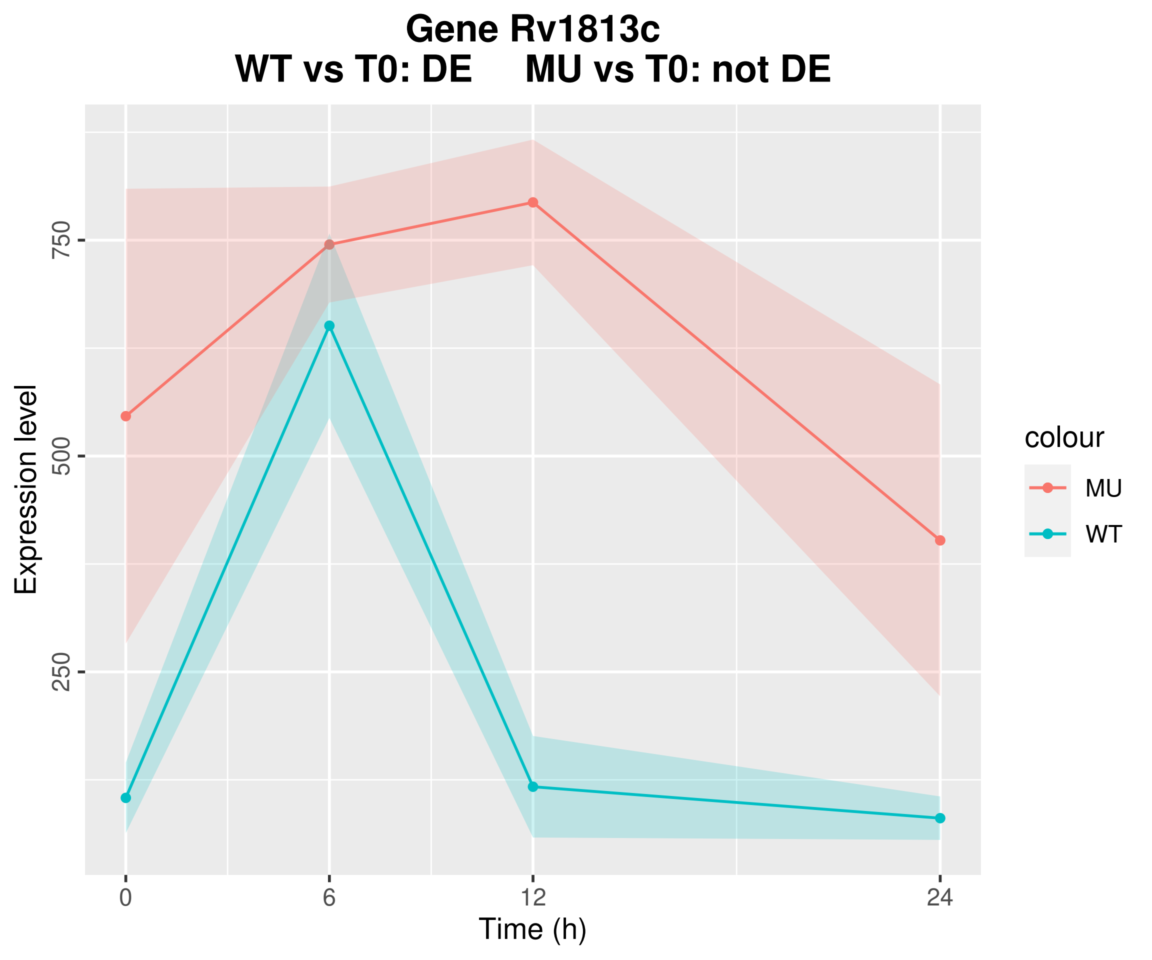

Gene Rv1813c
WT vs T0: DE   MU vs T0: not DE
Expression level
750
500
250
0
6
12
24
Time (h)
colour
MU
WT

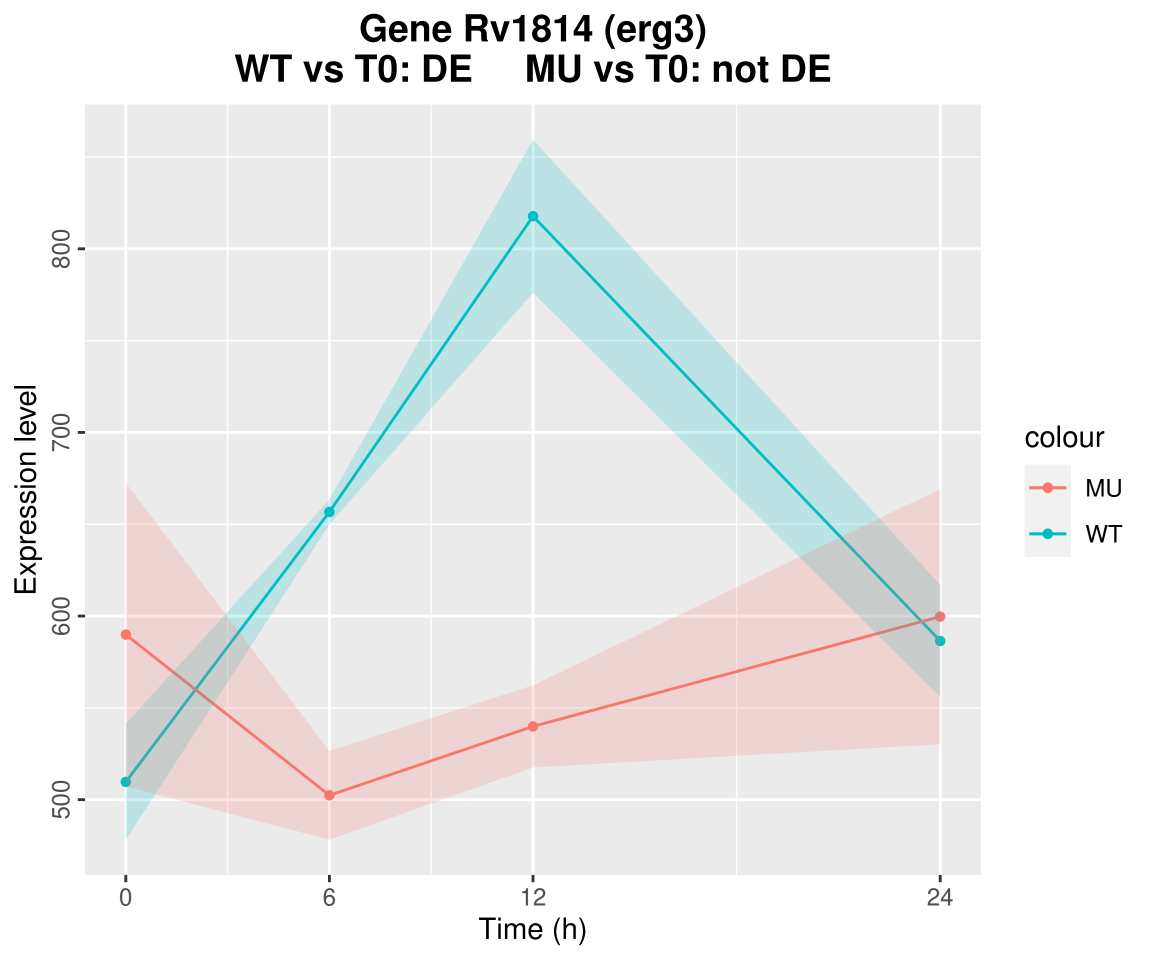
Gene Rv1814 (erg3)
WT vs T0: DE   MU vs T0: not DE
Expression level
800
700
600
500
0
6
12
24
Time (h)
colour
MU
WT

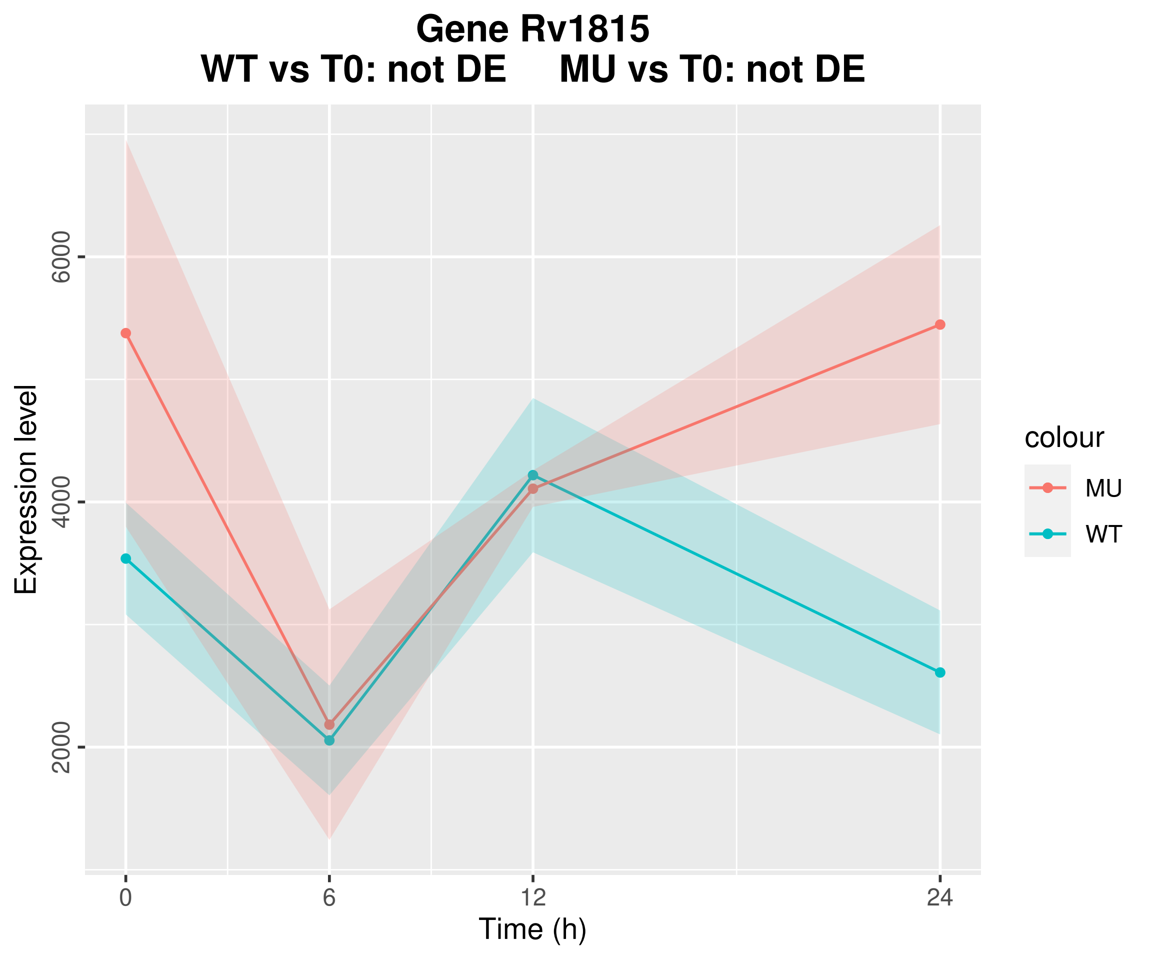

Gene Rv1815
WT vs T0: not DE   MU vs T0: not DE
Expression level
6000
4000
2000
0
6
12
24
Time (h)
colour
MU
WT

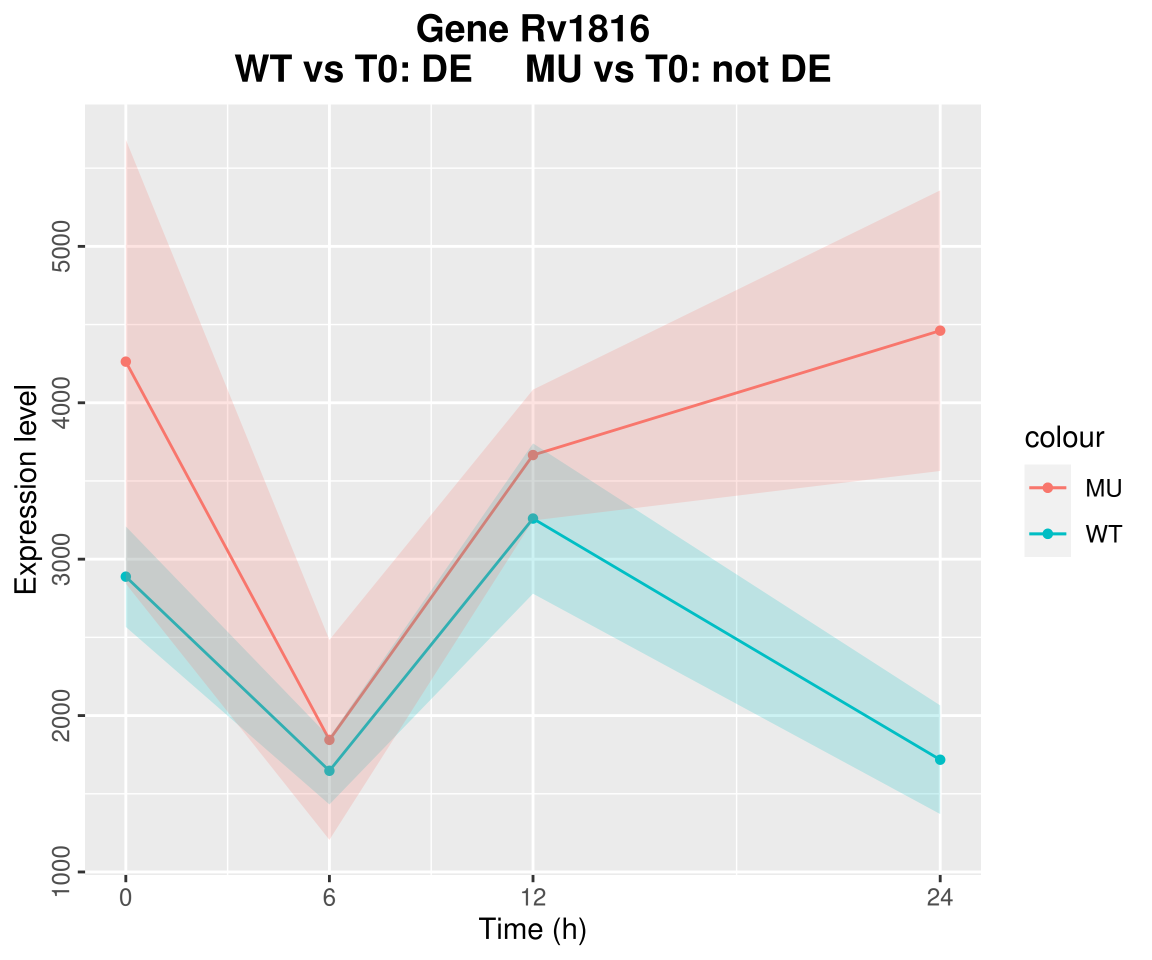

Gene Rv1816
WT vs T0: DE MU vs T0: not DE
Expression level
5000
4000
3000
2000
1000
0
6
12
24
Time (h)
colour
MU
WT

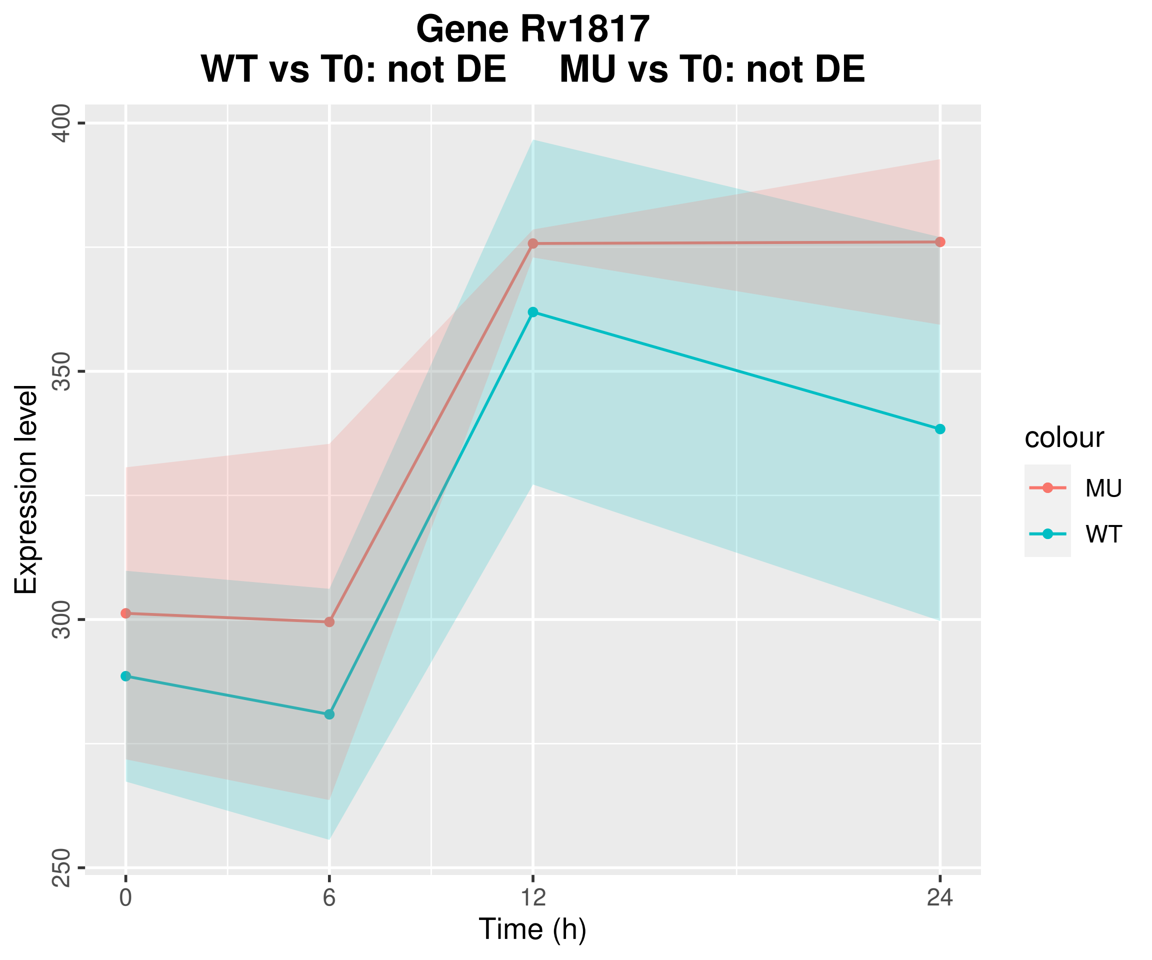
Gene Rv1817
WT vs T0: not DE   MU vs T0: not DE
Expression level
400
350
300
250
0
6
12
24
Time (h)
colour
MU
WT

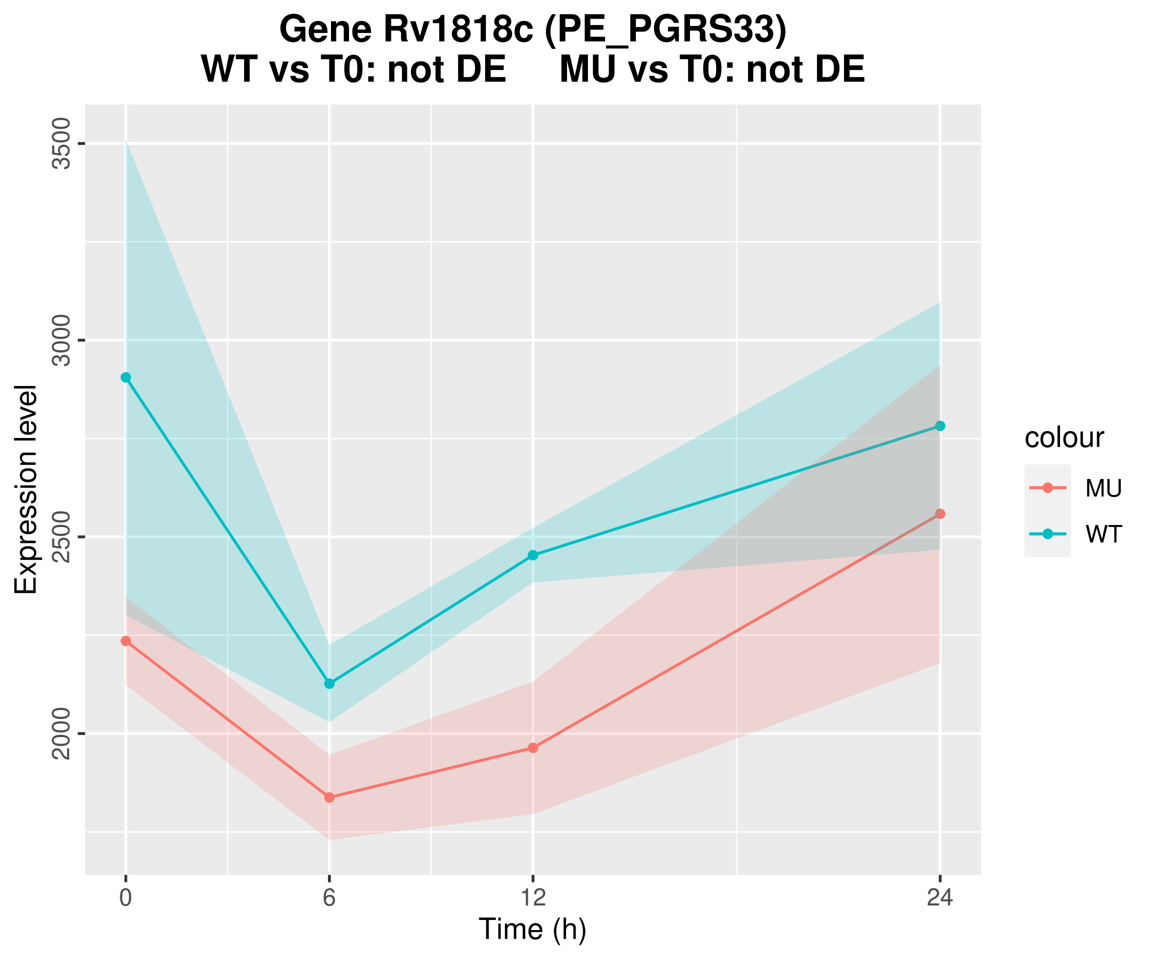

Gene Rv1818c (PE_PGRS33)
WT vs T0: not DE   MU vs T0: not DE
Expression level
3500
3000
2500
2000
0
6
12
24
Time (h)
colour
MU
WT

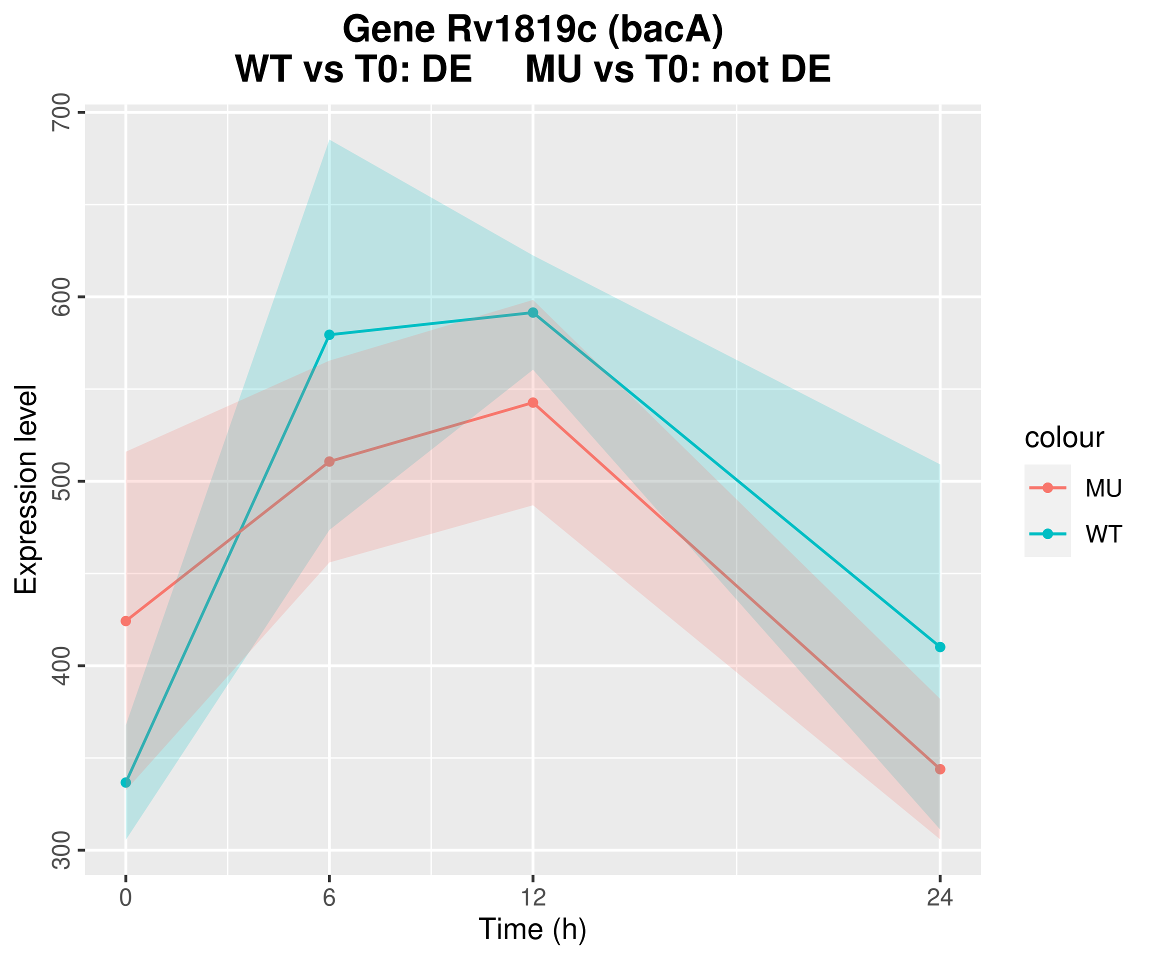
Gene Rv1819c (bacA)
WT vs T0: DE MU vs T0: not DE
700
600
500
400
300
Expression level
0
6
12
24
Time (h)
colour
MU
WT

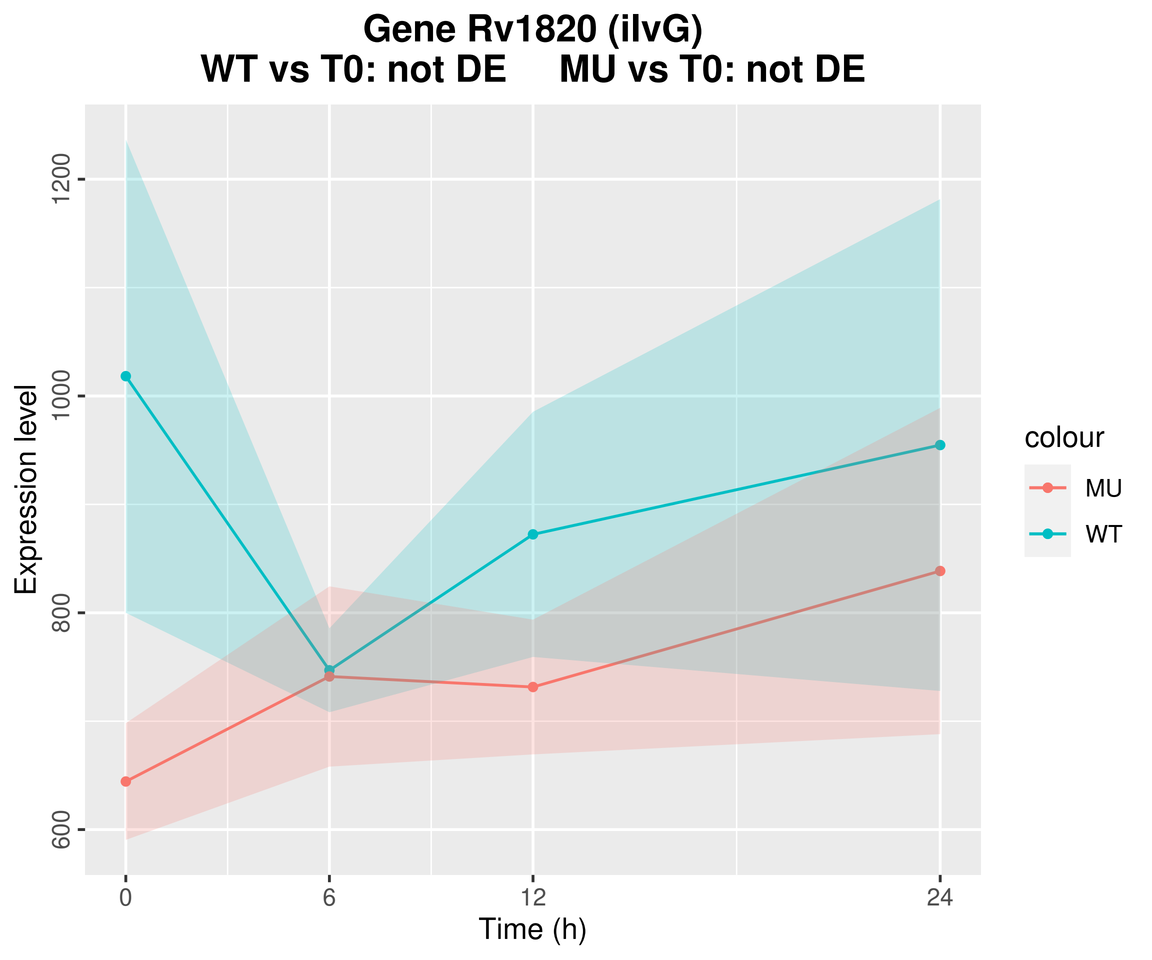
Gene Rv1820 (ilvG)
WT vs T0: not DE MU vs T0: not DE
Expression level
1200
1000
800
600
0
6
12
24
Time (h)
colour
MU
WT

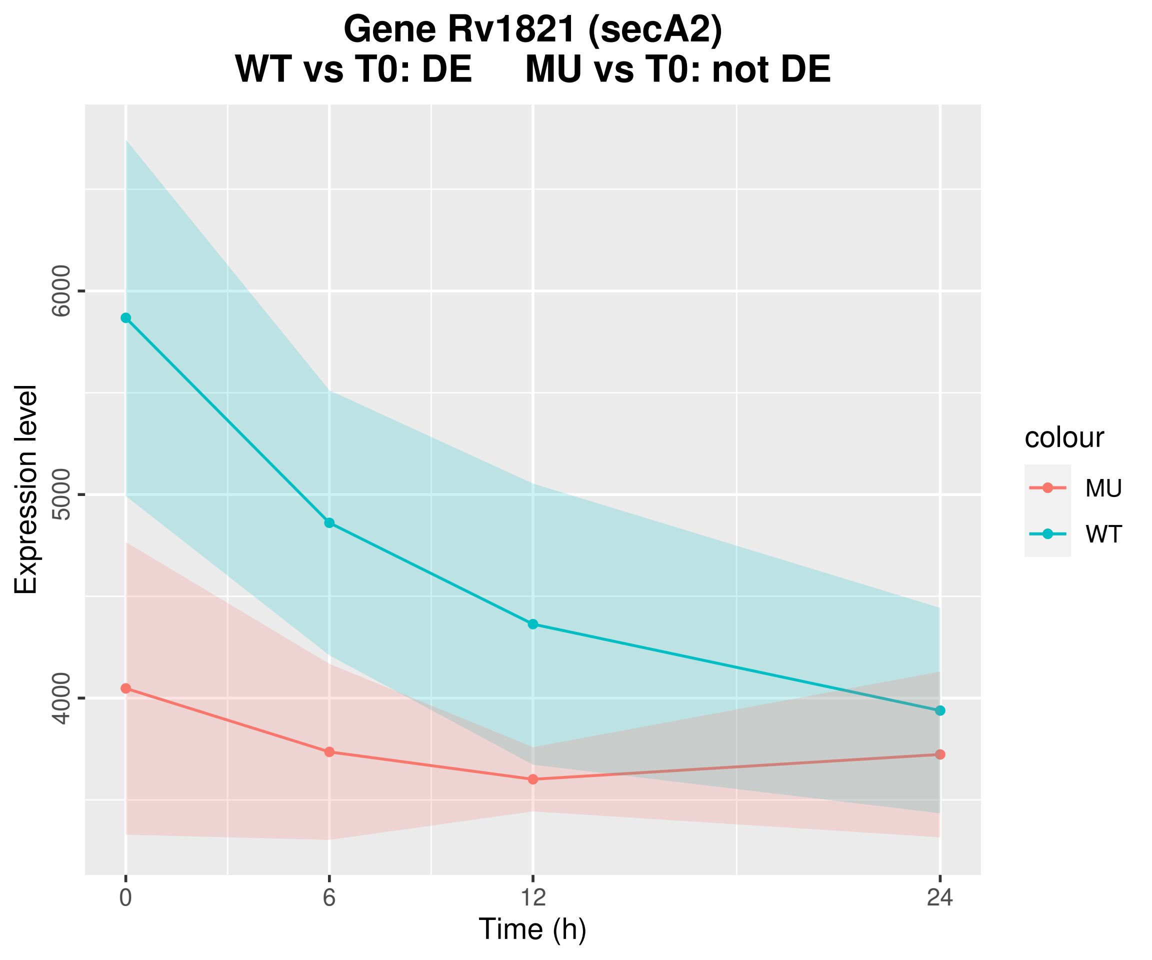
Gene Rv1821 (secA2)
WT vs T0: DE   MU vs T0: not DE
Expression level
6000
5000
4000
0
6
12
24
Time (h)
colour
MU
WT

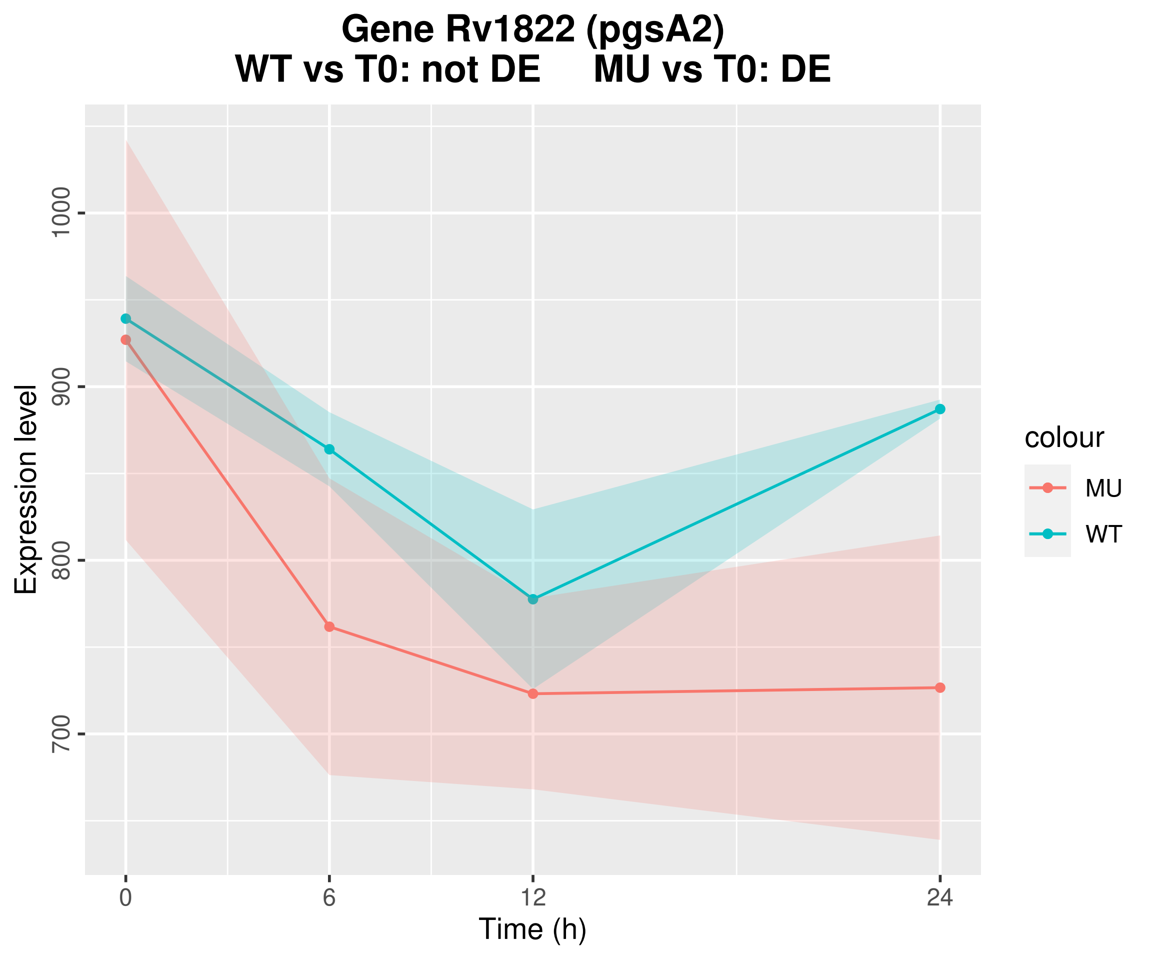
Gene Rv1822 (pgsA2)
WT vs T0: not DE MU vs T0: DE
Expression level
1000
900
800
700
0
6
12
24
Time (h)
colour
MU
WT

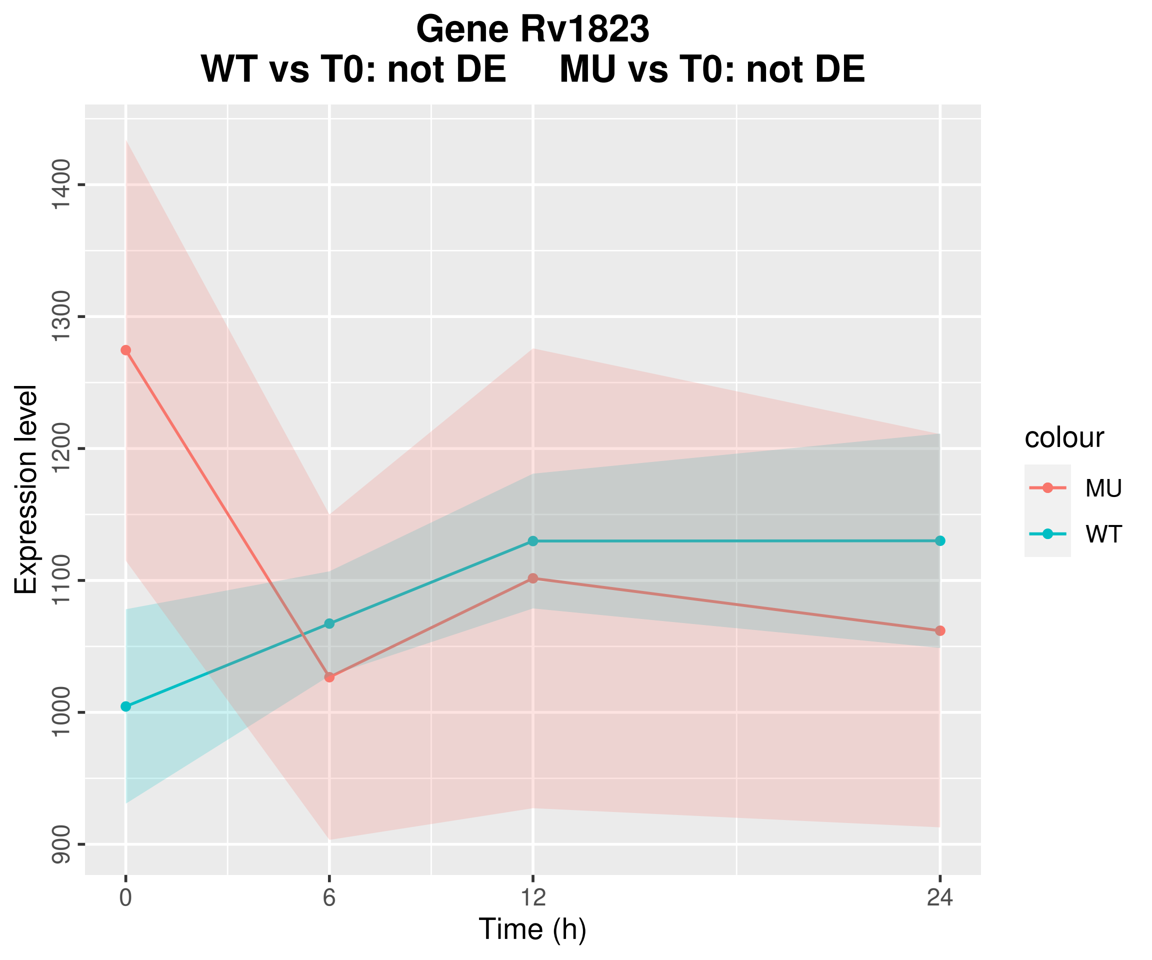
Gene Rv1823
WT vs T0: not DE   MU vs T0: not DE
Expression level
Time (h)
1400
1300
1200
1100
1000
900
0
6
12
24
colour
MU
WT

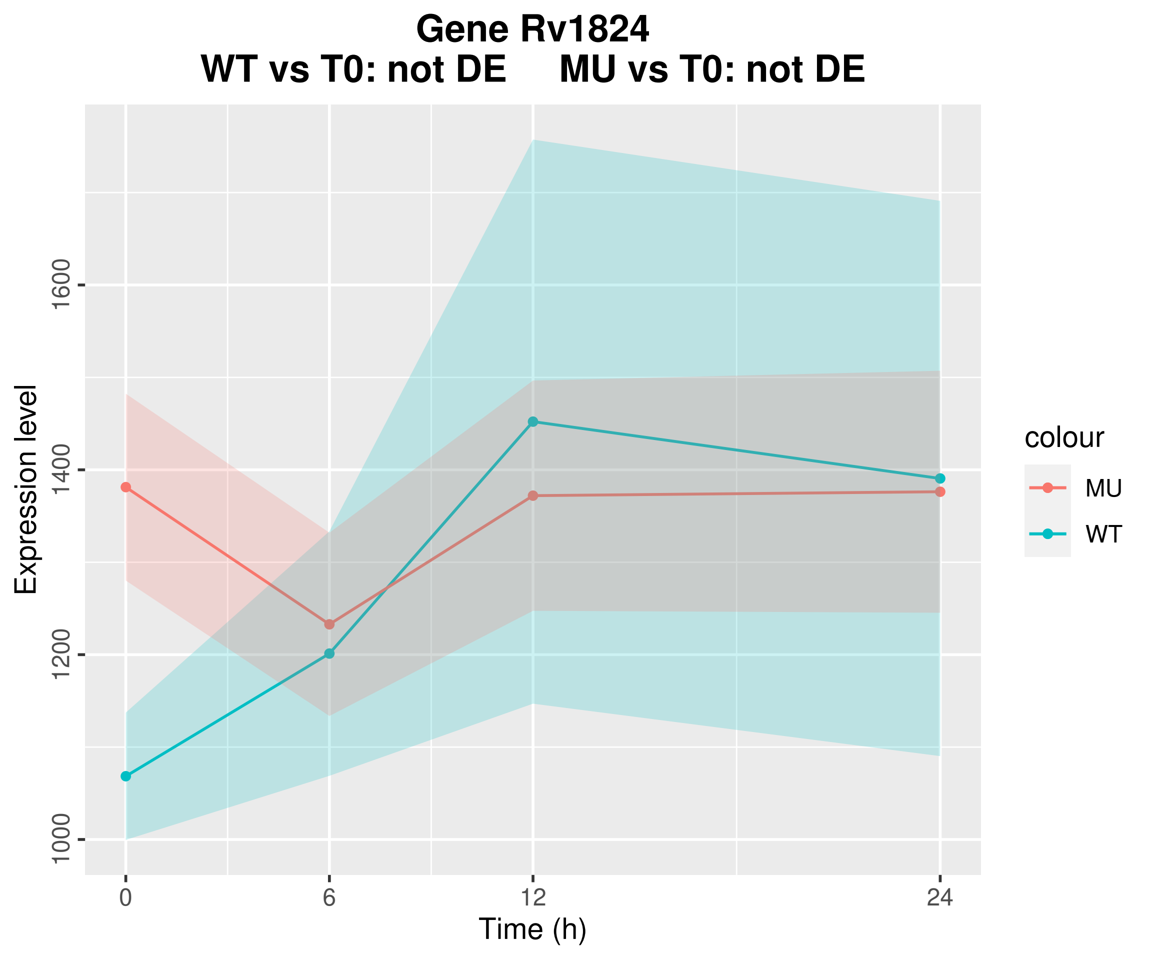
Gene Rv1824
WT vs T0: not DE MU vs T0: not DE
Expression level
1000
1200
1400
1600
0
6
12
24
Time (h)
colour
MU
WT

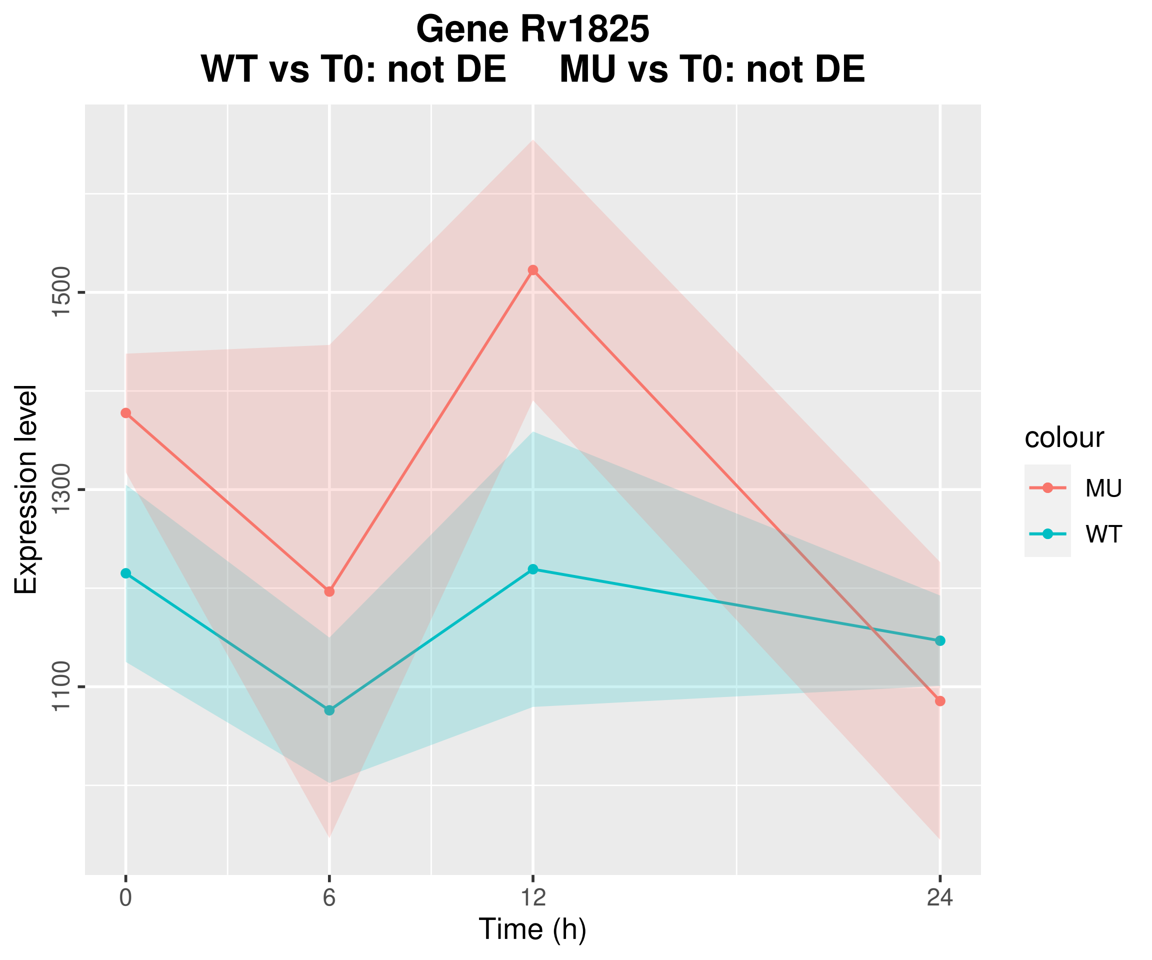
Gene Rv1825
WT vs T0: not DE MU vs T0: not DE
Expression level
1100
1300
1500
0
6
12
24
Time (h)
colour
MU
WT

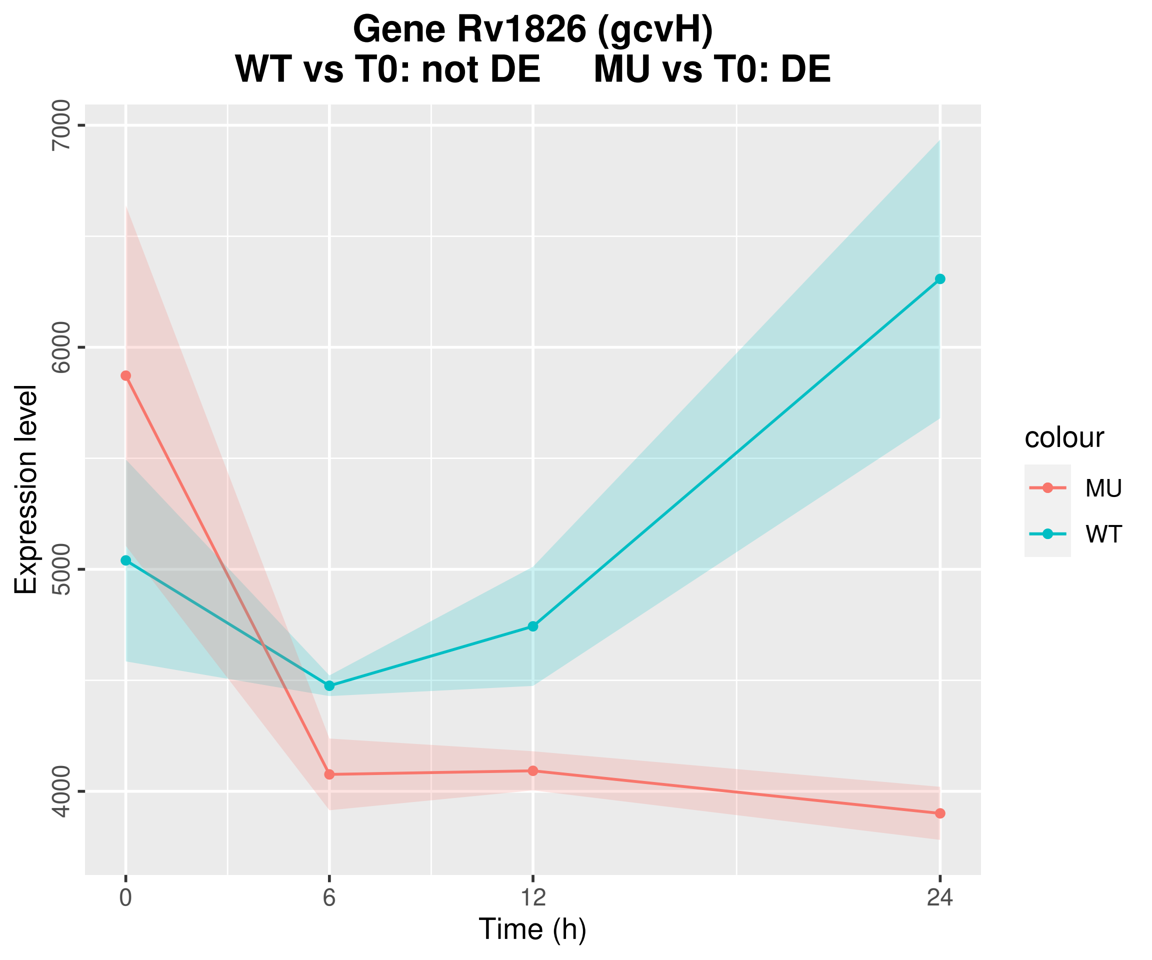
Gene Rv1826 (gcvH)
WT vs T0: not DE   MU vs T0: DE
7000
6000
5000
4000
Expression level
0
6
12
24
Time (h)
colour
MU
WT

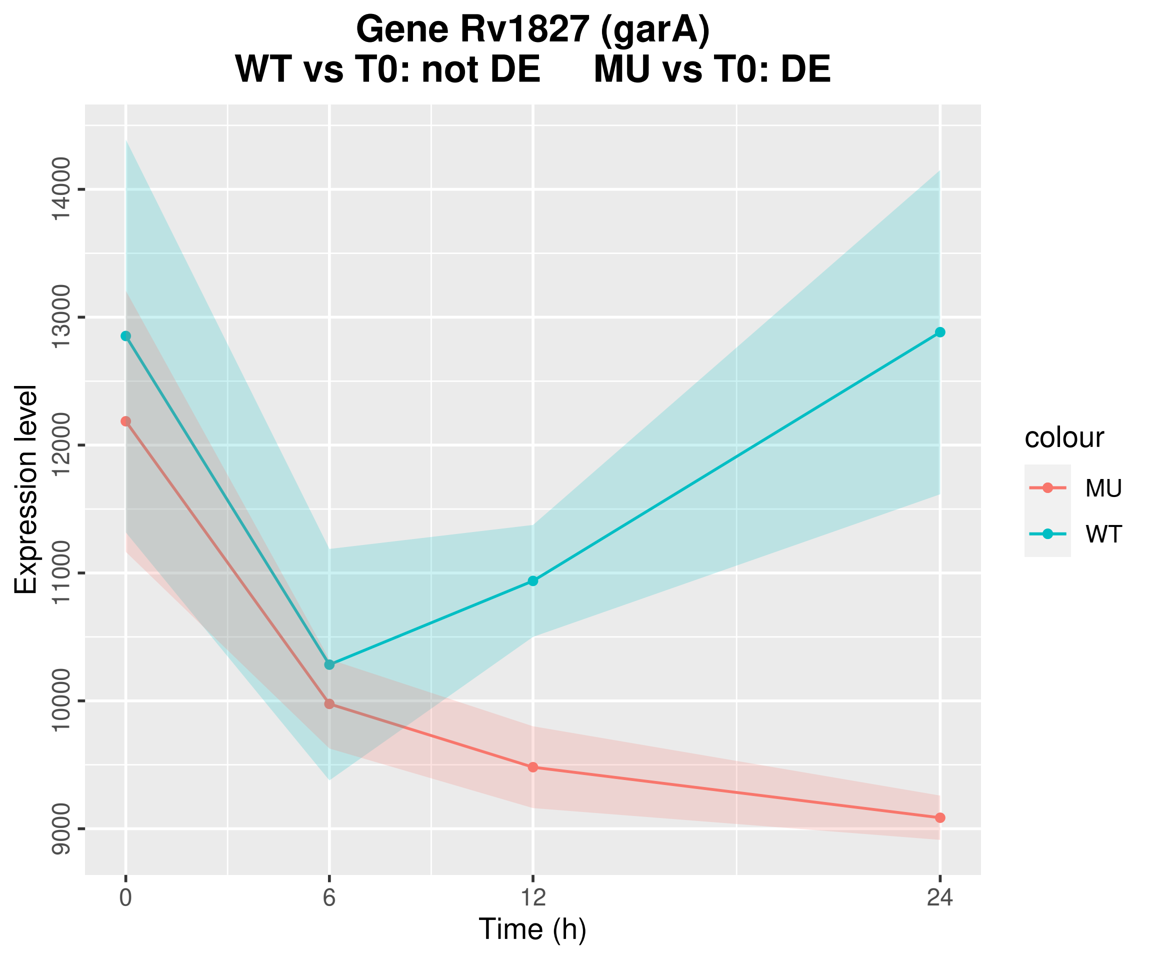
Gene Rv1827 (garA)
WT vs T0: not DE   MU vs T0: DE
Expression level
14000
13000
12000
11000
10000
9000
0
6
12
24
Time (h)
colour
MU
WT

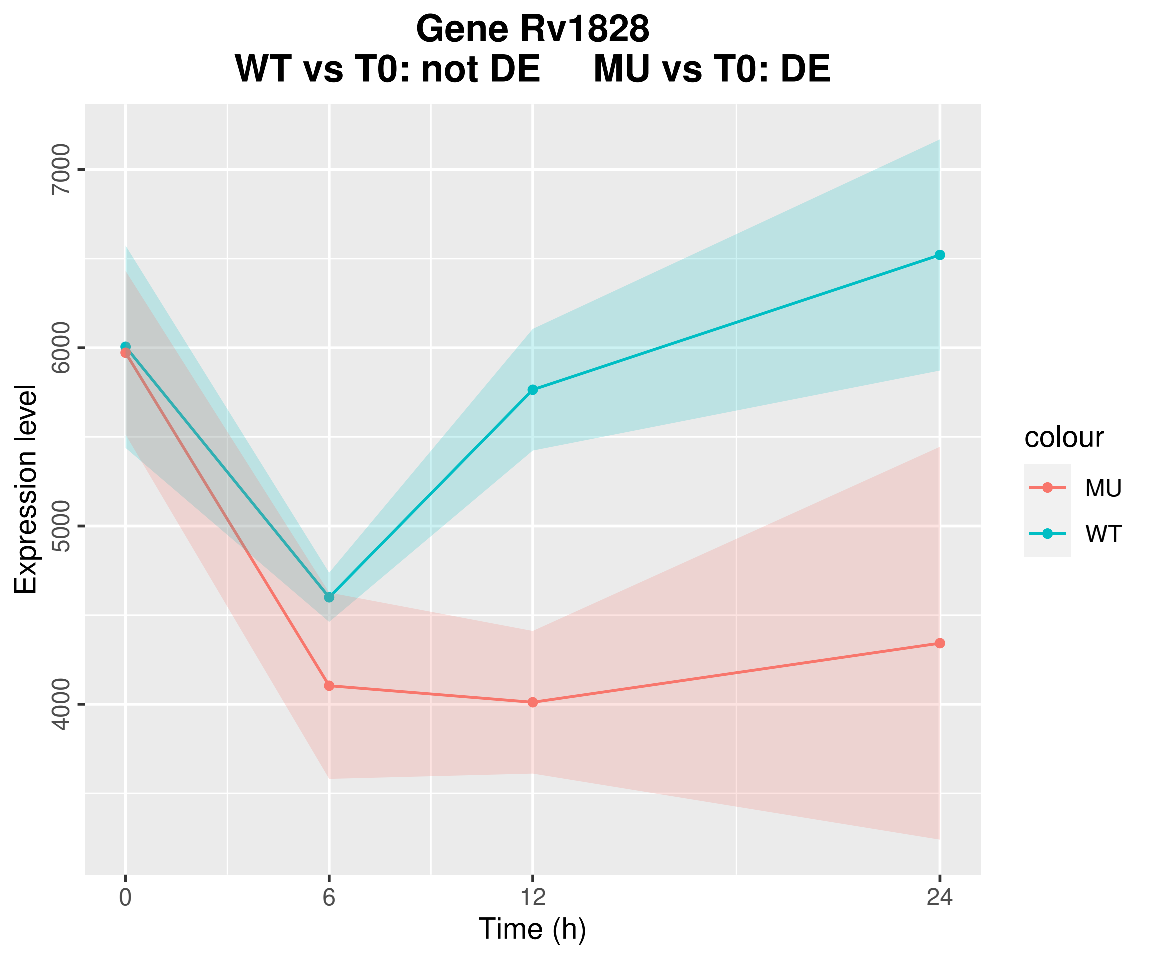
Gene Rv1828
WT vs T0: not DE MU vs T0: DE
Expression level
7000
6000
5000
4000
0
6
12
24
Time (h)
colour
MU
WT

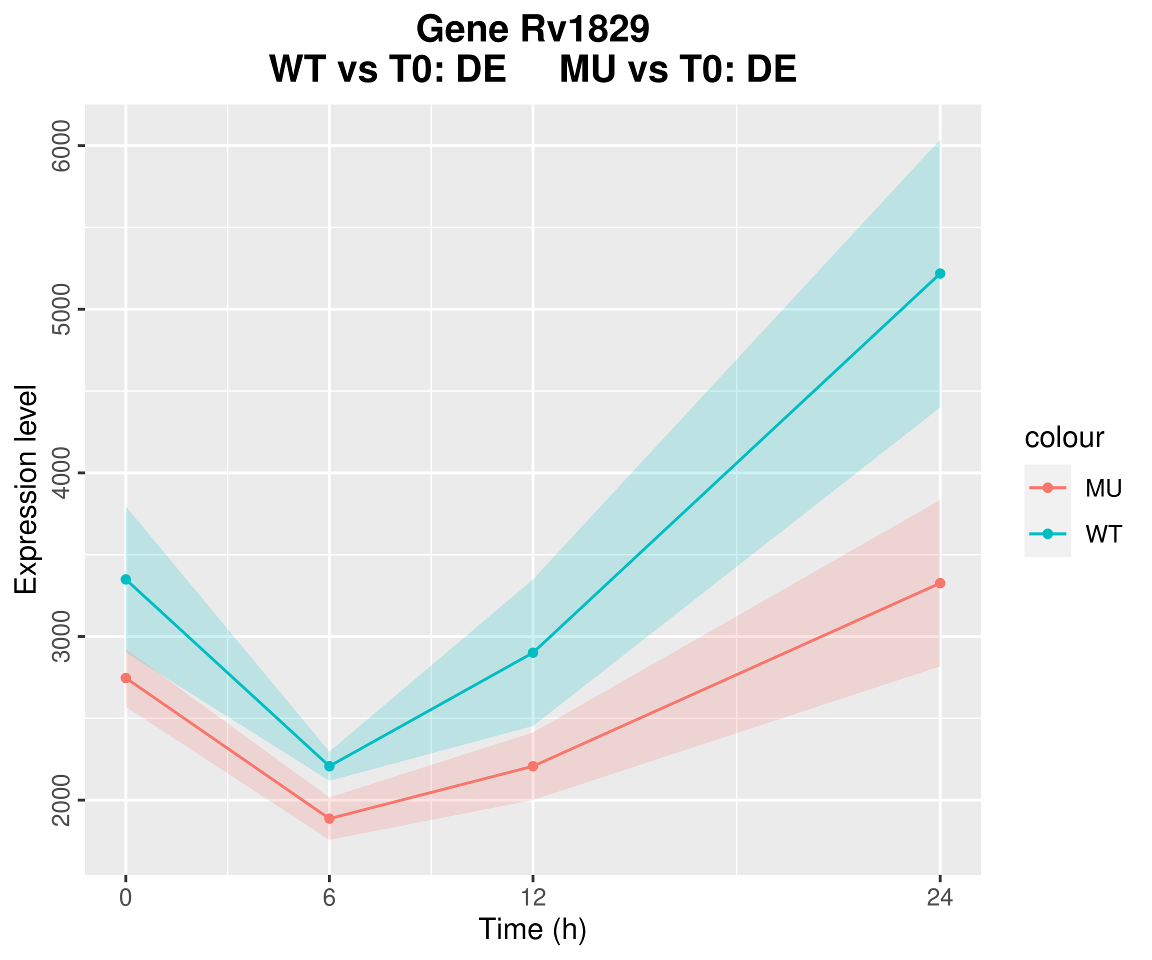
Gene Rv1829
WT vs T0: DE   MU vs T0: DE
Expression level
6000
5000
4000
3000
2000
0
6
12
24
Time (h)
colour
MU
WT

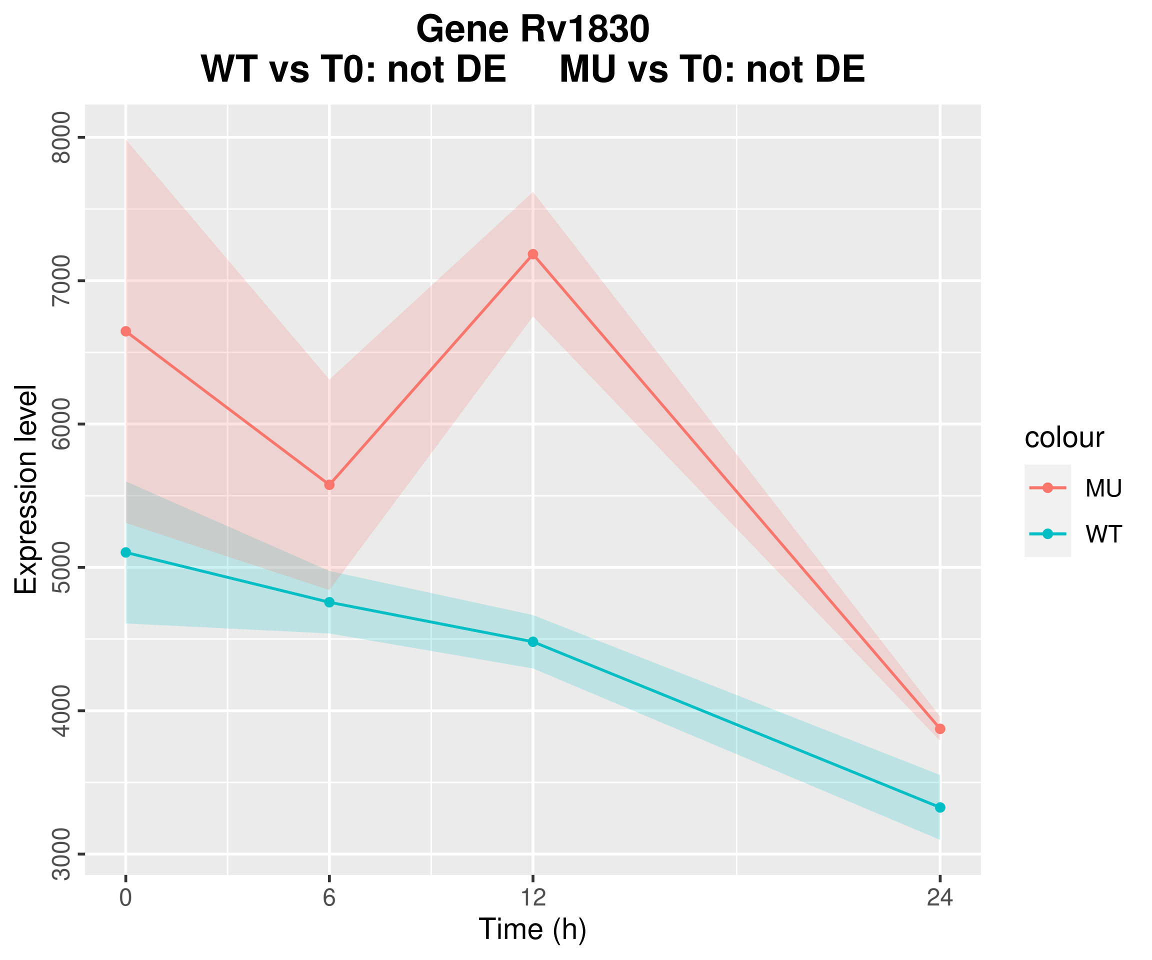
Gene Rv1830
WT vs T0: not DE   MU vs T0: not DE
Expression level
8000
7000
6000
5000
4000
3000
0
6
12
24
Time (h)
colour
MU
WT

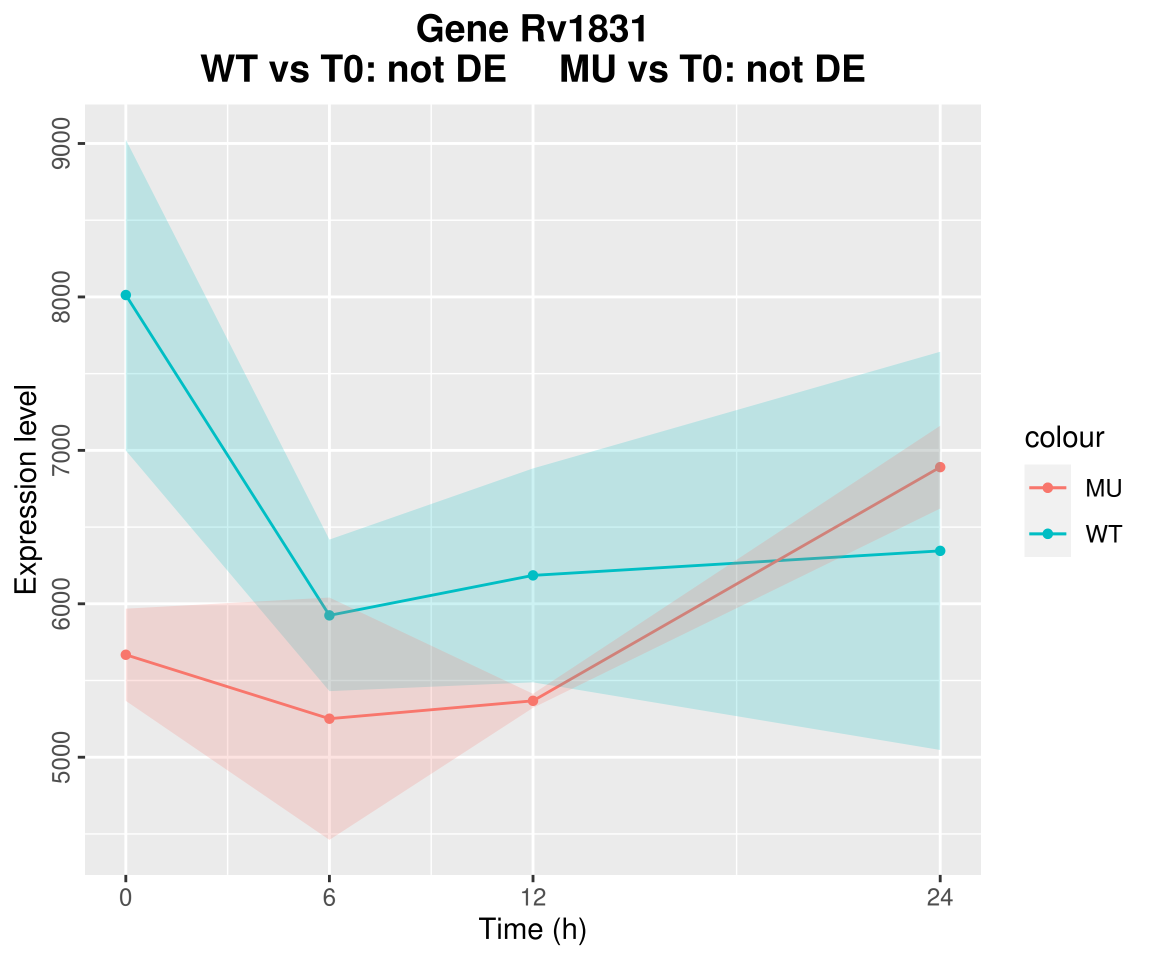

Gene Rv1831
WT vs T0: not DE   MU vs T0: not DE
Expression level
9000
8000
7000
6000
5000
0
6
12
24
Time (h)
colour
MU
WT

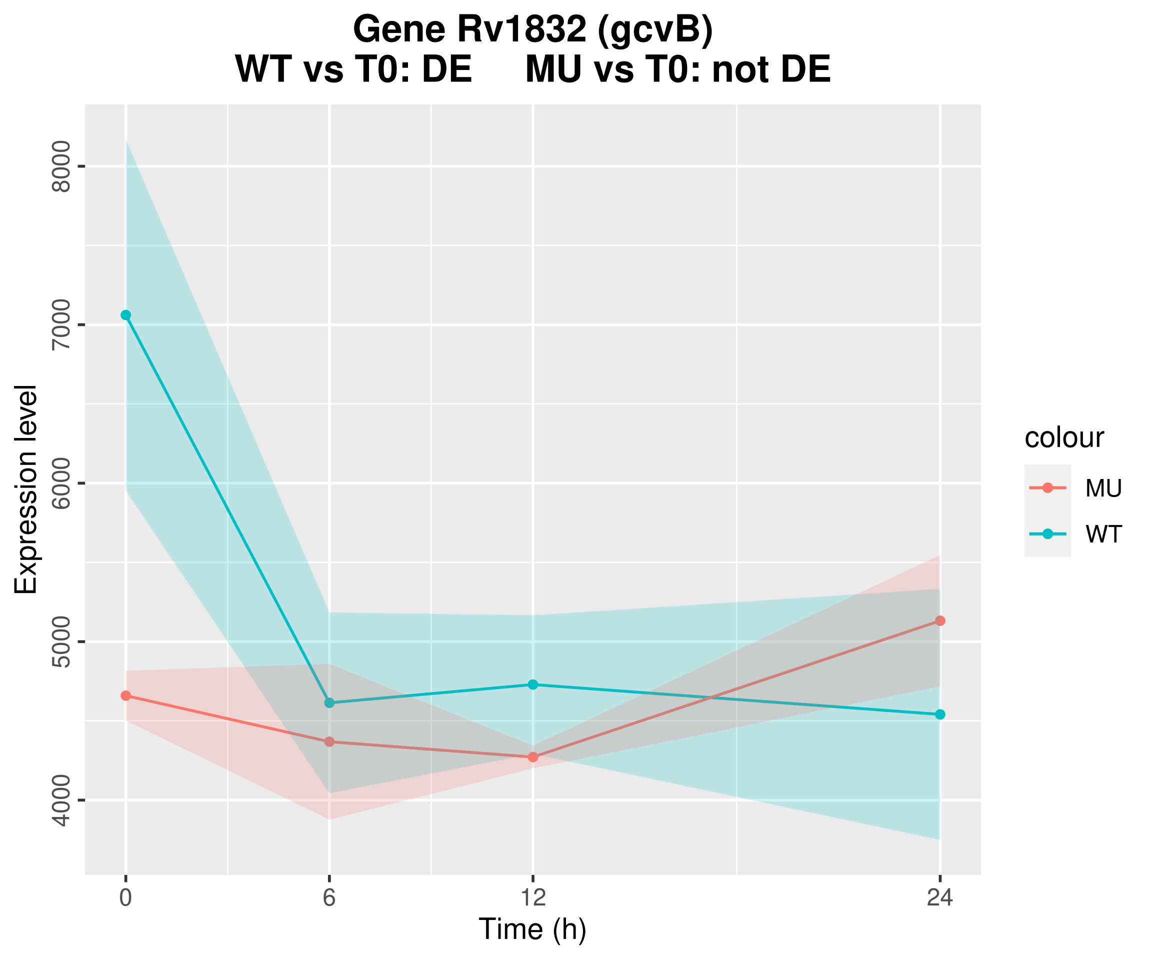

Gene Rv1832 (gcvB)
WT vs T0: DE MU vs T0: not DE
Expression level
8000
7000
6000
5000
4000
0
6
12
24
Time (h)
colour
MU
WT

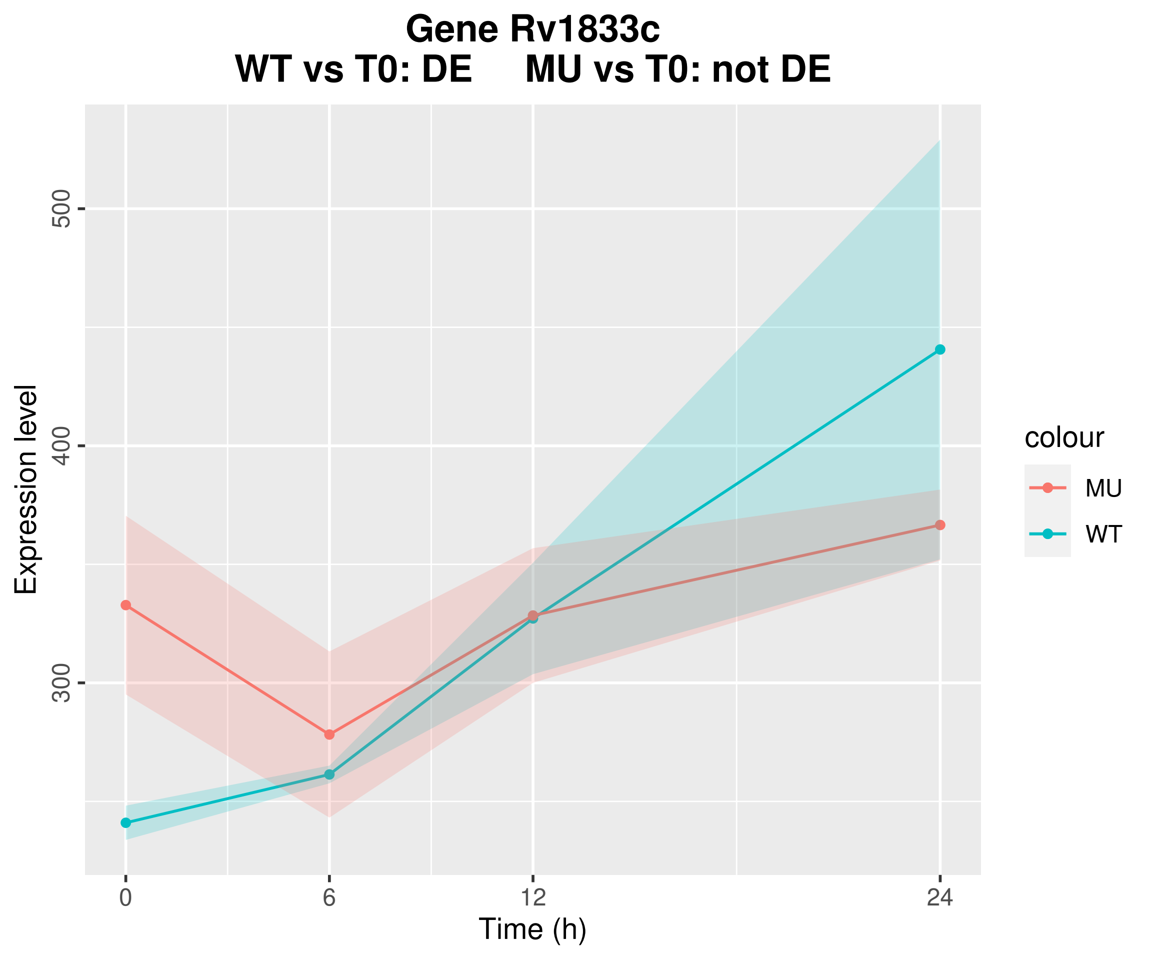

Gene Rv1833c
WT vs T0: DE MU vs T0: not DE
Expression level
500
400
300
0
6
12
24
Time (h)
colour
MU
WT

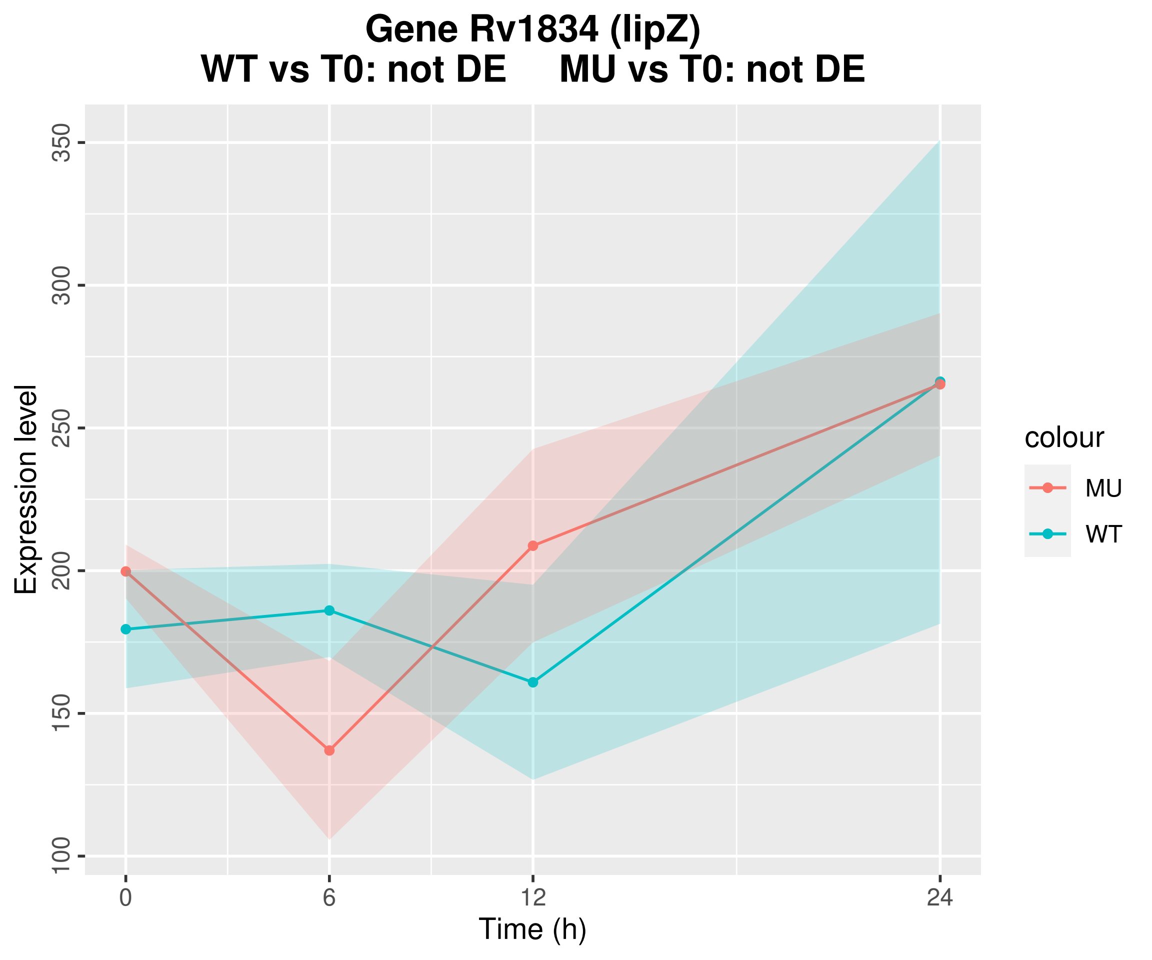

Gene Rv1834 (lipZ)
WT vs T0: not DE MU vs T0: not DE
Expression level
350
300
250
200
150
100
0
6
12
24
Time (h)
colour
MU
WT

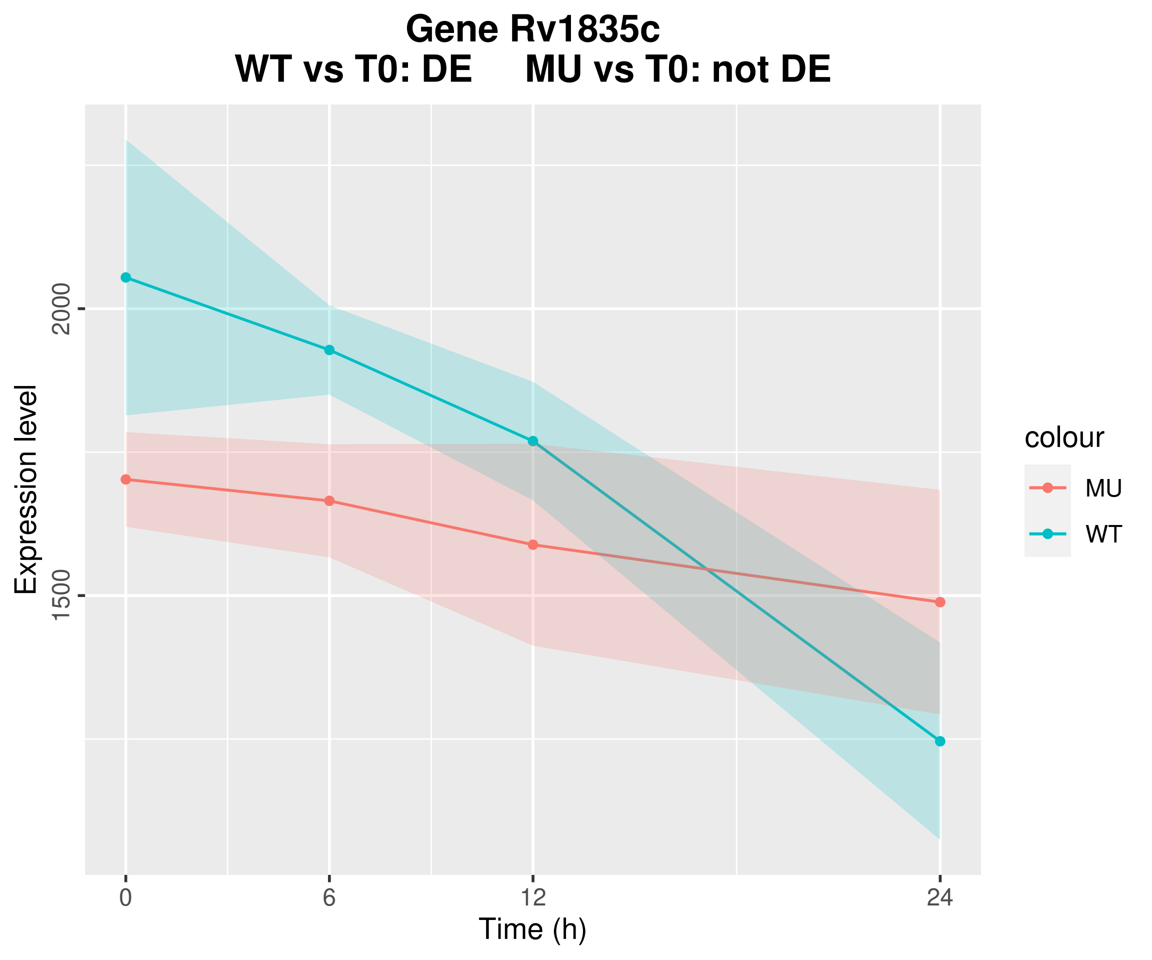
Gene Rv1835c
WT vs T0: DE   MU vs T0: not DE
Expression level
2000
1500
0
6
12
24
Time (h)
colour
MU
WT

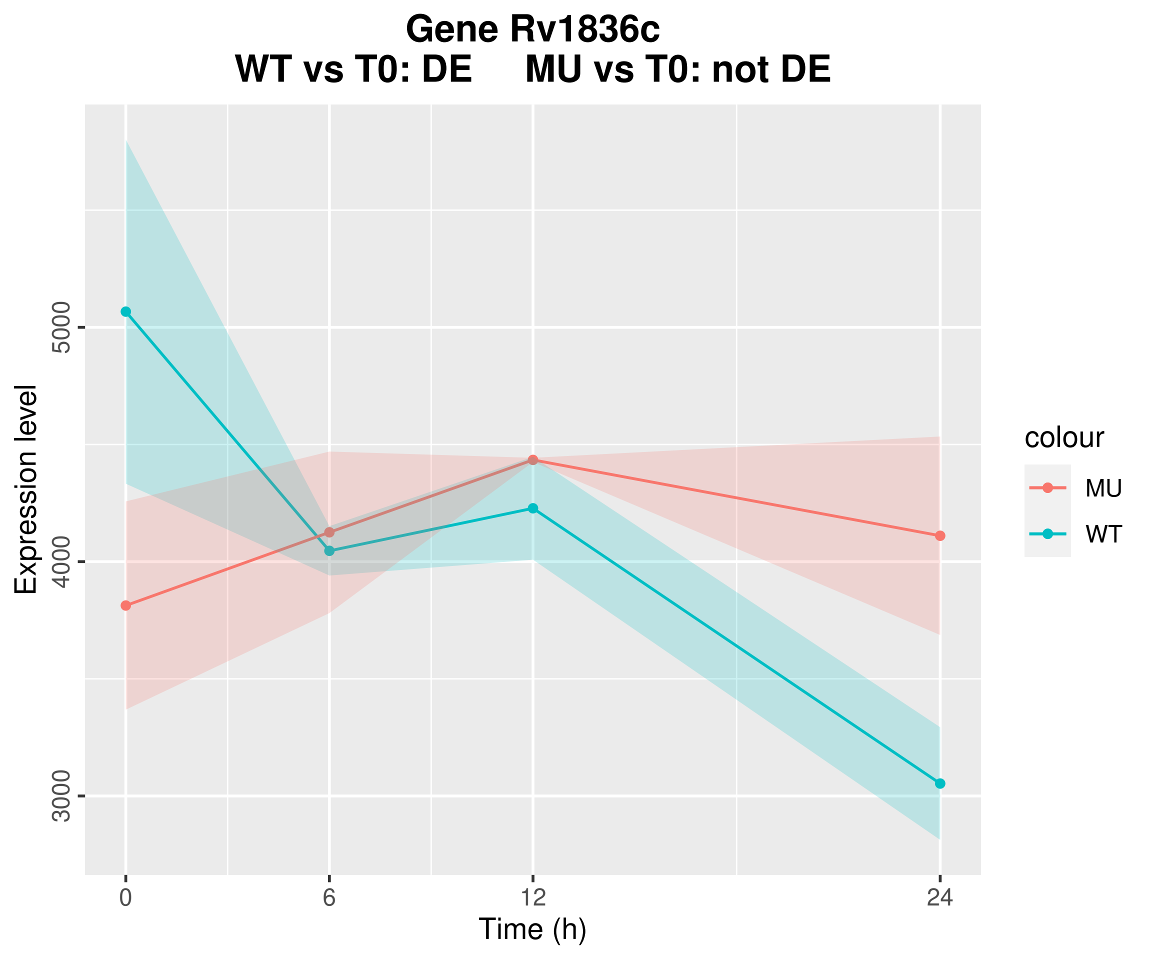
Gene Rv1836c
WT vs T0: DE   MU vs T0: not DE
Expression level
5000
4000
3000
0
6
12
24
Time (h)
colour
MU
WT

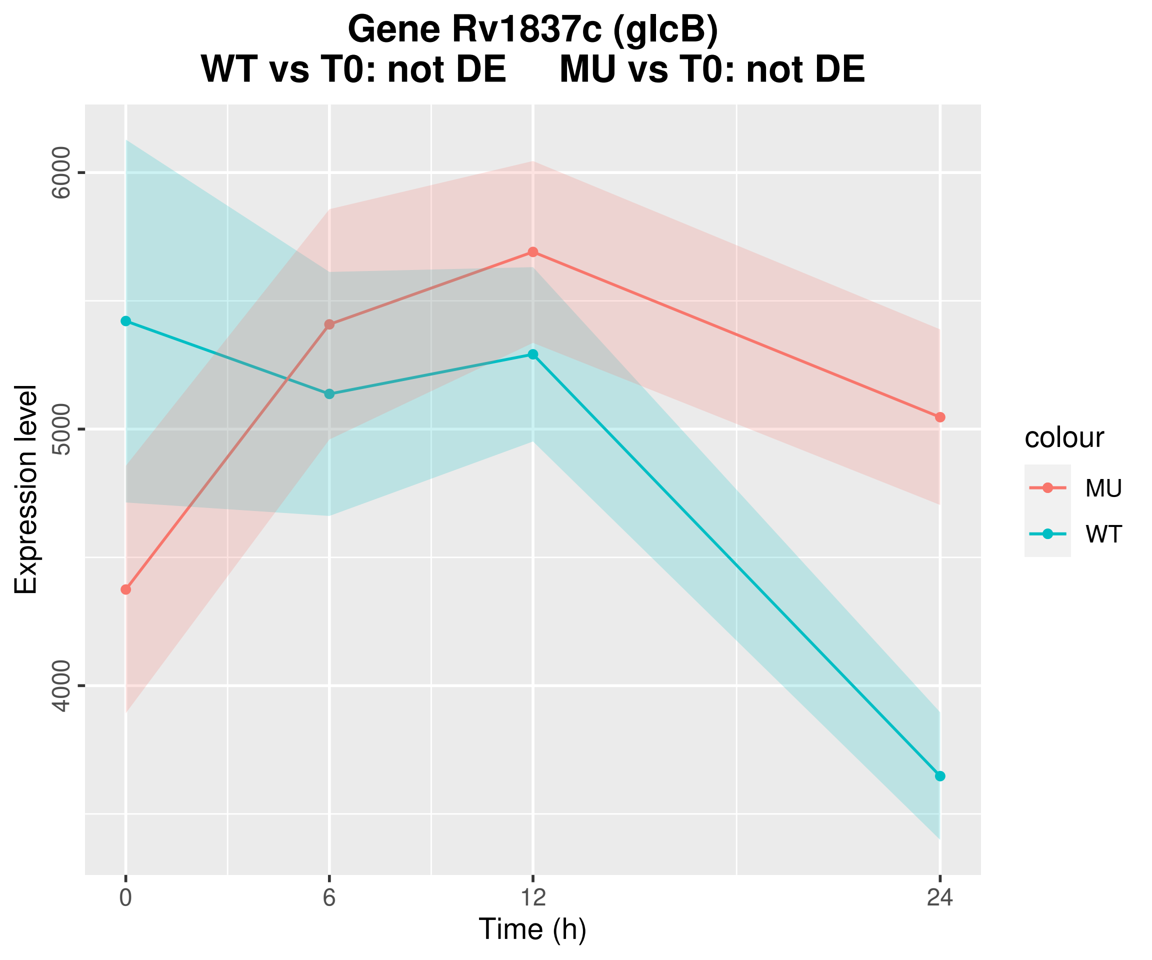

Gene Rv1837c (glcB)
WT vs T0: not DE MU vs T0: not DE
Expression level
6000
5000
4000
0
6
12
24
Time (h)
colour
MU
WT

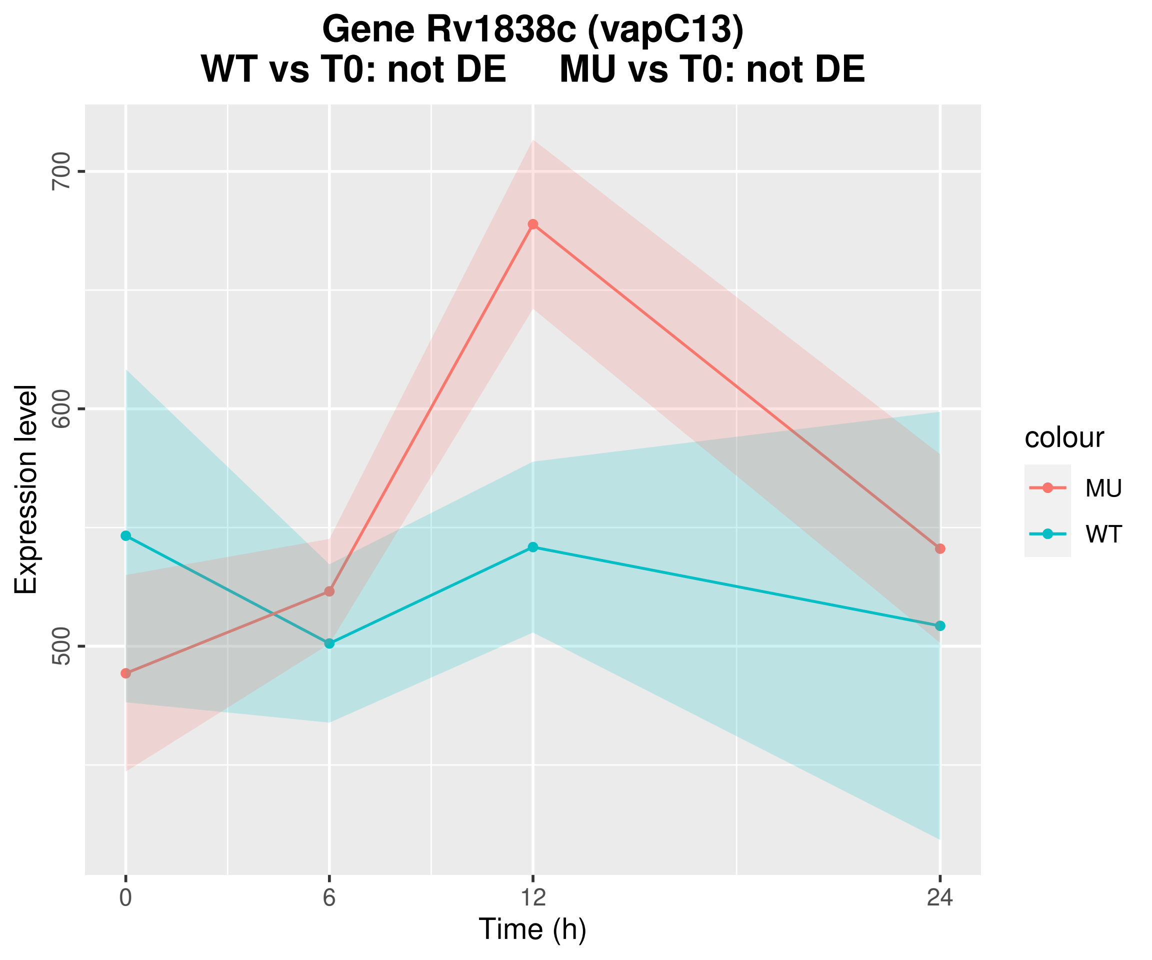
Gene Rv1838c (vapC13)
WT vs T0: not DE   MU vs T0: not DE
Expression level
700
600
500
Time (h)
0
6
12
24
colour
MU
WT

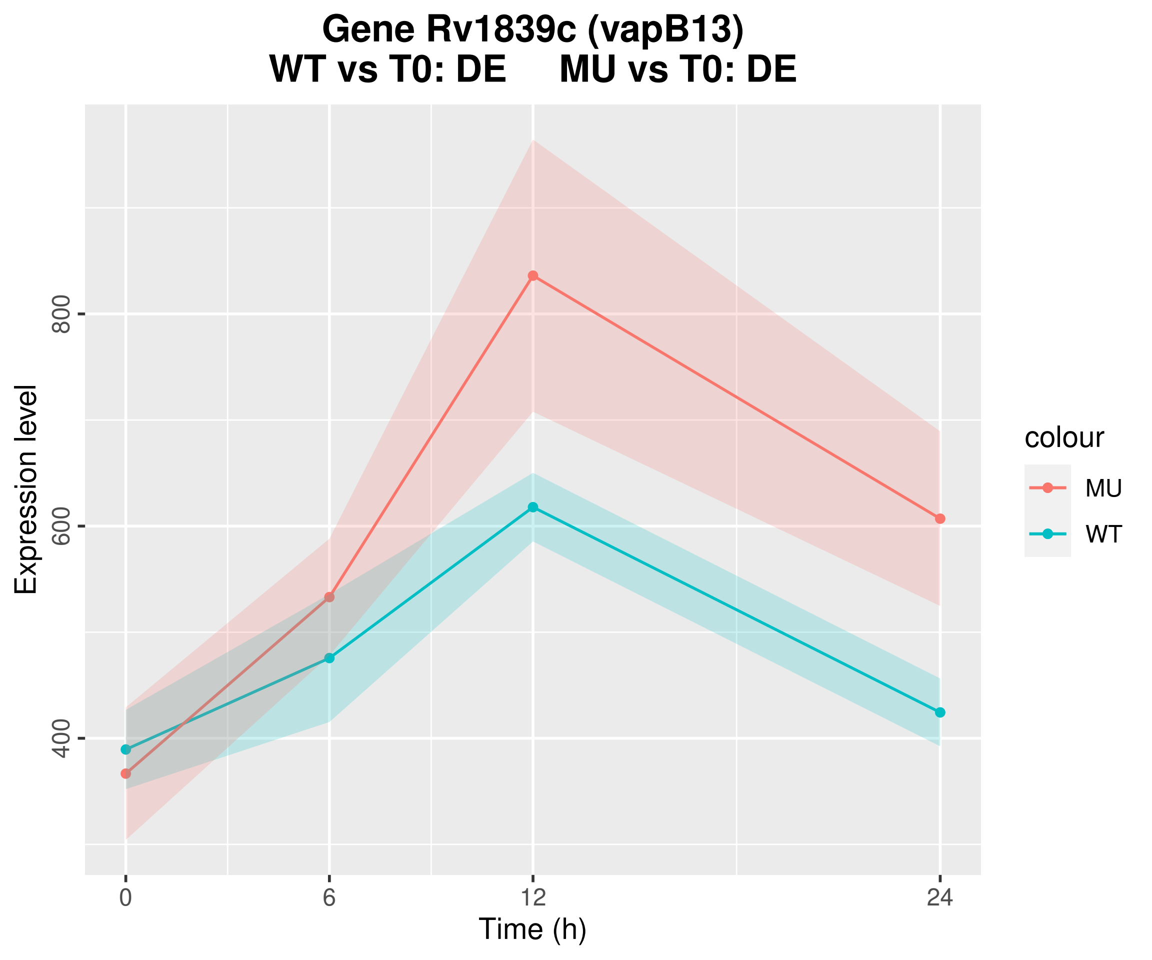
Gene Rv1839c (vapB13)
WT vs T0: DE MU vs T0: DE
Expression level
800
600
400
0
6
12
24
Time (h)
colour
MU
WT

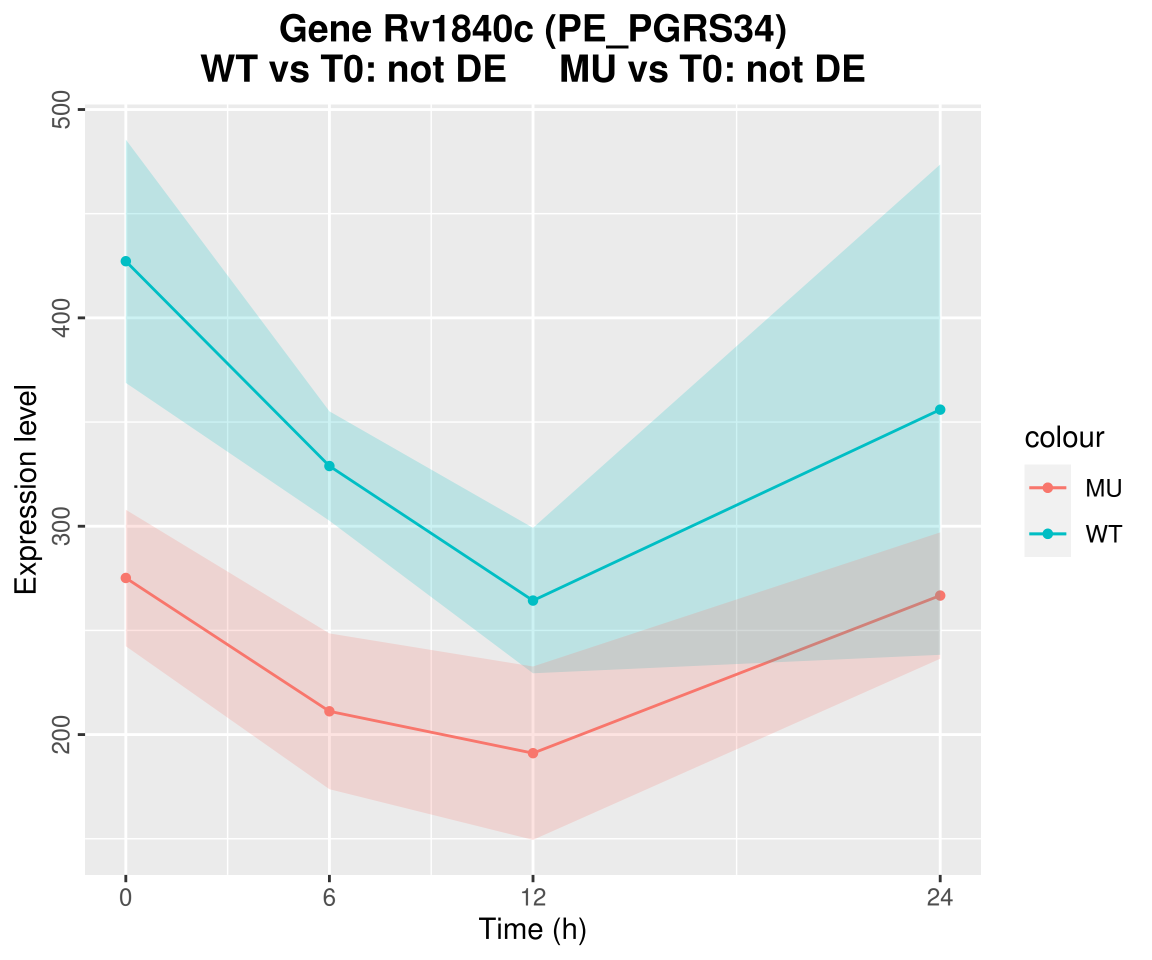
Gene Rv1840c (PE_PGRS34)
WT vs T0: not DE   MU vs T0: not DE
500
400
300
200
Expression level
0
6
12
24
Time (h)
colour
MU
WT

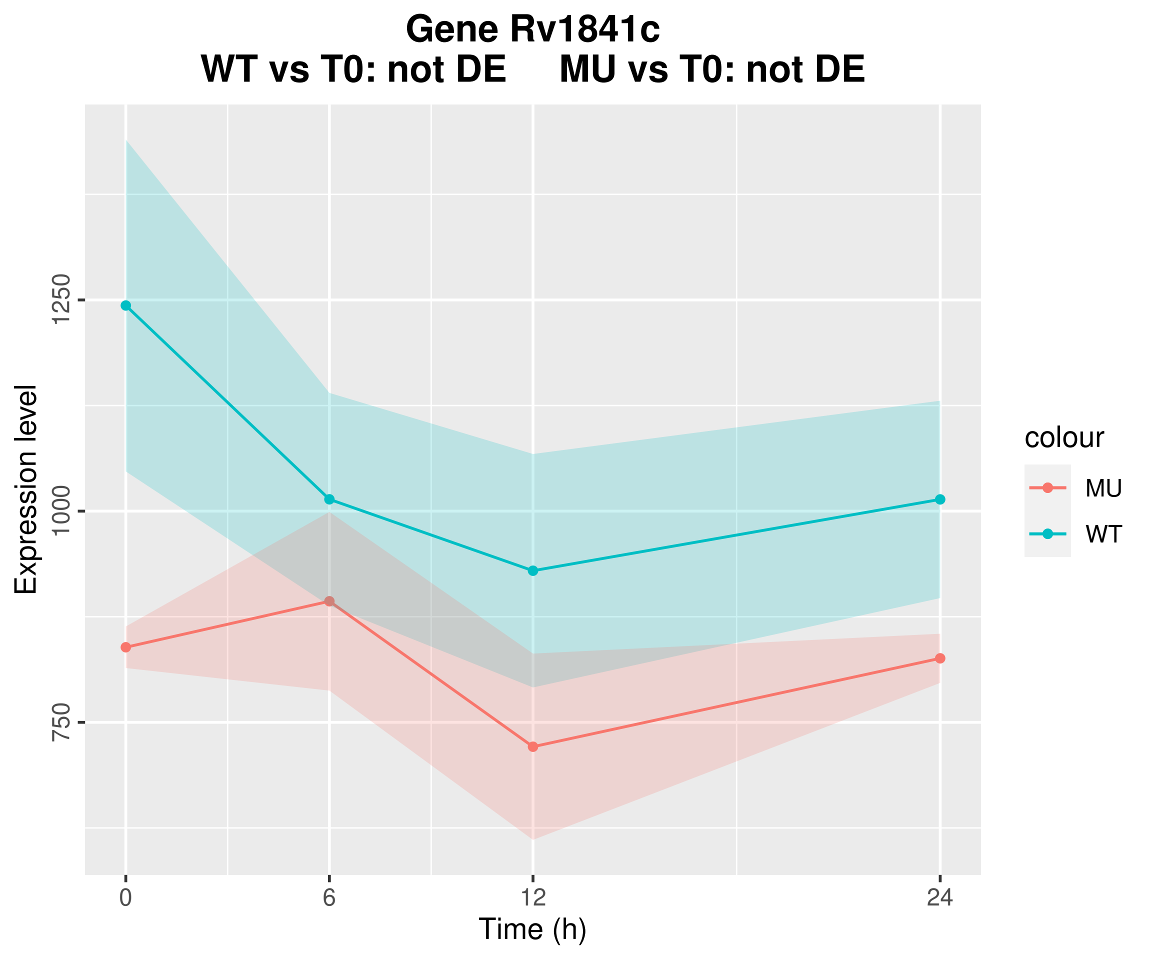

Gene Rv1841c
WT vs T0: not DE   MU vs T0: not DE
1250
1000
750
Expression level
0
6
12
24
Time (h)
colour
MU
WT

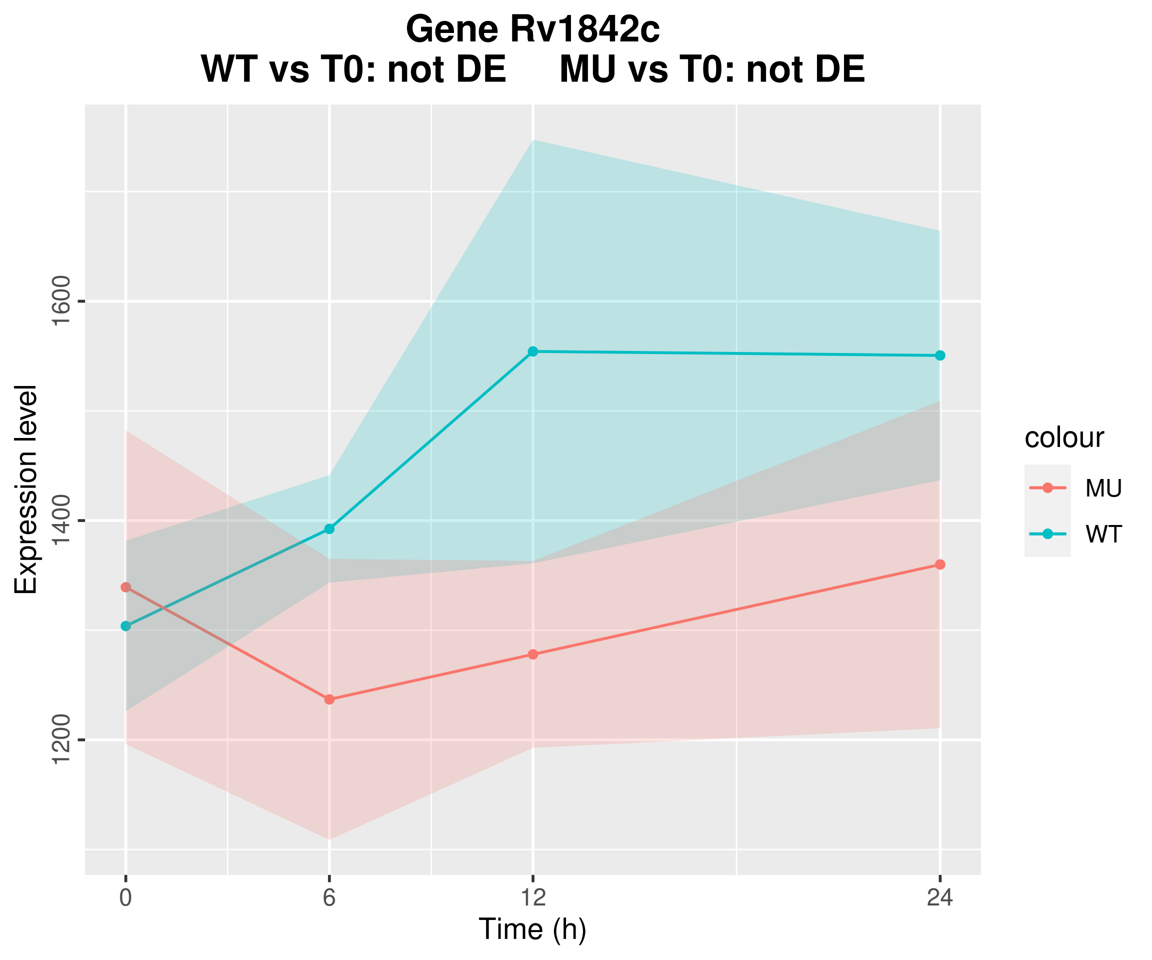

Gene Rv1842c
WT vs T0: not DE   MU vs T0: not DE
Expression level
1200
1400
1600
Time (h)
0
6
12
24
colour
MU
WT

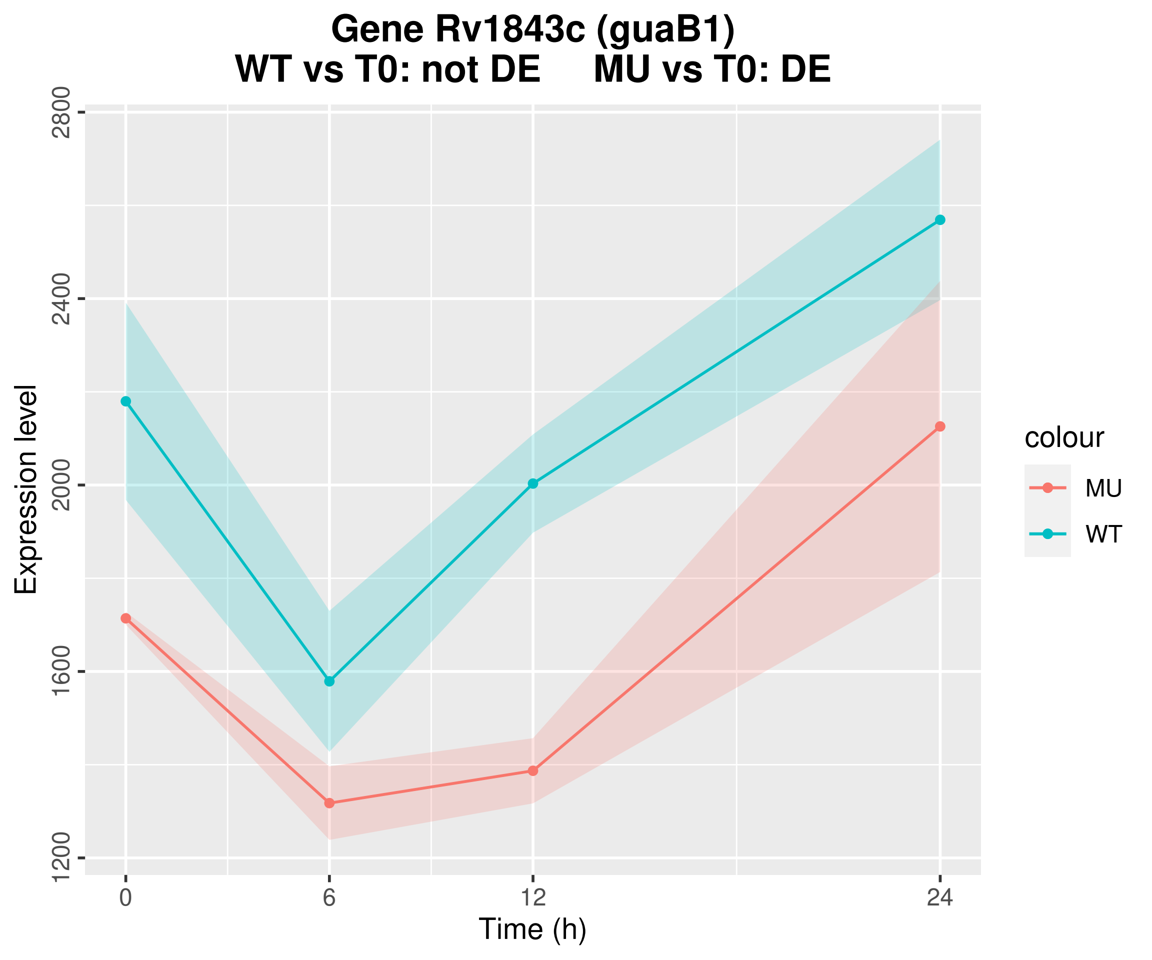
Gene Rv1843c (guaB1)
WT vs T0: not DE MU vs T0: DE
Expression level
2800
2400
2000
1600
1200
0
6
12
24
Time (h)
colour
MU
WT

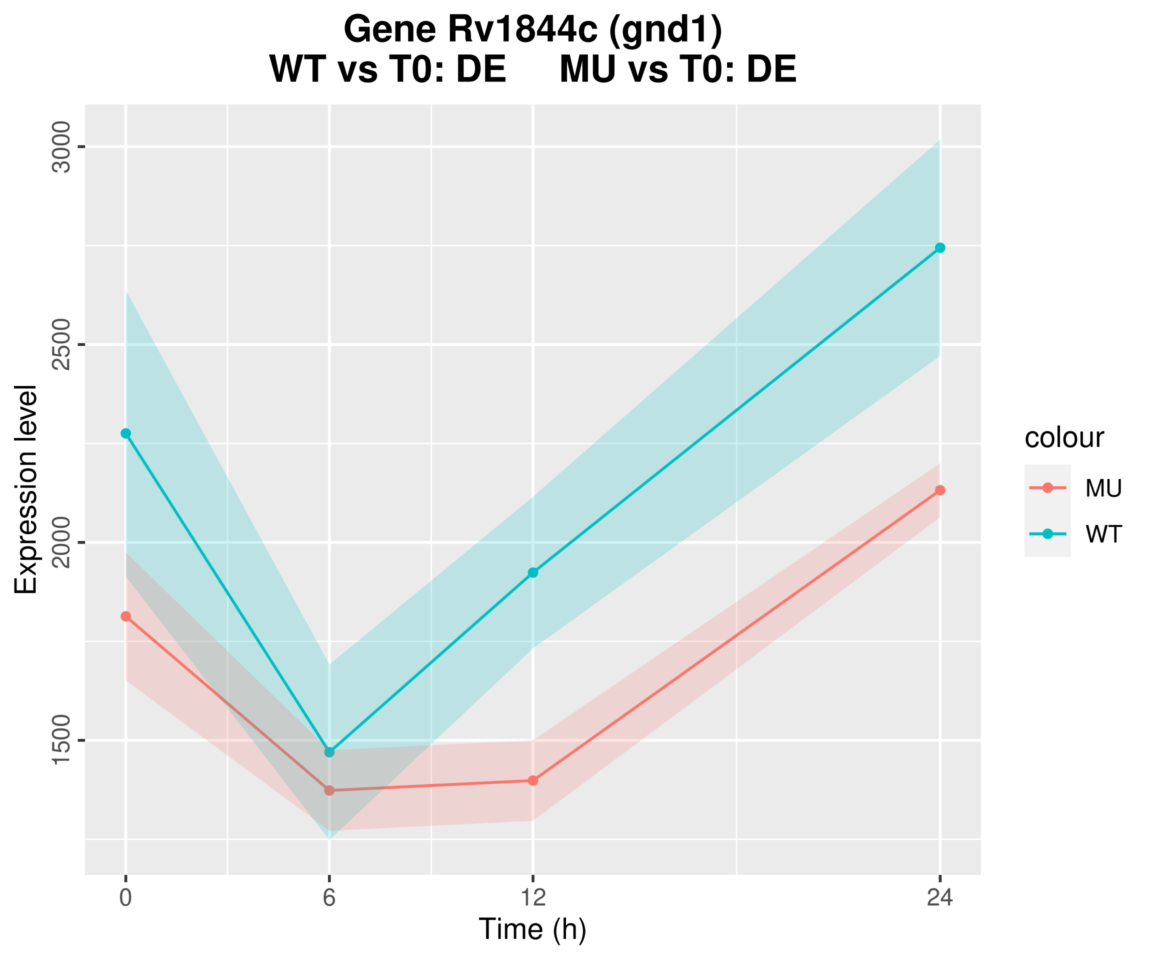
Gene Rv1844c (gnd1)
WT vs T0: DE MU vs T0: DE
3000
2500
2000
1500
Expression level
0
6
12
24
Time (h)
colour
MU
WT

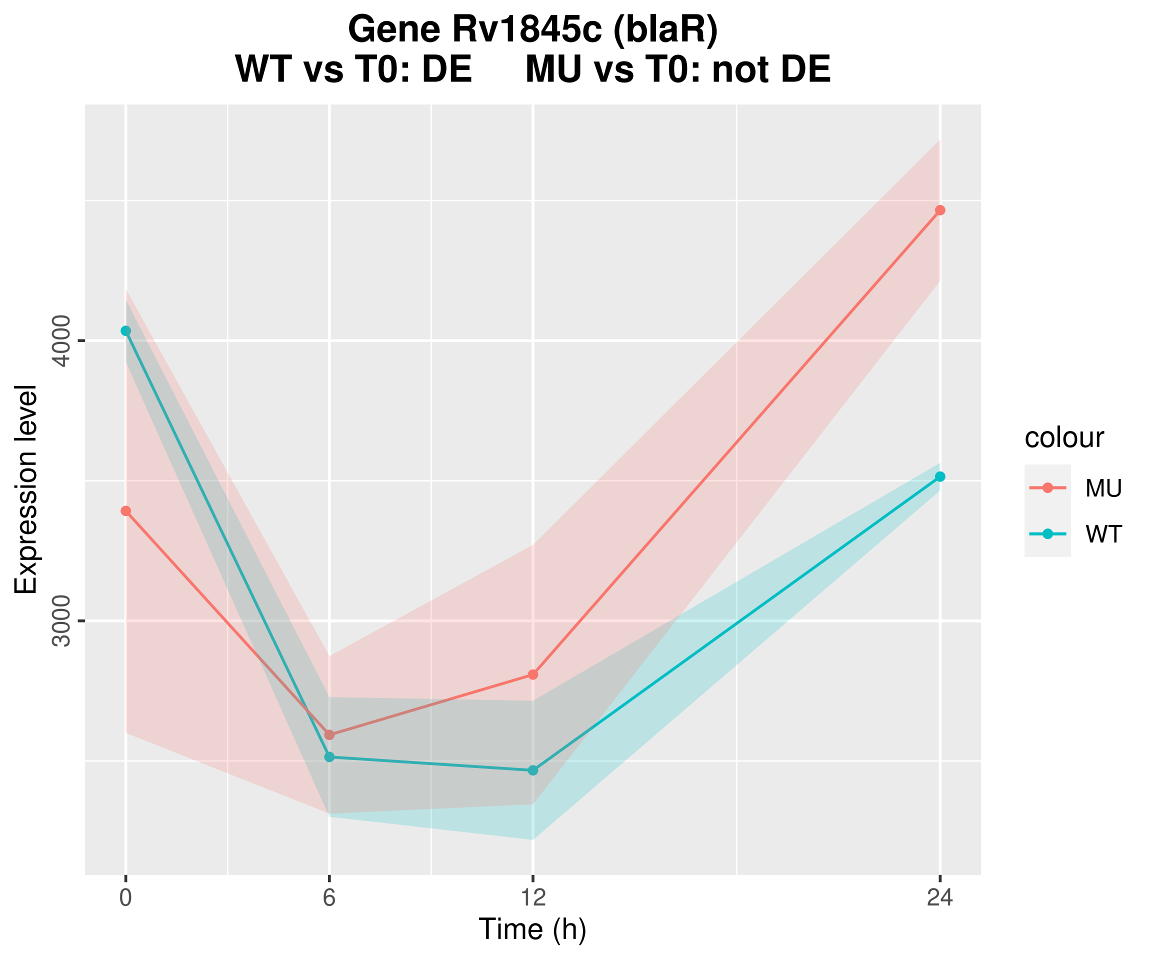

Gene Rv1845c (blaR)
WT vs T0: DE   MU vs T0: not DE
Expression level
4000
3000
0
6
12
24
Time (h)
colour
MU
WT

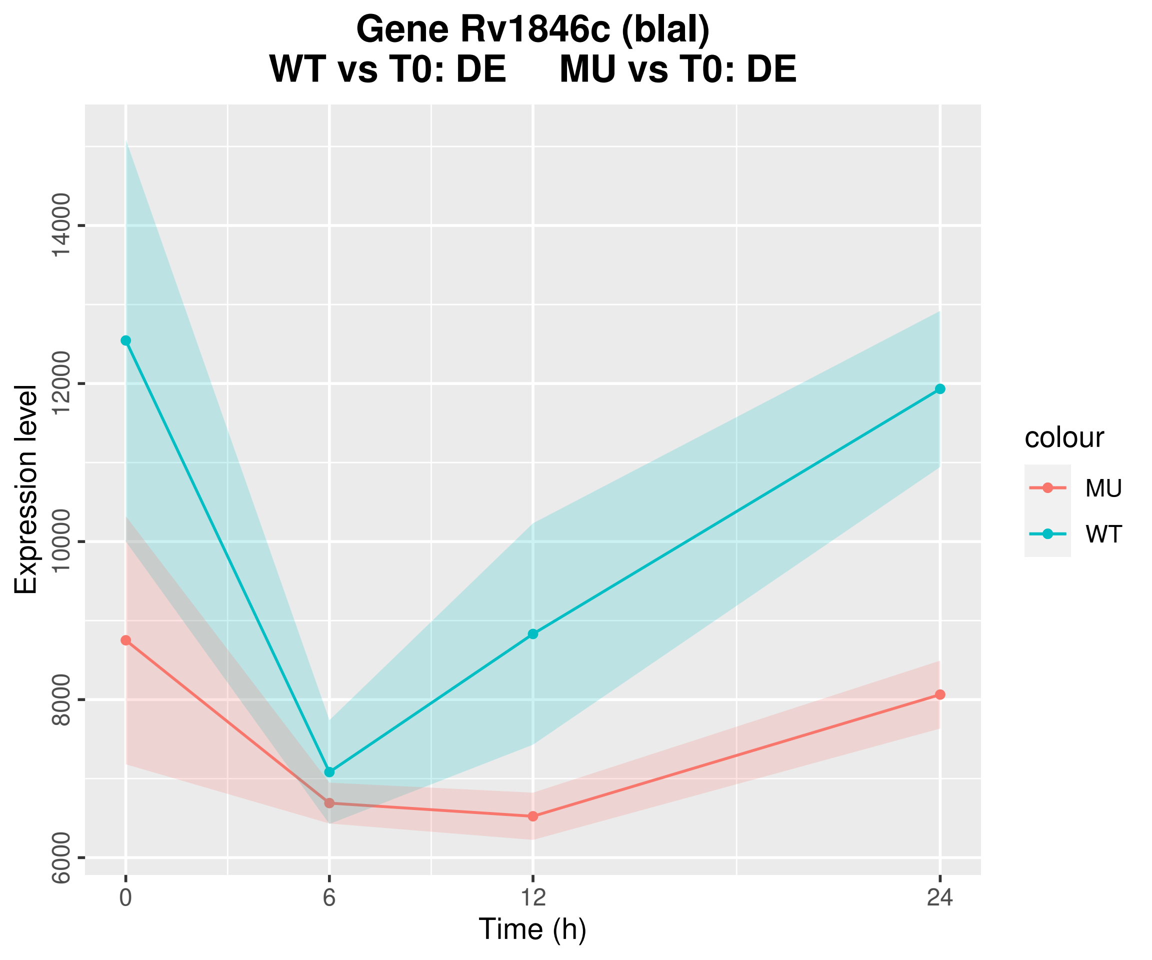

Gene Rv1846c (blaI)
WT vs T0: DE MU vs T0: DE
Expression level
14000
12000
10000
8000
6000
0
6
12
24
Time (h)
colour
MU
WT

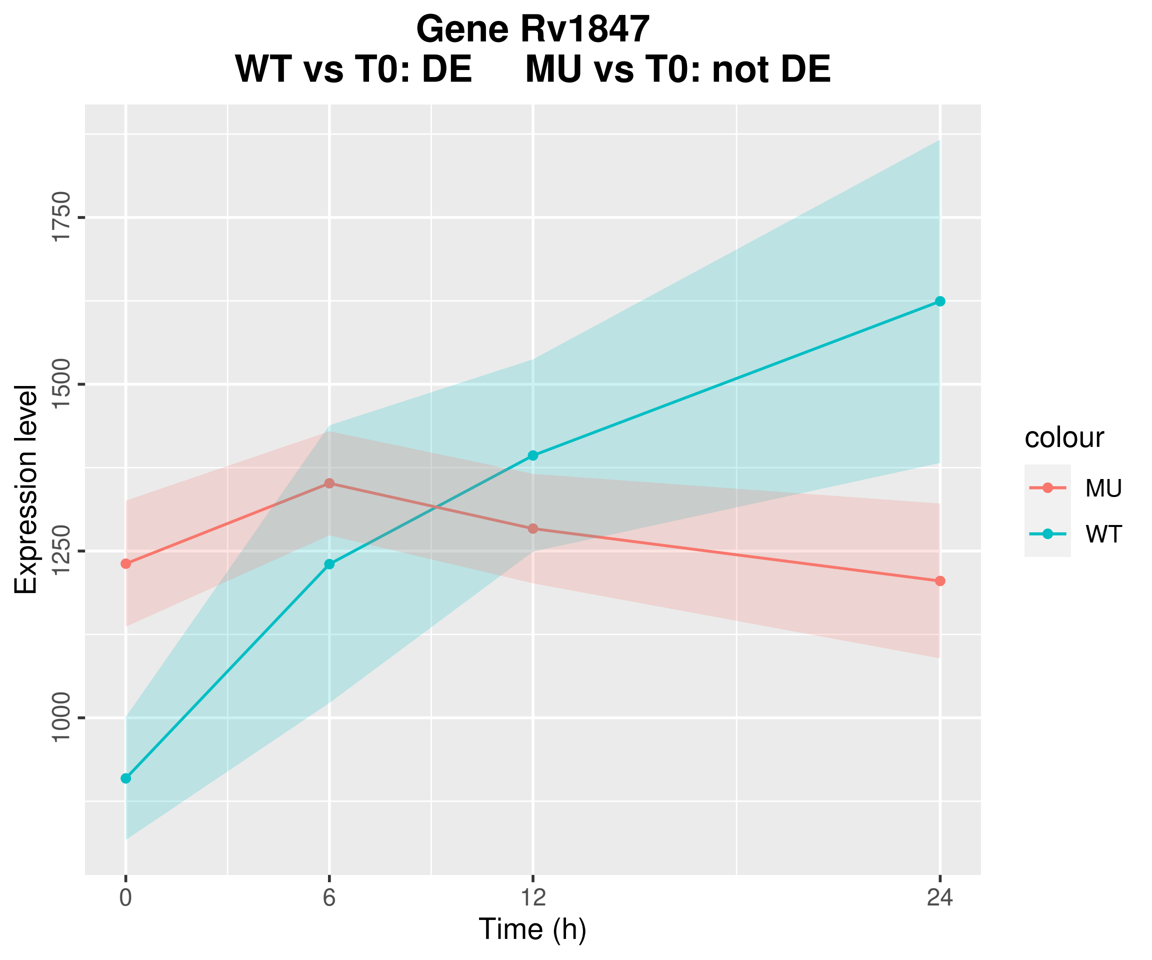

Gene Rv1847
WT vs T0: DE   MU vs T0: not DE
Expression level
1000
1250
1500
1750
0
6
12
24
Time (h)
colour
MU
WT

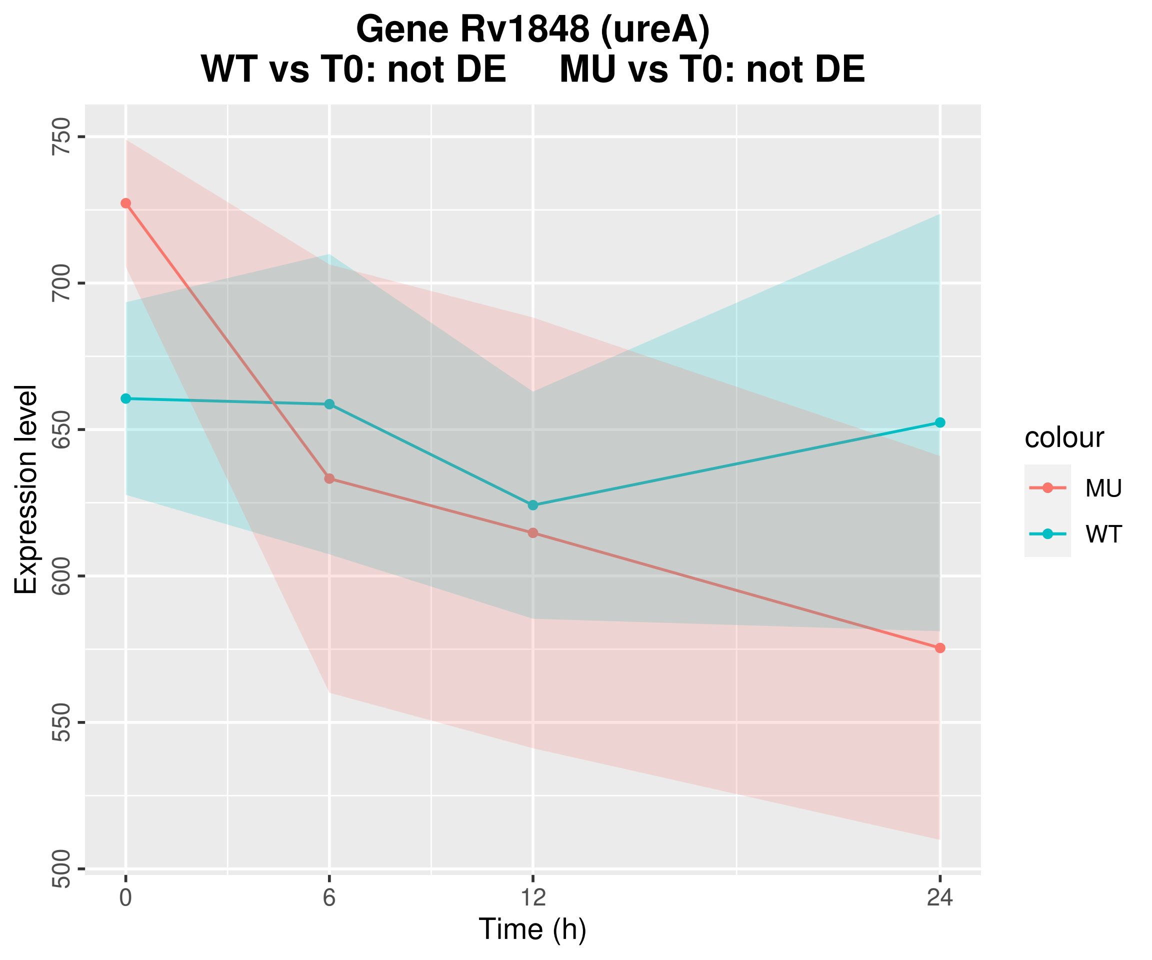
Gene Rv1848 (ureA)
WT vs T0: not DE   MU vs T0: not DE
Expression level
750
700
650
600
550
500
0
6
12
24
Time (h)
colour
MU
WT

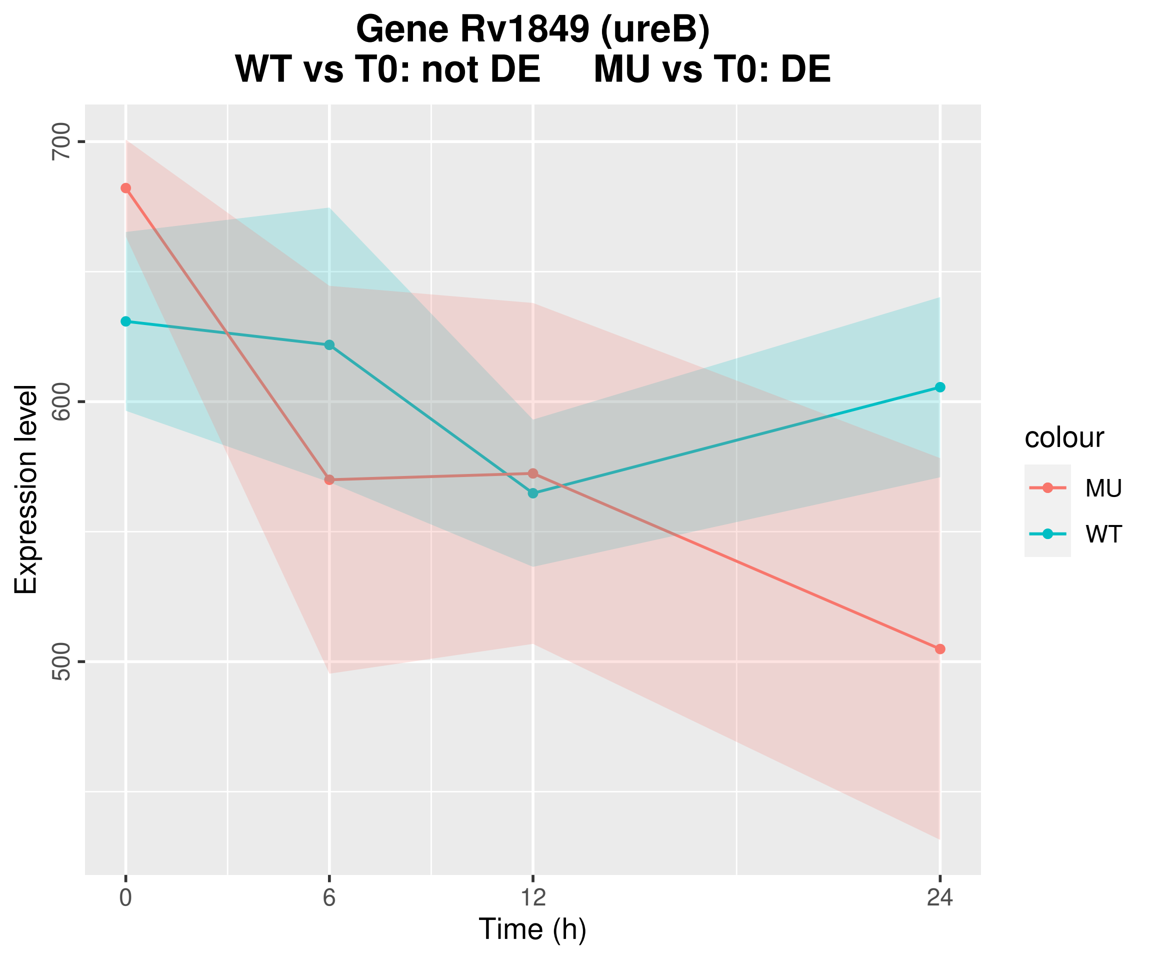

Gene Rv1849 (ureB)
WT vs T0: not DE MU vs T0: DE
700
600
500
Expression level
0
6
12
24
Time (h)
colour
MU
WT

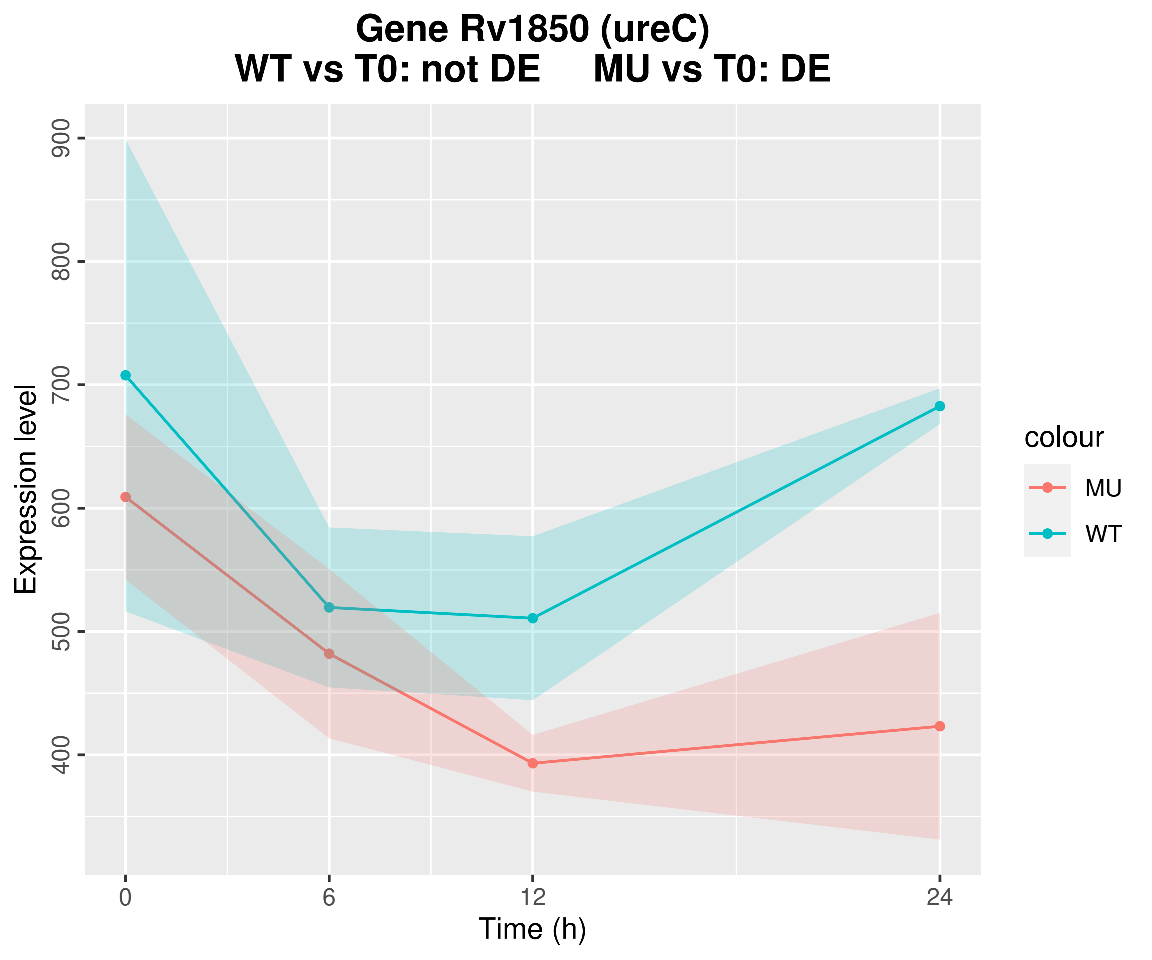
Gene Rv1850 (ureC)
WT vs T0: not DE MU vs T0: DE
Expression level
900
800
700
600
500
400
0
6
12
24
Time (h)
colour
MU
WT

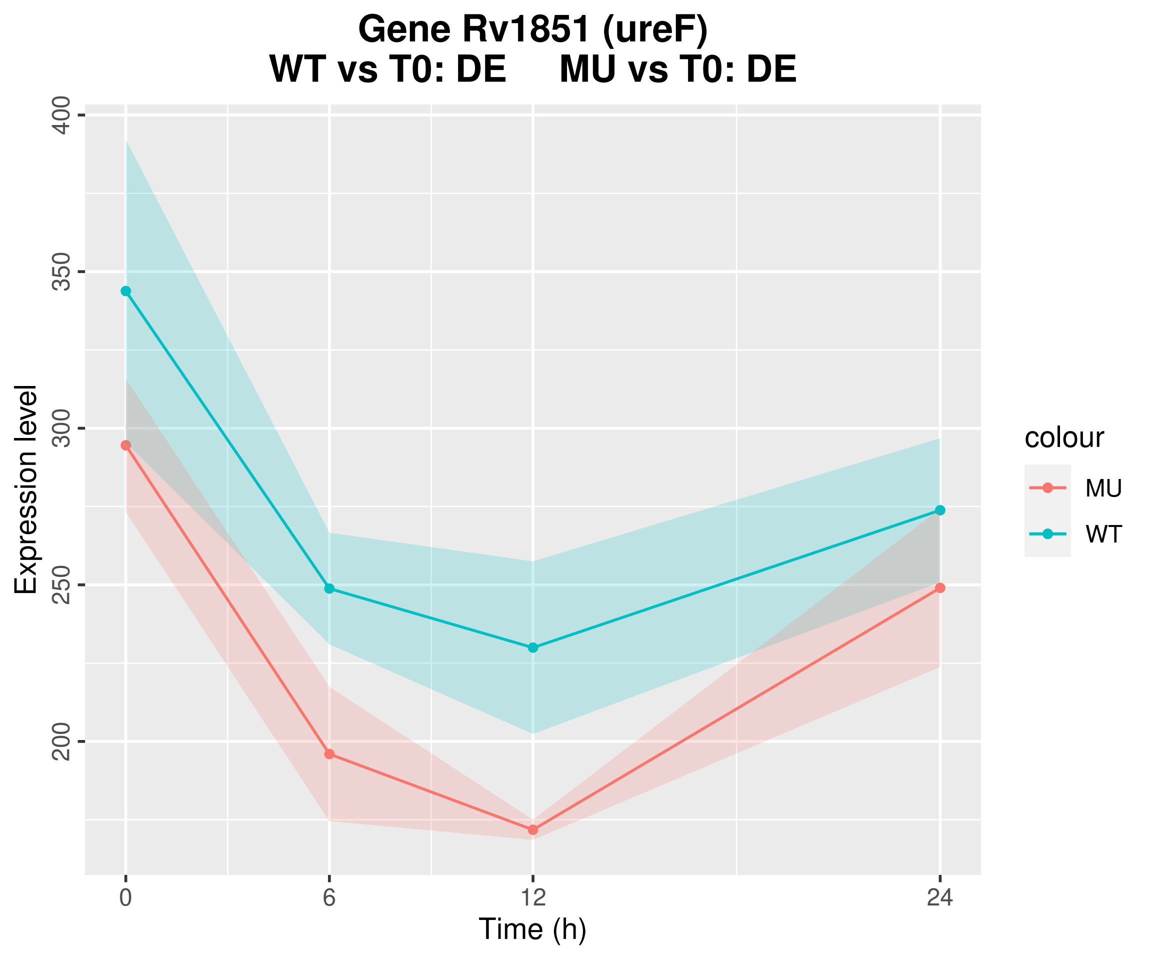

Gene Rv1851 (ureF)
WT vs T0: DE   MU vs T0: DE
Expression level
400
350
300
250
200
0
6
12
24
Time (h)
colour
MU
WT

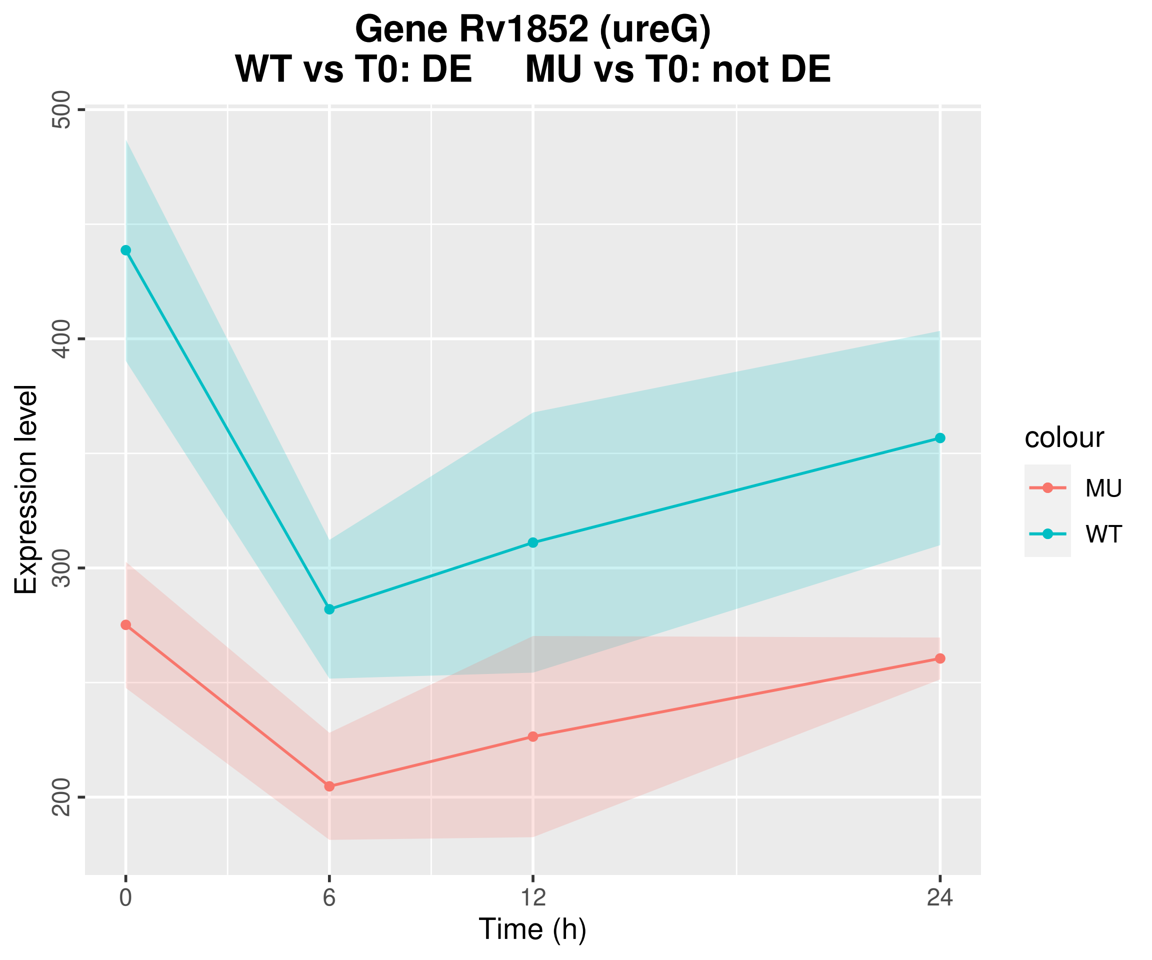
Gene Rv1852 (ureG)
WT vs T0: DE   MU vs T0: not DE
Expression level
500
400
300
200
0
6
12
24
Time (h)
colour
MU
WT

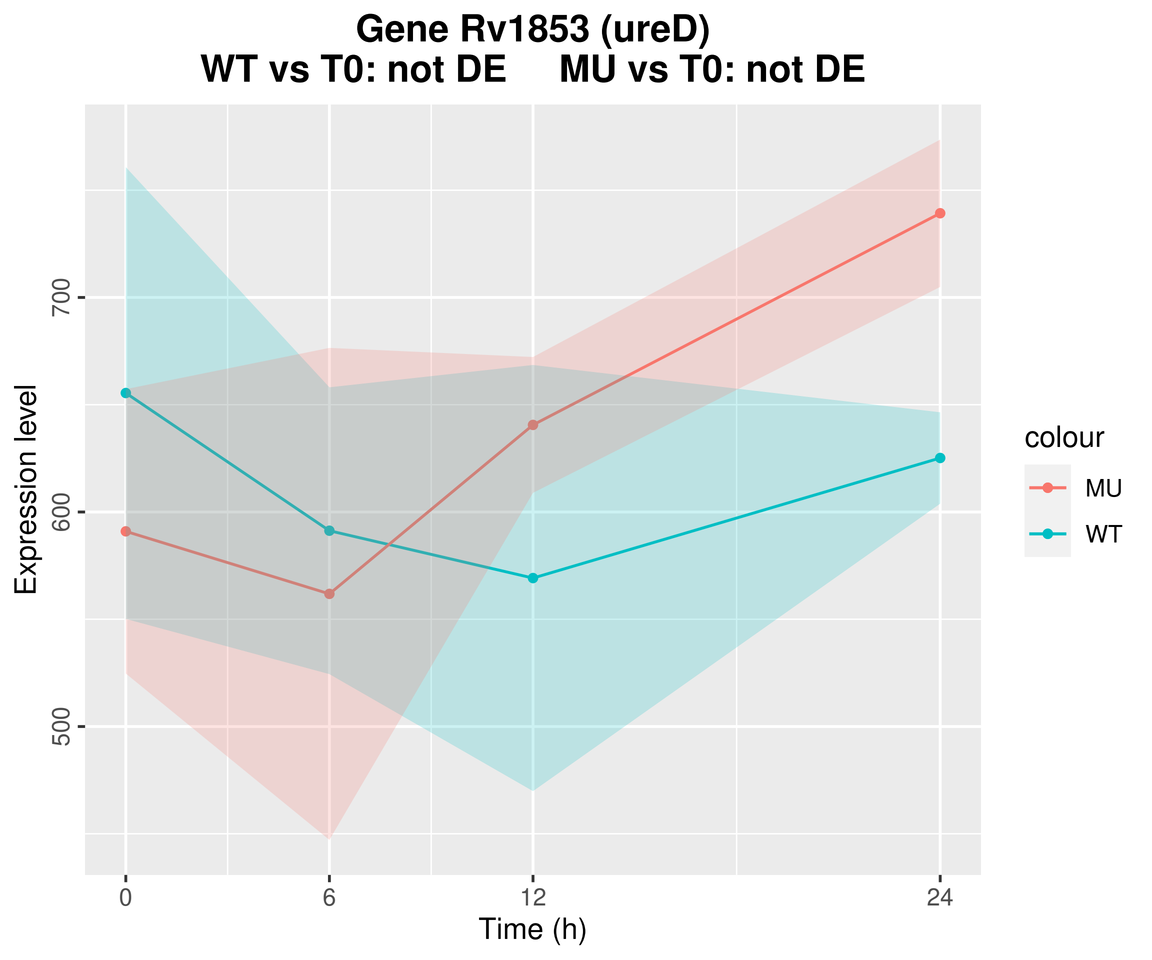
Gene Rv1853 (ureD)
WT vs T0: not DE   MU vs T0: not DE
Expression level
700
600
500
0
6
12
24
Time (h)
colour
MU
WT

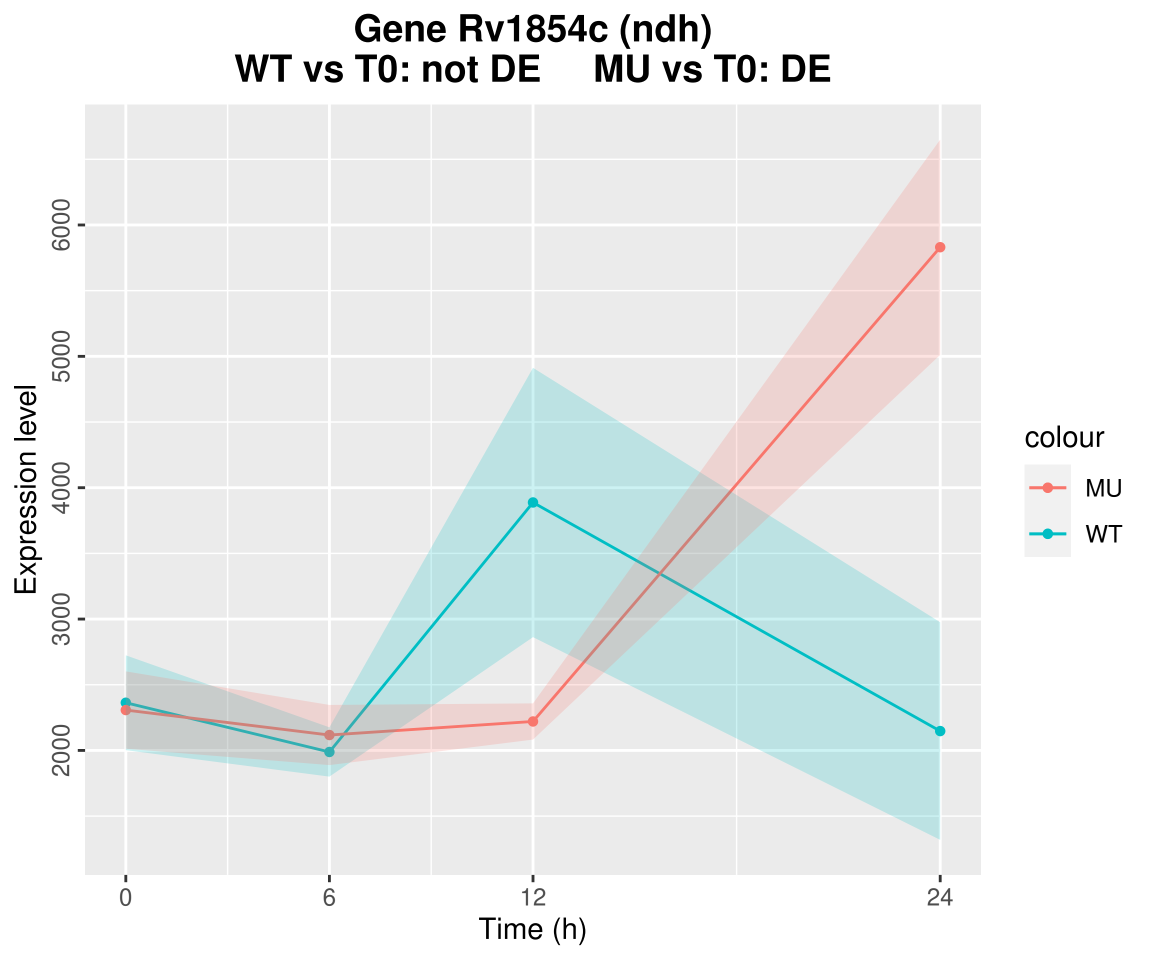
Gene Rv1854c (ndh)
WT vs T0: not DE   MU vs T0: DE
Expression level
6000
5000
4000
3000
2000
0
6
12
24
Time (h)
colour
MU
WT

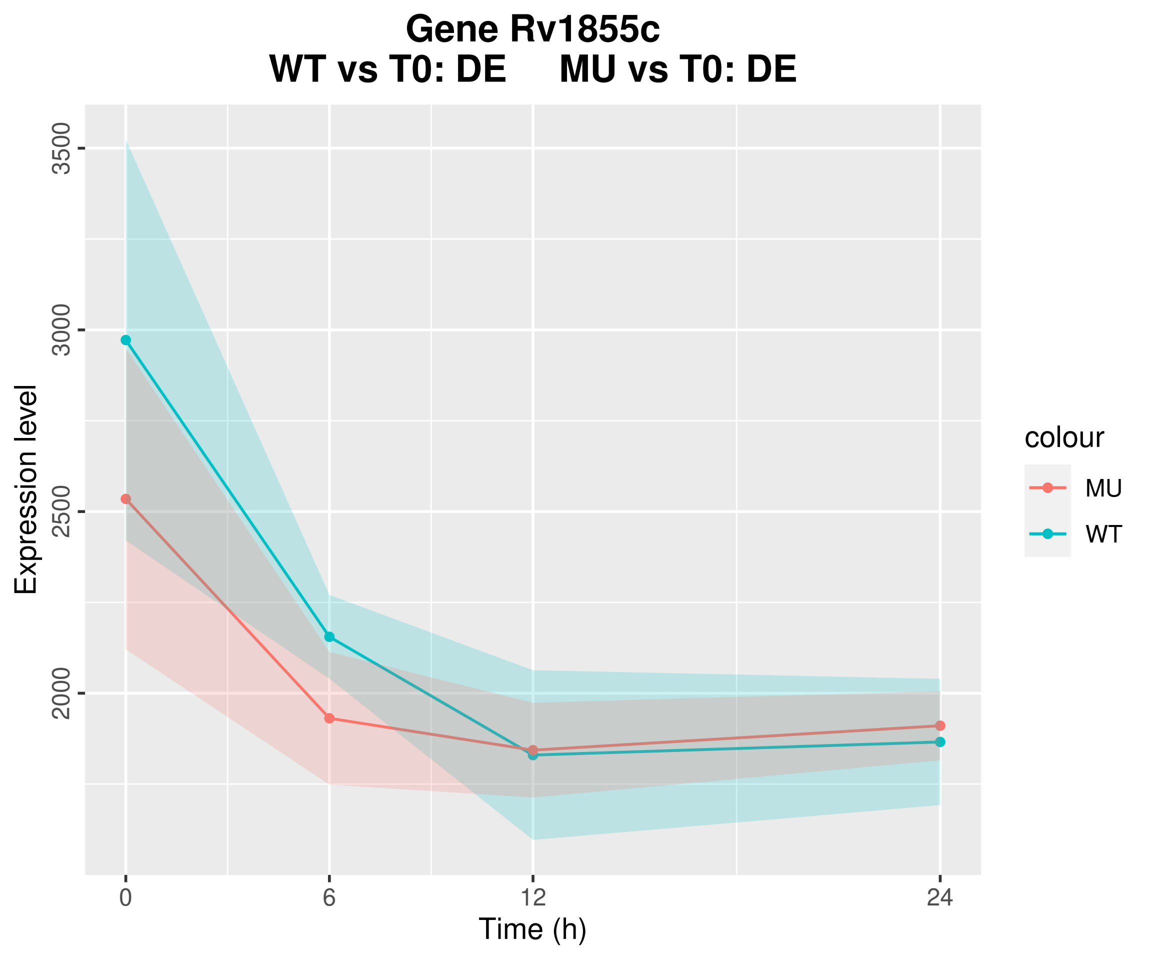

Gene Rv1855c
WT vs T0: DE MU vs T0: DE
Expression level
3500
3000
2500
2000
0
6
12
24
Time (h)
colour
MU
WT

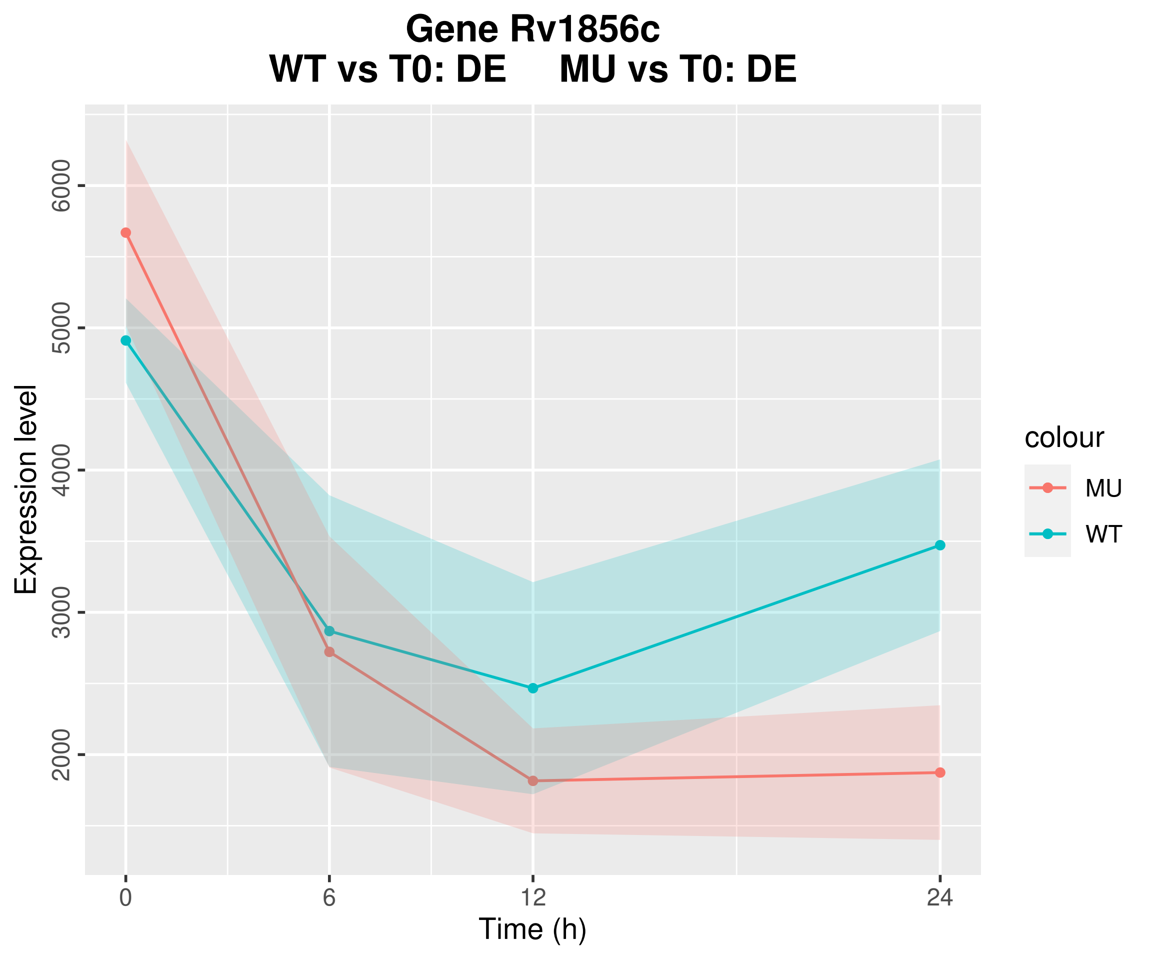
Gene Rv1856c
WT vs T0: DE MU vs T0: DE
Expression level
6000
5000
4000
3000
2000
0
6
12
24
Time (h)
colour
MU
WT

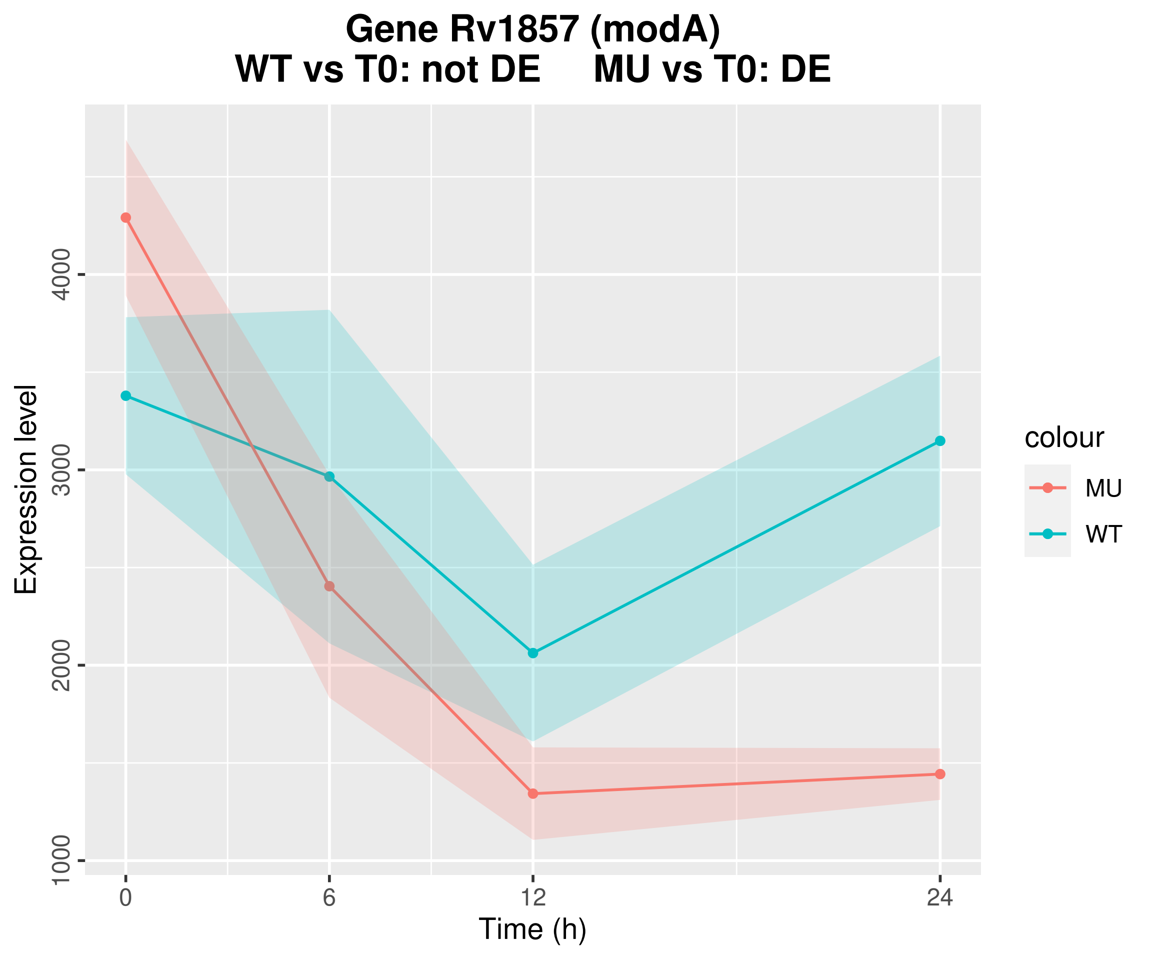
Gene Rv1857 (modA)
WT vs T0: not DE MU vs T0: DE
Expression level
1000
2000
3000
4000
0
6
12
24
Time (h)
colour
MU
WT

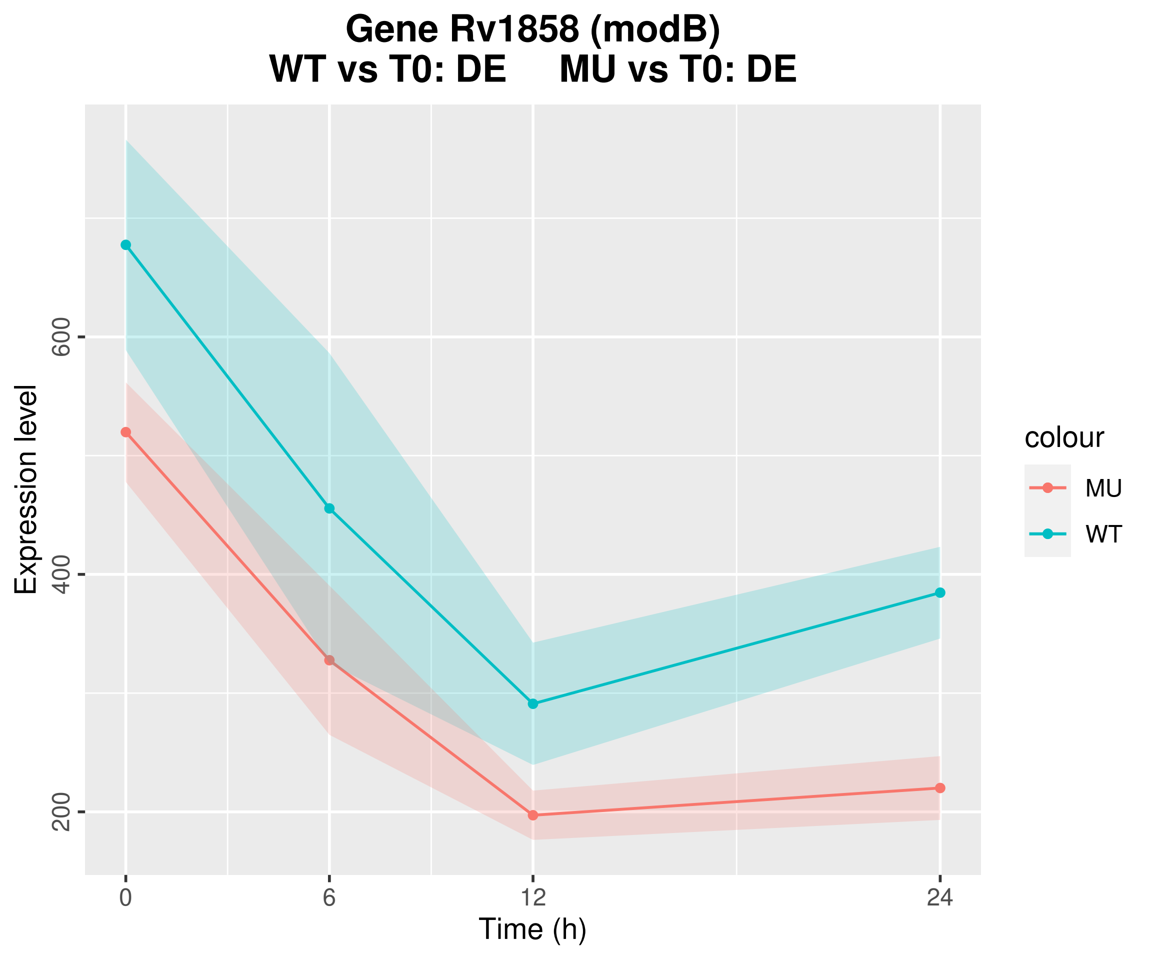
Gene Rv1858 (modB)
WT vs T0: DE   MU vs T0: DE
Expression level
600
400
200
0
6
12
24
Time (h)
colour
MU
WT

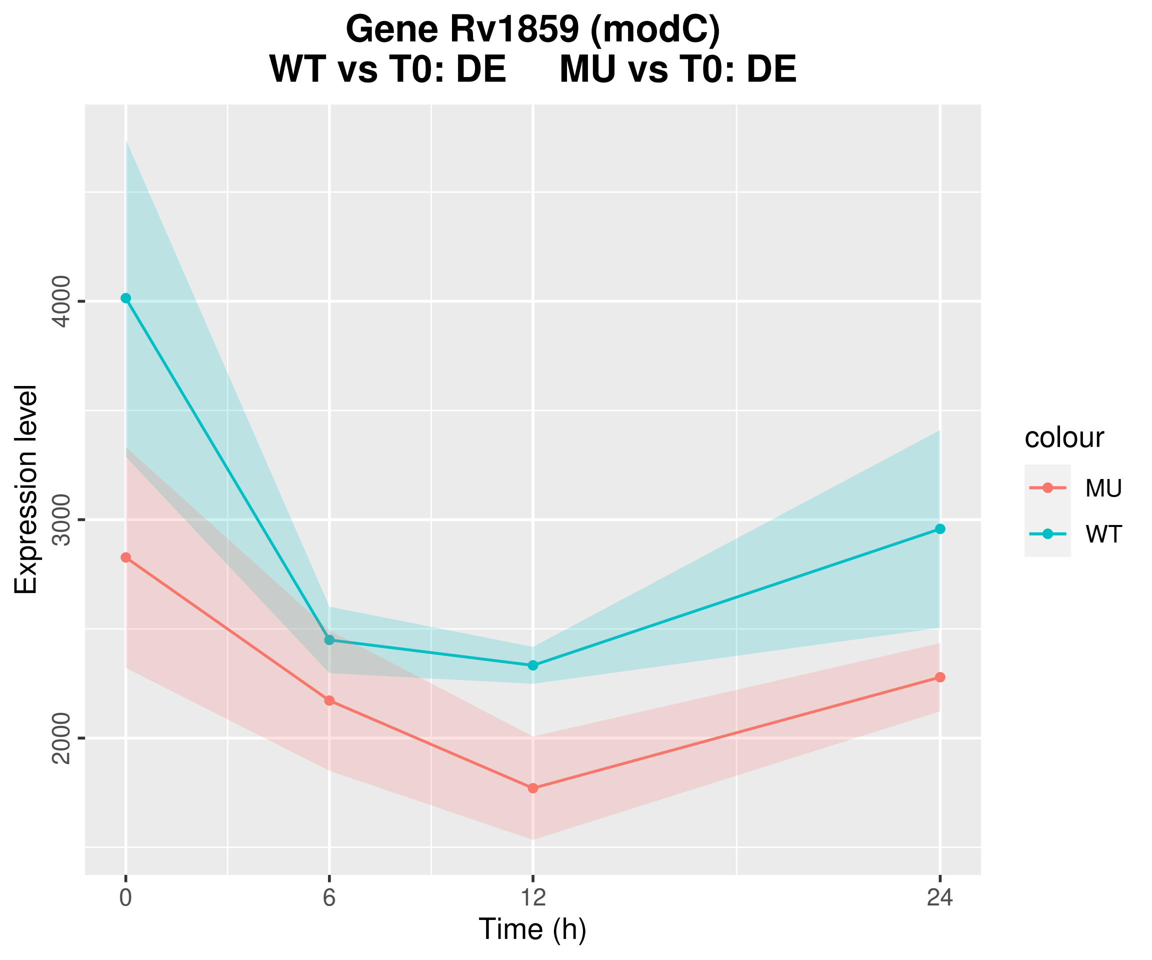
Gene Rv1859 (modC)
WT vs T0: DE MU vs T0: DE
Expression level
4000
3000
2000
0
6
12
24
Time (h)
colour
MU
WT

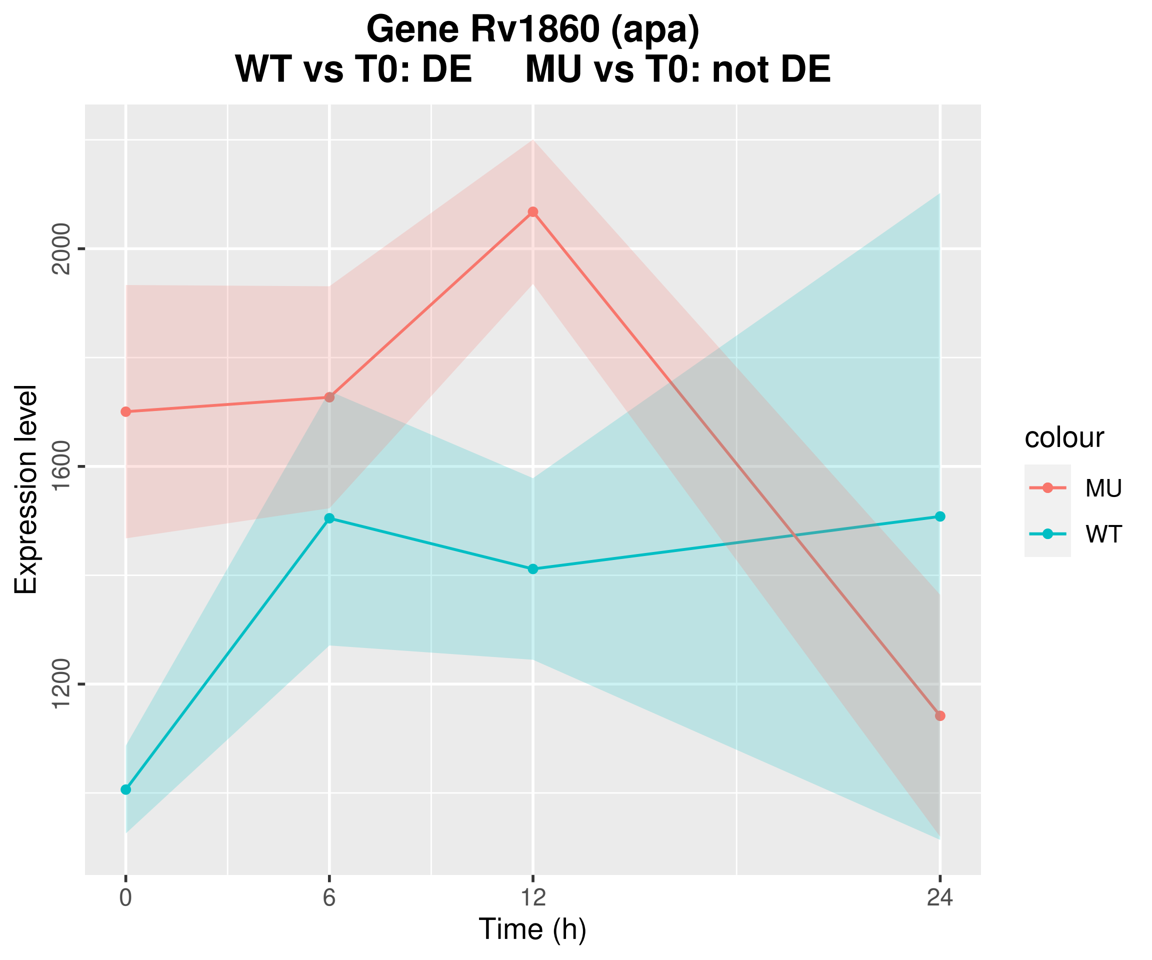
Gene Rv1860 (apa)
WT vs T0: DE   MU vs T0: not DE
Expression level
2000
1600
1200
0
6
12
24
Time (h)
colour
MU
WT

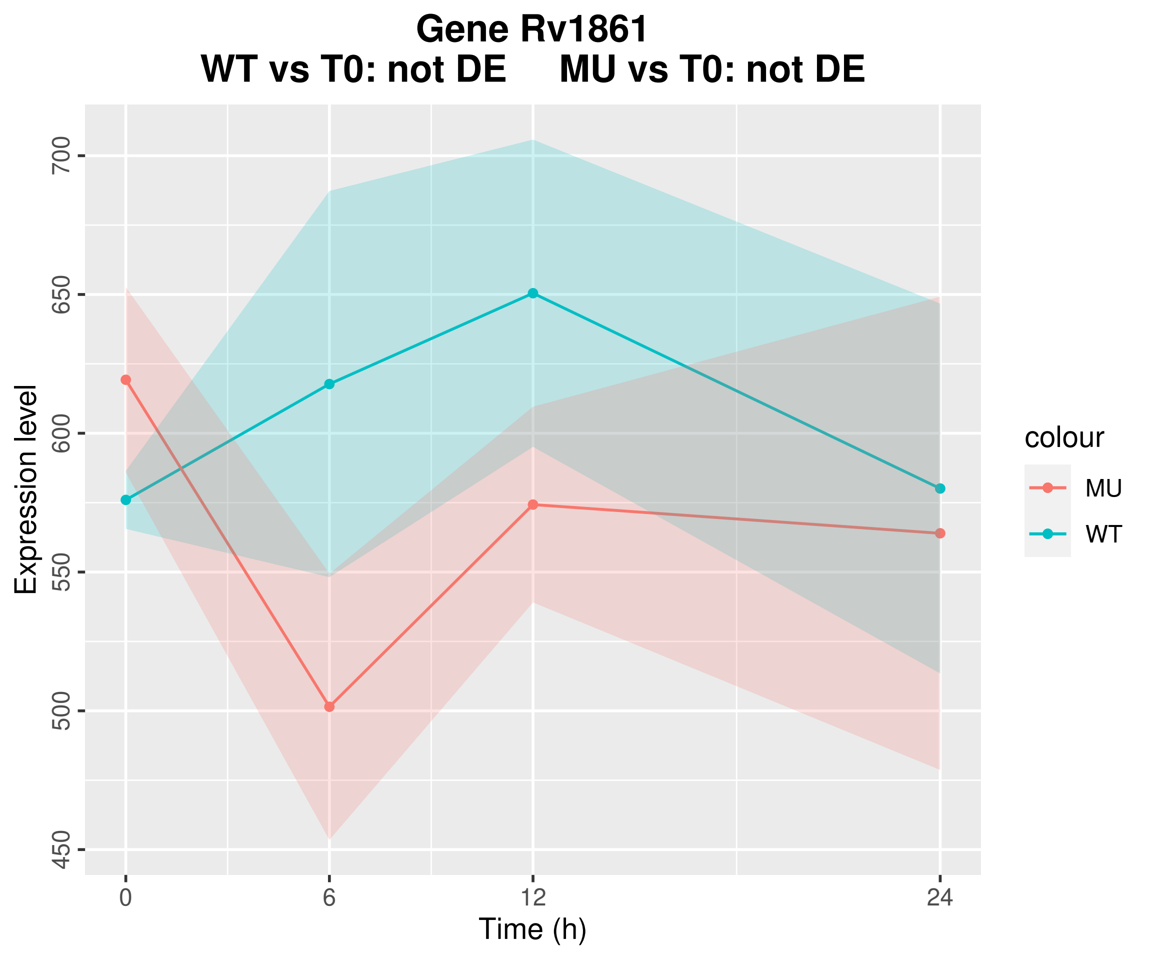
Gene Rv1861
WT vs T0: not DE MU vs T0: not DE
Expression level
450
500
550
600
650
700
0
6
12
24
Time (h)
colour
MU
WT

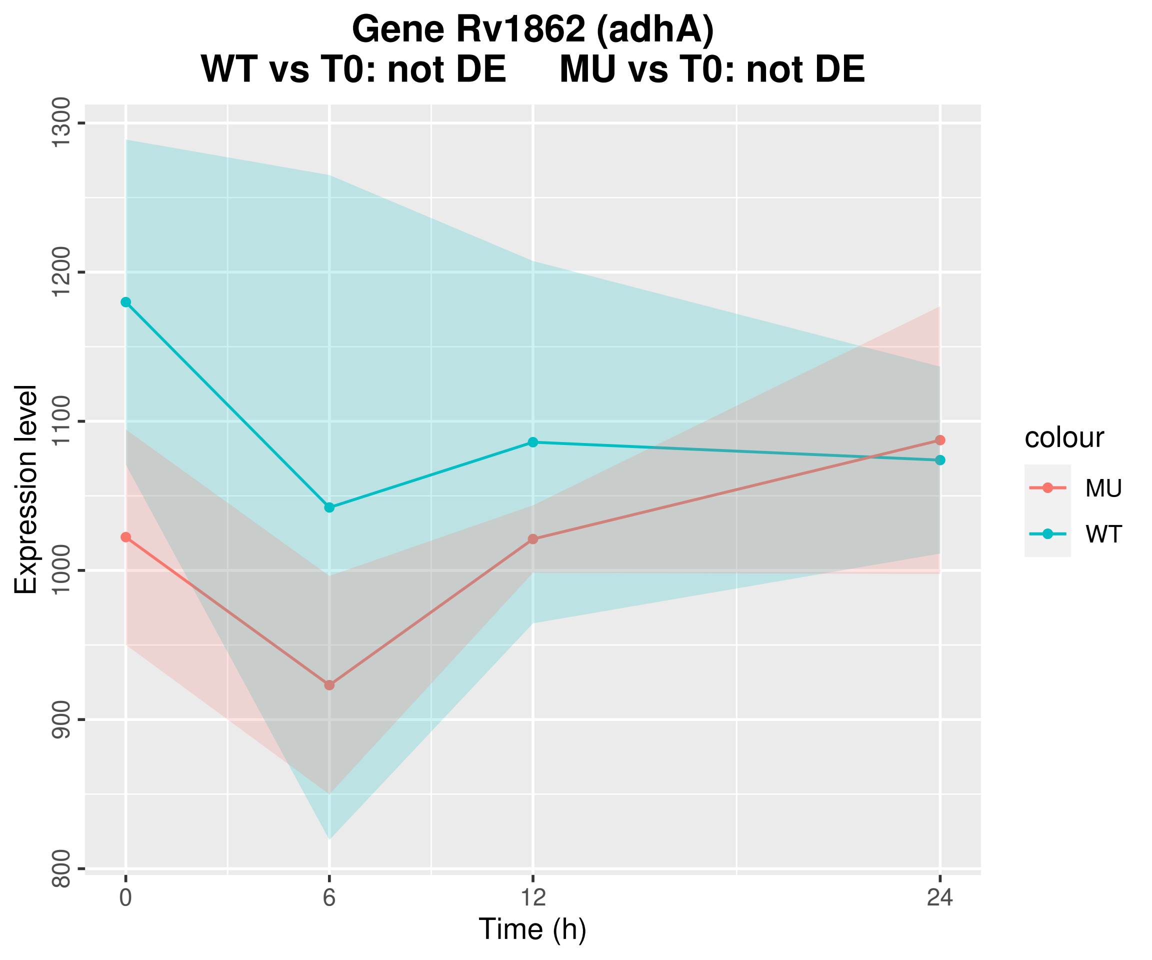

Gene Rv1862 (adhA)
WT vs T0: not DE MU vs T0: not DE
Expression level
1300
1200
1100
1000
900
800
0
6
12
24
Time (h)
colour
MU
WT

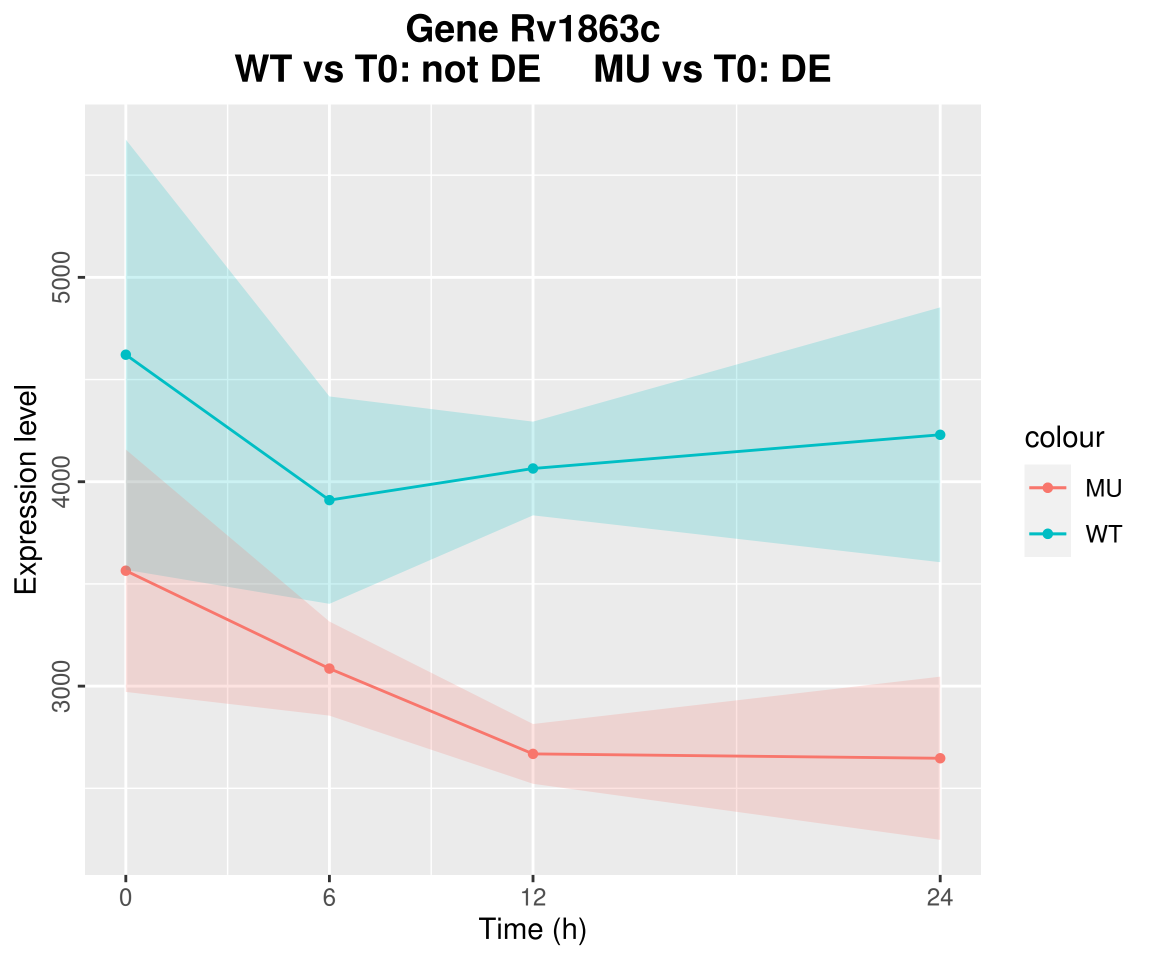
Gene Rv1863c
WT vs T0: not DE MU vs T0: DE
Expression level
5000
4000
3000
0
6
12
24
Time (h)
colour
MU
WT

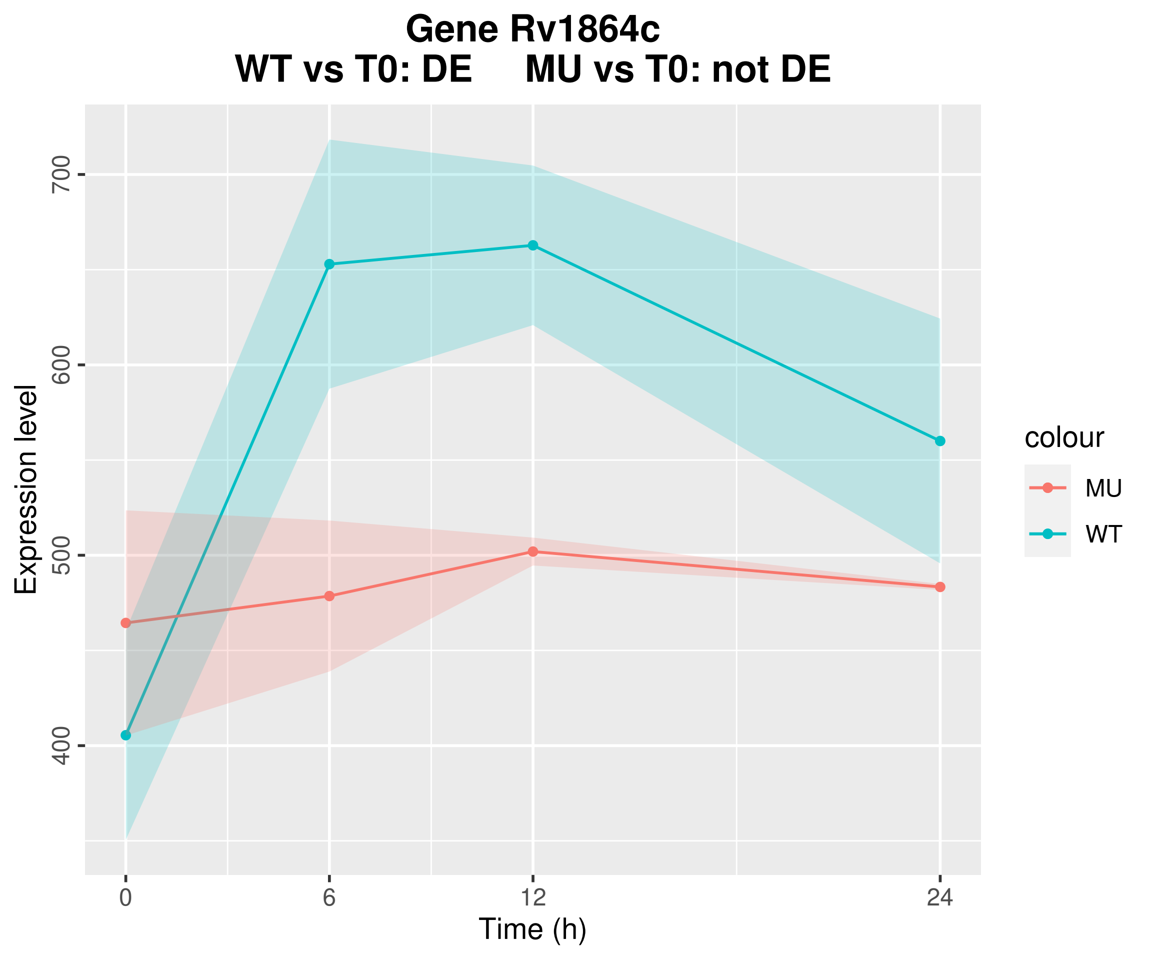
Gene Rv1864c
WT vs T0: DE   MU vs T0: not DE
Expression level
700
600
500
400
0
6
12
24
Time (h)
colour
MU
WT

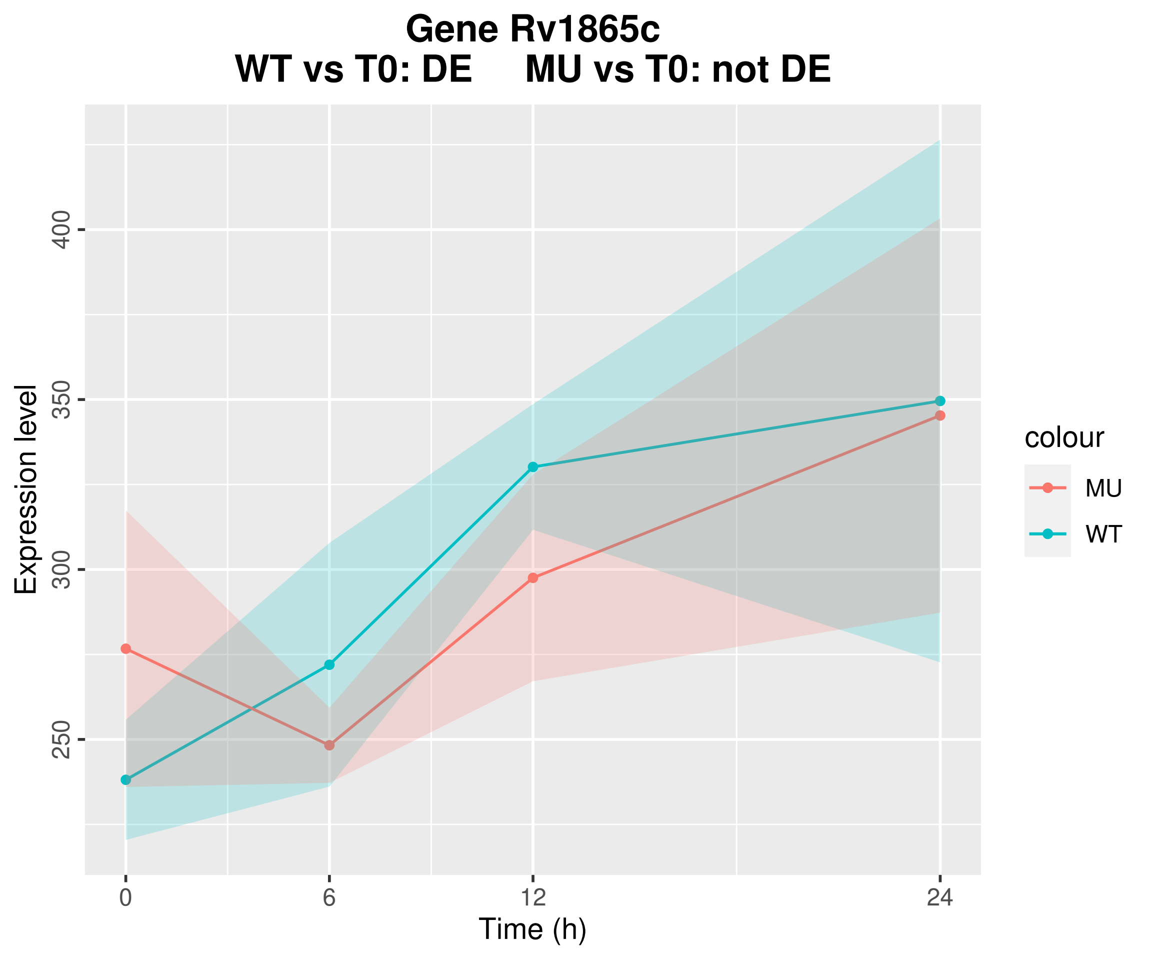
Gene Rv1865c
WT vs T0: DE MU vs T0: not DE
Expression level
250
300
350
400
0
6
12
24
Time (h)
colour
MU
WT

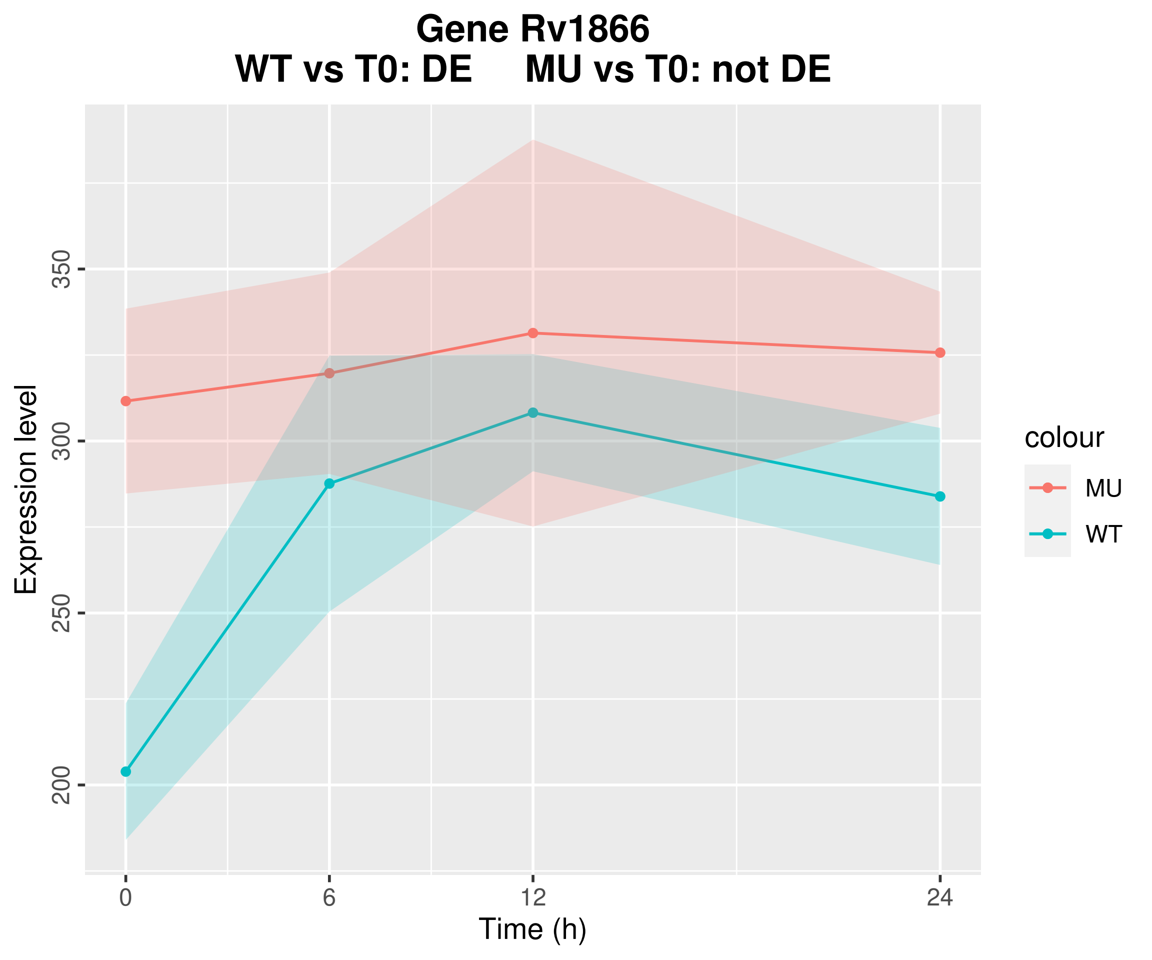
Gene Rv1866
WT vs T0: DE   MU vs T0: not DE
Expression level
350
300
250
200
0
6
12
24
Time (h)
colour
MU
WT

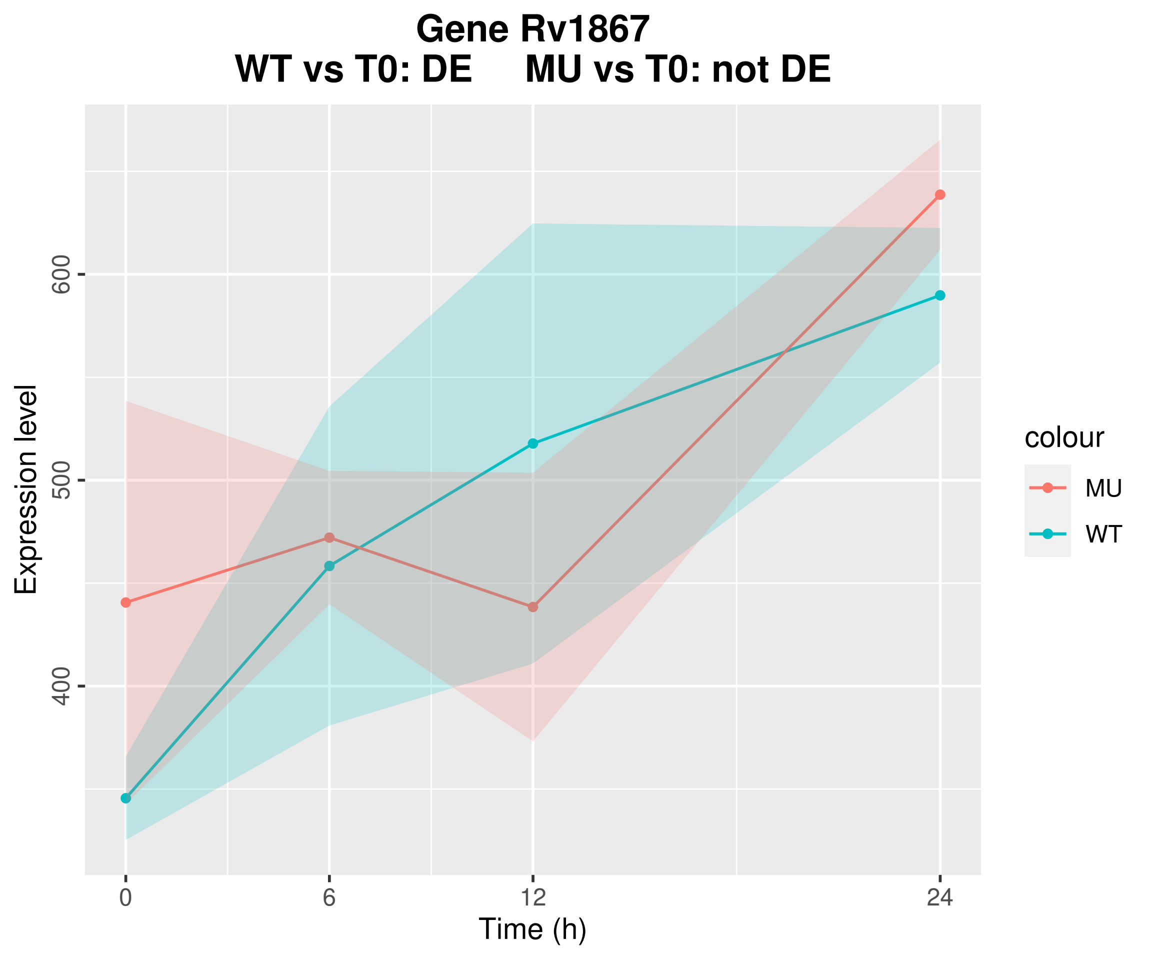

Gene Rv1867
WT vs T0: DE MU vs T0: not DE
Expression level
400
500
600
0
6
12
24
Time (h)
colour
MU
WT

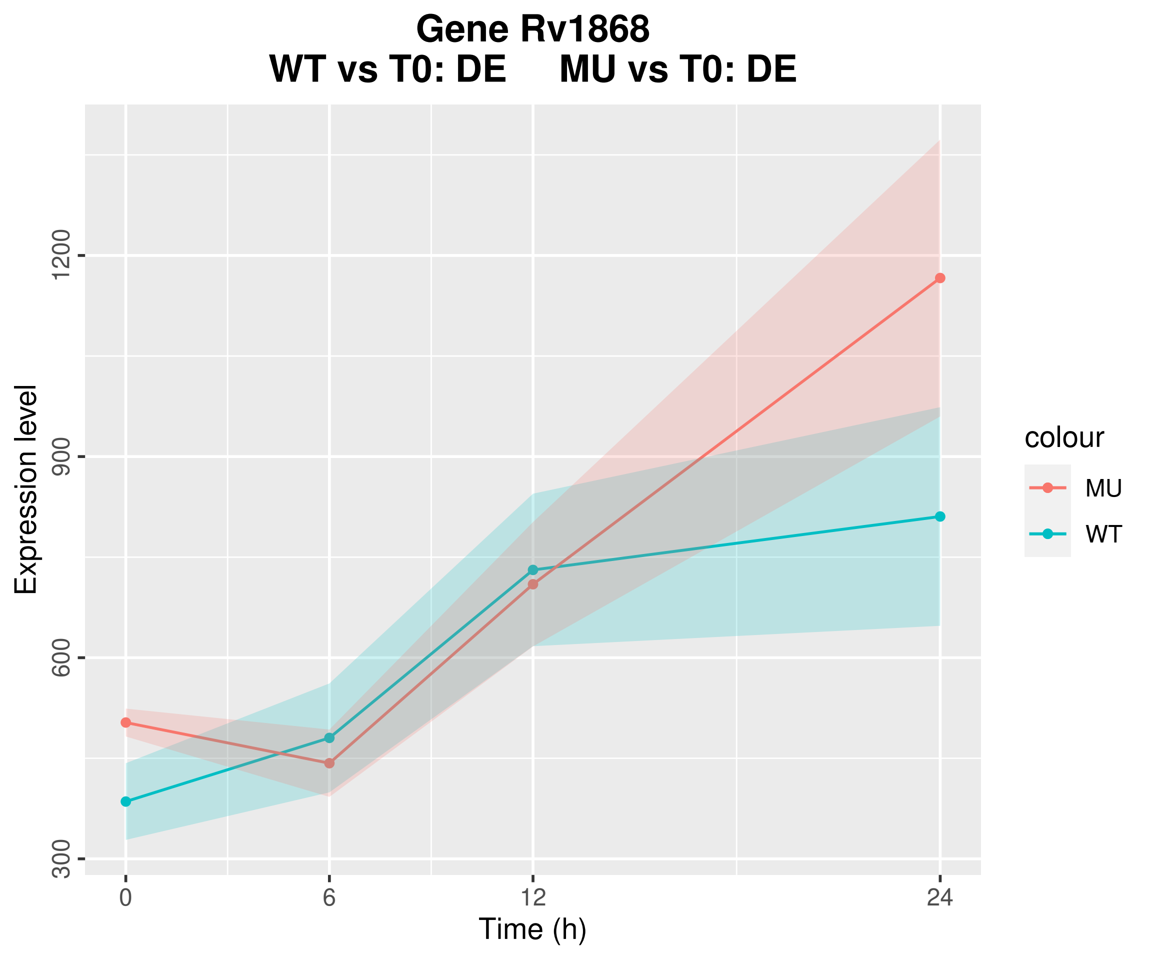

Gene Rv1868
WT vs T0: DE MU vs T0: DE
Expression level
1200
900
600
300
0
6
12
24
Time (h)
colour
MU
WT

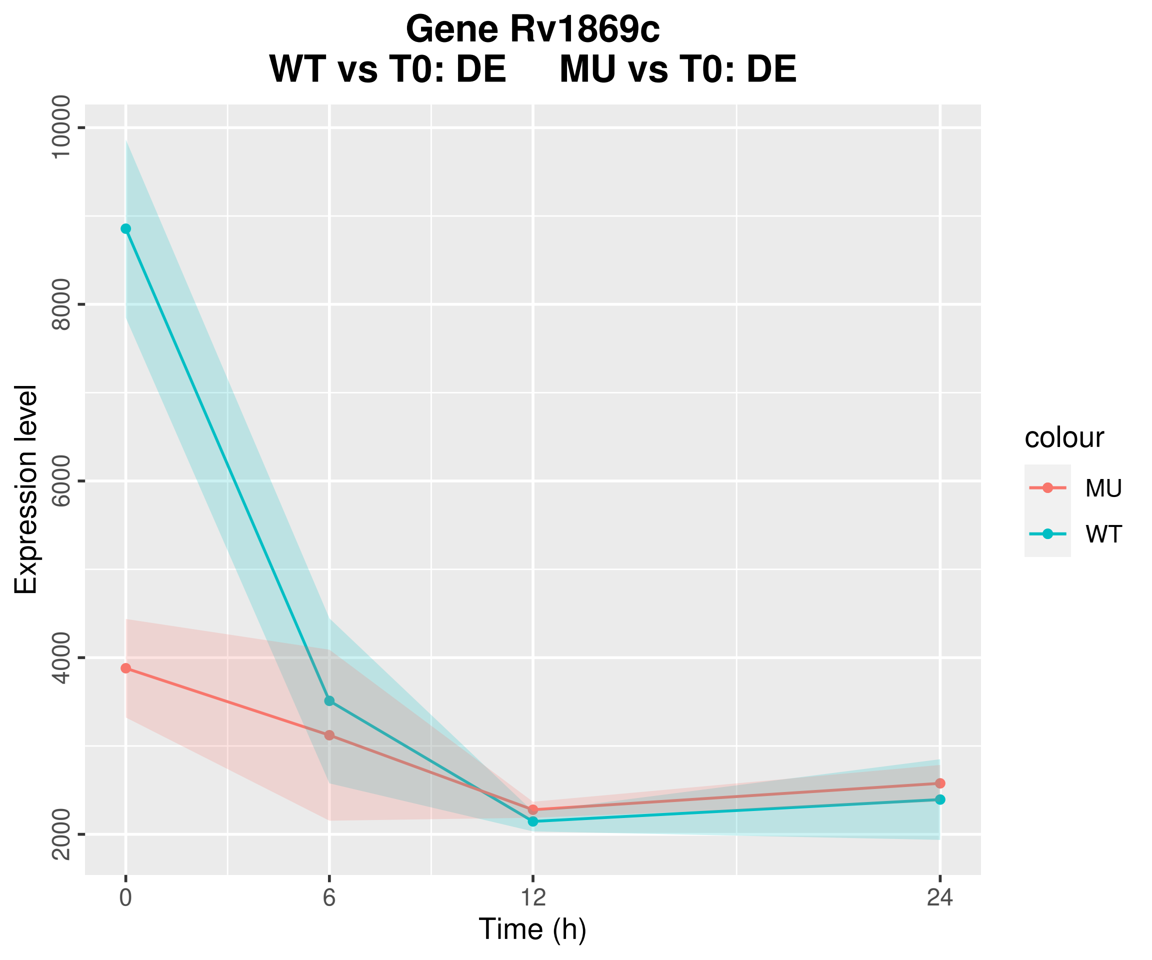
Gene Rv1869c
WT vs T0: DE   MU vs T0: DE
Expression level
10000
8000
6000
4000
2000
0
6
12
24
Time (h)
colour
MU
WT

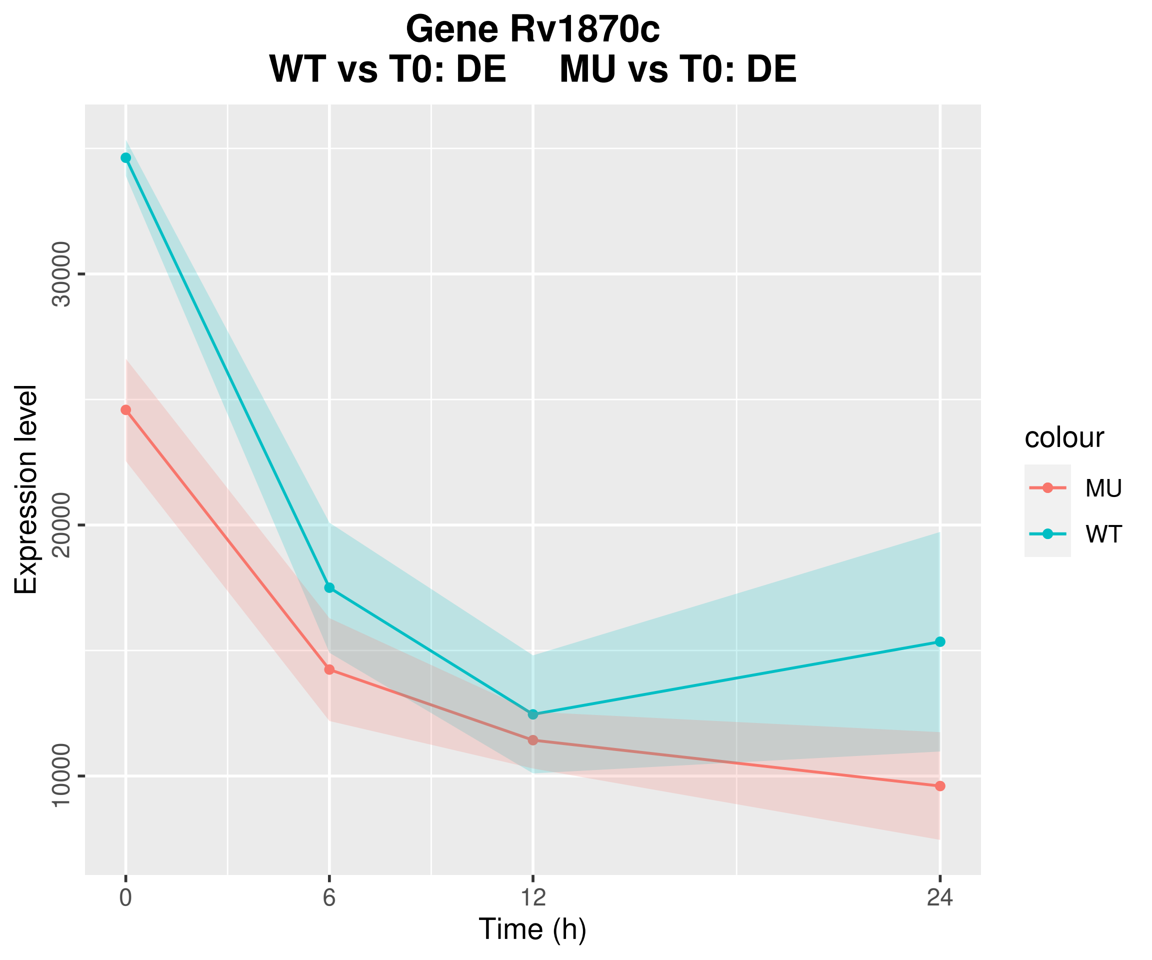
Gene Rv1870c
WT vs T0: DE MU vs T0: DE
Expression level
30000
20000
10000
0
6
12
24
Time (h)
colour
MU
WT

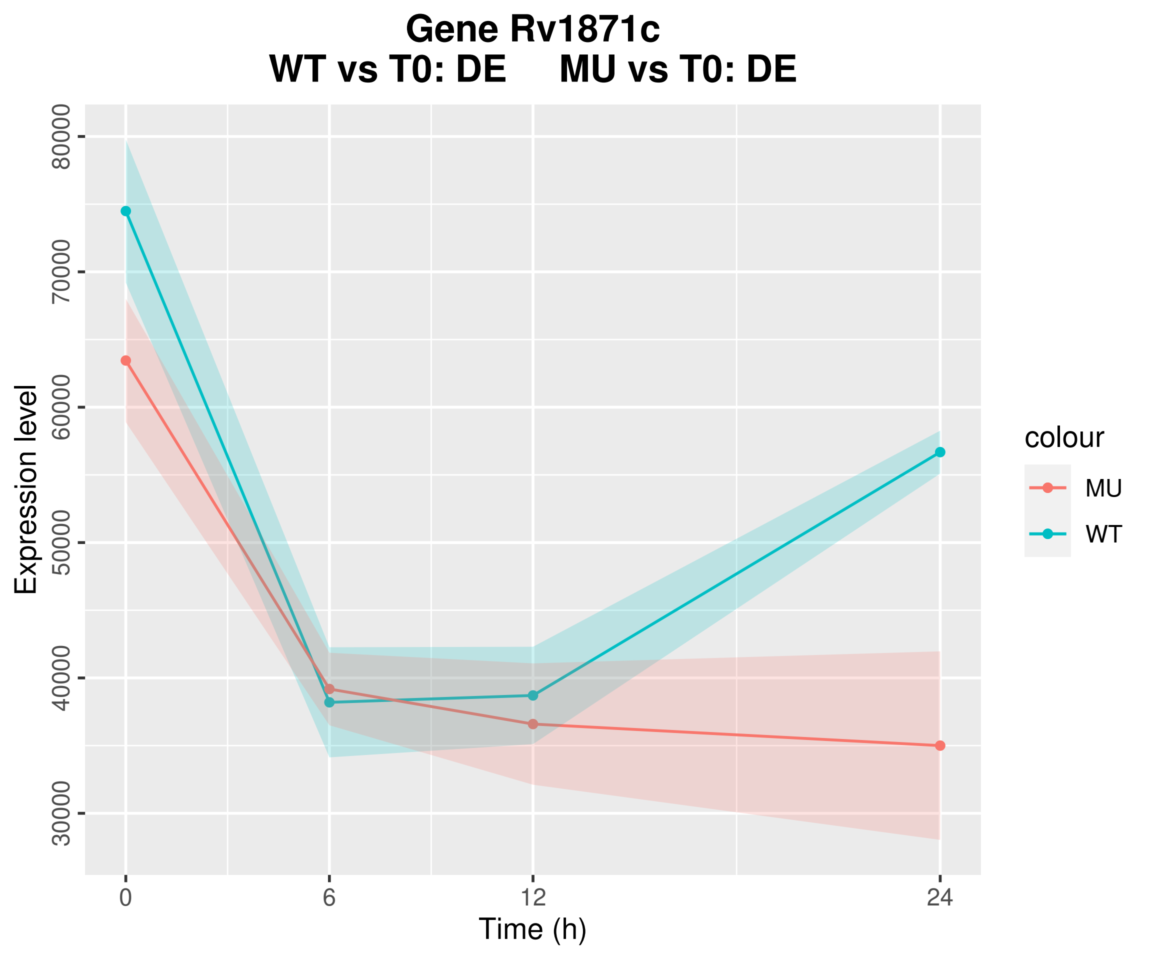

Gene Rv1871c
WT vs T0: DE MU vs T0: DE
Expression level
80000
70000
60000
50000
40000
30000
0
6
12
24
Time (h)
colour
MU
WT

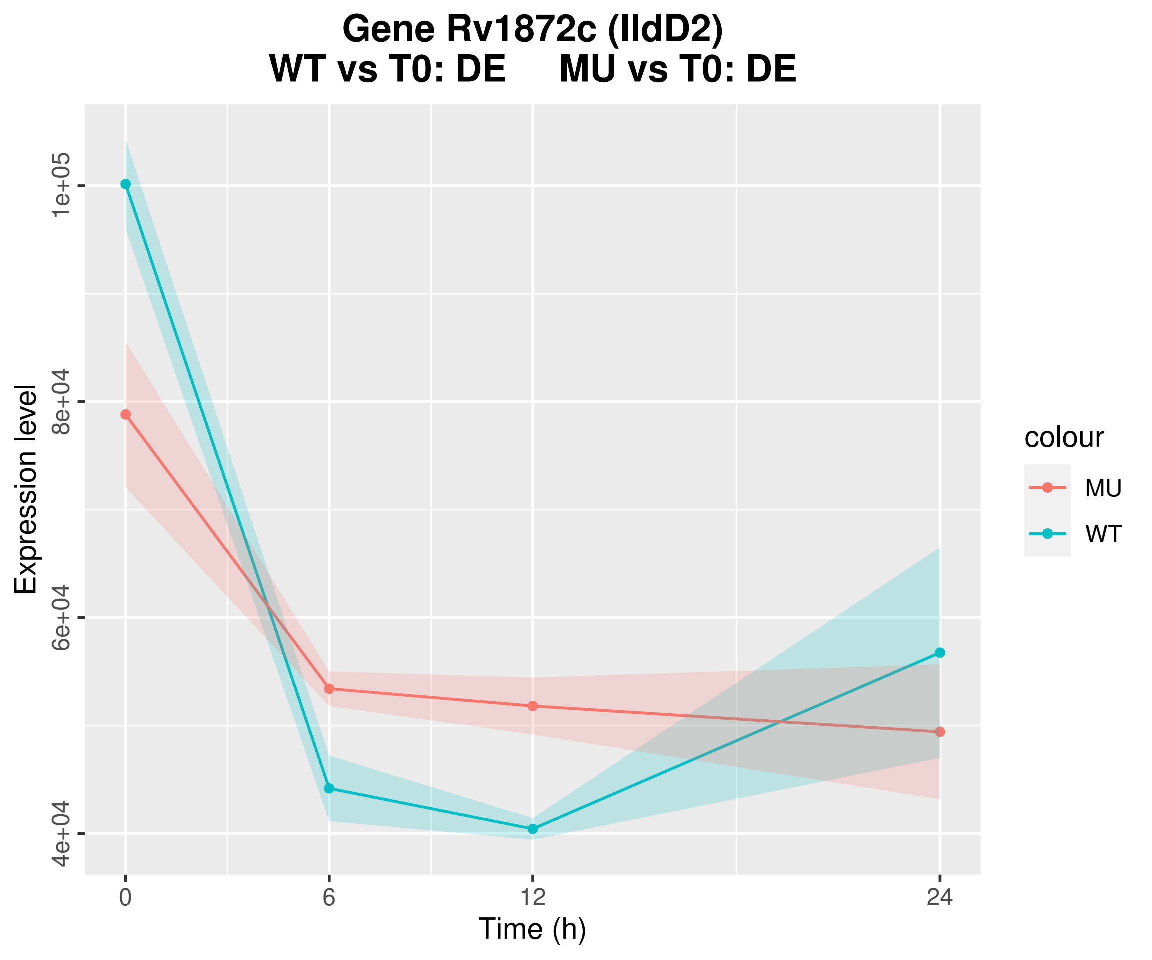

Gene Rv1872c (lldD2)
WT vs T0: DE MU vs T0: DE
Expression level
1e+05
8e+04
6e+04
4e+04
0
6
12
24
Time (h)
colour
MU
WT

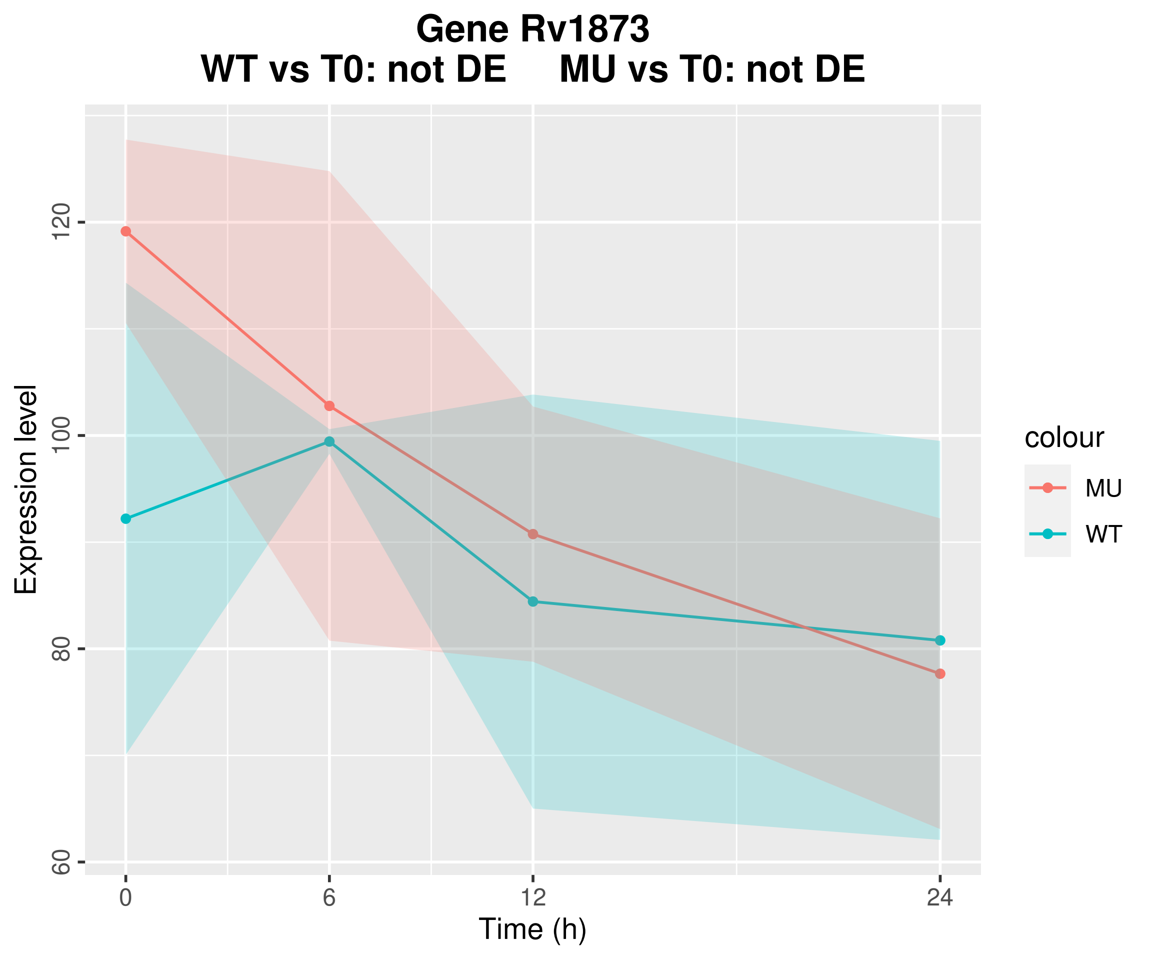

Gene Rv1873
WT vs T0: not DE MU vs T0: not DE
Expression level
120
100
80
60
0
6
12
24
Time (h)
colour
MU
WT

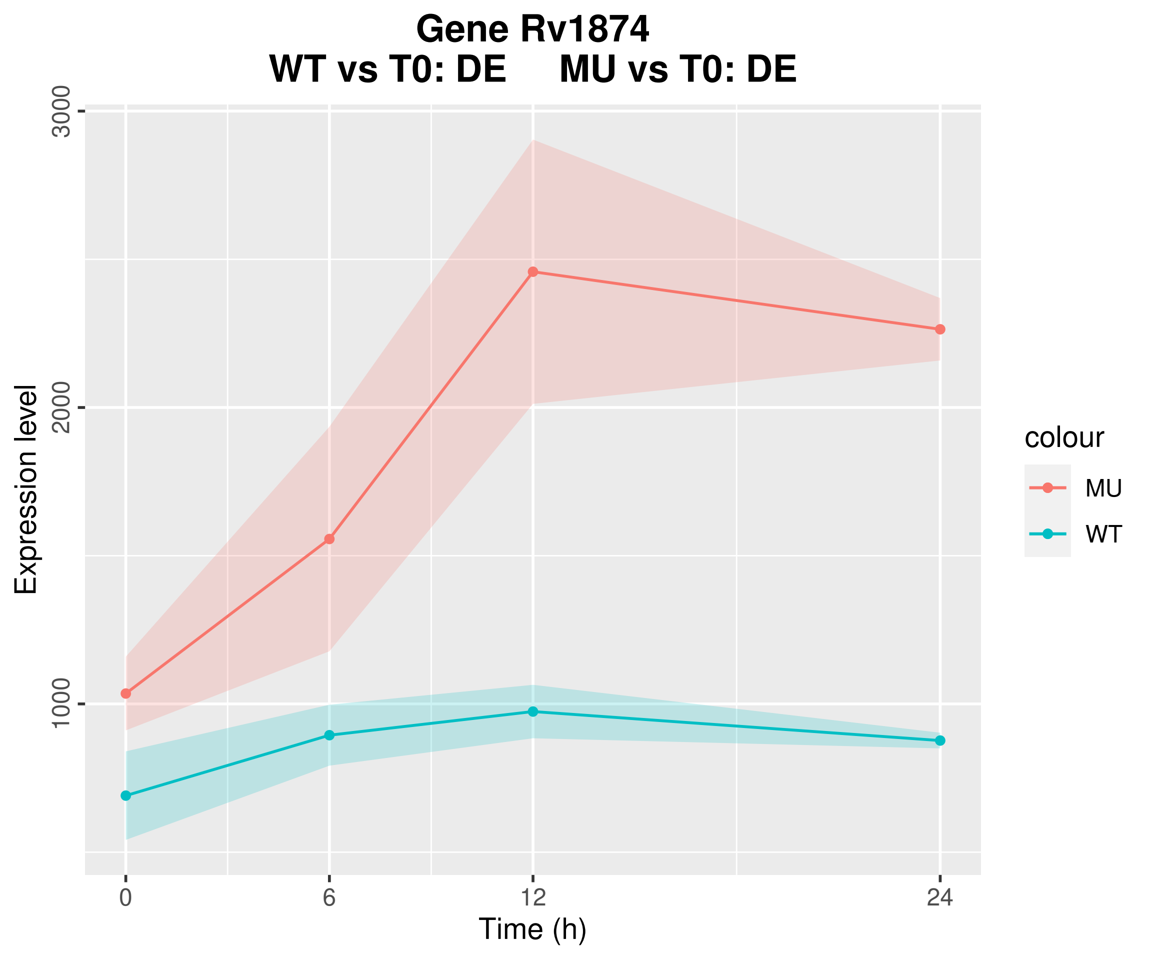

Gene Rv1874
WT vs T0: DE MU vs T0: DE
Expression level
3000
2000
1000
0
6
12
24
Time (h)
colour
MU
WT

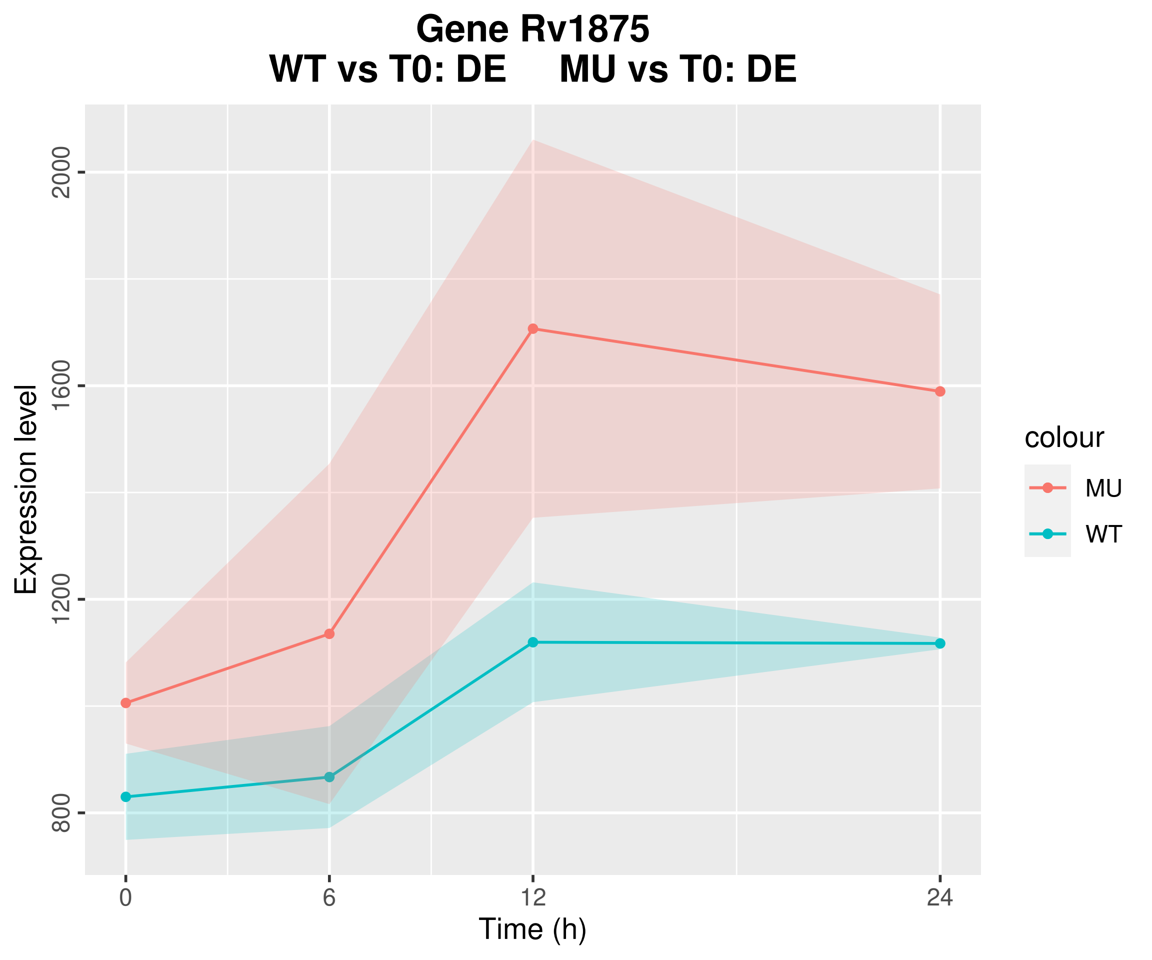
Gene Rv1875
WT vs T0: DE MU vs T0: DE
Expression level
2000
1600
1200
800
0
6
12
24
Time (h)
colour
MU
WT

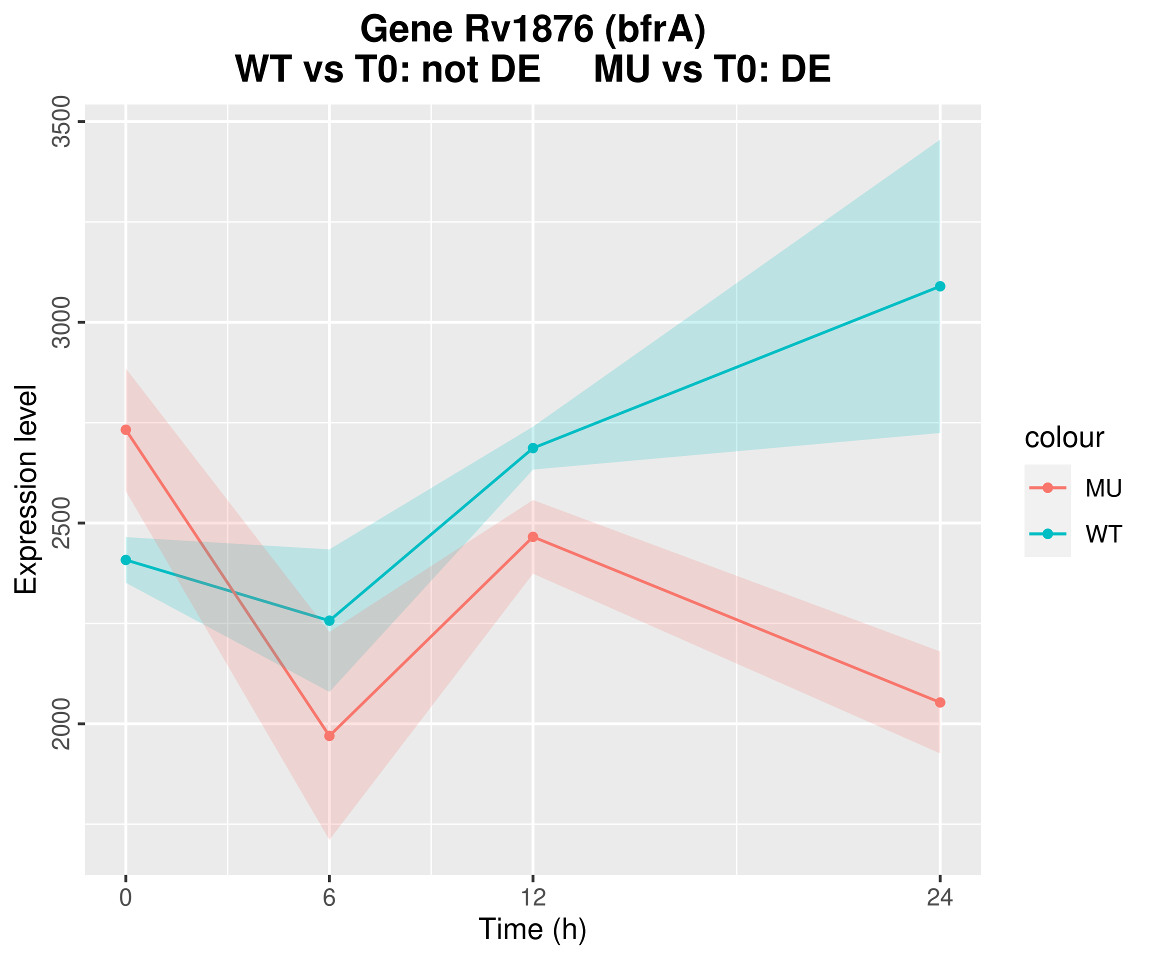

Gene Rv1876 (bfrA)
WT vs T0: not DE MU vs T0: DE
3500
3000
2500
2000
Expression level
0
6
12
24
Time (h)
colour
MU
WT

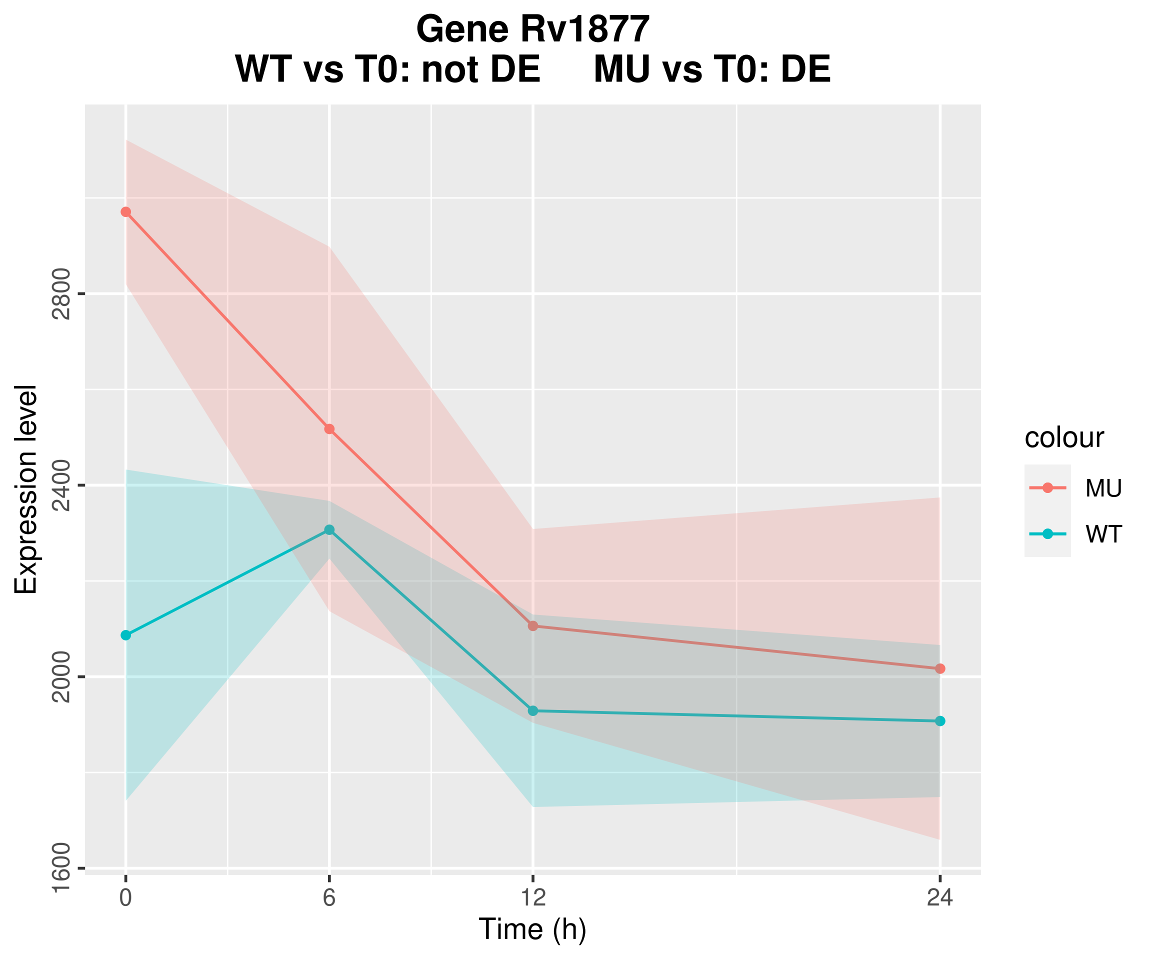

Gene Rv1877
WT vs T0: not DE MU vs T0: DE
Expression level
2800
2400
2000
1600
0
6
12
24
Time (h)
colour
MU
WT

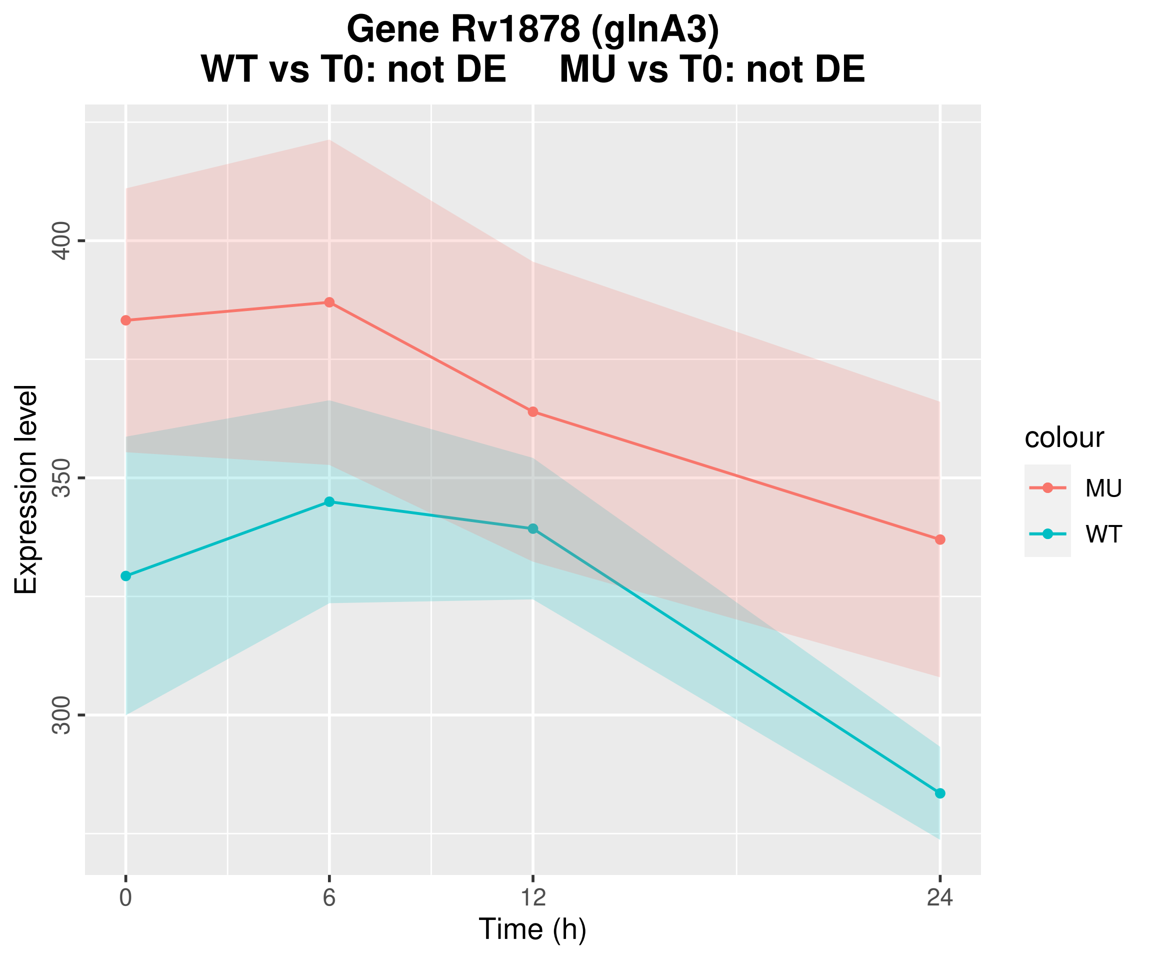

Gene Rv1878 (glnA3)
WT vs T0: not DE   MU vs T0: not DE
Expression level
400
350
300
0
6
12
24
Time (h)
colour
MU
WT

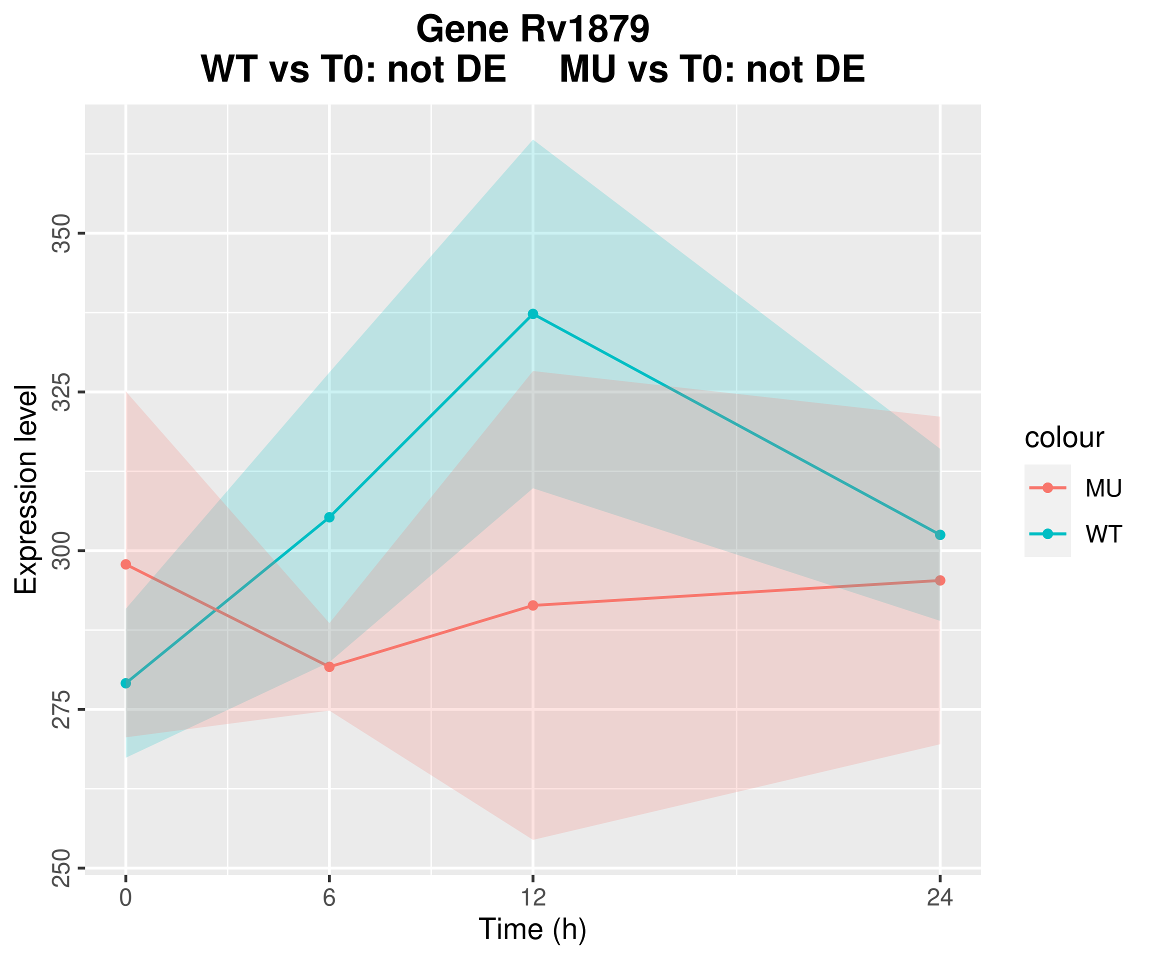
Gene Rv1879
WT vs T0: not DE   MU vs T0: not DE
Expression level
350
325
300
275
250
0
6
12
24
Time (h)
colour
MU
WT

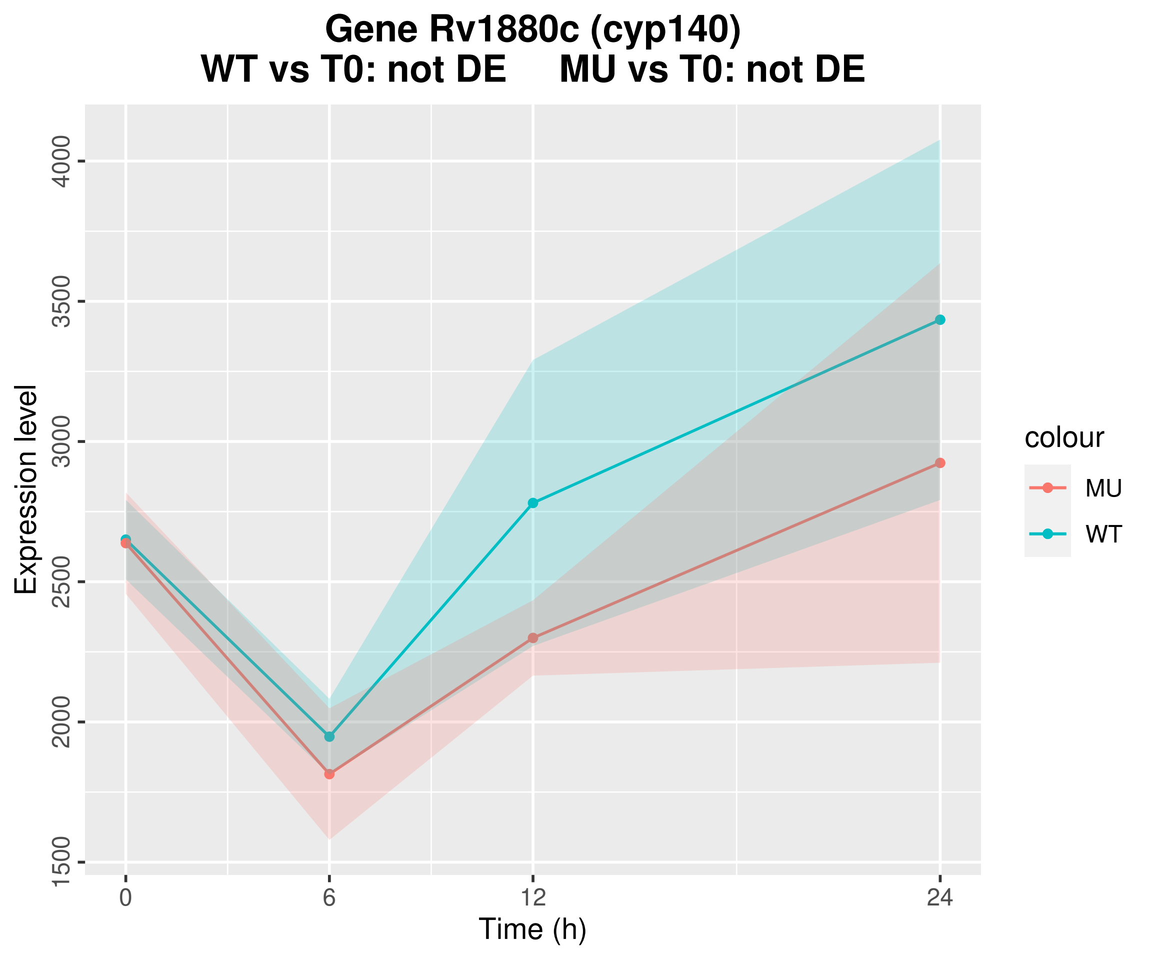
Gene Rv1880c (cyp140)
WT vs T0: not DE   MU vs T0: not DE
Expression level
Time (h)
1500
2000
2500
3000
3500
4000
0
6
12
24
colour
MU
WT

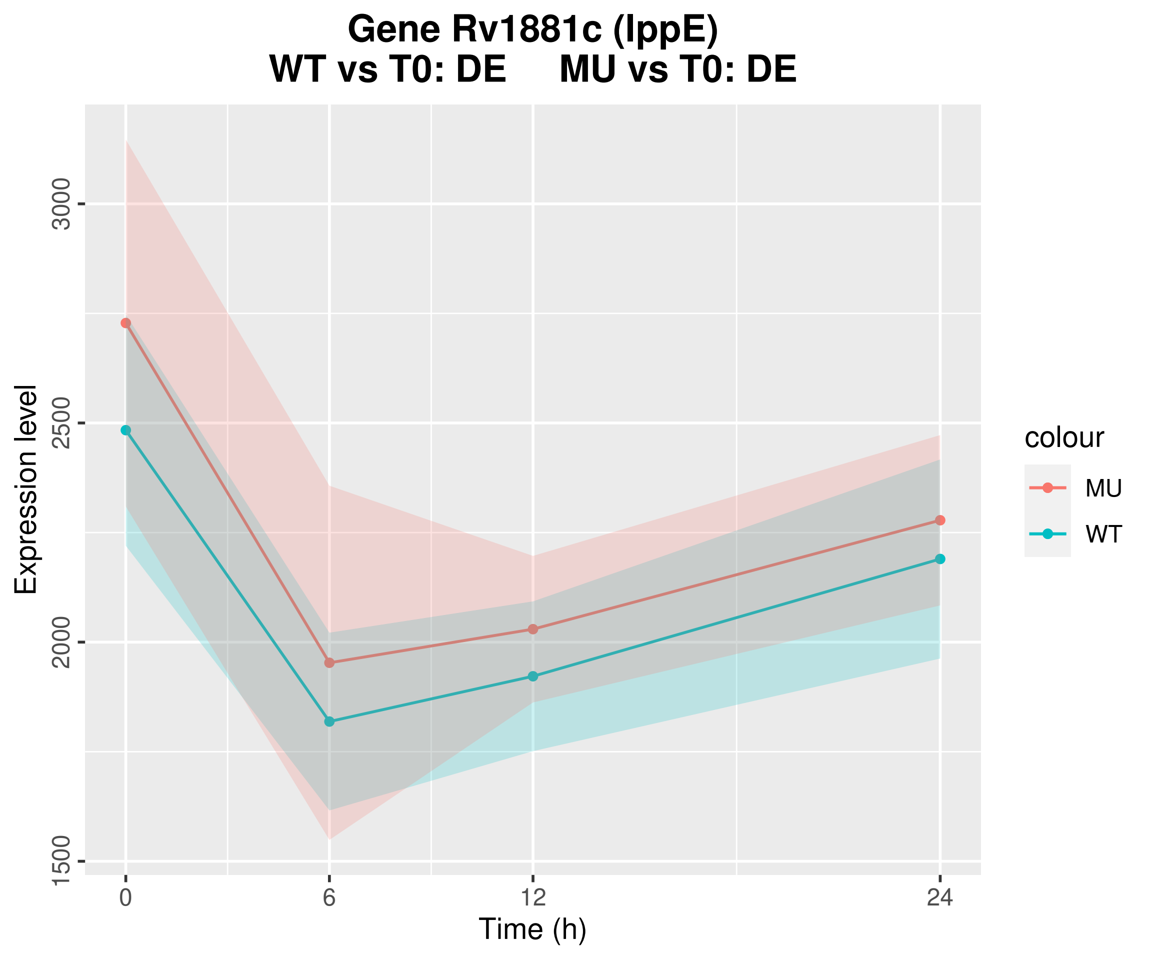

Gene Rv1881c (lppE)
WT vs T0: DE   MU vs T0: DE
Expression level
3000
2500
2000
1500
0
6
12
24
Time (h)
colour
MU
WT

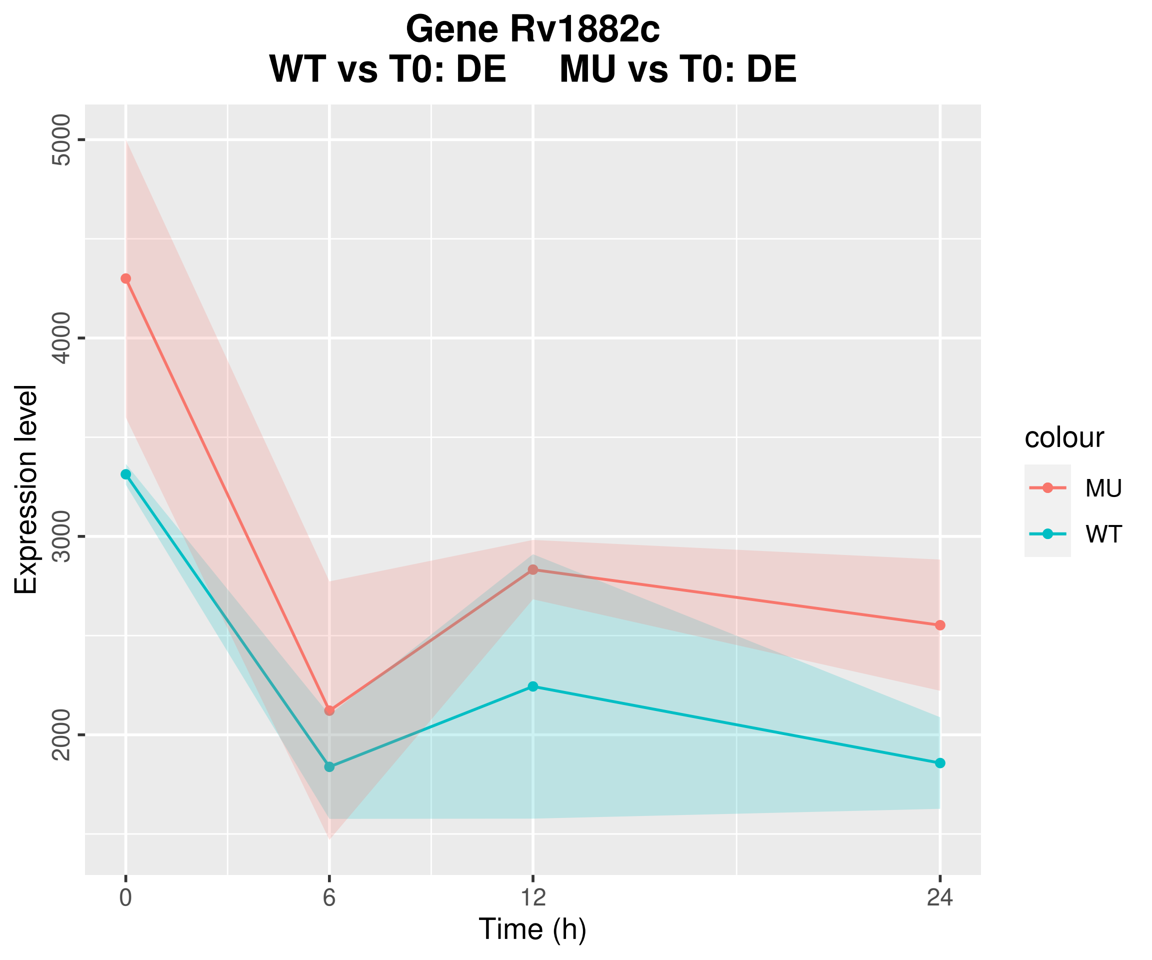

Gene Rv1882c
WT vs T0: DE MU vs T0: DE
Expression level
5000
4000
3000
2000
0
6
12
24
Time (h)
colour
MU
WT

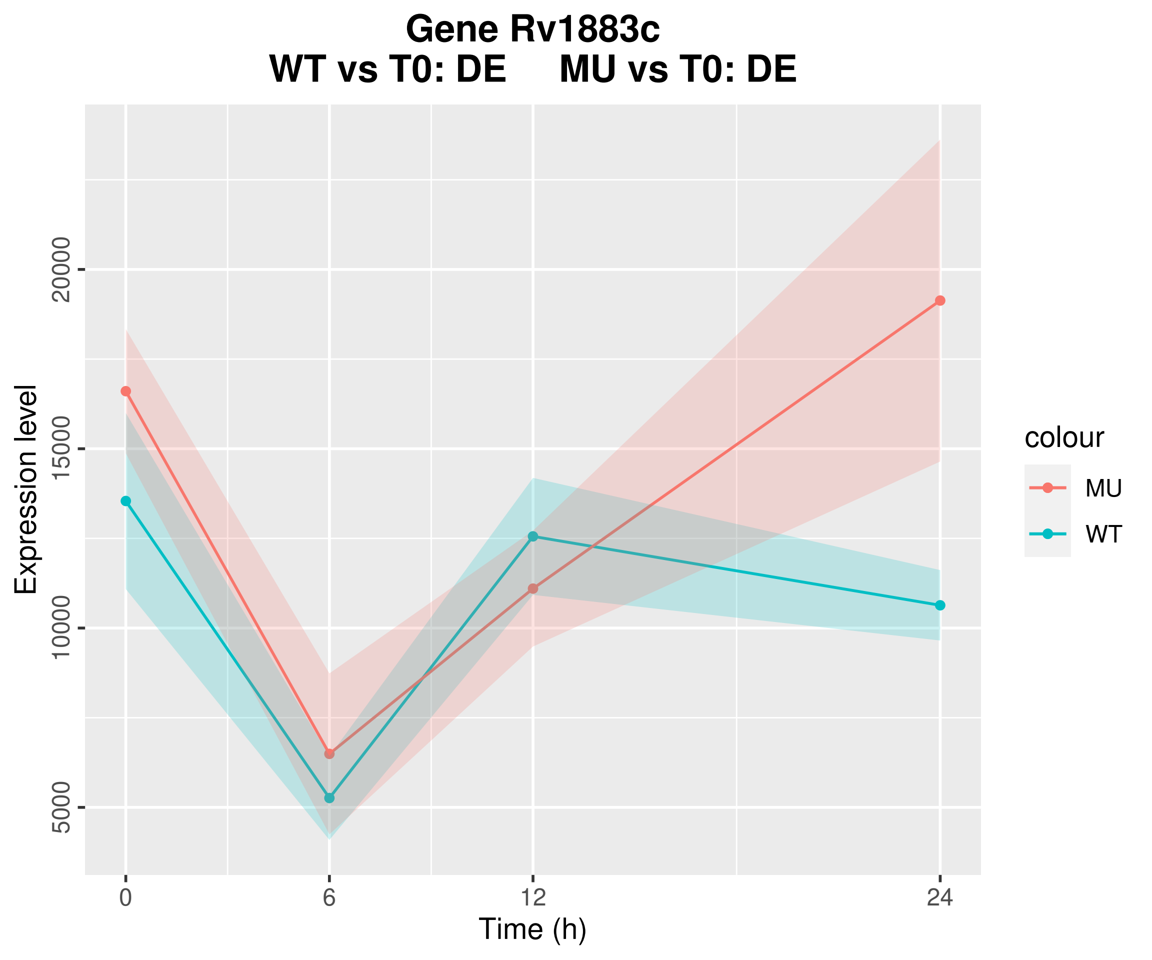

Gene Rv1883c
WT vs T0: DE MU vs T0: DE
Expression level
5000
10000
15000
20000
0
6
12
24
Time (h)
colour
MU
WT

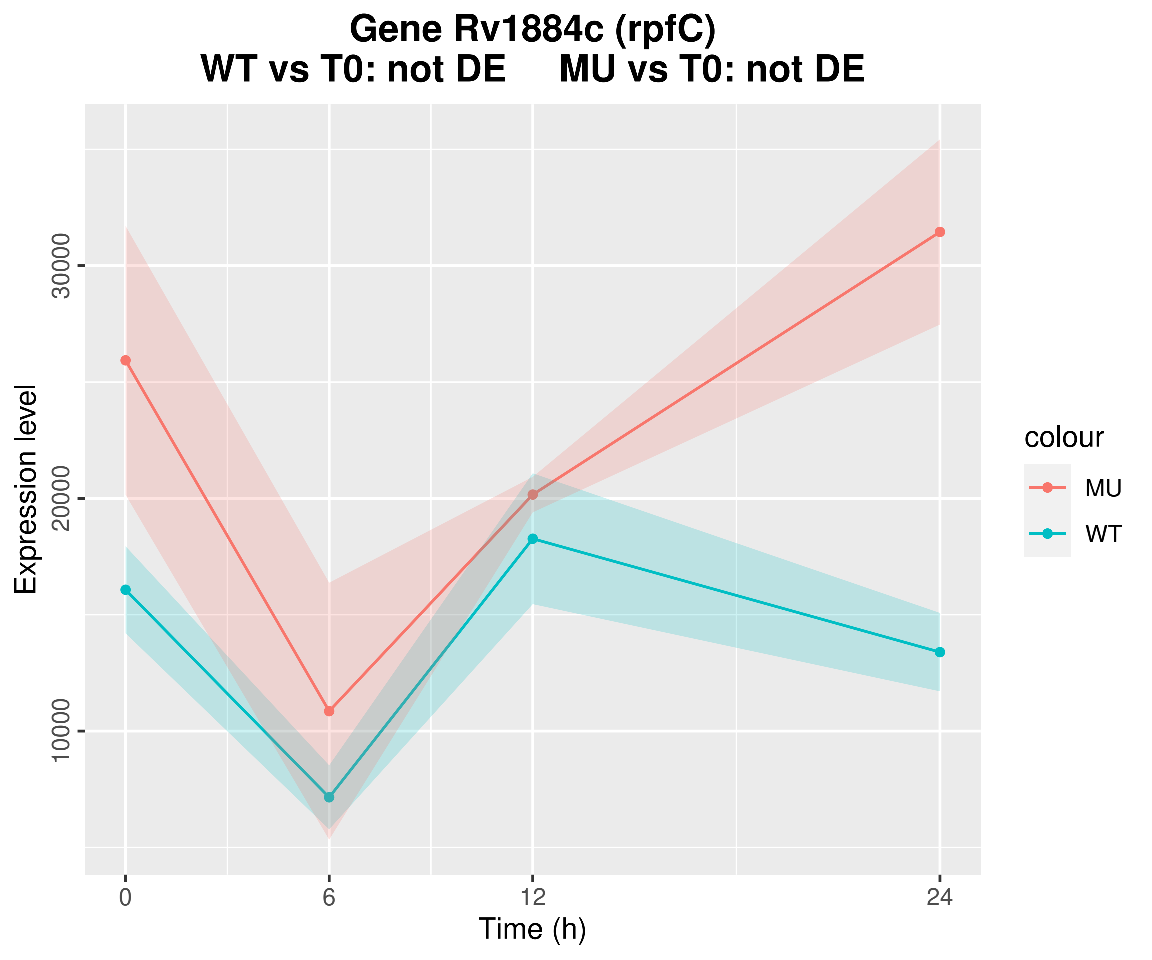
Gene Rv1884c (rpfC)
WT vs T0: not DE   MU vs T0: not DE
Expression level
30000
20000
10000
0
6
12
24
Time (h)
colour
MU
WT

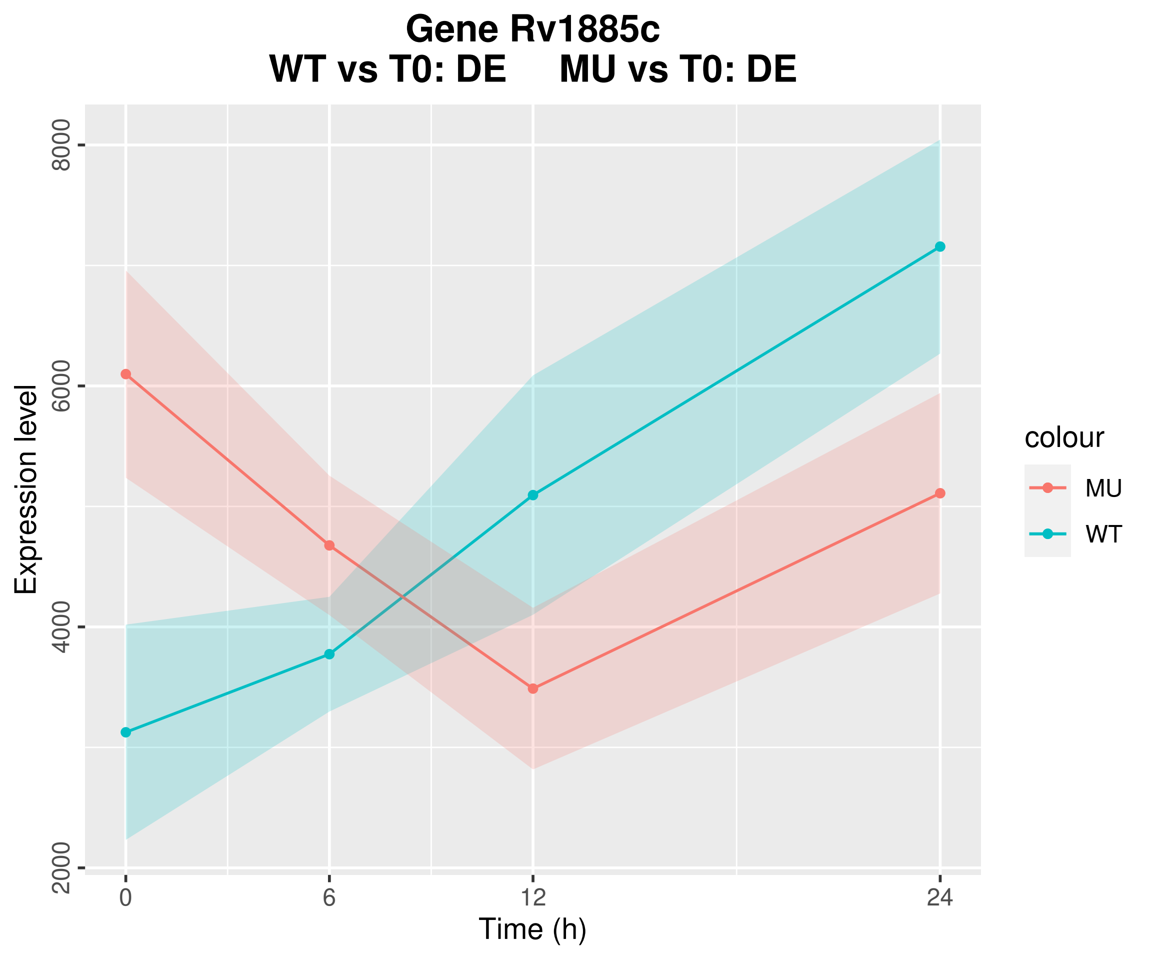
Gene Rv1885c
WT vs T0: DE   MU vs T0: DE
Expression level
8000
6000
4000
2000
0
6
12
24
Time (h)
colour
MU
WT

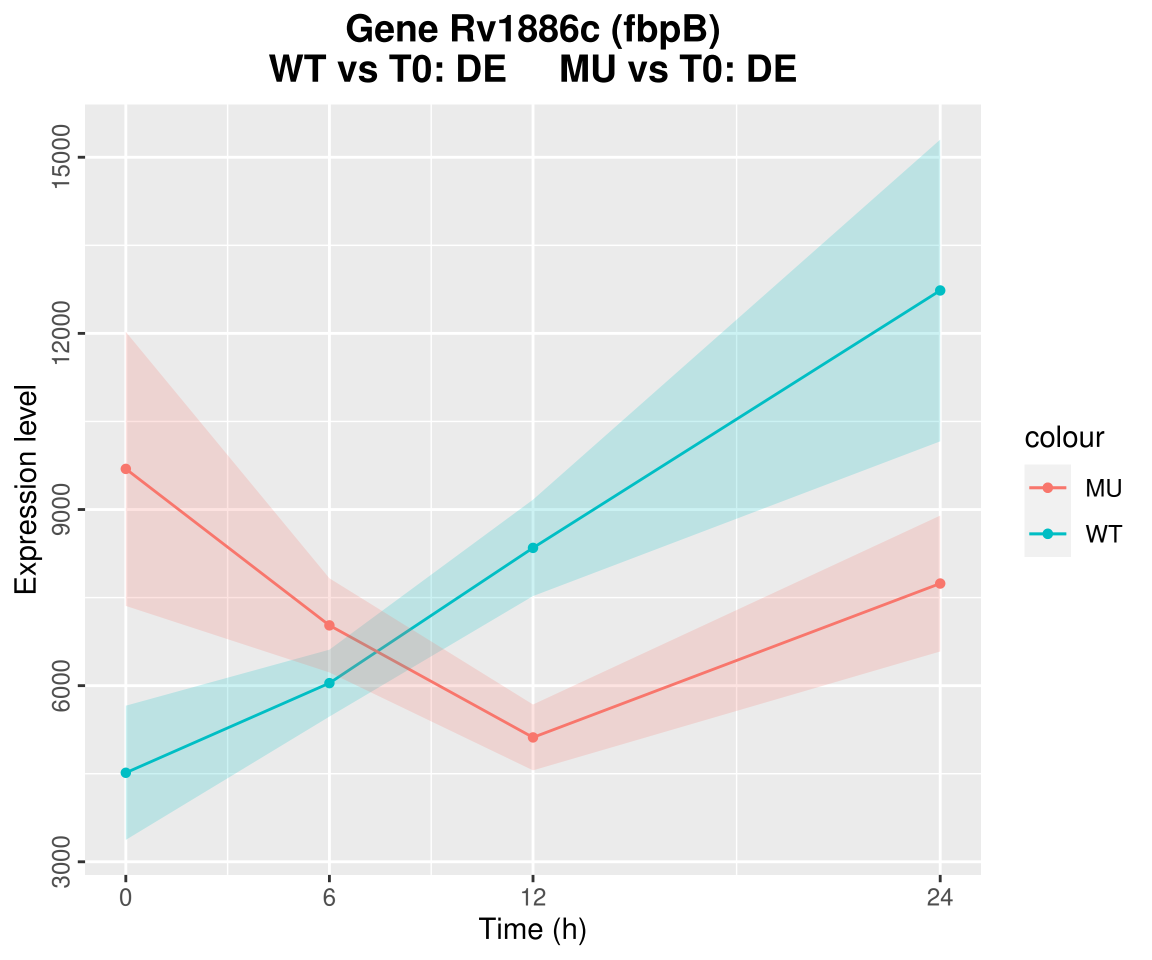

Gene Rv1886c (fbpB)
WT vs T0: DE MU vs T0: DE
Expression level
15000
12000
9000
6000
3000
0
6
12
24
Time (h)
colour
MU
WT

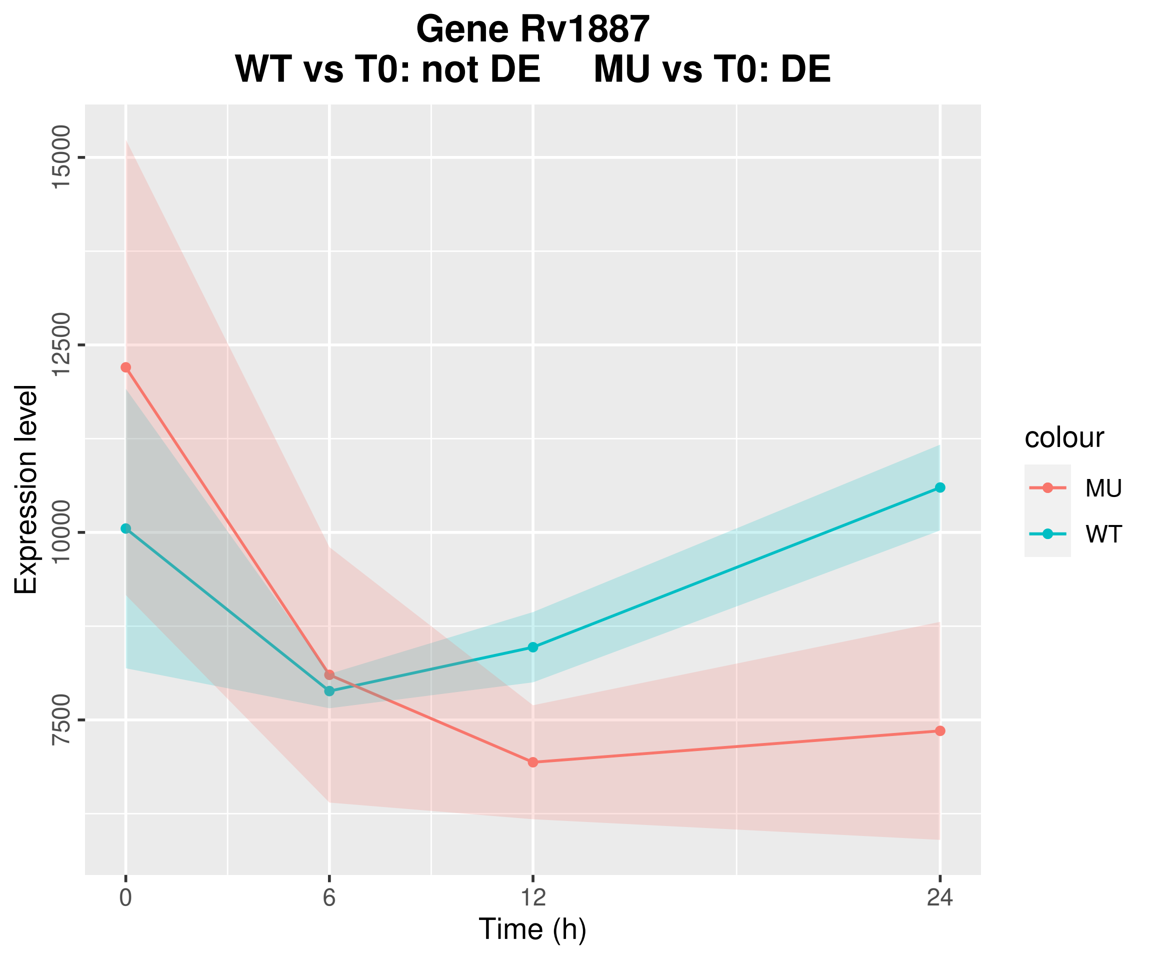
Gene Rv1887
WT vs T0: not DE   MU vs T0: DE
Expression level
15000
12500
10000
7500
0
6
12
24
Time (h)
colour
MU
WT

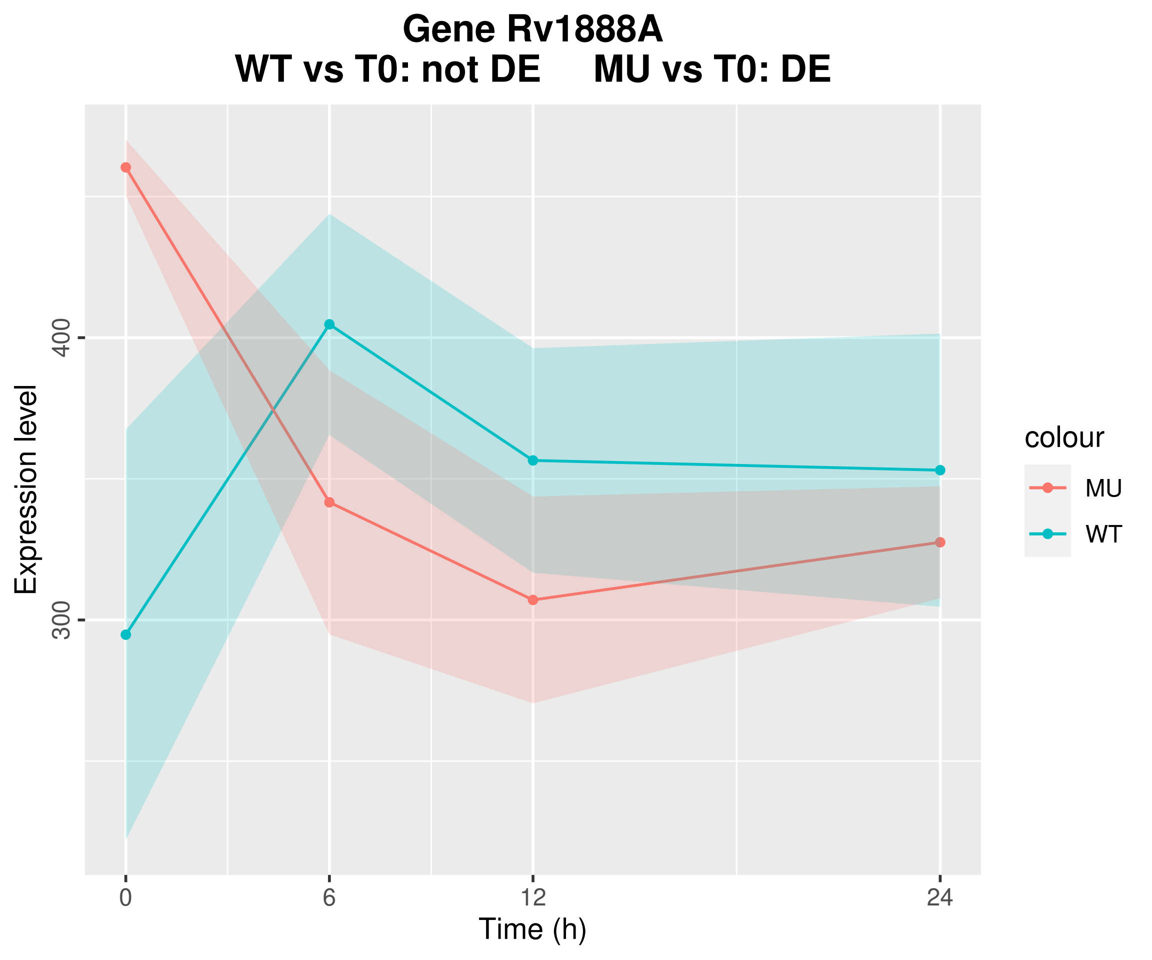

Gene Rv1888A
WT vs T0: not DE MU vs T0: DE
Expression level
400
300
0
6
12
24
Time (h)
colour
MU
WT

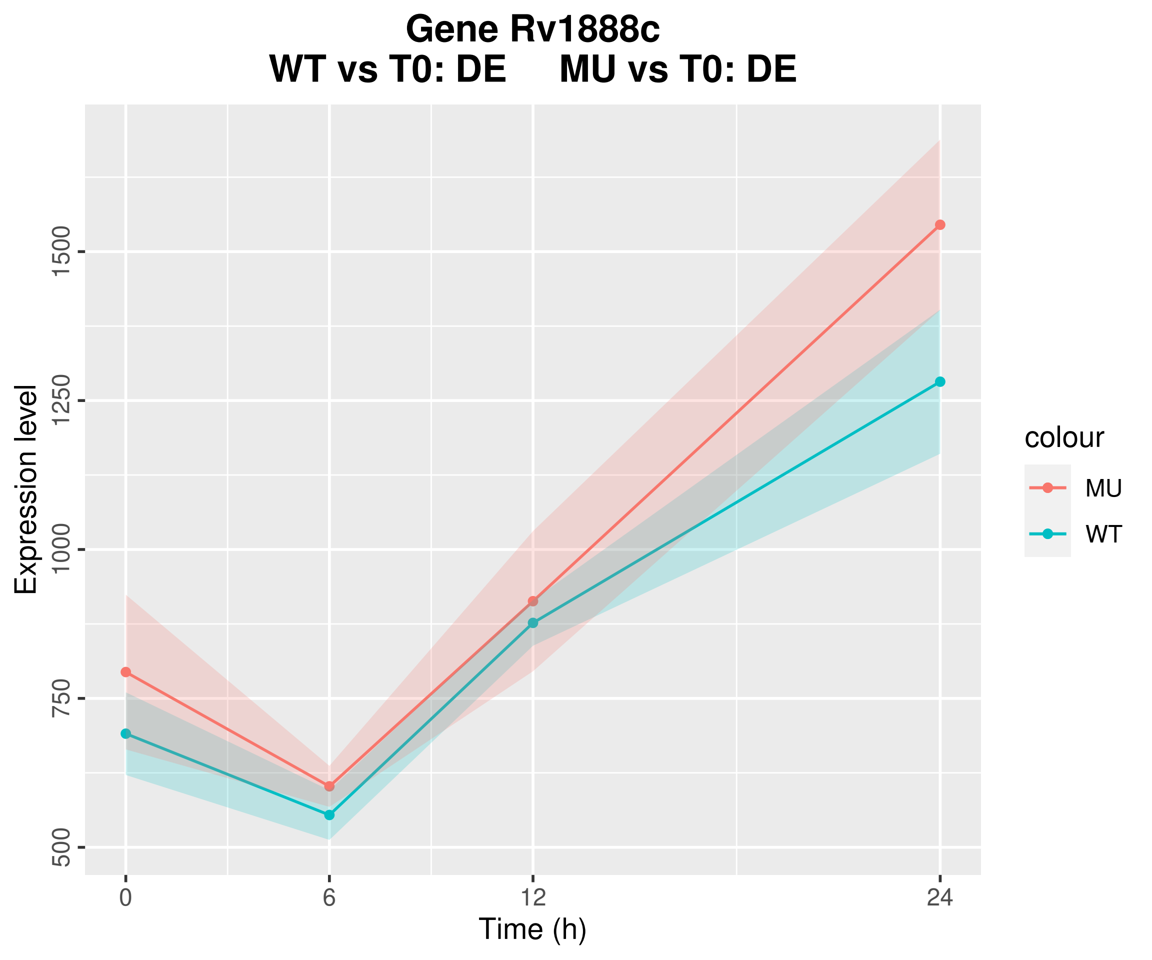
Gene Rv1888c
WT vs T0: DE   MU vs T0: DE
Expression level
1500
1250
1000
750
500
0
6
12
24
Time (h)
colour
MU
WT

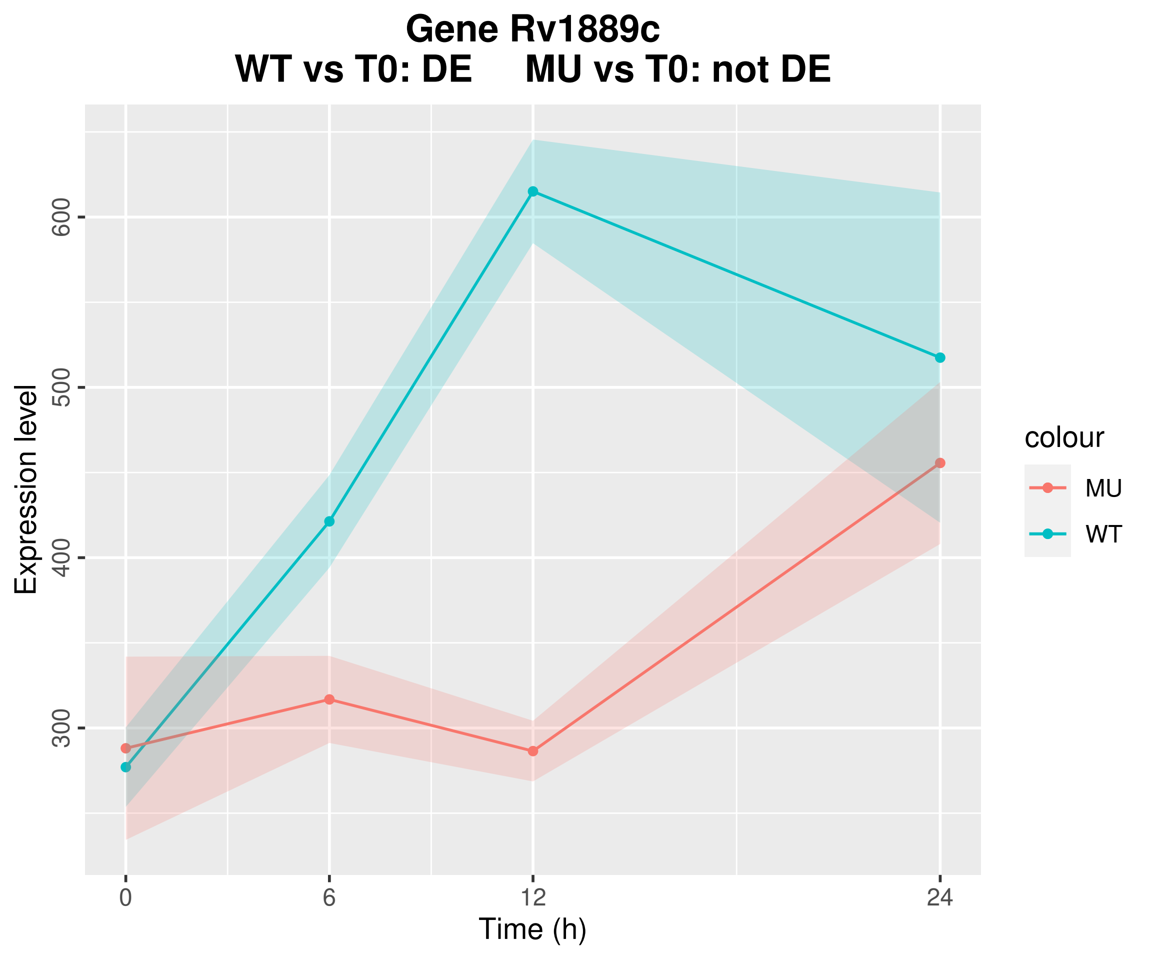
Gene Rv1889c
WT vs T0: DE   MU vs T0: not DE
Expression level
600
500
400
300
0
6
12
24
Time (h)
colour
MU
WT

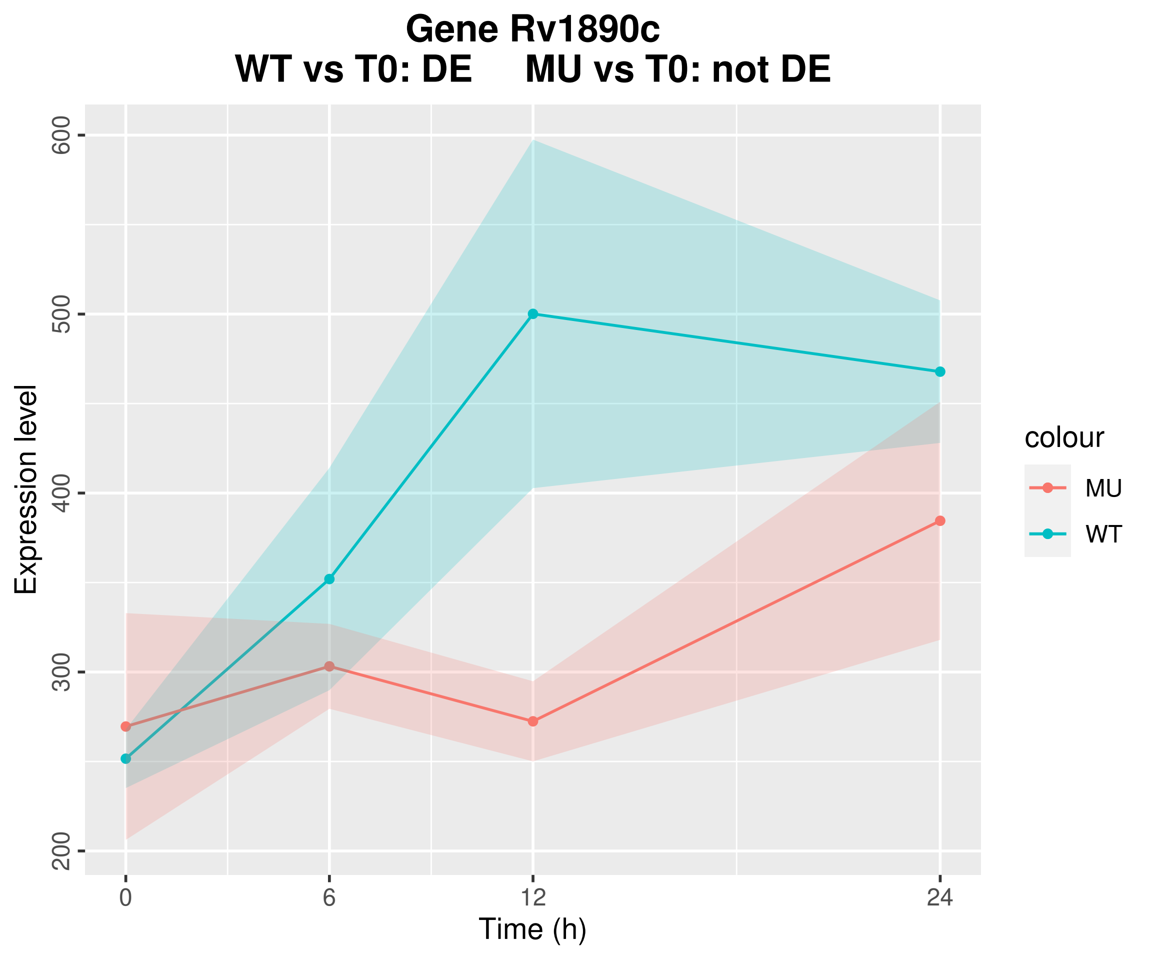

Gene Rv1890c
WT vs T0: DE   MU vs T0: not DE
Expression level
600
500
400
300
200
0
6
12
24
Time (h)
colour
MU
WT

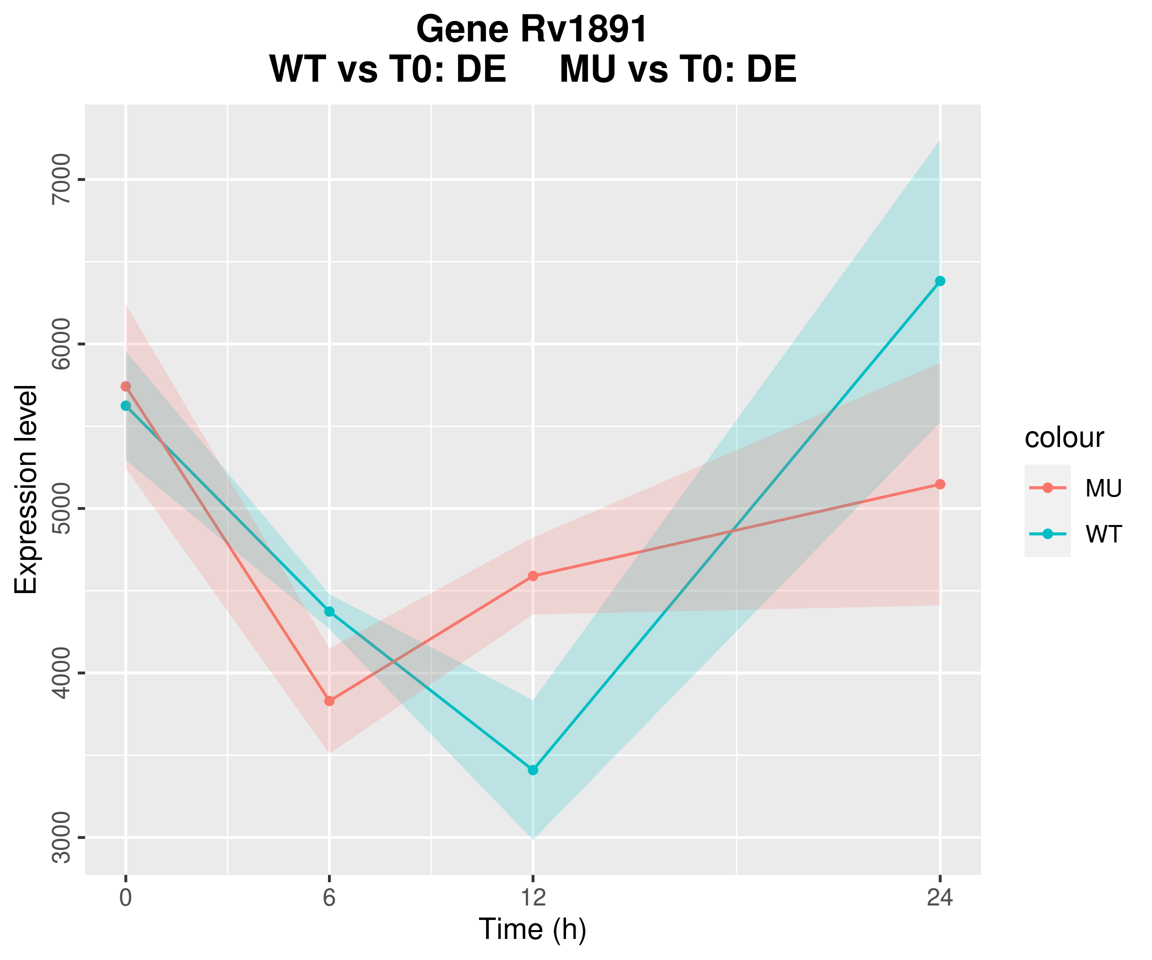

Gene Rv1891
WT vs T0: DE MU vs T0: DE
Expression level
7000
6000
5000
4000
3000
0
6
12
24
Time (h)
colour
MU
WT

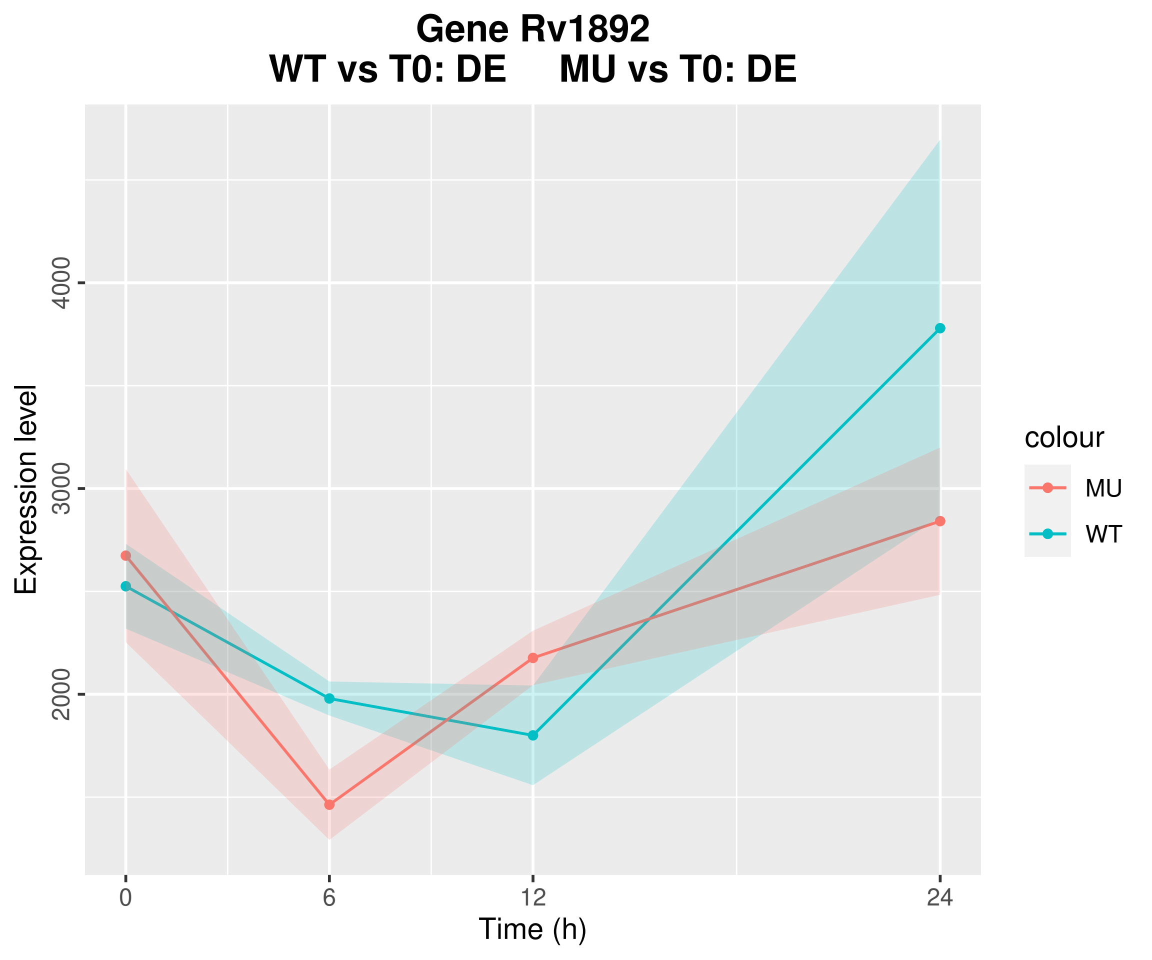
Gene Rv1892
WT vs T0: DE MU vs T0: DE
Expression level
4000
3000
2000
0
6
12
24
Time (h)
colour
MU
WT

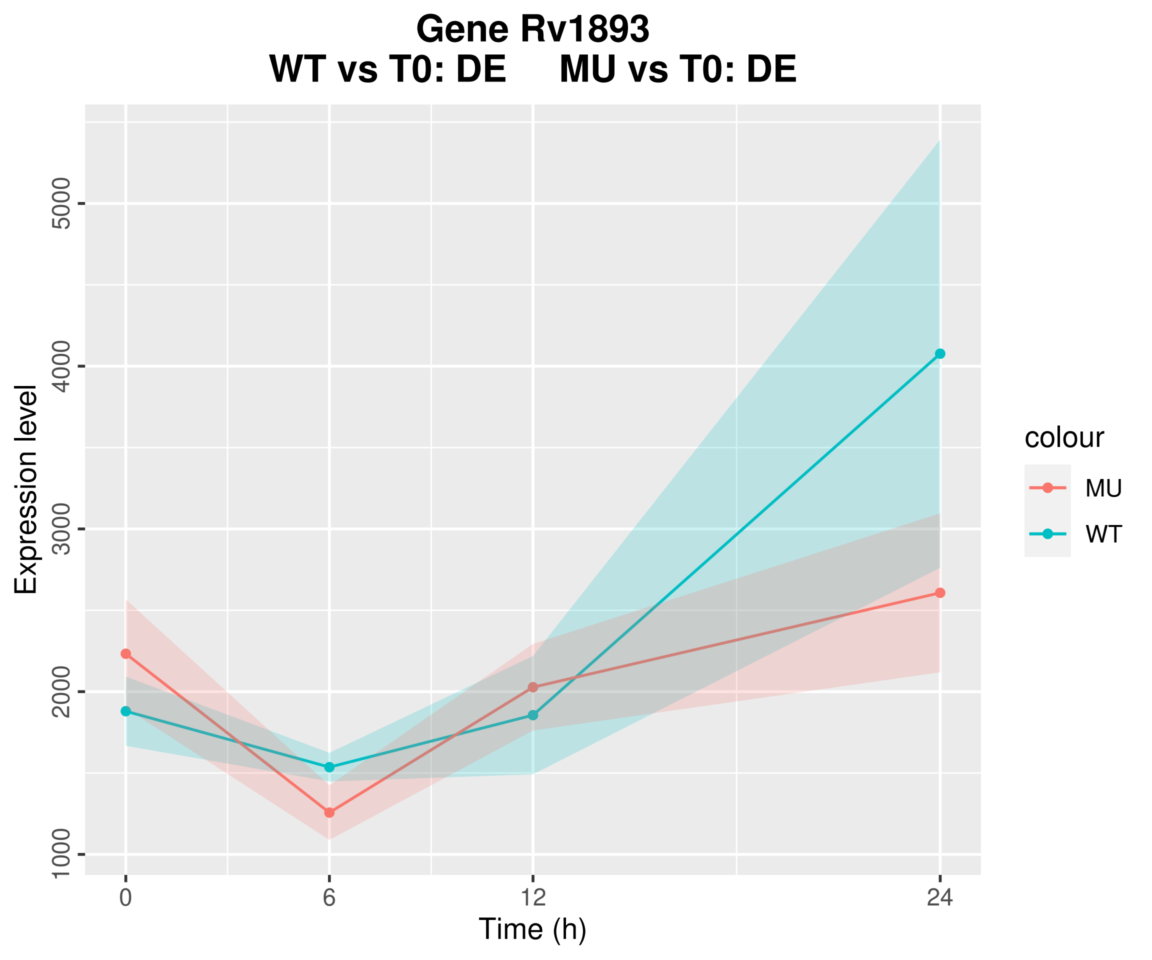
Gene Rv1893
WT vs T0: DE MU vs T0: DE
Expression level
1000
2000
3000
4000
5000
0
6
12
24
Time (h)
colour
MU
WT

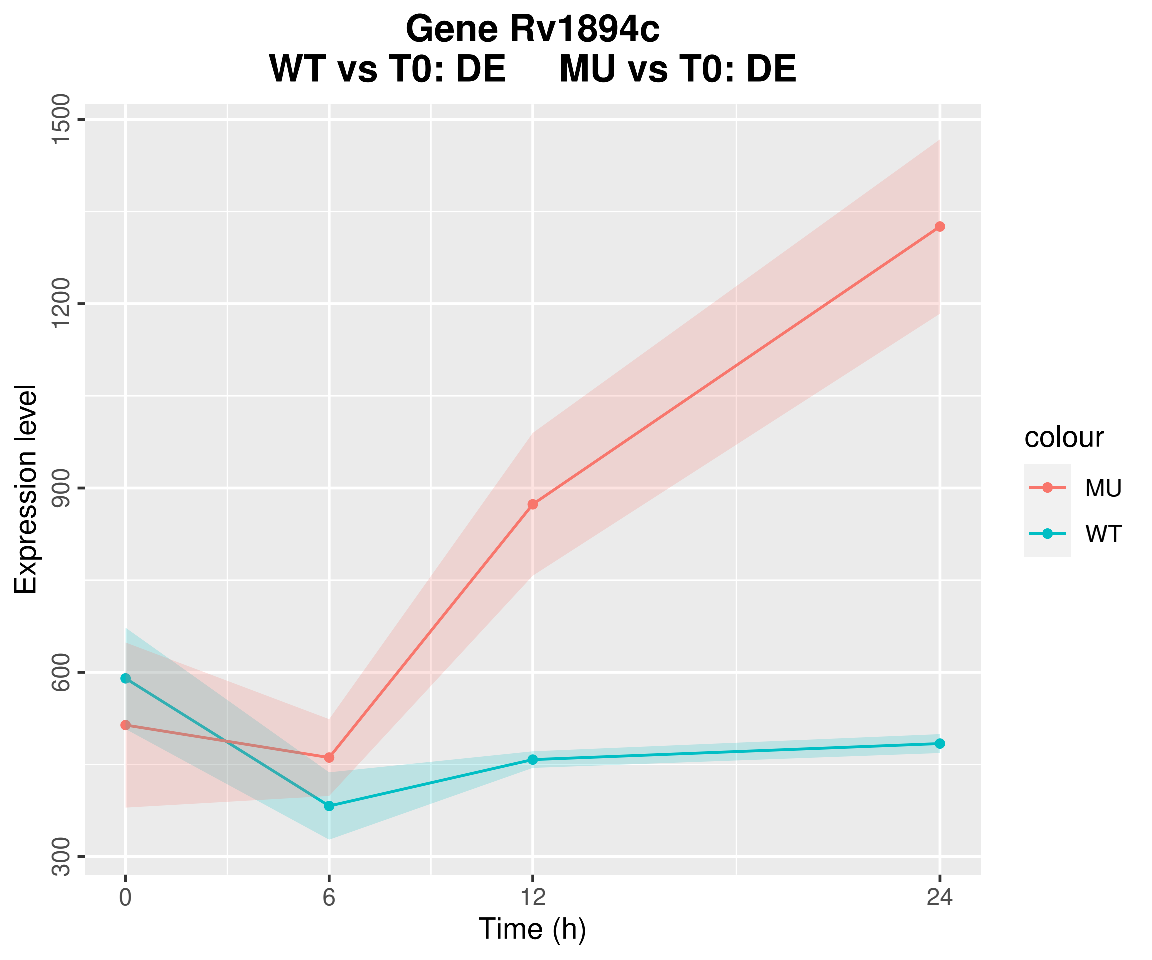

Gene Rv1894c
WT vs T0: DE MU vs T0: DE
Expression level
1500
1200
900
600
300
0
6
12
24
Time (h)
colour
MU
WT

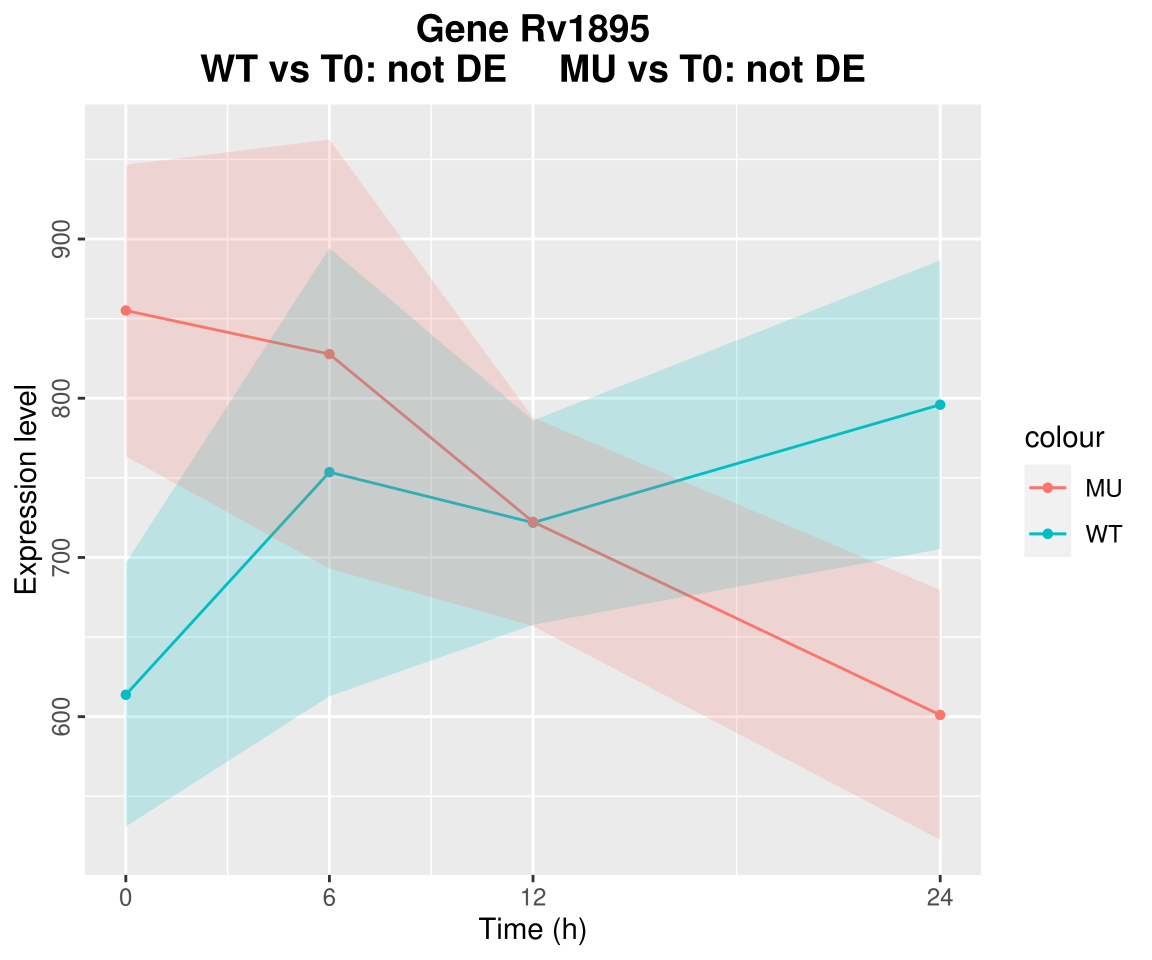
Gene Rv1895
WT vs T0: not DE MU vs T0: not DE
Expression level
900
800
700
600
0
6
12
24
Time (h)
colour
MU
WT

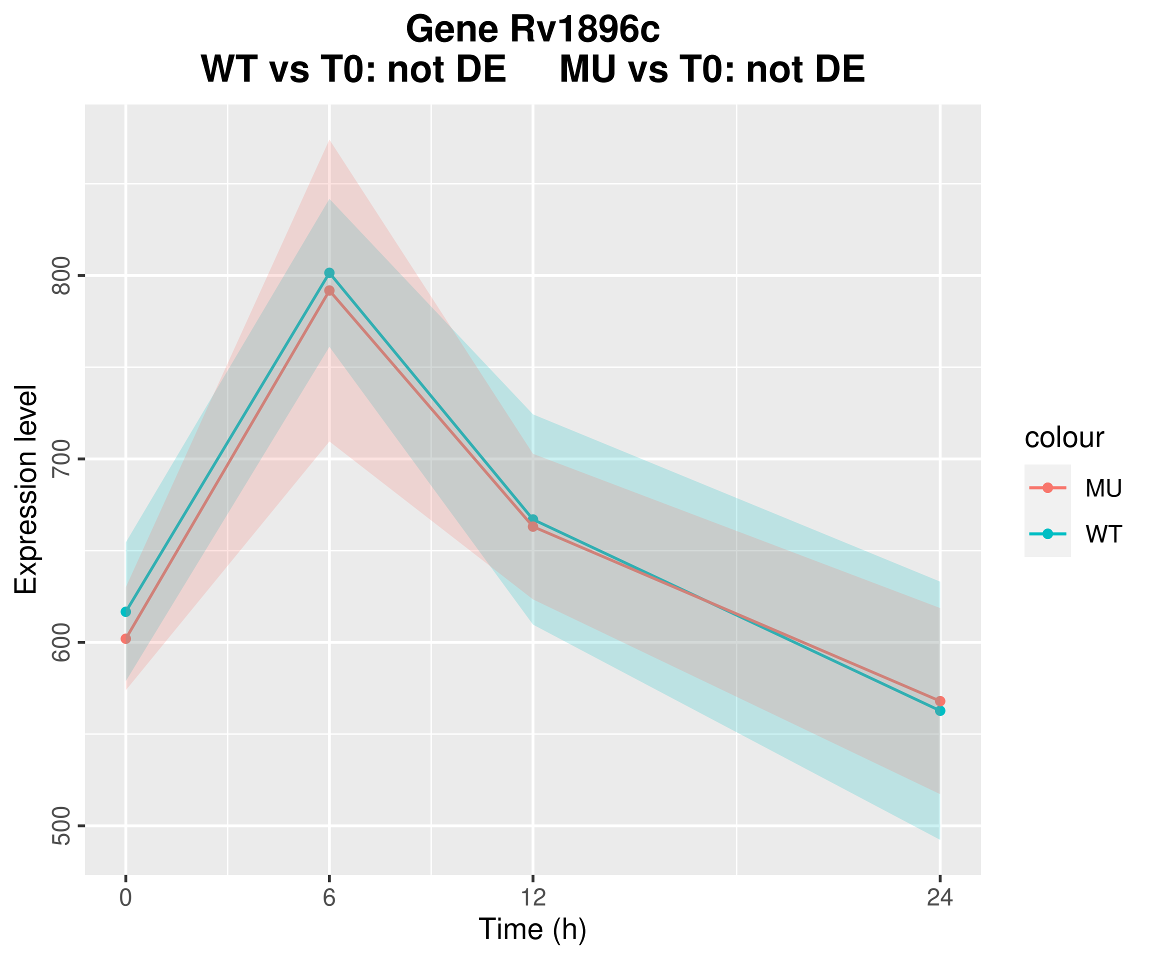
Gene Rv1896c
WT vs T0: not DE   MU vs T0: not DE
Expression level
800
700
600
500
0
6
12
24
Time (h)
colour
MU
WT

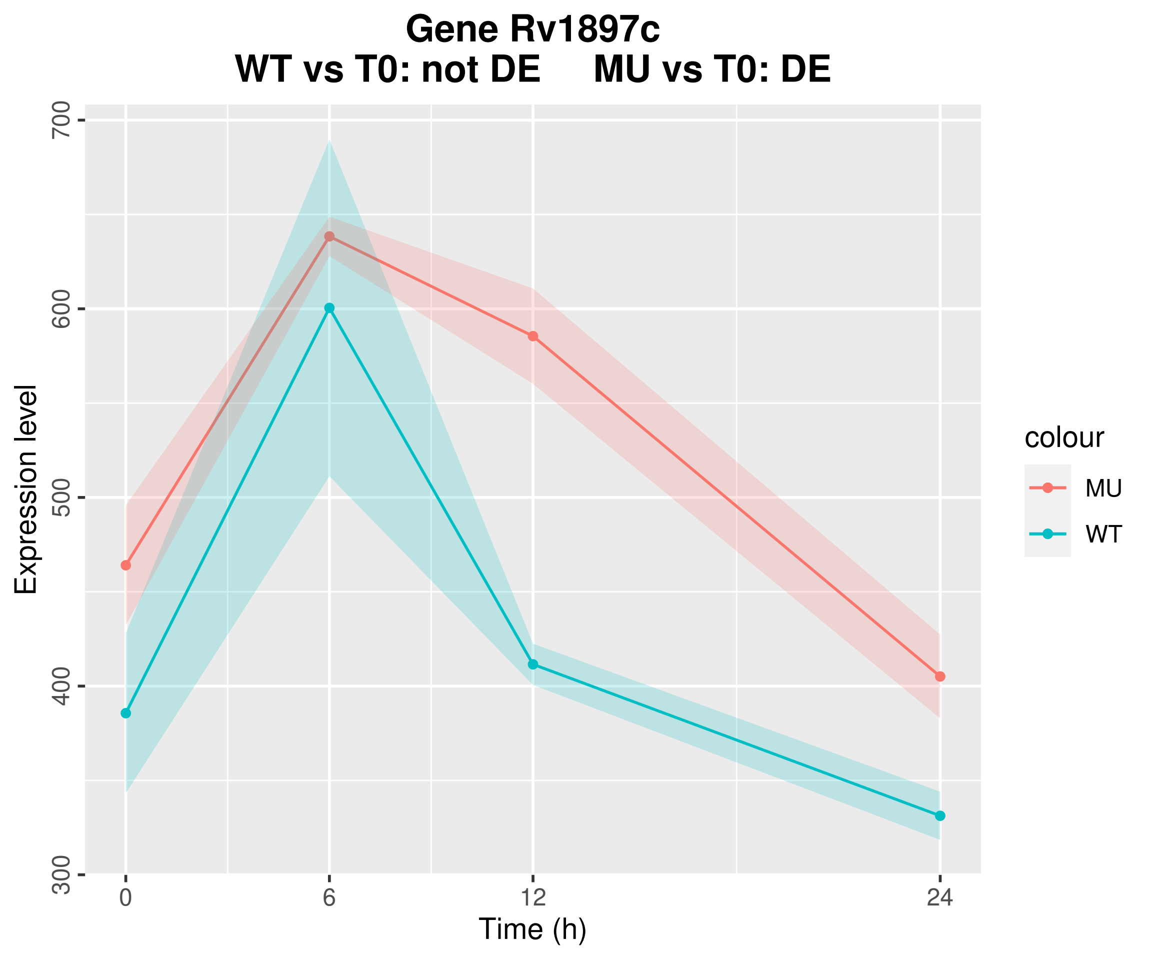
Gene Rv1897c
WT vs T0: not DE   MU vs T0: DE
Expression level
700
600
500
400
300
0
6
12
24
Time (h)
colour
MU
WT

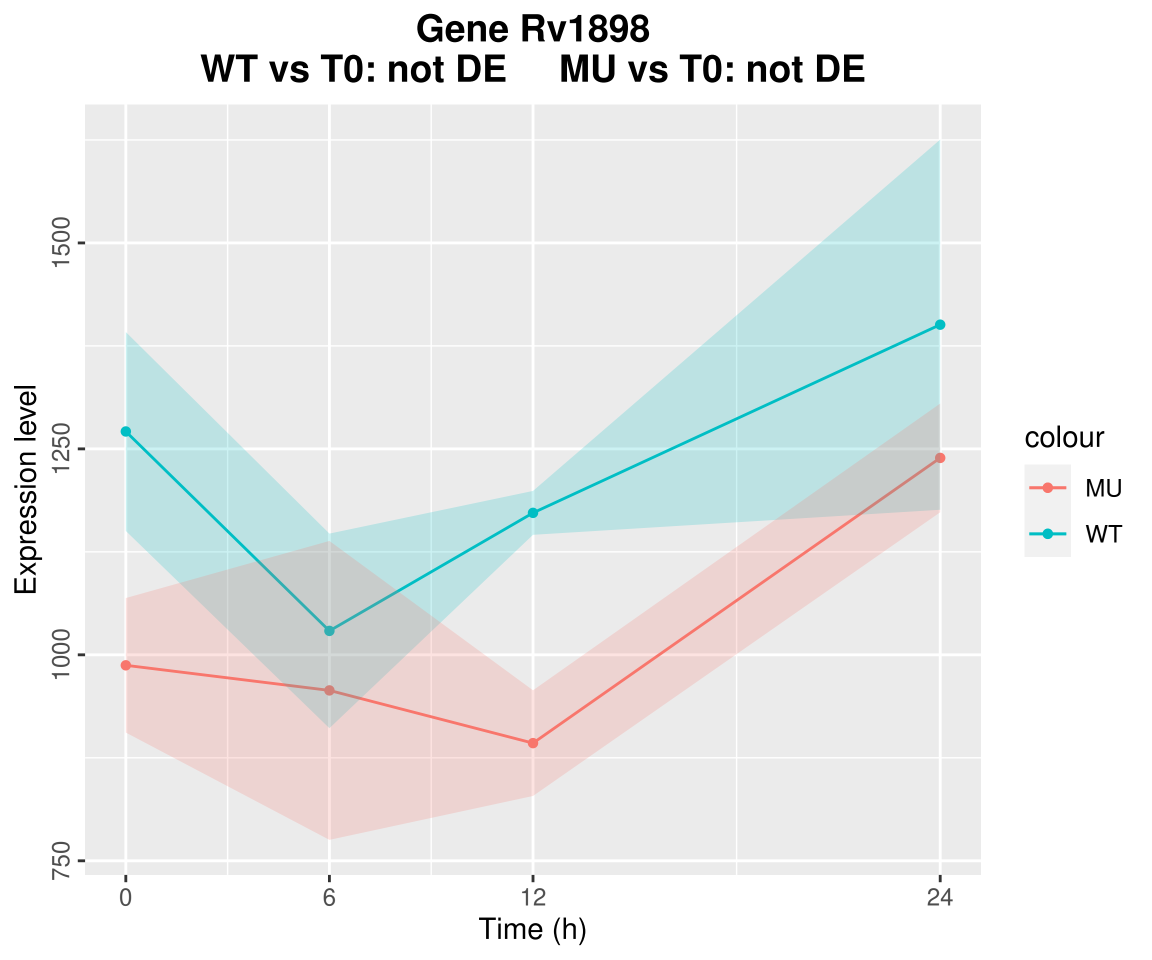

Gene Rv1898
WT vs T0: not DE MU vs T0: not DE
Expression level
1500
1250
1000
750
0
6
12
24
Time (h)
colour
MU
WT

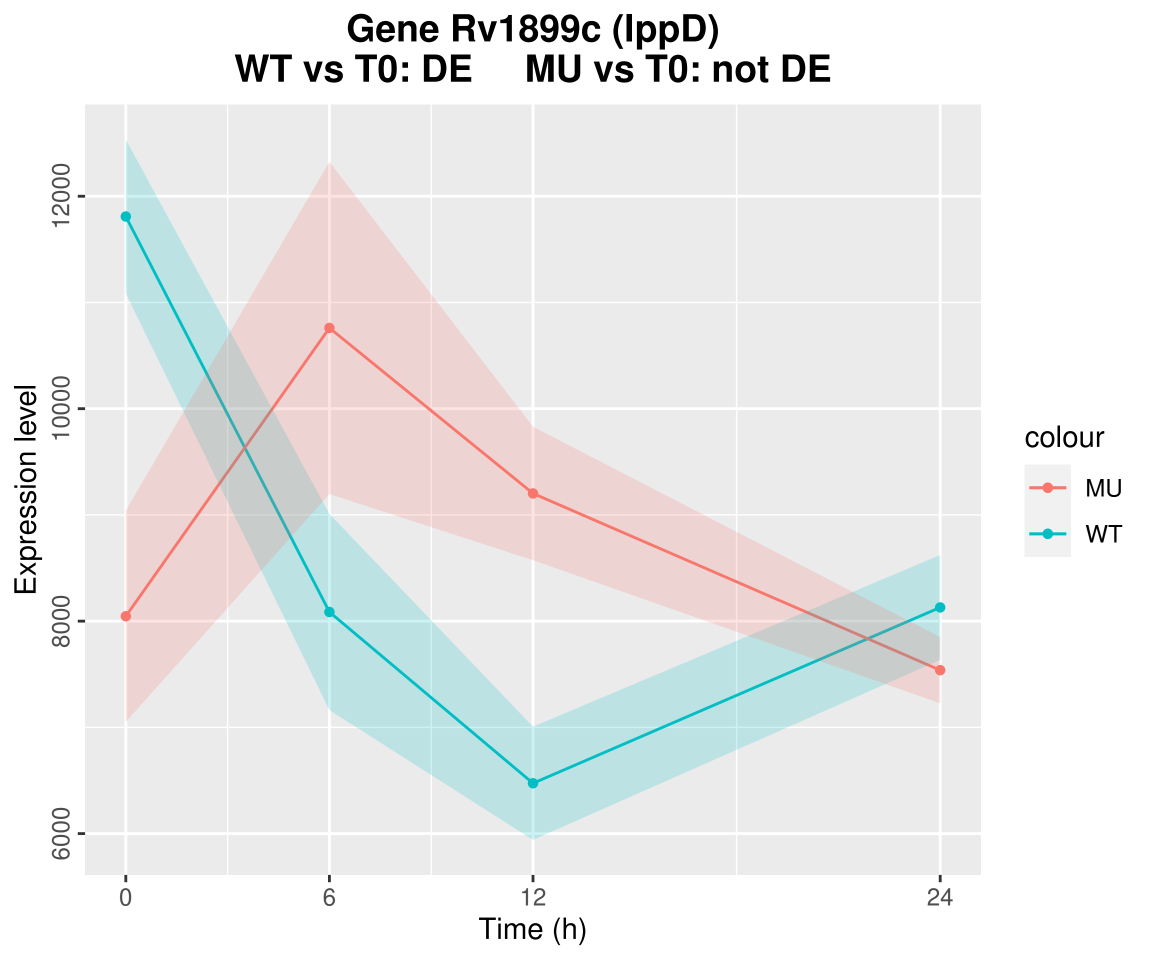

Gene Rv1899c (lppD)
WT vs T0: DE MU vs T0: not DE
Expression level
12000
10000
8000
6000
0
6
12
24
Time (h)
colour
MU
WT

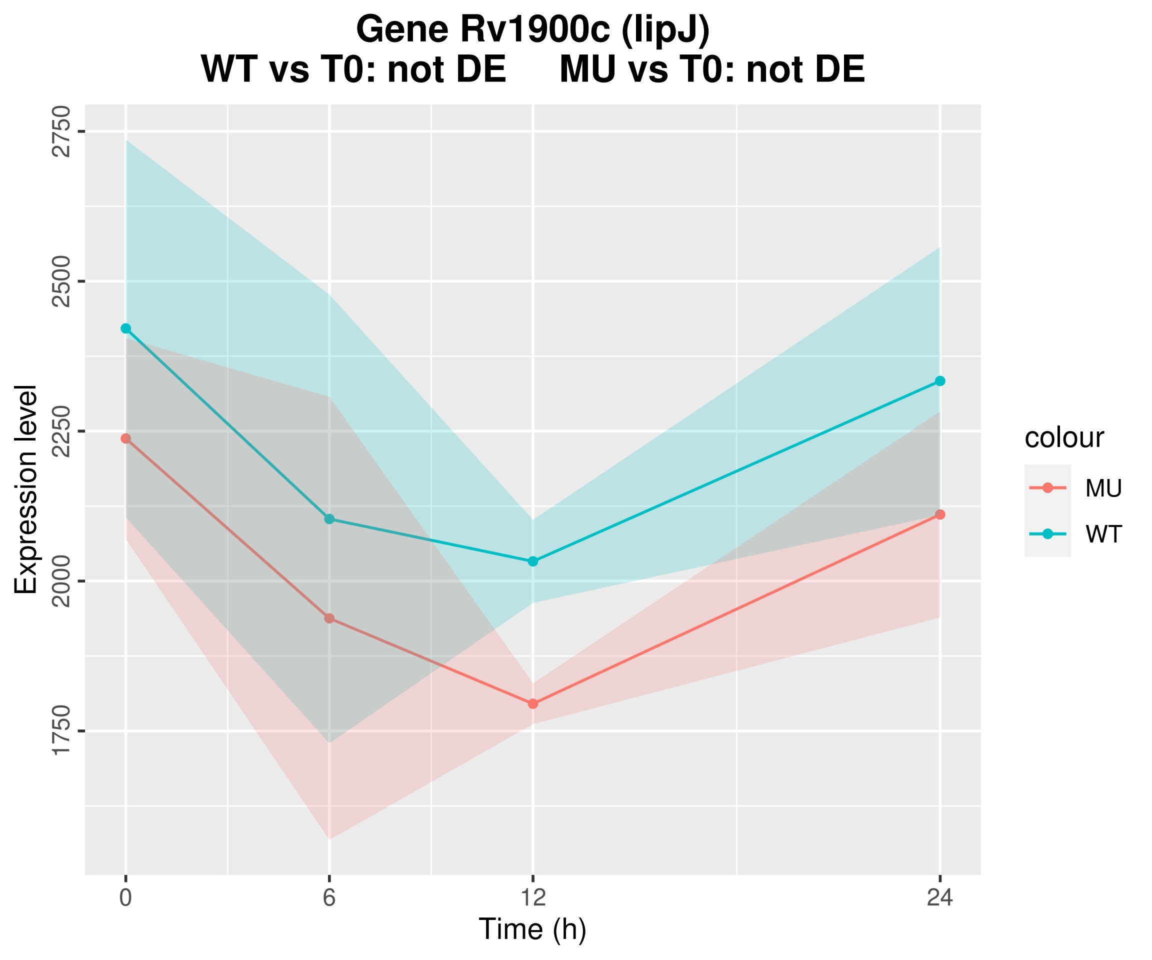
Gene Rv1900c (lipJ)
WT vs T0: not DE   MU vs T0: not DE
Expression level
2750
2500
2250
2000
1750
0
6
12
24
Time (h)
colour
MU
WT

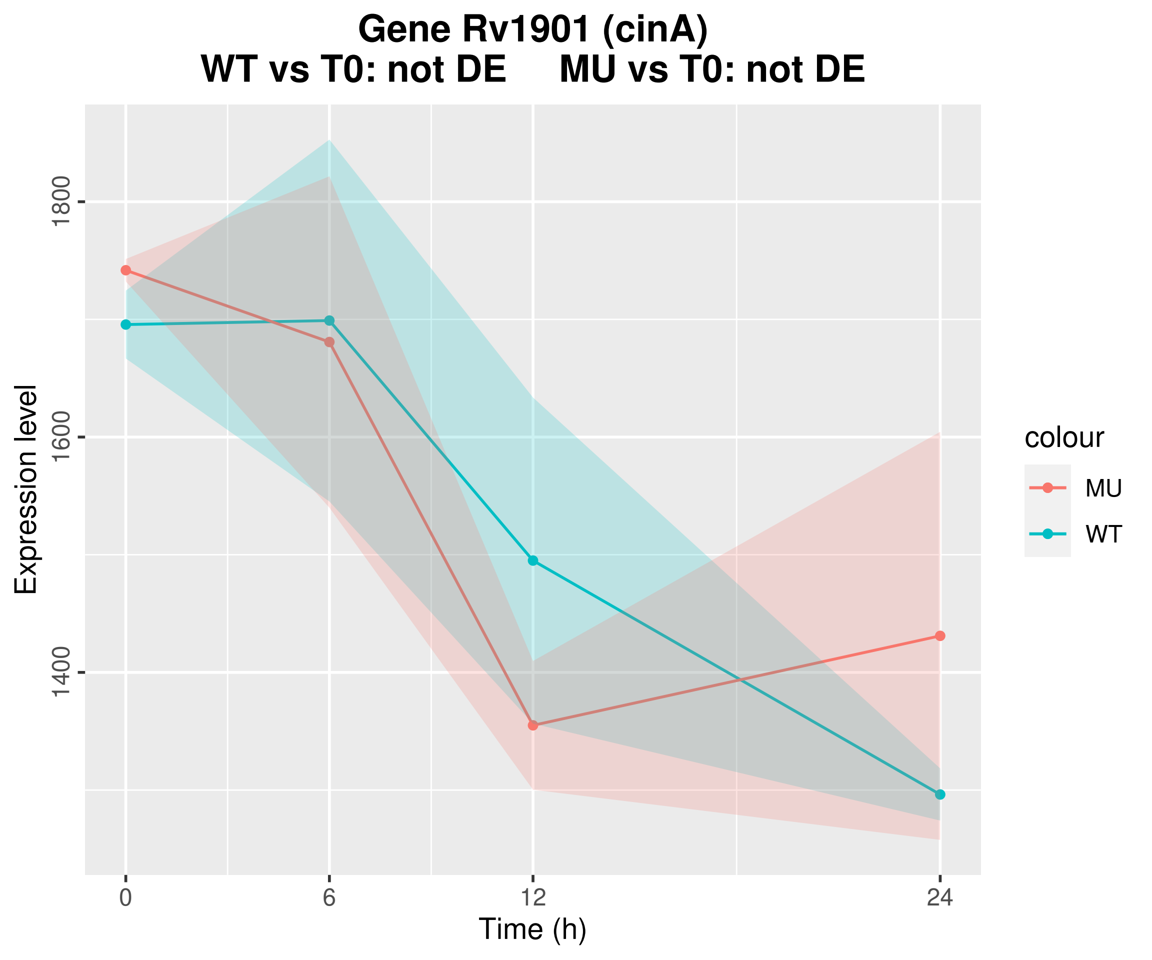

Gene Rv1901 (cinA)
WT vs T0: not DE   MU vs T0: not DE
1800
1600
1400
Expression level
0
6
12
24
Time (h)
colour
MU
WT

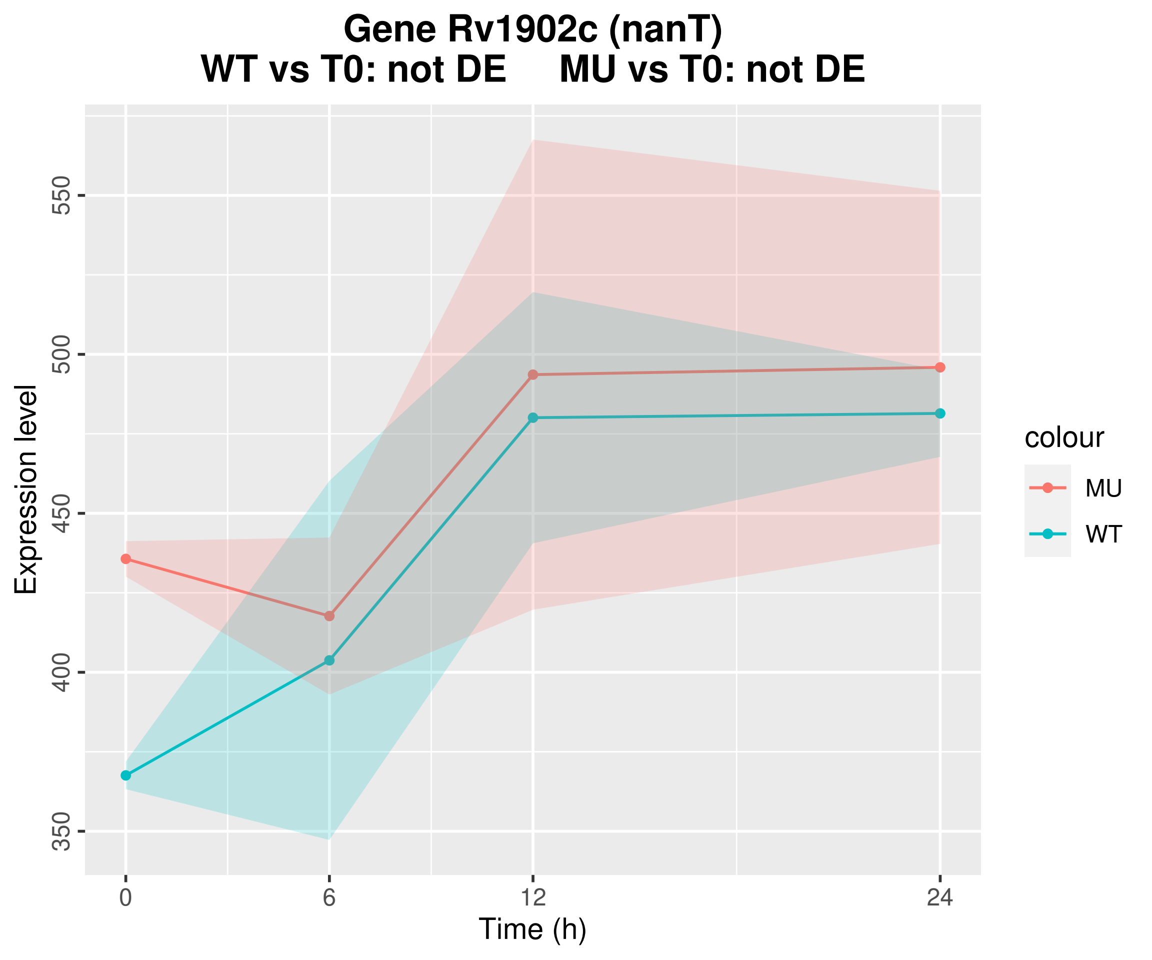
Gene Rv1902c (nanT)
WT vs T0: not DE   MU vs T0: not DE
Expression level
350
400
450
500
550
0
6
12
24
Time (h)
colour
MU
WT

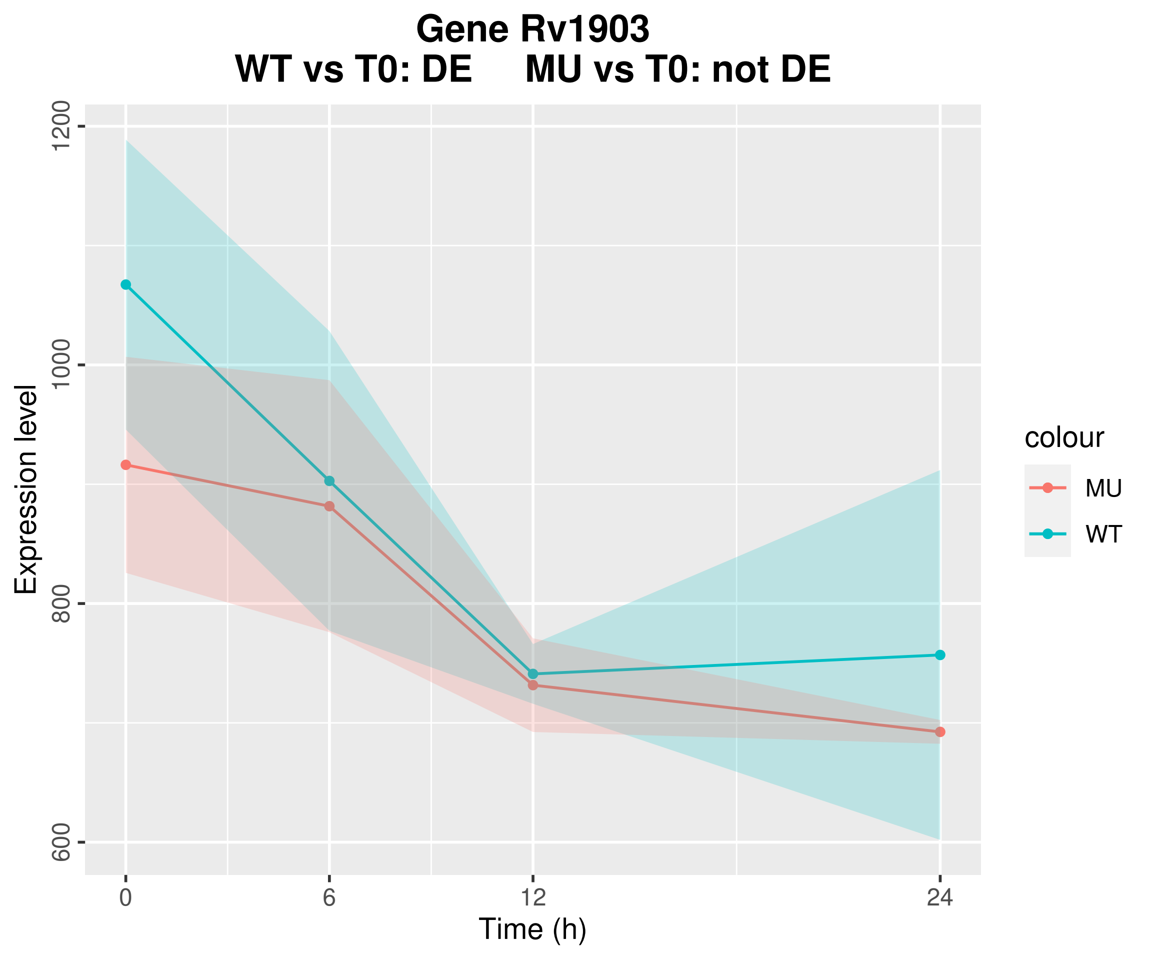

Gene Rv1903
WT vs T0: DE   MU vs T0: not DE
Expression level
1200
1000
800
600
0
6
12
24
Time (h)
colour
MU
WT

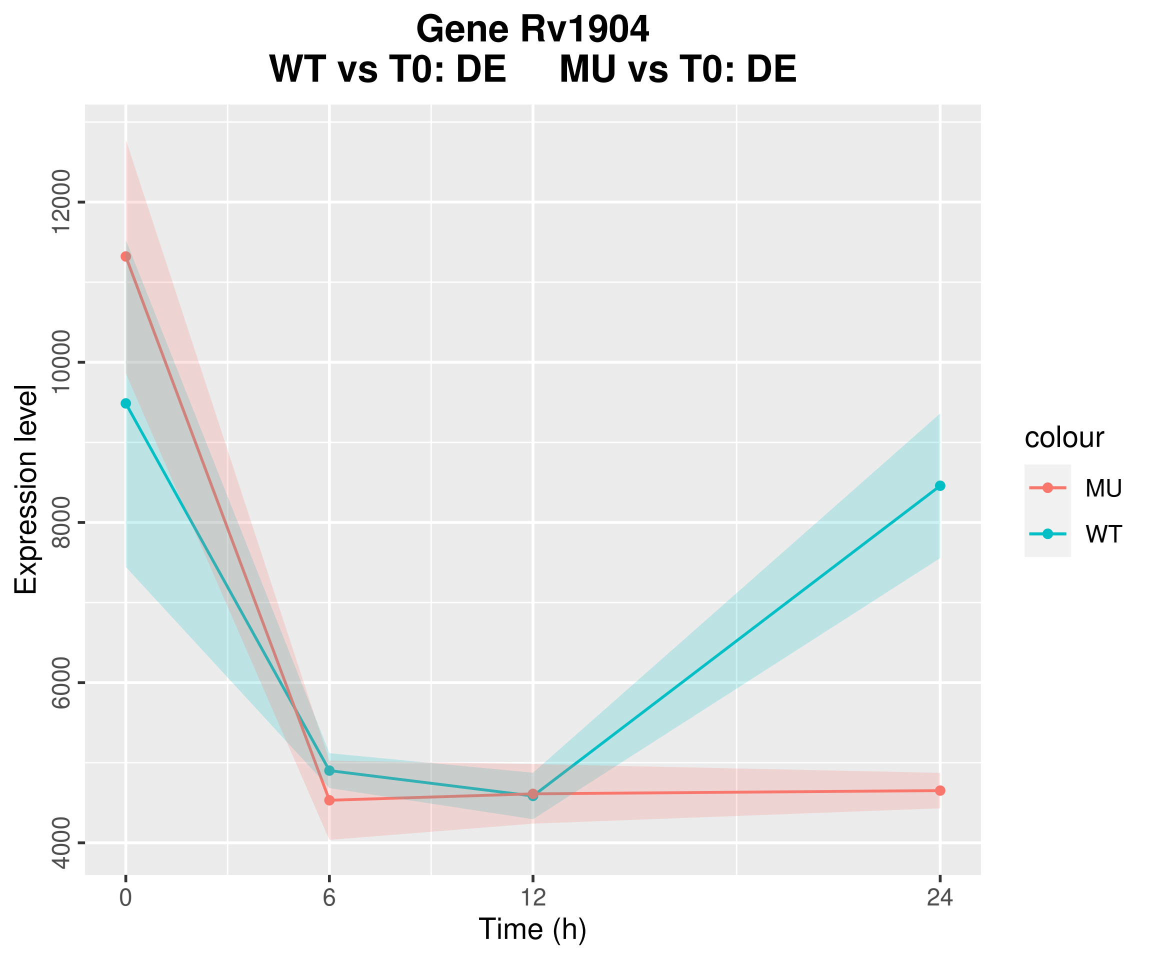

Gene Rv1904
WT vs T0: DE   MU vs T0: DE
Expression level
12000
10000
8000
6000
4000
0
6
12
24
Time (h)
colour
MU
WT

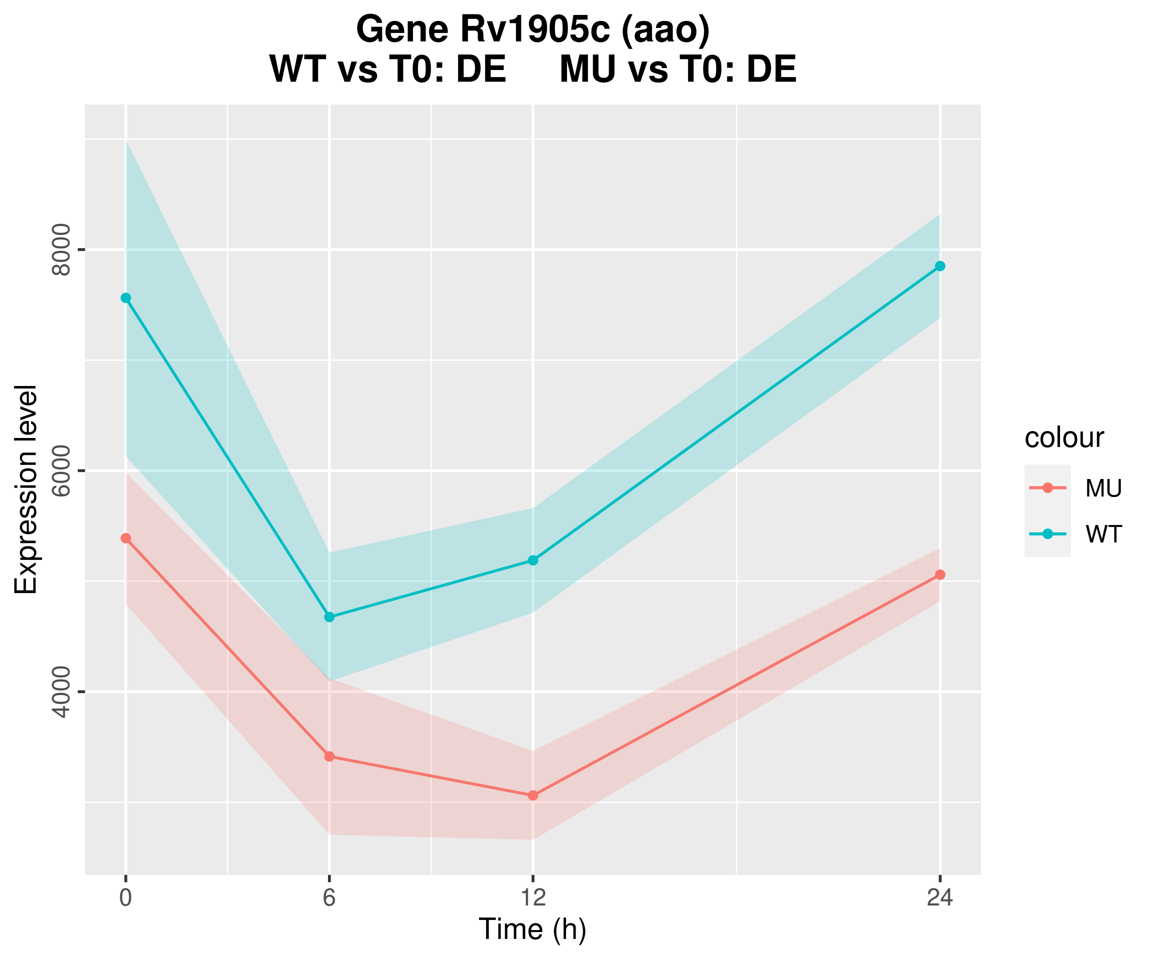
Gene Rv1905c (aao)
WT vs T0: DE MU vs T0: DE
Expression level
8000
6000
4000
0
6
12
24
Time (h)
colour
MU
WT

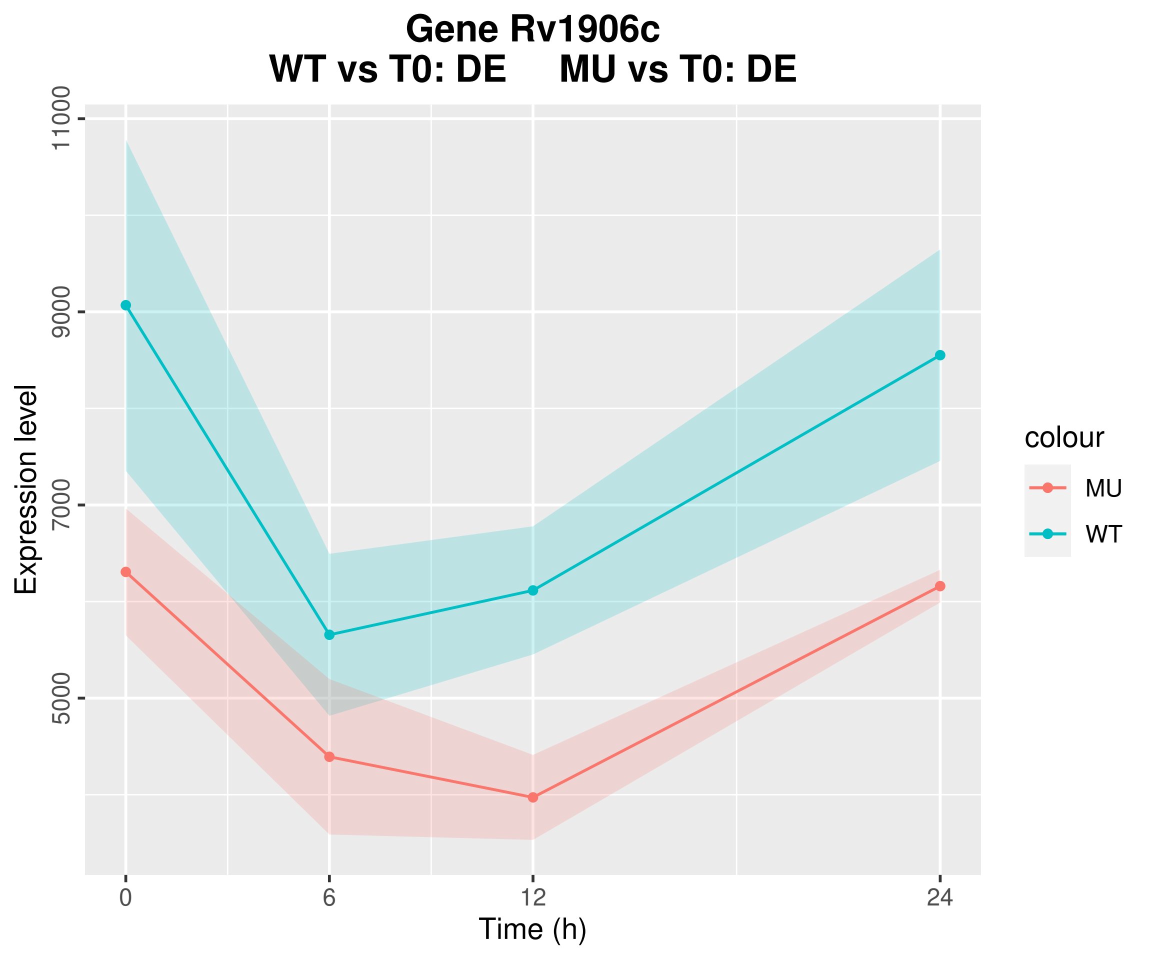

Gene Rv1906c
WT vs T0: DE MU vs T0: DE
Expression level
11000
9000
7000
5000
0
6
12
24
Time (h)
colour
MU
WT

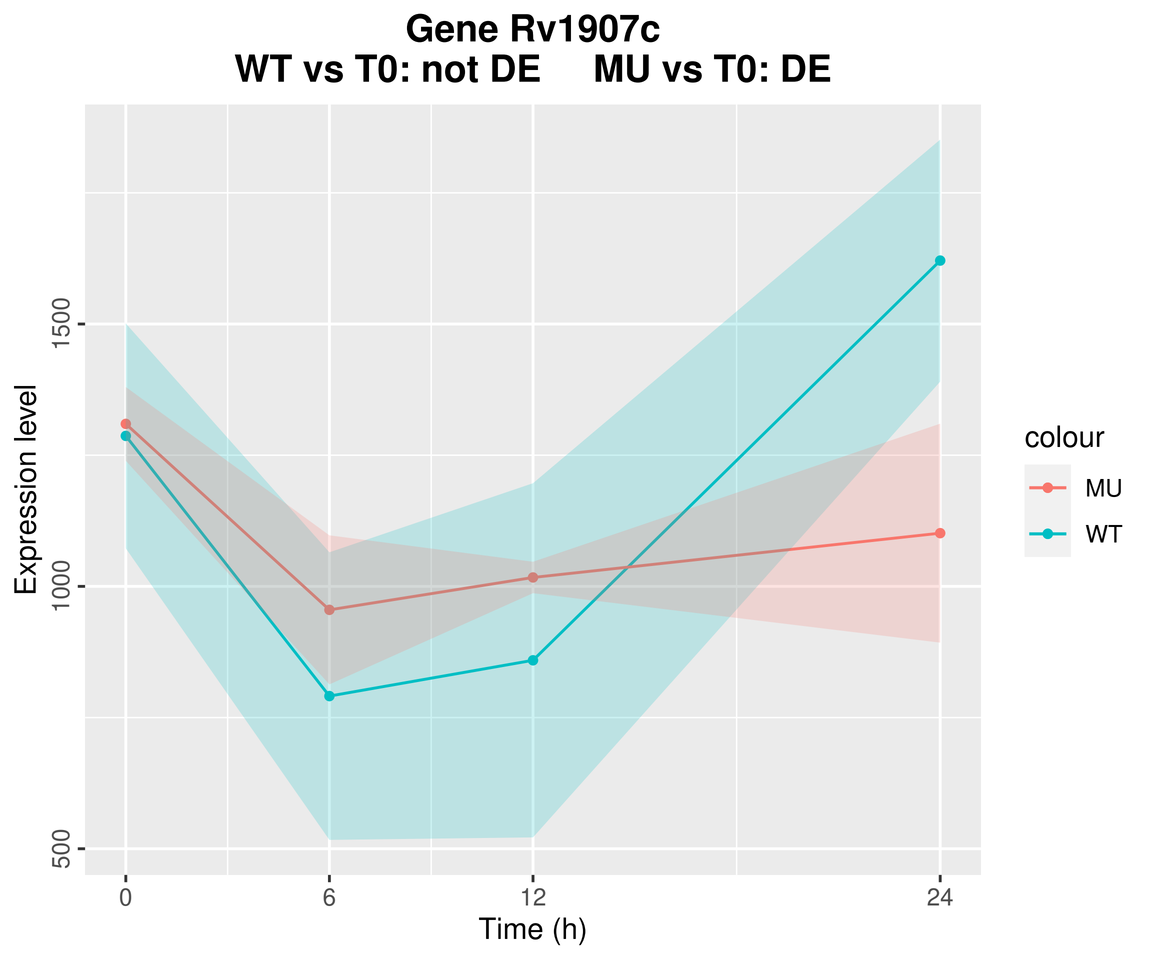

Gene Rv1907c
WT vs T0: not DE   MU vs T0: DE
Expression level
1500
1000
500
0
6
12
24
Time (h)
colour
MU
WT

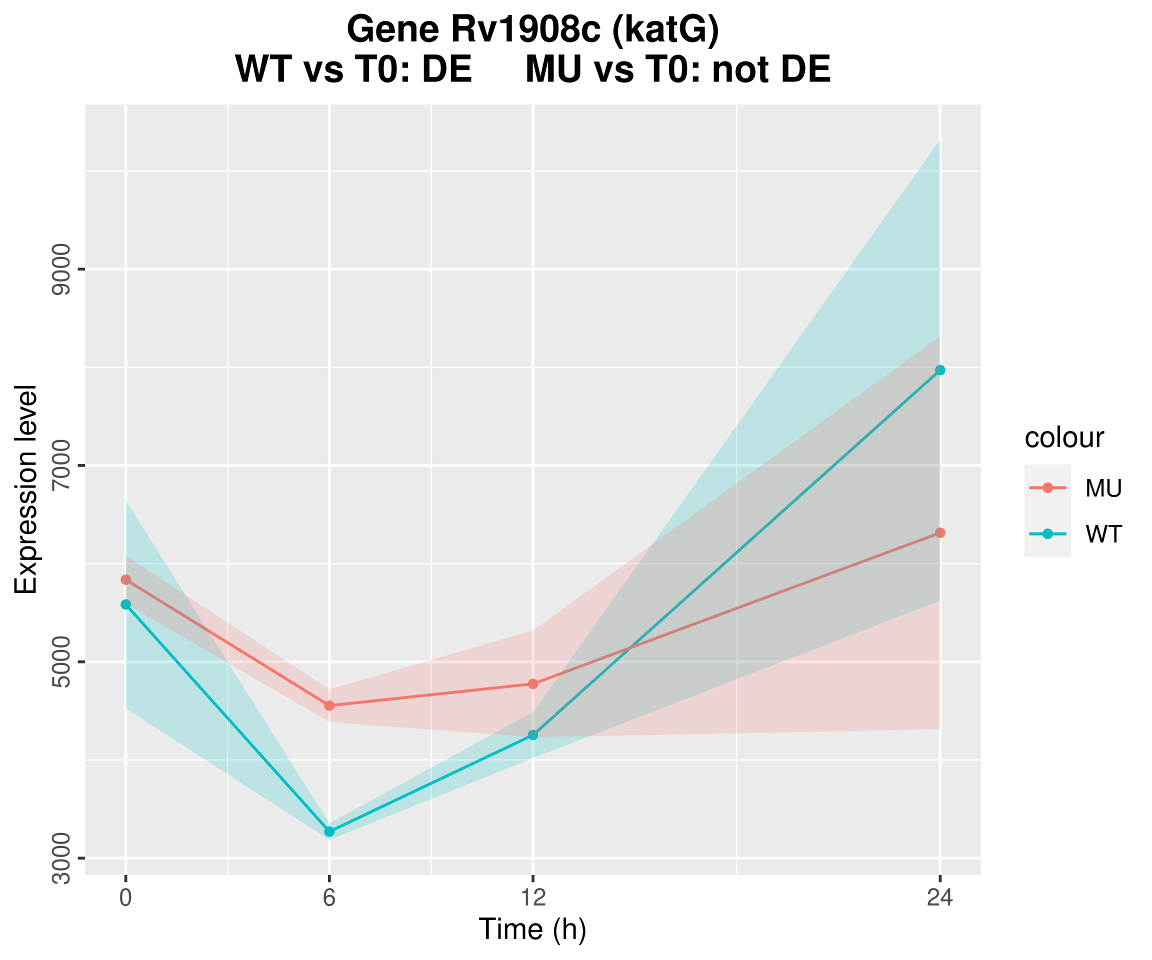

Gene Rv1908c (katG)
WT vs T0: DE MU vs T0: not DE
Expression level
9000
7000
5000
3000
0
6
12
24
Time (h)
colour
MU
WT

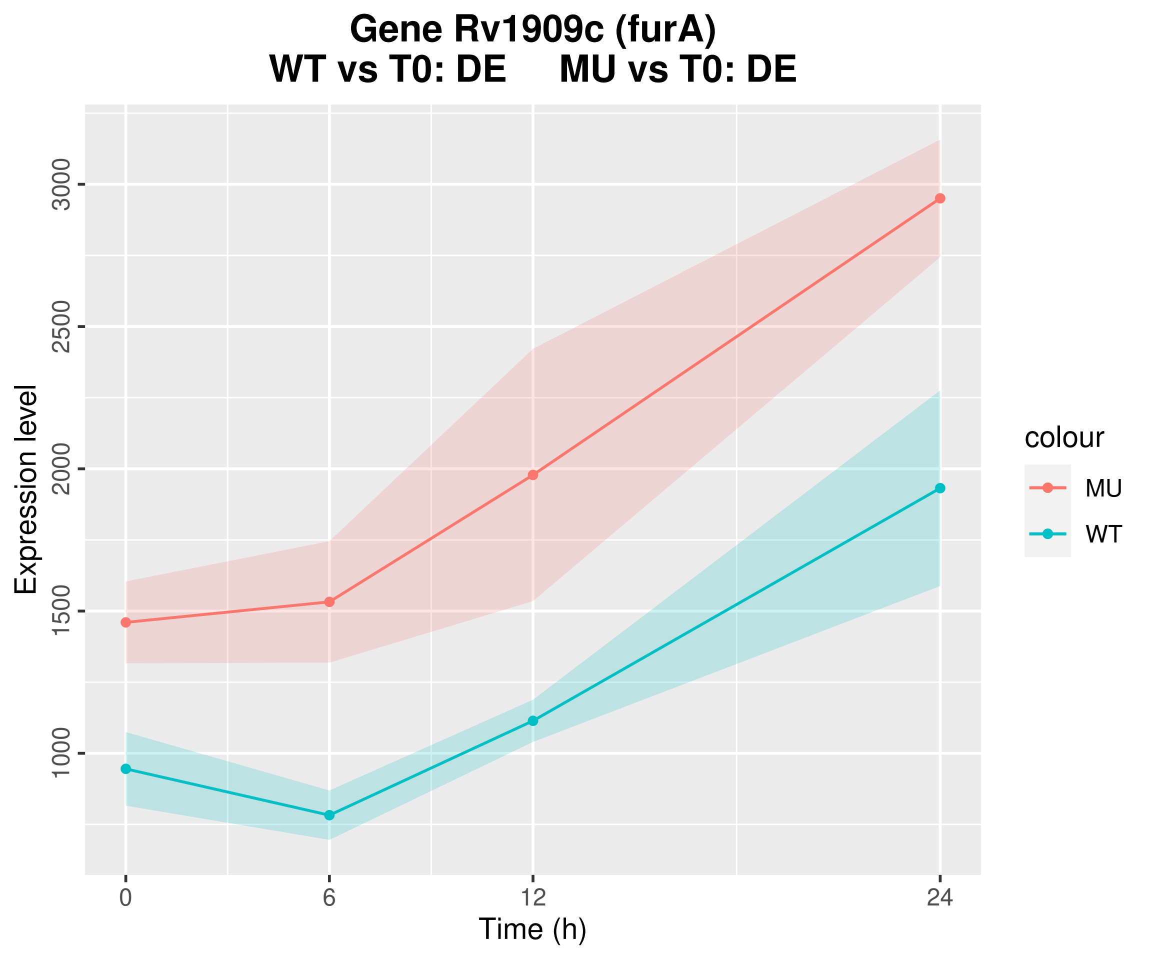
Gene Rv1909c (furA)
WT vs T0: DE   MU vs T0: DE
Expression level
3000
2500
2000
1500
1000
0
6
12
24
Time (h)
colour
MU
WT

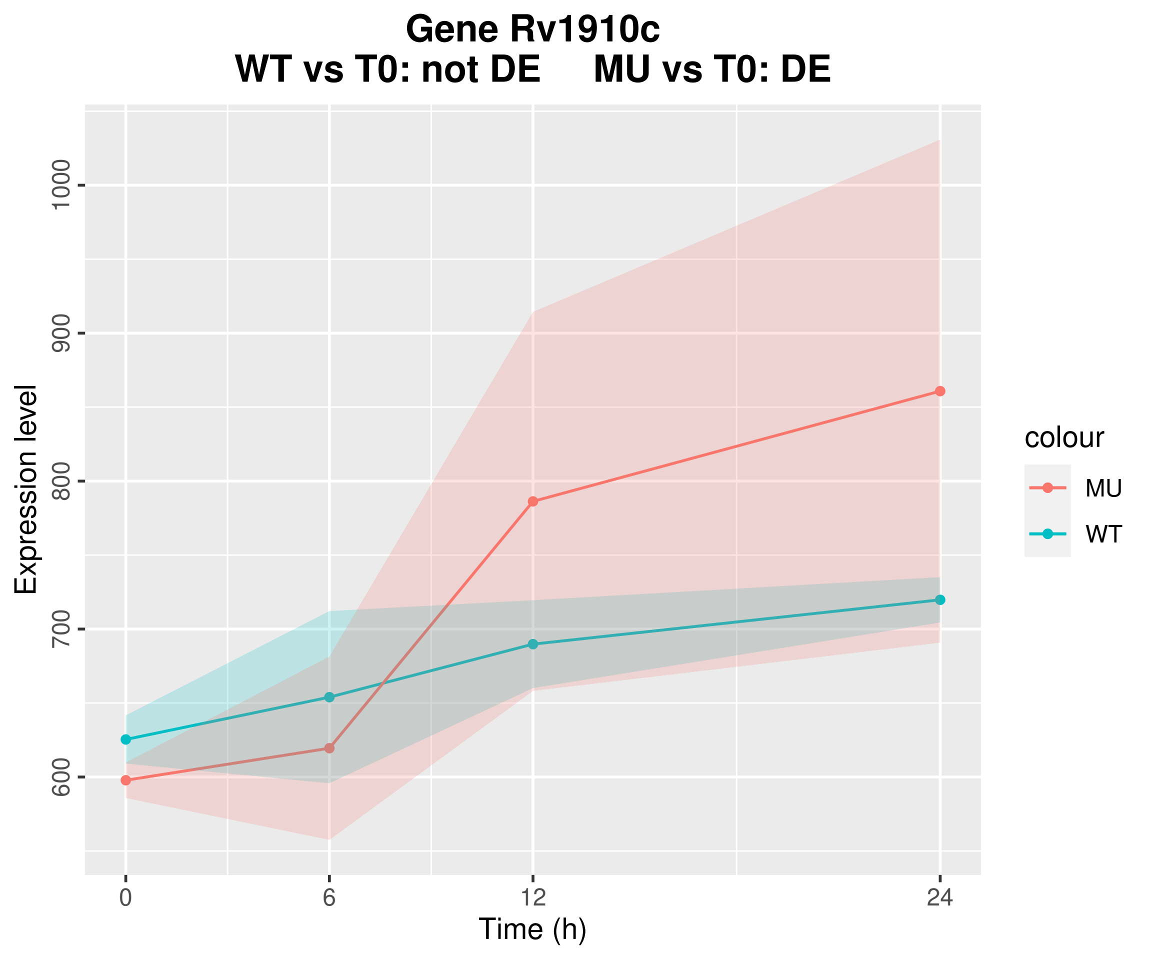

Gene Rv1910c
WT vs T0: not DE   MU vs T0: DE
Expression level
1000
900
800
700
600
0
6
12
24
Time (h)
colour
MU
WT

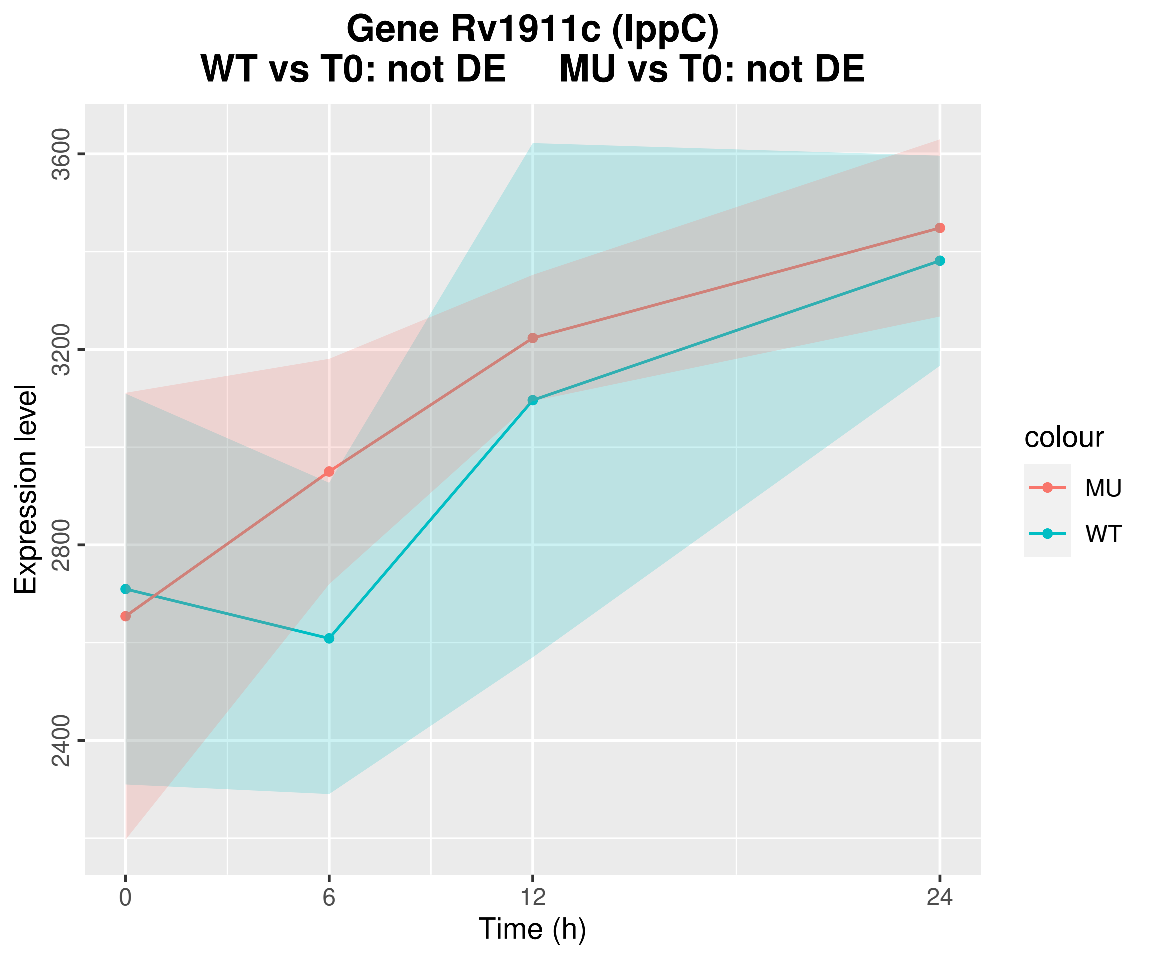

Gene Rv1911c (lppC)
WT vs T0: not DE   MU vs T0: not DE
3600
3200
2800
2400
Expression level
0
6
12
24
Time (h)
colour
MU
WT

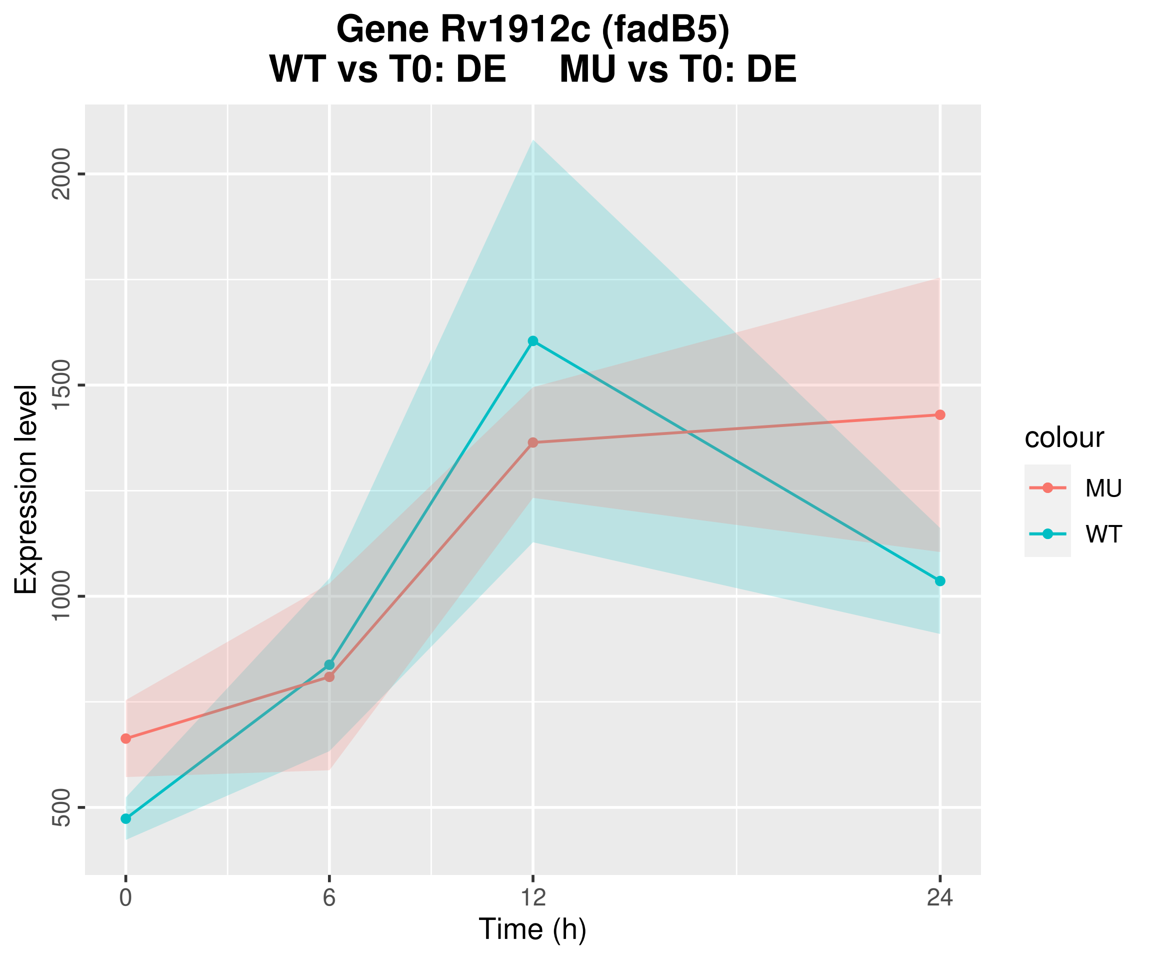

Gene Rv1912c (fadB5)
WT vs T0: DE MU vs T0: DE
Expression level
2000
1500
1000
500
0
6
12
24
Time (h)
colour
MU
WT

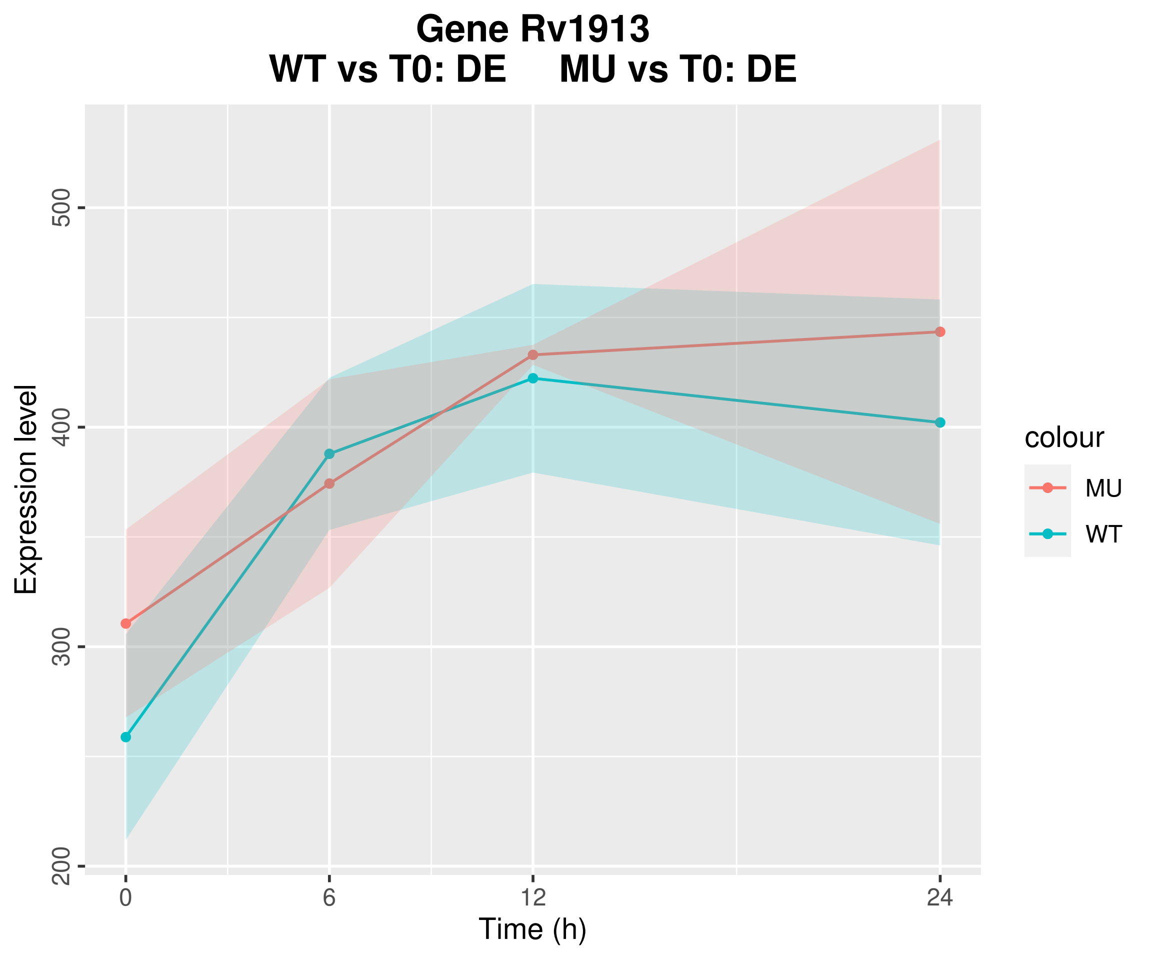

Gene Rv1913
WT vs T0: DE   MU vs T0: DE
Expression level
500
400
300
200
0
6
12
24
Time (h)
colour
MU
WT

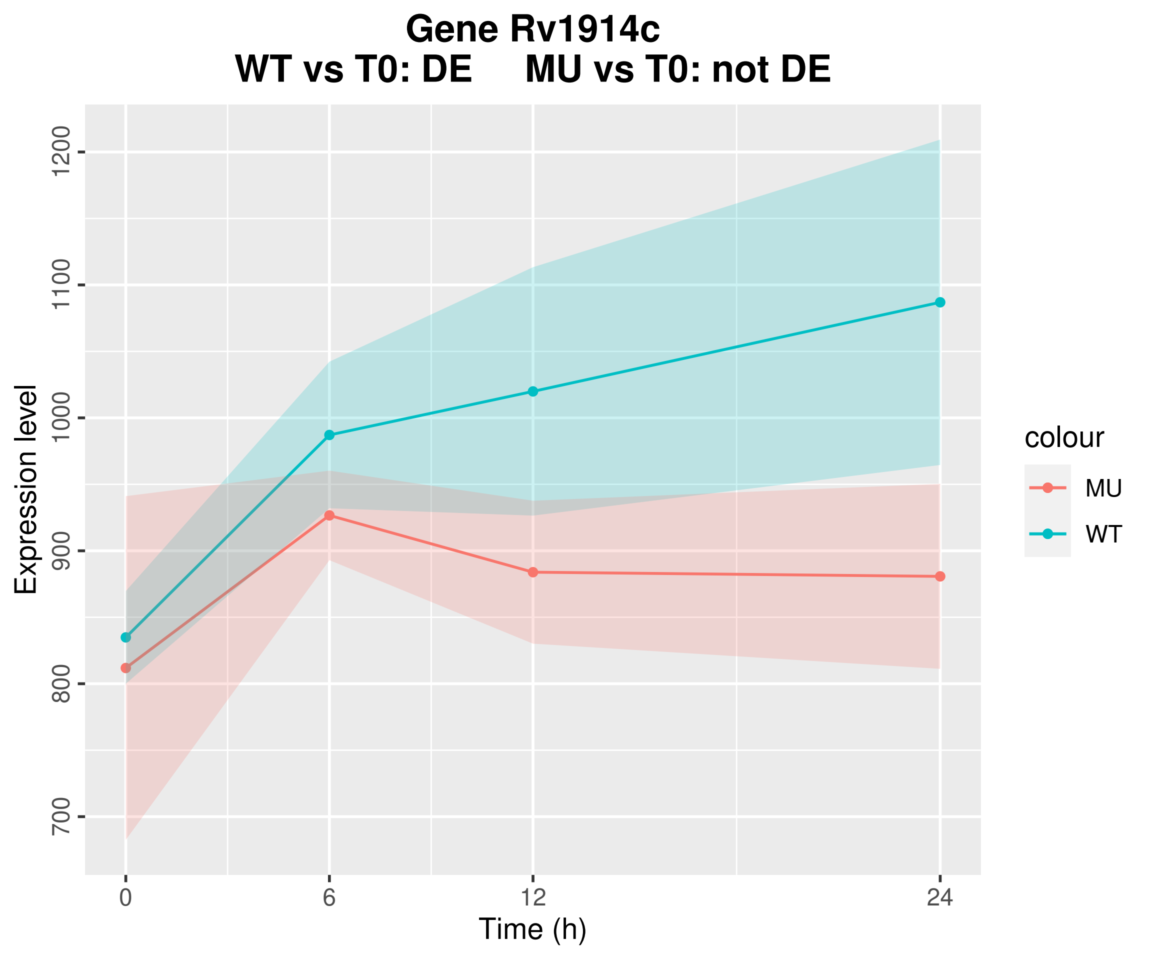
Gene Rv1914c
WT vs T0: DE   MU vs T0: not DE
Expression level
1200
1100
1000
900
800
700
0
6
12
24
Time (h)
colour
MU
WT

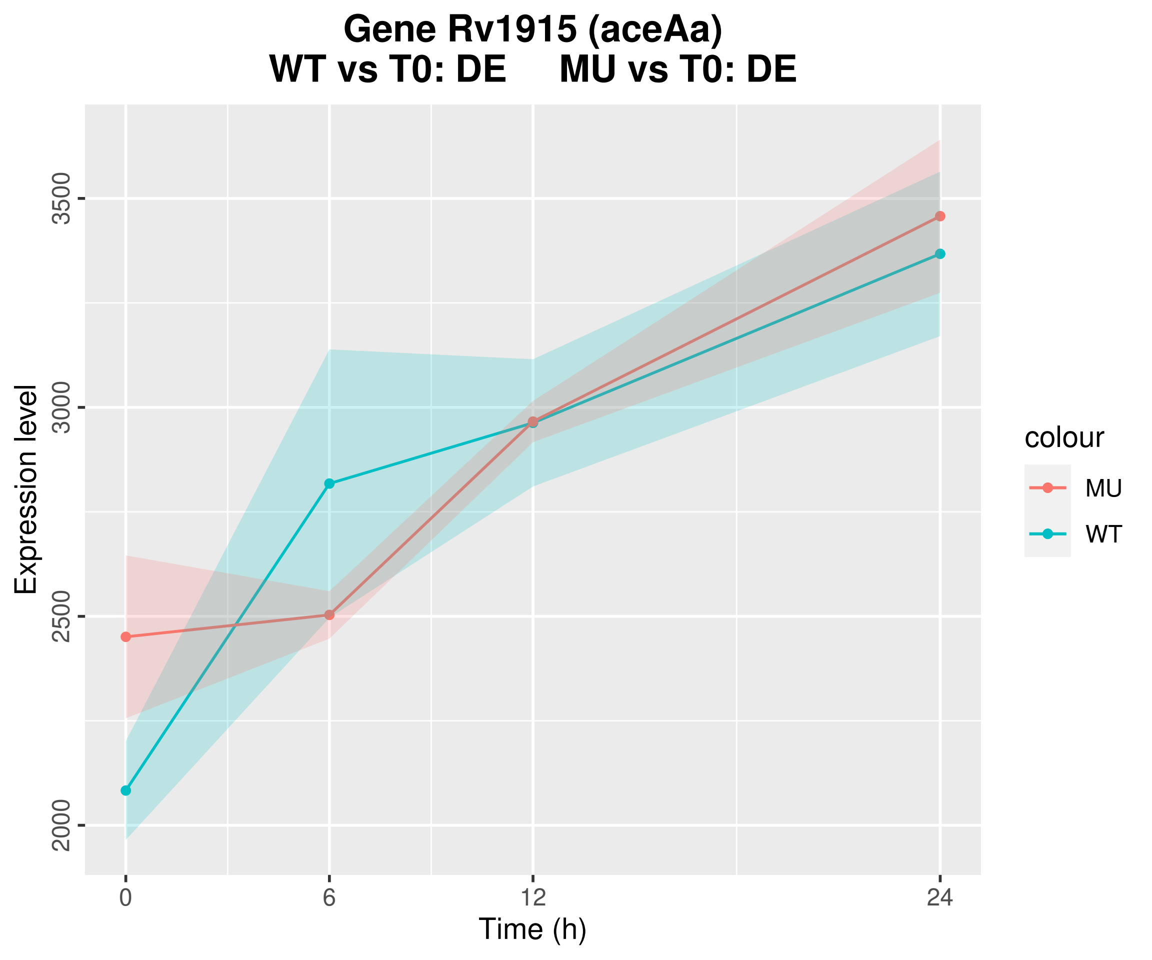

Gene Rv1915 (aceAa)
WT vs T0: DE MU vs T0: DE
Expression level
2000
2500
3000
3500
0
6
12
24
Time (h)
colour
MU
WT

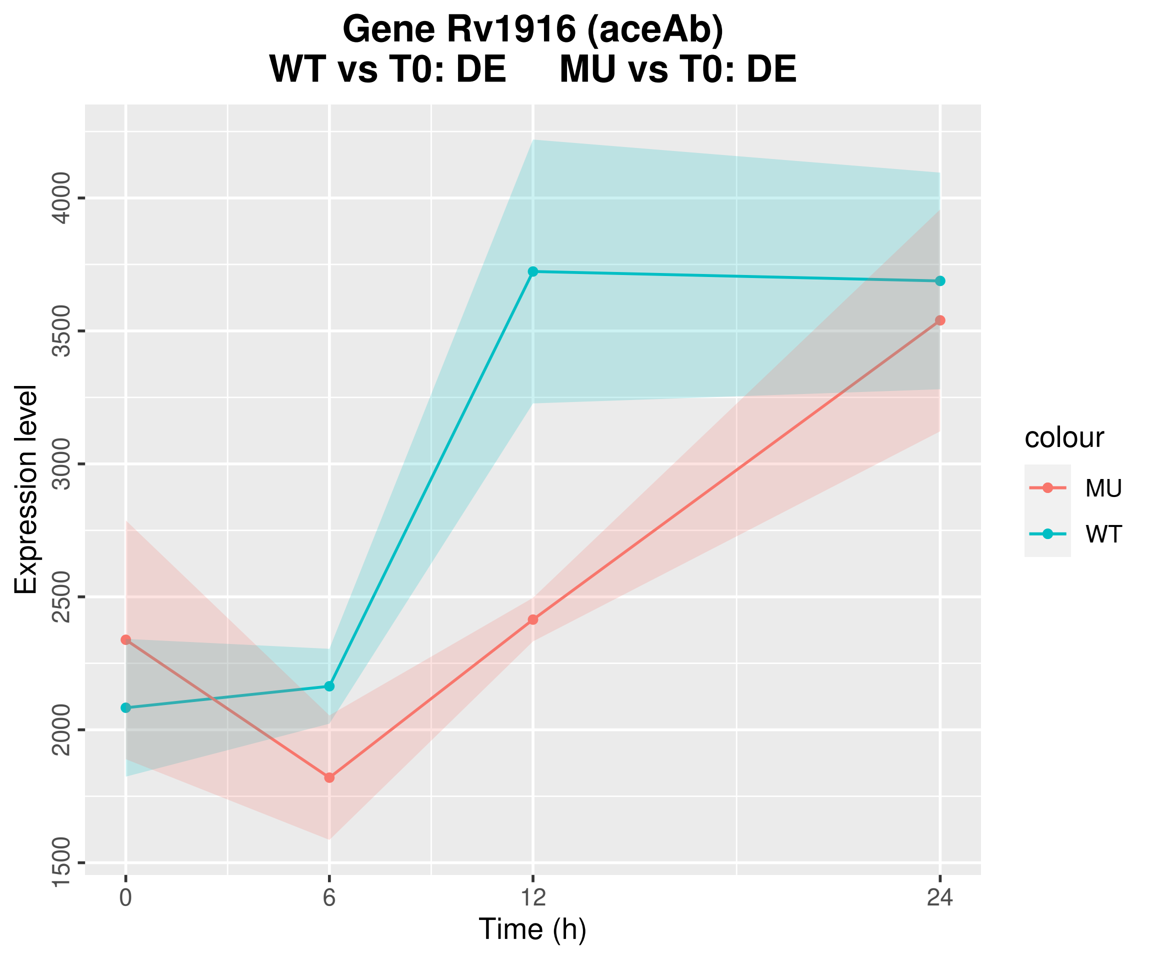

Gene Rv1916 (aceAb)
WT vs T0: DE MU vs T0: DE
Expression level
4000
3500
3000
2500
2000
1500
0
6
12
24
Time (h)
colour
MU
WT

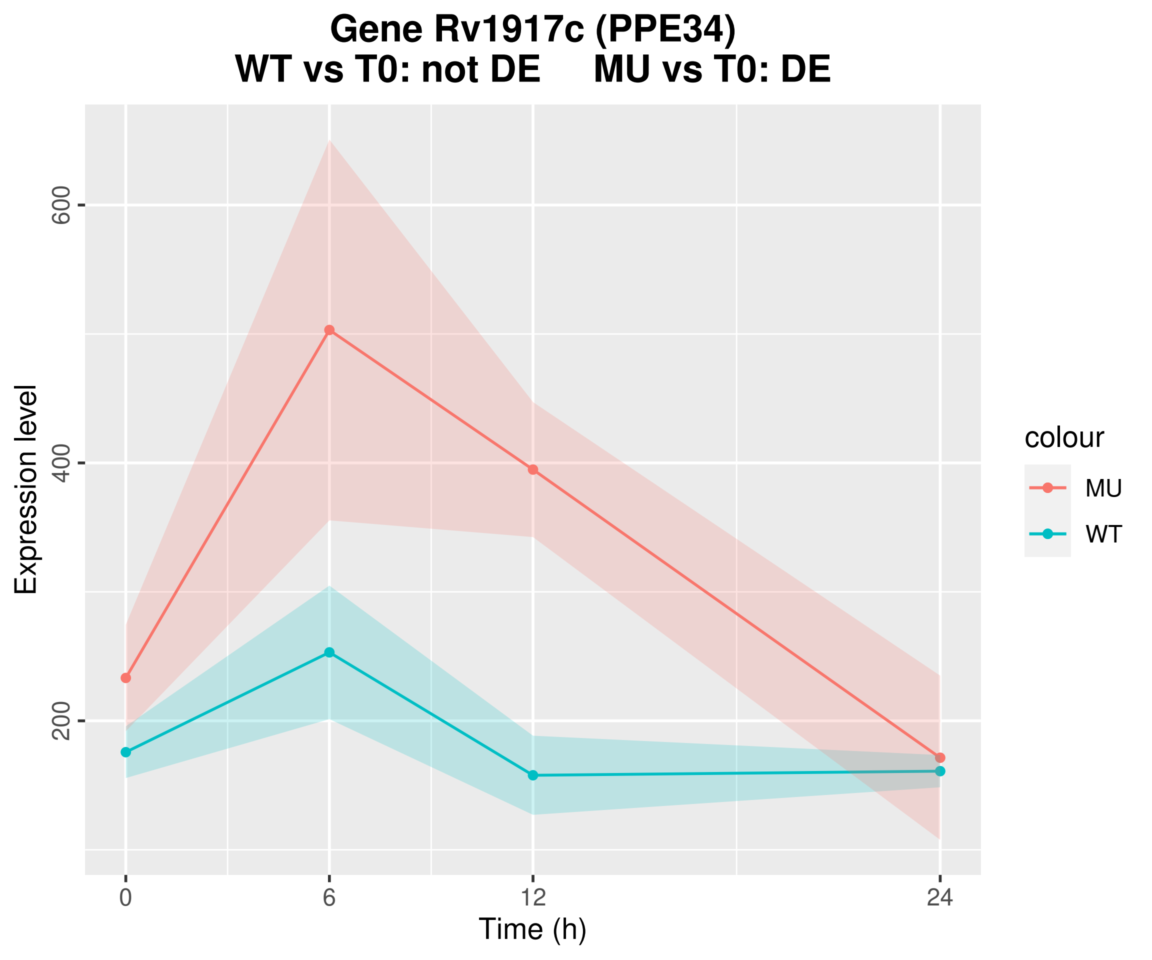

Gene Rv1917c (PPE34)
WT vs T0: not DE   MU vs T0: DE
600
400
200
Expression level
0
6
12
24
Time (h)
colour
MU
WT

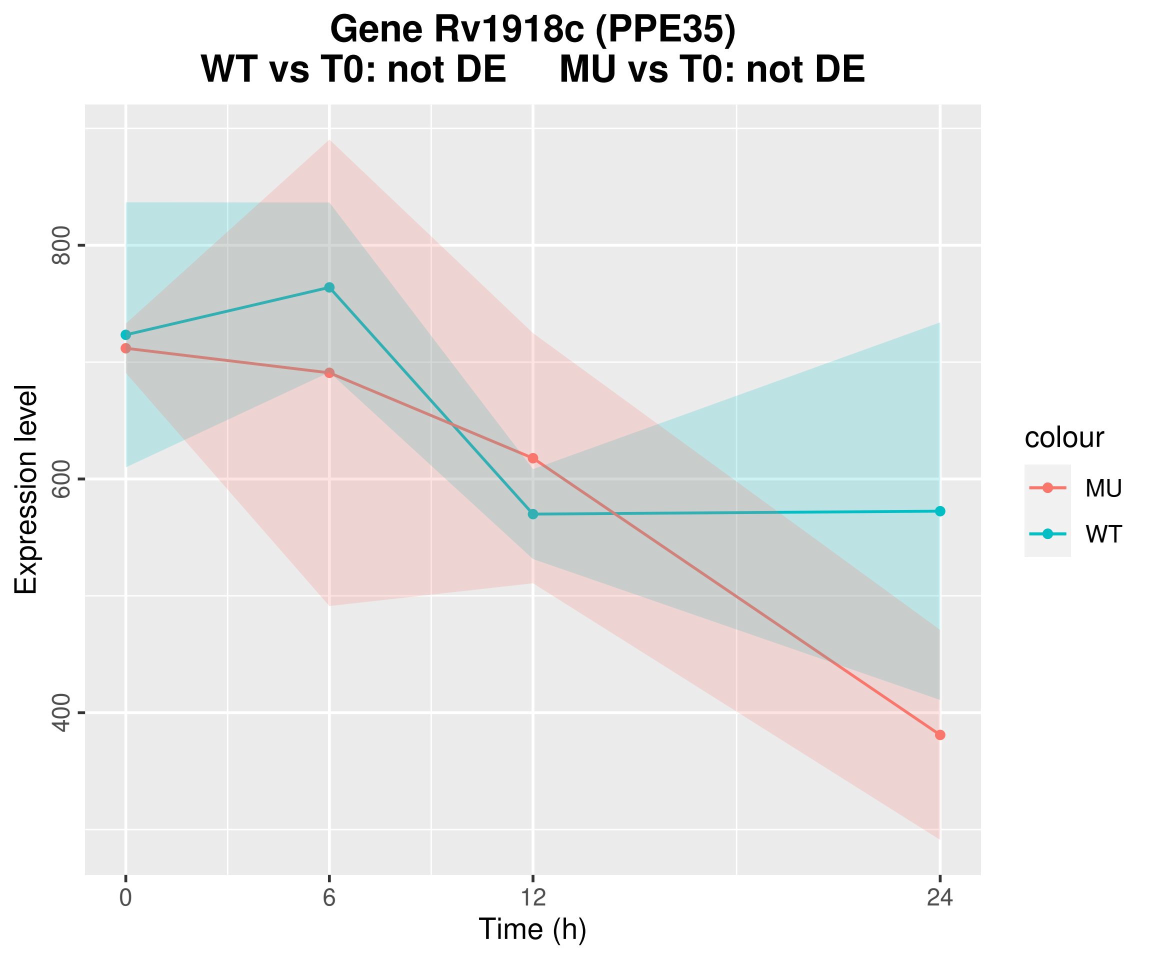
Gene Rv1918c (PPE35)
WT vs T0: not DE   MU vs T0: not DE
Expression level
800
600
400
0
6
12
24
Time (h)
colour
MU
WT

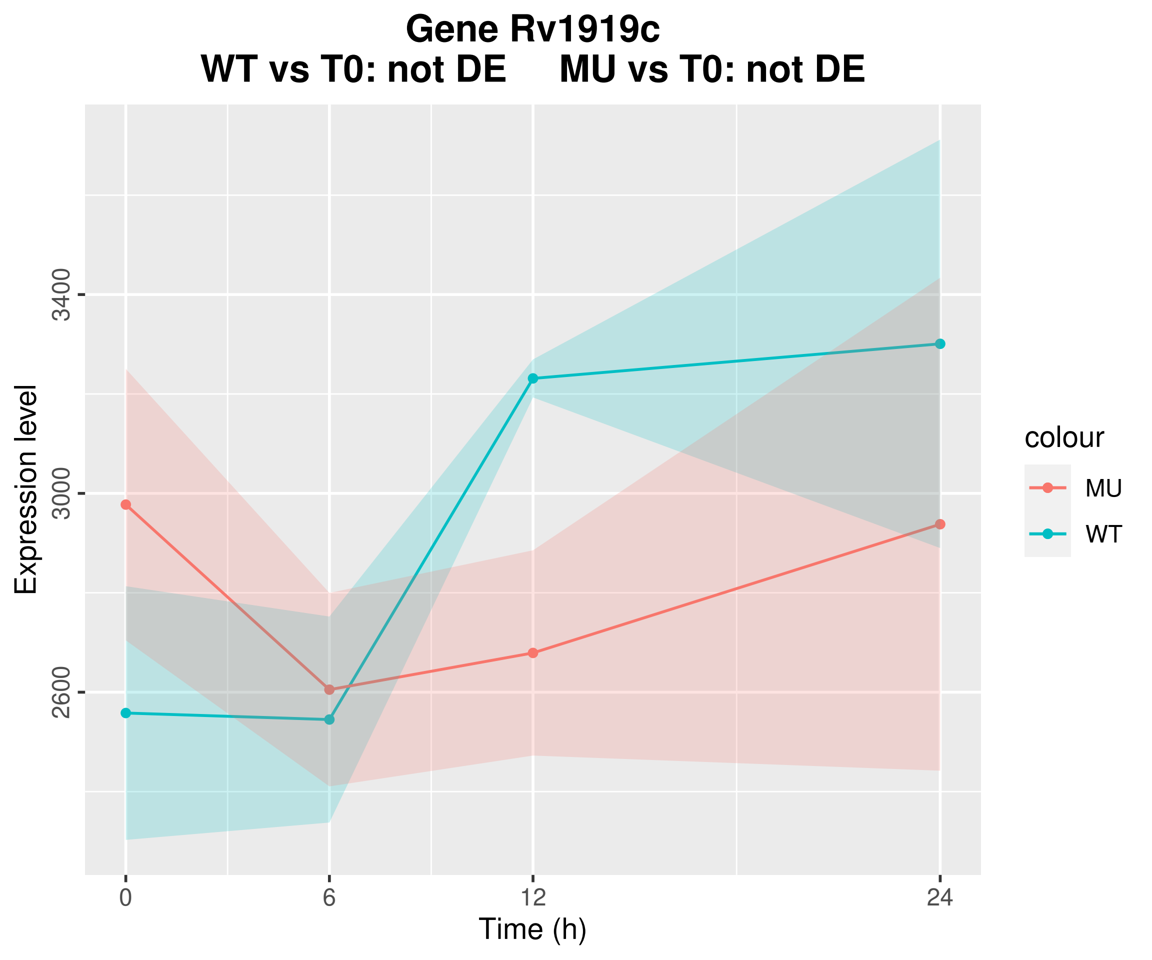

Gene Rv1919c
WT vs T0: not DE   MU vs T0: not DE
Expression level
3400
3000
2600
0
6
12
24
Time (h)
colour
MU
WT

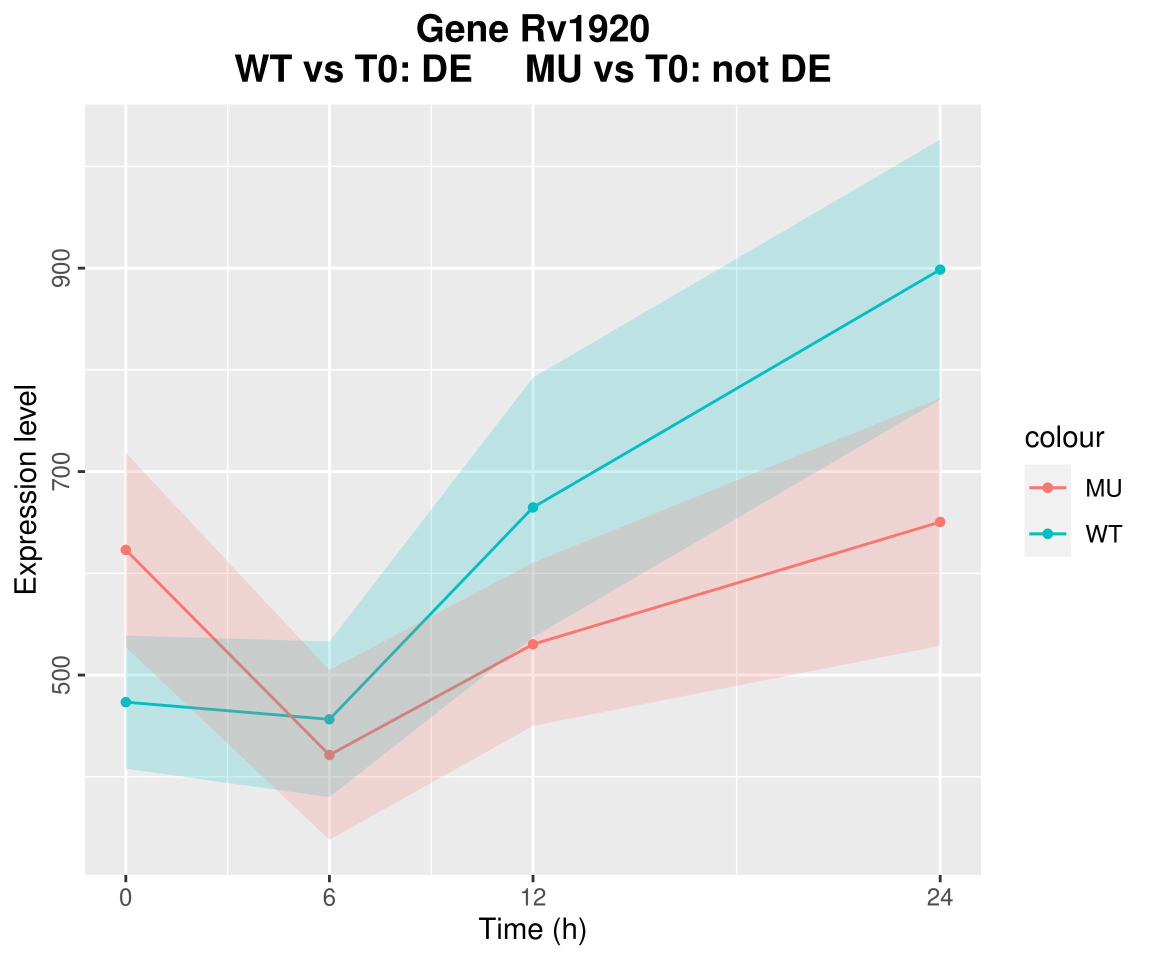

Gene Rv1920
WT vs T0: DE MU vs T0: not DE
Expression level
900
700
500
0
6
12
24
Time (h)
colour
MU
WT

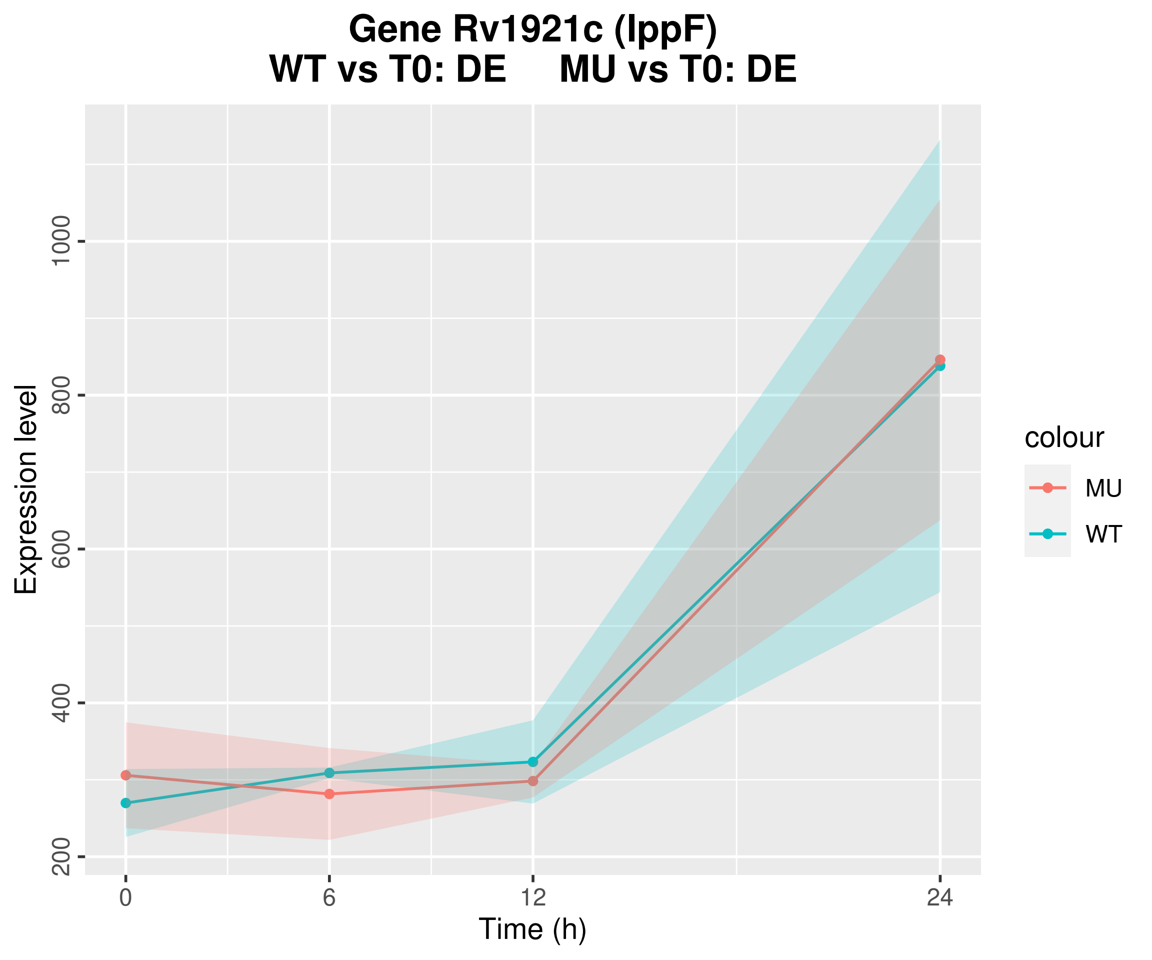
Gene Rv1921c (lppF)
WT vs T0: DE   MU vs T0: DE
Expression level
1000
800
600
400
200
0
6
12
24
Time (h)
colour
MU
WT

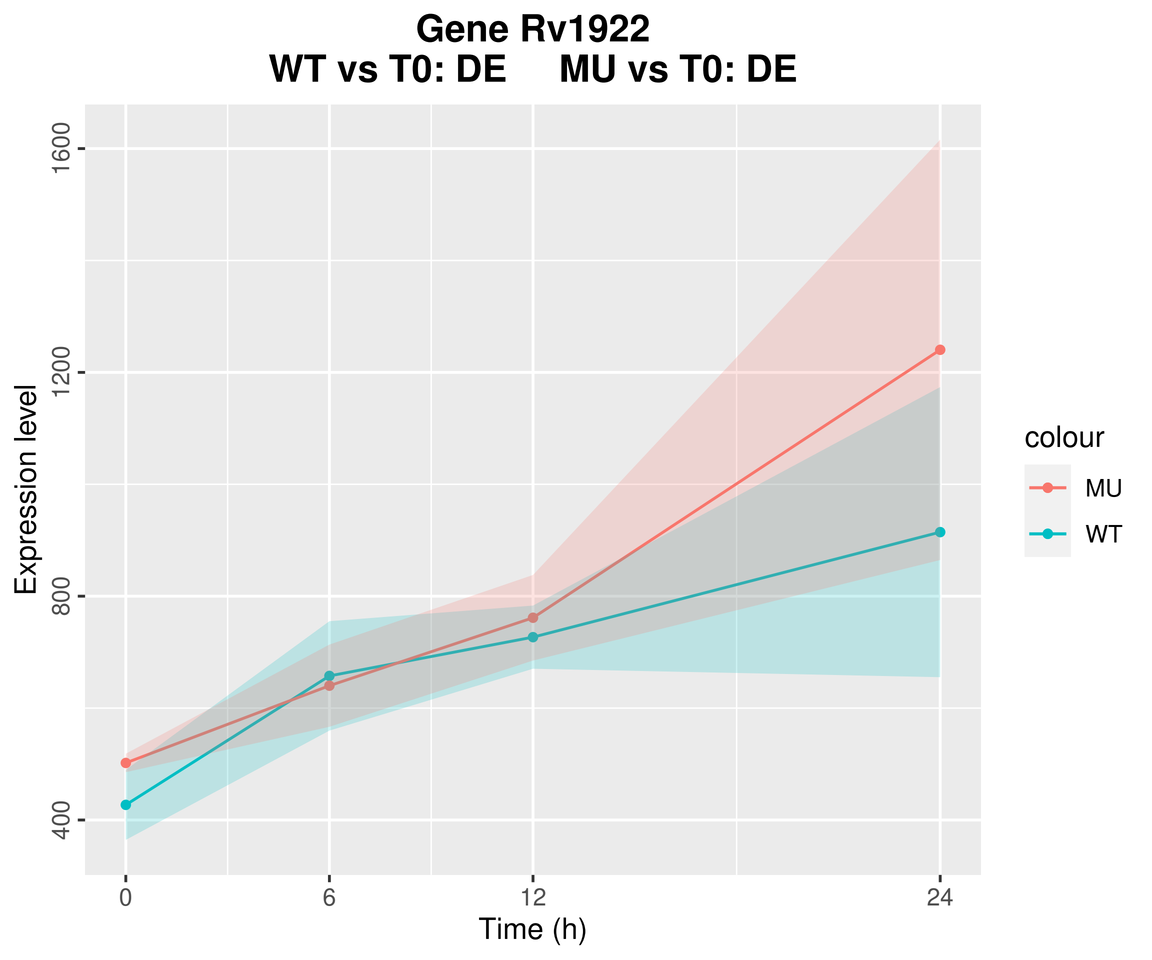

Gene Rv1922
WT vs T0: DE   MU vs T0: DE
Expression level
1600
1200
800
400
0
6
12
24
Time (h)
colour
MU
WT

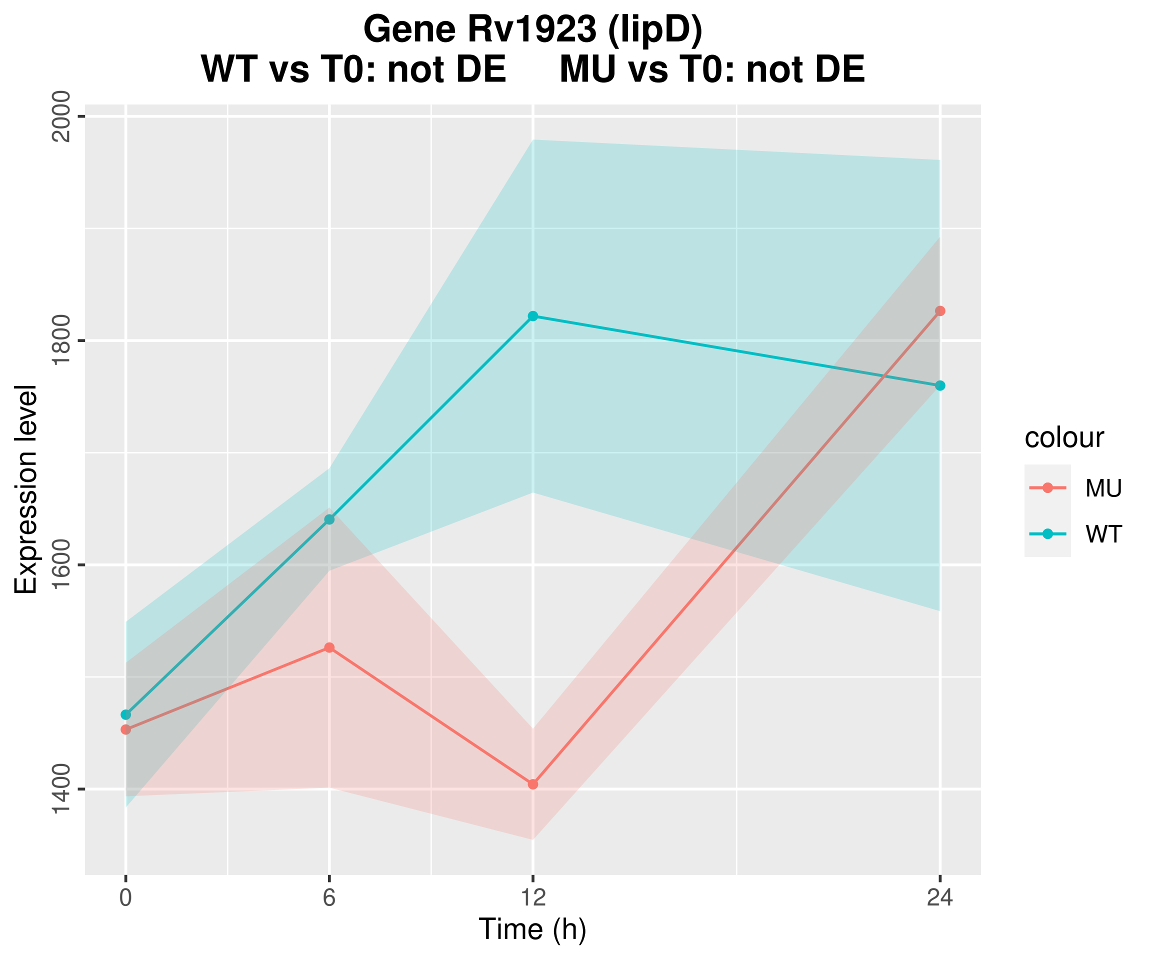
Gene Rv1923 (lipD)
WT vs T0: not DE   MU vs T0: not DE
Expression level
2000
1800
1600
1400
0
6
12
24
Time (h)
colour
MU
WT

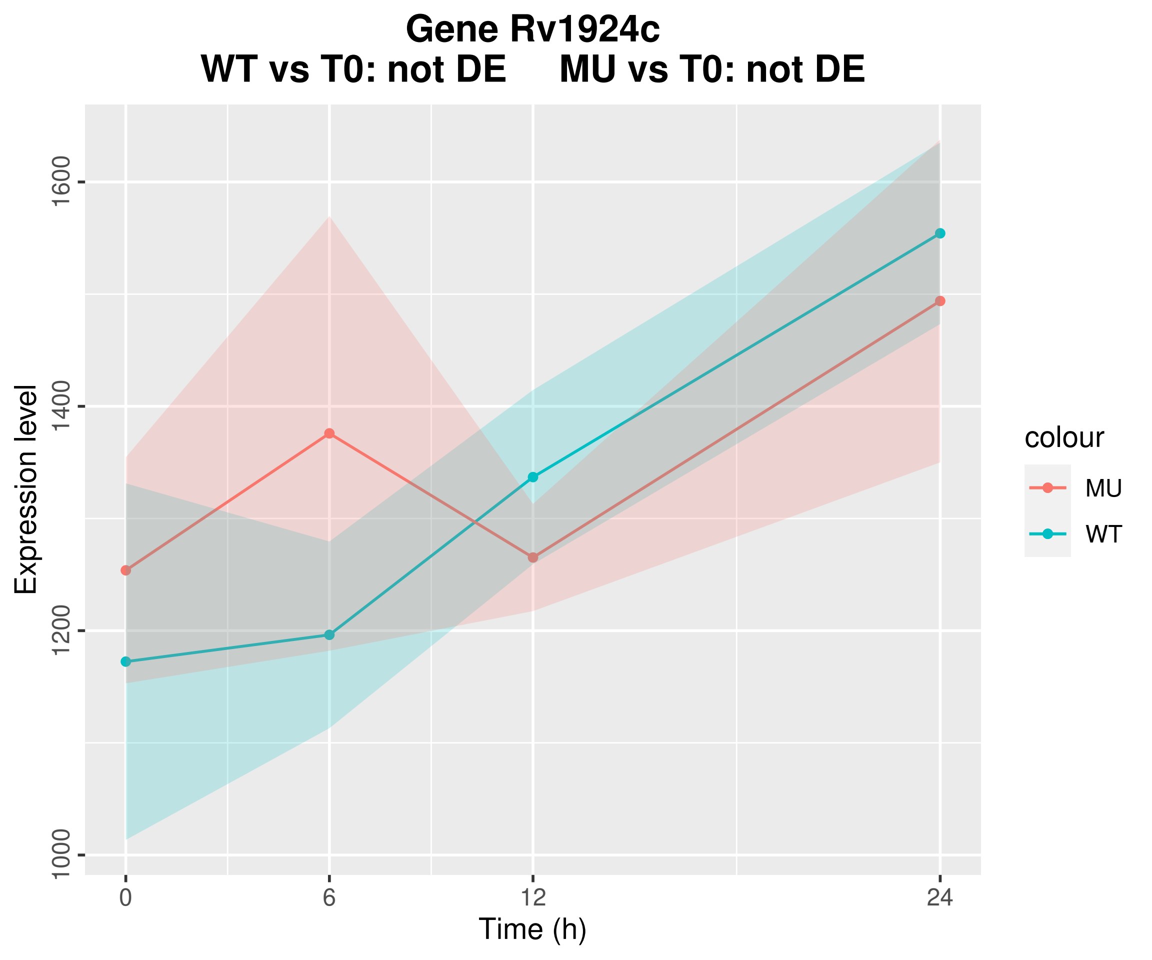
Gene Rv1924c
WT vs T0: not DE   MU vs T0: not DE
Expression level
1000
1200
1400
1600
0
6
12
24
Time (h)
colour
MU
WT

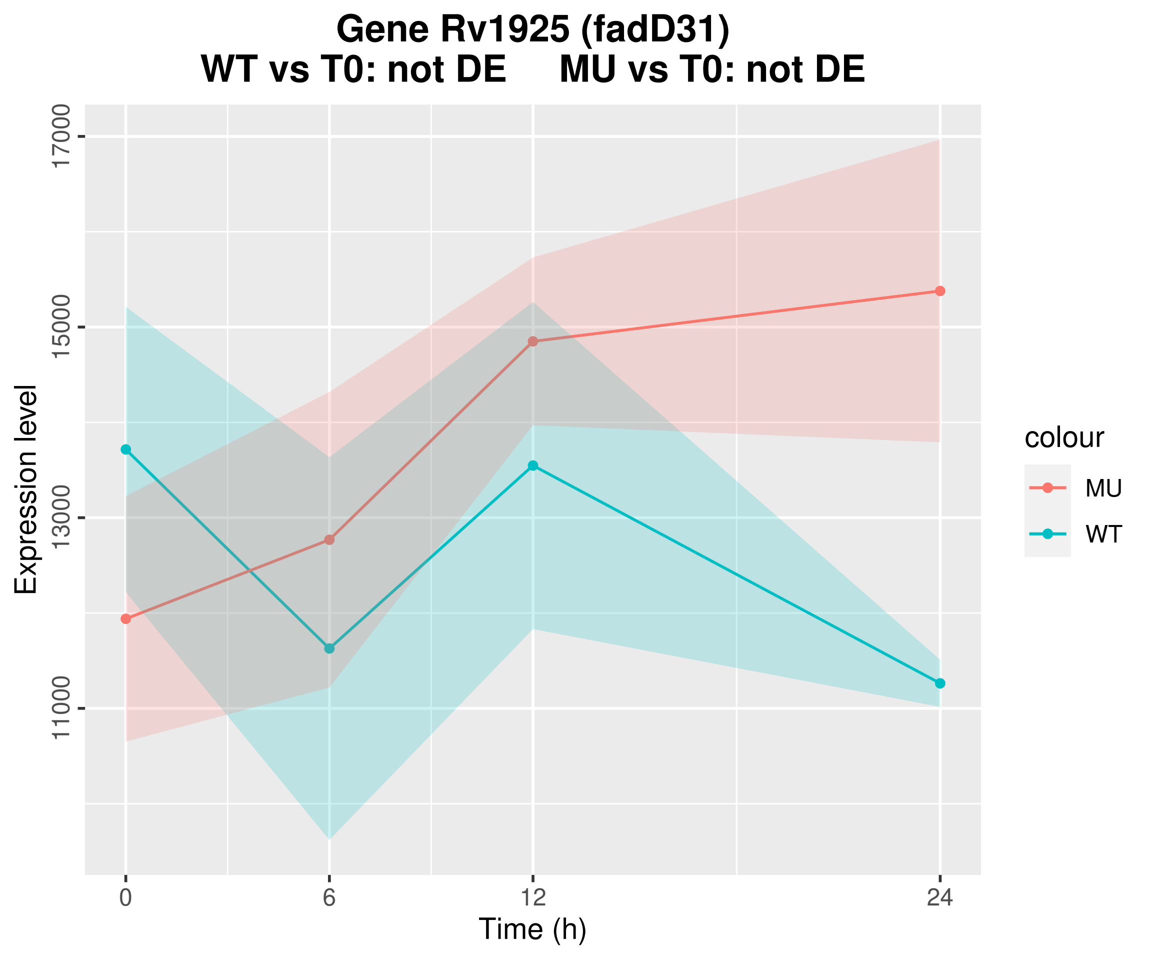

Gene Rv1925 (fadD31)
WT vs T0: not DE   MU vs T0: not DE
Expression level
11000
13000
15000
17000
0
6
12
24
Time (h)
colour
MU
WT

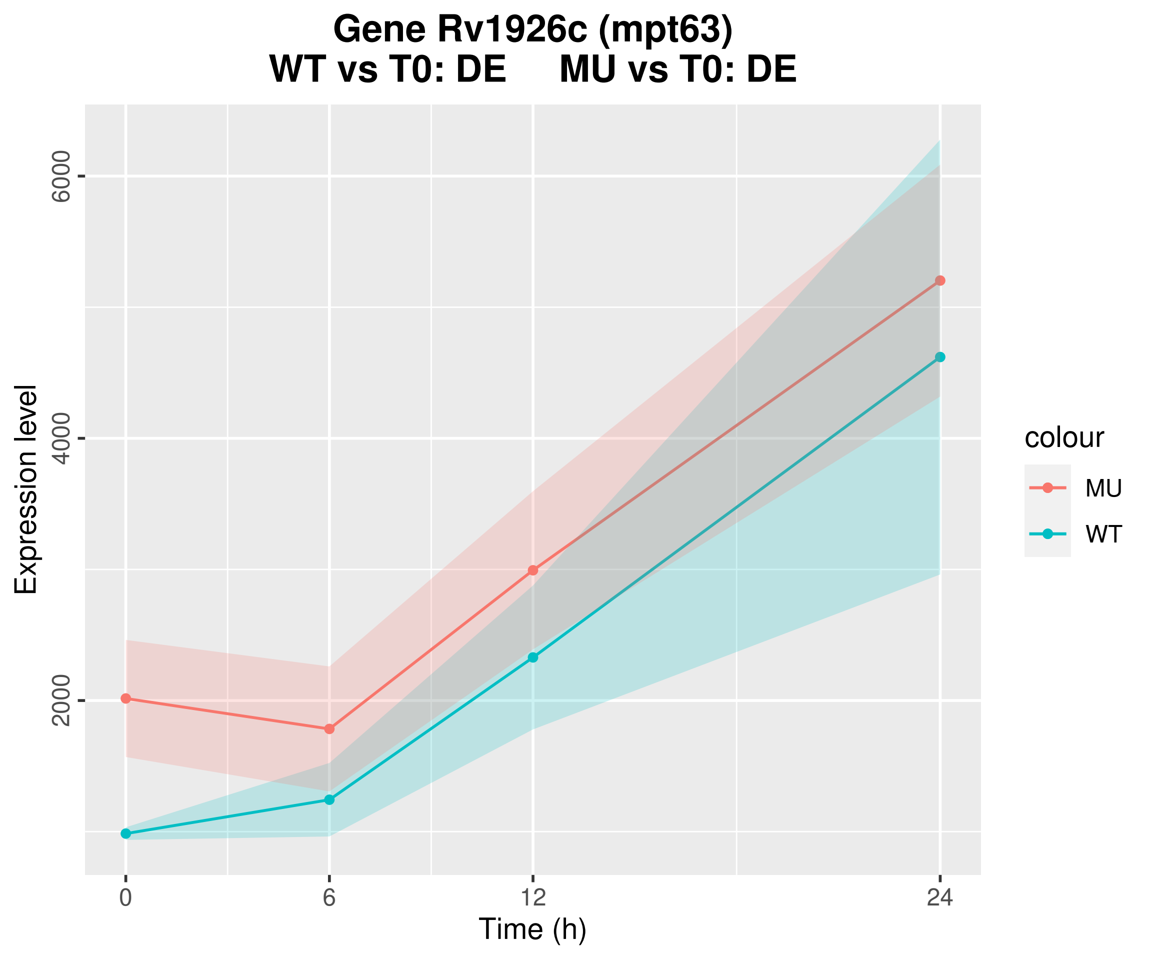

Gene Rv1926c (mpt63)
WT vs T0: DE MU vs T0: DE
6000
4000
2000
0
6
12
24
Time (h)
Expression level
colour
MU
WT

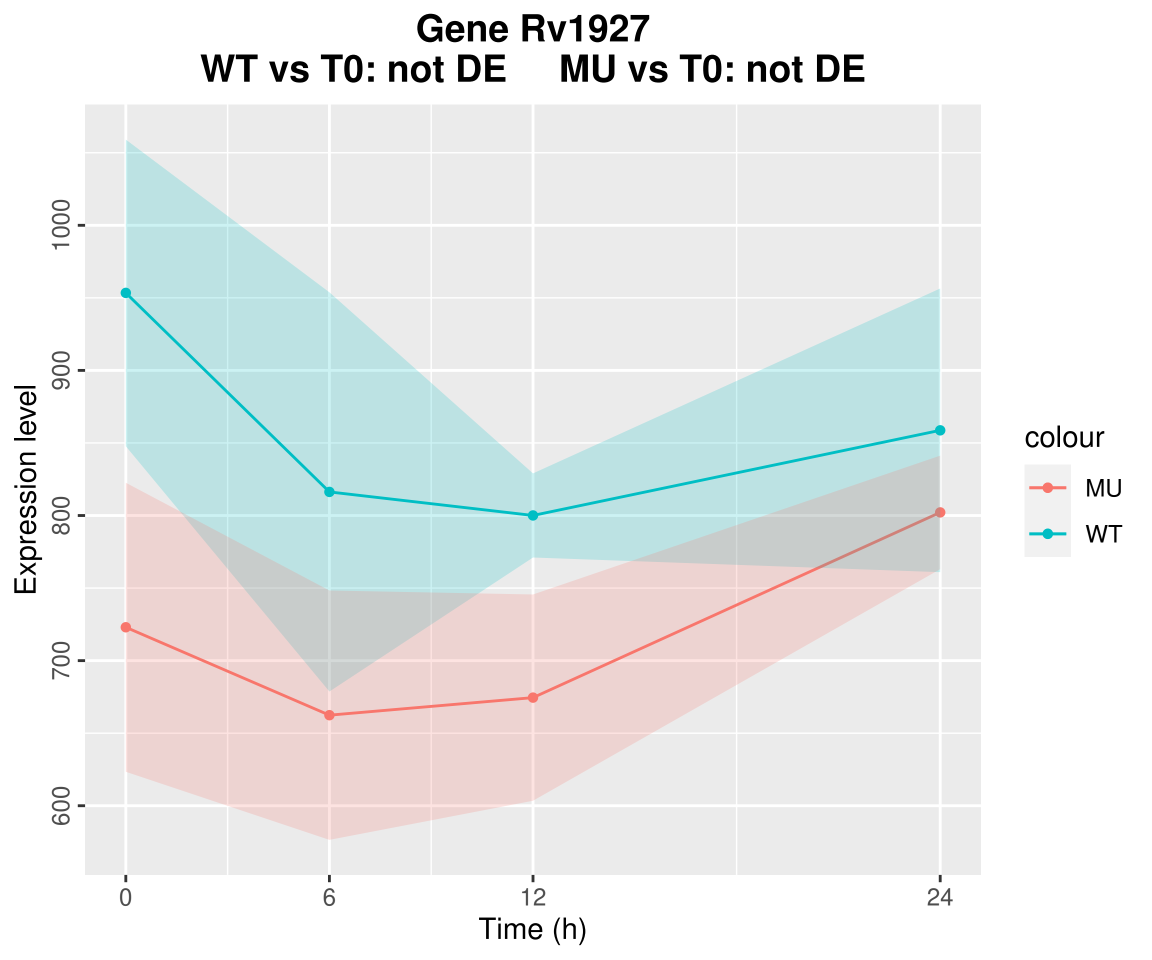
Gene Rv1927
WT vs T0: not DE   MU vs T0: not DE
Expression level
1000
900
800
700
600
0
6
12
24
Time (h)
colour
MU
WT

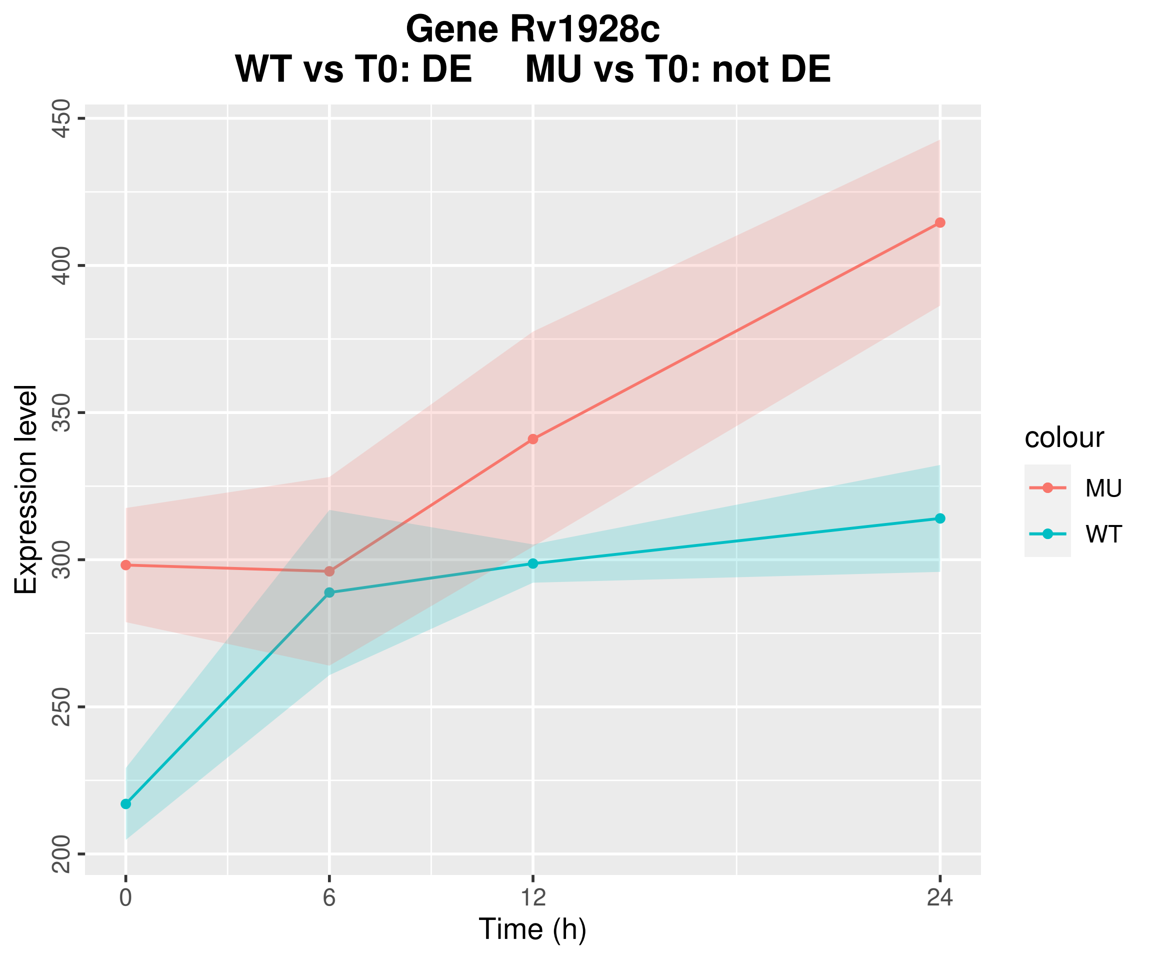

Gene Rv1928c
WT vs T0: DE   MU vs T0: not DE
Expression level
450
400
350
300
250
200
0
6
12
24
Time (h)
colour
MU
WT

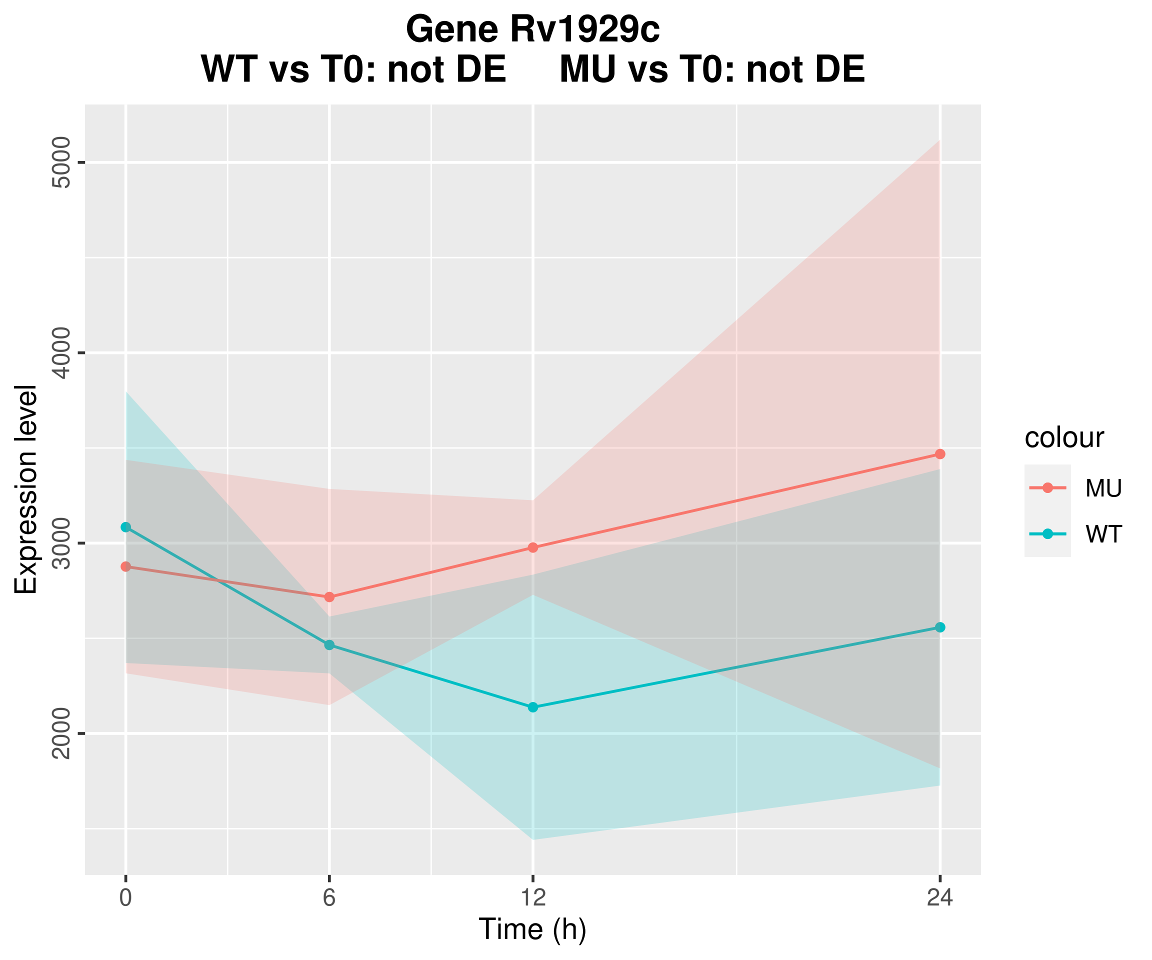

Gene Rv1929c
WT vs T0: not DE   MU vs T0: not DE
5000
4000
3000
2000
Expression level
0
6
12
24
Time (h)
colour
MU
WT

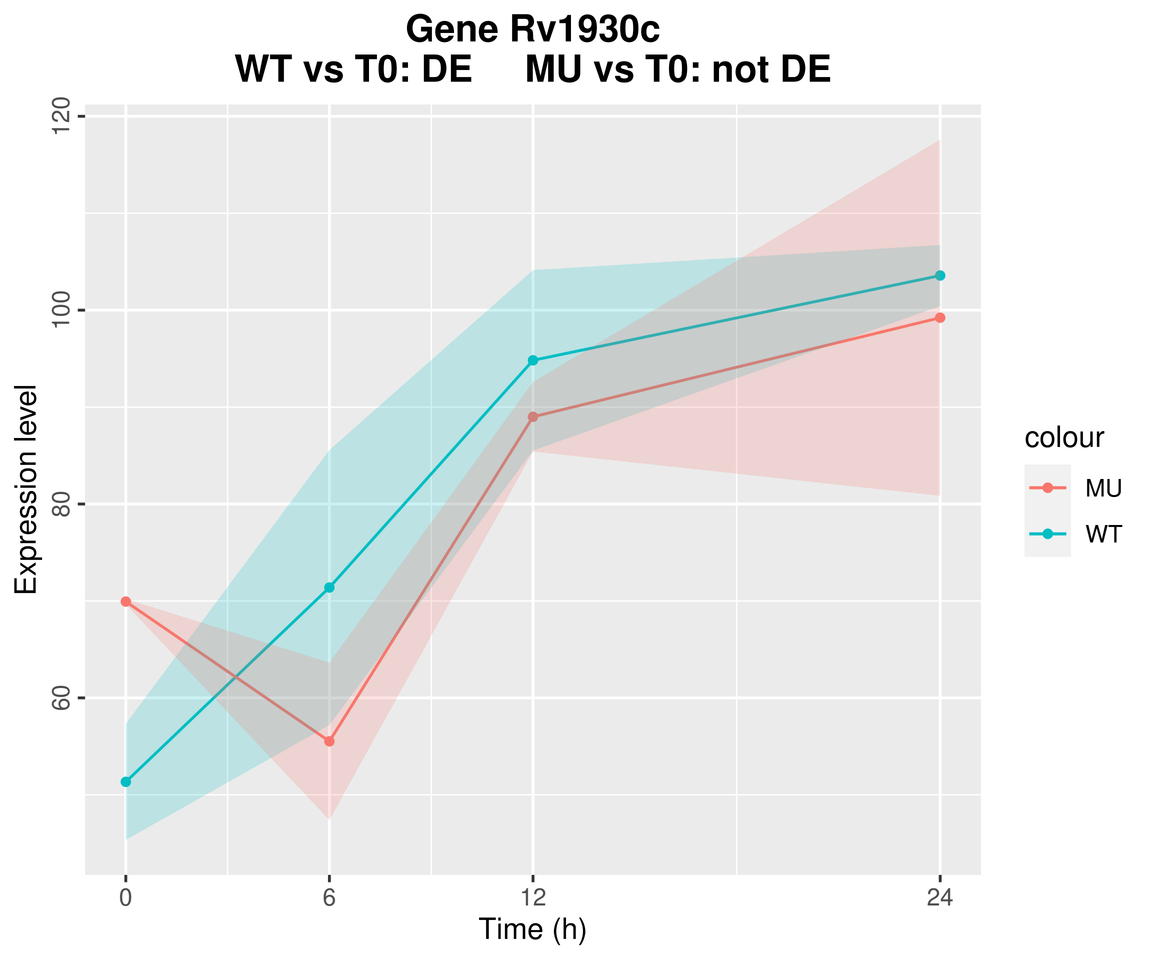
Gene Rv1930c
WT vs T0: DE MU vs T0: not DE
Expression level
120
100
80
60
0
6
12
24
Time (h)
colour
MU
WT

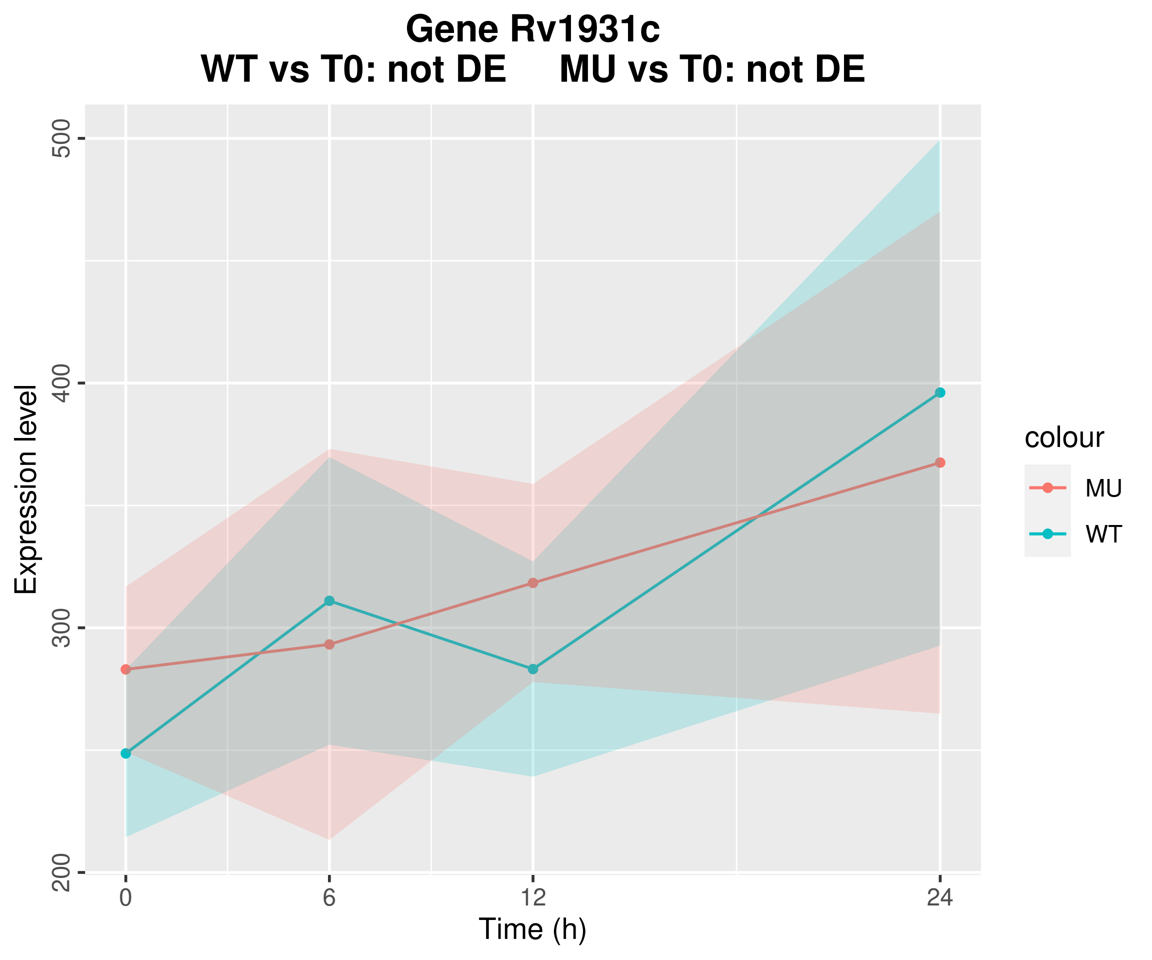
Gene Rv1931c
WT vs T0: not DE   MU vs T0: not DE
Expression level
500
400
300
200
0
6
12
24
Time (h)
colour
MU
WT

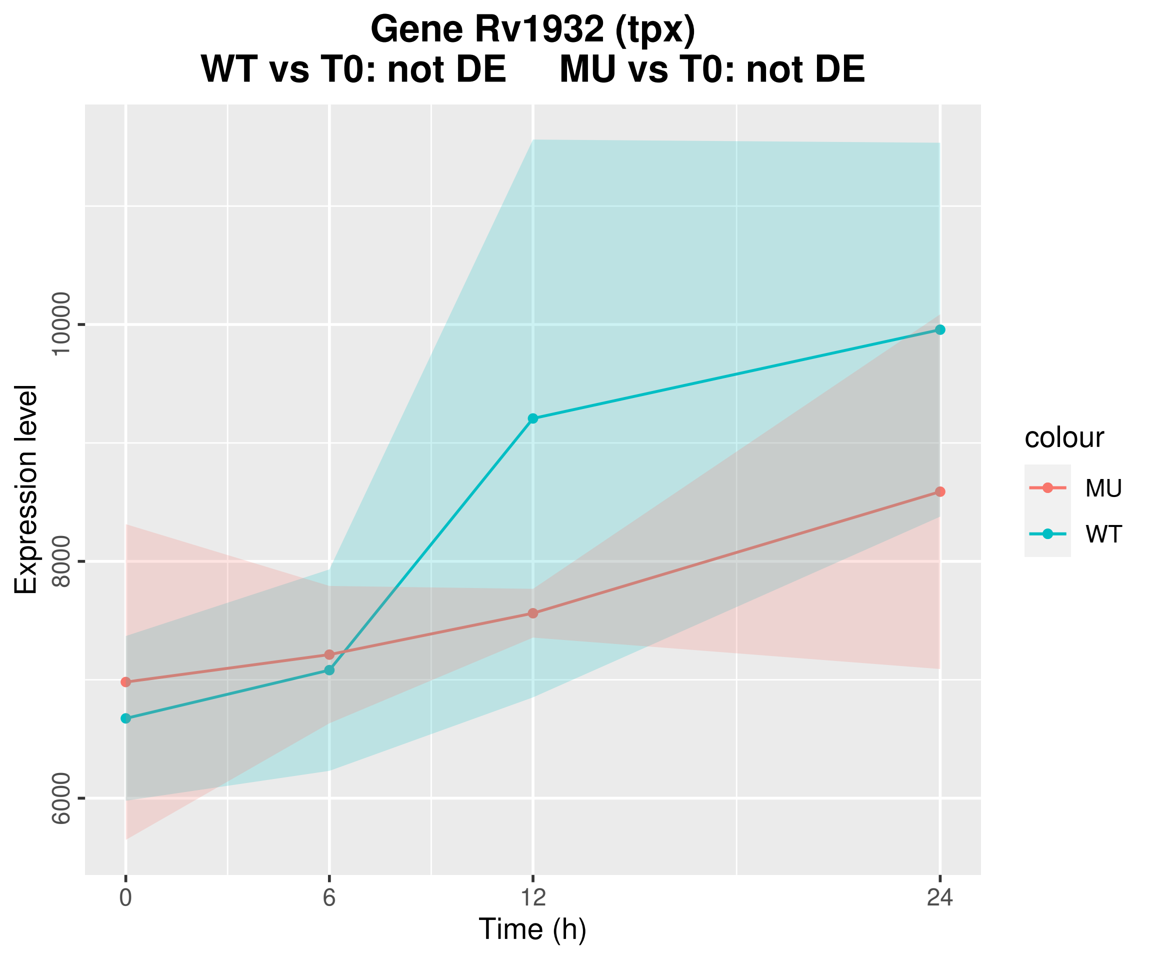
Gene Rv1932 (tpx)
WT vs T0: not DE   MU vs T0: not DE
Expression level
10000
8000
6000
0
6
12
24
Time (h)
colour
MU
WT

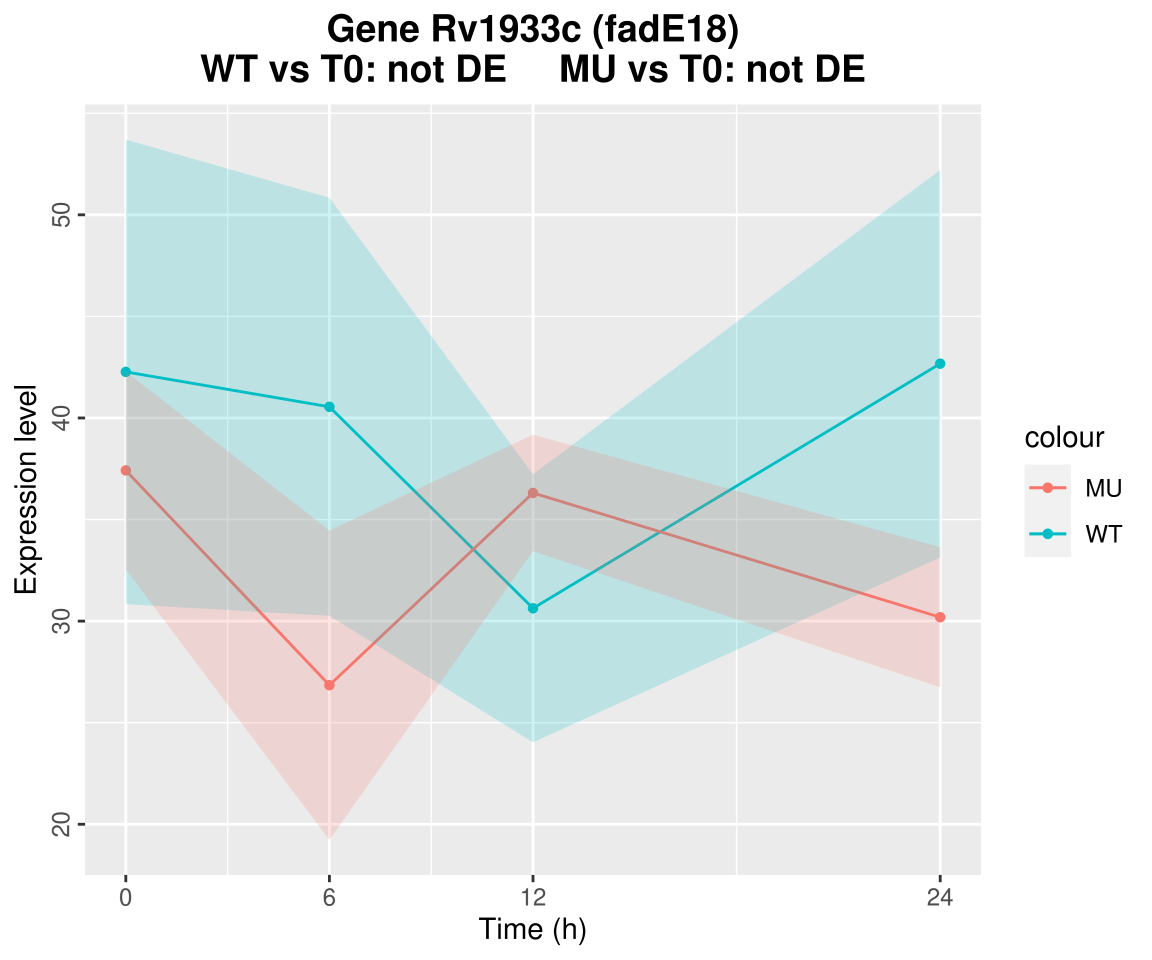

Gene Rv1933c (fadE18)
WT vs T0: not DE MU vs T0: not DE
Expression level
50
40
30
20
0
6
12
24
Time (h)
colour
MU
WT

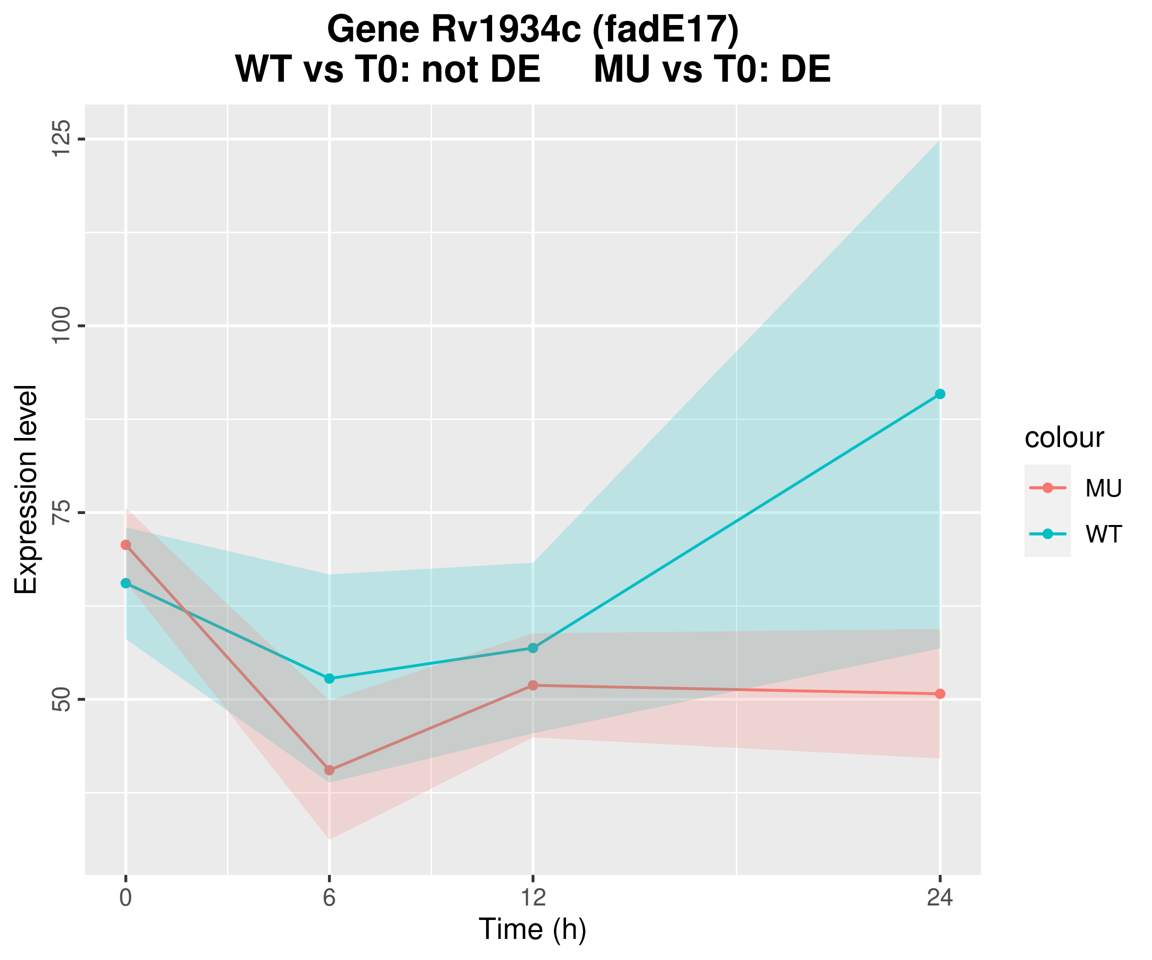
Gene Rv1934c (fadE17)
WT vs T0: not DE   MU vs T0: DE
125
100
75
50
Expression level
0
6
12
24
Time (h)
colour
MU
WT

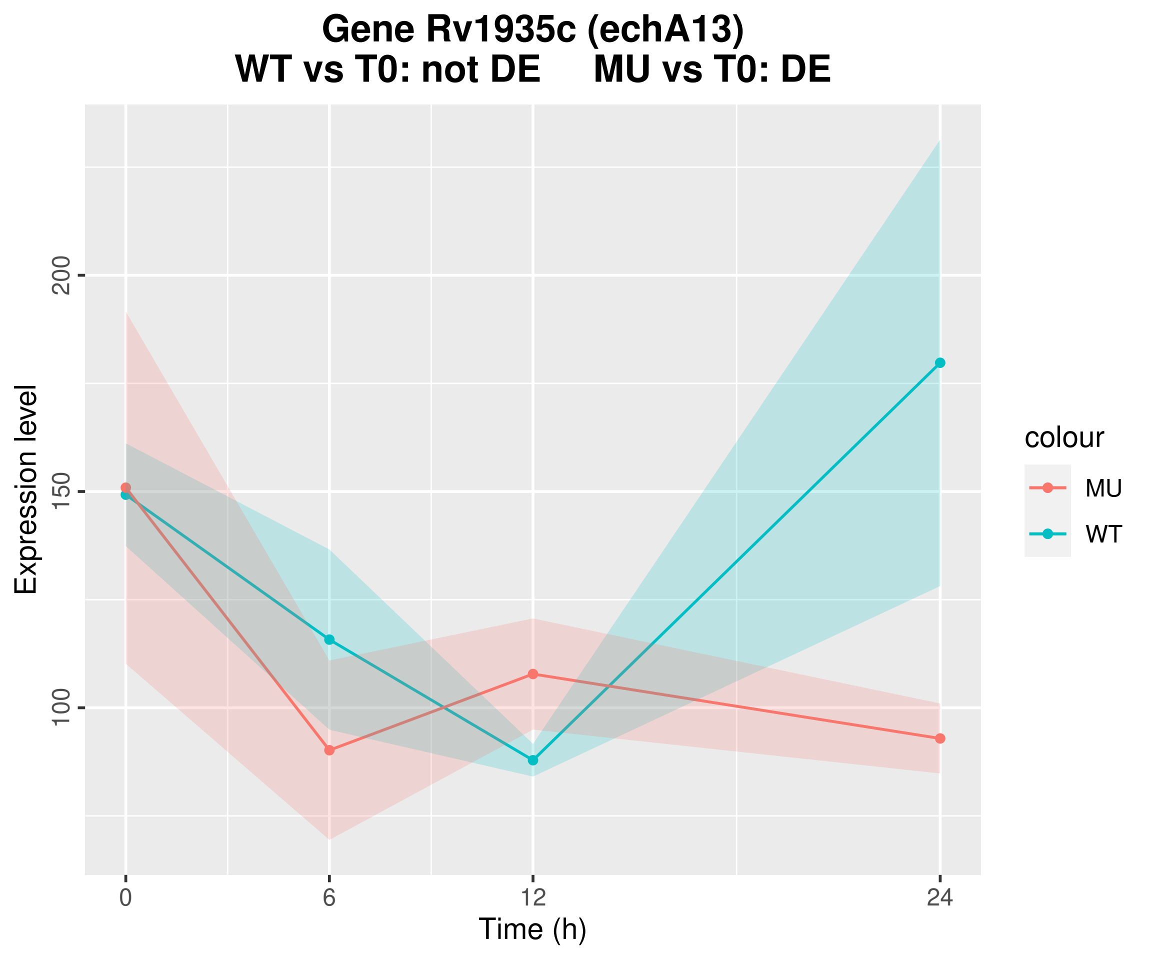

Gene Rv1935c (echA13)
WT vs T0: not DE MU vs T0: DE
Expression level
200
150
100
0
6
12
24
Time (h)
colour
MU
WT

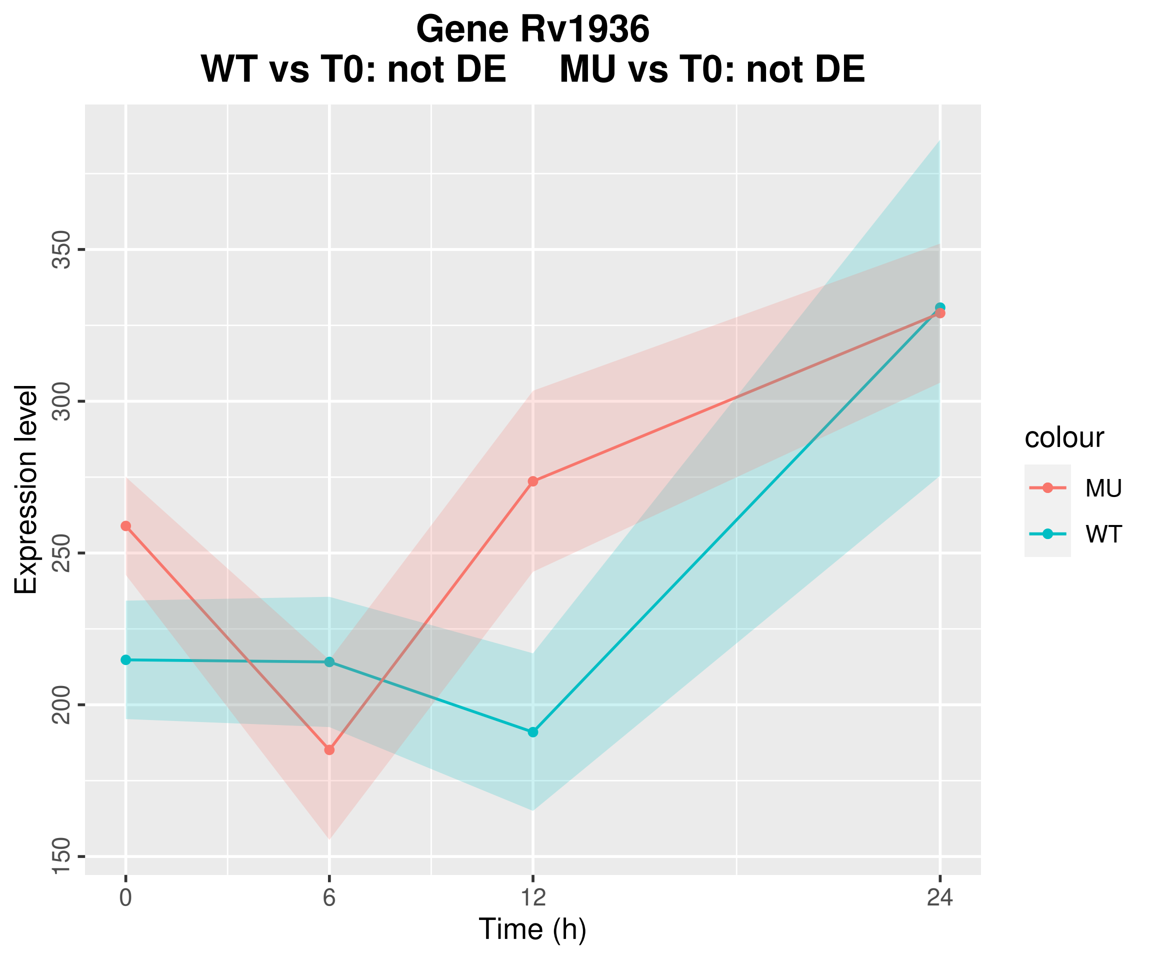

Gene Rv1936
WT vs T0: not DE   MU vs T0: not DE
Expression level
350
300
250
200
150
0
6
12
24
Time (h)
colour
MU
WT

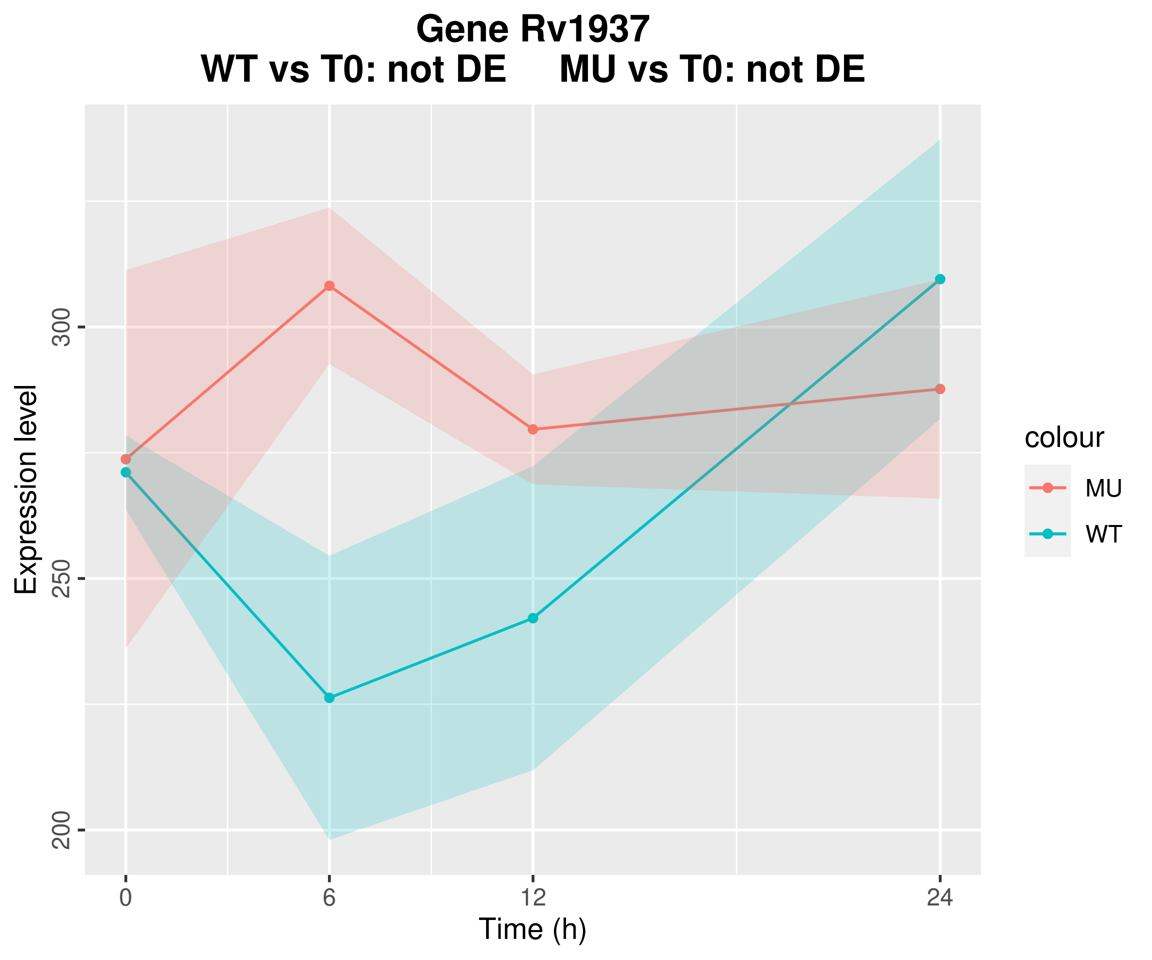
Gene Rv1937
WT vs T0: not DE   MU vs T0: not DE
Expression level
300
250
200
0
6
12
24
Time (h)
colour
MU
WT

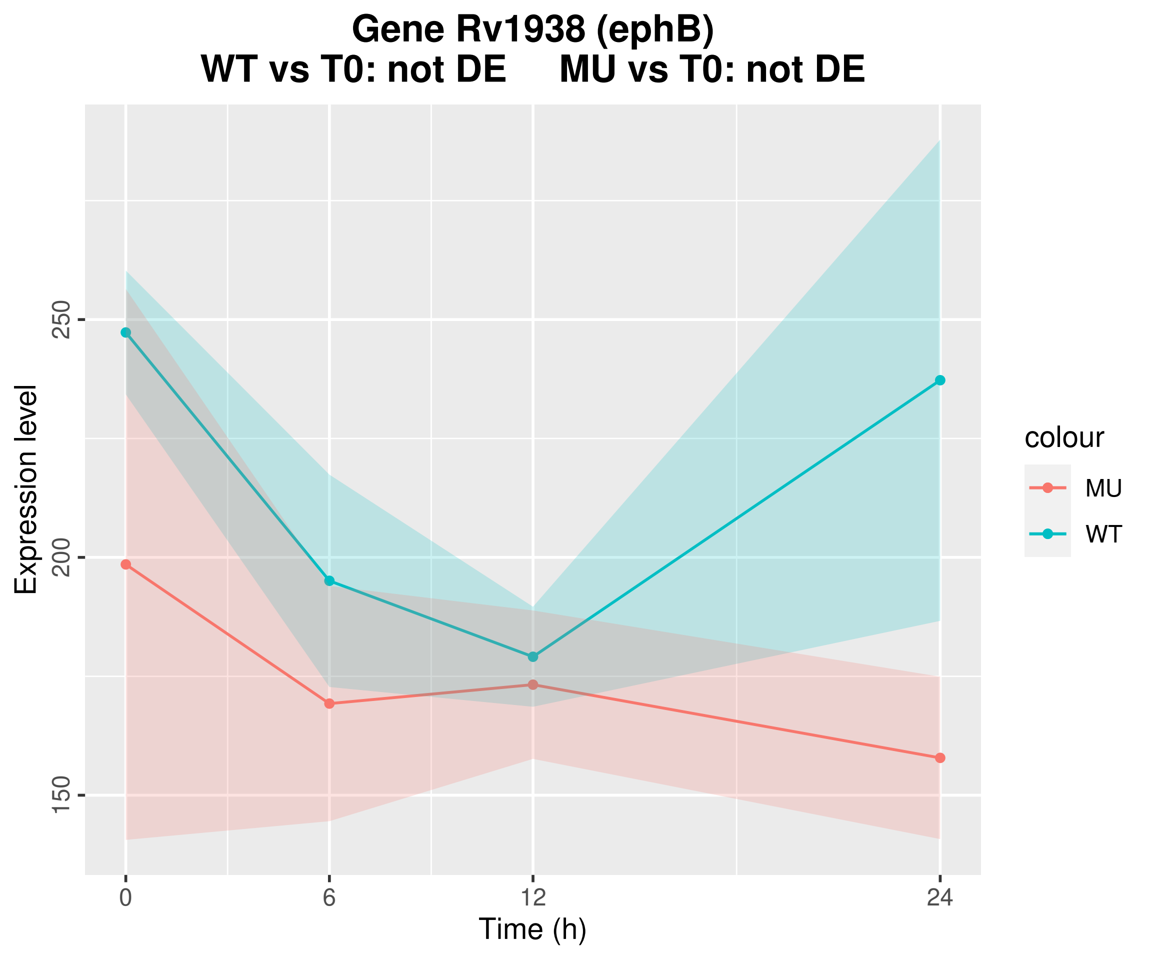
Gene Rv1938 (ephB)
WT vs T0: not DE MU vs T0: not DE
Expression level
250
200
150
0
6
12
24
Time (h)
colour
MU
WT

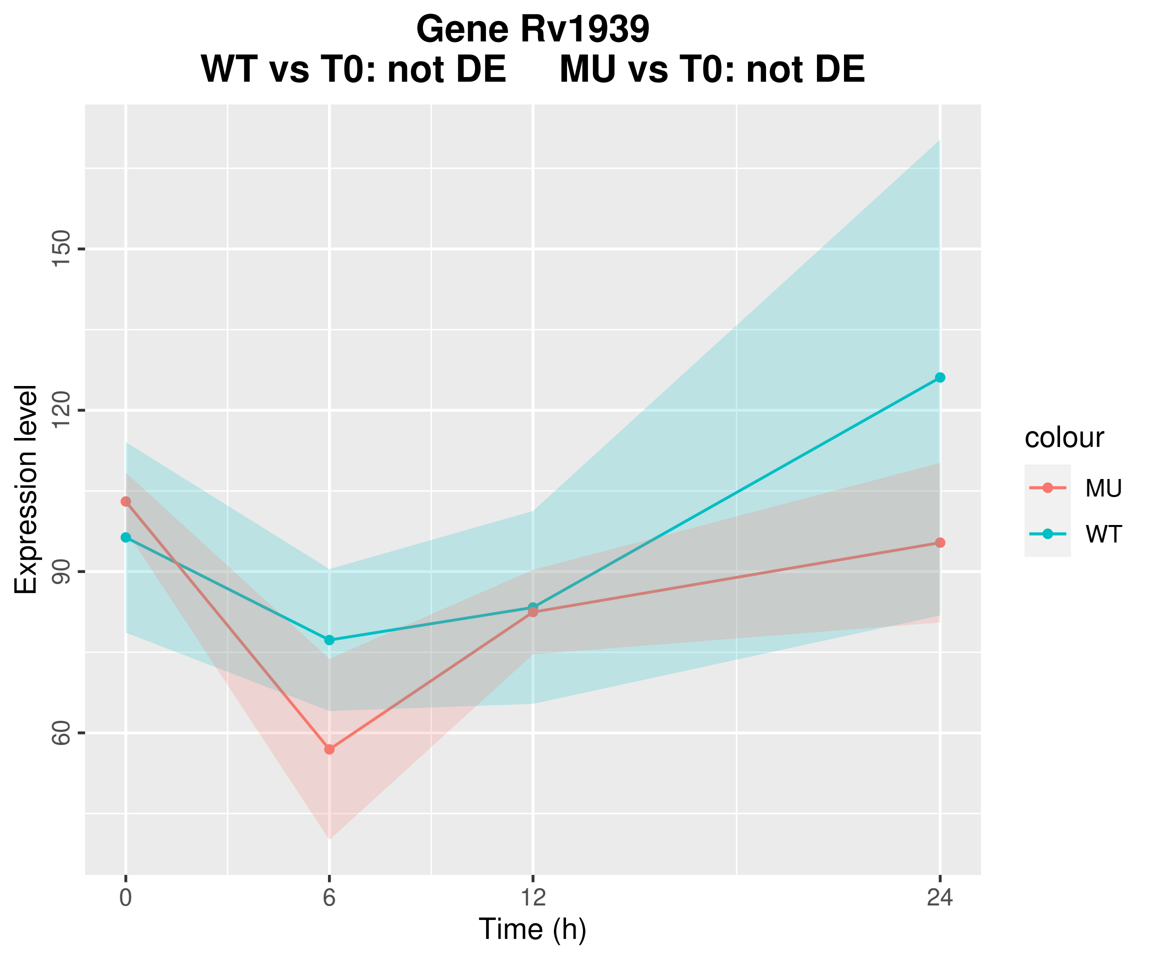
Gene Rv1939
WT vs T0: not DE   MU vs T0: not DE
Expression level
150
120
90
60
0
6
12
24
Time (h)
colour
MU
WT

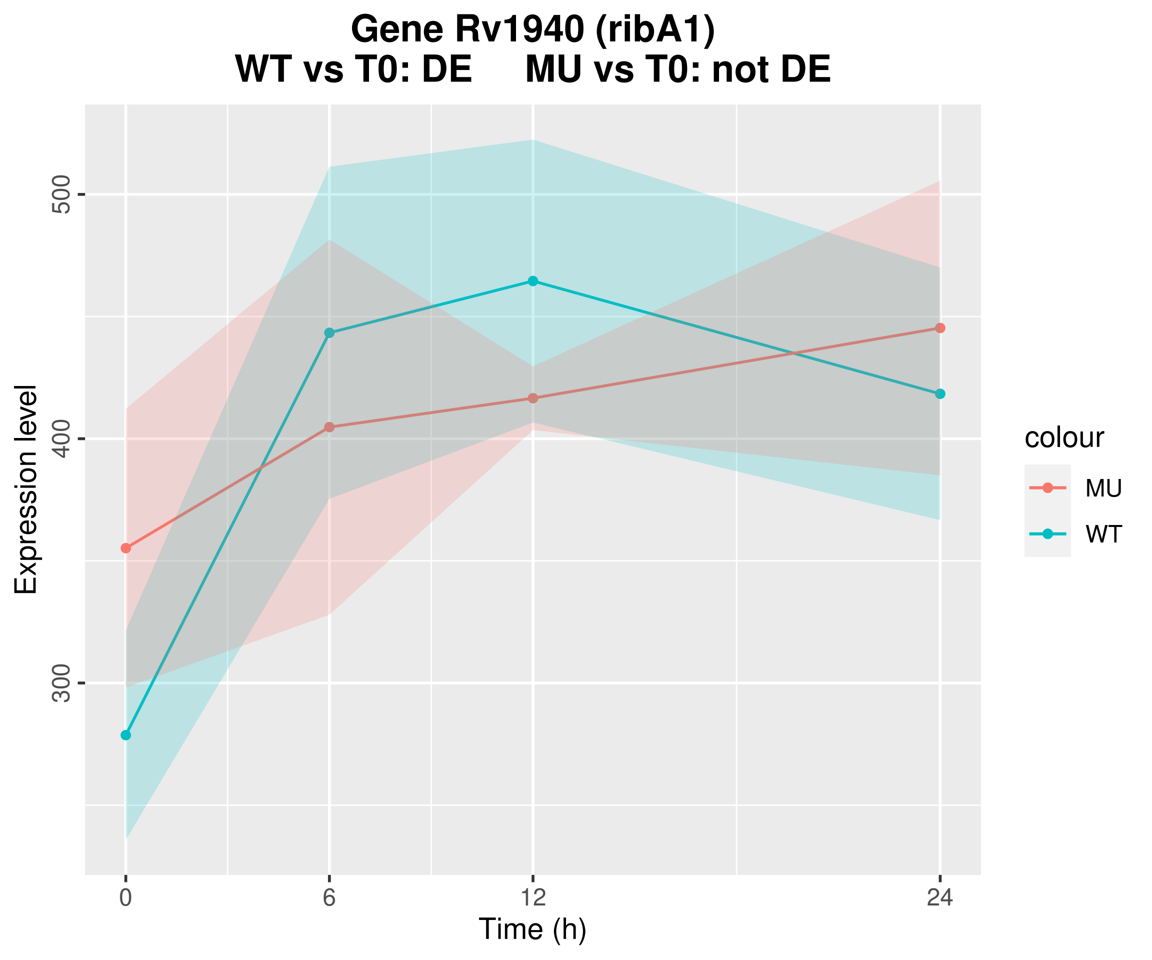

Gene Rv1940 (ribA1)
WT vs T0: DE   MU vs T0: not DE
Expression level
500
400
300
0
6
12
24
Time (h)
colour
MU
WT

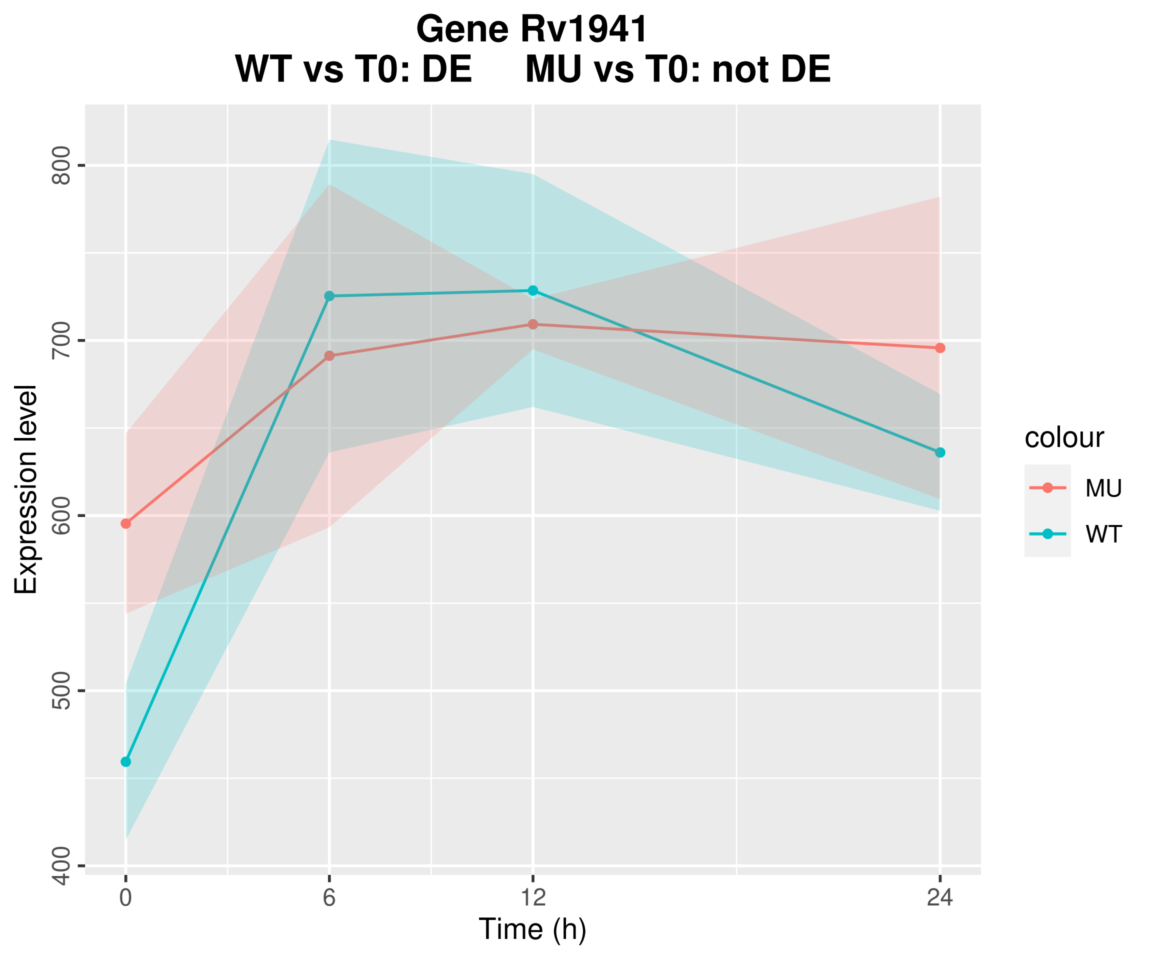
Gene Rv1941
WT vs T0: DE   MU vs T0: not DE
Expression level
800
700
600
500
400
0
6
12
24
Time (h)
colour
MU
WT

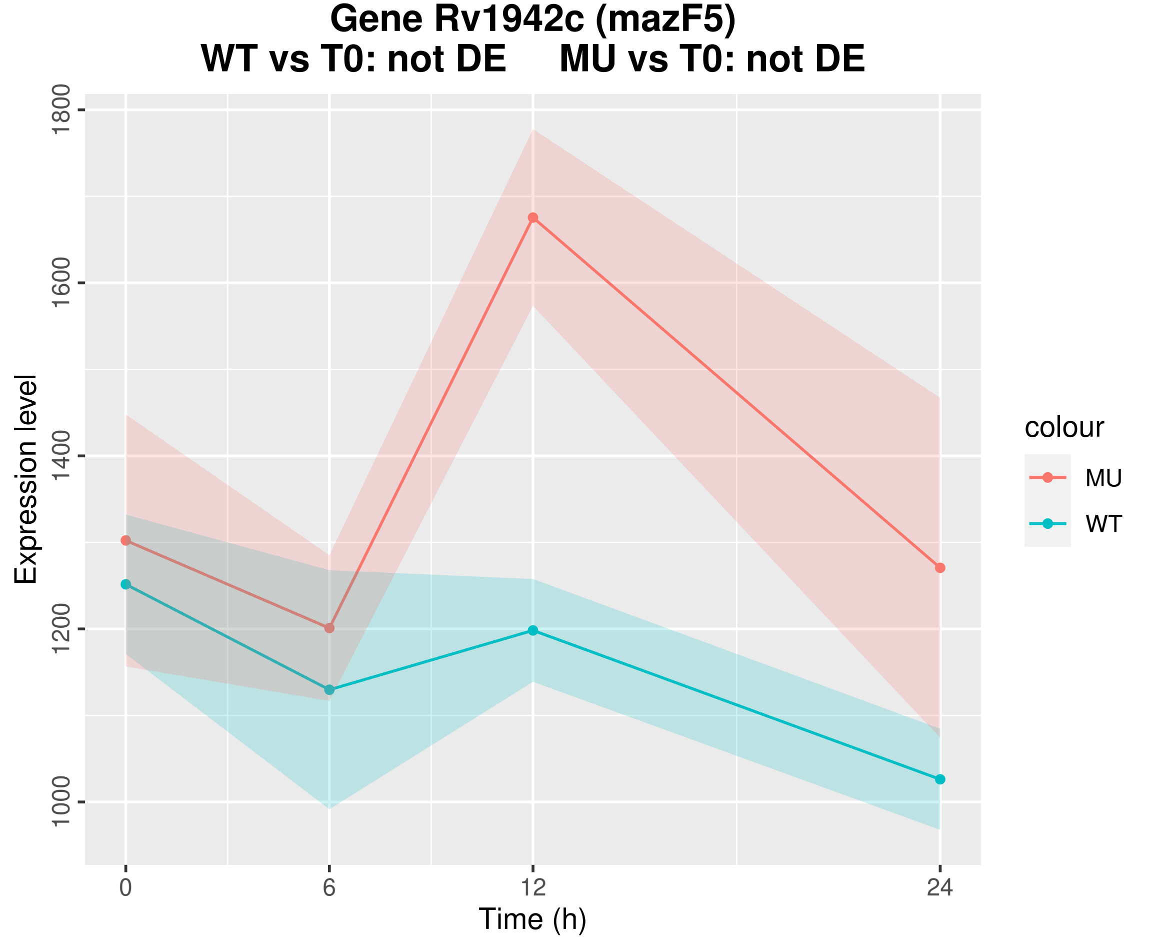

Gene Rv1942c (mazF5)
WT vs T0: not DE   MU vs T0: not DE
Expression level
1800
1600
1400
1200
1000
0
6
12
24
Time (h)
colour
MU
WT

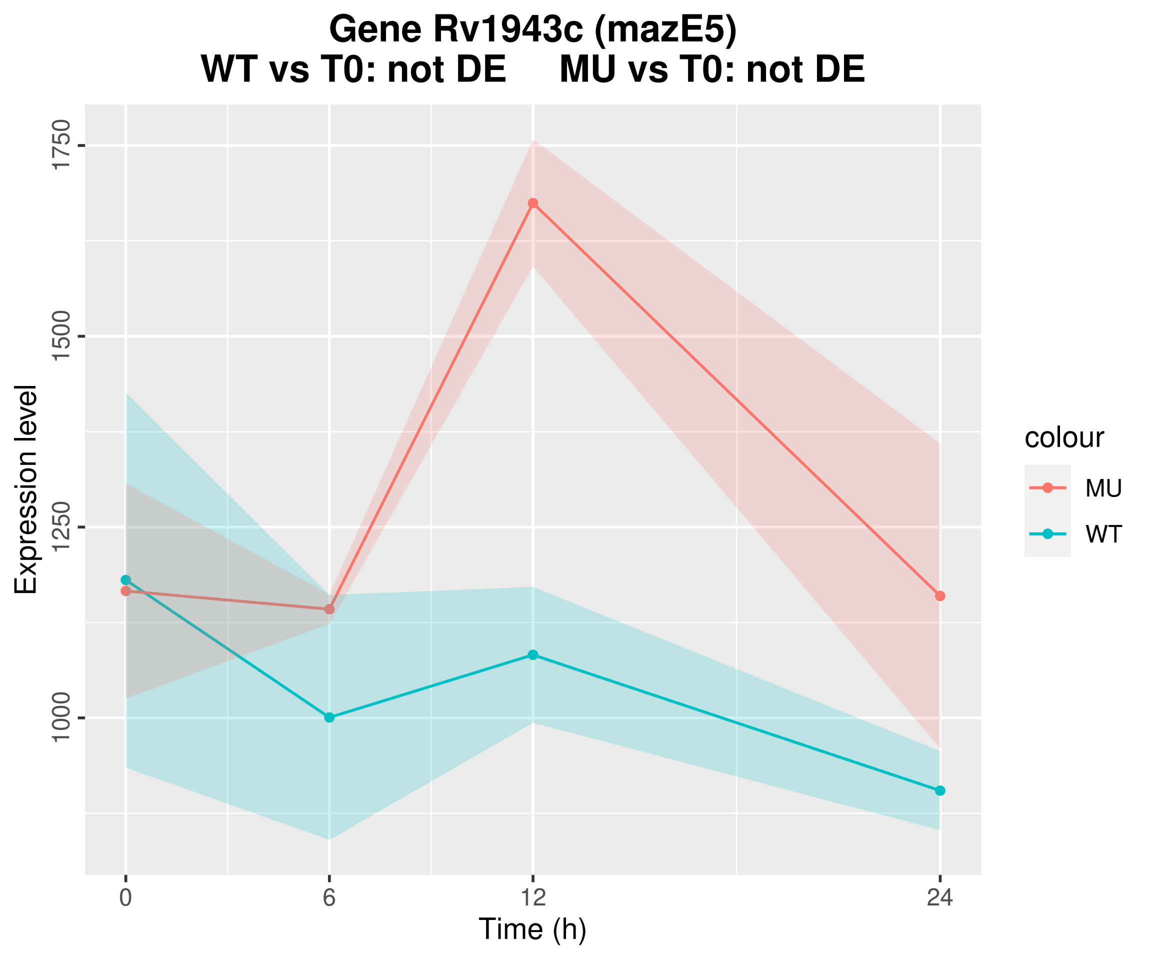

Gene Rv1943c (mazE5)
WT vs T0: not DE   MU vs T0: not DE
Expression level
1750
1500
1250
1000
0
6
12
24
Time (h)
colour
MU
WT

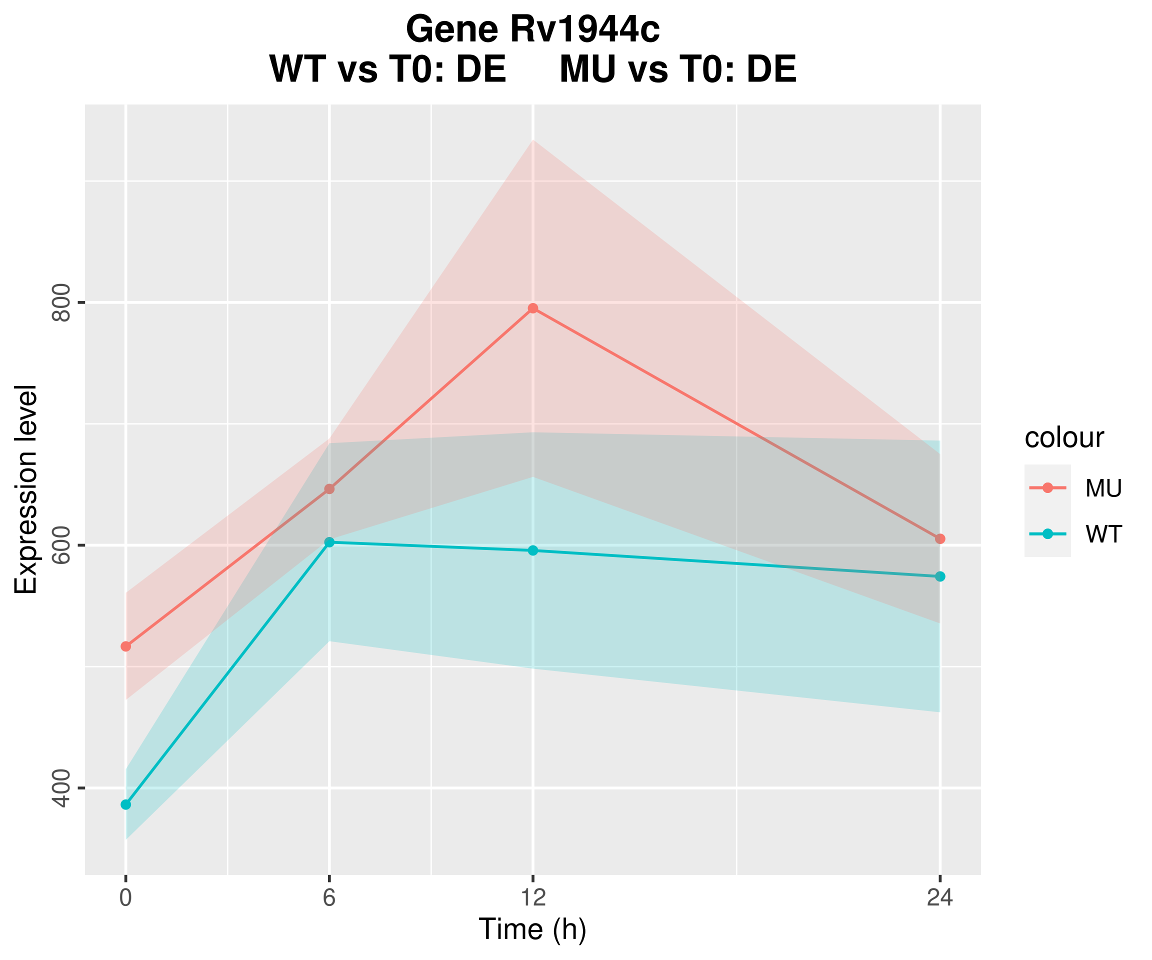

Gene Rv1944c
WT vs T0: DE MU vs T0: DE
Expression level
800
600
400
0
6
12
24
Time (h)
colour
MU
WT

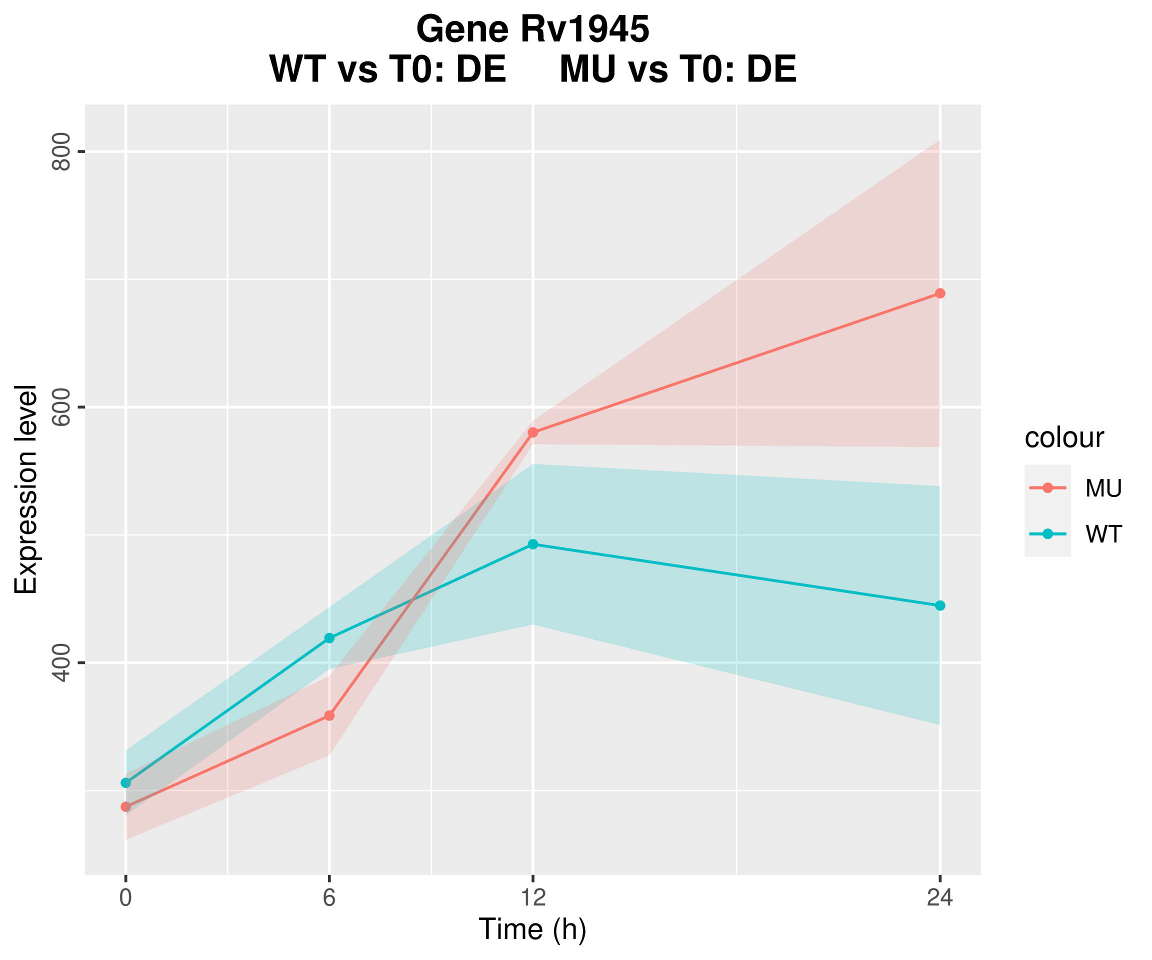

Gene Rv1945
WT vs T0: DE   MU vs T0: DE
800
600
400
Expression level
0
6
12
24
Time (h)
colour
MU
WT

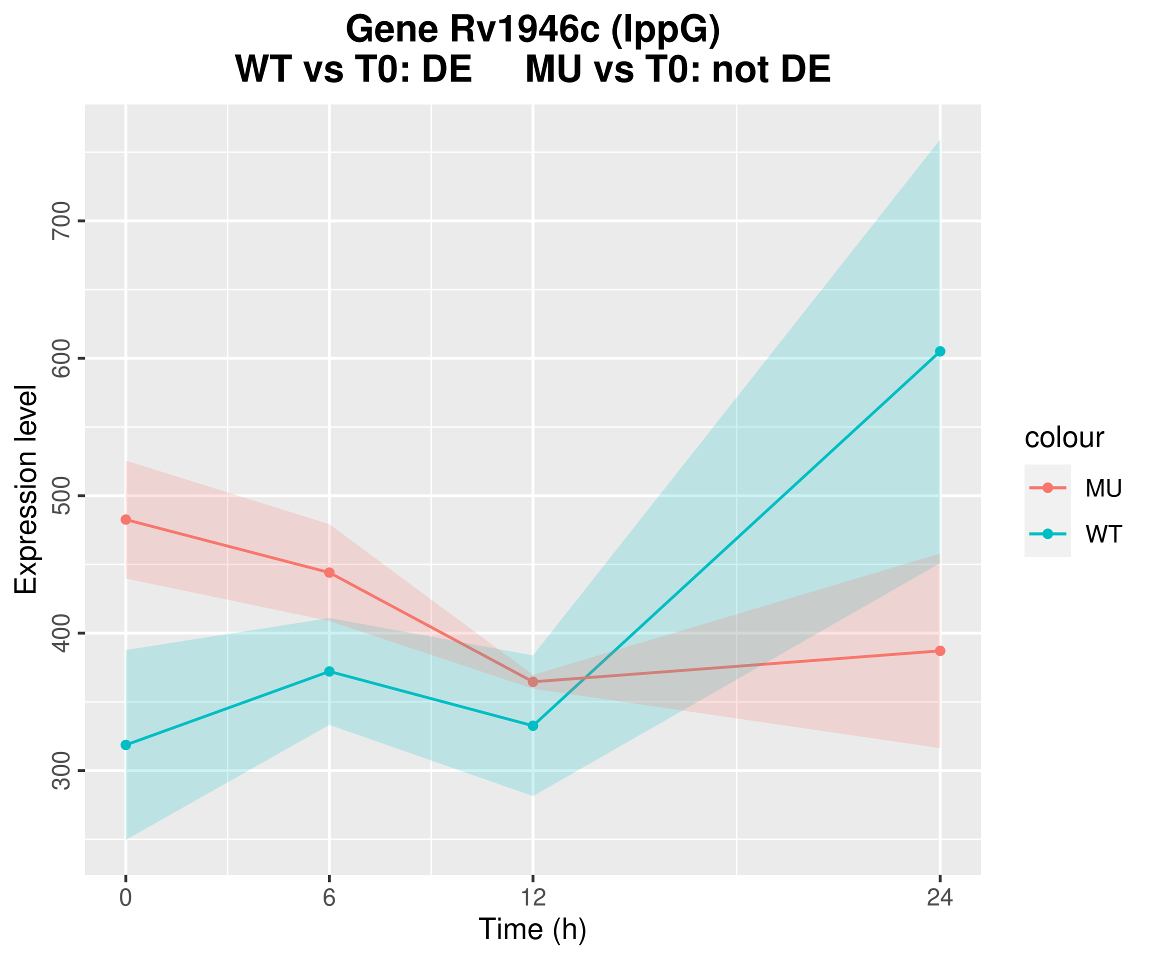

Gene Rv1946c (lppG)
WT vs T0: DE MU vs T0: not DE
Expression level
Time (h)
300
400
500
600
700
0
6
12
24
colour
MU
WT

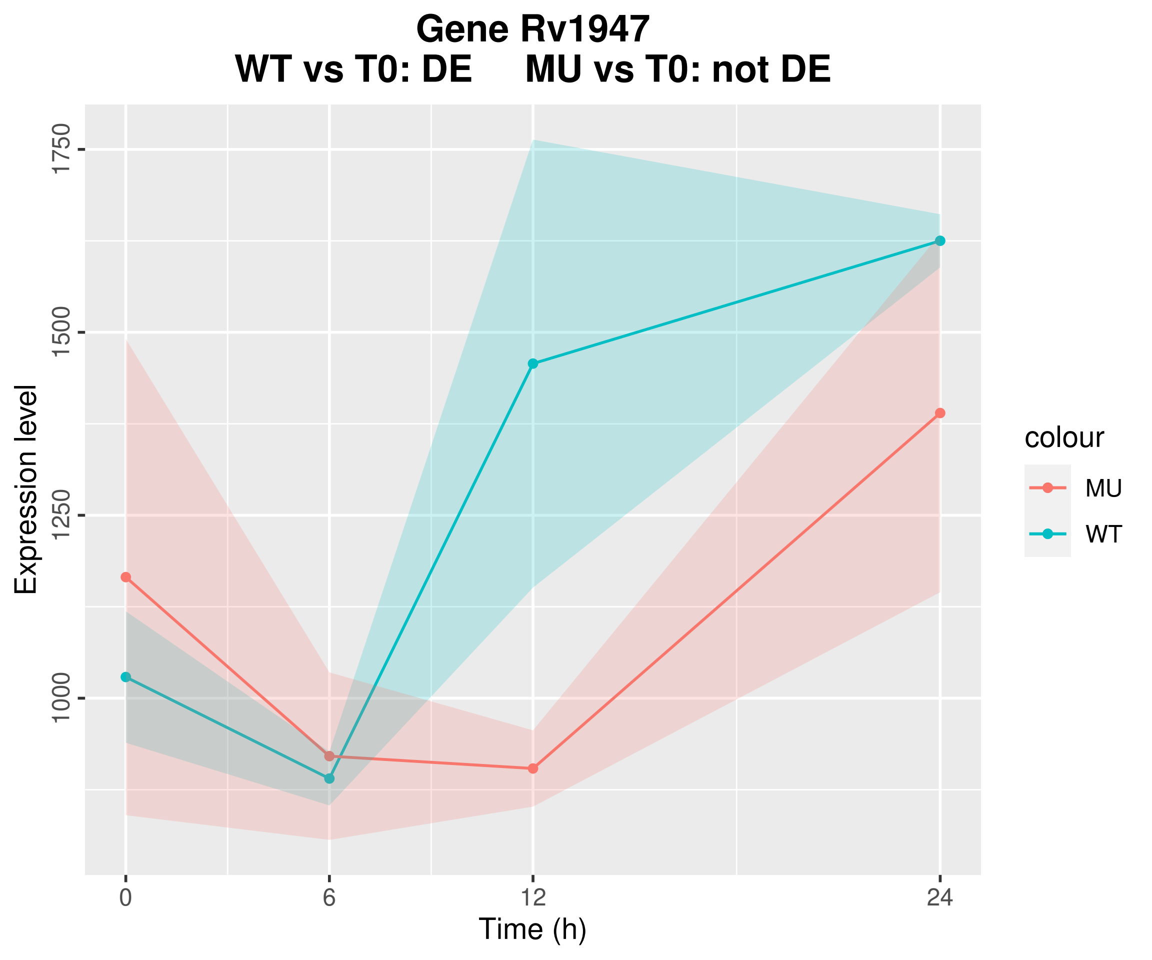

Gene Rv1947
WT vs T0: DE   MU vs T0: not DE
Expression level
1750
1500
1250
1000
0
6
12
24
Time (h)
colour
MU
WT

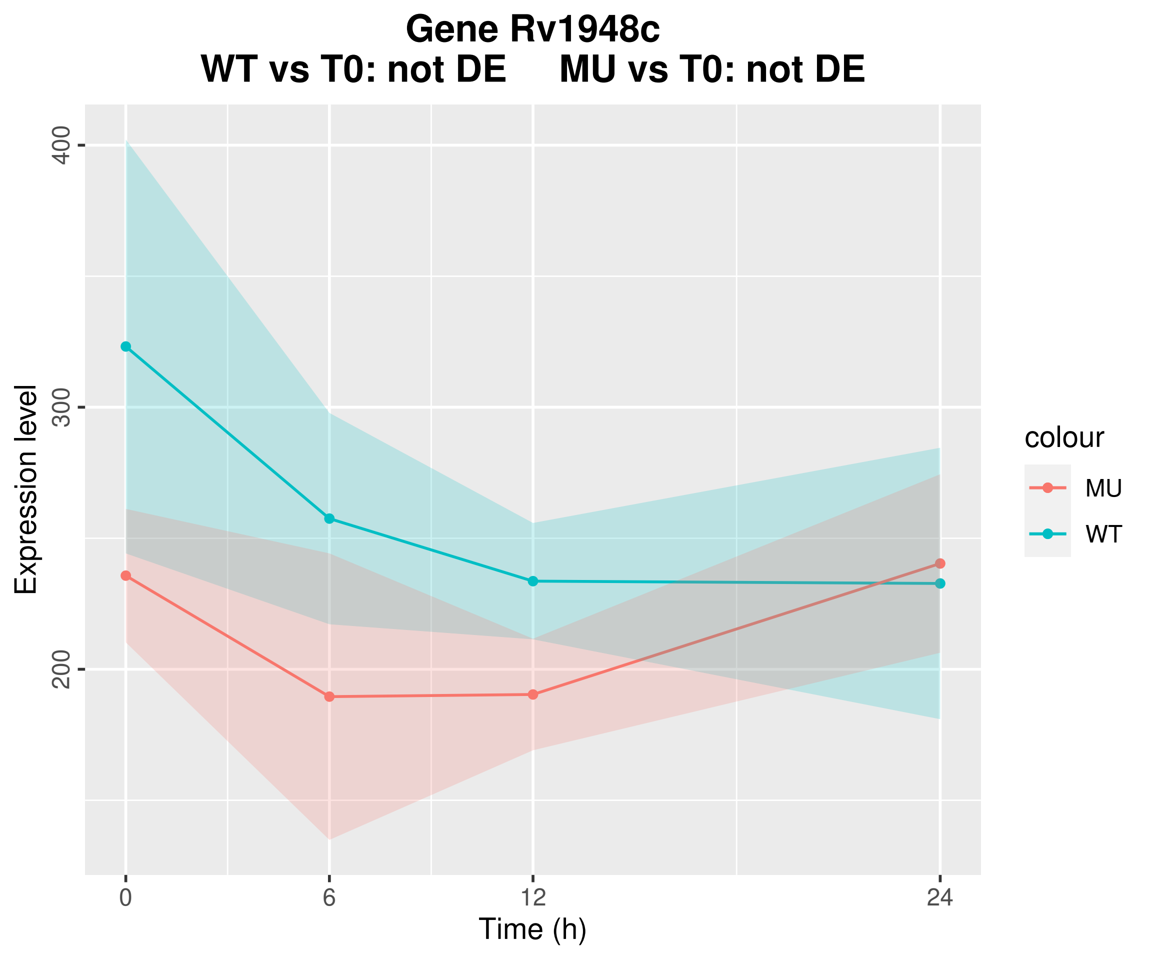
Gene Rv1948c
WT vs T0: not DE   MU vs T0: not DE
400
300
200
Expression level
0
6
12
24
Time (h)
colour
MU
WT

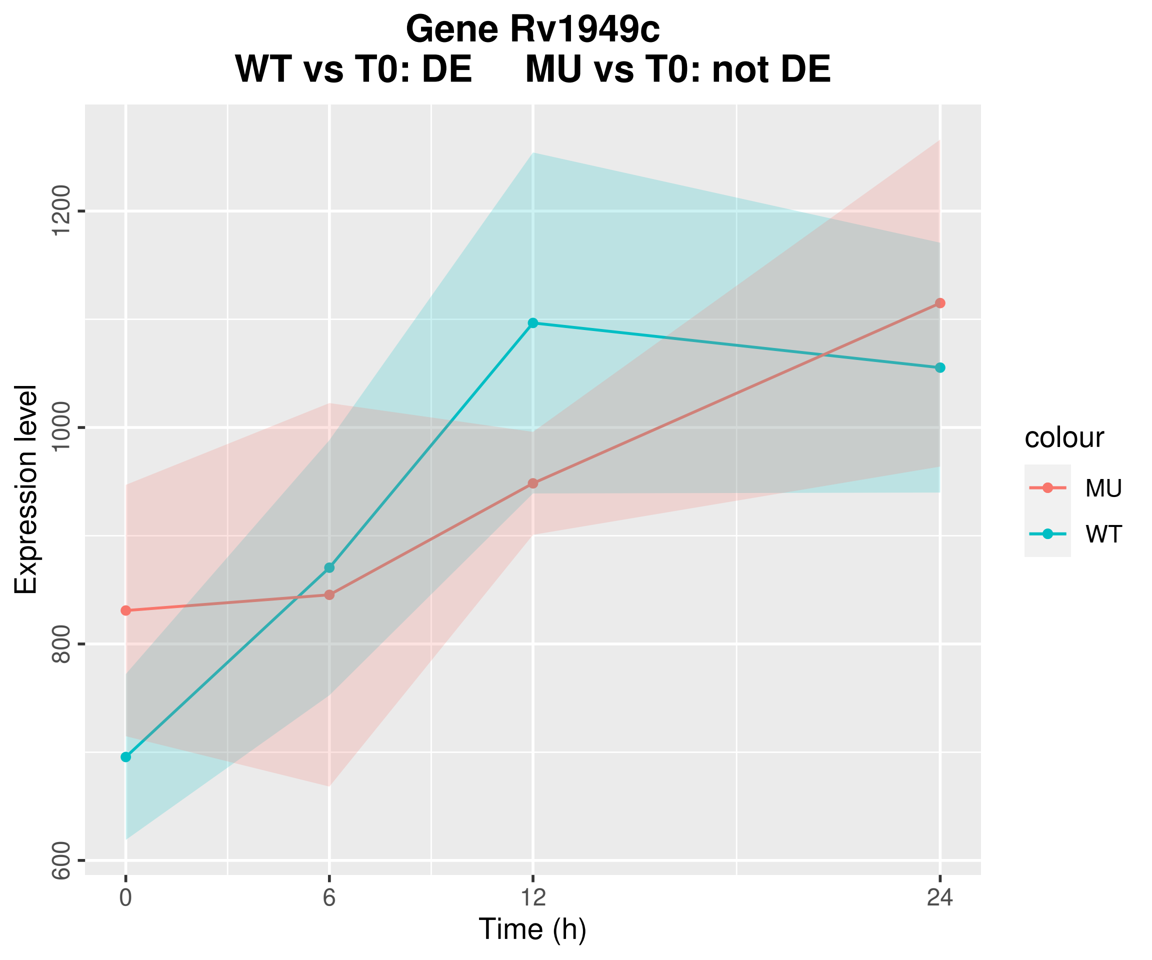
Gene Rv1949c
WT vs T0: DE MU vs T0: not DE
Expression level
600
800
1000
1200
0
6
12
24
Time (h)
colour
MU
WT

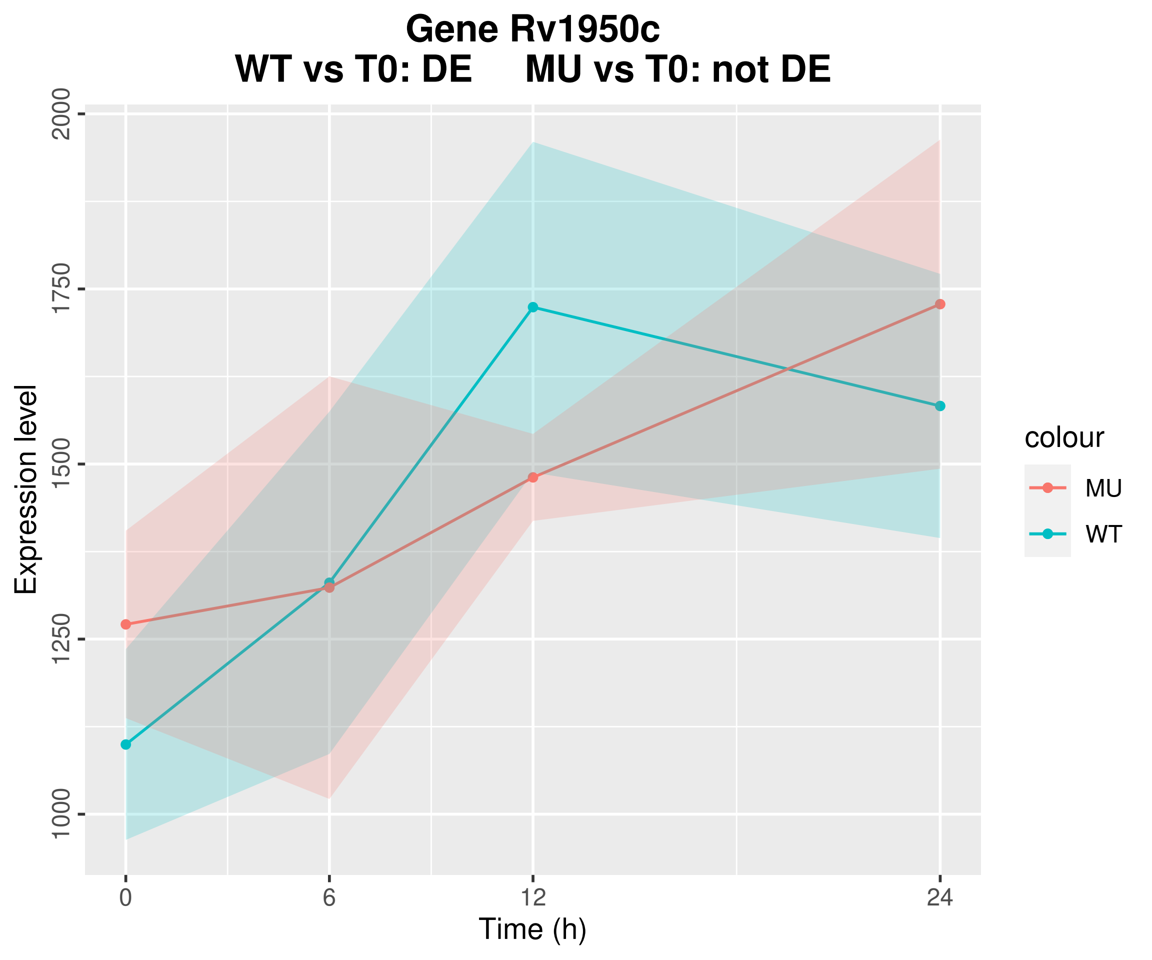

Gene Rv1950c
WT vs T0: DE MU vs T0: not DE
Expression level
2000
1750
1500
1250
1000
0
6
12
24
Time (h)
colour
MU
WT

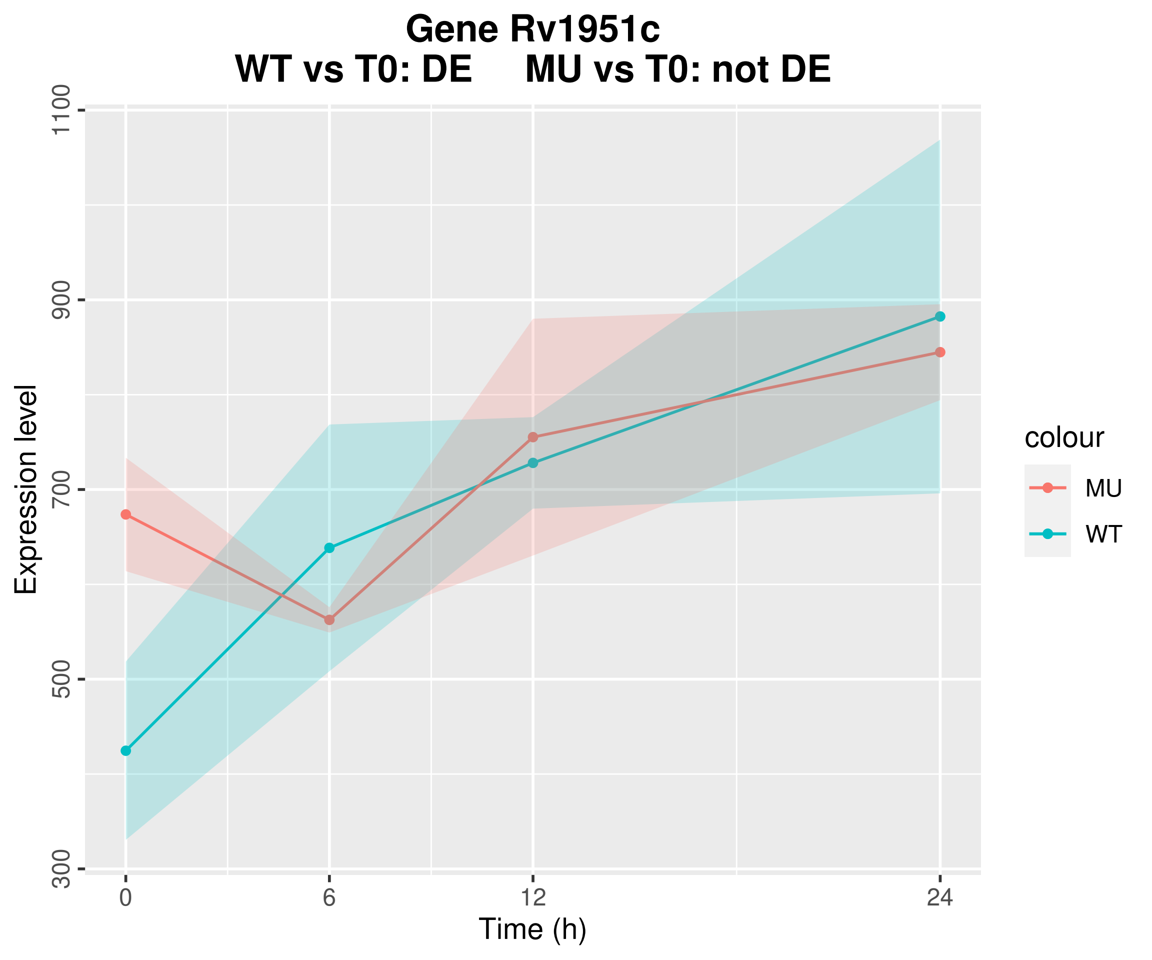

Gene Rv1951c
WT vs T0: DE MU vs T0: not DE
1100
900
700
500
300
Expression level
0
6
12
24
Time (h)
colour
MU
WT

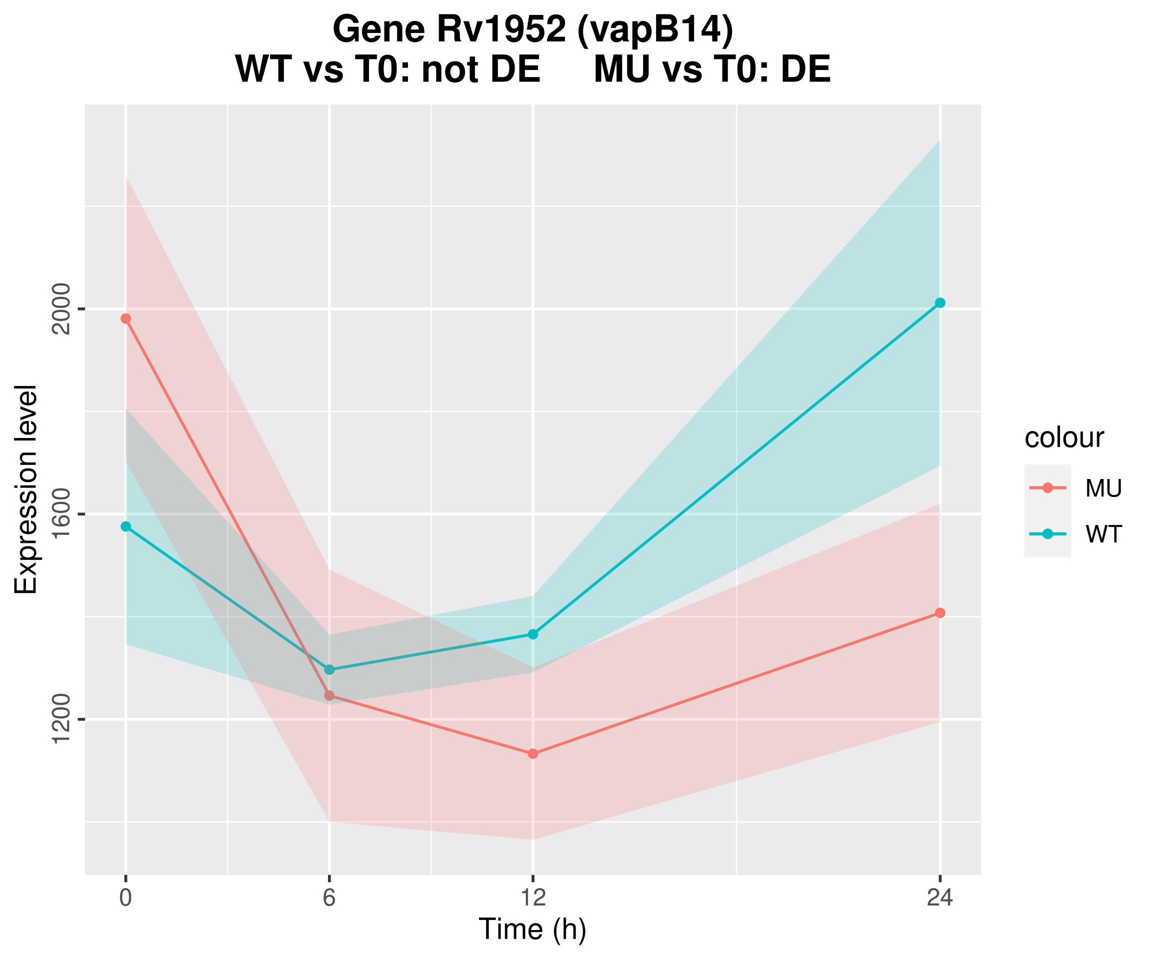

Gene Rv1952 (vapB14)
WT vs T0: not DE MU vs T0: DE
Expression level
2000
1600
1200
0
6
12
24
Time (h)
colour
MU
WT

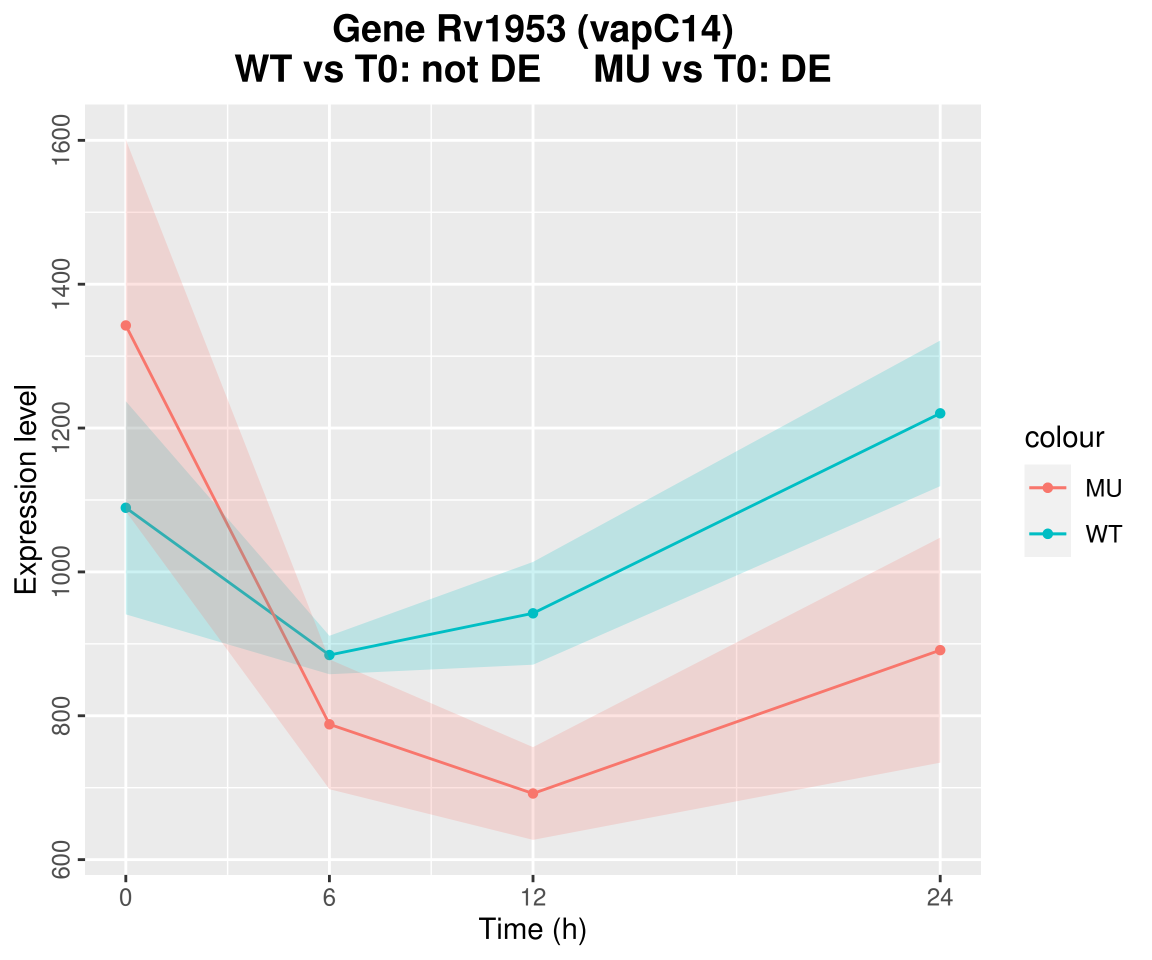
Gene Rv1953 (vapC14)
WT vs T0: not DE MU vs T0: DE
Expression level
1600
1400
1200
1000
800
600
0
6
12
24
Time (h)
colour
MU
WT

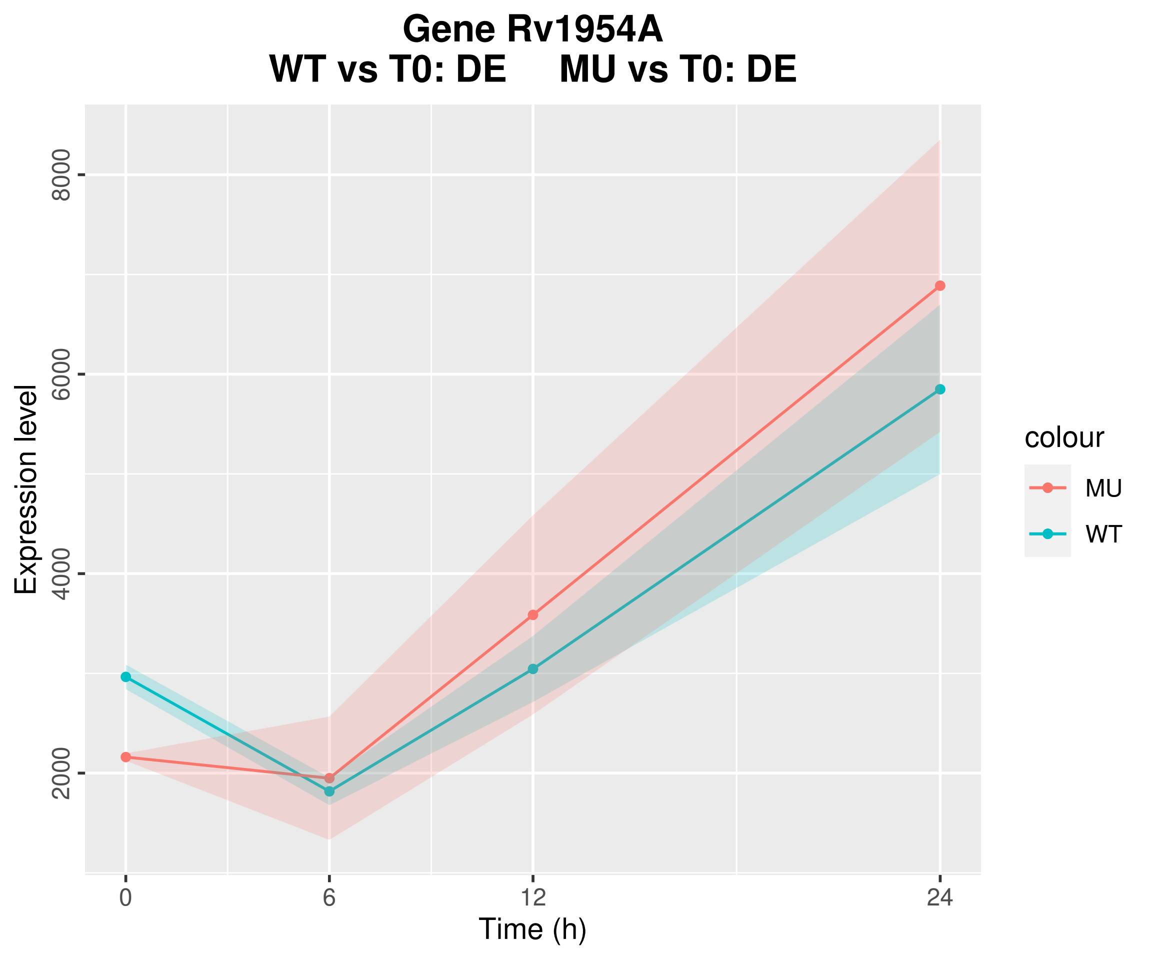

Gene Rv1954A
WT vs T0: DE MU vs T0: DE
Expression level
8000
6000
4000
2000
0
6
12
24
Time (h)
colour
MU
WT

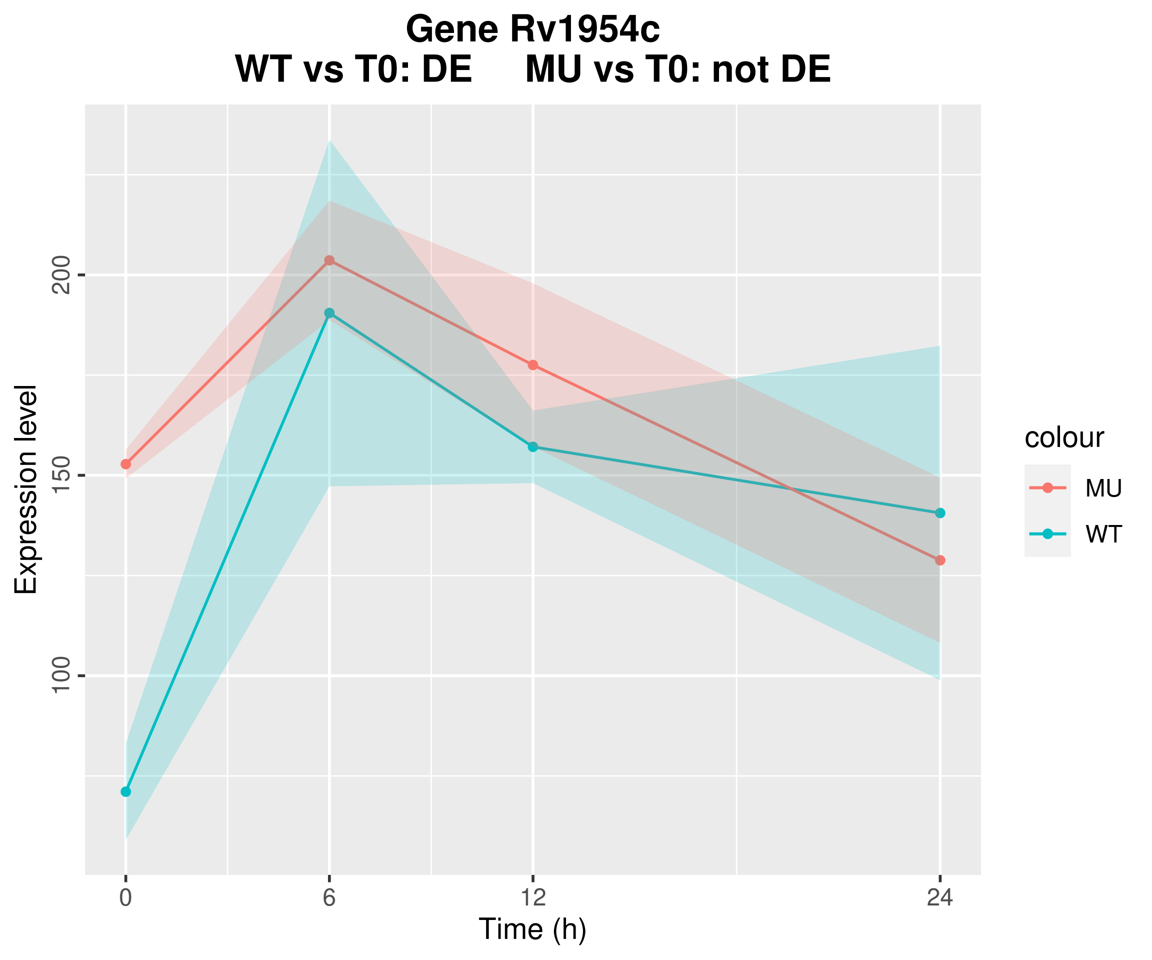

Gene Rv1954c
WT vs T0: DE   MU vs T0: not DE
Expression level
100
150
200
Time (h)
0
6
12
24
colour
MU
WT

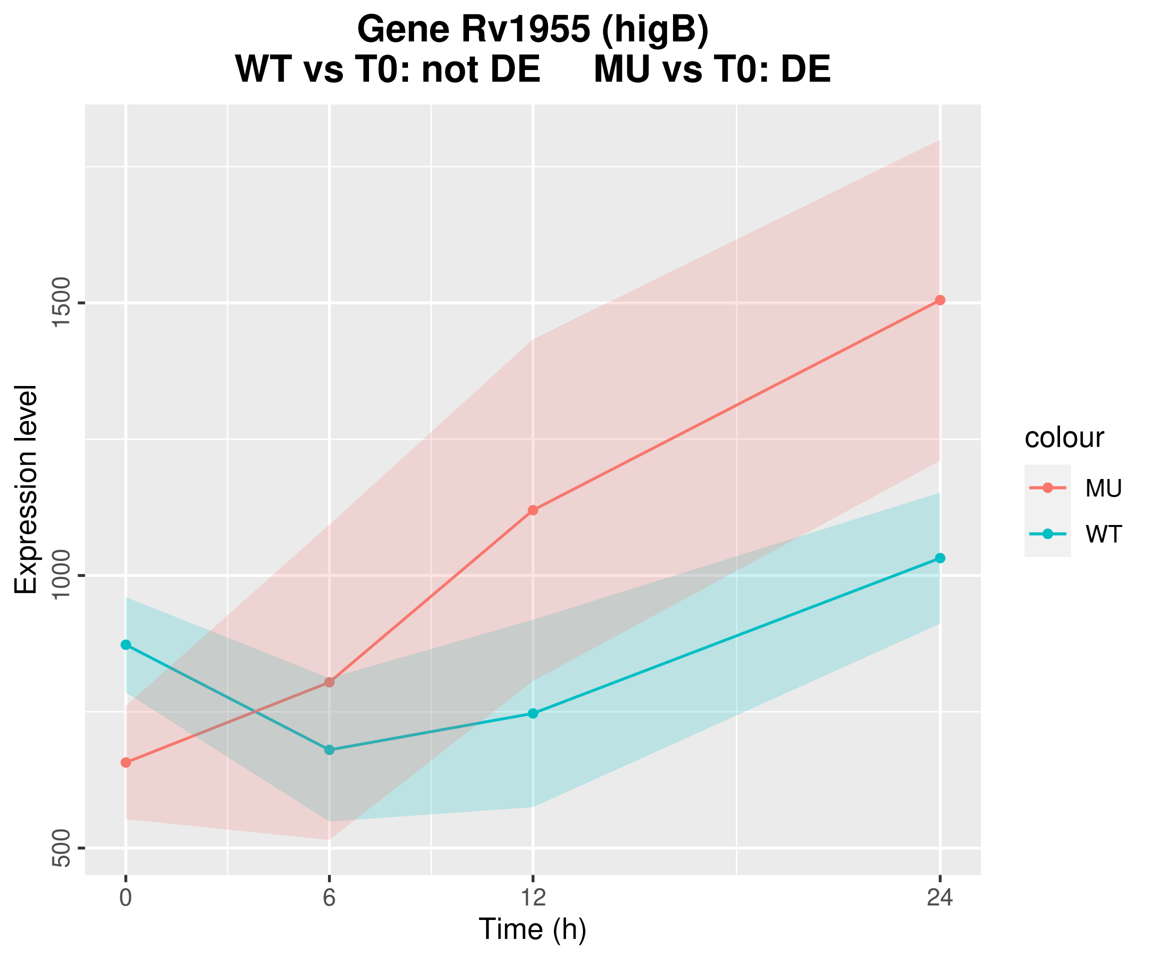

Gene Rv1955 (higB)
WT vs T0: not DE MU vs T0: DE
Expression level
1500
1000
500
0
6
12
24
Time (h)
colour
MU
WT

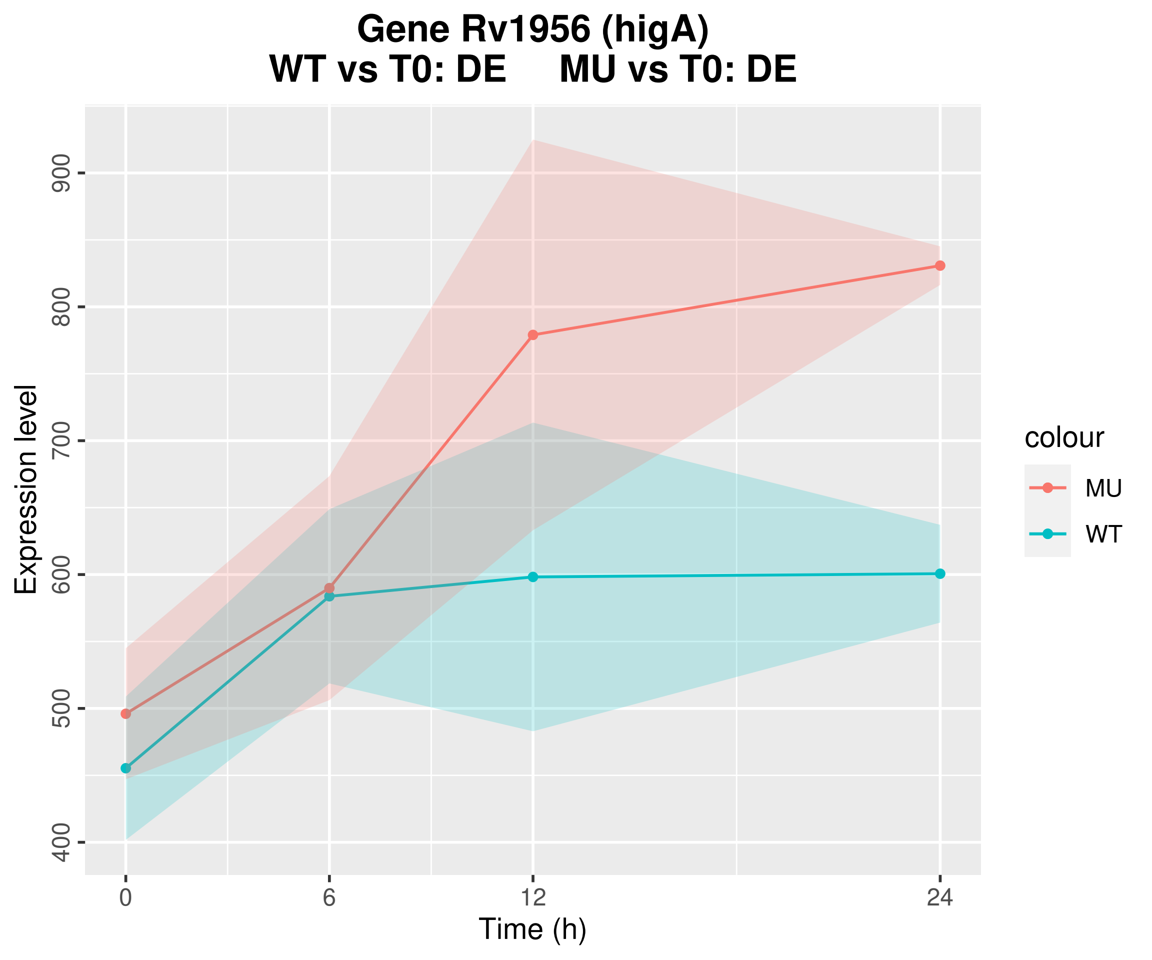
Gene Rv1956 (higA)
WT vs T0: DE MU vs T0: DE
Expression level
400
500
600
700
800
900
0
6
12
24
Time (h)
colour
MU
WT

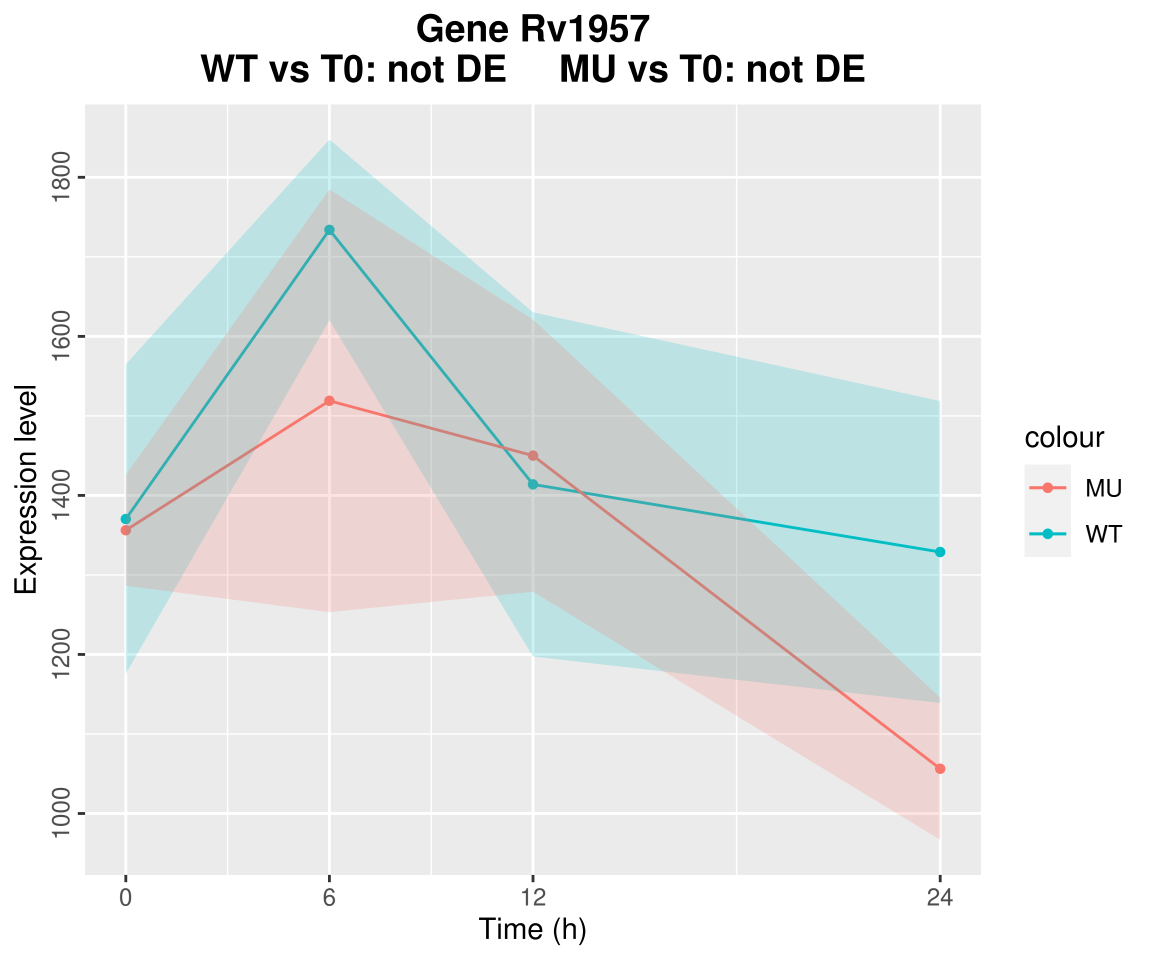
Gene Rv1957
WT vs T0: not DE   MU vs T0: not DE
Expression level
Time (h)
1800
1600
1400
1200
1000
0
6
12
24
colour
MU
WT

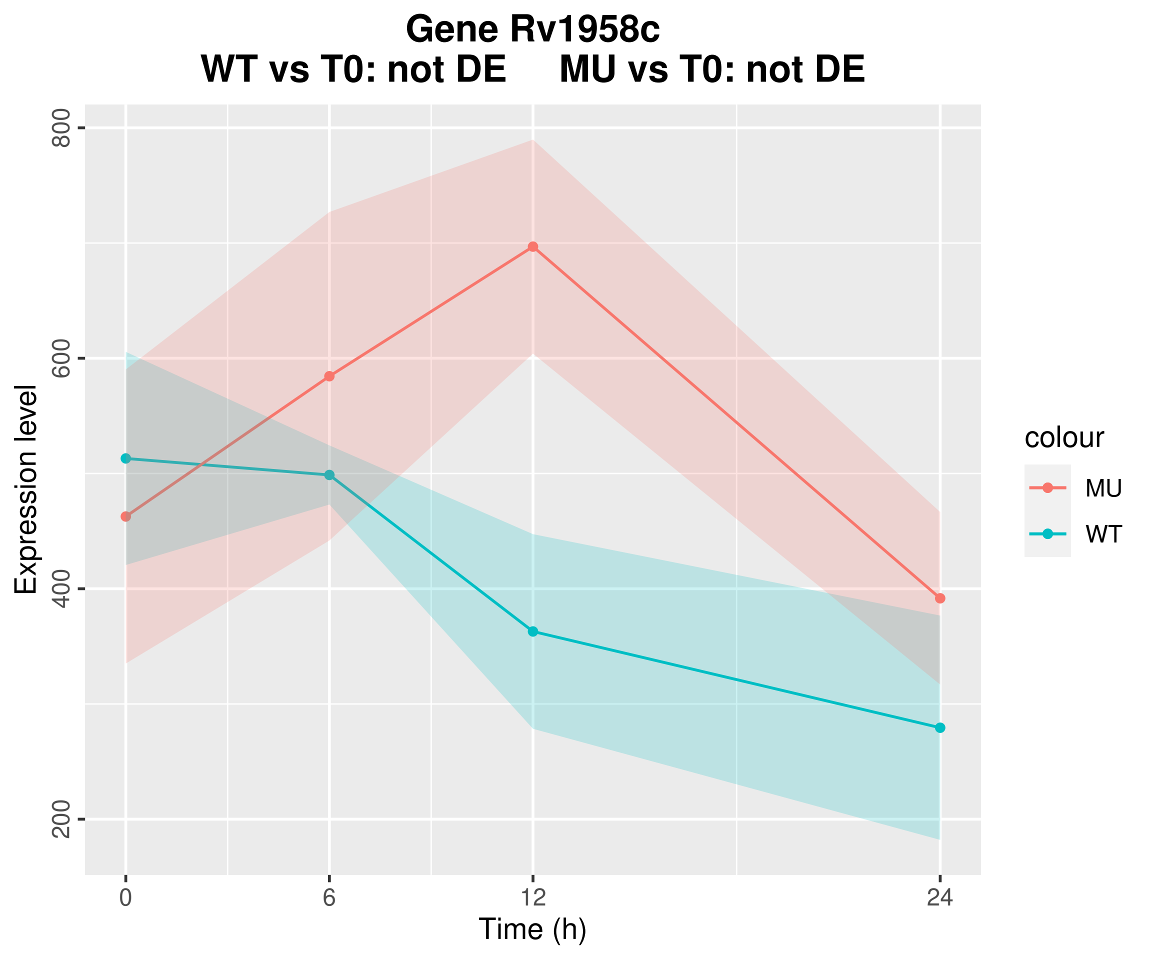
Gene Rv1958c
WT vs T0: not DE MU vs T0: not DE
800
600
400
200
Expression level
0
6
12
24
Time (h)
colour
MU
WT

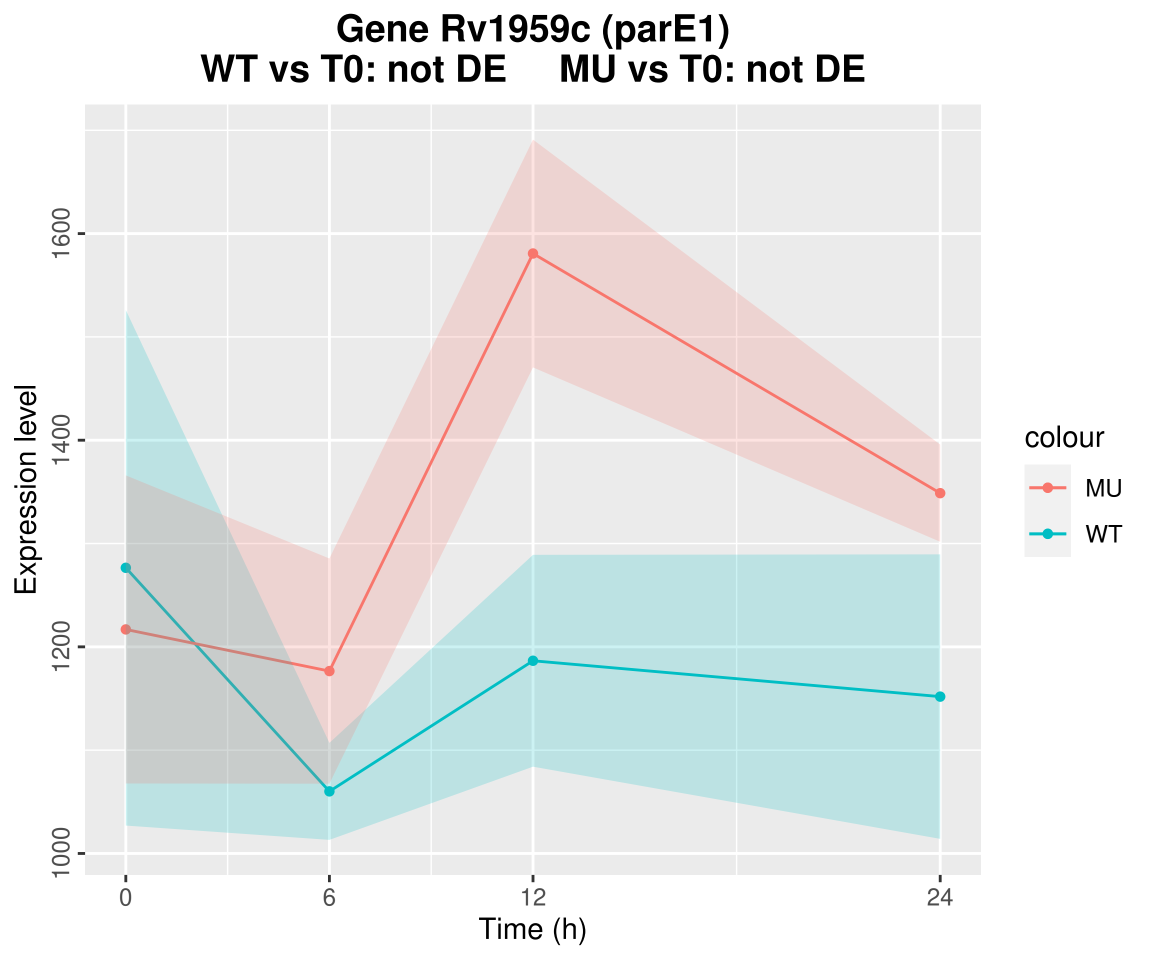
Gene Rv1959c (parE1)
WT vs T0: not DE   MU vs T0: not DE
Expression level
1000
1200
1400
1600
0
6
12
24
Time (h)
colour
MU
WT

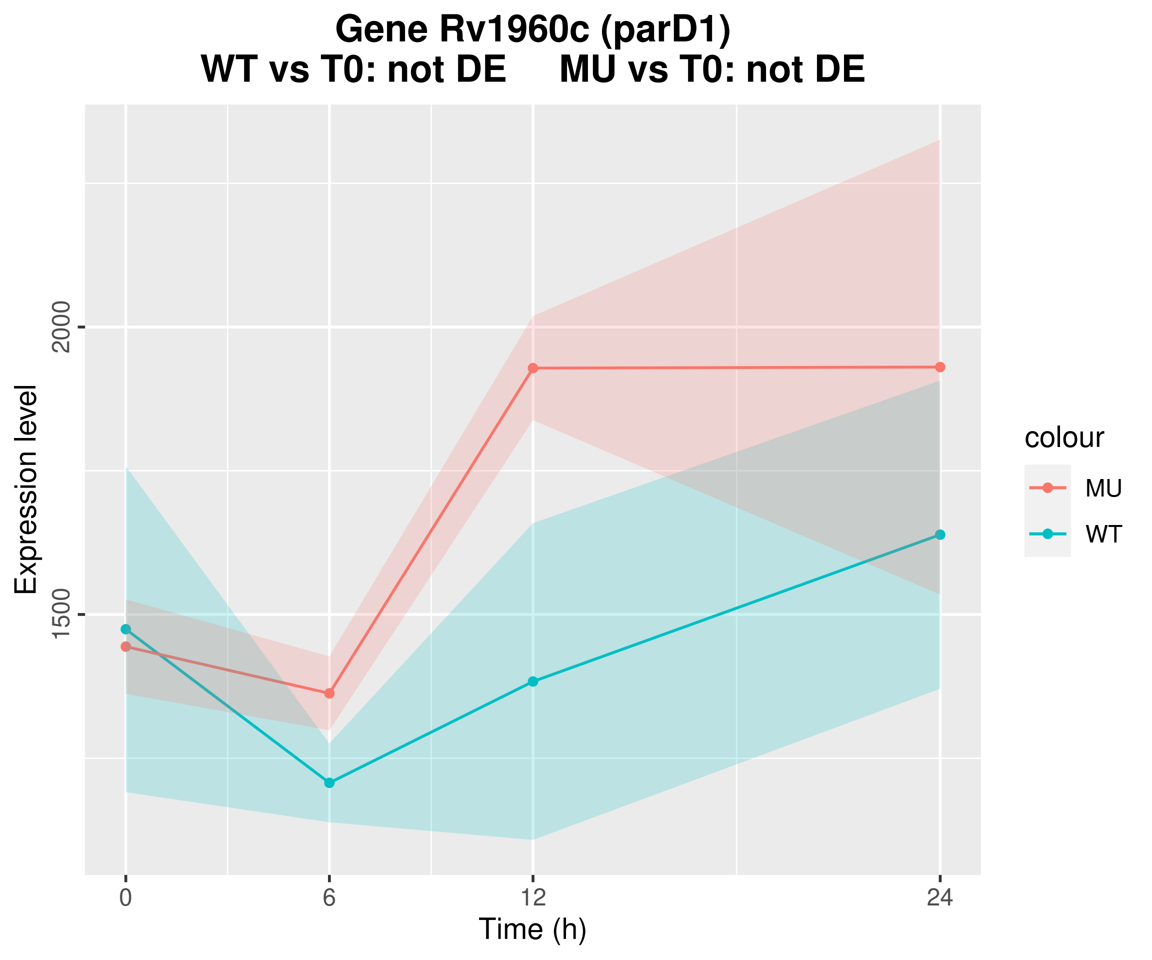
Gene Rv1960c (parD1)
WT vs T0: not DE   MU vs T0: not DE
Expression level
2000
1500
0
6
12
24
Time (h)
colour
MU
WT

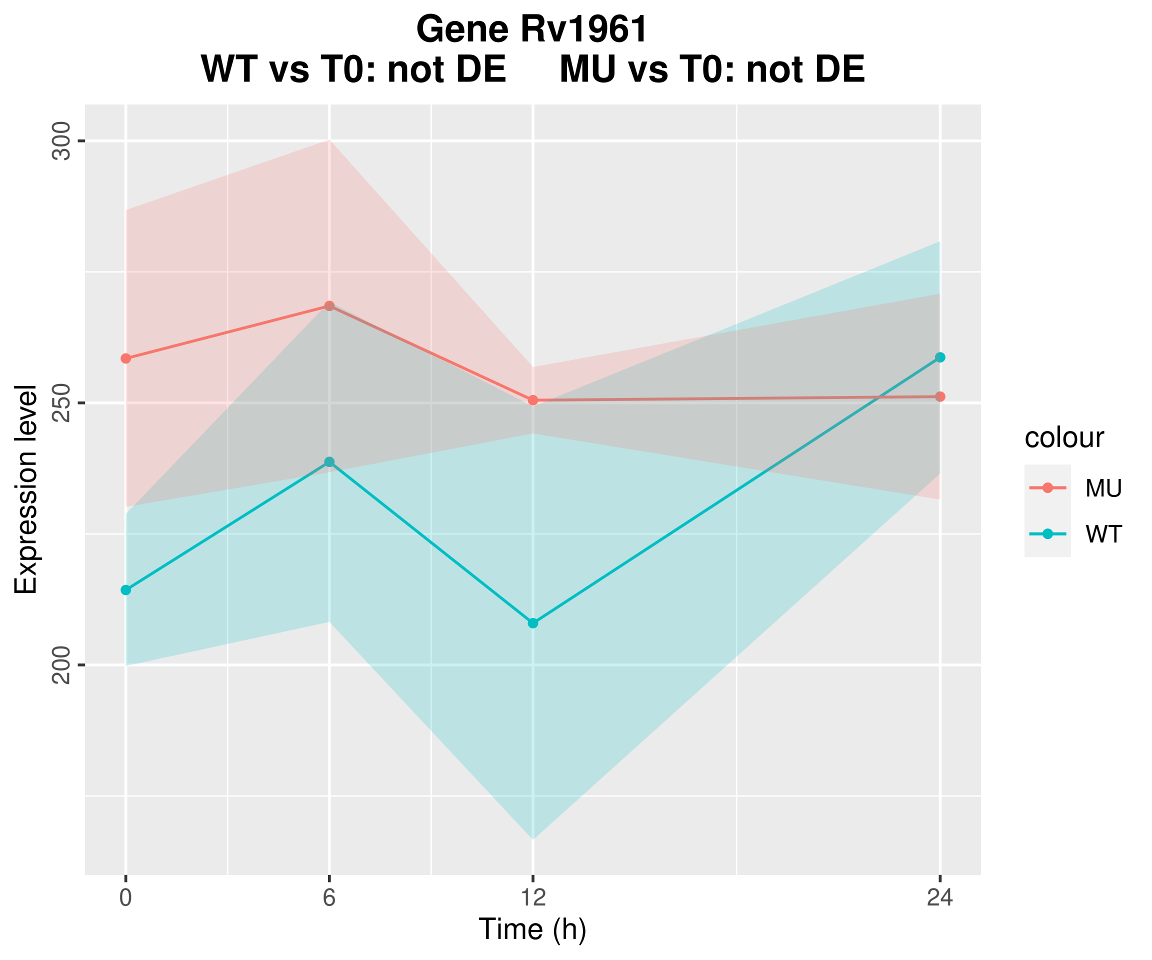

Gene Rv1961
WT vs T0: not DE   MU vs T0: not DE
300
250
200
Expression level
0
6
12
24
Time (h)
colour
MU
WT

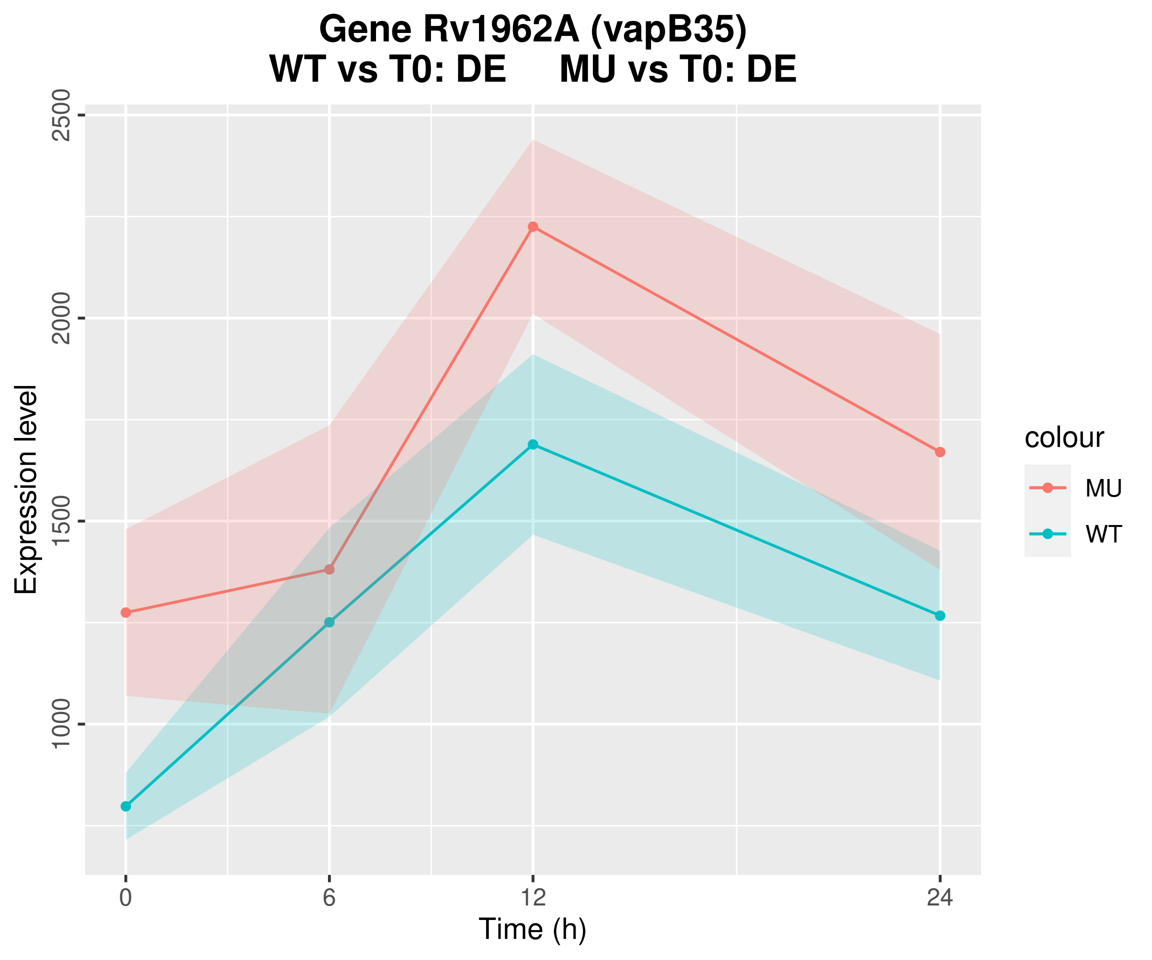

Gene Rv1962A (vapB35)
WT vs T0: DE   MU vs T0: DE
Expression level
2500
2000
1500
1000
0
6
12
24
Time (h)
colour
MU
WT

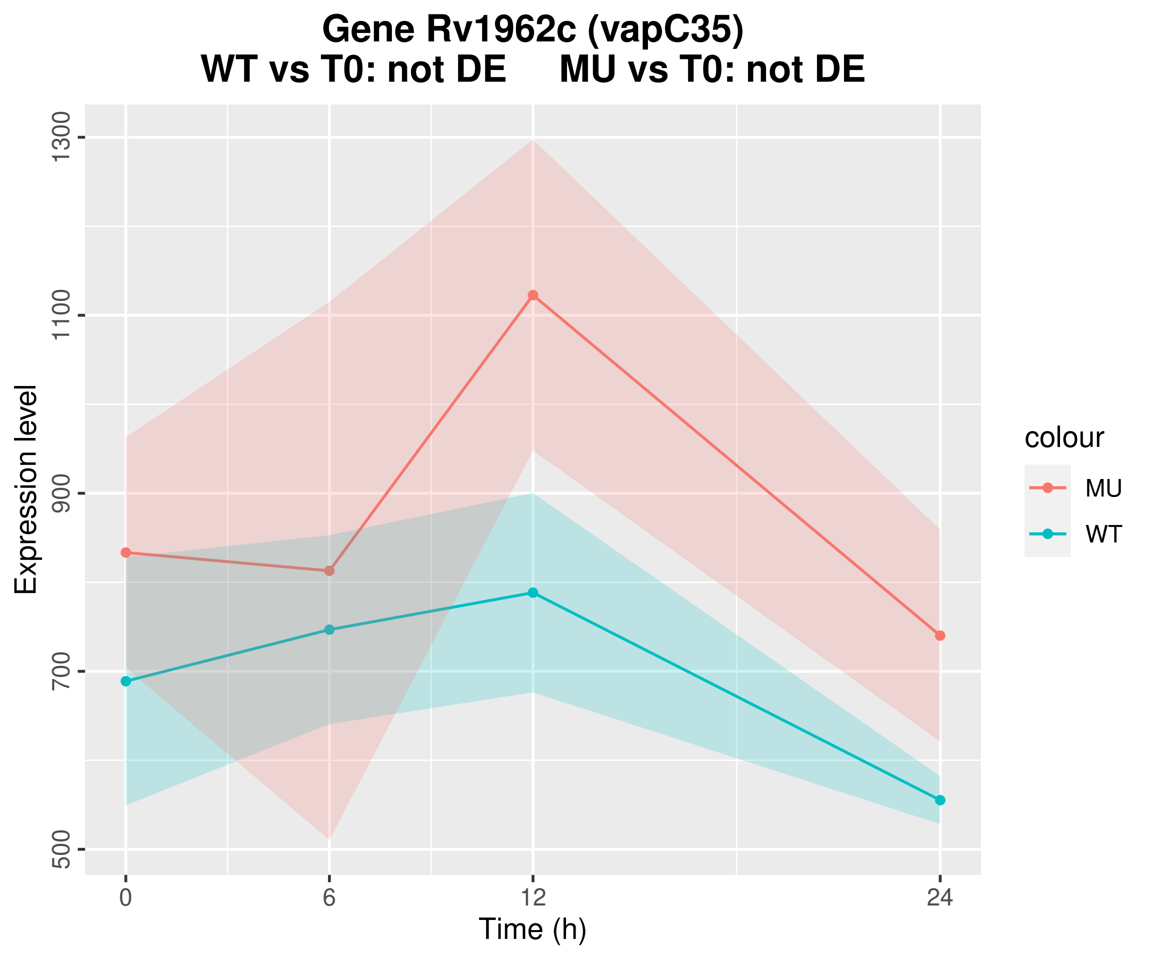
Gene Rv1962c (vapC35)
WT vs T0: not DE   MU vs T0: not DE
Expression level
1300
1100
900
700
500
0
6
12
24
Time (h)
colour
MU
WT

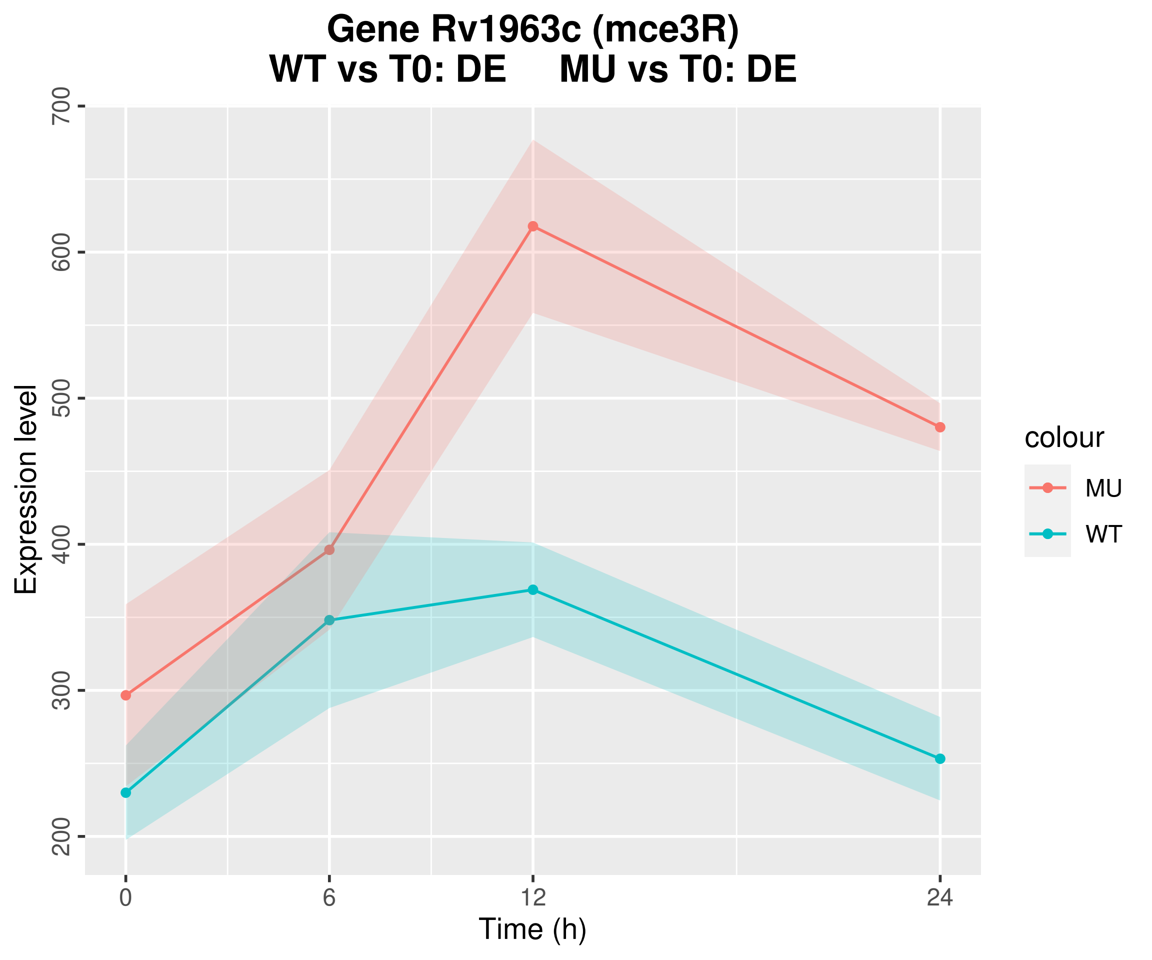

Gene Rv1963c (mce3R)
WT vs T0: DE   MU vs T0: DE
Expression level
700
600
500
400
300
200
0
6
12
24
Time (h)
colour
MU
WT

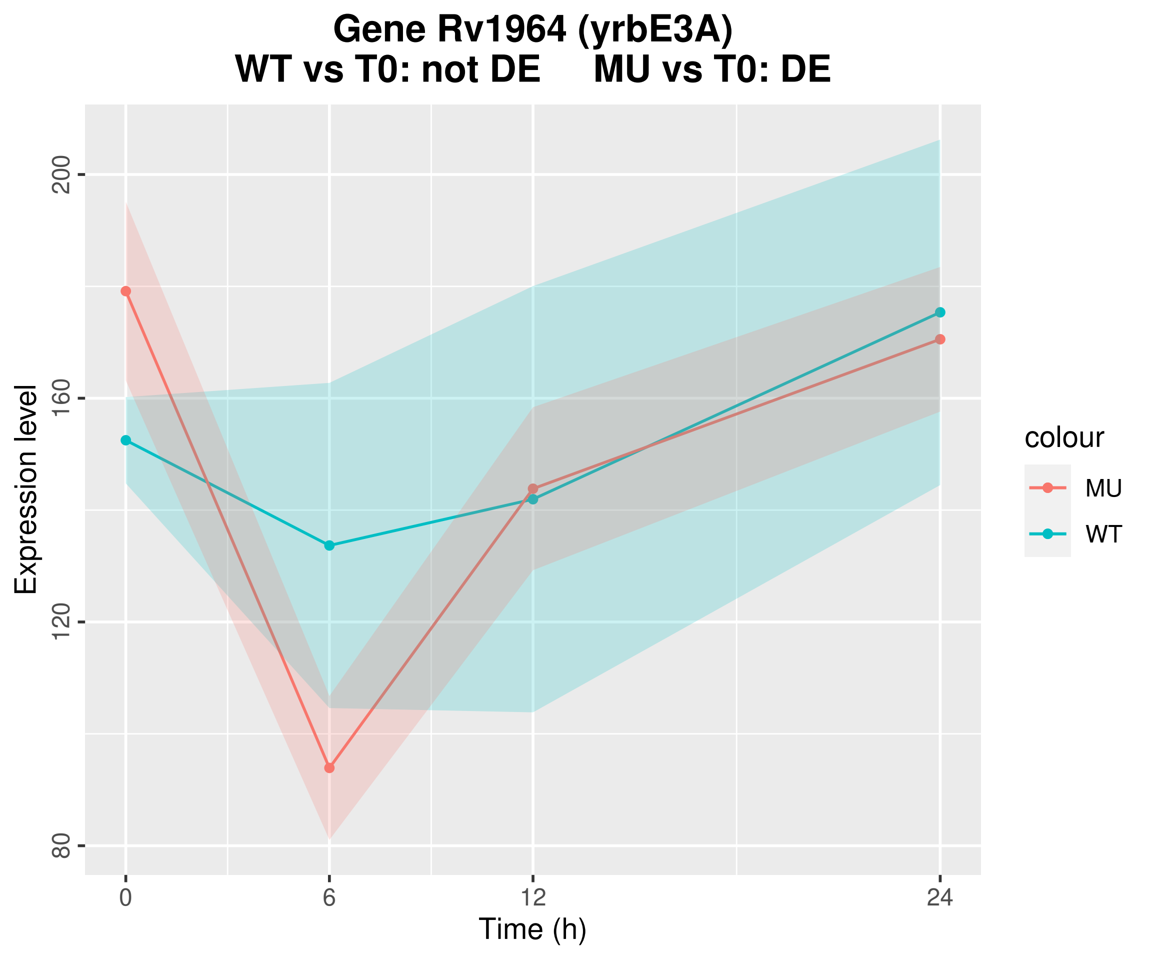
Gene Rv1964 (yrbE3A)
WT vs T0: not DE   MU vs T0: DE
Expression level
200
160
120
80
0
6
12
24
Time (h)
colour
MU
WT

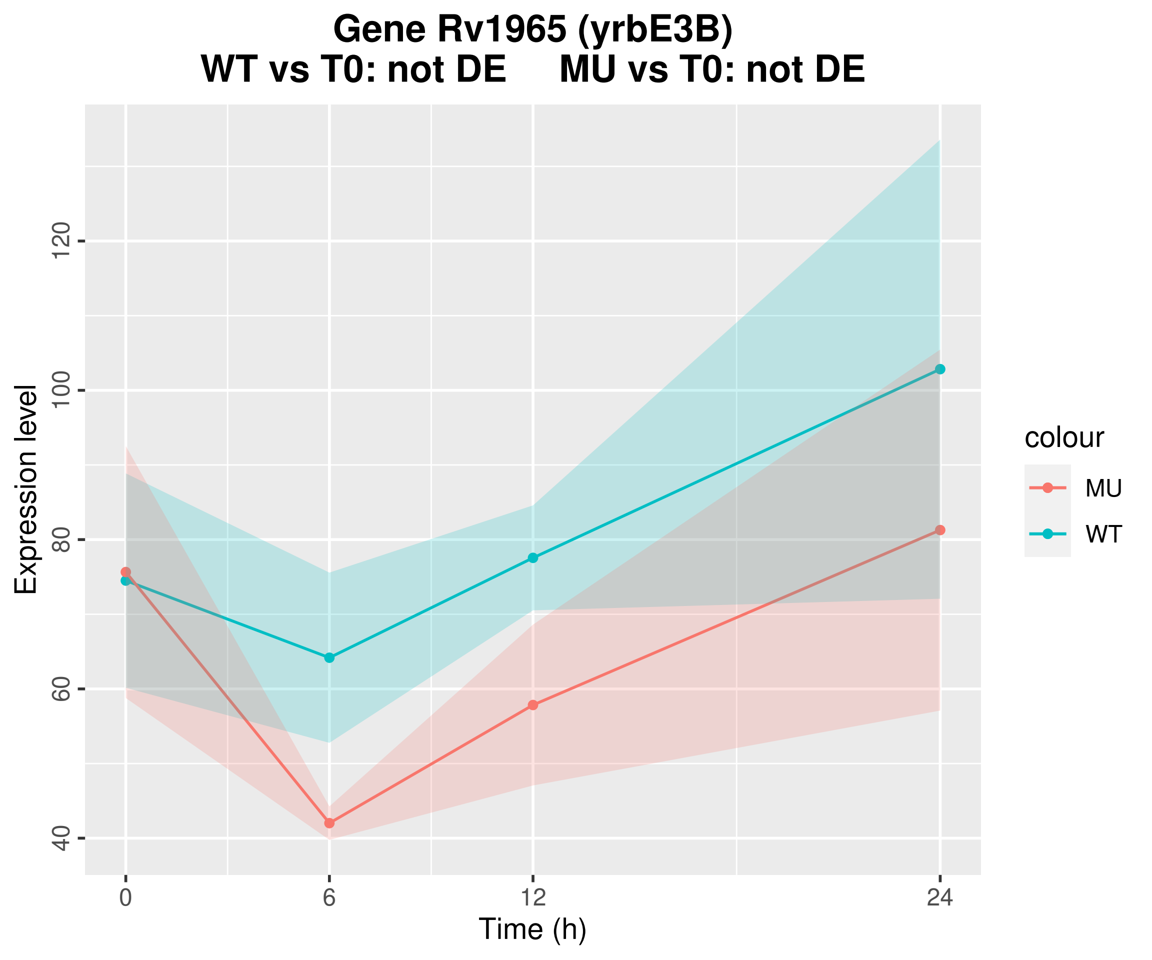

Gene Rv1965 (yrbE3B)
WT vs T0: not DE   MU vs T0: not DE
Expression level
120
100
80
60
40
0
6
12
24
Time (h)
colour
MU
WT

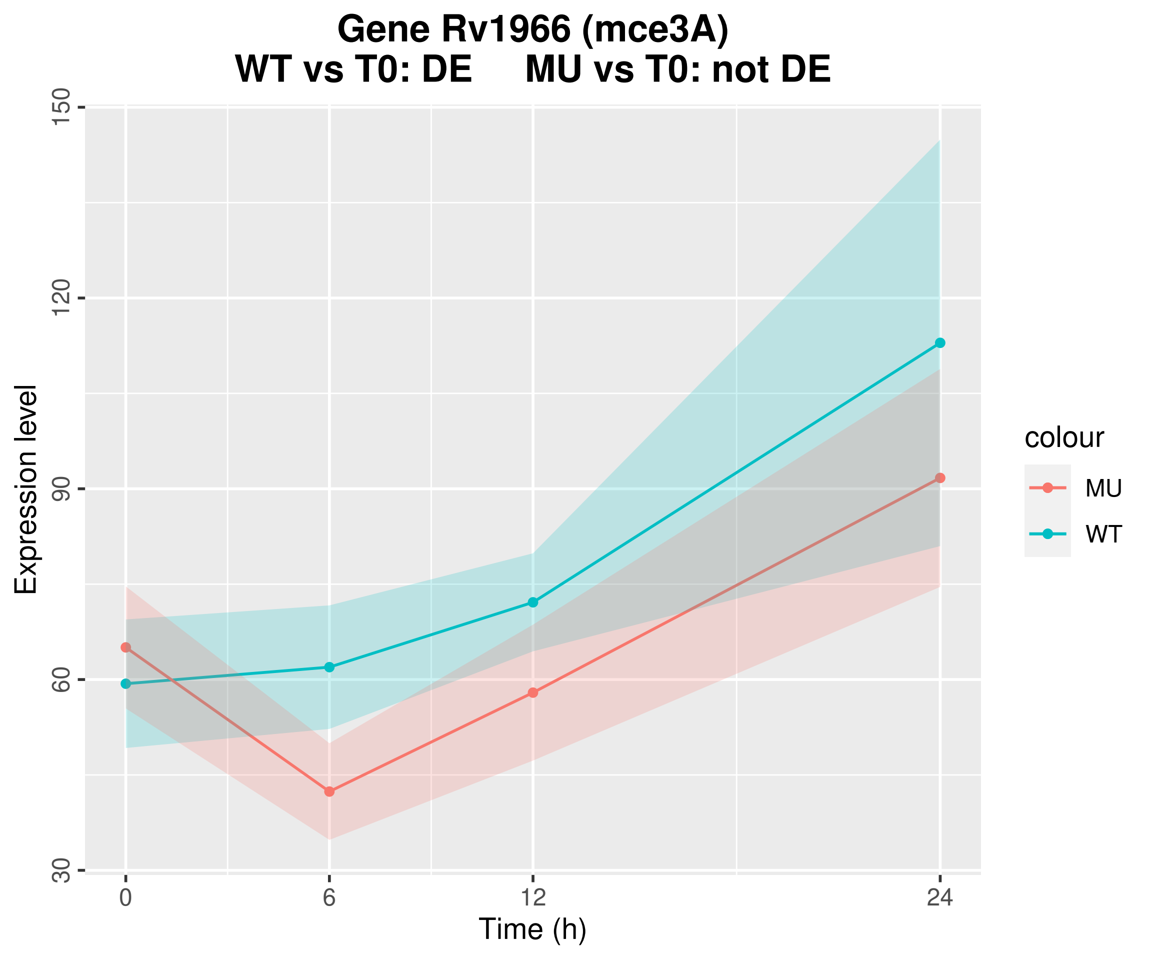

Gene Rv1966 (mce3A)
WT vs T0: DE MU vs T0: not DE
150
120
90
60
30
Expression level
0
6
12
24
Time (h)
colour
MU
WT

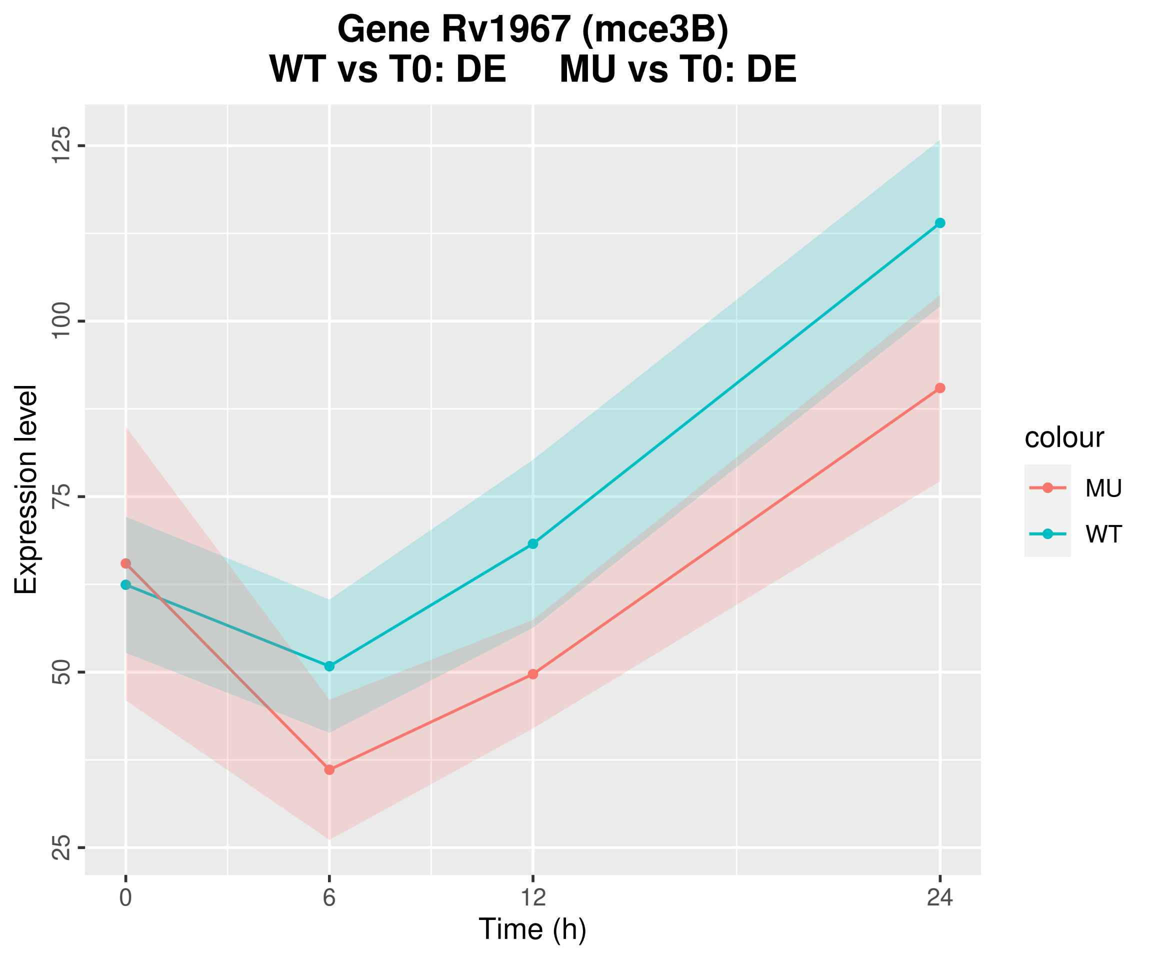

Gene Rv1967 (mce3B)
WT vs T0: DE   MU vs T0: DE
Expression level
125
100
75
50
25
0
6
12
24
Time (h)
colour
MU
WT

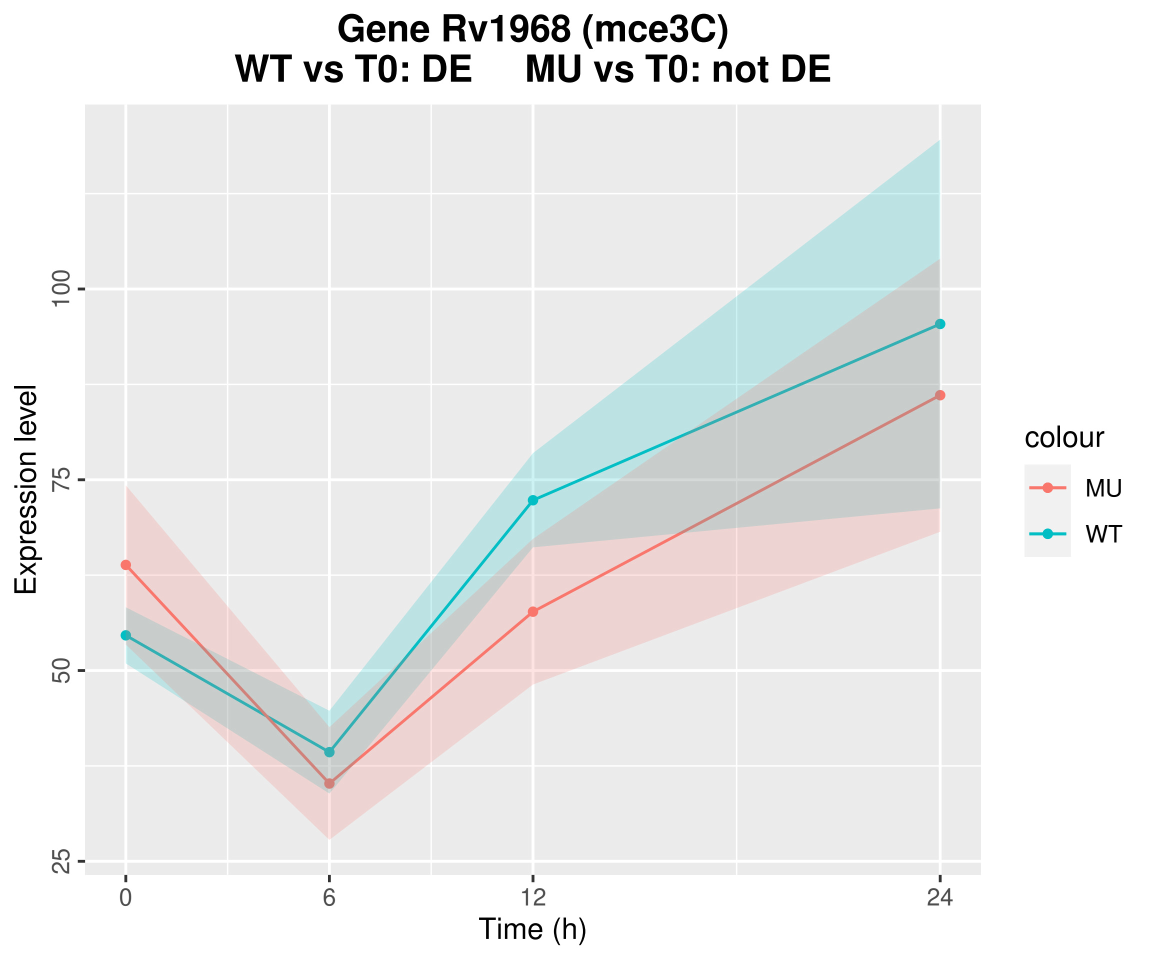
Gene Rv1968 (mce3C)
WT vs T0: DE MU vs T0: not DE
Expression level
100
75
50
25
0
6
12
24
Time (h)
colour
MU
WT

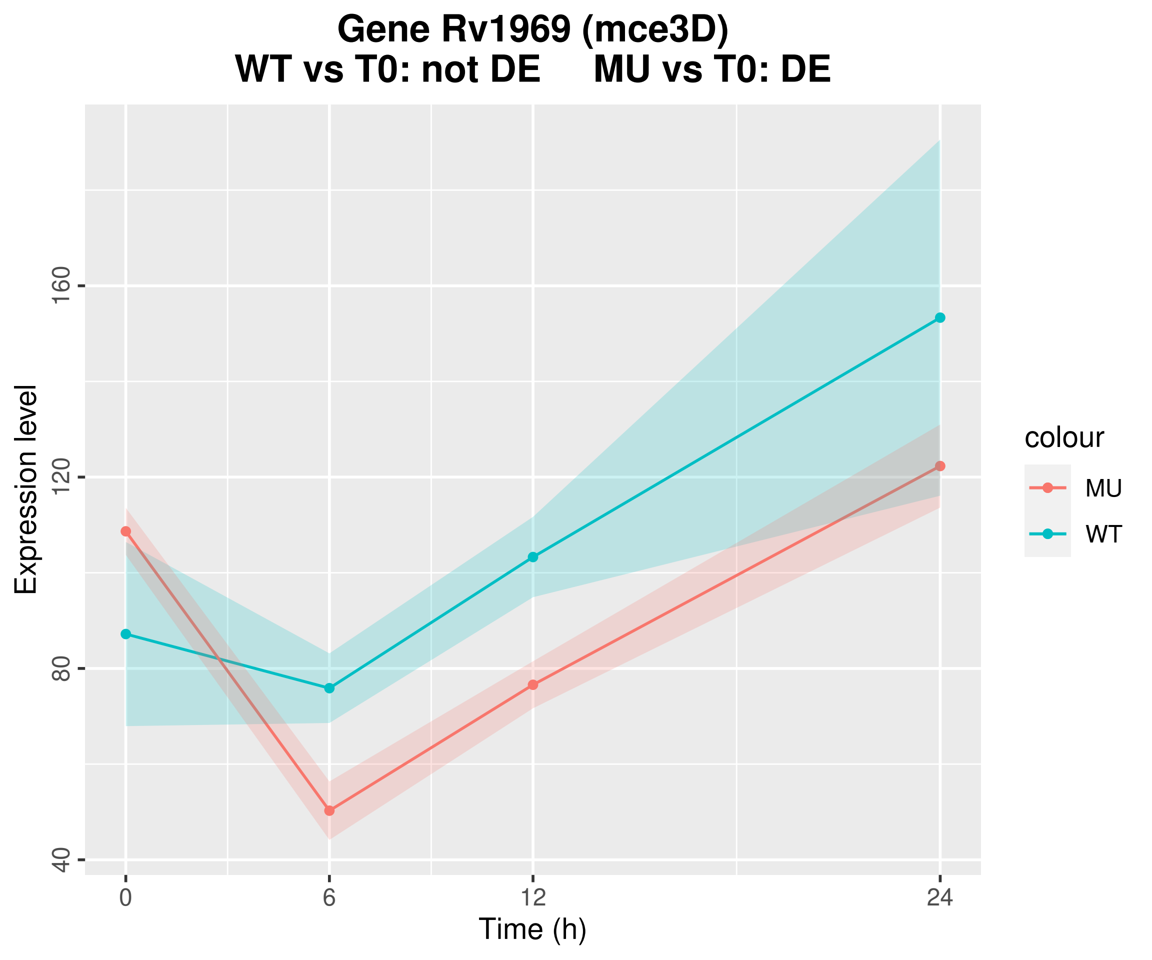
Gene Rv1969 (mce3D)
WT vs T0: not DE MU vs T0: DE
Expression level
160
120
80
40
0
6
12
24
Time (h)
colour
MU
WT

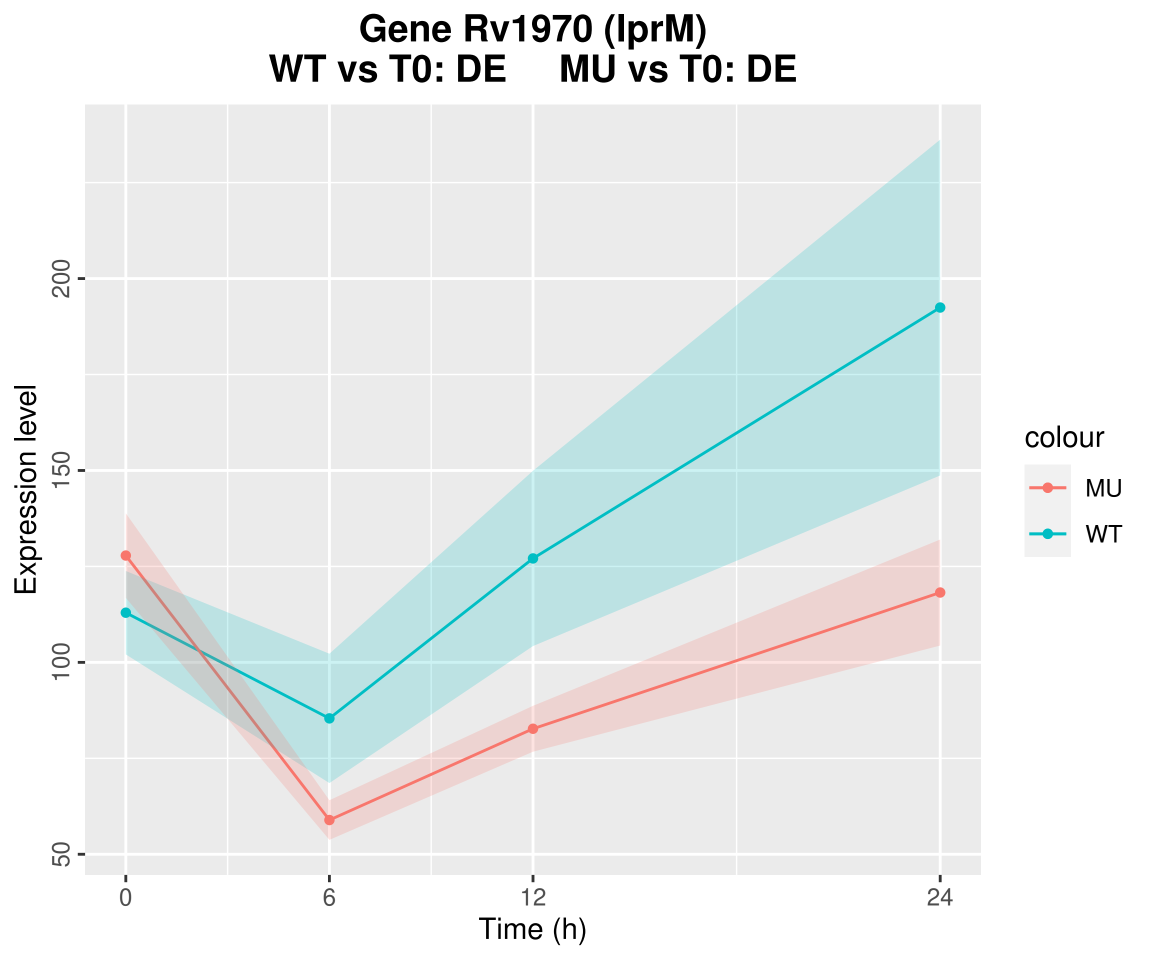
Gene Rv1970 (lprM)
WT vs T0: DE MU vs T0: DE
Expression level
200
150
100
50
0
6
12
24
Time (h)
colour
MU
WT

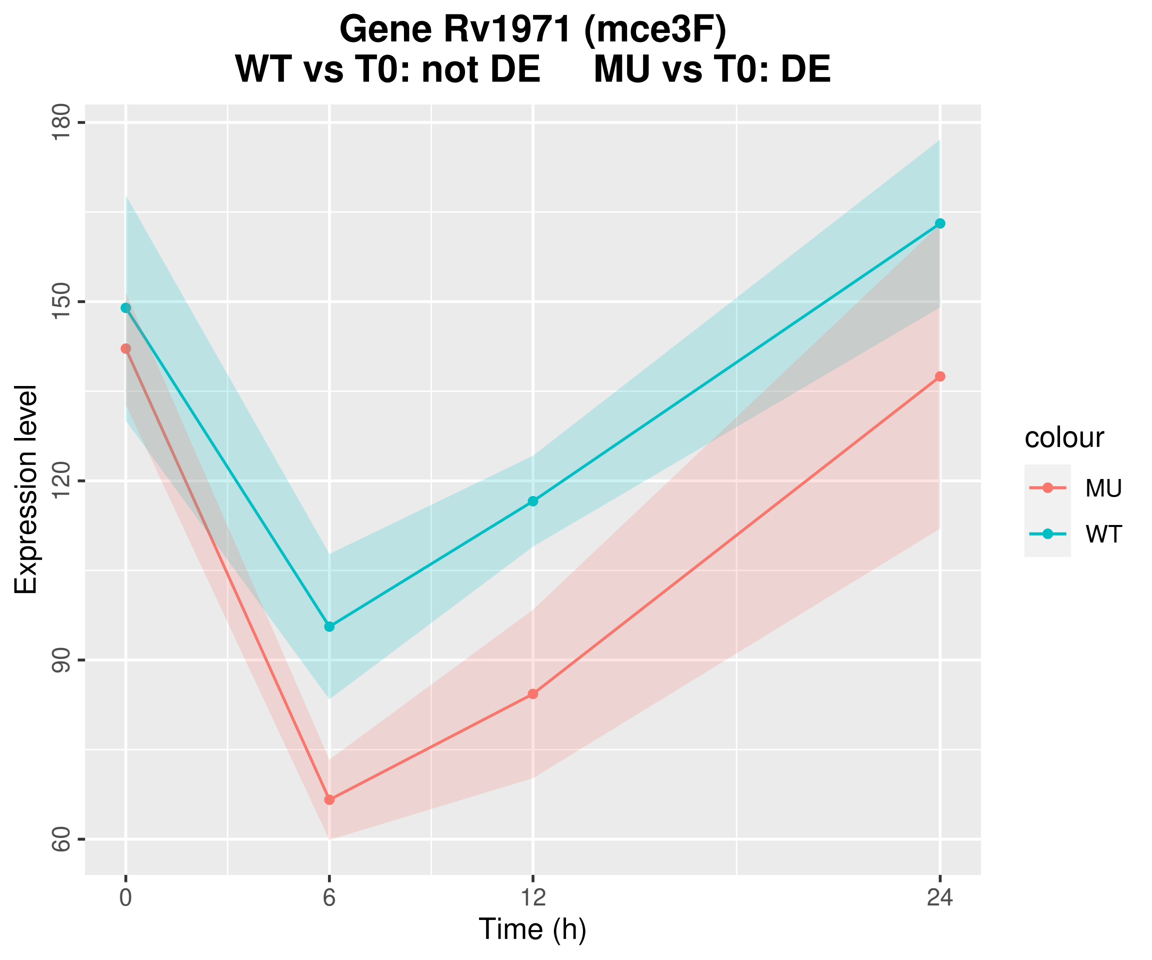
Gene Rv1971 (mce3F)
WT vs T0: not DE
MU vs T0: DE
Expression level
180
150
120
90
60
0
6
12
24
Time (h)
colour
MU
WT

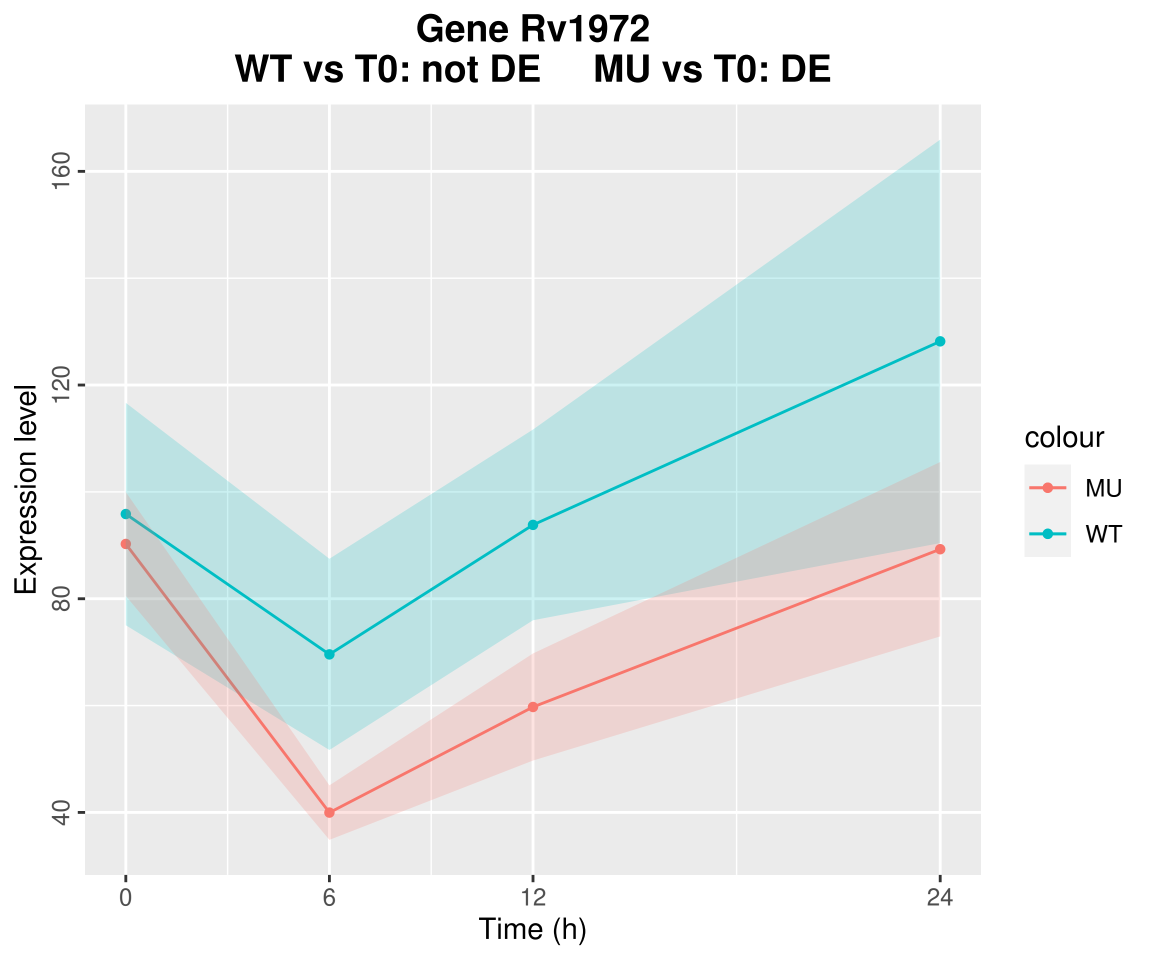
Gene Rv1972
WT vs T0: not DE   MU vs T0: DE
Expression level
160
120
80
40
0
6
12
24
Time (h)
colour
MU
WT

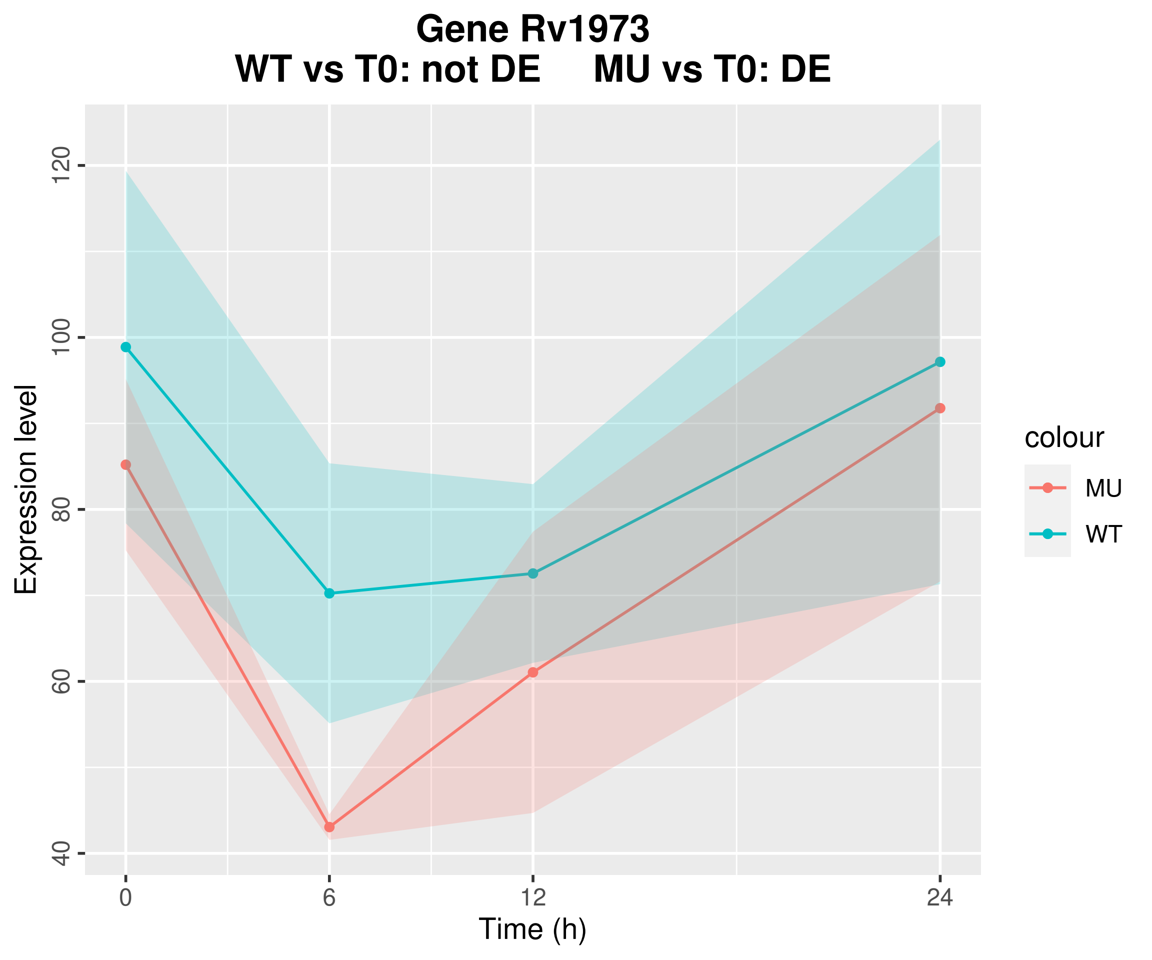

Gene Rv1973
WT vs T0: not DE MU vs T0: DE
Expression level
120
100
80
60
40
0
6
12
24
Time (h)
colour
MU
WT

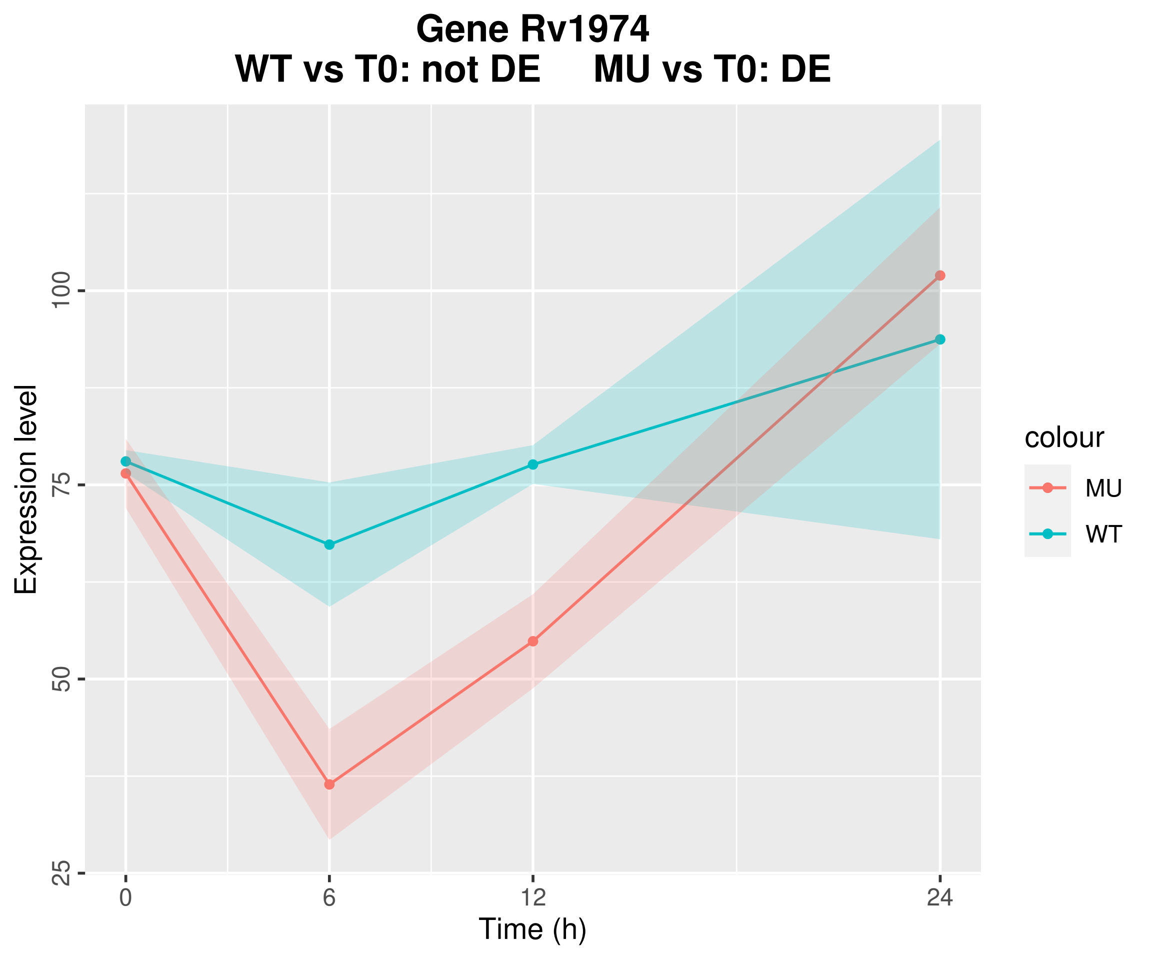

Gene Rv1974
WT vs T0: not DE   MU vs T0: DE
Expression level
100
75
50
25
0
6
12
24
Time (h)
colour
MU
WT

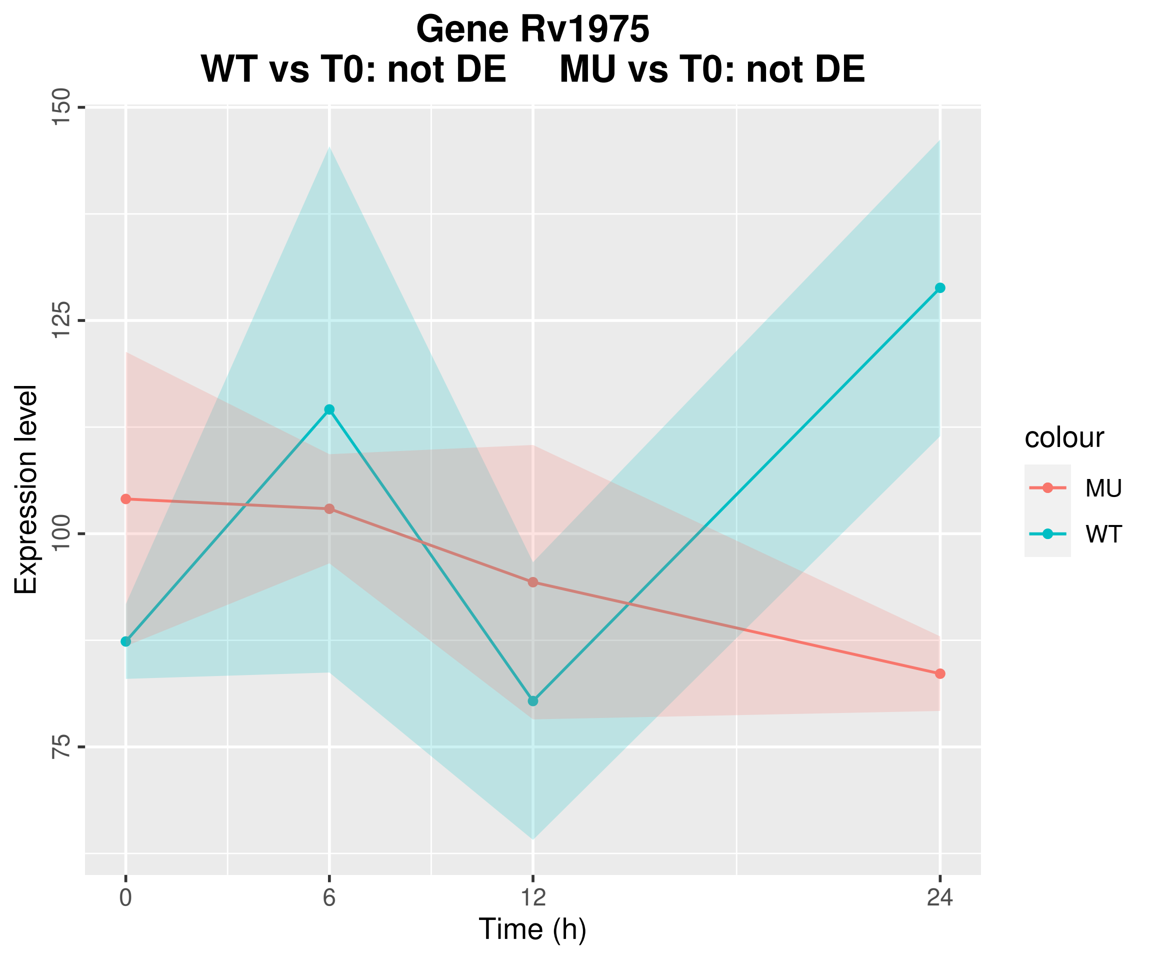

Gene Rv1975
WT vs T0: not DE MU vs T0: not DE
150
125
100
75
Expression level
0
6
12
24
Time (h)
colour
MU
WT

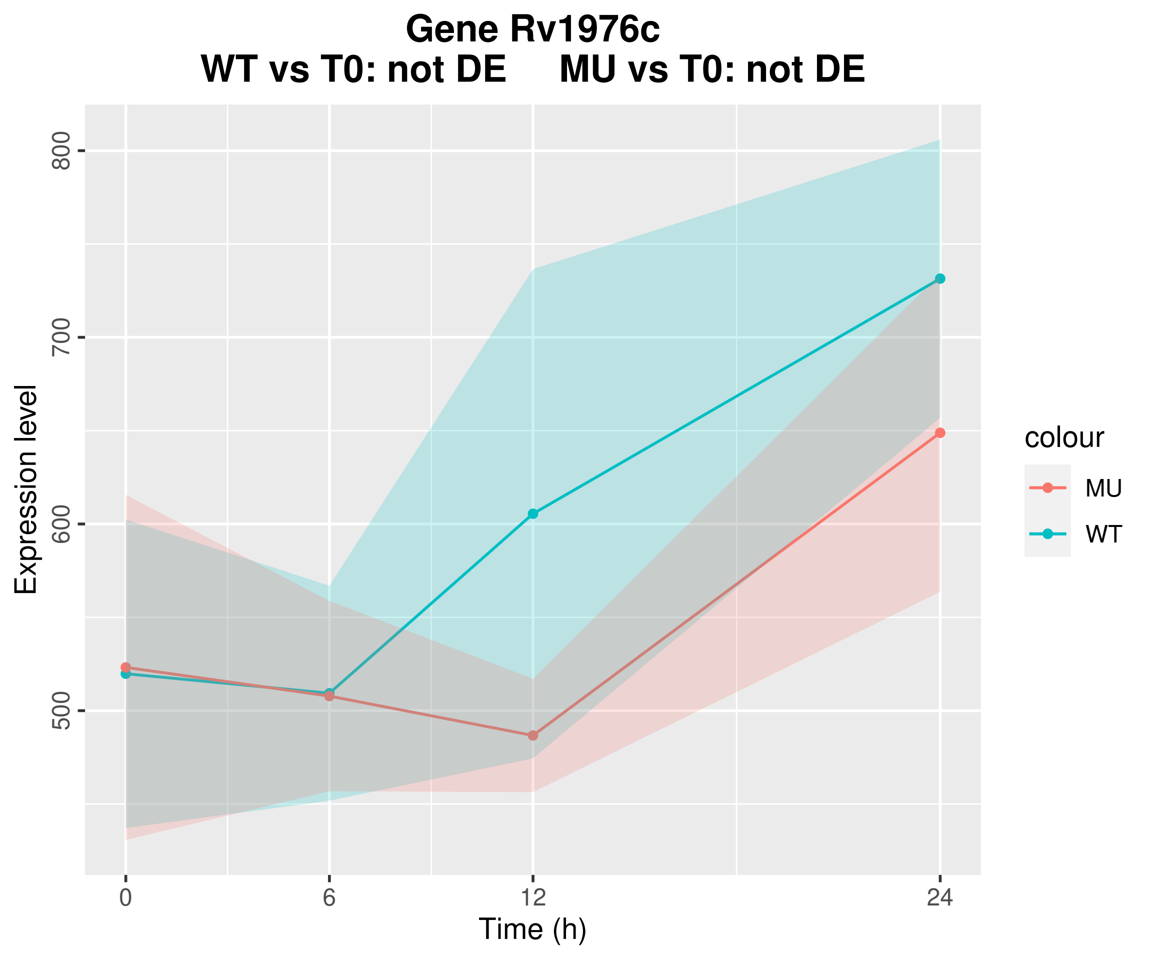
Gene Rv1976c
WT vs T0: not DE   MU vs T0: not DE
Expression level
800
700
600
500
0
6
12
24
Time (h)
colour
MU
WT

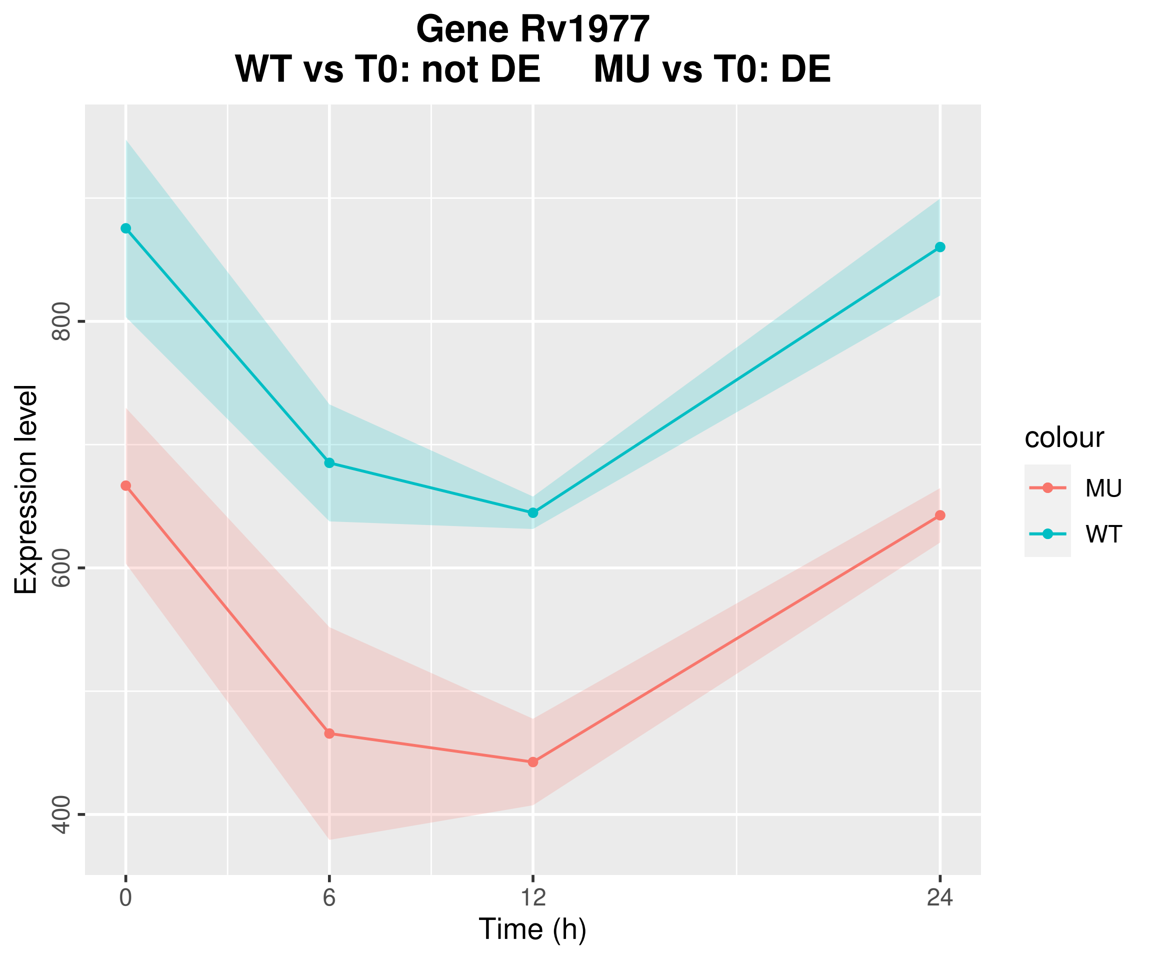

Gene Rv1977
WT vs T0: not DE MU vs T0: DE
Expression level
800
600
400
0
6
12
24
Time (h)
colour
MU
WT

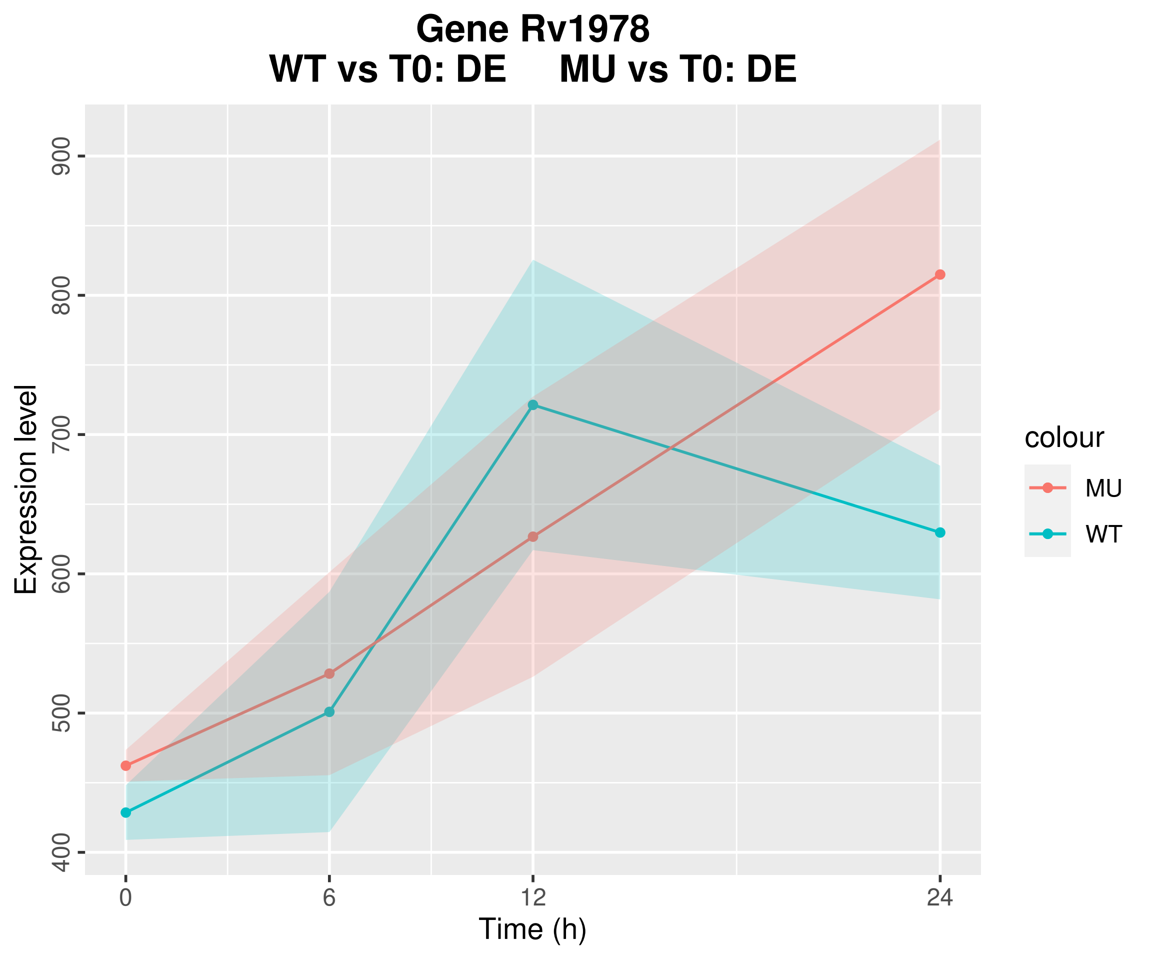
Gene Rv1978
WT vs T0: DE MU vs T0: DE
Expression level
900
800
700
600
500
400
0
6
12
24
Time (h)
colour
MU
WT

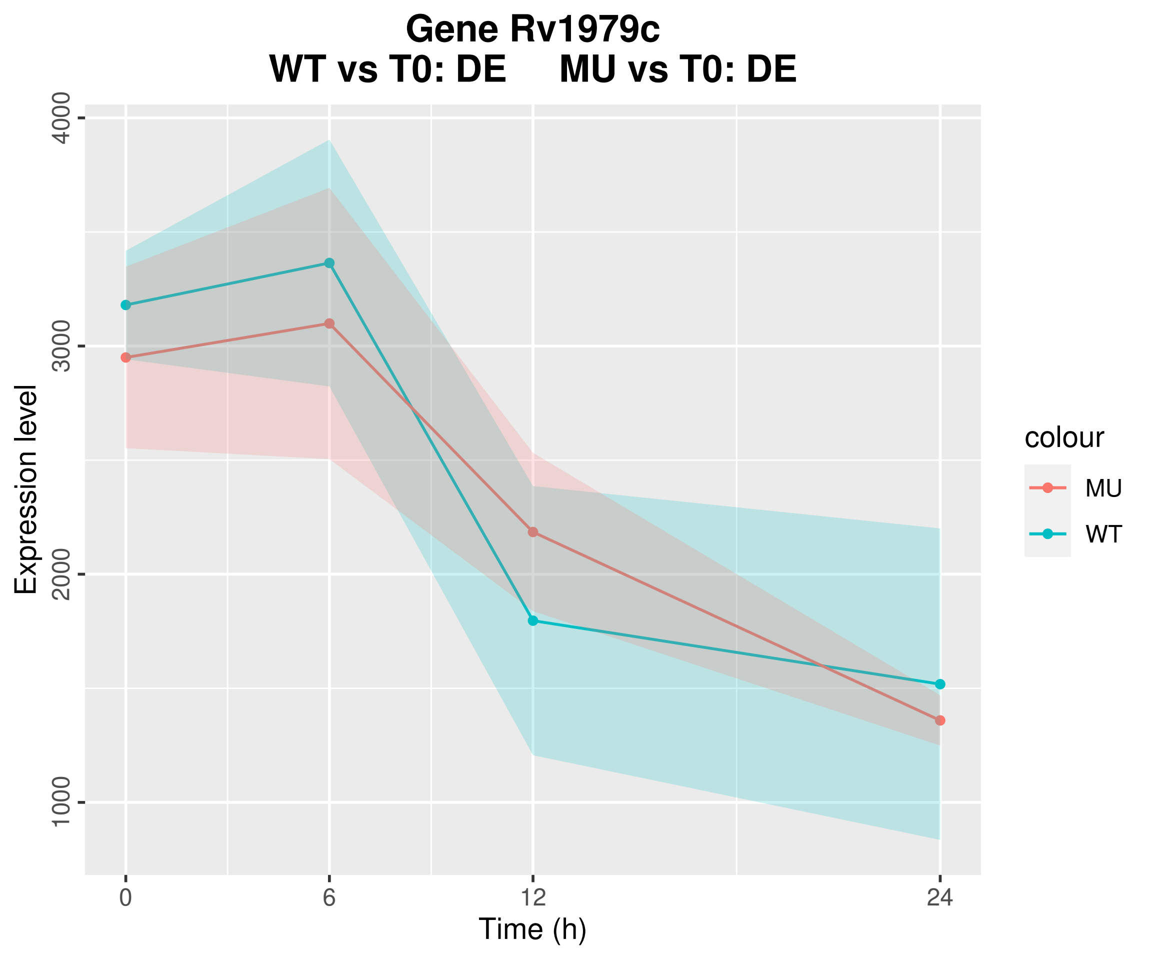
Gene Rv1979c
WT vs T0: DE   MU vs T0: DE
4000
3000
2000
1000
Expression level
0
6
12
24
Time (h)
colour
MU
WT

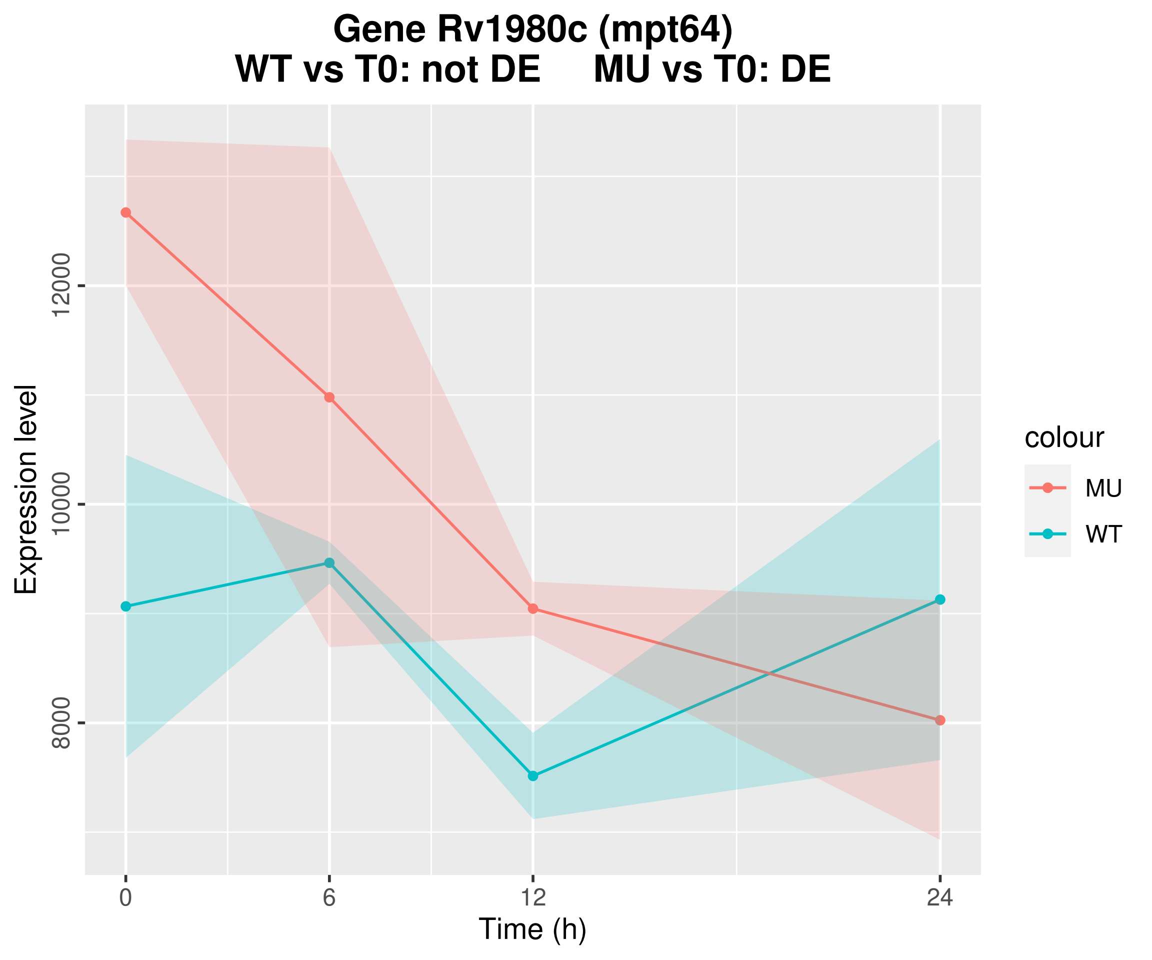
Gene Rv1980c (mpt64)
WT vs T0: not DE   MU vs T0: DE
Expression level
12000
10000
8000
0
6
12
24
Time (h)
colour
MU
WT

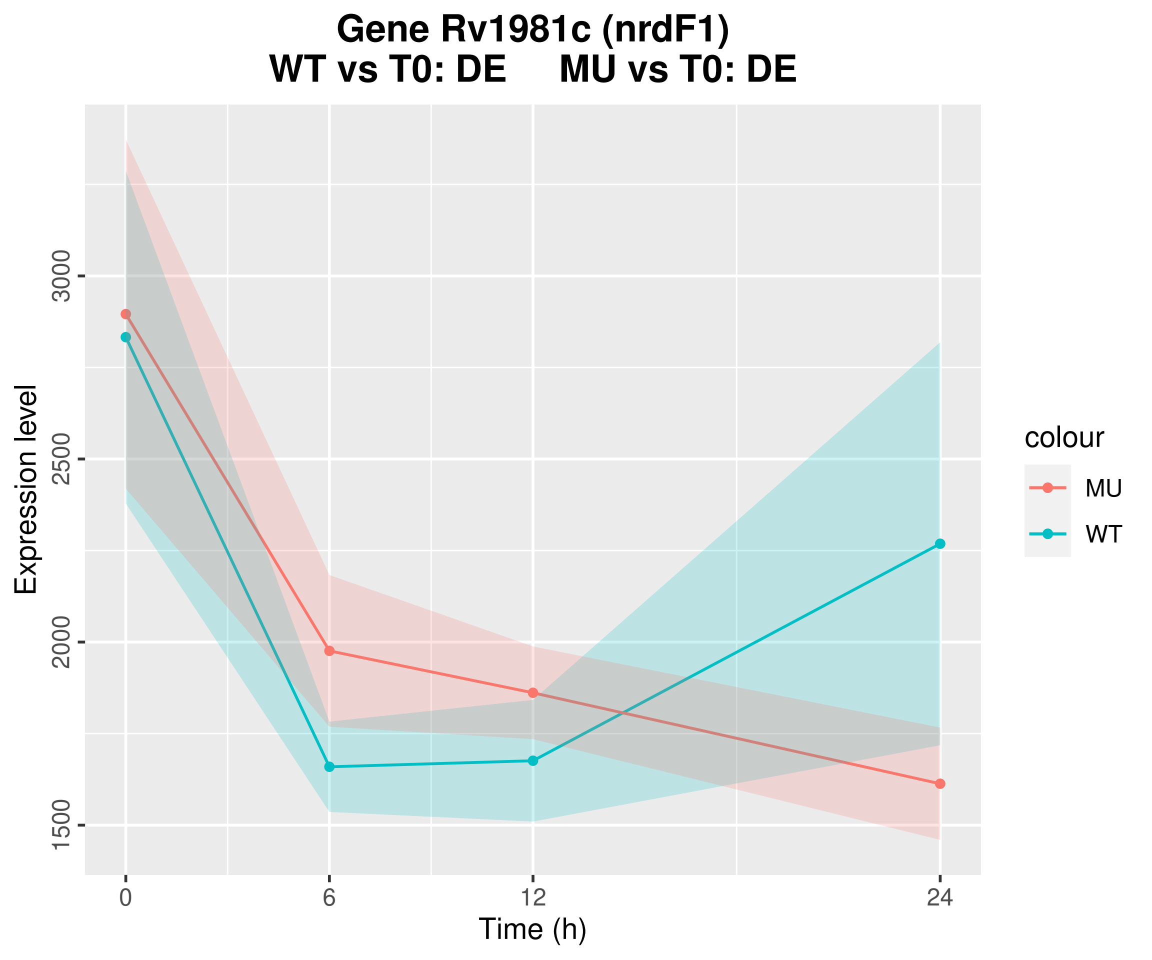

Gene Rv1981c (nrdF1)
WT vs T0: DE MU vs T0: DE
Expression level
3000
2500
2000
1500
0
6
12
24
Time (h)
colour
MU
WT

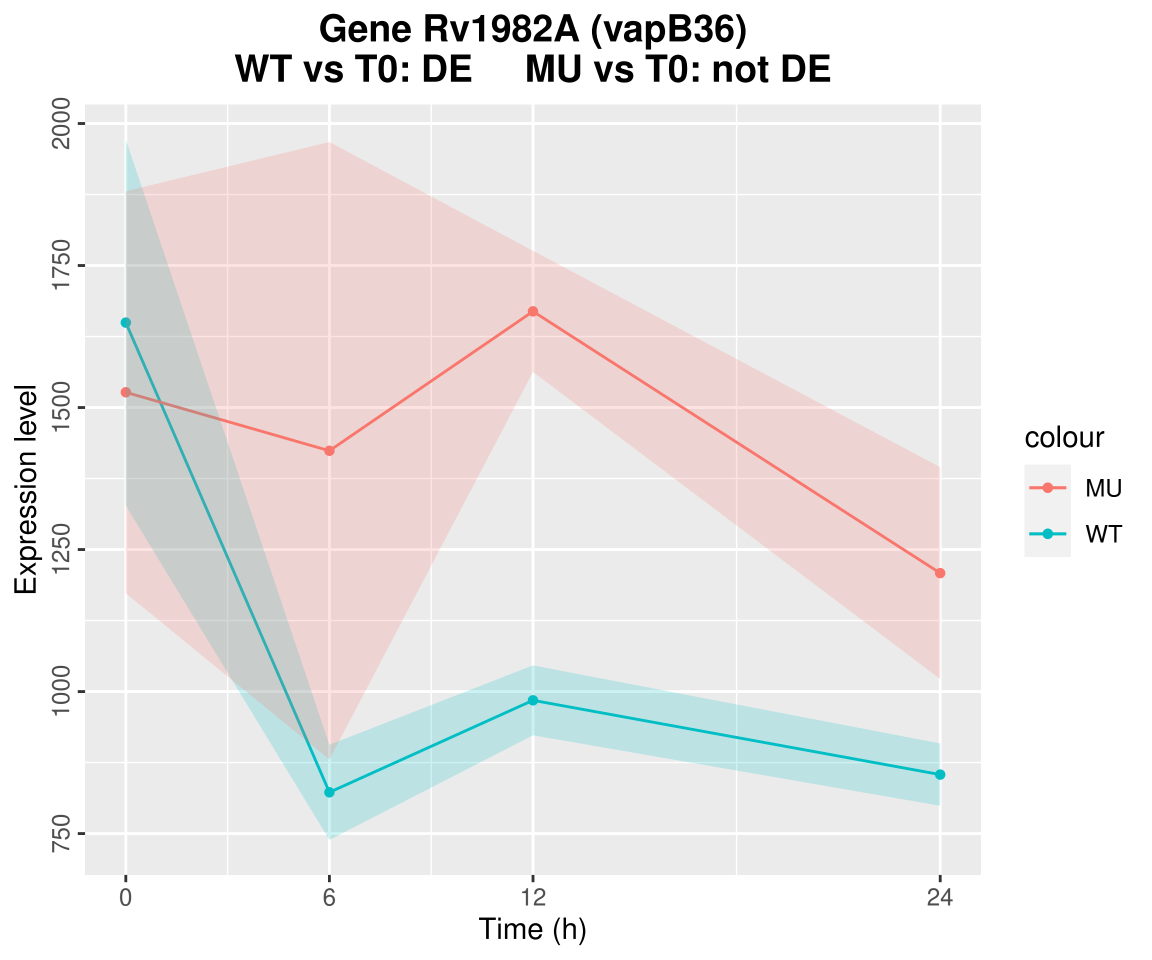

Gene Rv1982A (vapB36)
WT vs T0: DE MU vs T0: not DE
Expression level
2000
1750
1500
1250
1000
750
0
6
12
24
Time (h)
colour
MU
WT

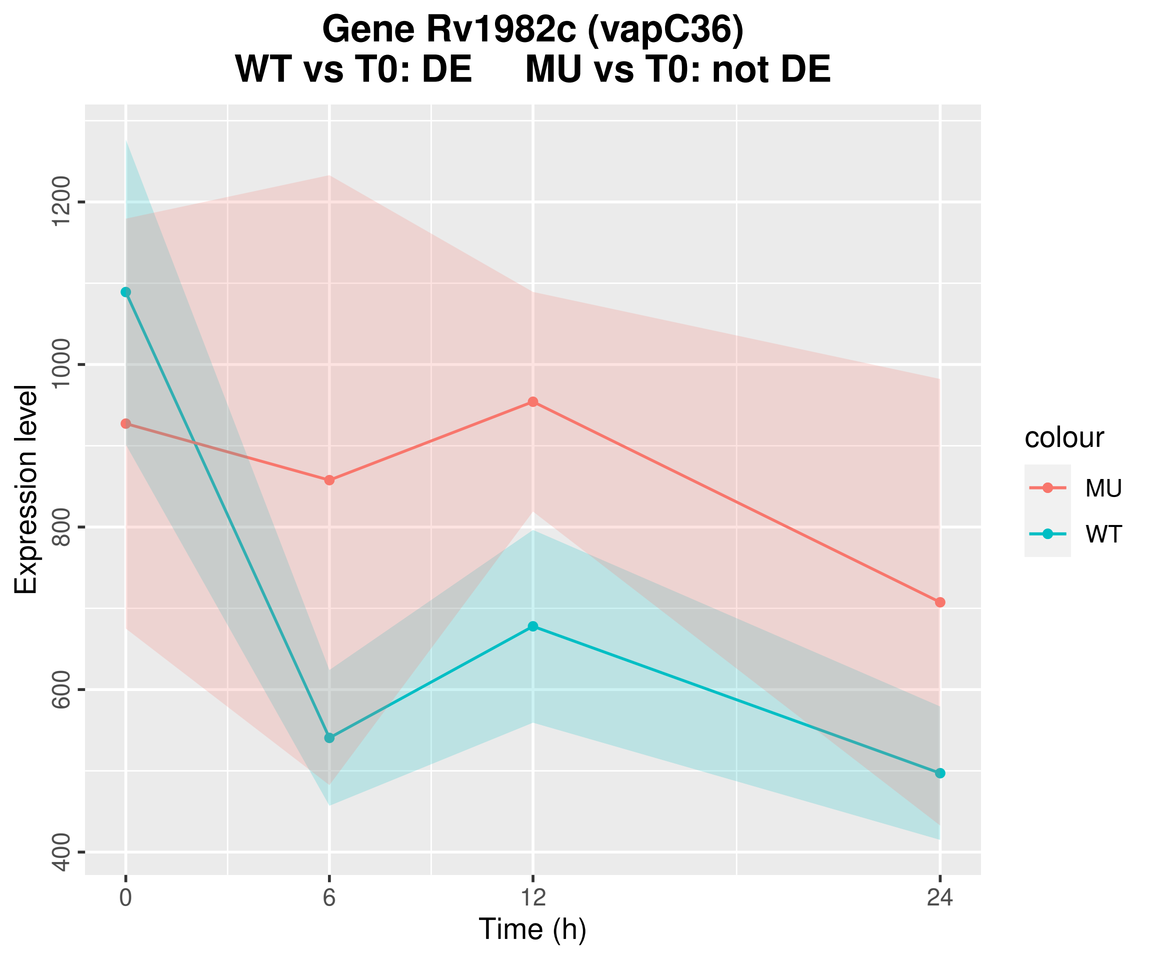
Gene Rv1982c (vapC36)
WT vs T0: DE   MU vs T0: not DE
Expression level
1200
1000
800
600
400
0
6
12
24
Time (h)
colour
MU
WT

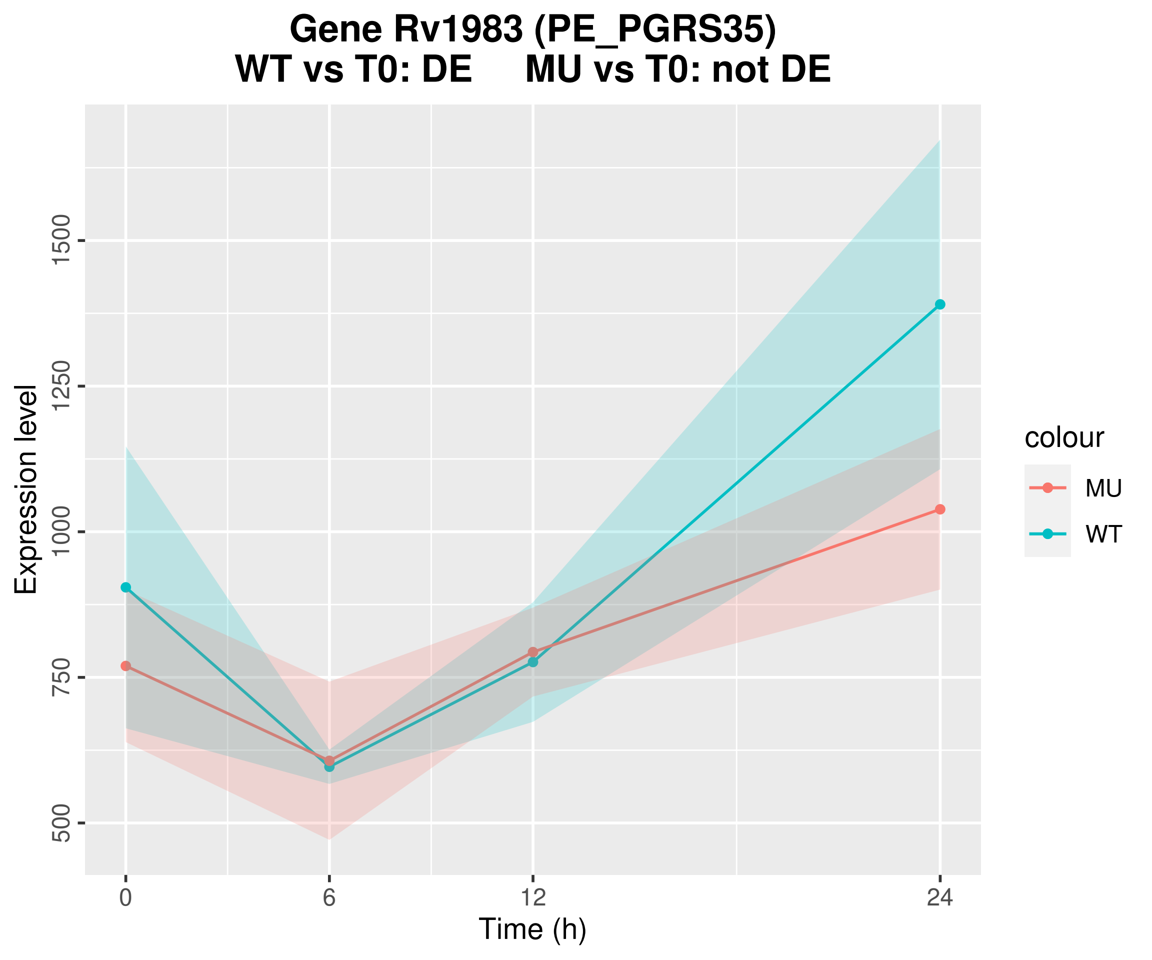

Gene Rv1983 (PE_PGRS35)
WT vs T0: DE MU vs T0: not DE
Expression level
1500
1250
1000
750
500
0
6
12
24
Time (h)
colour
MU
WT

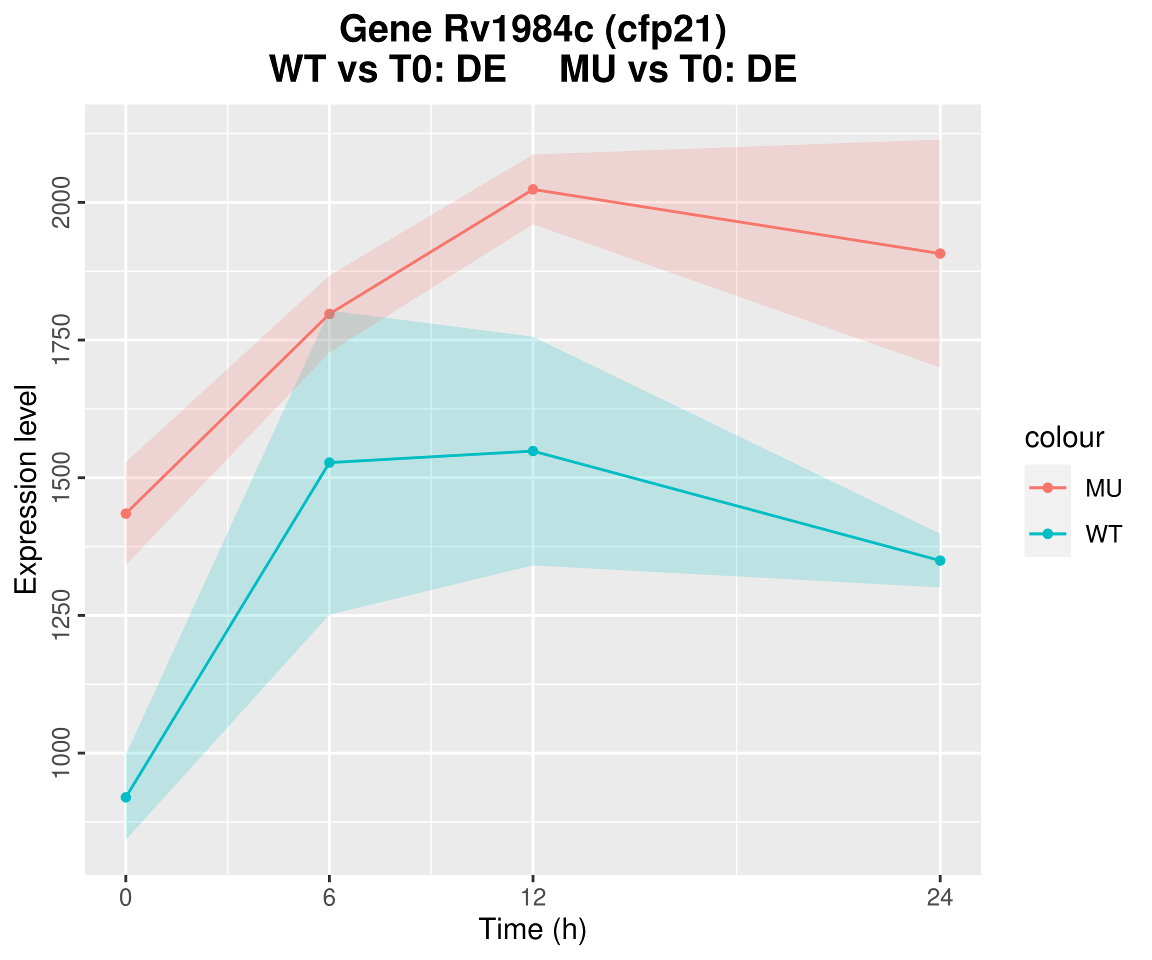
Gene Rv1984c (cfp21)
WT vs T0: DE   MU vs T0: DE
Expression level
2000
1750
1500
1250
1000
0
6
12
24
Time (h)
colour
MU
WT

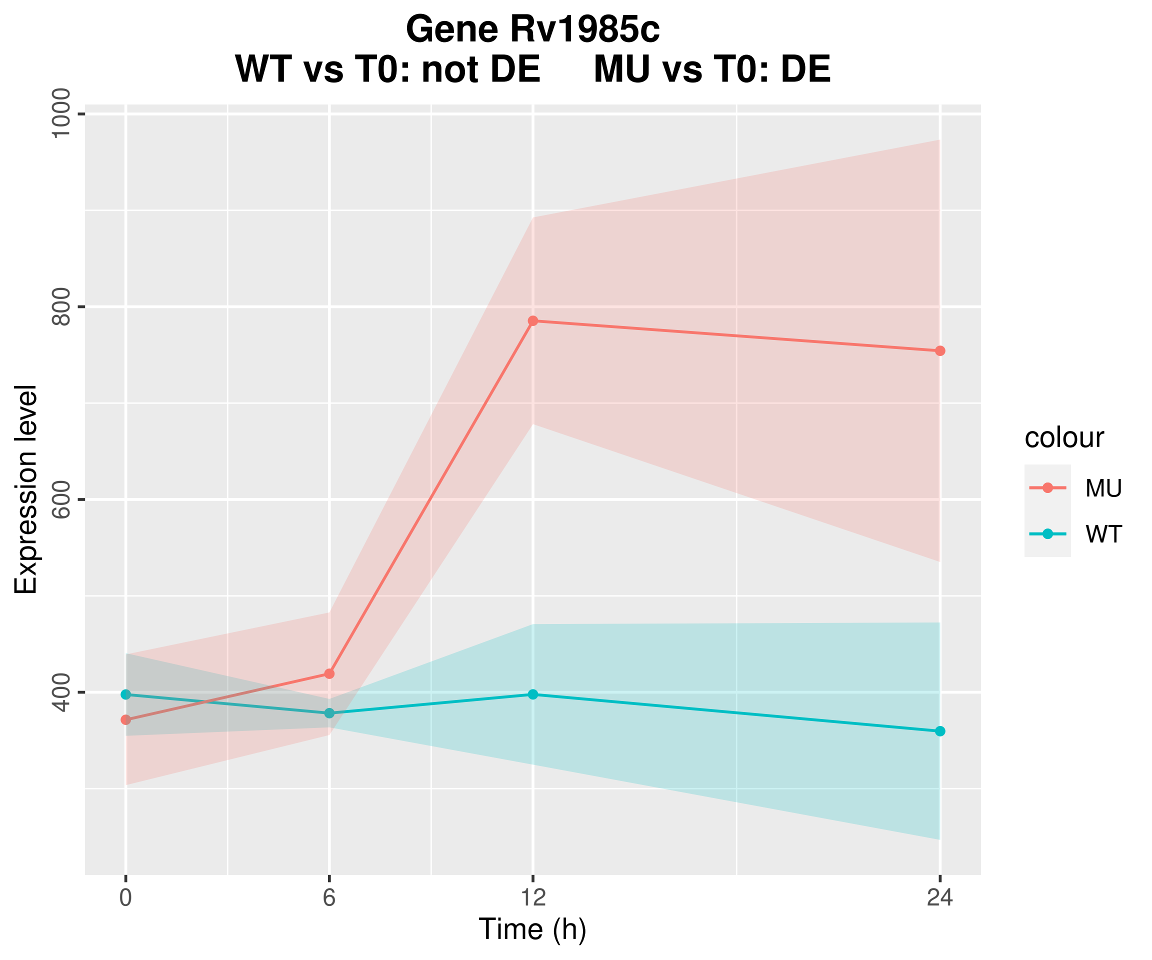
Gene Rv1985c
WT vs T0: not DE   MU vs T0: DE
1000
800
600
400
Expression level
0
6
12
24
Time (h)
colour
MU
WT

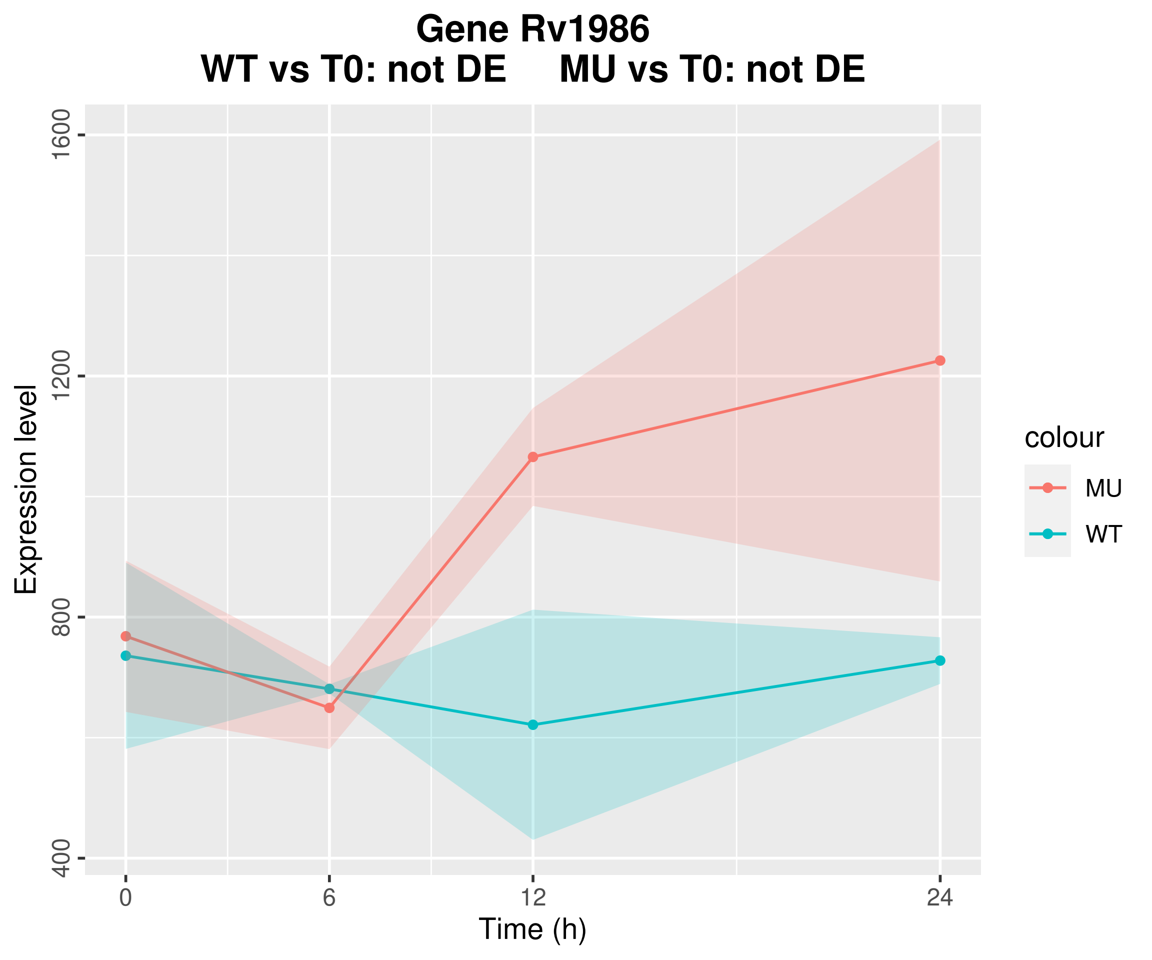

Gene Rv1986
WT vs T0: not DE MU vs T0: not DE
Expression level
1600
1200
800
400
0
6
12
24
Time (h)
colour
MU
WT

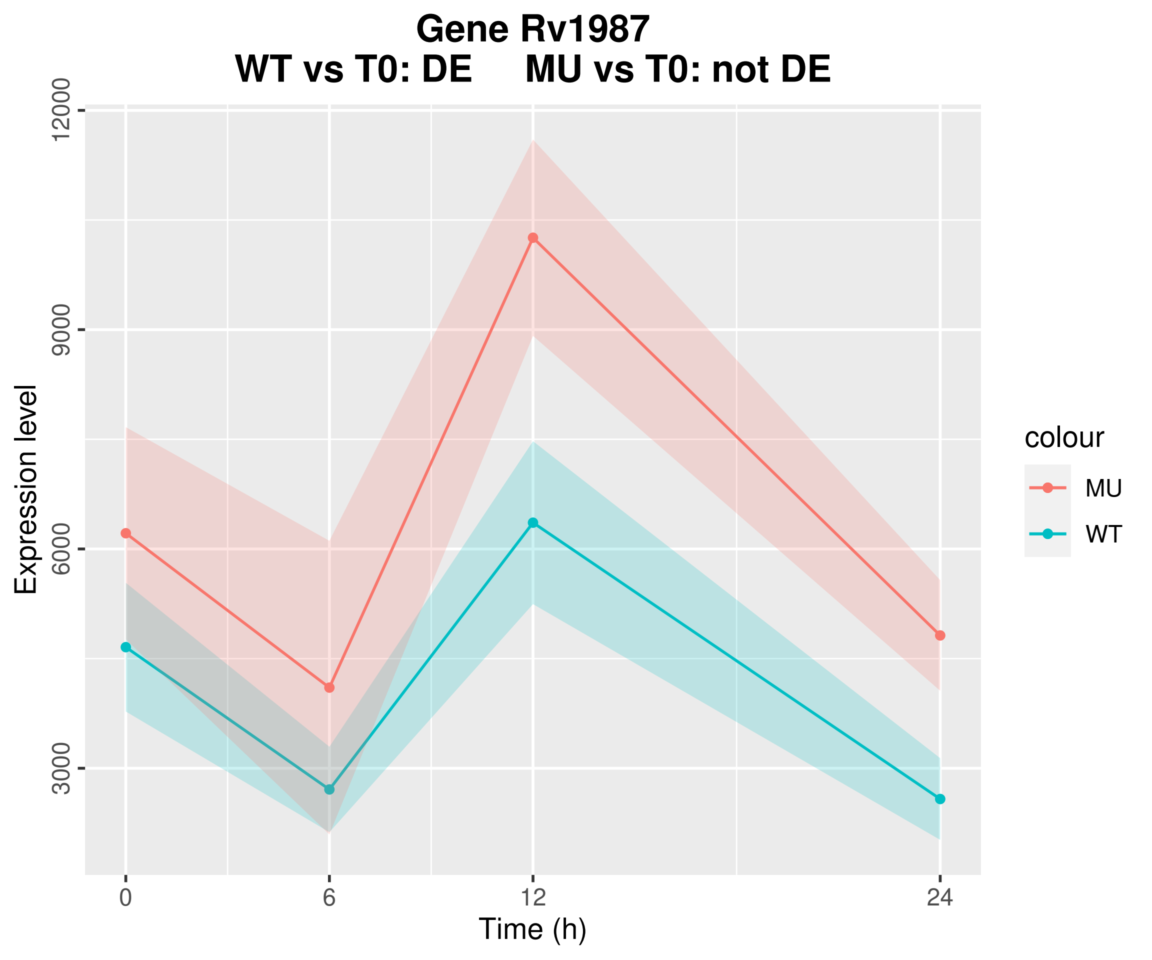

Gene Rv1987
WT vs T0: DE MU vs T0: not DE
Expression level
12000
9000
6000
3000
0
6
12
24
Time (h)
colour
MU
WT

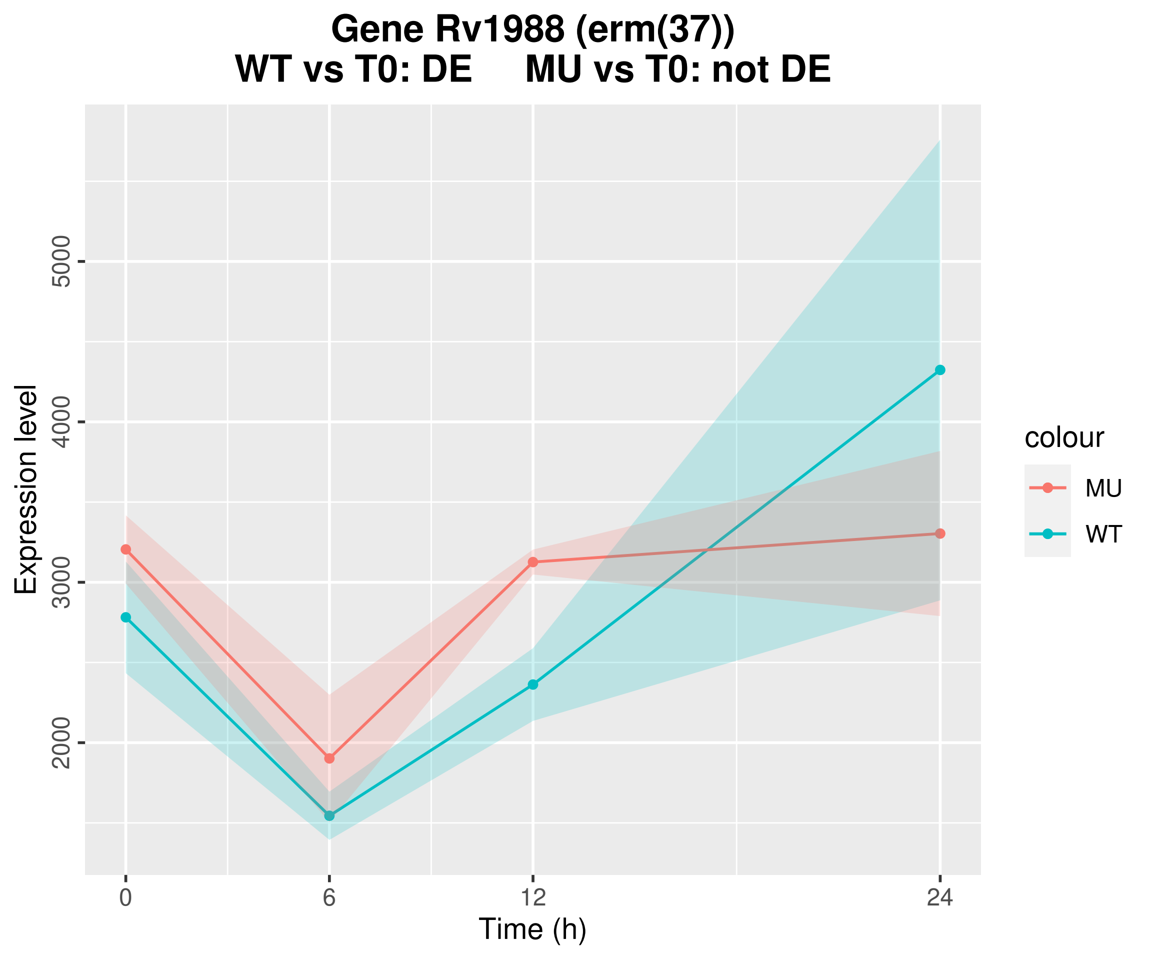
Gene Rv1988 (erm(37))
WT vs T0: DE   MU vs T0: not DE
Expression level
2000
3000
4000
5000
0
6
12
24
Time (h)
colour
MU
WT

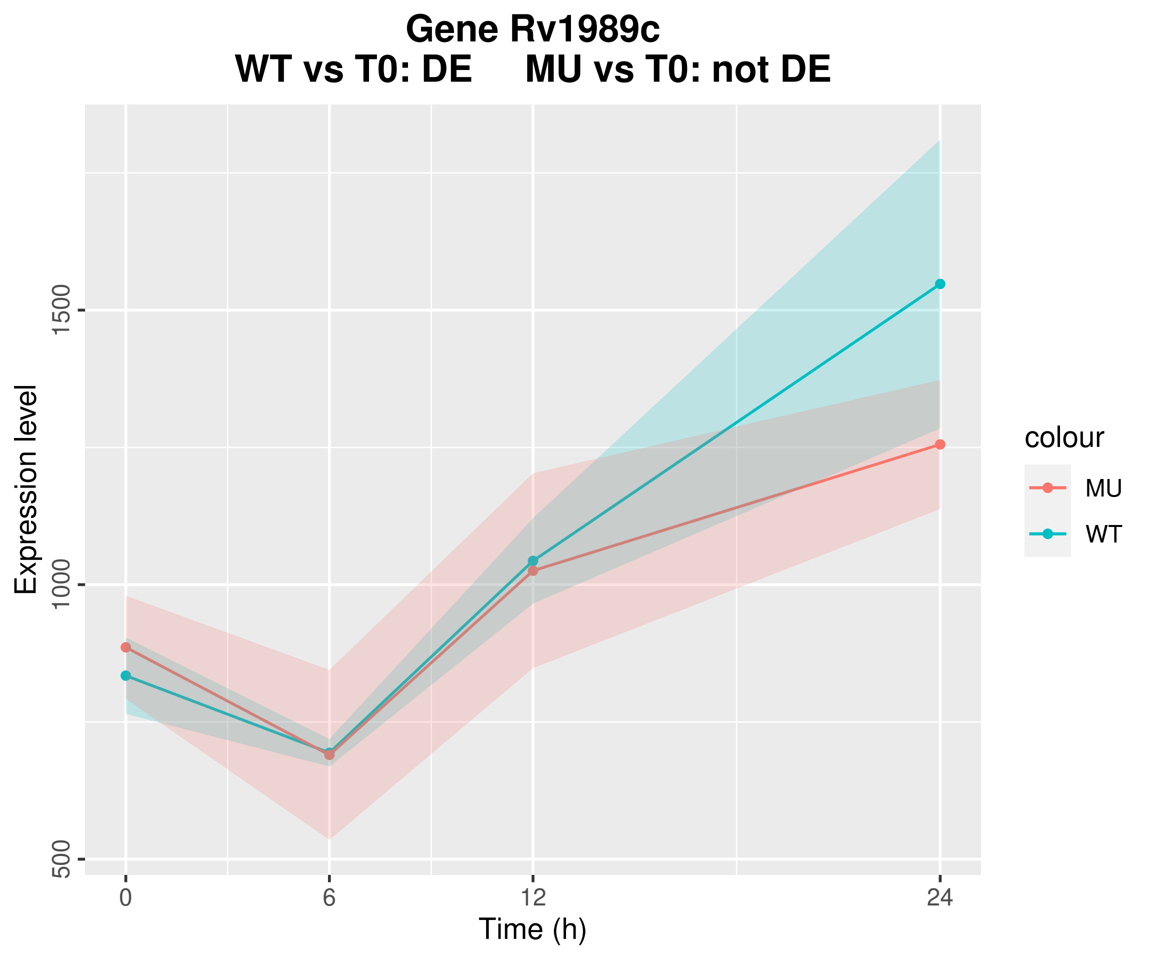

Gene Rv1989c
WT vs T0: DE MU vs T0: not DE
Expression level
1500
1000
500
0
6
12
24
Time (h)
colour
MU
WT

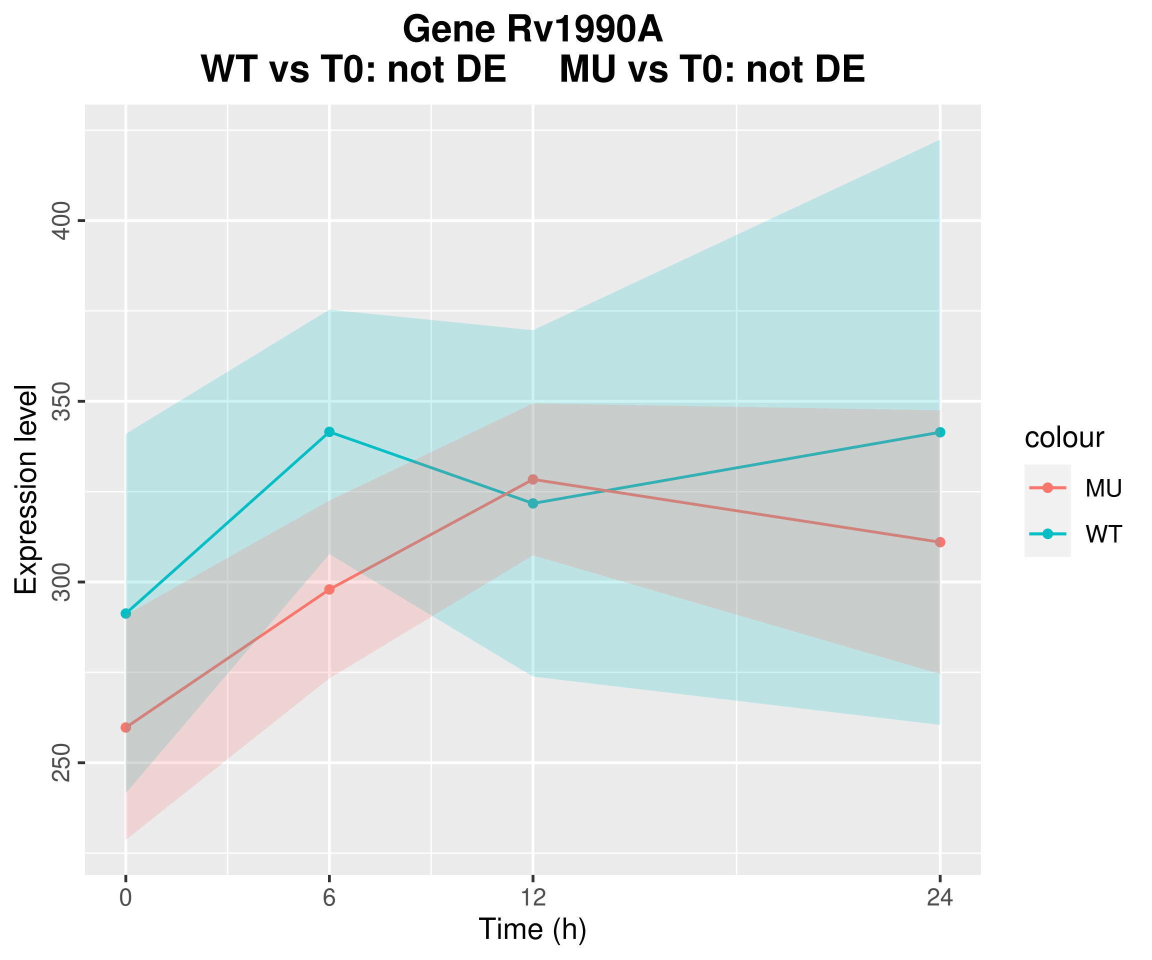
Gene Rv1990A
WT vs T0: not DE MU vs T0: not DE
Expression level
250
300
350
400
0
6
12
24
Time (h)
colour
MU
WT

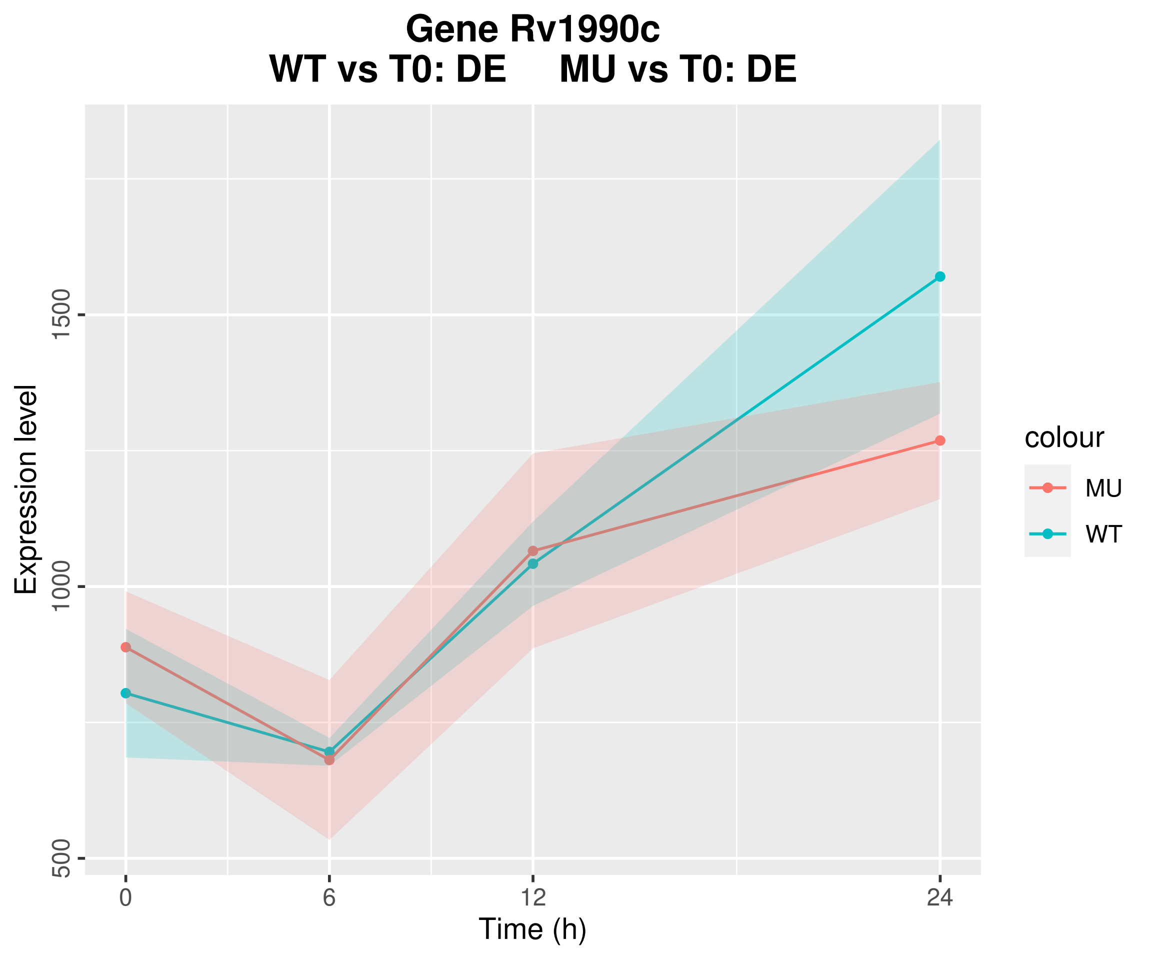
Gene Rv1990c
WT vs T0: DE MU vs T0: DE
Expression level
500
1000
1500
0
6
12
24
Time (h)
colour
MU
WT

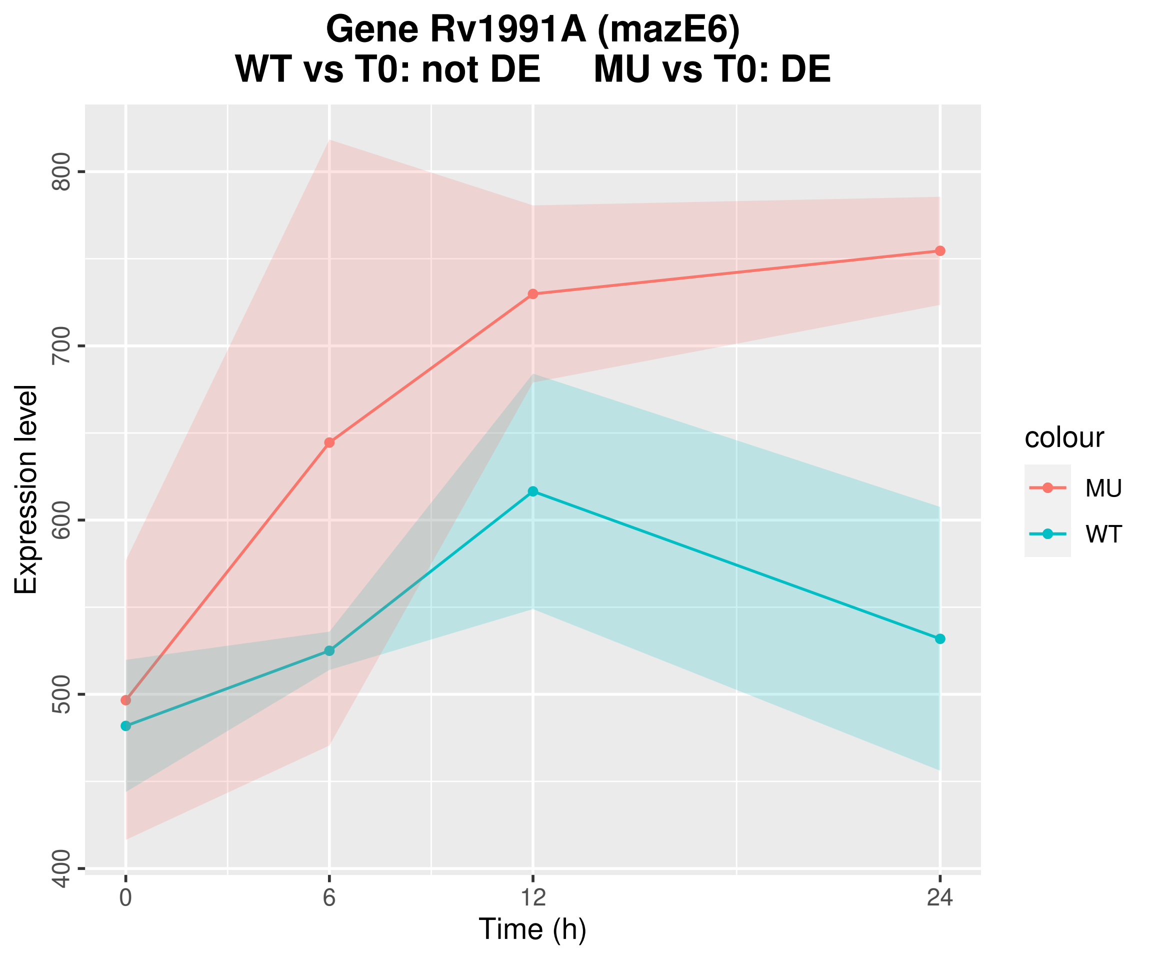
Gene Rv1991A (mazE6)
WT vs T0: not DE   MU vs T0: DE
Expression level
800
700
600
500
400
0
6
12
24
Time (h)
colour
MU
WT

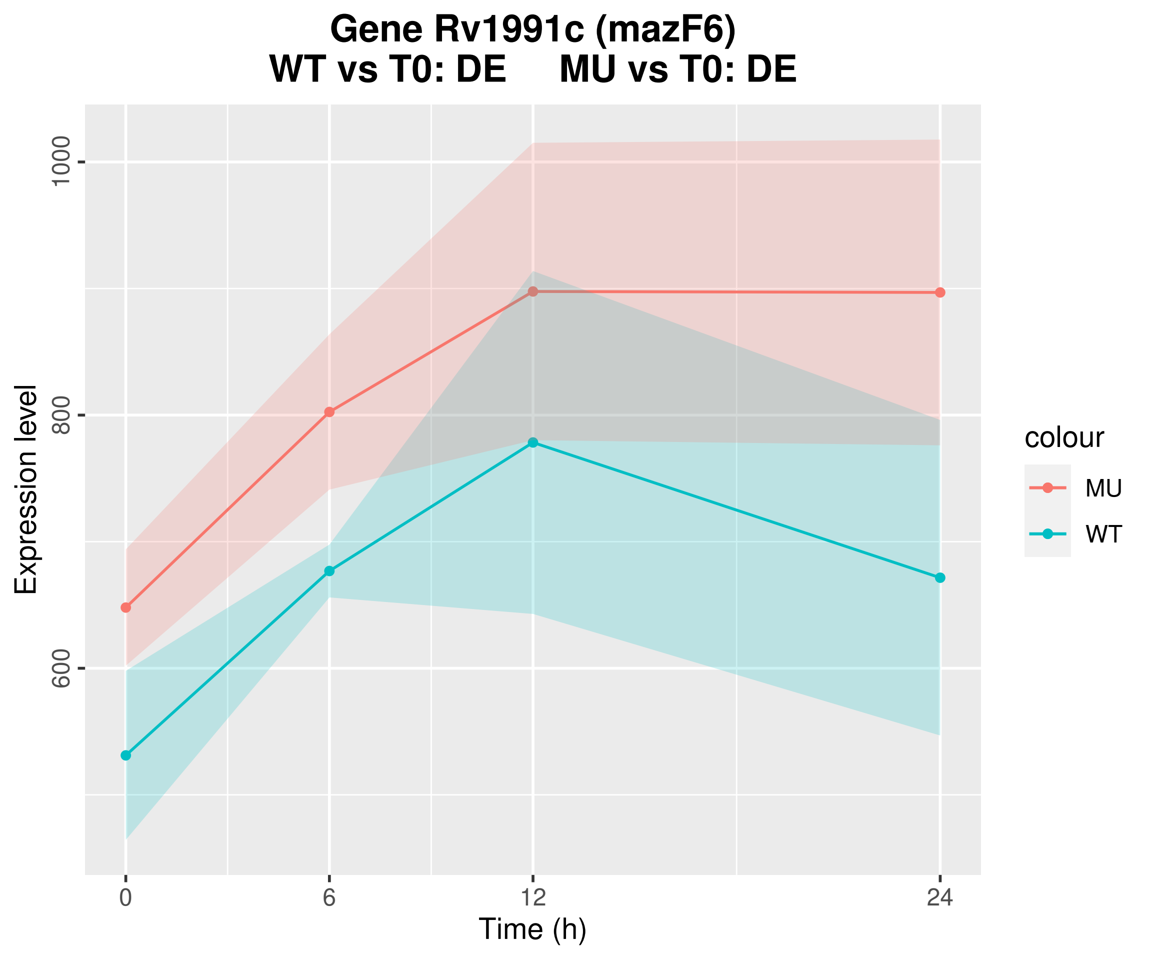

Gene Rv1991c (mazF6)
WT vs T0: DE MU vs T0: DE
1000
800
600
Expression level
0
6
12
24
Time (h)
colour
MU
WT

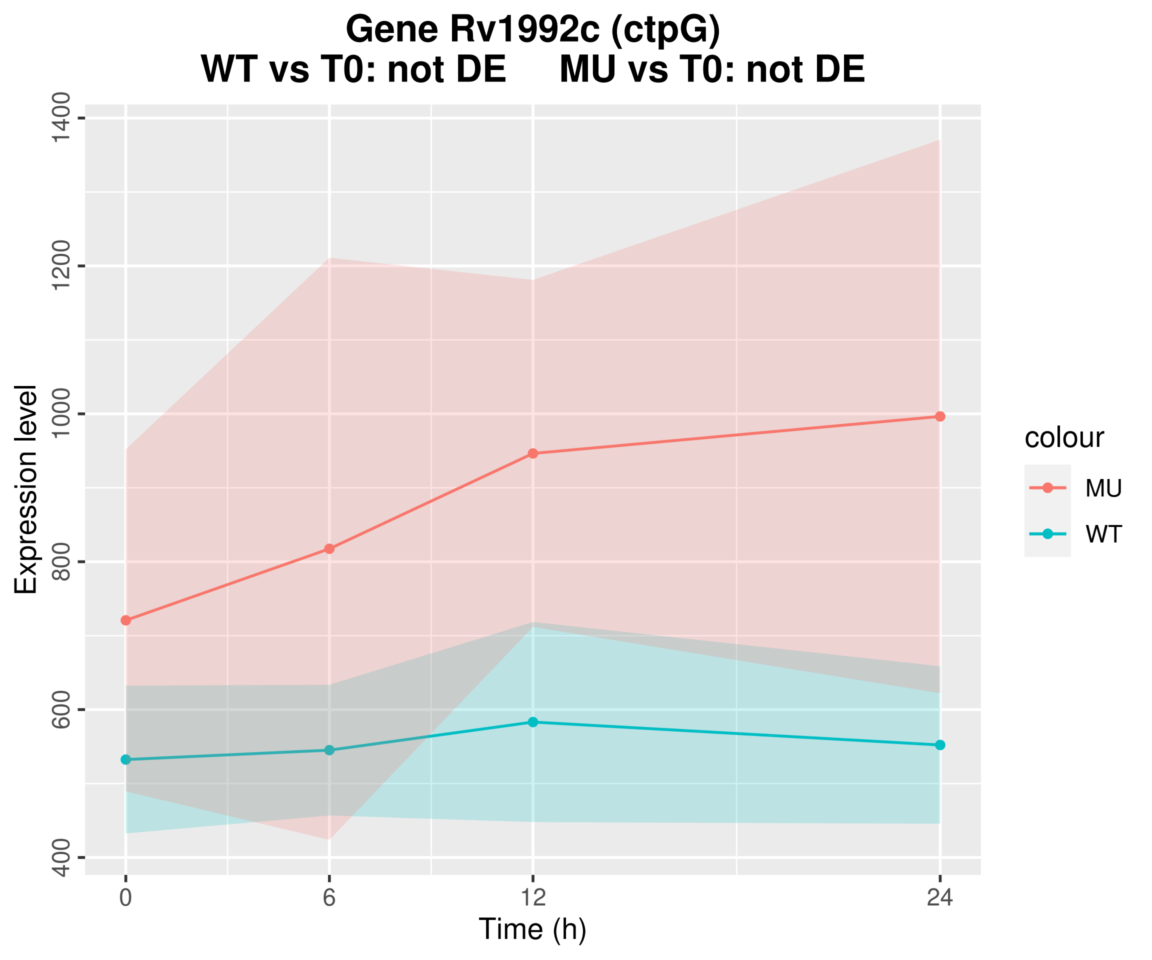

Gene Rv1992c (ctpG)
WT vs T0: not DE   MU vs T0: not DE
Expression level
1400
1200
1000
800
600
400
0
6
12
24
Time (h)
colour
MU
WT

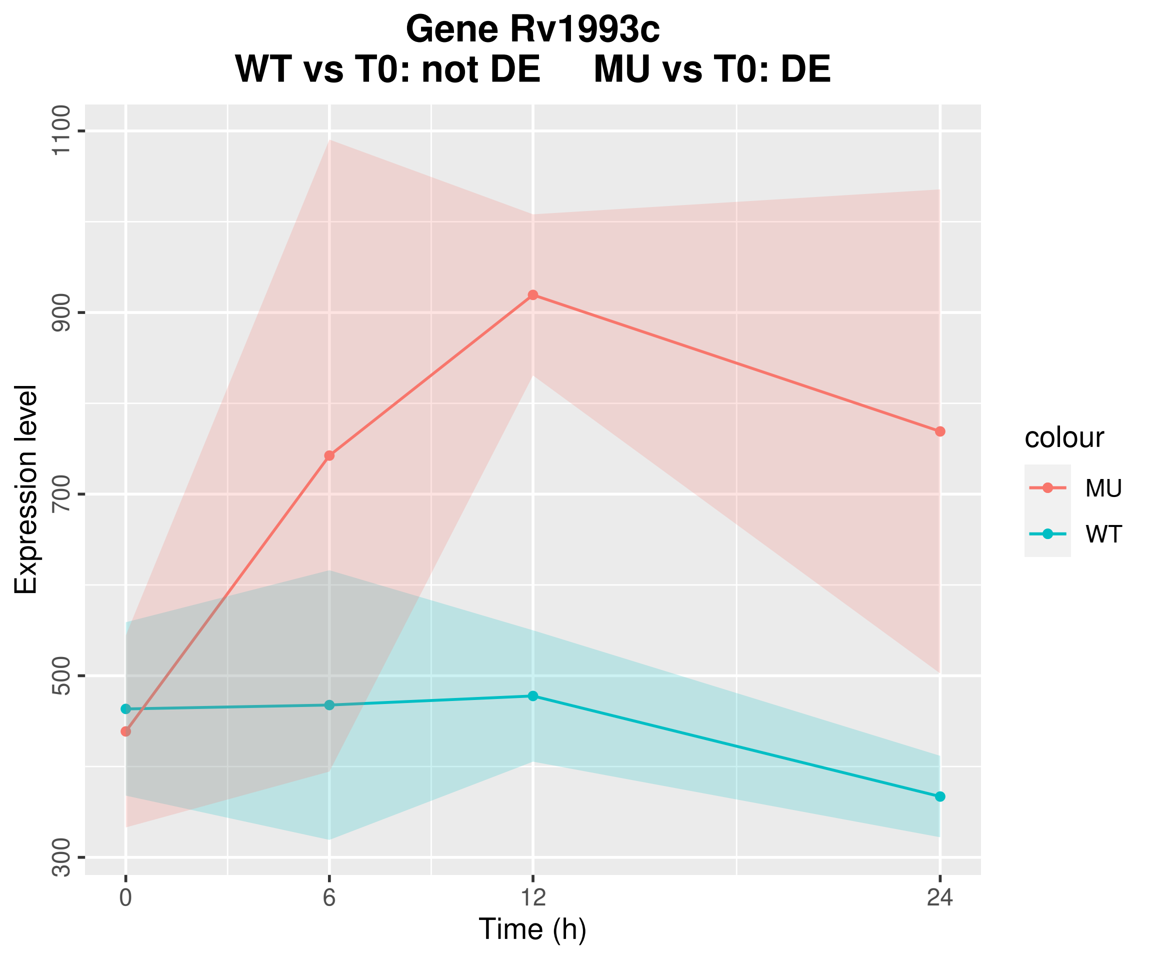

Gene Rv1993c
WT vs T0: not DE MU vs T0: DE
Expression level
1100
900
700
500
300
0
6
12
24
Time (h)
colour
MU
WT

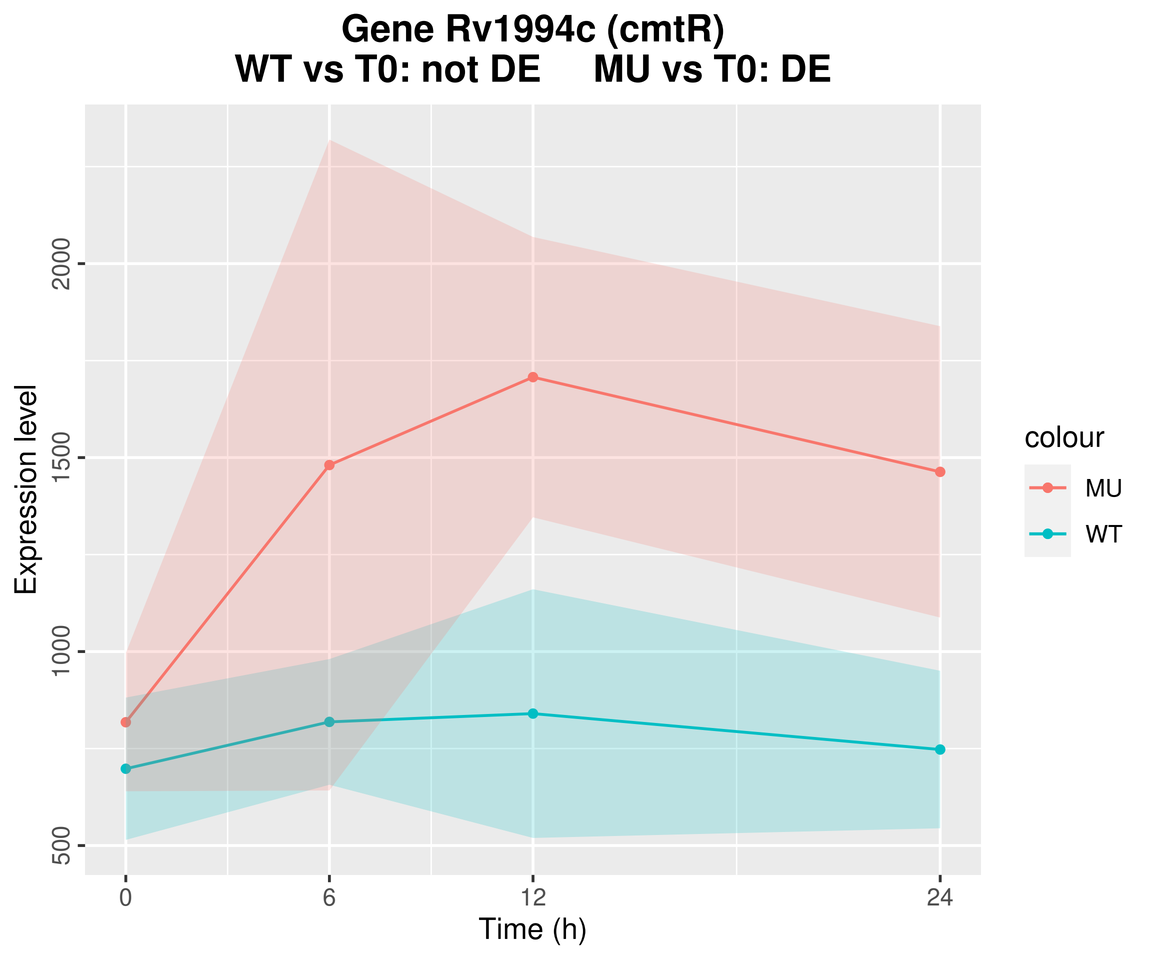
Gene Rv1994c (cmtR)
WT vs T0: not DE   MU vs T0: DE
Expression level
2000
1500
1000
500
0
6
12
24
Time (h)
colour
MU
WT

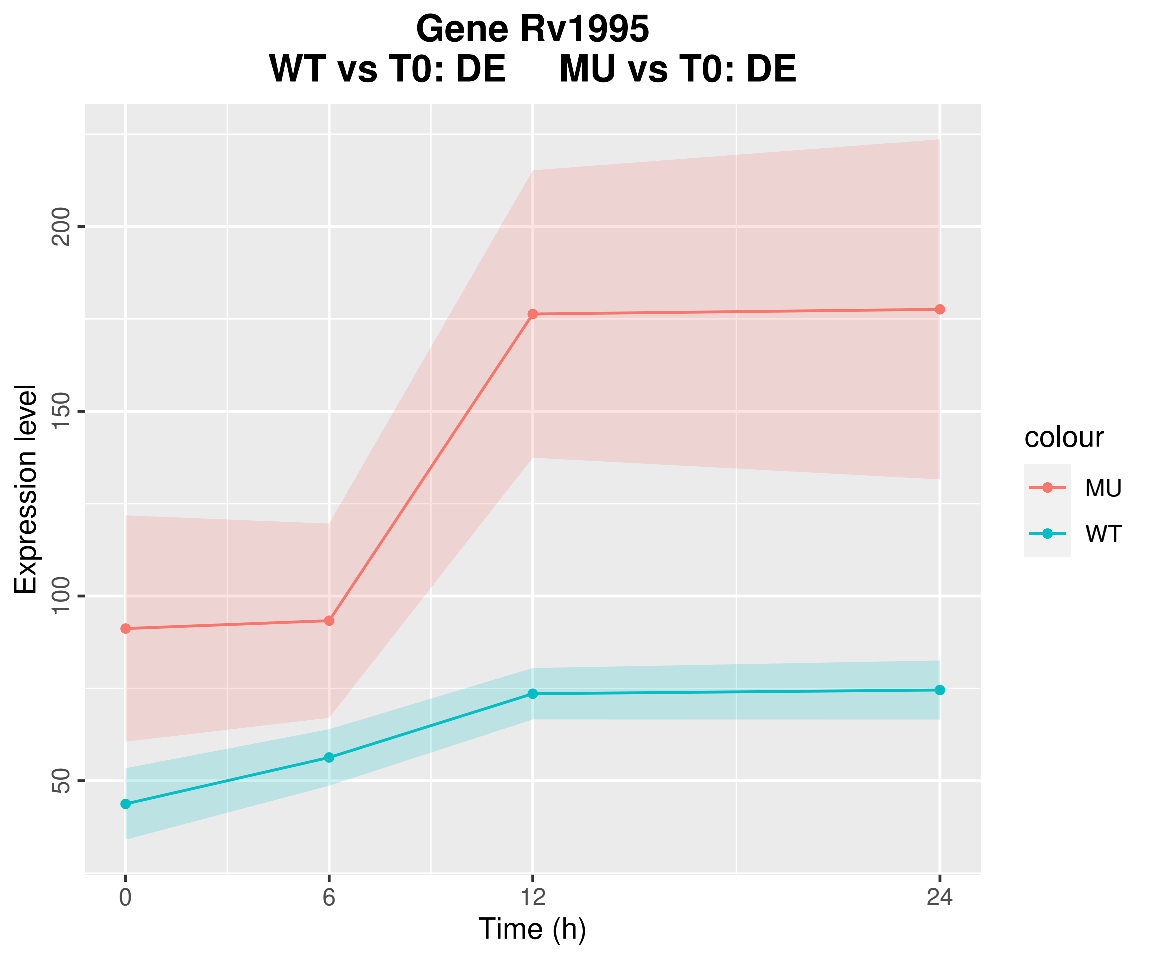

Gene Rv1995
WT vs T0: DE   MU vs T0: DE
Expression level
200
150
100
50
0
6
12
24
Time (h)
colour
MU
WT

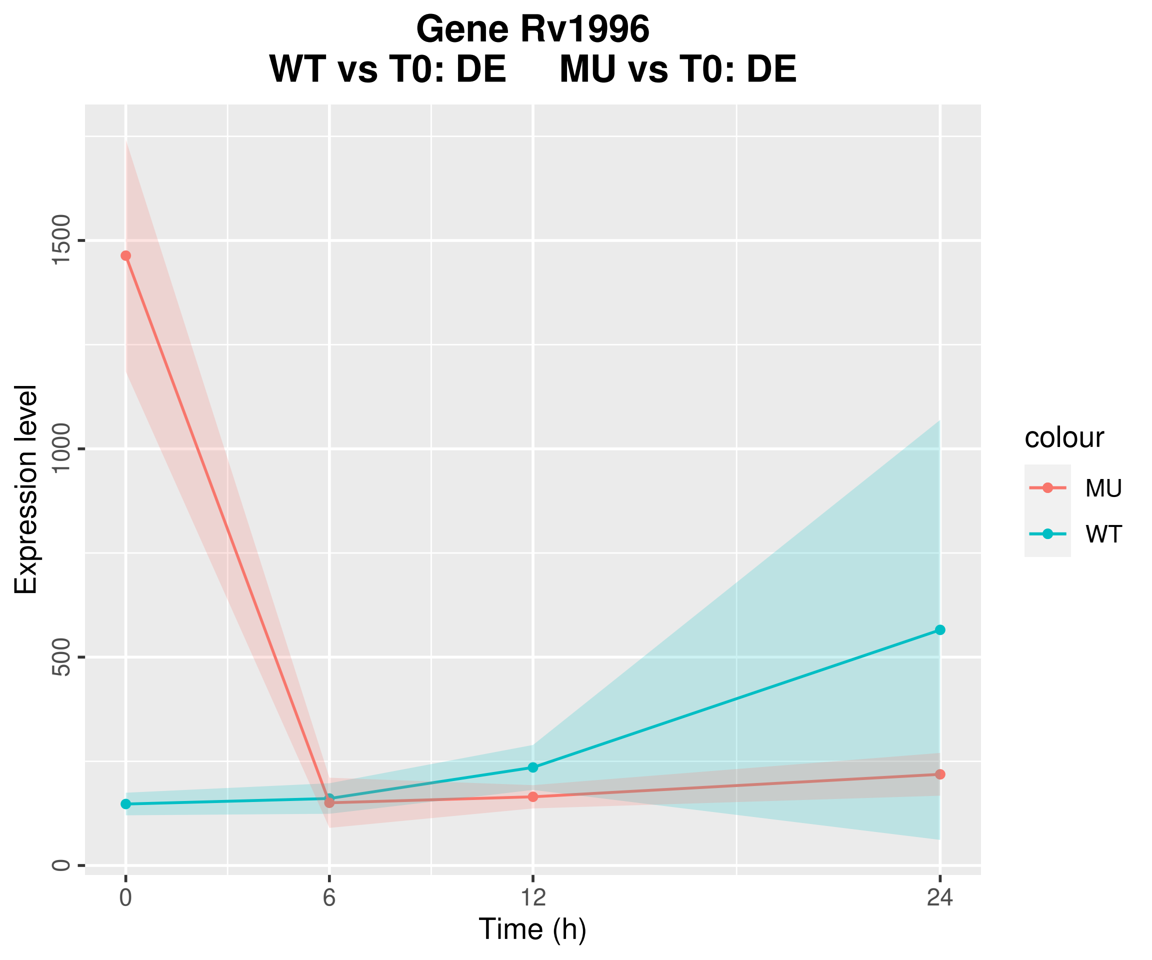

Gene Rv1996
WT vs T0: DE MU vs T0: DE
Expression level
1500
1000
500
0
0
6
12
24
Time (h)
colour
MU
WT

Gene Rv1997 (ctpF)
WT vs T0: DE   MU vs T0: DE

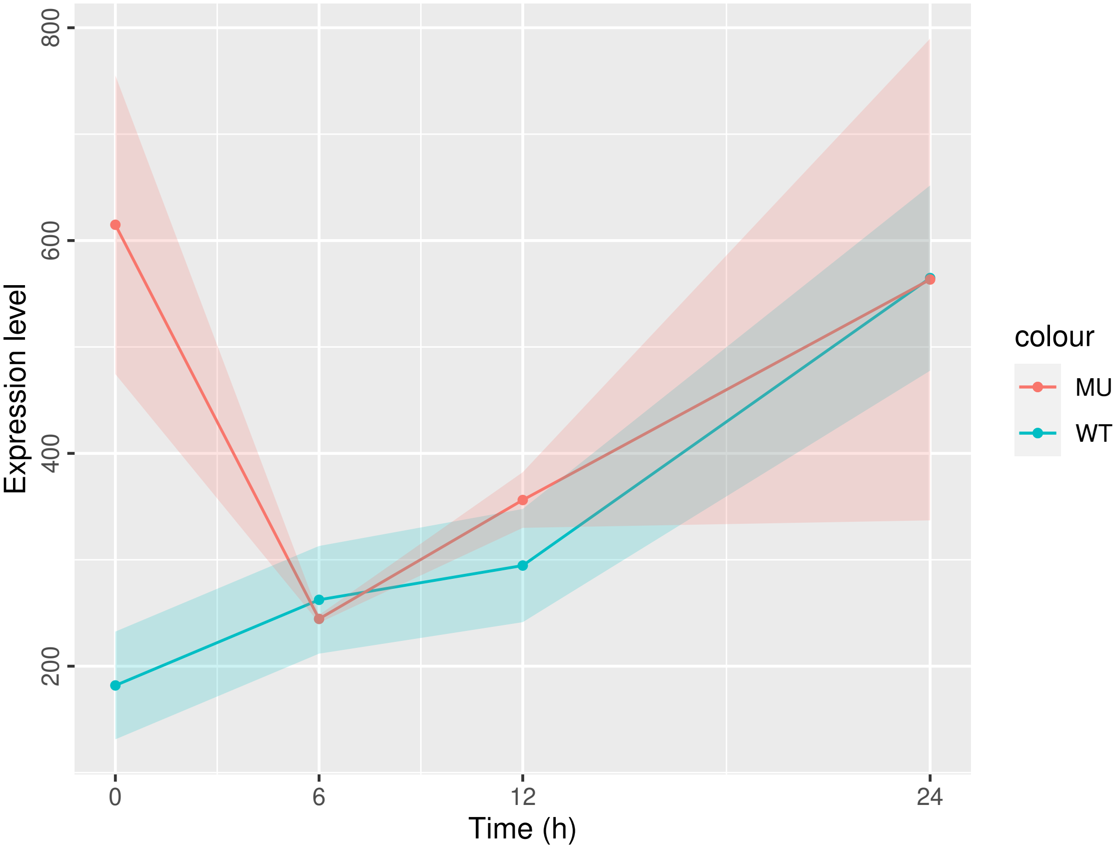

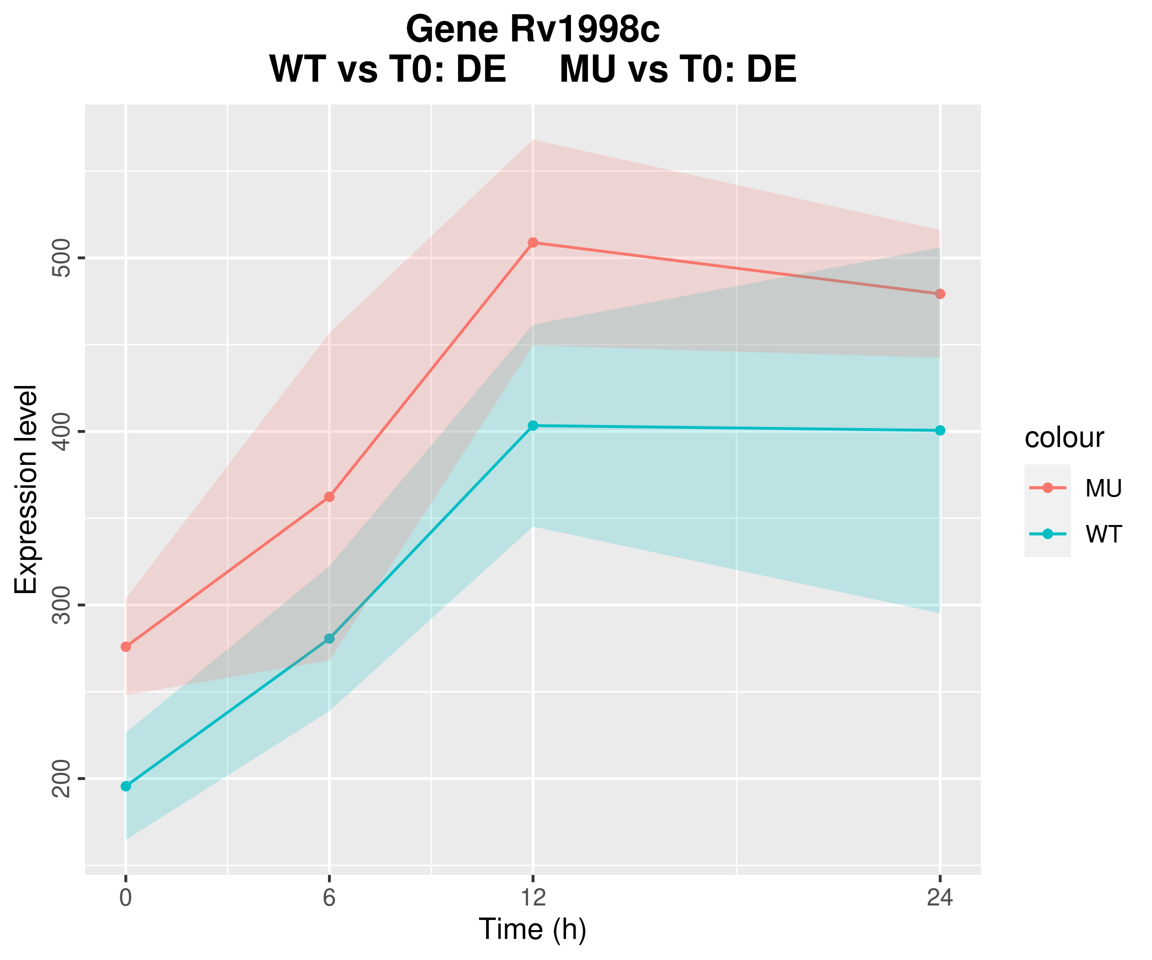

Gene Rv1998c
WT vs T0: DE   MU vs T0: DE
Expression level
200
300
400
500
0
6
12
24
Time (h)
colour
MU
WT

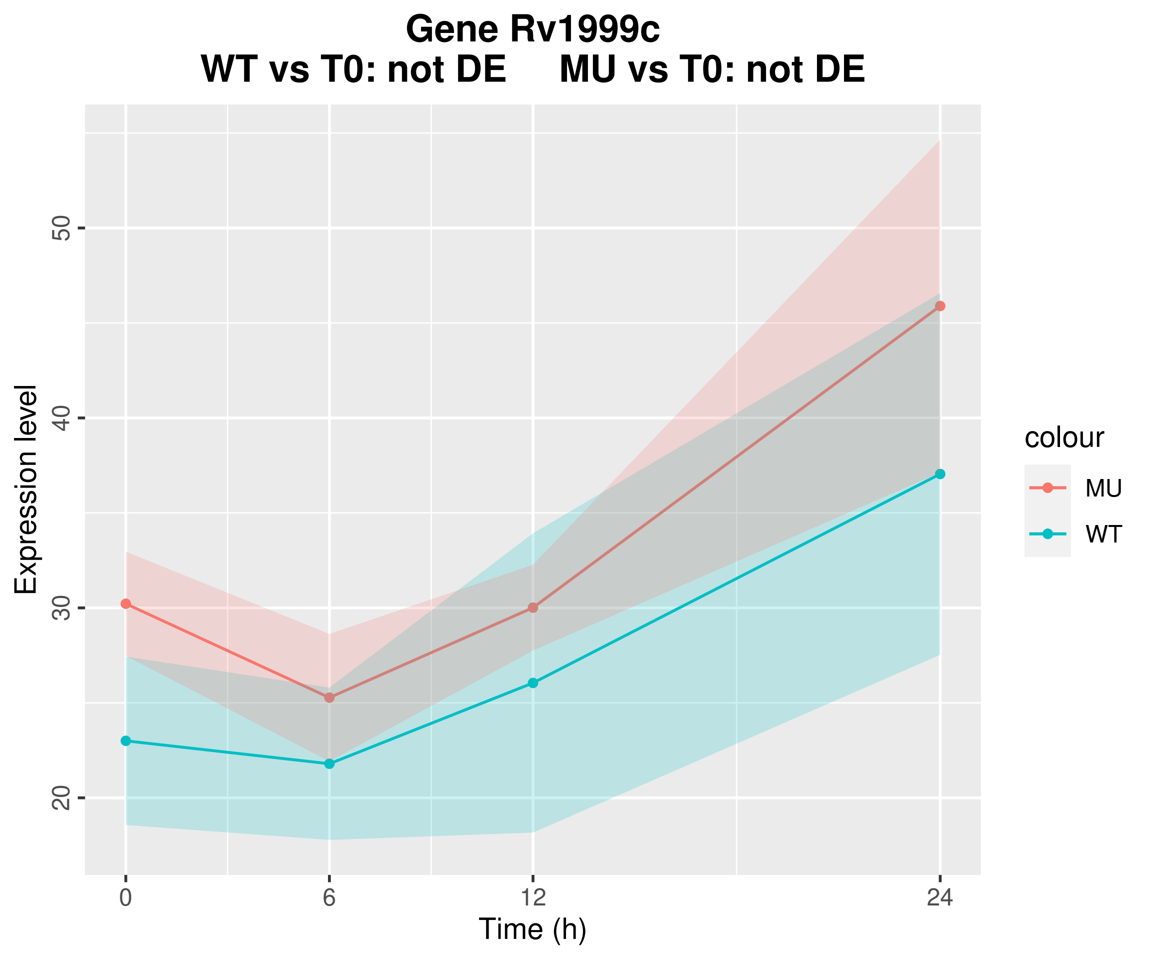

Gene Rv1999c
WT vs T0: not DE MU vs T0: not DE
Expression level
Time (h)
colour
MU
WT
20
30
40
50
0
6
12
24

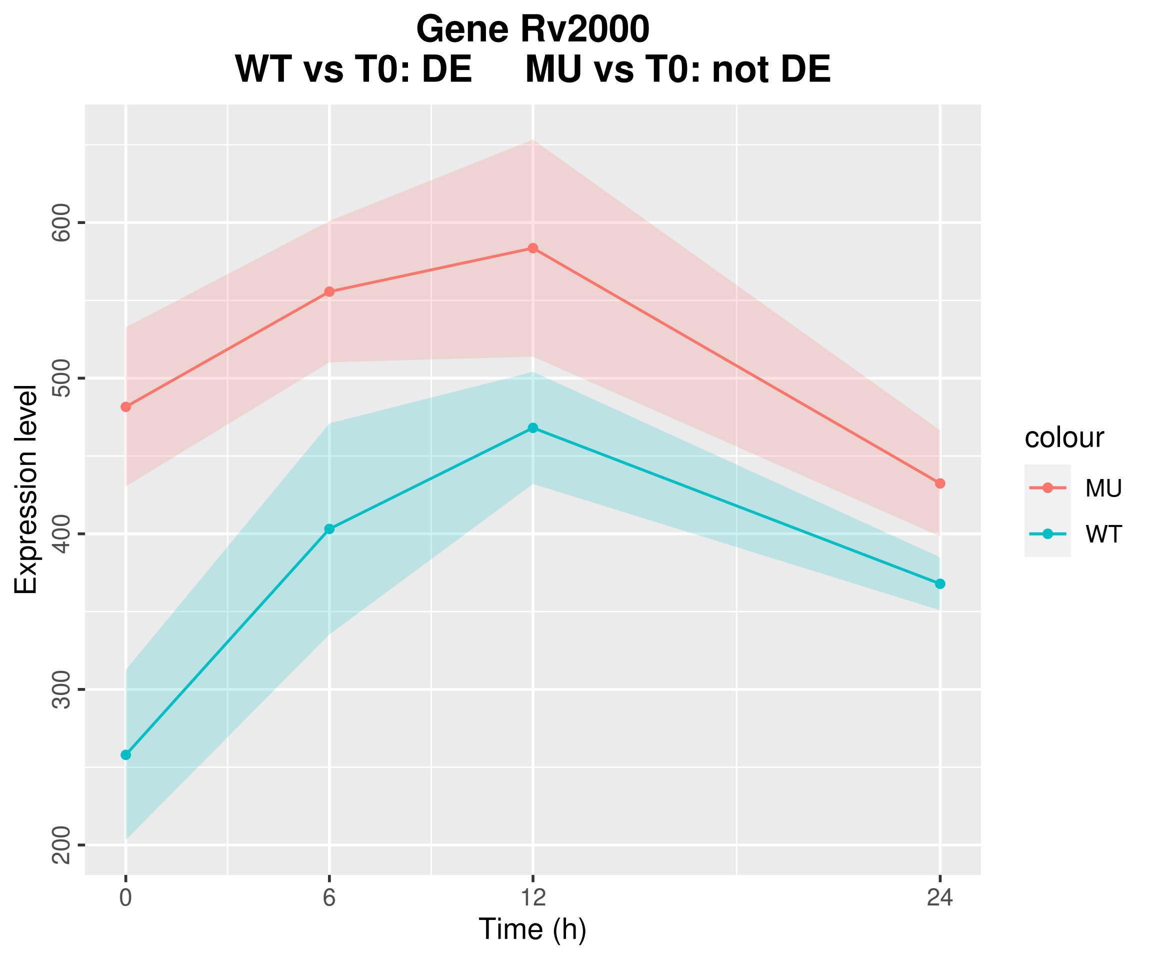

Gene Rv2000
WT vs T0: DE   MU vs T0: not DE
600
500
400
300
200
Expression level
0
6
12
24
Time (h)
colour
MU
WT

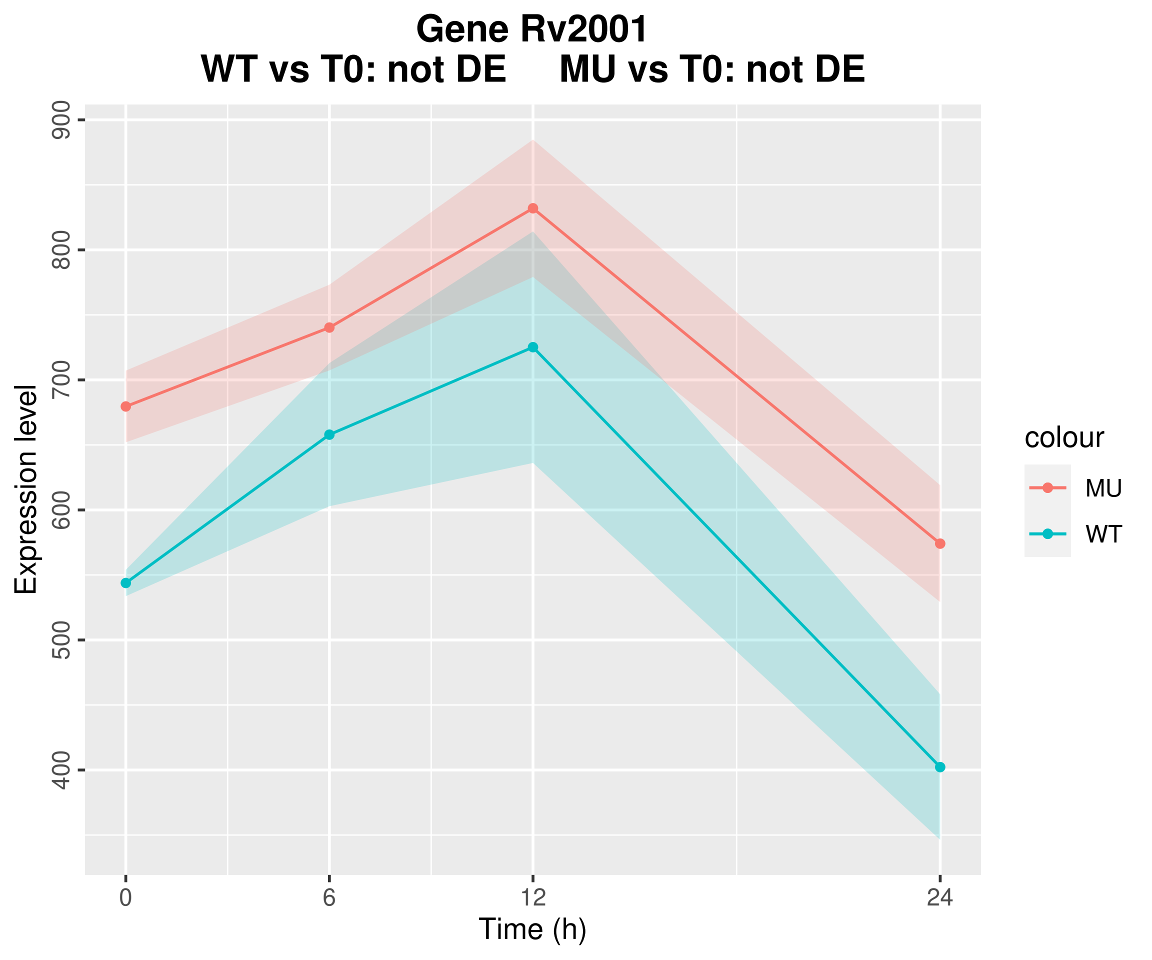

Gene Rv2001
WT vs T0: not DE MU vs T0: not DE
Expression level
900
800
700
600
500
400
0
6
12
24
Time (h)
colour
MU
WT

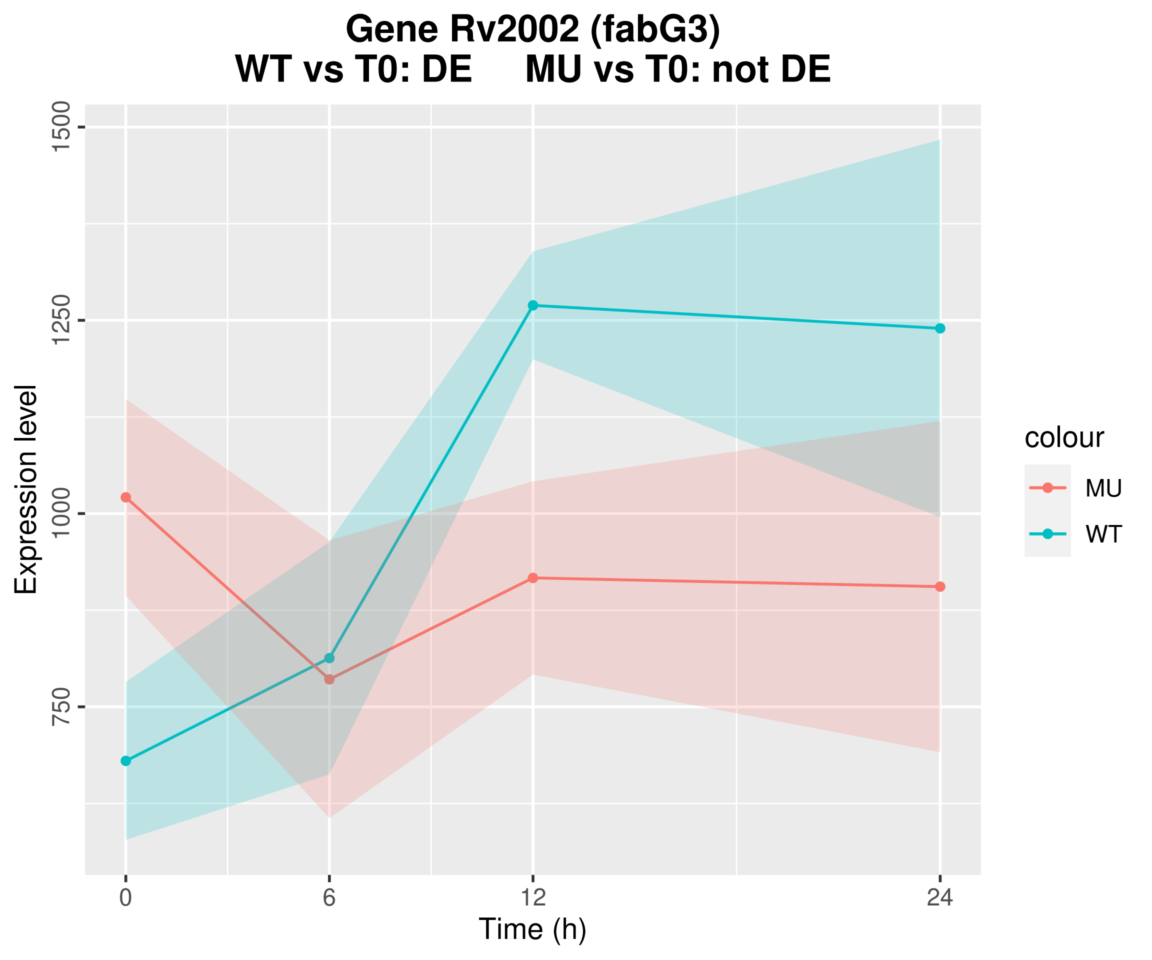
Gene Rv2002 (fabG3)
WT vs T0: DE MU vs T0: not DE
1500
1250
1000
750
Expression level
0
6
12
24
Time (h)
colour
MU
WT

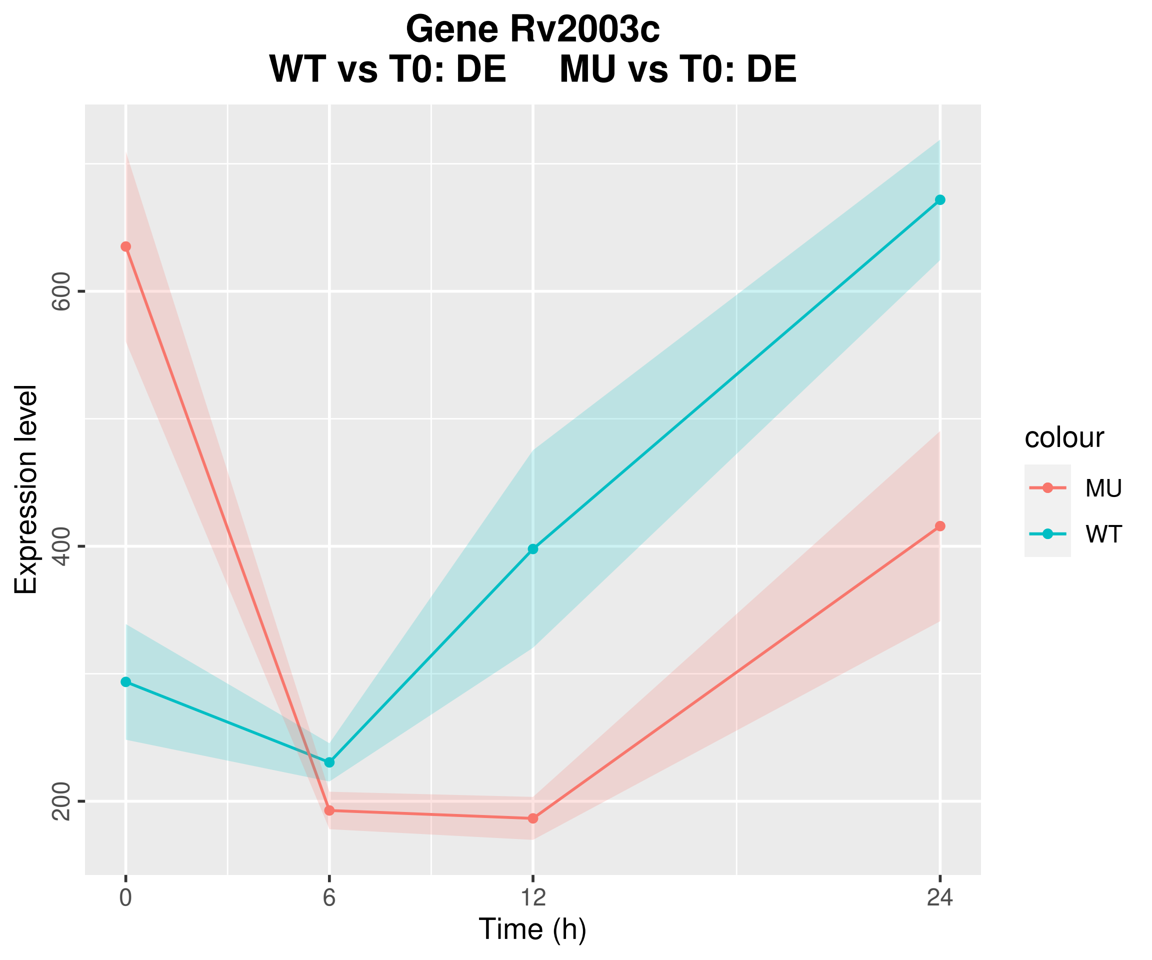

Gene Rv2003c
WT vs T0: DE MU vs T0: DE
Expression level
600
400
200
0
6
12
24
Time (h)
colour
MU
WT

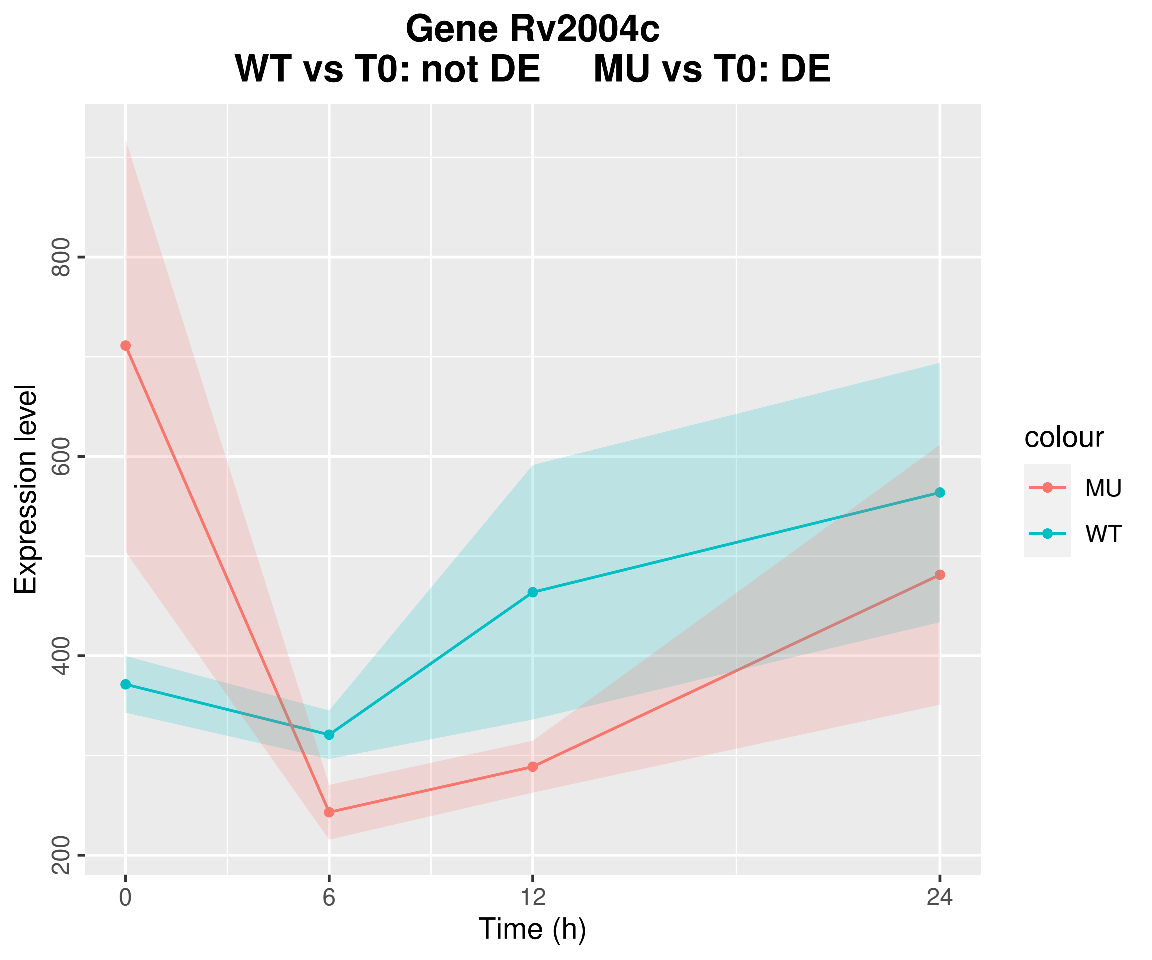
Gene Rv2004c
WT vs T0: not DE   MU vs T0: DE
Expression level
800
600
400
200
0
6
12
24
Time (h)
colour
MU
WT

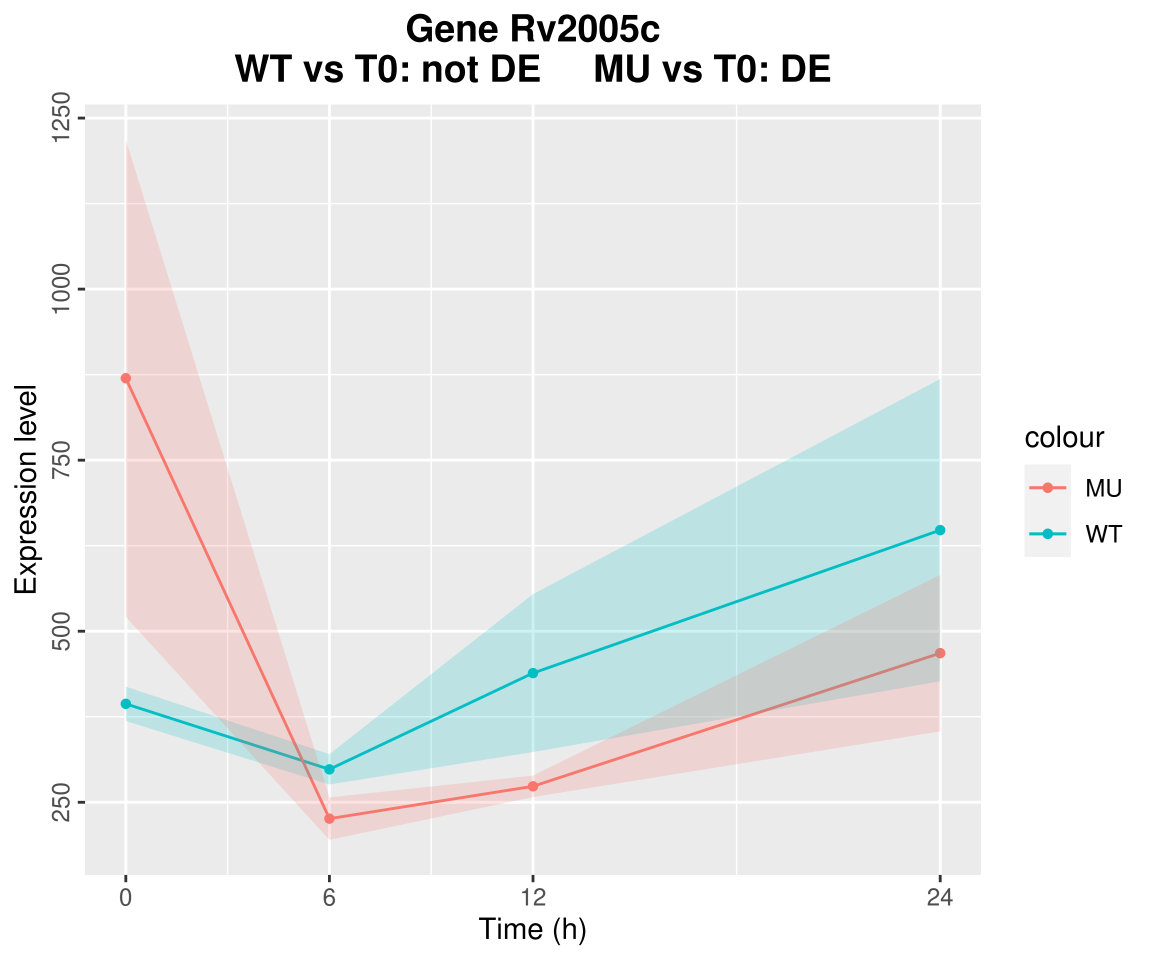
Gene Rv2005c
WT vs T0: not DE   MU vs T0: DE
Expression level
1250
1000
750
500
250
0
6
12
24
Time (h)
colour
MU
WT

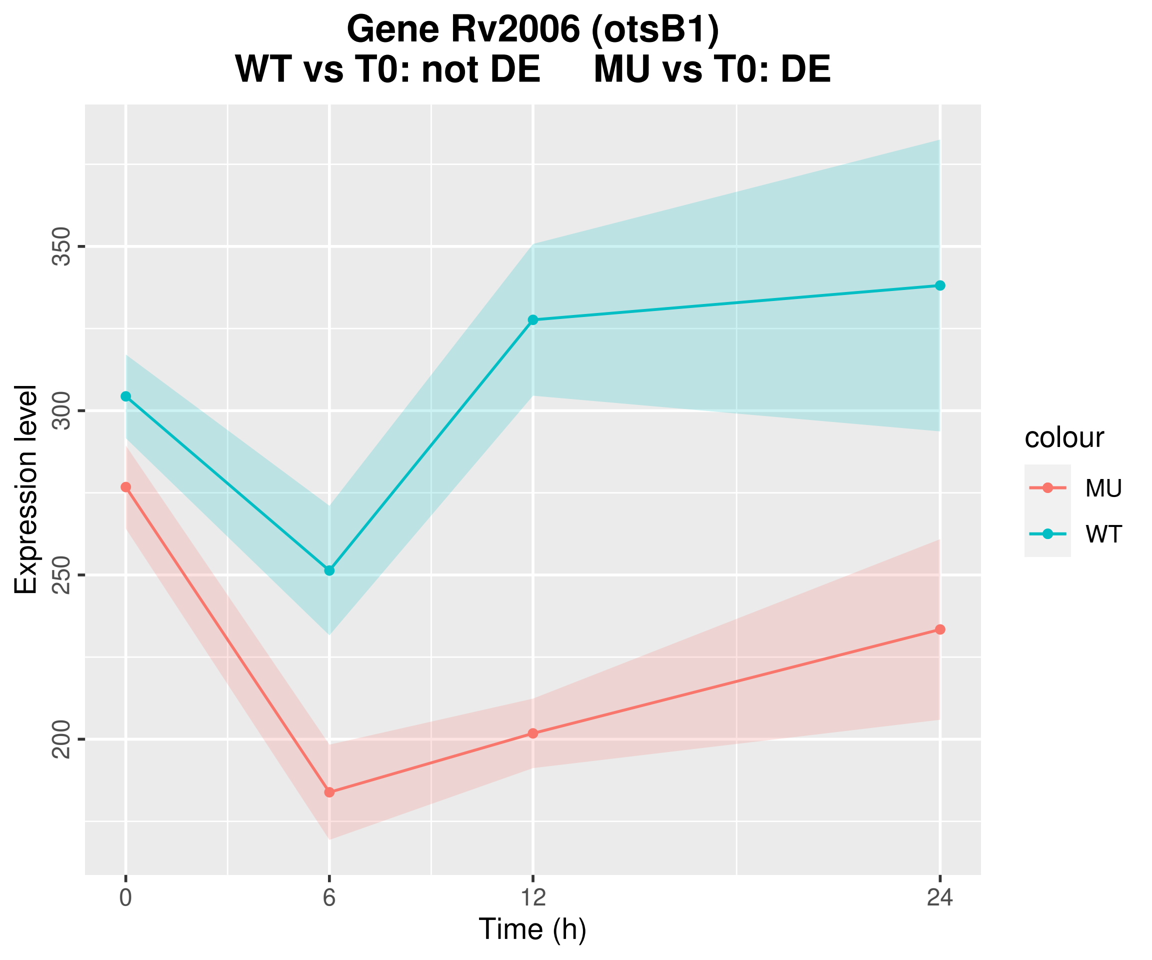

Gene Rv2006 (otsB1)
WT vs T0: not DE MU vs T0: DE
Expression level
350
300
250
200
0
6
12
24
Time (h)
colour
MU
WT

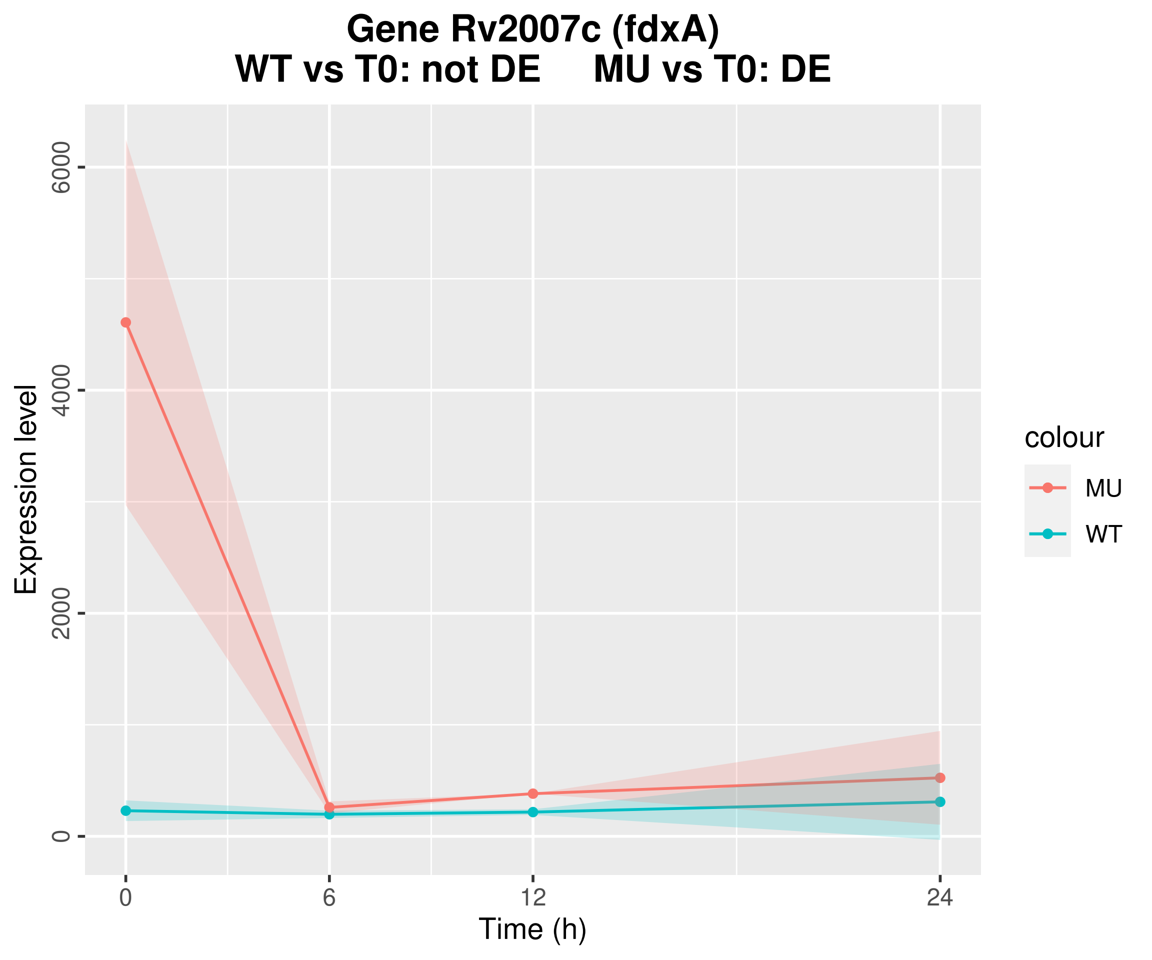
Gene Rv2007c (fdxA)
WT vs T0: not DE MU vs T0: DE
Expression level
6000
4000
2000
0
0
6
12
24
Time (h)
colour
MU
WT

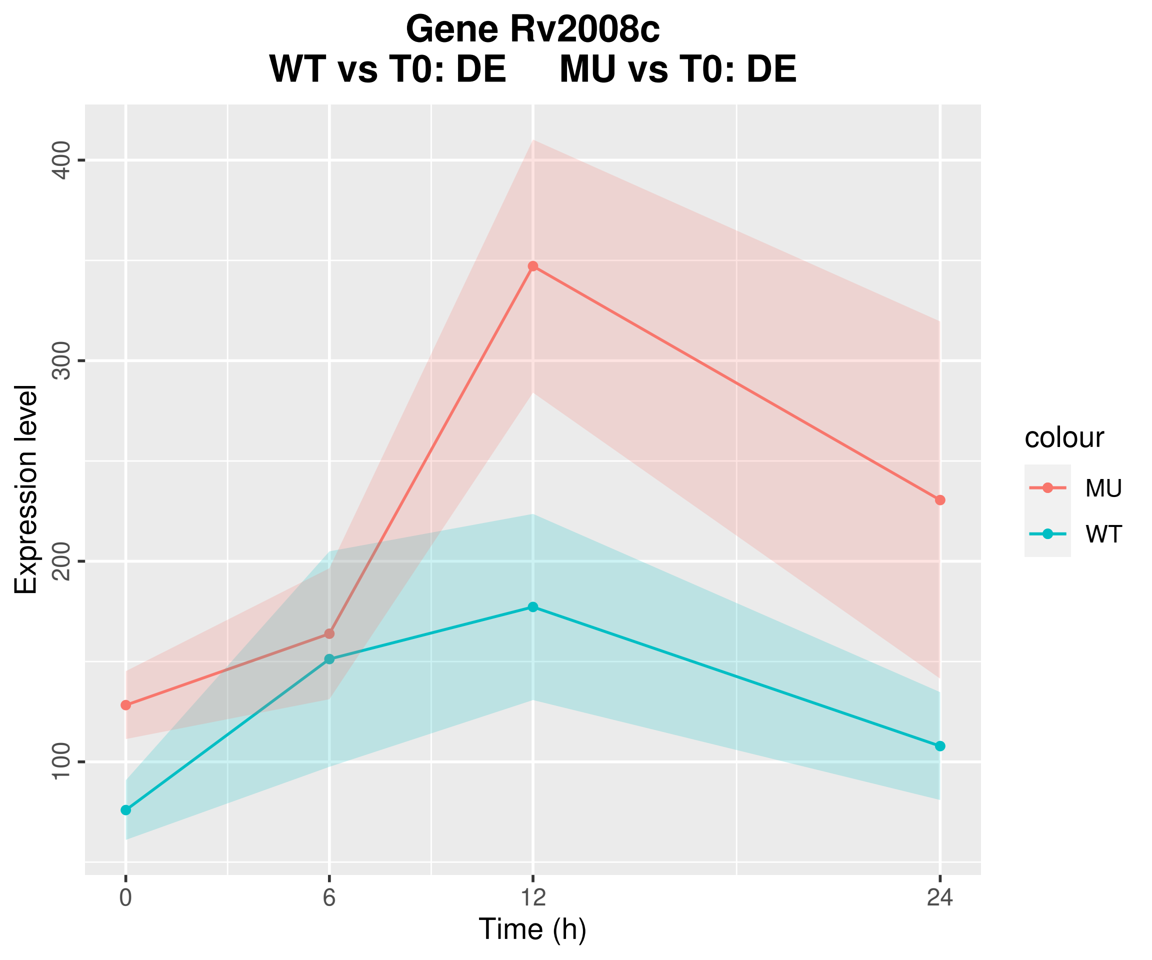

Gene Rv2008c
WT vs T0: DE MU vs T0: DE
Expression level
400
300
200
100
0
6
12
24
Time (h)
colour
MU
WT

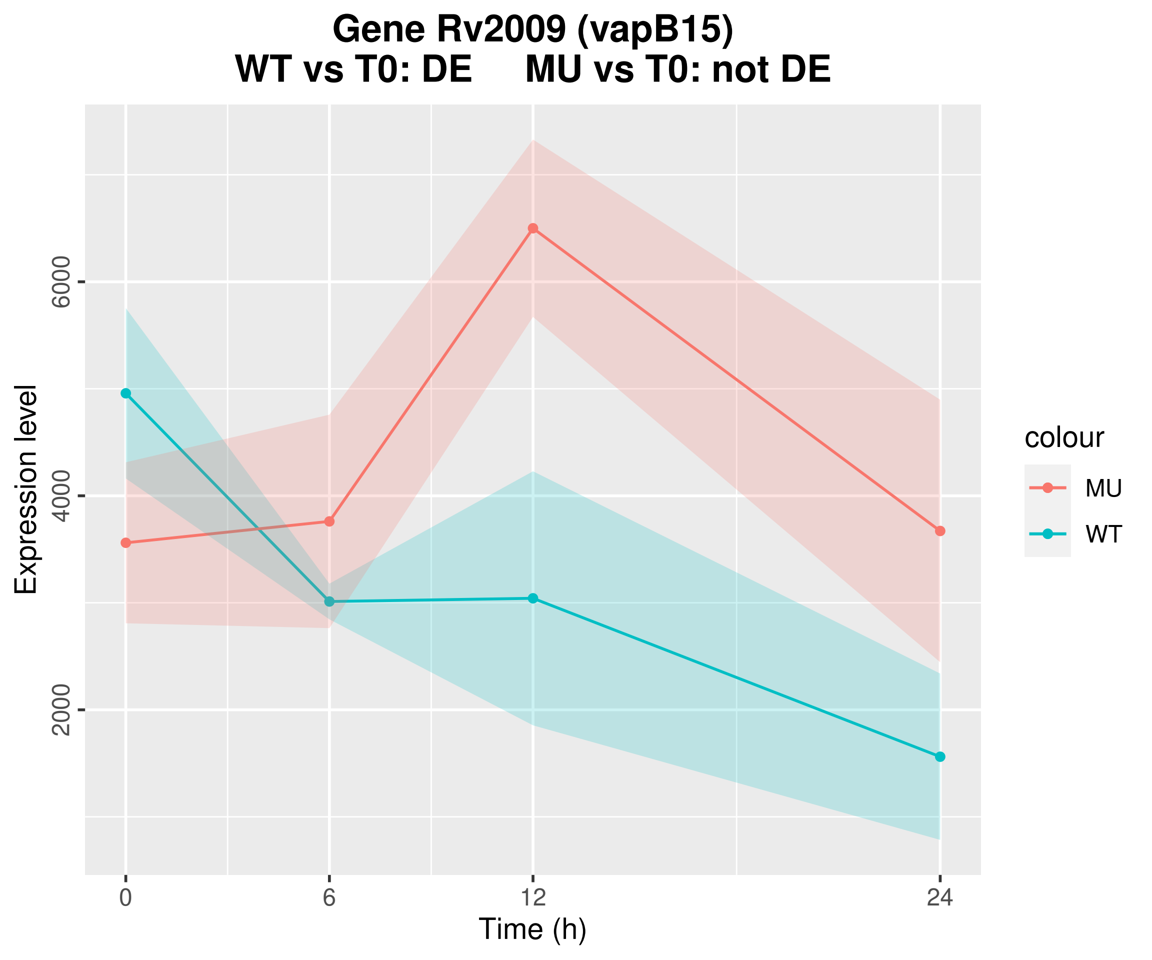

Gene Rv2009 (vapB15)
WT vs T0: DE   MU vs T0: not DE
Expression level
6000
4000
2000
0
6
12
24
Time (h)
colour
MU
WT

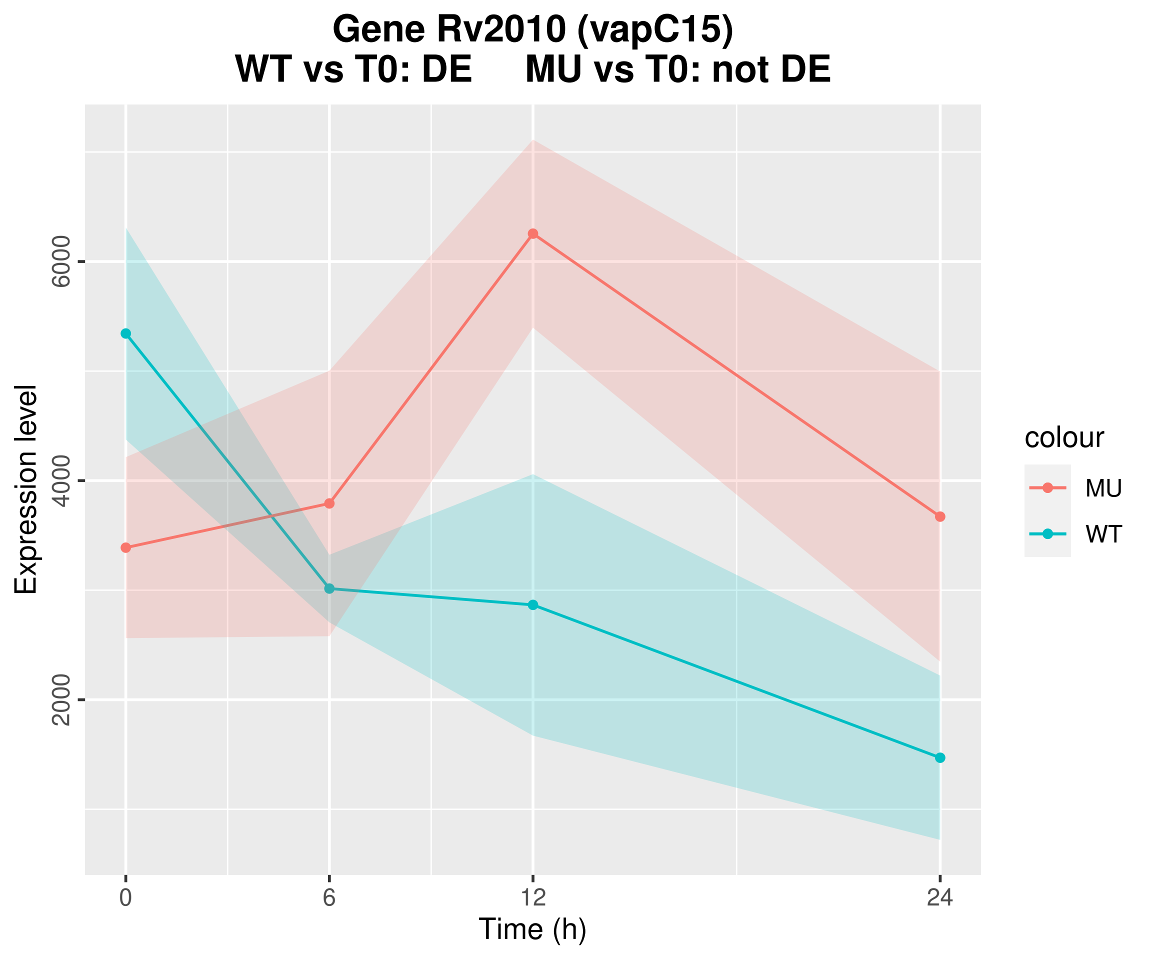
Gene Rv2010 (vapC15)
WT vs T0: DE   MU vs T0: not DE
Expression level
6000
4000
2000
0
6
12
24
Time (h)
colour
MU
WT

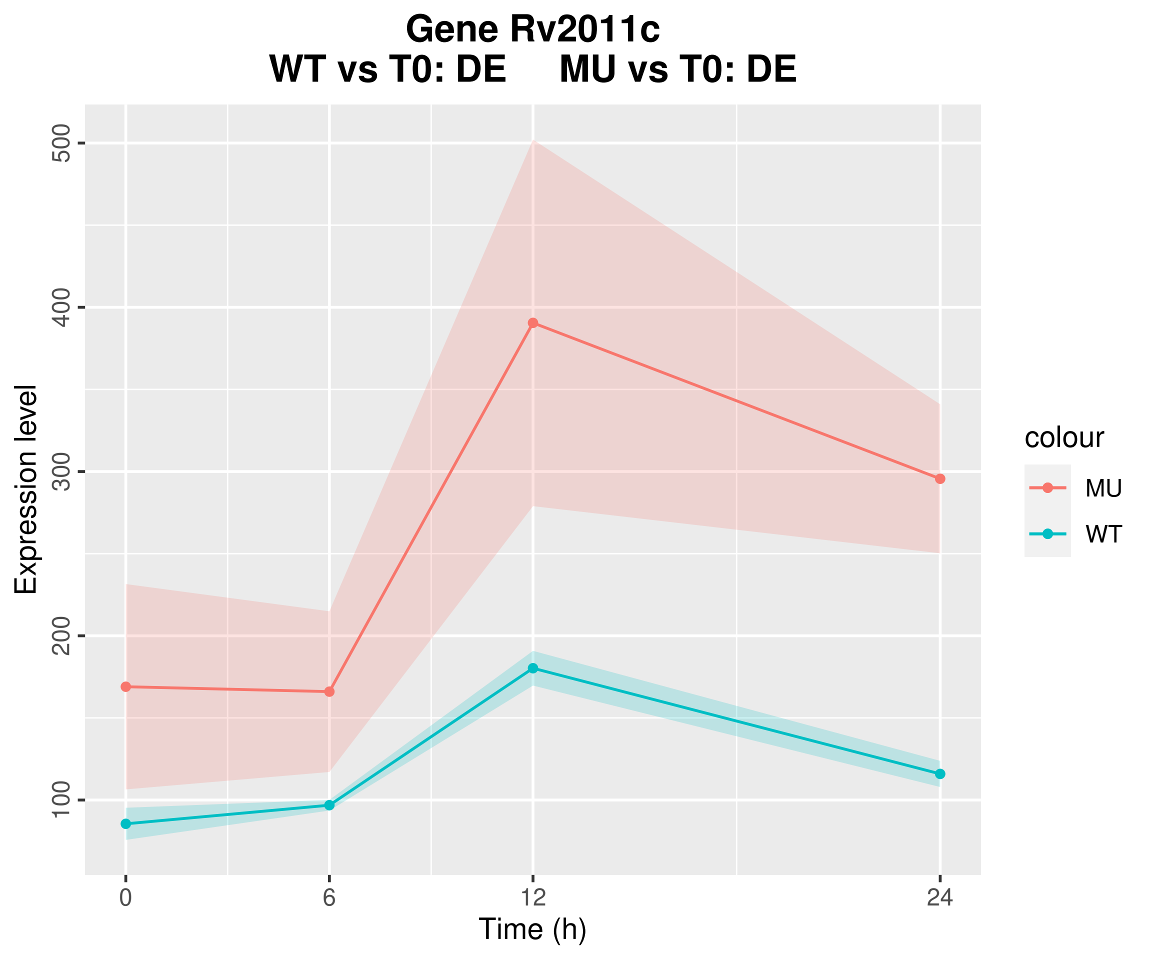
Gene Rv2011c
WT vs T0: DE MU vs T0: DE
Expression level
100
200
300
400
500
0
6
12
24
Time (h)
colour
MU
WT

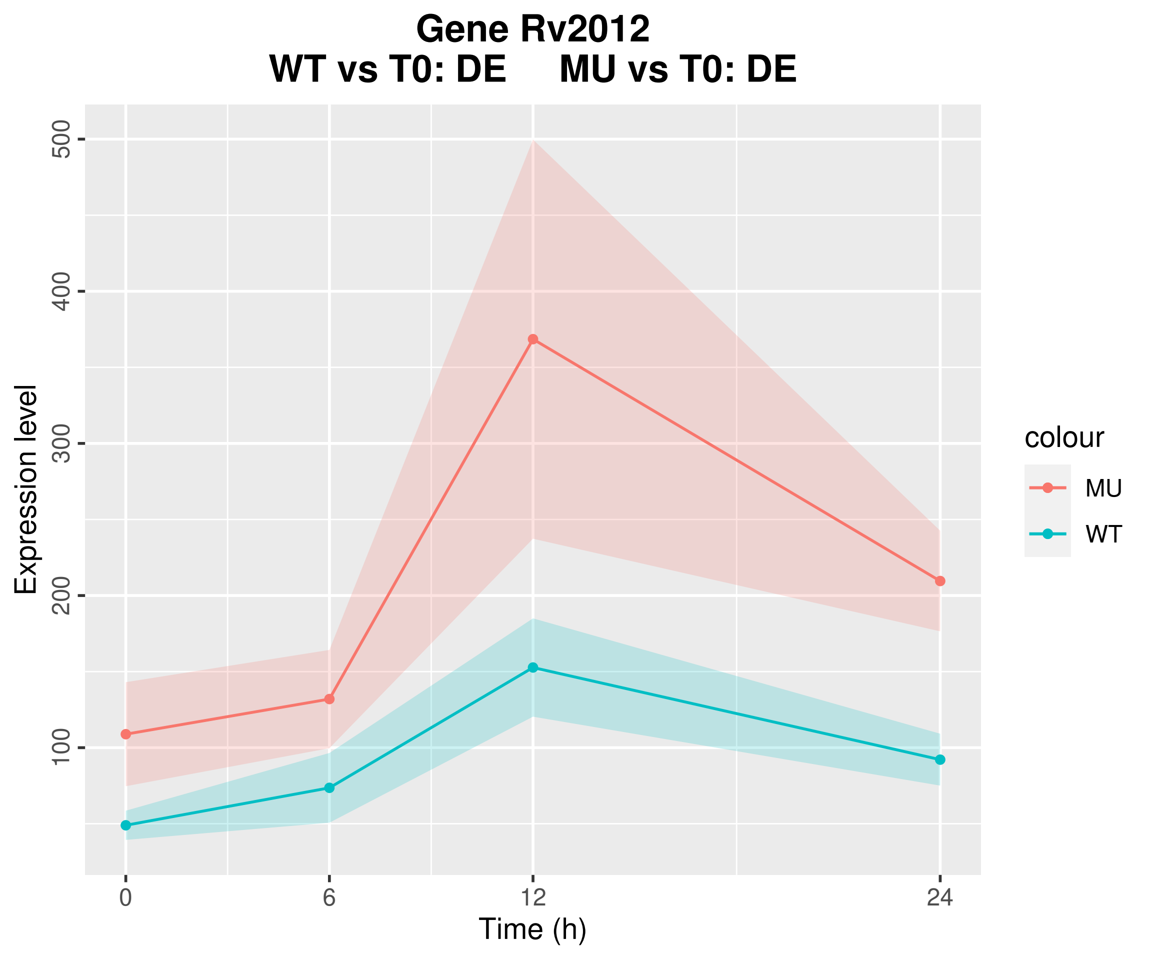
Gene Rv2012
WT vs T0: DE MU vs T0: DE
Expression level
500
400
300
200
100
0
6
12
24
Time (h)
colour
MU
WT

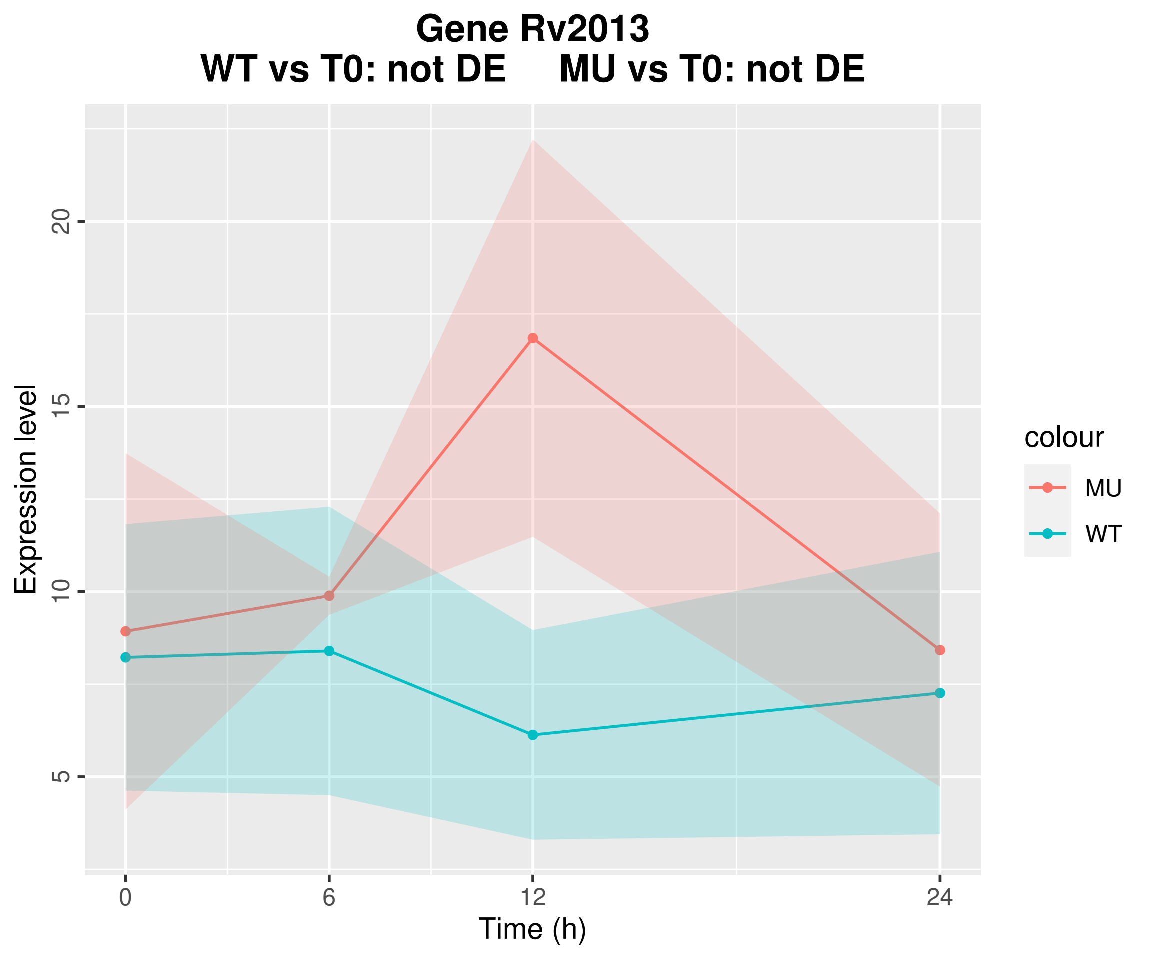
Gene Rv2013
WT vs T0: not DE   MU vs T0: not DE
Expression level
20
15
10
5
0
6
12
24
Time (h)
colour
MU
WT

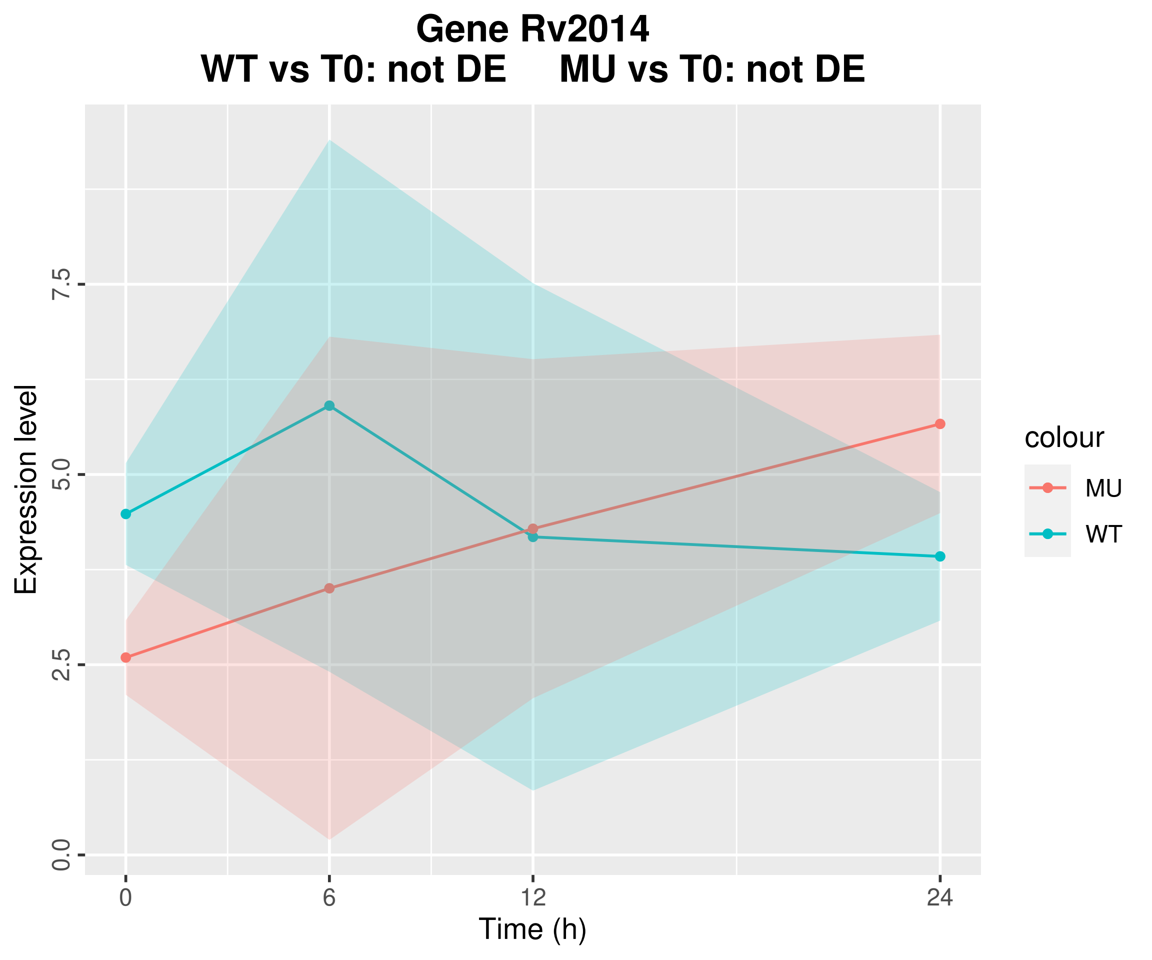
Gene Rv2014
WT vs T0: not DE   MU vs T0: not DE
Expression level
0.0
2.5
5.0
7.5
0
6
12
24
Time (h)
colour
MU
WT

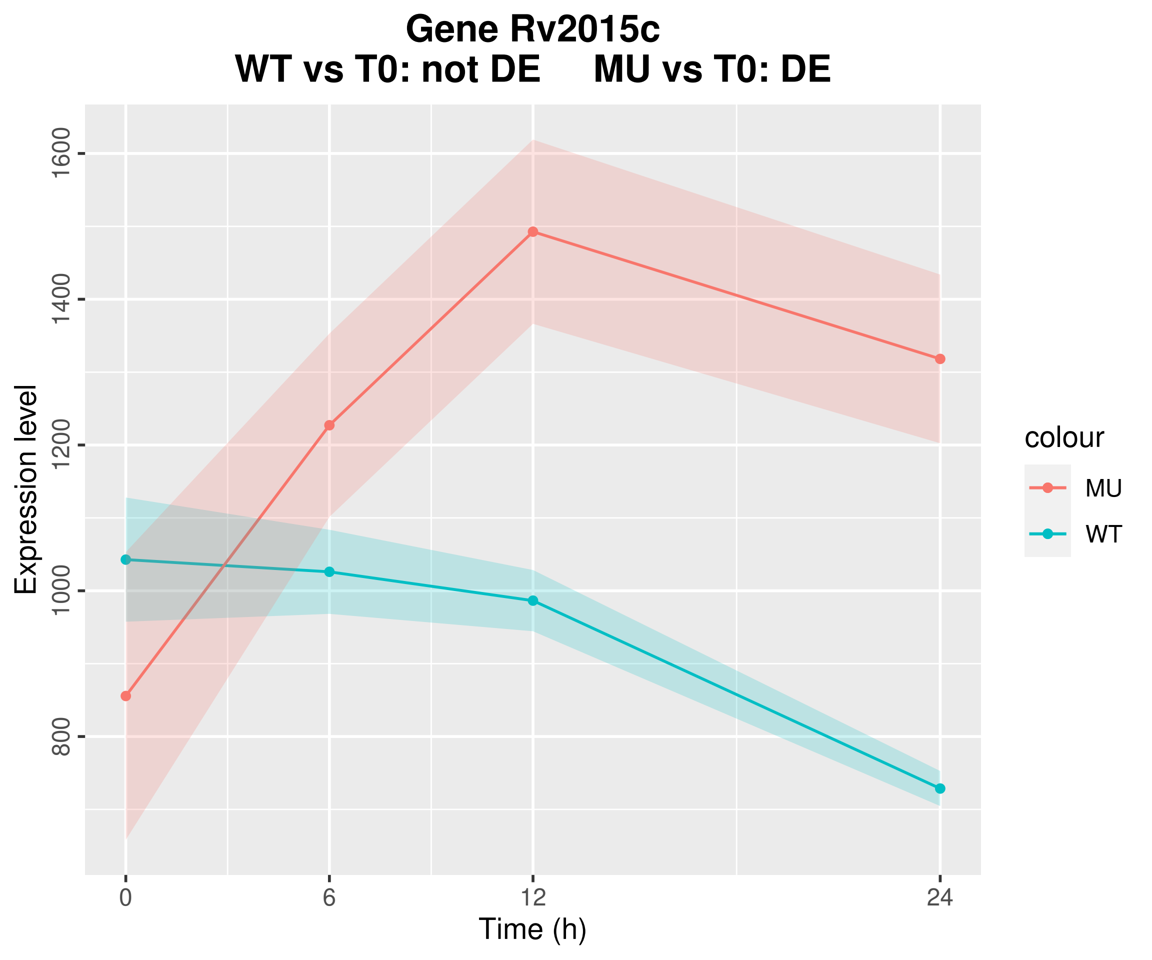

Gene Rv2015c
WT vs T0: not DE   MU vs T0: DE
Expression level
1600
1400
1200
1000
800
0
6
12
24
Time (h)
colour
MU
WT

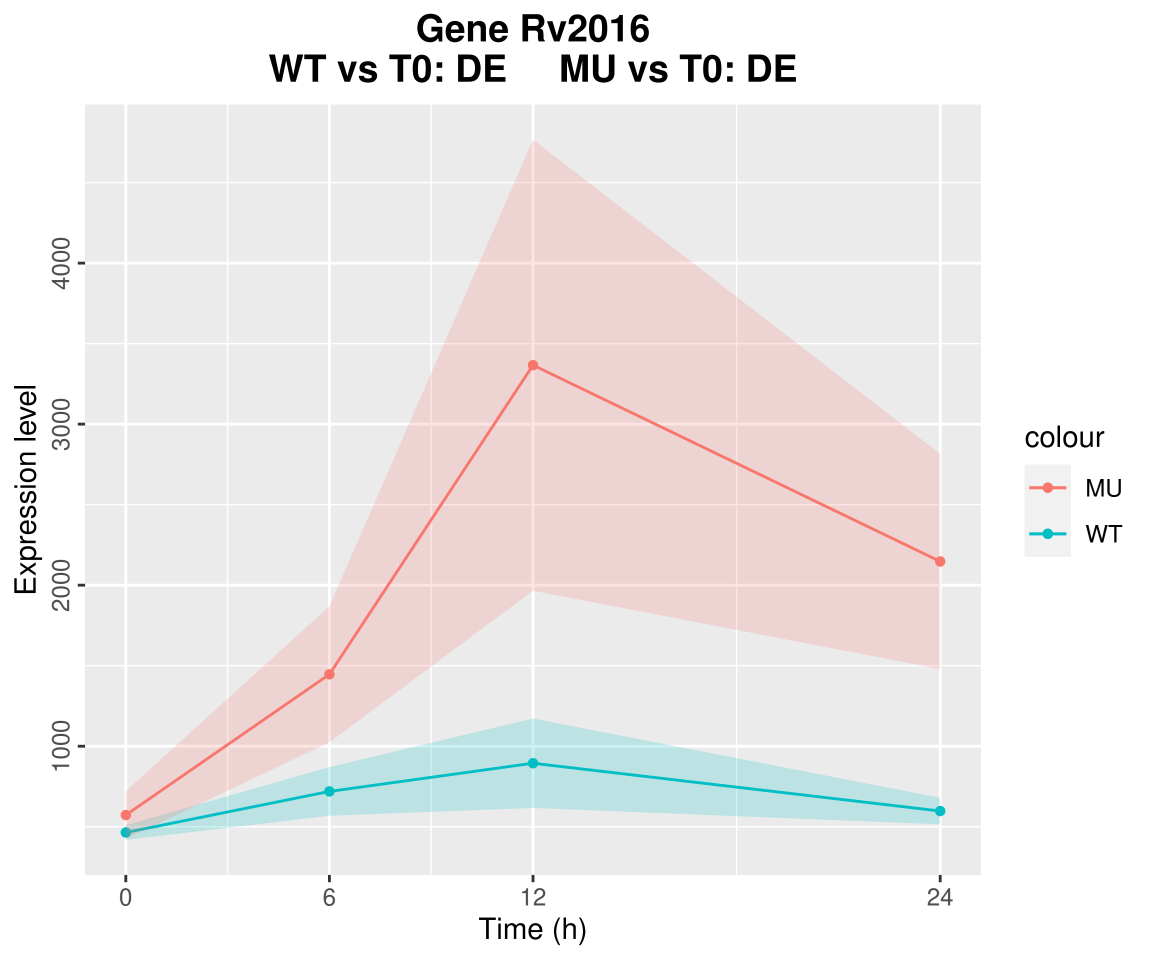
Gene Rv2016
WT vs T0: DE MU vs T0: DE
Expression level
4000
3000
2000
1000
0
6
12
24
Time (h)
colour
MU
WT

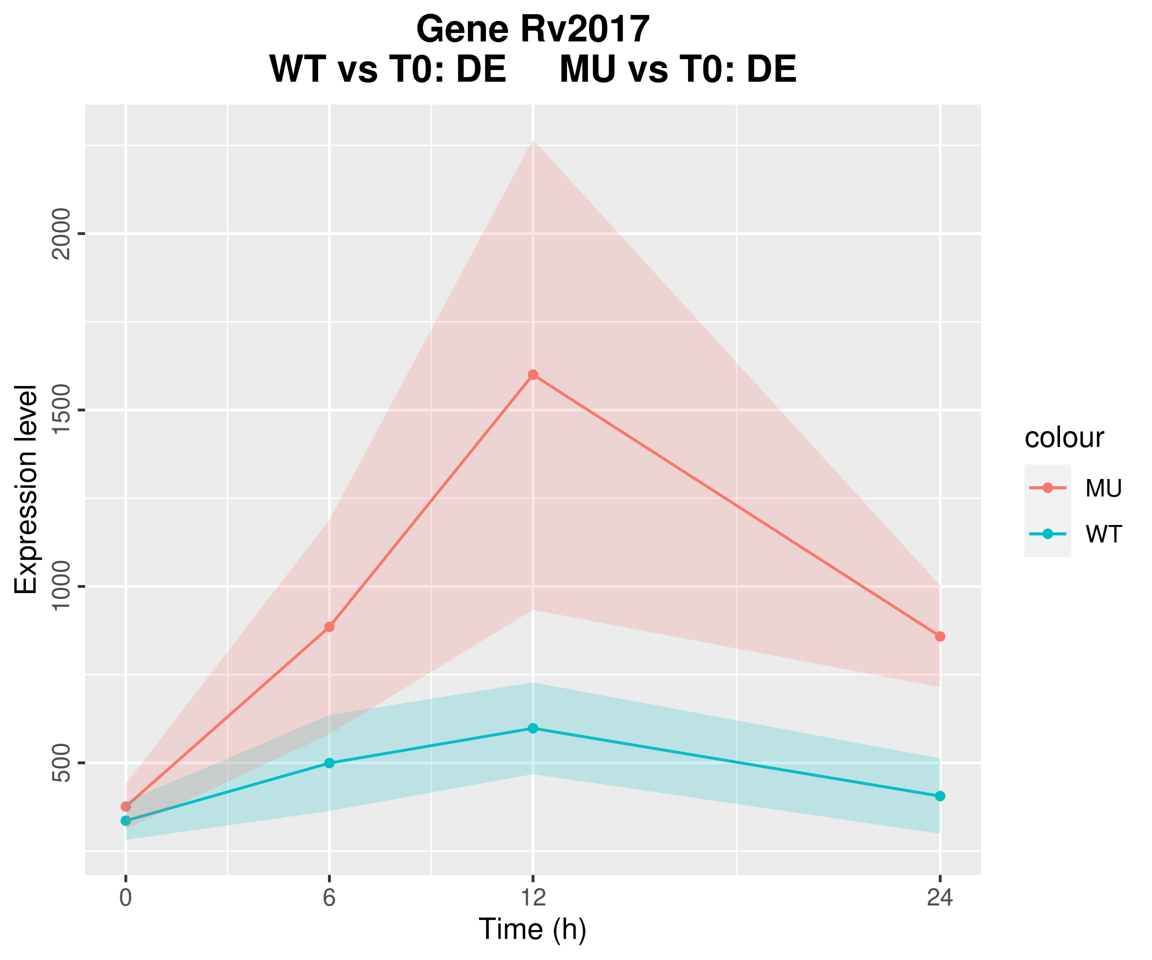
Gene Rv2017
WT vs T0: DE MU vs T0: DE
Expression level
2000
1500
1000
500
0
6
12
24
Time (h)
colour
MU
WT

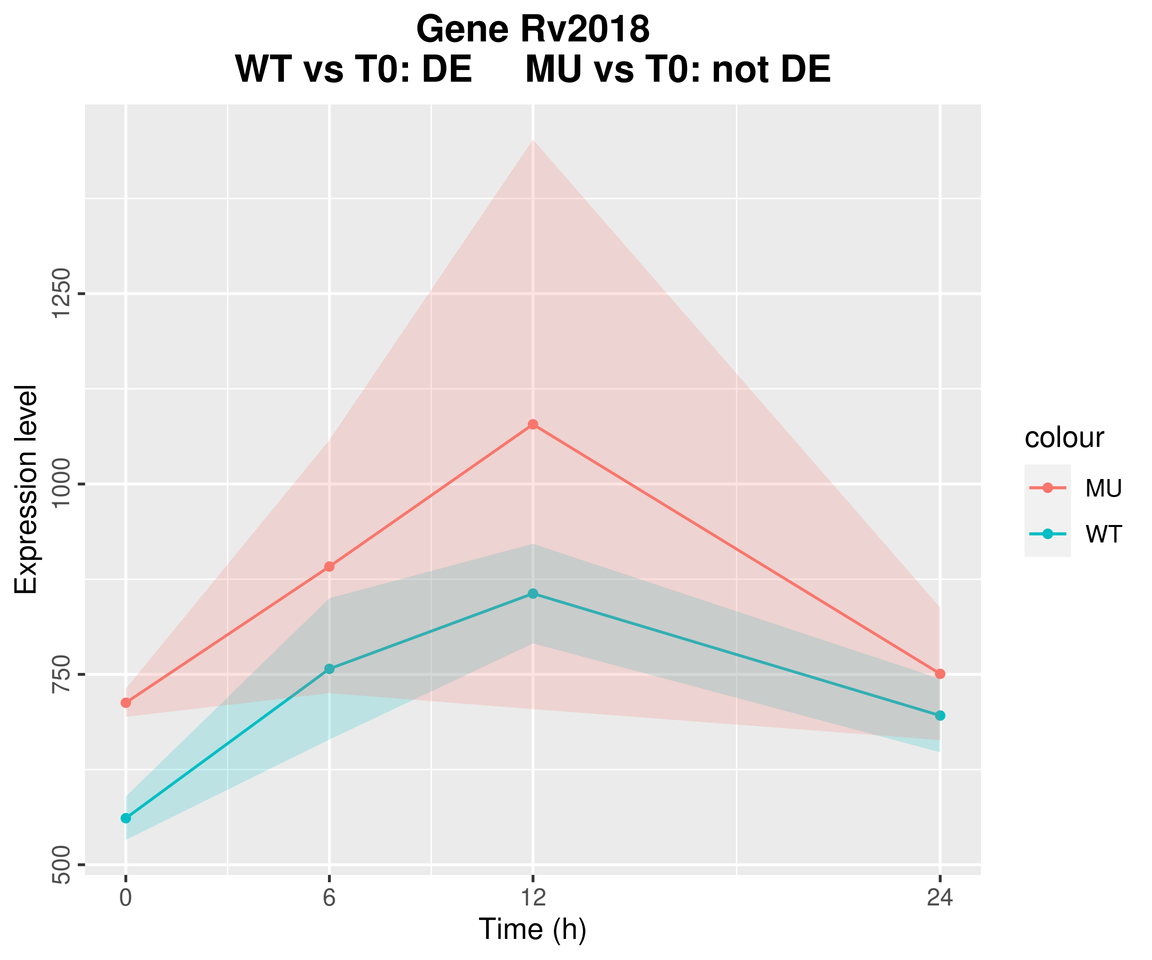

Gene Rv2018
WT vs T0: DE   MU vs T0: not DE
Expression level
1250
1000
750
500
0
6
12
24
Time (h)
colour
MU
WT

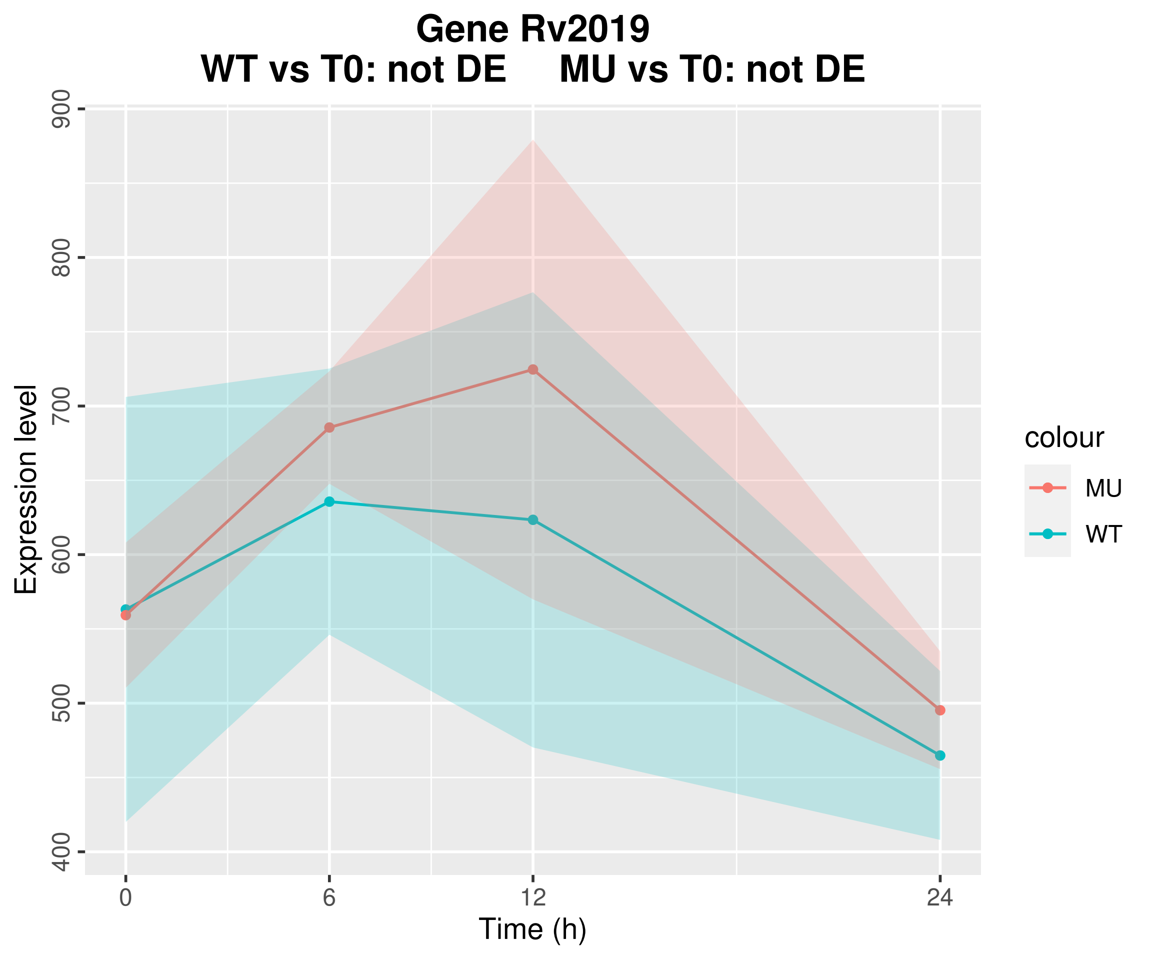
Gene Rv2019
WT vs T0: not DE   MU vs T0: not DE
Expression level
900
800
700
600
500
400
0
6
12
24
Time (h)
colour
MU
WT

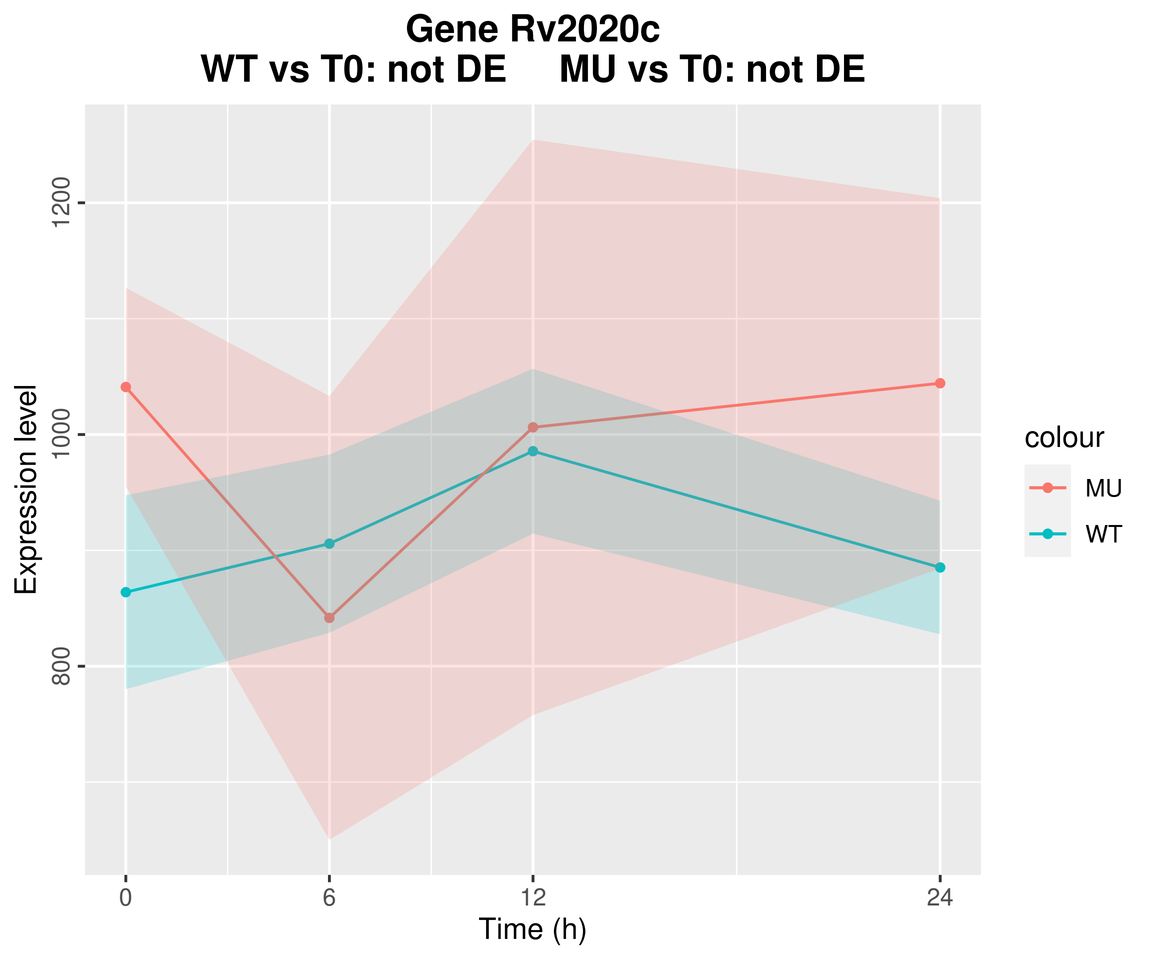
Gene Rv2020c
WT vs T0: not DE MU vs T0: not DE
Expression level
1200
1000
800
0
6
12
24
Time (h)
colour
MU
WT

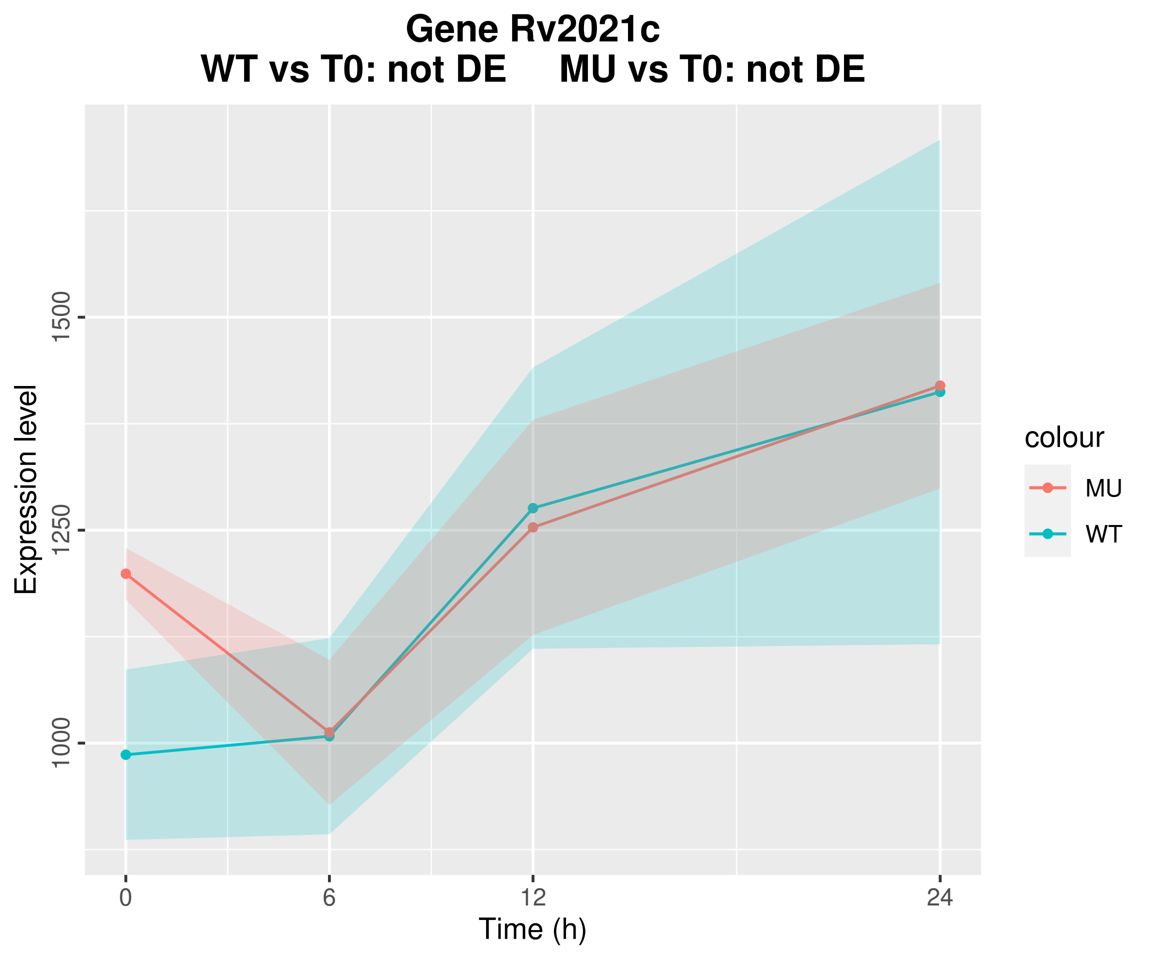
Gene Rv2021c
WT vs T0: not DE   MU vs T0: not DE
Expression level
1000
1250
1500
Time (h)
0
6
12
24
colour
MU
WT

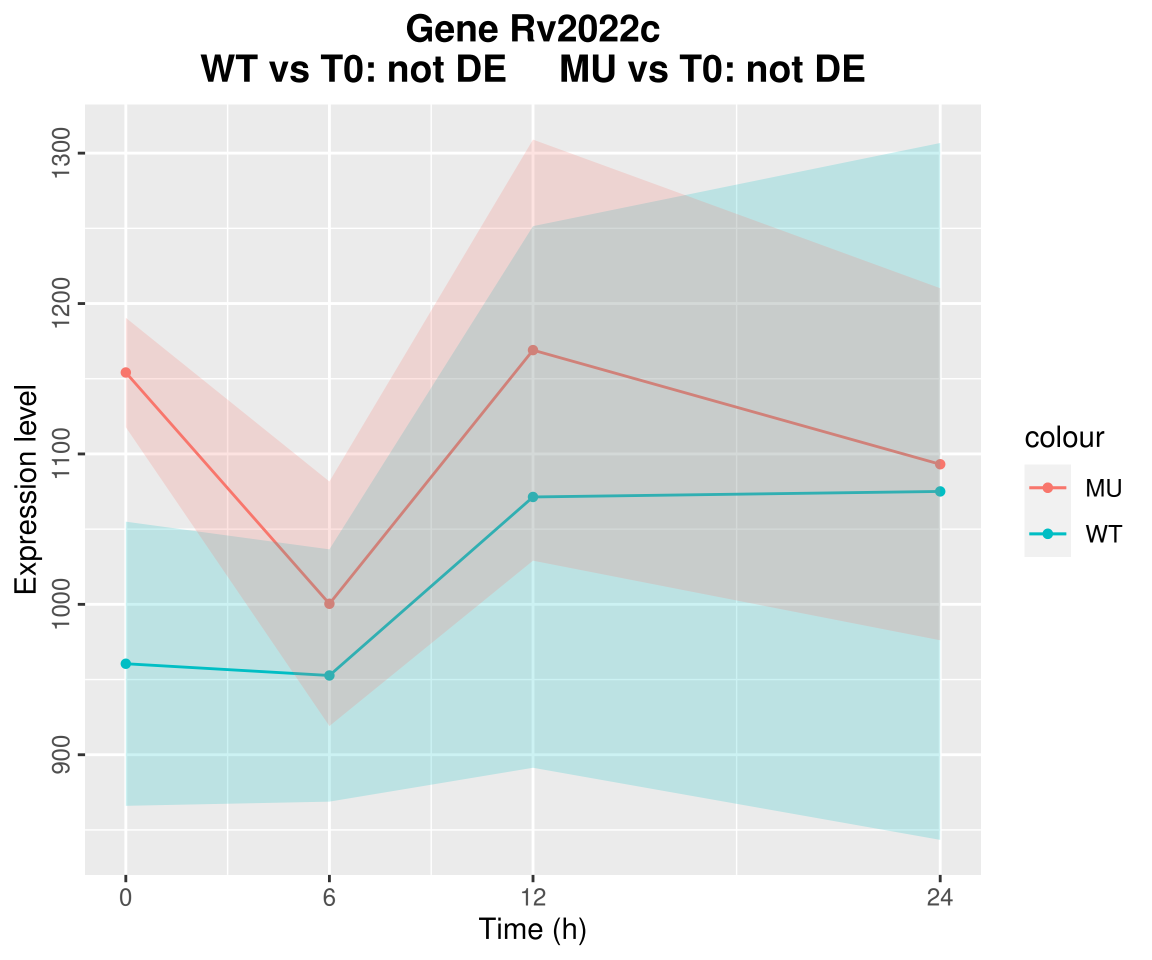

Gene Rv2022c
WT vs T0: not DE MU vs T0: not DE
1300
1200
1100
1000
900
Expression level
0
6
12
24
Time (h)
colour
MU
WT

Gene Rv2023A (biotype=pseudogene)
WT vs T0: DE   MU vs T0: DE

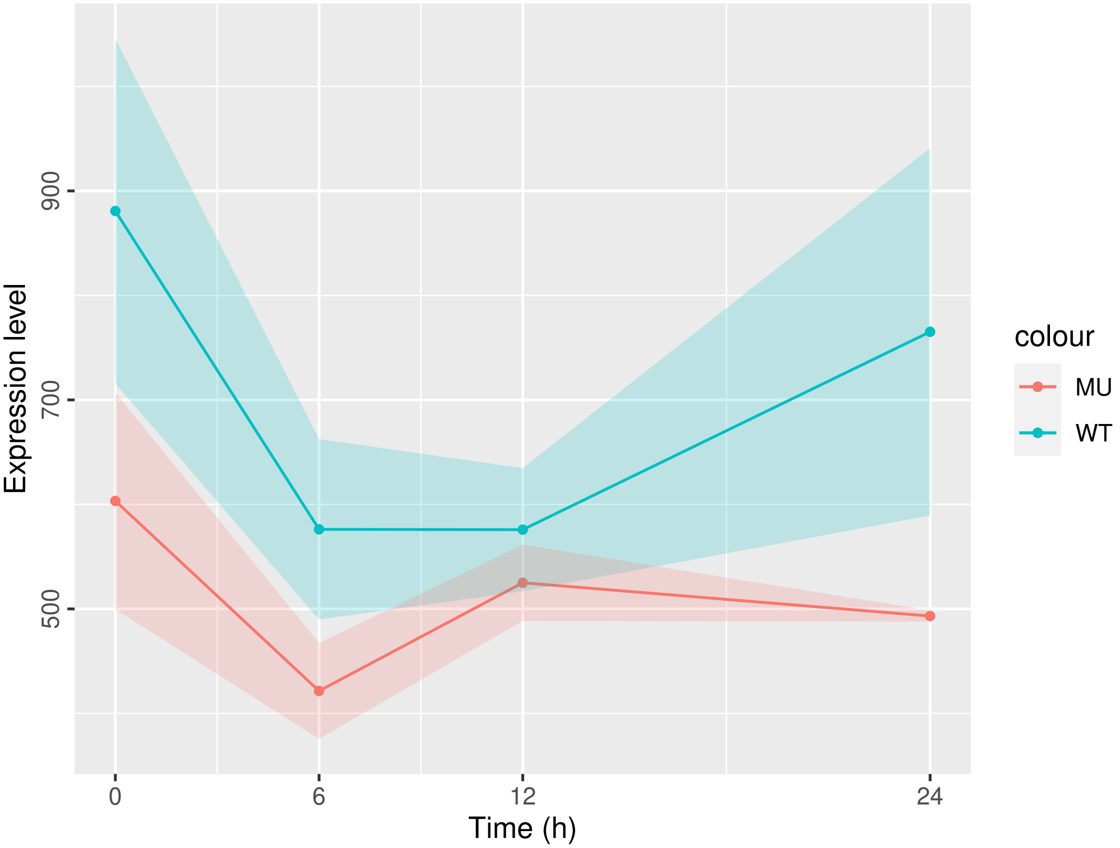

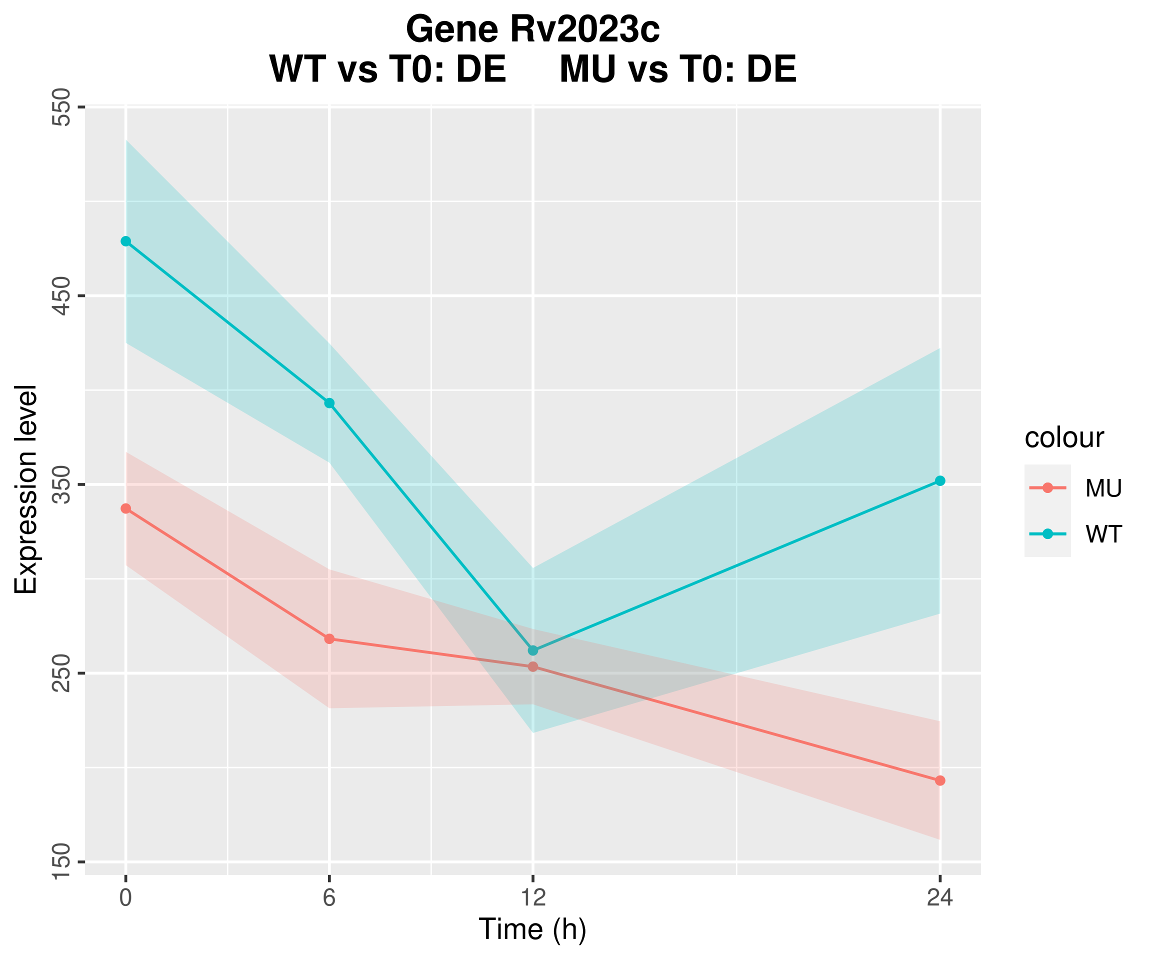

Gene Rv2023c
WT vs T0: DE   MU vs T0: DE
550
450
350
250
150
Expression level
0
6
12
24
Time (h)
colour
MU
WT

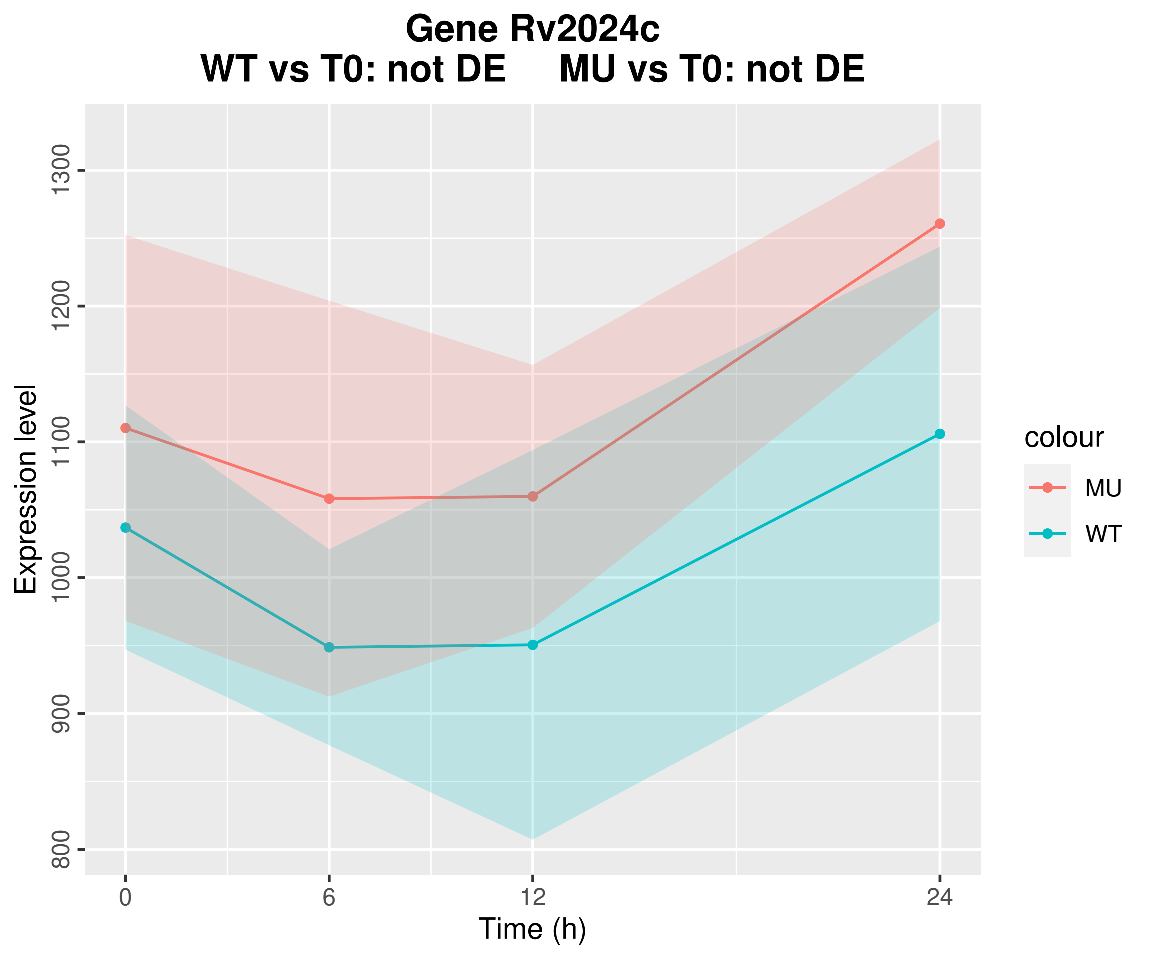

Gene Rv2024c
WT vs T0: not DE   MU vs T0: not DE
1300
1200
1100
1000
900
800
Expression level
0
6
12
24
Time (h)
colour
MU
WT

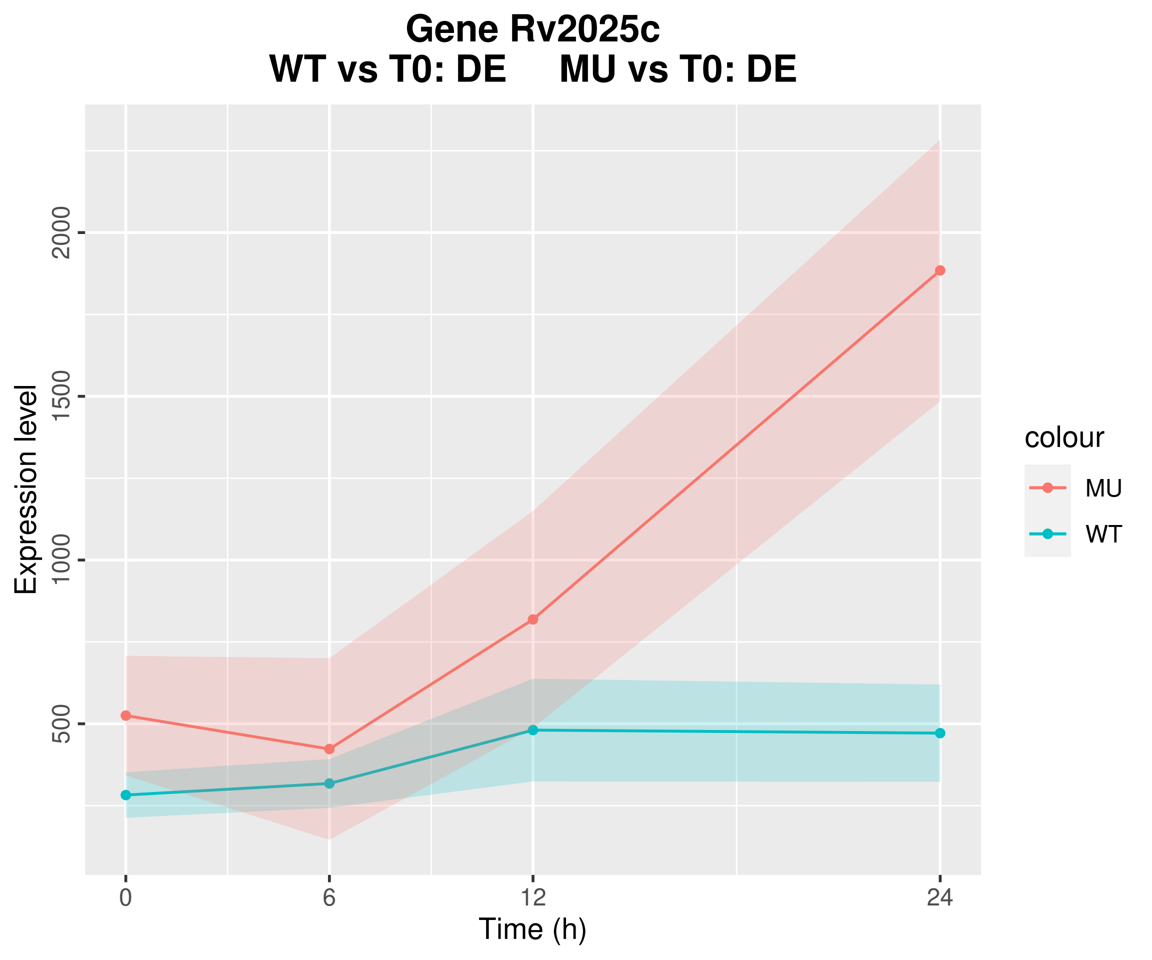

Gene Rv2025c
WT vs T0: DE MU vs T0: DE
Expression level
2000
1500
1000
500
0
6
12
24
Time (h)
colour
MU
WT

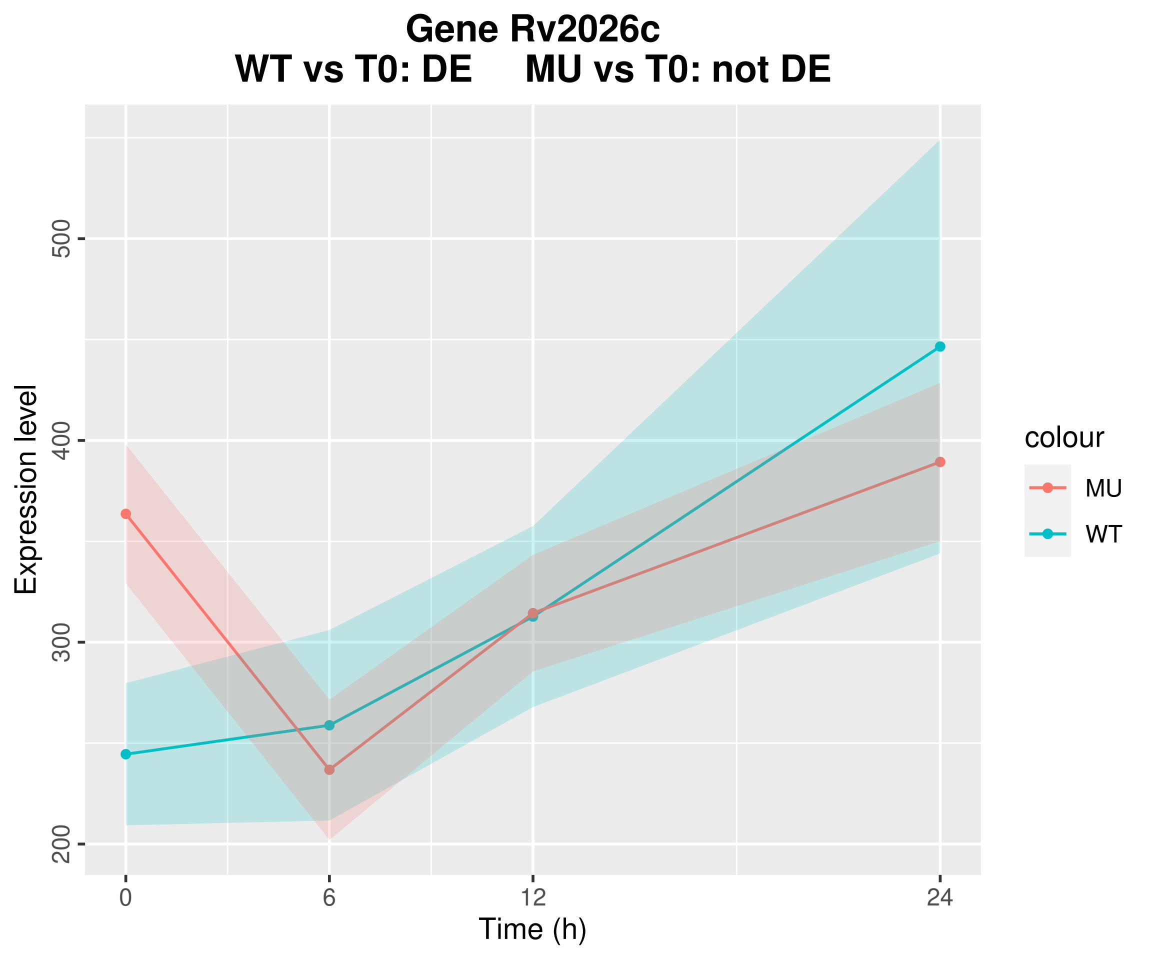

Gene Rv2026c
WT vs T0: DE MU vs T0: not DE
Expression level
500
400
300
200
0
6
12
24
Time (h)
colour
MU
WT

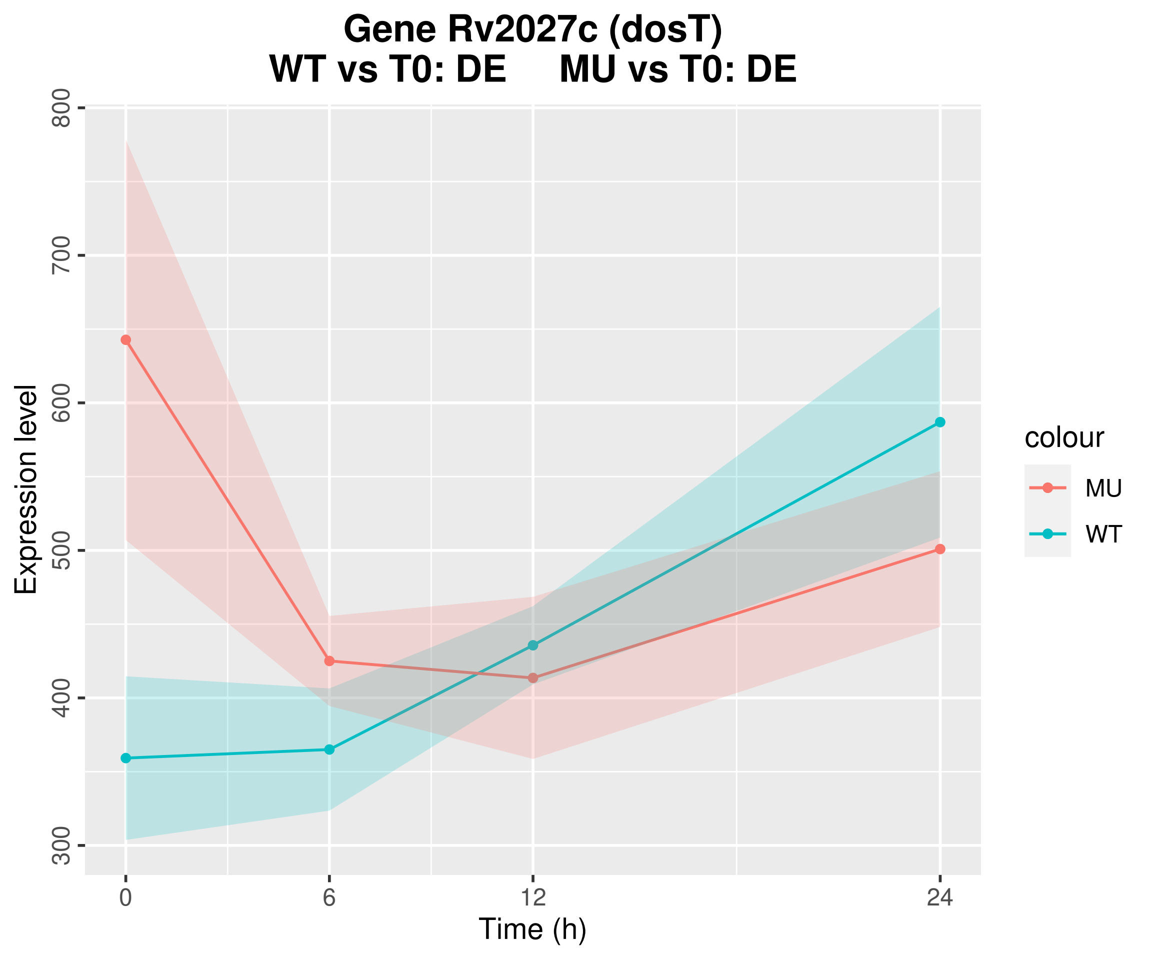
Gene Rv2027c (dosT)
WT vs T0: DE MU vs T0: DE
Expression level
800
700
600
500
400
300
0
6
12
24
Time (h)
colour
MU
WT

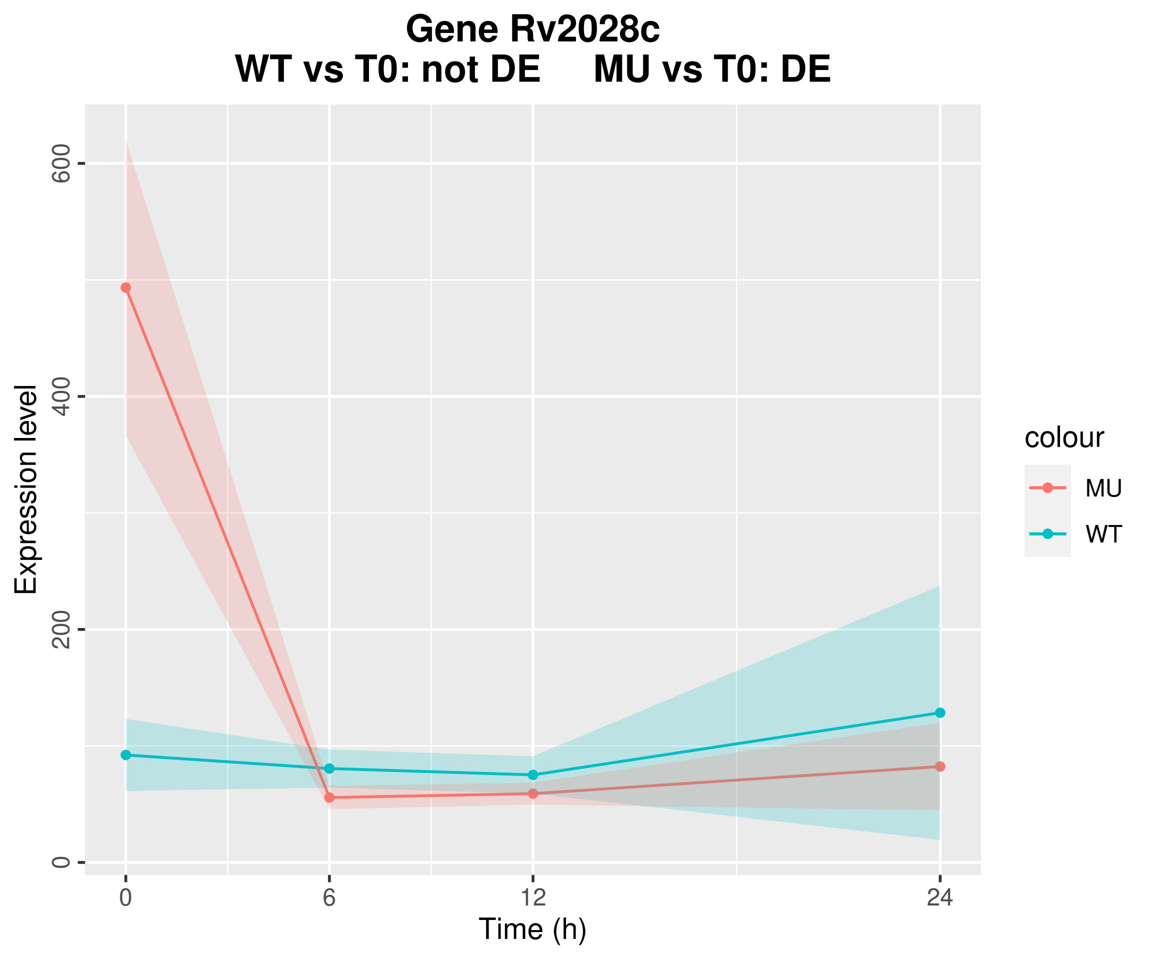

Gene Rv2028c
WT vs T0: not DE MU vs T0: DE
Expression level
600
400
200
0
0
6
12
24
Time (h)
colour
MU
WT

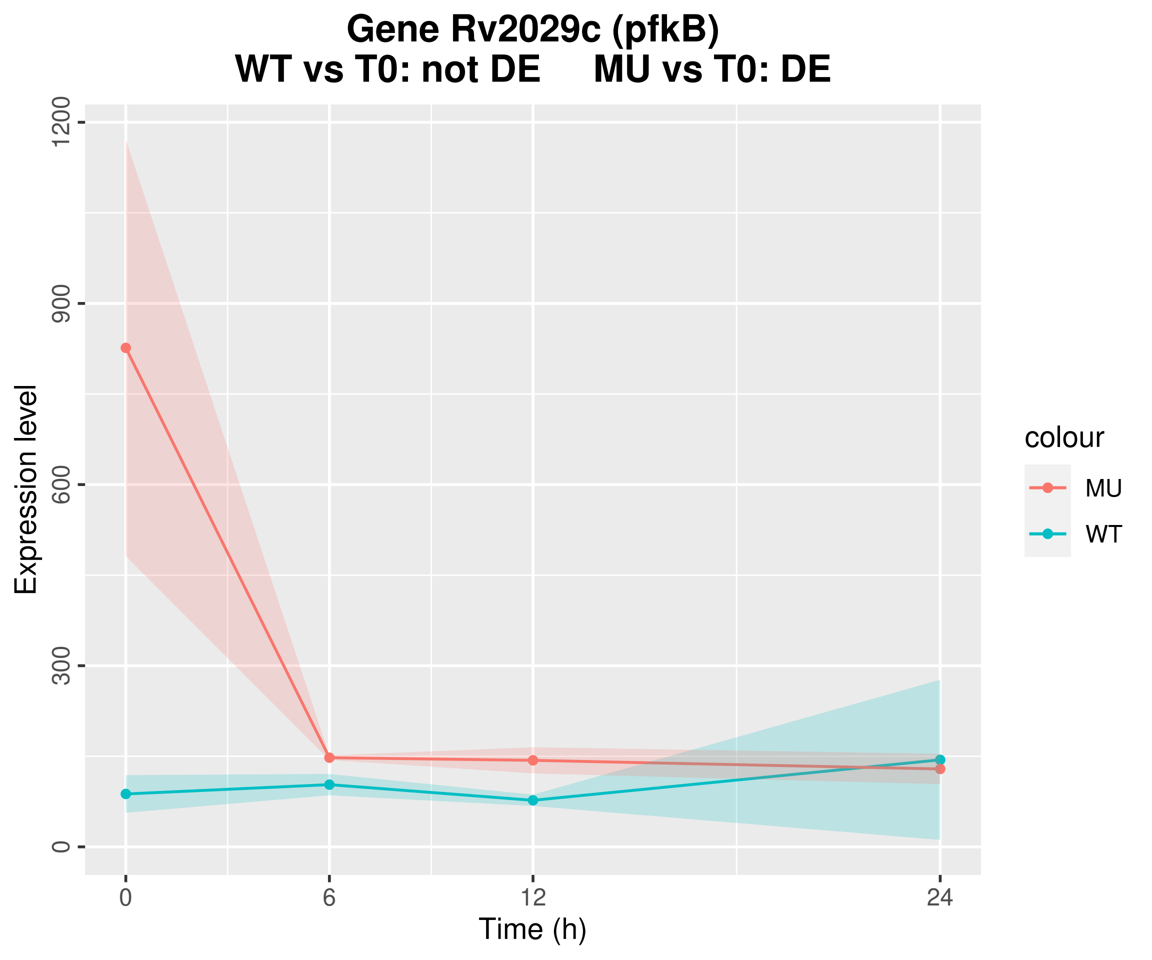
Gene Rv2029c (pfkB)
WT vs T0: not DE MU vs T0: DE
Expression level
1200
900
600
300
0
Time (h)
0
6
12
24
colour
MU
WT

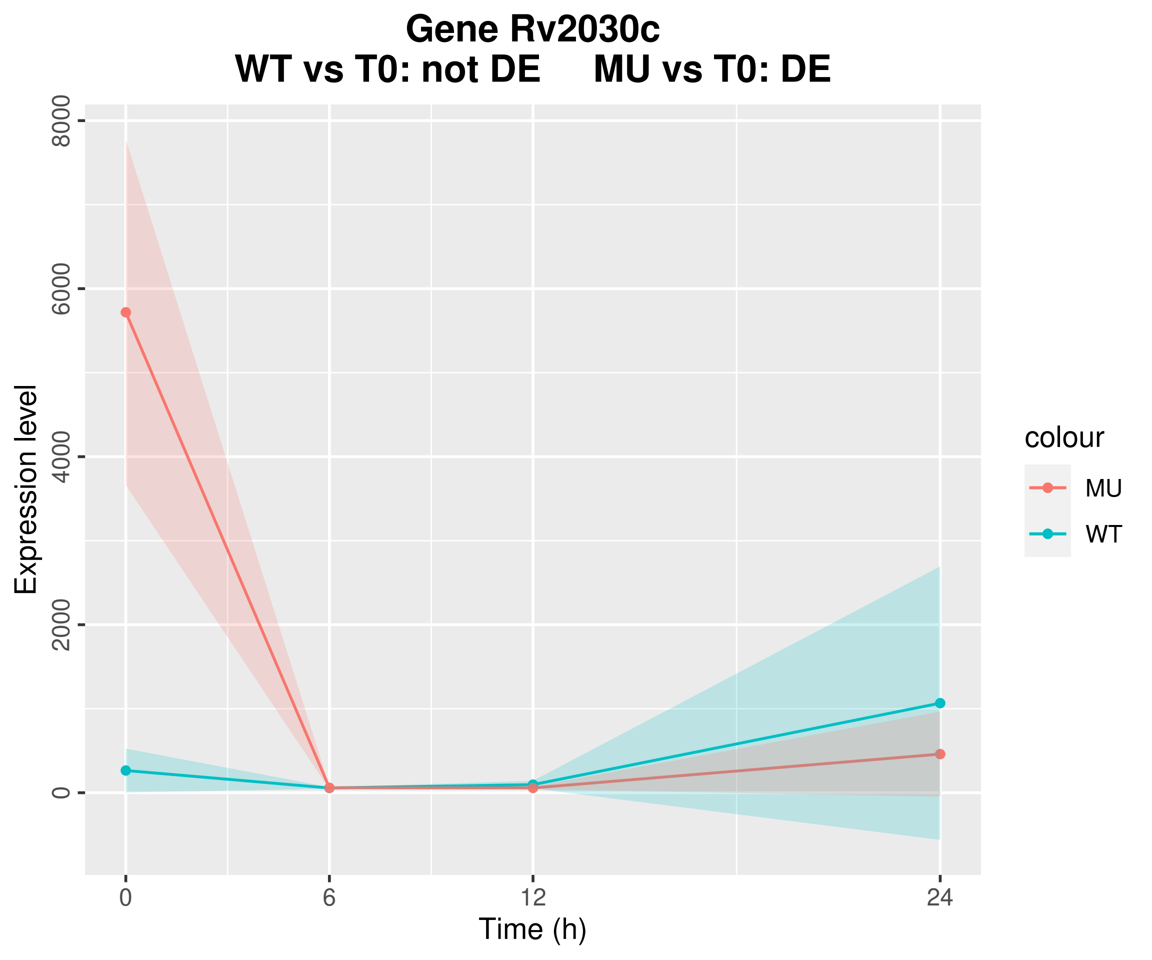

Gene Rv2030c
WT vs T0: not DE   MU vs T0: DE
Expression level
8000
6000
4000
2000
0
0
6
12
24
Time (h)
colour
MU
WT

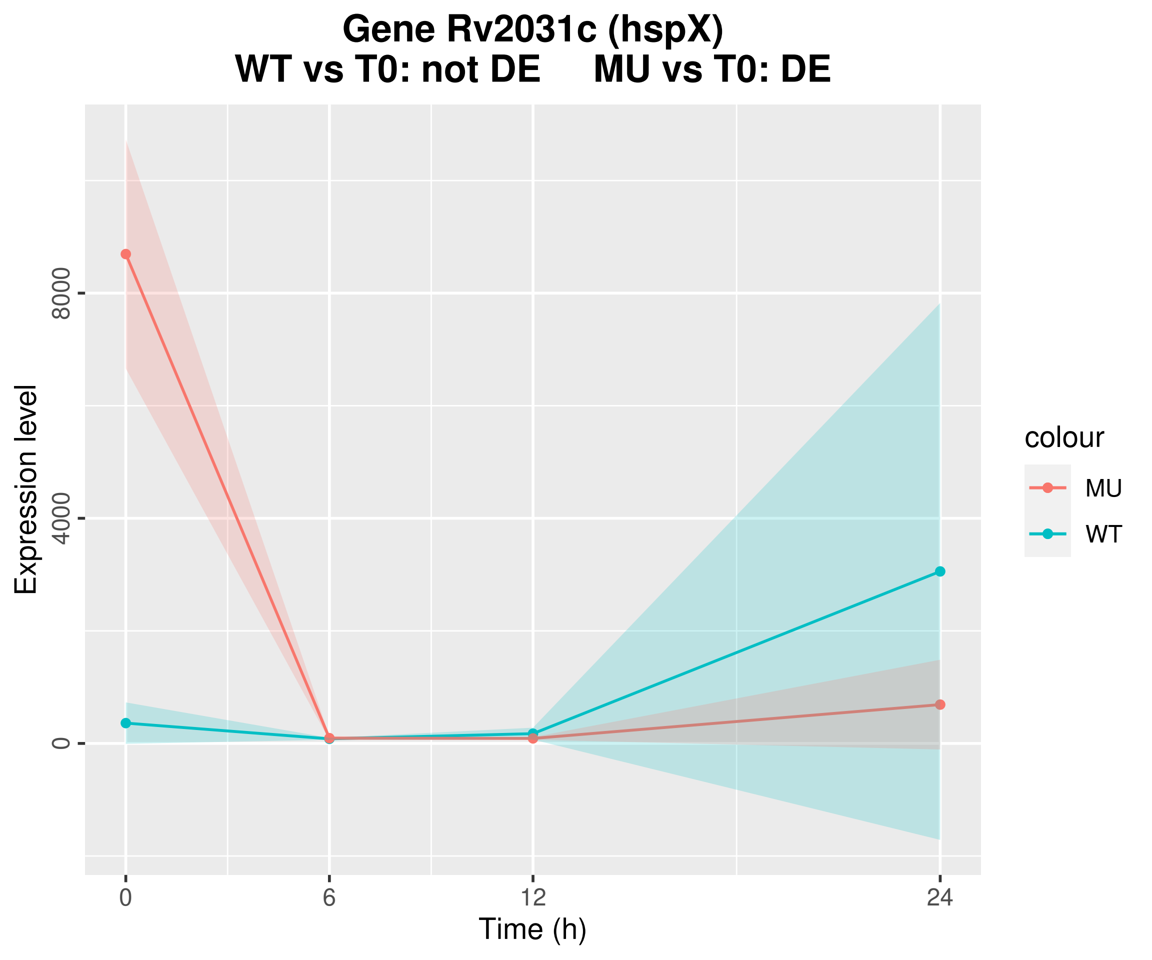
Gene Rv2031c (hspX)
WT vs T0: not DE MU vs T0: DE
Expression level
8000
4000
0
0
6
12
24
Time (h)
colour
MU
WT

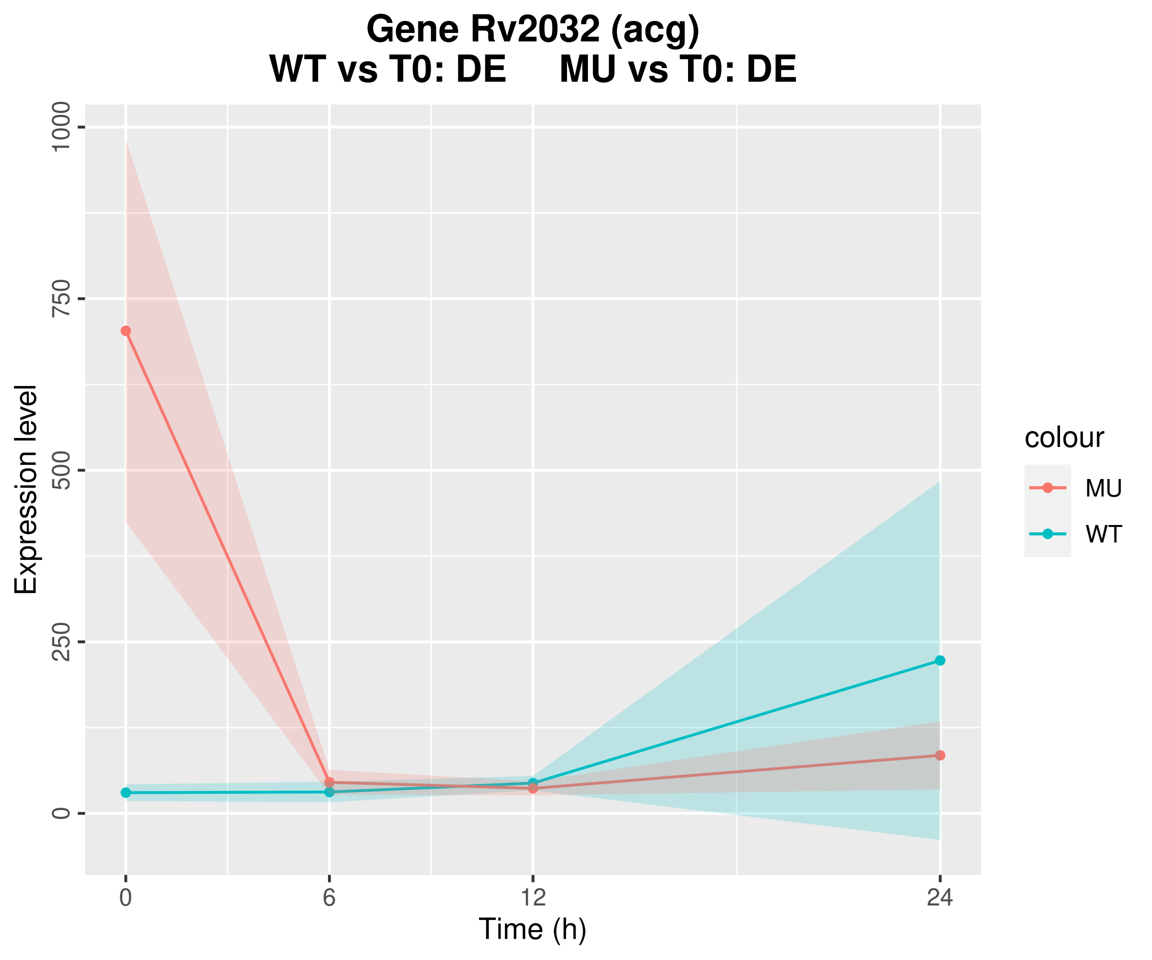

Gene Rv2032 (acg)
WT vs T0: DE MU vs T0: DE
Expression level
1000
750
500
250
0
0
6
12
24
Time (h)
colour
MU
WT

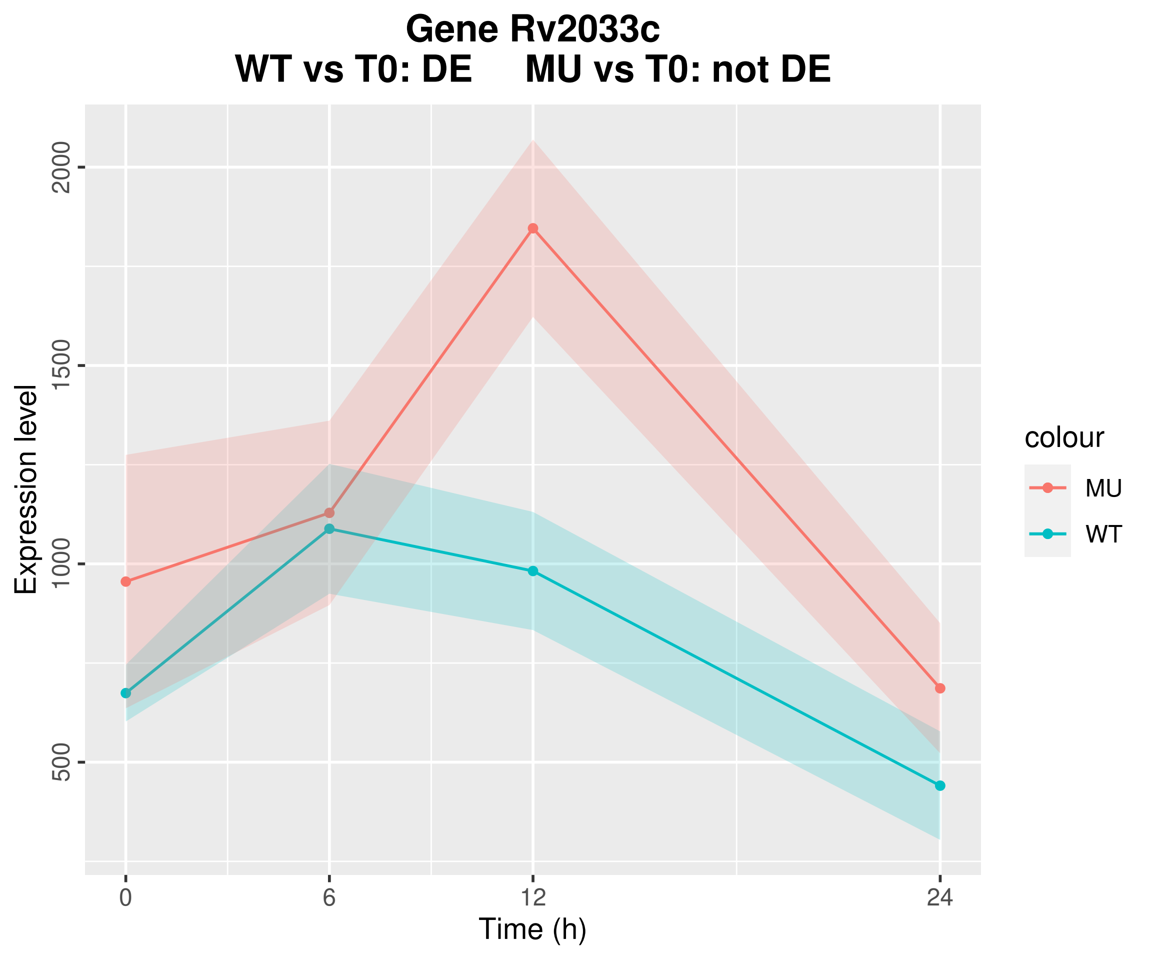

Gene Rv2033c
WT vs T0: DE   MU vs T0: not DE
Expression level
2000
1500
1000
500
0
6
12
24
Time (h)
colour
MU
WT

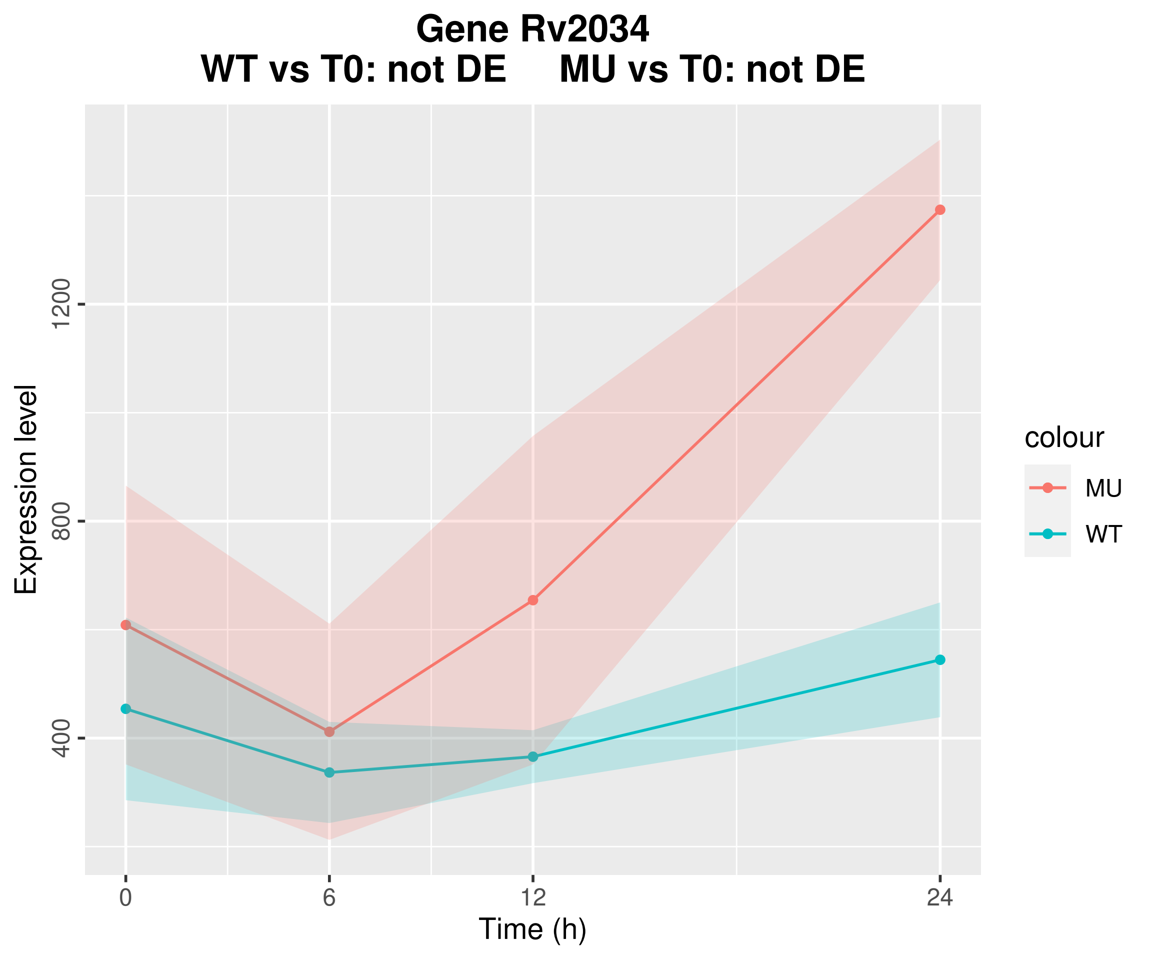
Gene Rv2034
WT vs T0: not DE MU vs T0: not DE
Expression level
1200
800
400
0
6
12
24
Time (h)
colour
MU
WT

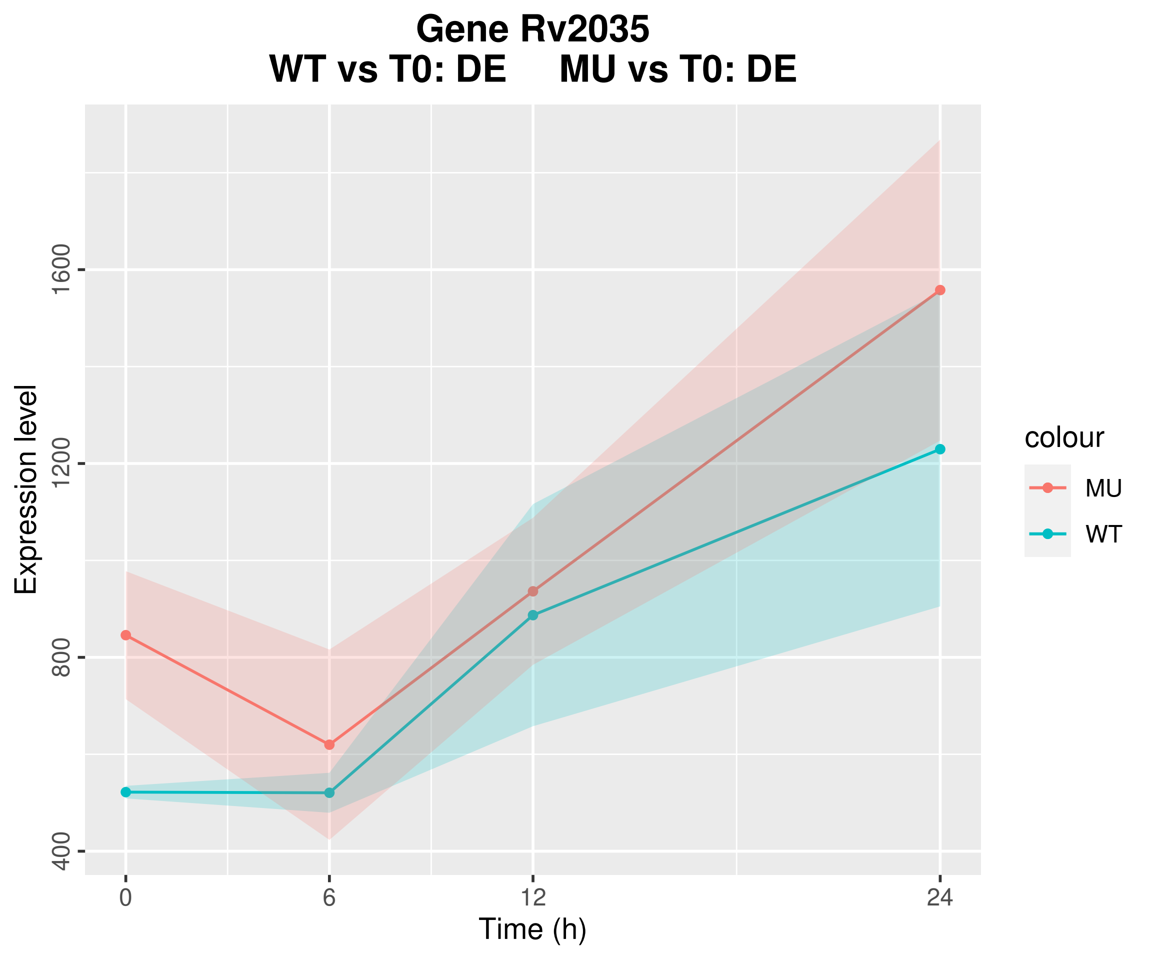

Gene Rv2035
WT vs T0: DE MU vs T0: DE
Expression level
1600
1200
800
400
0
6
12
24
Time (h)
colour
MU
WT

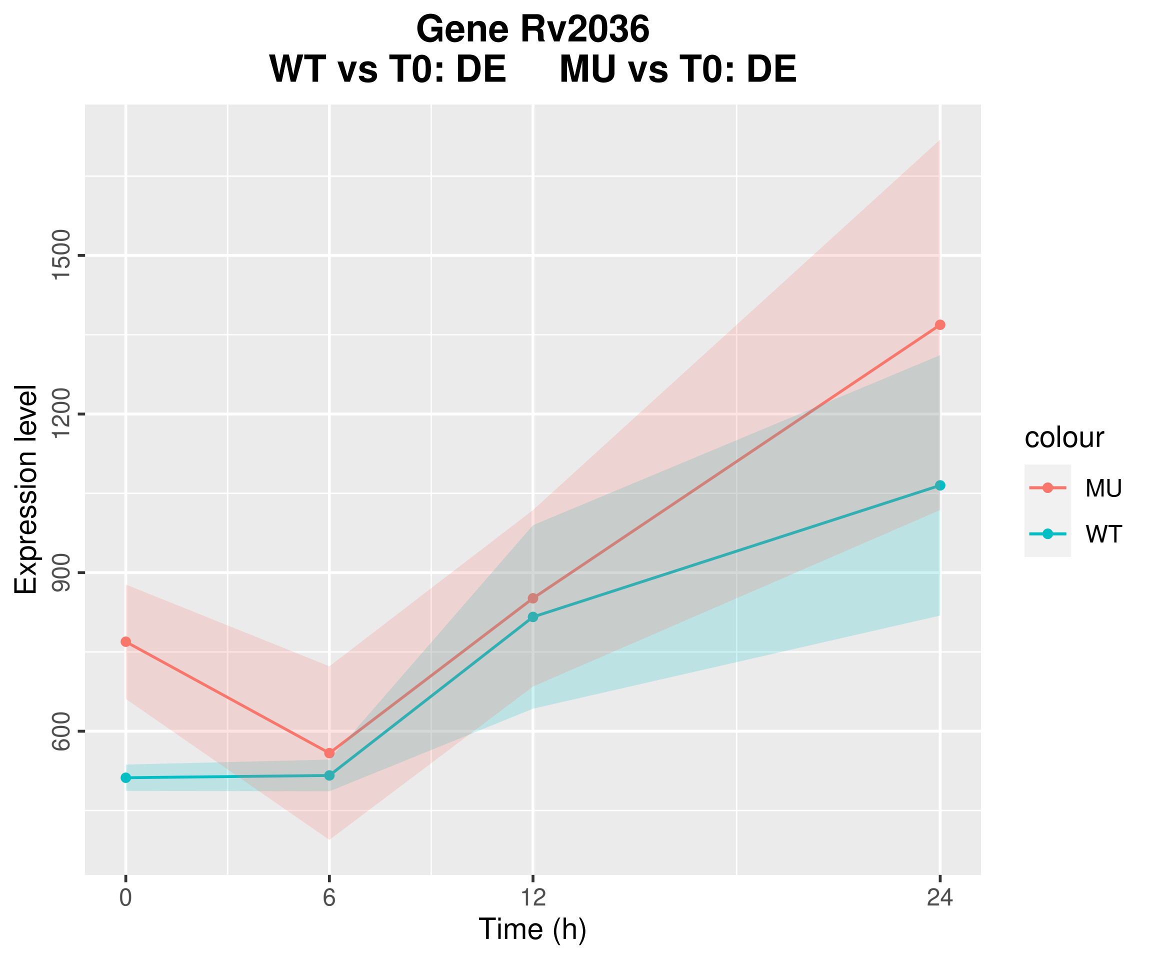
Gene Rv2036
WT vs T0: DE MU vs T0: DE
1500
1200
900
600
Expression level
0
6
12
24
Time (h)
colour
MU
WT

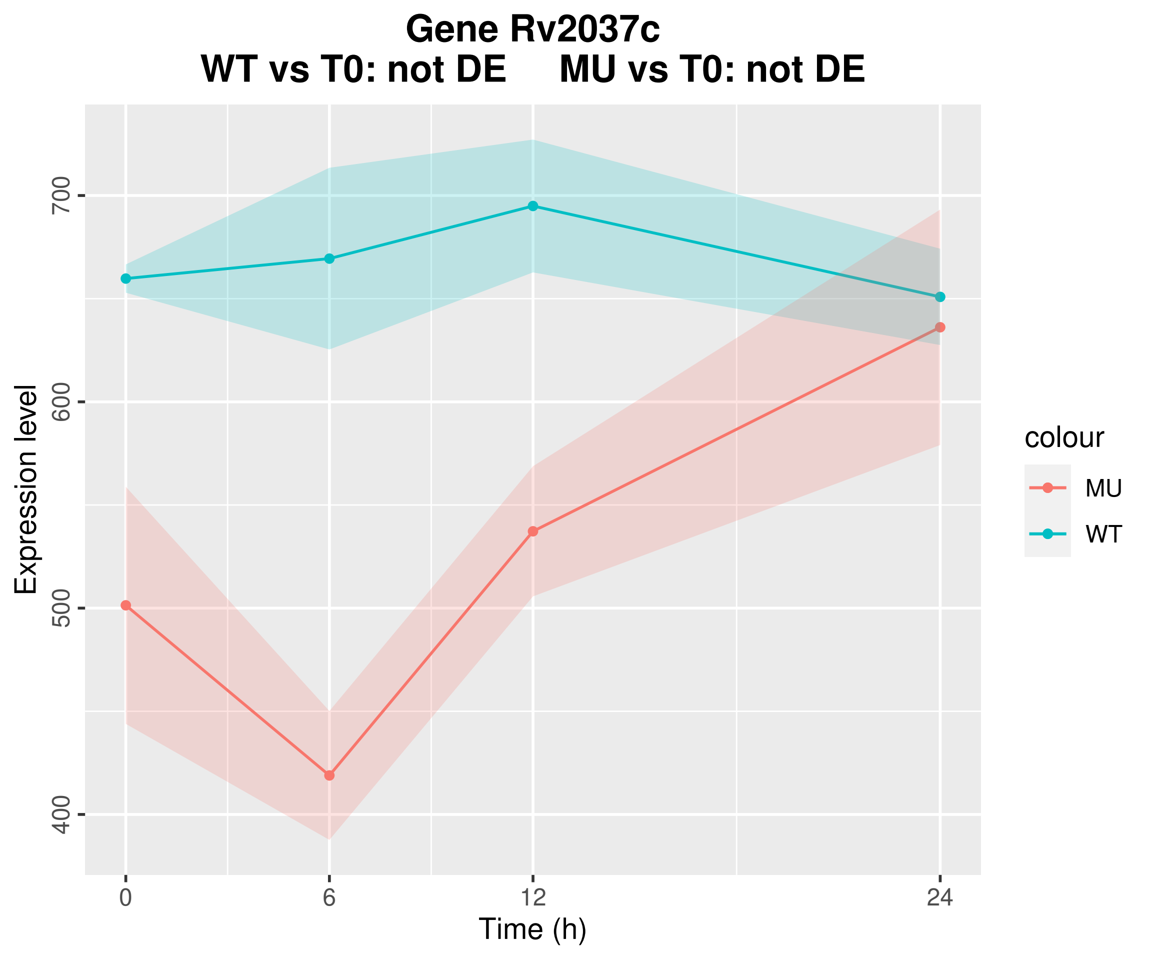

Gene Rv2037c
WT vs T0: not DE   MU vs T0: not DE
Expression level
700
600
500
400
0
6
12
24
Time (h)
colour
MU
WT

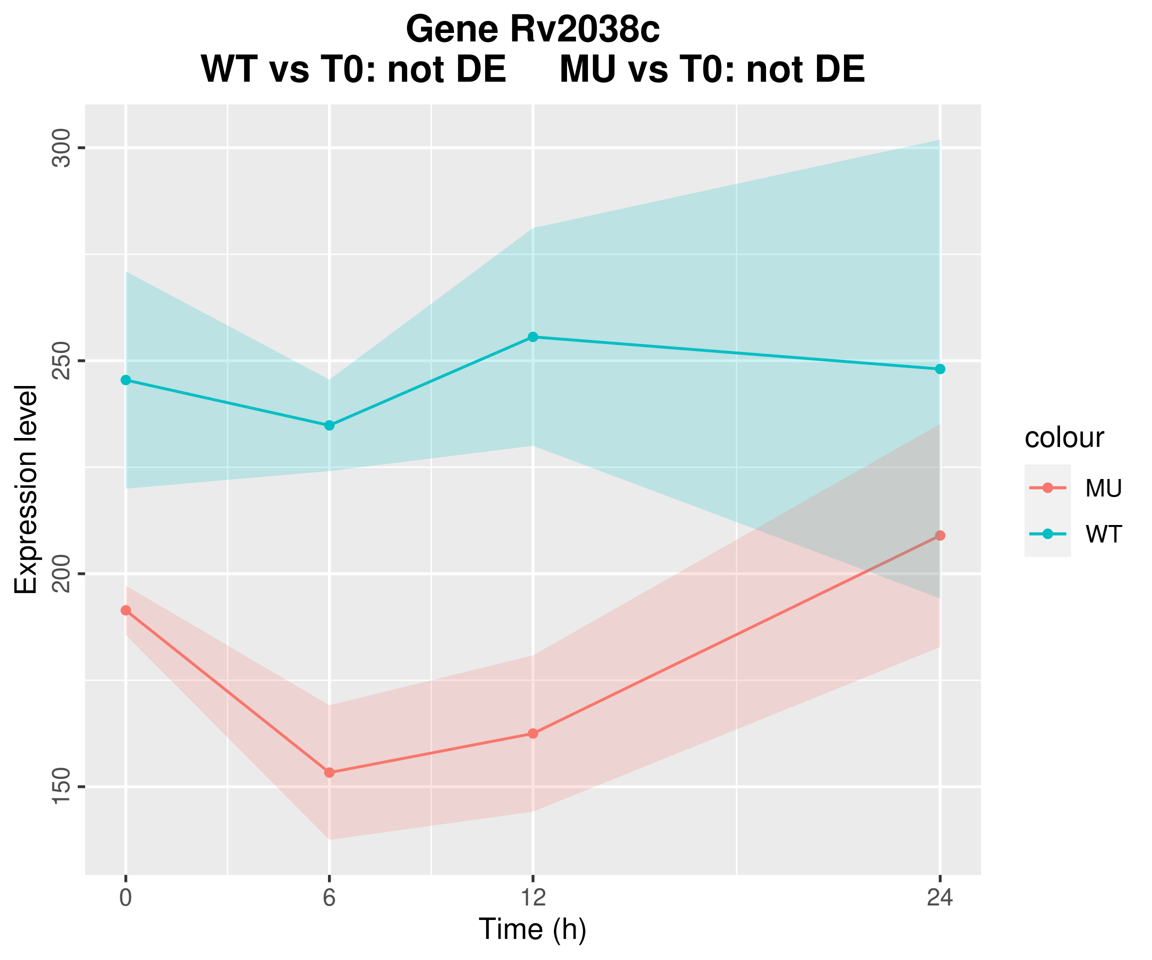
Gene Rv2038c
WT vs T0: not DE   MU vs T0: not DE
300
250
200
150
Expression level
0
6
12
24
Time (h)
colour
MU
WT

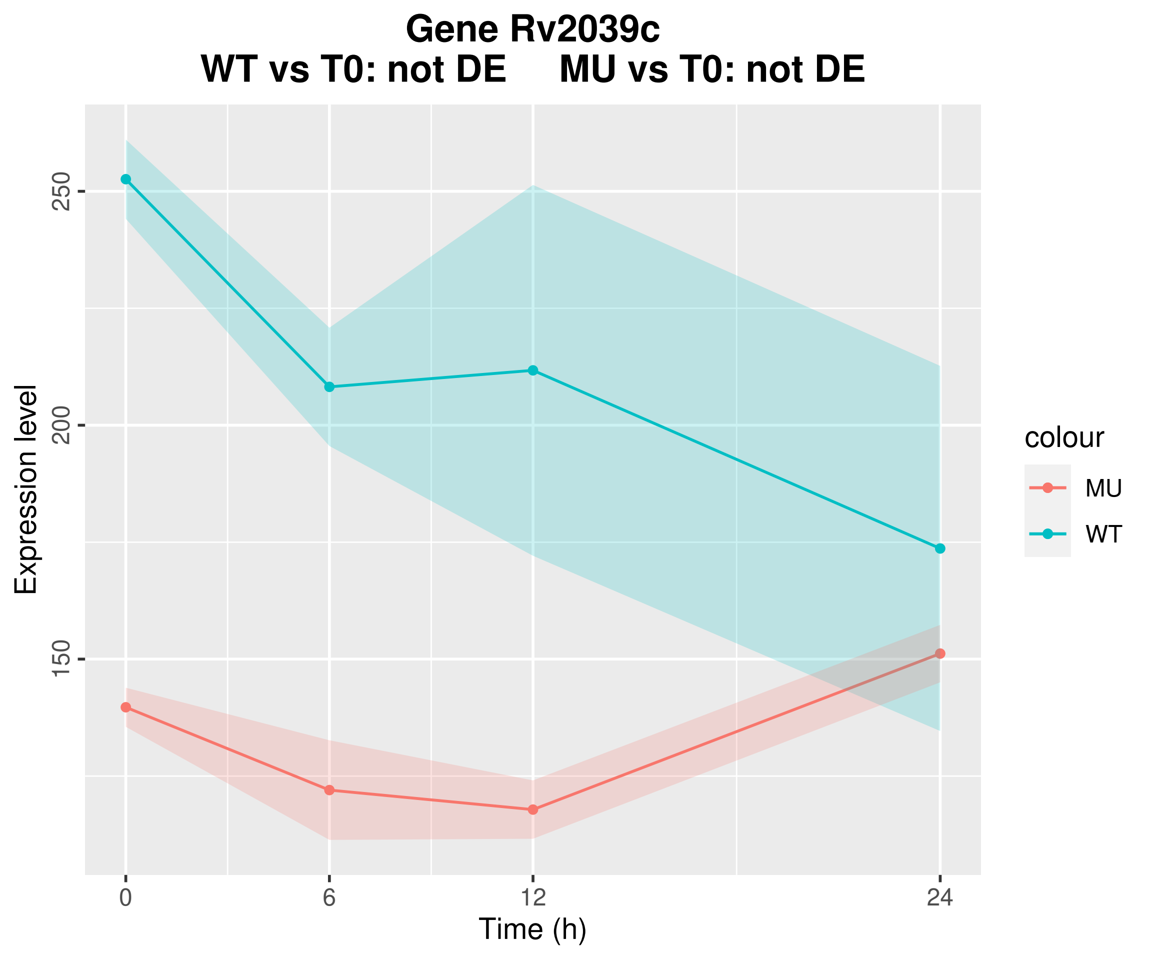
Gene Rv2039c
WT vs T0: not DE MU vs T0: not DE
Expression level
250
200
150
0
6
12
24
Time (h)
colour
MU
WT

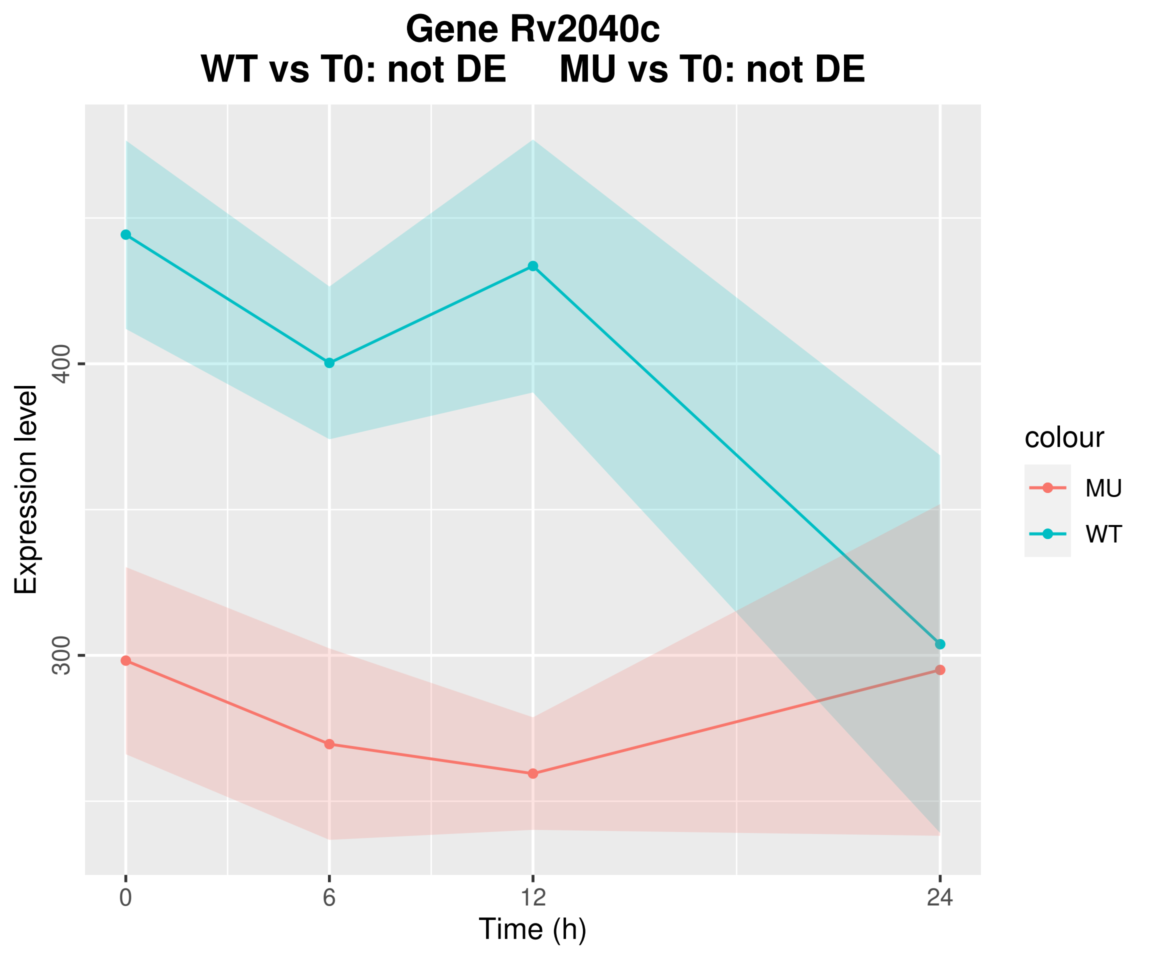

Gene Rv2040c
WT vs T0: not DE   MU vs T0: not DE
Expression level
400
300
0
6
12
24
Time (h)
colour
MU
WT

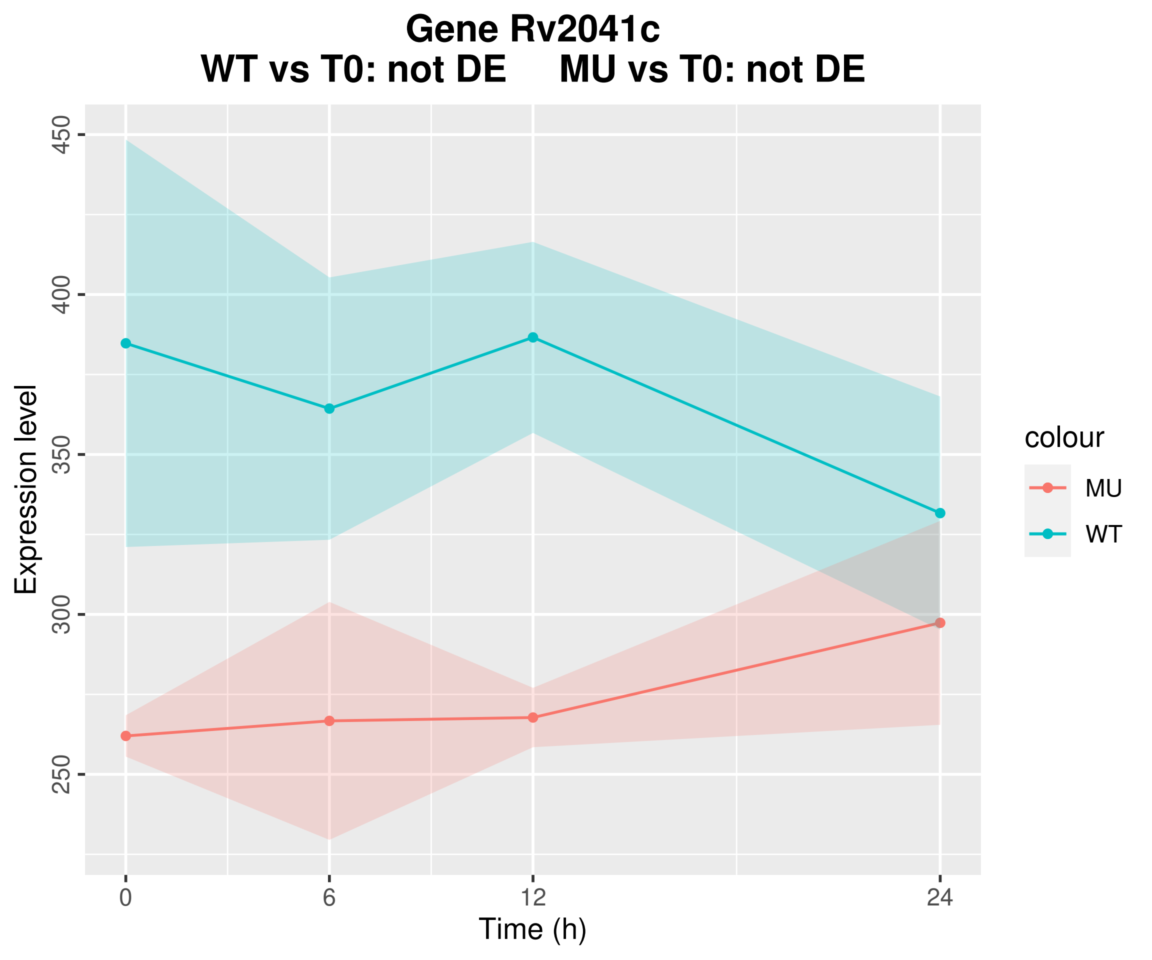
Gene Rv2041c
WT vs T0: not DE   MU vs T0: not DE
Expression level
450
400
350
300
250
0
6
12
24
Time (h)
colour
MU
WT

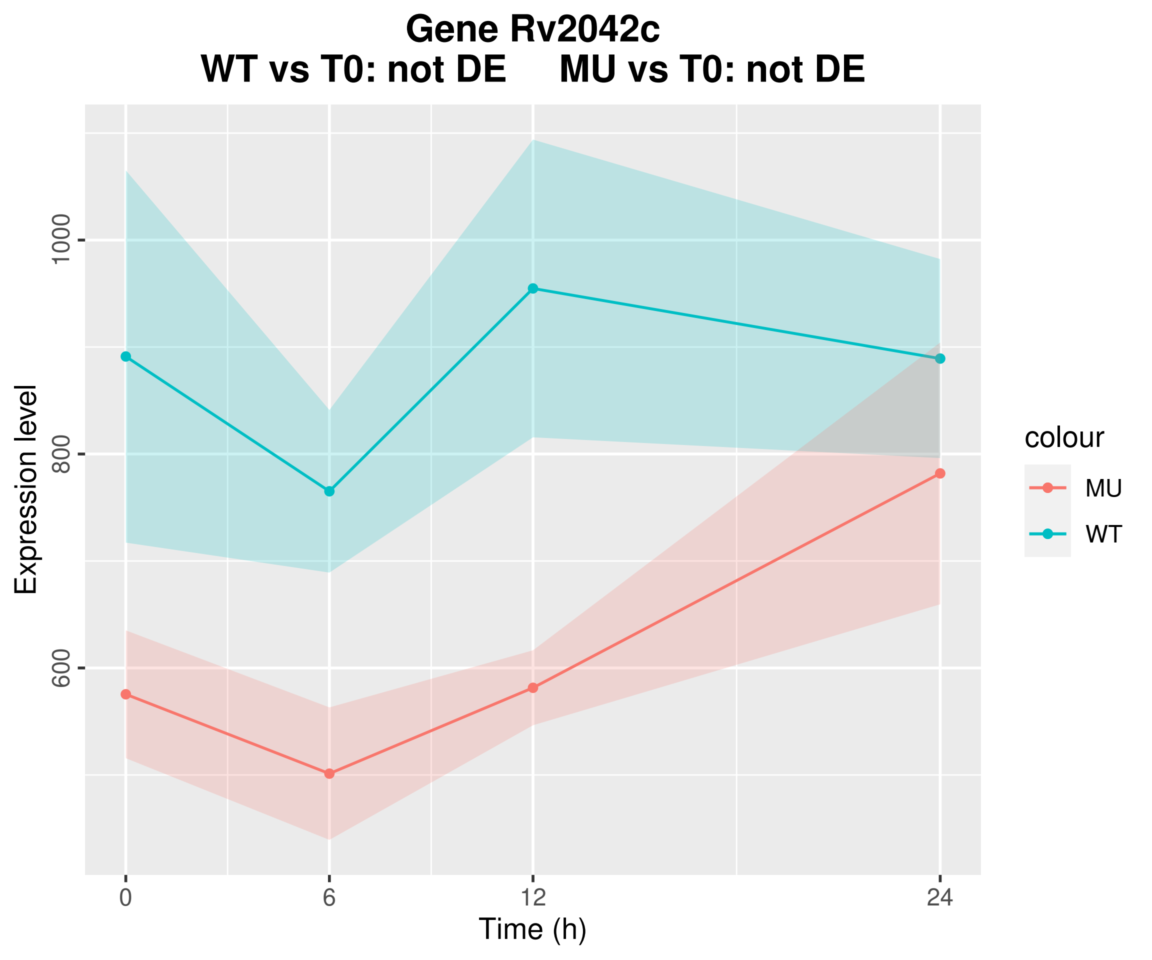

Gene Rv2042c
WT vs T0: not DE MU vs T0: not DE
Expression level
1000
800
600
0
6
12
24
Time (h)
colour
MU
WT

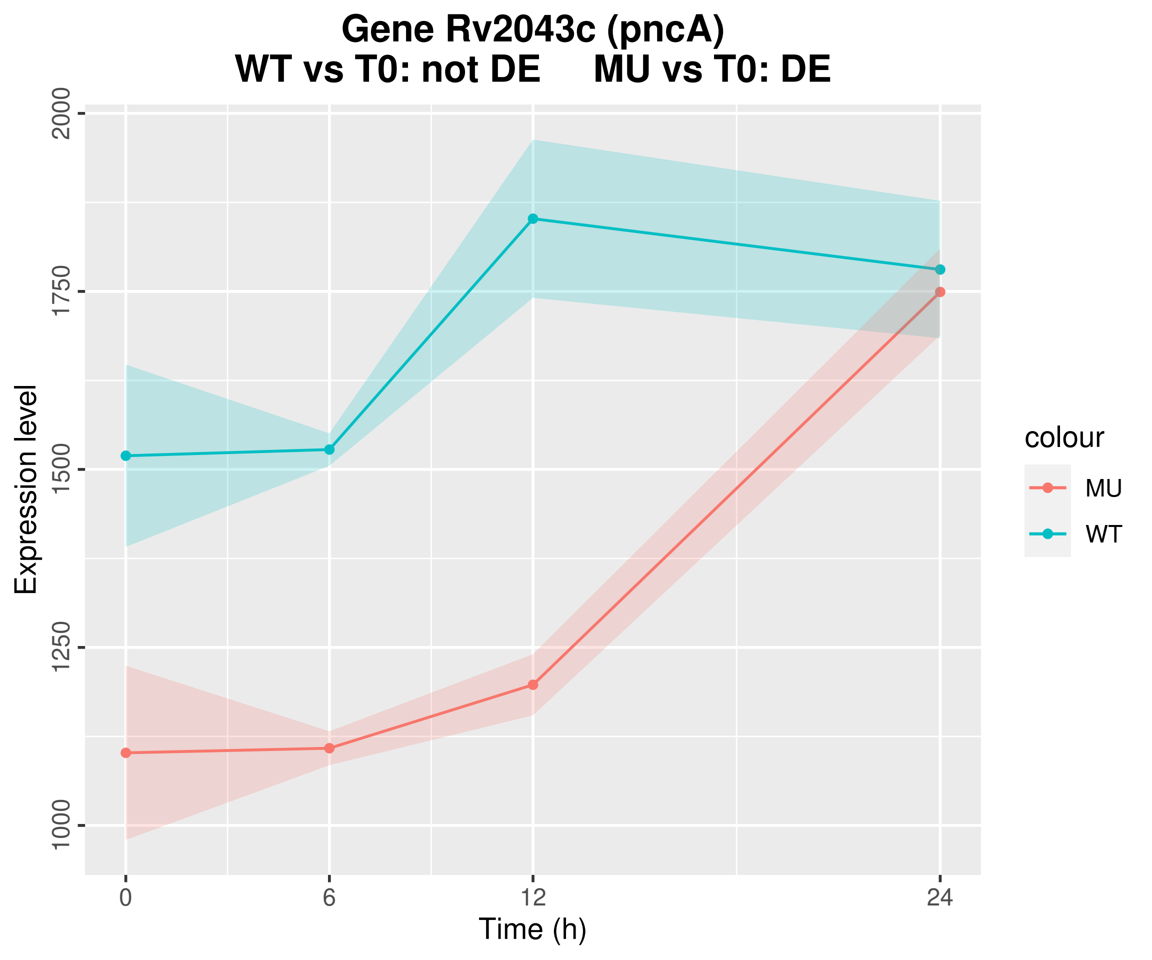
Gene Rv2043c (pncA)
WT vs T0: not DE   MU vs T0: DE
Expression level
2000
1750
1500
1250
1000
0
6
12
24
Time (h)
colour
MU
WT

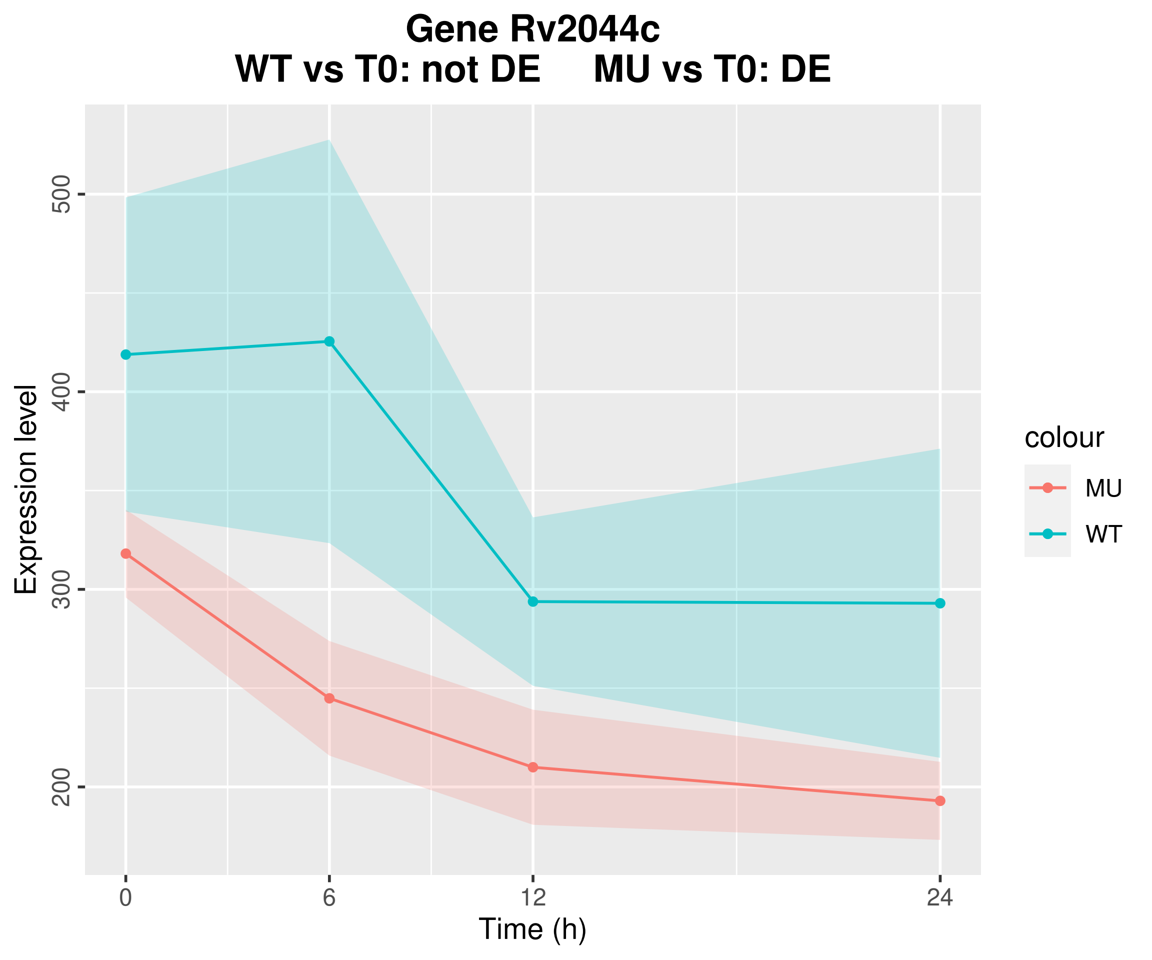

Gene Rv2044c
WT vs T0: not DE MU vs T0: DE
Expression level
500
400
300
200
0
6
12
24
Time (h)
colour
MU
WT

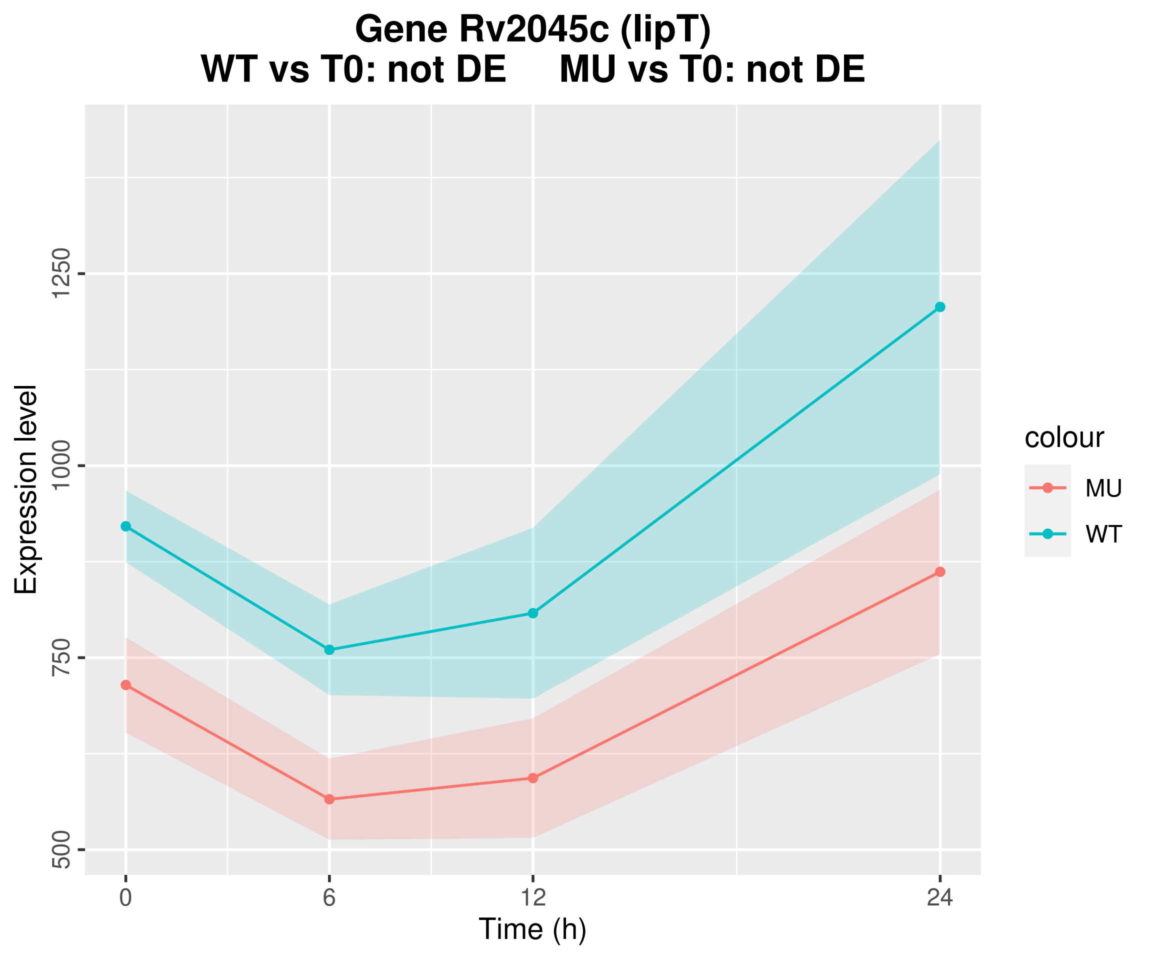

Gene Rv2045c (lipT)
WT vs T0: not DE   MU vs T0: not DE
Expression level
1250
1000
750
500
0
6
12
24
Time (h)
colour
MU
WT

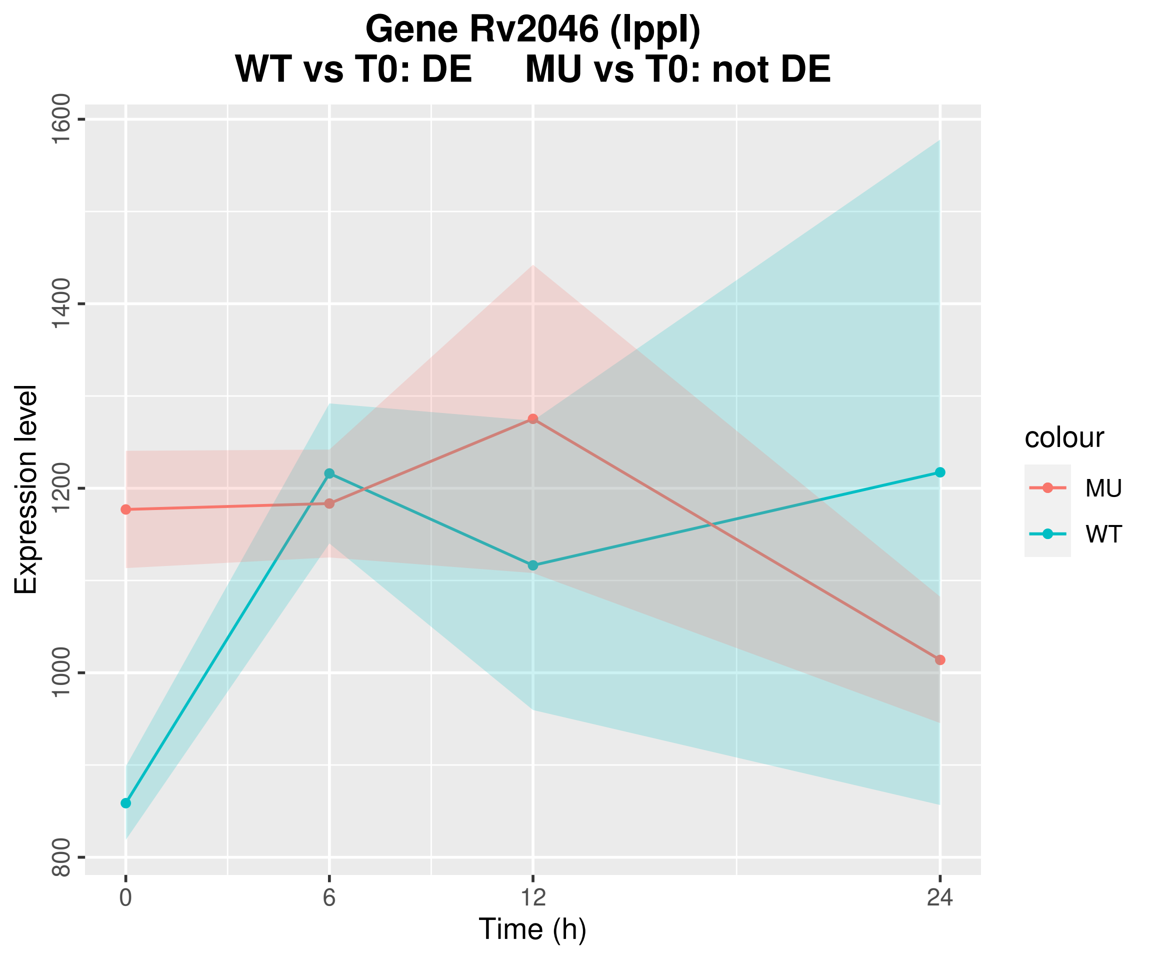

Gene Rv2046 (lppl)
WT vs T0: DE MU vs T0: not DE
Expression level
1600
1400
1200
1000
800
0
6
12
24
Time (h)
colour
MU
WT

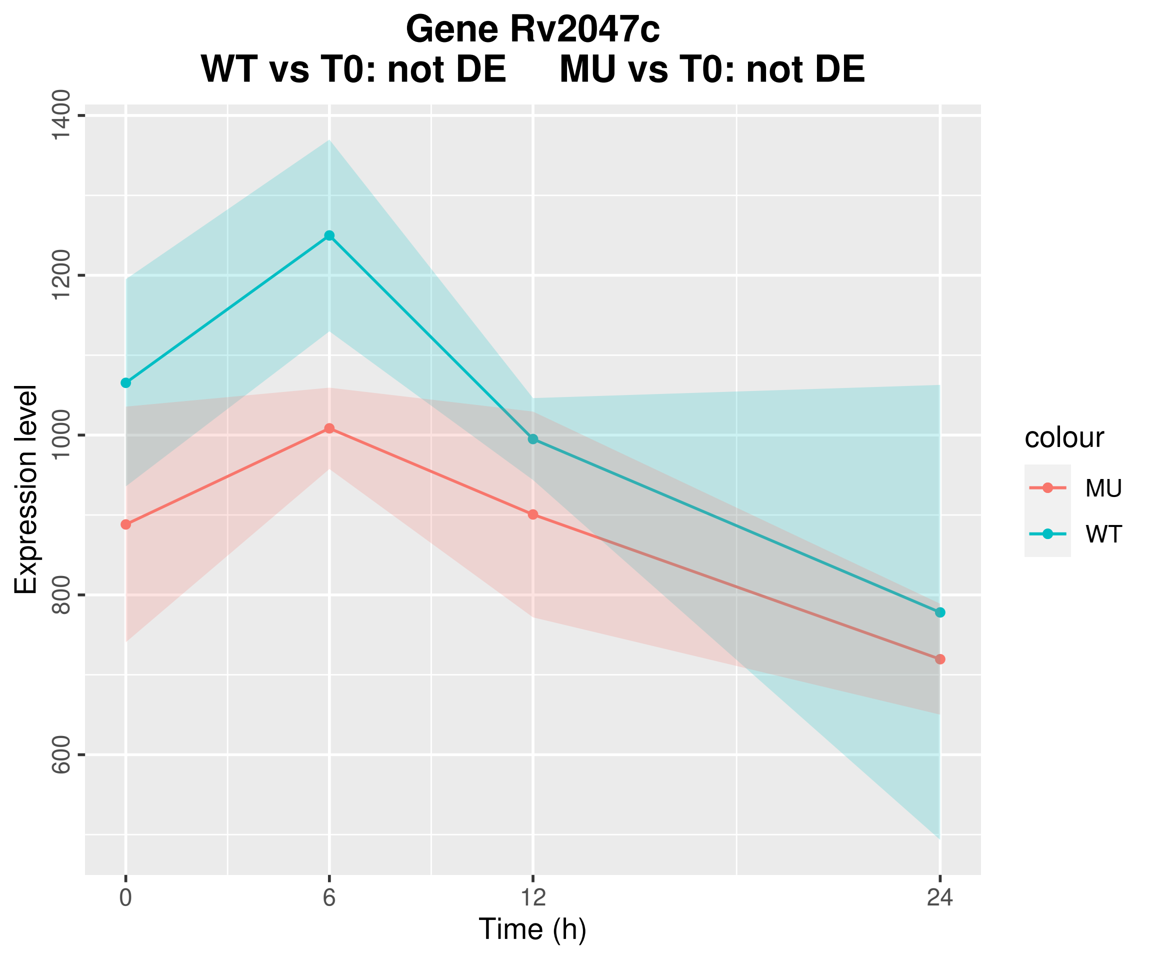
Gene Rv2047c
WT vs T0: not DE   MU vs T0: not DE
Expression level
600
800
1000
1200
1400
0
6
12
24
Time (h)
colour
MU
WT

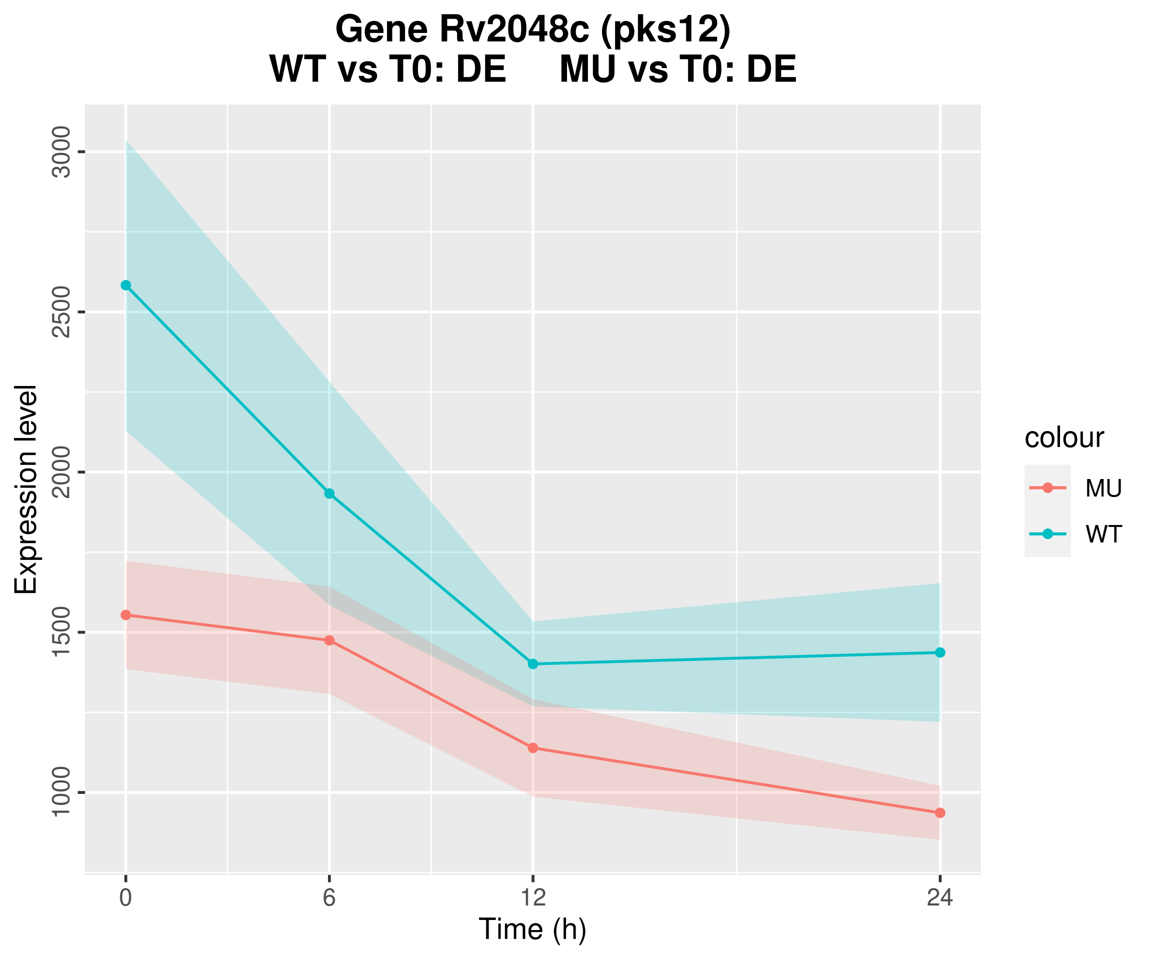

Gene Rv2048c (pks12)
WT vs T0: DE MU vs T0: DE
Expression level
3000
2500
2000
1500
1000
0
6
12
24
Time (h)
colour
MU
WT

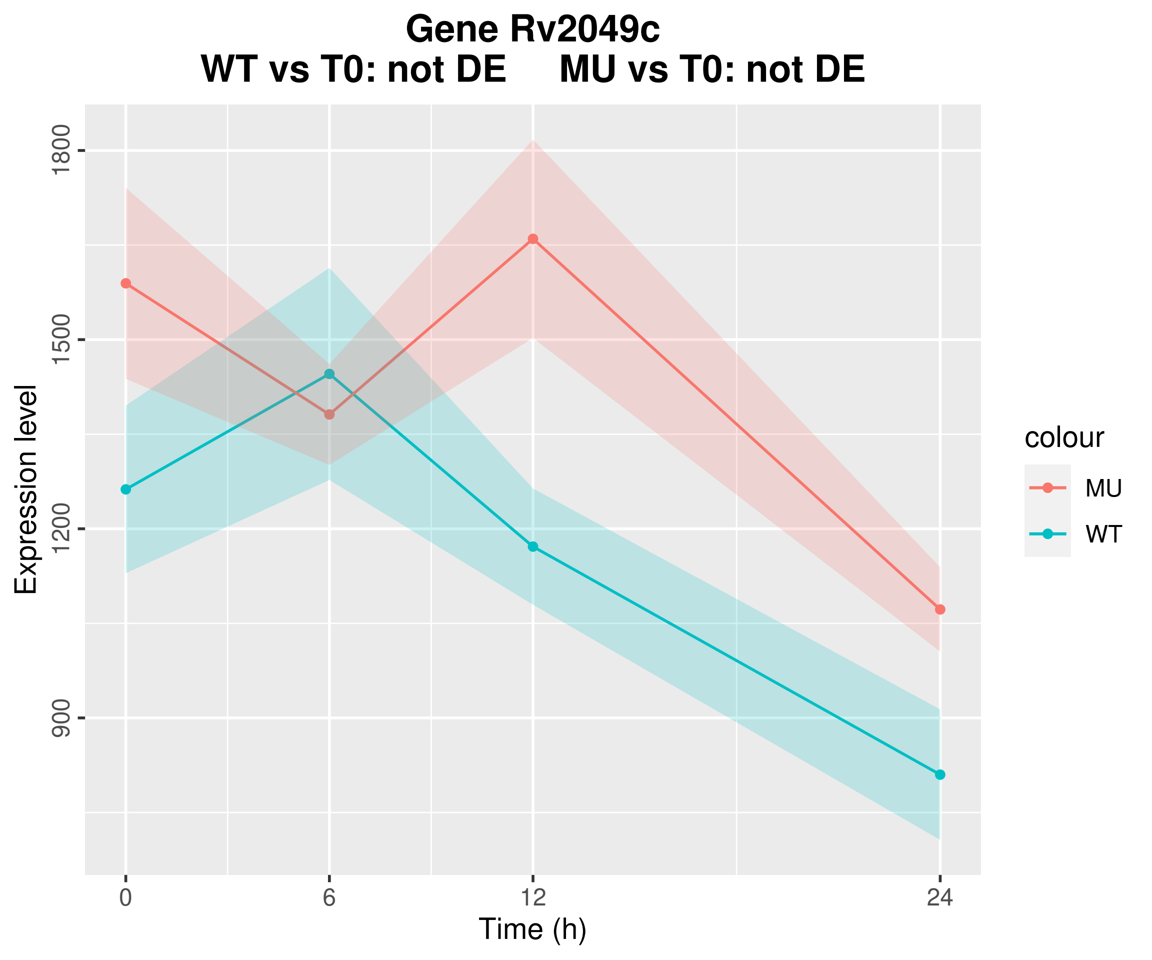
Gene Rv2049c
WT vs T0: not DE MU vs T0: not DE
Expression level
1800
1500
1200
900
0
6
12
24
Time (h)
colour
MU
WT

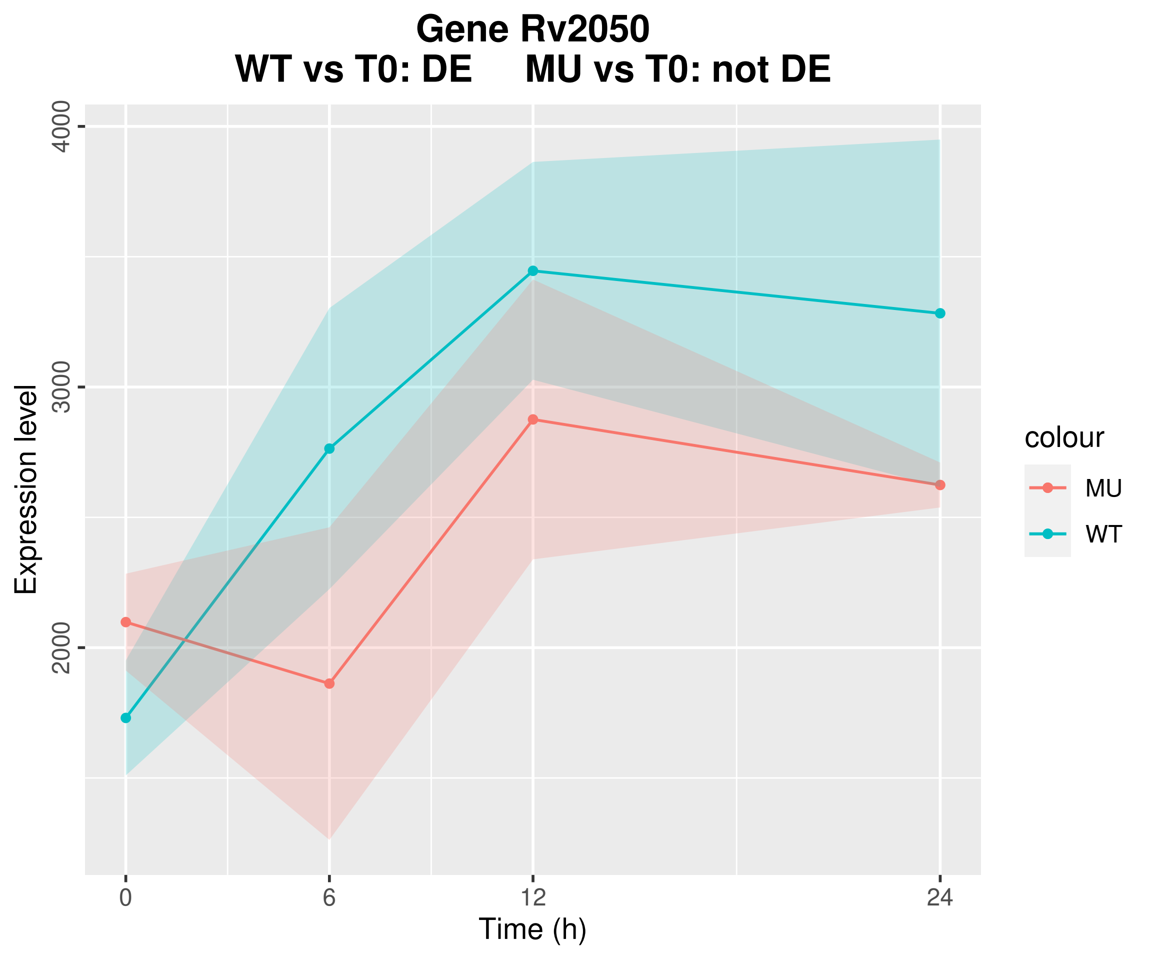

Gene Rv2050
WT vs T0: DE MU vs T0: not DE
Expression level
4000
3000
2000
0
6
12
24
Time (h)
colour
MU
WT

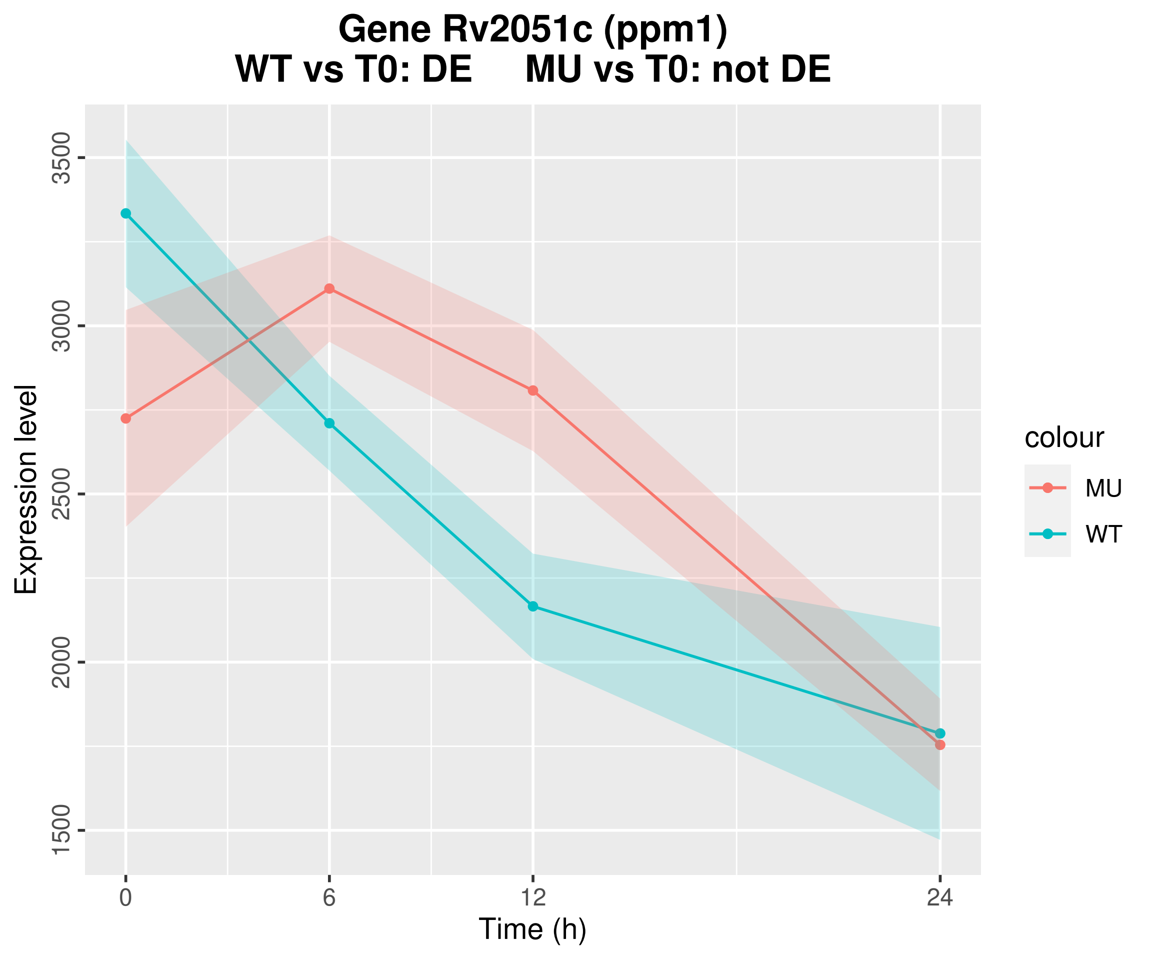
Gene Rv2051c (ppm1)
WT vs T0: DE MU vs T0: not DE
Expression level
3500
3000
2500
2000
1500
0
6
12
24
Time (h)
colour
MU
WT

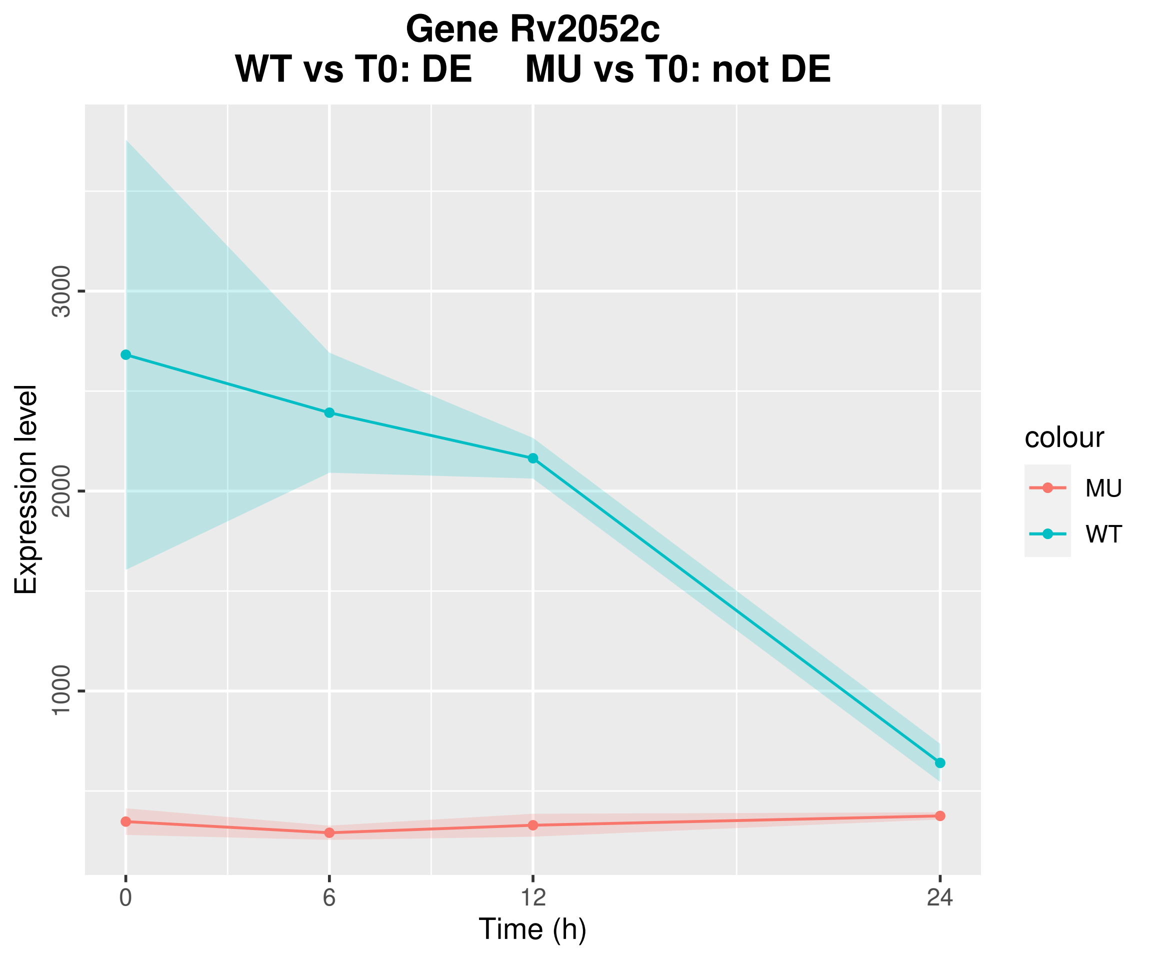
Gene Rv2052c
WT vs T0: DE MU vs T0: not DE
Expression level
3000
2000
1000
0
6
12
24
Time (h)
colour
MU
WT

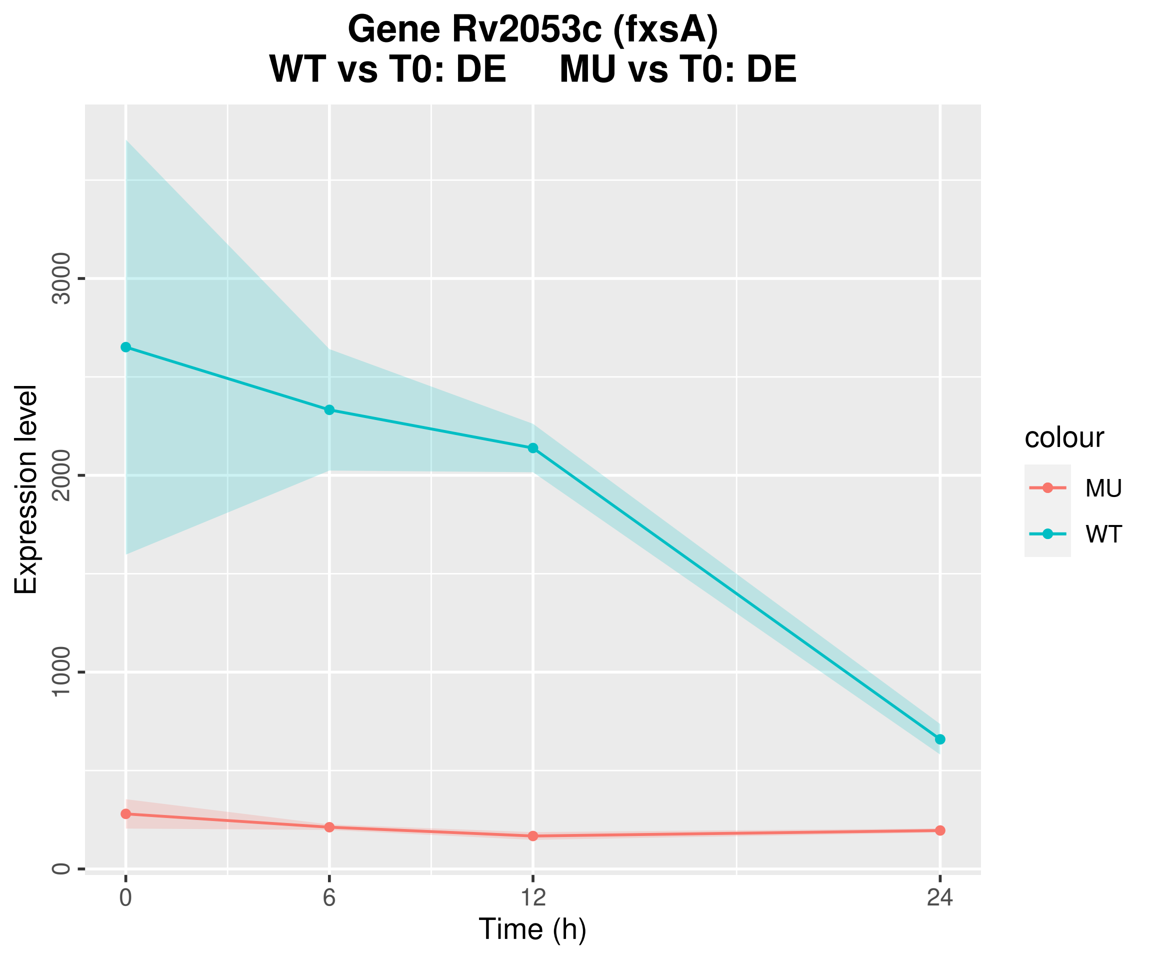

Gene Rv2053c (fxsA)
WT vs T0: DE MU vs T0: DE
Expression level
3000
2000
1000
0
0
6
12
24
Time (h)
colour
MU
WT

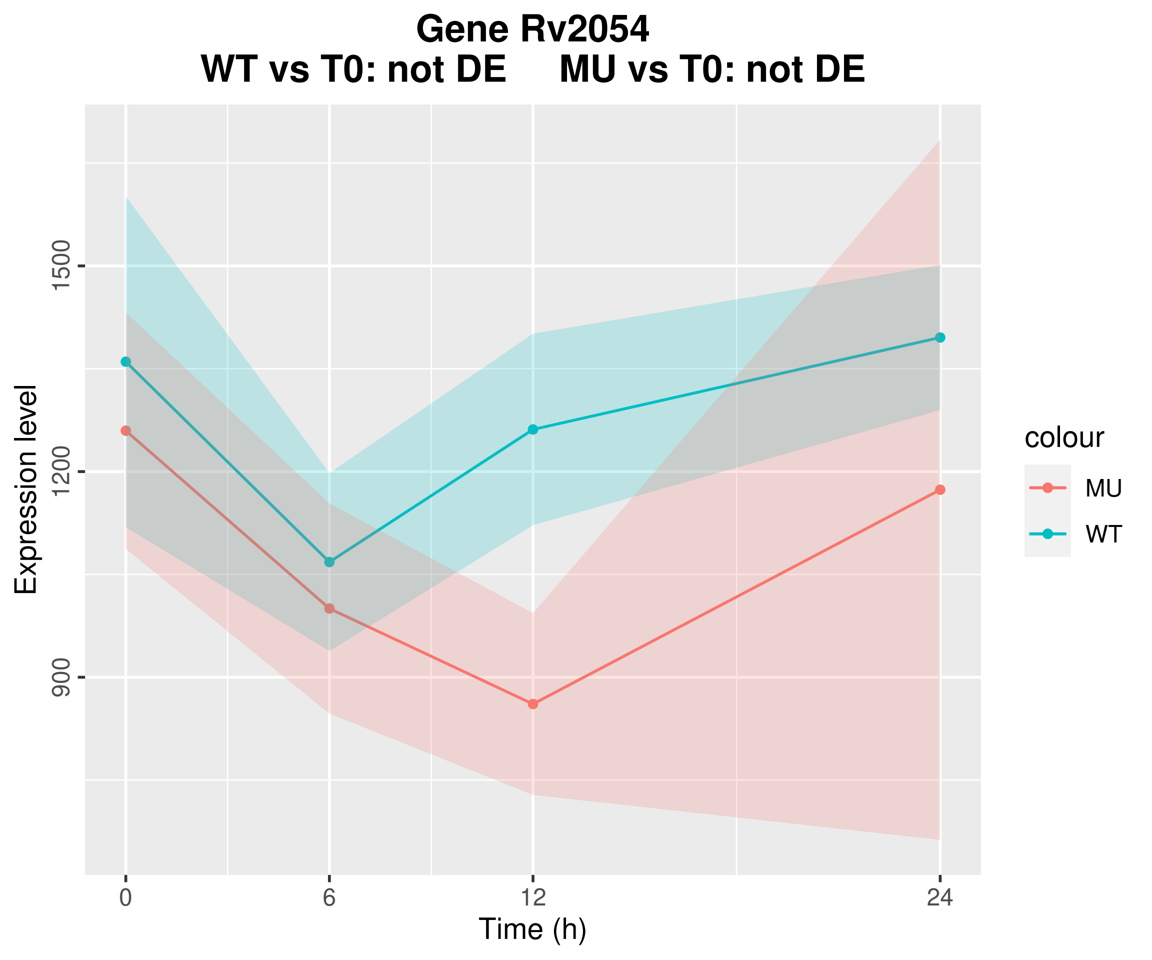
Gene Rv2054
WT vs T0: not DE   MU vs T0: not DE
Expression level
1500
1200
900
0
6
12
24
Time (h)
colour
MU
WT

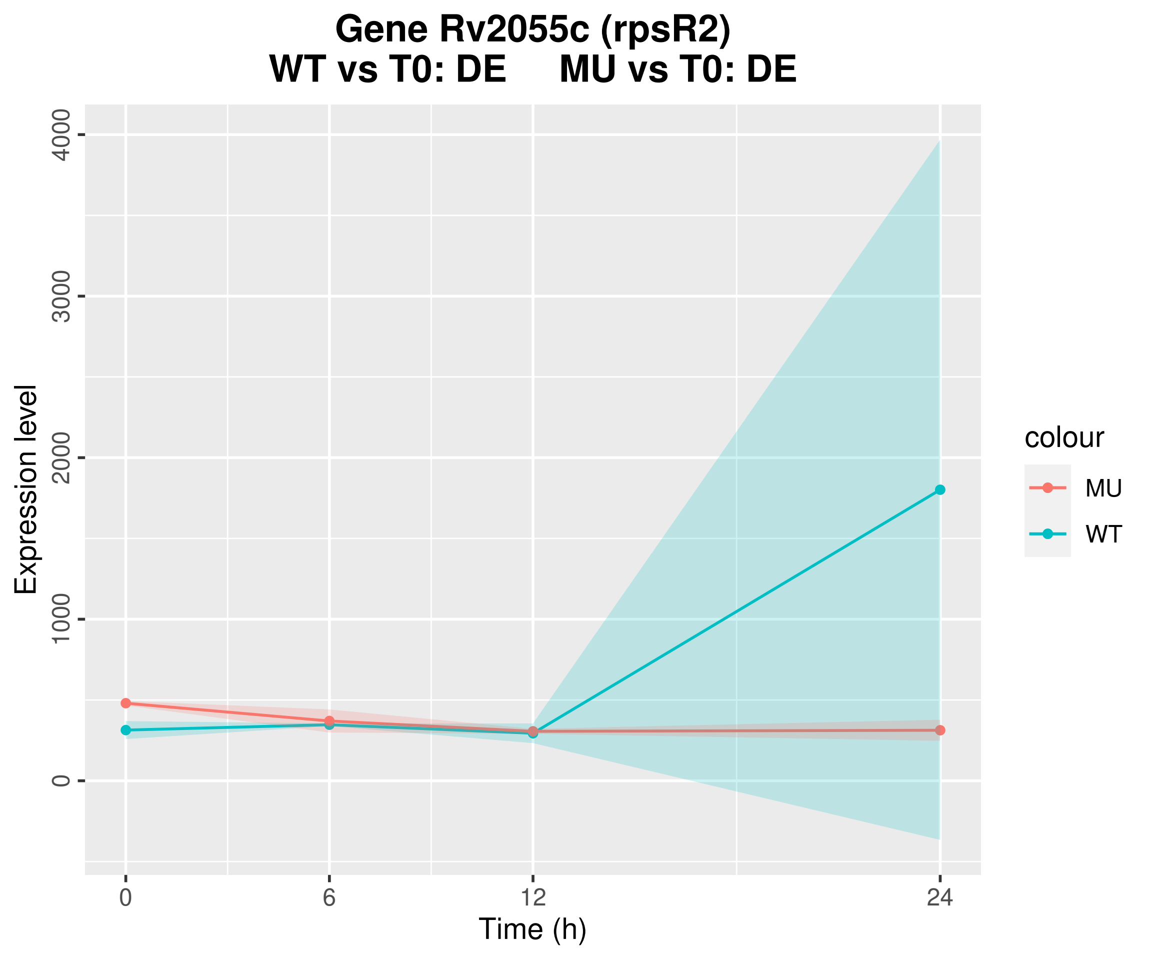
Gene Rv2055c (rpsR2)
WT vs T0: DE   MU vs T0: DE
Expression level
4000
3000
2000
1000
0
0
6
12
24
Time (h)
colour
MU
WT

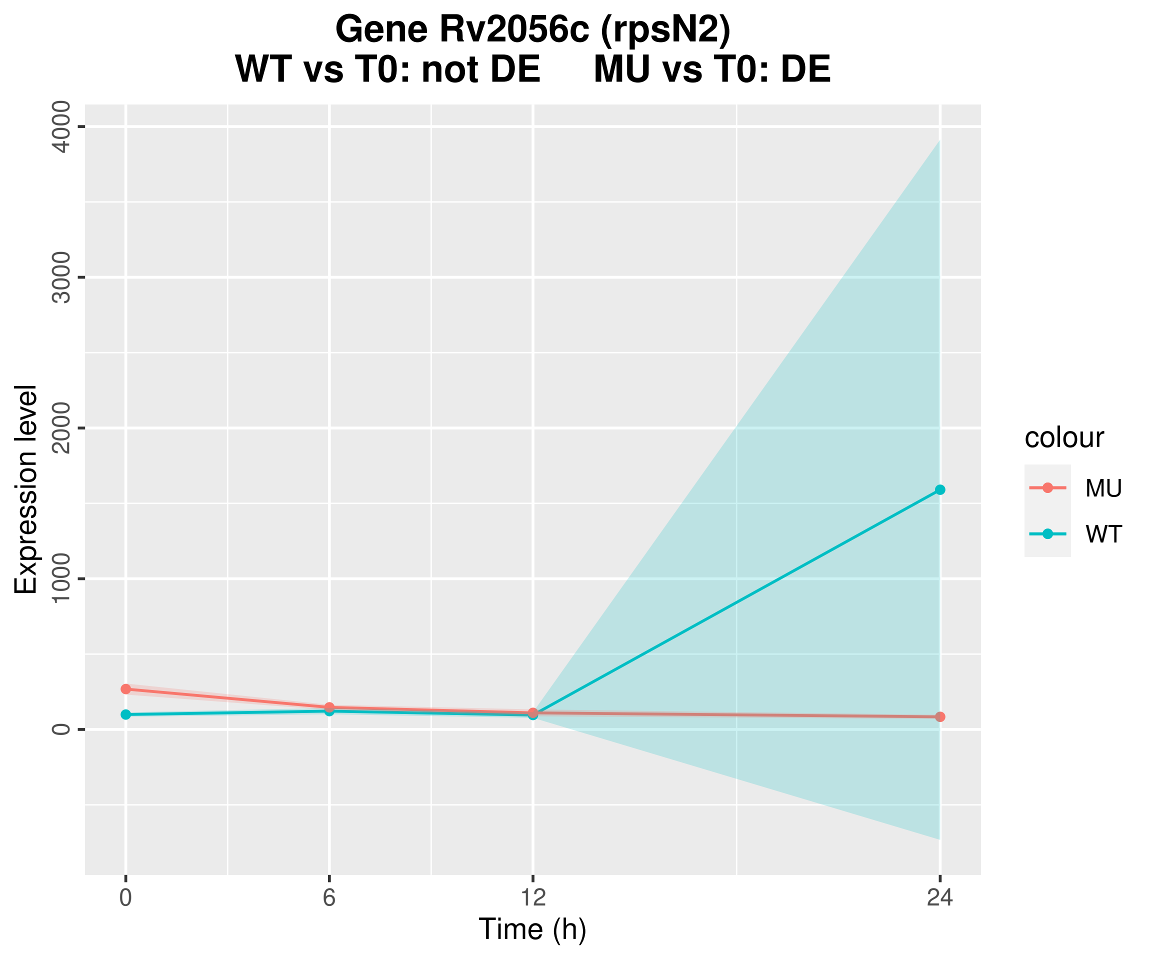
Gene Rv2056c (rpsN2)
WT vs T0: not DE   MU vs T0: DE
Expression level
4000
3000
2000
1000
0
0
6
12
24
Time (h)
colour
MU
WT

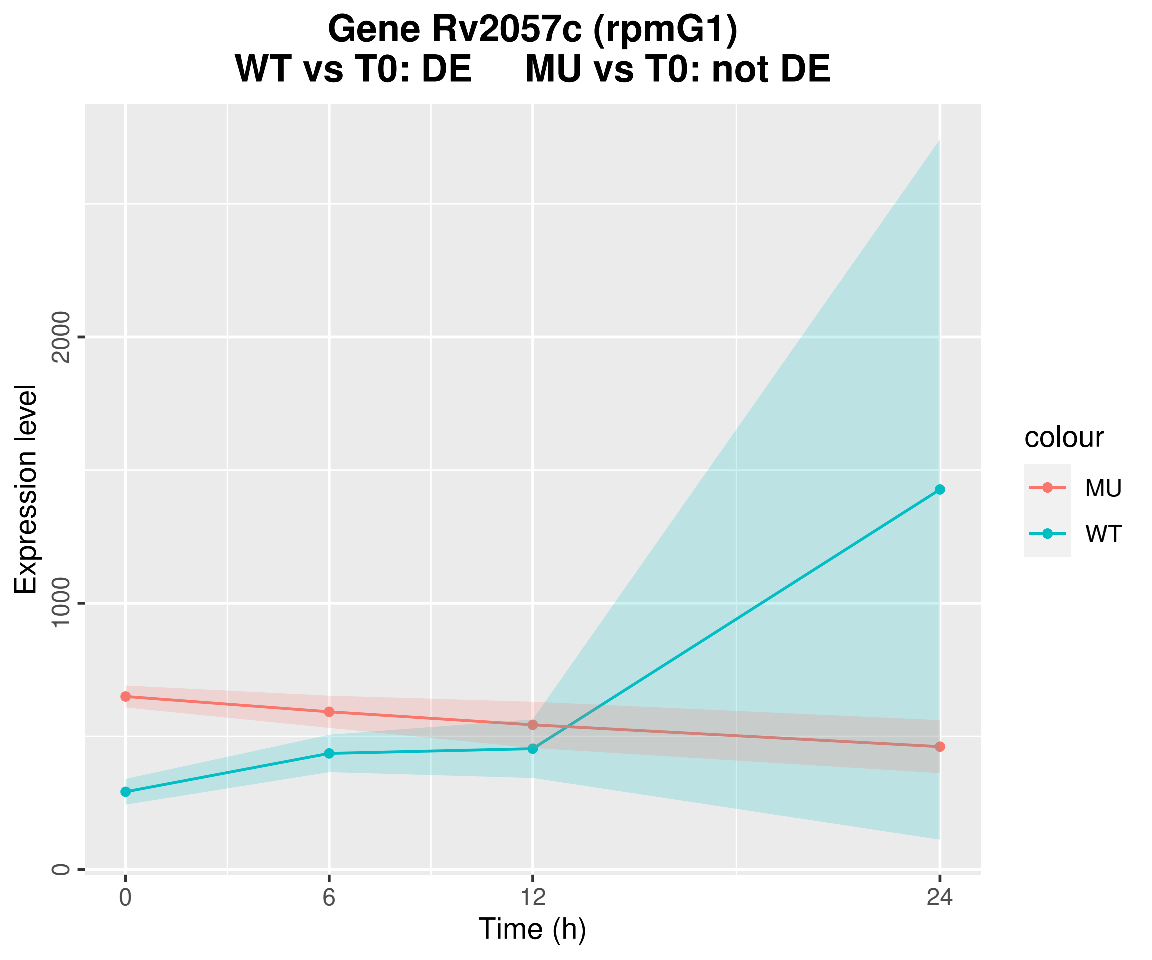
Gene Rv2057c (rpmG1)
WT vs T0: DE   MU vs T0: not DE
Expression level
2000
1000
0
0
6
12
24
Time (h)
colour
MU
WT

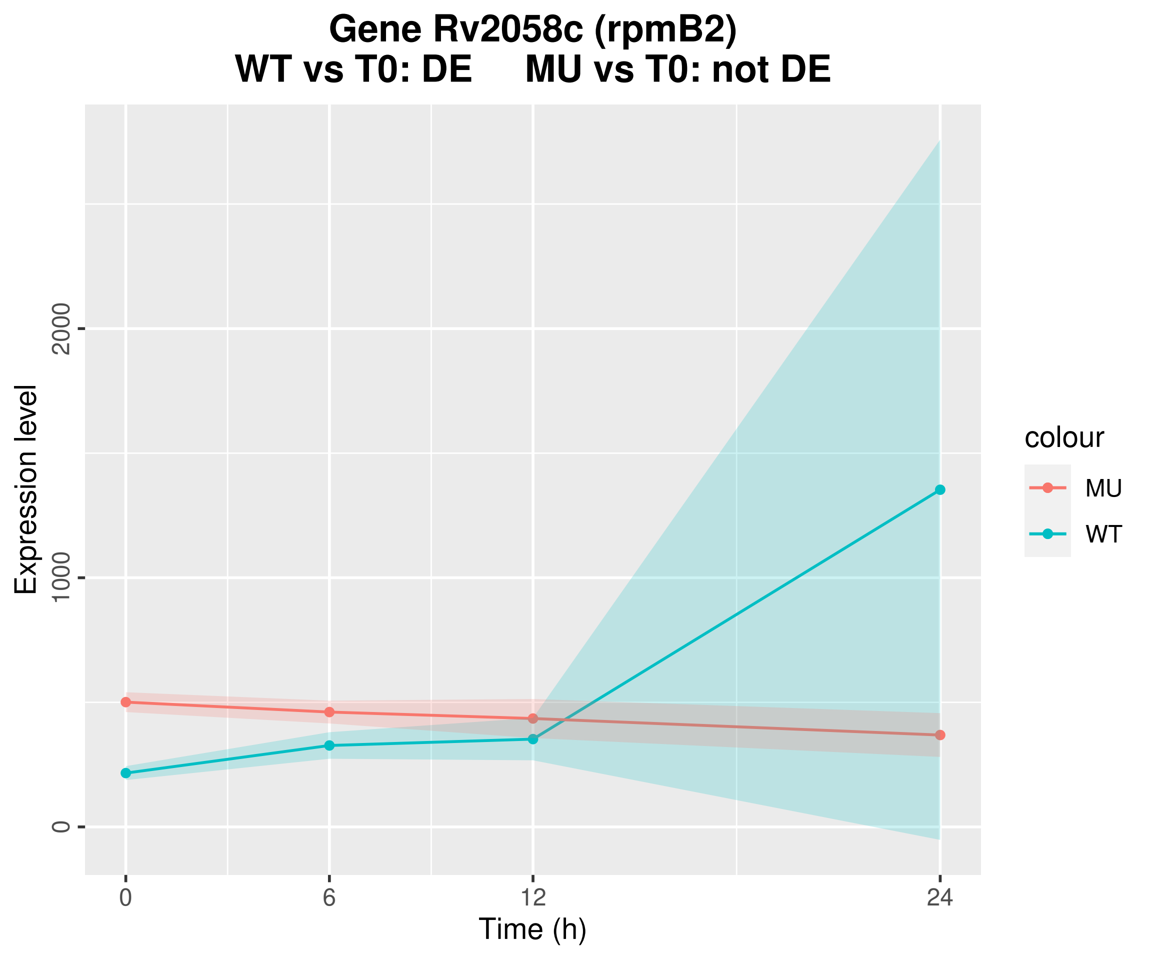

Gene Rv2058c (rpmB2)
WT vs T0: DE   MU vs T0: not DE
Expression level
0
1000
2000
0
6
12
24
Time (h)
colour
MU
WT

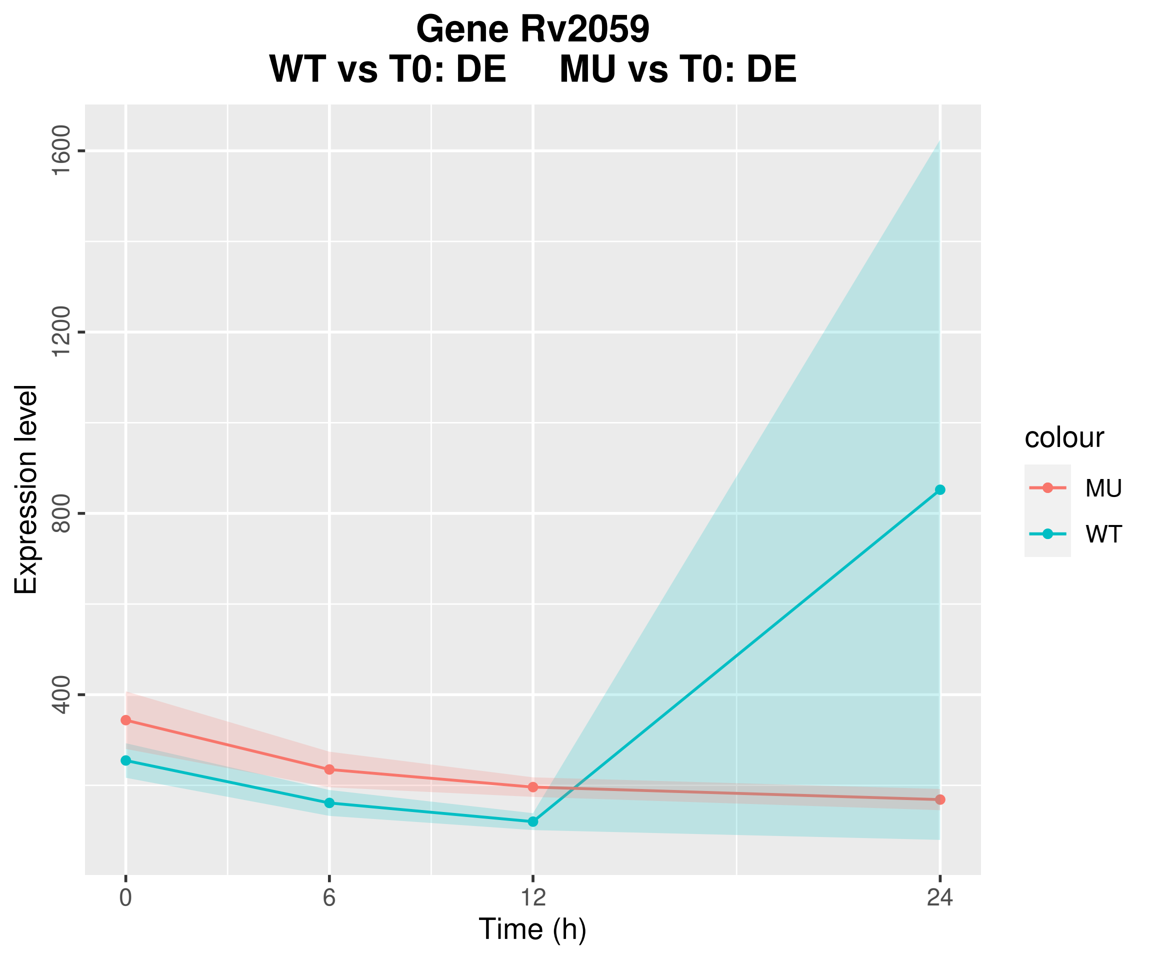

Gene Rv2059
WT vs T0: DE   MU vs T0: DE
Expression level
1600
1200
800
400
0
6
12
24
Time (h)
colour
MU
WT

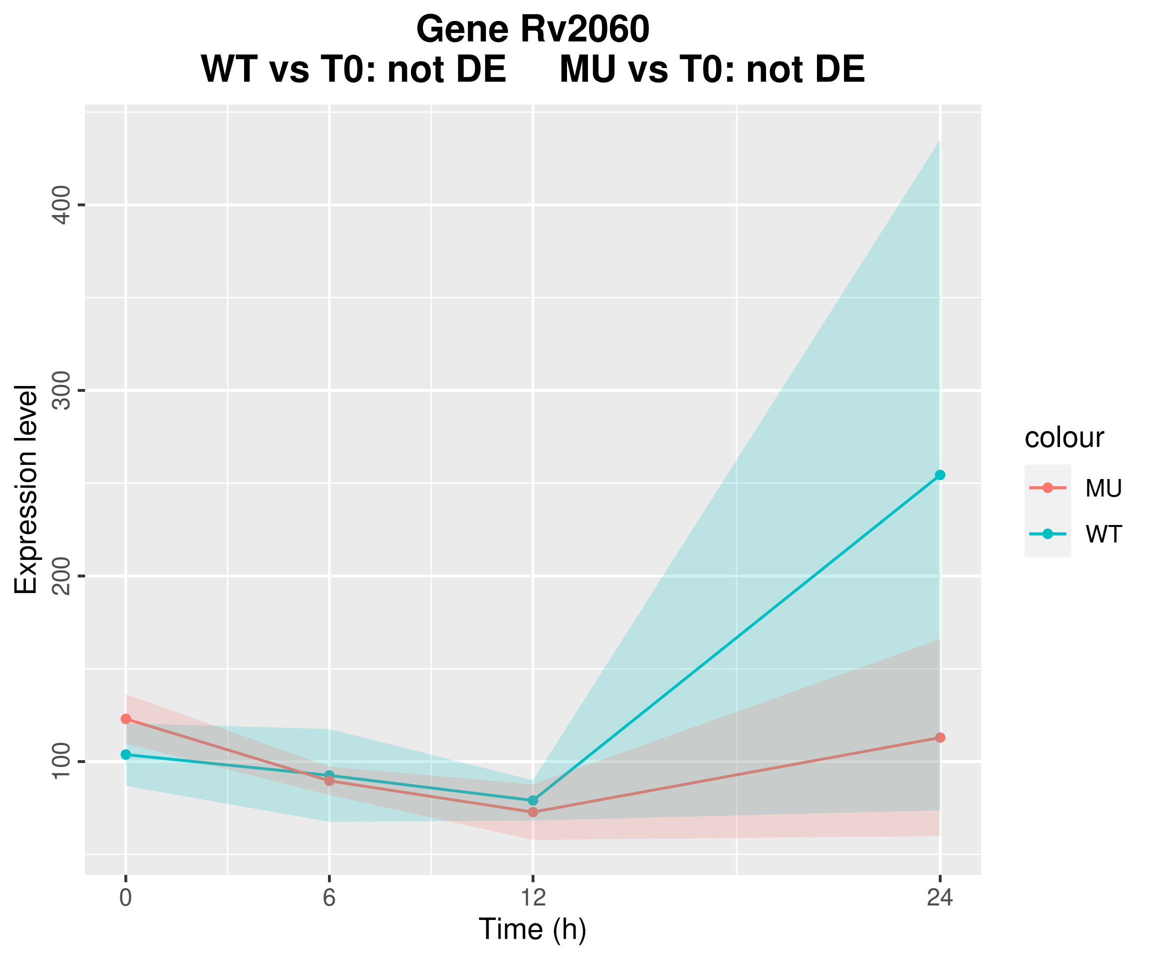
Gene Rv2060
WT vs T0: not DE MU vs T0: not DE
Expression level
400
300
200
100
0
6
12
24
Time (h)
colour
MU
WT

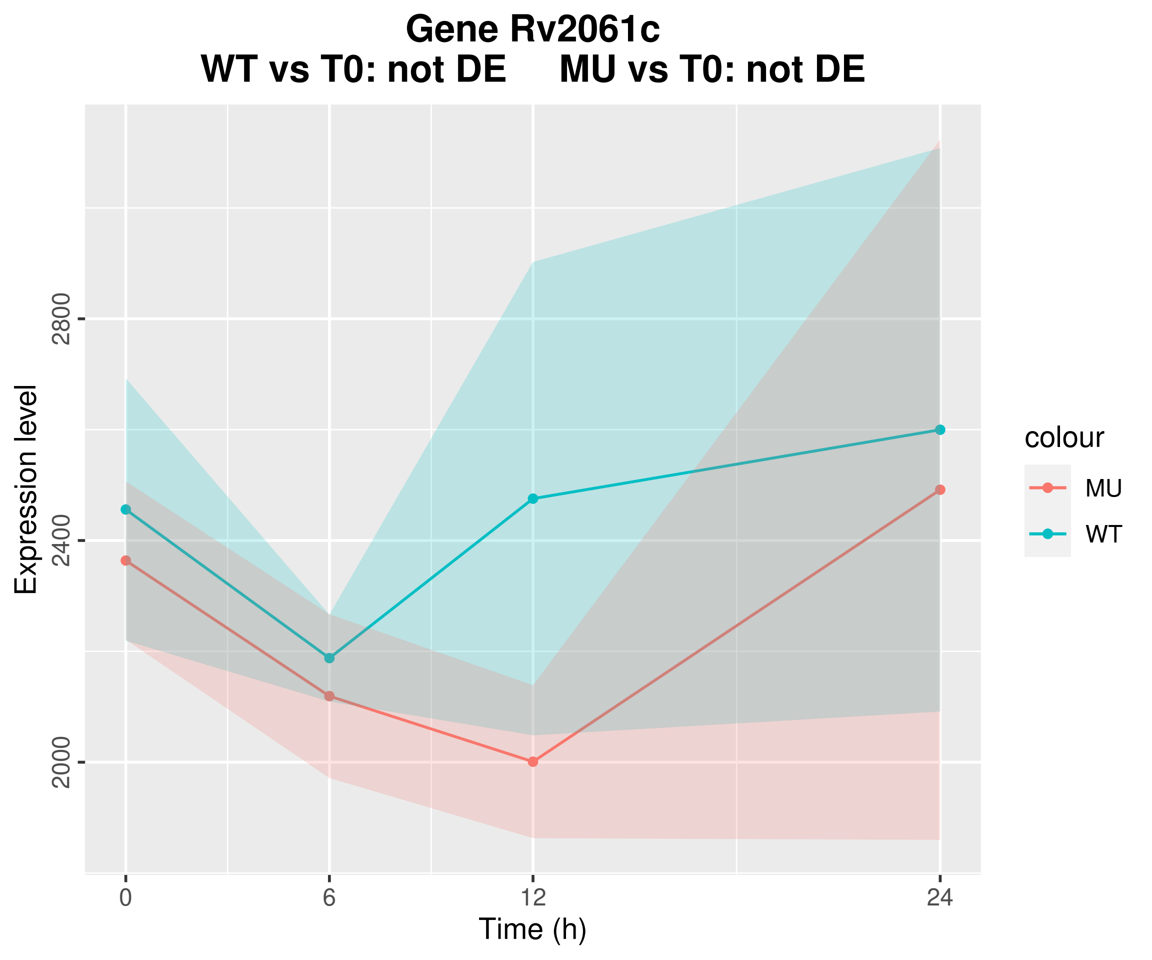
Gene Rv2061c
WT vs T0: not DE   MU vs T0: not DE
Expression level
2000
2400
2800
0
6
12
24
Time (h)
colour
MU
WT

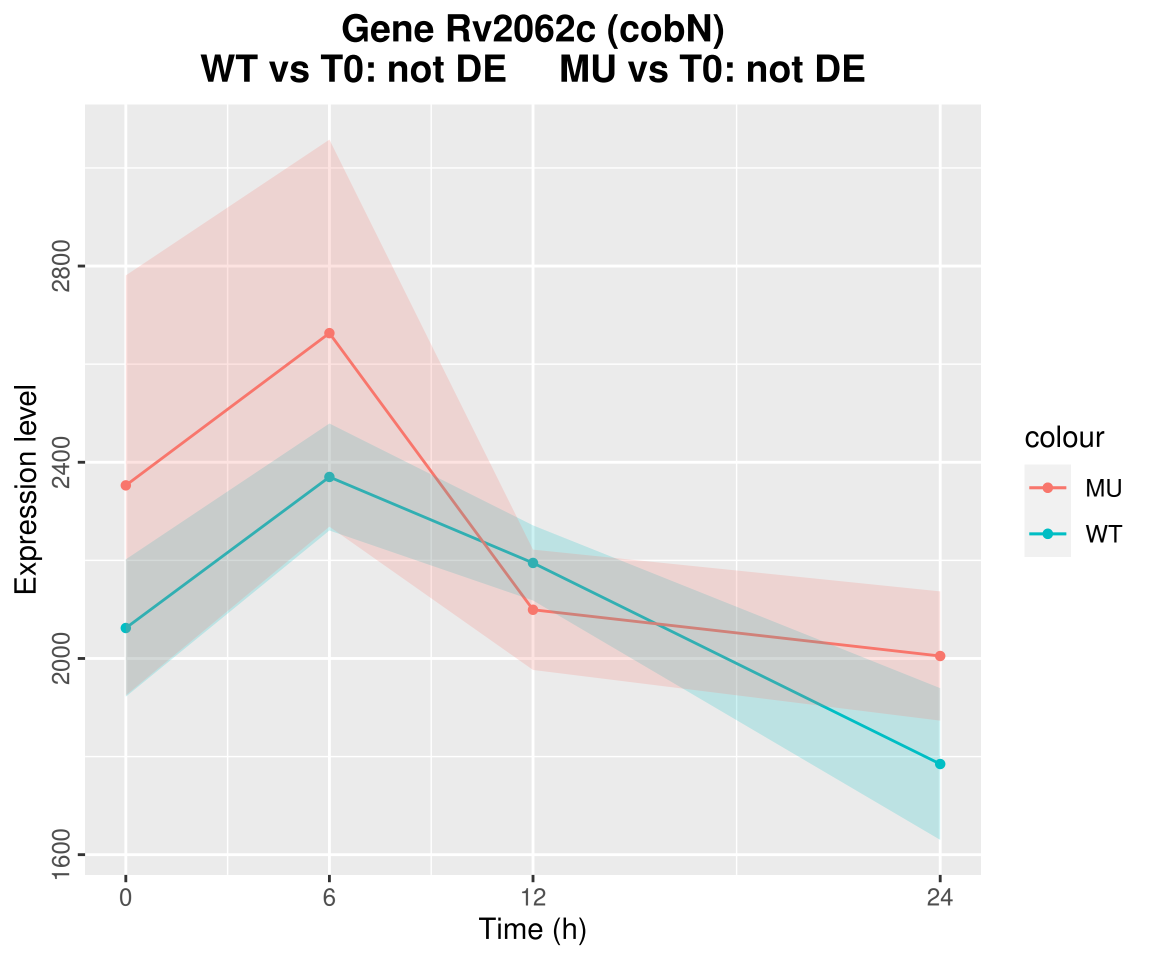
Gene Rv2062c (cobN)
WT vs T0: not DE   MU vs T0: not DE
Expression level
2800
2400
2000
1600
0
6
12
24
Time (h)
colour
MU
WT

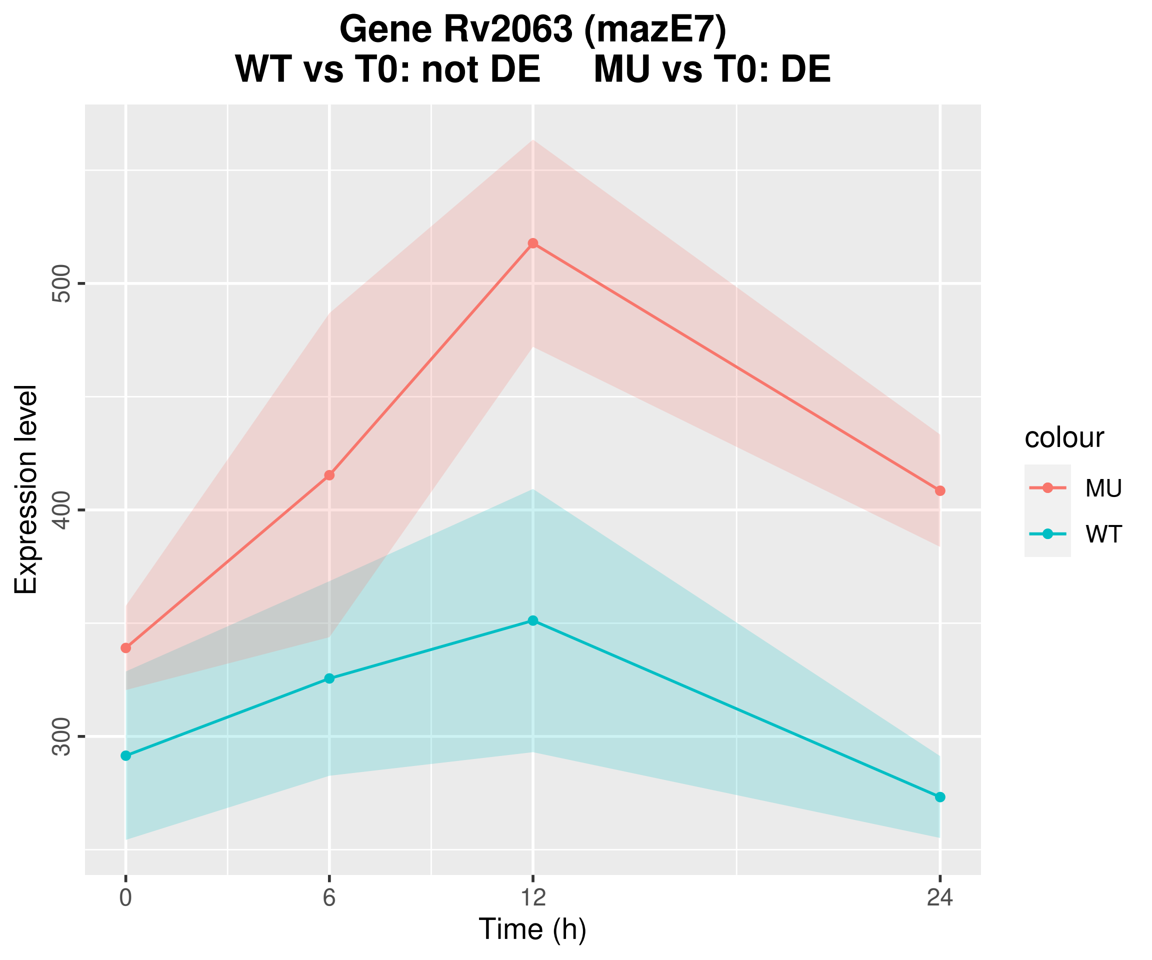

Gene Rv2063 (mazE7)
WT vs T0: not DE MU vs T0: DE
Expression level
500
400
300
0
6
12
24
Time (h)
colour
MU
WT

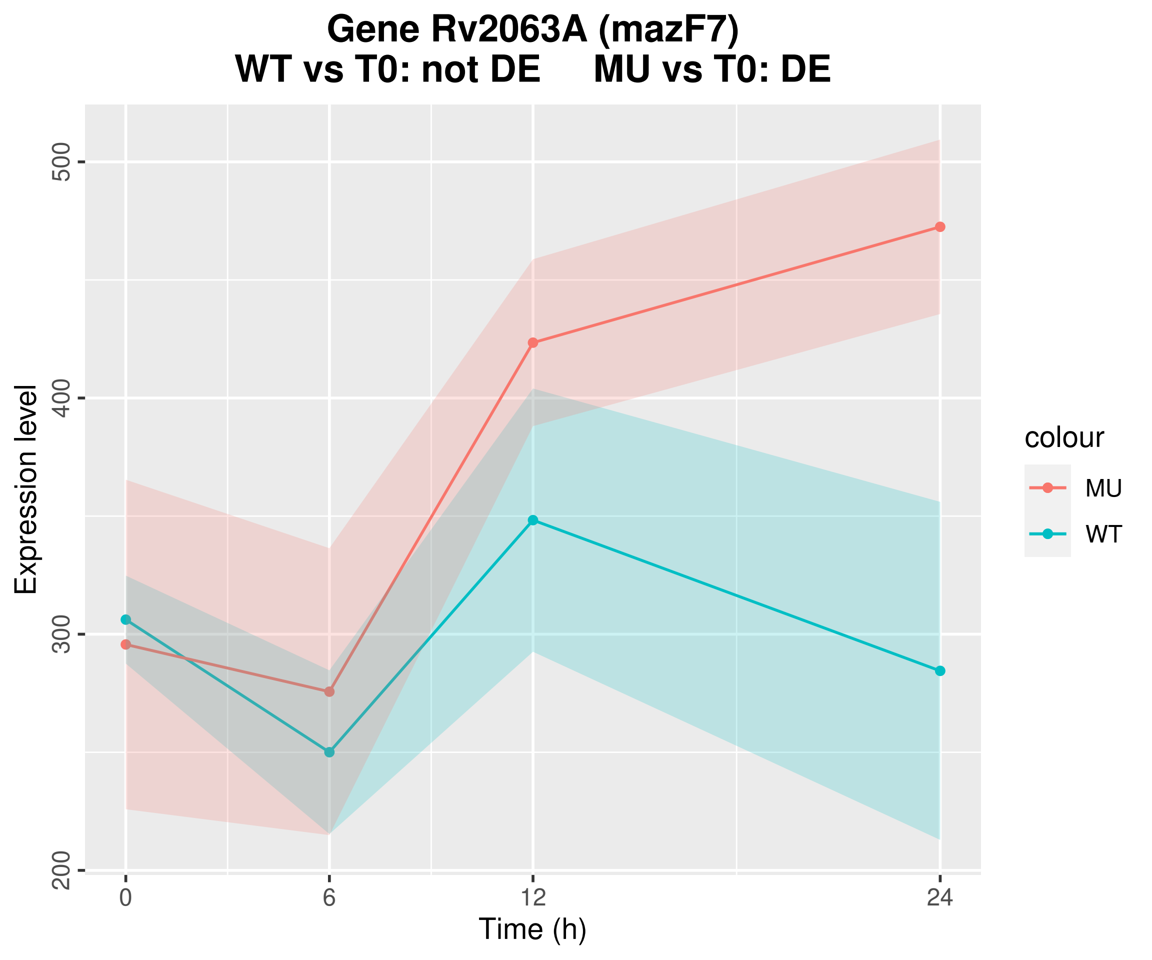

Gene Rv2063A (mazF7)
WT vs T0: not DE   MU vs T0: DE
Expression level
500
400
300
200
0
6
12
24
Time (h)
colour
MU
WT

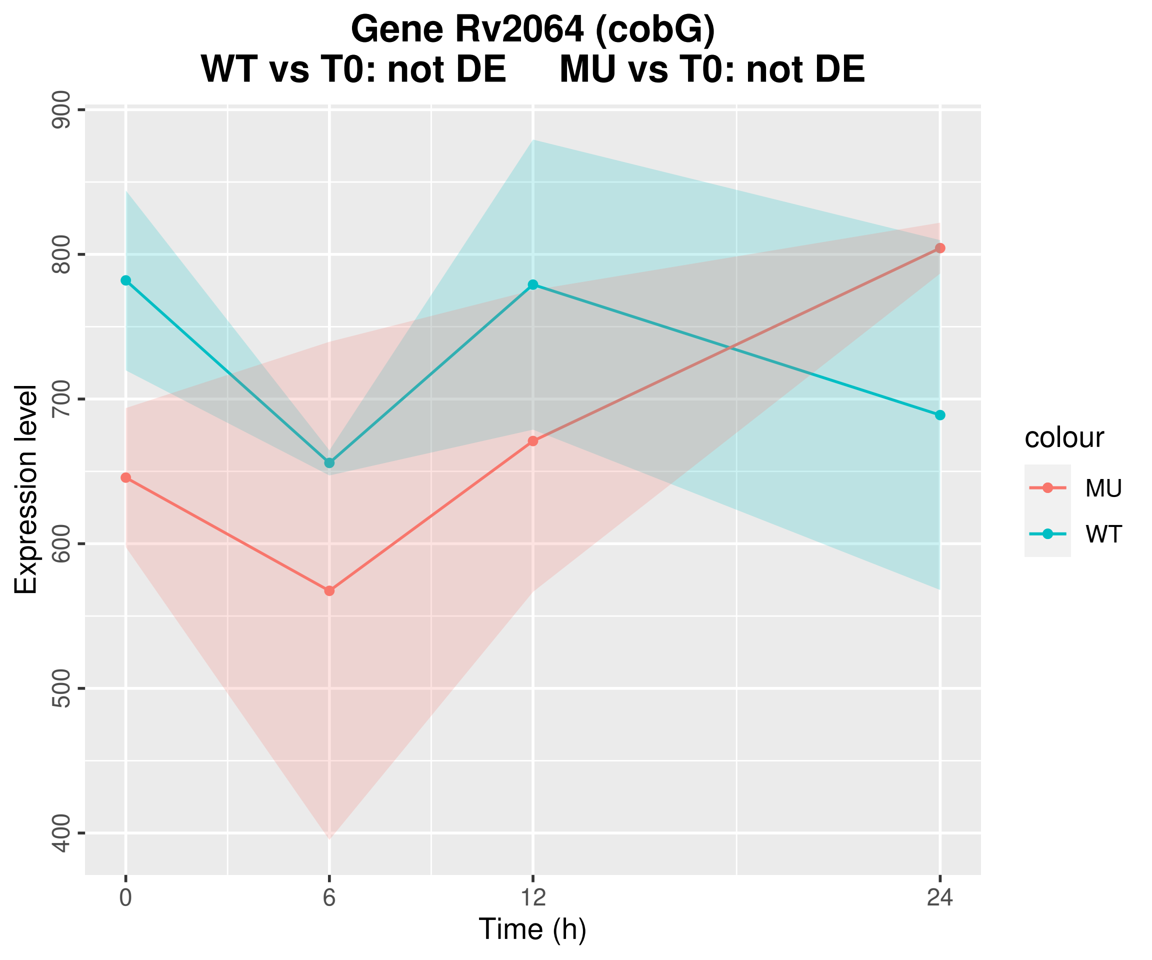
Gene Rv2064 (cobG)
WT vs T0: not DE   MU vs T0: not DE
Expression level
900
800
700
600
500
400
Time (h)
0
6
12
24
colour
MU
WT

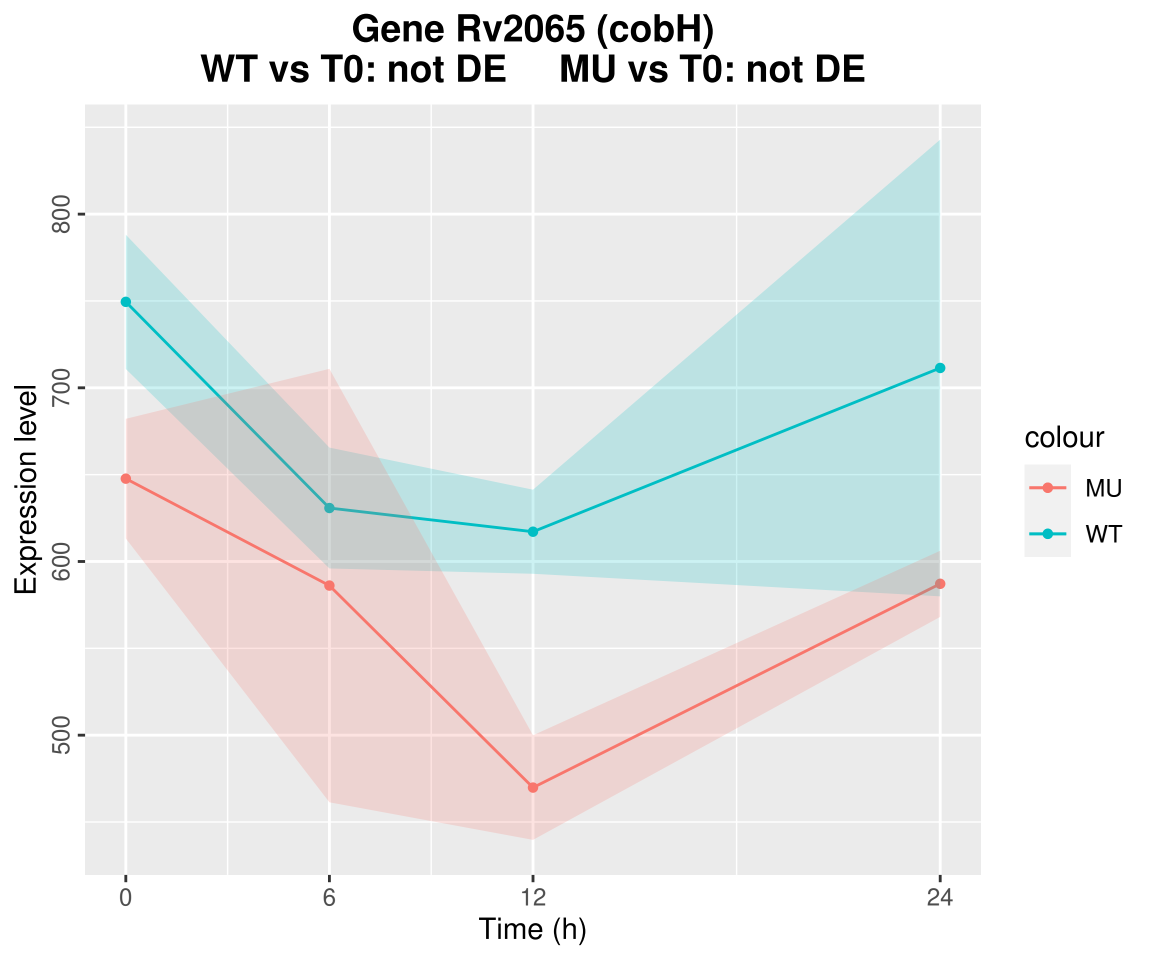

Gene Rv2065 (cobH)
WT vs T0: not DE   MU vs T0: not DE
Expression level
800
700
600
500
0
6
12
24
Time (h)
colour
MU
WT

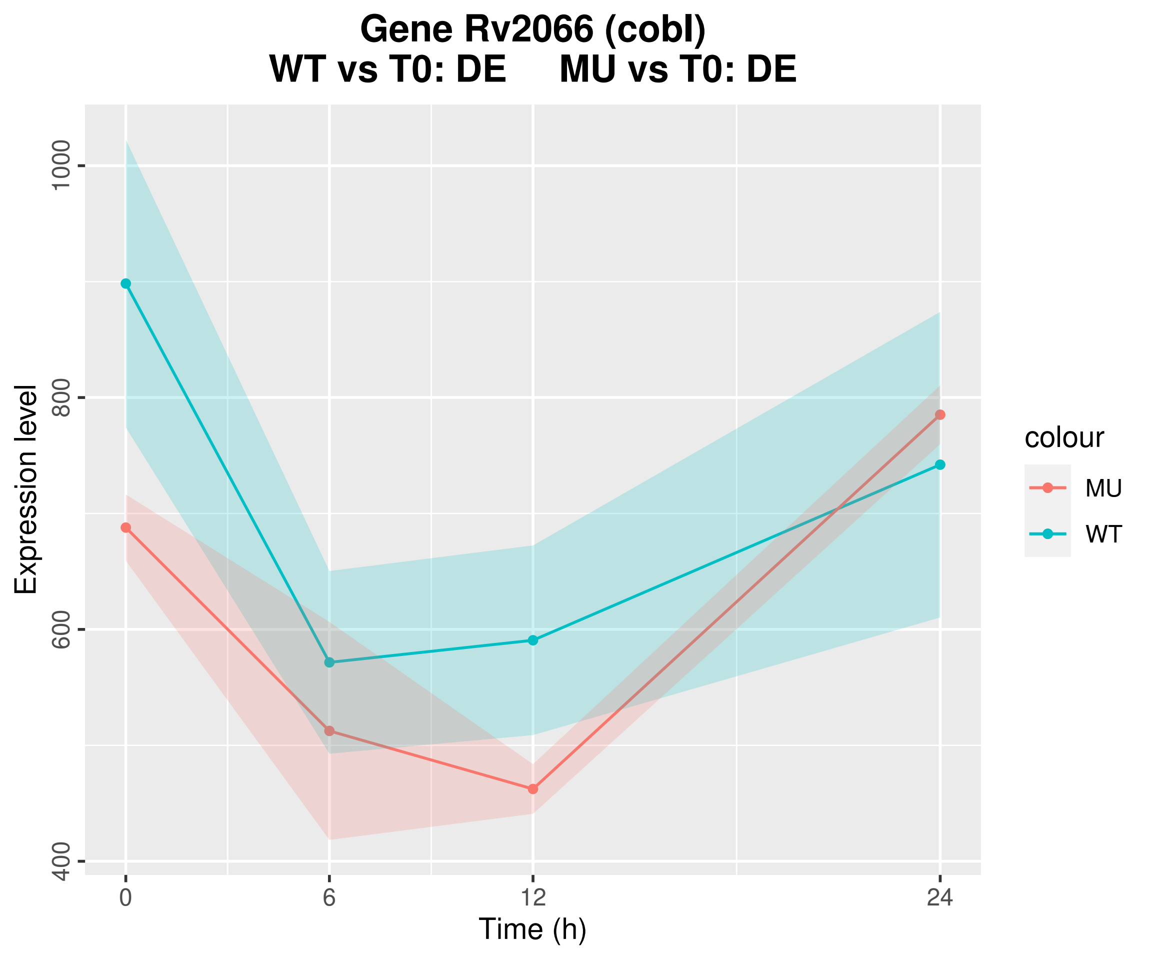
Gene Rv2066 (cobI)
WT vs T0: DE   MU vs T0: DE
Expression level
1000
800
600
400
0
6
12
24
Time (h)
colour
MU
WT

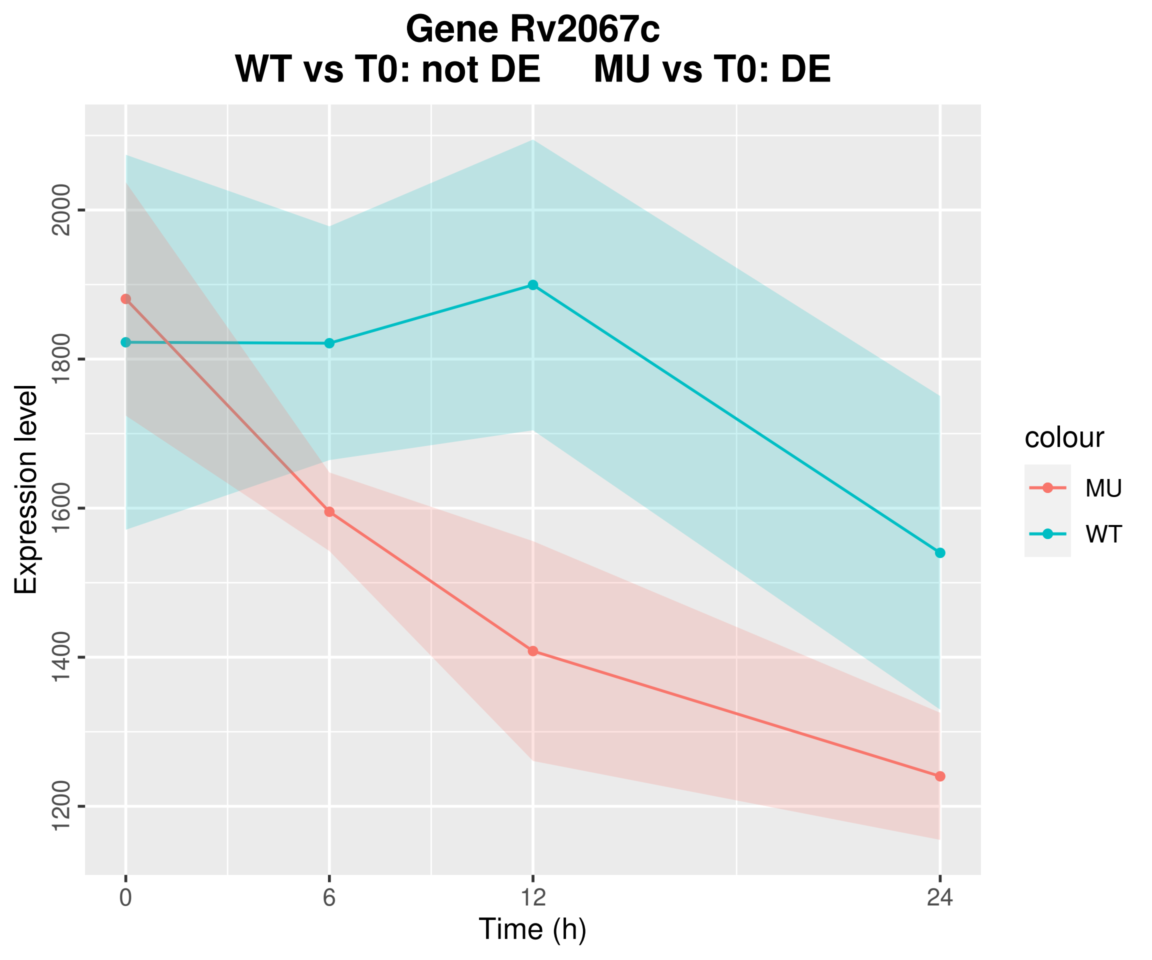
Gene Rv2067c
WT vs T0: not DE   MU vs T0: DE
Expression level
2000
1800
1600
1400
1200
0
6
12
24
Time (h)
colour
MU
WT

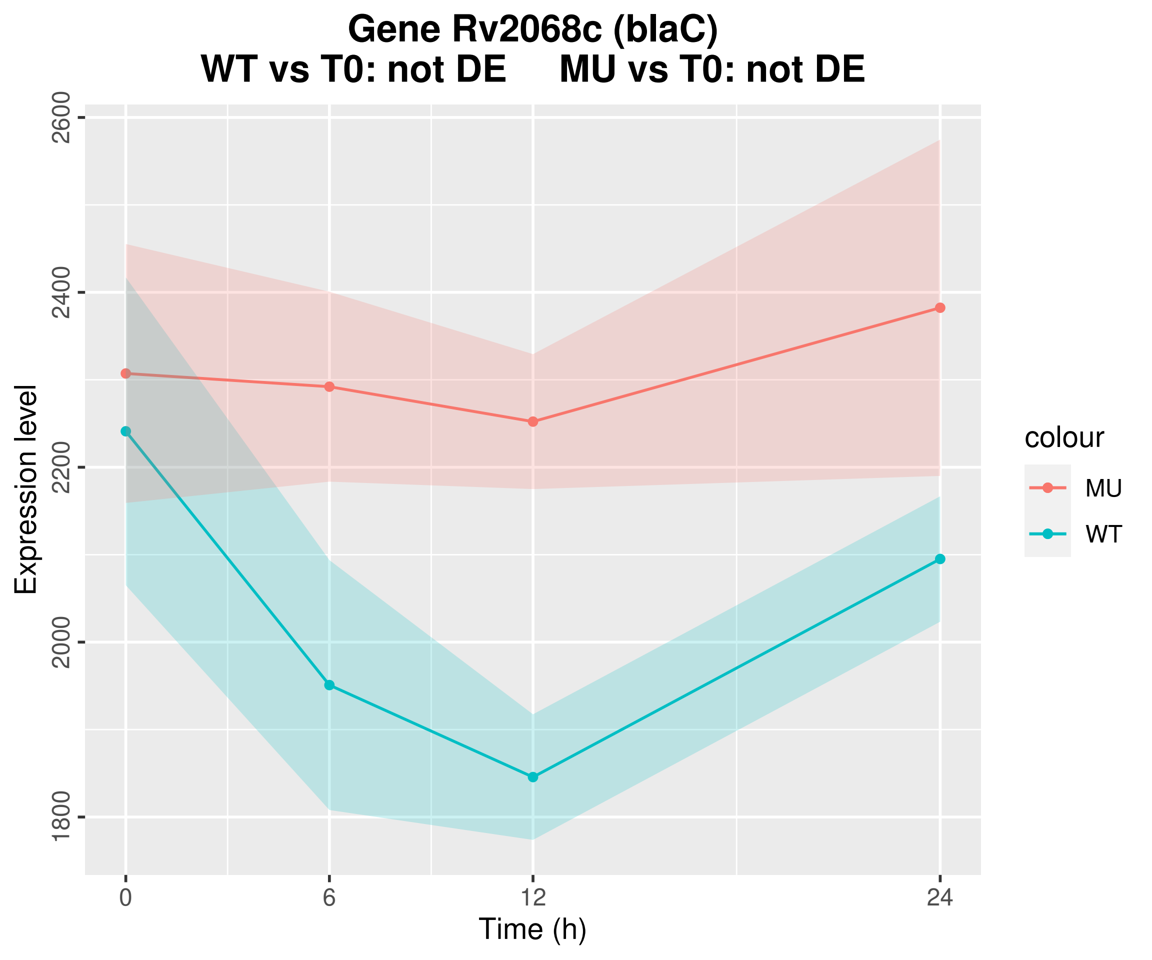
Gene Rv2068c (blaC)
WT vs T0: not DE   MU vs T0: not DE
Expression level
2600
2400
2200
2000
1800
0
6
12
24
Time (h)
colour
MU
WT

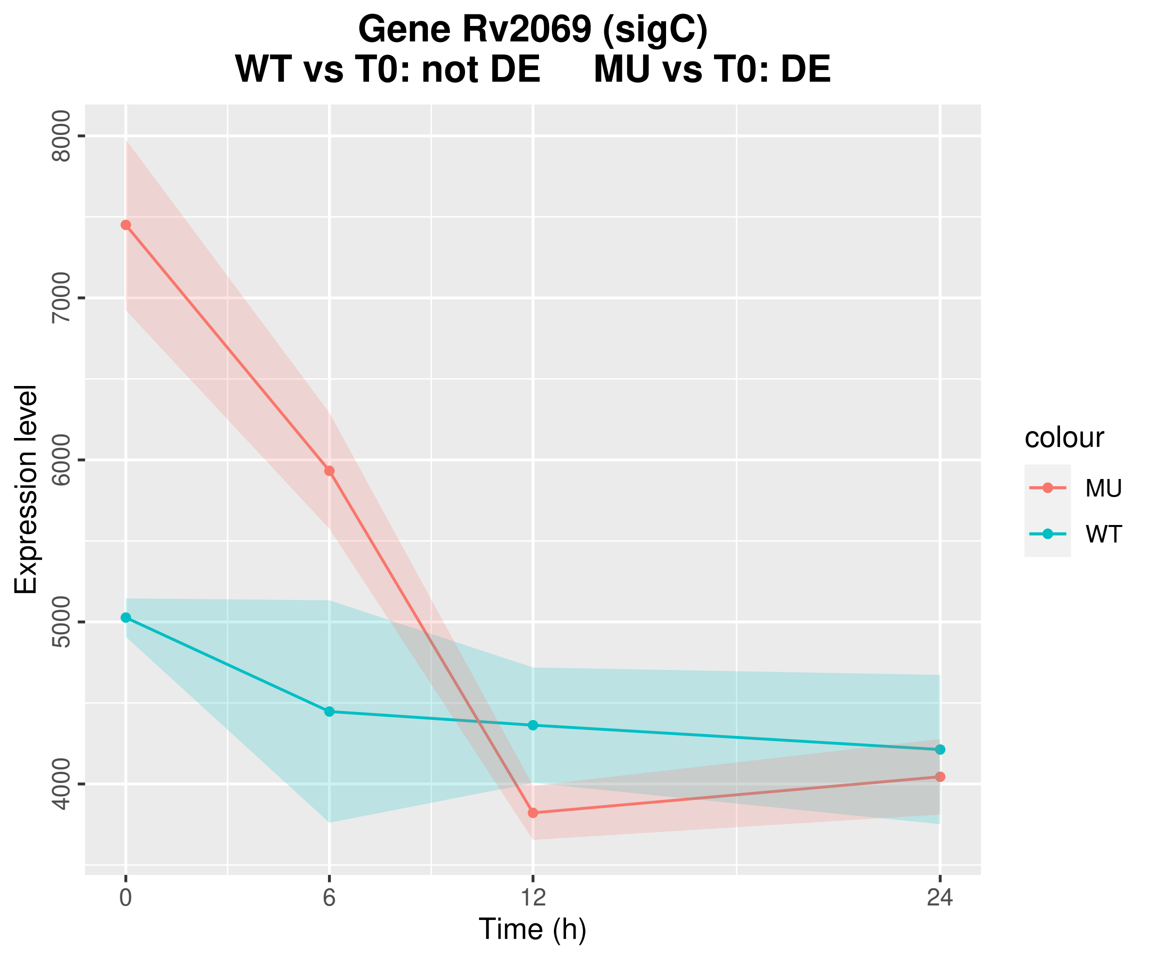
Gene Rv2069 (sigC)
WT vs T0: not DE   MU vs T0: DE
Expression level
8000
7000
6000
5000
4000
0
6
12
24
Time (h)
colour
MU
WT

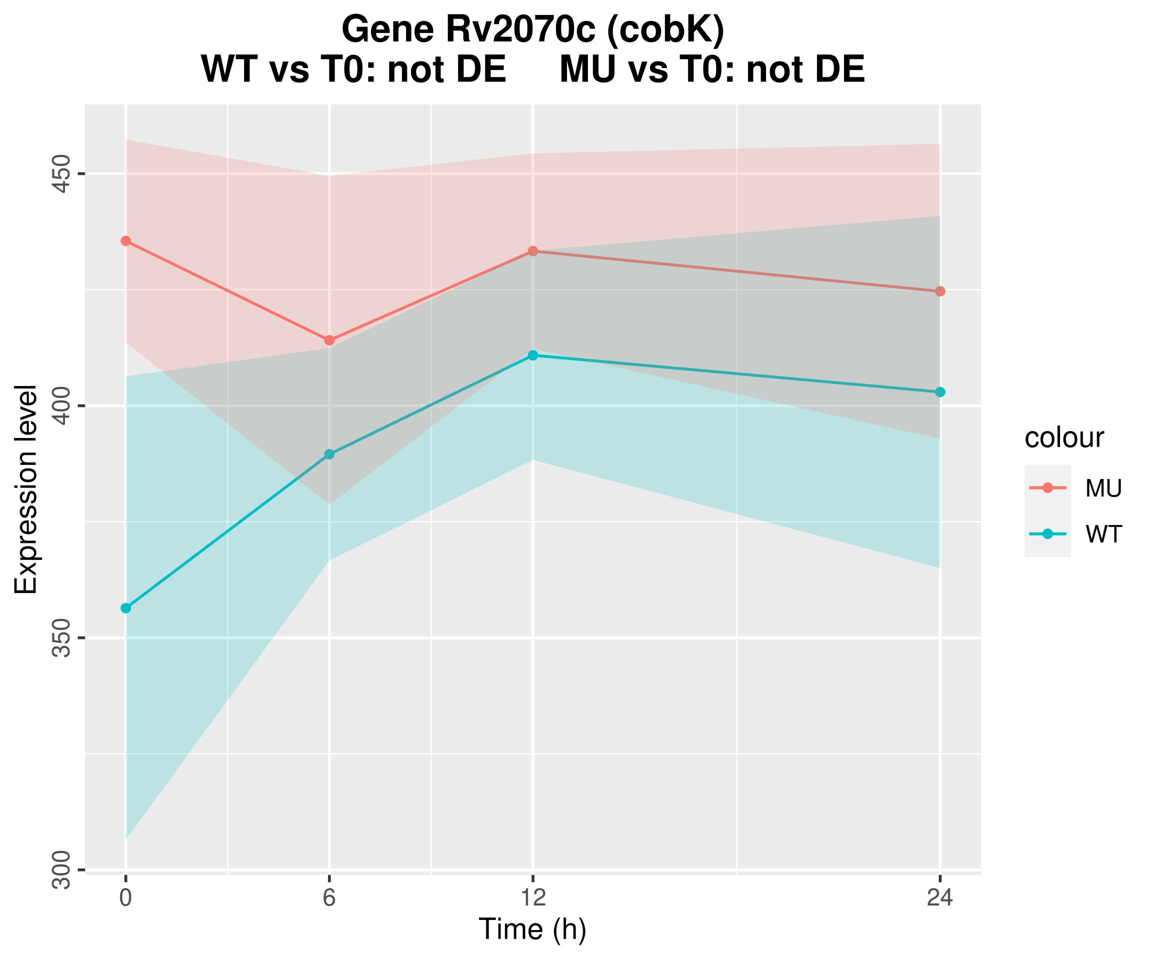
Gene Rv2070c (cobK)
WT vs T0: not DE   MU vs T0: not DE
Expression level
450
400
350
300
0
6
12
24
Time (h)
colour
MU
WT

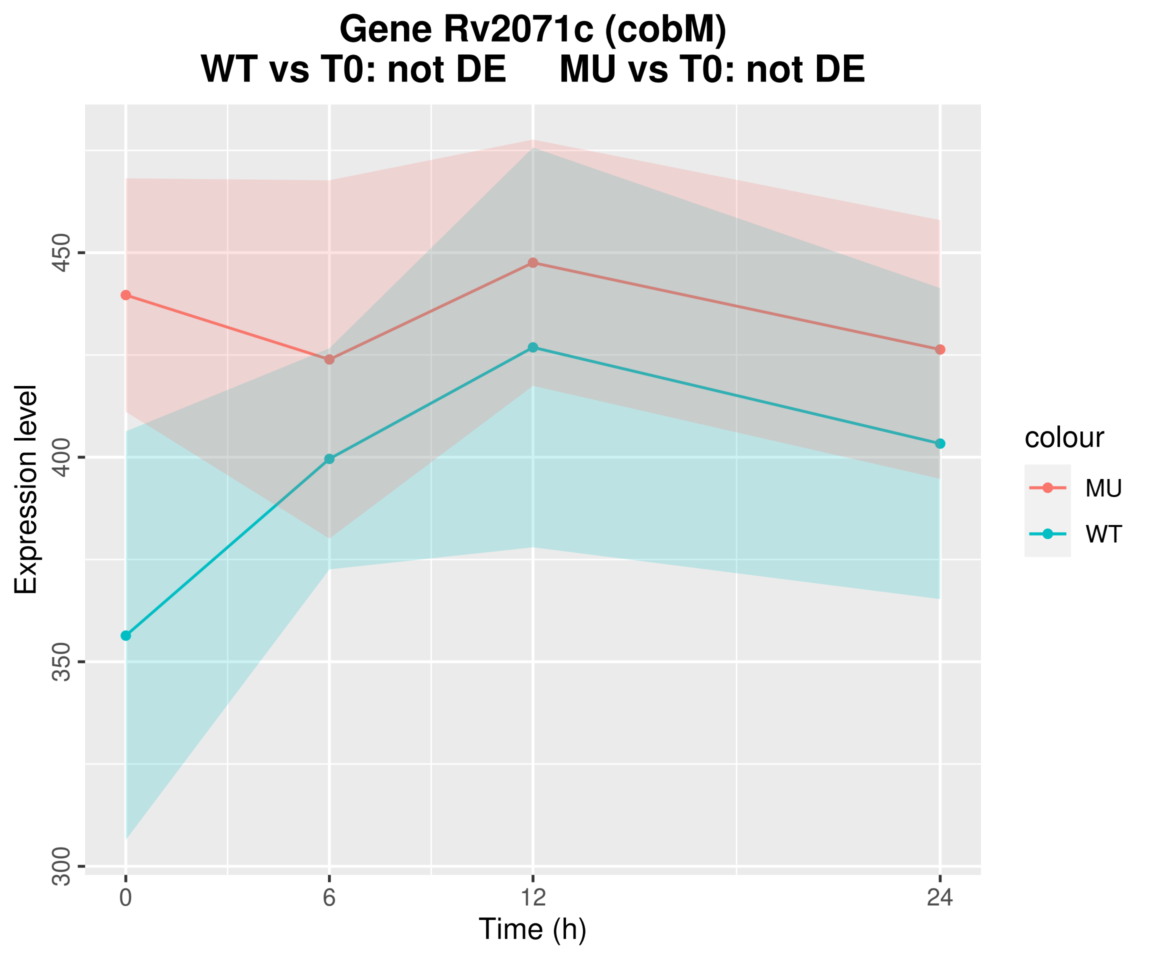
Gene Rv2071c (cobM)
WT vs T0: not DE   MU vs T0: not DE
Expression level
450
400
350
300
0
6
12
24
Time (h)
colour
MU
WT

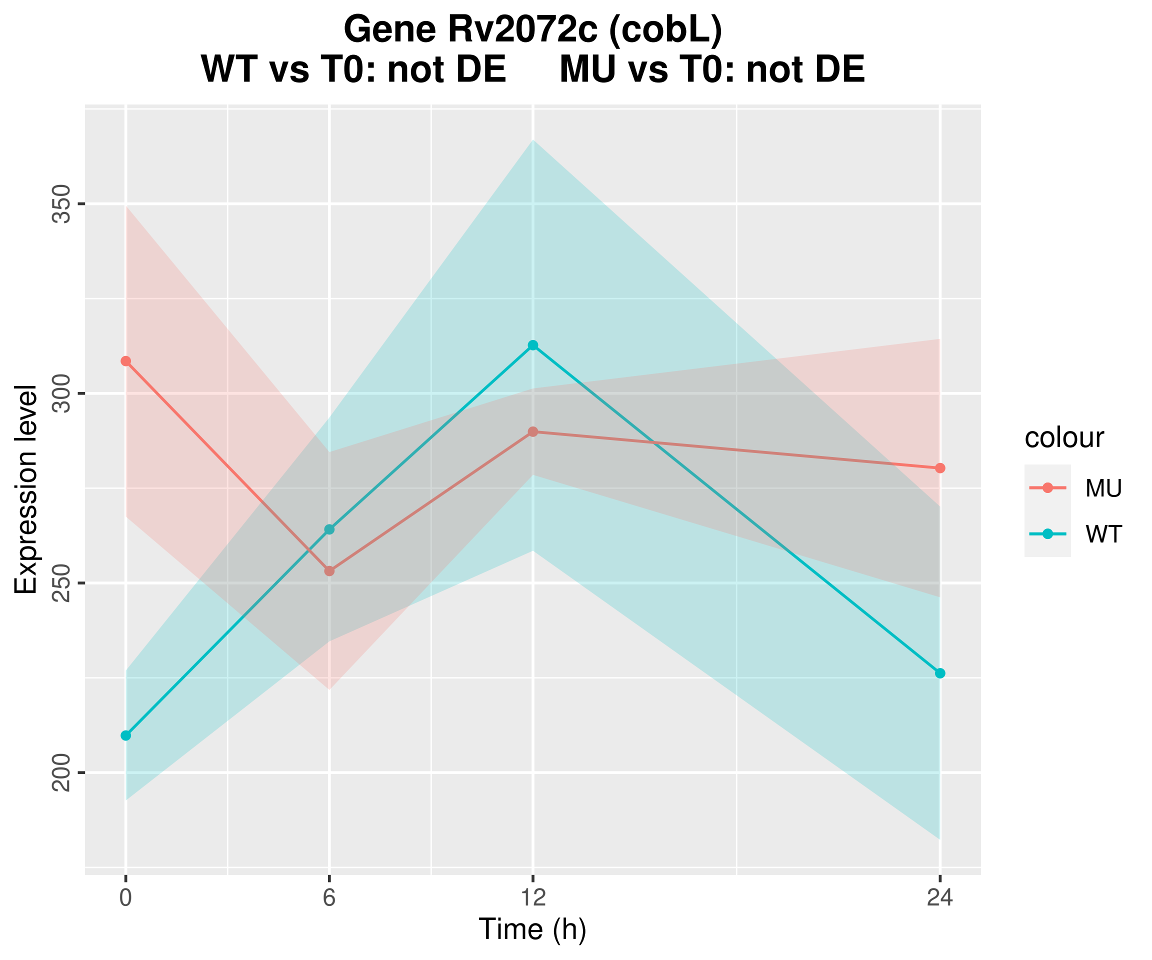

Gene Rv2072c (cobL)
WT vs T0: not DE MU vs T0: not DE
Expression level
350
300
250
200
0
6
12
24
Time (h)
colour
MU
WT

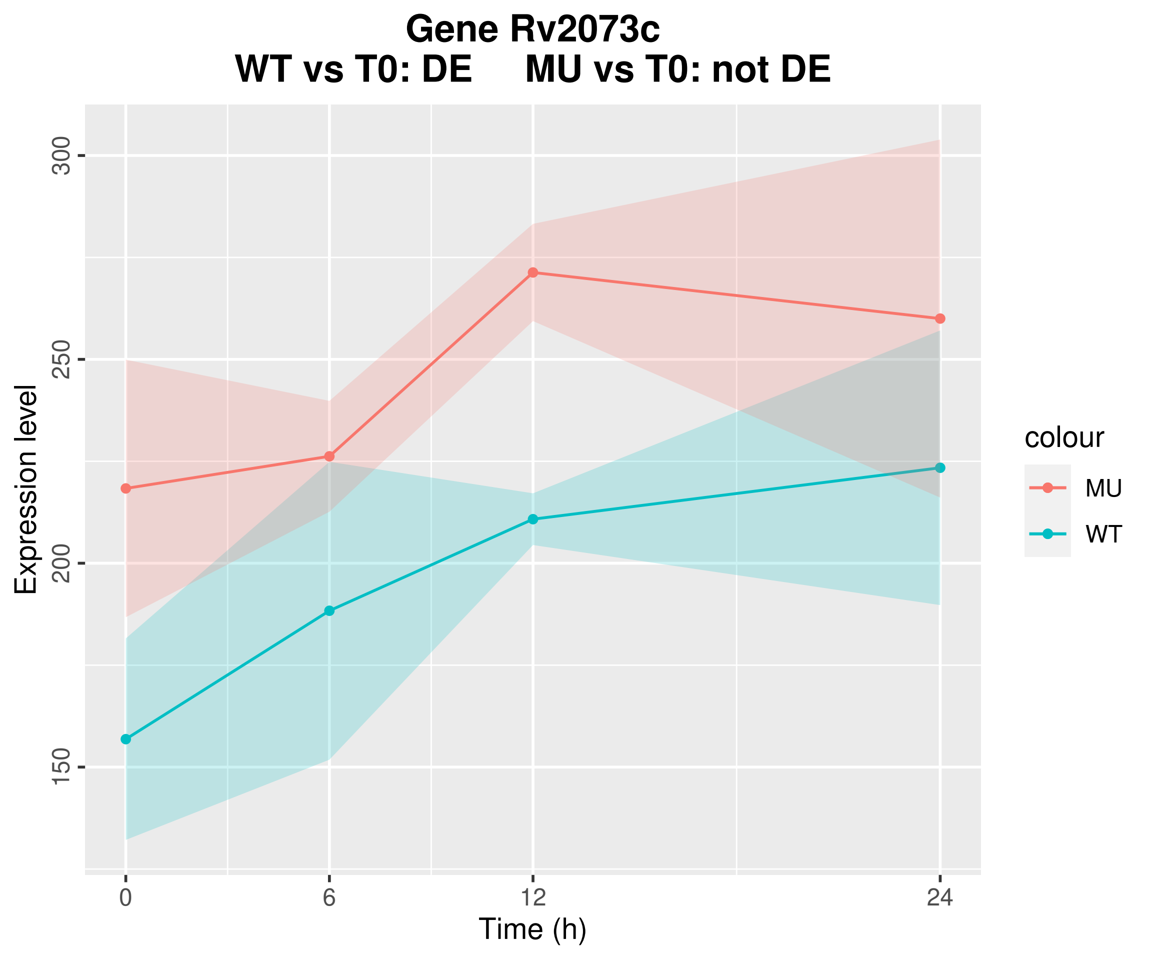
Gene Rv2073c
WT vs T0: DE MU vs T0: not DE
Expression level
300
250
200
150
0
6
12
24
Time (h)
colour
MU
WT

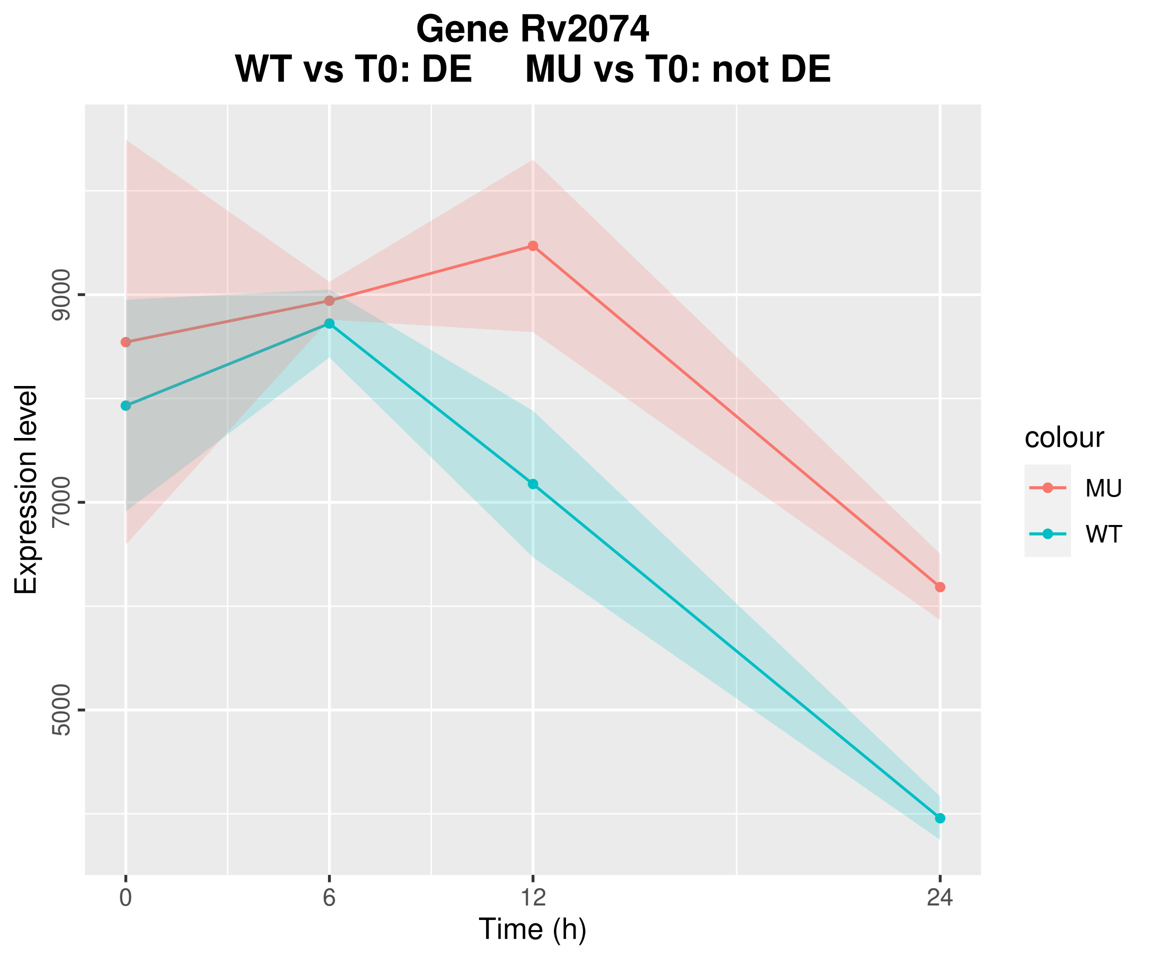

Gene Rv2074
WT vs T0: DE MU vs T0: not DE
Expression level
9000
7000
5000
0
6
12
24
Time (h)
colour
MU
WT

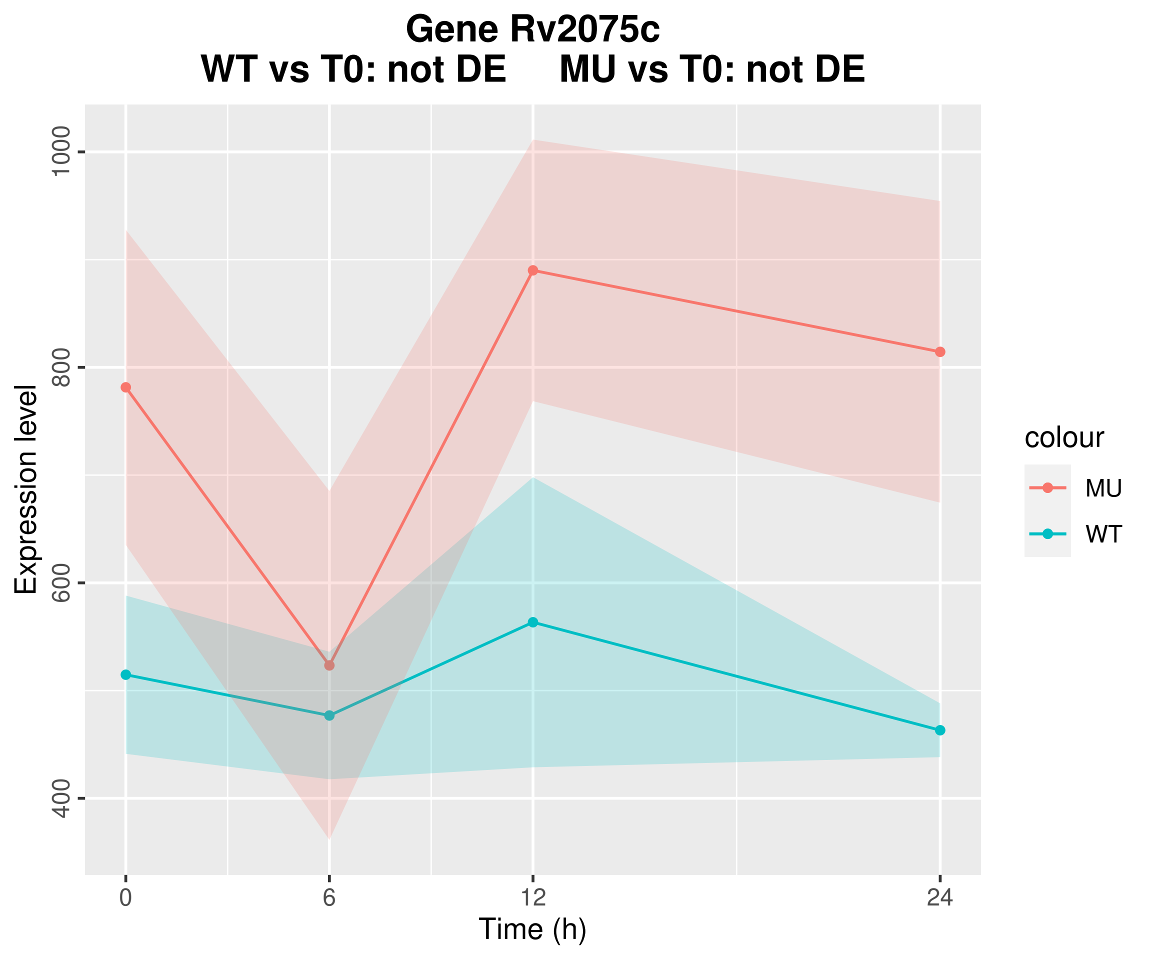

Gene Rv2075c
WT vs T0: not DE MU vs T0: not DE
Expression level
1000
800
600
400
0
6
12
24
Time (h)
colour
MU
WT

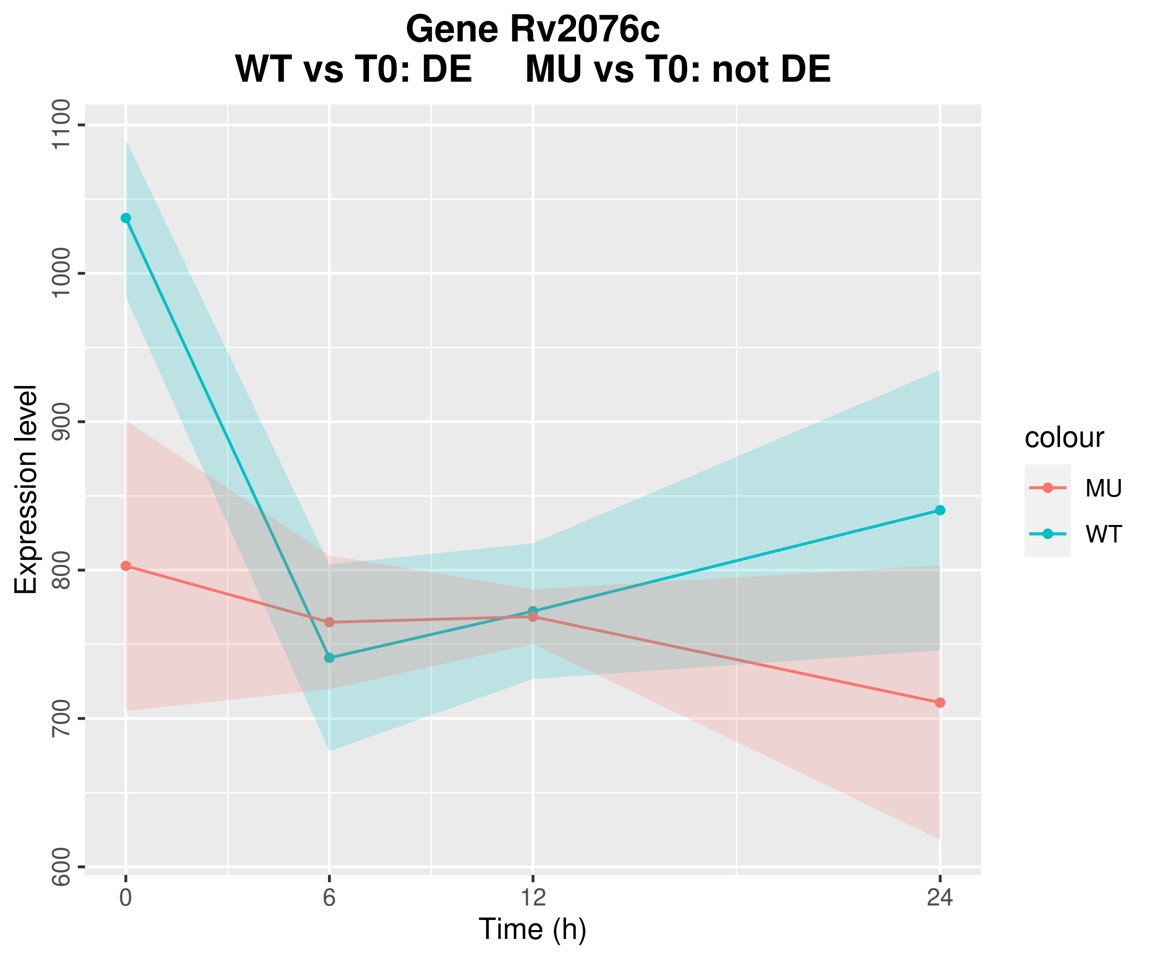

Gene Rv2076c
WT vs T0: DE   MU vs T0: not DE
Expression level
1100
1000
900
800
700
600
0
6
12
24
Time (h)
colour
MU
WT

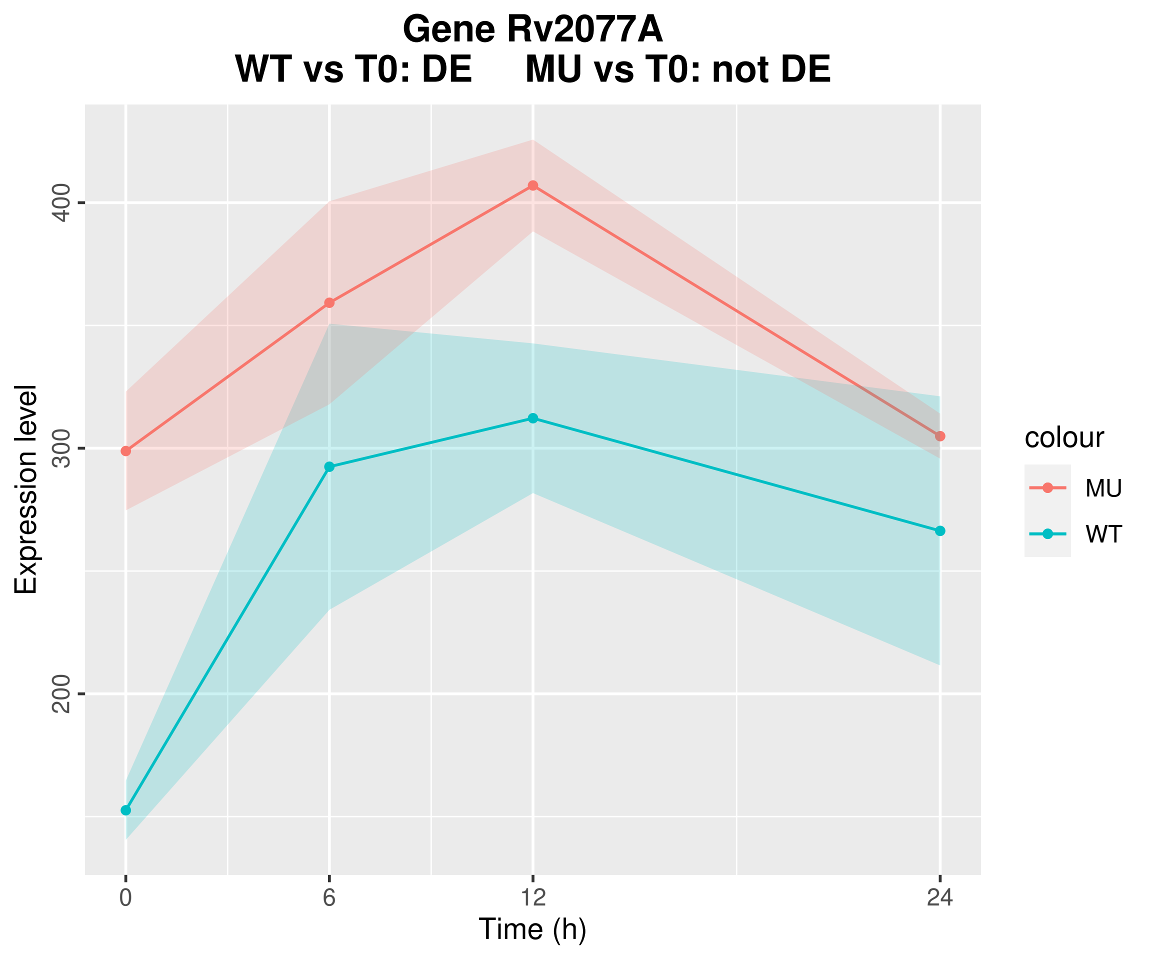
Gene Rv2077A
WT vs T0: DE   MU vs T0: not DE
Expression level
400
300
200
0
6
12
24
Time (h)
colour
MU
WT

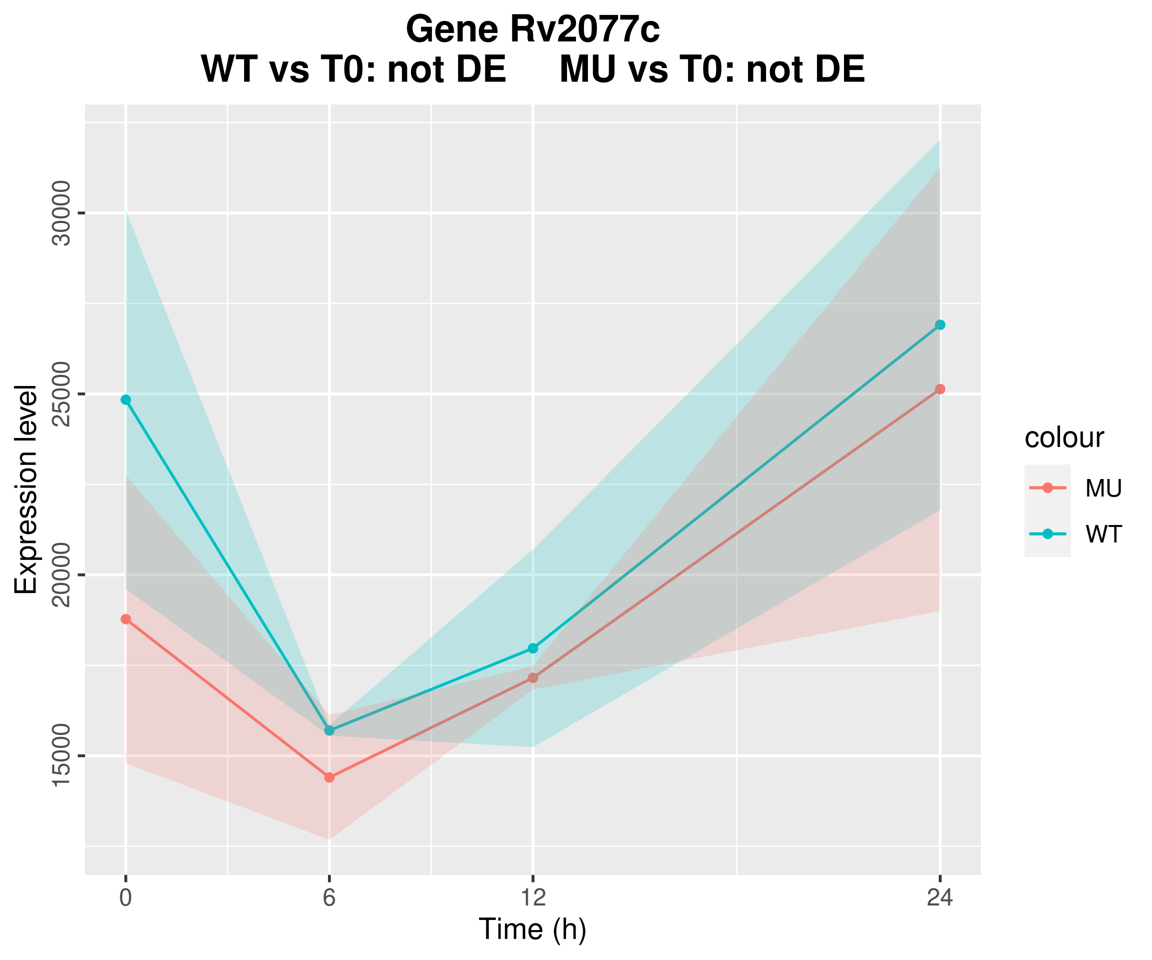

Gene Rv2077c
WT vs T0: not DE MU vs T0: not DE
Expression level
30000
25000
20000
15000
0
6
12
24
Time (h)
colour
MU
WT

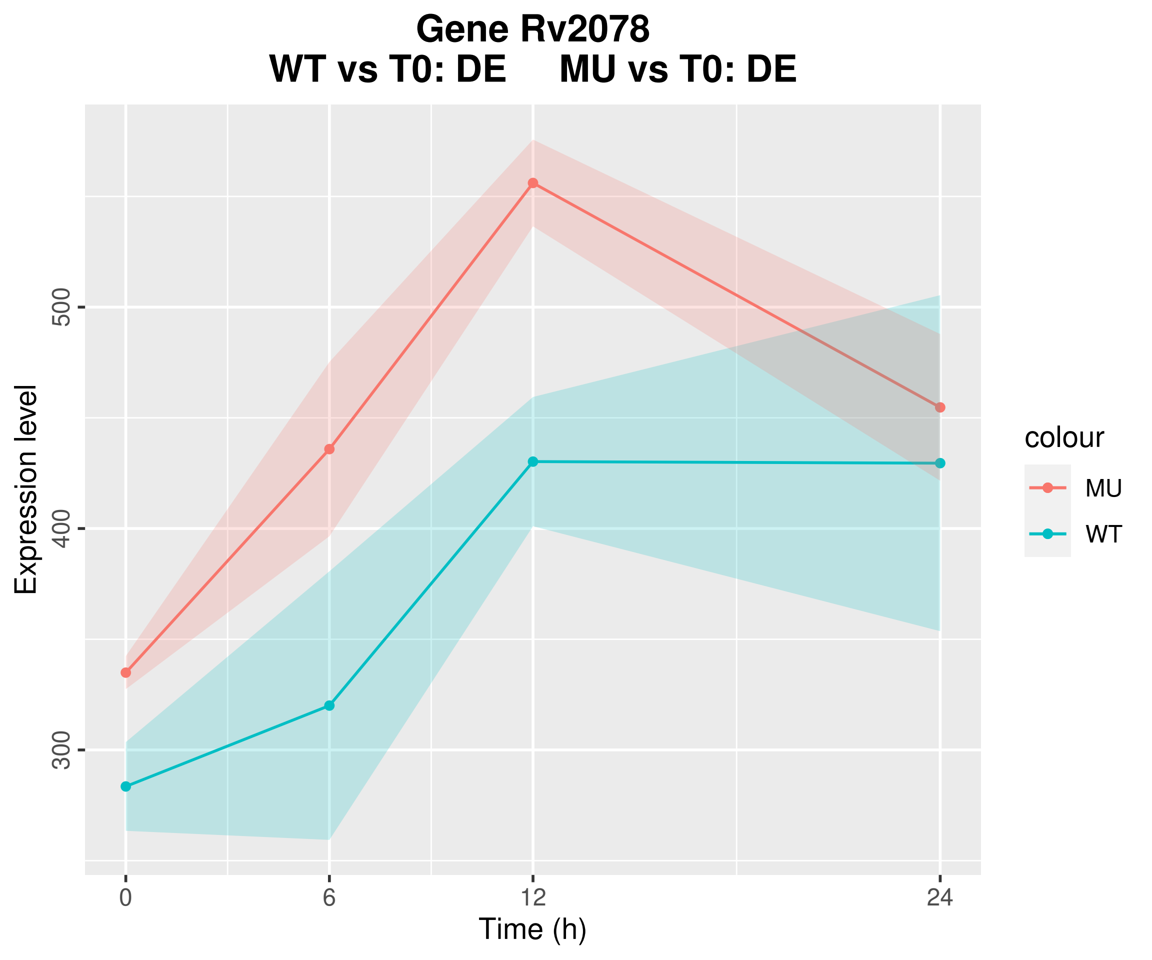
Gene Rv2078
WT vs T0: DE MU vs T0: DE
Expression level
300
400
500
0
6
12
24
Time (h)
colour
MU
WT

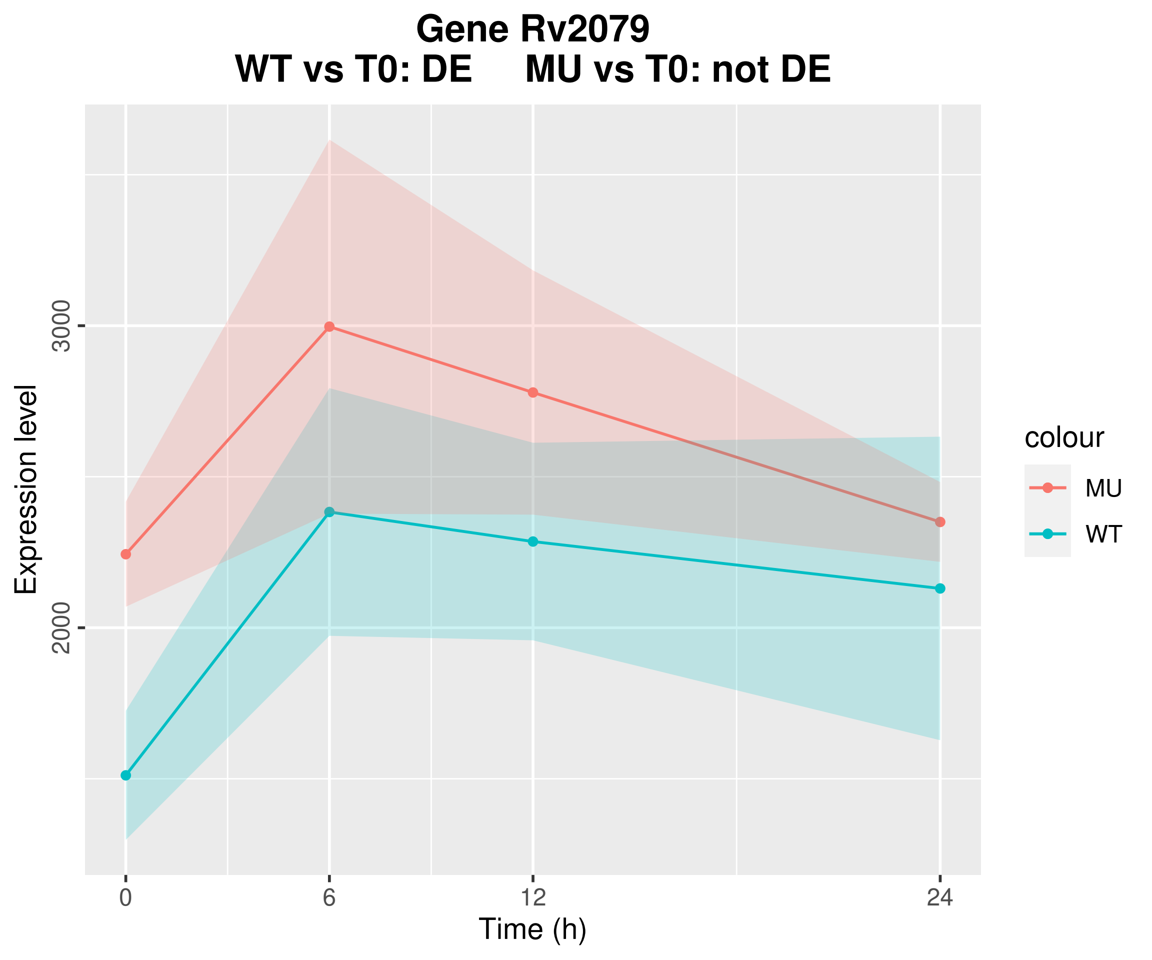

Gene Rv2079
WT vs T0: DE   MU vs T0: not DE
Expression level
3000
2000
0
6
12
24
Time (h)
colour
MU
WT

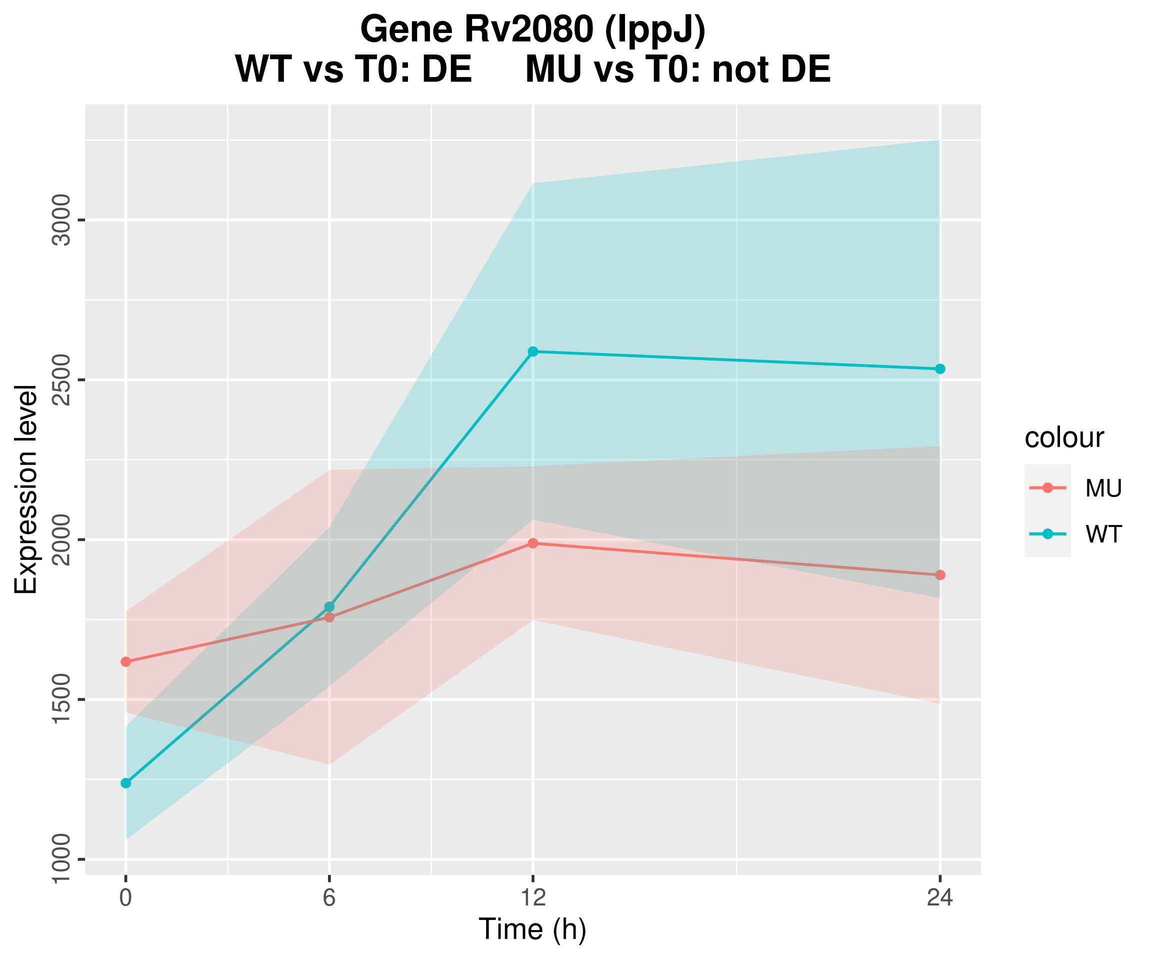
Gene Rv2080 (lppJ)
WT vs T0: DE   MU vs T0: not DE
Expression level
3000
2500
2000
1500
1000
0
6
12
24
Time (h)
colour
MU
WT

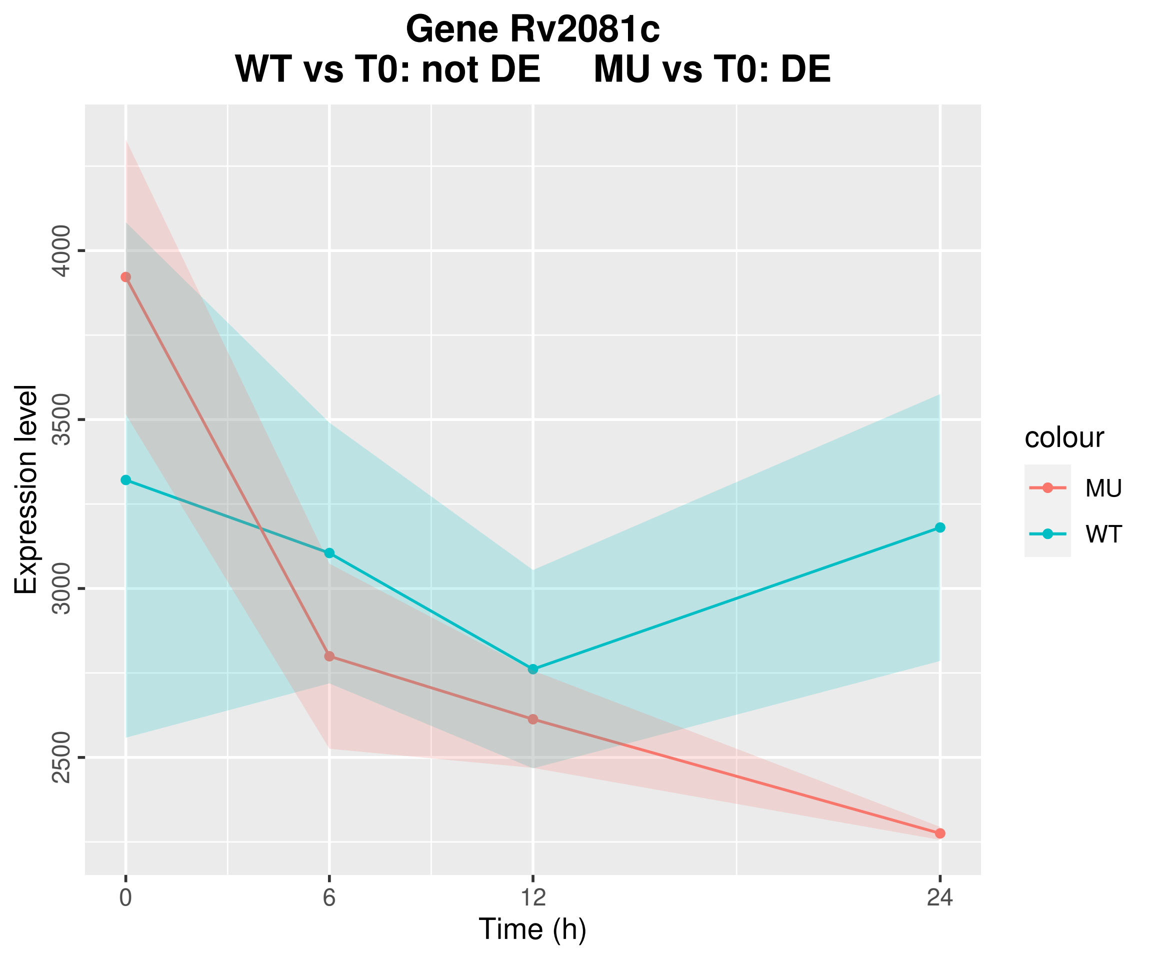

Gene Rv2081c
WT vs T0: not DE MU vs T0: DE
Expression level
4000
3500
3000
2500
0
6
12
24
Time (h)
colour
MU
WT

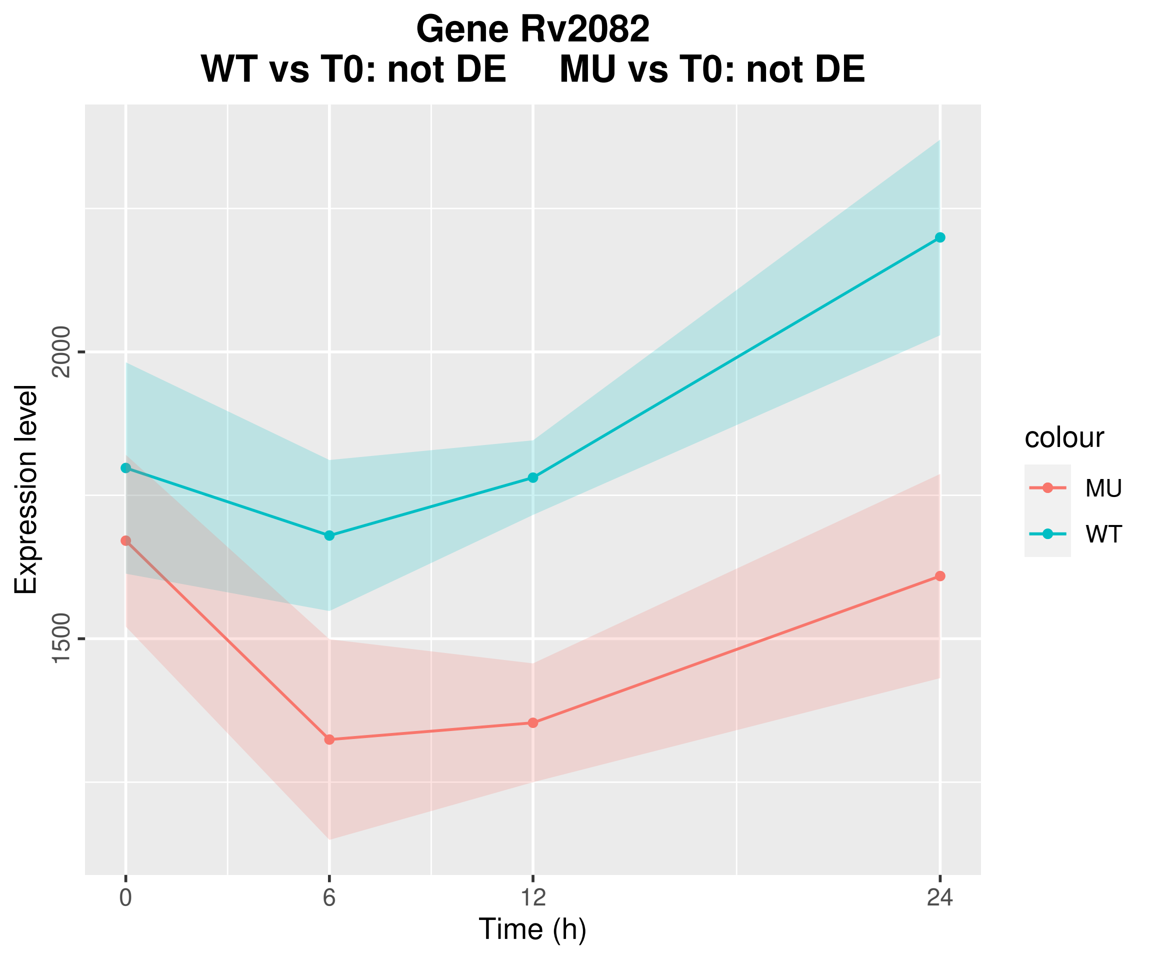
Gene Rv2082
WT vs T0: not DE MU vs T0: not DE
Expression level
2000
1500
0
6
12
24
Time (h)
colour
MU
WT

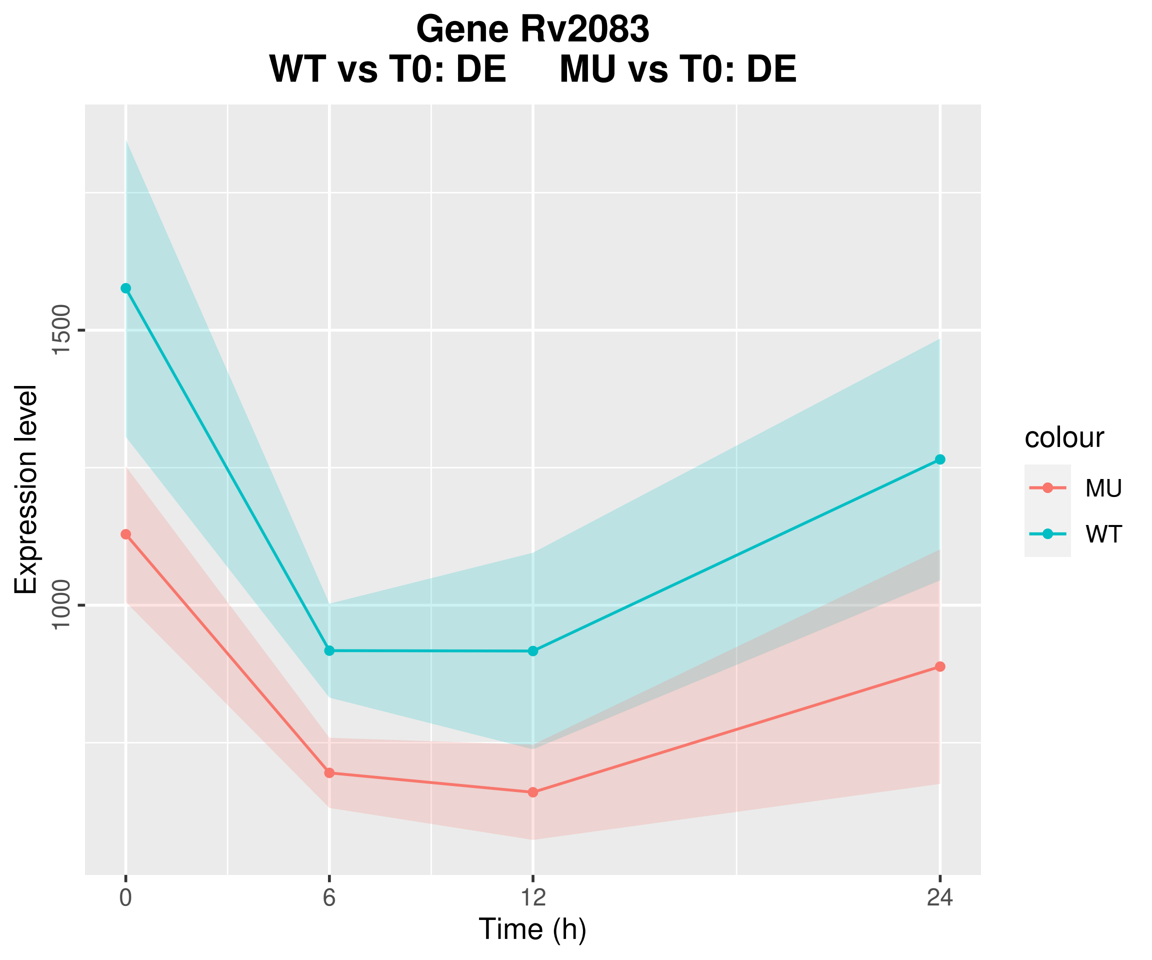
Gene Rv2083
WT vs T0: DE   MU vs T0: DE
Expression level
1500
1000
0
6
12
24
Time (h)
colour
MU
WT

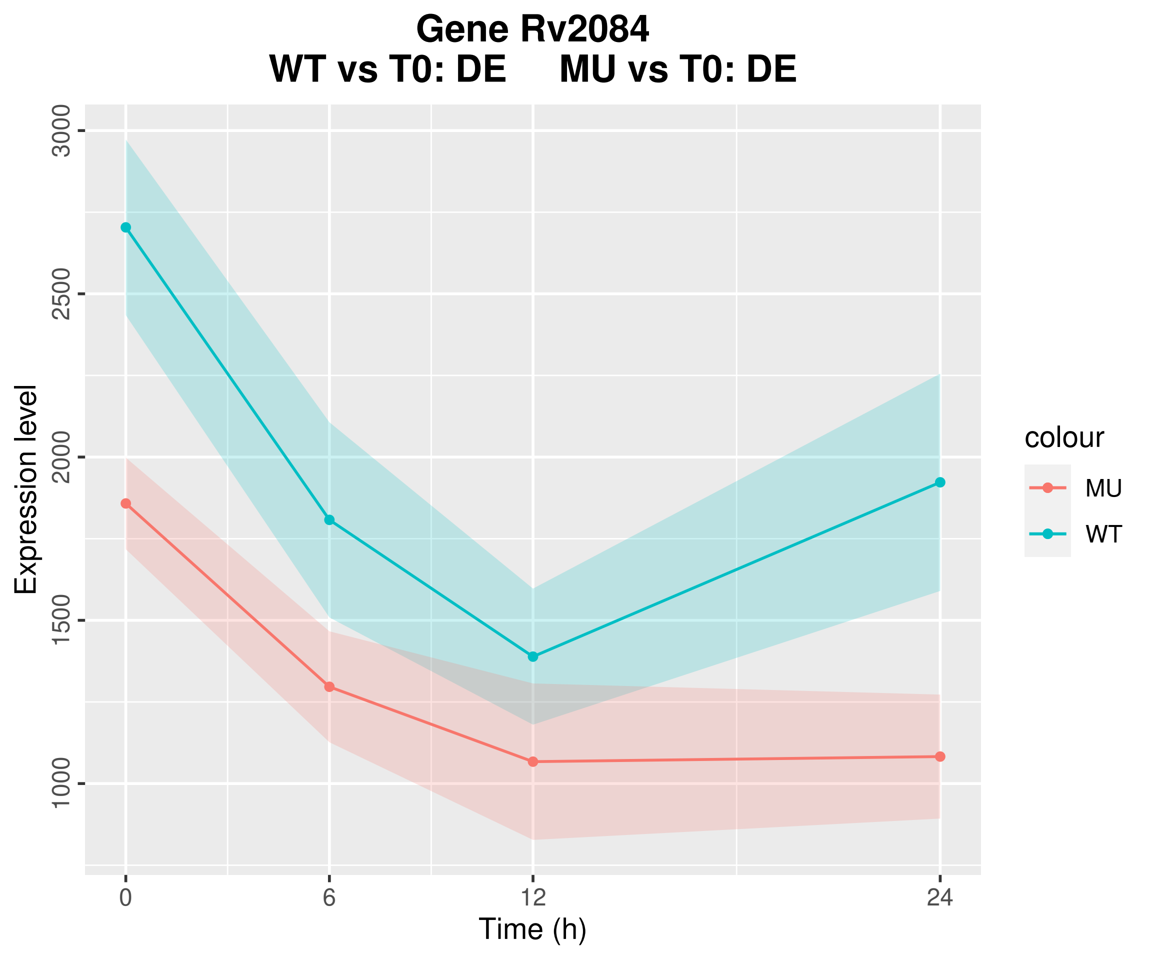
Gene Rv2084
WT vs T0: DE   MU vs T0: DE
Expression level
3000
2500
2000
1500
1000
0
6
12
24
Time (h)
colour
MU
WT

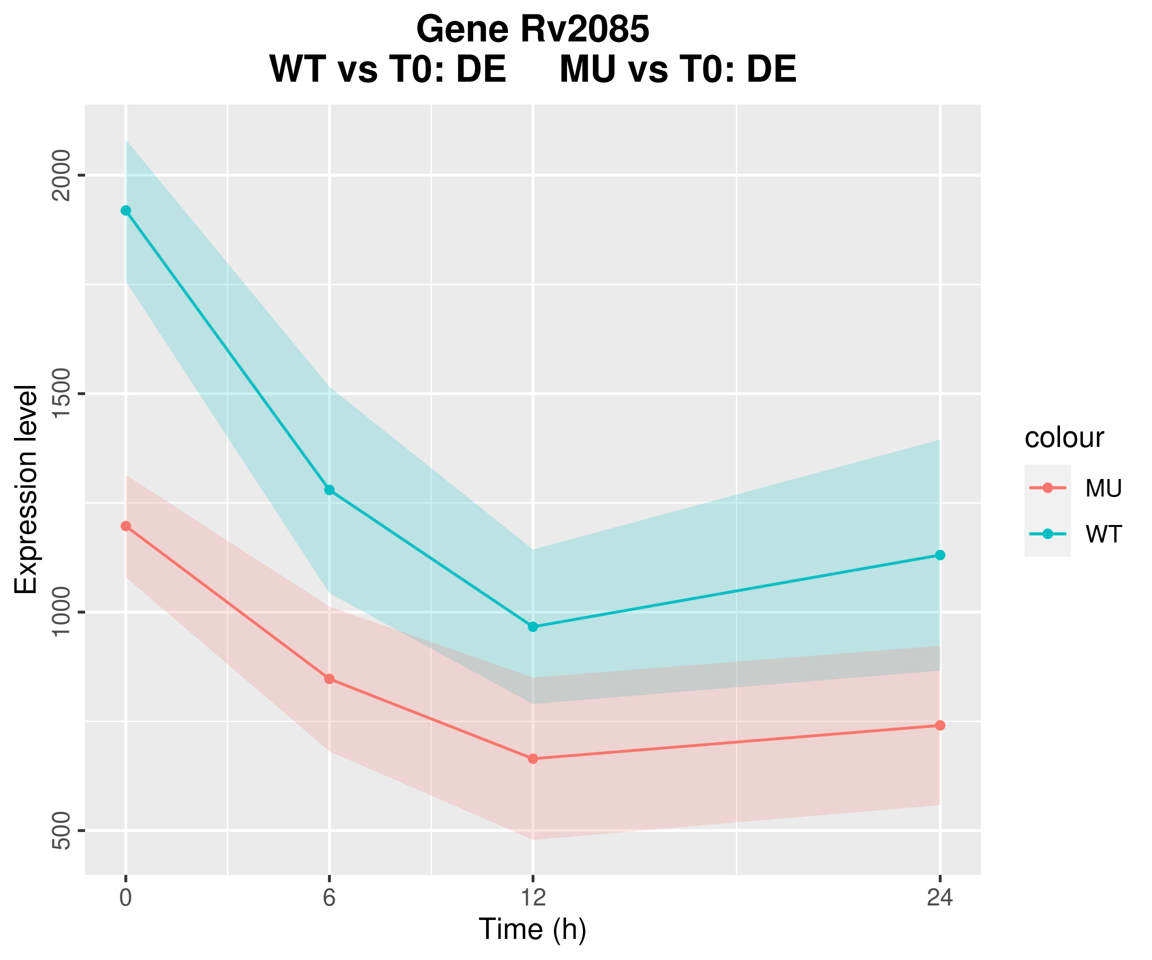

Gene Rv2085
WT vs T0: DE MU vs T0: DE
Expression level
2000
1500
1000
500
0
6
12
24
Time (h)
colour
MU
WT

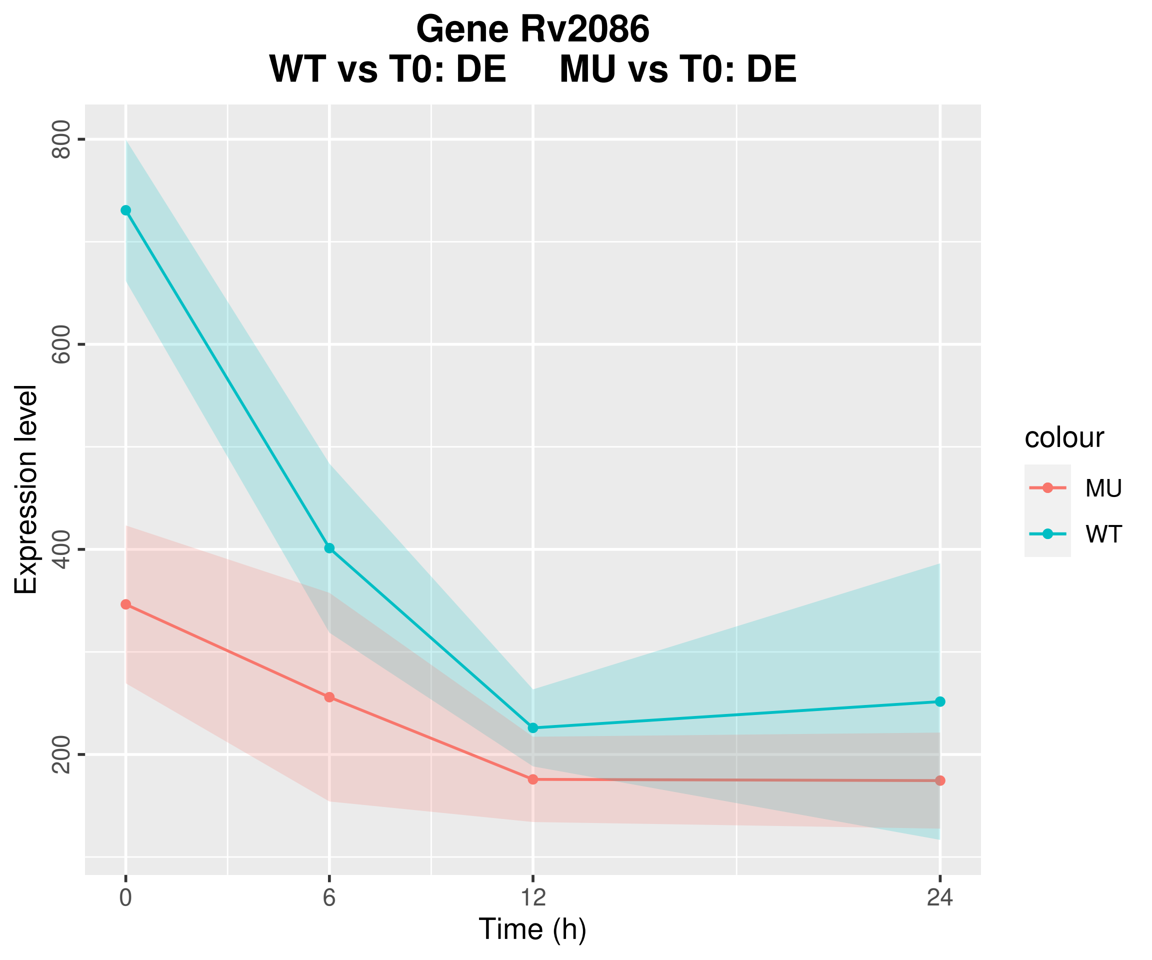
Gene Rv2086
WT vs T0: DE   MU vs T0: DE
Expression level
800
600
400
200
0
6
12
24
Time (h)
colour
MU
WT

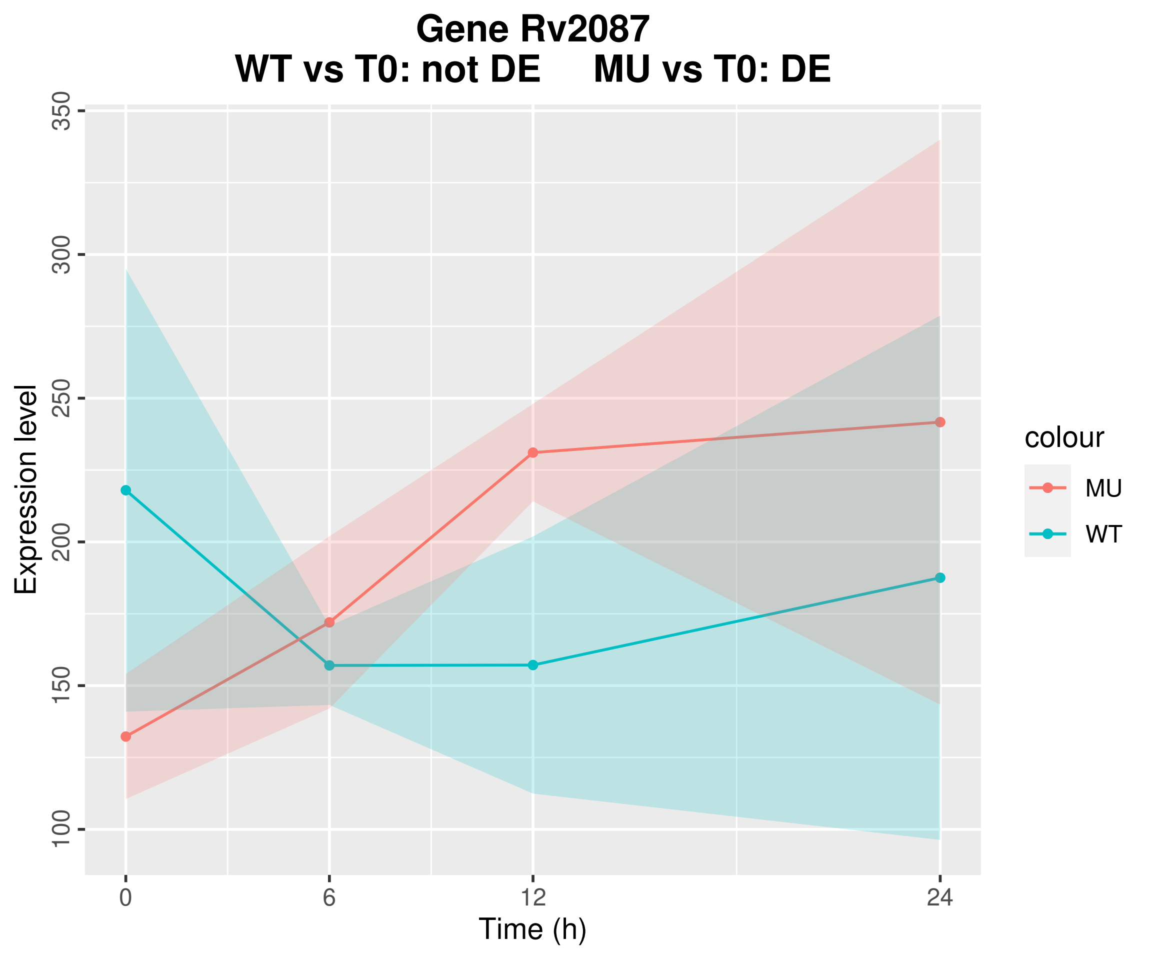

Gene Rv2087
WT vs T0: not DE MU vs T0: DE
Expression level
100
150
200
250
300
350
0
6
12
24
Time (h)
colour
MU
WT

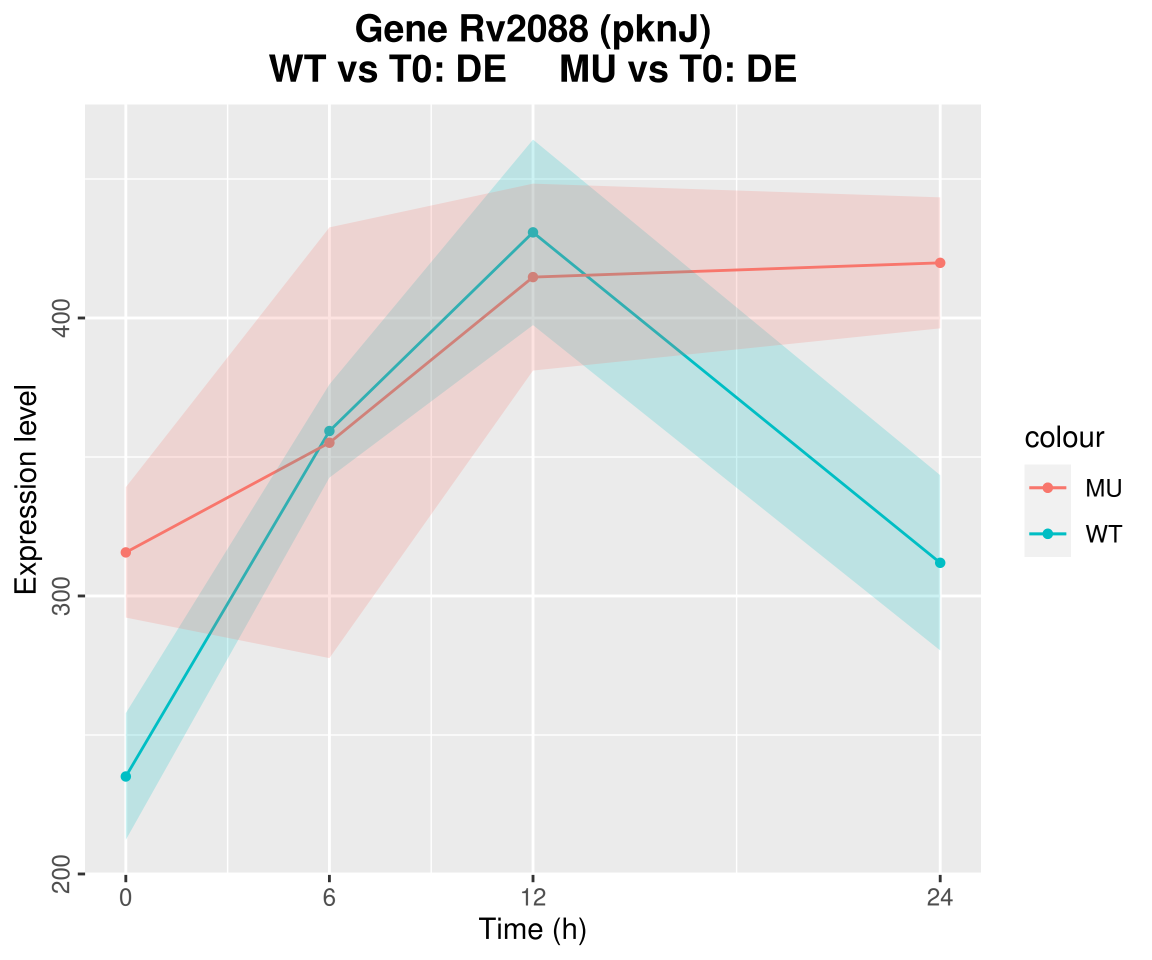

Gene Rv2088 (pknJ)
WT vs T0: DE   MU vs T0: DE
Expression level
400
300
200
0
6
12
24
Time (h)
colour
MU
WT

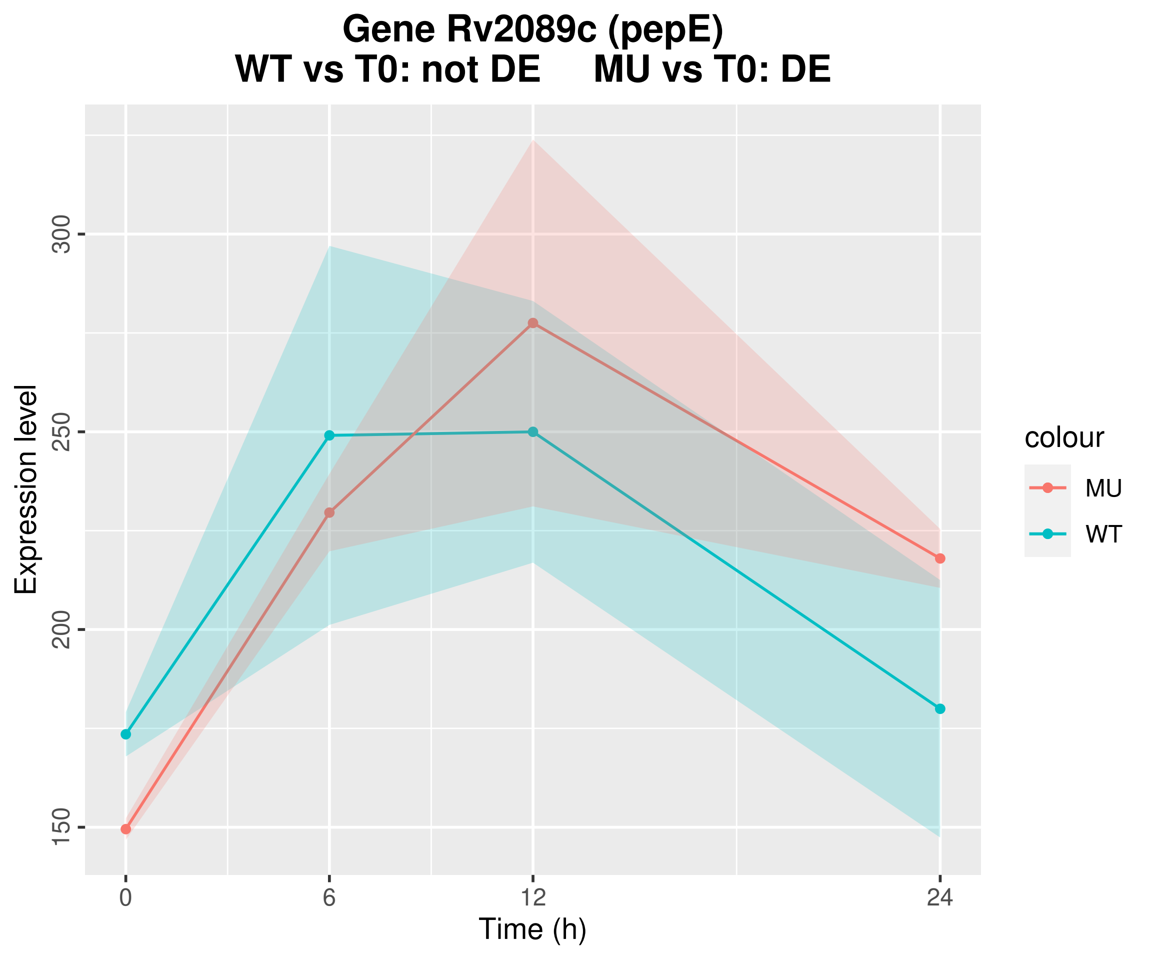

Gene Rv2089c (pepE)
WT vs T0: not DE   MU vs T0: DE
Expression level
300
250
200
150
0
6
12
24
Time (h)
colour
MU
WT

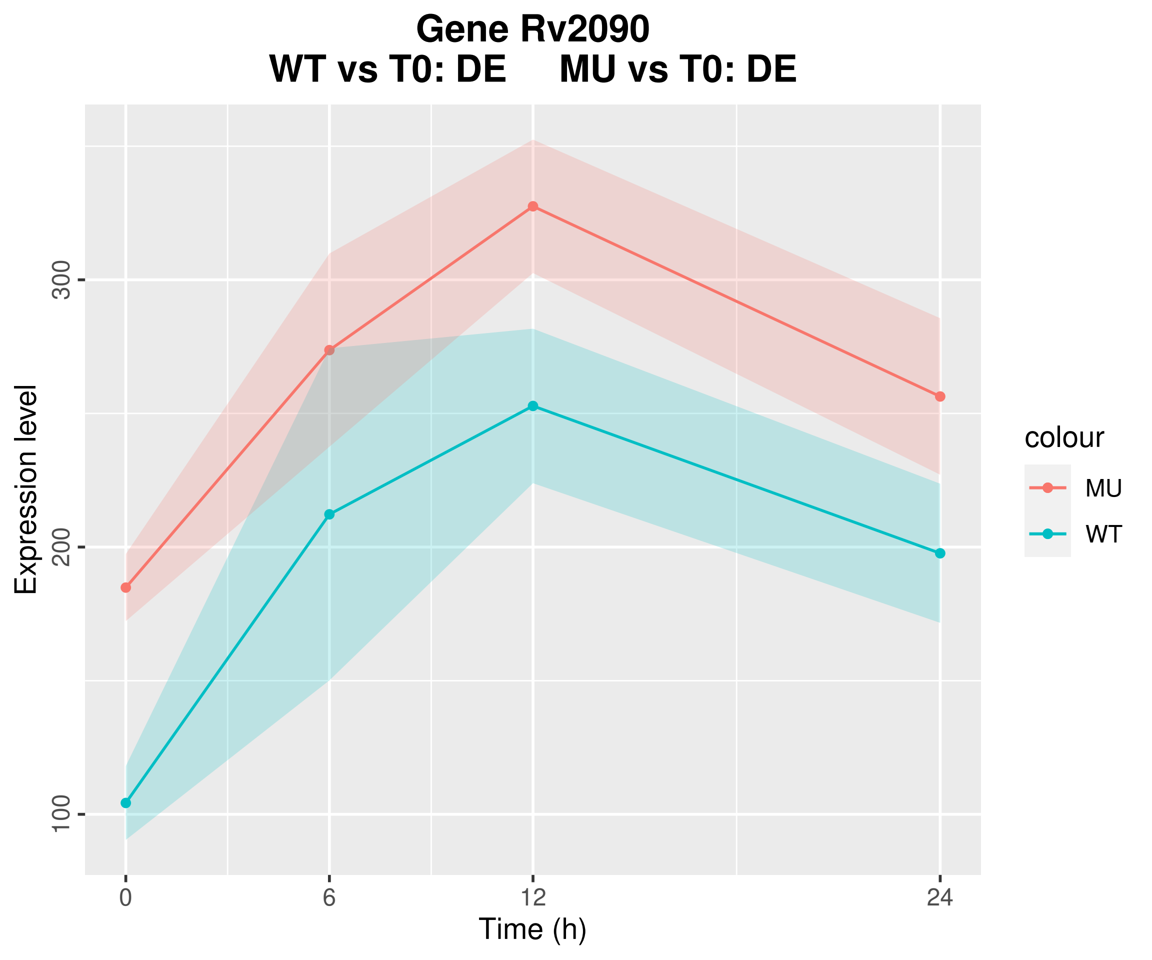

Gene Rv2090
WT vs T0: DE   MU vs T0: DE
Expression level
100
200
300
0
6
12
24
Time (h)
colour
MU
WT

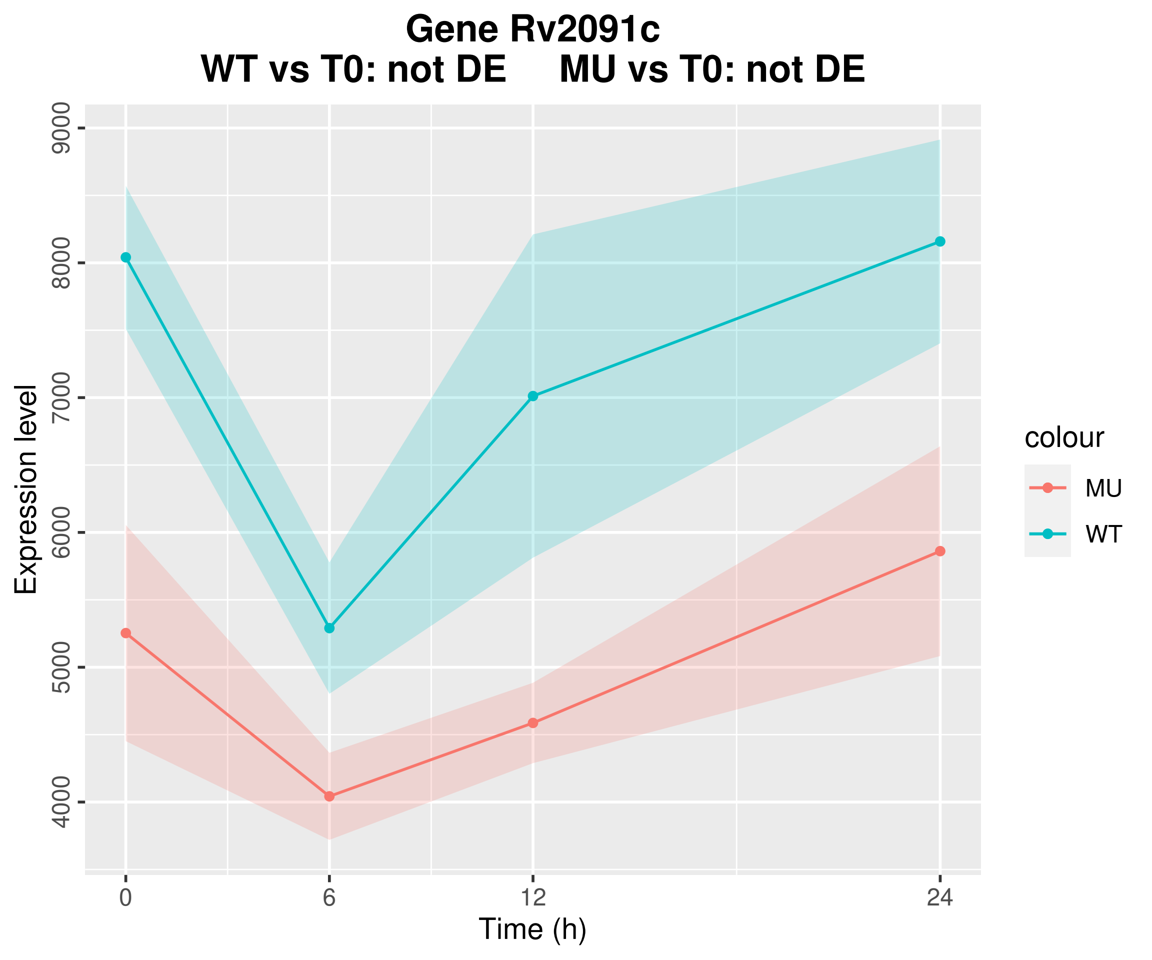
Gene Rv2091c
WT vs T0: not DE MU vs T0: not DE
Expression level
9000
8000
7000
6000
5000
4000
0
6
12
24
Time (h)
colour
MU
WT

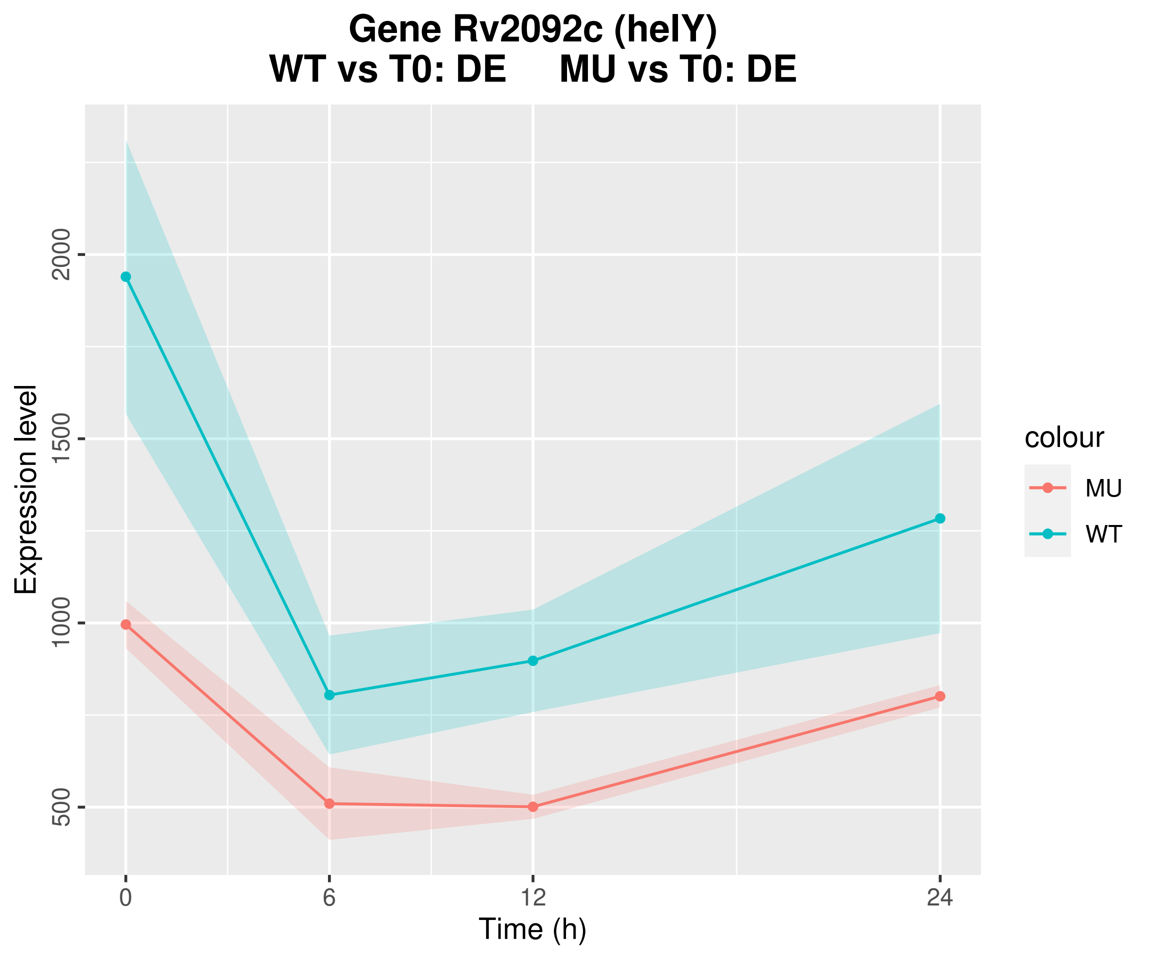

Gene Rv2092c (helY)
WT vs T0: DE MU vs T0: DE
Expression level
2000
1500
1000
500
0
6
12
24
Time (h)
colour
MU
WT

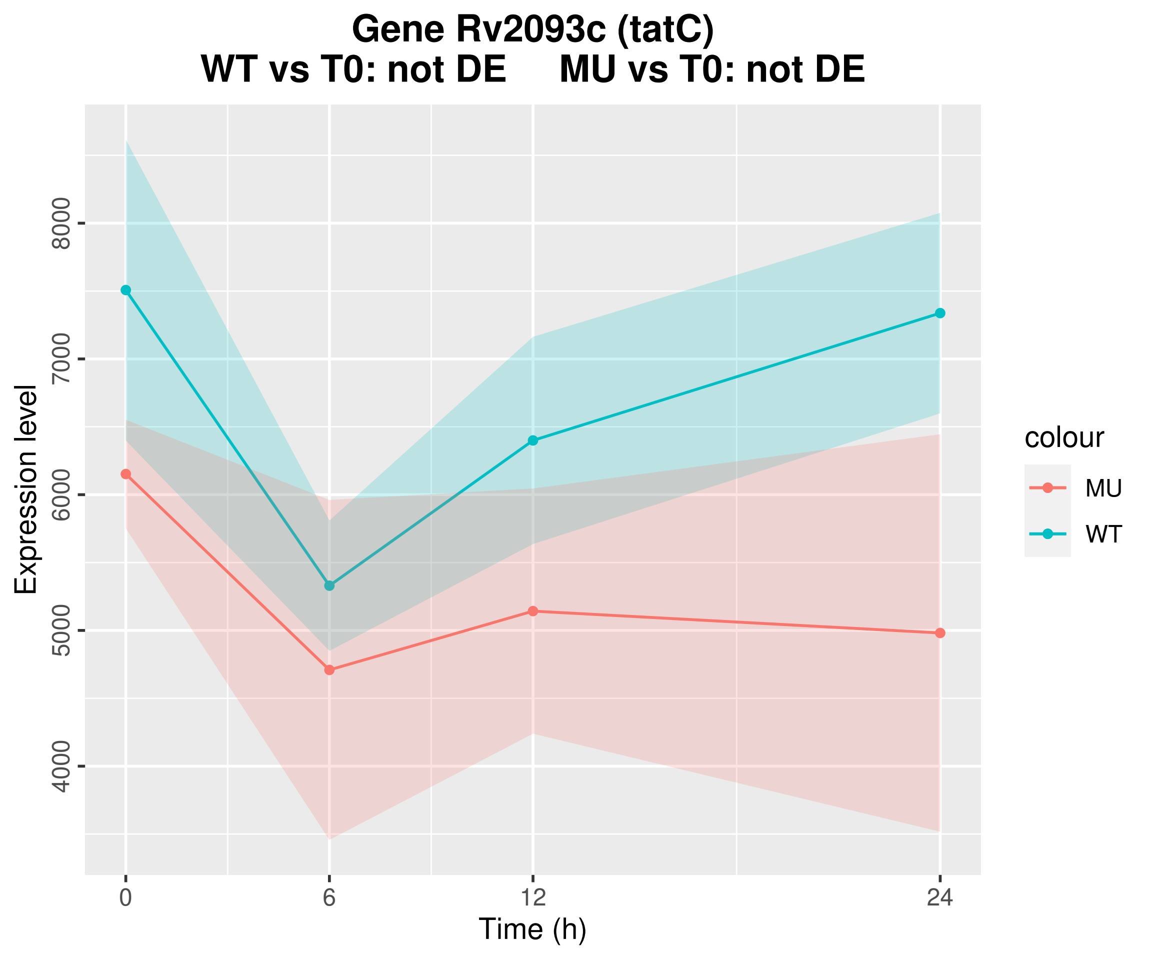

Gene Rv2093c (tatC)
WT vs T0: not DE   MU vs T0: not DE
Expression level
8000
7000
6000
5000
4000
0
6
12
24
Time (h)
colour
MU
WT

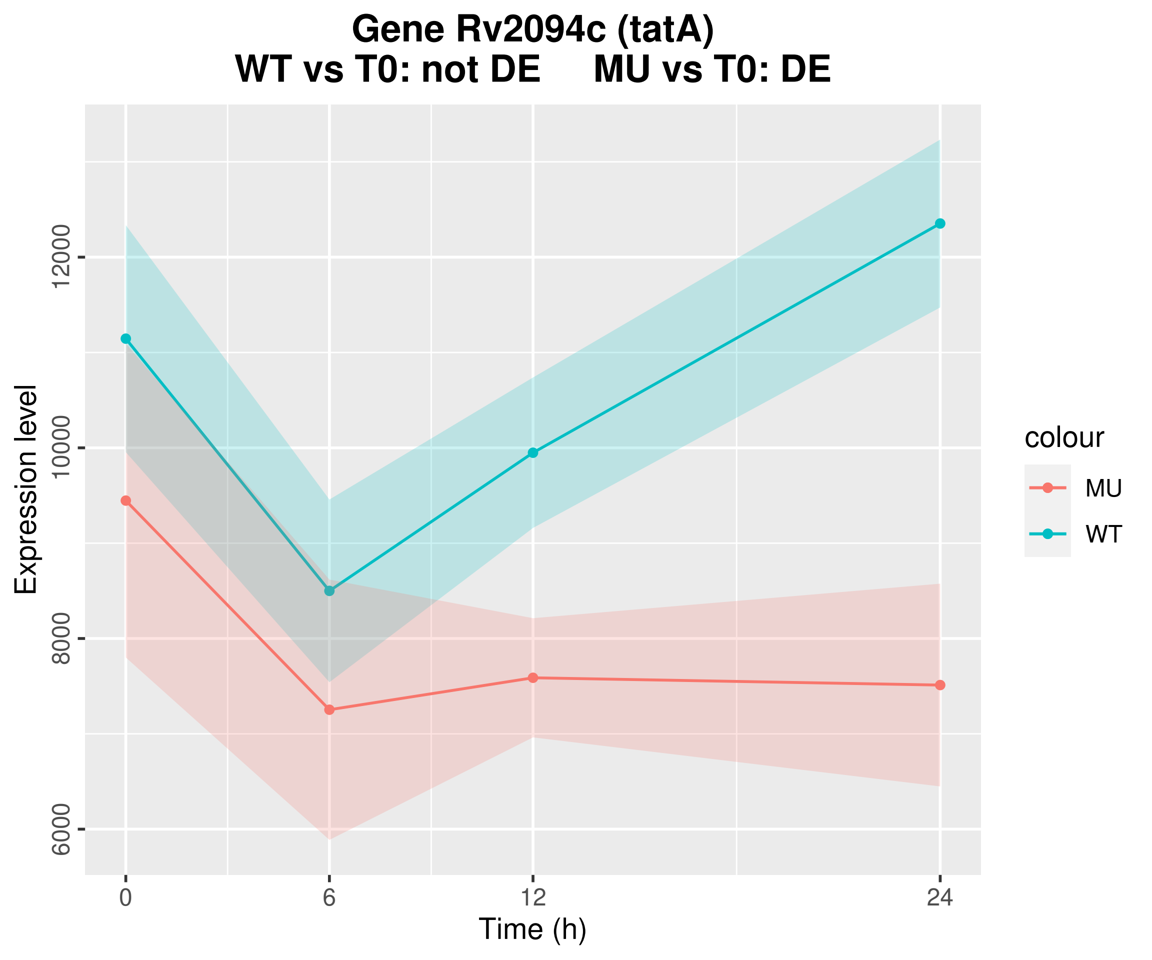

Gene Rv2094c (tatA)
WT vs T0: not DE MU vs T0: DE
Expression level
6000
8000
10000
12000
0
6
12
24
Time (h)
colour
MU
WT

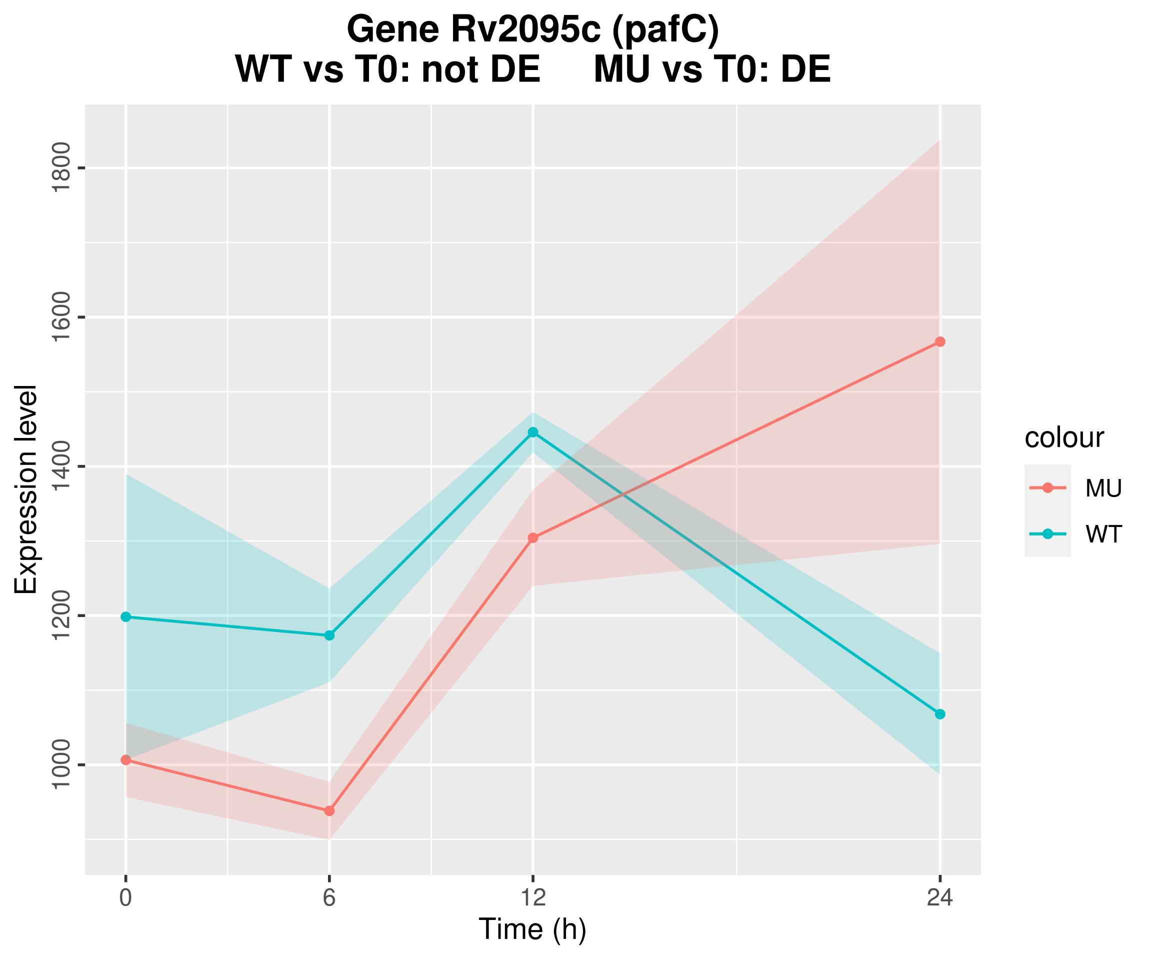

Gene Rv2095c (pafC)
WT vs T0: not DE MU vs T0: DE
Expression level
1000
1200
1400
1600
1800
0
6
12
24
Time (h)
colour
MU
WT

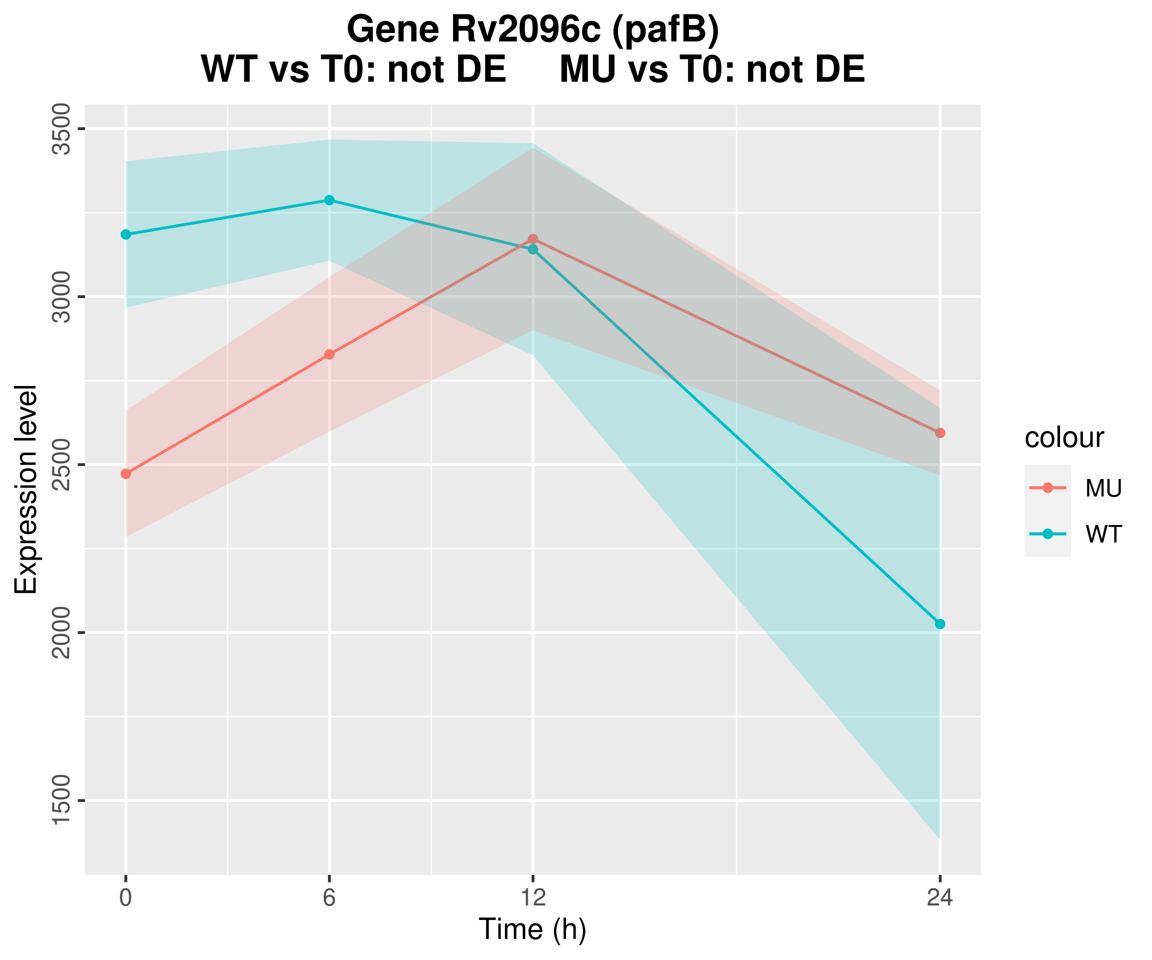
Gene Rv2096c (pafB)
WT vs T0: not DE   MU vs T0: not DE
Expression level
3500
3000
2500
2000
1500
0
6
12
24
Time (h)
colour
MU
WT

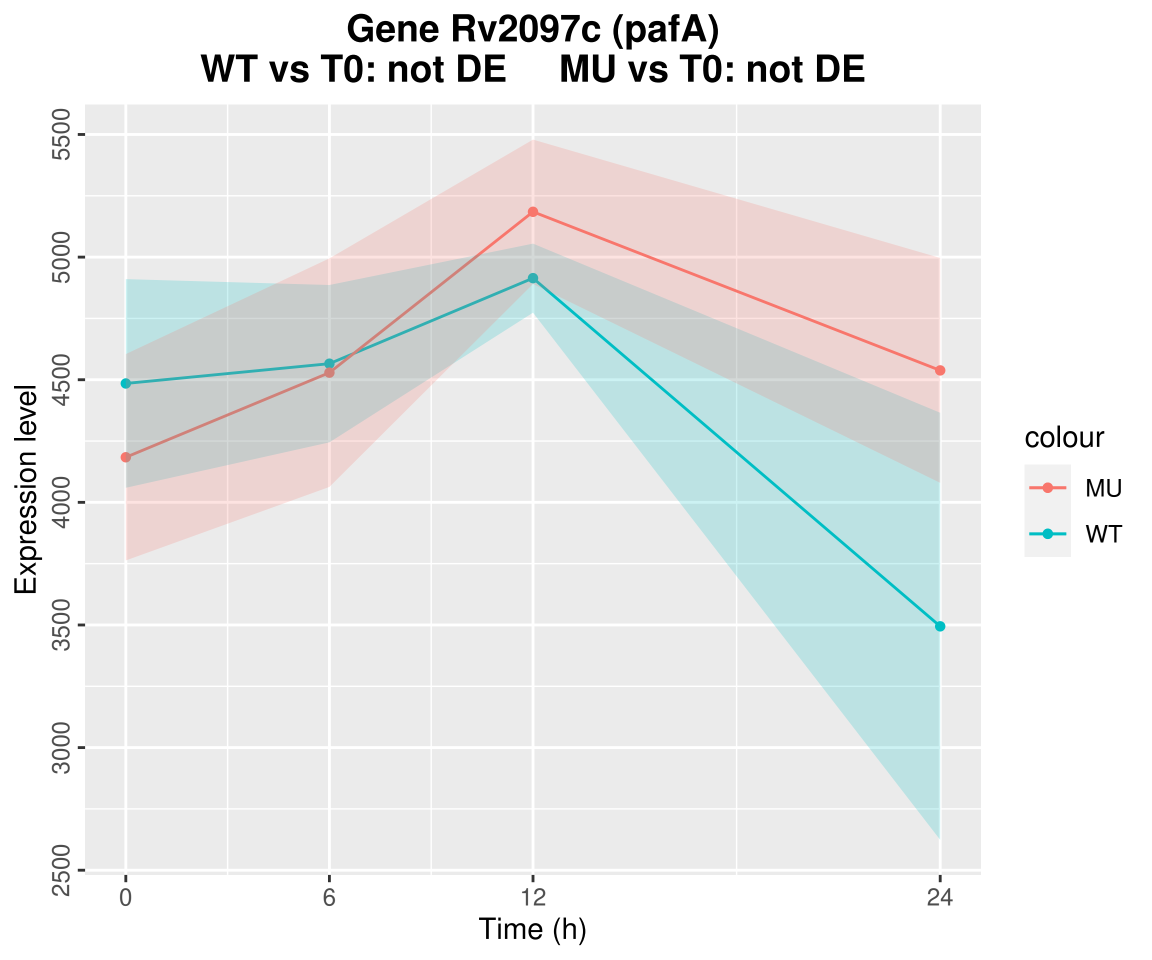
Gene Rv2097c (pafA)
WT vs T0: not DE MU vs T0: not DE
Expression level
5500
5000
4500
4000
3500
3000
2500
0
6
12
24
Time (h)
colour
MU
WT

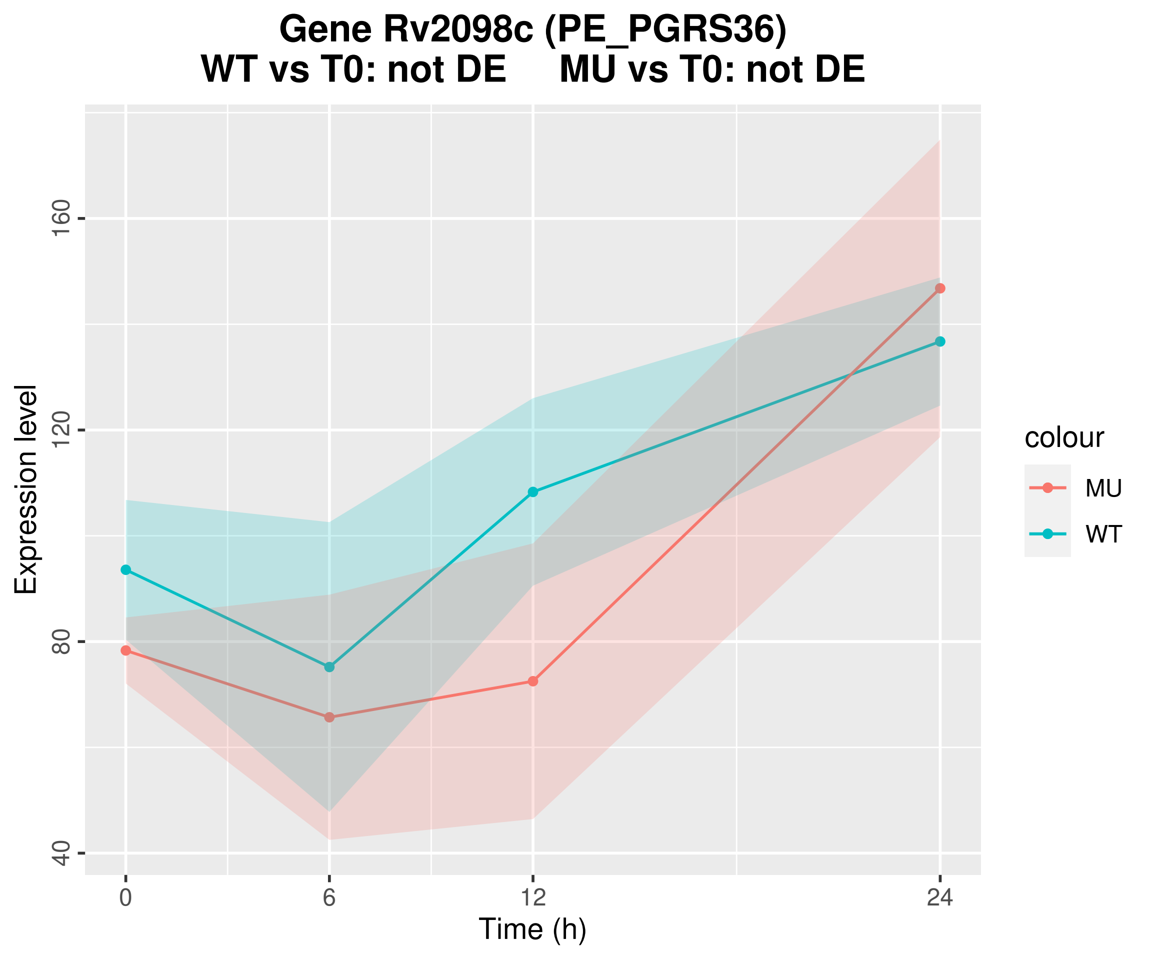

Gene Rv2098c (PE_PGRS36)
WT vs T0: not DE MU vs T0: not DE
Expression level
160
120
80
40
0
6
12
24
Time (h)
colour
MU
WT

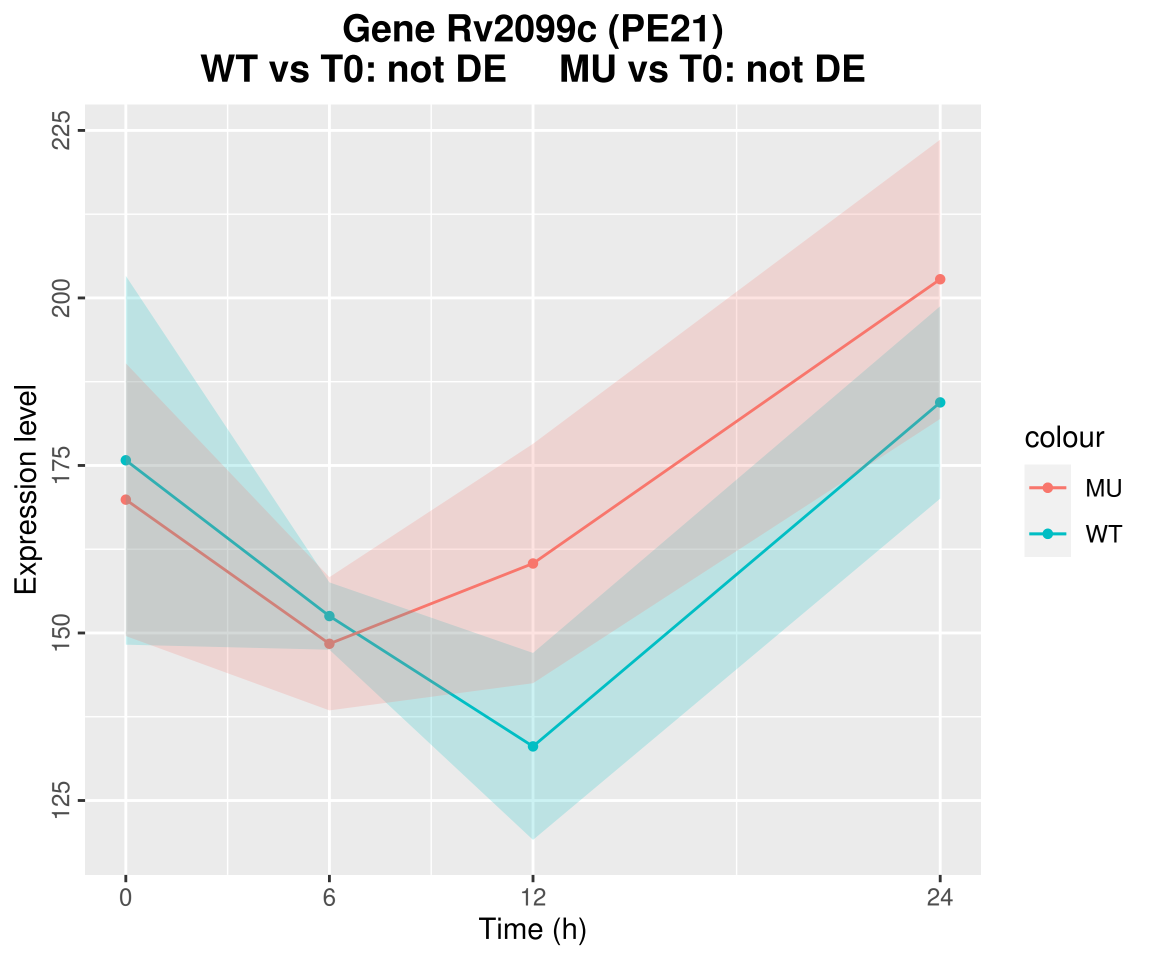
Gene Rv2099c (PE21)
WT vs T0: not DE   MU vs T0: not DE
Expression level
225
200
175
150
125
0
6
12
24
Time (h)
colour
MU
WT

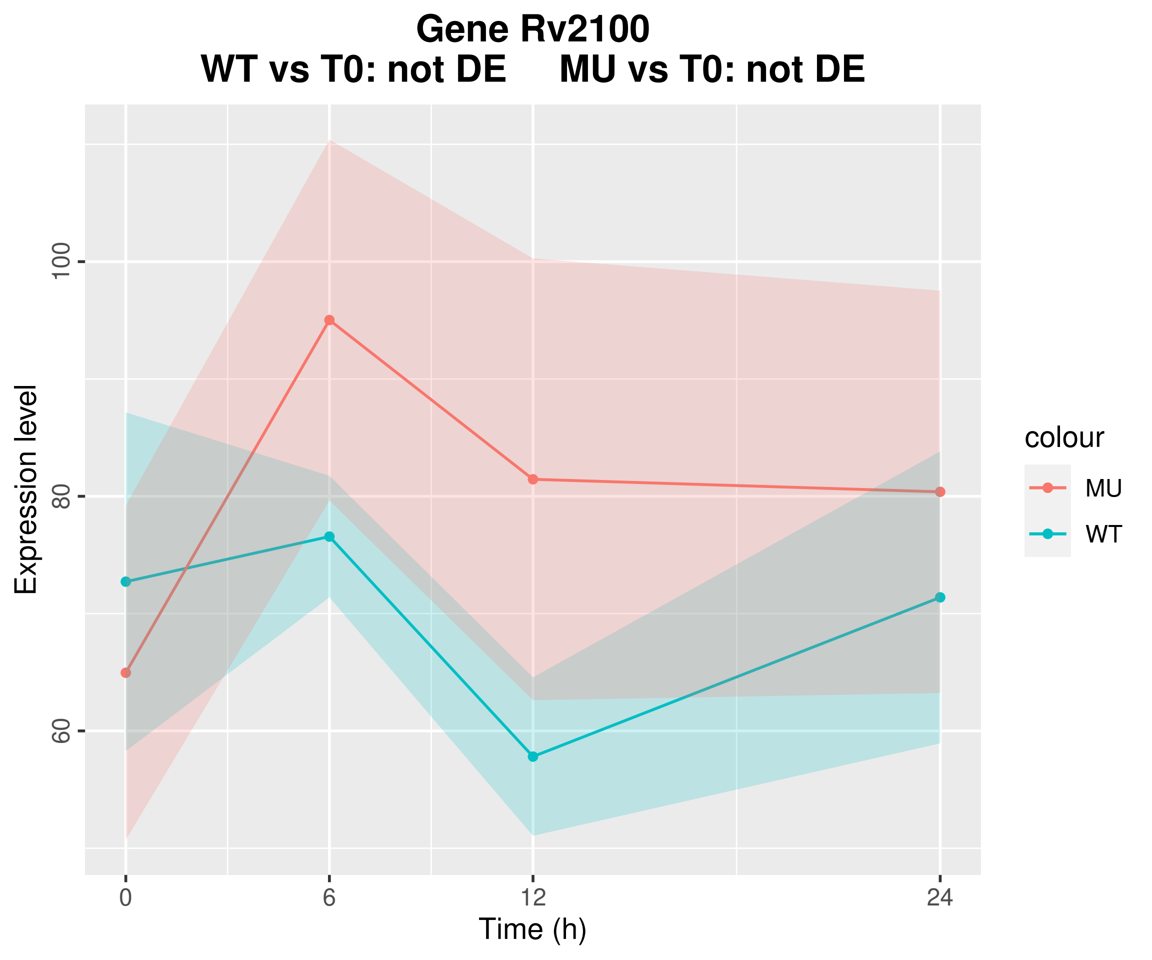

Gene Rv2100
WT vs T0: not DE   MU vs T0: not DE
Expression level
100
80
60
0
6
12
24
Time (h)
colour
MU
WT

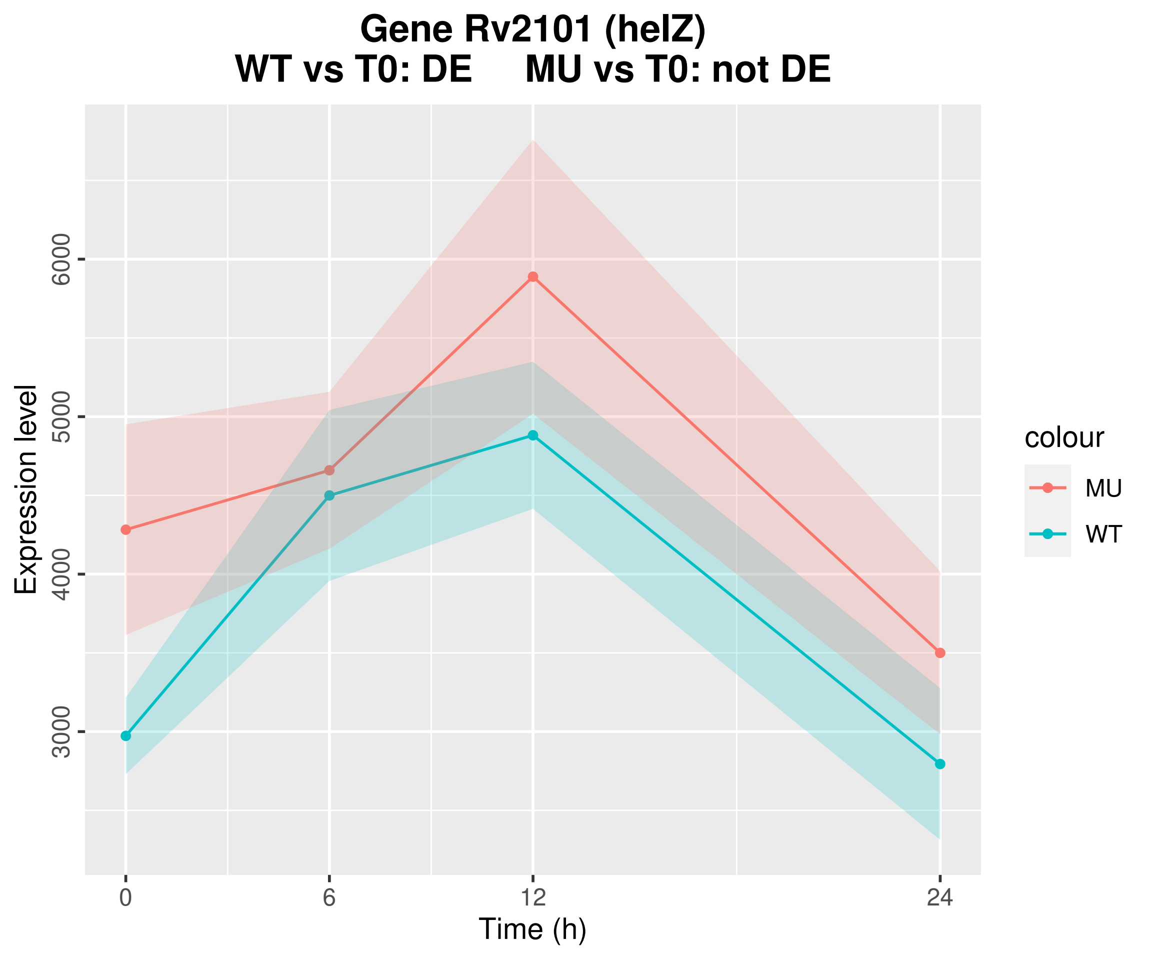
Gene Rv2101 (helZ)
WT vs T0: DE   MU vs T0: not DE
Expression level
6000
5000
4000
3000
0
6
12
24
Time (h)
colour
MU
WT

Gene Rv2102
WT vs T0: DE   MU vs T0: not DE

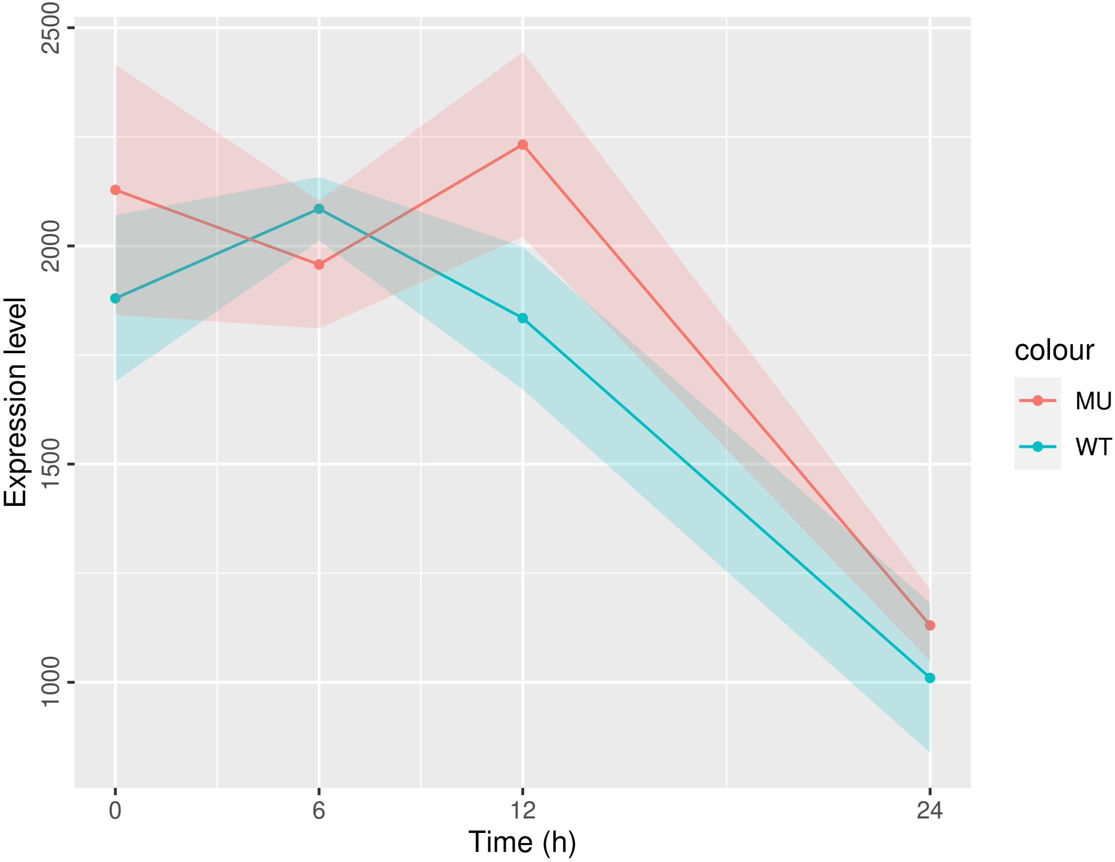

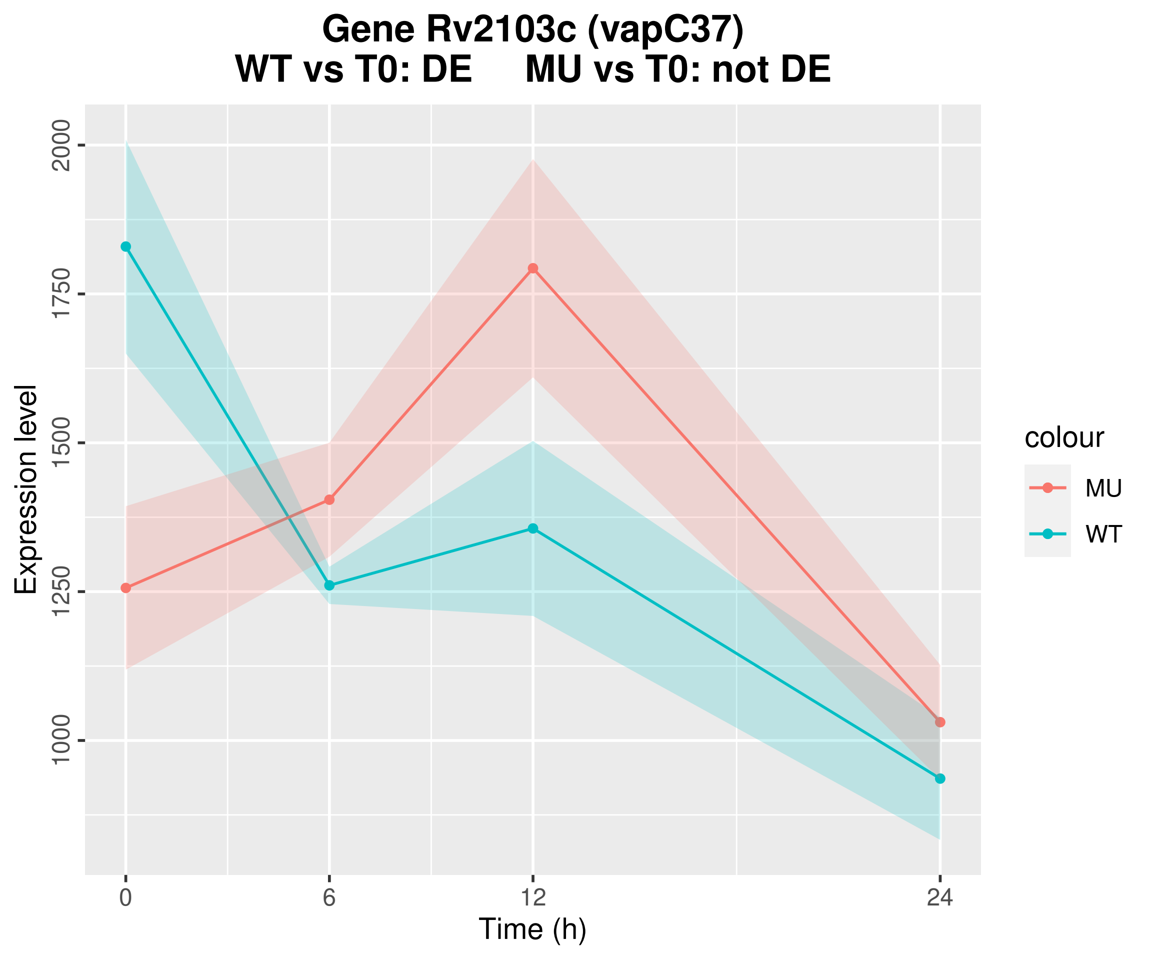
Gene Rv2103c (vapC37)
WT vs T0: DE   MU vs T0: not DE
Expression level
2000
1750
1500
1250
1000
0
6
12
24
Time (h)
colour
MU
WT

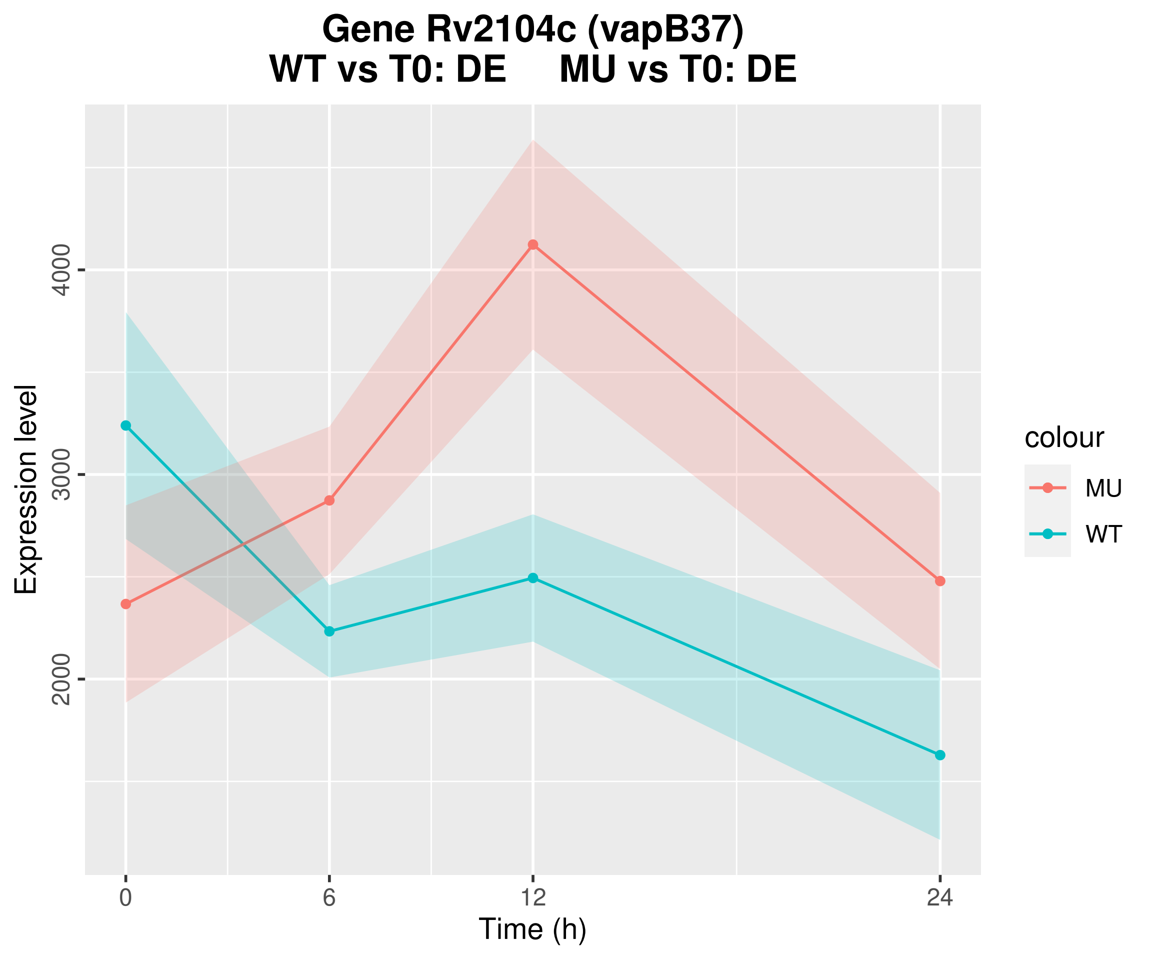

Gene Rv2104c (vapB37)
WT vs T0: DE MU vs T0: DE
Expression level
4000
3000
2000
0
6
12
24
Time (h)
colour
MU
WT

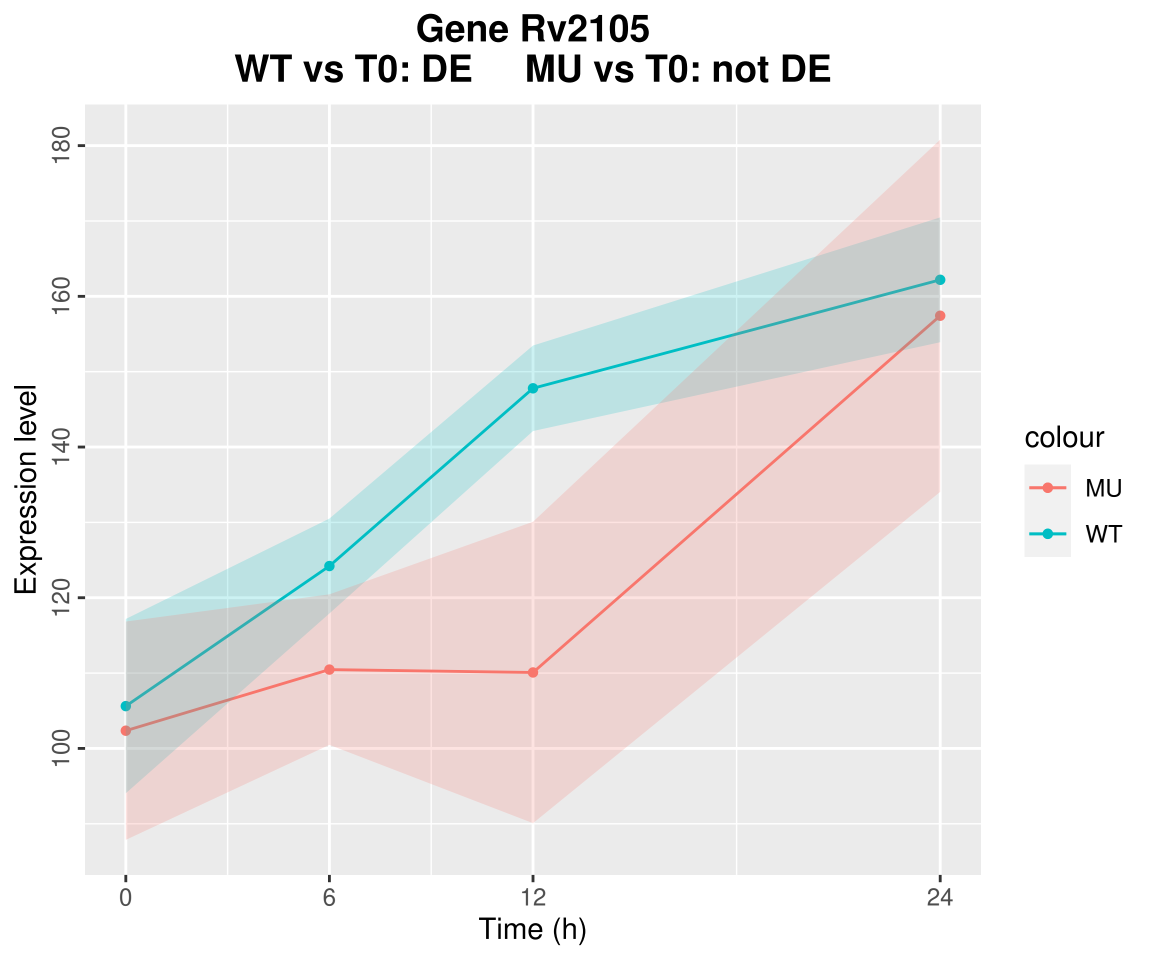
Gene Rv2105
WT vs T0: DE   MU vs T0: not DE
Expression level
180
160
140
120
100
0
6
12
24
Time (h)
colour
MU
WT

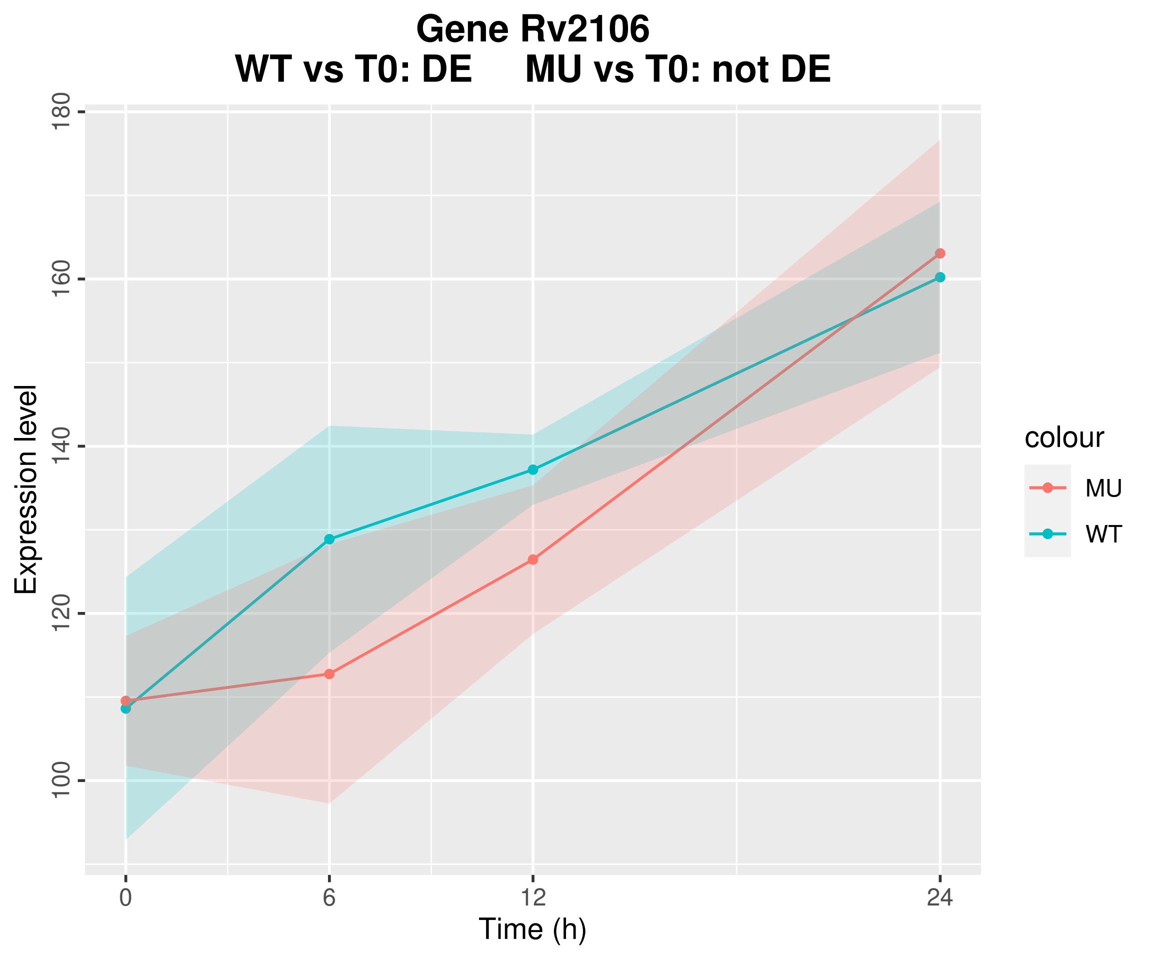
Gene Rv2106
WT vs T0: DE MU vs T0: not DE
180
160
140
120
100
Expression level
0
6
12
24
Time (h)
colour
MU
WT

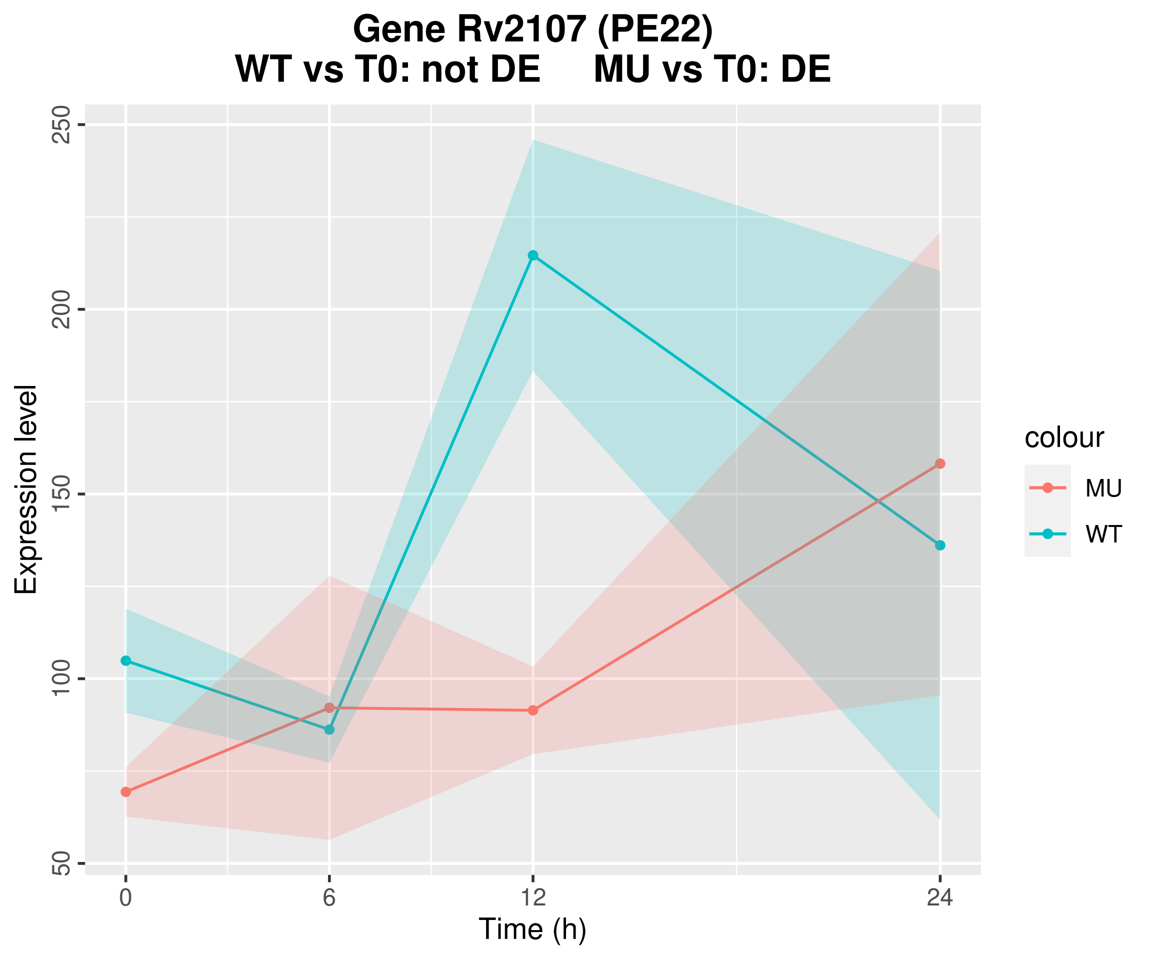
Gene Rv2107 (PE22)
WT vs T0: not DE   MU vs T0: DE
Expression level
250
200
150
100
50
0
6
12
24
Time (h)
colour
MU
WT

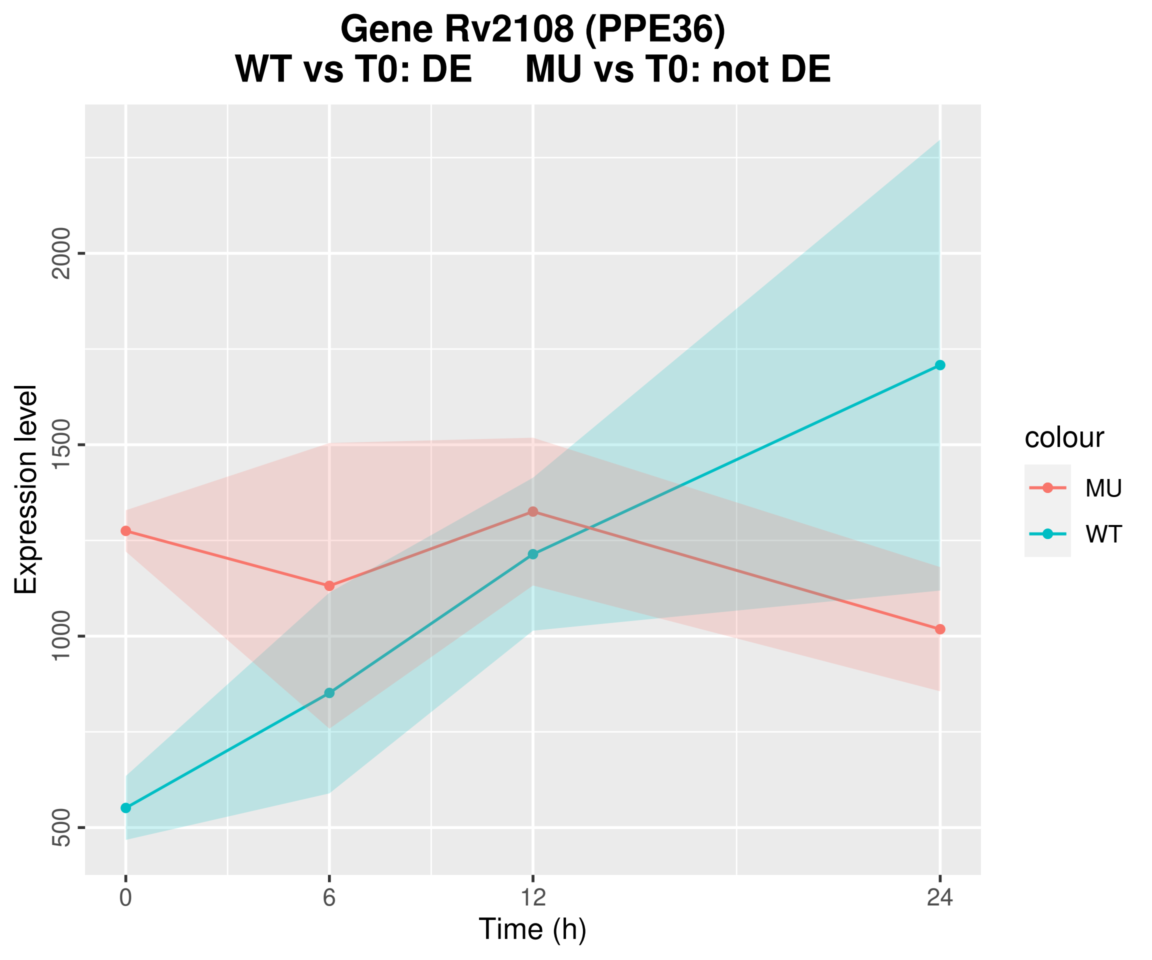
Gene Rv2108 (PPE36)
WT vs T0: DE   MU vs T0: not DE
Expression level
2000
1500
1000
500
0
6
12
24
Time (h)
colour
MU
WT

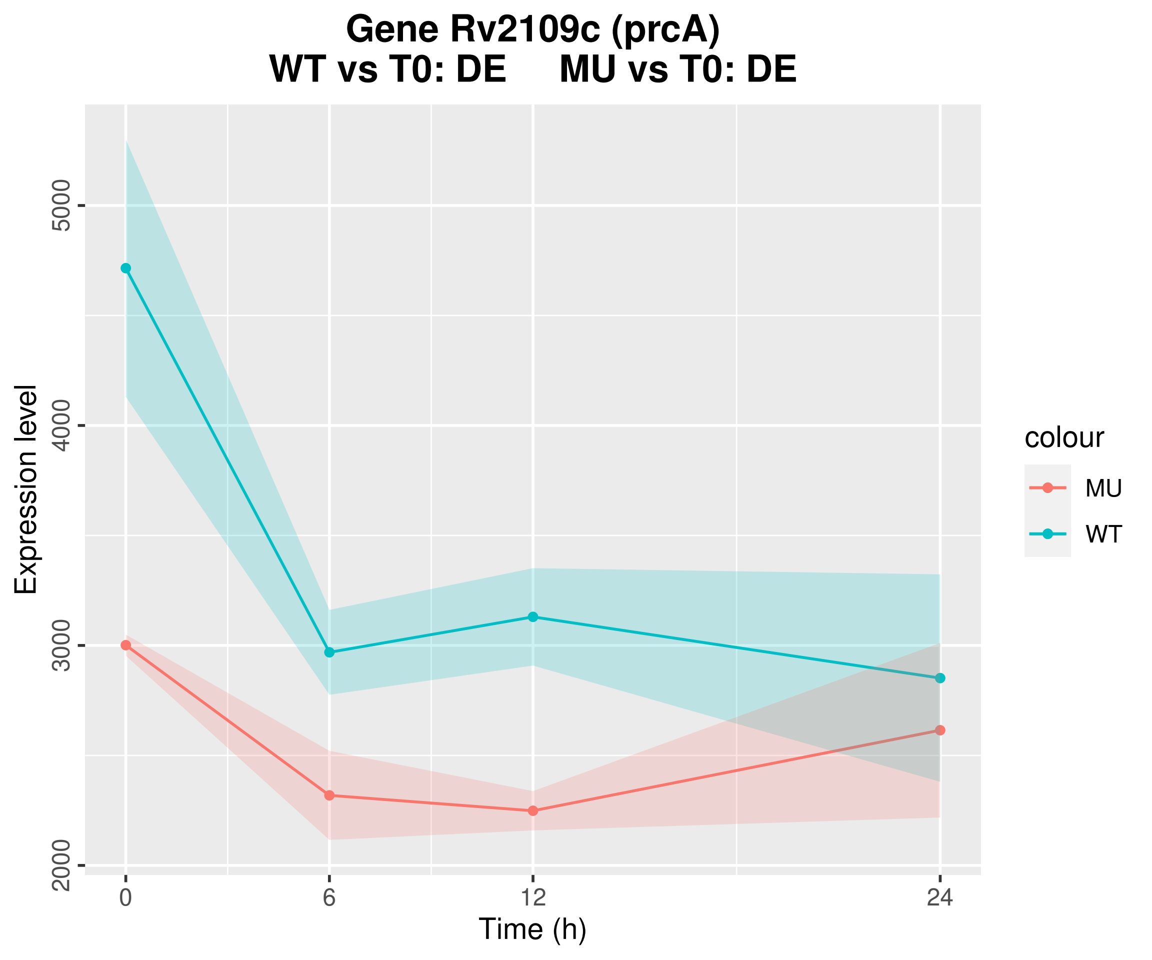

Gene Rv2109c (prcA)
WT vs T0: DE   MU vs T0: DE
Expression level
5000
4000
3000
2000
0
6
12
24
Time (h)
colour
MU
WT

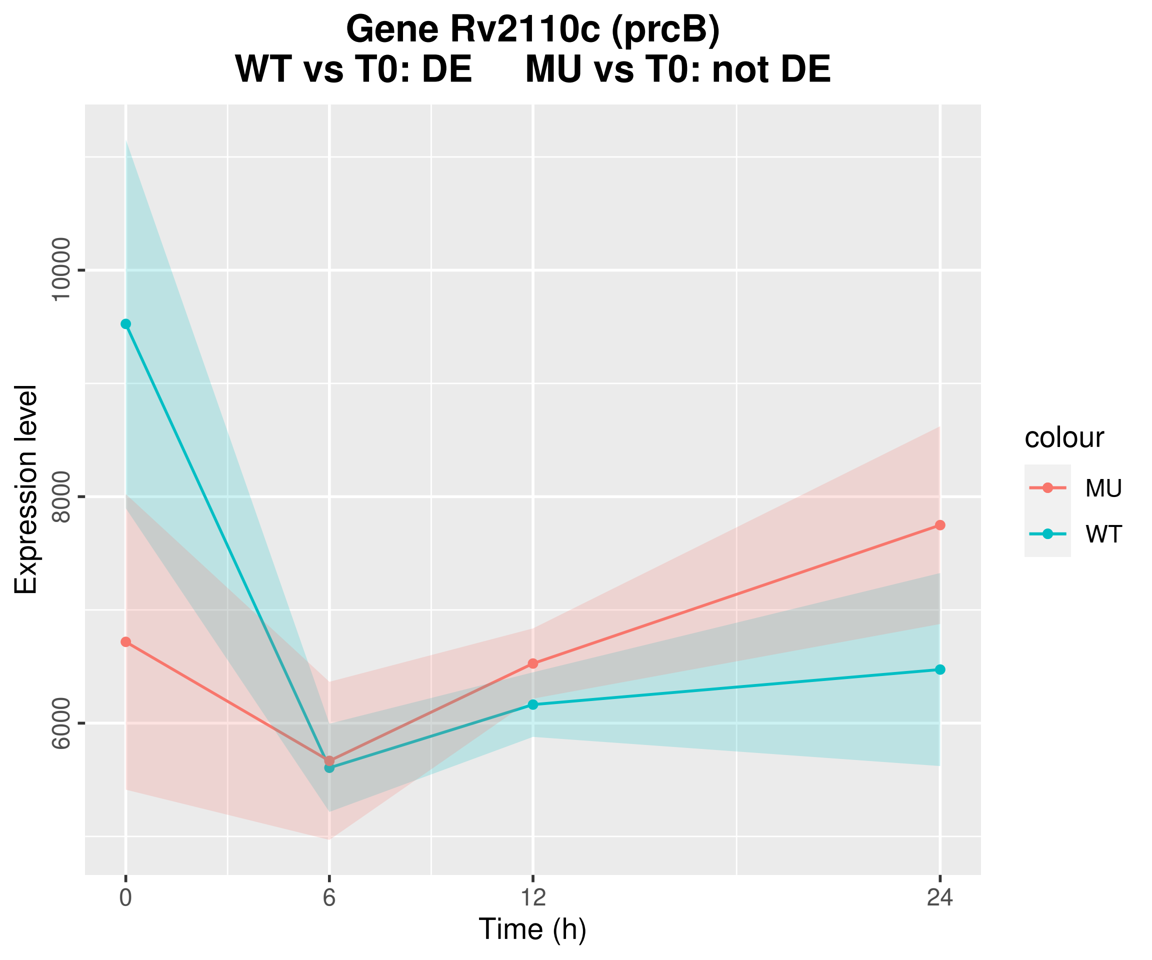

Gene Rv2110c (prcB)
WT vs T0: DE   MU vs T0: not DE
Expression level
10000
8000
6000
0
6
12
24
Time (h)
colour
MU
WT

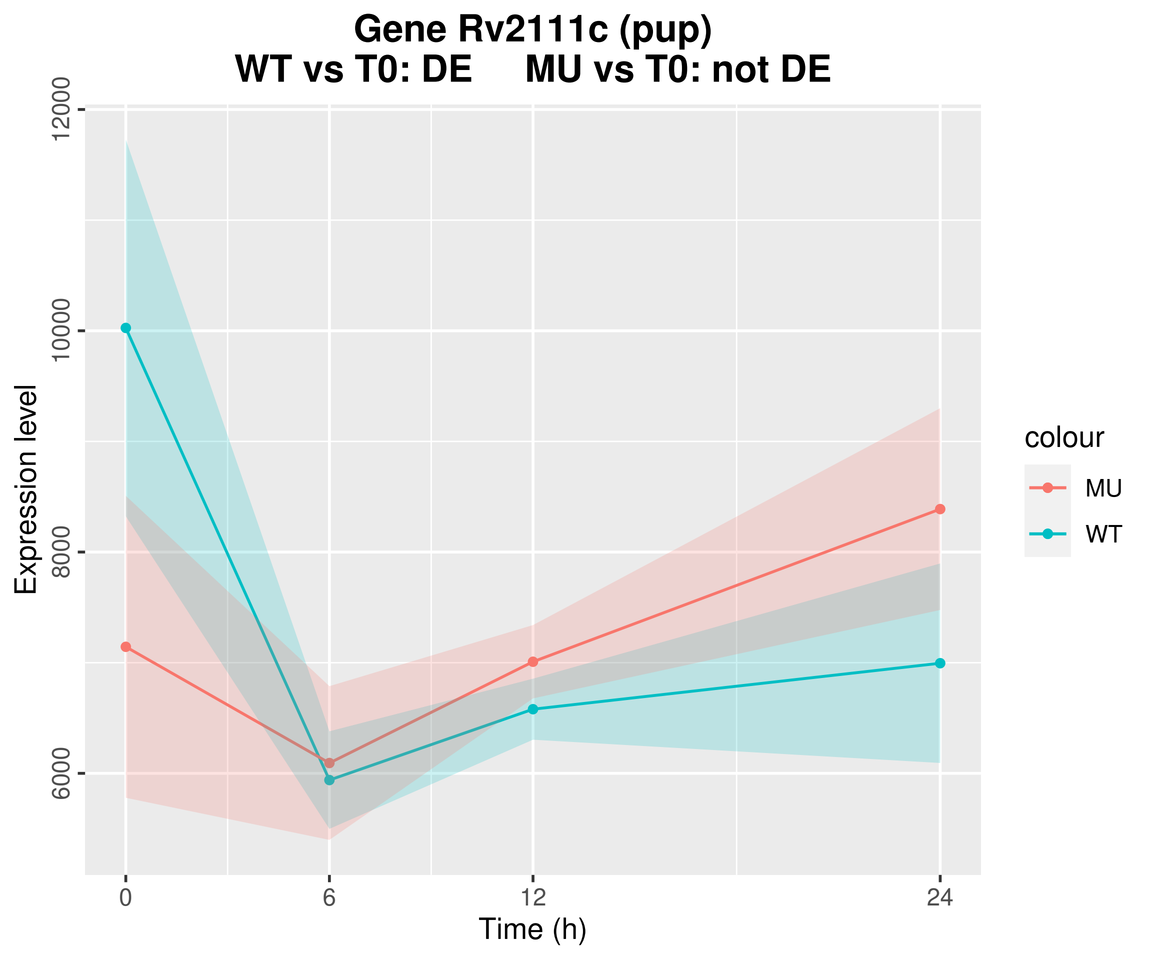

Gene Rv2111c (pup)
WT vs T0: DE   MU vs T0: not DE
Expression level
12000
10000
8000
6000
0
6
12
24
Time (h)
colour
MU
WT

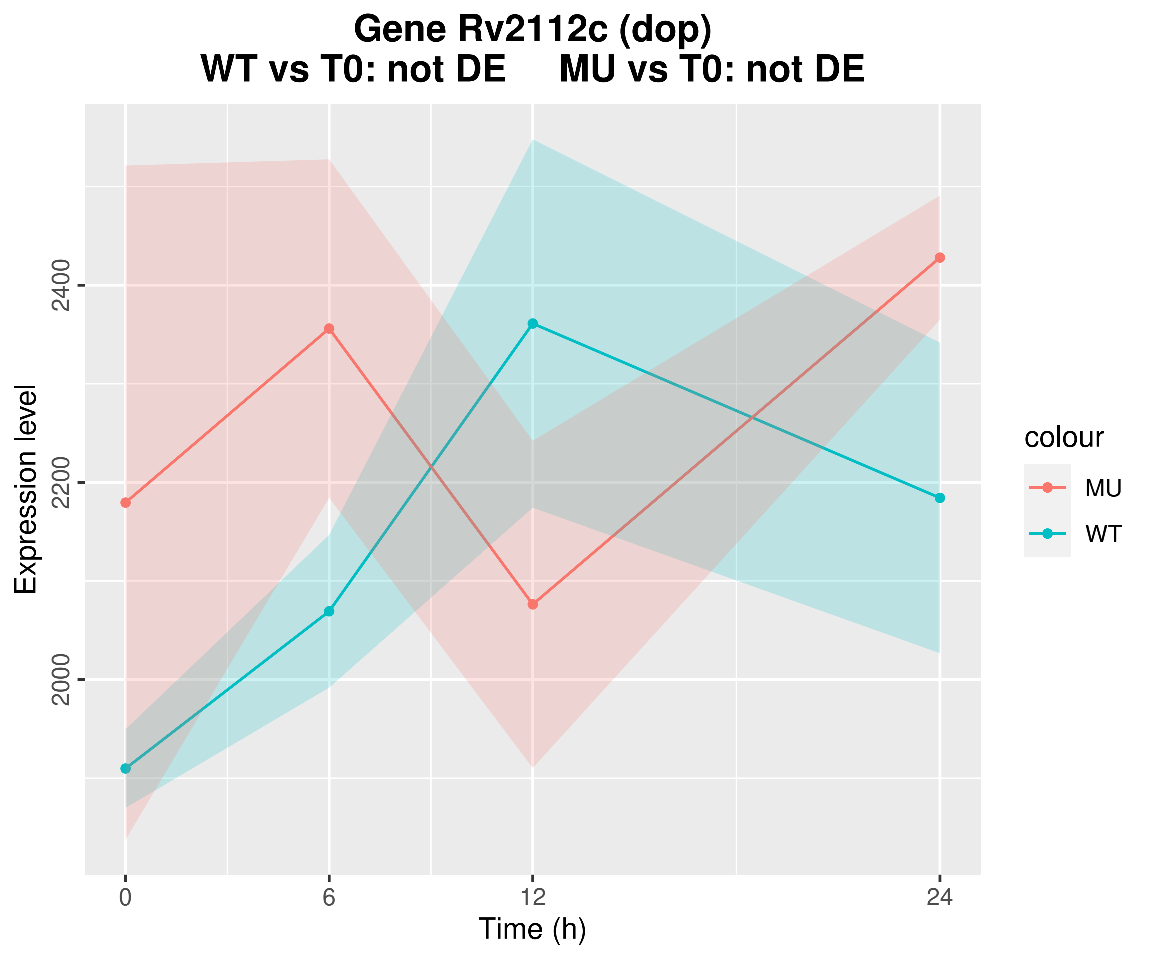

Gene Rv2112c (dop)
WT vs T0: not DE   MU vs T0: not DE
Expression level
2000
2200
2400
0
6
12
24
Time (h)
colour
MU
WT

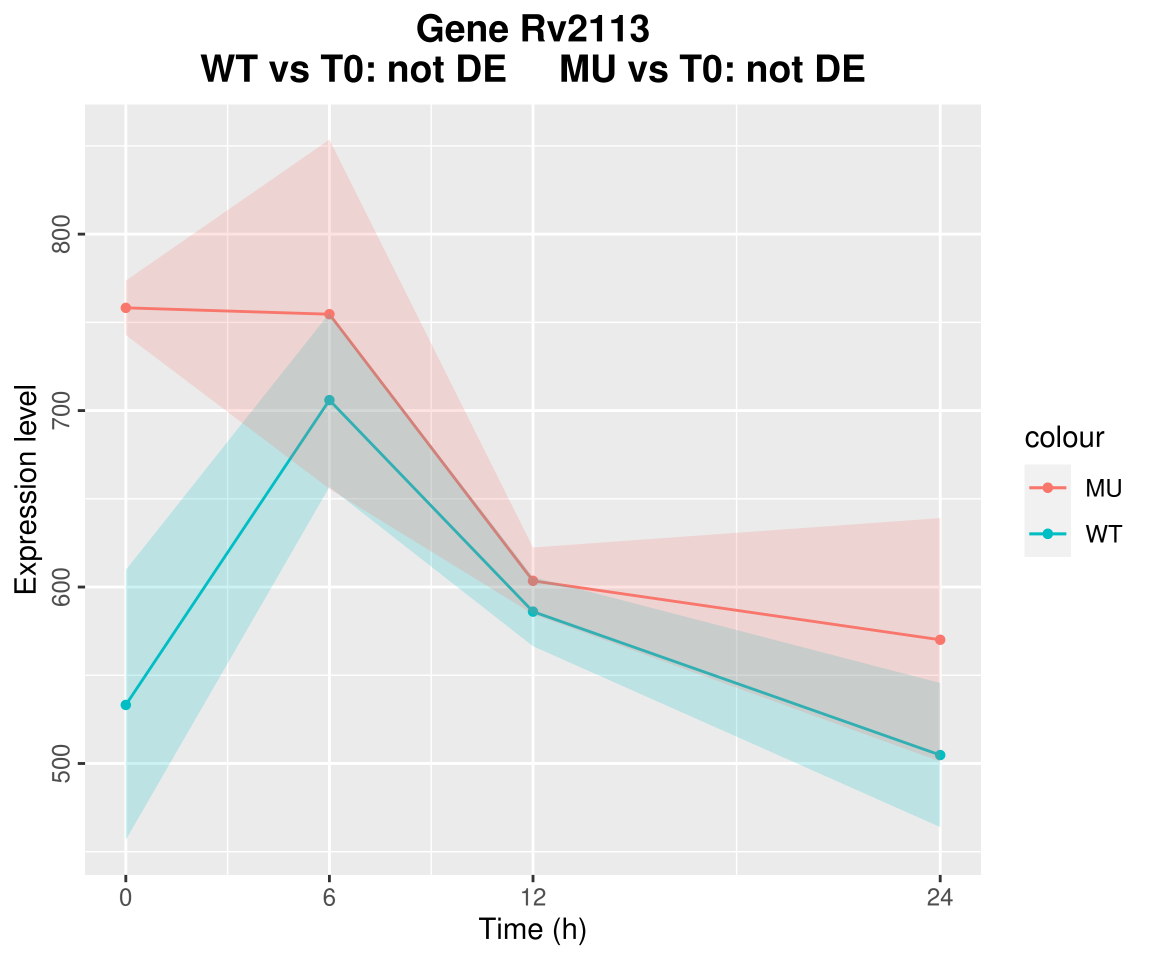
Gene Rv2113
WT vs T0: not DE   MU vs T0: not DE
Expression level
800
700
600
500
0
6
12
24
Time (h)
colour
MU
WT

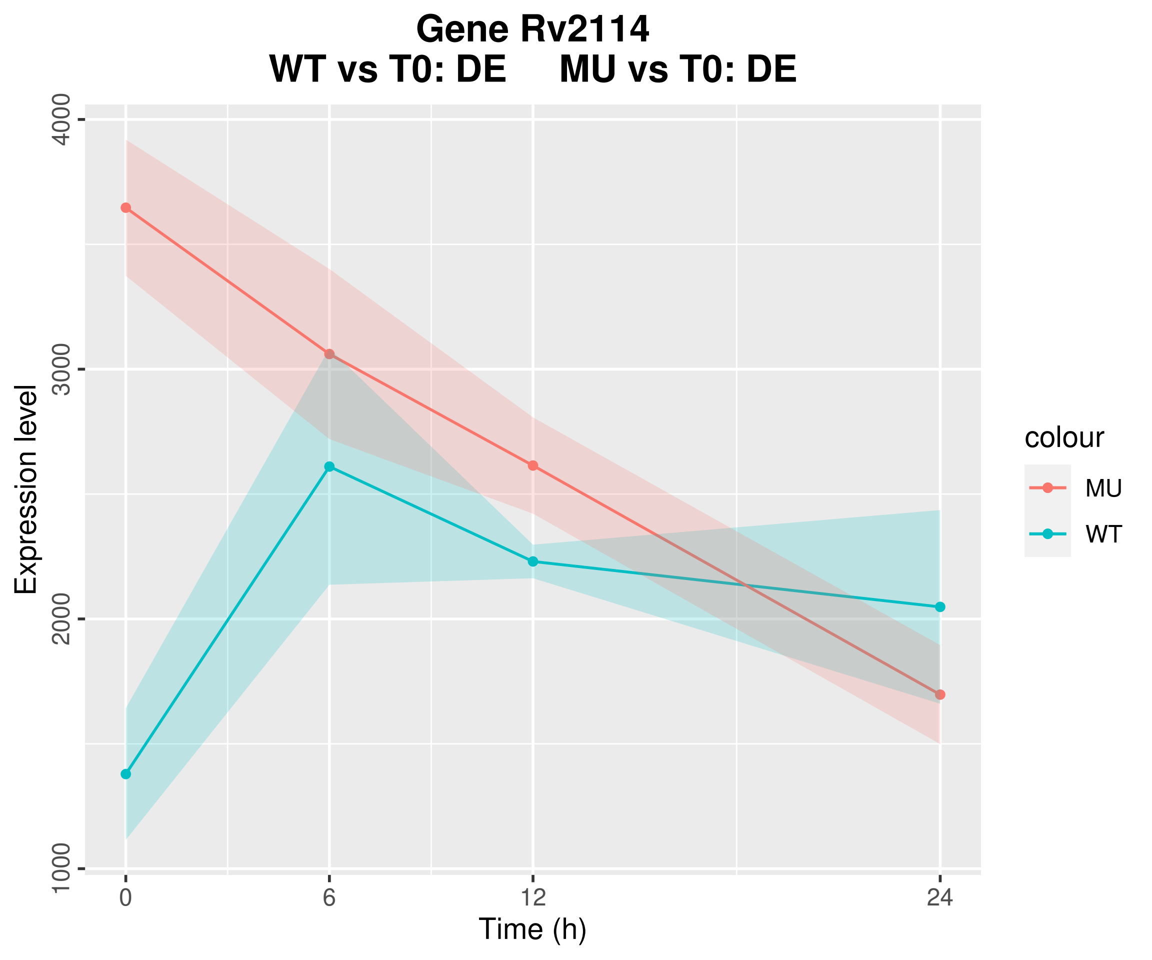

Gene Rv2114
WT vs T0: DE MU vs T0: DE
Expression level
4000
3000
2000
1000
0
6
12
24
Time (h)
colour
MU
WT

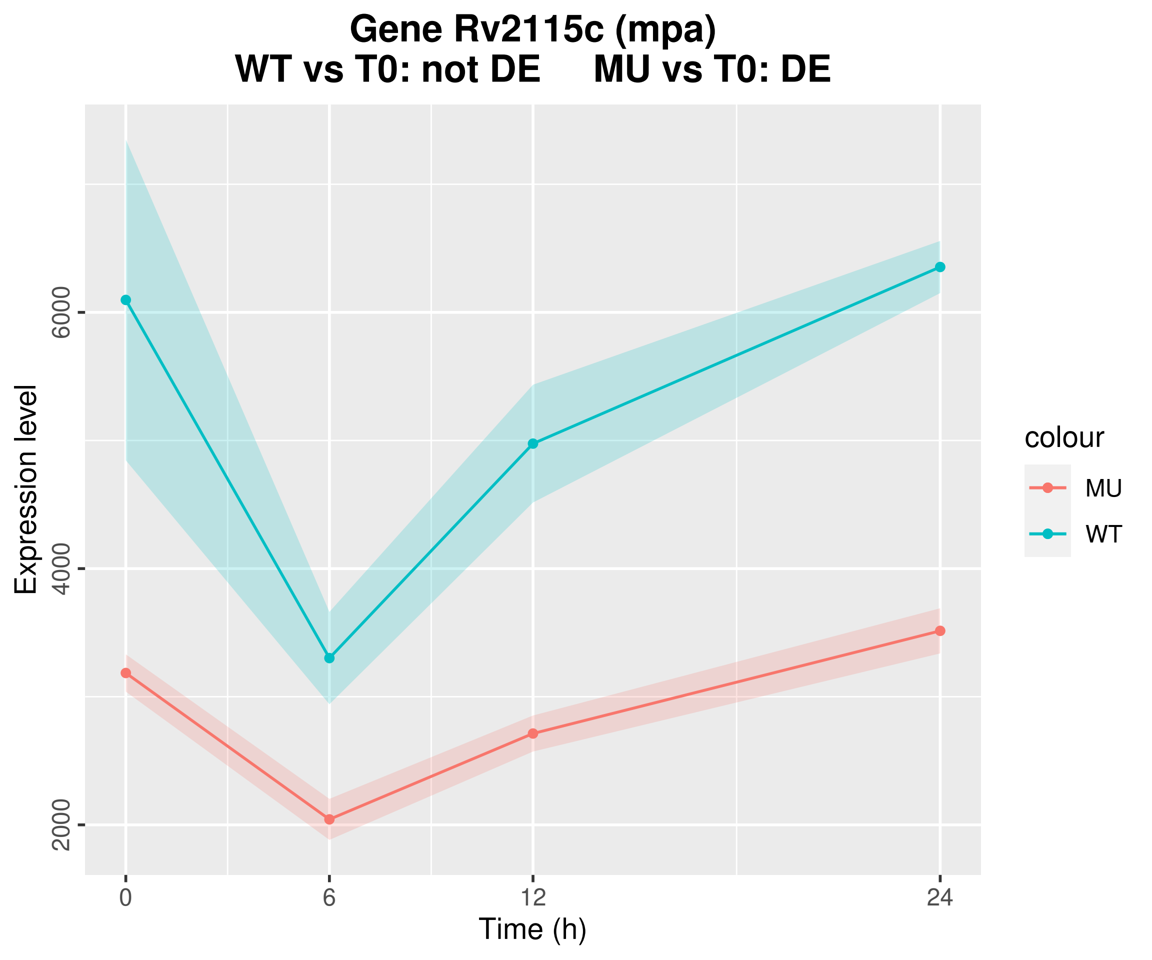
Gene Rv2115c (mpa)
WT vs T0: not DE MU vs T0: DE
6000
4000
2000
Expression level
0
6
12
24
Time (h)
colour
MU
WT

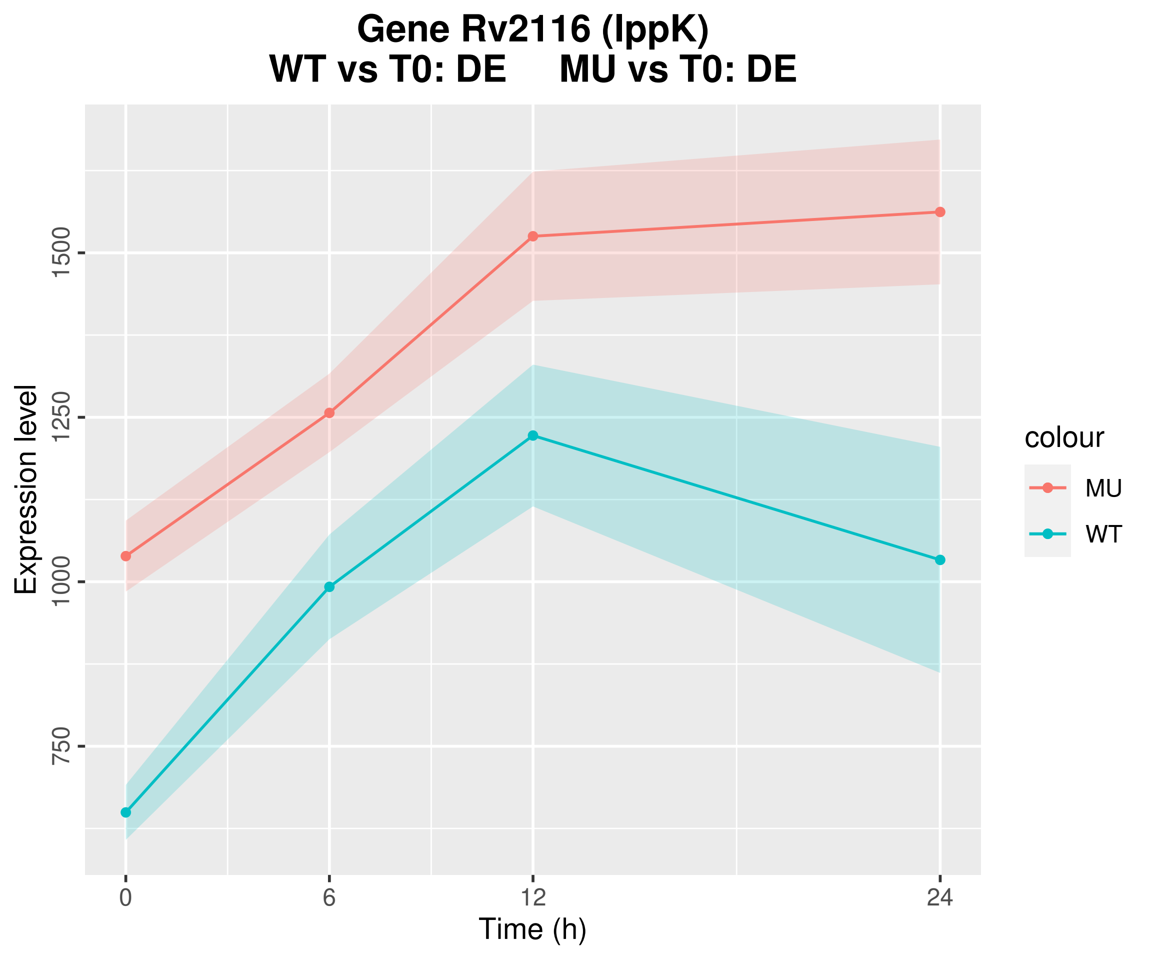
Gene Rv2116 (lppK)
WT vs T0: DE   MU vs T0: DE
Expression level
1500
1250
1000
750
0
6
12
24
Time (h)
colour
MU
WT

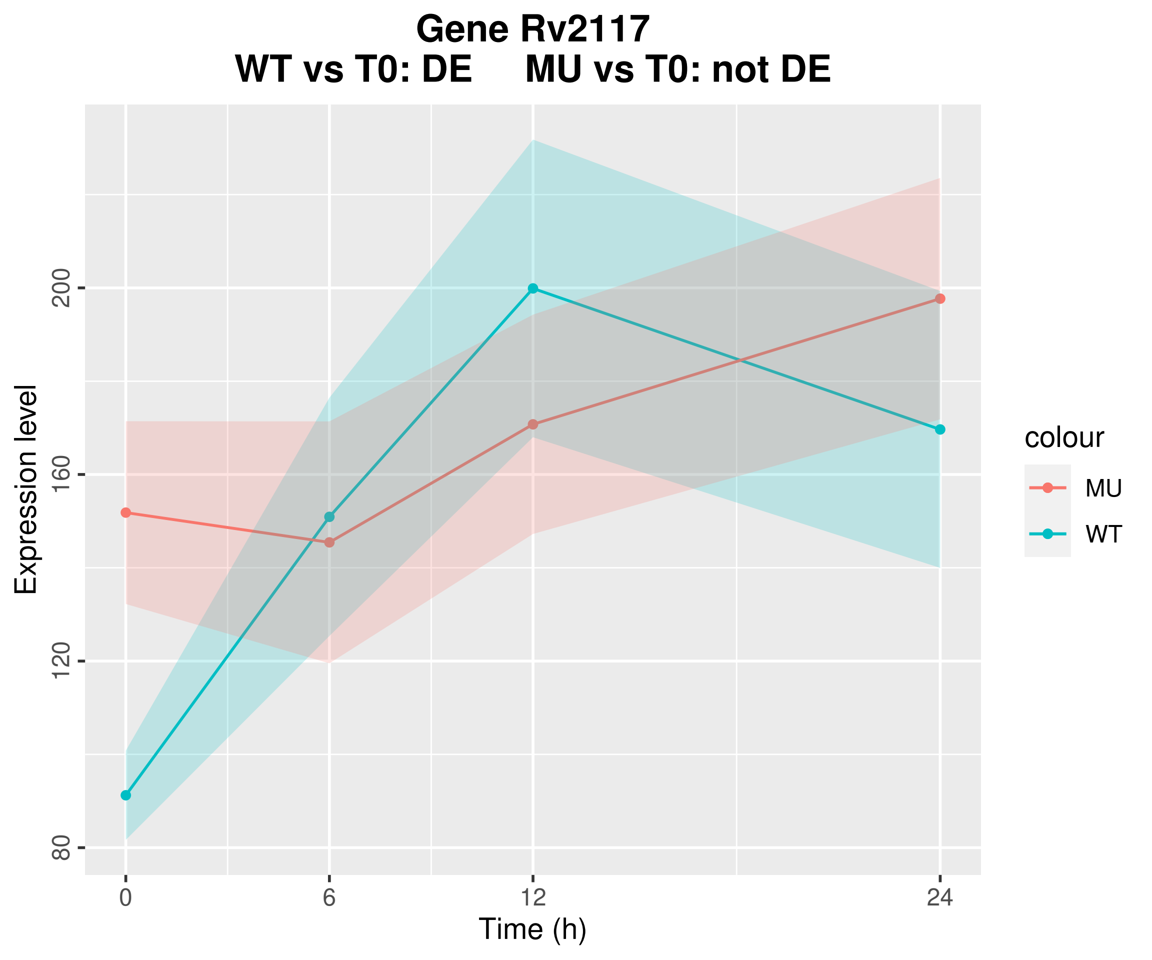
Gene Rv2117
WT vs T0: DE MU vs T0: not DE
Expression level
80
120
160
200
0
6
12
24
Time (h)
colour
MU
WT

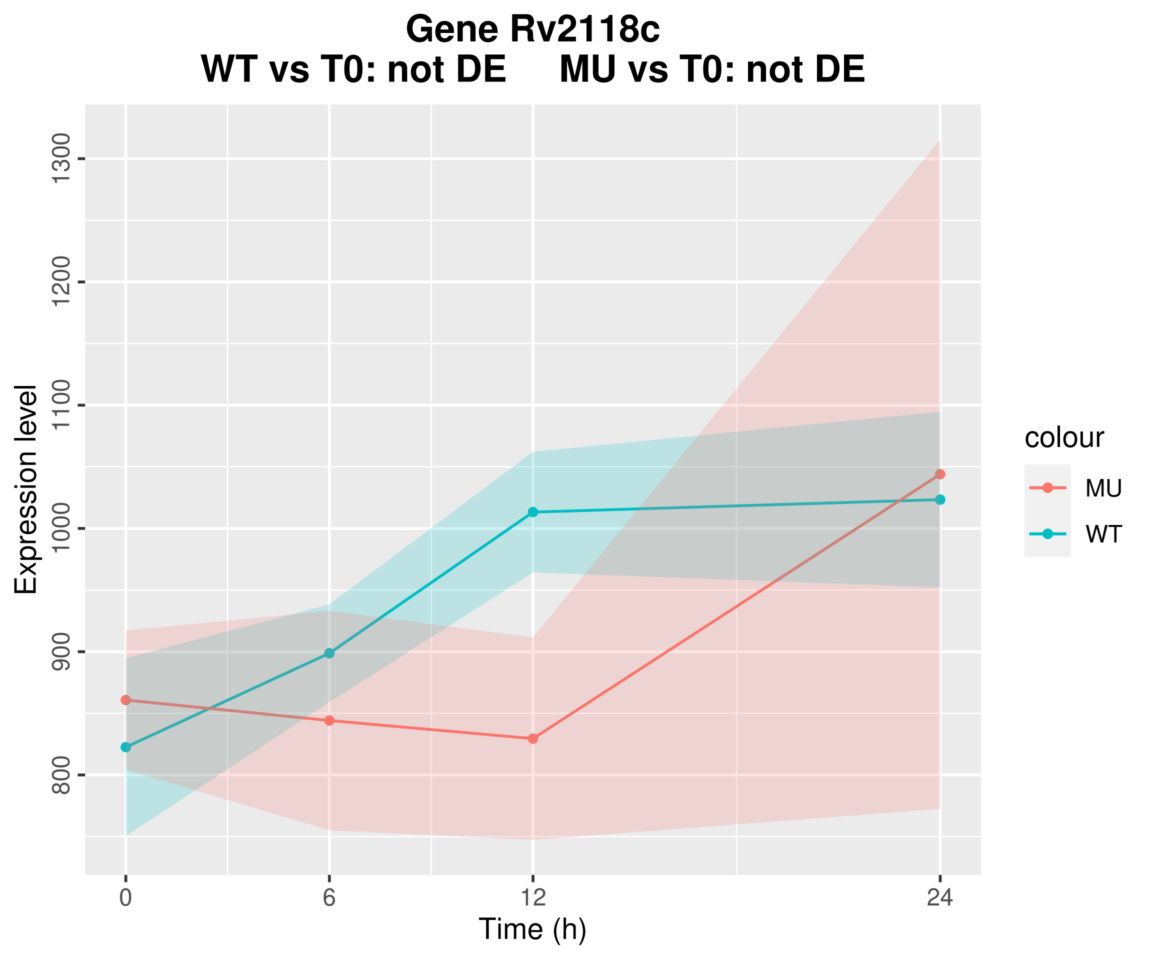

Gene Rv2118c
WT vs T0: not DE   MU vs T0: not DE
Expression level
800
900
1000
1100
1200
1300
0
6
12
24
Time (h)
colour
MU
WT

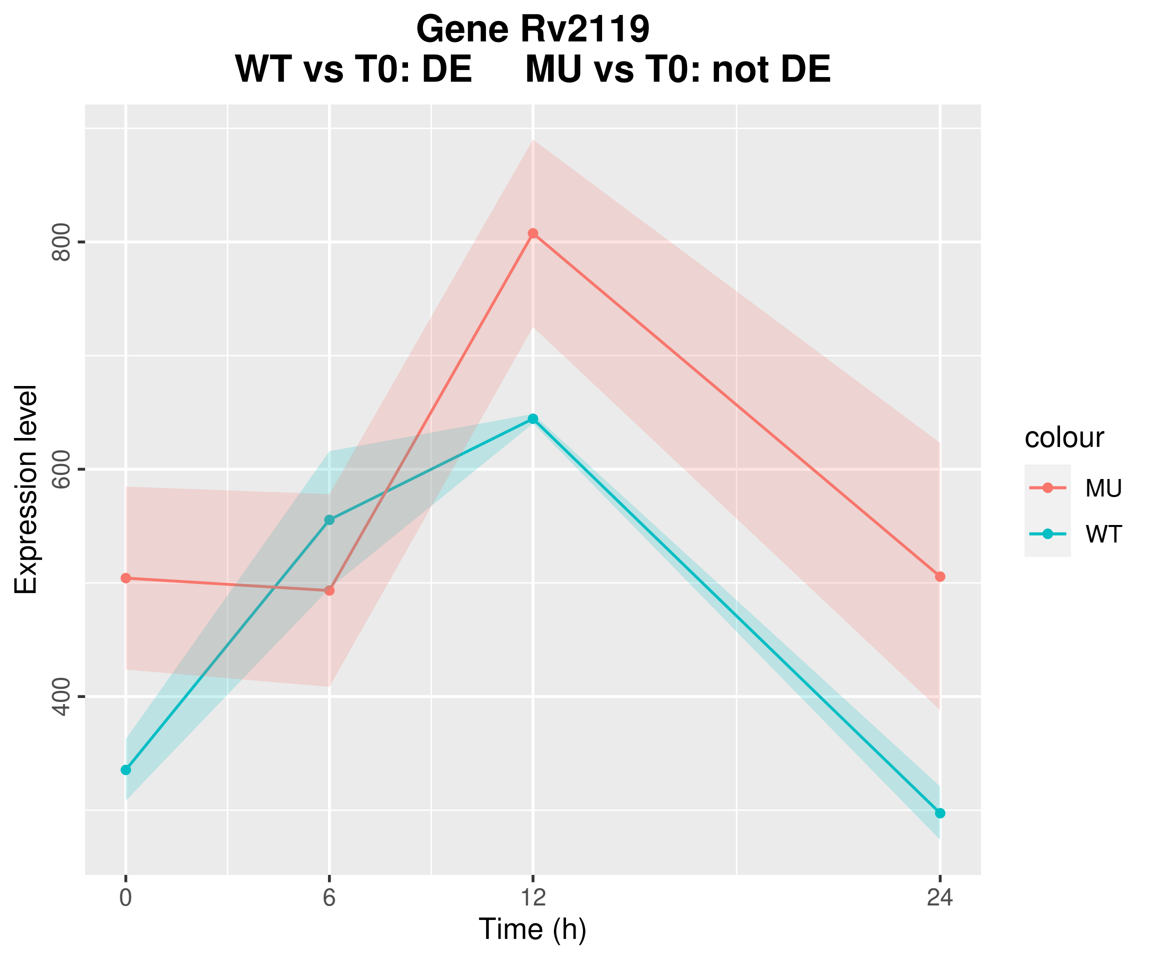

Gene Rv2119
WT vs T0: DE   MU vs T0: not DE
Expression level
800
600
400
0
6
12
24
Time (h)
colour
MU
WT

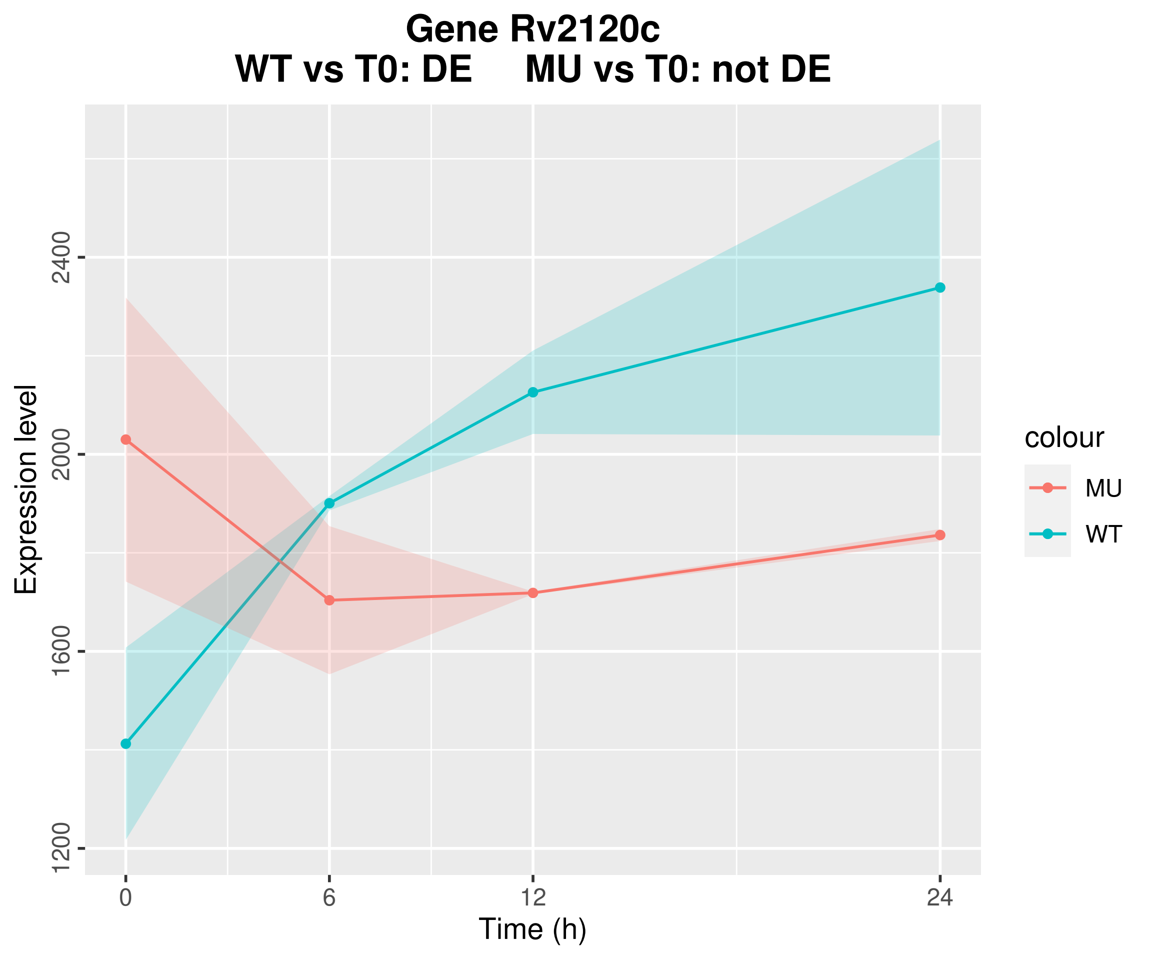
Gene Rv2120c
WT vs T0: DE MU vs T0: not DE
Expression level
2400
2000
1600
1200
0
6
12
24
Time (h)
colour
MU
WT

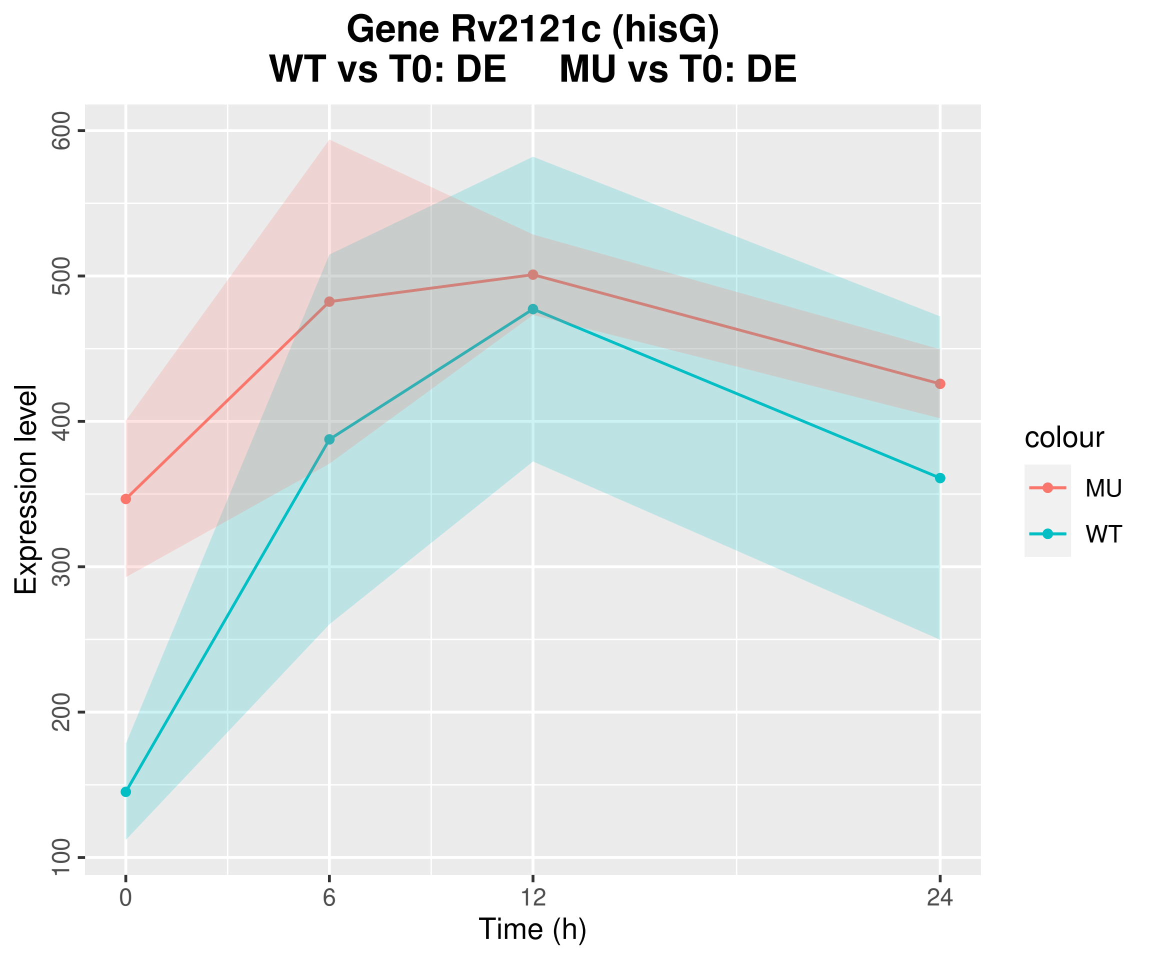

Gene Rv2121c (hisG)
WT vs T0: DE MU vs T0: DE
Expression level
100
200
300
400
500
600
0
6
12
24
Time (h)
colour
MU
WT

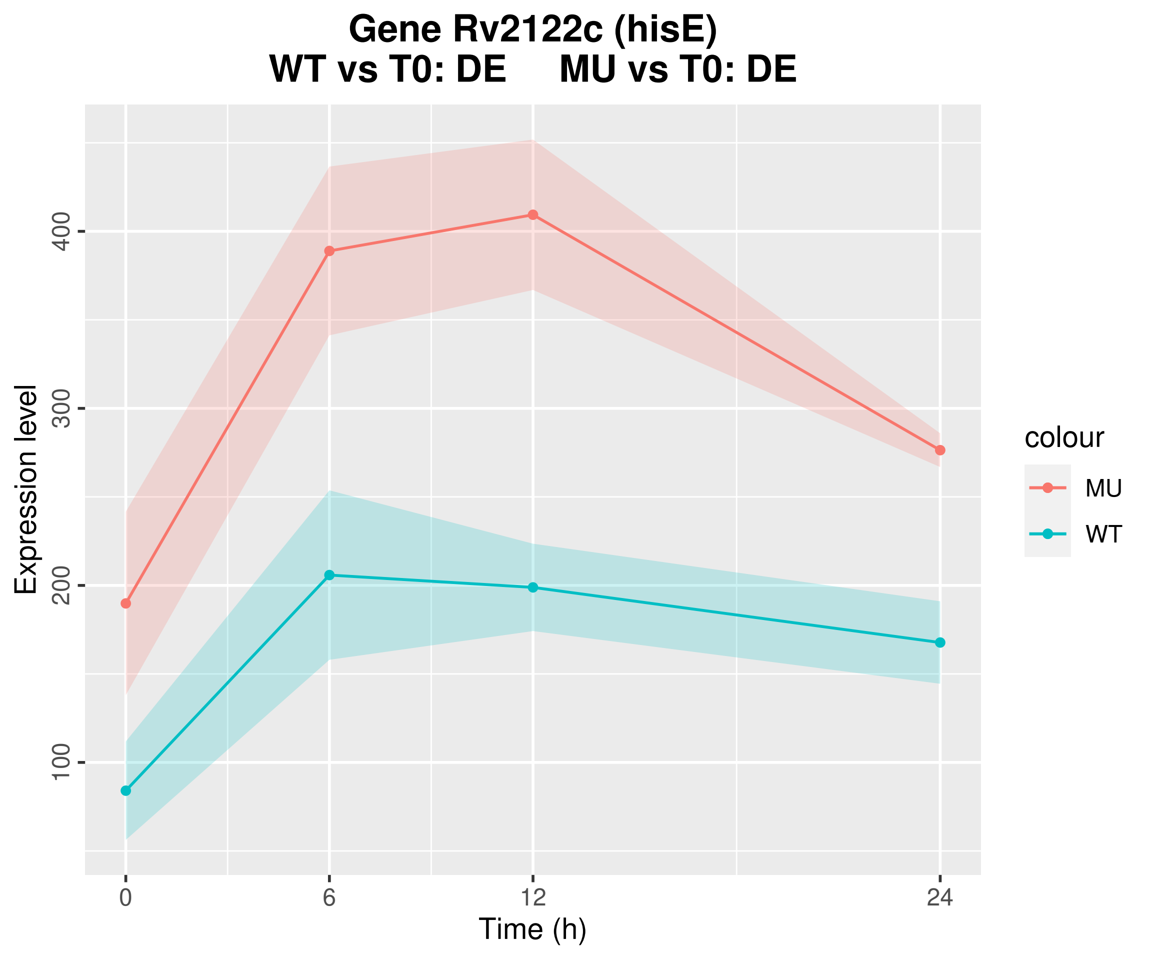
Gene Rv2122c (hisE)
WT vs T0: DE MU vs T0: DE
Expression level
400
300
200
100
0
6
12
24
Time (h)
colour
MU
WT

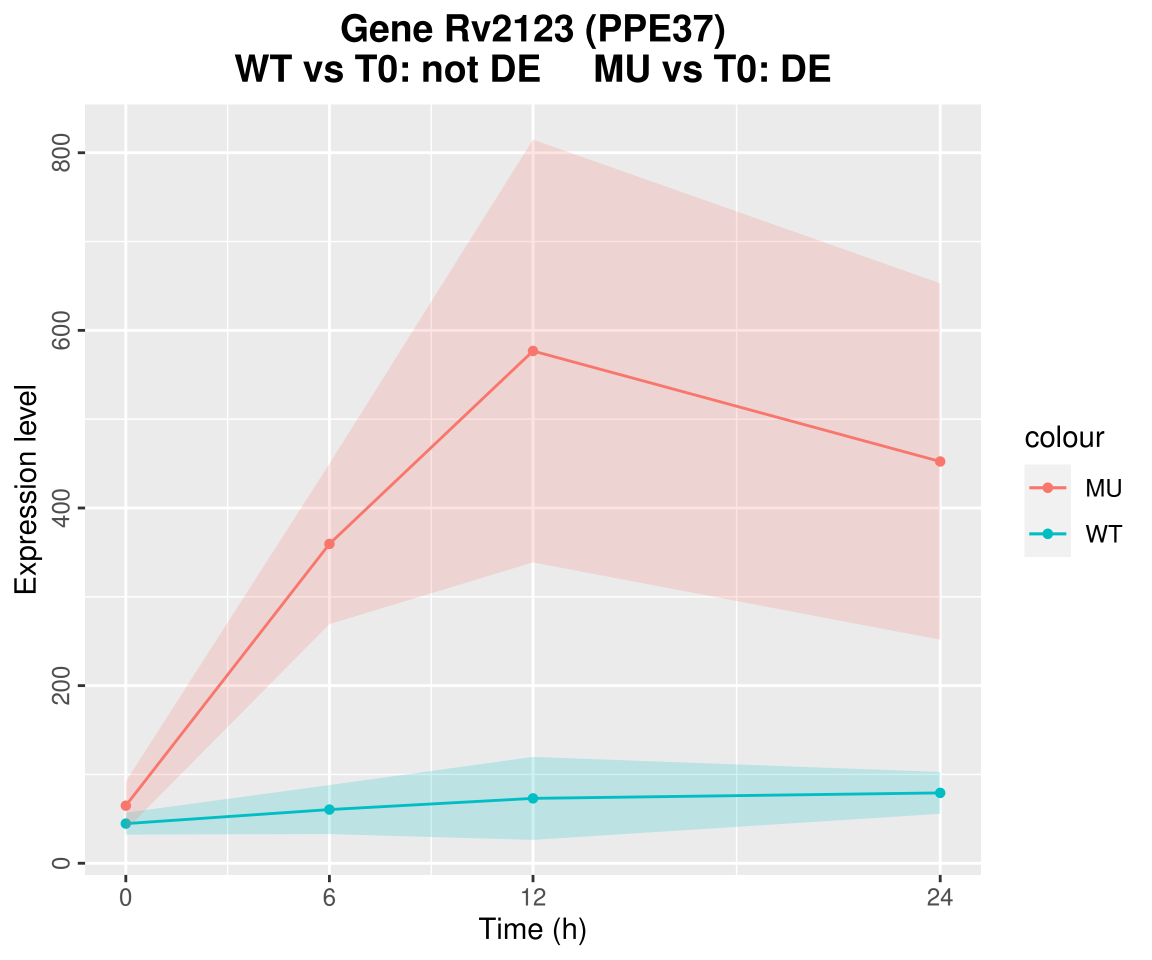
Gene Rv2123 (PPE37)
WT vs T0: not DE   MU vs T0: DE
Expression level
800
600
400
200
0
0
6
12
24
Time (h)
colour
MU
WT

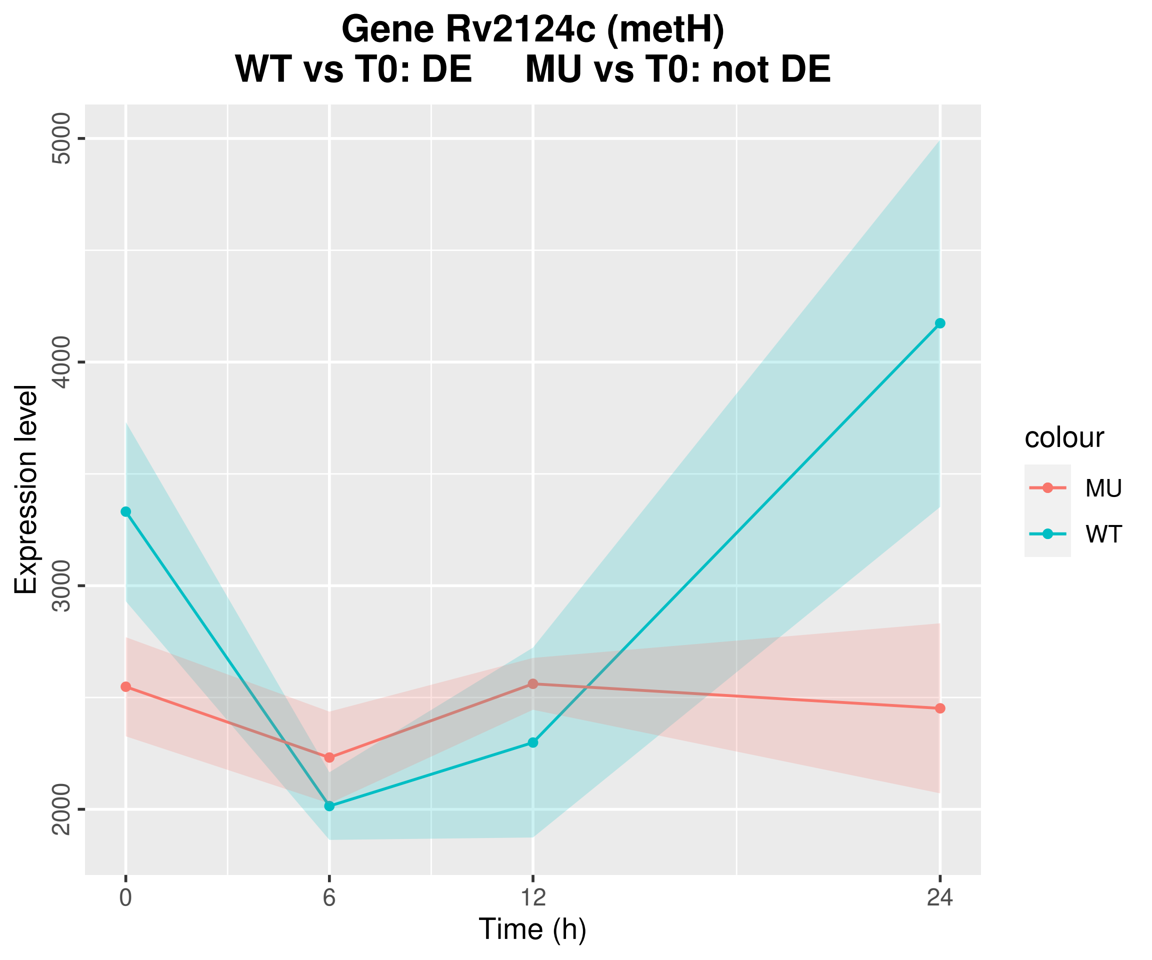

Gene Rv2124c (metH)
WT vs T0: DE   MU vs T0: not DE
Expression level
5000
4000
3000
2000
0
6
12
24
Time (h)
colour
MU
WT

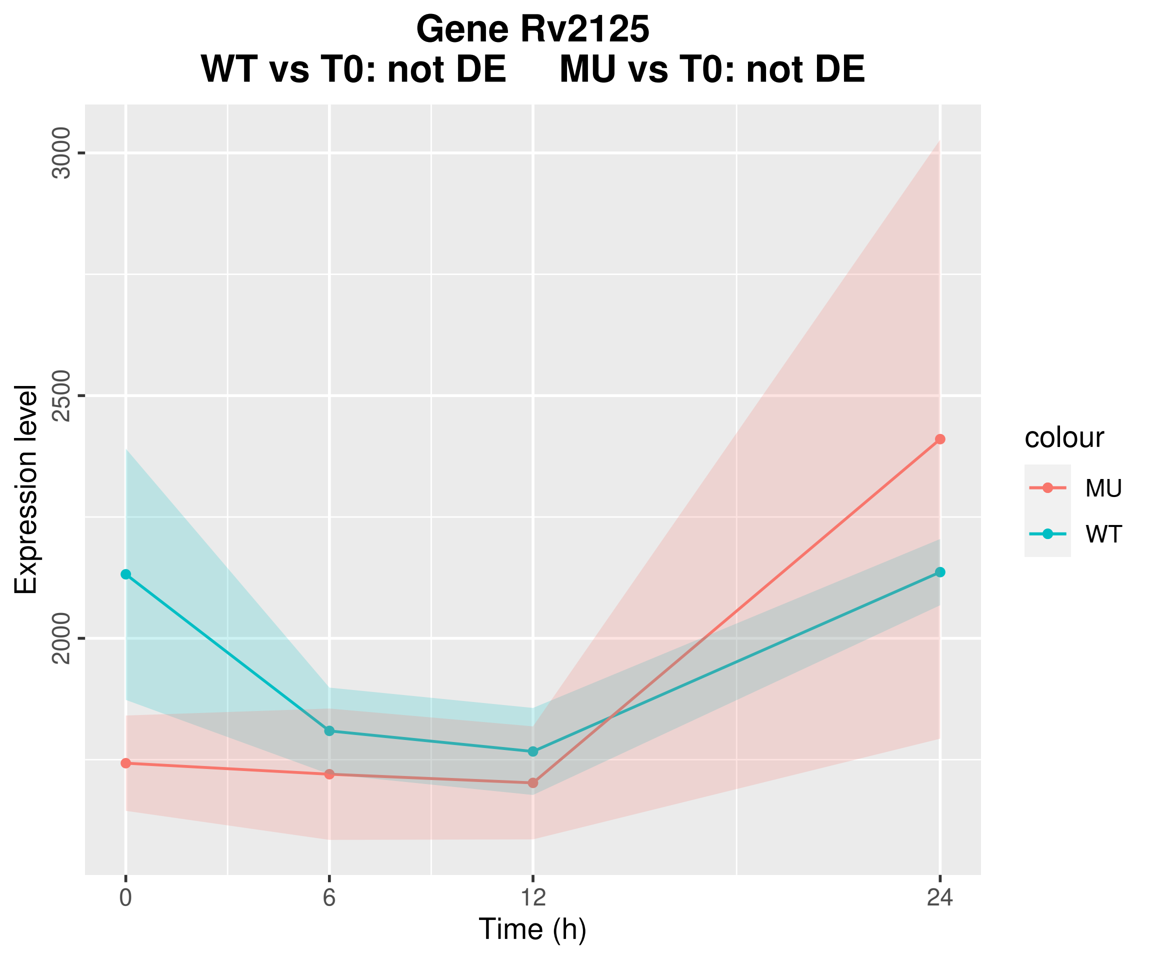
Gene Rv2125
WT vs T0: not DE   MU vs T0: not DE
3000
2500
2000
Expression level
0
6
12
24
Time (h)
colour
MU
WT

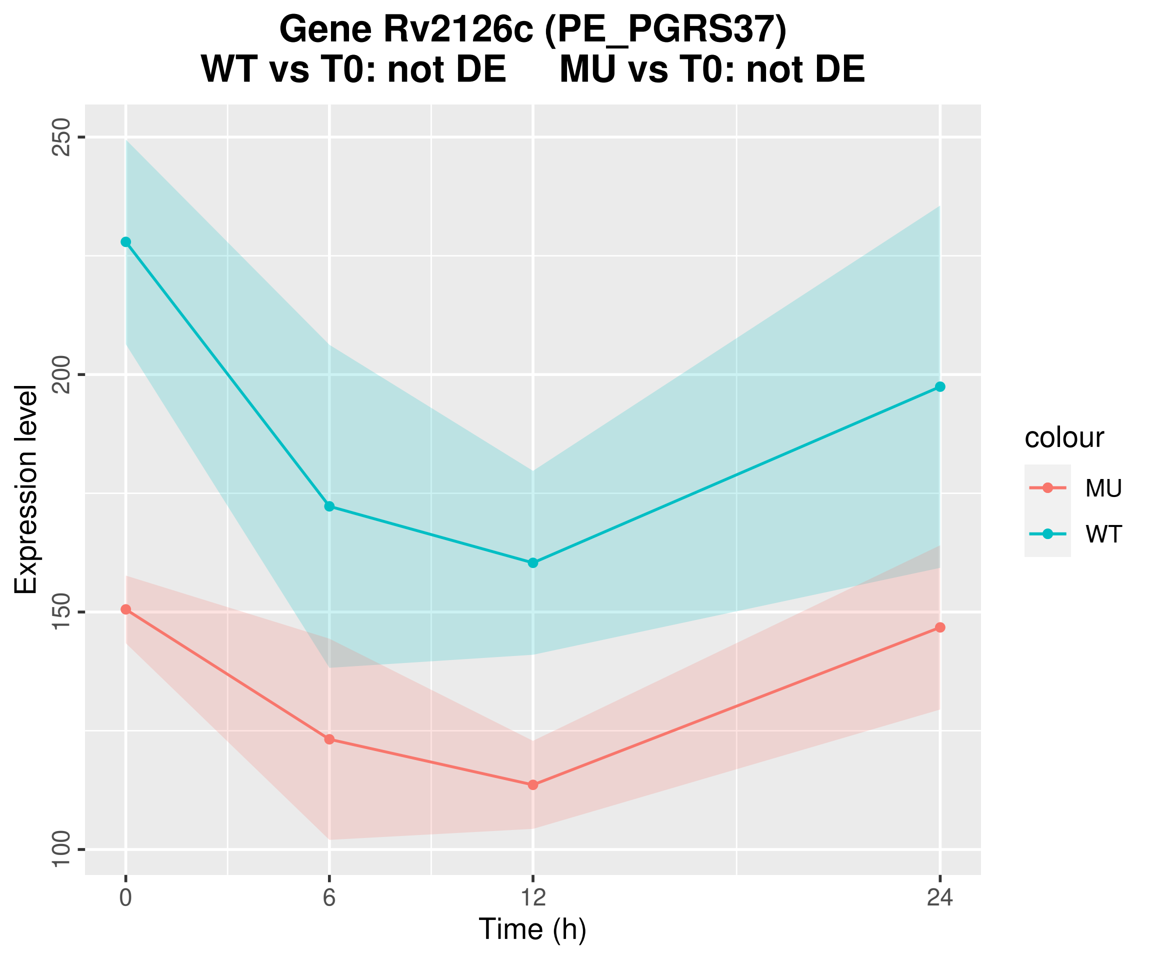

Gene Rv2126c (PE_PGRS37)
WT vs T0: not DE   MU vs T0: not DE
Expression level
250
200
150
100
0
6
12
24
Time (h)
colour
MU
WT

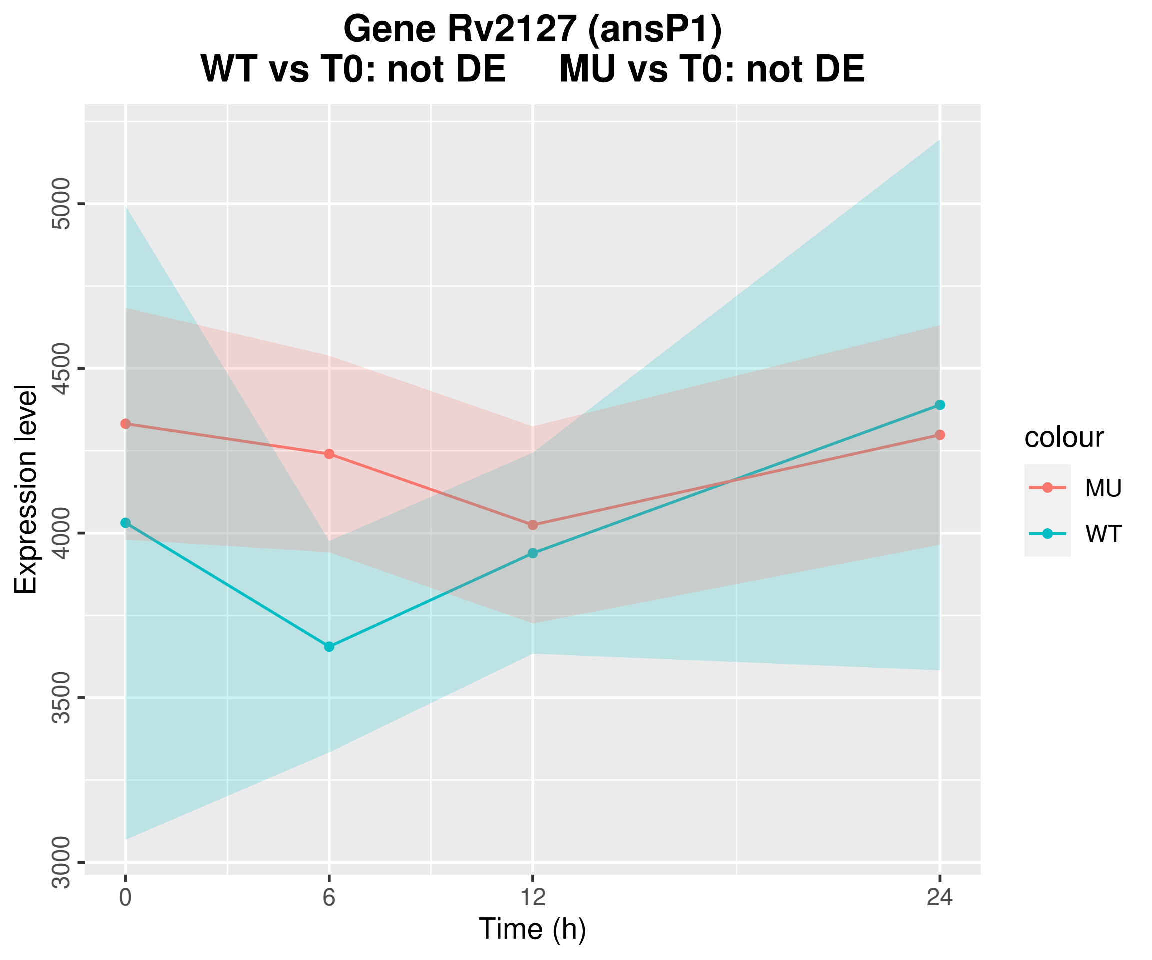

Gene Rv2127 (ansP1)
WT vs T0: not DE   MU vs T0: not DE
Expression level
5000
4500
4000
3500
3000
0
6
12
24
Time (h)
colour
MU
WT

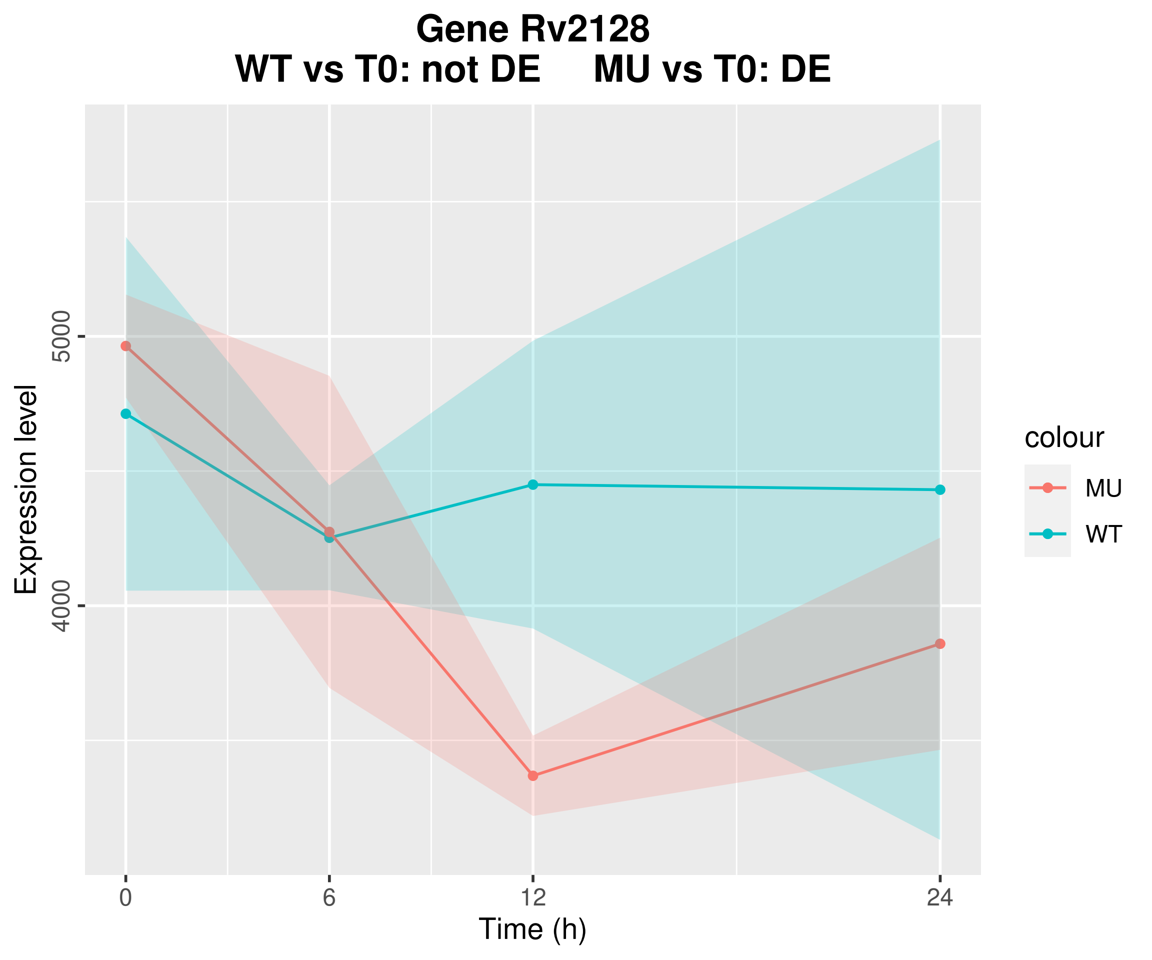
Gene Rv2128
WT vs T0: not DE   MU vs T0: DE
Expression level
5000
4000
0
6
12
24
Time (h)
colour
MU
WT

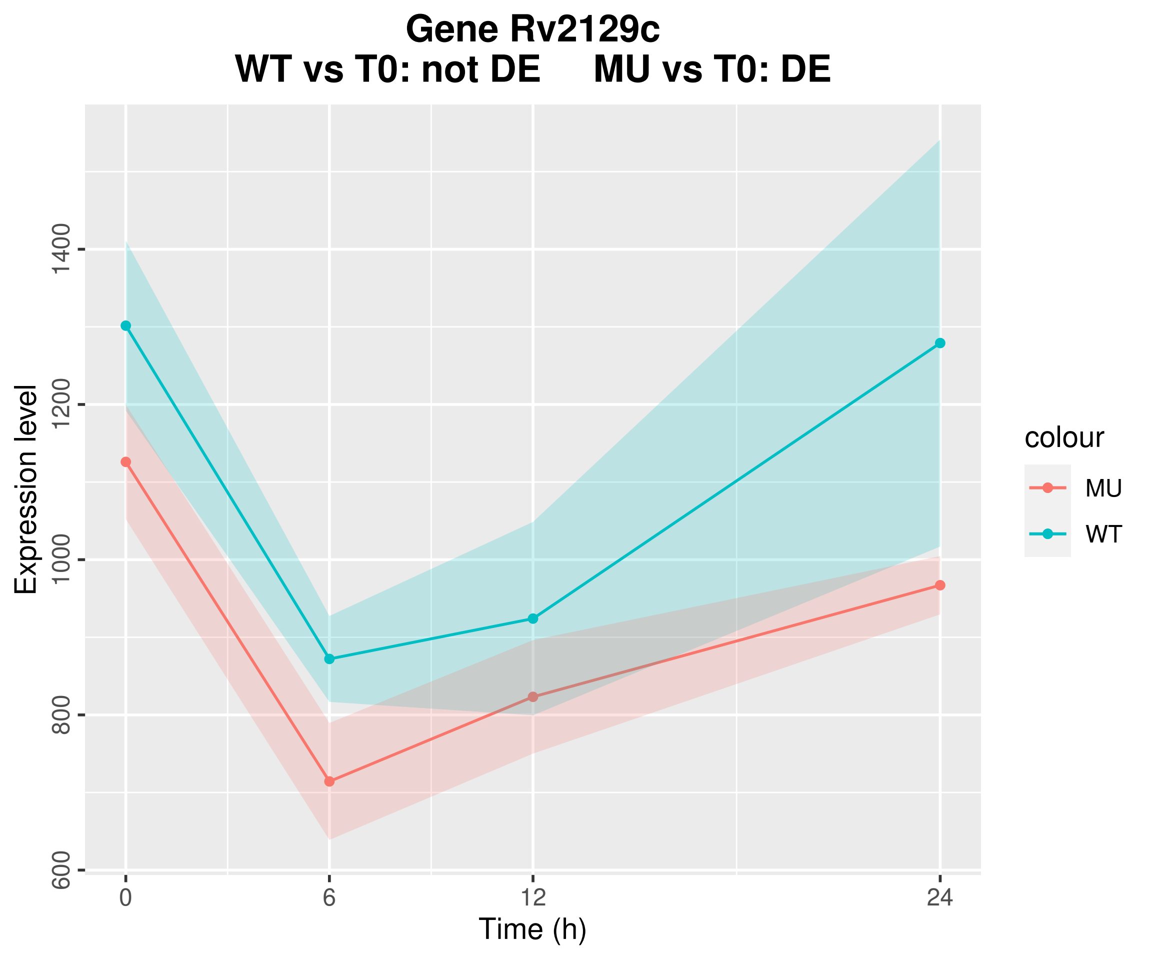
Gene Rv2129c
WT vs T0: not DE   MU vs T0: DE
Expression level
1400
1200
1000
800
600
0
6
12
24
Time (h)
colour
MU
WT

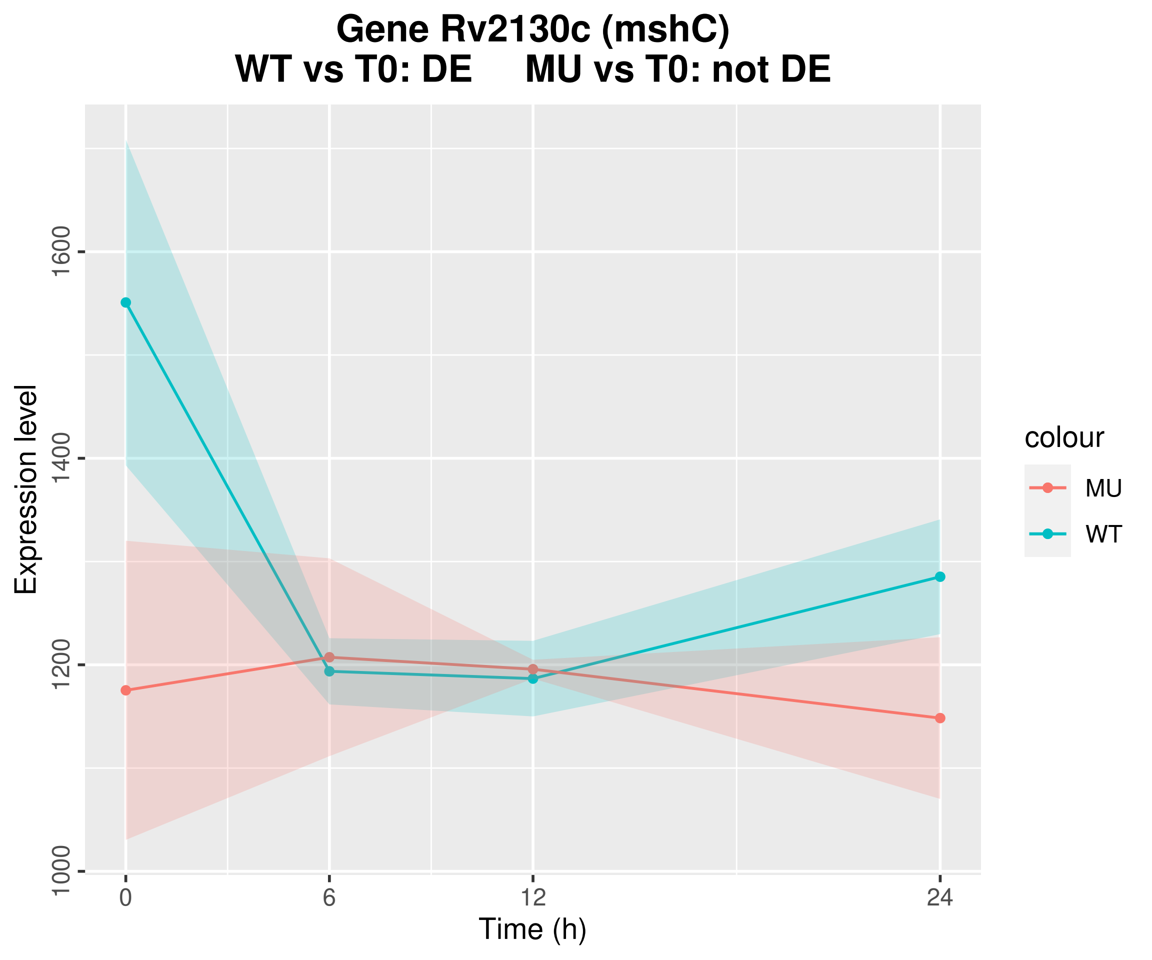

Gene Rv2130c (mshC)
WT vs T0: DE   MU vs T0: not DE
Expression level
1000
1200
1400
1600
0
6
12
24
Time (h)
colour
MU
WT

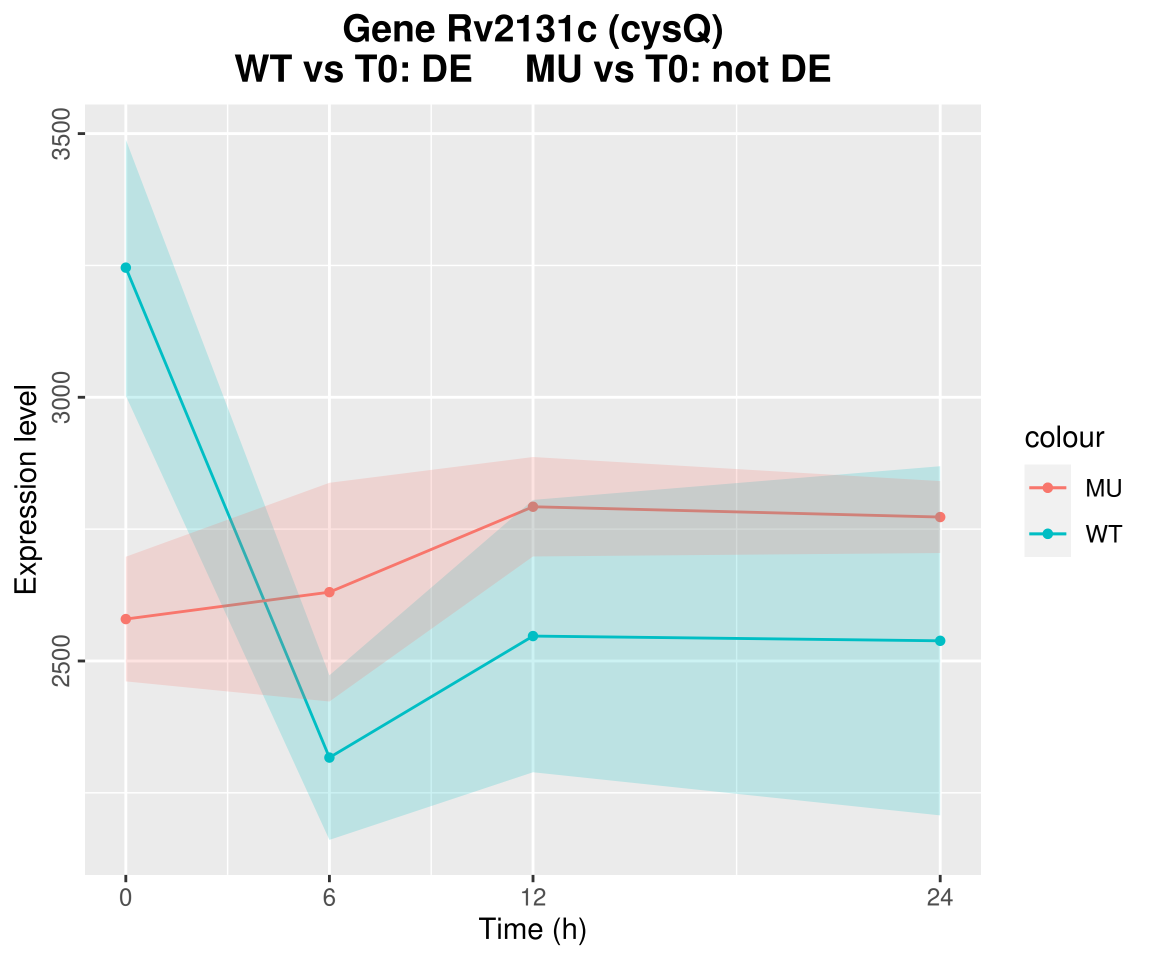

Gene Rv2131c (cysQ)
WT vs T0: DE MU vs T0: not DE
Expression level
3500
3000
2500
0
6
12
24
Time (h)
colour
MU
WT

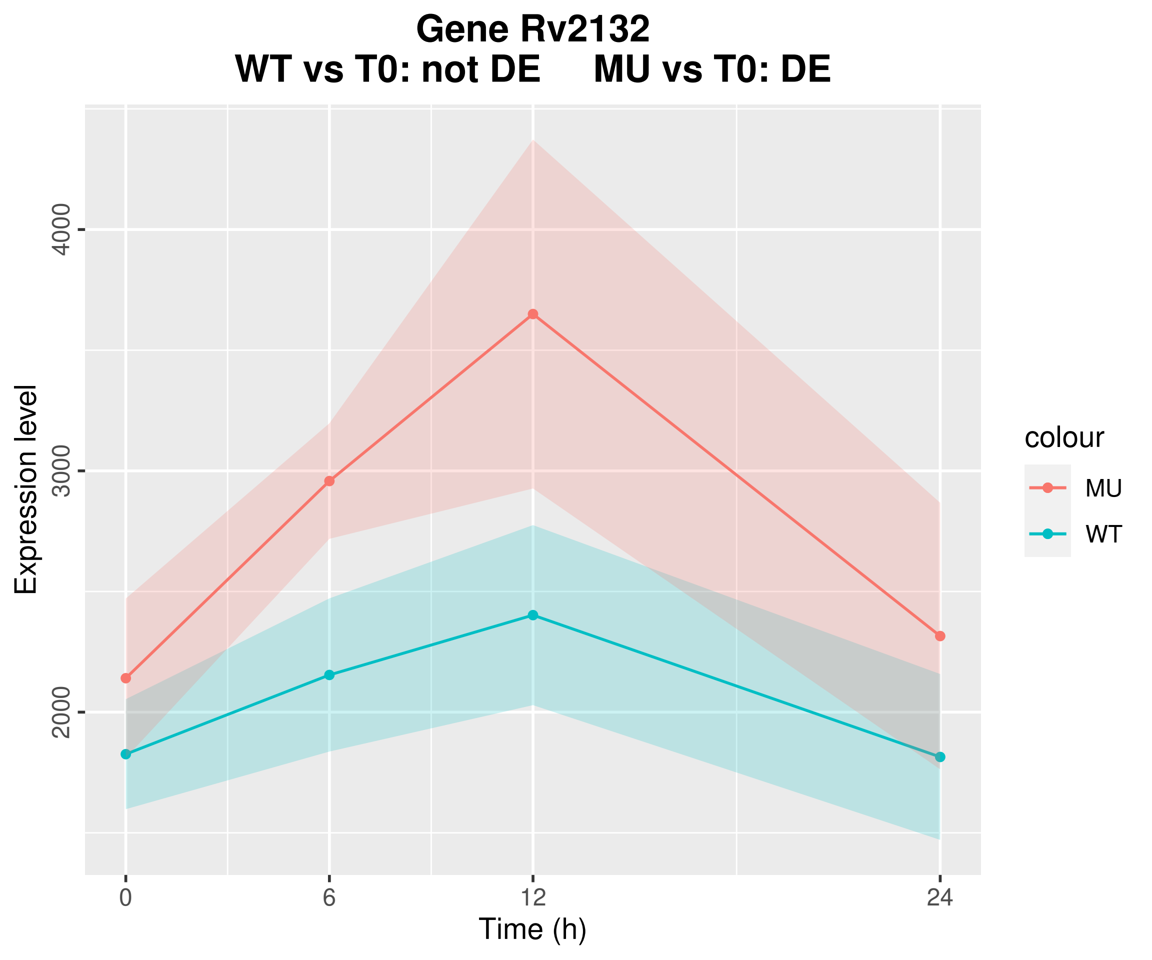
Gene Rv2132
WT vs T0: not DE MU vs T0: DE
Expression level
4000
3000
2000
0
6
12
24
Time (h)
colour
MU
WT

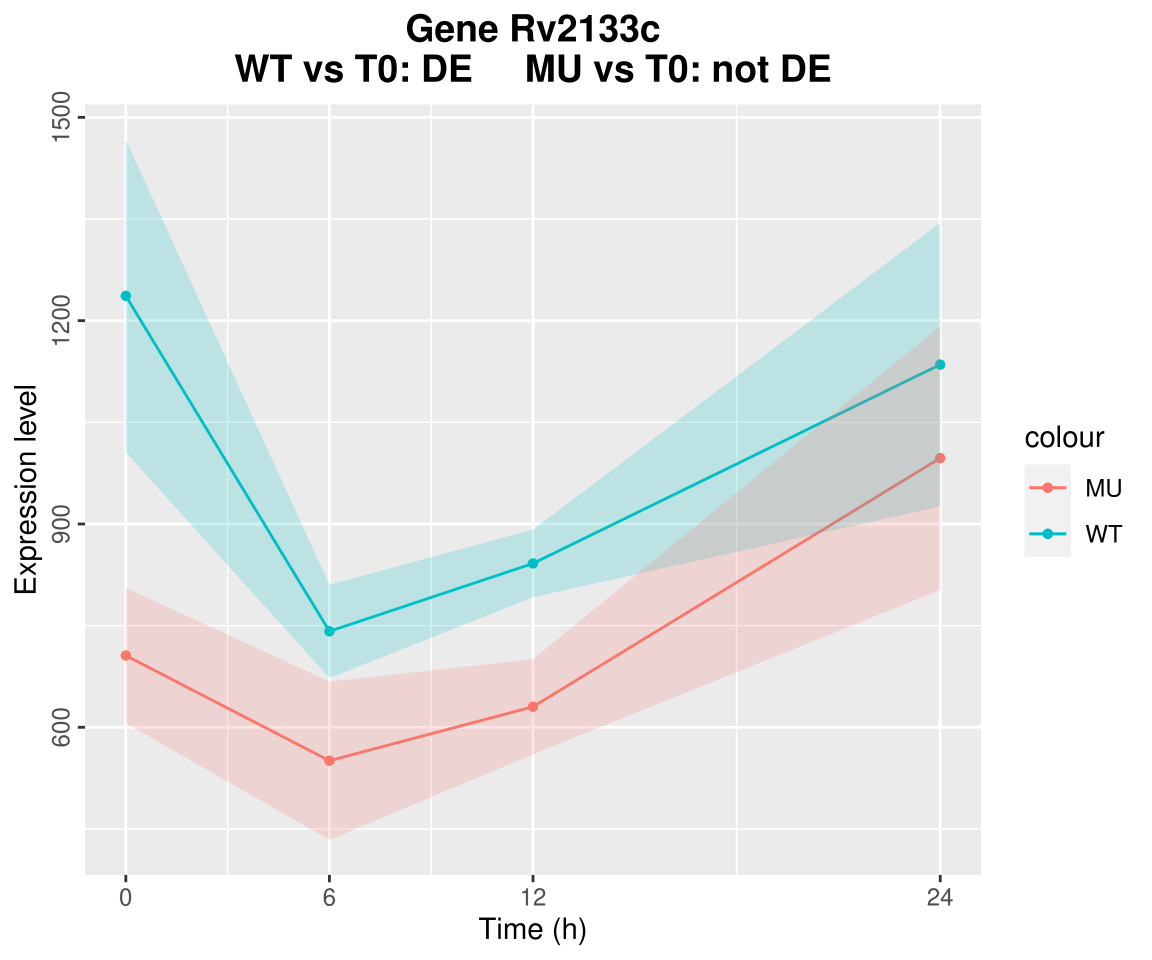
Gene Rv2133c
WT vs T0: DE MU vs T0: not DE
1500
1200
900
600
Expression level
0
6
12
24
Time (h)
colour
MU
WT

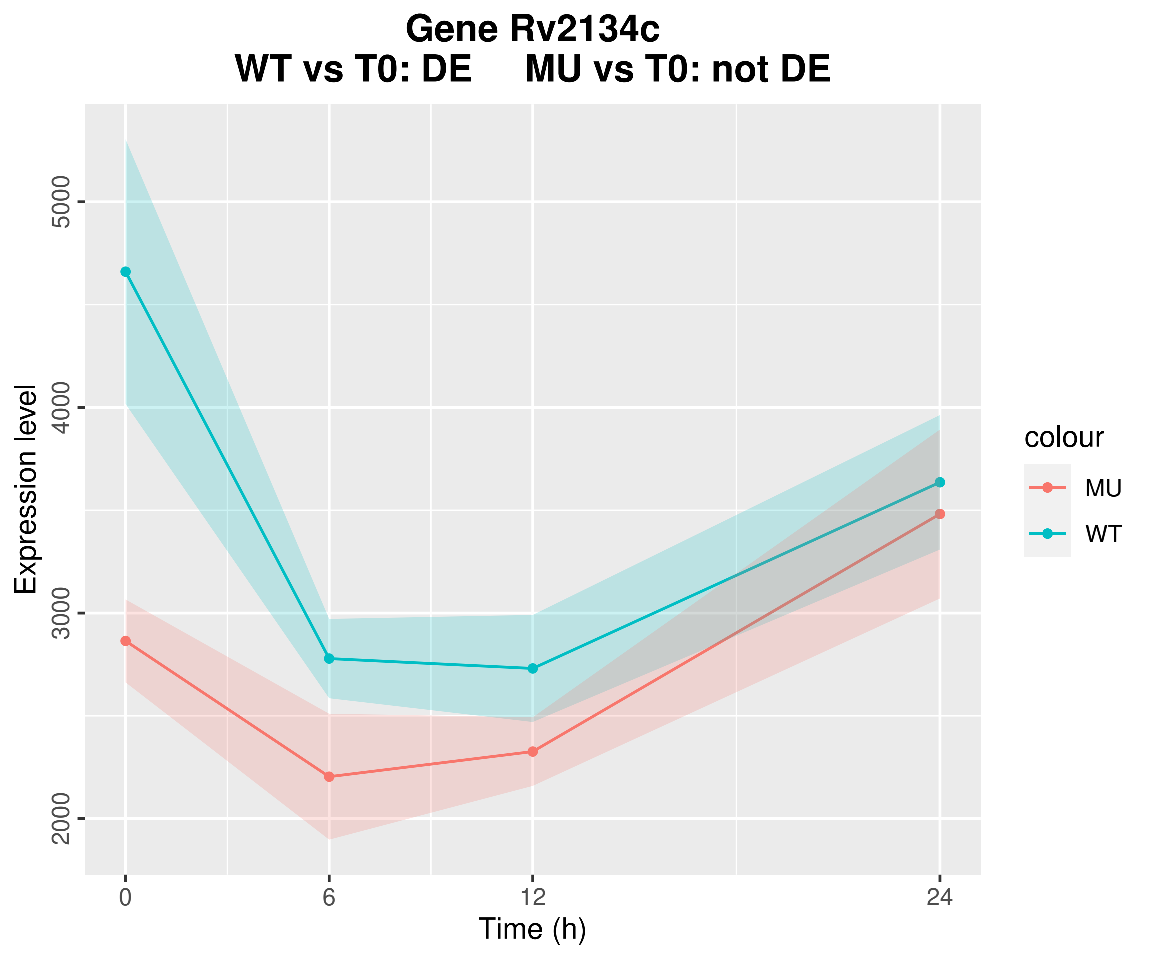

Gene Rv2134c
WT vs T0: DE   MU vs T0: not DE
Expression level
5000
4000
3000
2000
0
6
12
24
Time (h)
colour
MU
WT

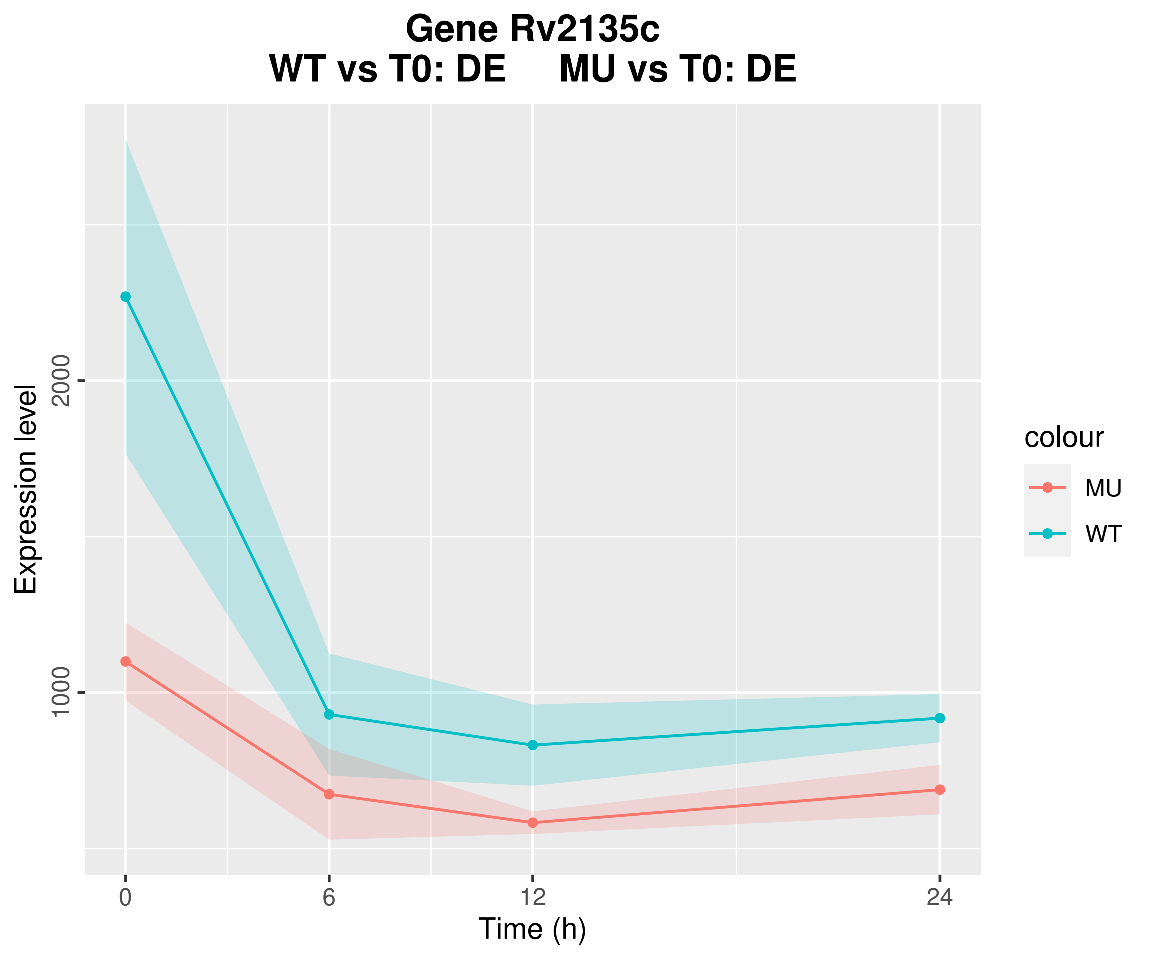

Gene Rv2135c
WT vs T0: DE MU vs T0: DE
Expression level
2000
1000
0
6
12
24
Time (h)
colour
MU
WT

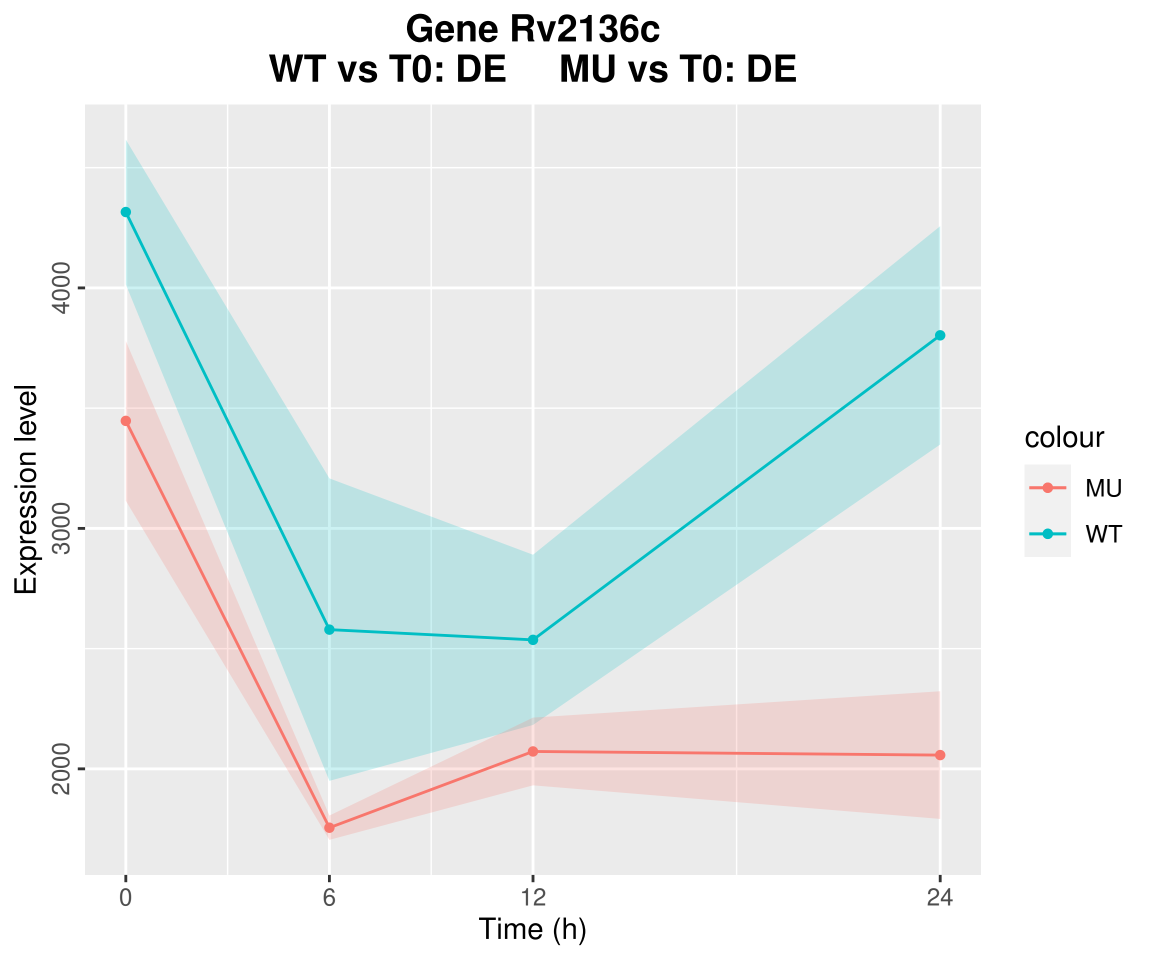
Gene Rv2136c
WT vs T0: DE MU vs T0: DE
Expression level
4000
3000
2000
0
6
12
24
Time (h)
colour
MU
WT

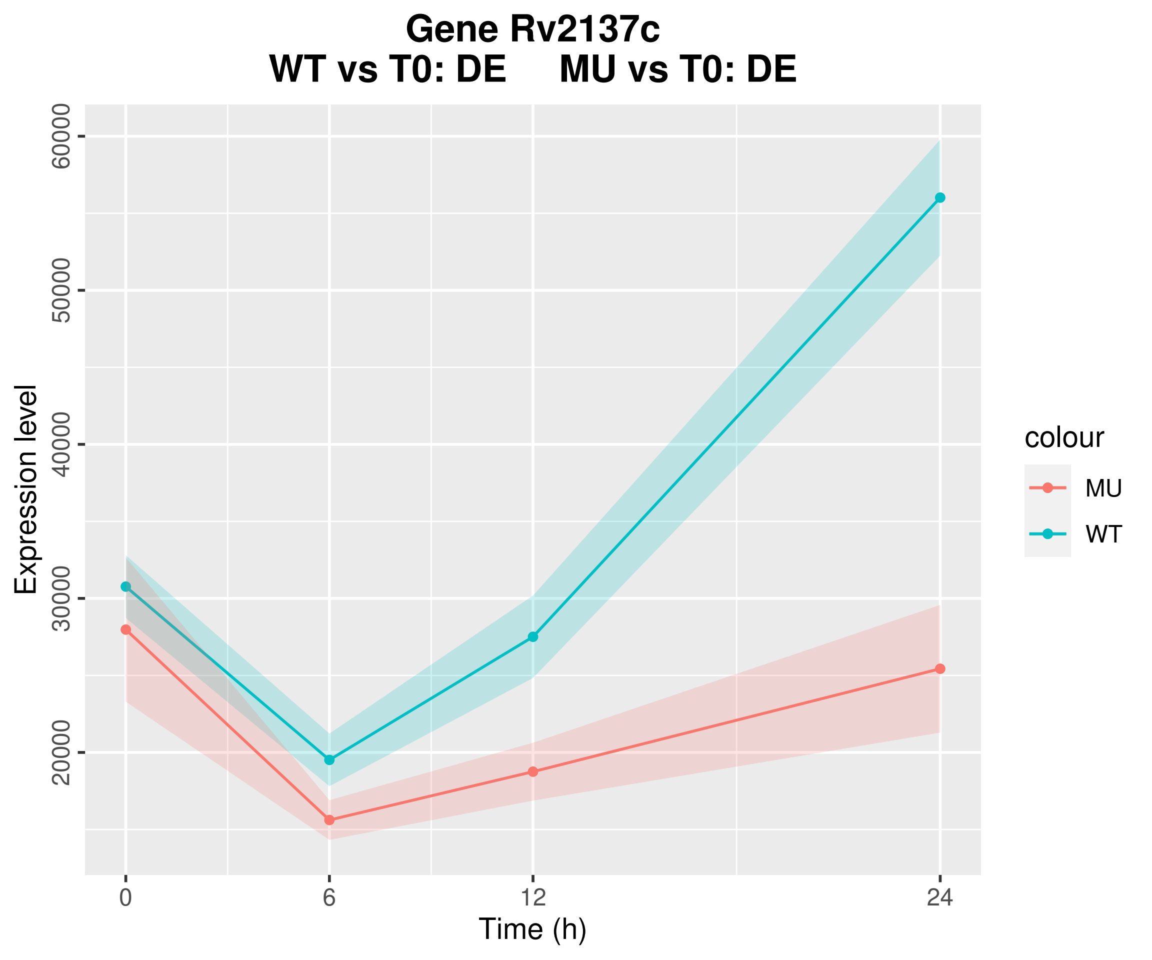

Gene Rv2137c
WT vs T0: DE MU vs T0: DE
Expression level
60000
50000
40000
30000
20000
0
6
12
24
Time (h)
colour
MU
WT

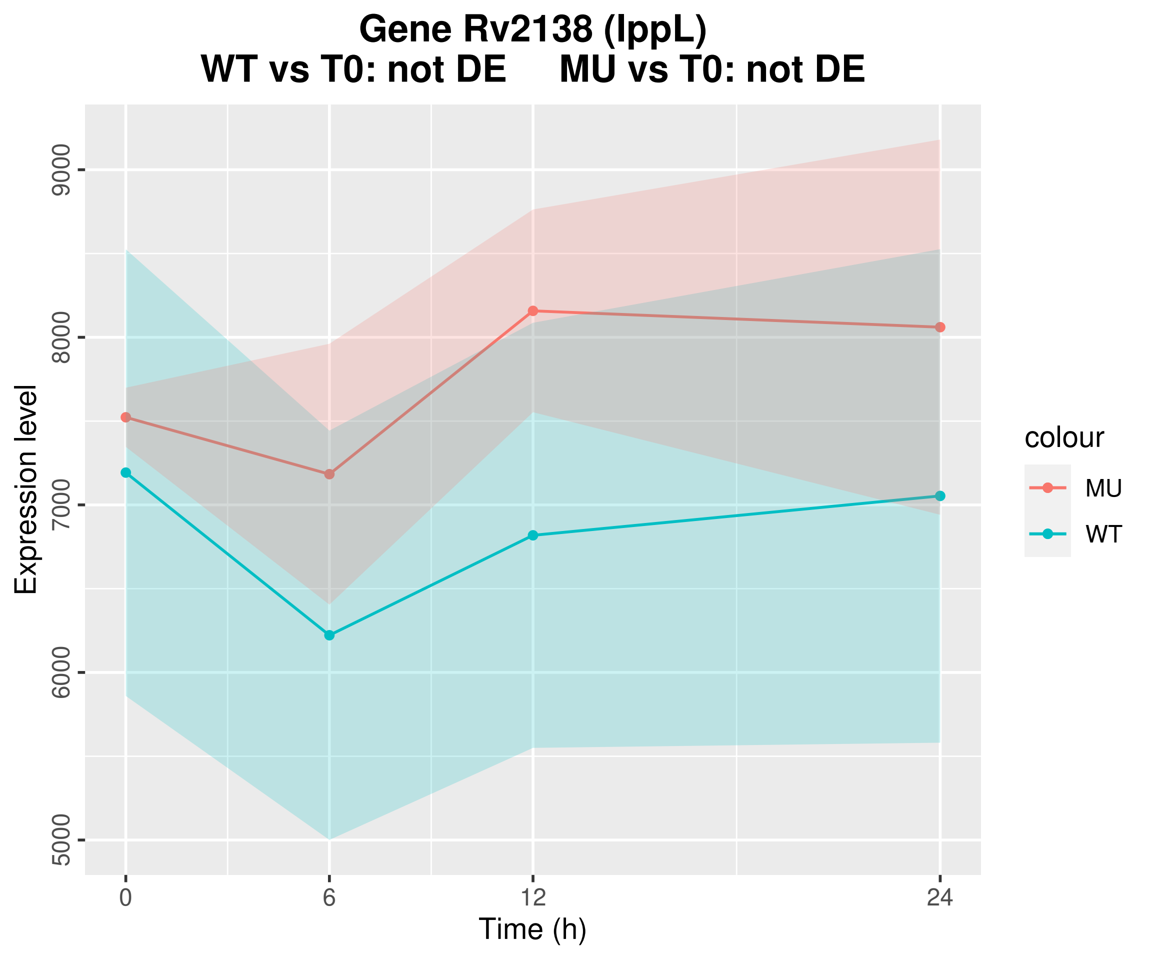
Gene Rv2138 (lppL)
WT vs T0: not DE   MU vs T0: not DE
Expression level
9000
8000
7000
6000
5000
0
6
12
24
Time (h)
colour
MU
WT

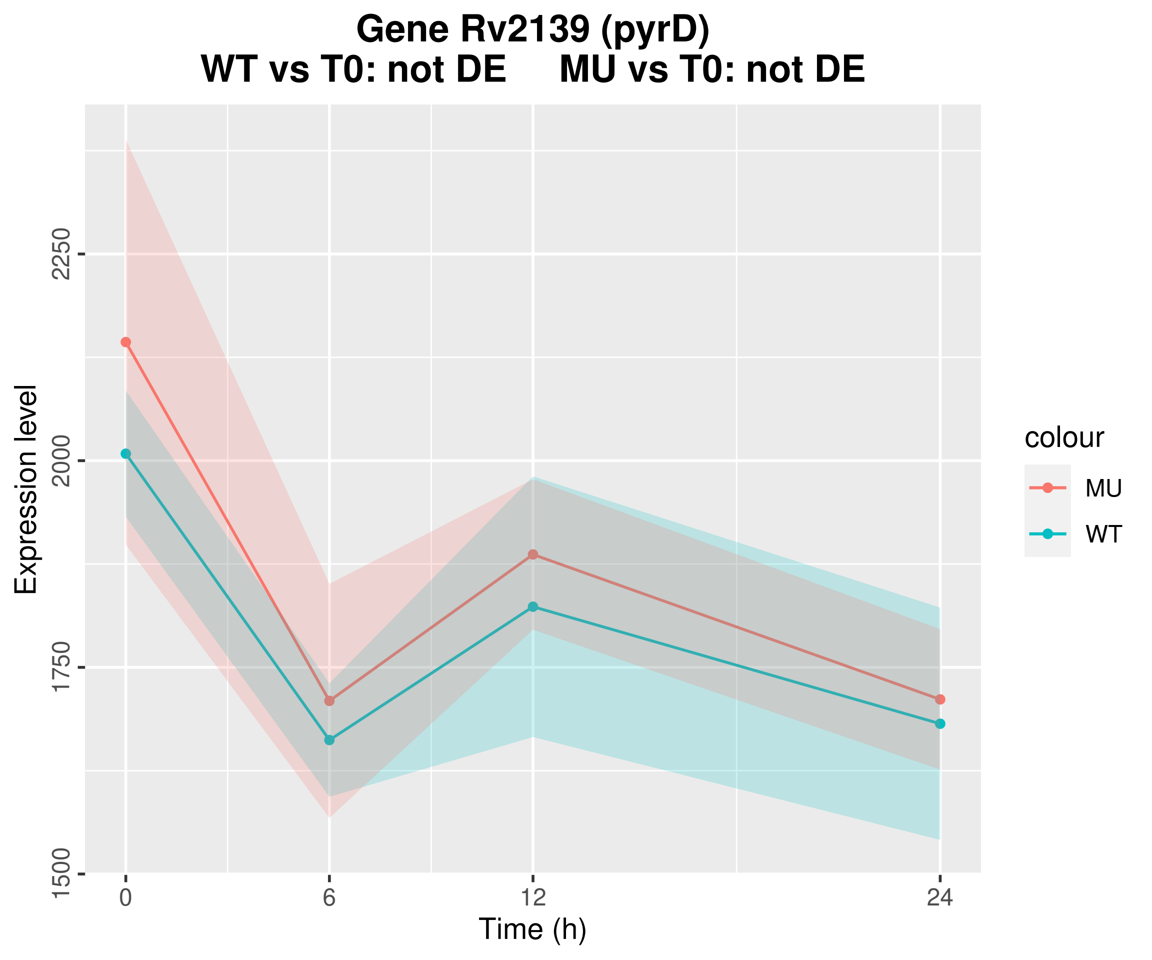
Gene Rv2139 (pyrD)
WT vs T0: not DE   MU vs T0: not DE
Expression level
2250
2000
1750
1500
0
6
12
24
Time (h)
colour
MU
WT

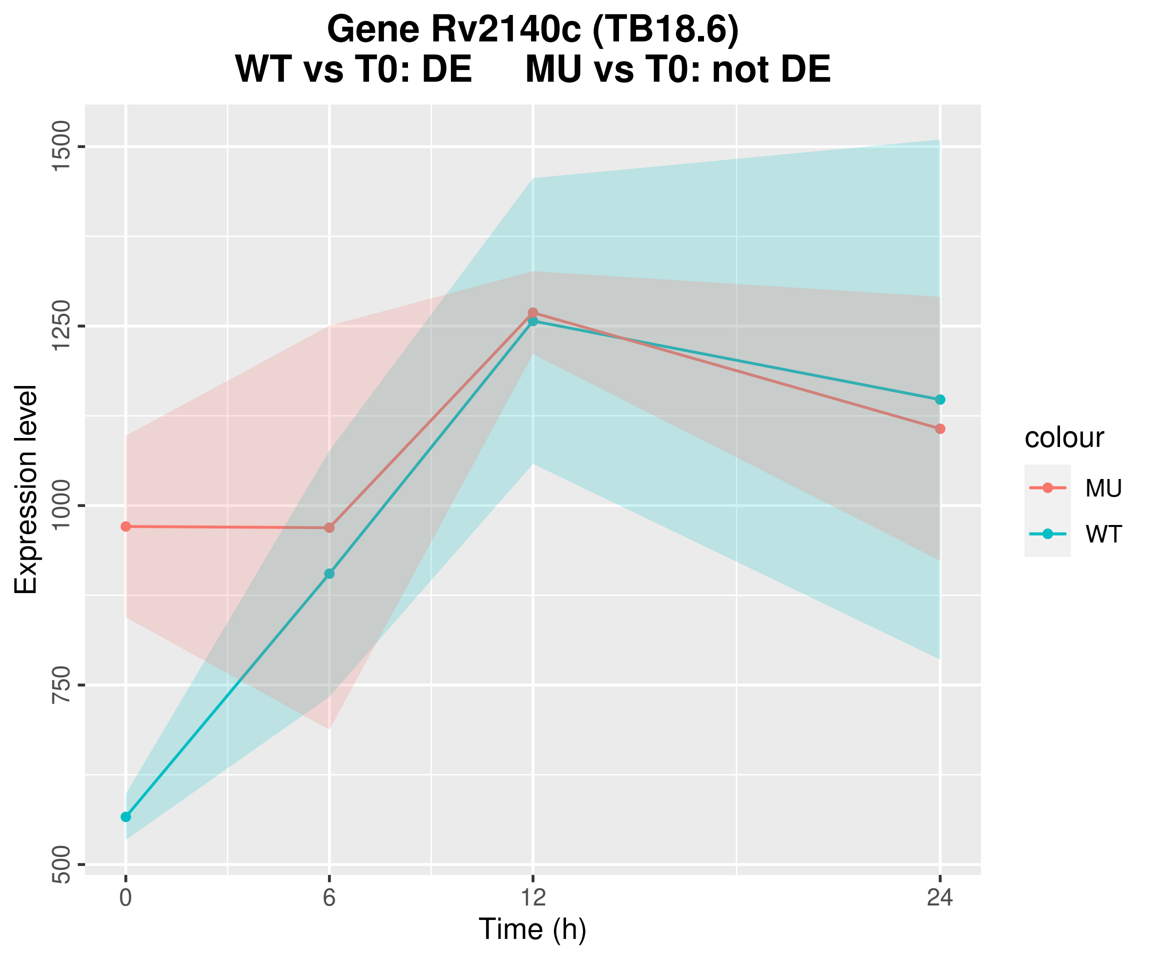
Gene Rv2140c (TB18.6)
WT vs T0: DE   MU vs T0: not DE
Expression level
1500
1250
1000
750
500
0
6
12
24
Time (h)
colour
MU
WT

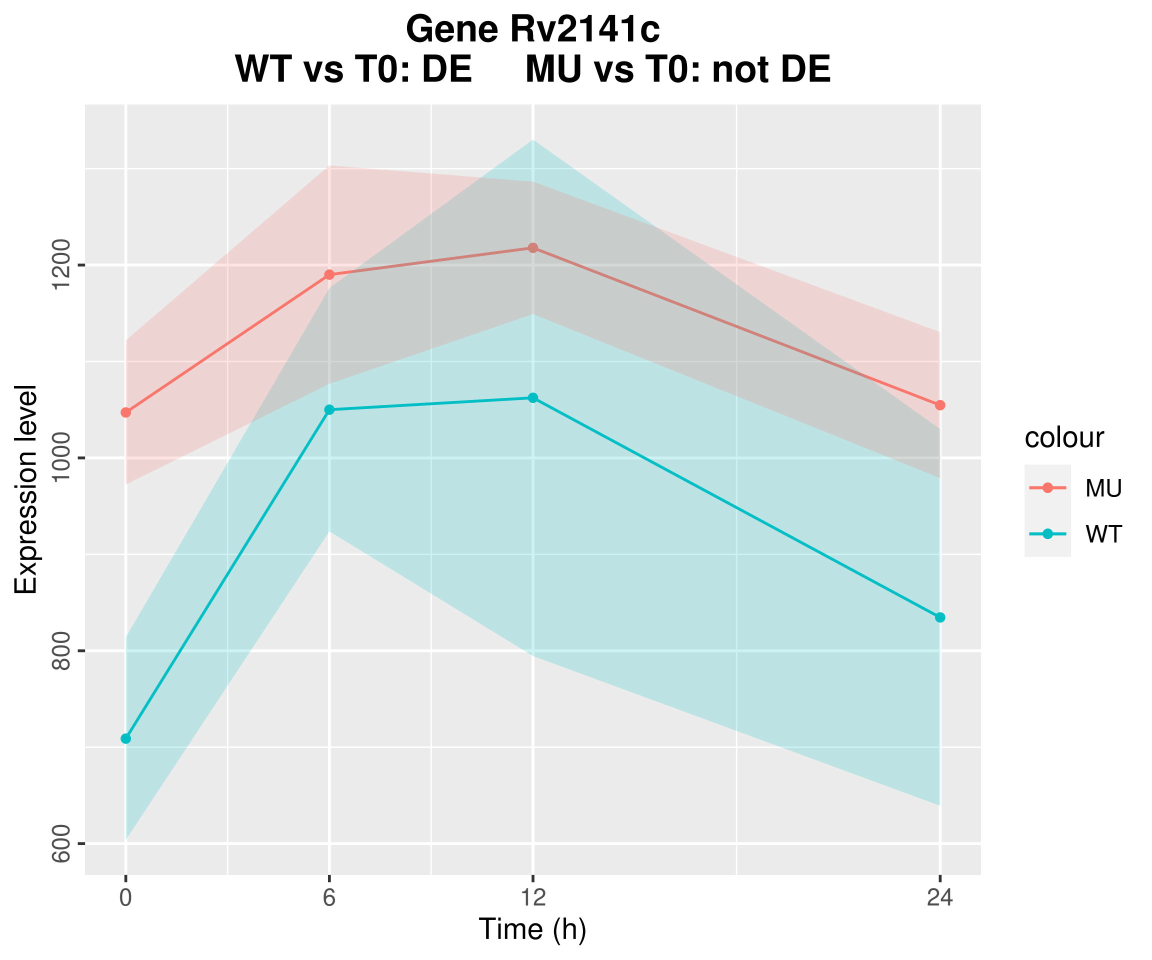

Gene Rv2141c
WT vs T0: DE MU vs T0: not DE
Expression level
600
800
1000
1200
0
6
12
24
Time (h)
colour
MU
WT

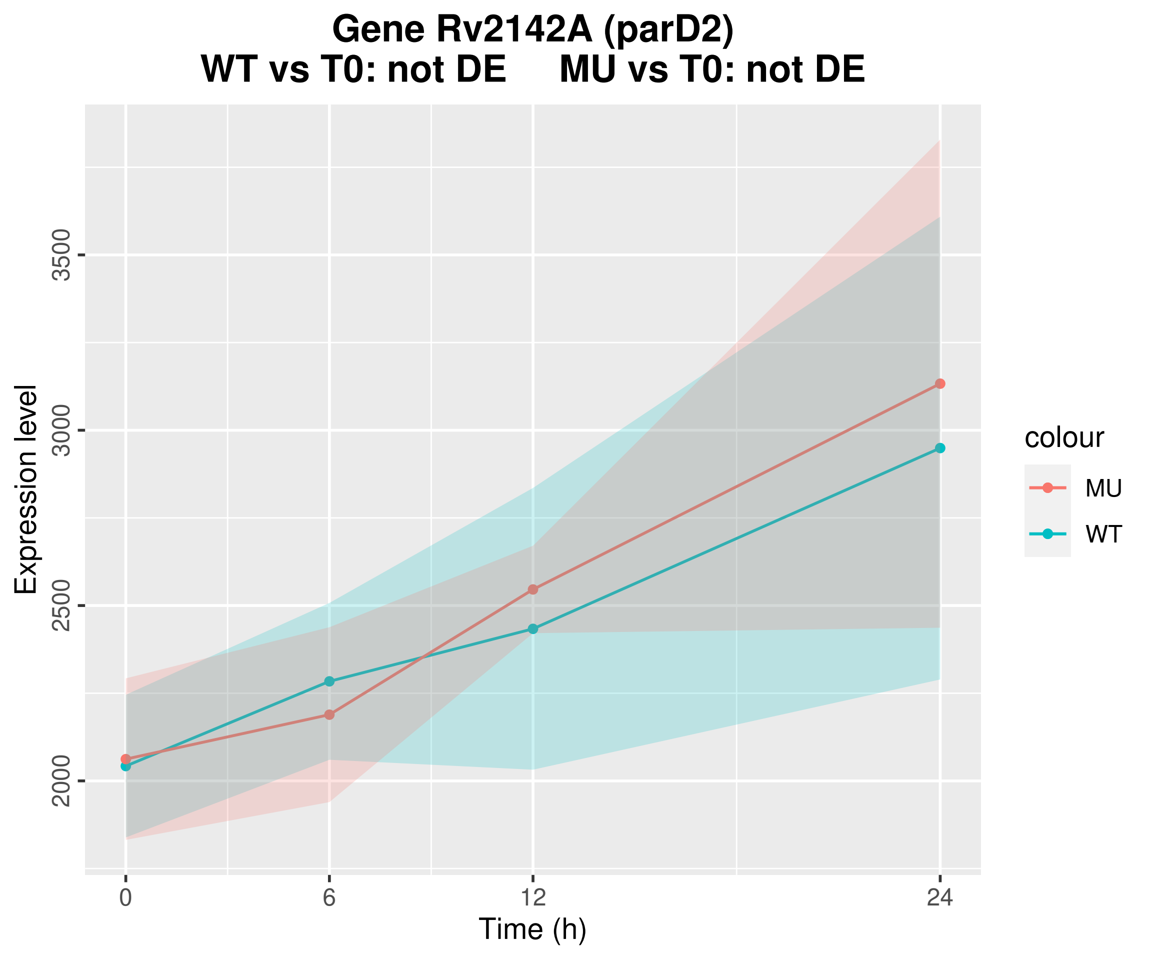

Gene Rv2142A (parD2)
WT vs T0: not DE MU vs T0: not DE
Expression level
3500
3000
2500
2000
0
6
12
24
Time (h)
colour
MU
WT

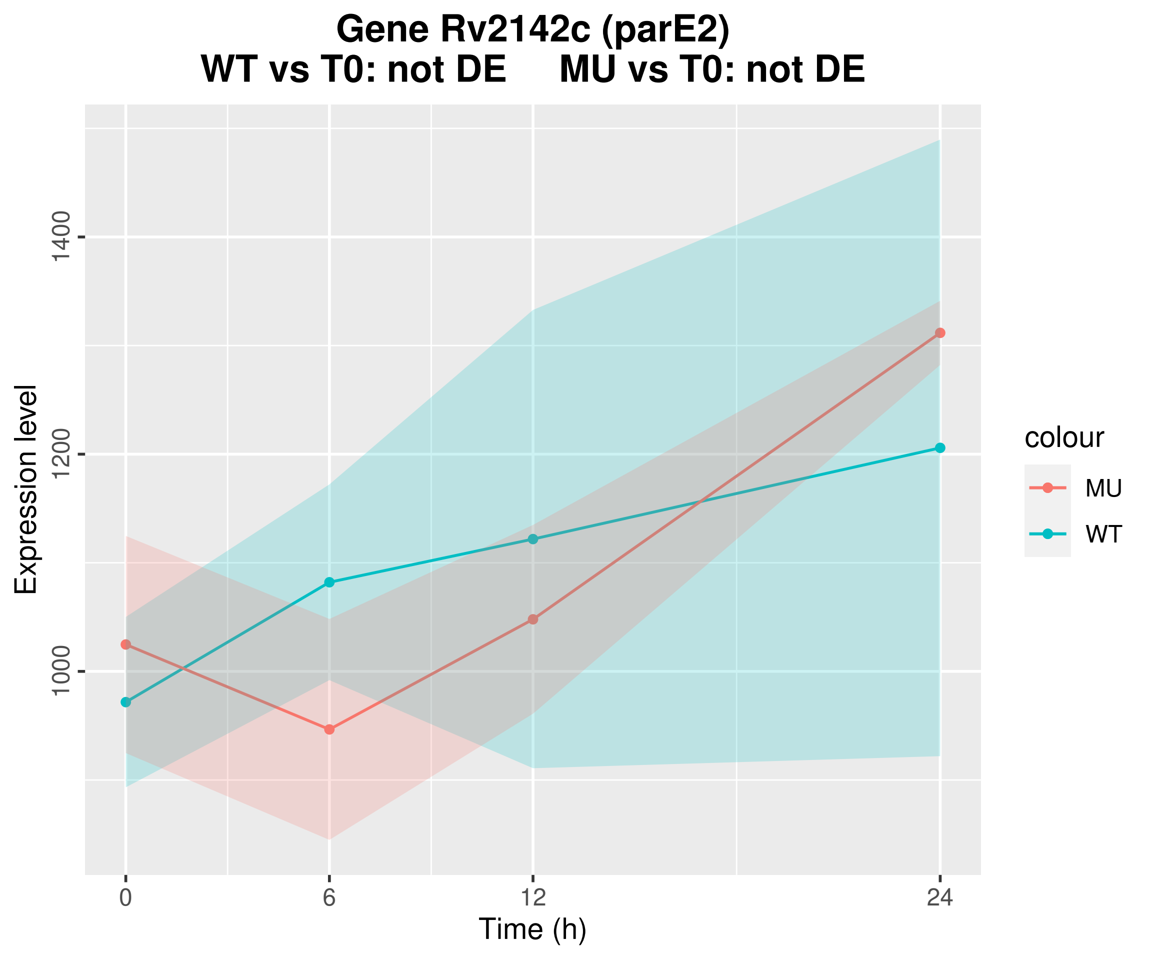
Gene Rv2142c (parE2)
WT vs T0: not DE   MU vs T0: not DE
1400
1200
1000
Expression level
0
6
12
24
Time (h)
colour
MU
WT

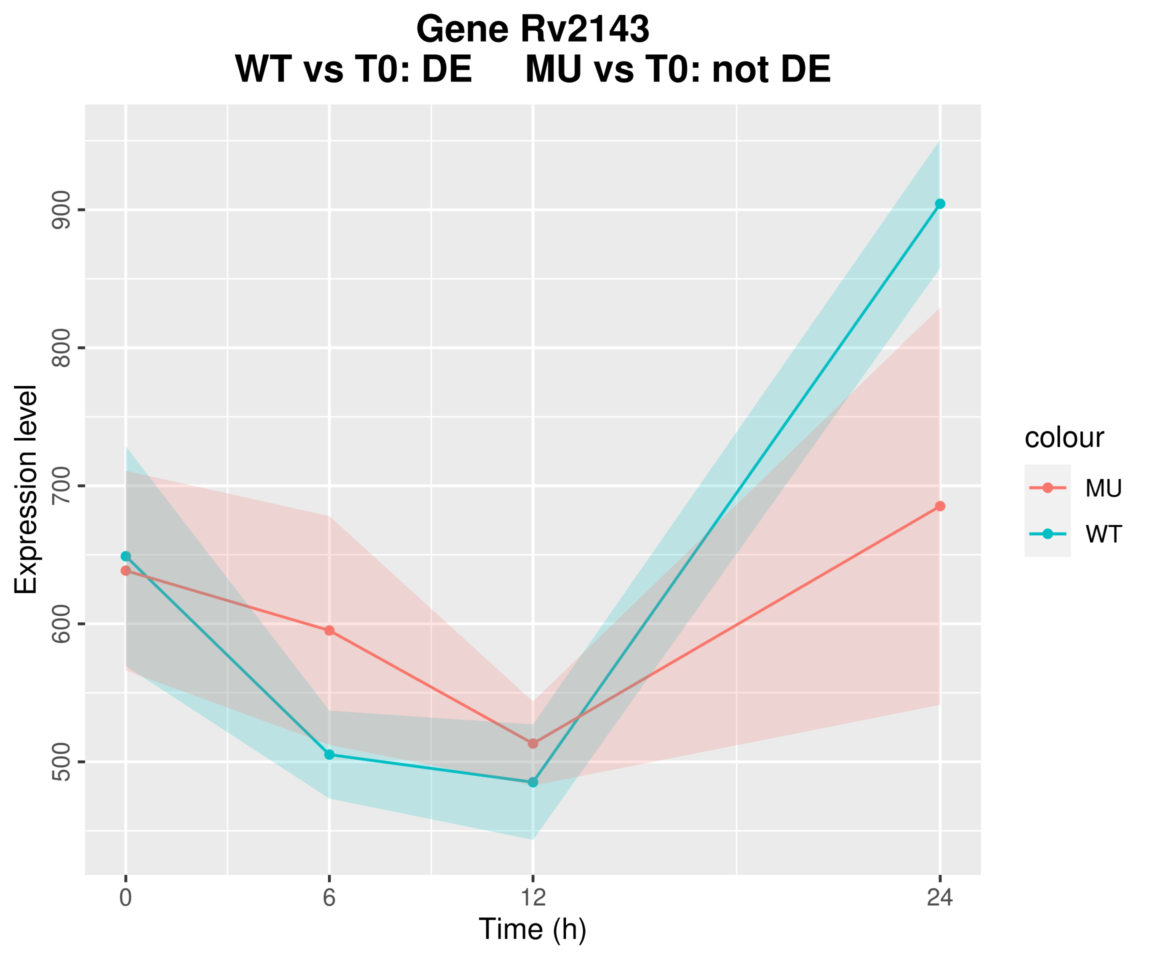
Gene Rv2143
WT vs T0: DE   MU vs T0: not DE
Expression level
900
800
700
600
500
0
6
12
24
Time (h)
colour
MU
WT

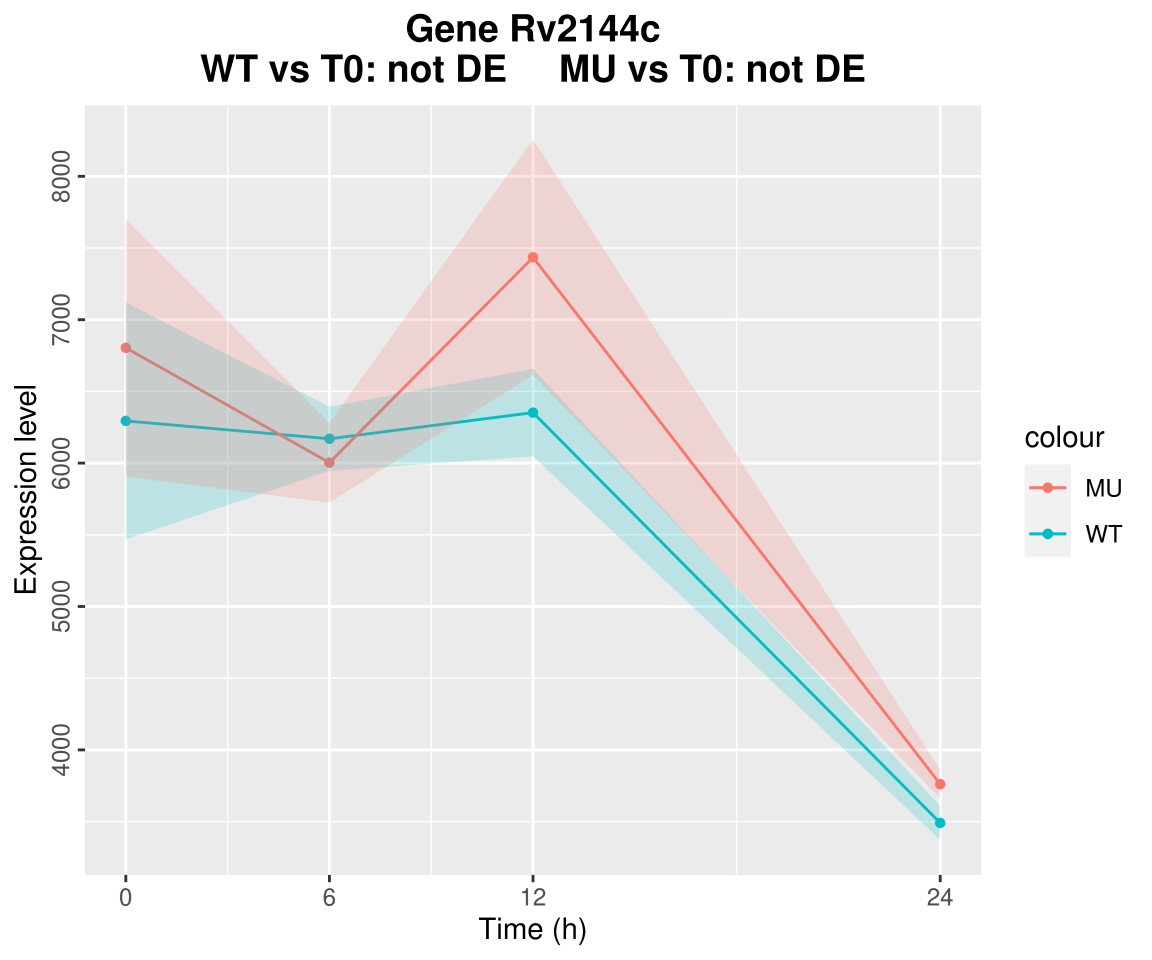

Gene Rv2144c
WT vs T0: not DE MU vs T0: not DE
Expression level
8000
7000
6000
5000
4000
0
6
12
24
Time (h)
colour
MU
WT

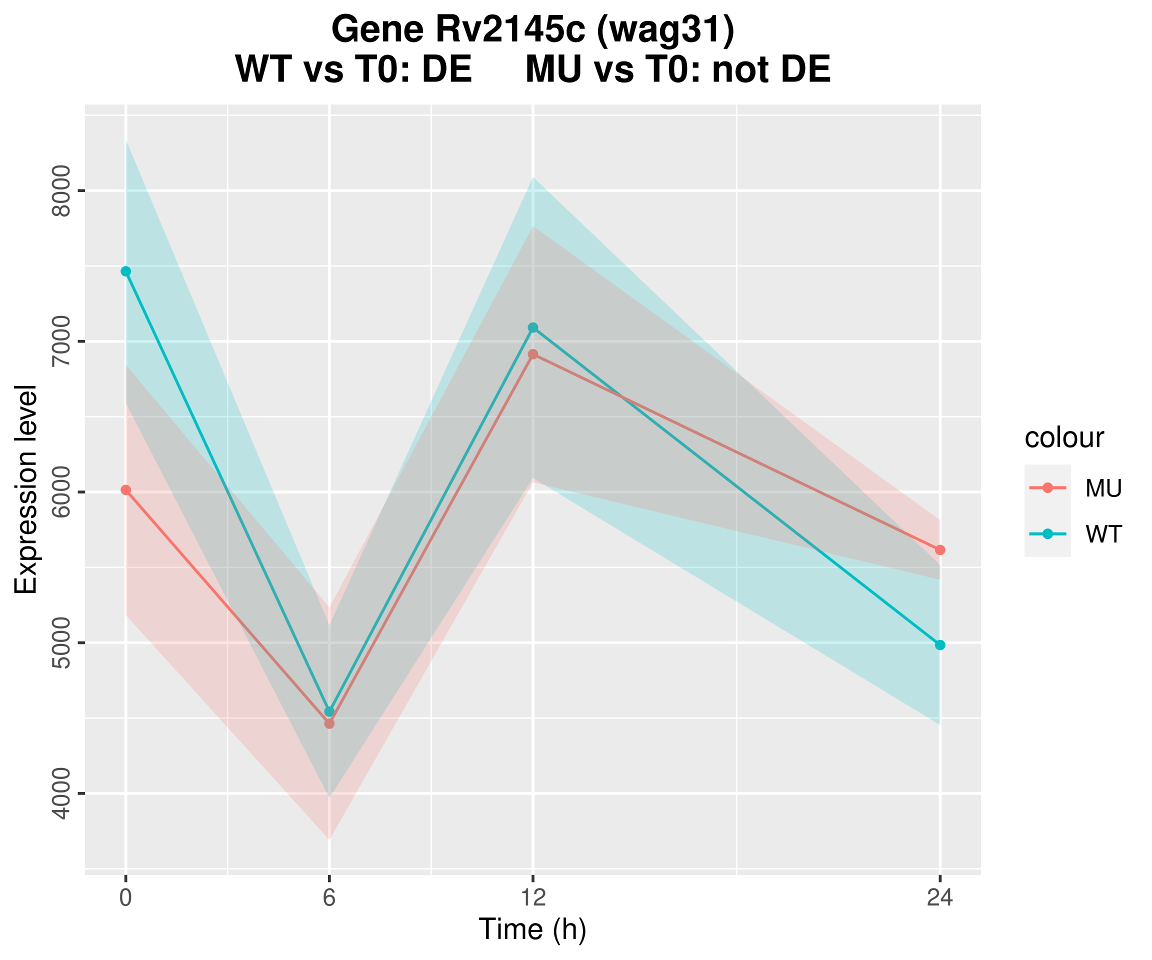

Gene Rv2145c (wag31)
WT vs T0: DE MU vs T0: not DE
Expression level
4000
5000
6000
7000
8000
0
6
12
24
Time (h)
colour
MU
WT

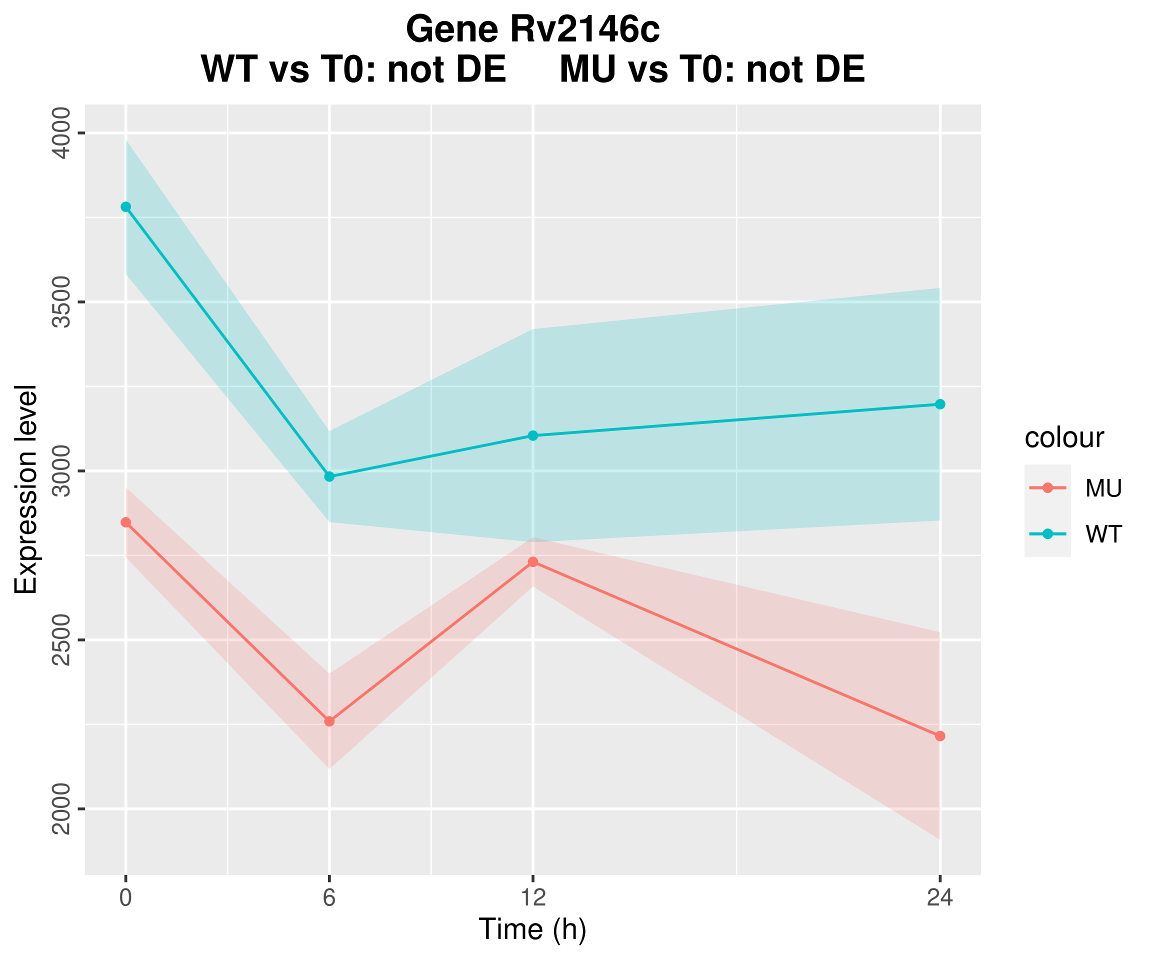

Gene Rv2146c
WT vs T0: not DE   MU vs T0: not DE
Expression level
4000
3500
3000
2500
2000
0
6
12
24
Time (h)
colour
MU
WT

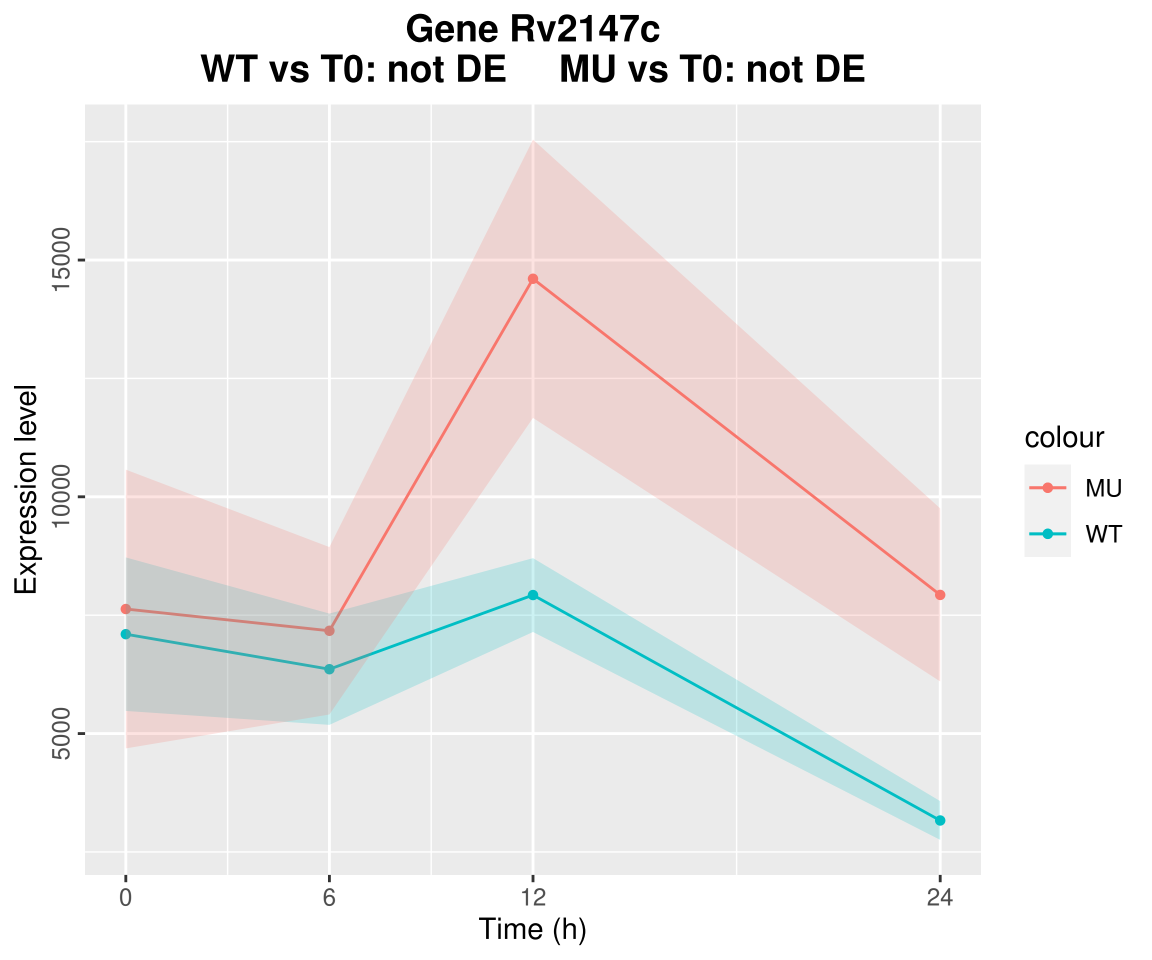

Gene Rv2147c
WT vs T0: not DE   MU vs T0: not DE
Expression level
15000
10000
5000
0
6
12
24
Time (h)
colour
MU
WT

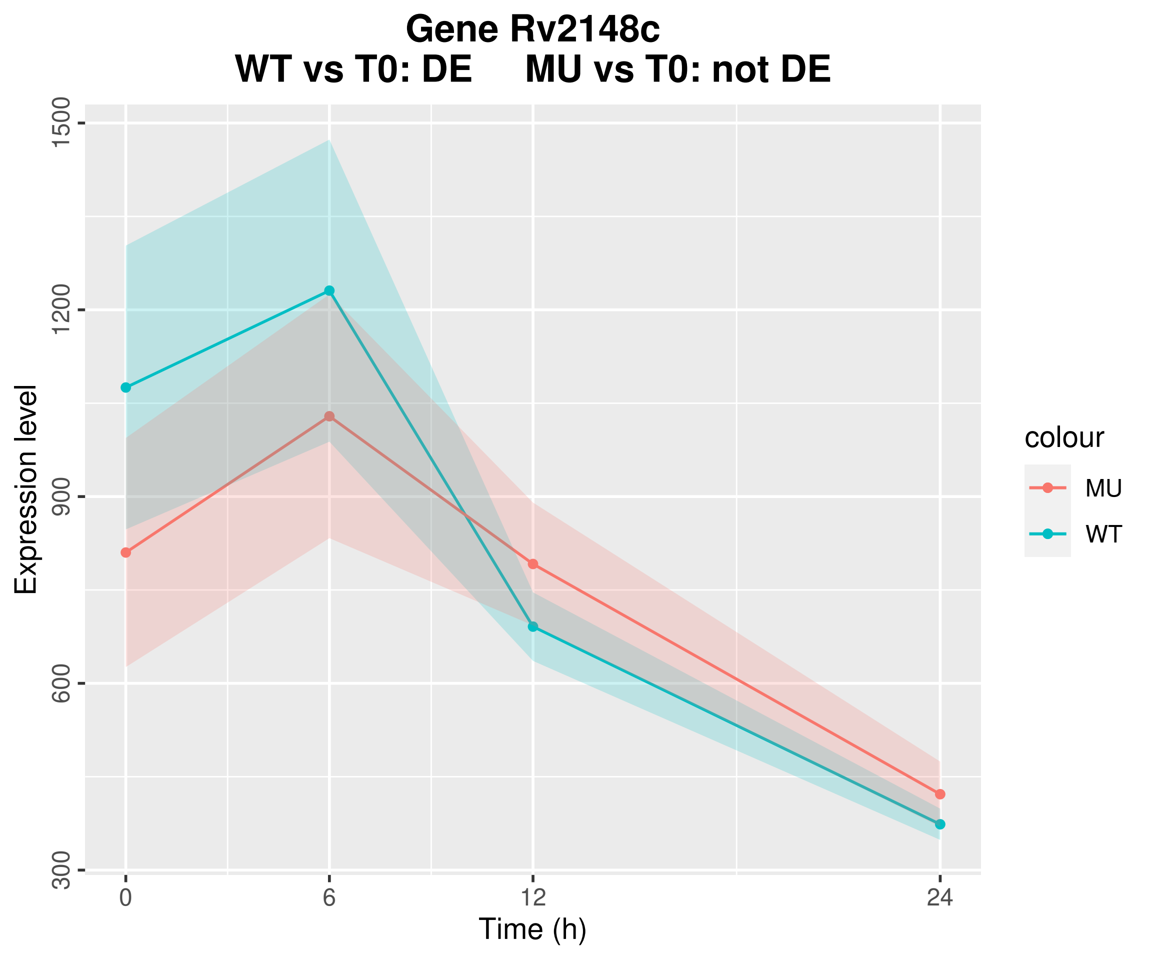
Gene Rv2148c
WT vs T0: DE   MU vs T0: not DE
Expression level
1500
1200
900
600
300
0
6
12
24
Time (h)
colour
MU
WT

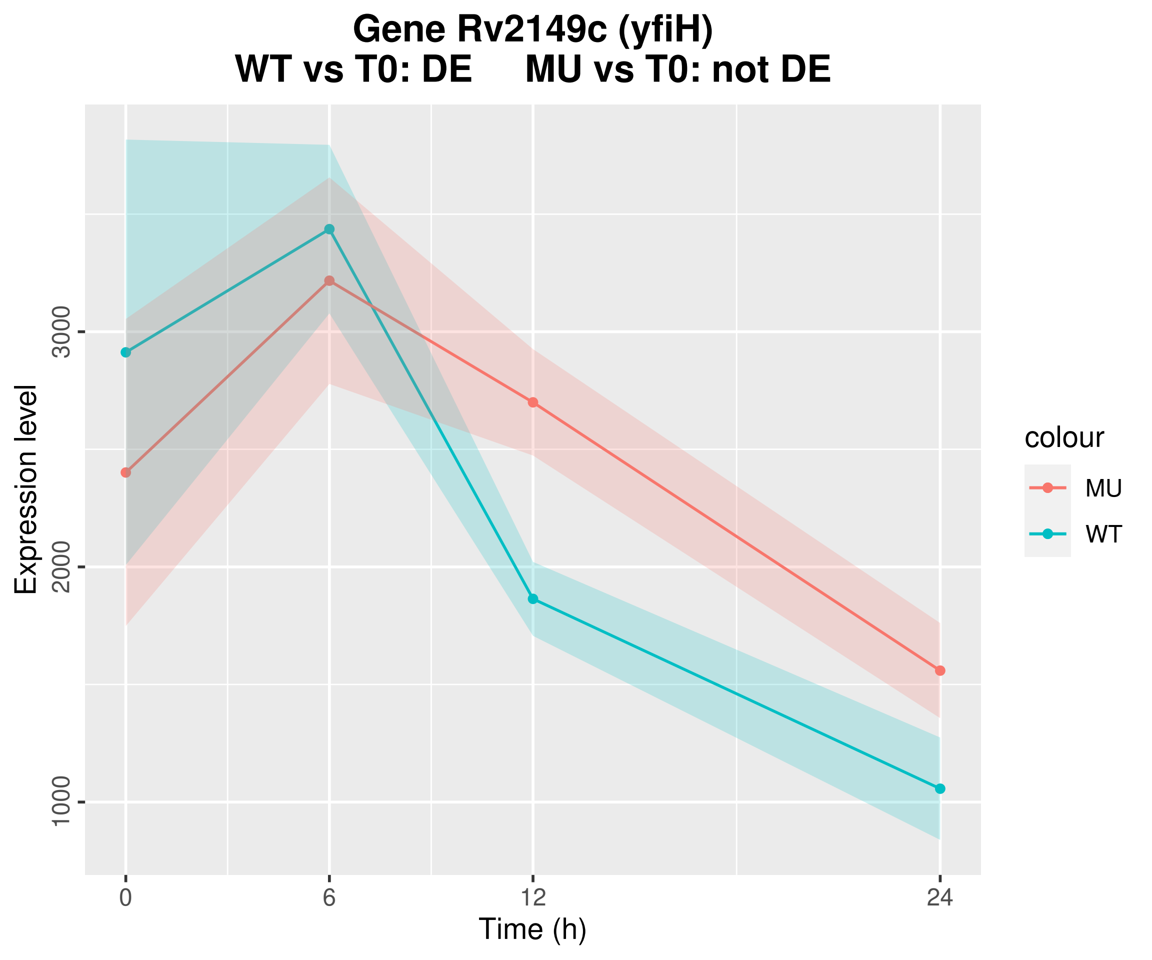
Gene Rv2149c (yfiH)
WT vs T0: DE   MU vs T0: not DE
Expression level
3000
2000
1000
0
6
12
24
Time (h)
colour
MU
WT

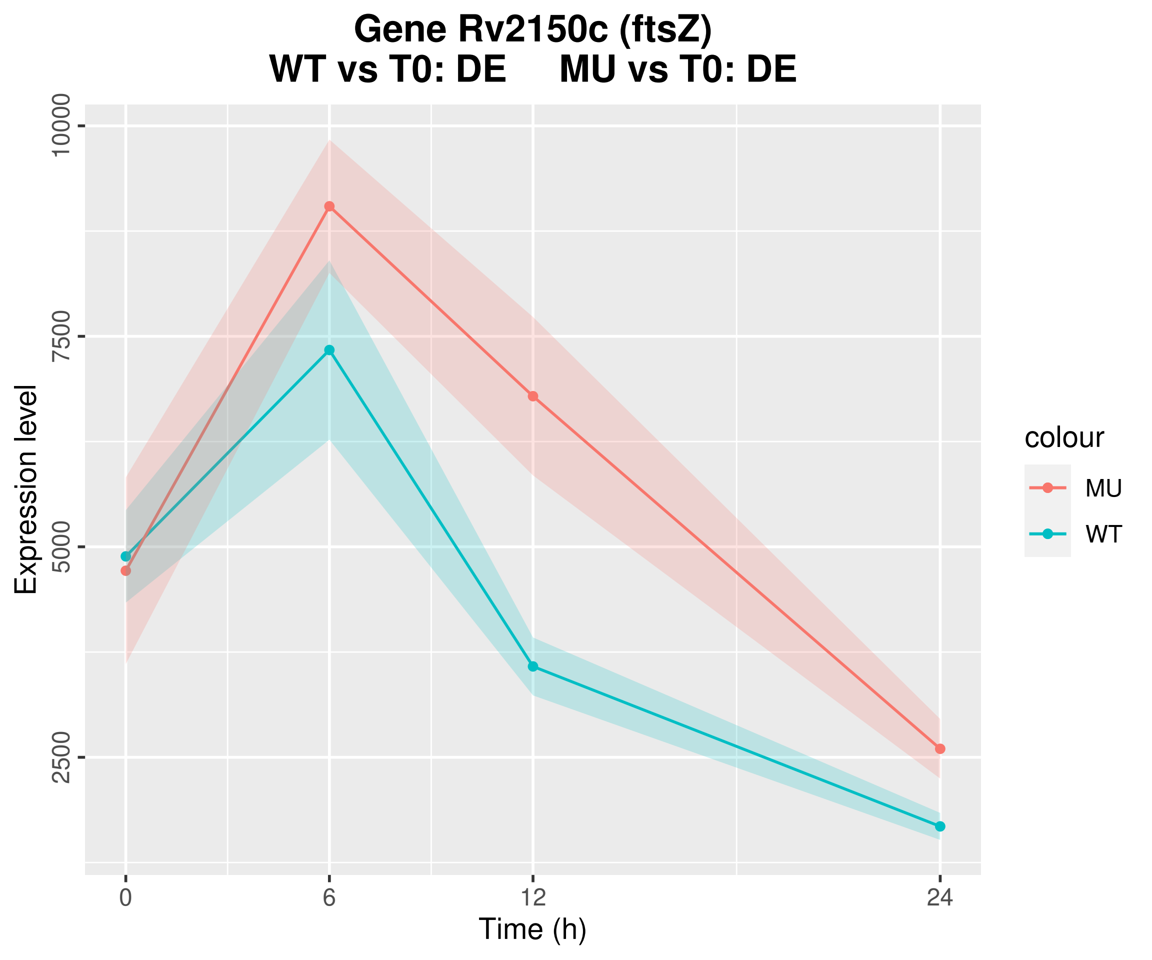
Gene Rv2150c (ftsZ)
WT vs T0: DE MU vs T0: DE
10000
7500
5000
2500
Expression level
0
6
12
24
Time (h)
colour
MU
WT

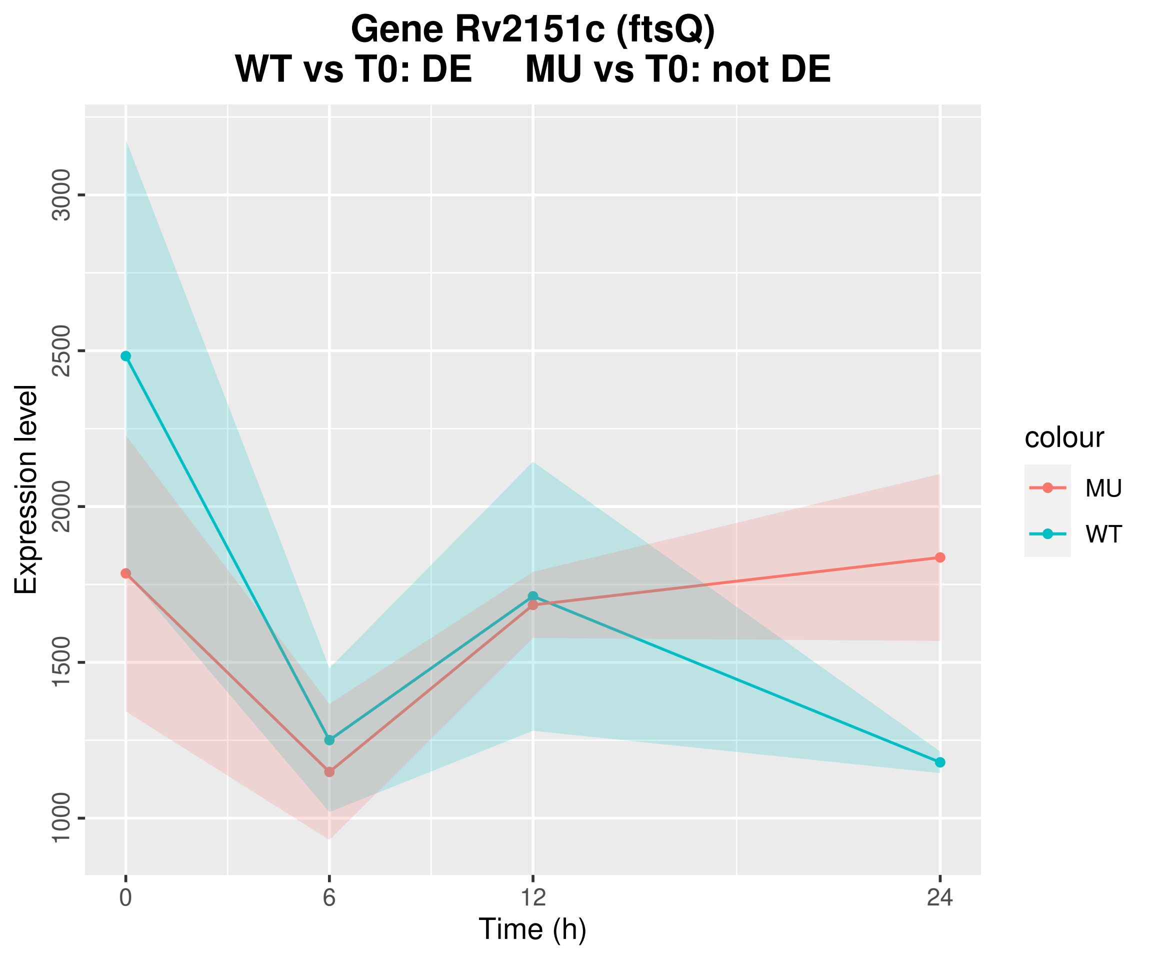
Gene Rv2151c (ftsQ)
WT vs T0: DE   MU vs T0: not DE
Expression level
3000
2500
2000
1500
1000
0
6
12
24
Time (h)
colour
MU
WT

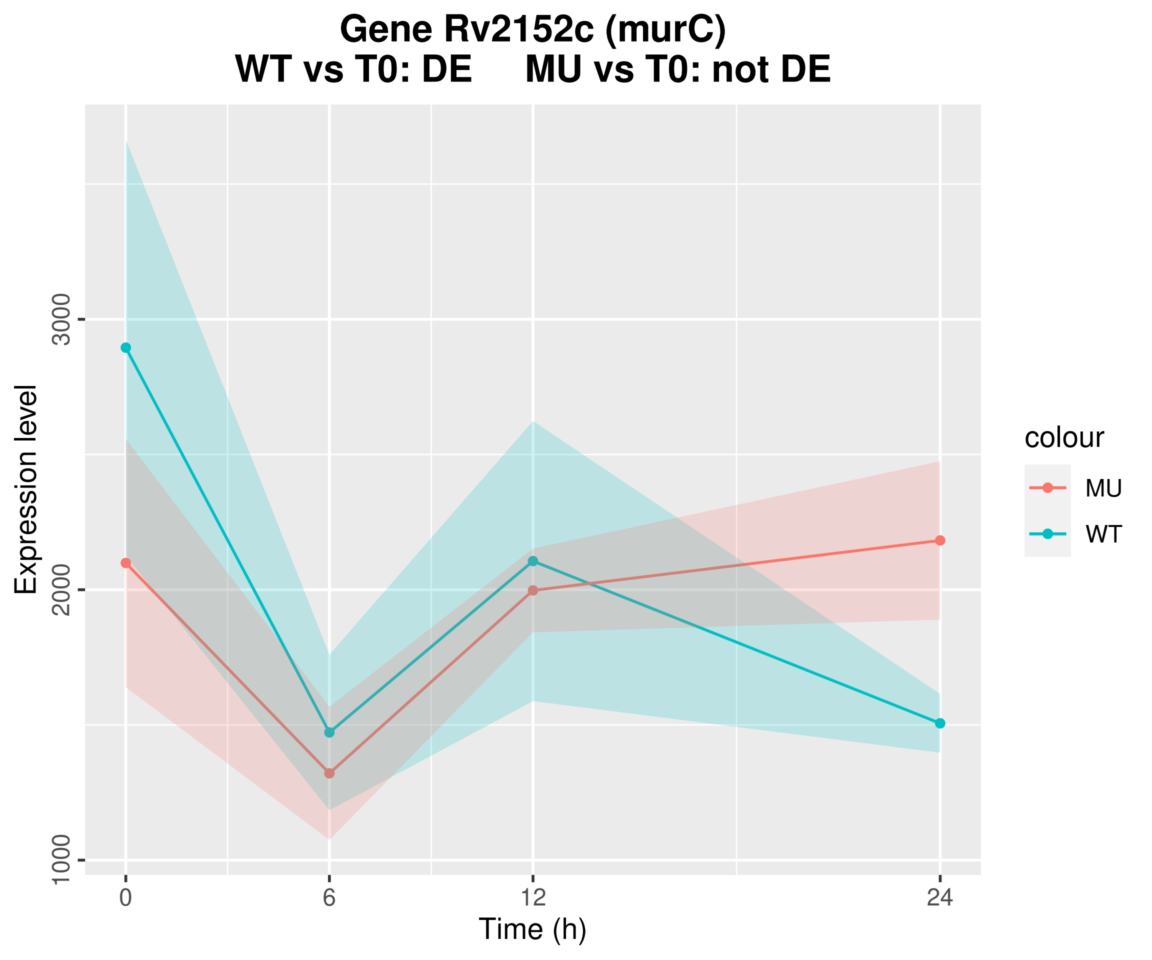

Gene Rv2152c (murC)
WT vs T0: DE MU vs T0: not DE
Expression level
3000
2000
1000
0
6
12
24
Time (h)
colour
MU
WT

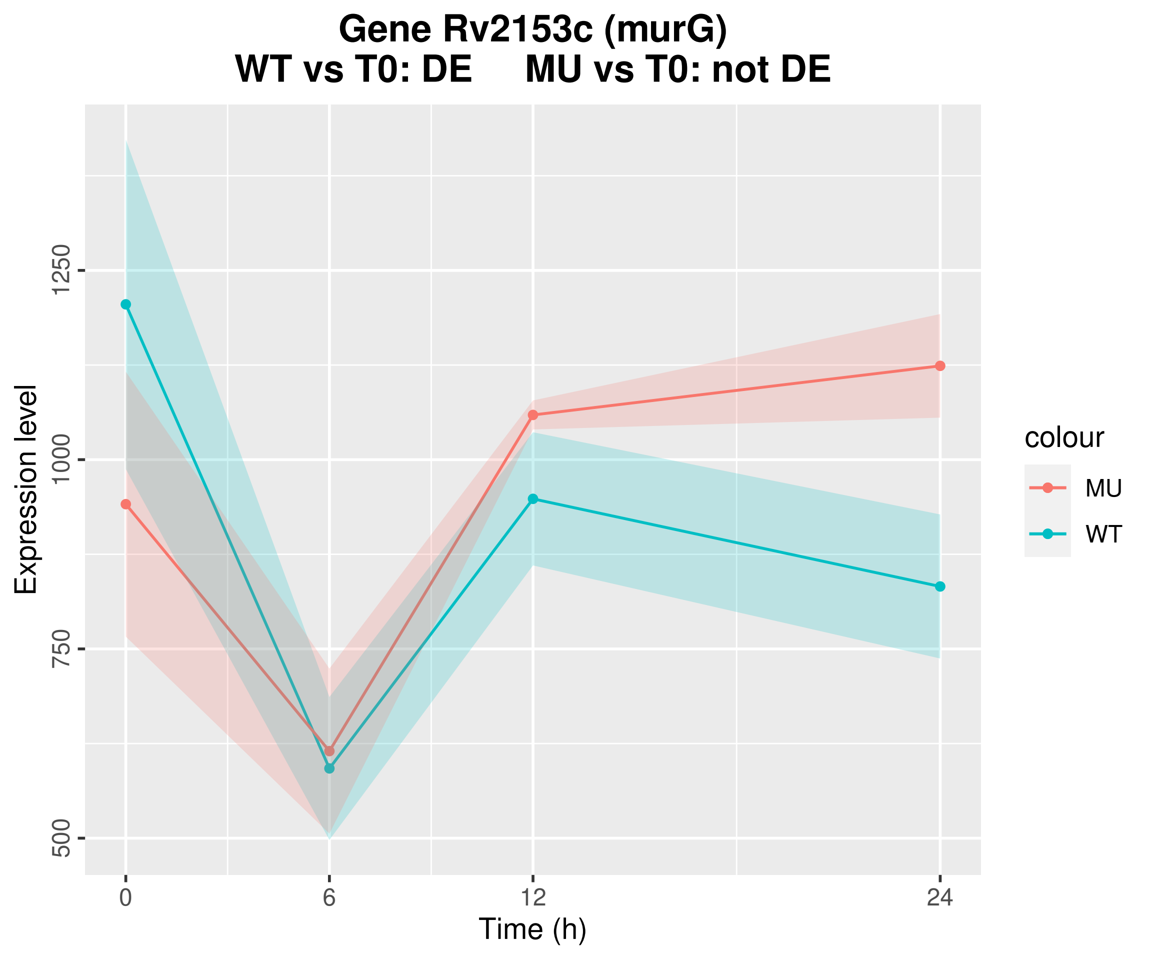
Gene Rv2153c (murG)
WT vs T0: DE   MU vs T0: not DE
Expression level
1250
1000
750
500
0
6
12
24
Time (h)
colour
MU
WT

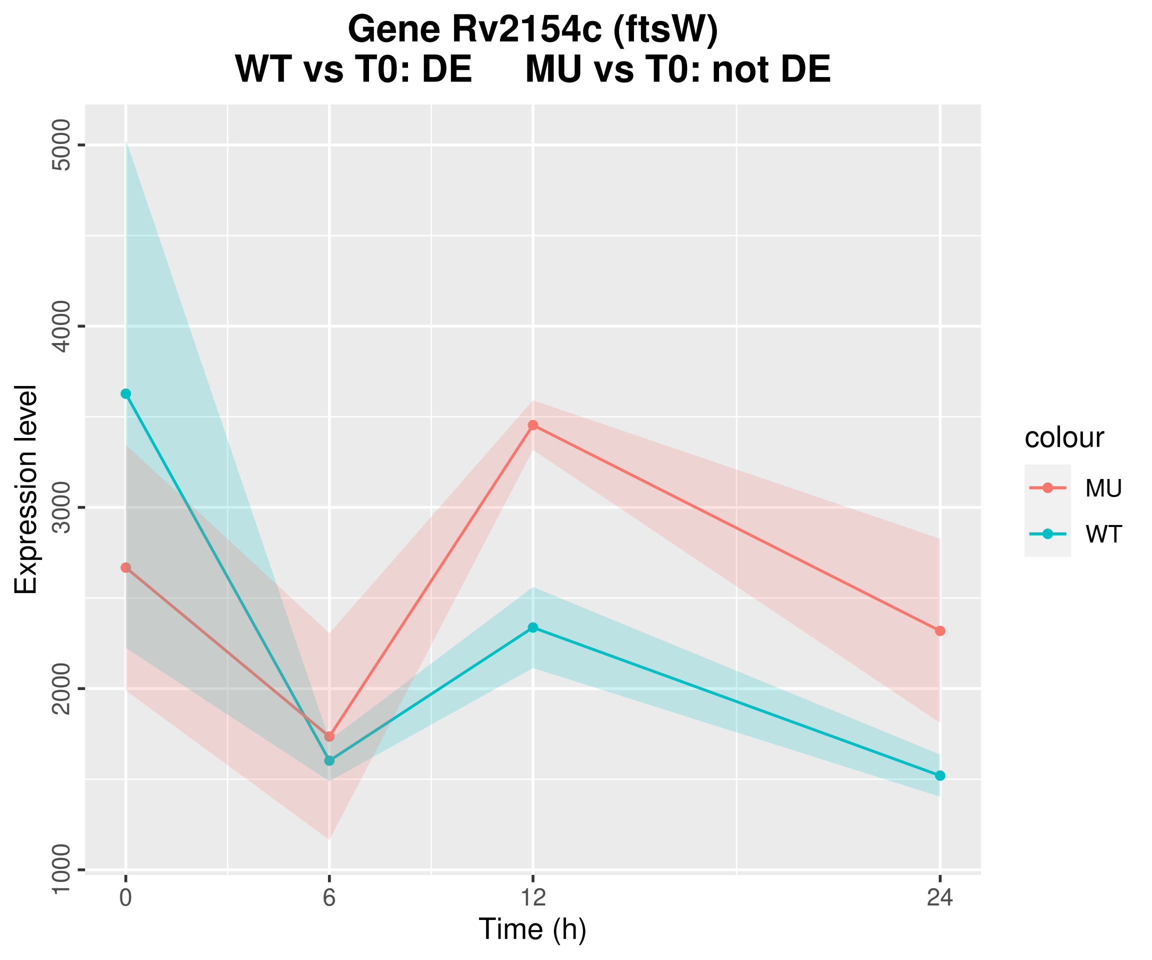
Gene Rv2154c (ftsW)
WT vs T0: DE   MU vs T0: not DE
5000
4000
3000
2000
1000
Expression level
0
6
12
24
Time (h)
colour
MU
WT

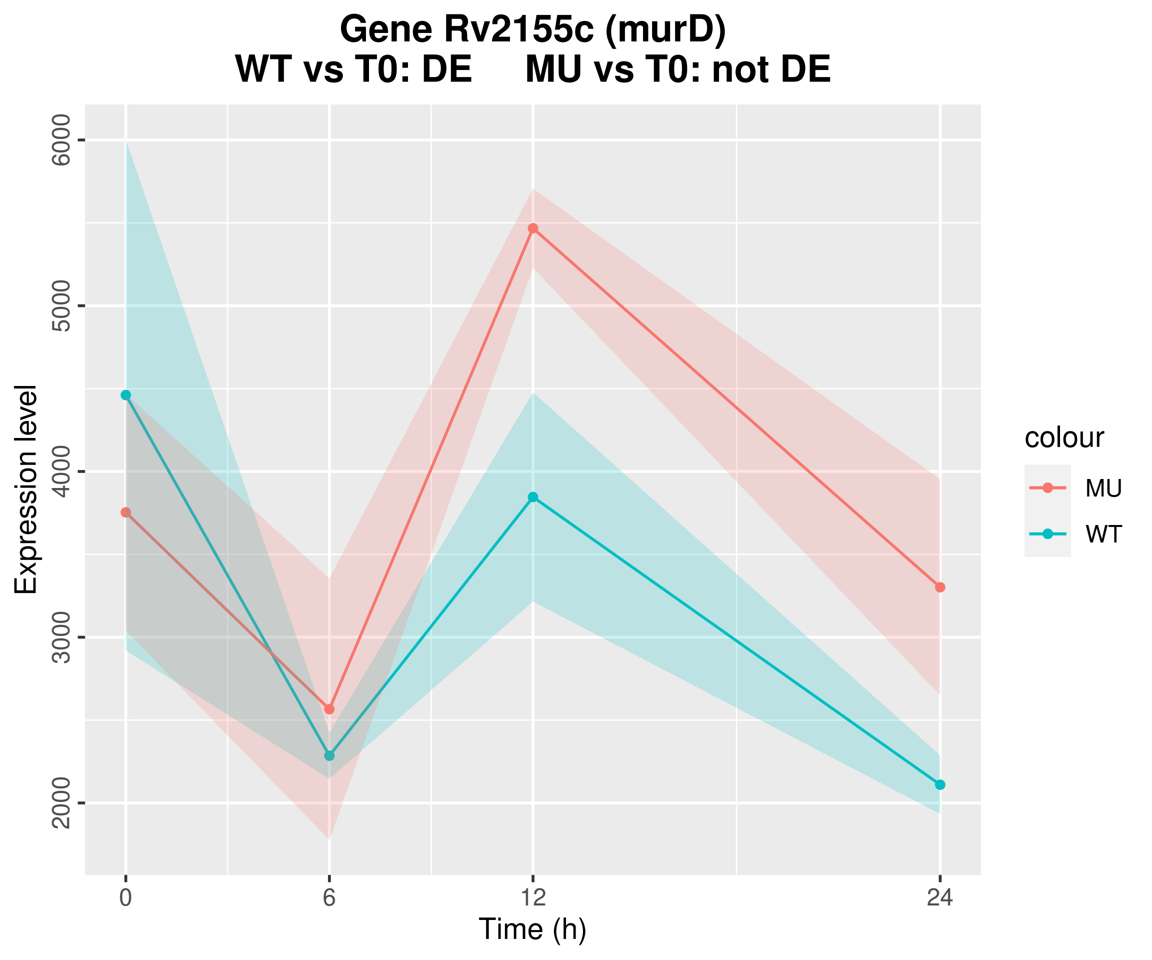

Gene Rv2155c (murD)
WT vs T0: DE   MU vs T0: not DE
Expression level
6000
5000
4000
3000
2000
Time (h)
0
6
12
24
colour
MU
WT

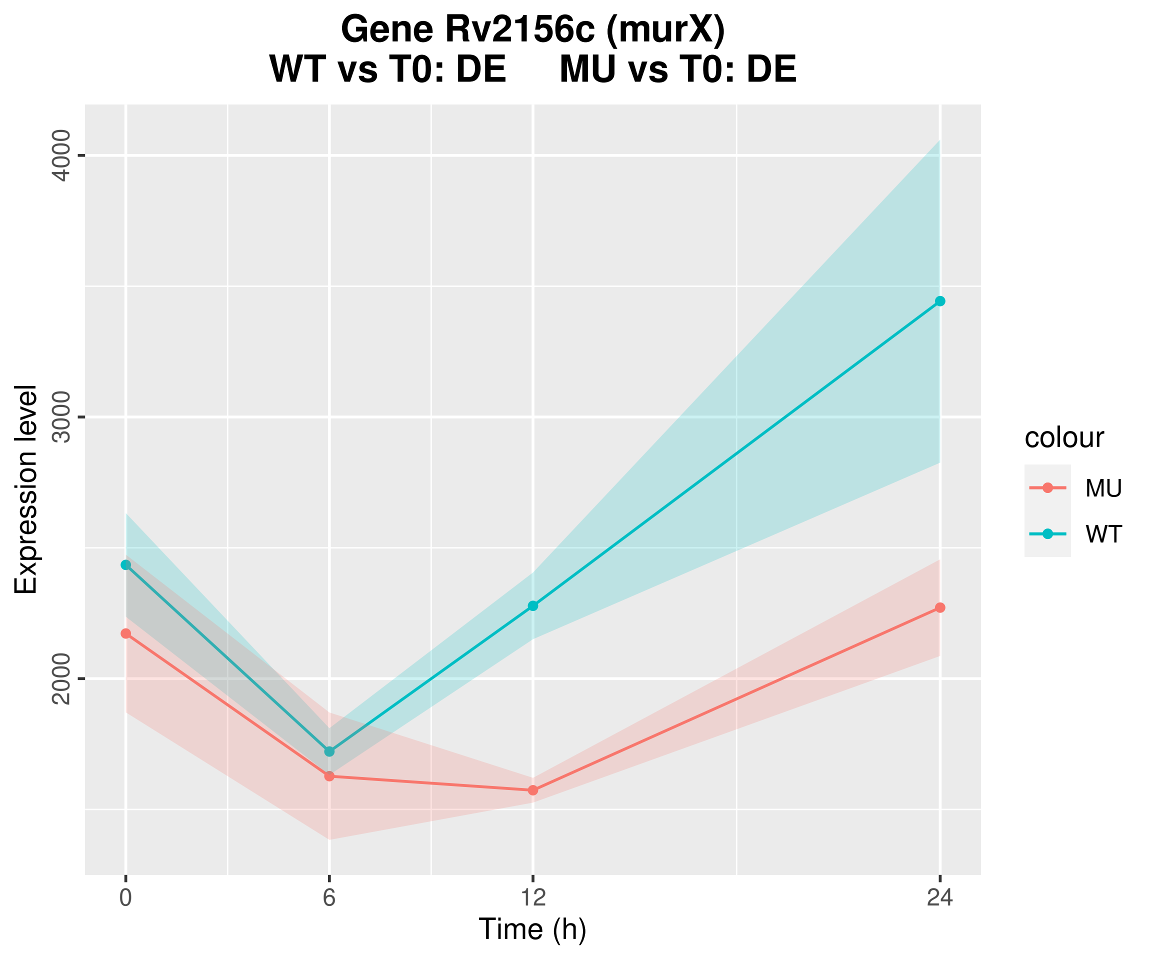
Gene Rv2156c (murX)
WT vs T0: DE MU vs T0: DE
Expression level
4000
3000
2000
0
6
12
24
Time (h)
colour
MU
WT

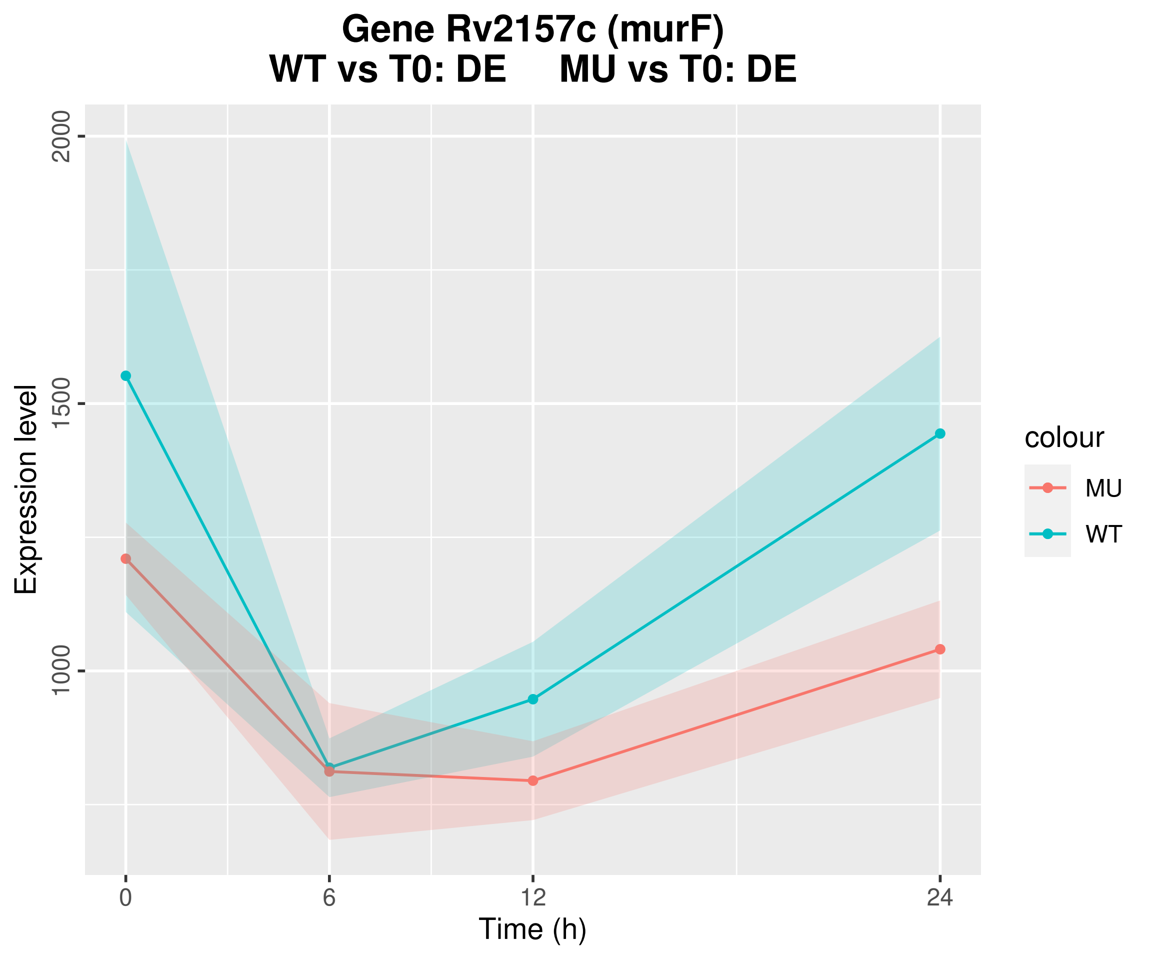

Gene Rv2157c (murF)
WT vs T0: DE MU vs T0: DE
Expression level
2000
1500
1000
0
6
12
24
Time (h)
colour
MU
WT

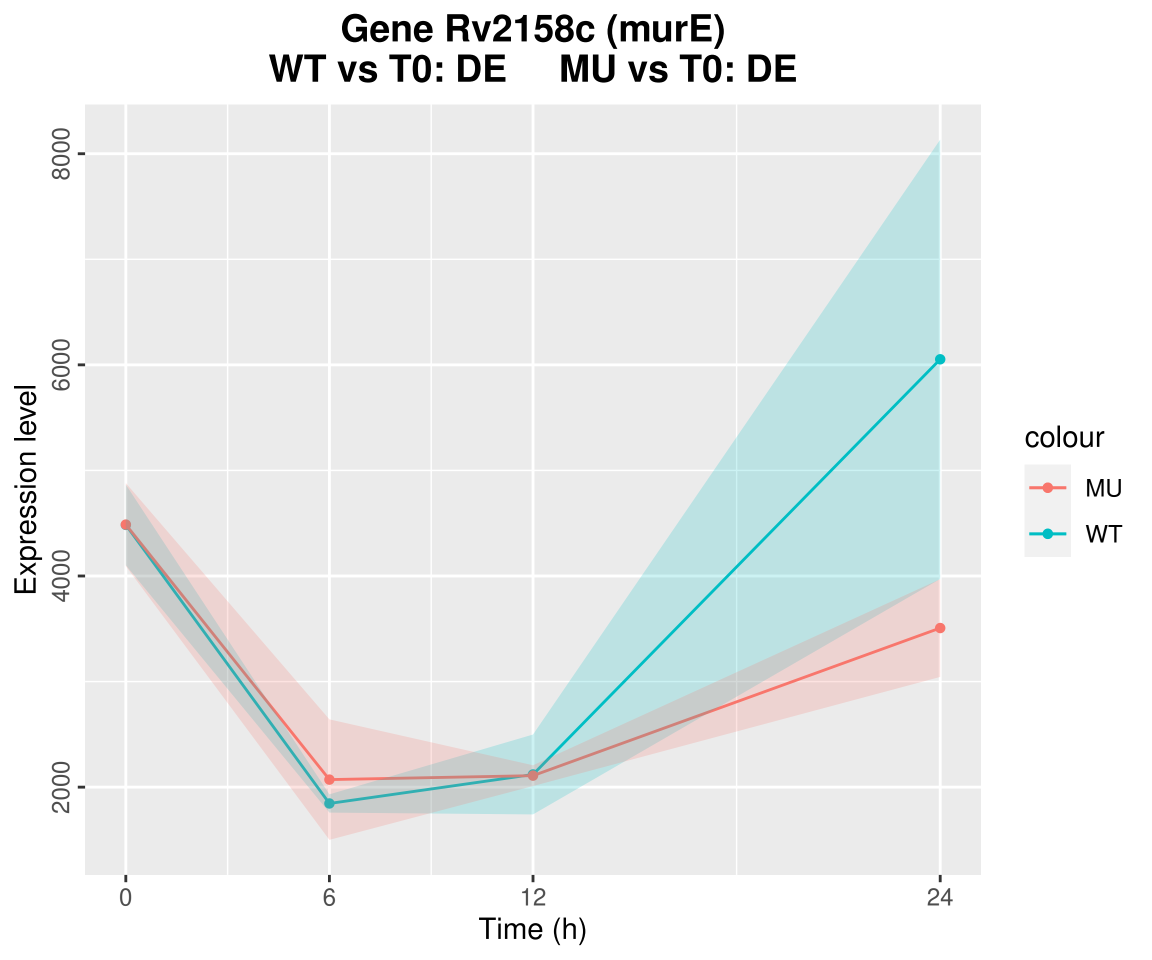

Gene Rv2158c (murE)
WT vs T0: DE MU vs T0: DE
Expression level
8000
6000
4000
2000
0
6
12
24
Time (h)
colour
MU
WT

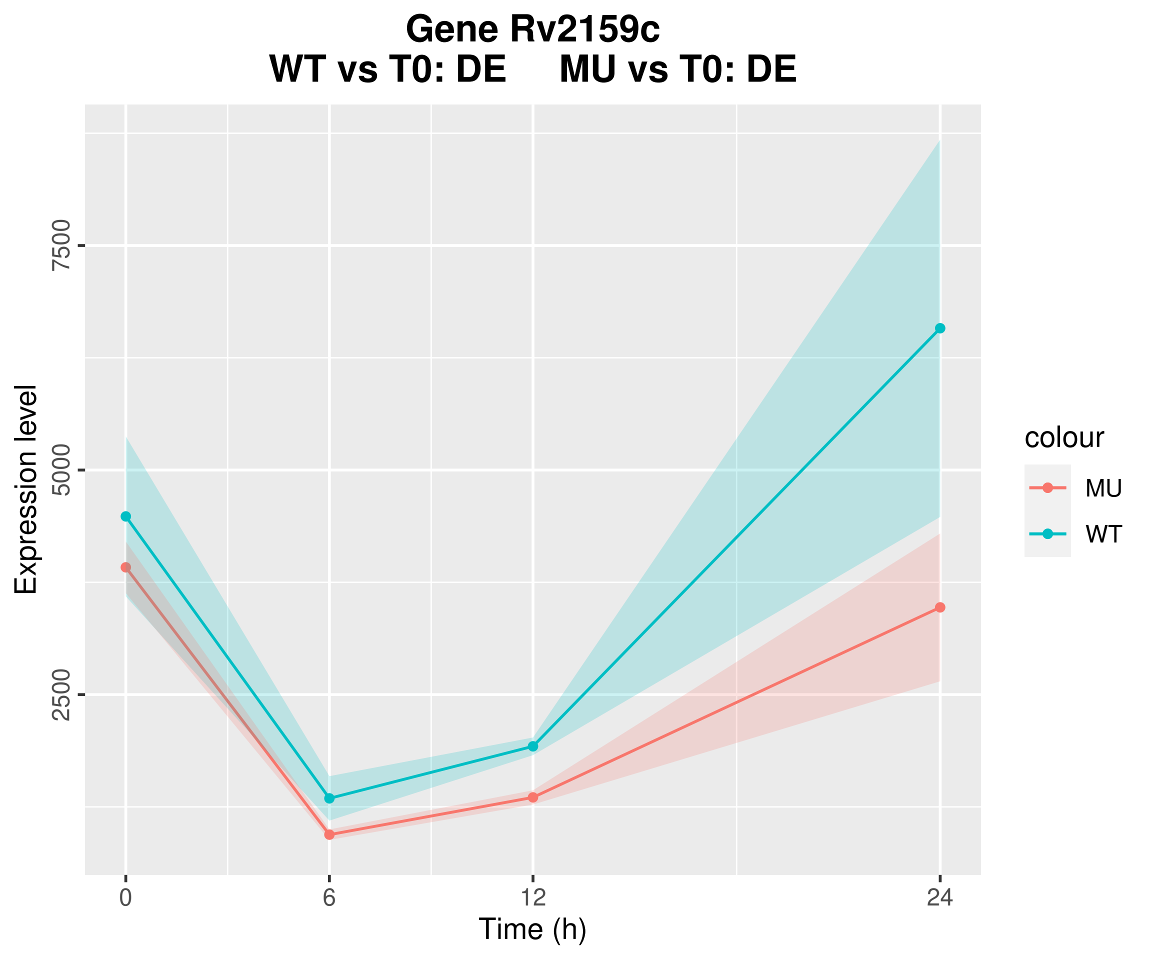
Gene Rv2159c
WT vs T0: DE MU vs T0: DE
Expression level
7500
5000
2500
0
6
12
24
Time (h)
colour
MU
WT

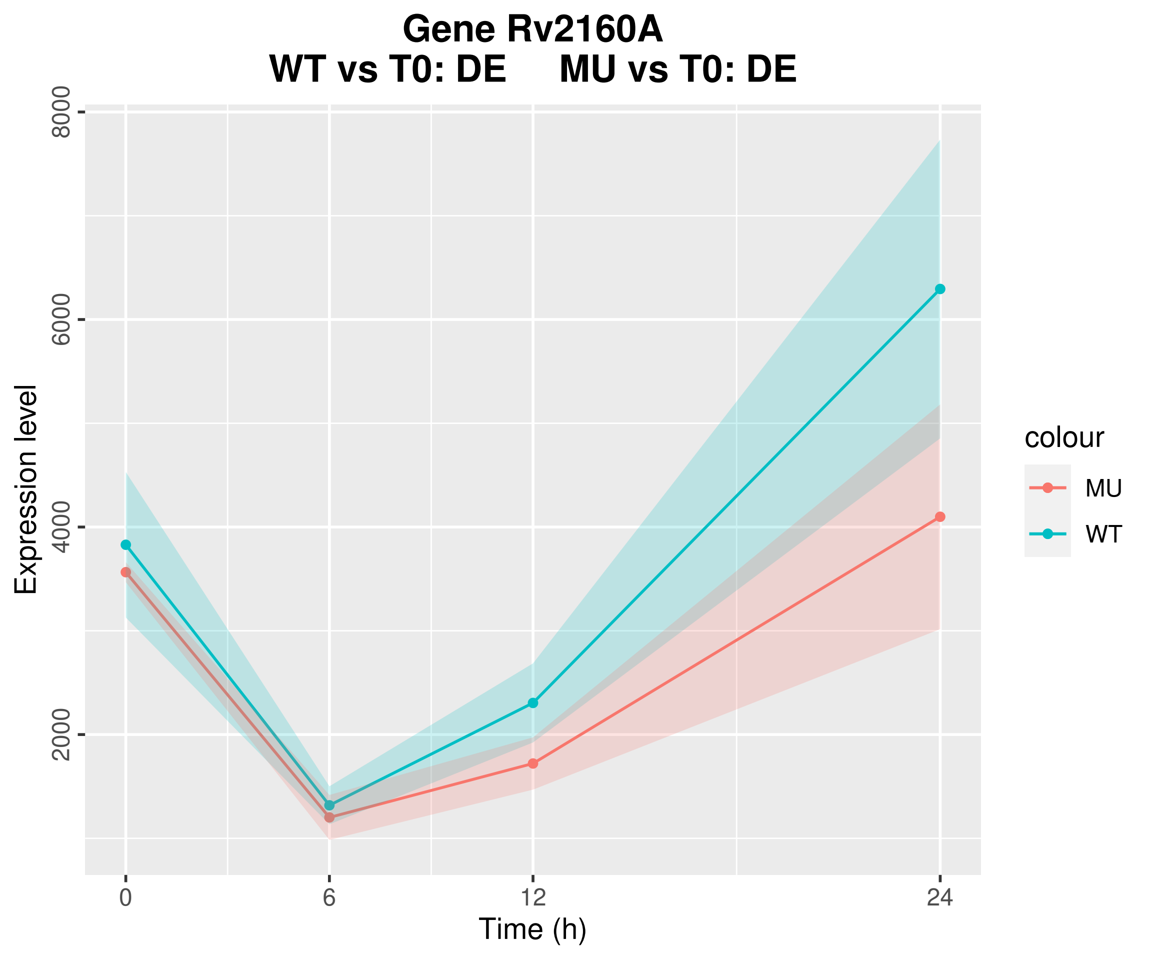

Gene Rv2160A
WT vs T0: DE MU vs T0: DE
8000
6000
4000
2000
Expression level
0
6
12
24
Time (h)
colour
MU
WT

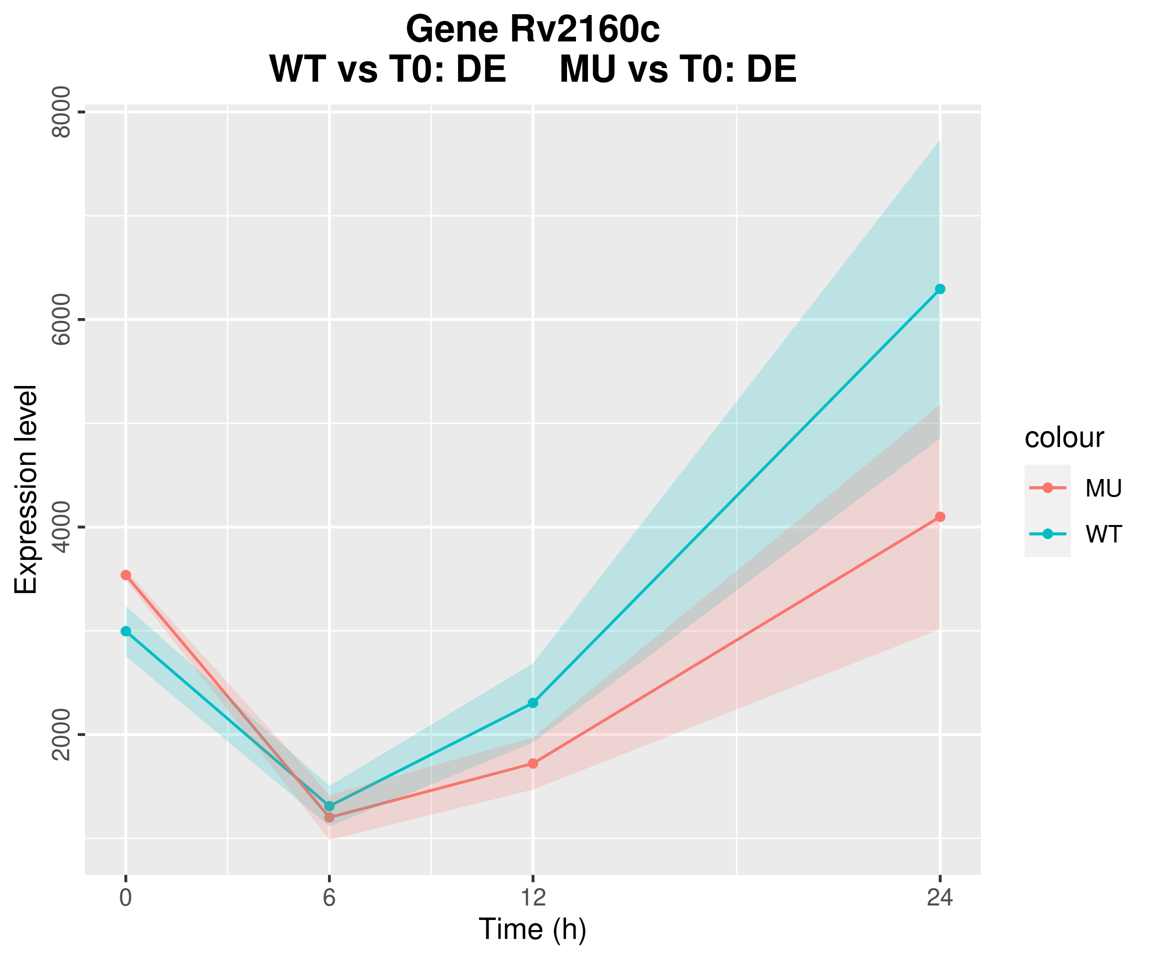
Gene Rv2160c
WT vs T0: DE MU vs T0: DE
Expression level
8000
6000
4000
2000
0
6
12
24
Time (h)
colour
MU
WT

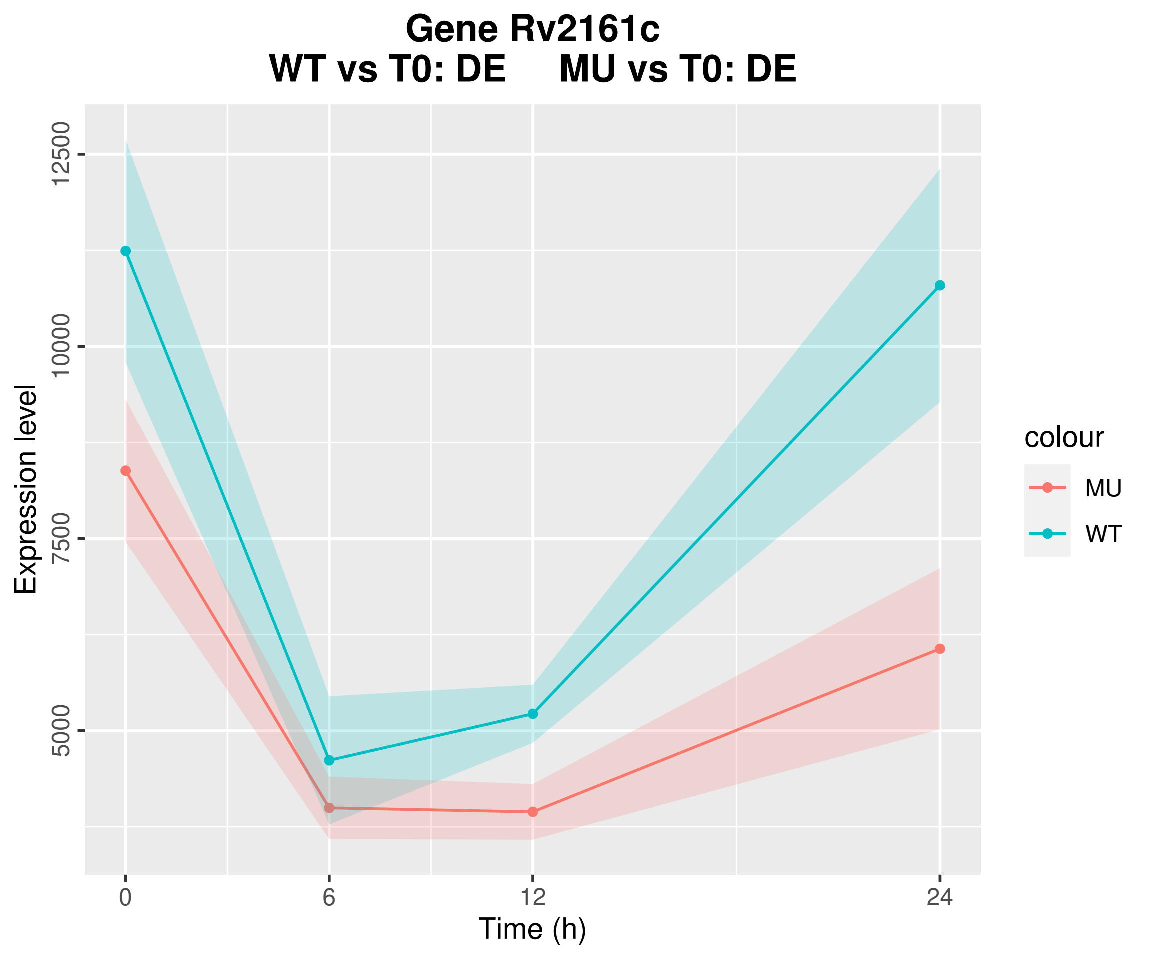

Gene Rv2161c
WT vs T0: DE MU vs T0: DE
Expression level
12500
10000
7500
5000
0
6
12
24
Time (h)
colour
MU
WT

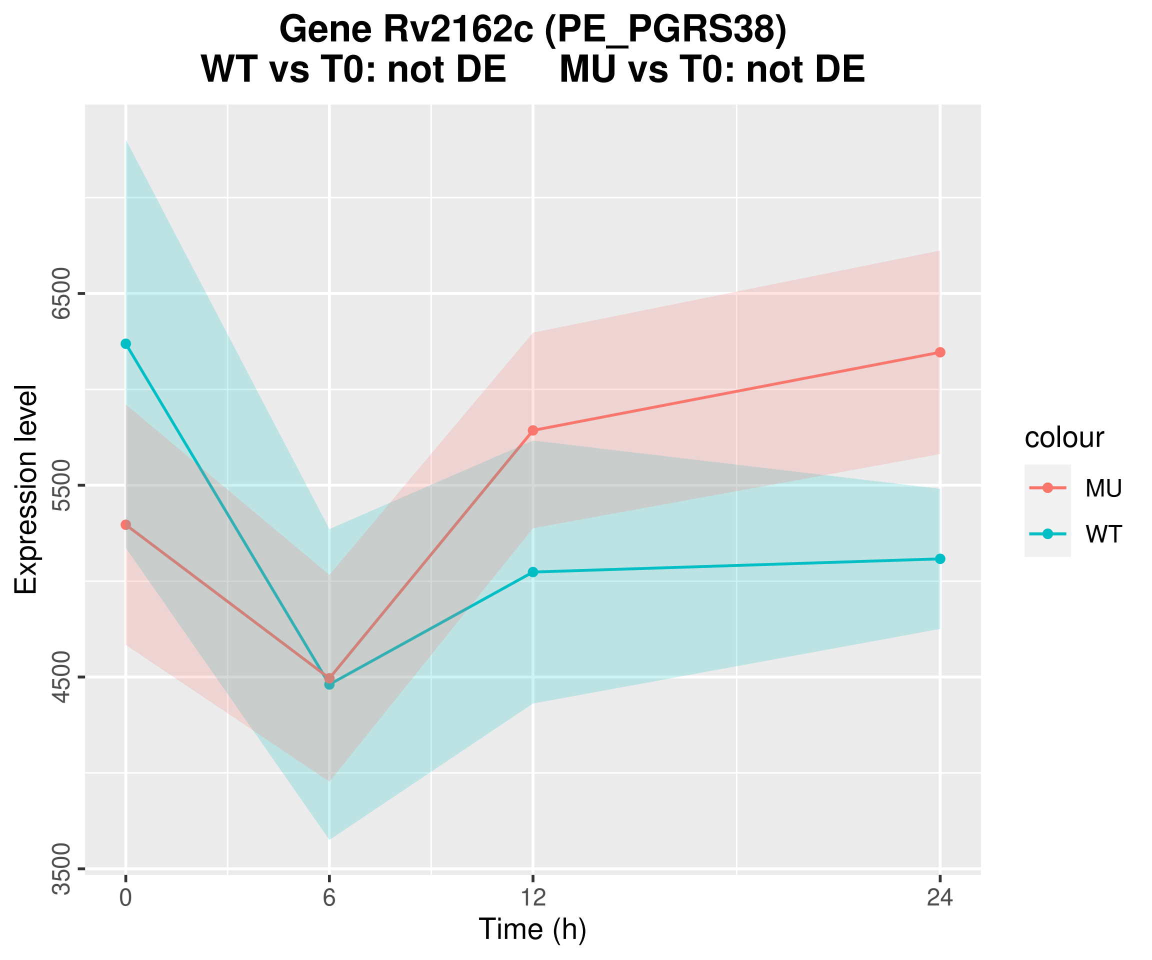
Gene Rv2162c (PE_PGRS38)
WT vs T0: not DE   MU vs T0: not DE
Expression level
6500
5500
4500
3500
0
6
12
24
Time (h)
colour
MU
WT

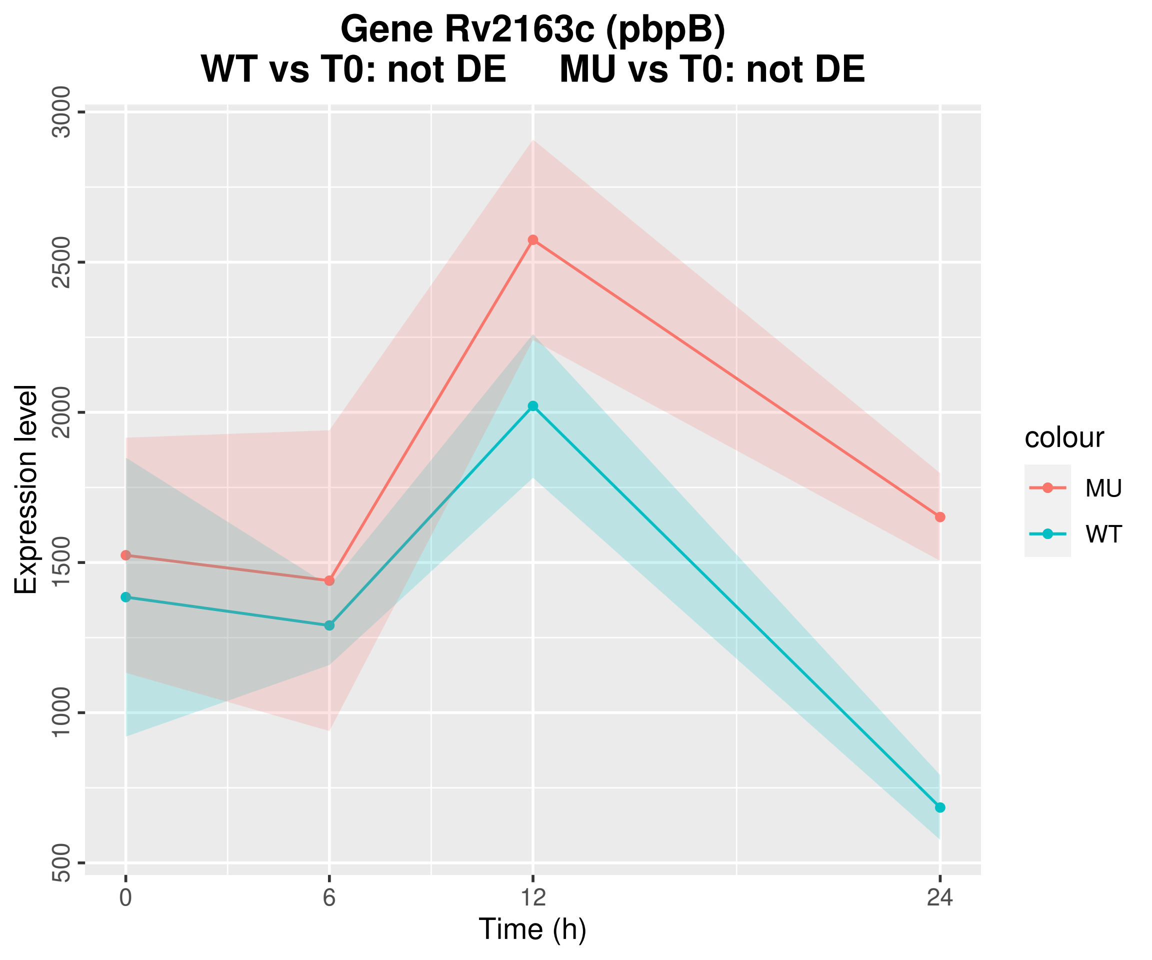

Gene Rv2163c (pbpB)
WT vs T0: not DE   MU vs T0: not DE
Expression level
500
1000
1500
2000
2500
3000
0
6
12
24
Time (h)
colour
MU
WT

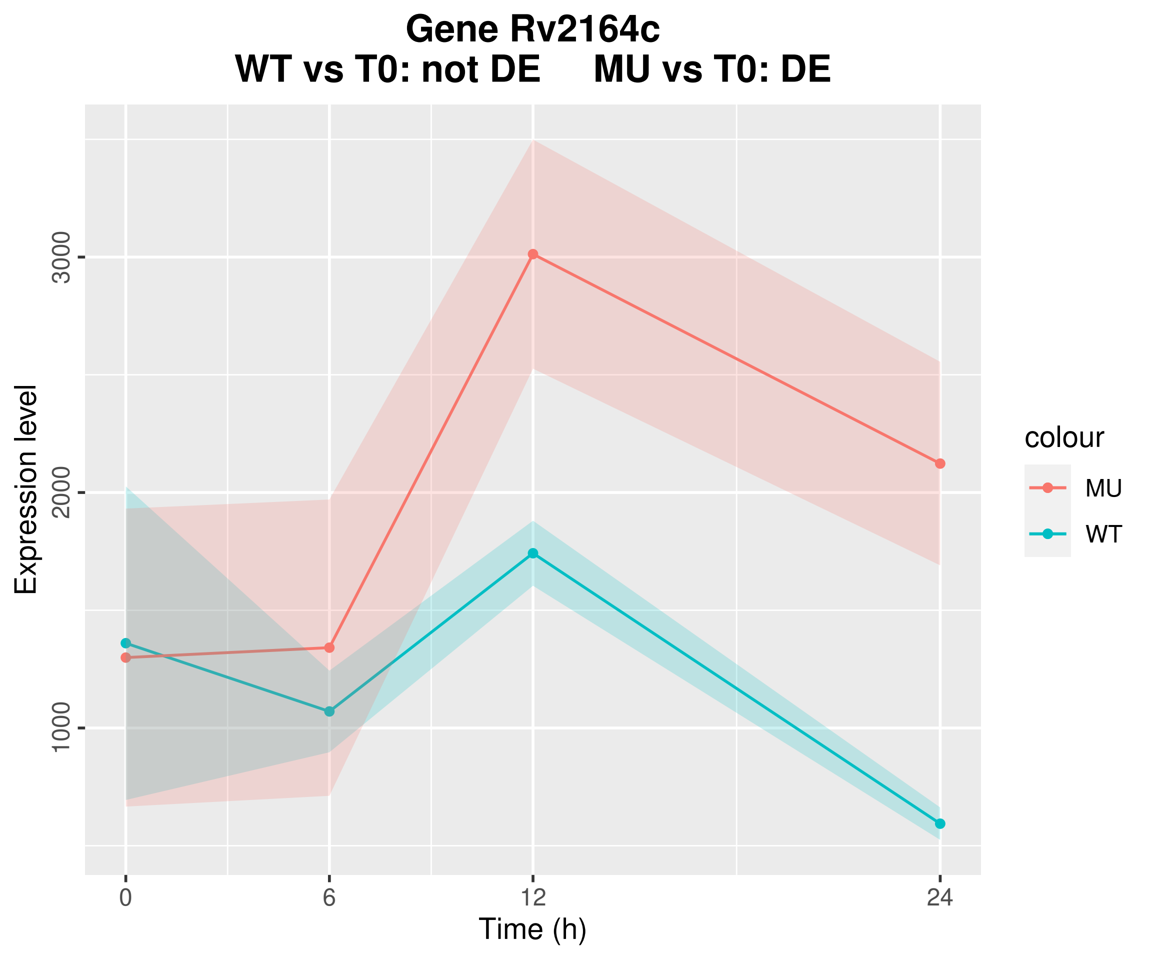

Gene Rv2164c
WT vs T0: not DE   MU vs T0: DE
Expression level
3000
2000
1000
0
6
12
24
Time (h)
colour
MU
WT

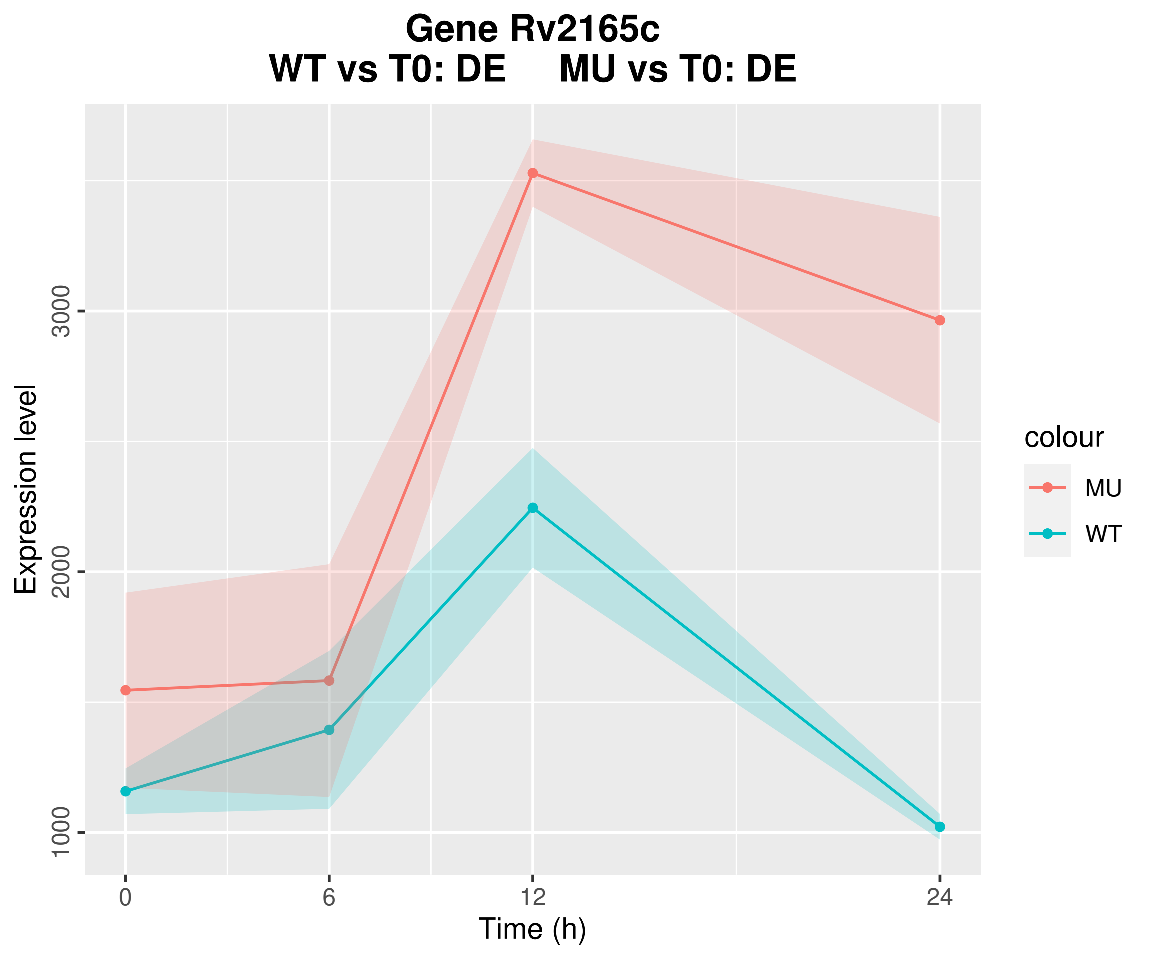
Gene Rv2165c
WT vs T0: DE MU vs T0: DE
Expression level
3000
2000
1000
0
6
12
24
Time (h)
colour
MU
WT

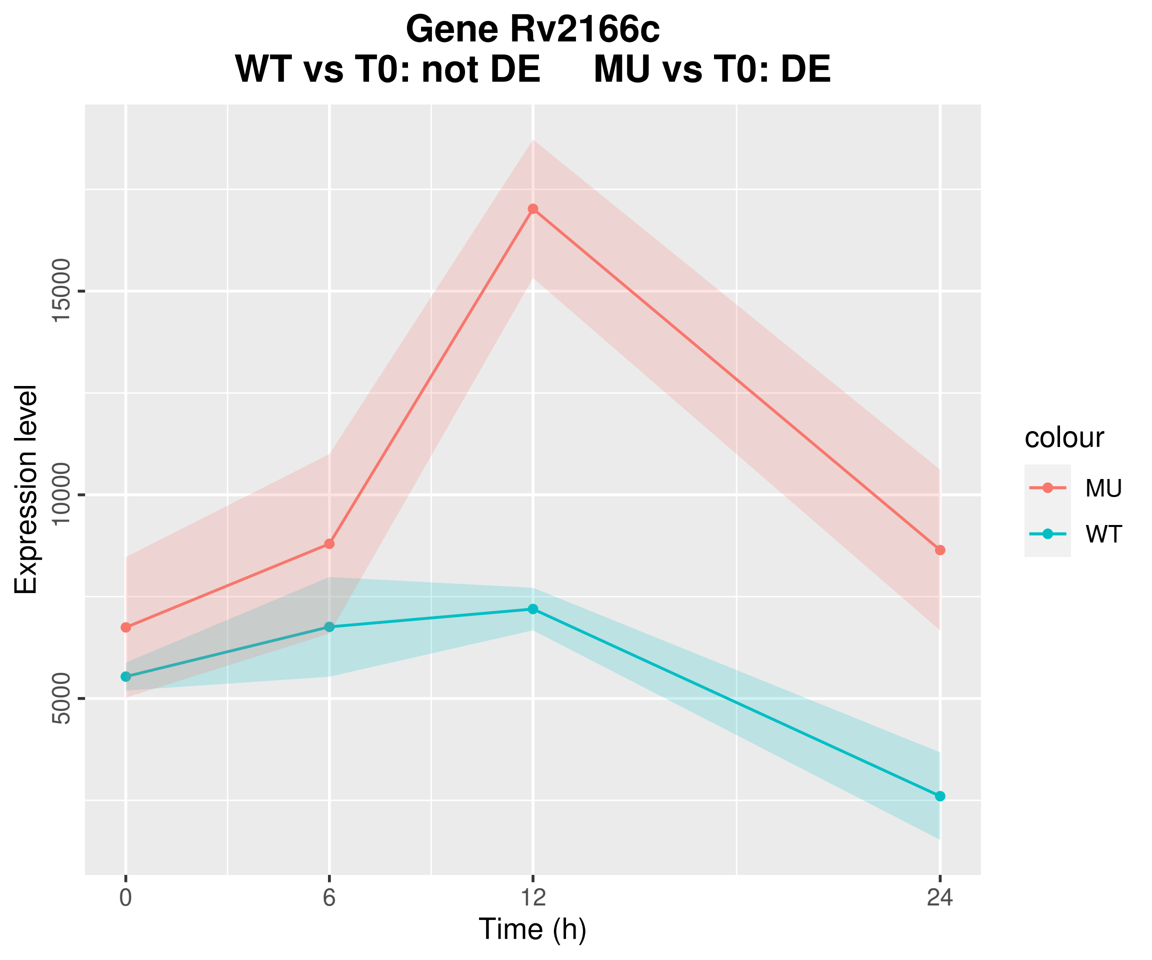
Gene Rv2166c
WT vs T0: not DE MU vs T0: DE
Expression level
15000
10000
5000
0
6
12
24
Time (h)
colour
MU
WT

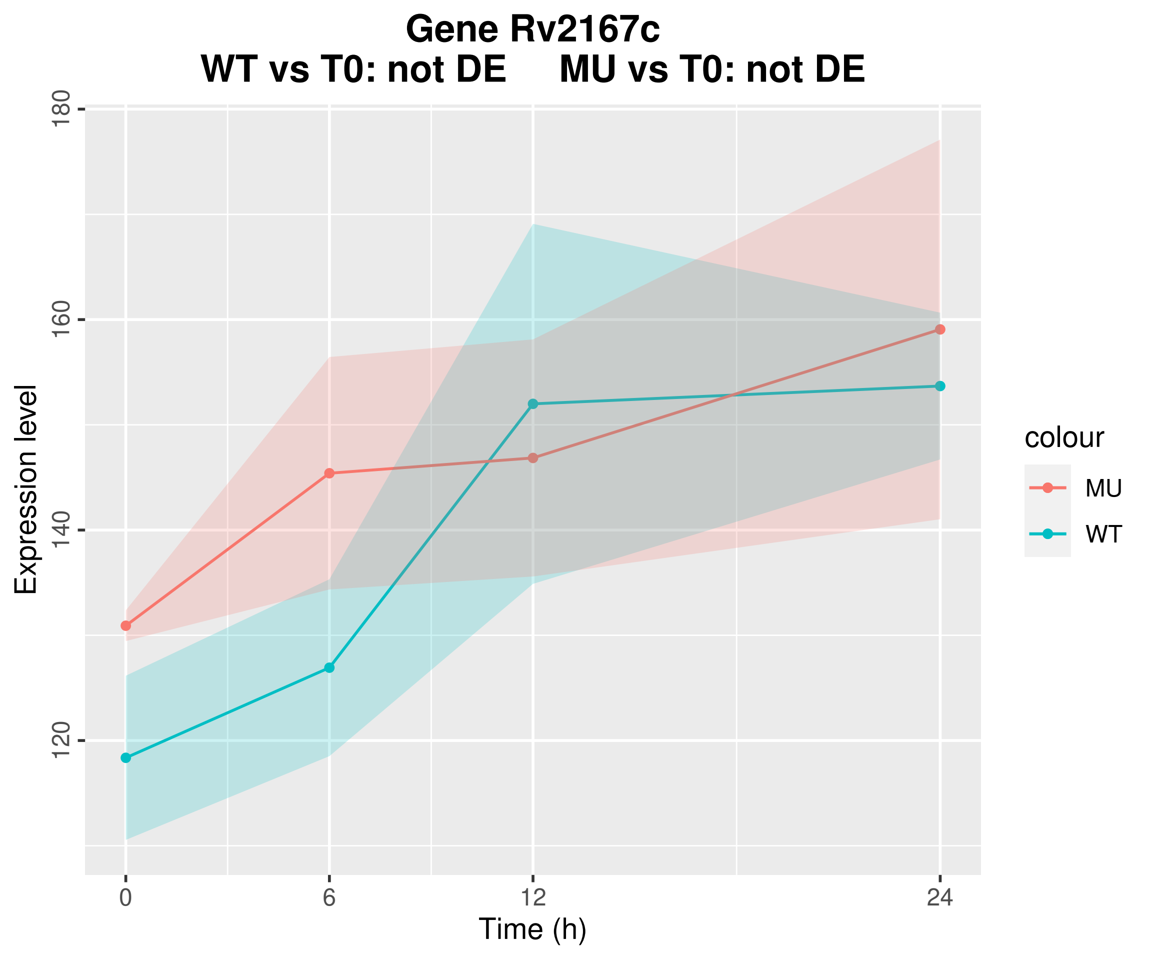
Gene Rv2167c
WT vs T0: not DE   MU vs T0: not DE
180
160
140
120
Expression level
0
6
12
24
Time (h)
colour
MU
WT

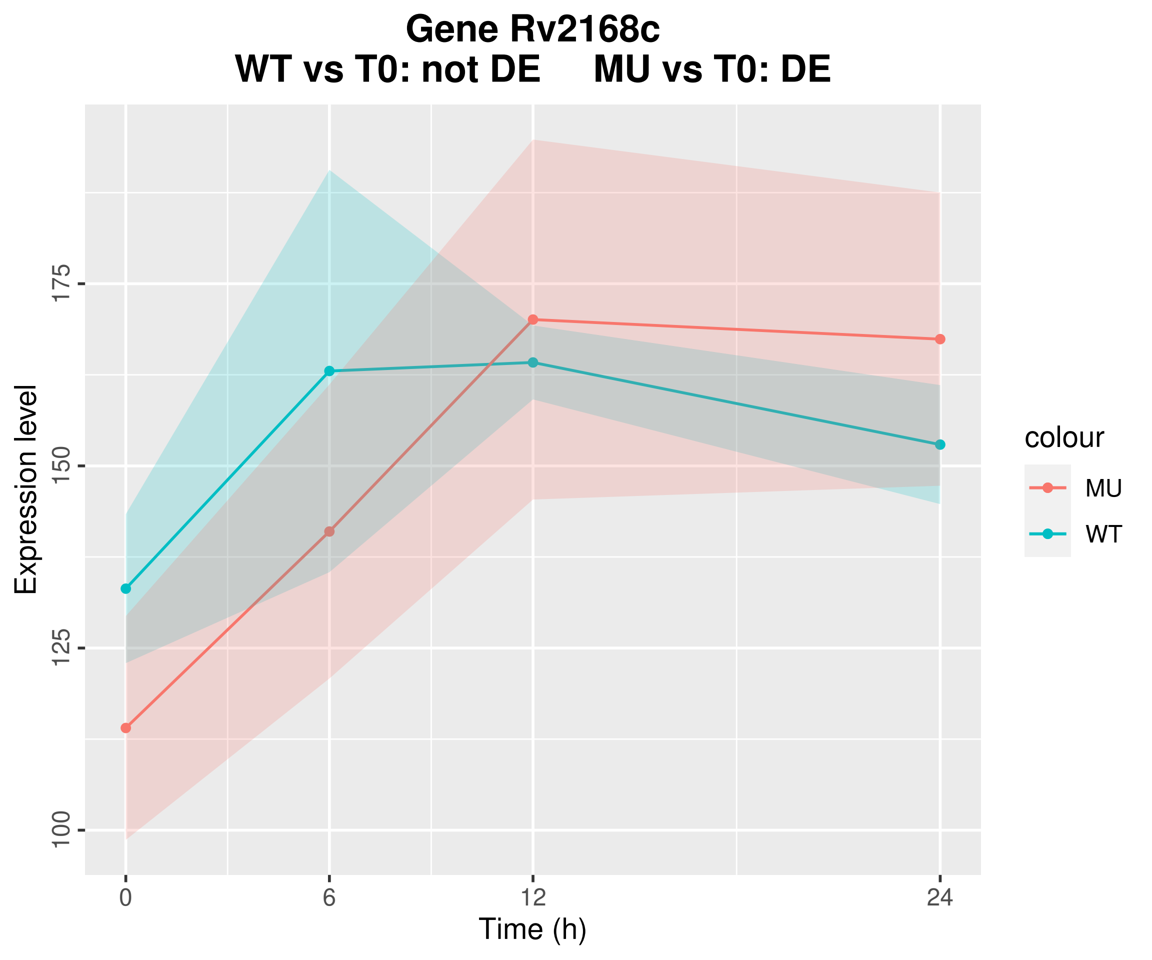
Gene Rv2168c
WT vs T0: not DE   MU vs T0: DE
Expression level
100
125
150
175
0
6
12
24
Time (h)
colour
MU
WT

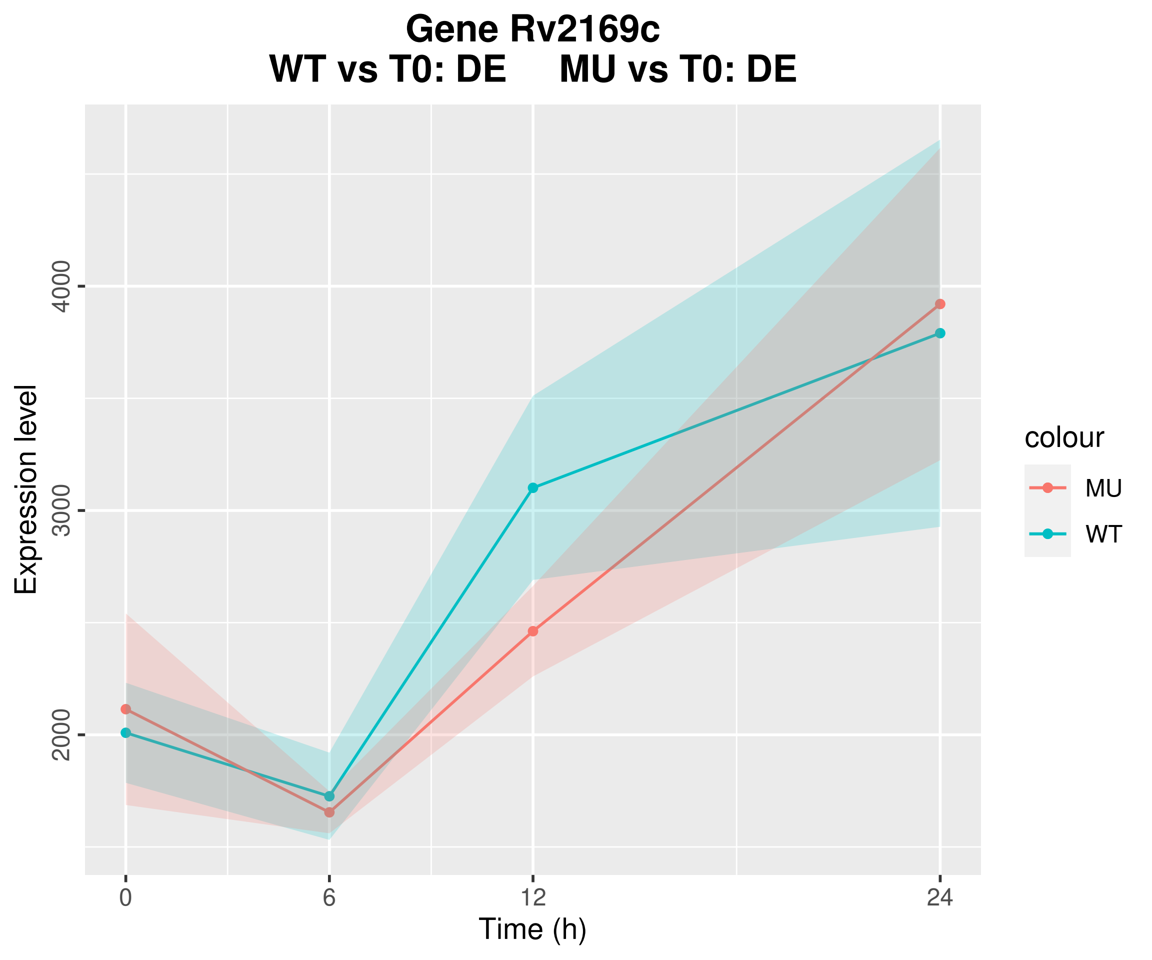

Gene Rv2169c
WT vs T0: DE   MU vs T0: DE
Expression level
4000
3000
2000
0
6
12
24
Time (h)
colour
MU
WT

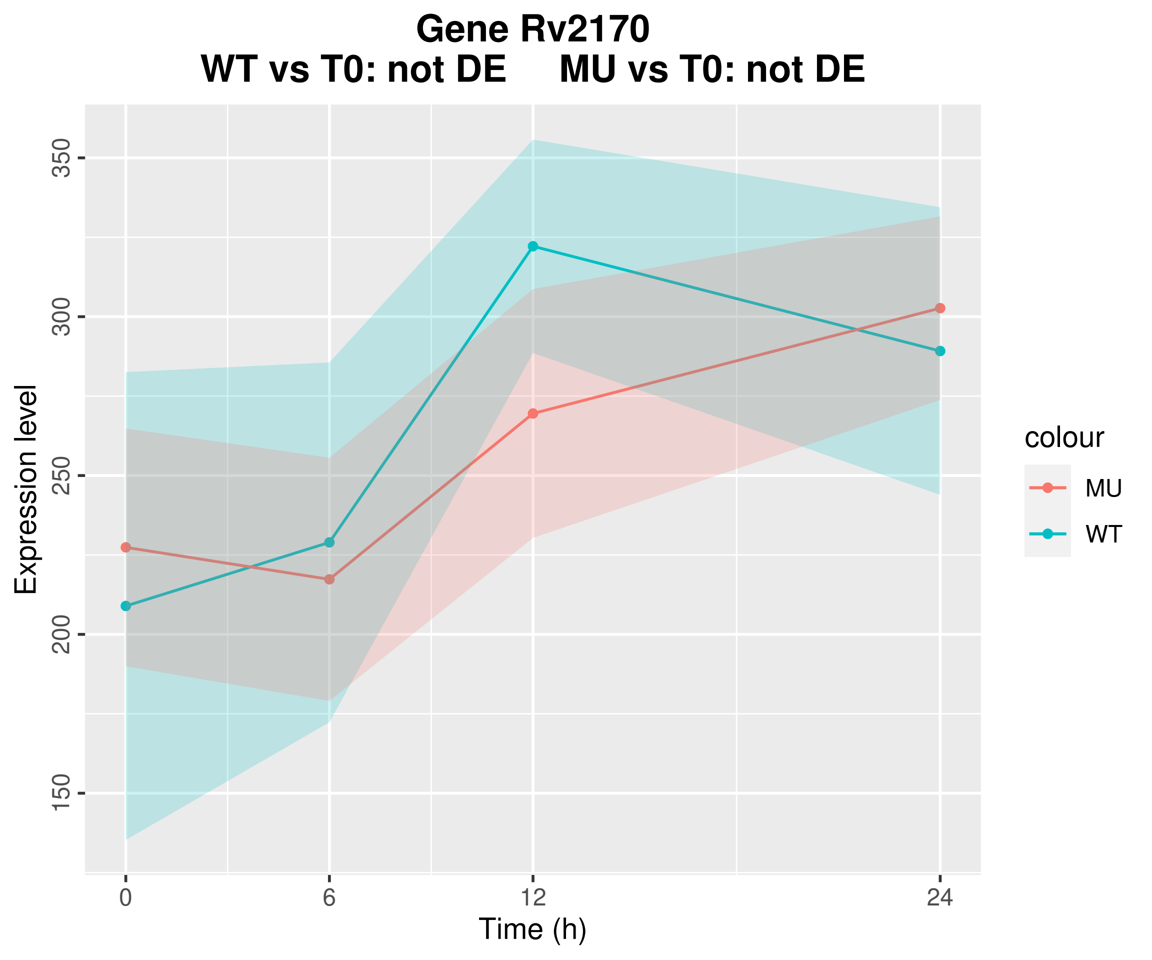

Gene Rv2170
WT vs T0: not DE   MU vs T0: not DE
350
300
250
200
150
Expression level
0
6
12
24
Time (h)
colour
MU
WT

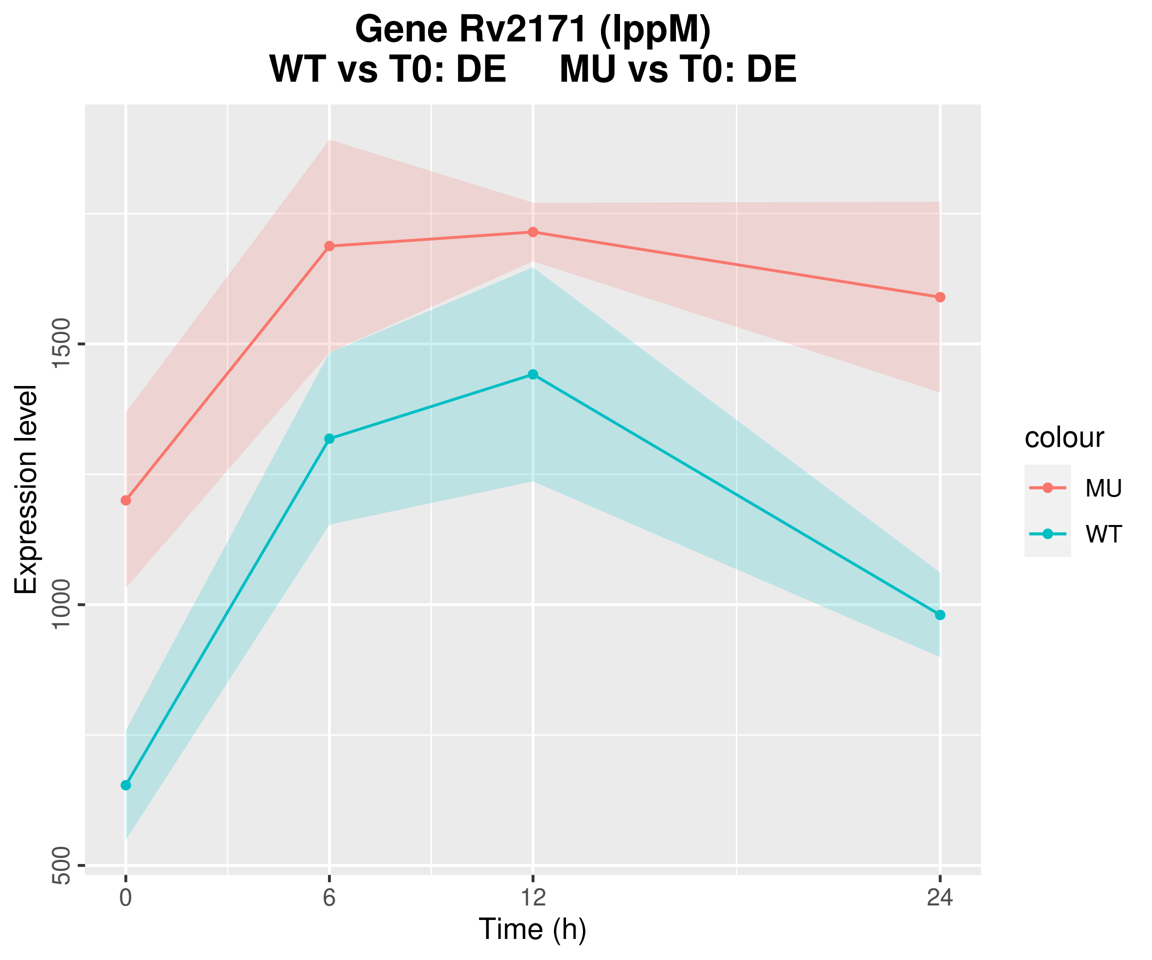

Gene Rv2171 (lppM)
WT vs T0: DE MU vs T0: DE
Expression level
1500
1000
500
0
6
12
24
Time (h)
colour
MU
WT

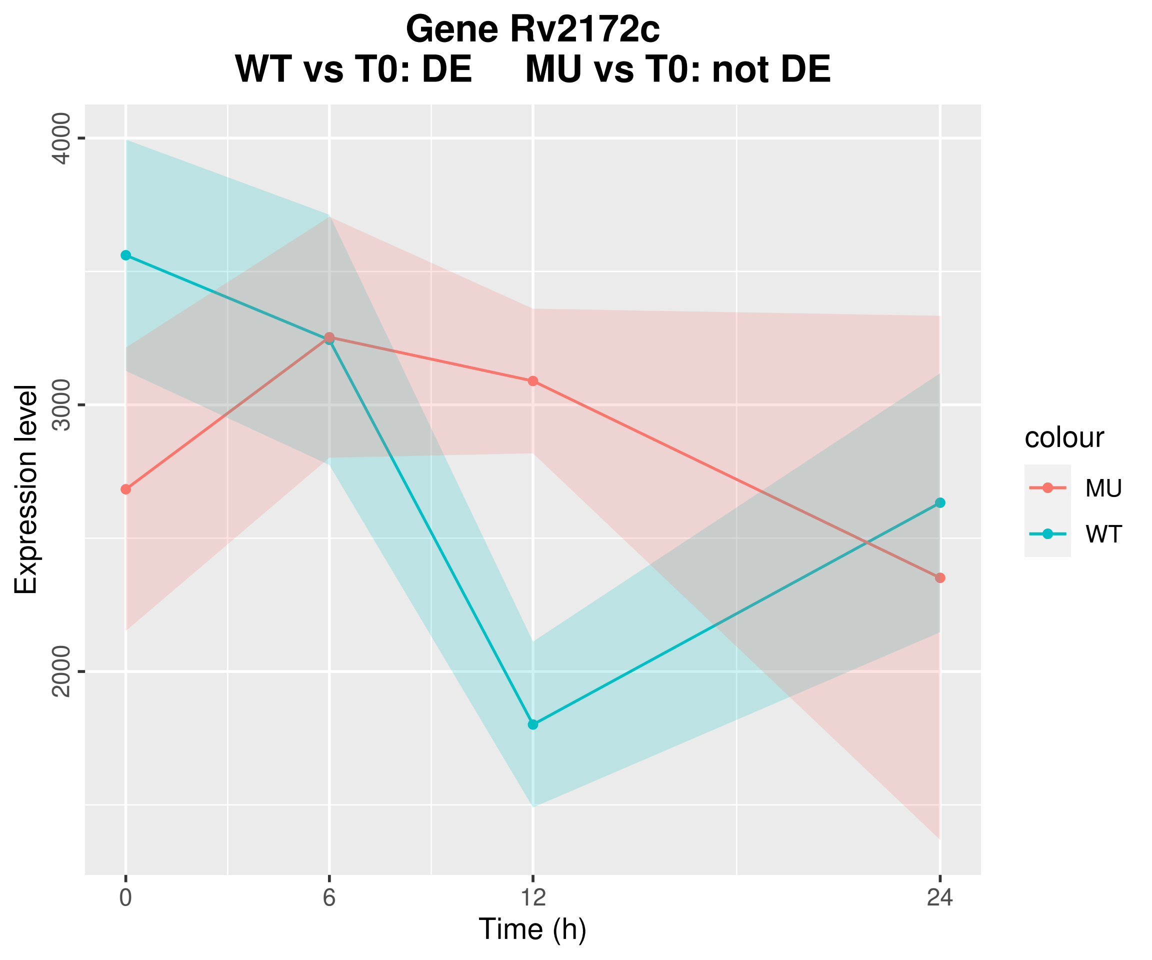
Gene Rv2172c
WT vs T0: DE   MU vs T0: not DE
Expression level
4000
3000
2000
0
6
12
24
Time (h)
colour
MU
WT

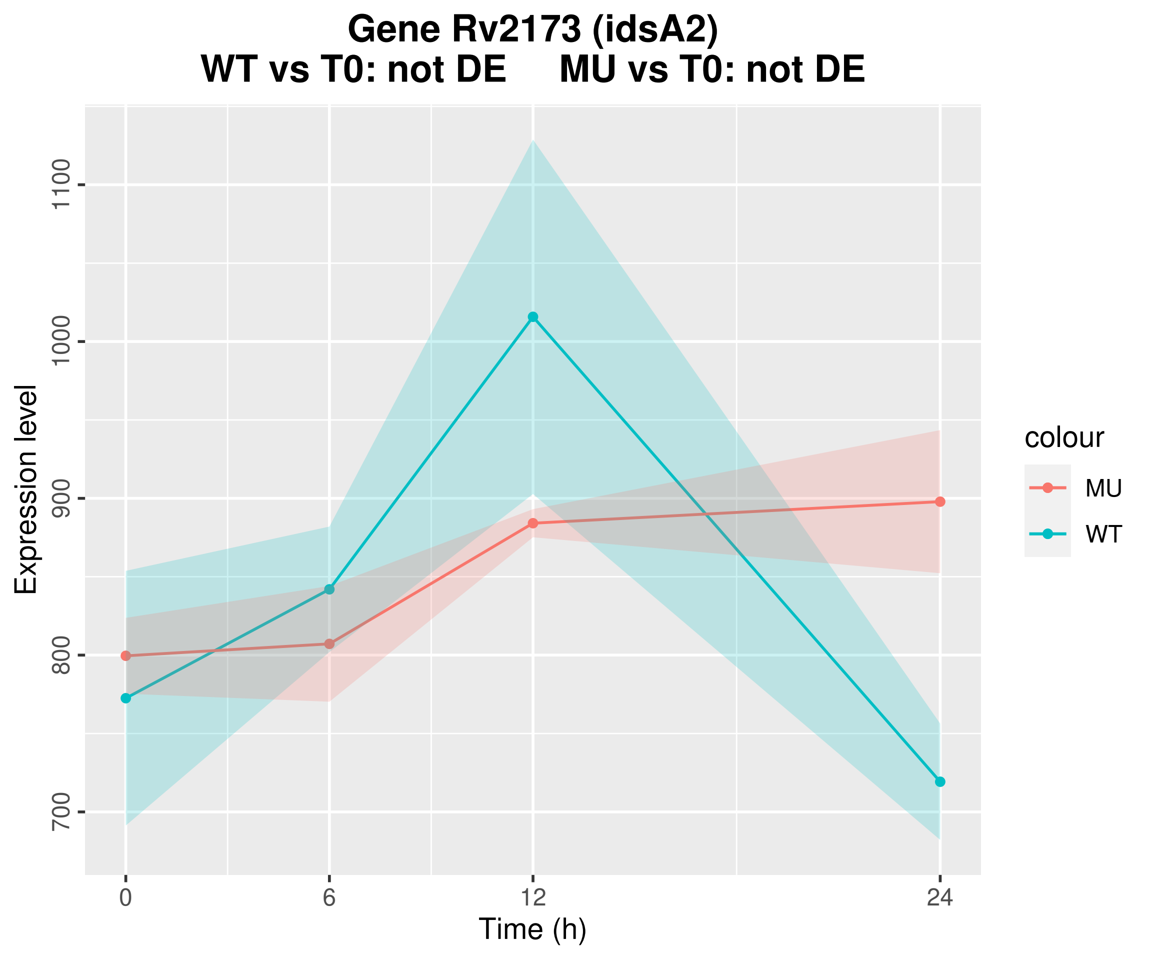
Gene Rv2173 (idsA2)
WT vs T0: not DE   MU vs T0: not DE
Expression level
1100
1000
900
800
700
0
6
12
24
Time (h)
colour
MU
WT

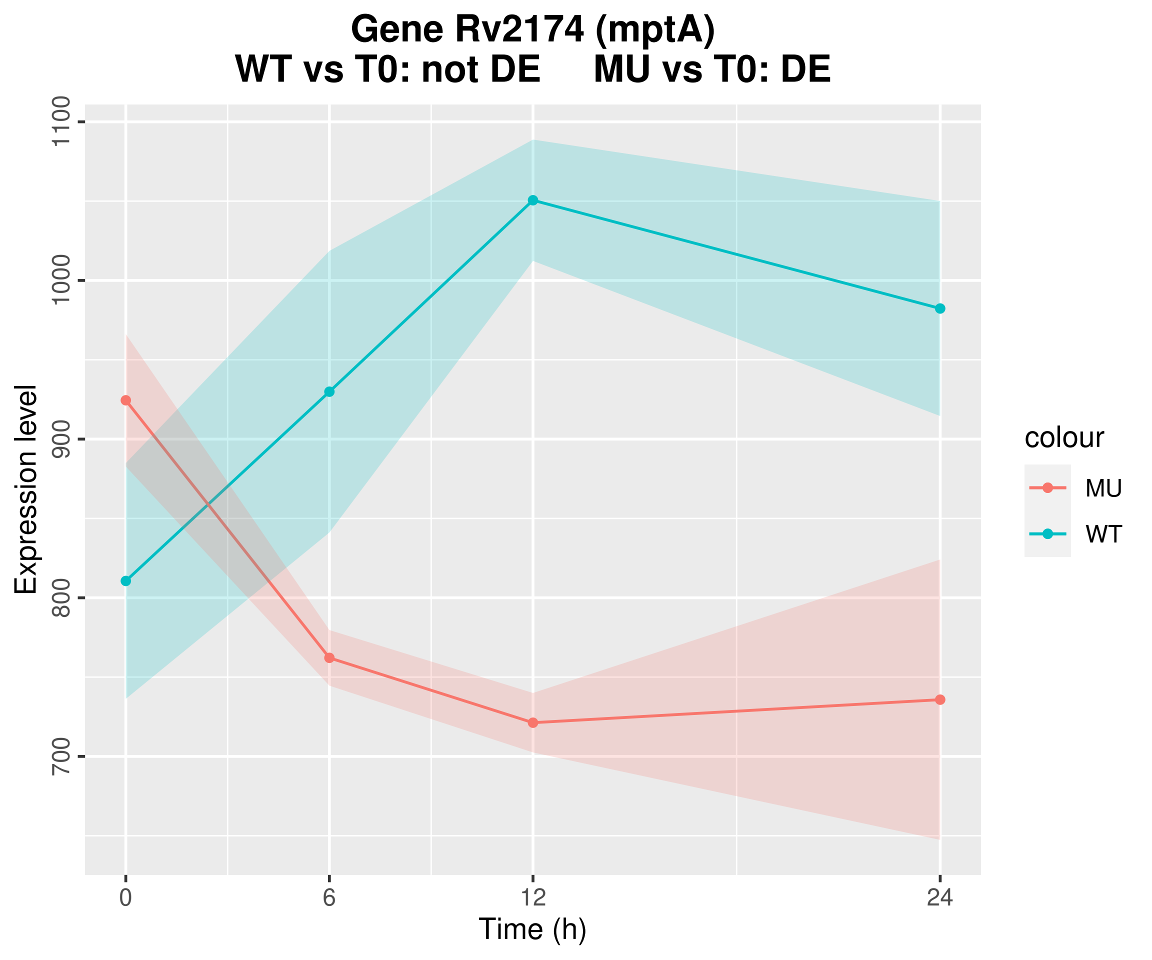
Gene Rv2174 (mptA)
WT vs T0: not DE   MU vs T0: DE
Expression level
1100
1000
900
800
700
0
6
12
24
Time (h)
colour
MU
WT

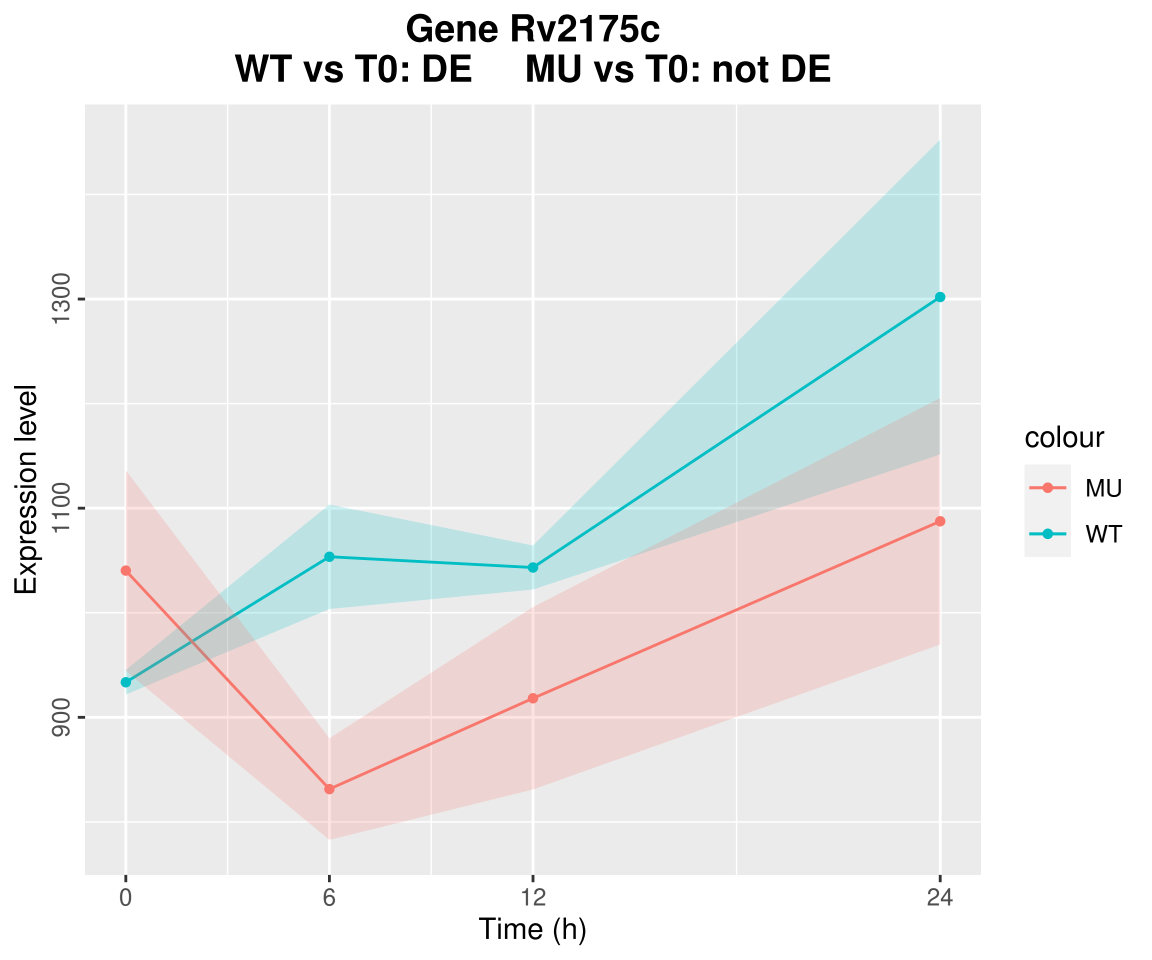

Gene Rv2175c
WT vs T0: DE   MU vs T0: not DE
Expression level
900
1100
1300
0
6
12
24
Time (h)
colour
MU
WT

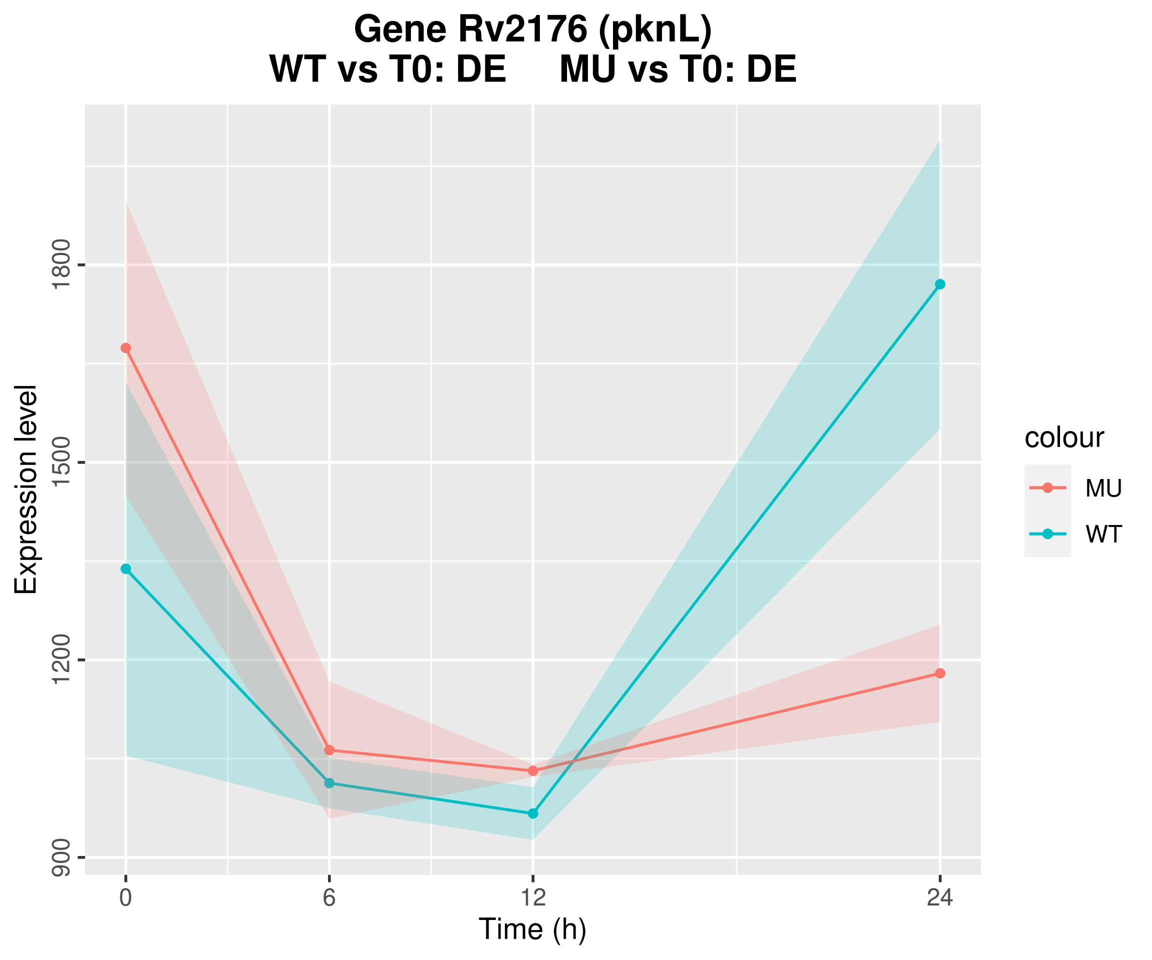

Gene Rv2176 (pknL)
WT vs T0: DE   MU vs T0: DE
Expression level
1800
1500
1200
900
0
6
12
24
Time (h)
colour
MU
WT

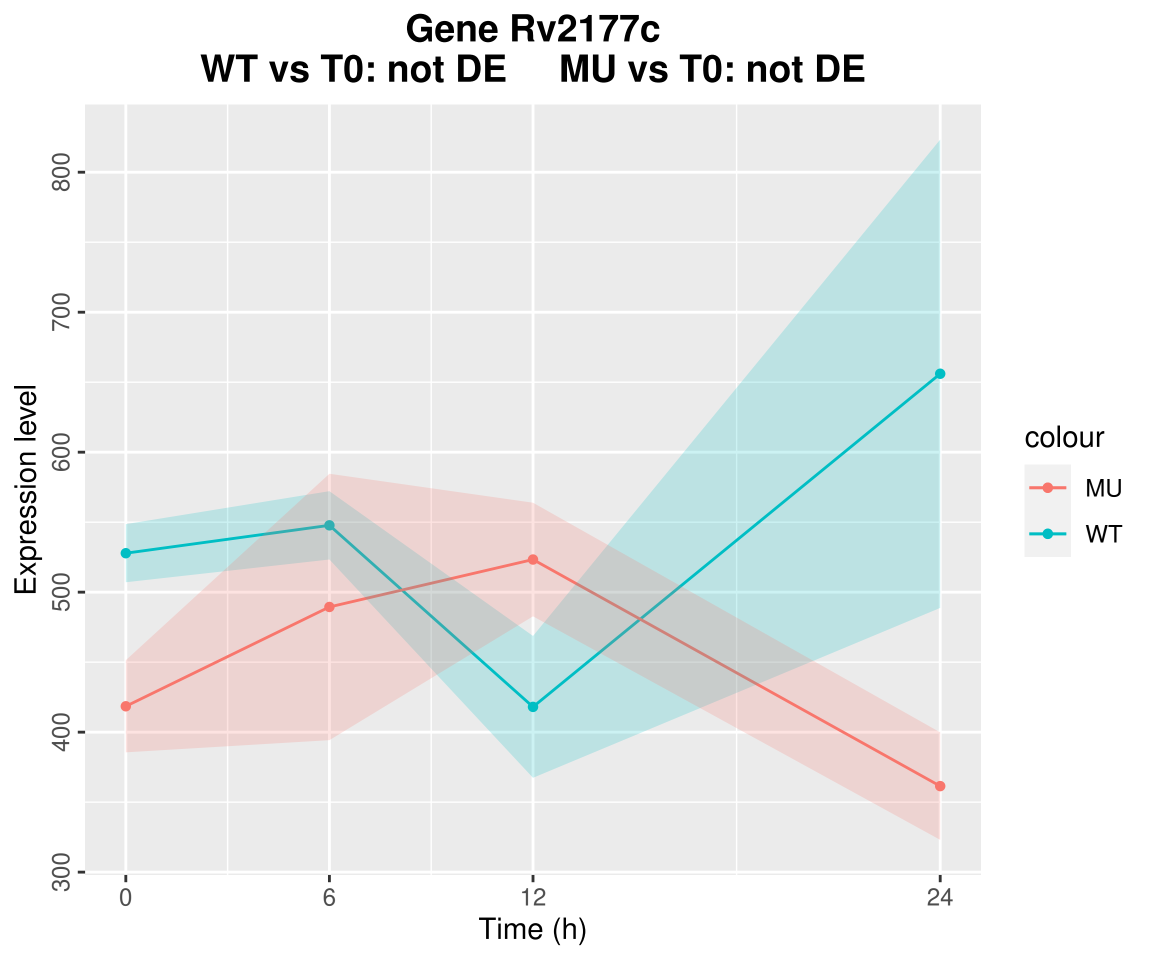
Gene Rv2177c
WT vs T0: not DE MU vs T0: not DE
Expression level
800
700
600
500
400
300
0
6
12
24
Time (h)
colour
MU
WT

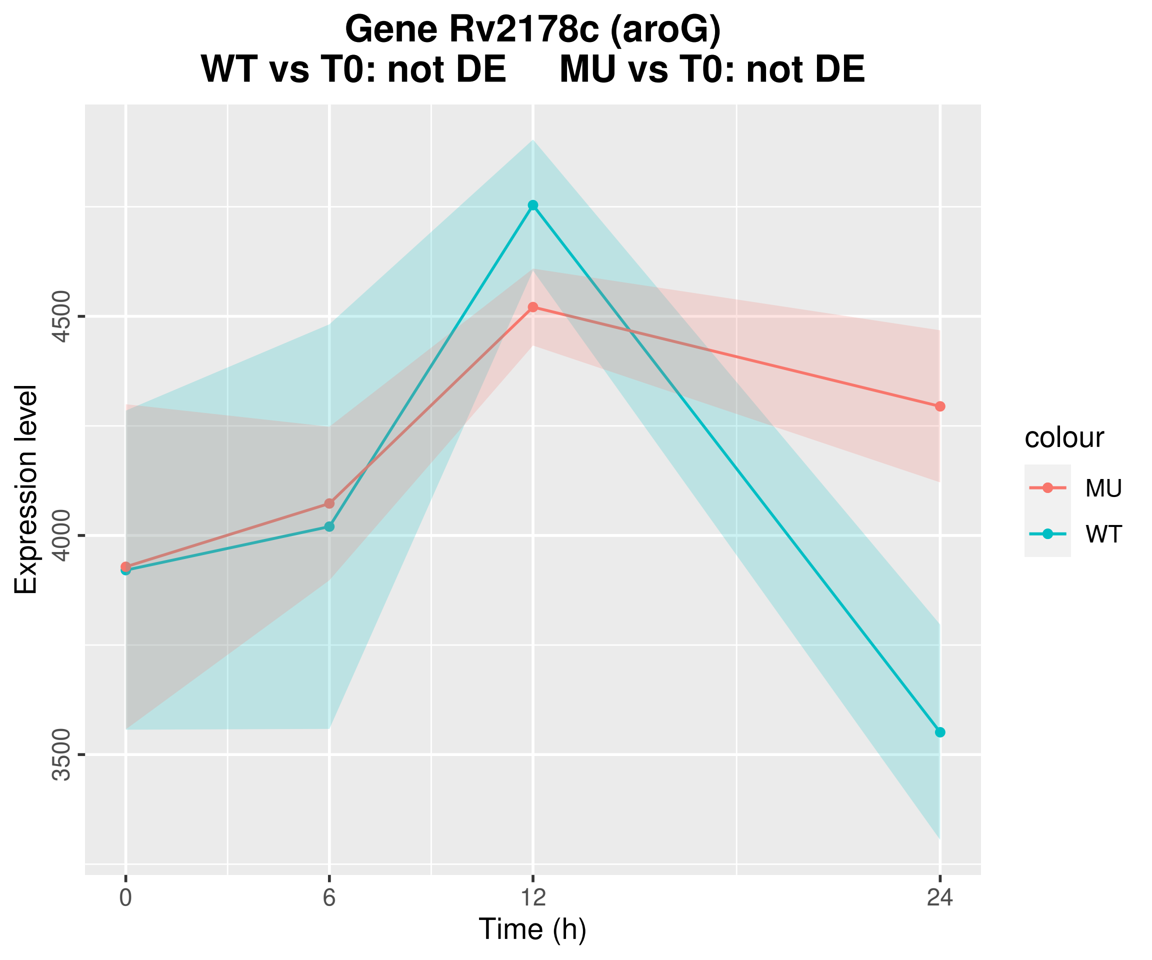

Gene Rv2178c (aroG)
WT vs T0: not DE
MU vs T0: not DE
Expression level
3500
4000
4500
0
6
12
24
Time (h)
colour
MU
WT

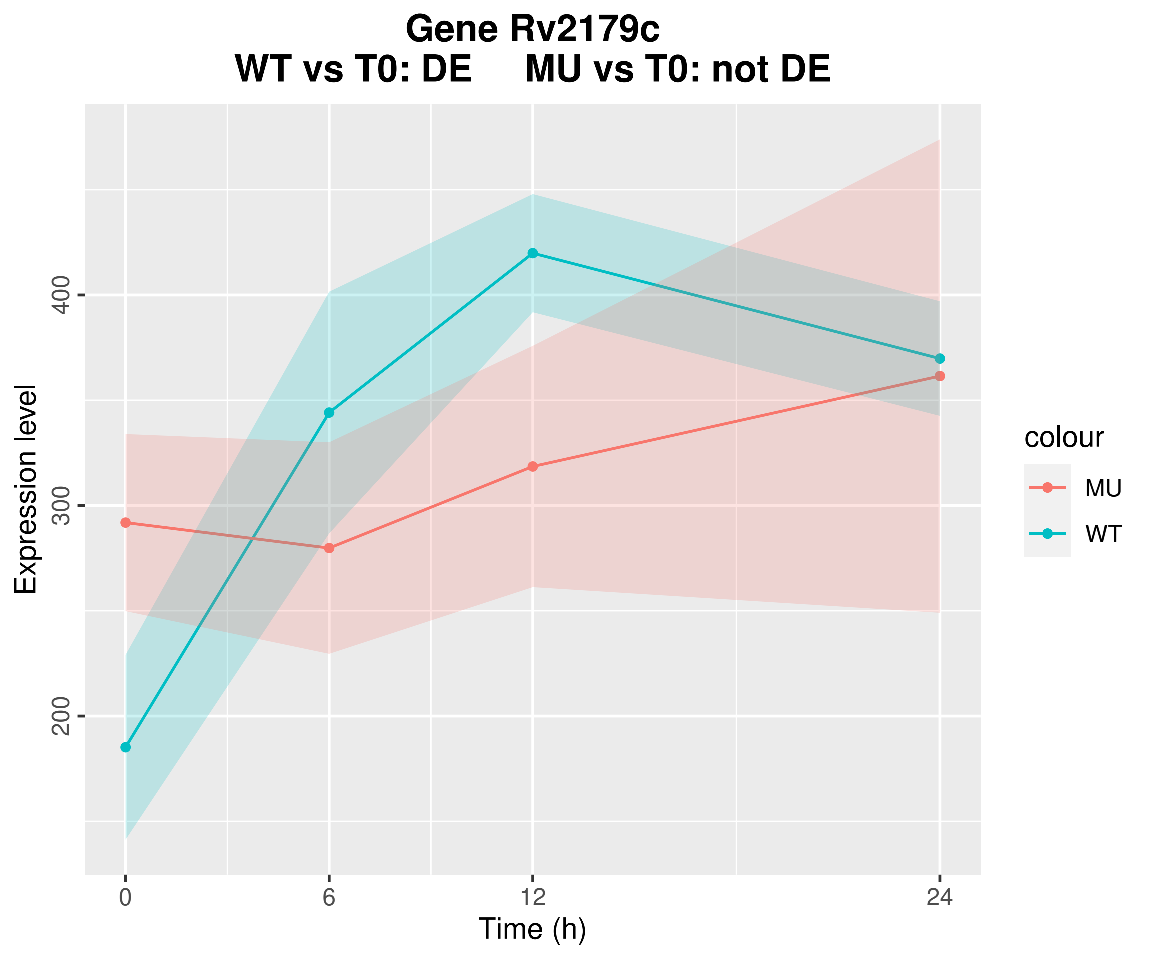
Gene Rv2179c
WT vs T0: DE   MU vs T0: not DE
Expression level
400
300
200
0
6
12
24
Time (h)
colour
MU
WT

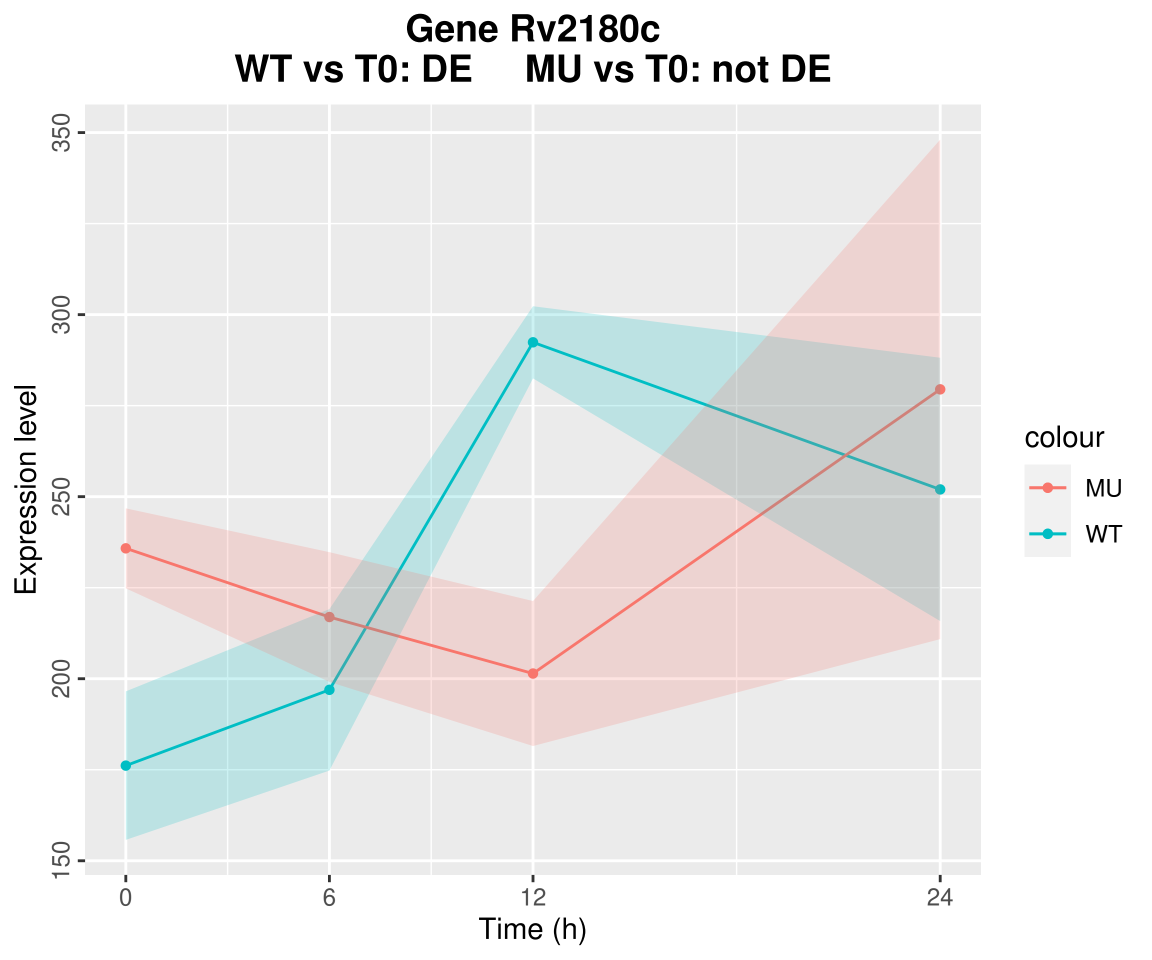

Gene Rv2180c
WT vs T0: DE   MU vs T0: not DE
Expression level
350
300
250
200
150
0
6
12
24
Time (h)
colour
MU
WT

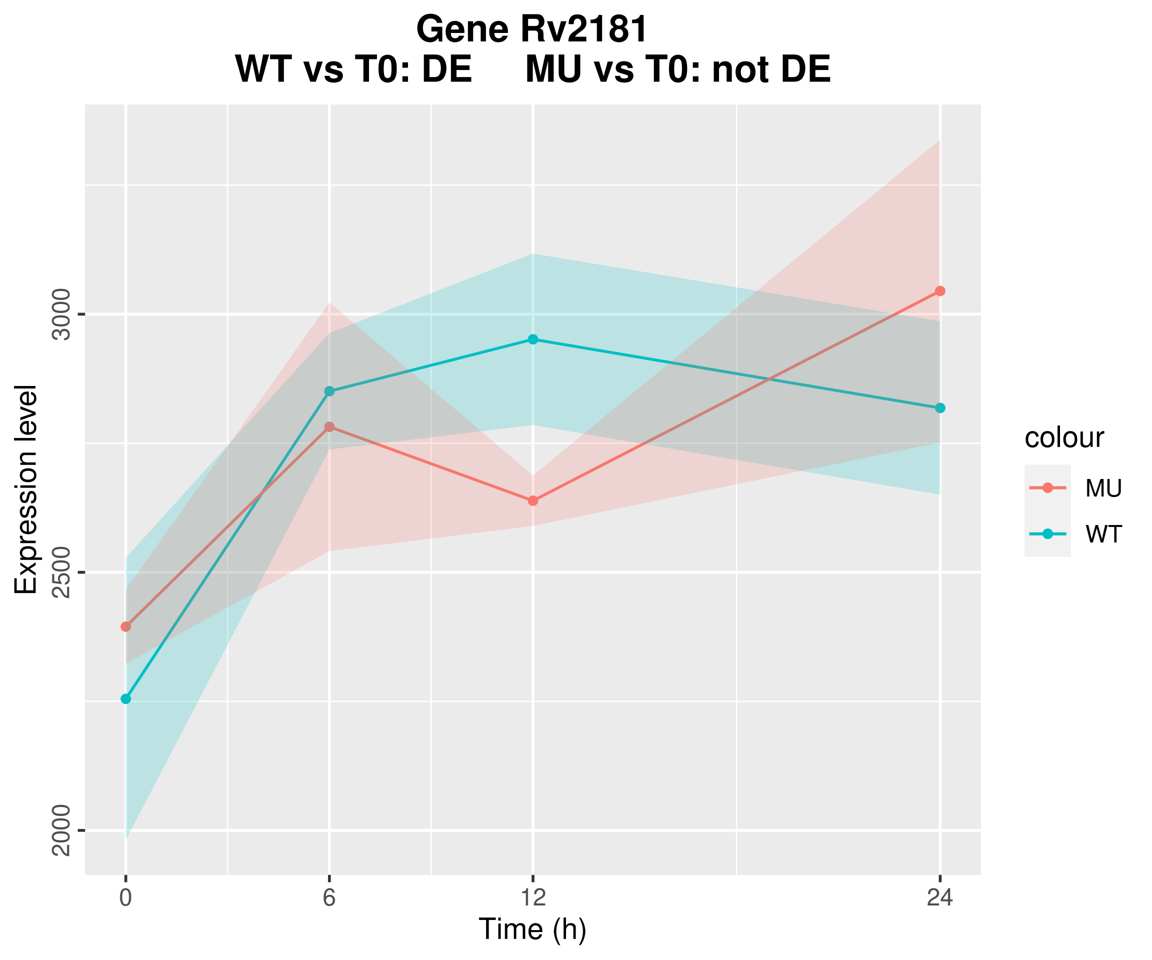
Gene Rv2181
WT vs T0: DE   MU vs T0: not DE
Expression level
3000
2500
2000
Time (h)
0
6
12
24
colour
MU
WT

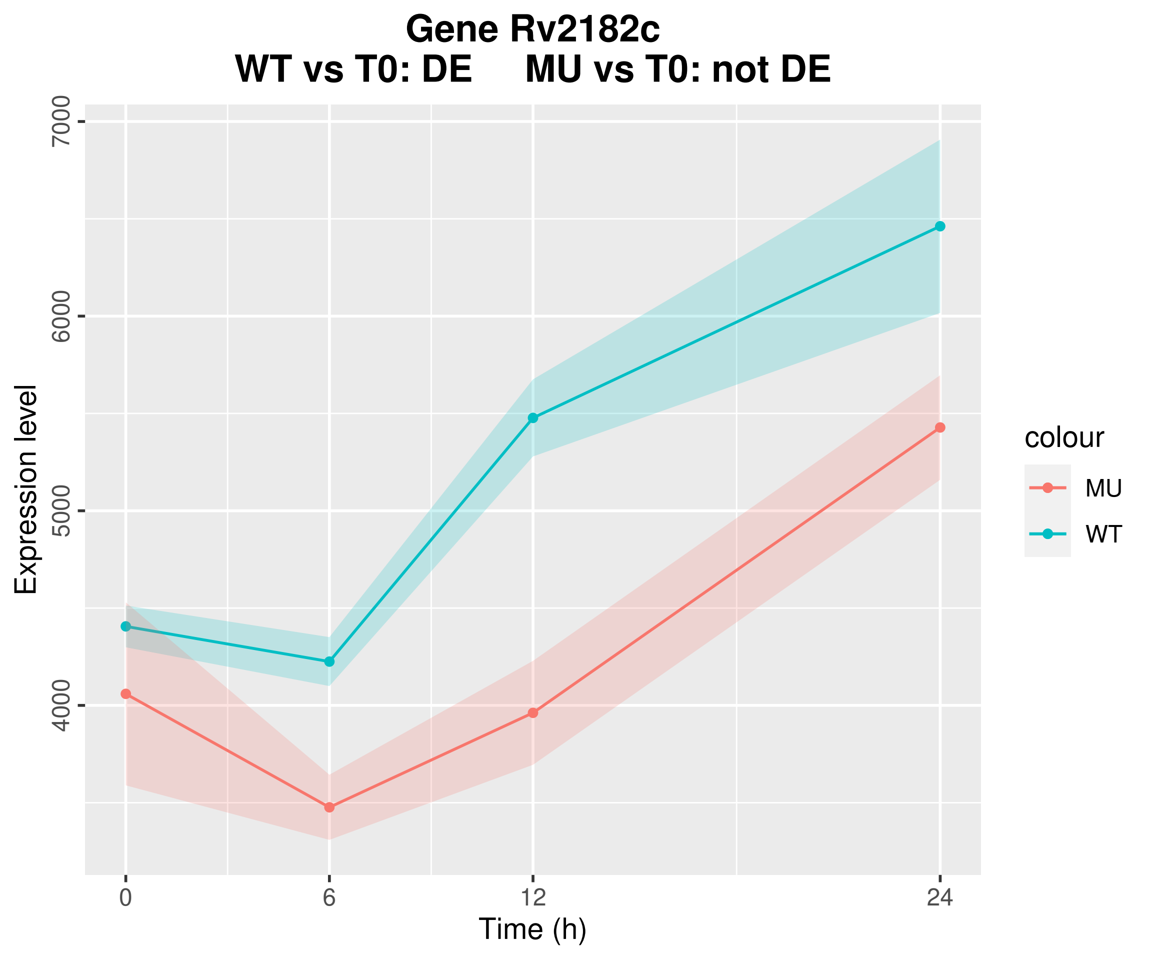

Gene Rv2182c
WT vs T0: DE   MU vs T0: not DE
7000
6000
5000
4000
Expression level
0
6
12
24
Time (h)
colour
MU
WT

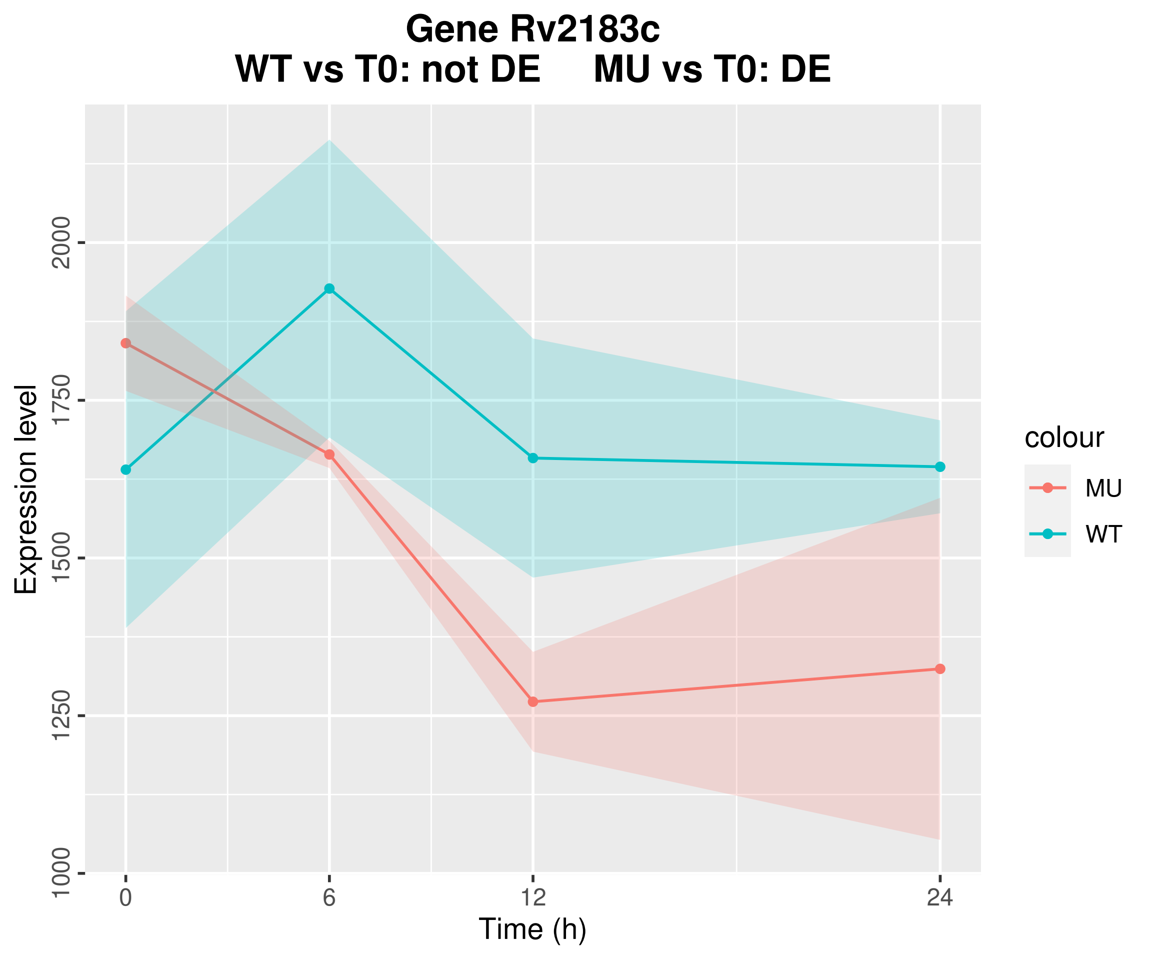

Gene Rv2183c
WT vs T0: not DE   MU vs T0: DE
Expression level
1000
1250
1500
1750
2000
0
6
12
24
Time (h)
colour
MU
WT

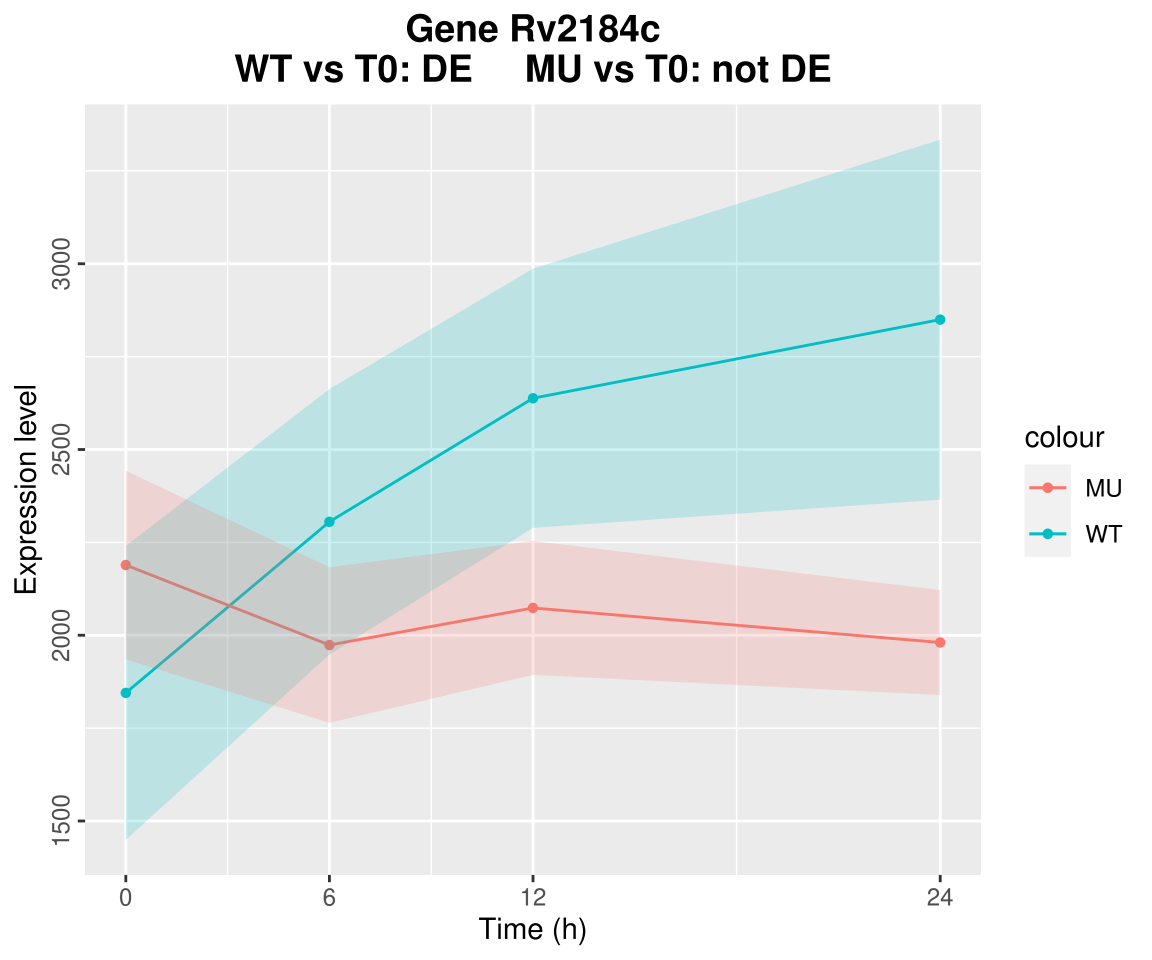

Gene Rv2184c
WT vs T0: DE   MU vs T0: not DE
Expression level
3000
2500
2000
1500
0
6
12
24
Time (h)
colour
MU
WT

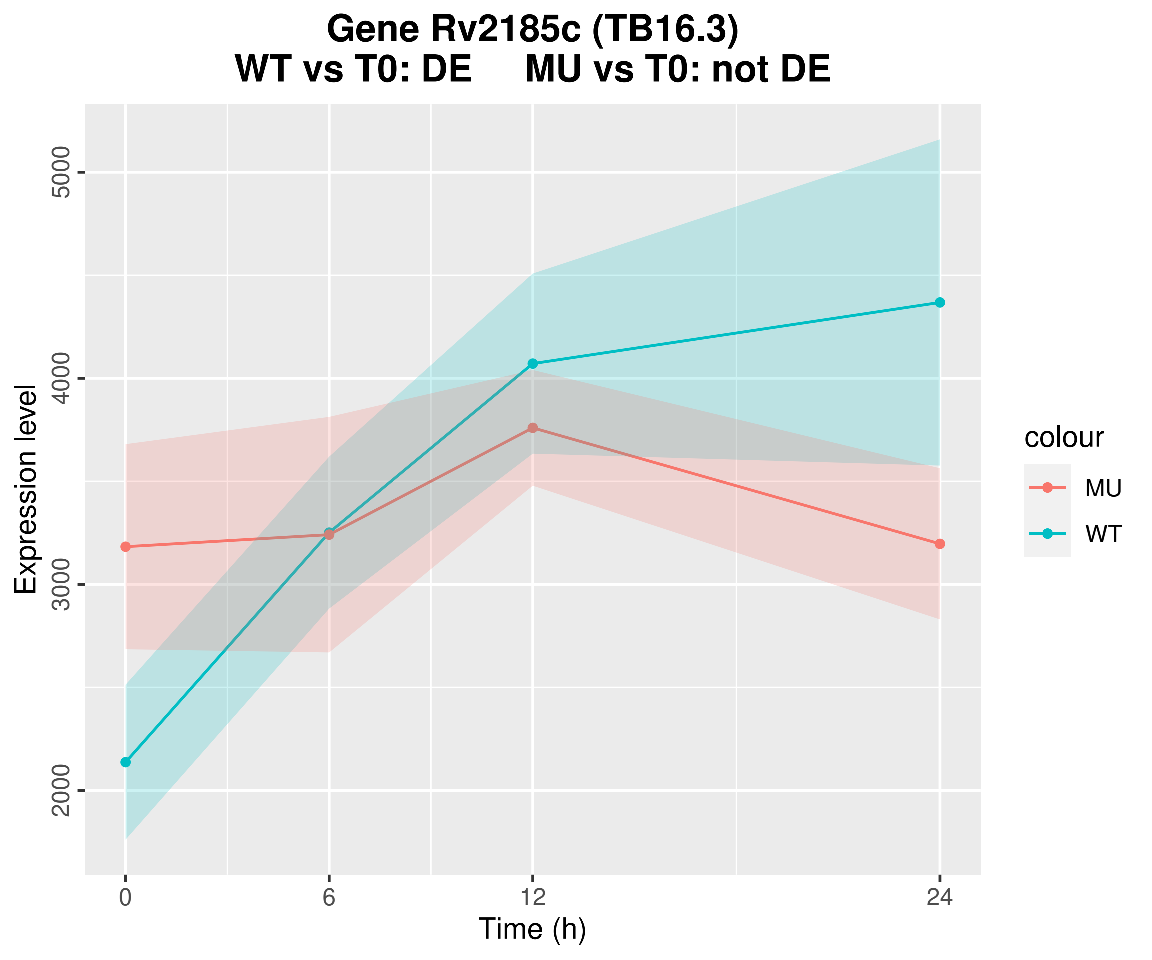

Gene Rv2185c (TB16.3)
WT vs T0: DE MU vs T0: not DE
Expression level
5000
4000
3000
2000
0
6
12
24
Time (h)
colour
MU
WT

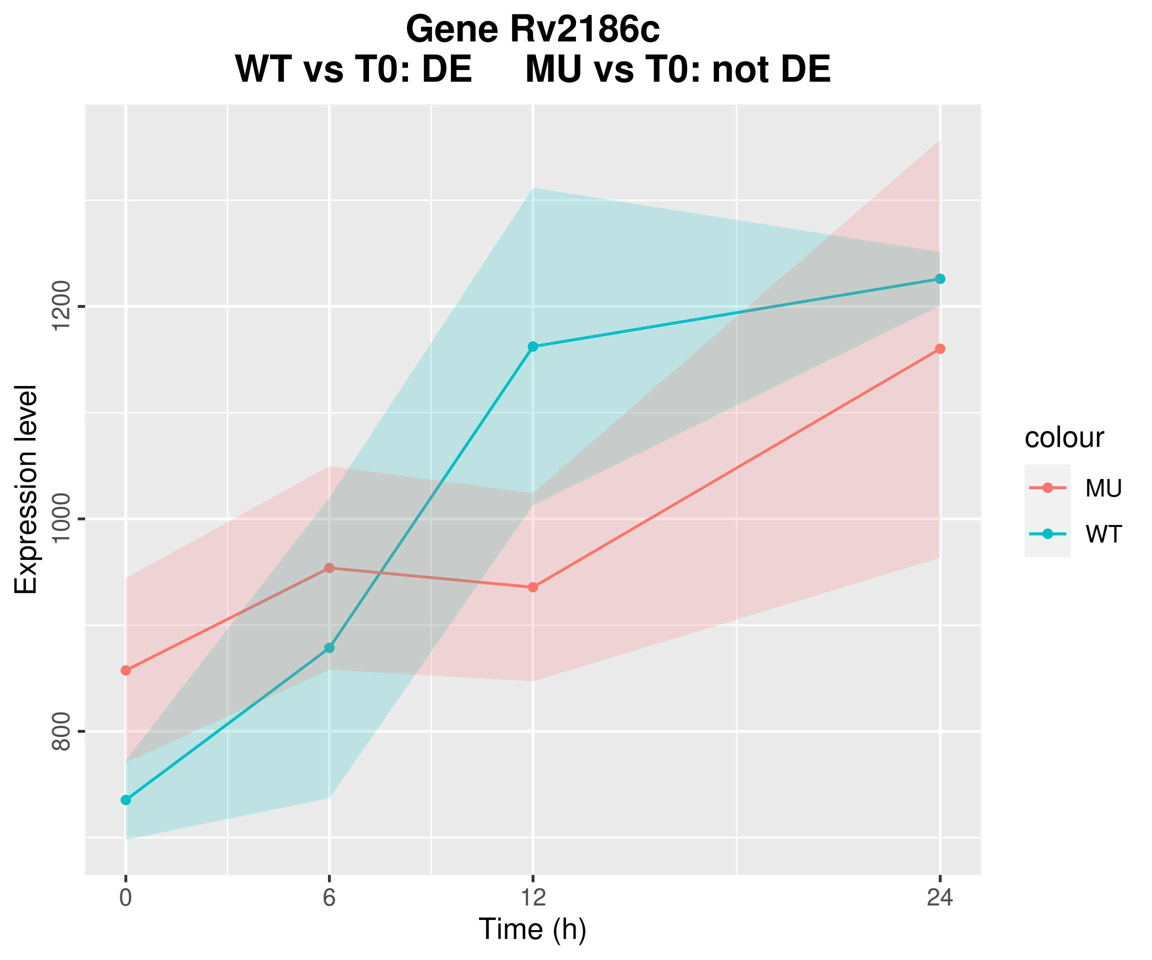
Gene Rv2186c
WT vs T0: DE   MU vs T0: not DE
Expression level
1200
1000
800
0
6
12
24
Time (h)
colour
MU
WT

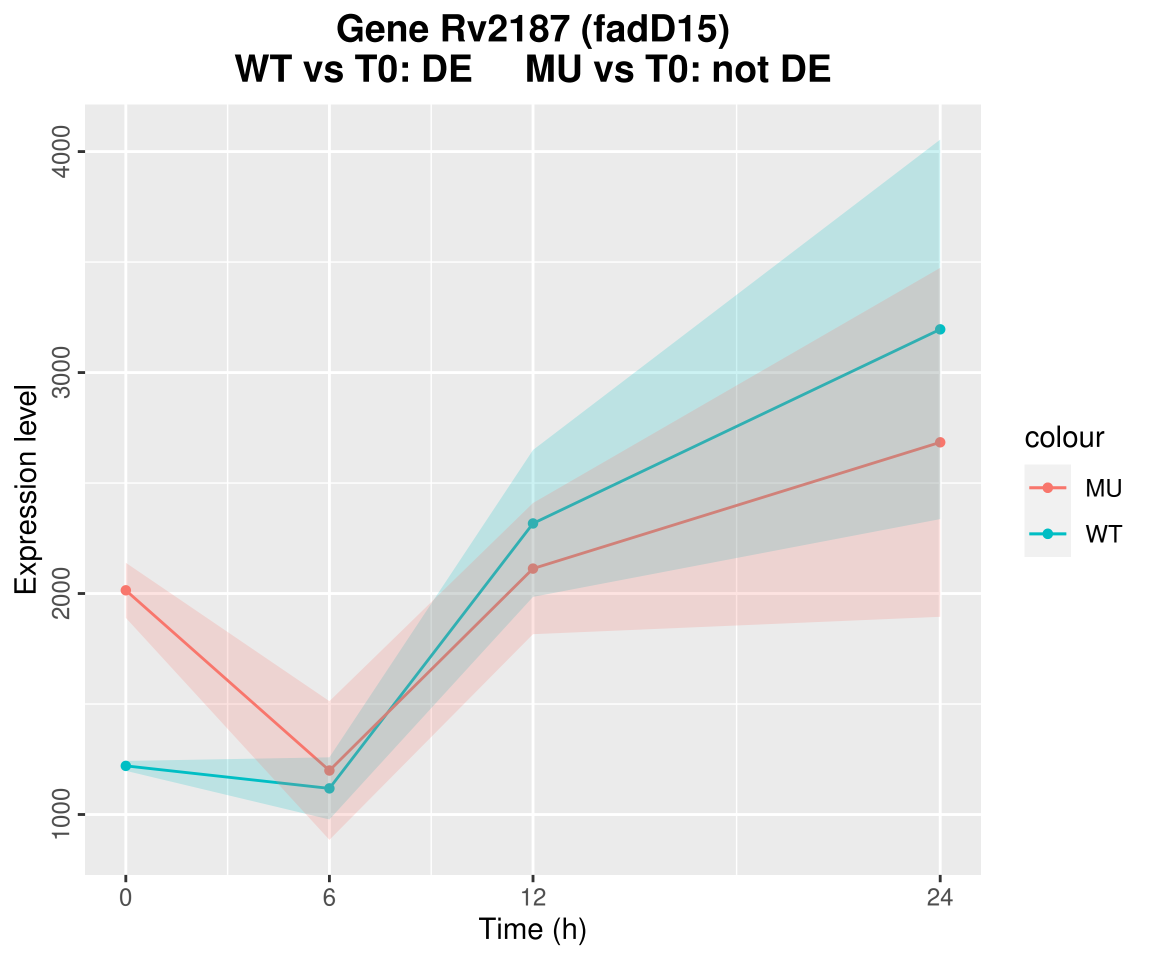
Gene Rv2187 (fadD15)
WT vs T0: DE   MU vs T0: not DE
Expression level
4000
3000
2000
1000
0
6
12
24
Time (h)
colour
MU
WT

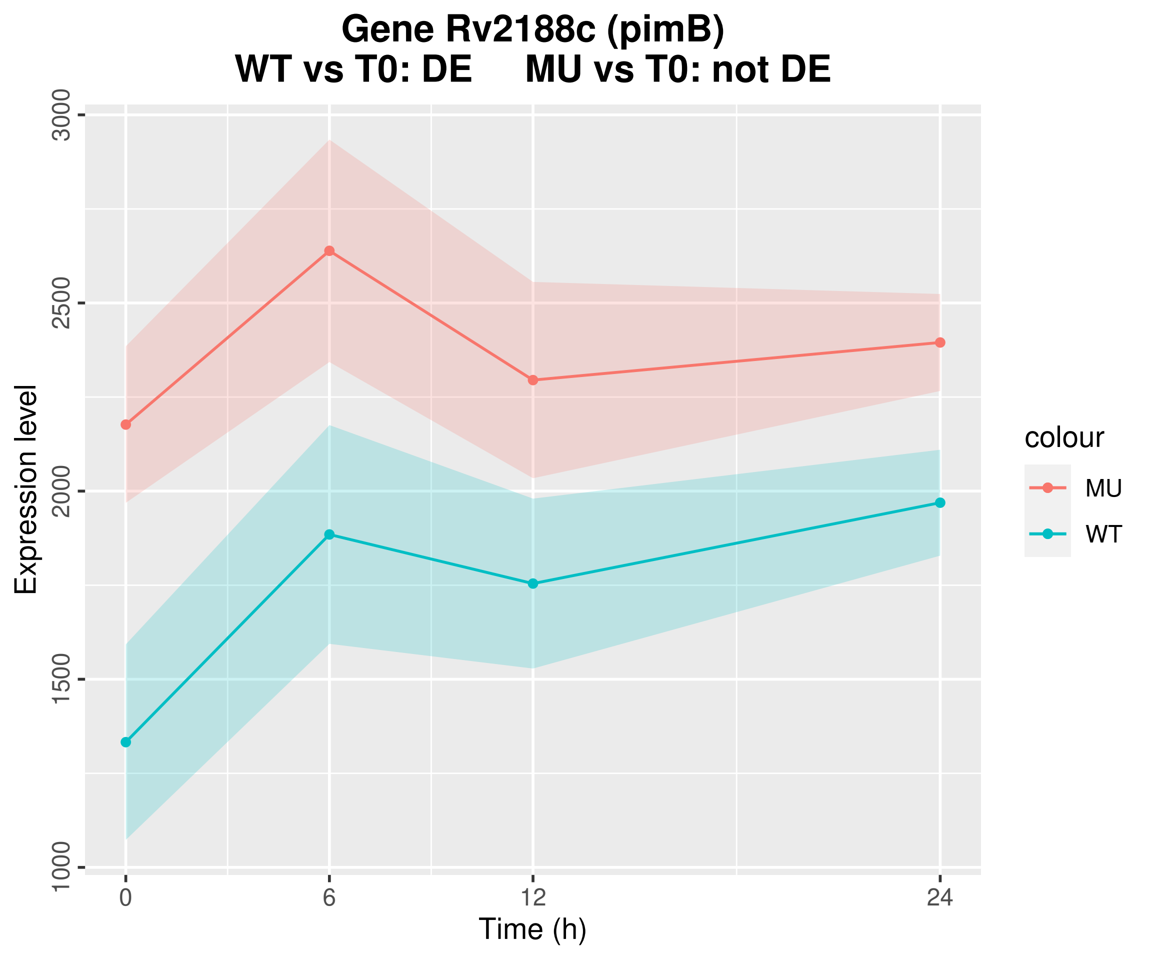
Gene Rv2188c (pimB)
WT vs T0: DE MU vs T0: not DE
Expression level
3000
2500
2000
1500
1000
0
6
12
24
Time (h)
colour
MU
WT

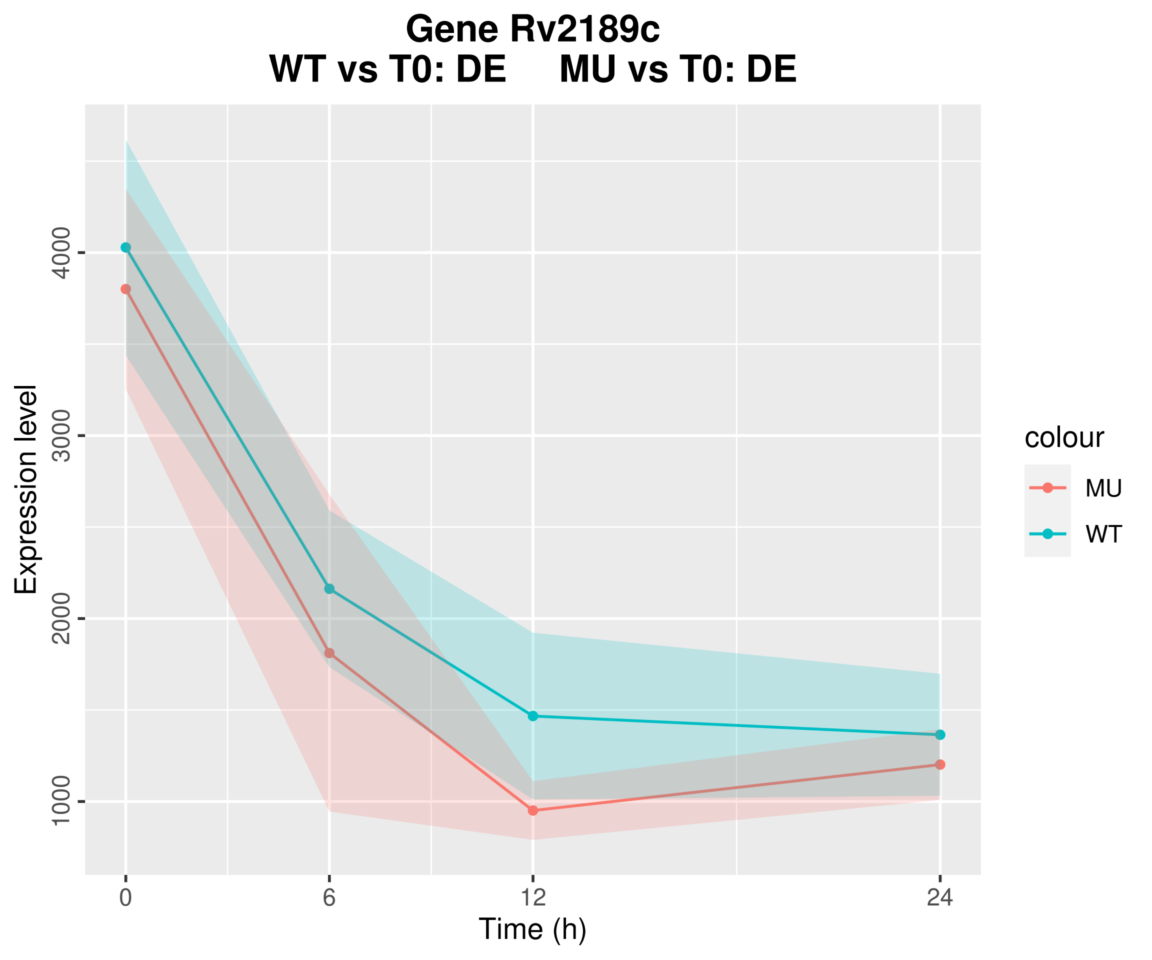

Gene Rv2189c
WT vs T0: DE MU vs T0: DE
Expression level
4000
3000
2000
1000
0
6
12
24
Time (h)
colour
MU
WT

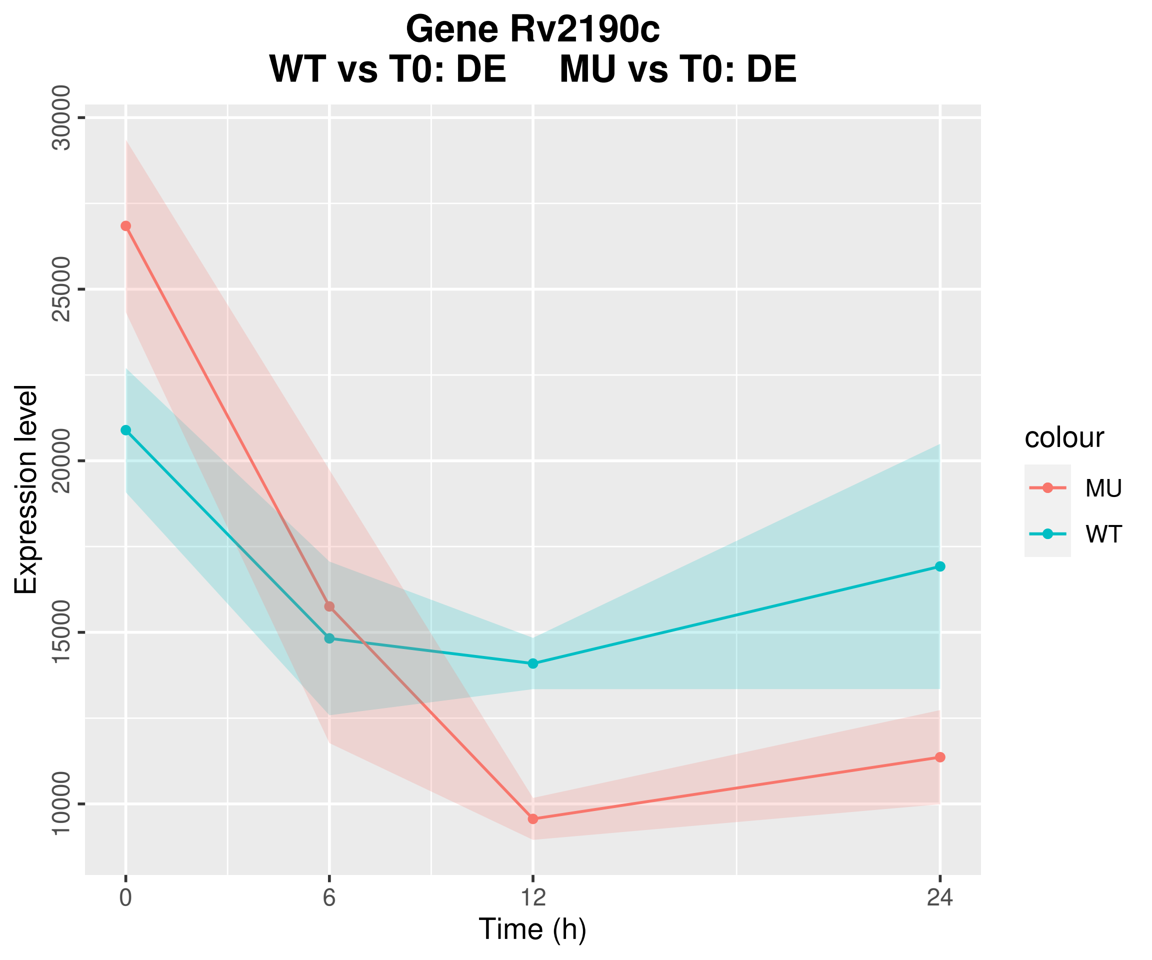
Gene Rv2190c
WT vs T0: DE   MU vs T0: DE
Expression level
30000
25000
20000
15000
10000
0
6
12
24
Time (h)
colour
MU
WT

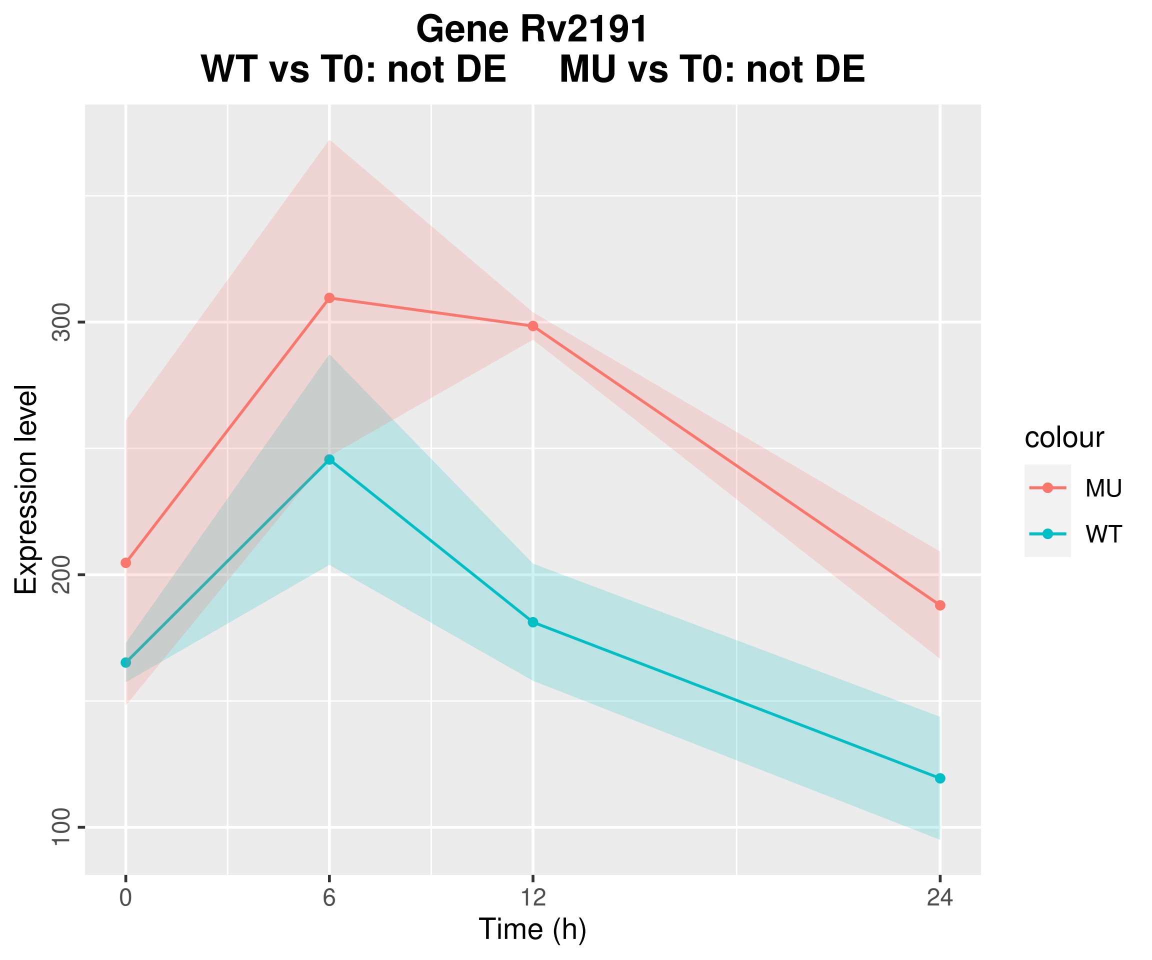
Gene Rv2191
WT vs T0: not DE MU vs T0: not DE
Expression level
300
200
100
0
6
12
24
Time (h)
colour
MU
WT

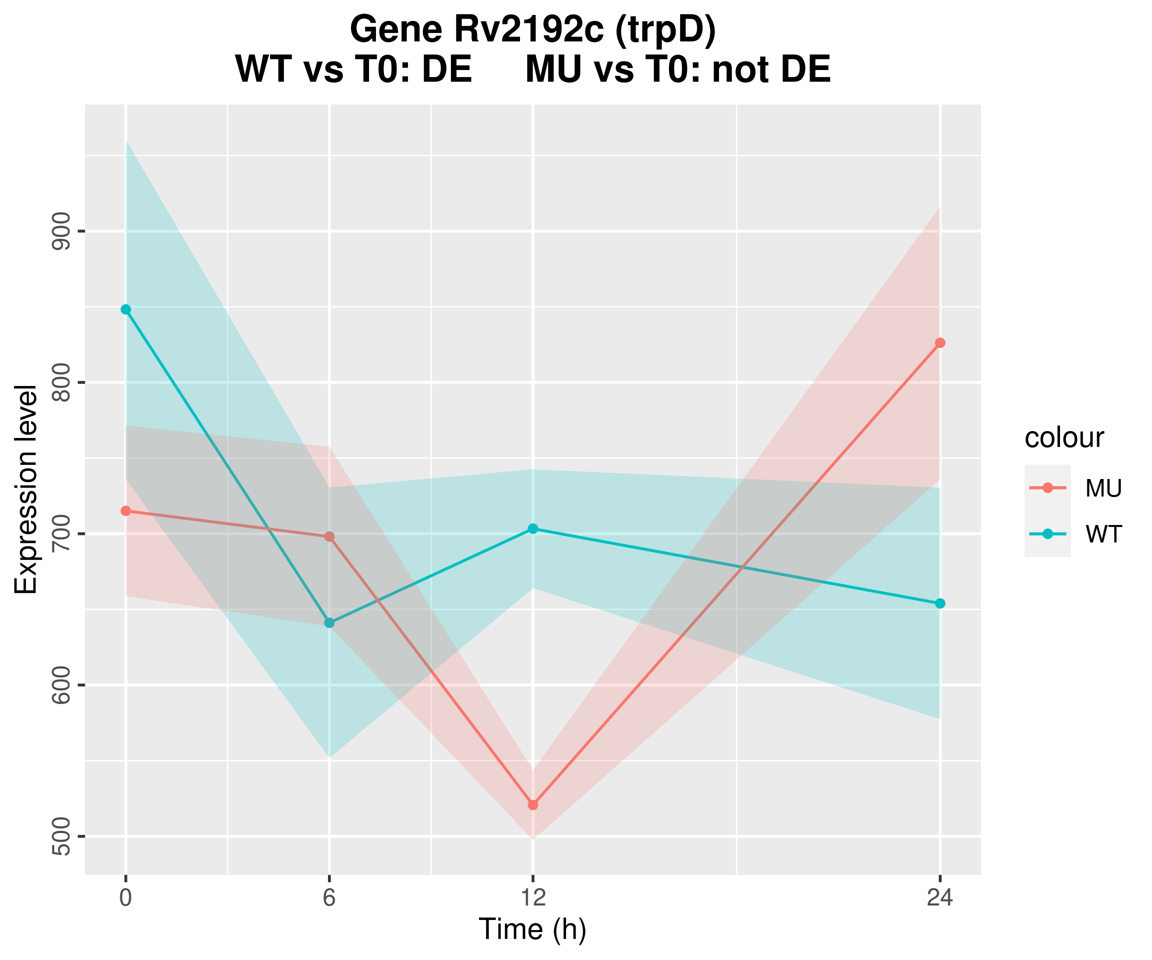
Gene Rv2192c (trpD)
WT vs T0: DE   MU vs T0: not DE
Expression level
500
600
700
800
900
0
6
12
24
Time (h)
colour
MU
WT

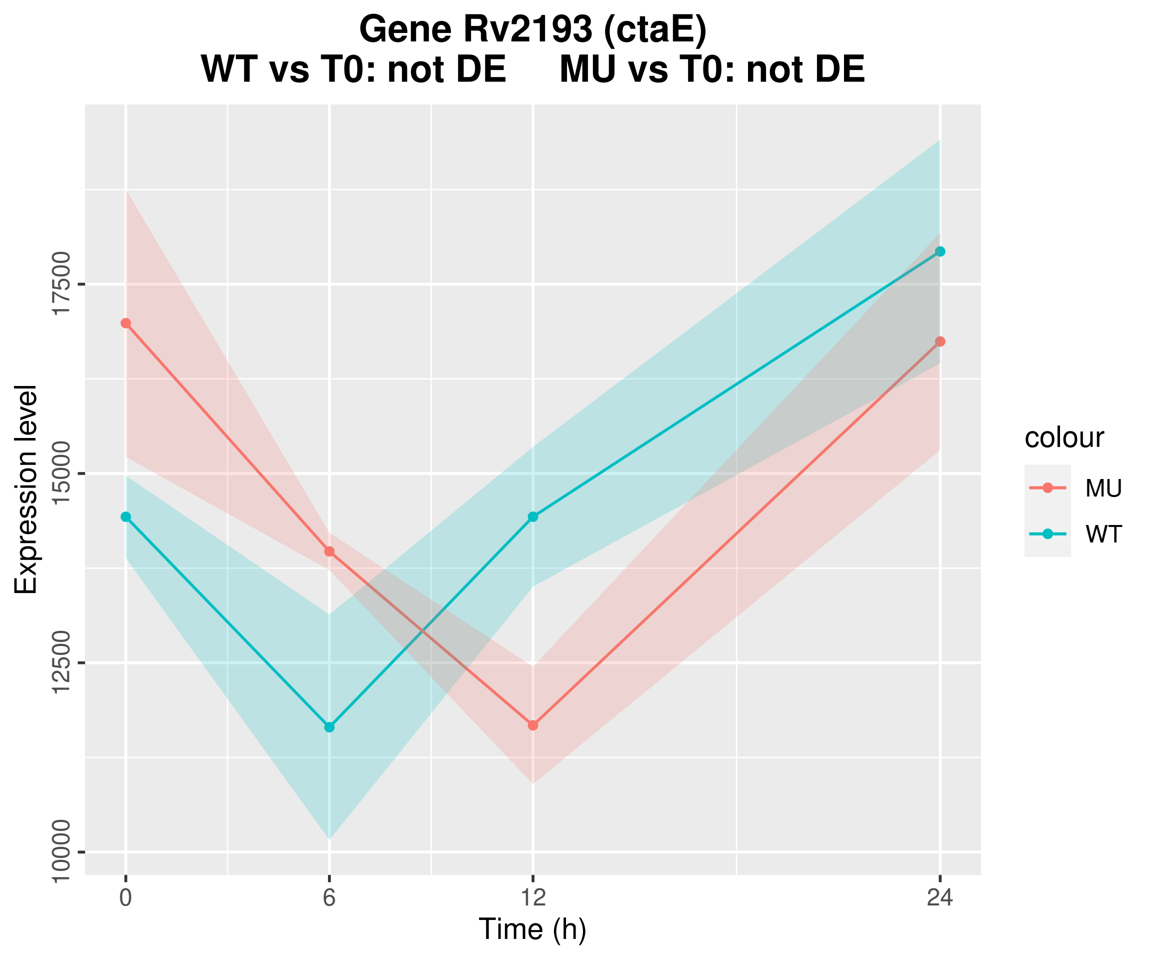
Gene Rv2193 (ctaE)
WT vs T0: not DE   MU vs T0: not DE
Expression level
17500
15000
12500
10000
0
6
12
24
Time (h)
colour
MU
WT

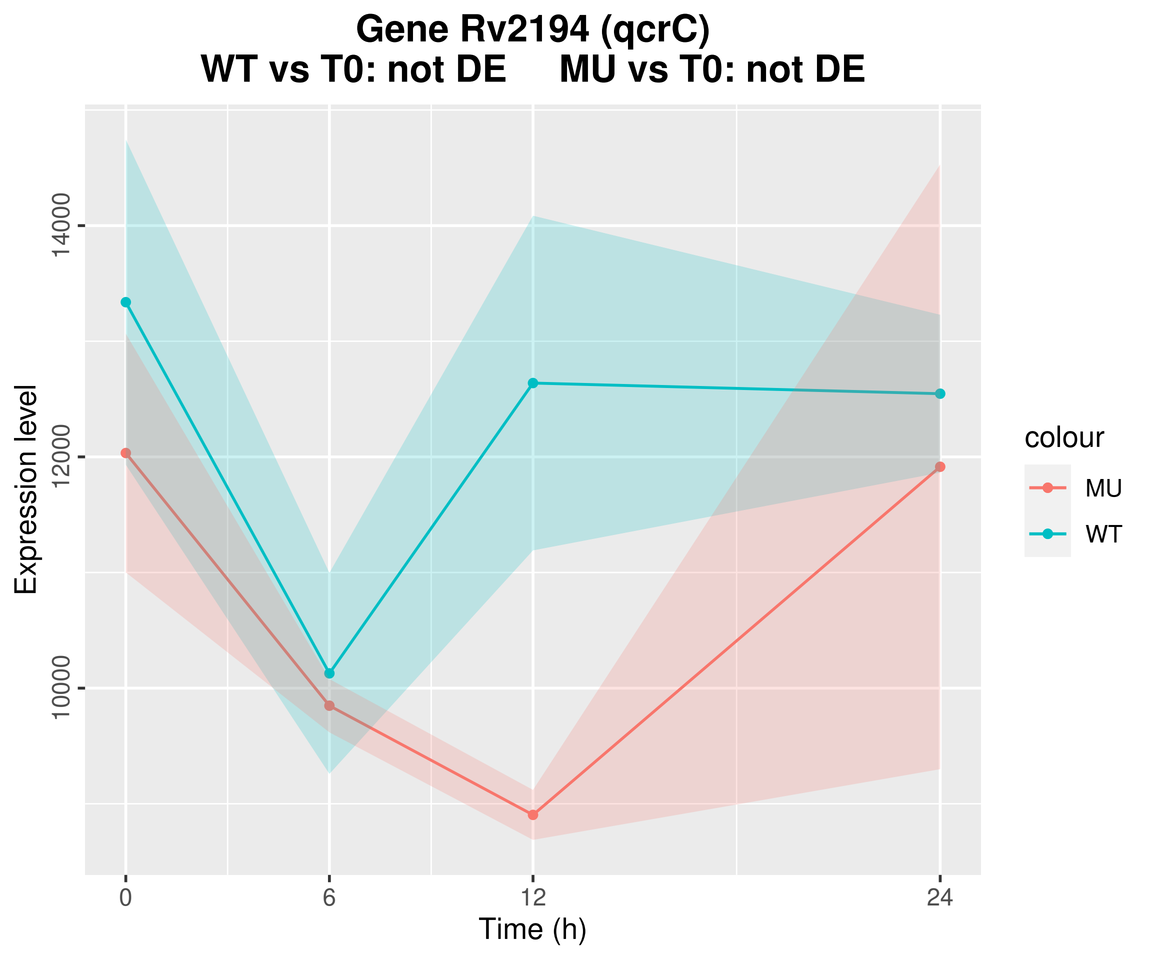
Gene Rv2194 (qcrC)
WT vs T0: not DE   MU vs T0: not DE
Expression level
14000
12000
10000
0
6
12
24
Time (h)
colour
MU
WT

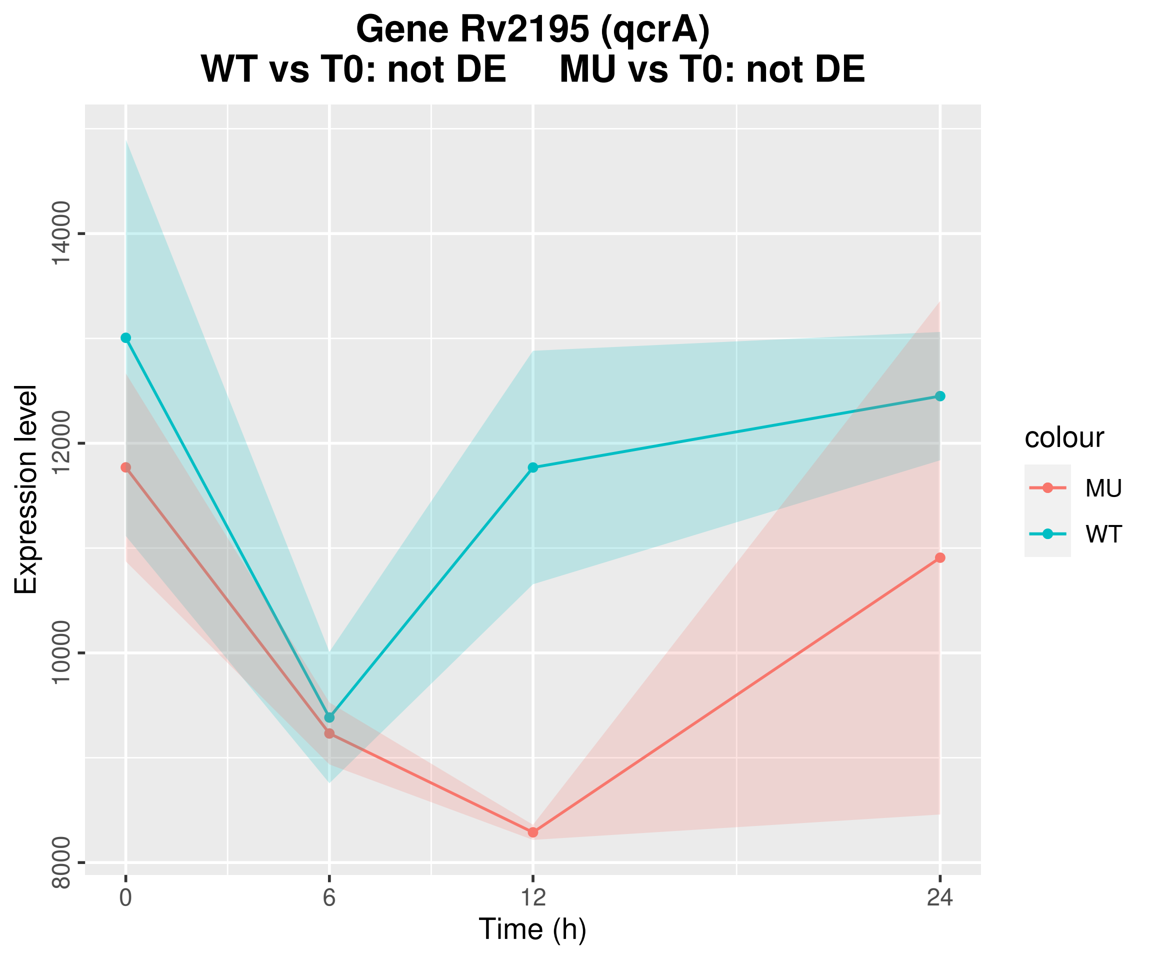
Gene Rv2195 (qcrA)
WT vs T0: not DE MU vs T0: not DE
Expression level
14000
12000
10000
8000
0
6
12
24
Time (h)
colour
MU
WT

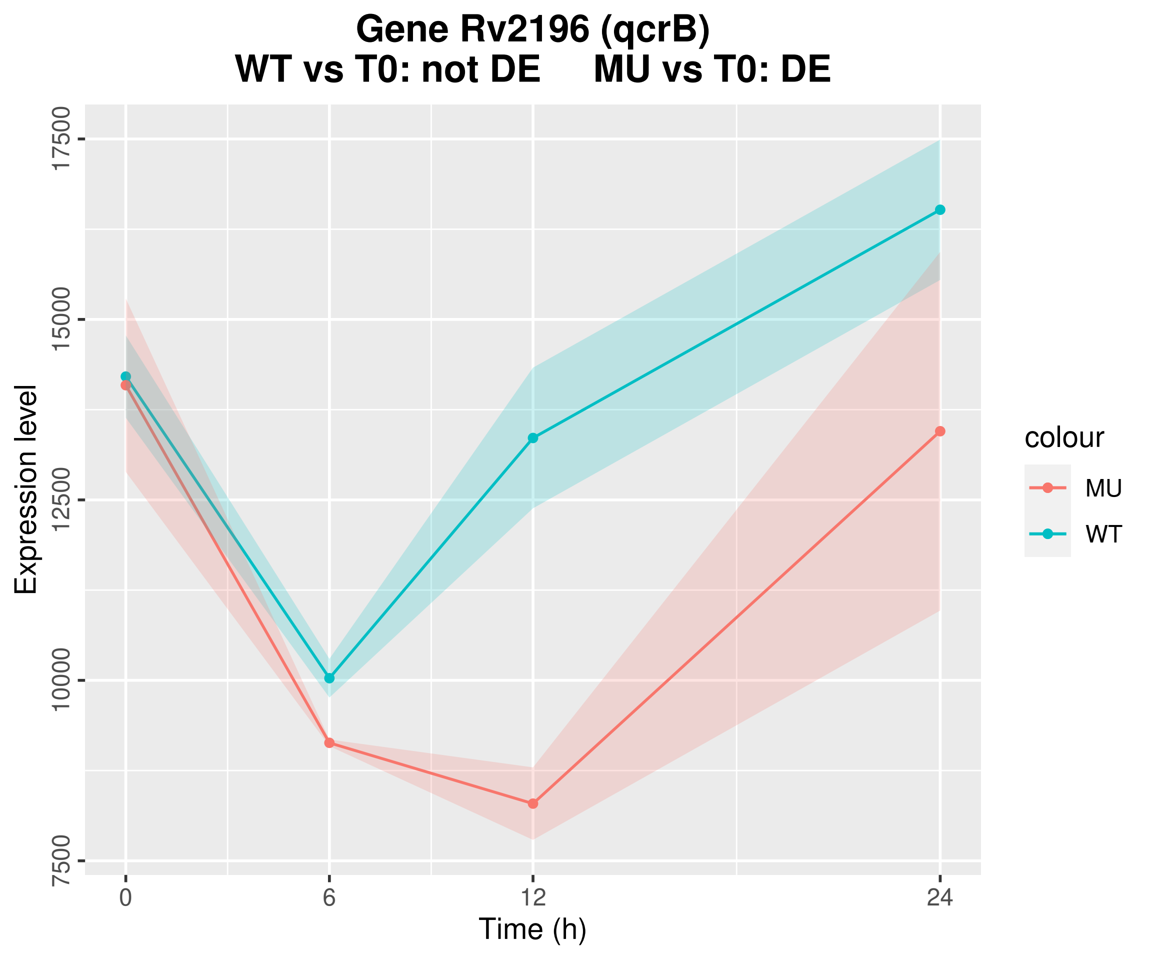

Gene Rv2196 (qcrB)
WT vs T0: not DE   MU vs T0: DE
Expression level
17500
15000
12500
10000
7500
0
6
12
24
Time (h)
colour
MU
WT

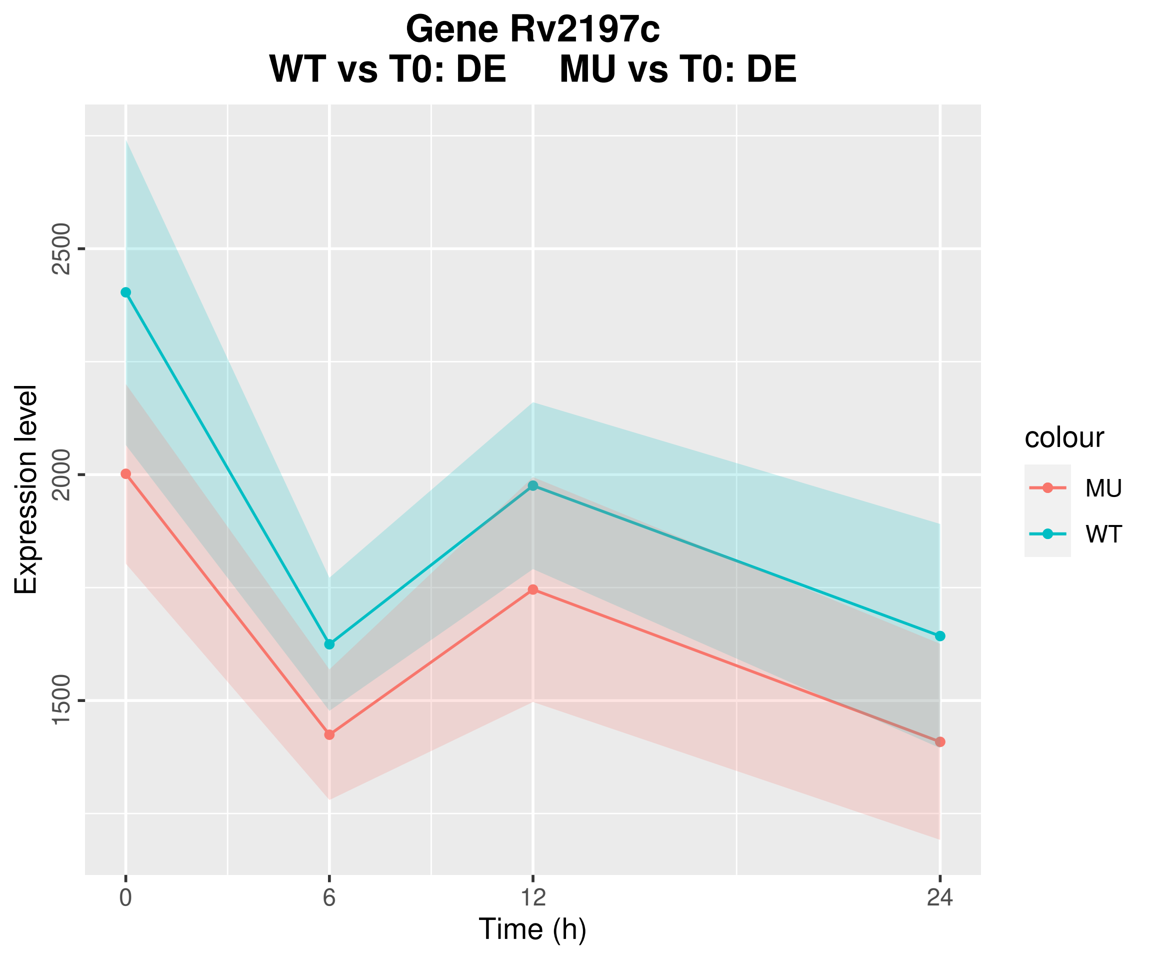
Gene Rv2197c
WT vs T0: DE MU vs T0: DE
Expression level
2500
2000
1500
0
6
12
24
Time (h)
colour
MU
WT

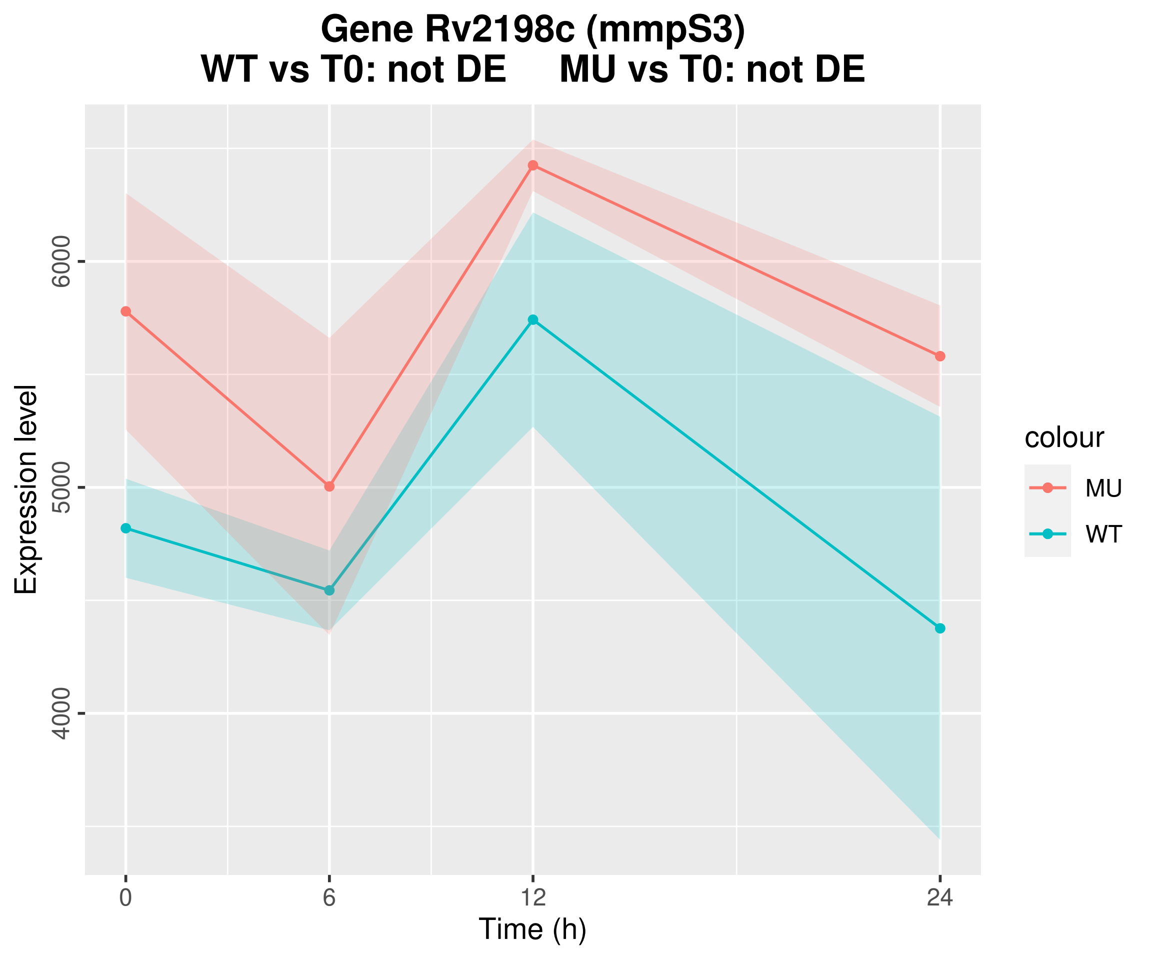
Gene Rv2198c (mmpS3)
WT vs T0: not DE   MU vs T0: not DE
Expression level
6000
5000
4000
0
6
12
24
Time (h)
colour
MU
WT

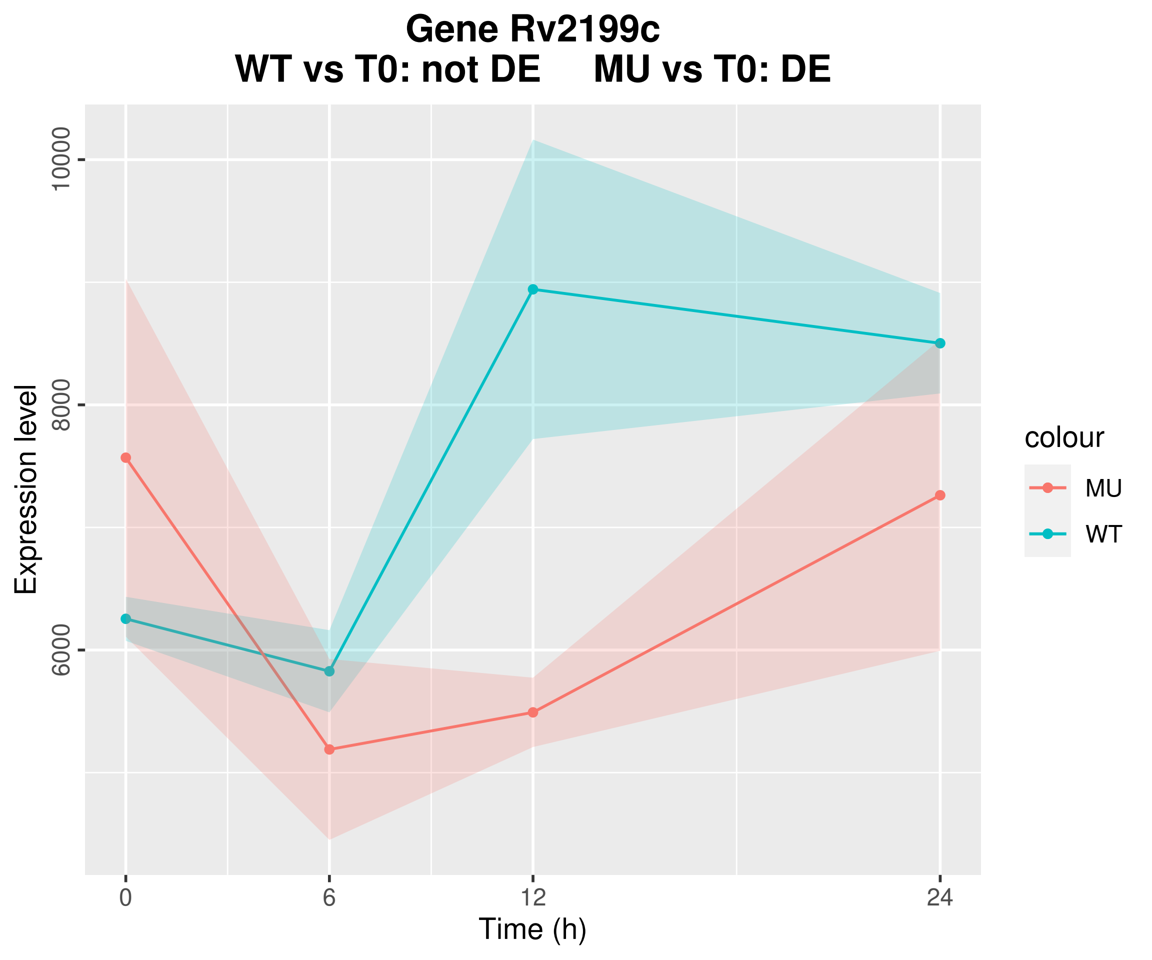
Gene Rv2199c
WT vs T0: not DE   MU vs T0: DE
Expression level
10000
8000
6000
0
6
12
24
Time (h)
colour
MU
WT

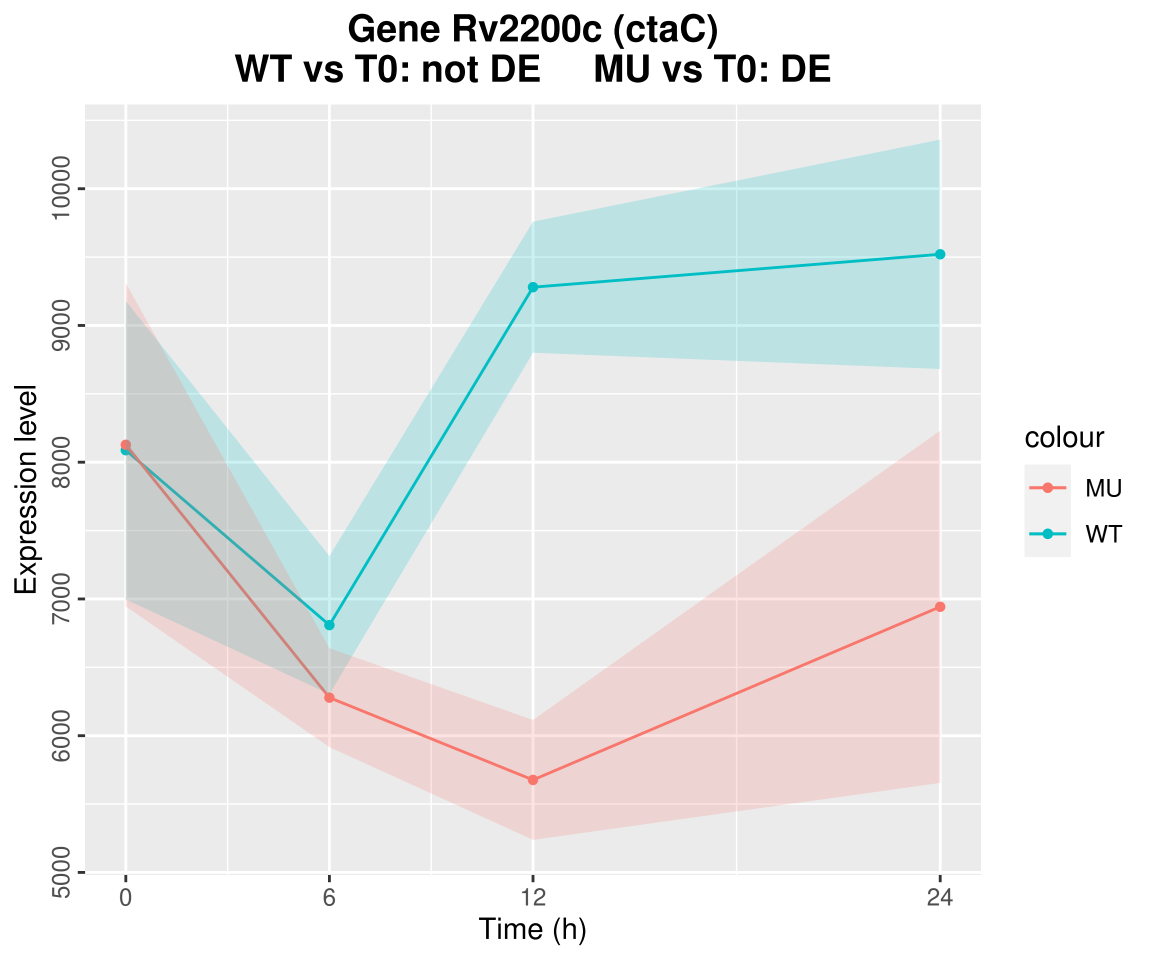

Gene Rv2200c (ctaC)
WT vs T0: not DE MU vs T0: DE
Expression level
10000
9000
8000
7000
6000
5000
0
6
12
24
Time (h)
colour
MU
WT

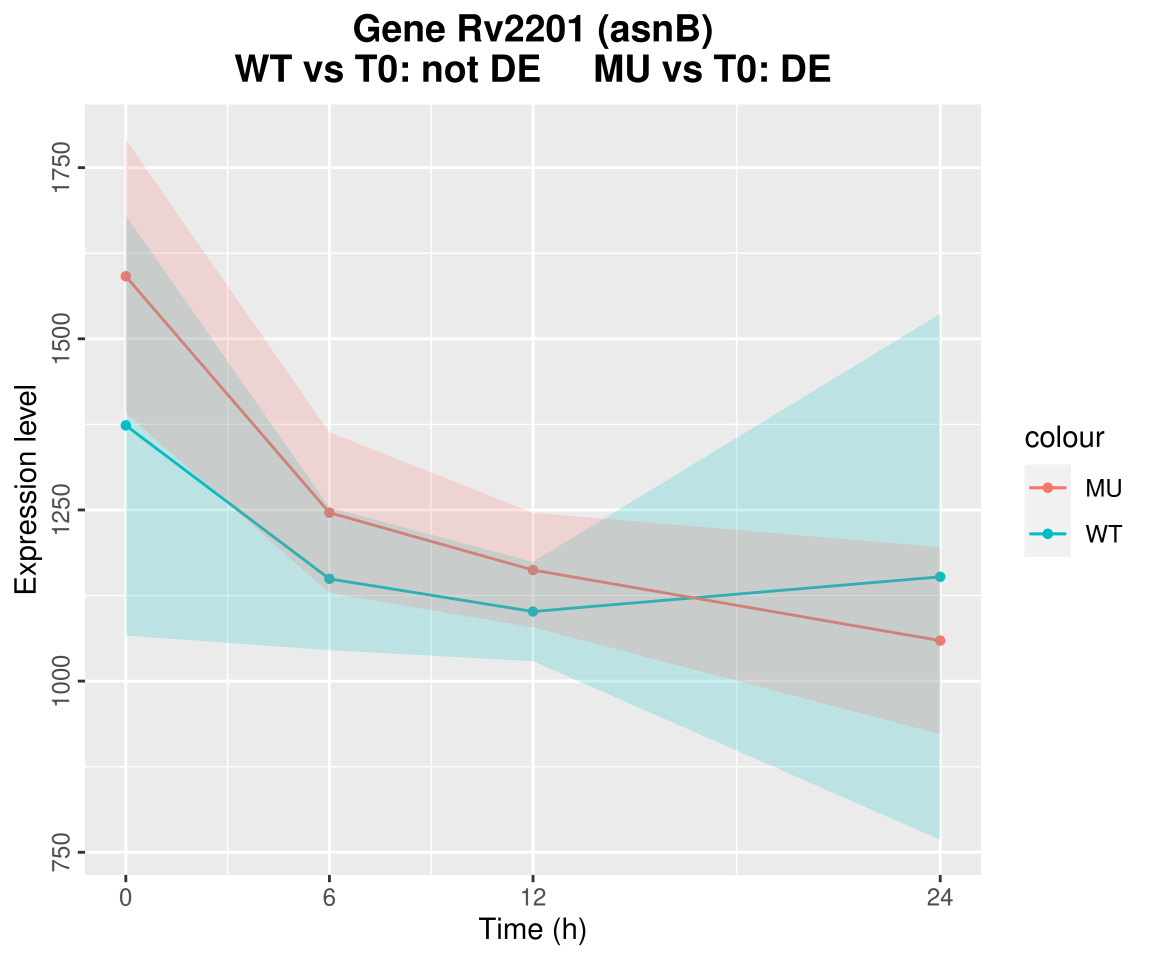

Gene Rv2201 (asnB)
WT vs T0: not DE   MU vs T0: DE
Expression level
1750
1500
1250
1000
750
0
6
12
24
Time (h)
colour
MU
WT

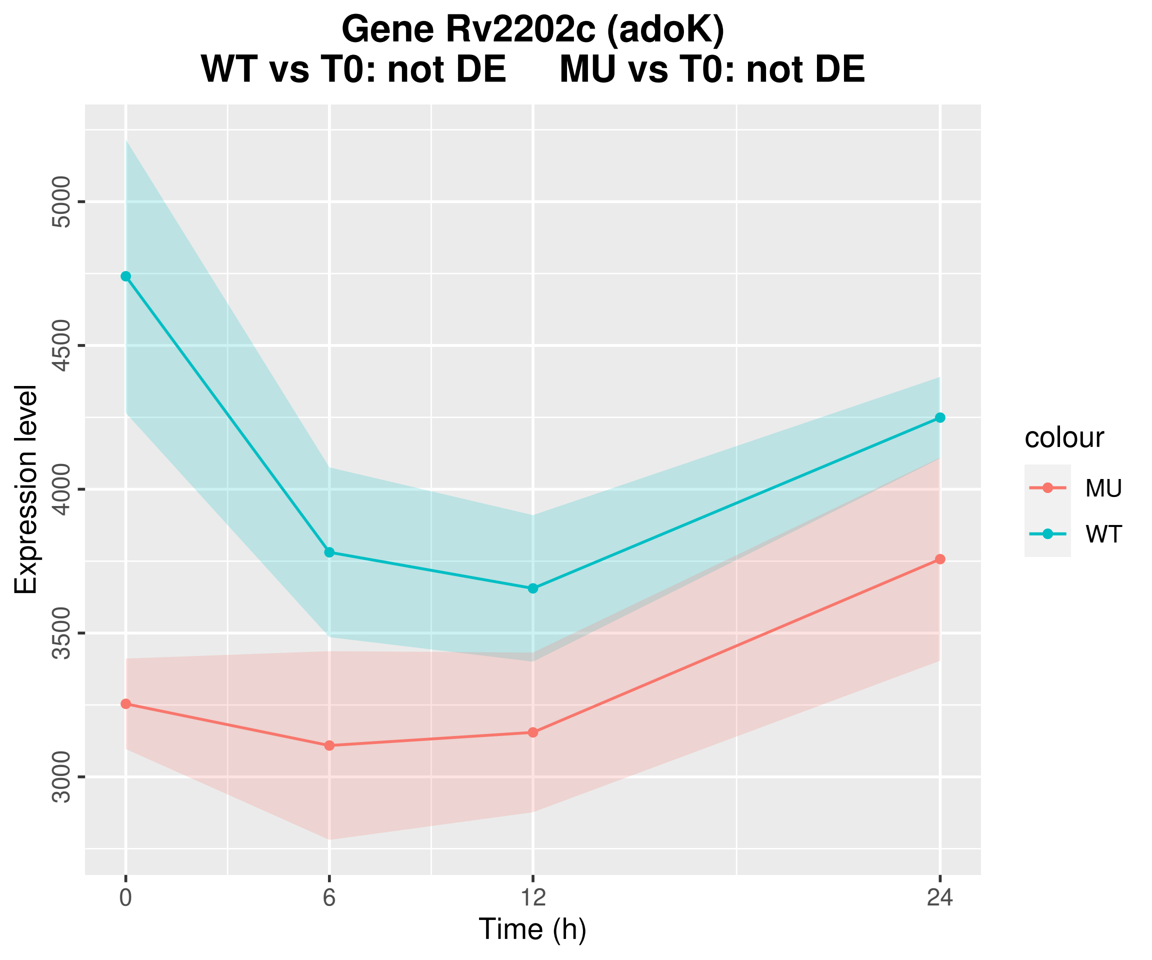
Gene Rv2202c (adoK)
WT vs T0: not DE   MU vs T0: not DE
Expression level
5000
4500
4000
3500
3000
0
6
12
24
Time (h)
colour
MU
WT

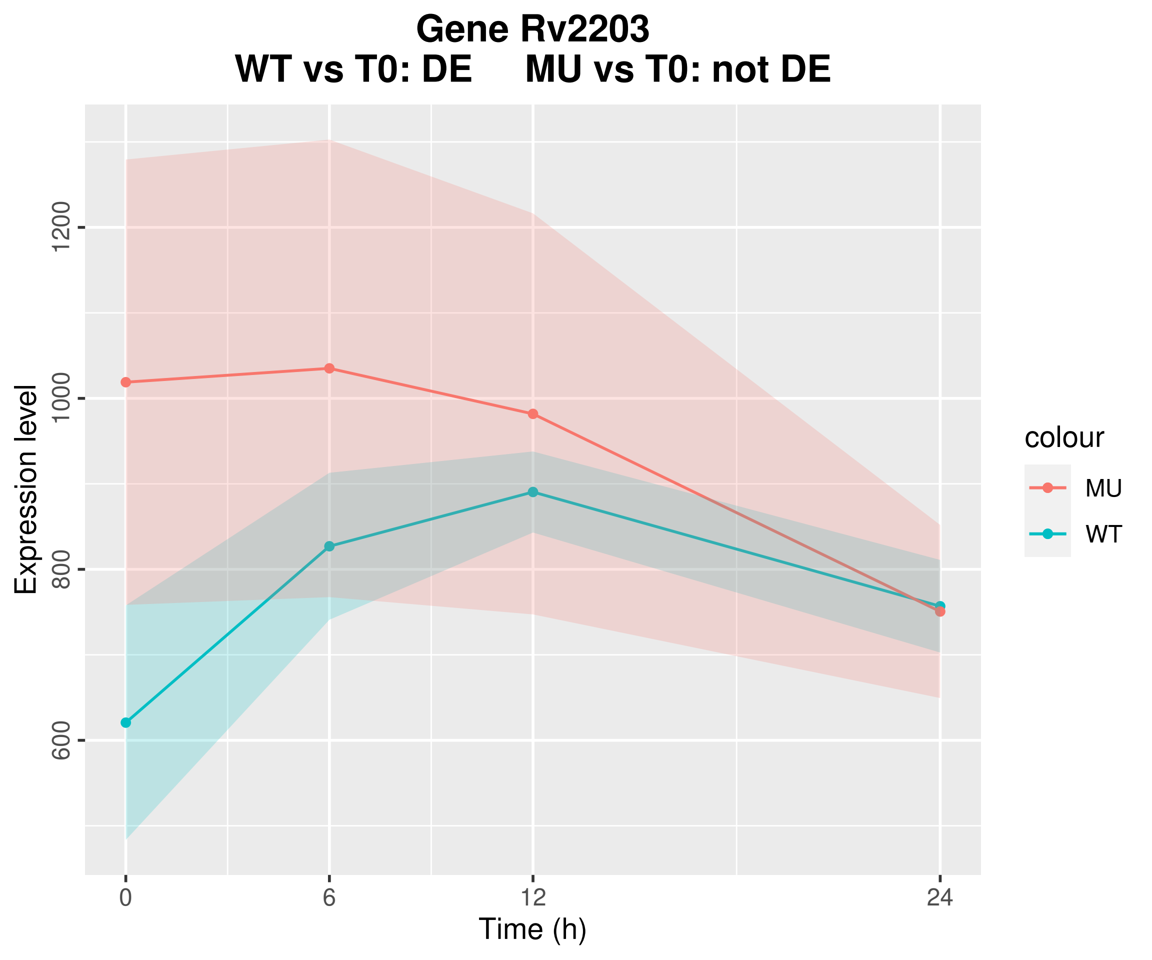

Gene Rv2203
WT vs T0: DE MU vs T0: not DE
Expression level
1200
1000
800
600
0
6
12
24
Time (h)
colour
MU
WT

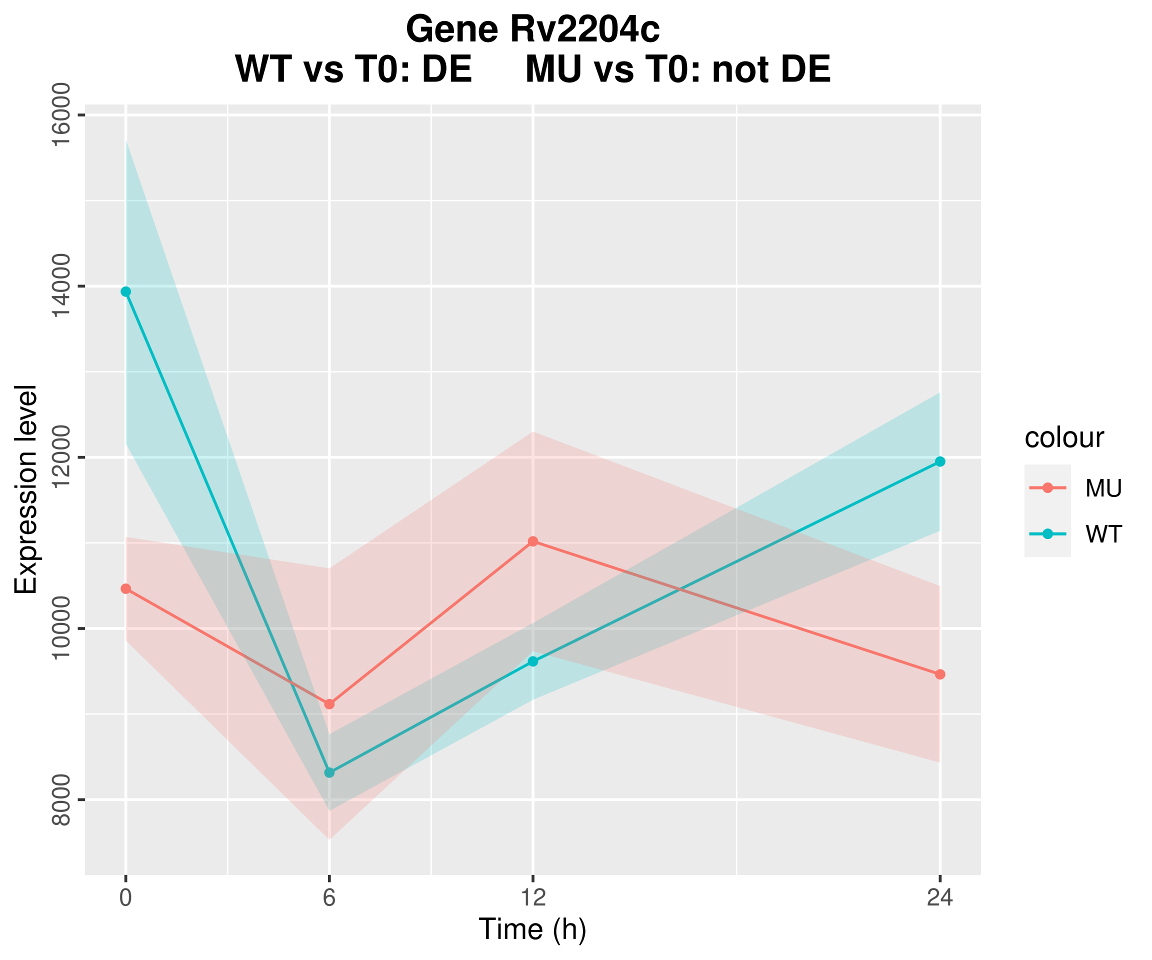
Gene Rv2204c
WT vs T0: DE   MU vs T0: not DE
Expression level
16000
14000
12000
10000
8000
0
6
12
24
Time (h)
colour
MU
WT

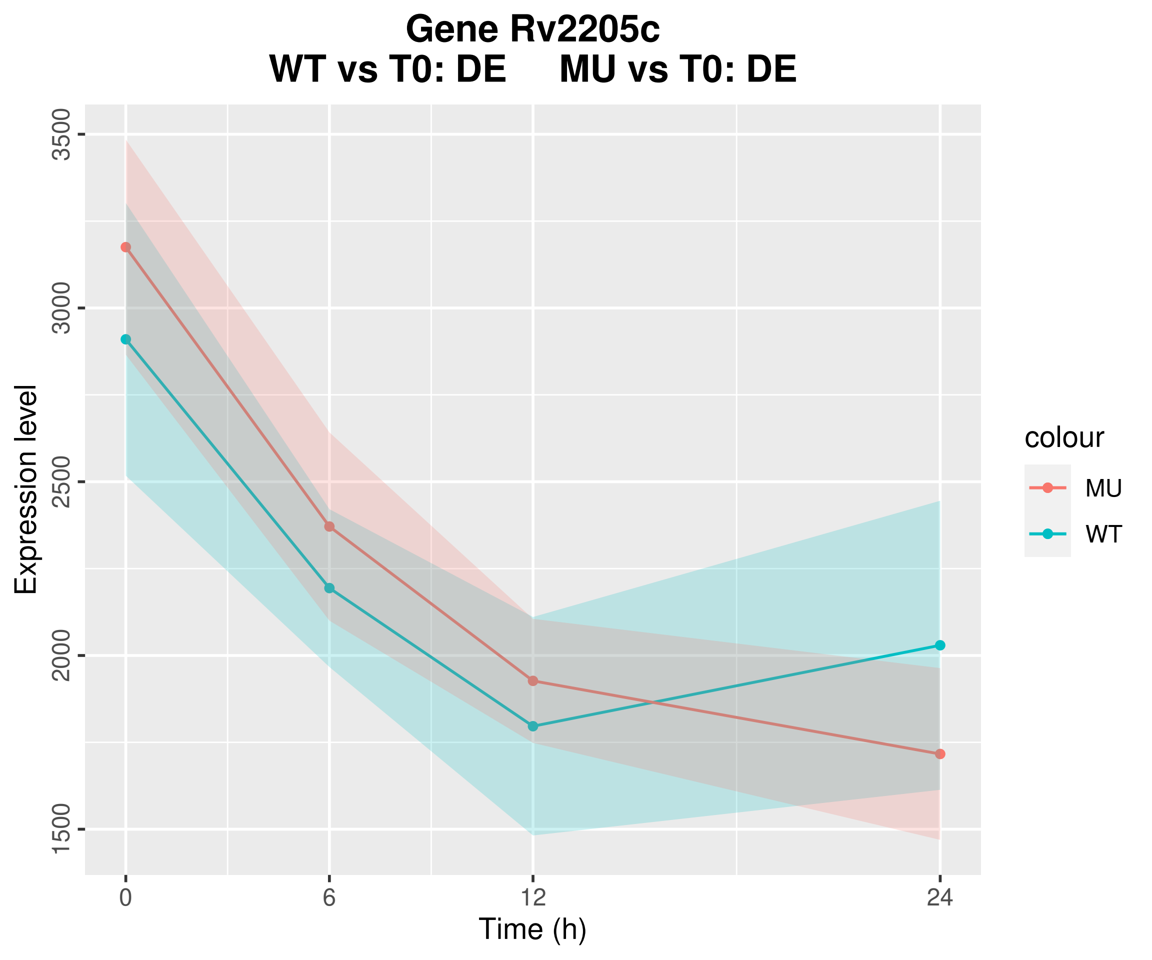

Gene Rv2205c
WT vs T0: DE MU vs T0: DE
Expression level
3500
3000
2500
2000
1500
0
6
12
24
Time (h)
colour
MU
WT

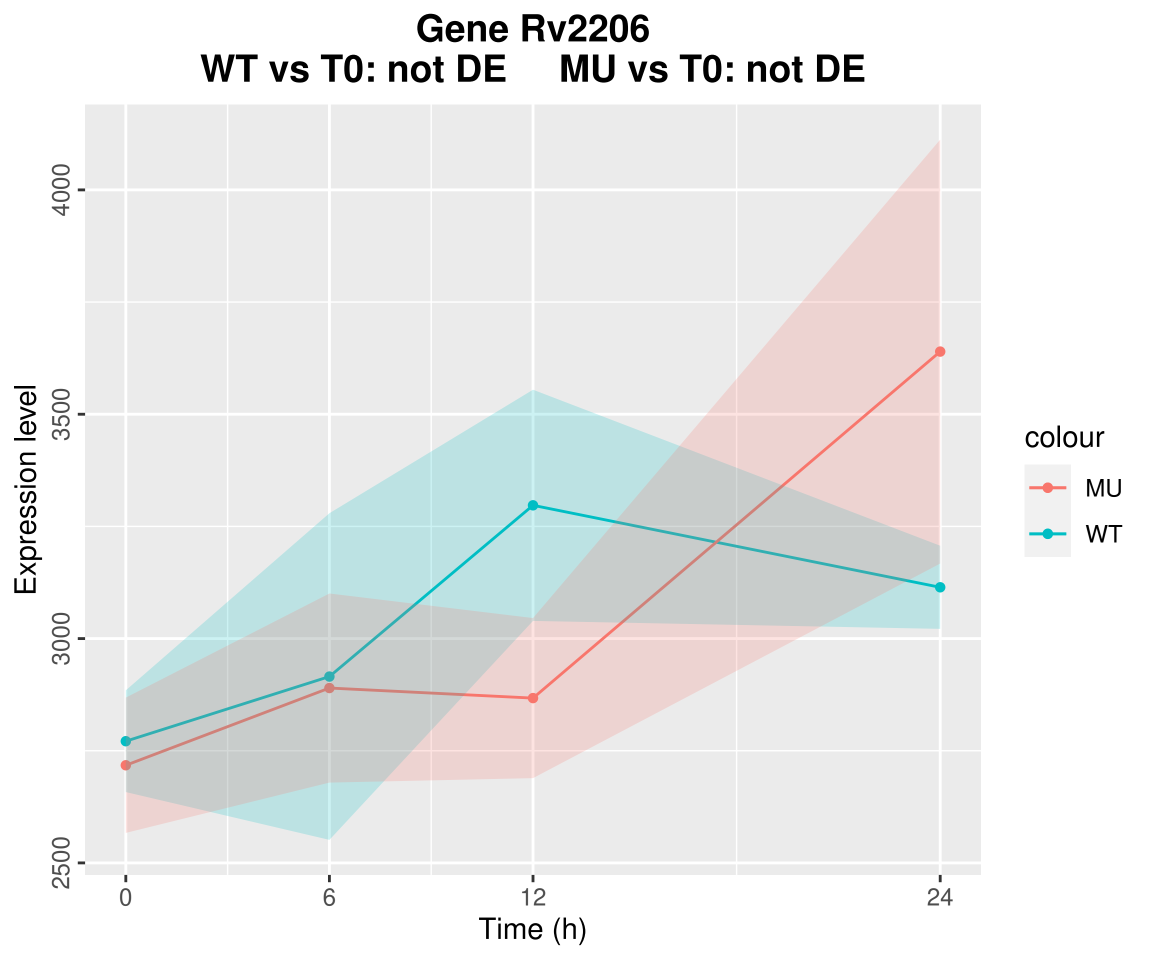

Gene Rv2206
WT vs T0: not DE   MU vs T0: not DE
Expression level
4000
3500
3000
2500
0
6
12
24
Time (h)
colour
MU
WT

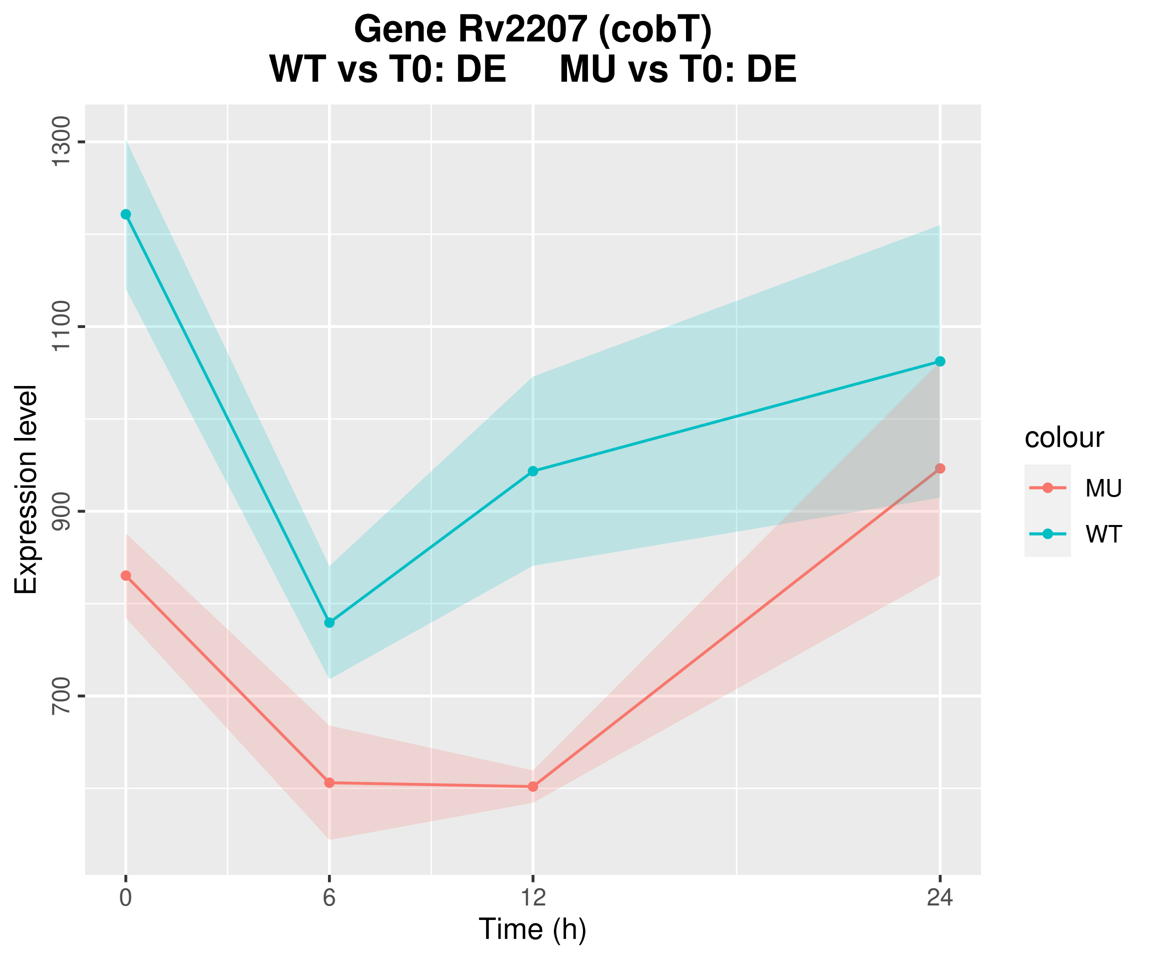

Gene Rv2207 (cobT)
WT vs T0: DE MU vs T0: DE
Expression level
1300
1100
900
700
0
6
12
24
Time (h)
colour
MU
WT

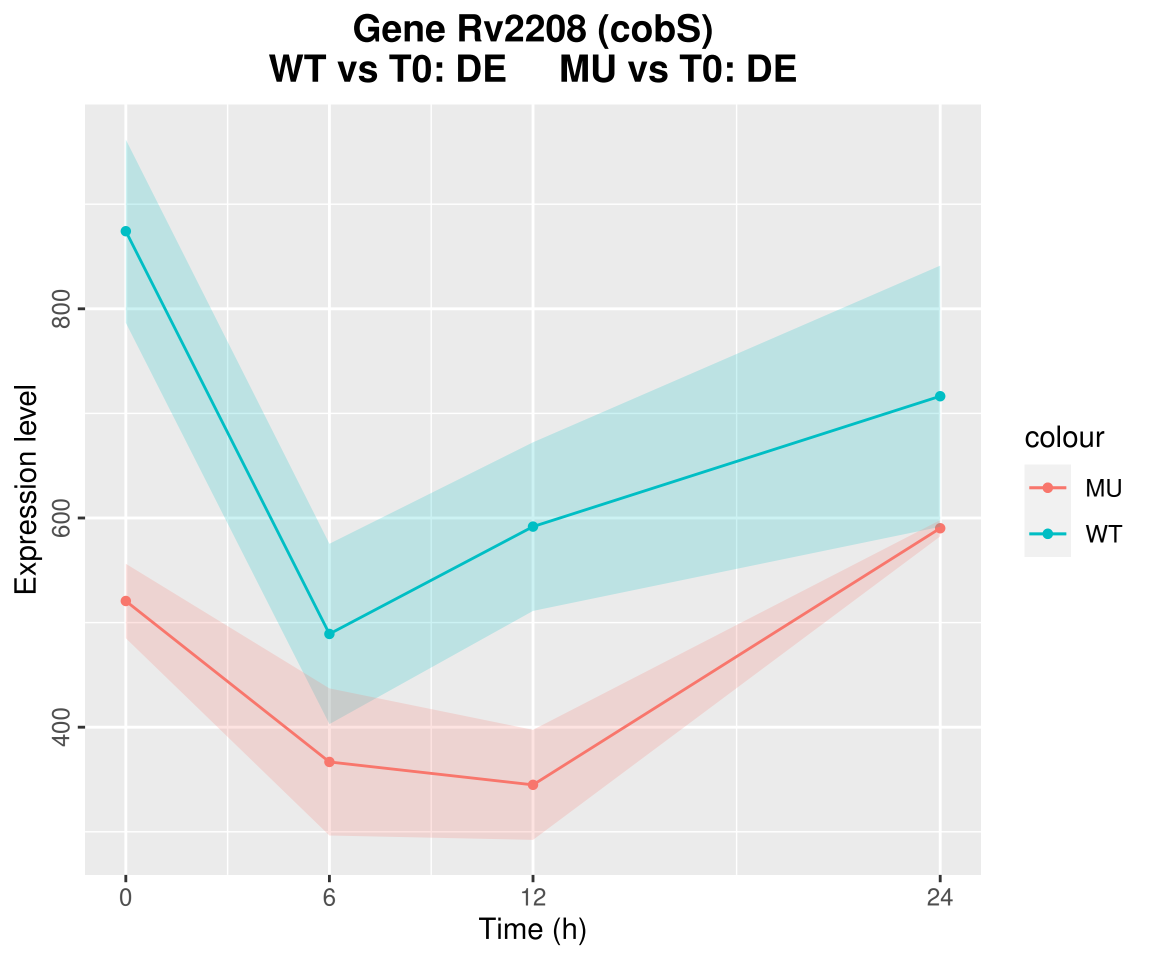
Gene Rv2208 (cobS)
WT vs T0: DE   MU vs T0: DE
Expression level
800
600
400
0
6
12
24
Time (h)
colour
MU
WT

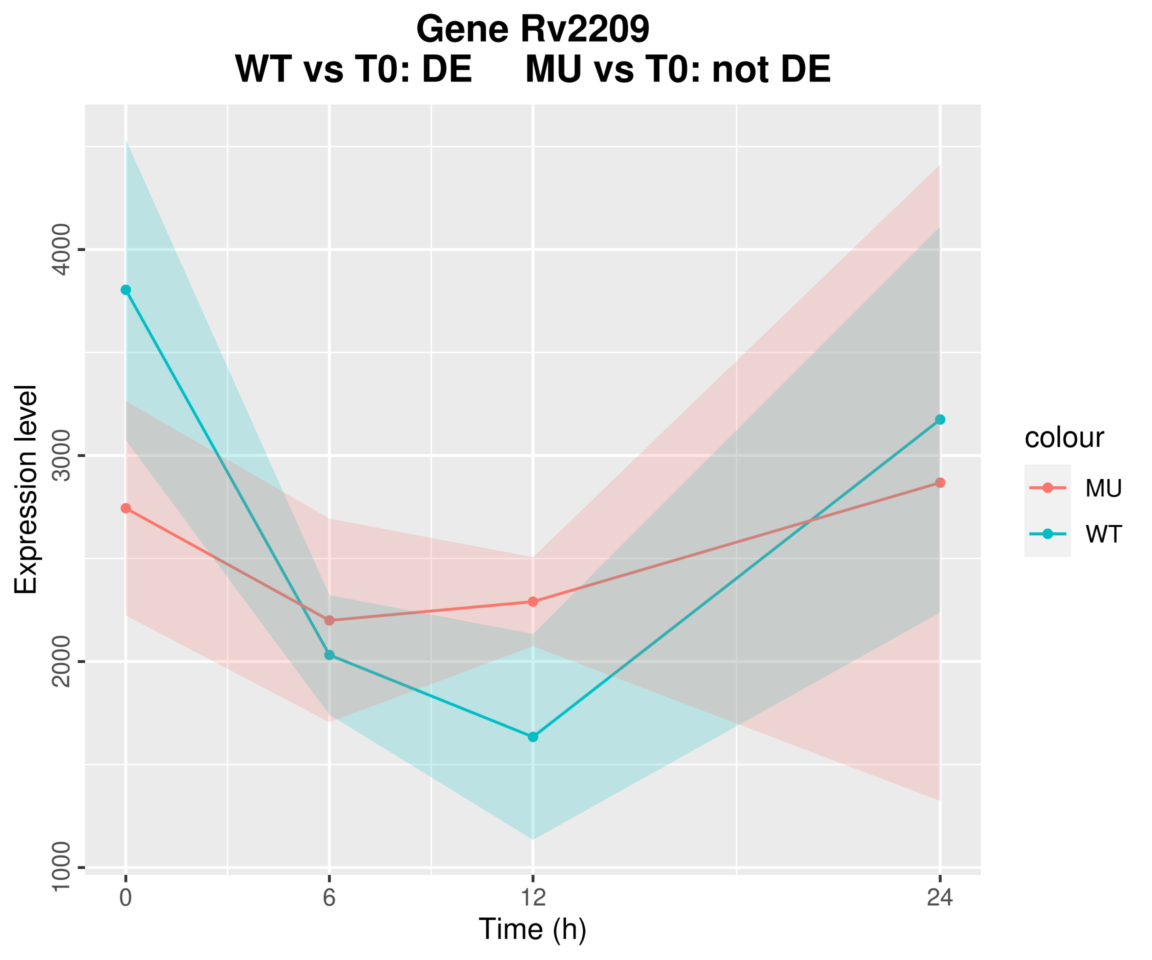

Gene Rv2209
WT vs T0: DE MU vs T0: not DE
Expression level
1000
2000
3000
4000
0
6
12
24
Time (h)
colour
MU
WT

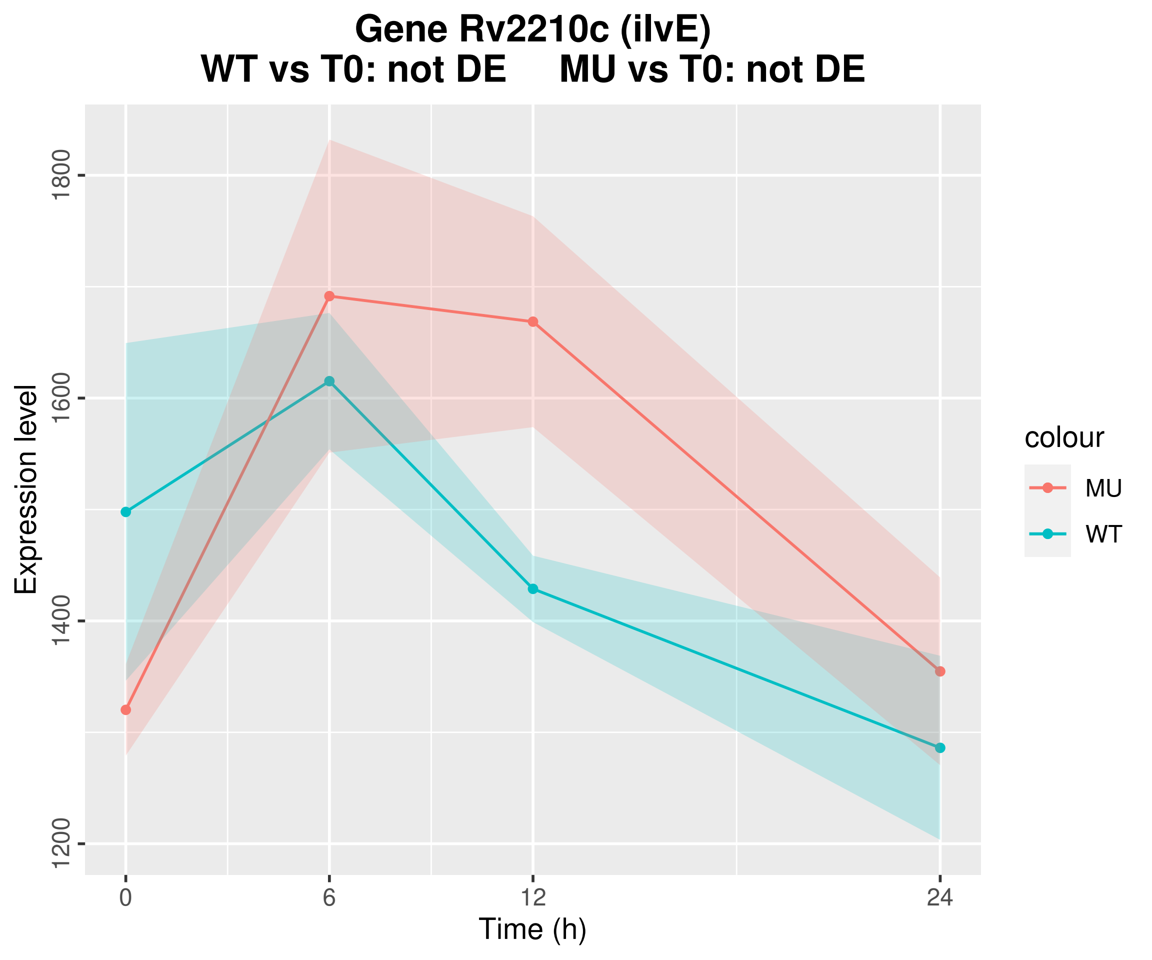
Gene Rv2210c (ilvE)
WT vs T0: not DE   MU vs T0: not DE
Expression level
1200
1400
1600
1800
0
6
12
24
Time (h)
colour
MU
WT

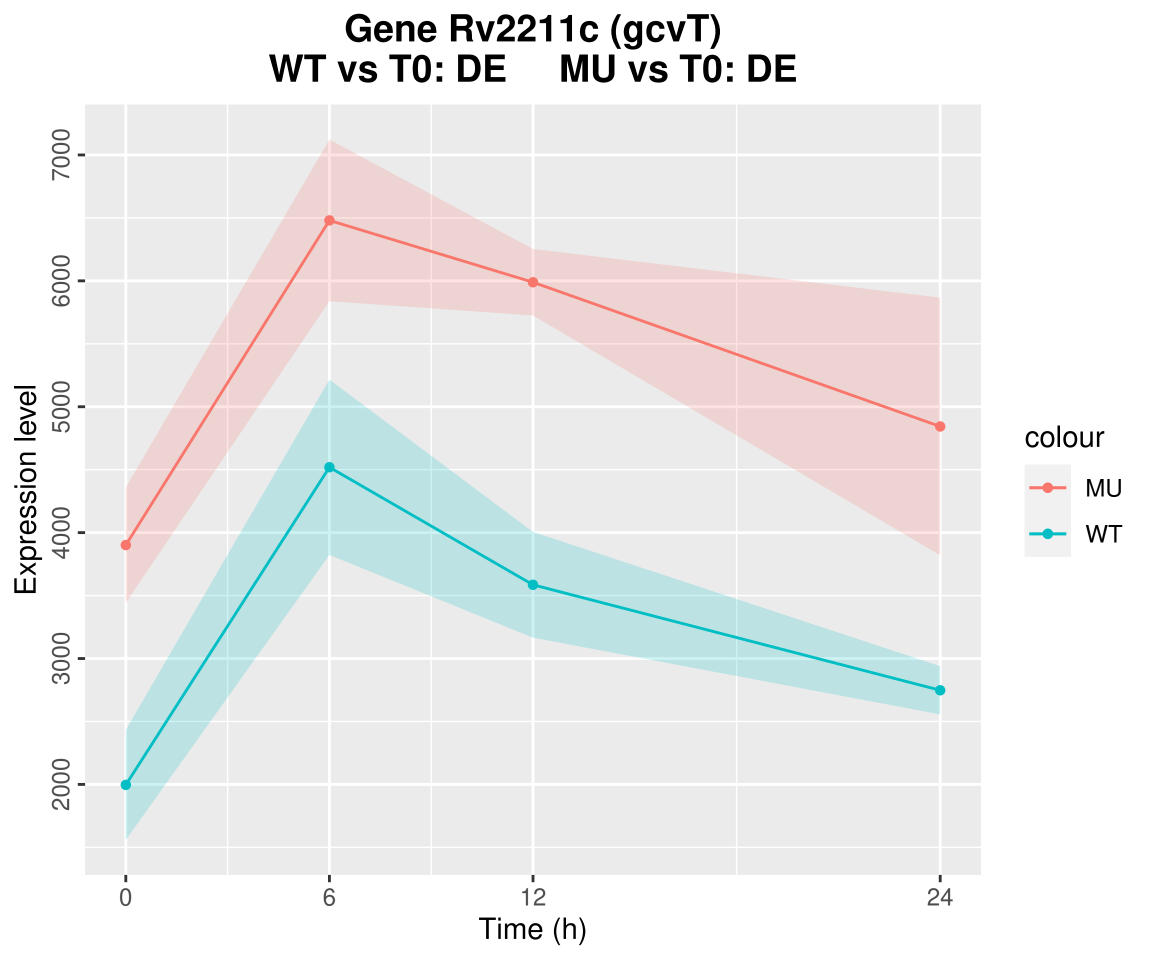
Gene Rv2211c (gcvT)
WT vs T0: DE   MU vs T0: DE
Expression level
7000
6000
5000
4000
3000
2000
0
6
12
24
Time (h)
colour
MU
WT

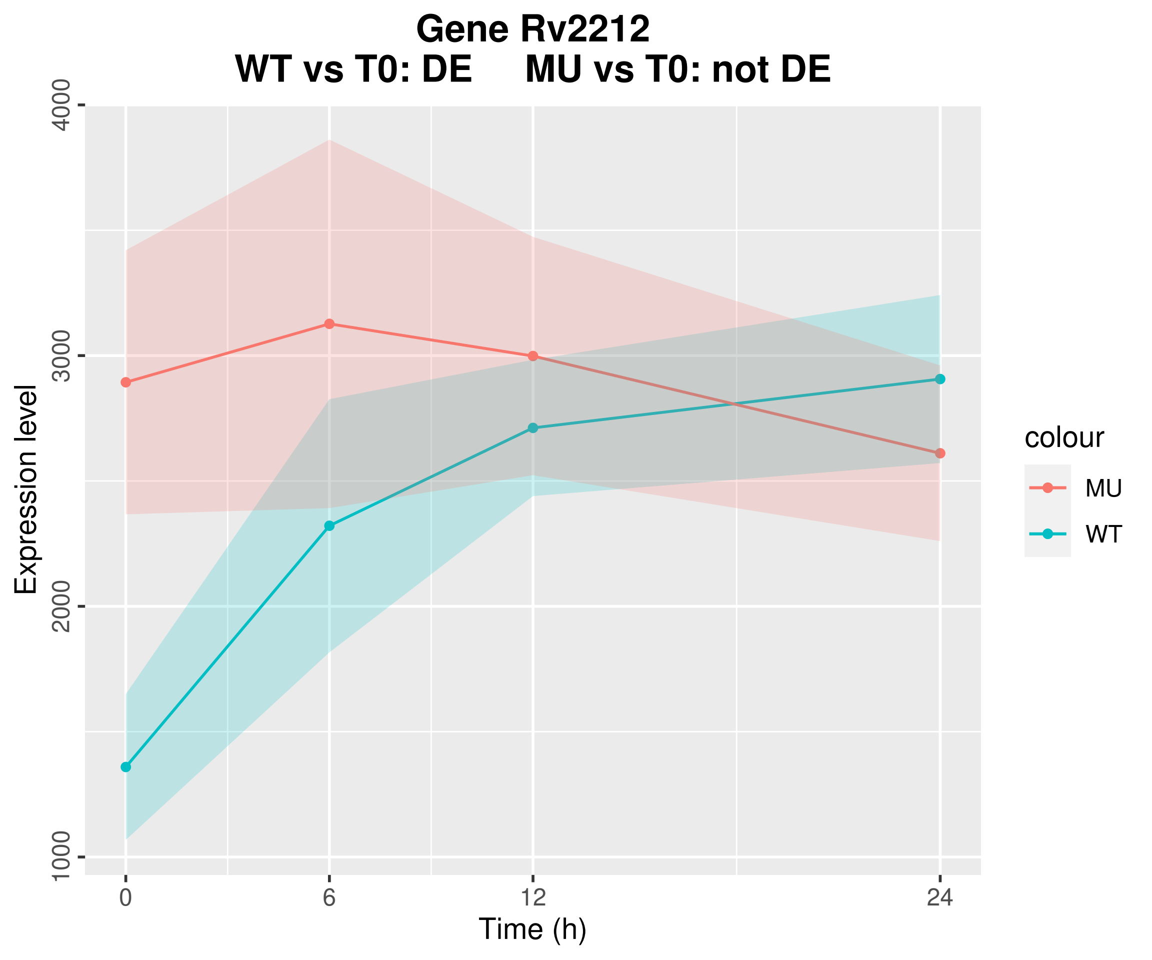
Gene Rv2212
WT vs T0: DE MU vs T0: not DE
Expression level
4000
3000
2000
1000
0
6
12
24
Time (h)
colour
MU
WT

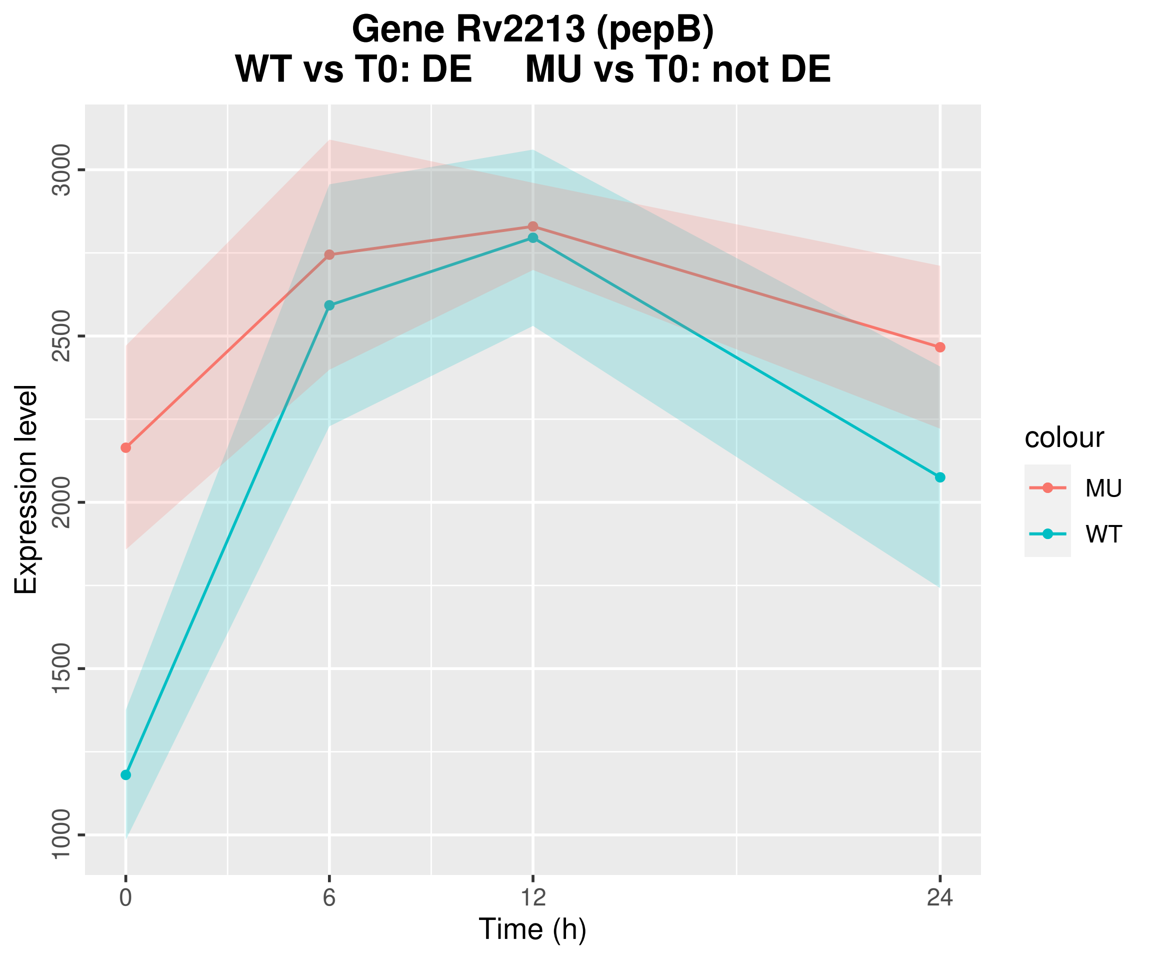
Gene Rv2213 (pepB)
WT vs T0: DE   MU vs T0: not DE
Expression level
3000
2500
2000
1500
1000
0
6
12
24
Time (h)
colour
MU
WT

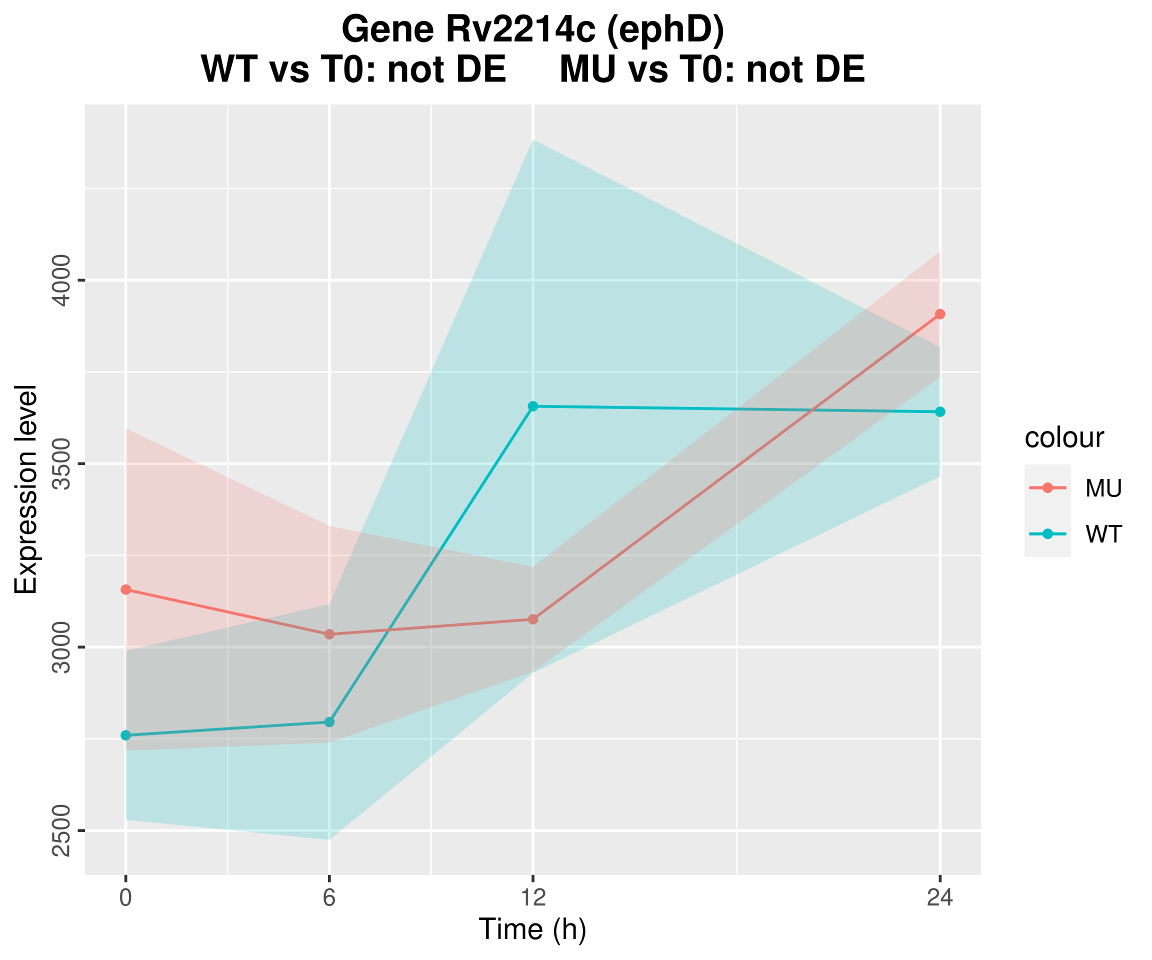

Gene Rv2214c (ephD)
WT vs T0: not DE   MU vs T0: not DE
Expression level
4000
3500
3000
2500
0
6
12
24
Time (h)
colour
MU
WT

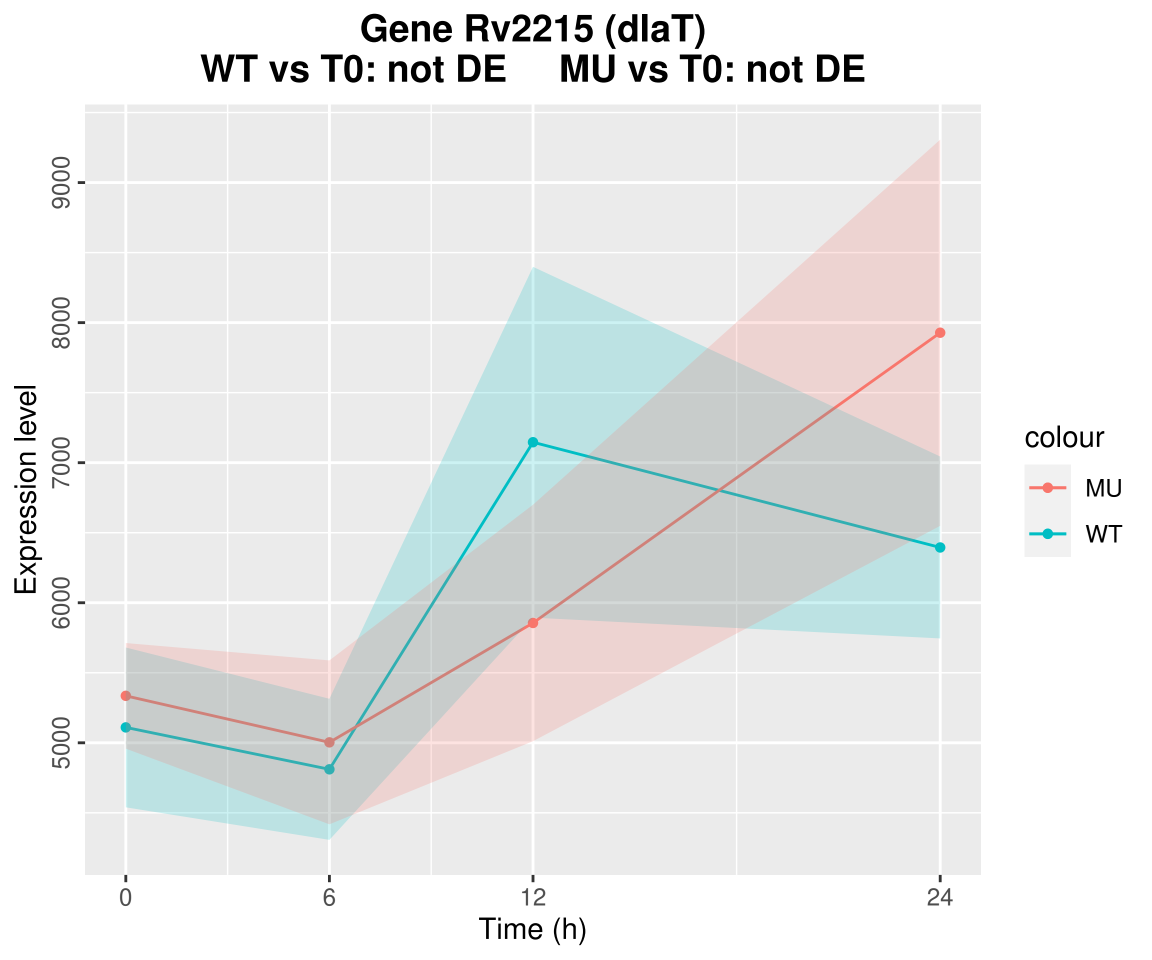

Gene Rv2215 (dlaT)
WT vs T0: not DE   MU vs T0: not DE
Expression level
9000
8000
7000
6000
5000
0
6
12
24
Time (h)
colour
MU
WT

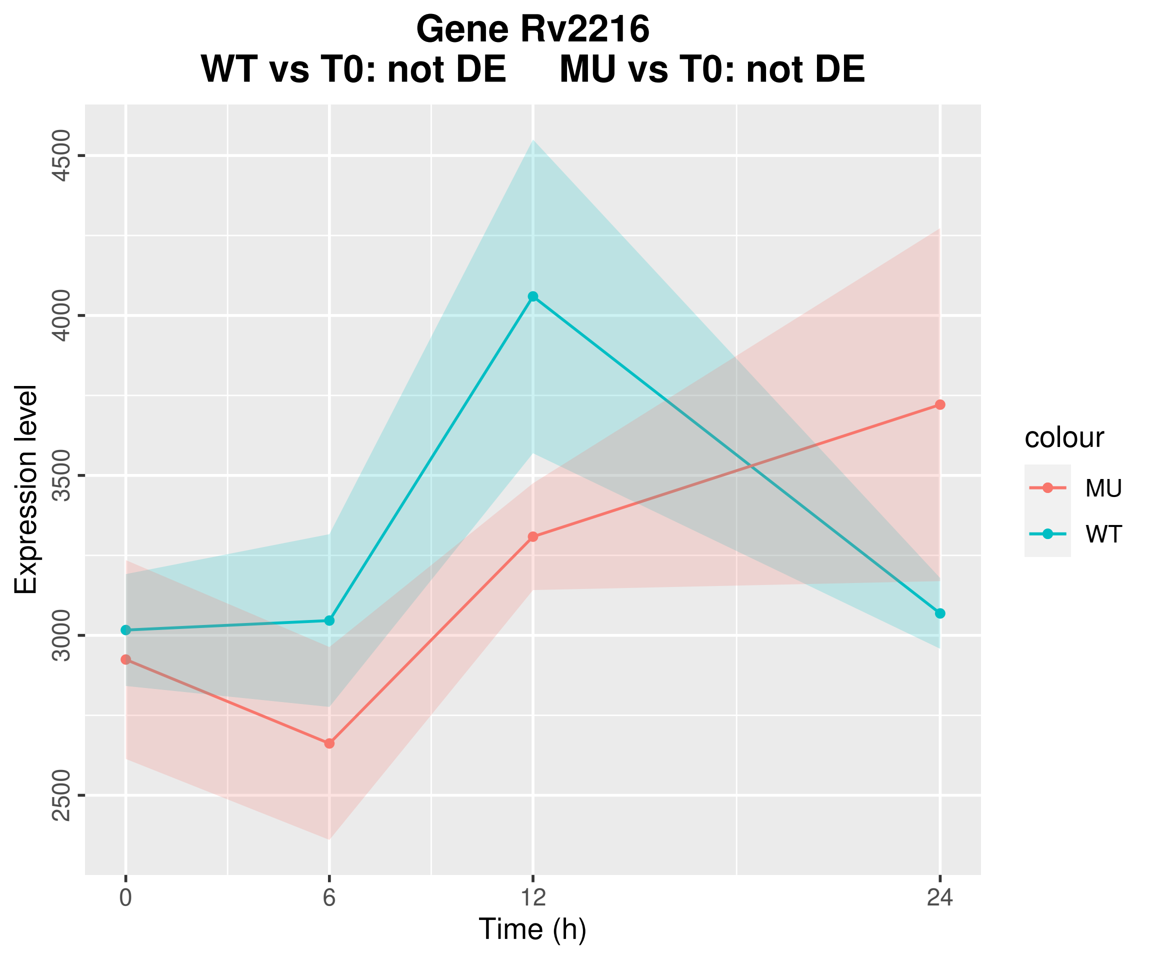
Gene Rv2216
WT vs T0: not DE MU vs T0: not DE
Expression level
4500
4000
3500
3000
2500
0
6
12
24
Time (h)
colour
MU
WT

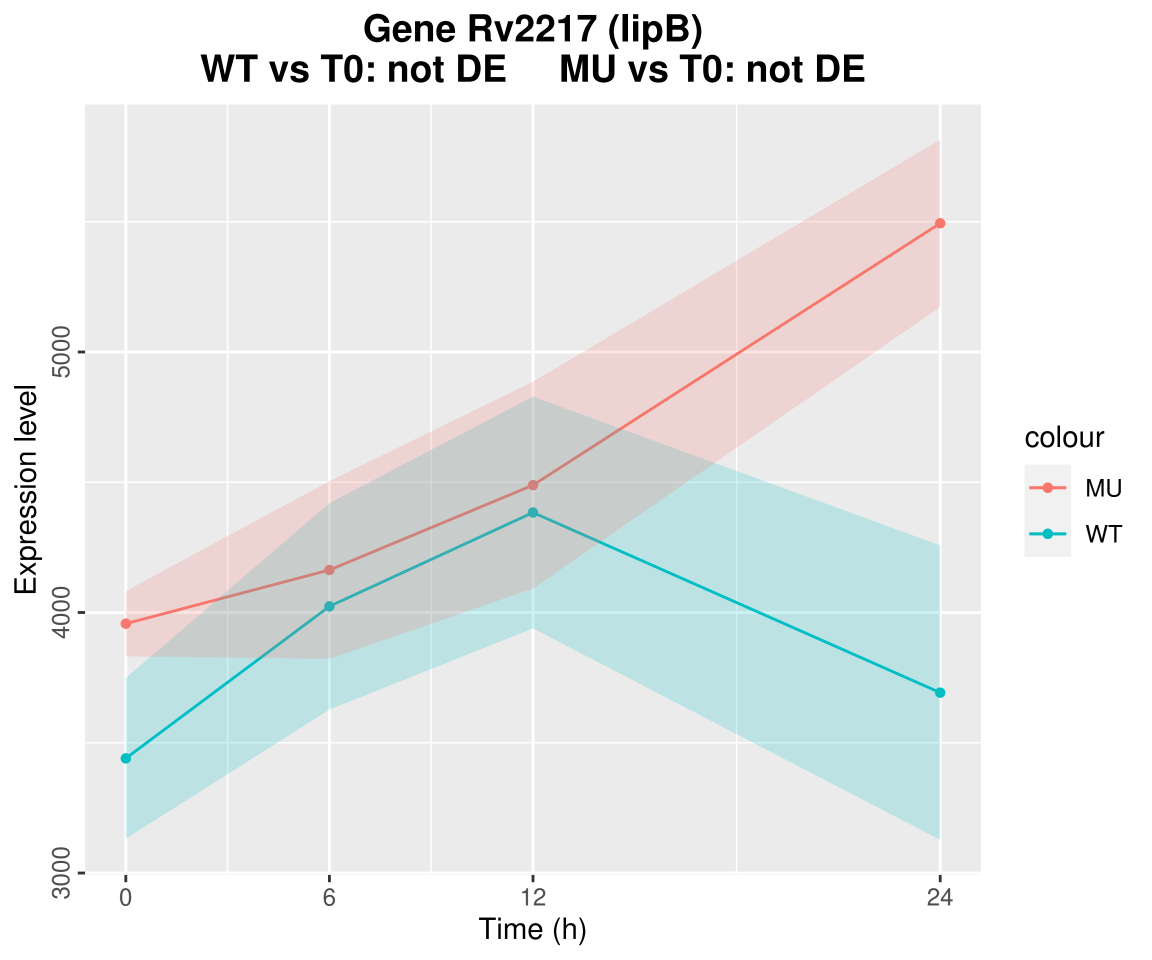

Gene Rv2217 (lipB)
WT vs T0: not DE MU vs T0: not DE
Expression level
5000
4000
3000
0
6
12
24
Time (h)
colour
MU
WT

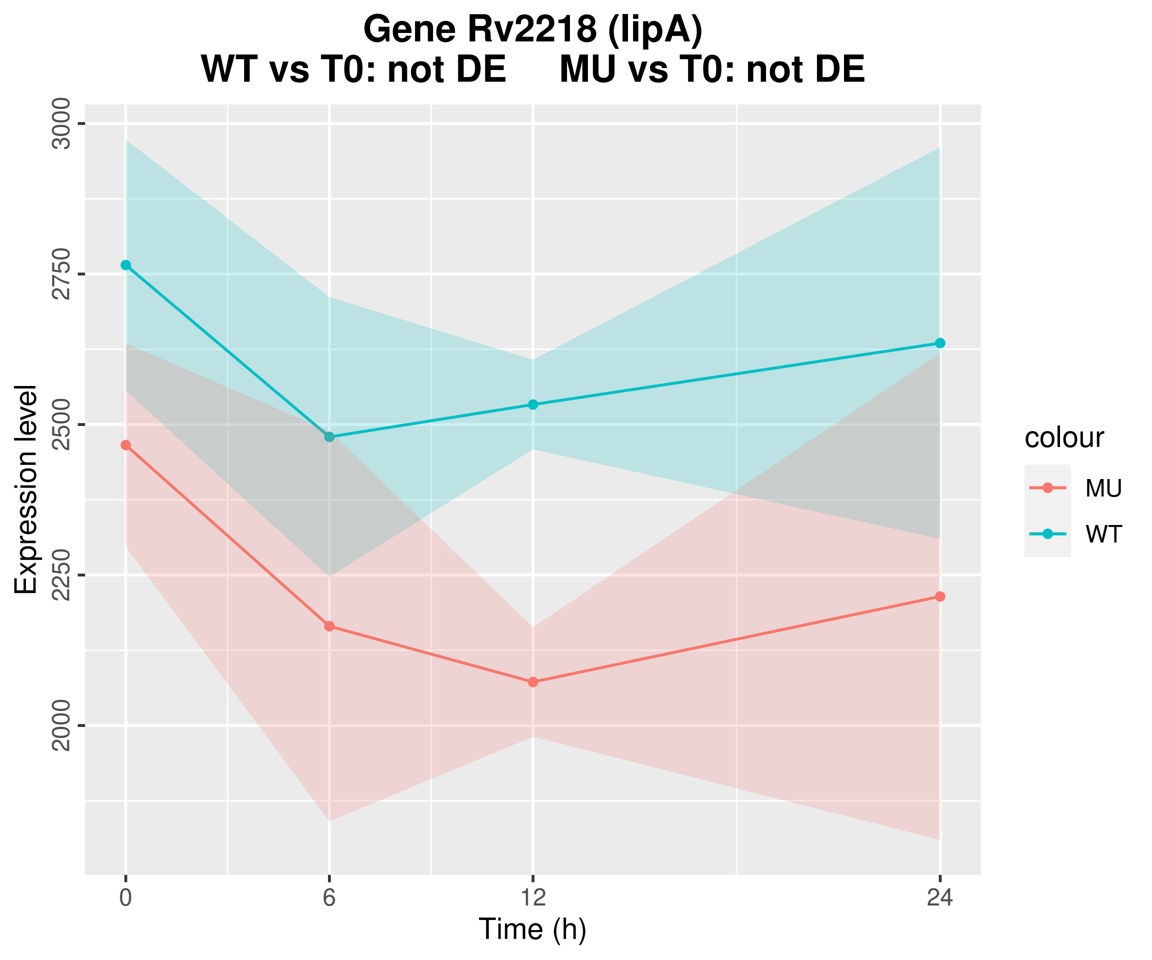
Gene Rv2218 (lipA)
WT vs T0: not DE   MU vs T0: not DE
Expression level
3000
2750
2500
2250
2000
0
6
12
24
Time (h)
colour
MU
WT

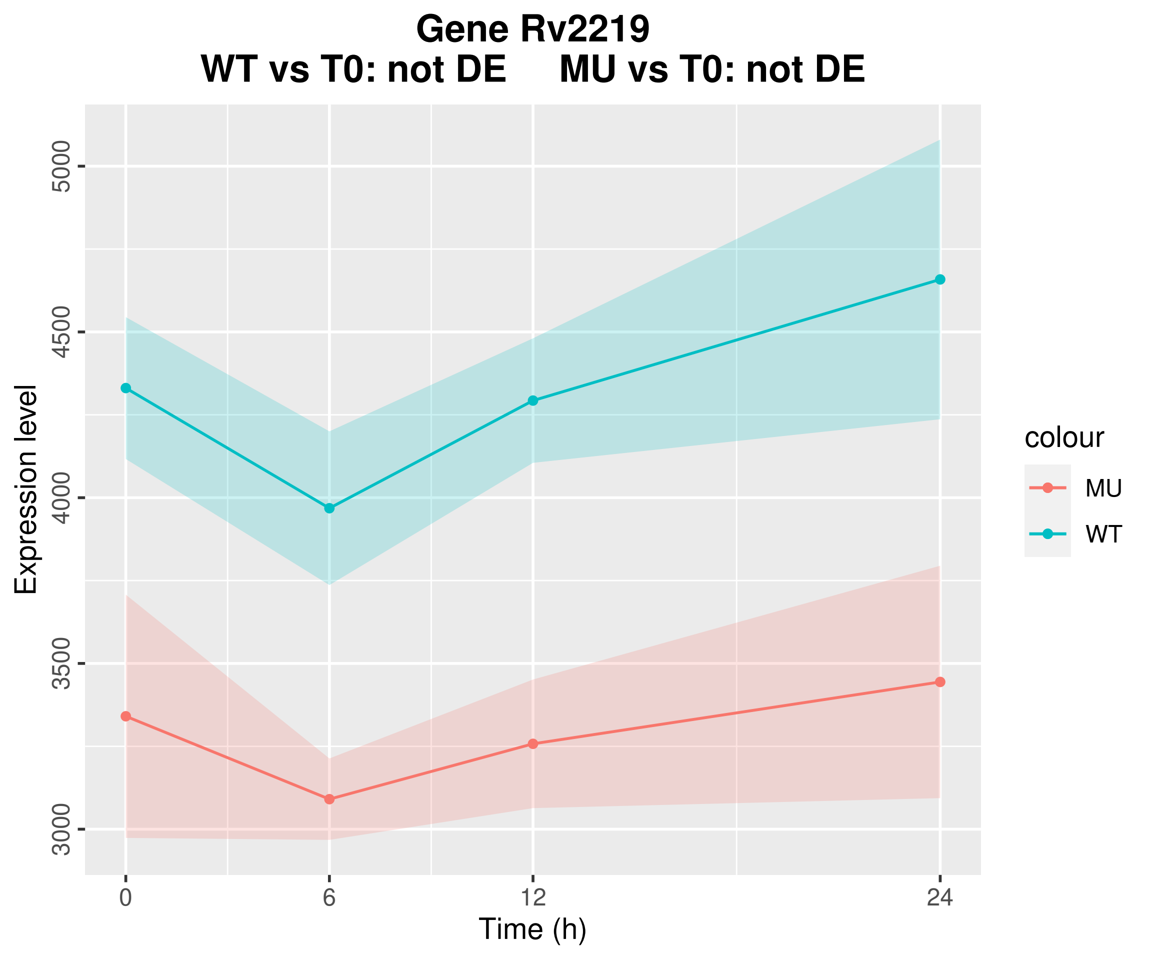
Gene Rv2219
WT vs T0: not DE   MU vs T0: not DE
Expression level
5000
4500
4000
3500
3000
Time (h)
0
6
12
24
colour
MU
WT

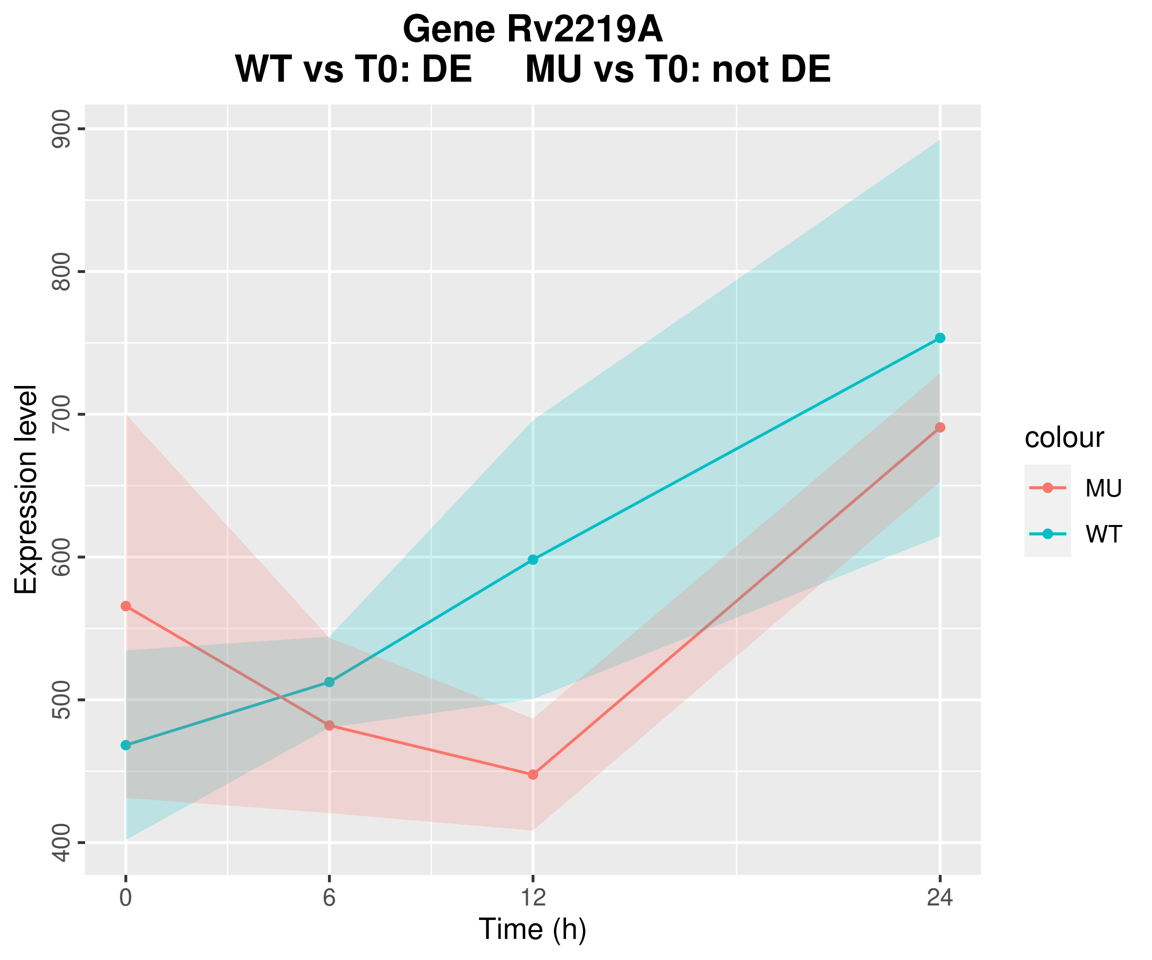

Gene Rv2219A
WT vs T0: DE MU vs T0: not DE
Expression level
900
800
700
600
500
400
0
6
12
24
Time (h)
colour
MU
WT

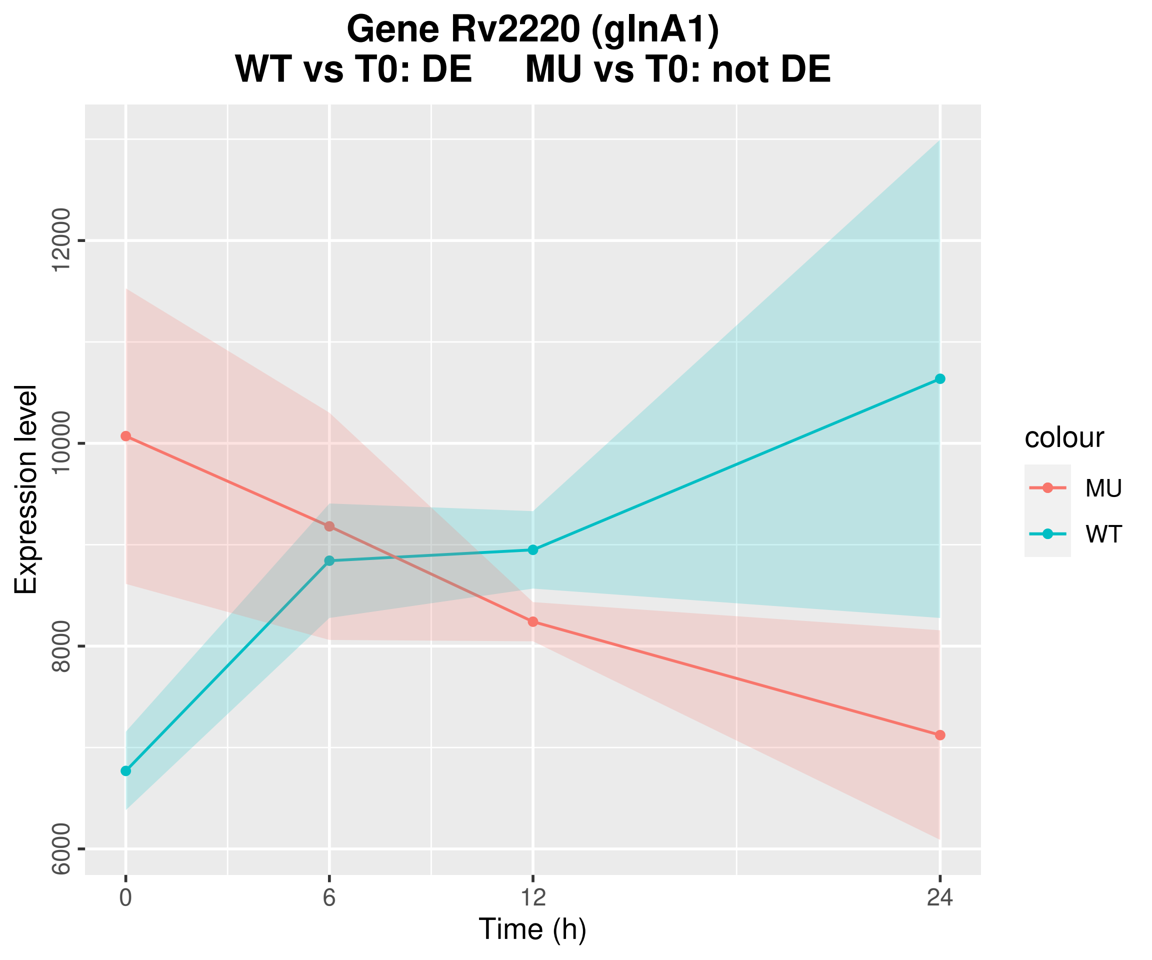
Gene Rv2220 (glnA1)
WT vs T0: DE   MU vs T0: not DE
Expression level
12000
10000
8000
6000
0
6
12
24
Time (h)
colour
MU
WT

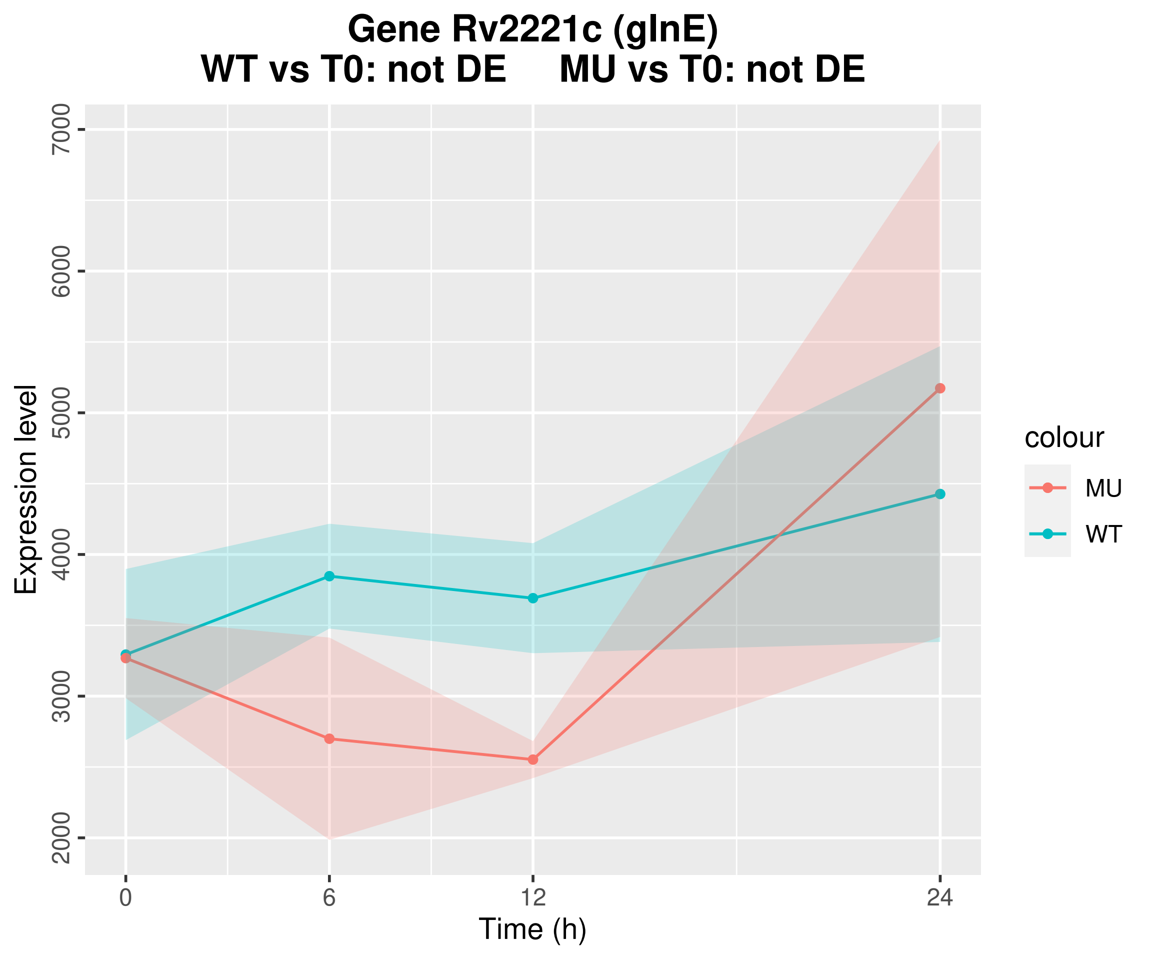

Gene Rv2221c (glnE)
WT vs T0: not DE   MU vs T0: not DE
Expression level
7000
6000
5000
4000
3000
2000
0
6
12
24
Time (h)
colour
MU
WT

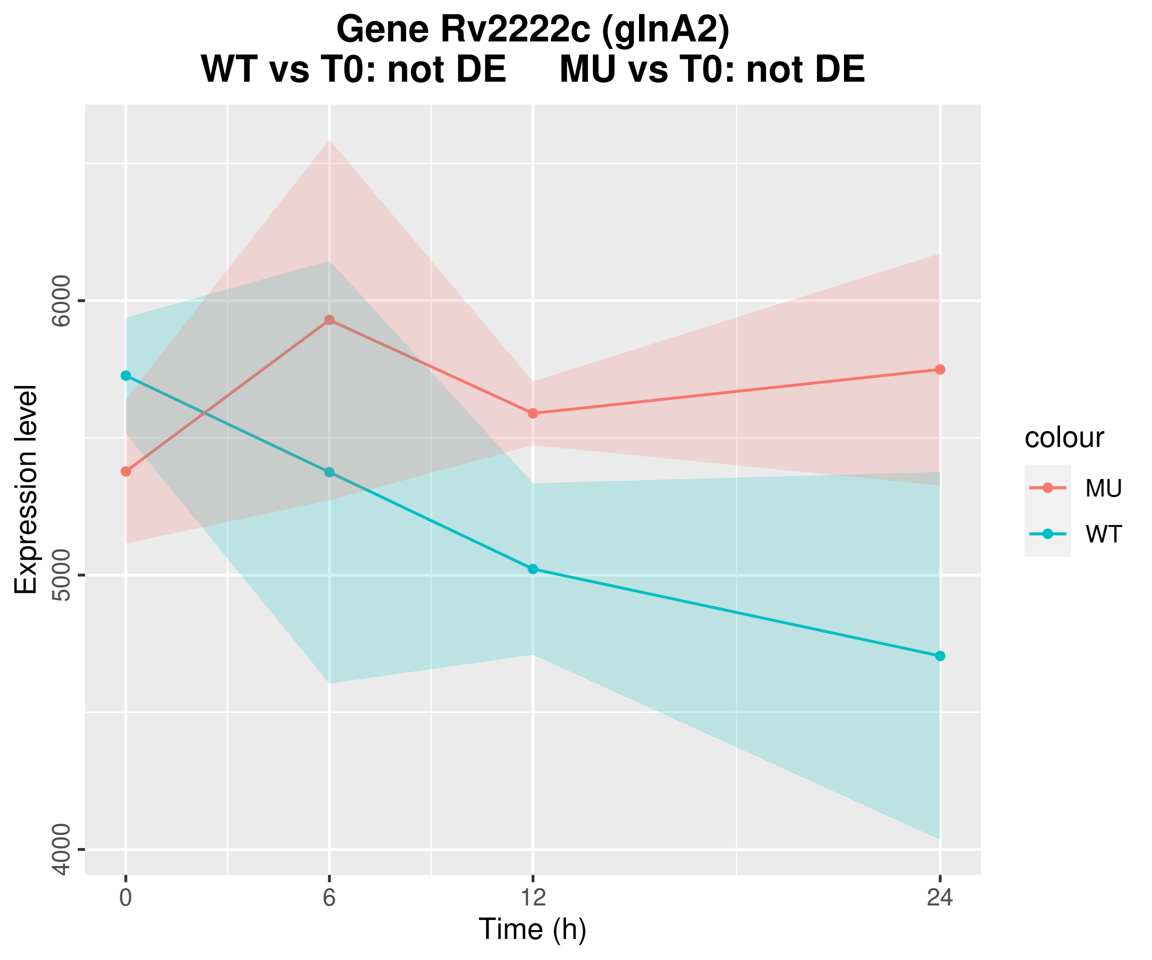

Gene Rv2222c (glnA2)
WT vs T0: not DE   MU vs T0: not DE
Expression level
6000
5000
4000
0
6
12
24
Time (h)
colour
MU
WT

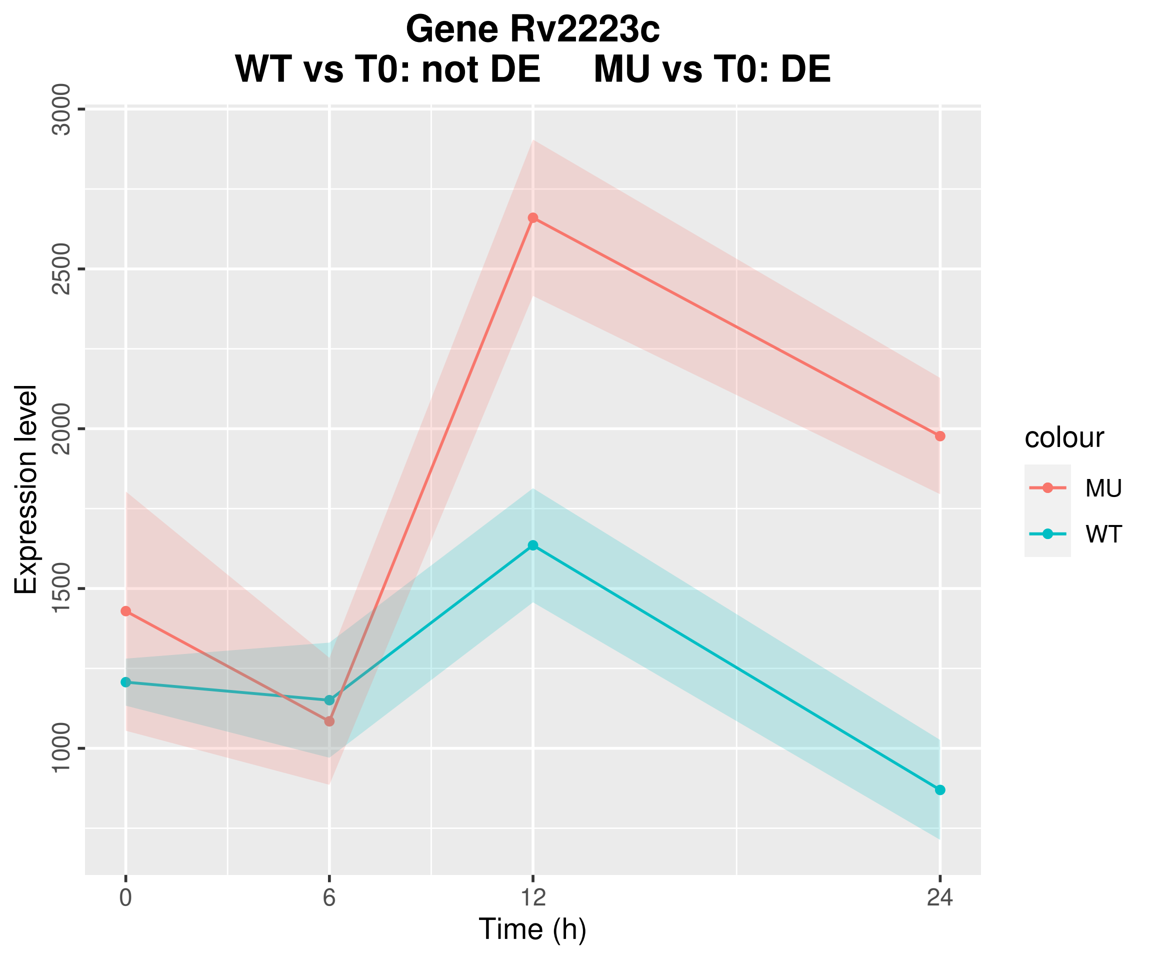
Gene Rv2223c
WT vs T0: not DE   MU vs T0: DE
Expression level
3000
2500
2000
1500
1000
0
6
12
24
Time (h)
colour
MU
WT

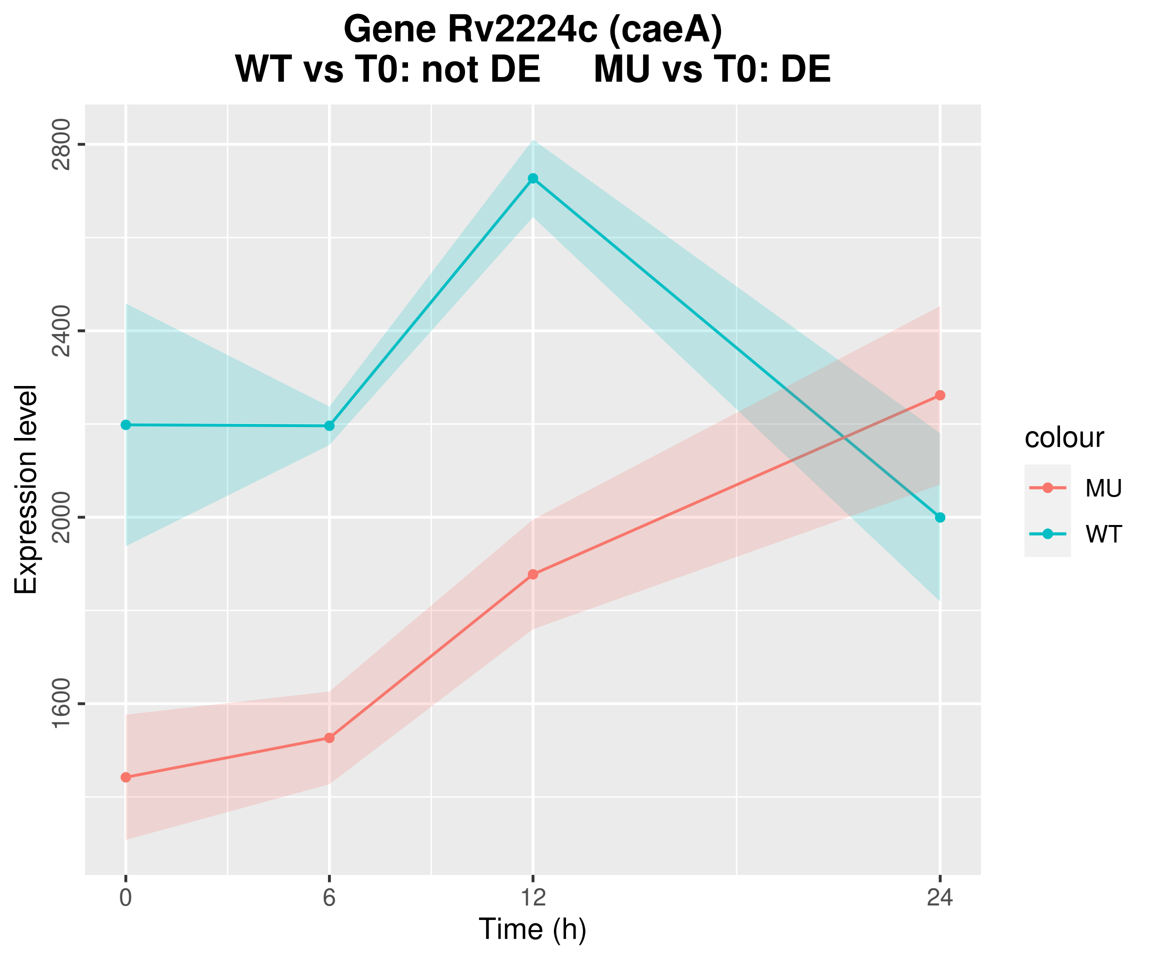
Gene Rv2224c (caeA)
WT vs T0: not DE   MU vs T0: DE
Expression level
2800
2400
2000
1600
0
6
12
24
Time (h)
colour
MU
WT

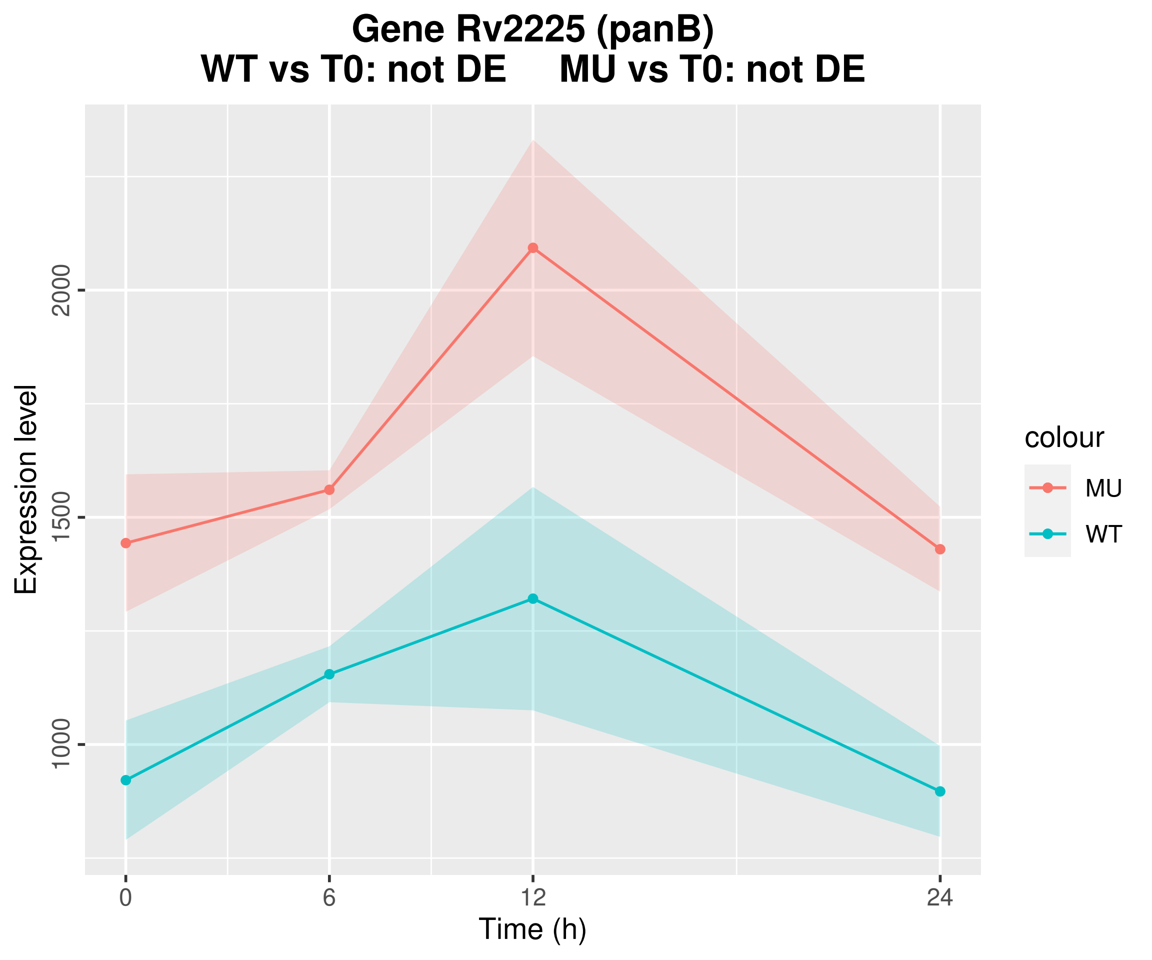
Gene Rv2225 (panB)
WT vs T0: not DE   MU vs T0: not DE
Expression level
2000
1500
1000
0
6
12
24
Time (h)
colour
MU
WT

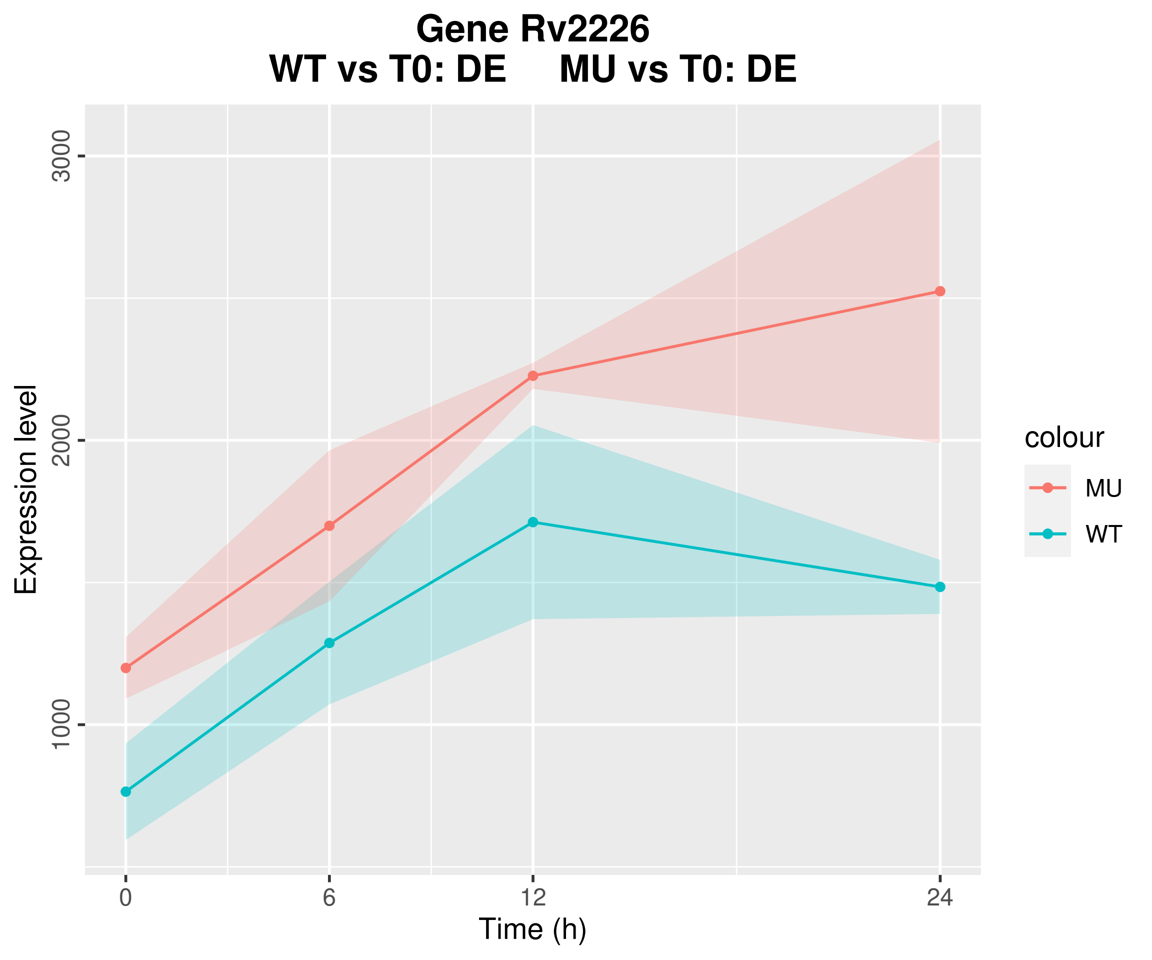
Gene Rv2226
WT vs T0: DE   MU vs T0: DE
Expression level
3000
2000
1000
0
6
12
24
Time (h)
colour
MU
WT

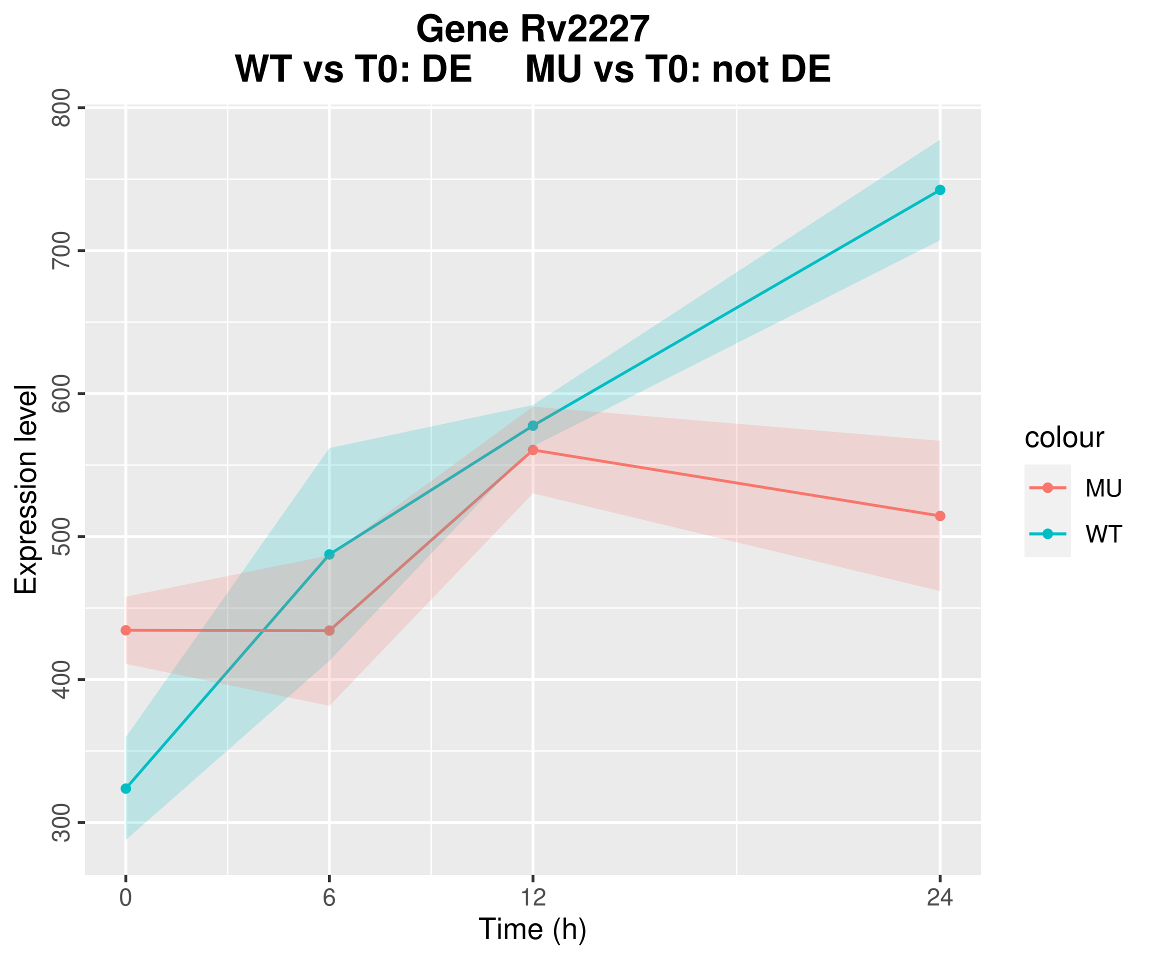

Gene Rv2227
WT vs T0: DE MU vs T0: not DE
Expression level
800
700
600
500
400
300
0
6
12
24
Time (h)
colour
MU
WT

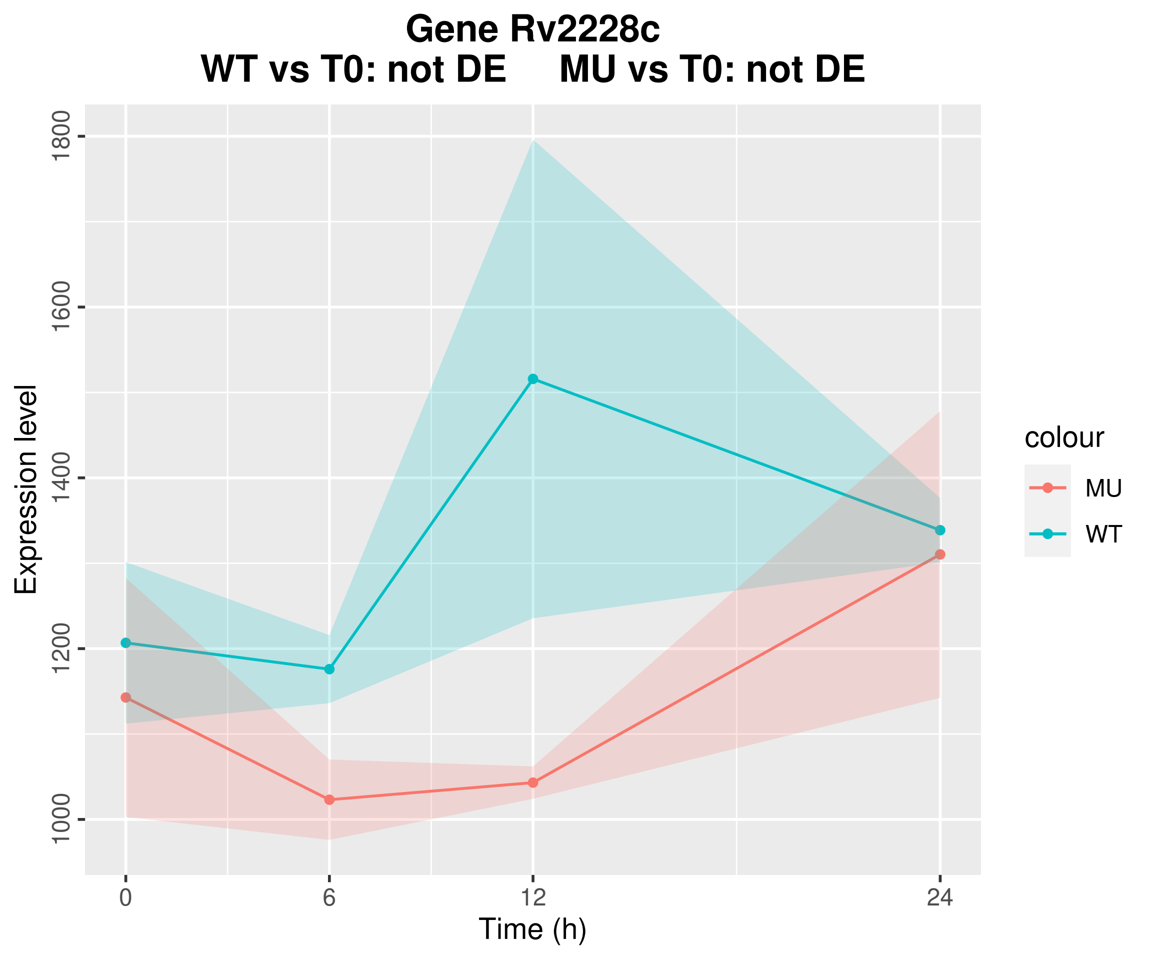

Gene Rv2228c
WT vs T0: not DE MU vs T0: not DE
Expression level
1000
1200
1400
1600
1800
0
6
12
24
Time (h)
colour
MU
WT

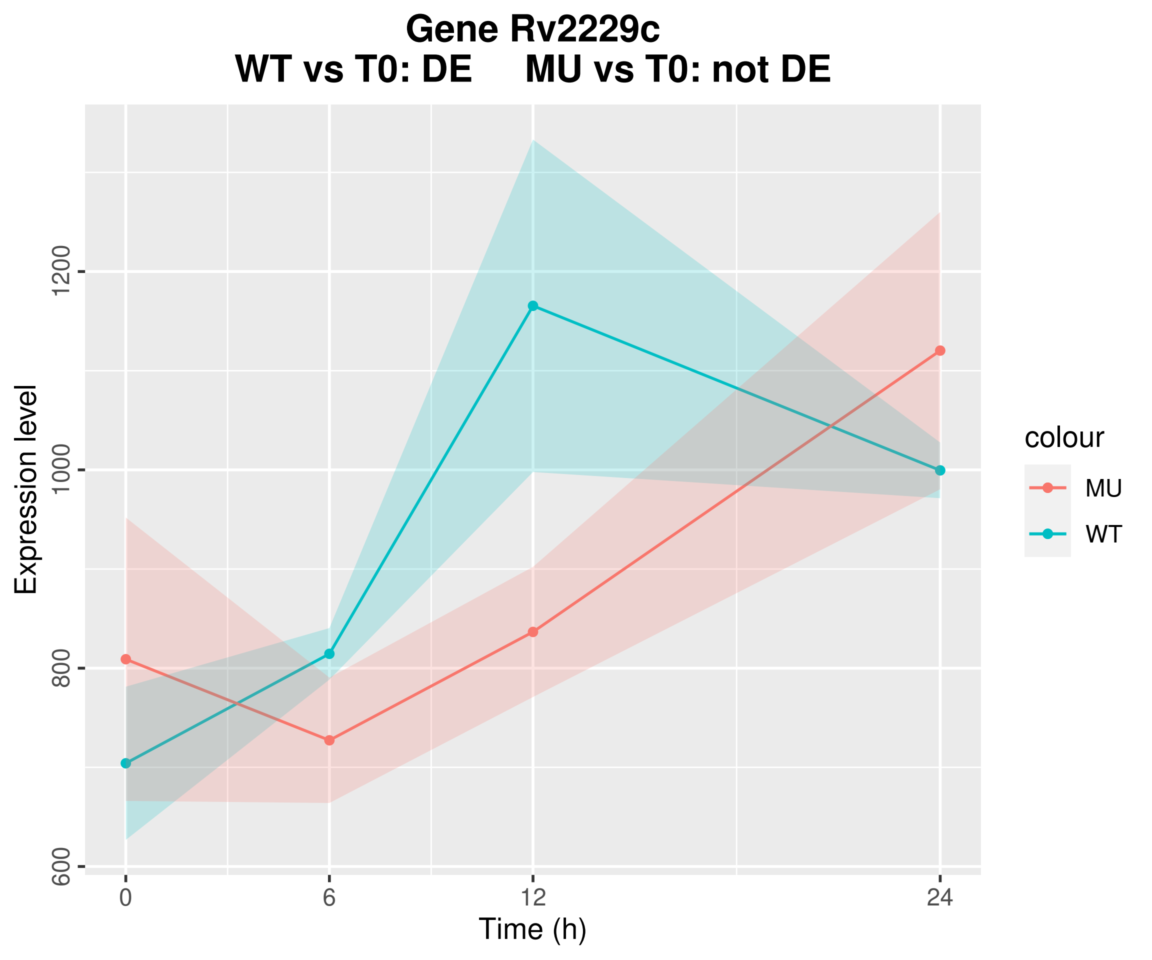

Gene Rv2229c
WT vs T0: DE MU vs T0: not DE
Expression level
1200
1000
800
600
0
6
12
24
Time (h)
colour
MU
WT

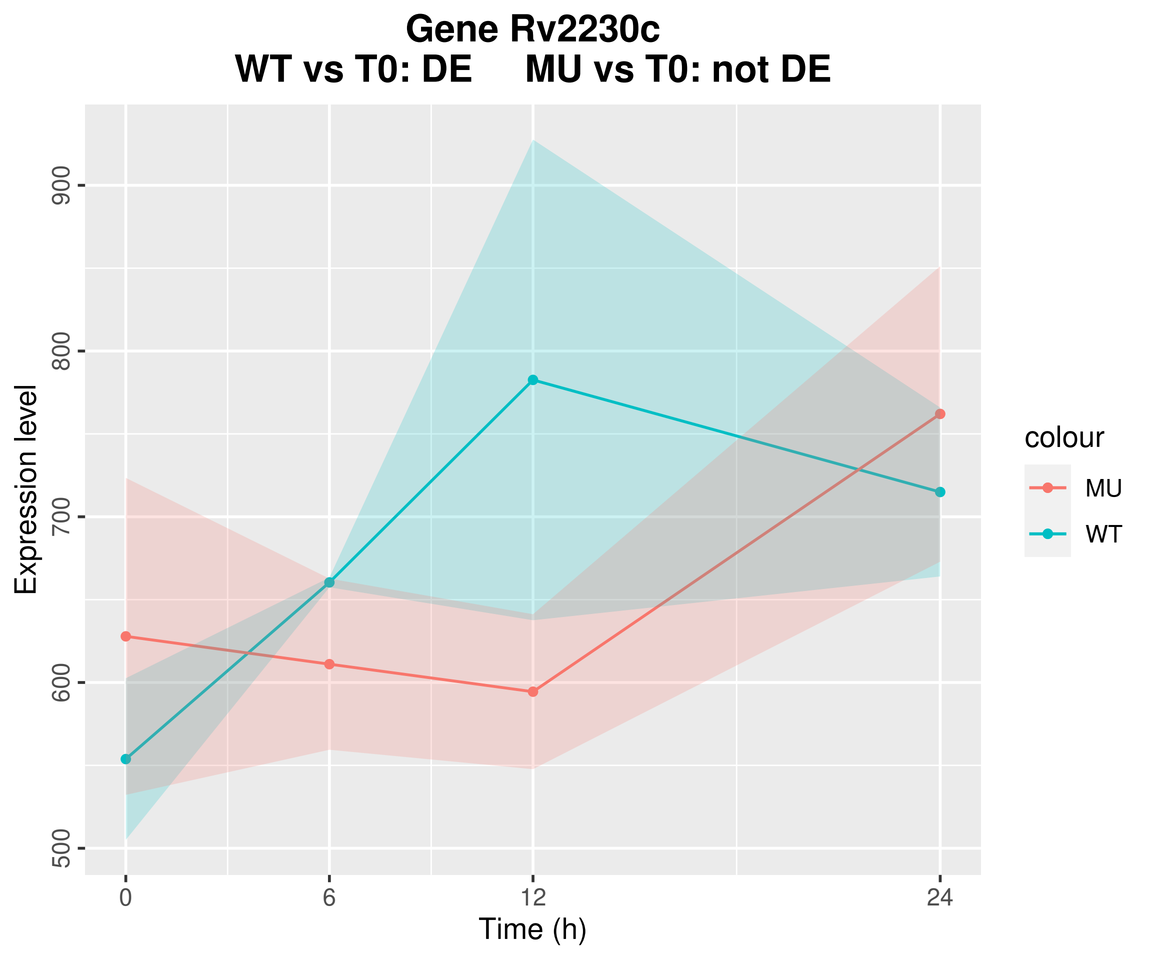

Gene Rv2230c
WT vs T0: DE MU vs T0: not DE
Expression level
900
800
700
600
500
0
6
12
24
Time (h)
colour
MU
WT

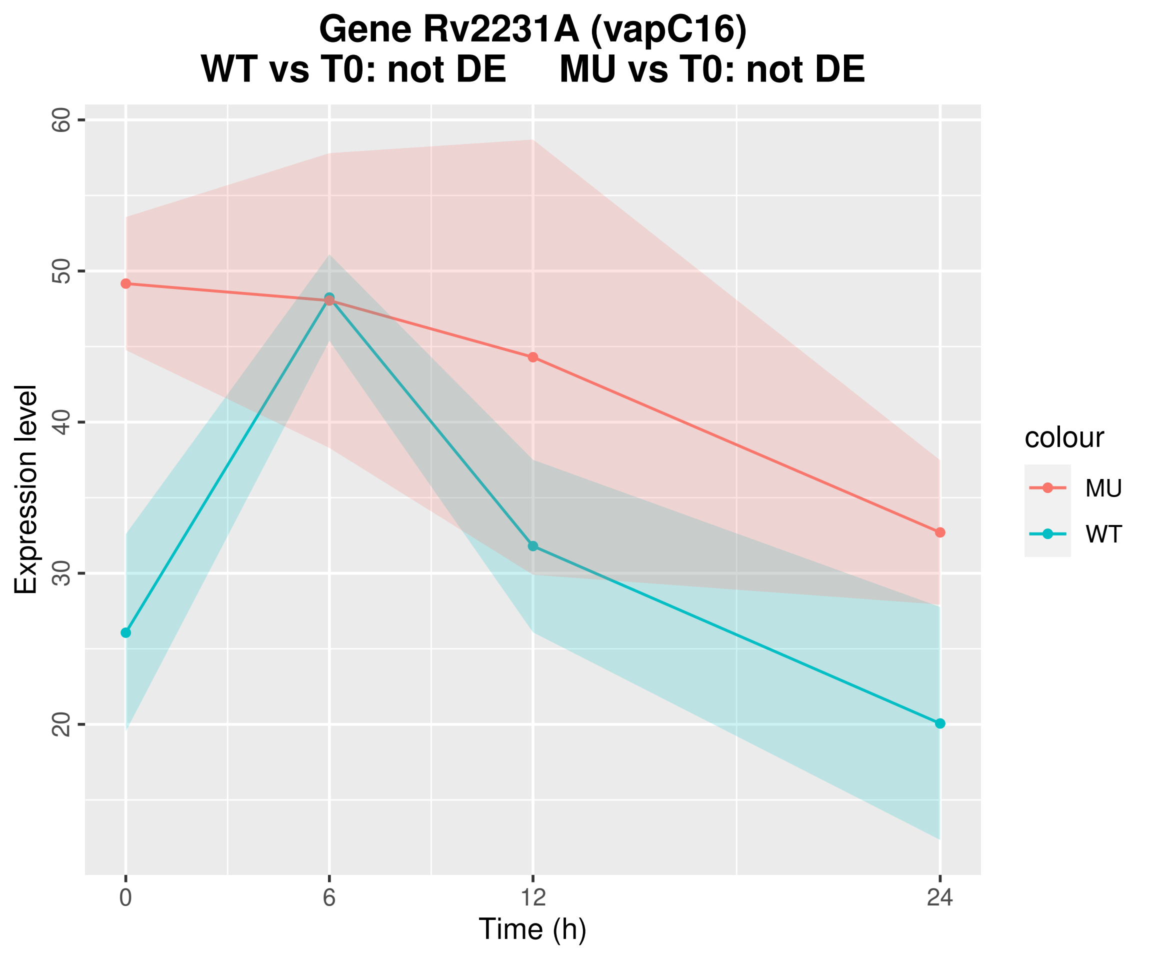

Gene Rv2231A (vapC16)
WT vs T0: not DE MU vs T0: not DE
60
50
40
30
20
Expression level
0
6
12
24
Time (h)
colour
MU
WT

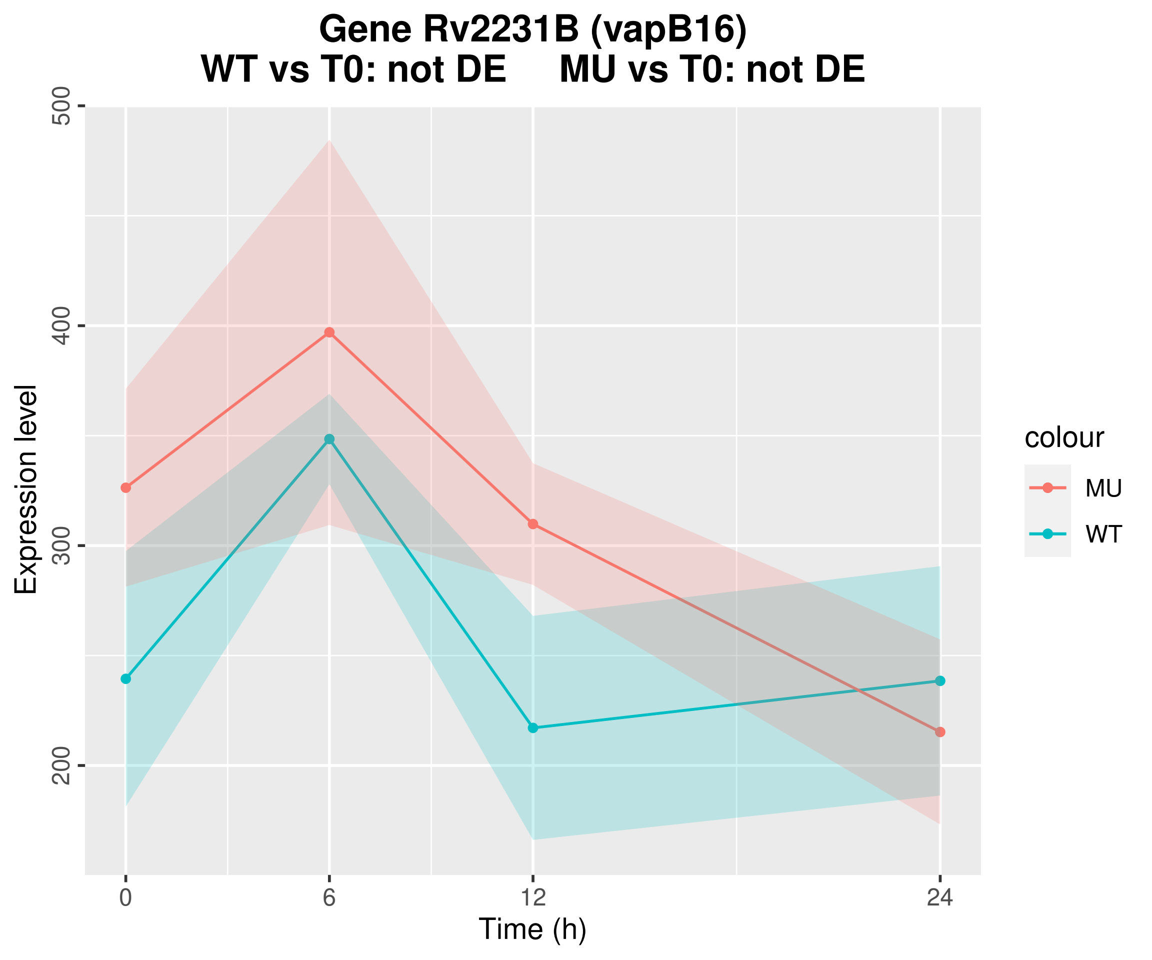
Gene Rv2231B (vapB16)
WT vs T0: not DE   MU vs T0: not DE
Expression level
500
400
300
200
0
6
12
24
Time (h)
colour
MU
WT

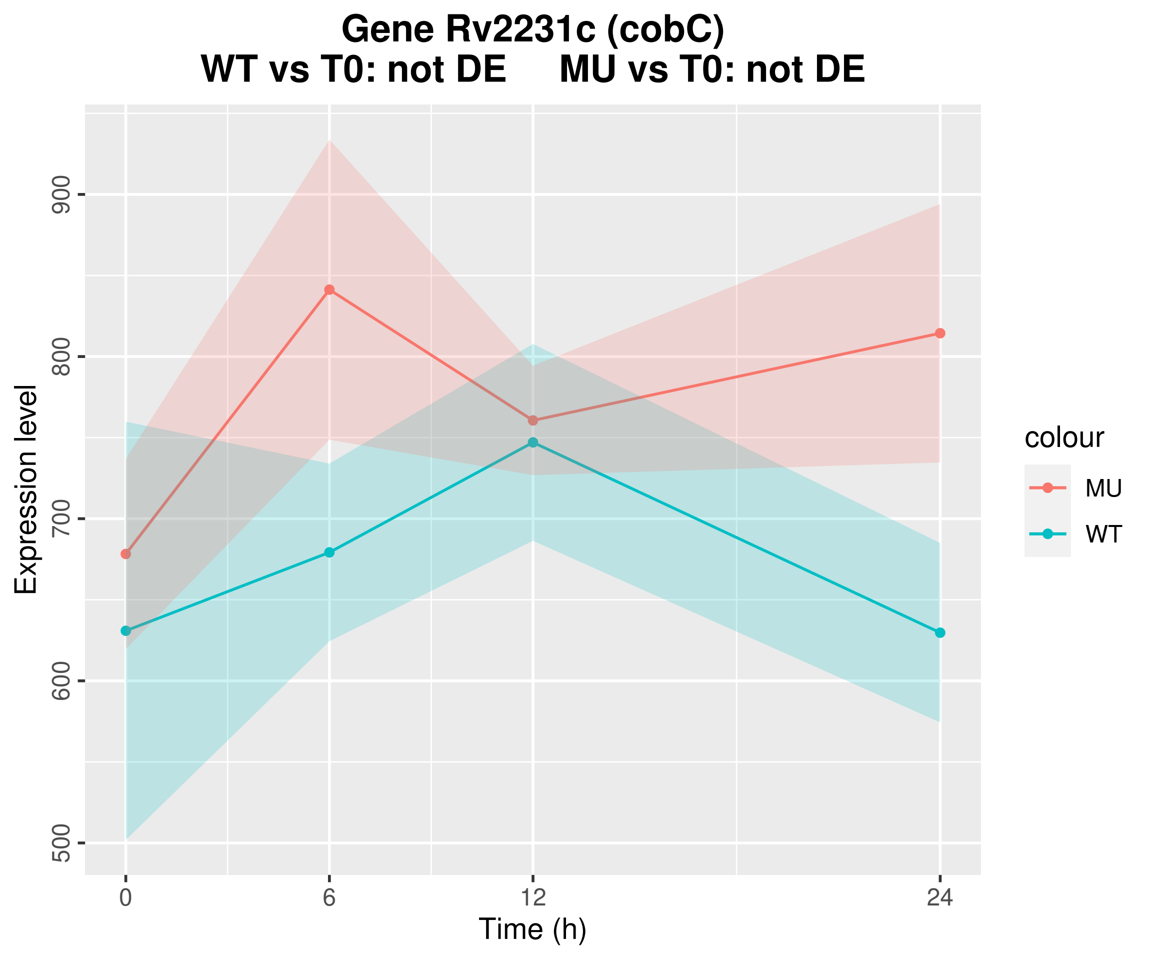
Gene Rv2231c (cobC)
WT vs T0: not DE   MU vs T0: not DE
Expression level
900
800
700
600
500
0
6
12
24
Time (h)
colour
MU
WT

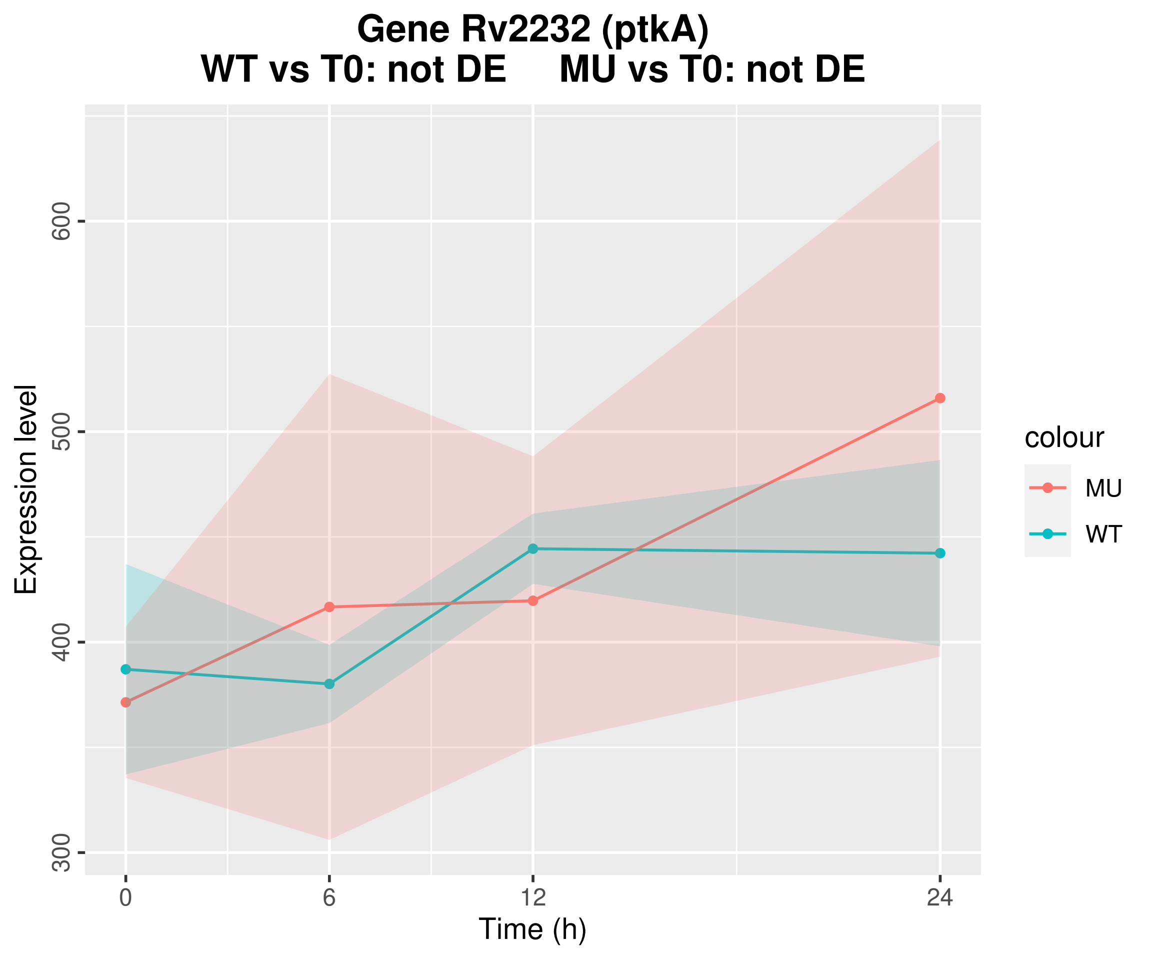

Gene Rv2232 (ptkA)
WT vs T0: not DE MU vs T0: not DE
Expression level
300
400
500
600
0
6
12
24
Time (h)
colour
MU
WT

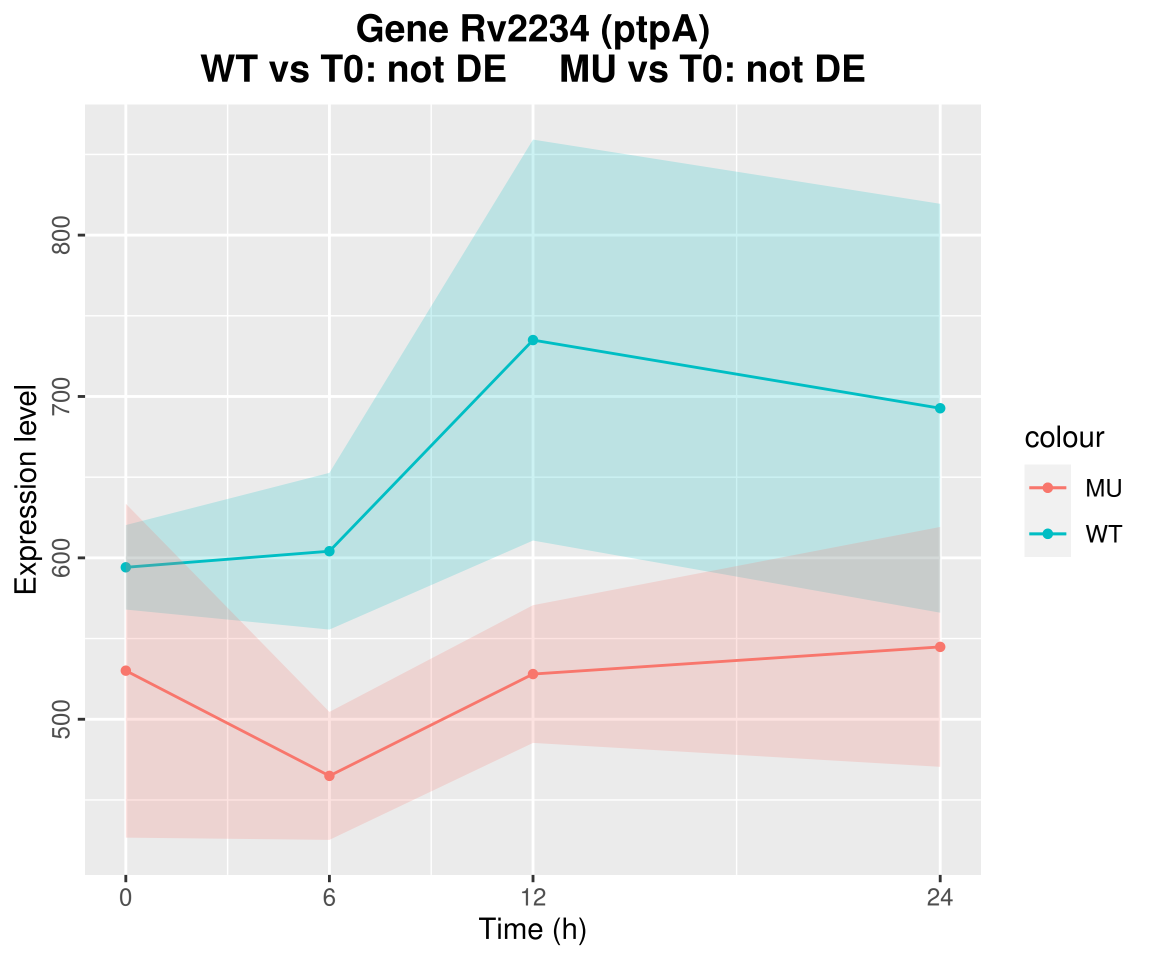

Gene Rv2234 (ptpA)
WT vs T0: not DE MU vs T0: not DE
Expression level
800
700
600
500
0
6
12
24
Time (h)
colour
MU
WT

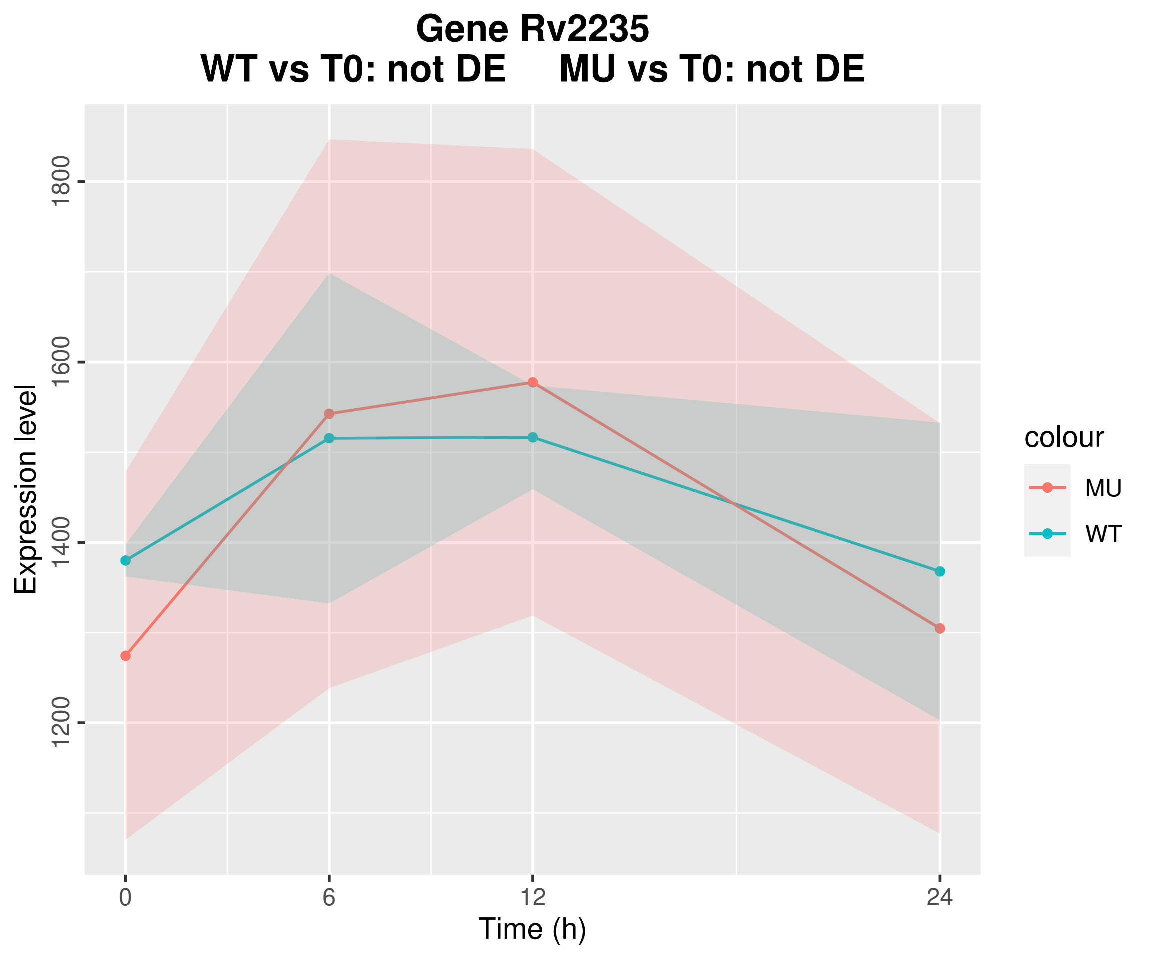

Gene Rv2235
WT vs T0: not DE   MU vs T0: not DE
Expression level
1200
1400
1600
1800
0
6
12
24
Time (h)
colour
MU
WT

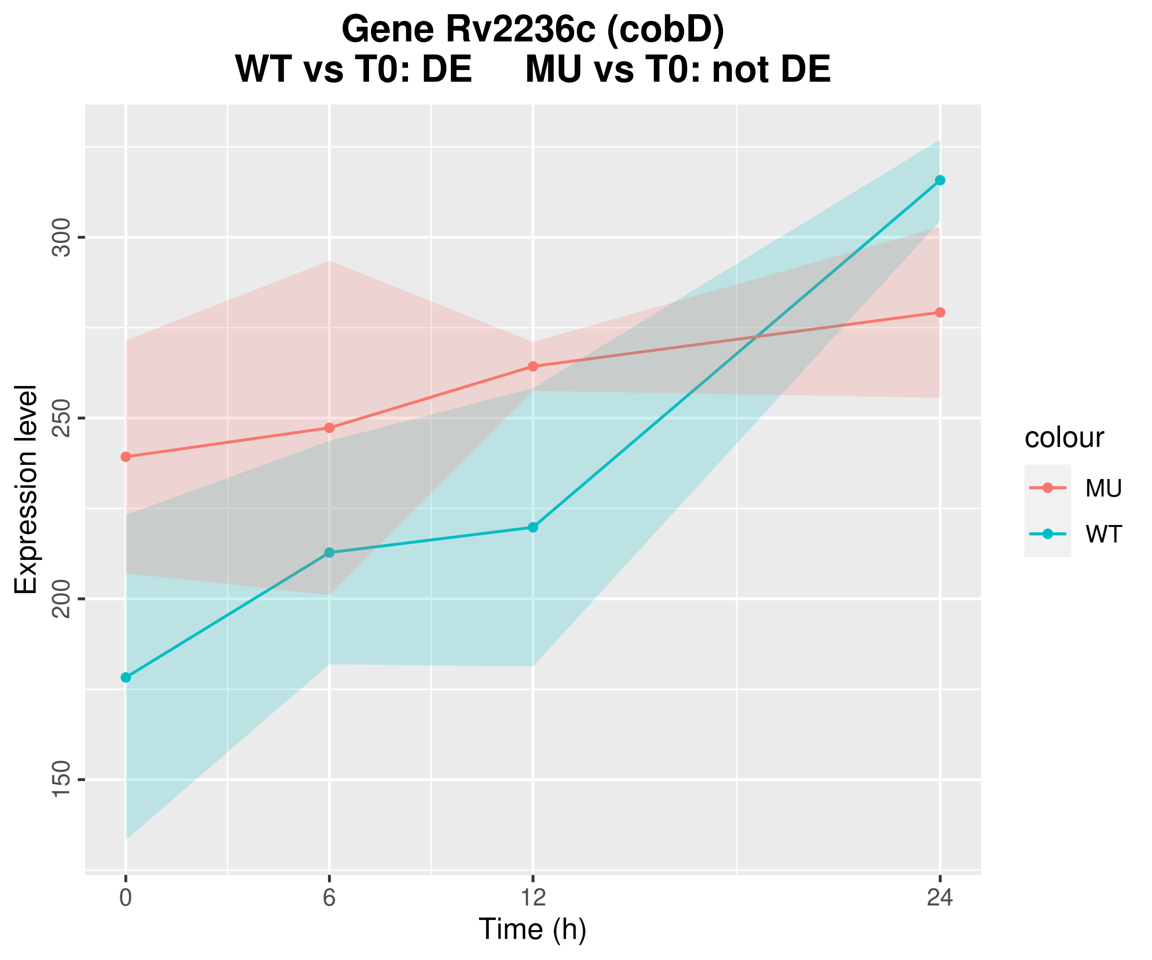

Gene Rv2236c (cobD)
WT vs T0: DE   MU vs T0: not DE
Expression level
300
250
200
150
0
6
12
24
Time (h)
colour
MU
WT

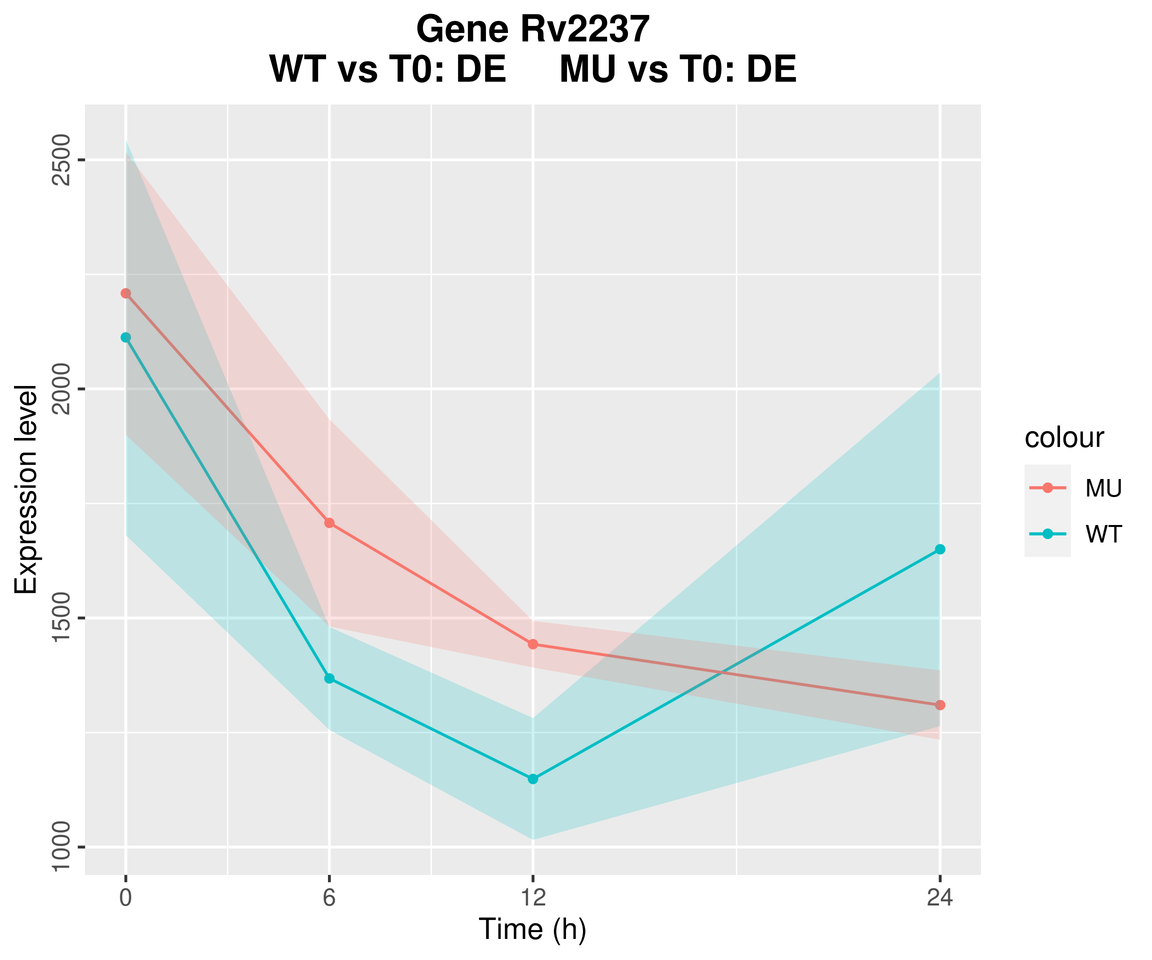

Gene Rv2237
WT vs T0: DE   MU vs T0: DE
Expression level
2500
2000
1500
1000
0
6
12
24
Time (h)
colour
MU
WT

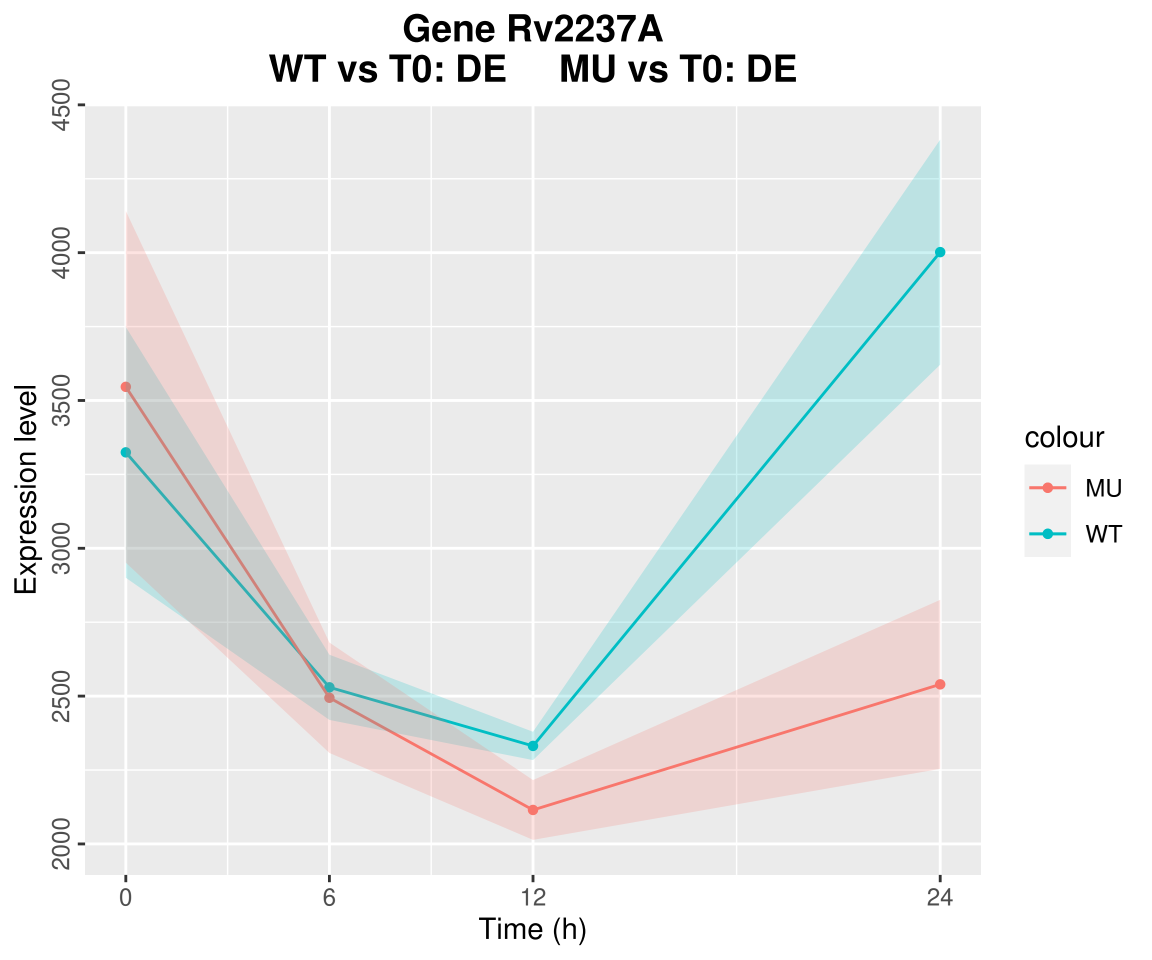
Gene Rv2237A
WT vs T0: DE MU vs T0: DE
Expression level
4500
4000
3500
3000
2500
2000
0
6
12
24
Time (h)
colour
MU
WT

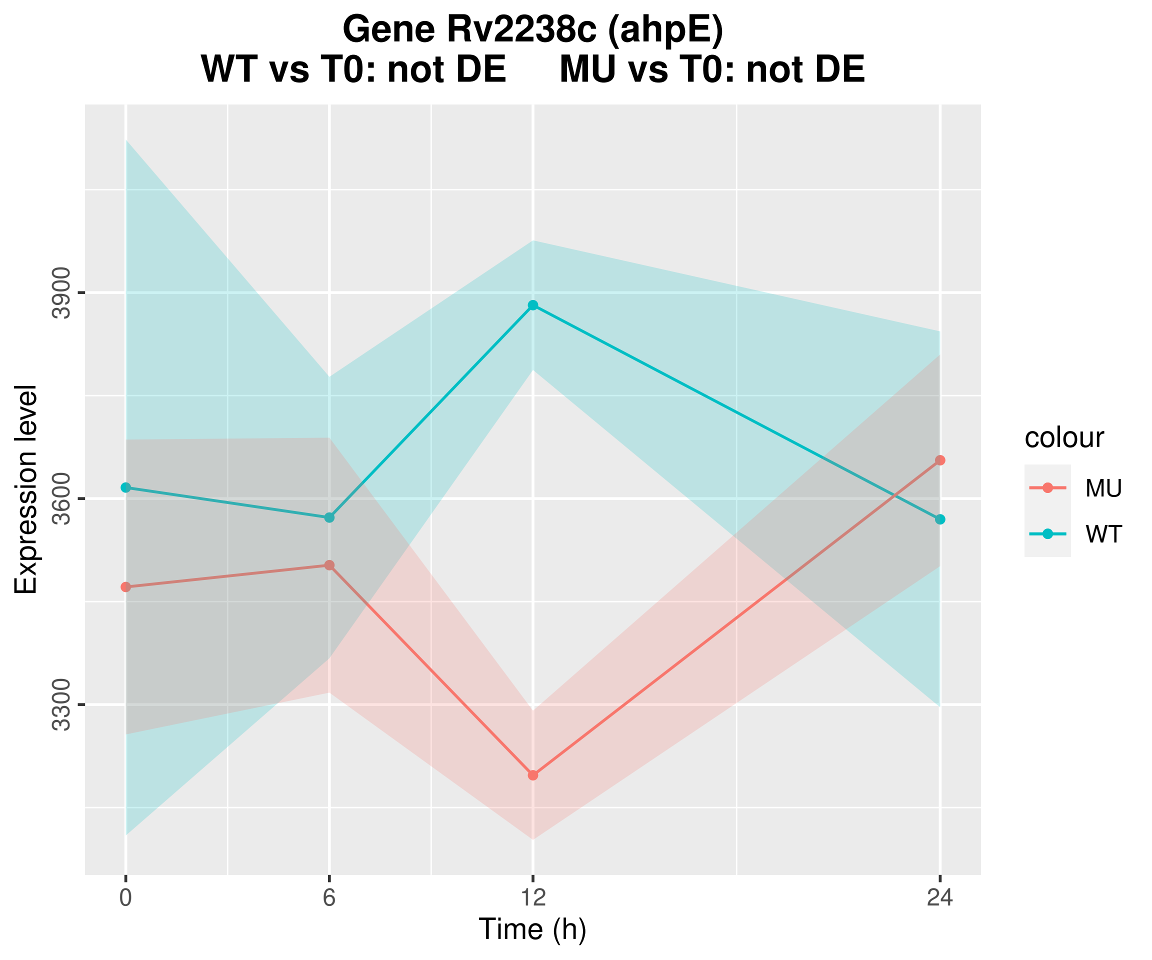

Gene Rv2238c (ahpE)
WT vs T0: not DE MU vs T0: not DE
Expression level
3900
3600
3300
0
6
12
24
Time (h)
colour
MU
WT

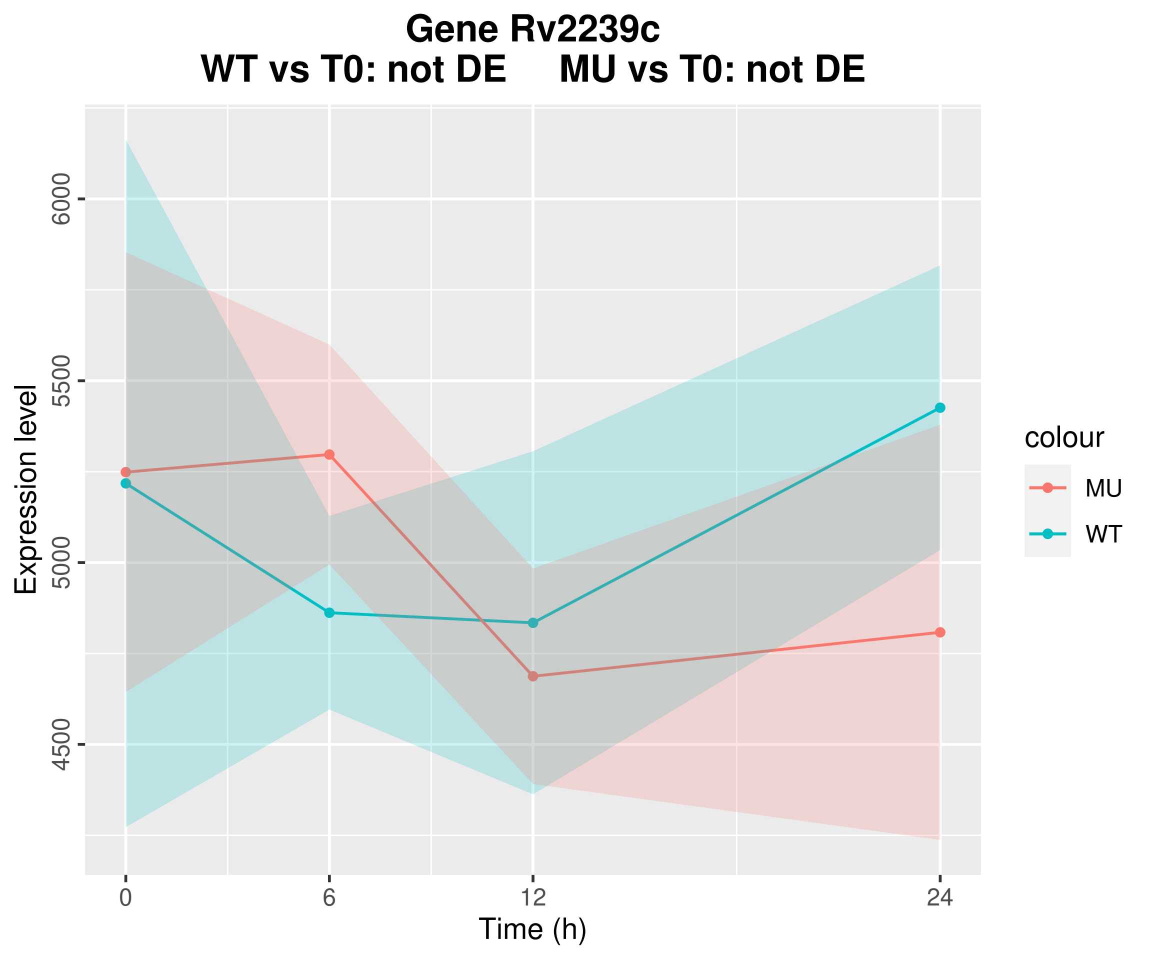

Gene Rv2239c
WT vs T0: not DE MU vs T0: not DE
Expression level
6000
5500
5000
4500
0
6
12
24
Time (h)
colour
MU
WT

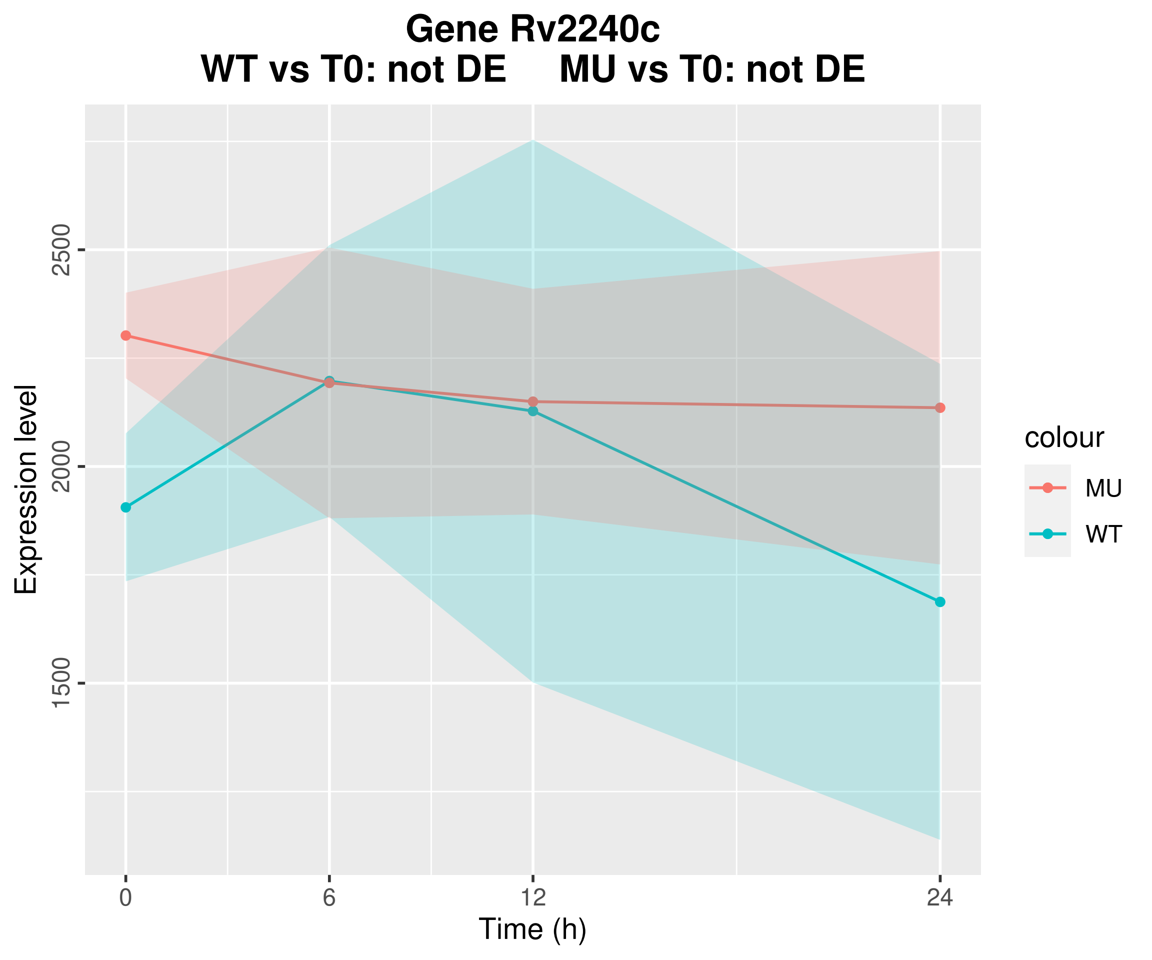

Gene Rv2240c
WT vs T0: not DE   MU vs T0: not DE
Expression level
2500
2000
1500
0
6
12
24
Time (h)
colour
MU
WT

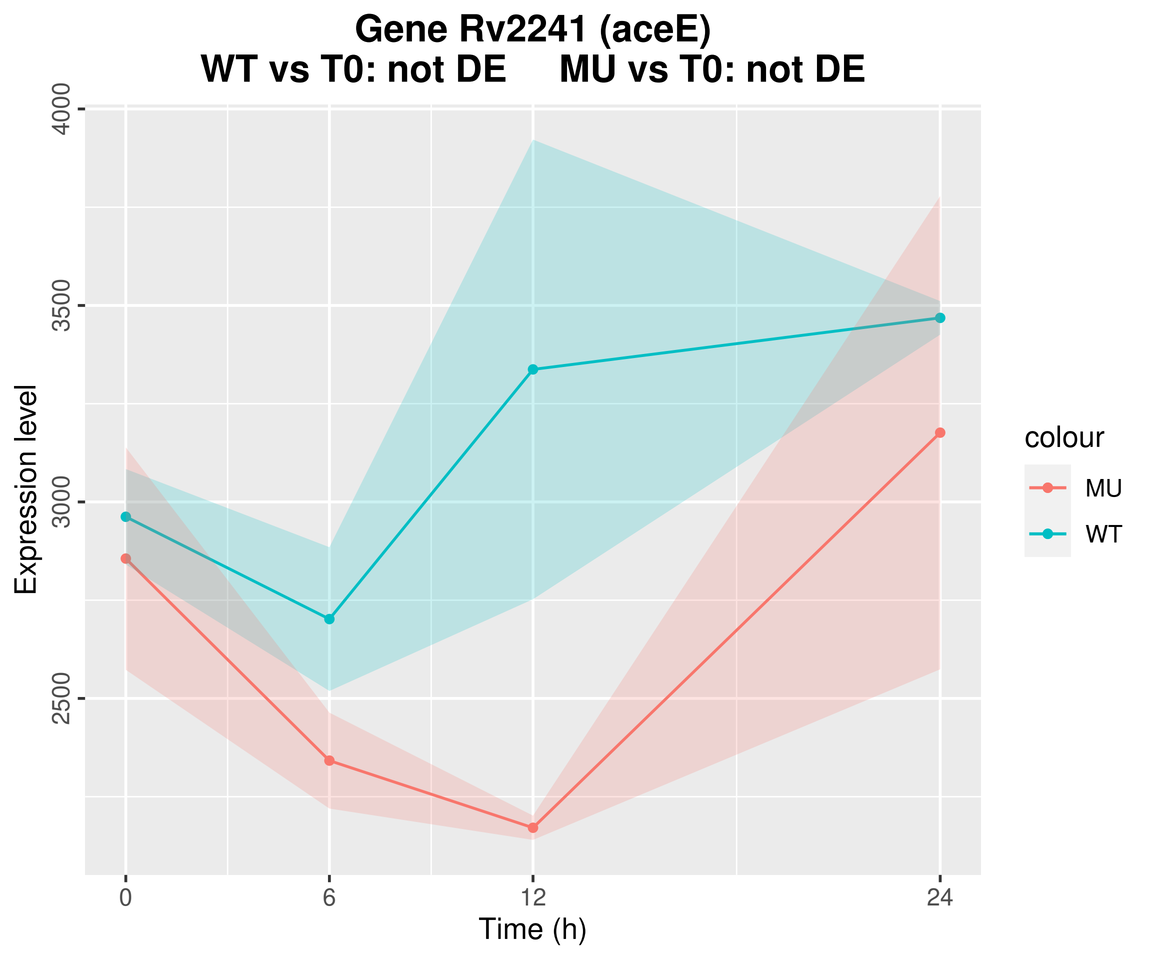

Gene Rv2241 (aceE)
WT vs T0: not DE   MU vs T0: not DE
4000
3500
3000
2500
Expression level
0
6
12
24
Time (h)
colour
MU
WT

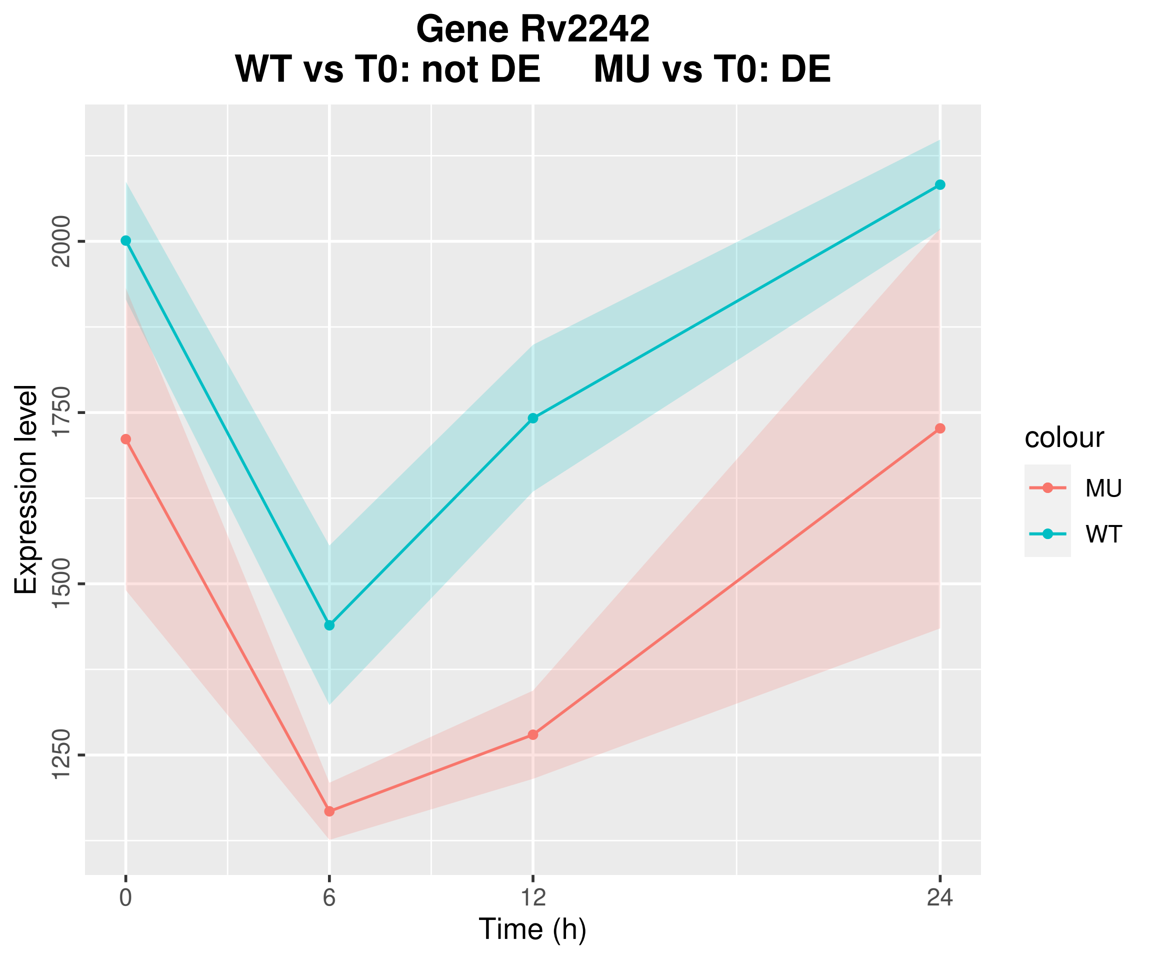

Gene Rv2242
WT vs T0: not DE MU vs T0: DE
Expression level
1250
1500
1750
2000
0
6
12
24
Time (h)
colour
MU
WT

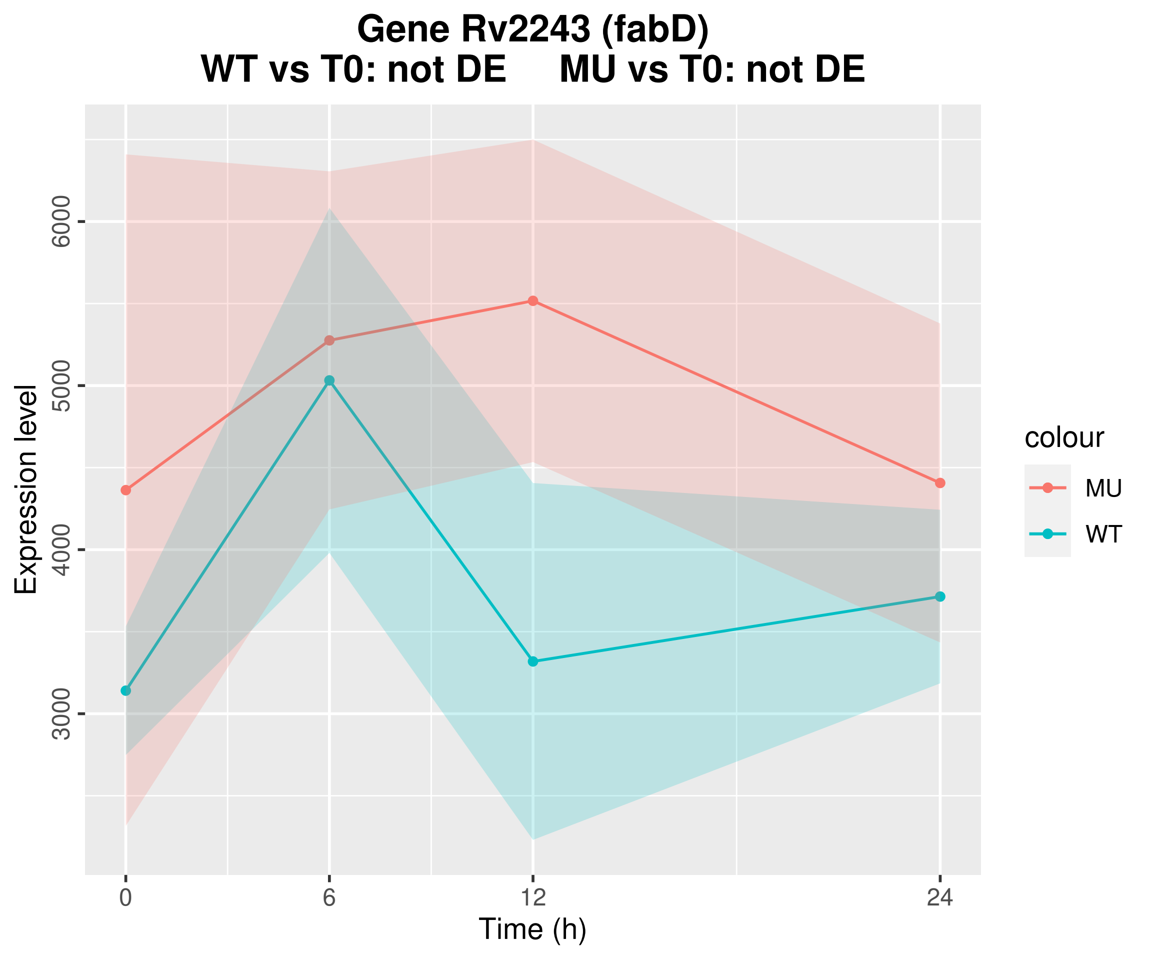
Gene Rv2243 (fabD)
WT vs T0: not DE   MU vs T0: not DE
Expression level
6000
5000
4000
3000
0
6
12
24
Time (h)
colour
MU
WT

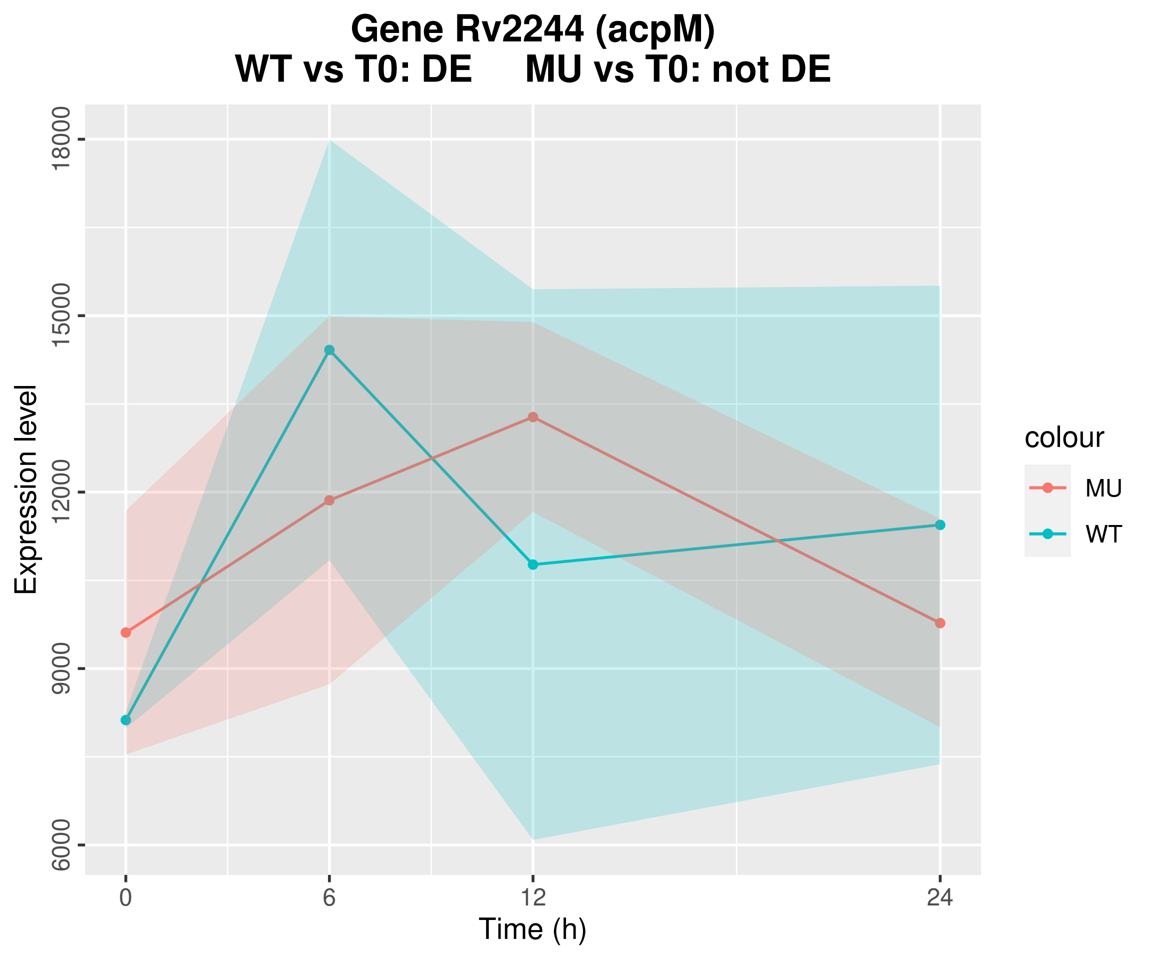

Gene Rv2244 (acpM)
WT vs T0: DE   MU vs T0: not DE
Expression level
18000
15000
12000
9000
6000
0
6
12
24
Time (h)
colour
MU
WT

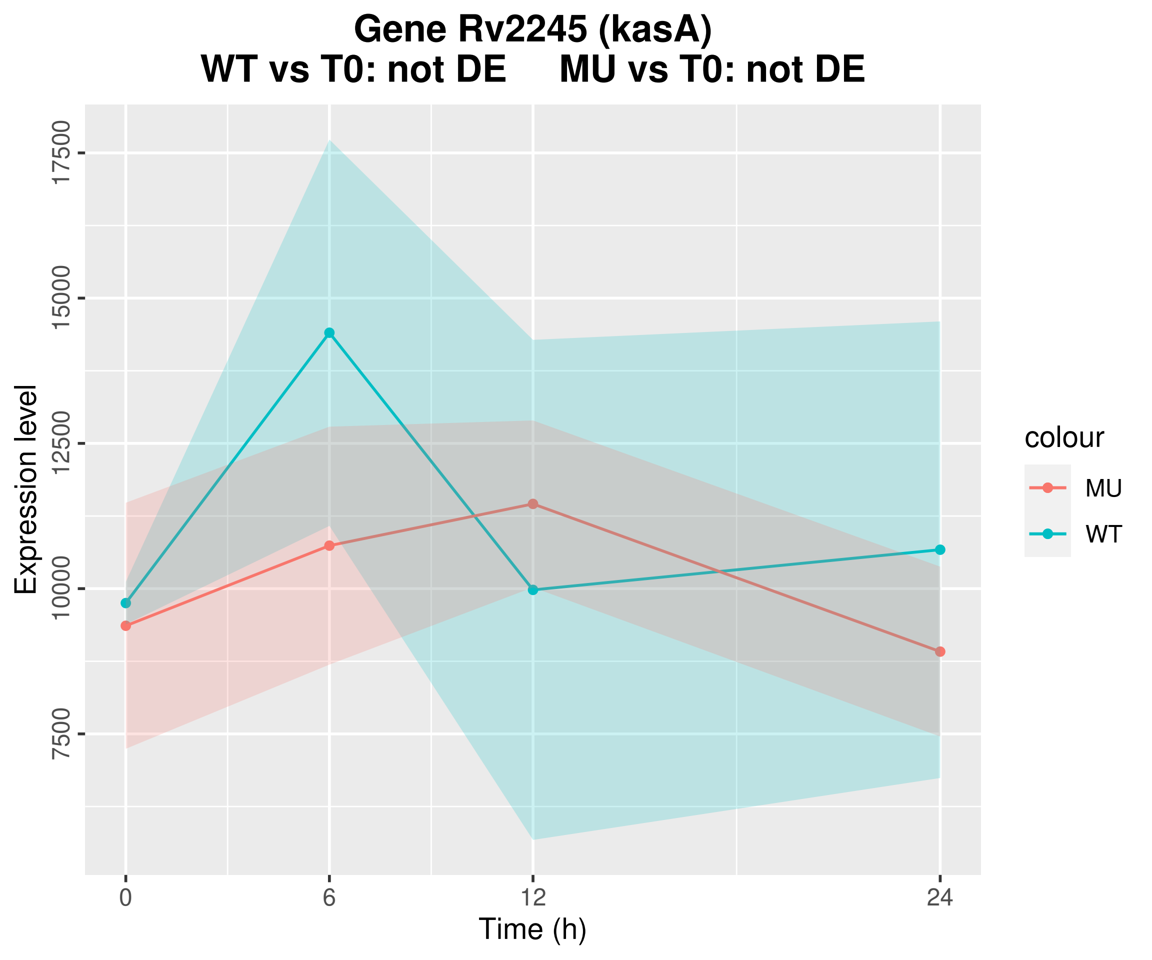

Gene Rv2245 (kasA)
WT vs T0: not DE   MU vs T0: not DE
17500
15000
12500
10000
7500
Expression level
0
6
12
24
Time (h)
colour
MU
WT

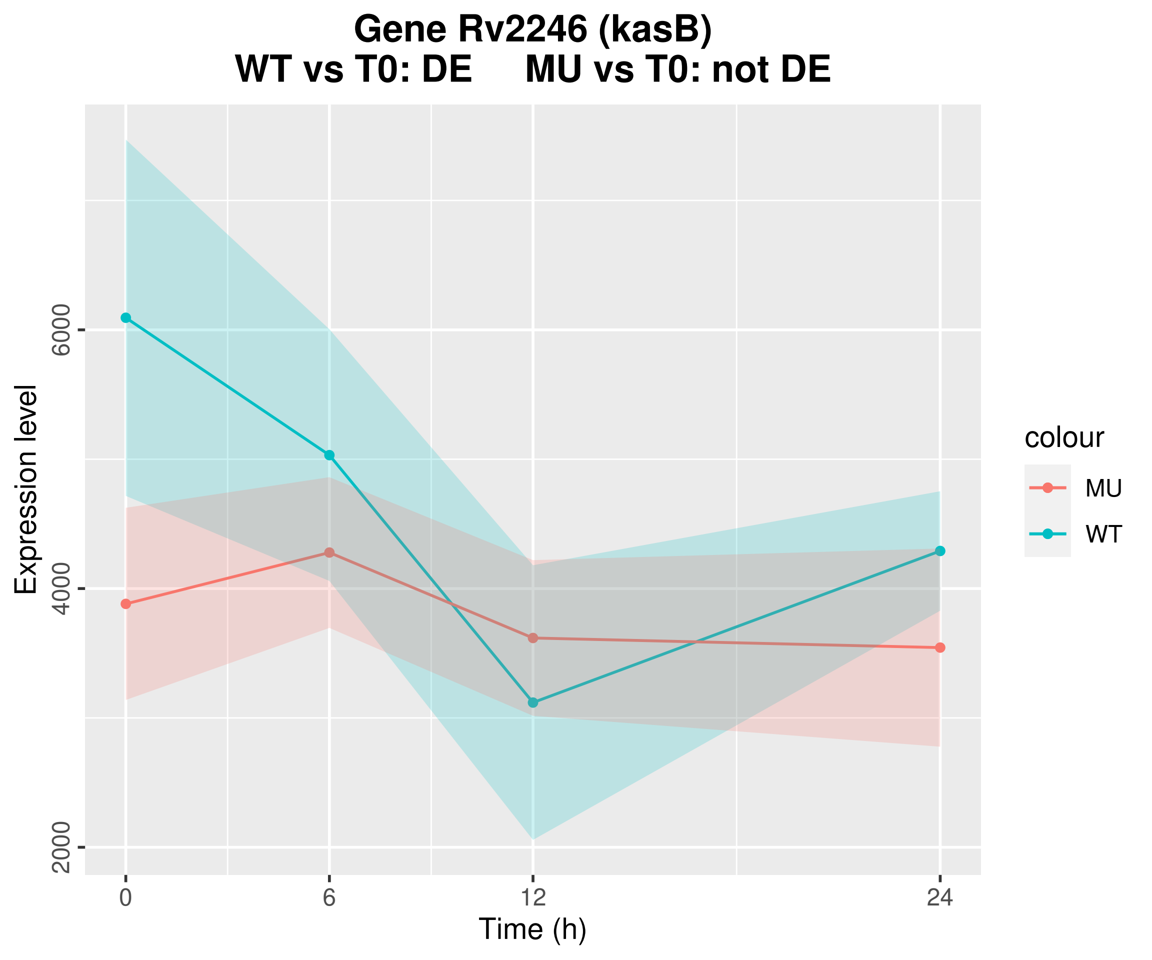
Gene Rv2246 (kasB)
WT vs T0: DE   MU vs T0: not DE
Expression level
2000
4000
6000
0
6
12
24
Time (h)
colour
MU
WT

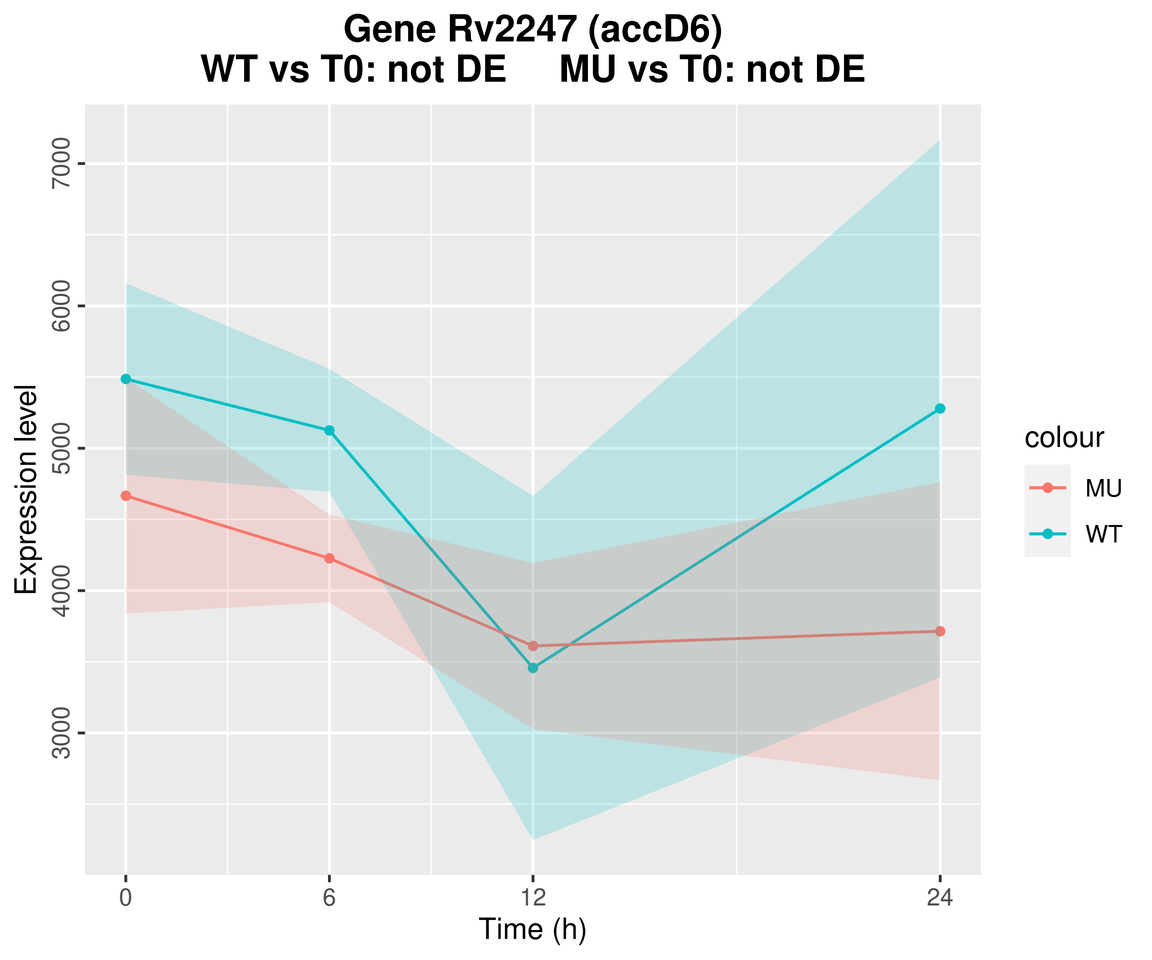
Gene Rv2247 (accD6)
WT vs T0: not DE   MU vs T0: not DE
Expression level
7000
6000
5000
4000
3000
0
6
12
24
Time (h)
colour
MU
WT

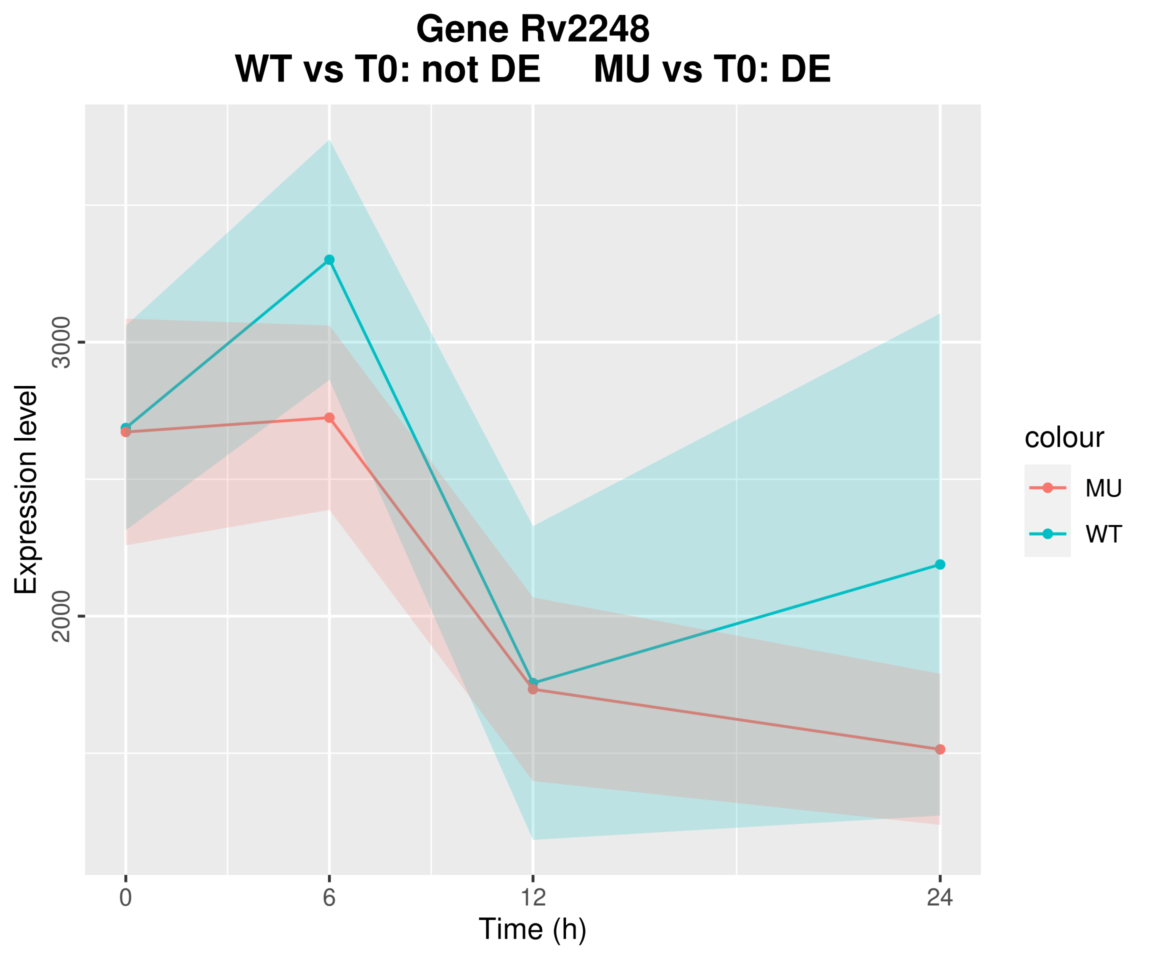

Gene Rv2248
WT vs T0: not DE   MU vs T0: DE
3000
2000
Expression level
0
6
12
24
Time (h)
colour
MU
WT

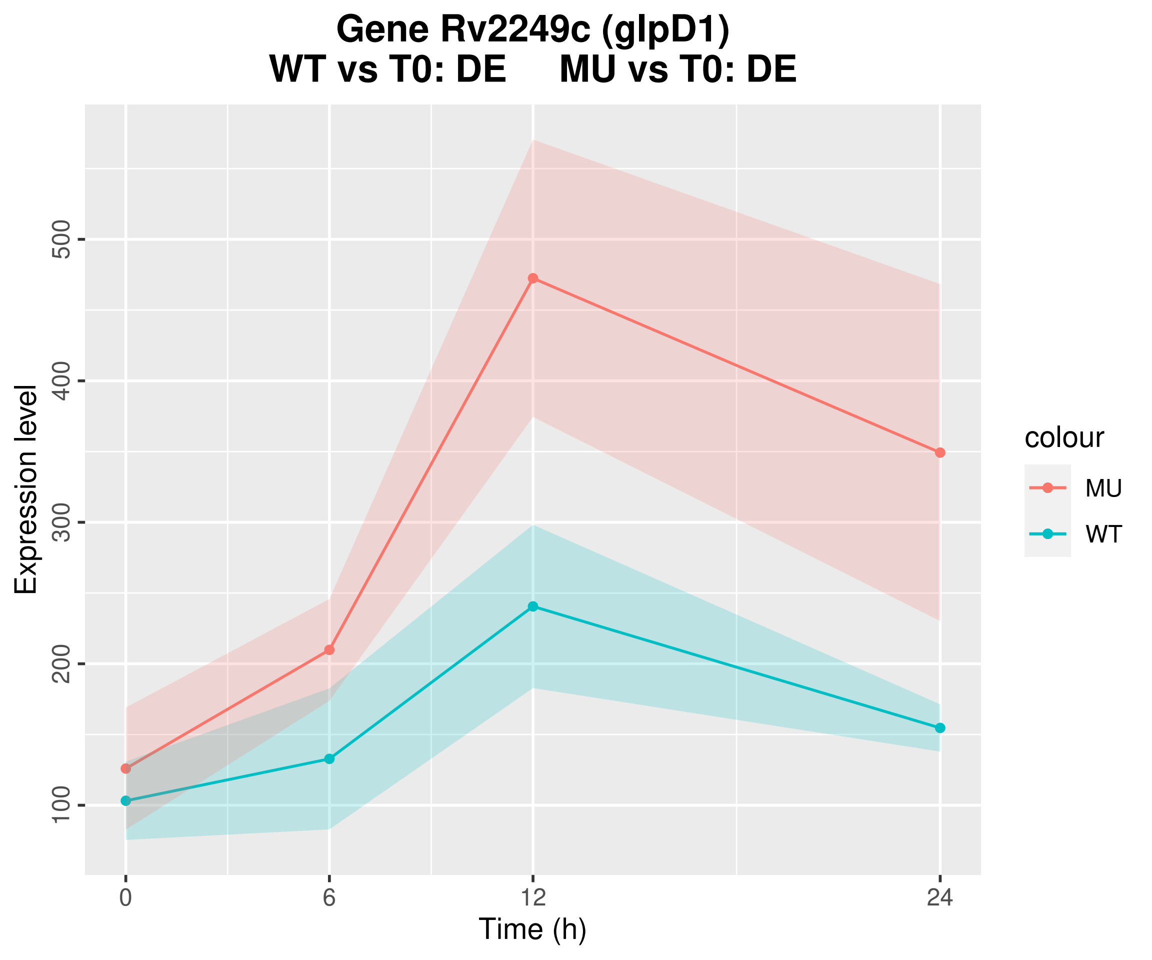

Gene Rv2249c (glpD1)
WT vs T0: DE   MU vs T0: DE
Expression level
500
400
300
200
100
0
6
12
24
Time (h)
colour
MU
WT

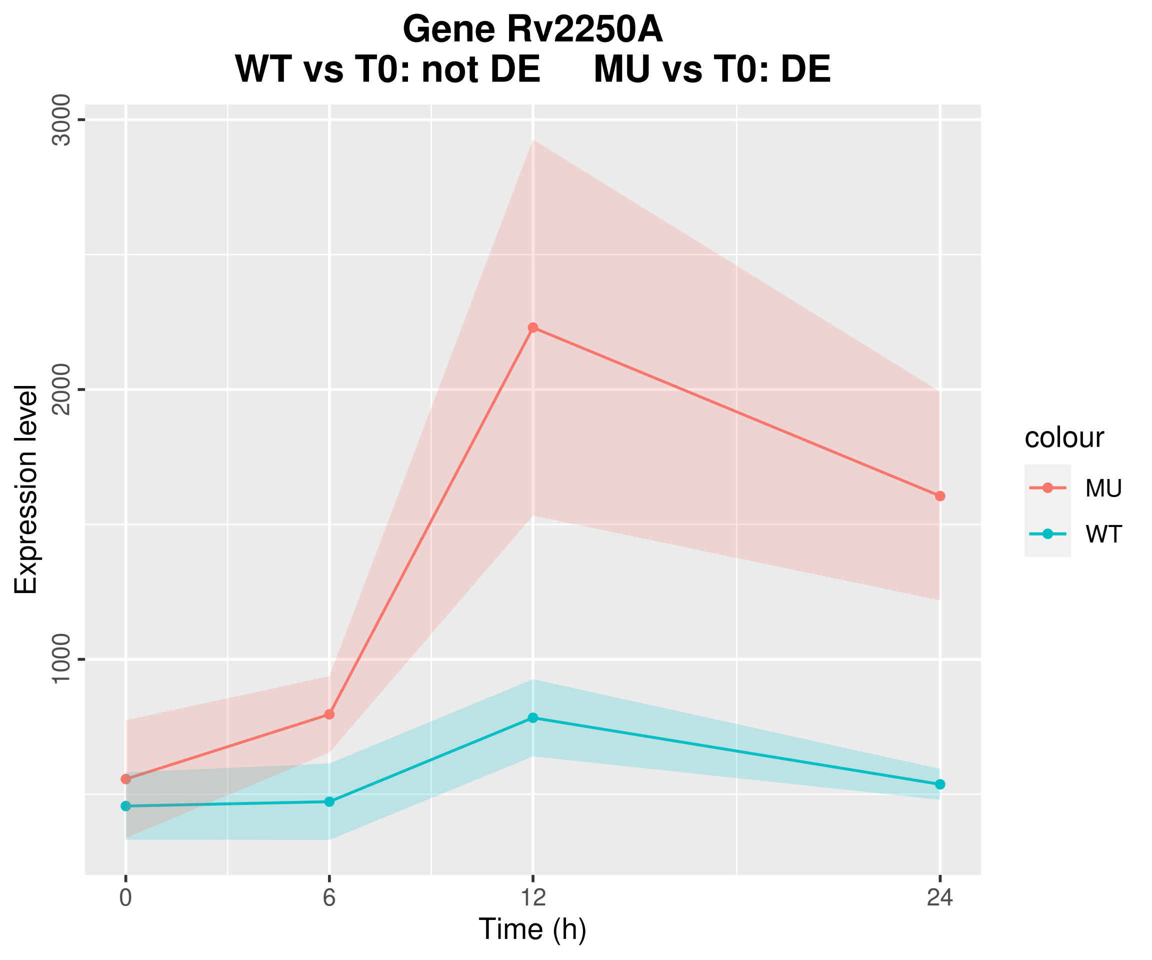

Gene Rv2250A
WT vs T0: not DE MU vs T0: DE
3000
2000
1000
Expression level
0
6
12
24
Time (h)
colour
MU
WT

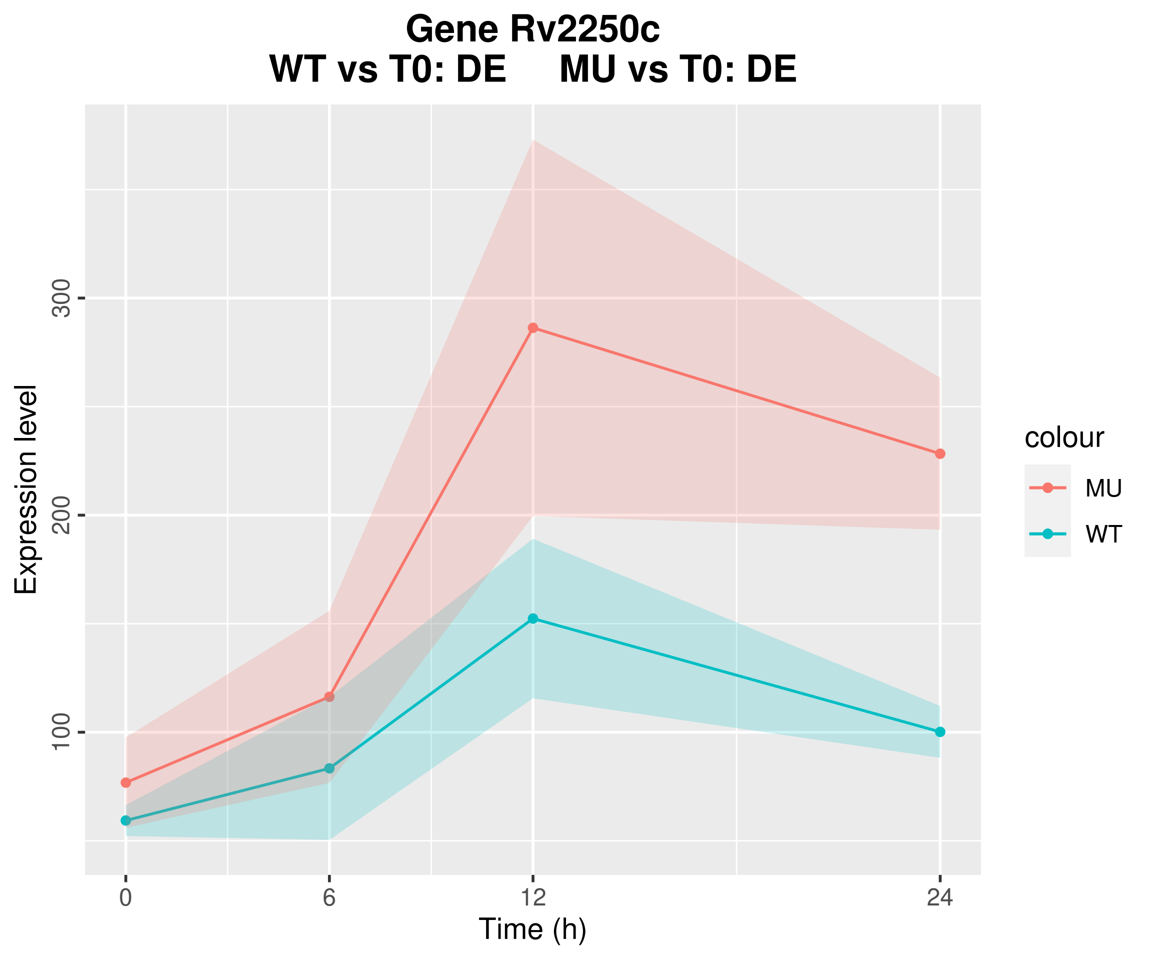

Gene Rv2250c
WT vs T0: DE MU vs T0: DE
Expression level
300
200
100
0
6
12
24
Time (h)
colour
MU
WT

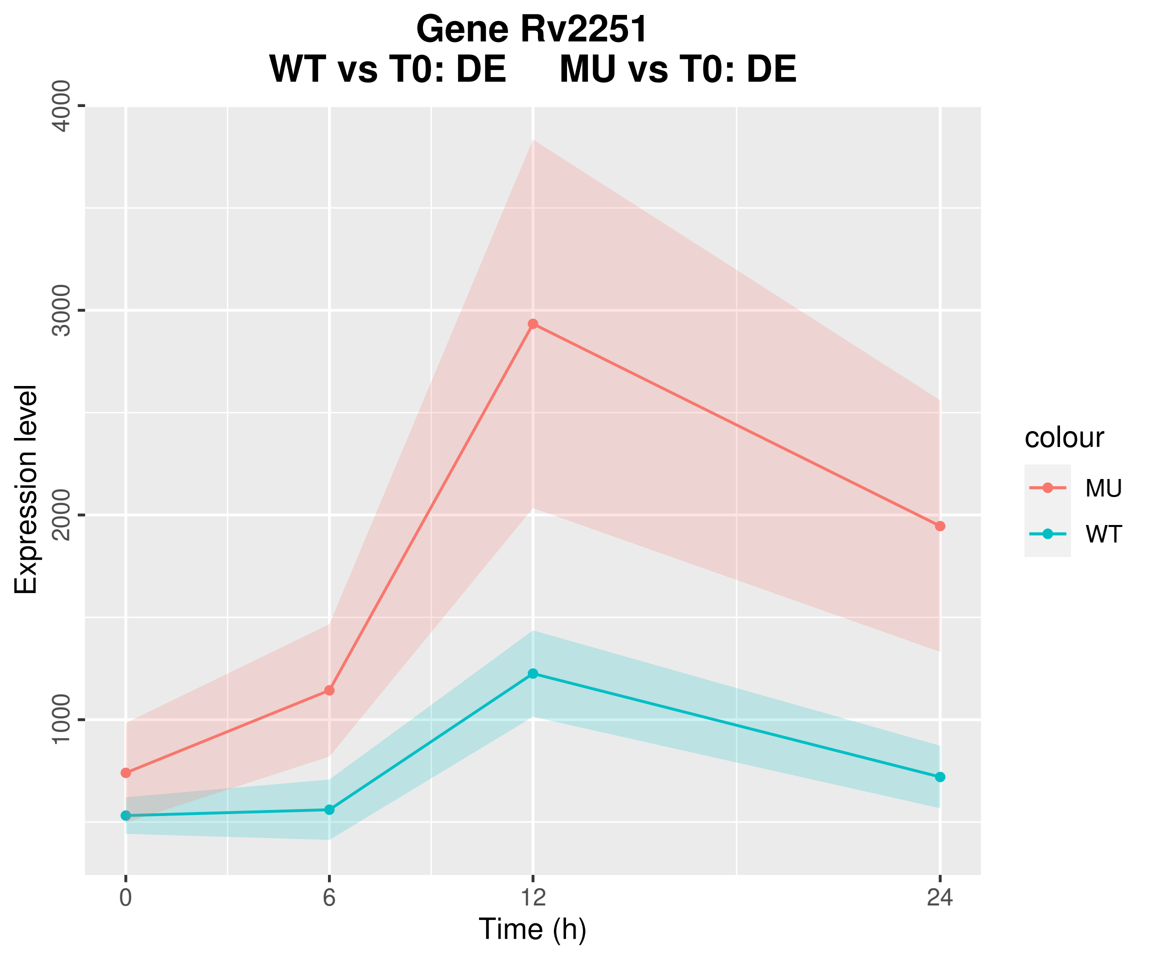

Gene Rv2251
WT vs T0: DE MU vs T0: DE
4000
3000
2000
1000
Expression level
0
6
12
24
Time (h)
colour
MU
WT

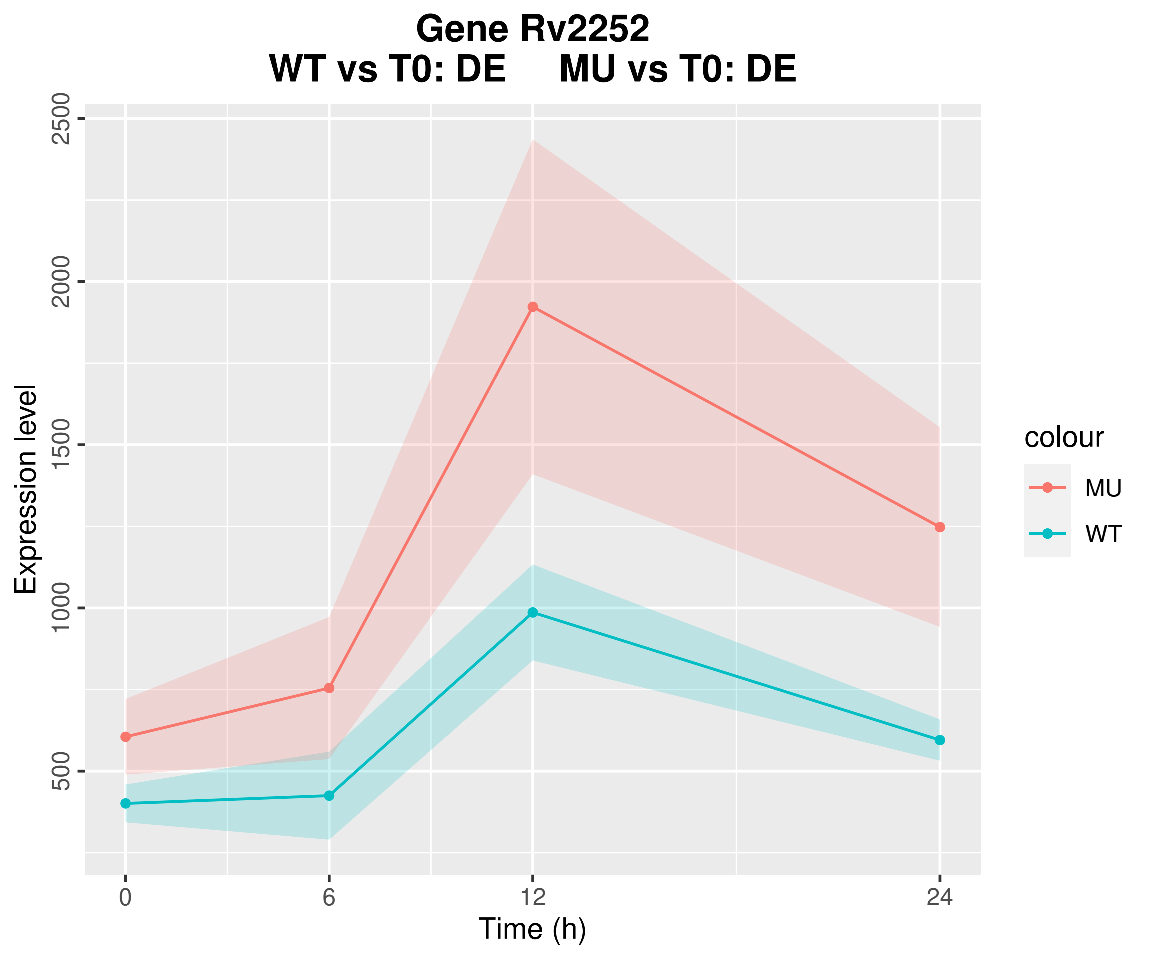
Gene Rv2252
WT vs T0: DE MU vs T0: DE
Expression level
2500
2000
1500
1000
500
0
6
12
24
Time (h)
colour
MU
WT

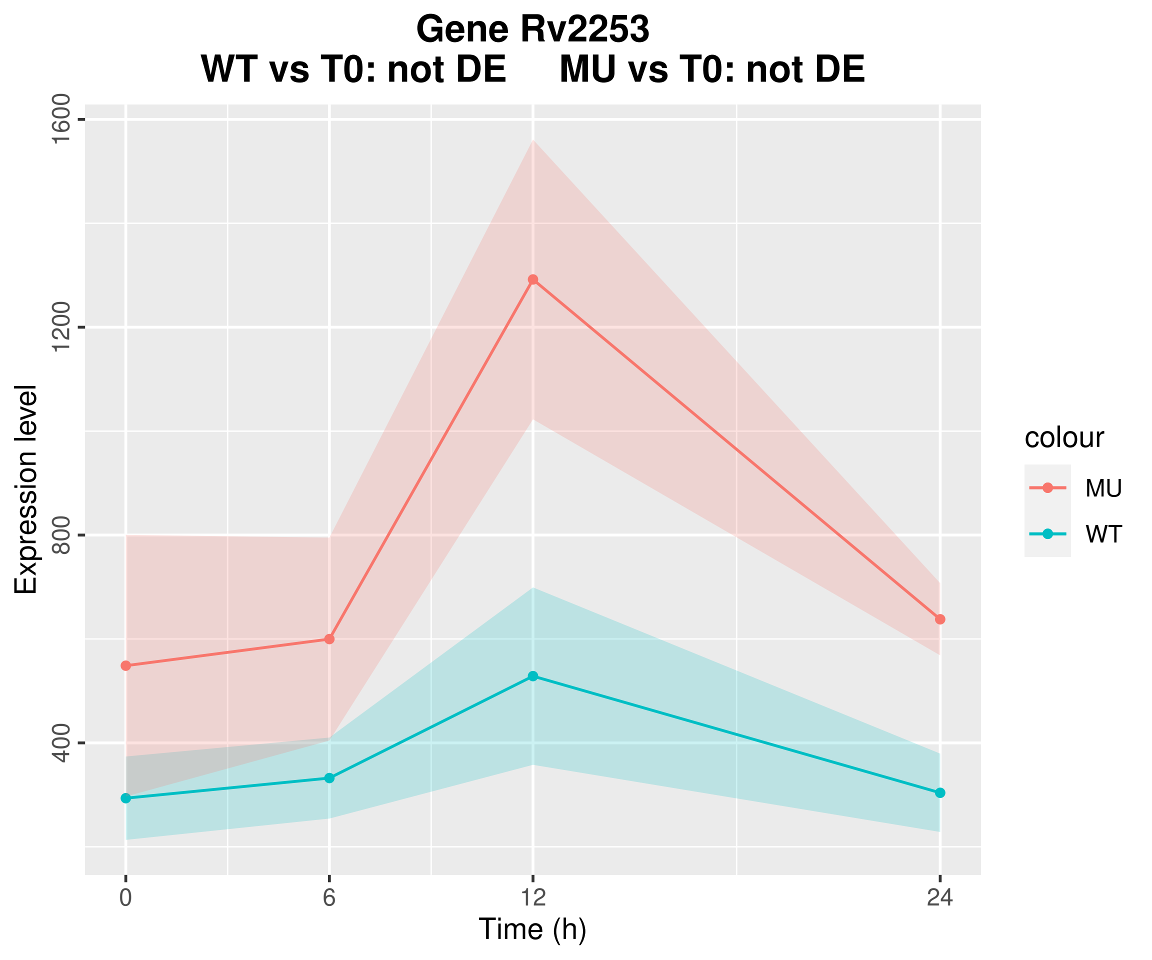

Gene Rv2253
WT vs T0: not DE   MU vs T0: not DE
1600
1200
800
400
Expression level
0
6
12
24
Time (h)
colour
MU
WT

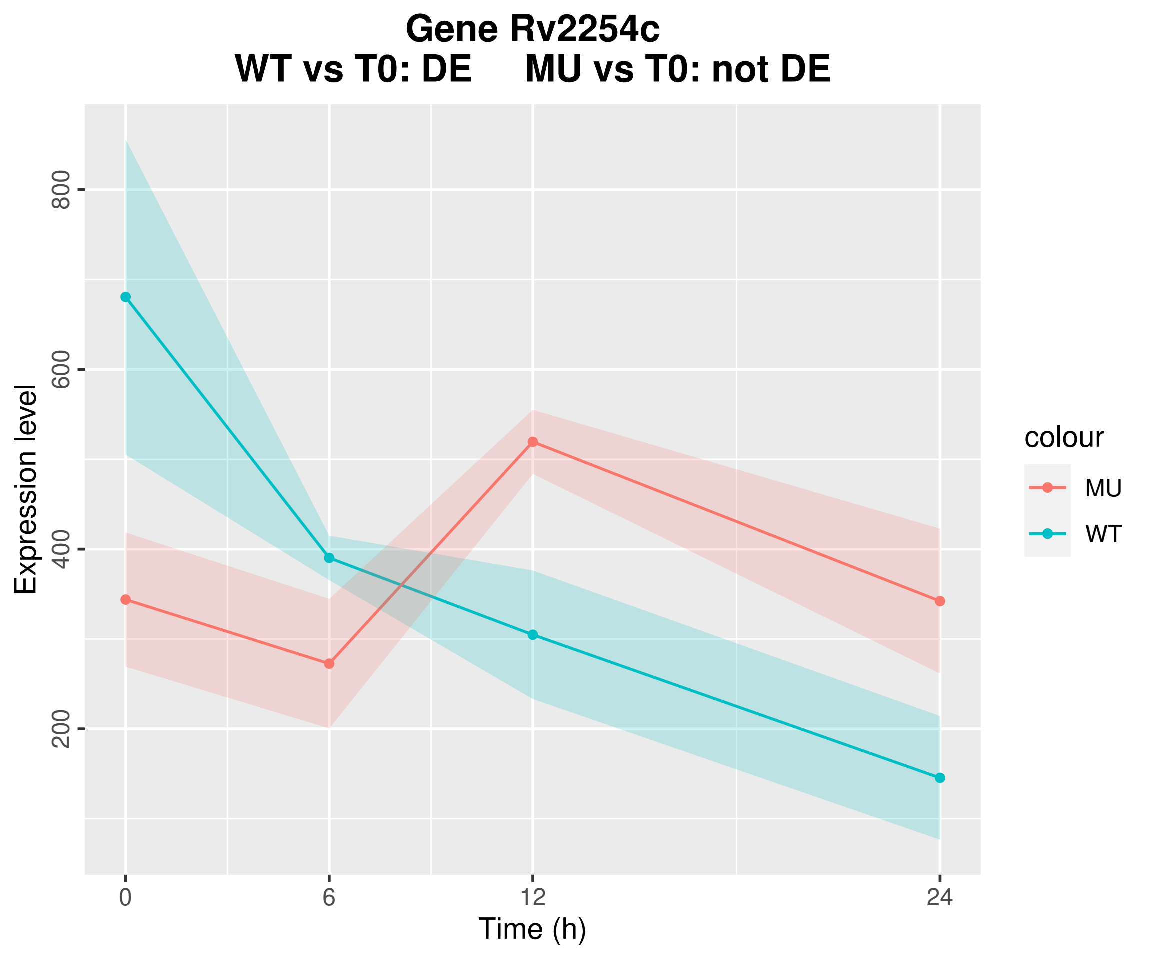
Gene Rv2254c
WT vs T0: DE   MU vs T0: not DE
Expression level
800
600
400
200
0
6
12
24
Time (h)
colour
MU
WT

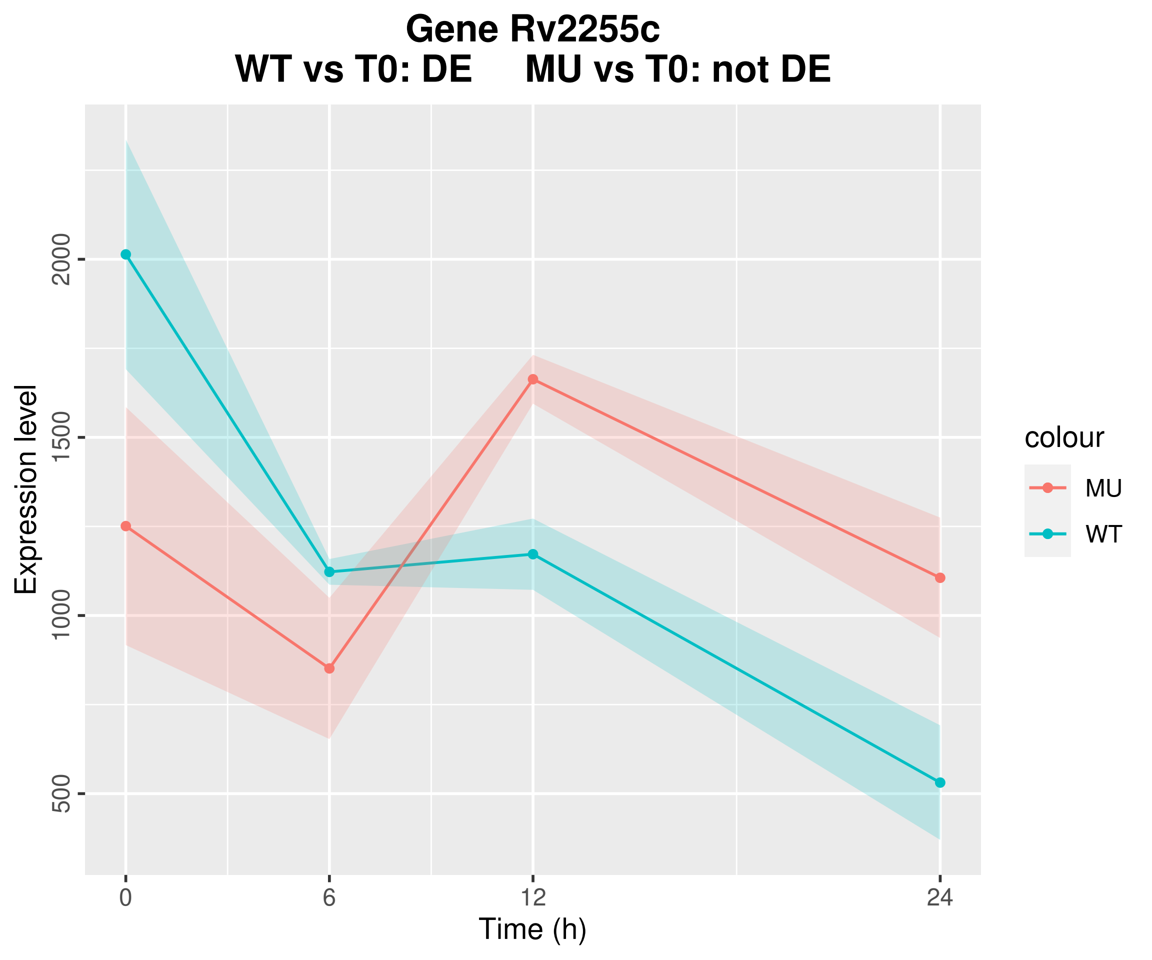
Gene Rv2255c
WT vs T0: DE   MU vs T0: not DE
Expression level
2000
1500
1000
500
0
6
12
24
Time (h)
colour
MU
WT

Gene Rv2256c
WT vs T0: DE   MU vs T0: not DE

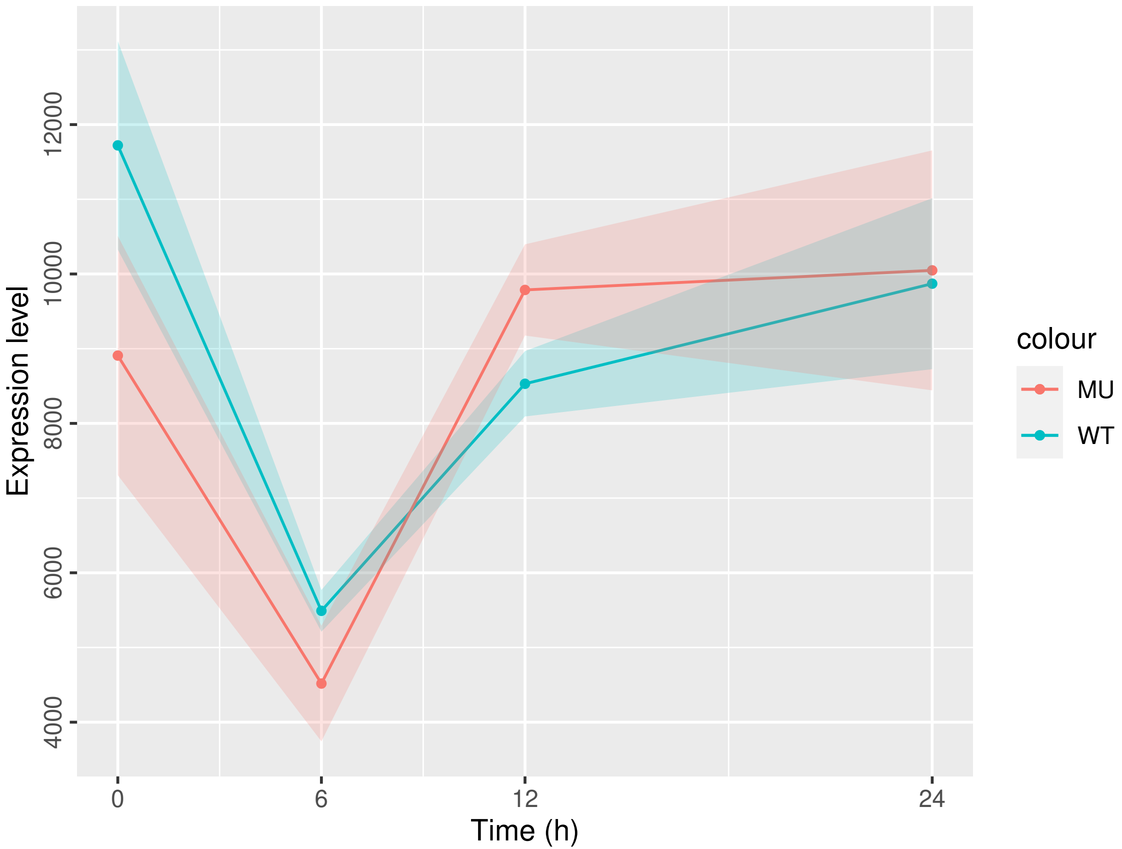

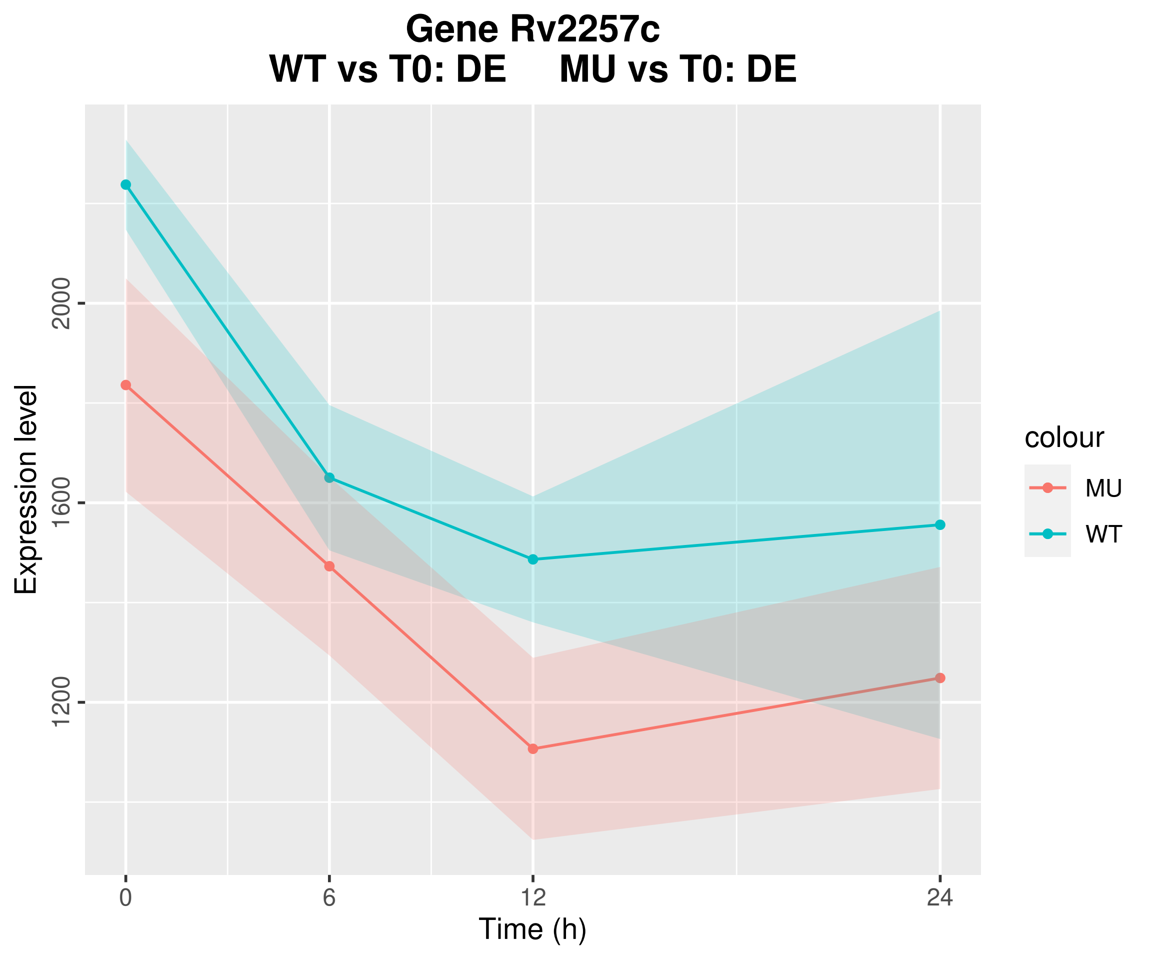

Gene Rv2257c
WT vs T0: DE   MU vs T0: DE
Expression level
2000
1600
1200
0
6
12
24
Time (h)
colour
MU
WT

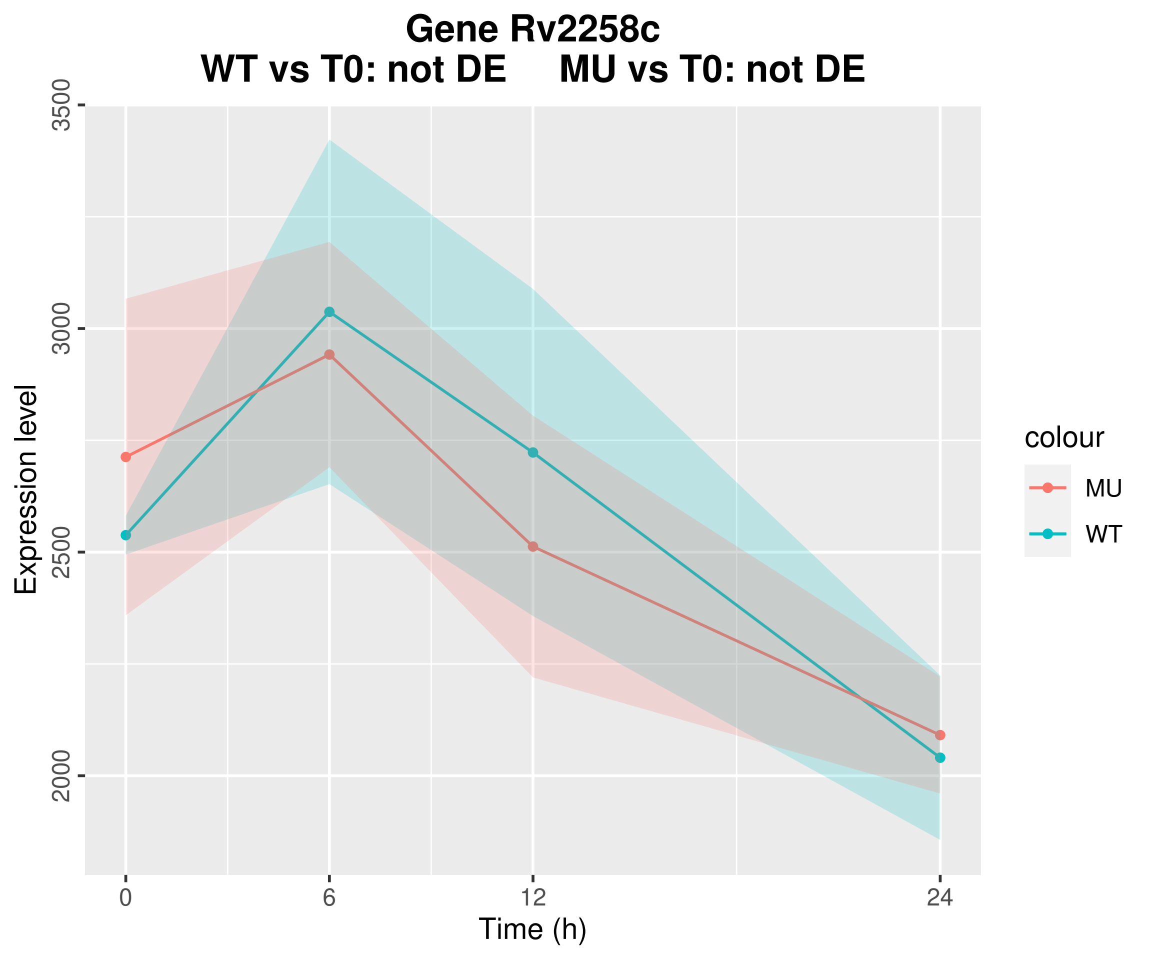

Gene Rv2258c
WT vs T0: not DE MU vs T0: not DE
3500
3000
2500
2000
Expression level
0
6
12
24
Time (h)
colour
MU
WT

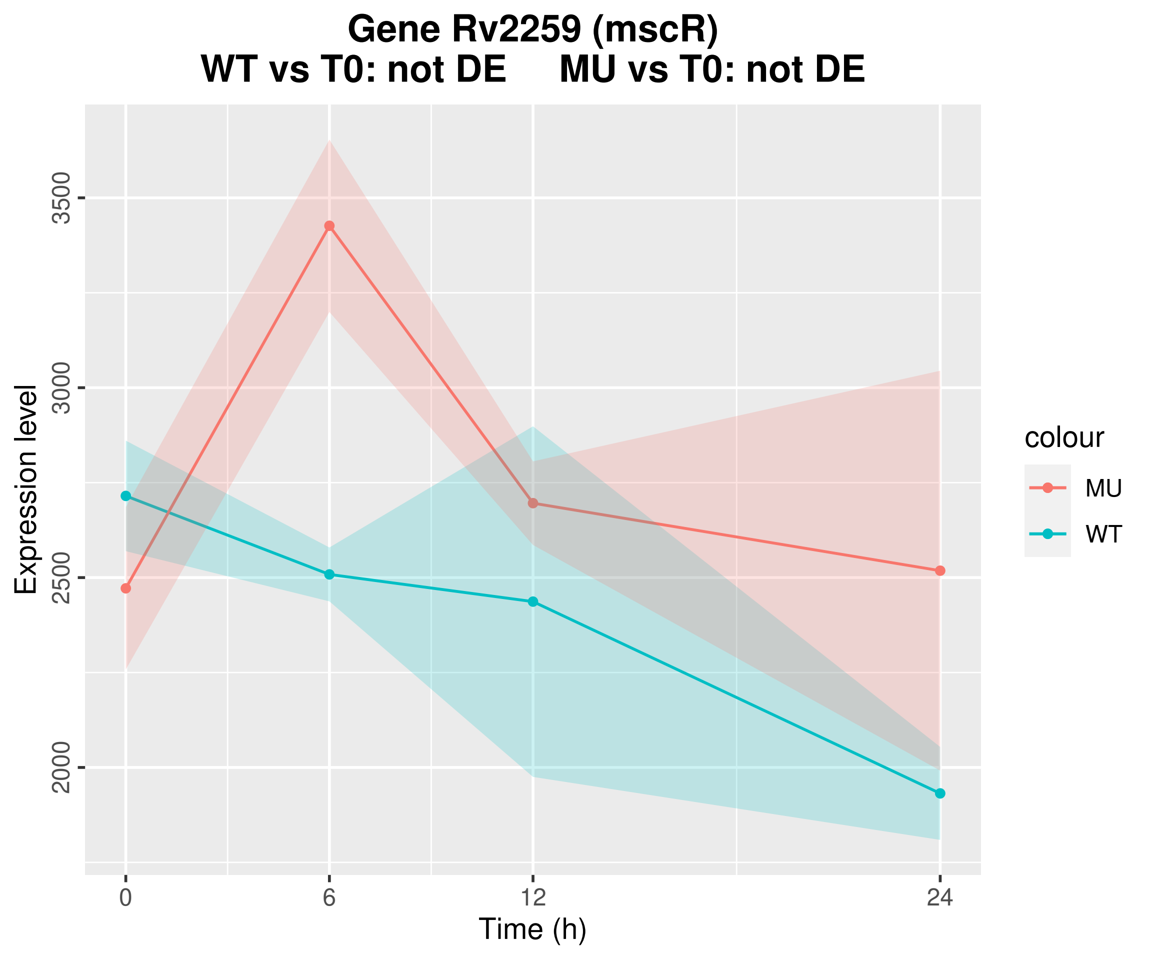

Gene Rv2259 (mscR)
WT vs T0: not DE MU vs T0: not DE
3500
3000
2500
2000
Expression level
0
6
12
24
Time (h)
colour
MU
WT

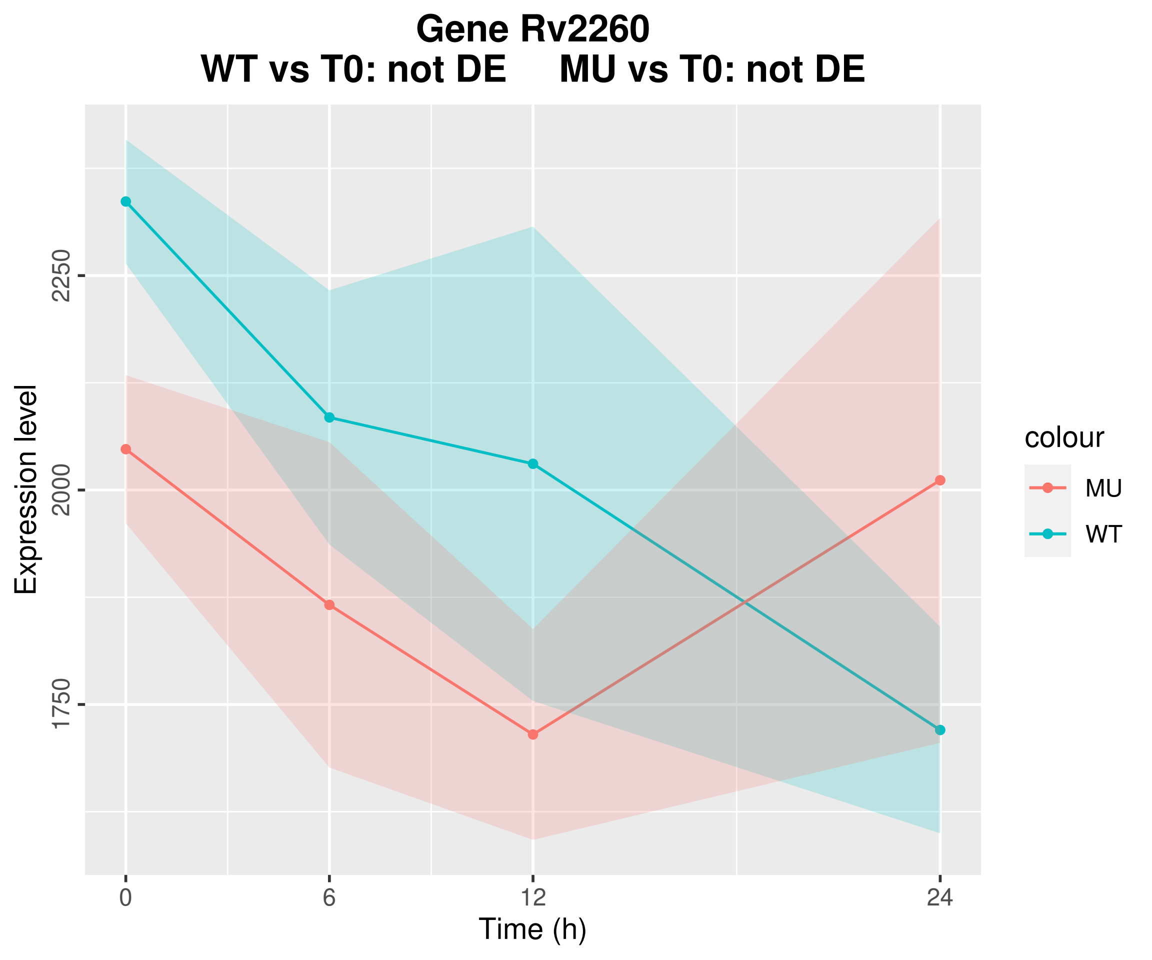
Gene Rv2260
WT vs T0: not DE   MU vs T0: not DE
Expression level
2250
2000
1750
0
6
12
24
Time (h)
colour
MU
WT

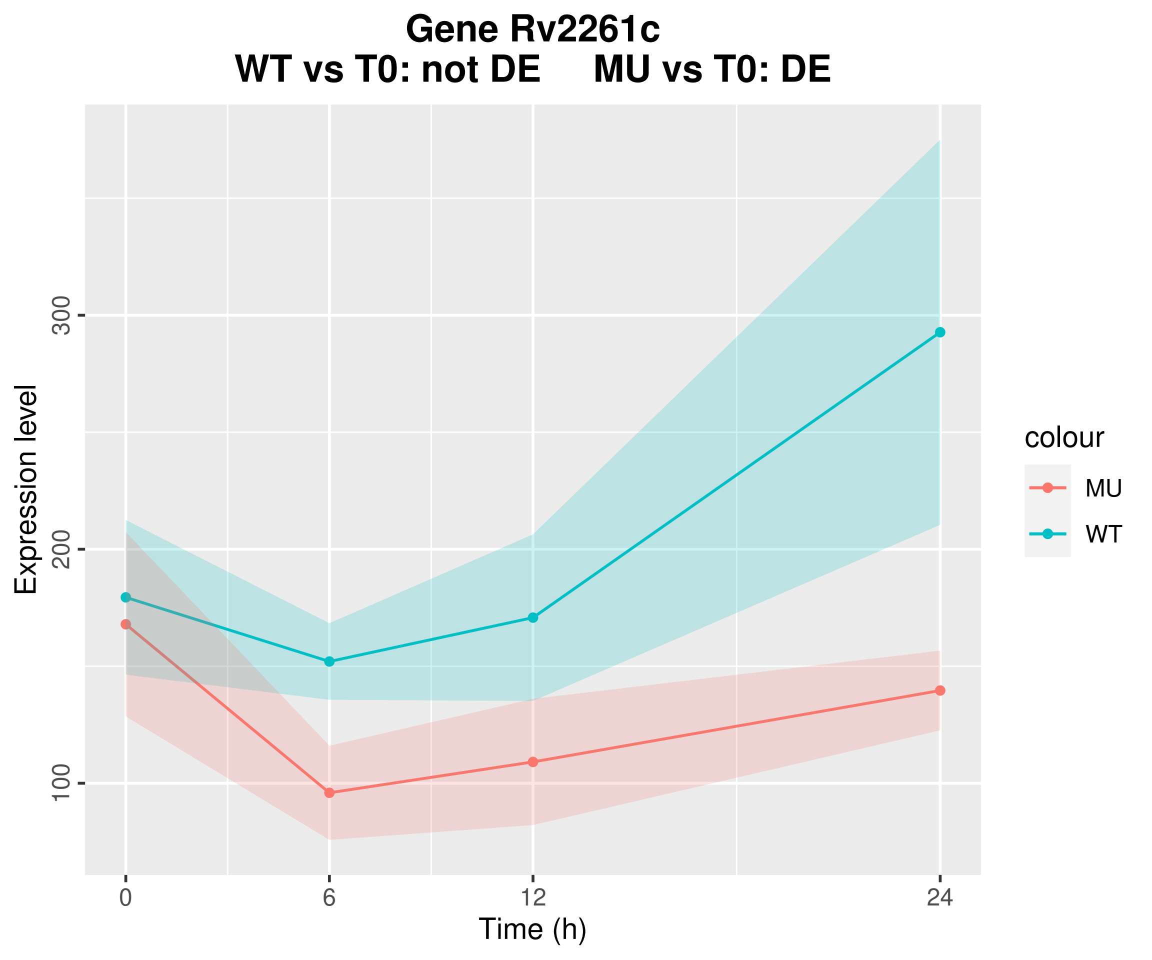

Gene Rv2261c
WT vs T0: not DE MU vs T0: DE
Expression level
300
200
100
0
6
12
24
Time (h)
colour
MU
WT

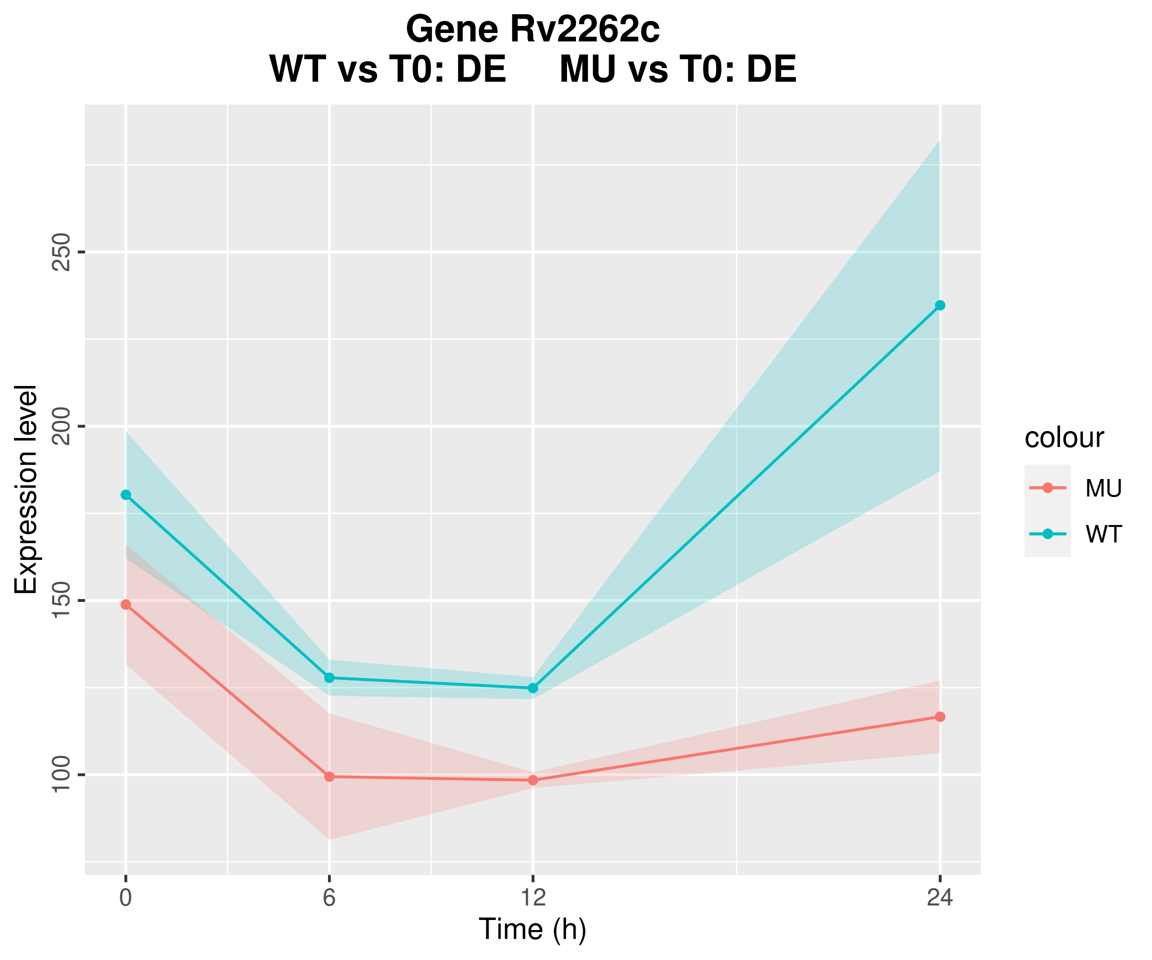

Gene Rv2262c
WT vs T0: DE MU vs T0: DE
Expression level
250
200
150
100
0
6
12
24
Time (h)
colour
MU
WT

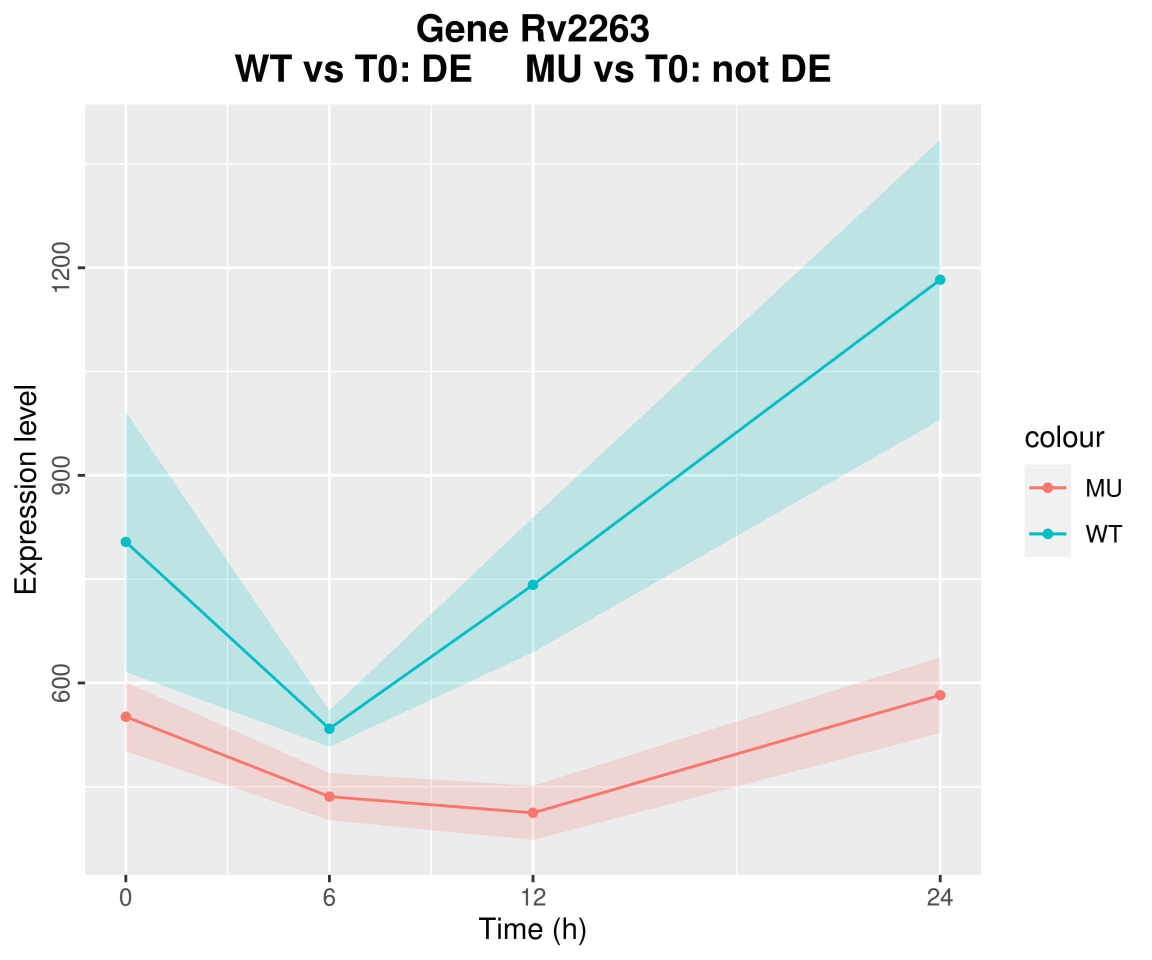
Gene Rv2263
WT vs T0: DE MU vs T0: not DE
Expression level
1200
900
600
0
6
12
24
Time (h)
colour
MU
WT

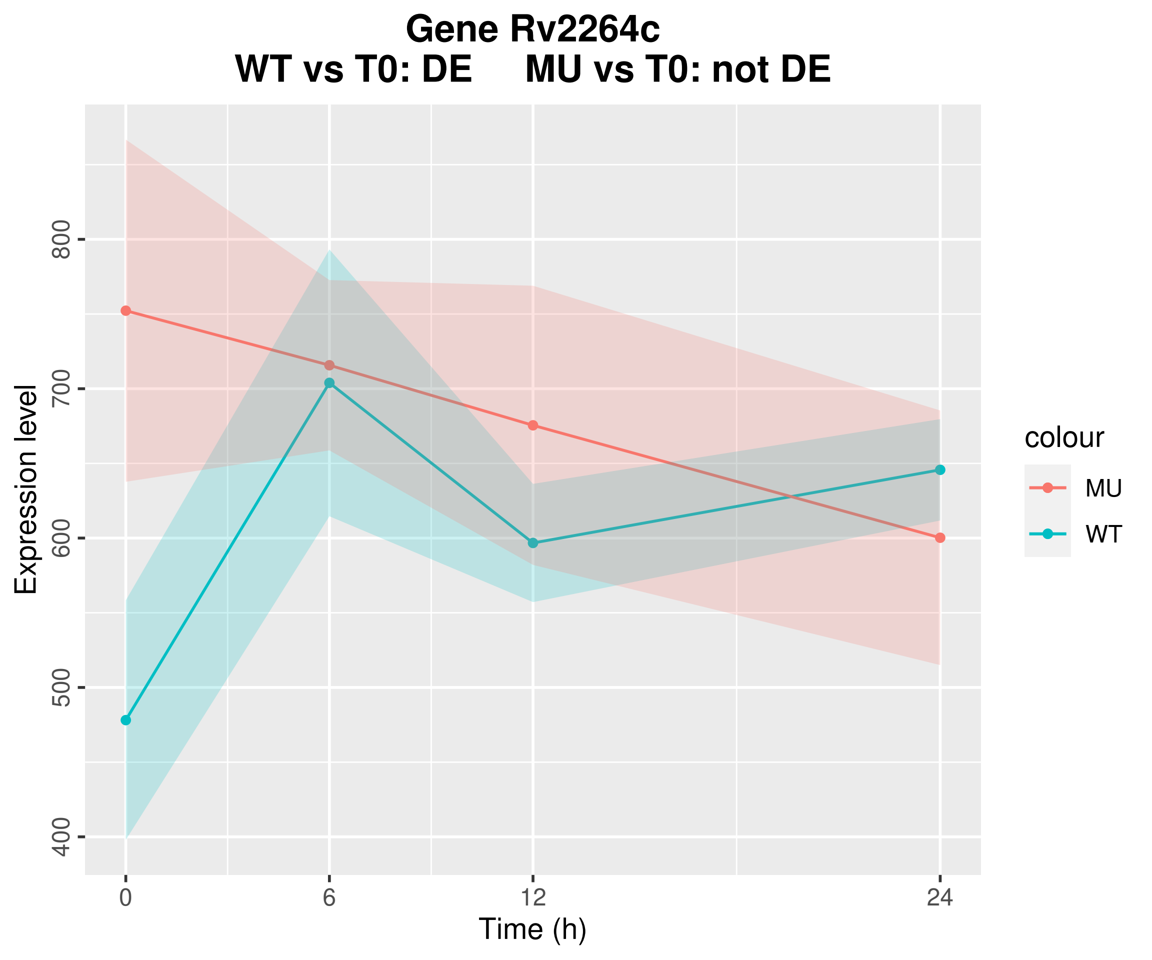
Gene Rv2264c
WT vs T0: DE   MU vs T0: not DE
Expression level
800
700
600
500
400
0
6
12
24
Time (h)
colour
MU
WT

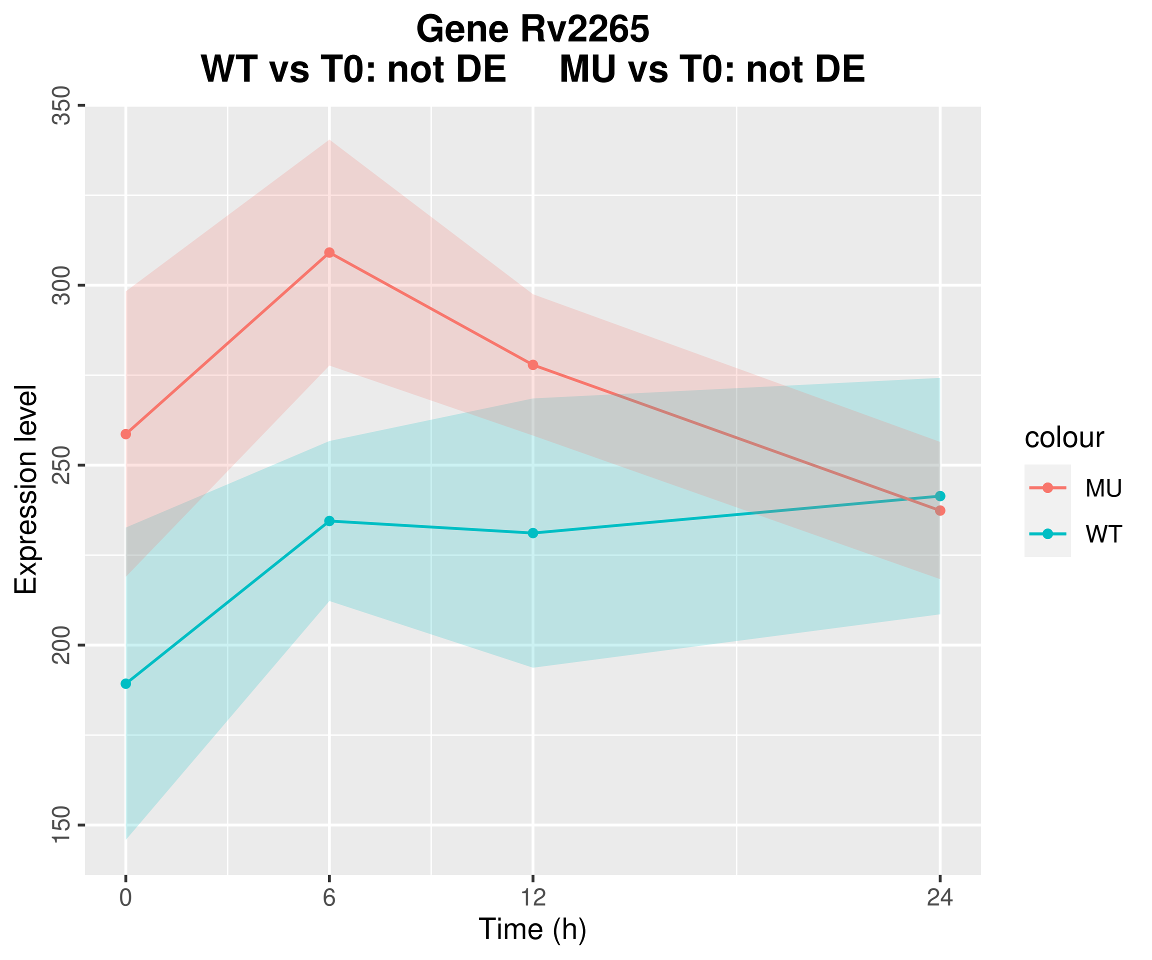
Gene Rv2265
WT vs T0: not DE MU vs T0: not DE
Expression level
350
300
250
200
150
0
6
12
24
Time (h)
colour
MU
WT

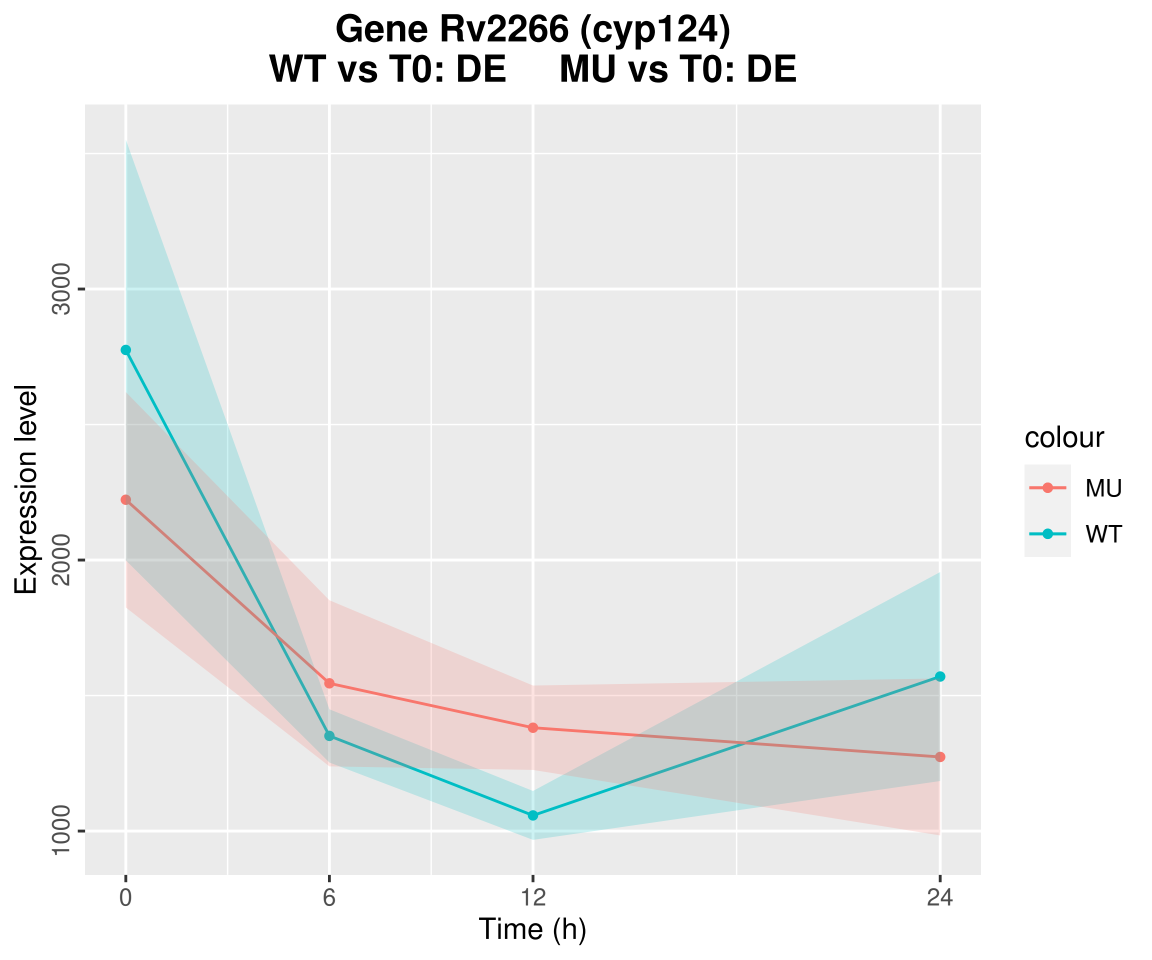
Gene Rv2266 (cyp124)
WT vs T0: DE   MU vs T0: DE
Expression level
3000
2000
1000
0
6
12
24
Time (h)
colour
MU
WT

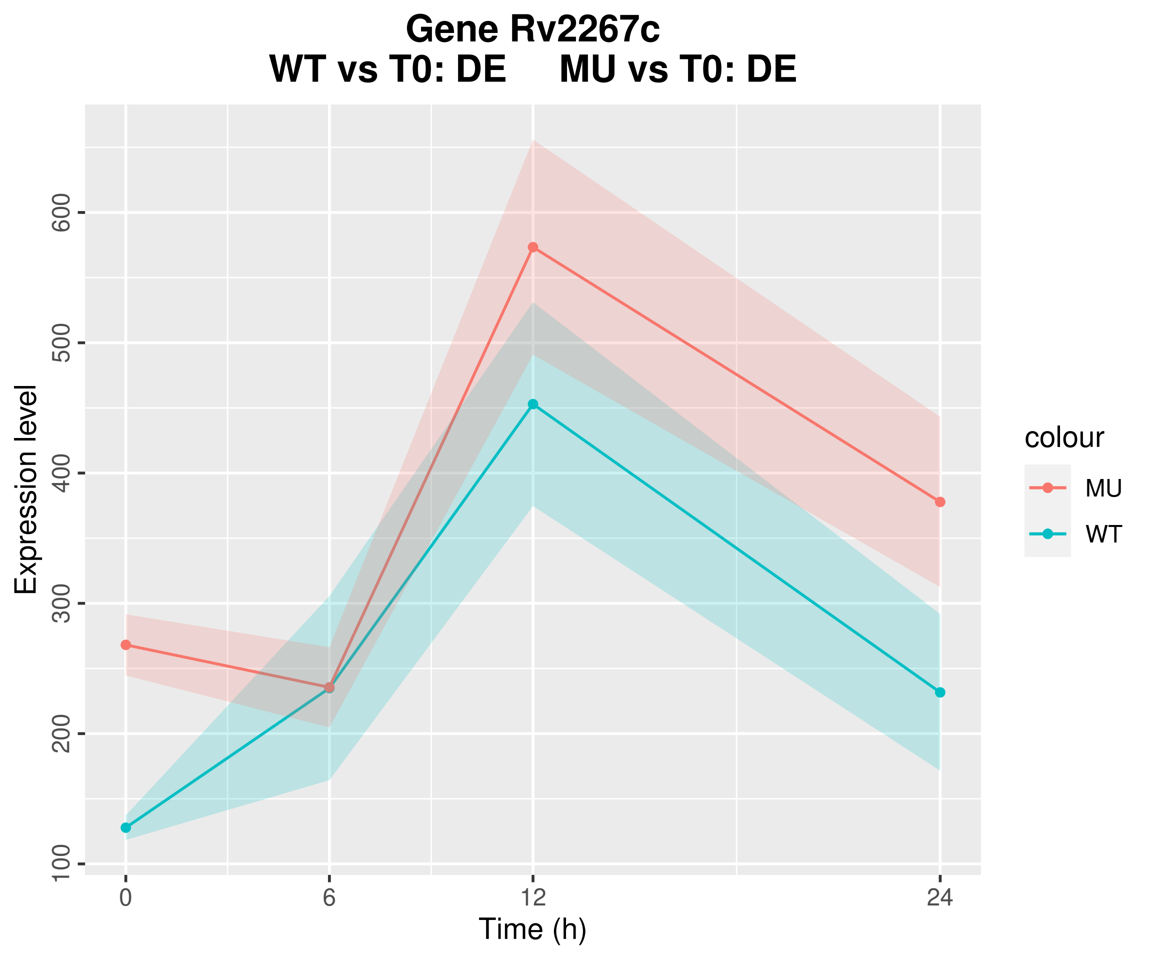

Gene Rv2267c
WT vs T0: DE MU vs T0: DE
Expression level
100
200
300
400
500
600
0
6
12
24
Time (h)
colour
MU
WT

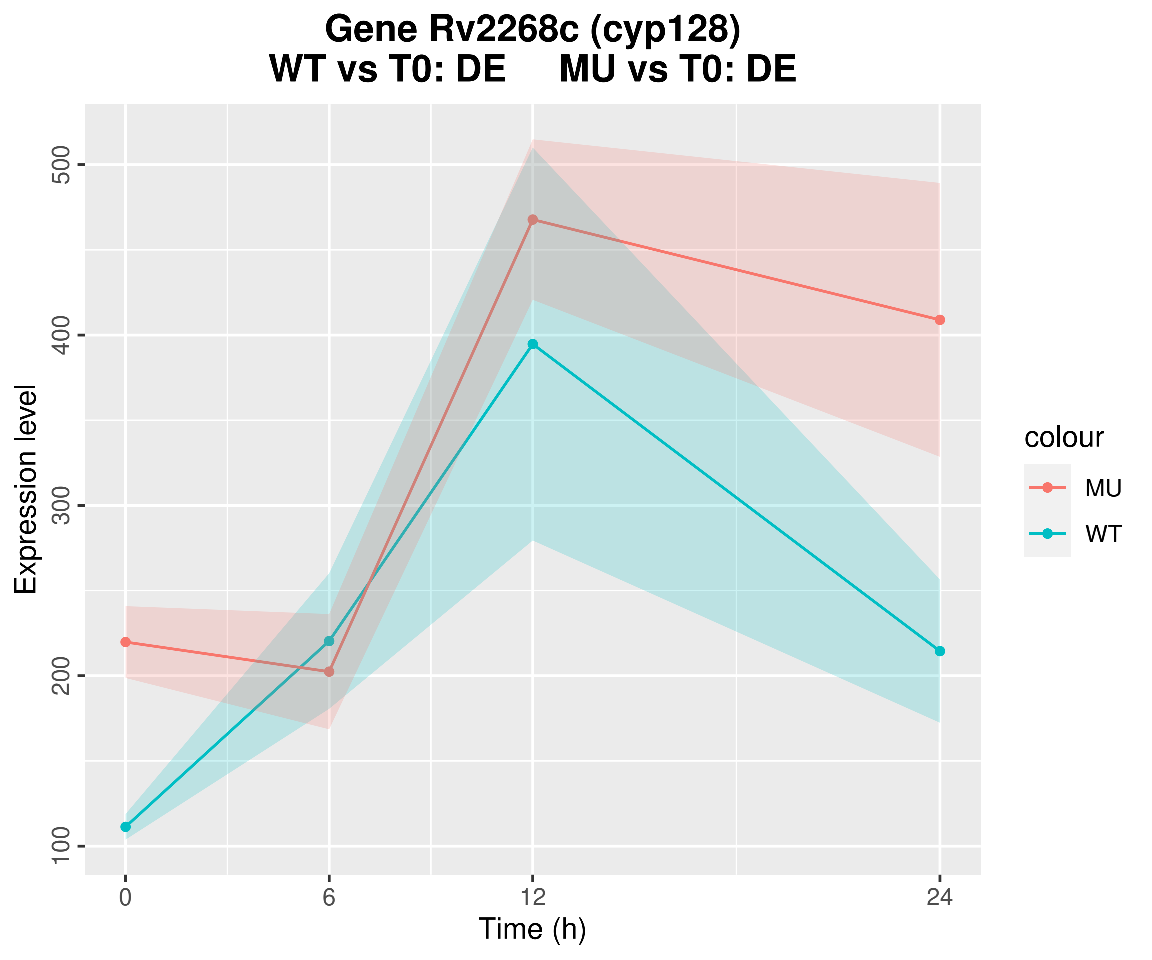
Gene Rv2268c (cyp128)
WT vs T0: DE MU vs T0: DE
Expression level
500
400
300
200
100
0
6
12
24
Time (h)
colour
MU
WT

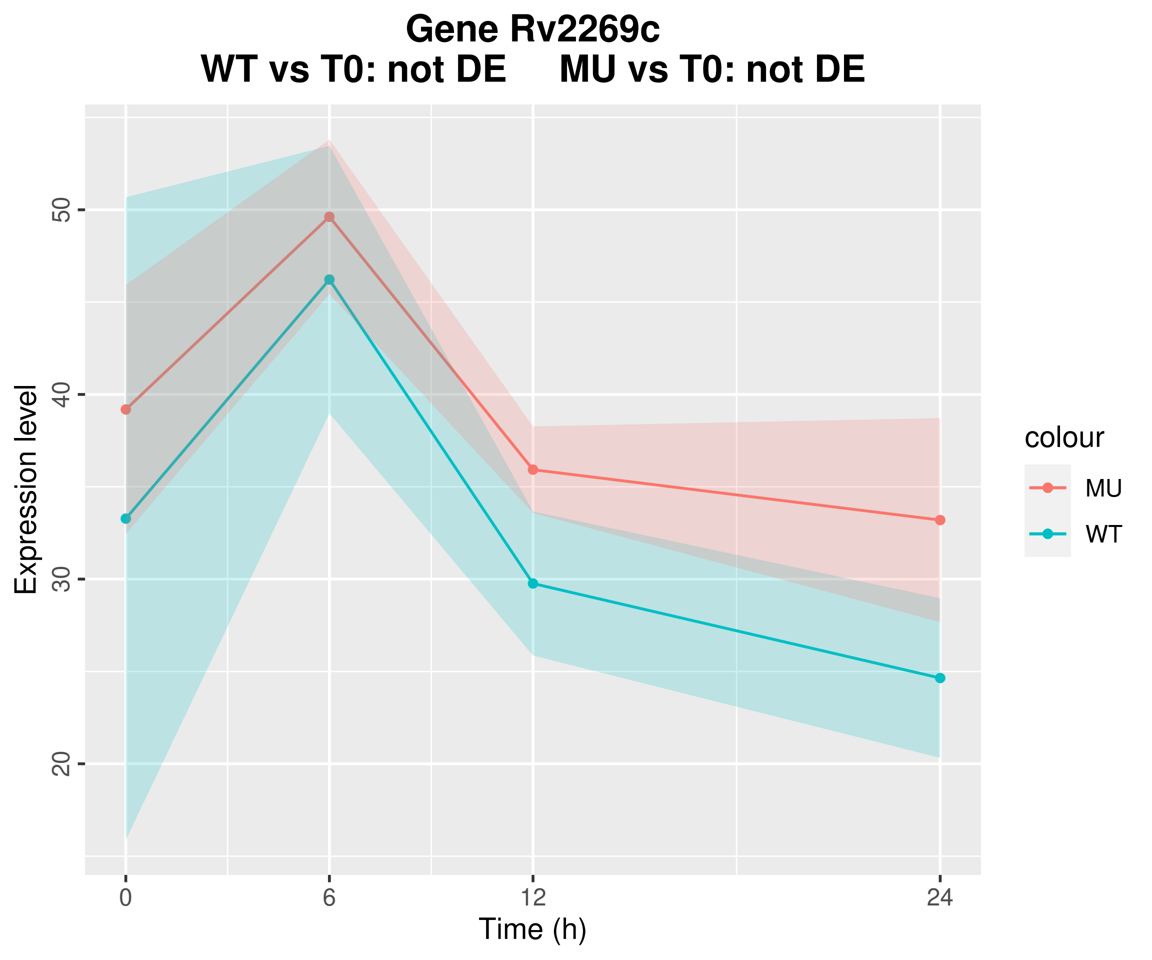
Gene Rv2269c
WT vs T0: not DE   MU vs T0: not DE
Expression level
Time (h)
0
6
12
24
20
30
40
50
colour
MU
WT

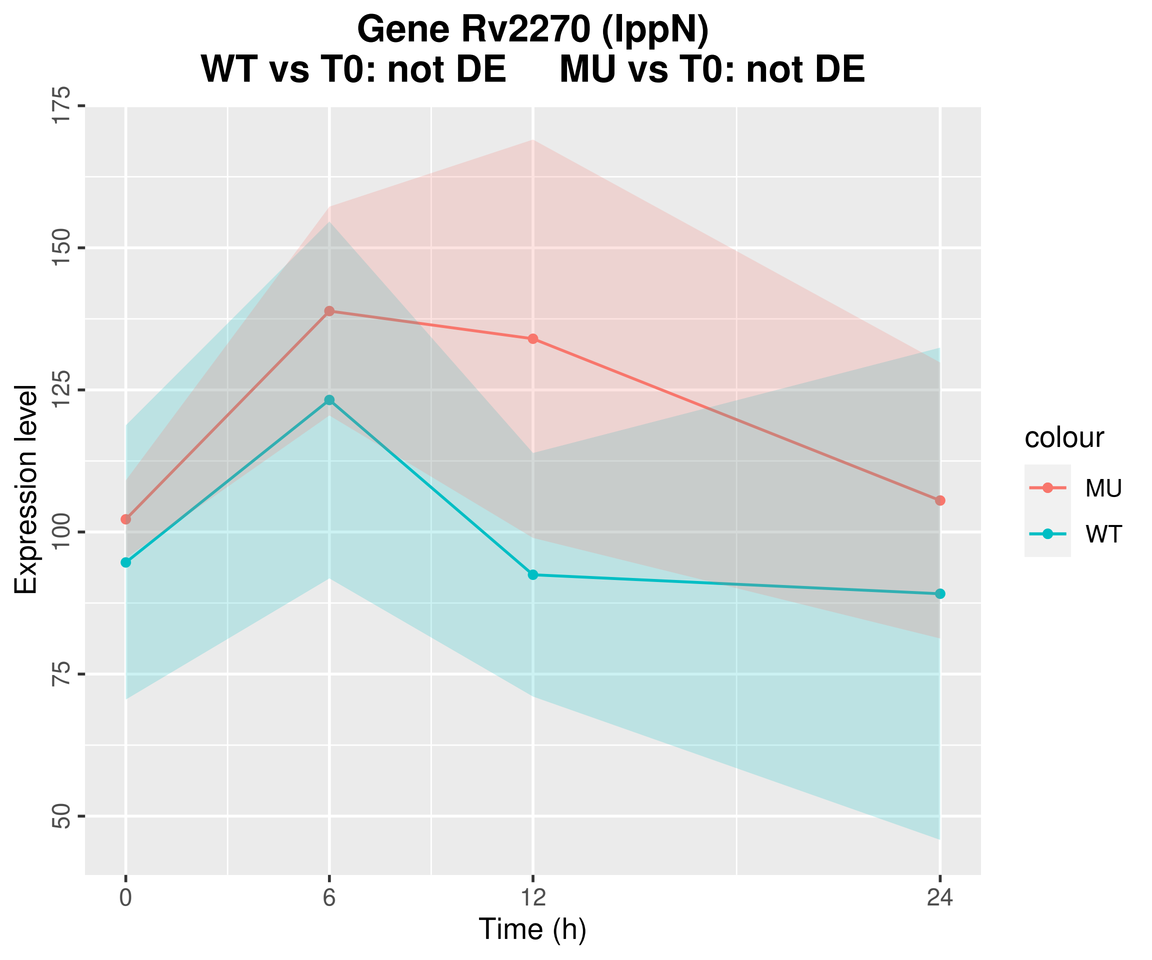
Gene Rv2270 (lppN)
WT vs T0: not DE MU vs T0: not DE
Expression level
175
150
125
100
75
50
0
6
12
24
Time (h)
colour
MU
WT

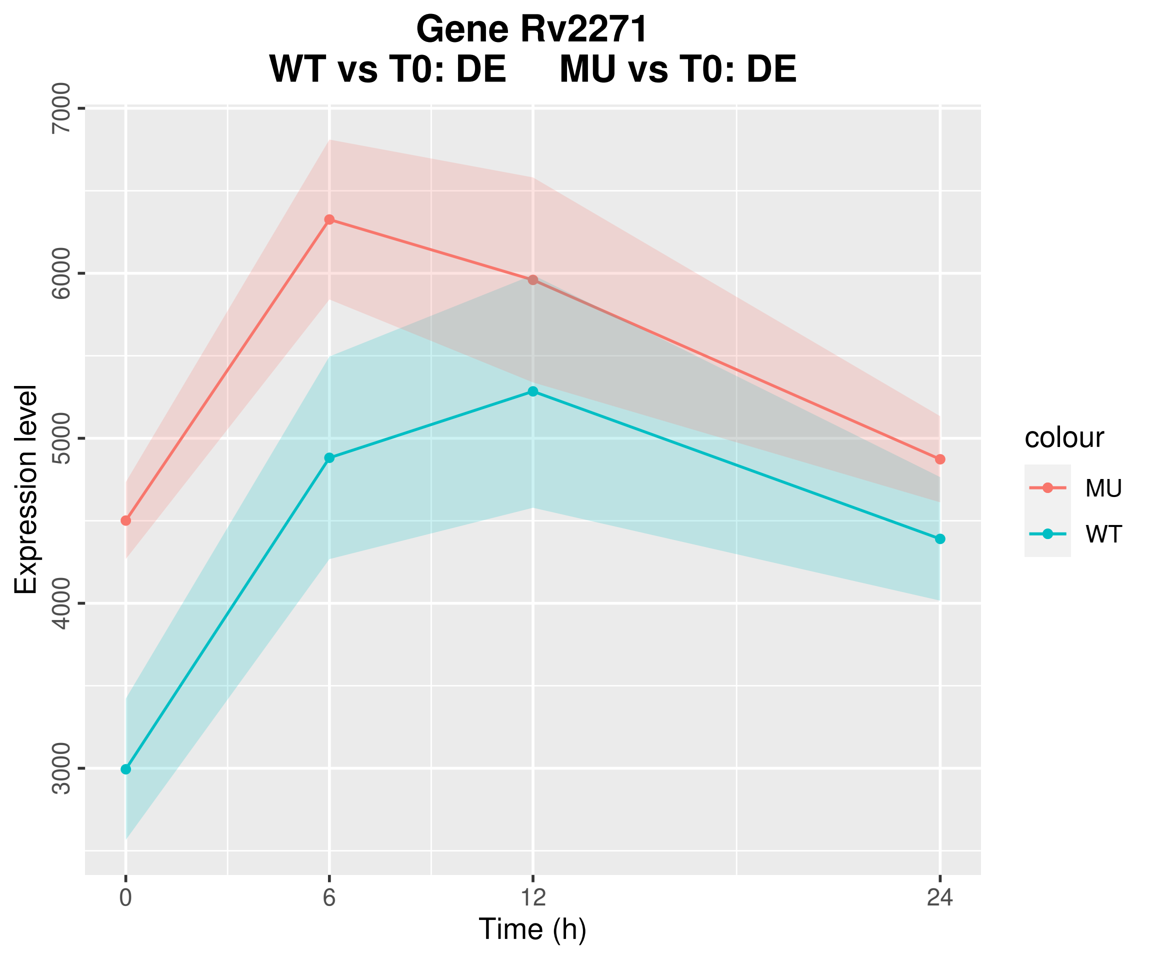
Gene Rv2271
WT vs T0: DE MU vs T0: DE
Expression level
7000
6000
5000
4000
3000
0
6
12
24
Time (h)
colour
MU
WT

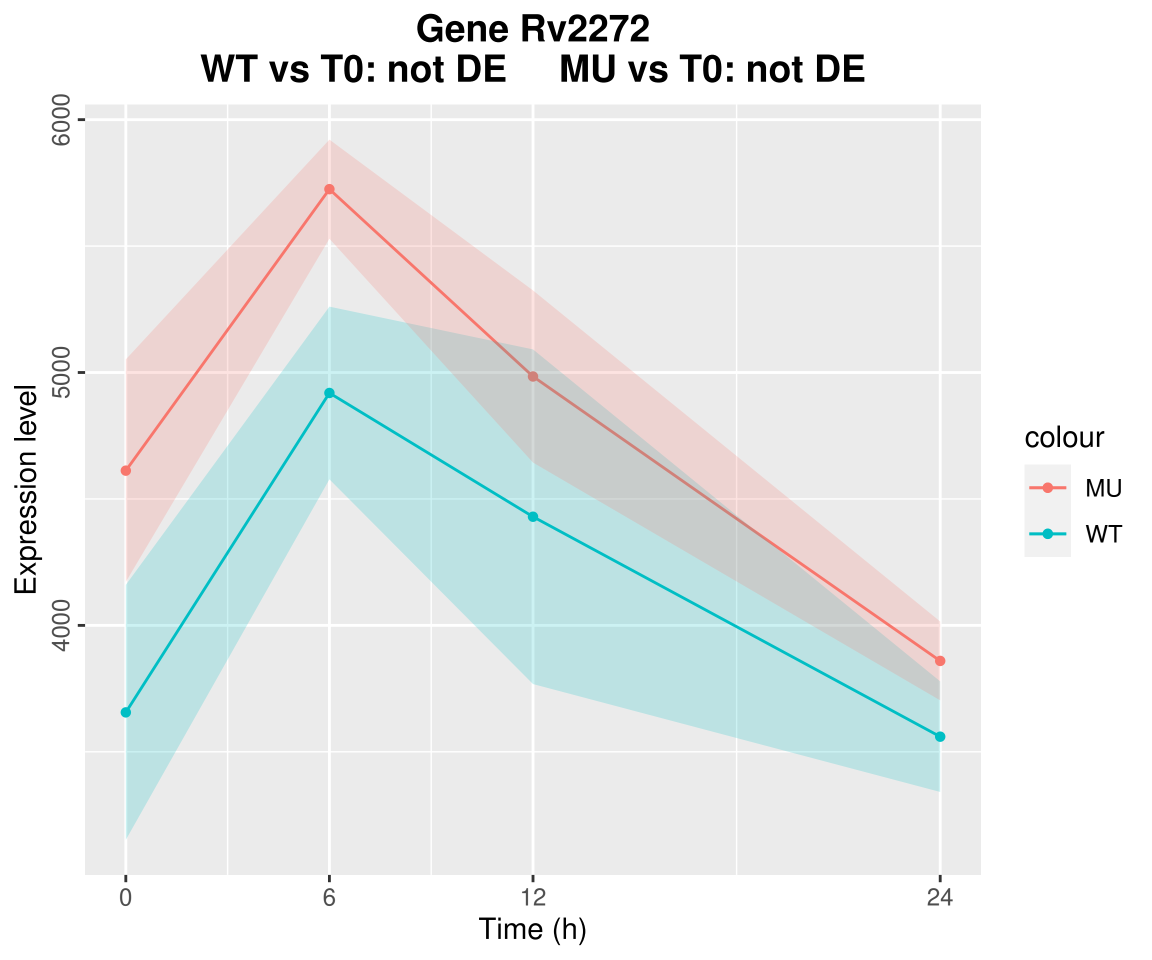

Gene Rv2272
WT vs T0: not DE MU vs T0: not DE
6000
5000
4000
Expression level
0
6
12
24
Time (h)
colour
MU
WT

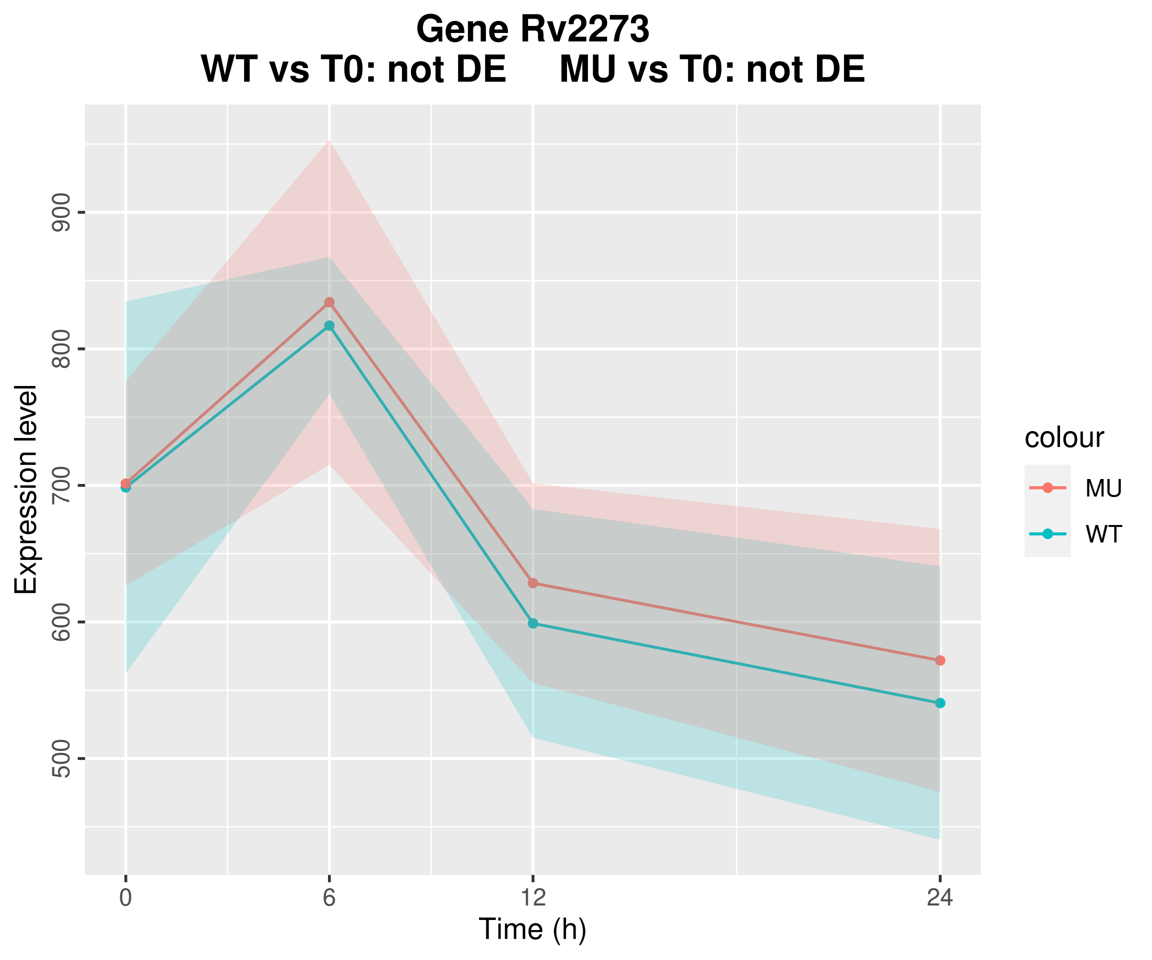
Gene Rv2273
WT vs T0: not DE MU vs T0: not DE
Expression level
900
800
700
600
500
0
6
12
24
Time (h)
colour
MU
WT

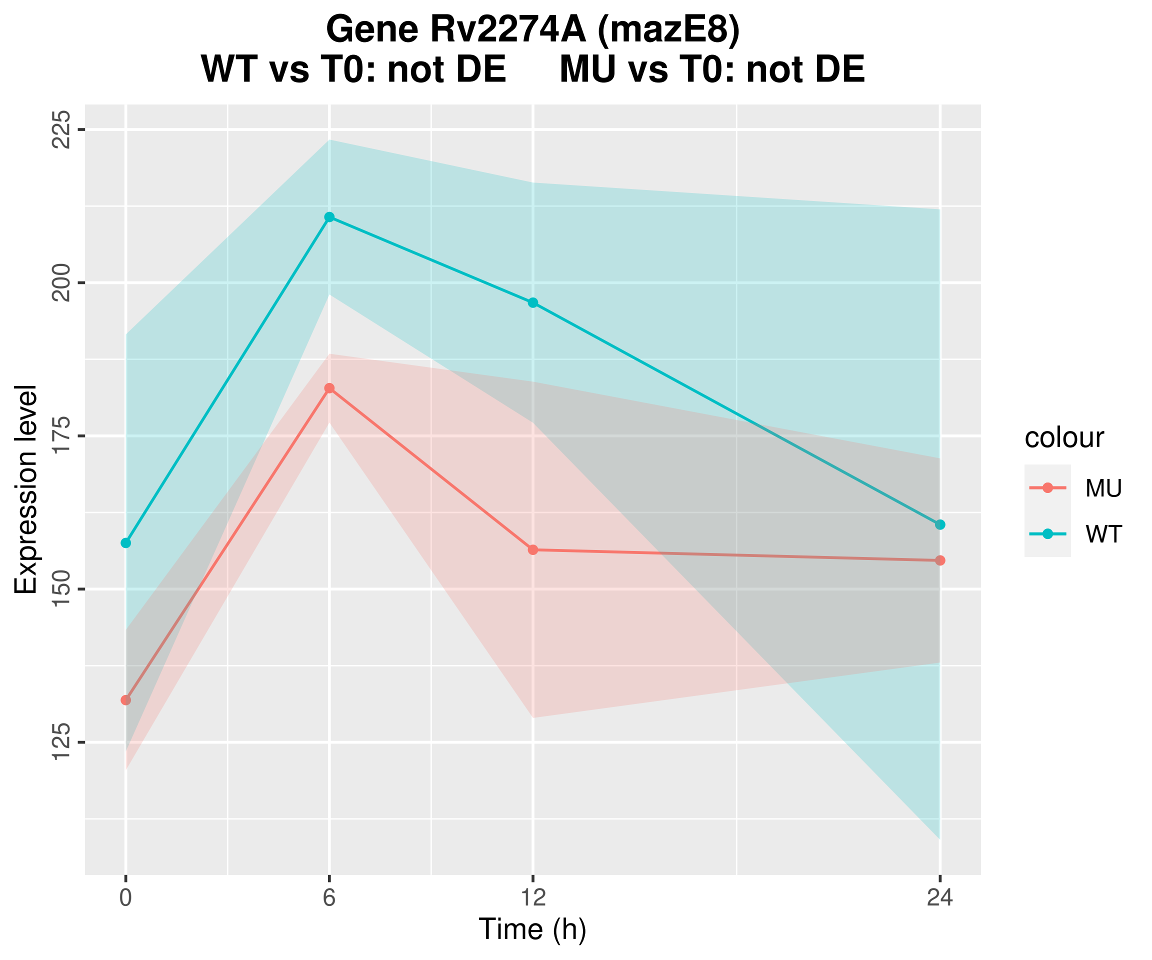

Gene Rv2274A (mazE8)
WT vs T0: not DE   MU vs T0: not DE
Expression level
225
200
175
150
125
0
6
12
24
Time (h)
colour
MU
WT

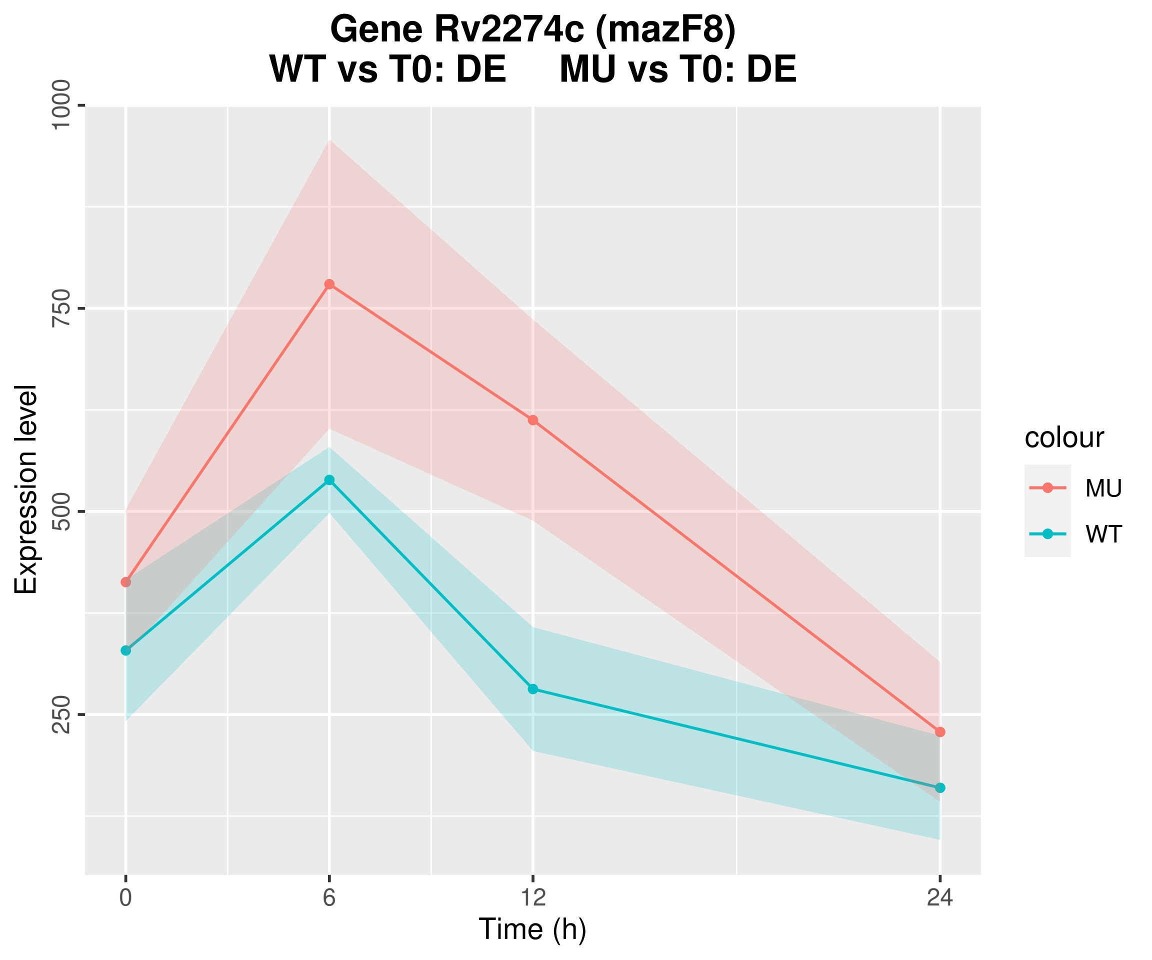

Gene Rv2274c (mazF8)
WT vs T0: DE MU vs T0: DE
1000
750
500
250
Expression level
0
6
12
24
Time (h)
colour
MU
WT

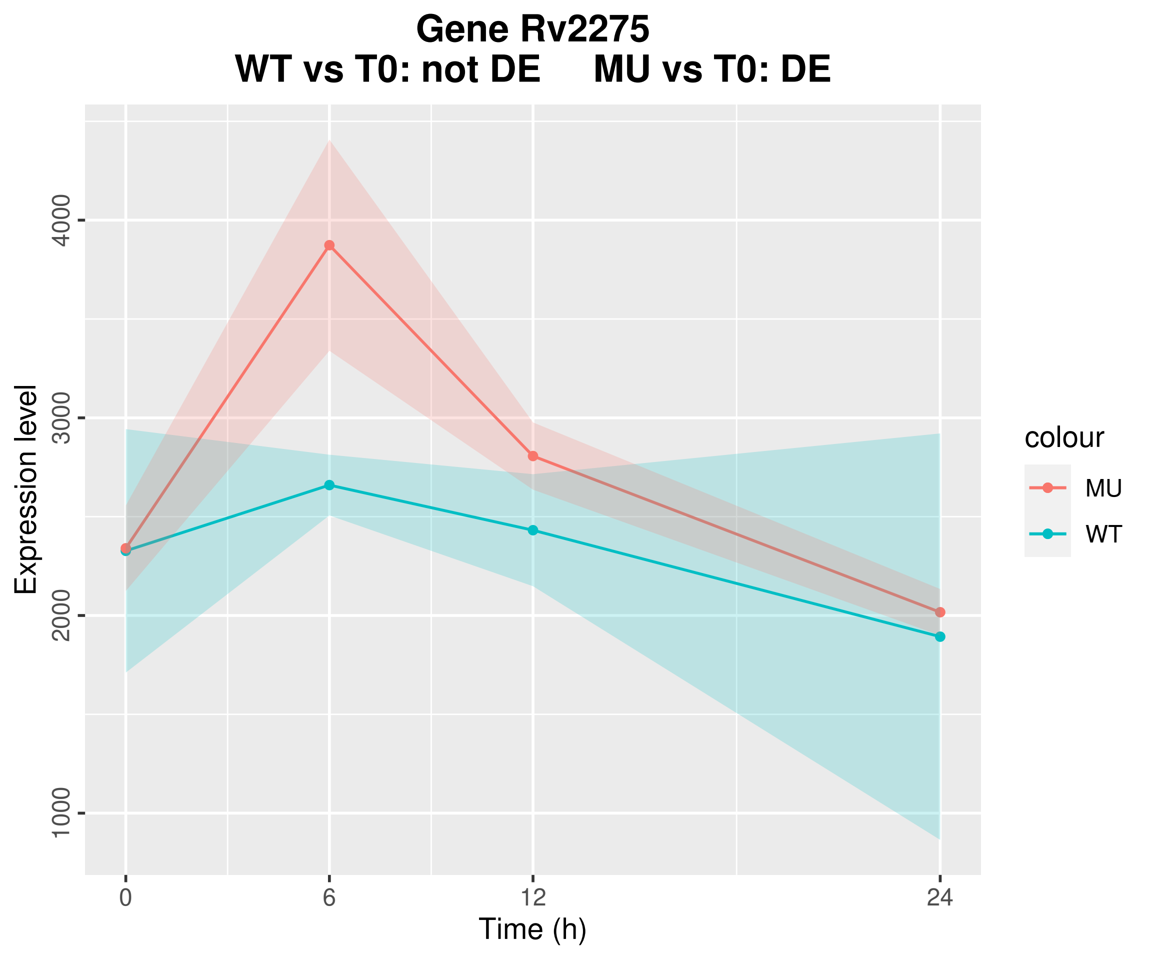
Gene Rv2275
WT vs T0: not DE   MU vs T0: DE
Expression level
4000
3000
2000
1000
0
6
12
24
Time (h)
colour
MU
WT

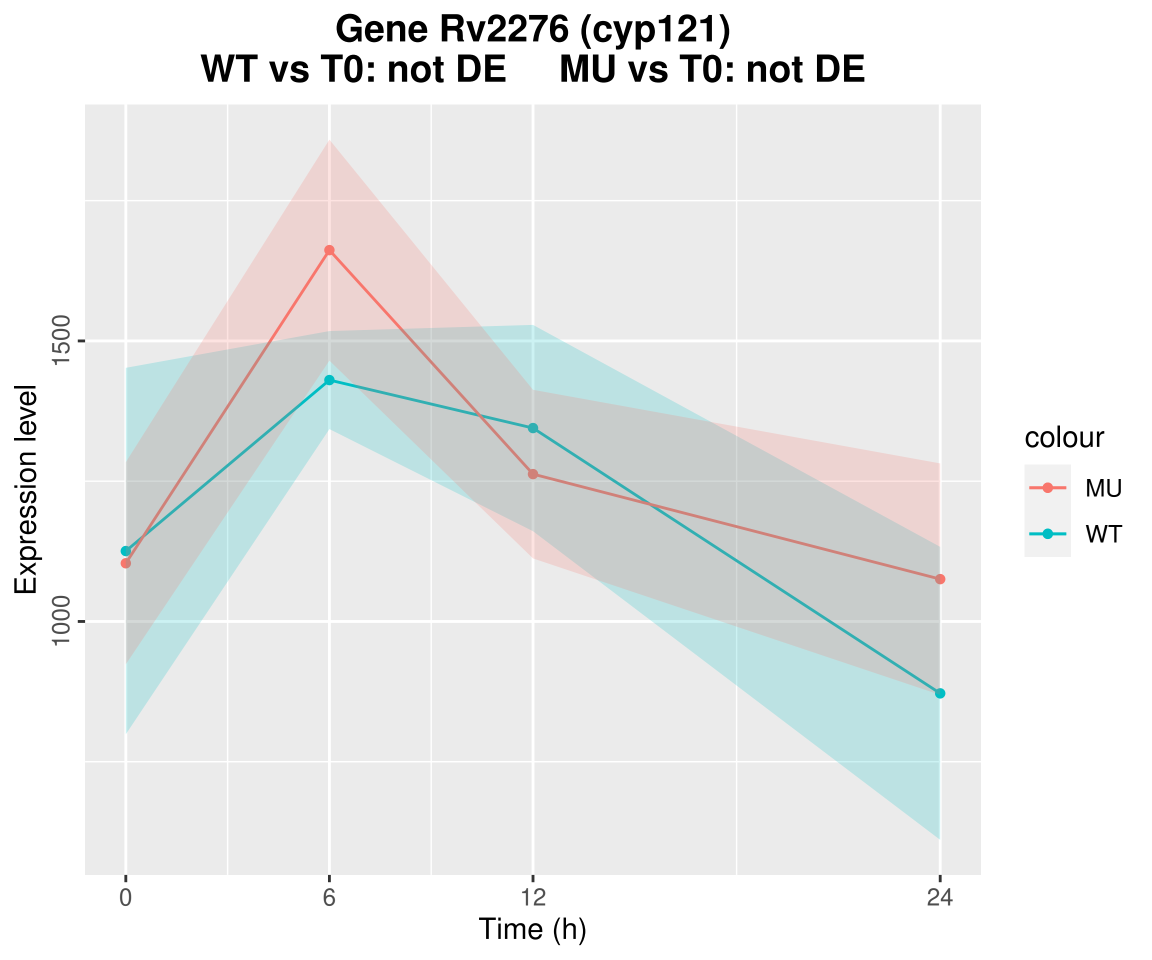
Gene Rv2276 (cyp121)
WT vs T0: not DE   MU vs T0: not DE
Expression level
1500
1000
0
6
12
24
Time (h)
colour
MU
WT

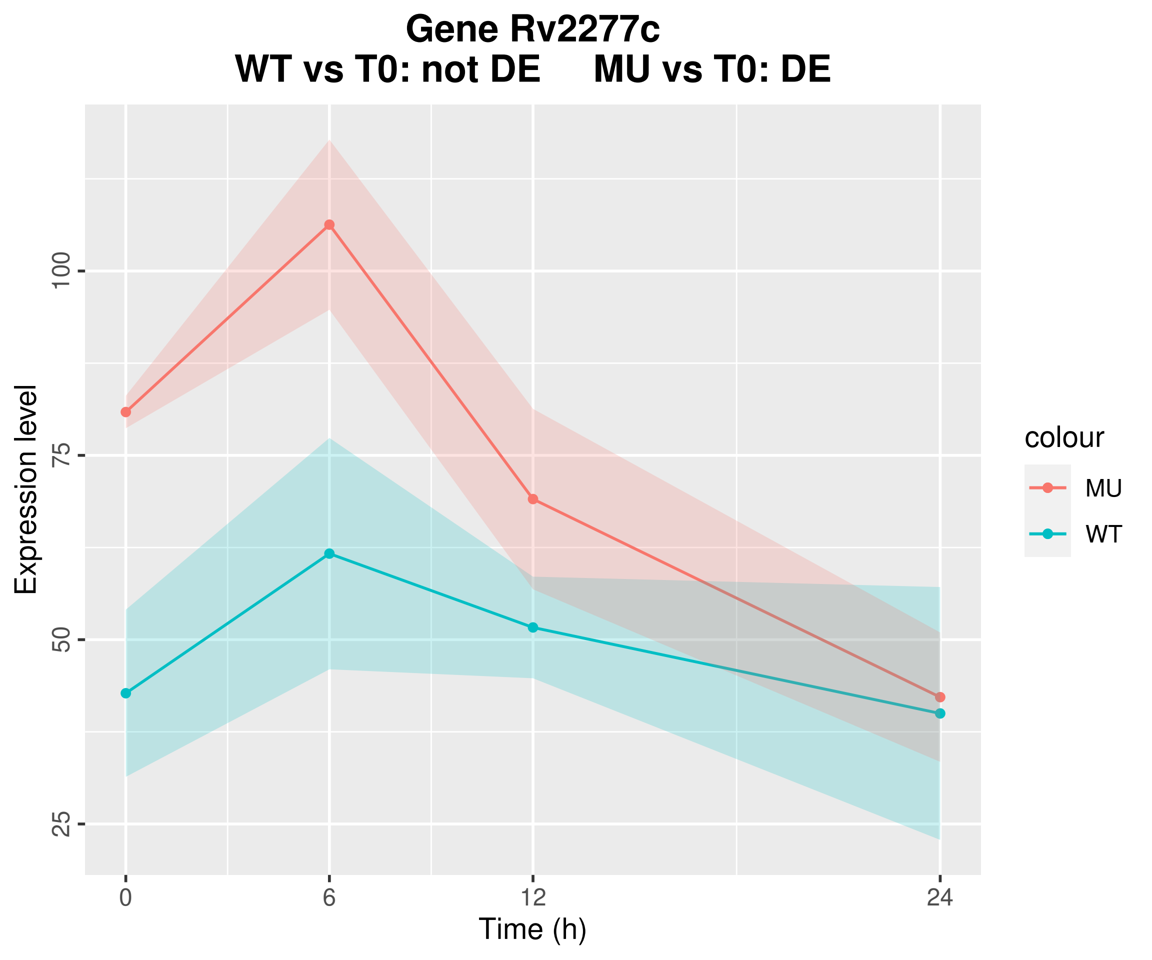

Gene Rv2277c
WT vs T0: not DE MU vs T0: DE
Expression level
100
75
50
25
0
6
12
24
Time (h)
colour
MU
WT

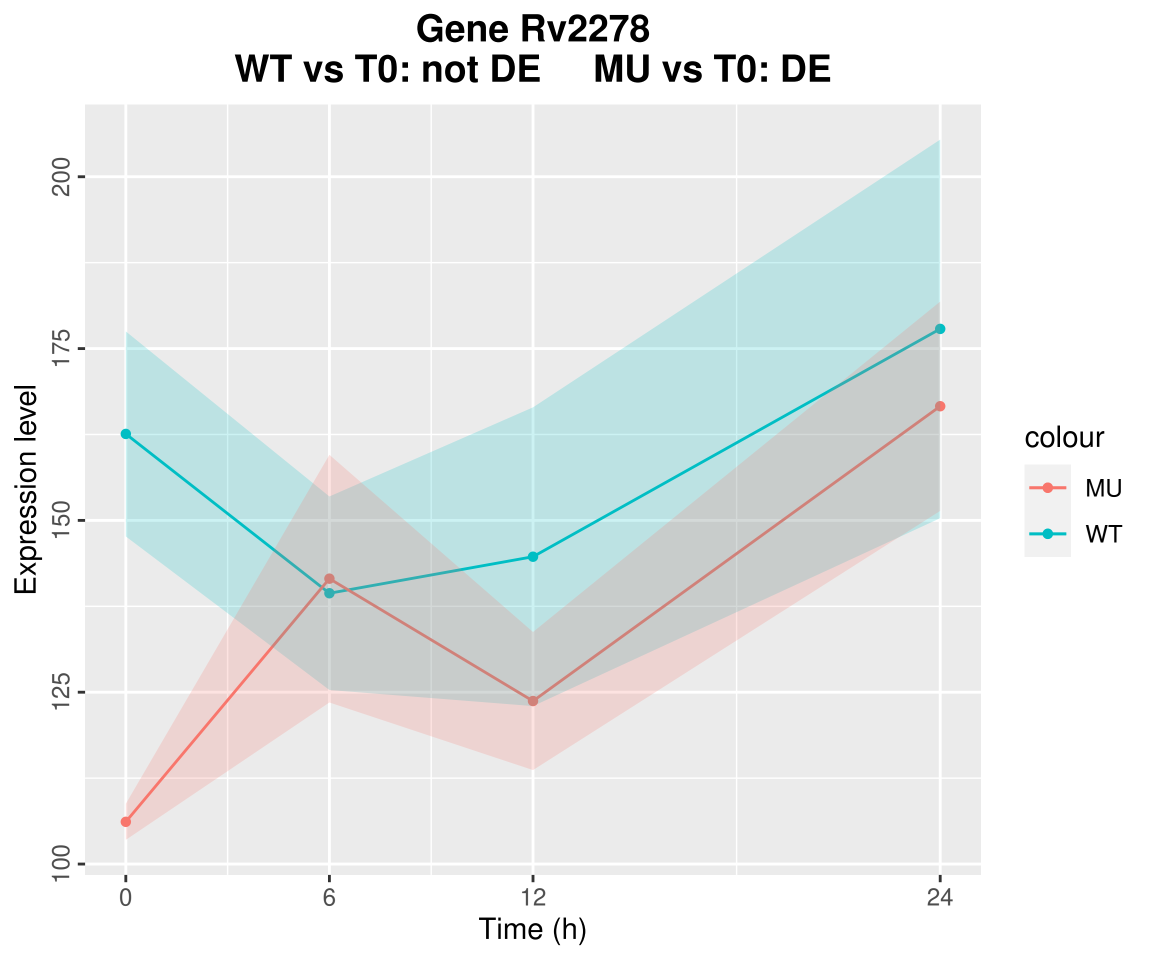

Gene Rv2278
WT vs T0: not DE MU vs T0: DE
Expression level
200
175
150
125
100
0
6
12
24
Time (h)
colour
MU
WT

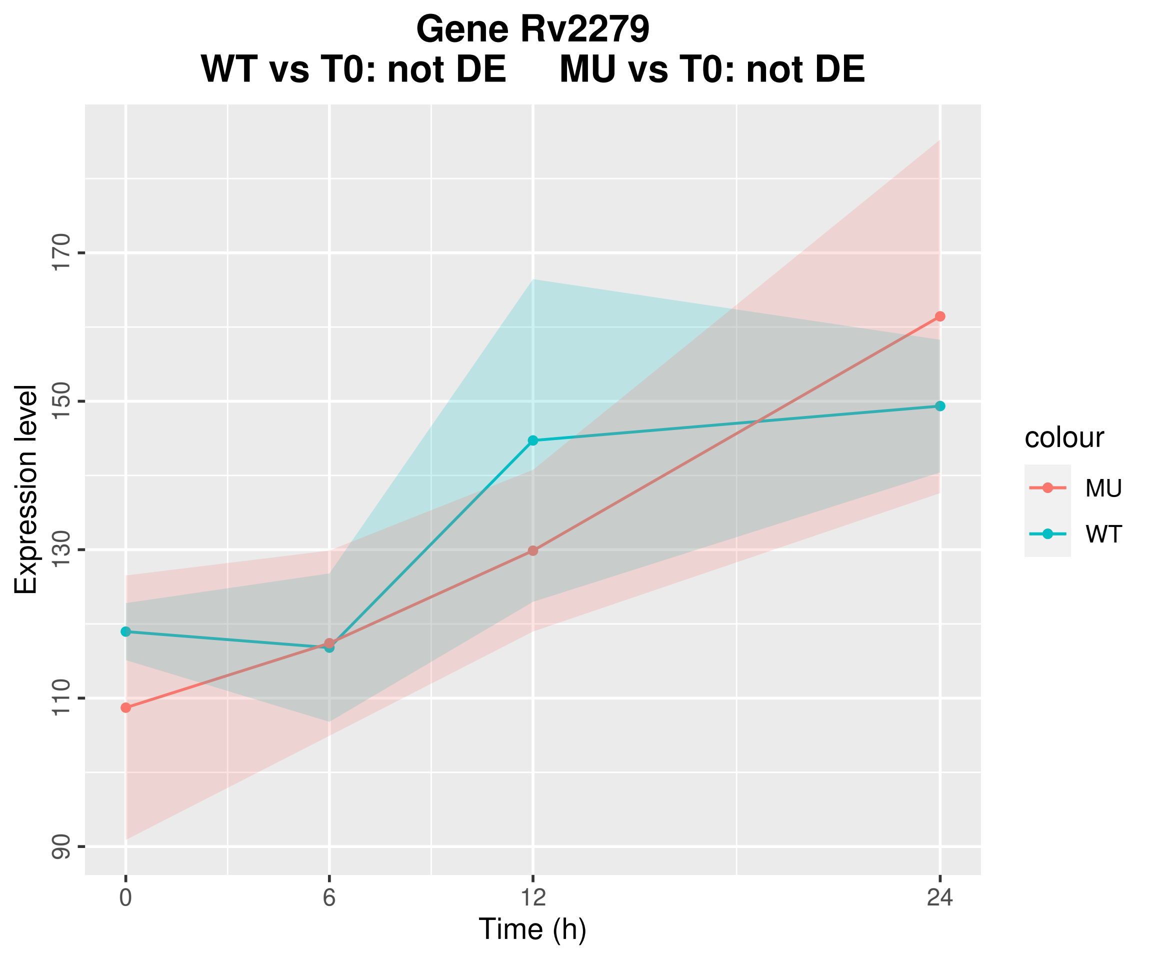

Gene Rv2279
WT vs T0: not DE   MU vs T0: not DE
Expression level
90
110
130
150
170
0
6
12
24
Time (h)
colour
MU
WT

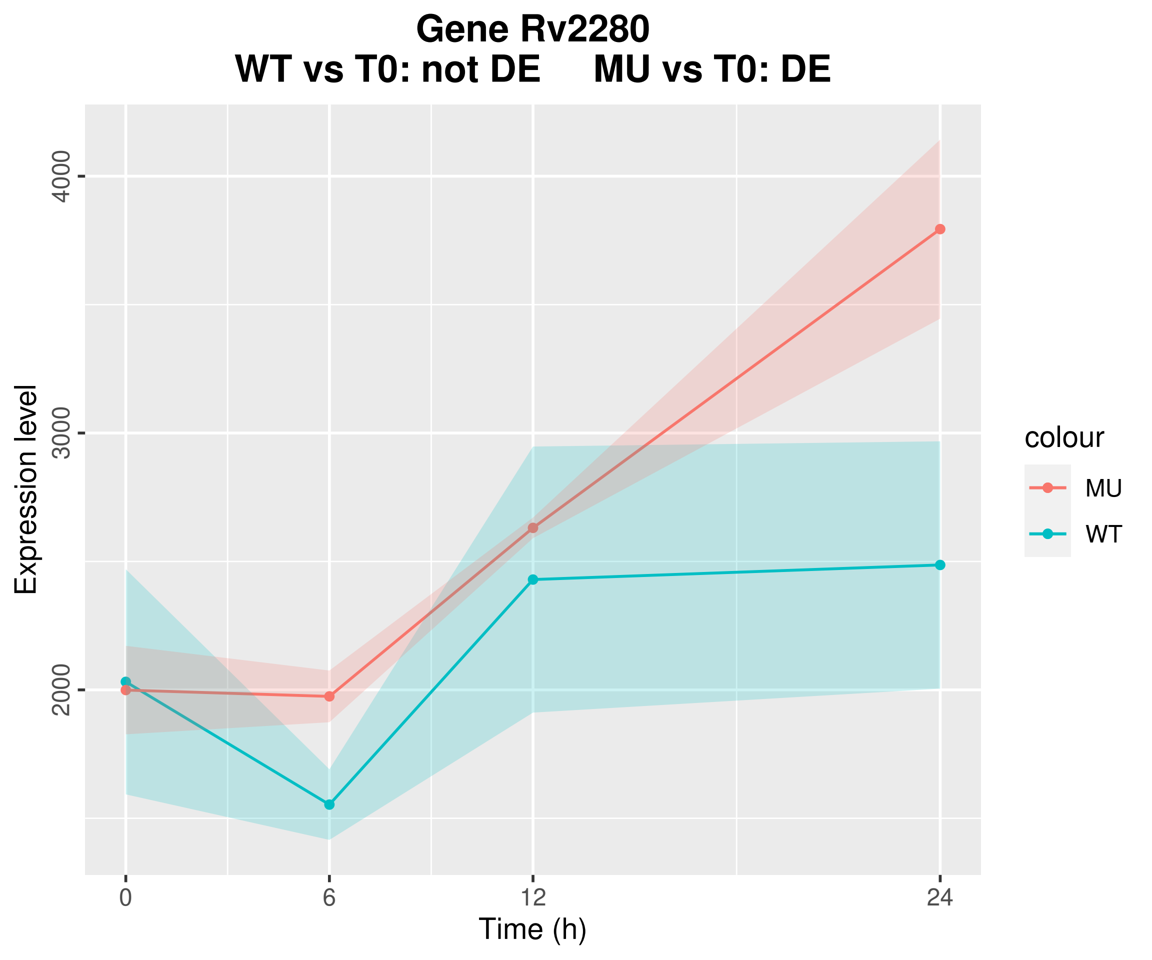
Gene Rv2280
WT vs T0: not DE   MU vs T0: DE
Expression level
4000
3000
2000
0
6
12
24
Time (h)
colour
MU
WT

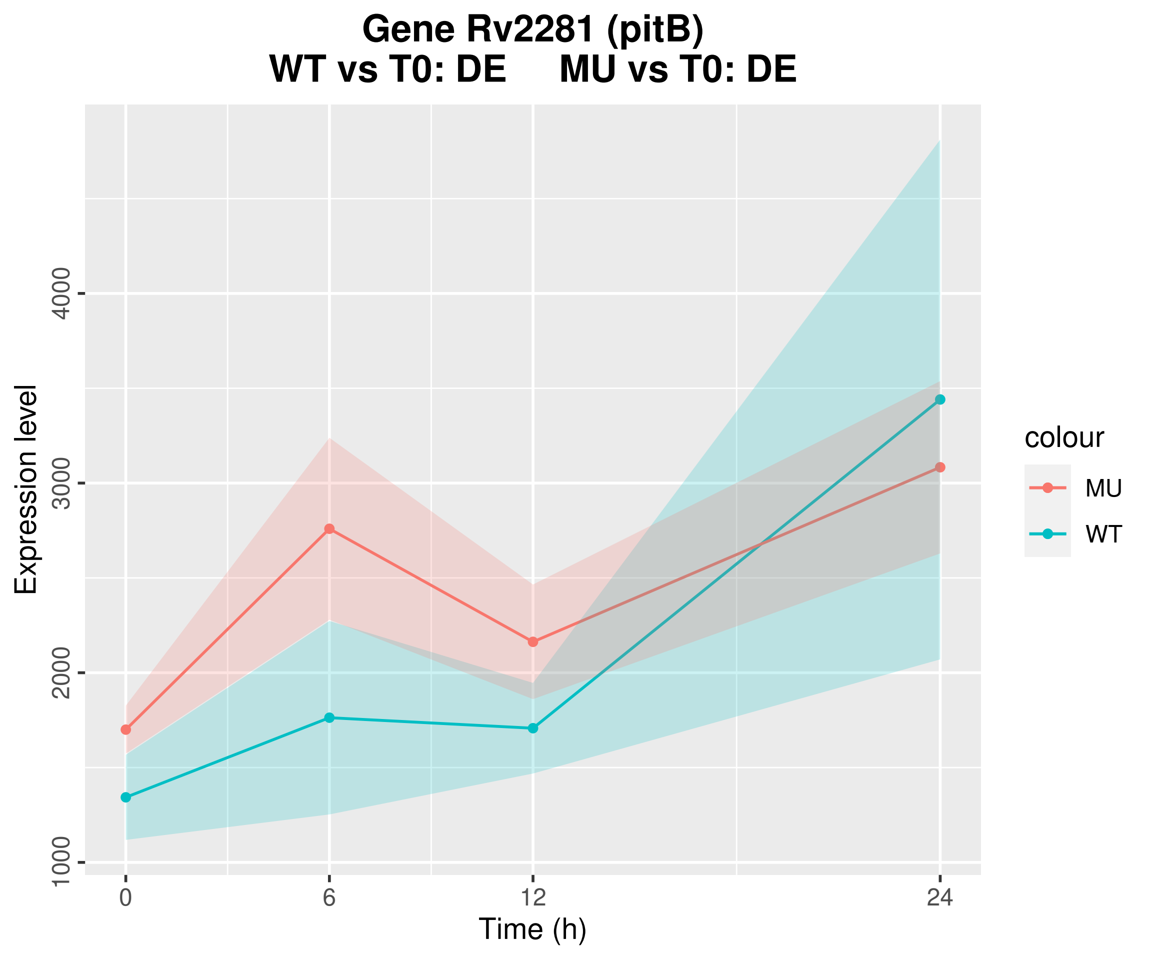

Gene Rv2281 (pitB)
WT vs T0: DE MU vs T0: DE
Expression level
1000
2000
3000
4000
0
6
12
24
Time (h)
colour
MU
WT

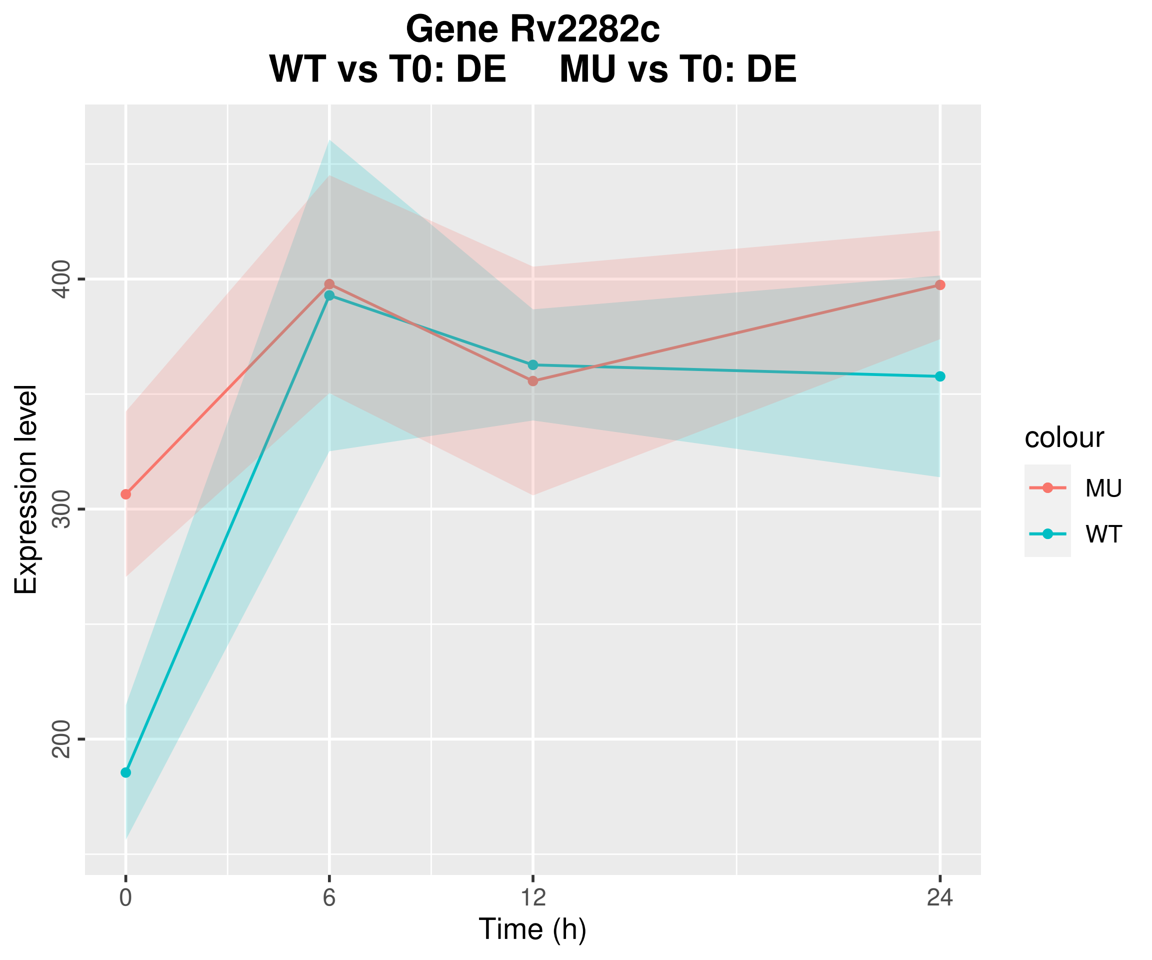

Gene Rv2282c
WT vs T0: DE MU vs T0: DE
Expression level
400
300
200
0
6
12
24
Time (h)
colour
MU
WT

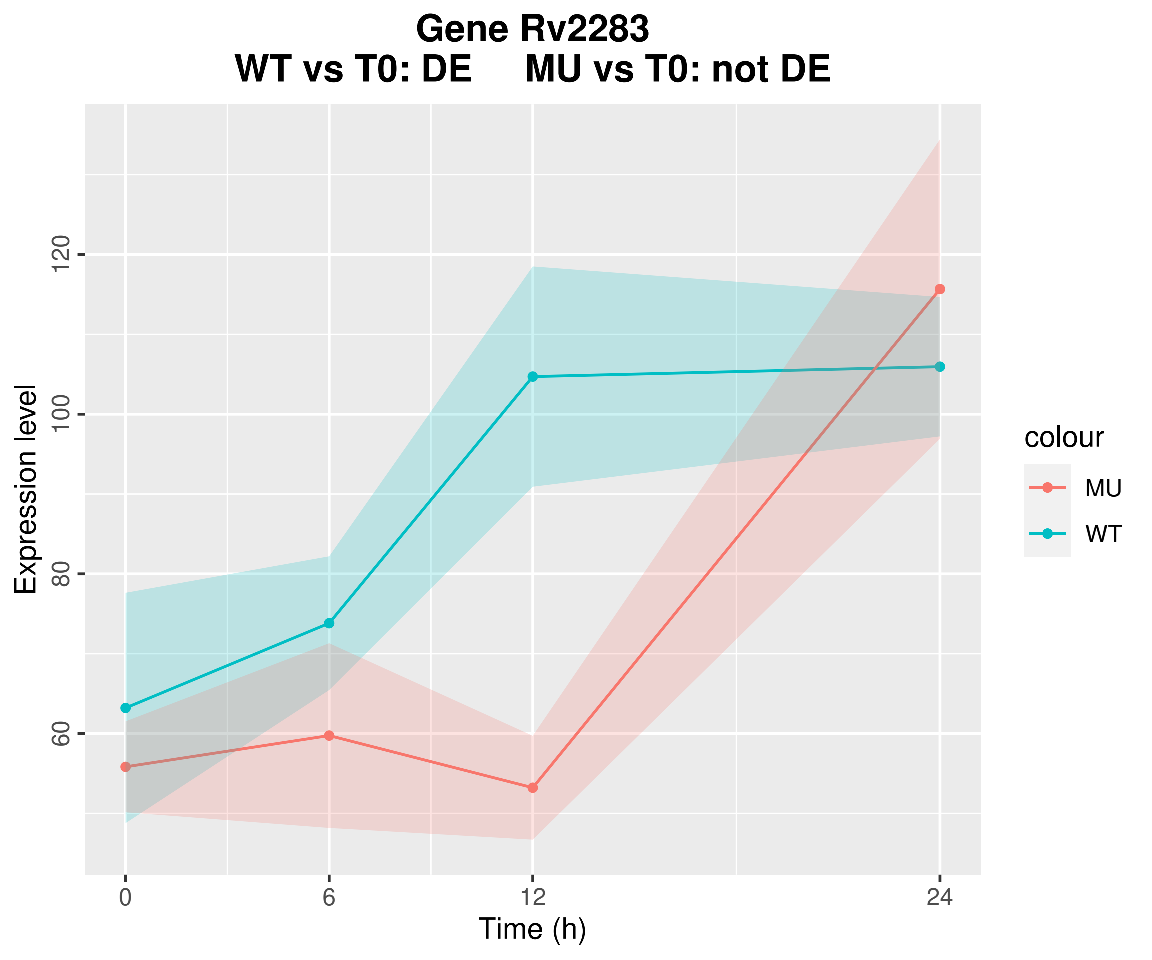

Gene Rv2283
WT vs T0: DE   MU vs T0: not DE
Expression level
120
100
80
60
Time (h)
0
6
12
24
colour
MU
WT

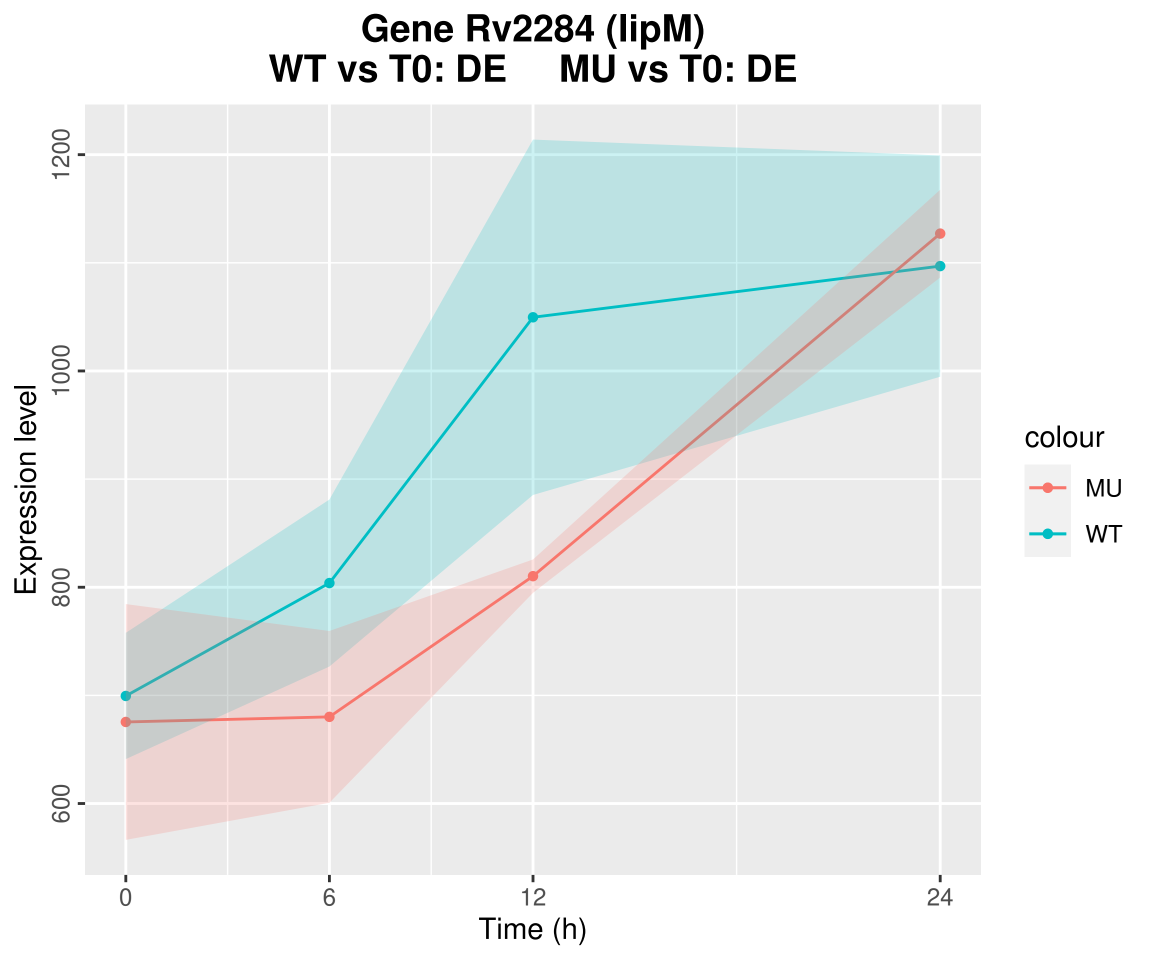

Gene Rv2284 (lipM)
WT vs T0: DE   MU vs T0: DE
Expression level
1200
1000
800
600
0
6
12
24
Time (h)
colour
MU
WT

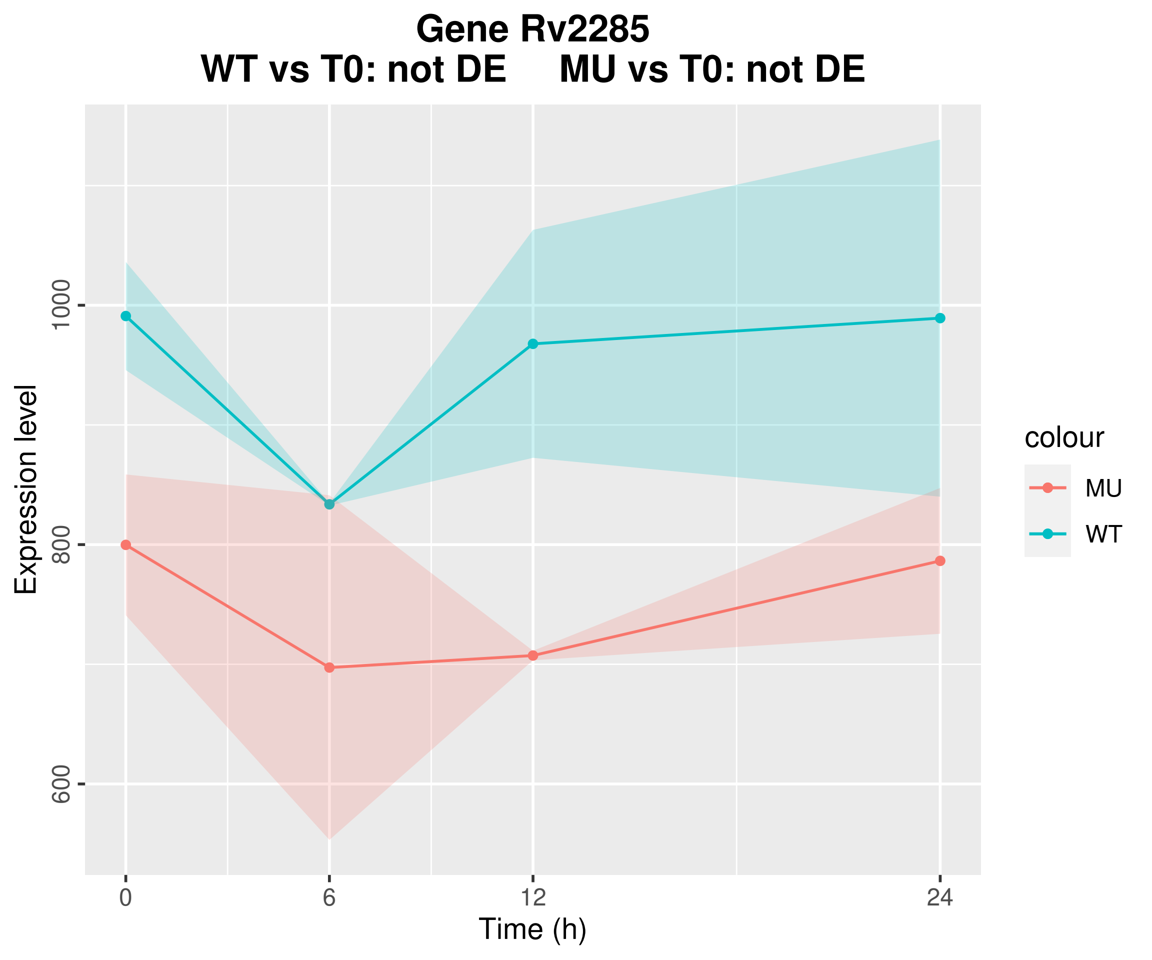

Gene Rv2285
WT vs T0: not DE   MU vs T0: not DE
1000
800
600
Expression level
0
6
12
24
Time (h)
colour
MU
WT

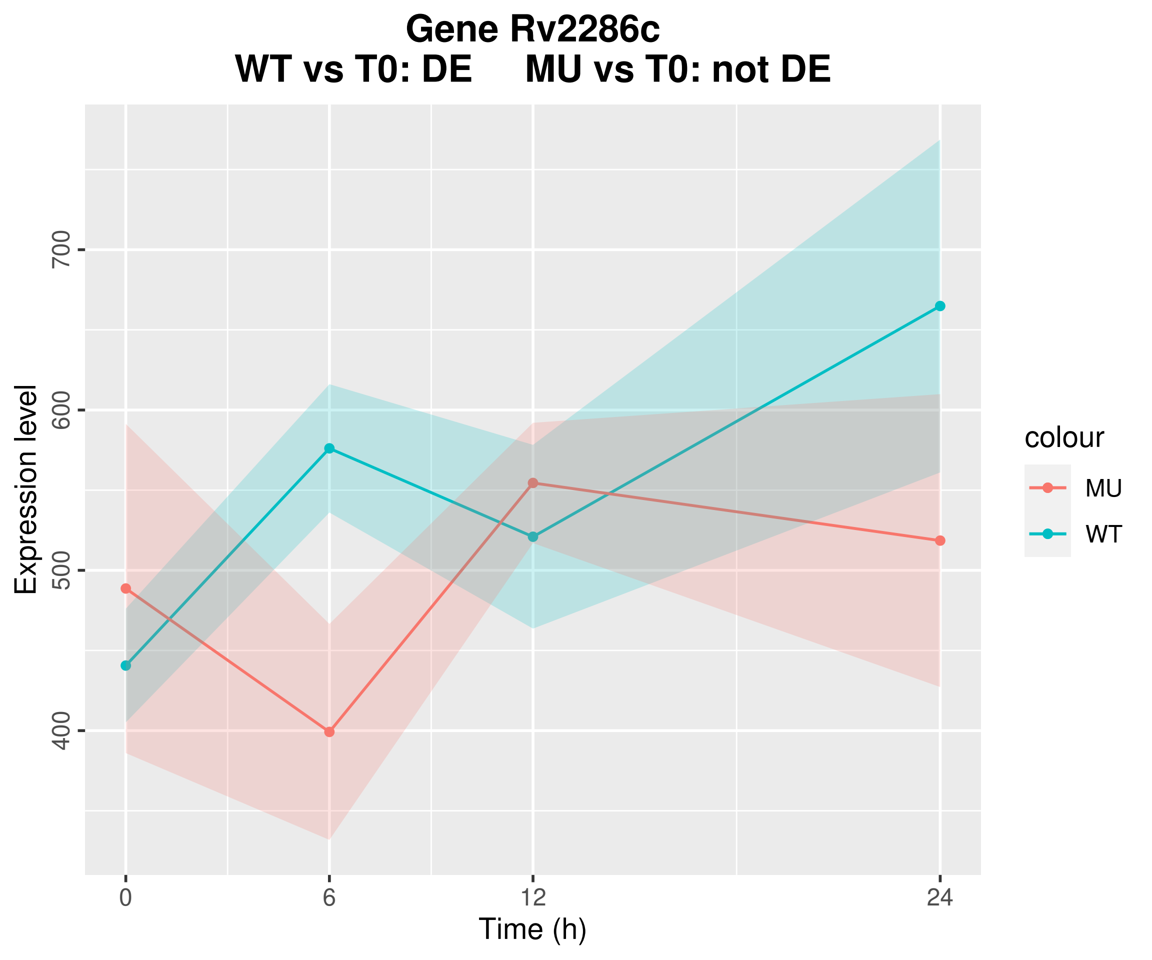
Gene Rv2286c
WT vs T0: DE MU vs T0: not DE
Expression level
700
600
500
400
0
6
12
24
Time (h)
colour
MU
WT

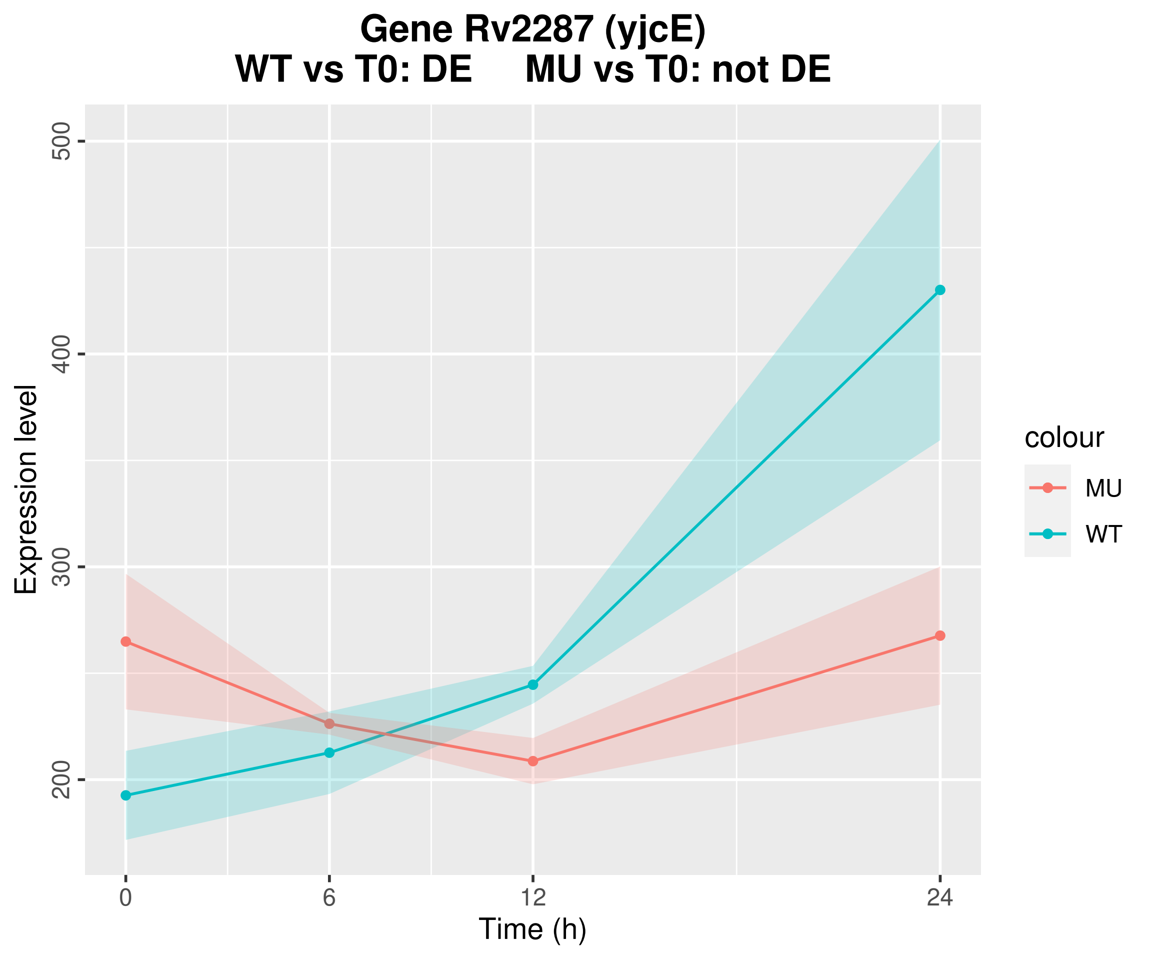
Gene Rv2287 (yjcE)
WT vs T0: DE   MU vs T0: not DE
Expression level
500
400
300
200
0
6
12
24
Time (h)
colour
MU
WT

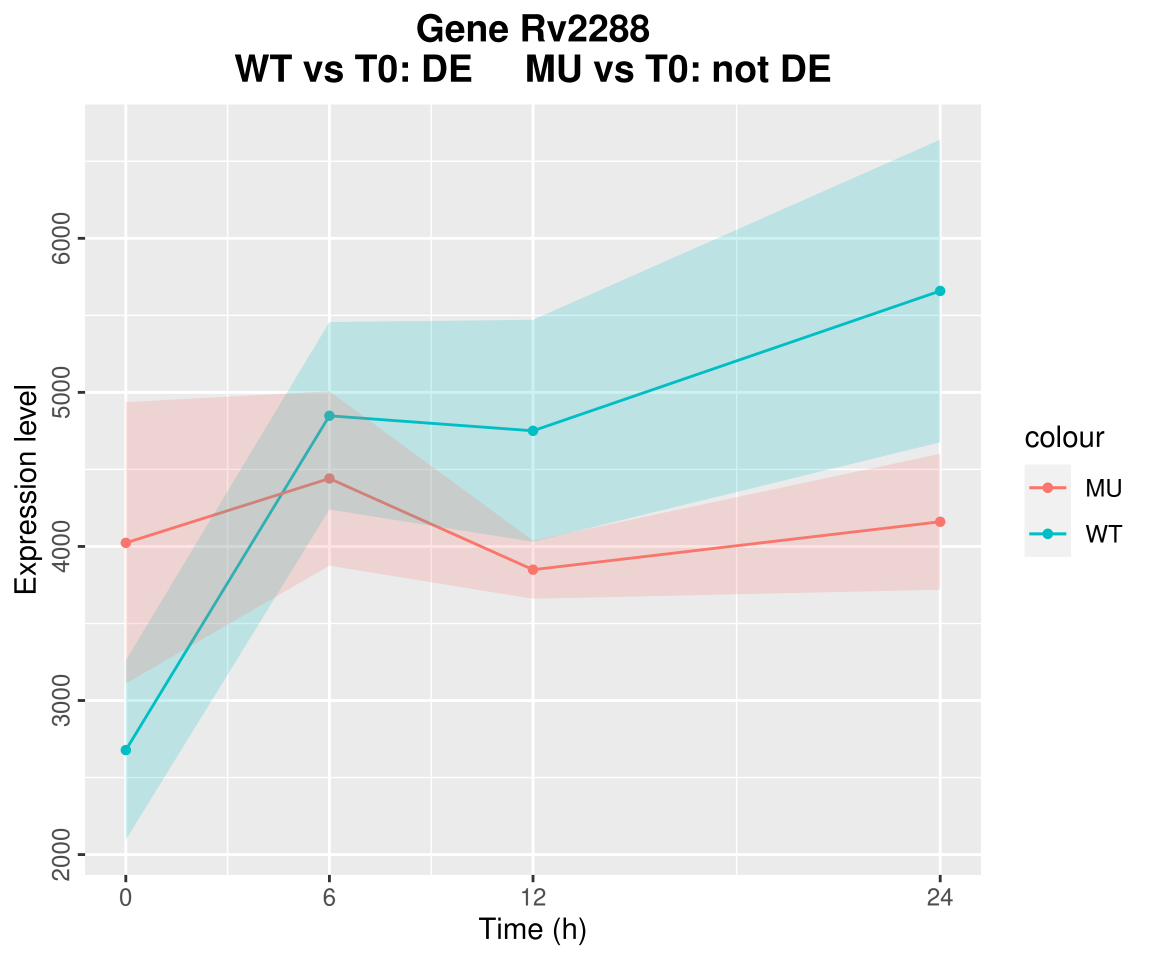
Gene Rv2288
WT vs T0: DE MU vs T0: not DE
Expression level
6000
5000
4000
3000
2000
0
6
12
24
Time (h)
colour
MU
WT

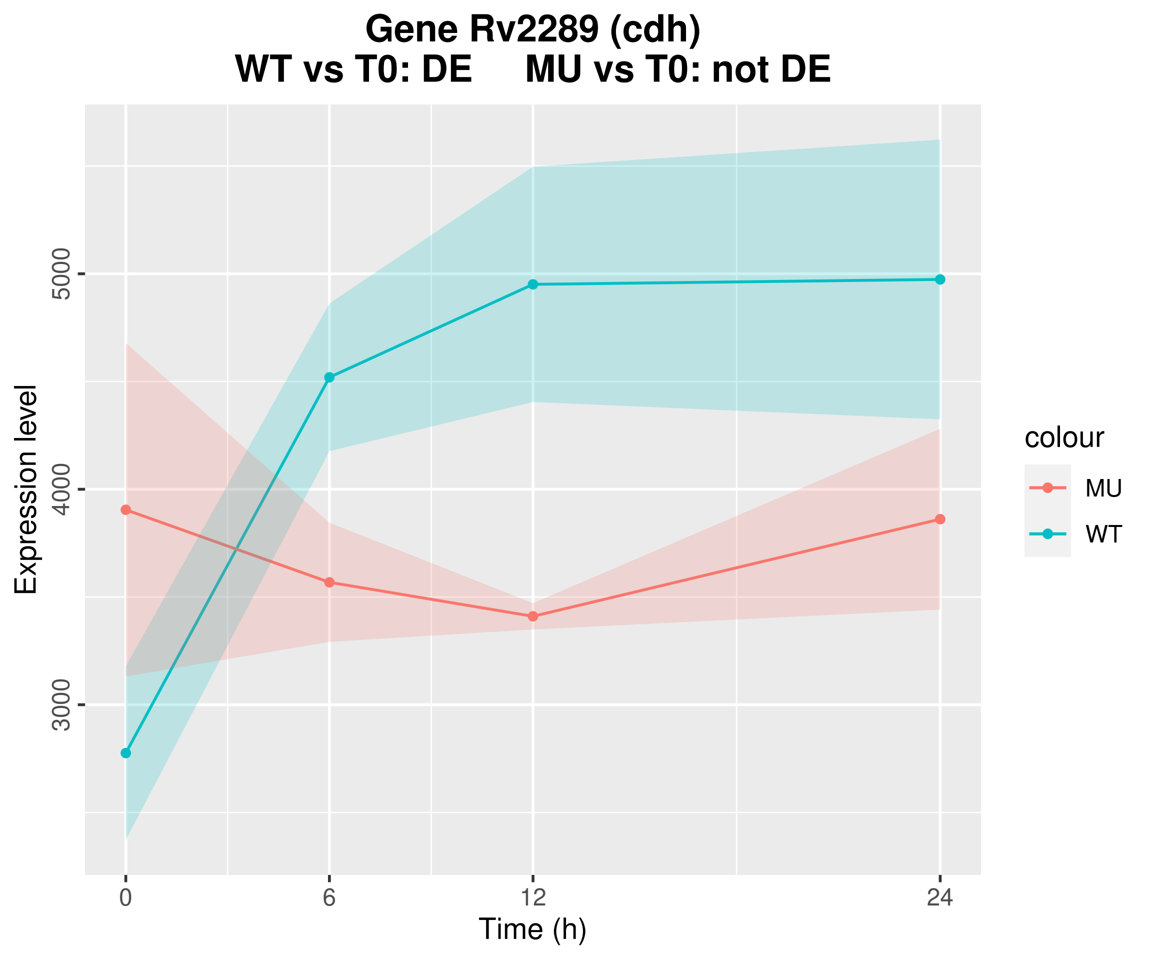
Gene Rv2289 (cdh)
WT vs T0: DE   MU vs T0: not DE
Expression level
5000
4000
3000
0
6
12
24
Time (h)
colour
MU
WT

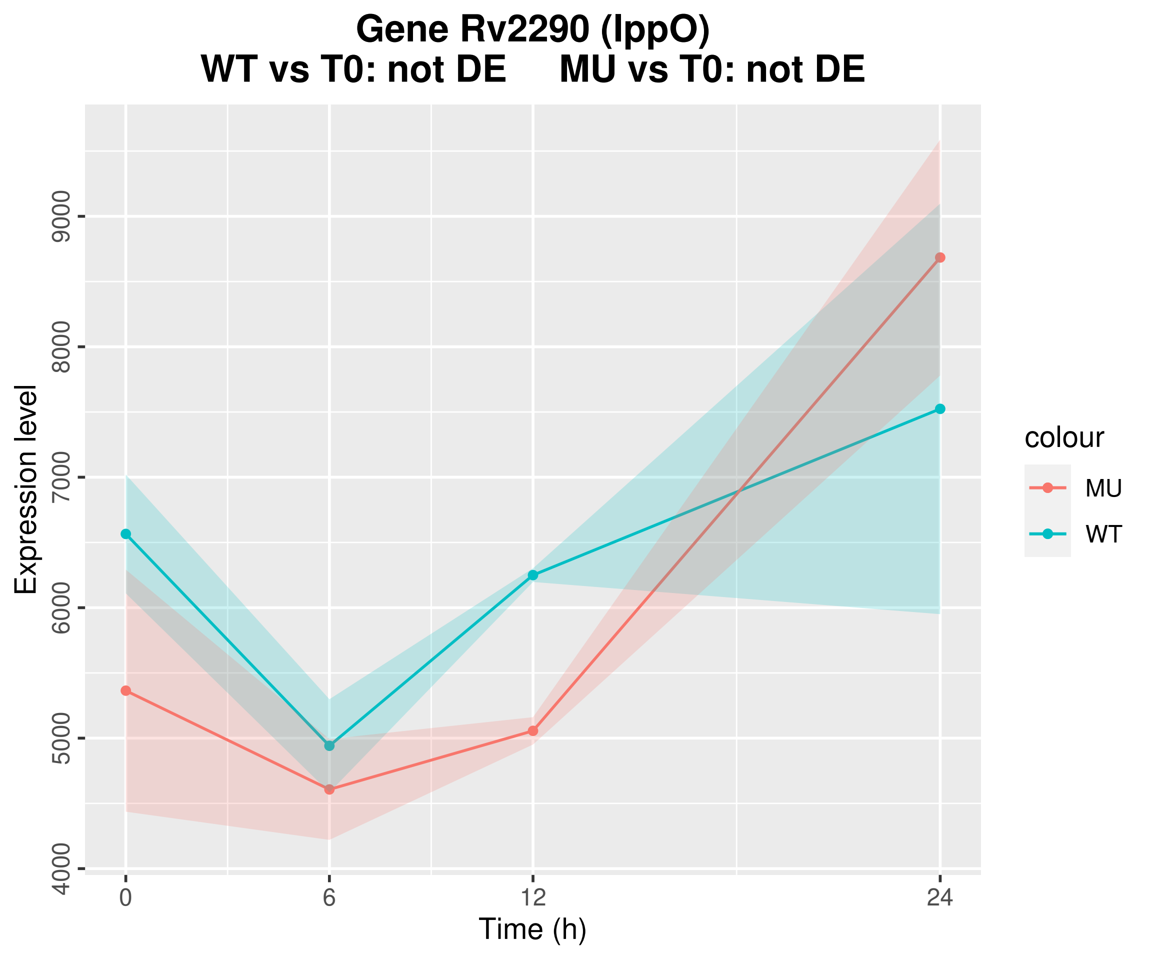

Gene Rv2290 (lppO)
WT vs T0: not DE MU vs T0: not DE
Expression level
9000
8000
7000
6000
5000
4000
0
6
12
24
Time (h)
colour
MU
WT

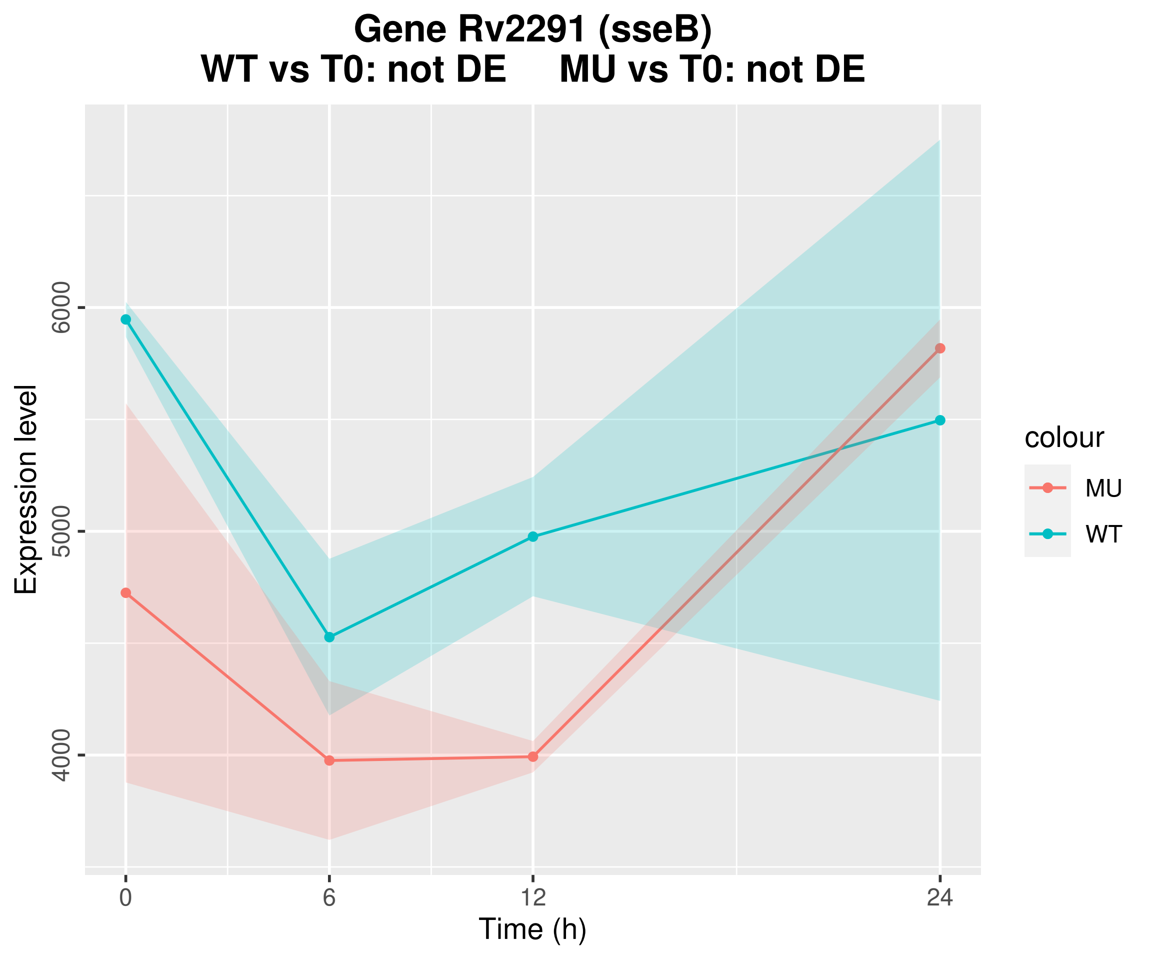

Gene Rv2291 (sseB)
WT vs T0: not DE   MU vs T0: not DE
Expression level
6000
5000
4000
0
6
12
24
Time (h)
colour
MU
WT

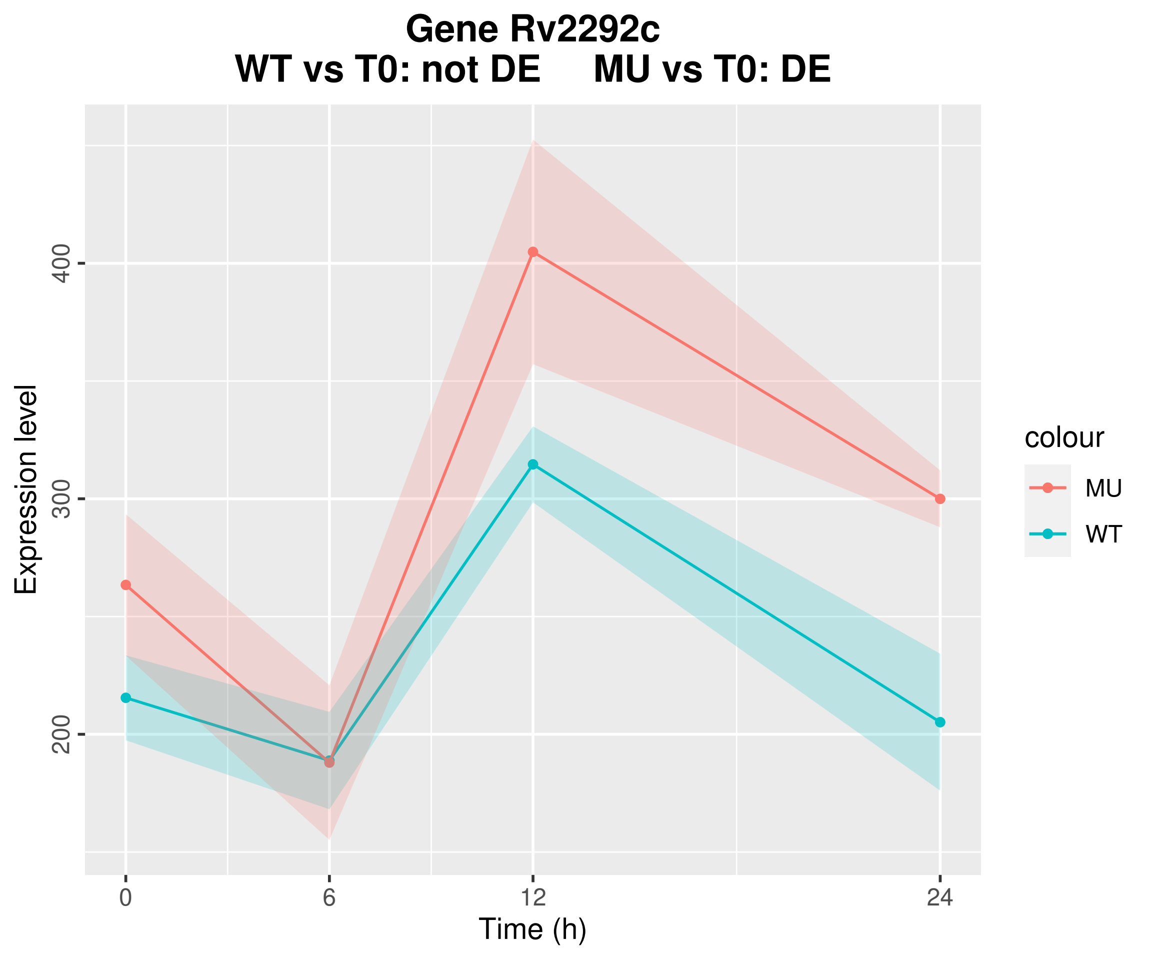
Gene Rv2292c
WT vs T0: not DE MU vs T0: DE
Expression level
400
300
200
0
6
12
24
Time (h)
colour
MU
WT

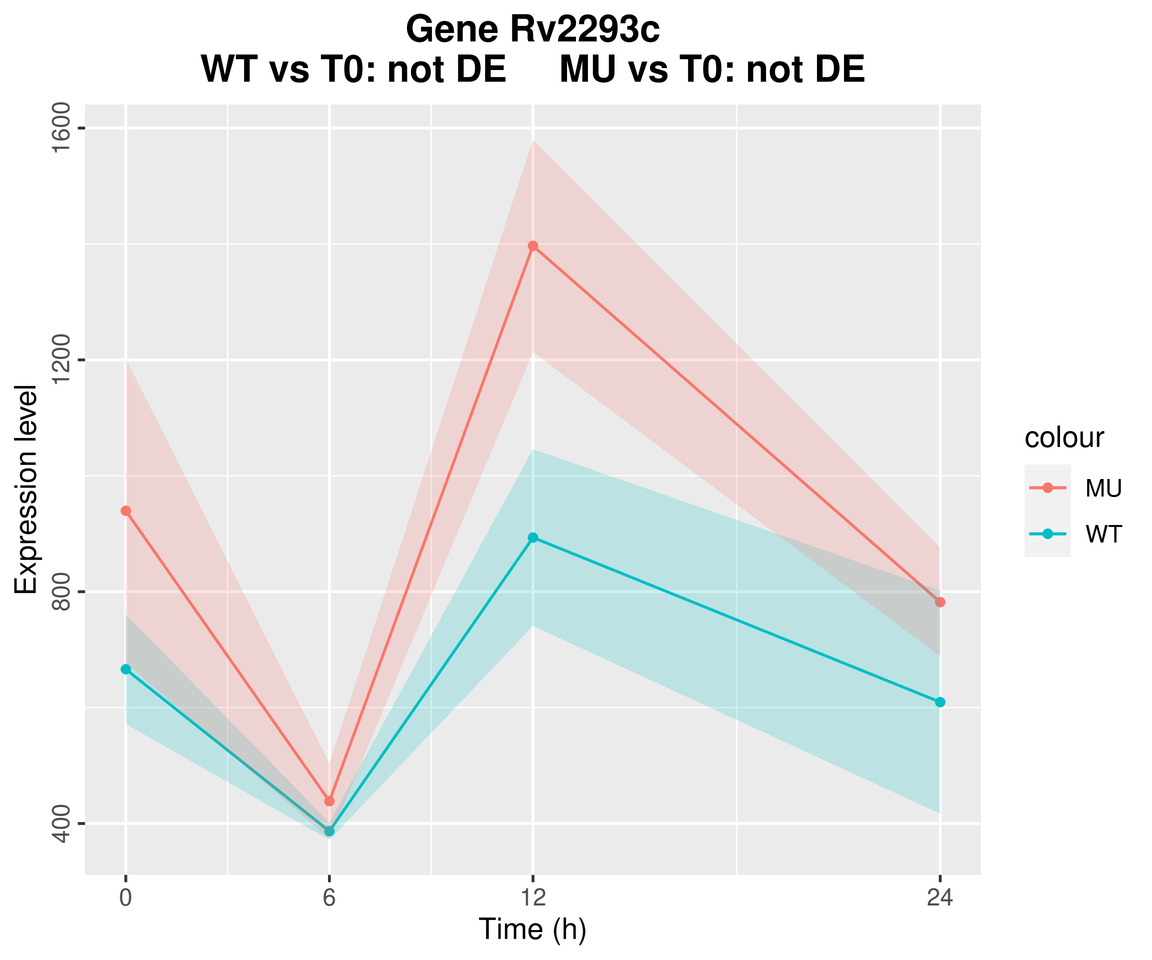
Gene Rv2293c
WT vs T0: not DE MU vs T0: not DE
Expression level
1600
1200
800
400
0
6
12
24
Time (h)
colour
MU
WT

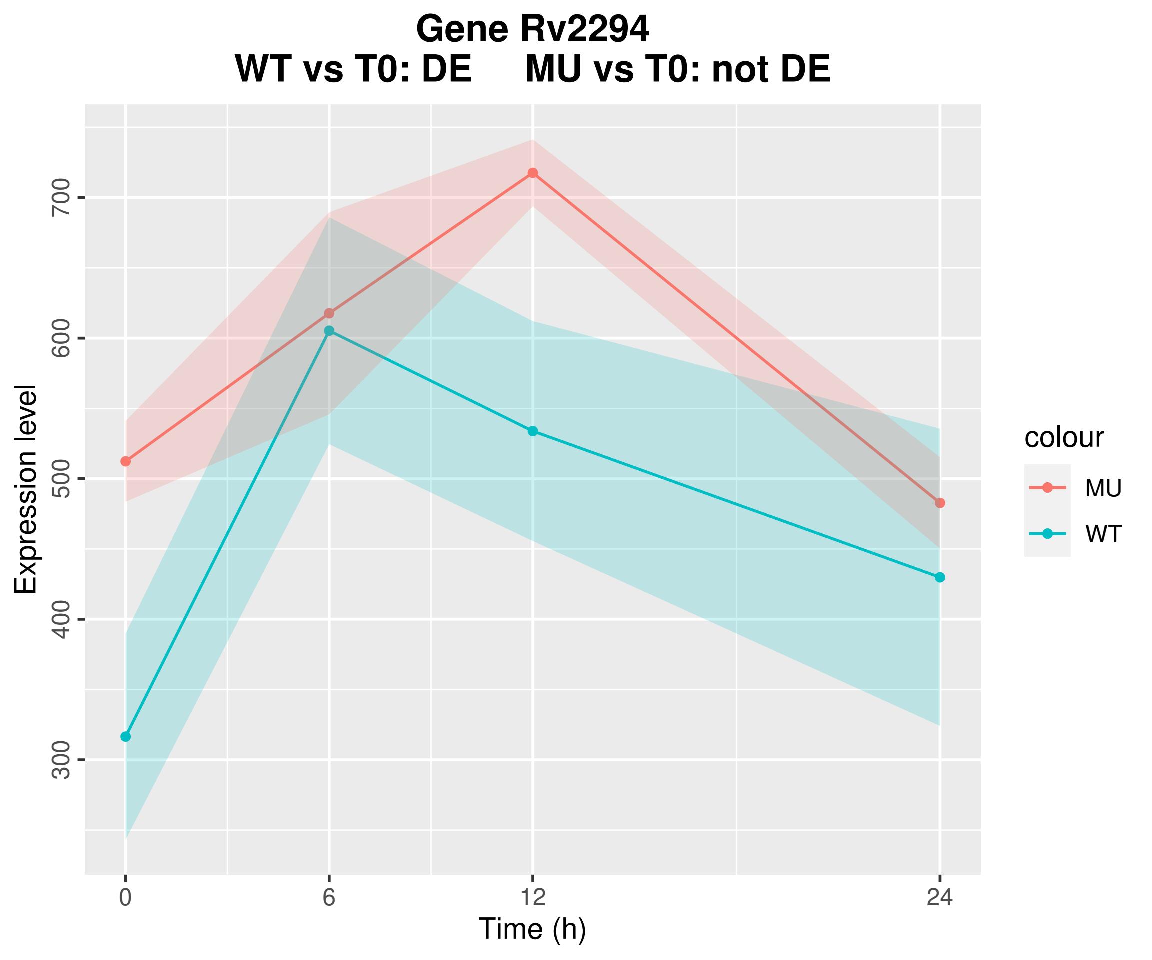

Gene Rv2294
WT vs T0: DE MU vs T0: not DE
Expression level
700
600
500
400
300
0
6
12
24
Time (h)
colour
MU
WT

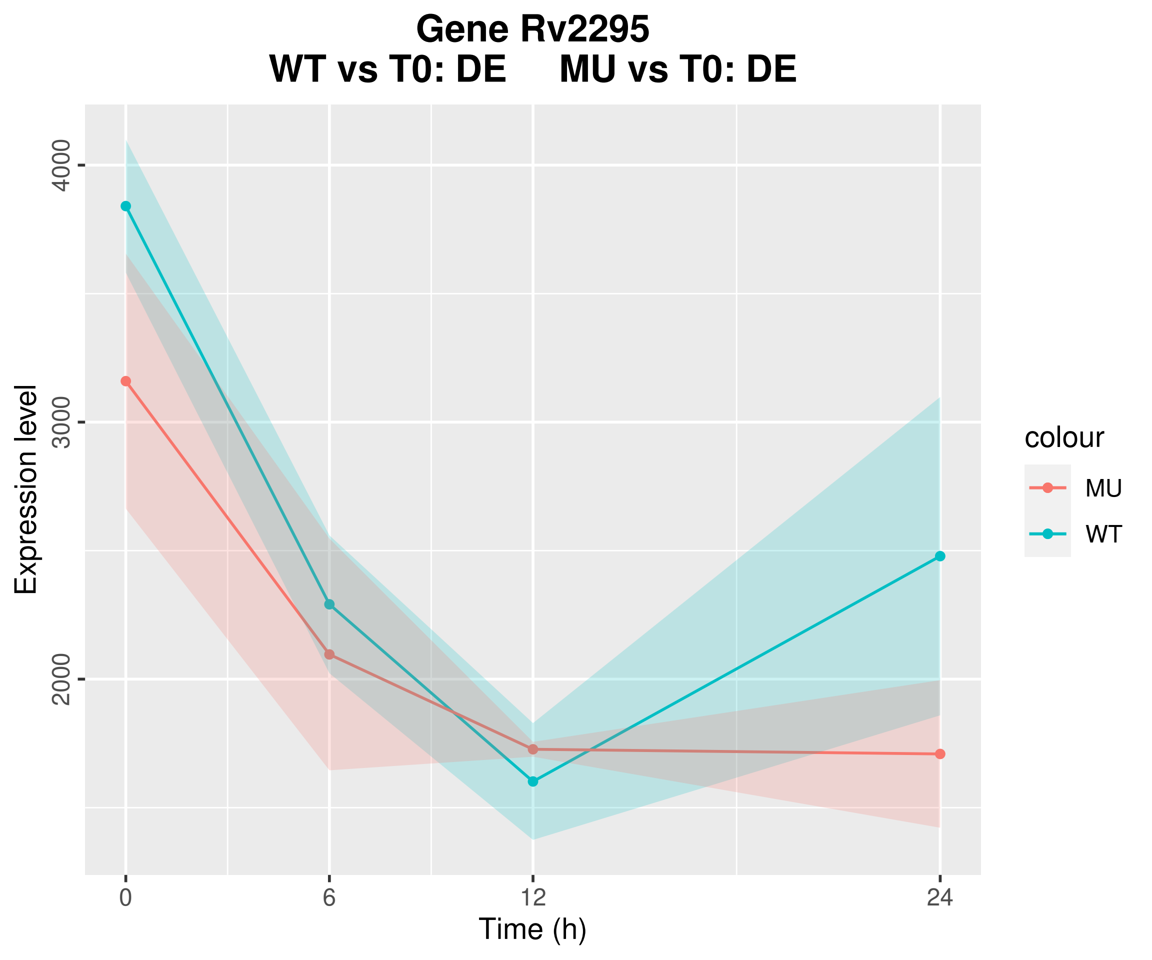

Gene Rv2295
WT vs T0: DE   MU vs T0: DE
4000
3000
2000
Expression level
0
6
12
24
Time (h)
colour
MU
WT

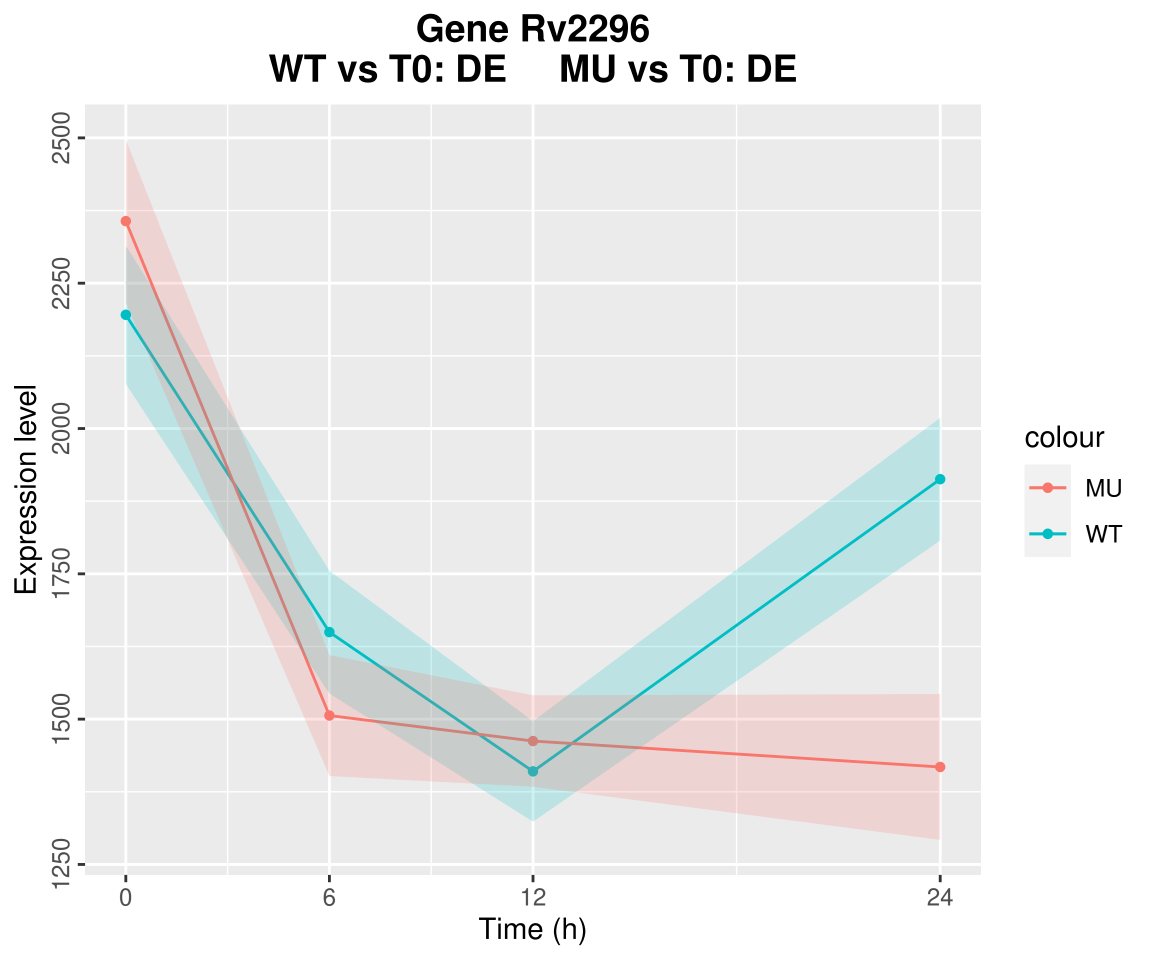

Gene Rv2296
WT vs T0: DE MU vs T0: DE
Expression level
2500
2250
2000
1750
1500
1250
0
6
12
24
Time (h)
colour
MU
WT

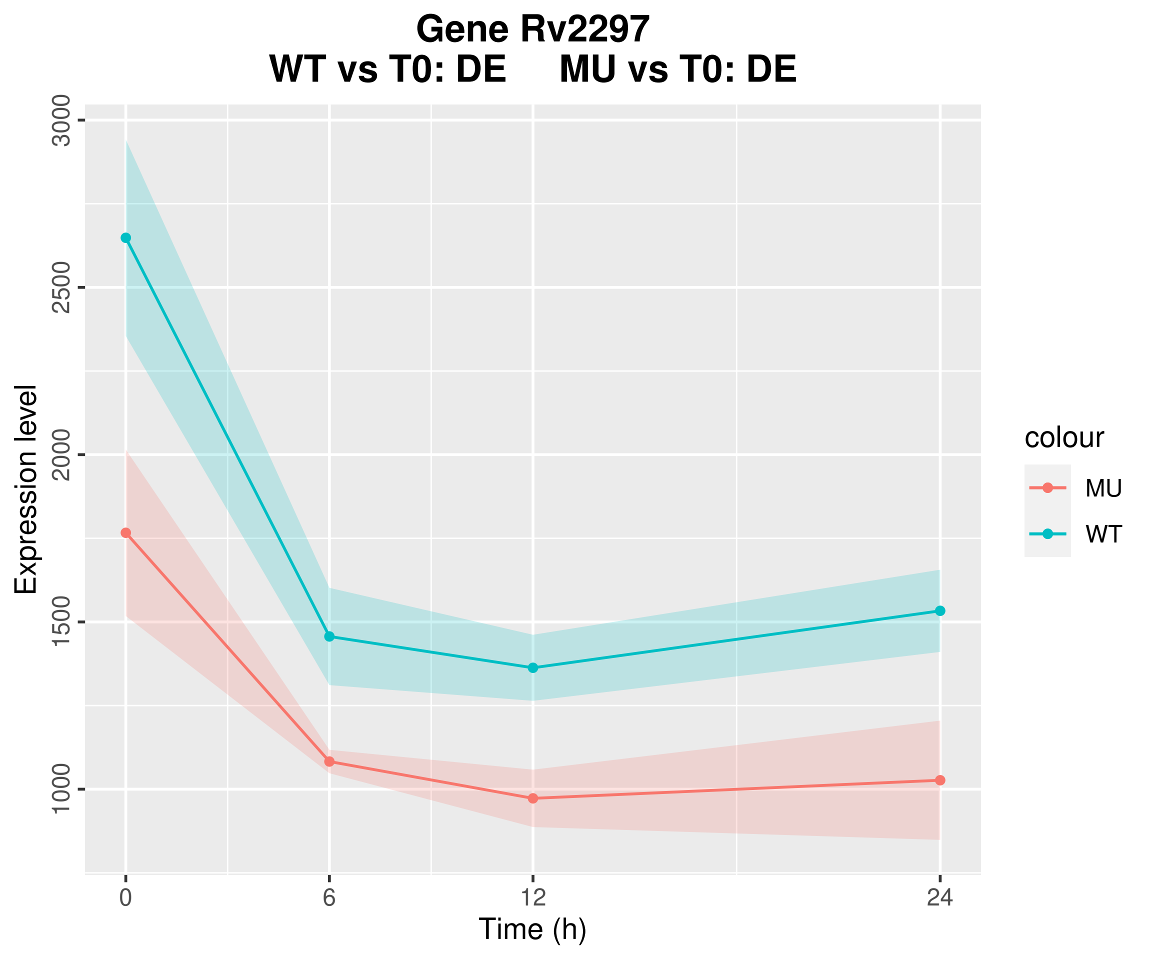
Gene Rv2297
WT vs T0: DE MU vs T0: DE
3000
2500
2000
1500
1000
Expression level
0
6
12
24
Time (h)
colour
MU
WT

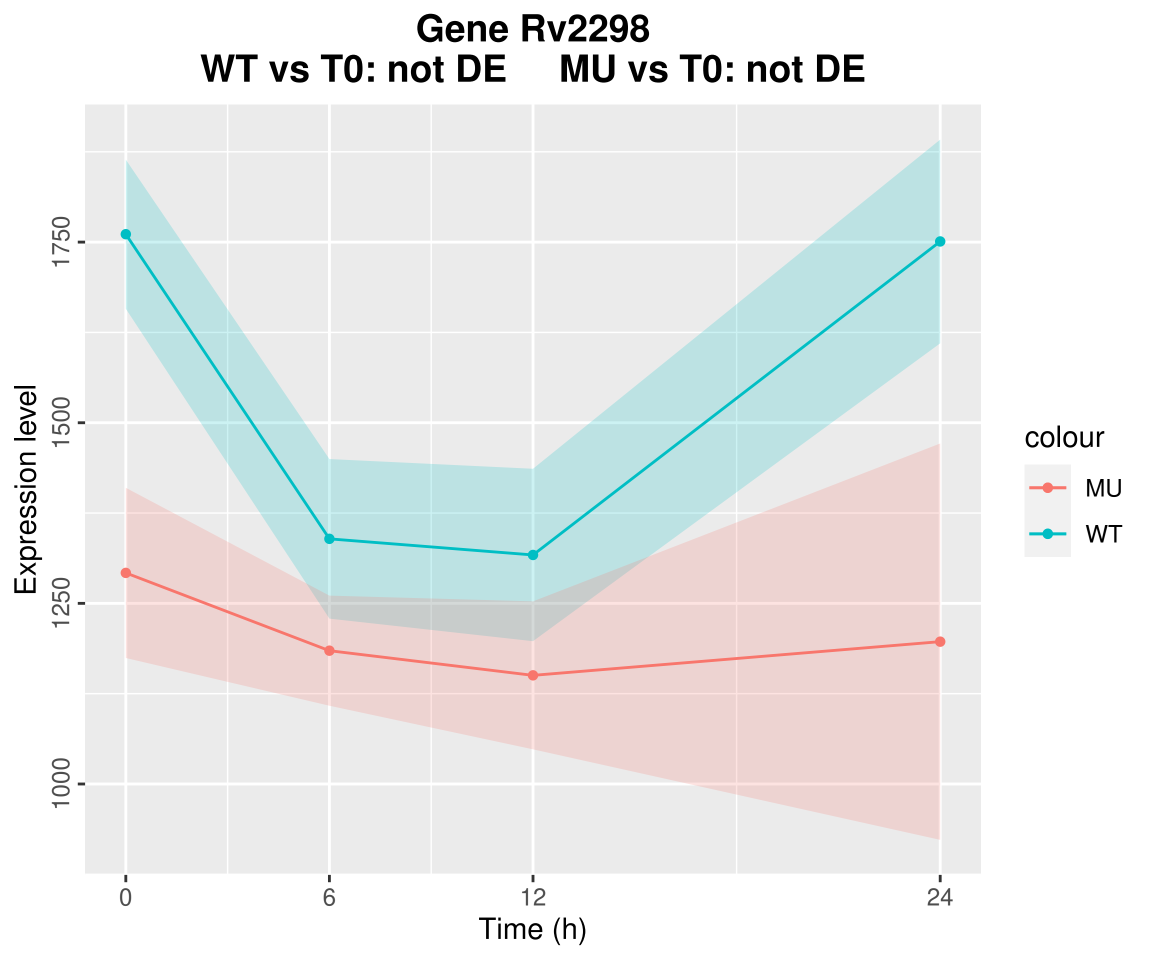
Gene Rv2298
WT vs T0: not DE MU vs T0: not DE
Expression level
1000
1250
1500
1750
Time (h)
0
6
12
24
colour
MU
WT

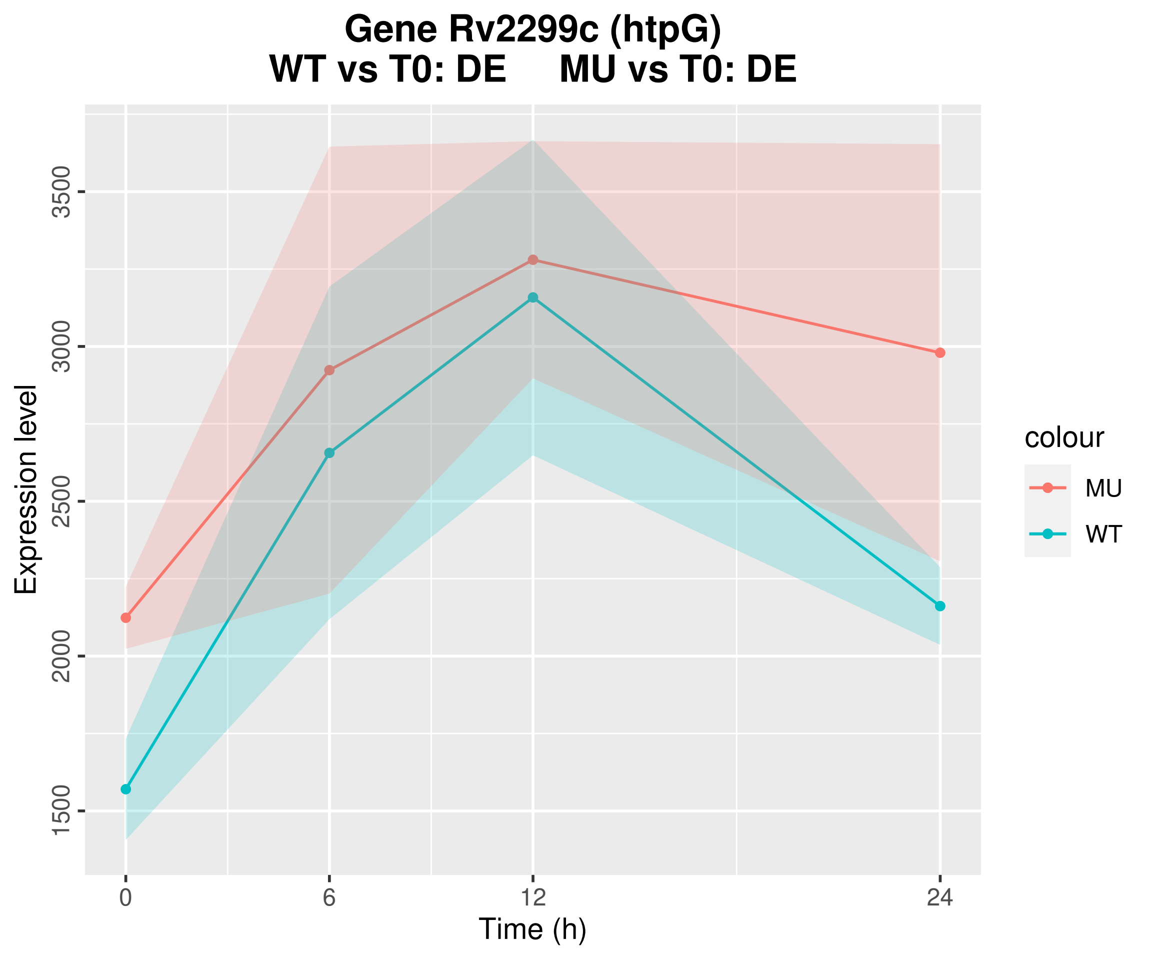
Gene Rv2299c (htpG)
WT vs T0: DE MU vs T0: DE
Expression level
3500
3000
2500
2000
1500
0
6
12
24
Time (h)
colour
MU
WT

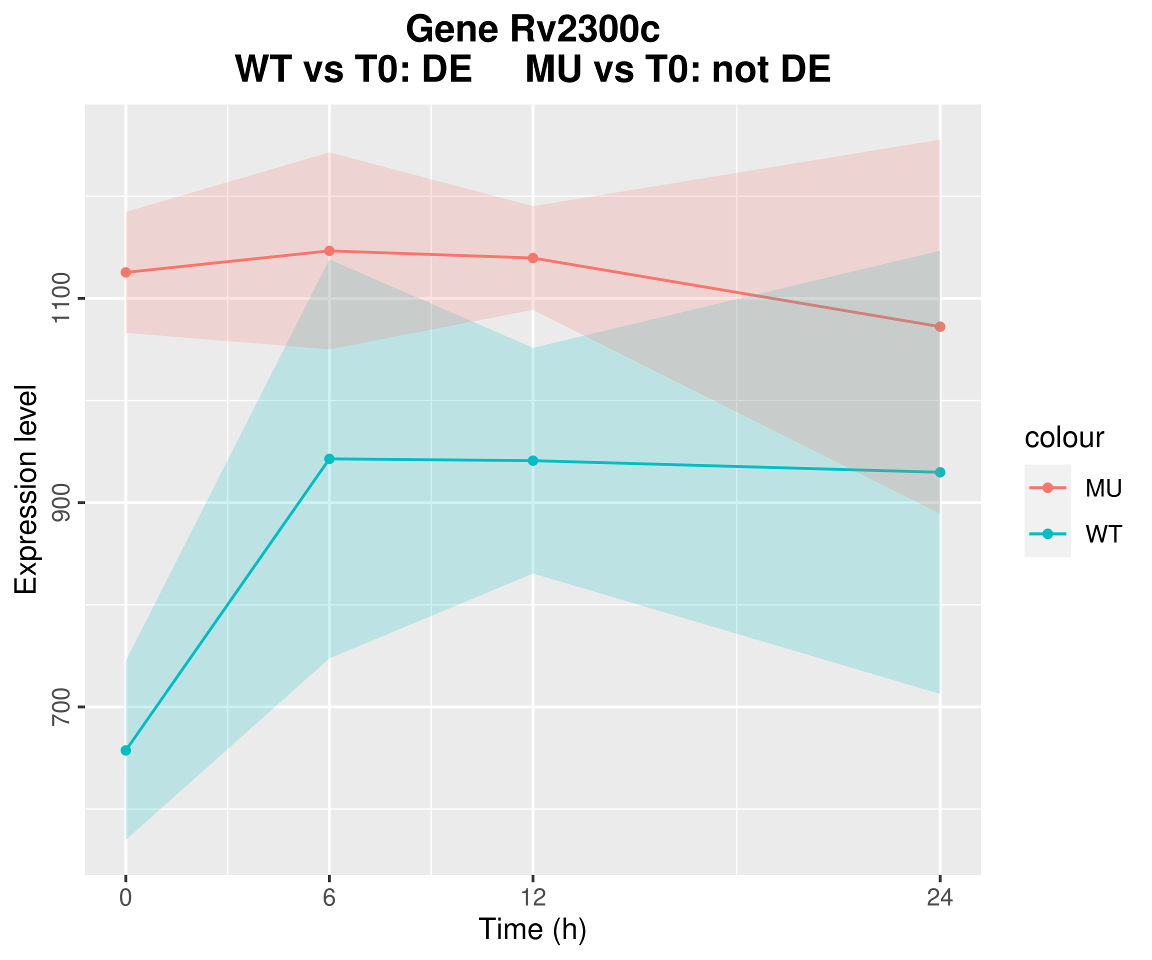
Gene Rv2300c
WT vs T0: DE MU vs T0: not DE
Expression level
1100
900
700
0
6
12
24
Time (h)
colour
MU
WT

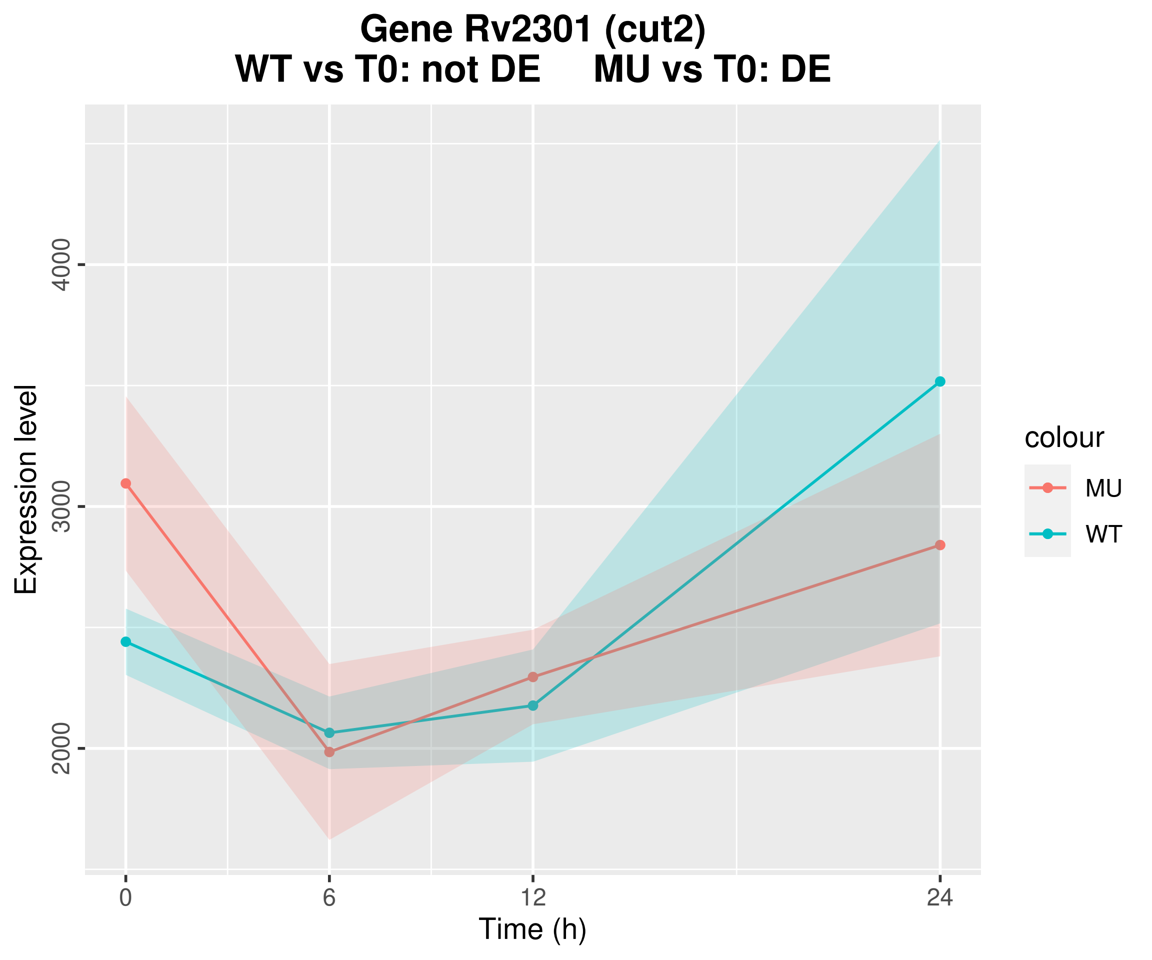

Gene Rv2301 (cut2)
WT vs T0: not DE   MU vs T0: DE
Expression level
4000
3000
2000
0
6
12
24
Time (h)
colour
MU
WT

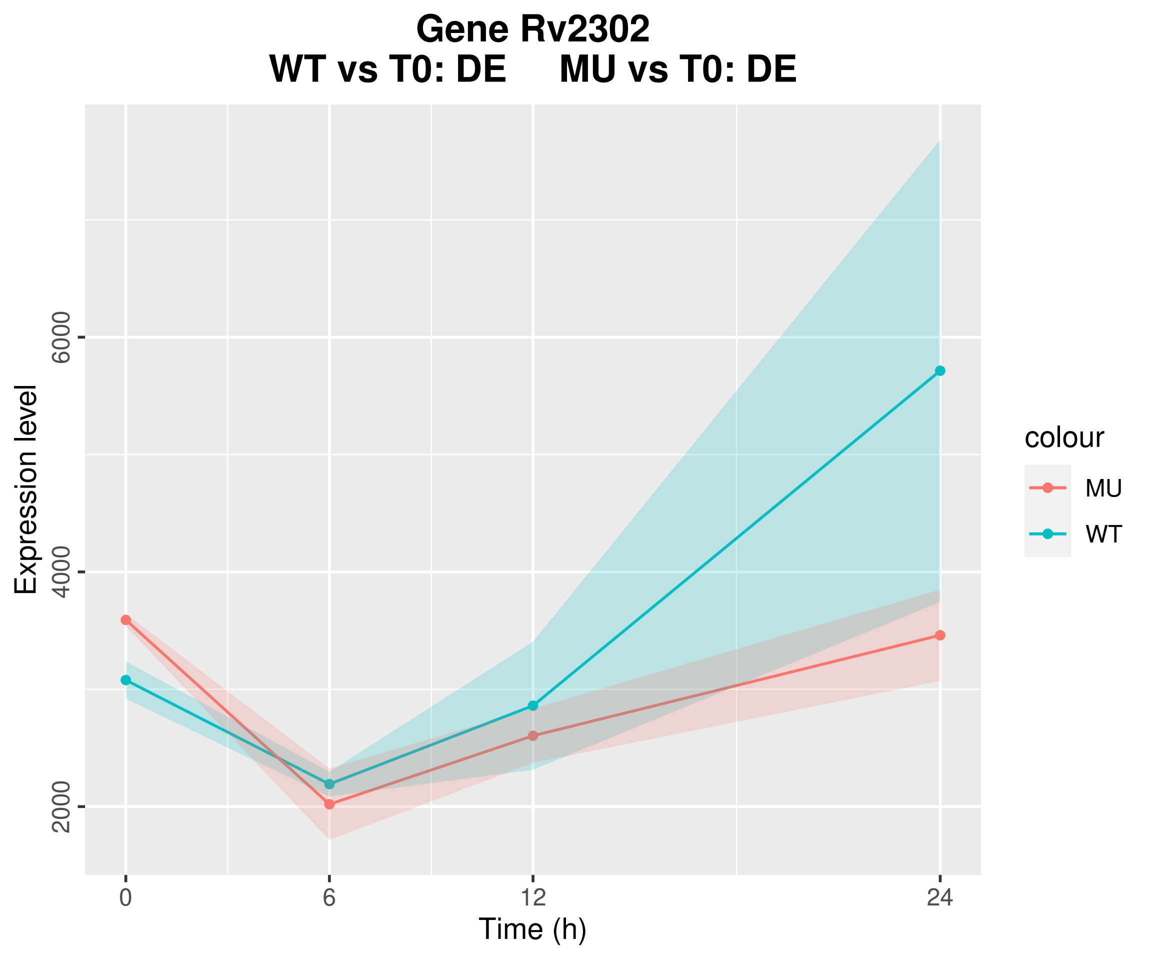

Gene Rv2302
WT vs T0: DE MU vs T0: DE
Expression level
2000
4000
6000
0
6
12
24
Time (h)
colour
MU
WT

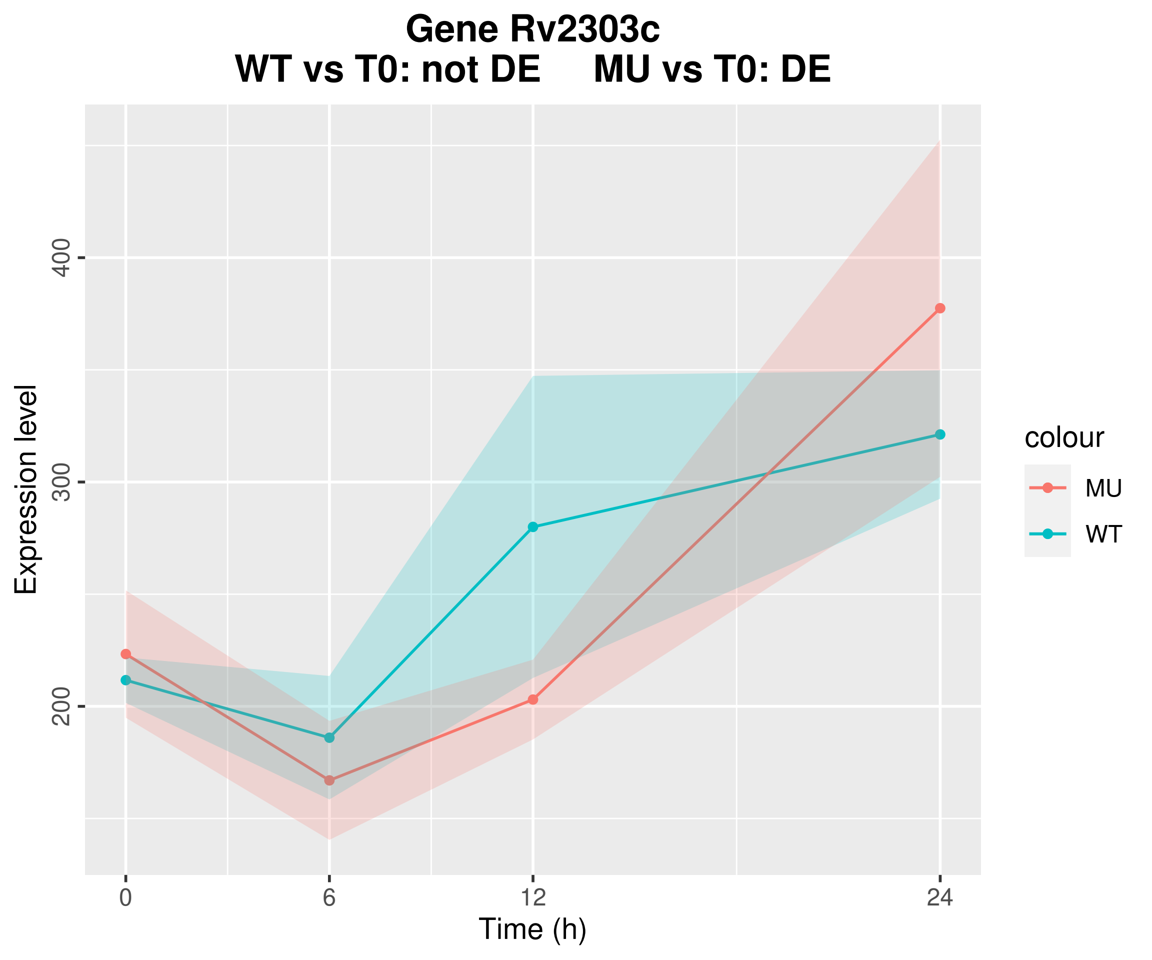
Gene Rv2303c
WT vs T0: not DE MU vs T0: DE
Expression level
400
300
200
0
6
12
24
Time (h)
colour
MU
WT

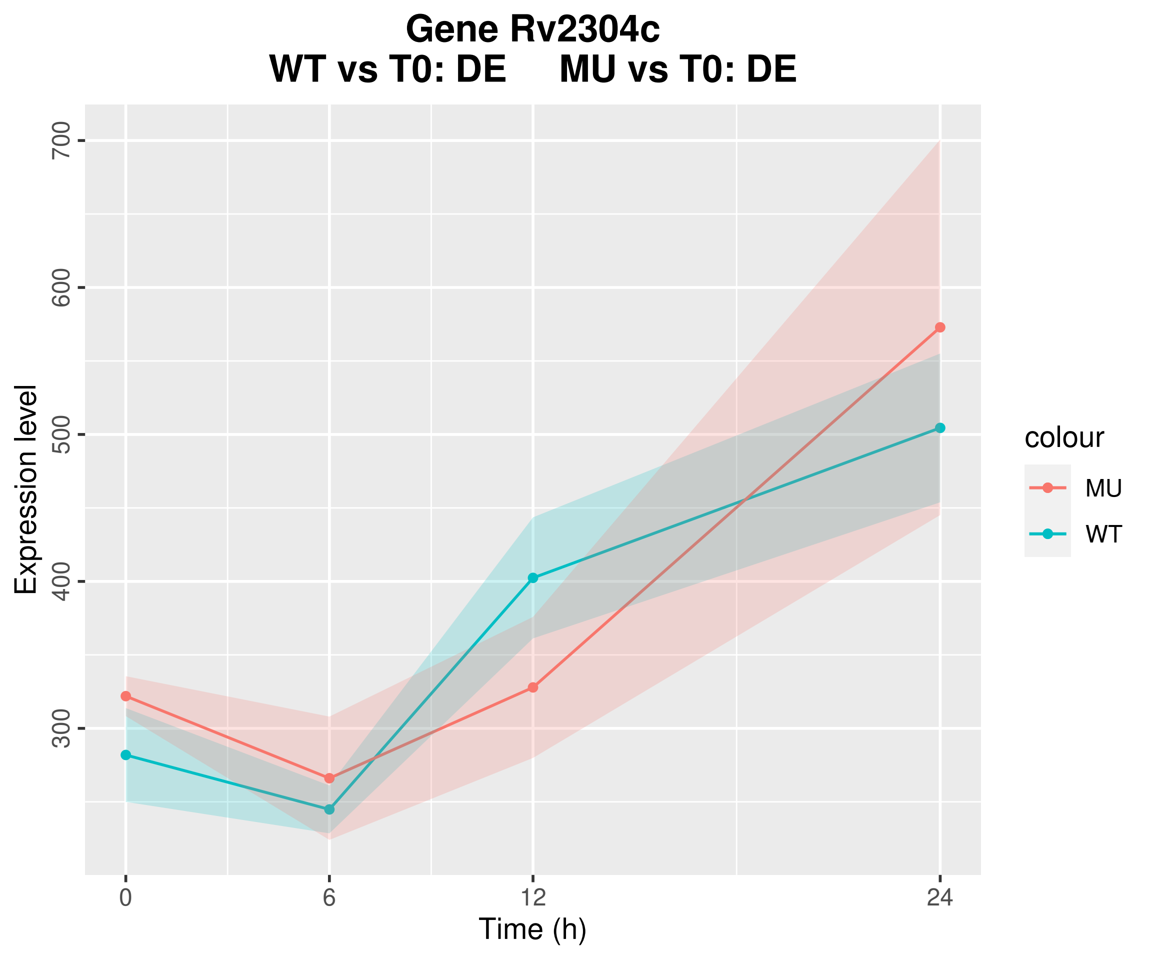
Gene Rv2304c
WT vs T0: DE MU vs T0: DE
Expression level
700
600
500
400
300
0
6
12
24
Time (h)
colour
MU
WT

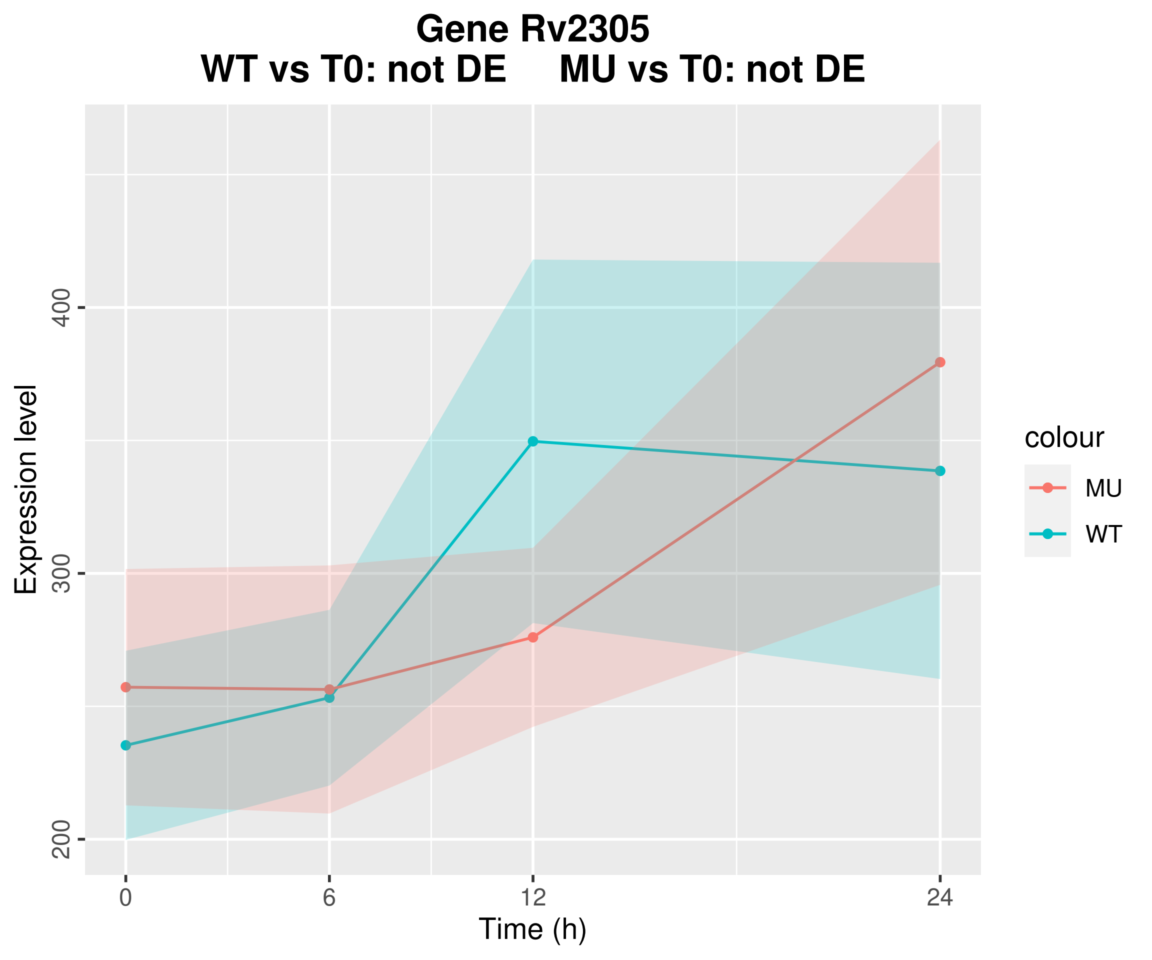

Gene Rv2305
WT vs T0: not DE MU vs T0: not DE
Expression level
400
300
200
0
6
12
24
Time (h)
colour
MU
WT

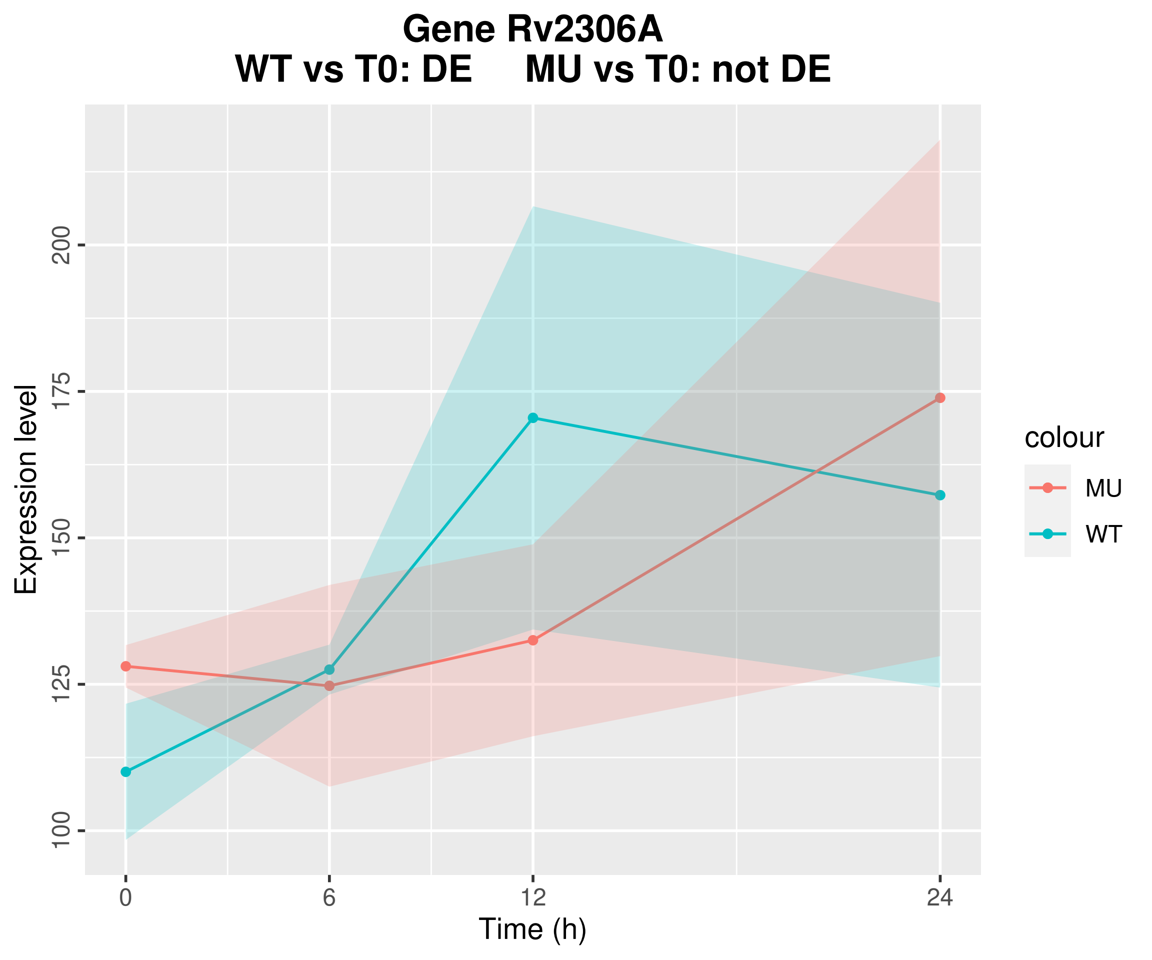

Gene Rv2306A
WT vs T0: DE   MU vs T0: not DE
Expression level
200
175
150
125
100
0
6
12
24
Time (h)
colour
MU
WT

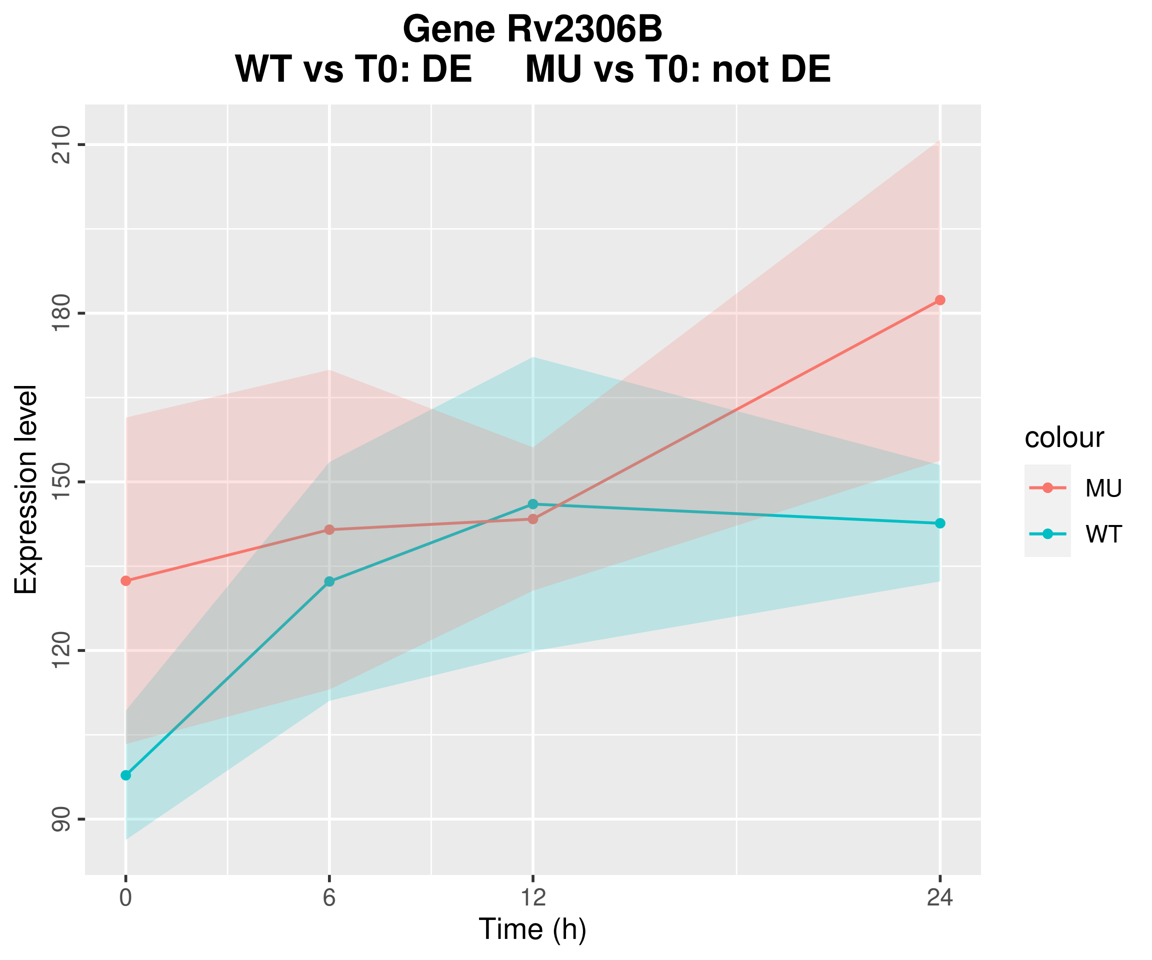

Gene Rv2306B
WT vs T0: DE MU vs T0: not DE
Expression level
210
180
150
120
90
0
6
12
24
Time (h)
colour
MU
WT

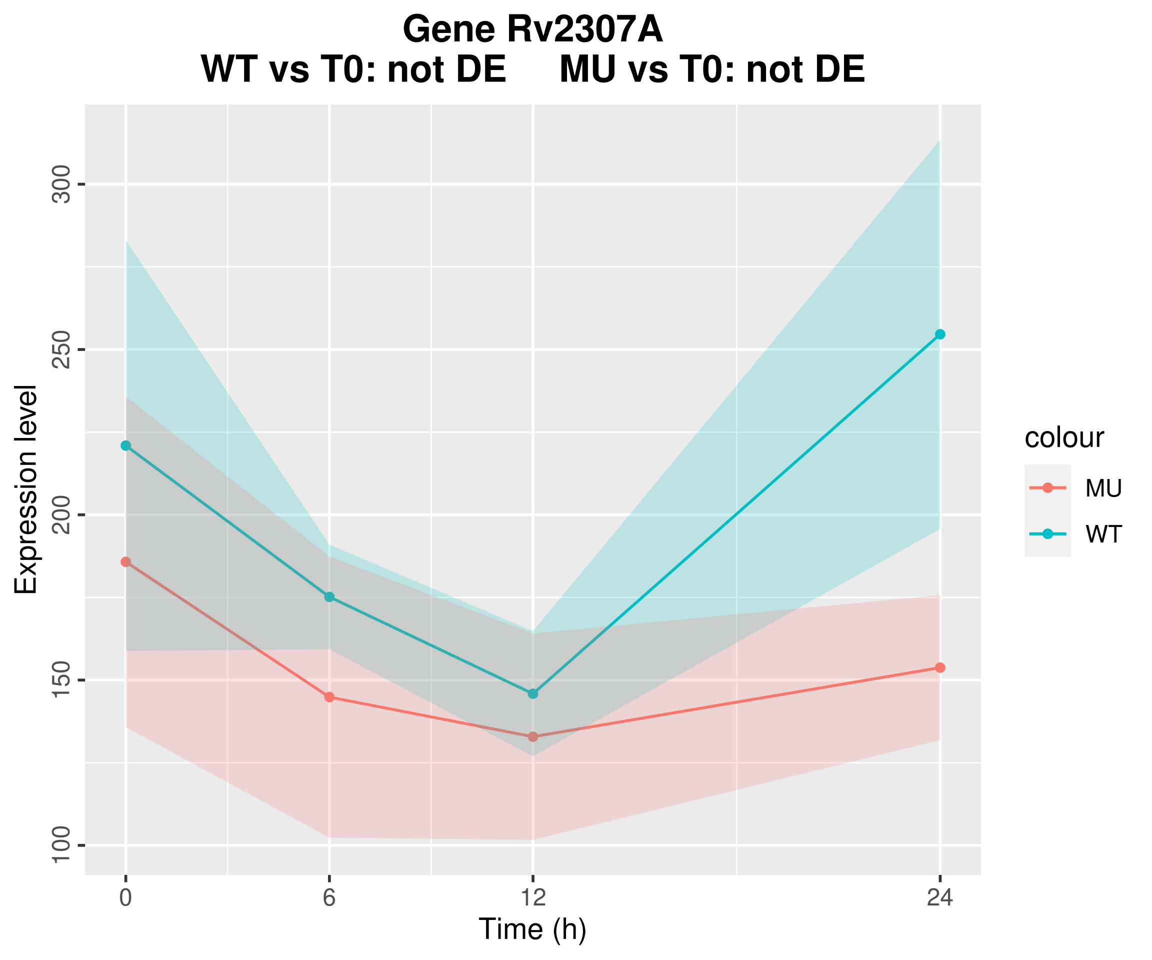
Gene Rv2307A
WT vs T0: not DE MU vs T0: not DE
Expression level
300
250
200
150
100
0
6
12
24
Time (h)
colour
MU
WT

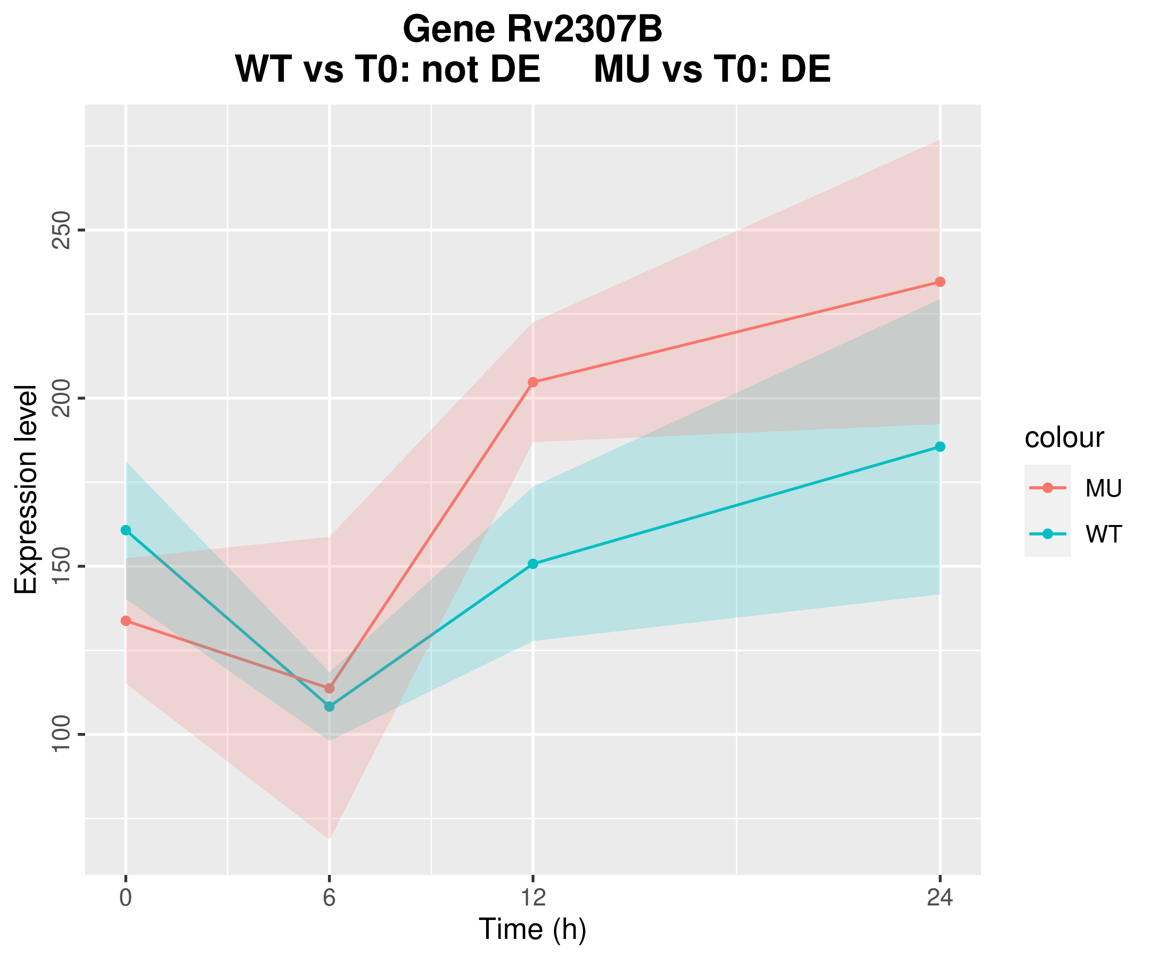
Gene Rv2307B
WT vs T0: not DE   MU vs T0: DE
Expression level
100
150
200
250
0
6
12
24
Time (h)
colour
MU
WT

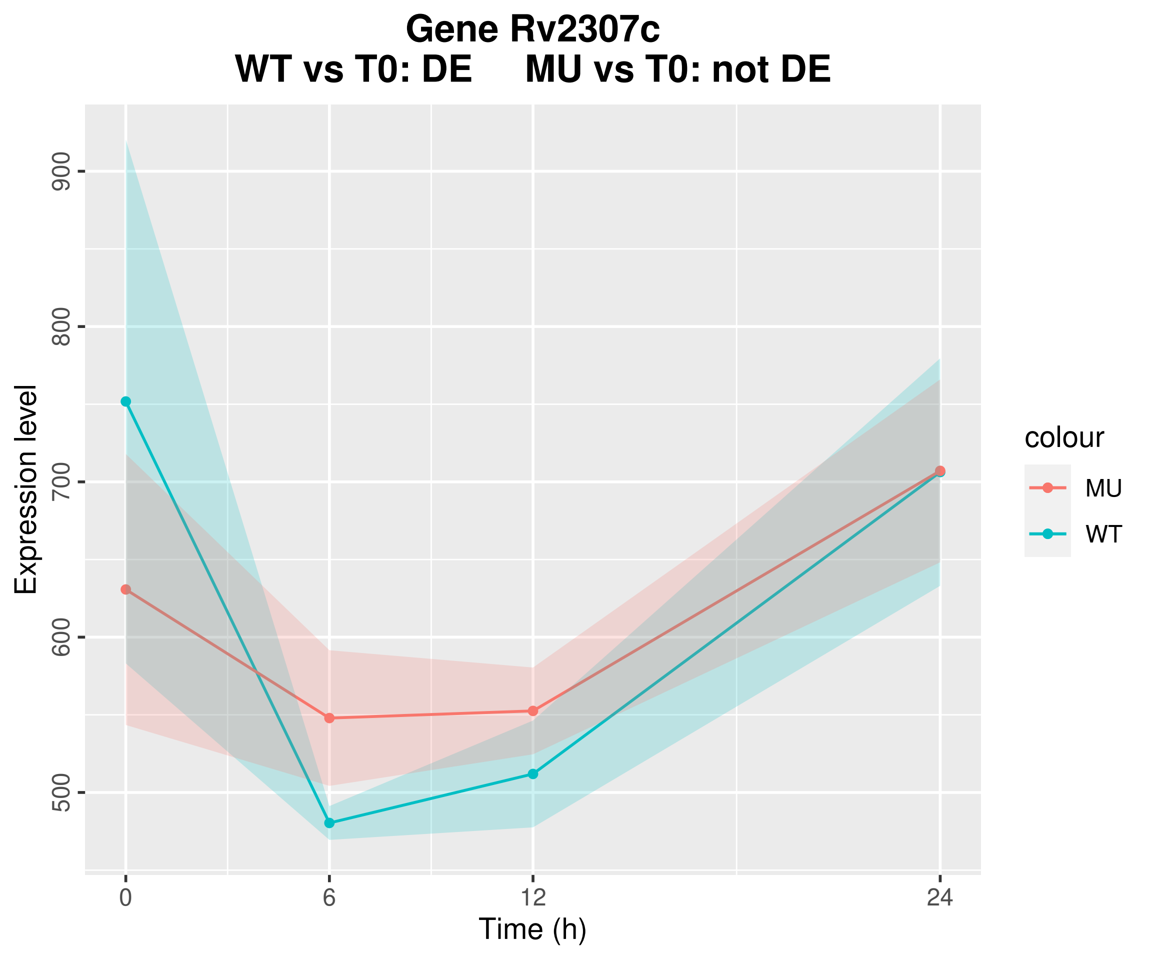

Gene Rv2307c
WT vs T0: DE   MU vs T0: not DE
Expression level
900
800
700
600
500
0
6
12
24
Time (h)
colour
MU
WT

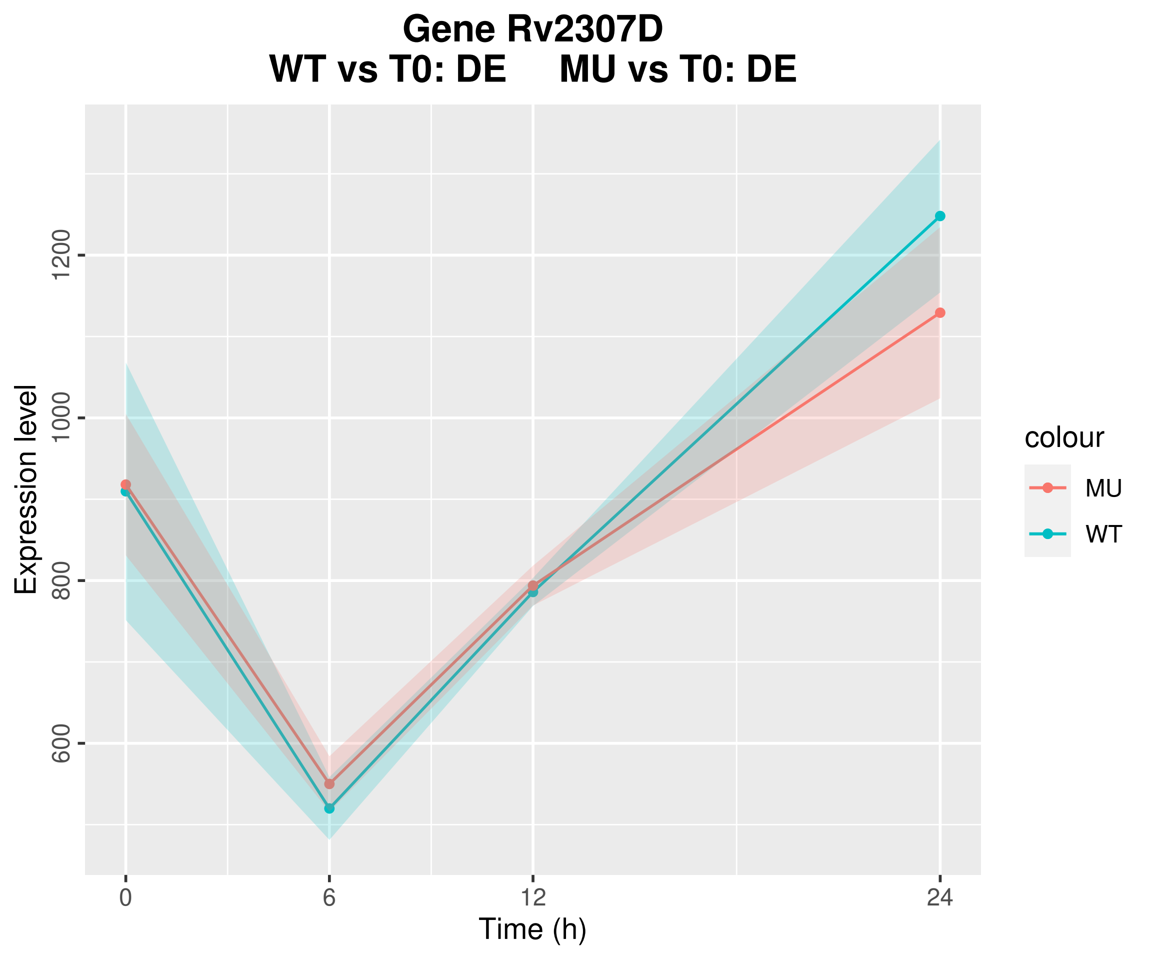

Gene Rv2307D
WT vs T0: DE MU vs T0: DE
Expression level
1200
1000
800
600
0
6
12
24
Time (h)
colour
MU
WT

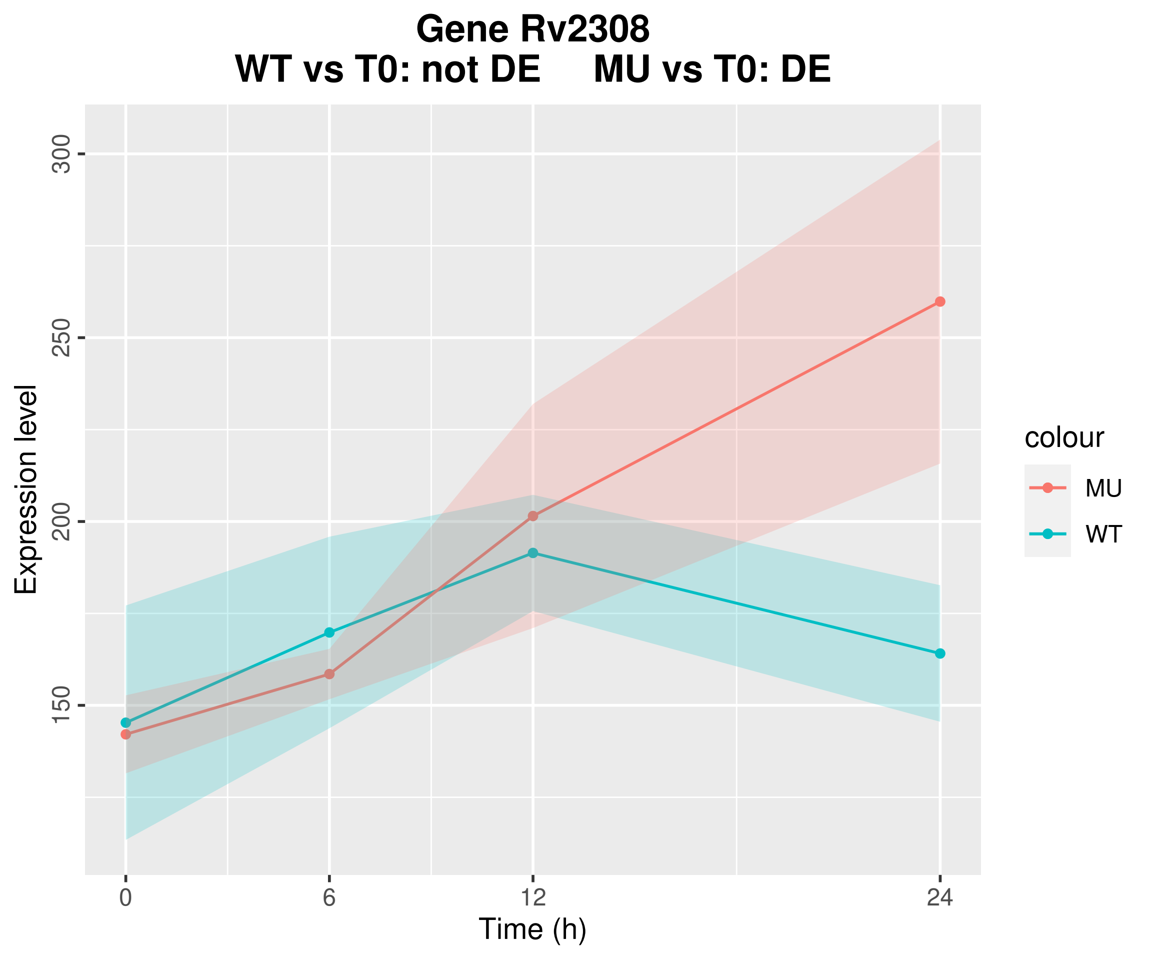
Gene Rv2308
WT vs T0: not DE MU vs T0: DE
Expression level
300
250
200
150
0
6
12
24
Time (h)
colour
MU
WT

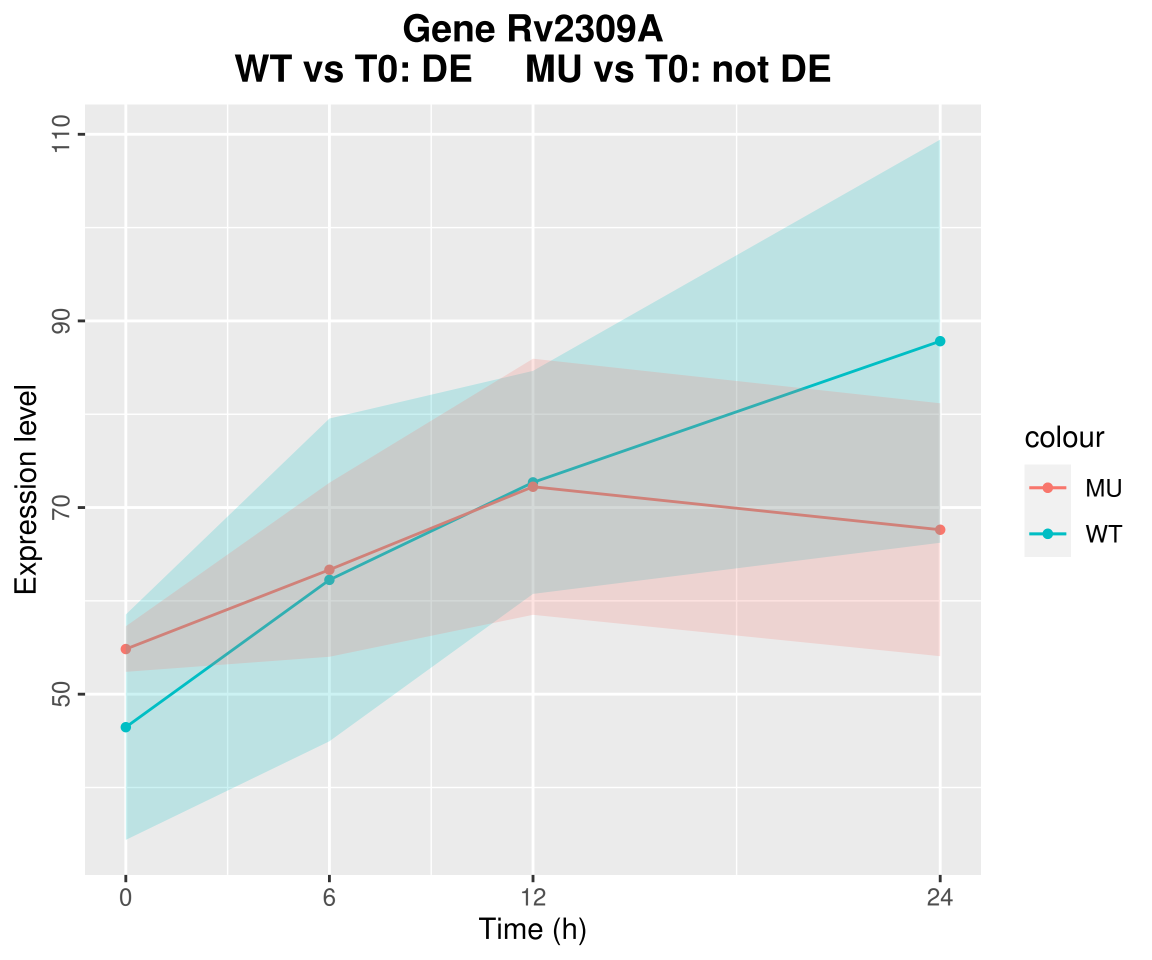
Gene Rv2309A
WT vs T0: DE MU vs T0: not DE
Expression level
Time (h)
0
6
12
24
50
70
90
110
colour
MU
WT

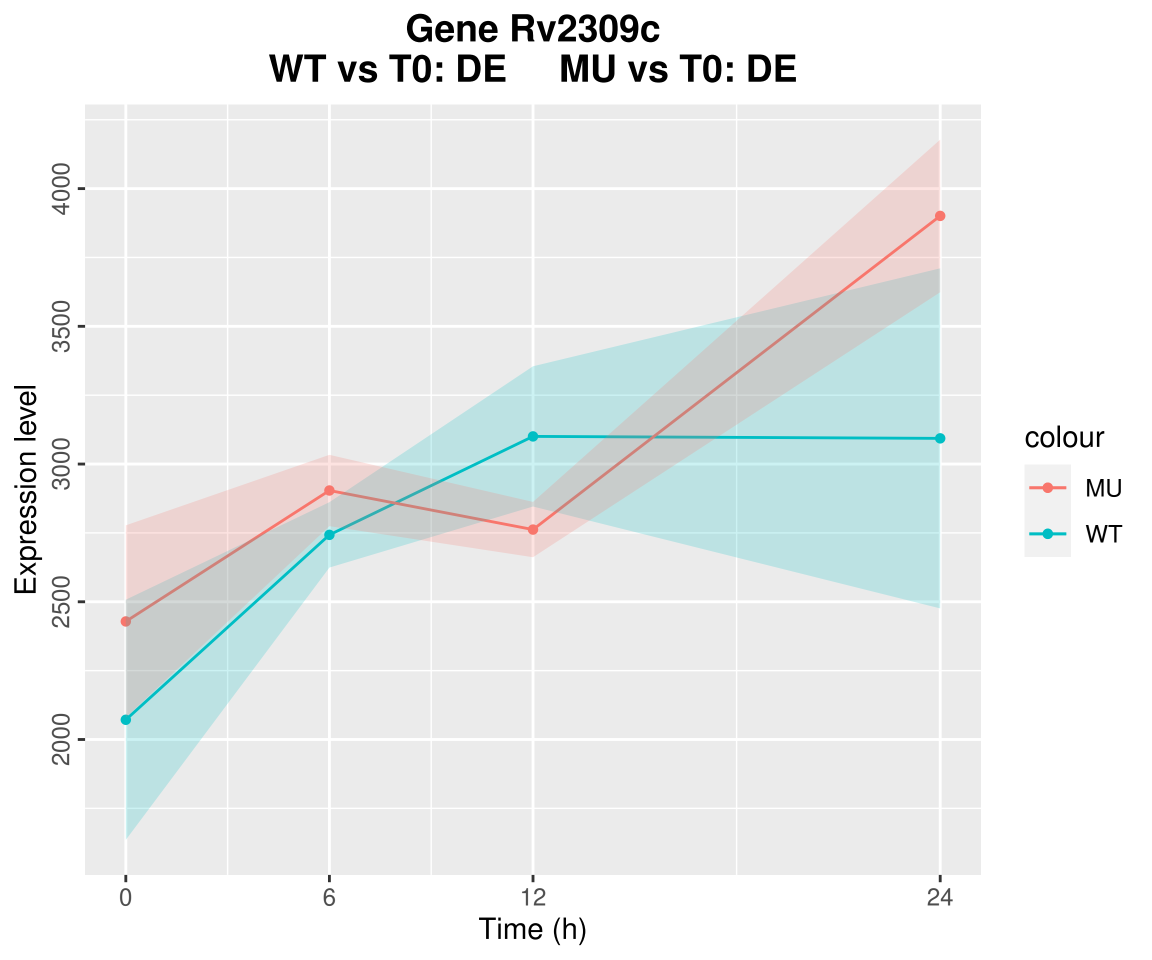
Gene Rv2309c
WT vs T0: DE MU vs T0: DE
Expression level
4000
3500
3000
2500
2000
0
6
12
24
Time (h)
colour
MU
WT

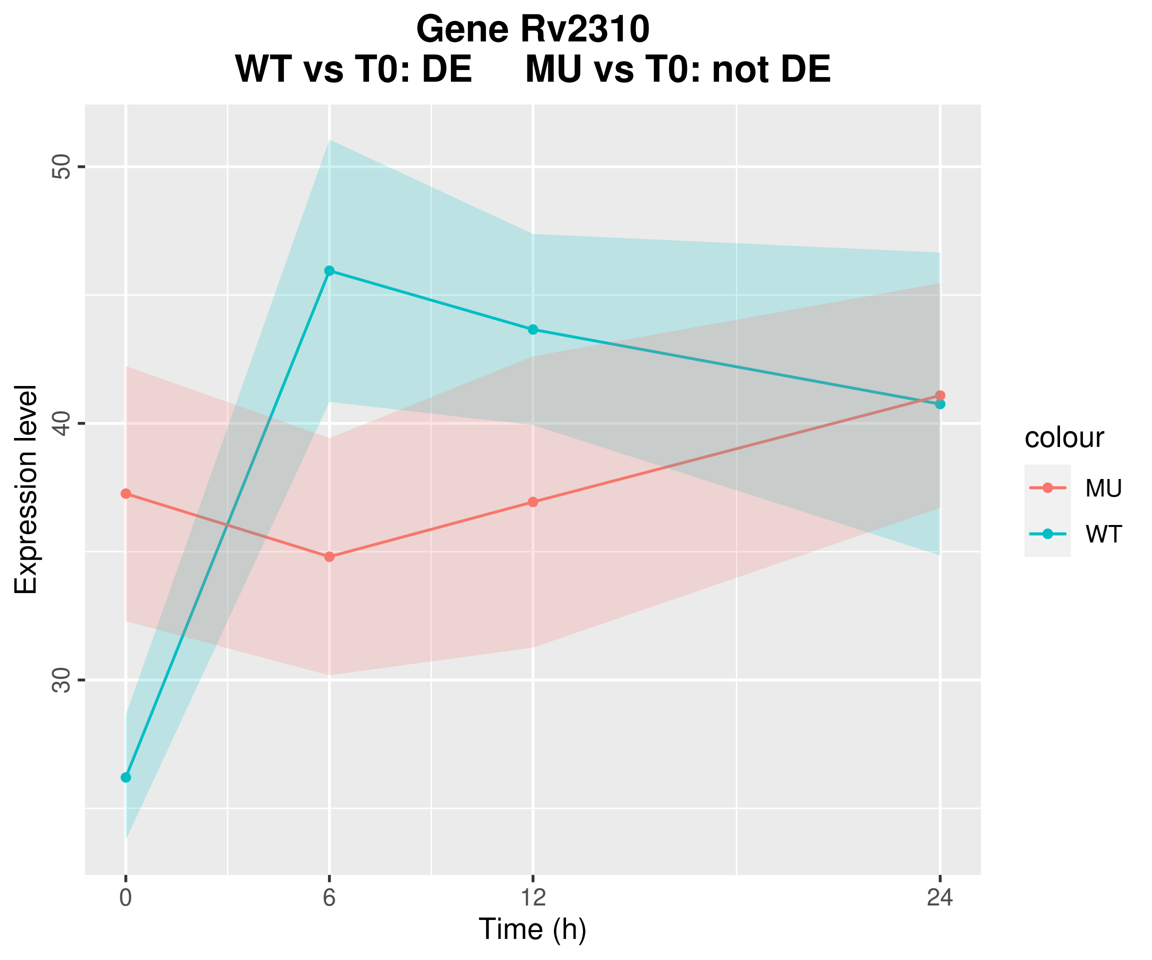

Gene Rv2310
WT vs T0: DE MU vs T0: not DE
50
40
30
Expression level
0
6
12
24
Time (h)
colour
MU
WT

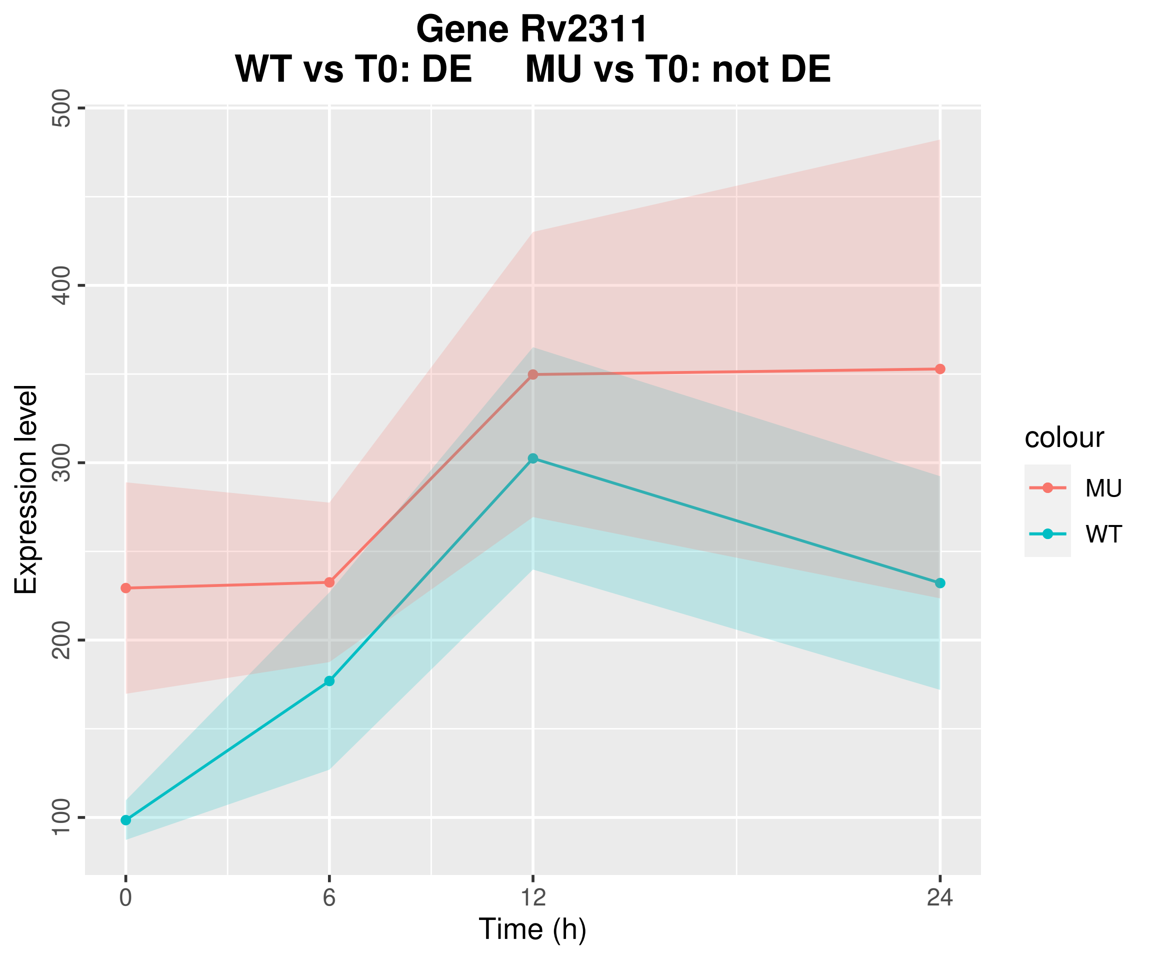

Gene Rv2311
WT vs T0: DE   MU vs T0: not DE
Expression level
100
200
300
400
500
Time (h)
0
6
12
24
colour
MU
WT

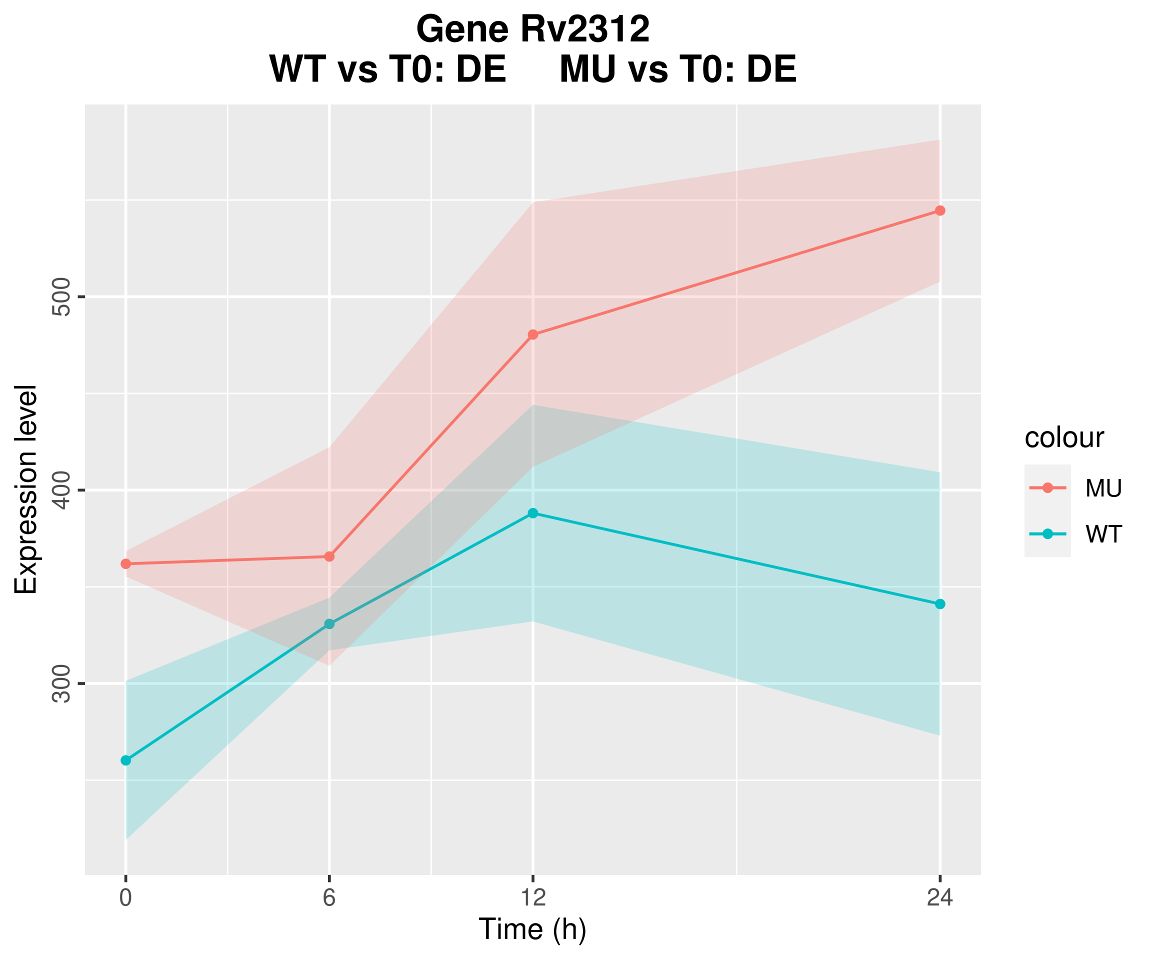
Gene Rv2312
WT vs T0: DE   MU vs T0: DE
Expression level
300
400
500
0
6
12
24
Time (h)
colour
MU
WT

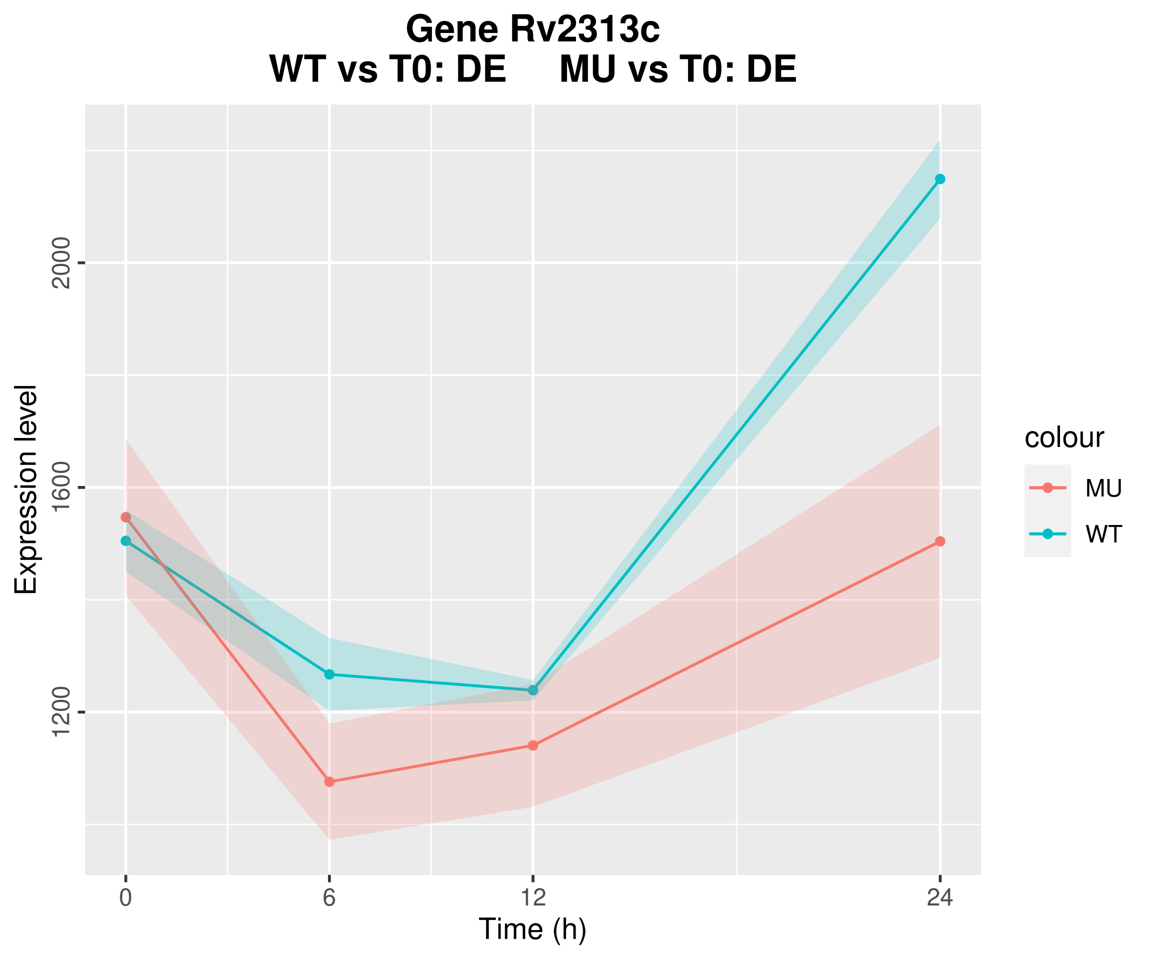
Gene Rv2313c
WT vs T0: DE   MU vs T0: DE
Expression level
2000
1600
1200
0
6
12
24
Time (h)
colour
MU
WT

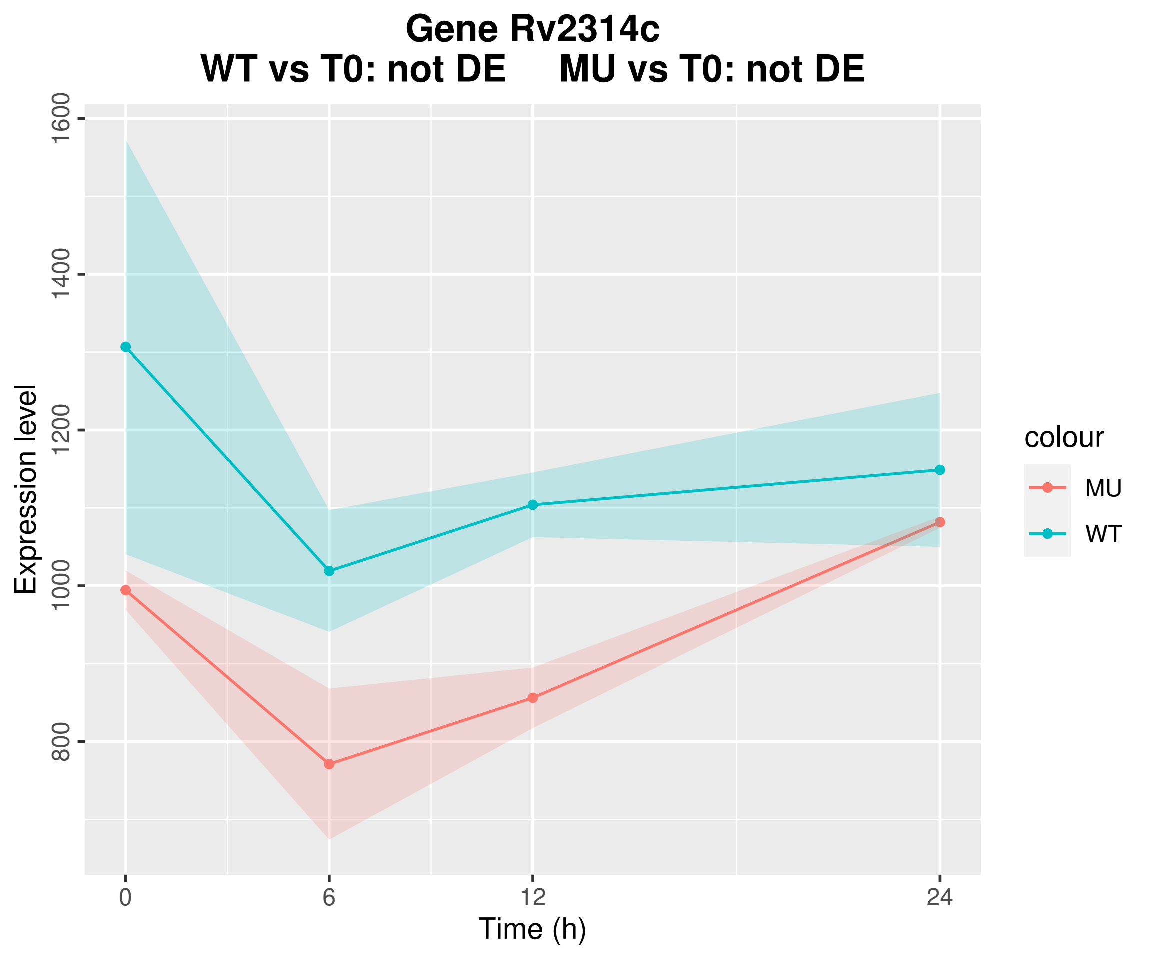
Gene Rv2314c
WT vs T0: not DE   MU vs T0: not DE
Expression level
1600
1400
1200
1000
800
0
6
12
24
Time (h)
colour
MU
WT

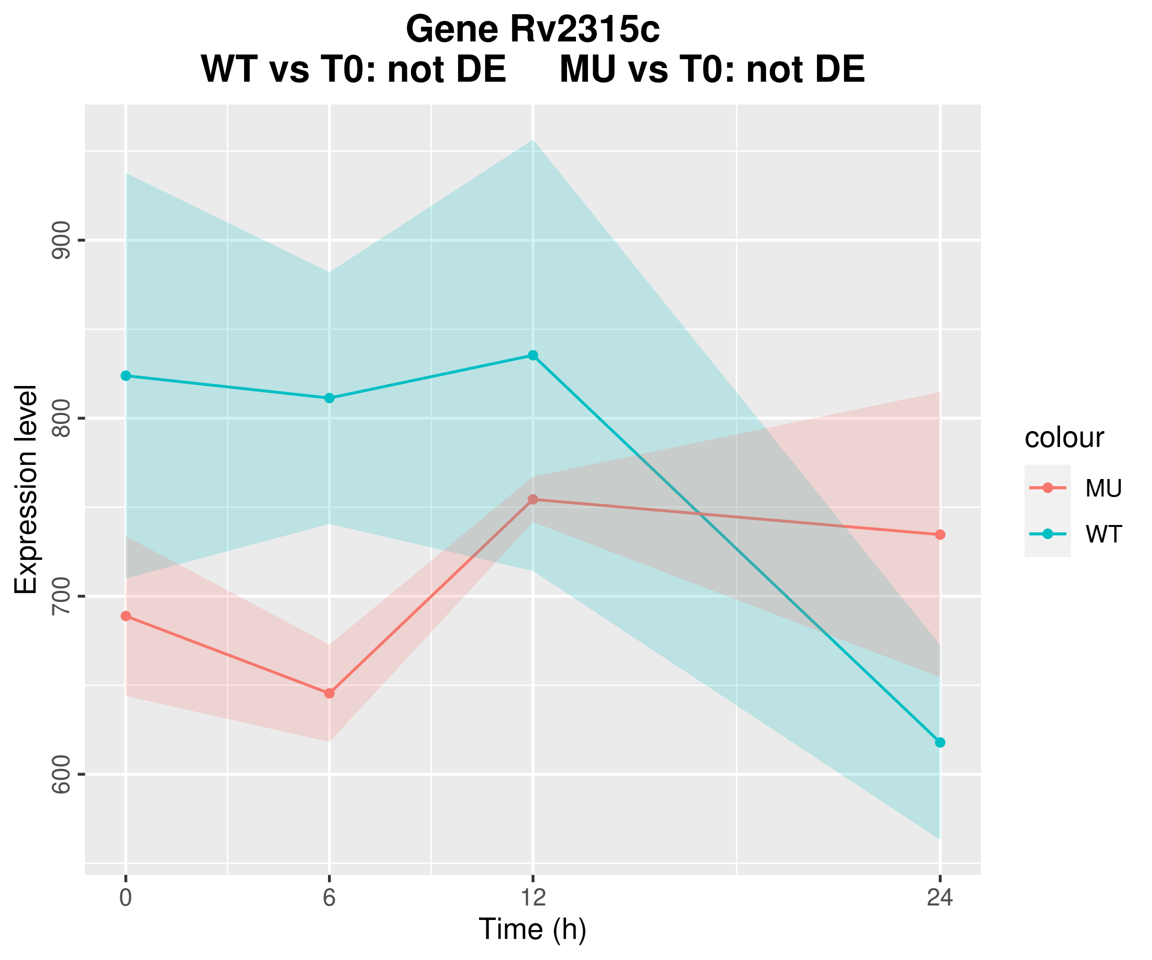
Gene Rv2315c
WT vs T0: not DE   MU vs T0: not DE
Expression level
900
800
700
600
0
6
12
24
Time (h)
colour
MU
WT

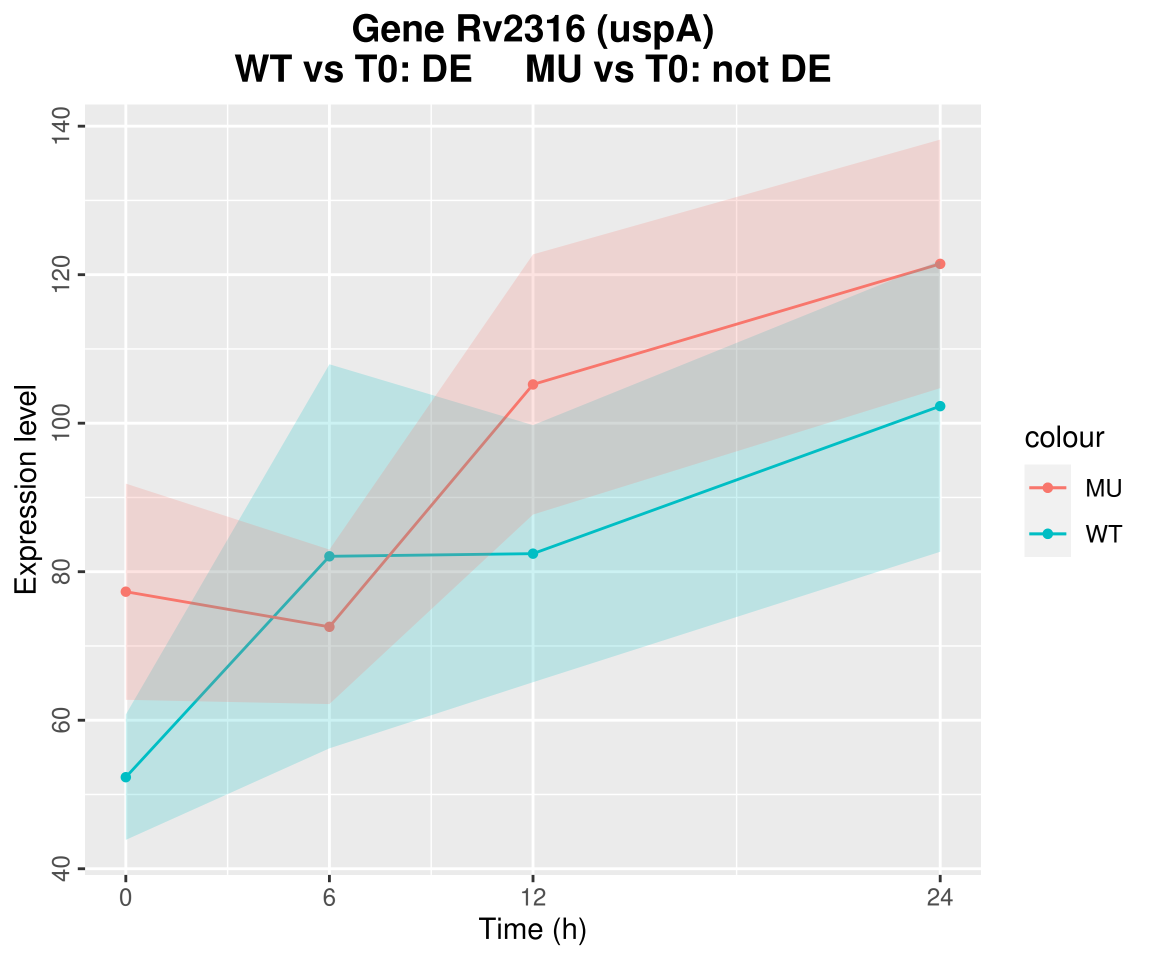

Gene Rv2316 (uspA)
WT vs T0: DE   MU vs T0: not DE
Expression level
140
120
100
80
60
40
0
6
12
24
Time (h)
colour
MU
WT

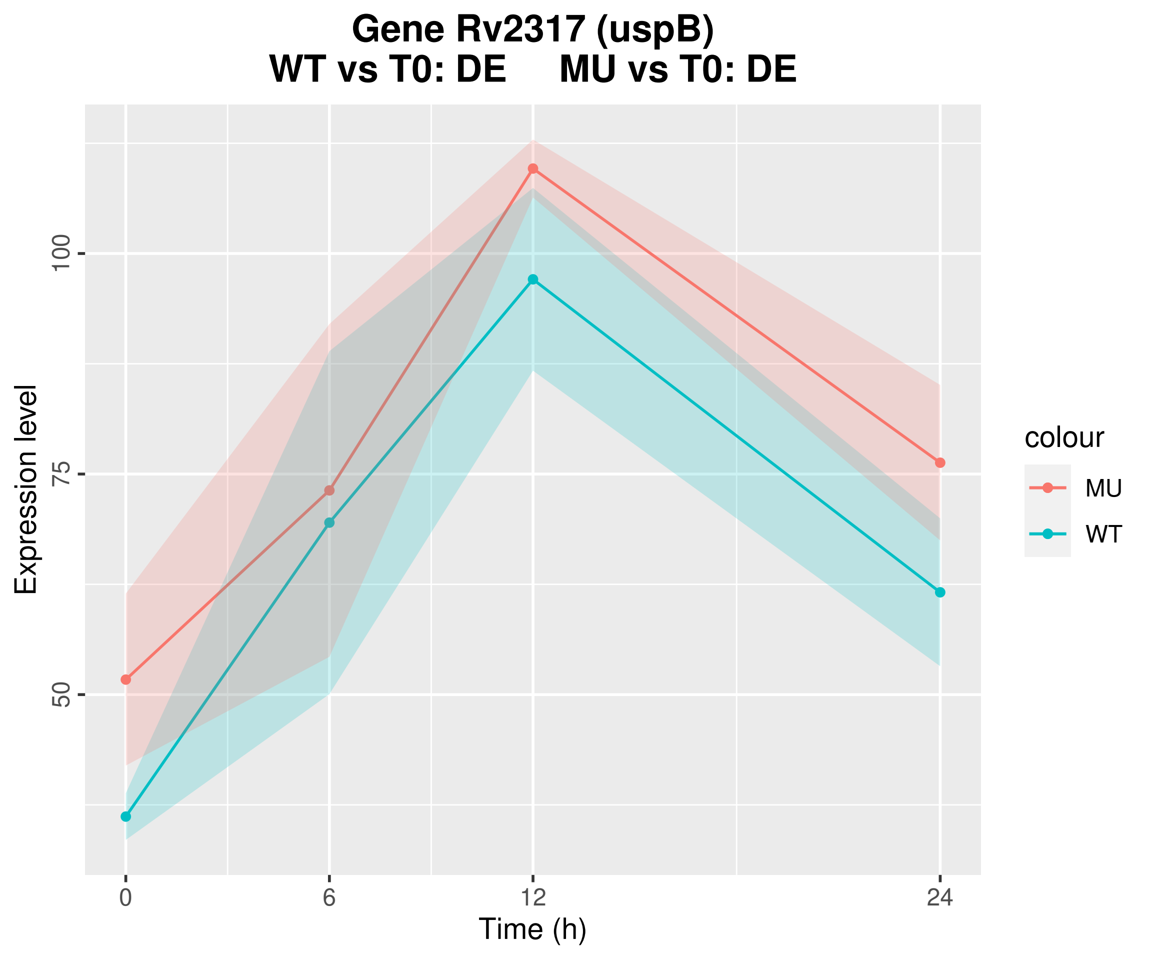
Gene Rv2317 (uspB)
WT vs T0: DE   MU vs T0: DE
Expression level
100
75
50
0
6
12
24
Time (h)
colour
MU
WT

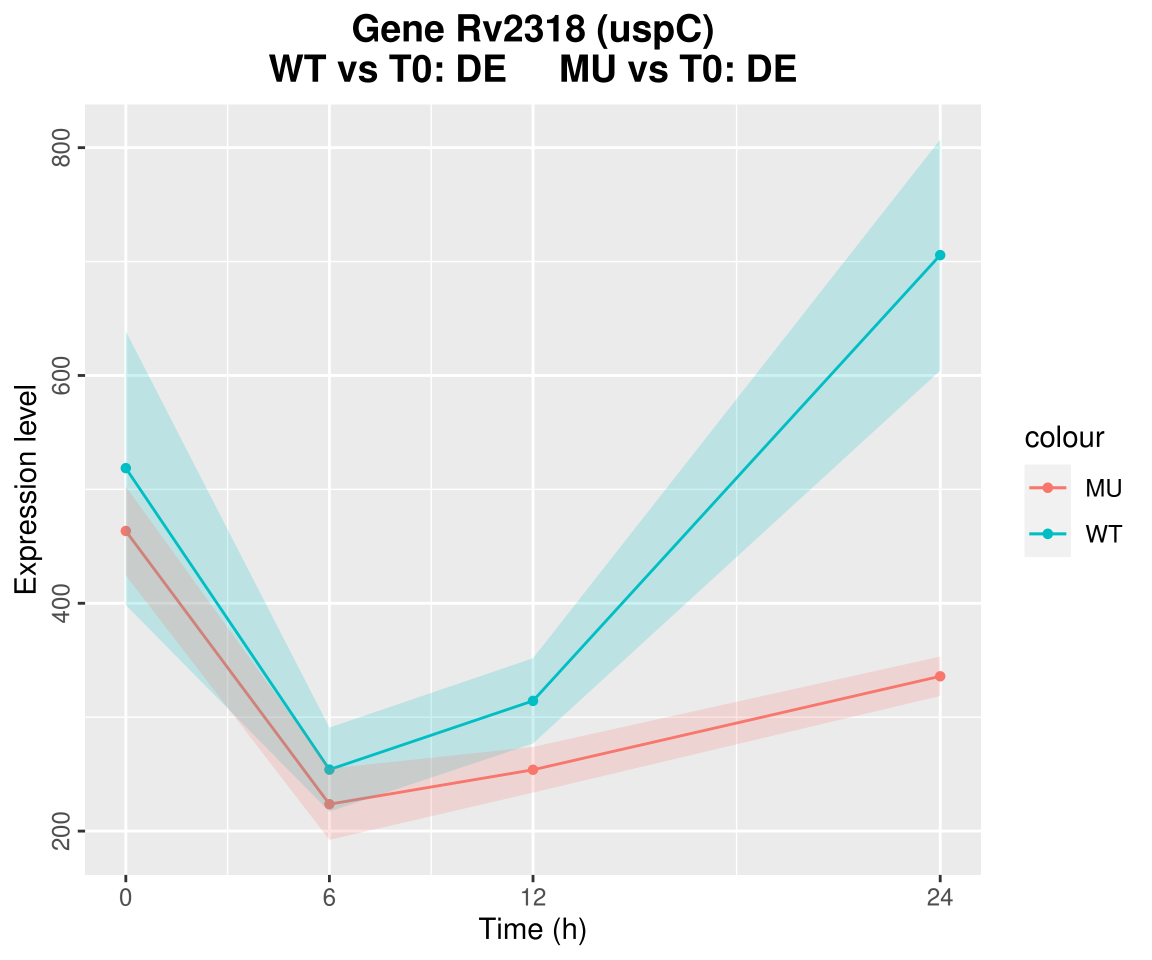
Gene Rv2318 (uspC)
WT vs T0: DE MU vs T0: DE
Expression level
800
600
400
200
0
6
12
24
Time (h)
colour
MU
WT

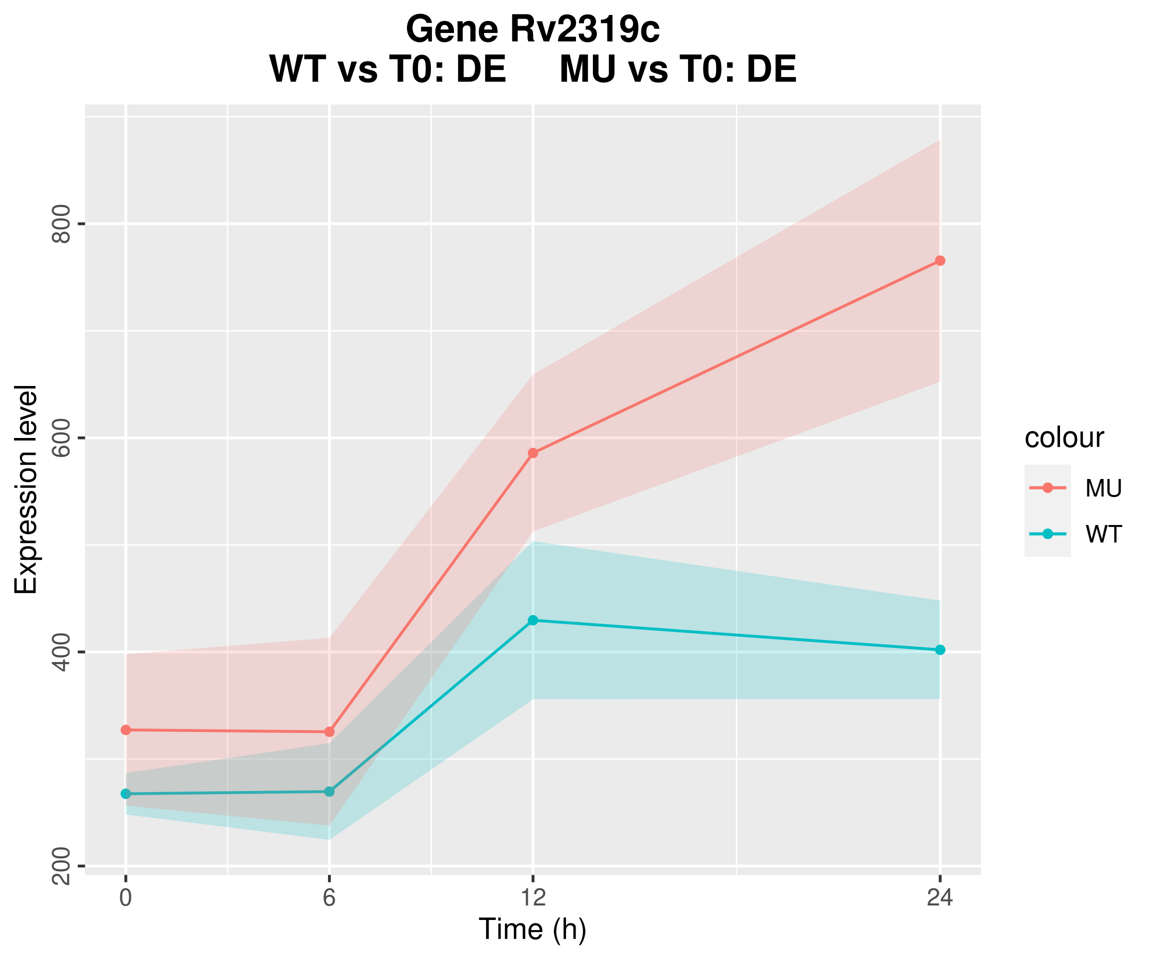
Gene Rv2319c
WT vs T0: DE MU vs T0: DE
Expression level
800
600
400
200
0
6
12
24
Time (h)
colour
MU
WT

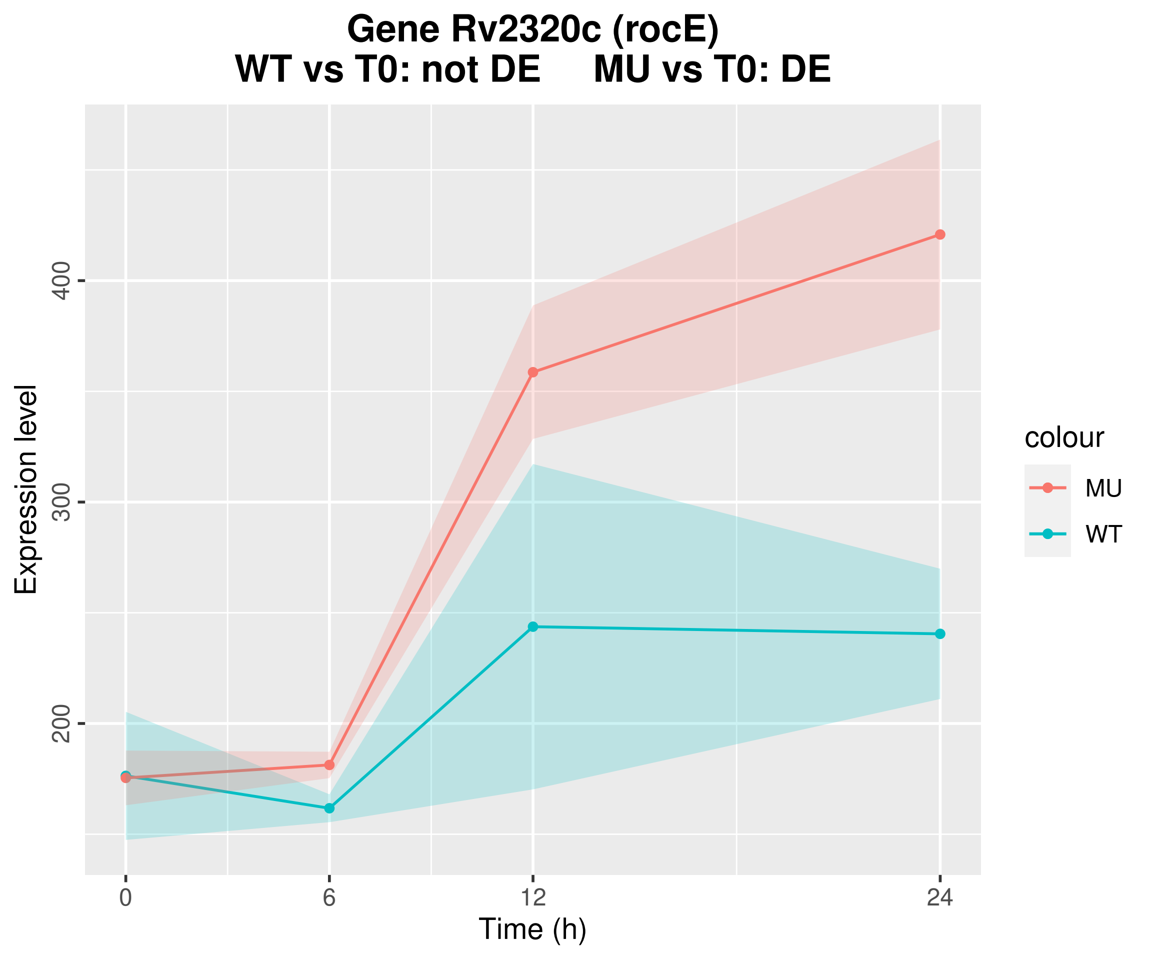

Gene Rv2320c (rocE)
WT vs T0: not DE   MU vs T0: DE
Expression level
400
300
200
0
6
12
24
Time (h)
colour
MU
WT

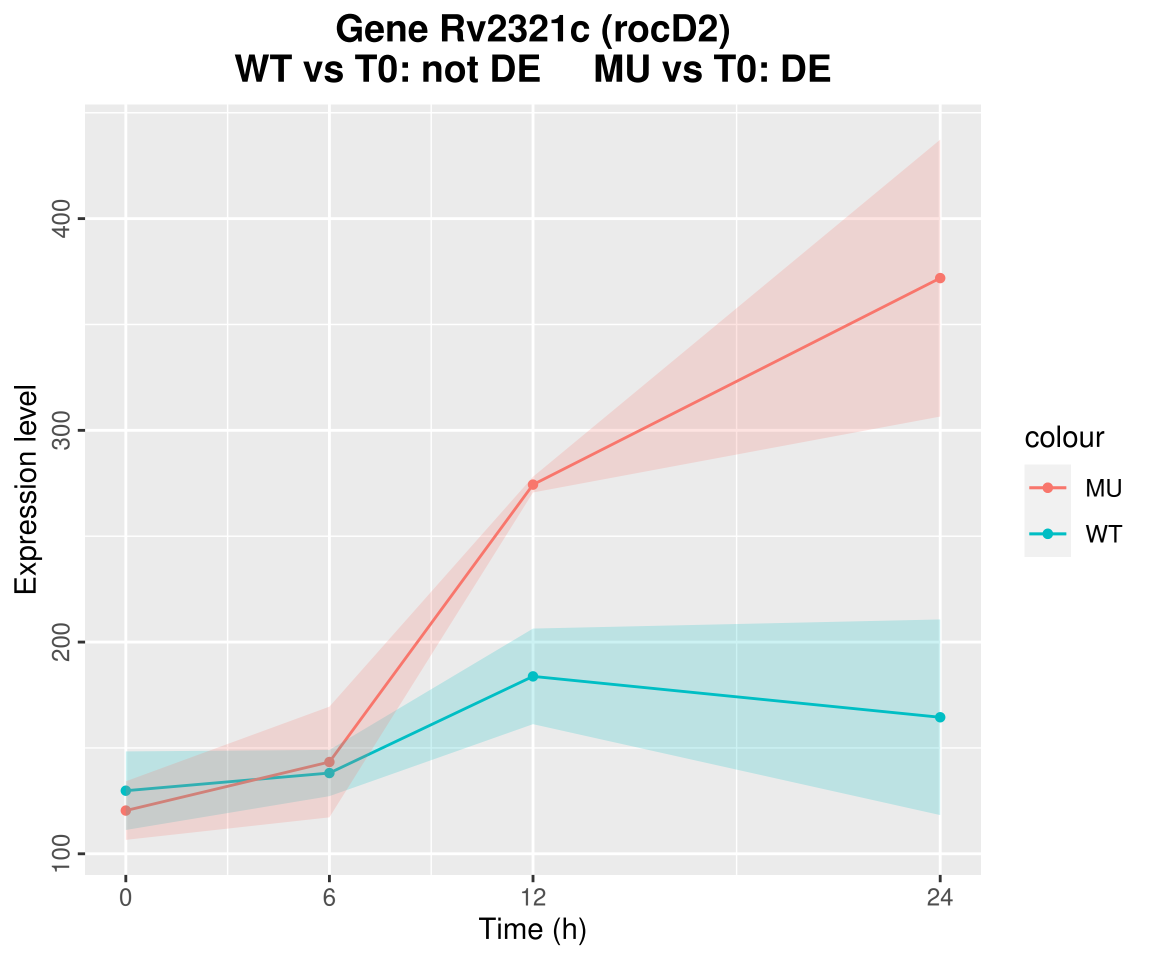
Gene Rv2321c (rocD2)
WT vs T0: not DE MU vs T0: DE
Expression level
400
300
200
100
0
6
12
24
Time (h)
colour
MU
WT

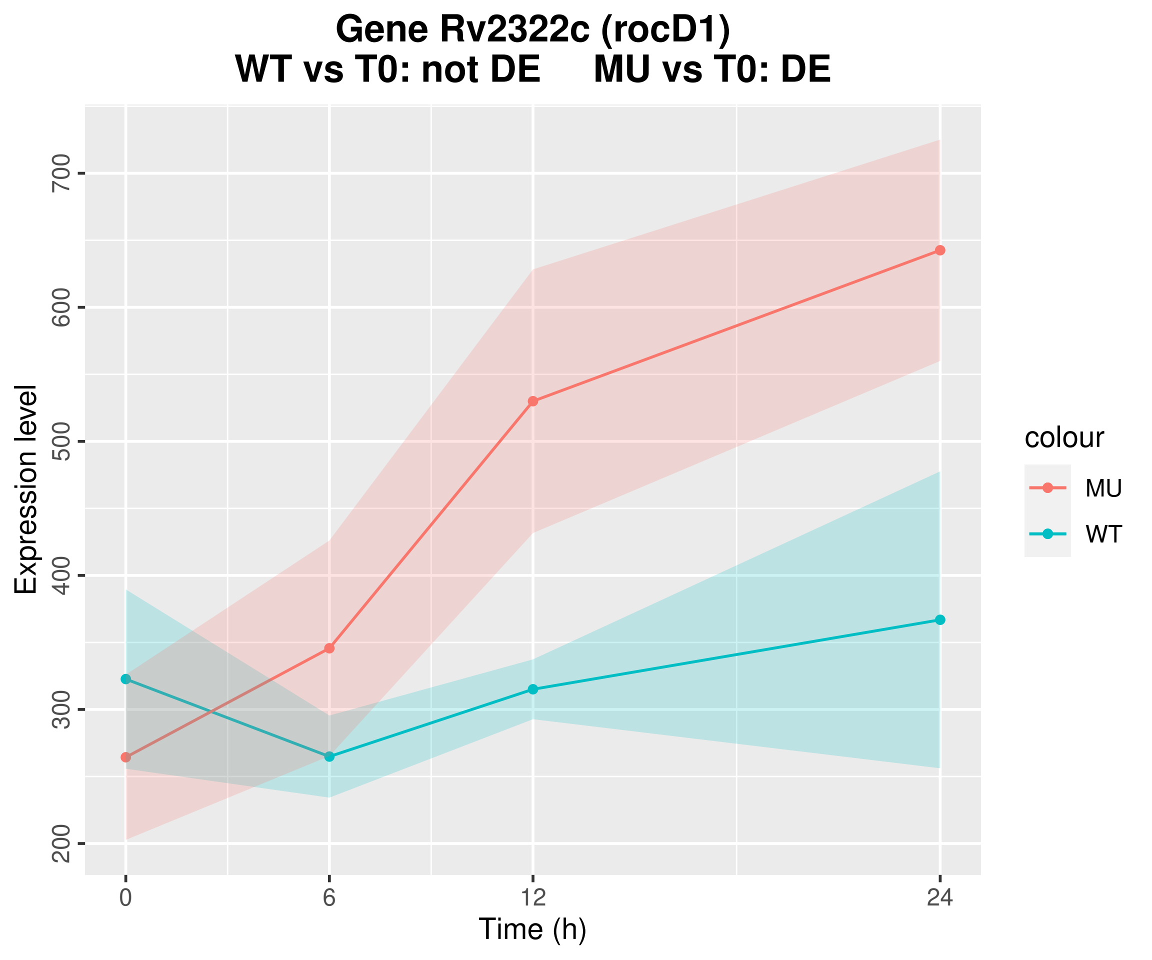
Gene Rv2322c (rocD1)
WT vs T0: not DE MU vs T0: DE
Expression level
200
300
400
500
600
700
0
6
12
24
Time (h)
colour
MU
WT

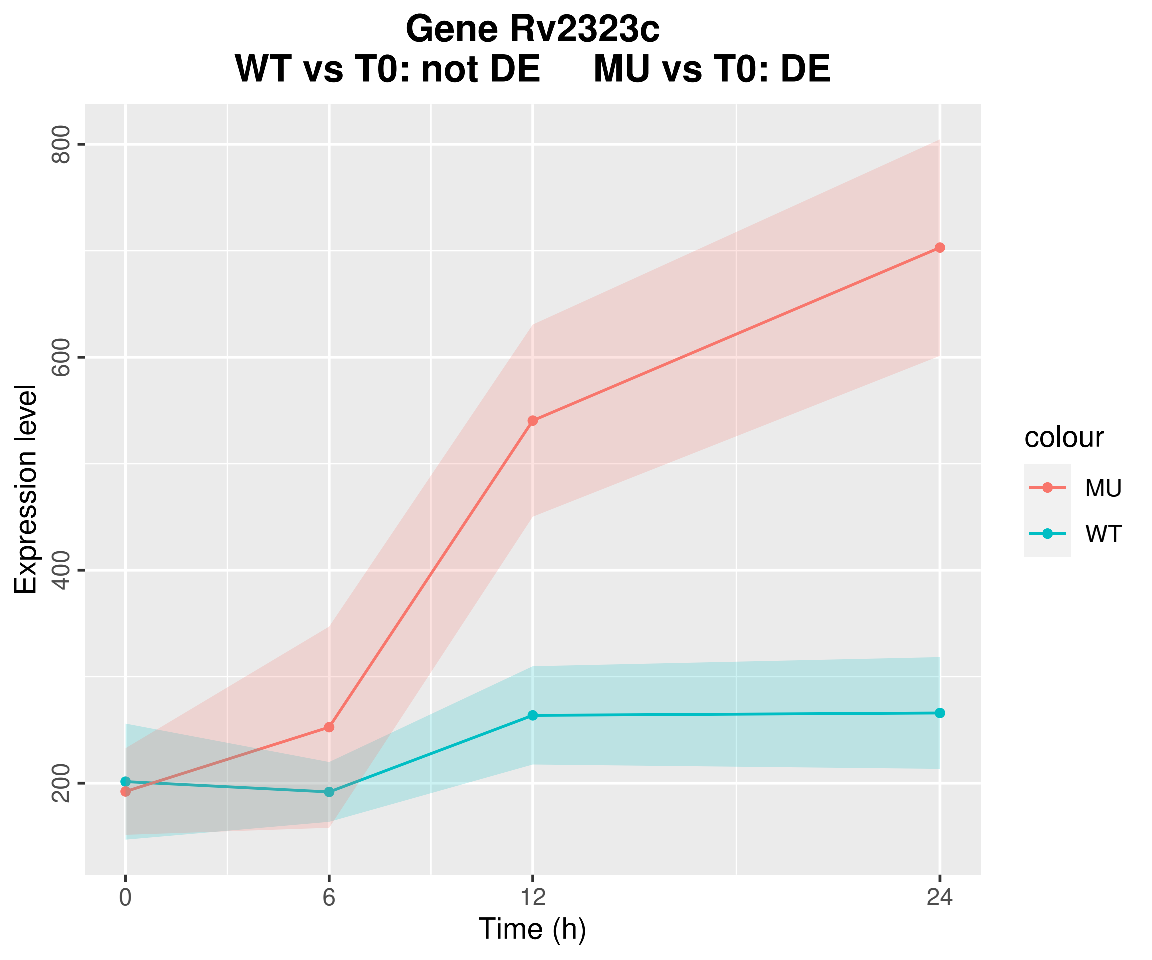
Gene Rv2323c
WT vs T0: not DE MU vs T0: DE
Expression level
200
400
600
800
0
6
12
24
Time (h)
colour
MU
WT

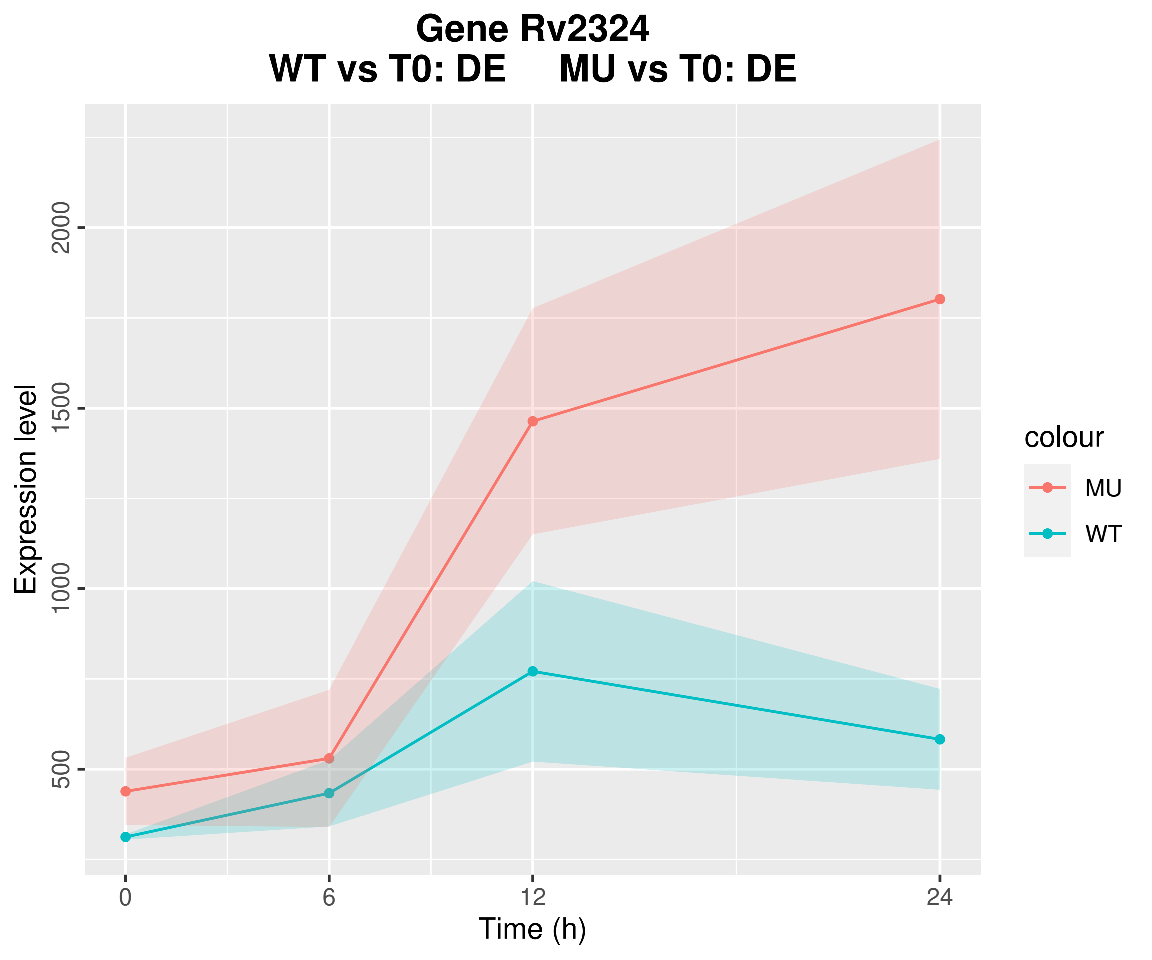

Gene Rv2324
WT vs T0: DE MU vs T0: DE
Expression level
2000
1500
1000
500
0
6
12
24
Time (h)
colour
MU
WT

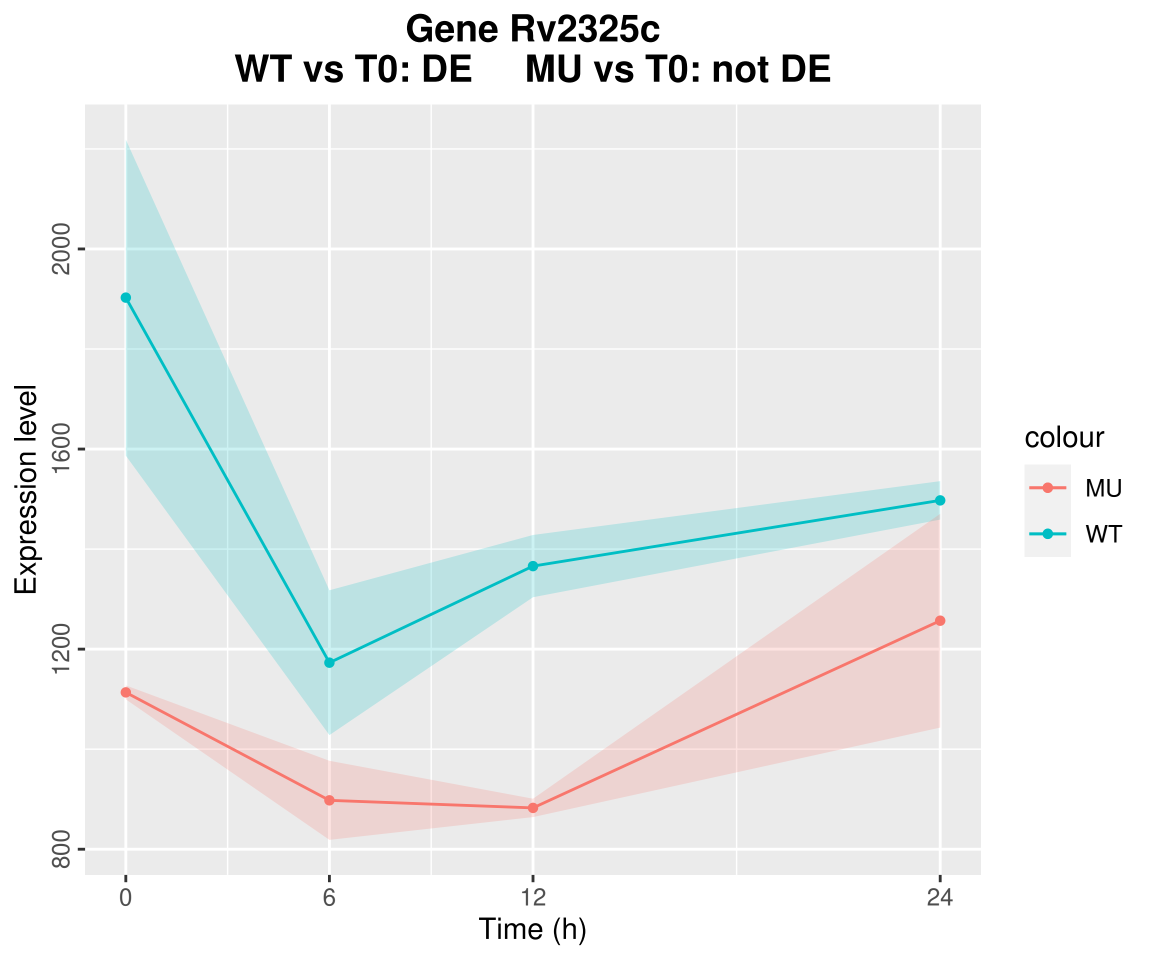
Gene Rv2325c
WT vs T0: DE   MU vs T0: not DE
Expression level
2000
1600
1200
800
0
6
12
24
Time (h)
colour
MU
WT

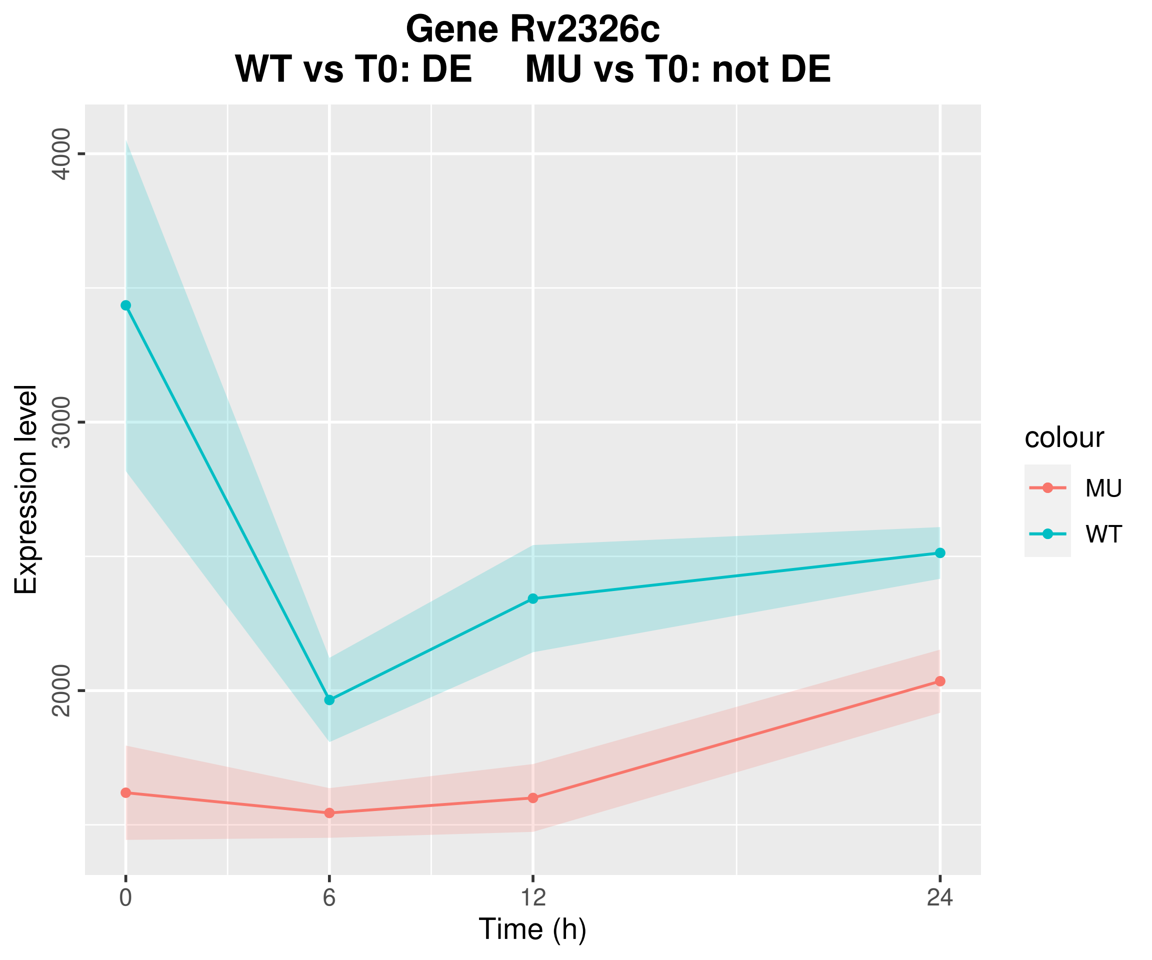
Gene Rv2326c
WT vs T0: DE   MU vs T0: not DE
4000
3000
2000
Expression level
0
6
12
24
Time (h)
colour
MU
WT

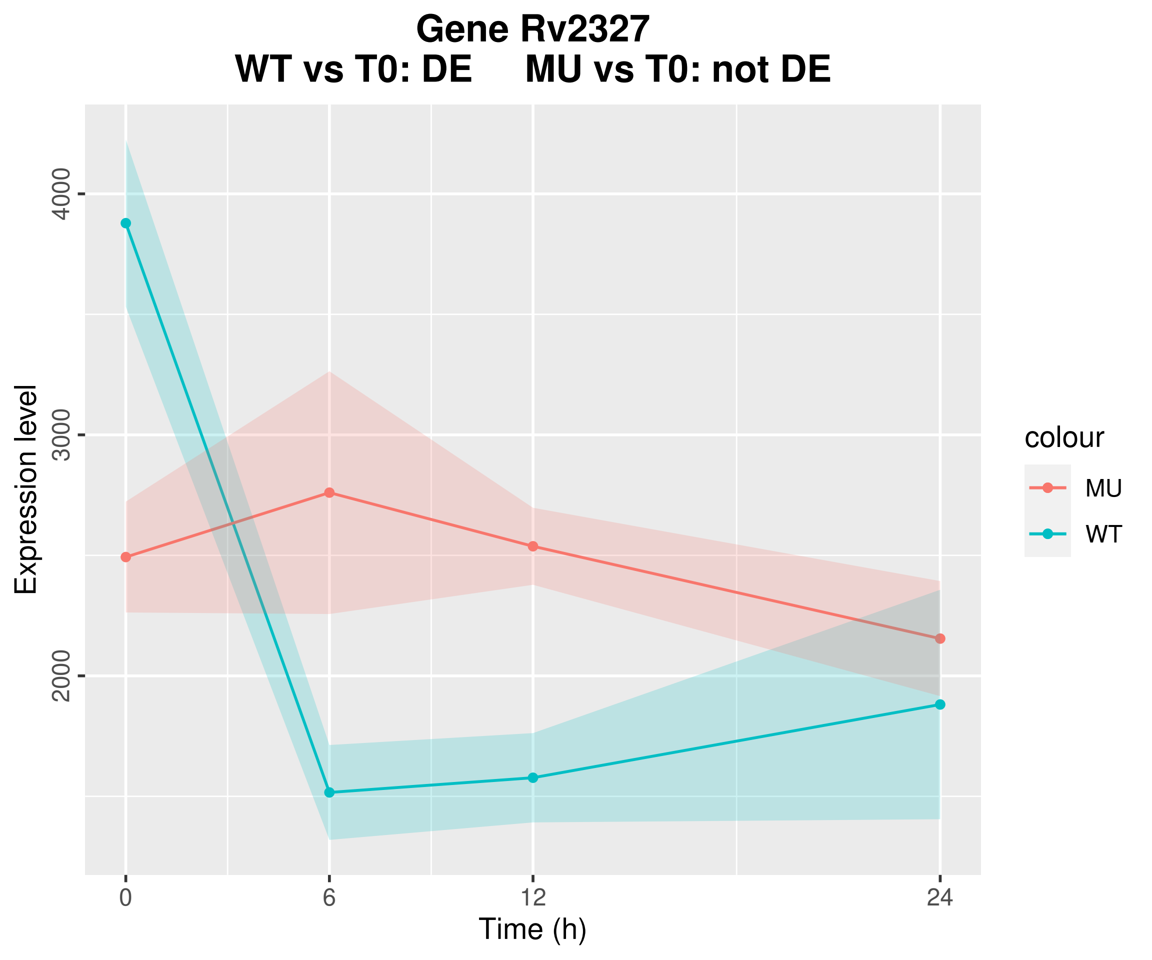
Gene Rv2327
WT vs T0: DE MU vs T0: not DE
Expression level
4000
3000
2000
0
6
12
24
Time (h)
colour
MU
WT

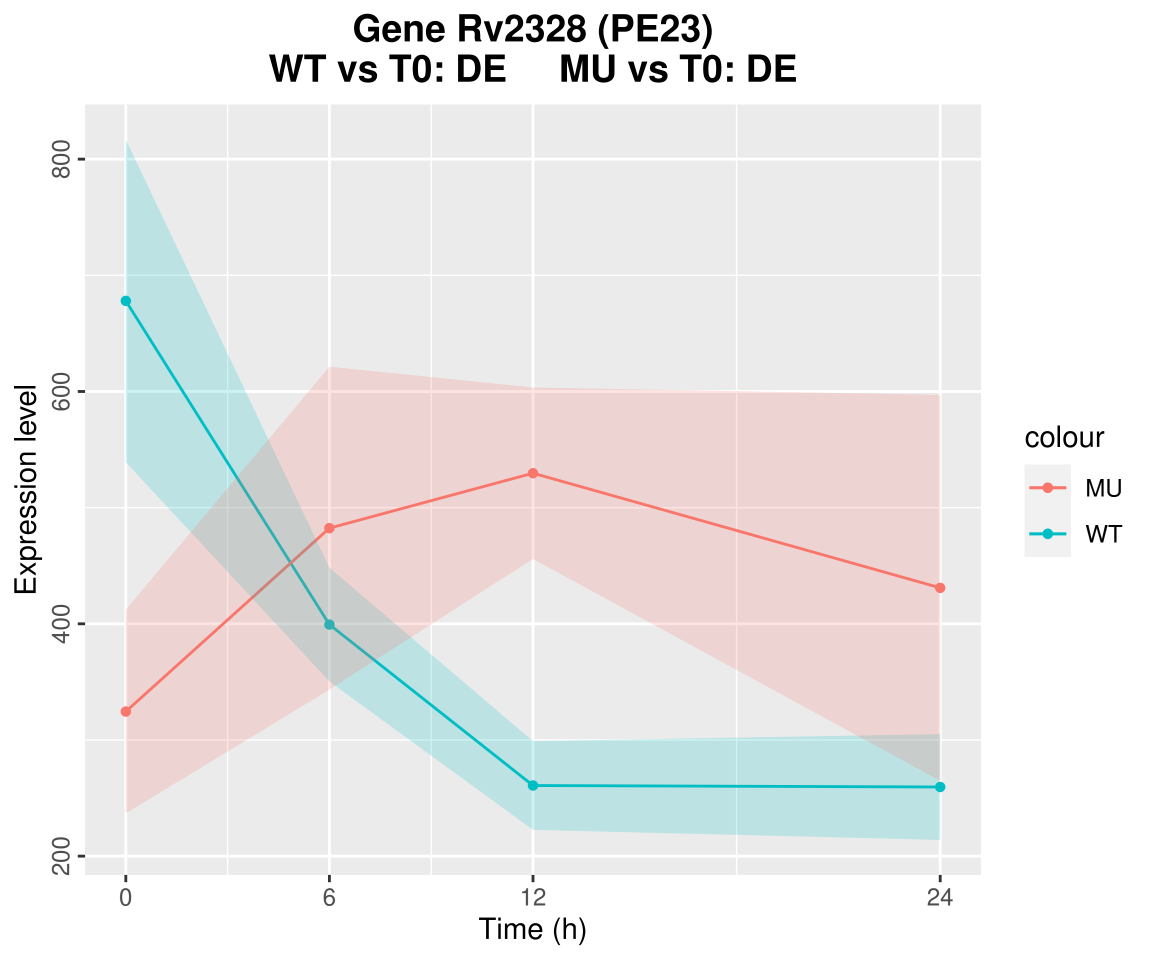

Gene Rv2328 (PE23)
WT vs T0: DE   MU vs T0: DE
Expression level
800
600
400
200
0
6
12
24
Time (h)
colour
MU
WT

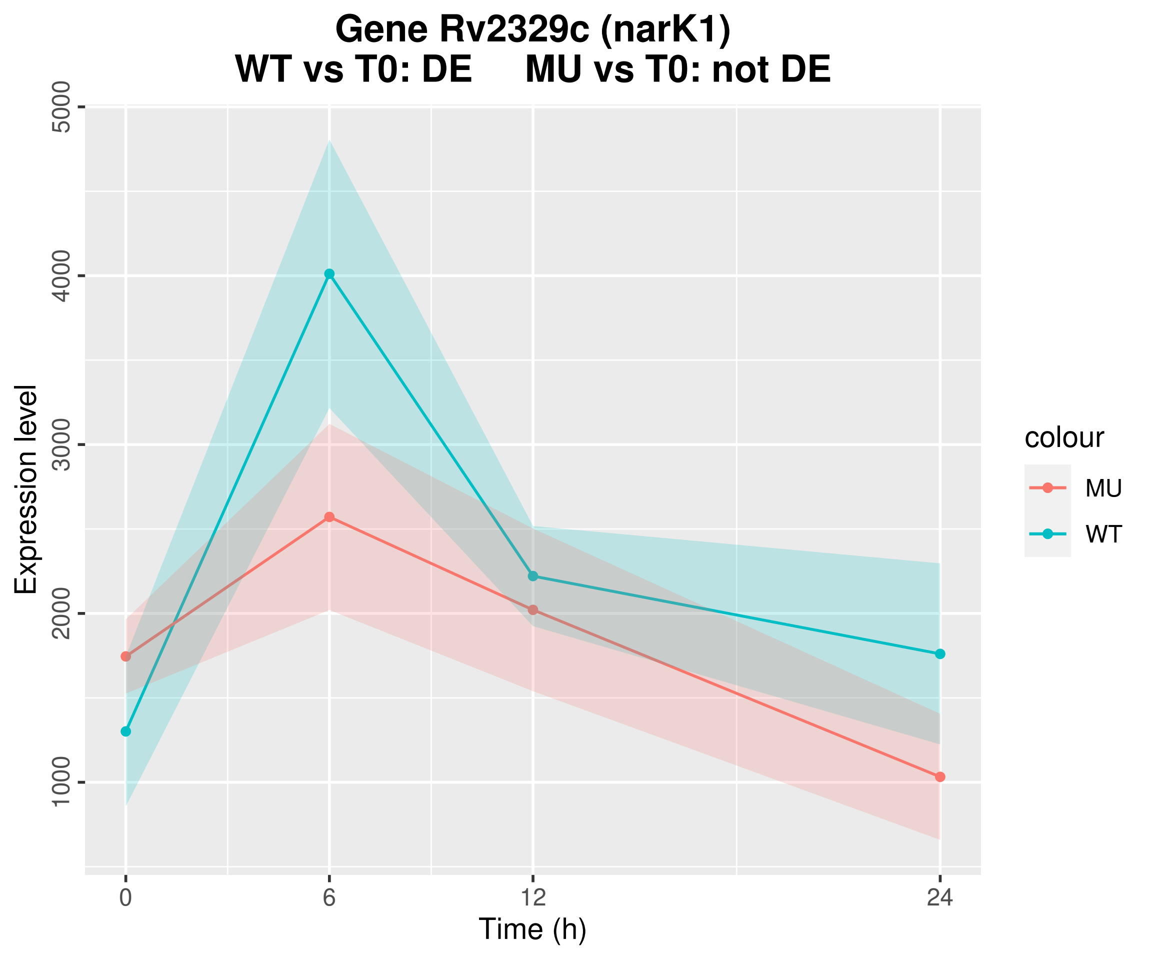
Gene Rv2329c (narK1)
WT vs T0: DE   MU vs T0: not DE
Expression level
5000
4000
3000
2000
1000
0
6
12
24
Time (h)
colour
MU
WT

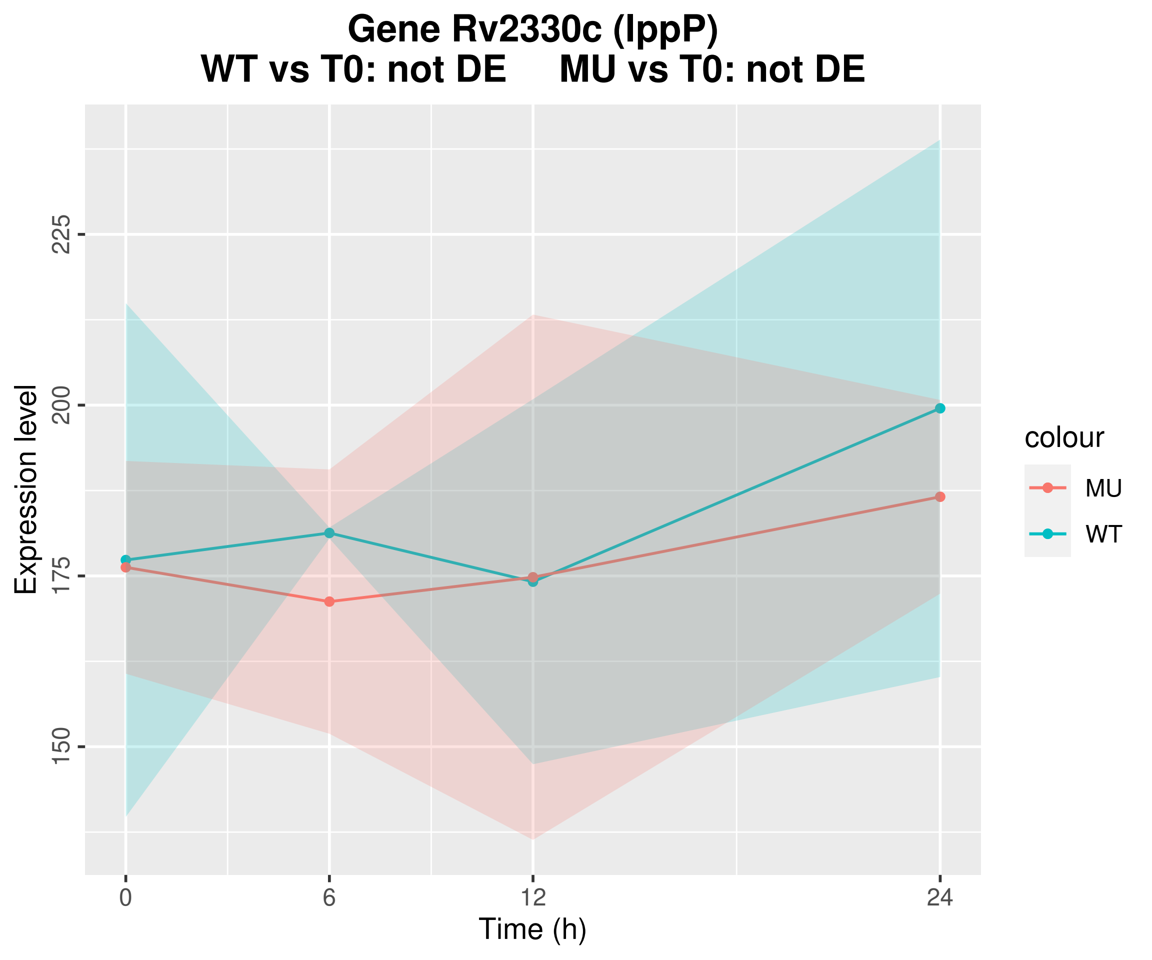

Gene Rv2330c (lppP)
WT vs T0: not DE MU vs T0: not DE
Expression level
225
200
175
150
0
6
12
24
Time (h)
colour
MU
WT

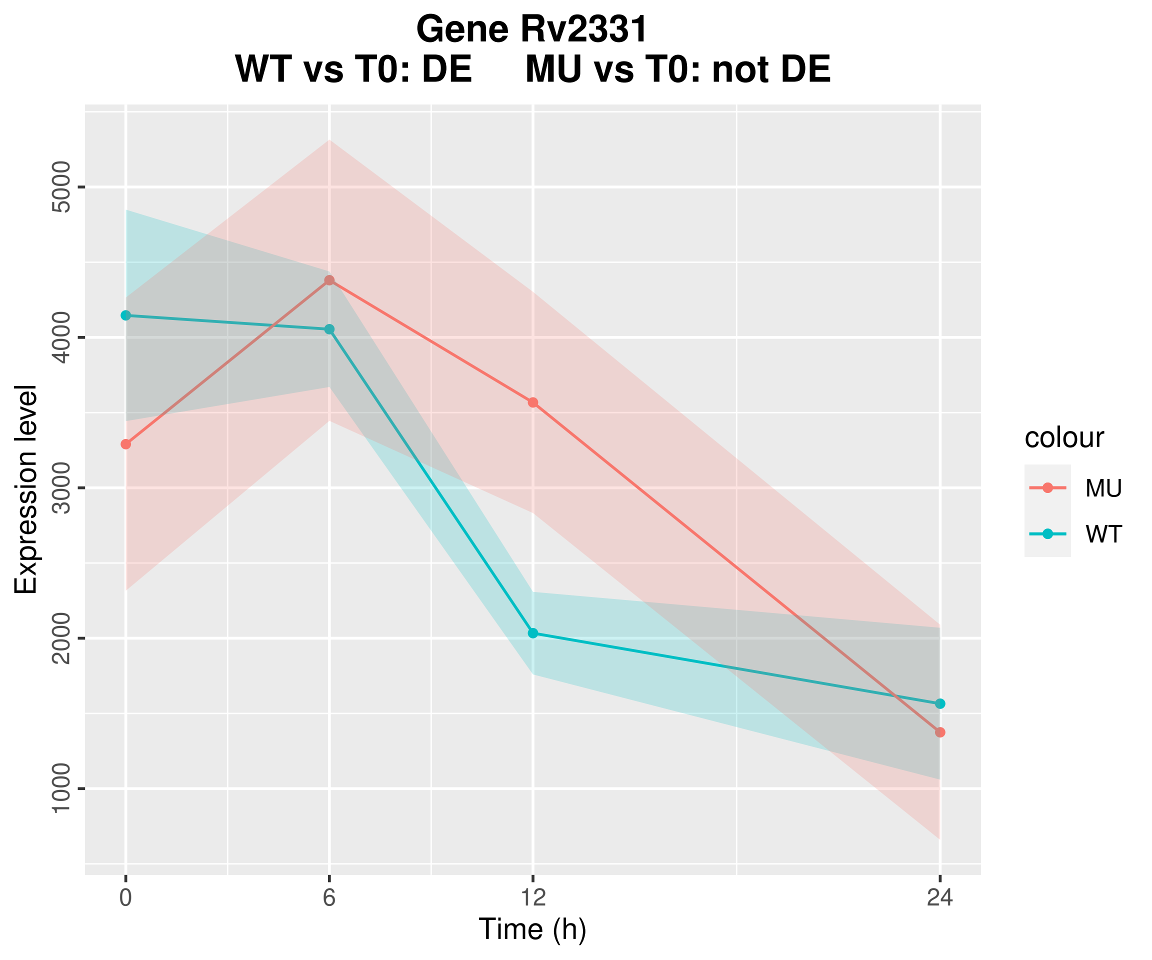

Gene Rv2331
WT vs T0: DE MU vs T0: not DE
Expression level
5000
4000
3000
2000
1000
0
6
12
24
Time (h)
colour
MU
WT

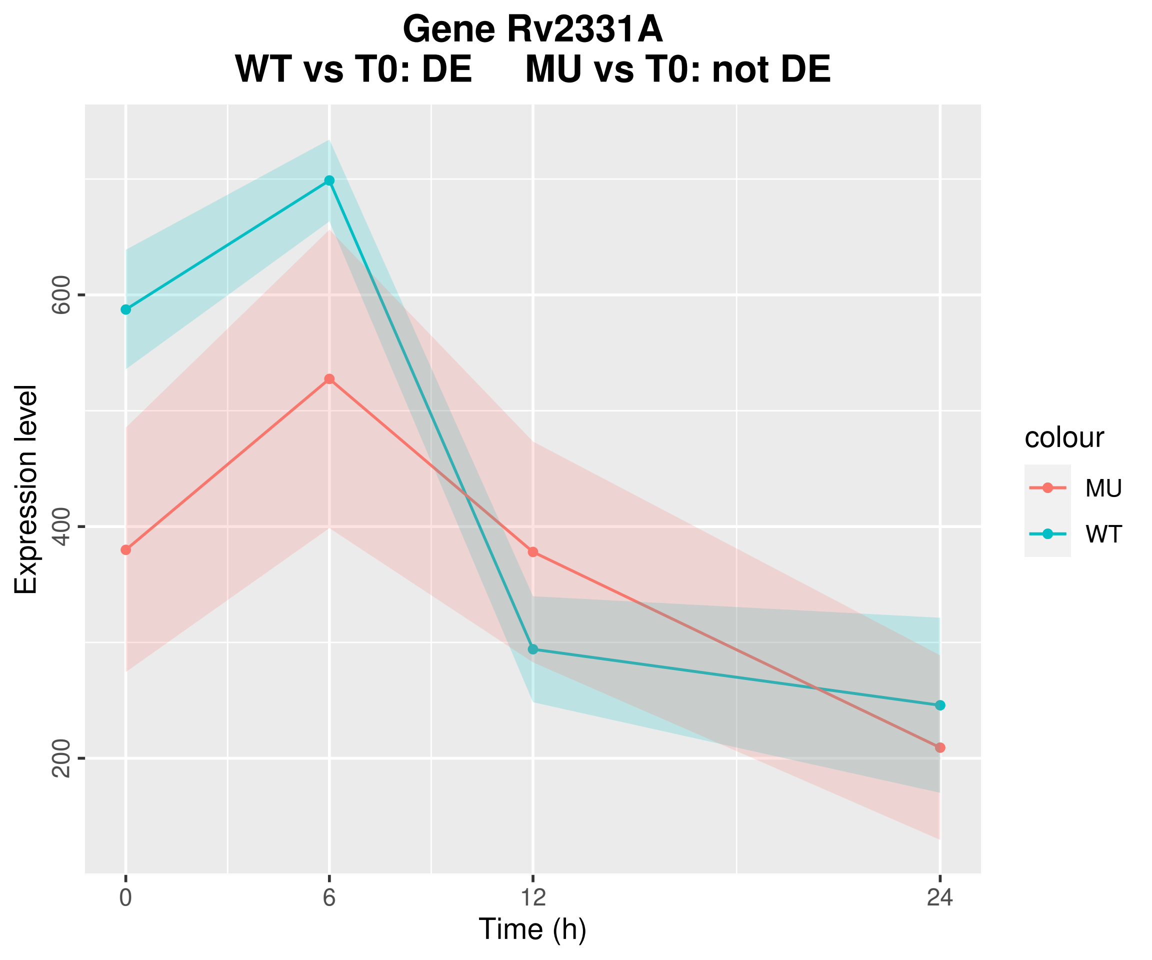
Gene Rv2331A
WT vs T0: DE MU vs T0: not DE
Expression level
600
400
200
0
6
12
24
Time (h)
colour
MU
WT

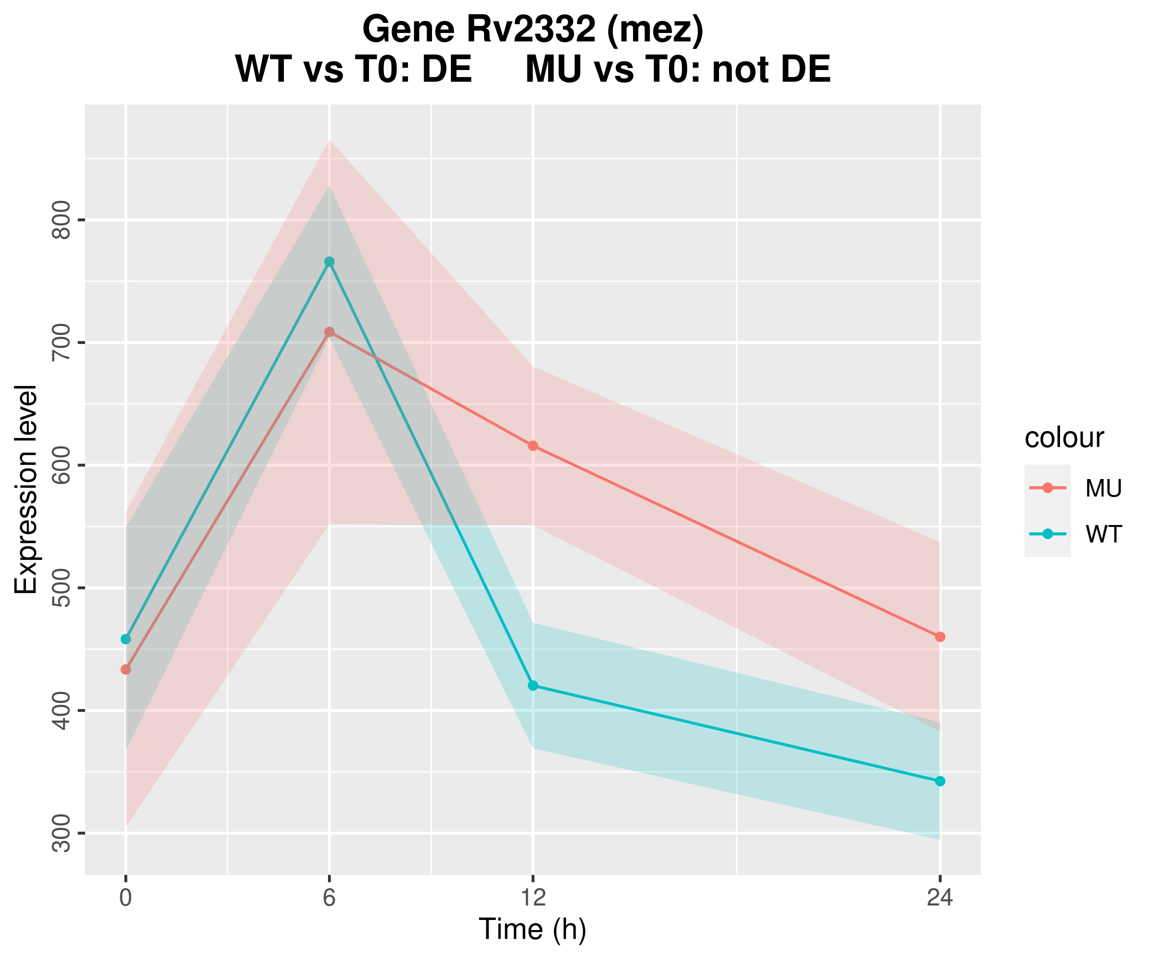
Gene Rv2332 (mez)
WT vs T0: DE   MU vs T0: not DE
Expression level
800
700
600
500
400
300
0
6
12
24
Time (h)
colour
MU
WT

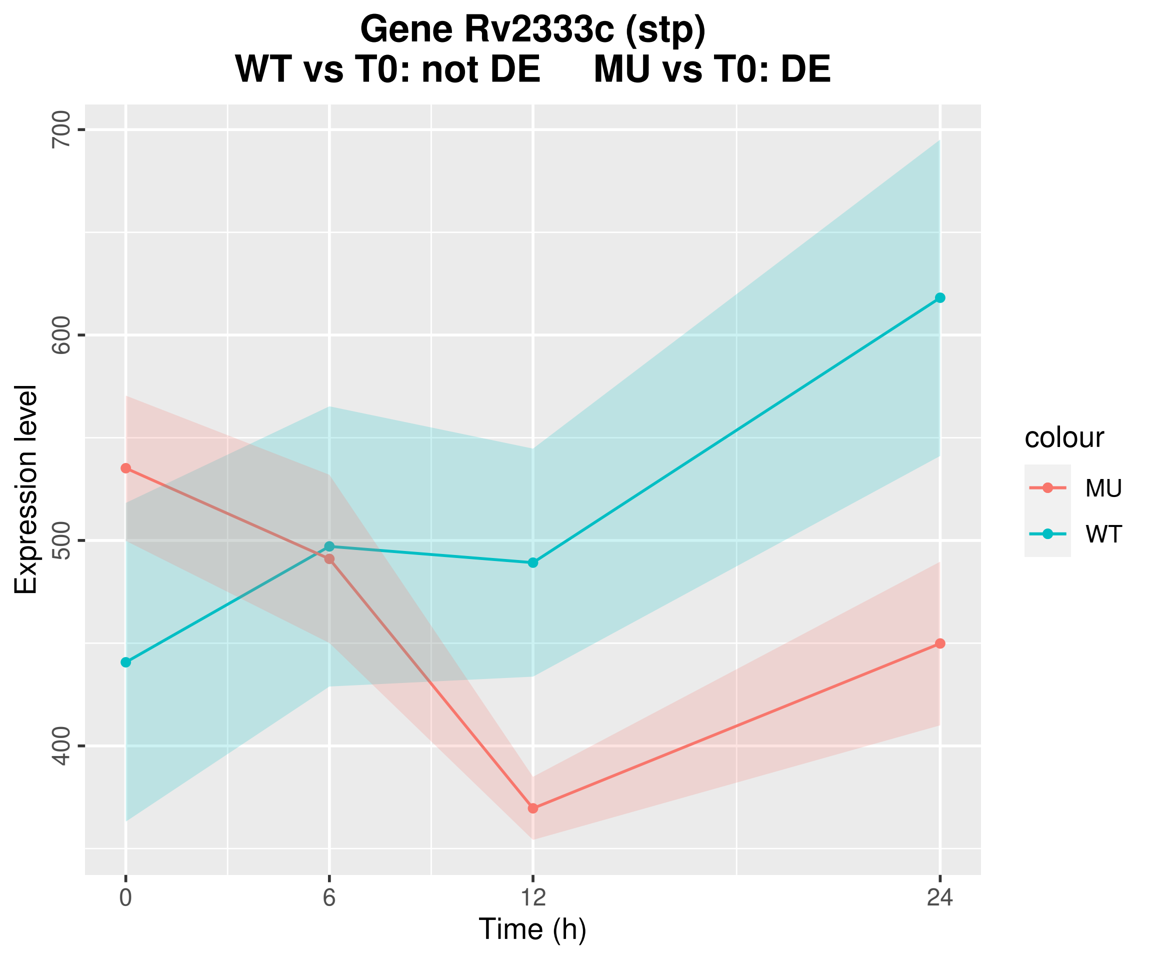

Gene Rv2333c (stp)
WT vs T0: not DE   MU vs T0: DE
Expression level
700
600
500
400
0
6
12
24
Time (h)
colour
MU
WT

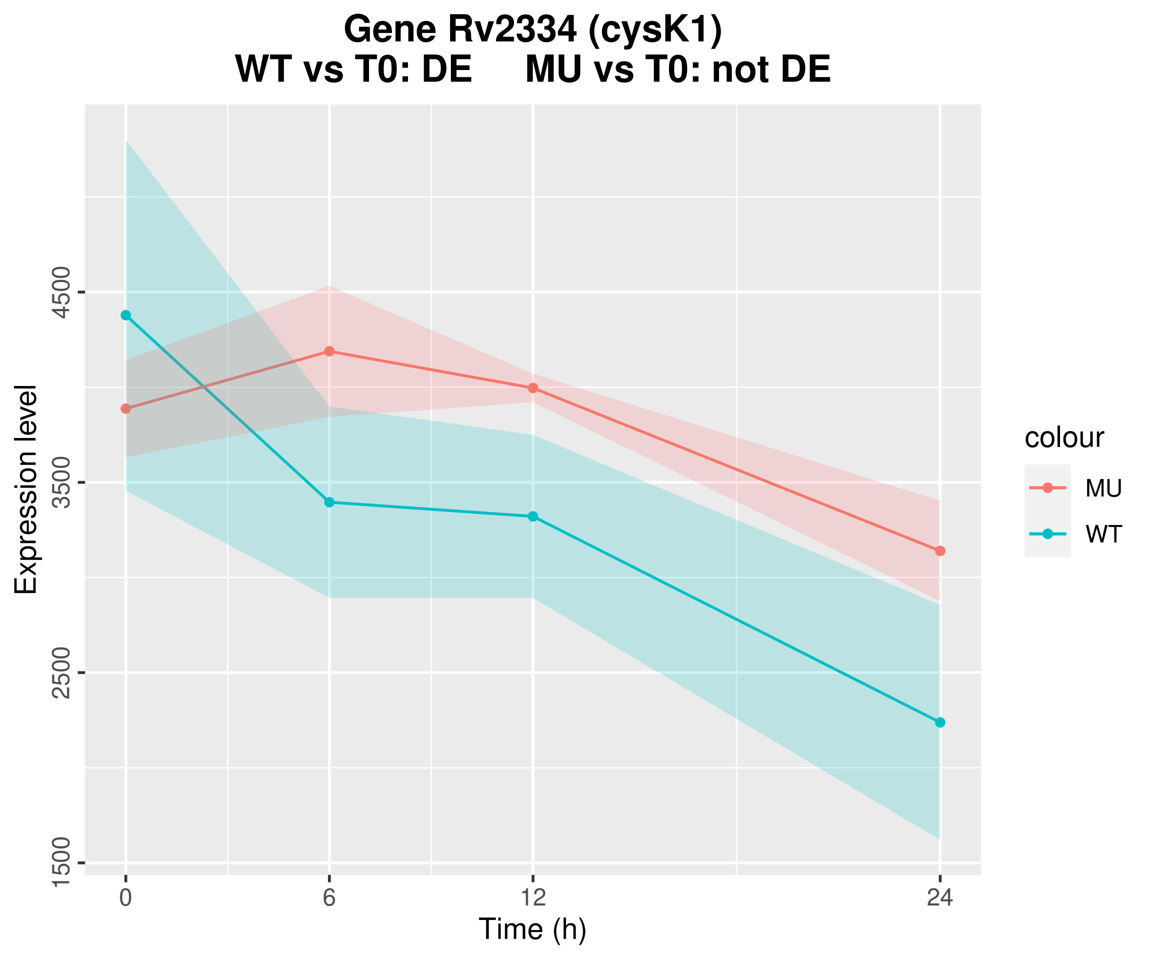

Gene Rv2334 (cysK1)
WT vs T0: DE MU vs T0: not DE
Expression level
4500
3500
2500
1500
0
6
12
24
Time (h)
colour
MU
WT

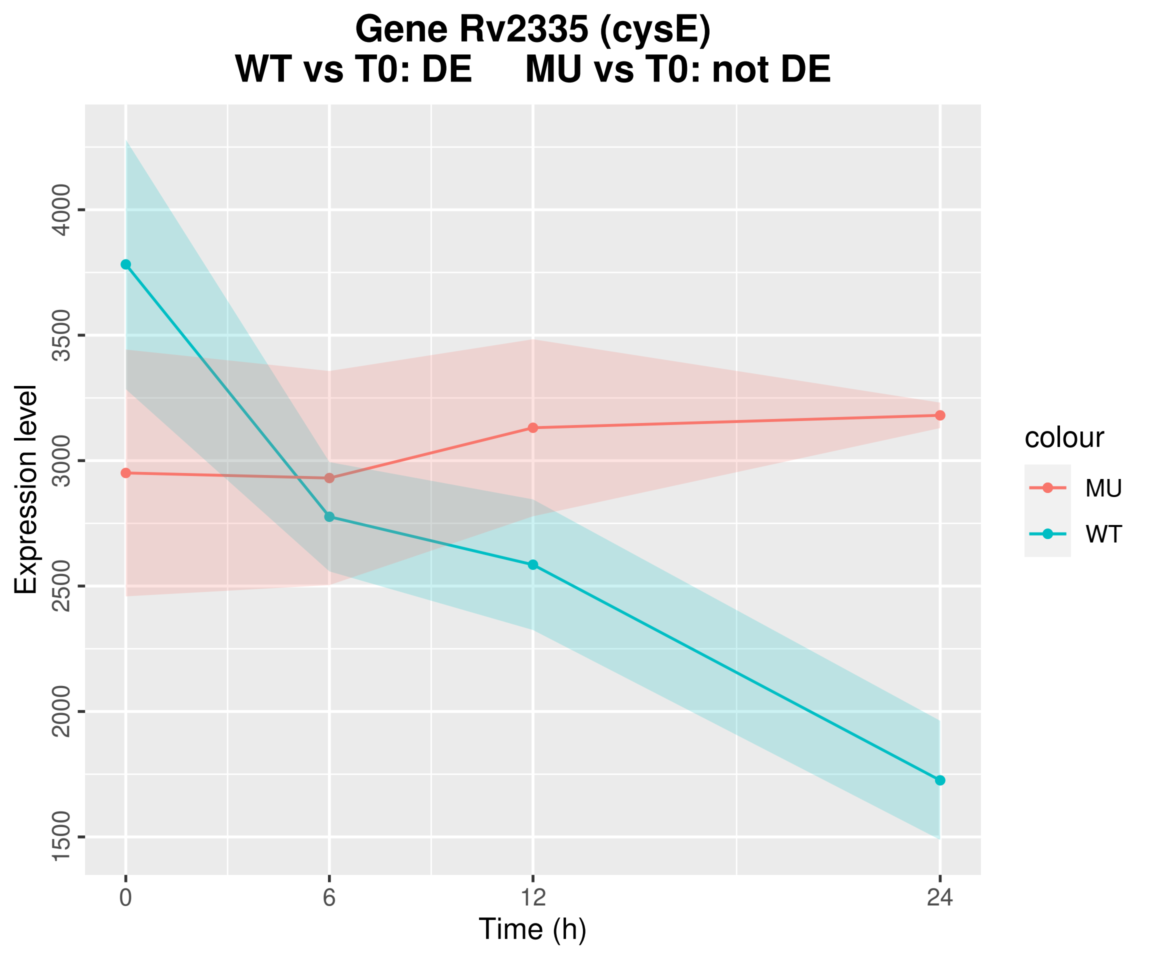
Gene Rv2335 (cysE)
WT vs T0: DE   MU vs T0: not DE
Expression level
4000
3500
3000
2500
2000
1500
0
6
12
24
Time (h)
colour
MU
WT

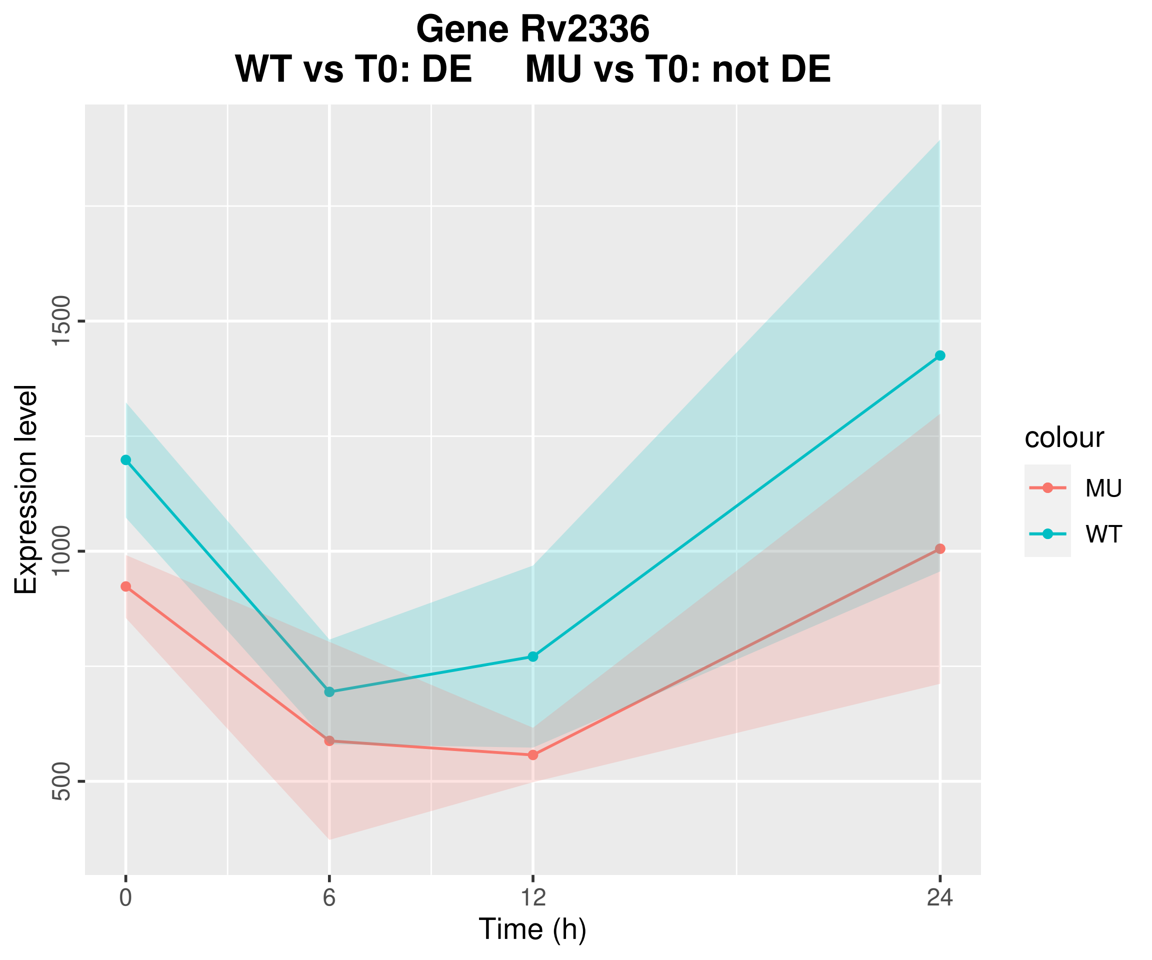
Gene Rv2336
WT vs T0: DE   MU vs T0: not DE
Expression level
1500
1000
500
0
6
12
24
Time (h)
colour
MU
WT

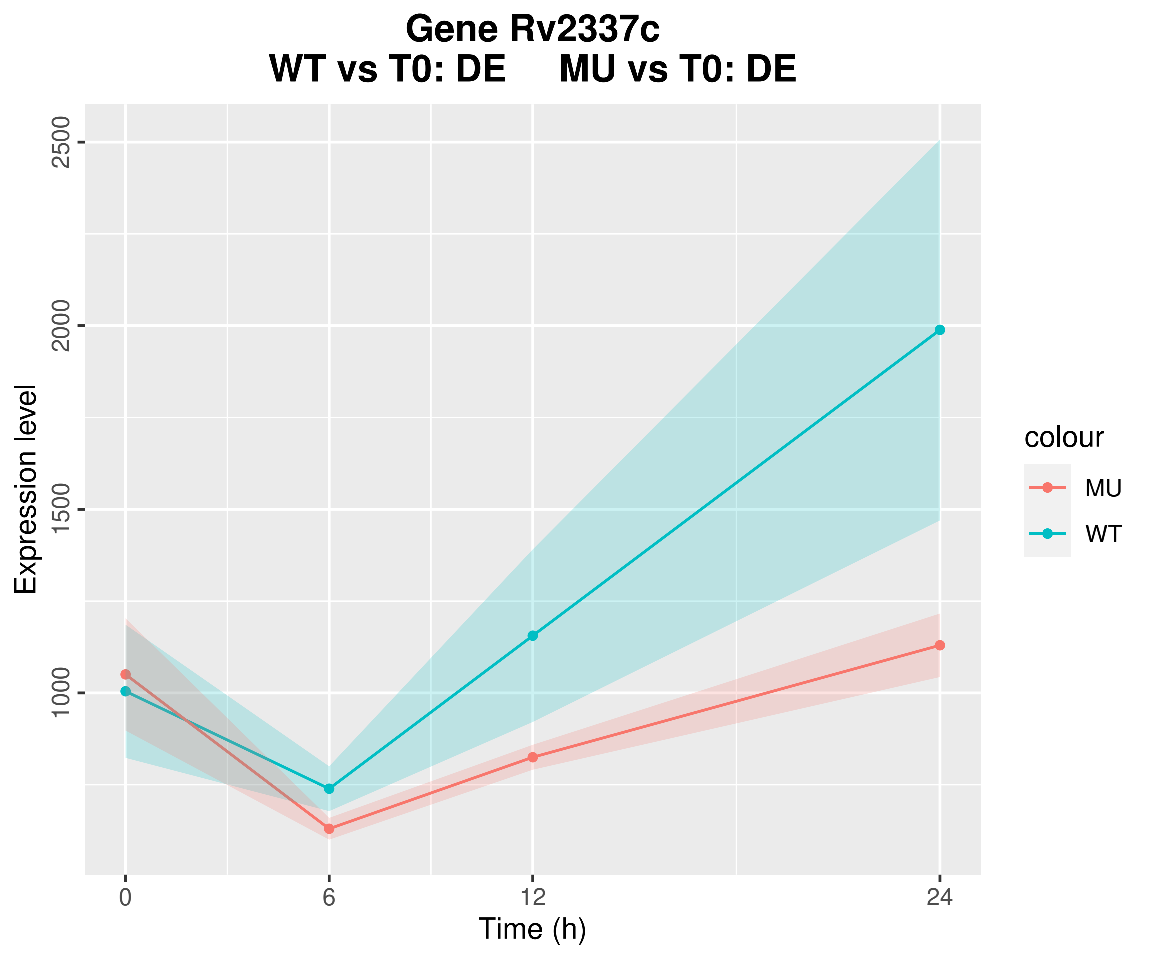

Gene Rv2337c
WT vs T0: DE   MU vs T0: DE
Expression level
2500
2000
1500
1000
0
6
12
24
Time (h)
colour
MU
WT

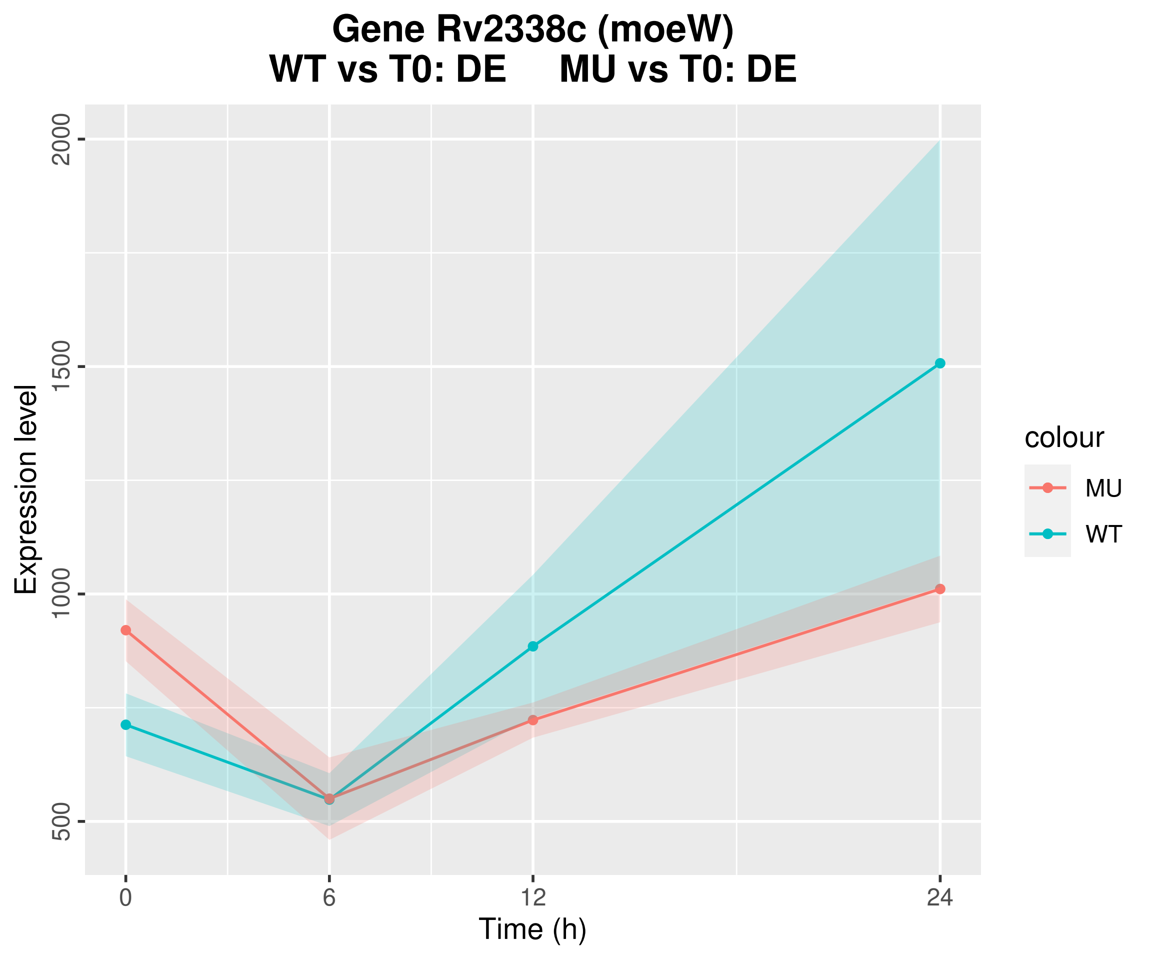
Gene Rv2338c (moeW)
WT vs T0: DE MU vs T0: DE
Expression level
2000
1500
1000
500
0
6
12
24
Time (h)
colour
MU
WT

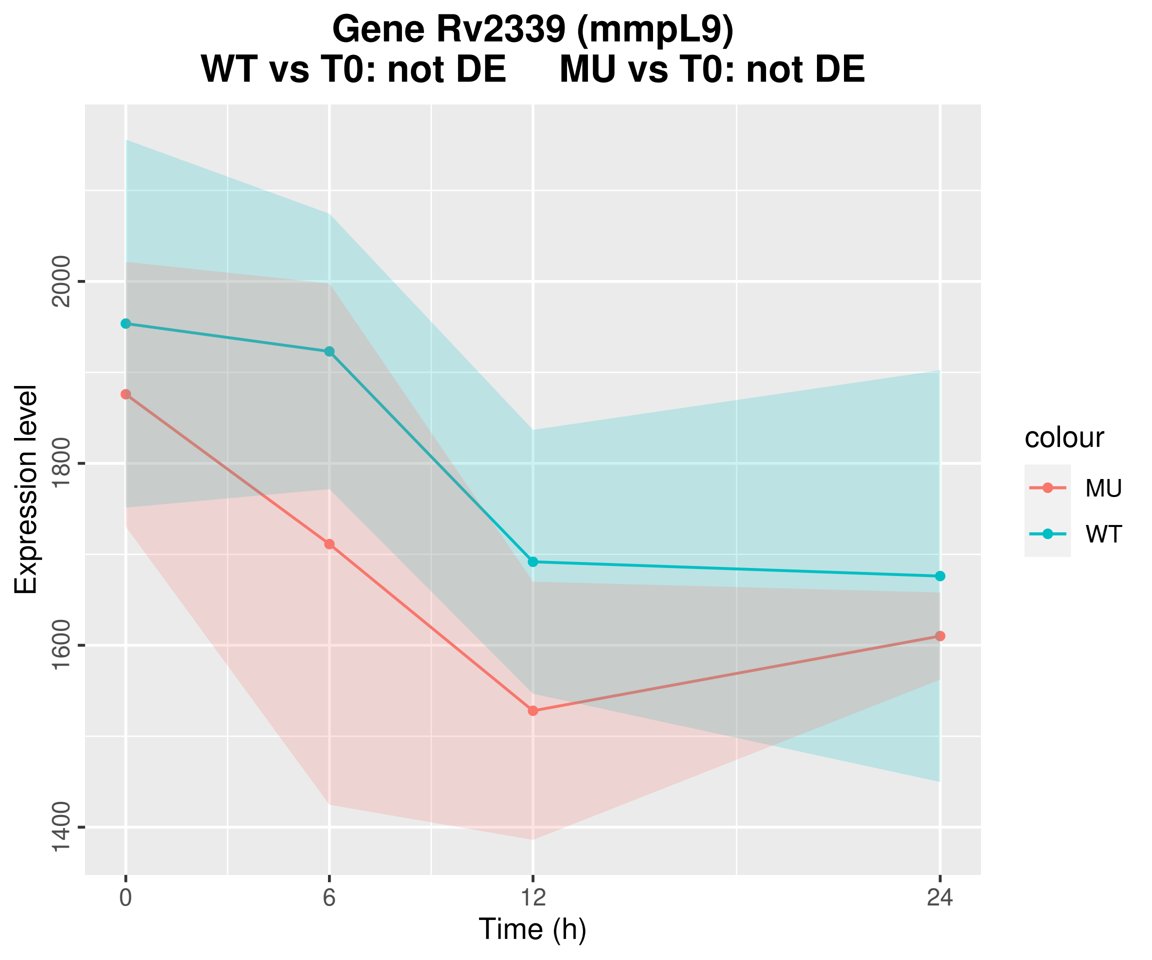

Gene Rv2339 (mmpL9)
WT vs T0: not DE   MU vs T0: not DE
Expression level
2000
1800
1600
1400
0
6
12
24
Time (h)
colour
MU
WT

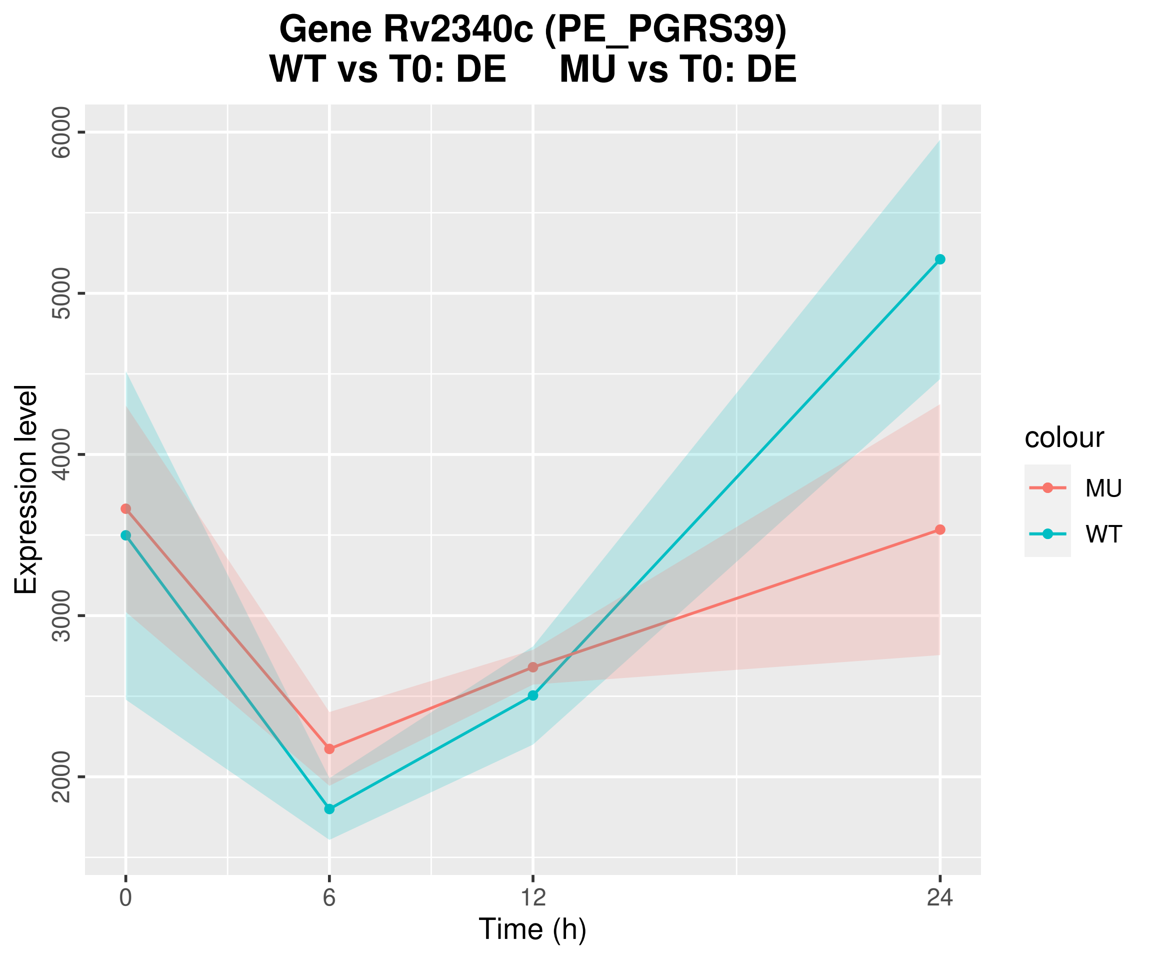
Gene Rv2340c (PE_PGRS39)
WT vs T0: DE   MU vs T0: DE
Expression level
6000
5000
4000
3000
2000
0
6
12
24
Time (h)
colour
MU
WT

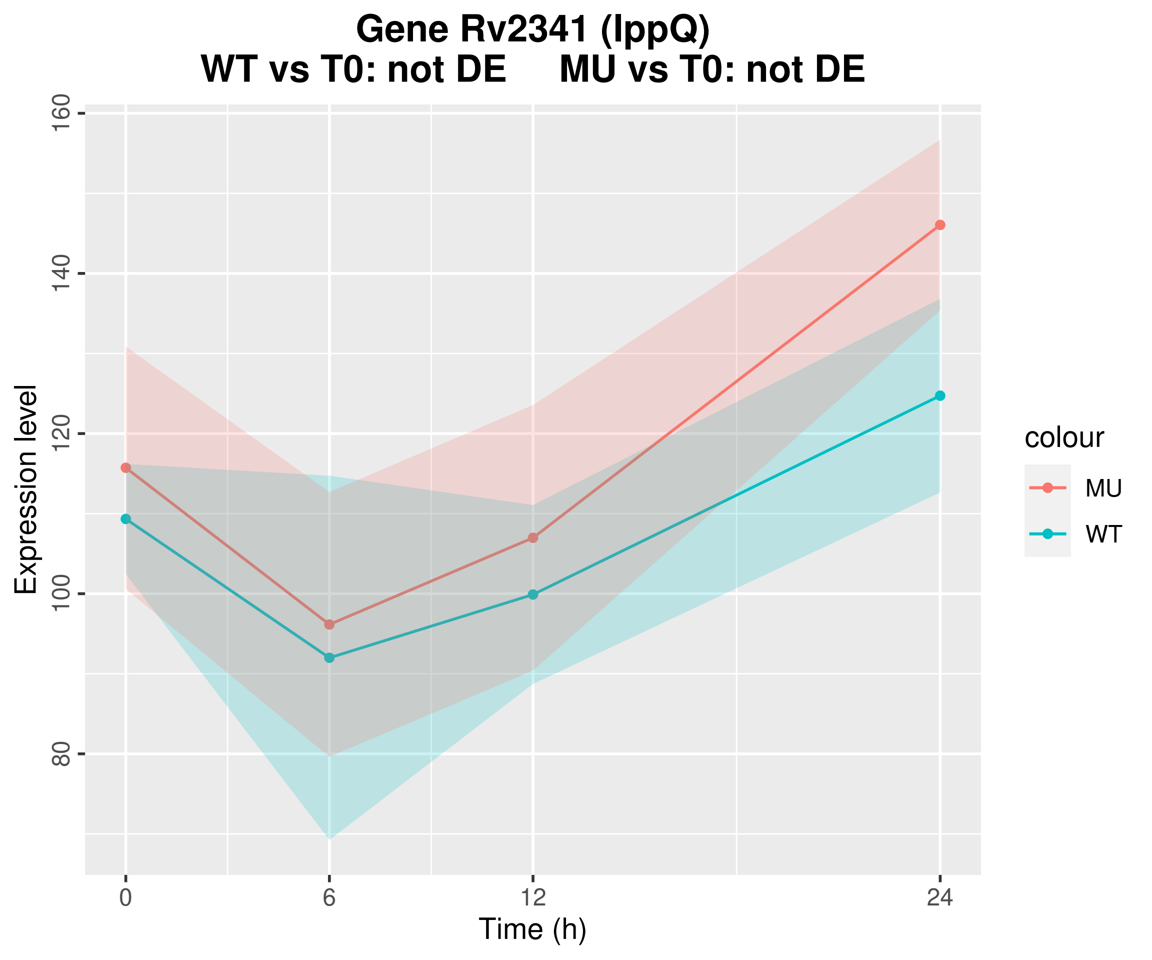

Gene Rv2341 (lppQ)
WT vs T0: not DE MU vs T0: not DE
Expression level
160
140
120
100
80
0
6
12
24
Time (h)
colour
MU
WT

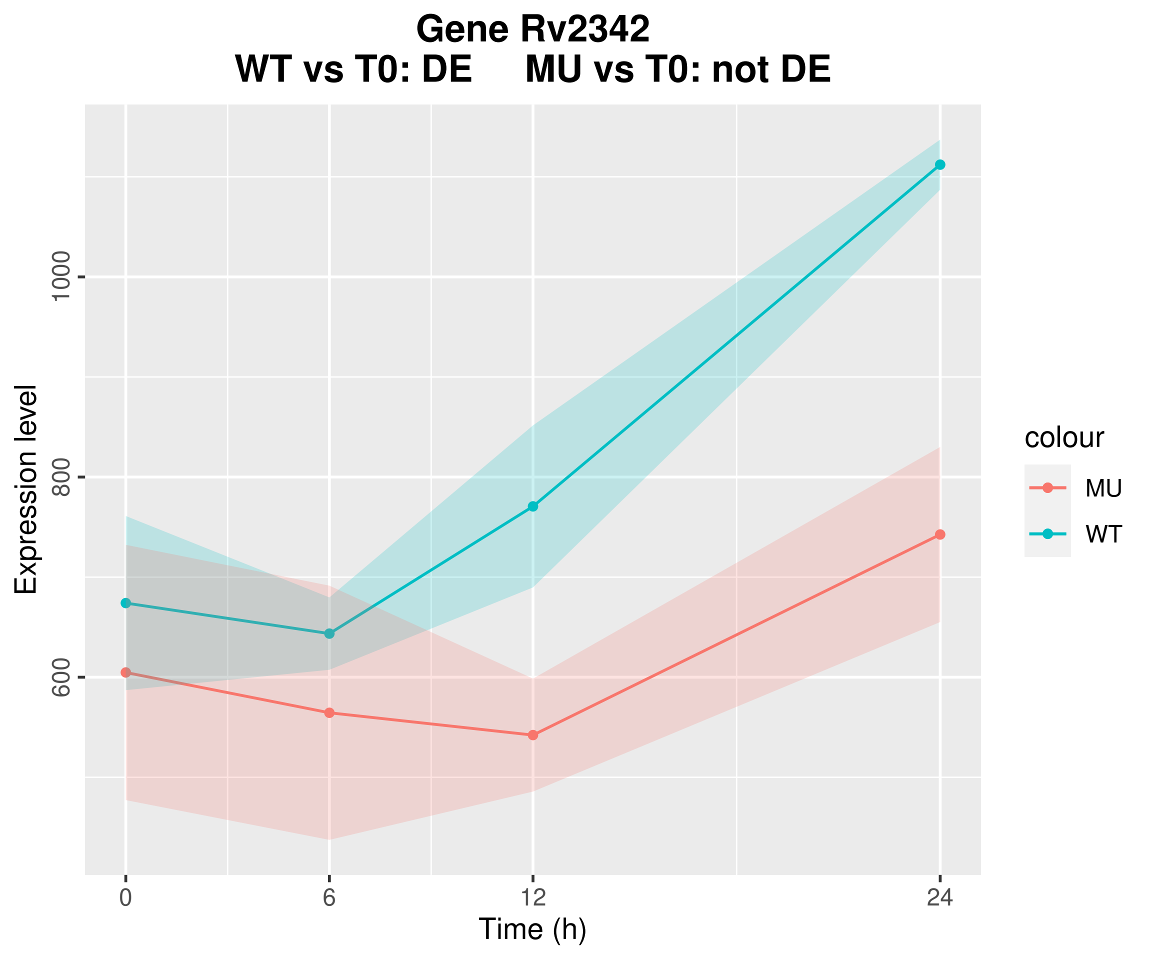
Gene Rv2342
WT vs T0: DE   MU vs T0: not DE
Expression level
1000
800
600
0
6
12
24
Time (h)
colour
MU
WT

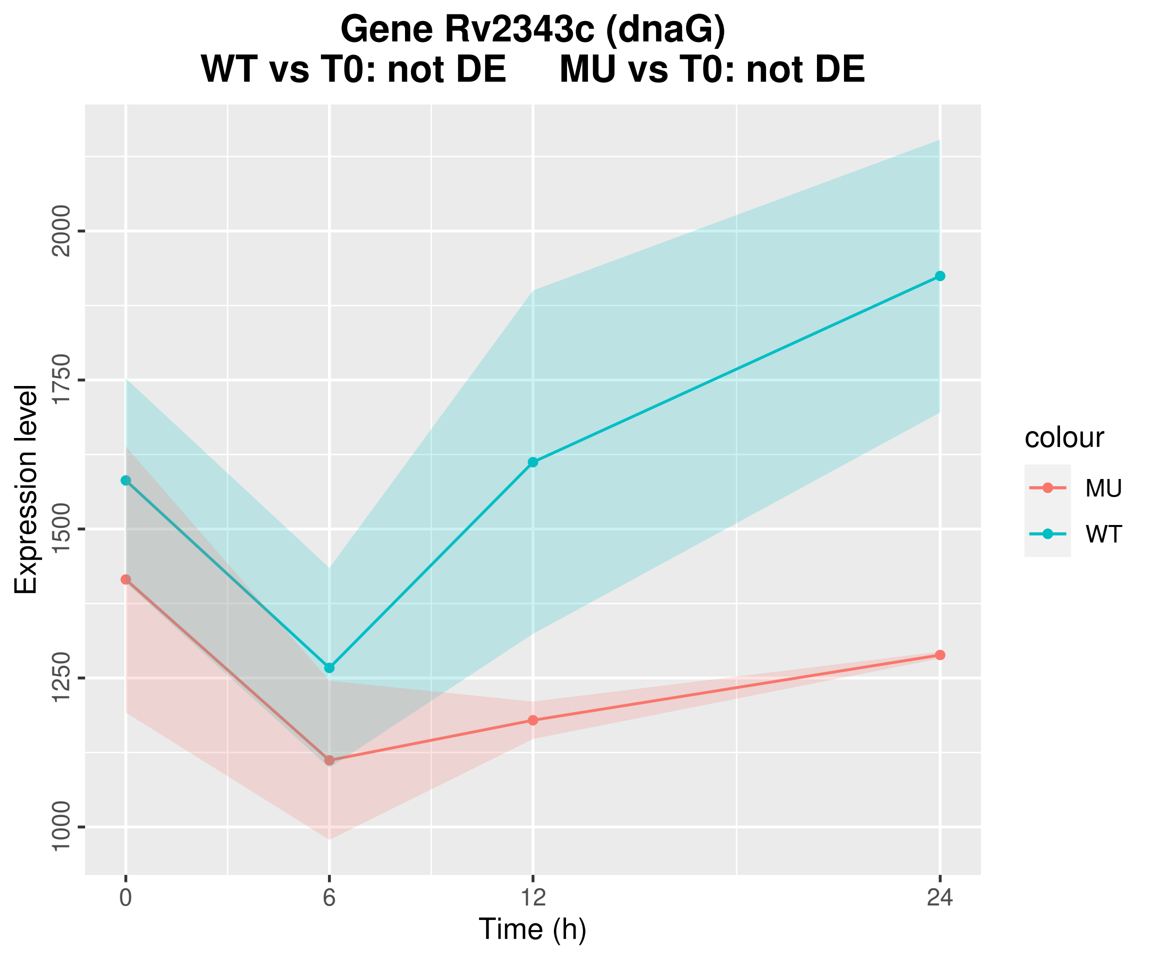
Gene Rv2343c (dnaG)
WT vs T0: not DE   MU vs T0: not DE
Expression level
2000
1750
1500
1250
1000
0
6
12
24
Time (h)
colour
MU
WT

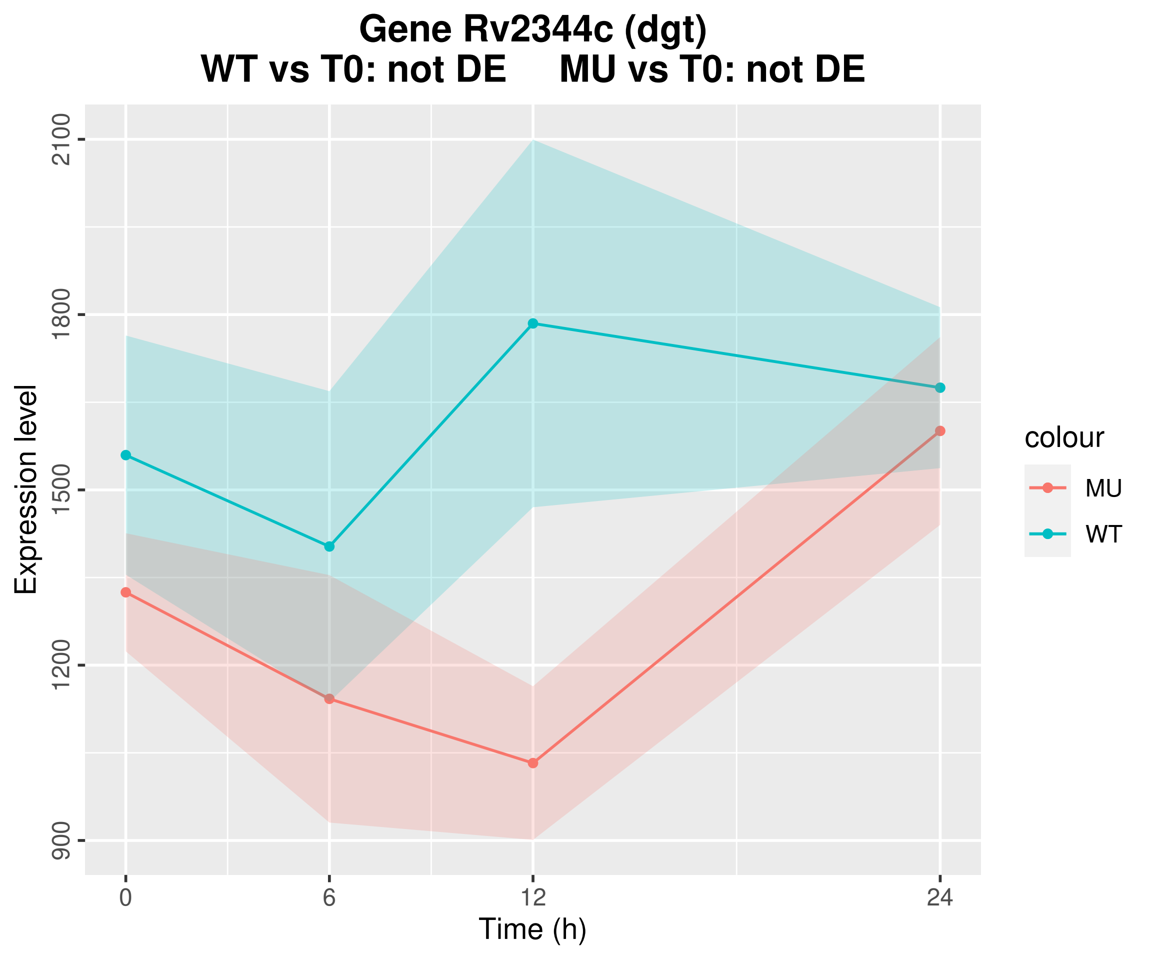

Gene Rv2344c (dgt)
WT vs T0: not DE   MU vs T0: not DE
Expression level
2100
1800
1500
1200
900
0
6
12
24
Time (h)
colour
MU
WT

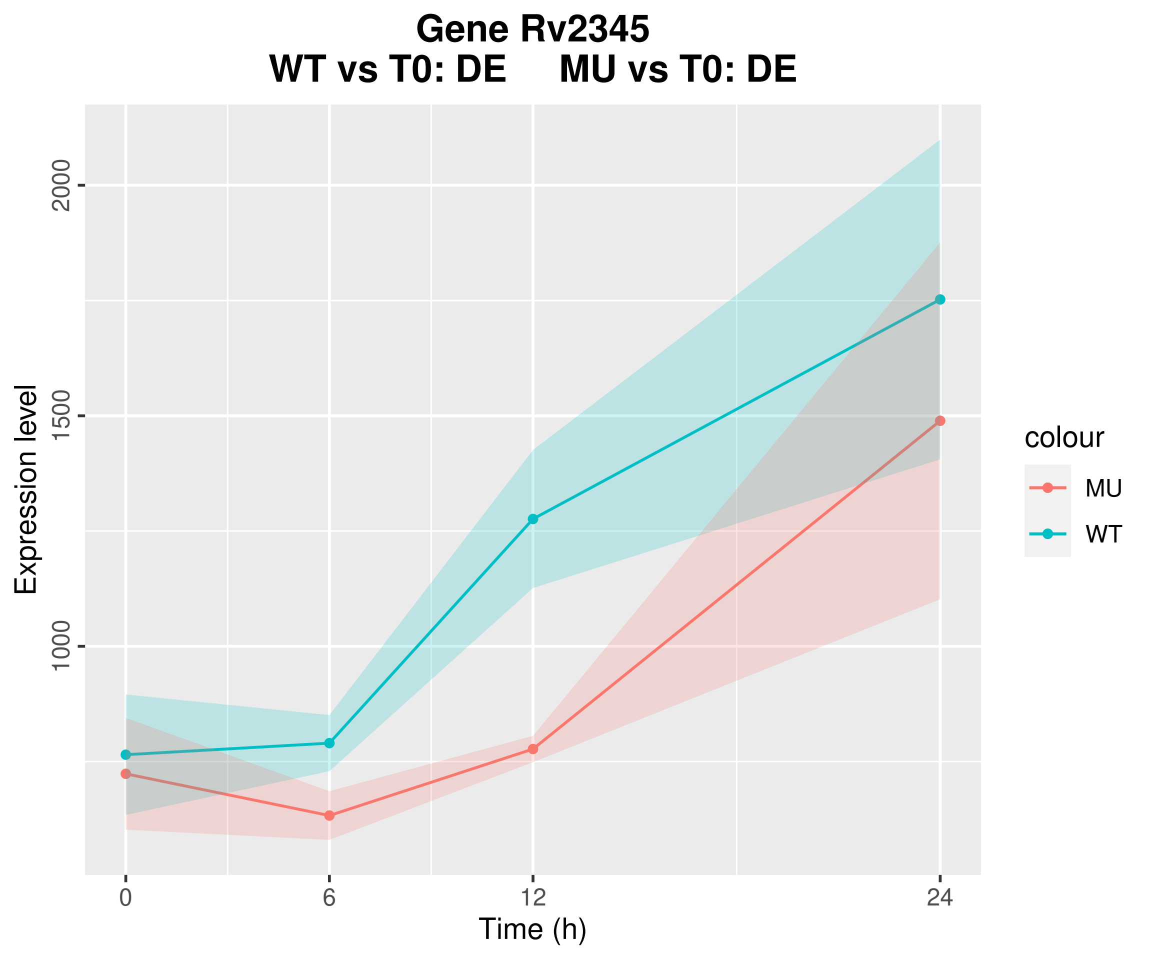

Gene Rv2345
WT vs T0: DE   MU vs T0: DE
2000
1500
1000
Expression level
0
6
12
24
Time (h)
colour
MU
WT

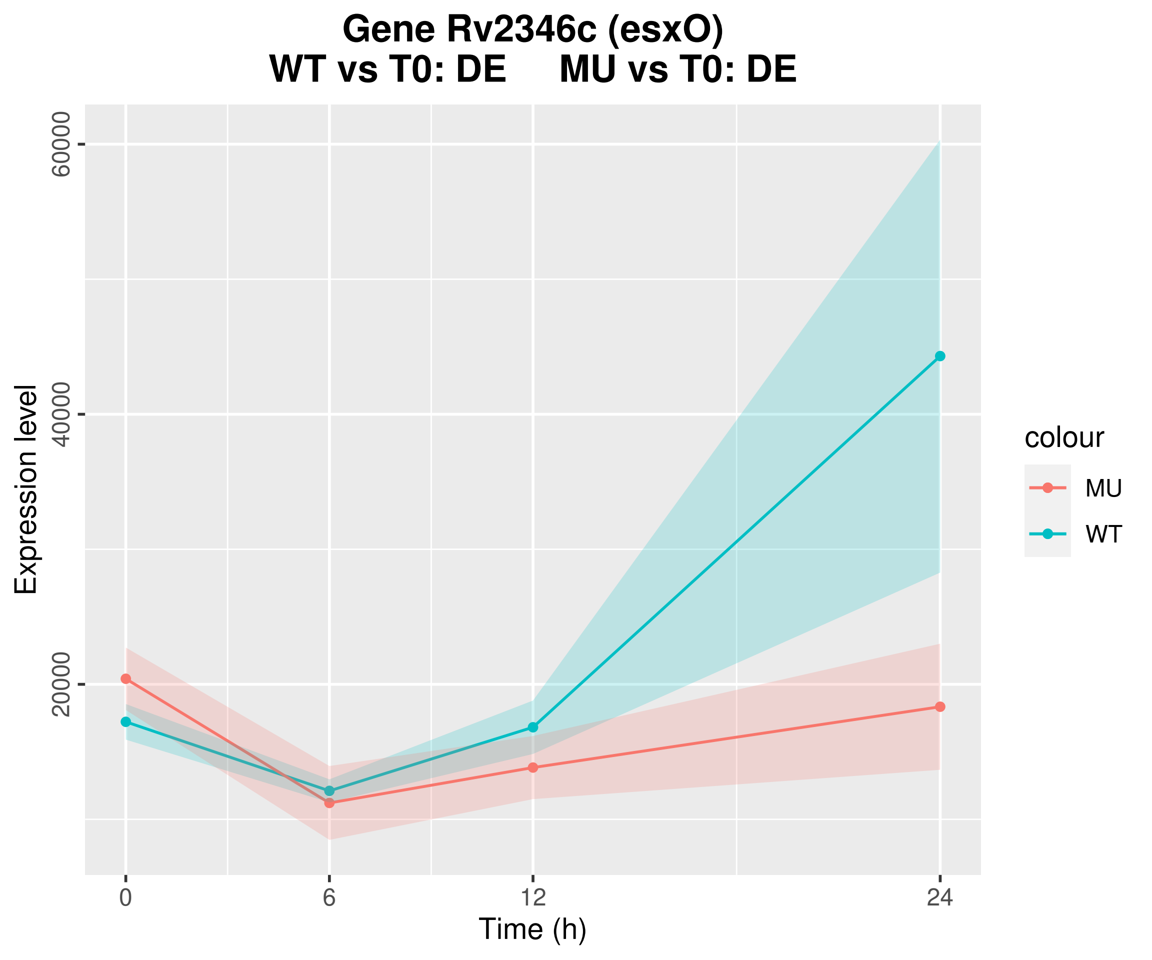

Gene Rv2346c (esxO)
WT vs T0: DE MU vs T0: DE
Expression level
60000
40000
20000
0
6
12
24
Time (h)
colour
MU
WT

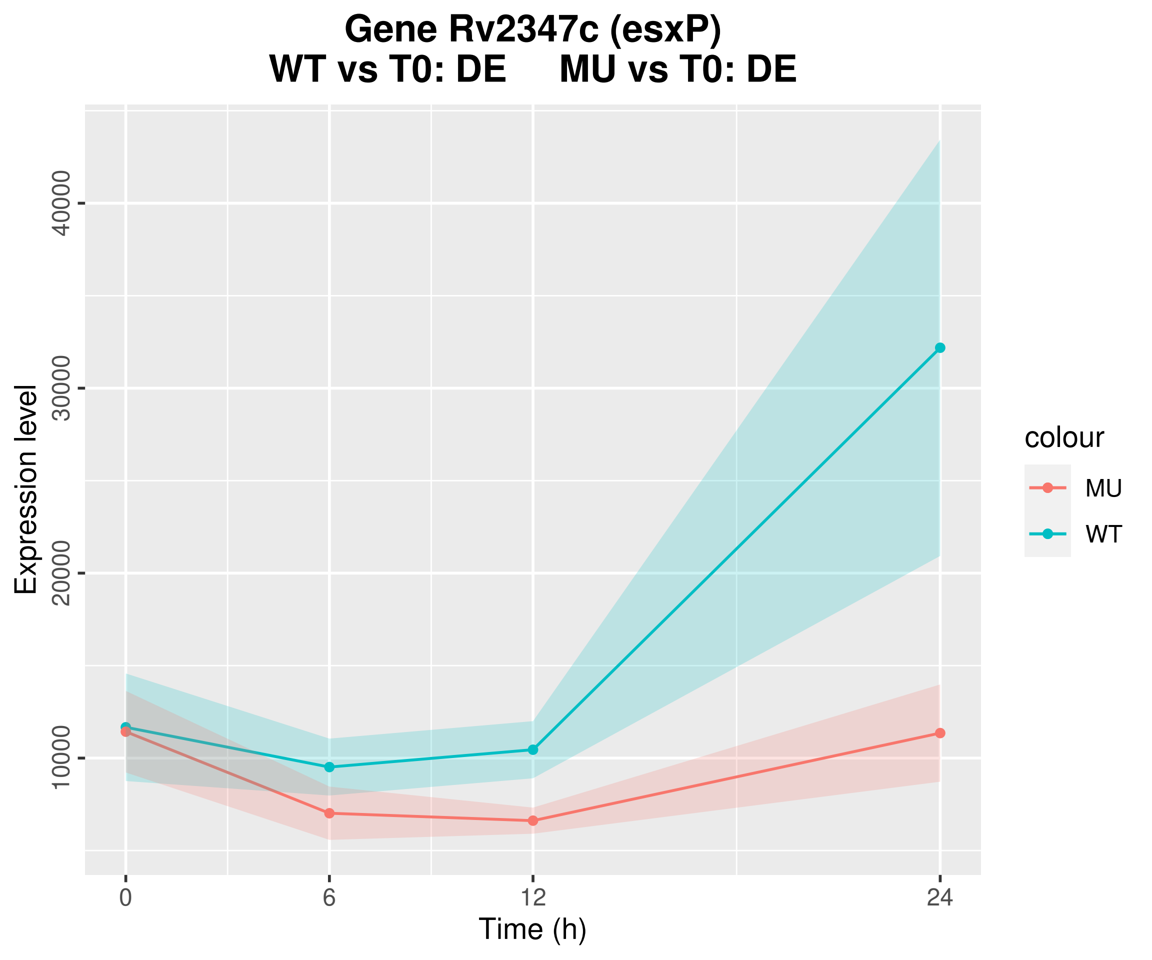
Gene Rv2347c (esxP)
WT vs T0: DE   MU vs T0: DE
Expression level
40000
30000
20000
10000
0
6
12
24
Time (h)
colour
MU
WT

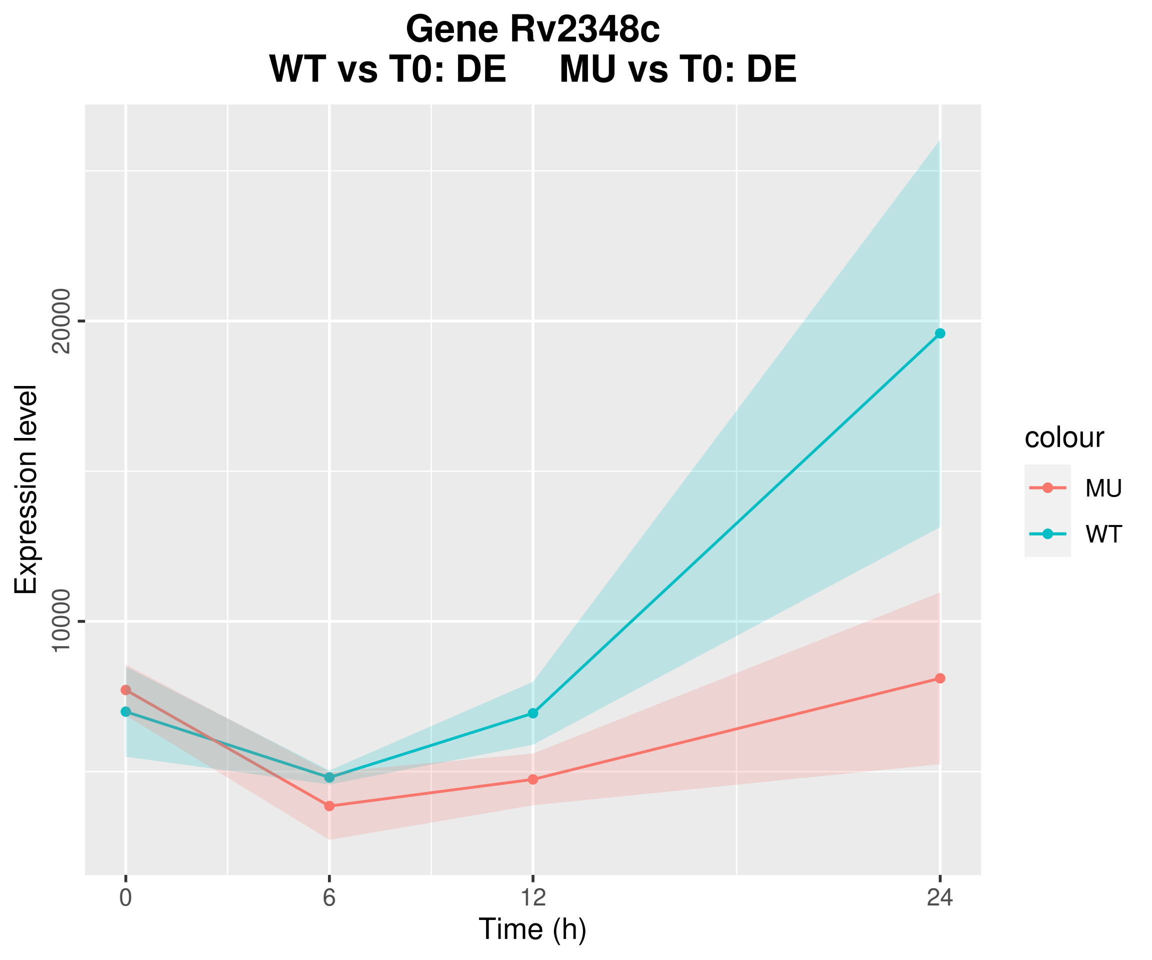

Gene Rv2348c
WT vs T0: DE MU vs T0: DE
Expression level
20000
10000
0
6
12
24
Time (h)
colour
MU
WT

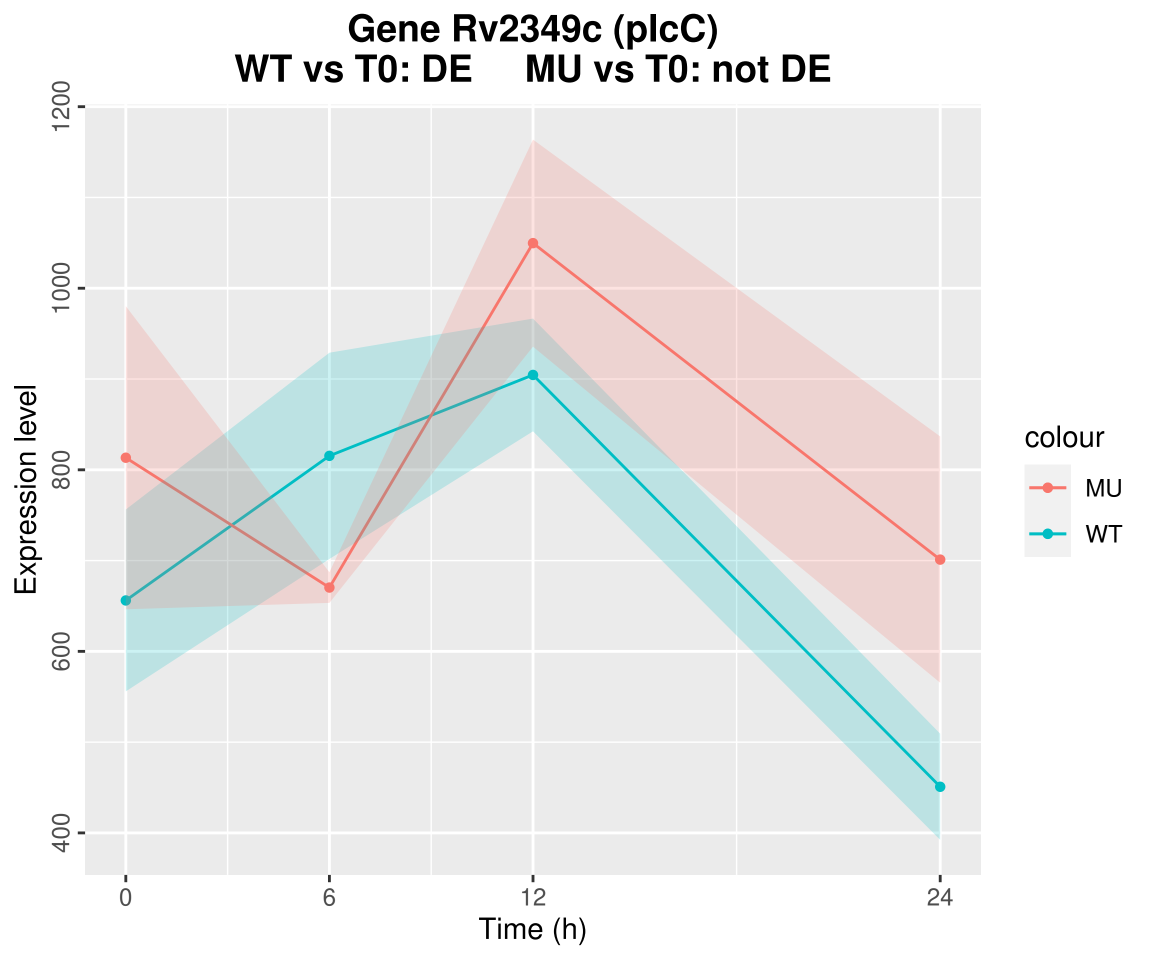
Gene Rv2349c (plcC)
WT vs T0: DE MU vs T0: not DE
Expression level
1200
1000
800
600
400
0
6
12
24
Time (h)
colour
MU
WT

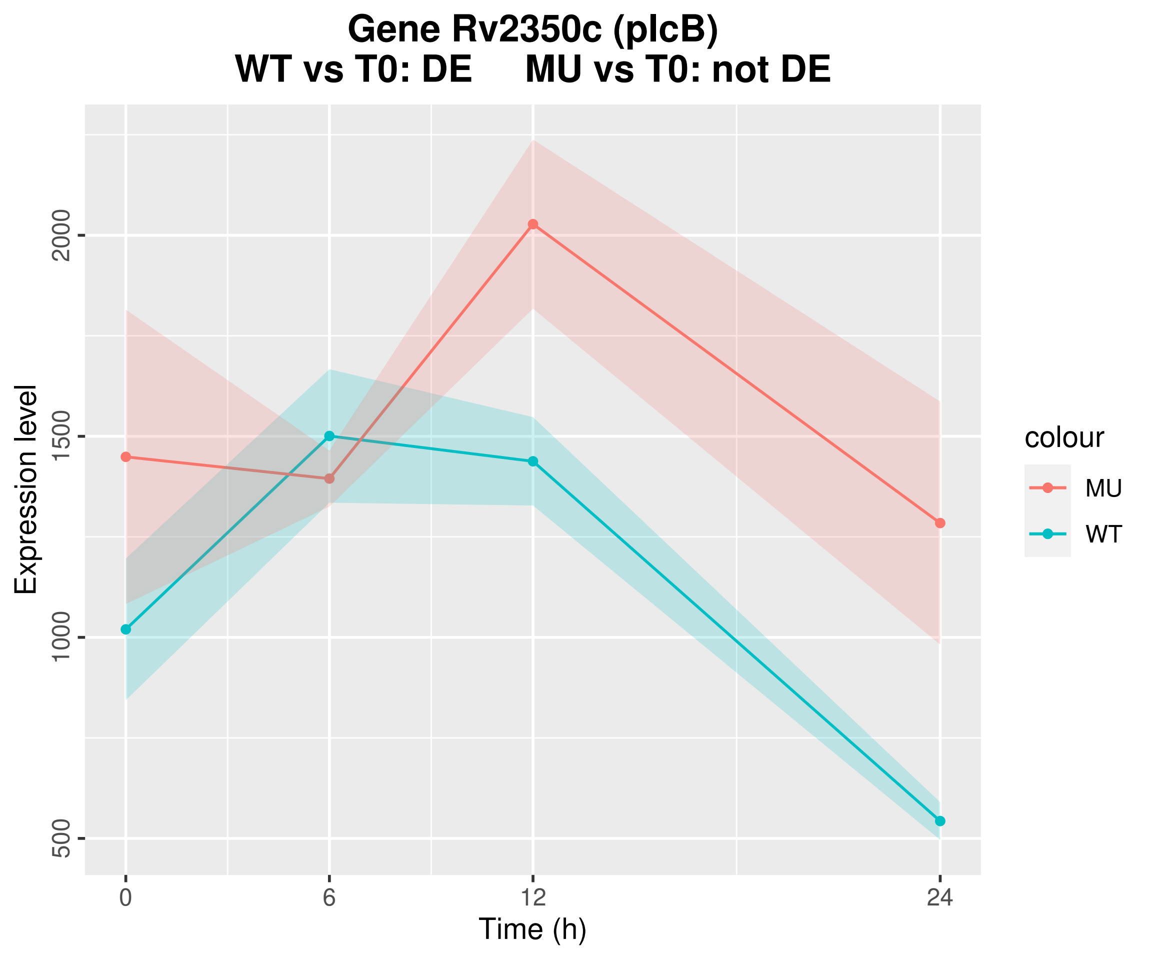

Gene Rv2350c (plcB)
WT vs T0: DE   MU vs T0: not DE
Expression level
2000
1500
1000
500
0
6
12
24
Time (h)
colour
MU
WT

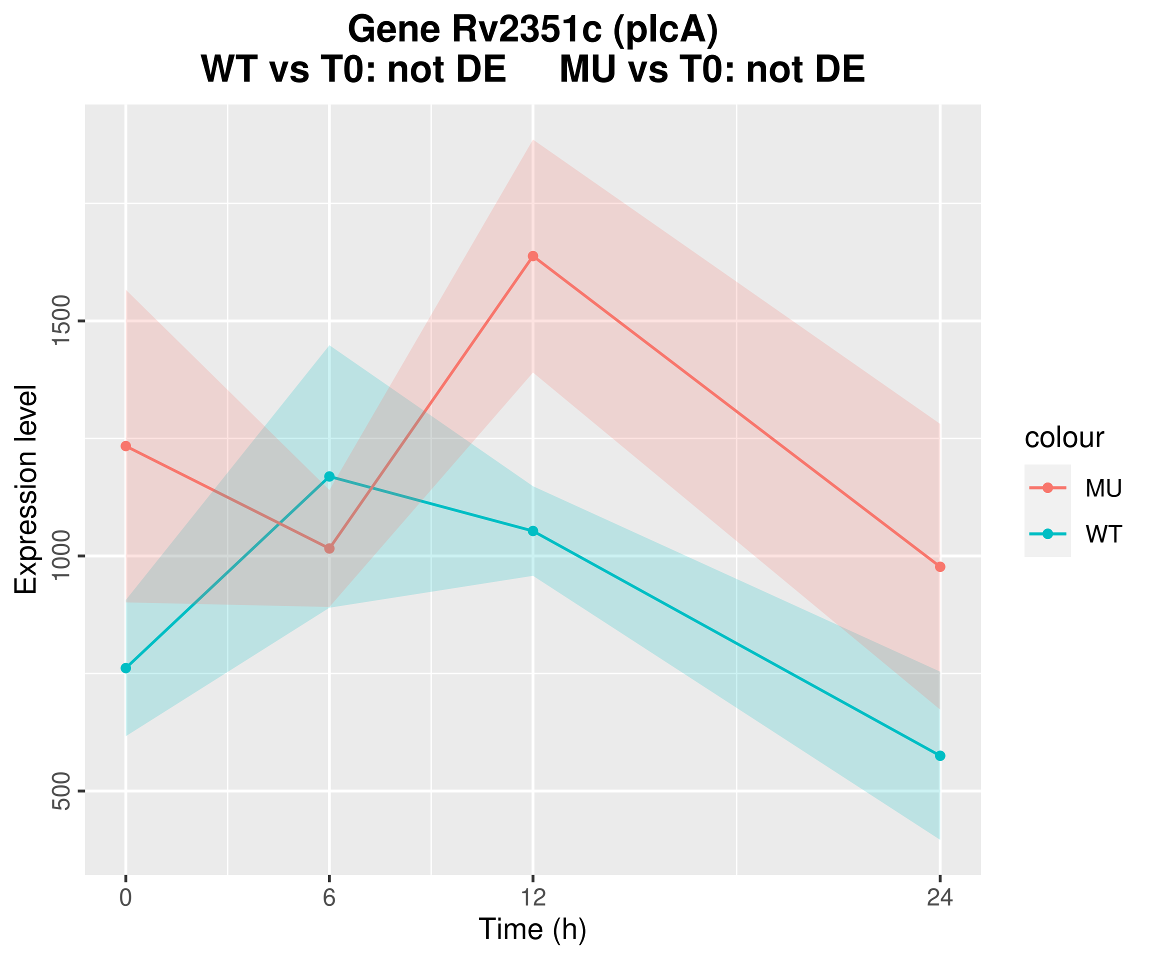
Gene Rv2351c (plcA)
WT vs T0: not DE   MU vs T0: not DE
Expression level
1500
1000
500
0
6
12
24
Time (h)
colour
MU
WT

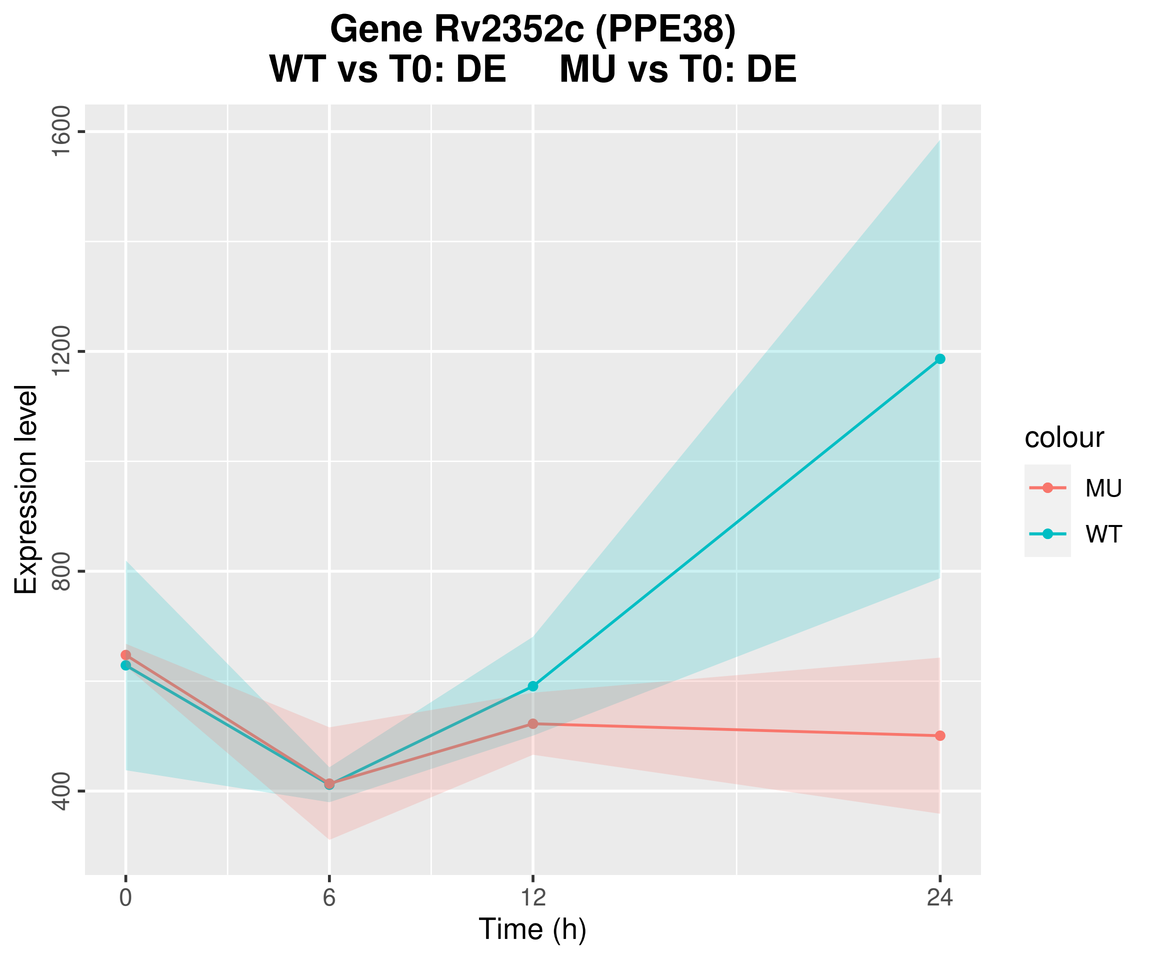

Gene Rv2352c (PPE38)
WT vs T0: DE MU vs T0: DE
Expression level
1600
1200
800
400
0
6
12
24
Time (h)
colour
MU
WT

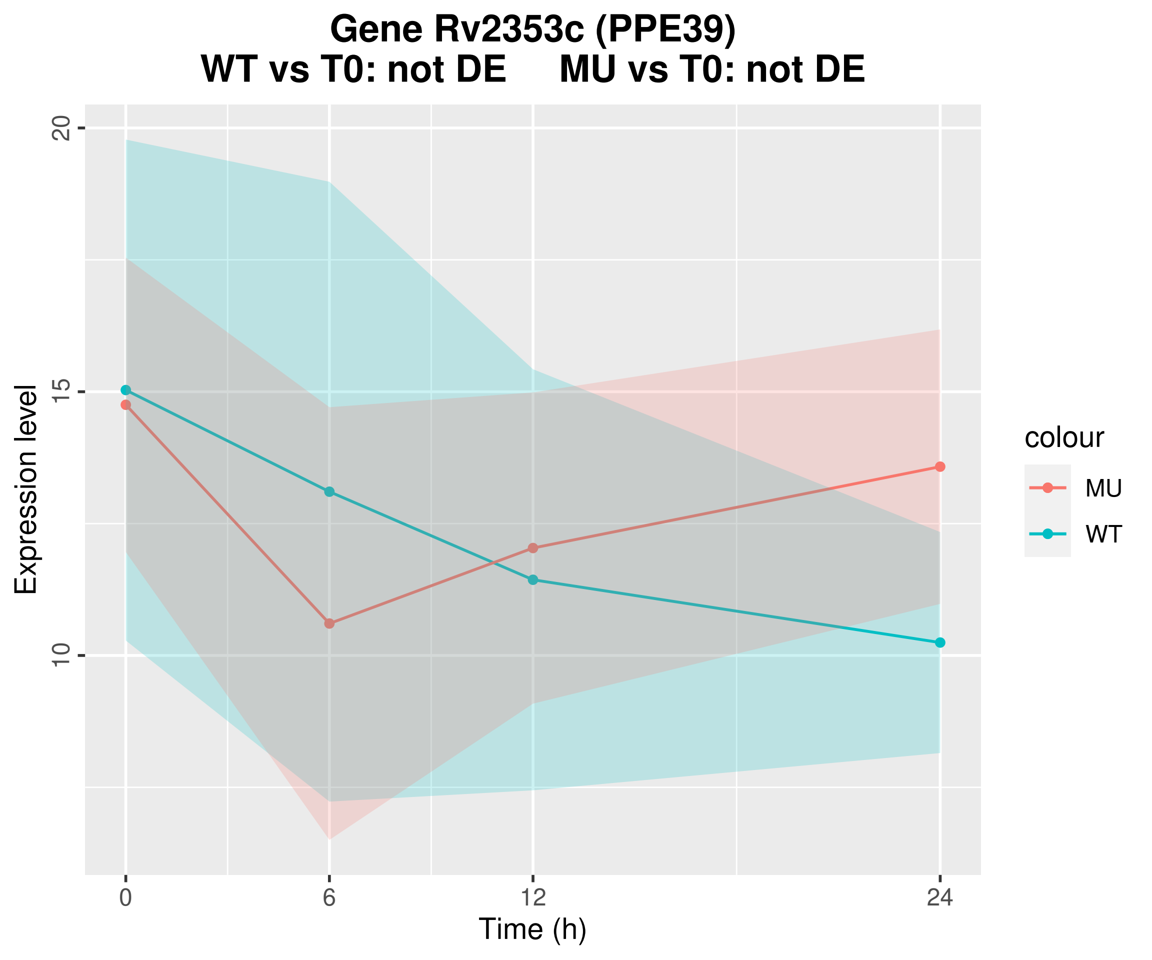

Gene Rv2353c (PPE39)
WT vs T0: not DE   MU vs T0: not DE
20
15
10
Expression level
0
6
12
24
Time (h)
colour
MU
WT

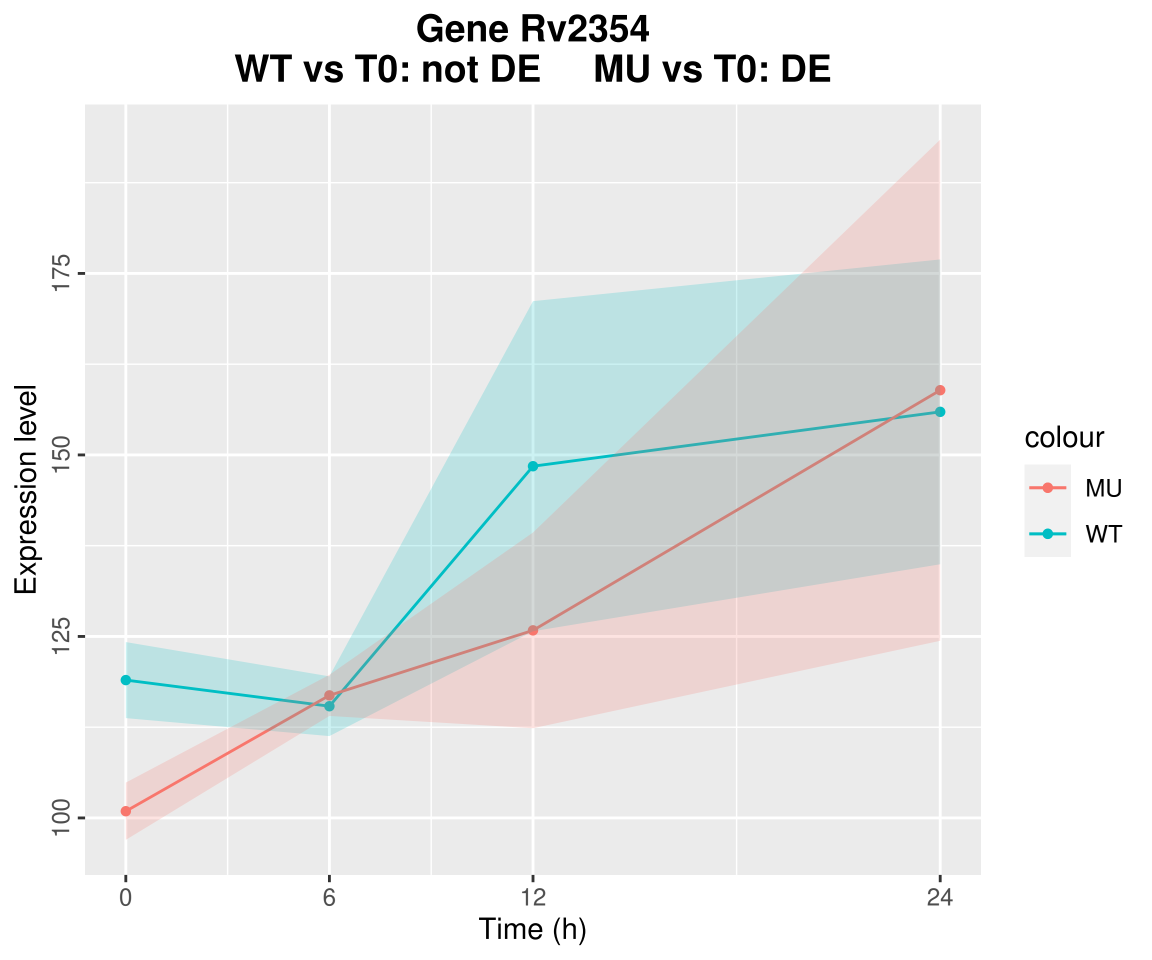

Gene Rv2354
WT vs T0: not DE MU vs T0: DE
Expression level
100
125
150
175
0
6
12
24
Time (h)
colour
MU
WT

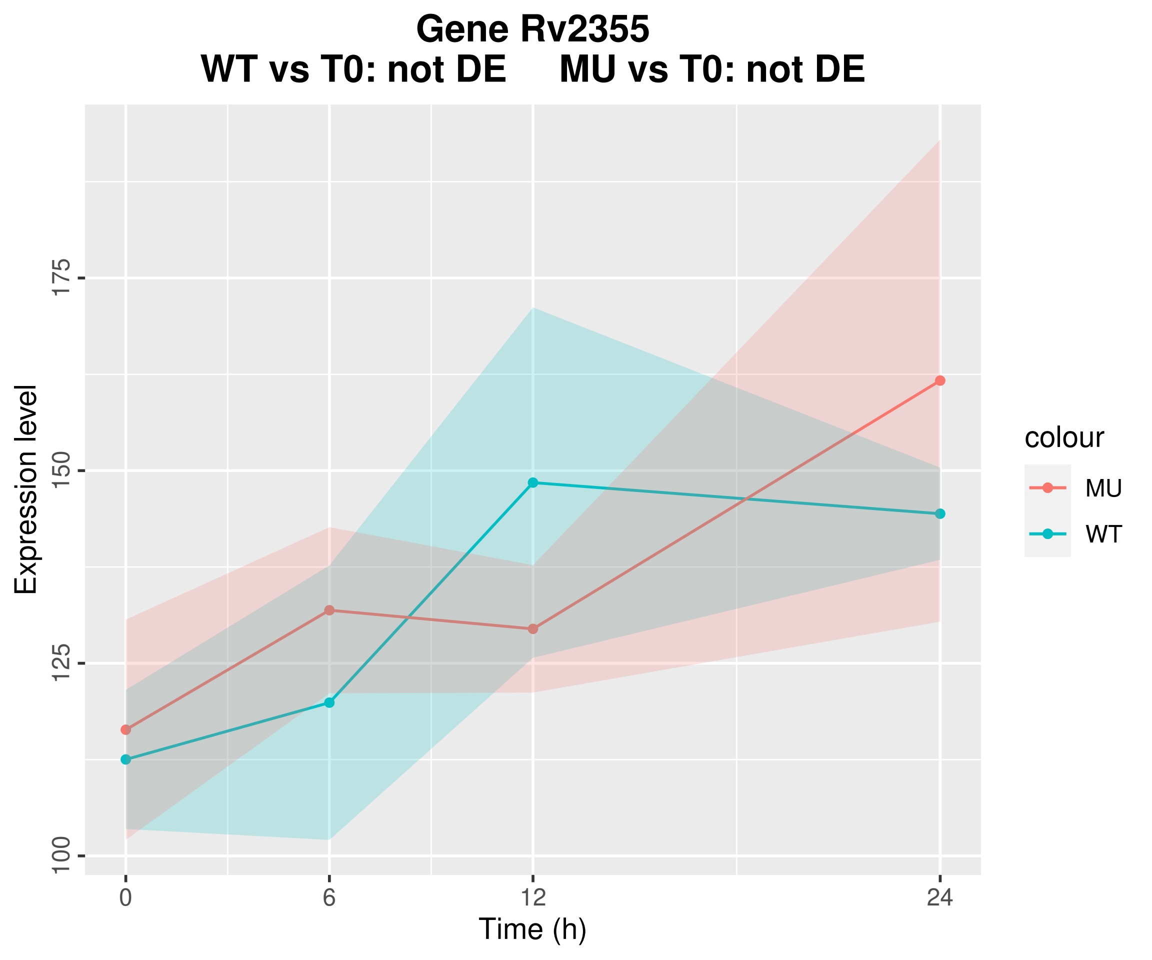

Gene Rv2355
WT vs T0: not DE   MU vs T0: not DE
Expression level
100
125
150
175
0
6
12
24
Time (h)
colour
MU
WT

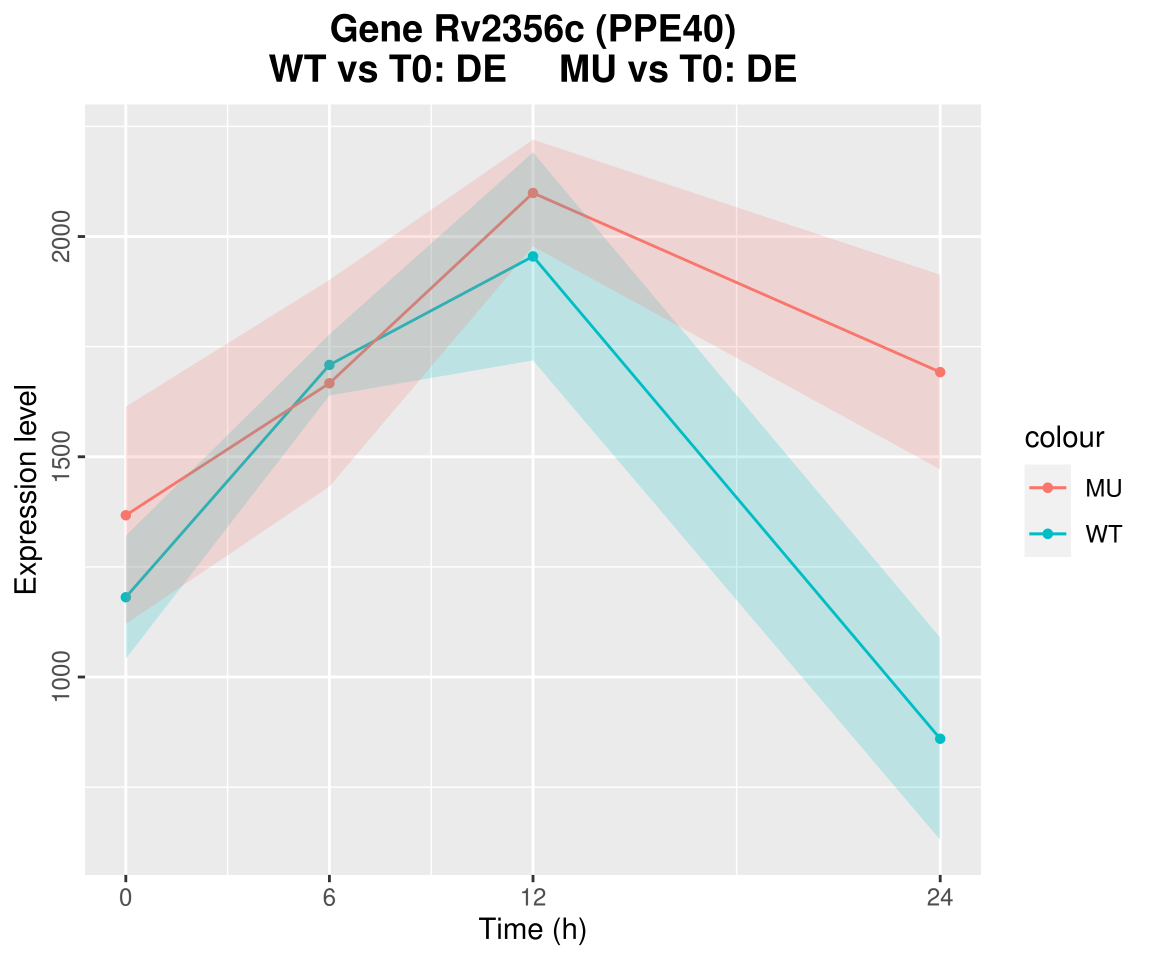

Gene Rv2356c (PPE40)
WT vs T0: DE   MU vs T0: DE
Expression level
Time (h)
0
6
12
24
1000
1500
2000
colour
MU
WT

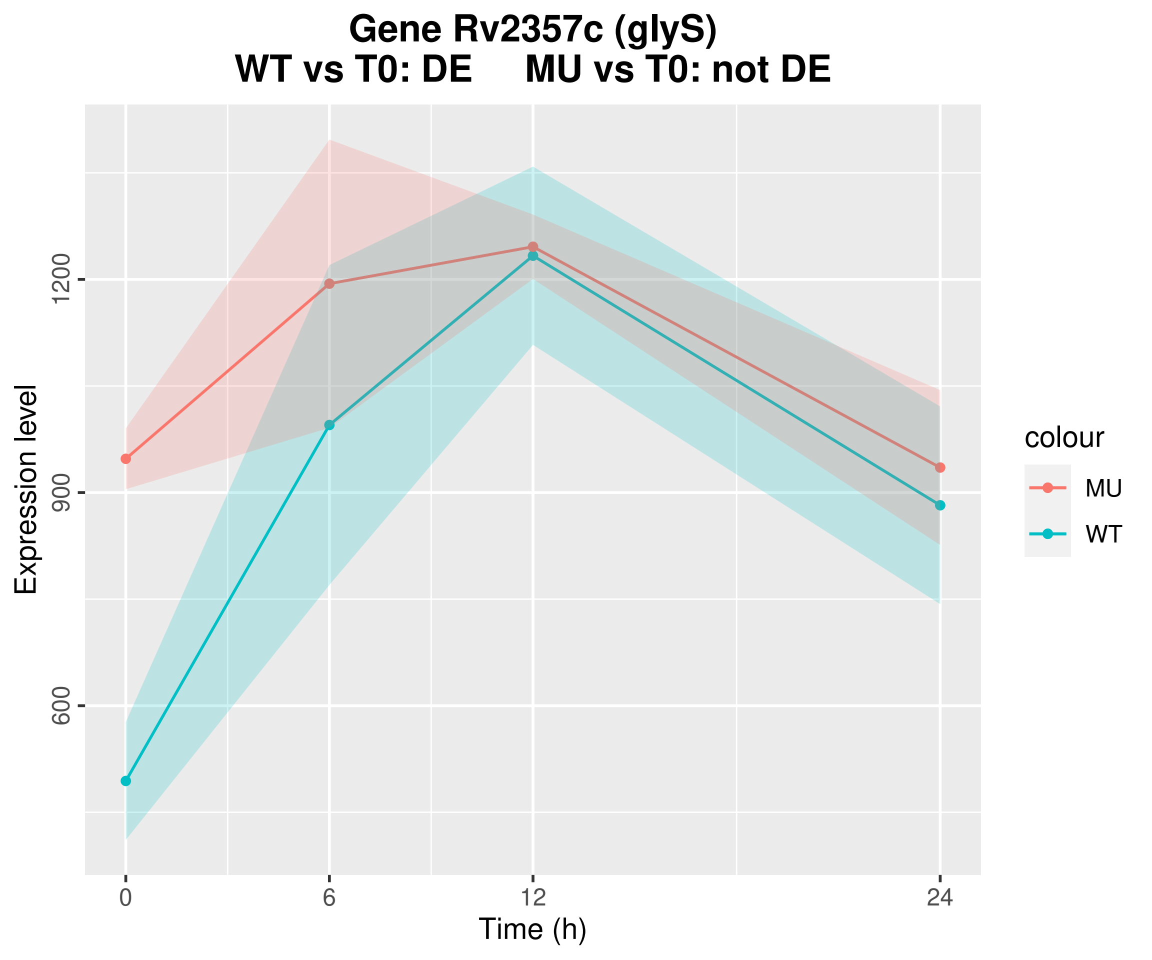
Gene Rv2357c (glyS)
WT vs T0: DE   MU vs T0: not DE
Expression level
1200
900
600
0
6
12
24
Time (h)
colour
MU
WT

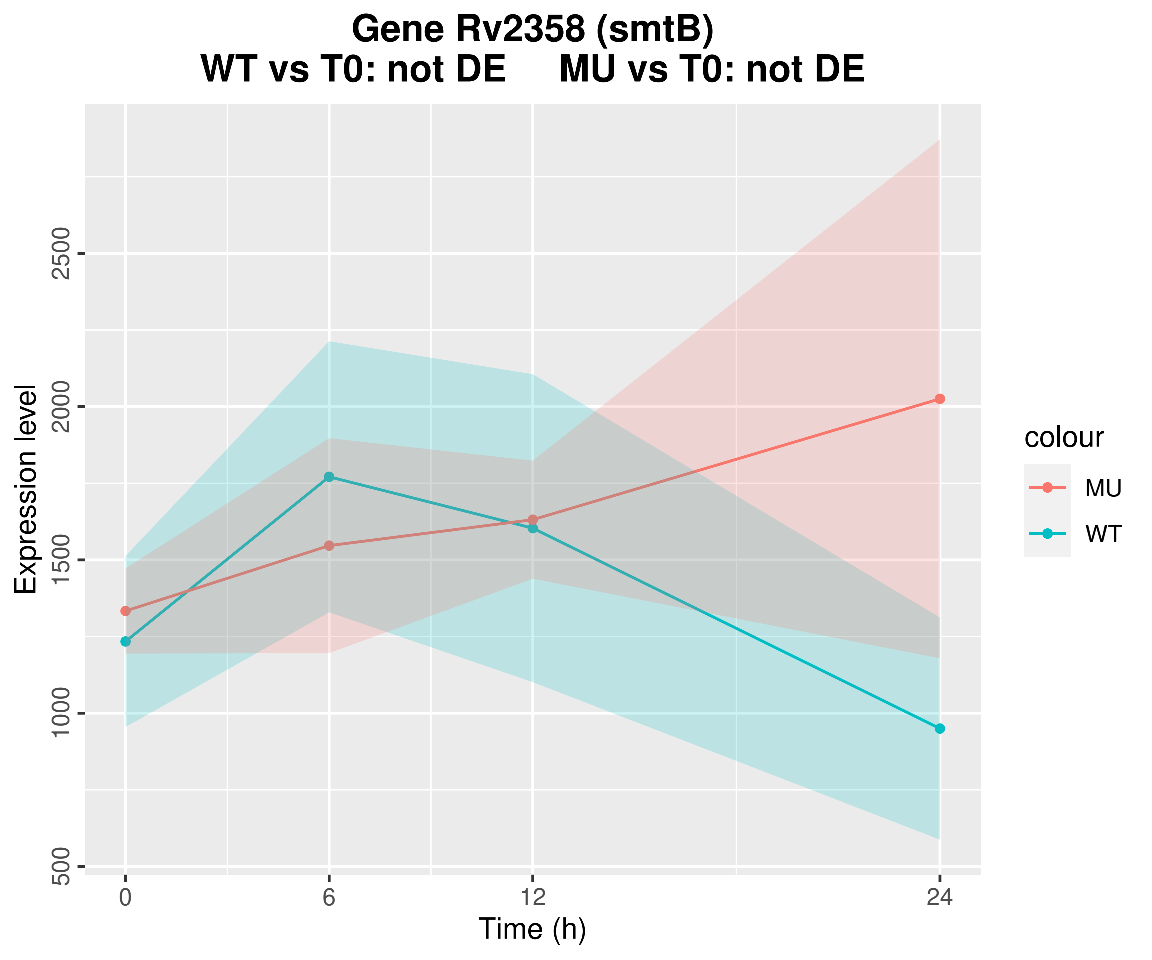
Gene Rv2358 (smtB)
WT vs T0: not DE   MU vs T0: not DE
Expression level
2500
2000
1500
1000
500
0
6
12
24
Time (h)
colour
MU
WT

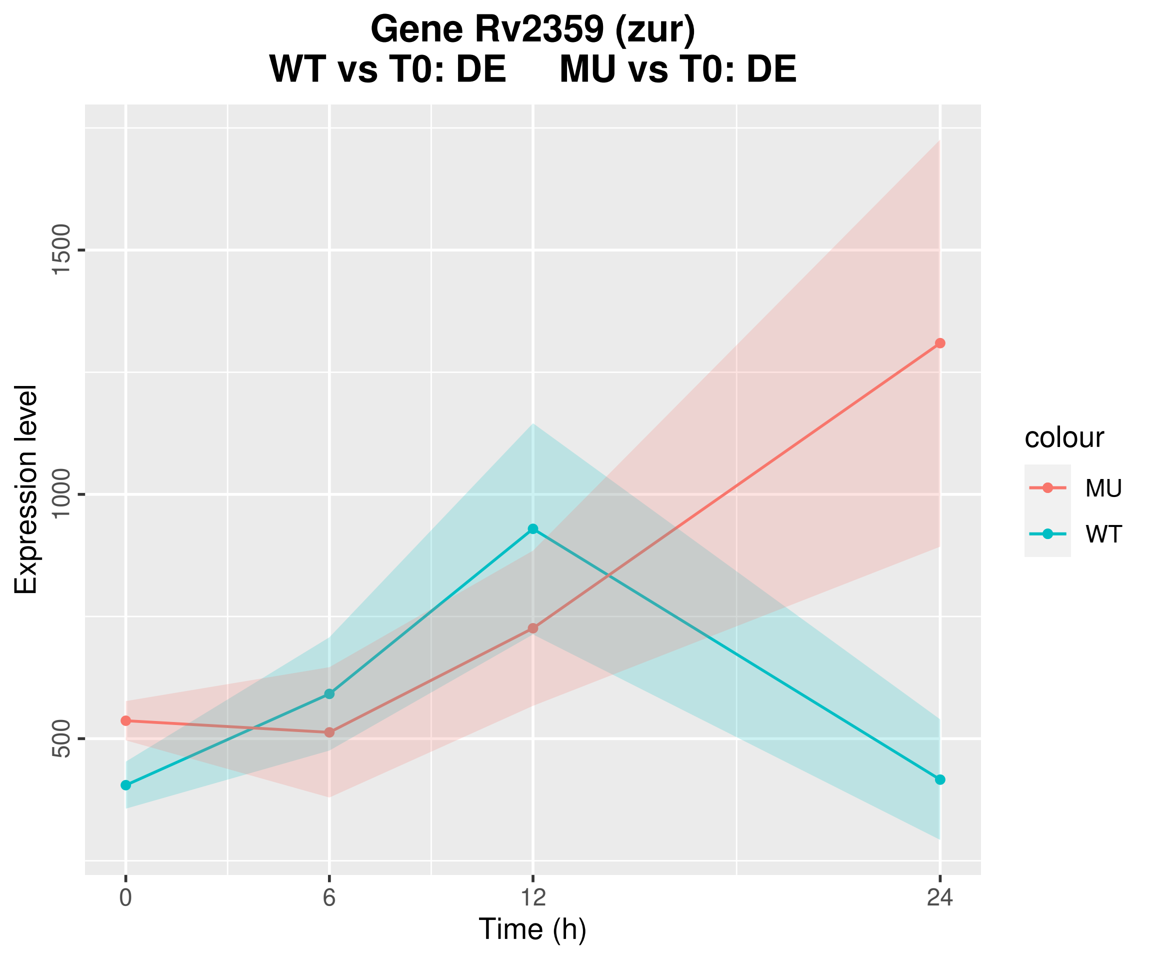
Gene Rv2359 (zur)
WT vs T0: DE   MU vs T0: DE
Expression level
1500
1000
500
0
6
12
24
Time (h)
colour
MU
WT

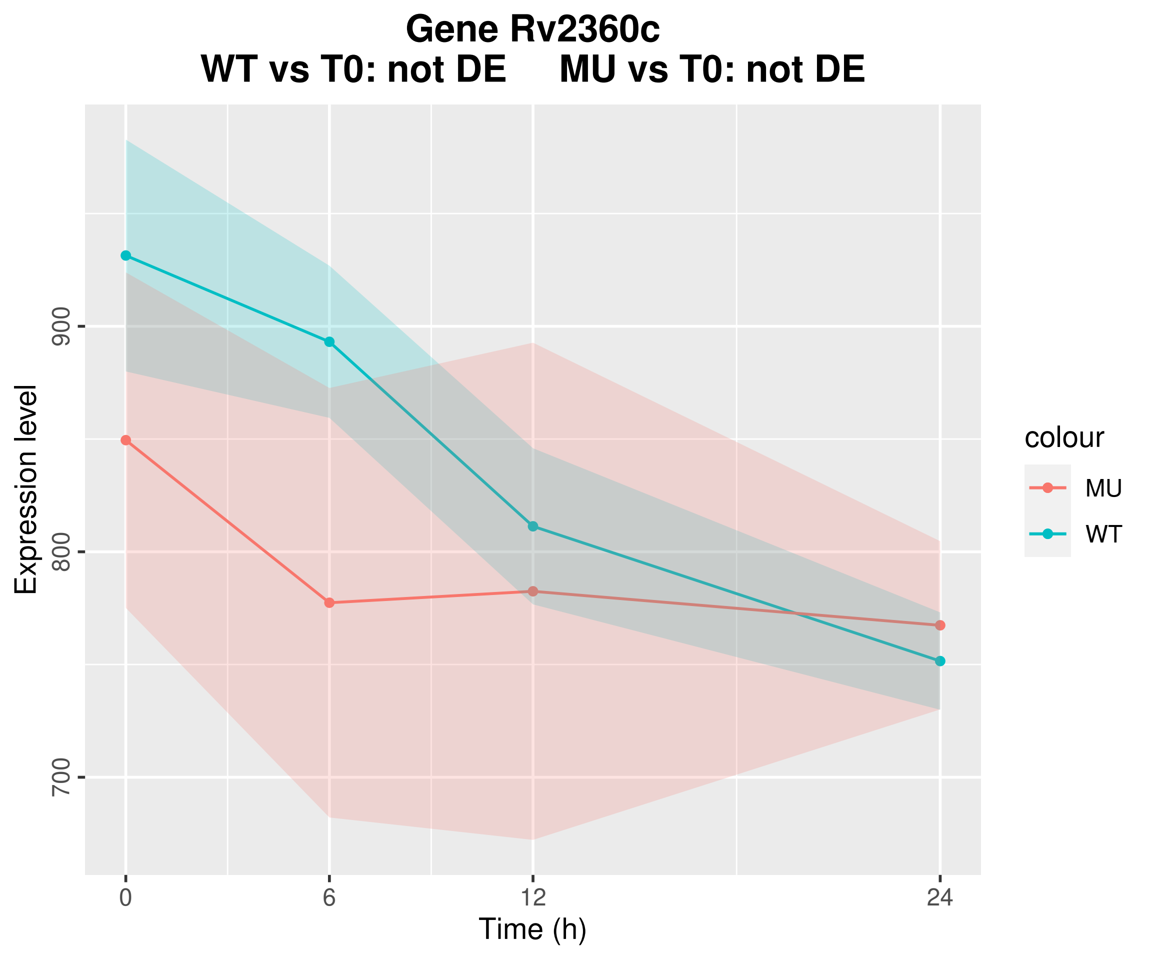
Gene Rv2360c
WT vs T0: not DE MU vs T0: not DE
Expression level
900
800
700
0
6
12
24
Time (h)
colour
MU
WT

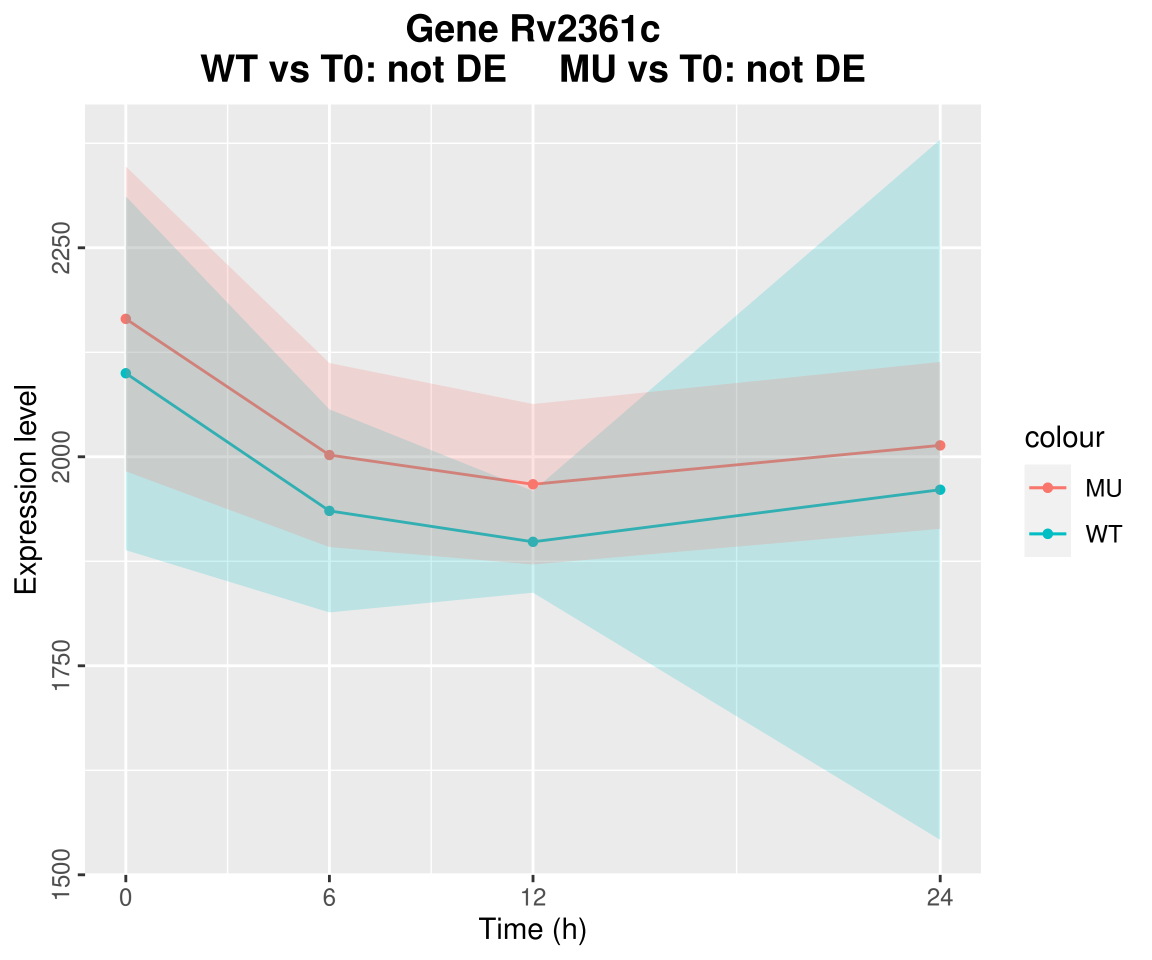

Gene Rv2361c
WT vs T0: not DE   MU vs T0: not DE
Expression level
2250
2000
1750
1500
0
6
12
24
Time (h)
colour
MU
WT

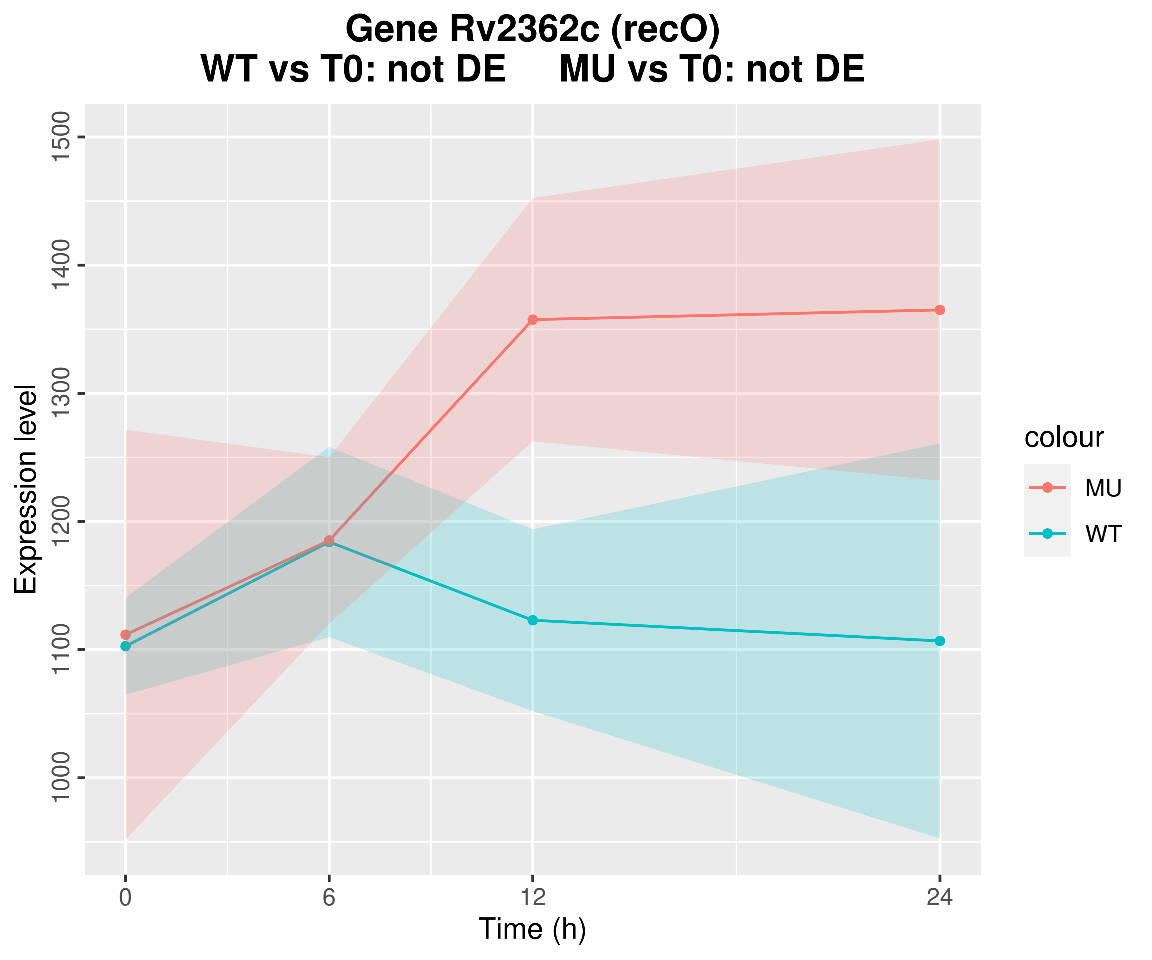

Gene Rv2362c (recO)
WT vs T0: not DE   MU vs T0: not DE
Expression level
1000
1100
1200
1300
1400
1500
0
6
12
24
Time (h)
colour
MU
WT

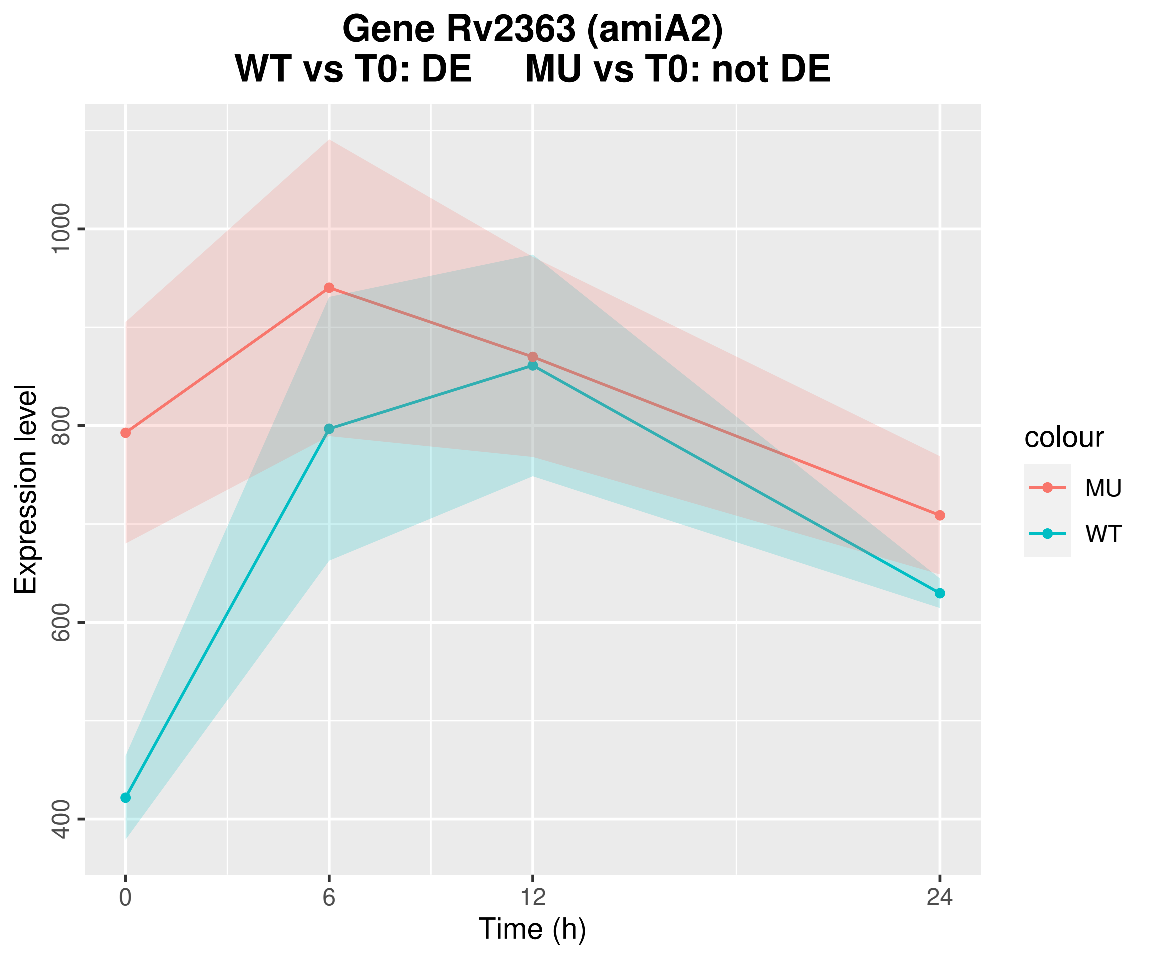

Gene Rv2363 (amiA2)
WT vs T0: DE   MU vs T0: not DE
Expression level
1000
800
600
400
0
6
12
24
Time (h)
colour
MU
WT

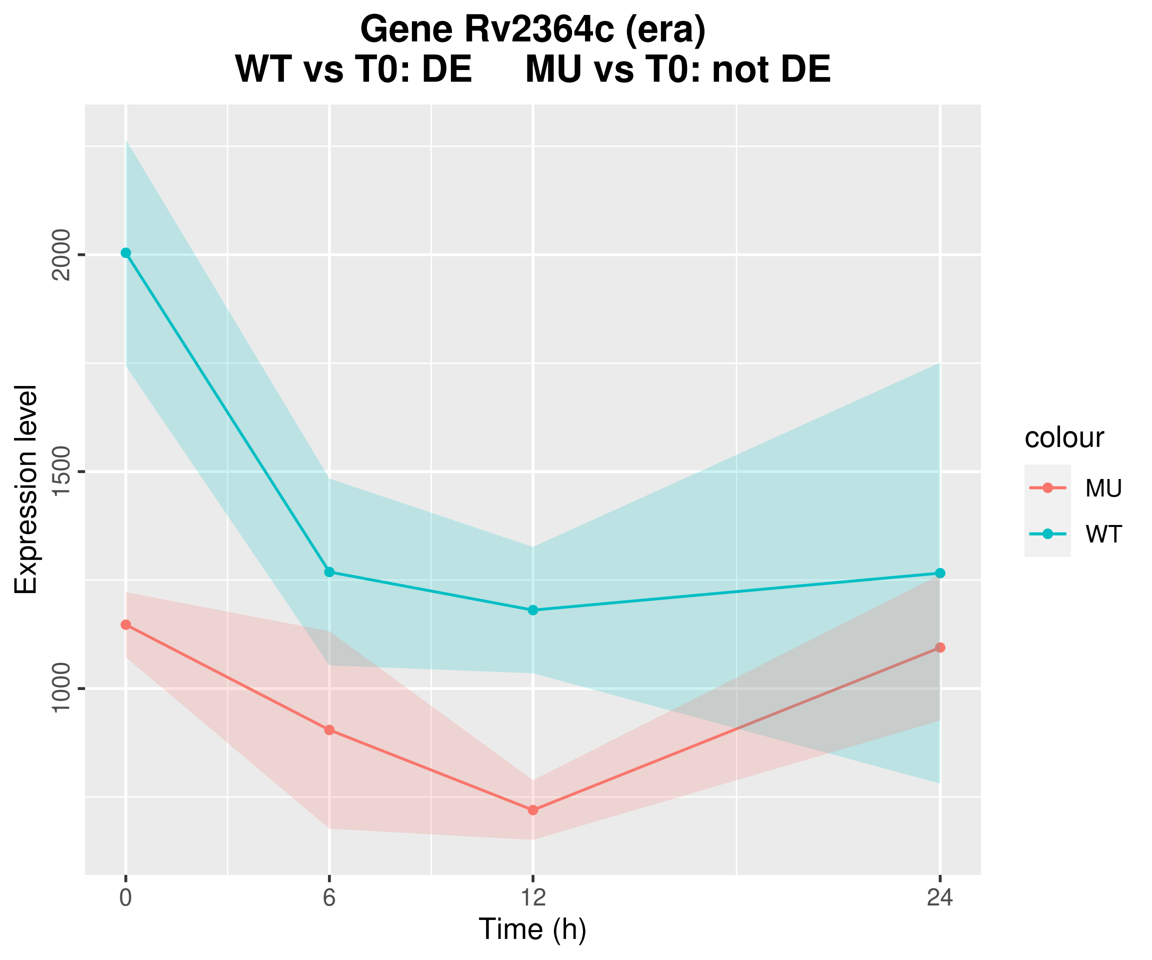
Gene Rv2364c (era)
WT vs T0: DE MU vs T0: not DE
Expression level
2000
1500
1000
0
6
12
24
Time (h)
colour
MU
WT

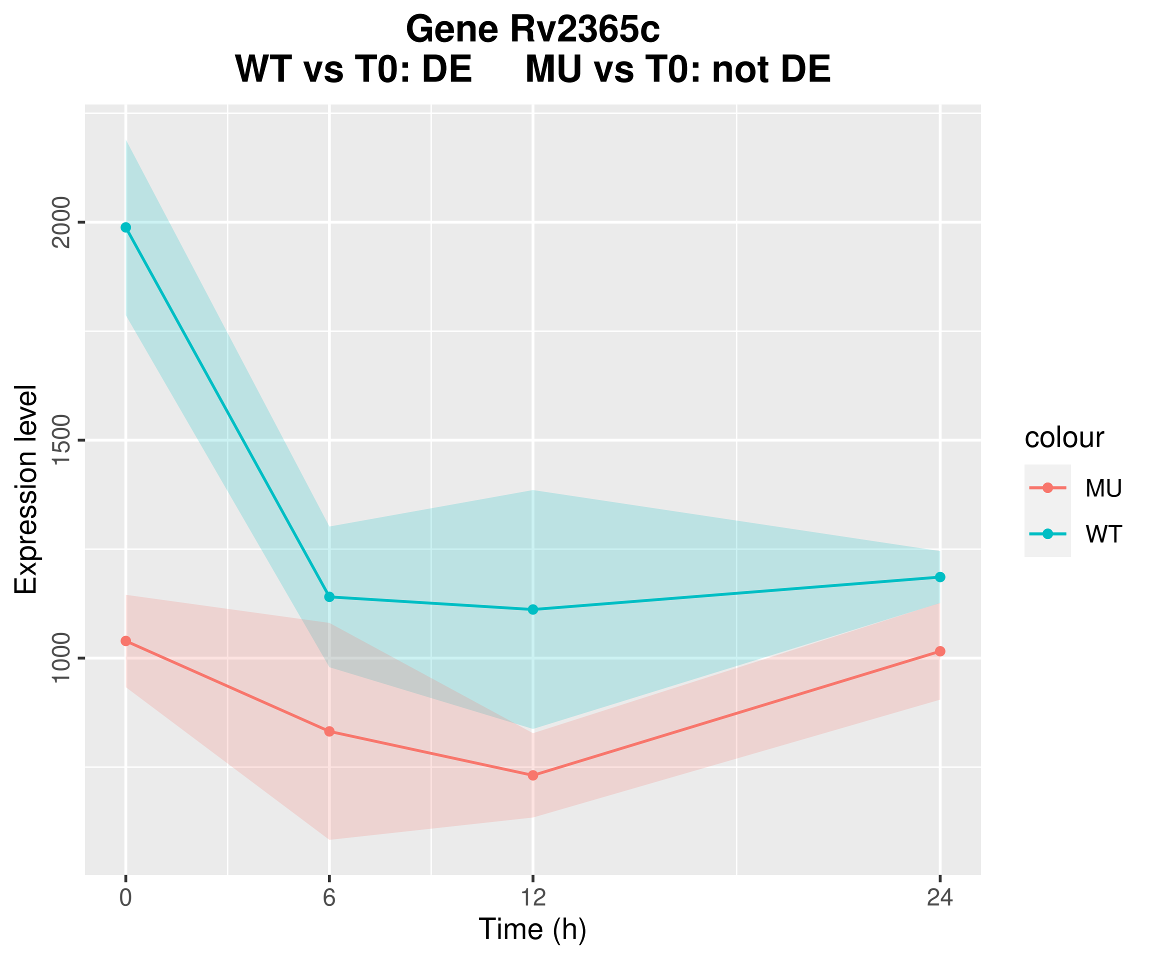

Gene Rv2365c
WT vs T0: DE   MU vs T0: not DE
Expression level
2000
1500
1000
0
6
12
24
Time (h)
colour
MU
WT

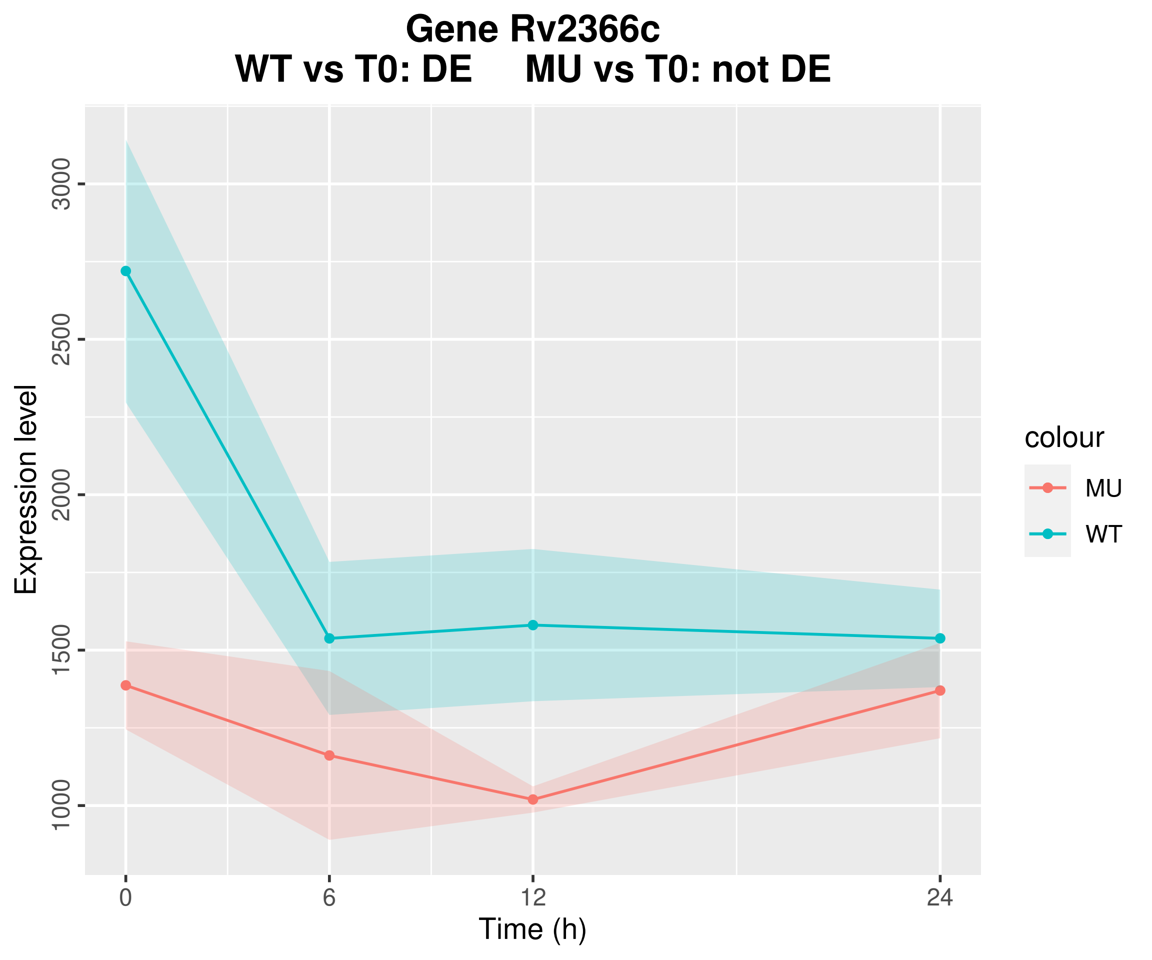
Gene Rv2366c
WT vs T0: DE MU vs T0: not DE
Expression level
3000
2500
2000
1500
1000
0
6
12
24
Time (h)
colour
MU
WT

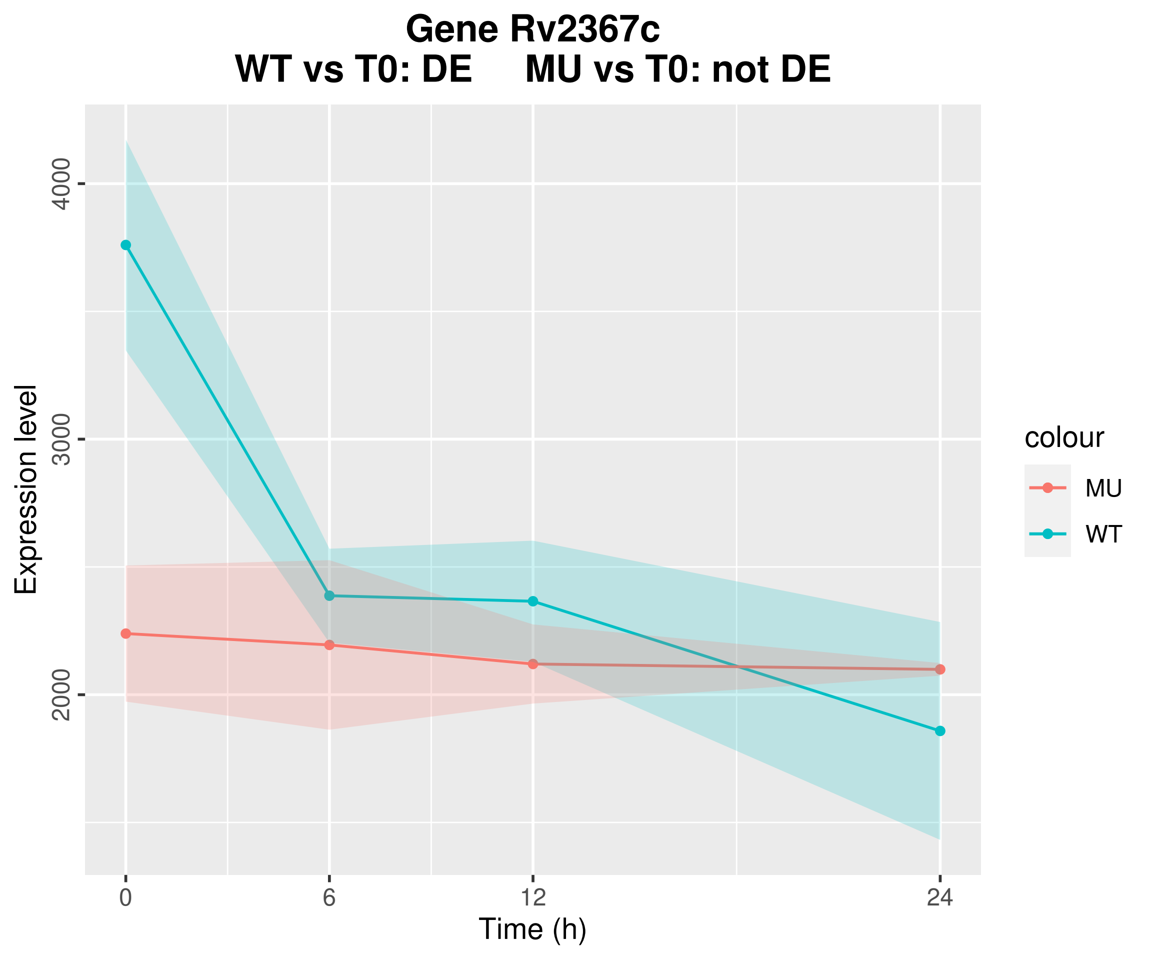
Gene Rv2367c
WT vs T0: DE   MU vs T0: not DE
Expression level
4000
3000
2000
0
6
12
24
Time (h)
colour
MU
WT

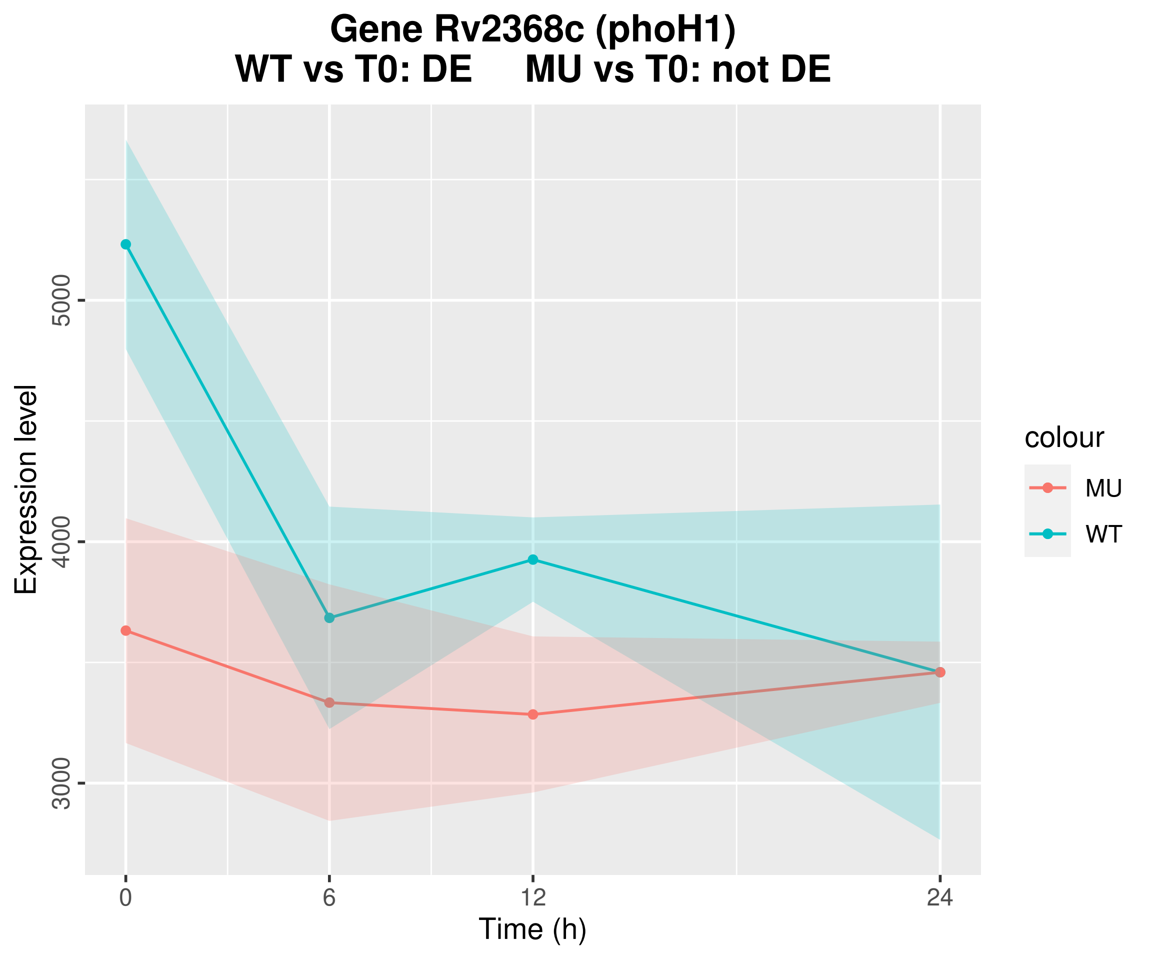
Gene Rv2368c (phoH1)
WT vs T0: DE   MU vs T0: not DE
Expression level
5000
4000
3000
0
6
12
24
Time (h)
colour
MU
WT

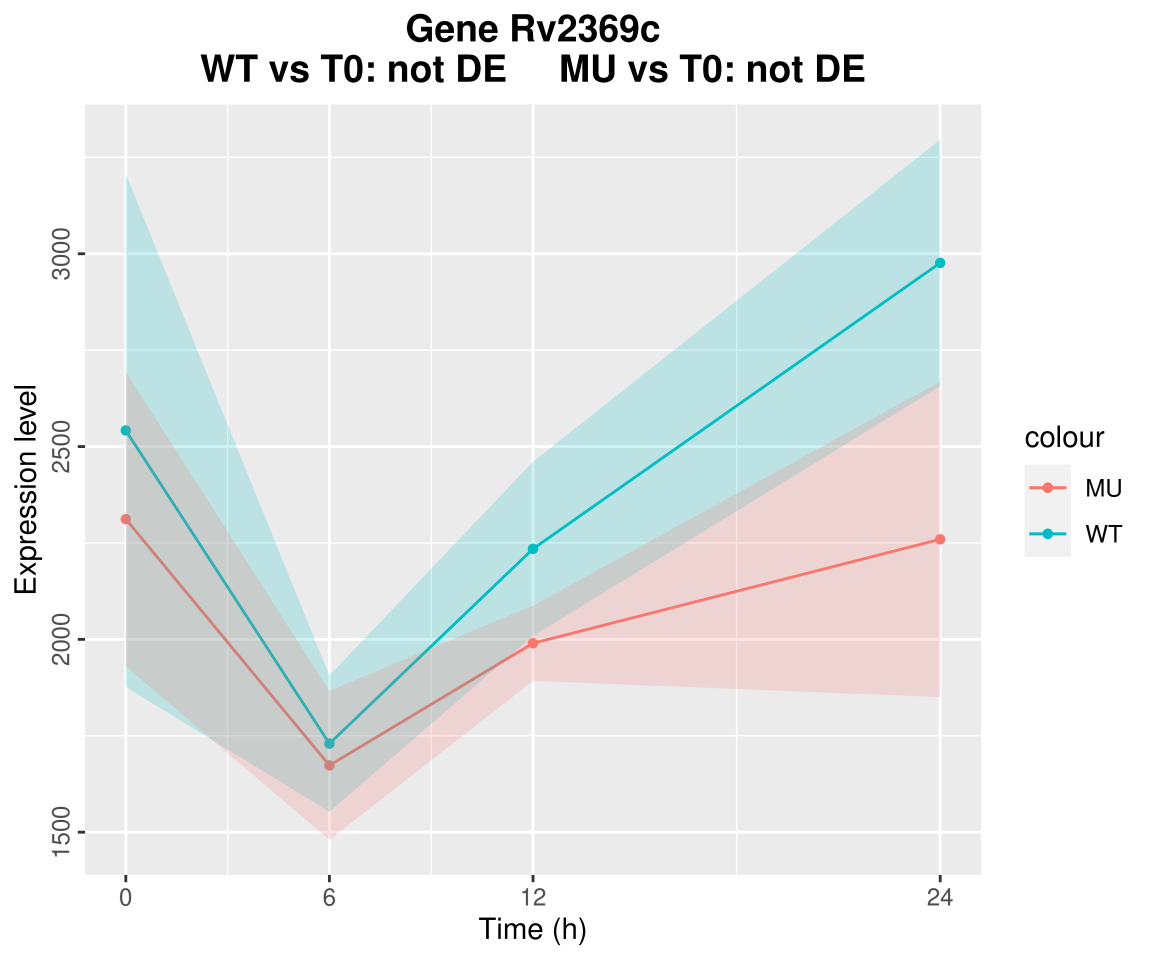

Gene Rv2369c
WT vs T0: not DE   MU vs T0: not DE
Expression level
1500
2000
2500
3000
Time (h)
0
6
12
24
colour
MU
WT

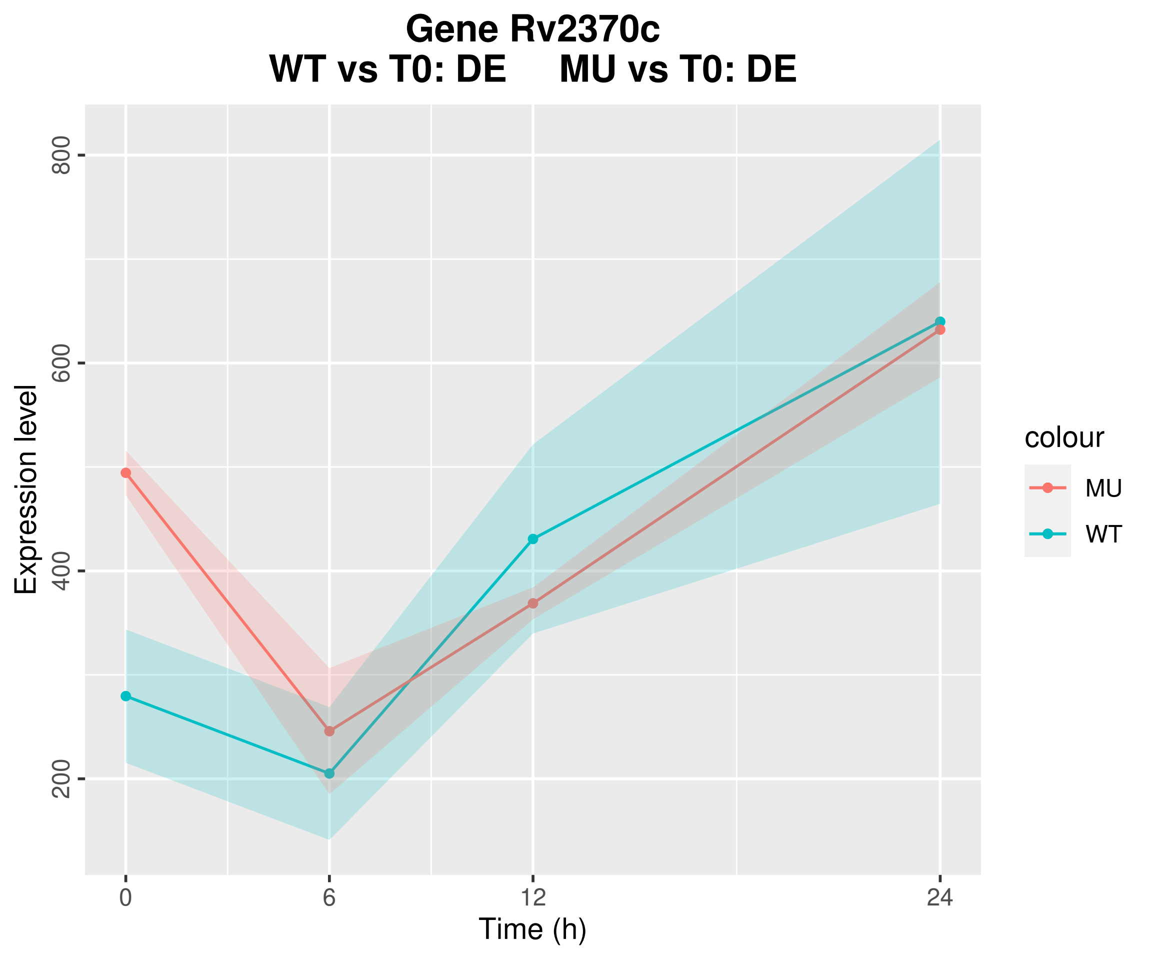

Gene Rv2370c
WT vs T0: DE   MU vs T0: DE
Expression level
800
600
400
200
0
6
12
24
Time (h)
colour
MU
WT

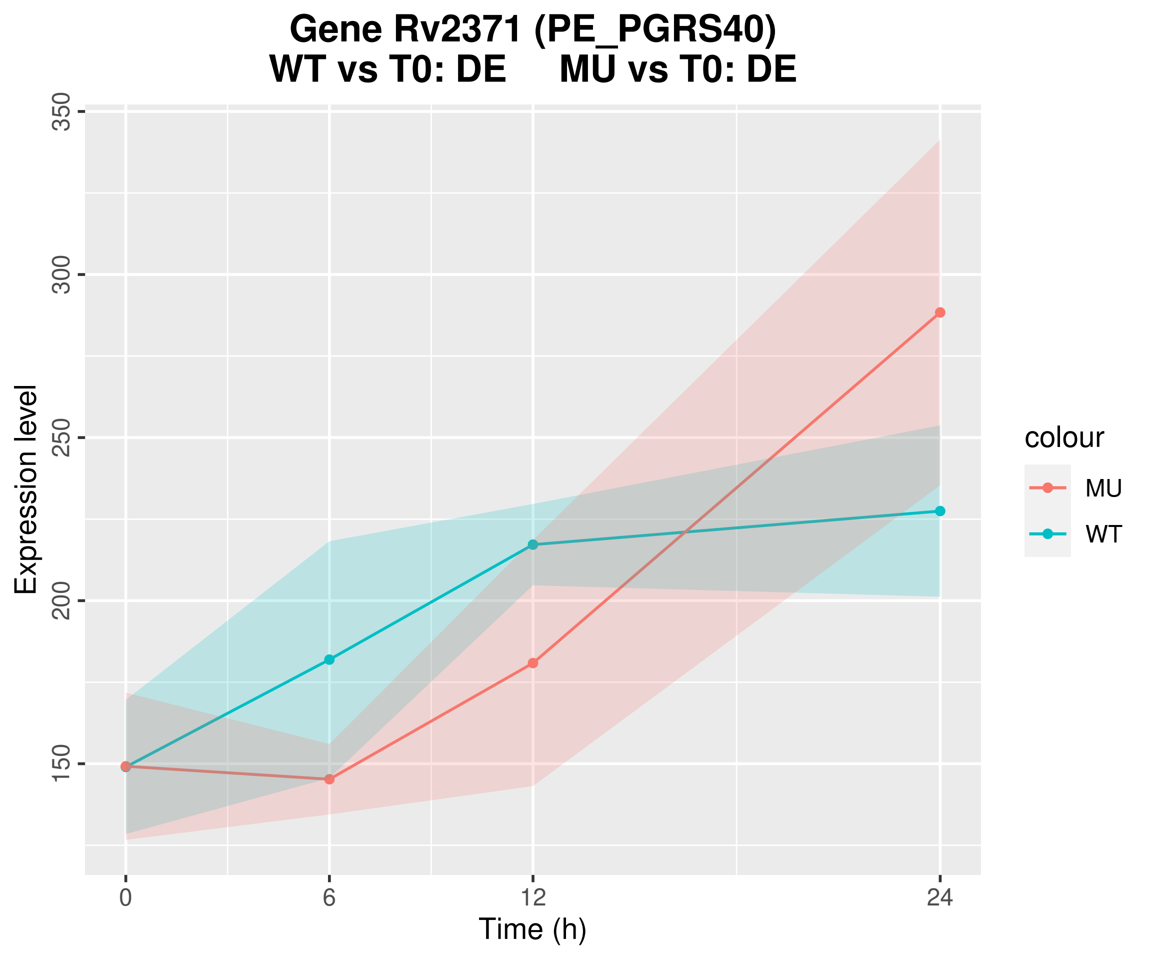

Gene Rv2371 (PE_PGRS40)
WT vs T0: DE   MU vs T0: DE
Expression level
350
300
250
200
150
0
6
12
24
Time (h)
colour
MU
WT

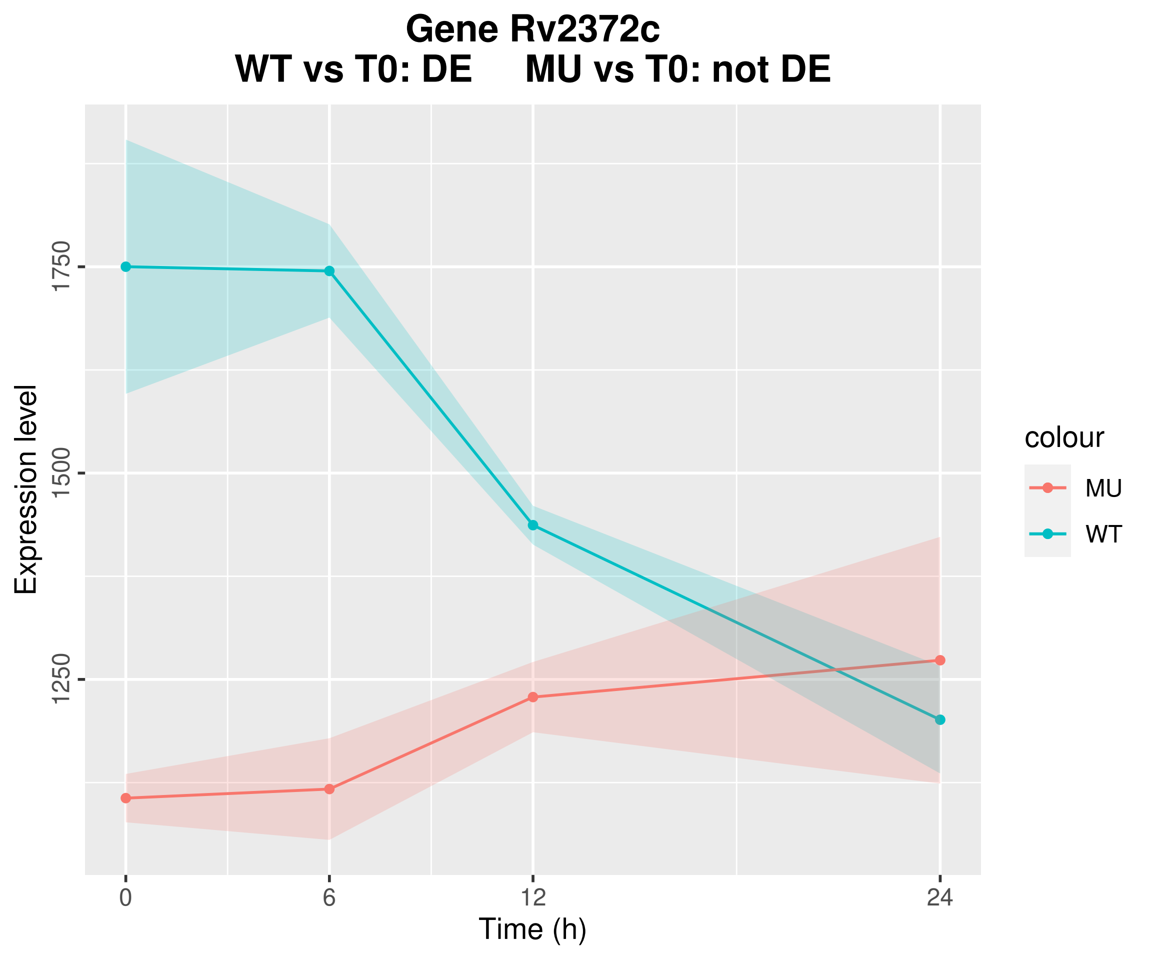

Gene Rv2372c
WT vs T0: DE   MU vs T0: not DE
Expression level
1750
1500
1250
0
6
12
24
Time (h)
colour
MU
WT

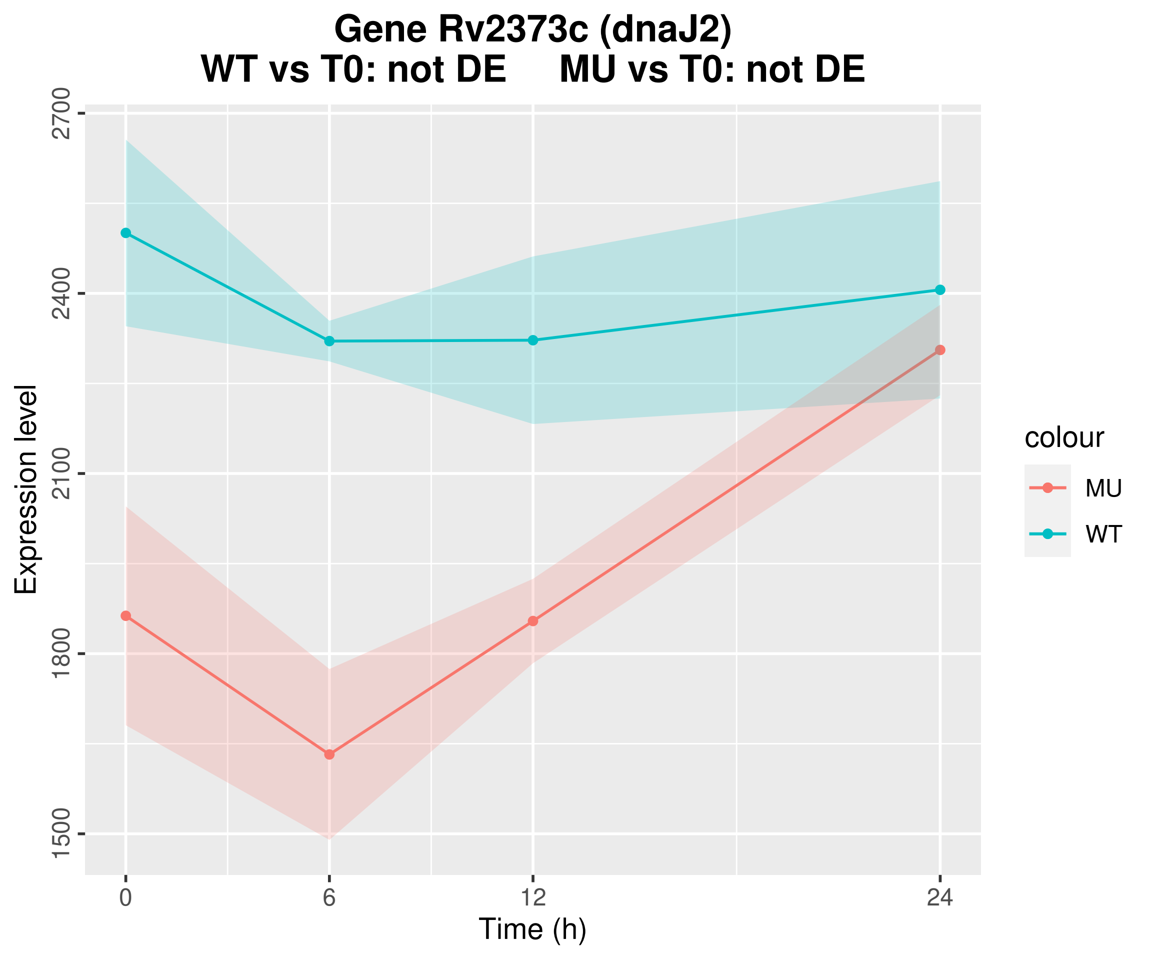
Gene Rv2373c (dnaJ2)
WT vs T0: not DE MU vs T0: not DE
Expression level
2700
2400
2100
1800
1500
0
6
12
24
Time (h)
colour
MU
WT

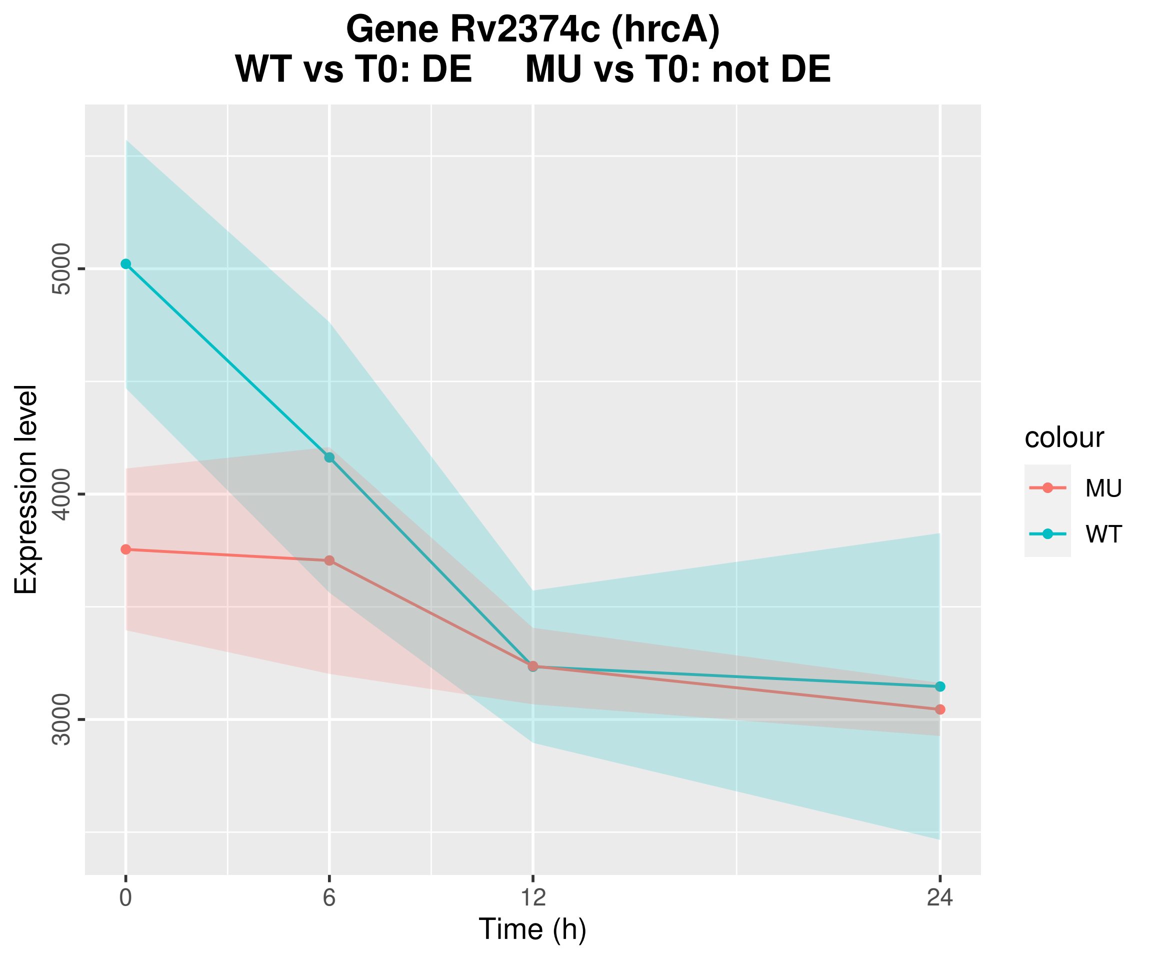
Gene Rv2374c (hrcA)
WT vs T0: DE   MU vs T0: not DE
Expression level
5000
4000
3000
Time (h)
0
6
12
24
colour
MU
WT

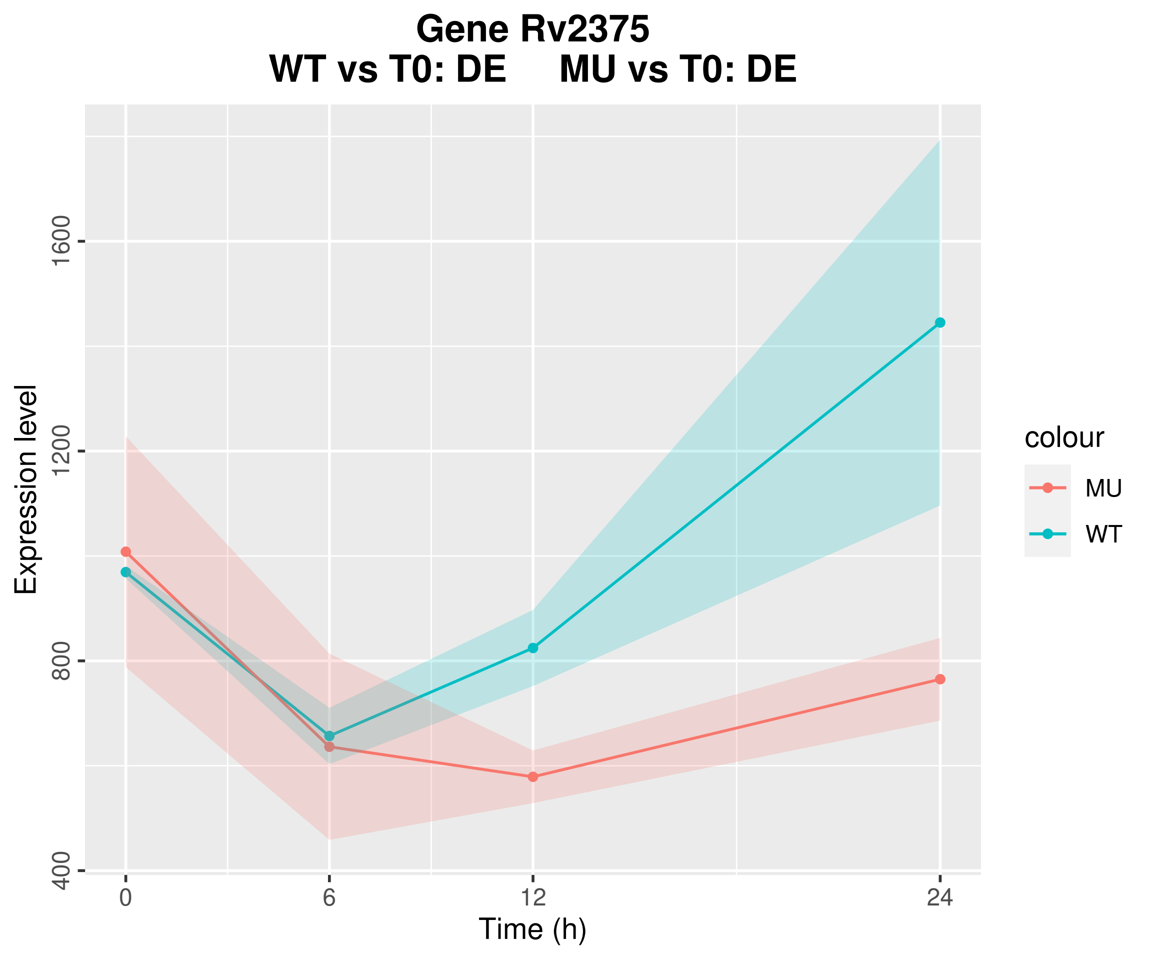

Gene Rv2375
WT vs T0: DE MU vs T0: DE
Expression level
400
800
1200
1600
0
6
12
24
Time (h)
colour
MU
WT

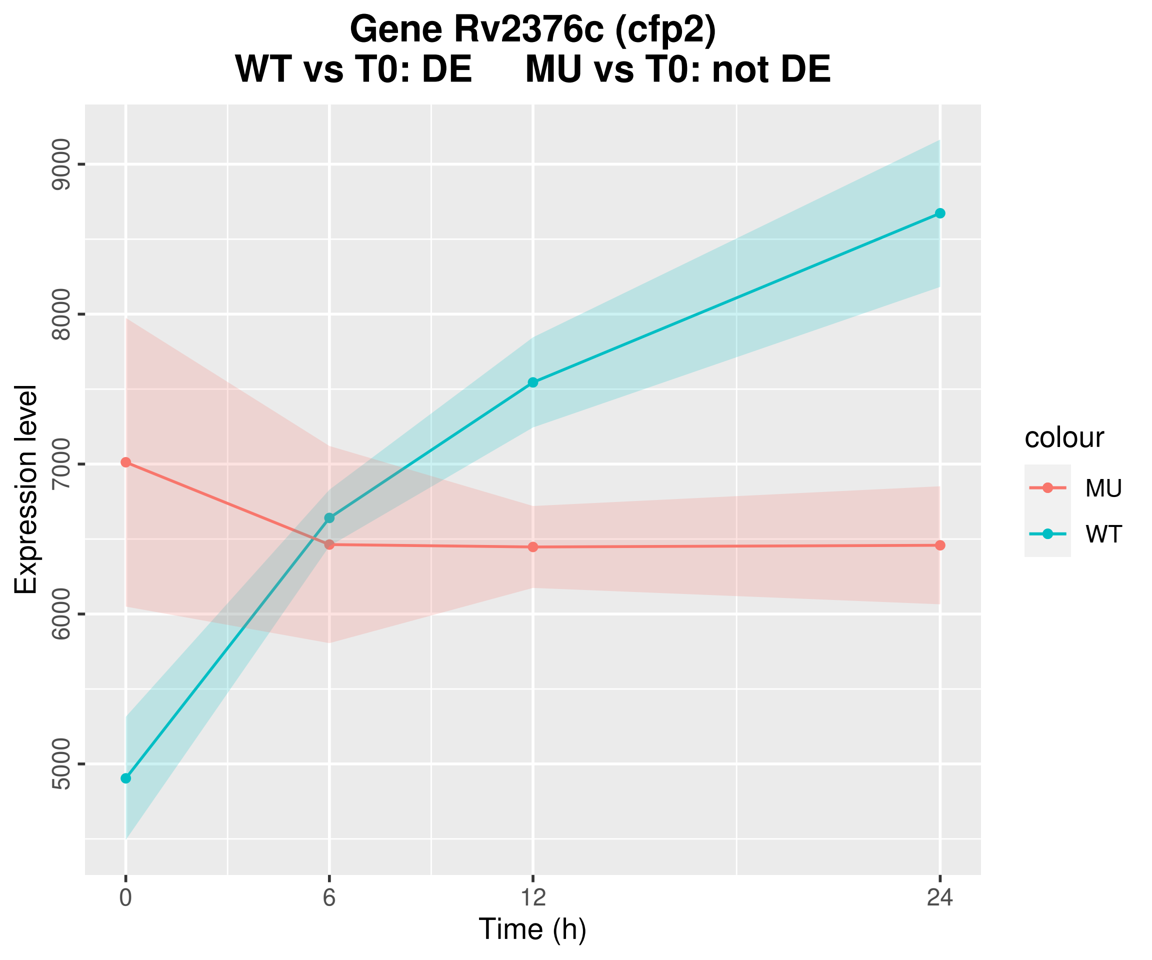
Gene Rv2376c (cfp2)
WT vs T0: DE   MU vs T0: not DE
Expression level
9000
8000
7000
6000
5000
0
6
12
24
Time (h)
colour
MU
WT

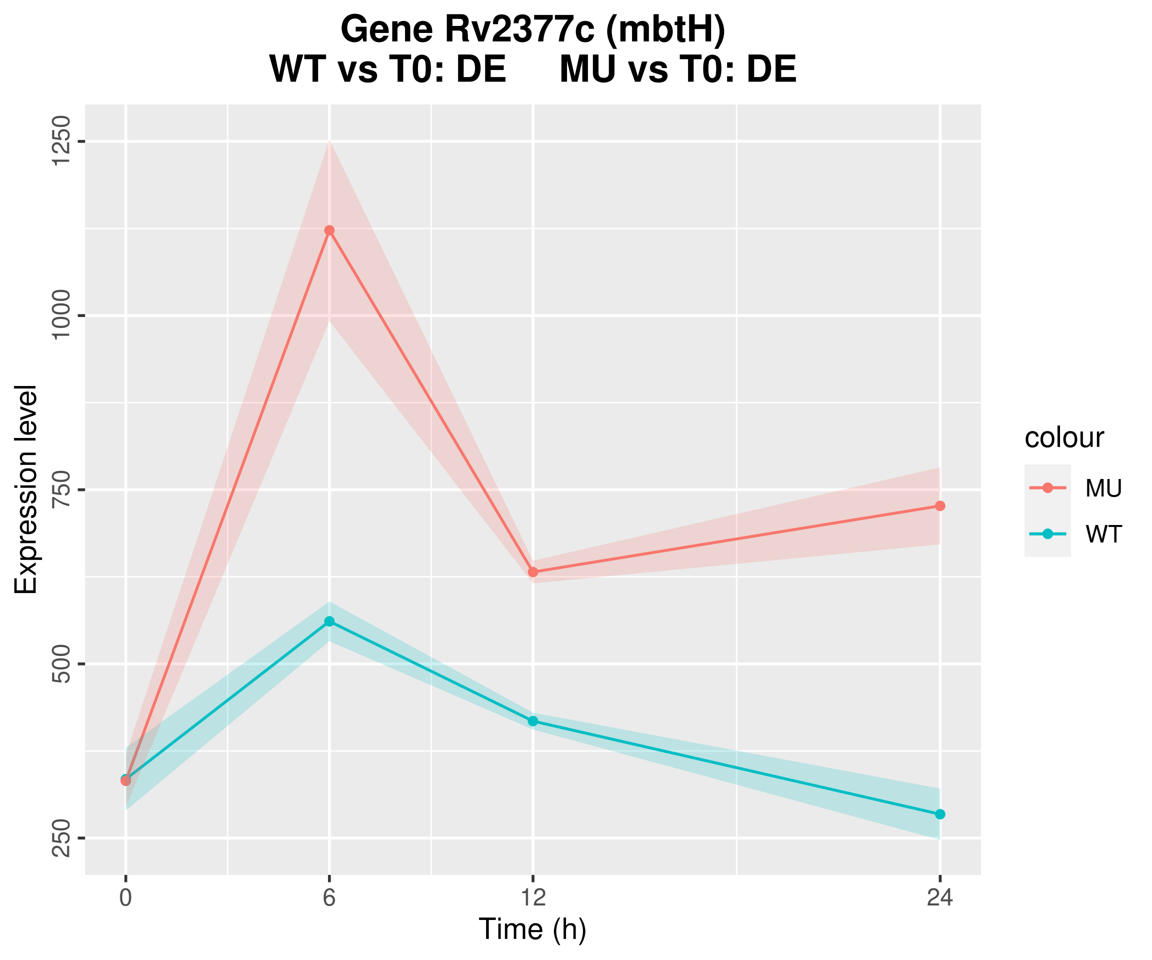

Gene Rv2377c (mbtH)
WT vs T0: DE MU vs T0: DE
Expression level
1250
1000
750
500
250
0
6
12
24
Time (h)
colour
MU
WT

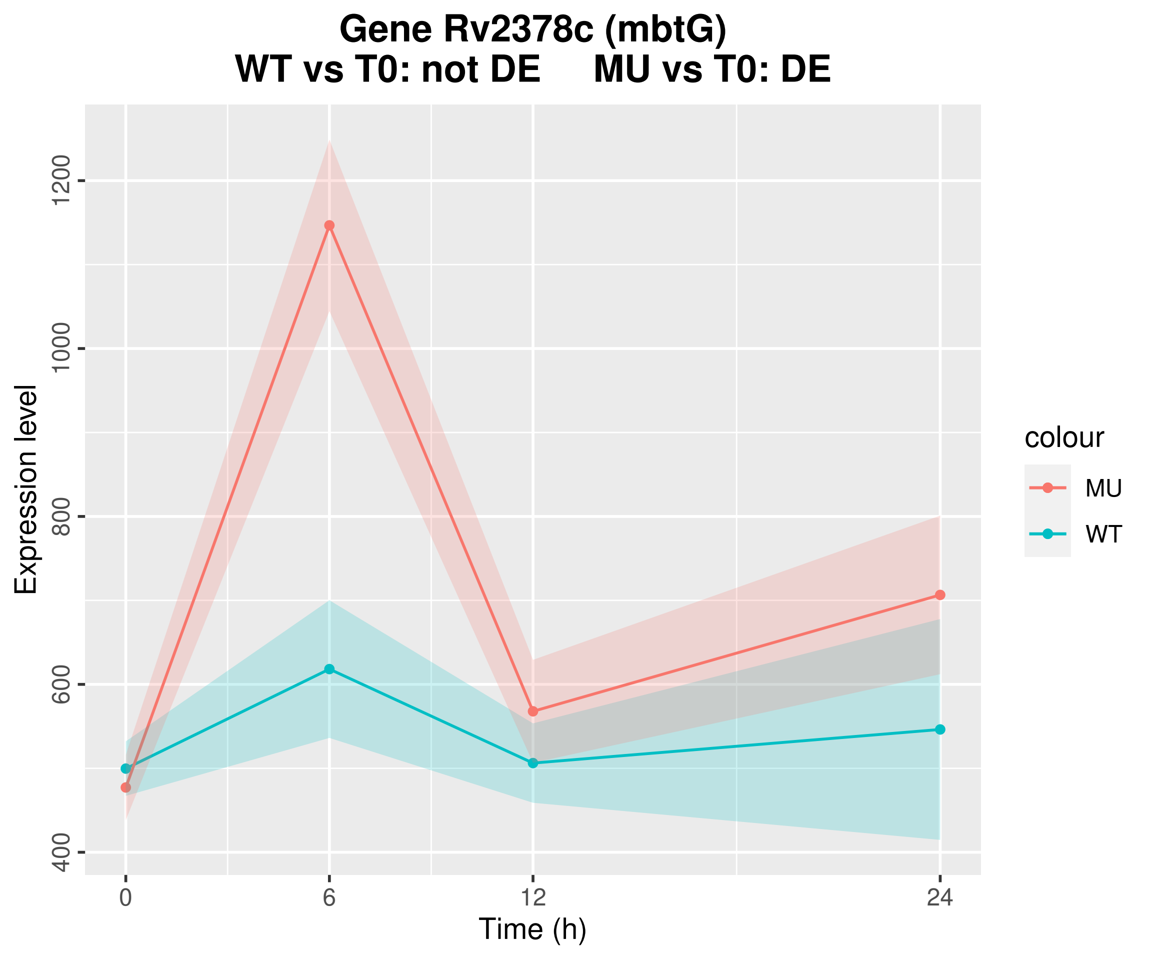
Gene Rv2378c (mbtG)
WT vs T0: not DE   MU vs T0: DE
Expression level
1200
1000
800
600
400
0
6
12
24
Time (h)
colour
MU
WT

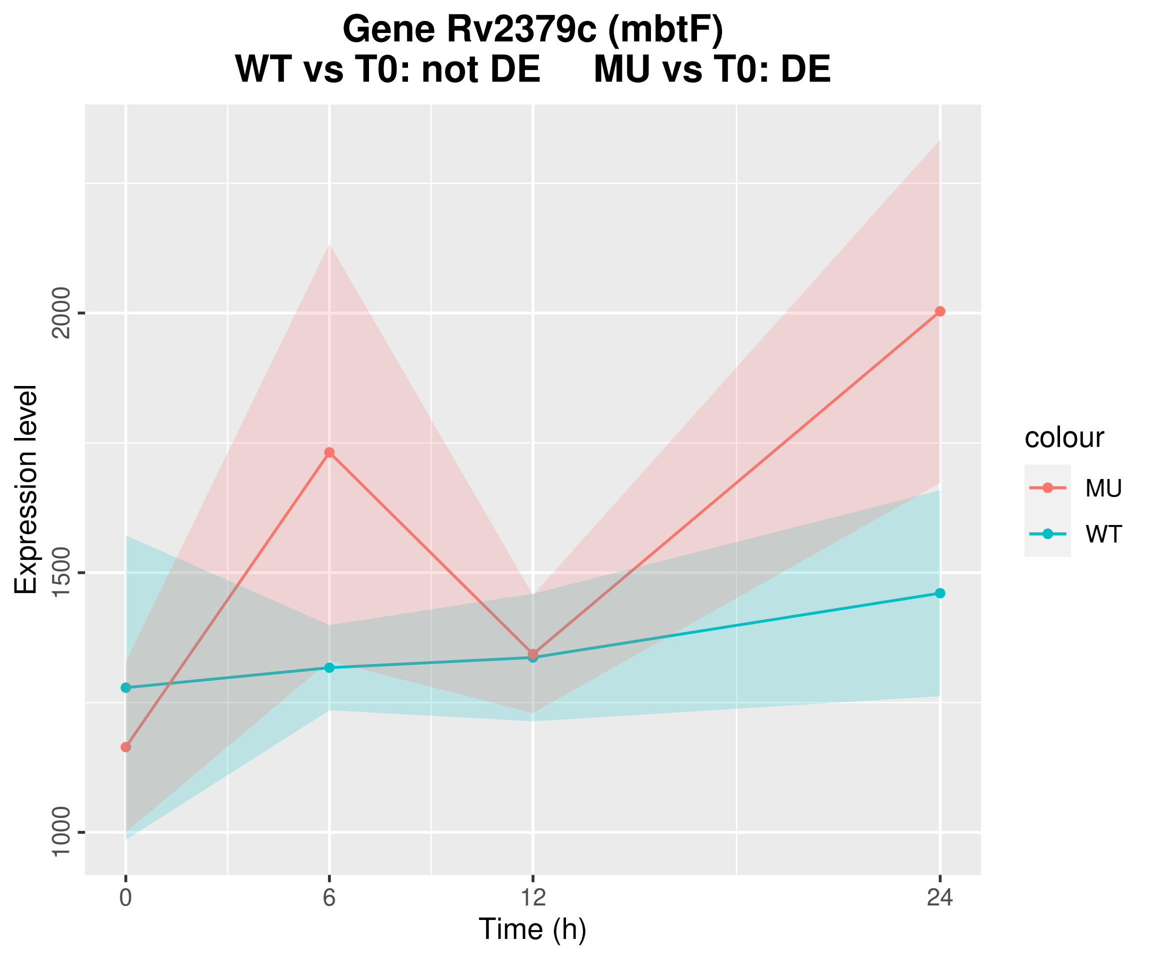

Gene Rv2379c (mbtF)
WT vs T0: not DE   MU vs T0: DE
Expression level
2000
1500
1000
0
6
12
24
Time (h)
colour
MU
WT

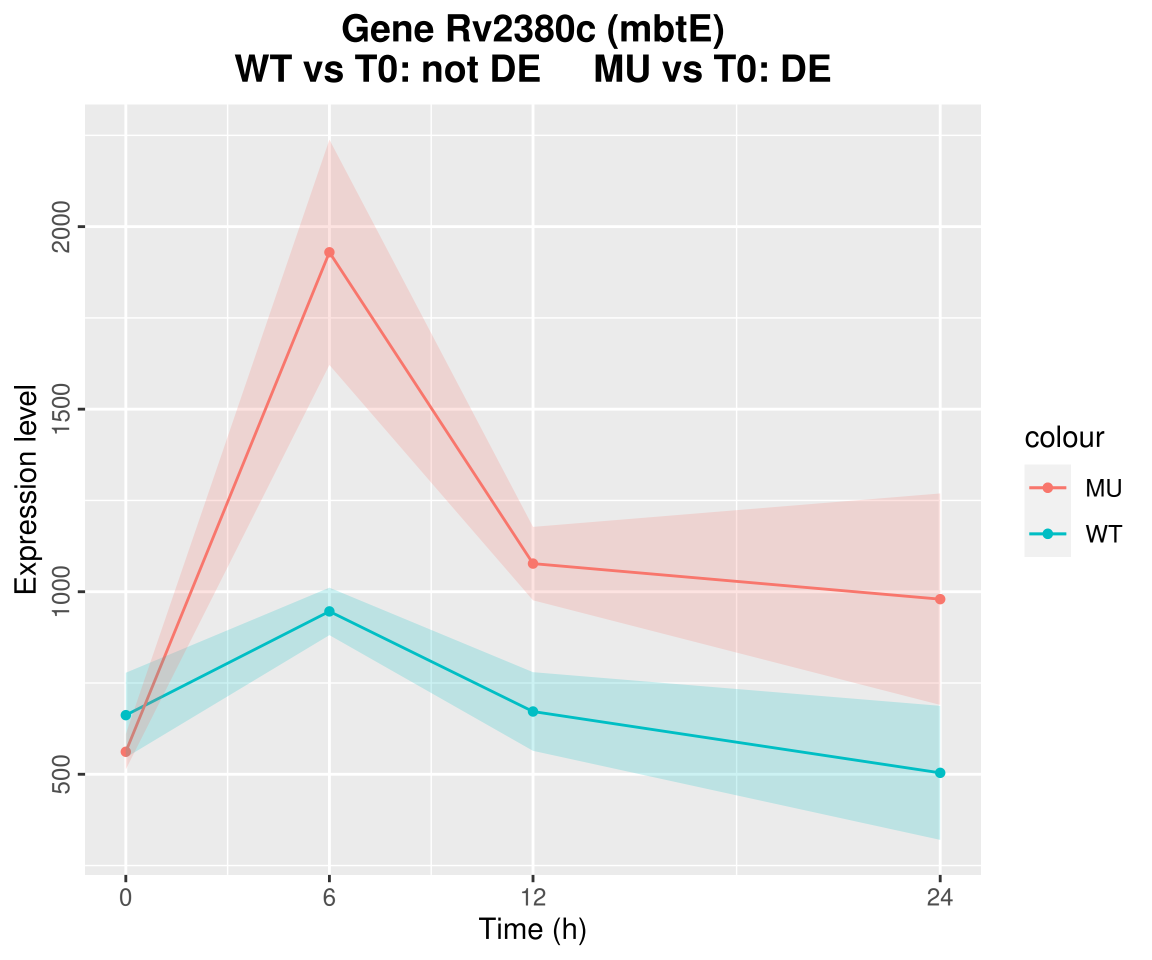
Gene Rv2380c (mbtE)
WT vs T0: not DE MU vs T0: DE
Expression level
2000
1500
1000
500
0
6
12
24
Time (h)
colour
MU
WT

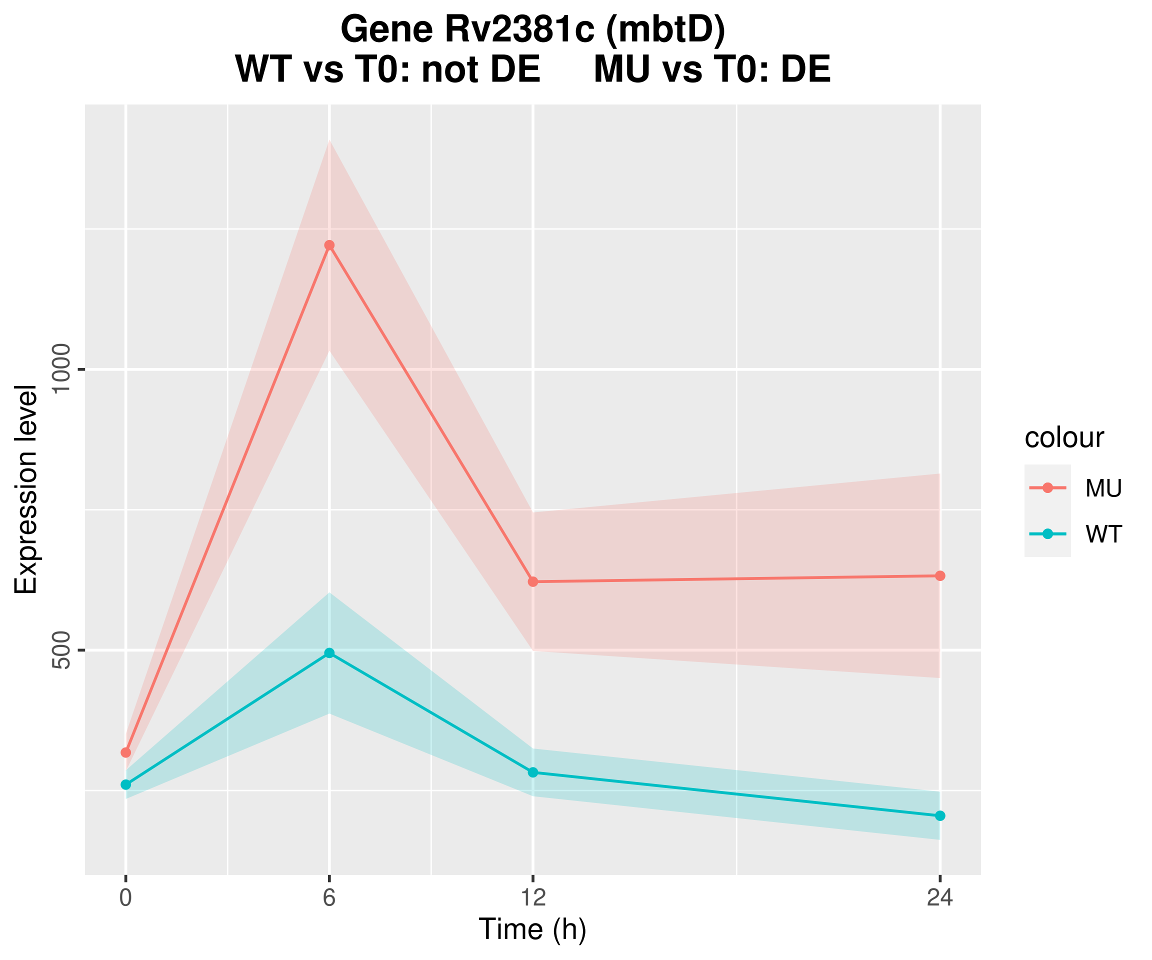

Gene Rv2381c (mbtD)
WT vs T0: not DE   MU vs T0: DE
Expression level
1000
500
0
6
12
24
Time (h)
colour
MU
WT

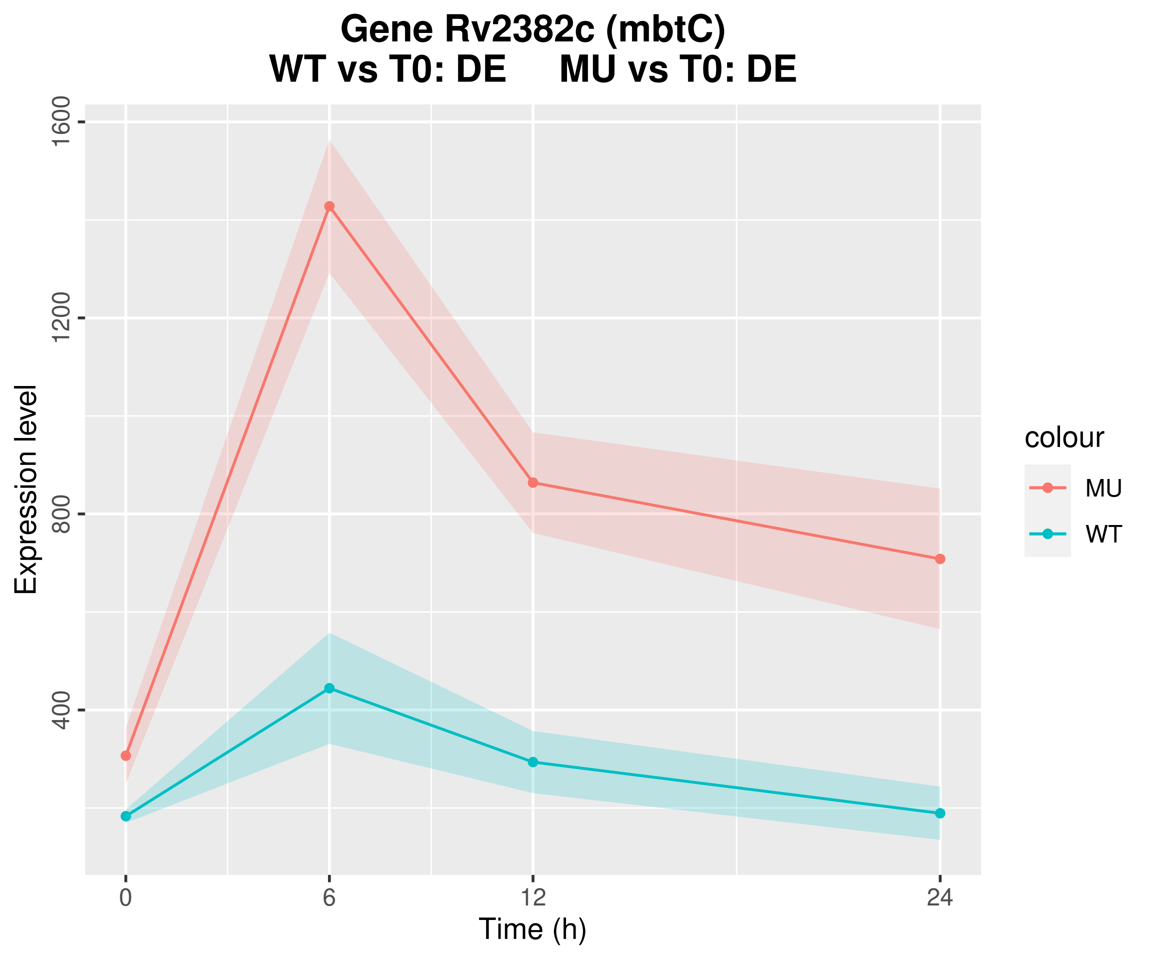

Gene Rv2382c (mbtC)
WT vs T0: DE MU vs T0: DE
1600
1200
800
400
Expression level
0
6
12
24
Time (h)
colour
MU
WT

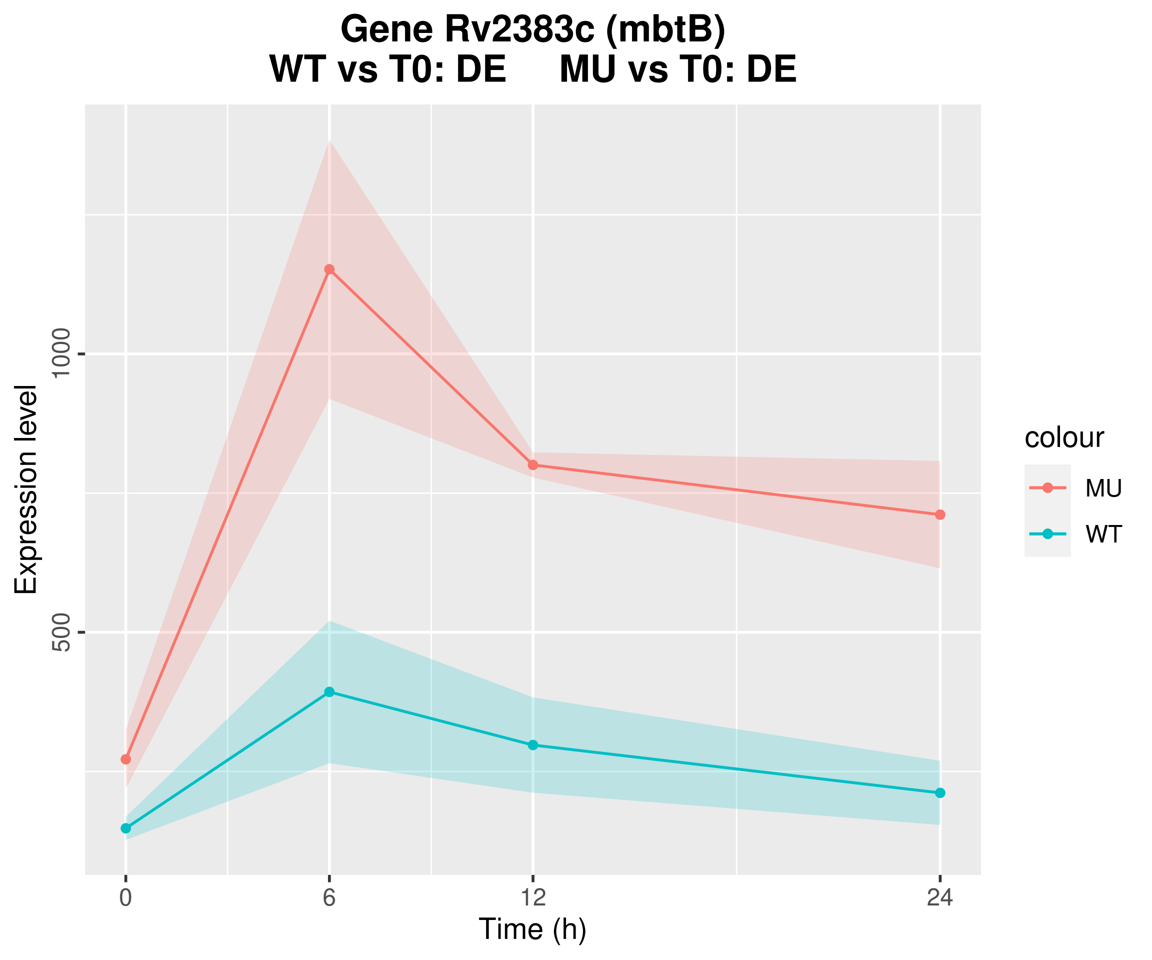

Gene Rv2383c (mbtB)
WT vs T0: DE MU vs T0: DE
Expression level
1000
500
0
6
12
24
Time (h)
colour
MU
WT

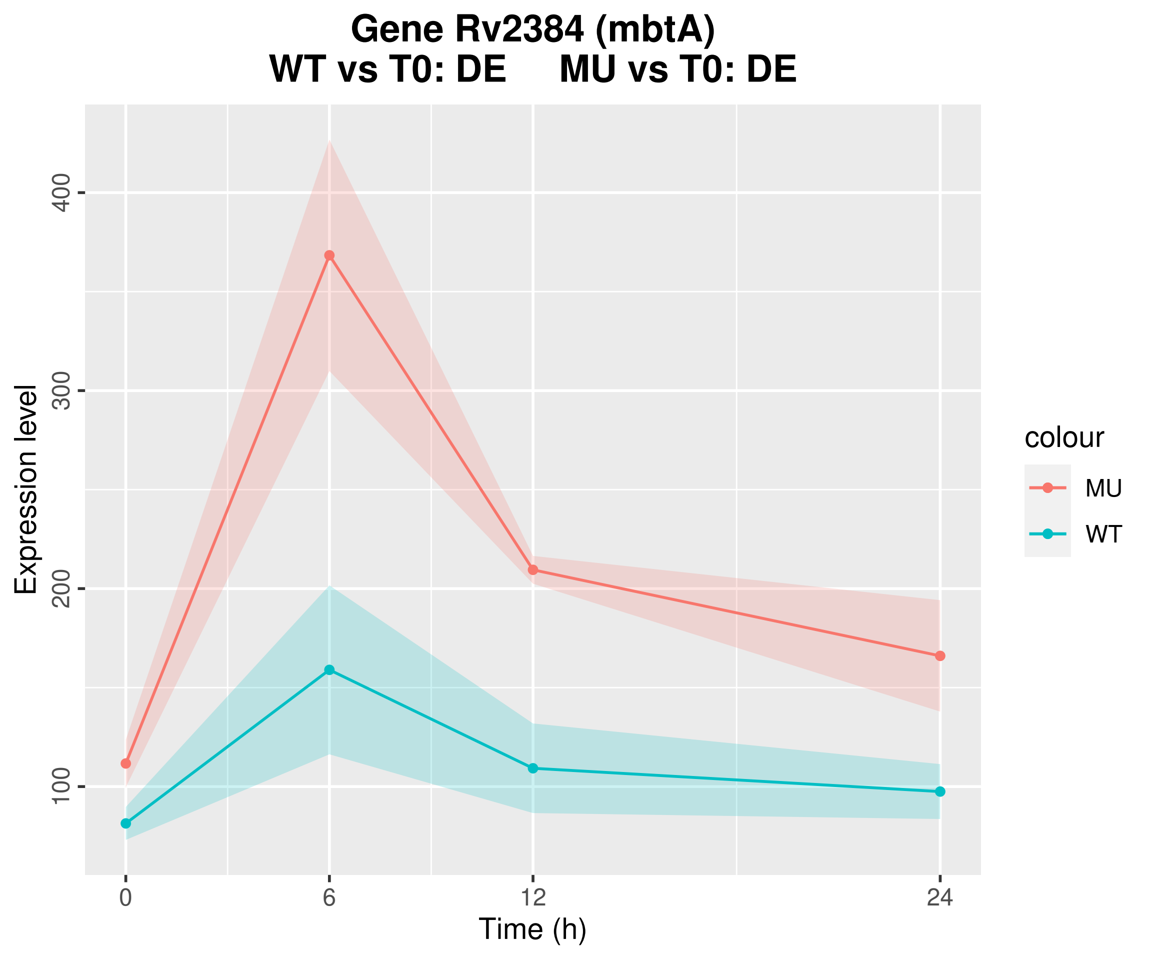

Gene Rv2384 (mbtA)
WT vs T0: DE   MU vs T0: DE
Expression level
400
300
200
100
0
6
12
24
Time (h)
colour
MU
WT

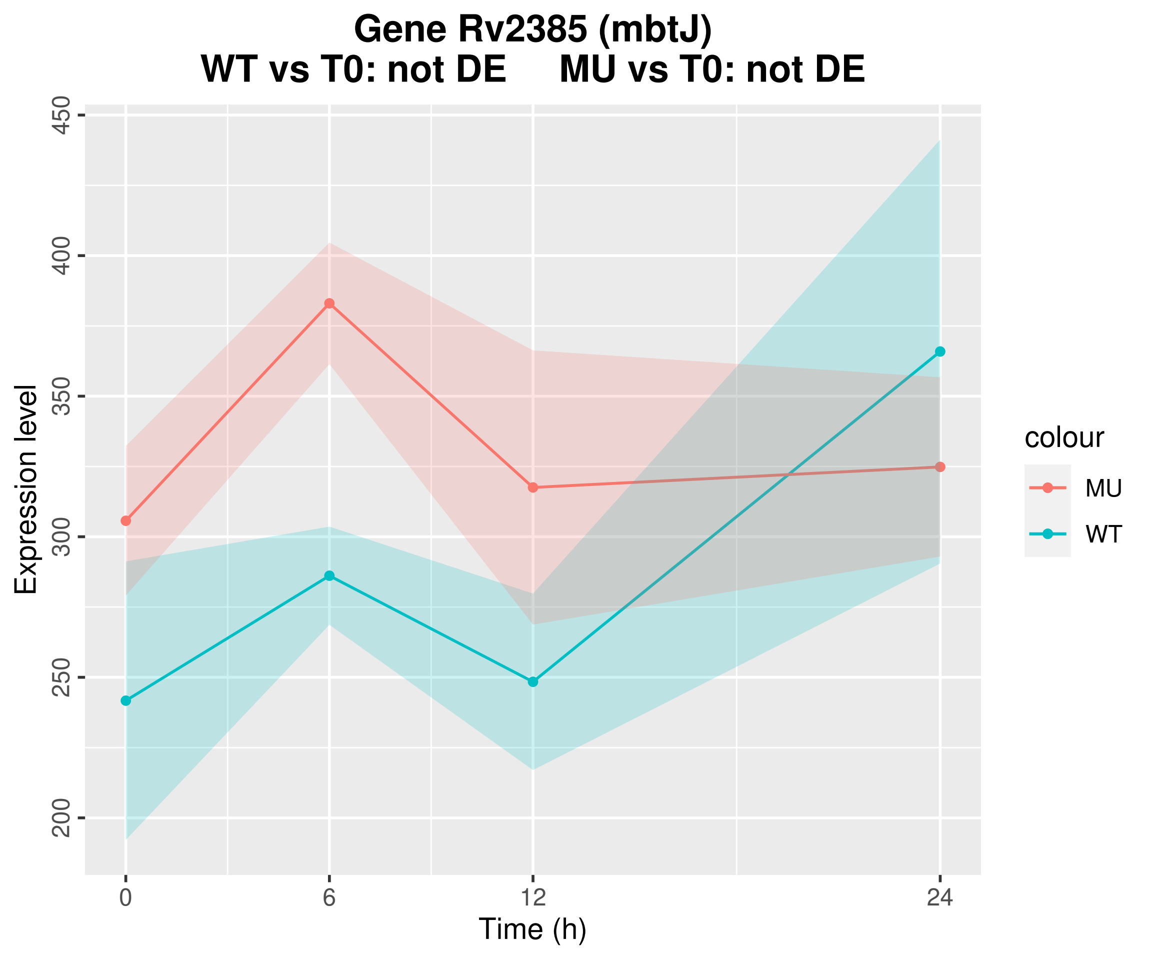
Gene Rv2385 (mbtJ)
WT vs T0: not DE   MU vs T0: not DE
Expression level
450
400
350
300
250
200
0
6
12
24
Time (h)
colour
MU
WT

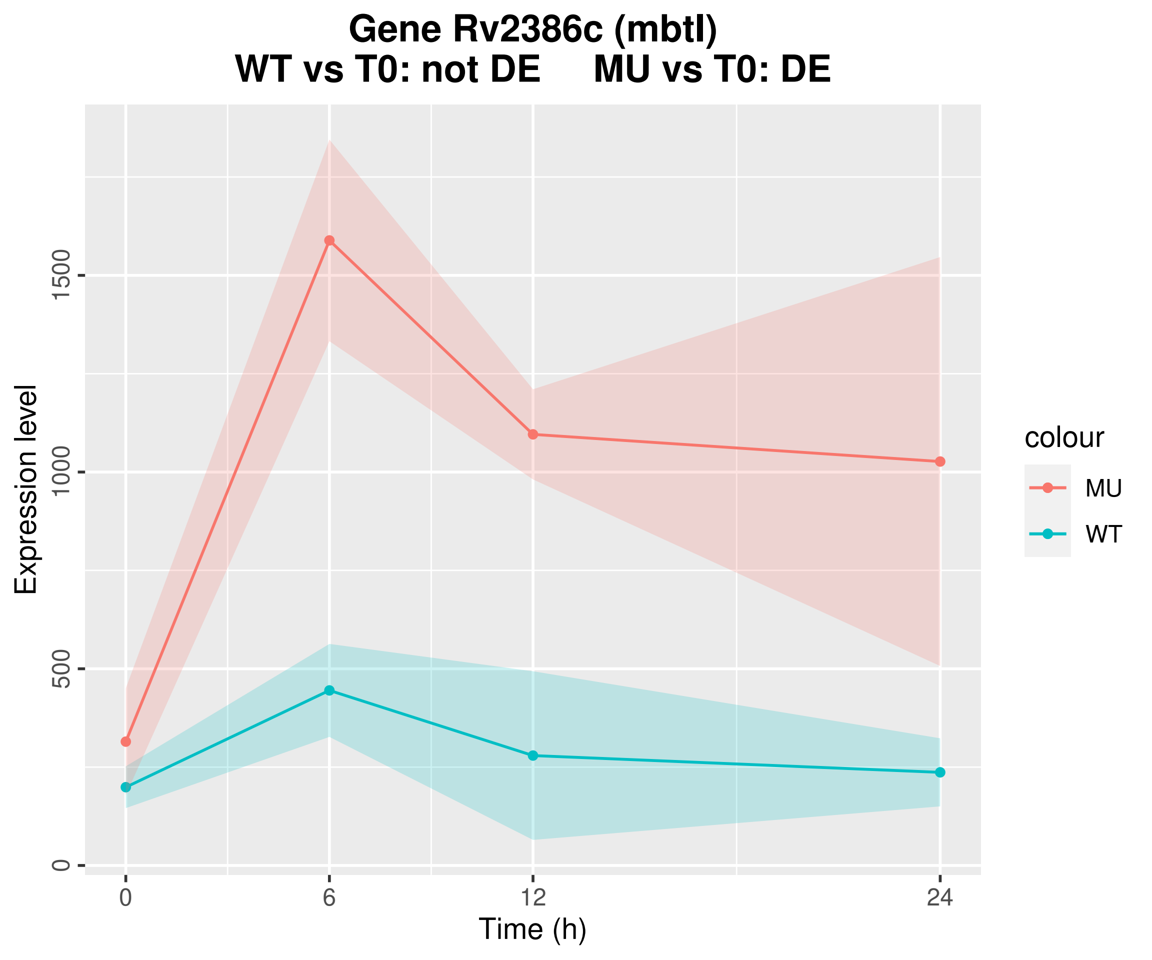

Gene Rv2386c (mbtI)
WT vs T0: not DE   MU vs T0: DE
Expression level
0
500
1000
1500
0
6
12
24
Time (h)
colour
MU
WT

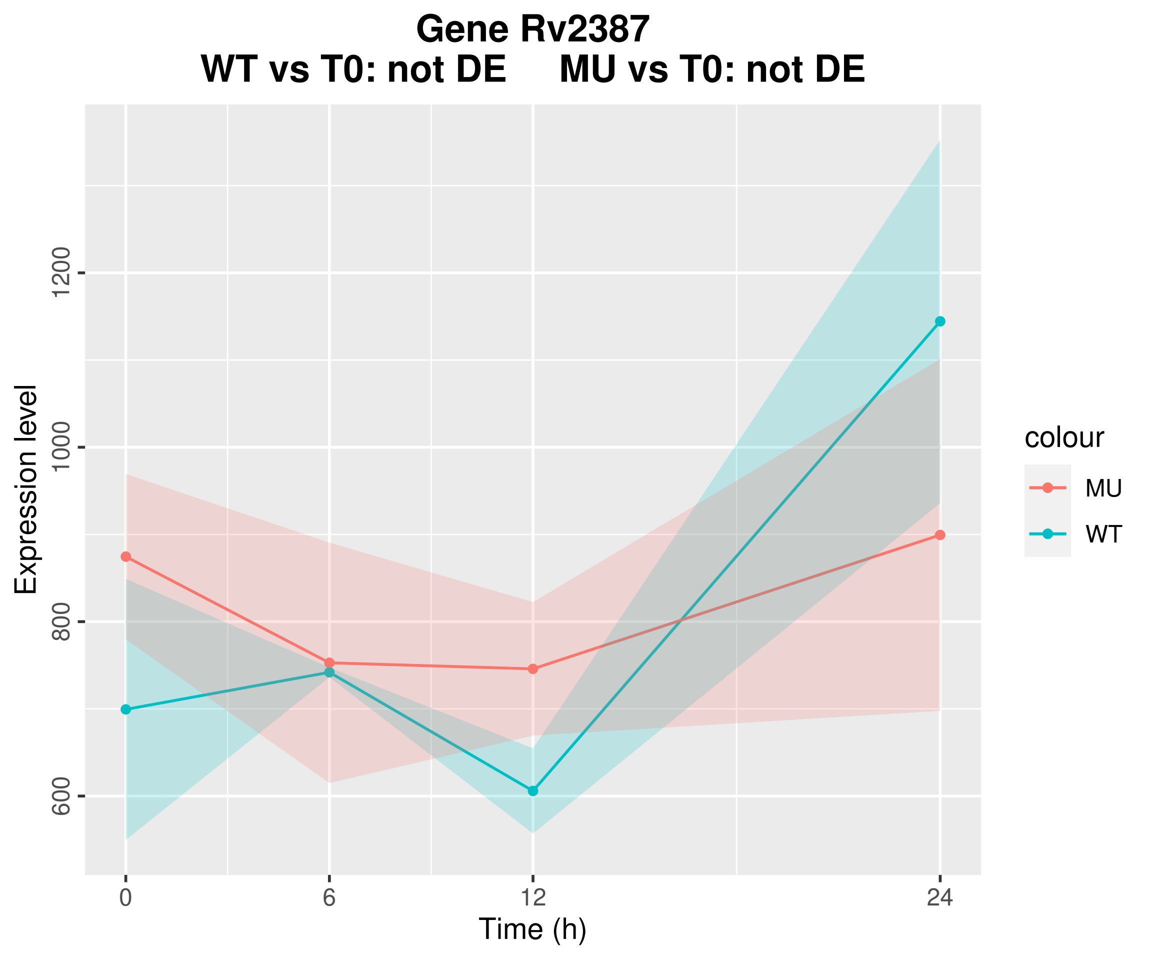

Gene Rv2387
WT vs T0: not DE   MU vs T0: not DE
Expression level
1200
1000
800
600
Time (h)
0
6
12
24
colour
MU
WT

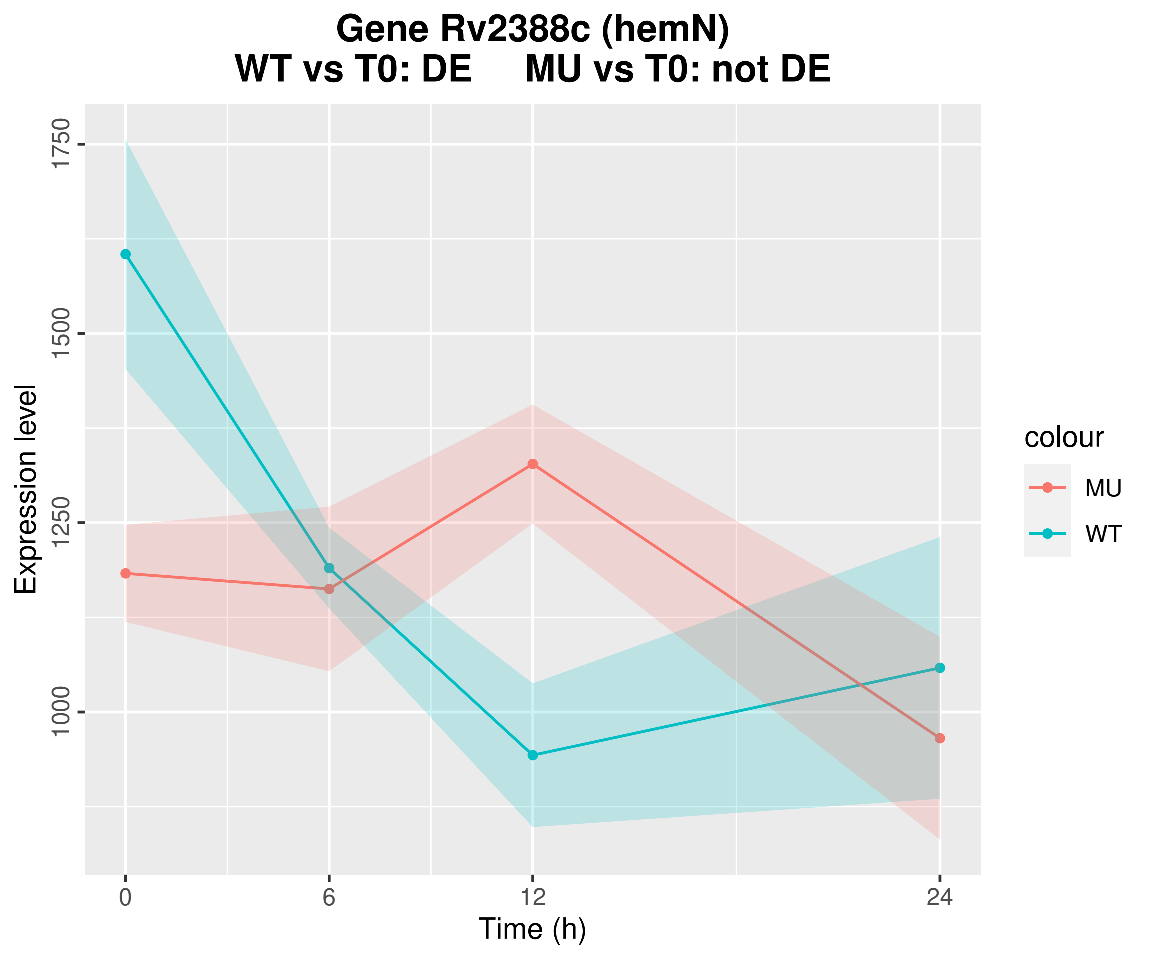

Gene Rv2388c (hemN)
WT vs T0: DE   MU vs T0: not DE
Expression level
1750
1500
1250
1000
0
6
12
24
Time (h)
colour
MU
WT

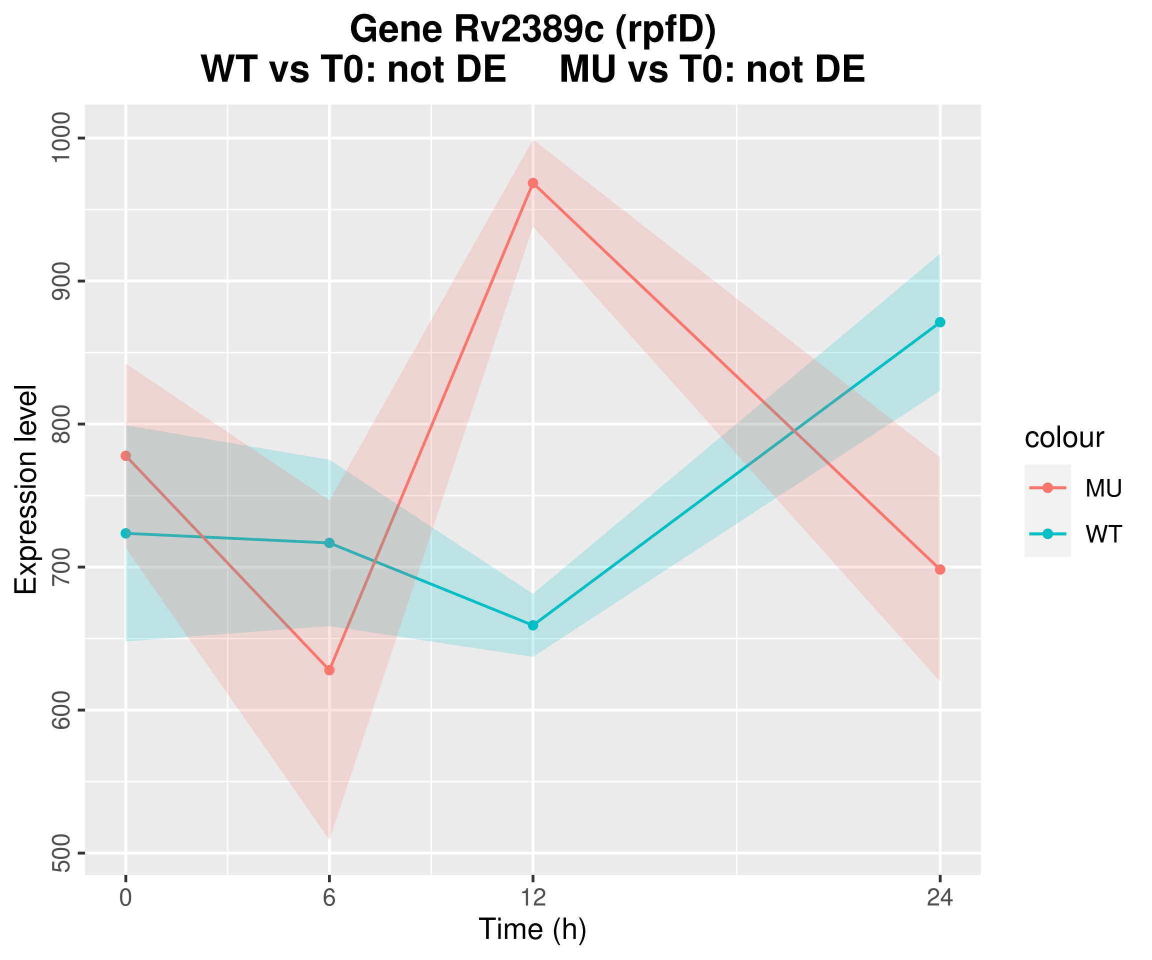
Gene Rv2389c (rpfD)
WT vs T0: not DE   MU vs T0: not DE
Expression level
1000
900
800
700
600
500
0
6
12
24
Time (h)
colour
MU
WT

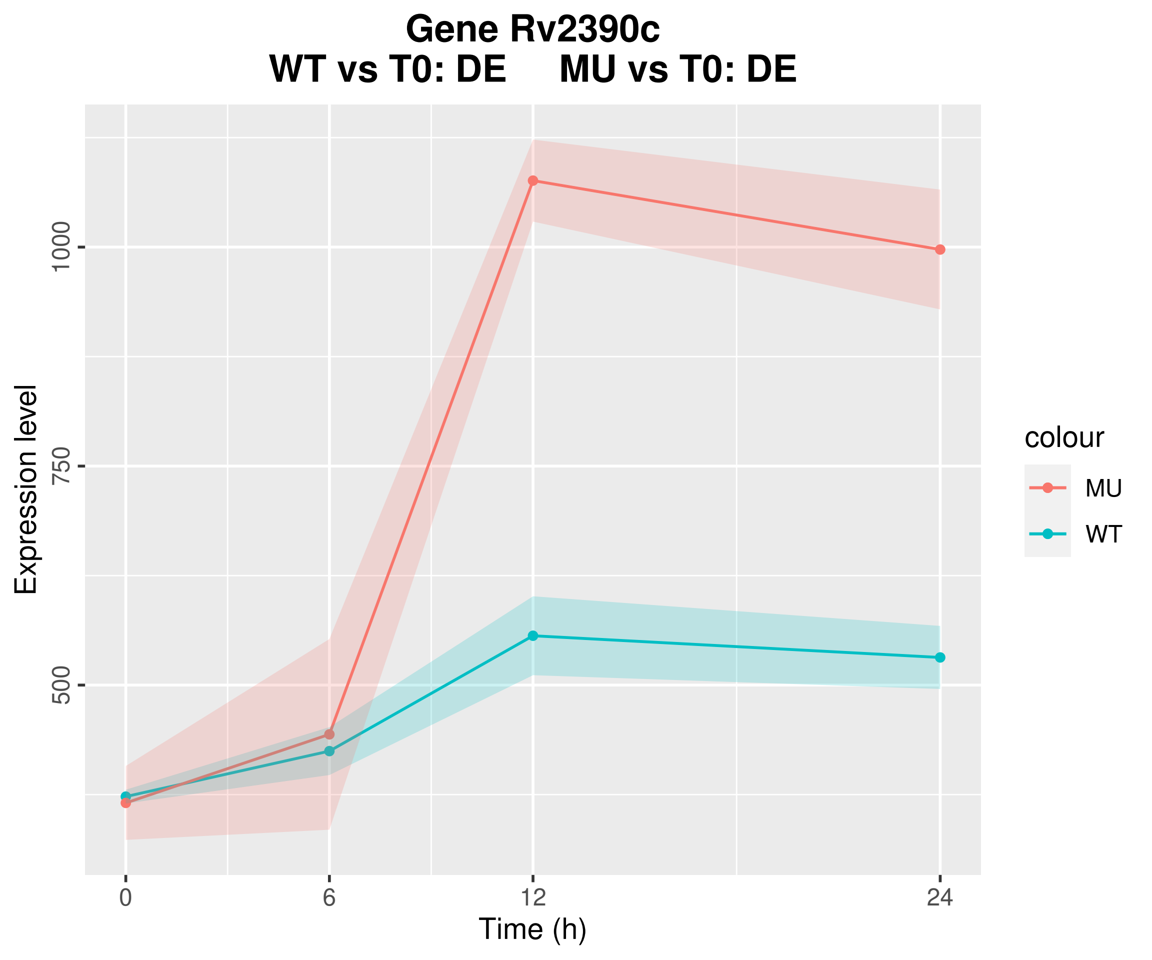
Gene Rv2390c
WT vs T0: DE MU vs T0: DE
Expression level
1000
750
500
0
6
12
24
Time (h)
colour
MU
WT

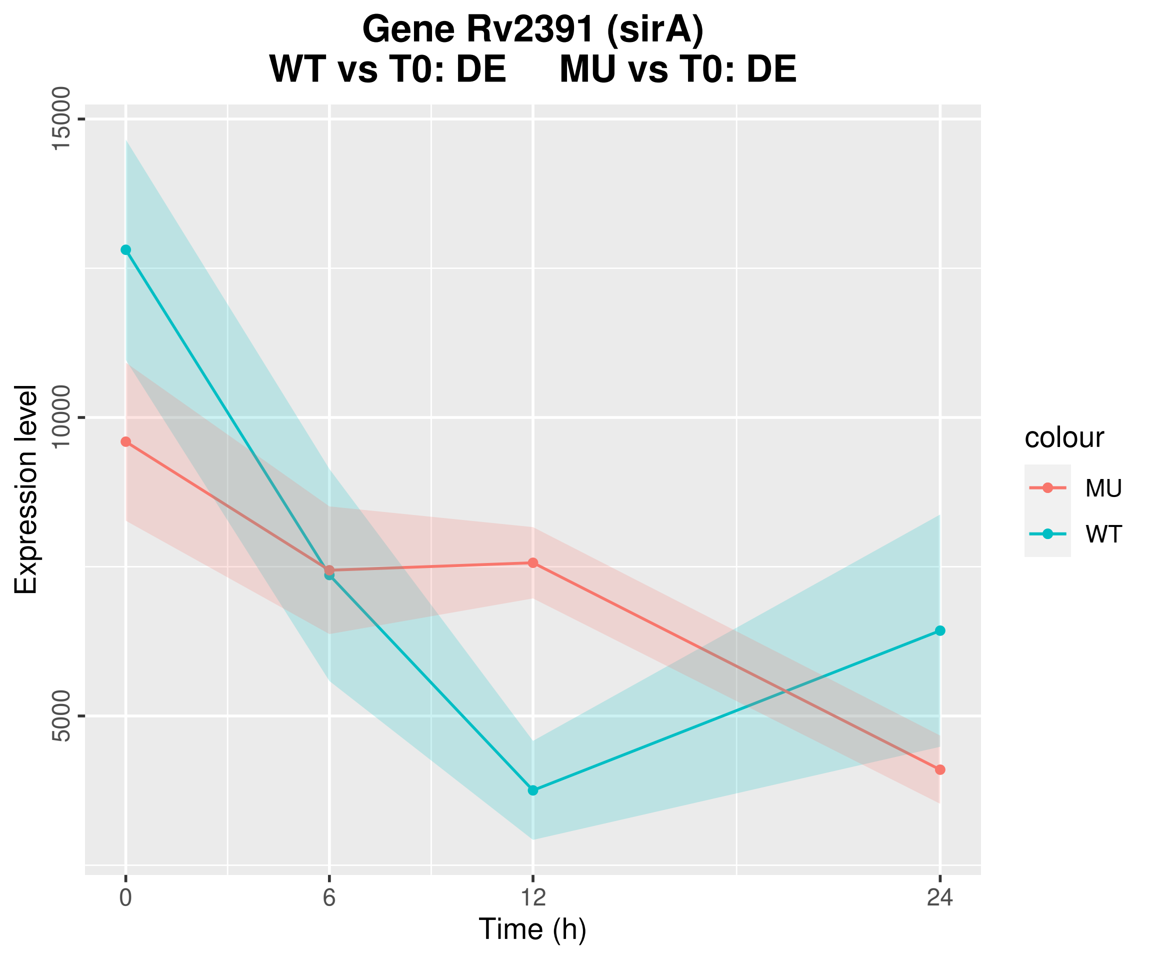

Gene Rv2391 (sirA)
WT vs T0: DE MU vs T0: DE
15000
10000
5000
Expression level
0
6
12
24
Time (h)
colour
MU
WT

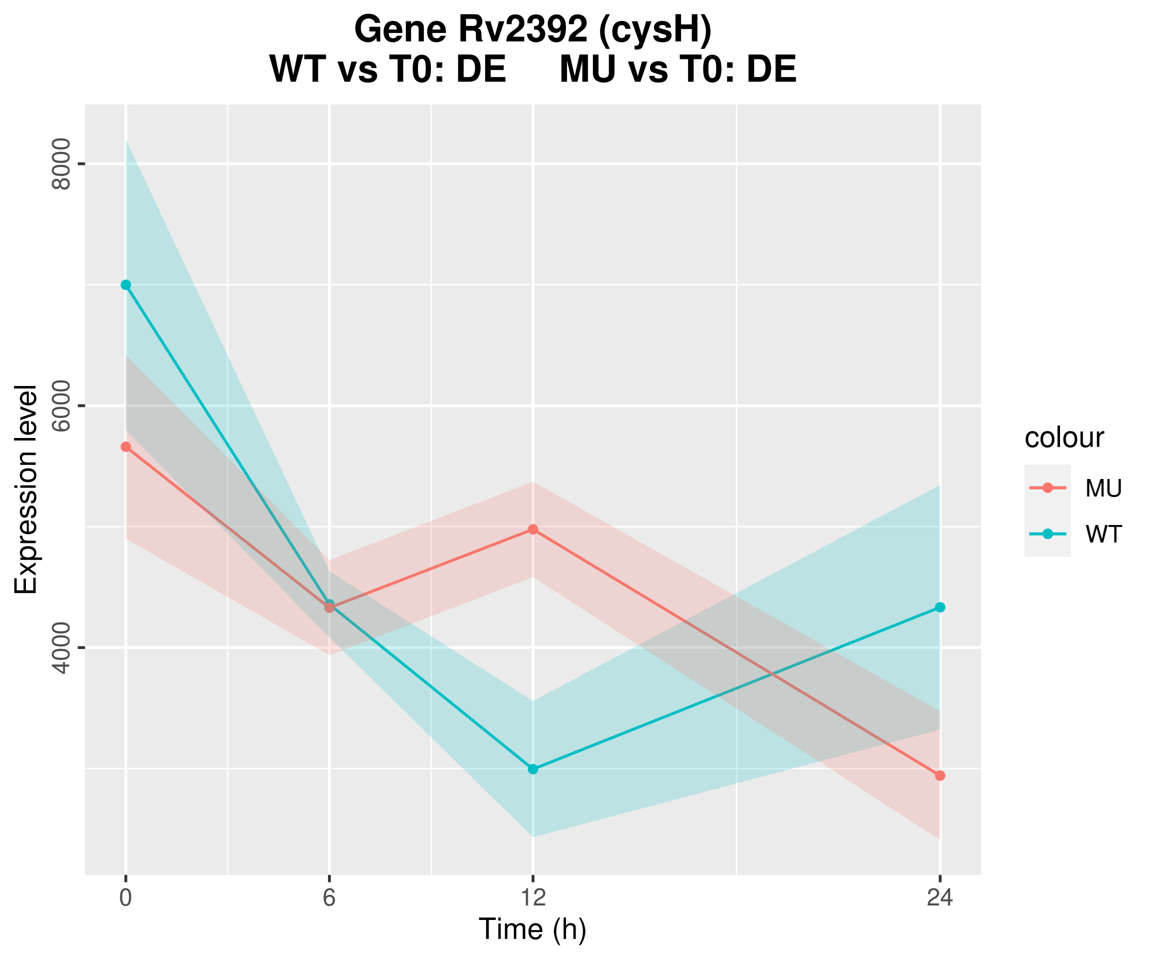
Gene Rv2392 (cysH)
WT vs T0: DE MU vs T0: DE
Expression level
8000
6000
4000
0
6
12
24
Time (h)
colour
MU
WT

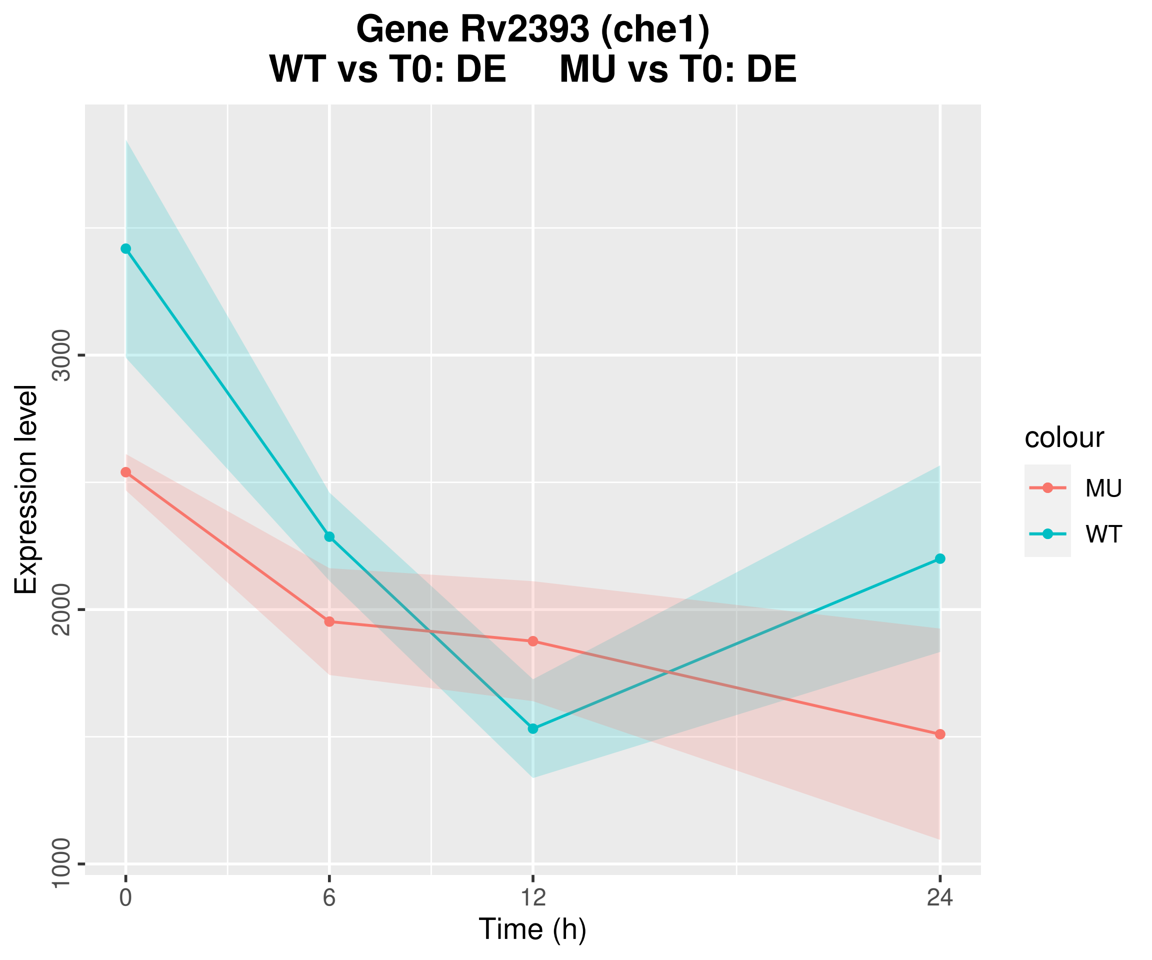

Gene Rv2393 (che1)
WT vs T0: DE   MU vs T0: DE
Expression level
3000
2000
1000
0
6
12
24
Time (h)
colour
MU
WT

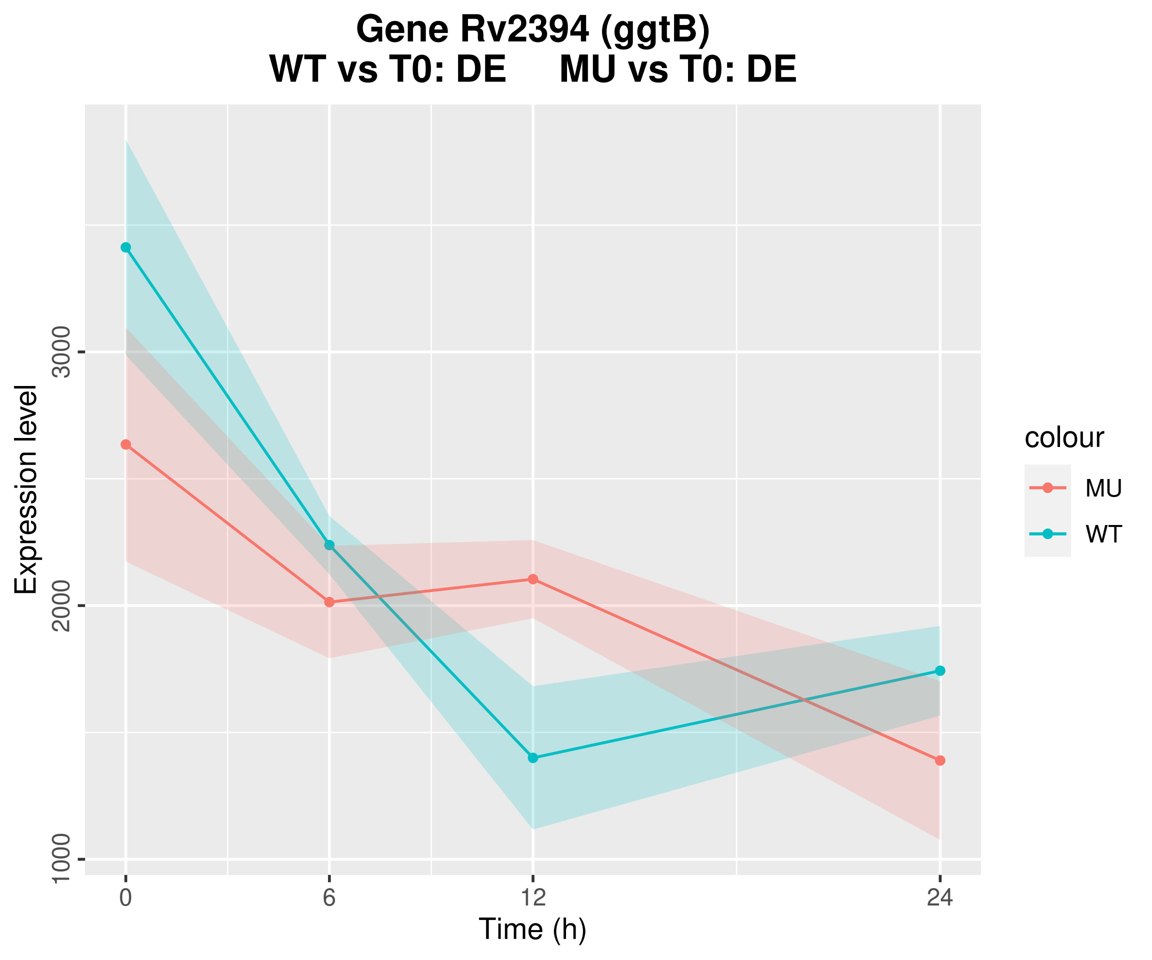
Gene Rv2394 (ggtB)
WT vs T0: DE MU vs T0: DE
Expression level
3000
2000
1000
0
6
12
24
Time (h)
colour
MU
WT

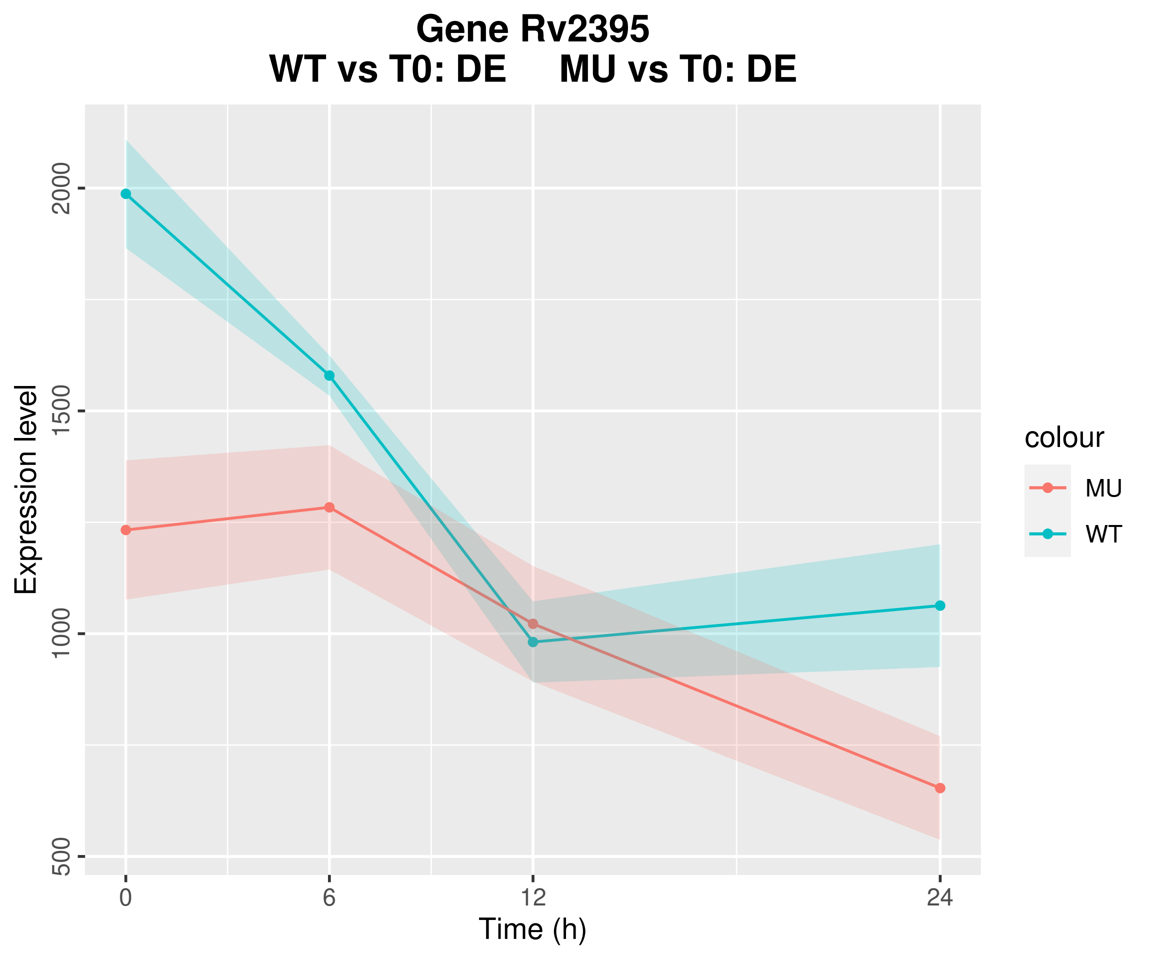
Gene Rv2395
WT vs T0: DE MU vs T0: DE
Expression level
2000
1500
1000
500
0
6
12
24
Time (h)
colour
MU
WT

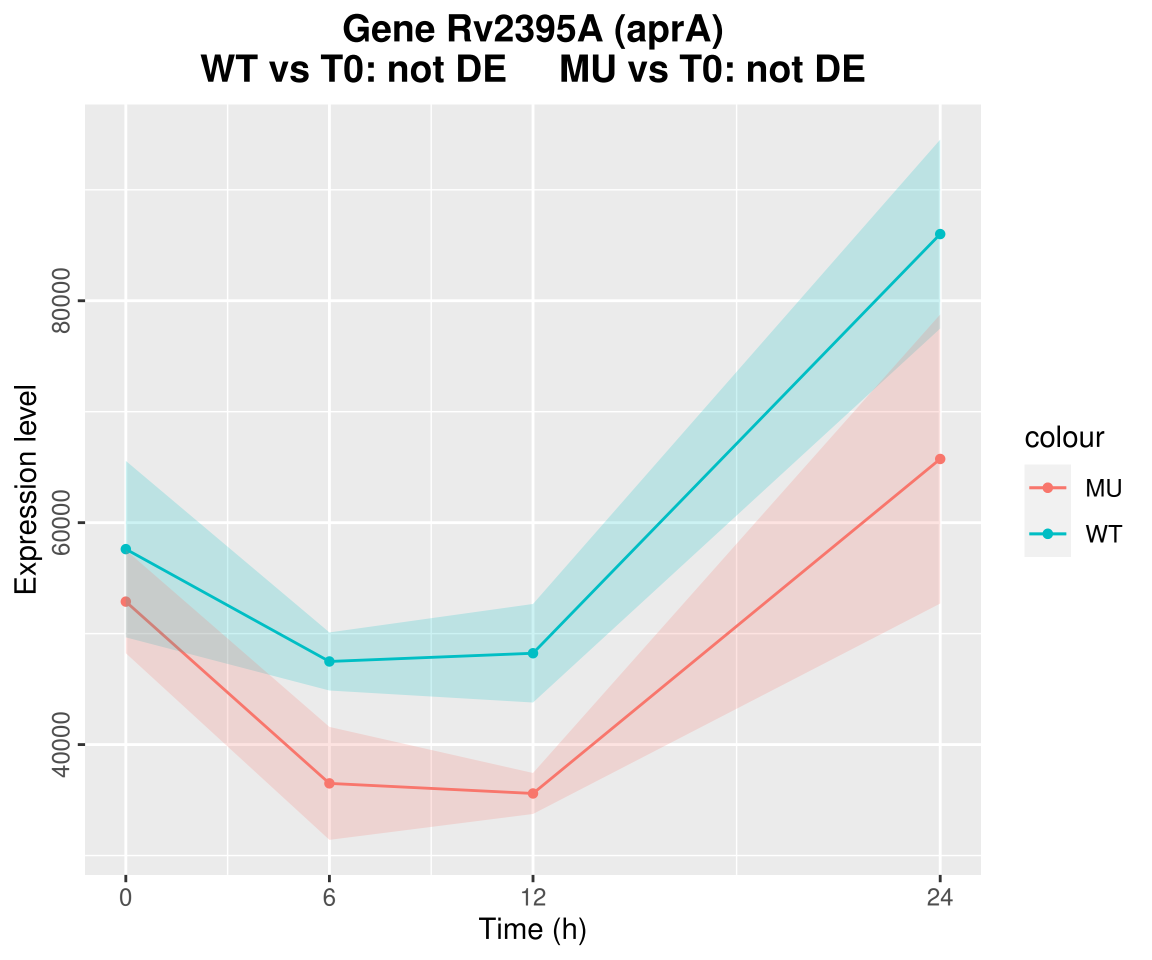
Gene Rv2395A (aprA)
WT vs T0: not DE   MU vs T0: not DE
Expression level
80000
60000
40000
0
6
12
24
Time (h)
colour
MU
WT

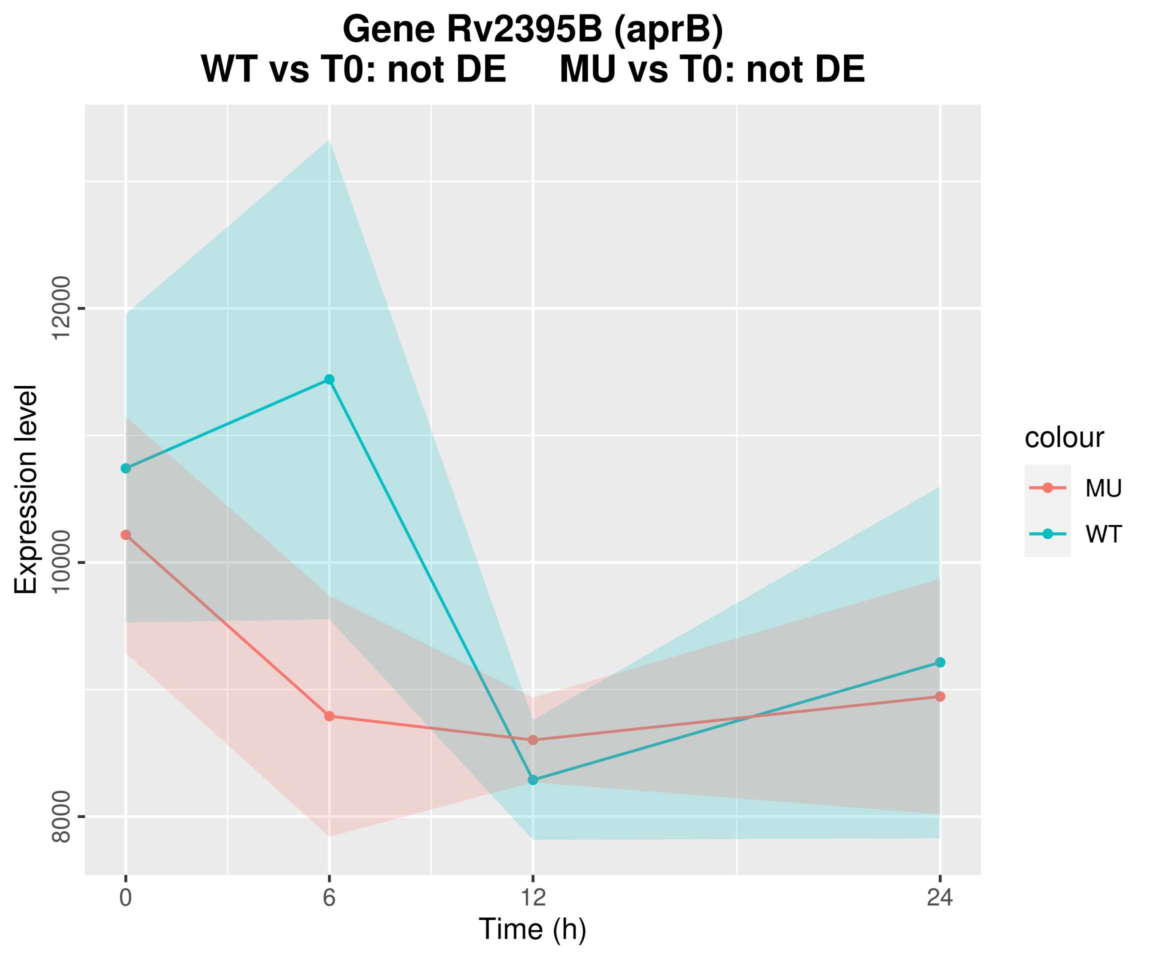

Gene Rv2395B (aprB)
WT vs T0: not DE   MU vs T0: not DE
Expression level
12000
10000
8000
0
6
12
24
Time (h)
colour
MU
WT

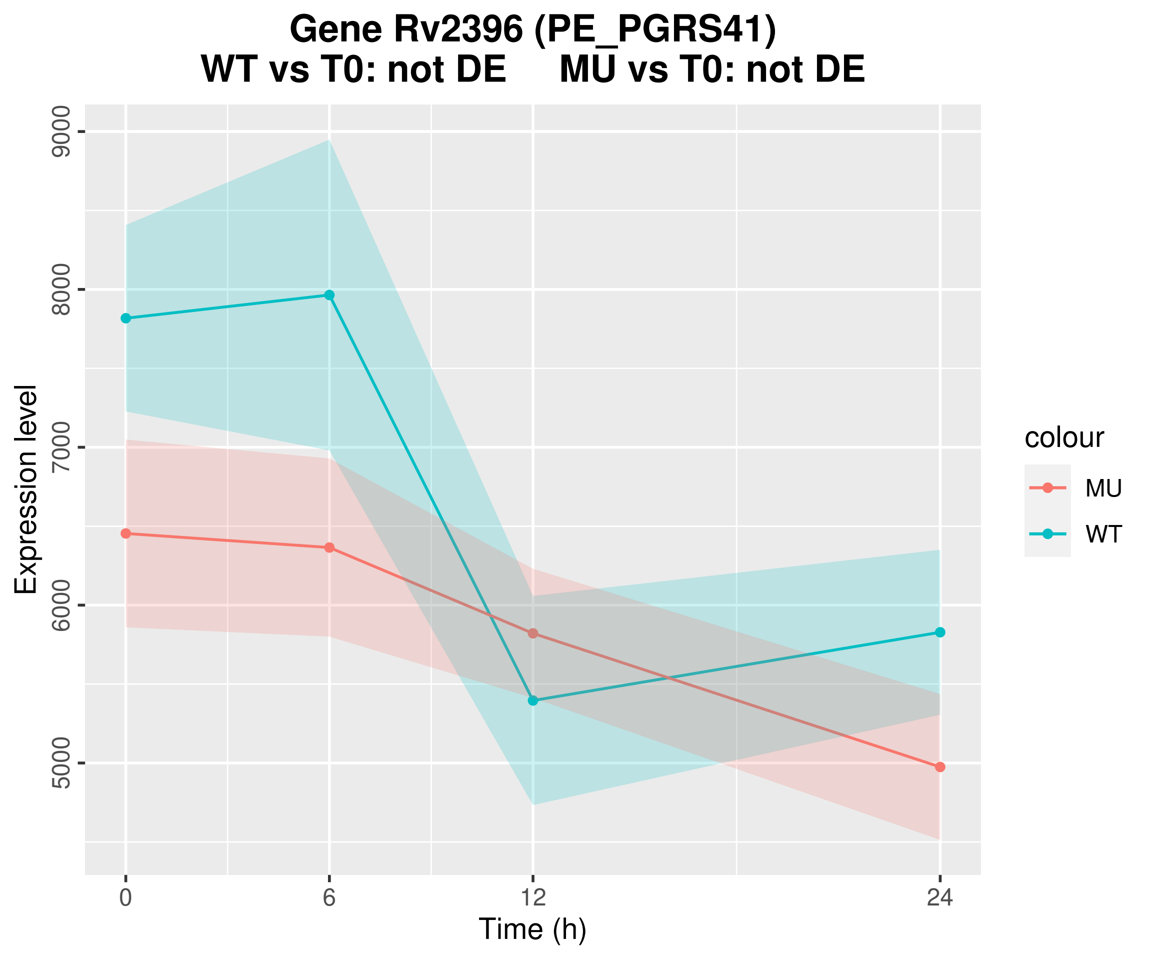

Gene Rv2396 (PE_PGRS41)
WT vs T0: not DE   MU vs T0: not DE
Expression level
9000
8000
7000
6000
5000
0
6
12
24
Time (h)
colour
MU
WT

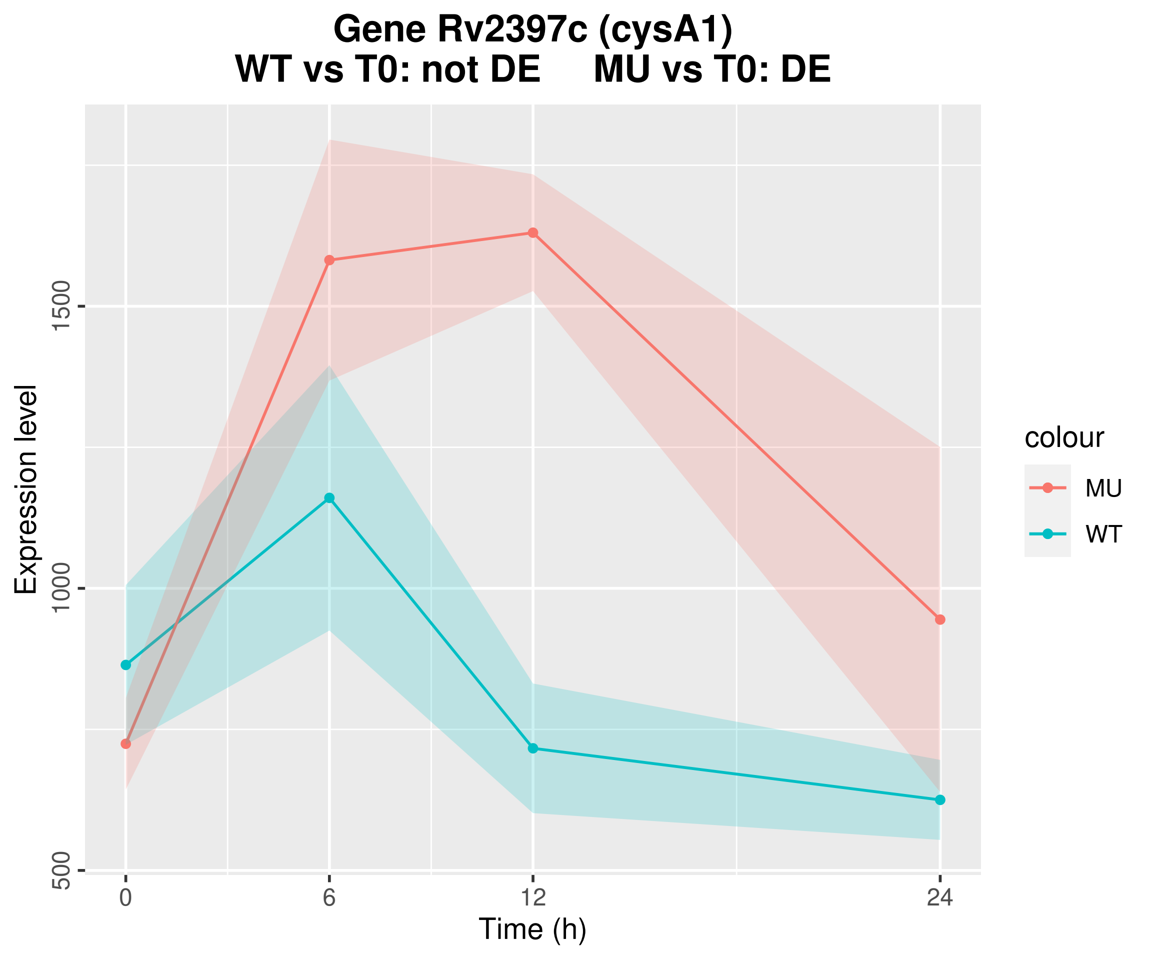

Gene Rv2397c (cysA1)
WT vs T0: not DE   MU vs T0: DE
Expression level
1500
1000
500
0
6
12
24
Time (h)
colour
MU
WT

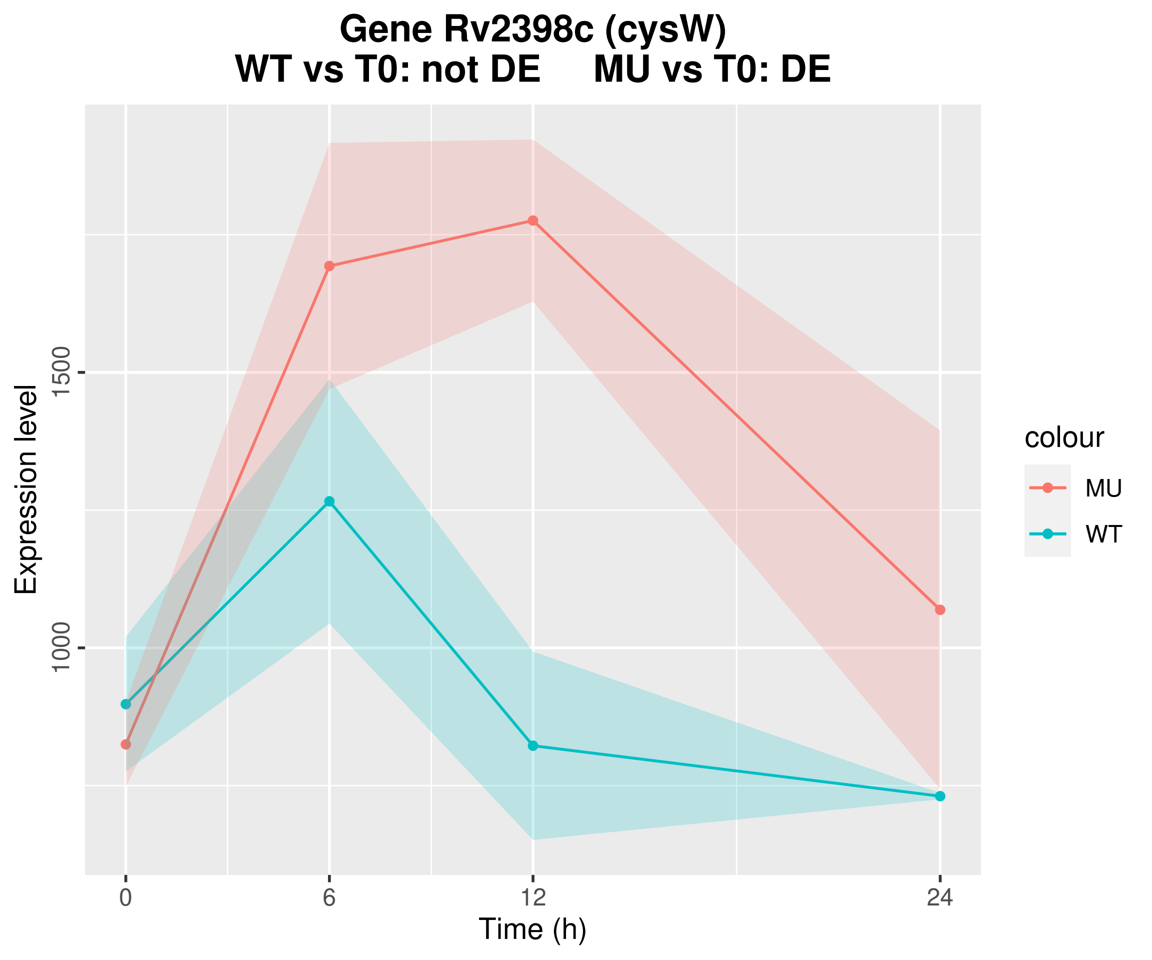
Gene Rv2398c (cysW)
WT vs T0: not DE
MU vs T0: DE
Expression level
1500
1000
0
6
12
24
Time (h)
colour
MU
WT

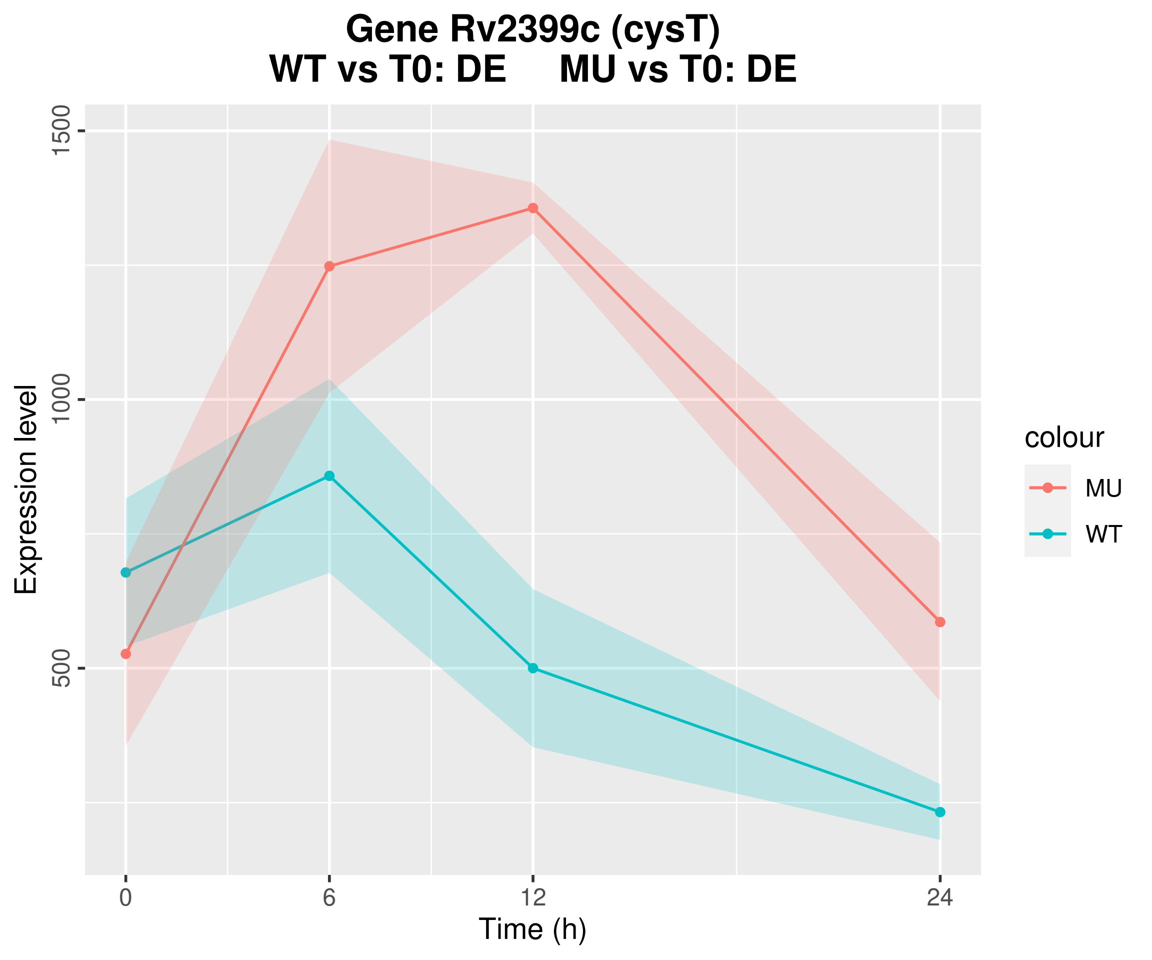
Gene Rv2399c (cysT)
WT vs T0: DE   MU vs T0: DE
1500
1000
500
Expression level
0
6
12
24
Time (h)
colour
MU
WT

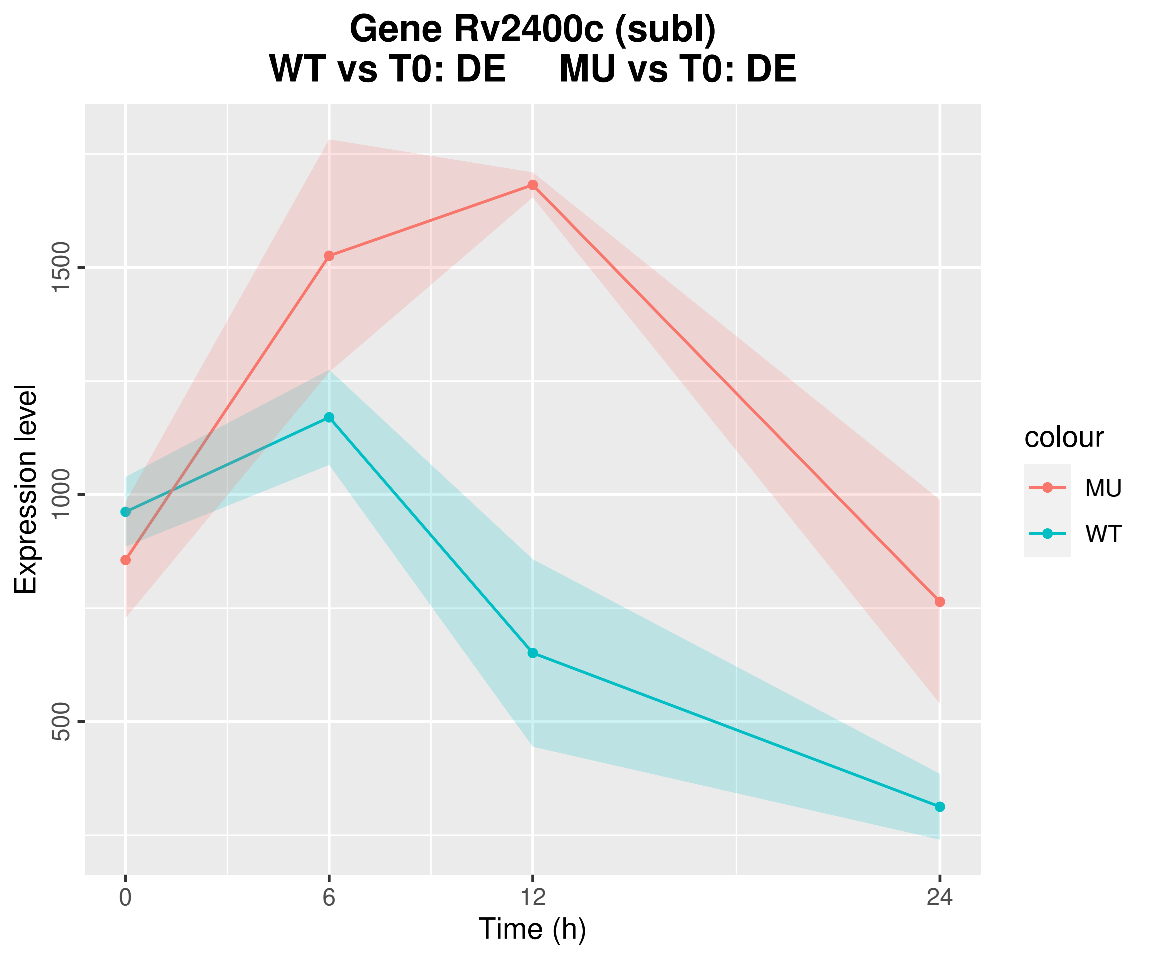
Gene Rv2400c (subI)
WT vs T0: DE MU vs T0: DE
Expression level
1500
1000
500
0
6
12
24
Time (h)
colour
MU
WT

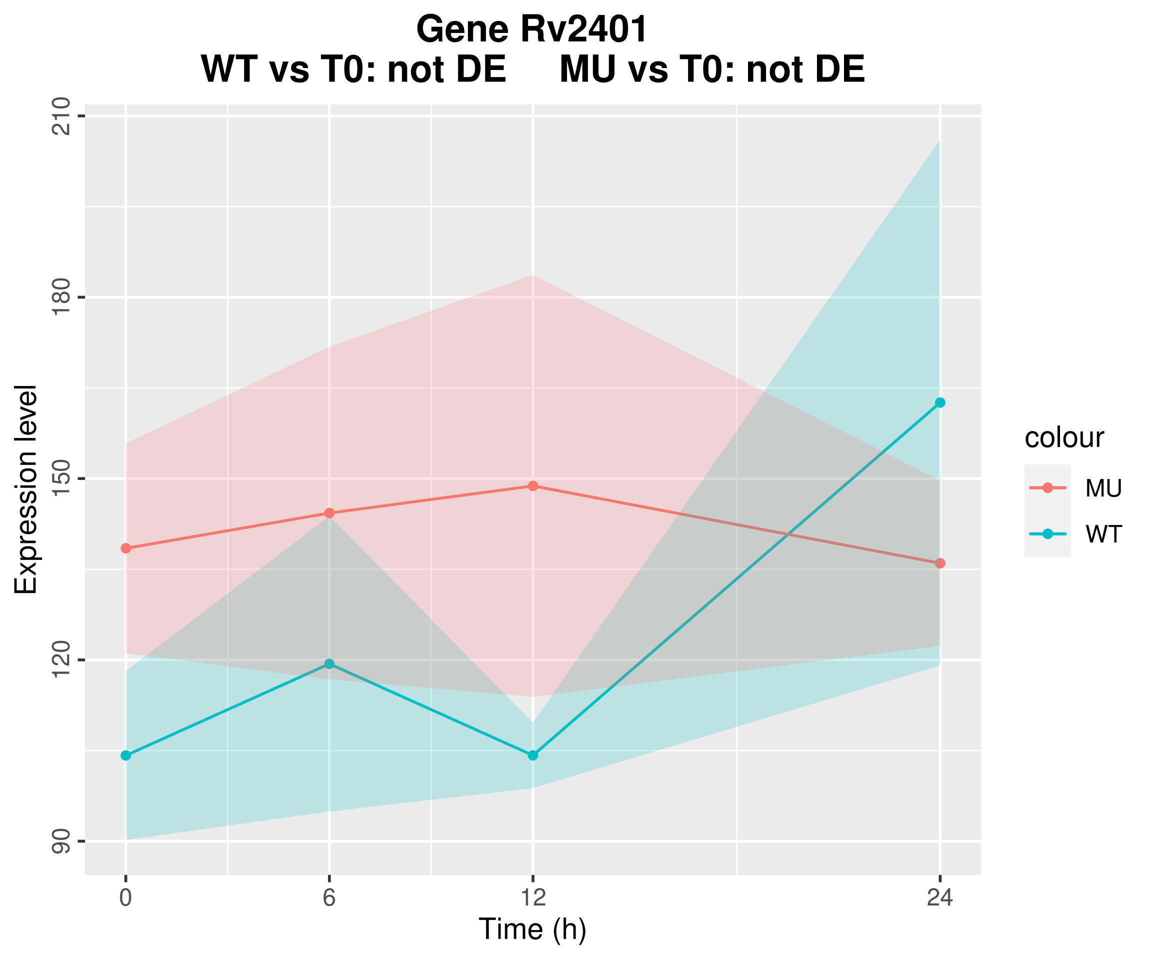
Gene Rv2401
WT vs T0: not DE MU vs T0: not DE
Expression level
210
180
150
120
90
0
6
12
24
Time (h)
colour
MU
WT

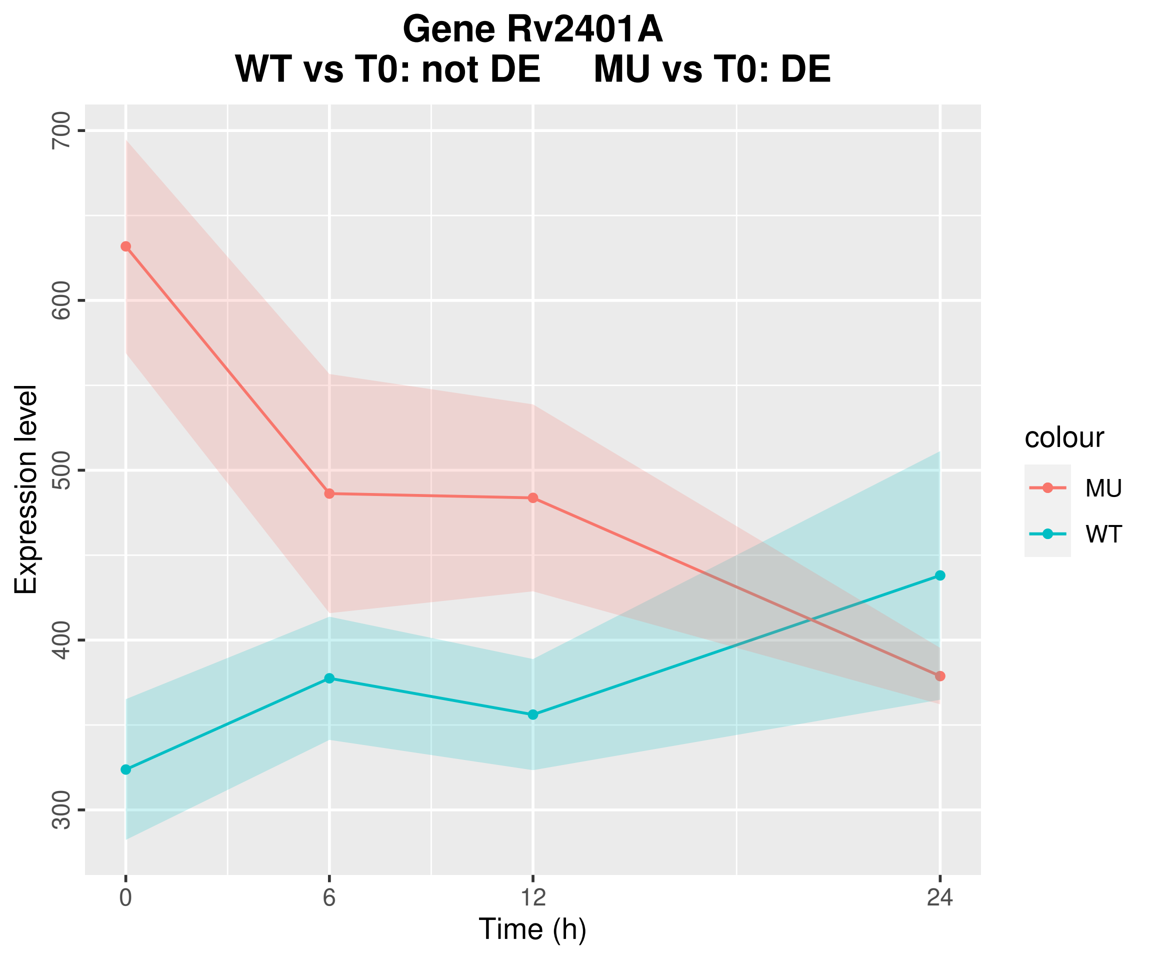

Gene Rv2401A
WT vs T0: not DE   MU vs T0: DE
Expression level
700
600
500
400
300
0
6
12
24
Time (h)
colour
MU
WT

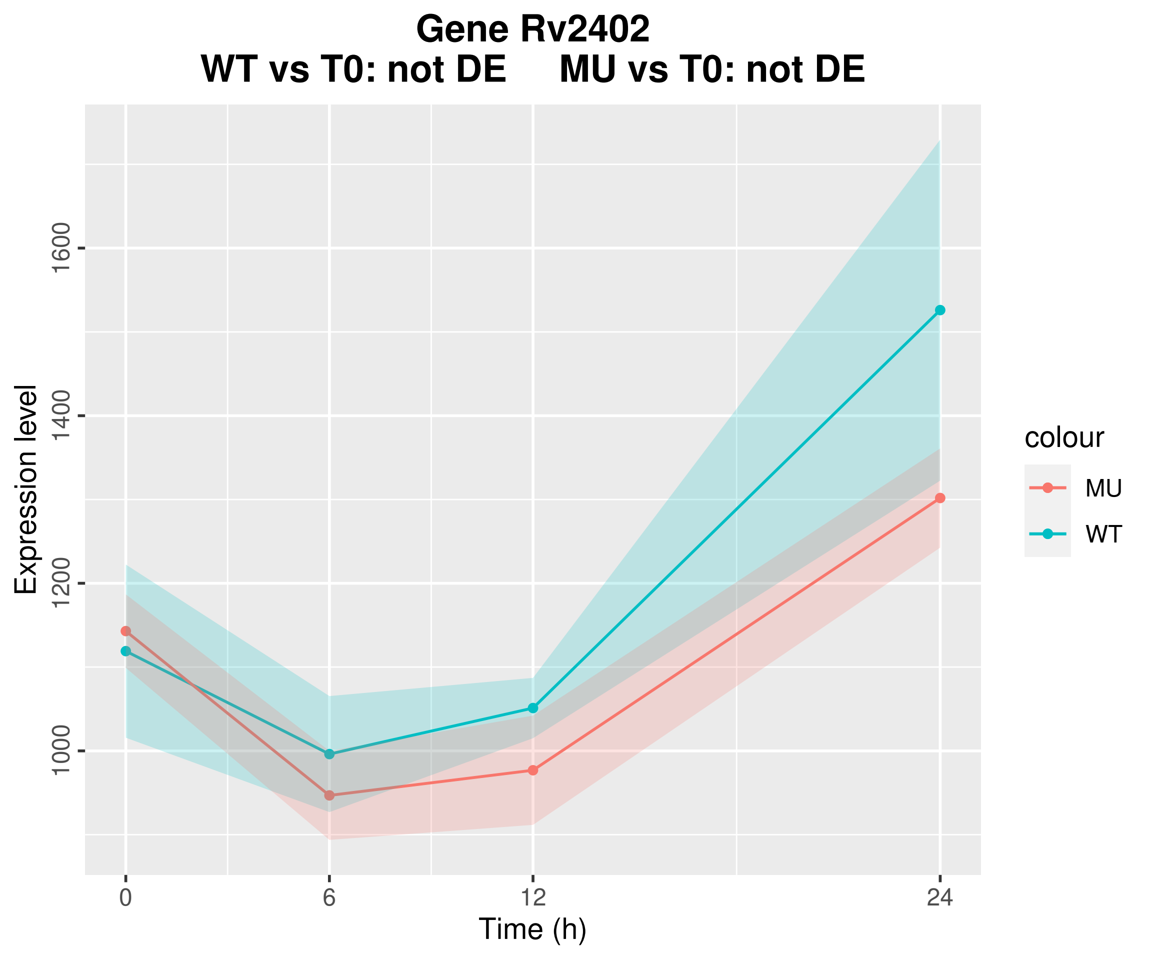

Gene Rv2402
WT vs T0: not DE   MU vs T0: not DE
Expression level
1000
1200
1400
1600
0
6
12
24
Time (h)
colour
MU
WT

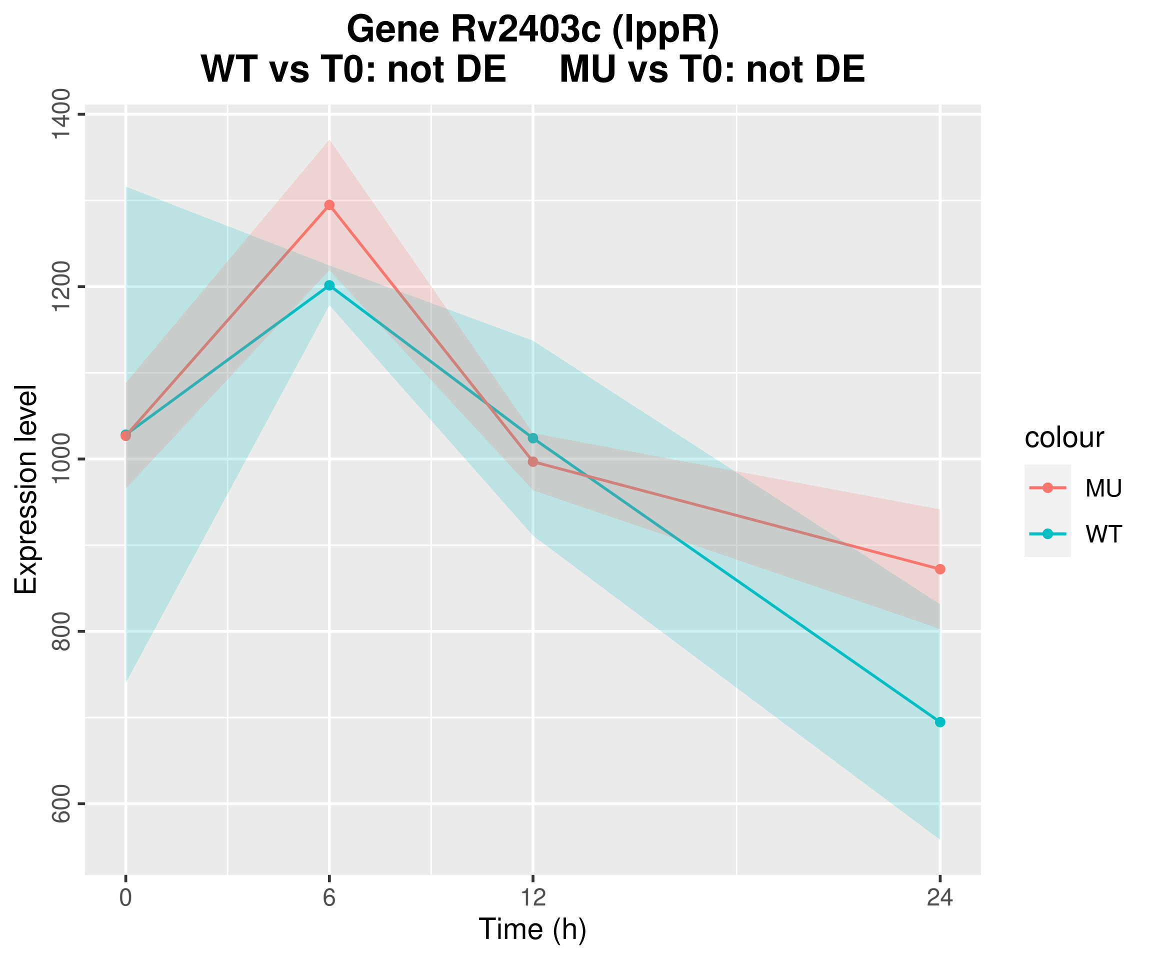

Gene Rv2403c (lppR)
WT vs T0: not DE MU vs T0: not DE
1400
1200
1000
800
600
Expression level
0
6
12
24
Time (h)
colour
MU
WT

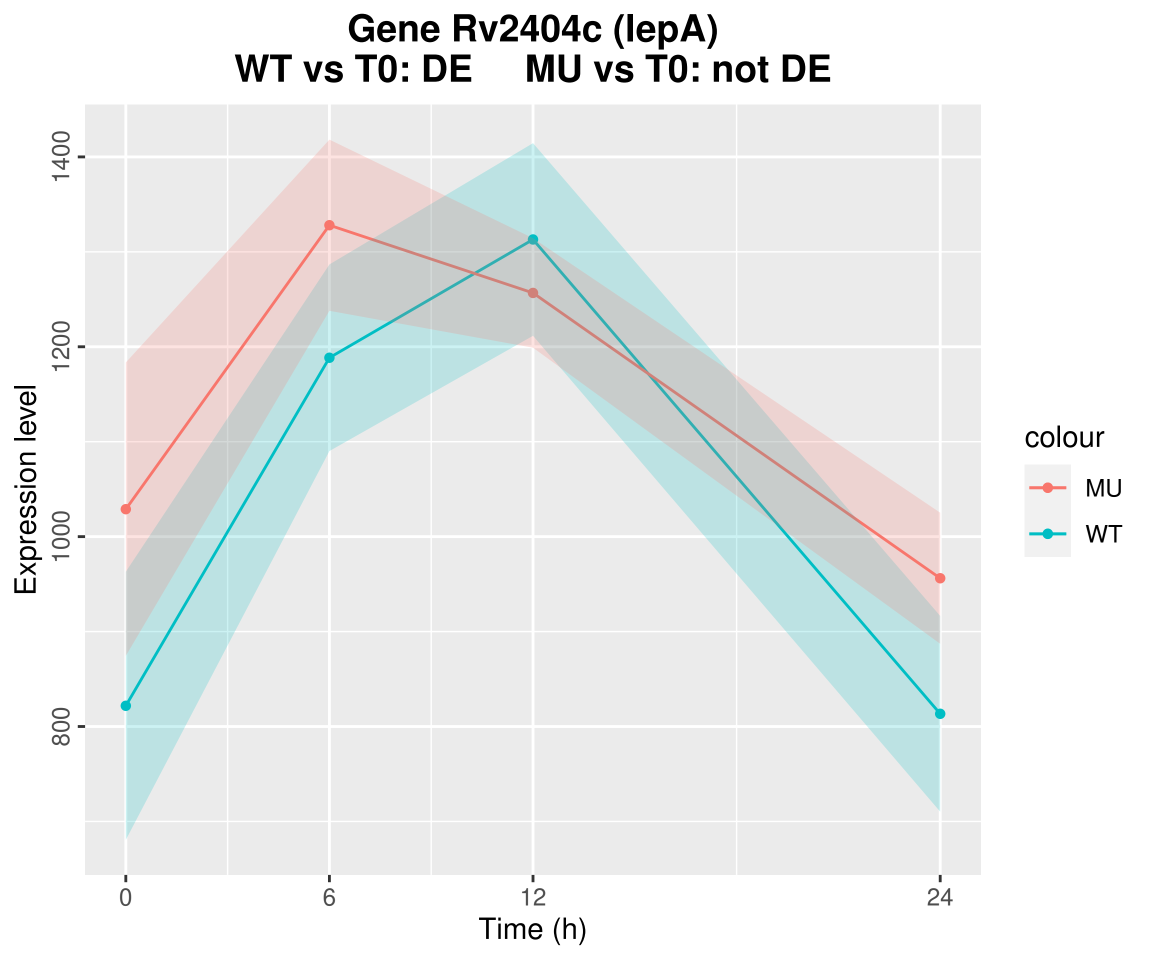

Gene Rv2404c (lepA)
WT vs T0: DE MU vs T0: not DE
1400
1200
1000
800
Expression level
0
6
12
24
Time (h)
colour
MU
WT

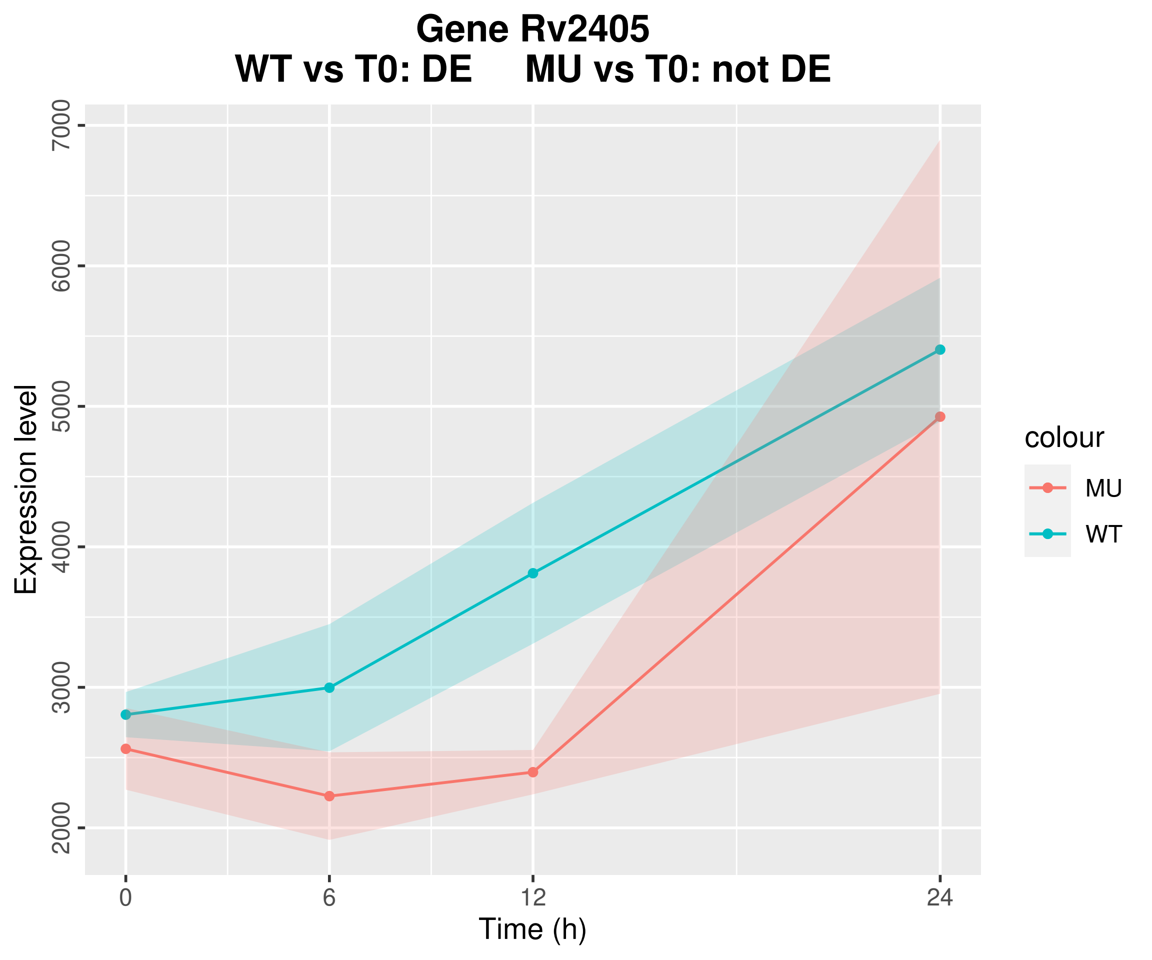
Gene Rv2405
WT vs T0: DE MU vs T0: not DE
Expression level
7000
6000
5000
4000
3000
2000
0
6
12
24
Time (h)
colour
MU
WT

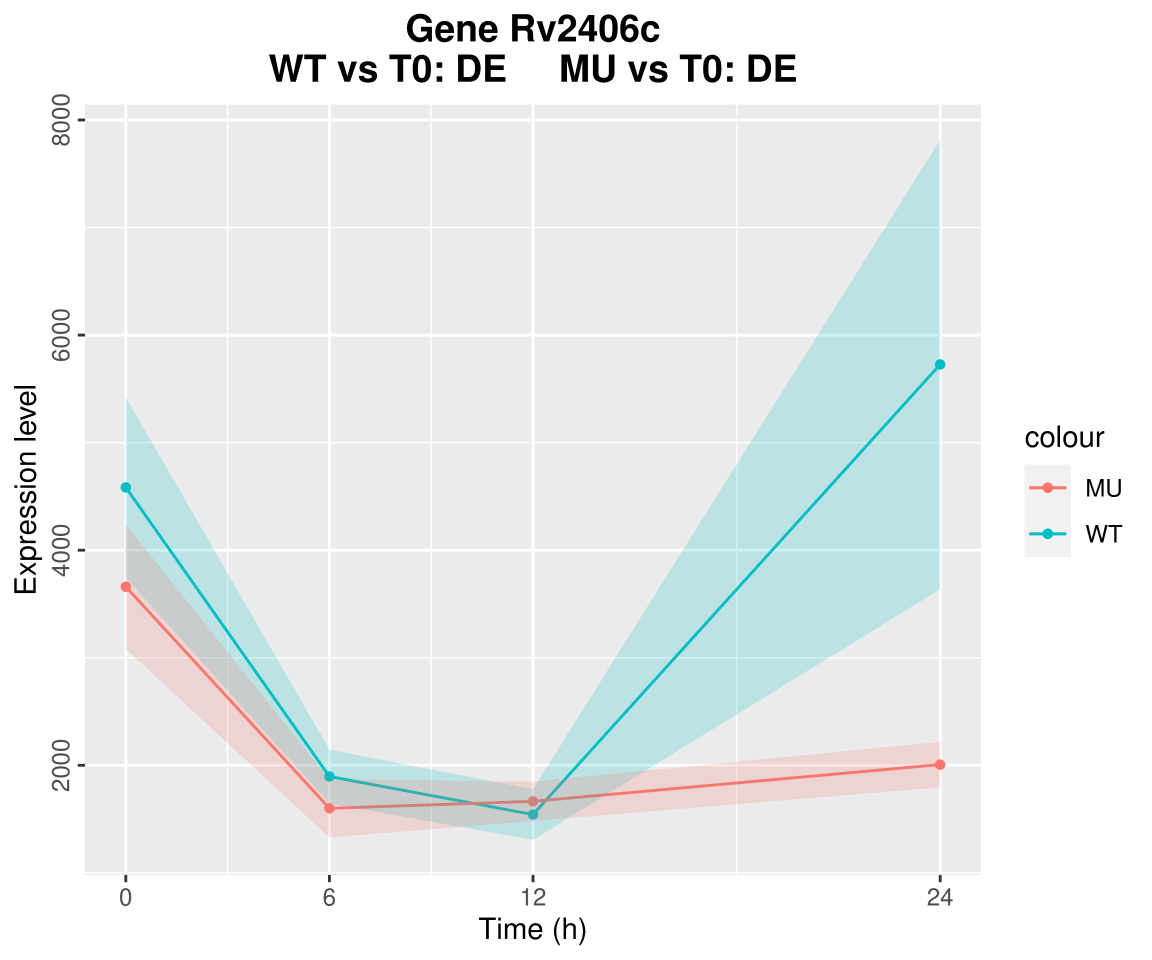

Gene Rv2406c
WT vs T0: DE MU vs T0: DE
Expression level
8000
6000
4000
2000
0
6
12
24
Time (h)
colour
MU
WT

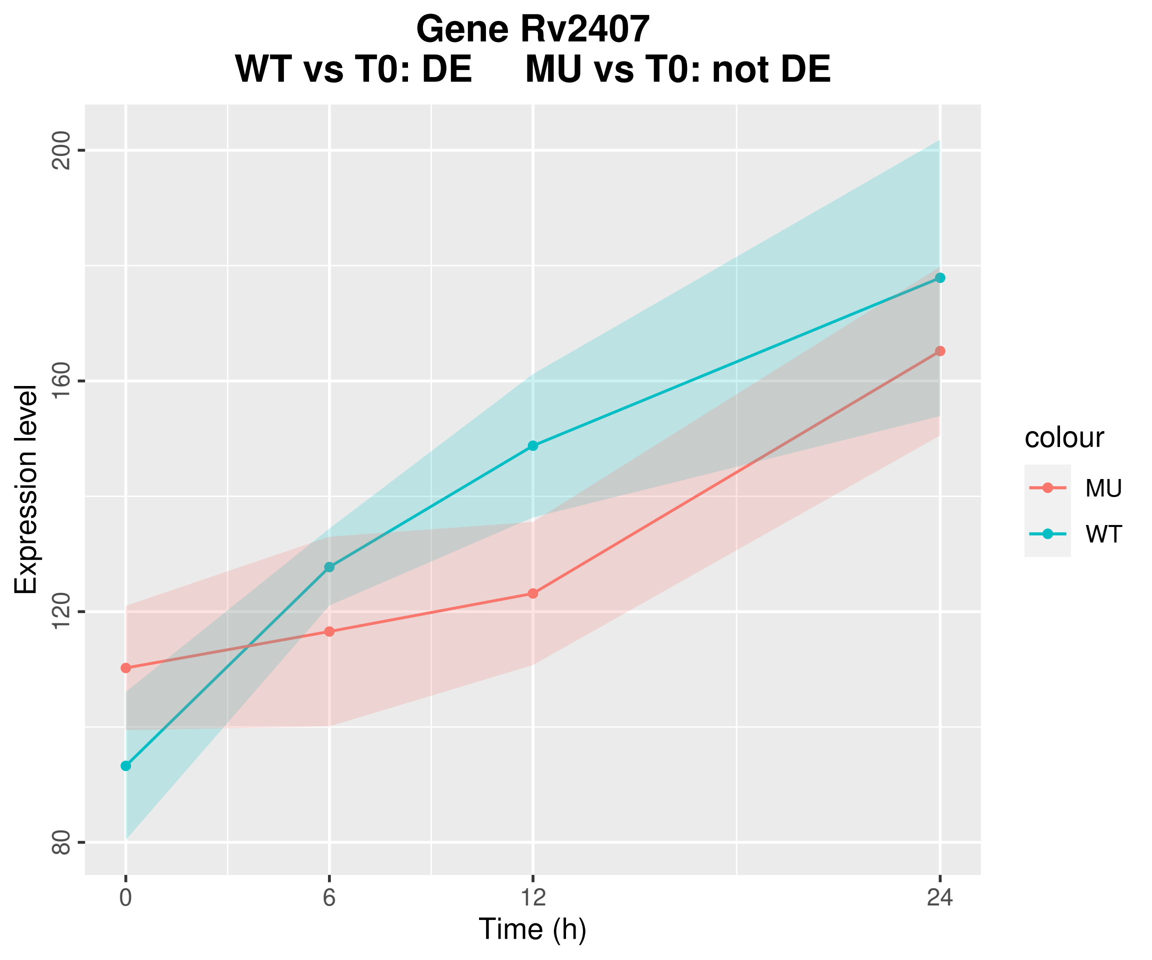
Gene Rv2407
WT vs T0: DE MU vs T0: not DE
200
160
120
80
Expression level
0
6
12
24
Time (h)
colour
MU
WT

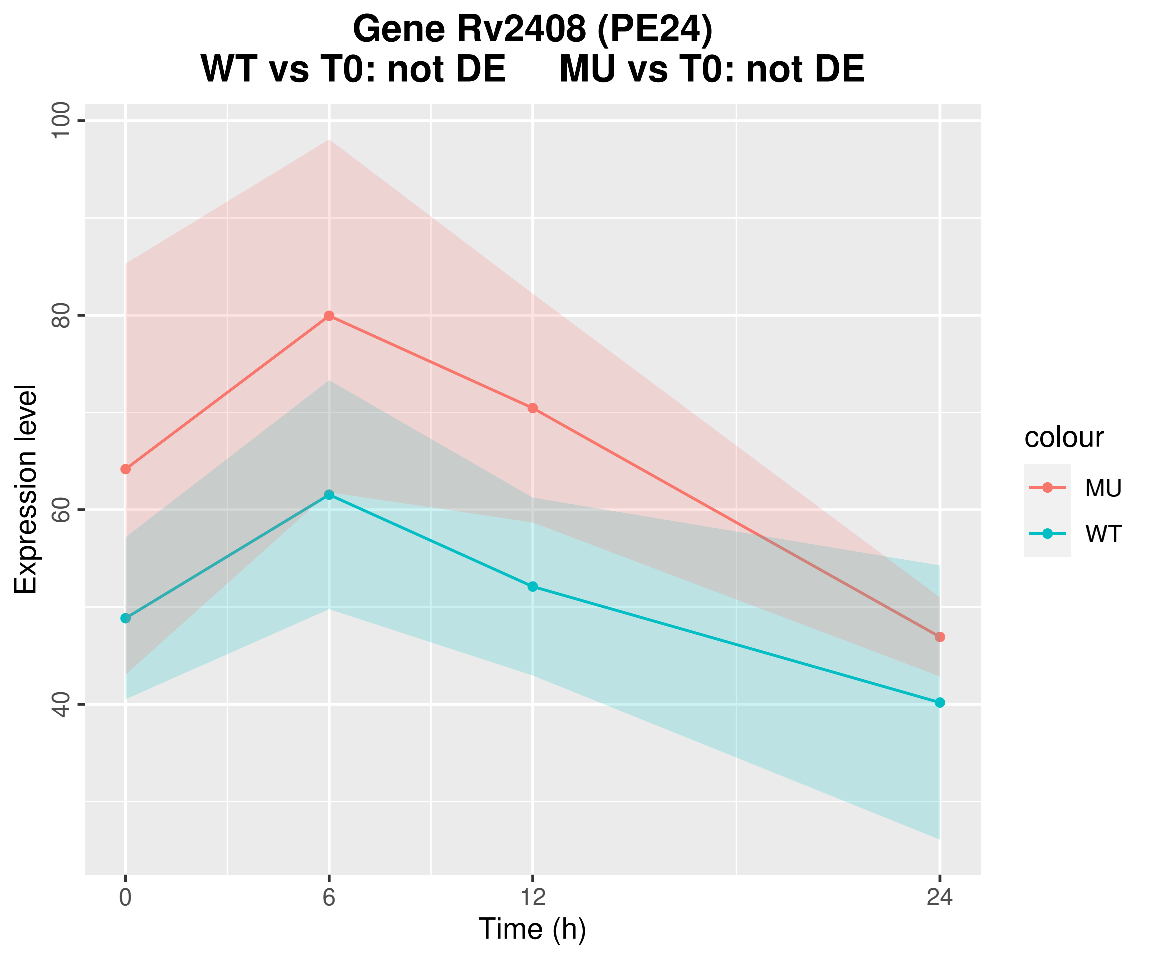
Gene Rv2408 (PE24)
WT vs T0: not DE   MU vs T0: not DE
Expression level
100
80
60
40
0
6
12
24
Time (h)
colour
MU
WT

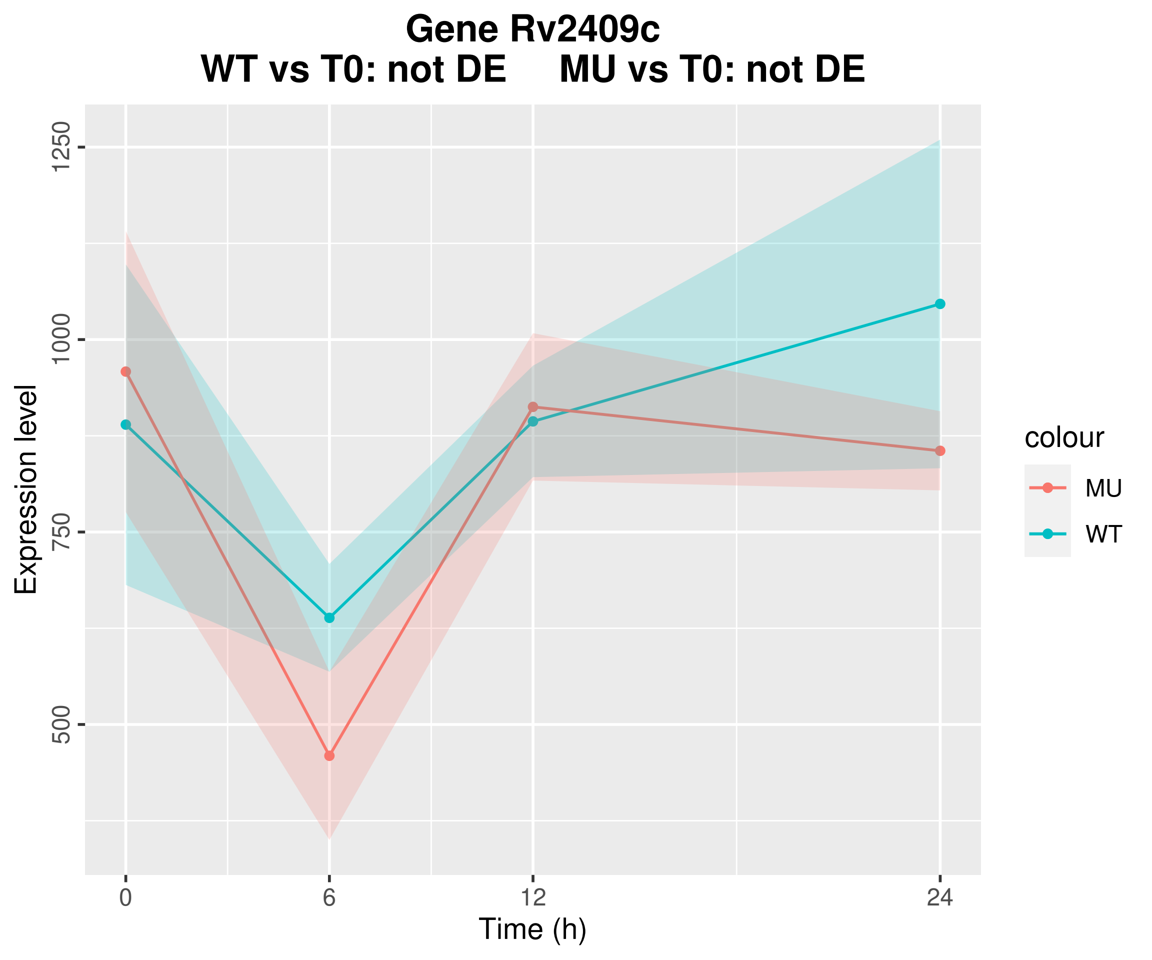

Gene Rv2409c
WT vs T0: not DE   MU vs T0: not DE
Expression level
1250
1000
750
500
Time (h)
0
6
12
24
colour
MU
WT

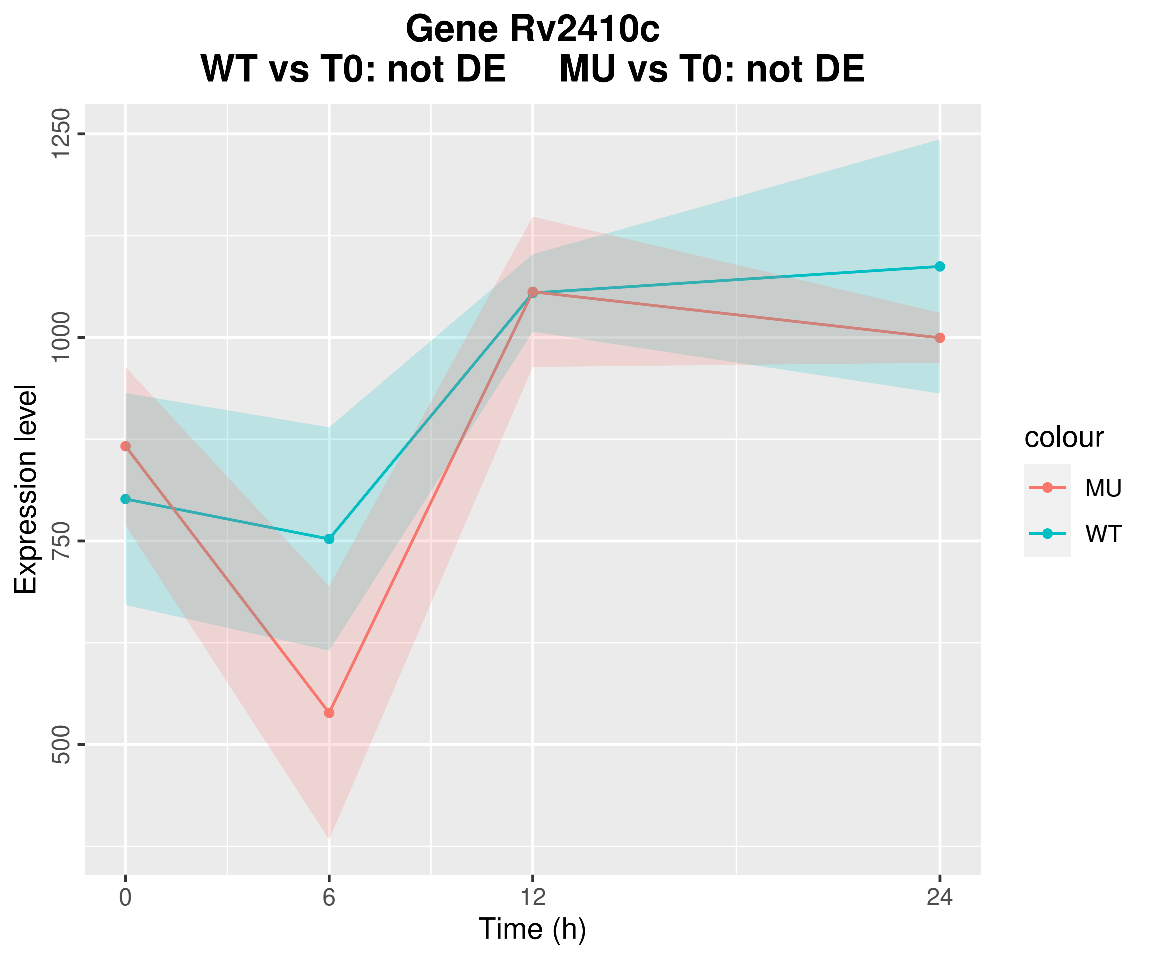
Gene Rv2410c
WT vs T0: not DE MU vs T0: not DE
Expression level
1250
1000
750
500
0
6
12
24
Time (h)
colour
MU
WT

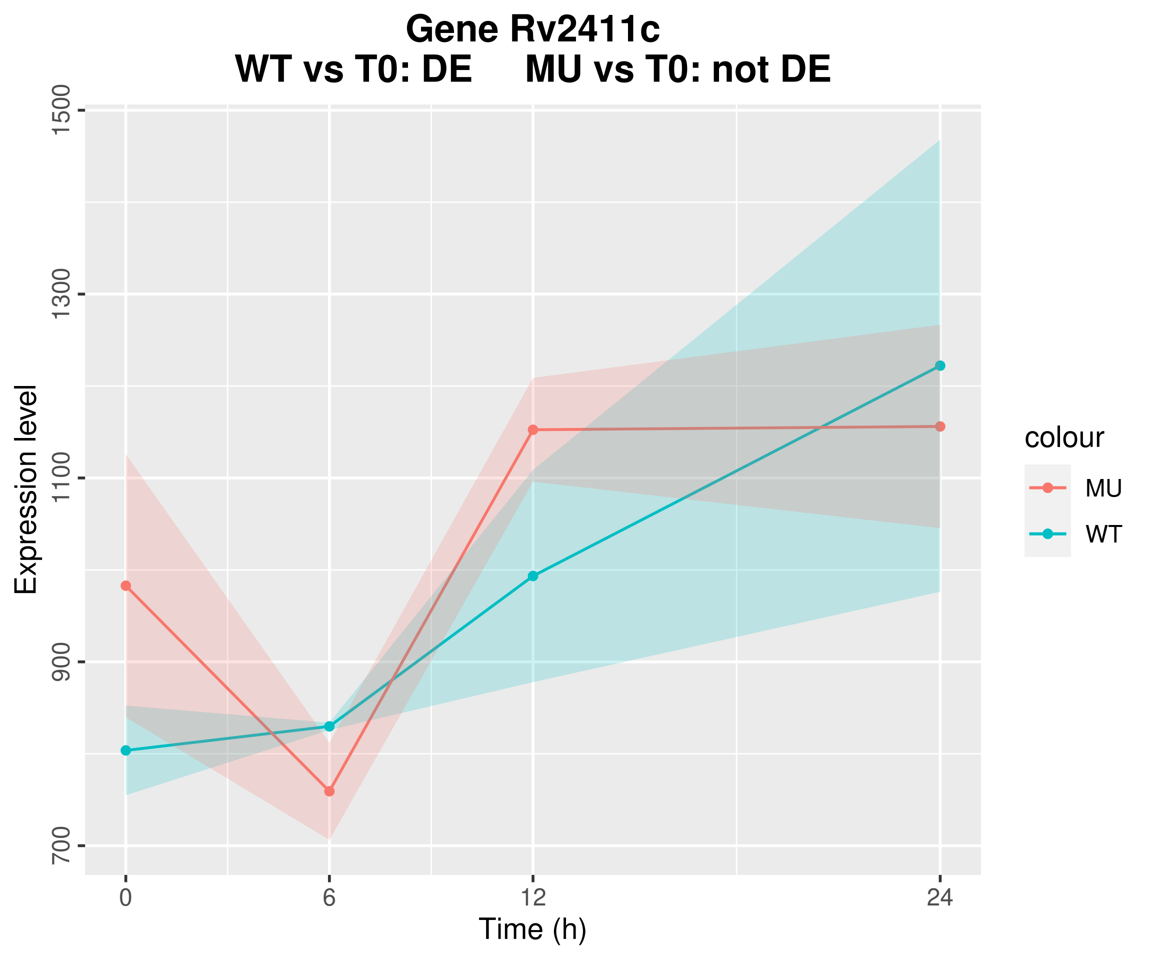

Gene Rv2411c
WT vs T0: DE MU vs T0: not DE
1500
1300
1100
900
700
Expression level
0
6
12
24
Time (h)
colour
MU
WT

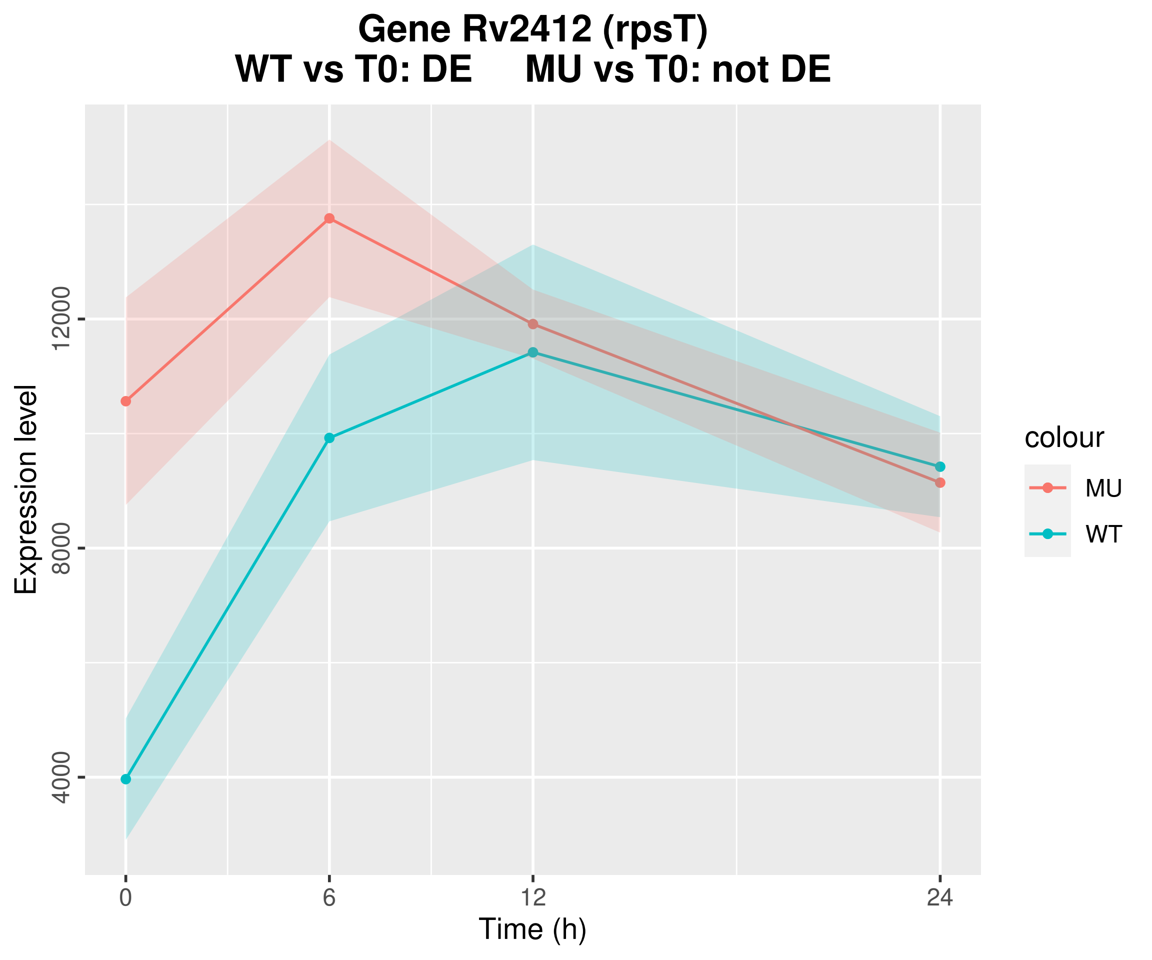
Gene Rv2412 (rpsT)
WT vs T0: DE MU vs T0: not DE
Expression level
12000
8000
4000
0
6
12
24
Time (h)
colour
MU
WT

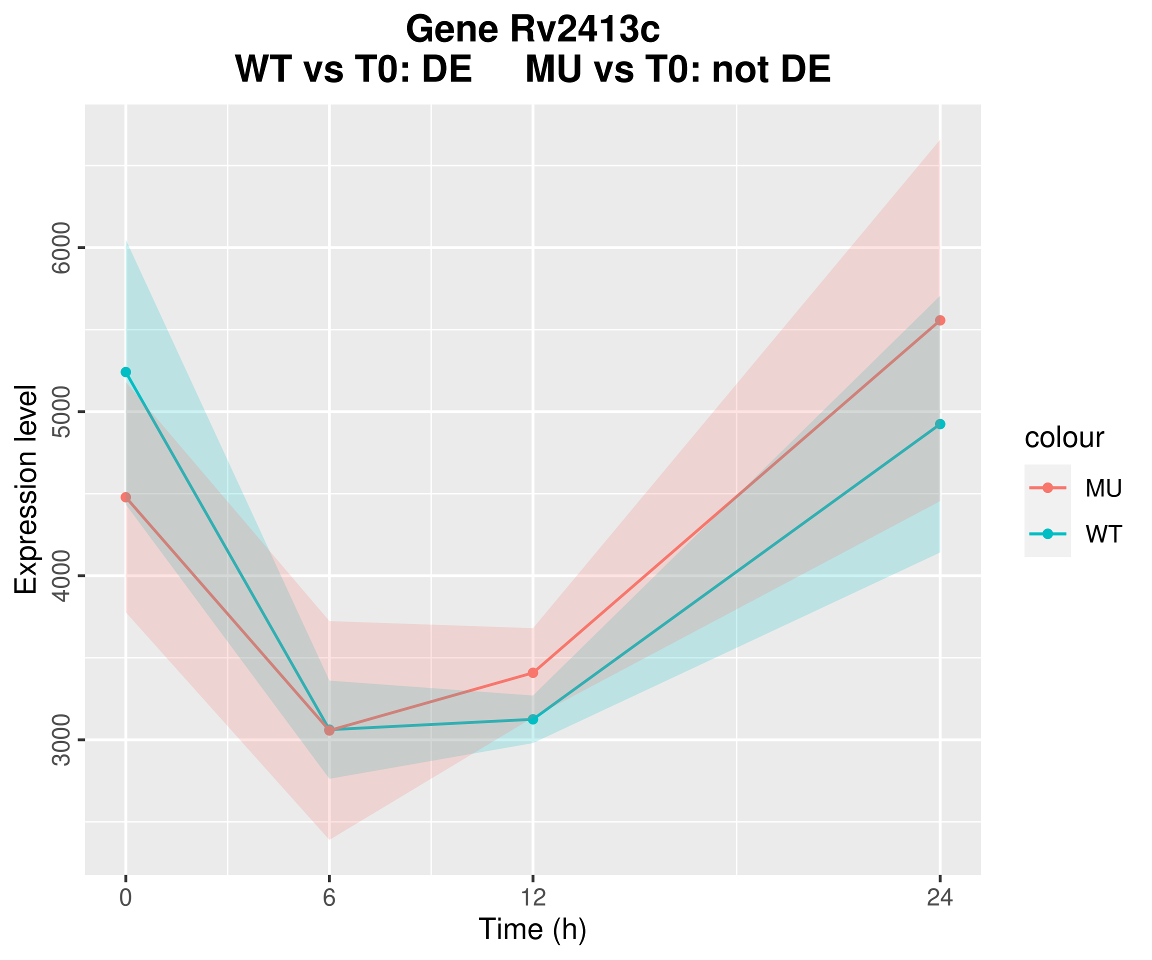

Gene Rv2413c
WT vs T0: DE   MU vs T0: not DE
Expression level
6000
5000
4000
3000
0
6
12
24
Time (h)
colour
MU
WT

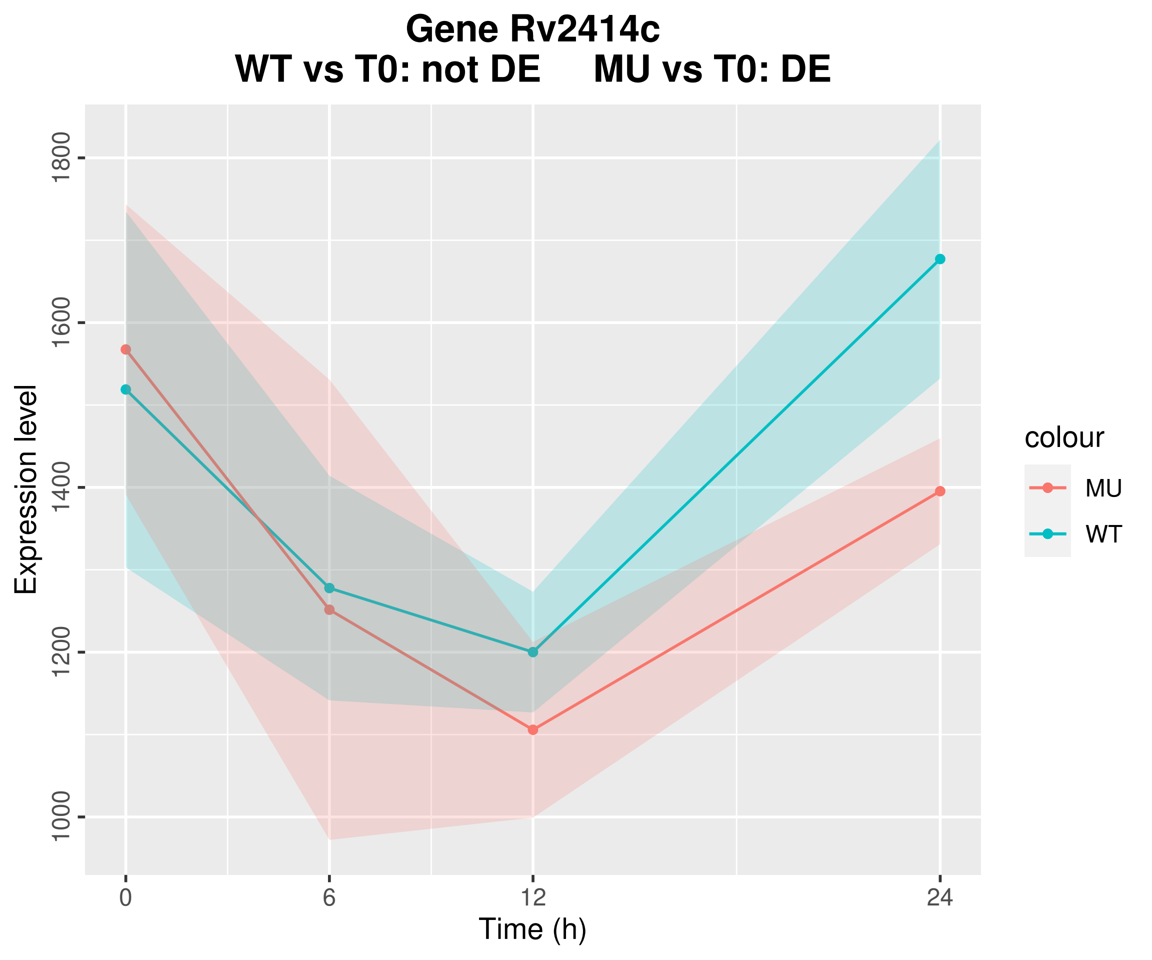
Gene Rv2414c
WT vs T0: not DE   MU vs T0: DE
Expression level
1000
1200
1400
1600
1800
0
6
12
24
Time (h)
colour
MU
WT

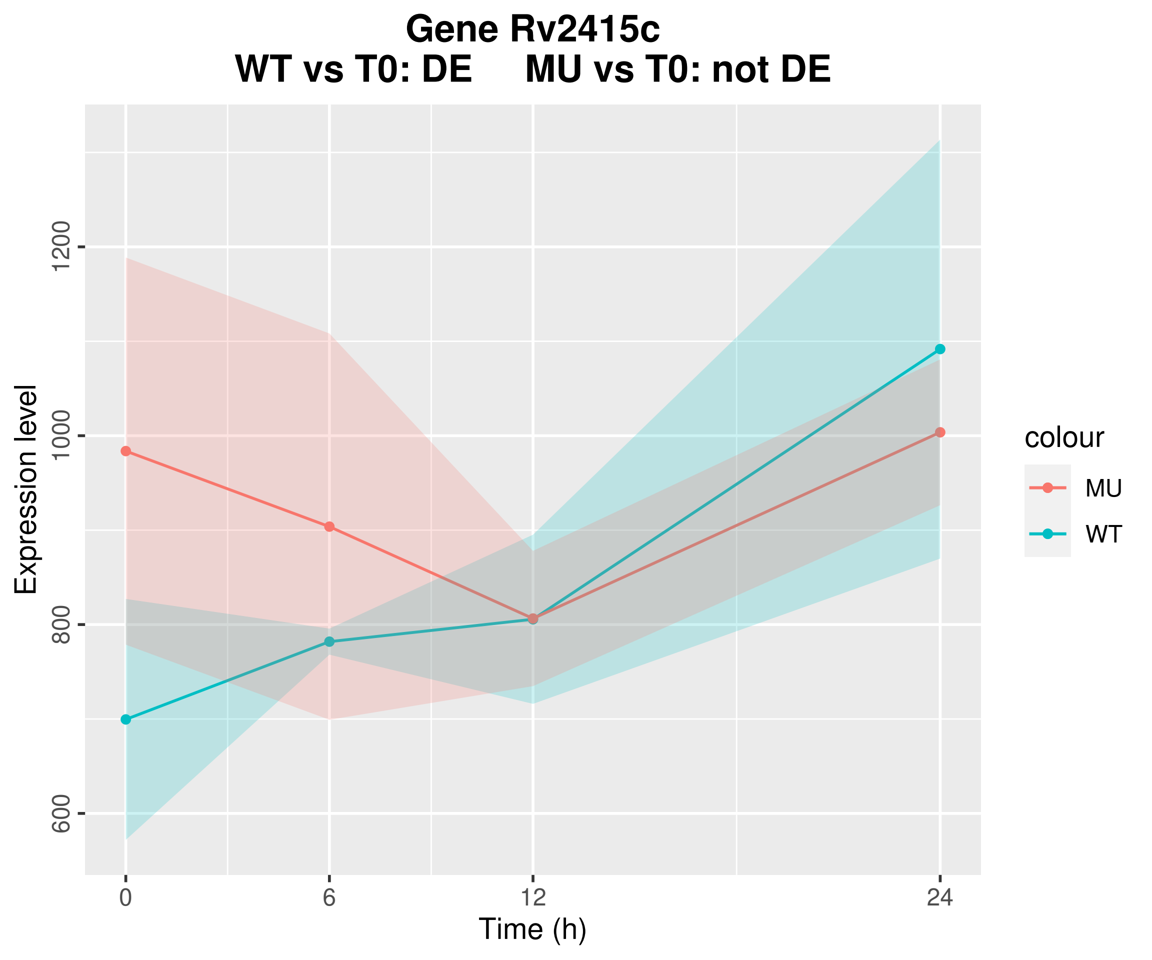
Gene Rv2415c
WT vs T0: DE   MU vs T0: not DE
Expression level
600
800
1000
1200
0
6
12
24
Time (h)
colour
MU
WT

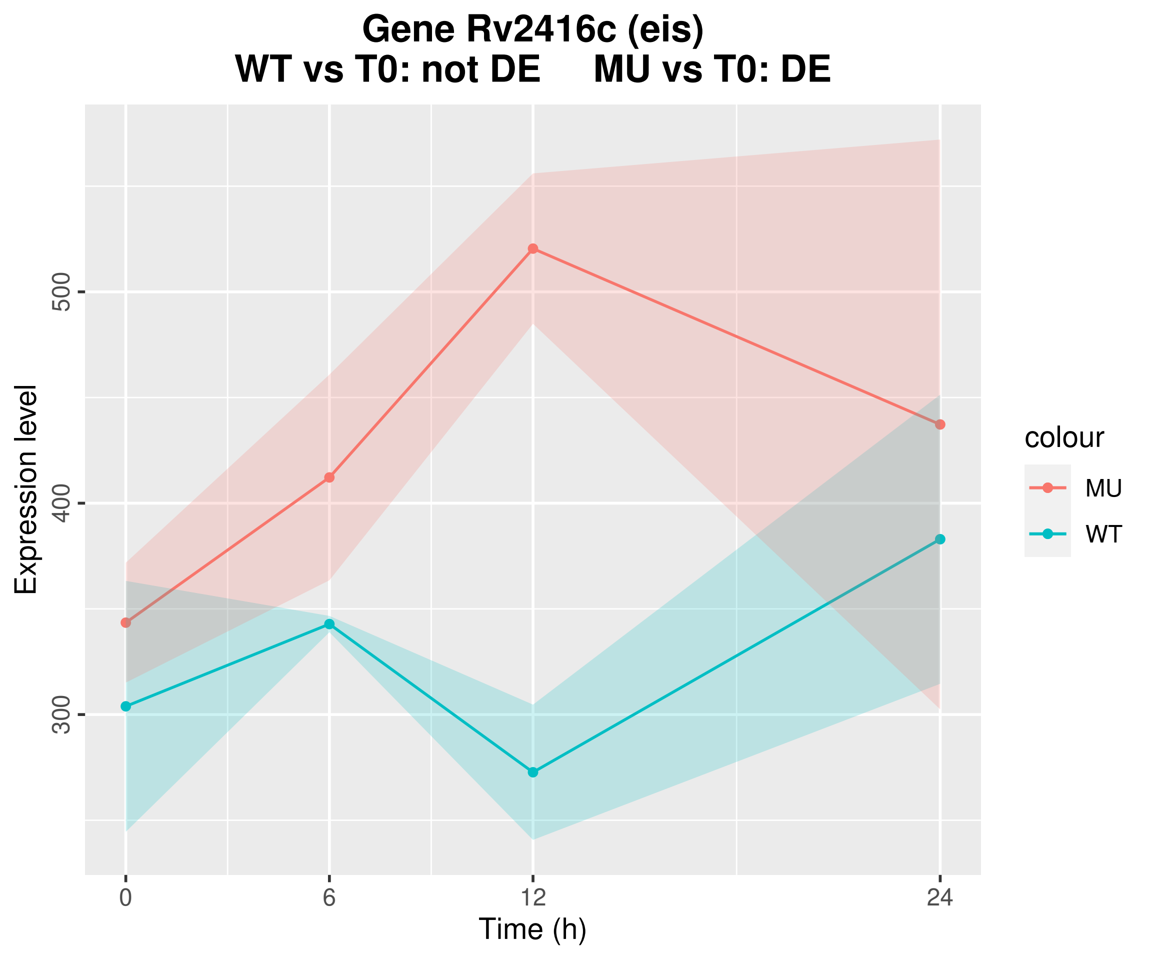
Gene Rv2416c (eis)
WT vs T0: not DE MU vs T0: DE
Expression level
500
400
300
0
6
12
24
Time (h)
colour
MU
WT

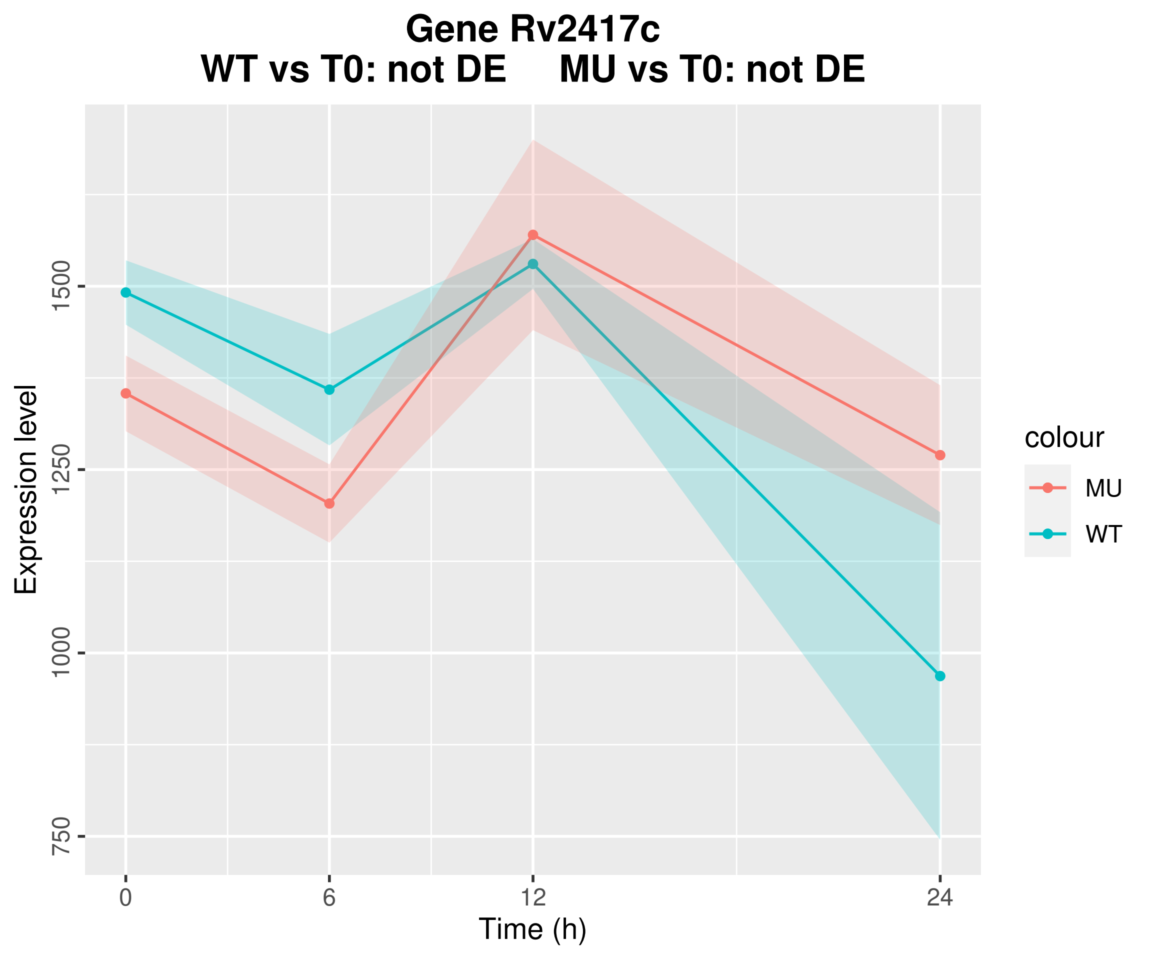

Gene Rv2417c
WT vs T0: not DE   MU vs T0: not DE
Expression level
1500
1250
1000
750
0
6
12
24
Time (h)
colour
MU
WT

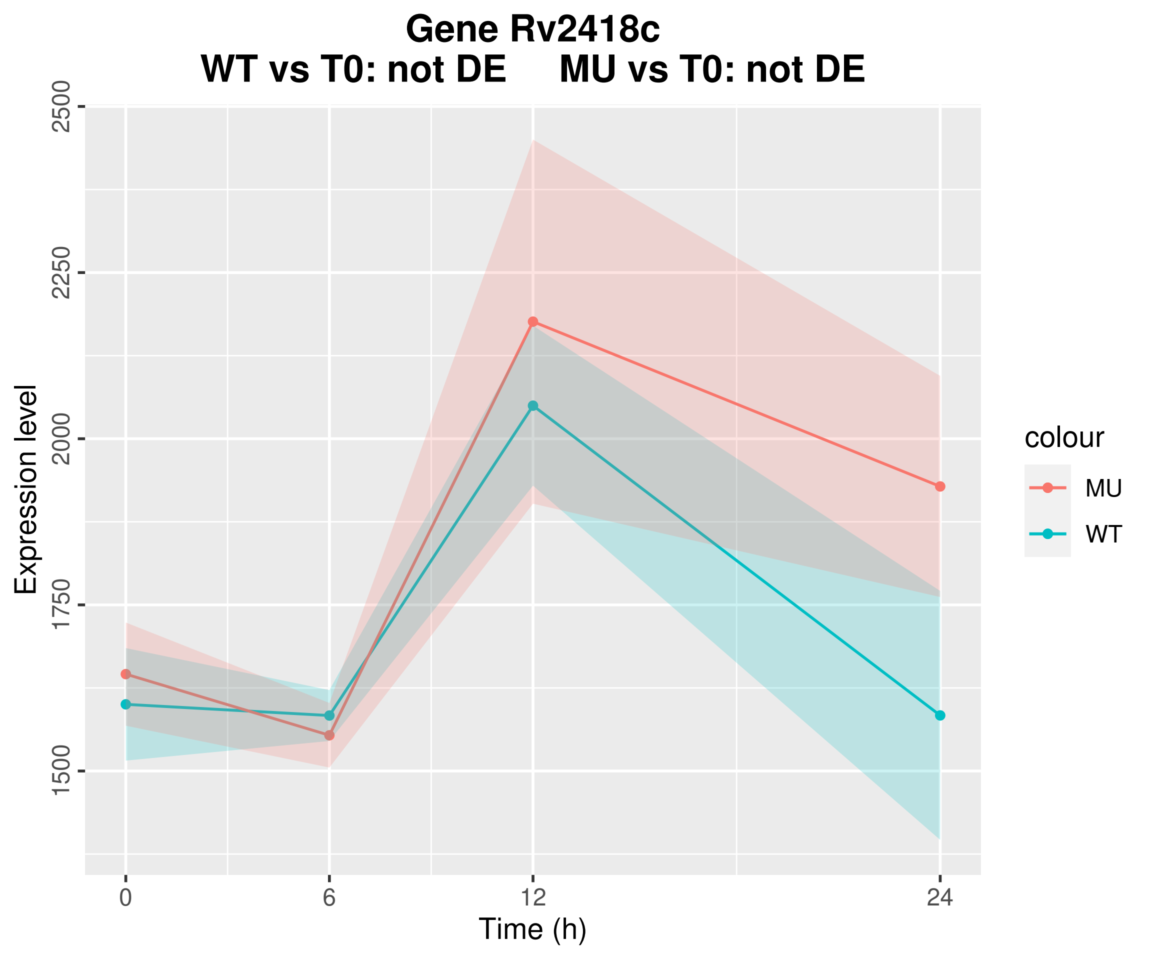

Gene Rv2418c
WT vs T0: not DE   MU vs T0: not DE
Expression level
2500
2250
2000
1750
1500
Time (h)
0
6
12
24
colour
MU
WT

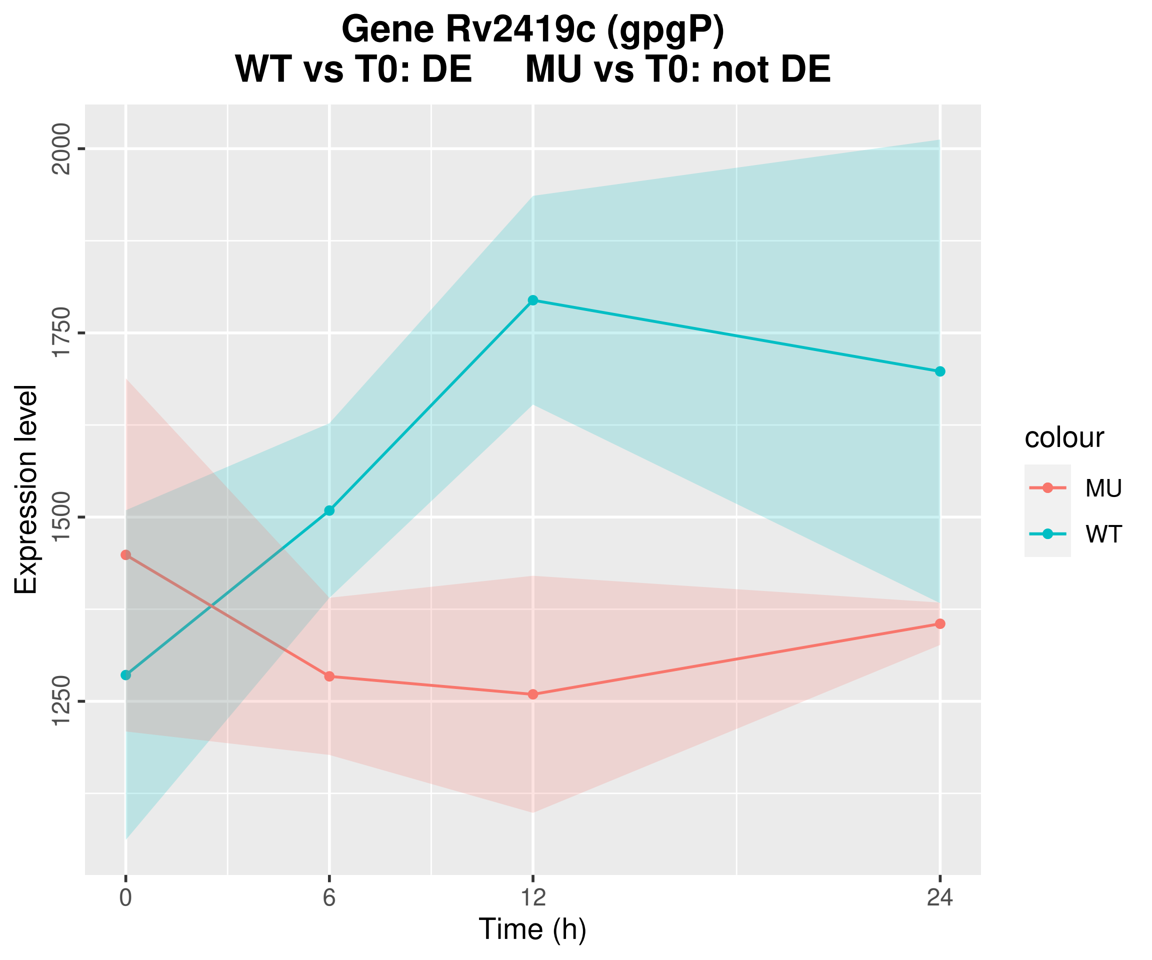

Gene Rv2419c (gpgP)
WT vs T0: DE   MU vs T0: not DE
2000
1750
1500
1250
Expression level
0
6
12
24
Time (h)
colour
MU
WT

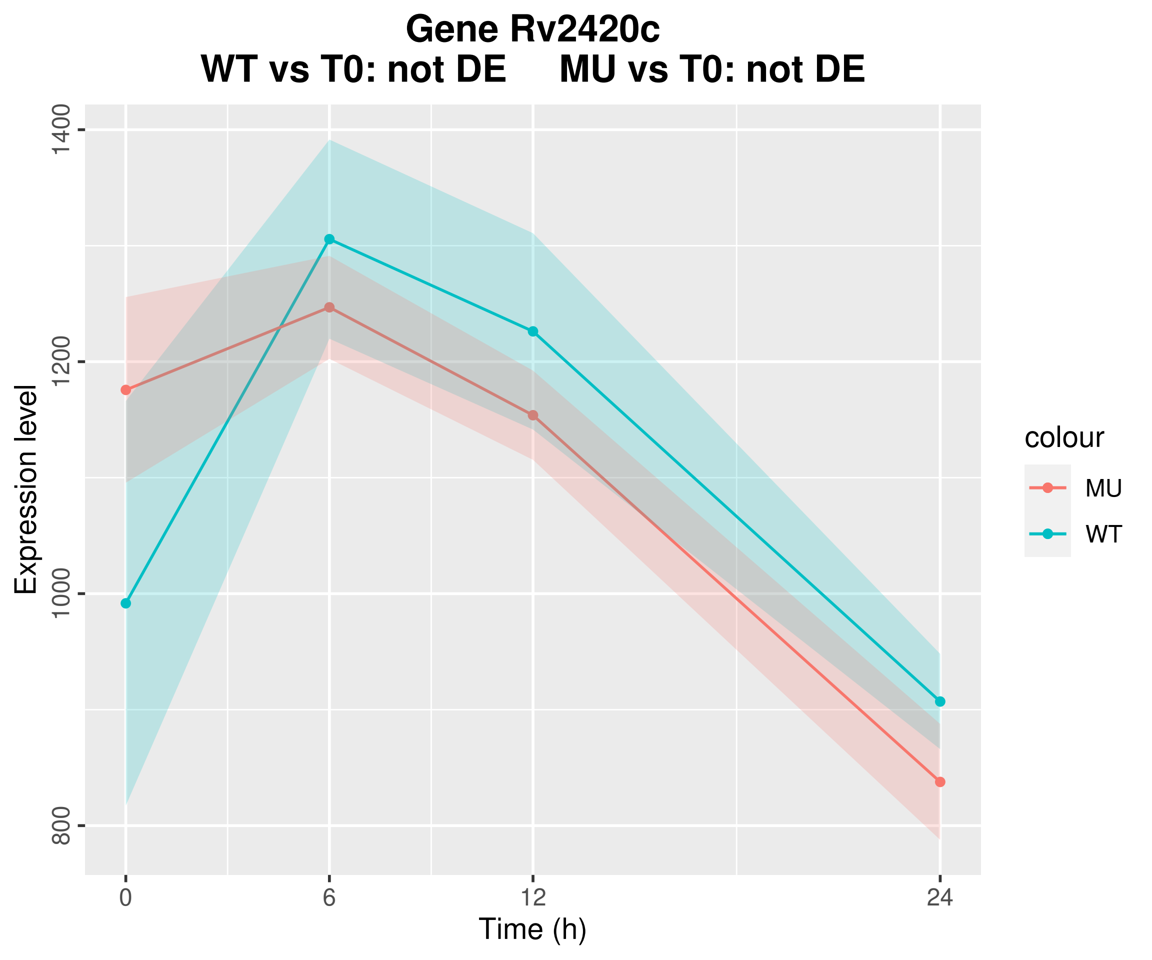

Gene Rv2420c
WT vs T0: not DE   MU vs T0: not DE
1400
1200
1000
800
Expression level
0
6
12
24
Time (h)
colour
MU
WT

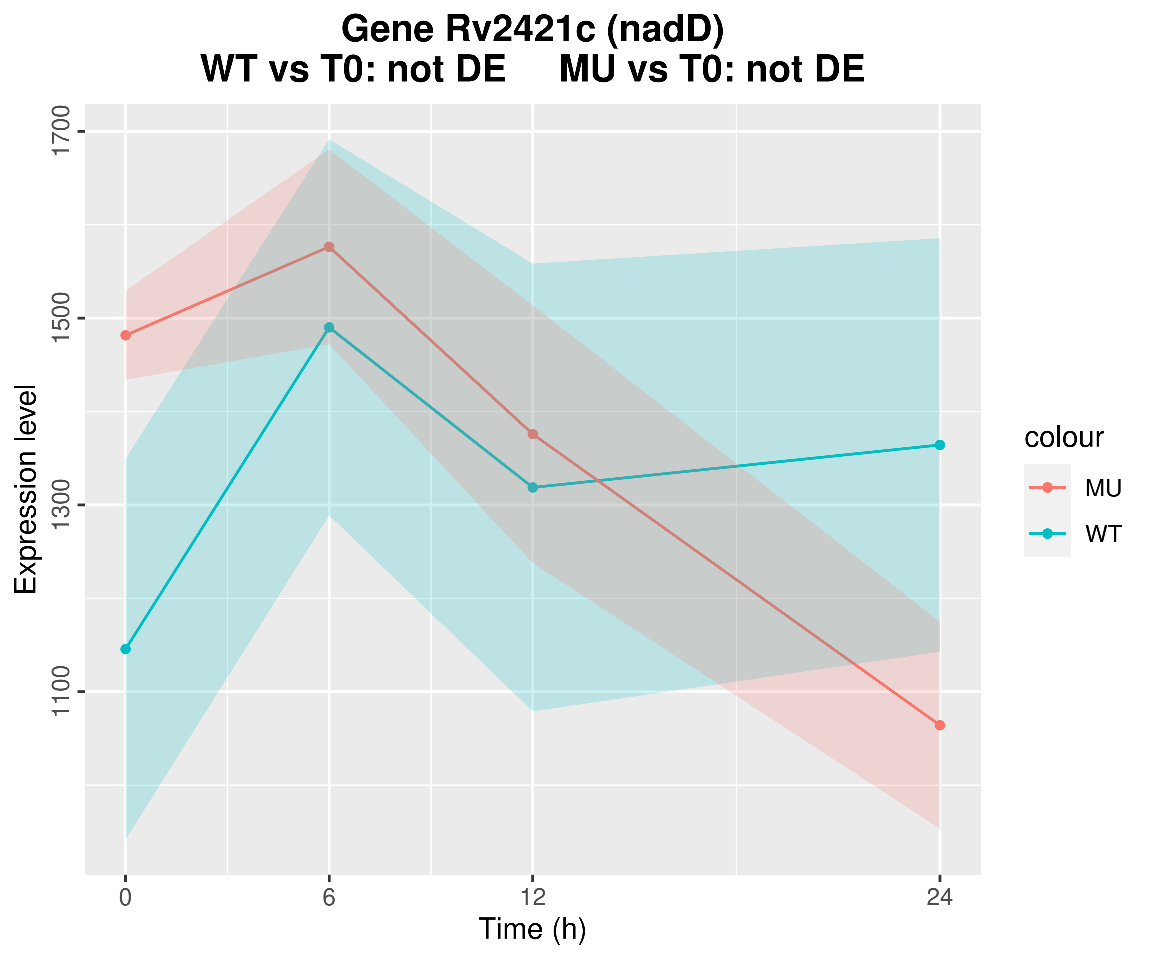

Gene Rv2421c (nadD)
WT vs T0: not DE   MU vs T0: not DE
Expression level
1700
1500
1300
1100
0
6
12
24
Time (h)
colour
MU
WT

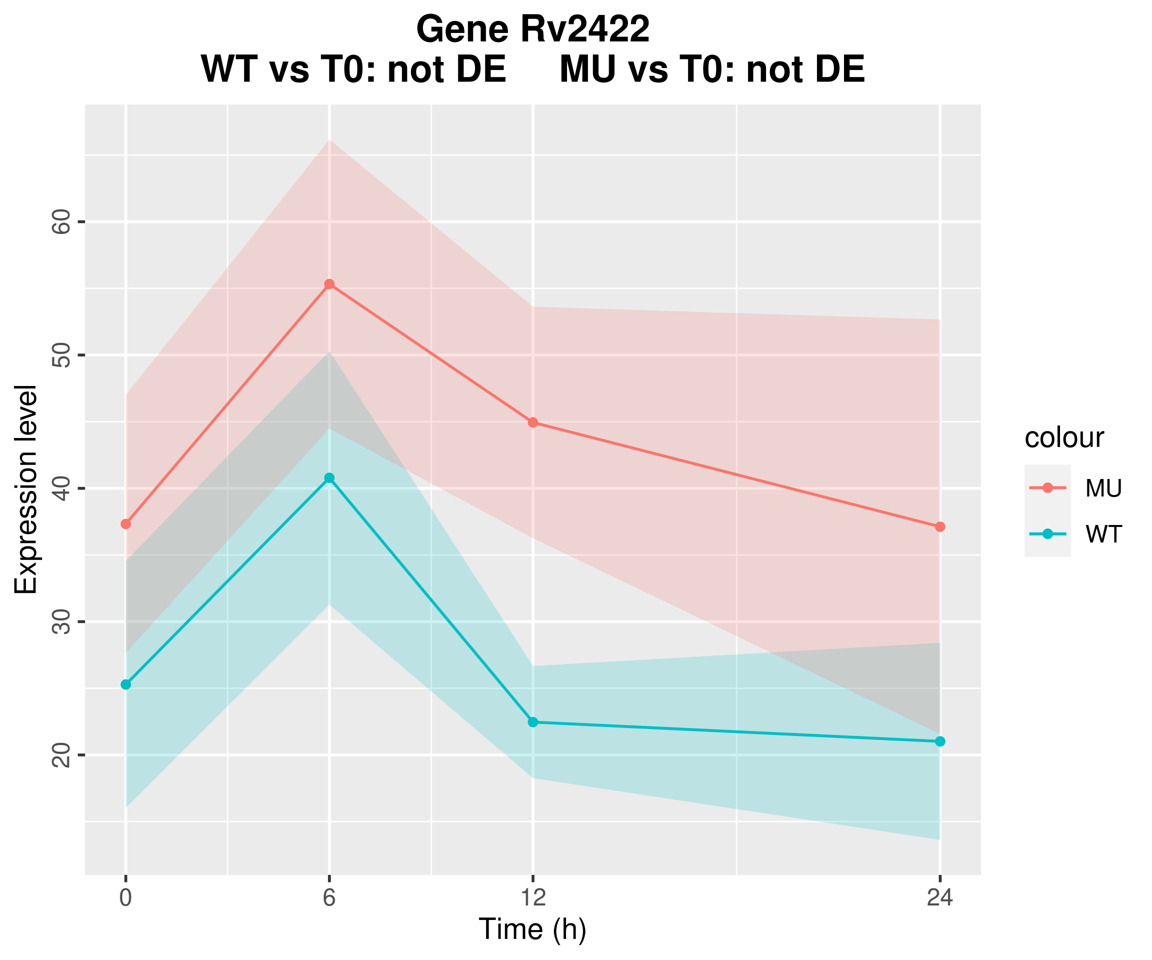

Gene Rv2422
WT vs T0: not DE MU vs T0: not DE
Expression level
60
50
40
30
20
0
6
12
24
Time (h)
colour
MU
WT

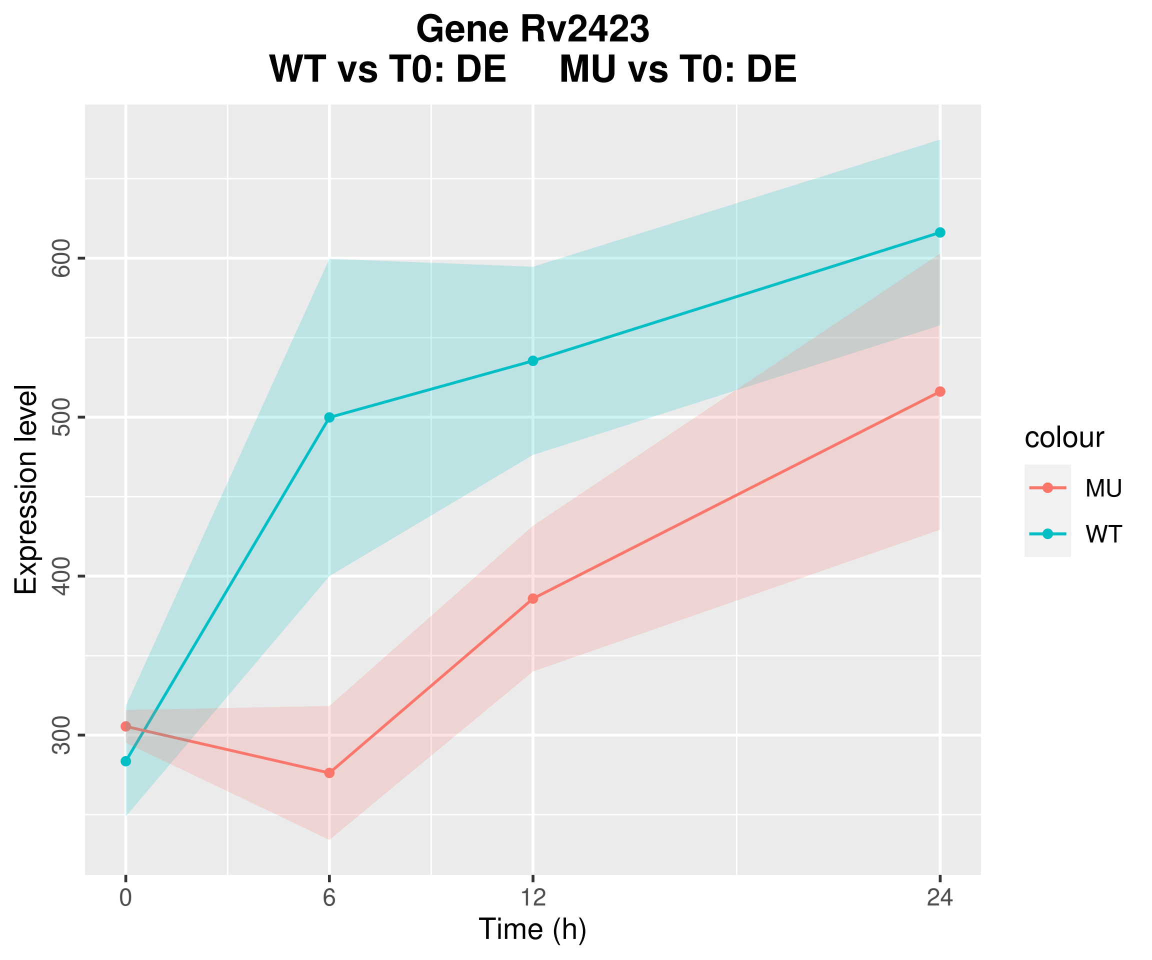
Gene Rv2423
WT vs T0: DE MU vs T0: DE
Expression level
600
500
400
300
0
6
12
24
Time (h)
colour
MU
WT

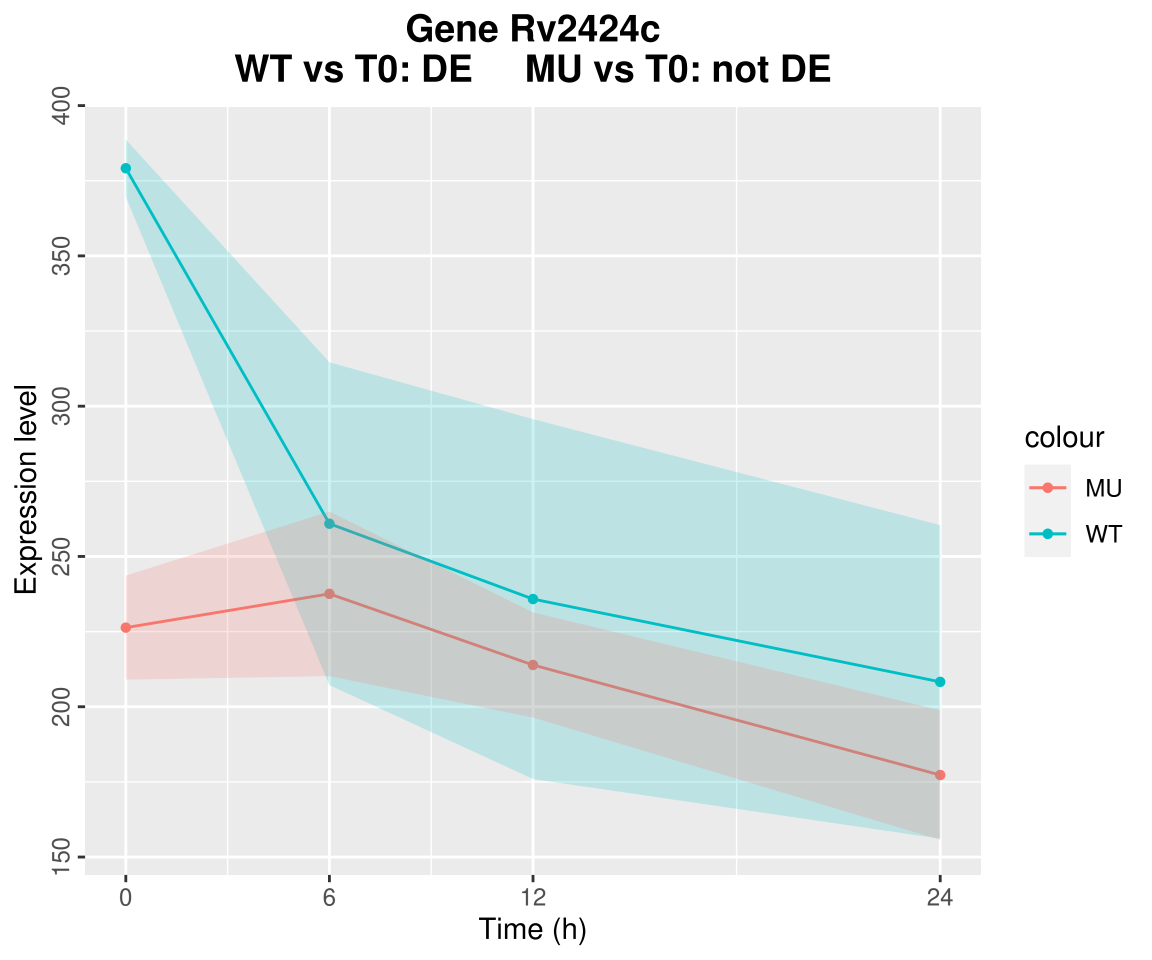

Gene Rv2424c
WT vs T0: DE   MU vs T0: not DE
400
350
300
250
200
150
Expression level
0
6
12
24
Time (h)
colour
MU
WT

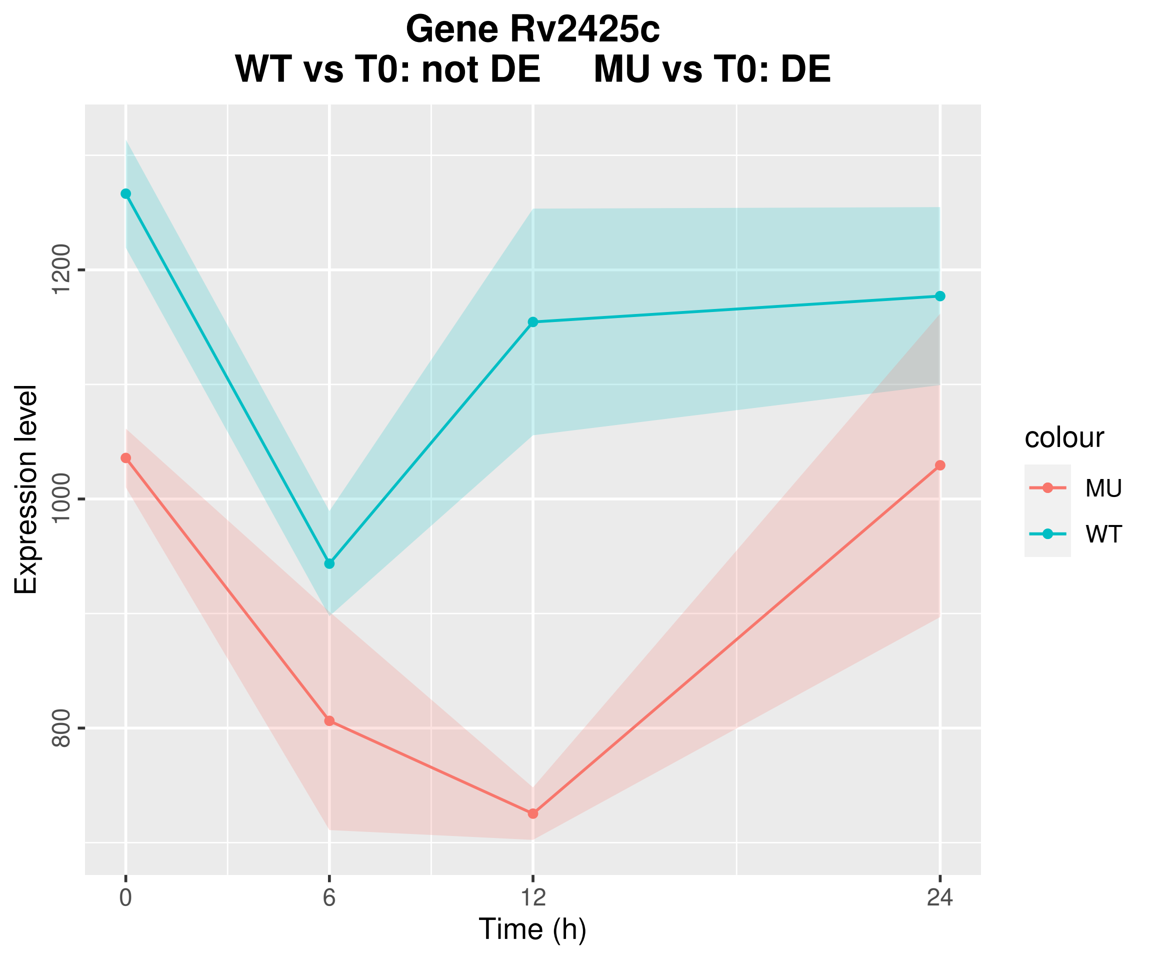
Gene Rv2425c
WT vs T0: not DE MU vs T0: DE
Expression level
800
1000
1200
Time (h)
0
6
12
24
colour
MU
WT

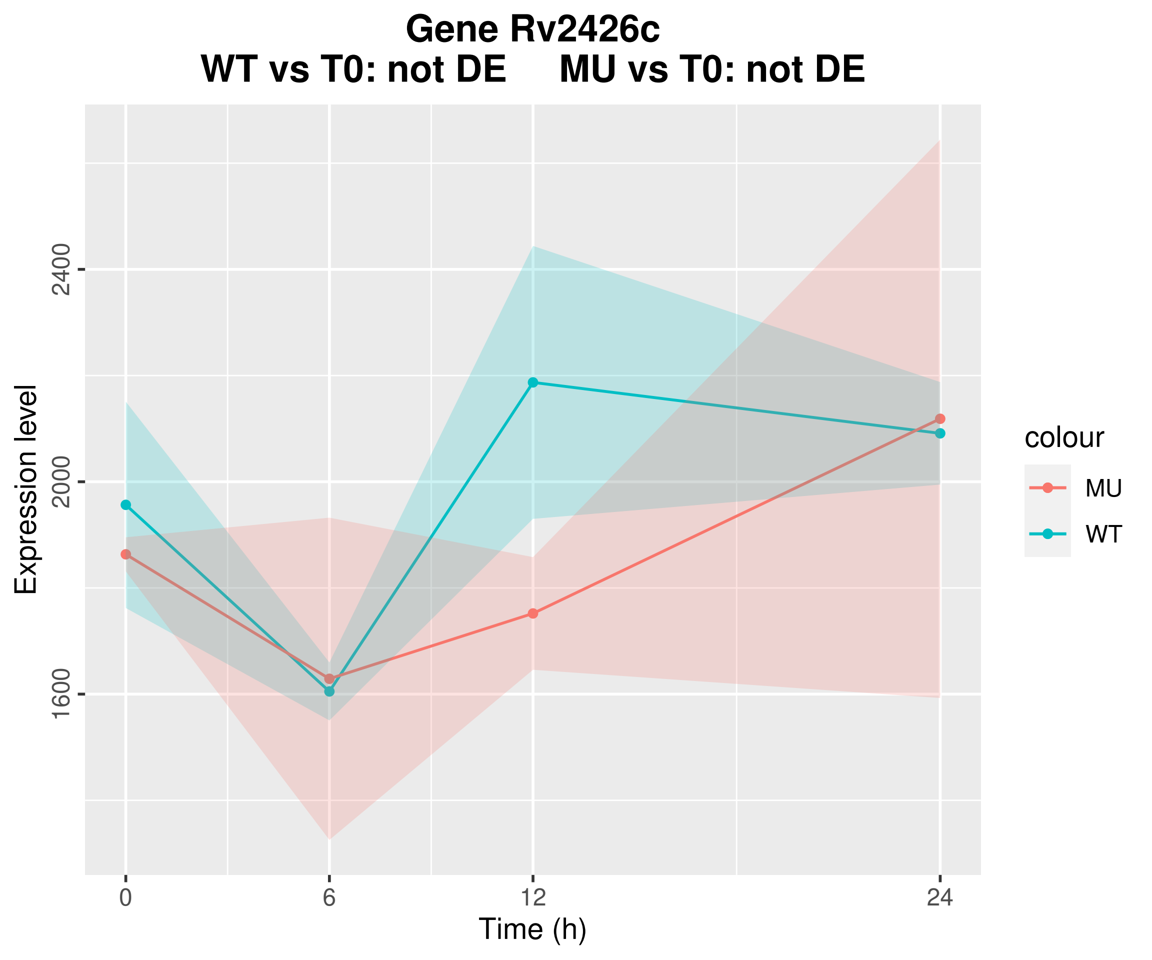

Gene Rv2426c
WT vs T0: not DE MU vs T0: not DE
Expression level
2400
2000
1600
0
6
12
24
Time (h)
colour
MU
WT

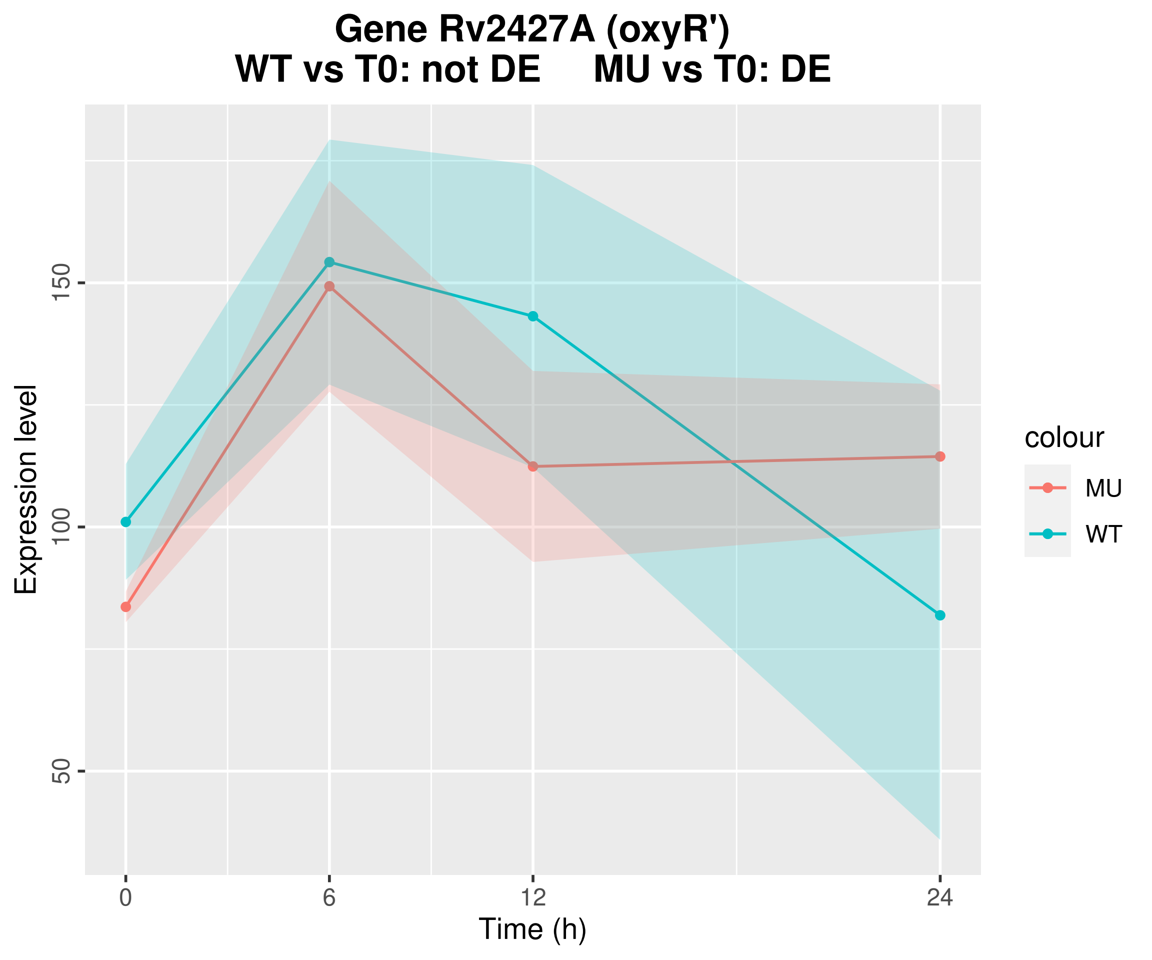
Gene Rv2427A (oxyR')
WT vs T0: not DE   MU vs T0: DE
Expression level
150
100
50
0
6
12
24
Time (h)
colour
MU
WT

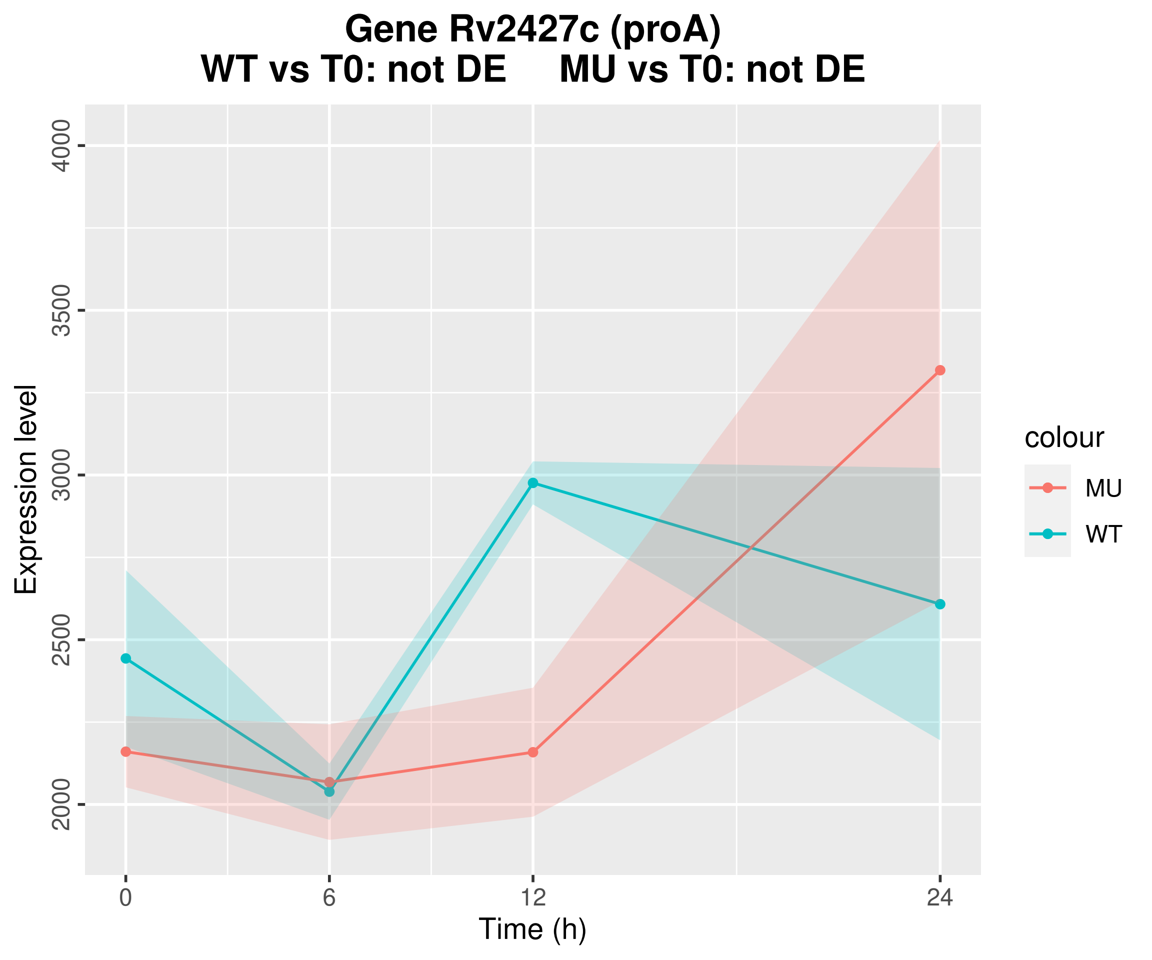

Gene Rv2427c (proA)
WT vs T0: not DE   MU vs T0: not DE
Expression level
4000
3500
3000
2500
2000
0
6
12
24
Time (h)
colour
MU
WT

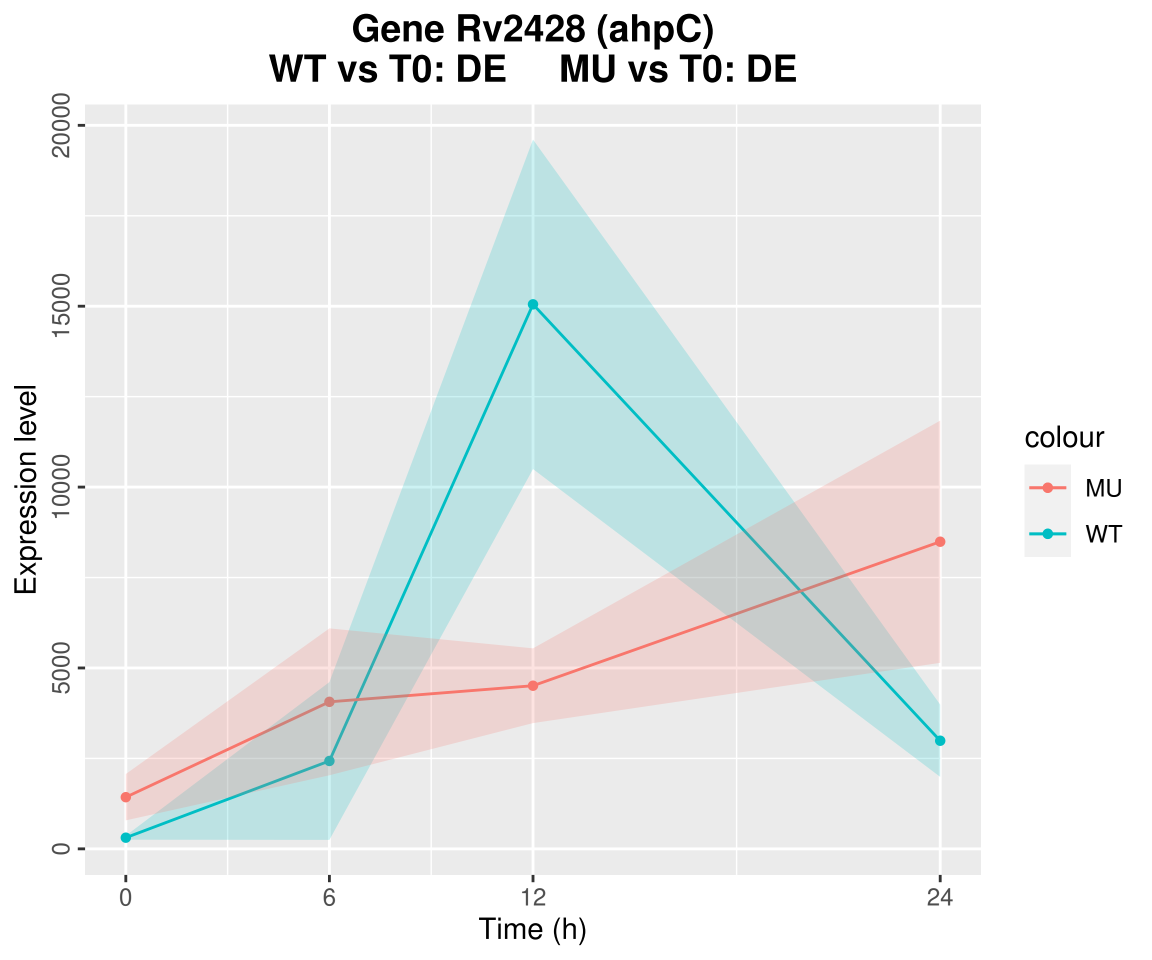

Gene Rv2428 (ahpC)
WT vs T0: DE MU vs T0: DE
Expression level
20000
15000
10000
5000
0
0
6
12
24
Time (h)
colour
MU
WT

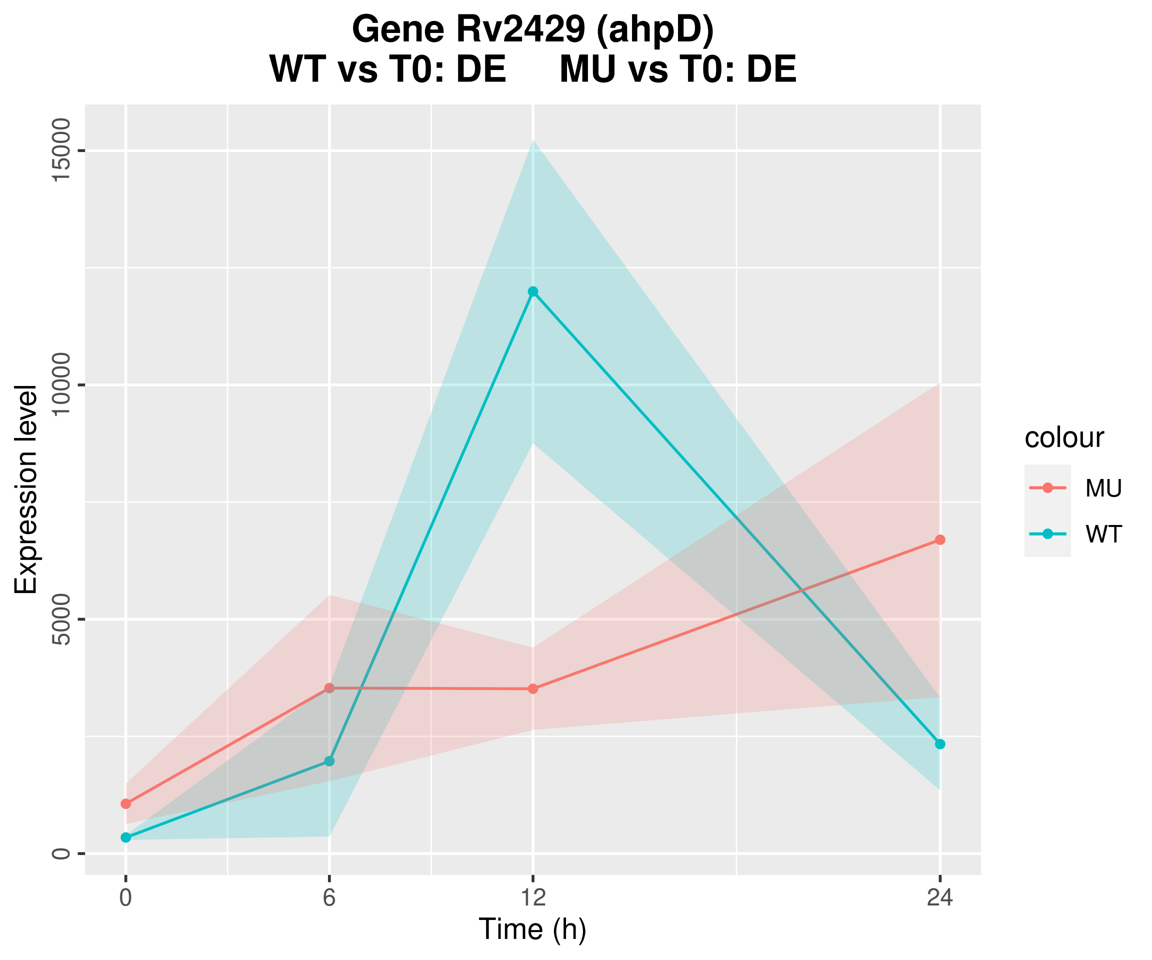

Gene Rv2429 (ahpD)
WT vs T0: DE   MU vs T0: DE
Expression level
15000
10000
5000
0
0
6
12
24
Time (h)
colour
MU
WT

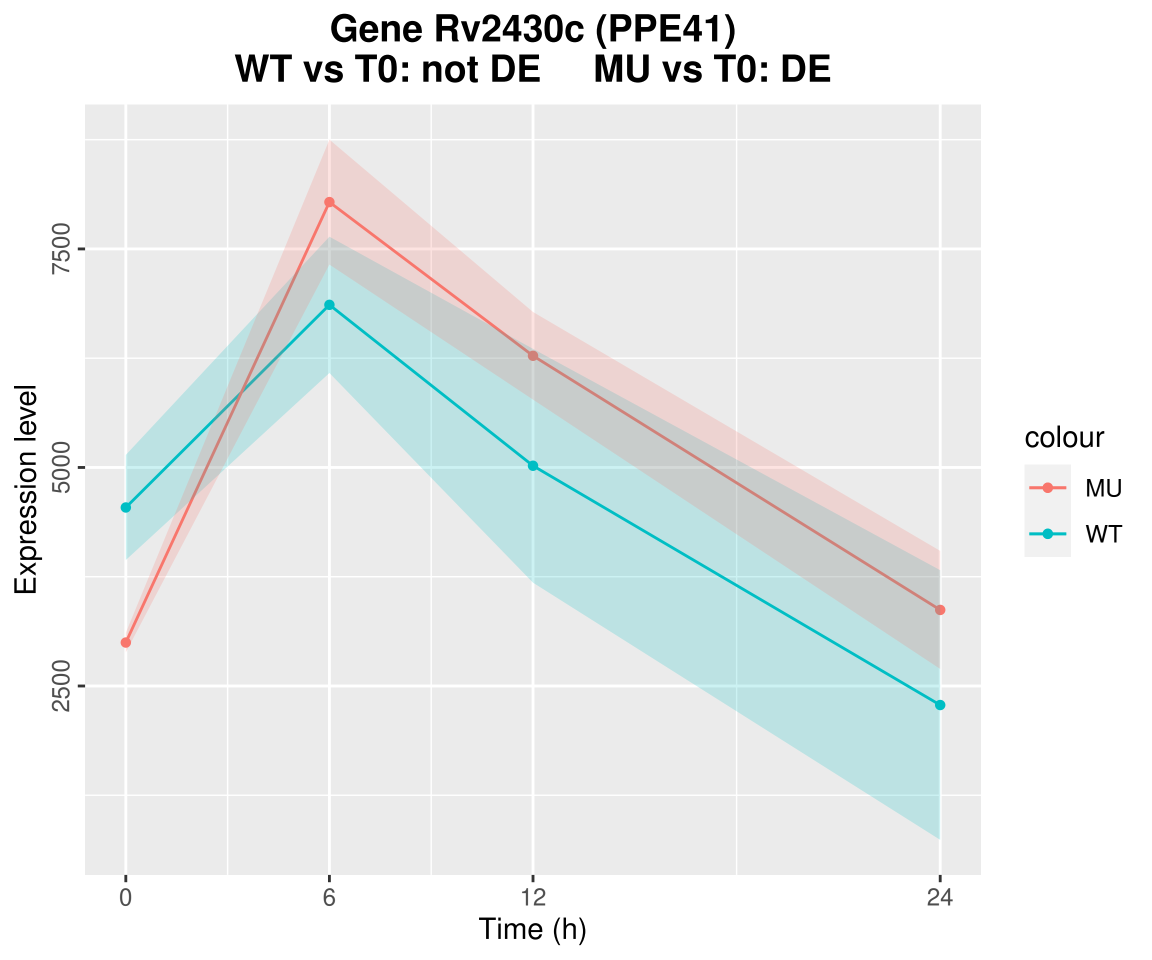

Gene Rv2430c (PPE41)
WT vs T0: not DE   MU vs T0: DE
Expression level
7500
5000
2500
0
6
12
24
Time (h)
colour
MU
WT

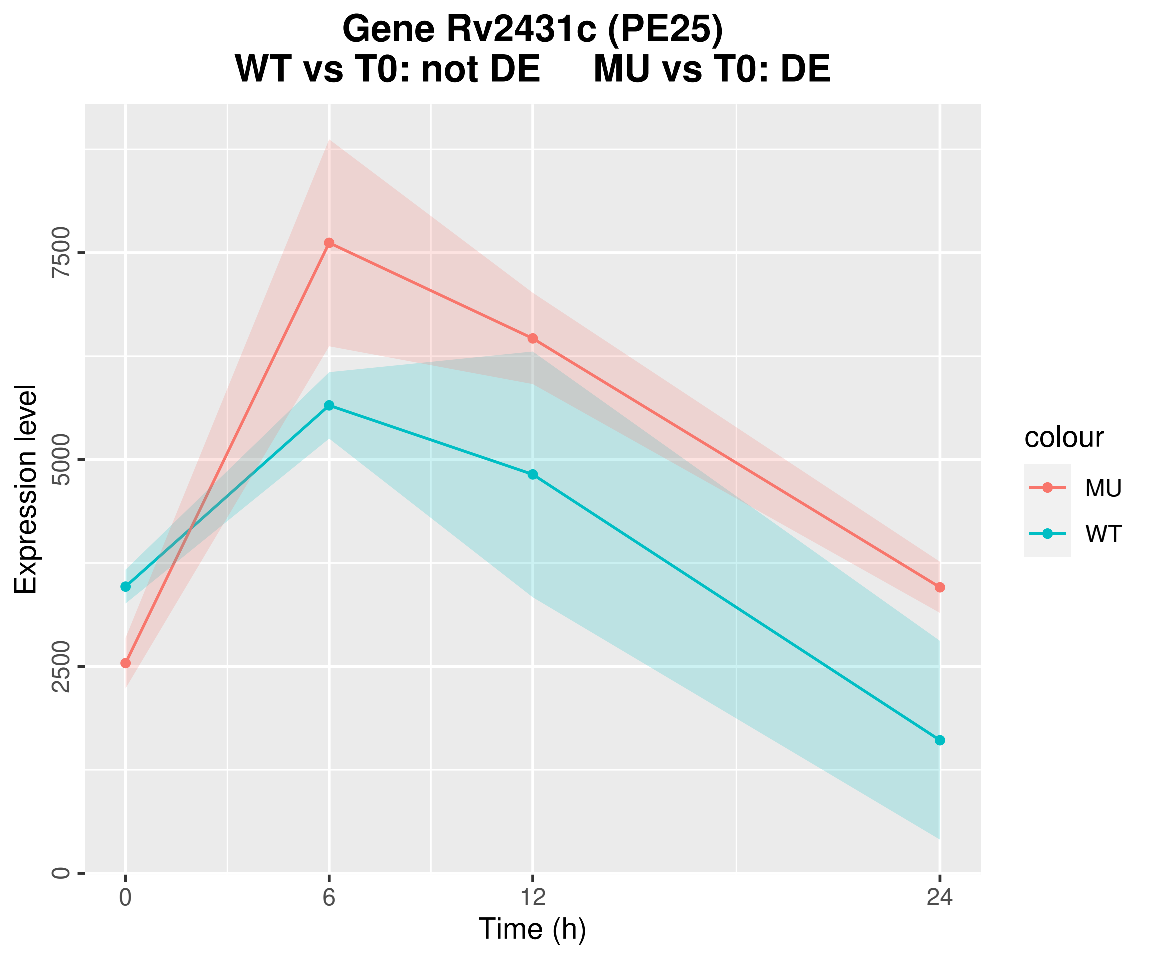
Gene Rv2431c (PE25)
WT vs T0: not DE MU vs T0: DE
Expression level
7500
5000
2500
0
0
6
12
24
Time (h)
colour
MU
WT

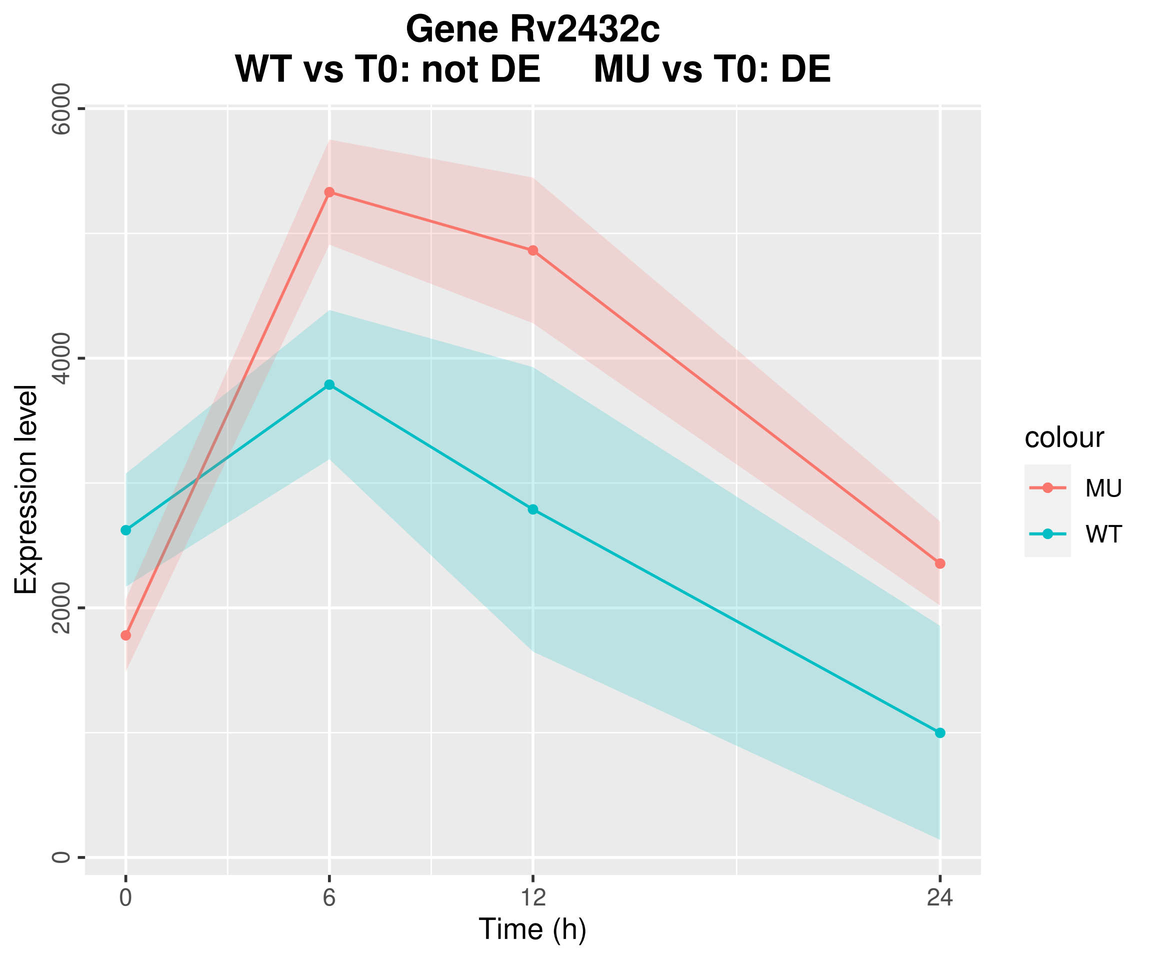

Gene Rv2432c
WT vs T0: not DE   MU vs T0: DE
6000
4000
2000
0
Expression level
0
6
12
24
Time (h)
colour
MU
WT

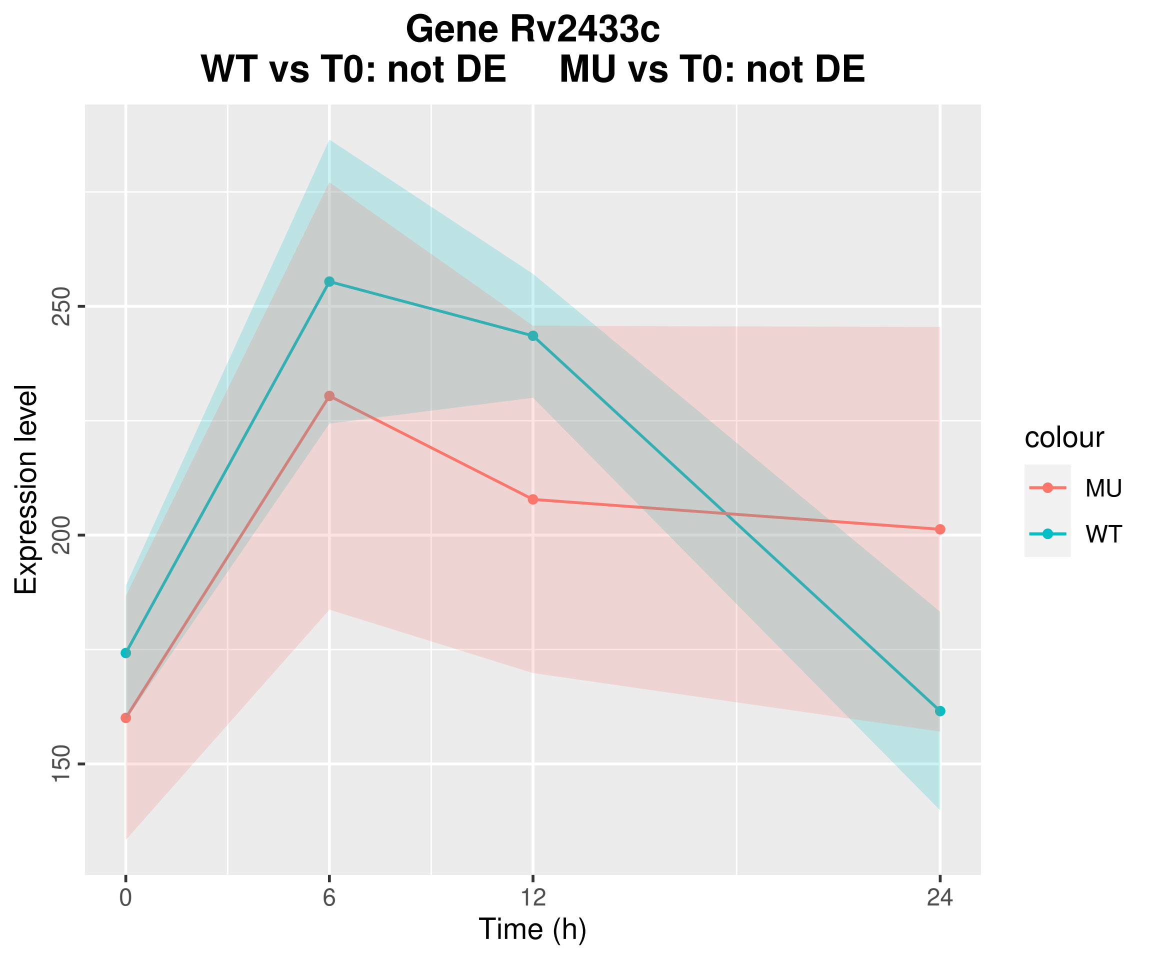

Gene Rv2433c
WT vs T0: not DE   MU vs T0: not DE
Expression level
250
200
150
0
6
12
24
Time (h)
colour
MU
WT

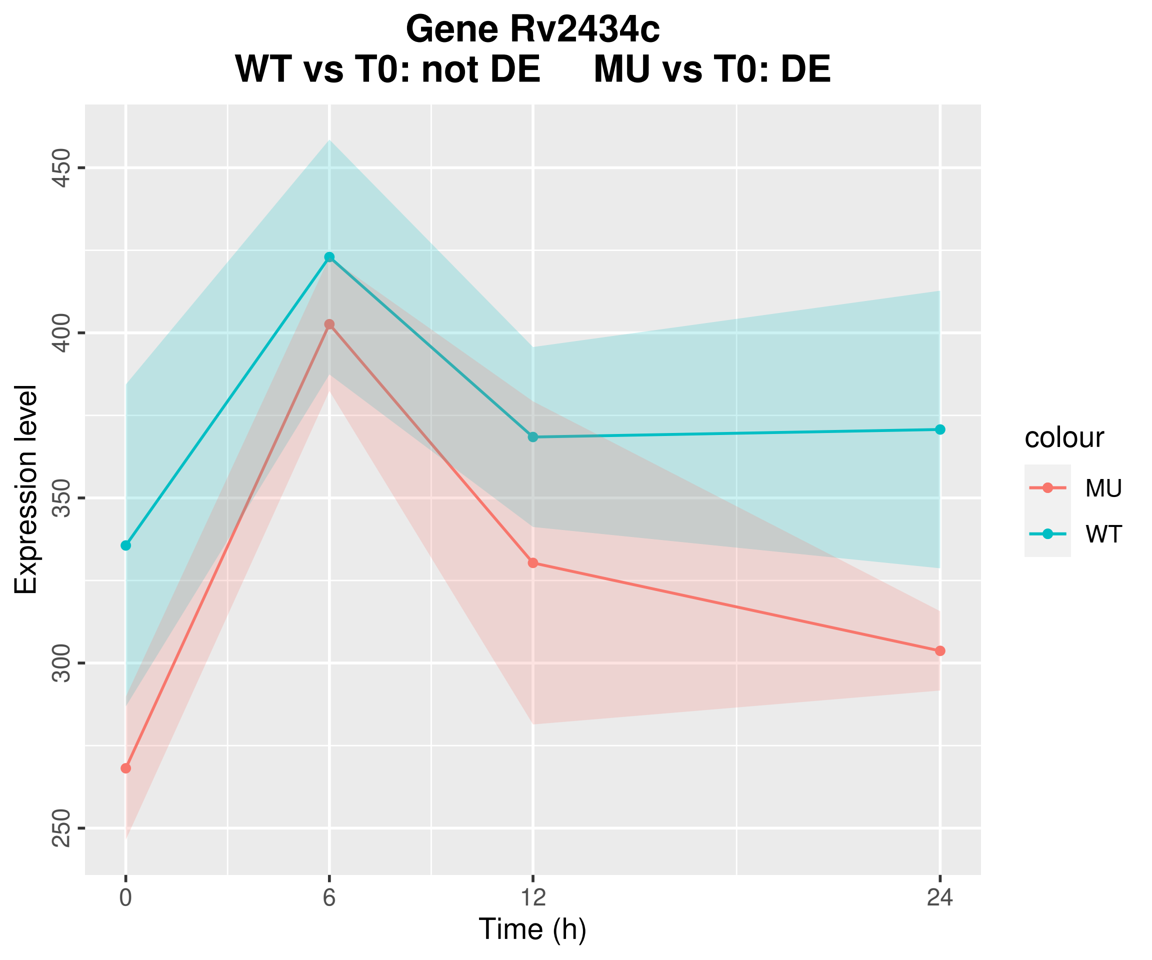
Gene Rv2434c
WT vs T0: not DE MU vs T0: DE
Expression level
450
400
350
300
250
0
6
12
24
Time (h)
colour
MU
WT

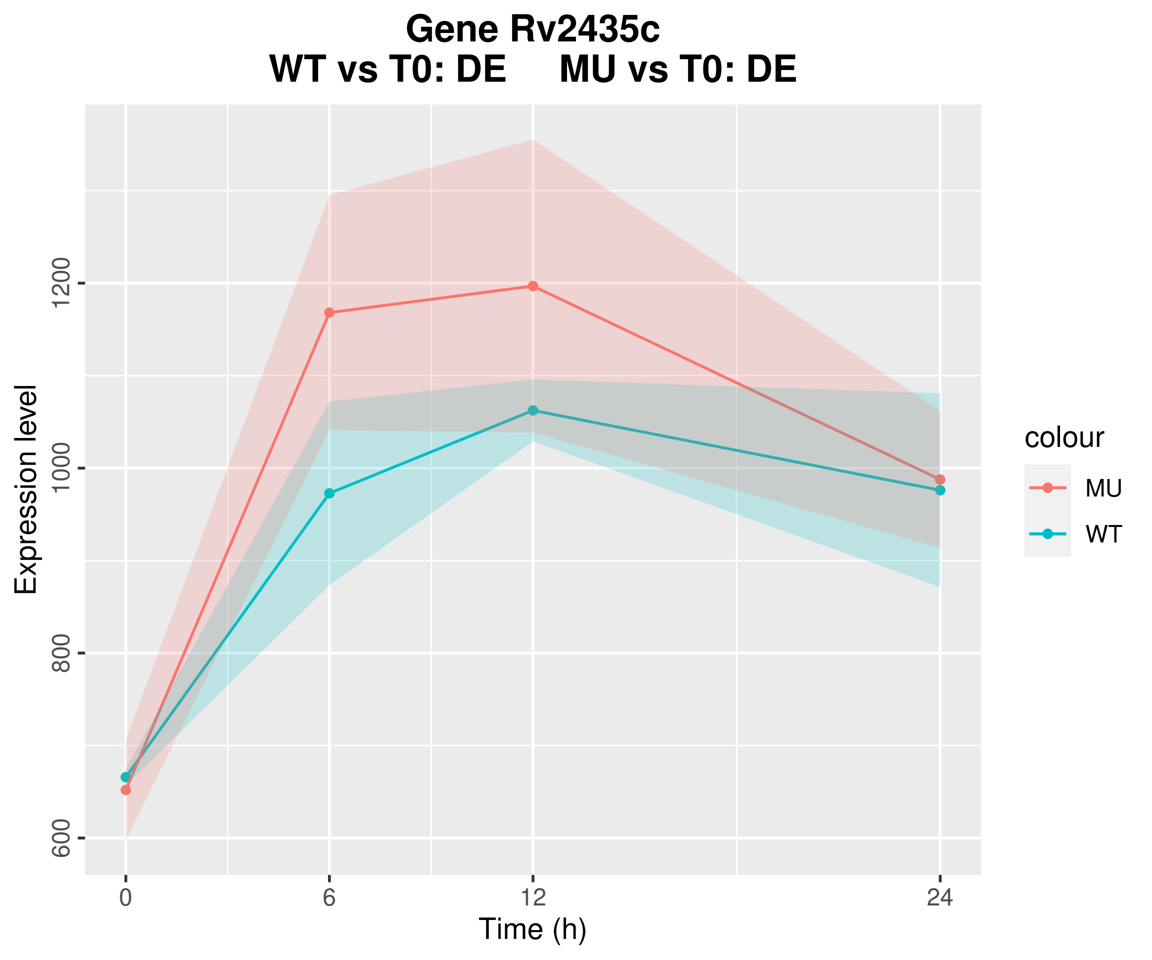

Gene Rv2435c
WT vs T0: DE MU vs T0: DE
Expression level
1200
1000
800
600
0
6
12
24
Time (h)
colour
MU
WT

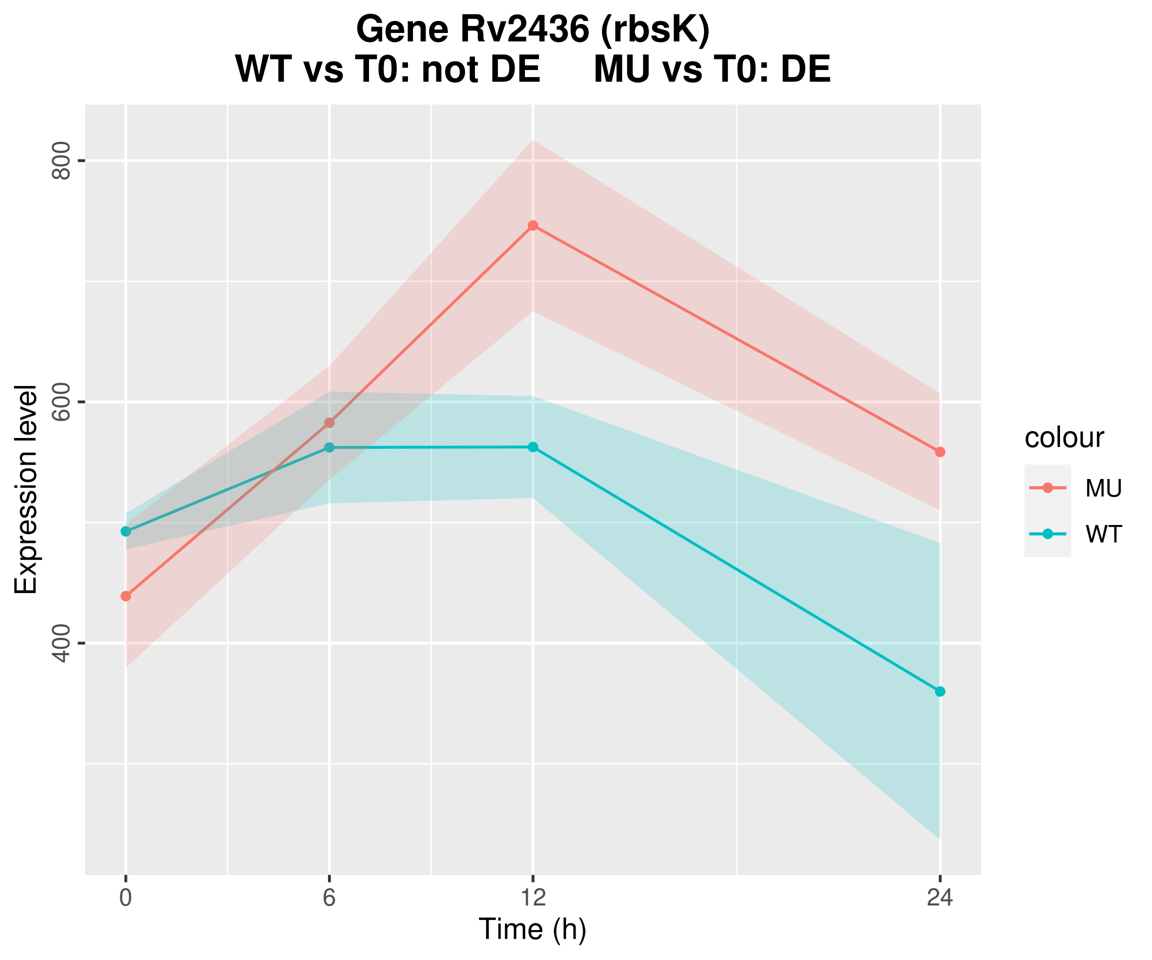

Gene Rv2436 (rbsK)
WT vs T0: not DE MU vs T0: DE
800
600
400
Expression level
0
6
12
24
Time (h)
colour
MU
WT

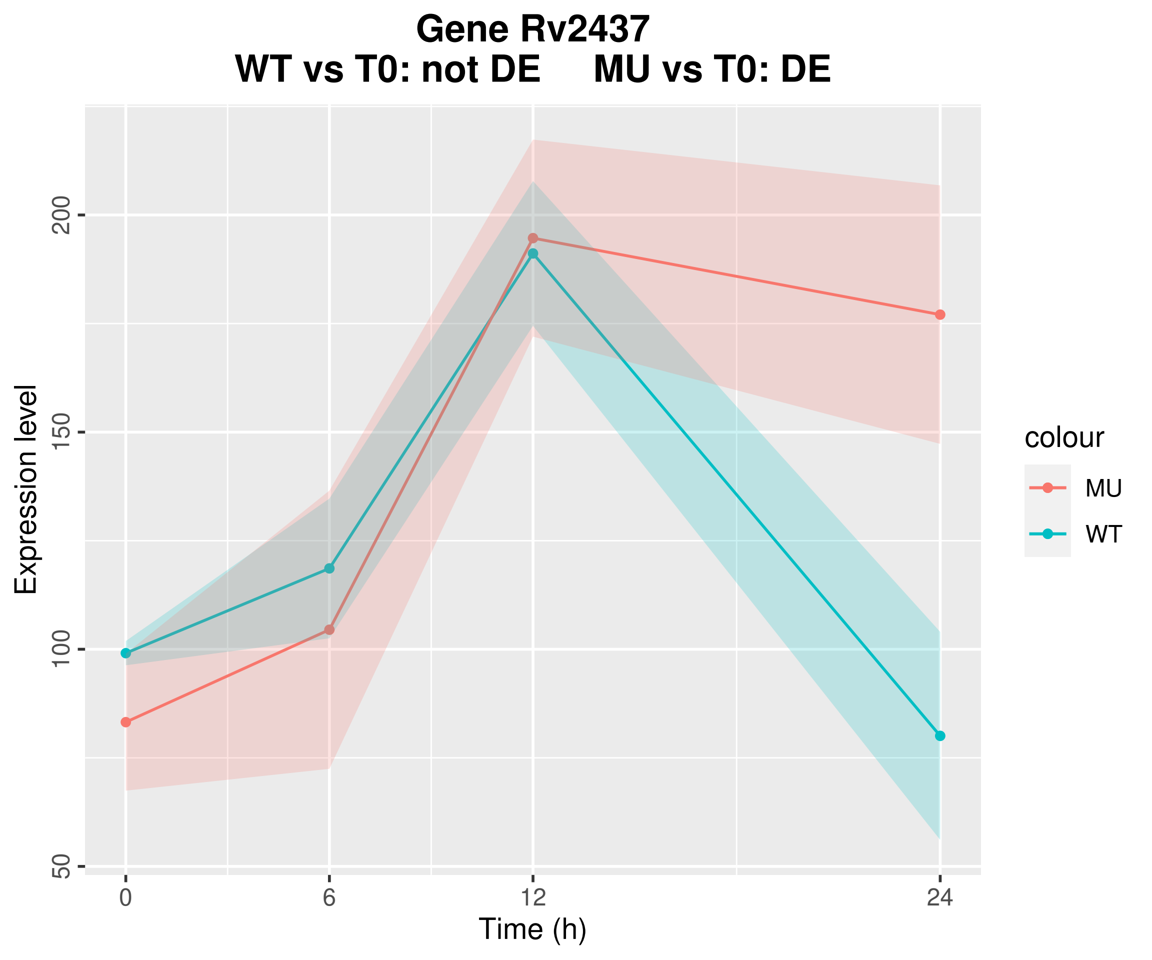

Gene Rv2437
WT vs T0: not DE MU vs T0: DE
Expression level
200
150
100
50
0
6
12
24
Time (h)
colour
MU
WT

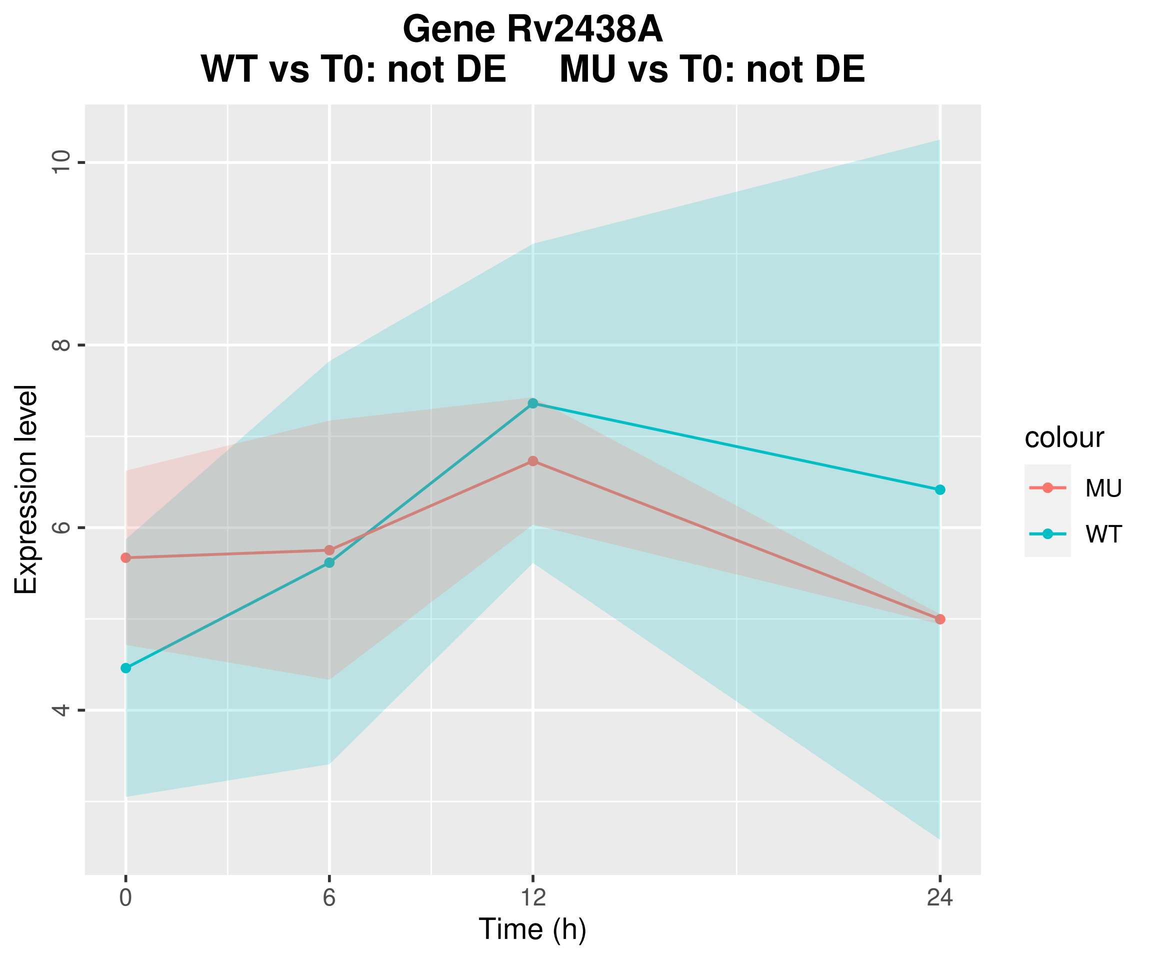

Gene Rv2438A
WT vs T0: not DE   MU vs T0: not DE
Expression level
10
8
6
4
0
6
12
24
Time (h)
colour
MU
WT

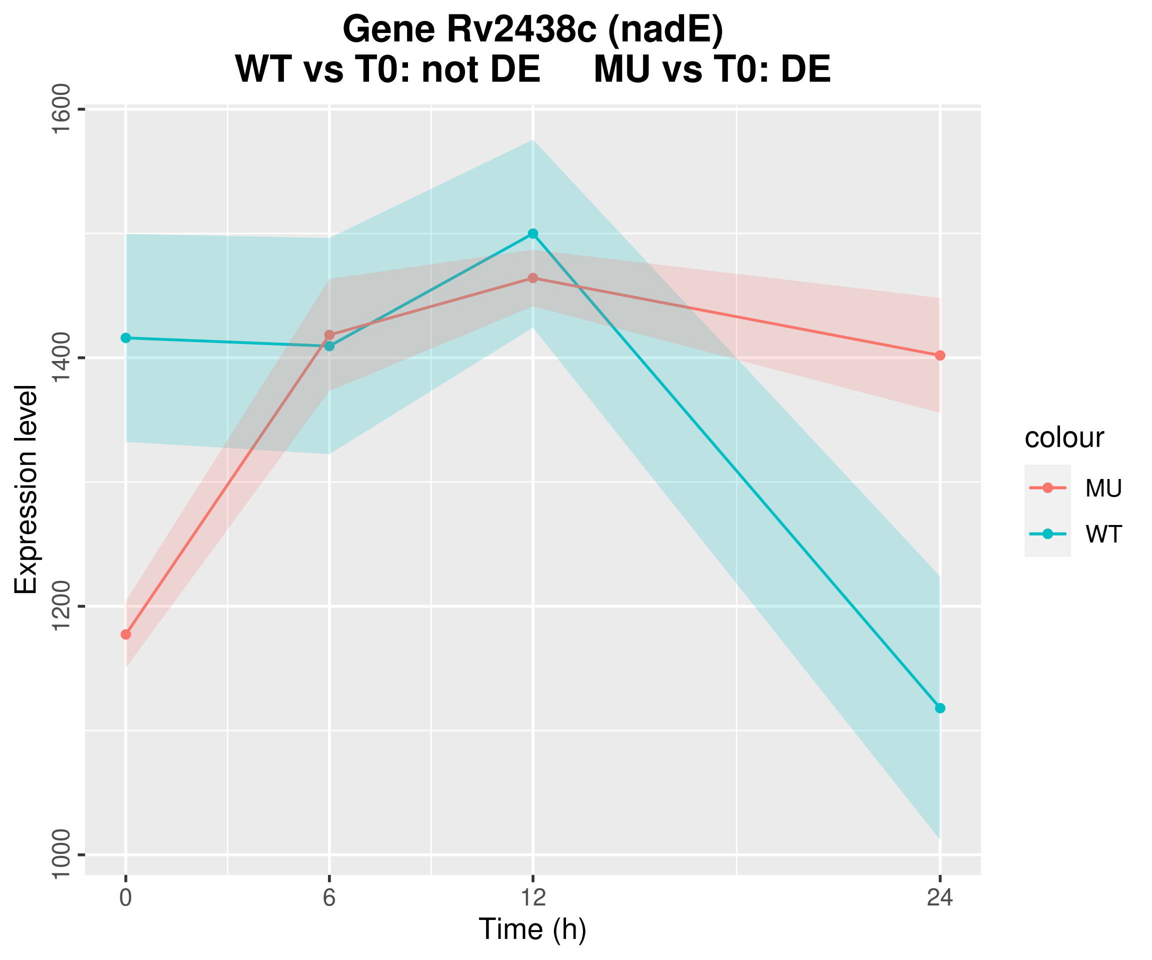

Gene Rv2438c (nadE)
WT vs T0: not DE MU vs T0: DE
1600
1400
1200
1000
Expression level
0
6
12
24
Time (h)
colour
MU
WT

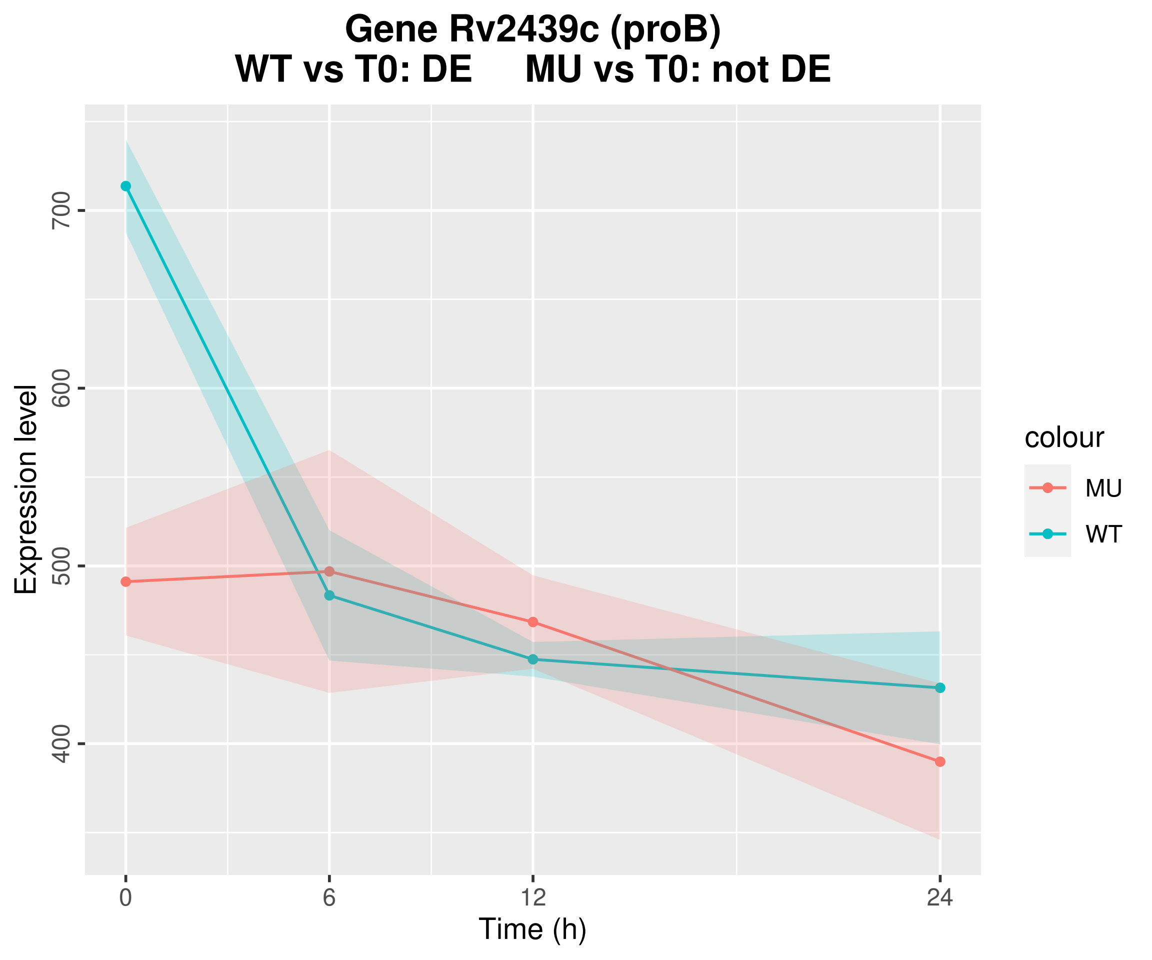

Gene Rv2439c (proB)
WT vs T0: DE   MU vs T0: not DE
Expression level
700
600
500
400
0
6
12
24
Time (h)
colour
MU
WT

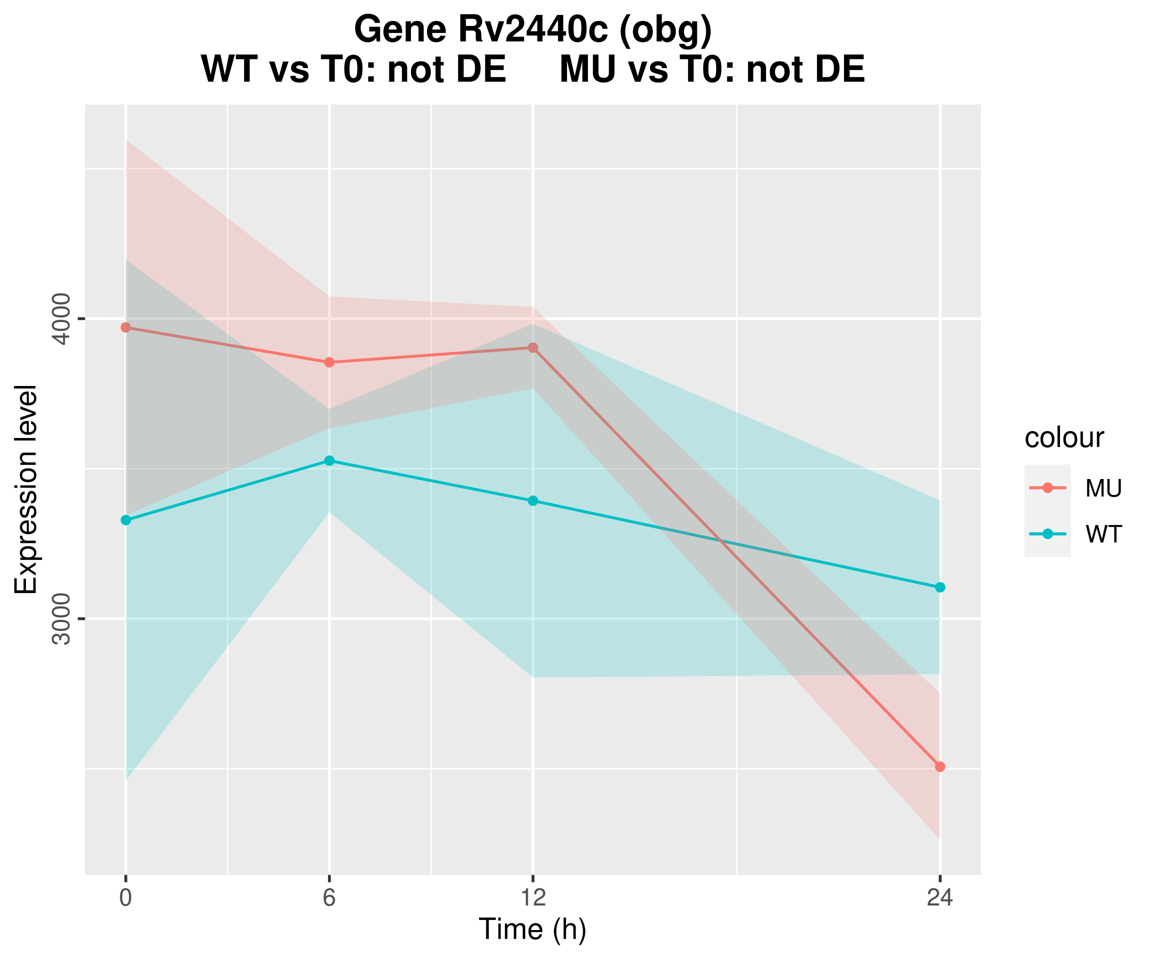
Gene Rv2440c (obg)
WT vs T0: not DE MU vs T0: not DE
Expression level
4000
3000
0
6
12
24
Time (h)
colour
MU
WT

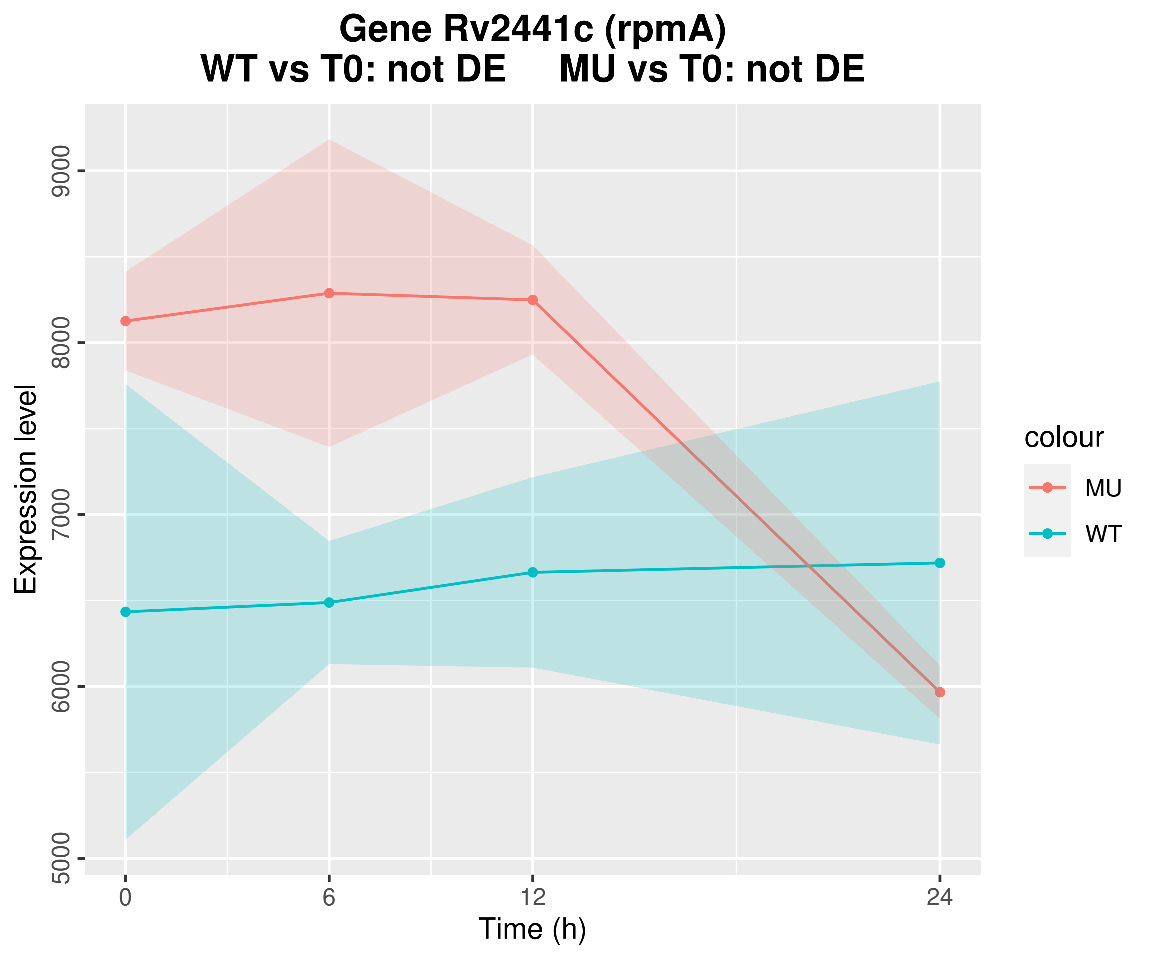
Gene Rv2441c (rpmA)
WT vs T0: not DE MU vs T0: not DE
Expression level
5000
6000
7000
8000
9000
0
6
12
24
Time (h)
colour
MU
WT

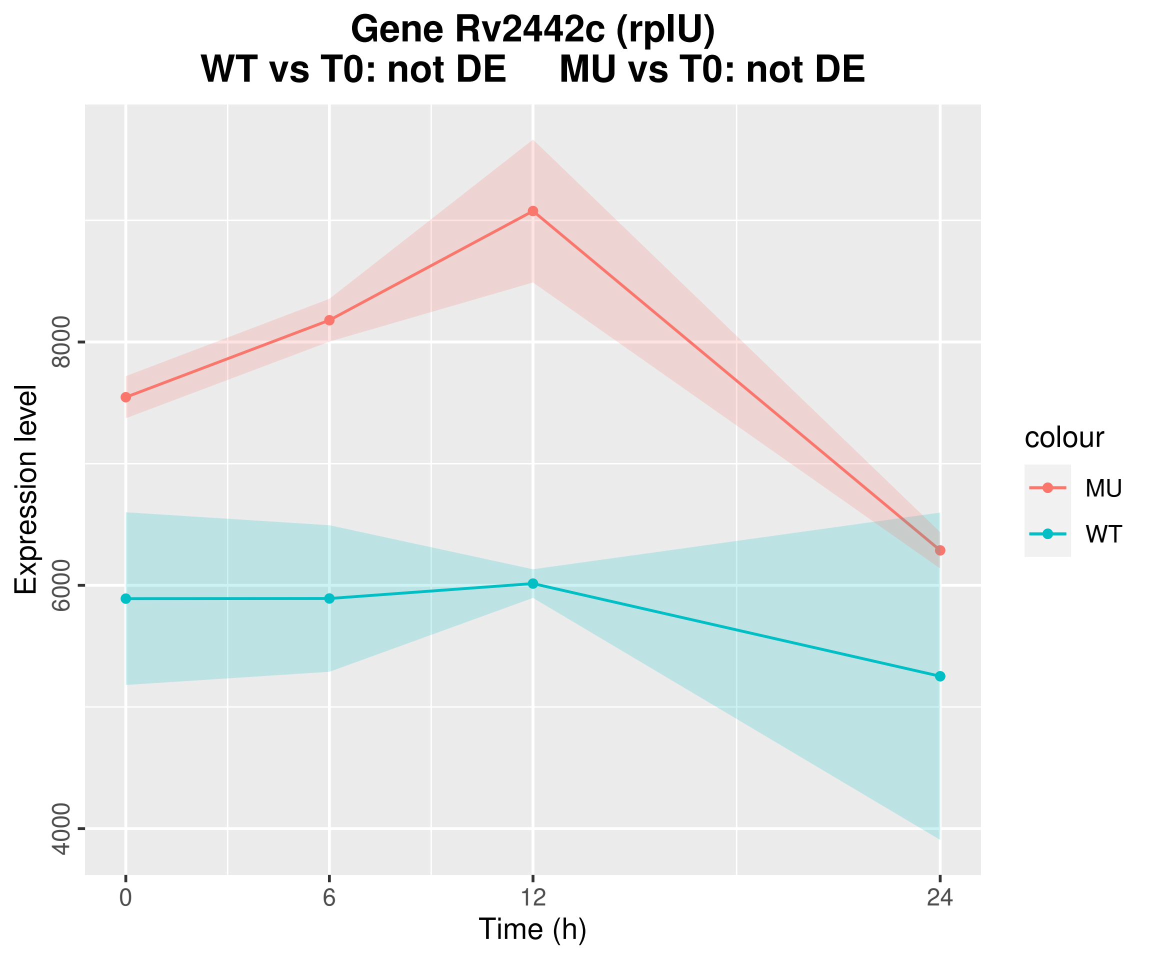
Gene Rv2442c (rplU)
WT vs T0: not DE MU vs T0: not DE
Expression level
8000
6000
4000
0
6
12
24
Time (h)
colour
MU
WT

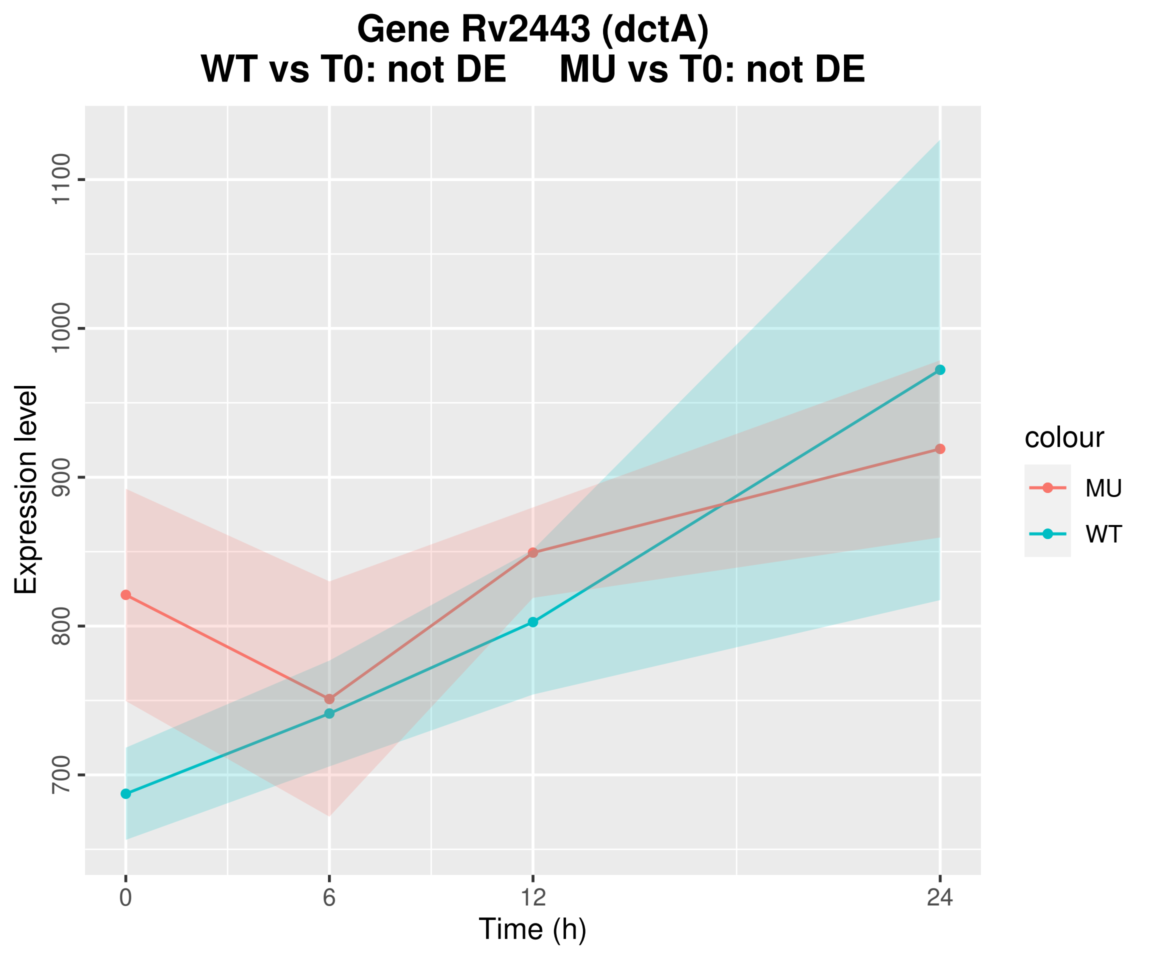
Gene Rv2443 (dctA)
WT vs T0: not DE   MU vs T0: not DE
Expression level
1100
1000
900
800
700
0
6
12
24
Time (h)
colour
MU
WT

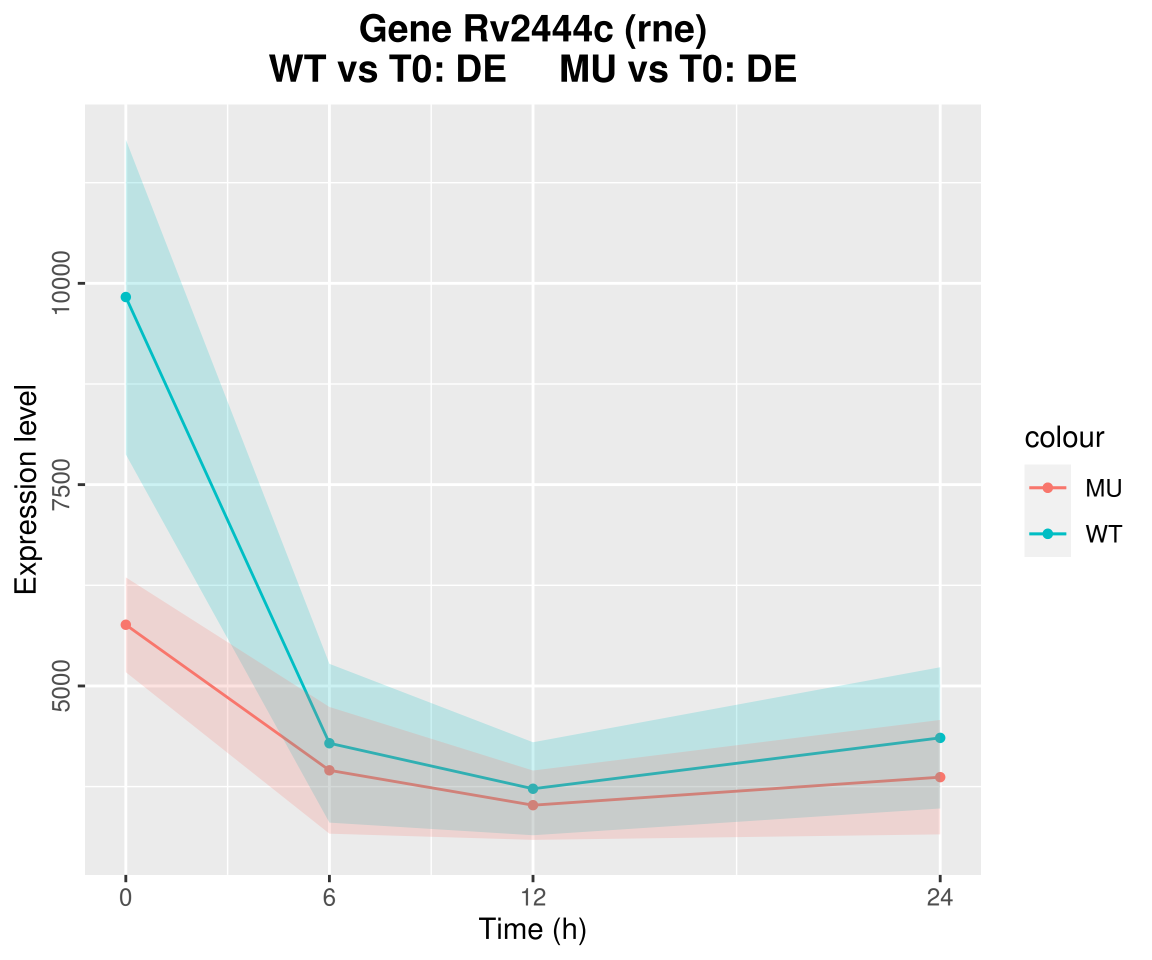

Gene Rv2444c (rne)
WT vs T0: DE   MU vs T0: DE
Expression level
10000
7500
5000
0
6
12
24
Time (h)
colour
MU
WT

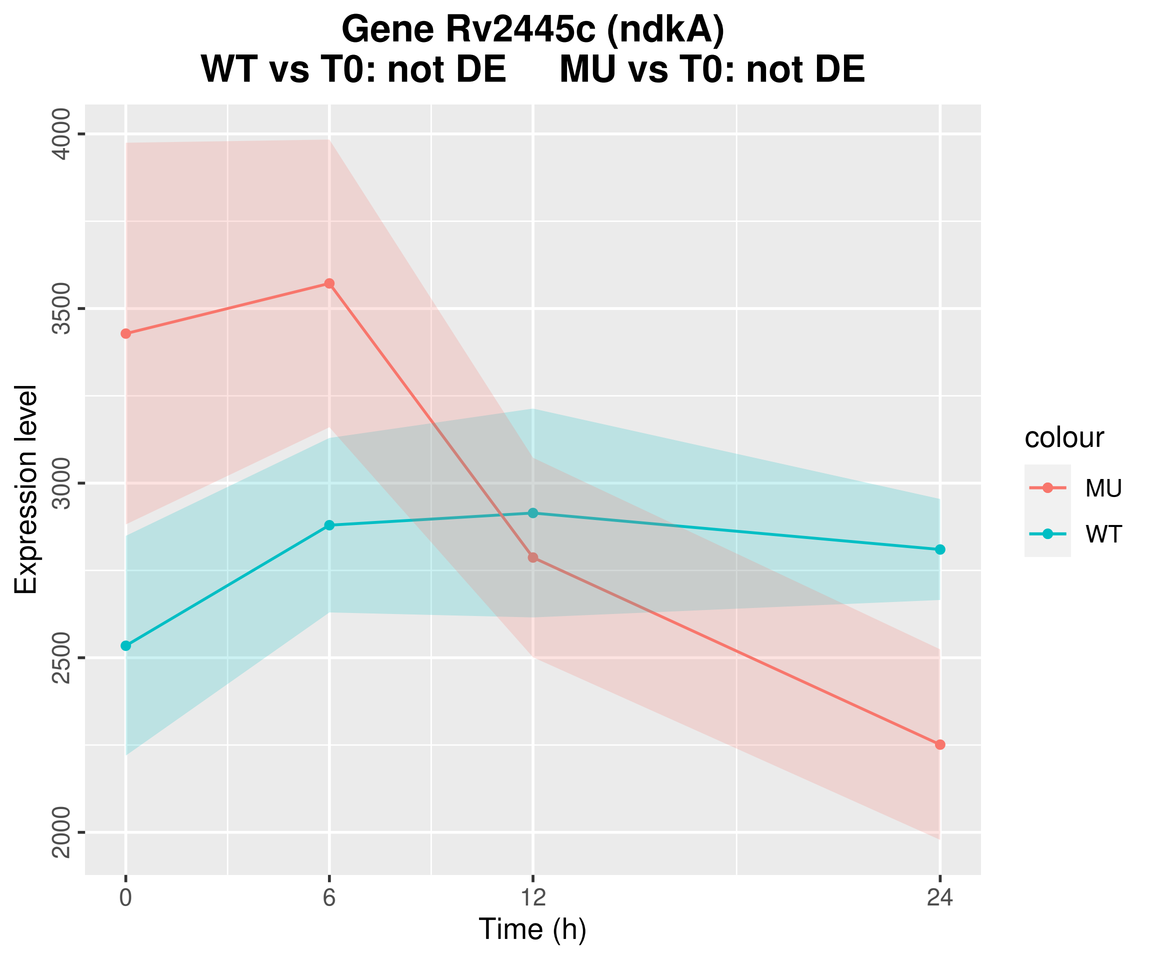
Gene Rv2445c (ndkA)
WT vs T0: not DE MU vs T0: not DE
Expression level
4000
3500
3000
2500
2000
0
6
12
24
Time (h)
colour
MU
WT

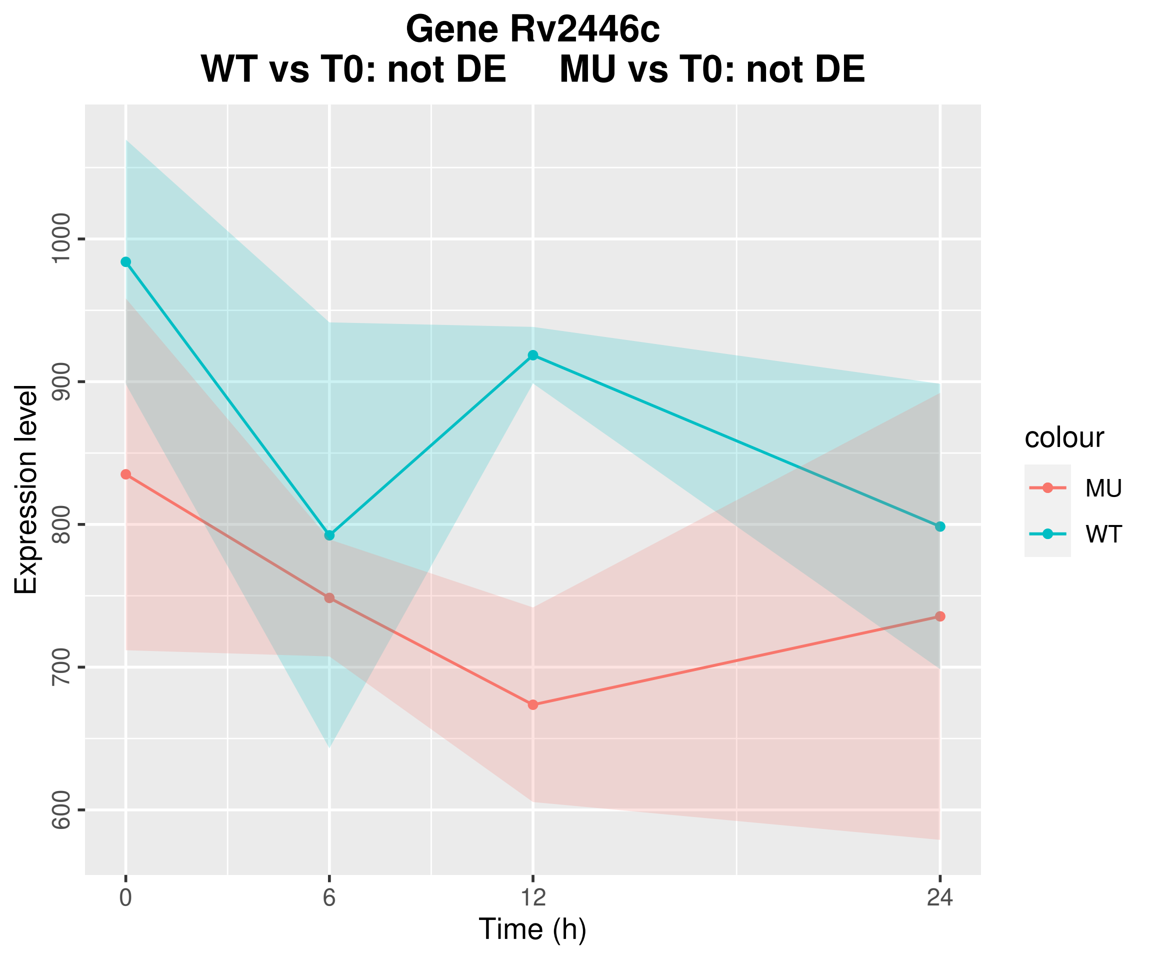
Gene Rv2446c
WT vs T0: not DE   MU vs T0: not DE
Expression level
1000
900
800
700
600
Time (h)
0
6
12
24
colour
MU
WT

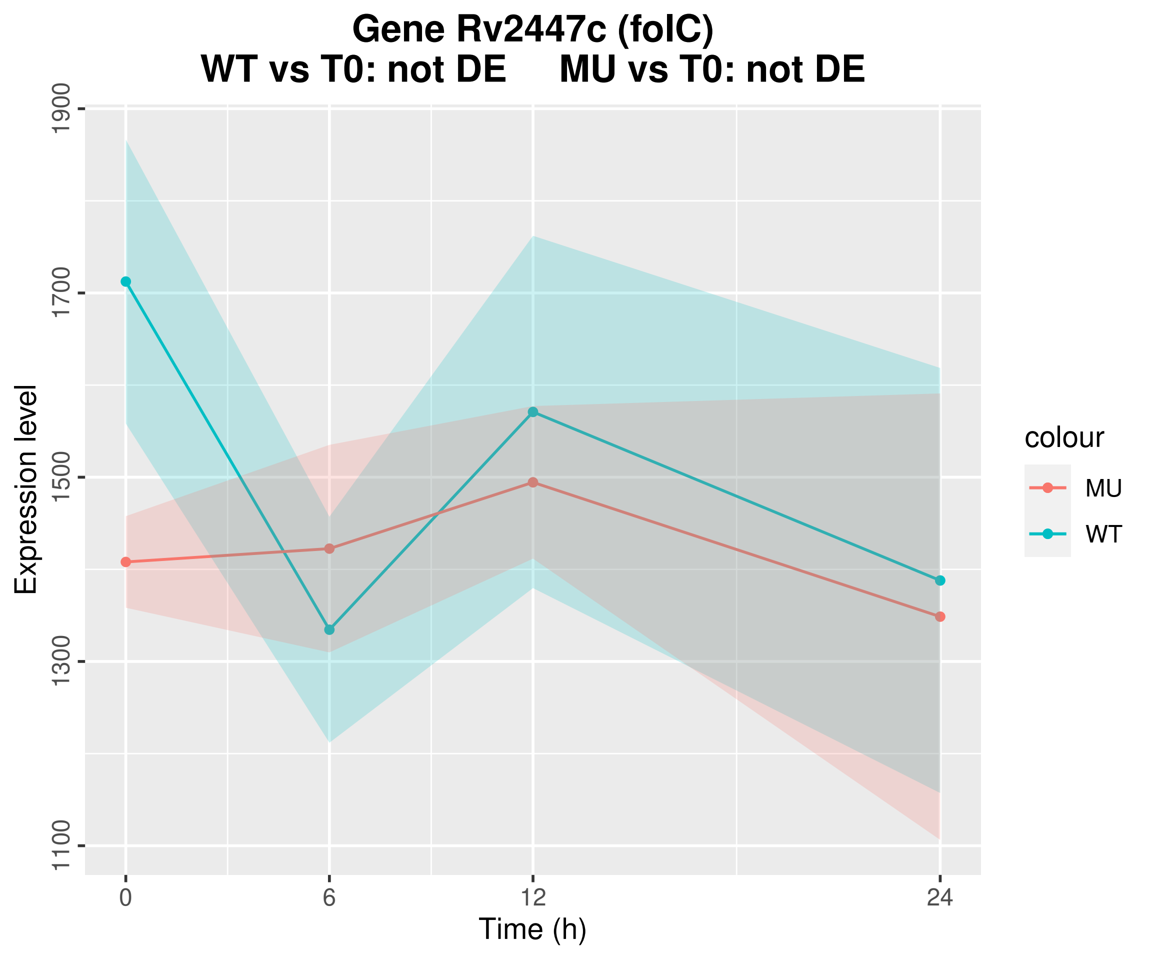

Gene Rv2447c (folC)
WT vs T0: not DE   MU vs T0: not DE
1900
1700
1500
1300
1100
Expression level
0
6
12
24
Time (h)
colour
MU
WT

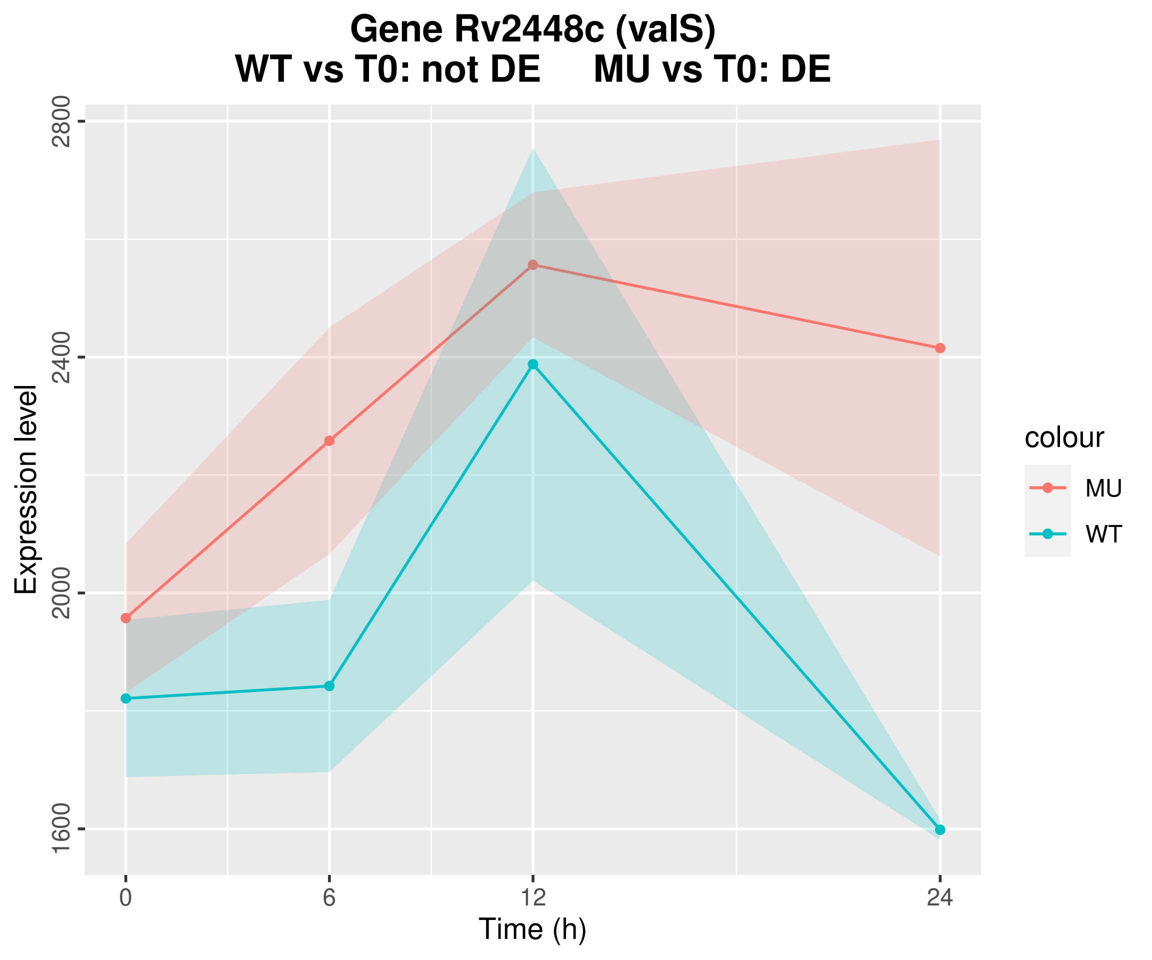

Gene Rv2448c (valS)
WT vs T0: not DE MU vs T0: DE
2800
2400
2000
1600
Expression level
0
6
12
24
Time (h)
colour
MU
WT

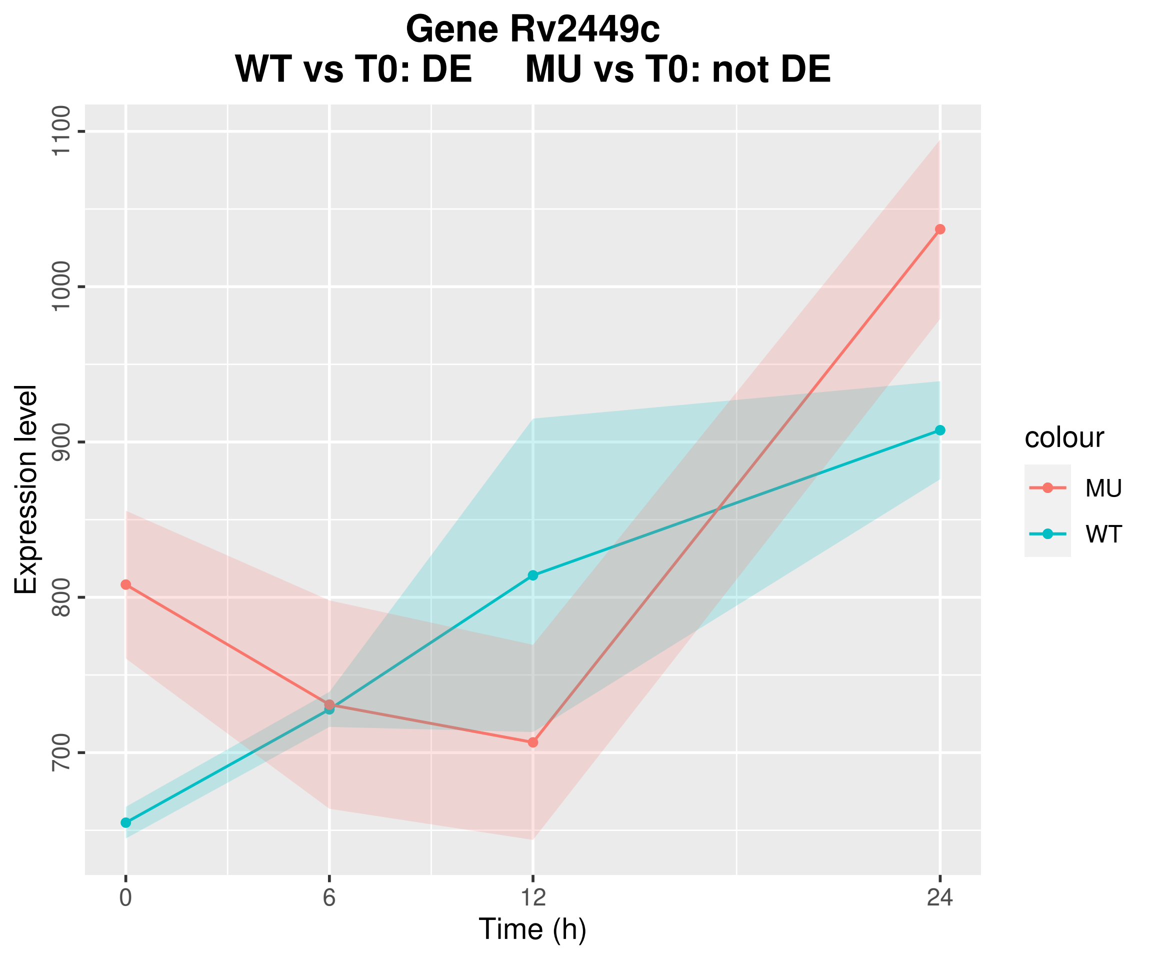
Gene Rv2449c
WT vs T0: DE   MU vs T0: not DE
Expression level
1100
1000
900
800
700
0
6
12
24
Time (h)
colour
MU
WT

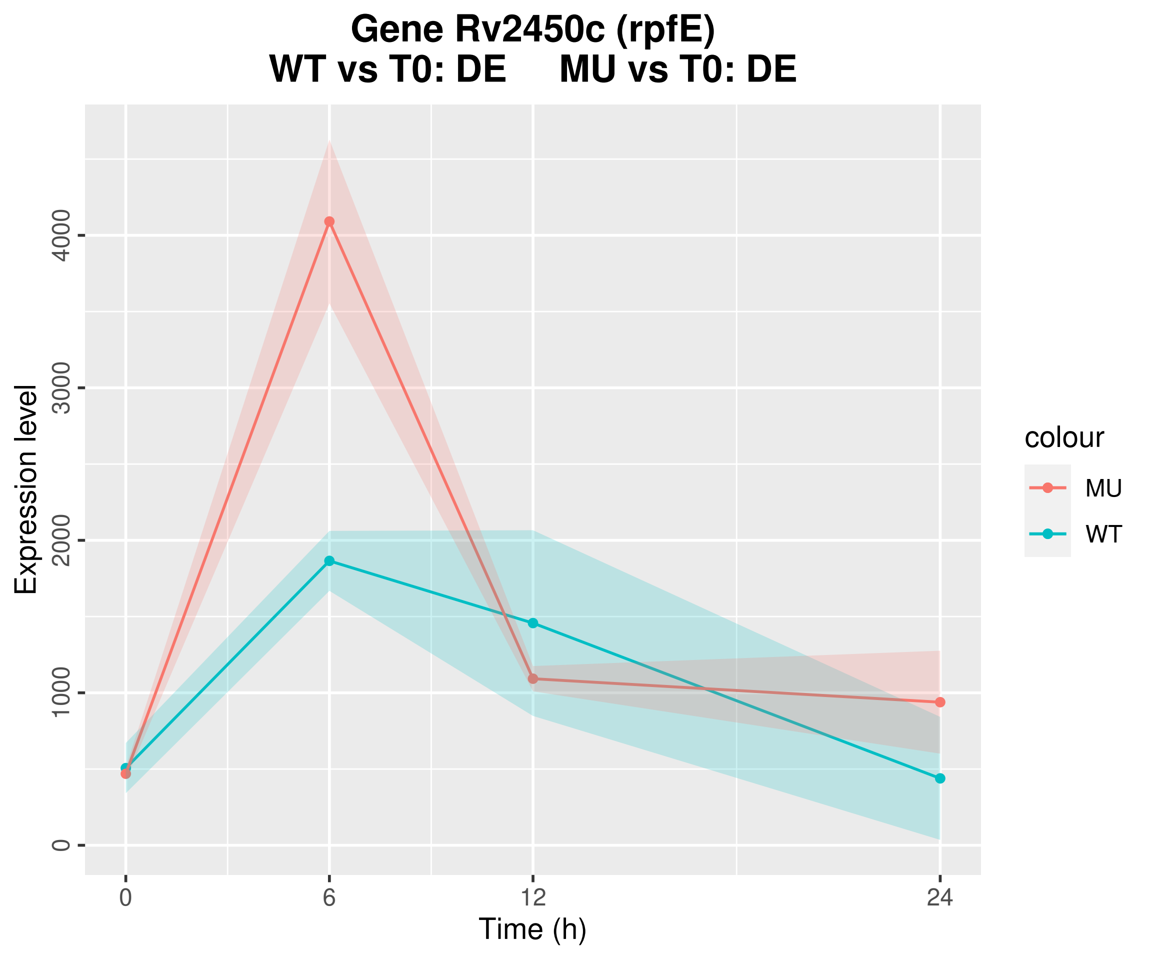
Gene Rv2450c (rpfE)
WT vs T0: DE   MU vs T0: DE
Expression level
0
1000
2000
3000
4000
Time (h)
0
6
12
24
colour
MU
WT

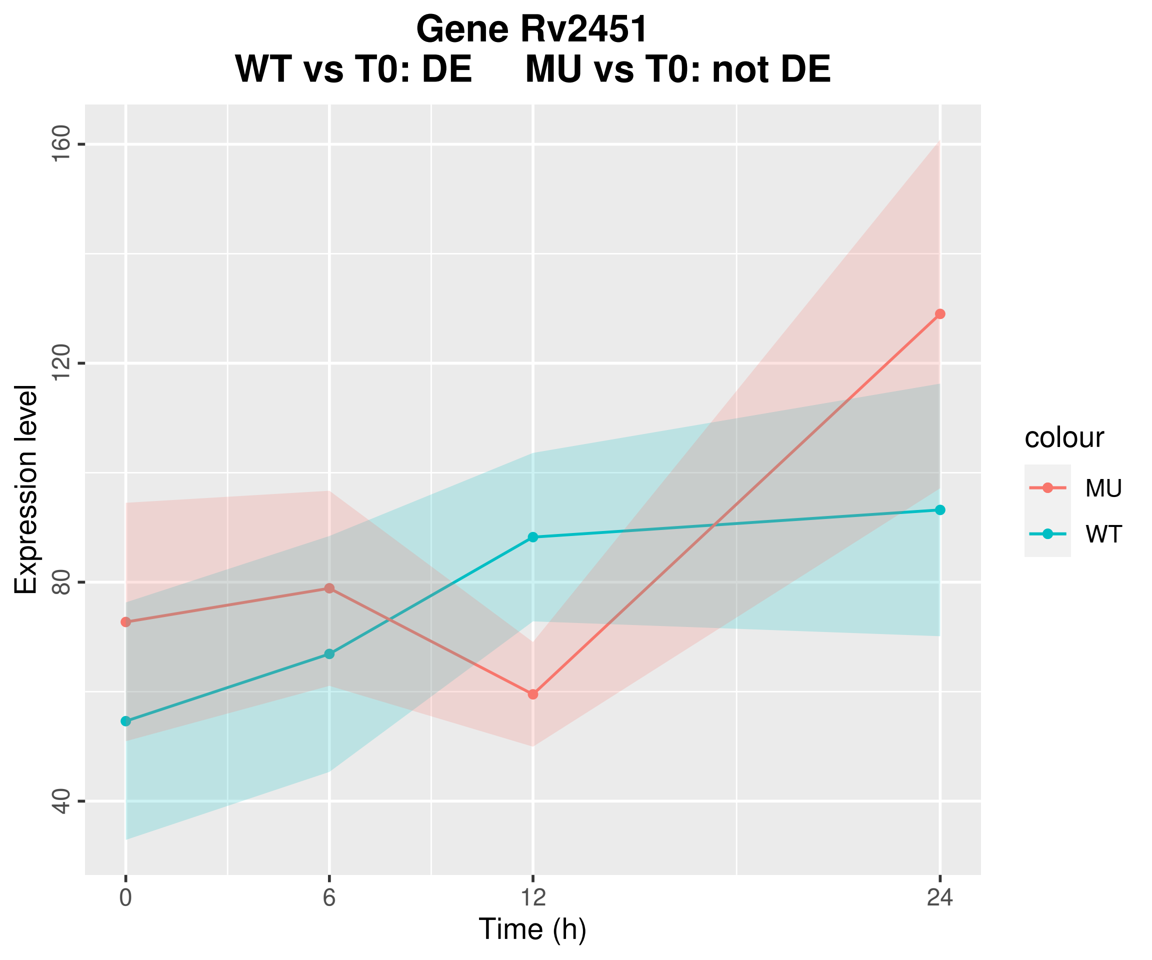
Gene Rv2451
WT vs T0: DE   MU vs T0: not DE
Expression level
160
120
80
40
0
6
12
24
Time (h)
colour
MU
WT

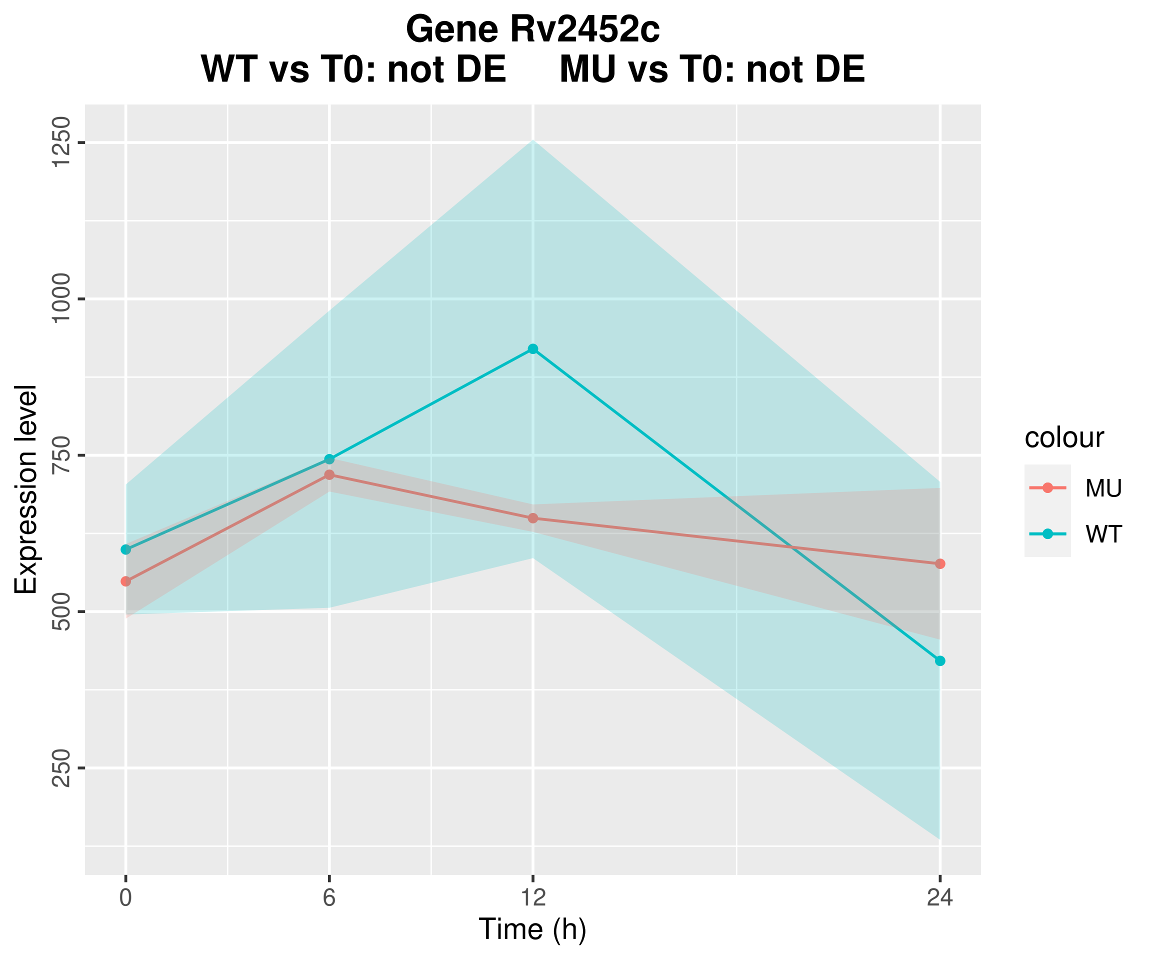

Gene Rv2452c
WT vs T0: not DE   MU vs T0: not DE
Expression level
1250
1000
750
500
250
0
6
12
24
Time (h)
colour
MU
WT

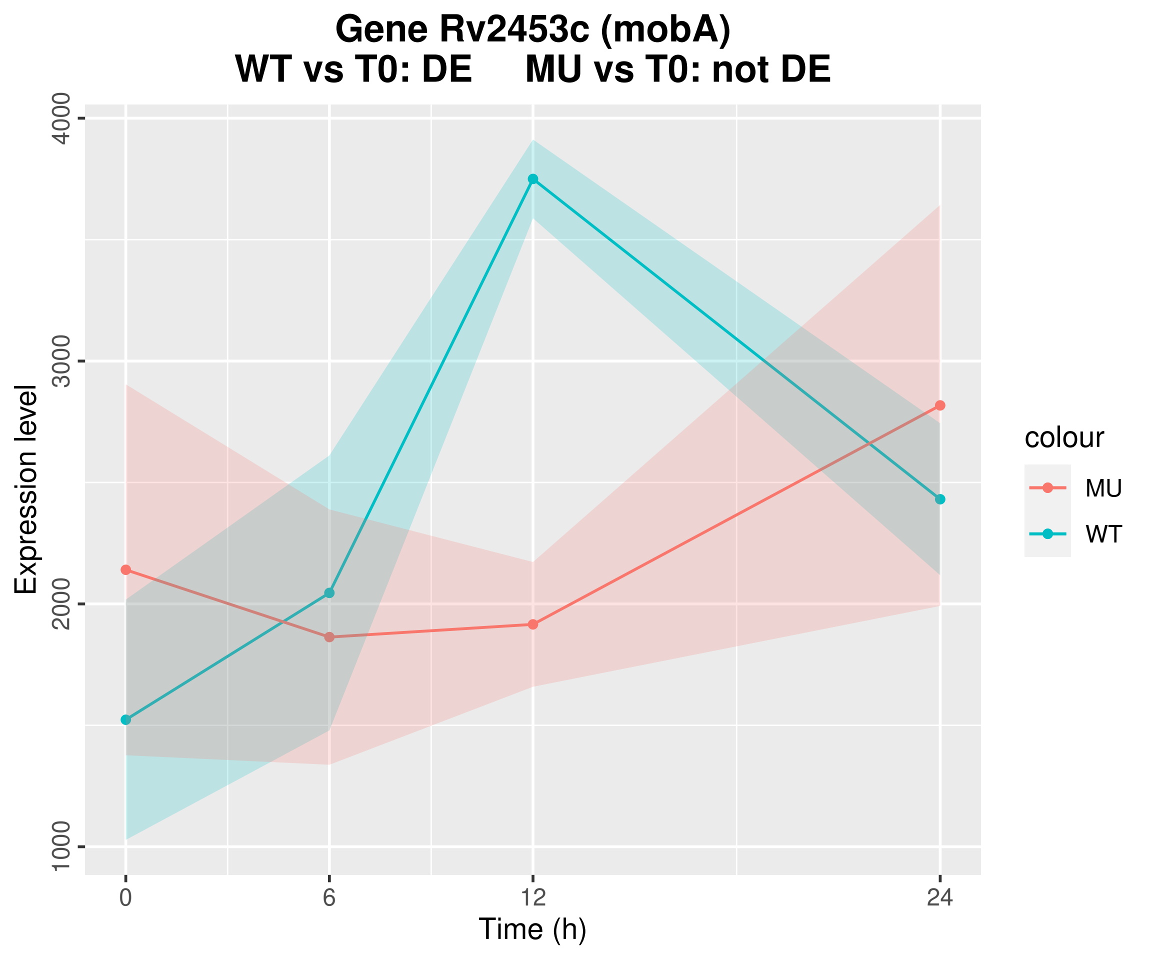

Gene Rv2453c (mobA)
WT vs T0: DE   MU vs T0: not DE
Expression level
4000
3000
2000
1000
0
6
12
24
Time (h)
colour
MU
WT

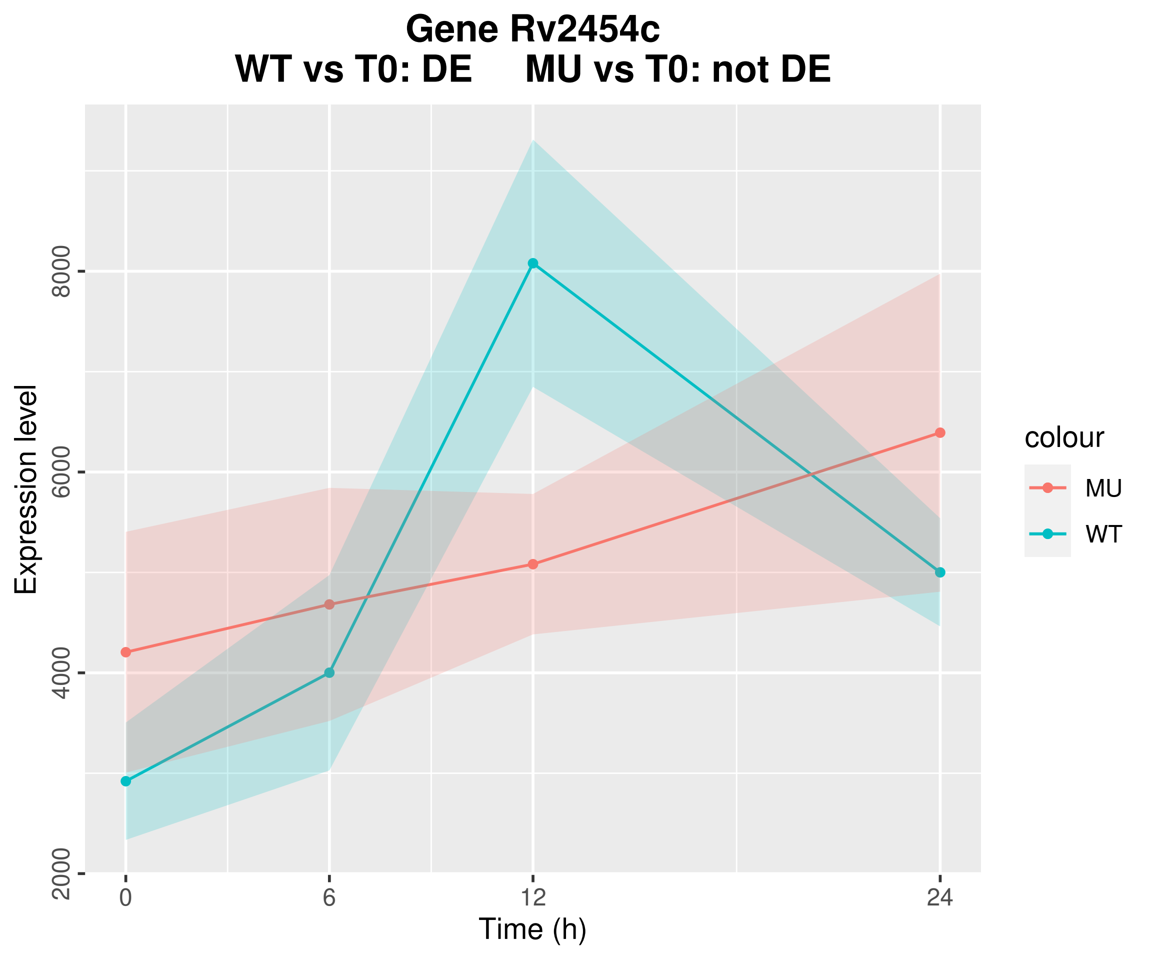
Gene Rv2454c
WT vs T0: DE   MU vs T0: not DE
Expression level
2000
4000
6000
8000
0
6
12
24
Time (h)
colour
MU
WT

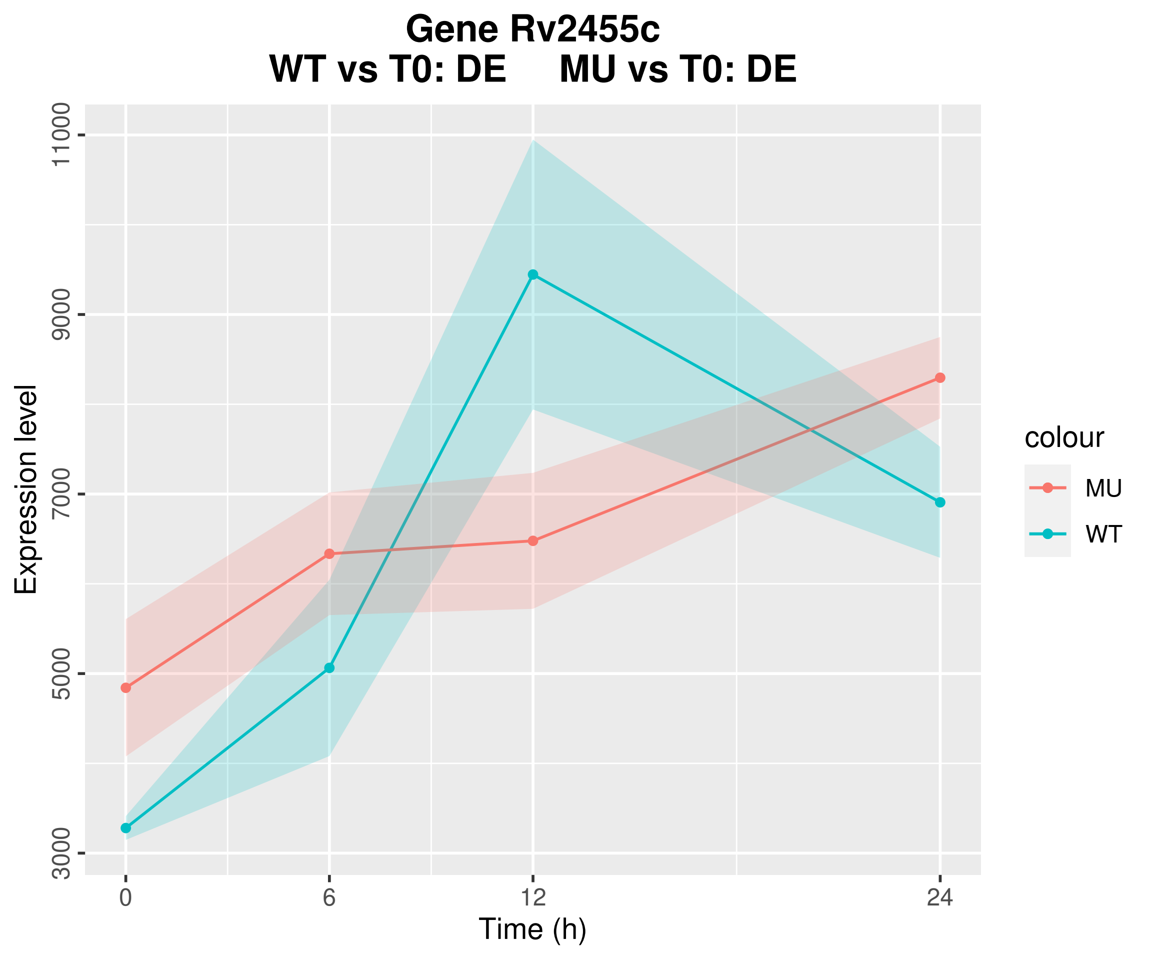

Gene Rv2455c
WT vs T0: DE   MU vs T0: DE
Expression level
Time (h)
3000
5000
7000
9000
11000
0
6
12
24
colour
MU
WT

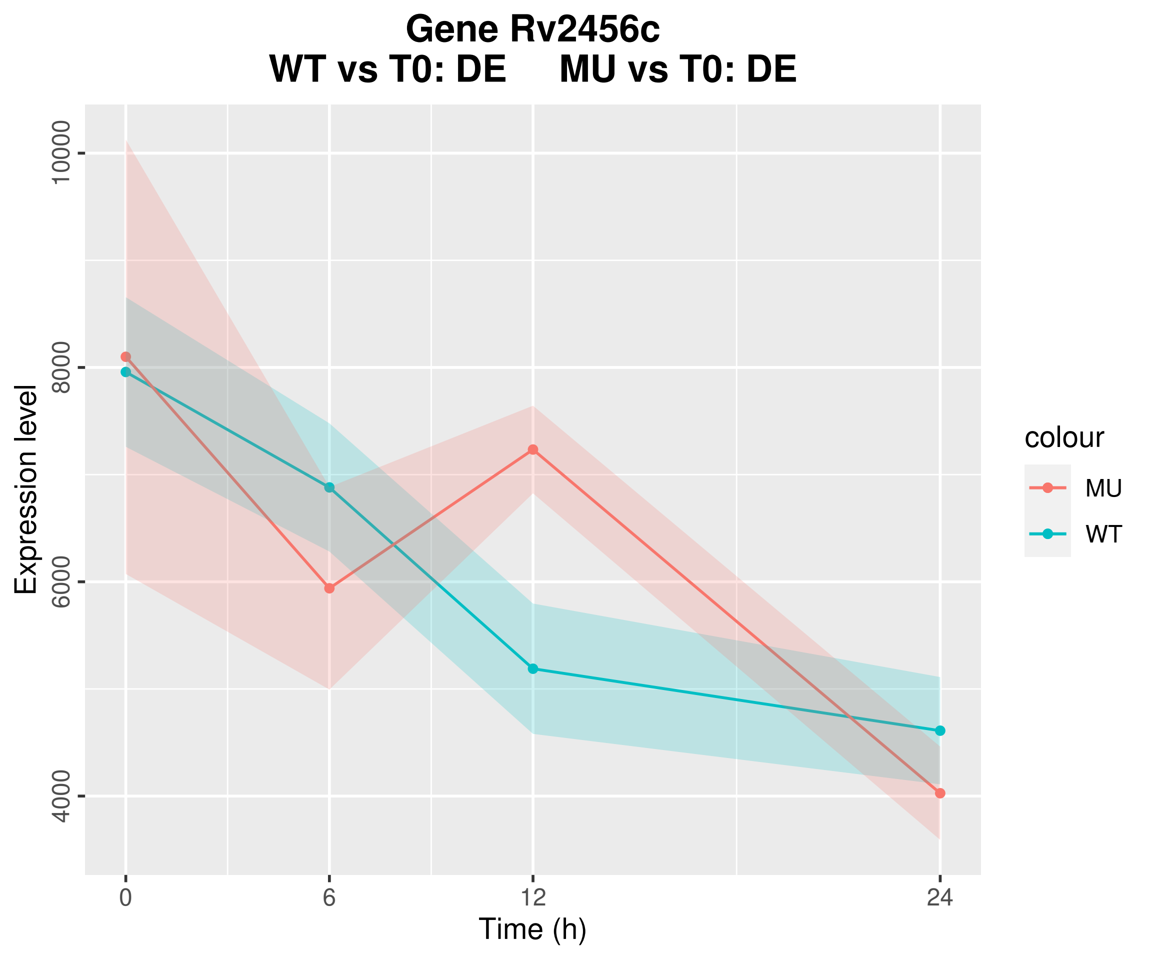

Gene Rv2456c
WT vs T0: DE   MU vs T0: DE
Expression level
10000
8000
6000
4000
0
6
12
24
Time (h)
colour
MU
WT

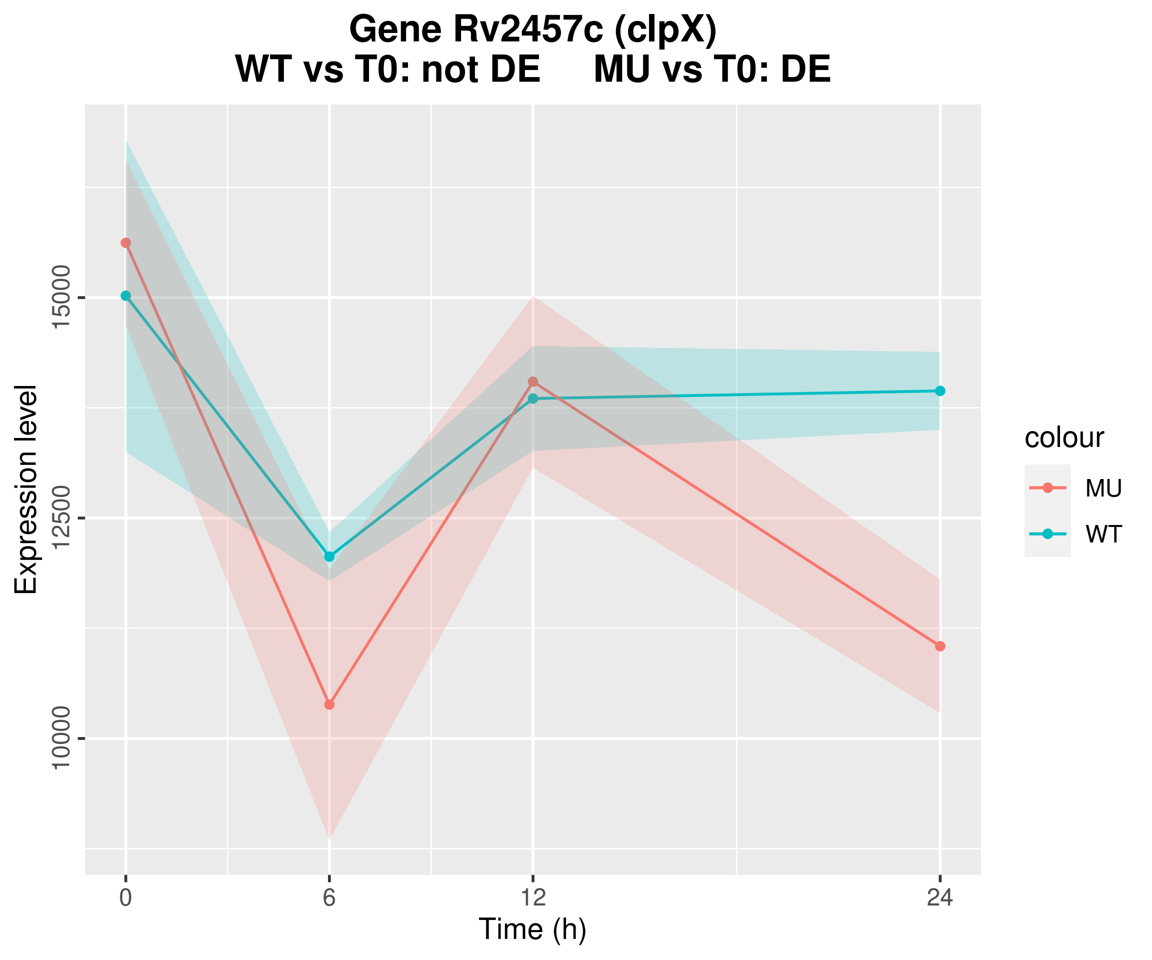
Gene Rv2457c (clpX)
WT vs T0: not DE   MU vs T0: DE
Expression level
15000
12500
10000
0
6
12
24
Time (h)
colour
MU
WT

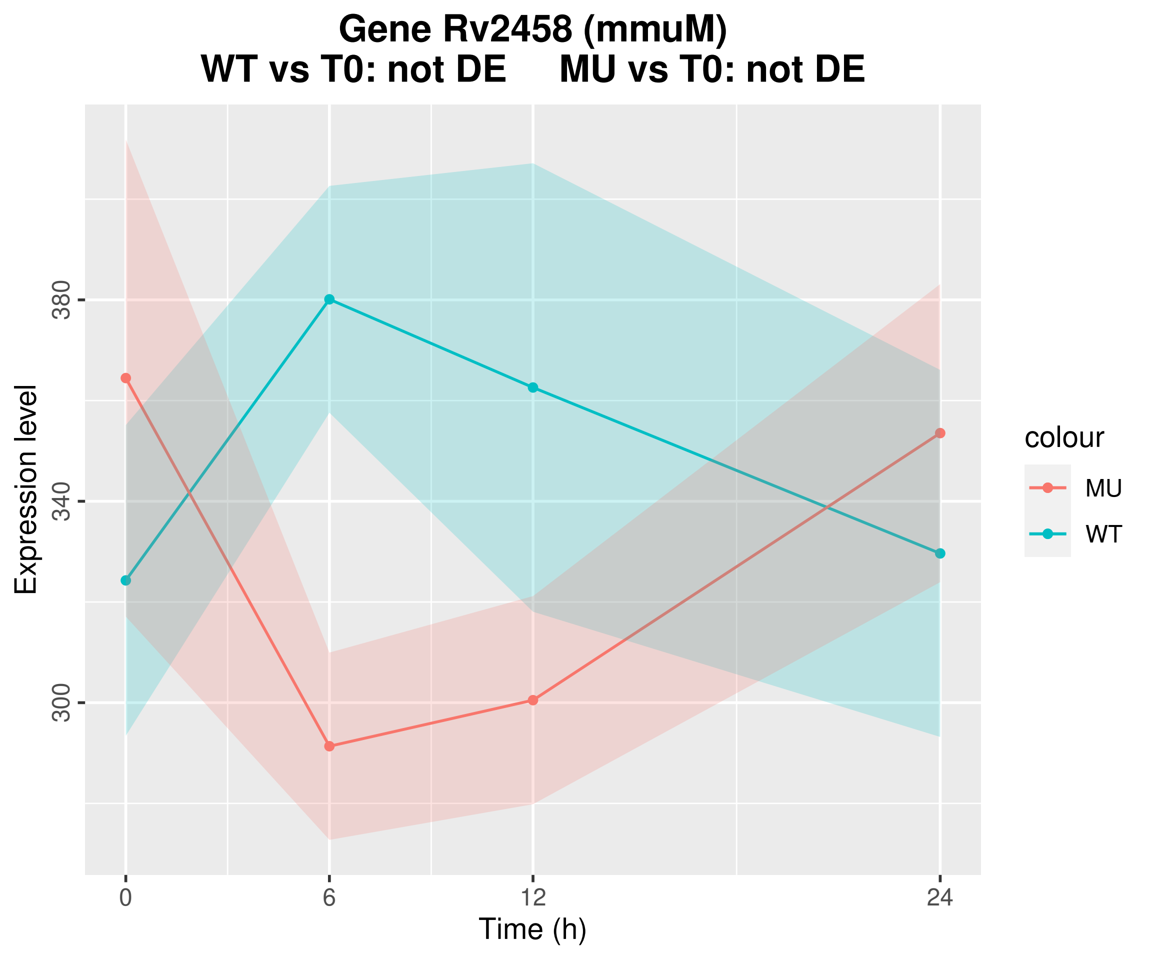
Gene Rv2458 (mmuM)
WT vs T0: not DE MU vs T0: not DE
Expression level
300
340
380
0
6
12
24
Time (h)
colour
MU
WT

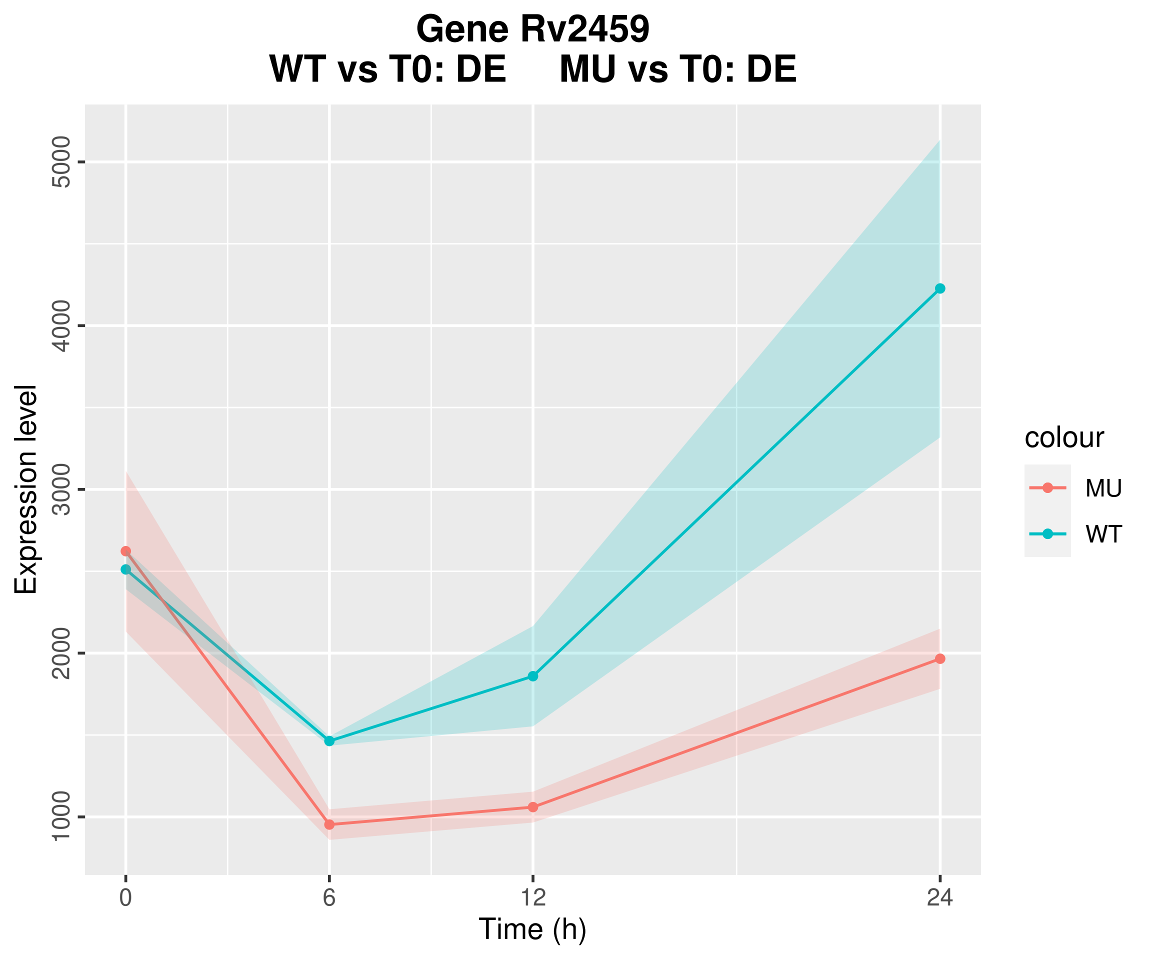

Gene Rv2459
WT vs T0: DE MU vs T0: DE
Expression level
1000
2000
3000
4000
5000
0
6
12
24
Time (h)
colour
MU
WT

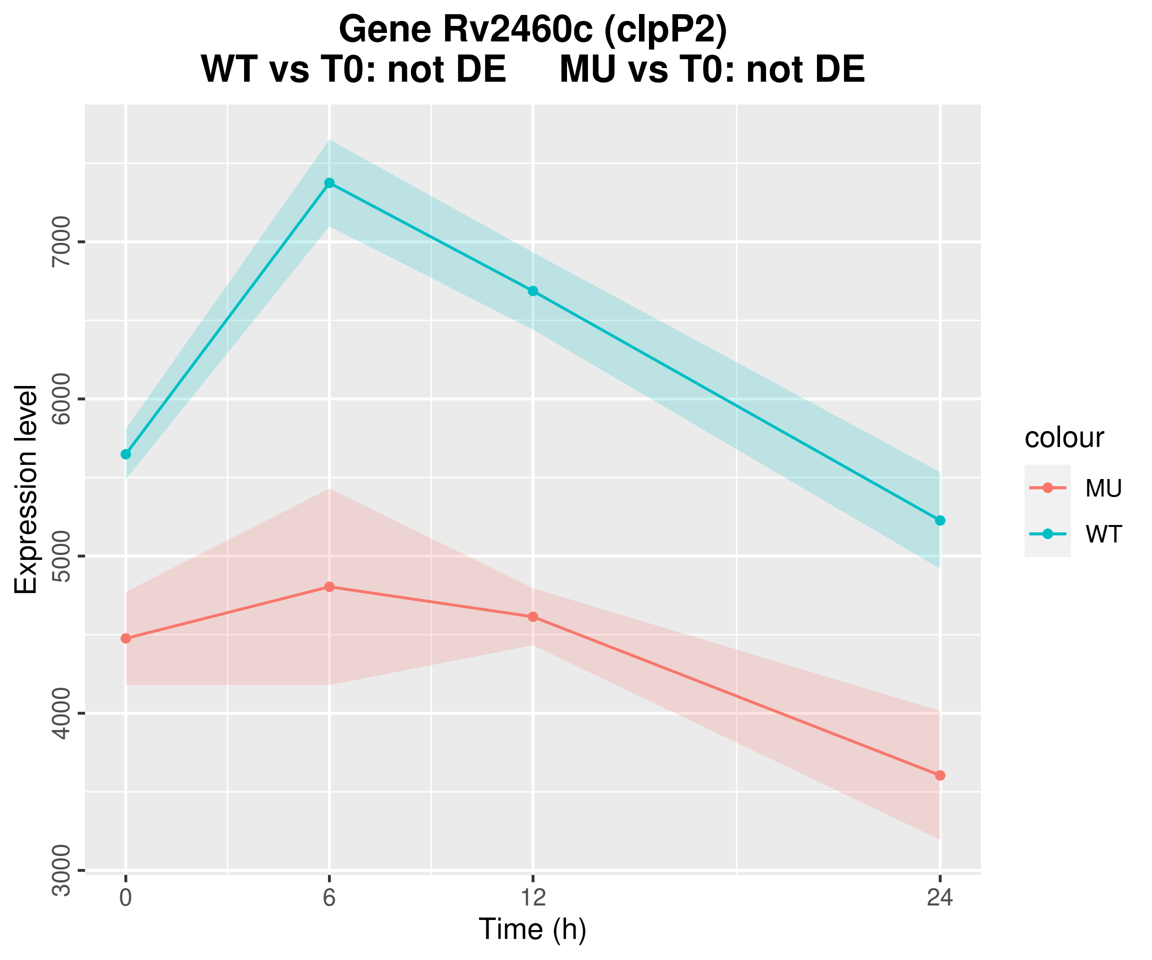

Gene Rv2460c (clpP2)
WT vs T0: not DE   MU vs T0: not DE
Expression level
3000
4000
5000
6000
7000
0
6
12
24
Time (h)
colour
MU
WT

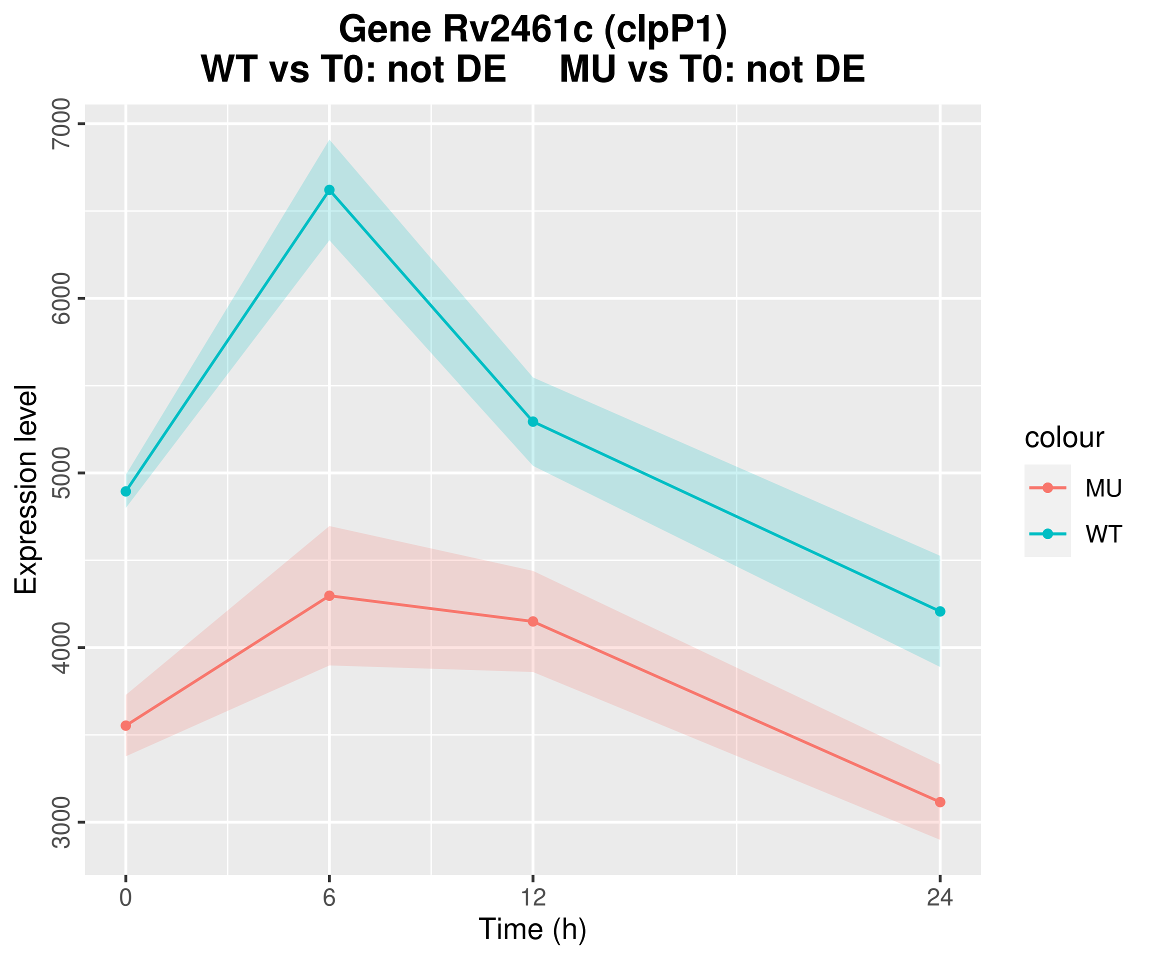
Gene Rv2461c (clpP1)
WT vs T0: not DE   MU vs T0: not DE
Expression level
7000
6000
5000
4000
3000
0
6
12
24
Time (h)
colour
MU
WT

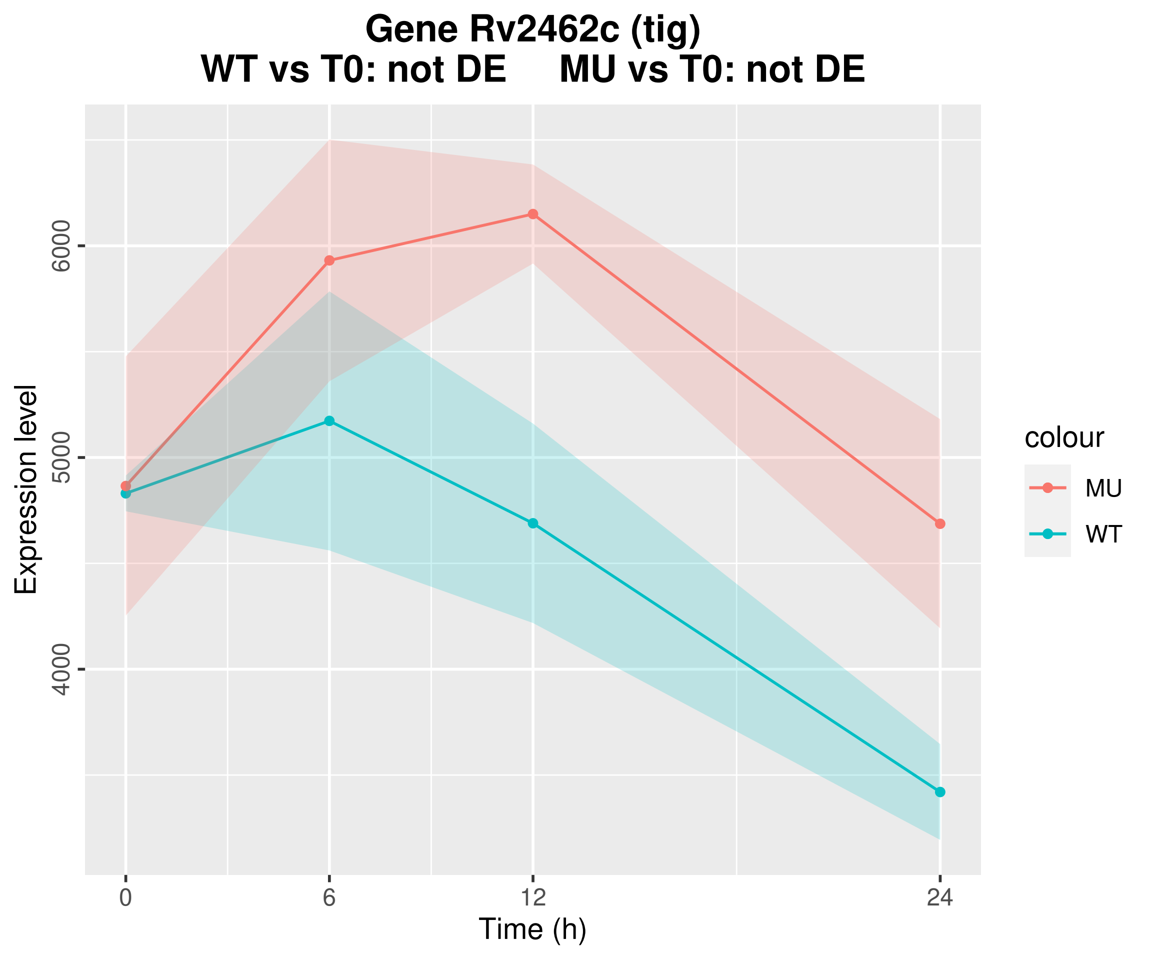
Gene Rv2462c (tig)
WT vs T0: not DE   MU vs T0: not DE
Expression level
6000
5000
4000
0
6
12
24
Time (h)
colour
MU
WT

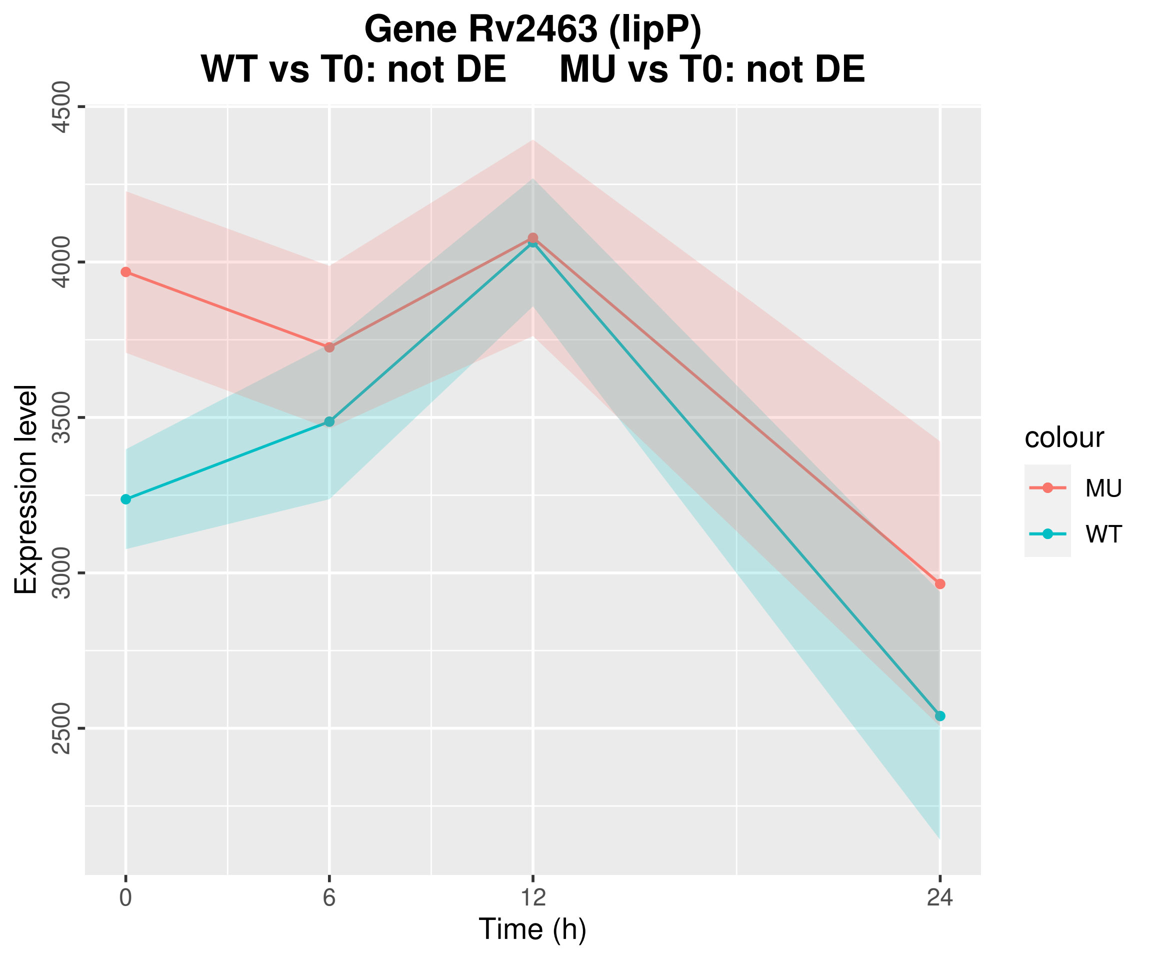
Gene Rv2463 (lipP)
WT vs T0: not DE   MU vs T0: not DE
Expression level
4500
4000
3500
3000
2500
0
6
12
24
Time (h)
colour
MU
WT

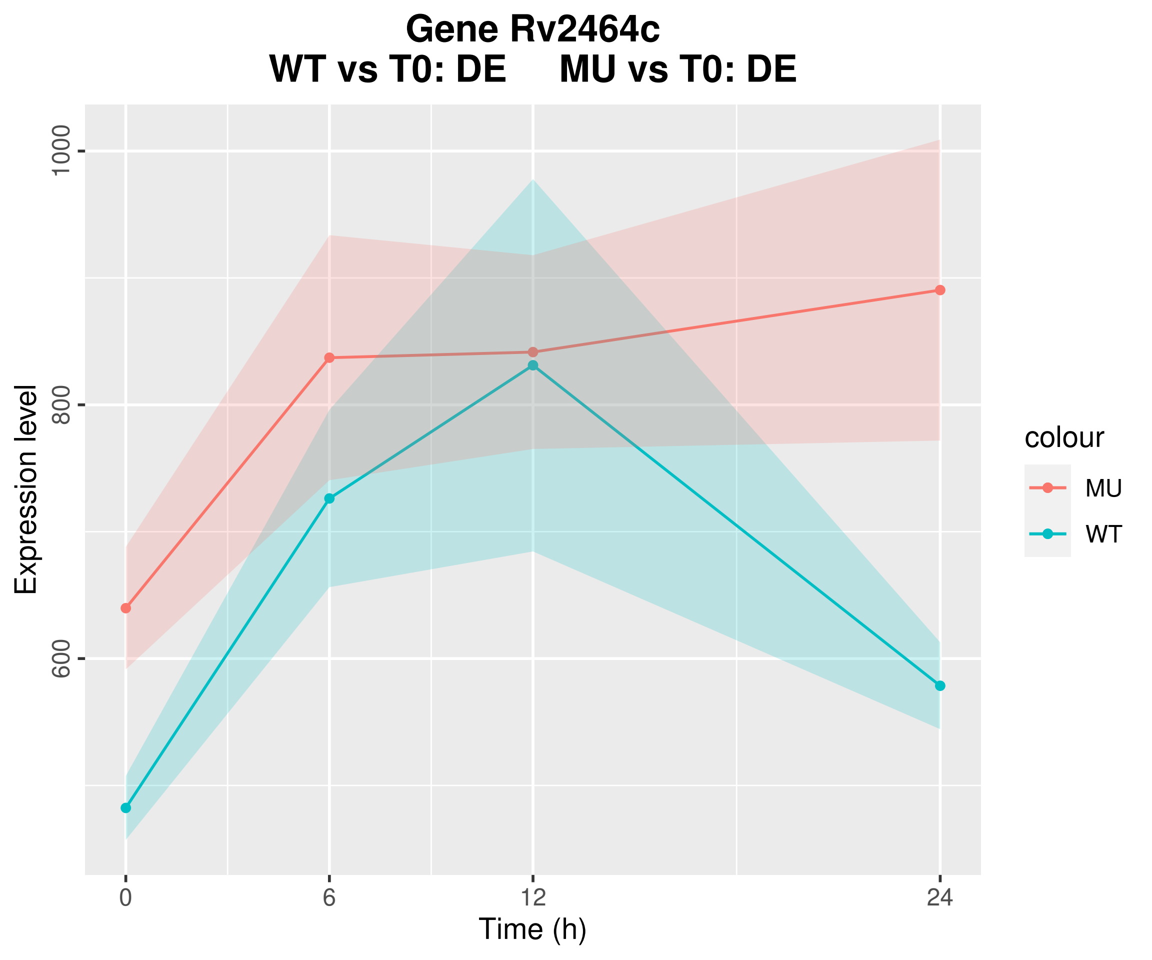

Gene Rv2464c
WT vs T0: DE   MU vs T0: DE
Expression level
1000
800
600
0
6
12
24
Time (h)
colour
MU
WT

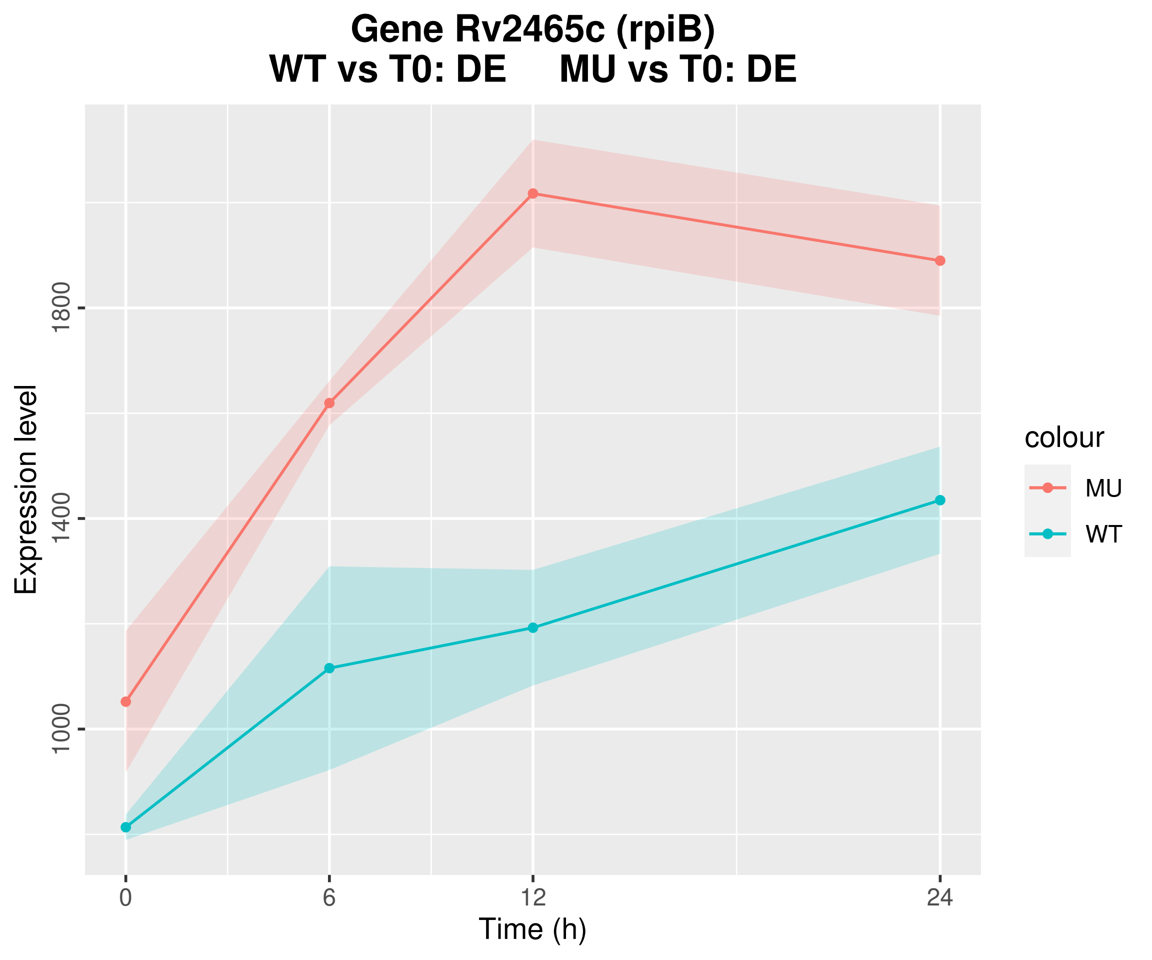
Gene Rv2465c (rpiB)
WT vs T0: DE   MU vs T0: DE
Expression level
1000
1400
1800
0
6
12
24
Time (h)
colour
MU
WT

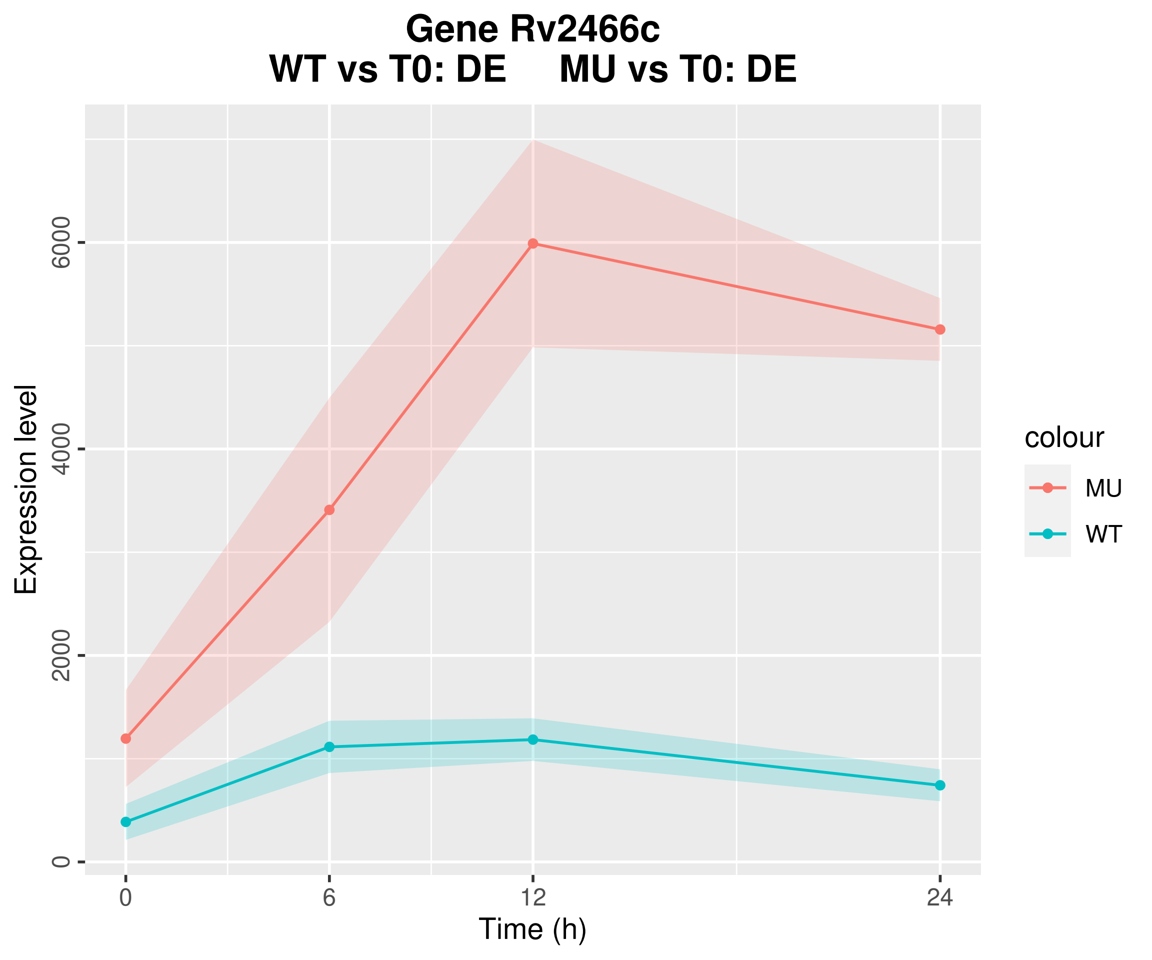

Gene Rv2466c
WT vs T0: DE   MU vs T0: DE
Expression level
0
2000
4000
6000
0
6
12
24
Time (h)
colour
MU
WT

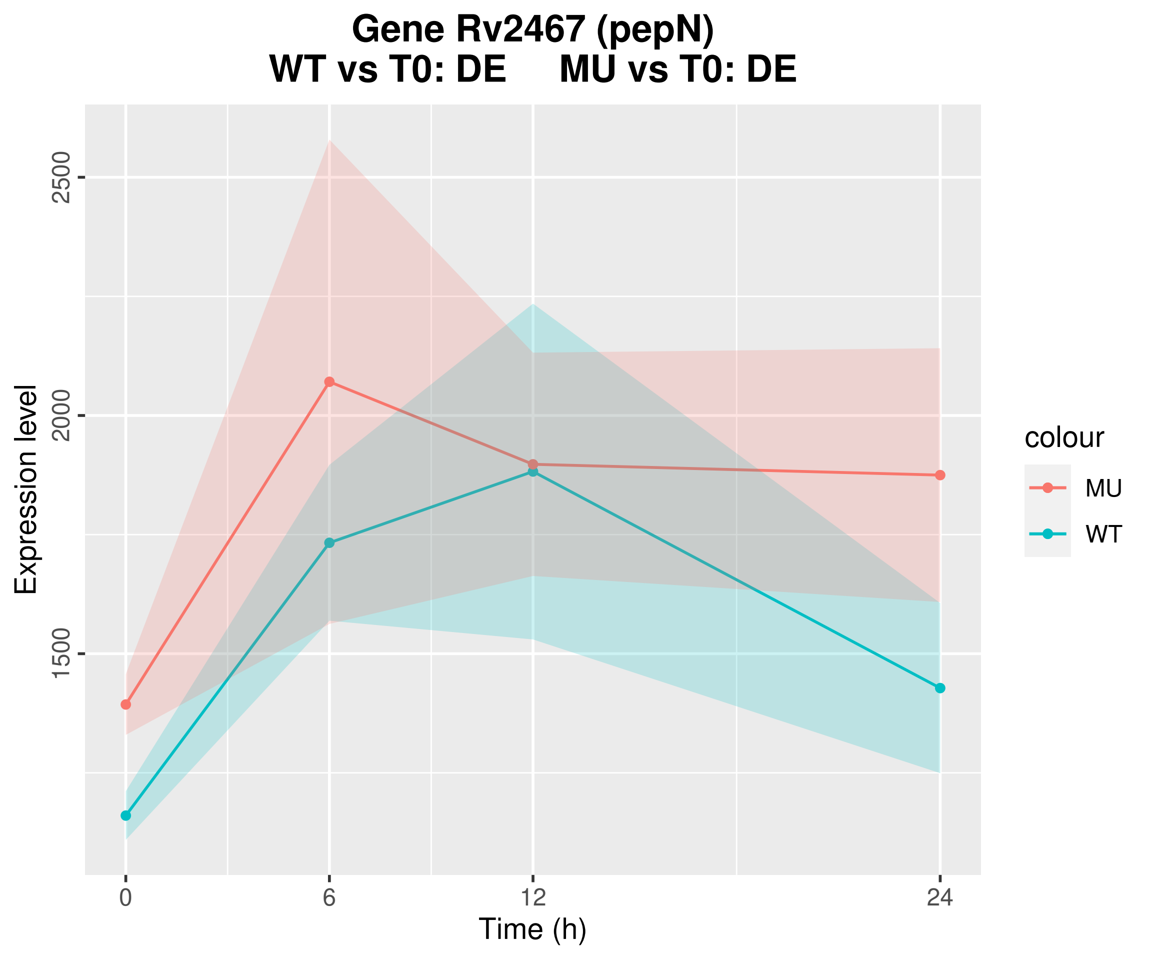
Gene Rv2467 (pepN)
WT vs T0: DE   MU vs T0: DE
Expression level
2500
2000
1500
0
6
12
24
Time (h)
colour
MU
WT

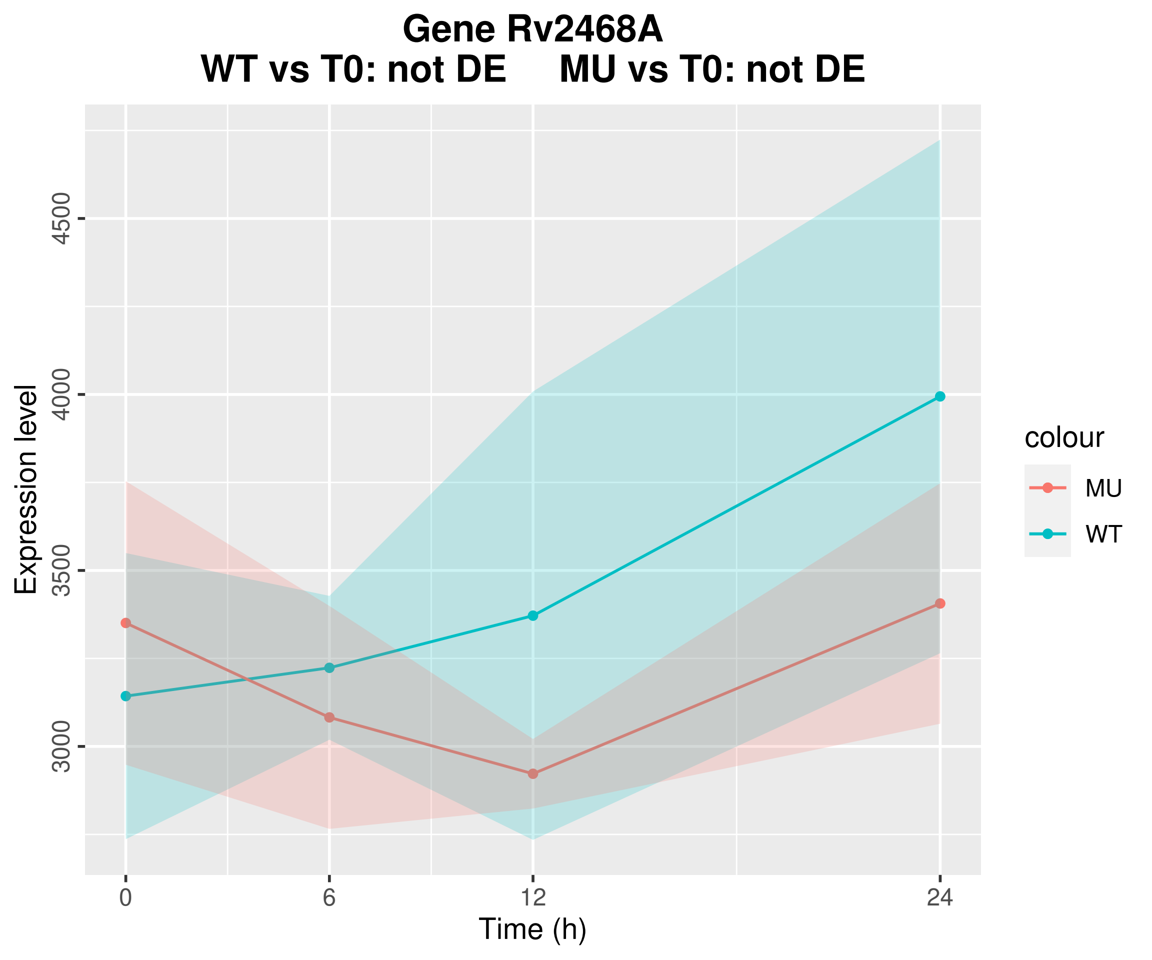

Gene Rv2468A
WT vs T0: not DE   MU vs T0: not DE
Expression level
4500
4000
3500
3000
0
6
12
24
Time (h)
colour
MU
WT

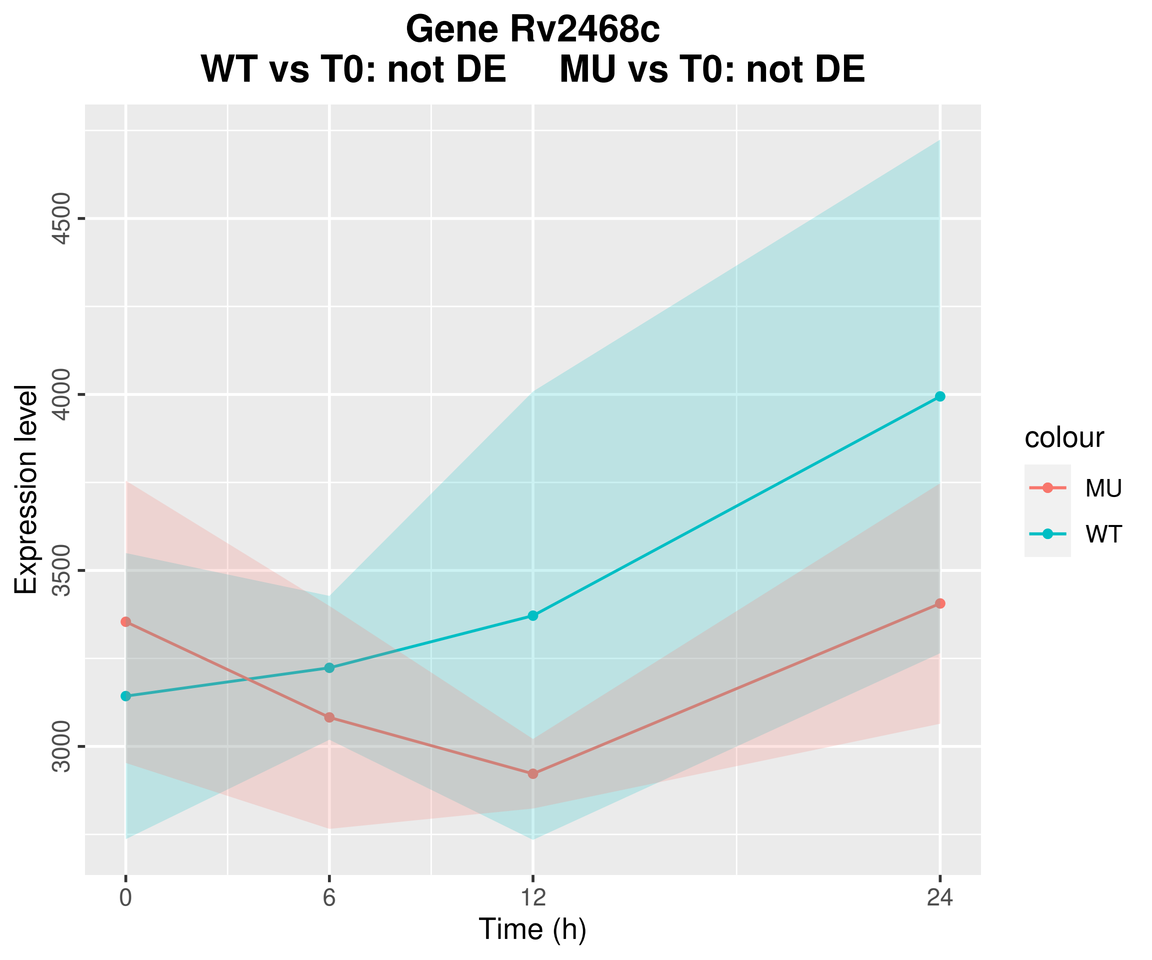
Gene Rv2468c
WT vs T0: not DE   MU vs T0: not DE
Expression level
4500
4000
3500
3000
0
6
12
24
Time (h)
colour
MU
WT

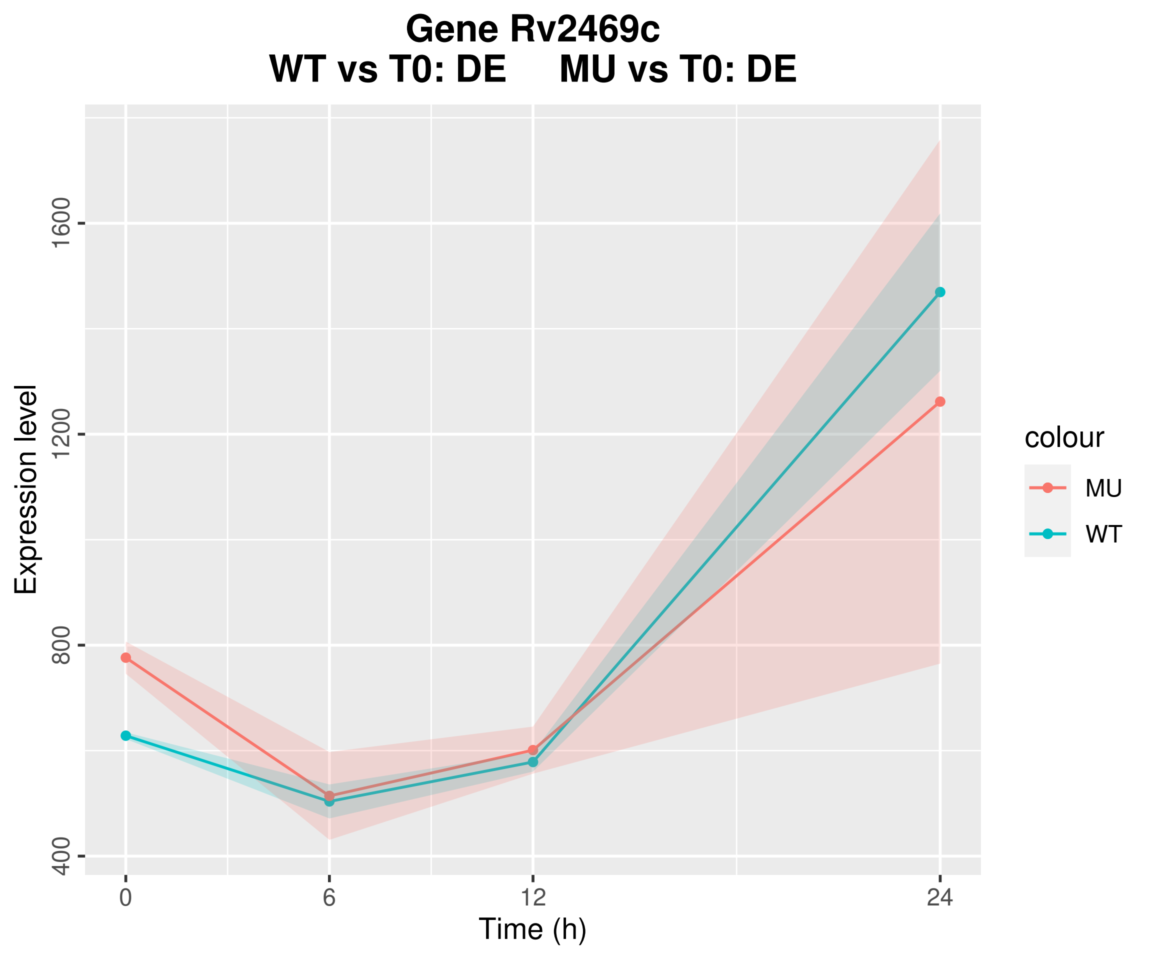
Gene Rv2469c
WT vs T0: DE   MU vs T0: DE
Expression level
1600
1200
800
400
0
6
12
24
Time (h)
colour
MU
WT

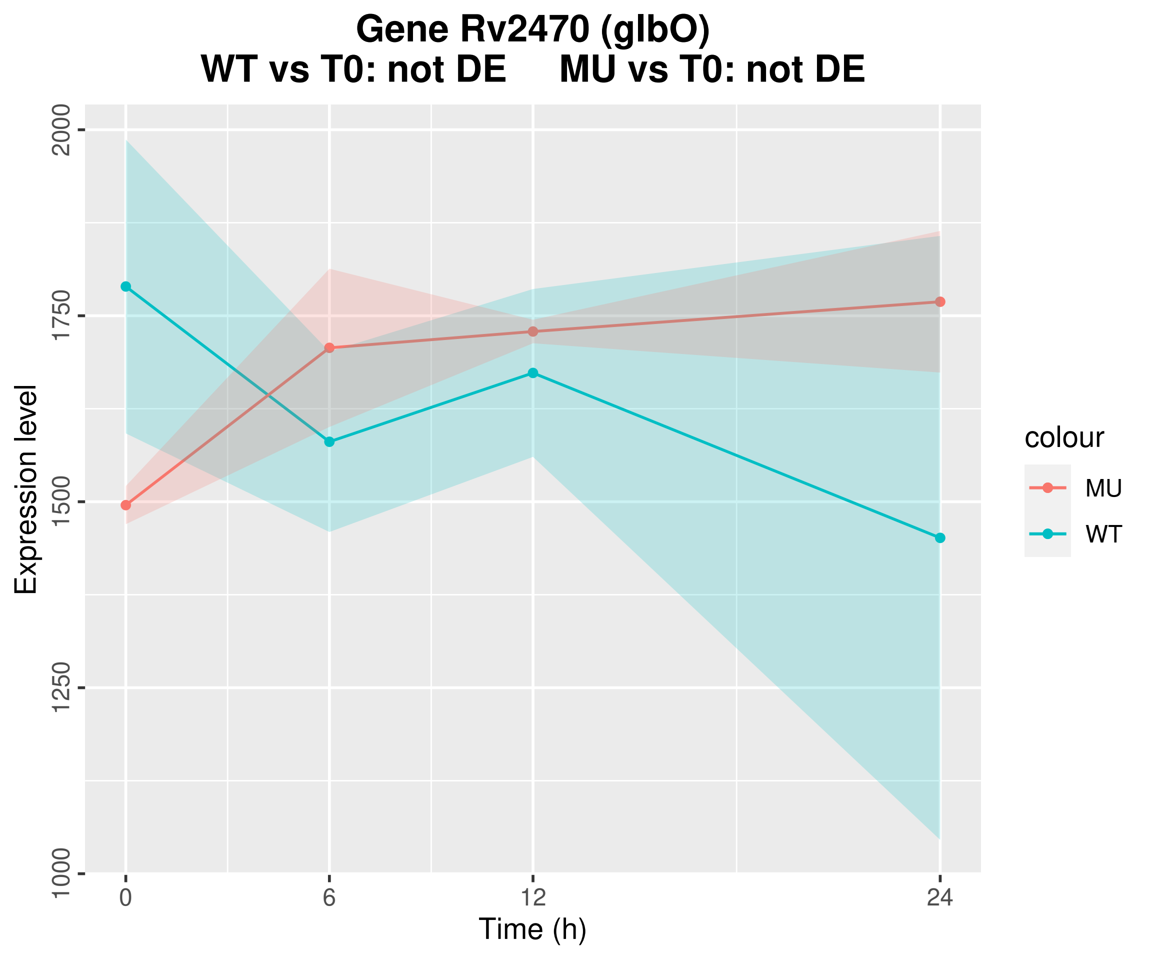
Gene Rv2470 (glbO)
WT vs T0: not DE   MU vs T0: not DE
Expression level
2000
1750
1500
1250
1000
0
6
12
24
Time (h)
colour
MU
WT

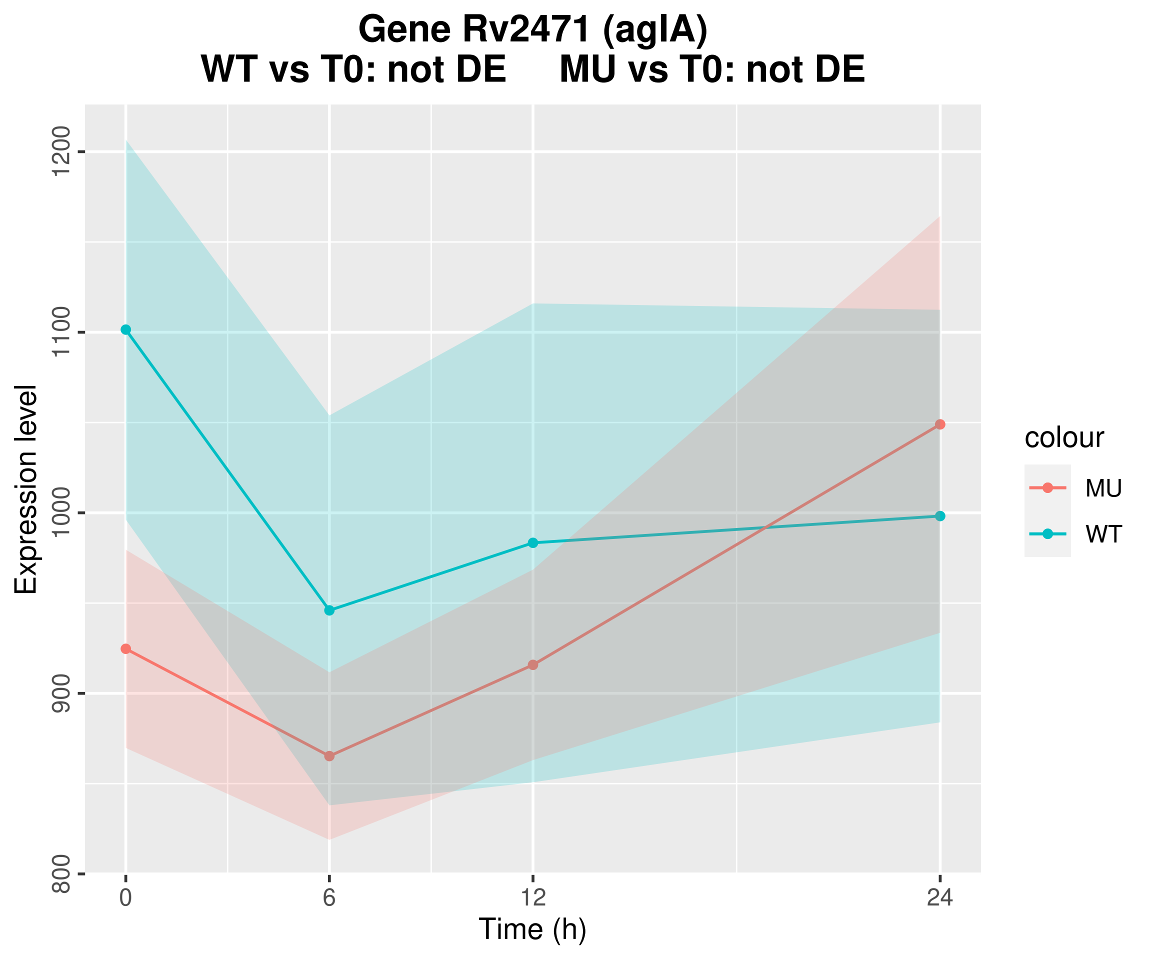
Gene Rv2471 (aglA)
WT vs T0: not DE   MU vs T0: not DE
Expression level
1200
1100
1000
900
800
0
6
12
24
Time (h)
colour
MU
WT

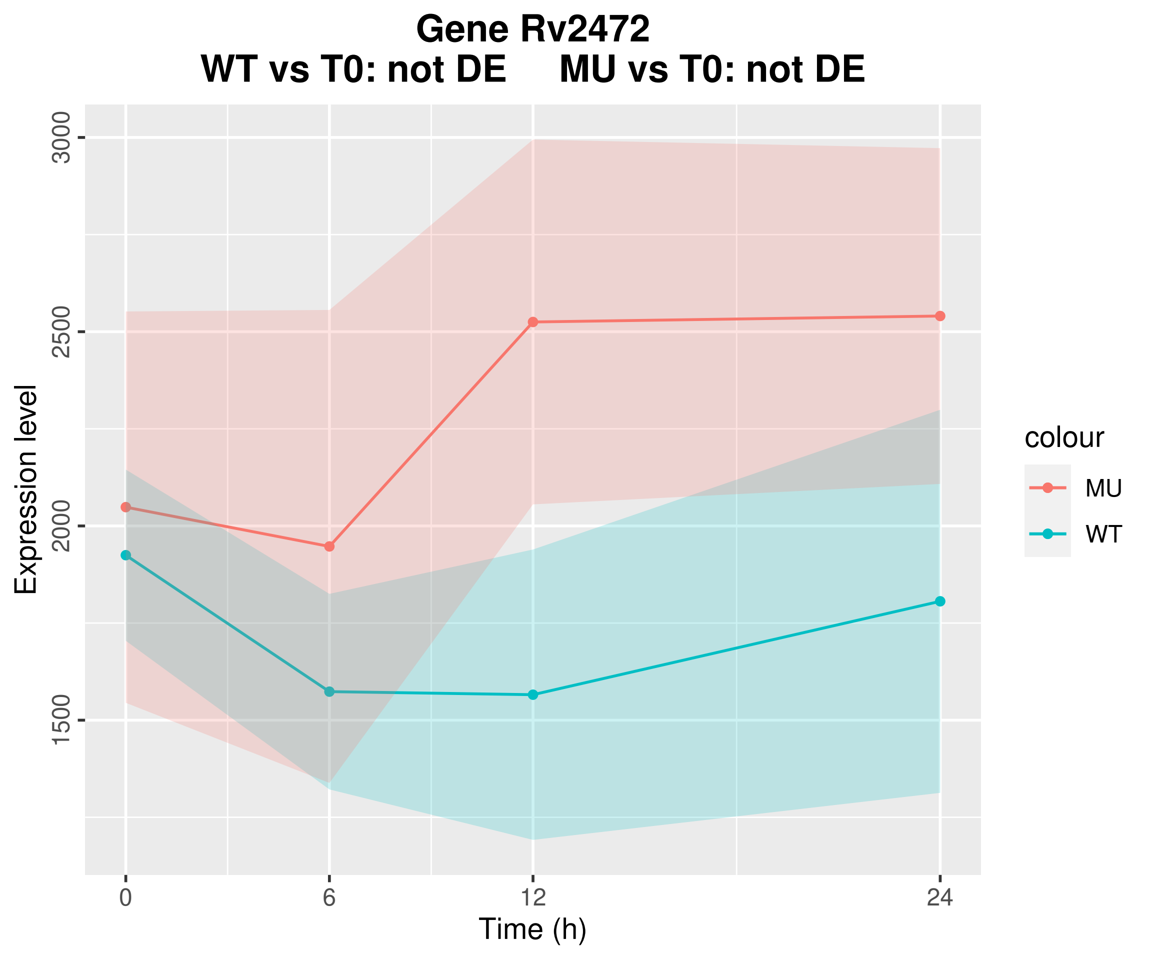
Gene Rv2472
WT vs T0: not DE   MU vs T0: not DE
Expression level
3000
2500
2000
1500
0
6
12
24
Time (h)
colour
MU
WT

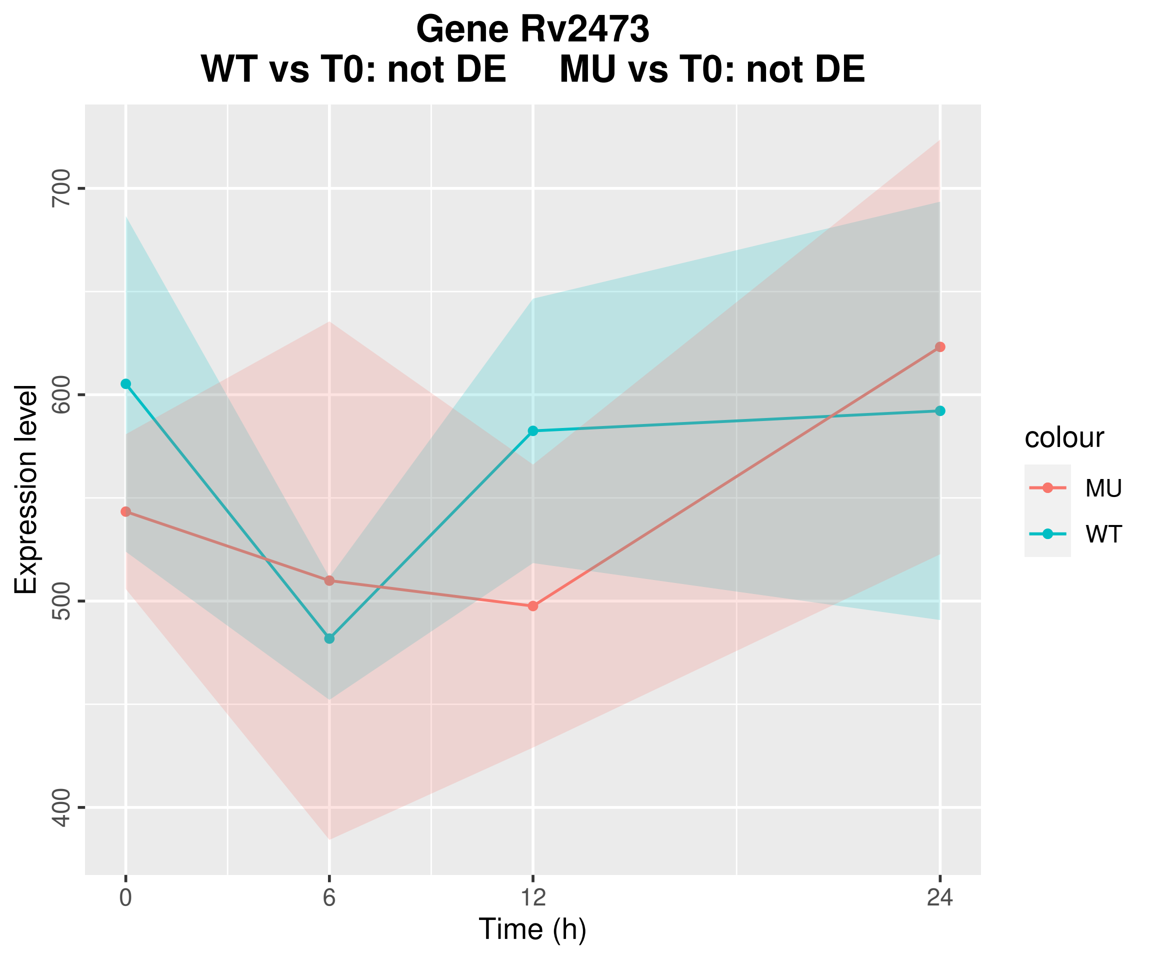
Gene Rv2473
WT vs T0: not DE   MU vs T0: not DE
Expression level
700
600
500
400
0
6
12
24
Time (h)
colour
MU
WT

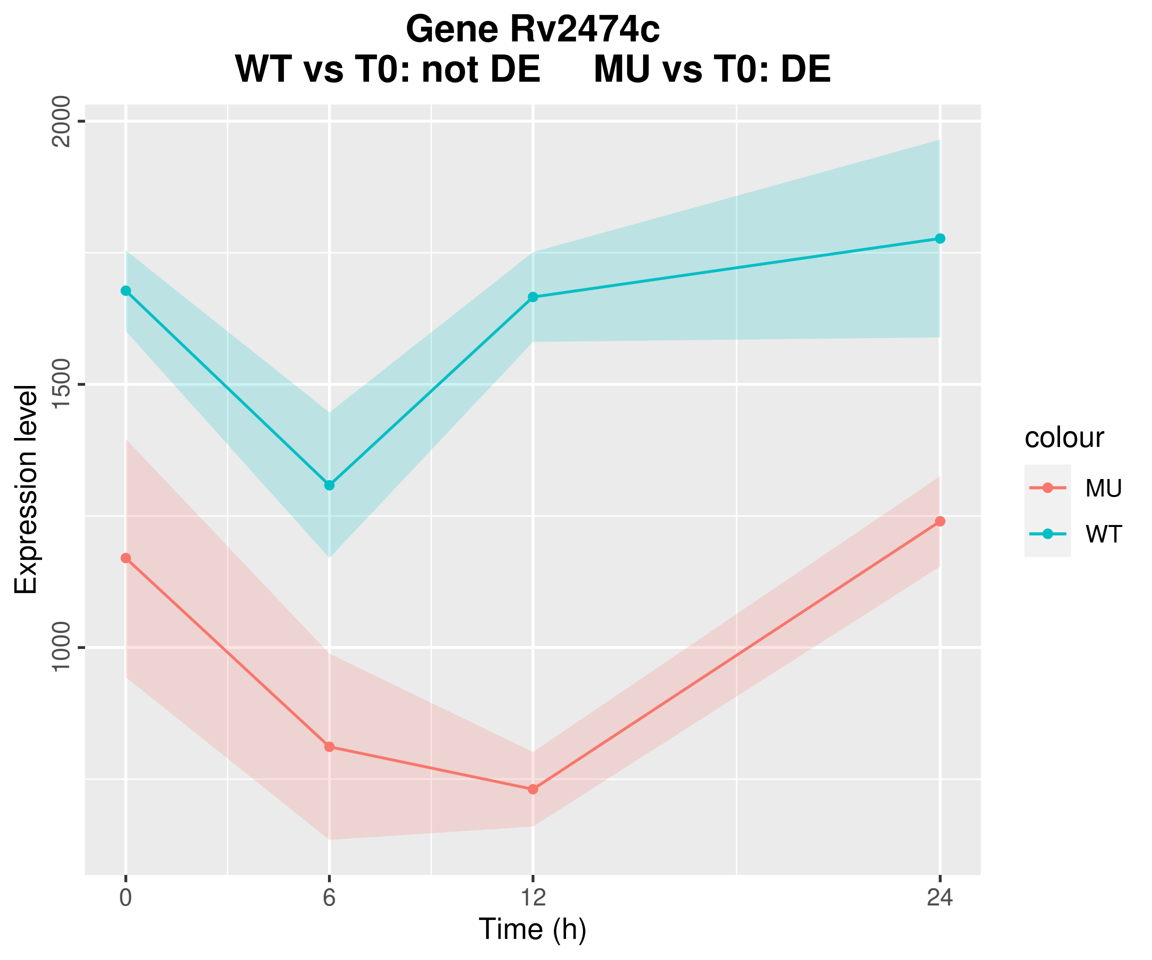

Gene Rv2474c
WT vs T0: not DE MU vs T0: DE
2000
1500
1000
Expression level
0
6
12
24
Time (h)
colour
MU
WT

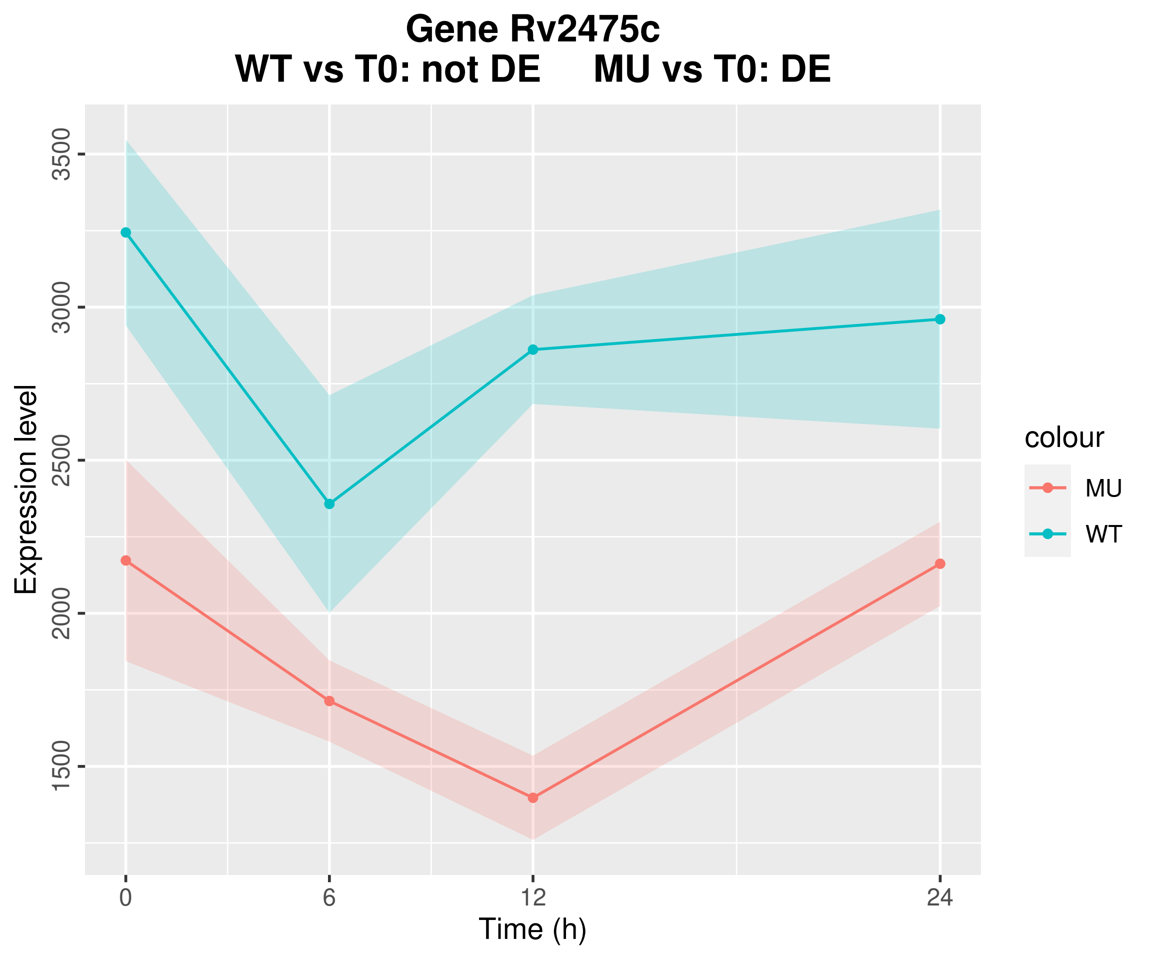
Gene Rv2475c
WT vs T0: not DE MU vs T0: DE
Expression level
3500
3000
2500
2000
1500
0
6
12
24
Time (h)
colour
MU
WT

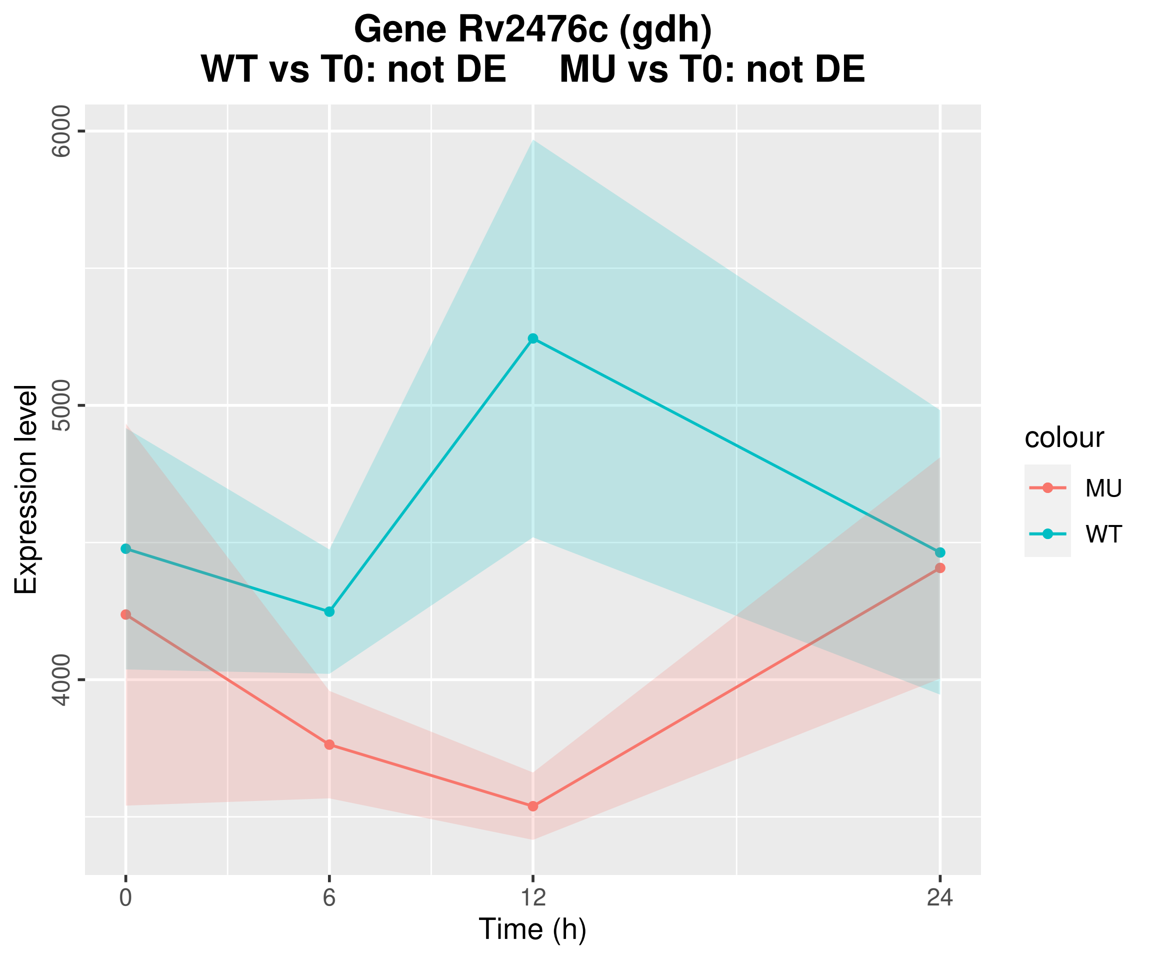

Gene Rv2476c (gdh)
WT vs T0: not DE MU vs T0: not DE
Expression level
6000
5000
4000
0
6
12
24
Time (h)
colour
MU
WT

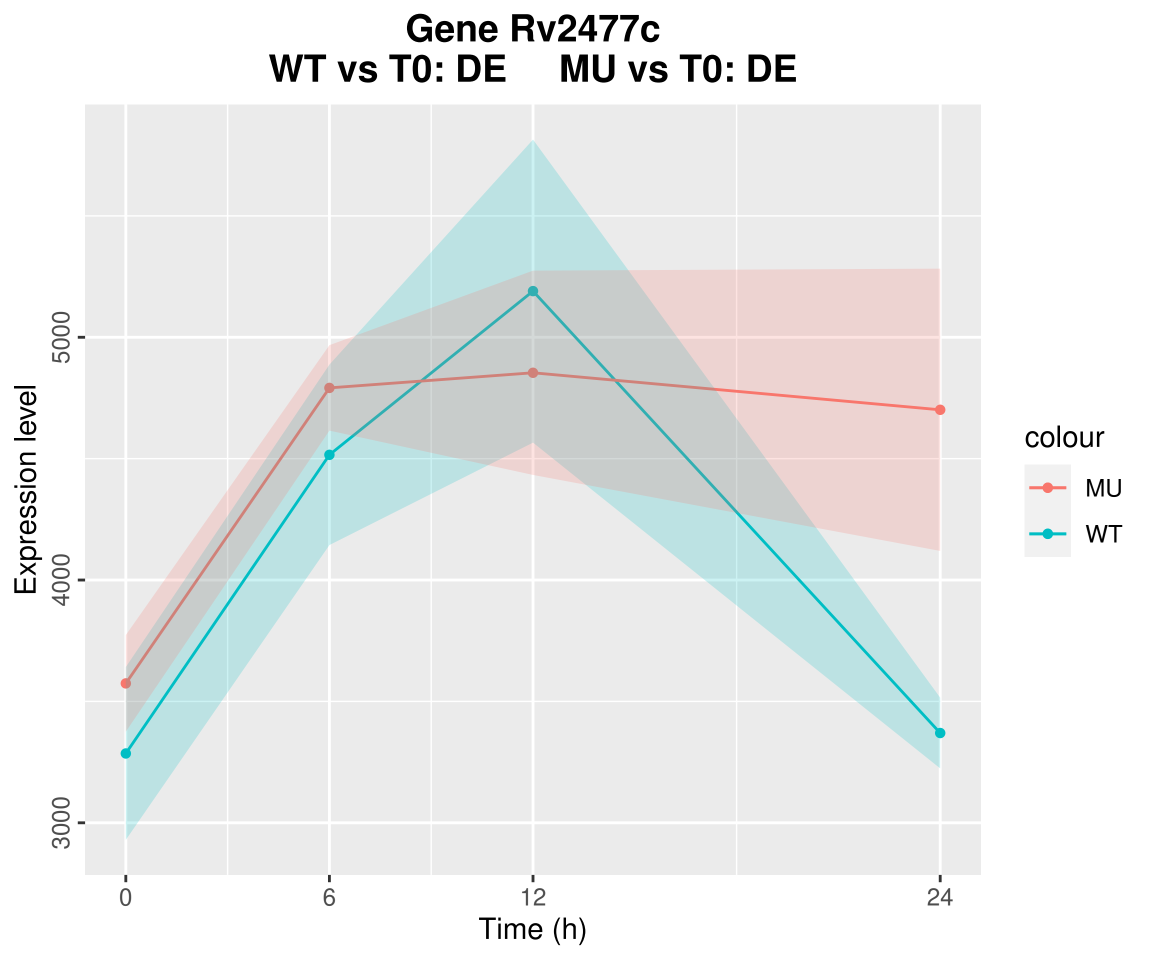

Gene Rv2477c
WT vs T0: DE MU vs T0: DE
Expression level
3000
4000
5000
0
6
12
24
Time (h)
colour
MU
WT

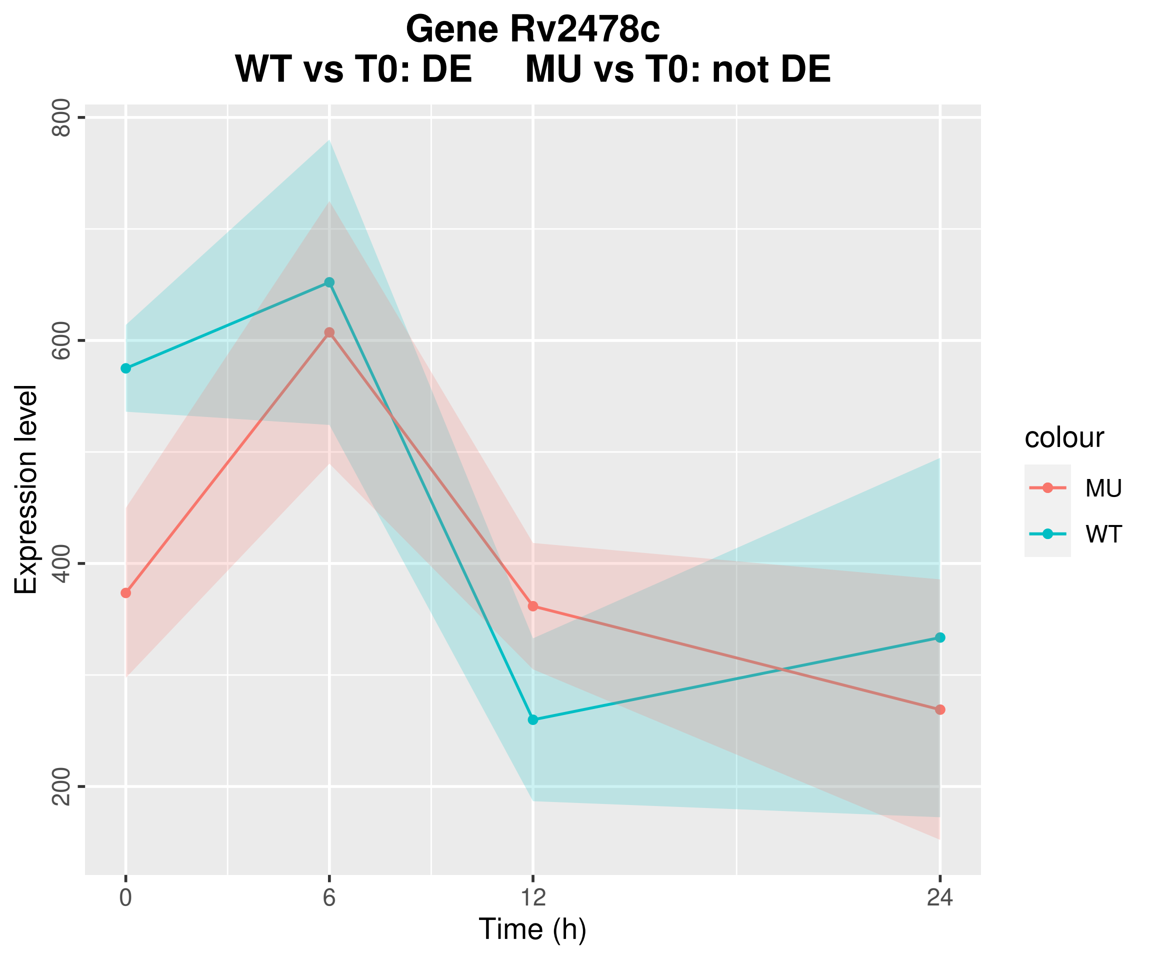

Gene Rv2478c
WT vs T0: DE MU vs T0: not DE
Expression level
800
600
400
200
0
6
12
24
Time (h)
colour
MU
WT

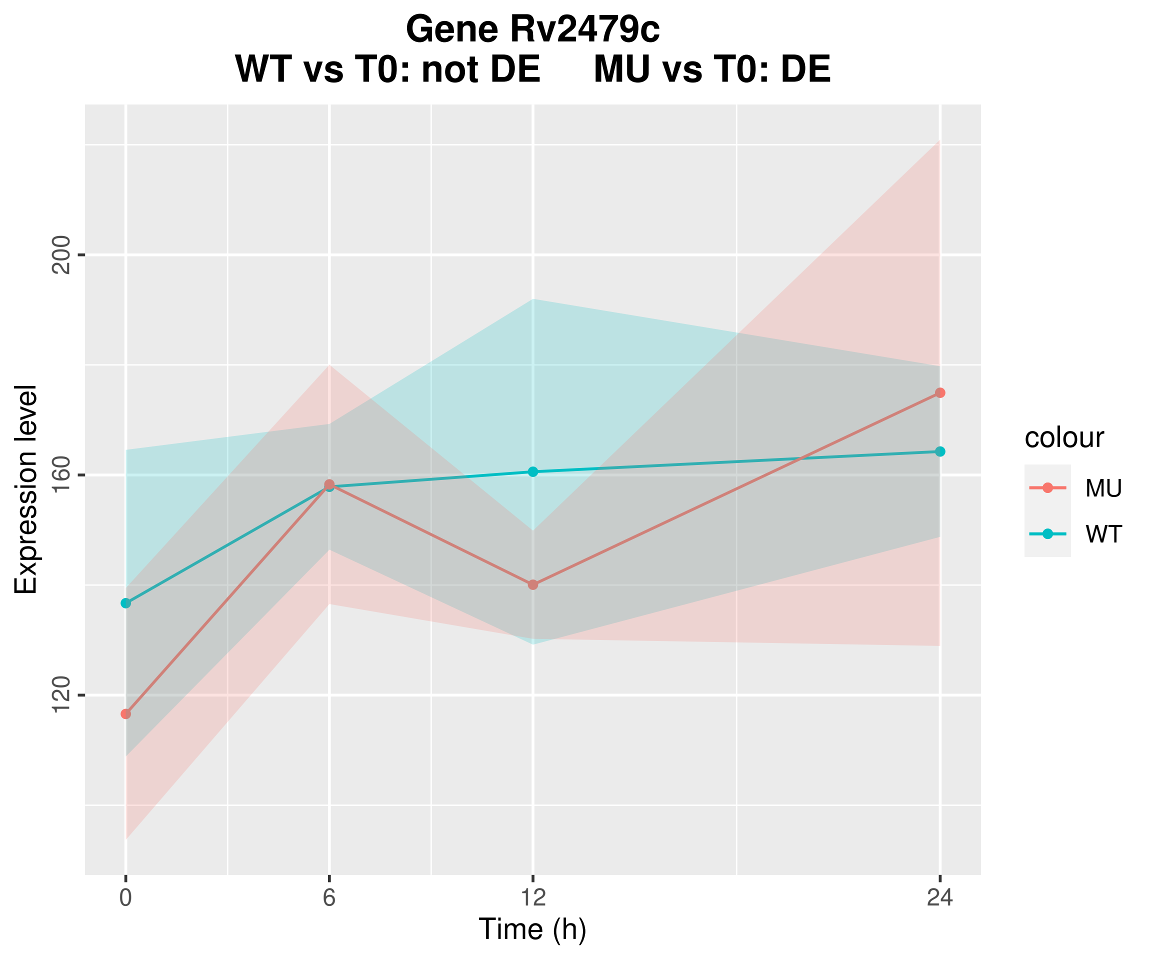

Gene Rv2479c
WT vs T0: not DE MU vs T0: DE
Expression level
200
160
120
0
6
12
24
Time (h)
colour
MU
WT

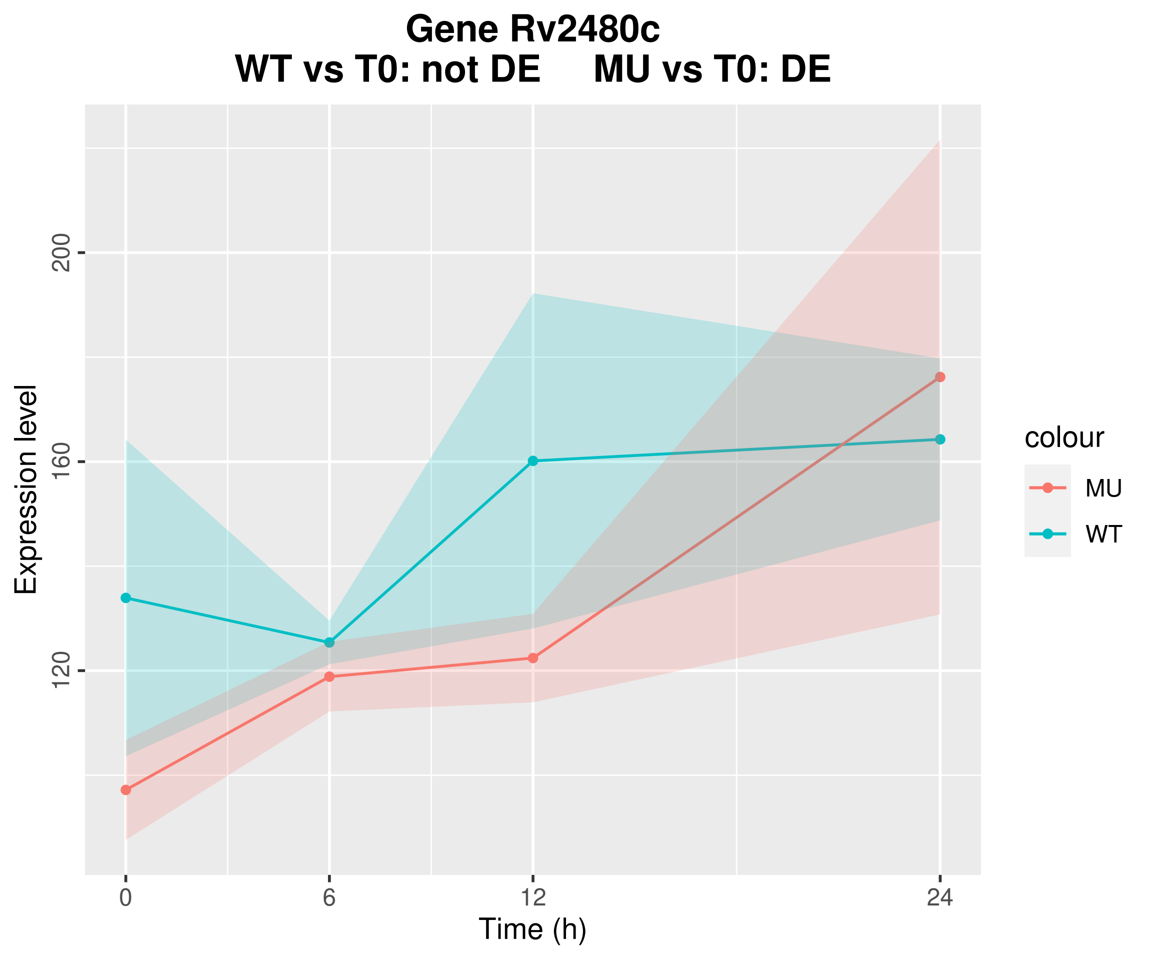

Gene Rv2480c
WT vs T0: not DE MU vs T0: DE
Expression level
200
160
120
0
6
12
24
Time (h)
colour
MU
WT

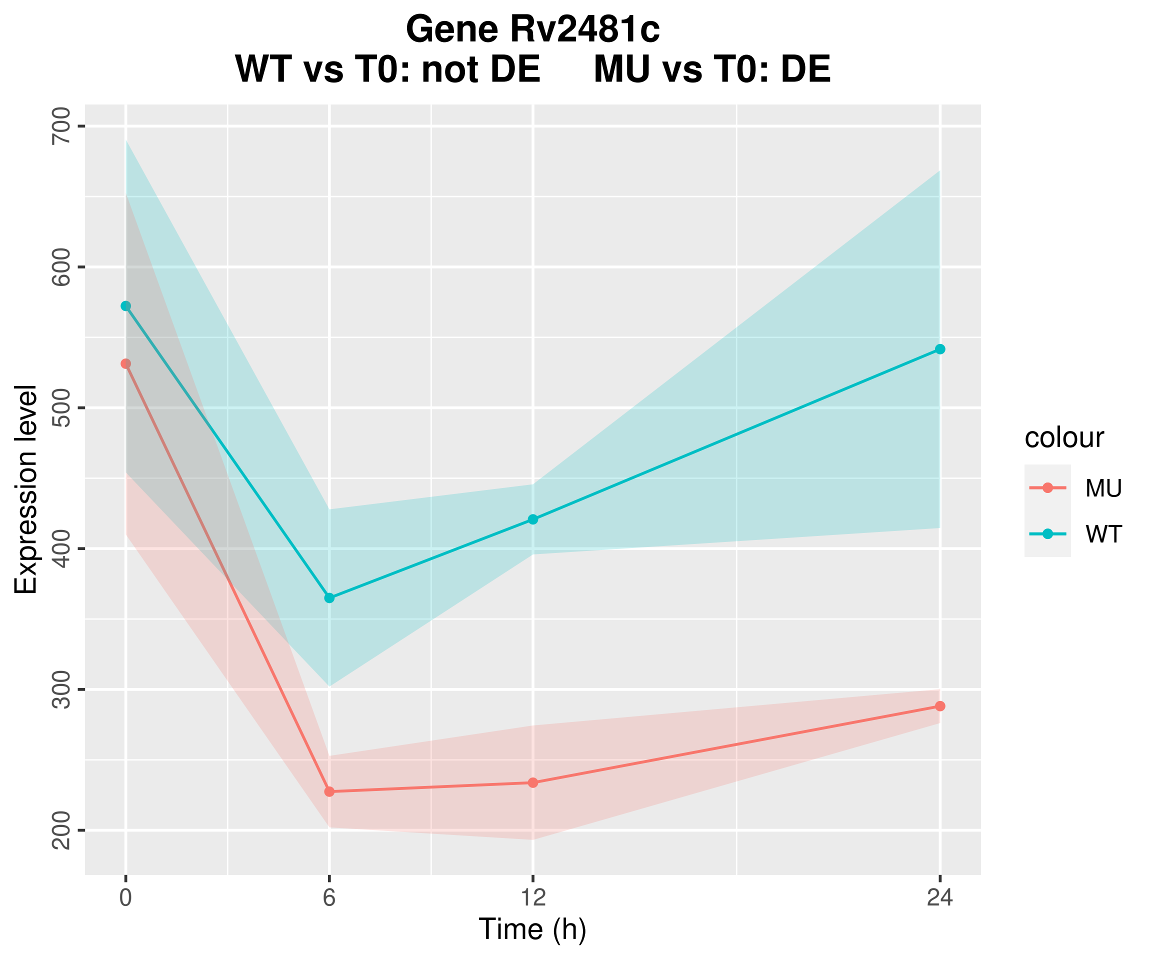

Gene Rv2481c
WT vs T0: not DE MU vs T0: DE
Expression level
700
600
500
400
300
200
0
6
12
24
Time (h)
colour
MU
WT

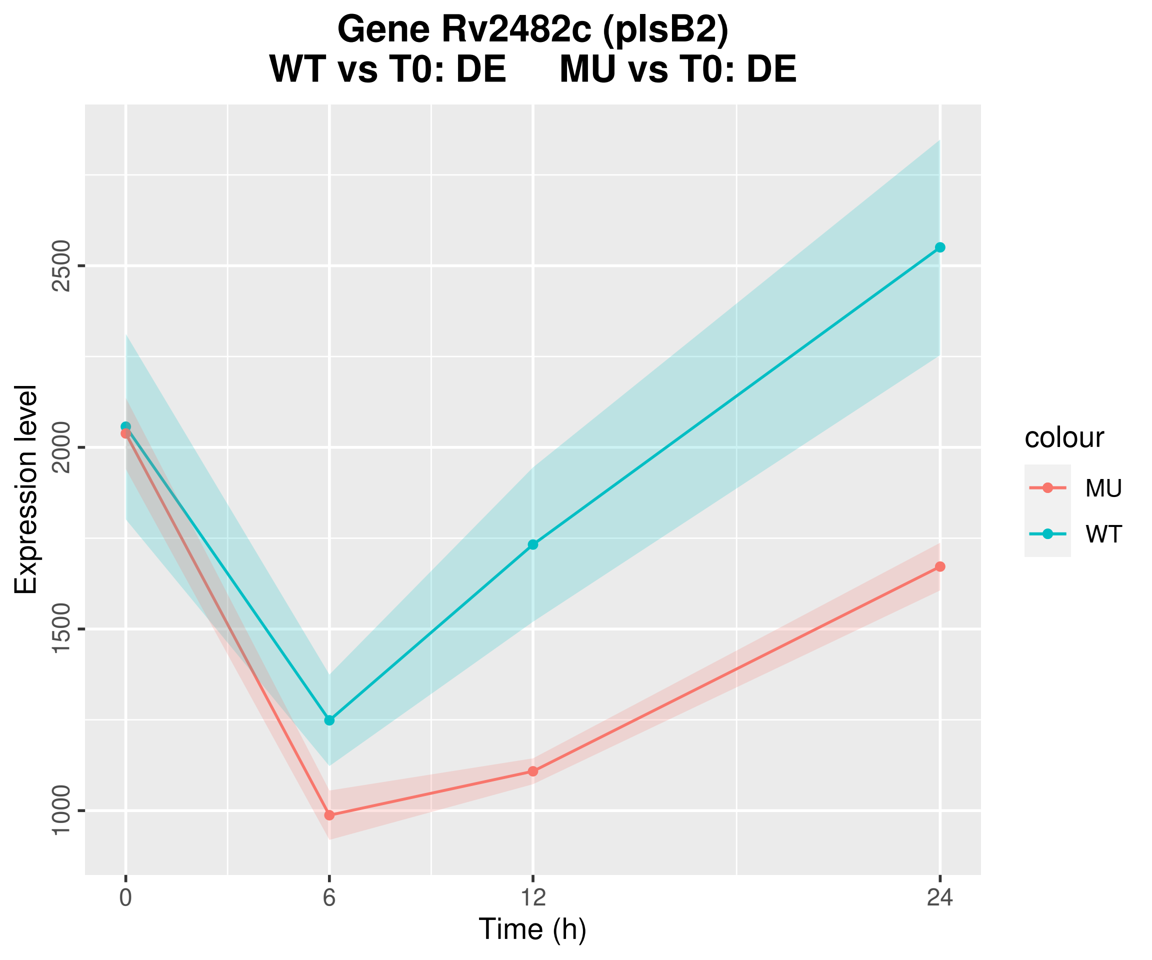
Gene Rv2482c (plsB2)
WT vs T0: DE   MU vs T0: DE
Expression level
2500
2000
1500
1000
0
6
12
24
Time (h)
colour
MU
WT

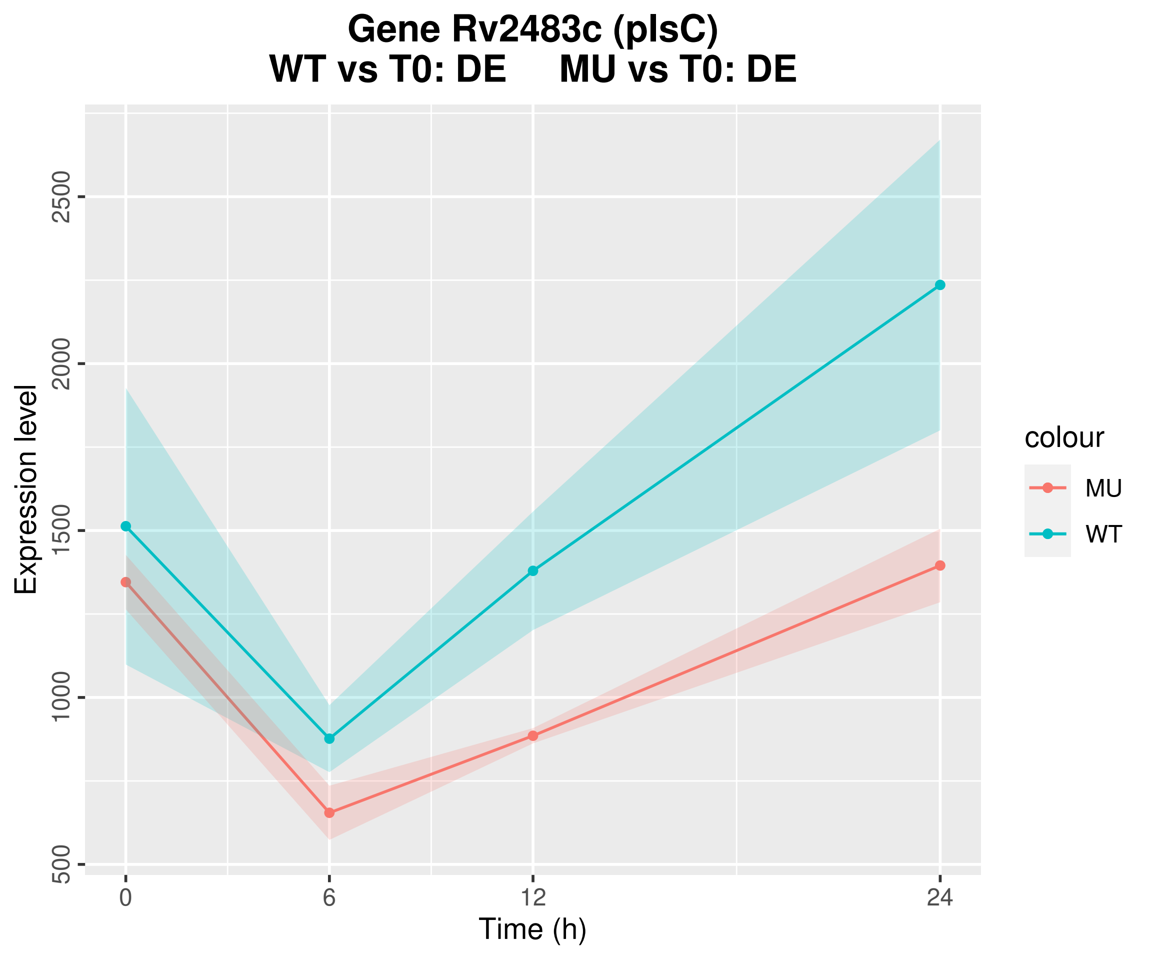
Gene Rv2483c (plsC)
WT vs T0: DE   MU vs T0: DE
Expression level
2500
2000
1500
1000
500
0
6
12
24
Time (h)
colour
MU
WT

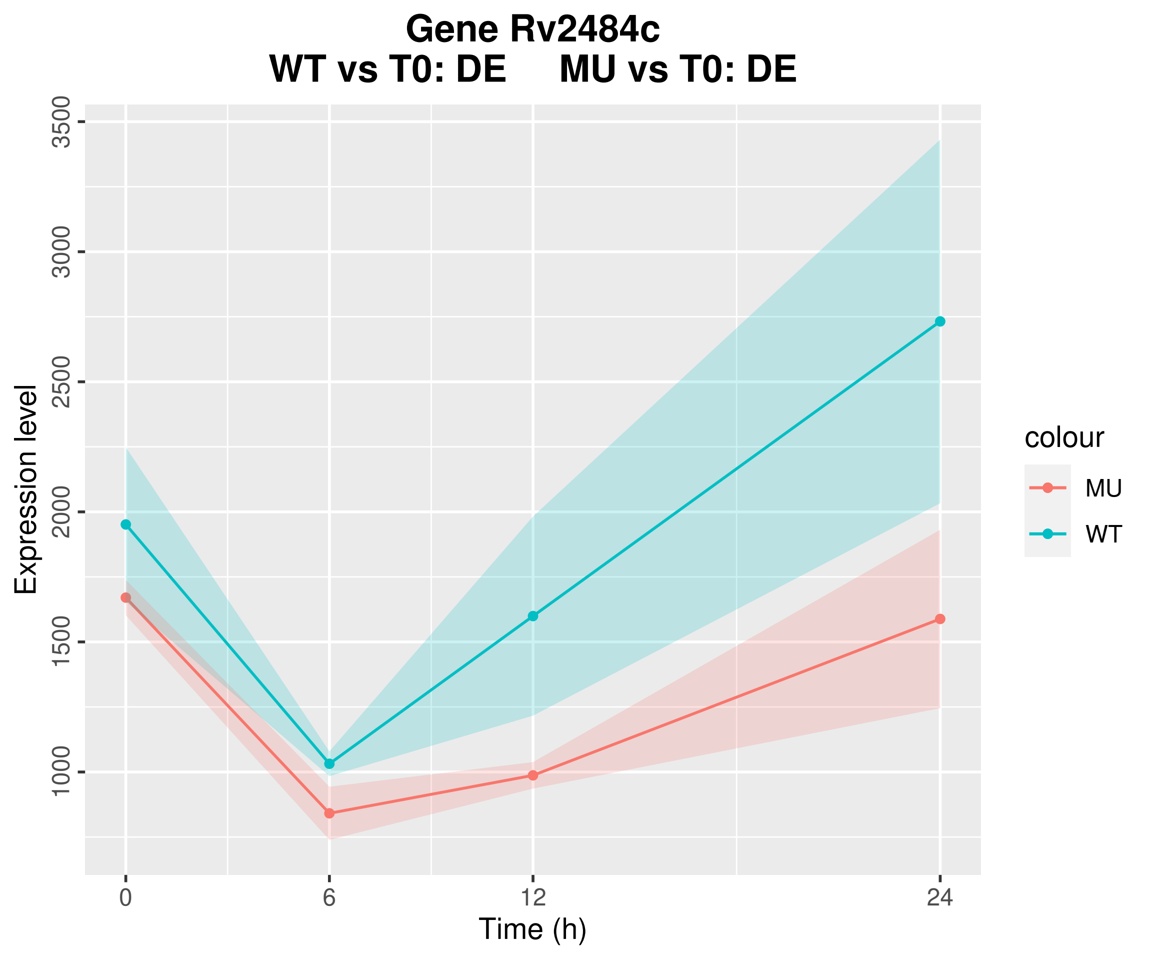

Gene Rv2484c
WT vs T0: DE   MU vs T0: DE
Expression level
3500
3000
2500
2000
1500
1000
0
6
12
24
Time (h)
colour
MU
WT

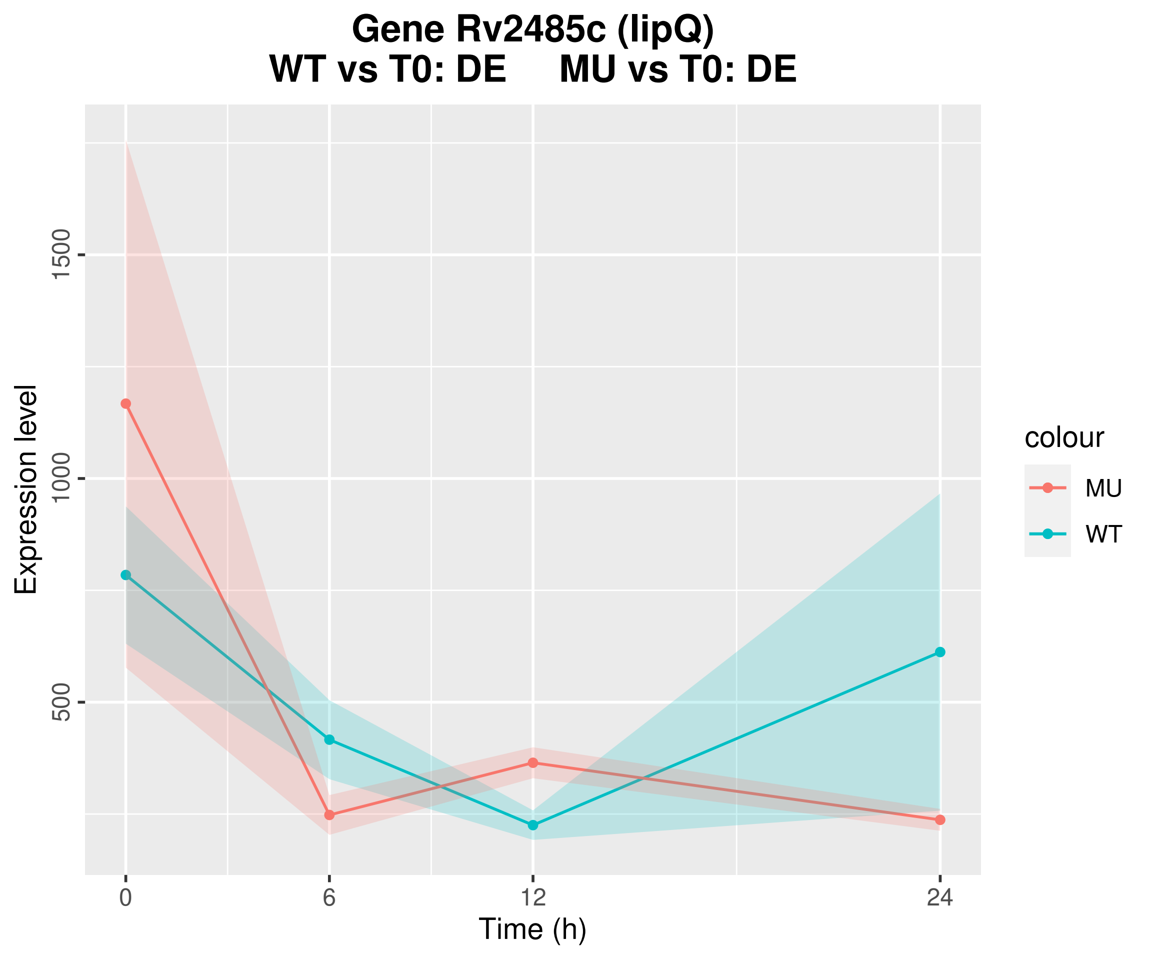
Gene Rv2485c (lipQ)
WT vs T0: DE   MU vs T0: DE
Expression level
1500
1000
500
0
6
12
24
Time (h)
colour
MU
WT

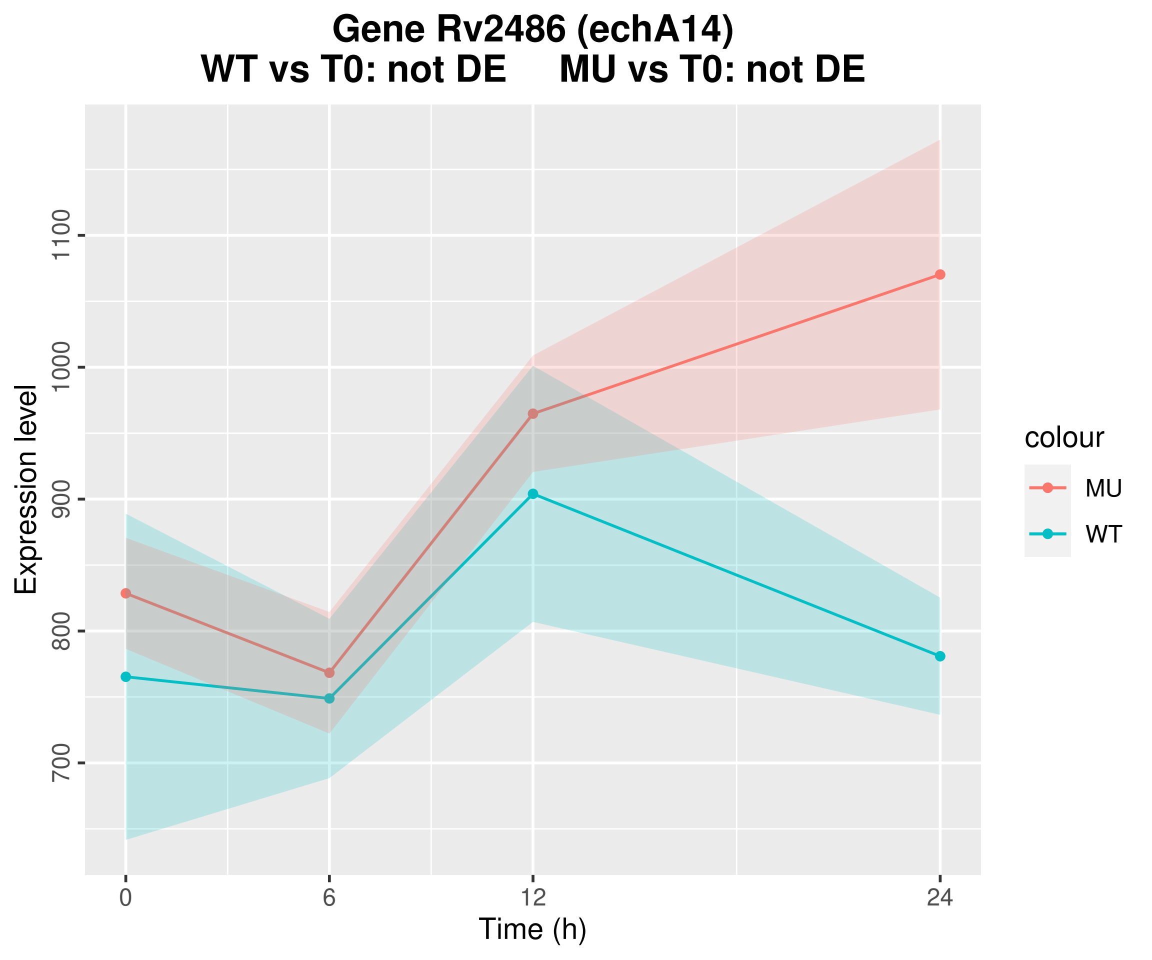
Gene Rv2486 (echA14)
WT vs T0: not DE   MU vs T0: not DE
Expression level
700
800
900
1000
1100
0
6
12
24
Time (h)
colour
MU
WT

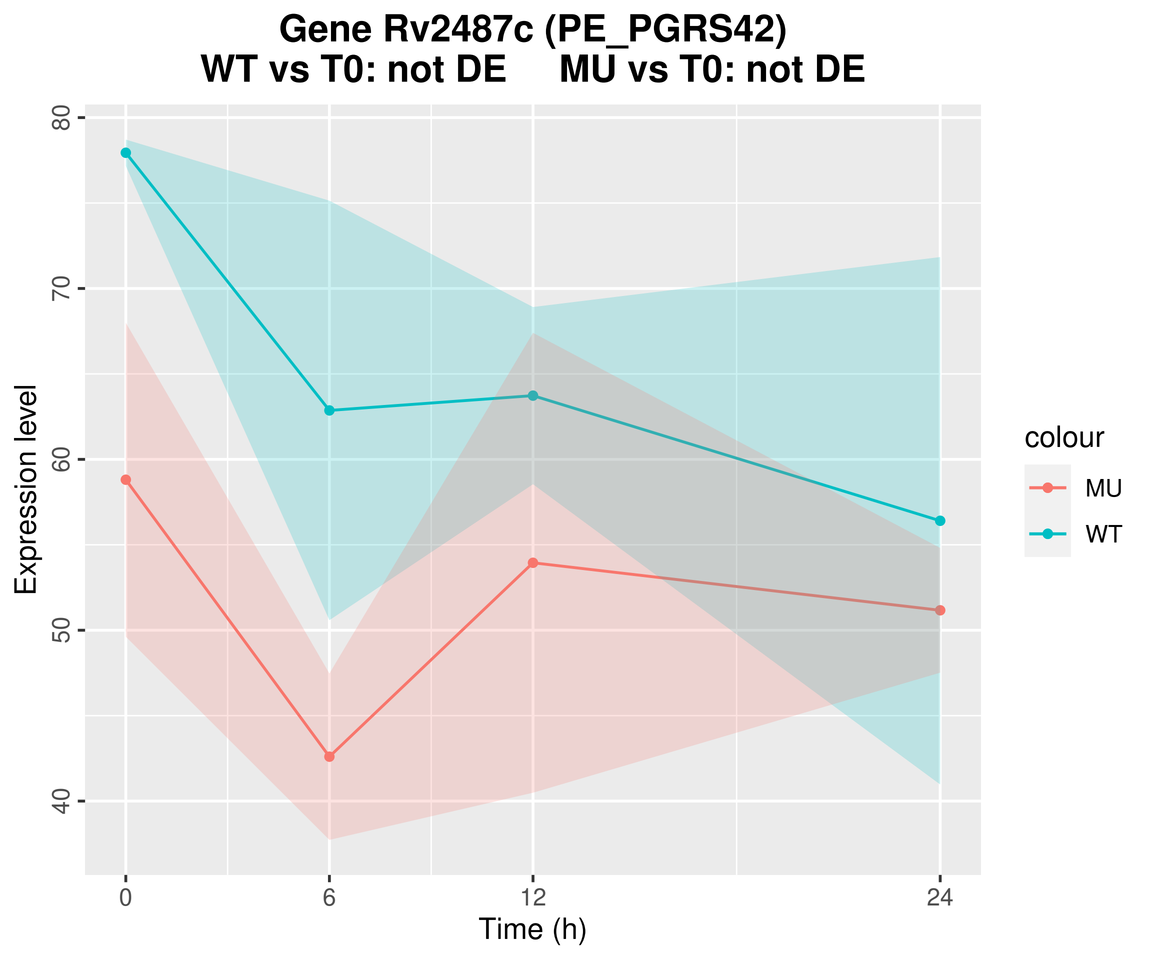
Gene Rv2487c (PE_PGRS42)
WT vs T0: not DE   MU vs T0: not DE
Expression level
80
70
60
50
40
0
6
12
24
Time (h)
colour
MU
WT

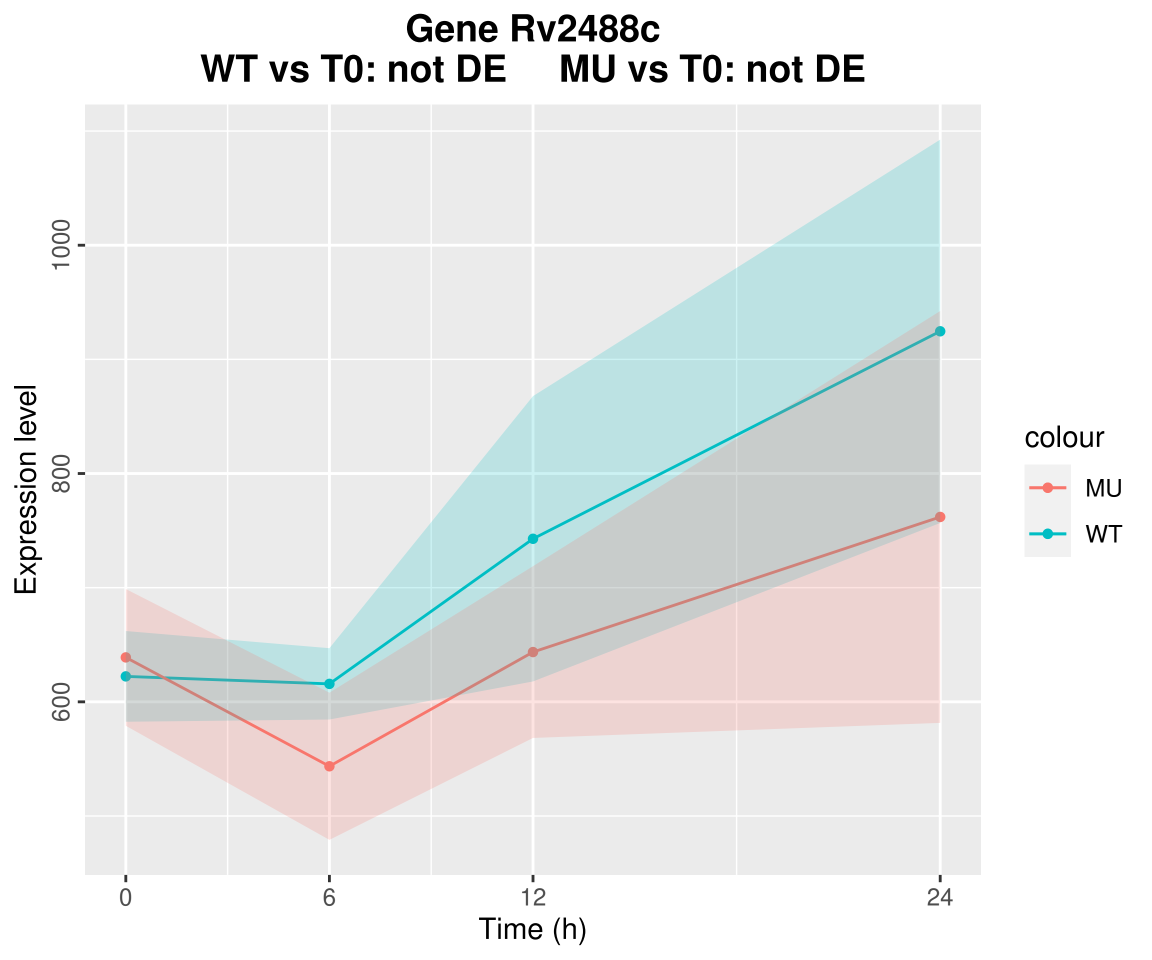
Gene Rv2488c
WT vs T0: not DE MU vs T0: not DE
Expression level
1000
800
600
0
6
12
24
Time (h)
colour
MU
WT

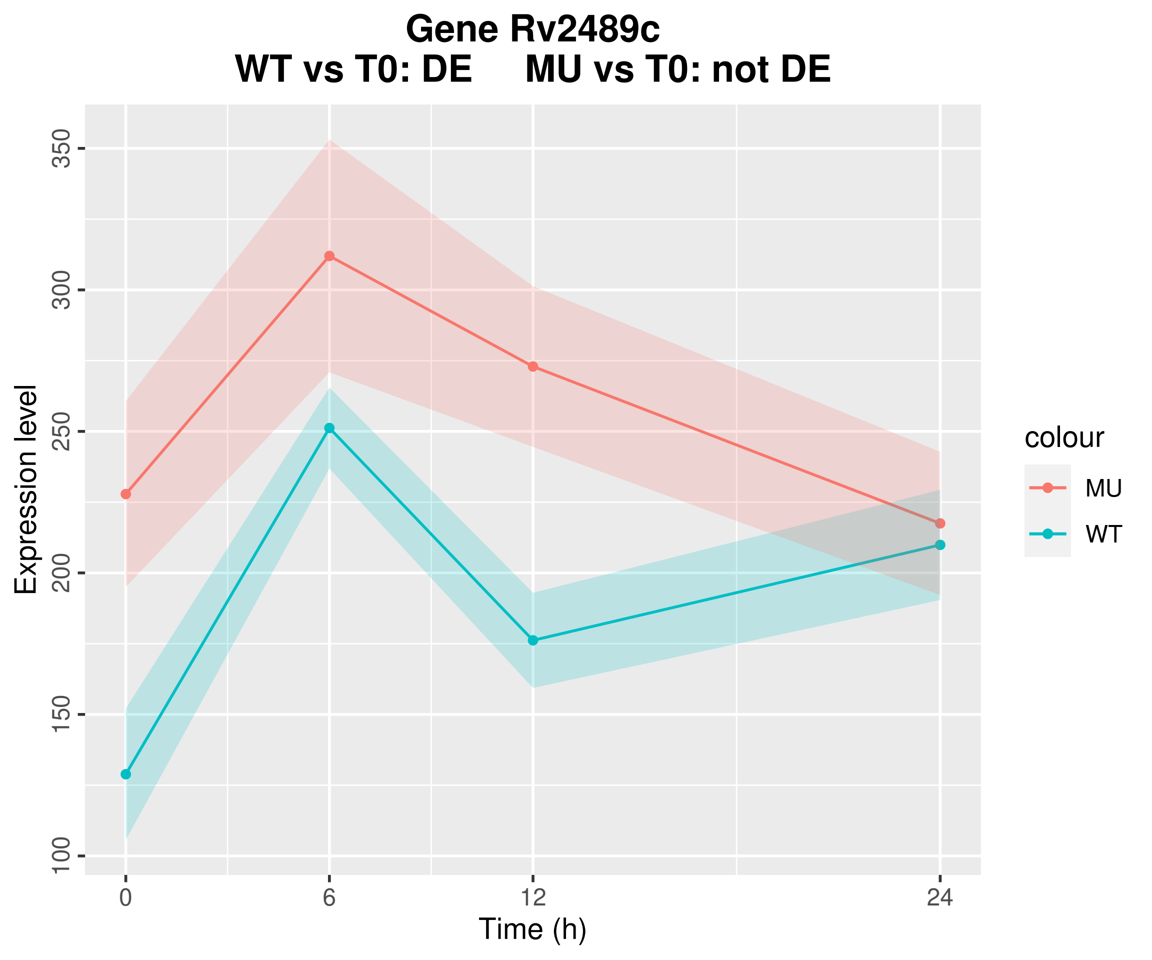

Gene Rv2489c
WT vs T0: DE MU vs T0: not DE
Expression level
350
300
250
200
150
100
0
6
12
24
Time (h)
colour
MU
WT

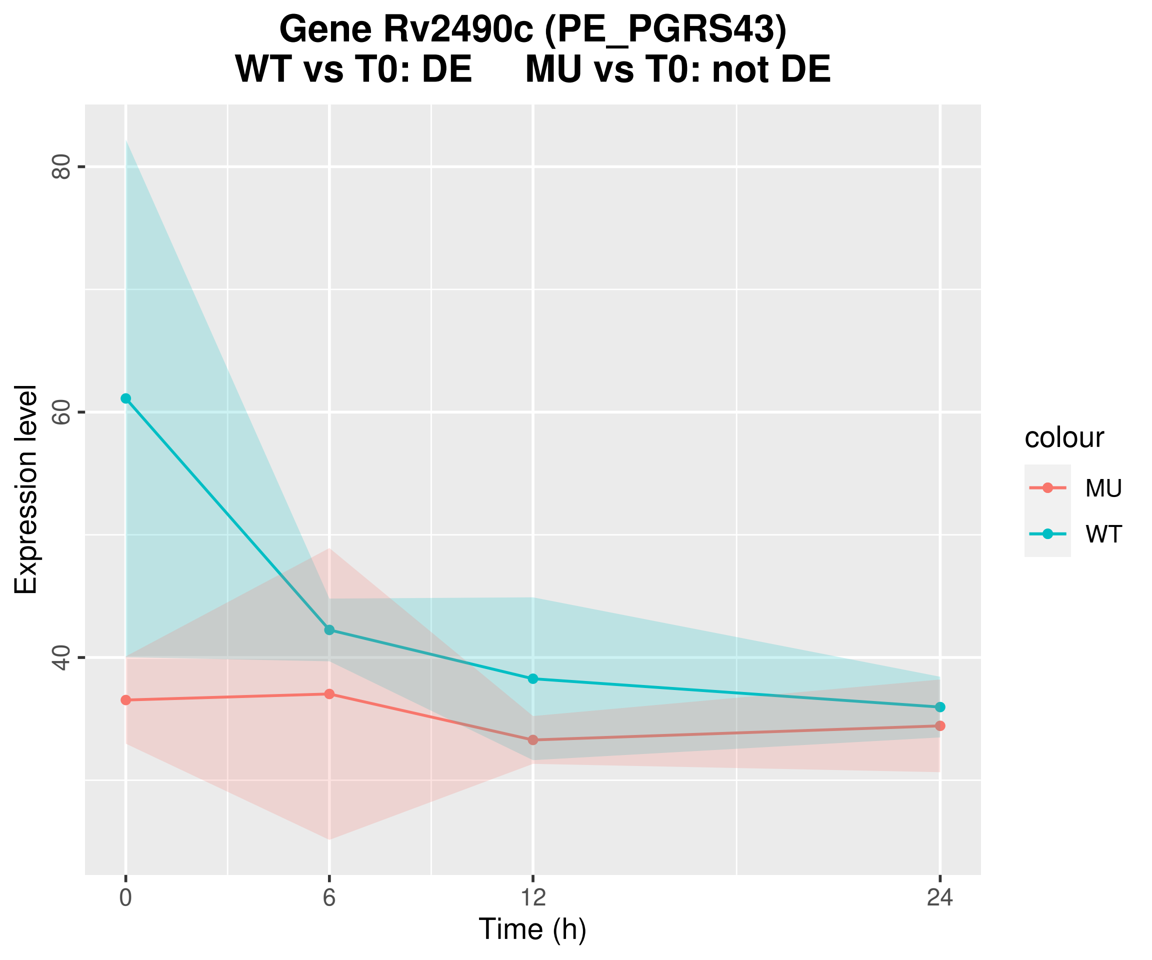

Gene Rv2490c (PE_PGRS43)
WT vs T0: DE   MU vs T0: not DE
Expression level
80
60
40
0
6
12
24
Time (h)
colour
MU
WT

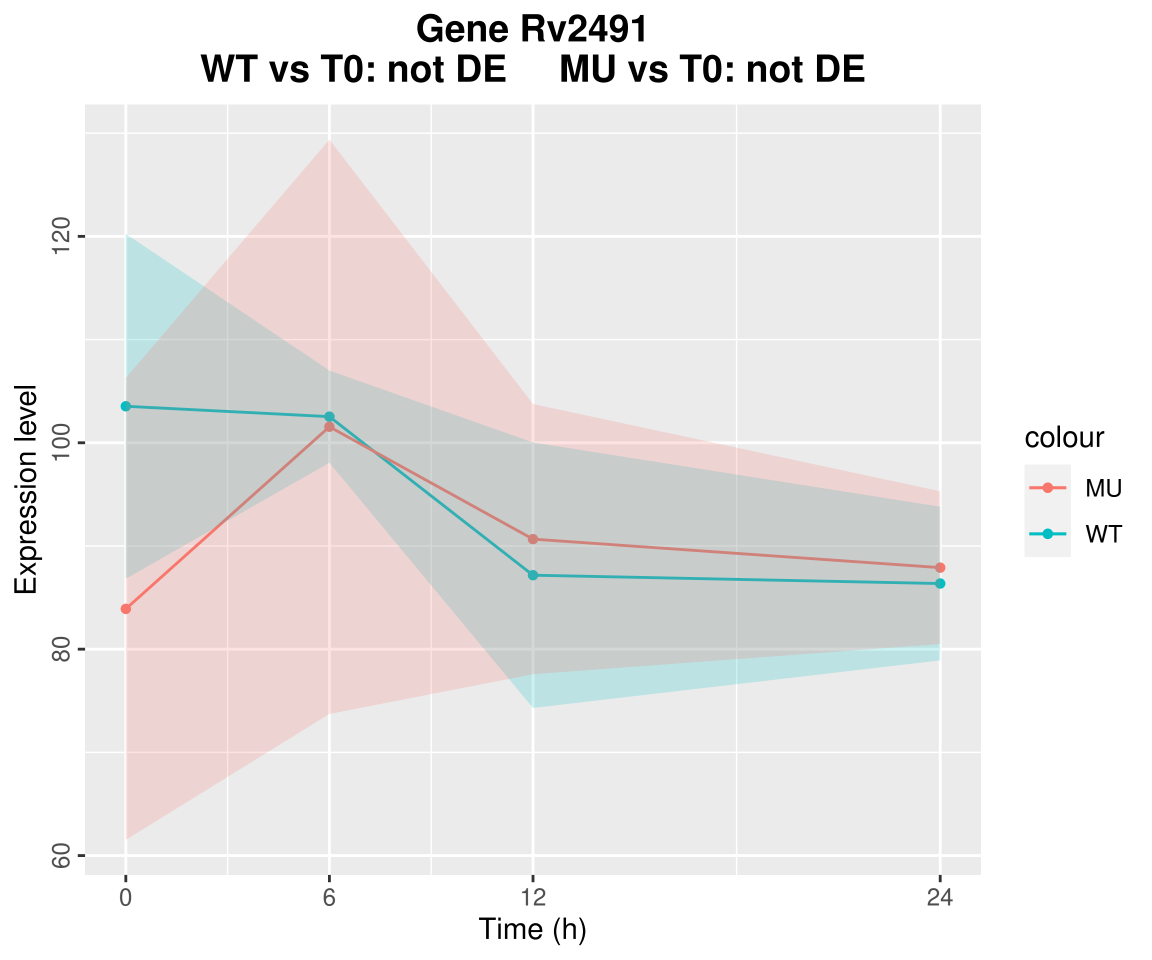

Gene Rv2491
WT vs T0: not DE   MU vs T0: not DE
Expression level
120
100
80
60
0
6
12
24
Time (h)
colour
MU
WT

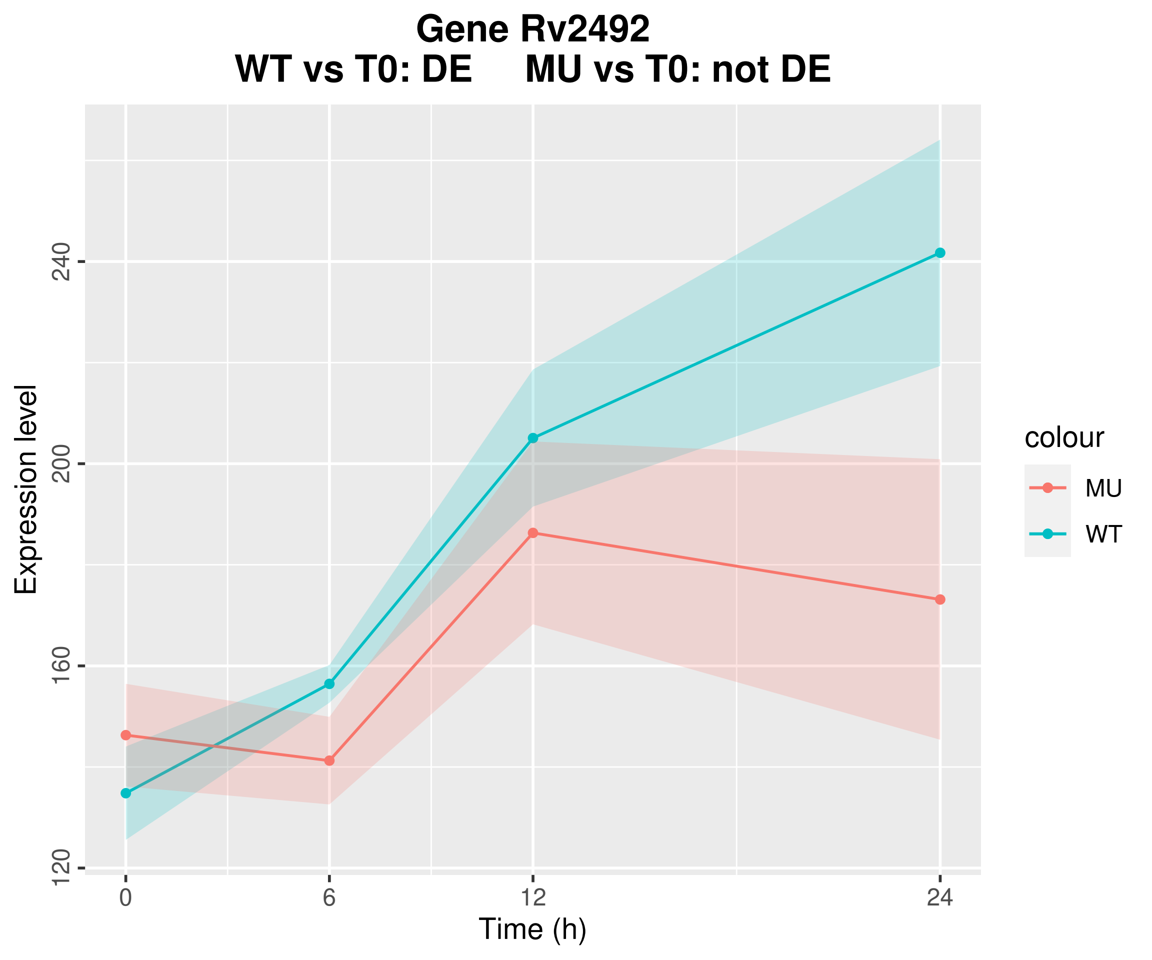

Gene Rv2492
WT vs T0: DE   MU vs T0: not DE
Expression level
120
160
200
240
0
6
12
24
Time (h)
colour
MU
WT

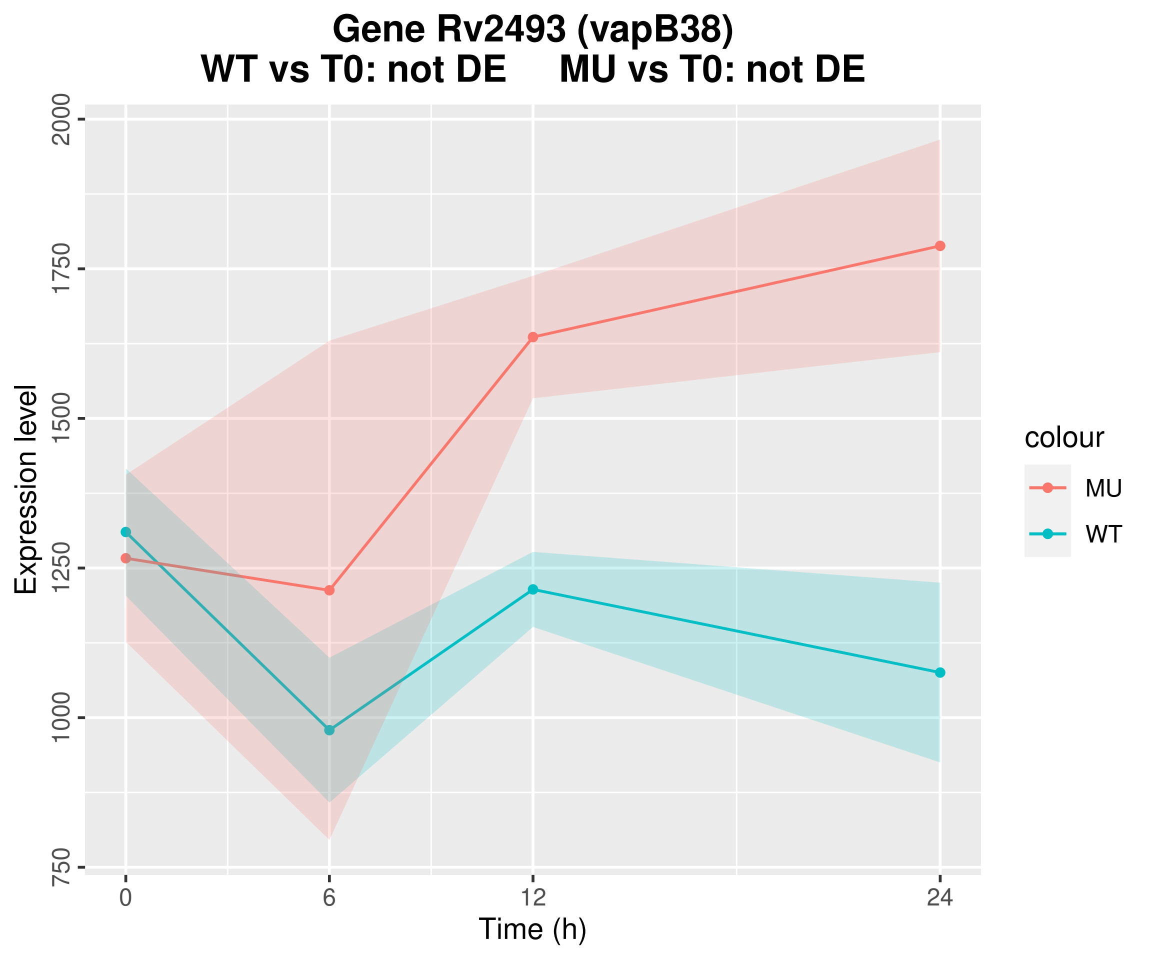
Gene Rv2493 (vapB38)
WT vs T0: not DE   MU vs T0: not DE
Expression level
2000
1750
1500
1250
1000
750
0
6
12
24
Time (h)
colour
MU
WT

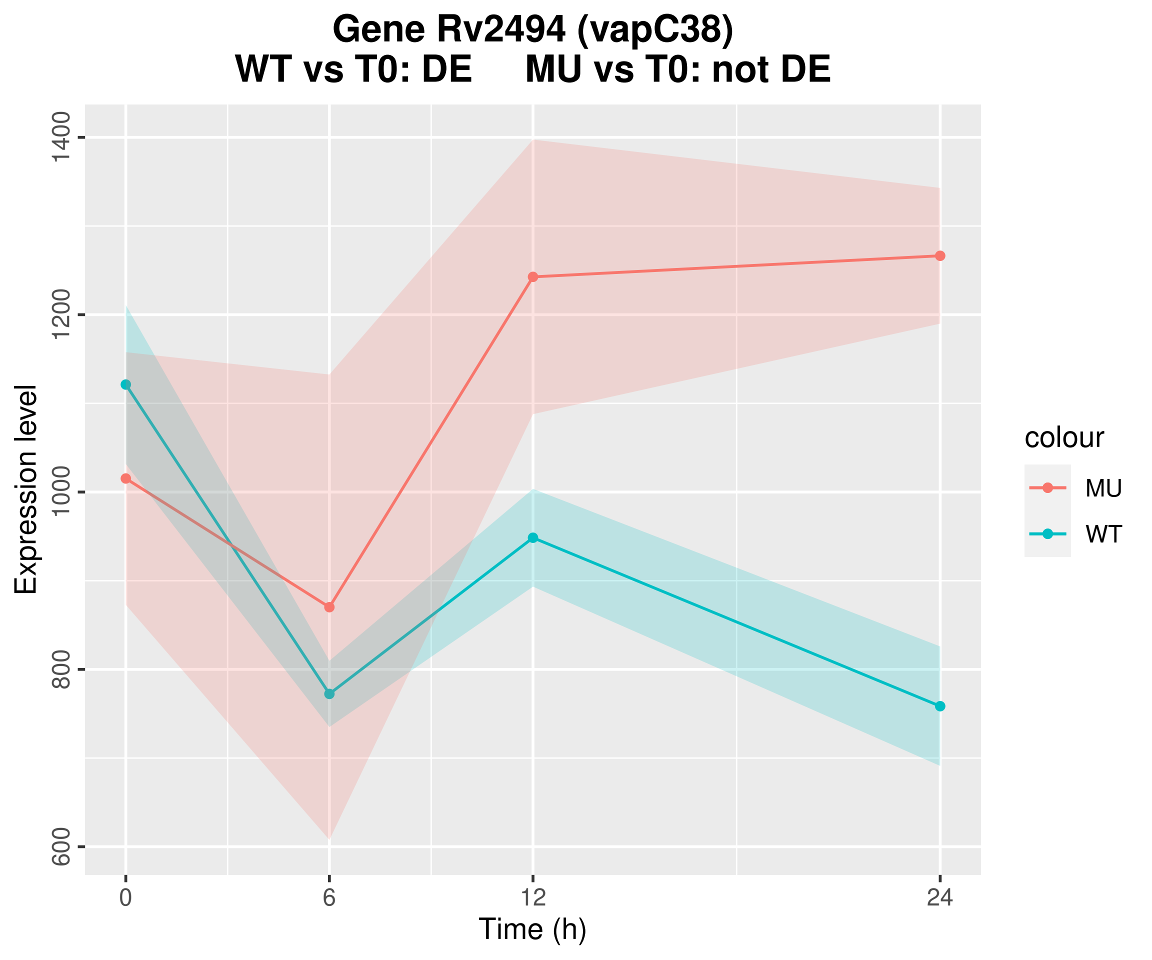
Gene Rv2494 (vapC38)
WT vs T0: DE   MU vs T0: not DE
Expression level
1400
1200
1000
800
600
0
6
12
24
Time (h)
colour
MU
WT

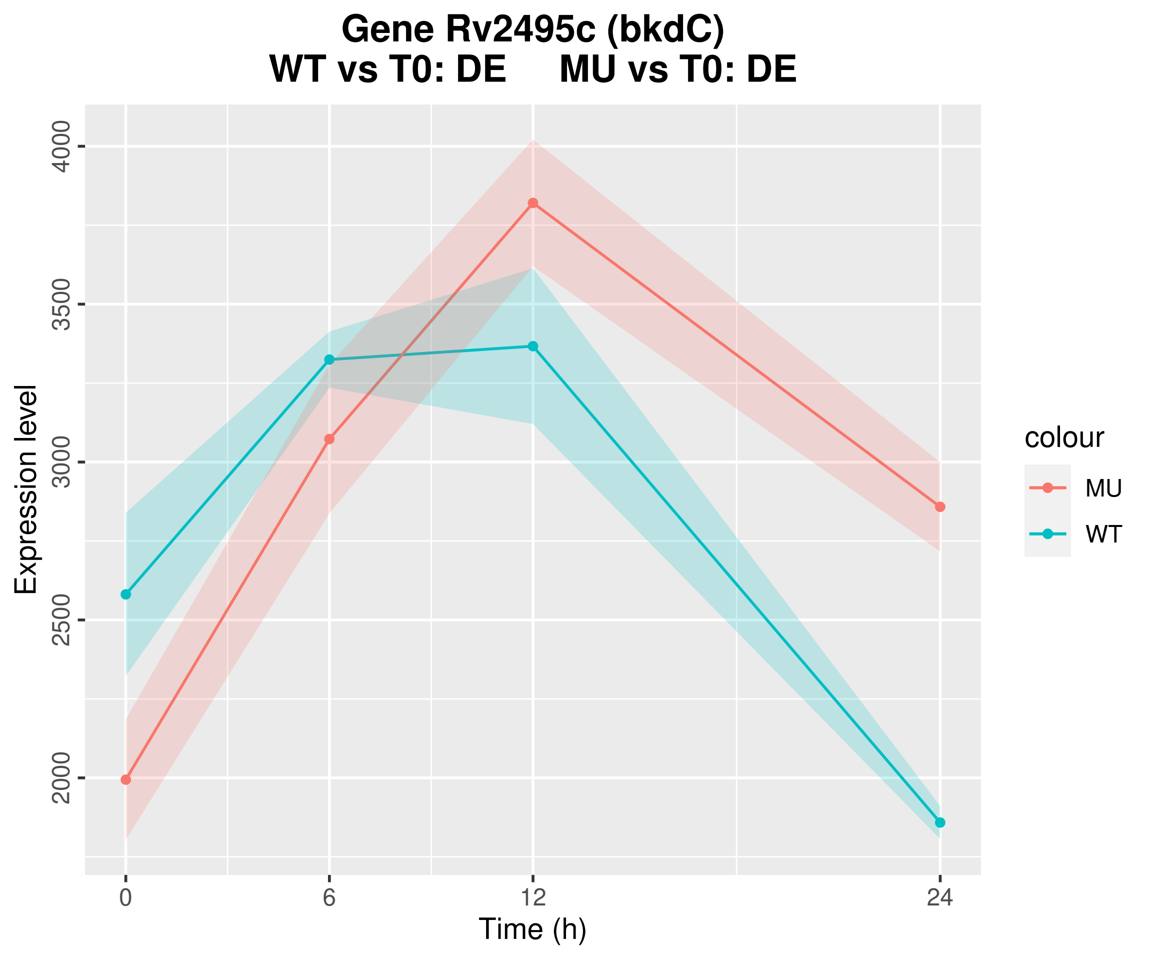
Gene Rv2495c (bkdC)
WT vs T0: DE MU vs T0: DE
Expression level
4000
3500
3000
2500
2000
0
6
12
24
Time (h)
colour
MU
WT

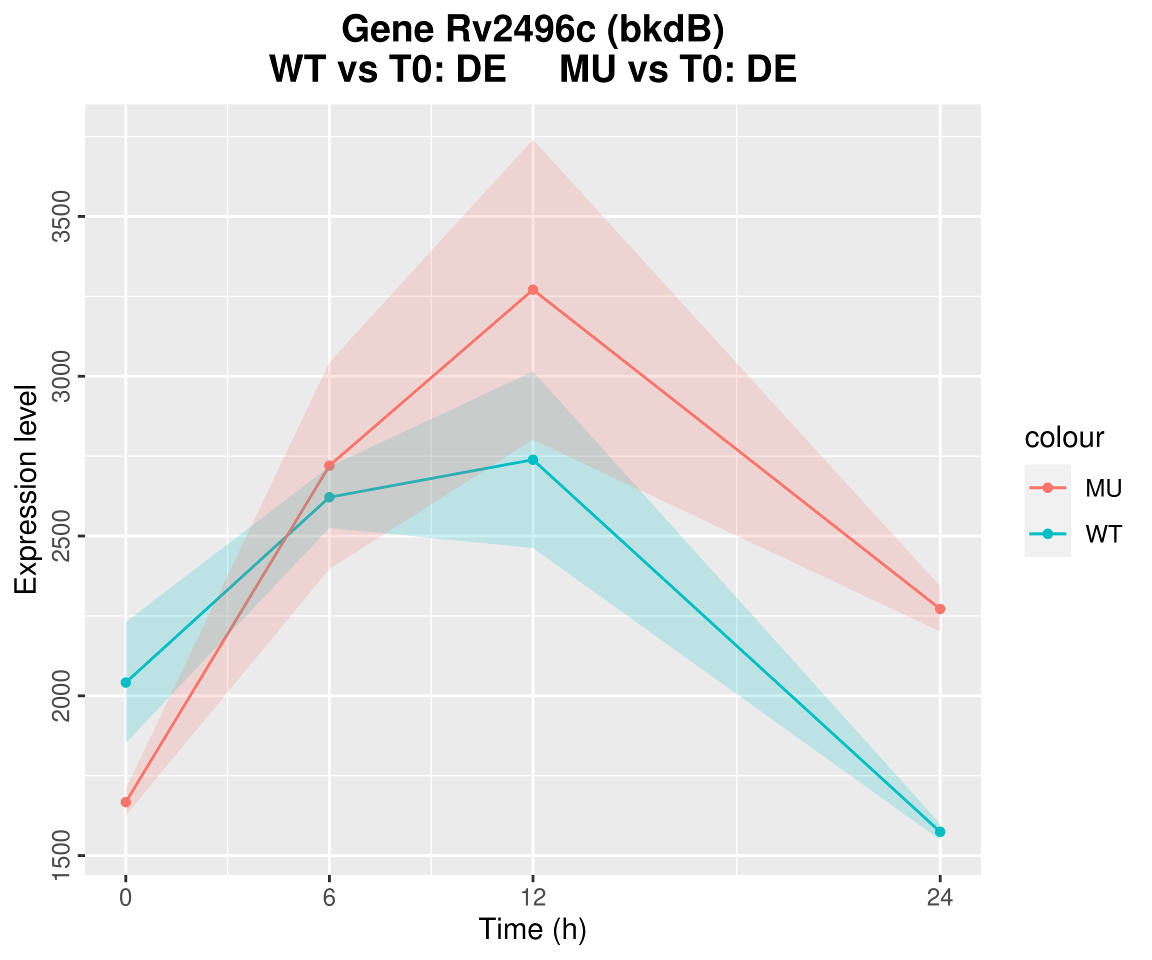

Gene Rv2496c (bkdB)
WT vs T0: DE MU vs T0: DE
Expression level
3500
3000
2500
2000
1500
0
6
12
24
Time (h)
colour
MU
WT

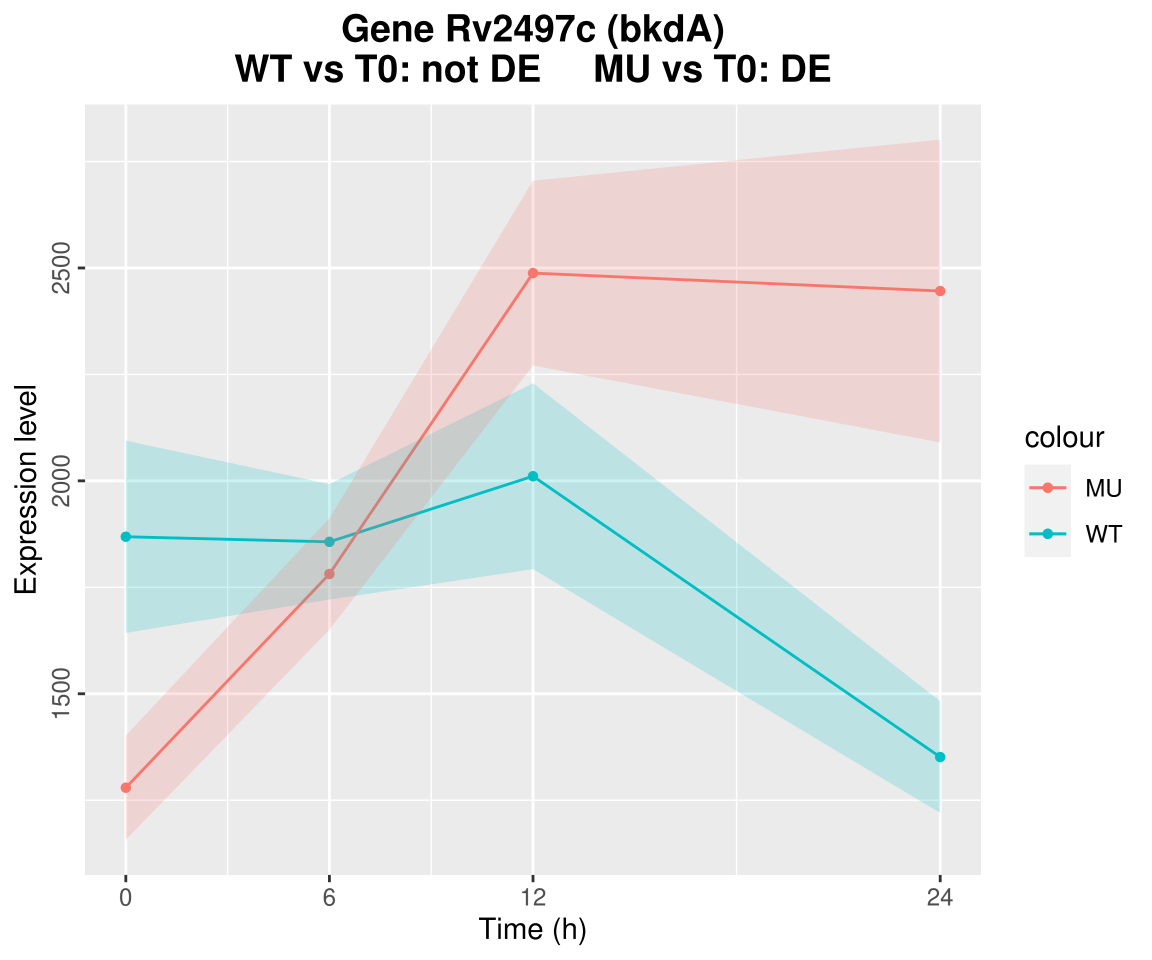

Gene Rv2497c (bkdA)
WT vs T0: not DE MU vs T0: DE
Expression level
2500
2000
1500
0
6
12
24
Time (h)
colour
MU
WT

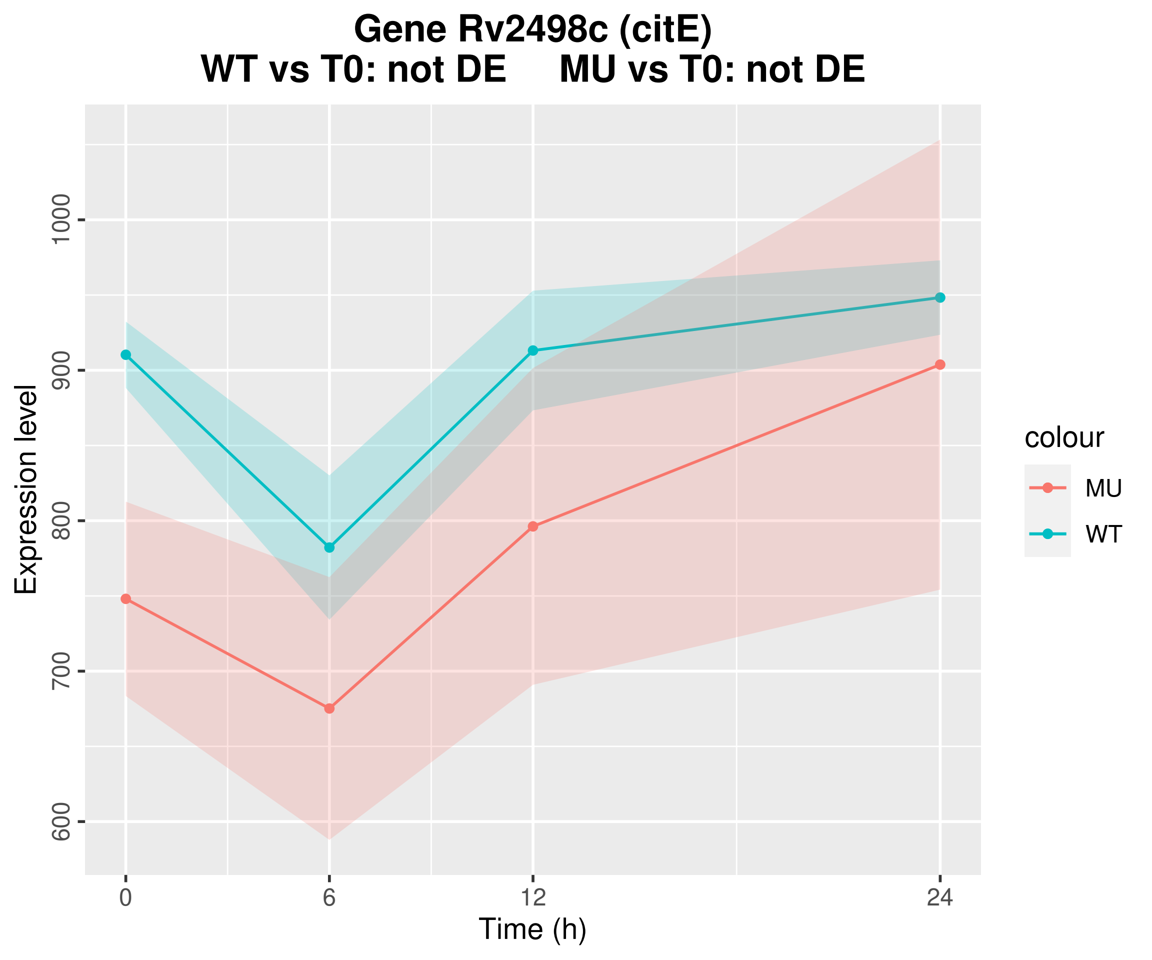

Gene Rv2498c (citE)
WT vs T0: not DE MU vs T0: not DE
Expression level
600
700
800
900
1000
0
6
12
24
Time (h)
colour
MU
WT

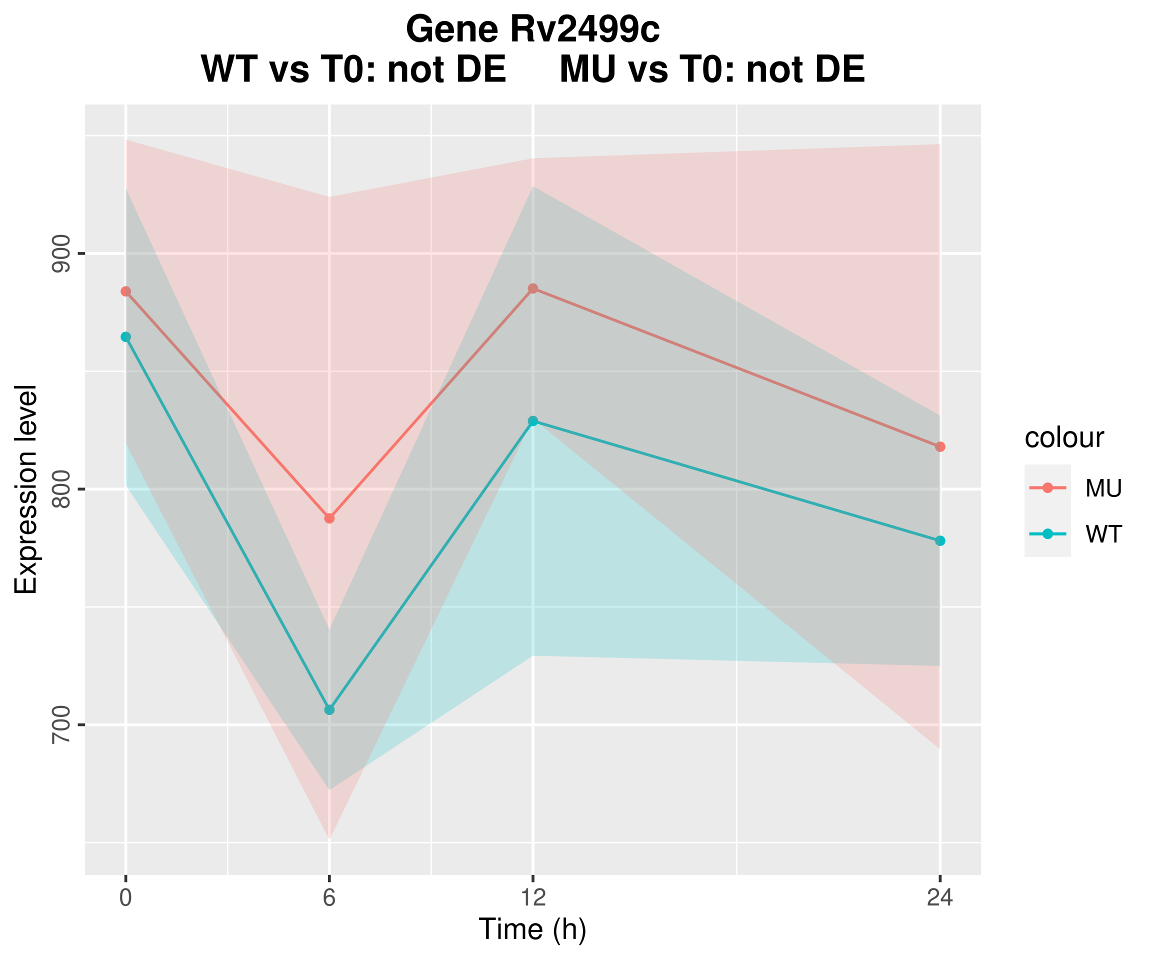

Gene Rv2499c
WT vs T0: not DE   MU vs T0: not DE
900
800
700
Expression level
0
6
12
24
Time (h)
colour
MU
WT

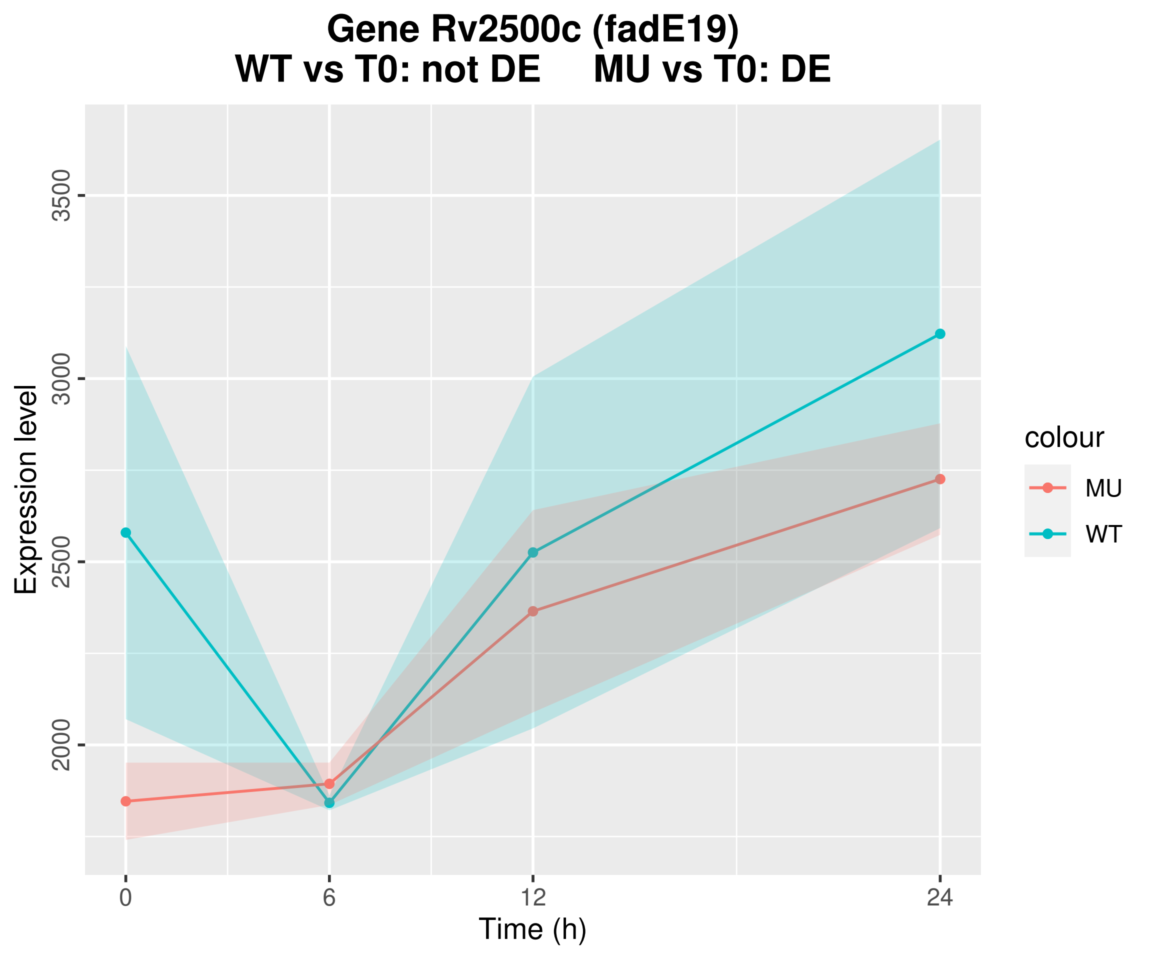
Gene Rv2500c (fadE19)
WT vs T0: not DE MU vs T0: DE
Expression level
3500
3000
2500
2000
0
6
12
24
Time (h)
colour
MU
WT

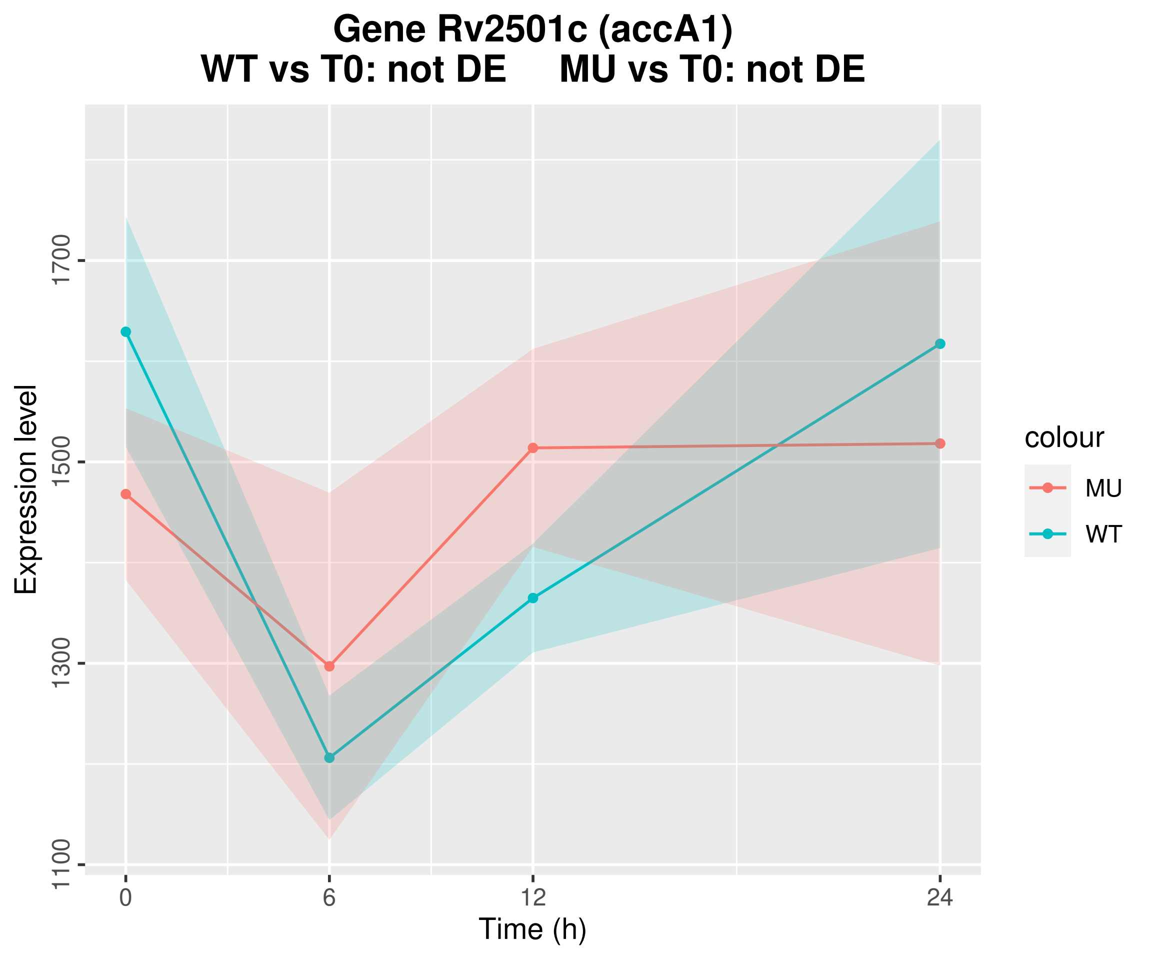

Gene Rv2501c (accA1)
WT vs T0: not DE   MU vs T0: not DE
Expression level
1100
1300
1500
1700
0
6
12
24
Time (h)
colour
MU
WT

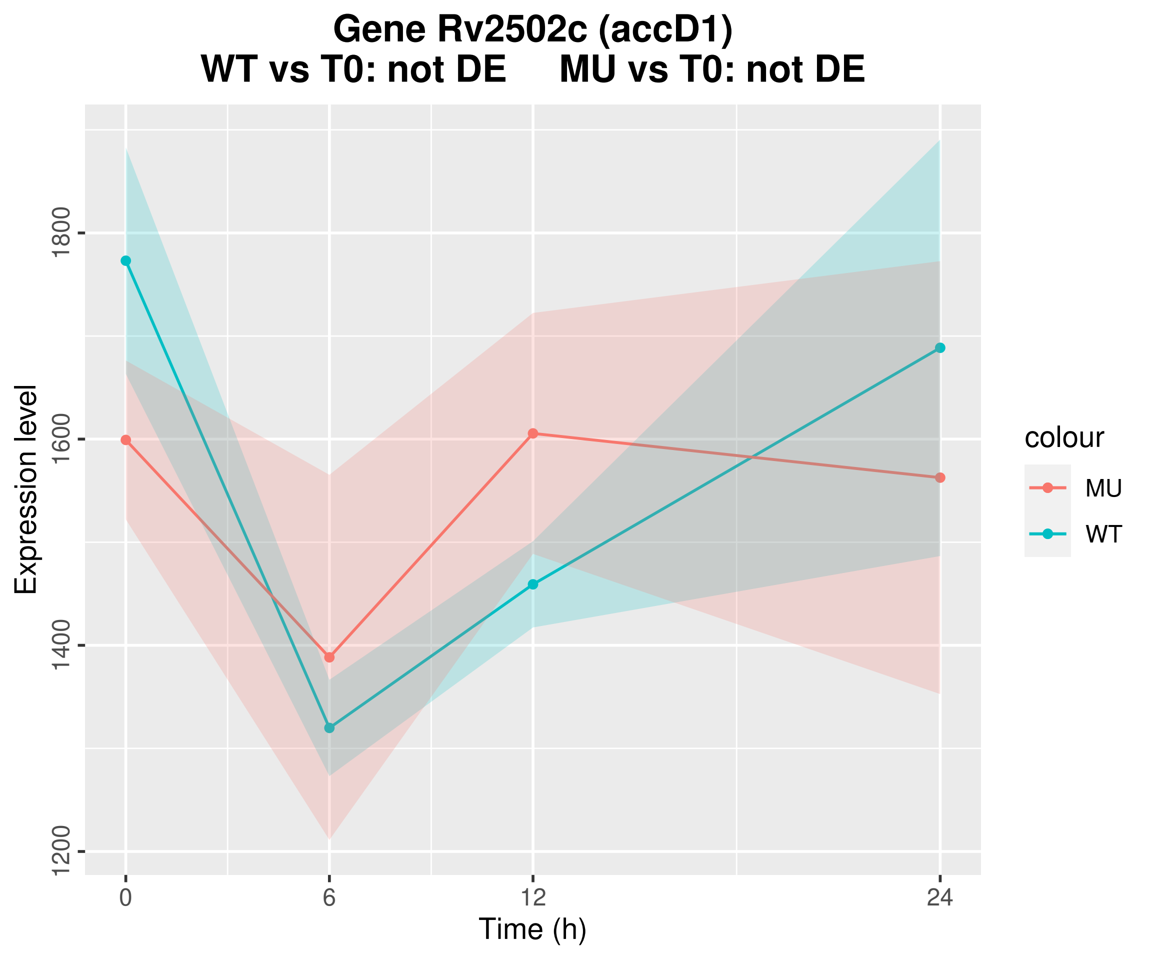

Gene Rv2502c (accD1)
WT vs T0: not DE   MU vs T0: not DE
Expression level
1200
1400
1600
1800
0
6
12
24
Time (h)
colour
MU
WT

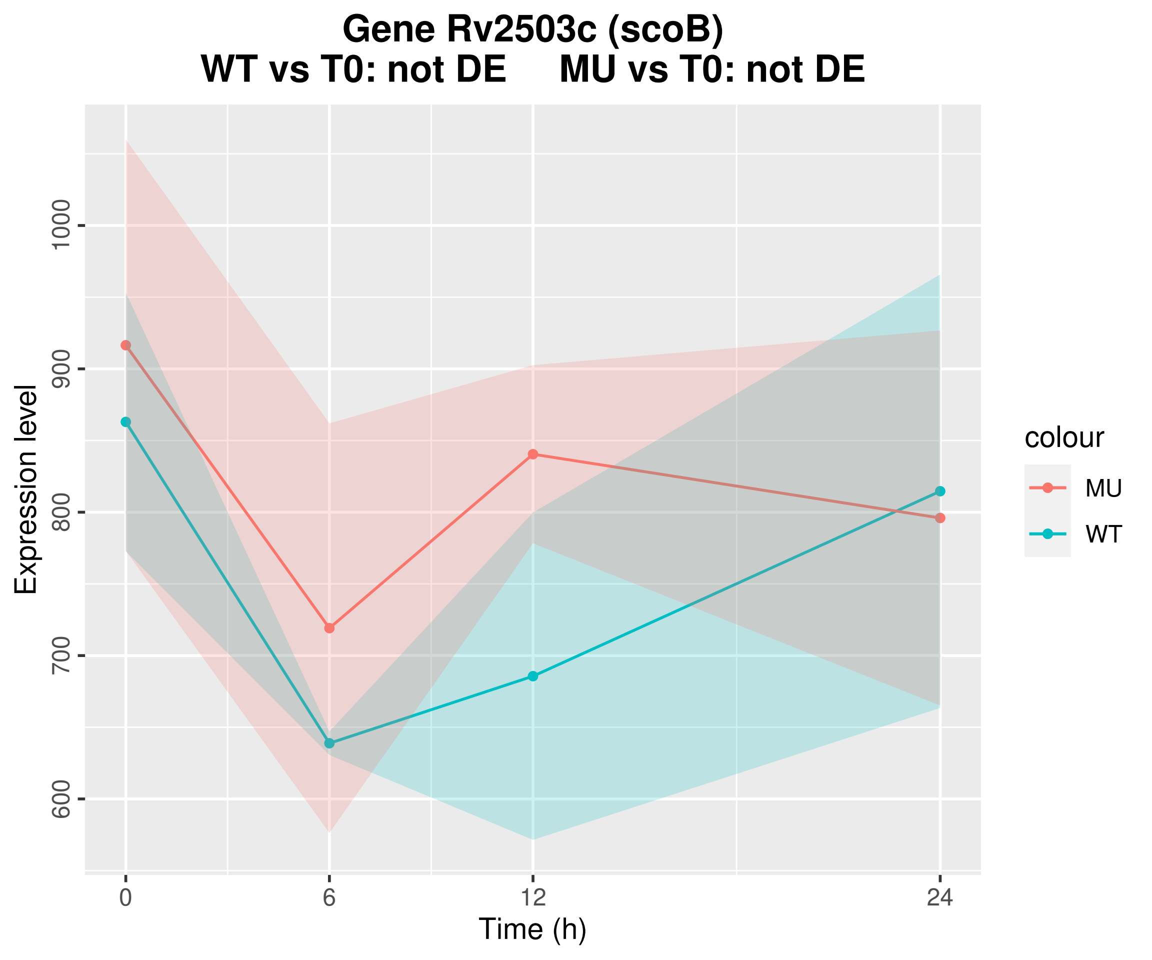

Gene Rv2503c (scoB)
WT vs T0: not DE   MU vs T0: not DE
Expression level
1000
900
800
700
600
0
6
12
24
Time (h)
colour
MU
WT

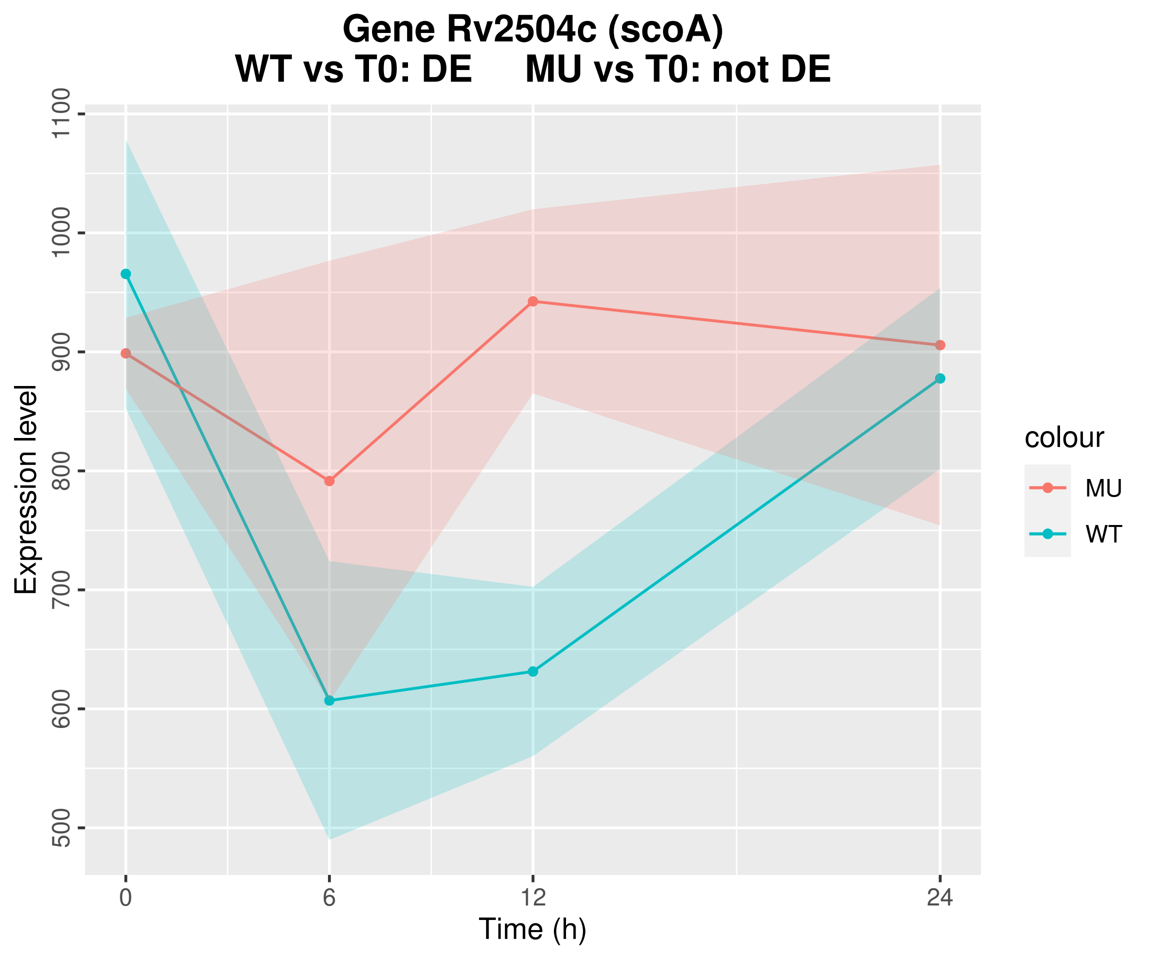

Gene Rv2504c (scoA)
WT vs T0: DE   MU vs T0: not DE
Expression level
1100
1000
900
800
700
600
500
0
6
12
24
Time (h)
colour
MU
WT

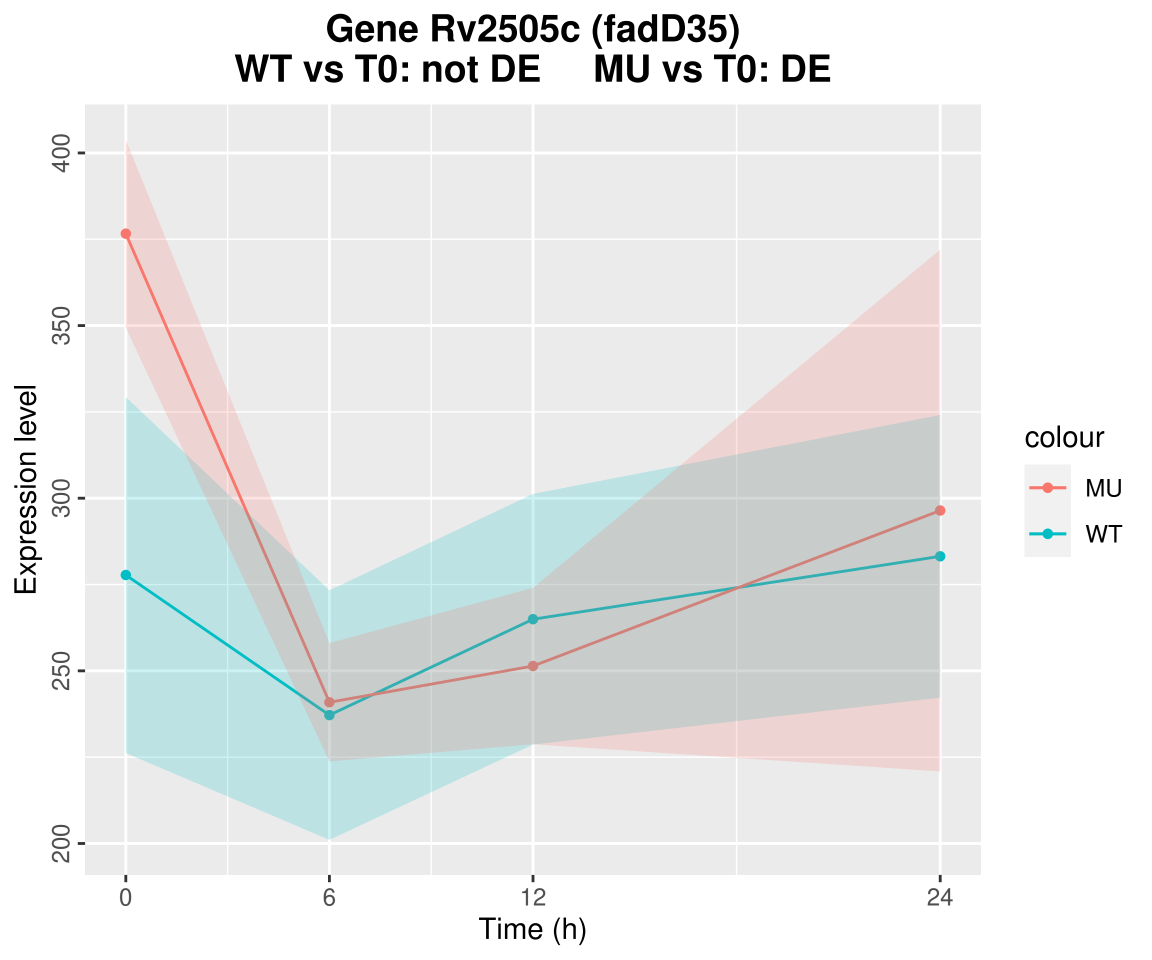
Gene Rv2505c (fadD35)
WT vs T0: not DE   MU vs T0: DE
Expression level
400
350
300
250
200
0
6
12
24
Time (h)
colour
MU
WT

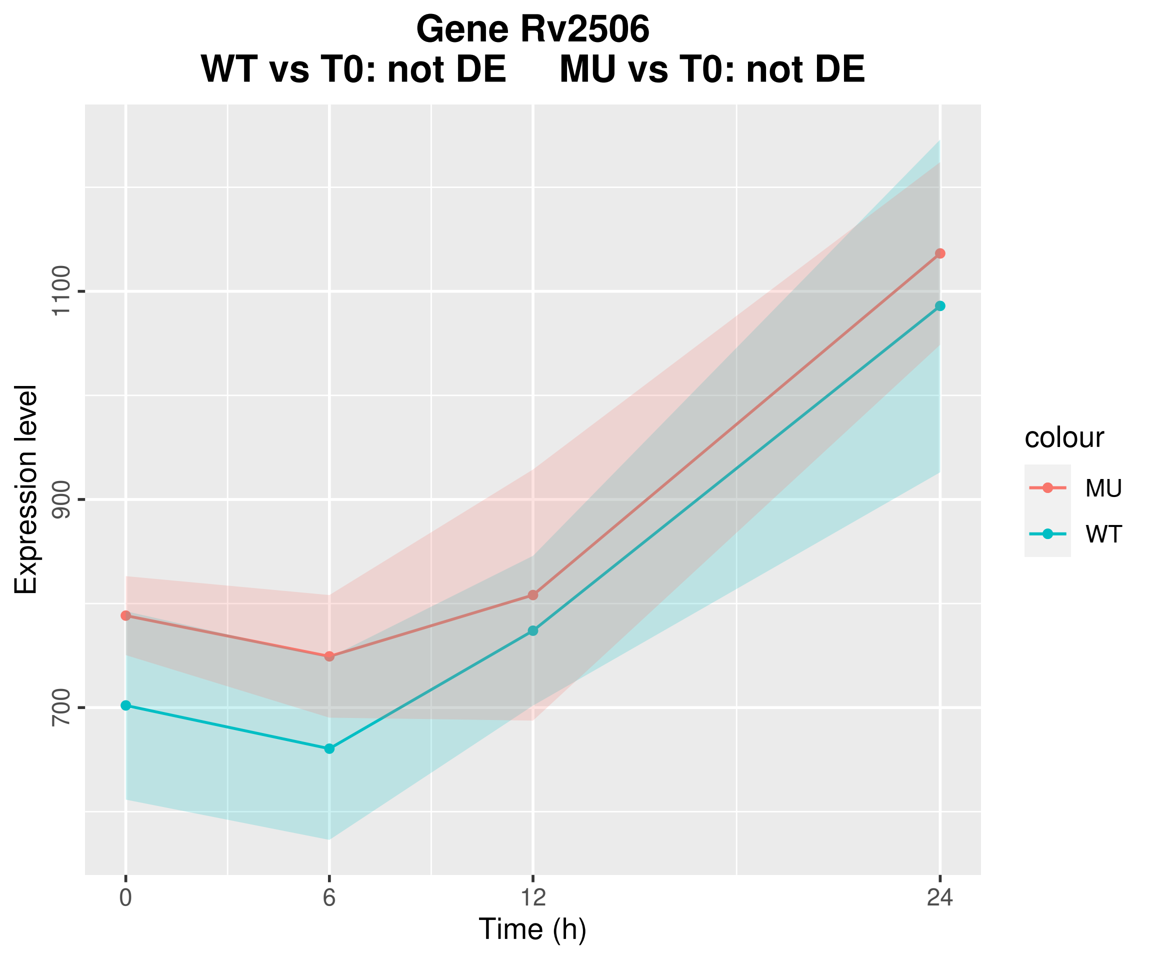
Gene Rv2506
WT vs T0: not DE   MU vs T0: not DE
Expression level
1100
900
700
0
6
12
24
Time (h)
colour
MU
WT

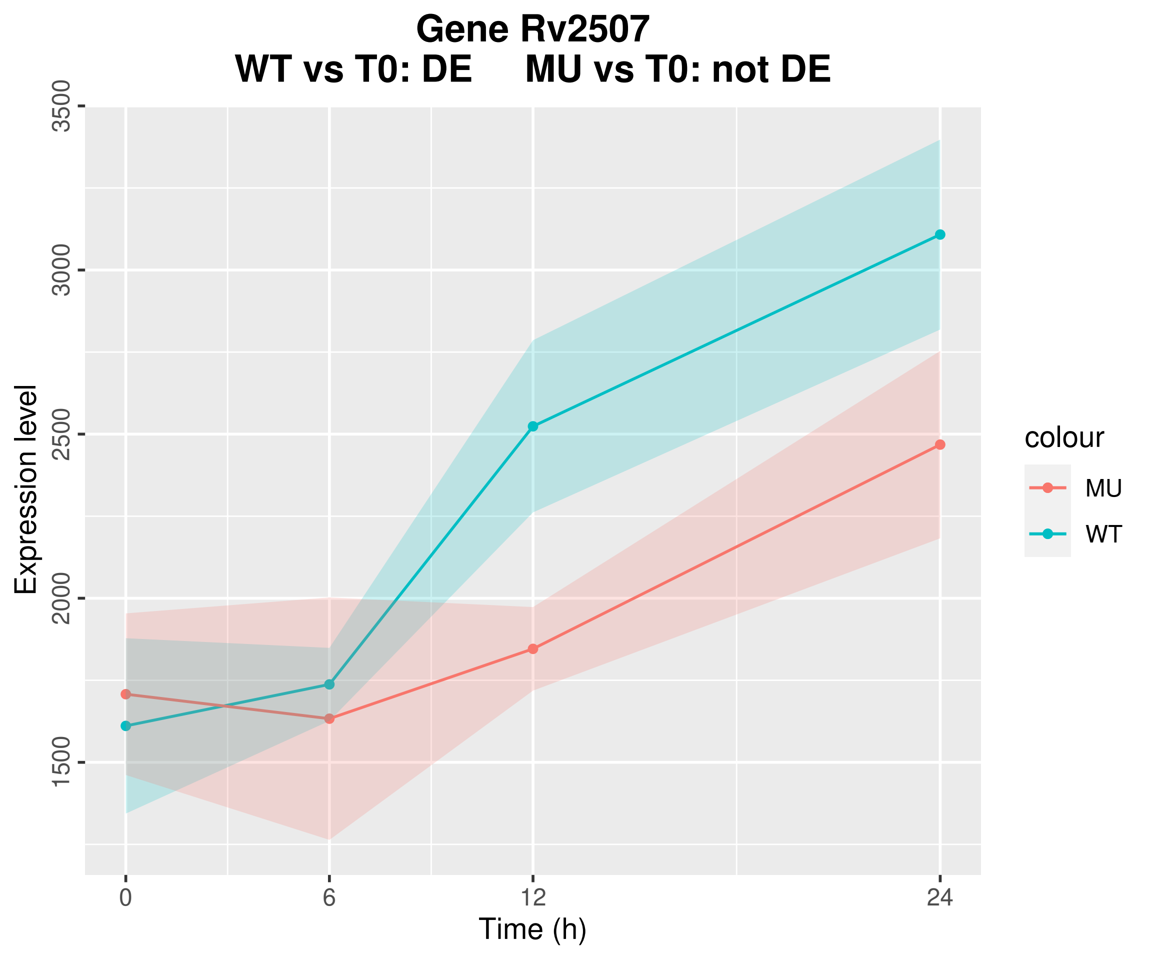

Gene Rv2507
WT vs T0: DE MU vs T0: not DE
Expression level
3500
3000
2500
2000
1500
0
6
12
24
Time (h)
colour
MU
WT

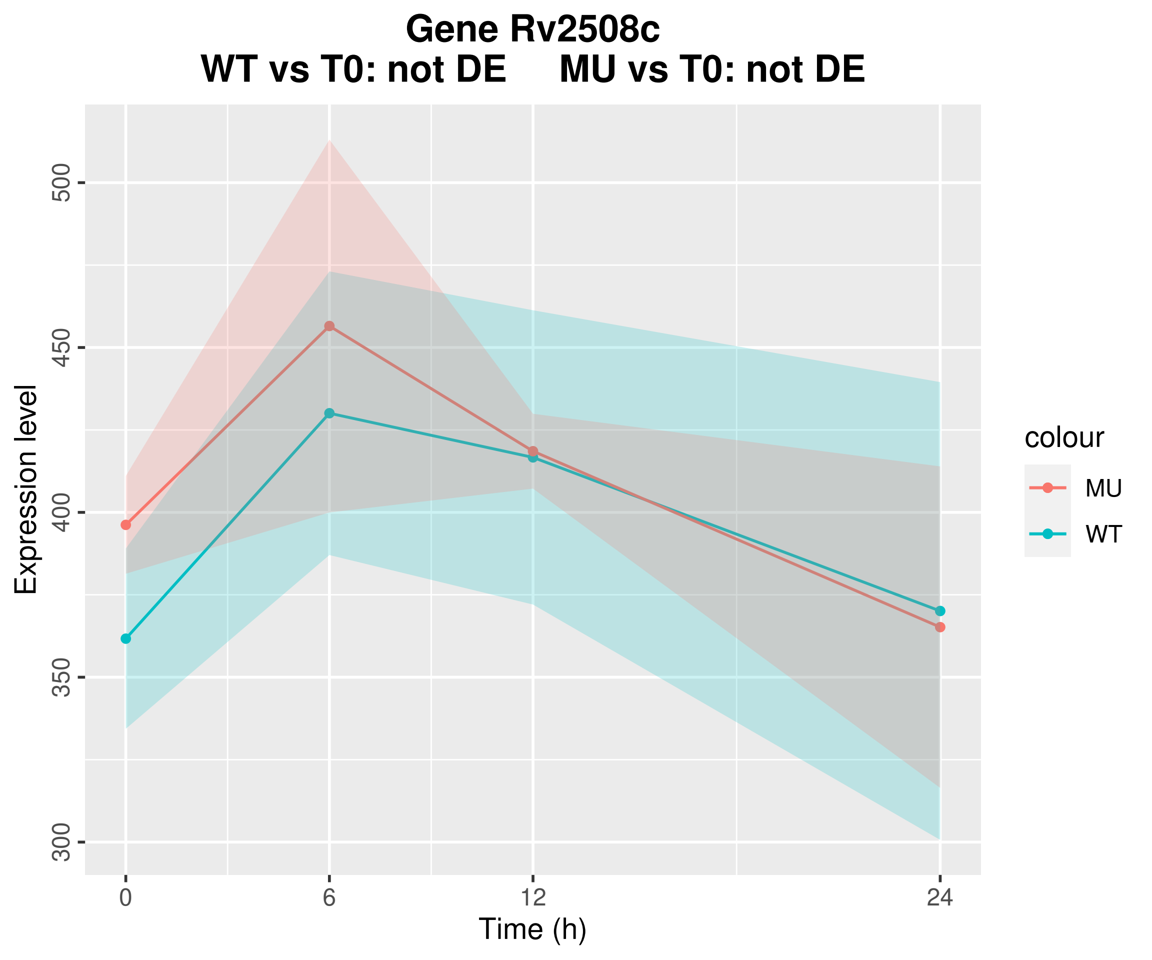

Gene Rv2508c
WT vs T0: not DE MU vs T0: not DE
Expression level
500
450
400
350
300
0
6
12
24
Time (h)
colour
MU
WT

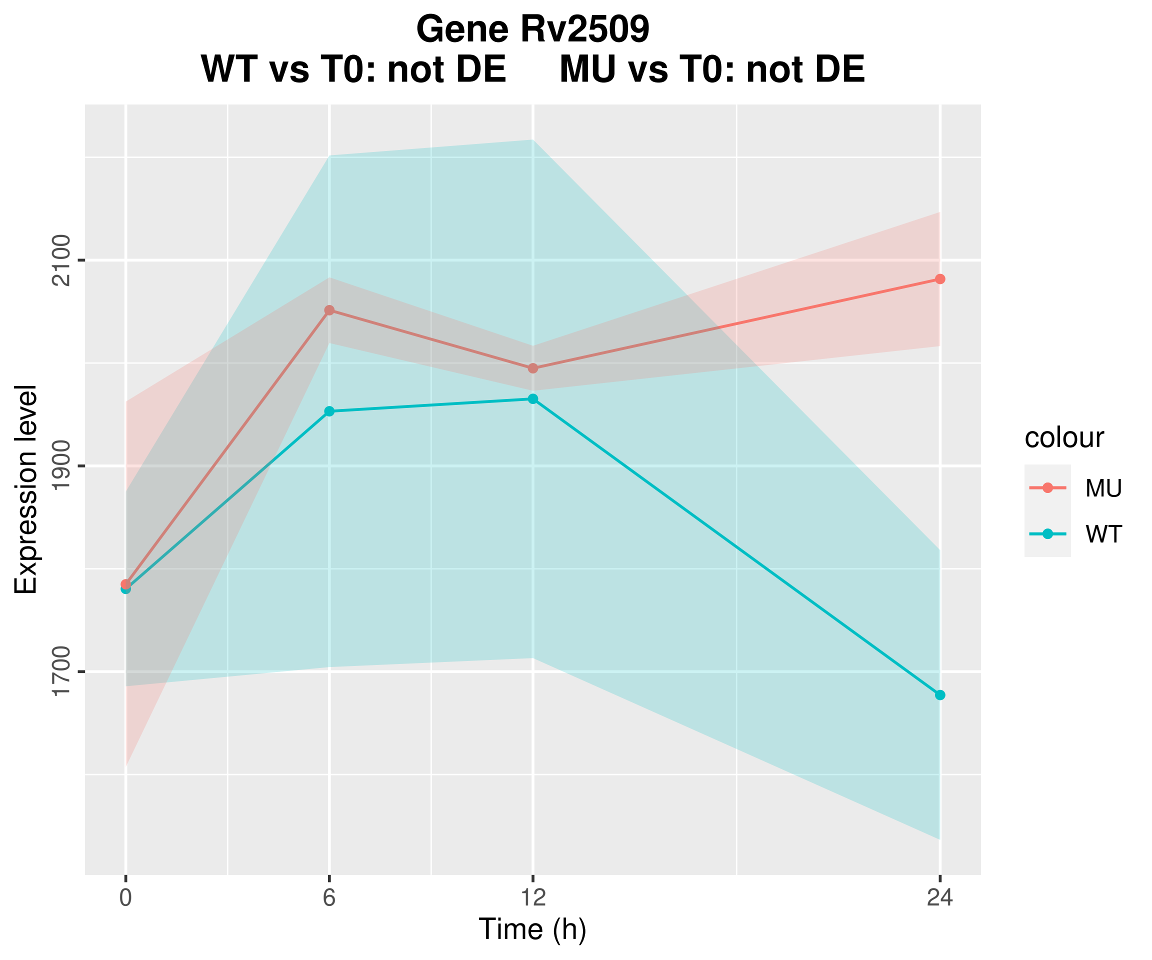

Gene Rv2509
WT vs T0: not DE   MU vs T0: not DE
Expression level
2100
1900
1700
0
6
12
24
Time (h)
colour
MU
WT

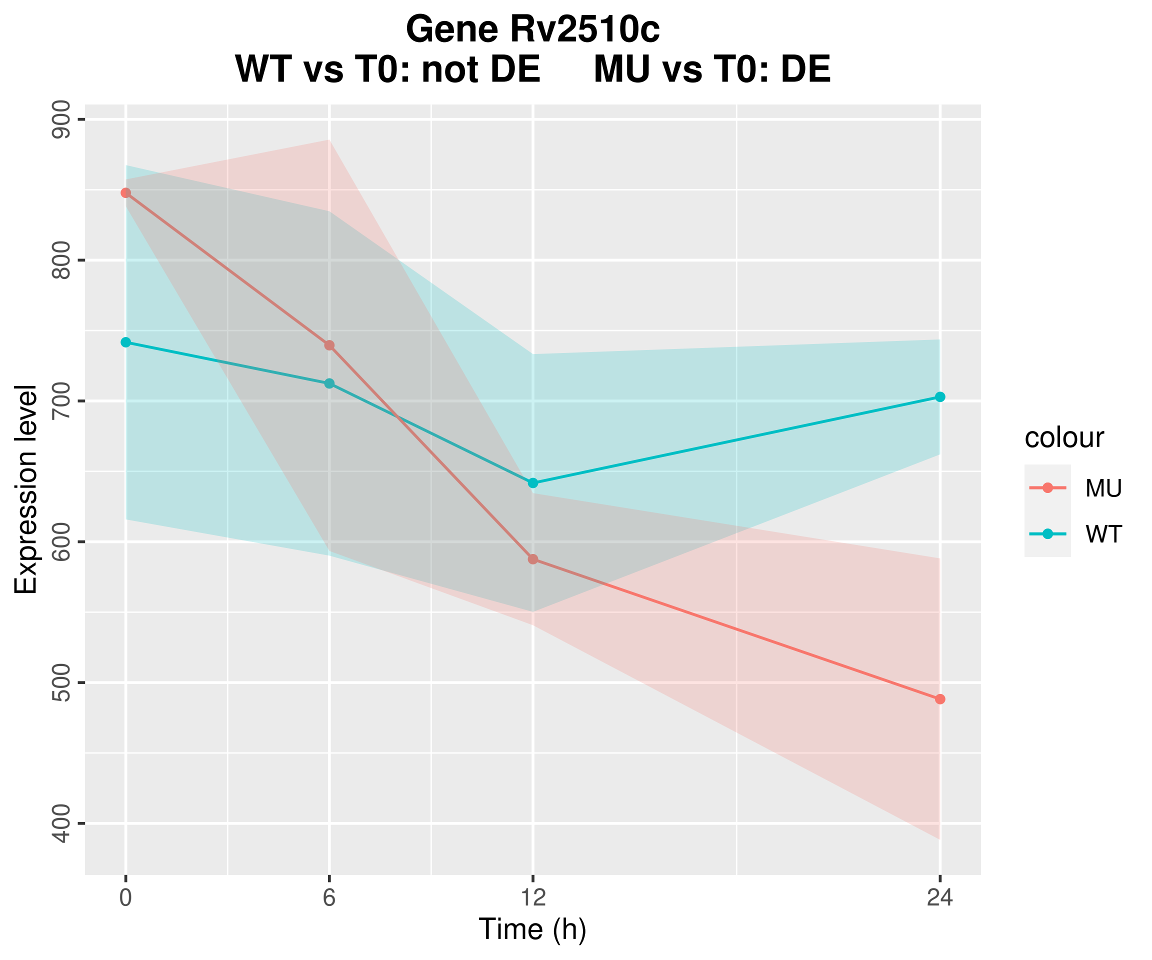
Gene Rv2510c
WT vs T0: not DE MU vs T0: DE
900
800
700
600
500
400
Expression level
0
6
12
24
Time (h)
colour
MU
WT

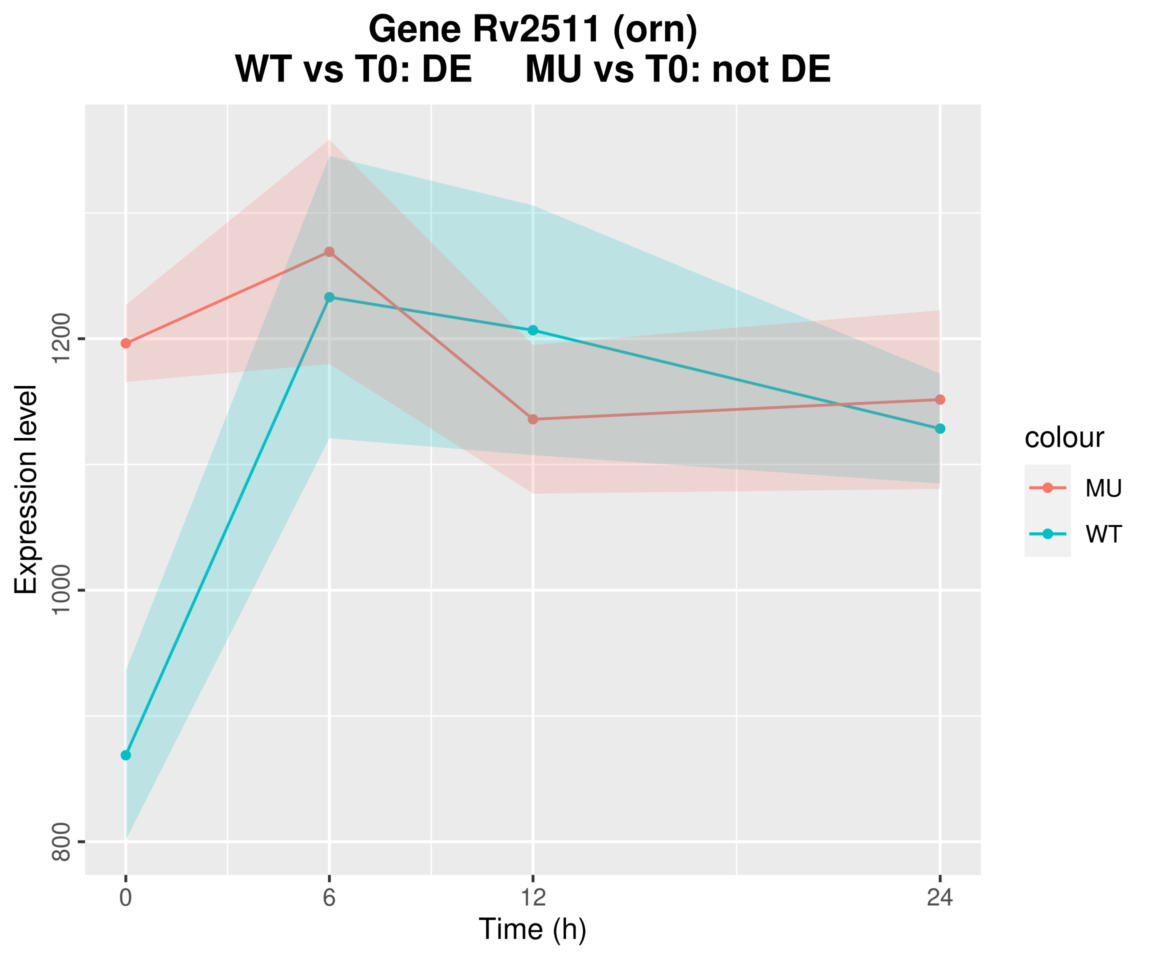

Gene Rv2511 (orn)
WT vs T0: DE MU vs T0: not DE
Expression level
800
1000
1200
0
6
12
24
Time (h)
colour
MU
WT

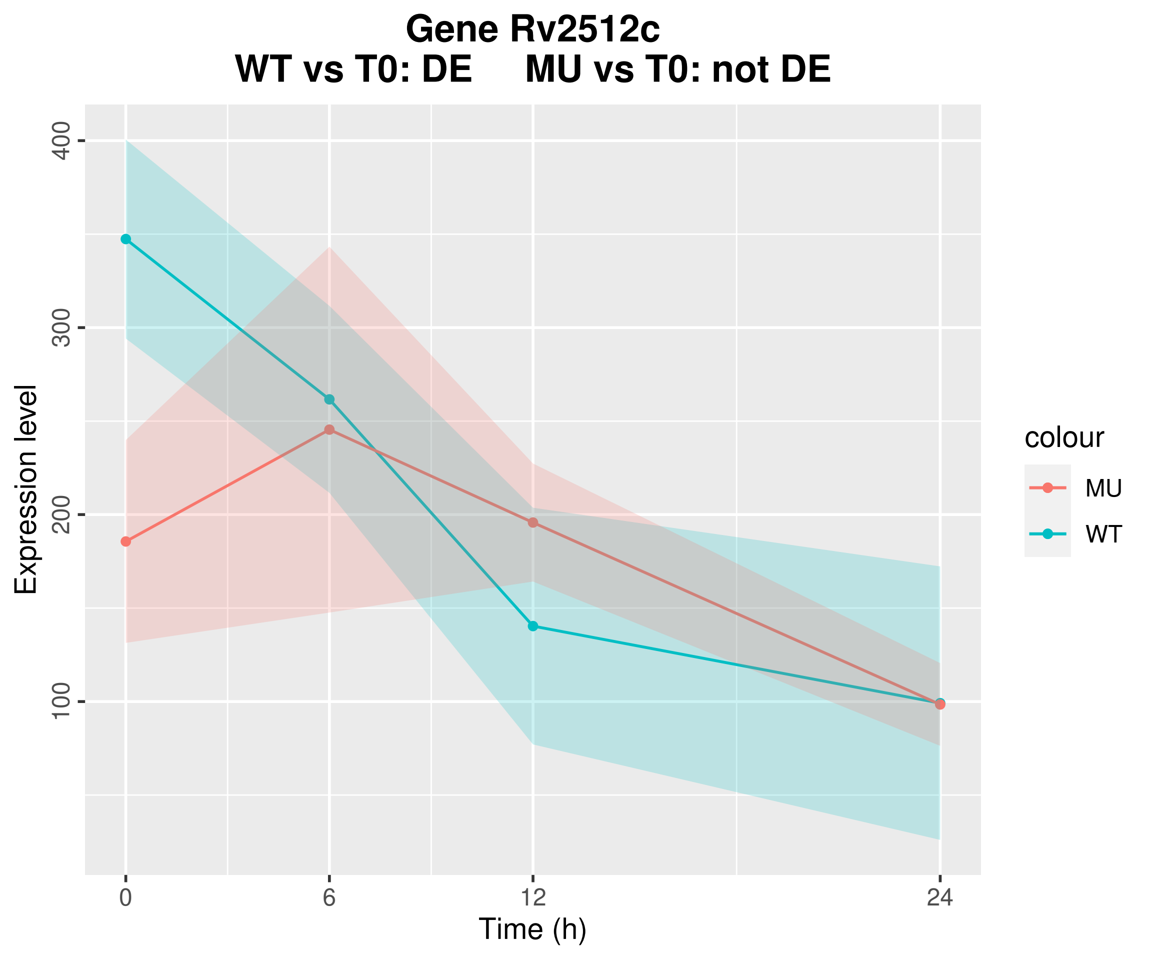

Gene Rv2512c
WT vs T0: DE MU vs T0: not DE
Expression level
400
300
200
100
0
6
12
24
Time (h)
colour
MU
WT

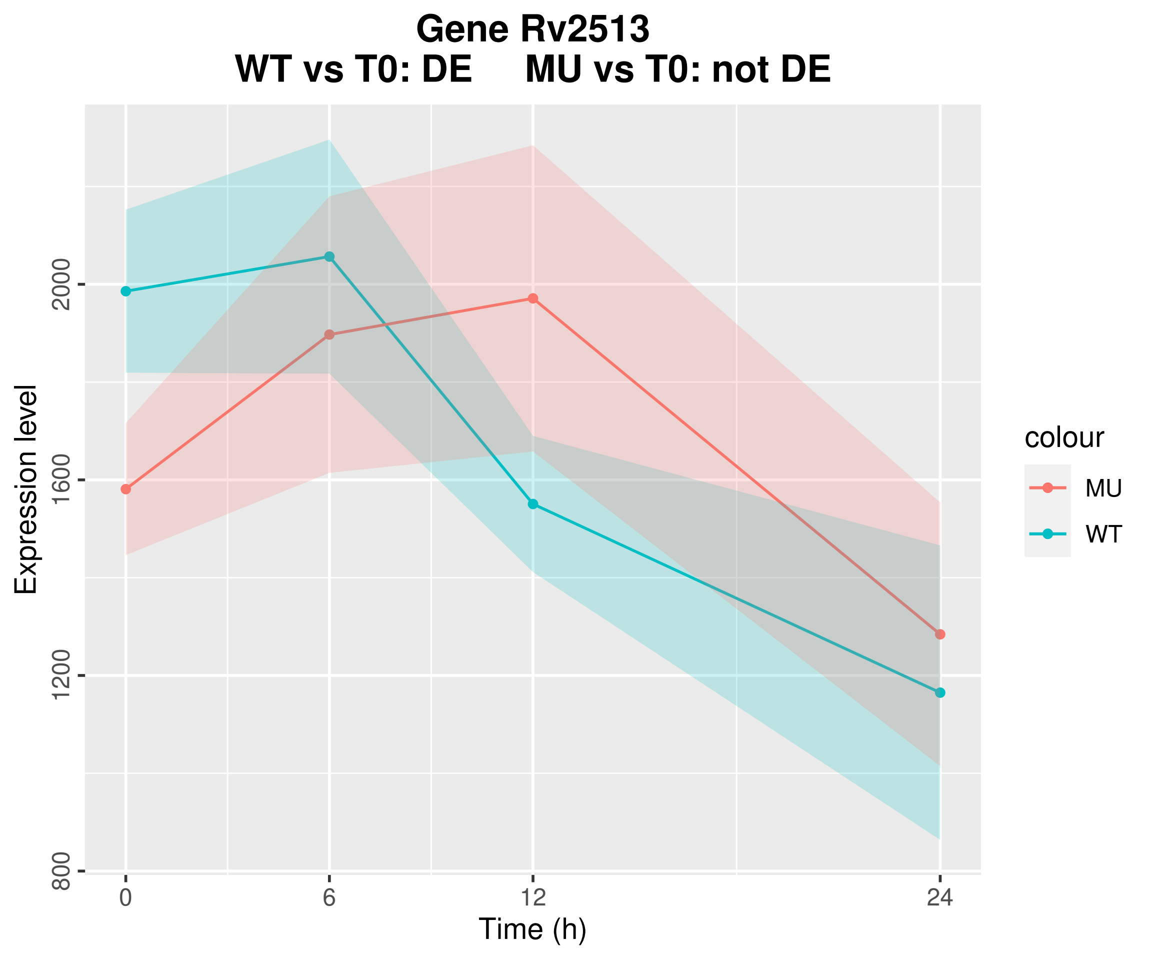
Gene Rv2513
WT vs T0: DE   MU vs T0: not DE
Expression level
2000
1600
1200
800
0
6
12
24
Time (h)
colour
MU
WT

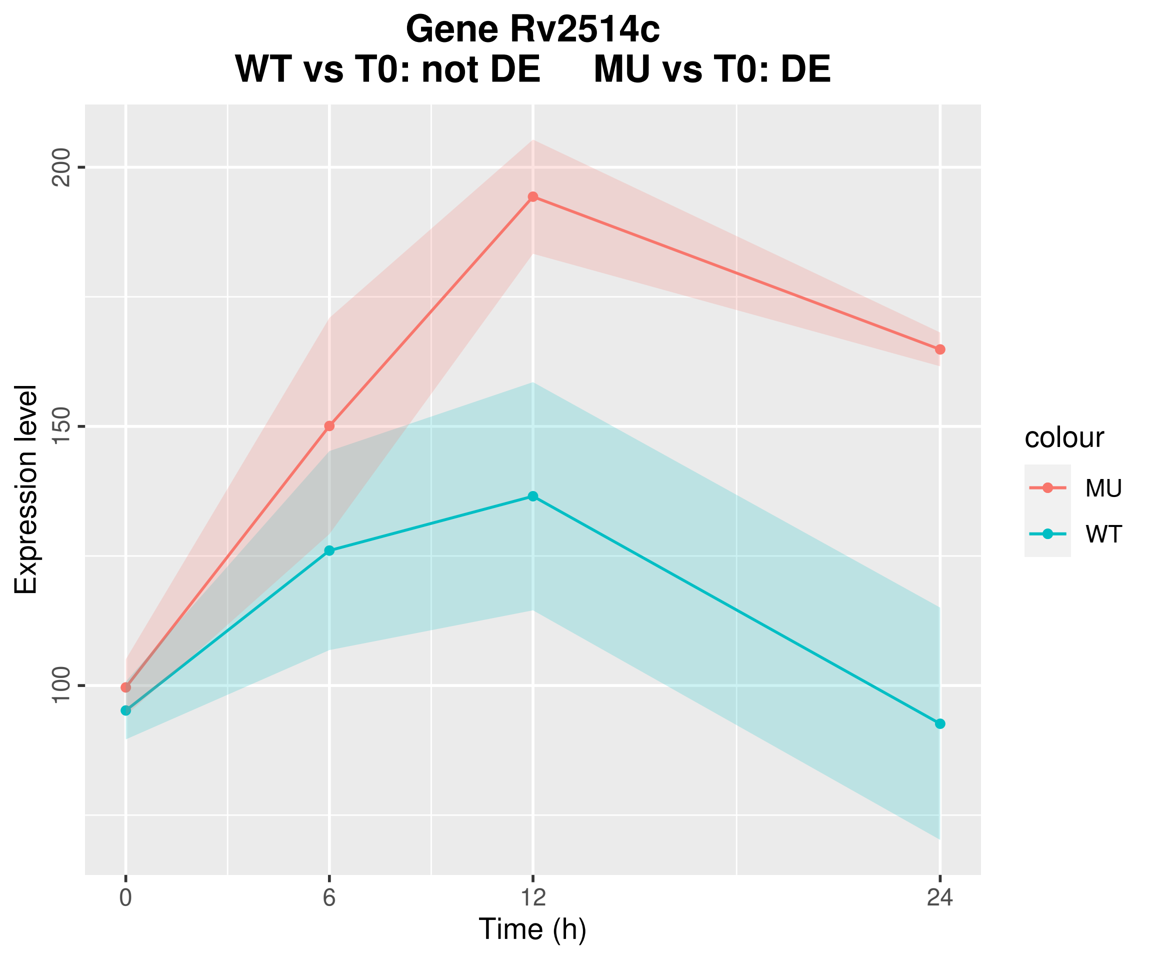
Gene Rv2514c
WT vs T0: not DE   MU vs T0: DE
Expression level
200
150
100
0
6
12
24
Time (h)
colour
MU
WT

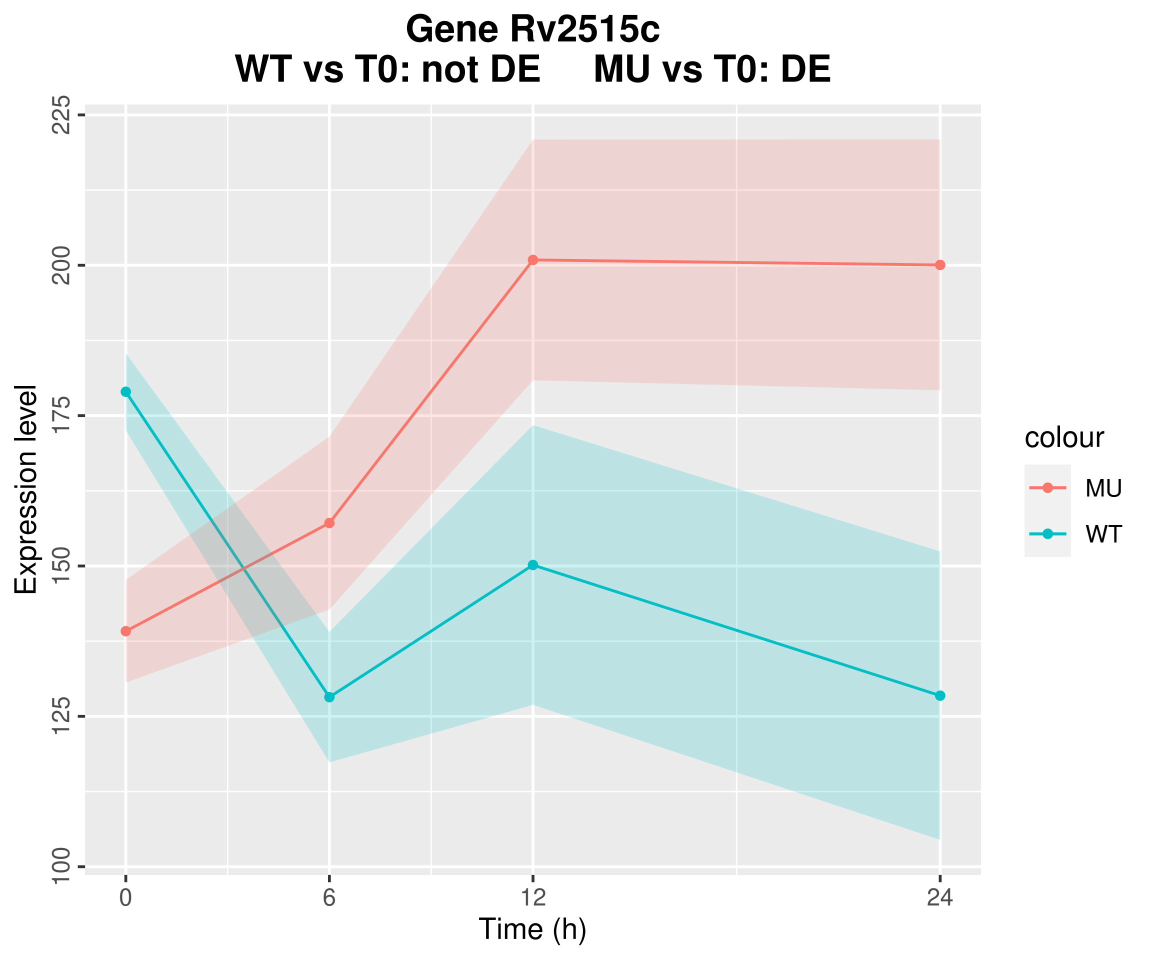
Gene Rv2515c
WT vs T0: not DE   MU vs T0: DE
Expression level
225
200
175
150
125
100
0
6
12
24
Time (h)
colour
MU
WT

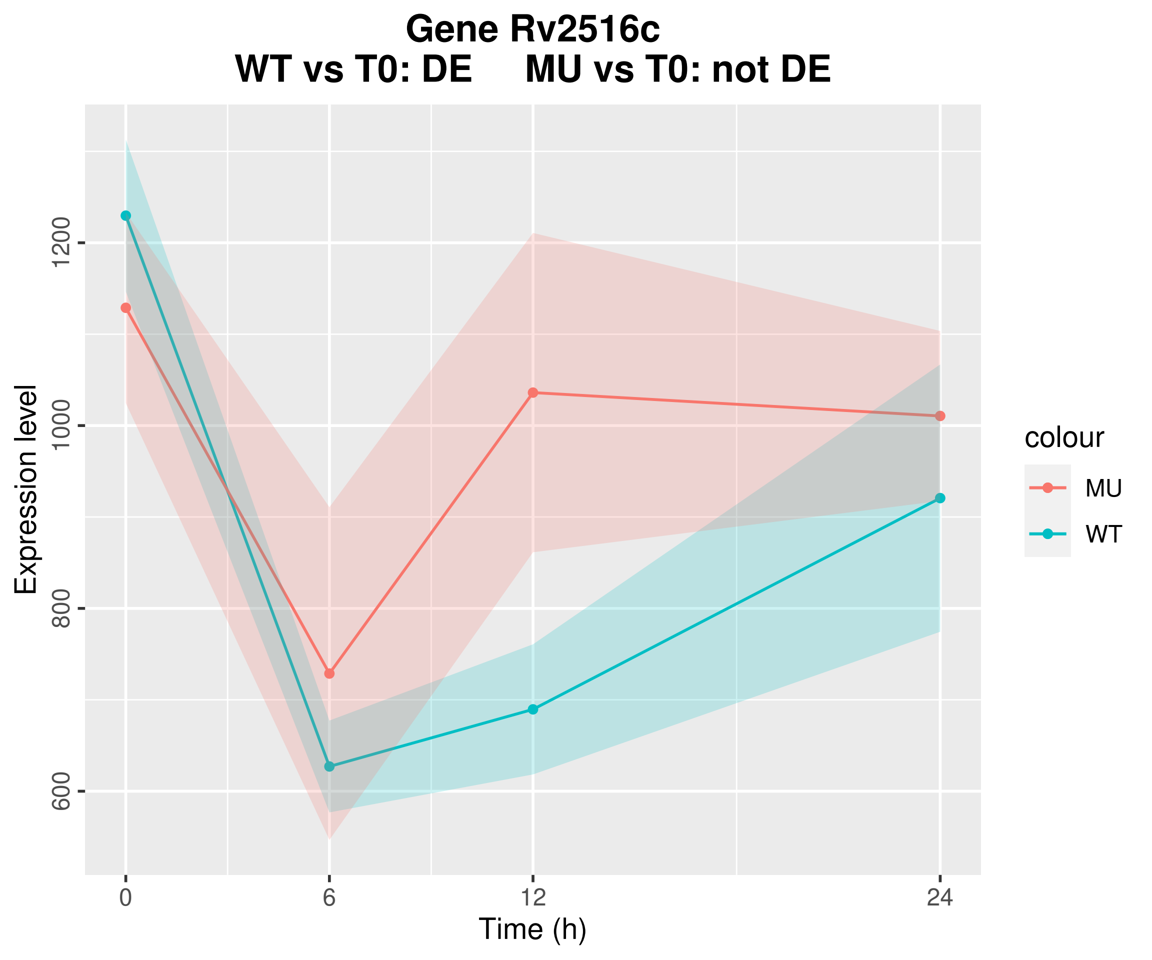

Gene Rv2516c
WT vs T0: DE   MU vs T0: not DE
Expression level
1200
1000
800
600
0
6
12
24
Time (h)
colour
MU
WT

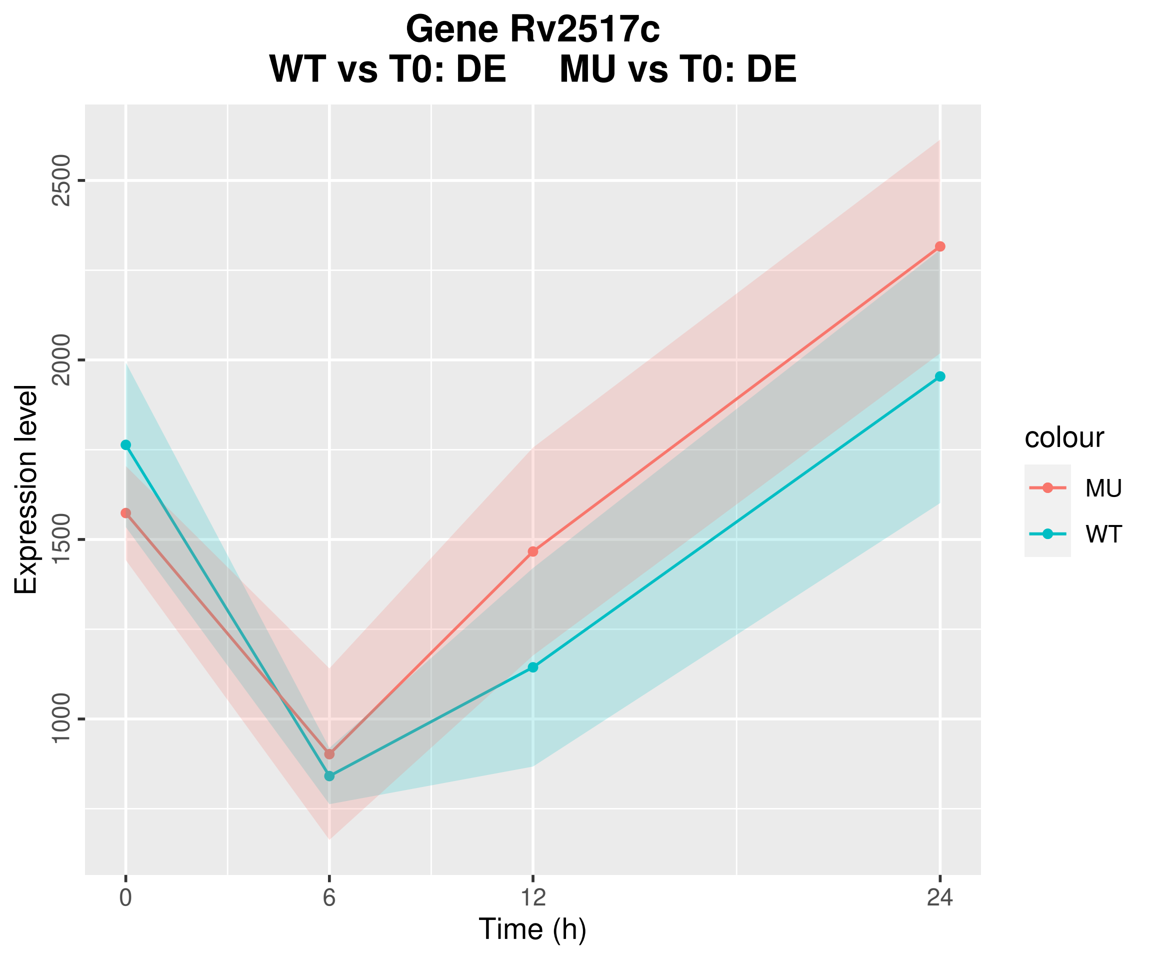

Gene Rv2517c
WT vs T0: DE   MU vs T0: DE
Expression level
2500
2000
1500
1000
0
6
12
24
Time (h)
colour
MU
WT

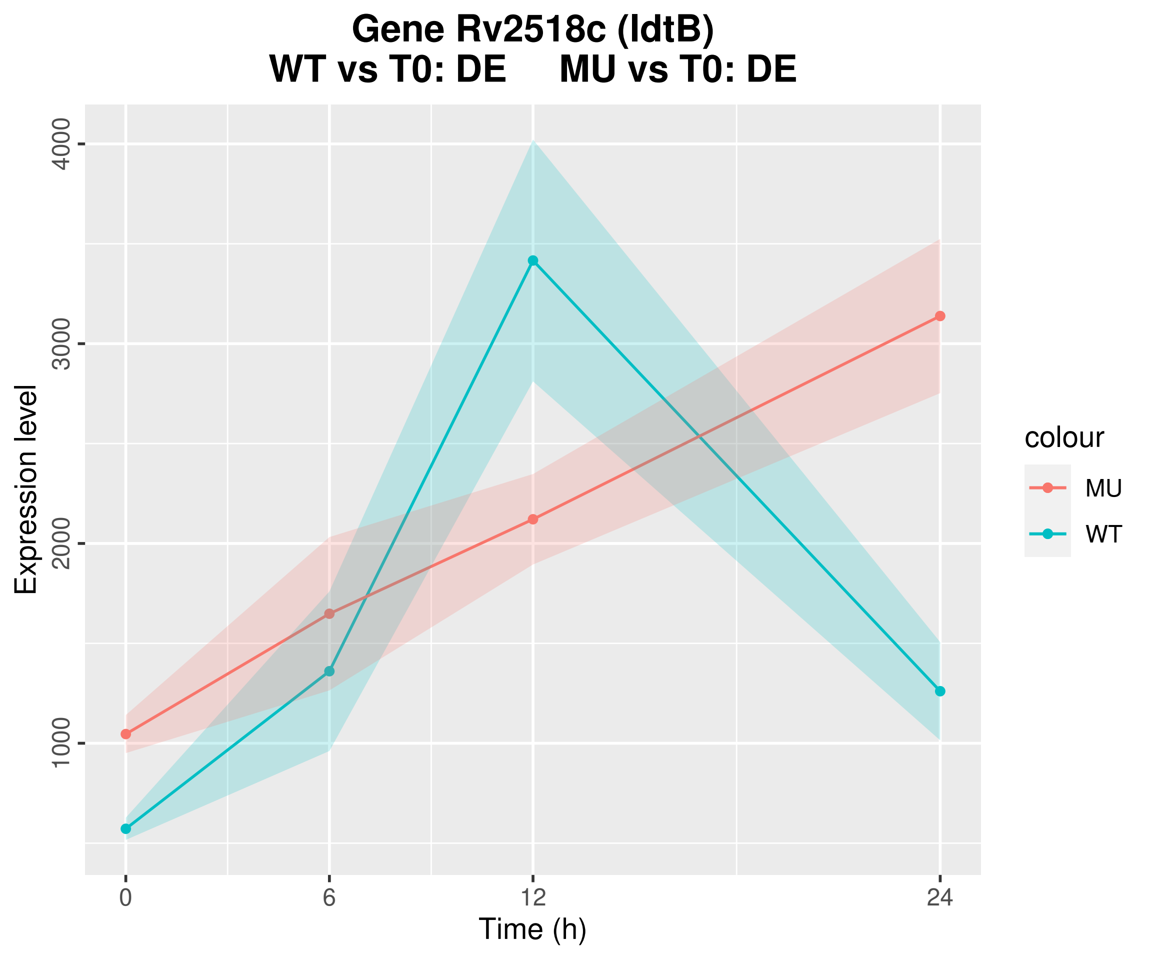

Gene Rv2518c (ldtB)
WT vs T0: DE MU vs T0: DE
Expression level
4000
3000
2000
1000
0
6
12
24
Time (h)
colour
MU
WT

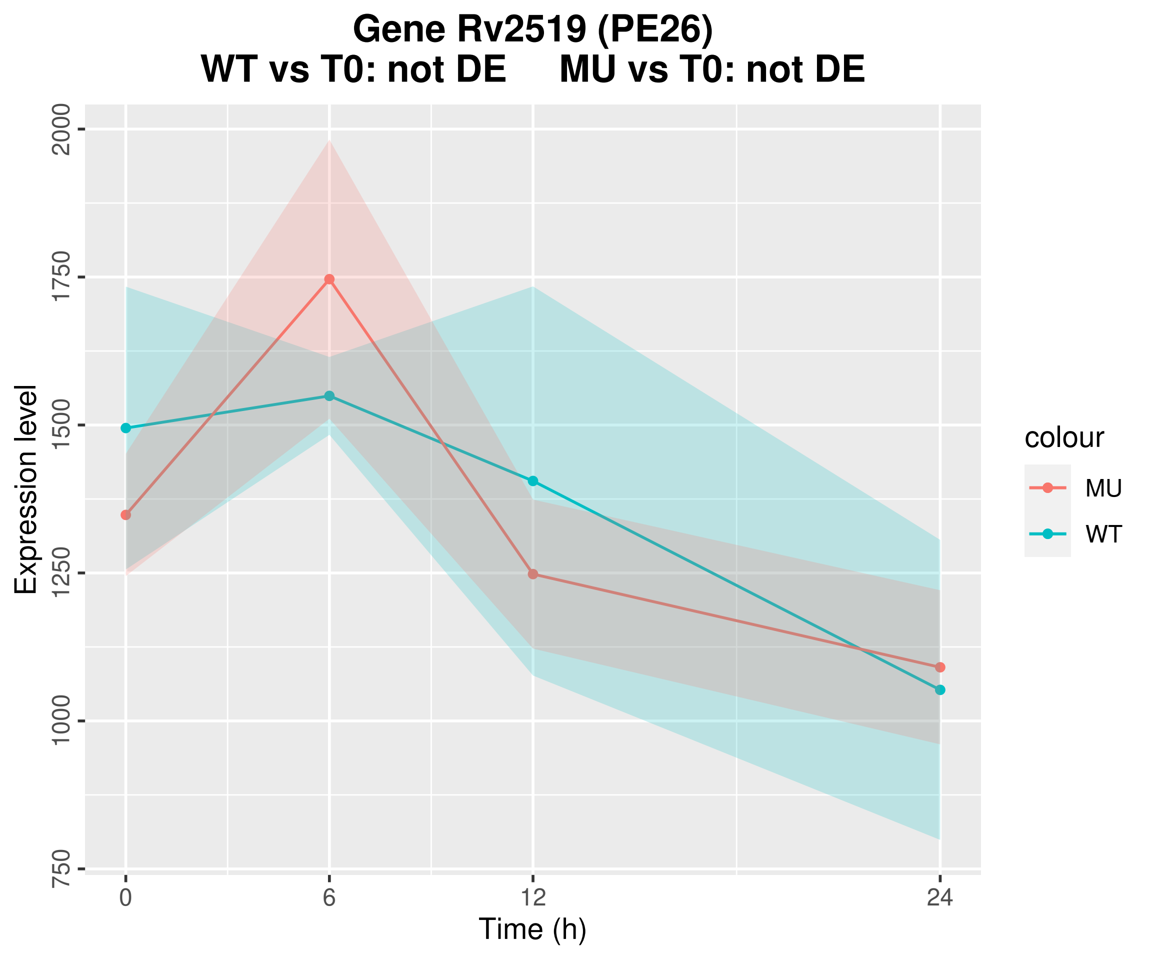
Gene Rv2519 (PE26)
WT vs T0: not DE   MU vs T0: not DE
Expression level
2000
1750
1500
1250
1000
750
0
6
12
24
Time (h)
colour
MU
WT

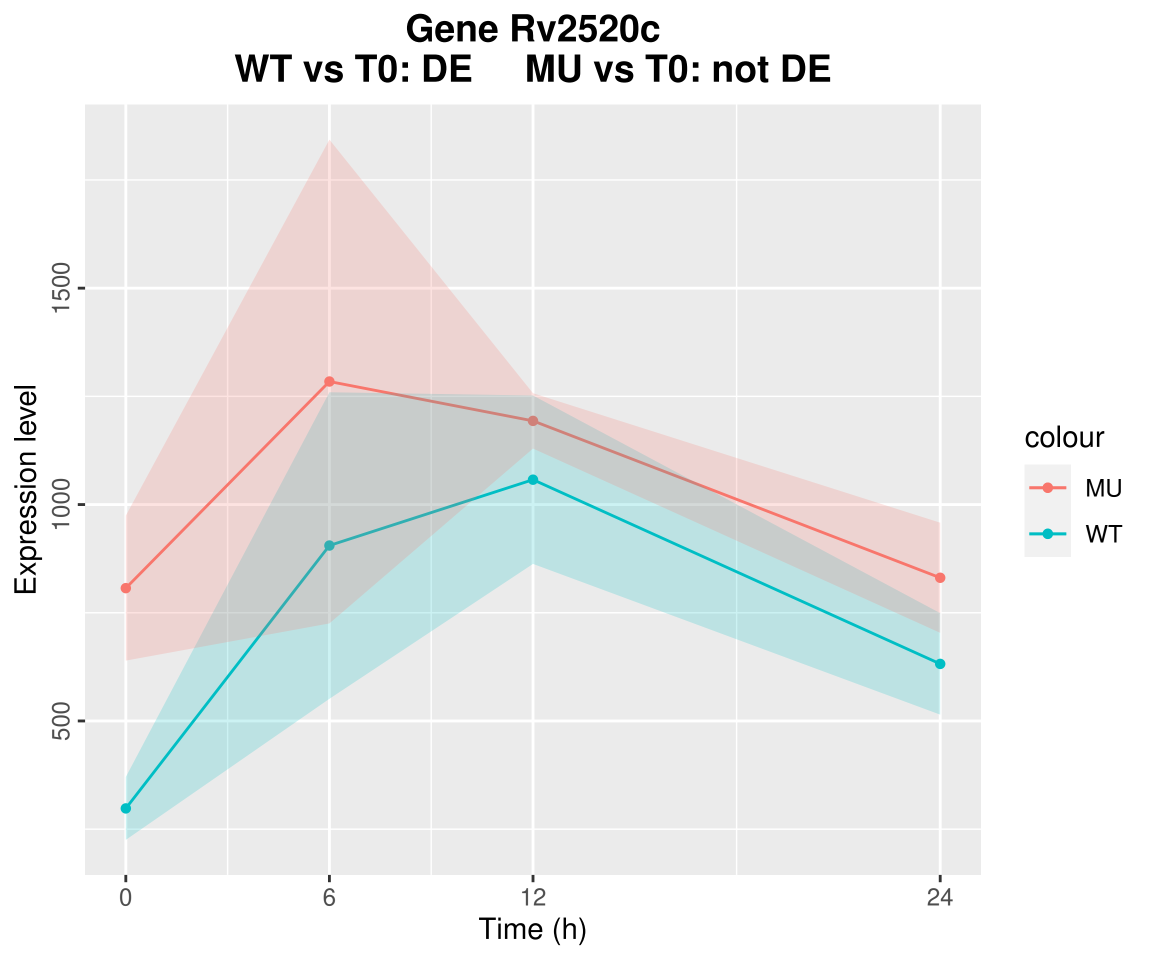

Gene Rv2520c
WT vs T0: DE MU vs T0: not DE
Expression level
1500
1000
500
0
6
12
24
Time (h)
colour
MU
WT

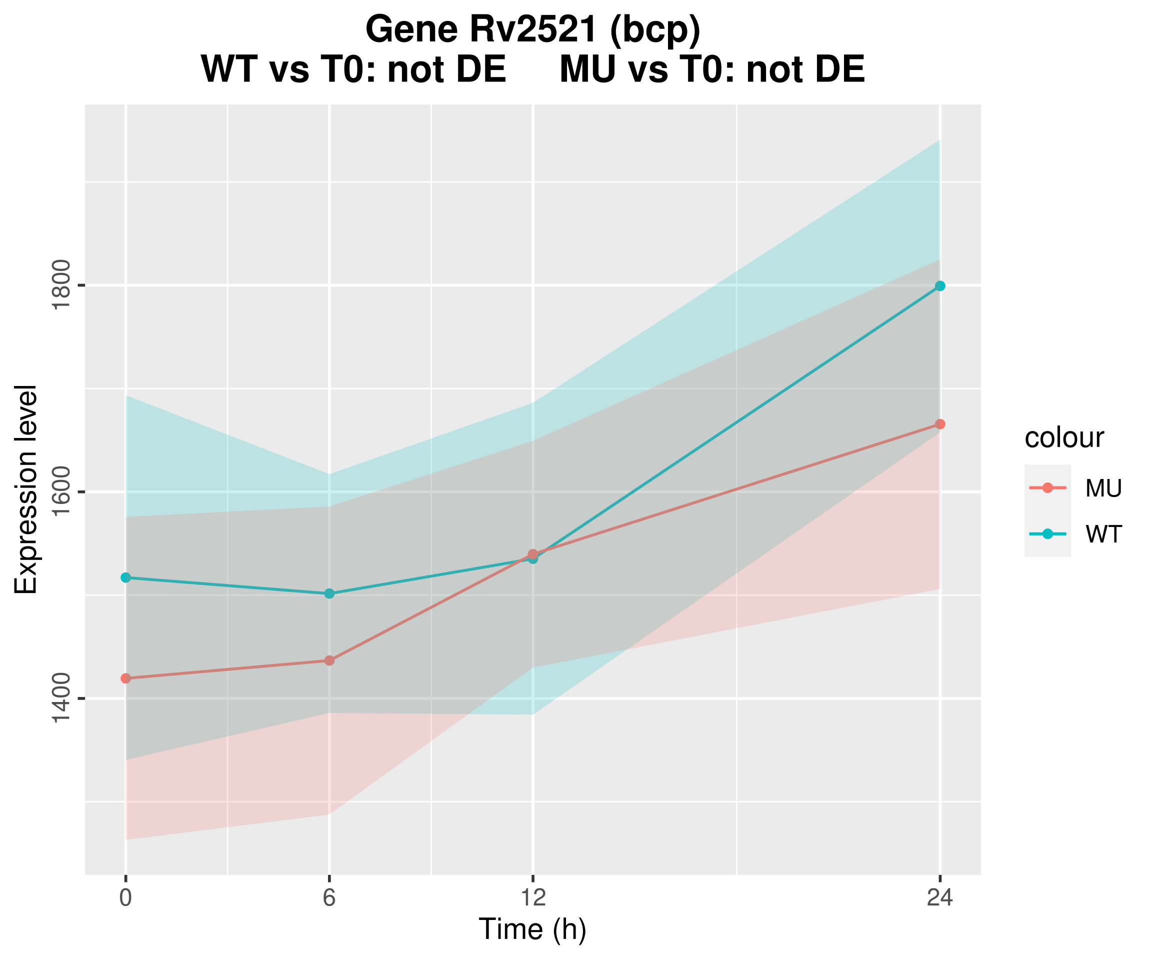

Gene Rv2521 (bcp)
WT vs T0: not DE   MU vs T0: not DE
Expression level
1400
1600
1800
0
6
12
24
Time (h)
colour
MU
WT

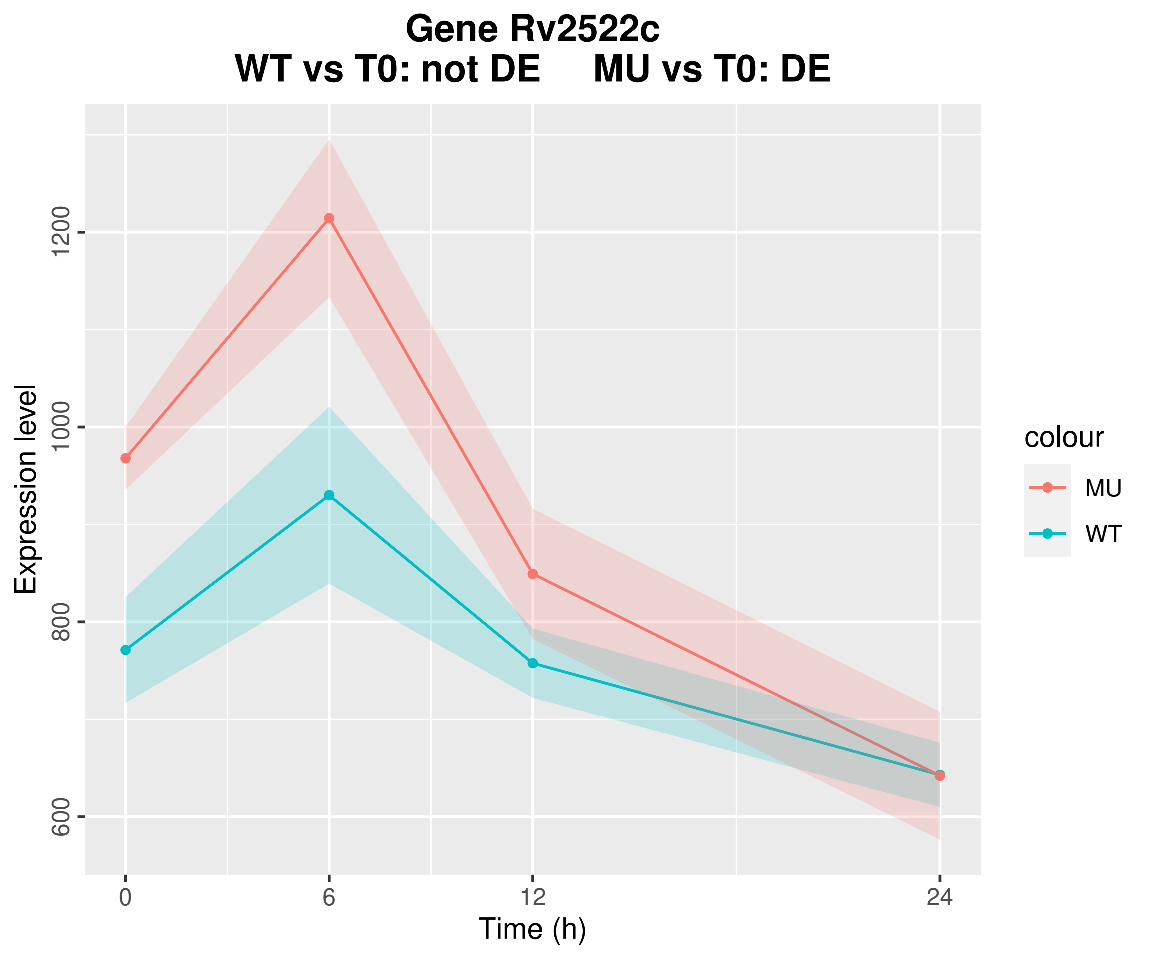
Gene Rv2522c
WT vs T0: not DE MU vs T0: DE
Expression level
1200
1000
800
600
0
6
12
24
Time (h)
colour
MU
WT

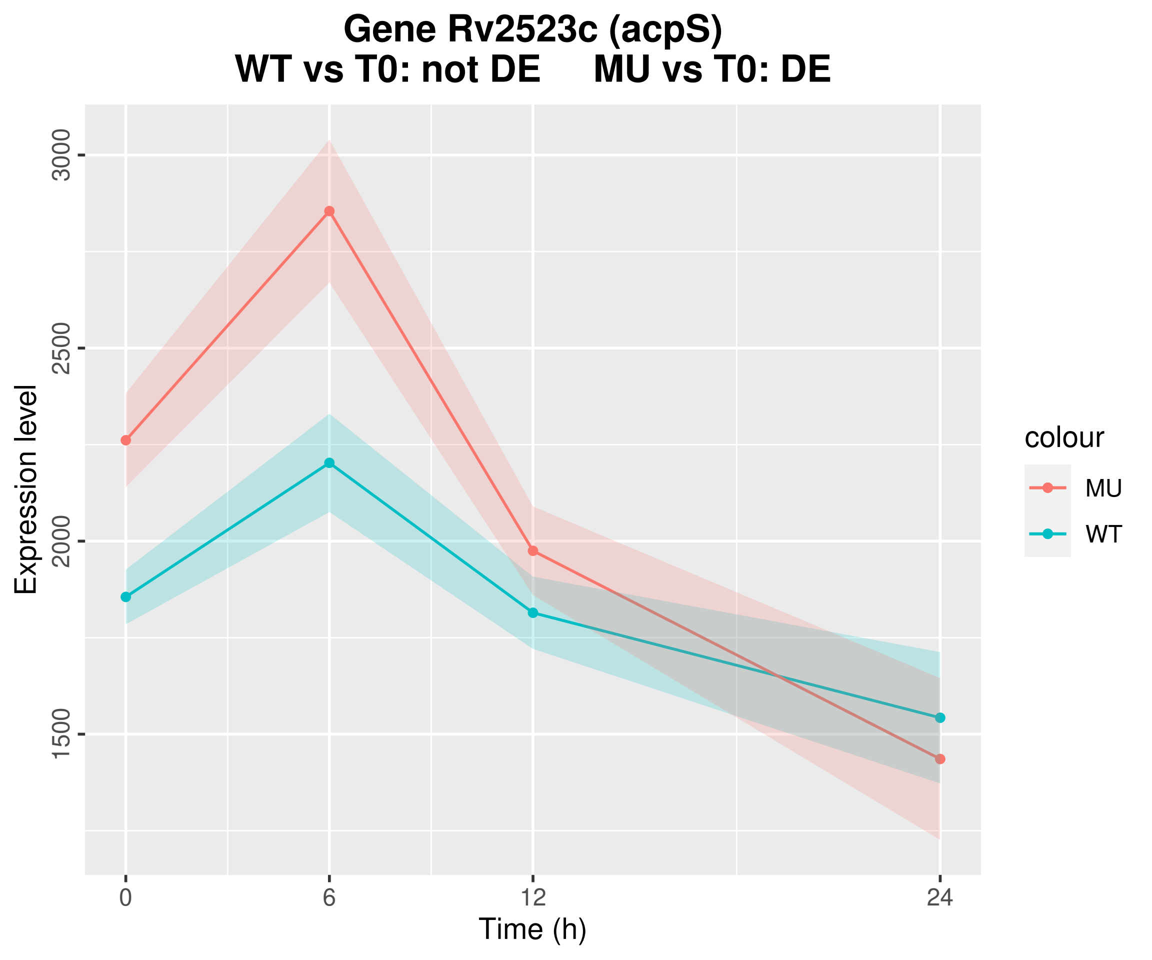

Gene Rv2523c (acpS)
WT vs T0: not DE MU vs T0: DE
Expression level
3000
2500
2000
1500
0
6
12
24
Time (h)
colour
MU
WT

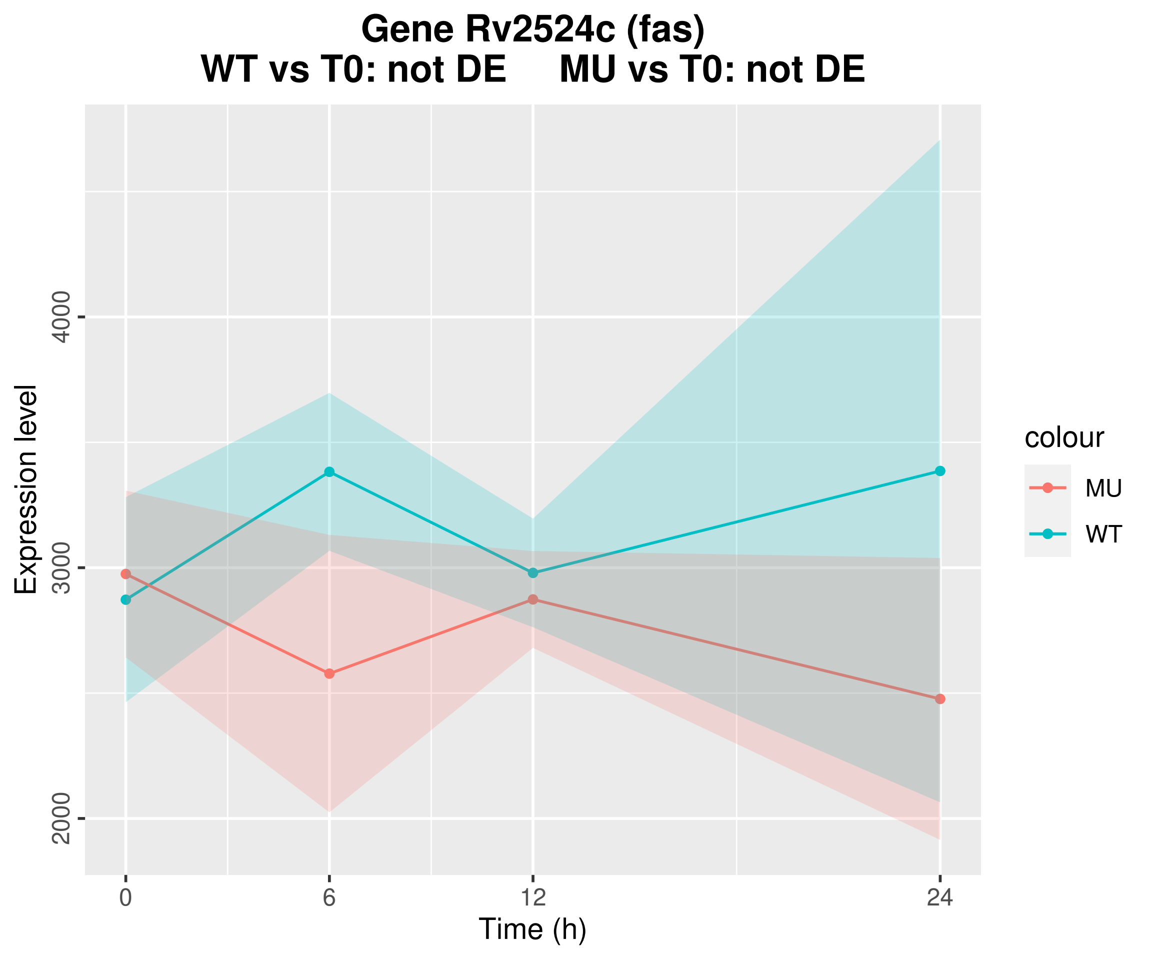

Gene Rv2524c (fas)
WT vs T0: not DE MU vs T0: not DE
Expression level
4000
3000
2000
0
6
12
24
Time (h)
colour
MU
WT

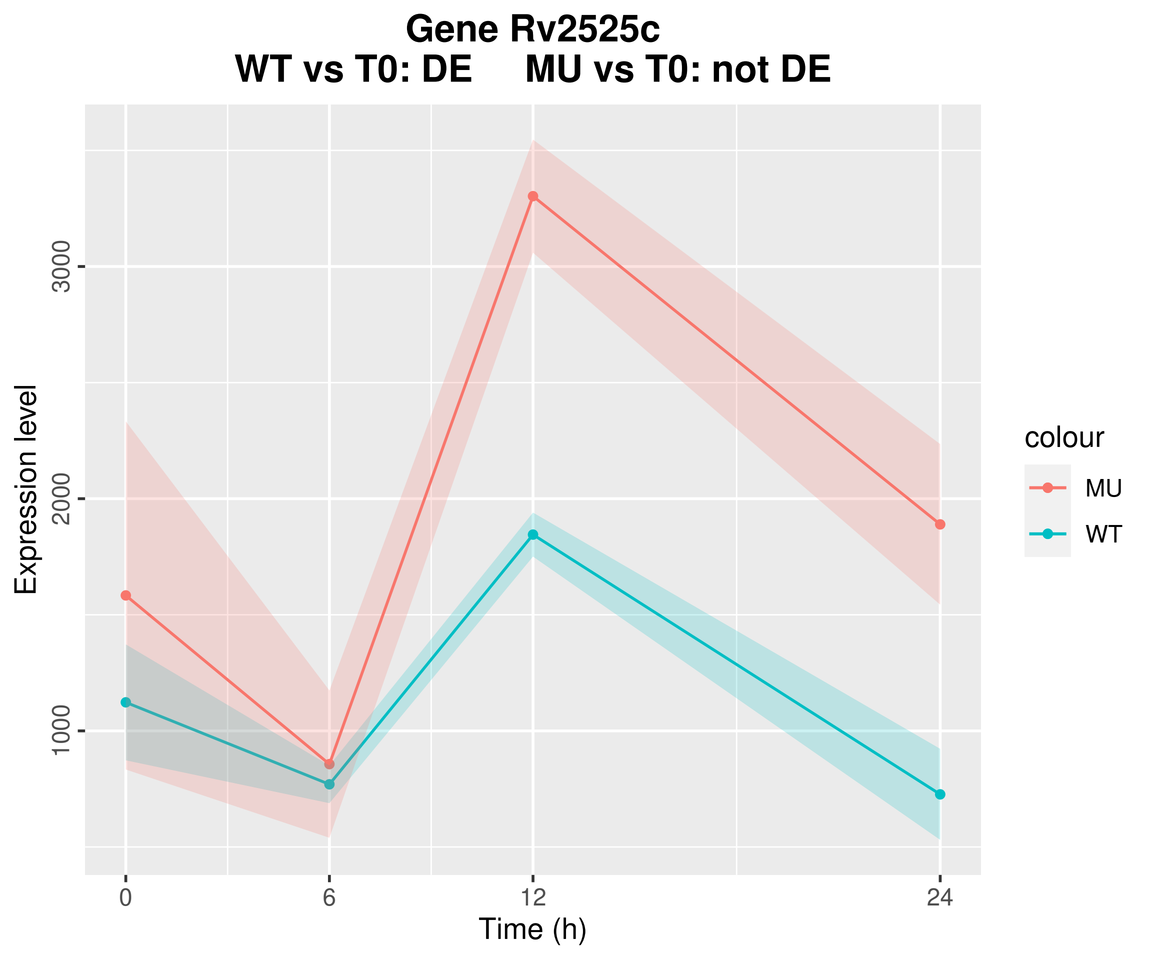

Gene Rv2525c
WT vs T0: DE   MU vs T0: not DE
Expression level
3000
2000
1000
0
6
12
24
Time (h)
colour
MU
WT

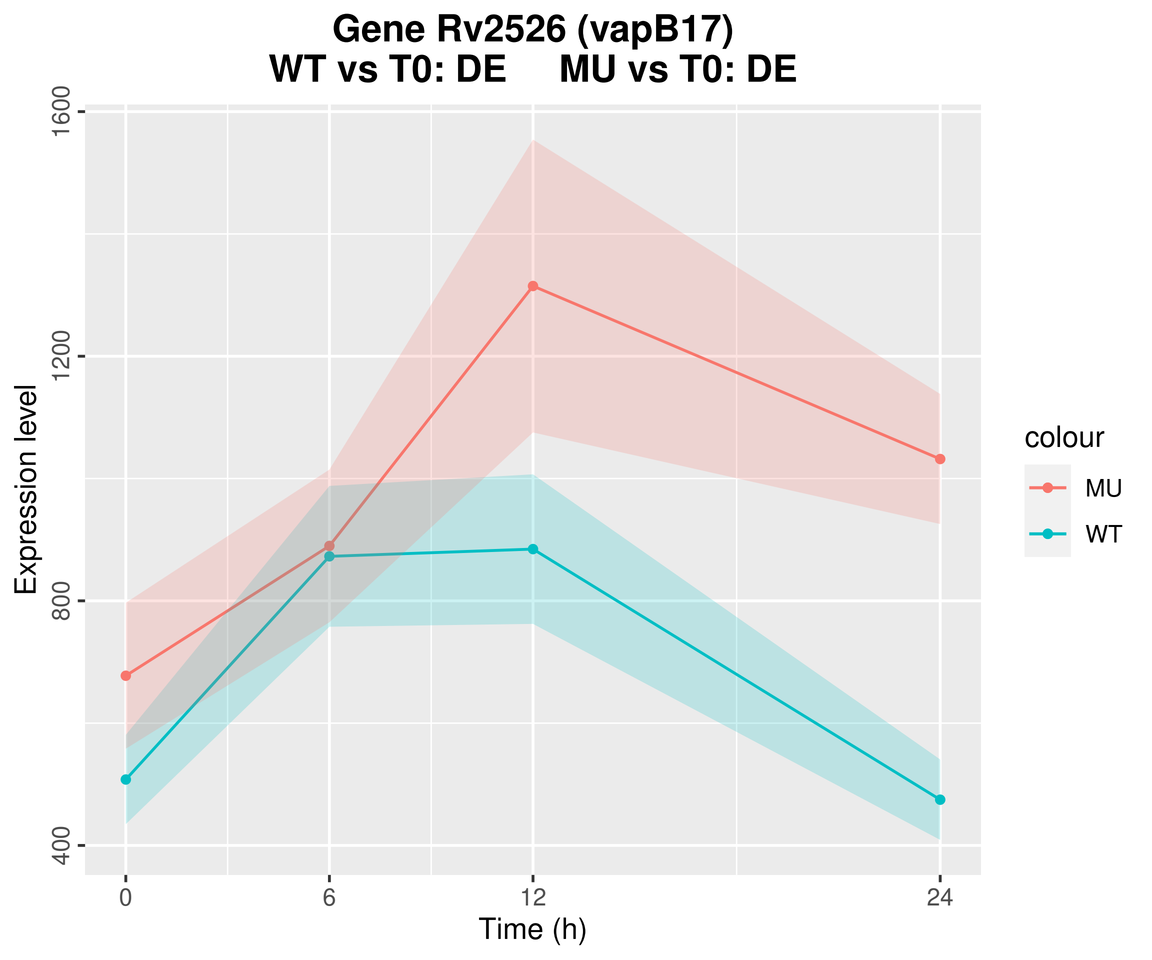

Gene Rv2526 (vapB17)
WT vs T0: DE   MU vs T0: DE
Expression level
1600
1200
800
400
0
6
12
24
Time (h)
colour
MU
WT

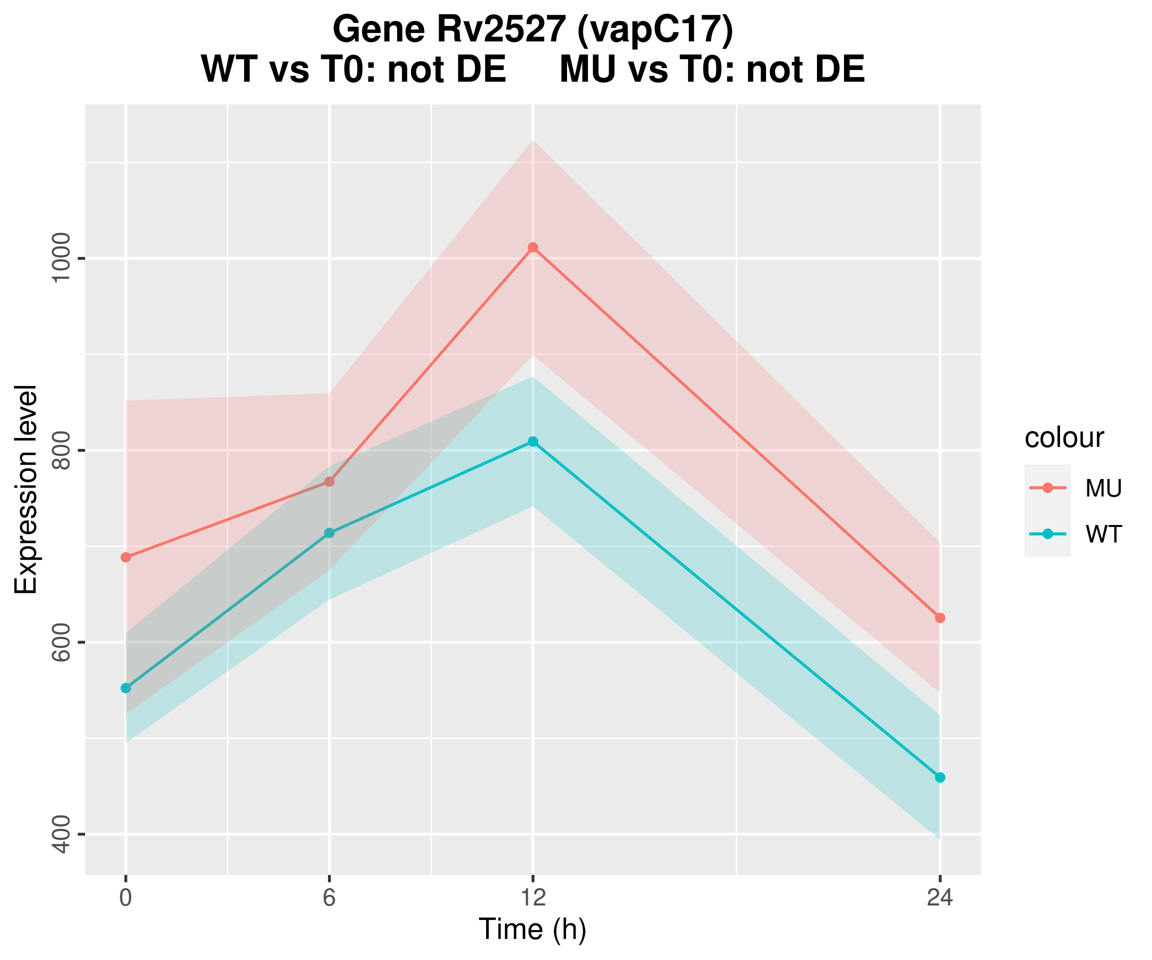
Gene Rv2527 (vapC17)
WT vs T0: not DE   MU vs T0: not DE
Expression level
400
600
800
1000
0
6
12
24
Time (h)
colour
MU
WT

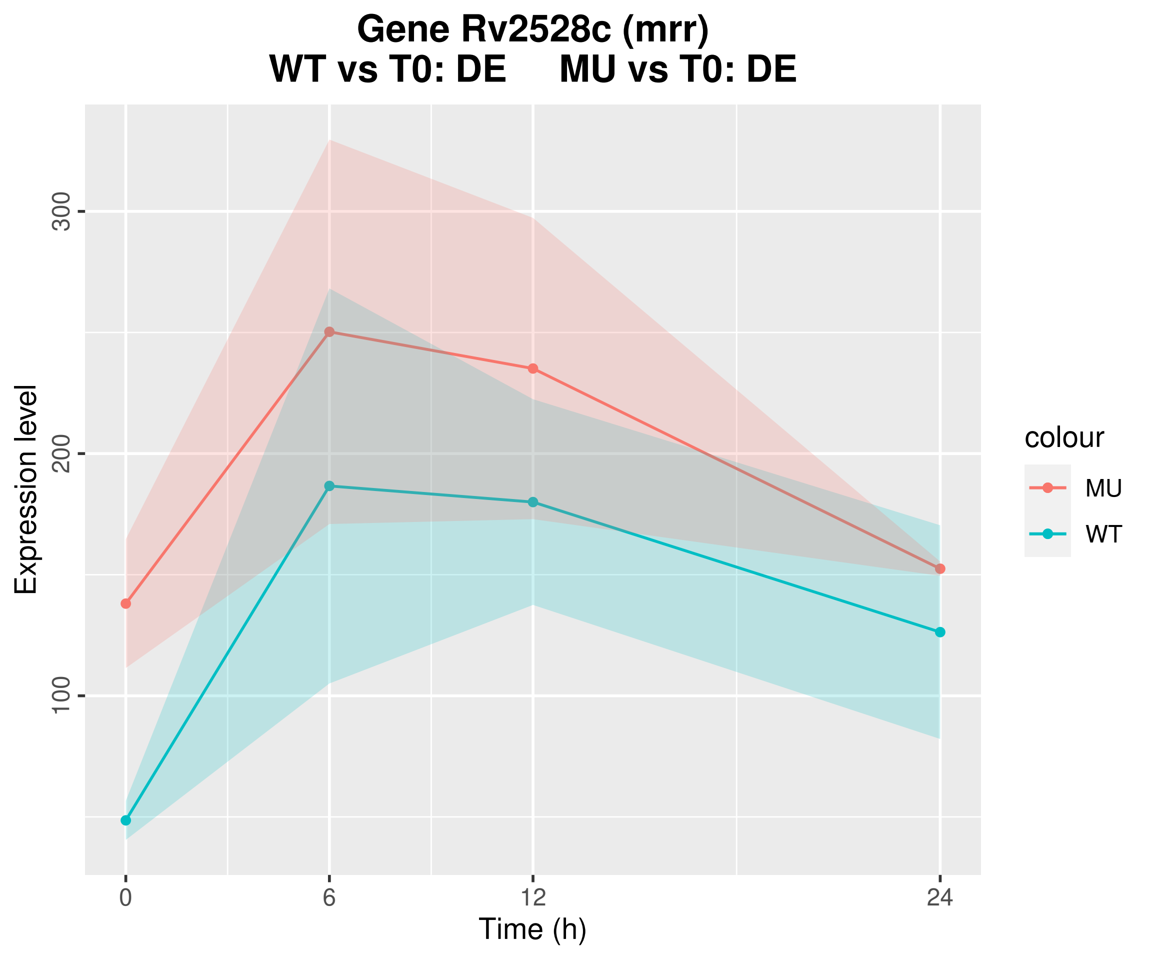
Gene Rv2528c (mrr)
WT vs T0: DE MU vs T0: DE
Expression level
300
200
100
0
6
12
24
Time (h)
colour
MU
WT

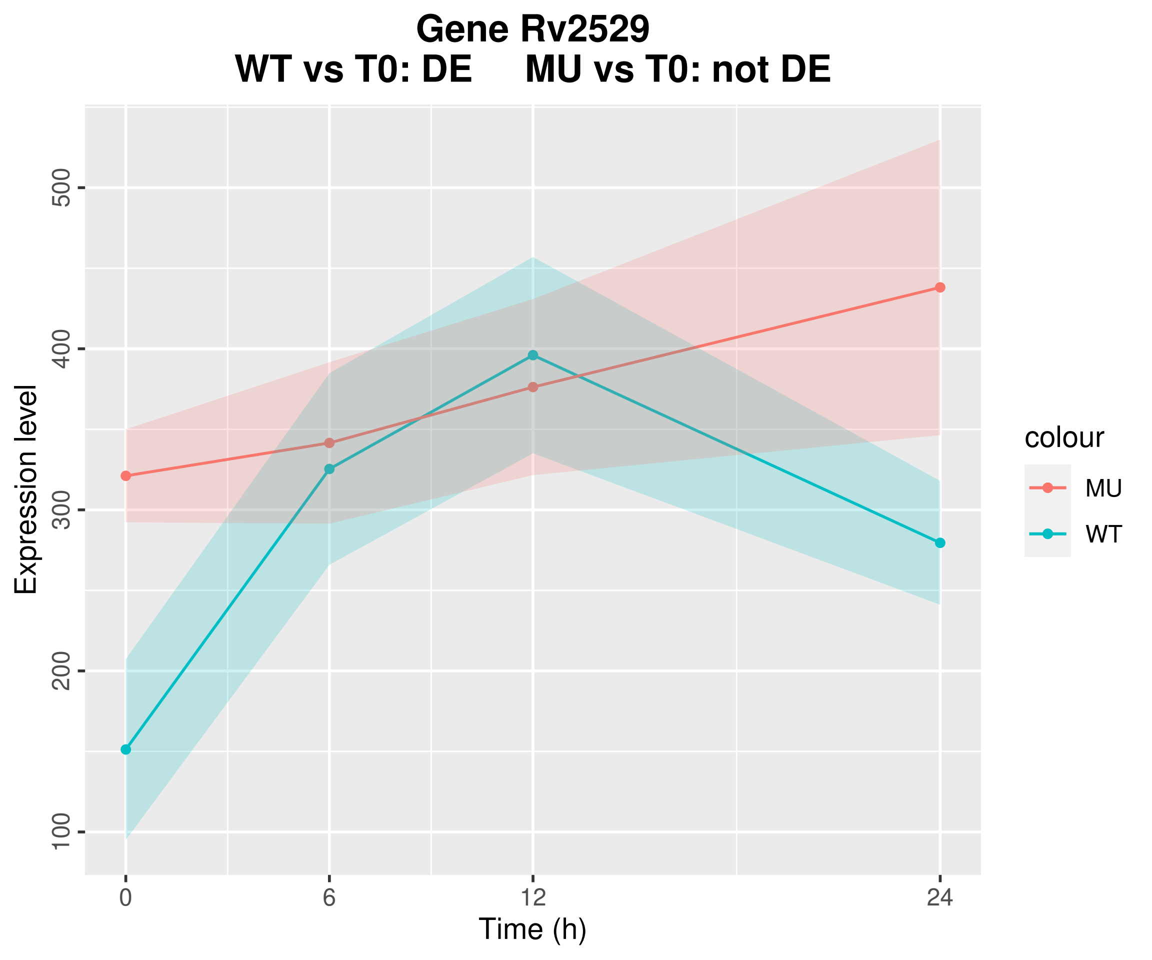
Gene Rv2529
WT vs T0: DE MU vs T0: not DE
Expression level
500
400
300
200
100
0
6
12
24
Time (h)
colour
MU
WT

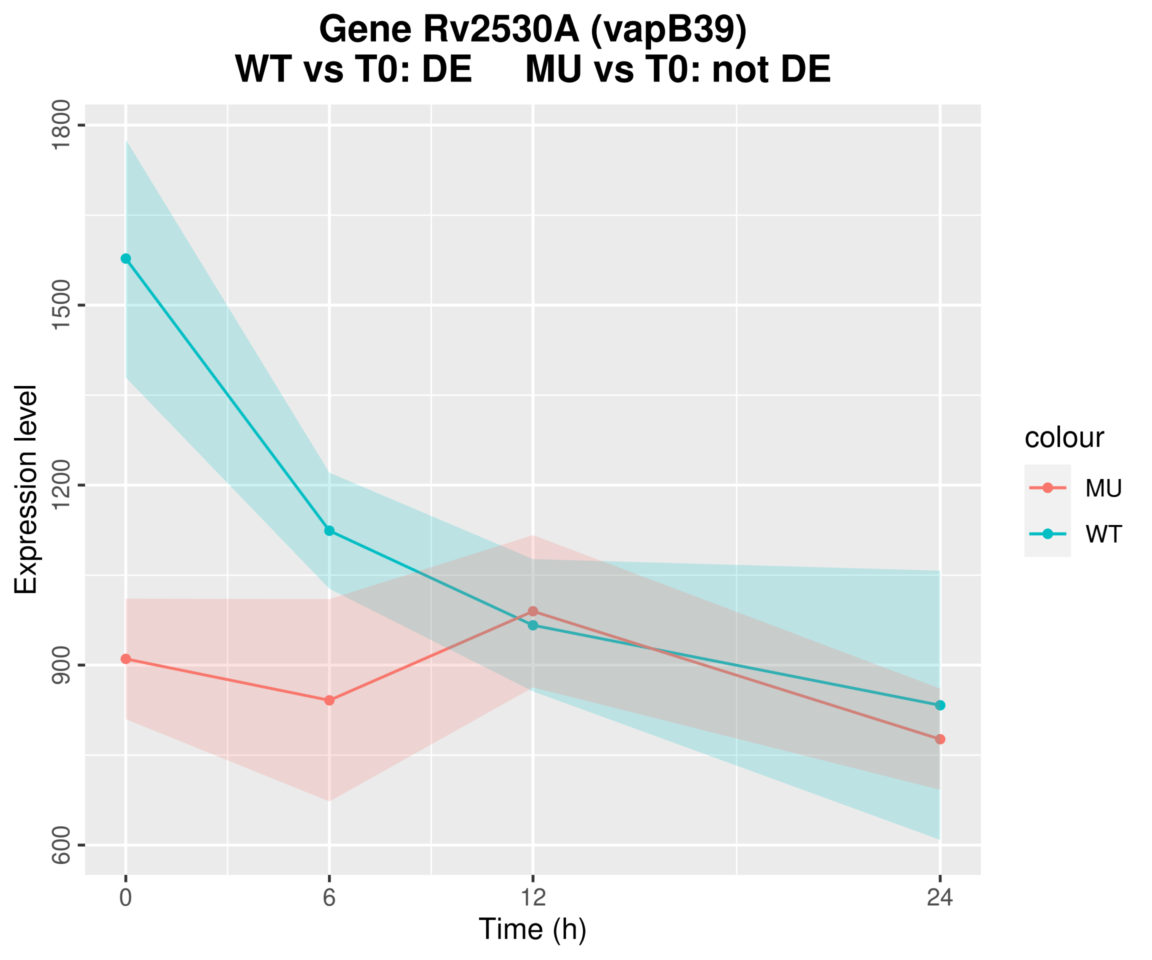

Gene Rv2530A (vapB39)
WT vs T0: DE MU vs T0: not DE
1800
1500
1200
900
600
Expression level
0
6
12
24
Time (h)
colour
MU
WT

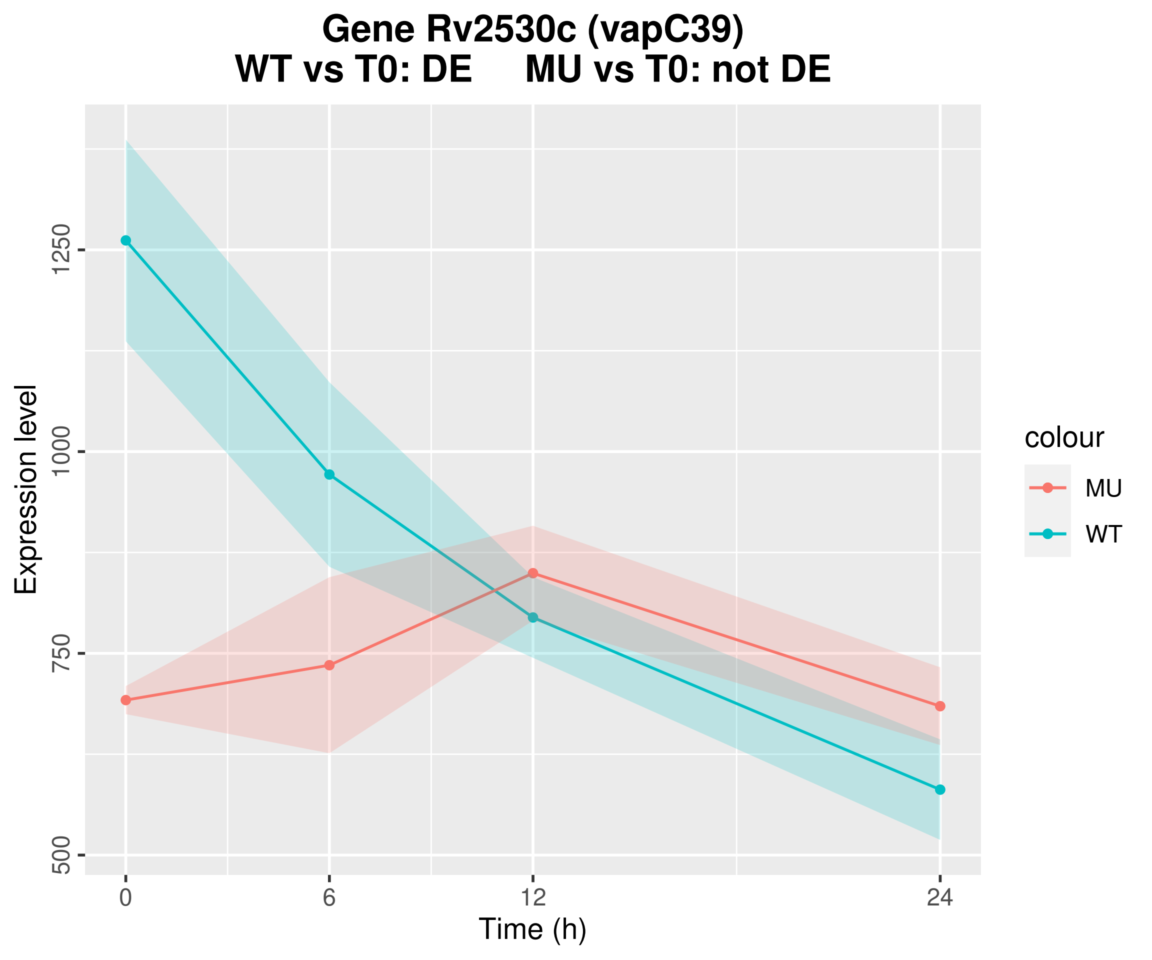

Gene Rv2530c (vapC39)
WT vs T0: DE   MU vs T0: not DE
Expression level
1250
1000
750
500
0
6
12
24
Time (h)
colour
MU
WT

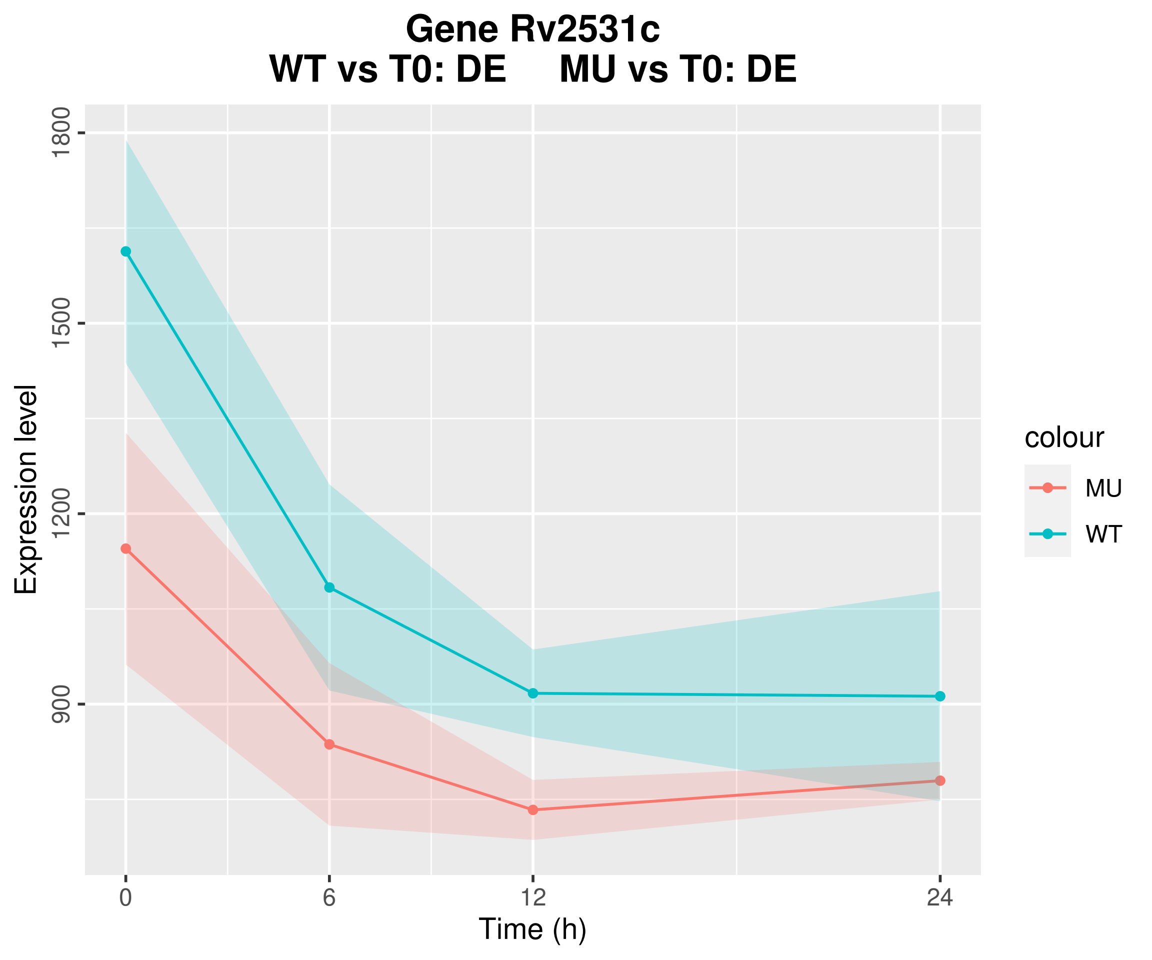

Gene Rv2531c
WT vs T0: DE MU vs T0: DE
Expression level
1800
1500
1200
900
0
6
12
24
Time (h)
colour
MU
WT

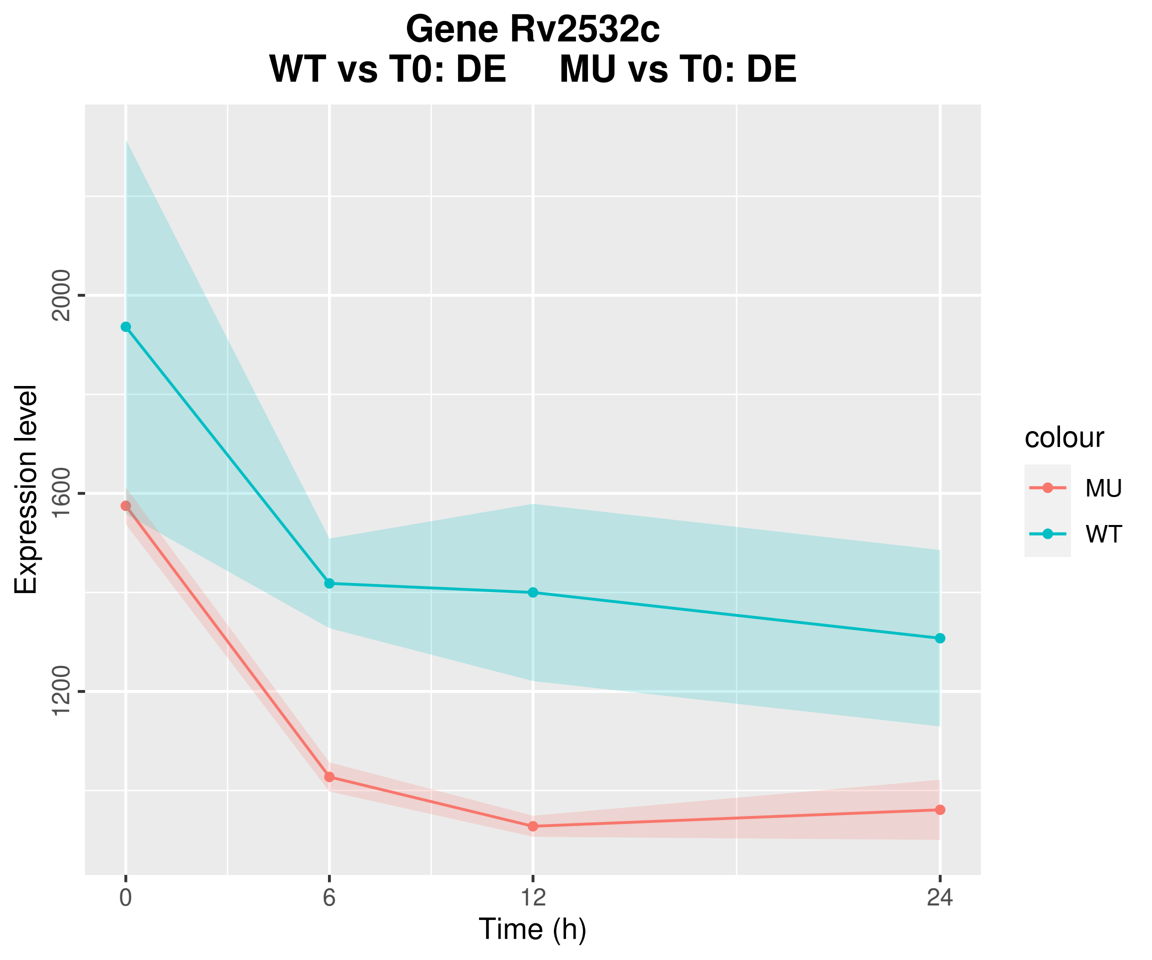
Gene Rv2532c
WT vs T0: DE MU vs T0: DE
Expression level
2000
1600
1200
0
6
12
24
Time (h)
colour
MU
WT

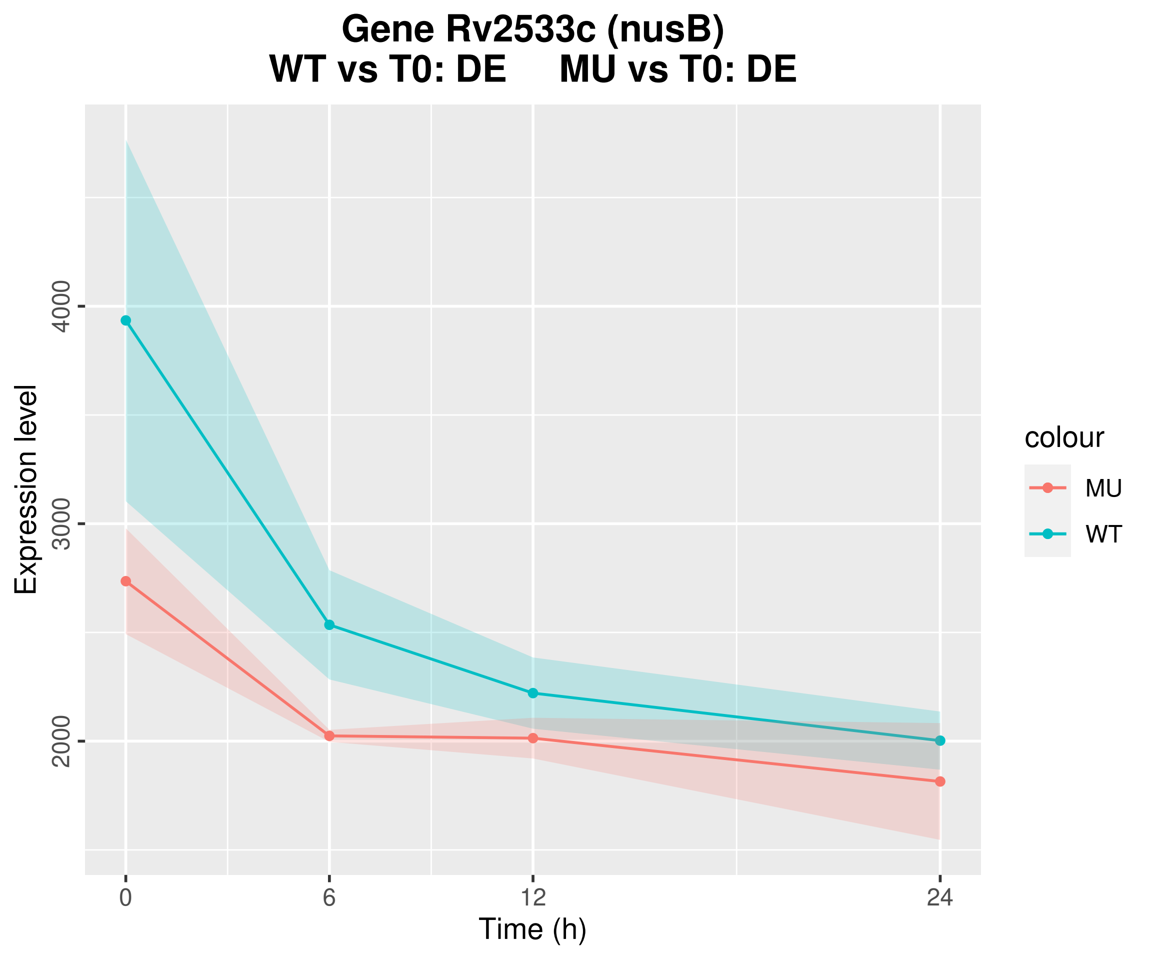
Gene Rv2533c (nusB)
WT vs T0: DE MU vs T0: DE
Expression level
4000
3000
2000
Time (h)
0
6
12
24
colour
MU
WT

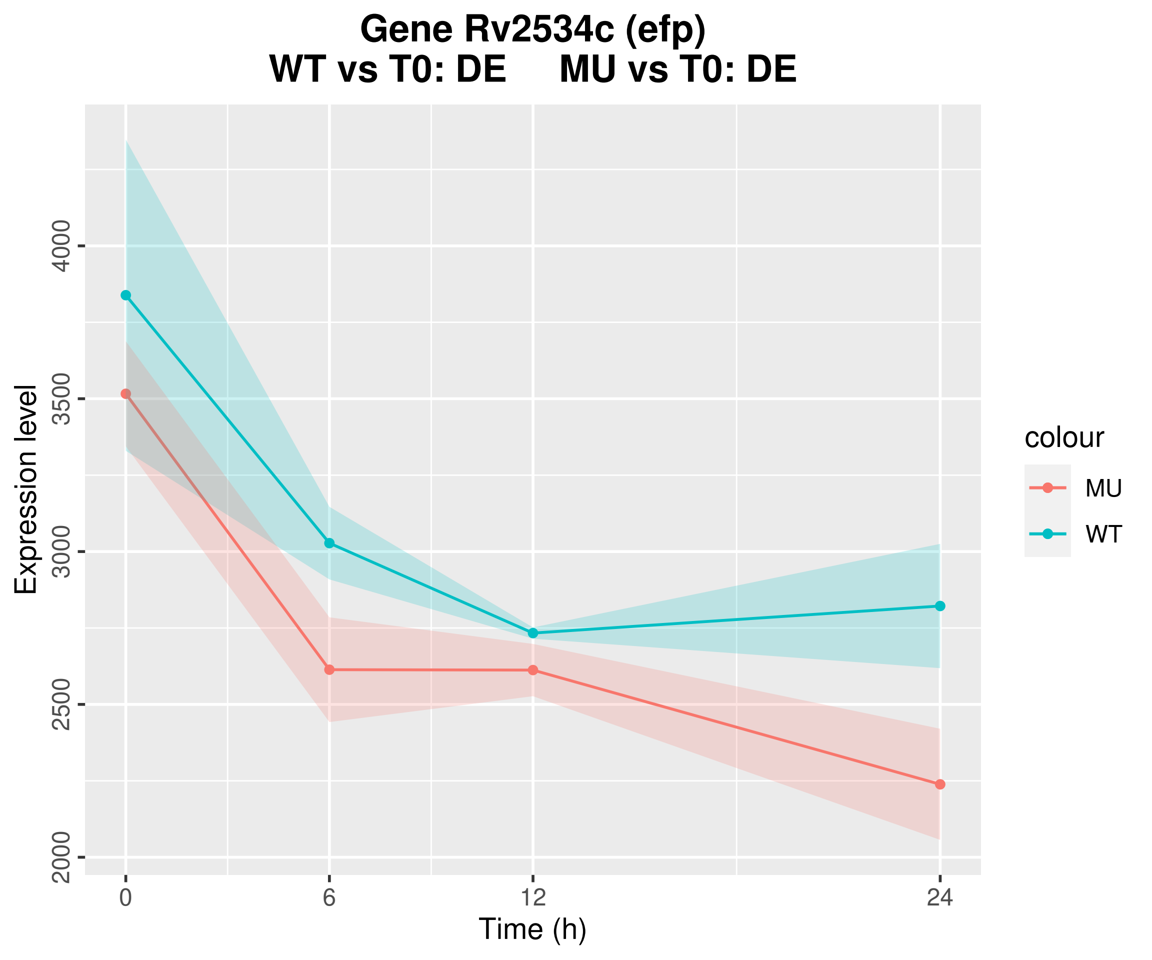
Gene Rv2534c (efp)
WT vs T0: DE   MU vs T0: DE
Expression level
2000
2500
3000
3500
4000
0
6
12
24
Time (h)
colour
MU
WT

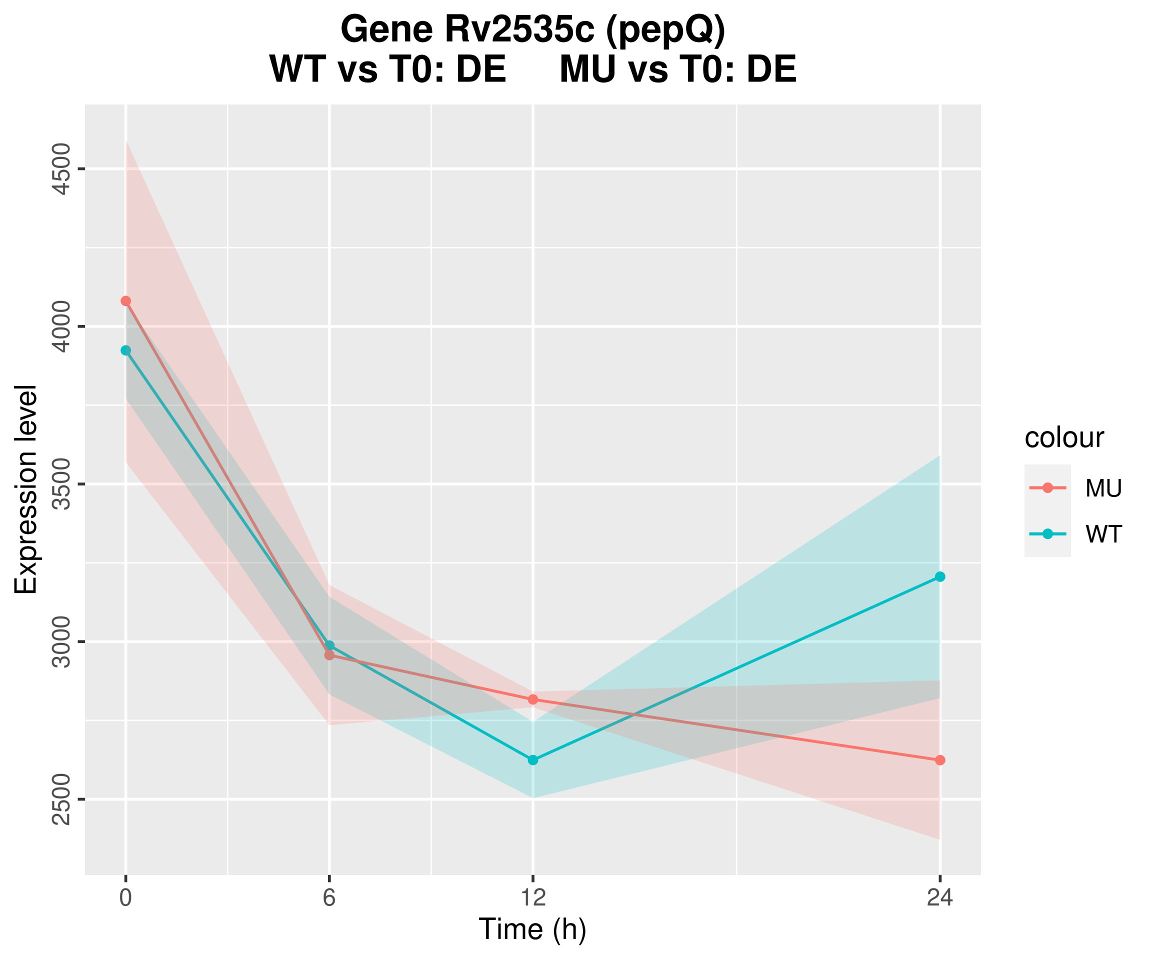

Gene Rv2535c (pepQ)
WT vs T0: DE MU vs T0: DE
Expression level
2500
3000
3500
4000
4500
0
6
12
24
Time (h)
colour
MU
WT

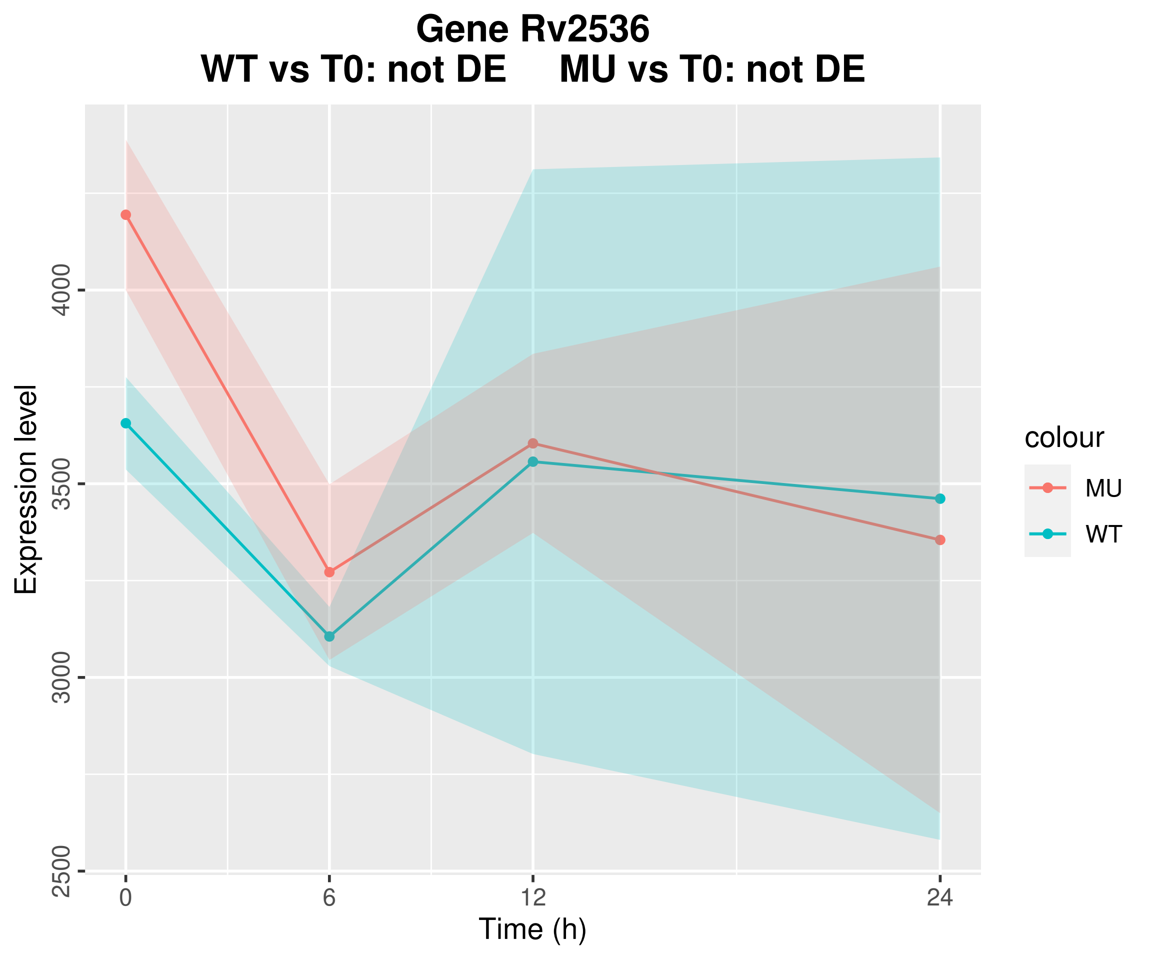
Gene Rv2536
WT vs T0: not DE   MU vs T0: not DE
Expression level
4000
3500
3000
2500
0
6
12
24
Time (h)
colour
MU
WT

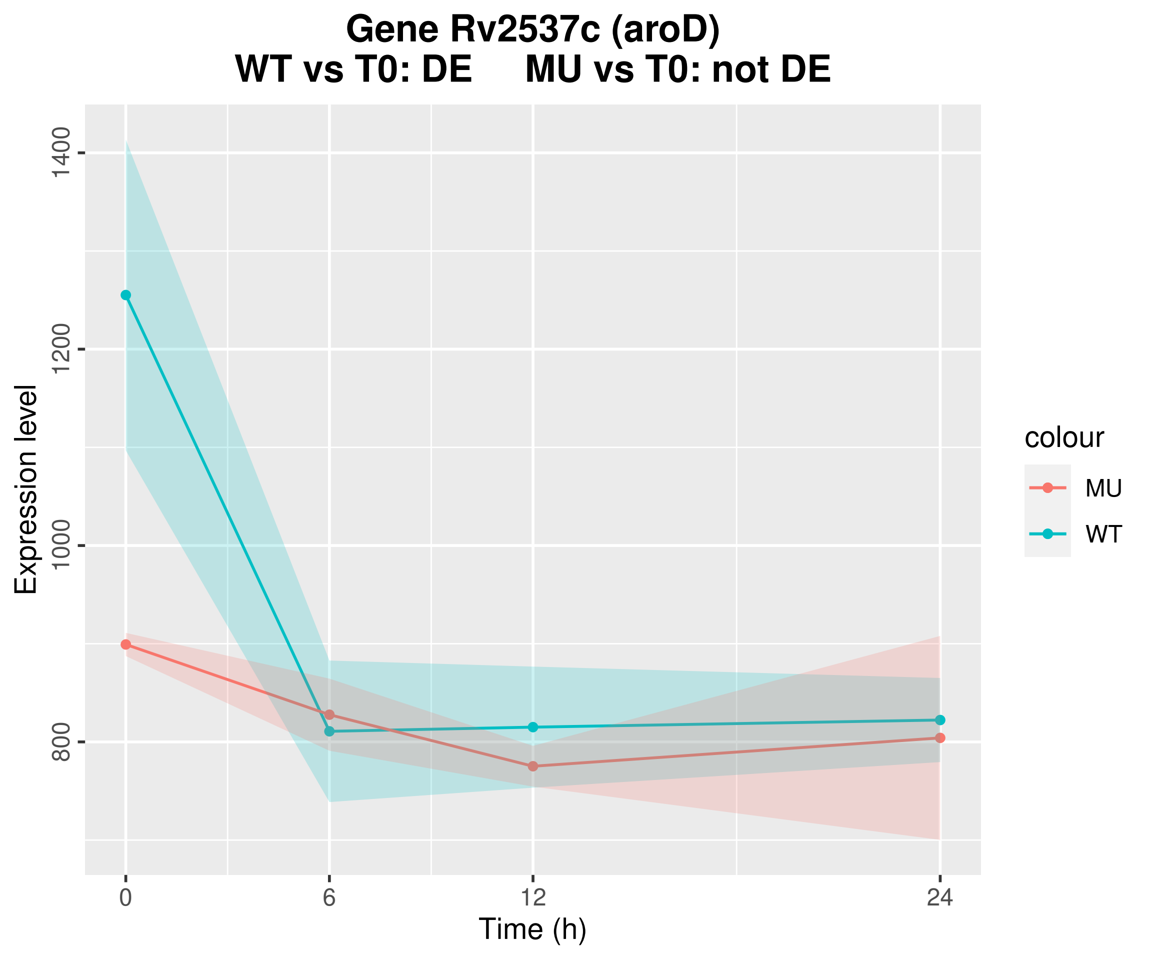

Gene Rv2537c (aroD)
WT vs T0: DE   MU vs T0: not DE
Expression level
1400
1200
1000
800
0
6
12
24
Time (h)
colour
MU
WT

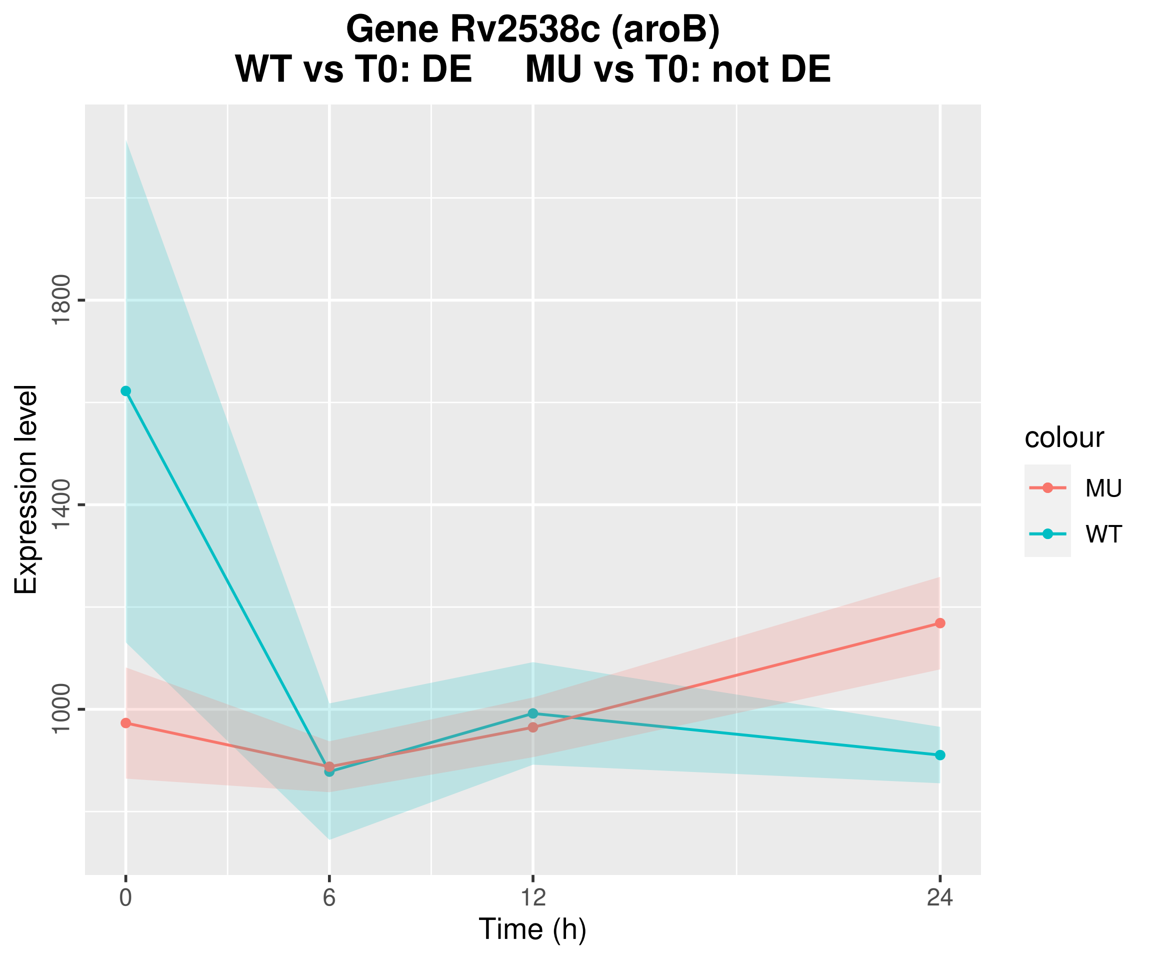
Gene Rv2538c (aroB)
WT vs T0: DE MU vs T0: not DE
Expression level
1800
1400
1000
0
6
12
24
Time (h)
colour
MU
WT

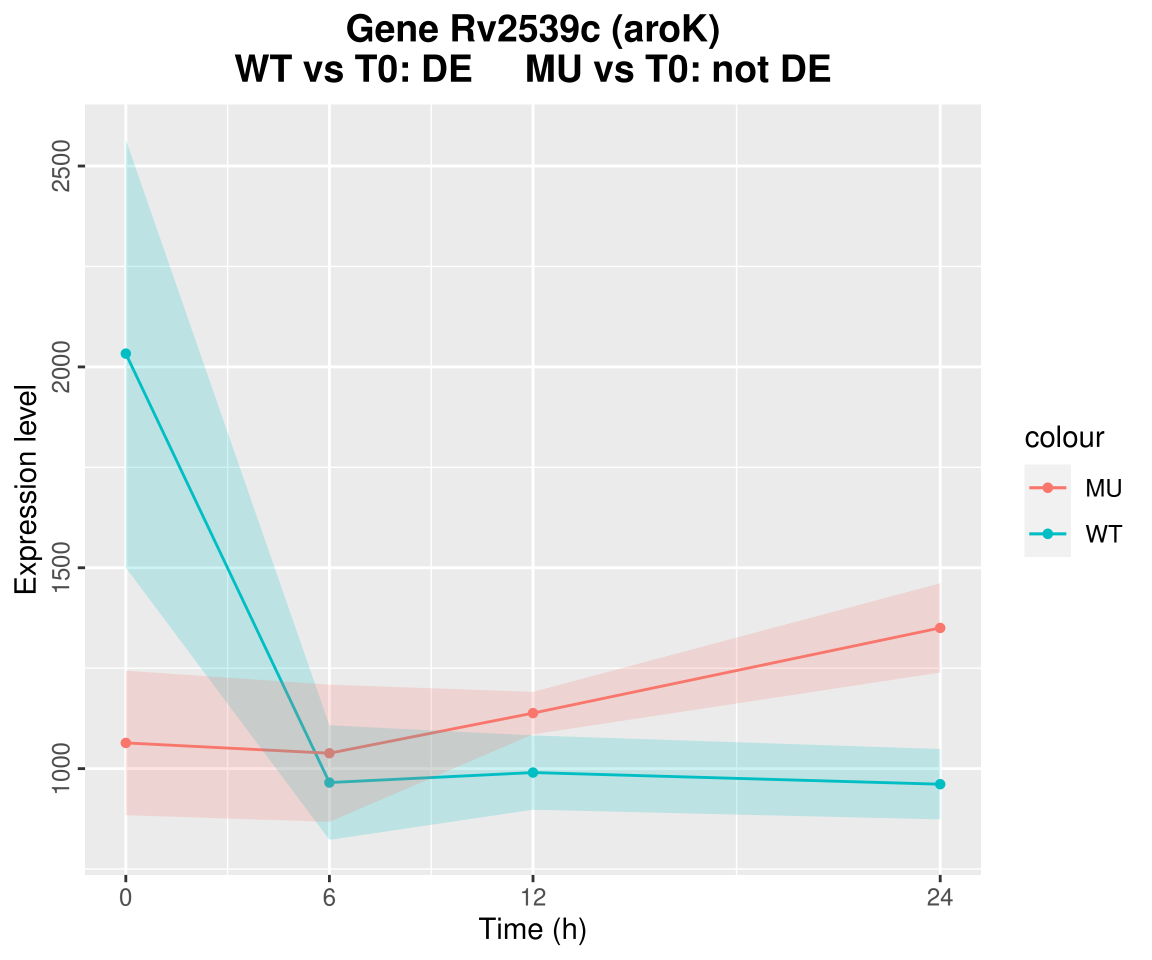
Gene Rv2539c (aroK)
WT vs T0: DE   MU vs T0: not DE
Expression level
2500
2000
1500
1000
0
6
12
24
Time (h)
colour
MU
WT

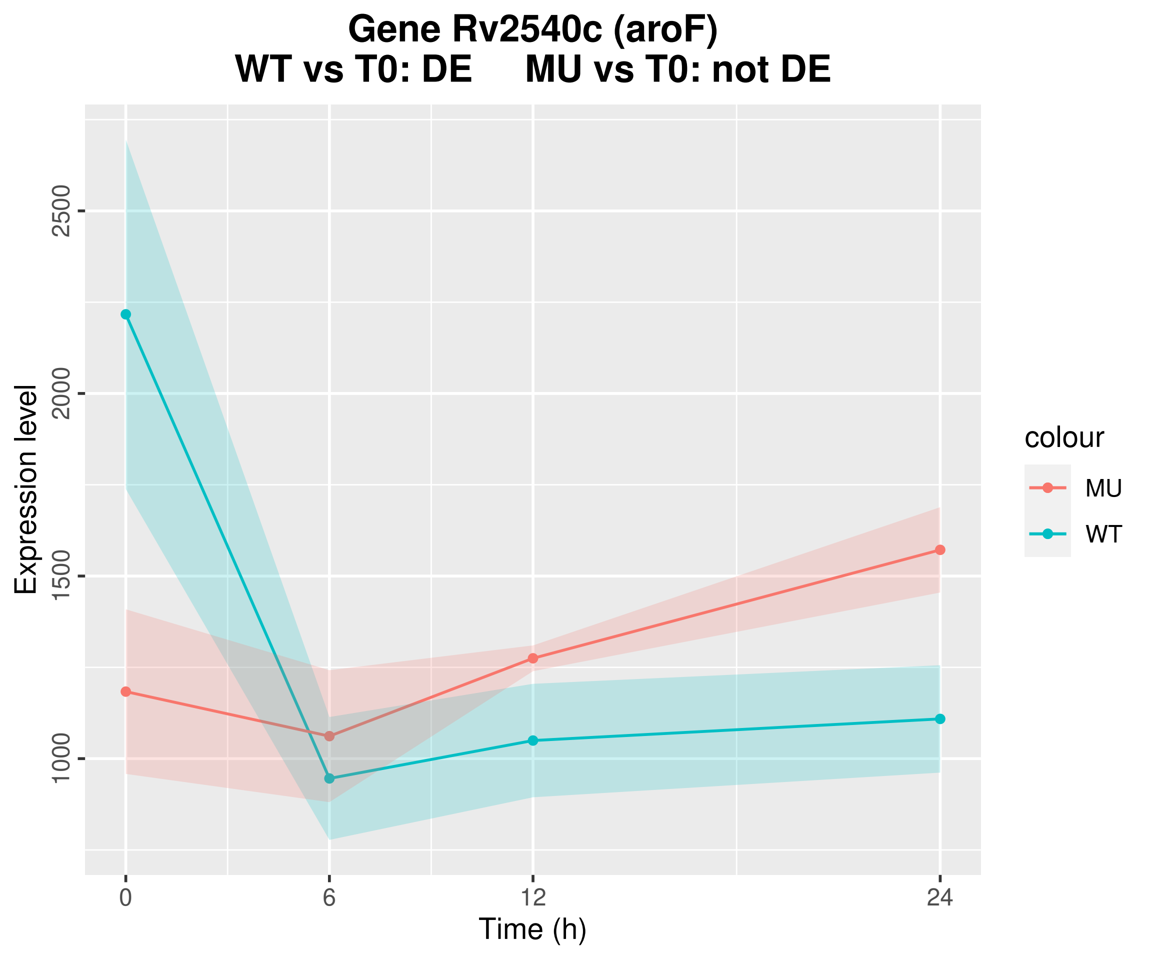

Gene Rv2540c (aroF)
WT vs T0: DE   MU vs T0: not DE
Expression level
2500
2000
1500
1000
0
6
12
24
Time (h)
colour
MU
WT

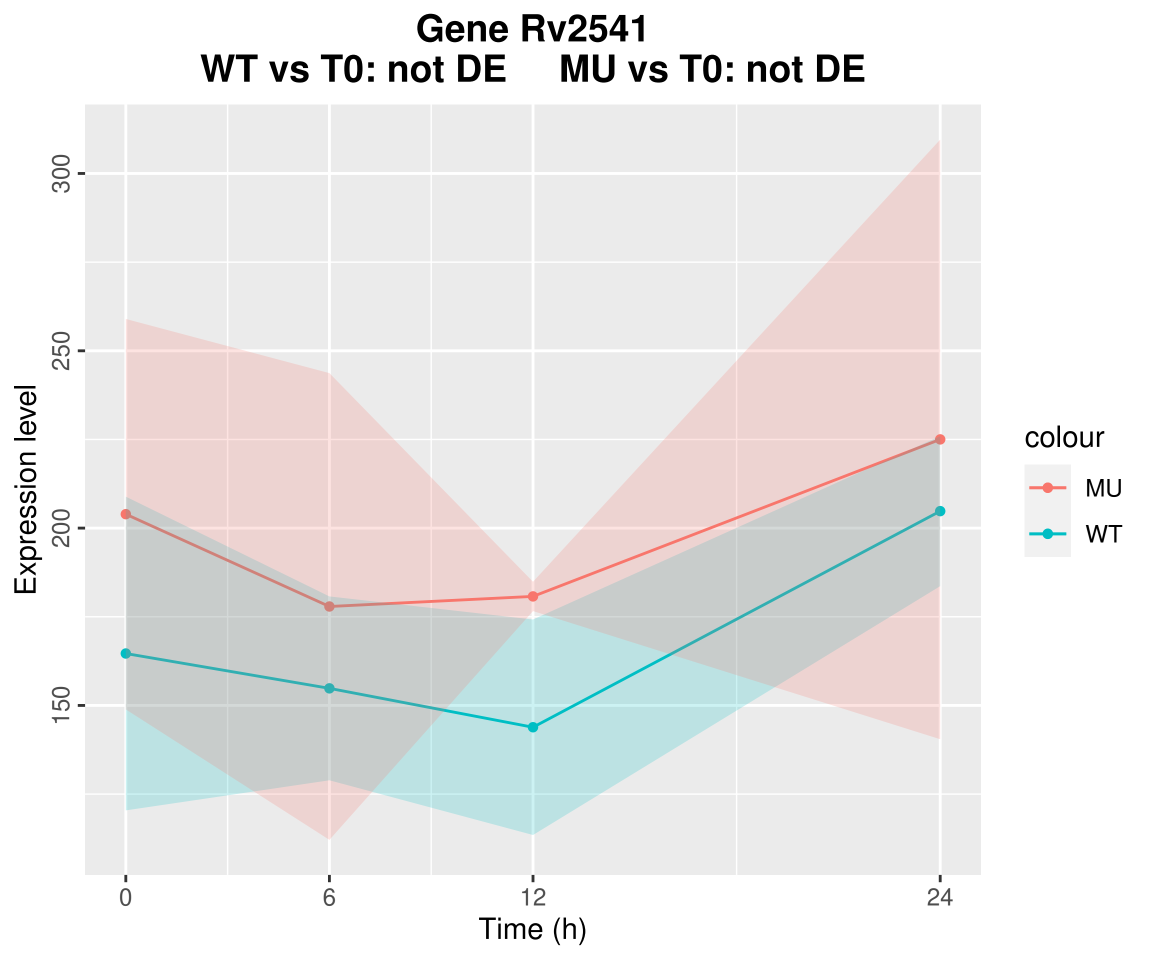
Gene Rv2541
WT vs T0: not DE MU vs T0: not DE
Expression level
300
250
200
150
0
6
12
24
Time (h)
colour
MU
WT

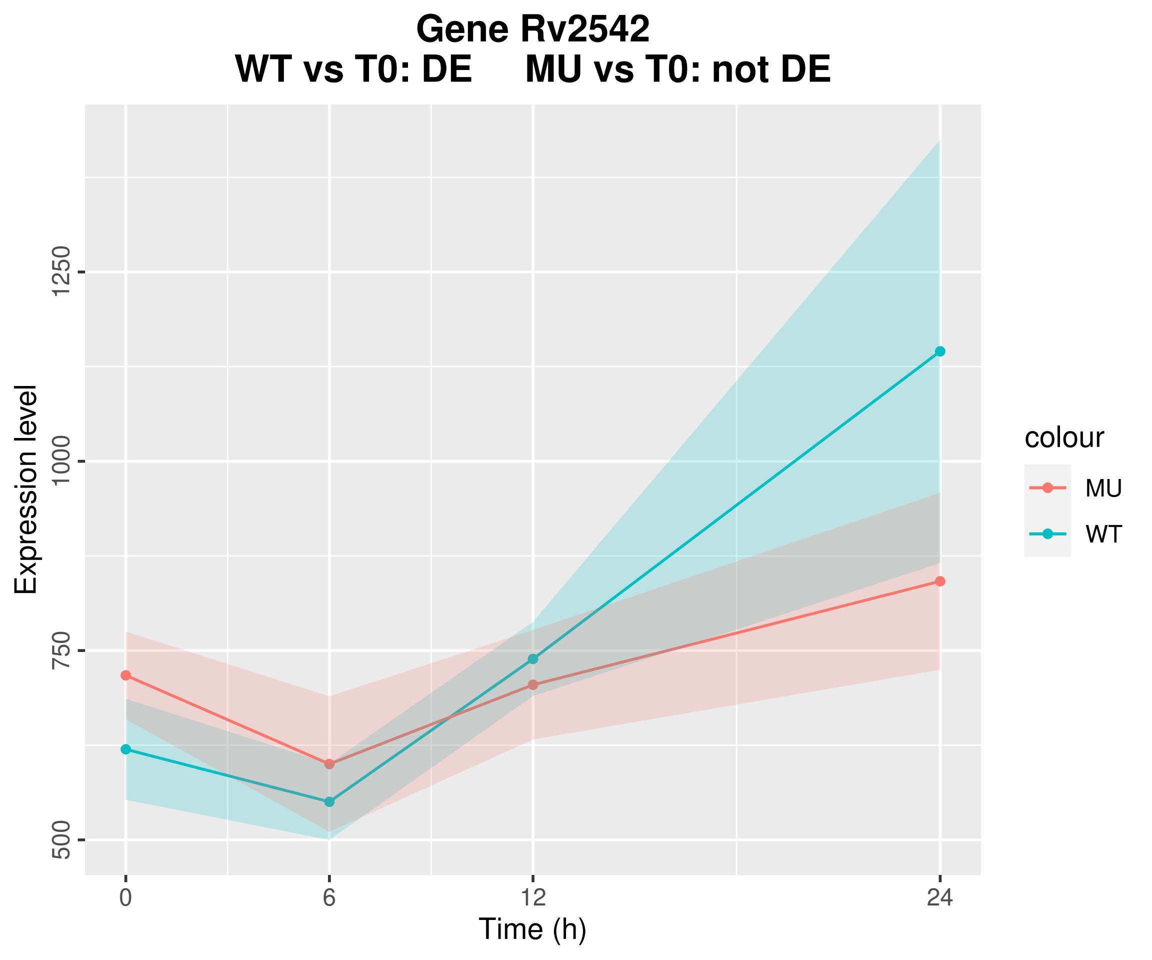
Gene Rv2542
WT vs T0: DE   MU vs T0: not DE
Expression level
500
750
1000
1250
0
6
12
24
Time (h)
colour
MU
WT

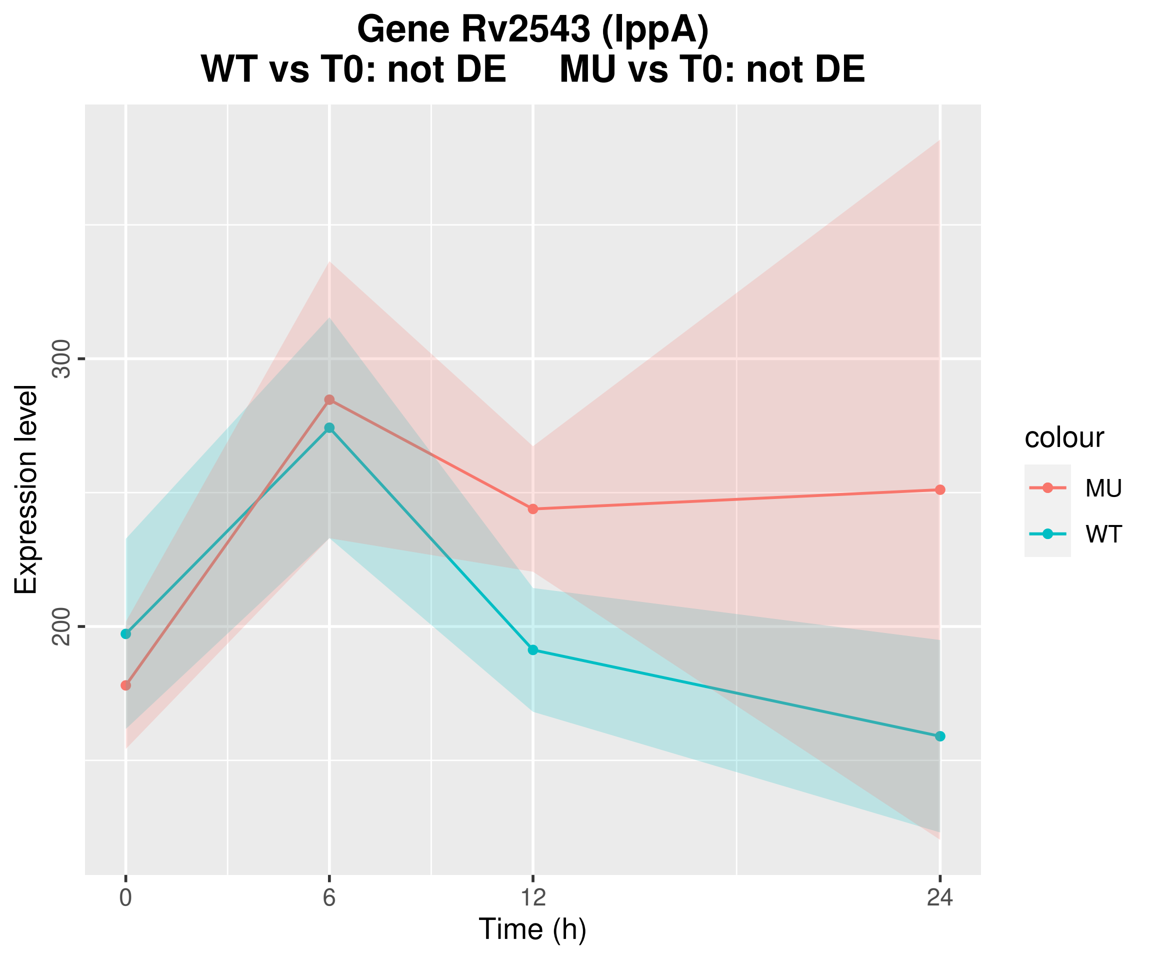
Gene Rv2543 (lppA)
WT vs T0: not DE   MU vs T0: not DE
Expression level
300
200
0
6
12
24
Time (h)
colour
MU
WT

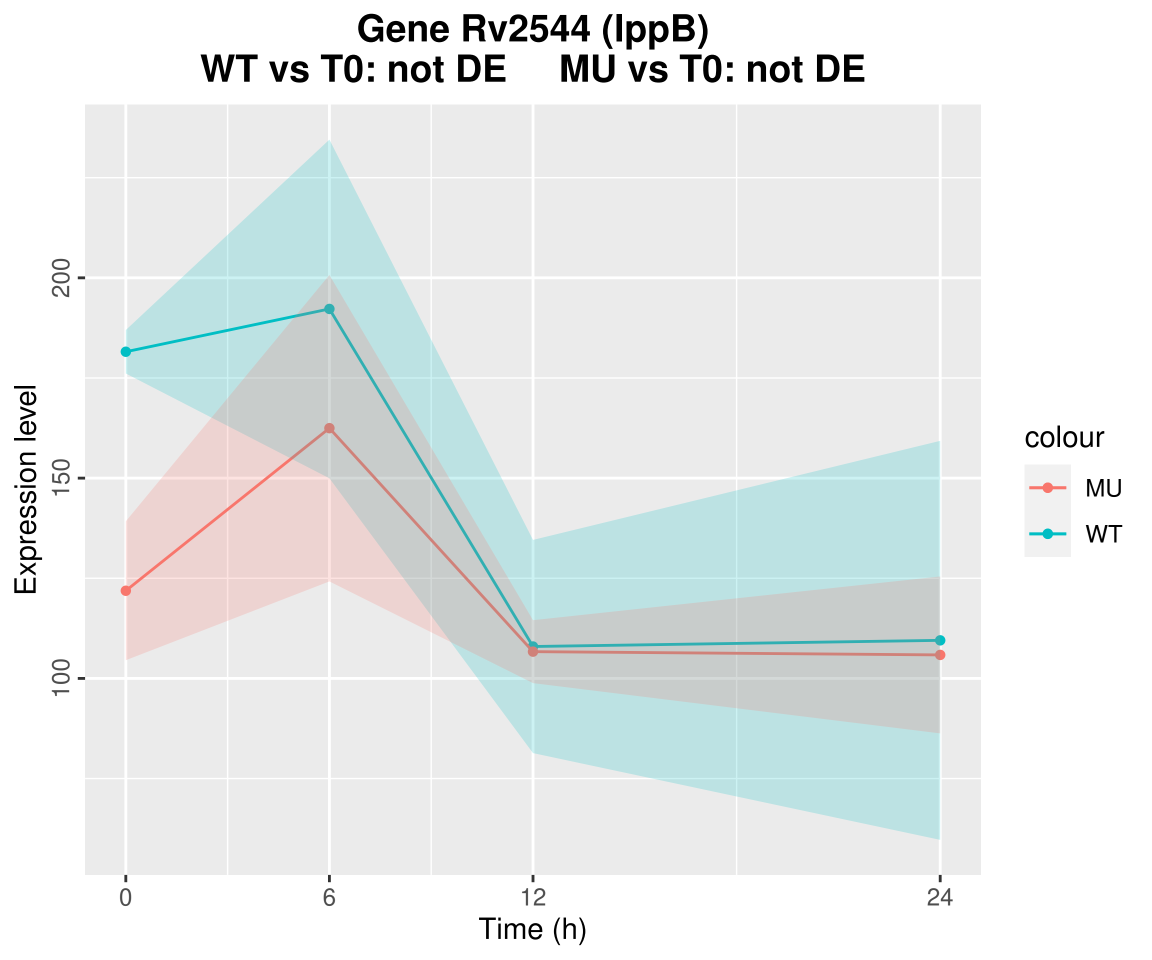

Gene Rv2544 (lppB)
WT vs T0: not DE   MU vs T0: not DE
Expression level
200
150
100
0
6
12
24
Time (h)
colour
MU
WT

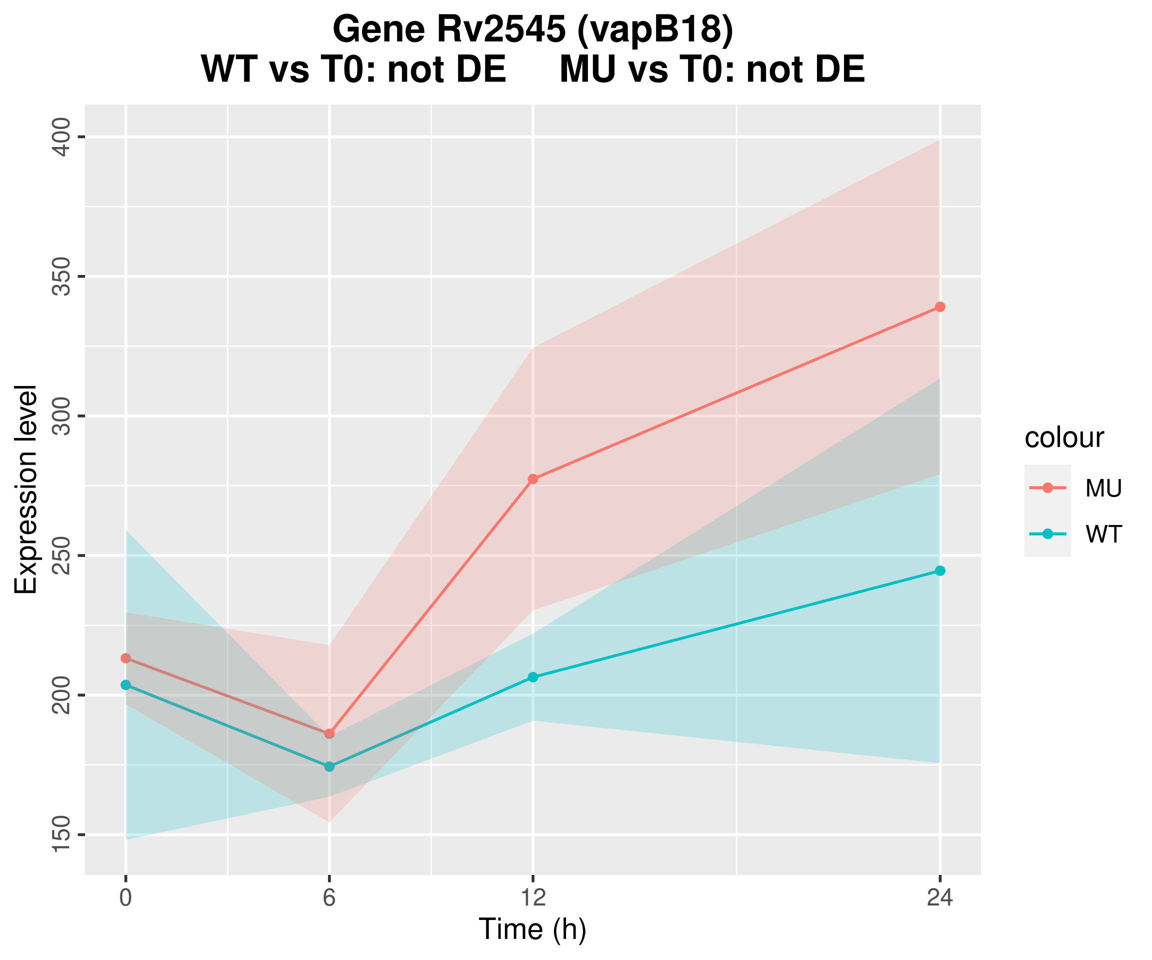
Gene Rv2545 (vapB18)
WT vs T0: not DE   MU vs T0: not DE
Expression level
400
350
300
250
200
150
0
6
12
24
Time (h)
colour
MU
WT

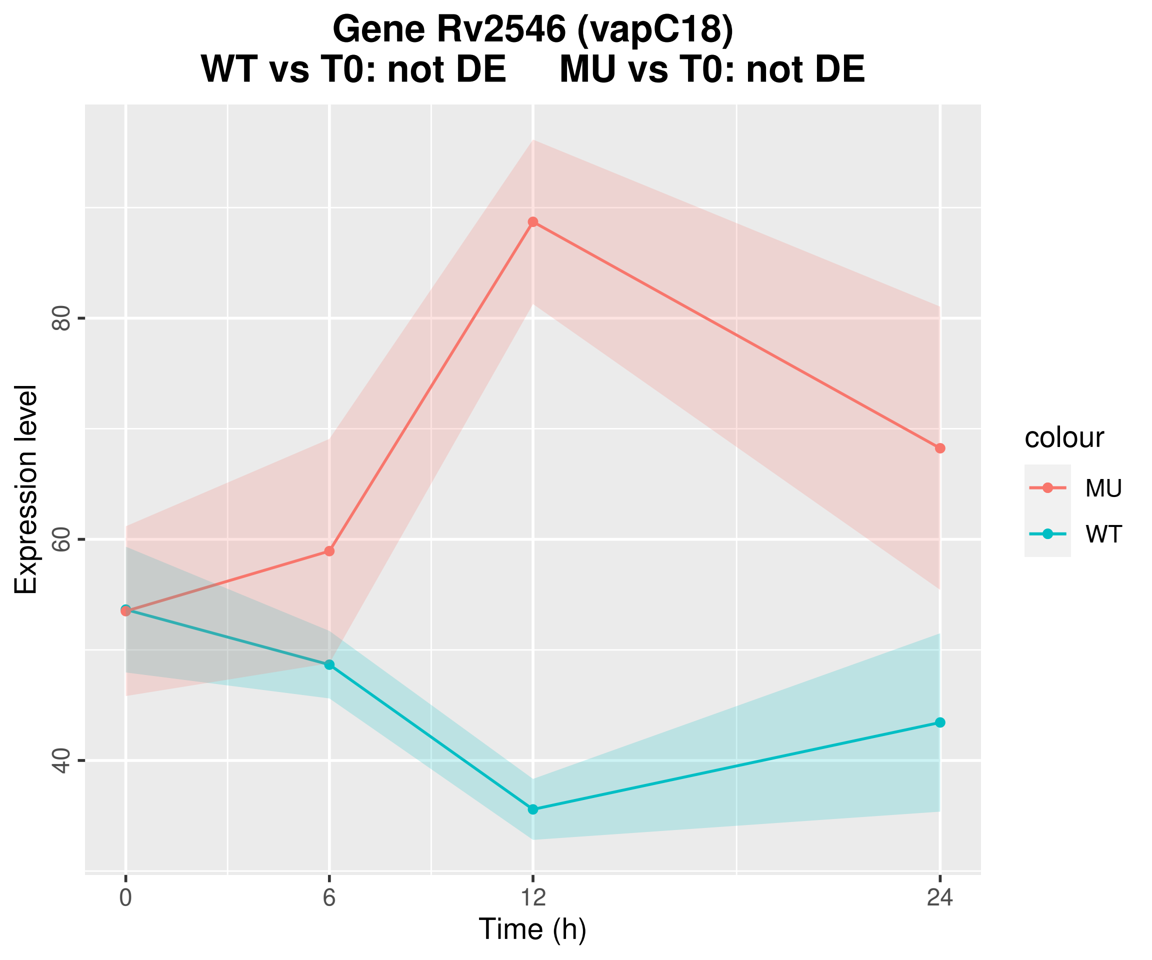

Gene Rv2546 (vapC18)
WT vs T0: not DE MU vs T0: not DE
Expression level
40
60
80
0
6
12
24
Time (h)
colour
MU
WT

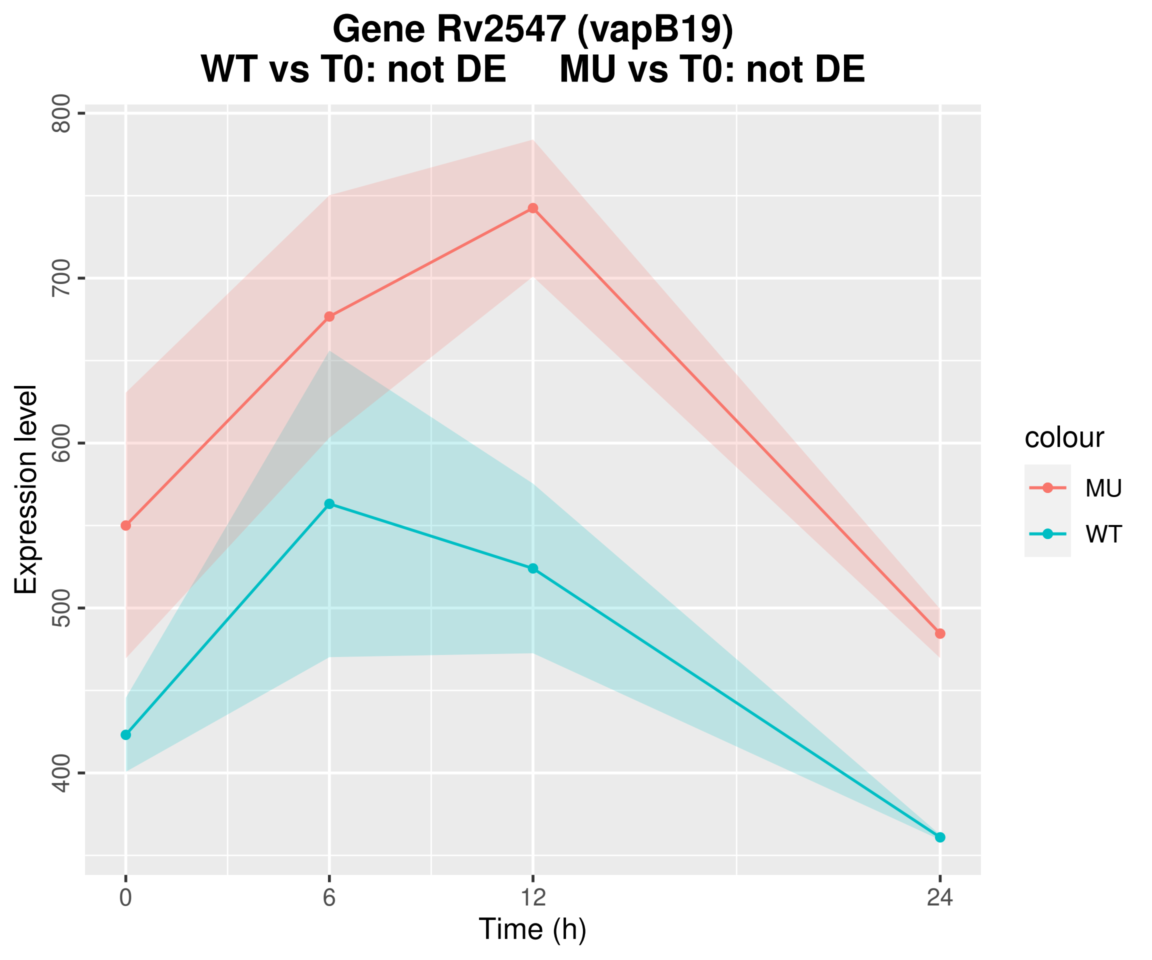

Gene Rv2547 (vapB19)
WT vs T0: not DE MU vs T0: not DE
Expression level
800
700
600
500
400
0
6
12
24
Time (h)
colour
MU
WT

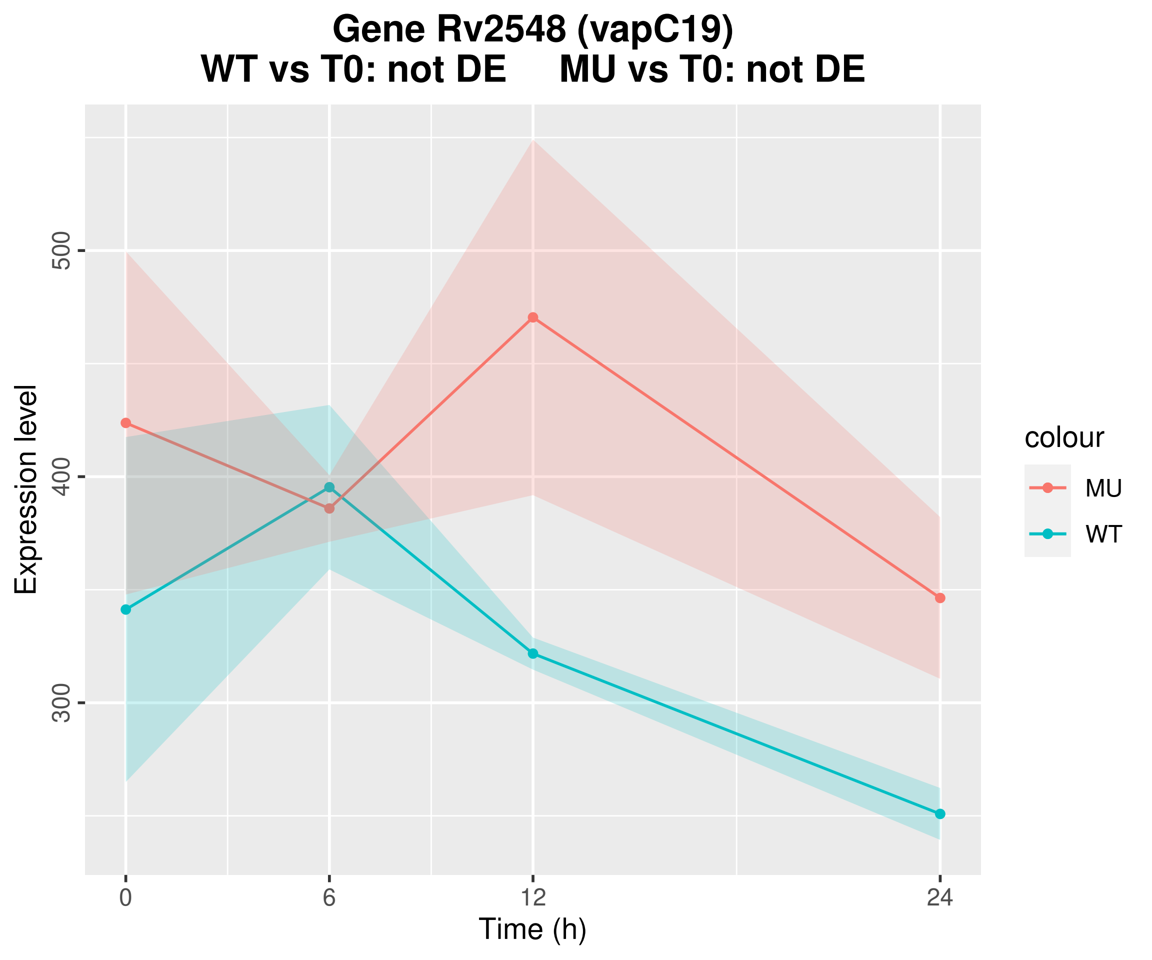

Gene Rv2548 (vapC19)
WT vs T0: not DE   MU vs T0: not DE
Expression level
500
400
300
0
6
12
24
Time (h)
colour
MU
WT

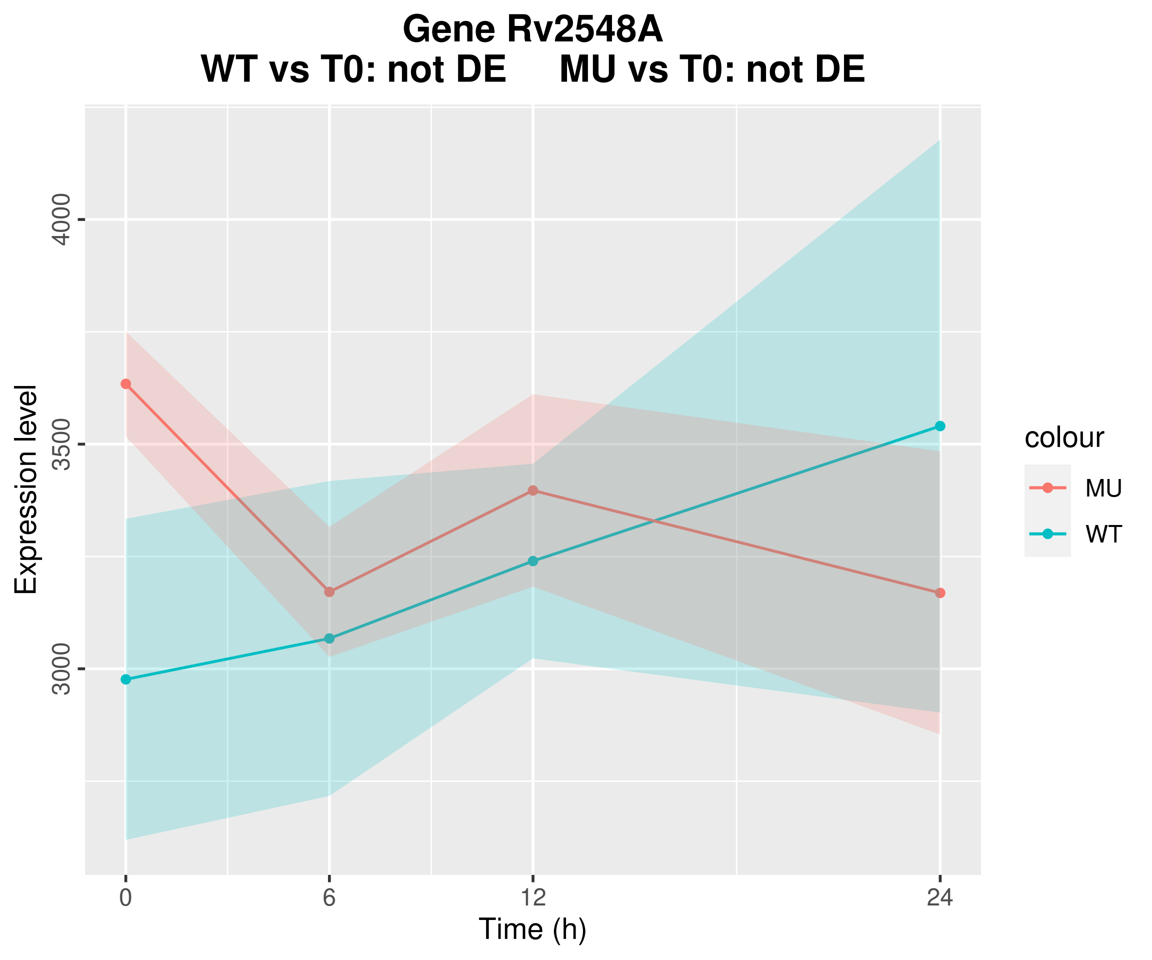
Gene Rv2548A
WT vs T0: not DE   MU vs T0: not DE
Expression level
4000
3500
3000
0
6
12
24
Time (h)
colour
MU
WT

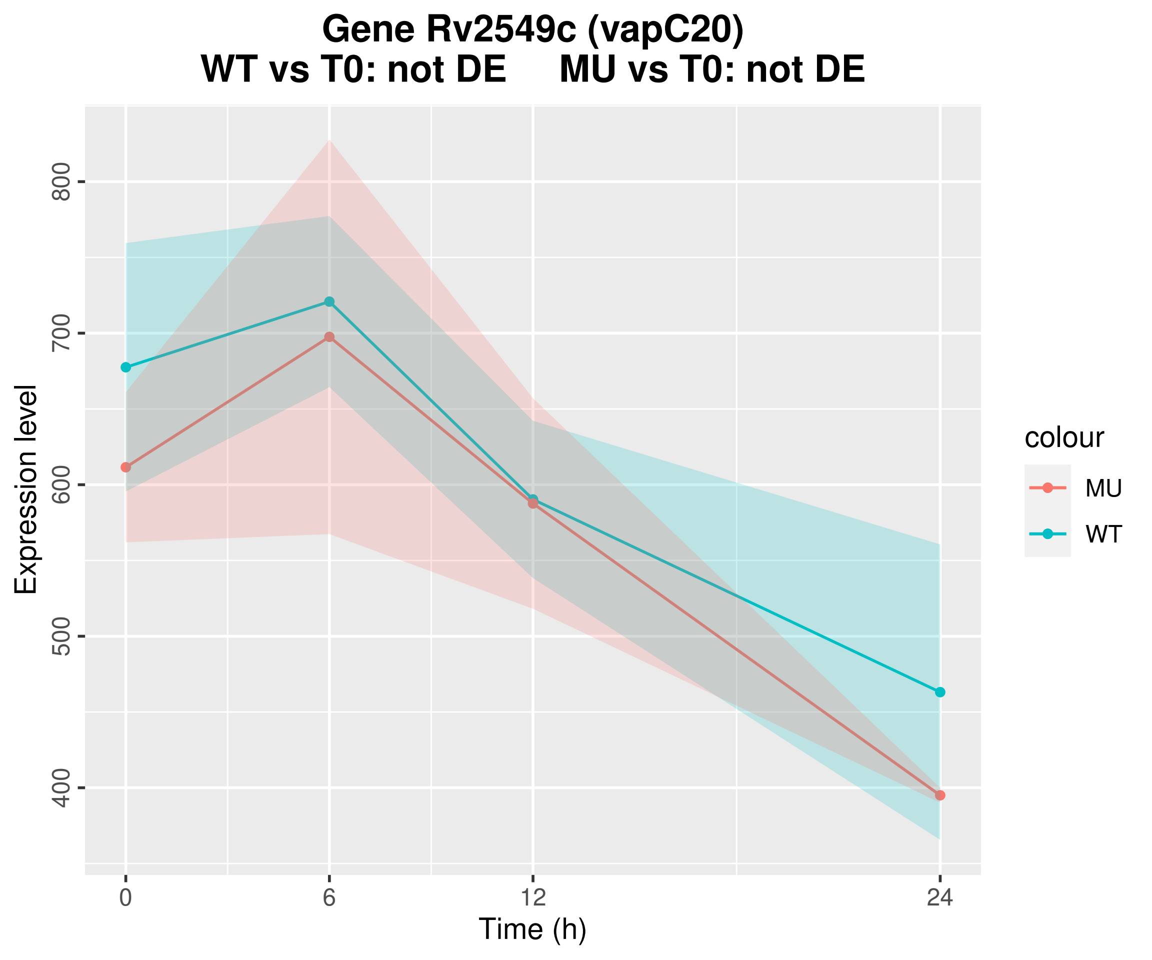

Gene Rv2549c (vapC20)
WT vs T0: not DE MU vs T0: not DE
Expression level
800
700
600
500
400
0
6
12
24
Time (h)
colour
MU
WT

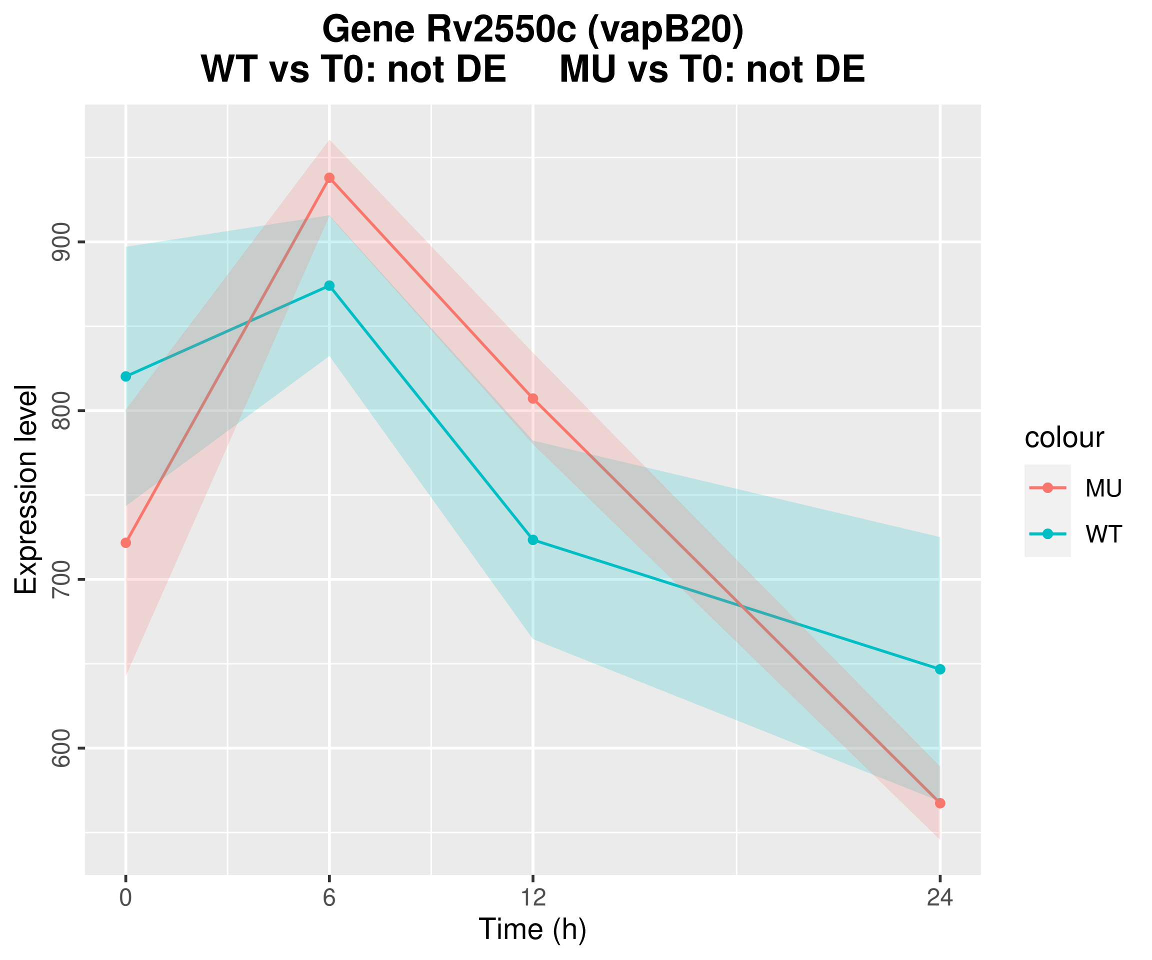

Gene Rv2550c (vapB20)
WT vs T0: not DE   MU vs T0: not DE
Expression level
900
800
700
600
0
6
12
24
Time (h)
colour
MU
WT

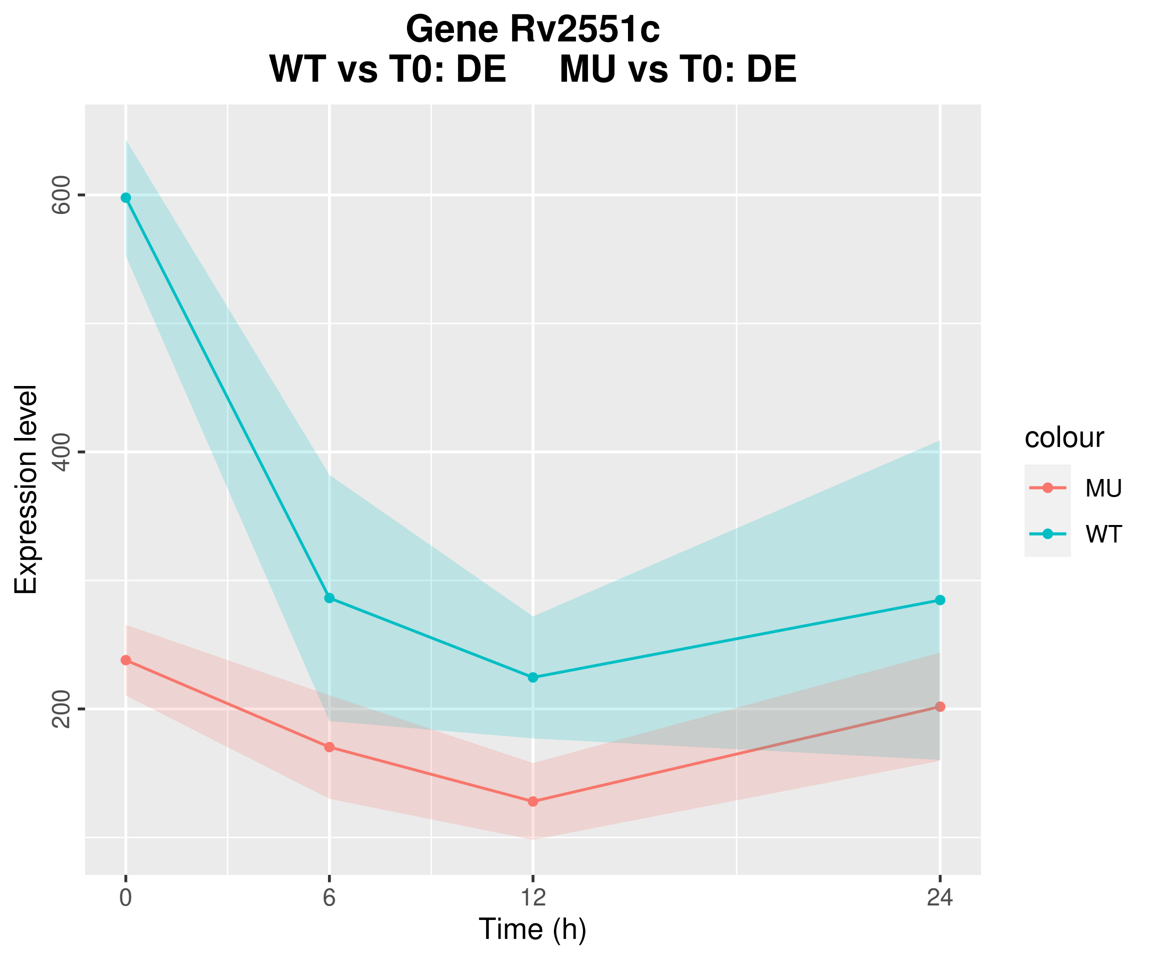
Gene Rv2551c
WT vs T0: DE   MU vs T0: DE
Expression level
600
400
200
0
6
12
24
Time (h)
colour
MU
WT

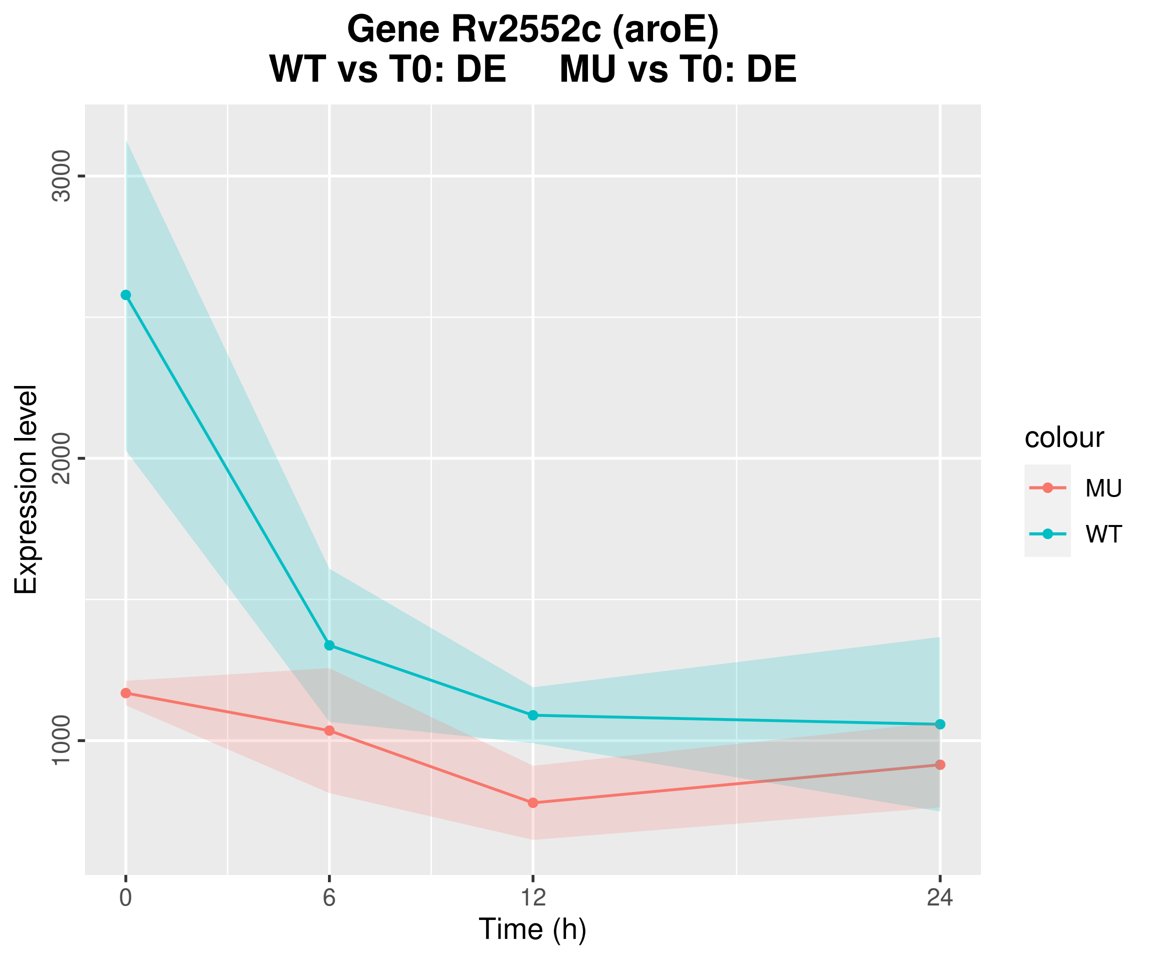

Gene Rv2552c (aroE)
WT vs T0: DE MU vs T0: DE
Expression level
3000
2000
1000
0
6
12
24
Time (h)
colour
MU
WT

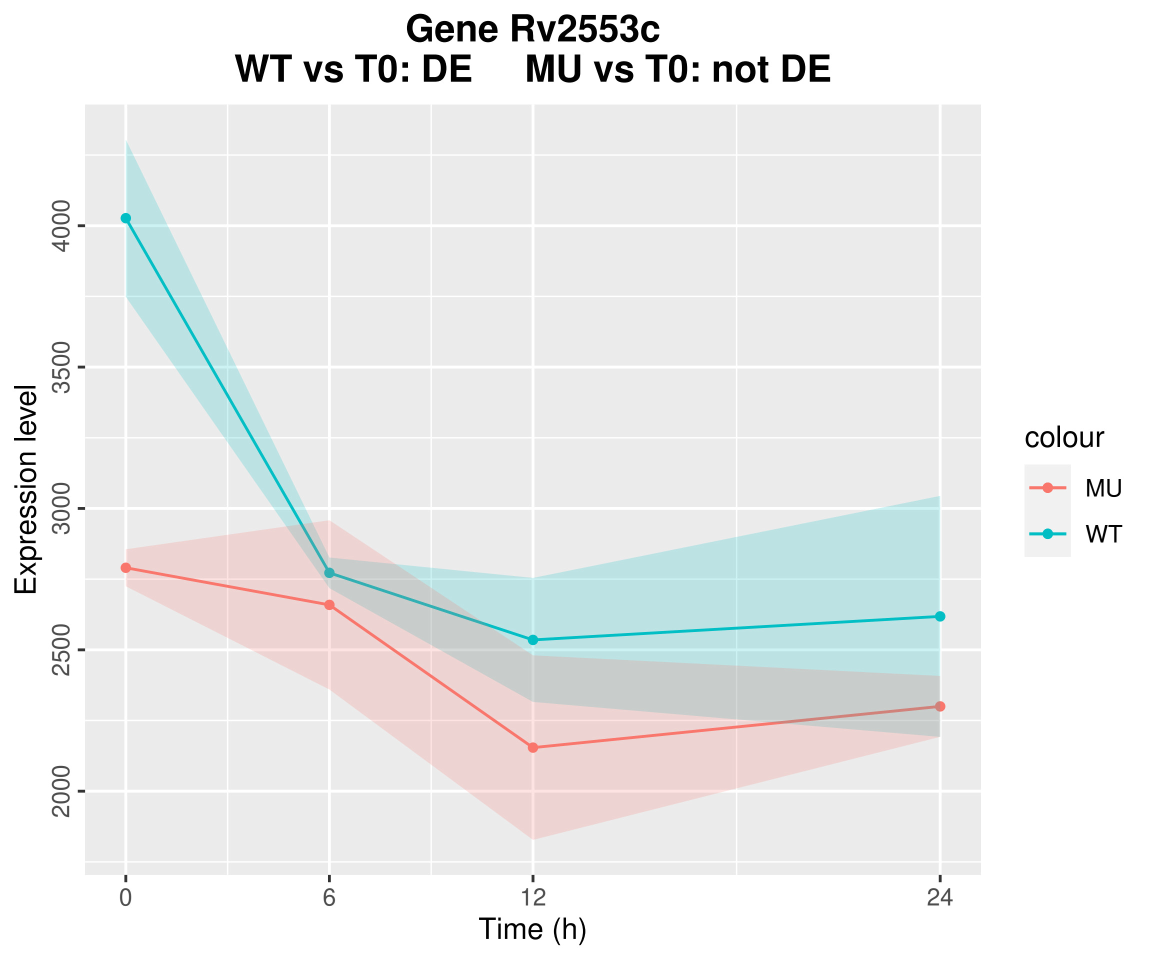

Gene Rv2553c
WT vs T0: DE MU vs T0: not DE
Expression level
4000
3500
3000
2500
2000
0
6
12
24
Time (h)
colour
MU
WT

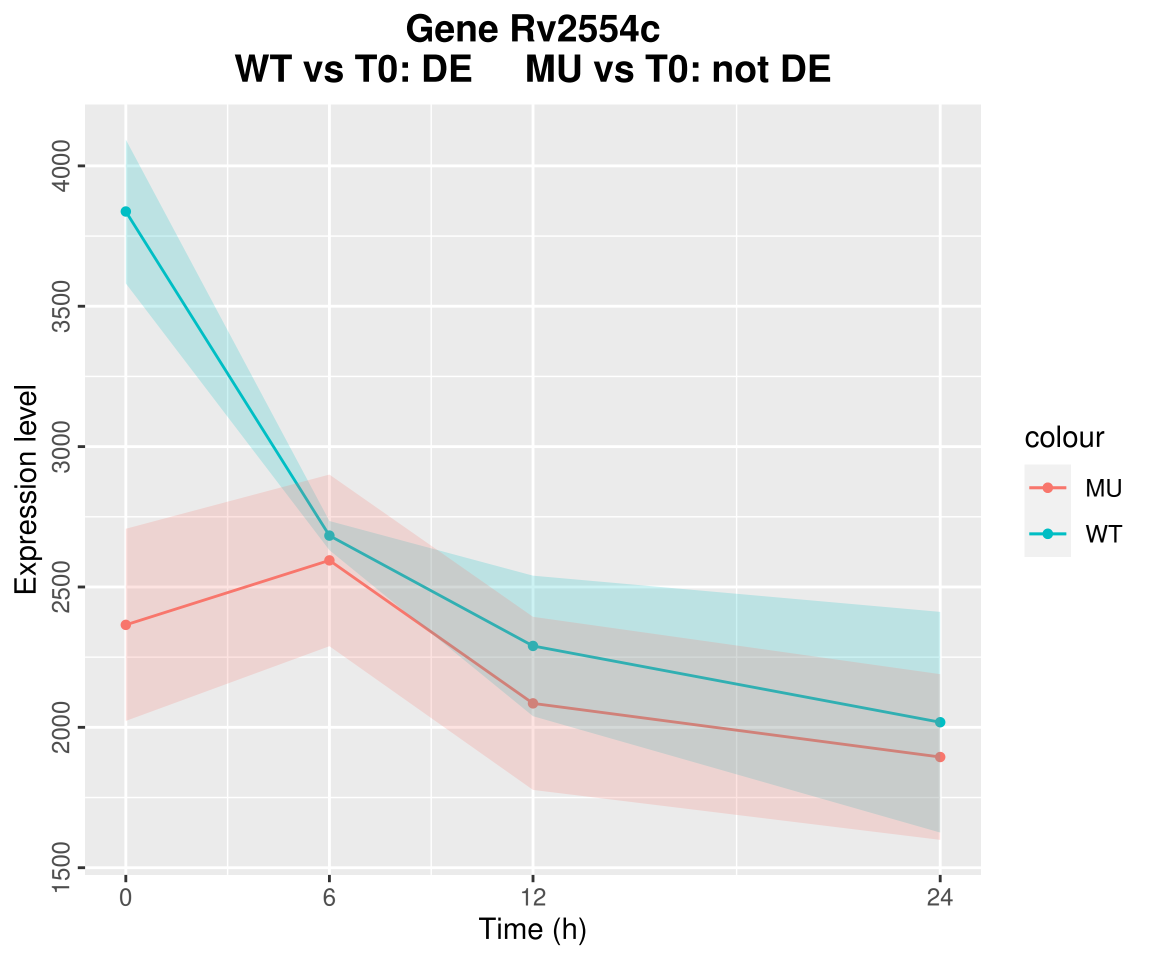
Gene Rv2554c
WT vs T0: DE   MU vs T0: not DE
Expression level
4000
3500
3000
2500
2000
1500
0
6
12
24
Time (h)
colour
MU
WT

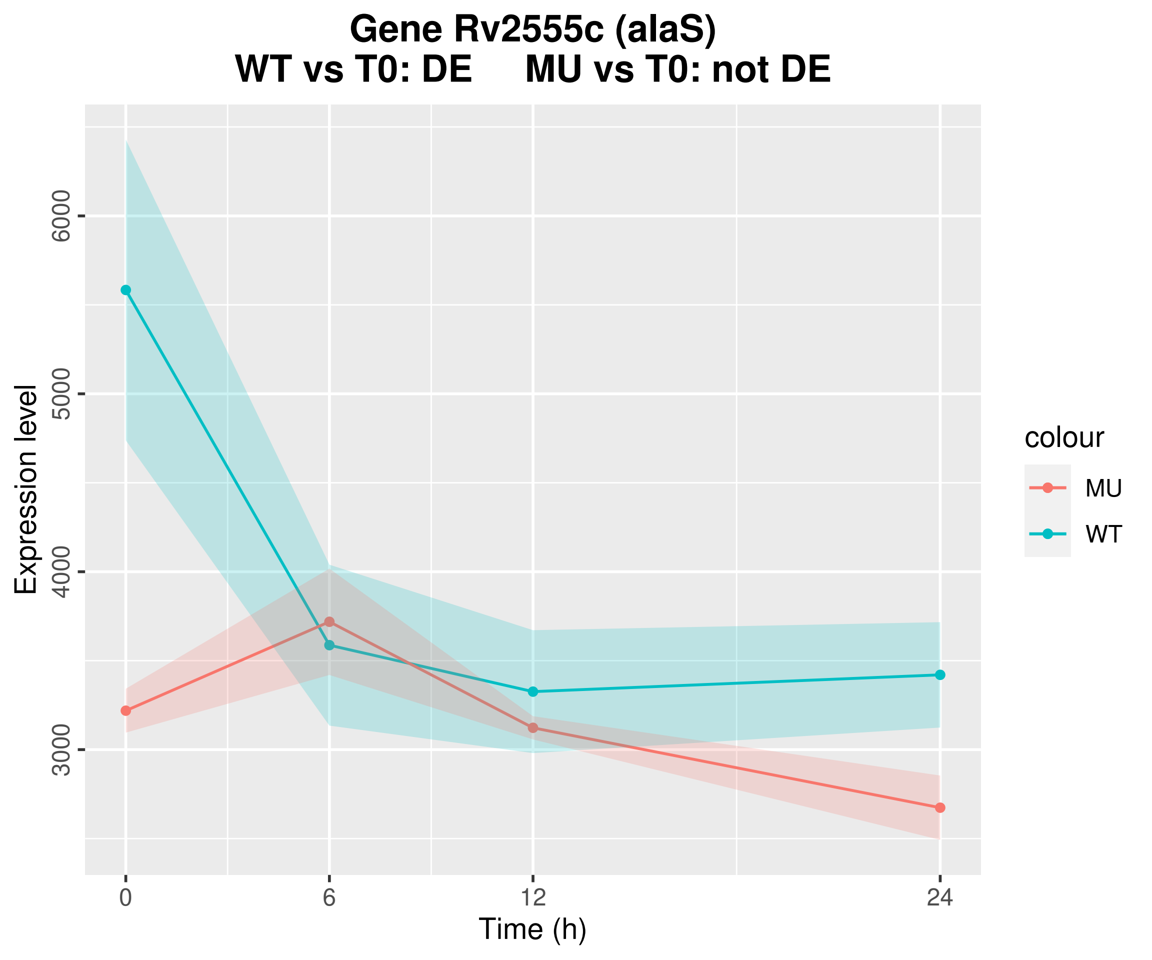
Gene Rv2555c (alaS)
WT vs T0: DE   MU vs T0: not DE
Expression level
6000
5000
4000
3000
0
6
12
24
Time (h)
colour
MU
WT

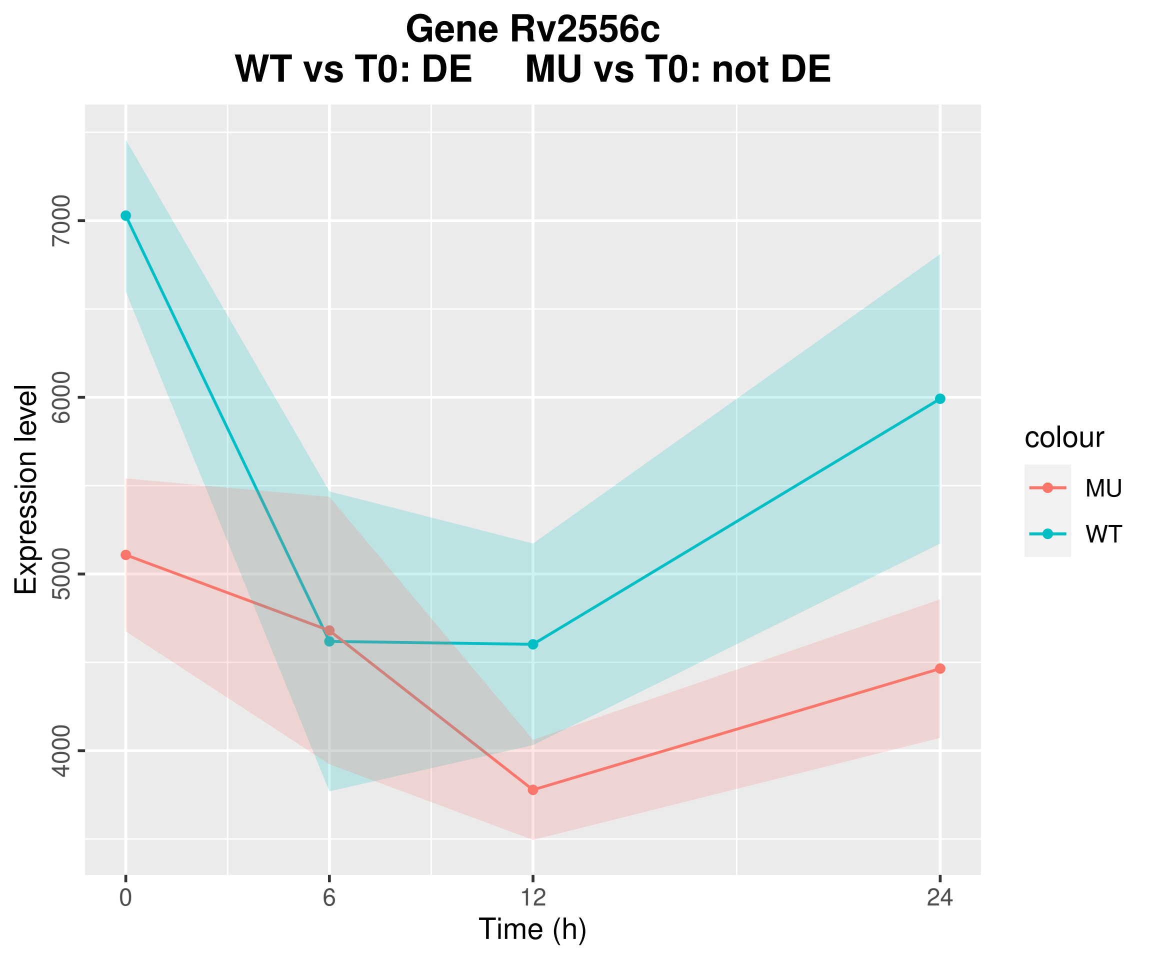

Gene Rv2556c
WT vs T0: DE MU vs T0: not DE
Expression level
7000
6000
5000
4000
0
6
12
24
Time (h)
colour
MU
WT

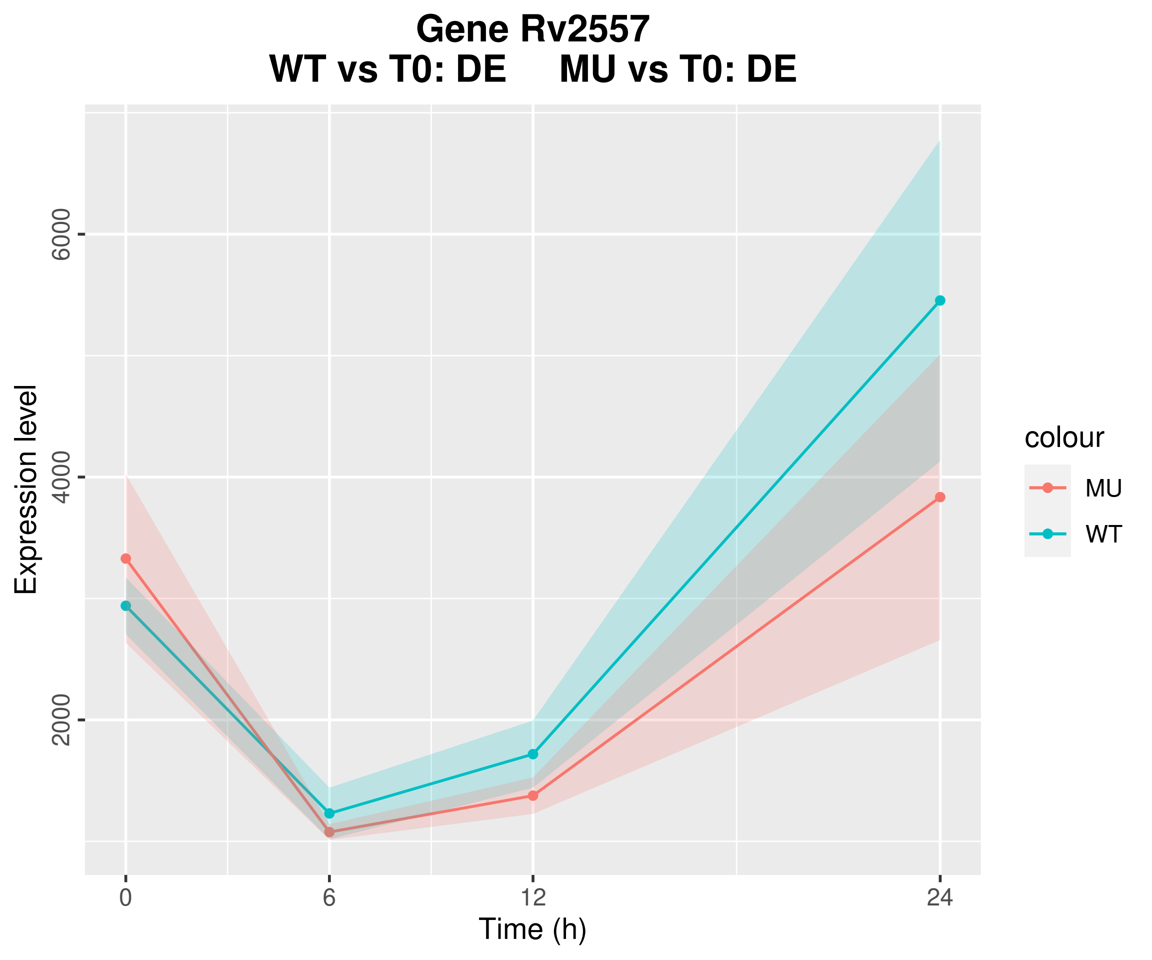
Gene Rv2557
WT vs T0: DE MU vs T0: DE
Expression level
6000
4000
2000
0
6
12
24
Time (h)
colour
MU
WT

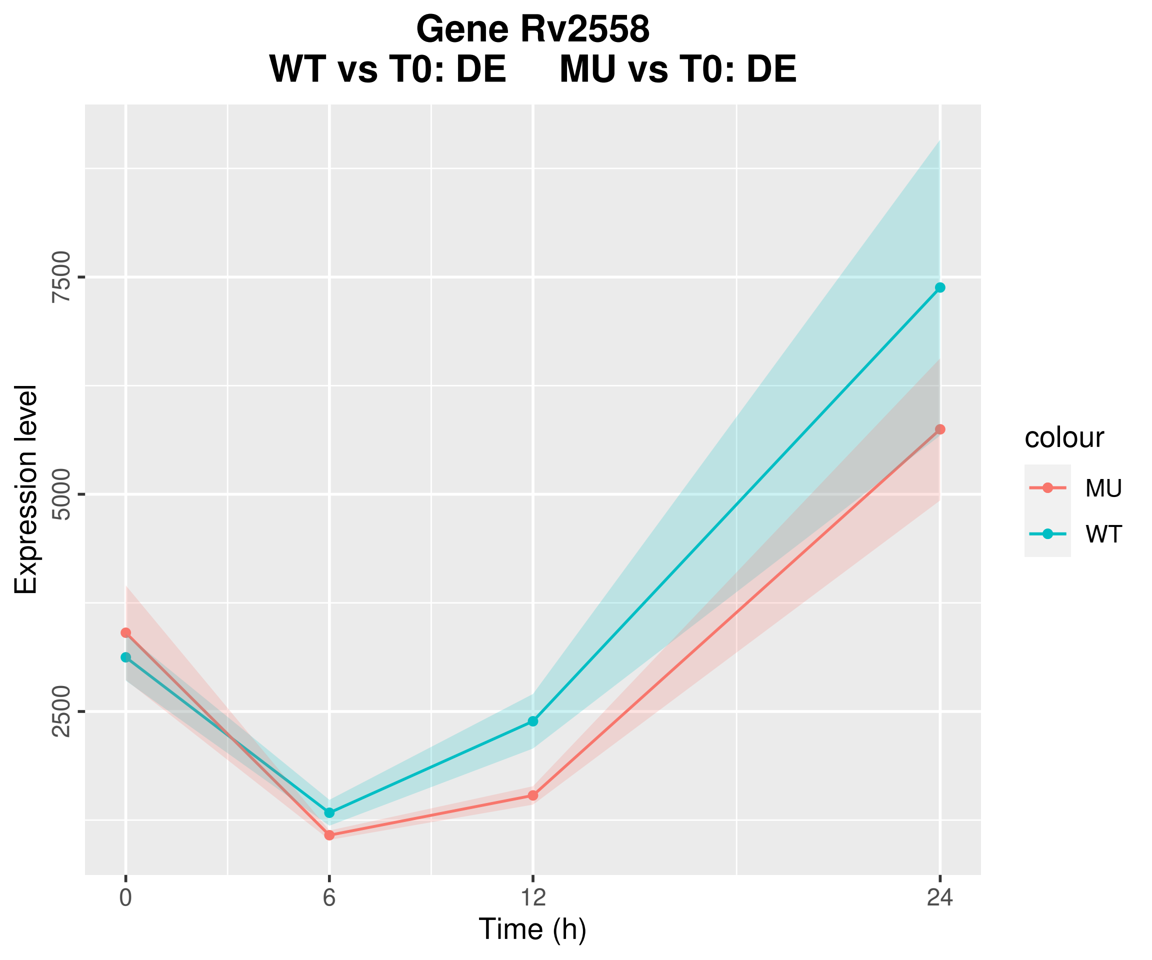
Gene Rv2558
WT vs T0: DE MU vs T0: DE
Expression level
7500
5000
2500
0
6
12
24
Time (h)
colour
MU
WT

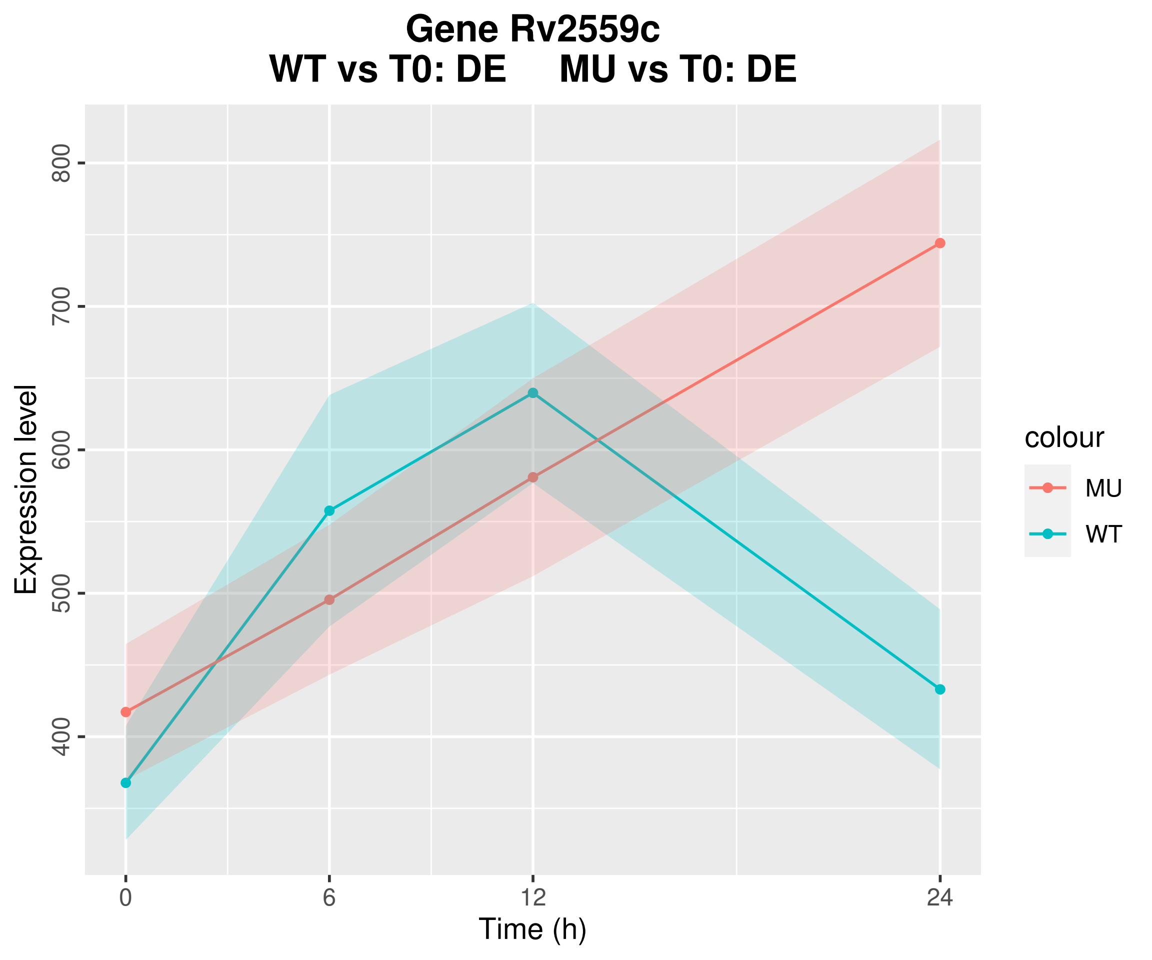

Gene Rv2559c
WT vs T0: DE MU vs T0: DE
Expression level
800
700
600
500
400
0
6
12
24
Time (h)
colour
MU
WT

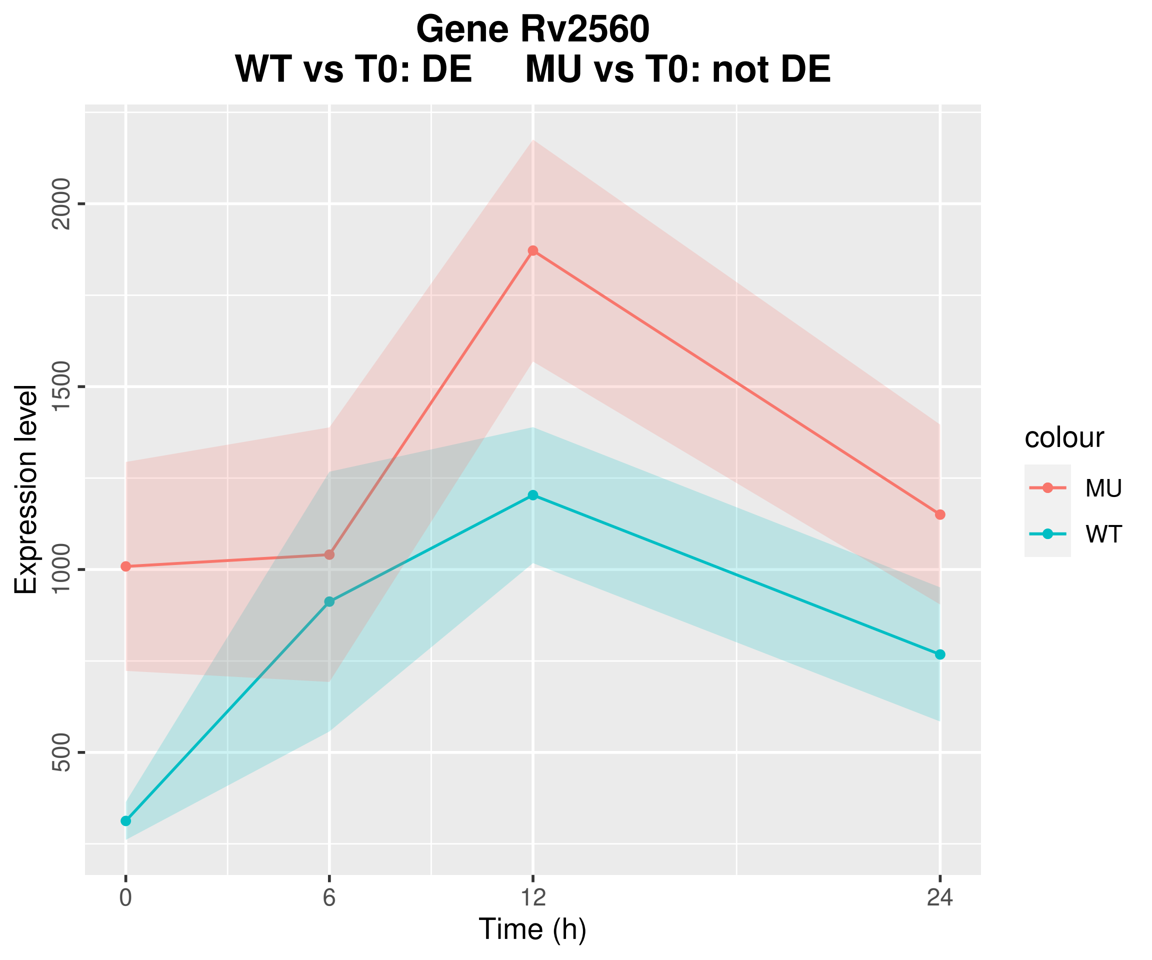
Gene Rv2560
WT vs T0: DE   MU vs T0: not DE
Expression level
2000
1500
1000
500
0
6
12
24
Time (h)
colour
MU
WT

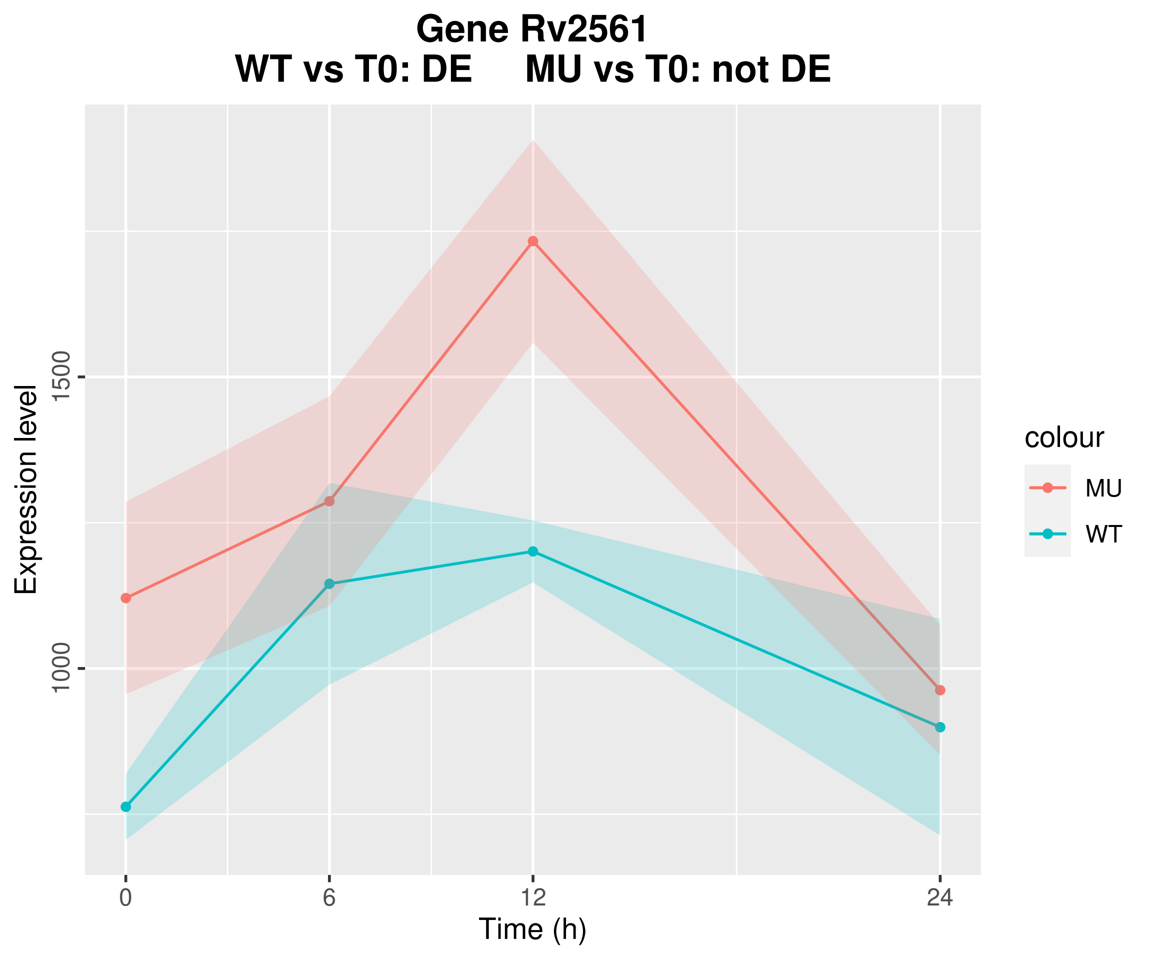

Gene Rv2561
WT vs T0: DE   MU vs T0: not DE
Expression level
1500
1000
Time (h)
0
6
12
24
colour
MU
WT

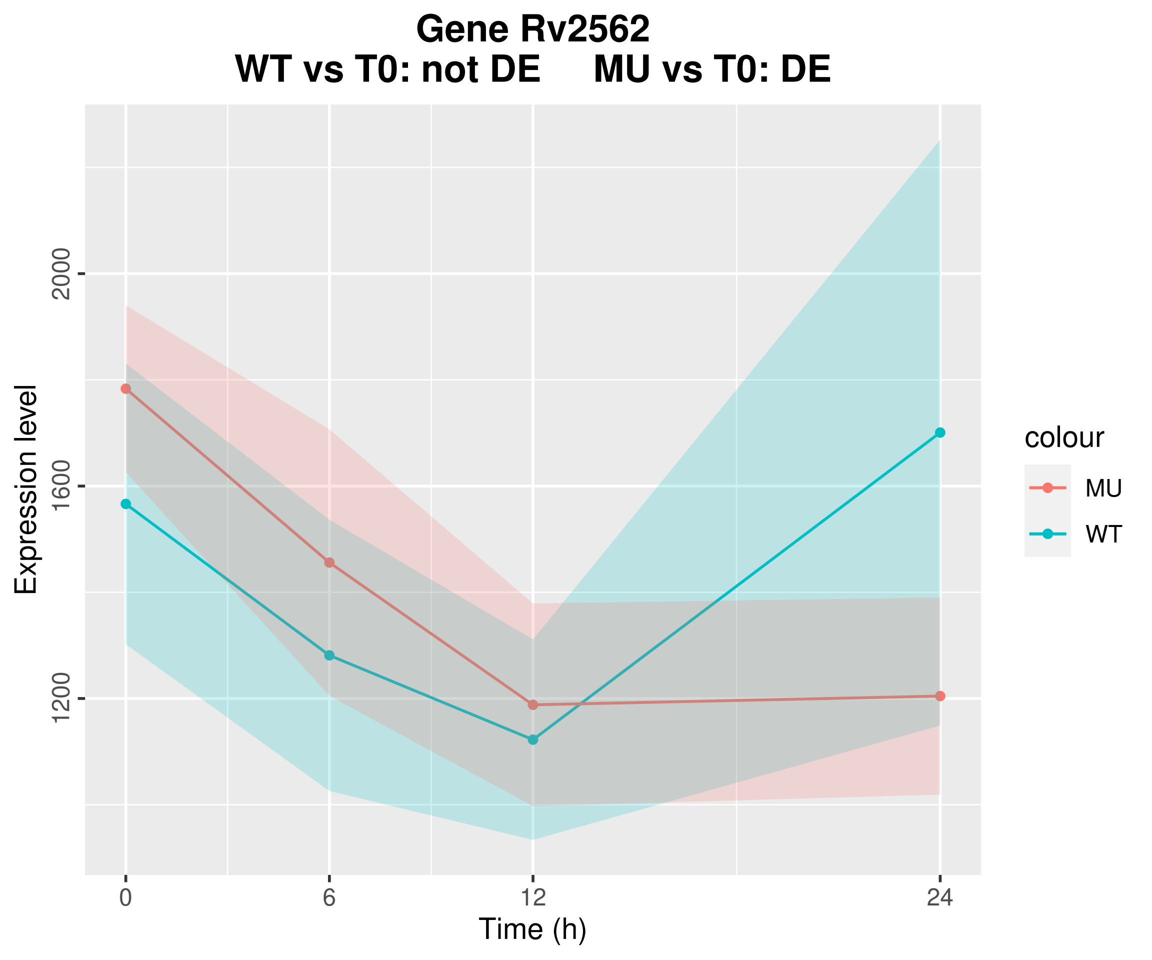
Gene Rv2562
WT vs T0: not DE MU vs T0: DE
Expression level
2000
1600
1200
0
6
12
24
Time (h)
colour
MU
WT

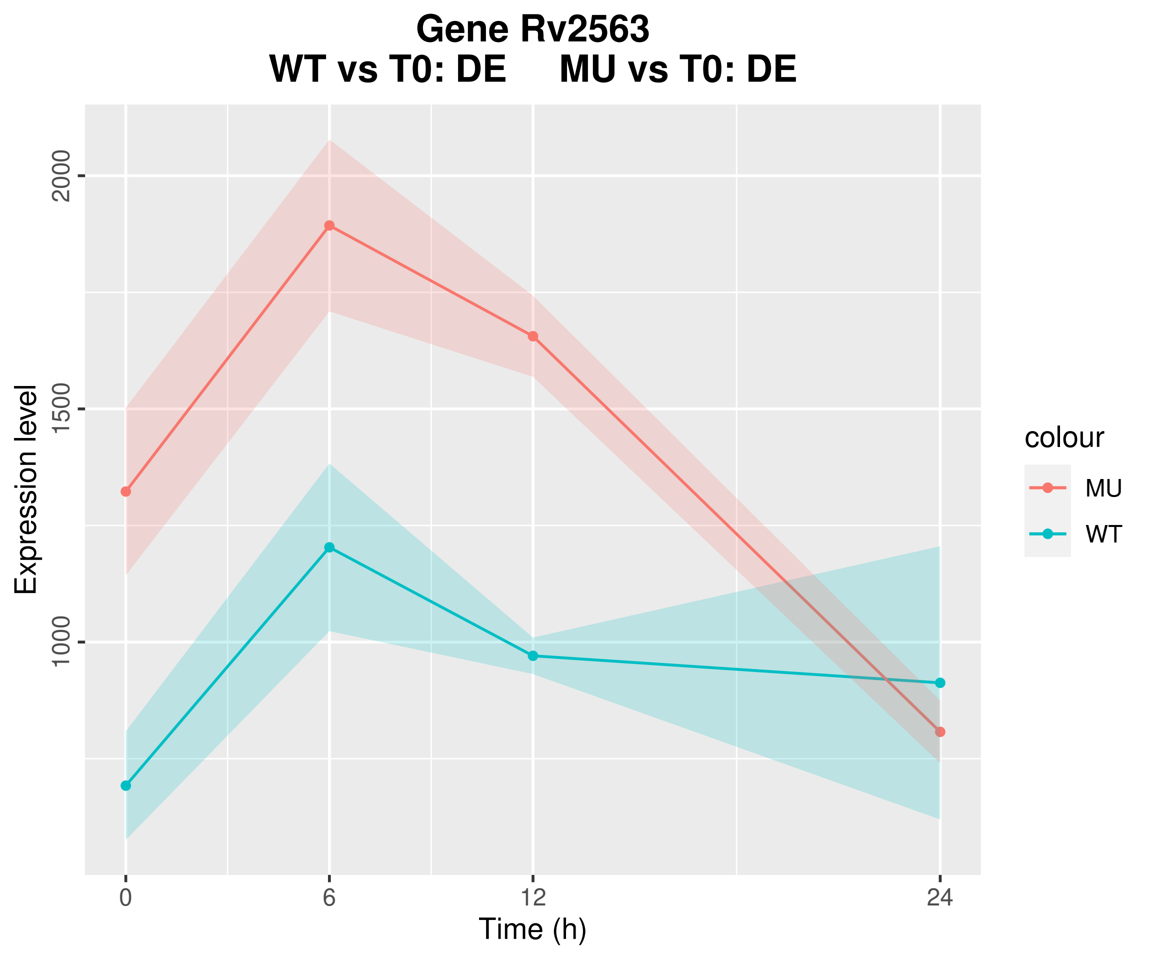
Gene Rv2563
WT vs T0: DE   MU vs T0: DE
Expression level
2000
1500
1000
0
6
12
24
Time (h)
colour
MU
WT

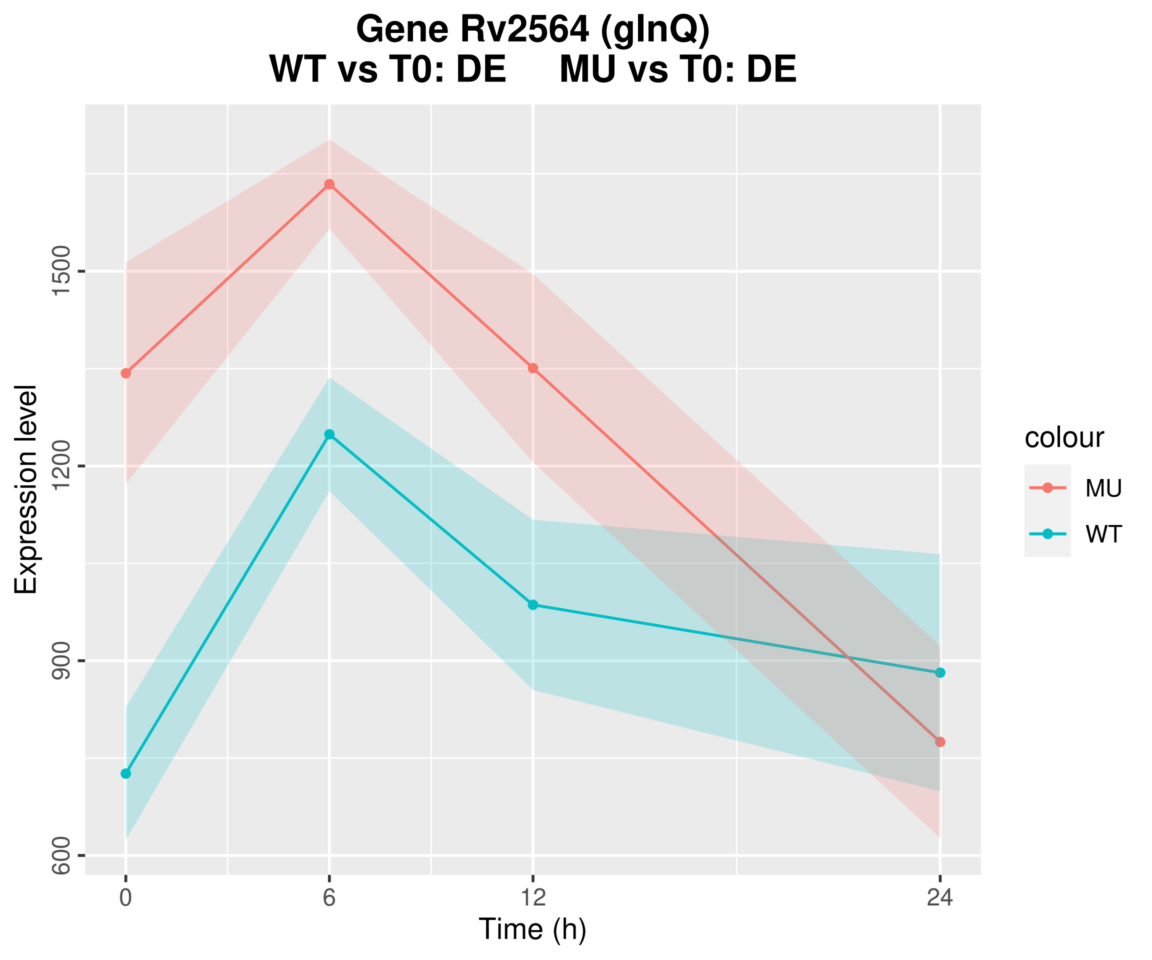
Gene Rv2564 (glnQ)
WT vs T0: DE   MU vs T0: DE
Expression level
1500
1200
900
600
0
6
12
24
Time (h)
colour
MU
WT

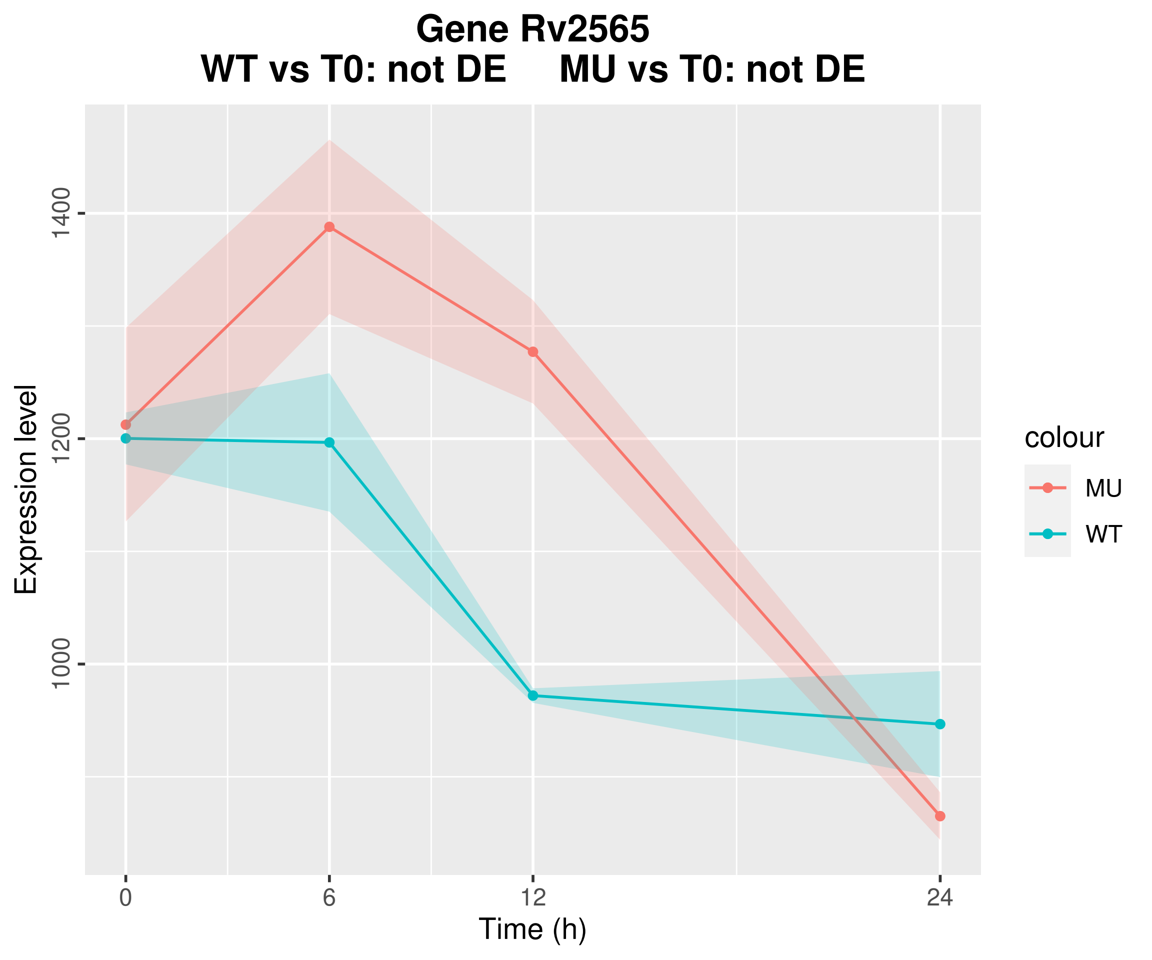

Gene Rv2565
WT vs T0: not DE   MU vs T0: not DE
1400
1200
1000
Expression level
0
6
12
24
Time (h)
colour
MU
WT

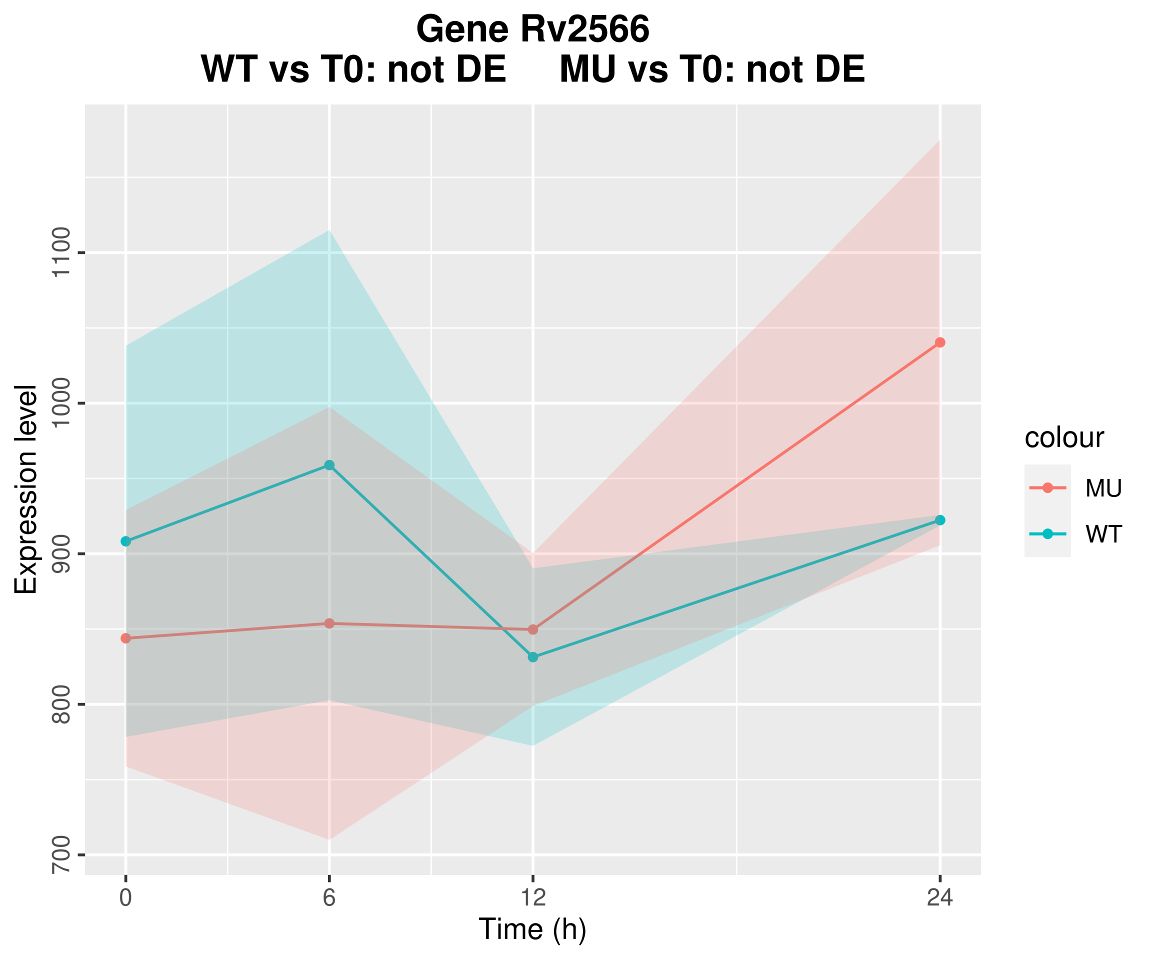

Gene Rv2566
WT vs T0: not DE MU vs T0: not DE
Expression level
700
800
900
1000
1100
0
6
12
24
Time (h)
colour
MU
WT

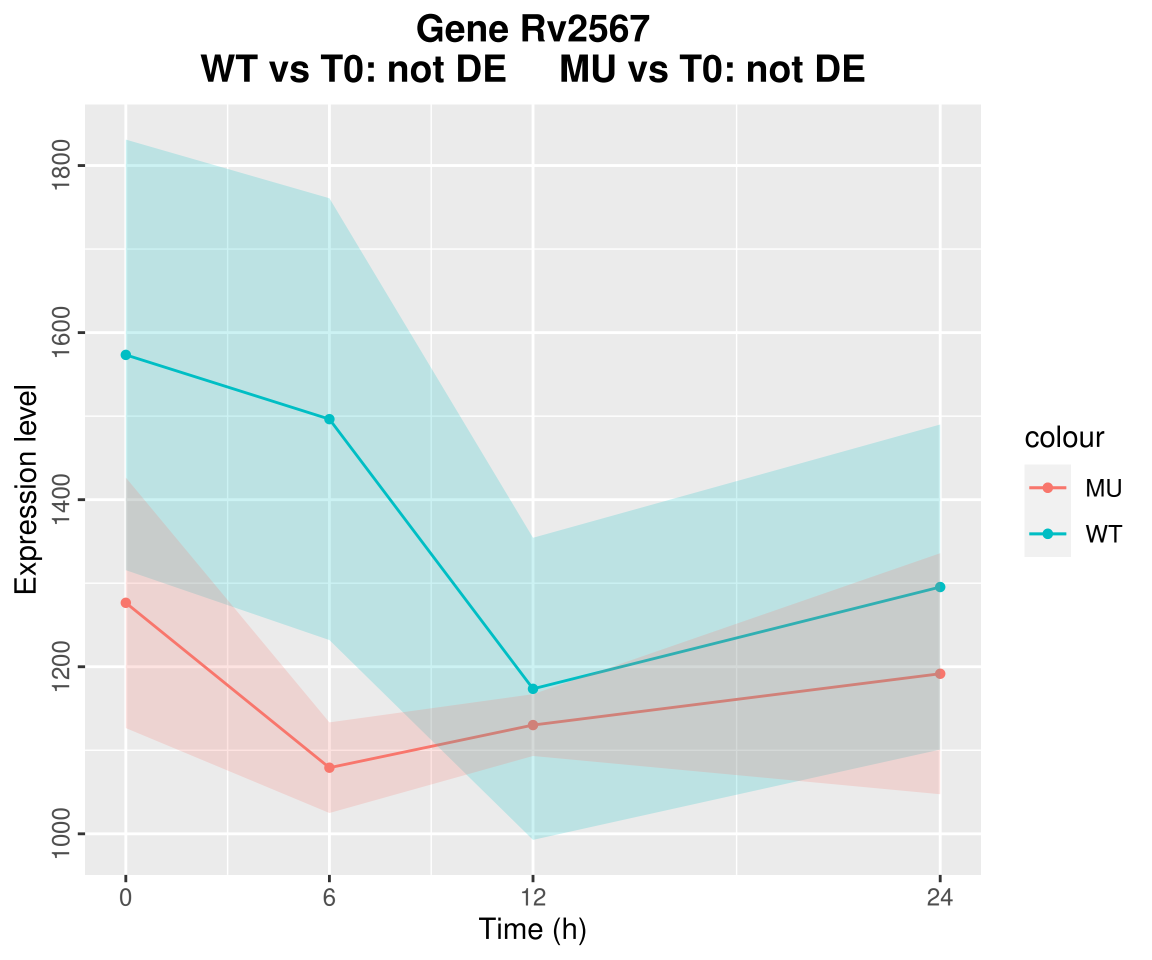
Gene Rv2567
WT vs T0: not DE   MU vs T0: not DE
Expression level
1000
1200
1400
1600
1800
0
6
12
24
Time (h)
colour
MU
WT

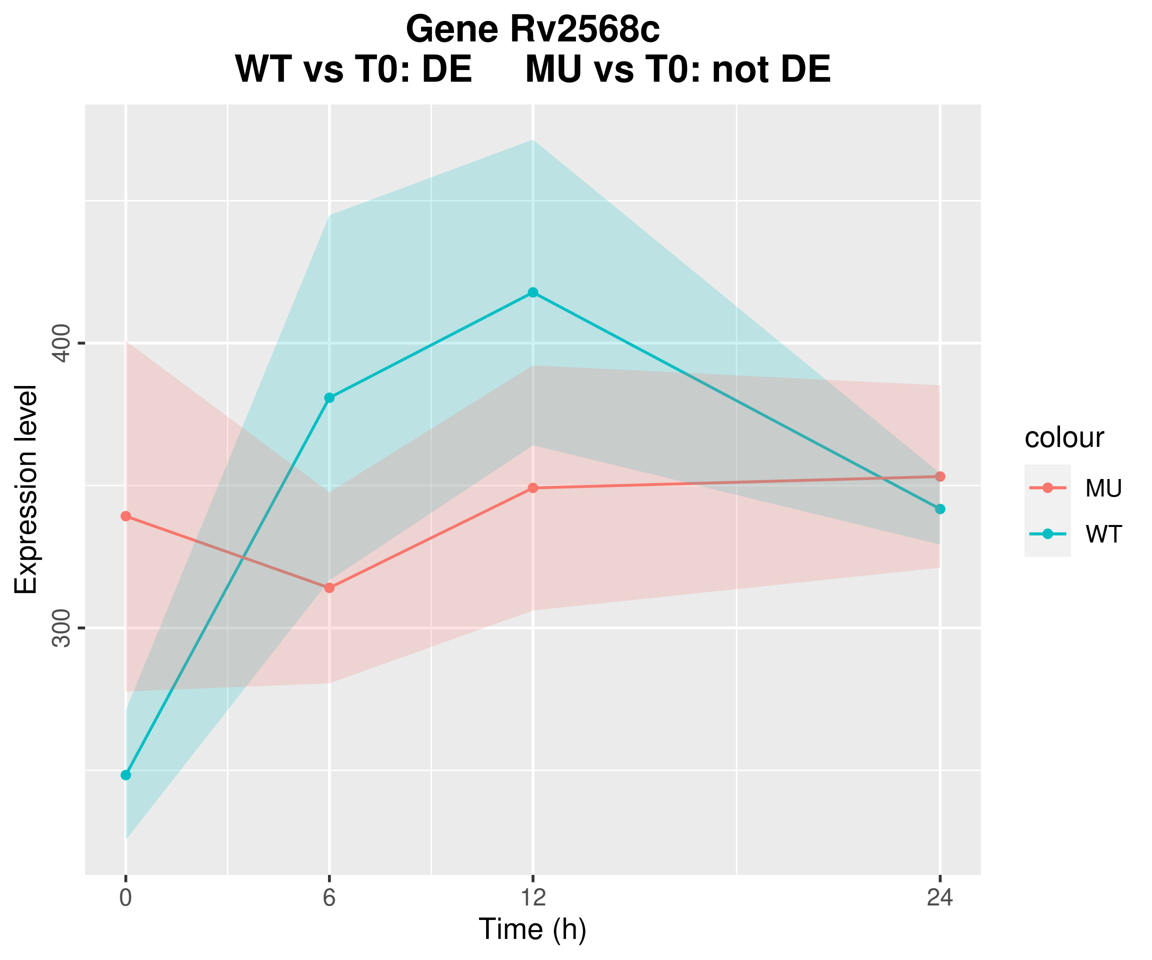

Gene Rv2568c
WT vs T0: DE MU vs T0: not DE
Expression level
400
300
0
6
12
24
Time (h)
colour
MU
WT

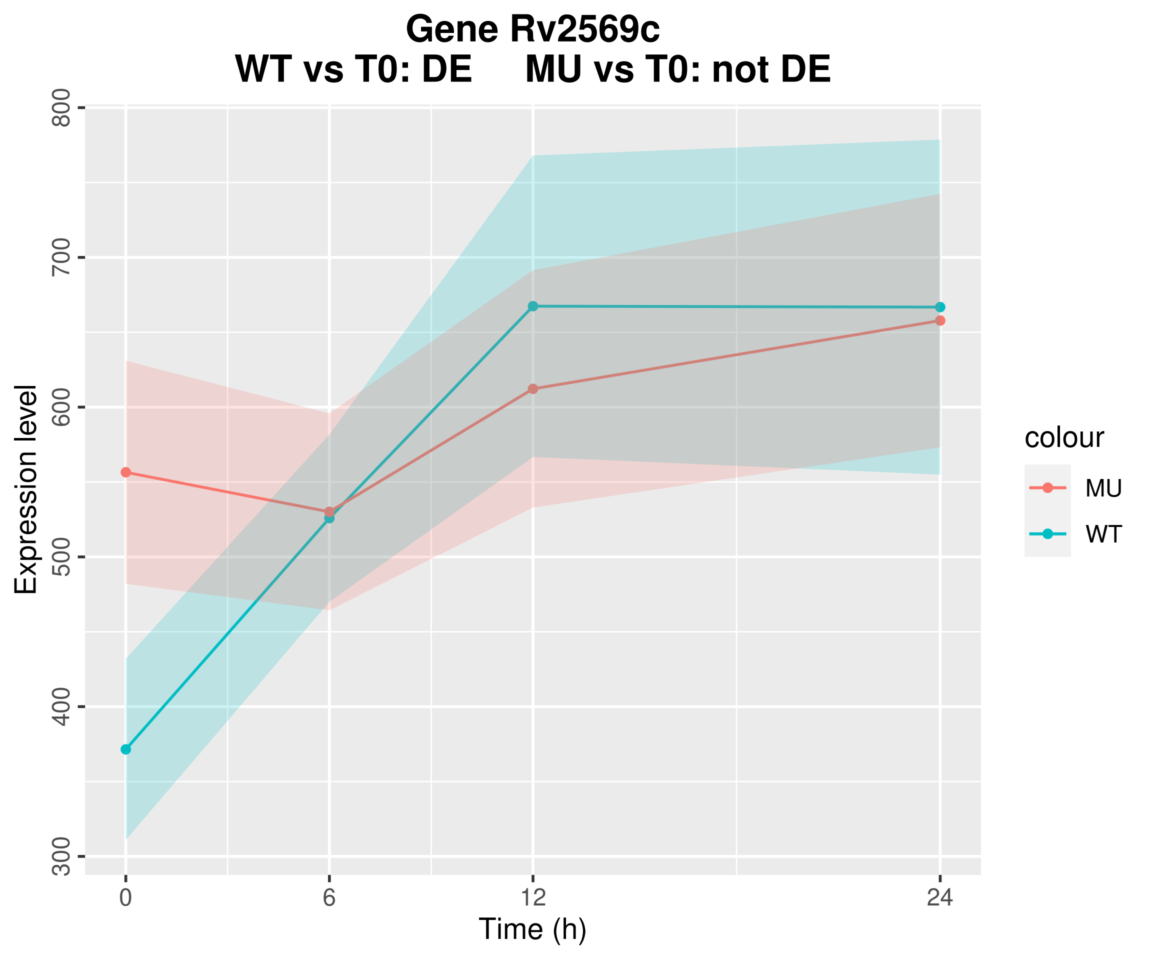
Gene Rv2569c
WT vs T0: DE MU vs T0: not DE
800
700
600
500
400
300
Expression level
0
6
12
24
Time (h)
colour
MU
WT

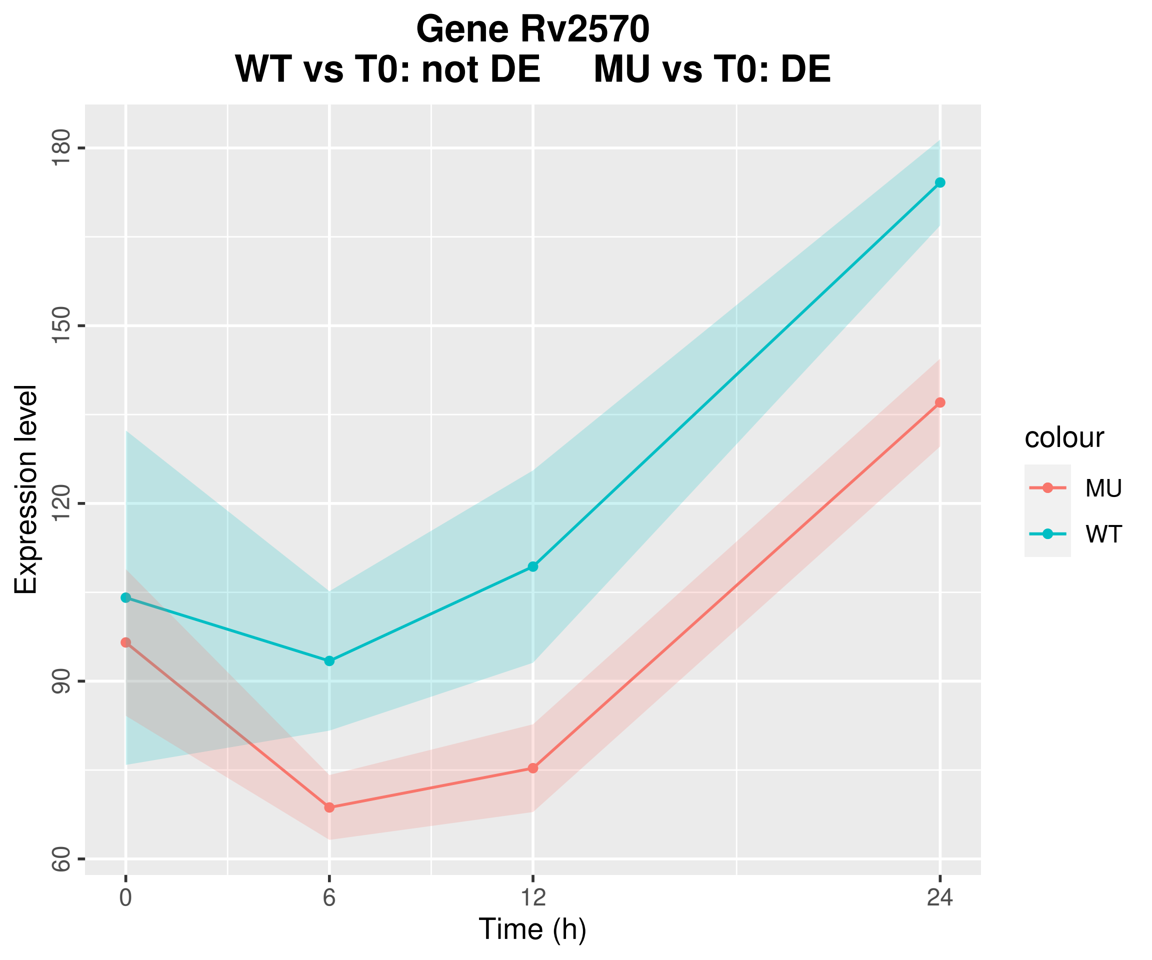
Gene Rv2570
WT vs T0: not DE   MU vs T0: DE
180
150
120
90
60
Expression level
0
6
12
24
Time (h)
colour
MU
WT

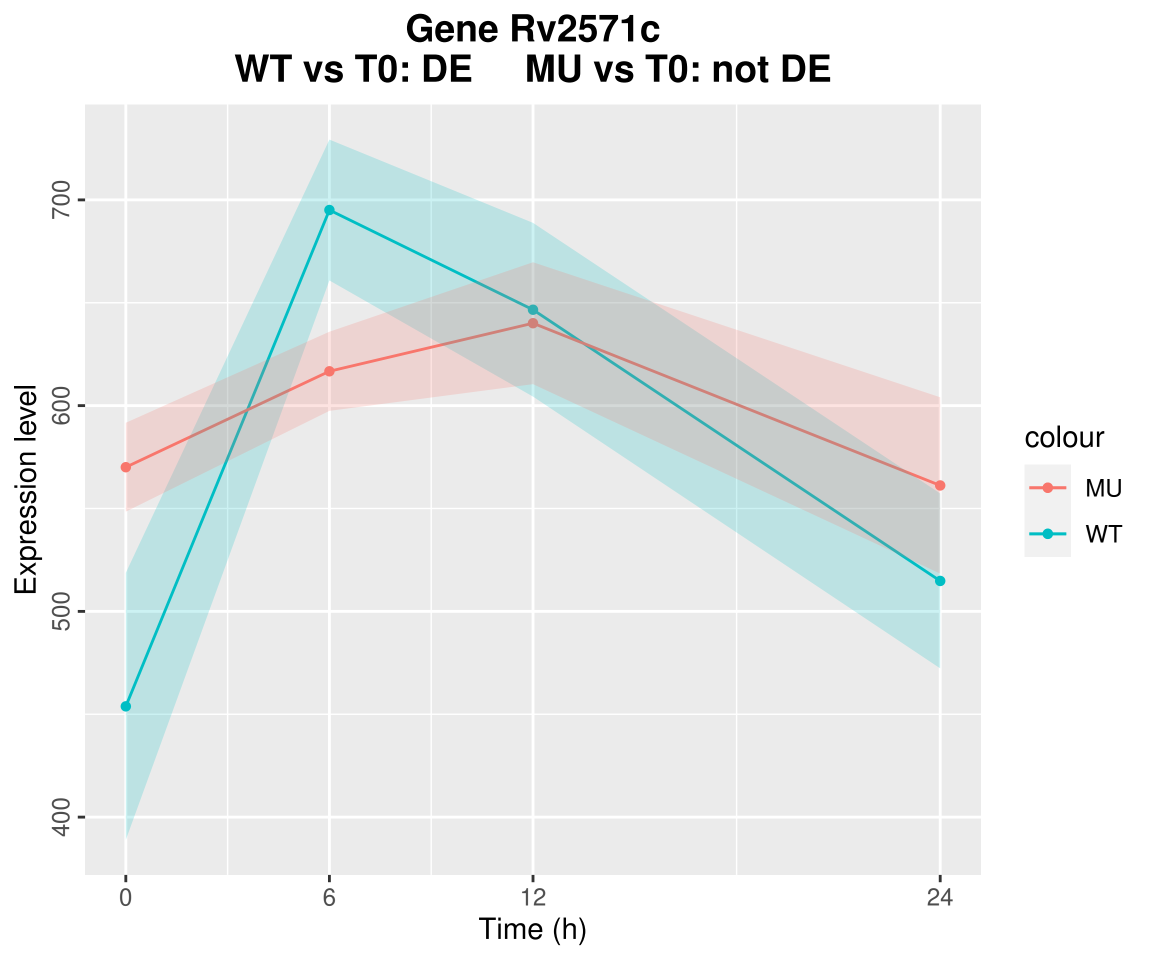
Gene Rv2571c
WT vs T0: DE   MU vs T0: not DE
Expression level
700
600
500
400
0
6
12
24
Time (h)
colour
MU
WT

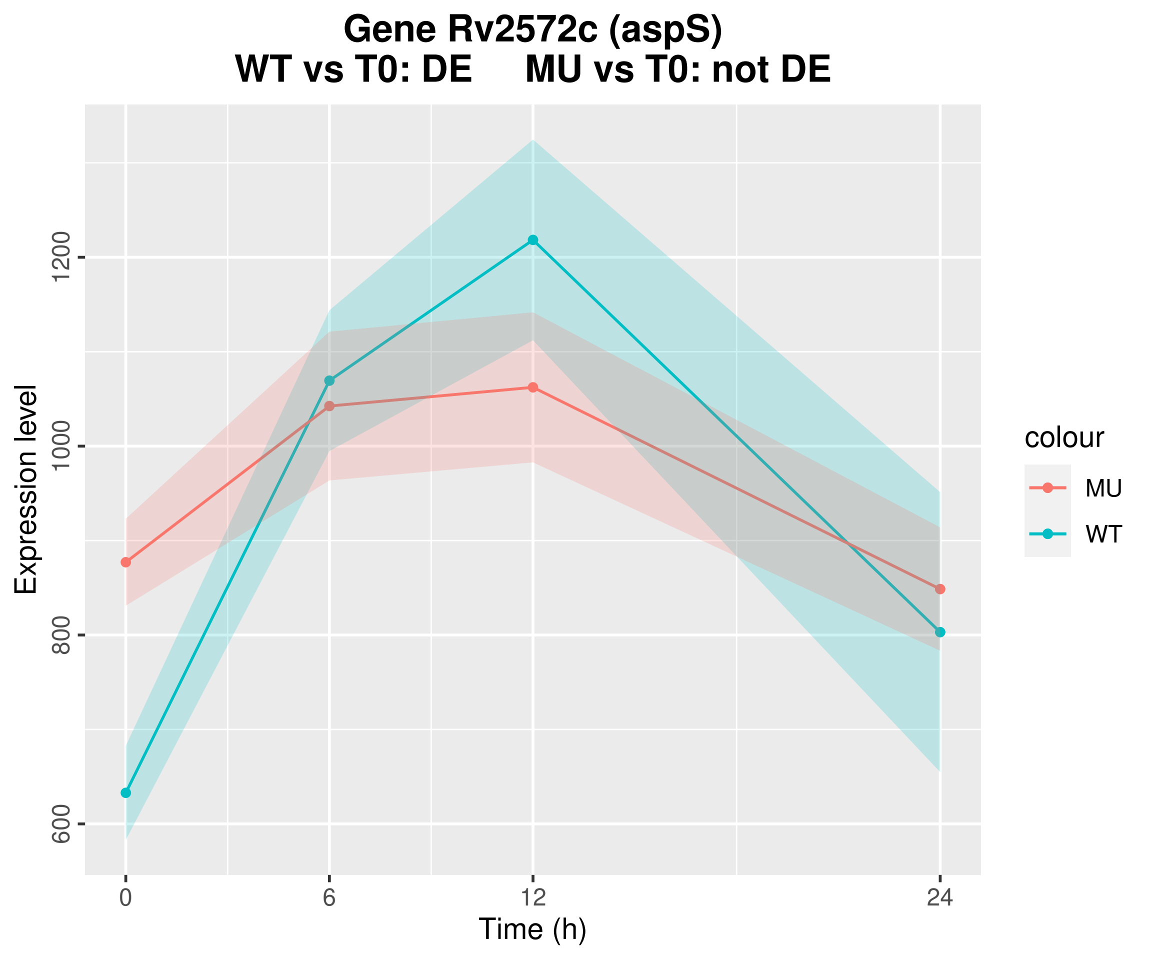
Gene Rv2572c (aspS)
WT vs T0: DE   MU vs T0: not DE
Expression level
600
800
1000
1200
0
6
12
24
Time (h)
colour
MU
WT

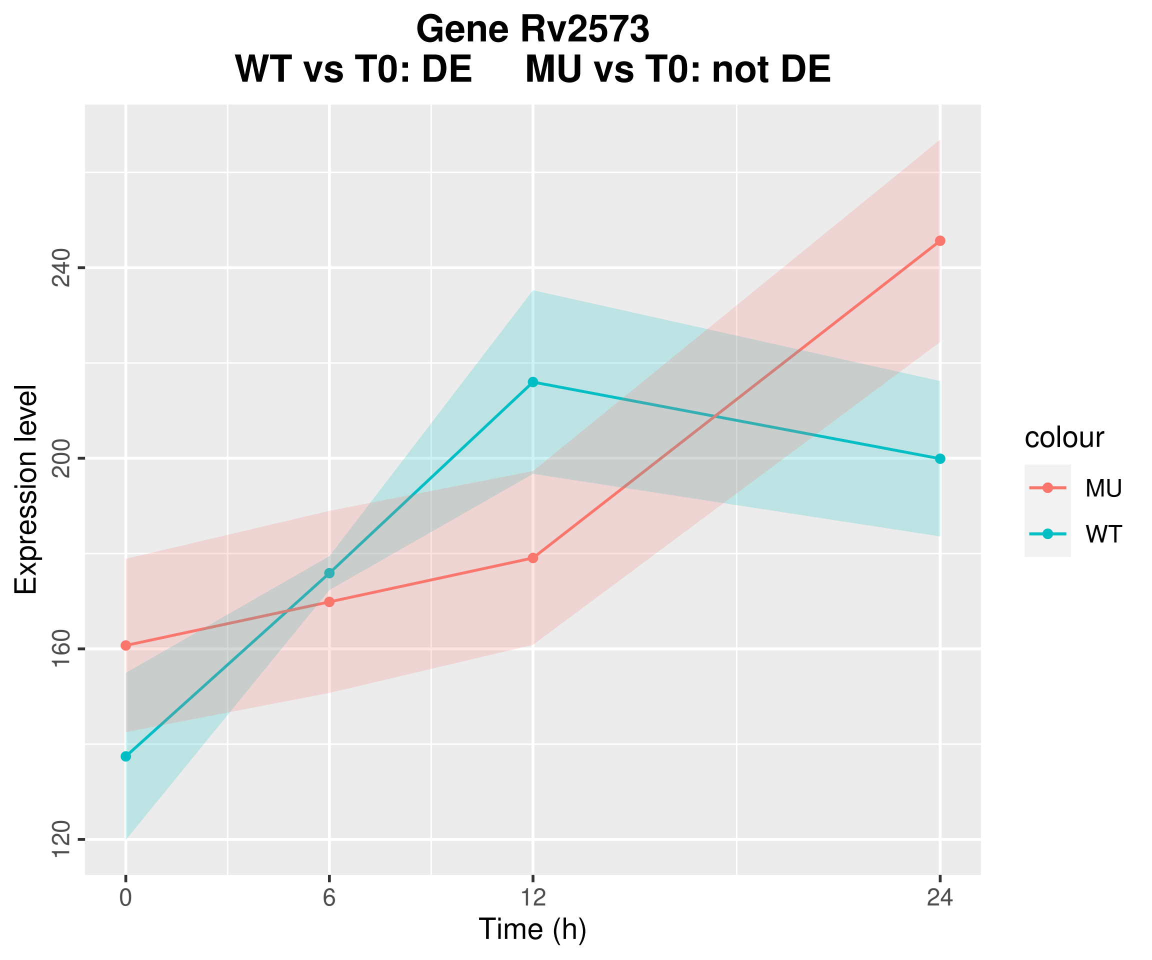
Gene Rv2573
WT vs T0: DE   MU vs T0: not DE
Expression level
120
160
200
240
0
6
12
24
Time (h)
colour
MU
WT

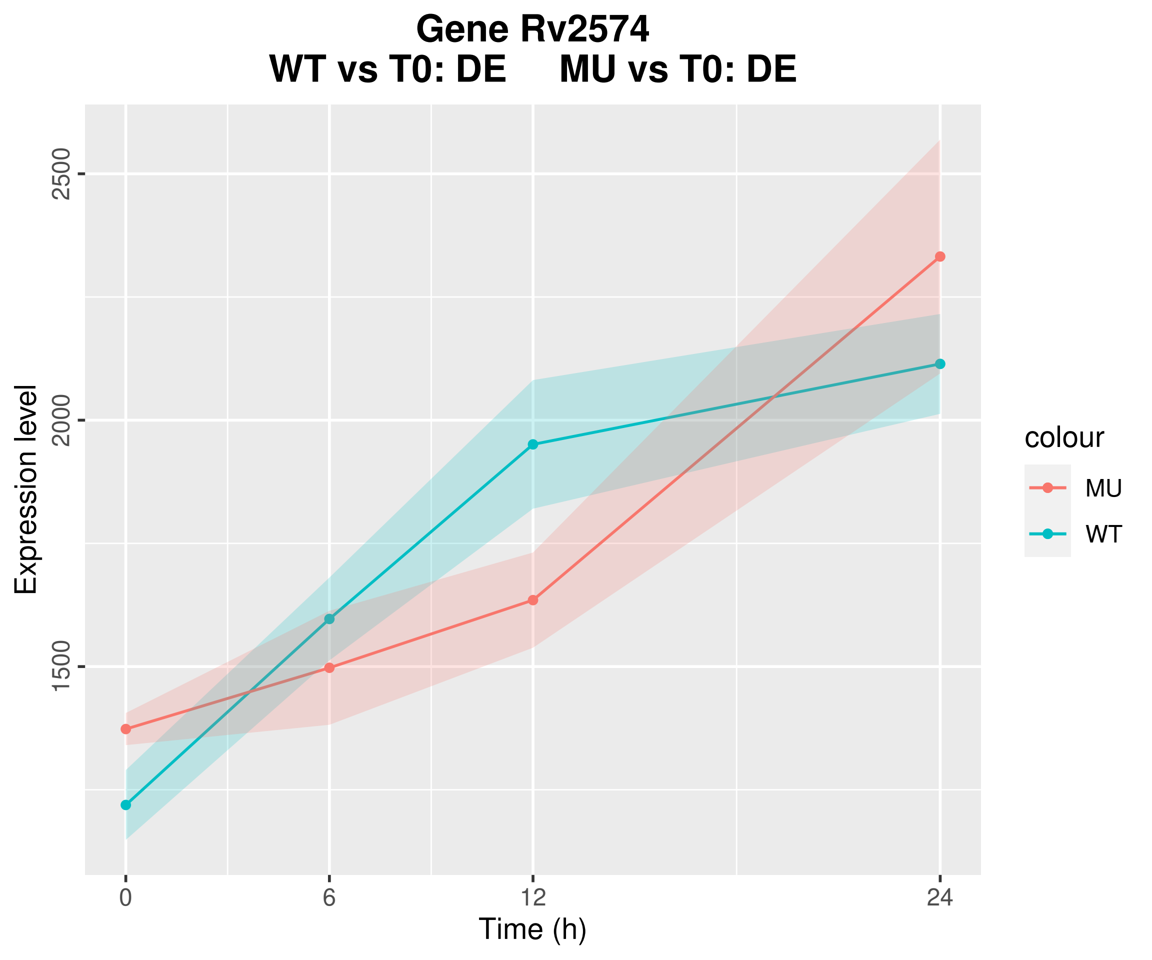

Gene Rv2574
WT vs T0: DE   MU vs T0: DE
Expression level
2500
2000
1500
0
6
12
24
Time (h)
colour
MU
WT

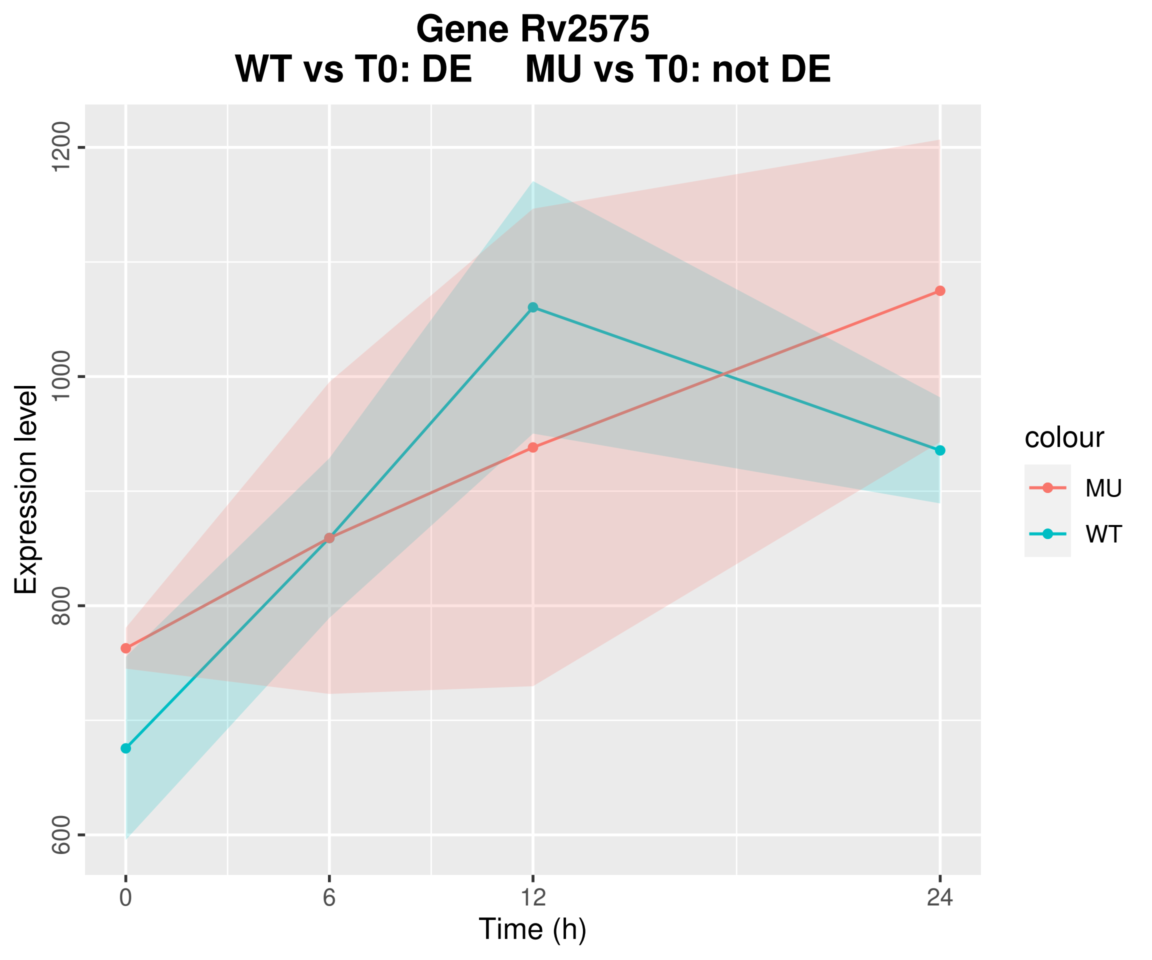

Gene Rv2575
WT vs T0: DE MU vs T0: not DE
Expression level
1200
1000
800
600
0
6
12
24
Time (h)
colour
MU
WT

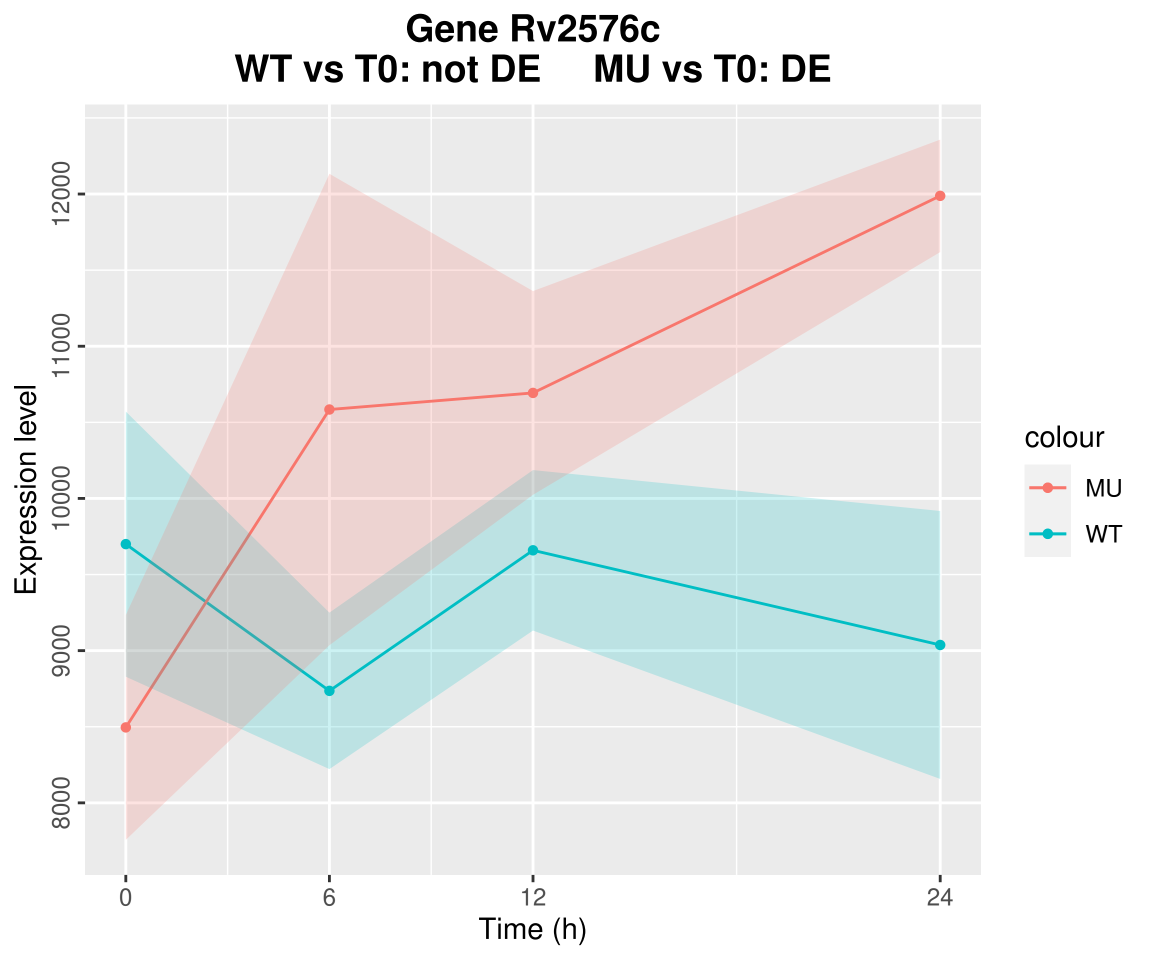
Gene Rv2576c
WT vs T0: not DE   MU vs T0: DE
Expression level
8000
9000
10000
11000
12000
Time (h)
0
6
12
24
colour
MU
WT

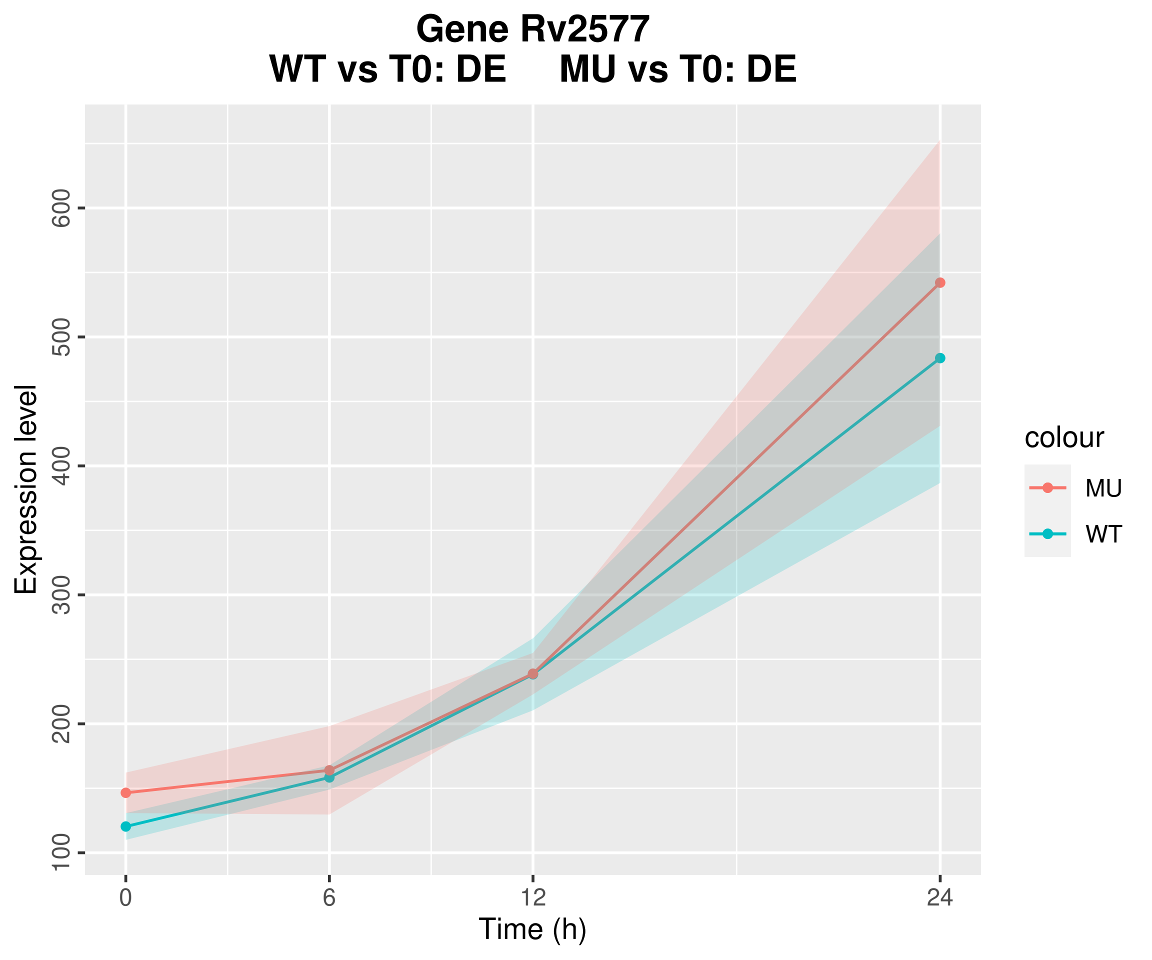
Gene Rv2577
WT vs T0: DE MU vs T0: DE
Expression level
100
200
300
400
500
600
0
6
12
24
Time (h)
colour
MU
WT

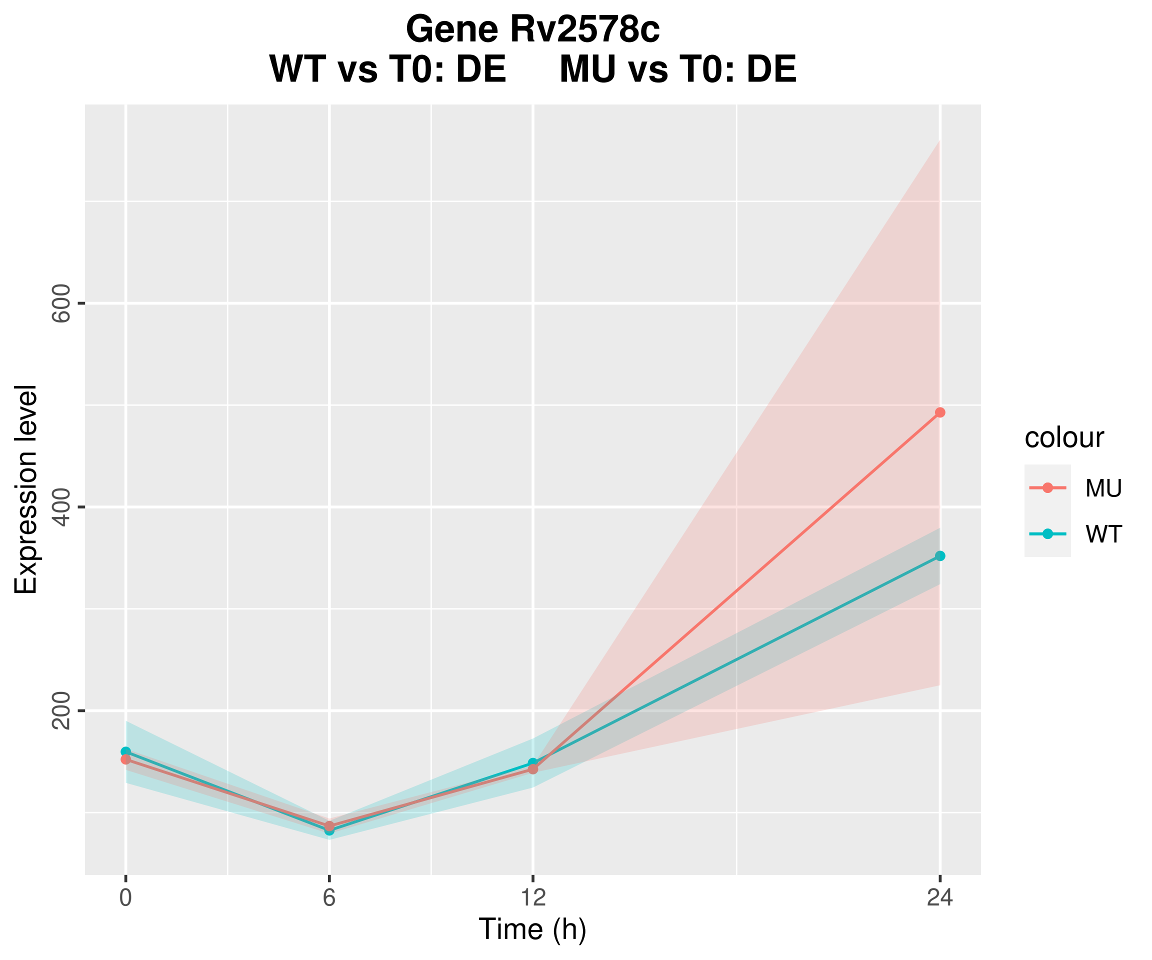

Gene Rv2578c
WT vs T0: DE MU vs T0: DE
Expression level
600
400
200
0
6
12
24
Time (h)
colour
MU
WT

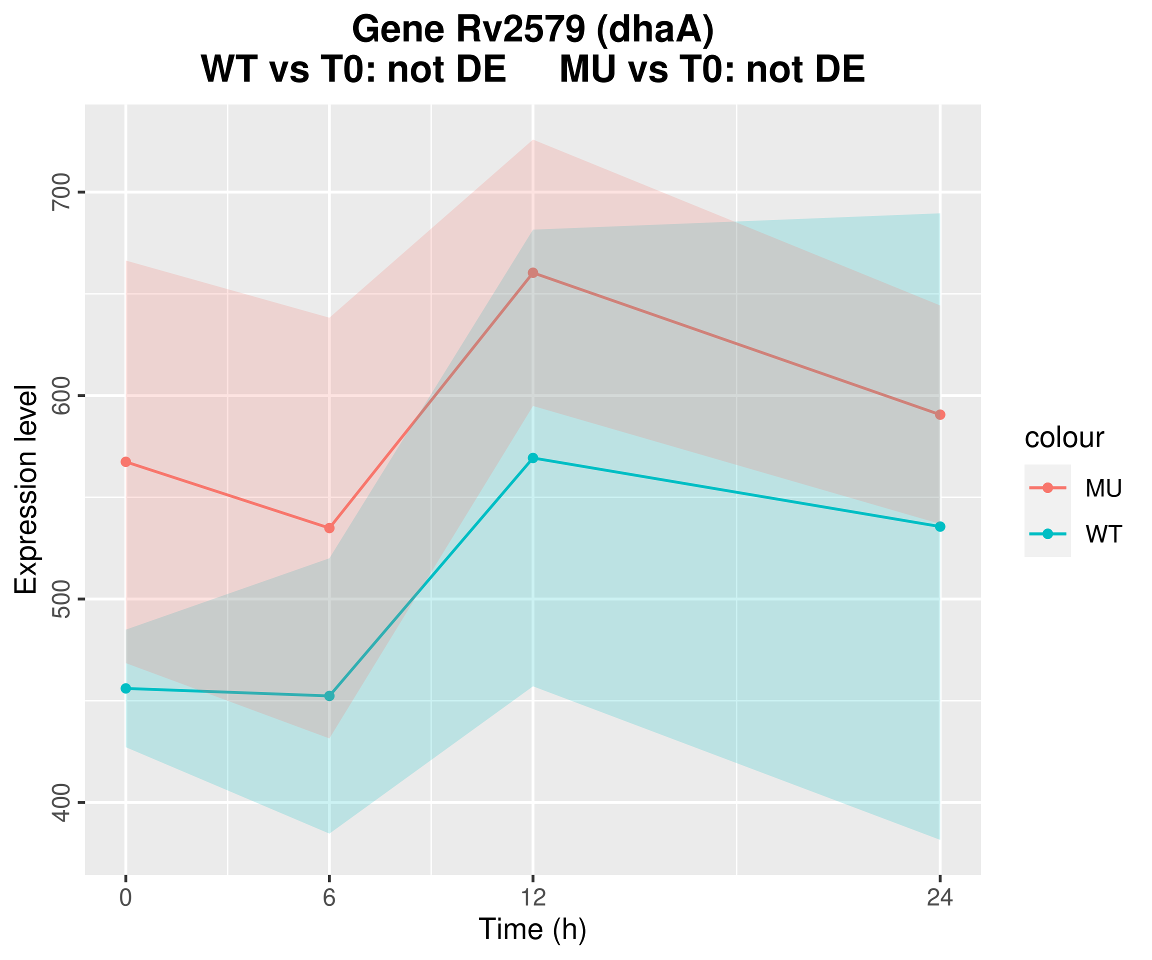

Gene Rv2579 (dhaA)
WT vs T0: not DE   MU vs T0: not DE
Expression level
700
600
500
400
0
6
12
24
Time (h)
colour
MU
WT

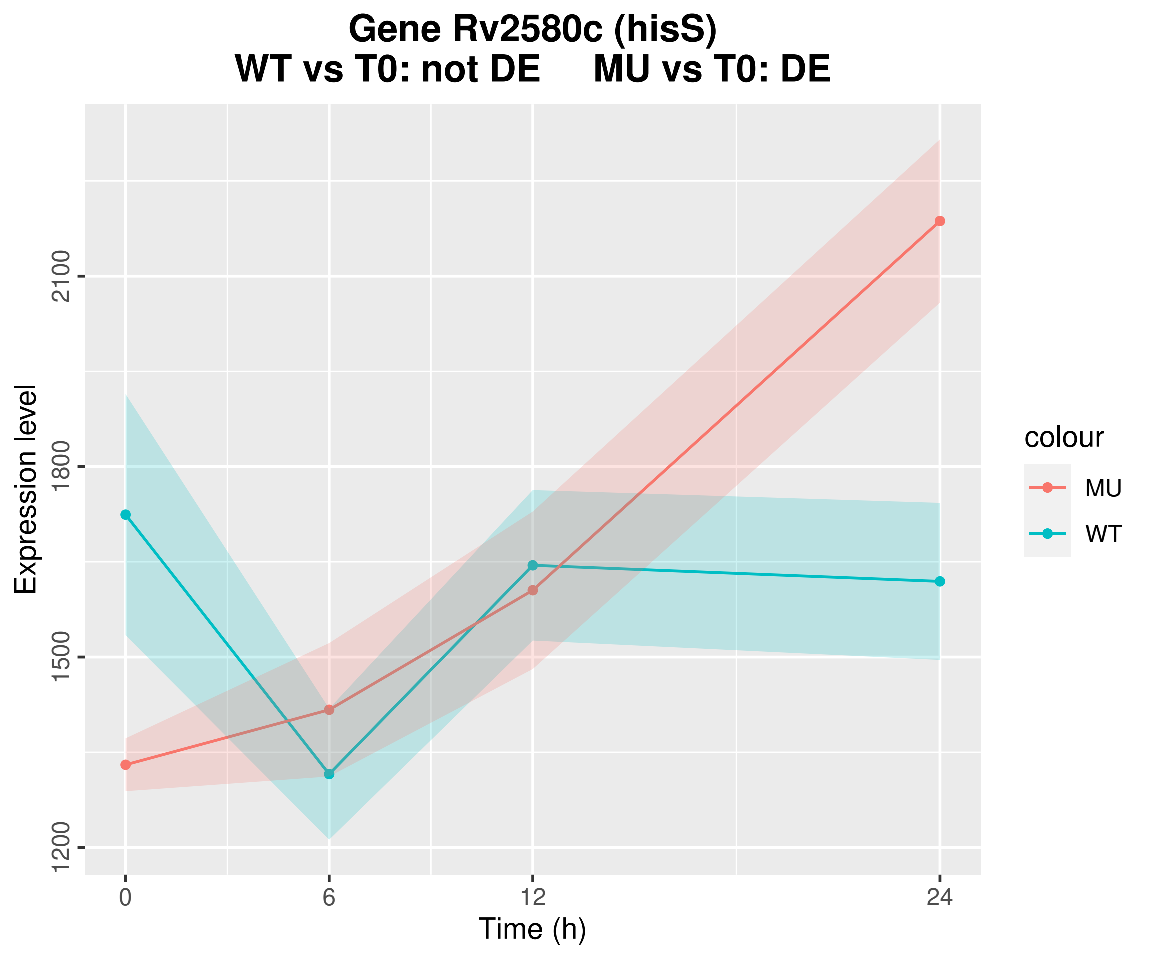

Gene Rv2580c (hisS)
WT vs T0: not DE MU vs T0: DE
Expression level
2100
1800
1500
1200
0
6
12
24
Time (h)
colour
MU
WT

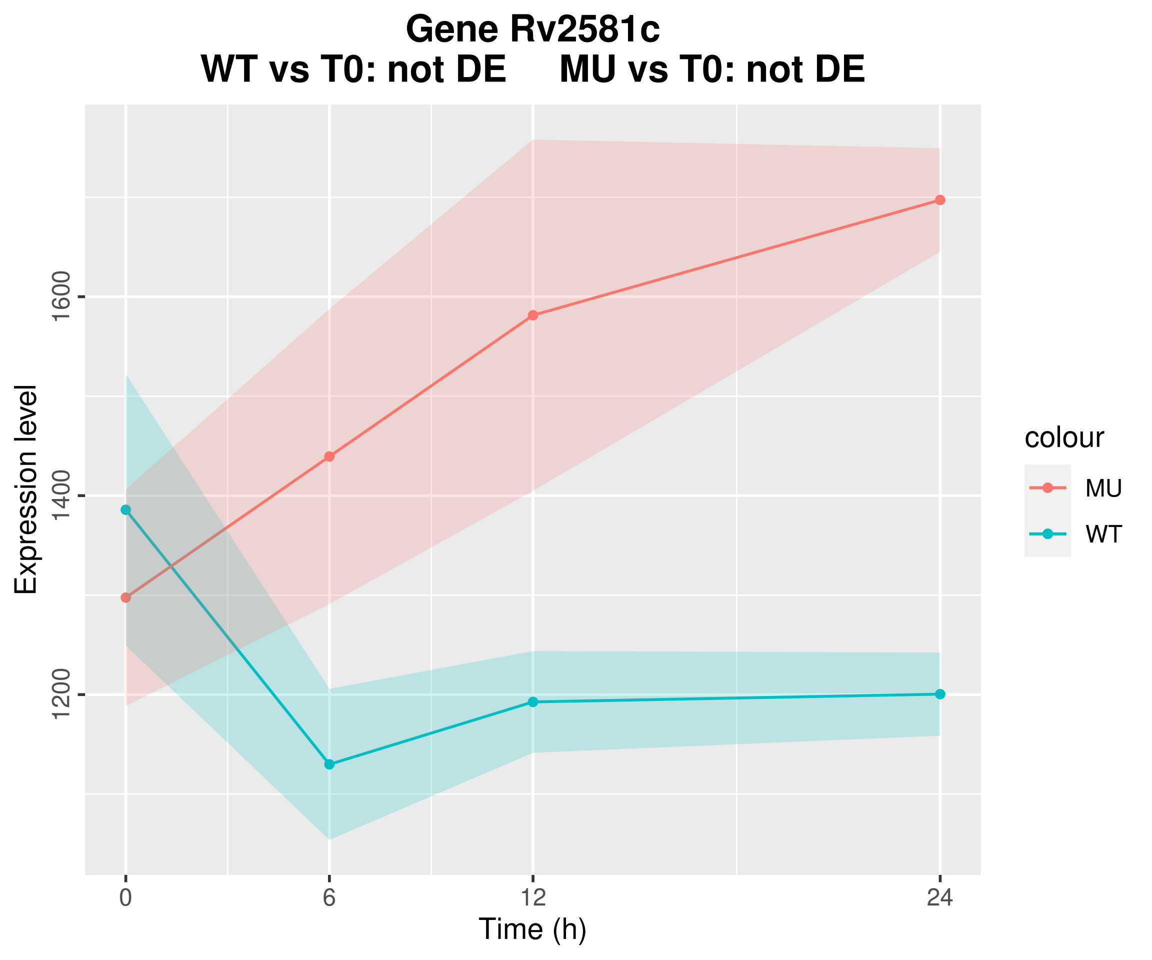

Gene Rv2581c
WT vs T0: not DE MU vs T0: not DE
Expression level
1200
1400
1600
0
6
12
24
Time (h)
colour
MU
WT

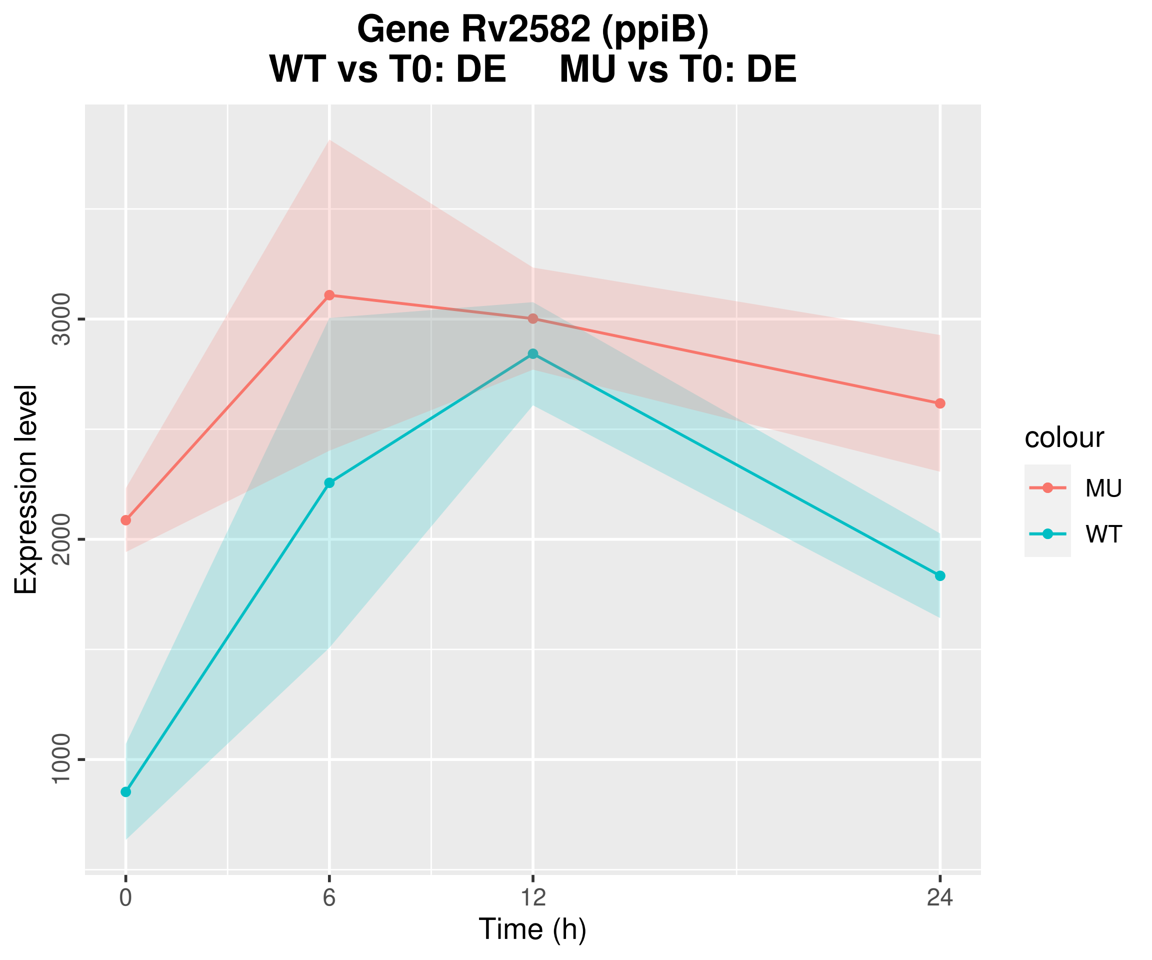
Gene Rv2582 (ppiB)
WT vs T0: DE   MU vs T0: DE
Expression level
1000
2000
3000
0
6
12
24
Time (h)
colour
MU
WT

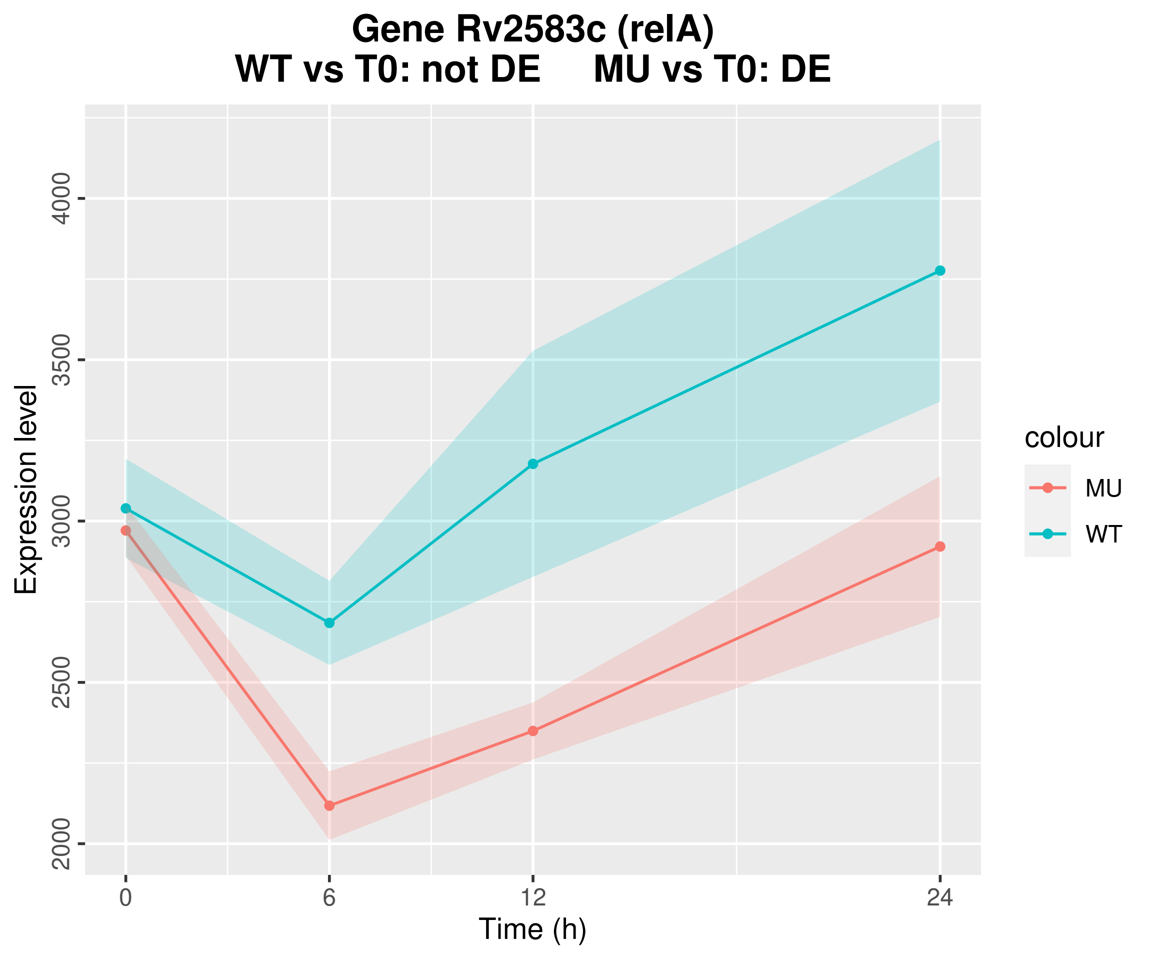
Gene Rv2583c (relA)
WT vs T0: not DE   MU vs T0: DE
Expression level
4000
3500
3000
2500
2000
0
6
12
24
Time (h)
colour
MU
WT

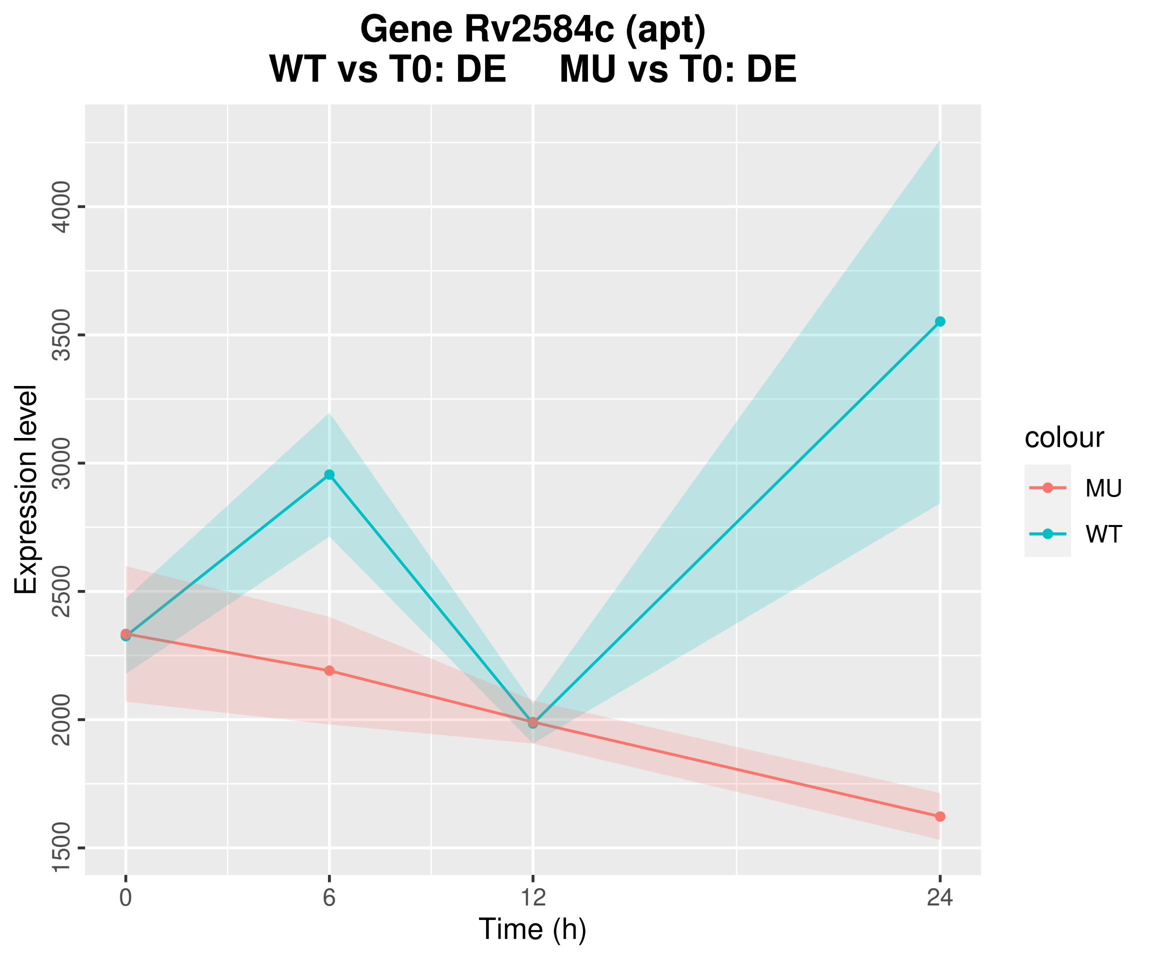
Gene Rv2584c (apt)
WT vs T0: DE MU vs T0: DE
Expression level
4000
3500
3000
2500
2000
1500
0
6
12
24
Time (h)
colour
MU
WT

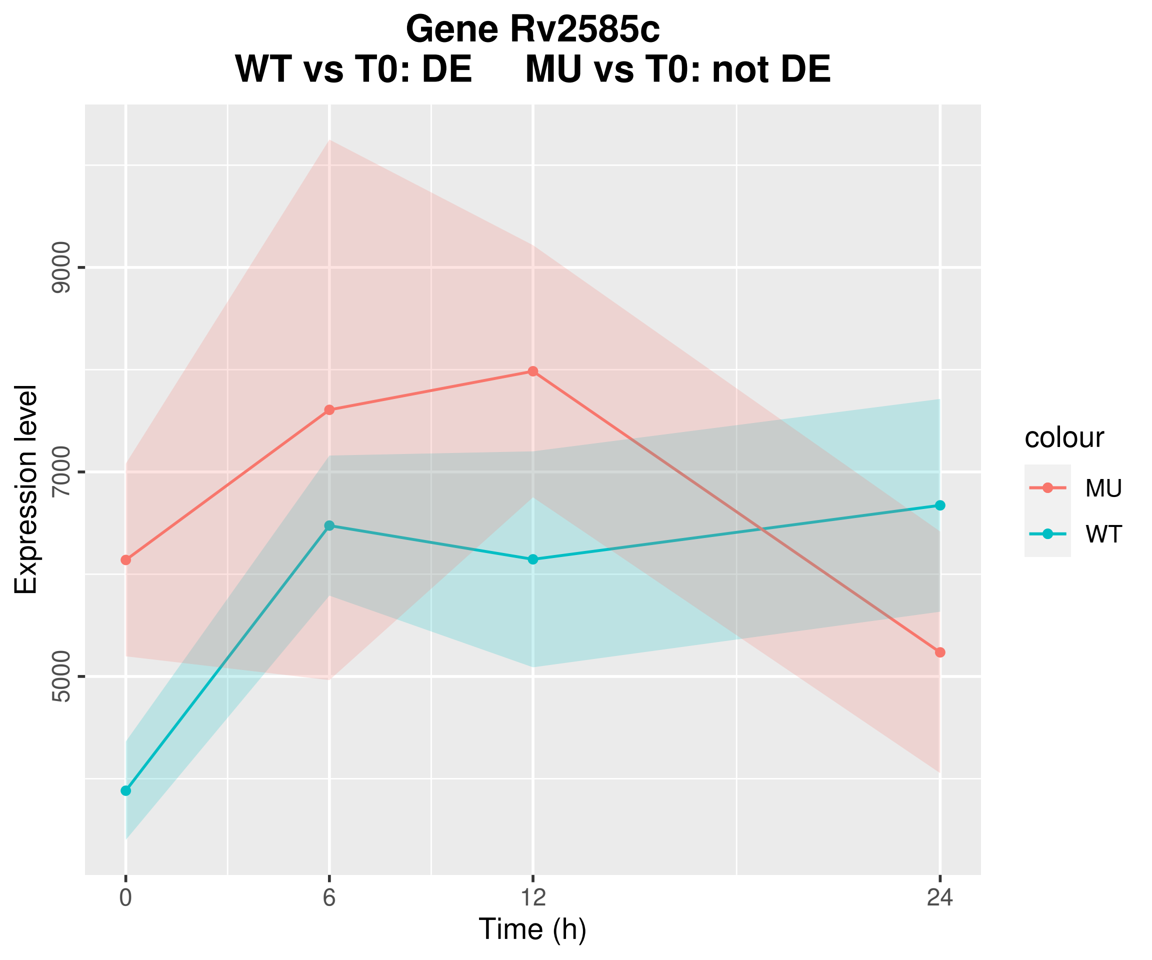

Gene Rv2585c
WT vs T0: DE   MU vs T0: not DE
Expression level
9000
7000
5000
0
6
12
24
Time (h)
colour
MU
WT

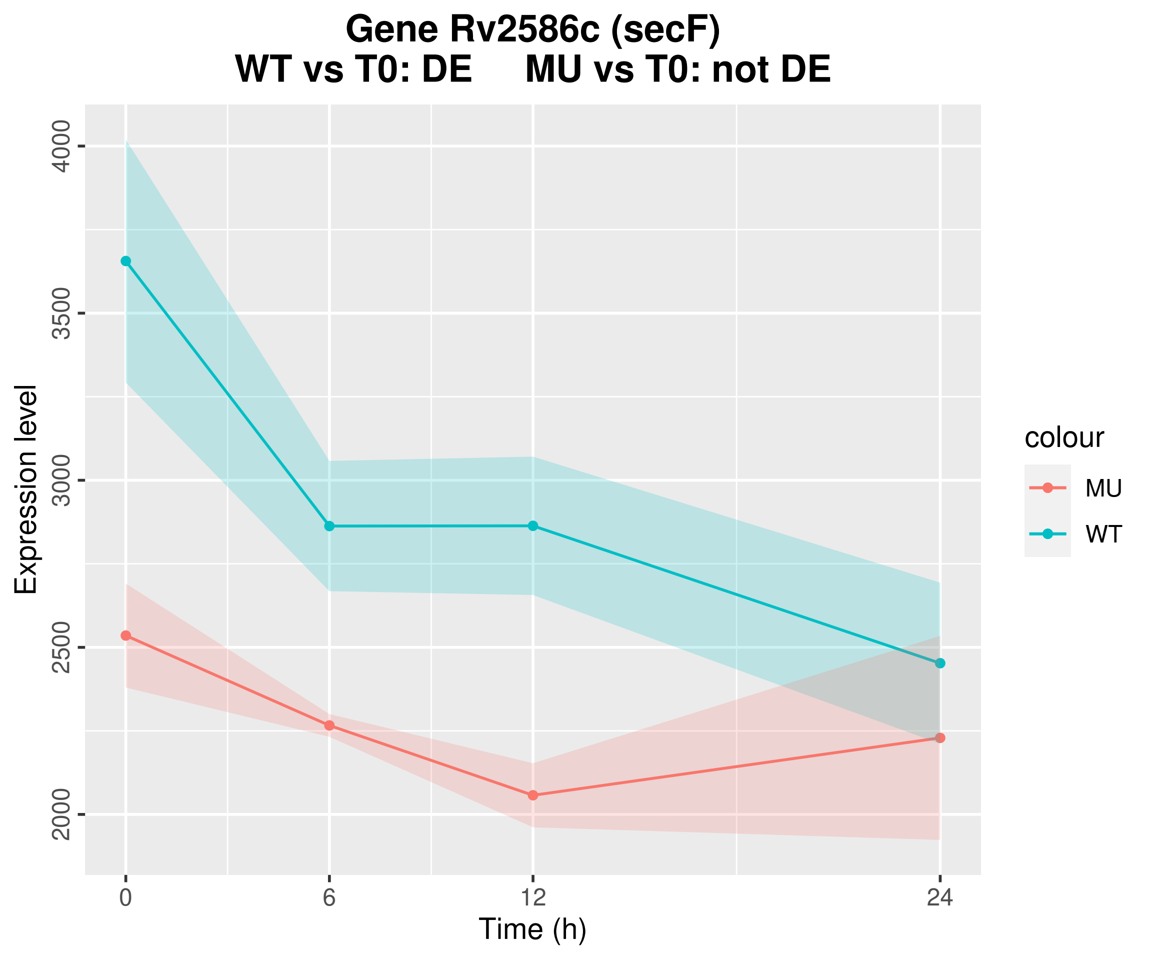
Gene Rv2586c (secF)
WT vs T0: DE MU vs T0: not DE
Expression level
4000
3500
3000
2500
2000
0
6
12
24
Time (h)
colour
MU
WT

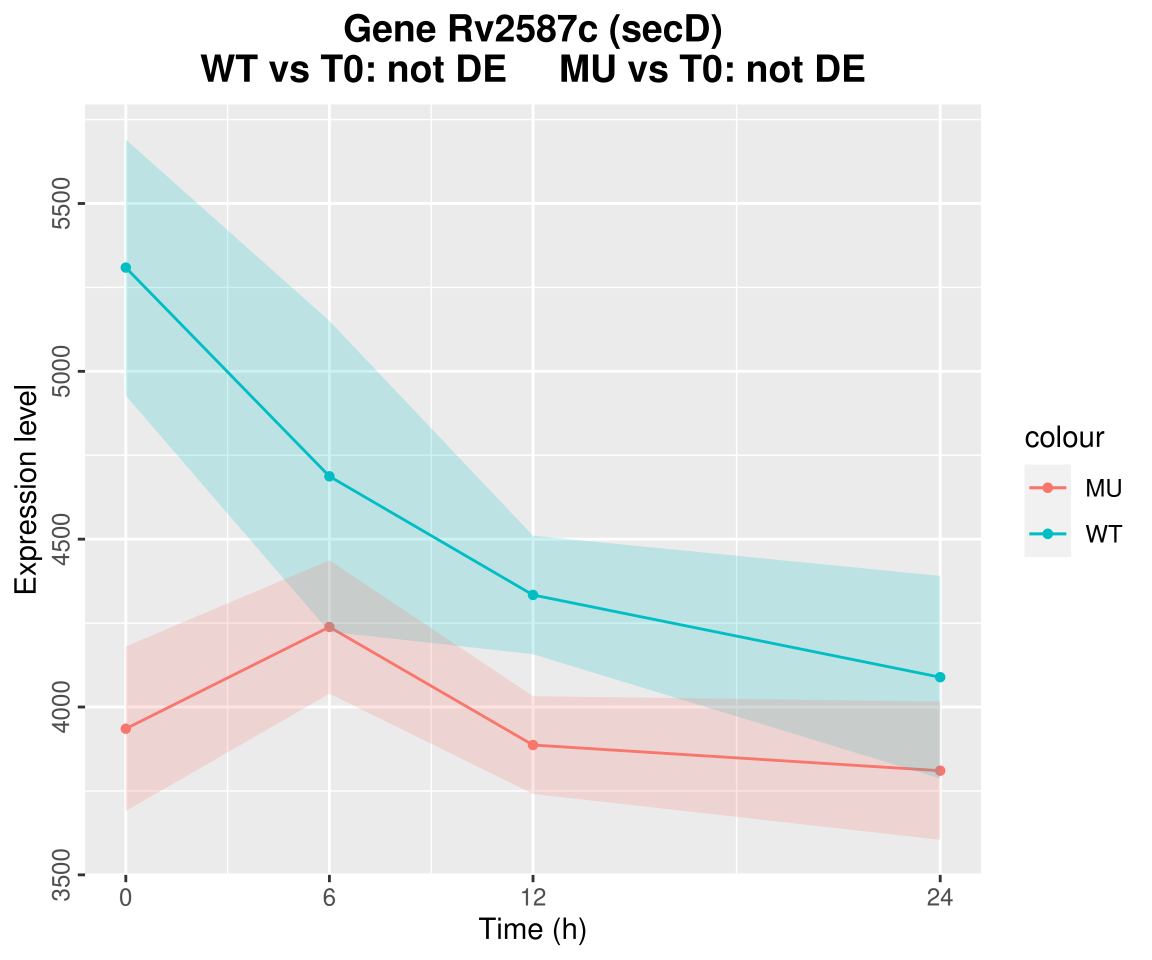
Gene Rv2587c (secD)
WT vs T0: not DE   MU vs T0: not DE
Expression level
5500
5000
4500
4000
3500
0
6
12
24
Time (h)
colour
MU
WT

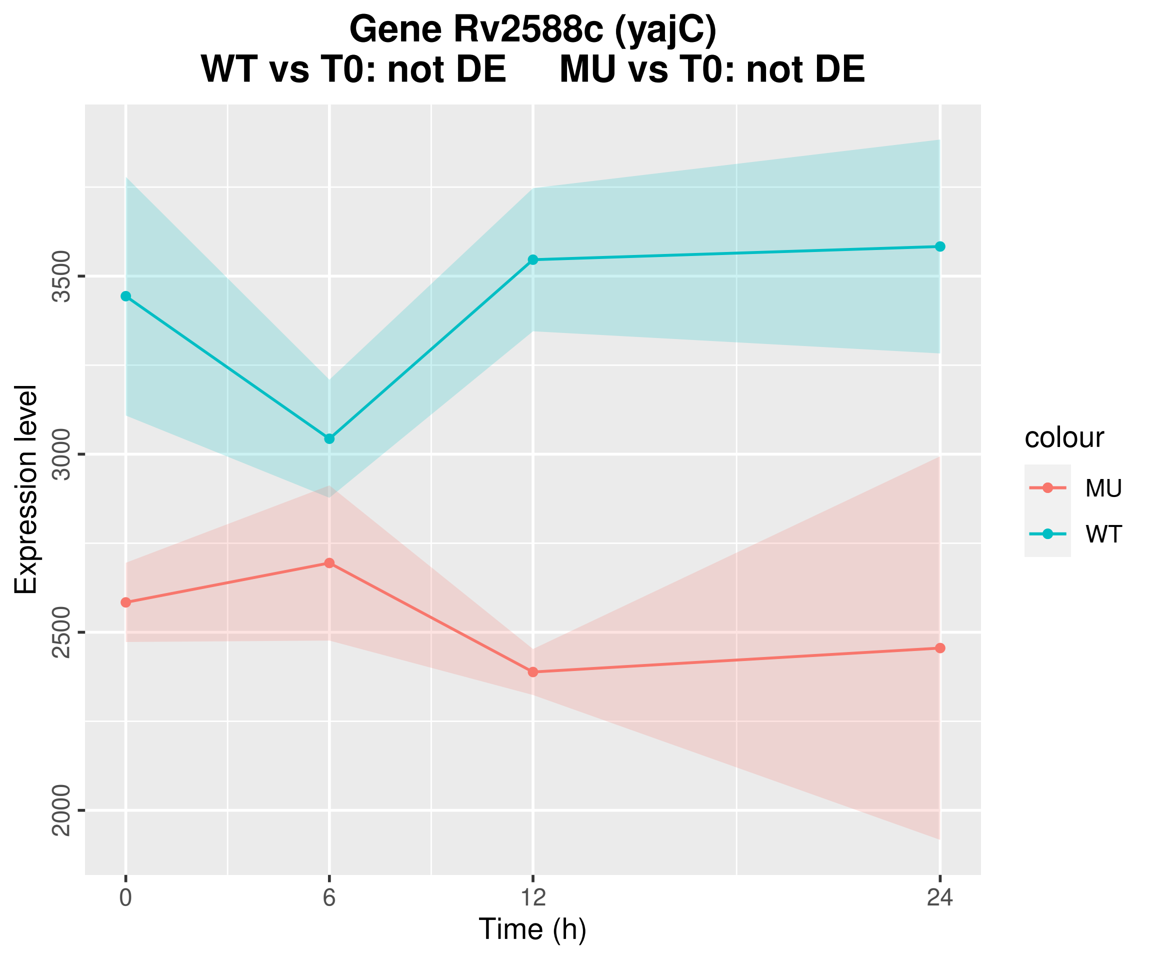

Gene Rv2588c (yajC)
WT vs T0: not DE   MU vs T0: not DE
Expression level
3500
3000
2500
2000
0
6
12
24
Time (h)
colour
MU
WT

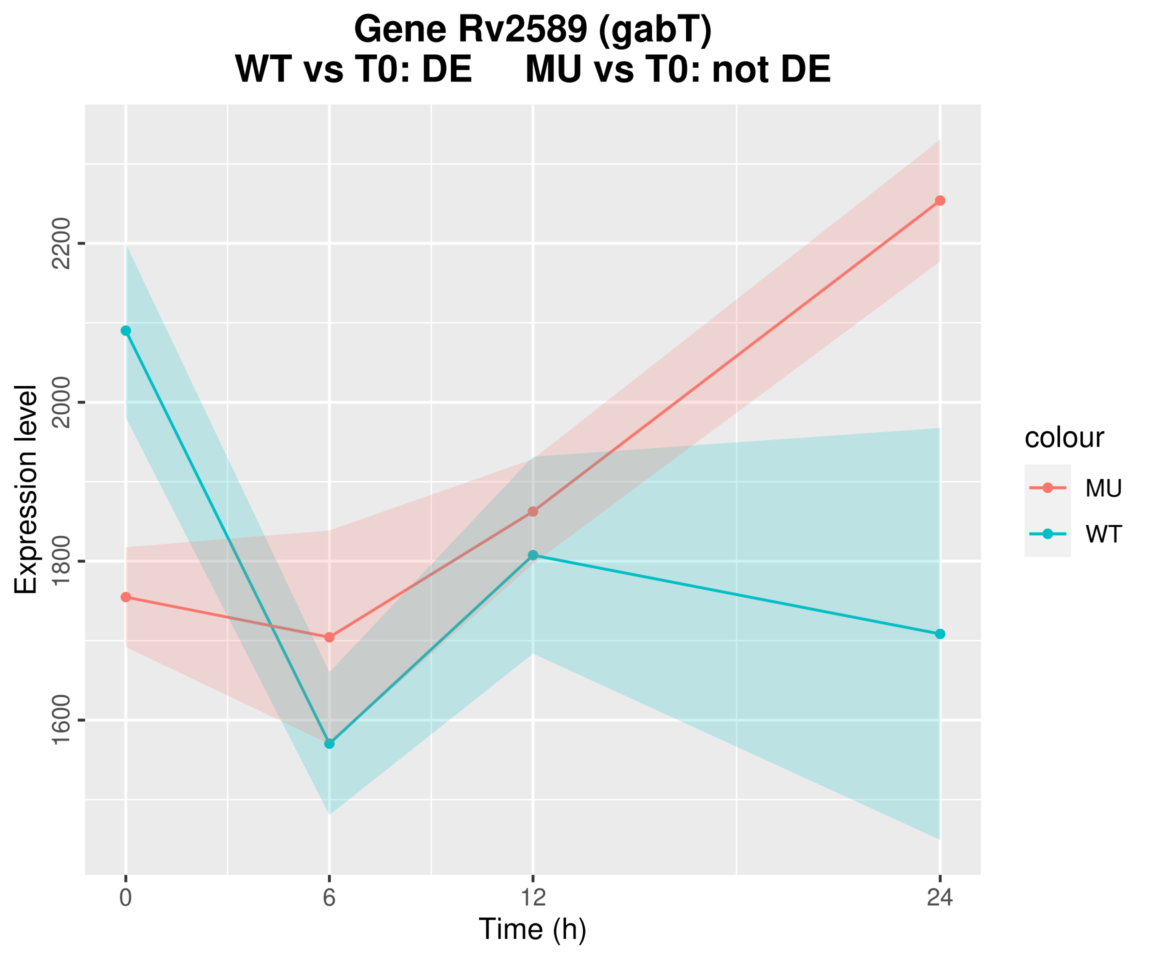

Gene Rv2589 (gabT)
WT vs T0: DE   MU vs T0: not DE
Expression level
2200
2000
1800
1600
0
6
12
24
Time (h)
colour
MU
WT

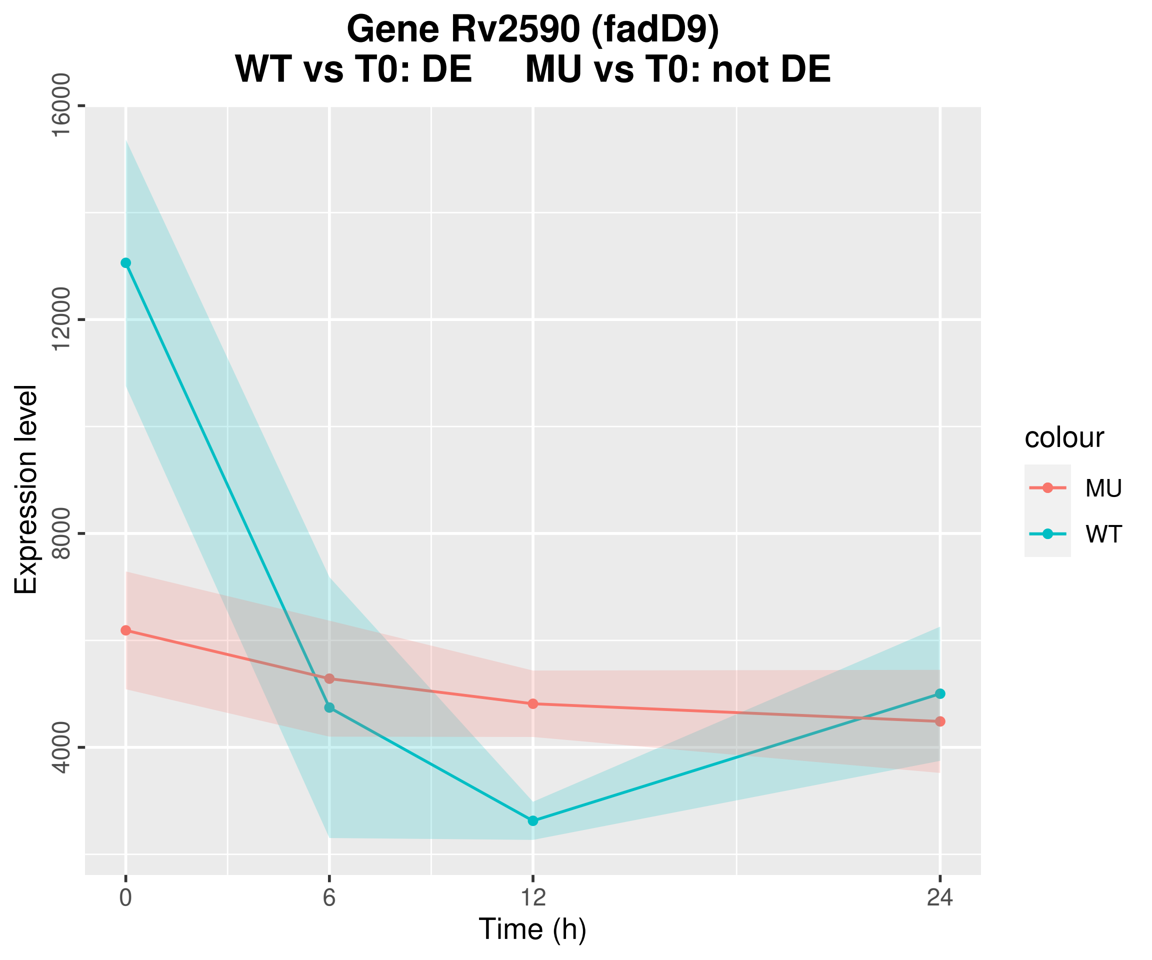

Gene Rv2590 (fadD9)
WT vs T0: DE MU vs T0: not DE
Expression level
16000
12000
8000
4000
0
6
12
24
Time (h)
colour
MU
WT

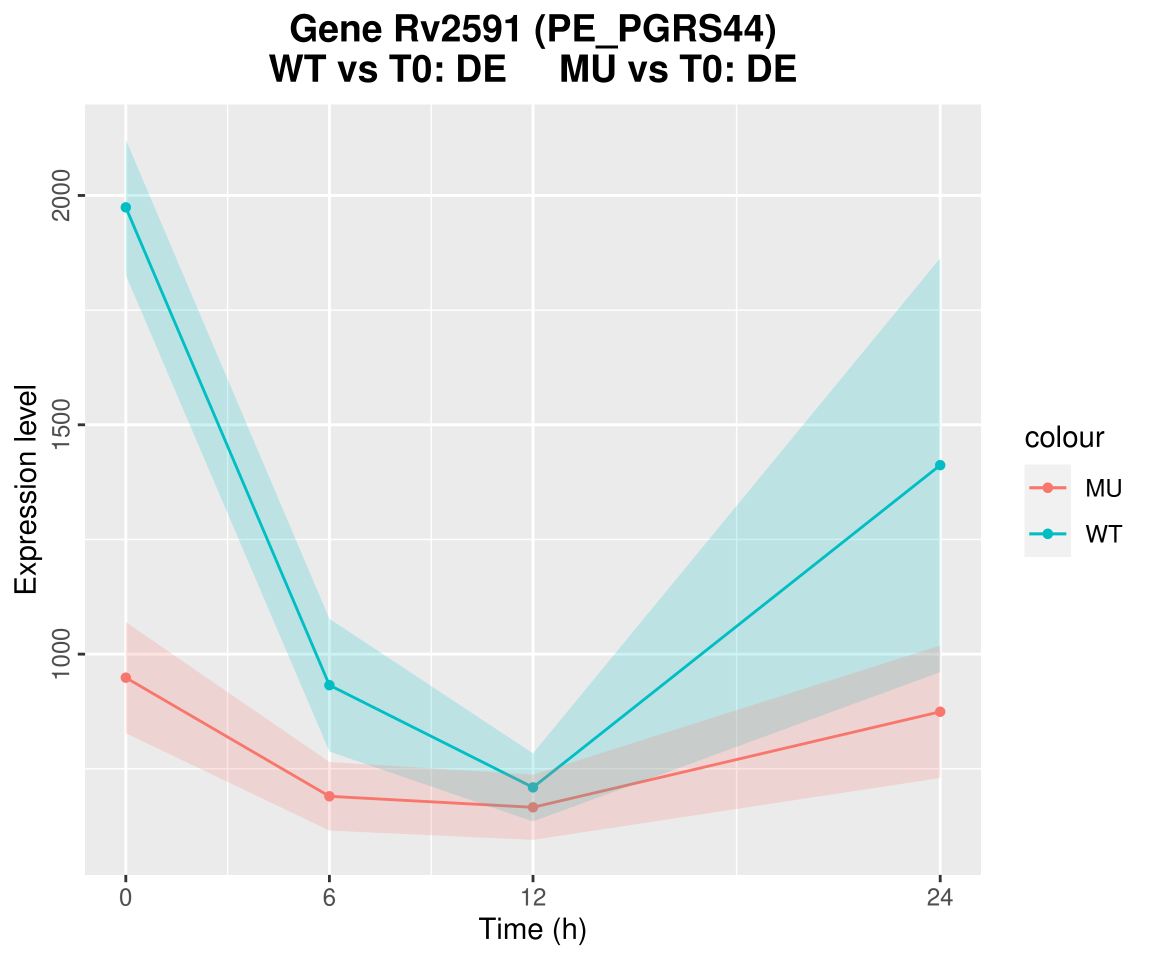
Gene Rv2591 (PE_PGRS44)
WT vs T0: DE   MU vs T0: DE
Expression level
2000
1500
1000
0
6
12
24
Time (h)
colour
MU
WT

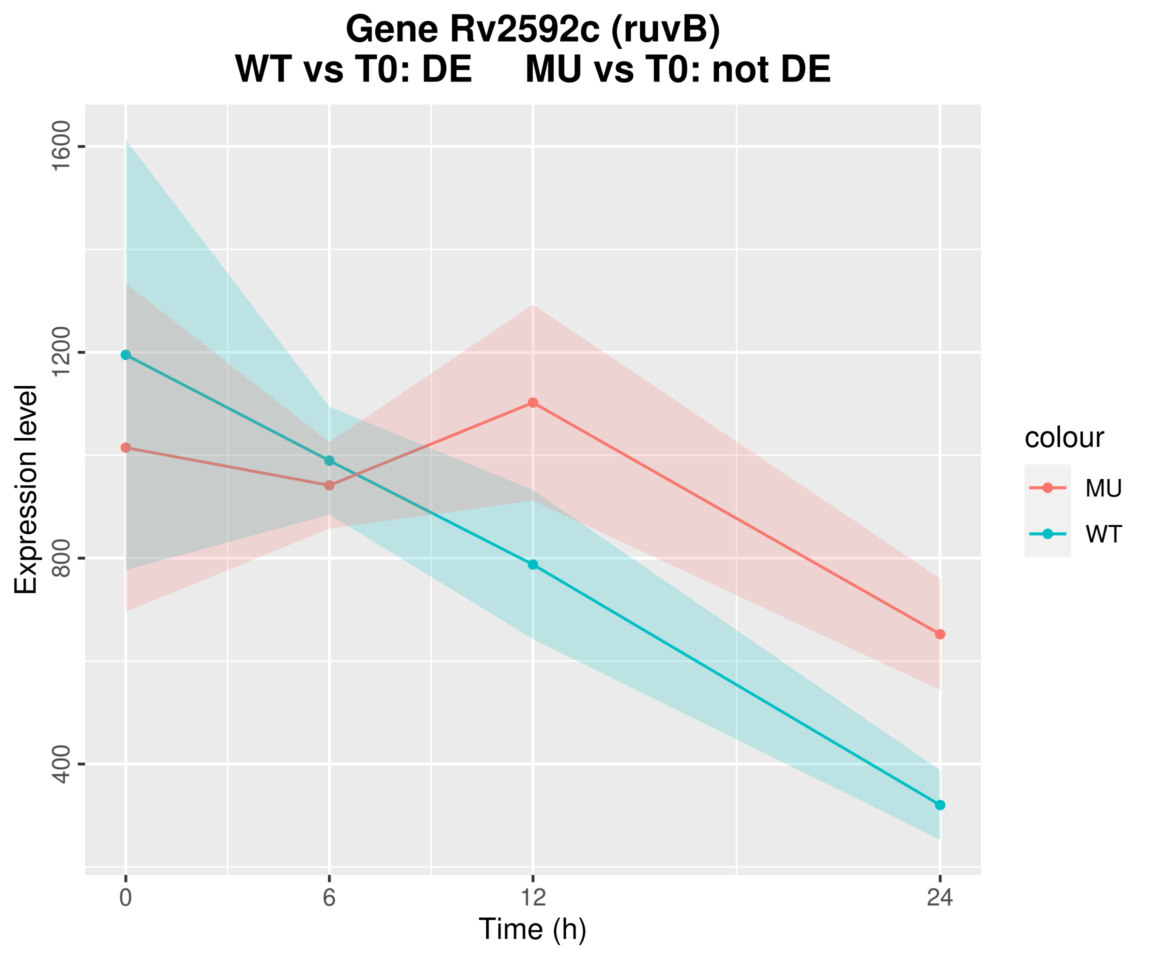
Gene Rv2592c (ruvB)
WT vs T0: DE   MU vs T0: not DE
Expression level
1600
1200
800
400
Time (h)
0
6
12
24
colour
MU
WT

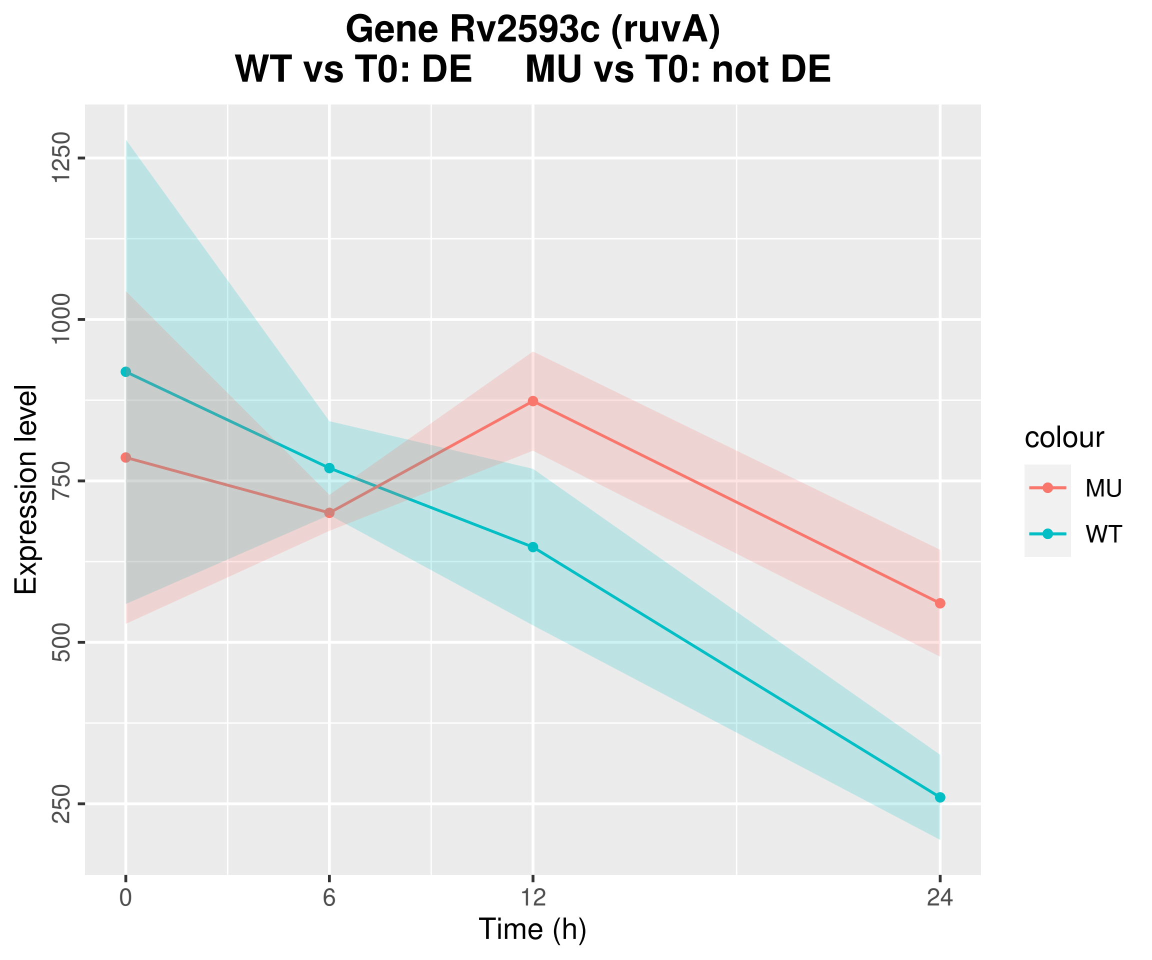

Gene Rv2593c (ruvA)
WT vs T0: DE   MU vs T0: not DE
Expression level
1250
1000
750
500
250
0
6
12
24
Time (h)
colour
MU
WT

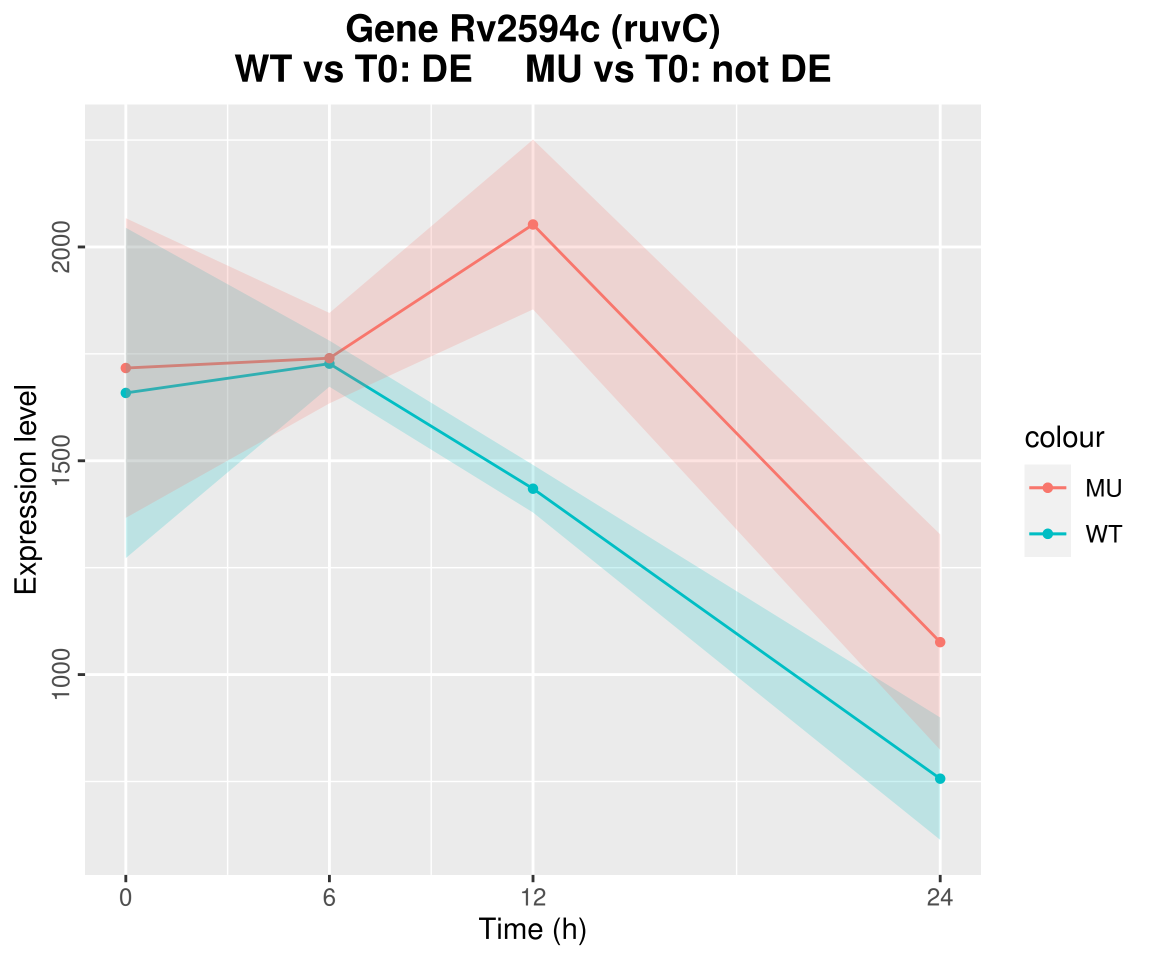

Gene Rv2594c (ruvC)
WT vs T0: DE   MU vs T0: not DE
Expression level
2000
1500
1000
0
6
12
24
Time (h)
colour
MU
WT

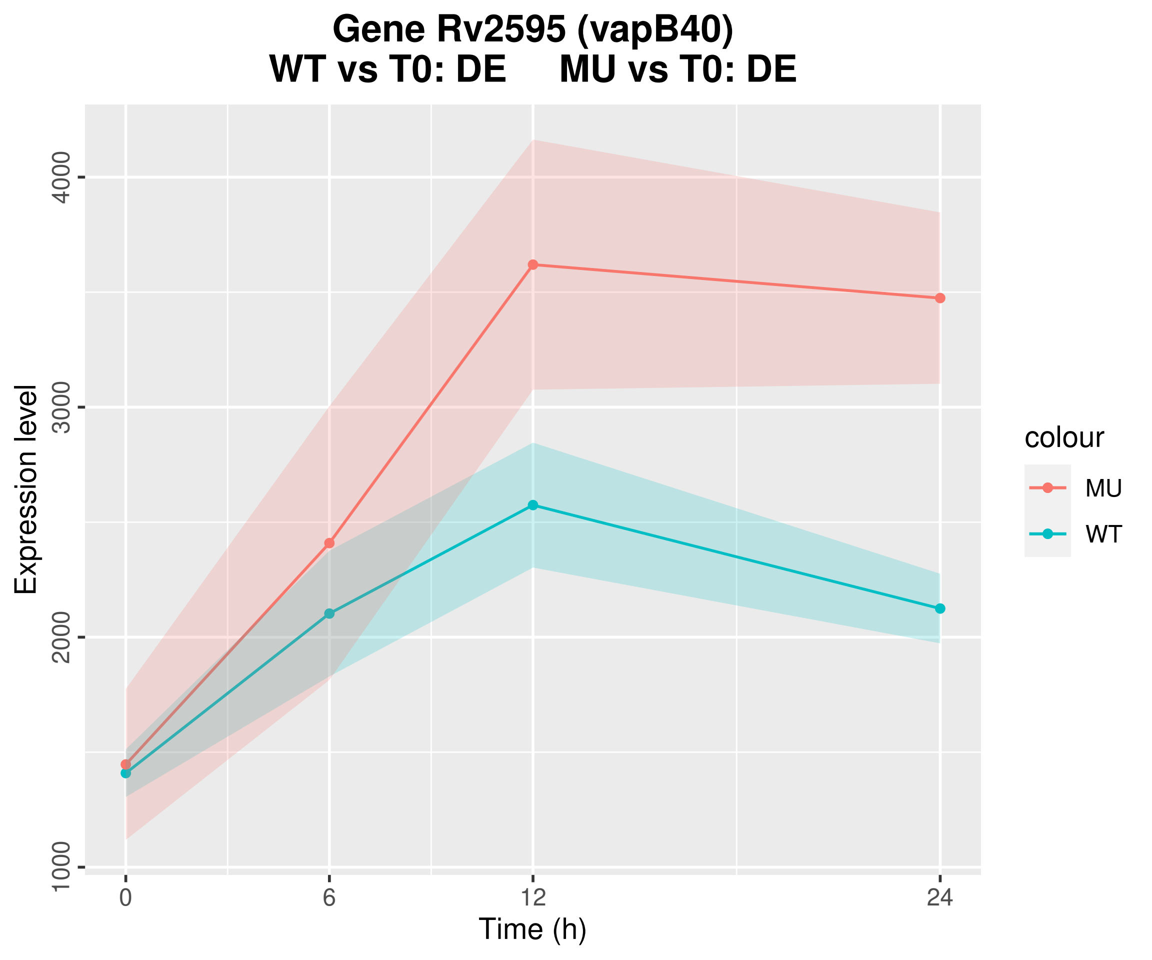

Gene Rv2595 (vapB40)
WT vs T0: DE MU vs T0: DE
Expression level
1000
2000
3000
4000
0
6
12
24
Time (h)
colour
MU
WT

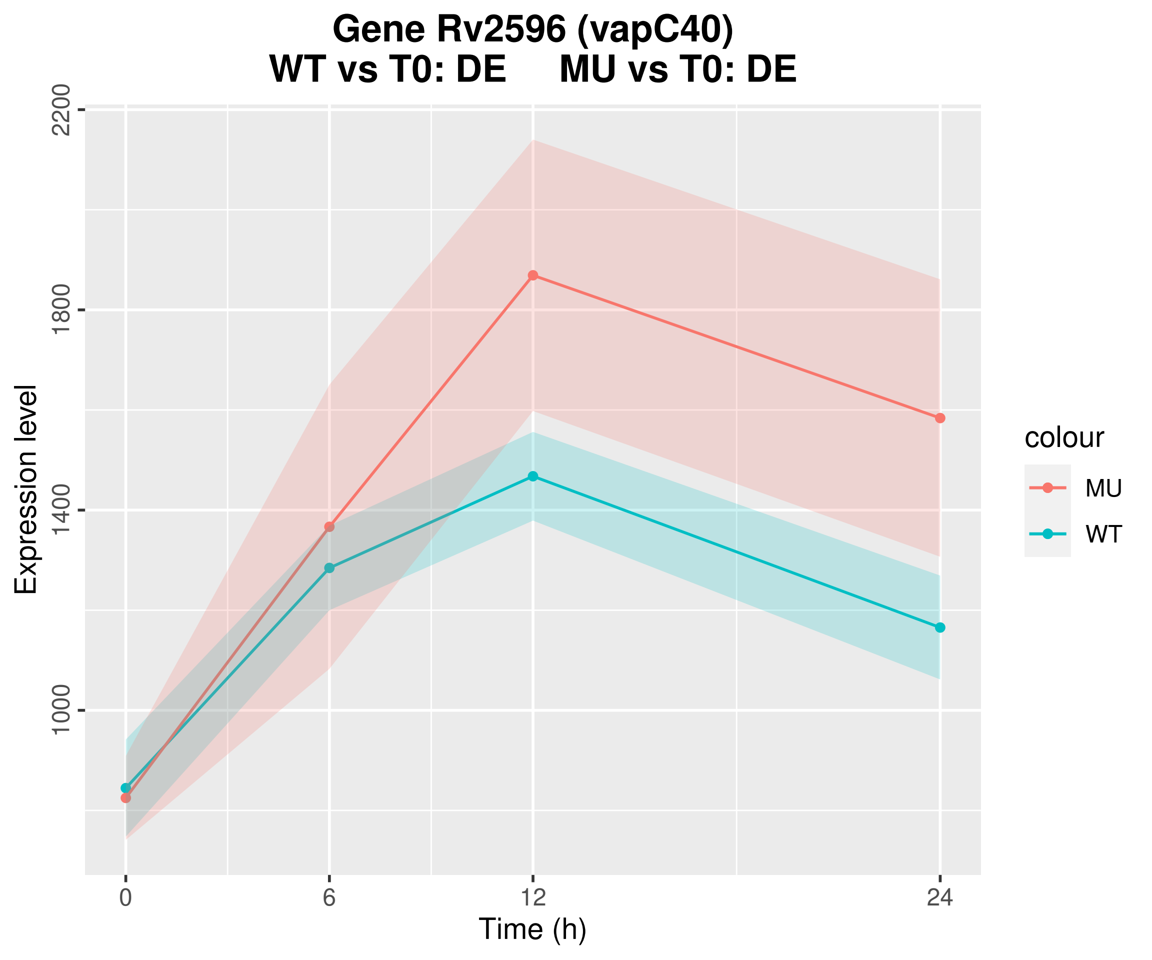

Gene Rv2596 (vapC40)
WT vs T0: DE   MU vs T0: DE
2200
1800
1400
1000
Expression level
0
6
12
24
Time (h)
colour
MU
WT

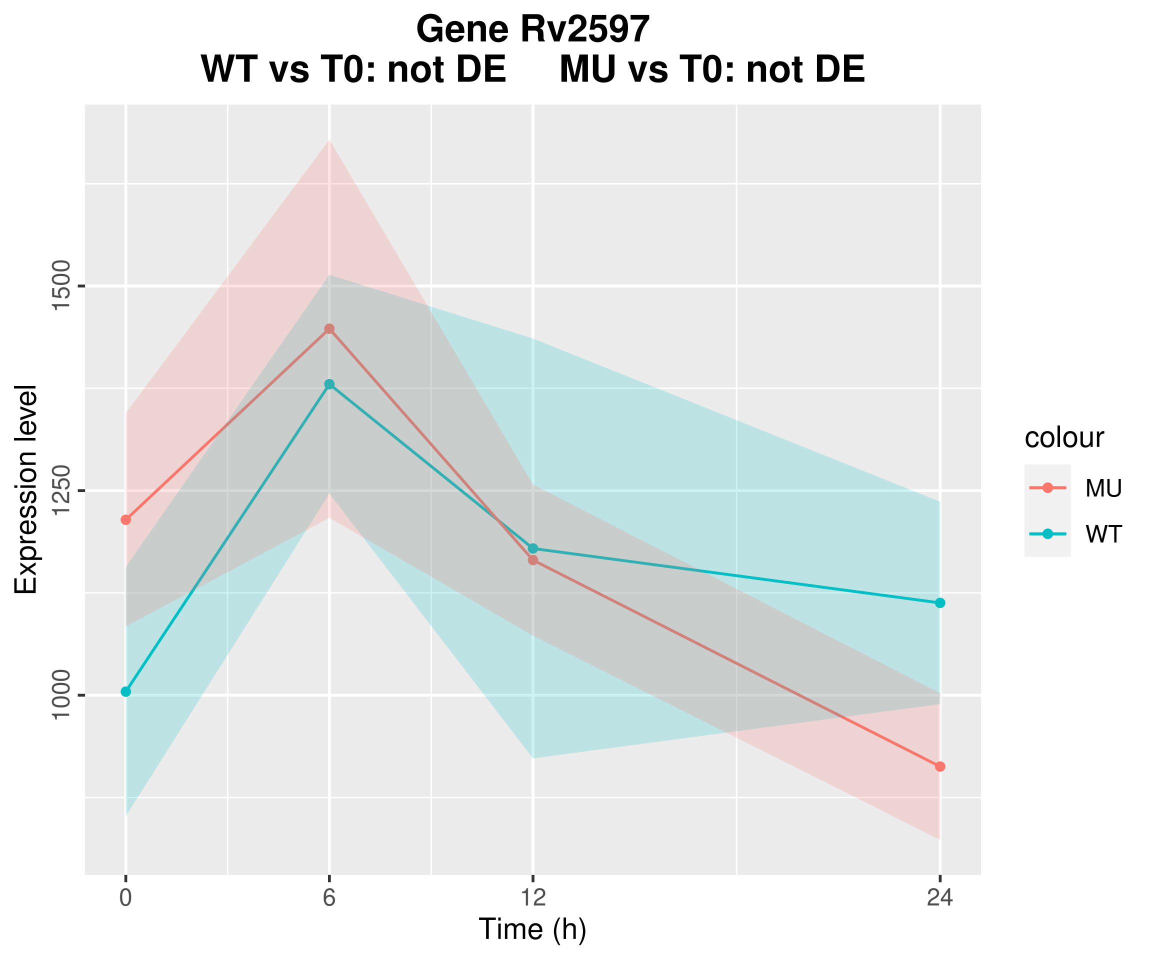

Gene Rv2597
WT vs T0: not DE   MU vs T0: not DE
Expression level
1500
1250
1000
0
6
12
24
Time (h)
colour
MU
WT

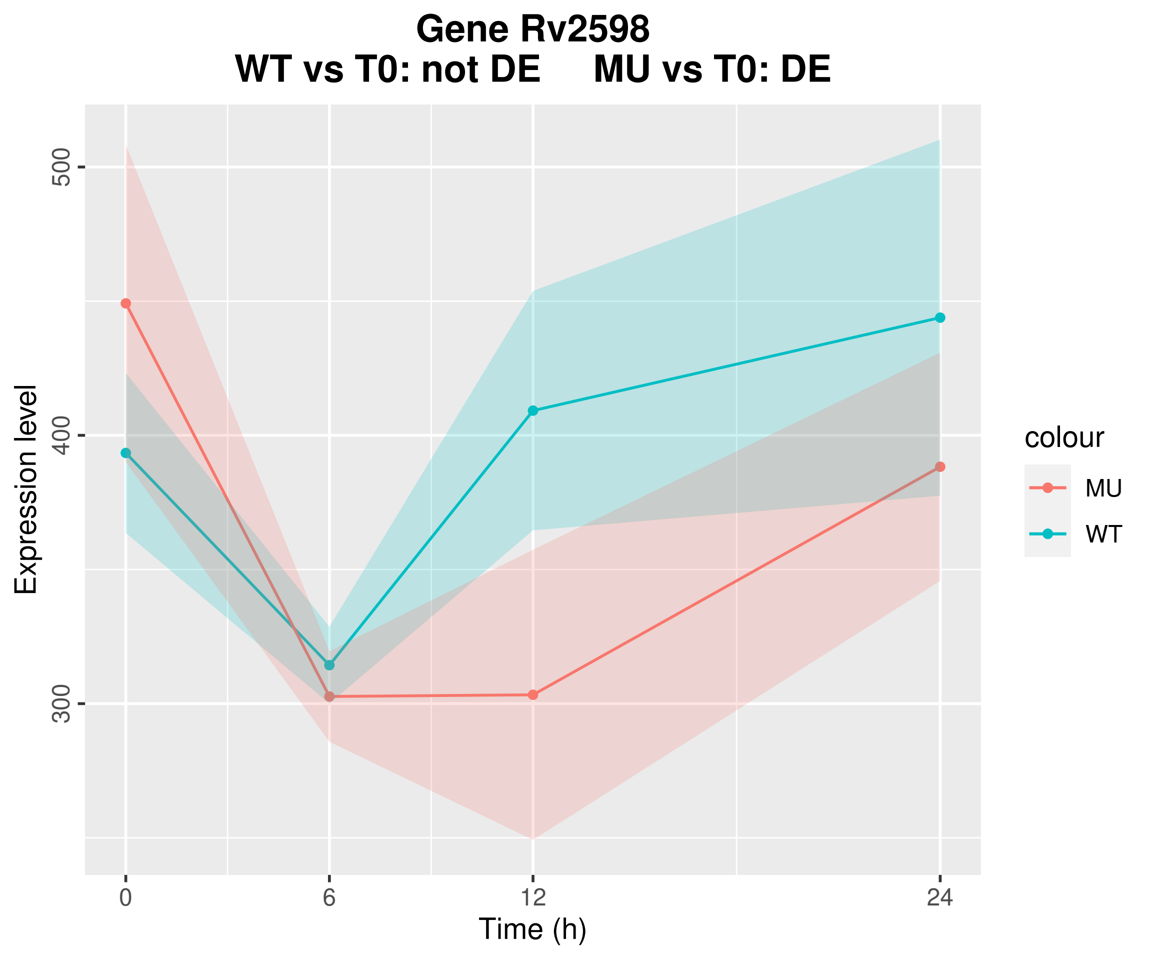

Gene Rv2598
WT vs T0: not DE MU vs T0: DE
Expression level
500
400
300
0
6
12
24
Time (h)
colour
MU
WT

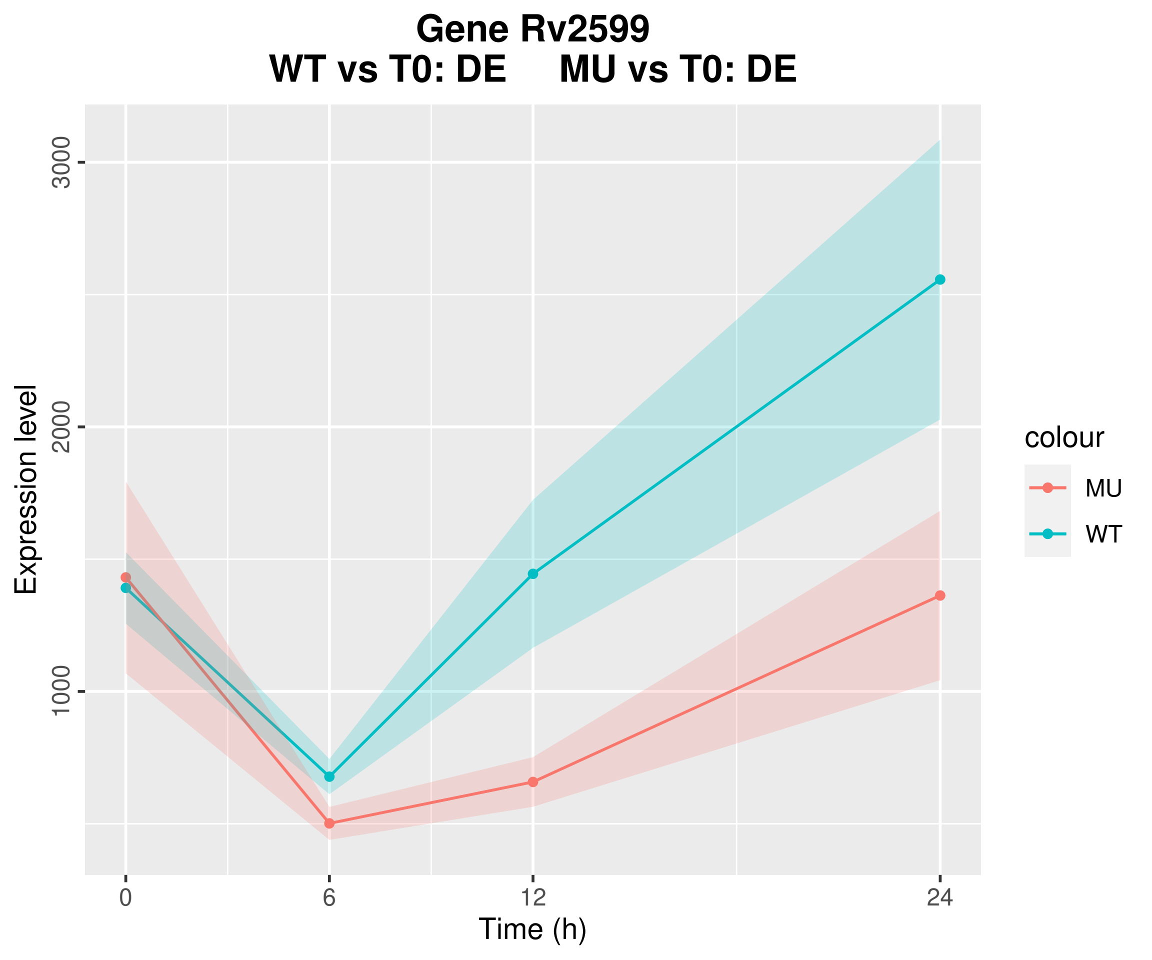

Gene Rv2599
WT vs T0: DE   MU vs T0: DE
Expression level
3000
2000
1000
0
6
12
24
Time (h)
colour
MU
WT

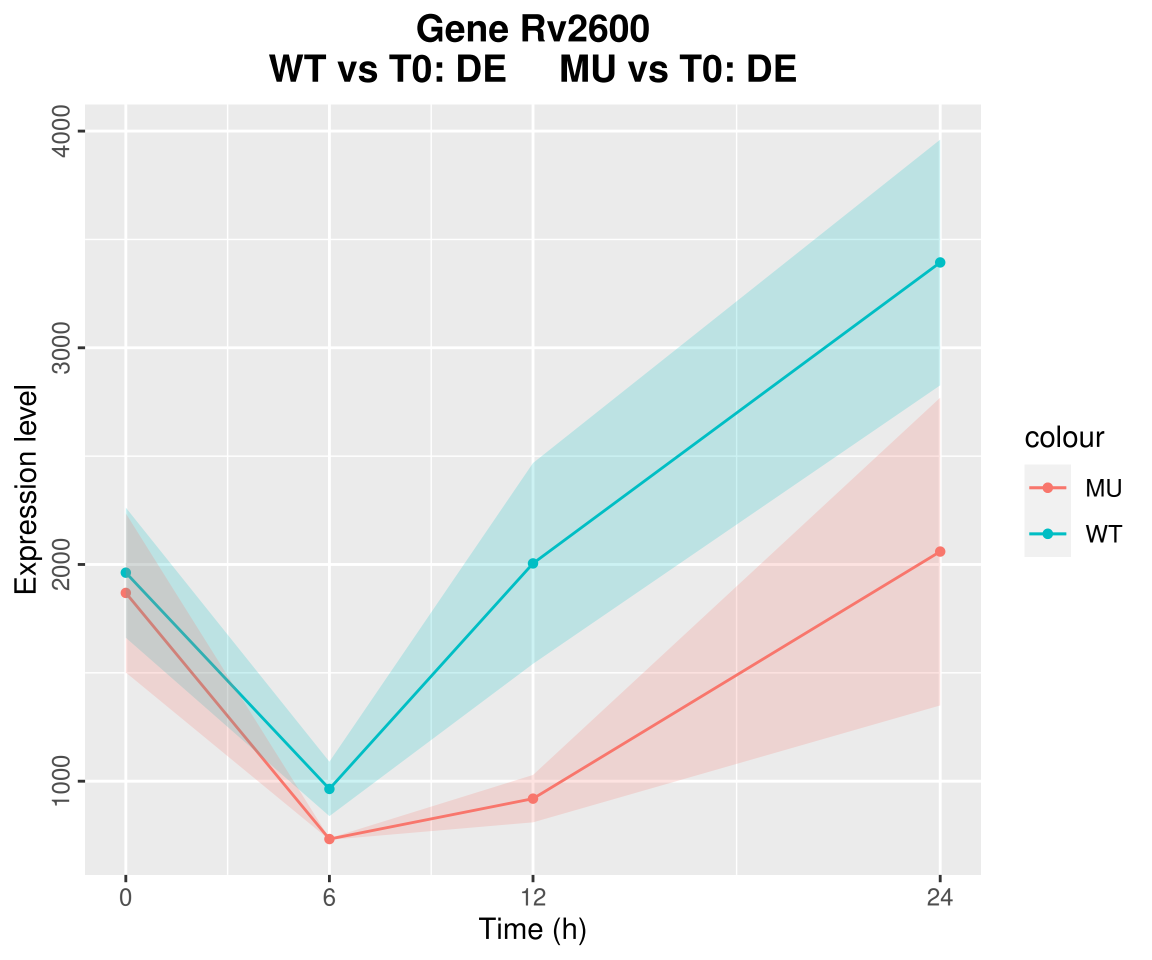

Gene Rv2600
WT vs T0: DE   MU vs T0: DE
Expression level
4000
3000
2000
1000
0
6
12
24
Time (h)
colour
MU
WT

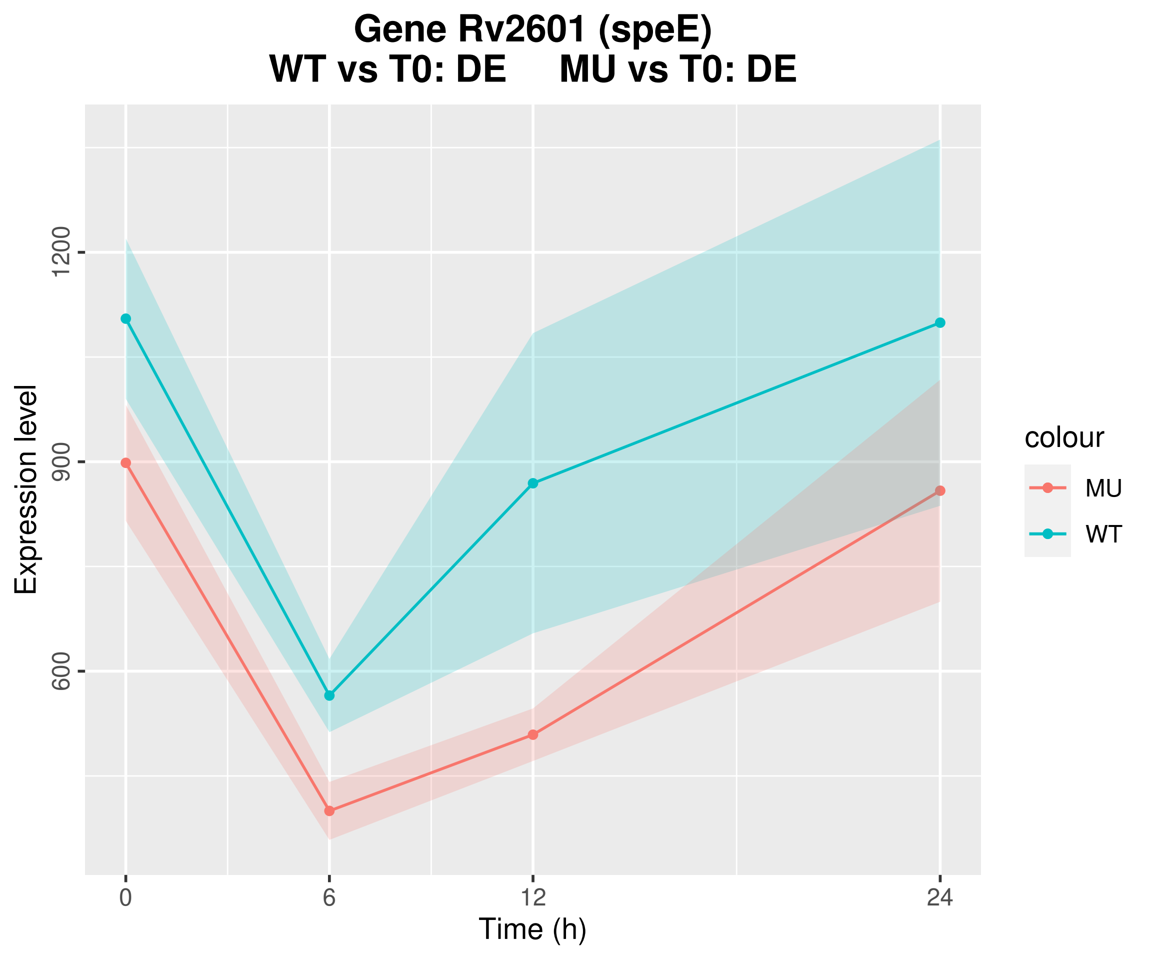

Gene Rv2601 (speE)
WT vs T0: DE   MU vs T0: DE
Expression level
1200
900
600
0
6
12
24
Time (h)
colour
MU
WT

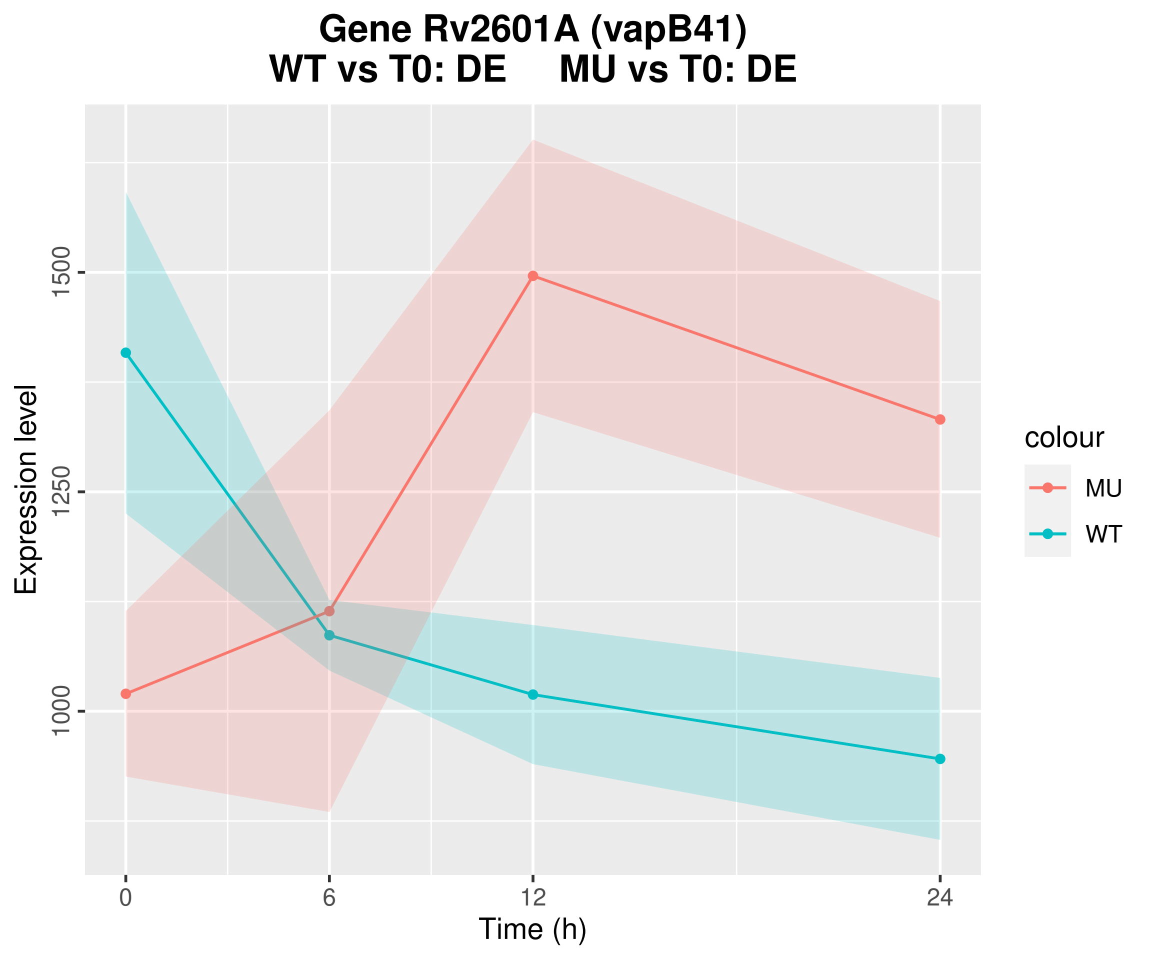

Gene Rv2601A (vapB41)
WT vs T0: DE   MU vs T0: DE
Expression level
1500
1250
1000
0
6
12
24
Time (h)
colour
MU
WT

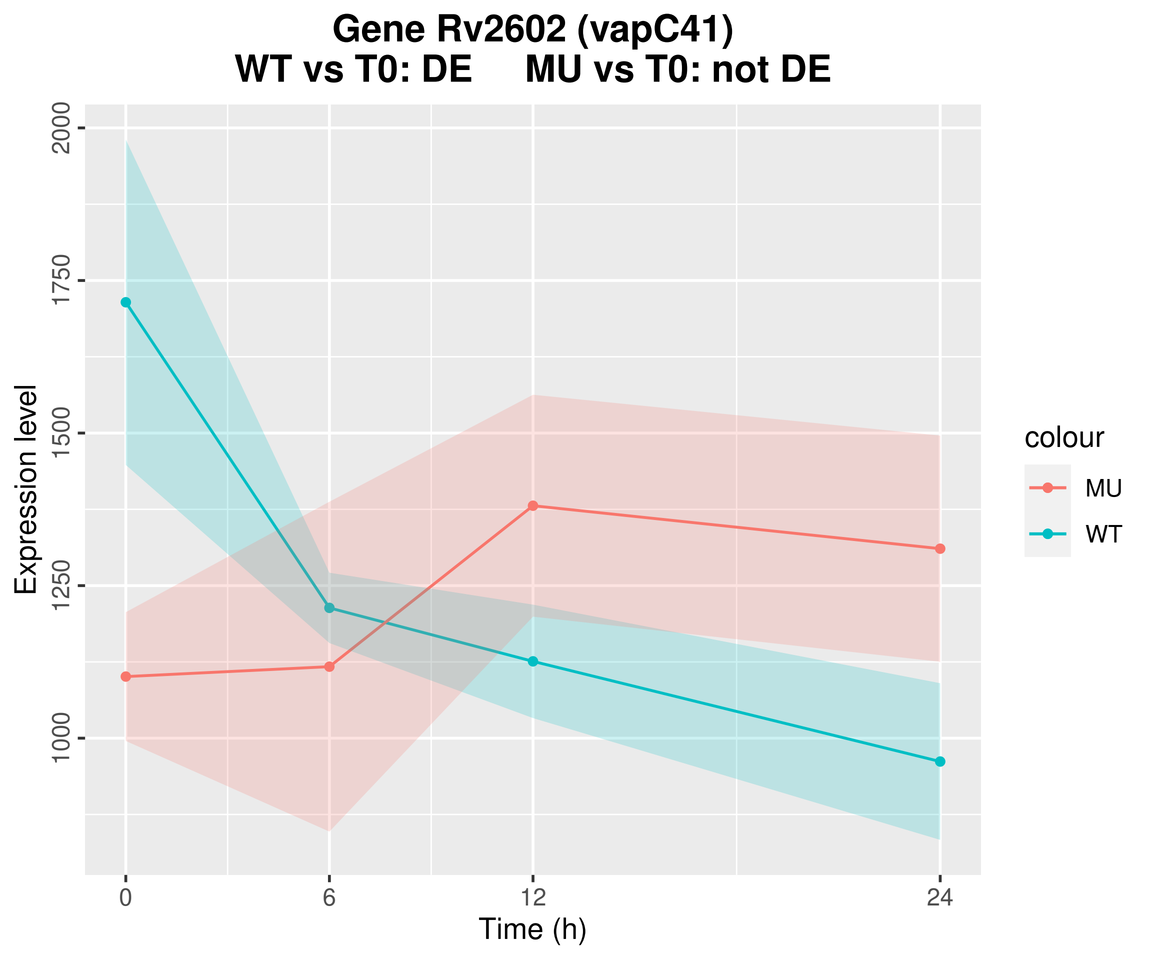

Gene Rv2602 (vapC41)
WT vs T0: DE MU vs T0: not DE
Expression level
2000
1750
1500
1250
1000
0
6
12
24
Time (h)
colour
MU
WT

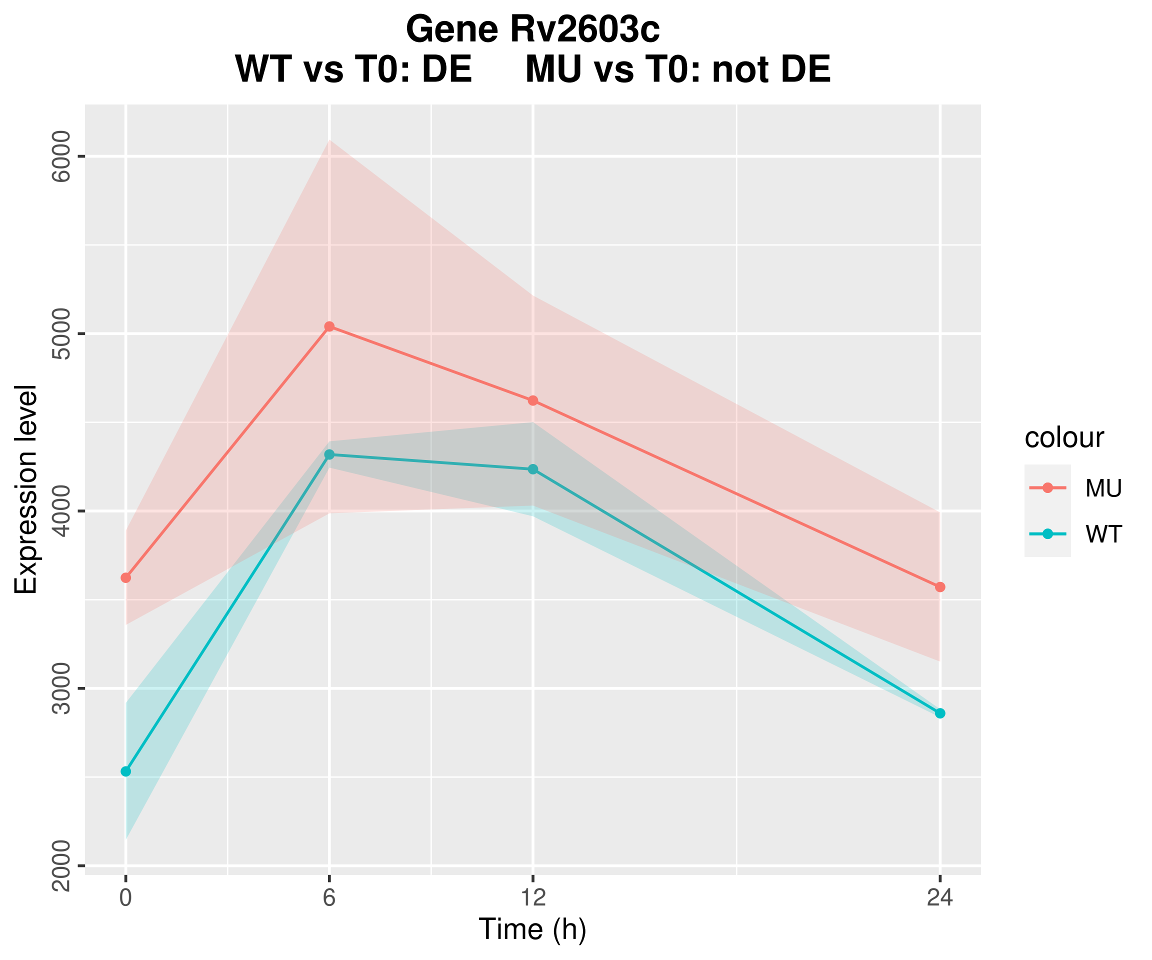

Gene Rv2603c
WT vs T0: DE MU vs T0: not DE
Expression level
6000
5000
4000
3000
2000
0
6
12
24
Time (h)
colour
MU
WT

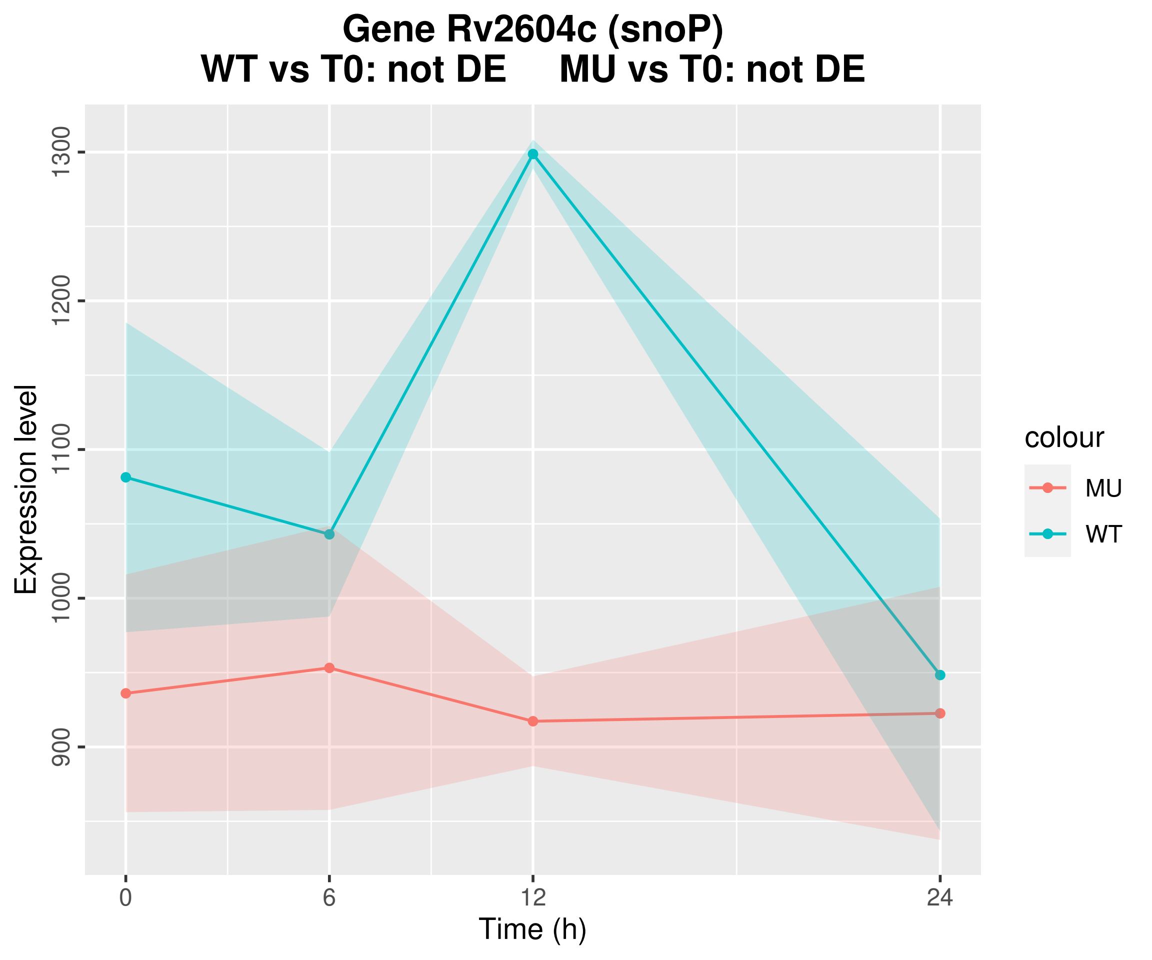
Gene Rv2604c (snoP)
WT vs T0: not DE   MU vs T0: not DE
Expression level
1300
1200
1100
1000
900
0
6
12
24
Time (h)
colour
MU
WT

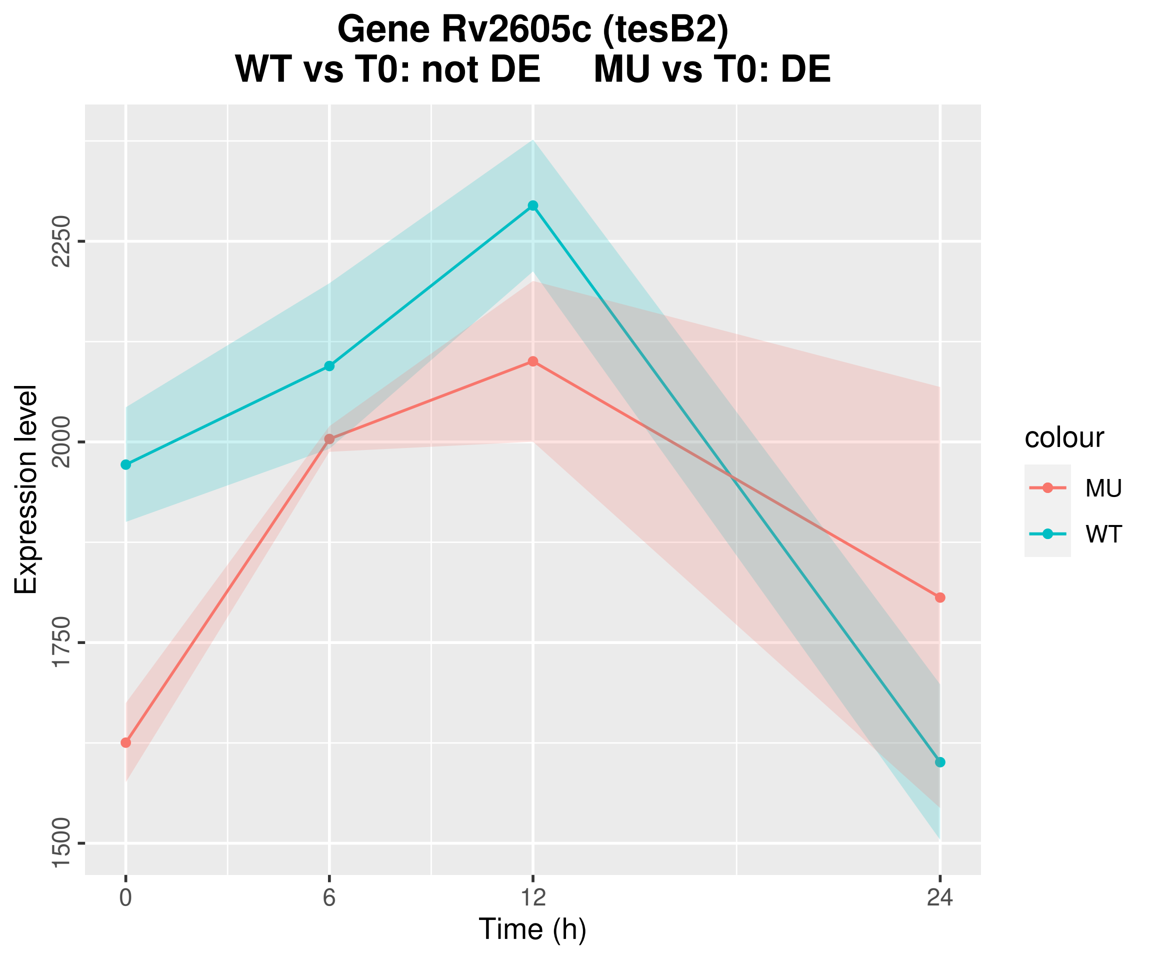
Gene Rv2605c (tesB2)
WT vs T0: not DE   MU vs T0: DE
Expression level
2250
2000
1750
1500
0
6
12
24
Time (h)
colour
MU
WT

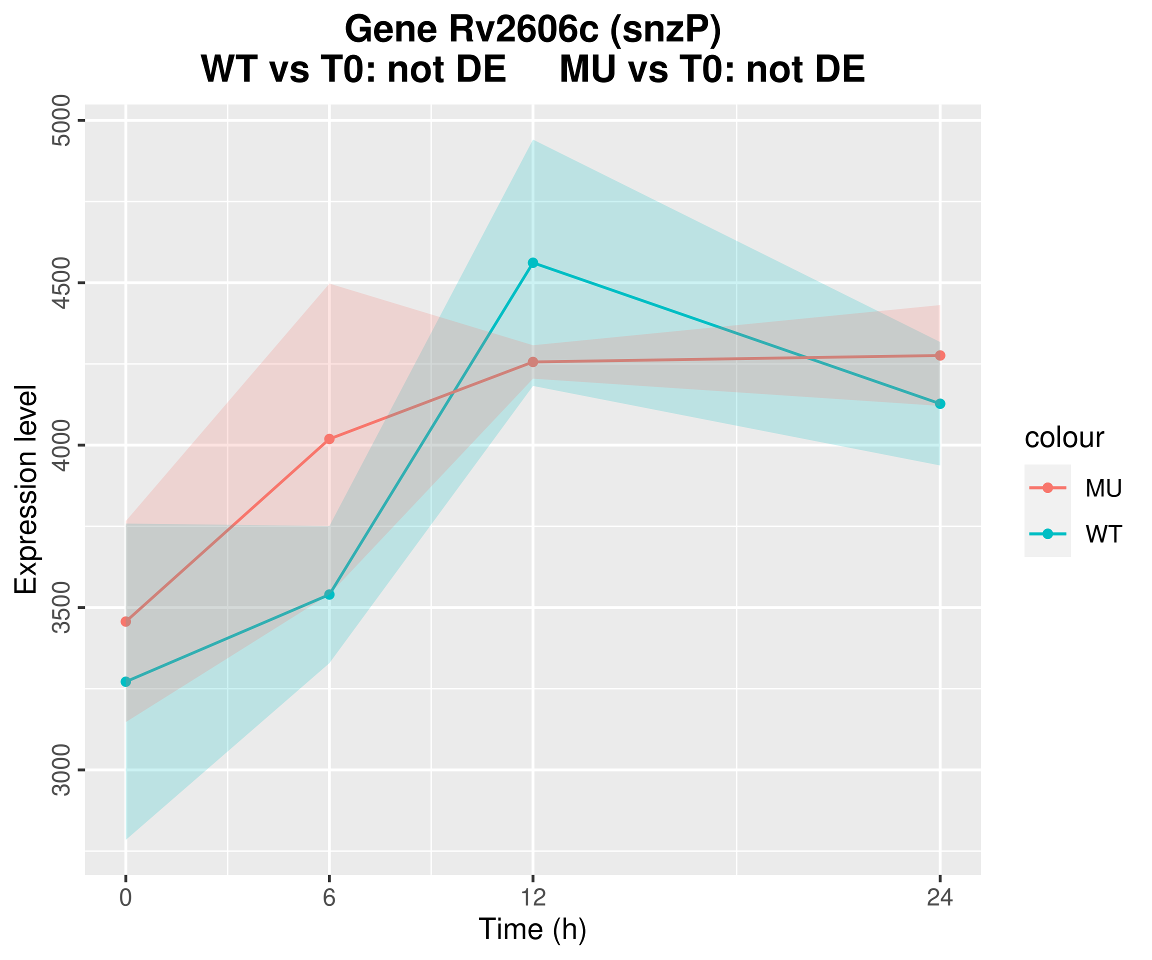

Gene Rv2606c (snzP)
WT vs T0: not DE MU vs T0: not DE
Expression level
5000
4500
4000
3500
3000
0
6
12
24
Time (h)
colour
MU
WT

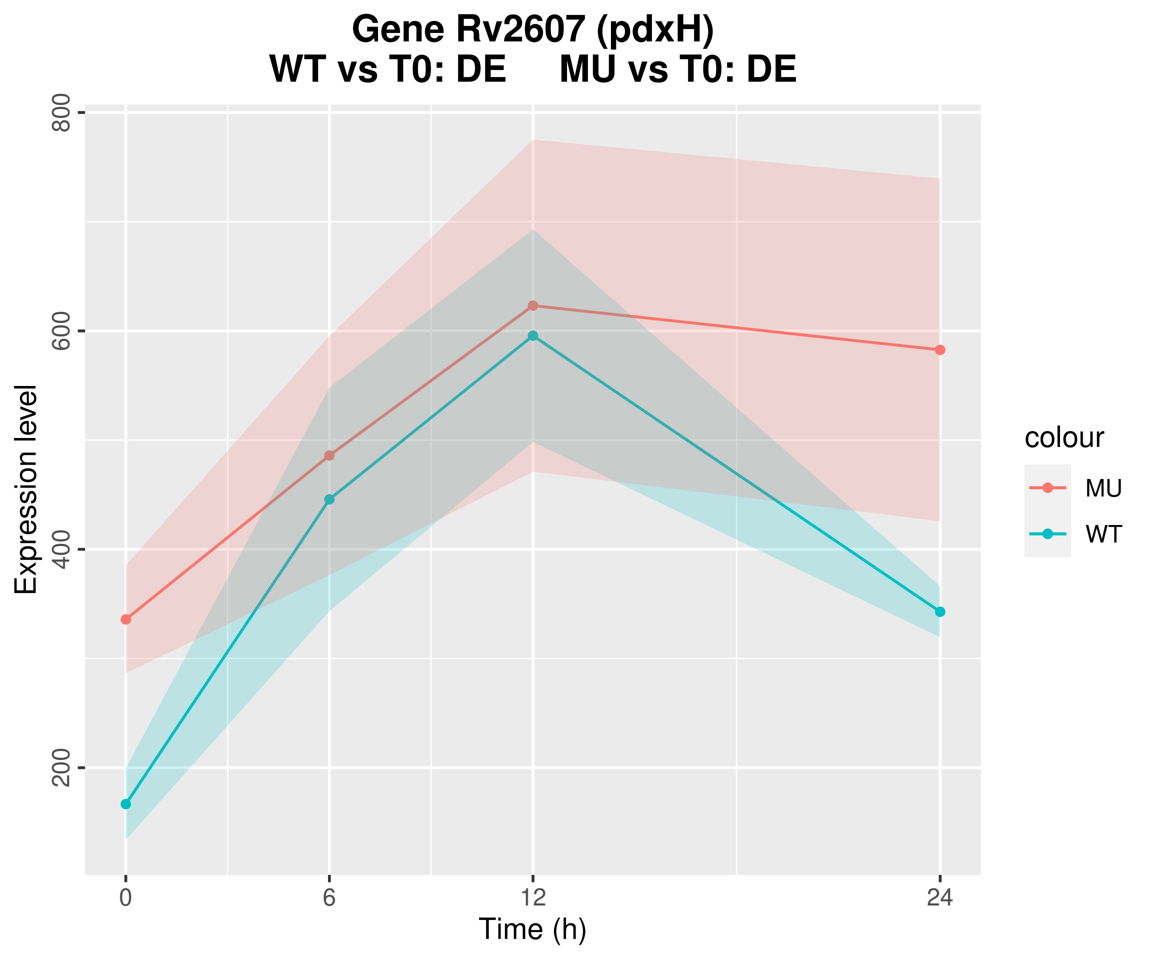

Gene Rv2607 (pdxH)
WT vs T0: DE MU vs T0: DE
Expression level
800
600
400
200
0
6
12
24
Time (h)
colour
MU
WT

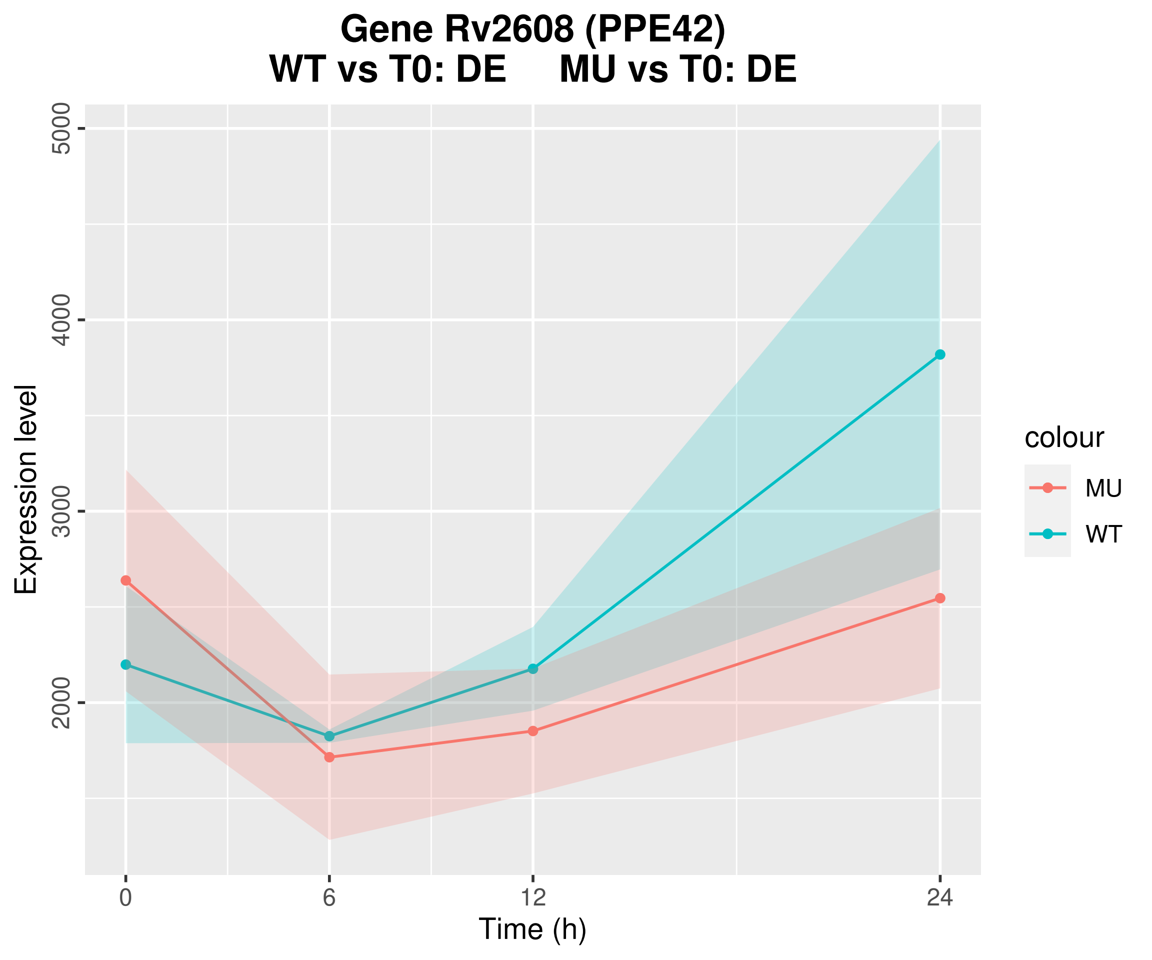

Gene Rv2608 (PPE42)
WT vs T0: DE MU vs T0: DE
Expression level
5000
4000
3000
2000
0
6
12
24
Time (h)
colour
MU
WT

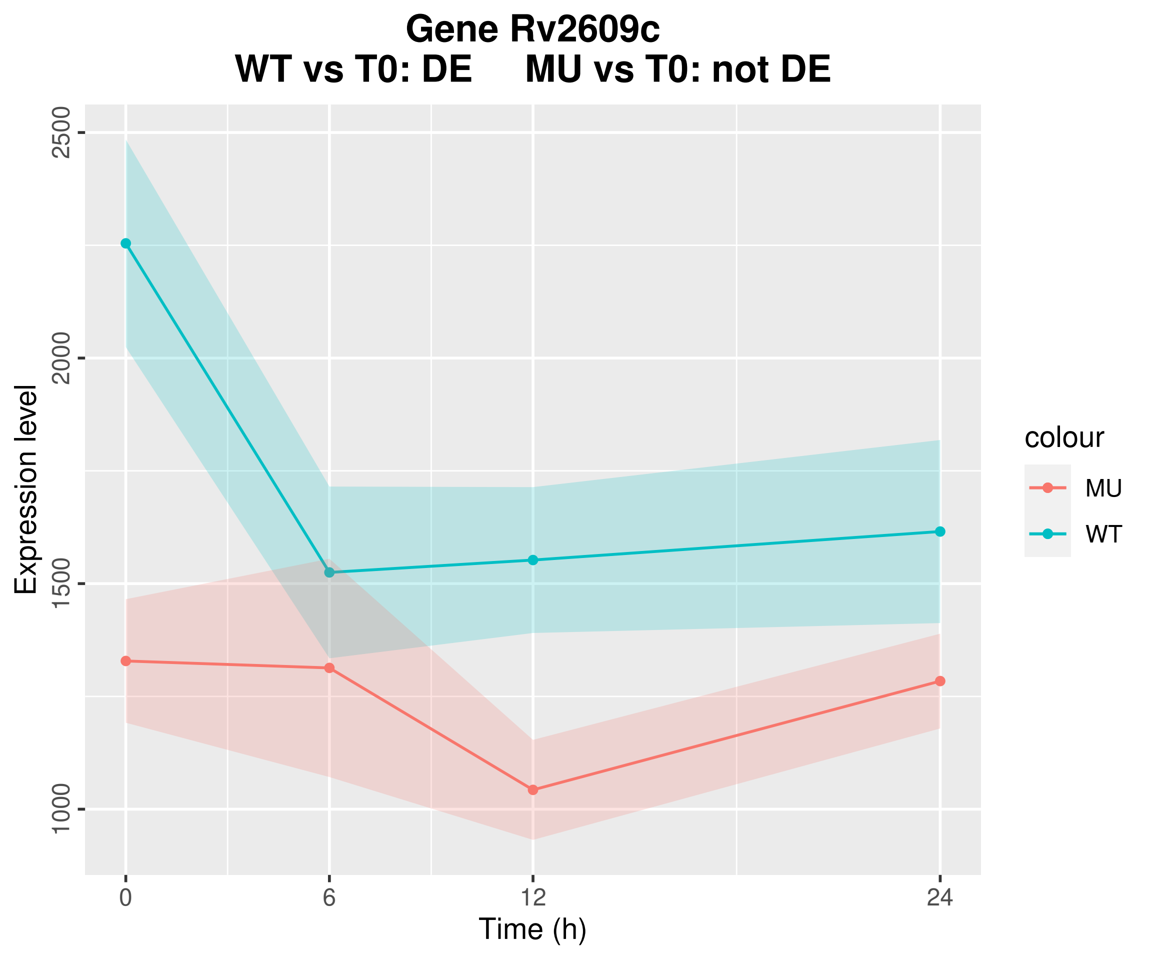

Gene Rv2609c
WT vs T0: DE MU vs T0: not DE
Expression level
2500
2000
1500
1000
0
6
12
24
Time (h)
colour
MU
WT

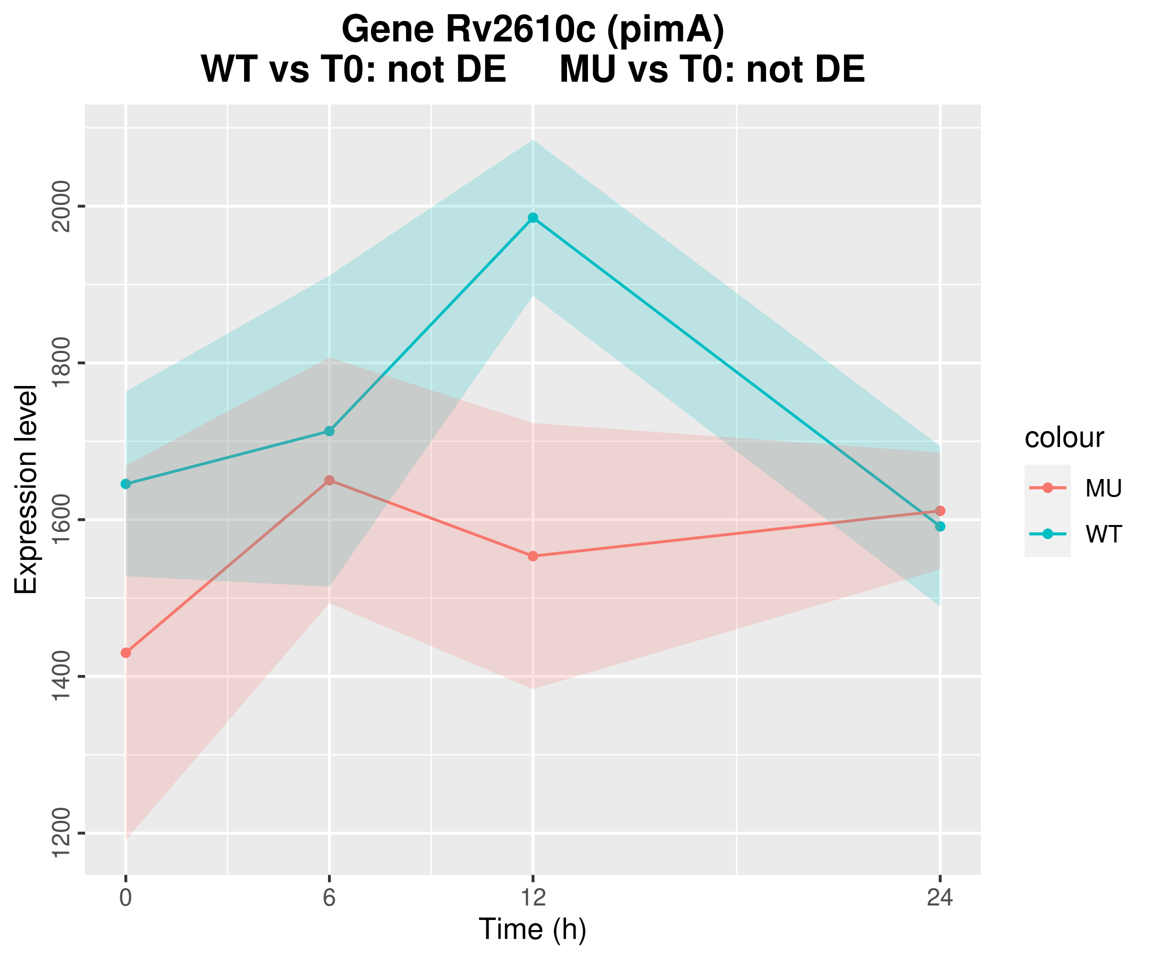
Gene Rv2610c (pimA)
WT vs T0: not DE   MU vs T0: not DE
Expression level
2000
1800
1600
1400
1200
Time (h)
0
6
12
24
colour
MU
WT

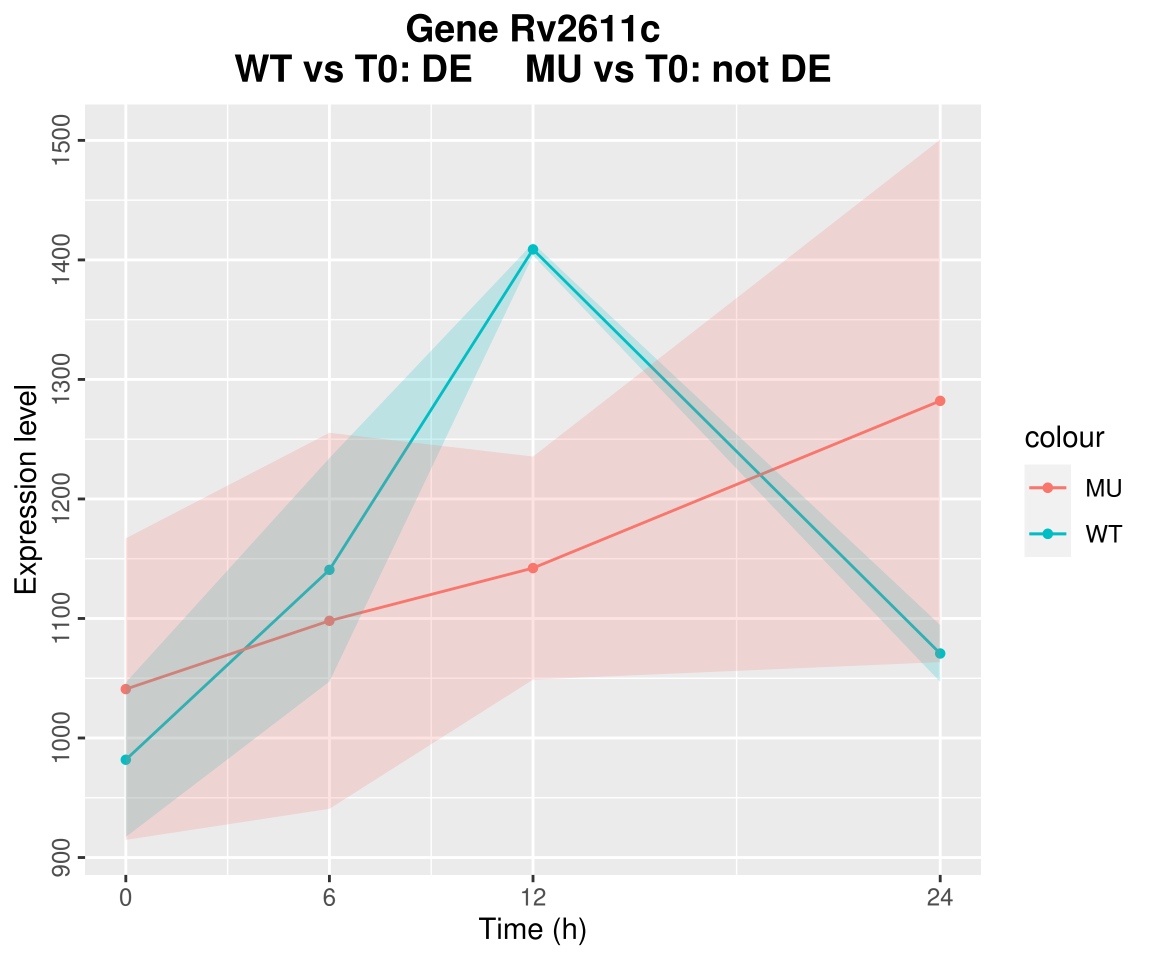
Gene Rv2611c
WT vs T0: DE   MU vs T0: not DE
Expression level
1500
1400
1300
1200
1100
1000
900
0
6
12
24
Time (h)
colour
MU
WT

Gene Rv2612c (pgsA1)
WT vs T0: not DE   MU vs T0: not DE

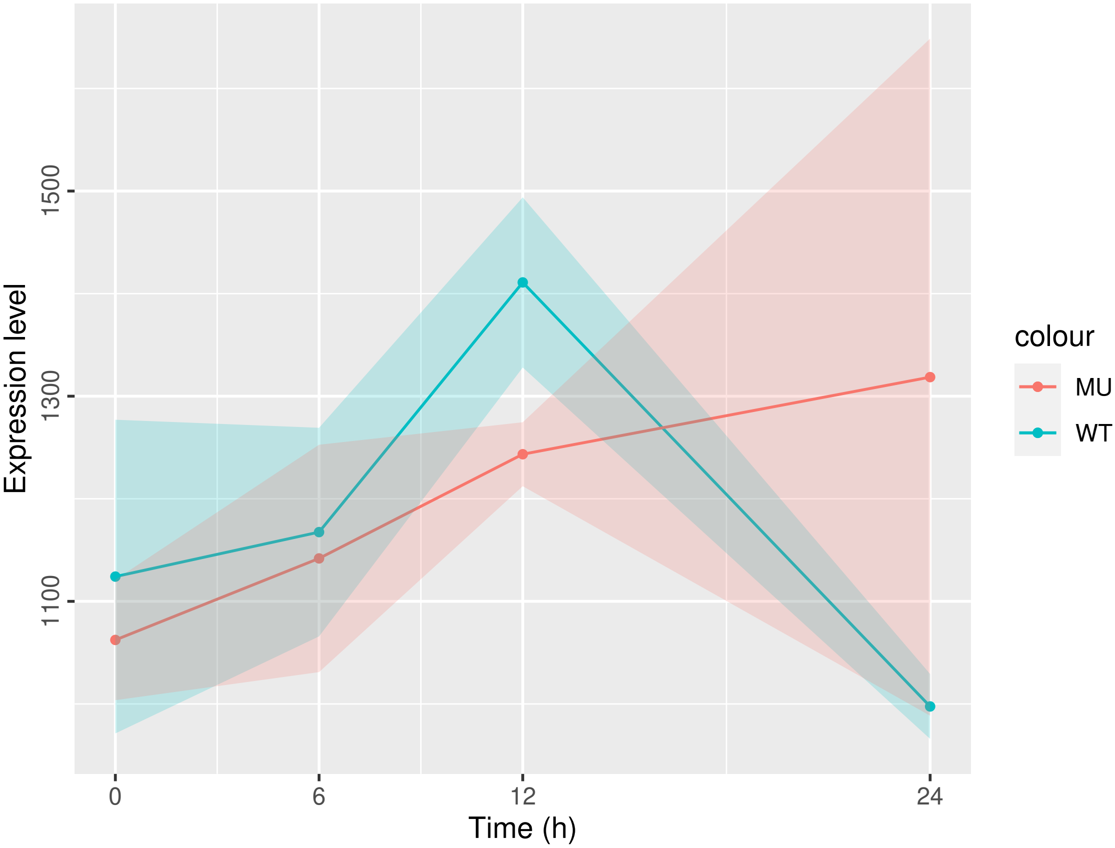

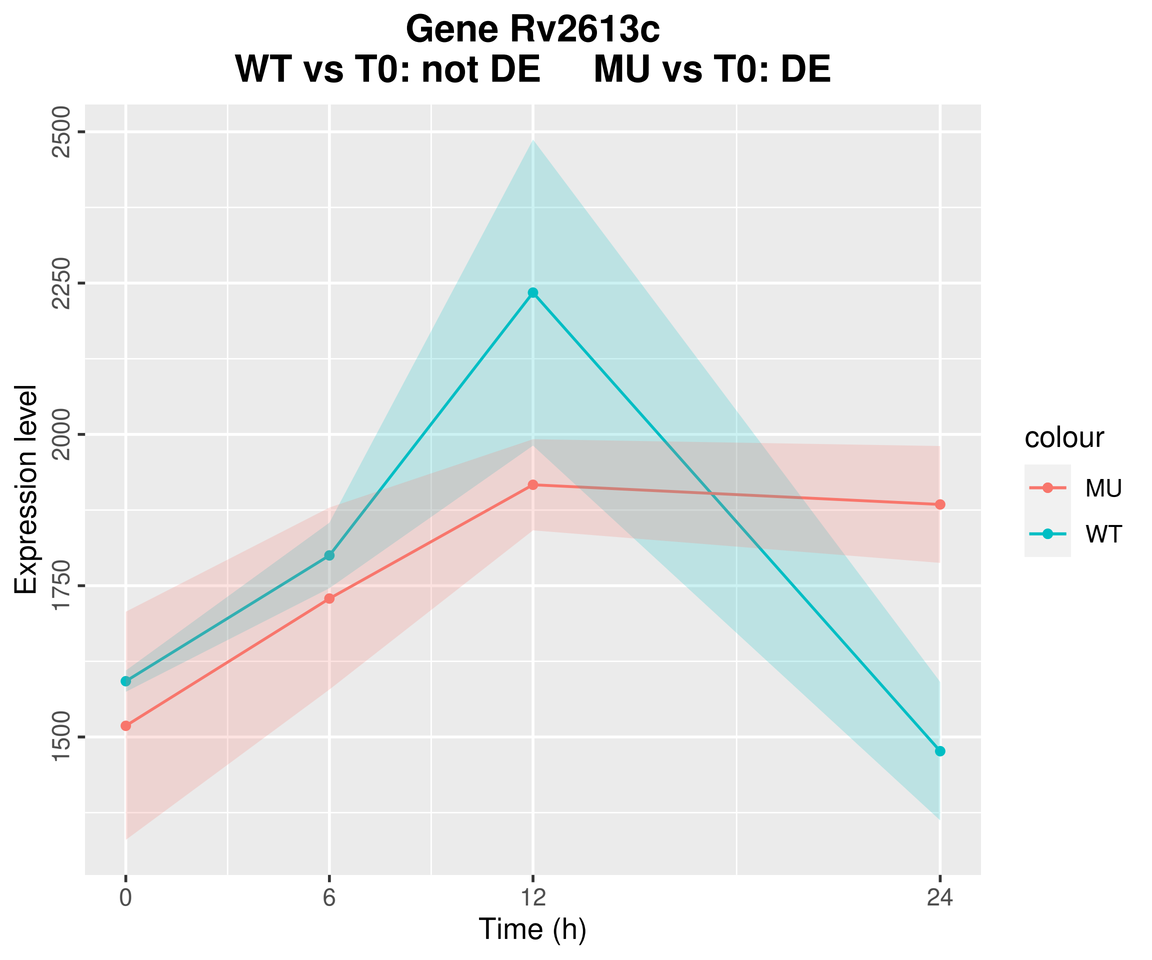

Gene Rv2613c
WT vs T0: not DE MU vs T0: DE
Expression level
2500
2250
2000
1750
1500
0
6
12
24
Time (h)
colour
MU
WT

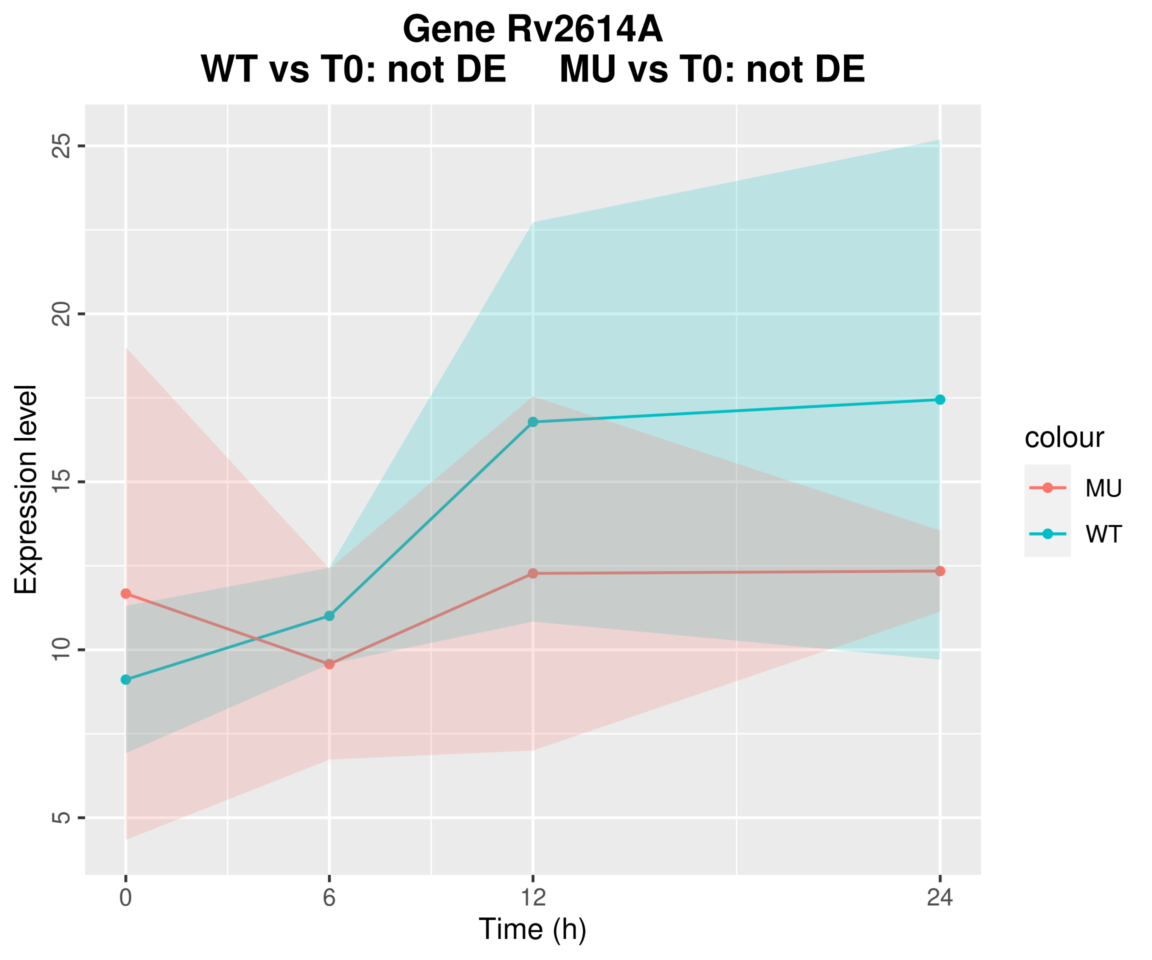
Gene Rv2614A
WT vs T0: not DE   MU vs T0: not DE
Expression level
Time (h)
0
6
12
24
5
10
15
20
25
colour
MU
WT

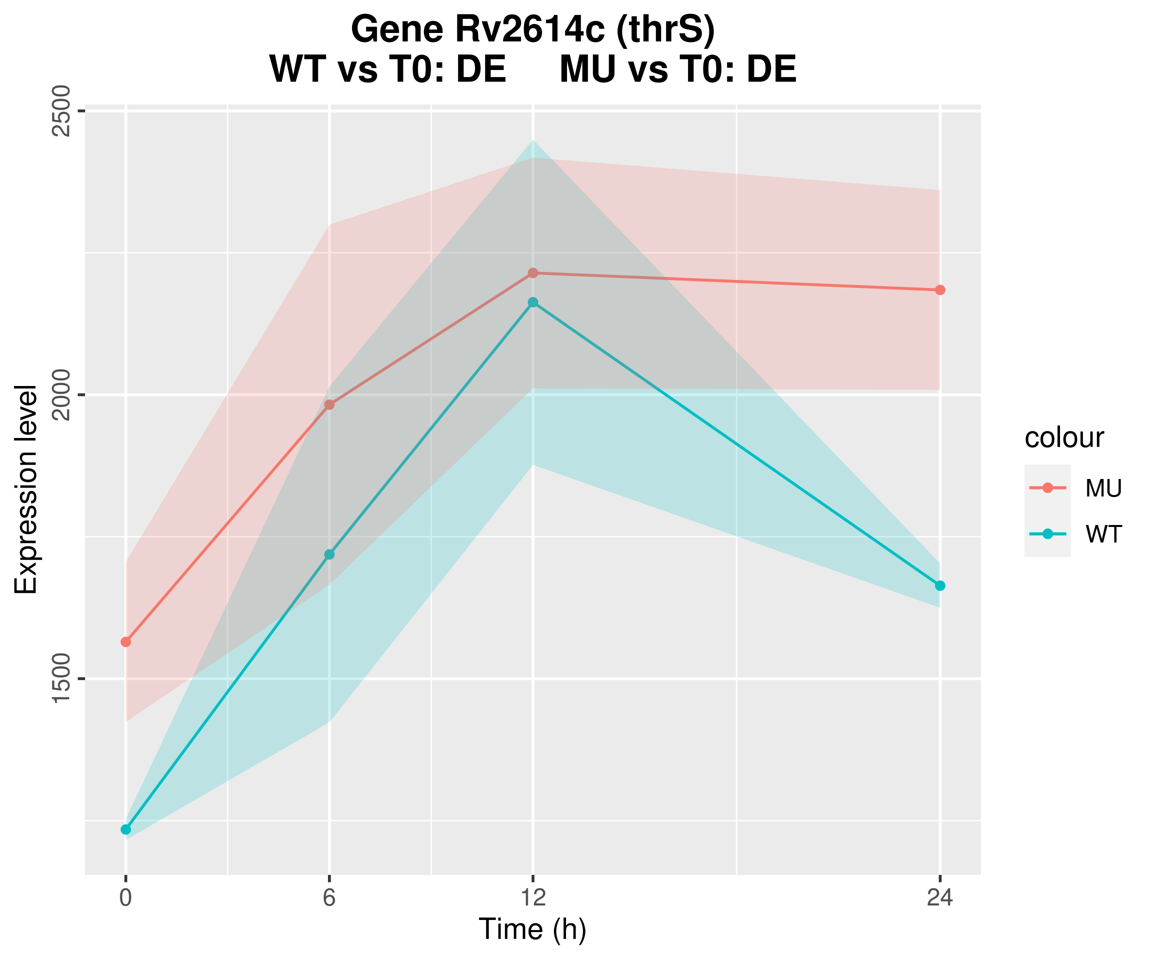
Gene Rv2614c (thrS)
WT vs T0: DE MU vs T0: DE
Expression level
2500
2000
1500
0
6
12
24
Time (h)
colour
MU
WT

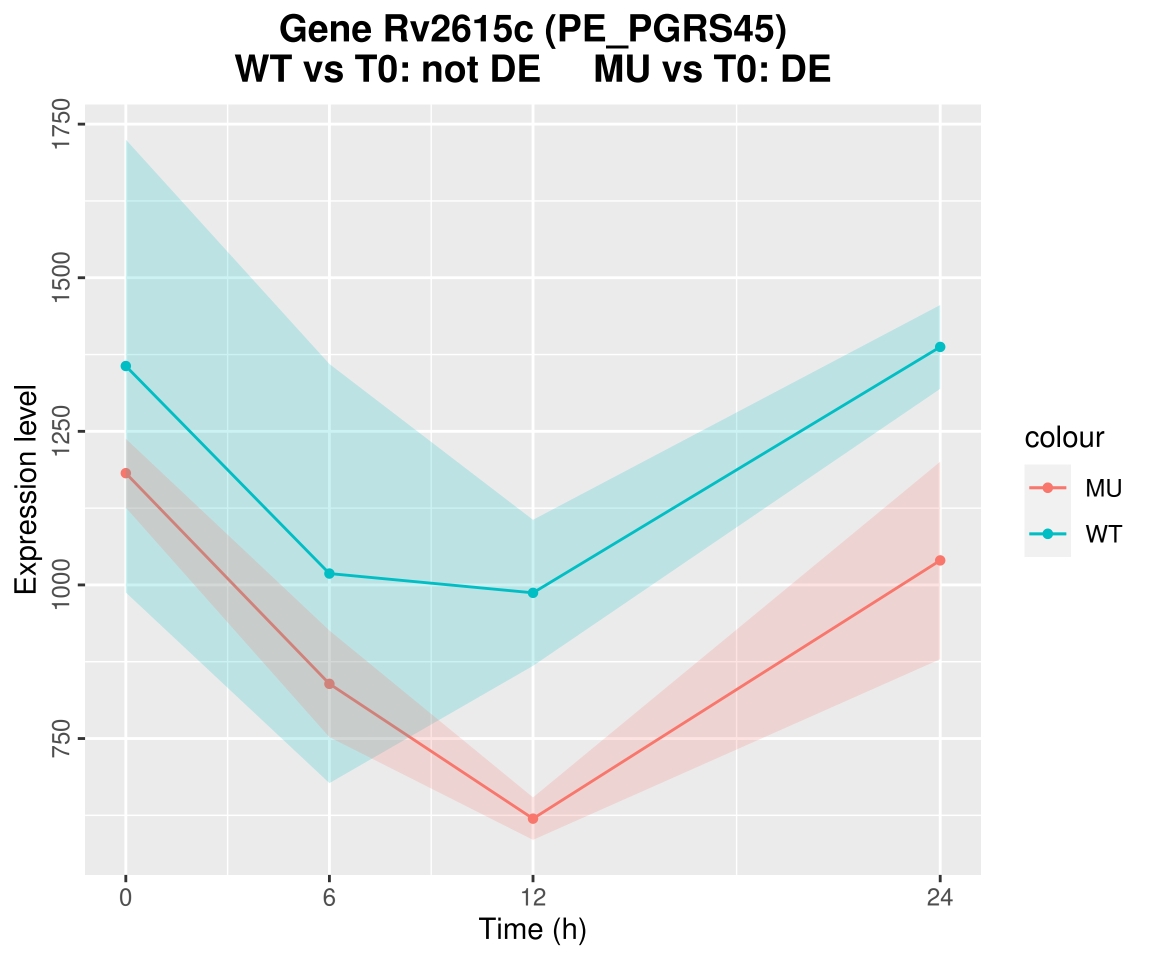

Gene Rv2615c (PE_PGRS45)
WT vs T0: not DE MU vs T0: DE
Expression level
1750
1500
1250
1000
750
0
6
12
24
Time (h)
colour
MU
WT

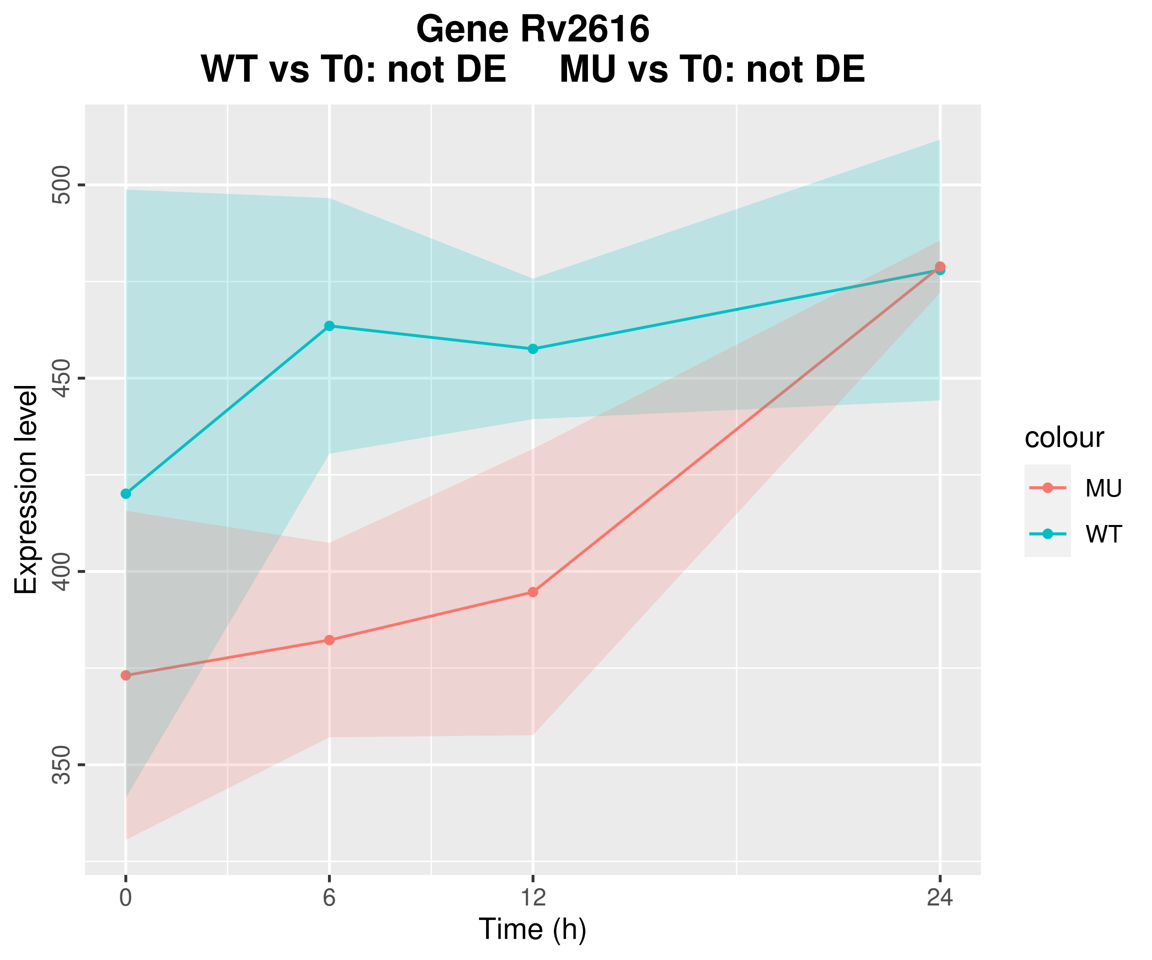
Gene Rv2616
WT vs T0: not DE   MU vs T0: not DE
Expression level
500
450
400
350
Time (h)
0
6
12
24
colour
MU
WT

Gene Rv2617c
WT vs T0: not DE   MU vs T0: not DE

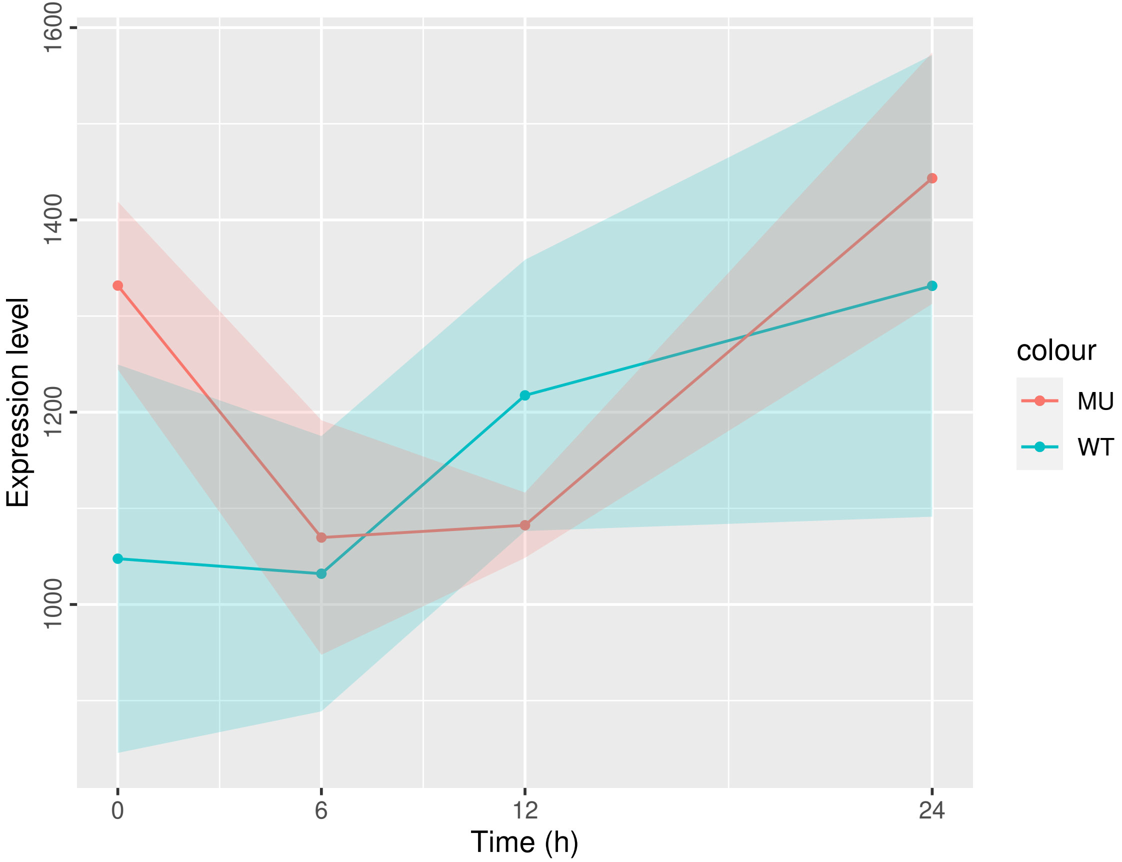

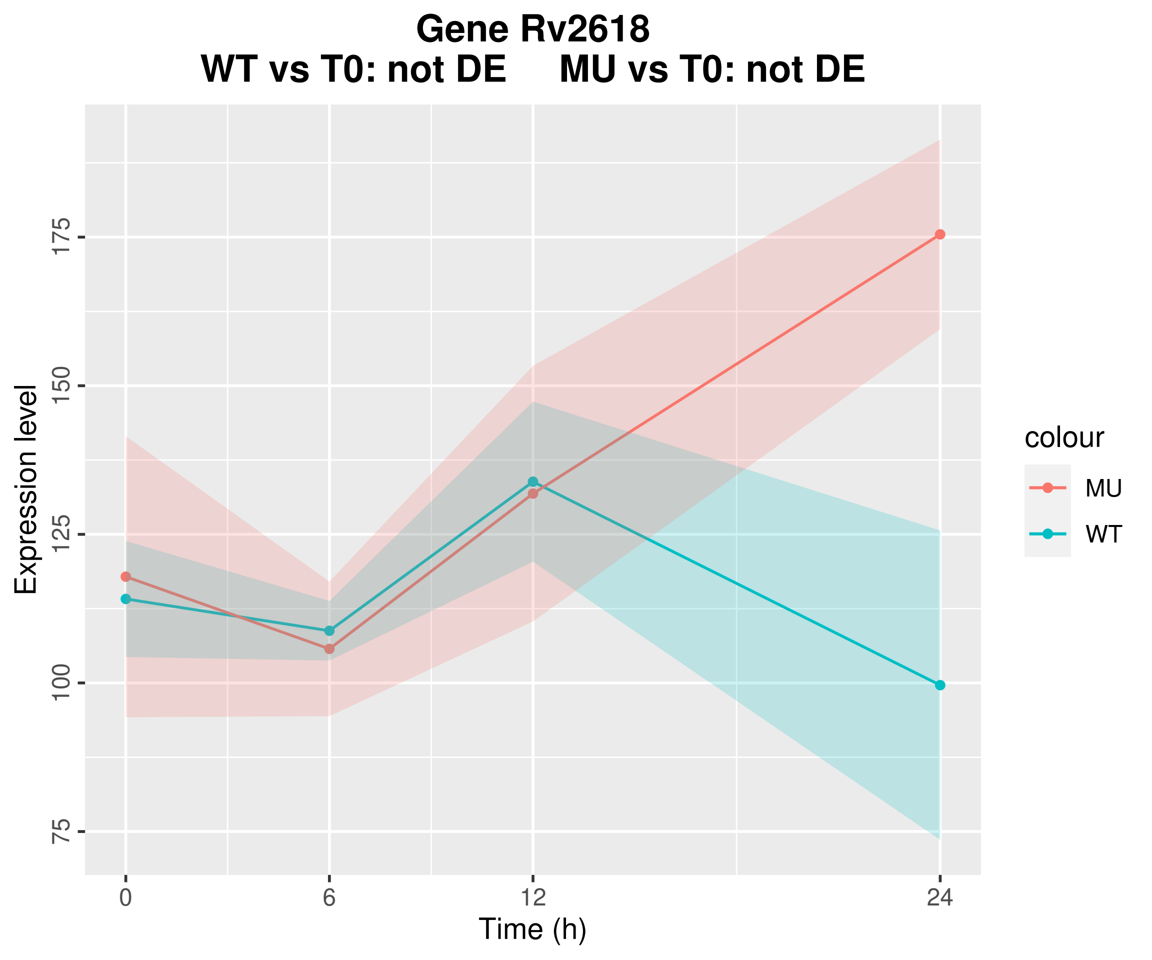

Gene Rv2618
WT vs T0: not DE MU vs T0: not DE
Expression level
75
100
125
150
175
0
6
12
24
Time (h)
colour
MU
WT

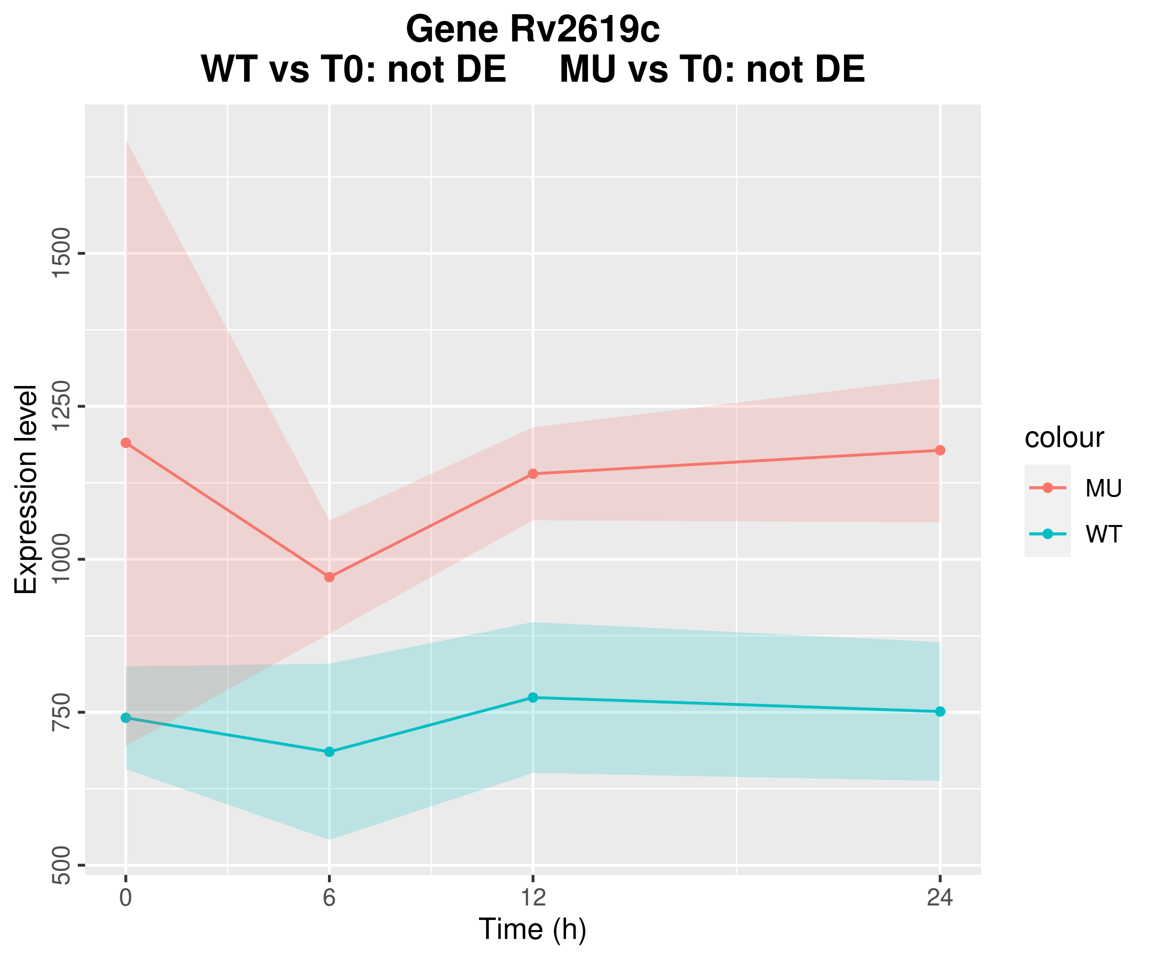

Gene Rv2619c
WT vs T0: not DE MU vs T0: not DE
Expression level
1500
1250
1000
750
500
0
6
12
24
Time (h)
colour
MU
WT

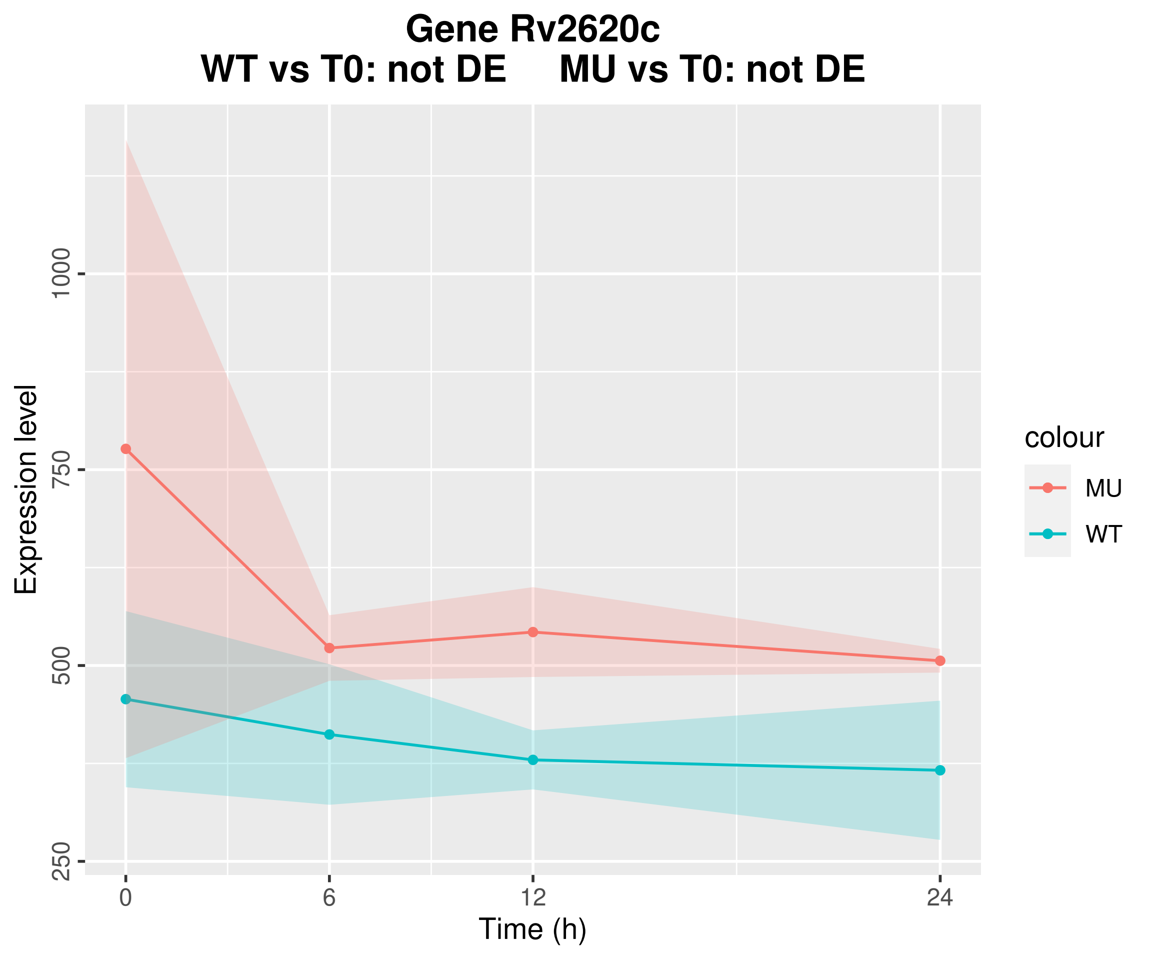
Gene Rv2620c
WT vs T0: not DE   MU vs T0: not DE
Expression level
1000
750
500
250
0
6
12
24
Time (h)
colour
MU
WT

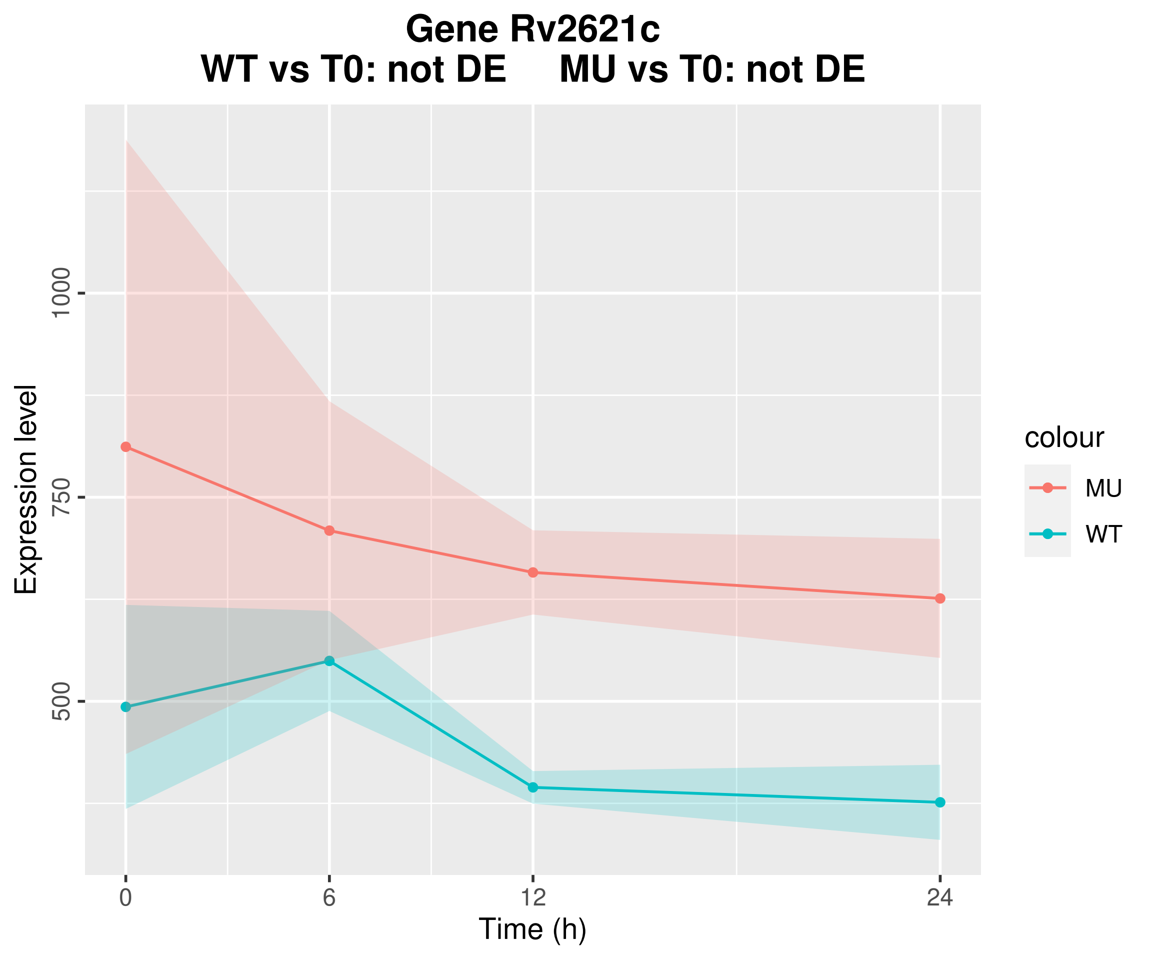

Gene Rv2621c
WT vs T0: not DE MU vs T0: not DE
Expression level
1000
750
500
0
6
12
24
Time (h)
colour
MU
WT

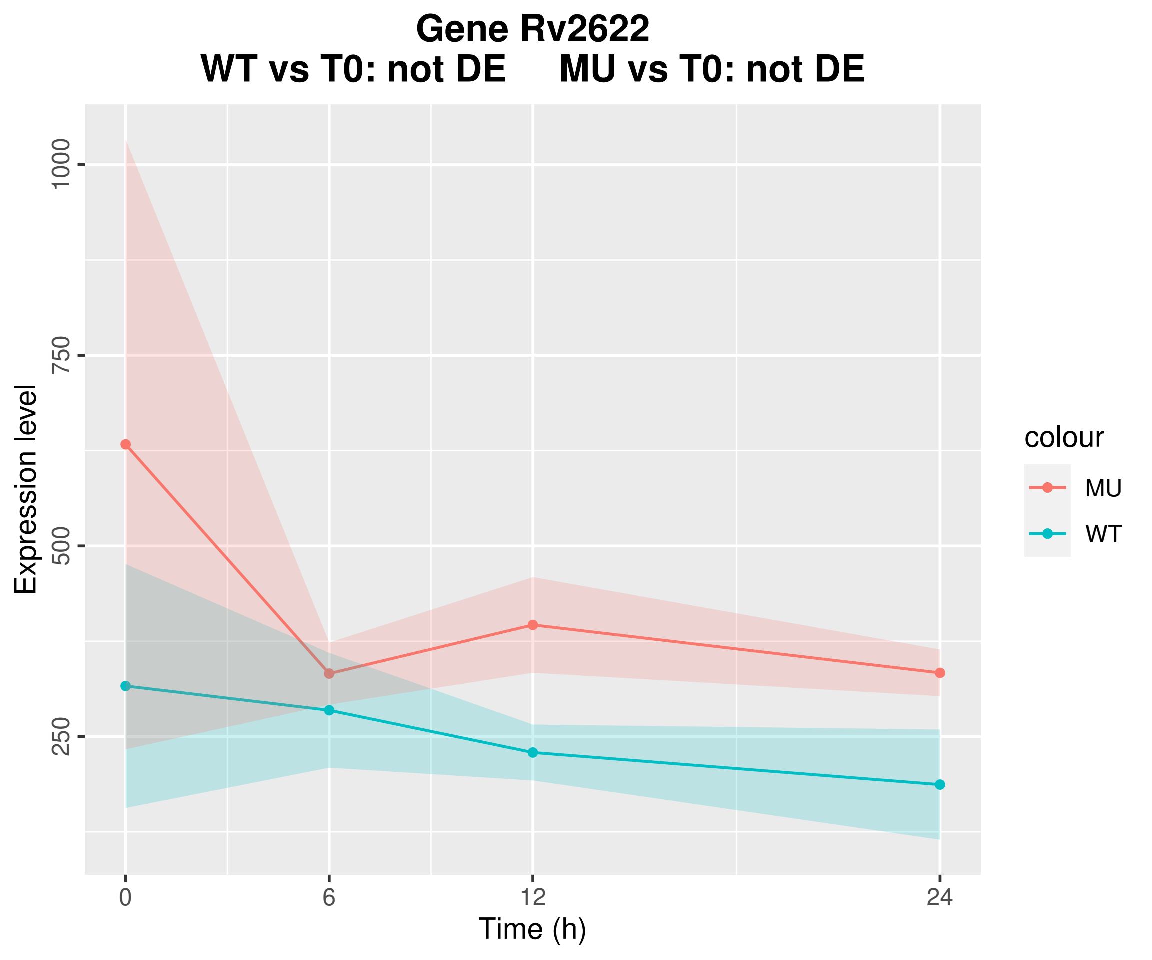

Gene Rv2622
WT vs T0: not DE   MU vs T0: not DE
Expression level
1000
750
500
250
0
6
12
24
Time (h)
colour
MU
WT

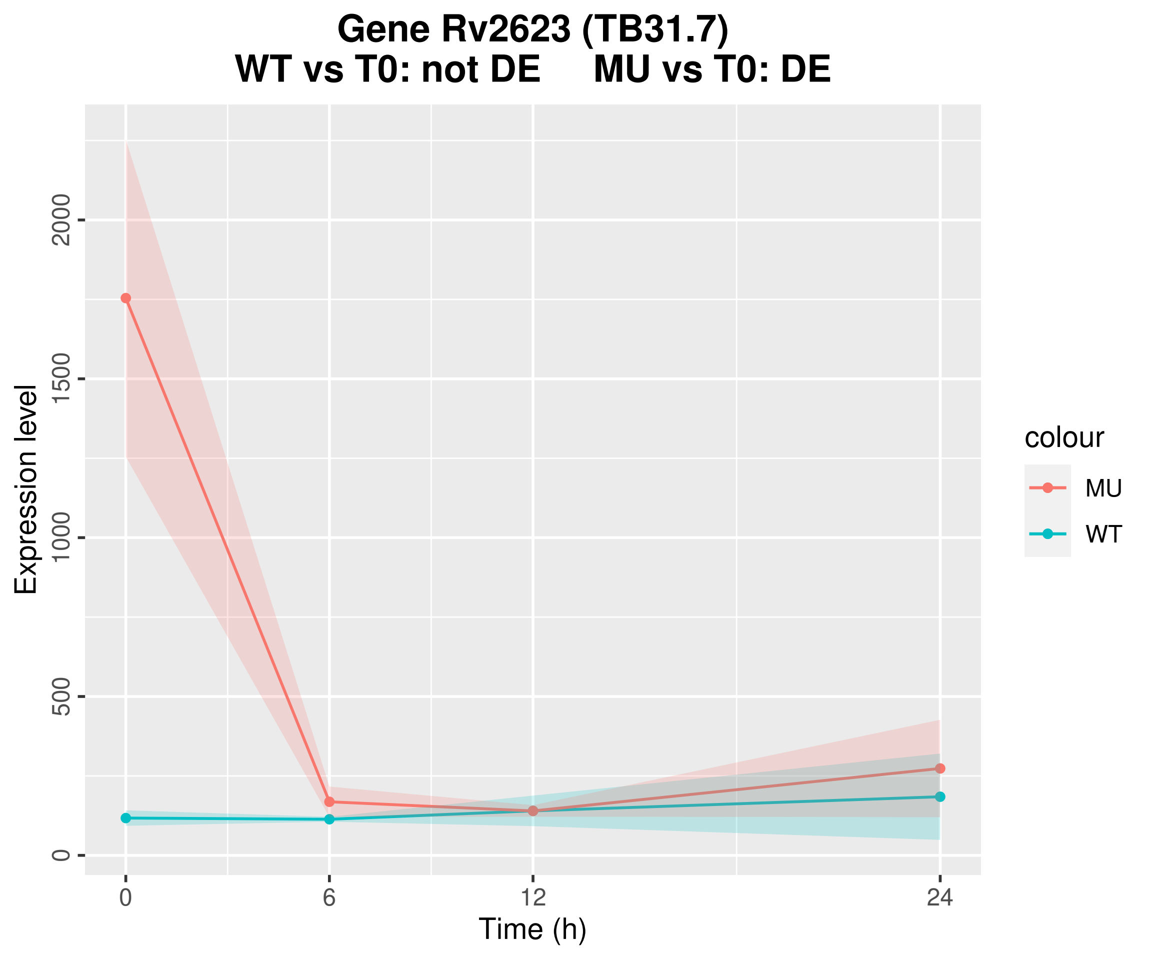

Gene Rv2623 (TB31.7)
WT vs T0: not DE   MU vs T0: DE
Expression level
0
500
1000
1500
2000
Time (h)
0
6
12
24
colour
MU
WT

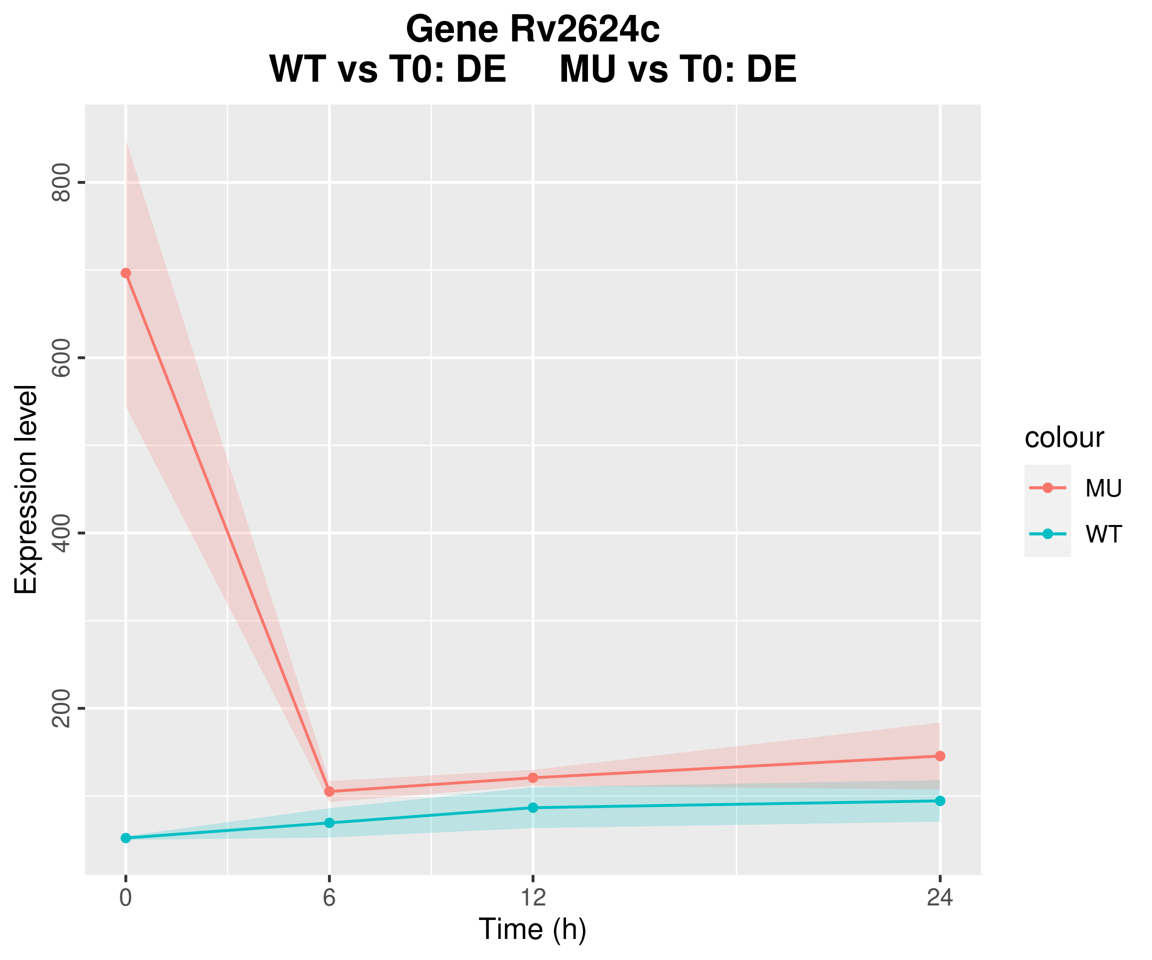

Gene Rv2624c
WT vs T0: DE   MU vs T0: DE
Expression level
800
600
400
200
0
6
12
24
Time (h)
colour
MU
WT

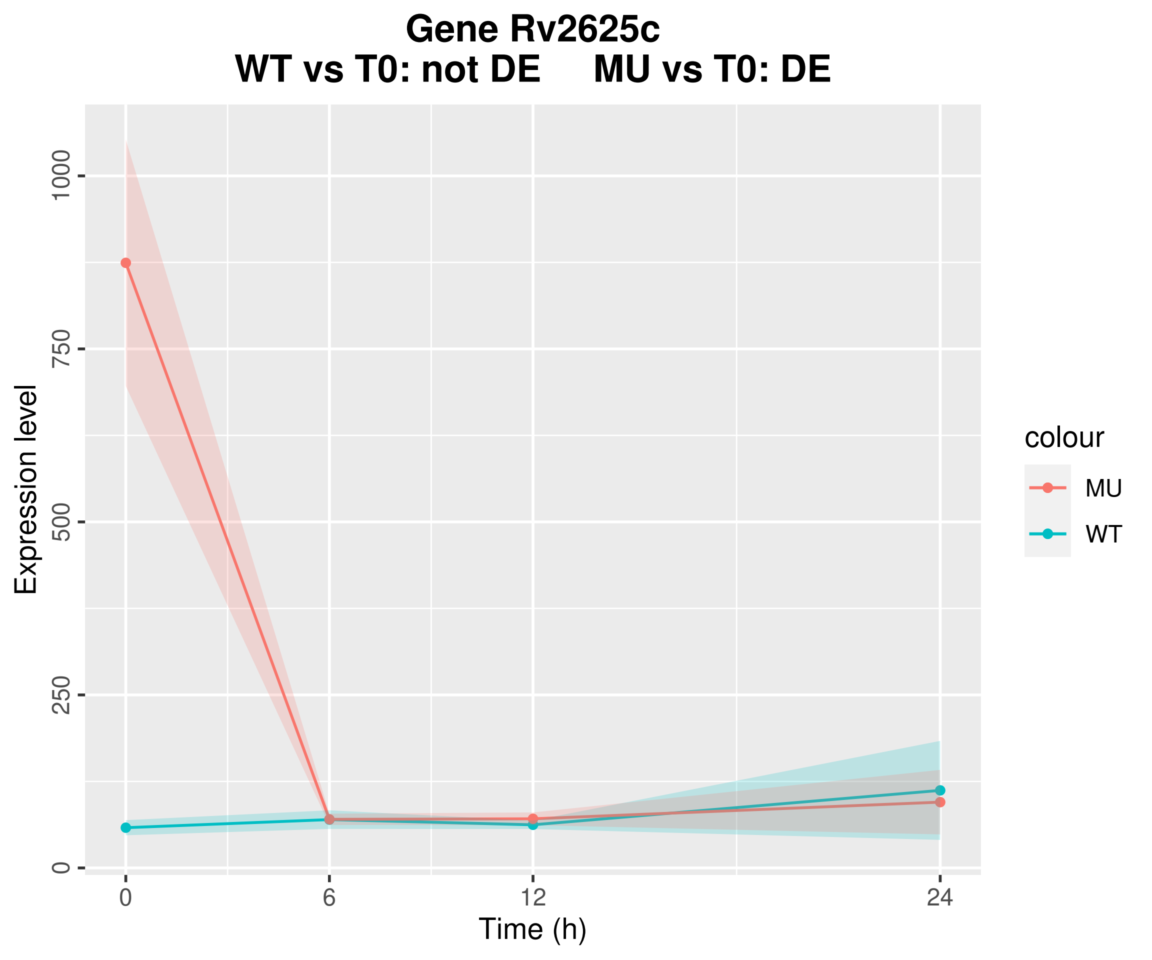

Gene Rv2625c
WT vs T0: not DE   MU vs T0: DE
Expression level
1000
750
500
250
0
0
6
12
24
Time (h)
colour
MU
WT

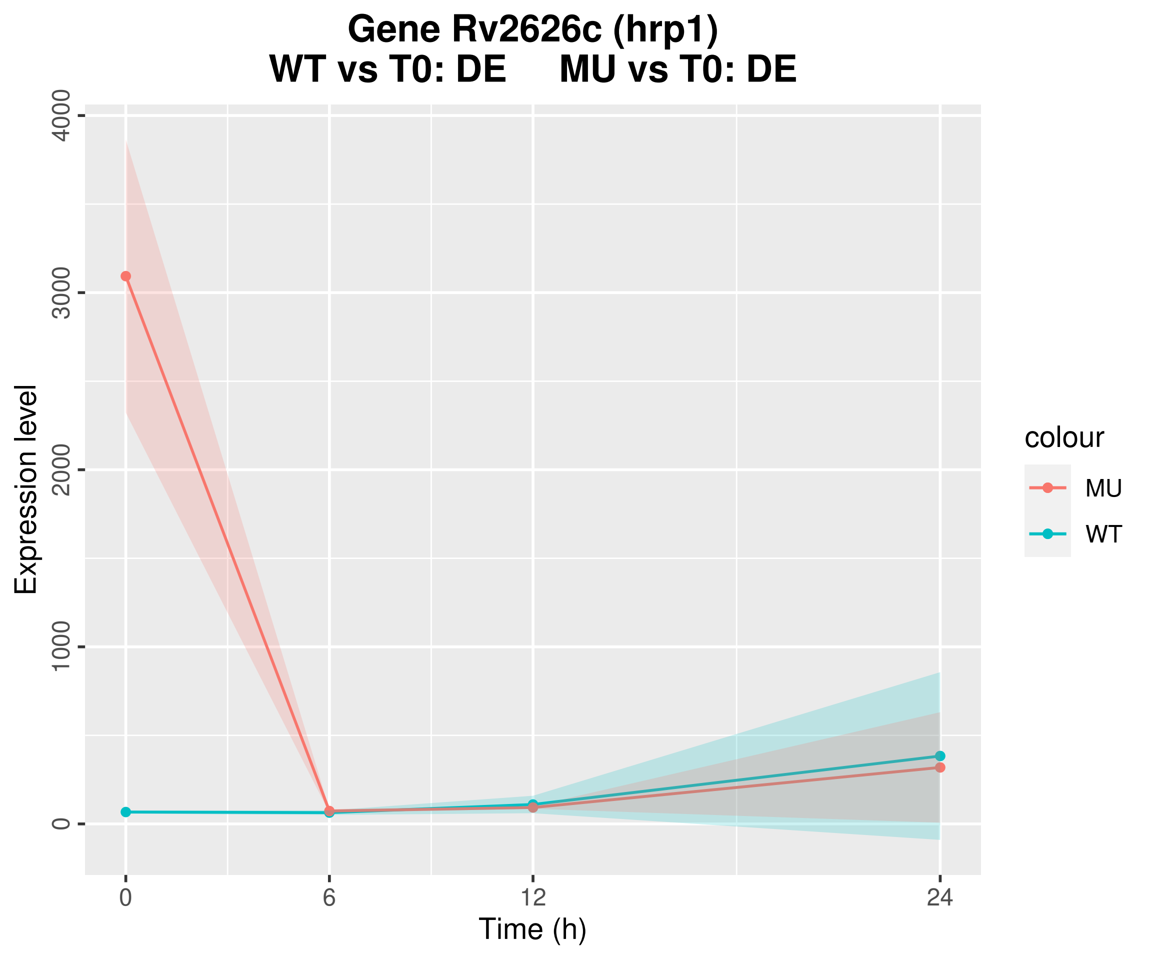

Gene Rv2626c (hrp1)
WT vs T0: DE MU vs T0: DE
4000
3000
2000
1000
0
Expression level
0
6
12
24
Time (h)
colour
MU
WT

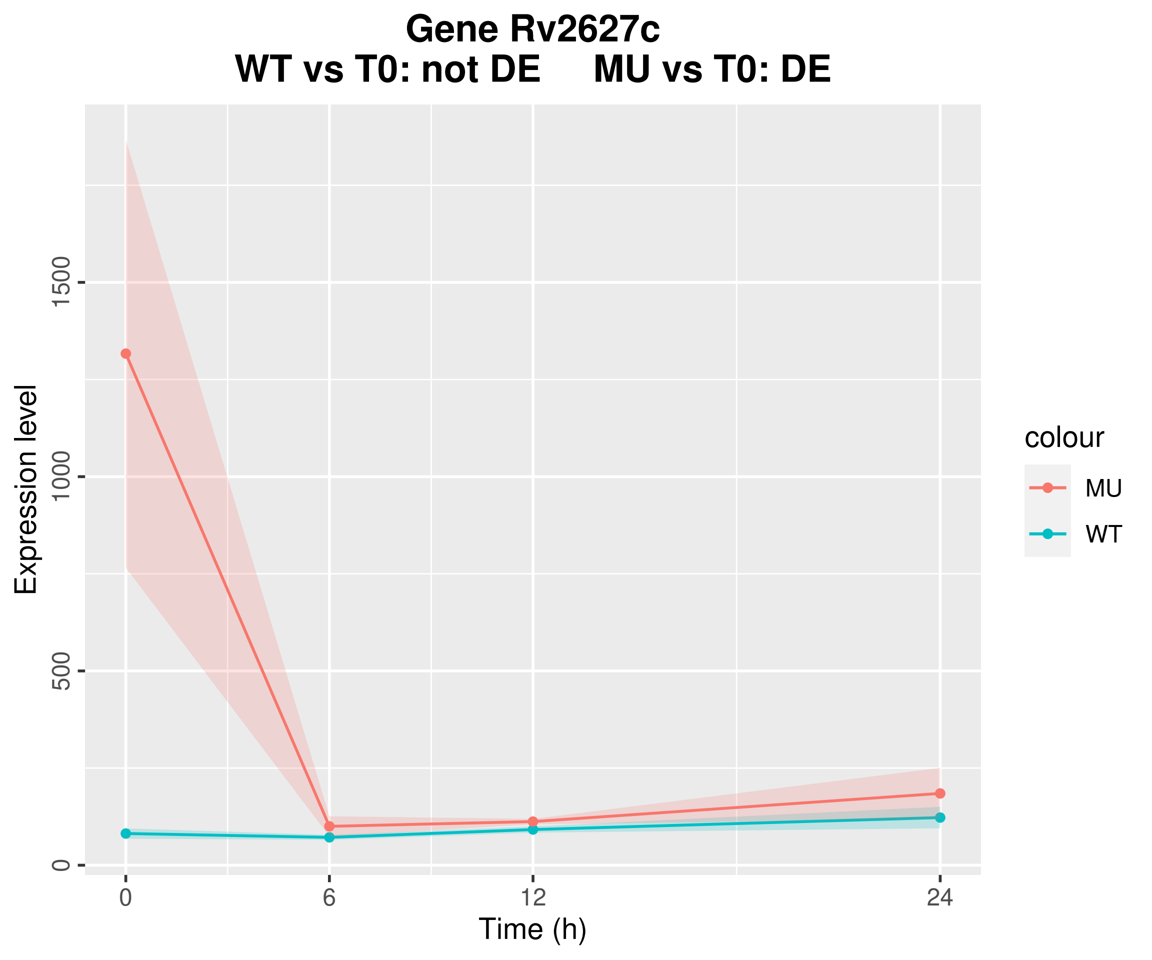

Gene Rv2627c
WT vs T0: not DE MU vs T0: DE
Expression level
1500
1000
500
0
0
6
12
24
Time (h)
colour
MU
WT

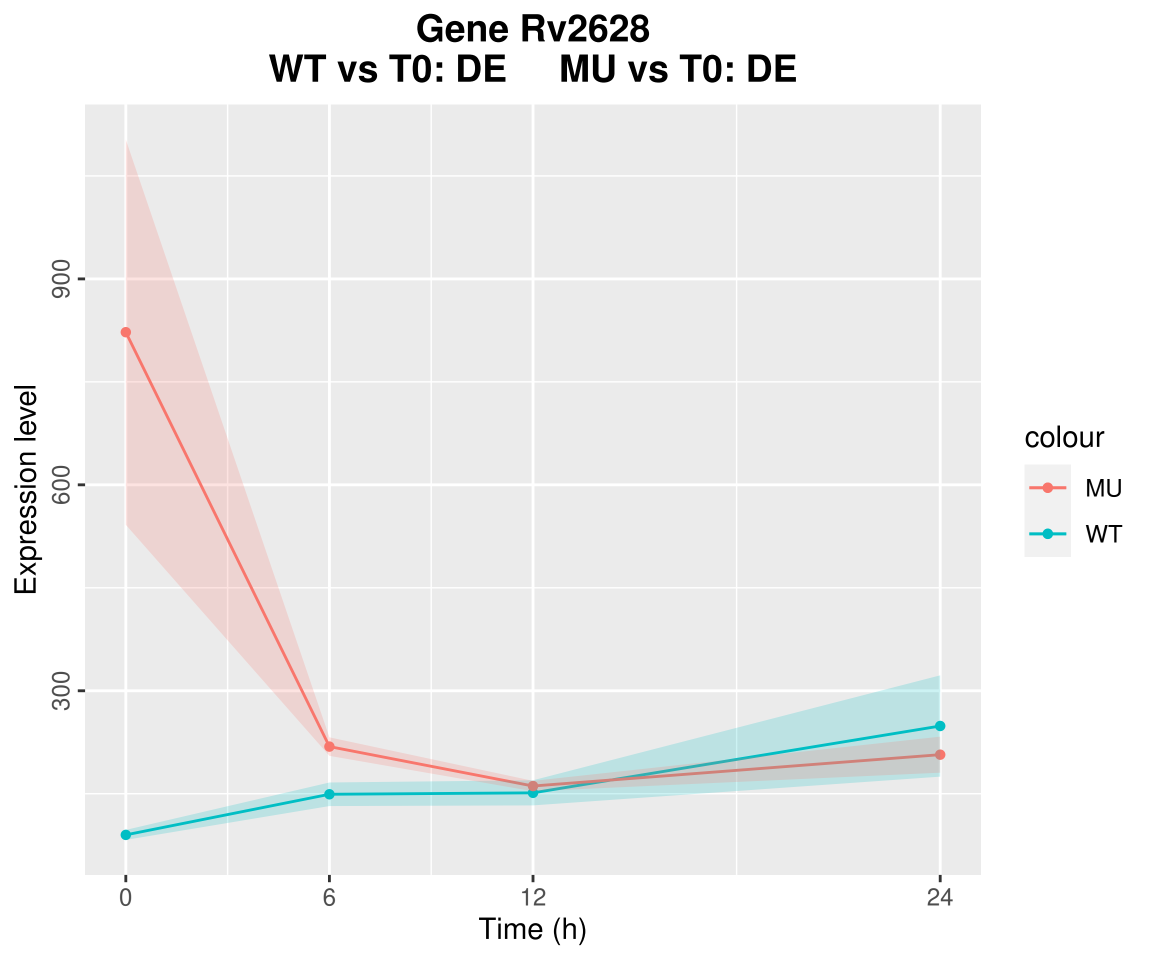
Gene Rv2628
WT vs T0: DE MU vs T0: DE
Expression level
900
600
300
0
6
12
24
Time (h)
colour
MU
WT

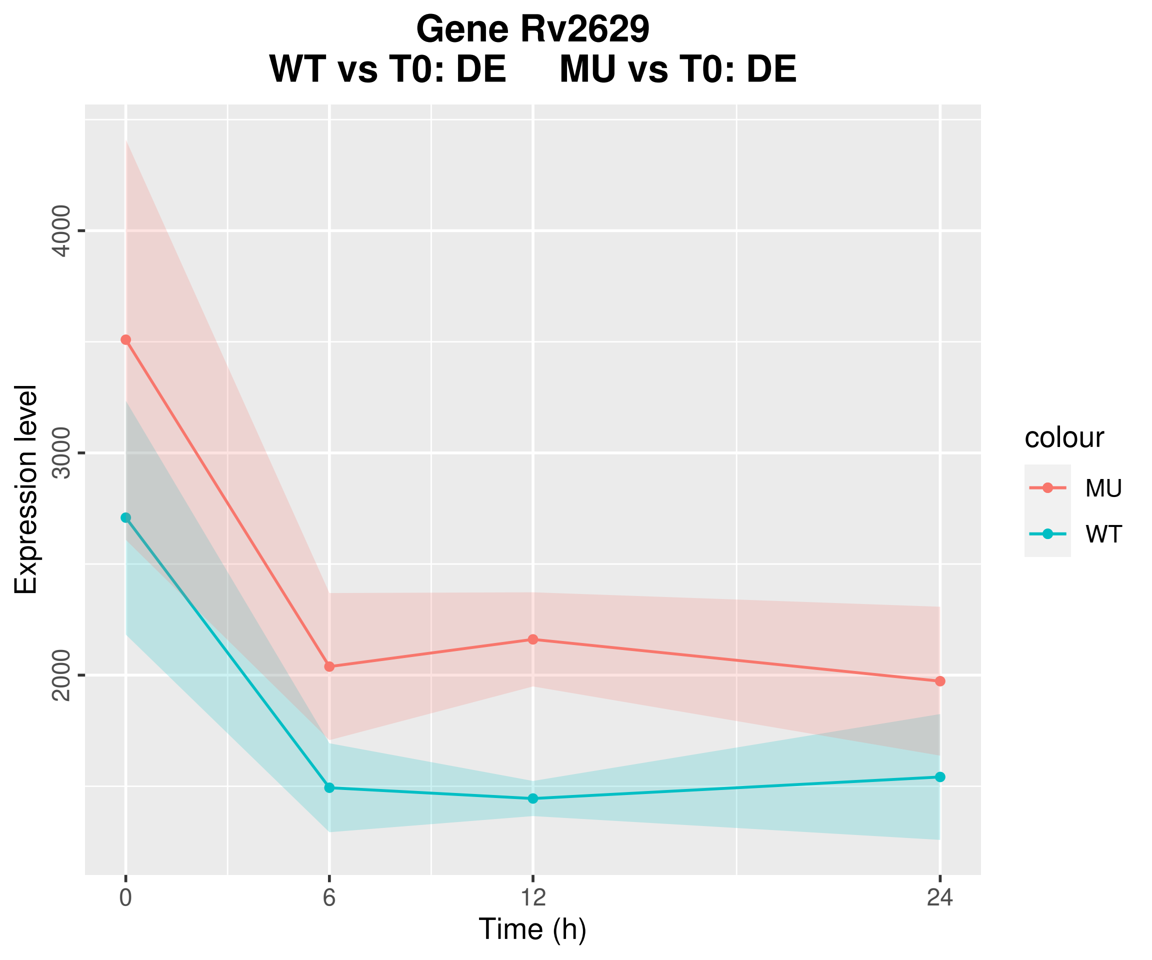

Gene Rv2629
WT vs T0: DE   MU vs T0: DE
Expression level
4000
3000
2000
0
6
12
24
Time (h)
colour
MU
WT

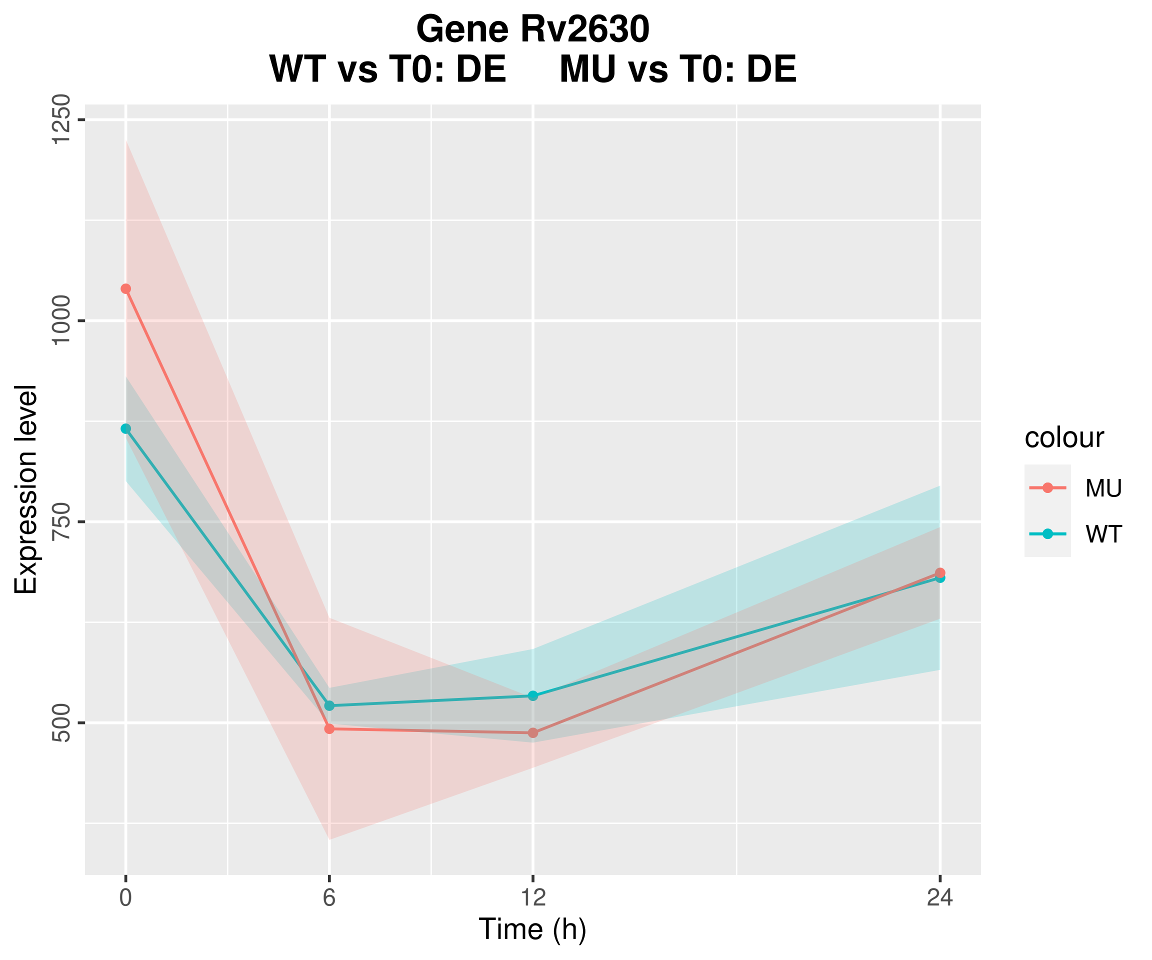

Gene Rv2630
WT vs T0: DE MU vs T0: DE
1250
1000
750
500
Expression level
0
6
12
24
Time (h)
colour
MU
WT

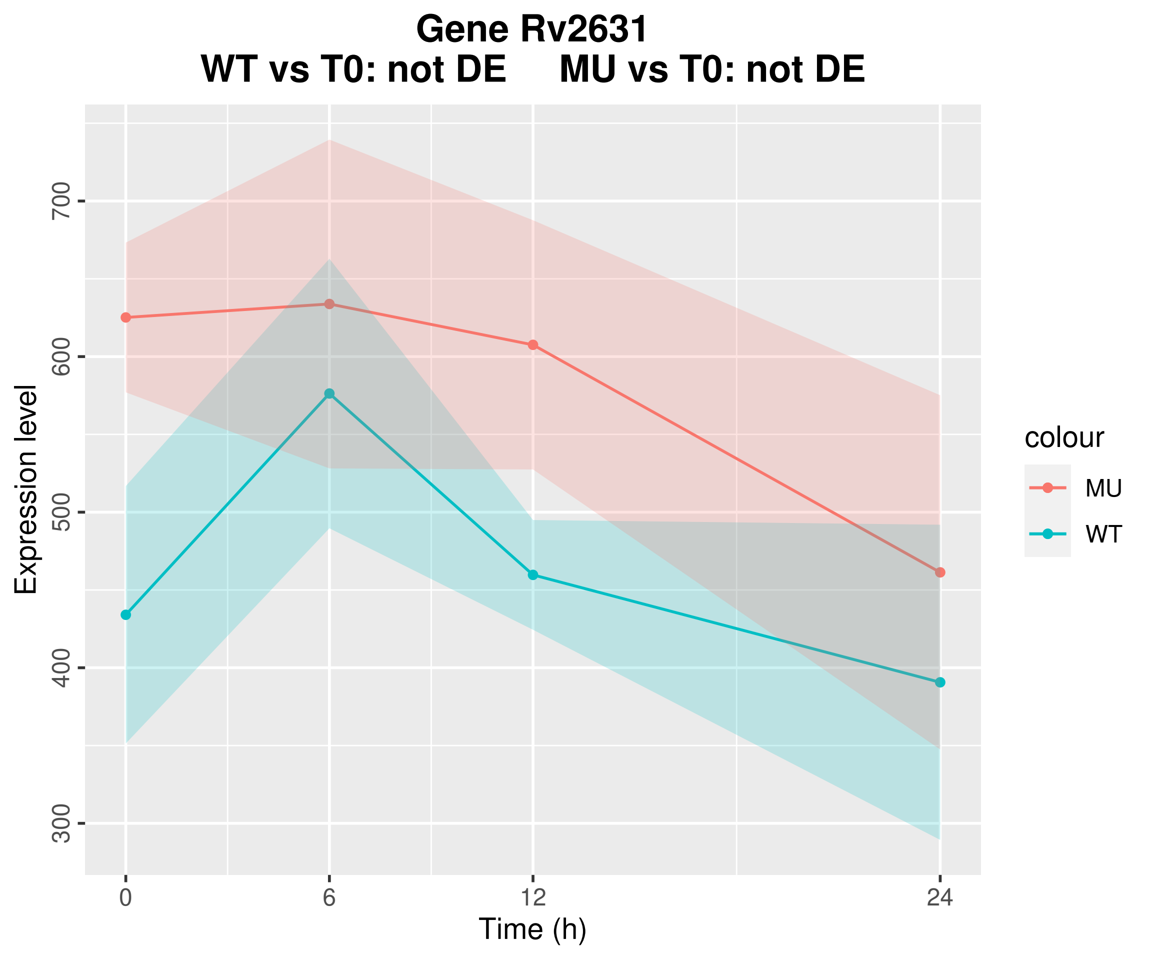
Gene Rv2631
WT vs T0: not DE MU vs T0: not DE
Expression level
700
600
500
400
300
0
6
12
24
Time (h)
colour
MU
WT

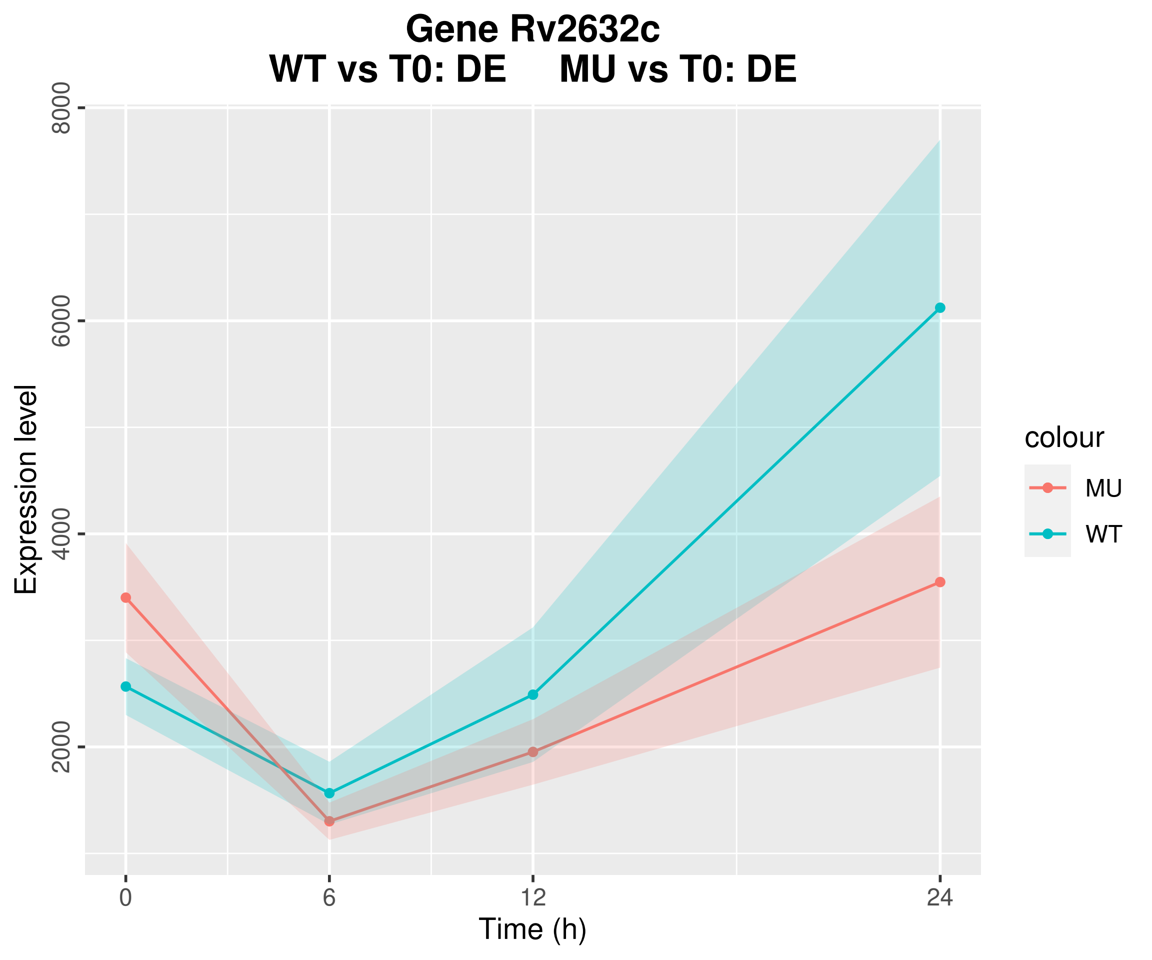

Gene Rv2632c
WT vs T0: DE MU vs T0: DE
8000
6000
4000
2000
Expression level
0
6
12
24
Time (h)
colour
MU
WT

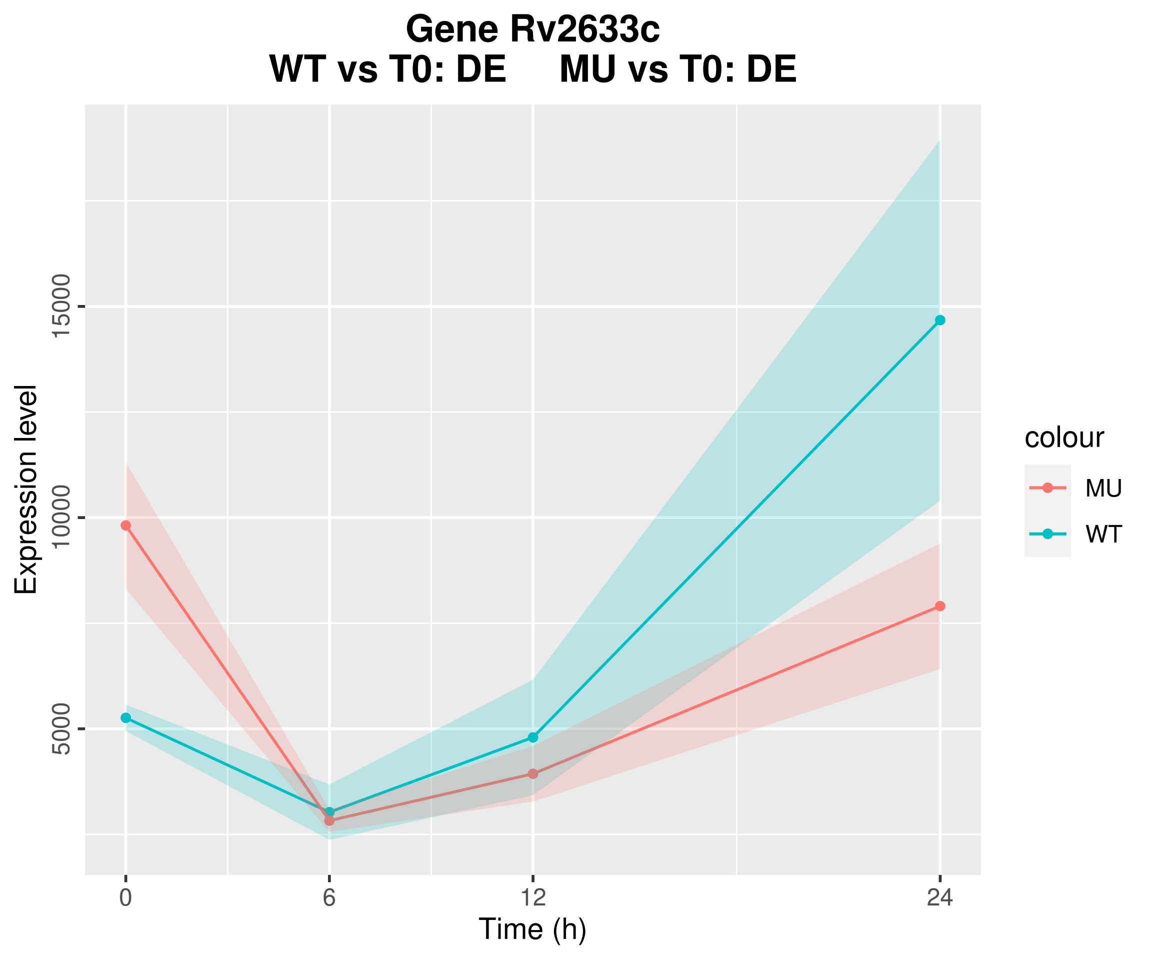
Gene Rv2633c
WT vs T0: DE MU vs T0: DE
Expression level
15000
10000
5000
0
6
12
24
Time (h)
colour
MU
WT

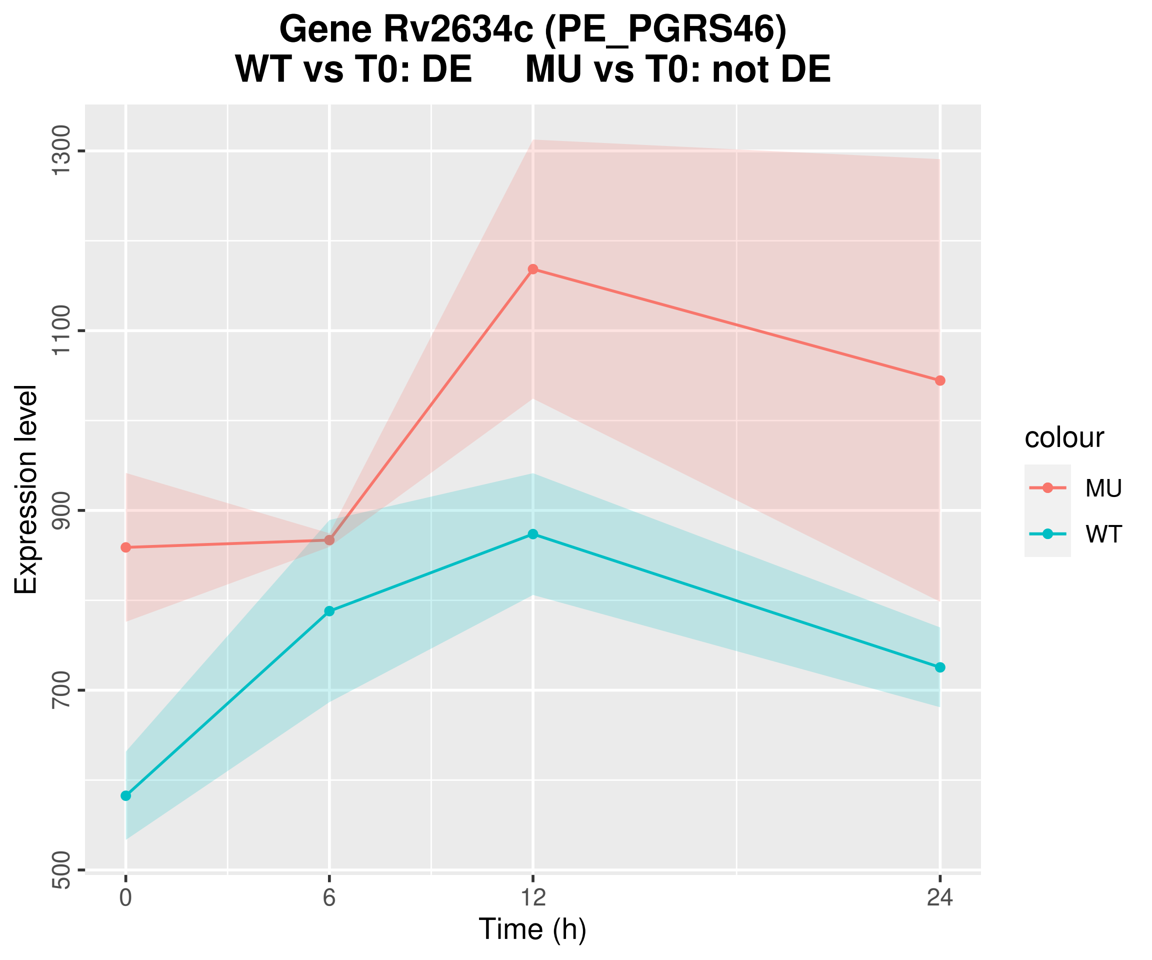

Gene Rv2634c (PE_PGRS46)
WT vs T0: DE   MU vs T0: not DE
Expression level
1300
1100
900
700
500
0
6
12
24
Time (h)
colour
MU
WT

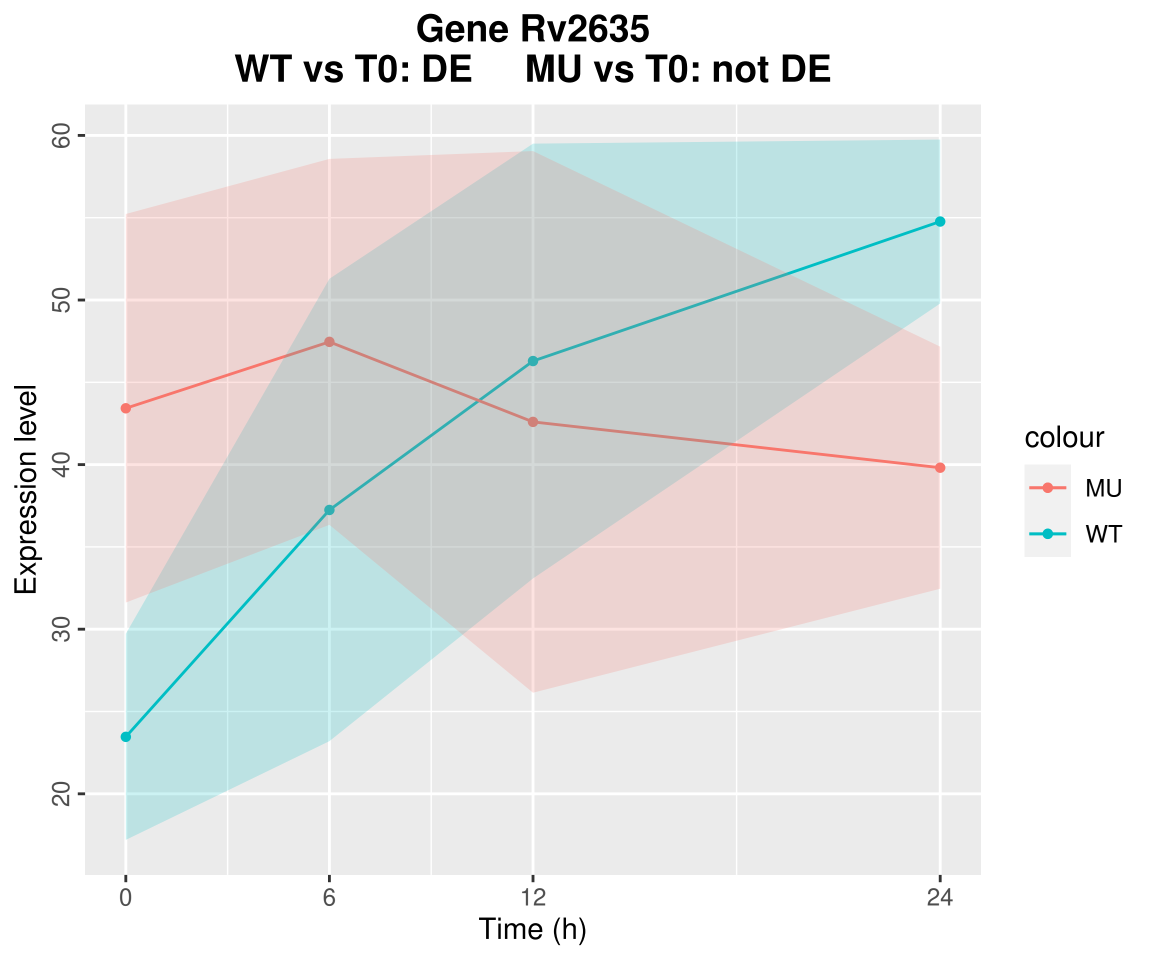

Gene Rv2635
WT vs T0: DE MU vs T0: not DE
Expression level
60
50
40
30
20
0
6
12
24
Time (h)
colour
MU
WT

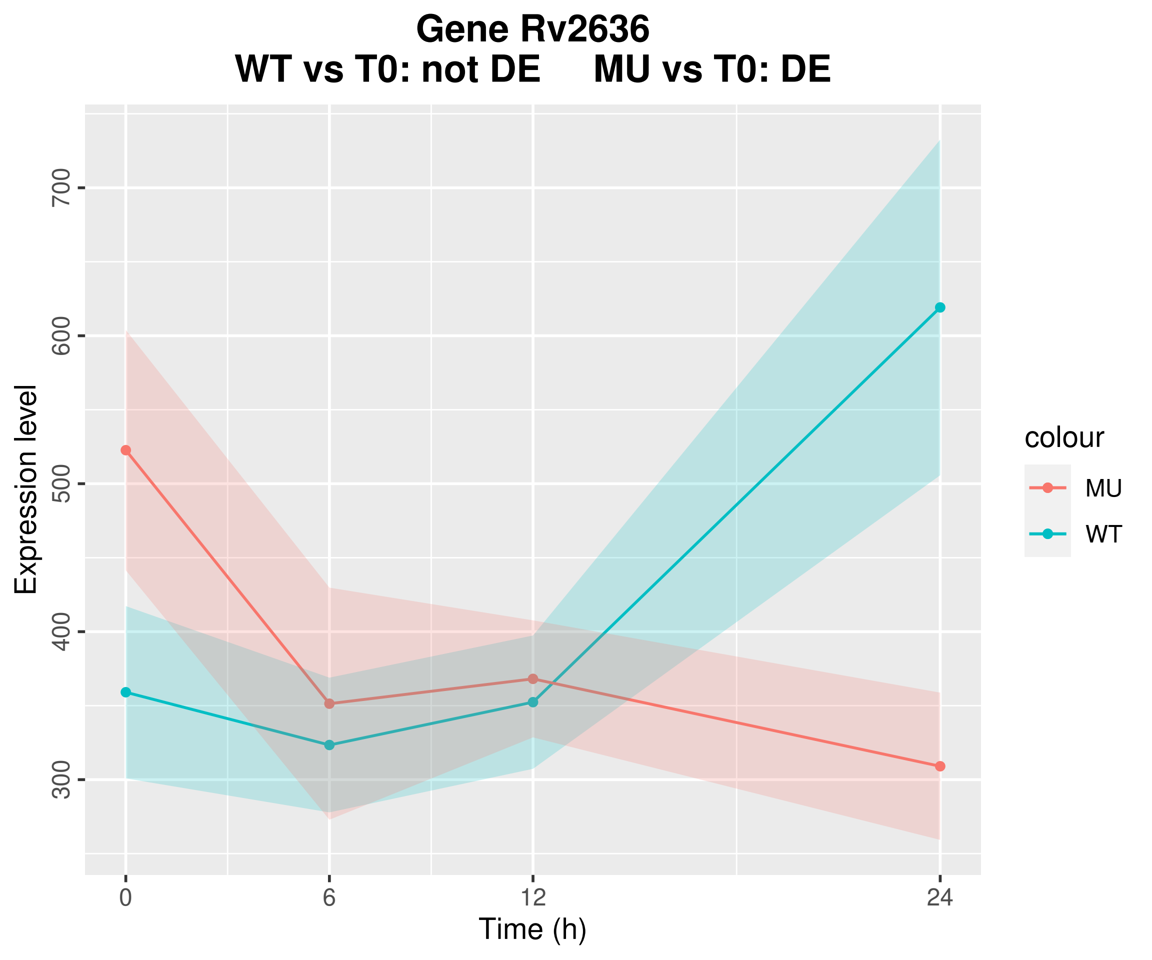
Gene Rv2636
WT vs T0: not DE   MU vs T0: DE
Expression level
300
400
500
600
700
0
6
12
24
Time (h)
colour
MU
WT

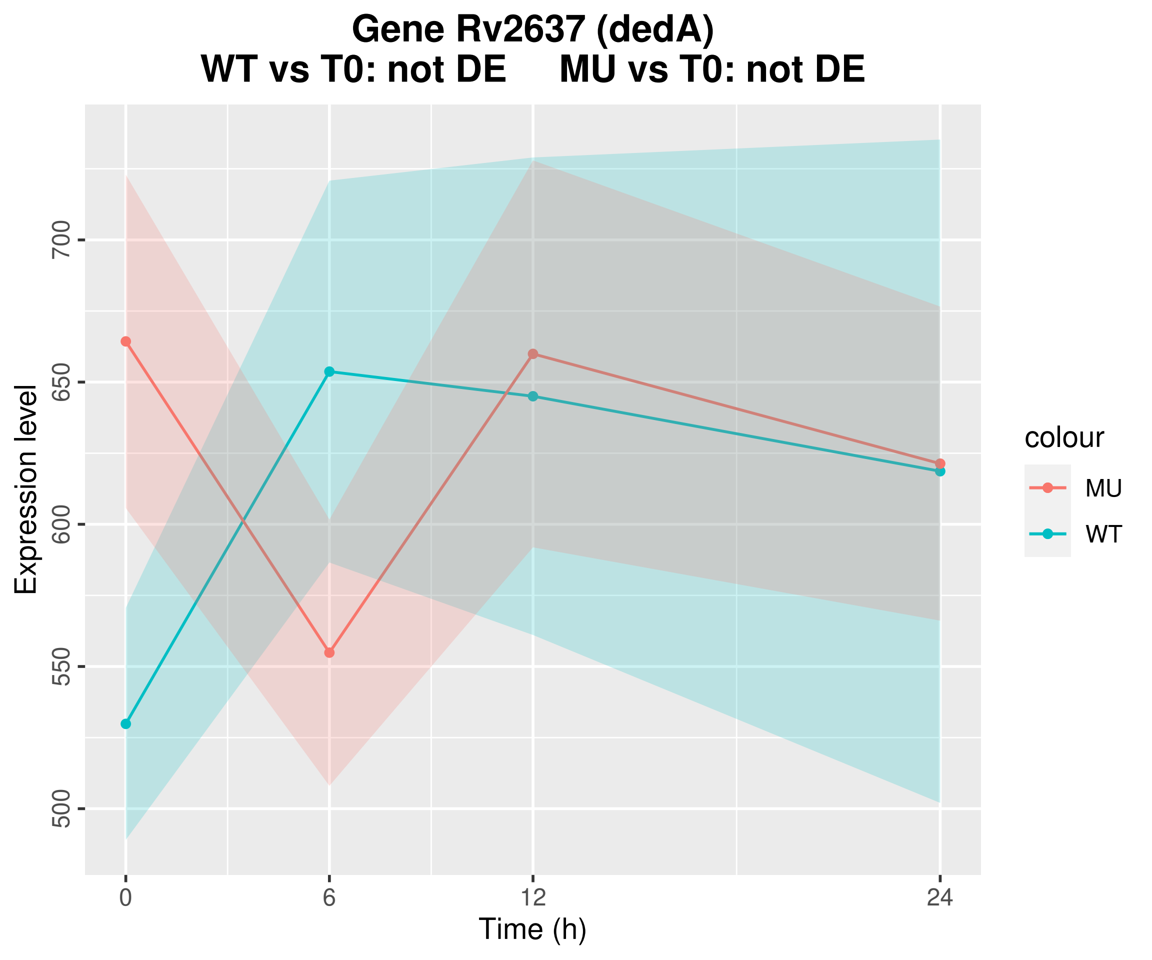
Gene Rv2637 (dedA)
WT vs T0: not DE   MU vs T0: not DE
Expression level
700
650
600
550
500
0
6
12
24
Time (h)
colour
MU
WT

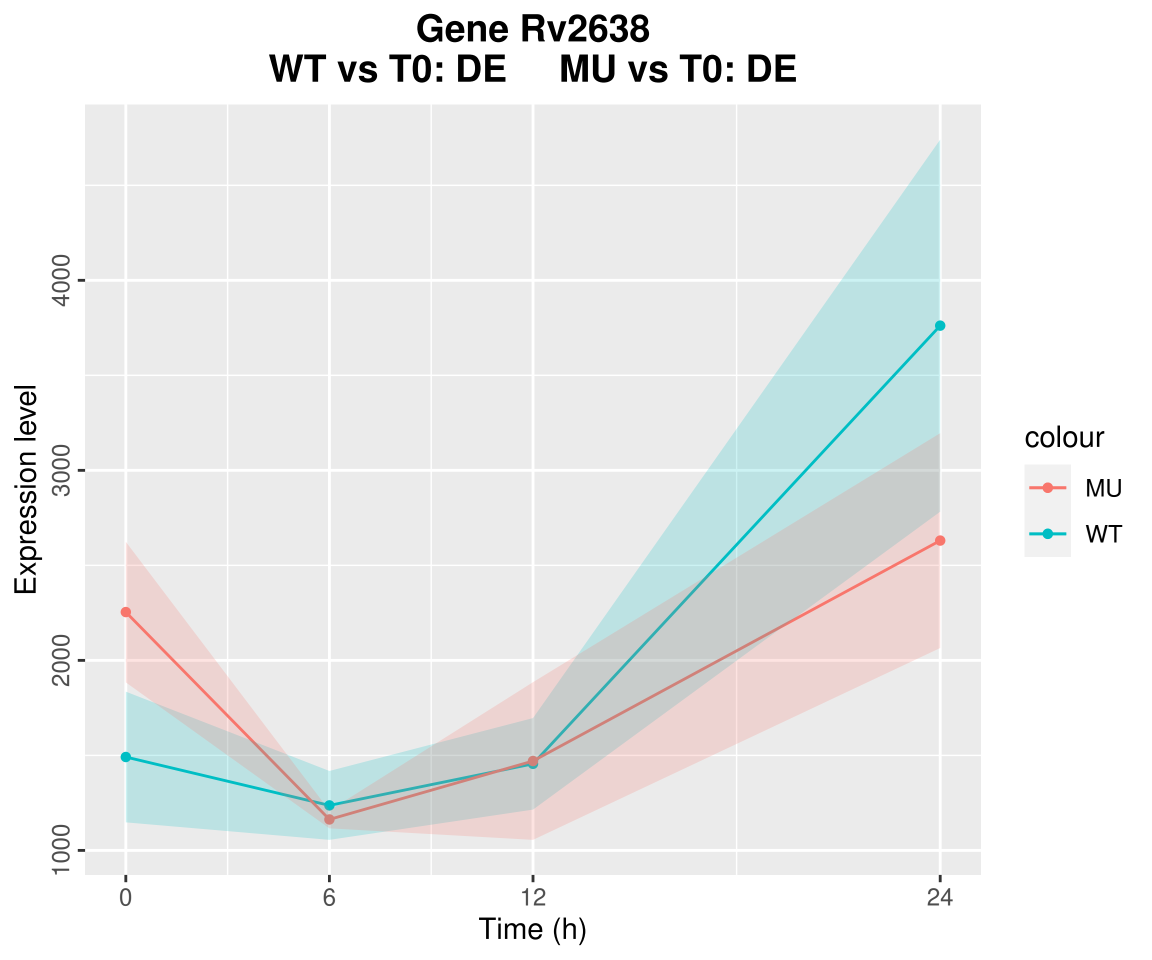
Gene Rv2638
WT vs T0: DE   MU vs T0: DE
Expression level
4000
3000
2000
1000
0
6
12
24
Time (h)
colour
MU
WT

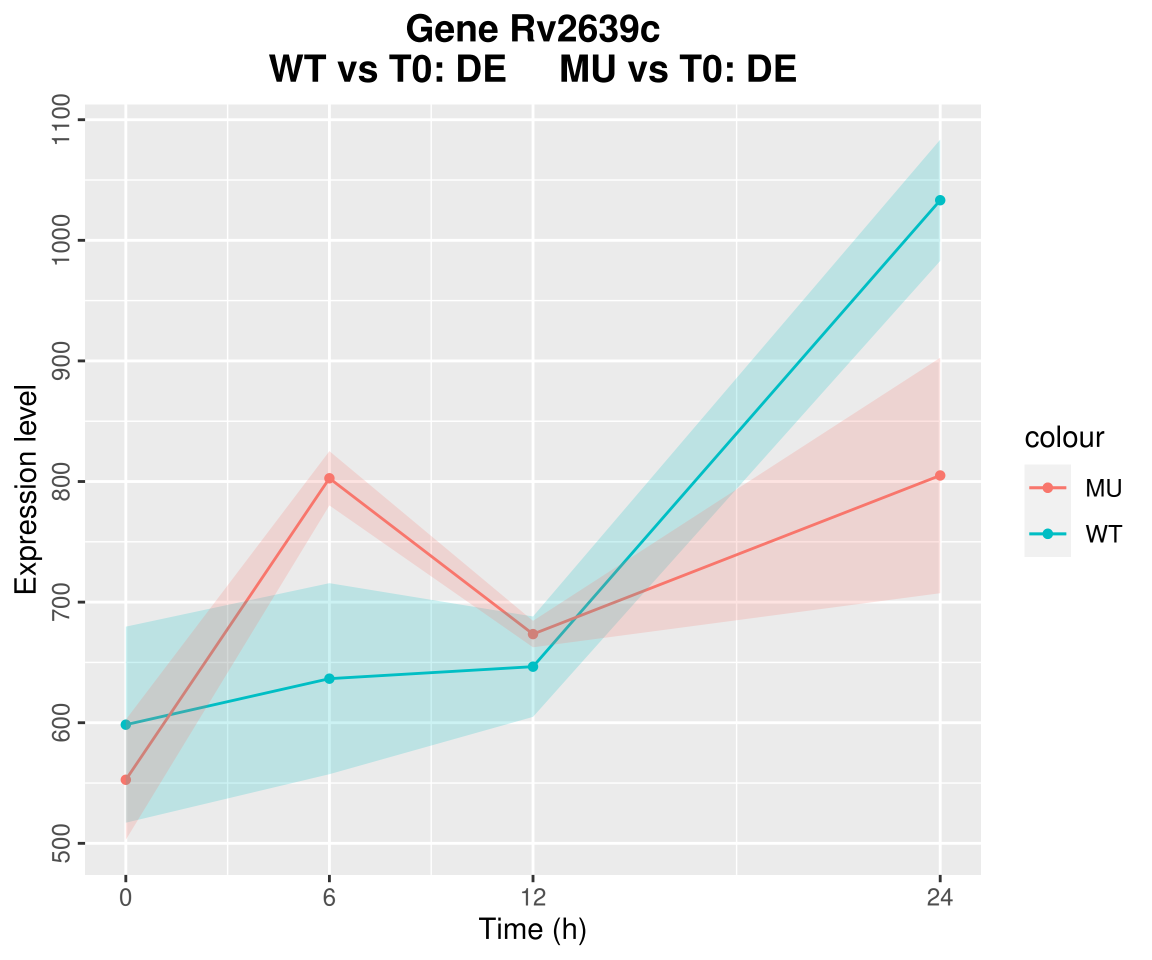
Gene Rv2639c
WT vs T0: DE MU vs T0: DE
Expression level
1100
1000
900
800
700
600
500
0
6
12
24
Time (h)
colour
MU
WT

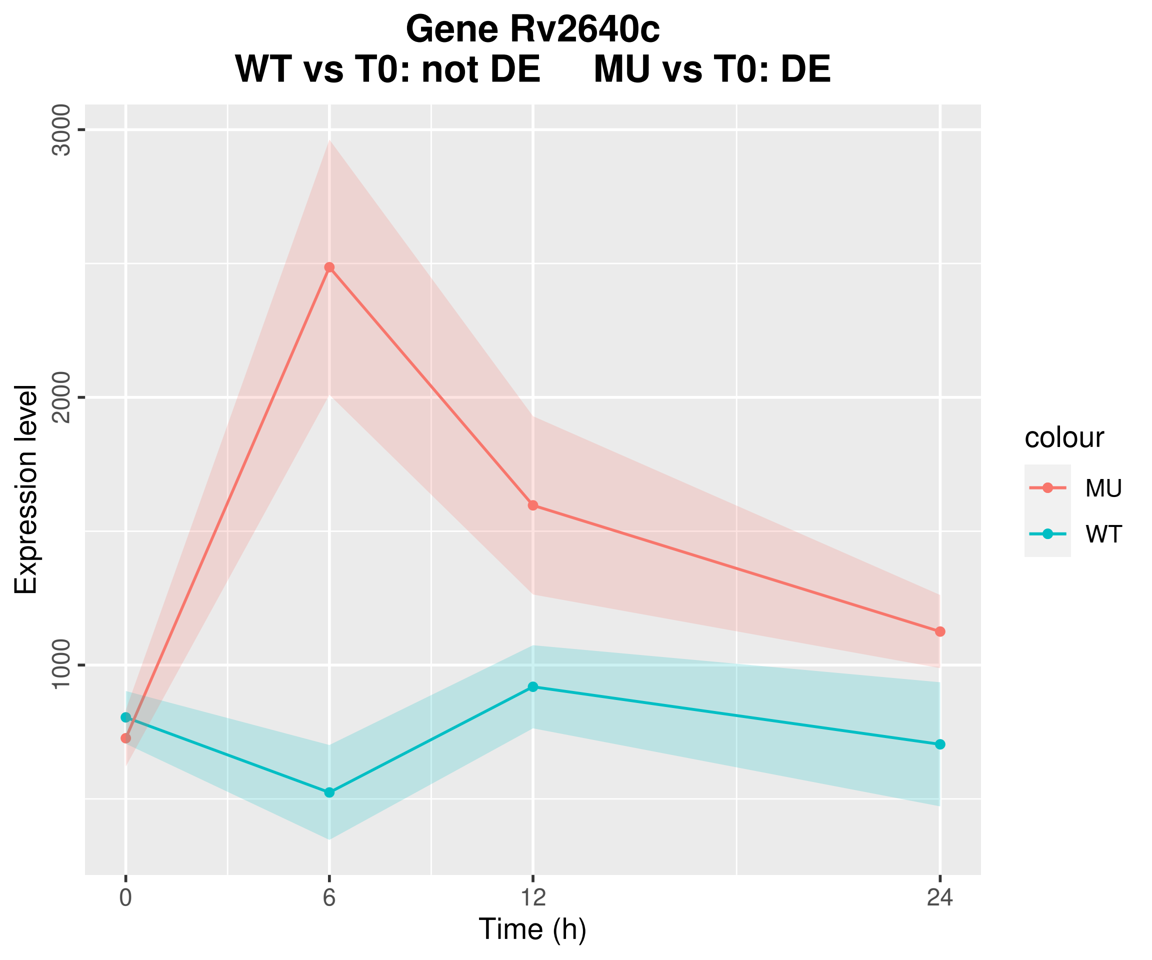

Gene Rv2640c
WT vs T0: not DE MU vs T0: DE
Expression level
3000
2000
1000
0
6
12
24
Time (h)
colour
MU
WT

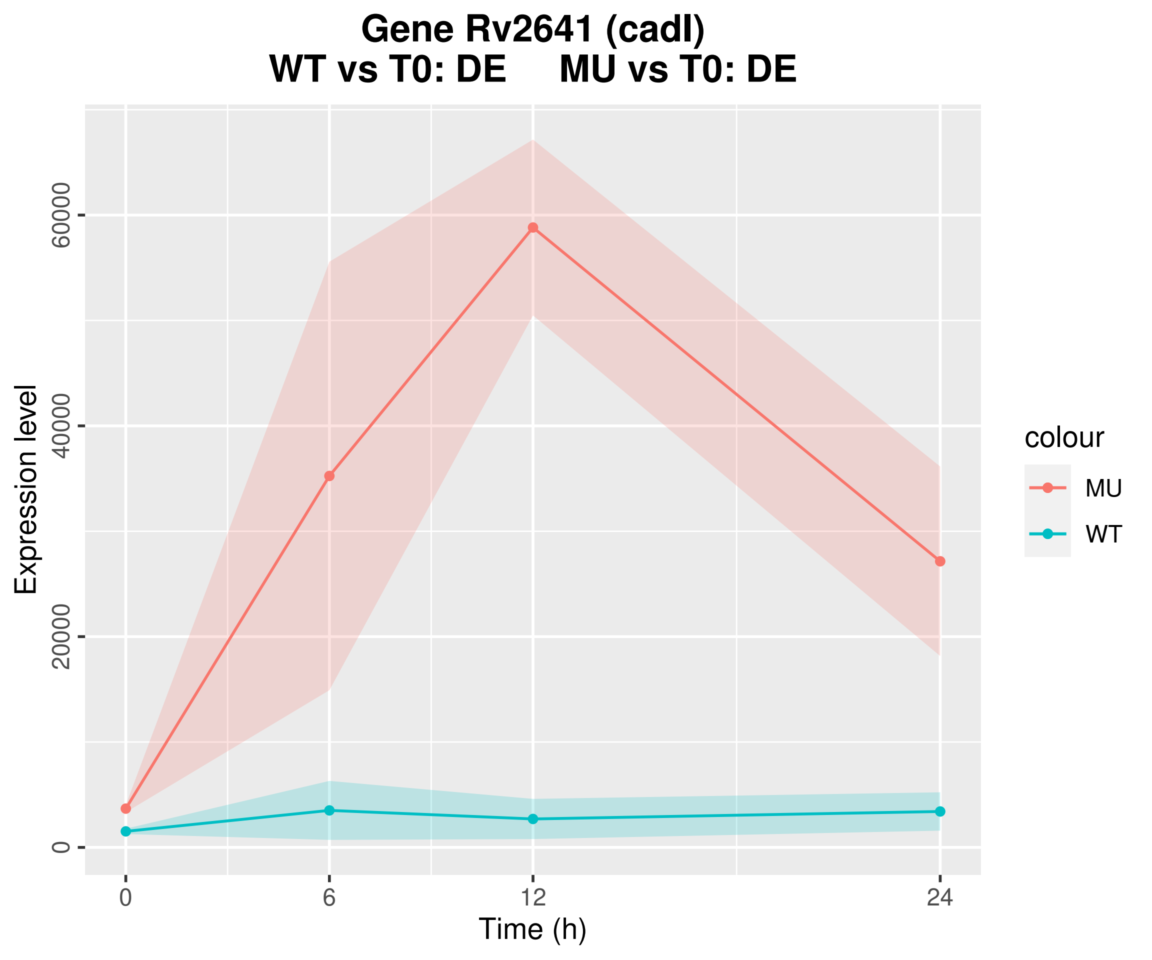

Gene Rv2641 (cadI)
WT vs T0: DE MU vs T0: DE
Expression level
0
20000
40000
60000
0
6
12
24
Time (h)
colour
MU
WT

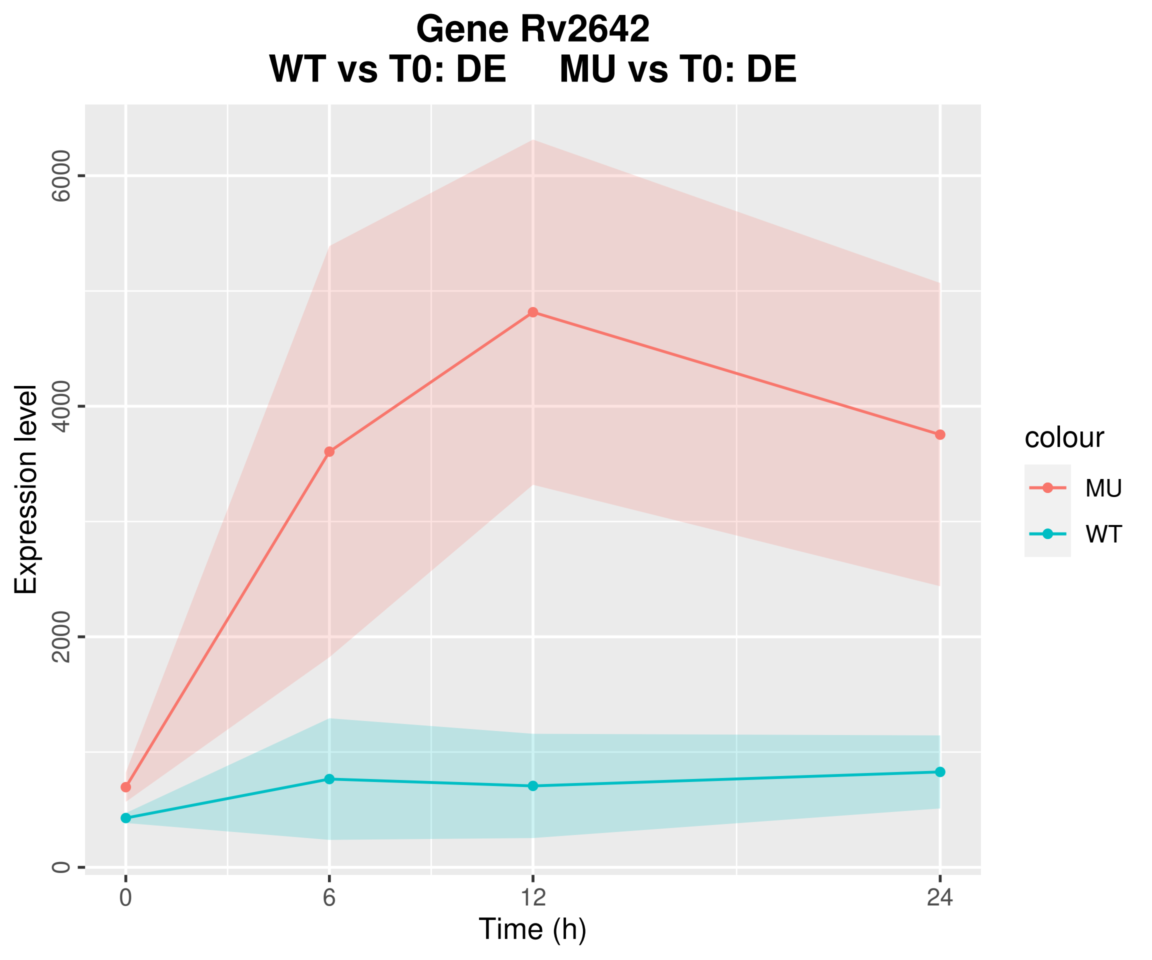
Gene Rv2642
WT vs T0: DE MU vs T0: DE
Expression level
6000
4000
2000
0
0
6
12
24
Time (h)
colour
MU
WT

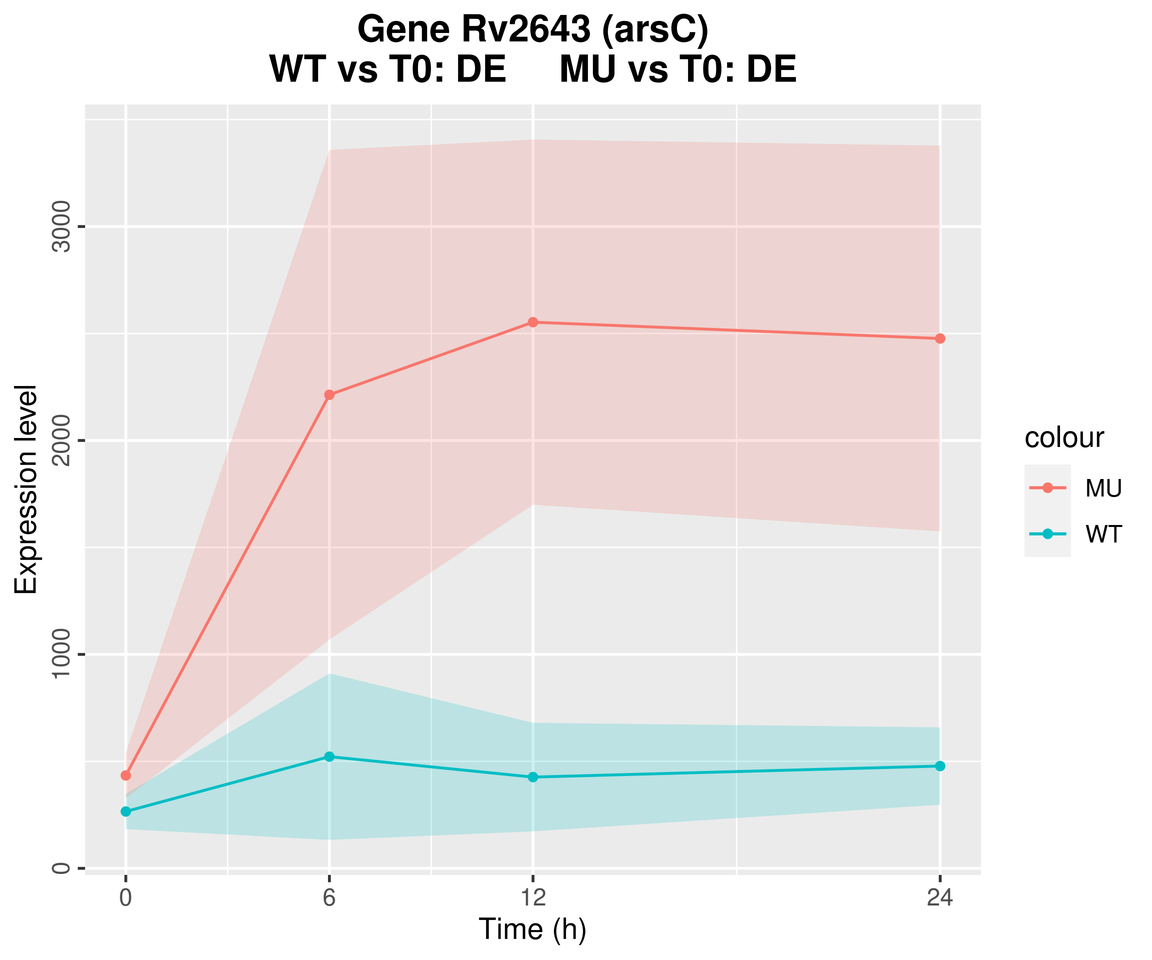
Gene Rv2643 (arsC)
WT vs T0: DE   MU vs T0: DE
Expression level
3000
2000
1000
0
0
6
12
24
Time (h)
colour
MU
WT

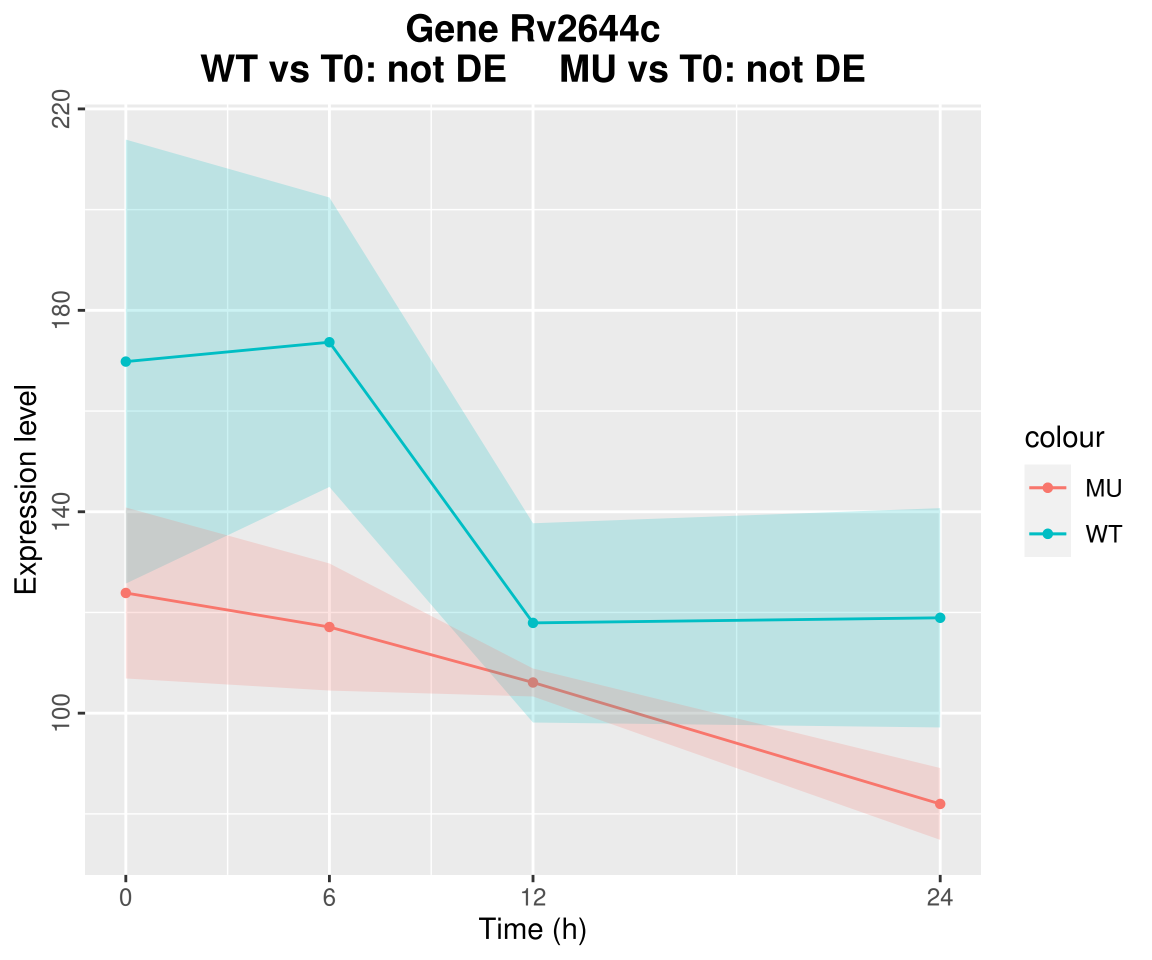
Gene Rv2644c
WT vs T0: not DE MU vs T0: not DE
220
180
140
100
Expression level
0
6
12
24
Time (h)
colour
MU
WT

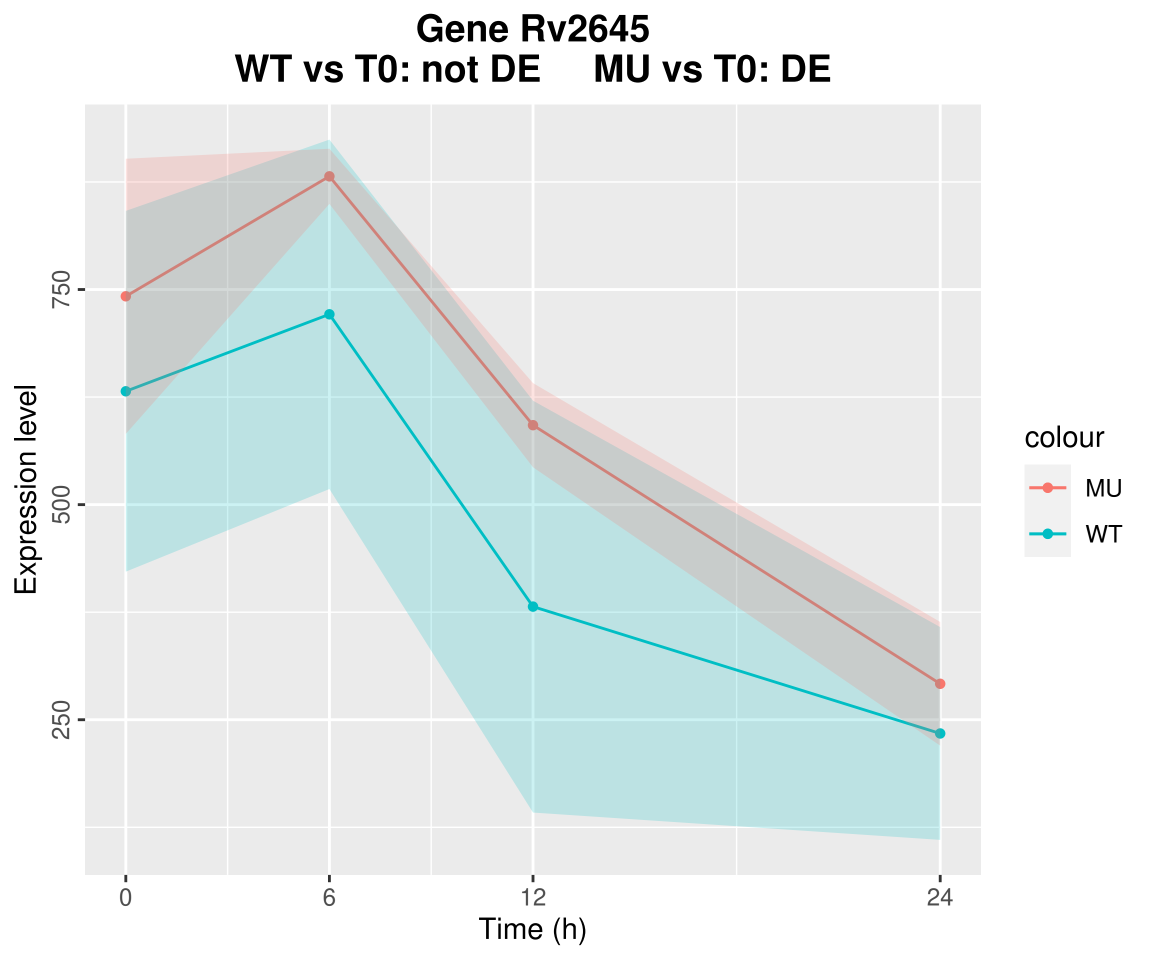
Gene Rv2645
WT vs T0: not DE   MU vs T0: DE
Expression level
750
500
250
0
6
12
24
Time (h)
colour
MU
WT

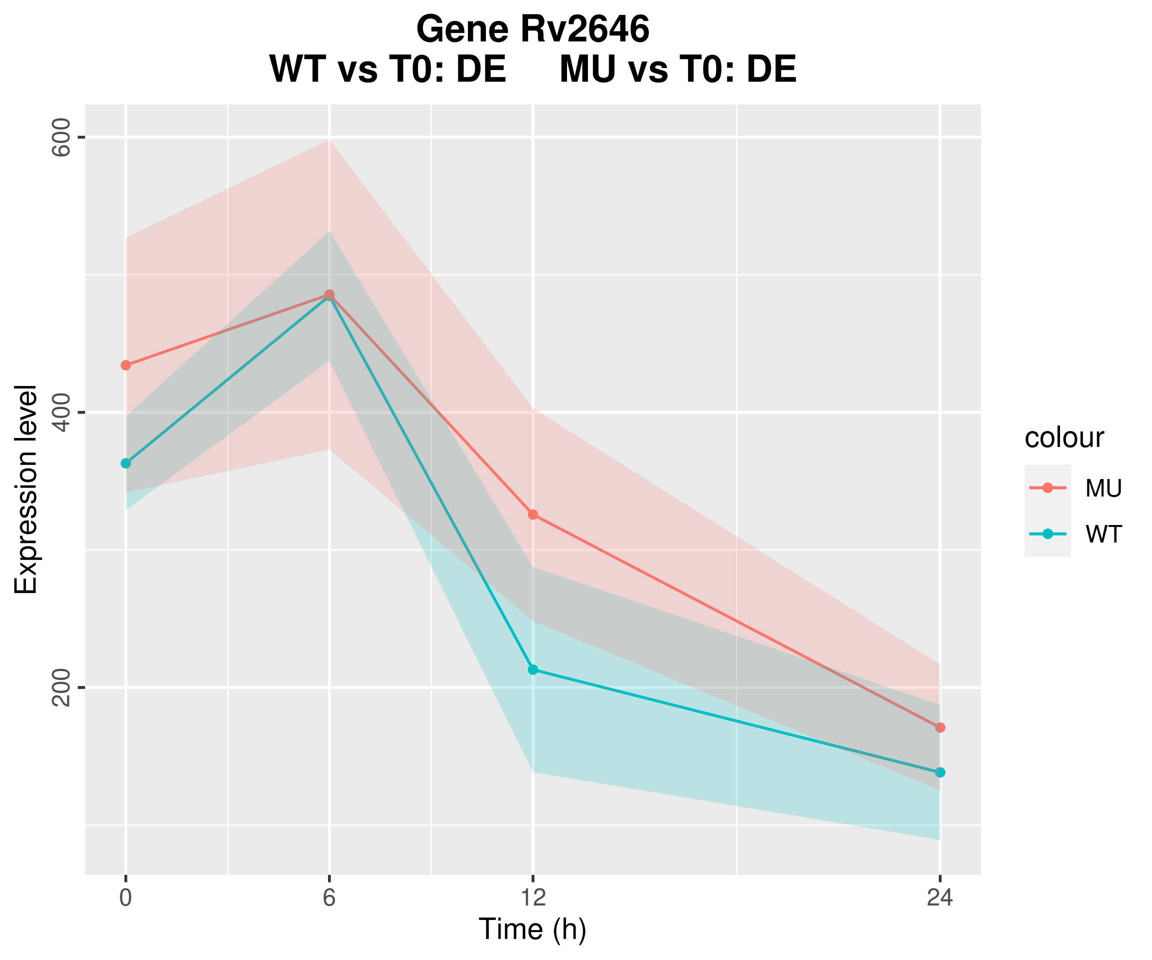

Gene Rv2646
WT vs T0: DE   MU vs T0: DE
600
400
200
Expression level
0
6
12
24
Time (h)
colour
MU
WT

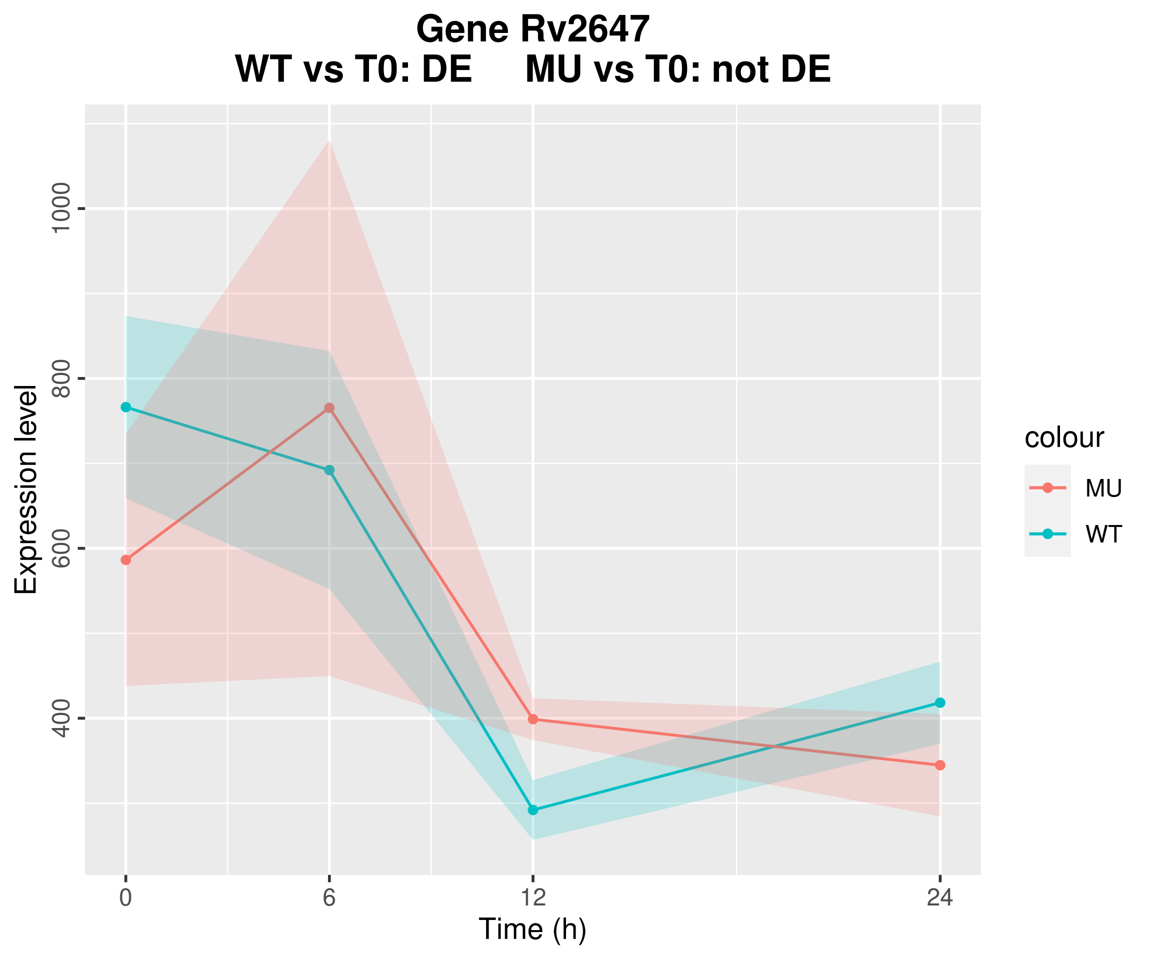
Gene Rv2647
WT vs T0: DE MU vs T0: not DE
Expression level
1000
800
600
400
0
6
12
24
Time (h)
colour
MU
WT

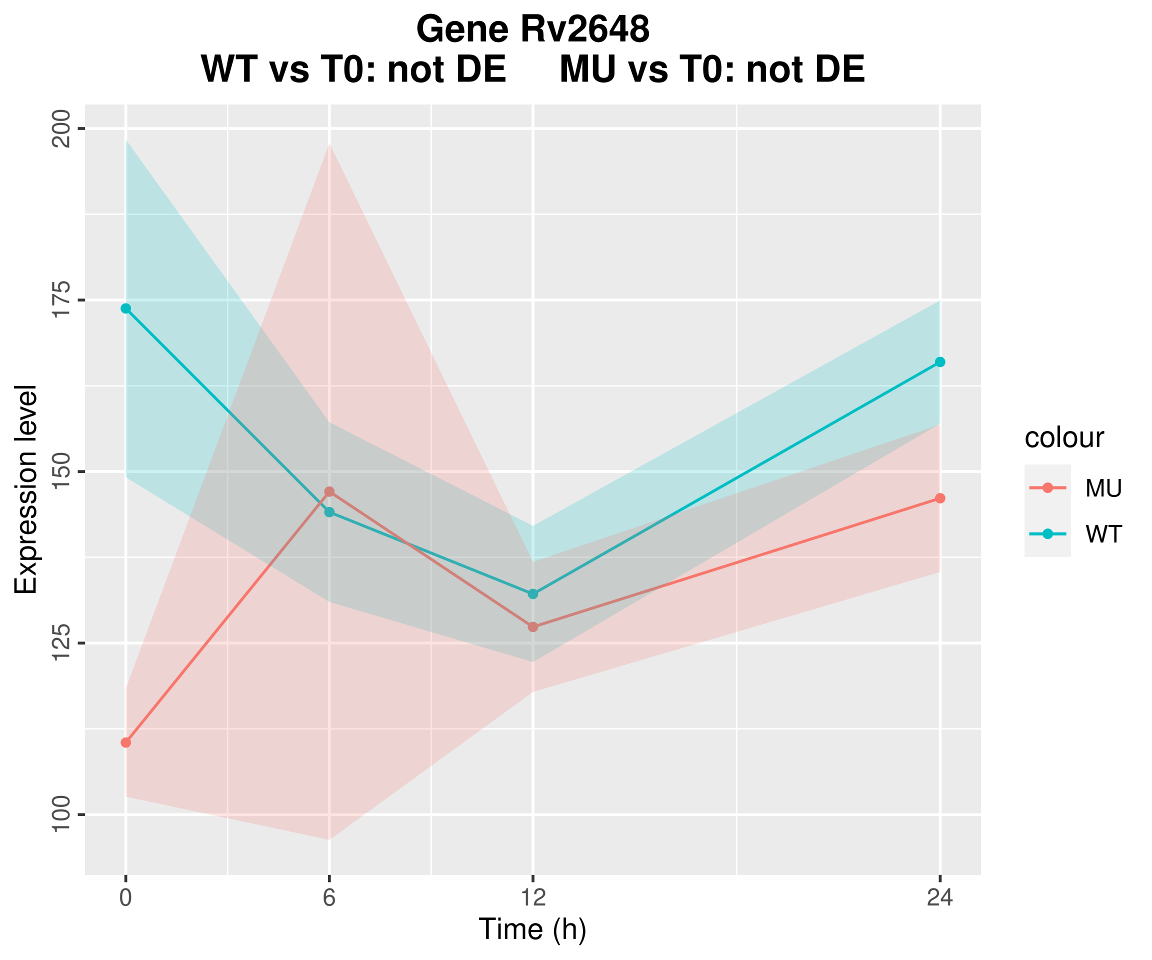

Gene Rv2648
WT vs T0: not DE   MU vs T0: not DE
Expression level
200
175
150
125
100
0
6
12
24
Time (h)
colour
MU
WT

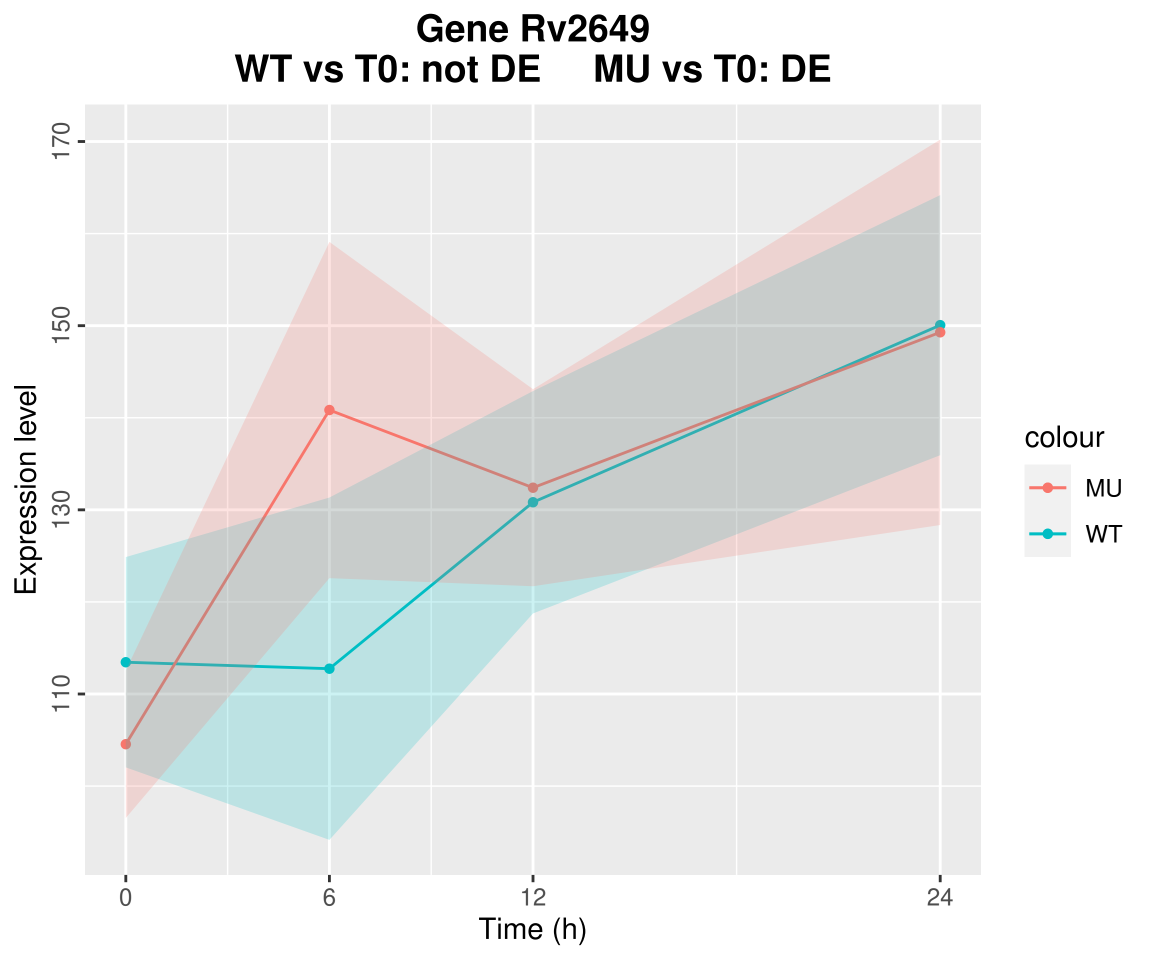
Gene Rv2649
WT vs T0: not DE MU vs T0: DE
Expression level
170
150
130
110
0
6
12
24
Time (h)
colour
MU
WT

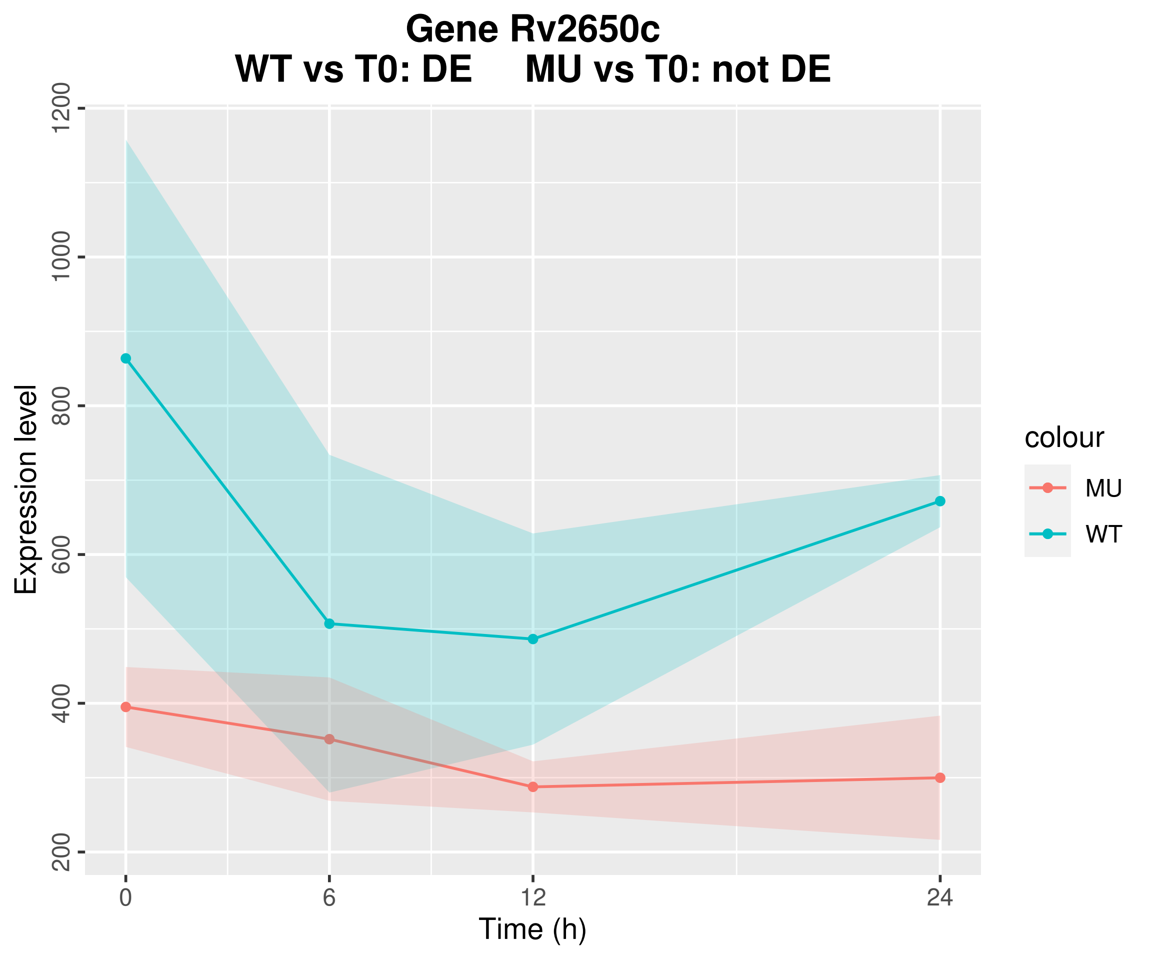
Gene Rv2650c
WT vs T0: DE   MU vs T0: not DE
Expression level
1200
1000
800
600
400
200
0
6
12
24
Time (h)
colour
MU
WT

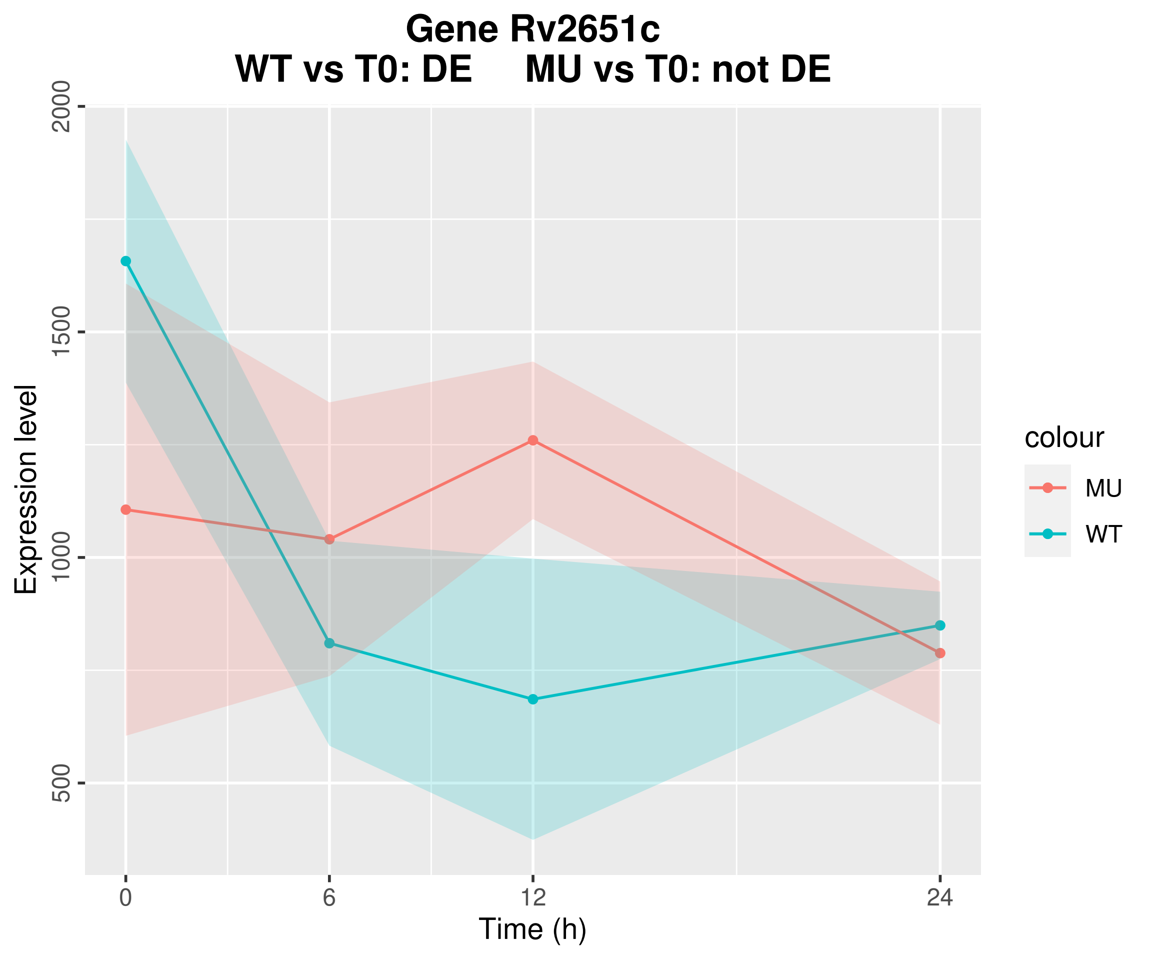
Gene Rv2651c
WT vs T0: DE   MU vs T0: not DE
Expression level
2000
1500
1000
500
0
6
12
24
Time (h)
colour
MU
WT

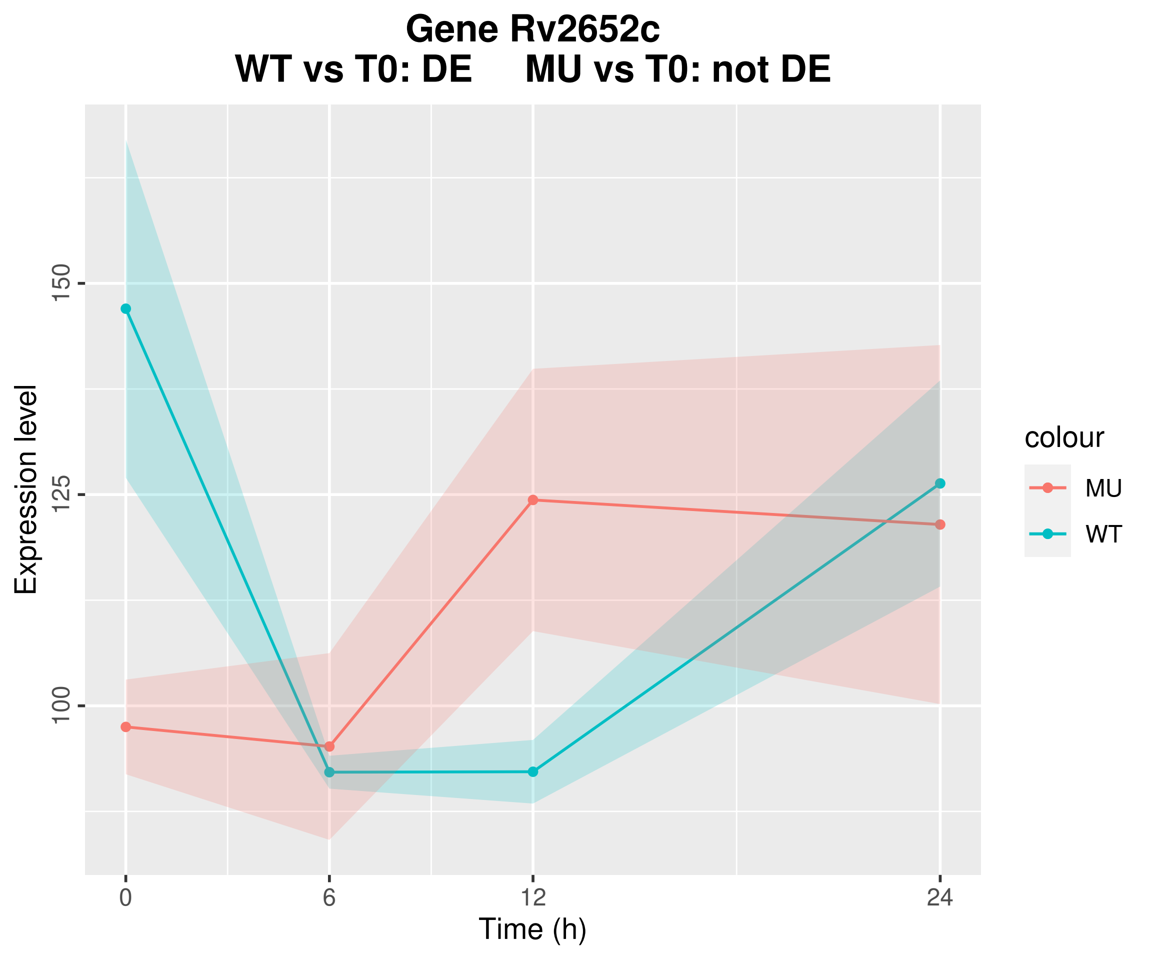

Gene Rv2652c
WT vs T0: DE   MU vs T0: not DE
150
125
100
Expression level
0
6
12
24
Time (h)
colour
MU
WT

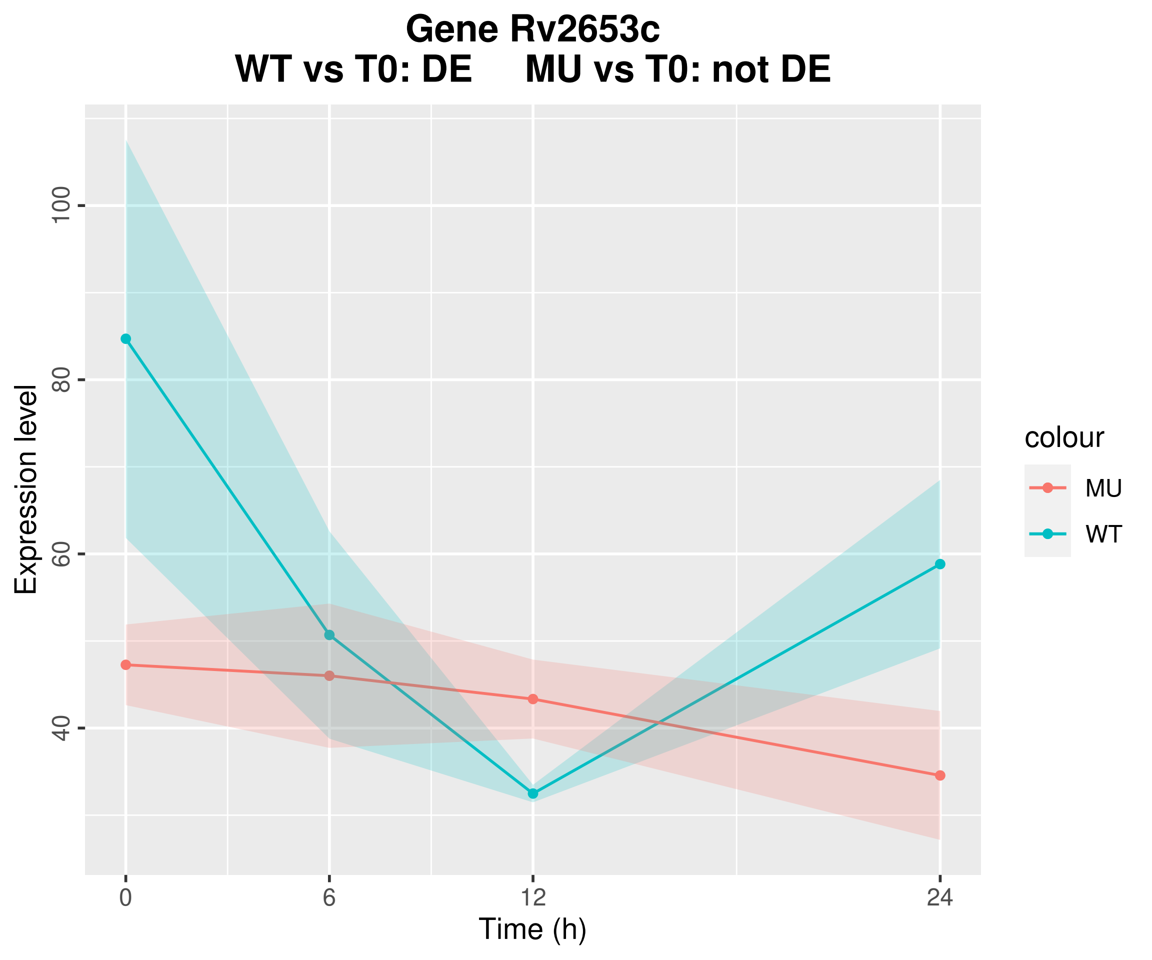
Gene Rv2653c
WT vs T0: DE   MU vs T0: not DE
Expression level
100
80
60
40
0
6
12
24
Time (h)
colour
MU
WT

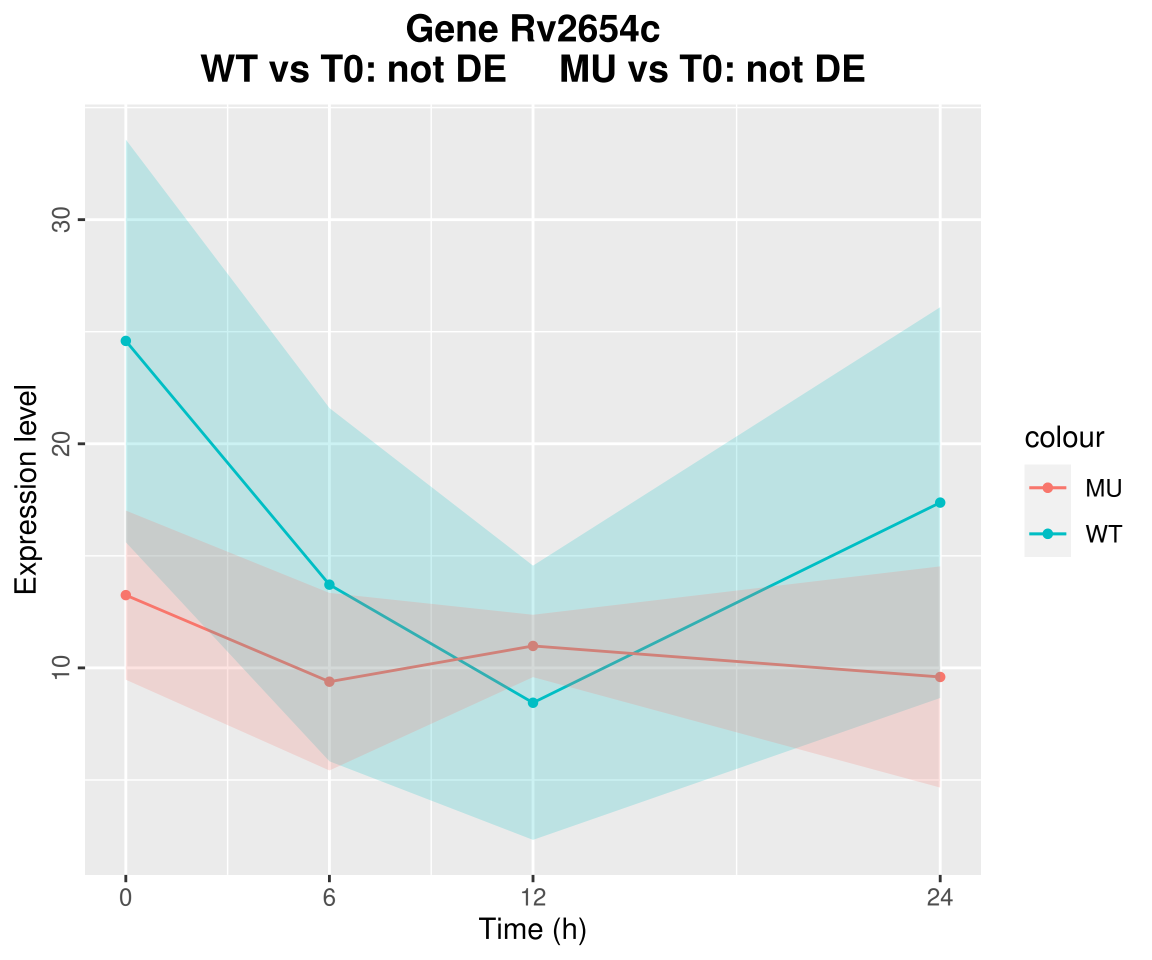
Gene Rv2654c
WT vs T0: not DE   MU vs T0: not DE
30
20
10
0
6
12
24
Time (h)
Expression level
colour
MU
WT

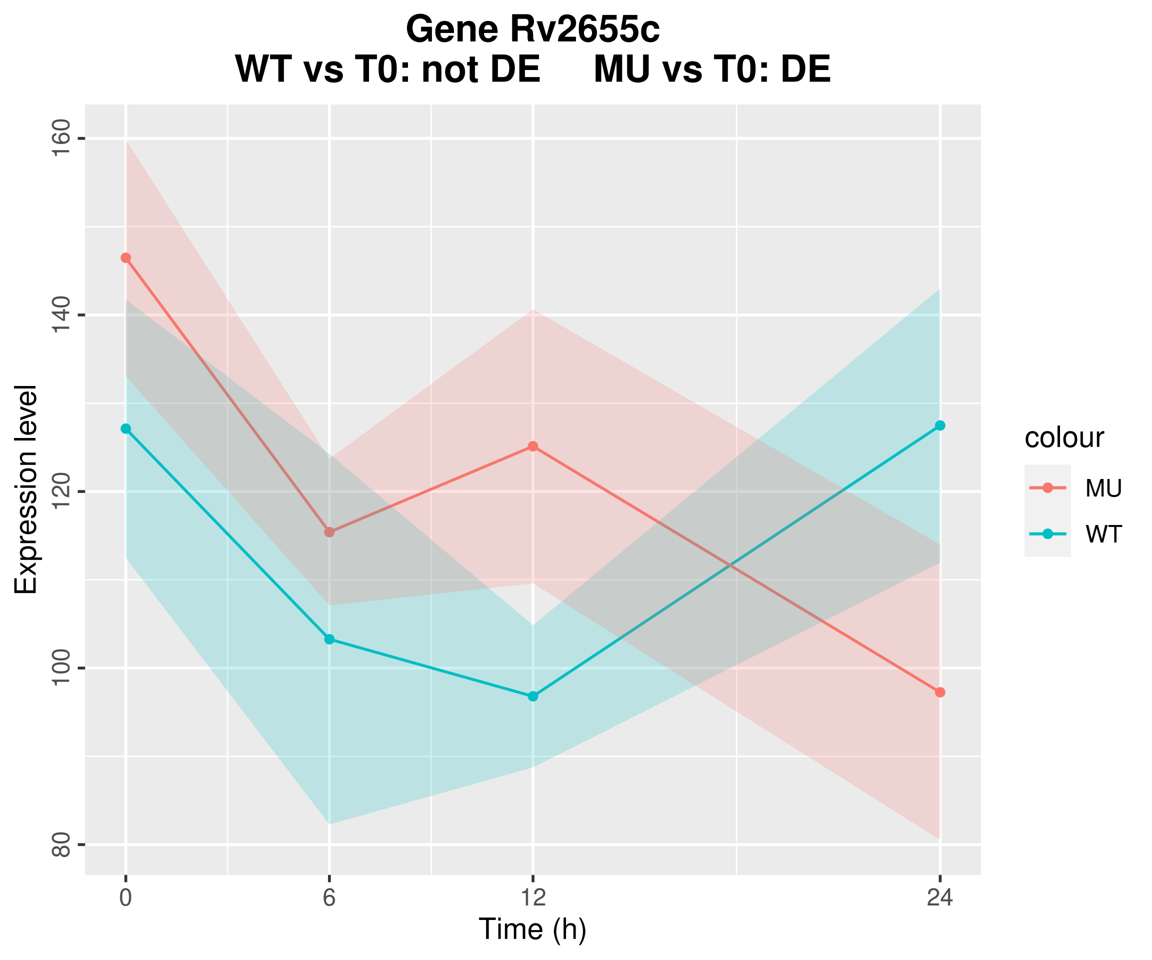

Gene Rv2655c
WT vs T0: not DE MU vs T0: DE
Expression level
160
140
120
100
80
0
6
12
24
Time (h)
colour
MU
WT

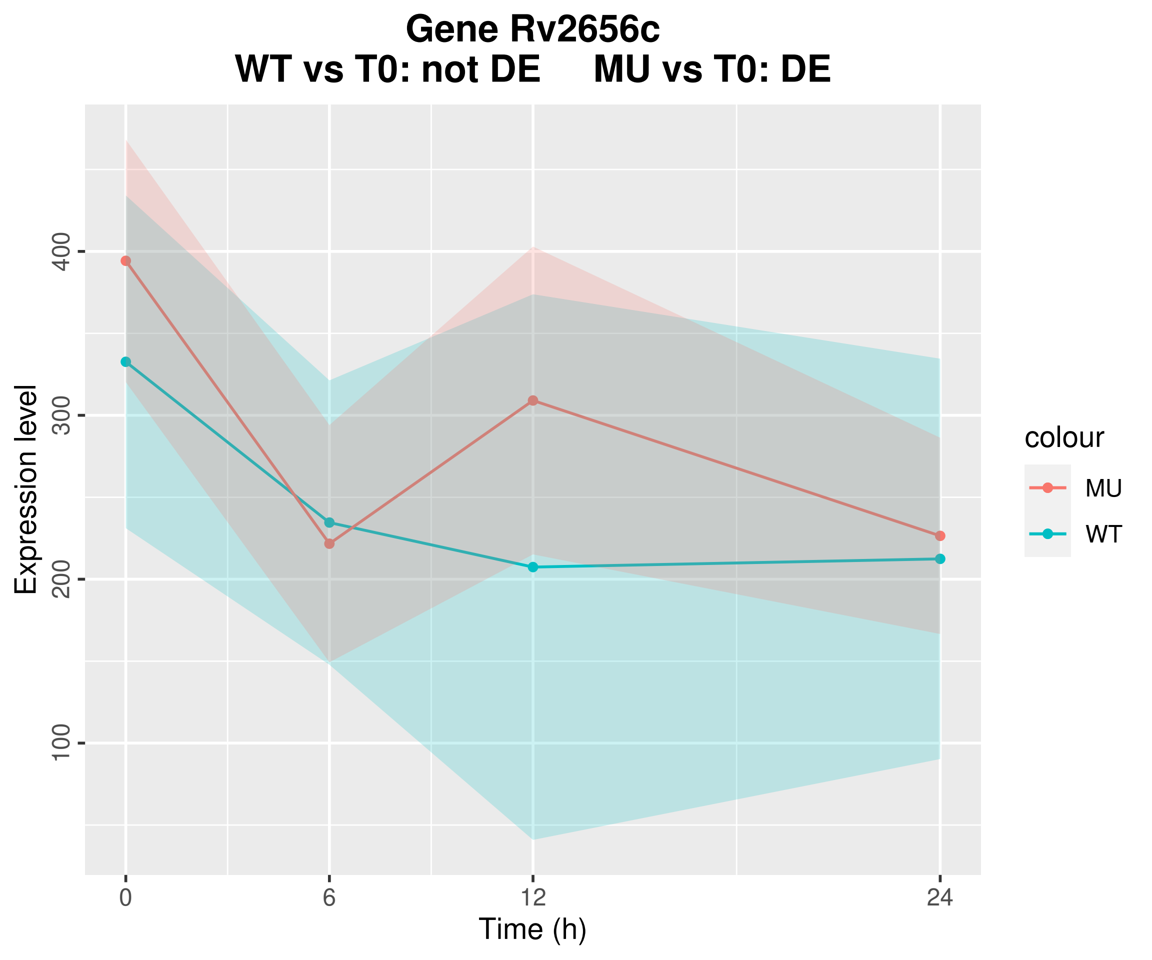
Gene Rv2656c
WT vs T0: not DE   MU vs T0: DE
Expression level
100
200
300
400
0
6
12
24
Time (h)
colour
MU
WT

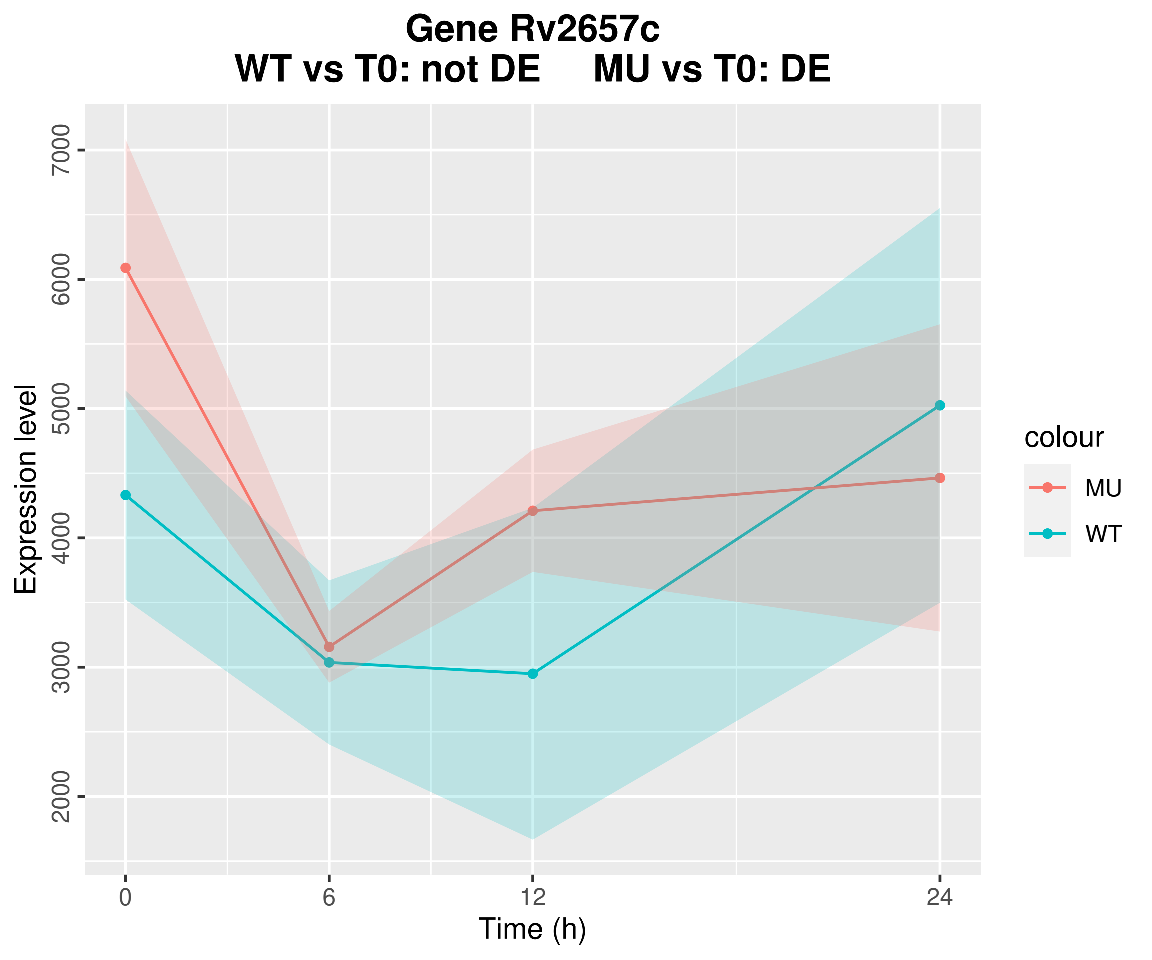

Gene Rv2657c
WT vs T0: not DE   MU vs T0: DE
Expression level
7000
6000
5000
4000
3000
2000
0
6
12
24
Time (h)
colour
MU
WT

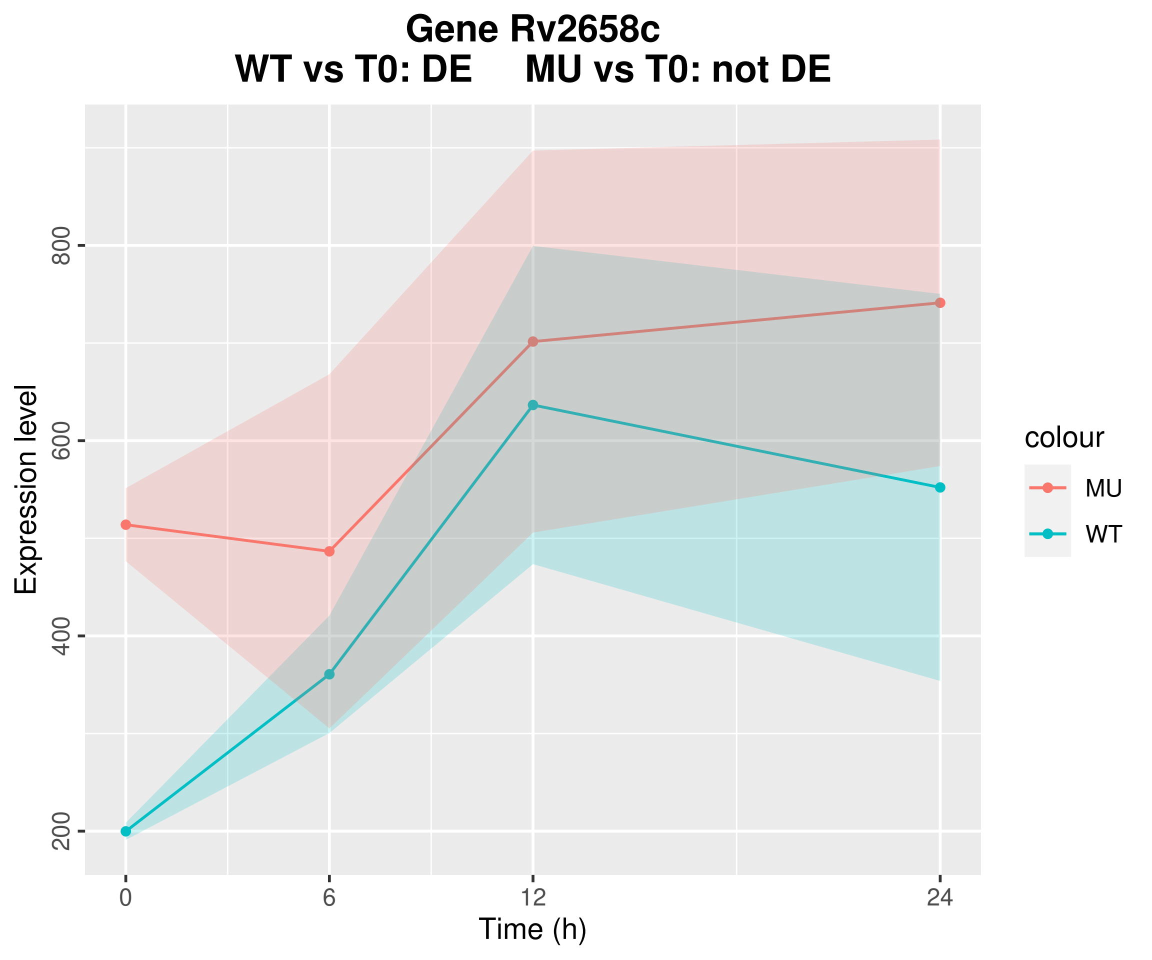
Gene Rv2658c
WT vs T0: DE MU vs T0: not DE
Expression level
200
400
600
800
0
6
12
24
Time (h)
colour
MU
WT

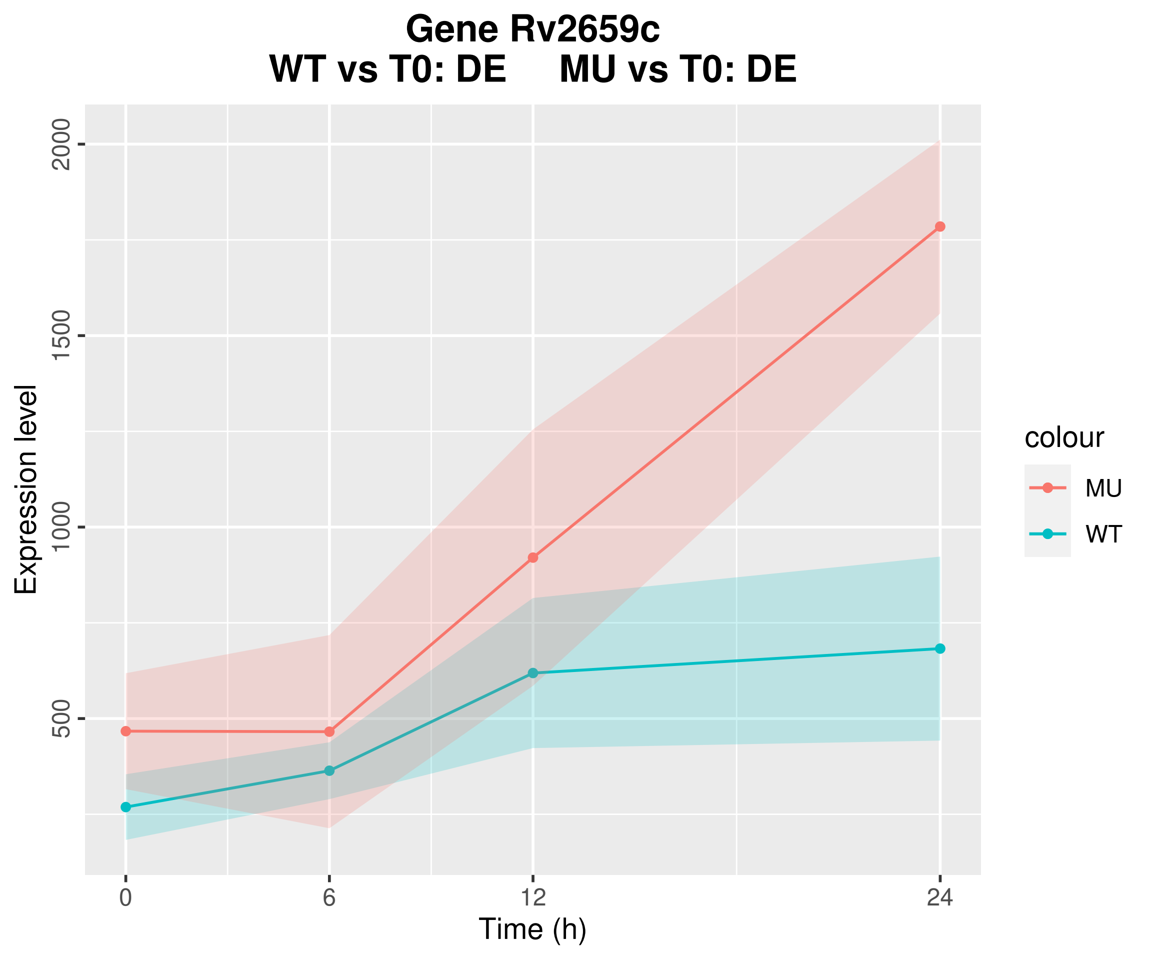
Gene Rv2659c
WT vs T0: DE MU vs T0: DE
Expression level
2000
1500
1000
500
0
6
12
24
Time (h)
colour
MU
WT

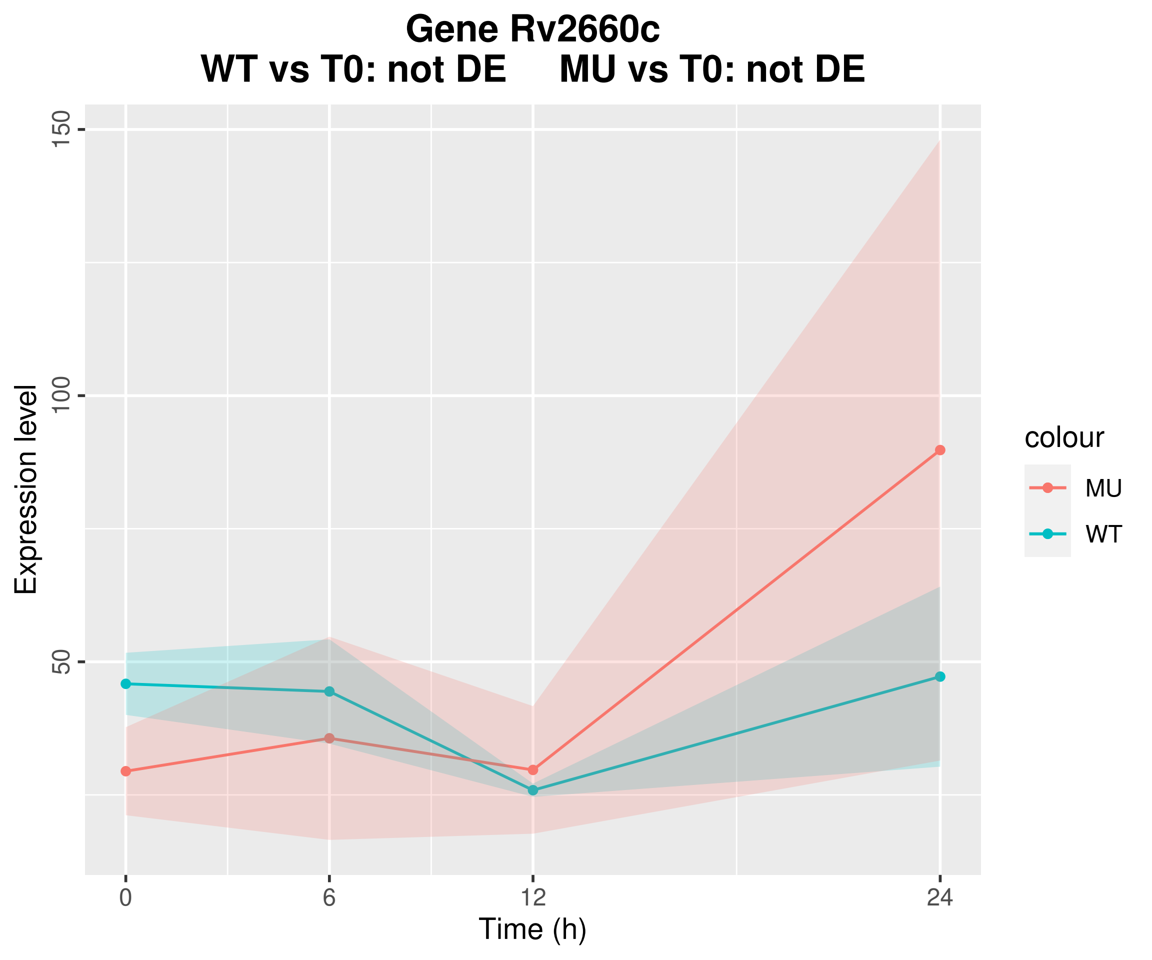

Gene Rv2660c
WT vs T0: not DE   MU vs T0: not DE
Expression level
150
100
50
0
6
12
24
Time (h)
colour
MU
WT

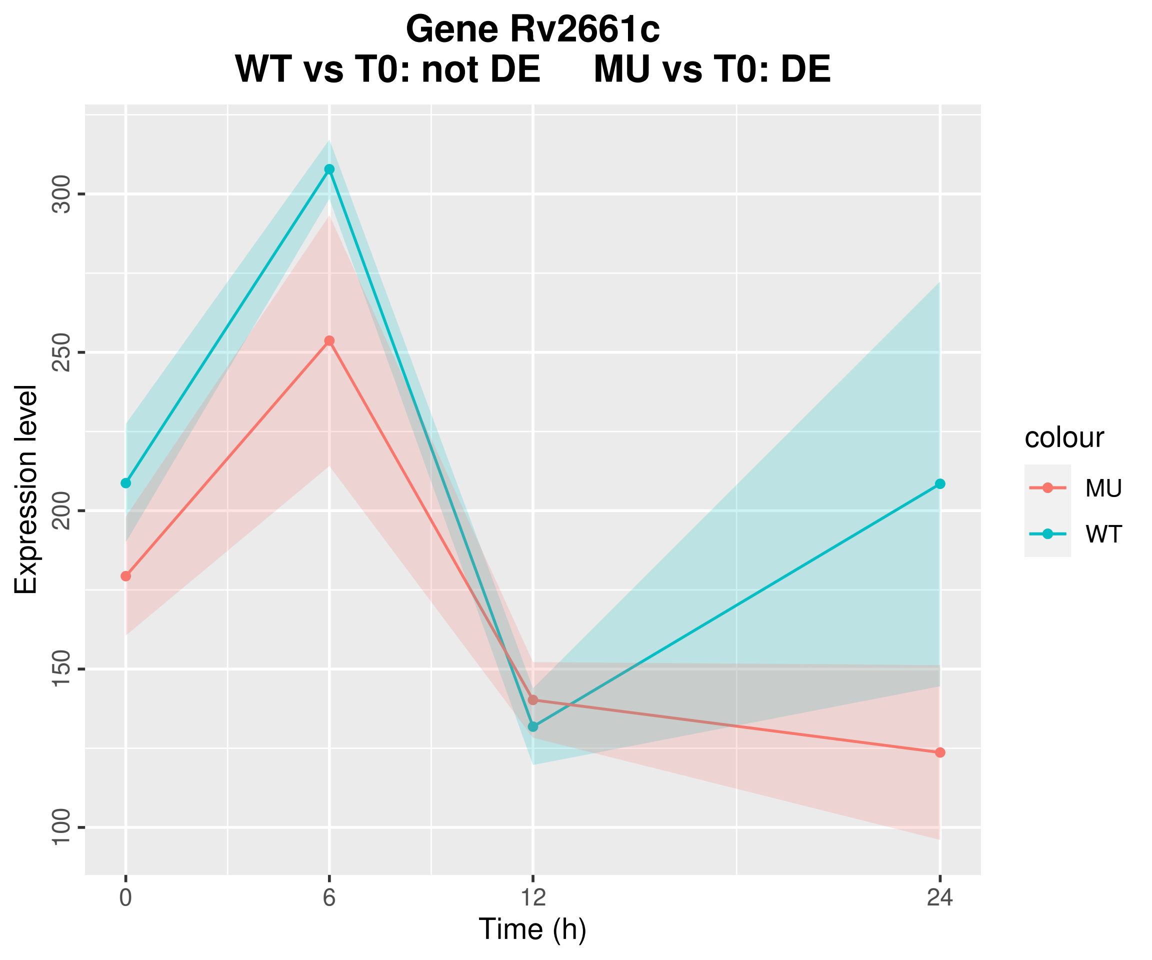

Gene Rv2661c
WT vs T0: not DE MU vs T0: DE
Expression level
300
250
200
150
100
0
6
12
24
Time (h)
colour
MU
WT

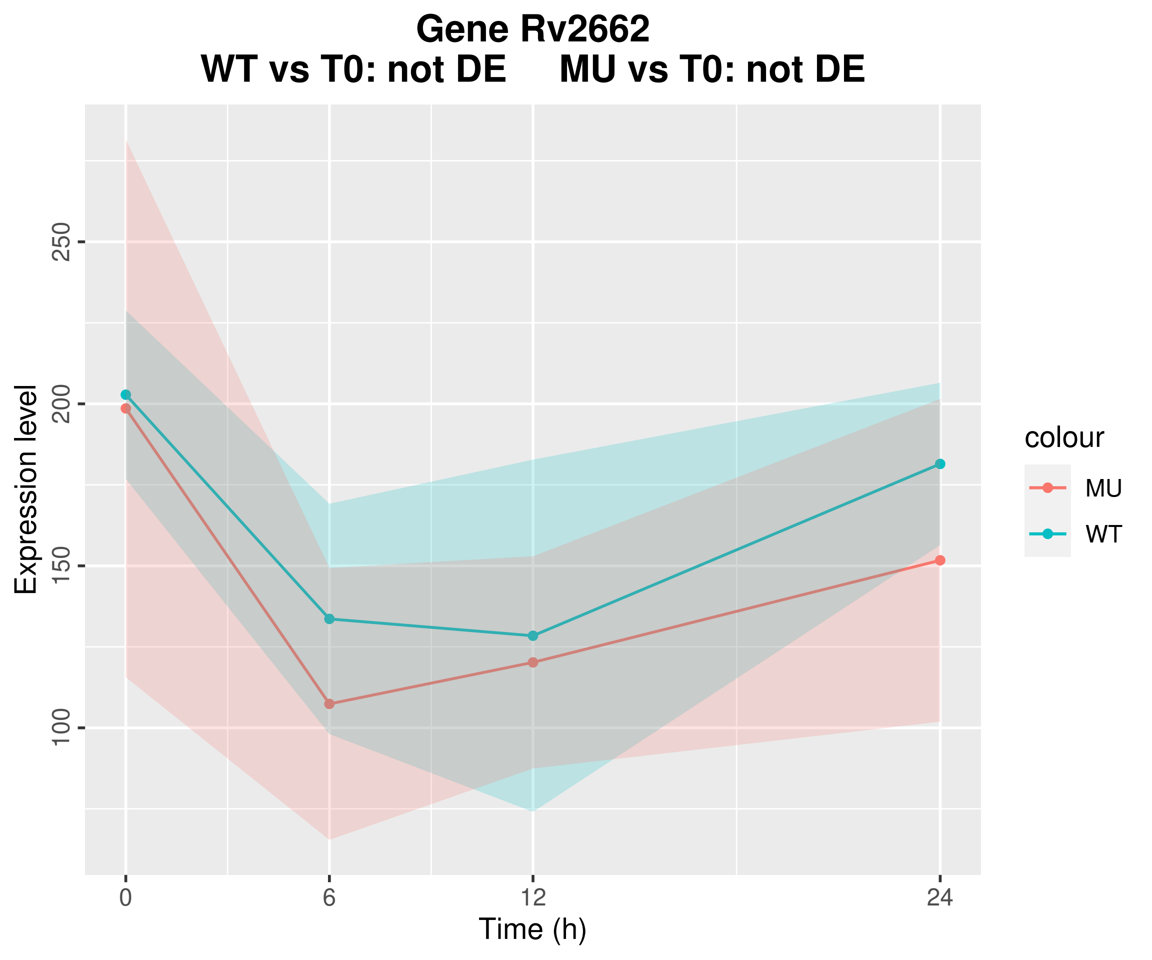

Gene Rv2662
WT vs T0: not DE   MU vs T0: not DE
Expression level
250
200
150
100
0
6
12
24
Time (h)
colour
MU
WT

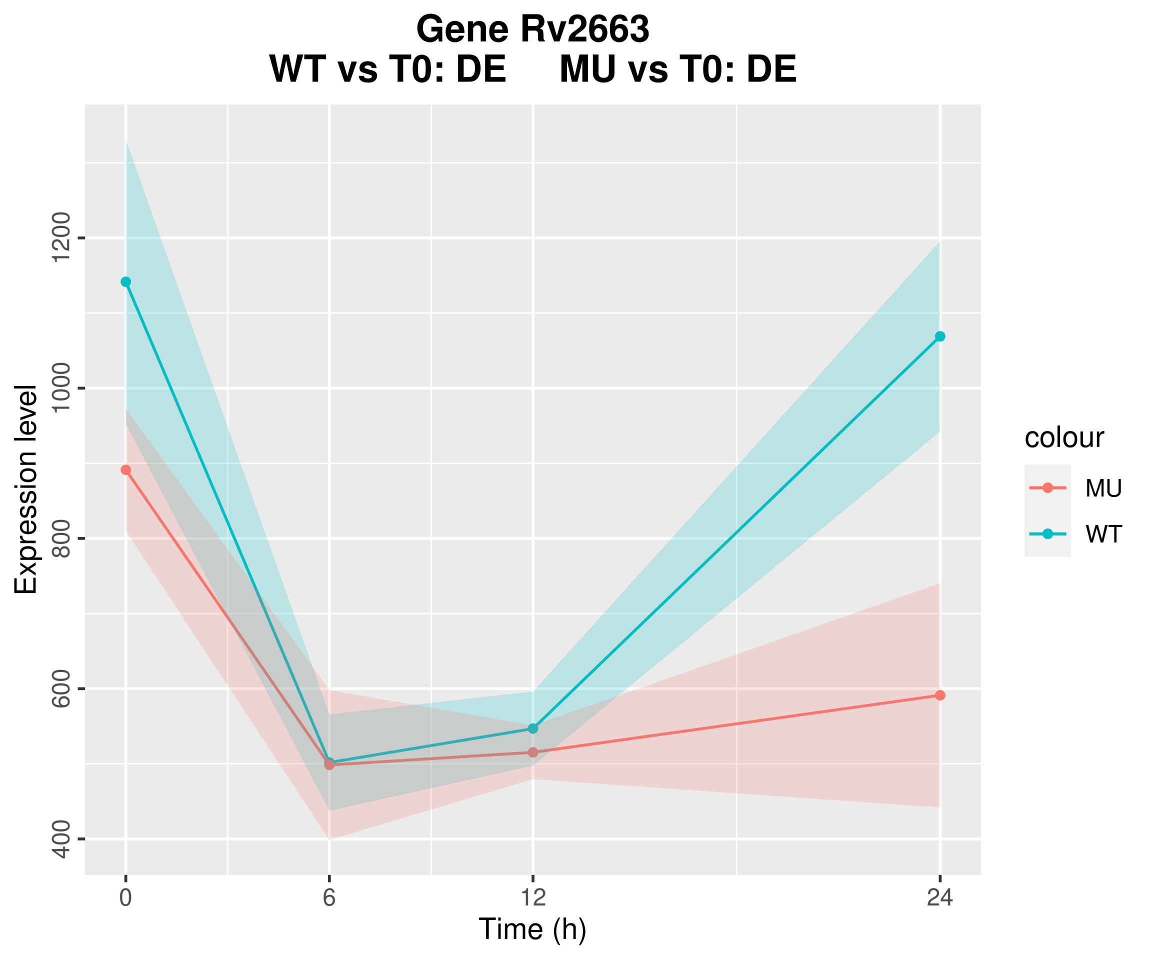

Gene Rv2663
WT vs T0: DE   MU vs T0: DE
Expression level
1200
1000
800
600
400
0
6
12
24
Time (h)
colour
MU
WT

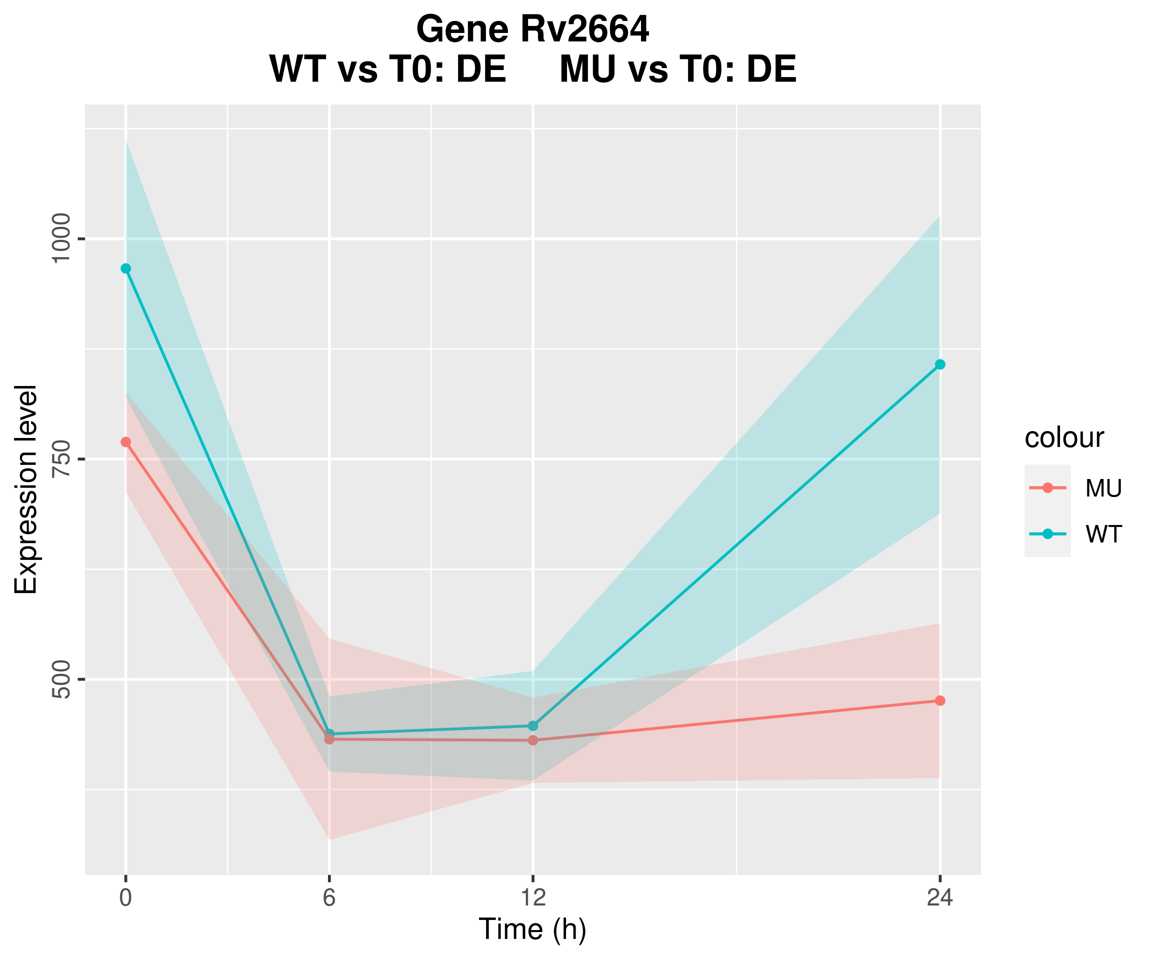

Gene Rv2664
WT vs T0: DE   MU vs T0: DE
1000
750
500
Expression level
0
6
12
24
Time (h)
colour
MU
WT

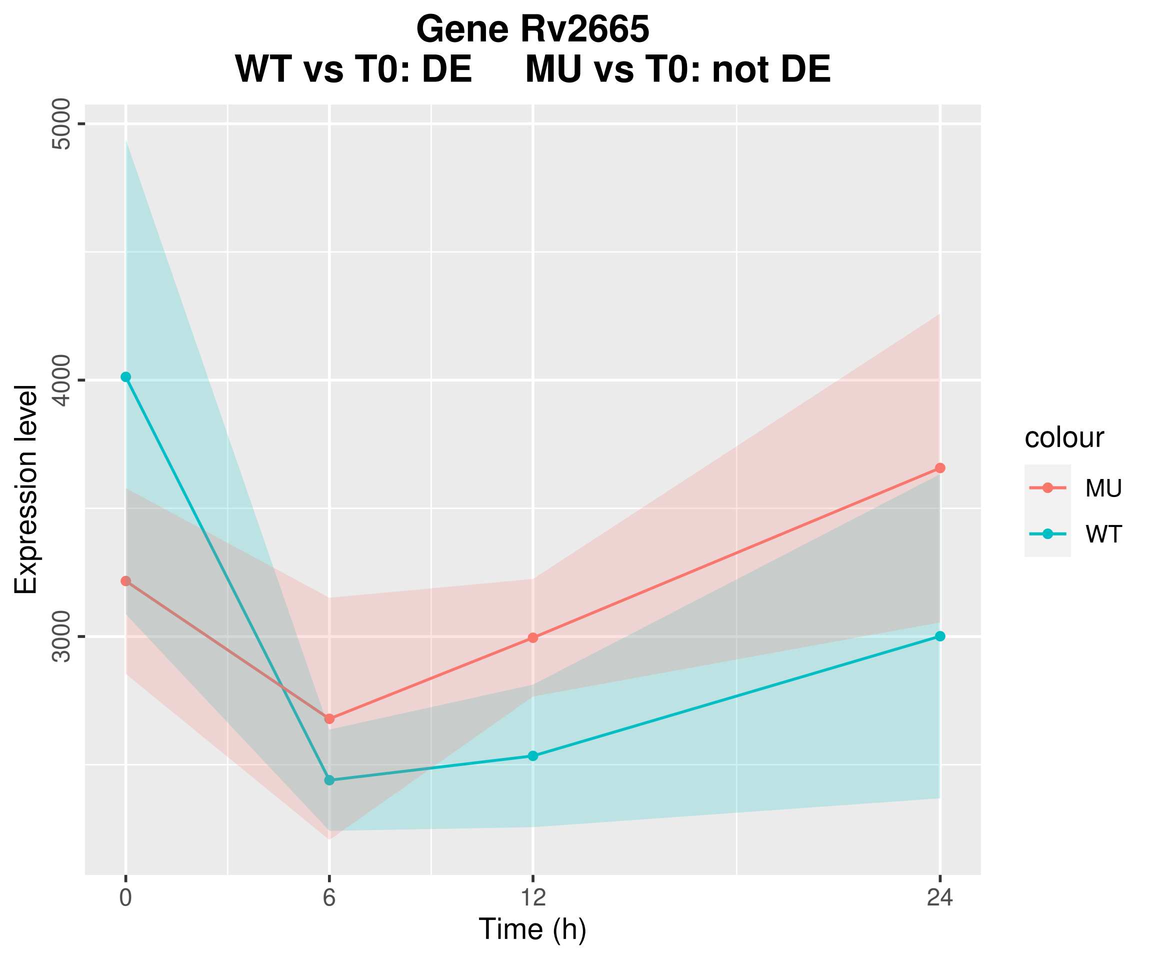
Gene Rv2665
WT vs T0: DE   MU vs T0: not DE
Expression level
5000
4000
3000
0
6
12
24
Time (h)
colour
MU
WT

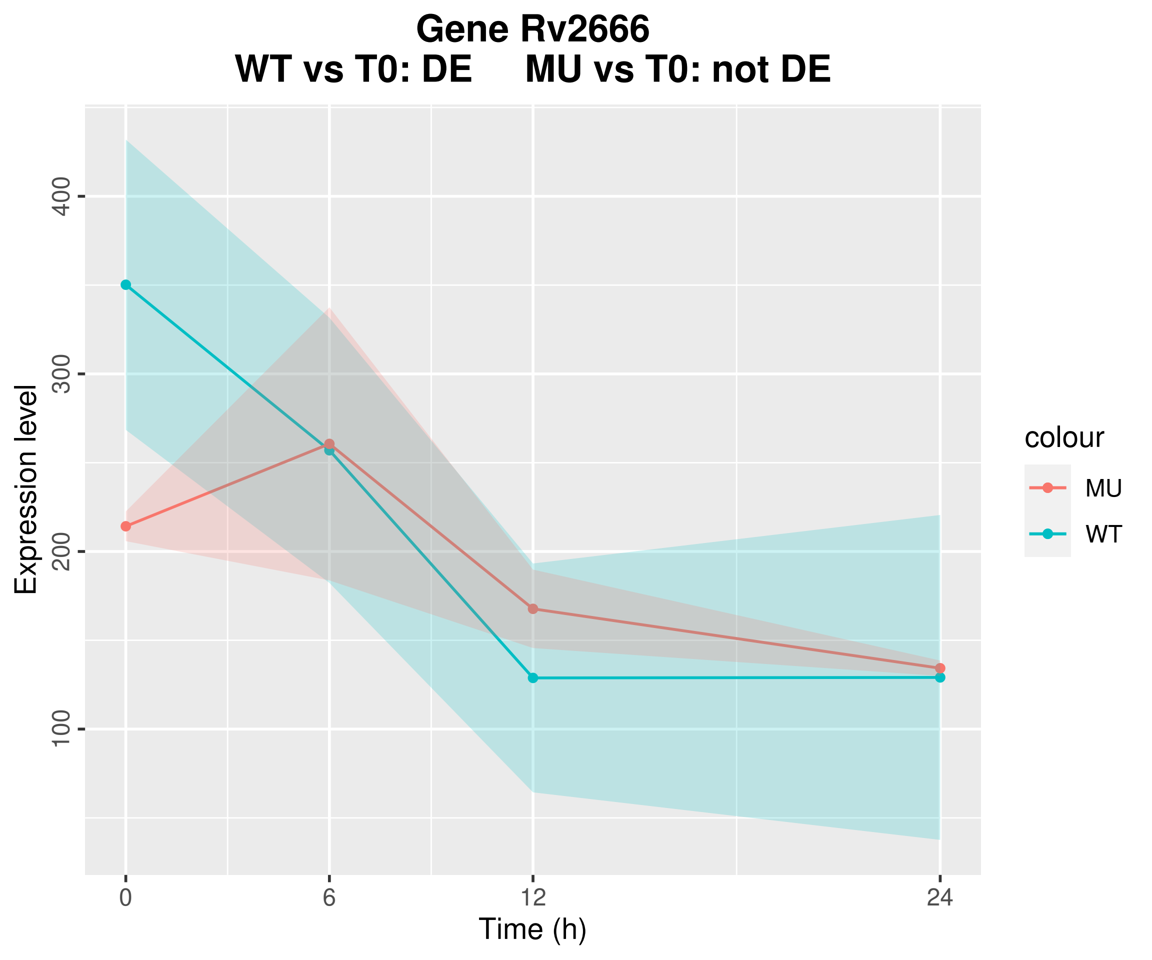
Gene Rv2666
WT vs T0: DE   MU vs T0: not DE
Expression level
400
300
200
100
0
6
12
24
Time (h)
colour
MU
WT

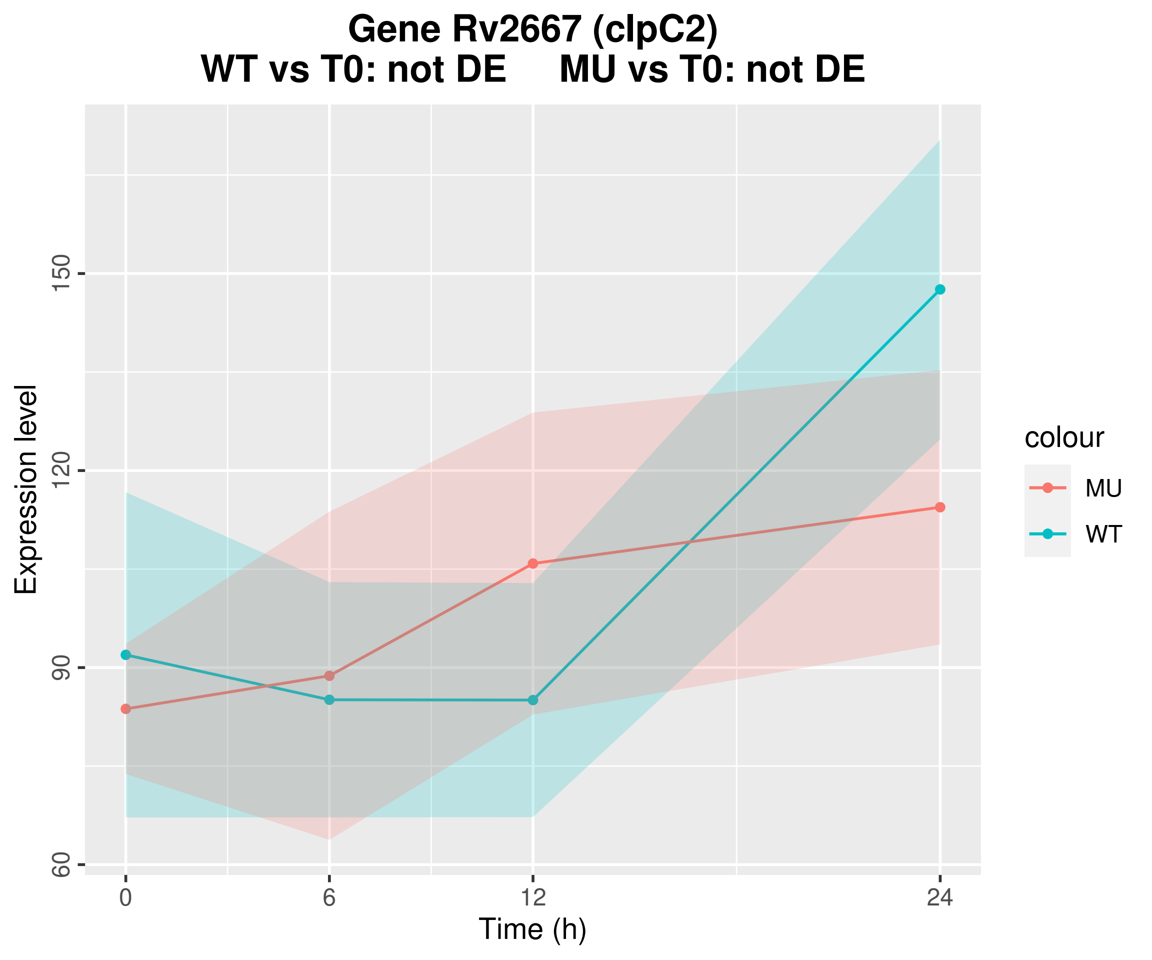
Gene Rv2667 (clpC2)
WT vs T0: not DE   MU vs T0: not DE
Expression level
150
120
90
60
0
6
12
24
Time (h)
colour
MU
WT

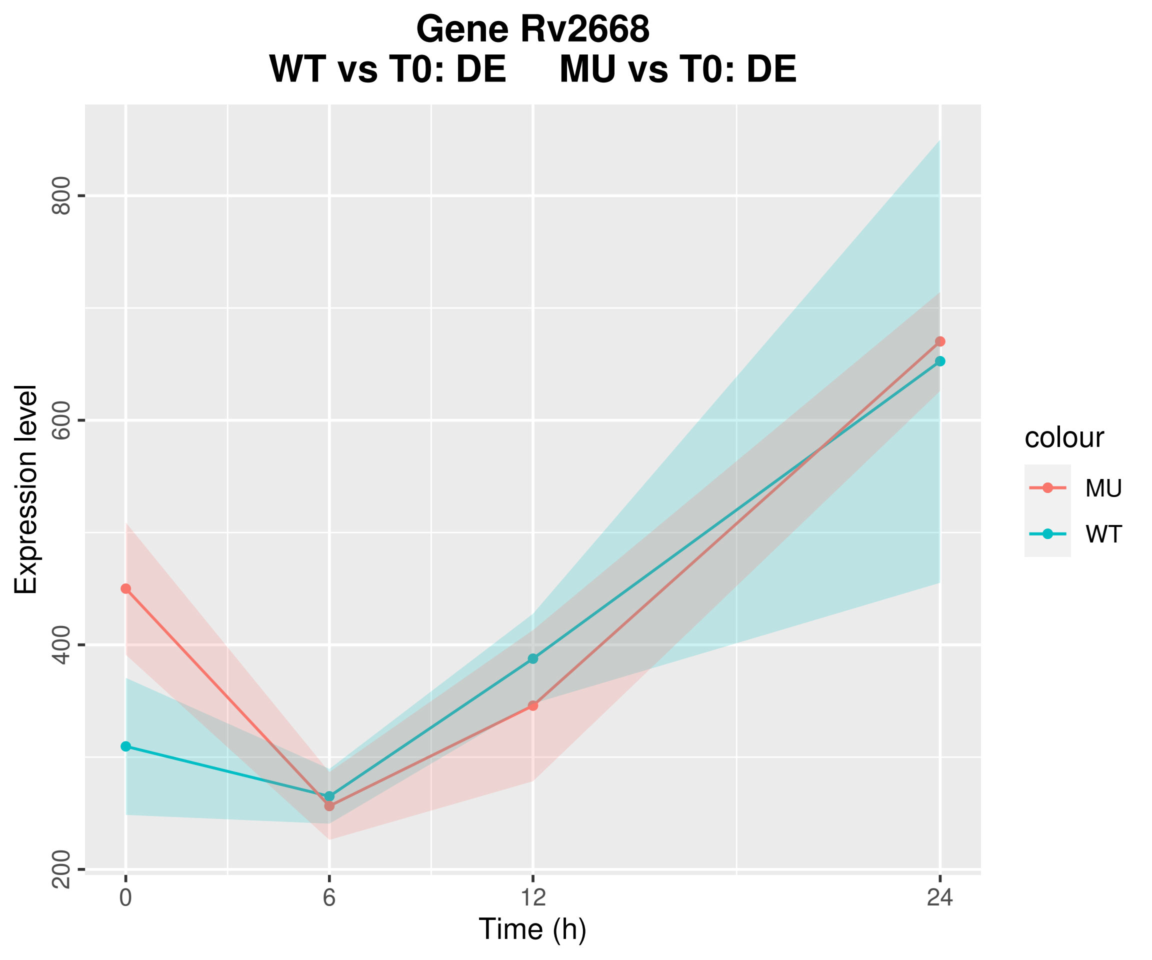
Gene Rv2668
WT vs T0: DE MU vs T0: DE
Expression level
800
600
400
200
0
6
12
24
Time (h)
colour
MU
WT

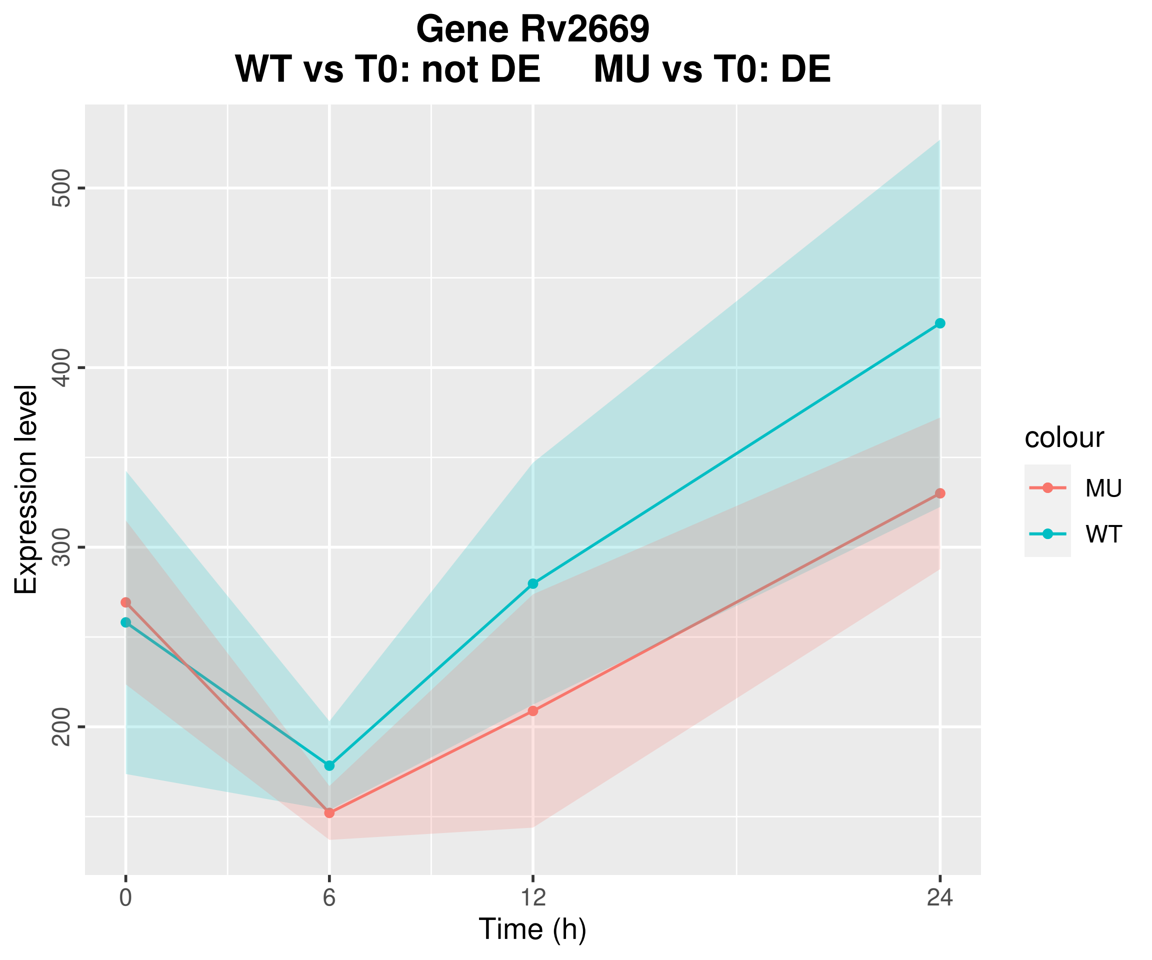
Gene Rv2669
WT vs T0: not DE MU vs T0: DE
Expression level
500
400
300
200
0
6
12
24
Time (h)
colour
MU
WT

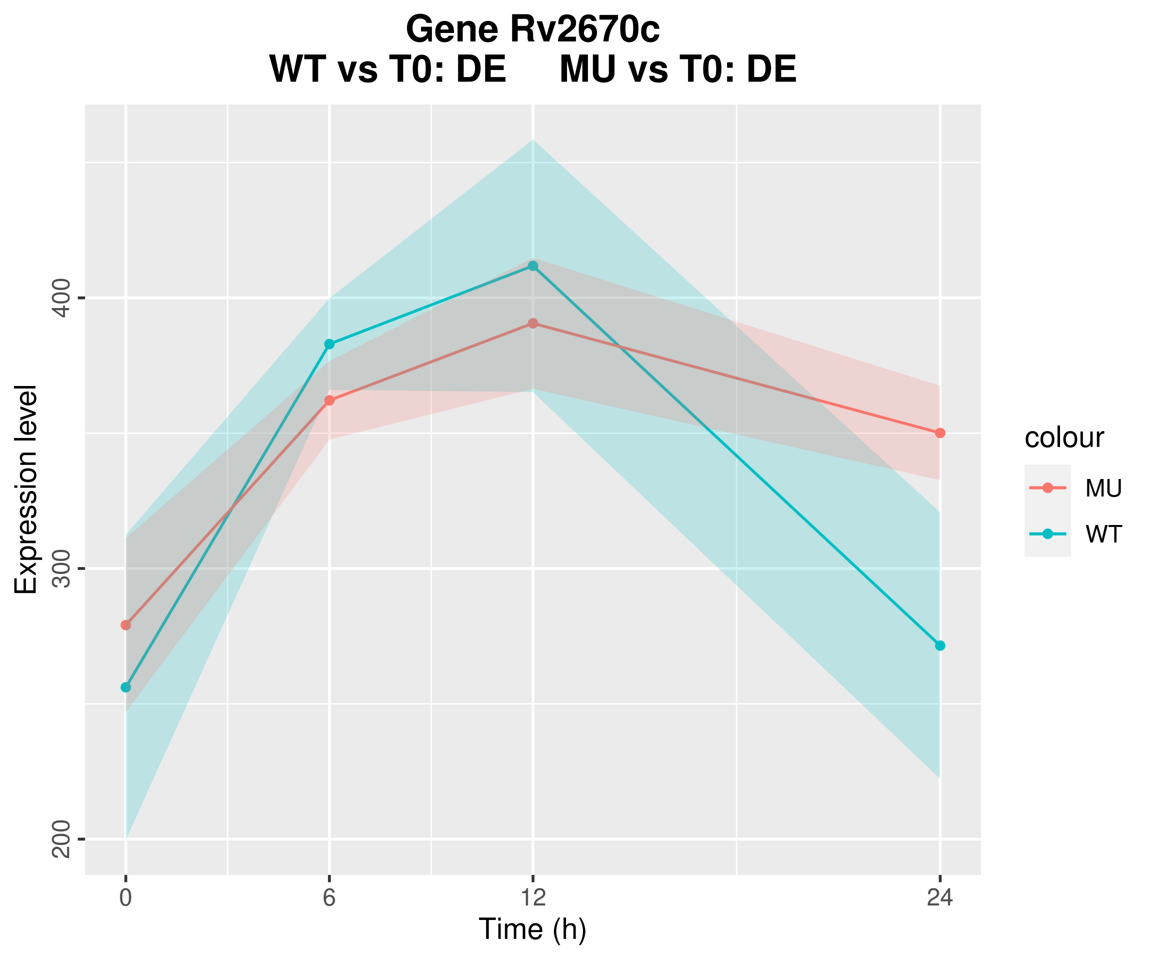
Gene Rv2670c
WT vs T0: DE MU vs T0: DE
Expression level
400
300
200
0
6
12
24
Time (h)
colour
MU
WT

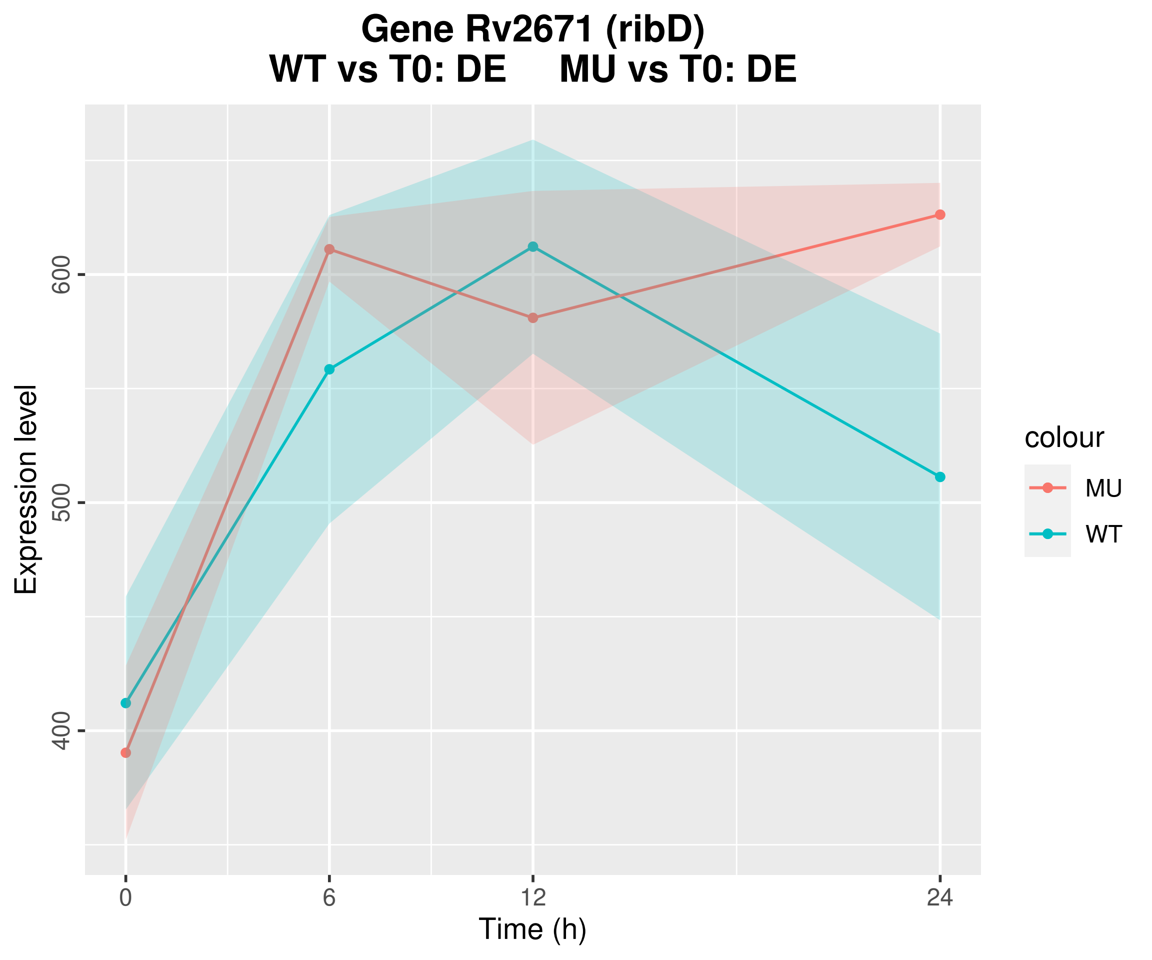
Gene Rv2671 (ribD)
WT vs T0: DE   MU vs T0: DE
Expression level
600
500
400
0
6
12
24
Time (h)
colour
MU
WT

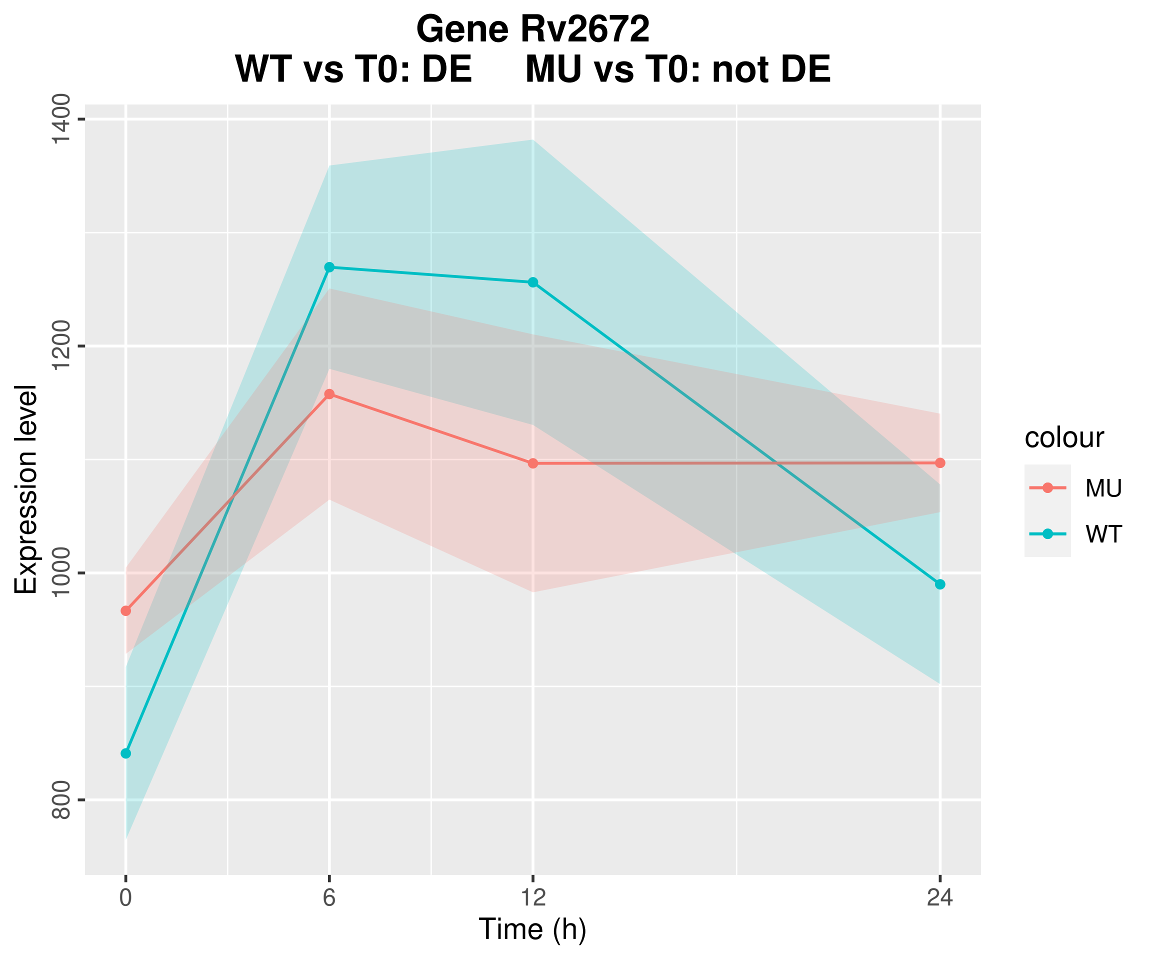

Gene Rv2672
WT vs T0: DE   MU vs T0: not DE
Expression level
1400
1200
1000
800
0
6
12
24
Time (h)
colour
MU
WT

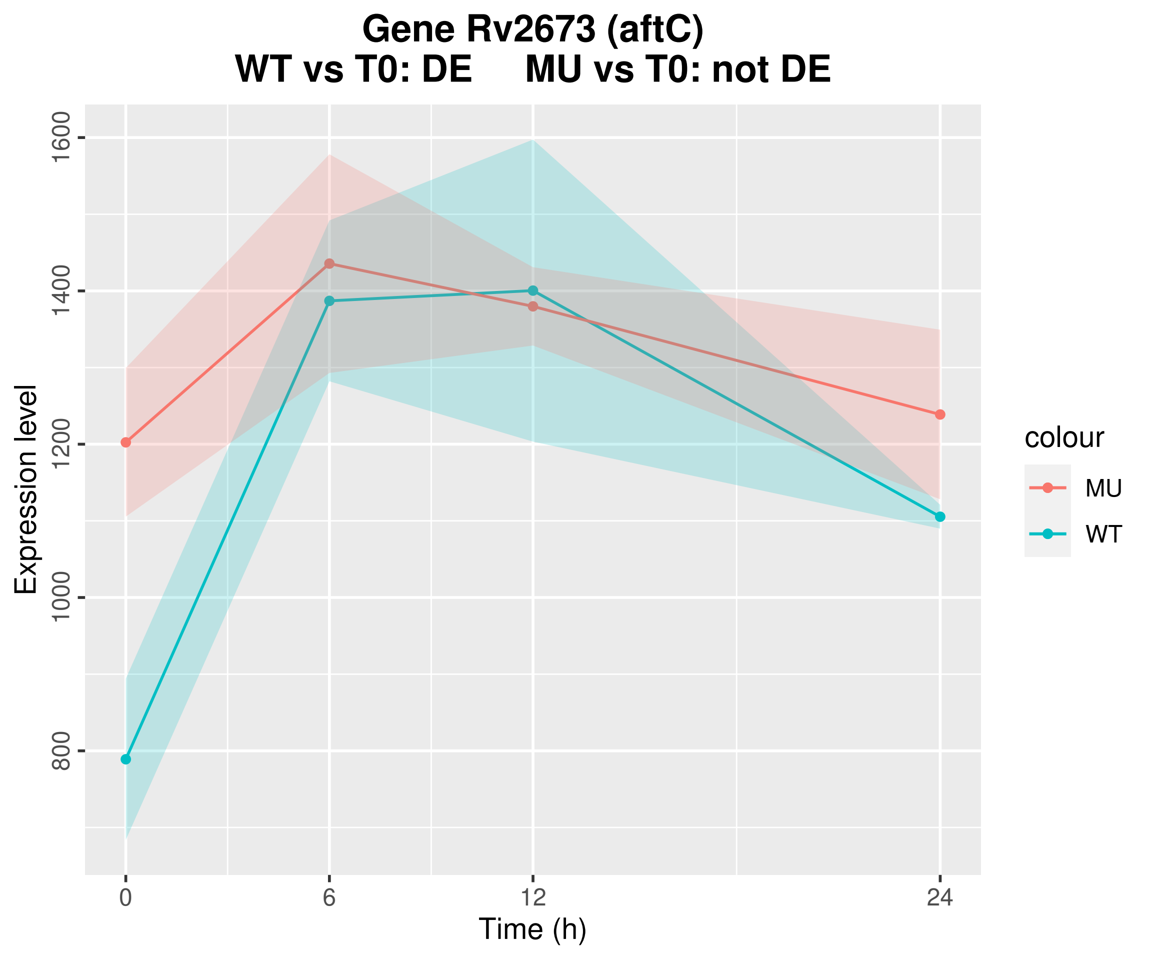

Gene Rv2673 (aftC)
WT vs T0: DE   MU vs T0: not DE
Expression level
1600
1400
1200
1000
800
0
6
12
24
Time (h)
colour
MU
WT

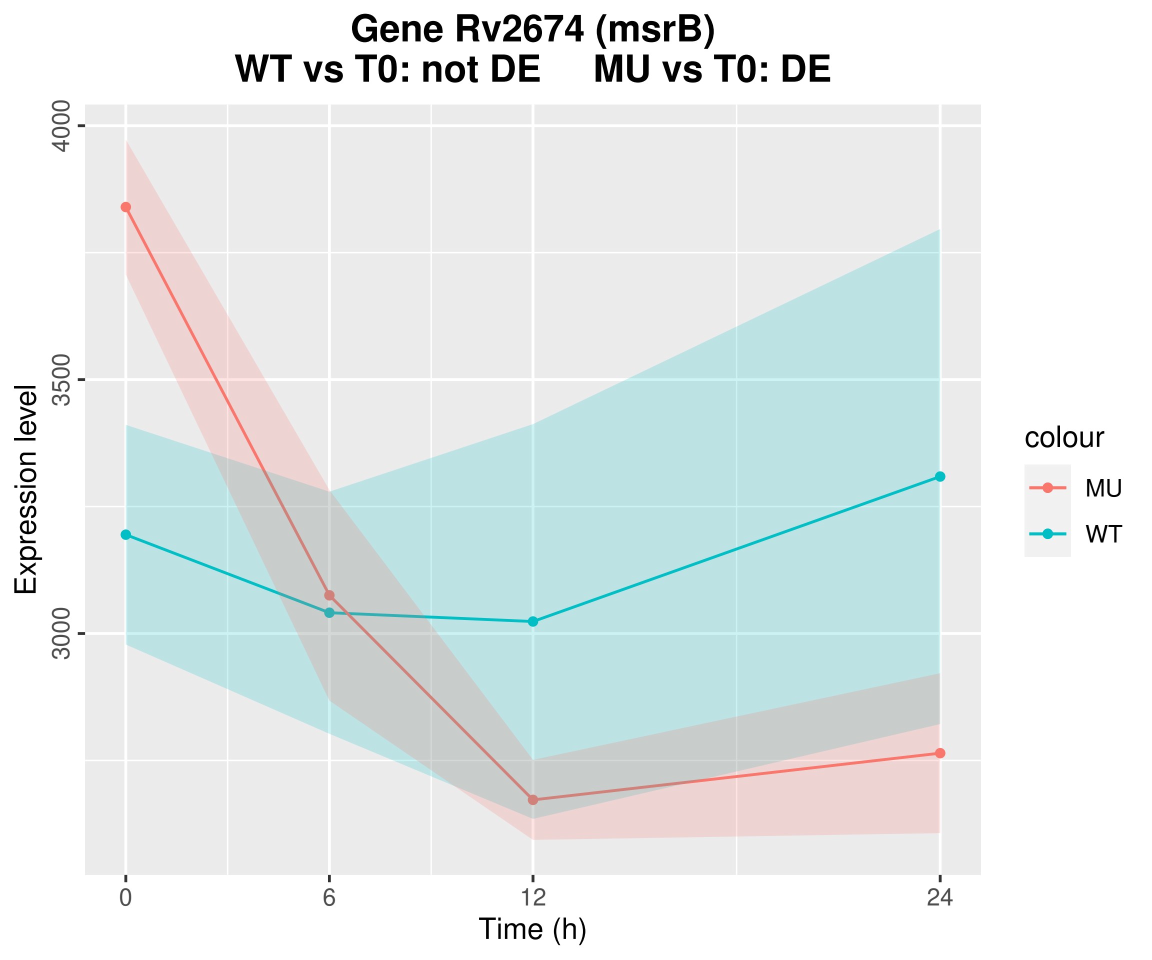

Gene Rv2674 (msrB)
WT vs T0: not DE MU vs T0: DE
4000
3500
3000
Expression level
0
6
12
24
Time (h)
colour
MU
WT

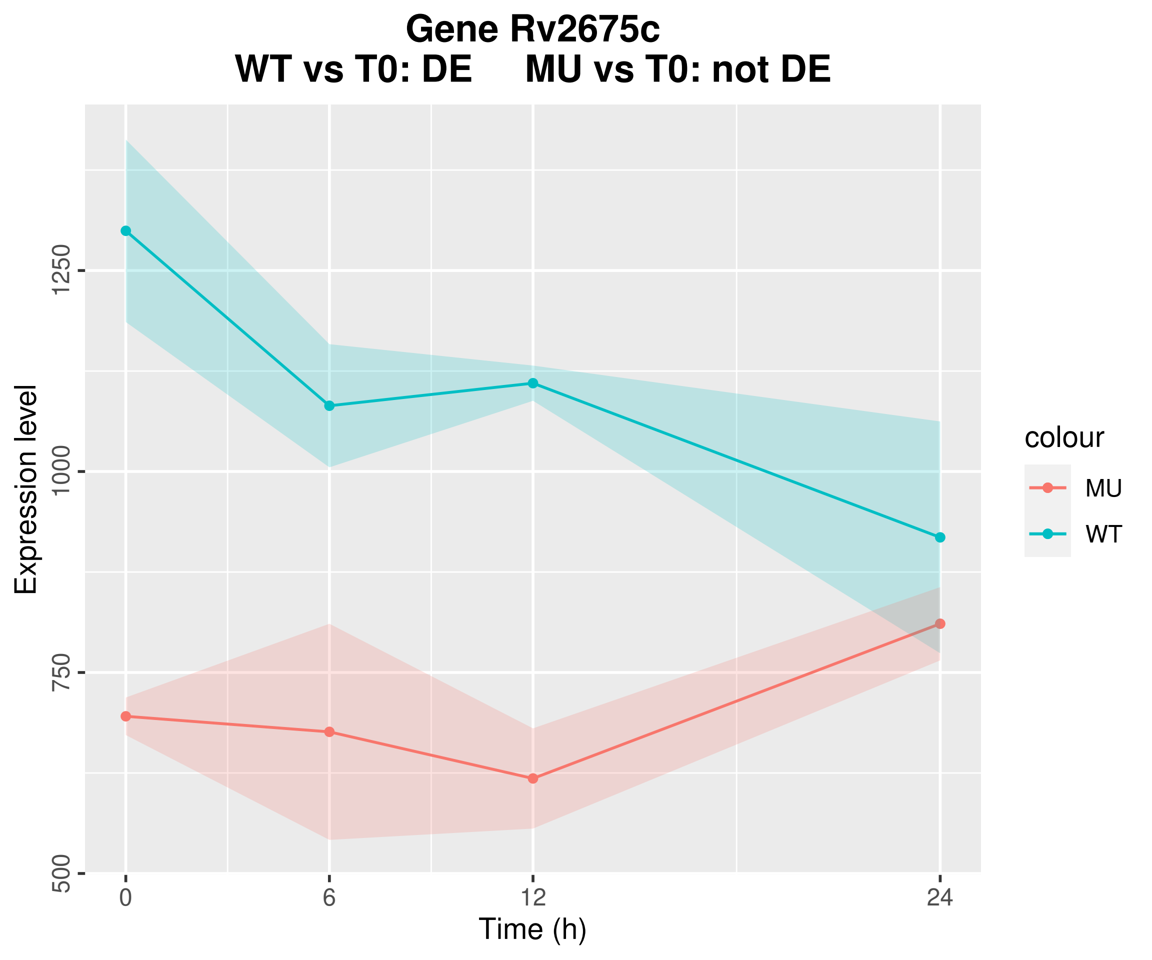

Gene Rv2675c
WT vs T0: DE MU vs T0: not DE
Expression level
1250
1000
750
500
0
6
12
24
Time (h)
colour
MU
WT

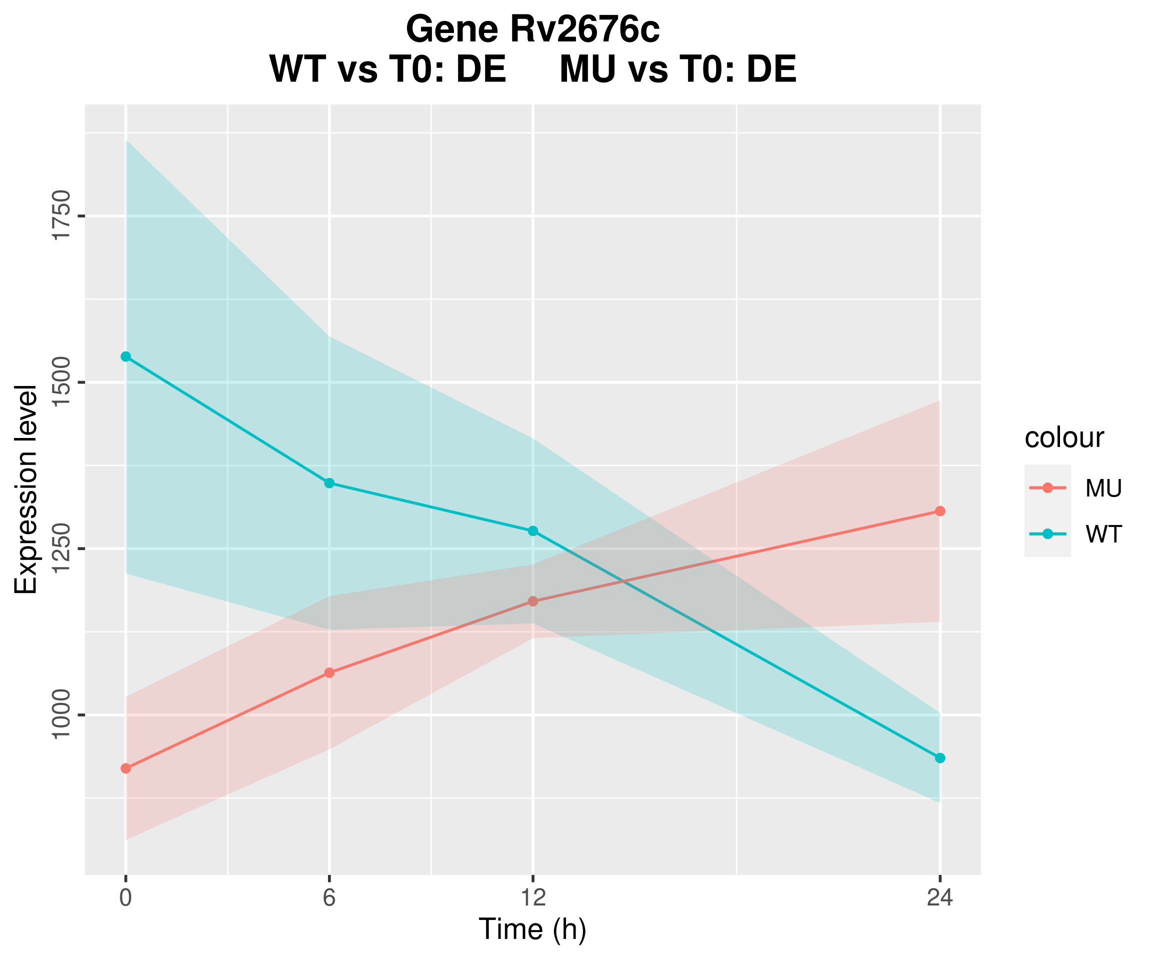

Gene Rv2676c
WT vs T0: DE   MU vs T0: DE
Expression level
1750
1500
1250
1000
0
6
12
24
Time (h)
colour
MU
WT

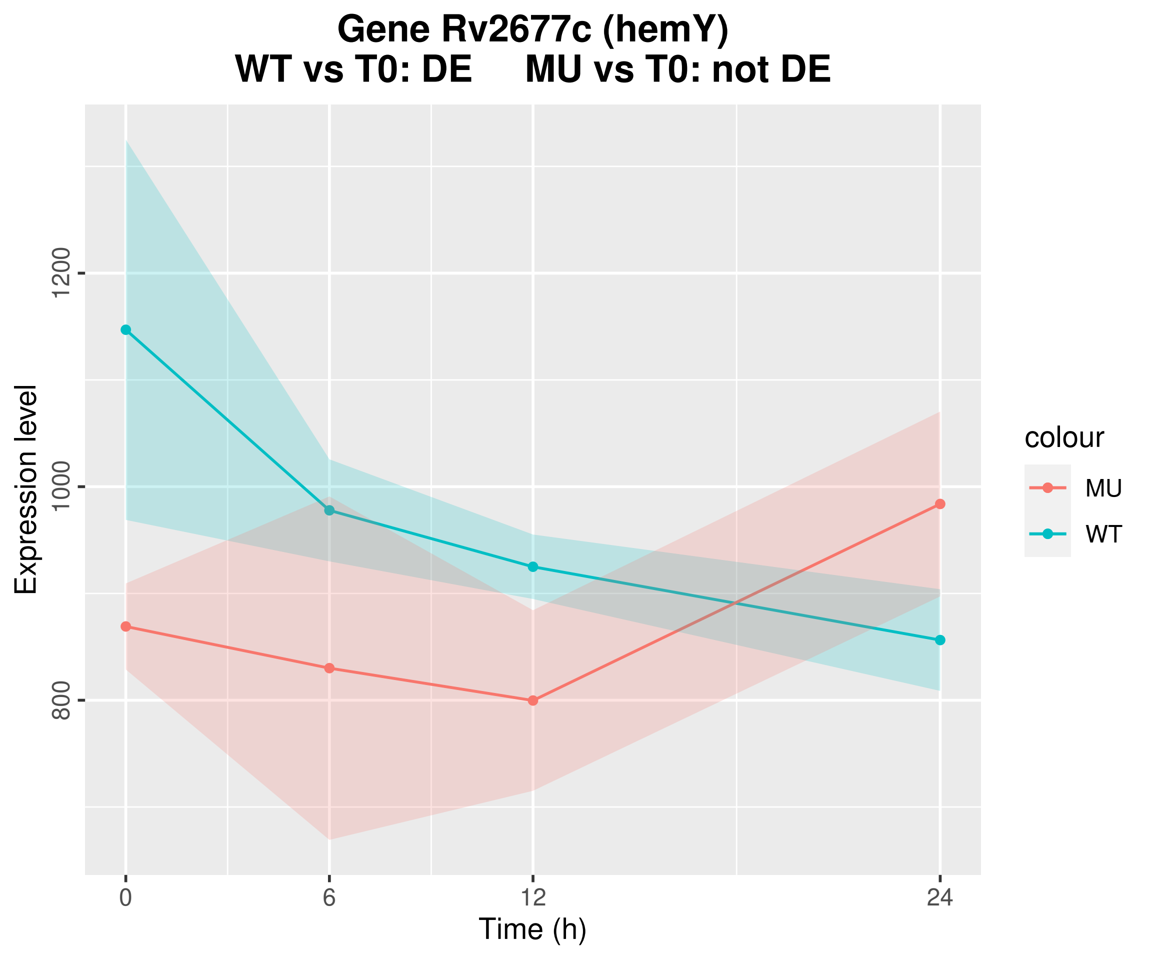
Gene Rv2677c (hemY)
WT vs T0: DE MU vs T0: not DE
Expression level
1200
1000
800
0
6
12
24
Time (h)
colour
MU
WT

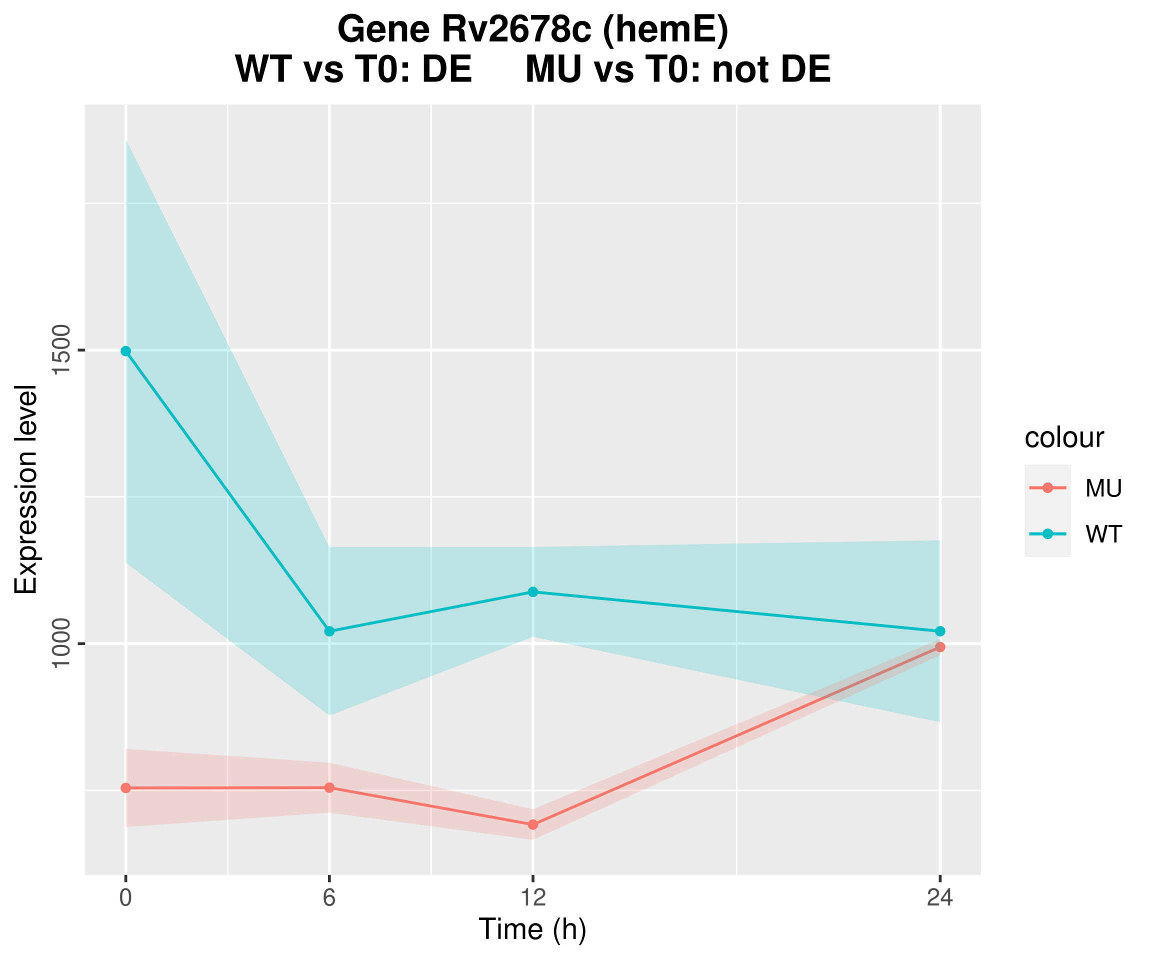
Gene Rv2678c (hemE)
WT vs T0: DE MU vs T0: not DE
Expression level
1500
1000
0
6
12
24
Time (h)
colour
MU
WT

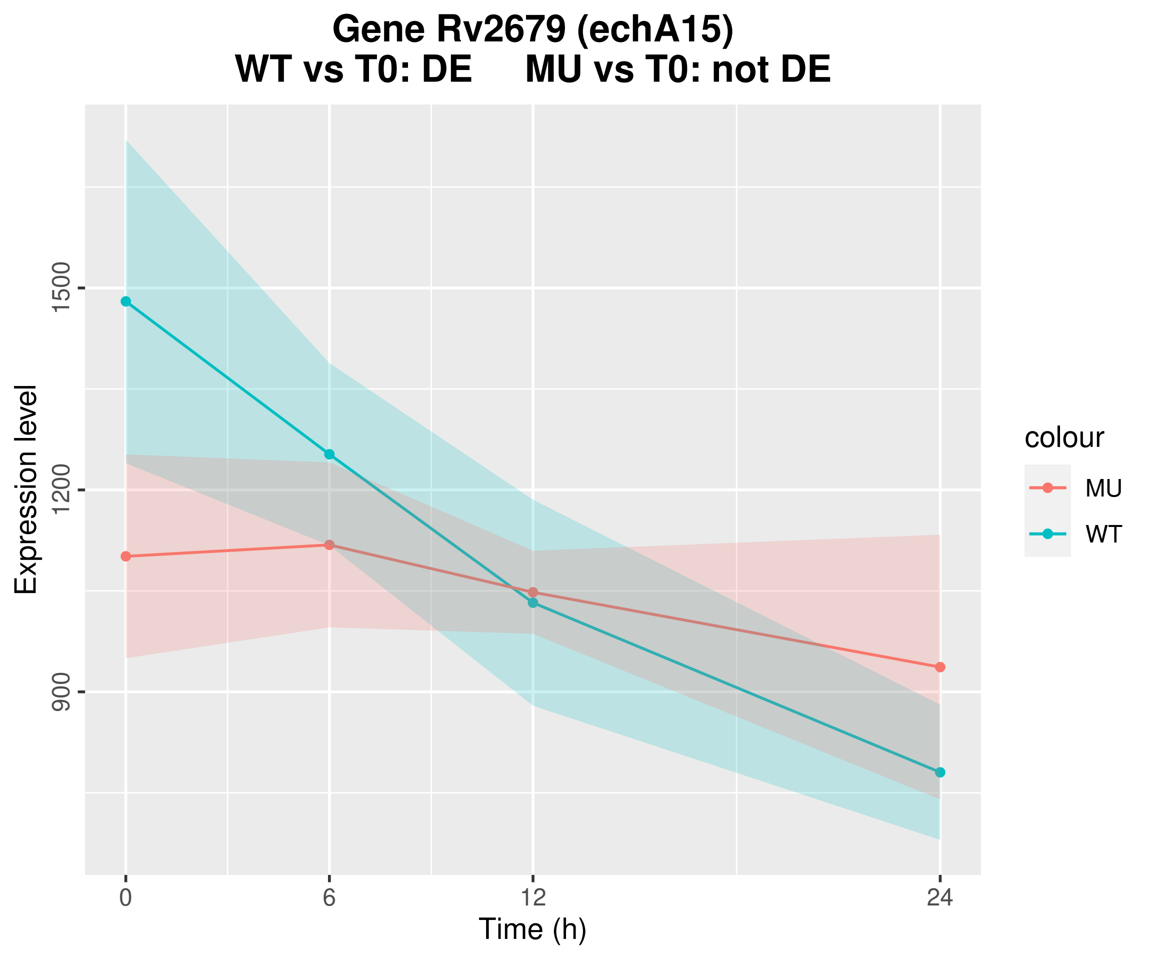
Gene Rv2679 (echA15)
WT vs T0: DE MU vs T0: not DE
Expression level
1500
1200
900
0
6
12
24
Time (h)
colour
MU
WT

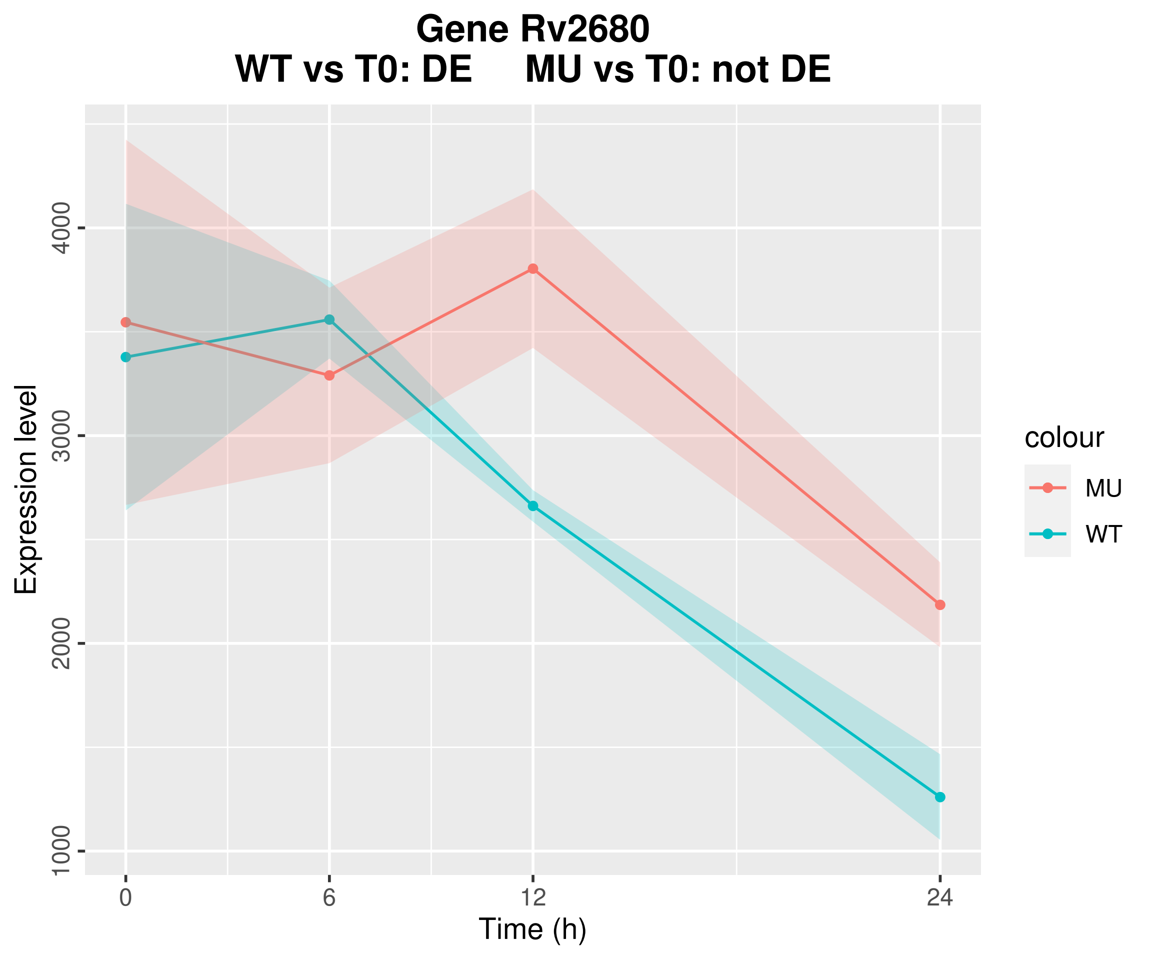
Gene Rv2680
WT vs T0: DE   MU vs T0: not DE
Expression level
4000
3000
2000
1000
0
6
12
24
Time (h)
colour
MU
WT

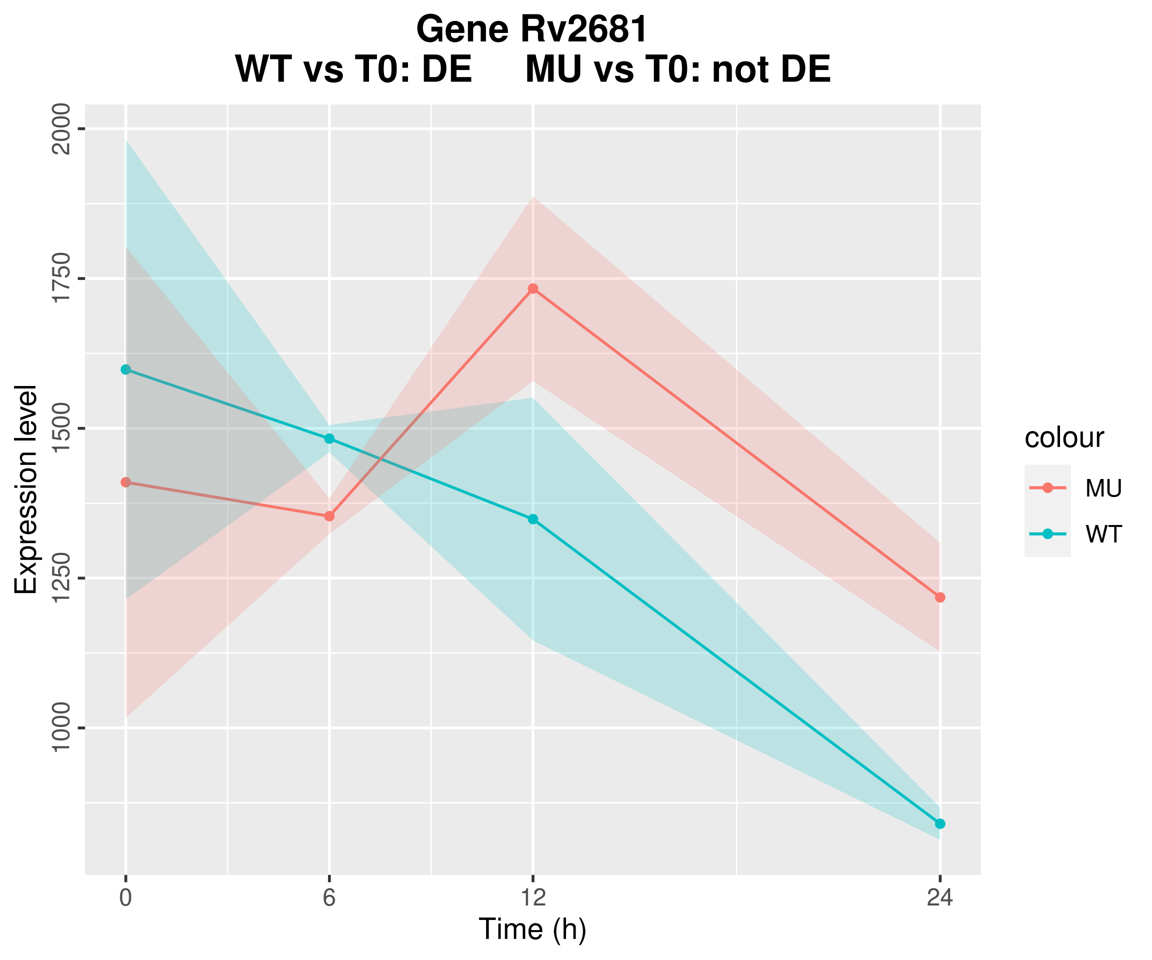
Gene Rv2681
WT vs T0: DE   MU vs T0: not DE
Expression level
2000
1750
1500
1250
1000
0
6
12
24
Time (h)
colour
MU
WT

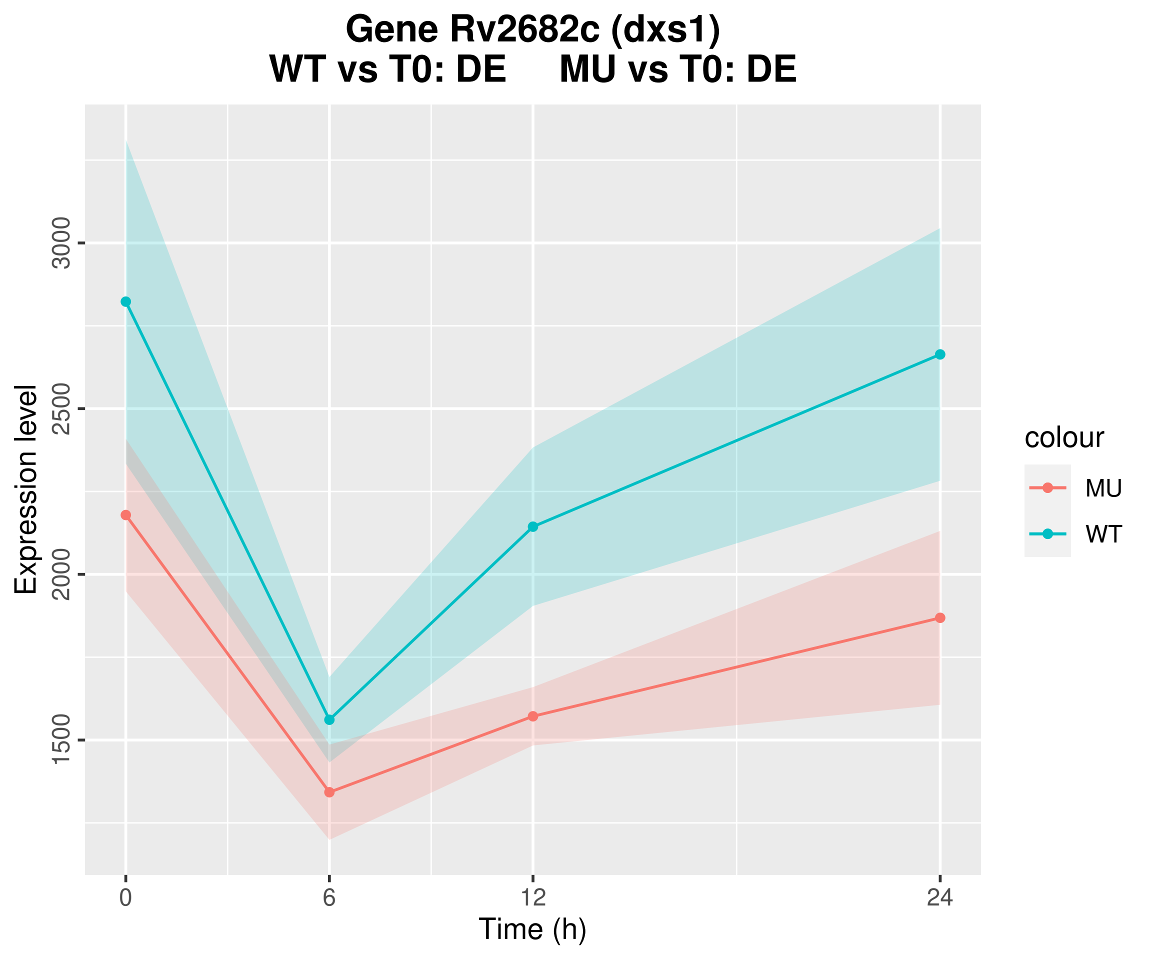
Gene Rv2682c (dxs1)
WT vs T0: DE   MU vs T0: DE
Expression level
3000
2500
2000
1500
0
6
12
24
Time (h)
colour
MU
WT

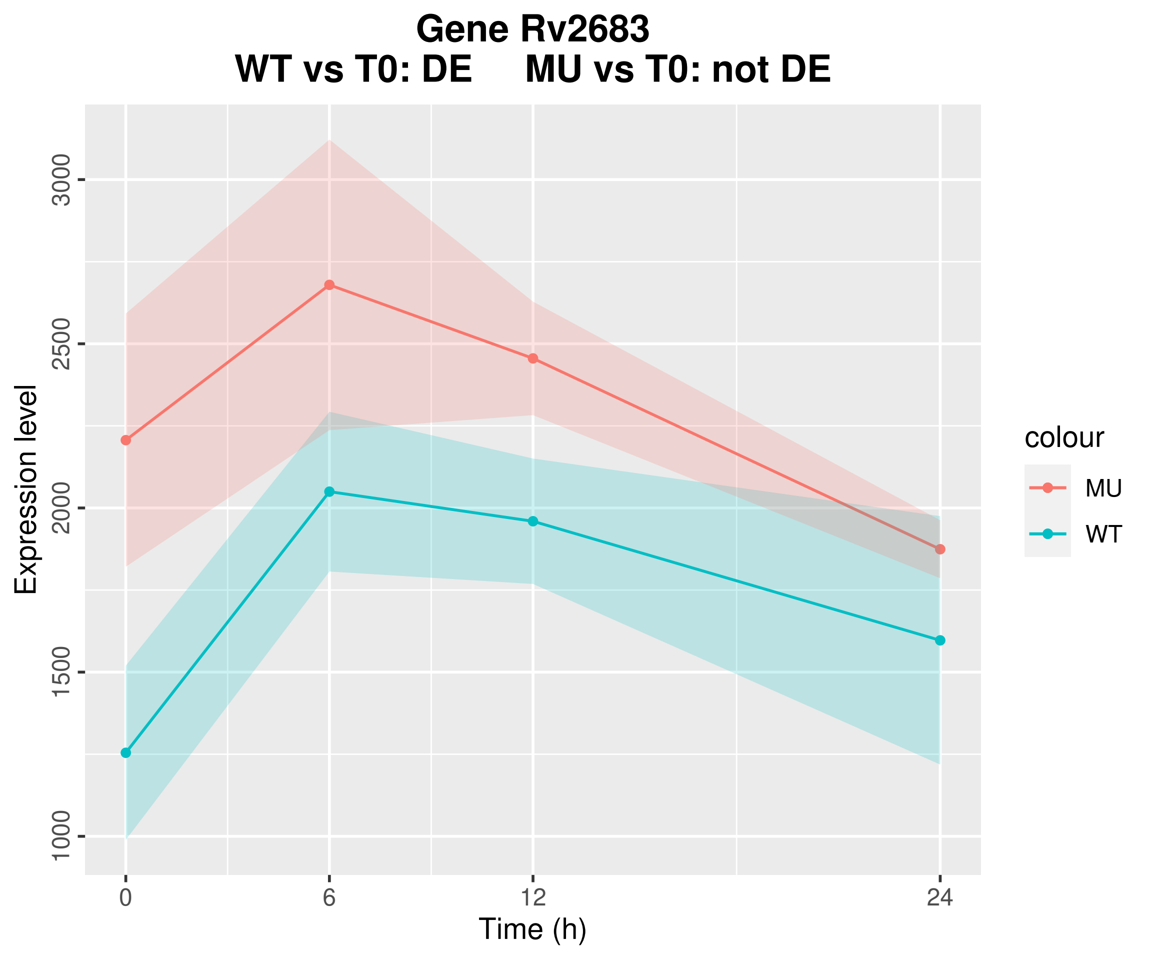
Gene Rv2683
WT vs T0: DE   MU vs T0: not DE
Expression level
3000
2500
2000
1500
1000
0
6
12
24
Time (h)
colour
MU
WT

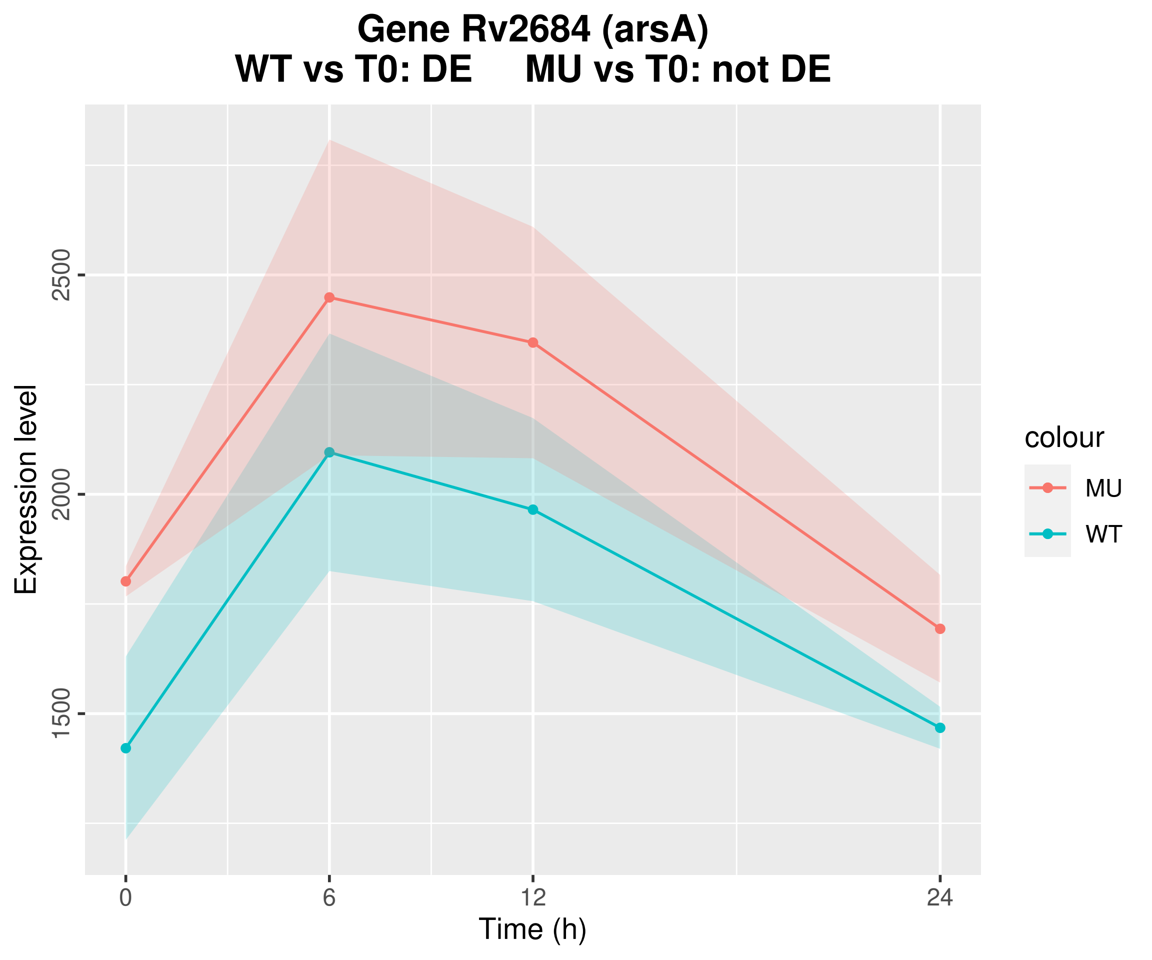
Gene Rv2684 (arsA)
WT vs T0: DE   MU vs T0: not DE
Expression level
2500
2000
1500
0
6
12
24
Time (h)
colour
MU
WT

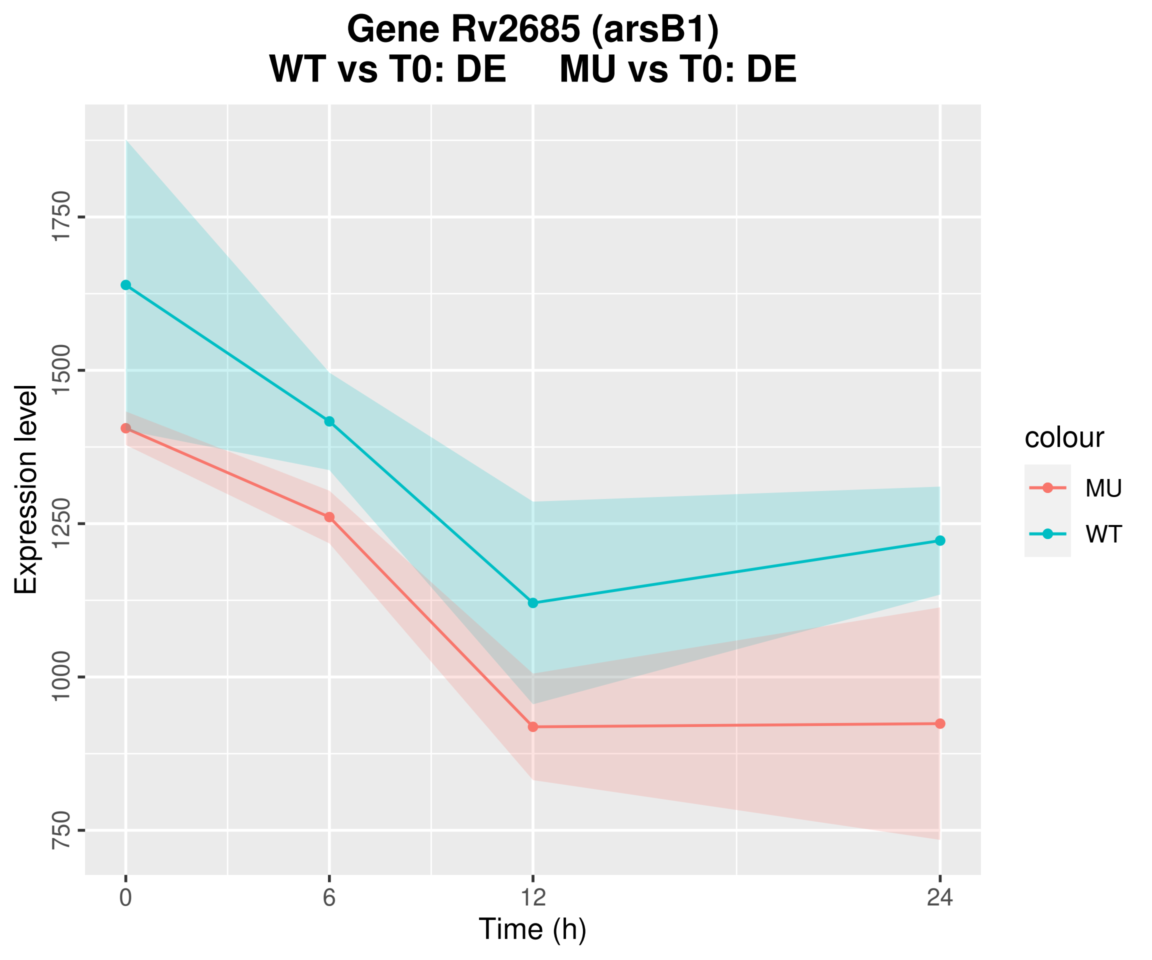

Gene Rv2685 (arsB1)
WT vs T0: DE   MU vs T0: DE
Expression level
1750
1500
1250
1000
750
0
6
12
24
Time (h)
colour
MU
WT

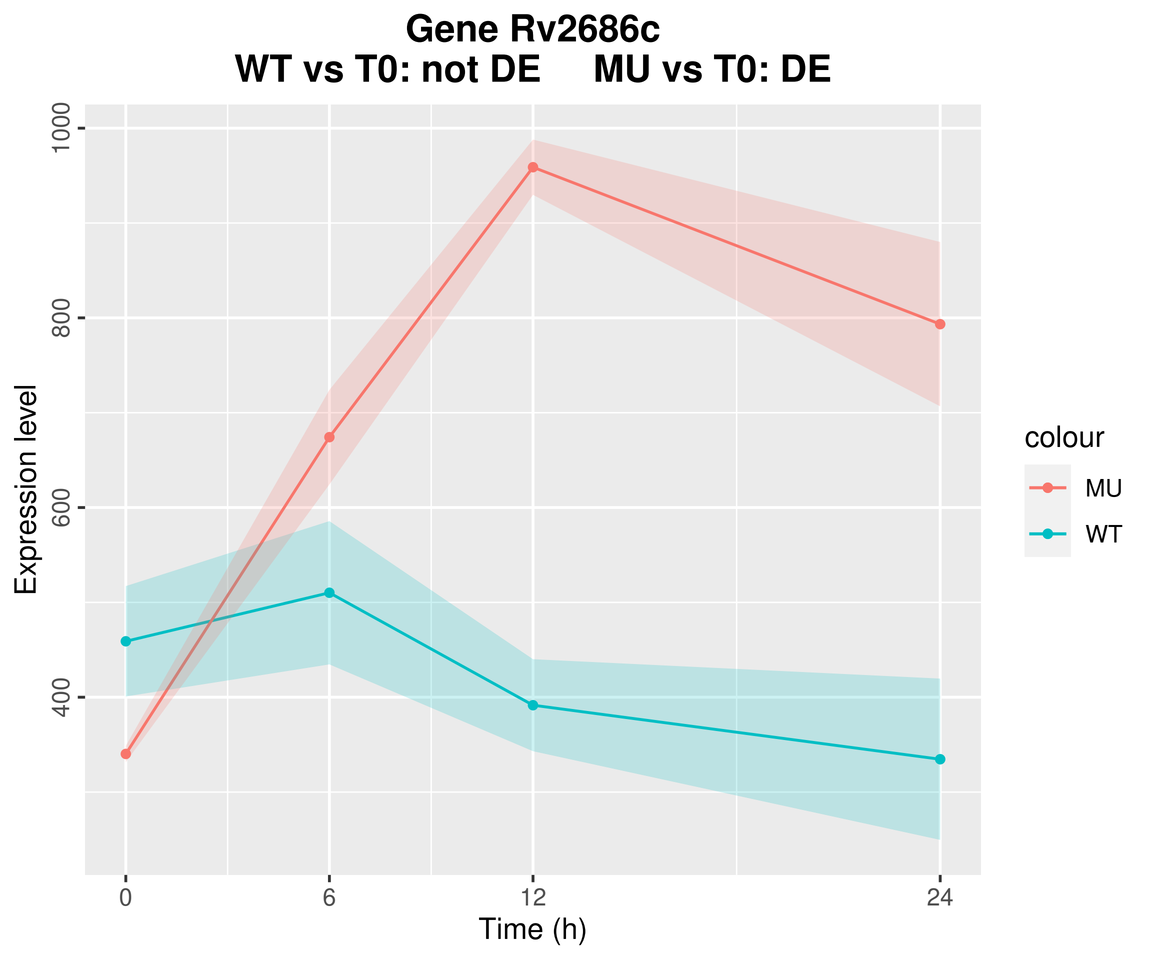

Gene Rv2686c
WT vs T0: not DE MU vs T0: DE
1000
800
600
400
Expression level
0
6
12
24
Time (h)
colour
MU
WT

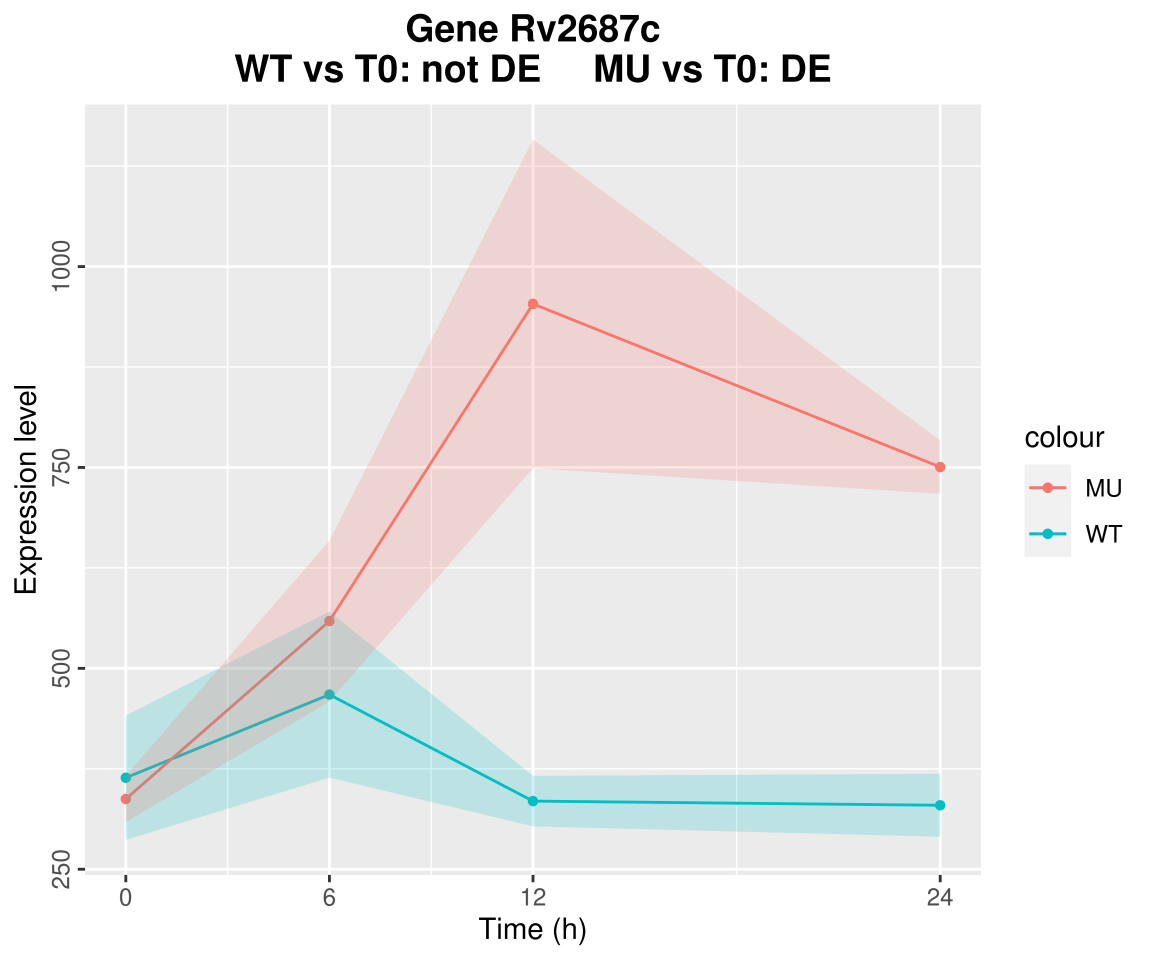

Gene Rv2687c
WT vs T0: not DE MU vs T0: DE
Expression level
1000
750
500
250
0
6
12
24
Time (h)
colour
MU
WT

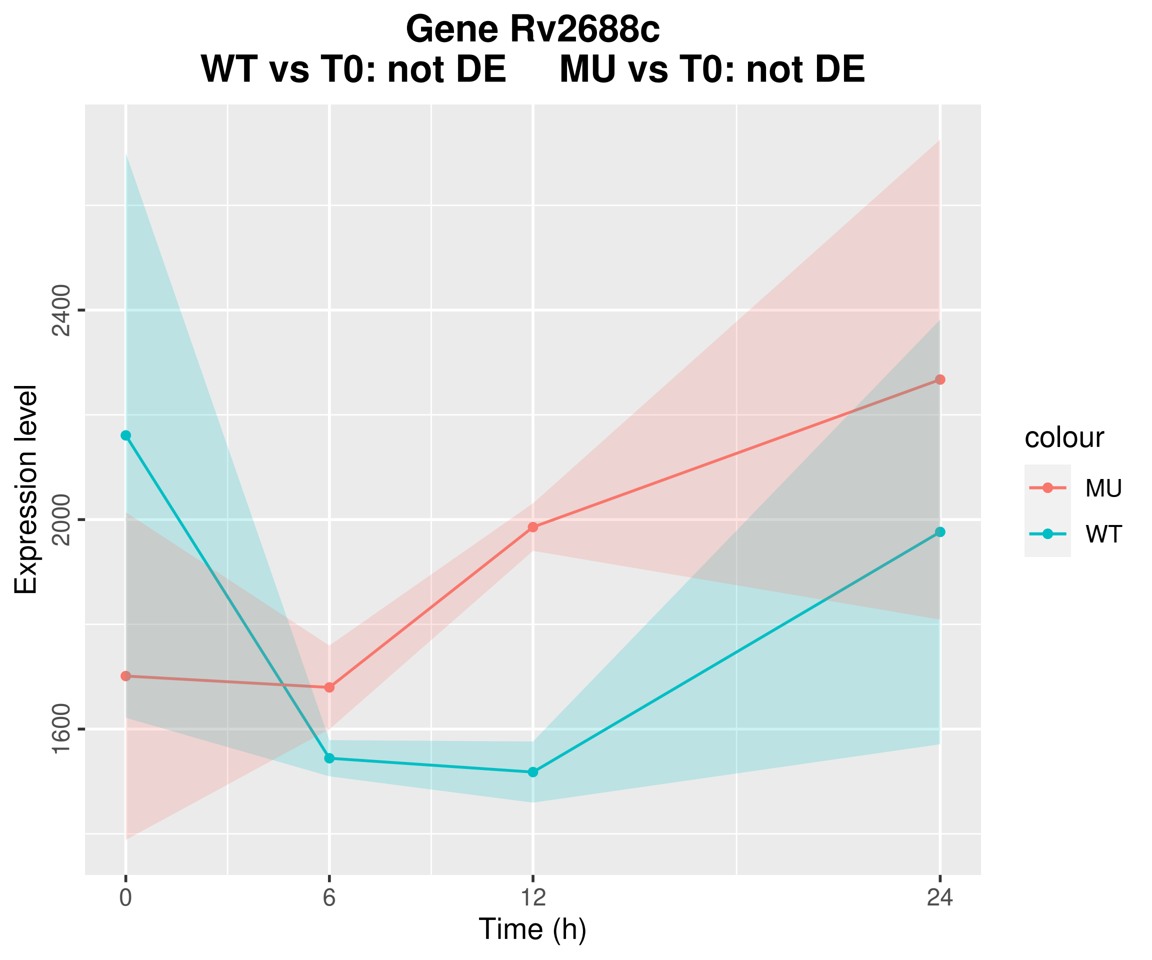
Gene Rv2688c
WT vs T0: not DE   MU vs T0: not DE
Expression level
2400
2000
1600
0
6
12
24
Time (h)
colour
MU
WT

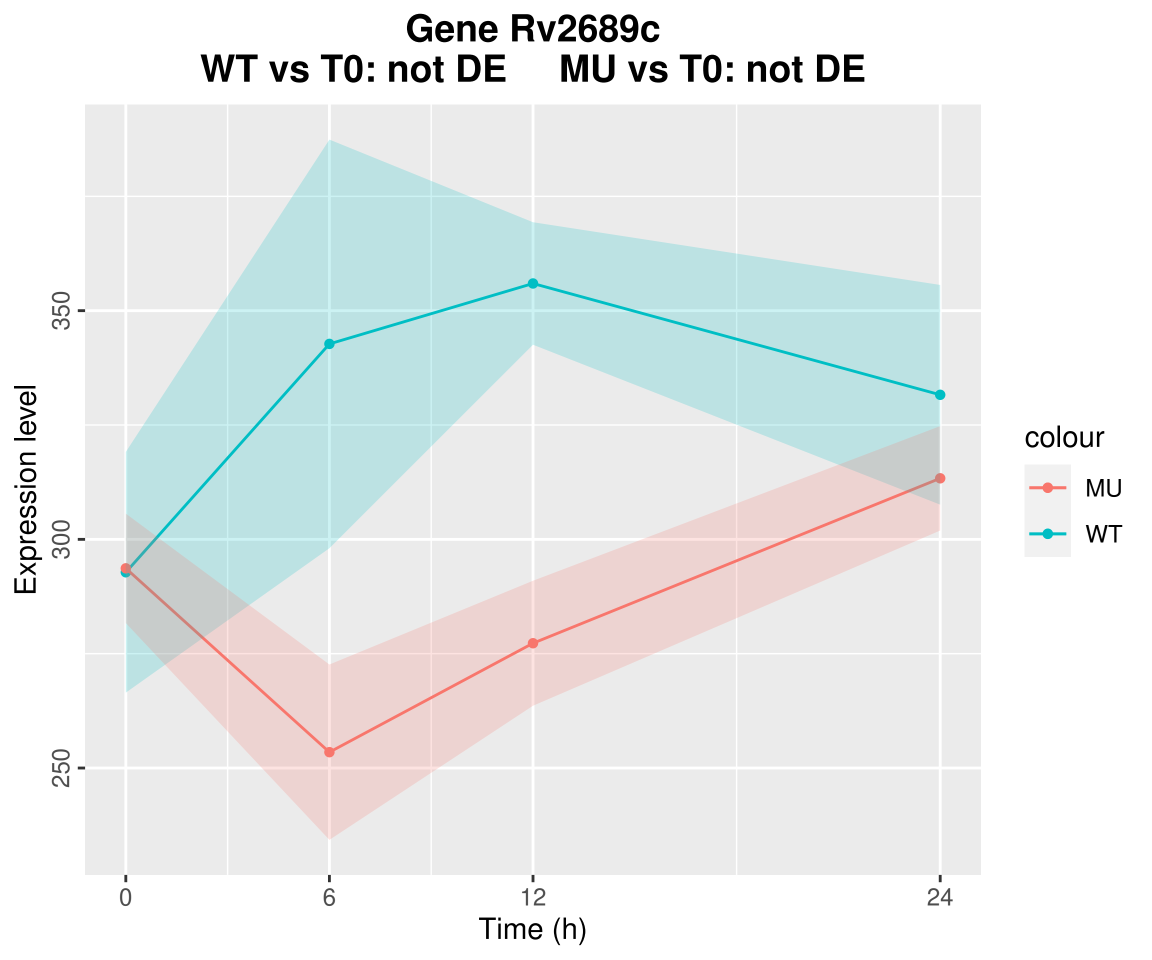

Gene Rv2689c
WT vs T0: not DE   MU vs T0: not DE
Expression level
350
300
250
0
6
12
24
Time (h)
colour
MU
WT

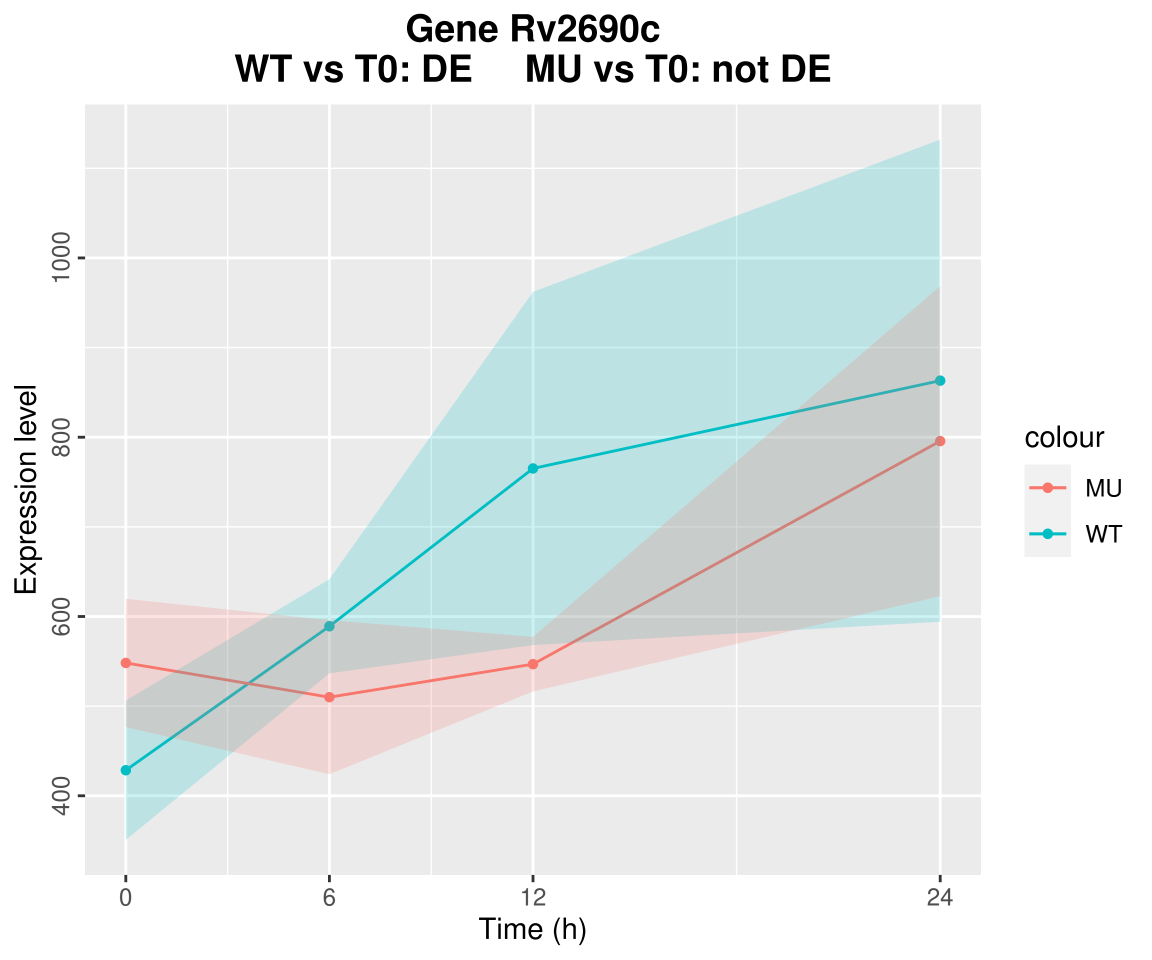

Gene Rv2690c
WT vs T0: DE MU vs T0: not DE
Expression level
400
600
800
1000
0
6
12
24
Time (h)
colour
MU
WT

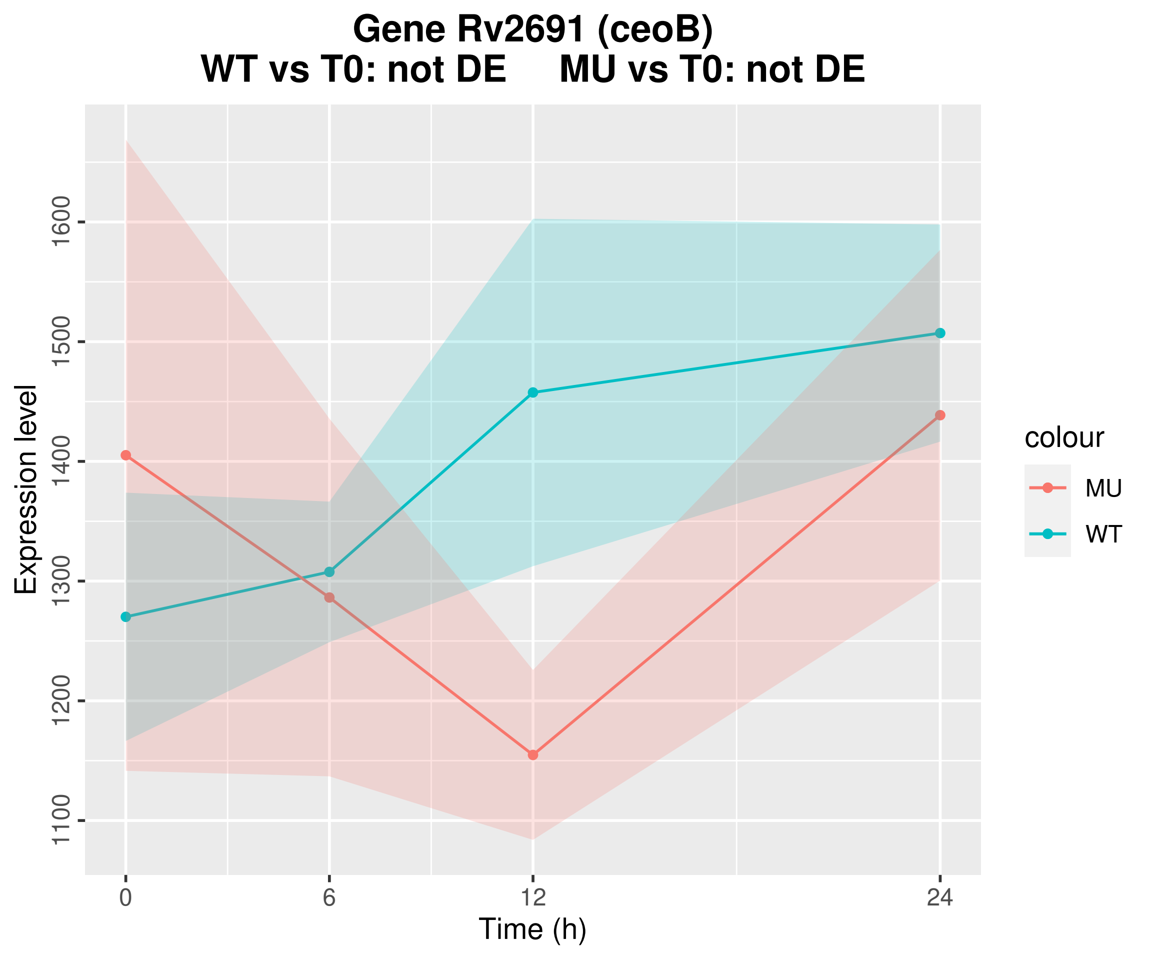
Gene Rv2691 (ceoB)
WT vs T0: not DE MU vs T0: not DE
Expression level
1100
1200
1300
1400
1500
1600
0
6
12
24
Time (h)
colour
MU
WT

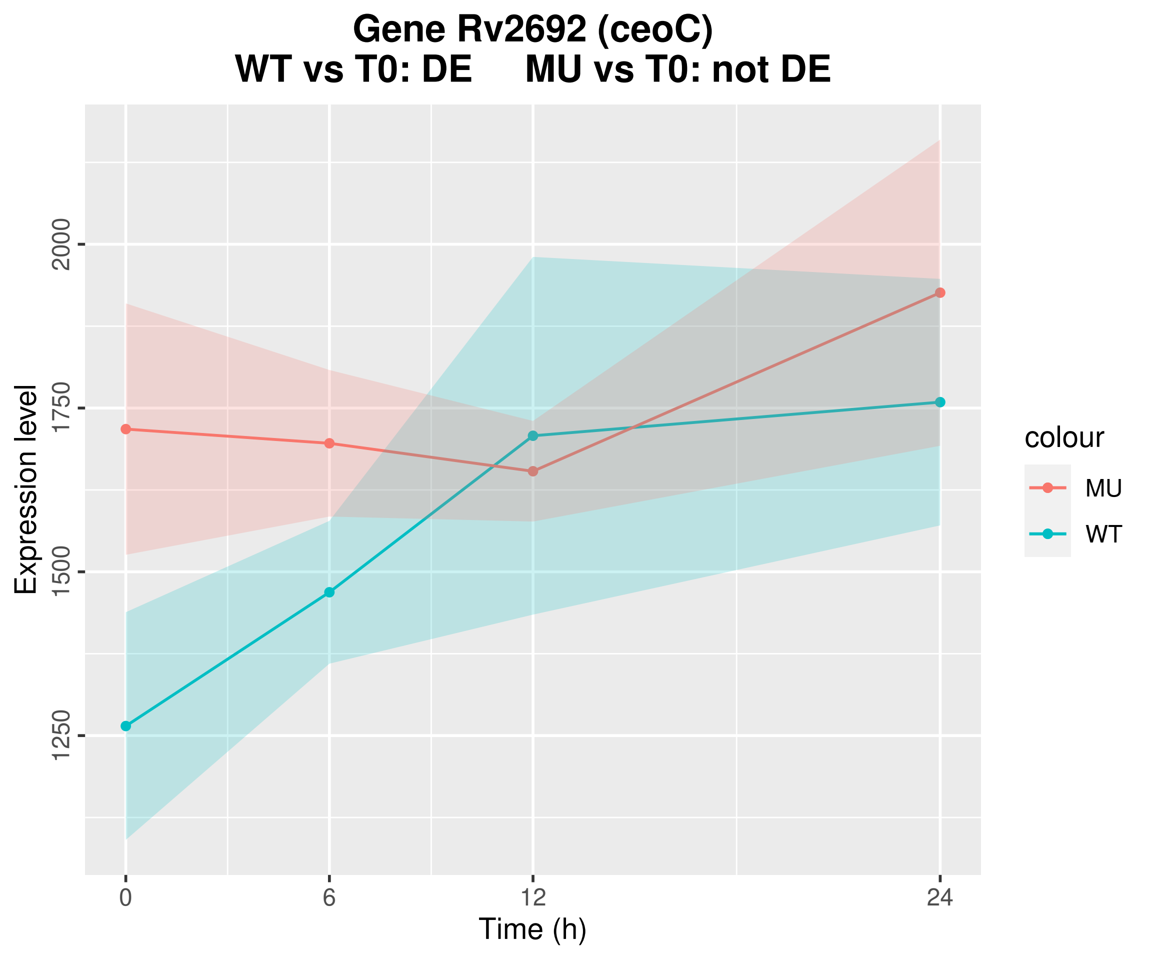

Gene Rv2692 (ceoC)
WT vs T0: DE MU vs T0: not DE
Expression level
1250
1500
1750
2000
0
6
12
24
Time (h)
colour
MU
WT

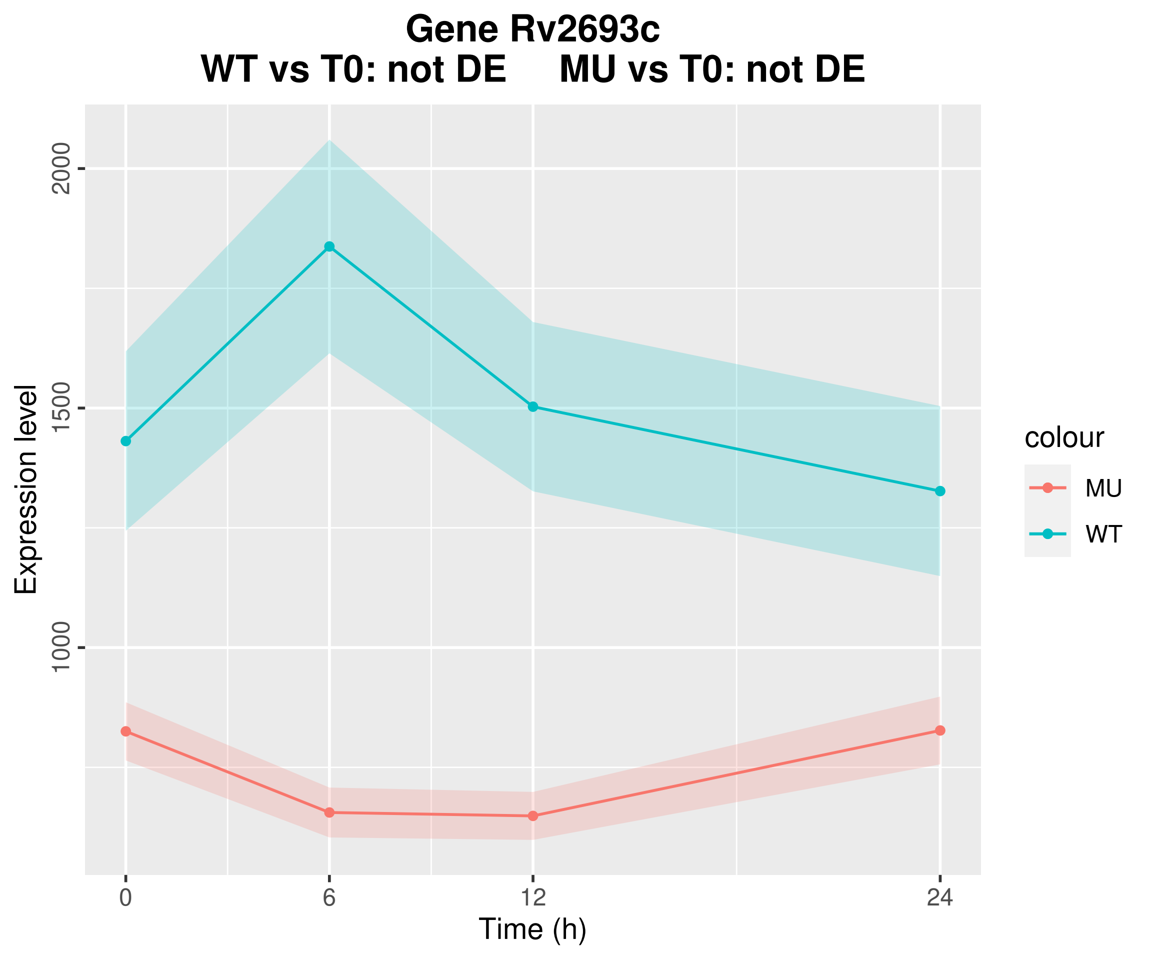
Gene Rv2693c
WT vs T0: not DE   MU vs T0: not DE
2000
1500
1000
Expression level
0
6
12
24
Time (h)
colour
MU
WT

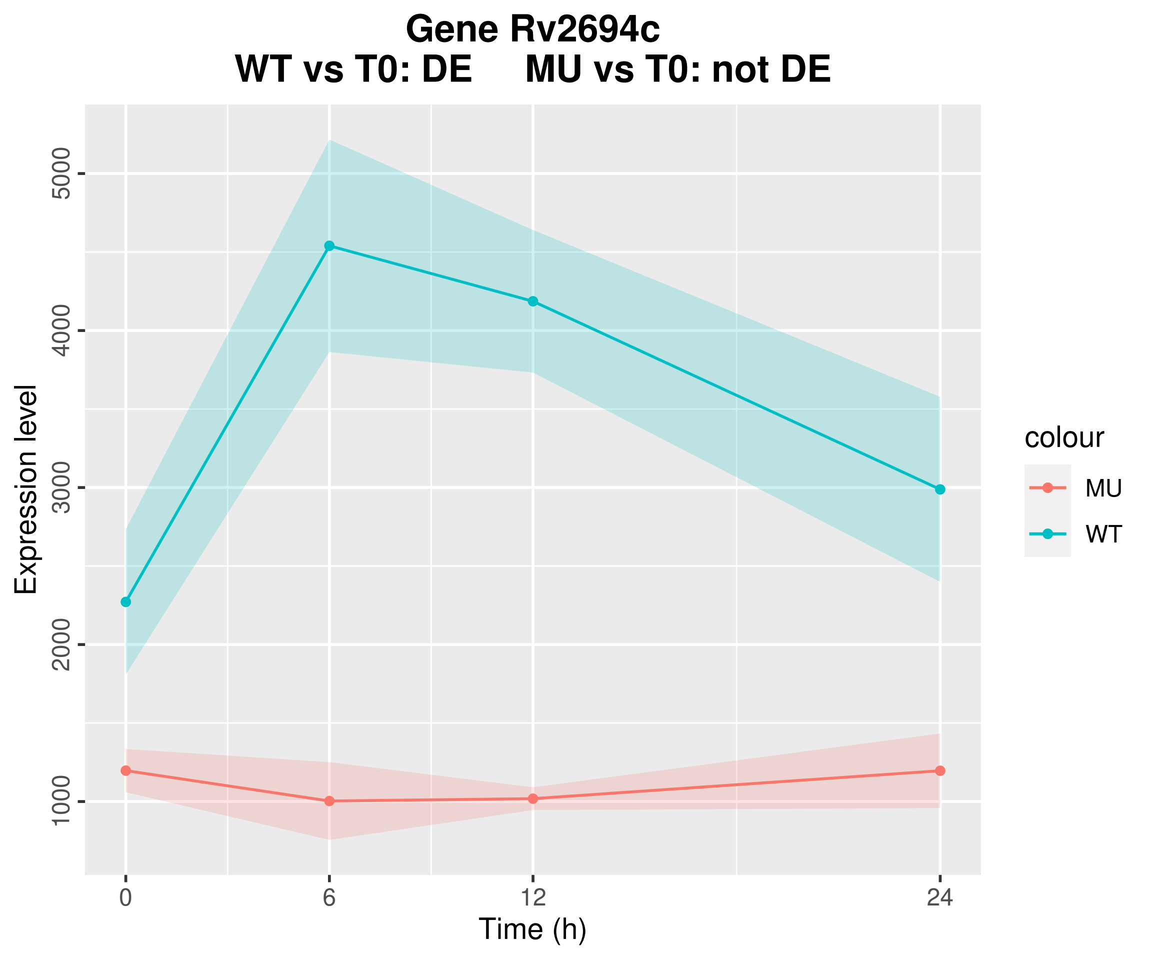

Gene Rv2694c
WT vs T0: DE MU vs T0: not DE
Expression level
5000
4000
3000
2000
1000
0
6
12
24
Time (h)
colour
MU
WT

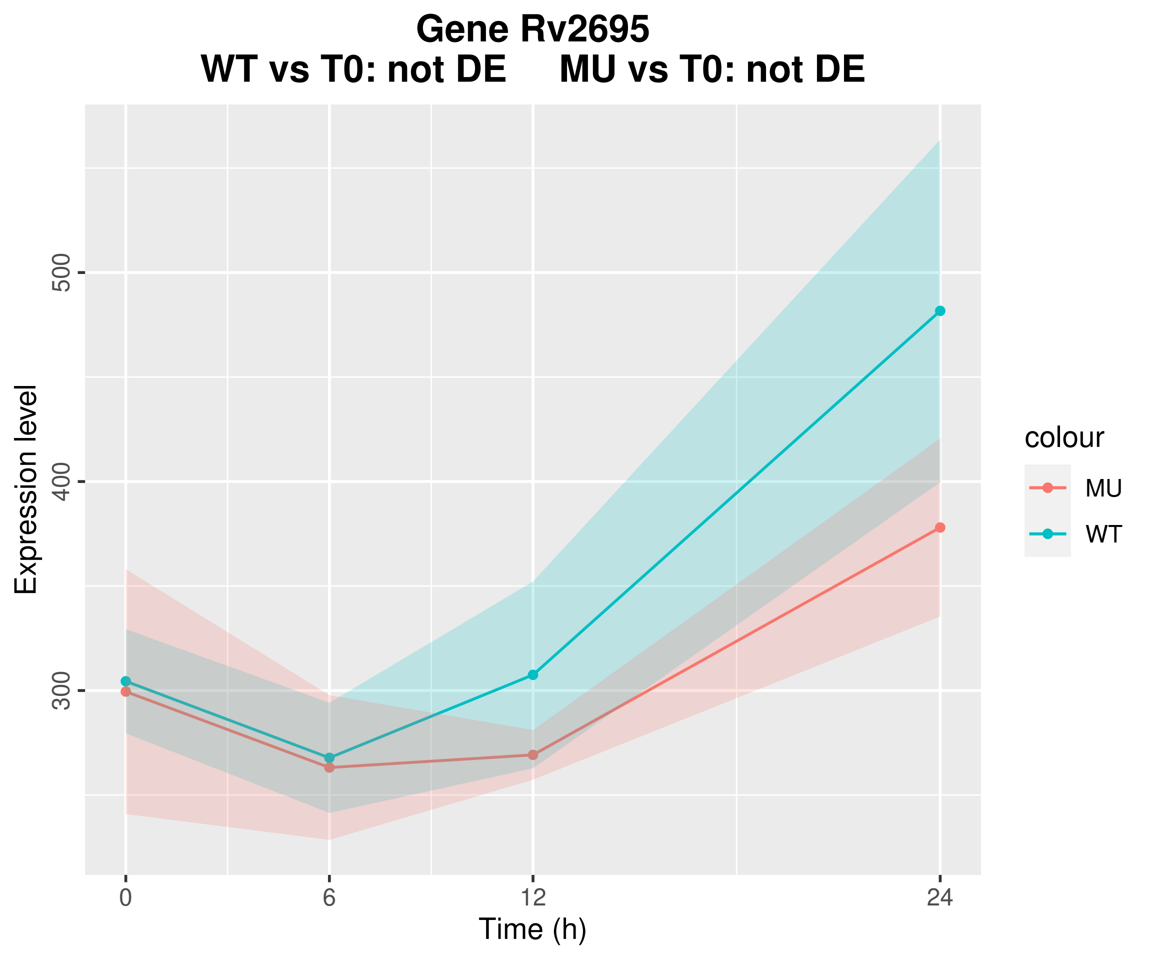

Gene Rv2695
WT vs T0: not DE MU vs T0: not DE
Expression level
500
400
300
0
6
12
24
Time (h)
colour
MU
WT

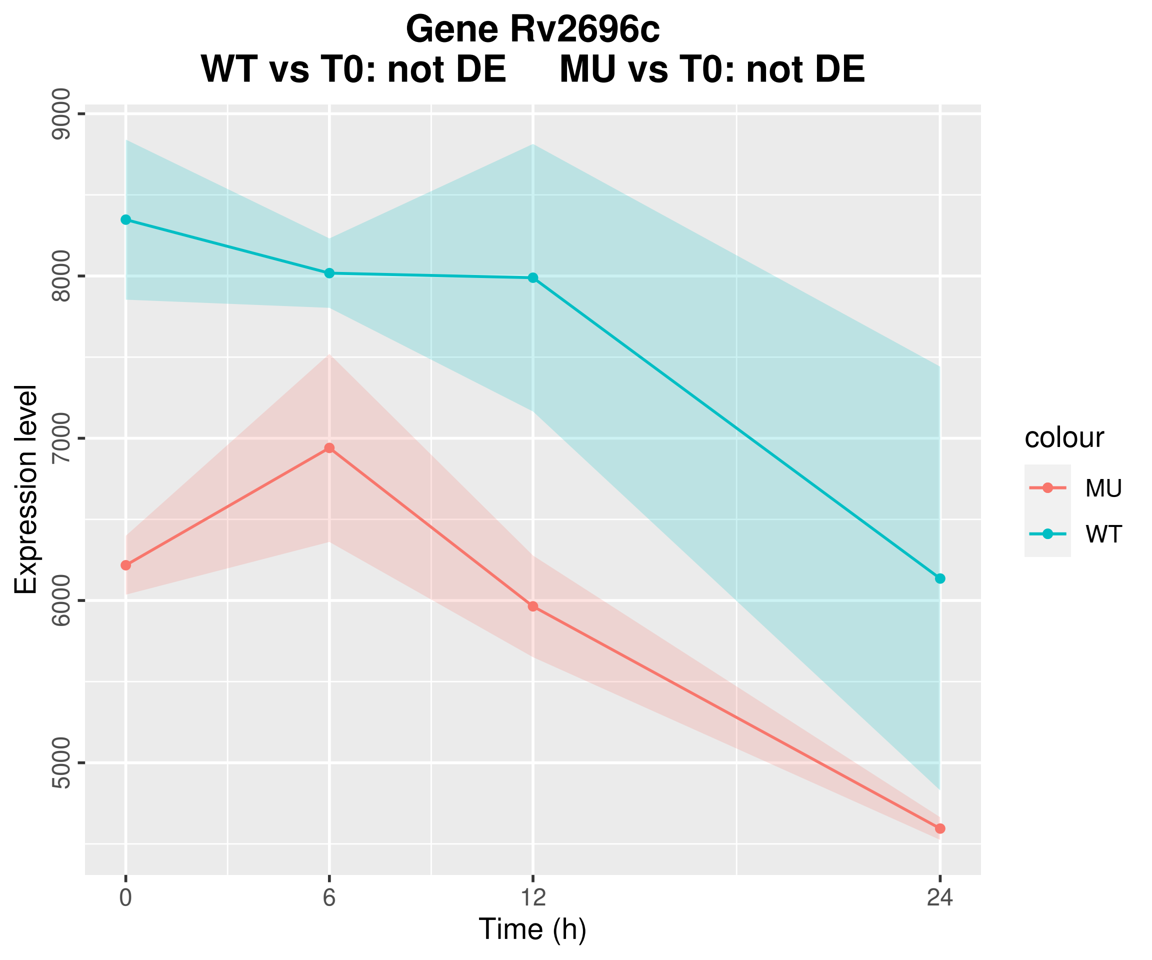

Gene Rv2696c
WT vs T0: not DE   MU vs T0: not DE
9000
8000
7000
6000
5000
Expression level
0
6
12
24
Time (h)
colour
MU
WT

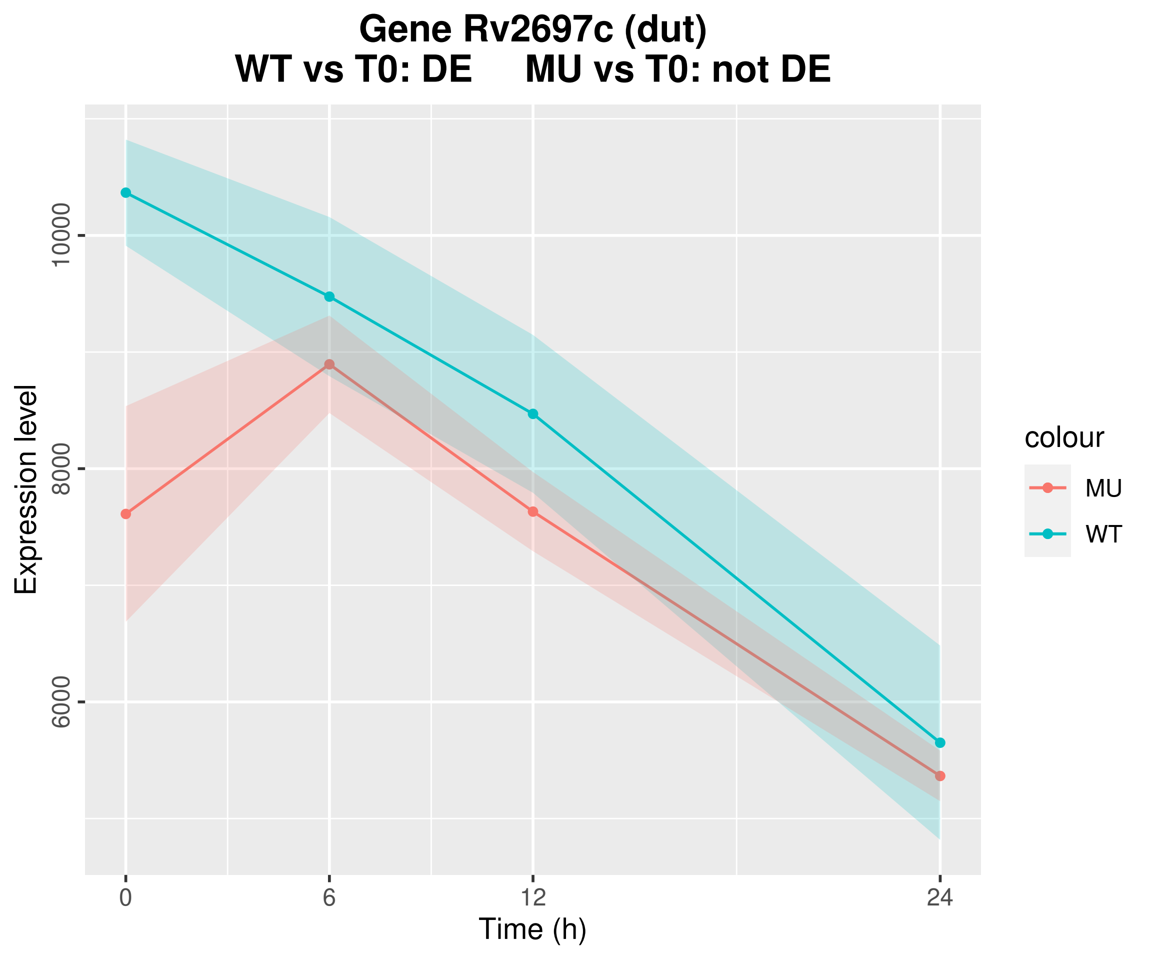
Gene Rv2697c (dut)
WT vs T0: DE   MU vs T0: not DE
Expression level
10000
8000
6000
0
6
12
24
Time (h)
colour
MU
WT

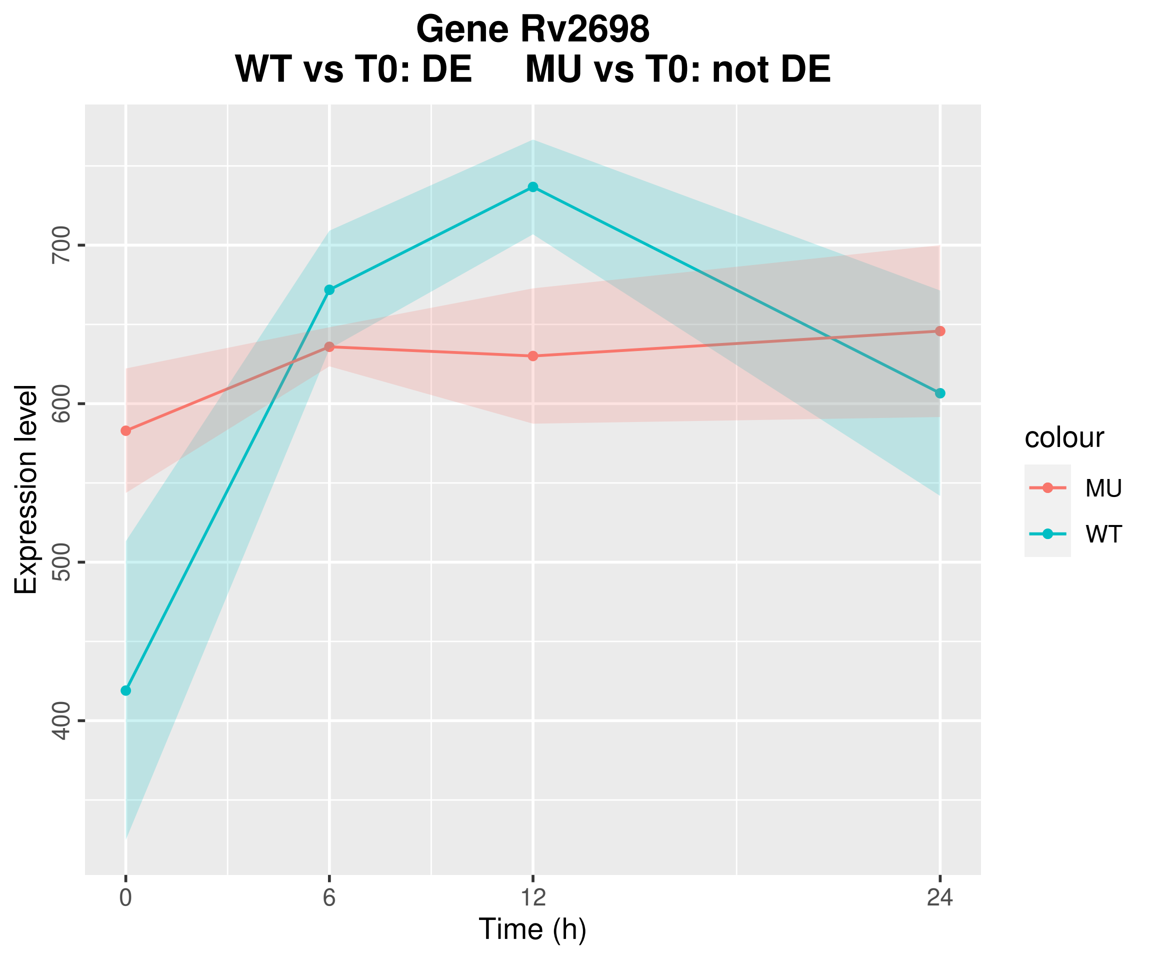
Gene Rv2698
WT vs T0: DE   MU vs T0: not DE
Expression level
700
600
500
400
0
6
12
24
Time (h)
colour
MU
WT

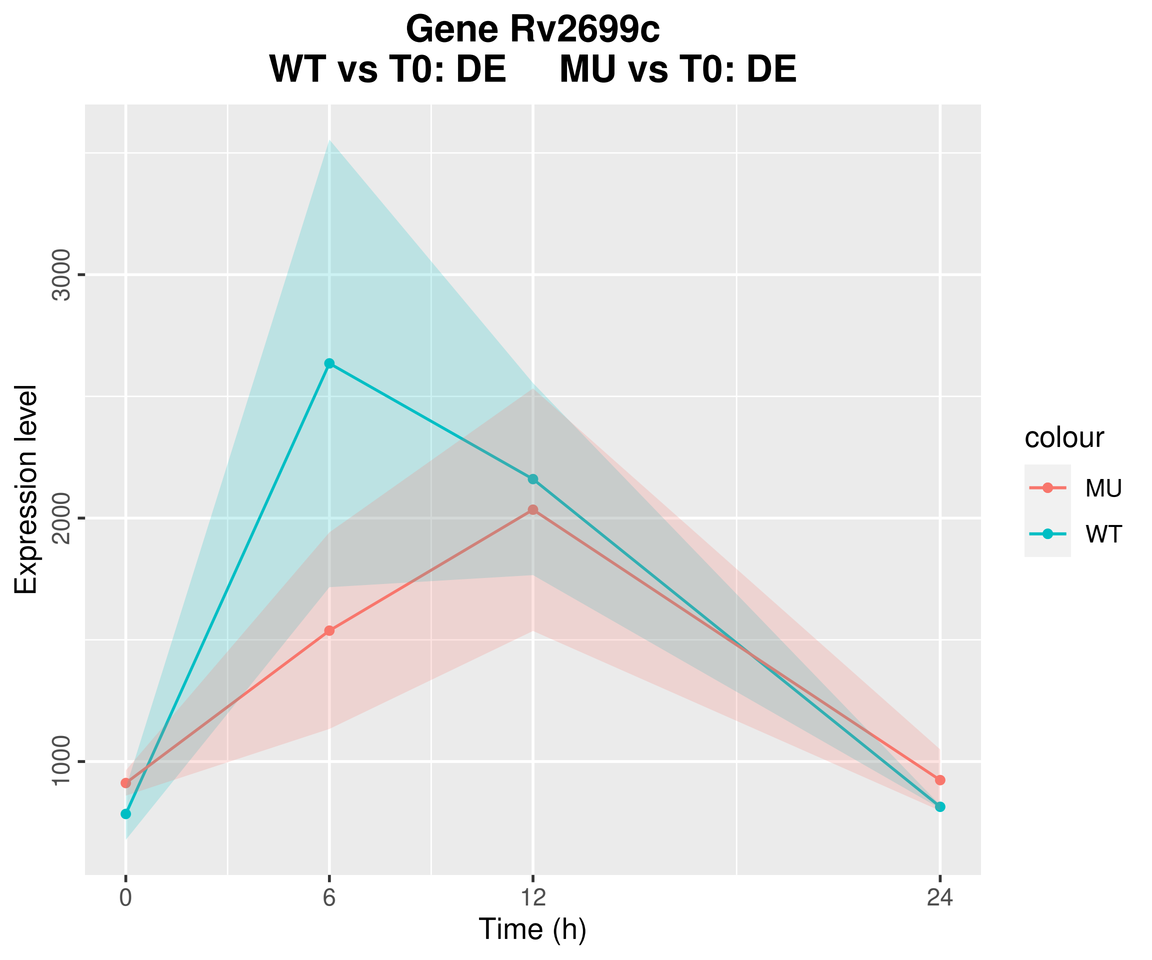
Gene Rv2699c
WT vs T0: DE MU vs T0: DE
Expression level
3000
2000
1000
0
6
12
24
Time (h)
colour
MU
WT

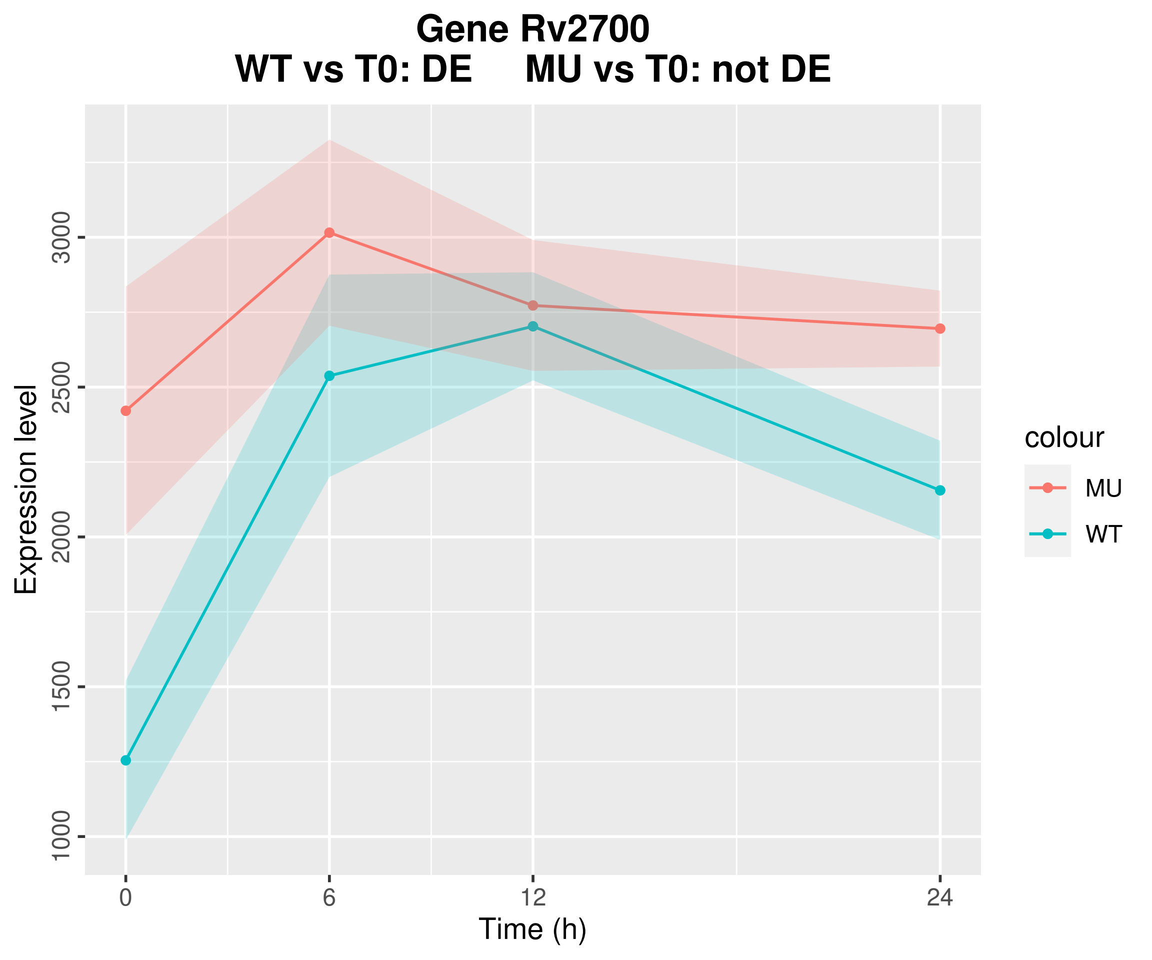
Gene Rv2700
WT vs T0: DE   MU vs T0: not DE
Expression level
3000
2500
2000
1500
1000
0
6
12
24
Time (h)
colour
MU
WT

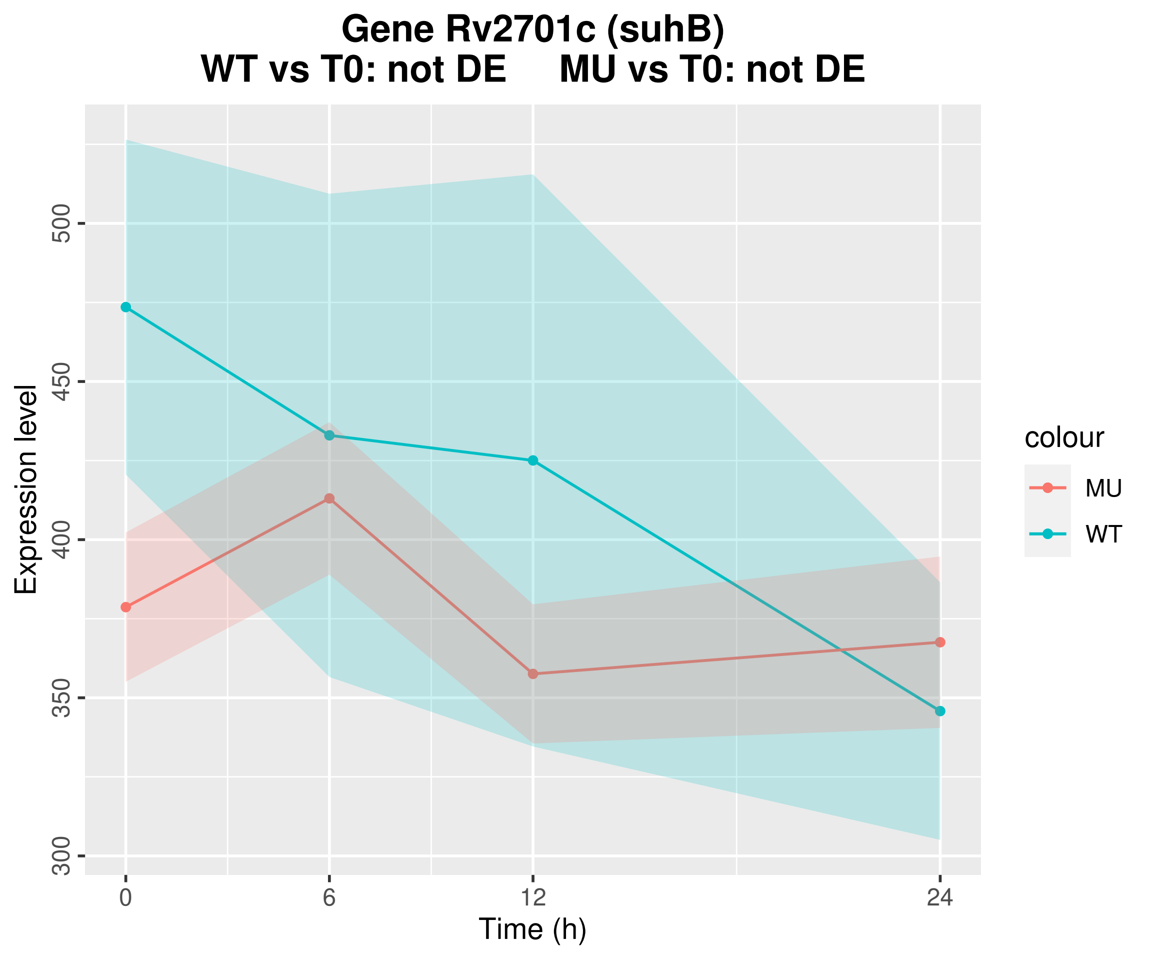
Gene Rv2701c (suhB)
WT vs T0: not DE   MU vs T0: not DE
Expression level
500
450
400
350
300
0
6
12
24
Time (h)
colour
MU
WT

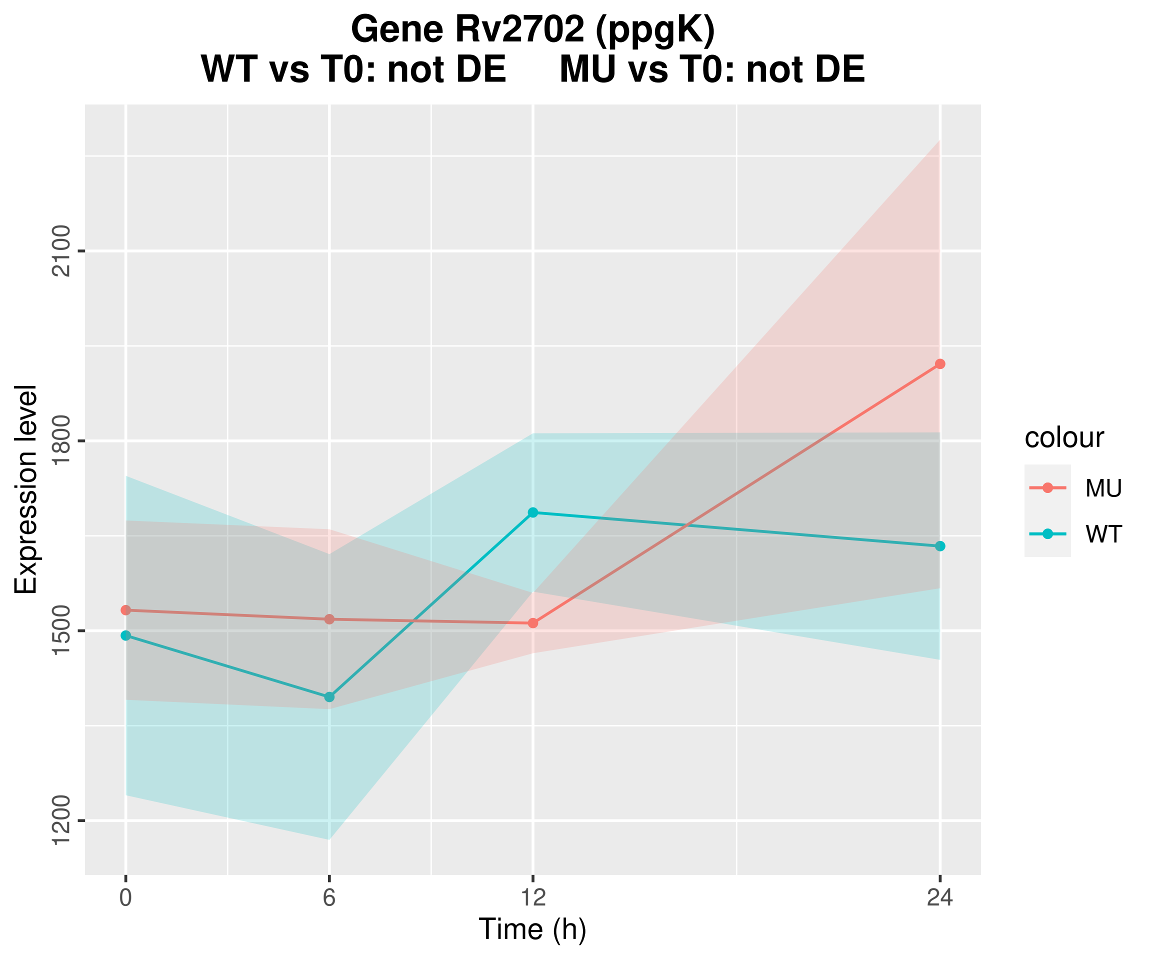
Gene Rv2702 (ppgK)
WT vs T0: not DE MU vs T0: not DE
Expression level
2100
1800
1500
1200
0
6
12
24
Time (h)
colour
MU
WT

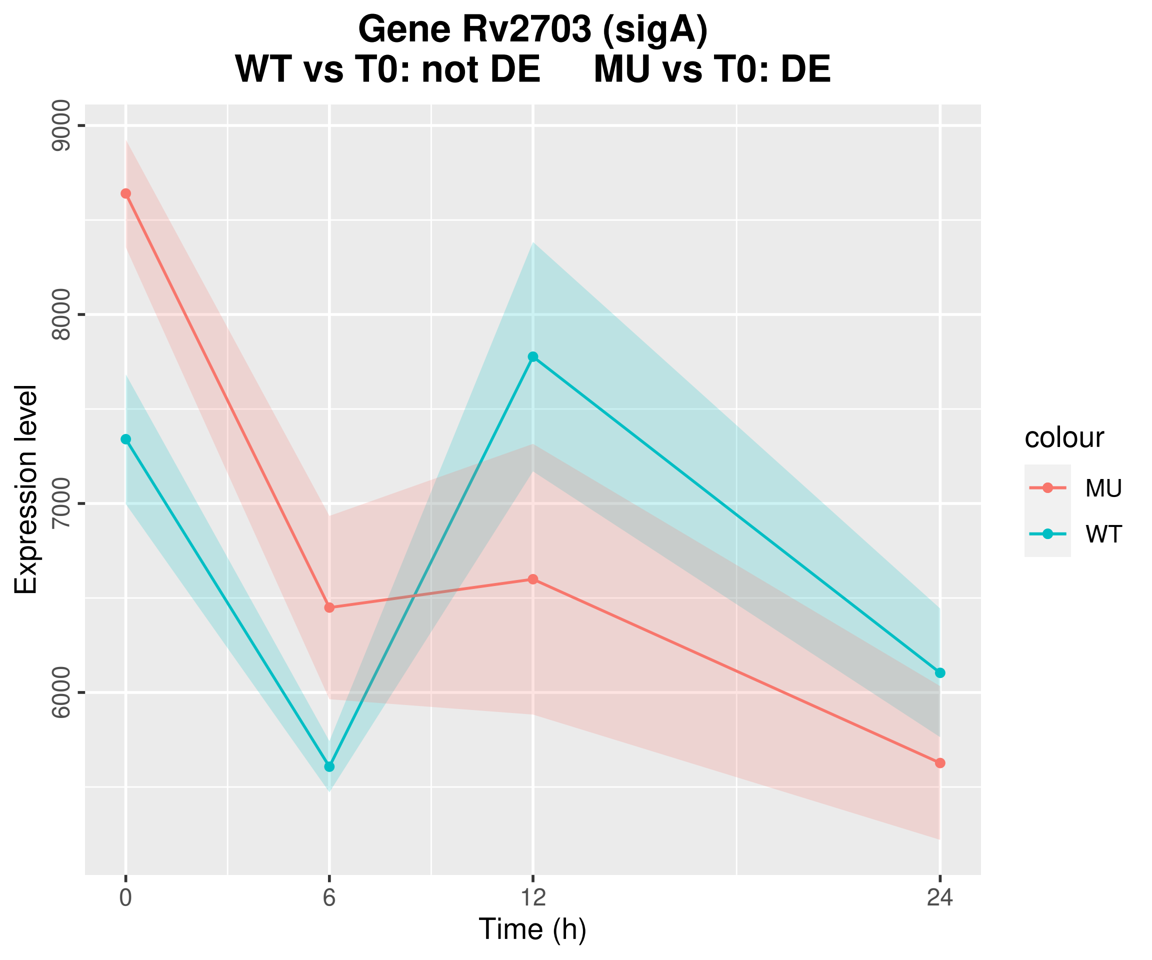
Gene Rv2703 (sigA)
WT vs T0: not DE MU vs T0: DE
Expression level
9000
8000
7000
6000
0
6
12
24
Time (h)
colour
MU
WT

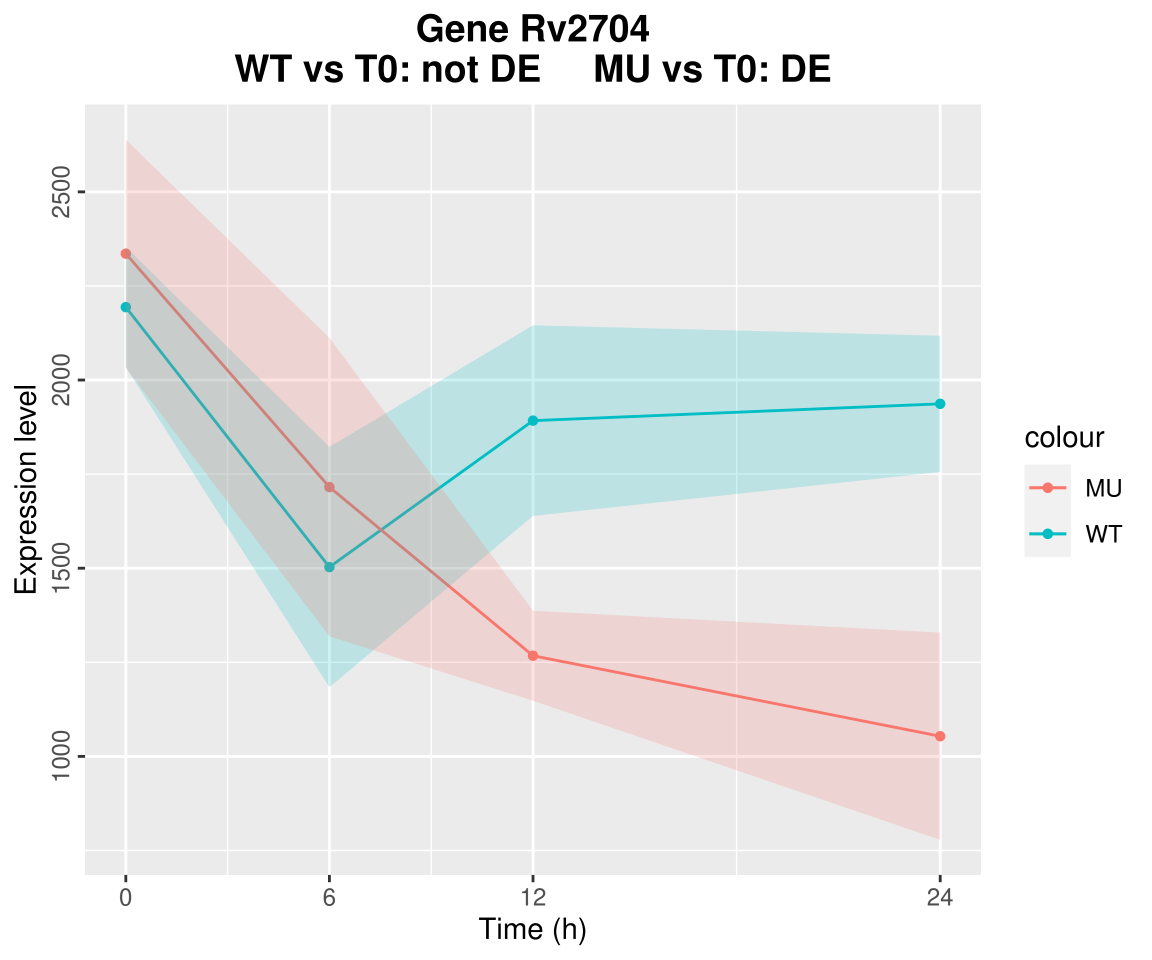

Gene Rv2704
WT vs T0: not DE MU vs T0: DE
Expression level
2500
2000
1500
1000
0
6
12
24
Time (h)
colour
MU
WT

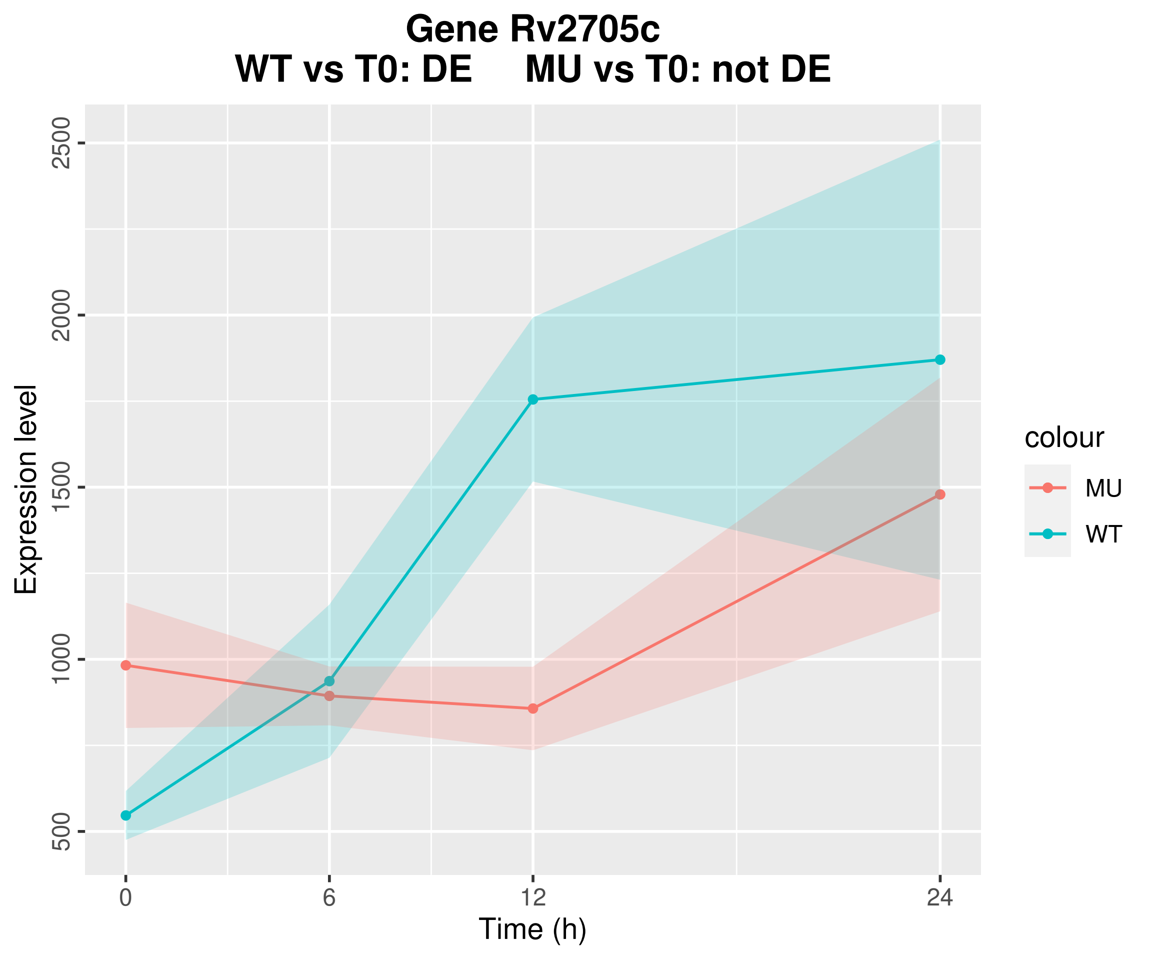
Gene Rv2705c
WT vs T0: DE MU vs T0: not DE
Expression level
2500
2000
1500
1000
500
0
6
12
24
Time (h)
colour
MU
WT

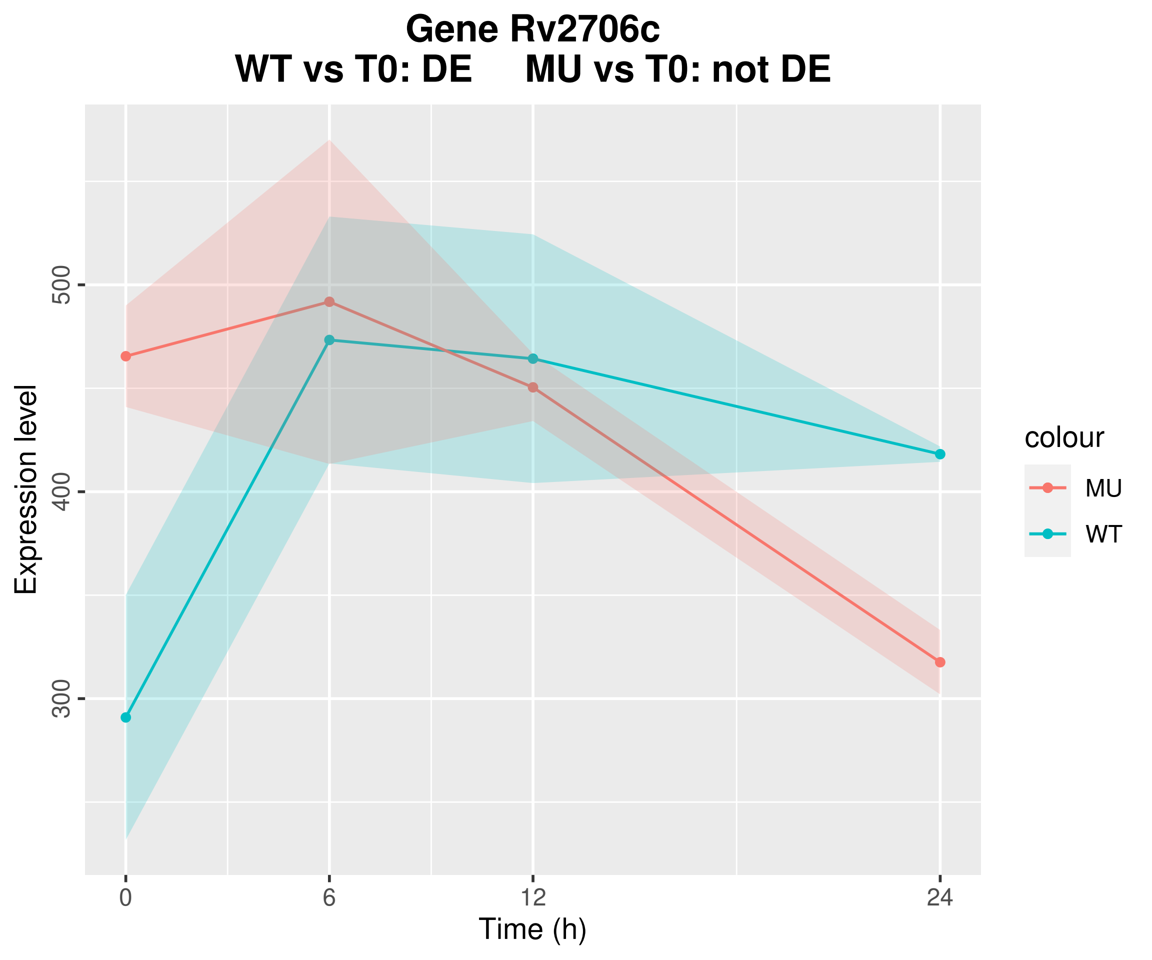
Gene Rv2706c
WT vs T0: DE MU vs T0: not DE
Expression level
500
400
300
0
6
12
24
Time (h)
colour
MU
WT

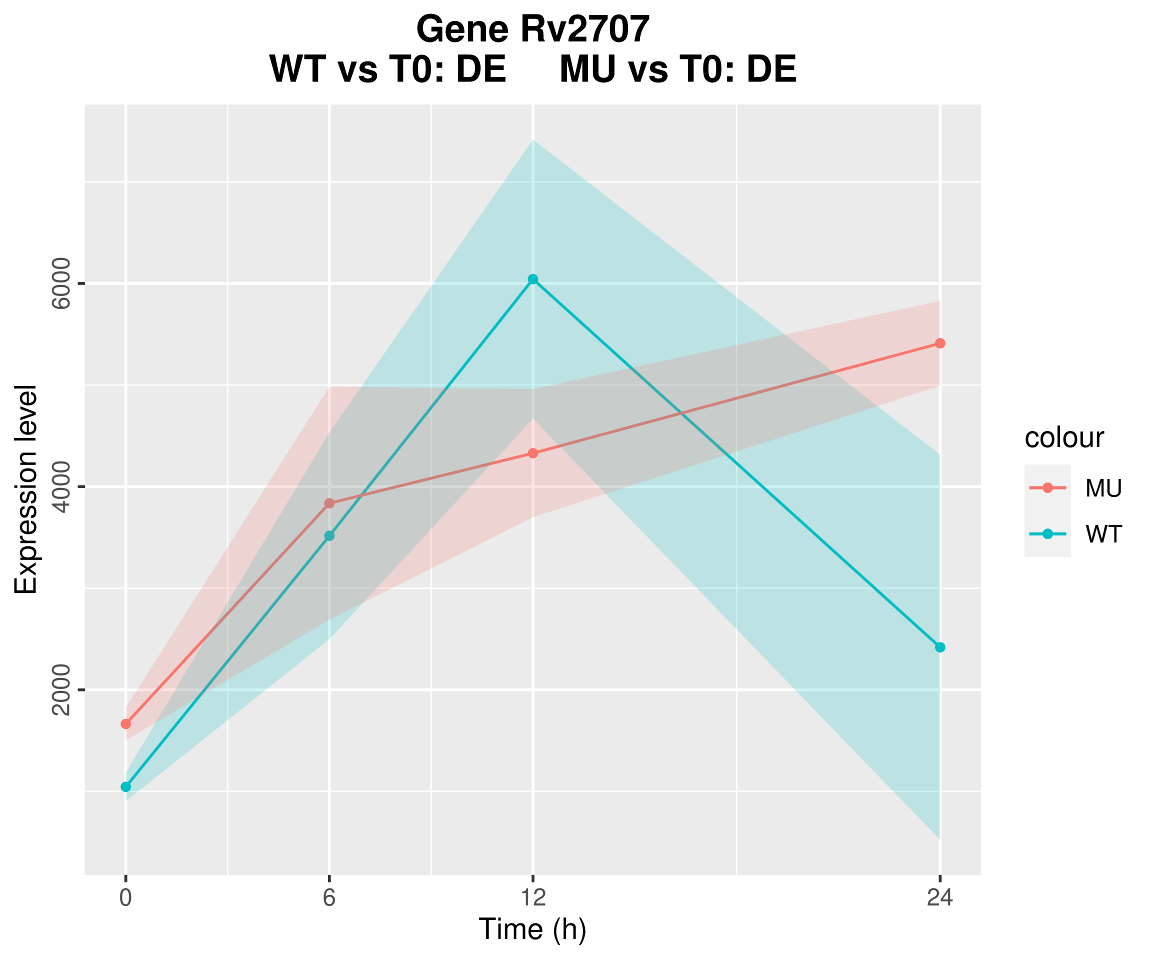
Gene Rv2707
WT vs T0: DE MU vs T0: DE
Expression level
6000
4000
2000
0
6
12
24
Time (h)
colour
MU
WT

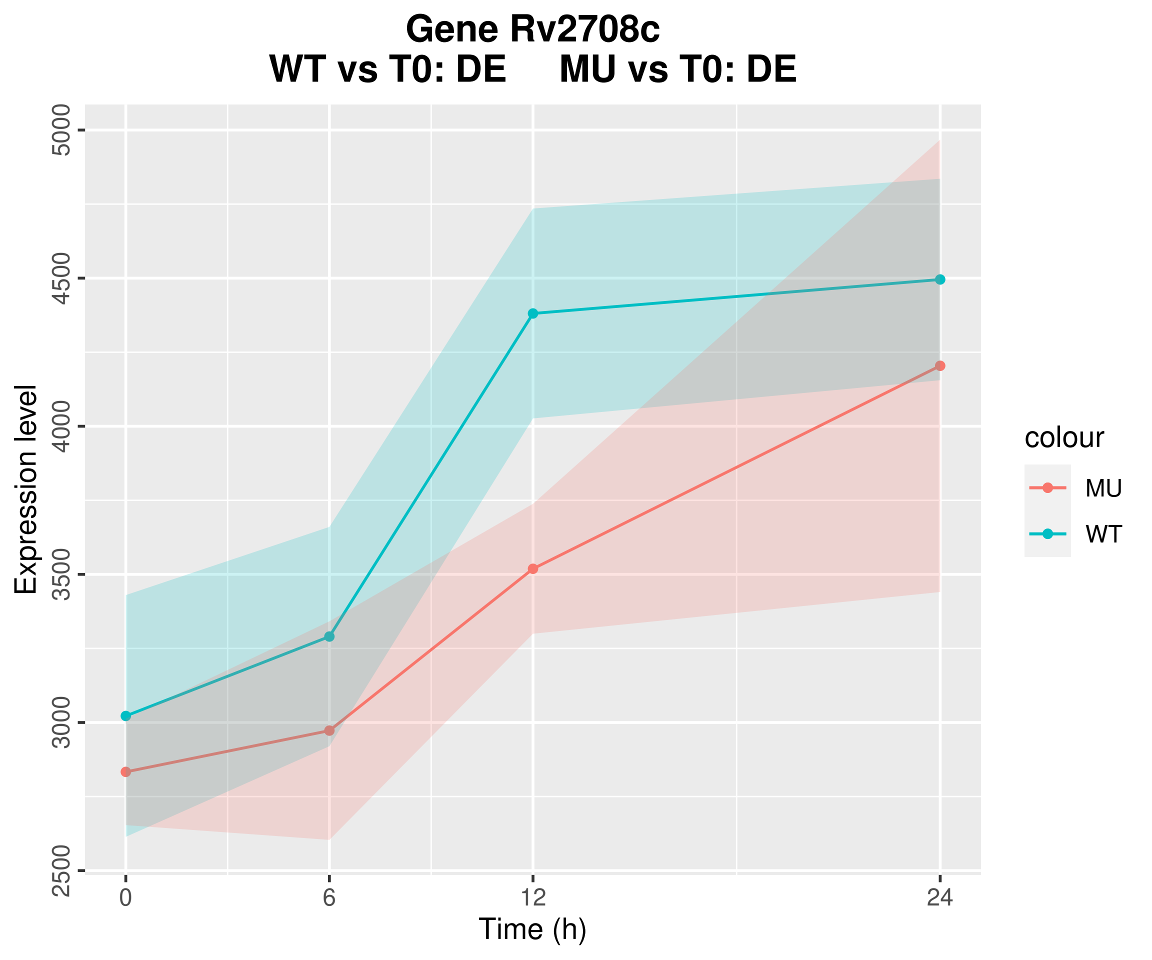

Gene Rv2708c
WT vs T0: DE MU vs T0: DE
Expression level
5000
4500
4000
3500
3000
2500
0
6
12
24
Time (h)
colour
MU
WT

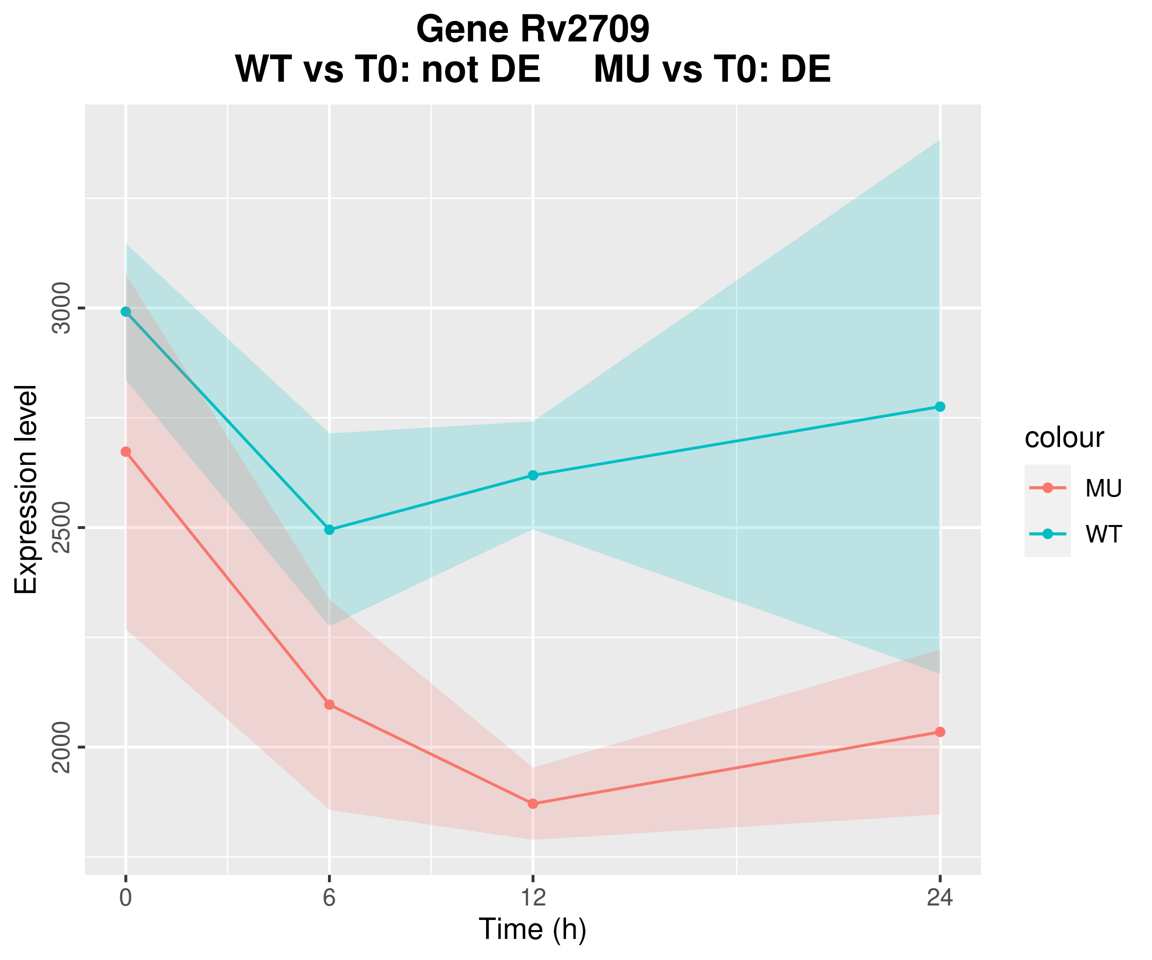
Gene Rv2709
WT vs T0: not DE MU vs T0: DE
Expression level
3000
2500
2000
0
6
12
24
Time (h)
colour
MU
WT

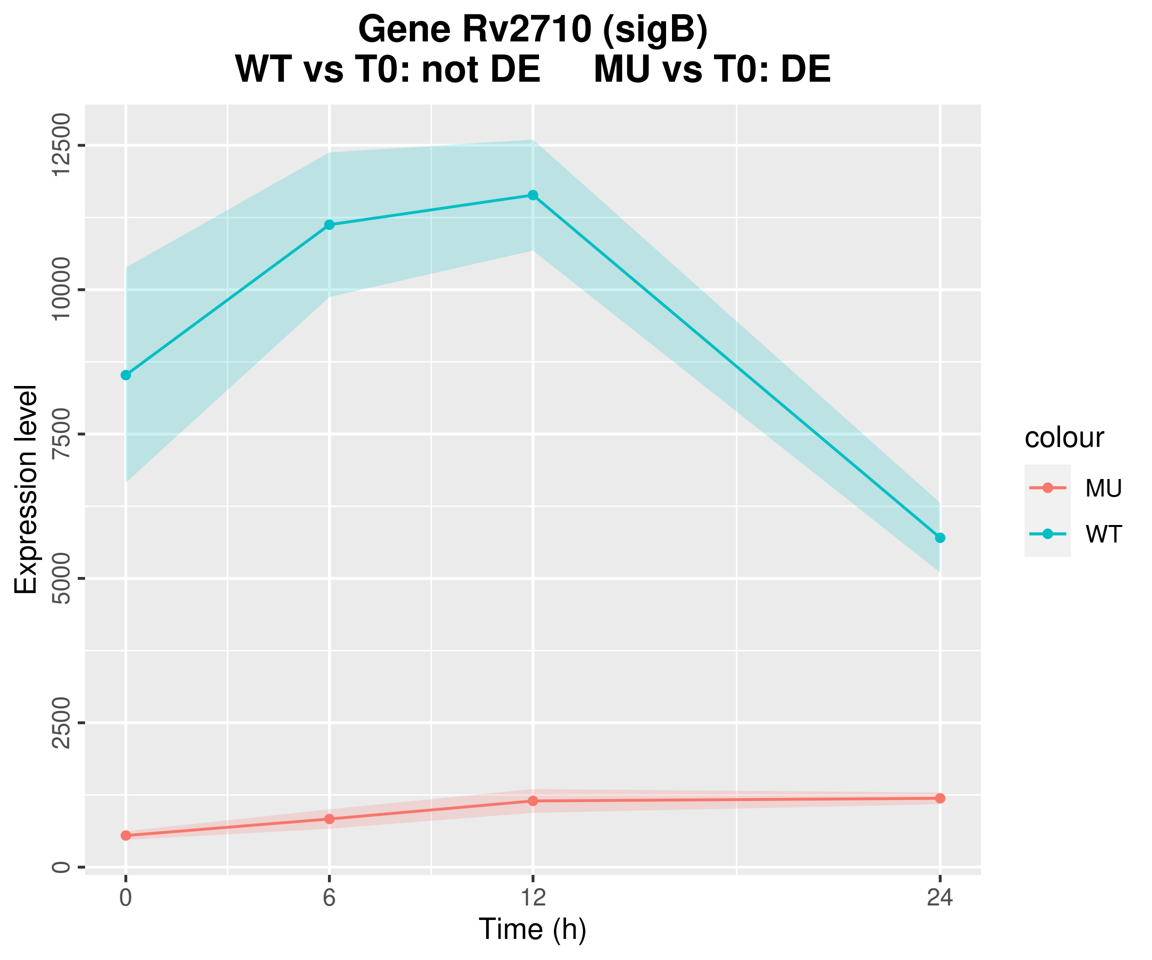

Gene Rv2710 (sigB)
WT vs T0: not DE MU vs T0: DE
Expression level
12500
10000
7500
5000
2500
0
0
6
12
24
Time (h)
colour
MU
WT

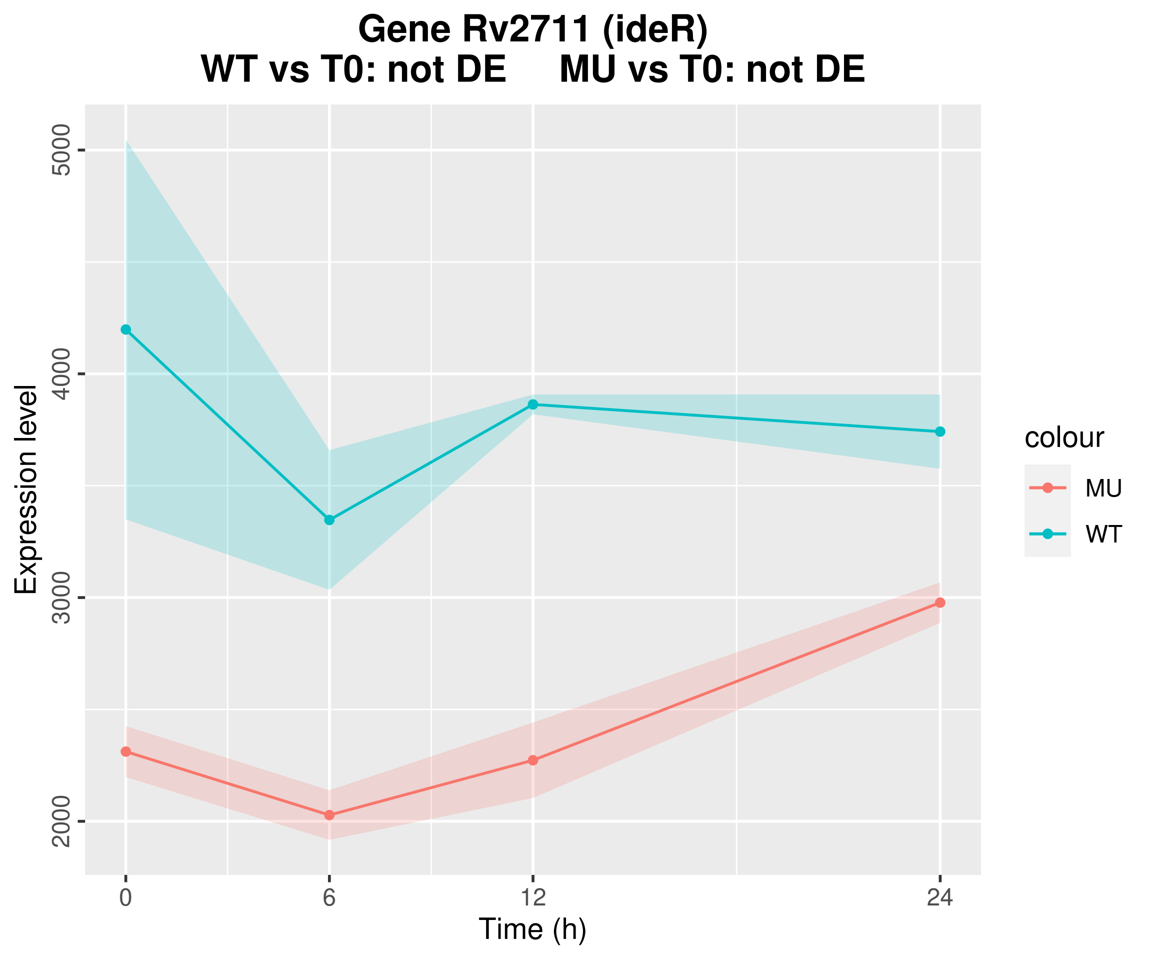
Gene Rv2711 (ideR)
WT vs T0: not DE   MU vs T0: not DE
Expression level
5000
4000
3000
2000
0
6
12
24
Time (h)
colour
MU
WT

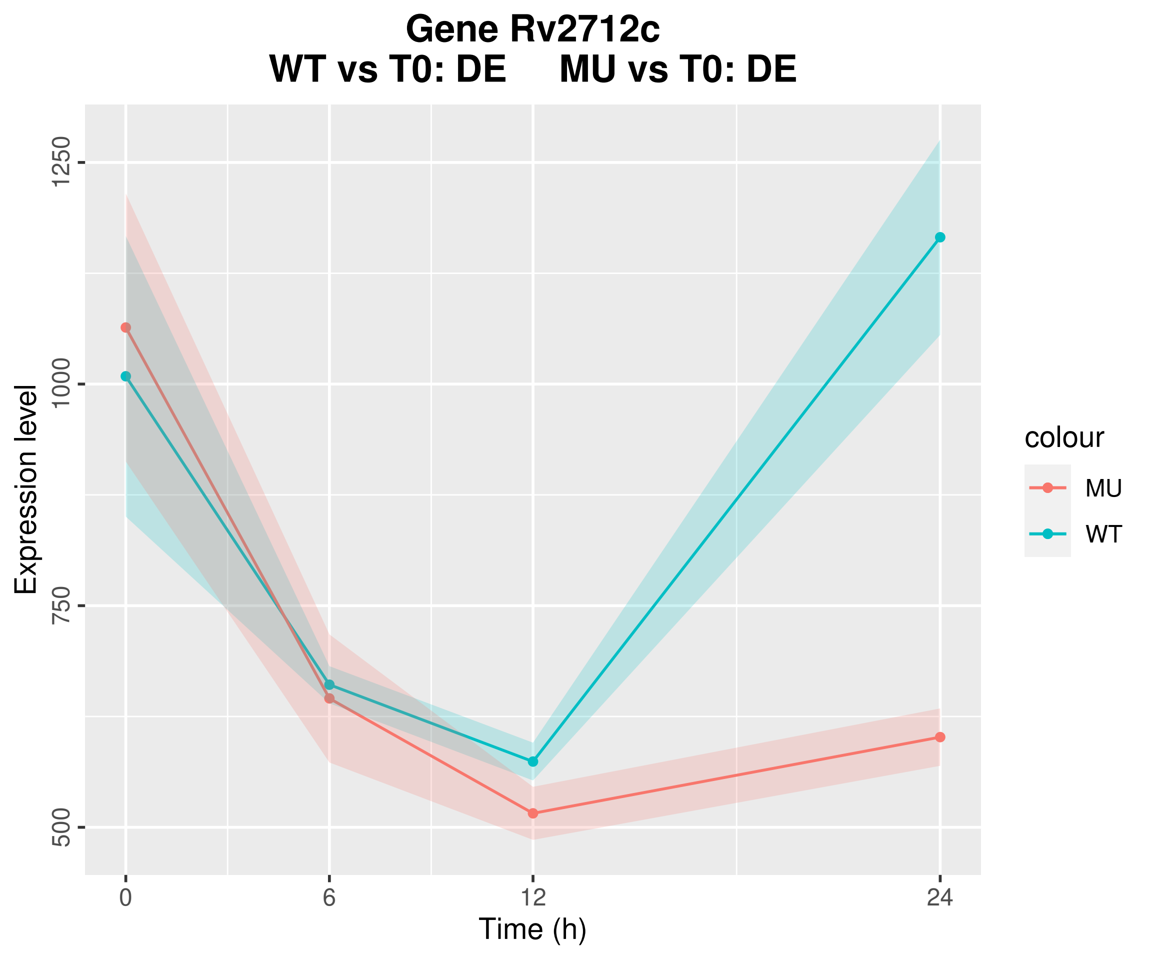

Gene Rv2712c
WT vs T0: DE MU vs T0: DE
Expression level
1250
1000
750
500
0
6
12
24
Time (h)
colour
MU
WT

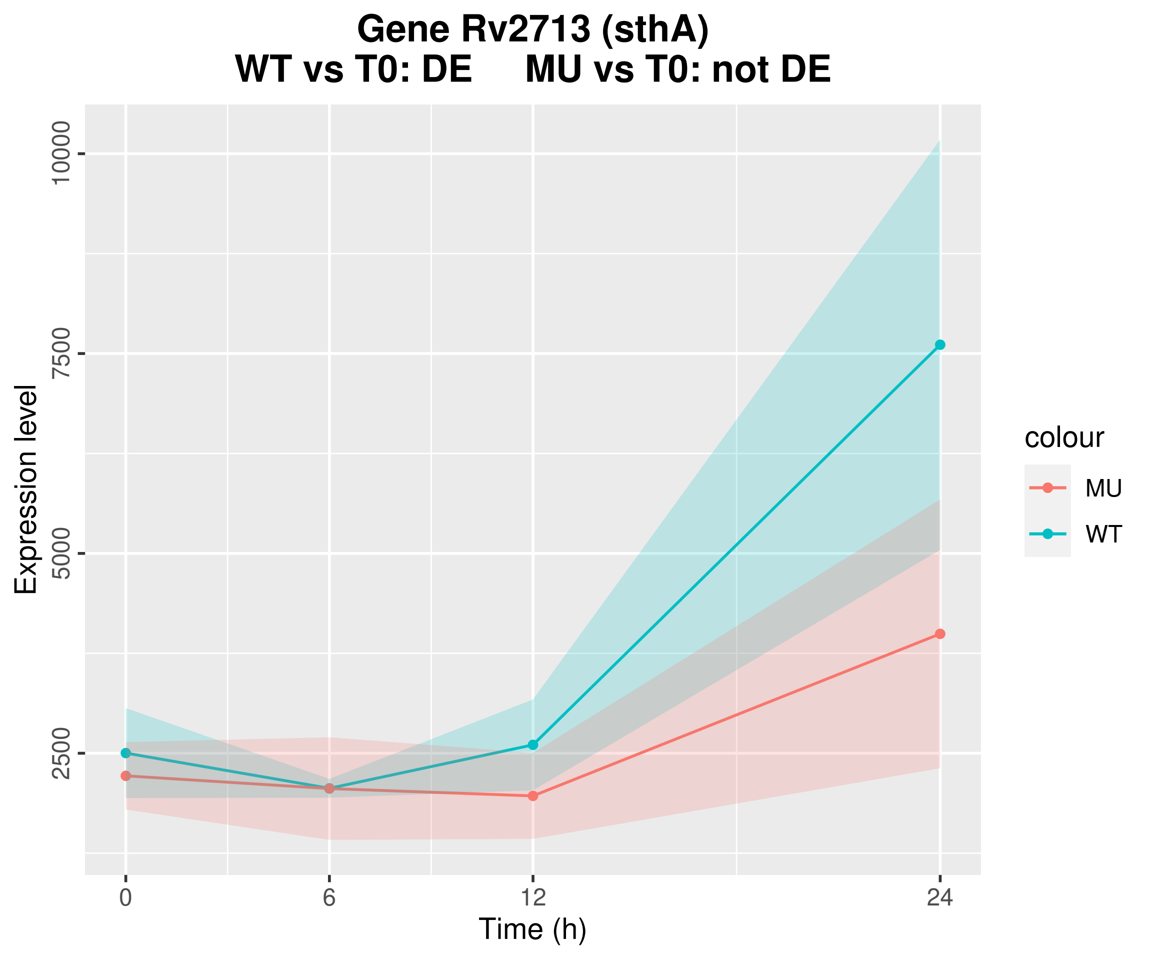

Gene Rv2713 (sthA)
WT vs T0: DE   MU vs T0: not DE
10000
7500
5000
2500
Expression level
0
6
12
24
Time (h)
colour
MU
WT

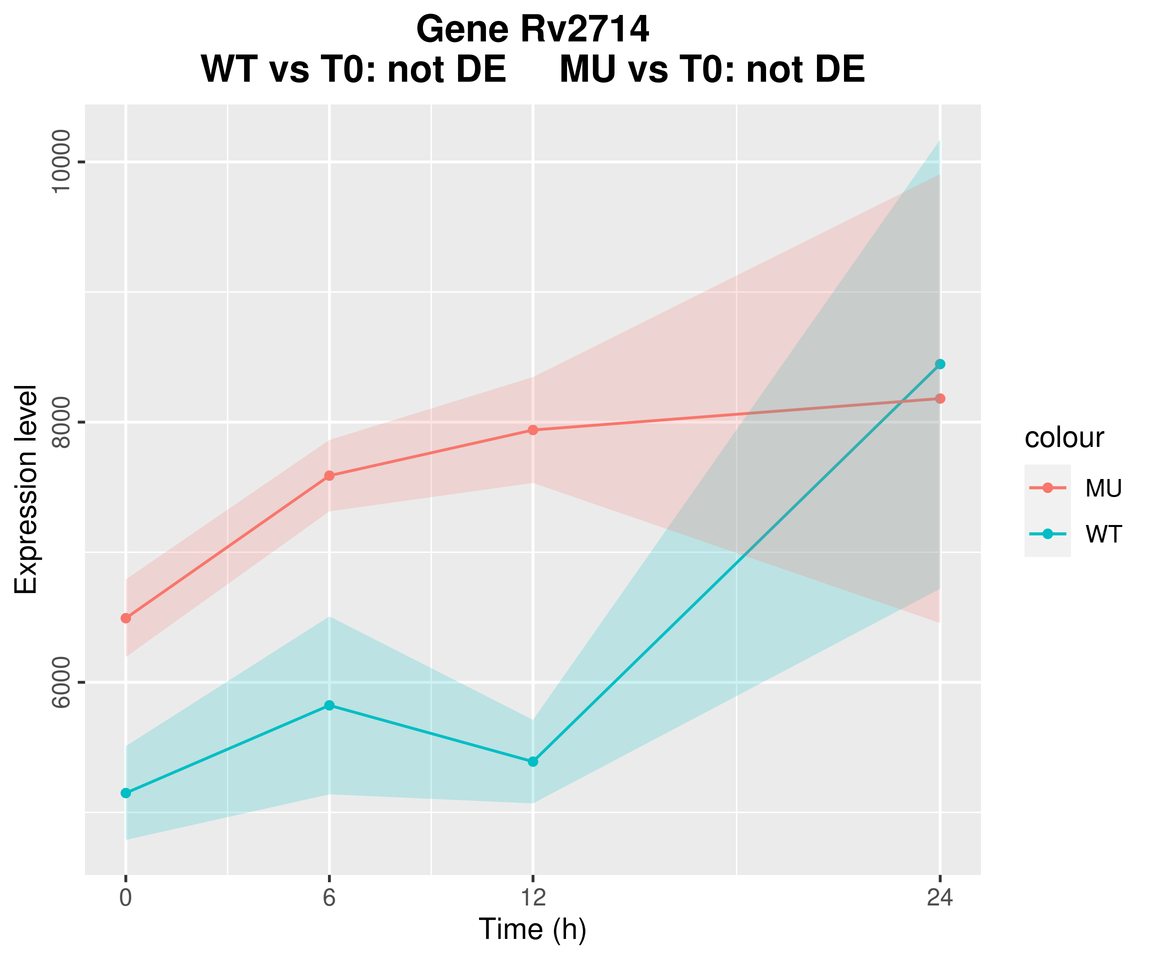

Gene Rv2714
WT vs T0: not DE   MU vs T0: not DE
10000
8000
6000
Expression level
0
6
12
24
Time (h)
colour
MU
WT

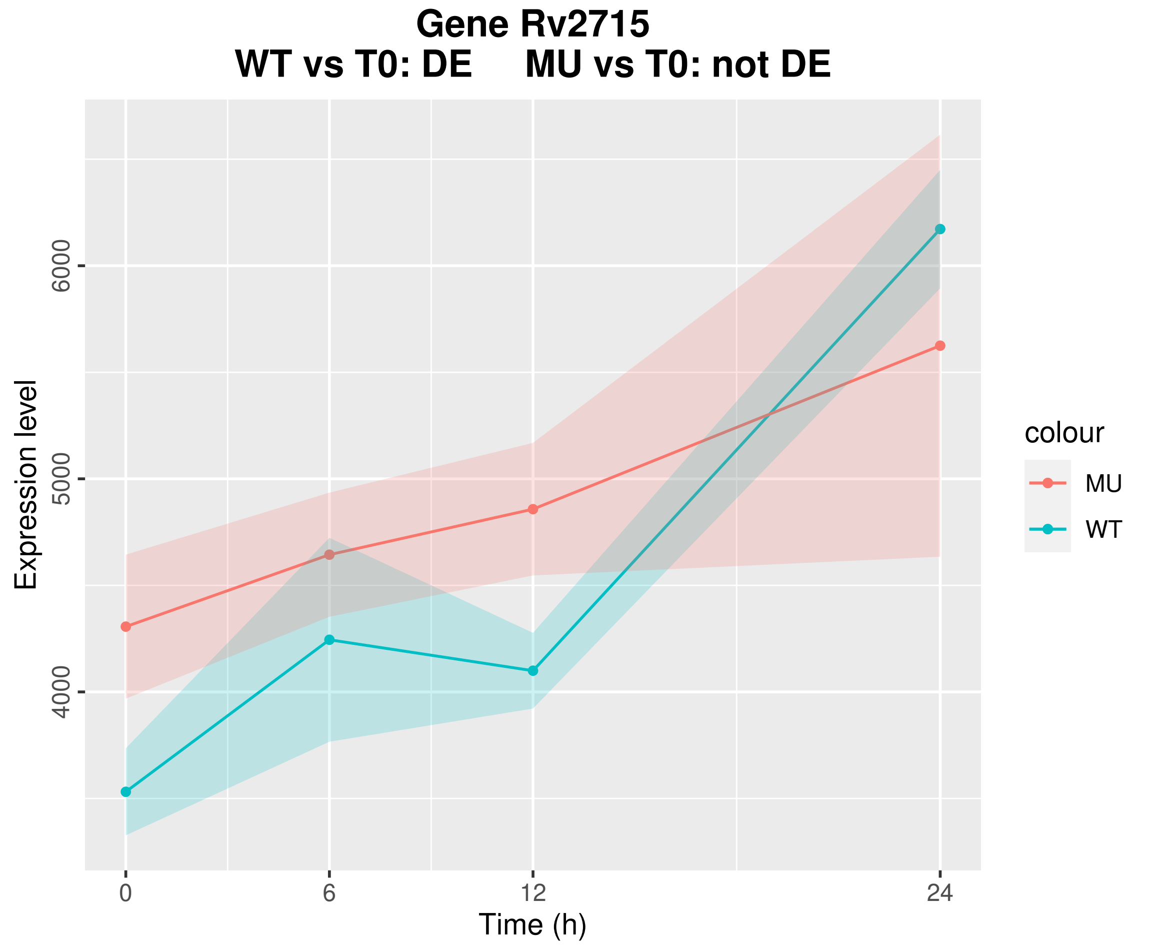

Gene Rv2715
WT vs T0: DE   MU vs T0: not DE
Expression level
6000
5000
4000
0
6
12
24
Time (h)
colour
MU
WT

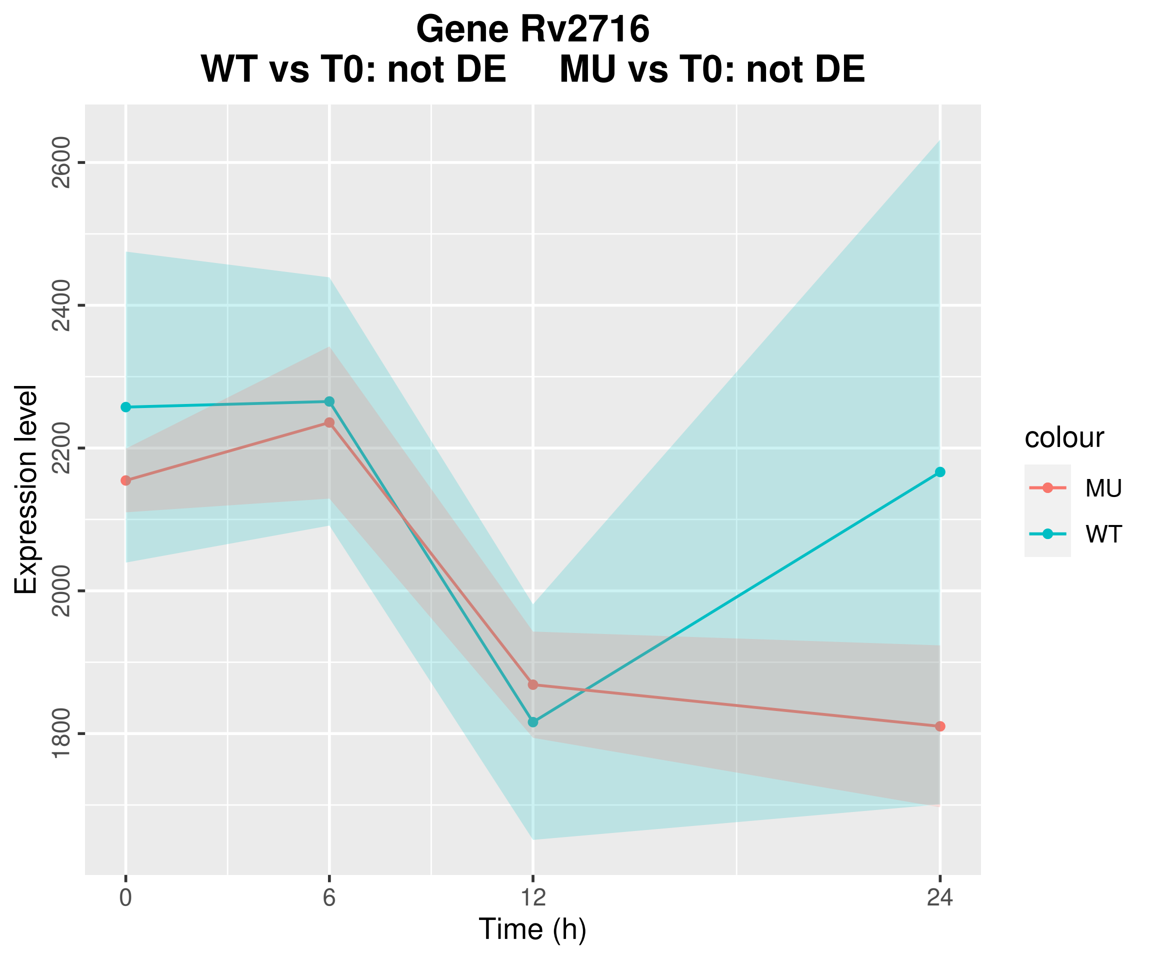
Gene Rv2716
WT vs T0: not DE MU vs T0: not DE
Expression level
2600
2400
2200
2000
1800
0
6
12
24
Time (h)
colour
MU
WT

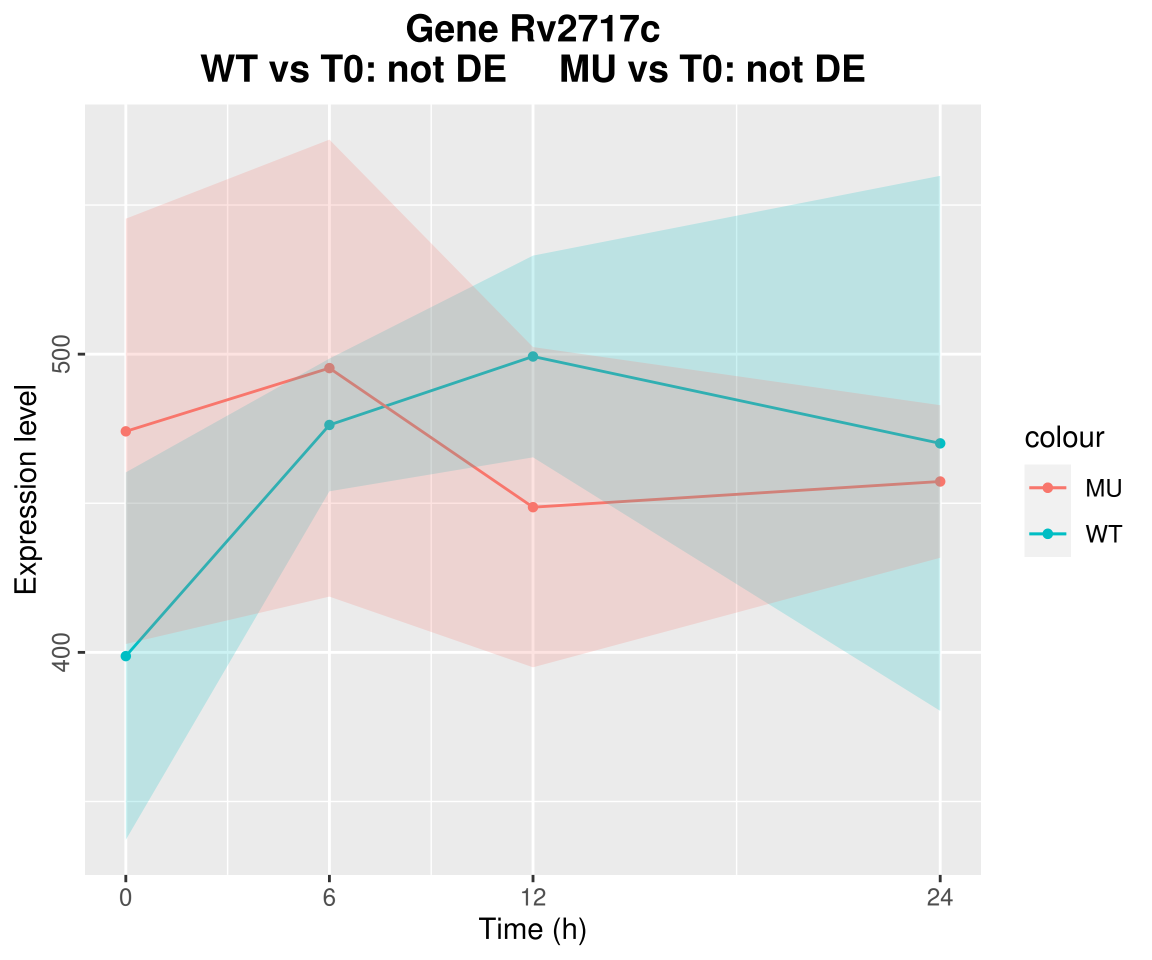
Gene Rv2717c
WT vs T0: not DE   MU vs T0: not DE
Expression level
500
400
0
6
12
24
Time (h)
colour
MU
WT

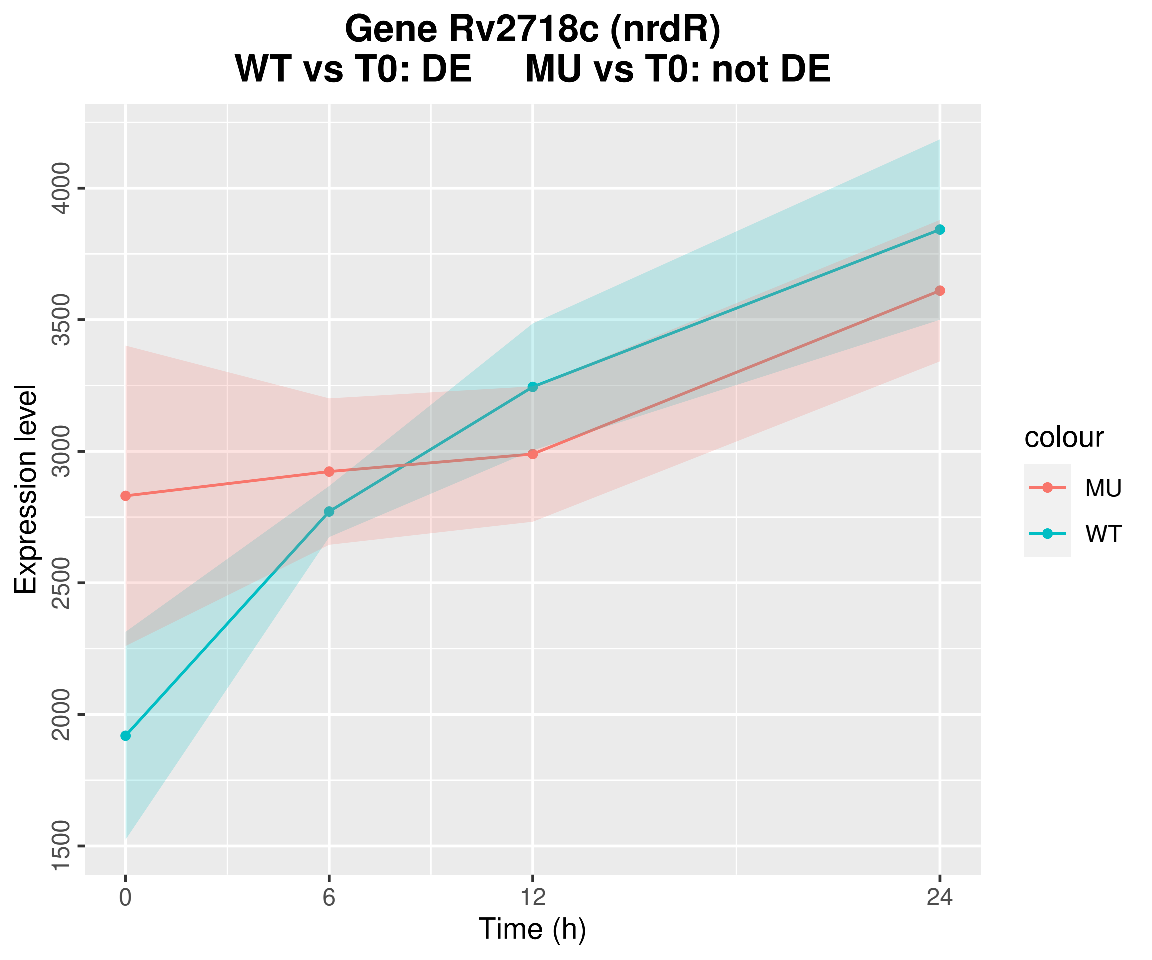
Gene Rv2718c (nrdR)
WT vs T0: DE   MU vs T0: not DE
Expression level
Time (h)
1500
2000
2500
3000
3500
4000
0
6
12
24
colour
MU
WT

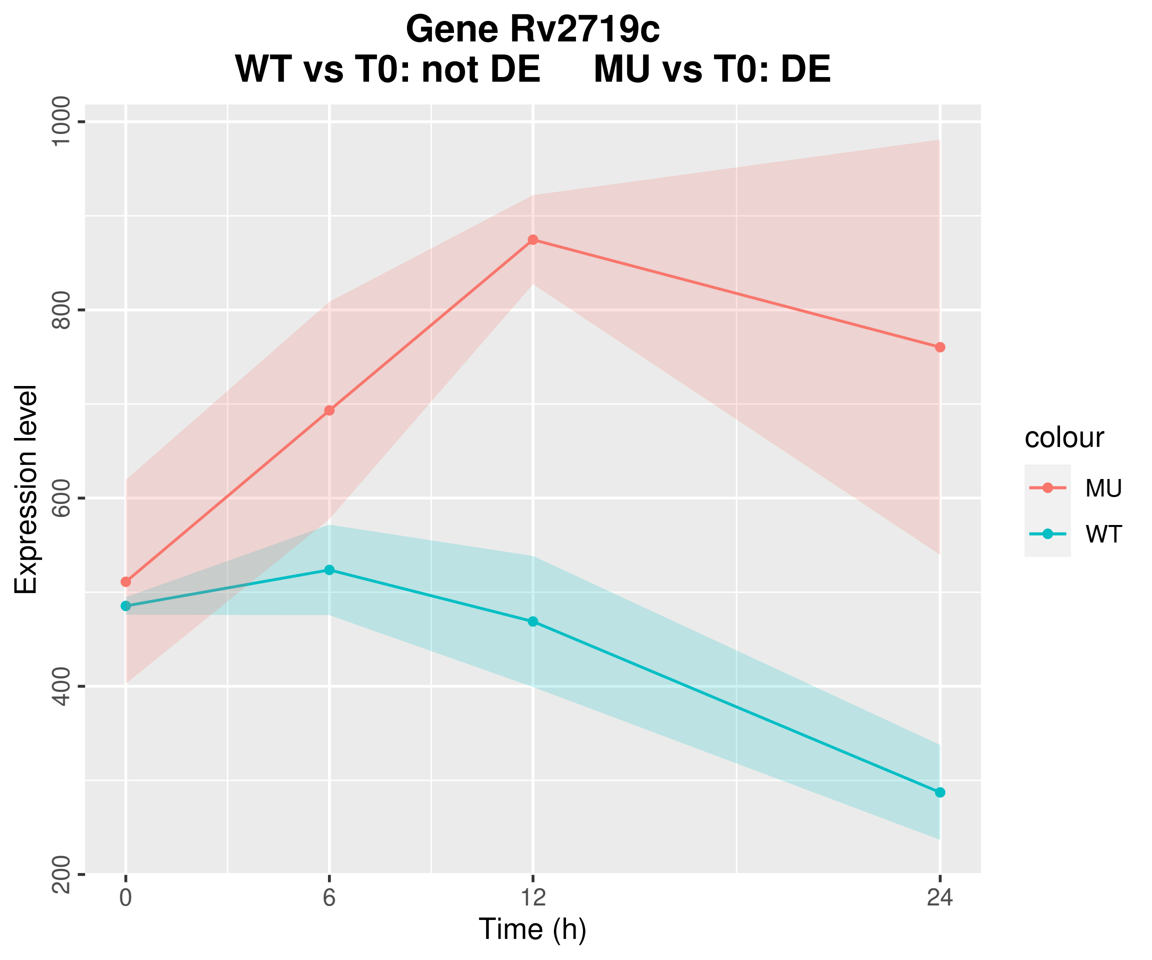

Gene Rv2719c
WT vs T0: not DE   MU vs T0: DE
Expression level
1000
800
600
400
200
0
6
12
24
Time (h)
colour
MU
WT

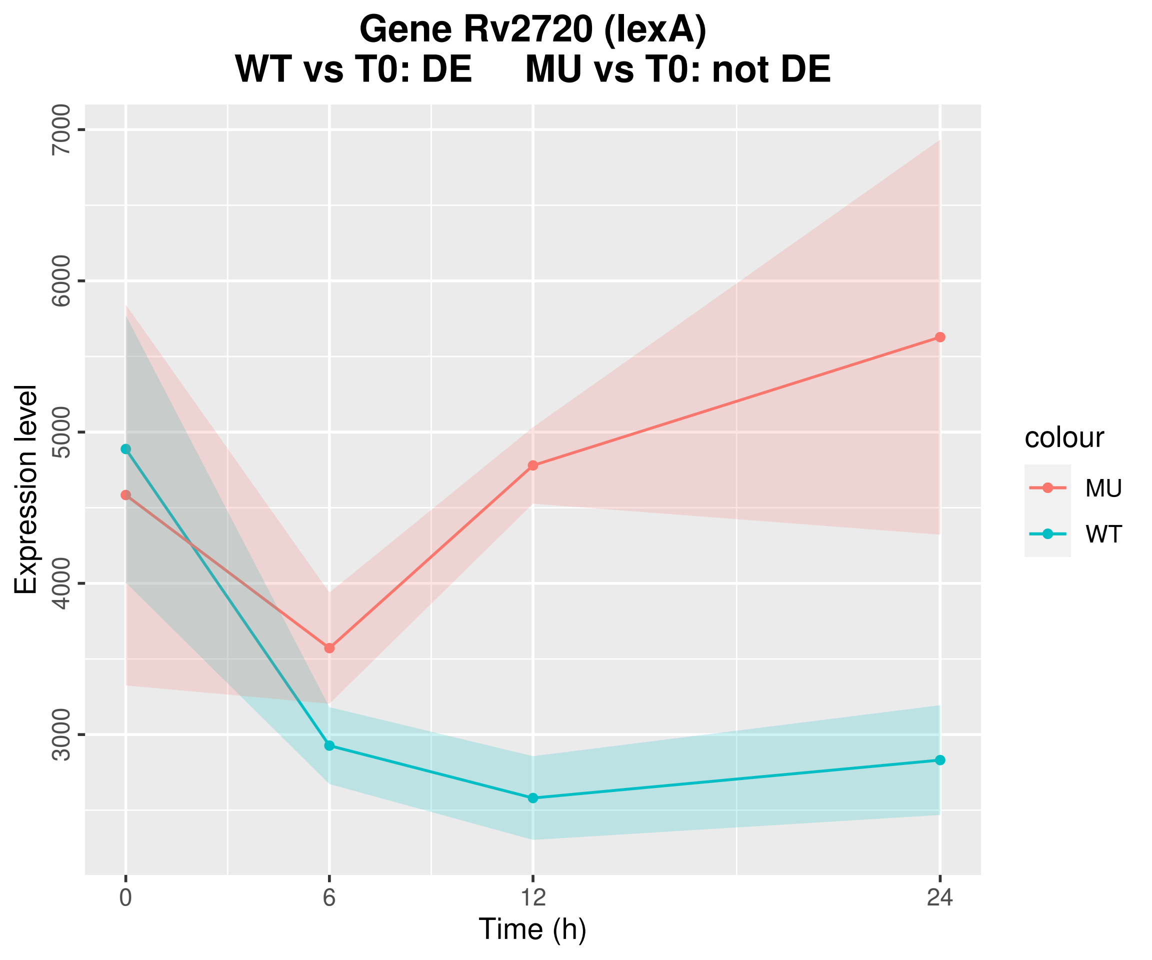
Gene Rv2720 (lexA)
WT vs T0: DE MU vs T0: not DE
Expression level
3000
4000
5000
6000
7000
0
6
12
24
Time (h)
colour
MU
WT

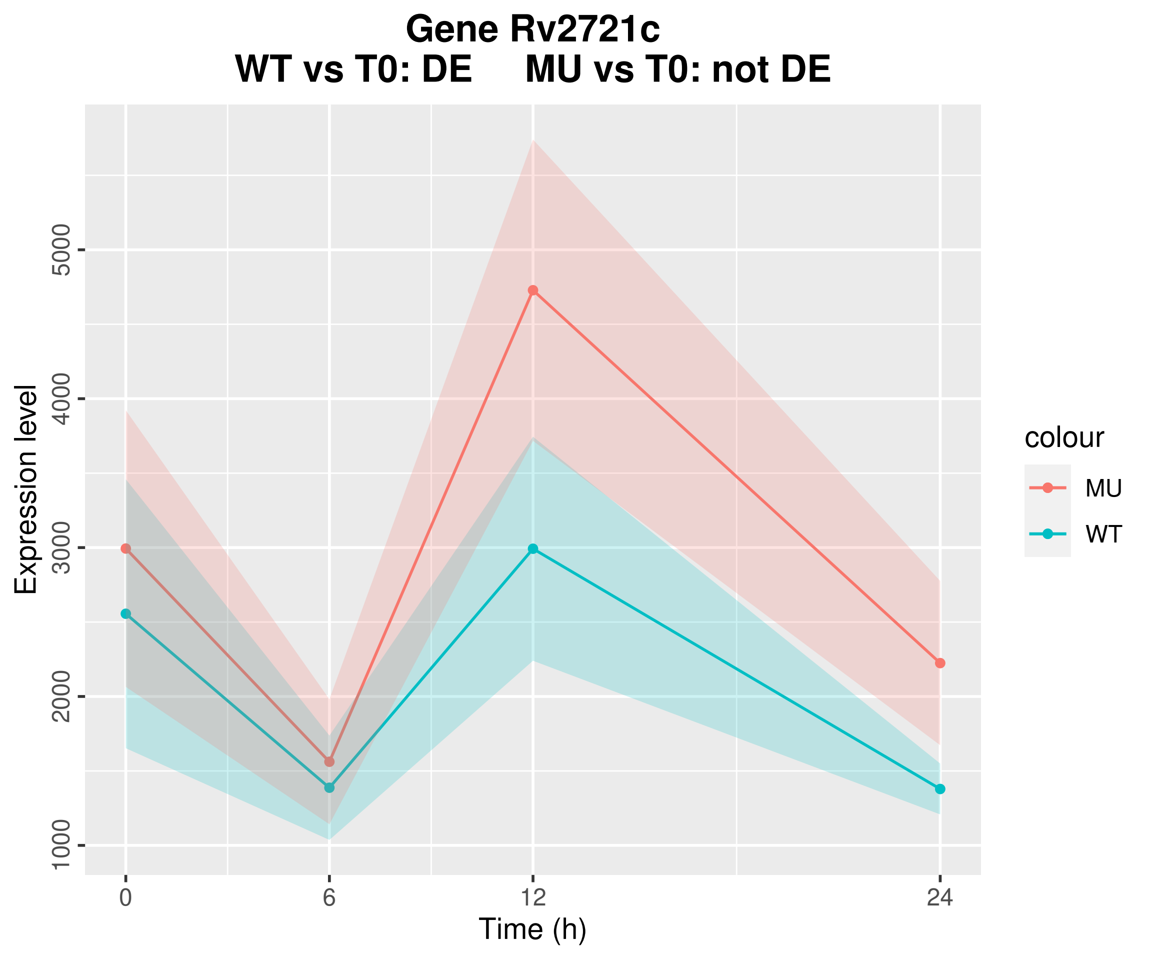
Gene Rv2721c
WT vs T0: DE MU vs T0: not DE
Expression level
5000
4000
3000
2000
1000
0
6
12
24
Time (h)
colour
MU
WT

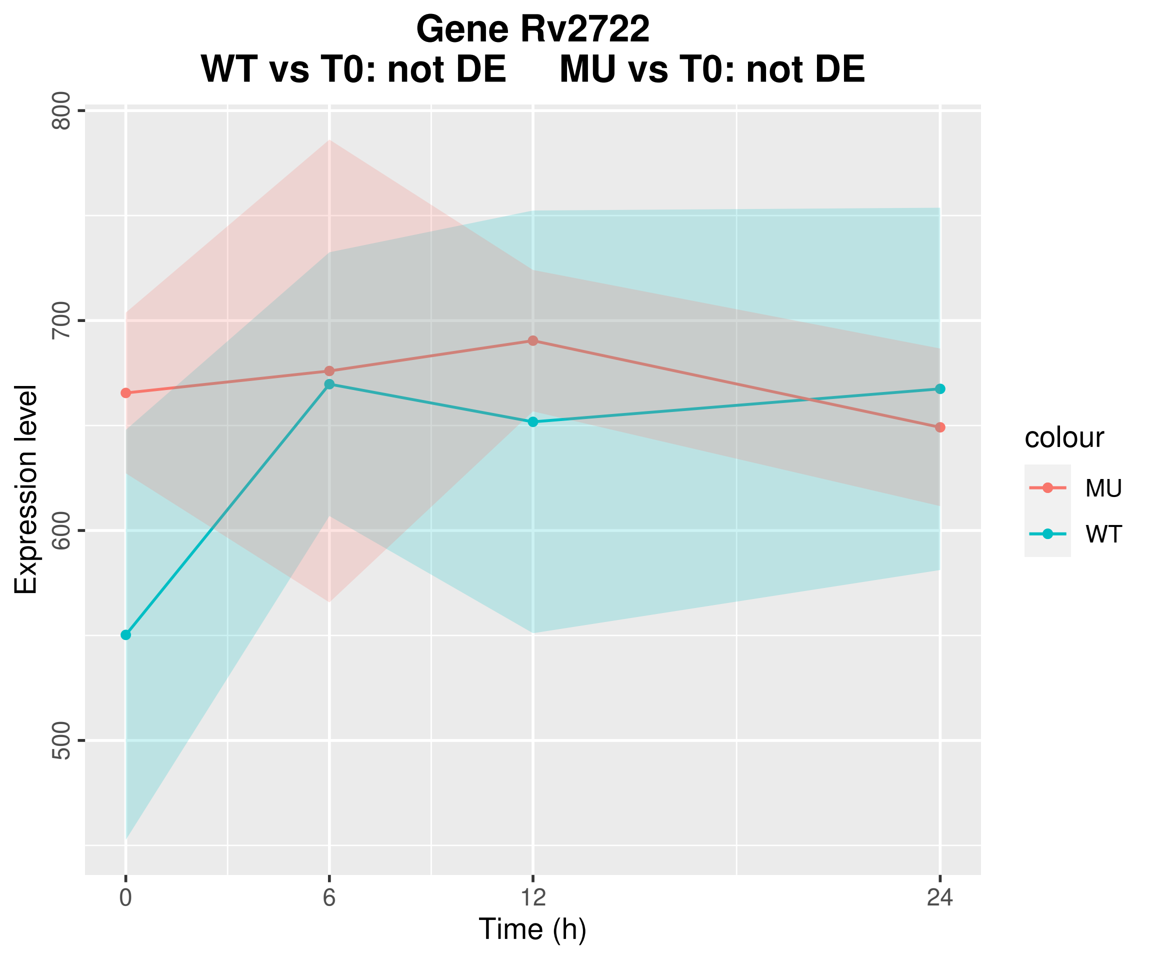
Gene Rv2722
WT vs T0: not DE   MU vs T0: not DE
Expression level
800
700
600
500
0
6
12
24
Time (h)
colour
MU
WT

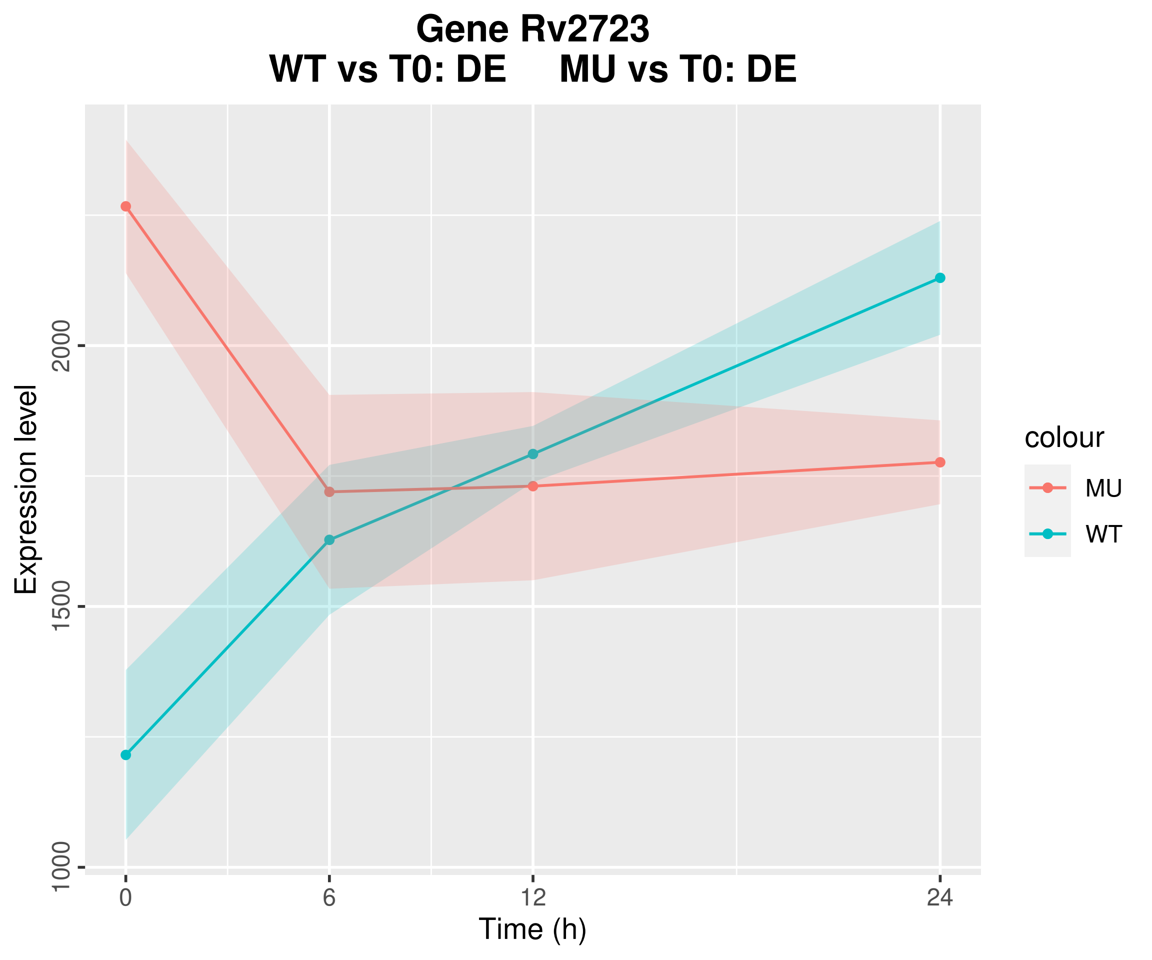
Gene Rv2723
WT vs T0: DE MU vs T0: DE
Expression level
1000
1500
2000
0
6
12
24
Time (h)
colour
MU
WT

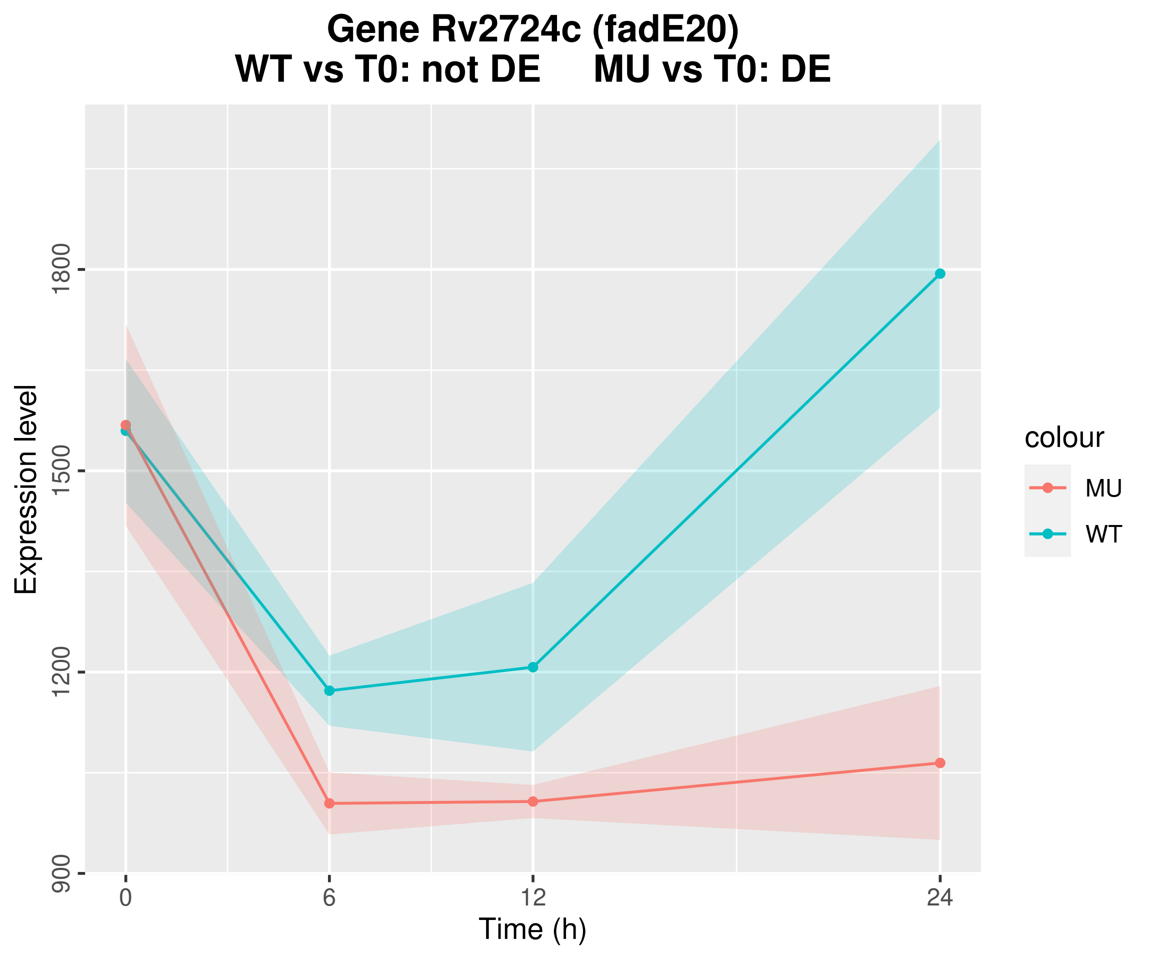

Gene Rv2724c (fadE20)
WT vs T0: not DE   MU vs T0: DE
Expression level
1800
1500
1200
900
0
6
12
24
Time (h)
colour
MU
WT

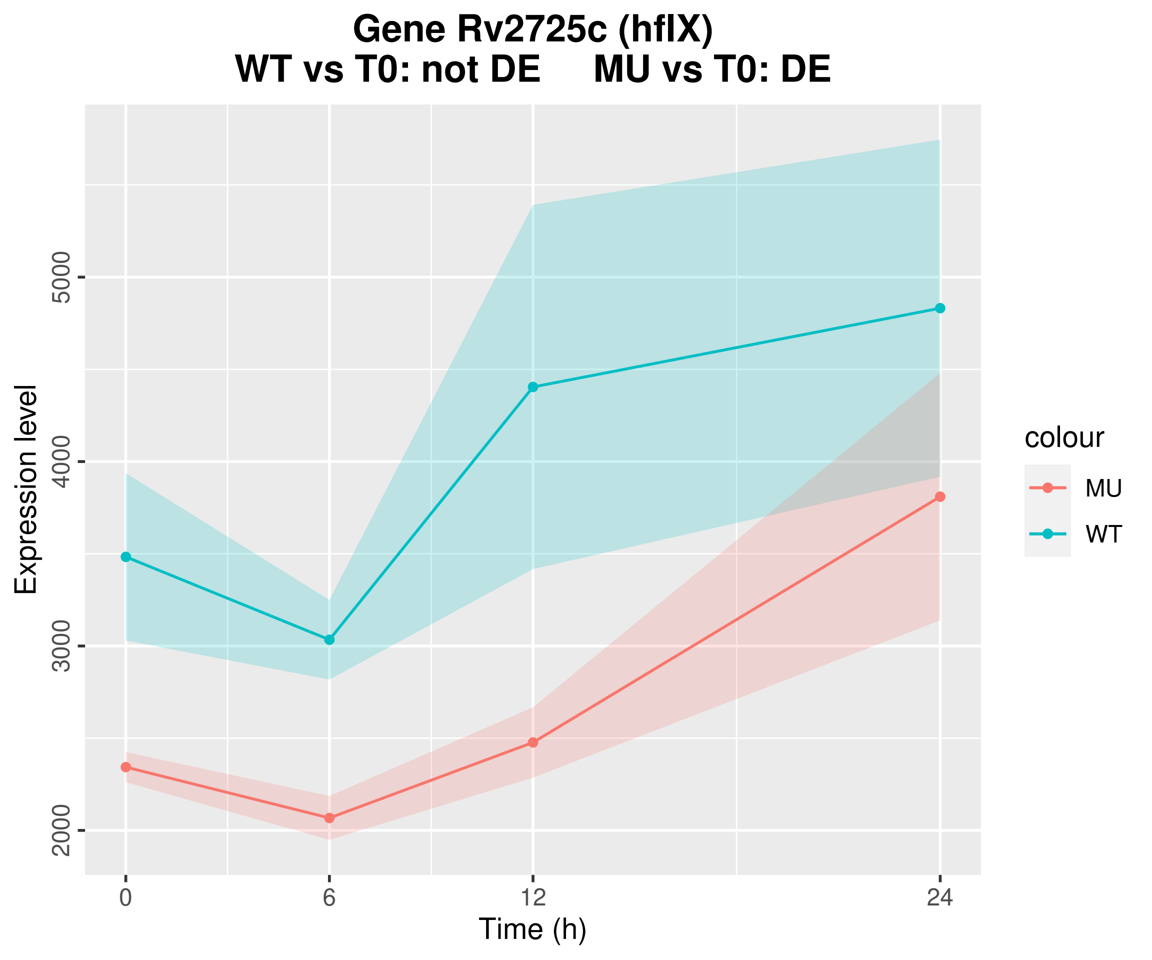

Gene Rv2725c (hflX)
WT vs T0: not DE   MU vs T0: DE
Expression level
5000
4000
3000
2000
0
6
12
24
Time (h)
colour
MU
WT

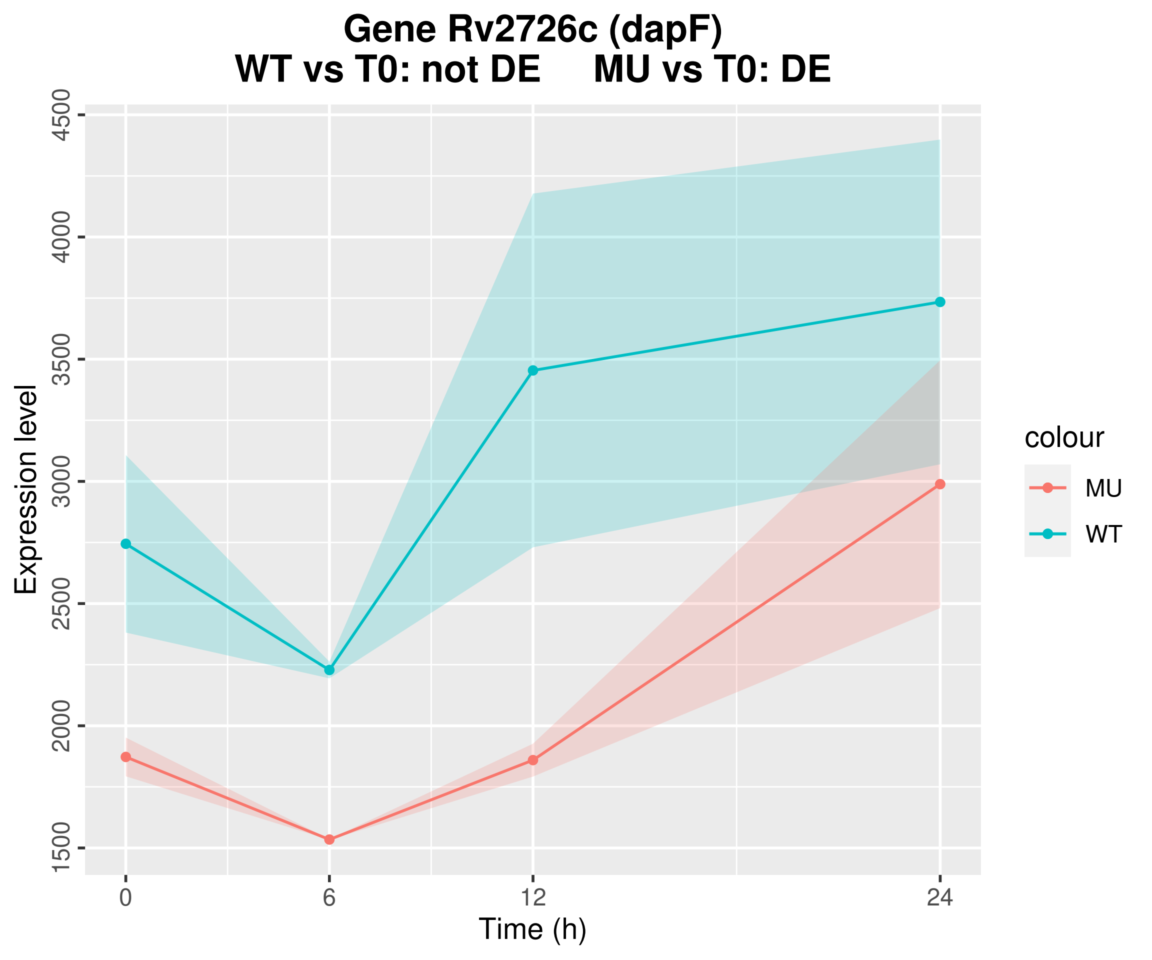
Gene Rv2726c (dapF)
WT vs T0: not DE MU vs T0: DE
Expression level
4500
4000
3500
3000
2500
2000
1500
0
6
12
24
Time (h)
colour
MU
WT

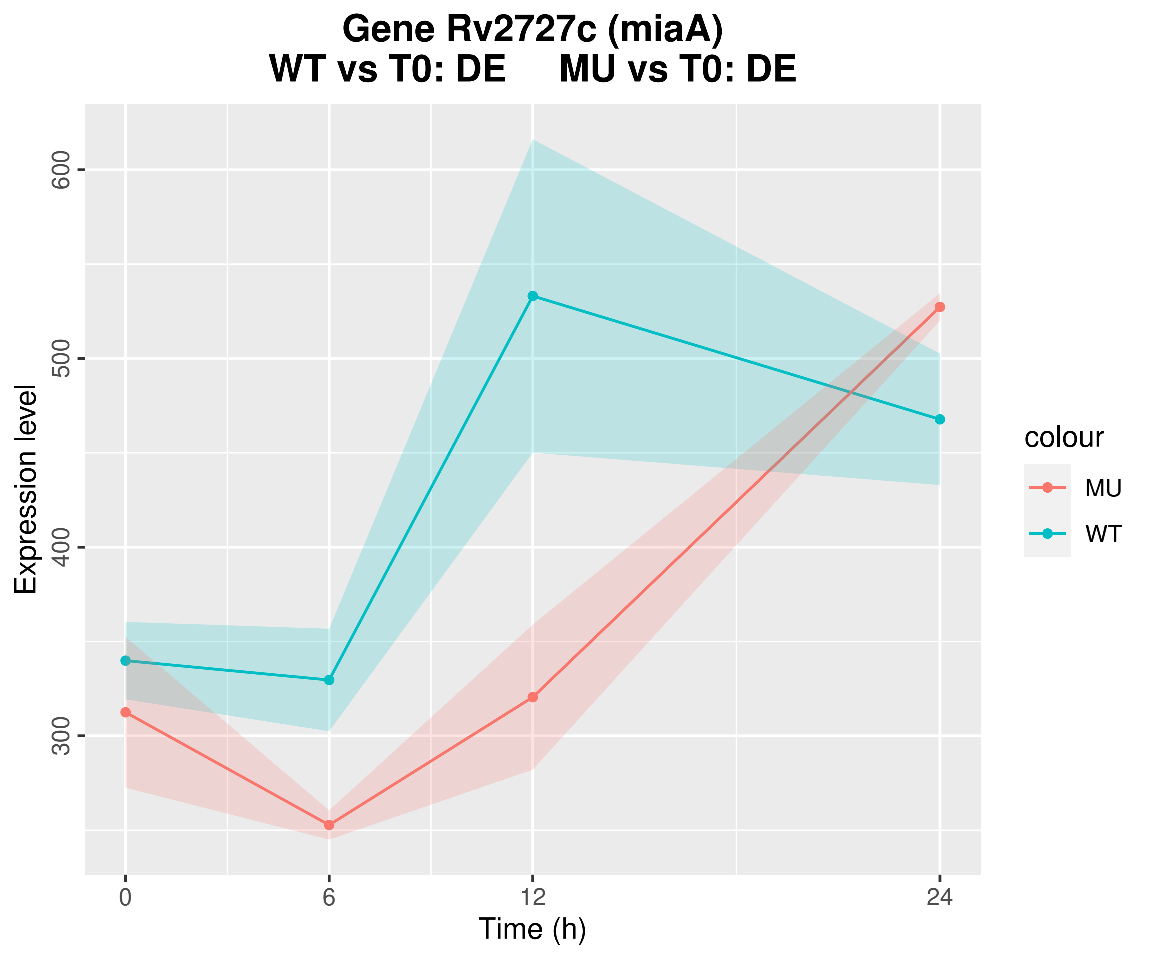
Gene Rv2727c (miaA)
WT vs T0: DE   MU vs T0: DE
Expression level
600
500
400
300
0
6
12
24
Time (h)
colour
MU
WT

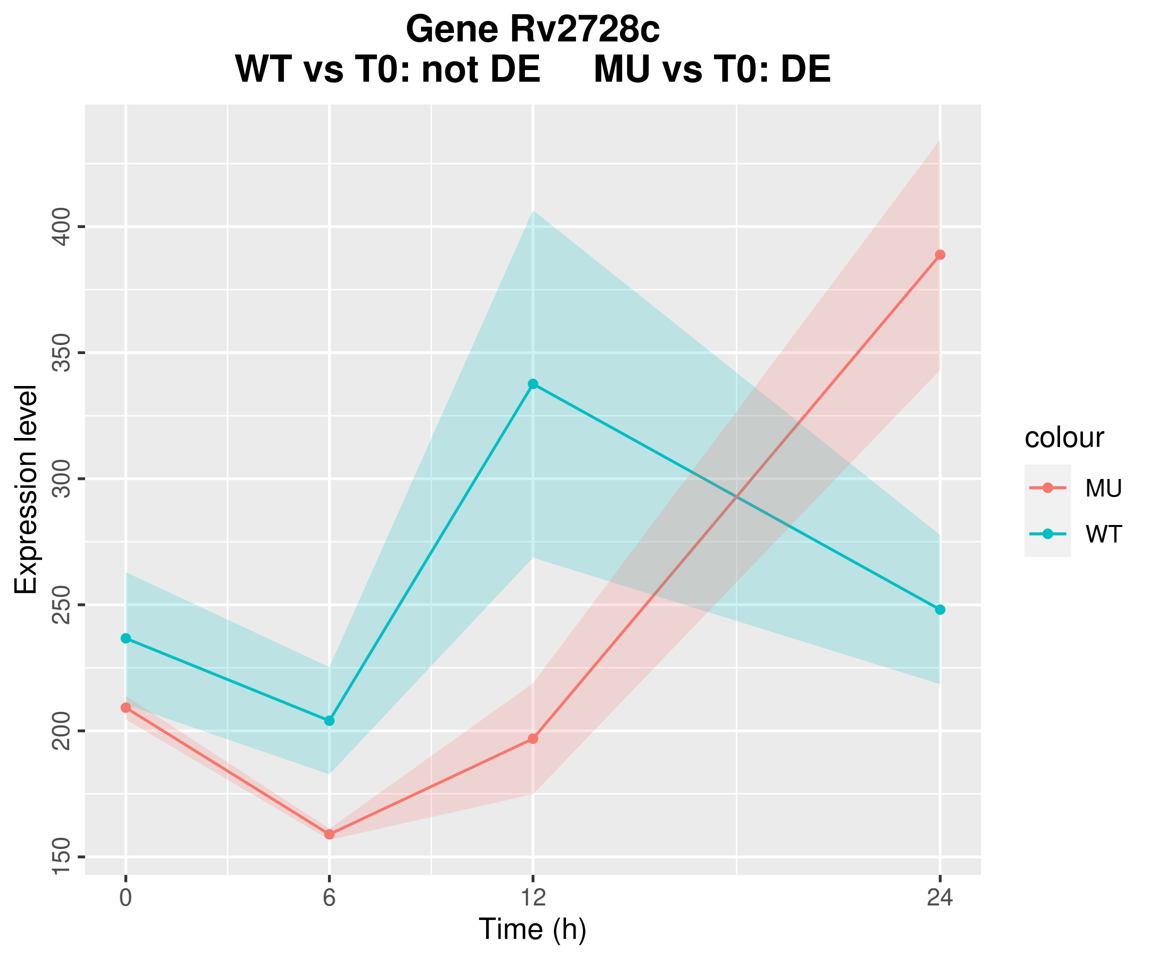
Gene Rv2728c
WT vs T0: not DE   MU vs T0: DE
Expression level
400
350
300
250
200
150
0
6
12
24
Time (h)
colour
MU
WT

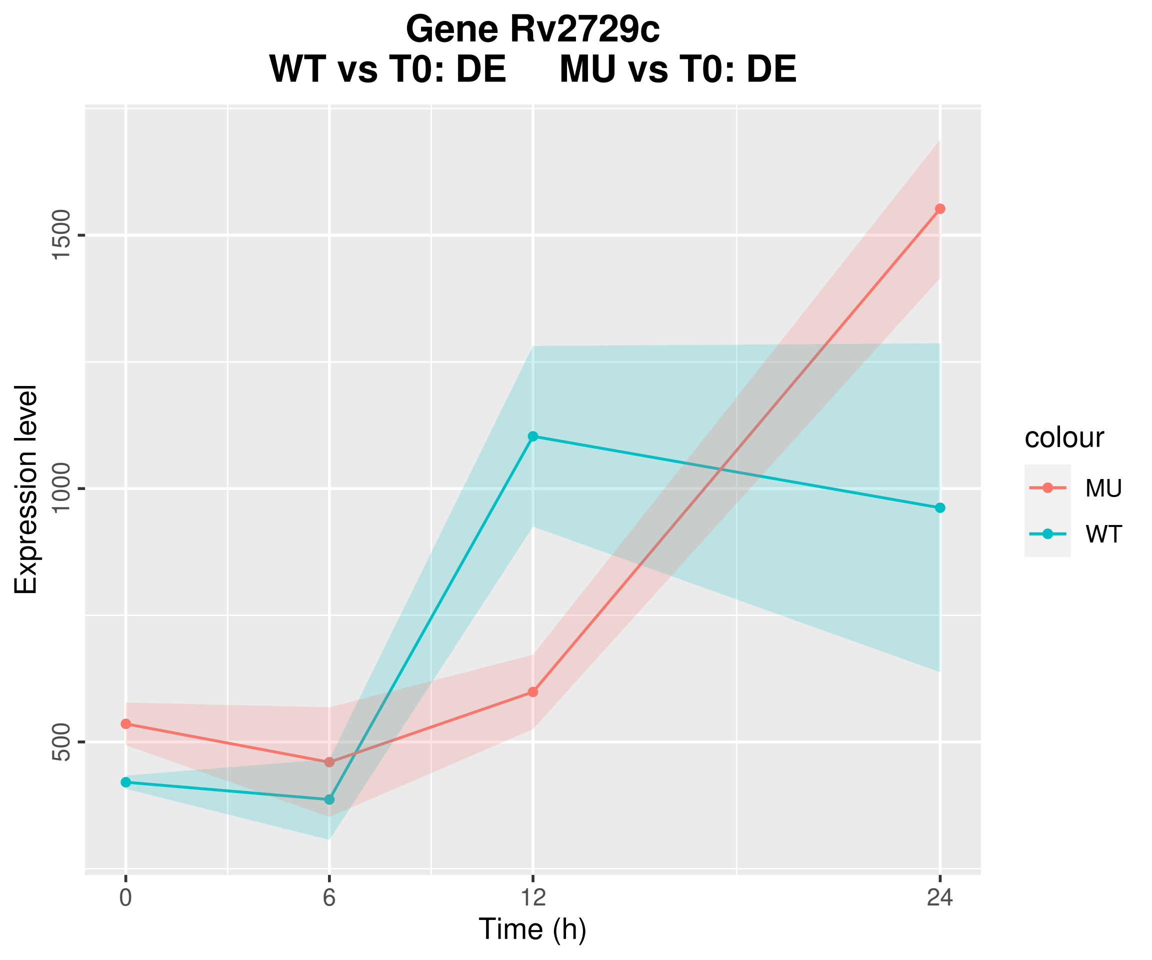

Gene Rv2729c
WT vs T0: DE   MU vs T0: DE
Expression level
1500
1000
500
0
6
12
24
Time (h)
colour
MU
WT

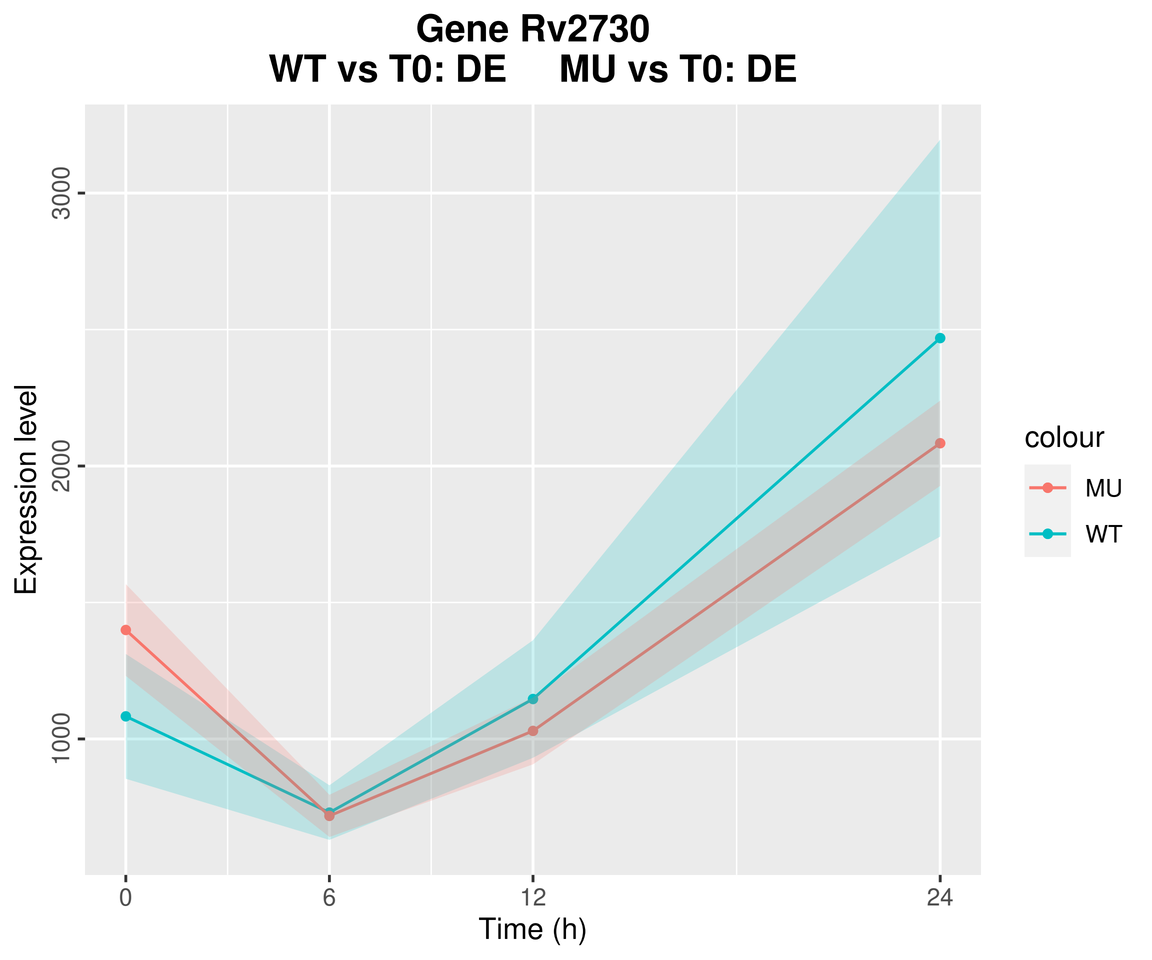

Gene Rv2730
WT vs T0: DE MU vs T0: DE
Expression level
3000
2000
1000
0
6
12
24
Time (h)
colour
MU
WT

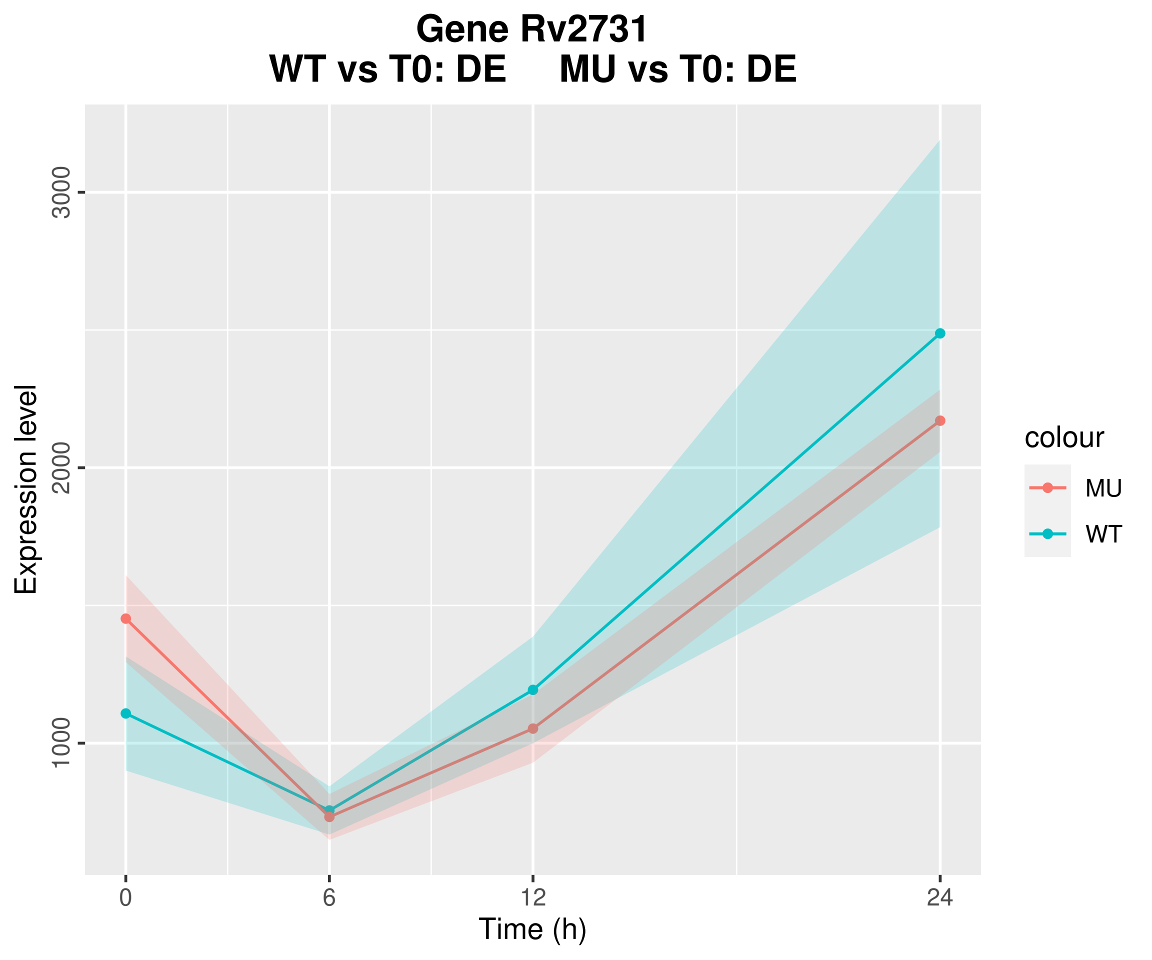
Gene Rv2731
WT vs T0: DE   MU vs T0: DE
Expression level
3000
2000
1000
0
6
12
24
Time (h)
colour
MU
WT

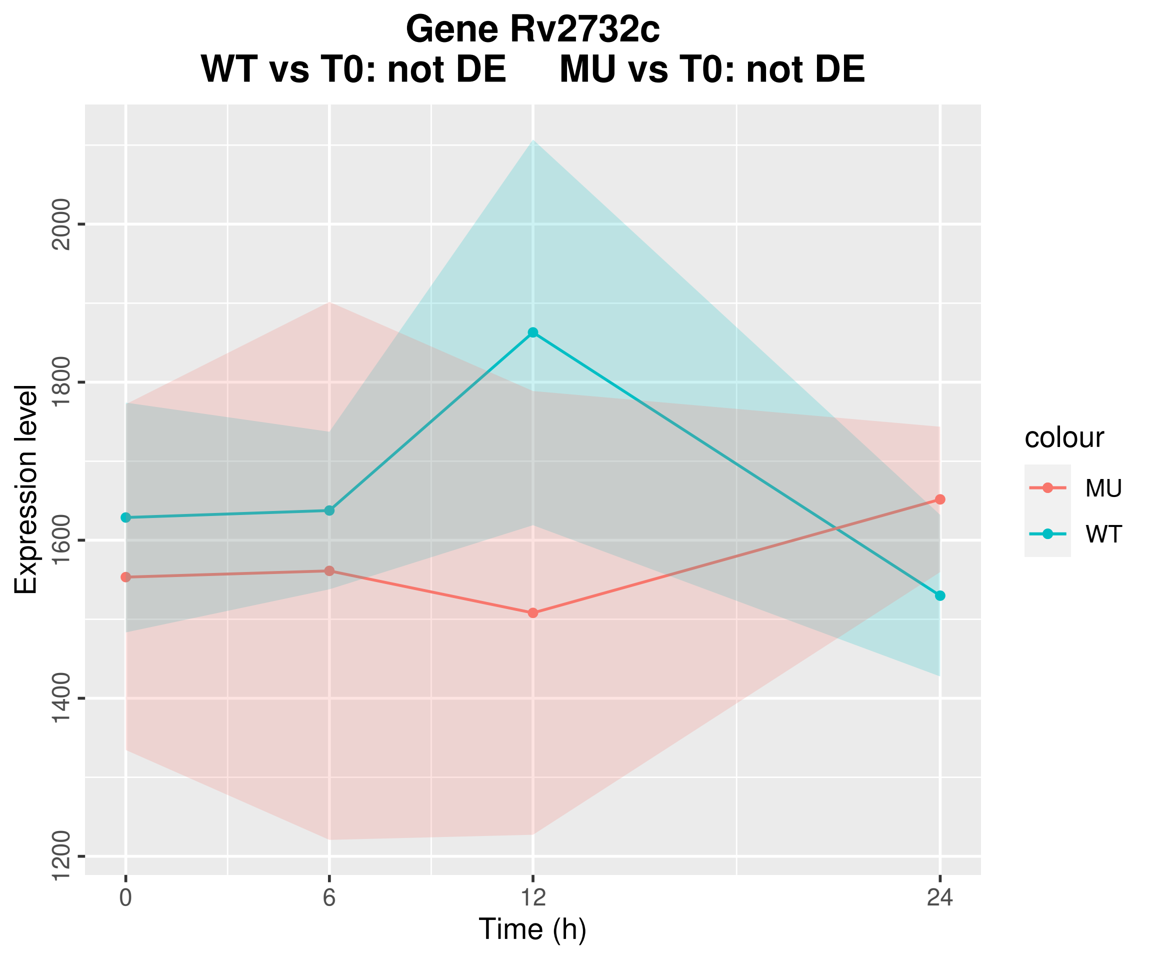
Gene Rv2732c
WT vs T0: not DE MU vs T0: not DE
Expression level
1200
1400
1600
1800
2000
0
6
12
24
Time (h)
colour
MU
WT

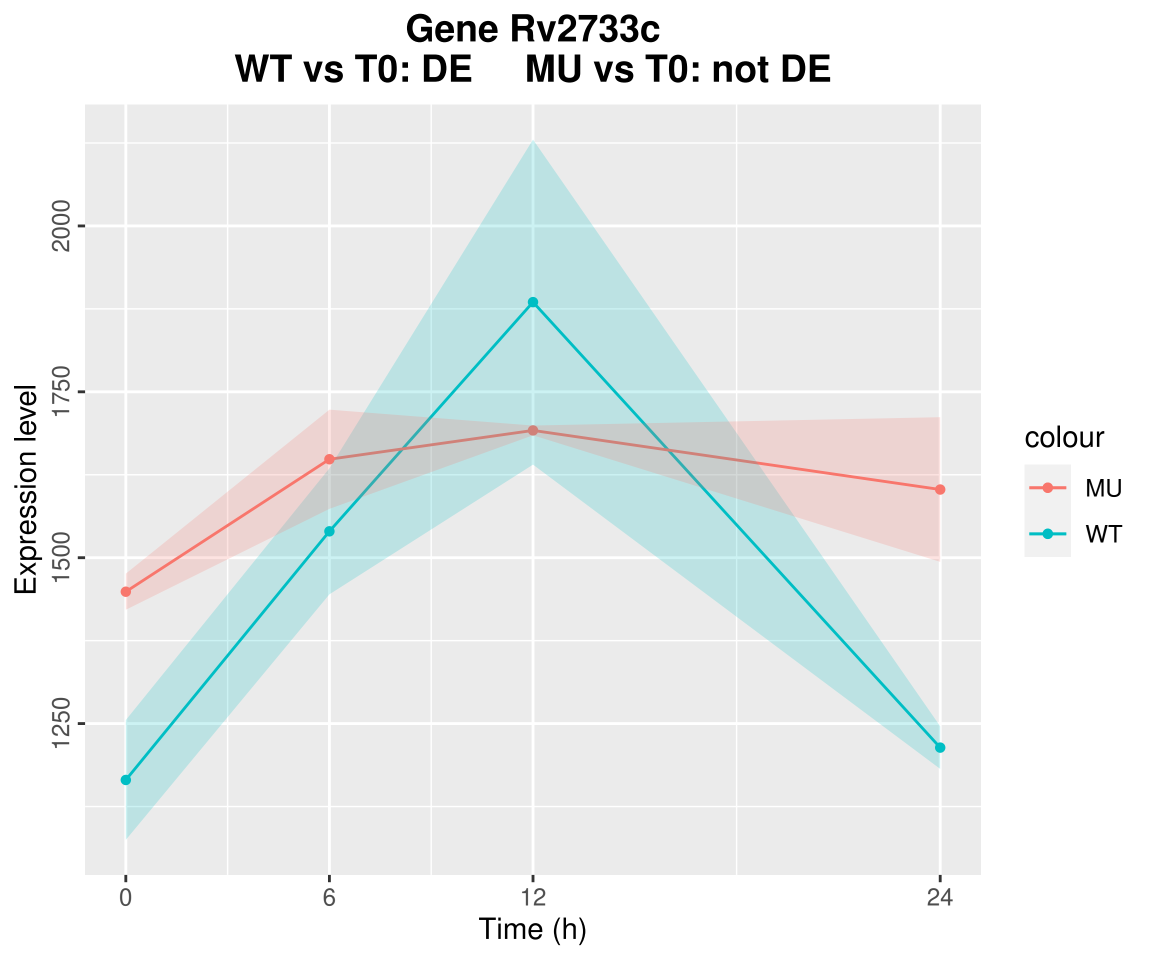
Gene Rv2733c
WT vs T0: DE   MU vs T0: not DE
Expression level
2000
1750
1500
1250
0
6
12
24
Time (h)
colour
MU
WT

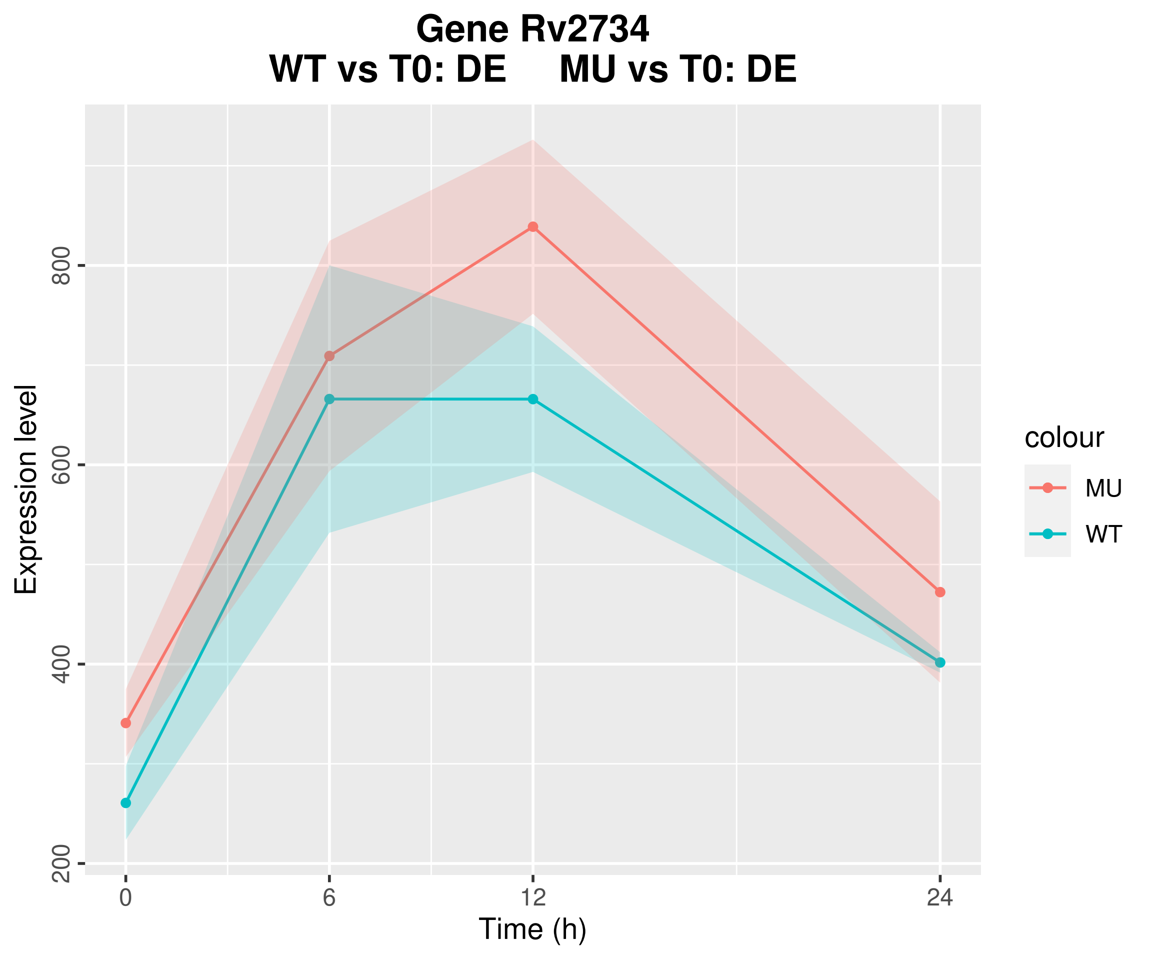
Gene Rv2734
WT vs T0: DE MU vs T0: DE
Expression level
800
600
400
200
0
6
12
24
Time (h)
colour
MU
WT

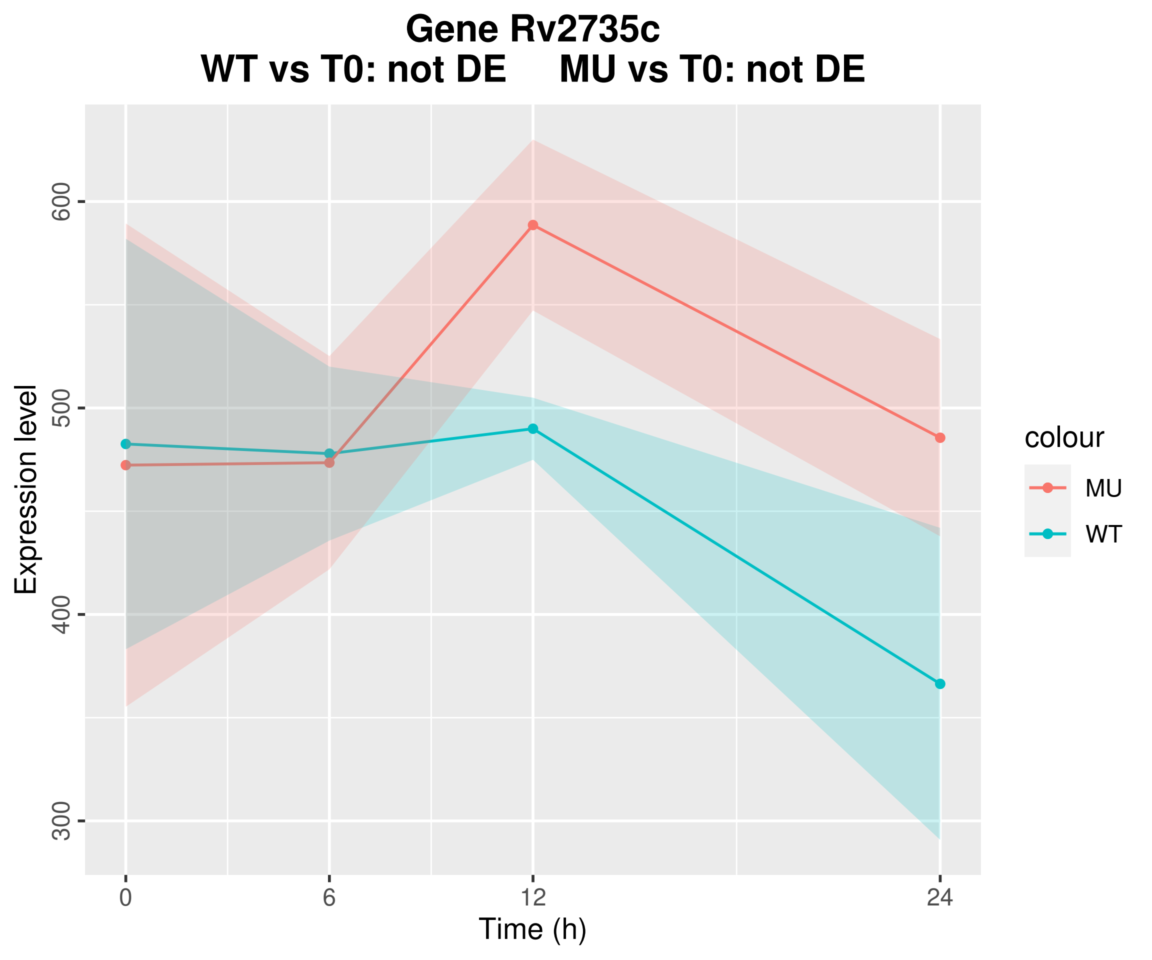
Gene Rv2735c
WT vs T0: not DE   MU vs T0: not DE
Expression level
600
500
400
300
0
6
12
24
Time (h)
colour
MU
WT

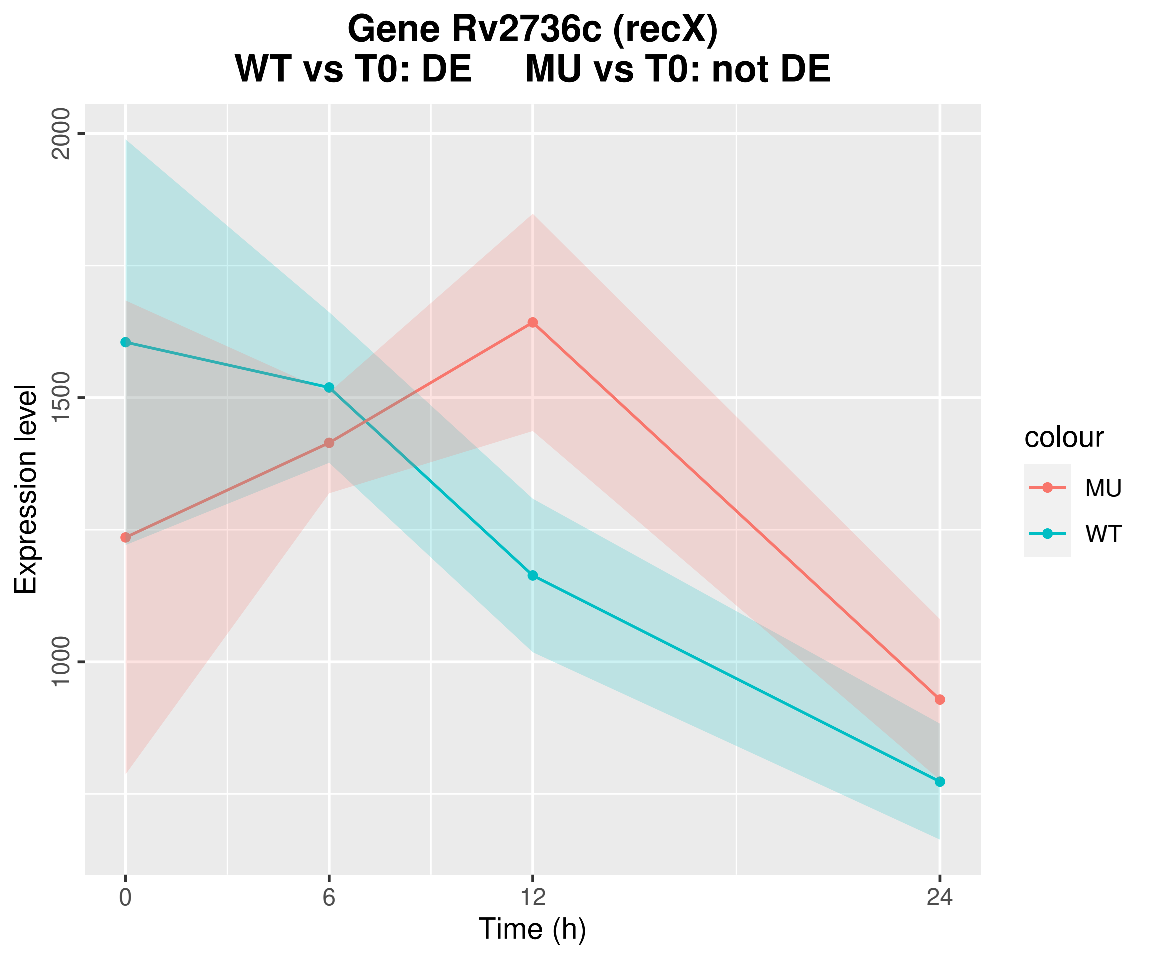
Gene Rv2736c (recX)
WT vs T0: DE   MU vs T0: not DE
2000
1500
1000
Expression level
0
6
12
24
Time (h)
colour
MU
WT

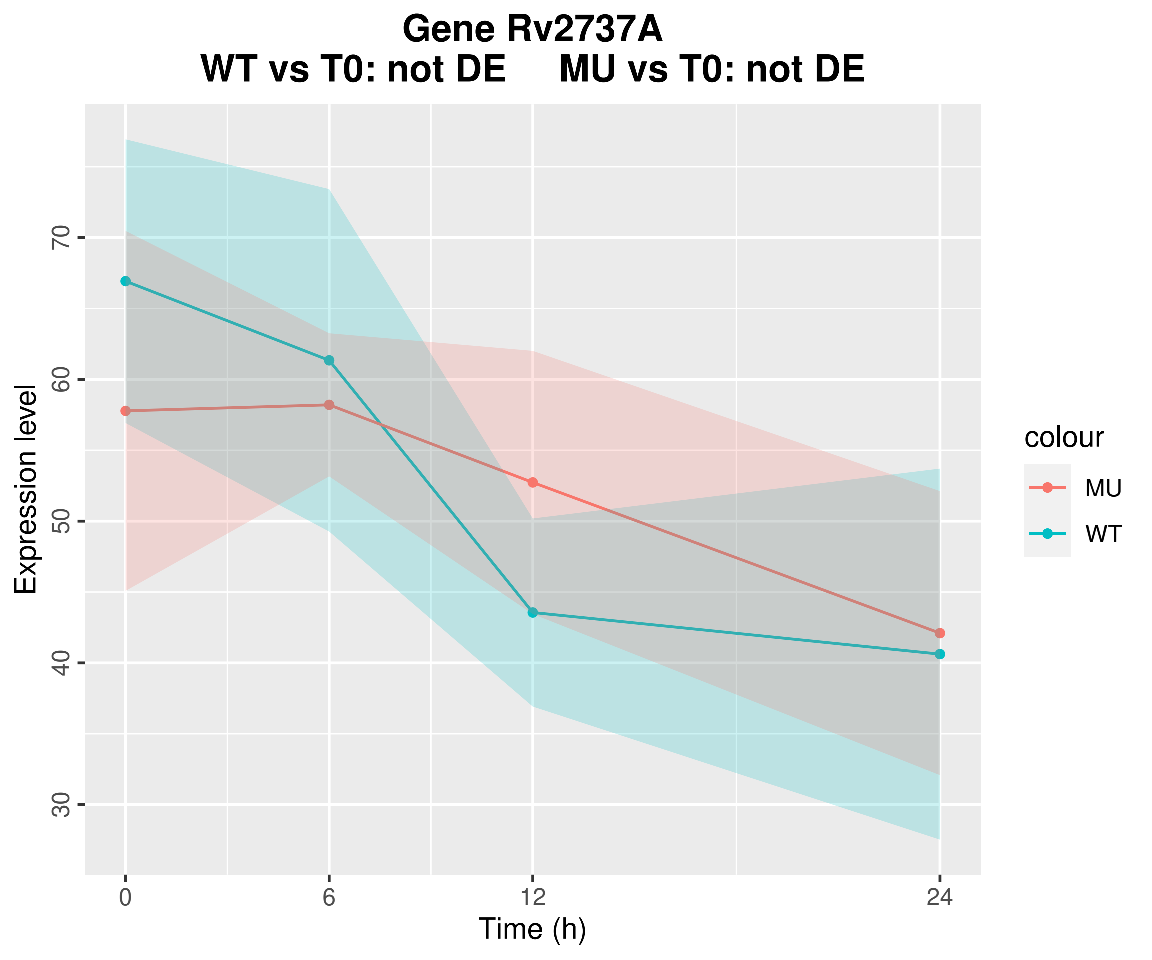
Gene Rv2737A
WT vs T0: not DE   MU vs T0: not DE
Expression level
30
40
50
60
70
0
6
12
24
Time (h)
colour
MU
WT

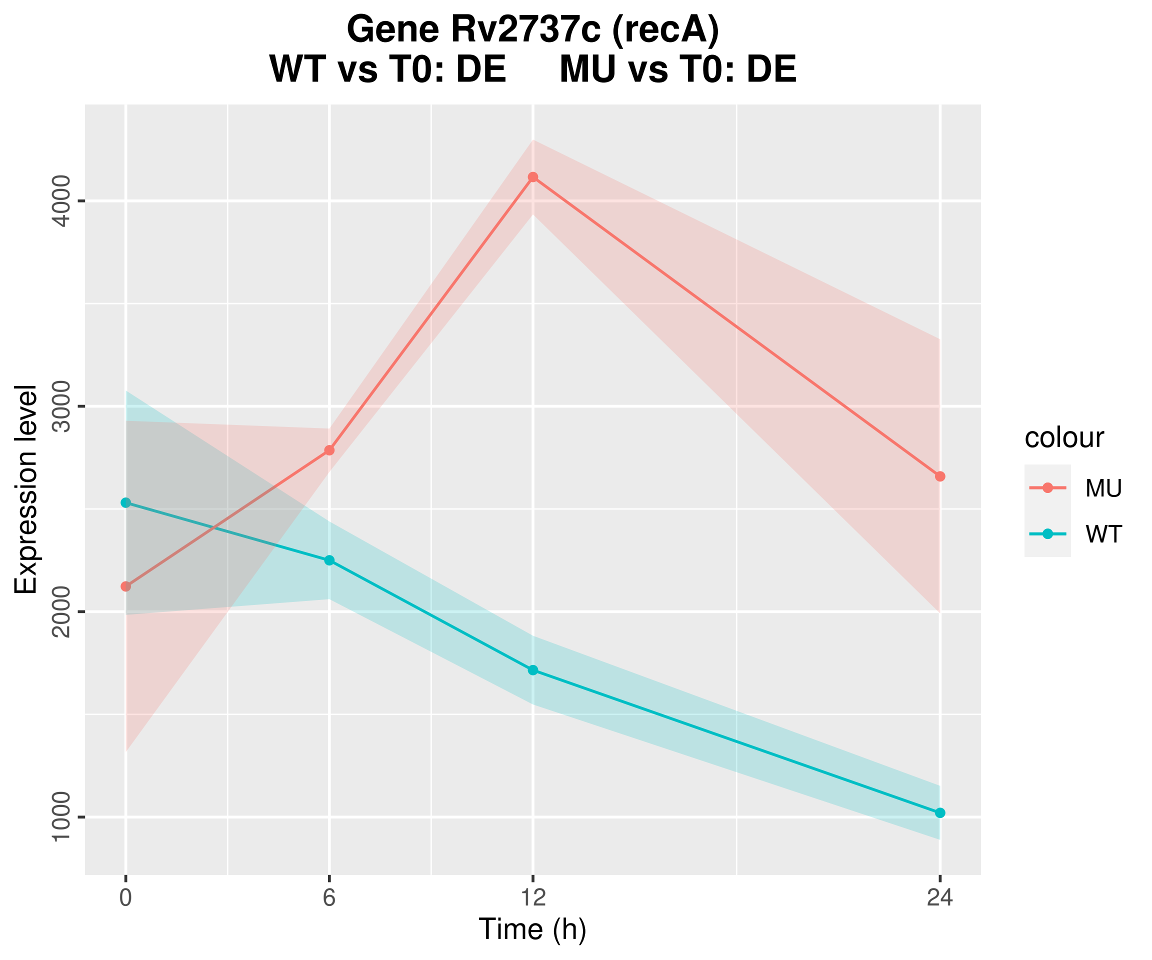
Gene Rv2737c (recA)
WT vs T0: DE MU vs T0: DE
Expression level
4000
3000
2000
1000
0
6
12
24
Time (h)
colour
MU
WT

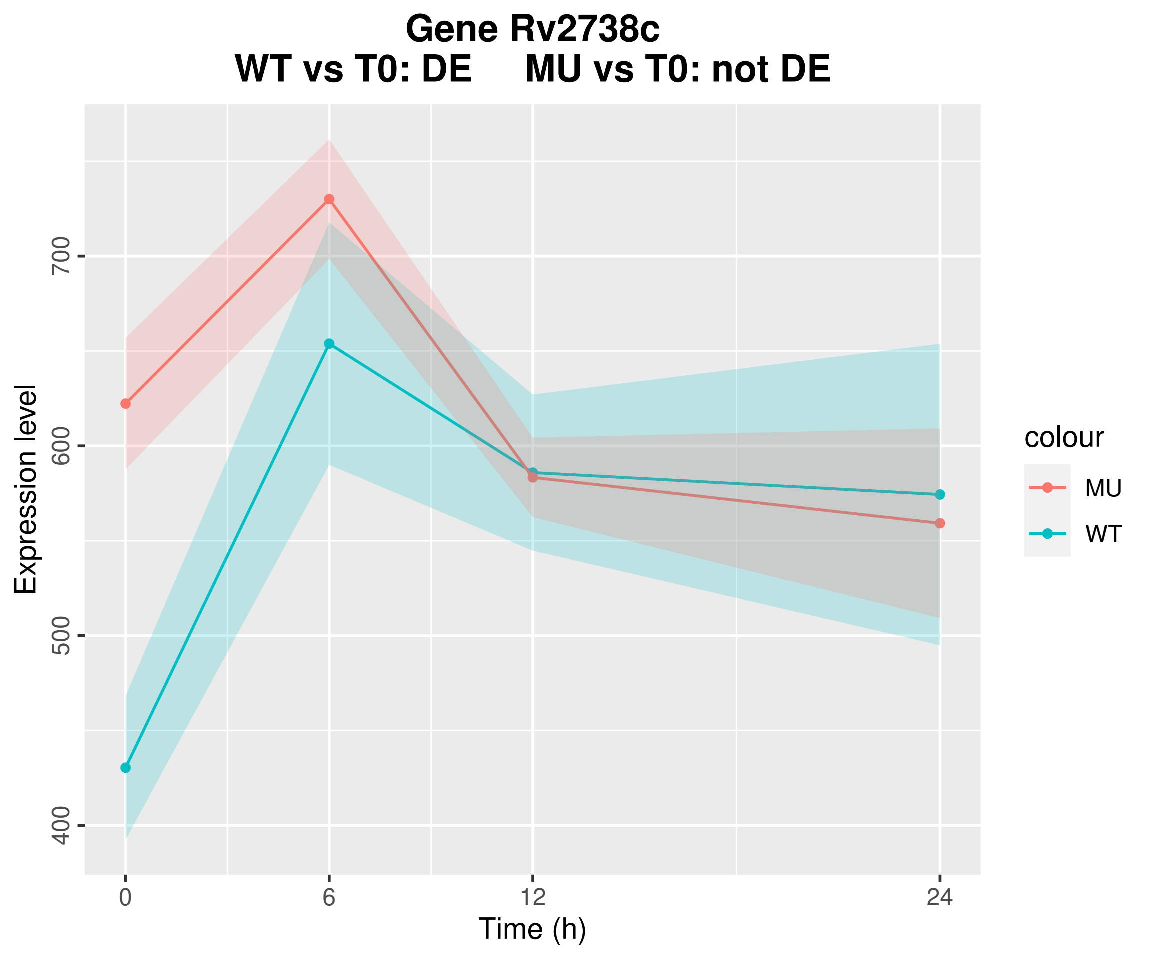
Gene Rv2738c
WT vs T0: DE   MU vs T0: not DE
Expression level
700
600
500
400
0
6
12
24
Time (h)
colour
MU
WT

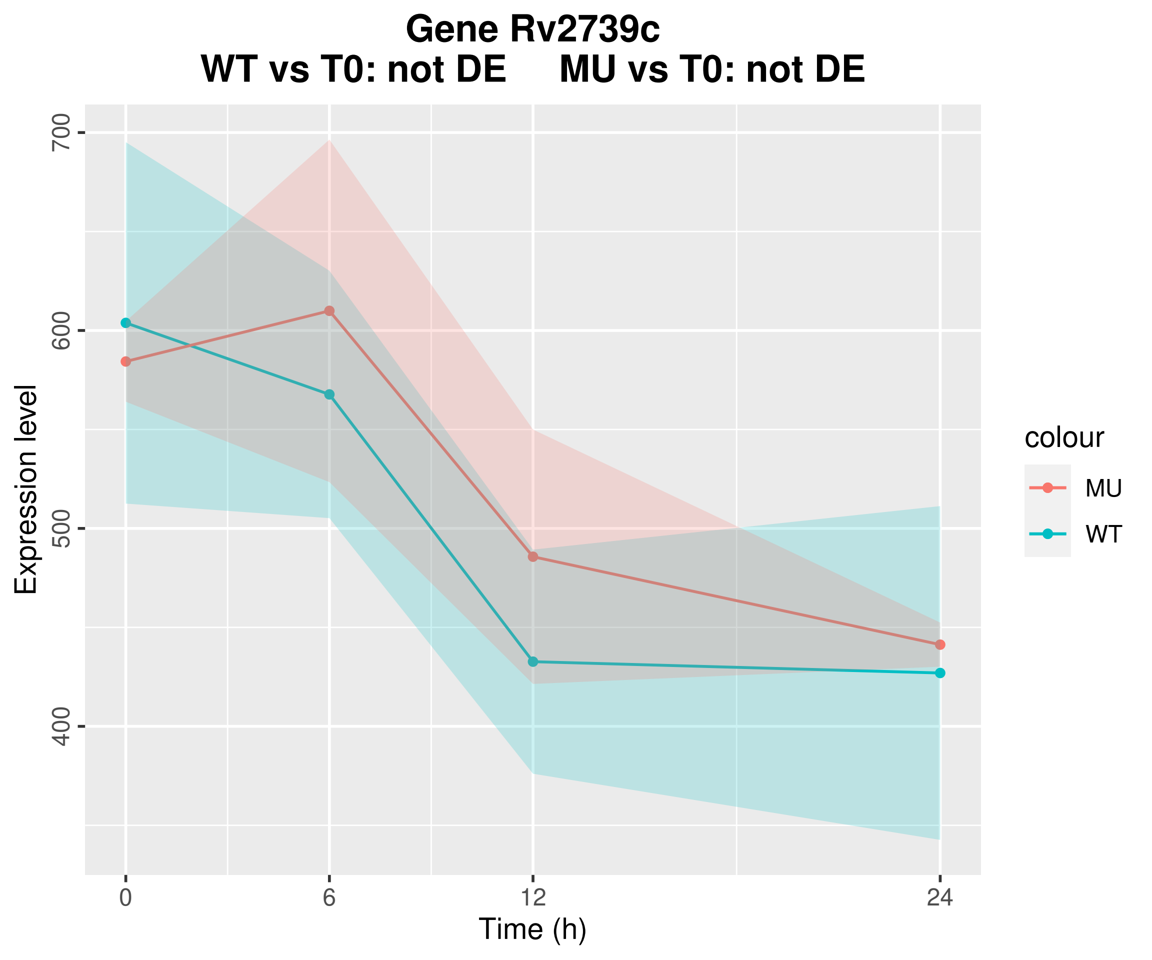

Gene Rv2739c
WT vs T0: not DE MU vs T0: not DE
Expression level
700
600
500
400
0
6
12
24
Time (h)
colour
MU
WT

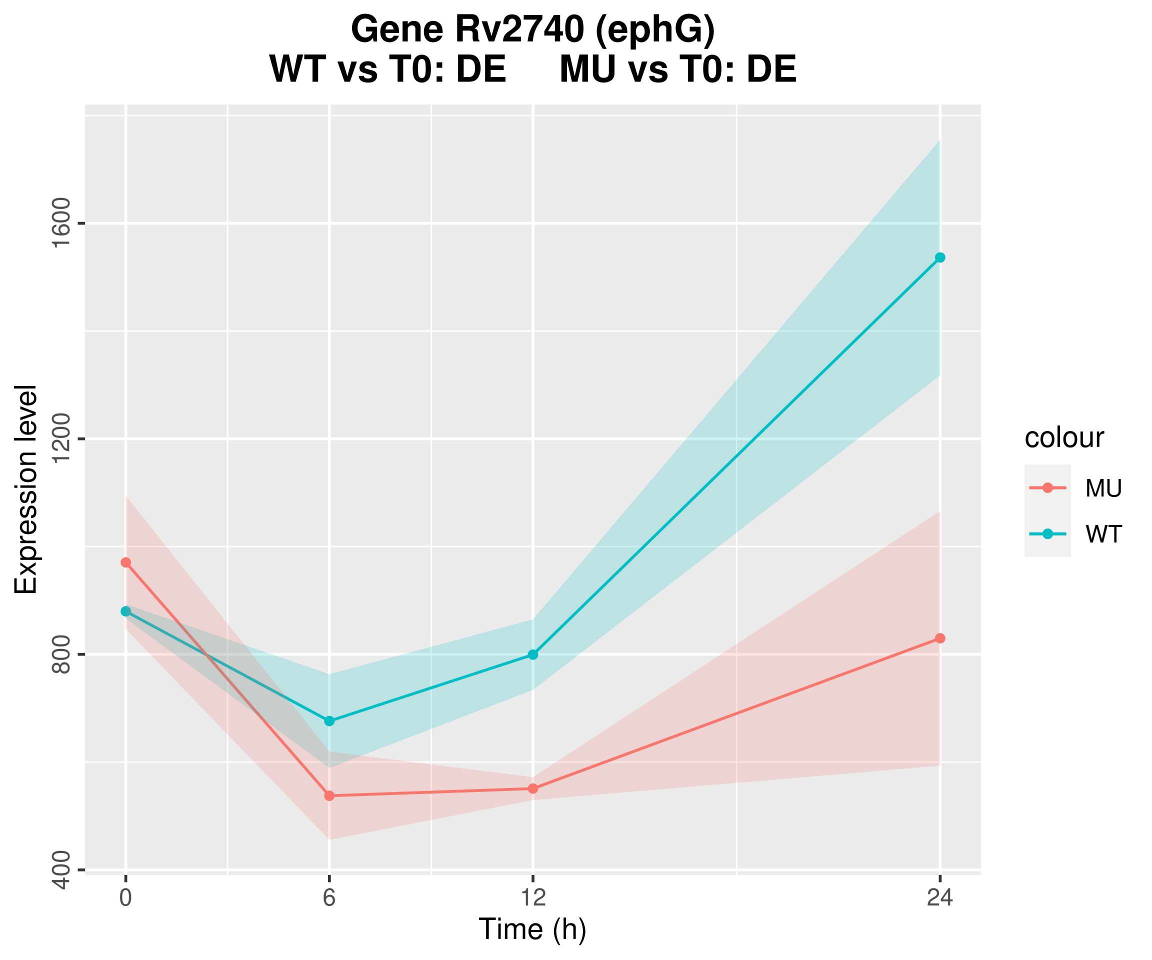
Gene Rv2740 (ephG)
WT vs T0: DE MU vs T0: DE
Expression level
1600
1200
800
400
0
6
12
24
Time (h)
colour
MU
WT

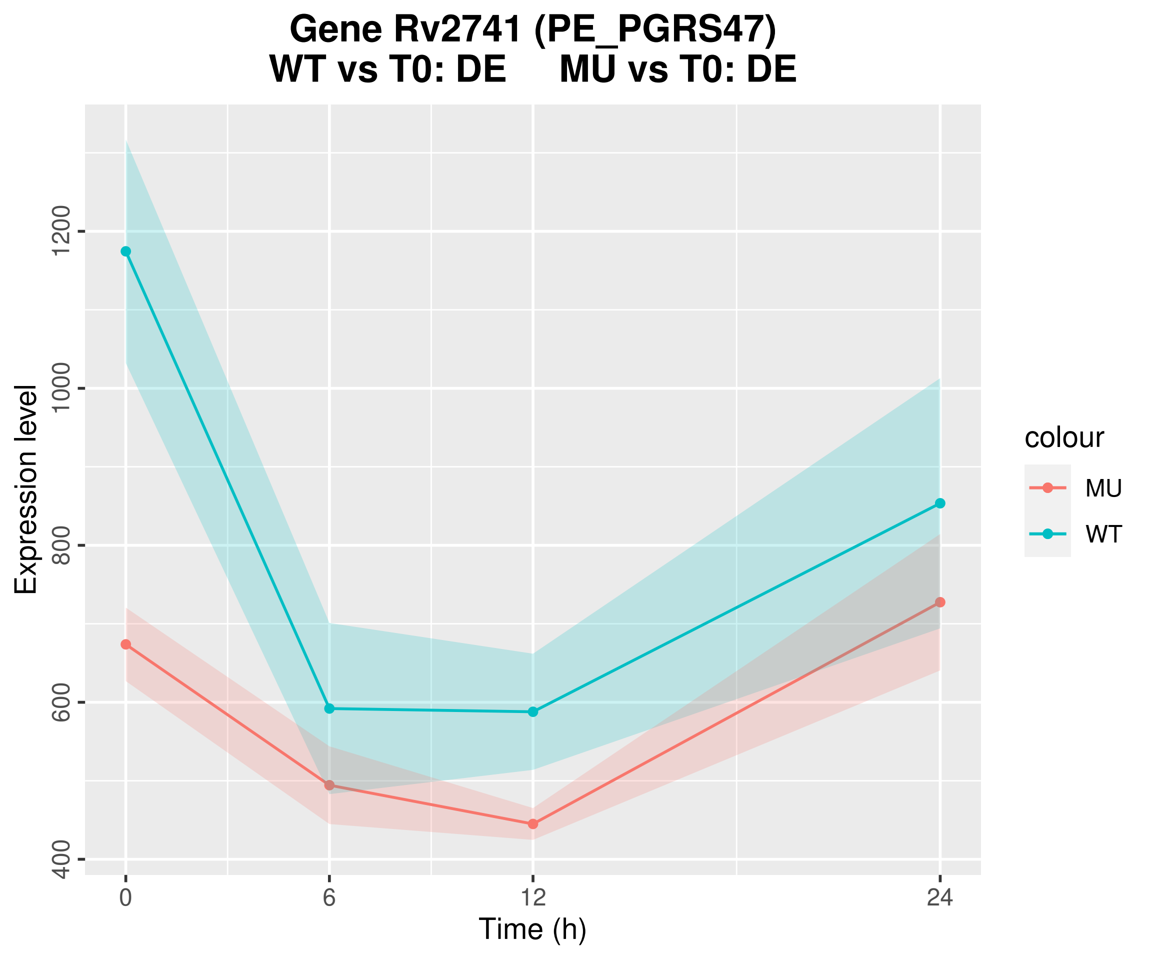
Gene Rv2741 (PE_PGRS47)
WT vs T0: DE MU vs T0: DE
Expression level
1200
1000
800
600
400
0
6
12
24
Time (h)
colour
MU
WT

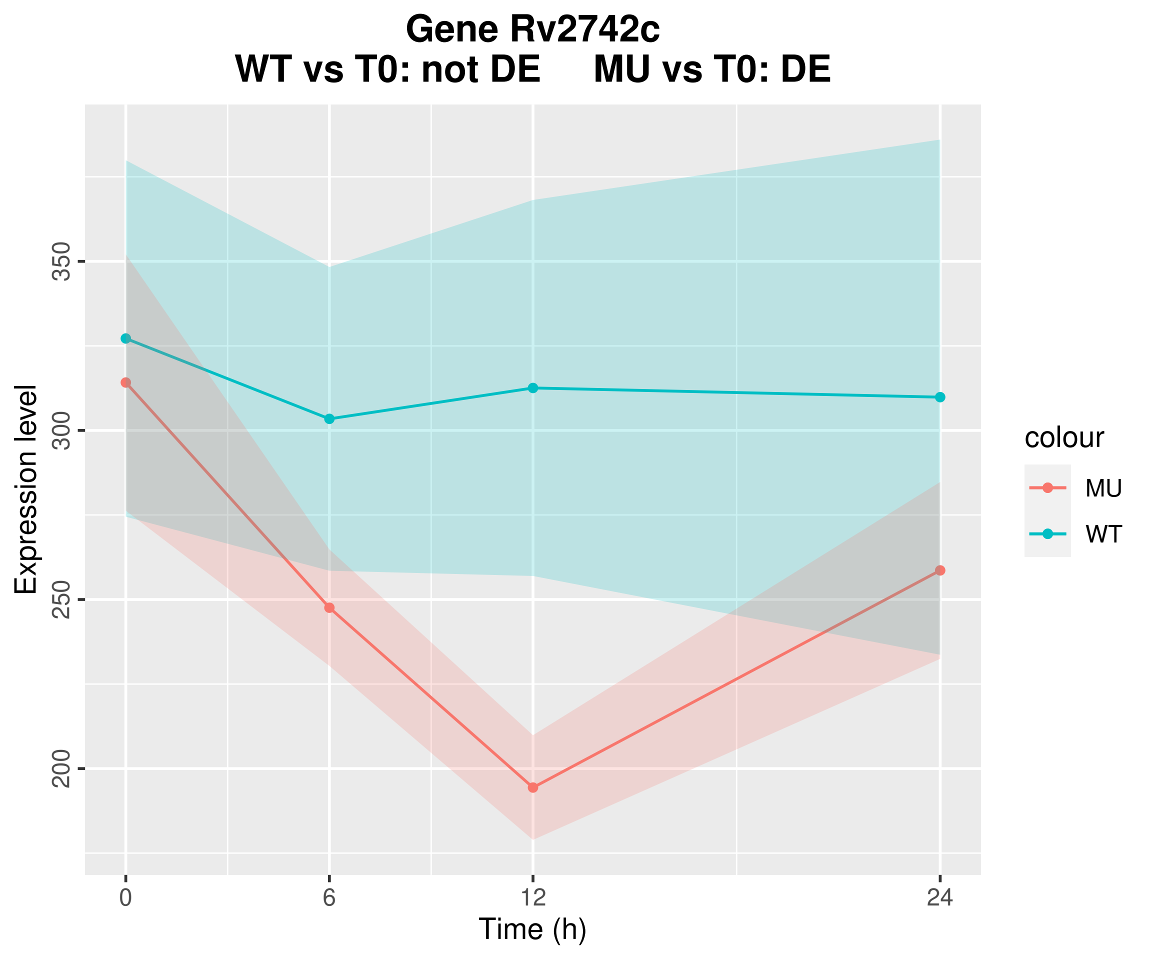
Gene Rv2742c
WT vs T0: not DE   MU vs T0: DE
Expression level
350
300
250
200
0
6
12
24
Time (h)
colour
MU
WT

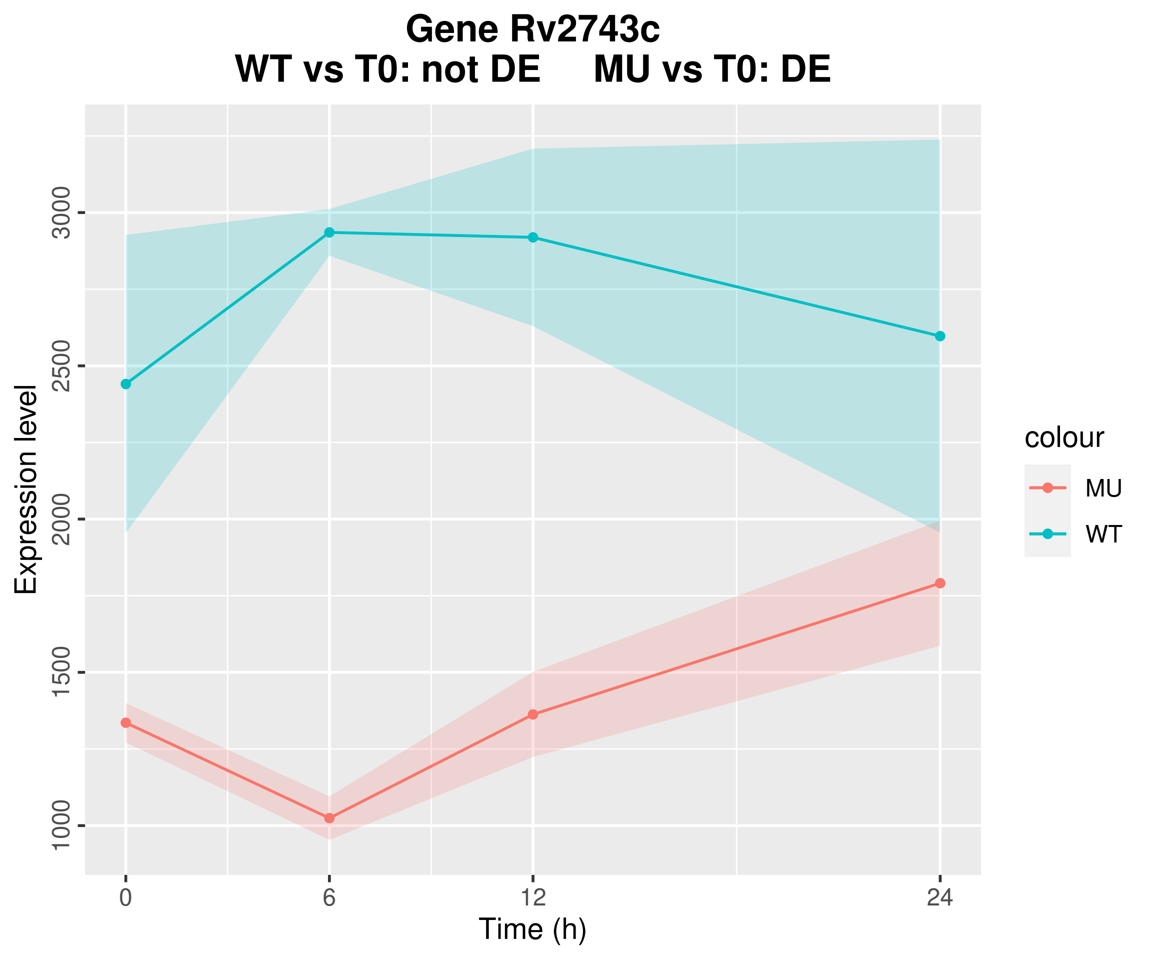

Gene Rv2743c
WT vs T0: not DE   MU vs T0: DE
Expression level
3000
2500
2000
1500
1000
0
6
12
24
Time (h)
colour
MU
WT

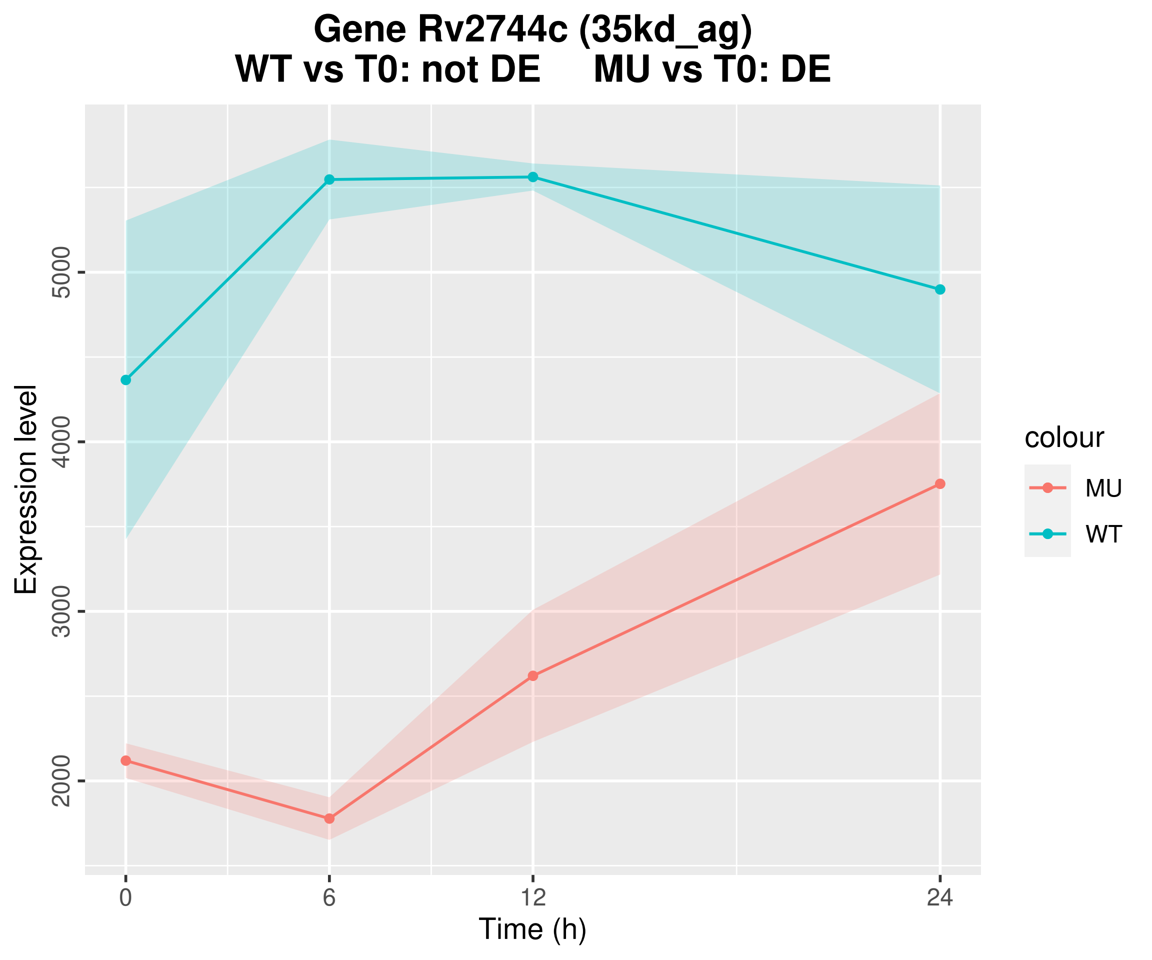
Gene Rv2744c (35kd_ag)
WT vs T0: not DE   MU vs T0: DE
Expression level
5000
4000
3000
2000
0
6
12
24
Time (h)
colour
MU
WT

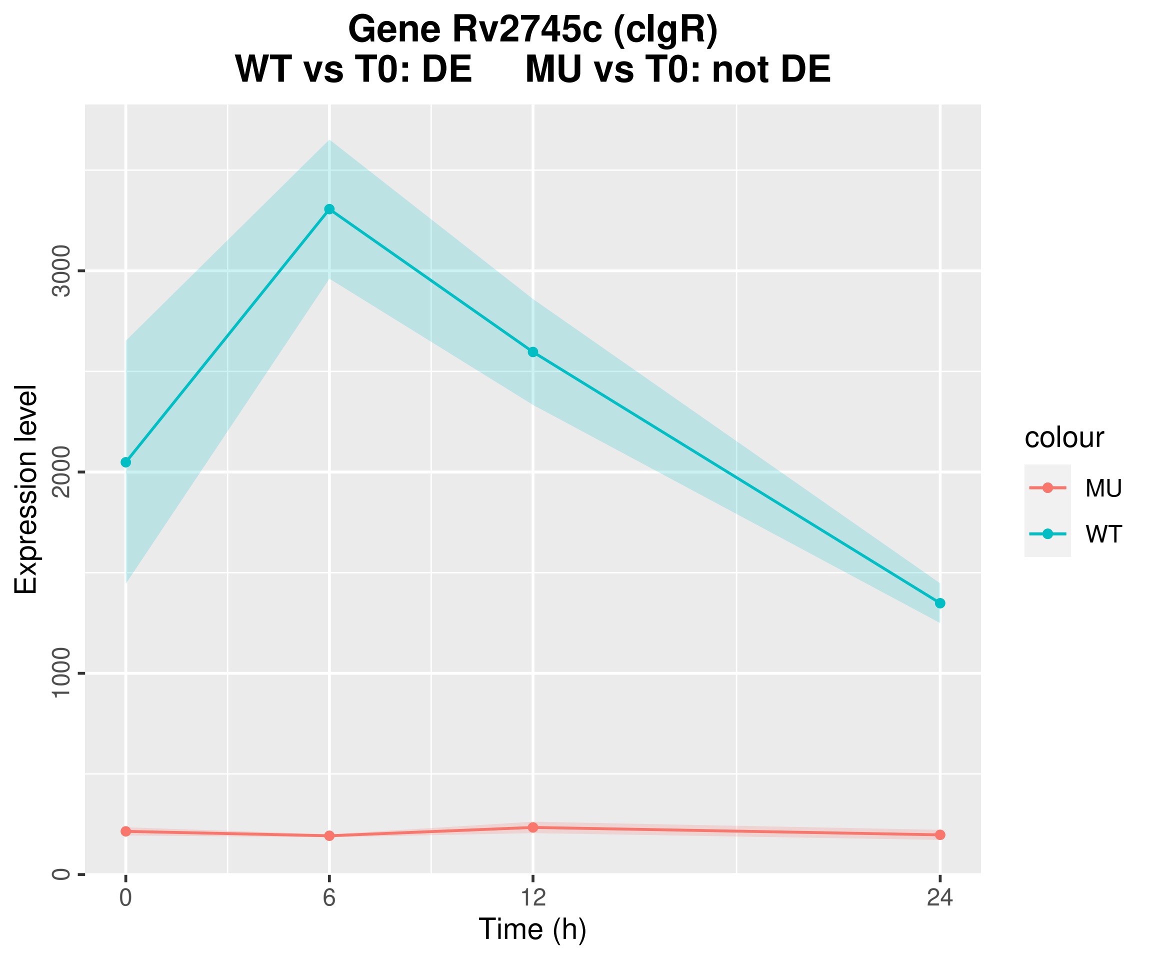
Gene Rv2745c (clgR)
WT vs T0: DE   MU vs T0: not DE
Expression level
3000
2000
1000
0
0
6
12
24
Time (h)
colour
MU
WT

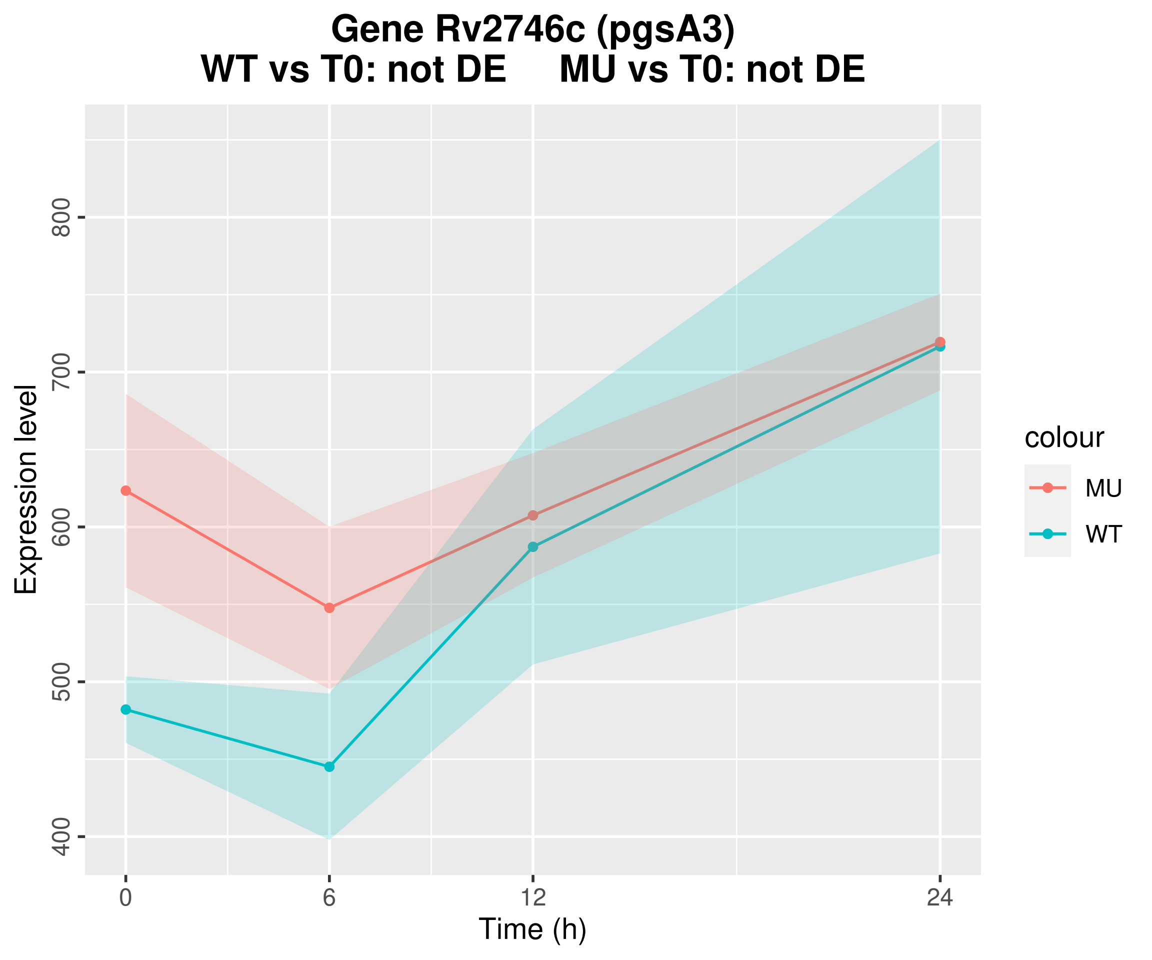
Gene Rv2746c (pgsA3)
WT vs T0: not DE MU vs T0: not DE
Expression level
800
700
600
500
400
0
6
12
24
Time (h)
colour
MU
WT

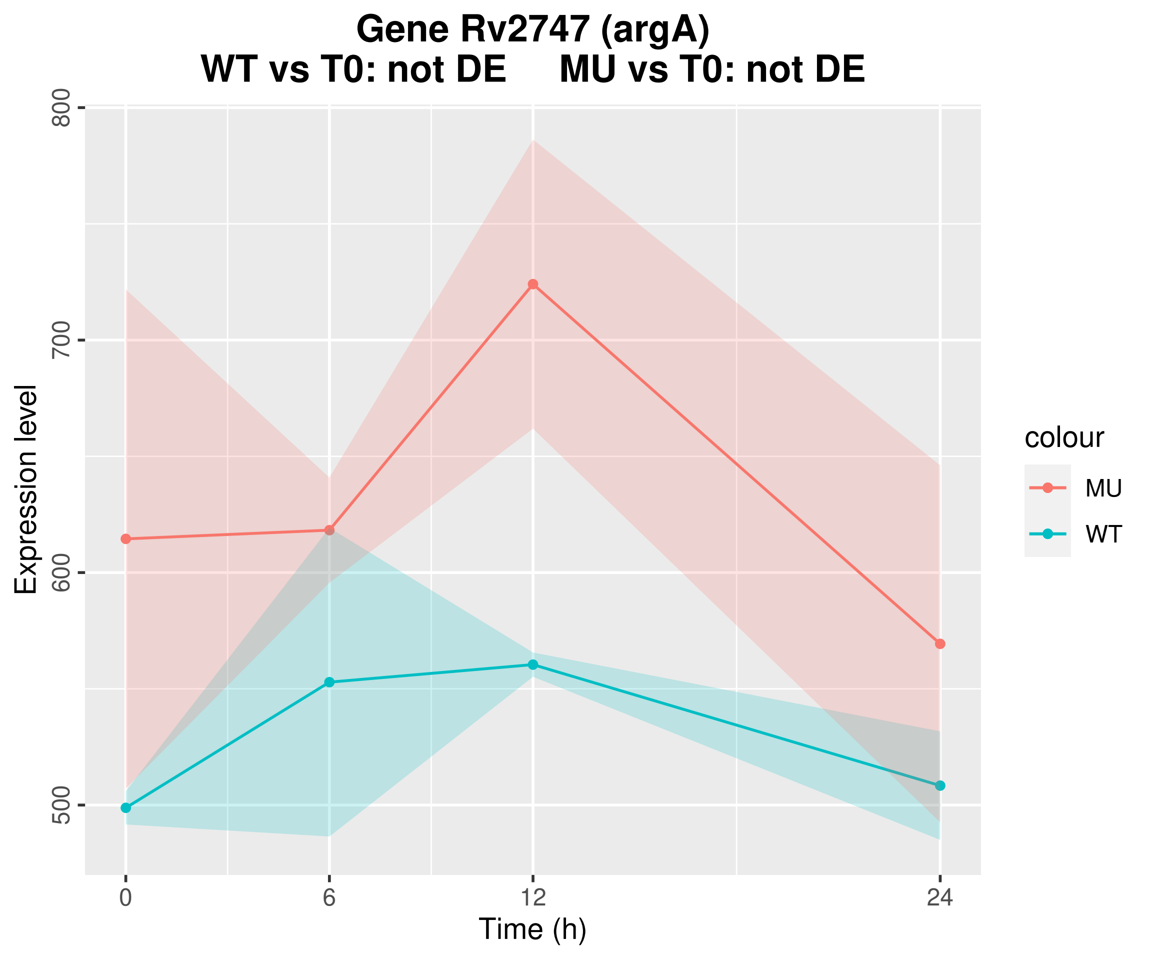

Gene Rv2747 (argA)
WT vs T0: not DE MU vs T0: not DE
800
700
600
500
Expression level
0
6
12
24
Time (h)
colour
MU
WT

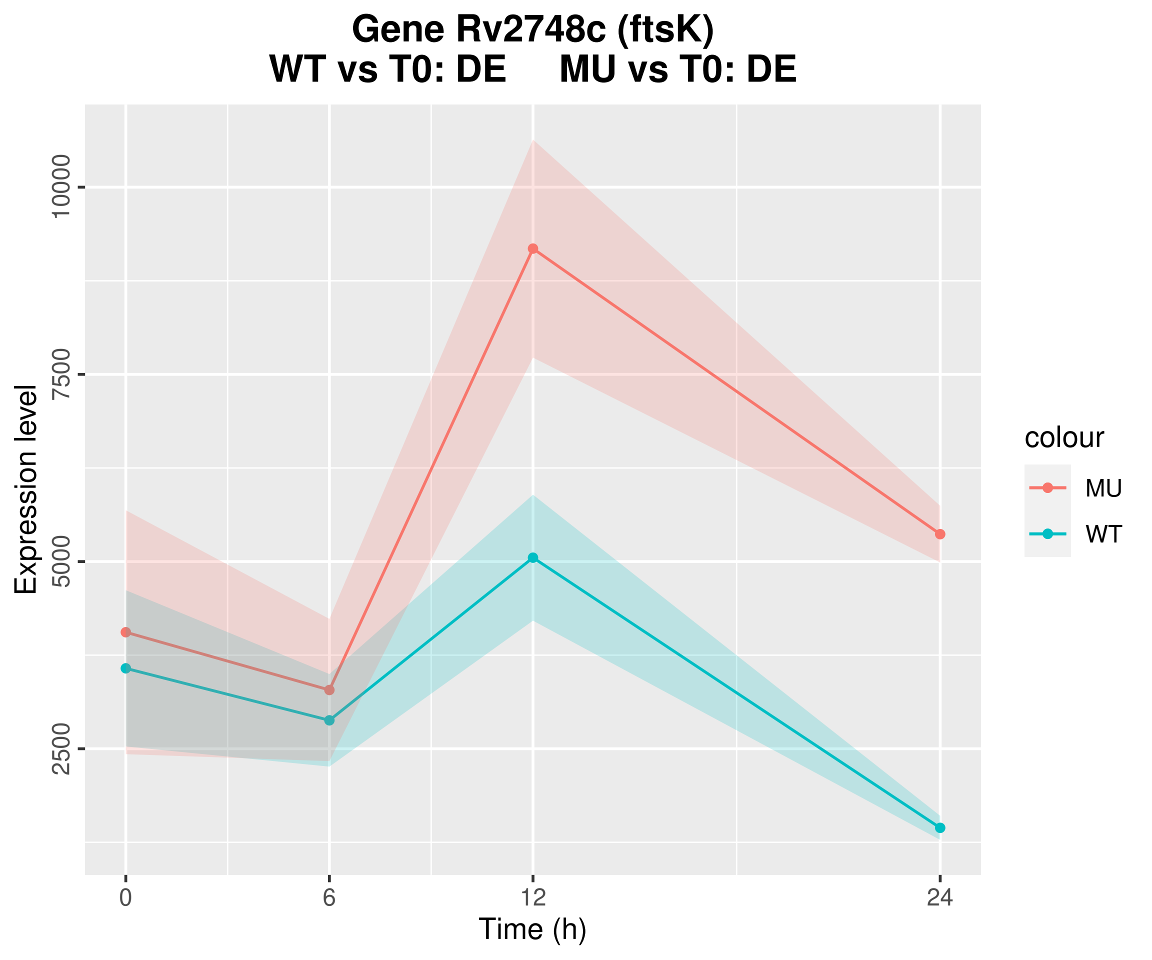

Gene Rv2748c (ftsK)
WT vs T0: DE MU vs T0: DE
Expression level
10000
7500
5000
2500
0
6
12
24
Time (h)
colour
MU
WT

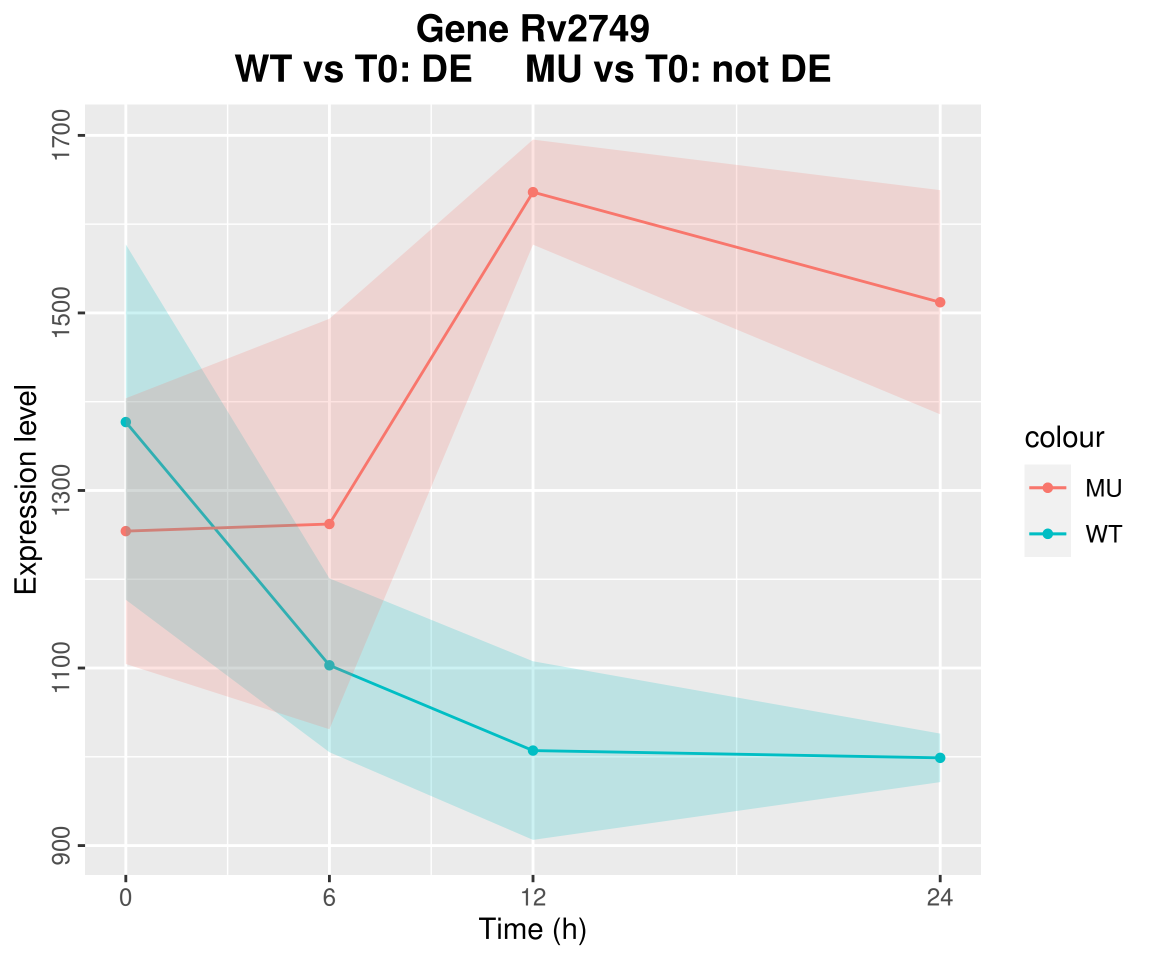

Gene Rv2749
WT vs T0: DE   MU vs T0: not DE
Expression level
1700
1500
1300
1100
900
0
6
12
24
Time (h)
colour
MU
WT

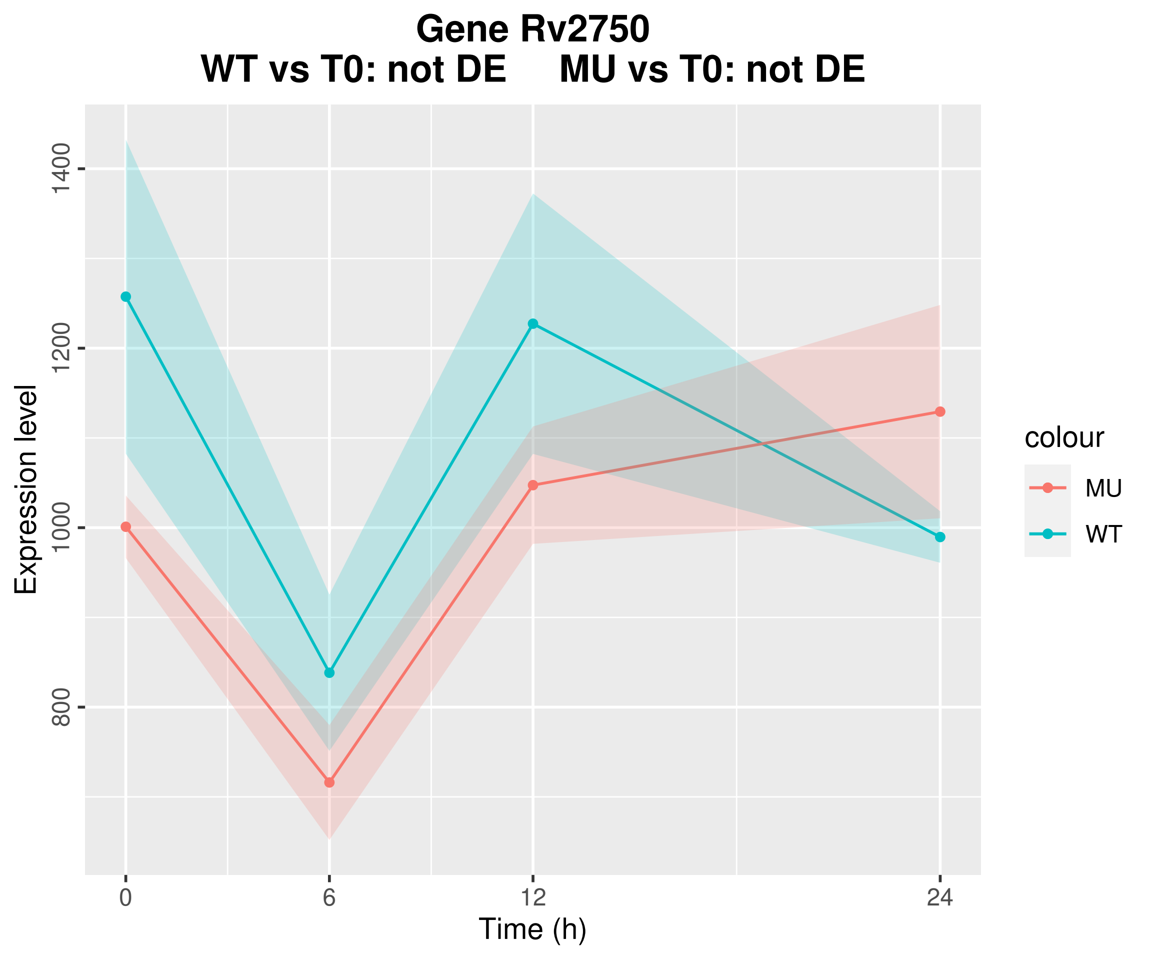

Gene Rv2750
WT vs T0: not DE MU vs T0: not DE
Expression level
1400
1200
1000
800
0
6
12
24
Time (h)
colour
MU
WT

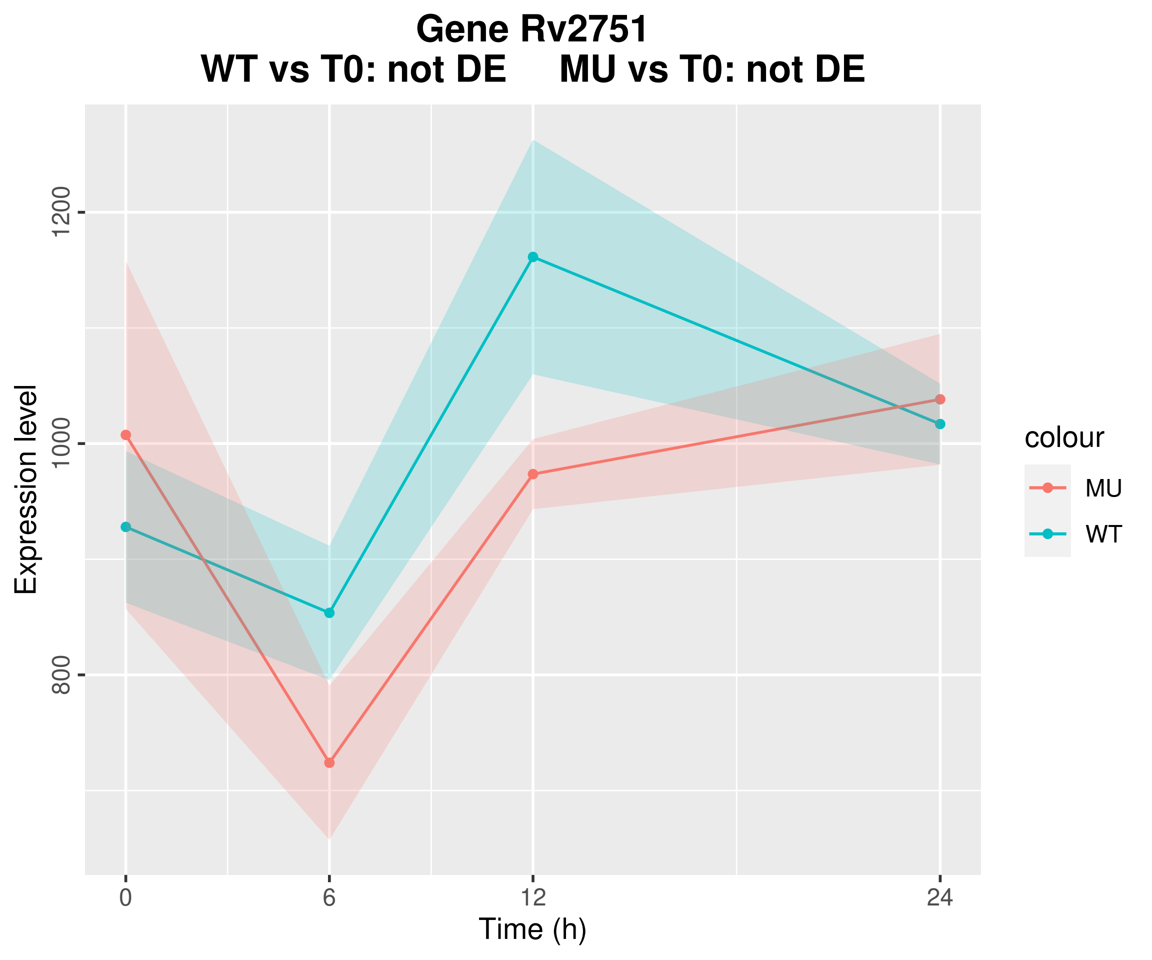

Gene Rv2751
WT vs T0: not DE   MU vs T0: not DE
Expression level
1200
1000
800
Time (h)
0
6
12
24
colour
MU
WT

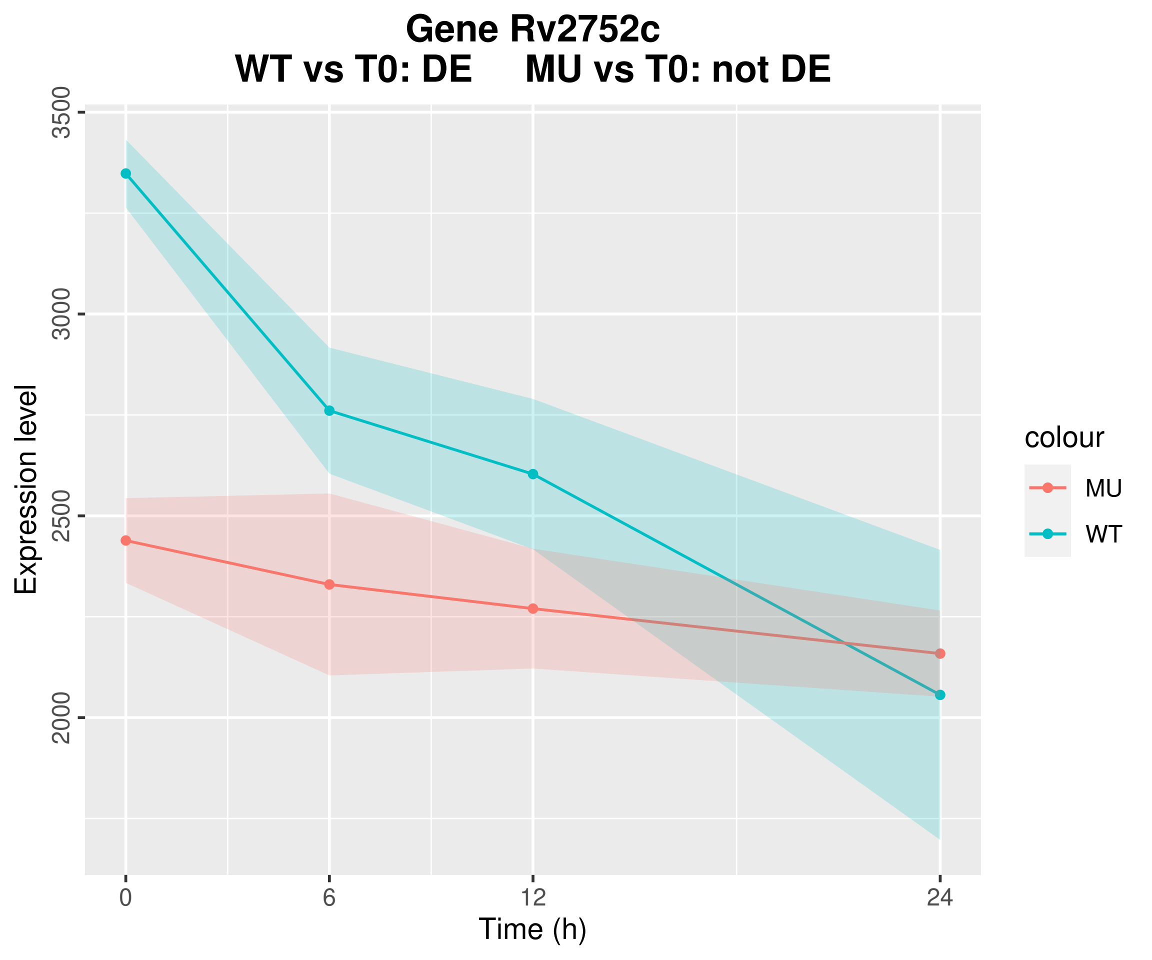
Gene Rv2752c
WT vs T0: DE   MU vs T0: not DE
3500
3000
2500
2000
Expression level
0
6
12
24
Time (h)
colour
MU
WT

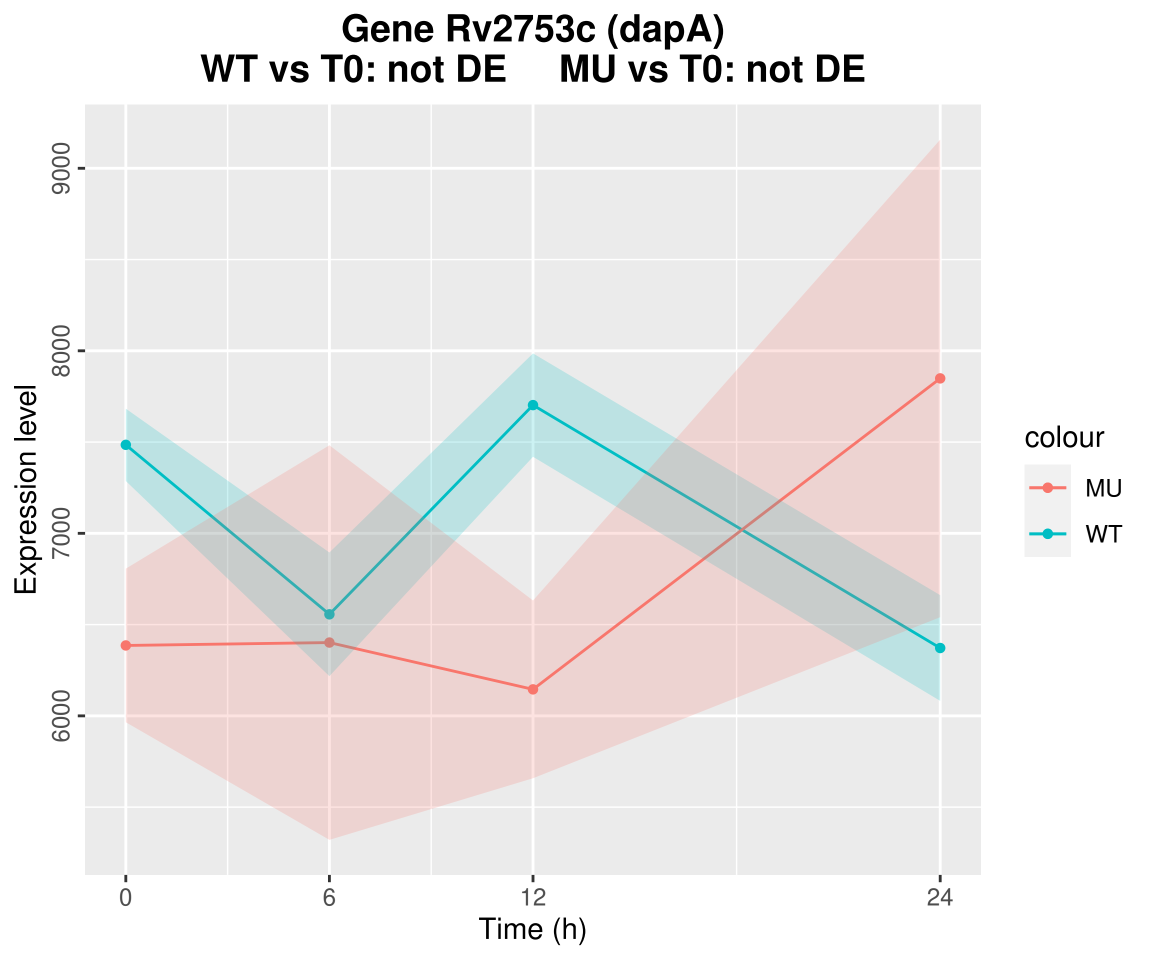
Gene Rv2753c (dapA)
WT vs T0: not DE MU vs T0: not DE
9000
8000
7000
6000
Expression level
0
6
12
24
Time (h)
colour
MU
WT

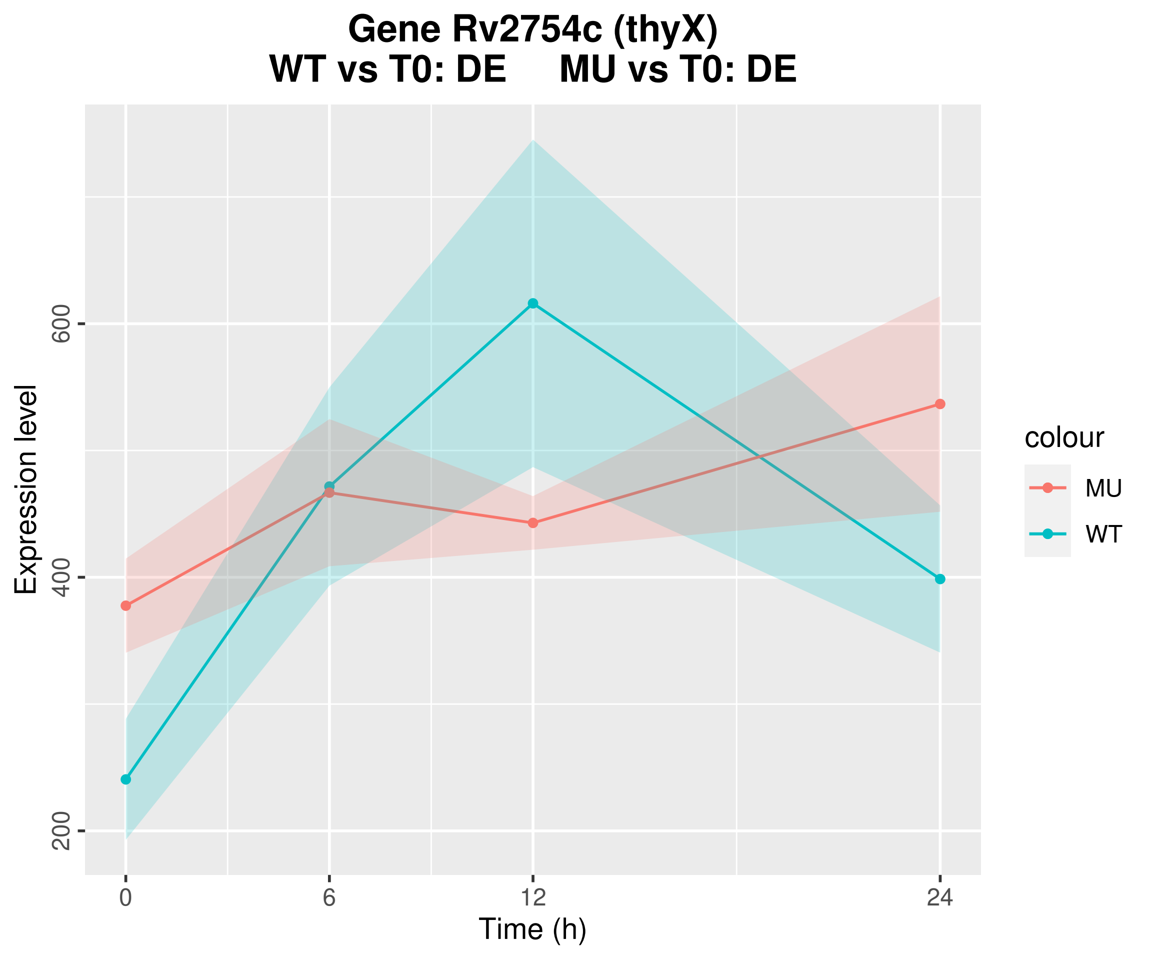

Gene Rv2754c (thyX)
WT vs T0: DE   MU vs T0: DE
Expression level
200
400
600
0
6
12
24
Time (h)
colour
MU
WT

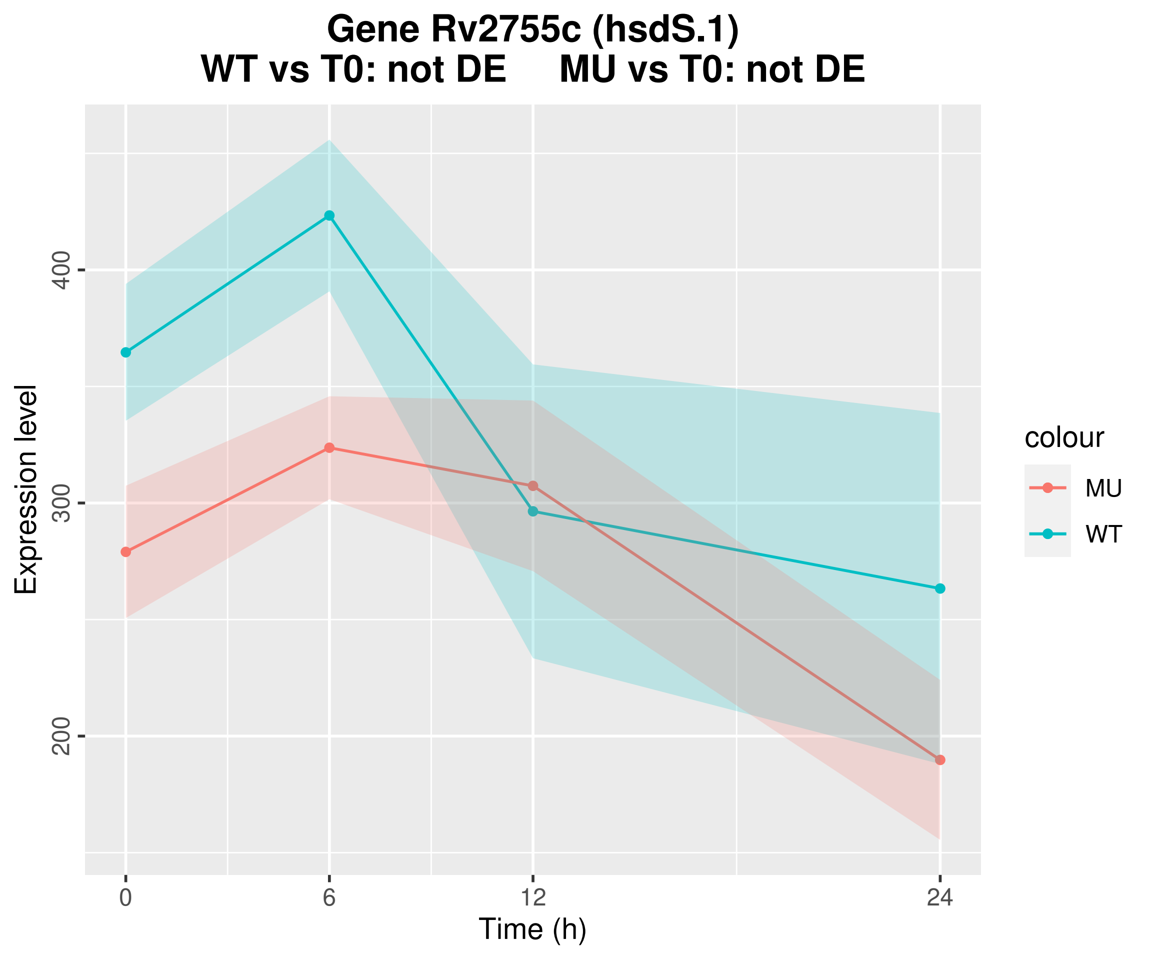
Gene Rv2755c (hsdS.1)
WT vs T0: not DE   MU vs T0: not DE
Expression level
400
300
200
0
6
12
24
Time (h)
colour
MU
WT

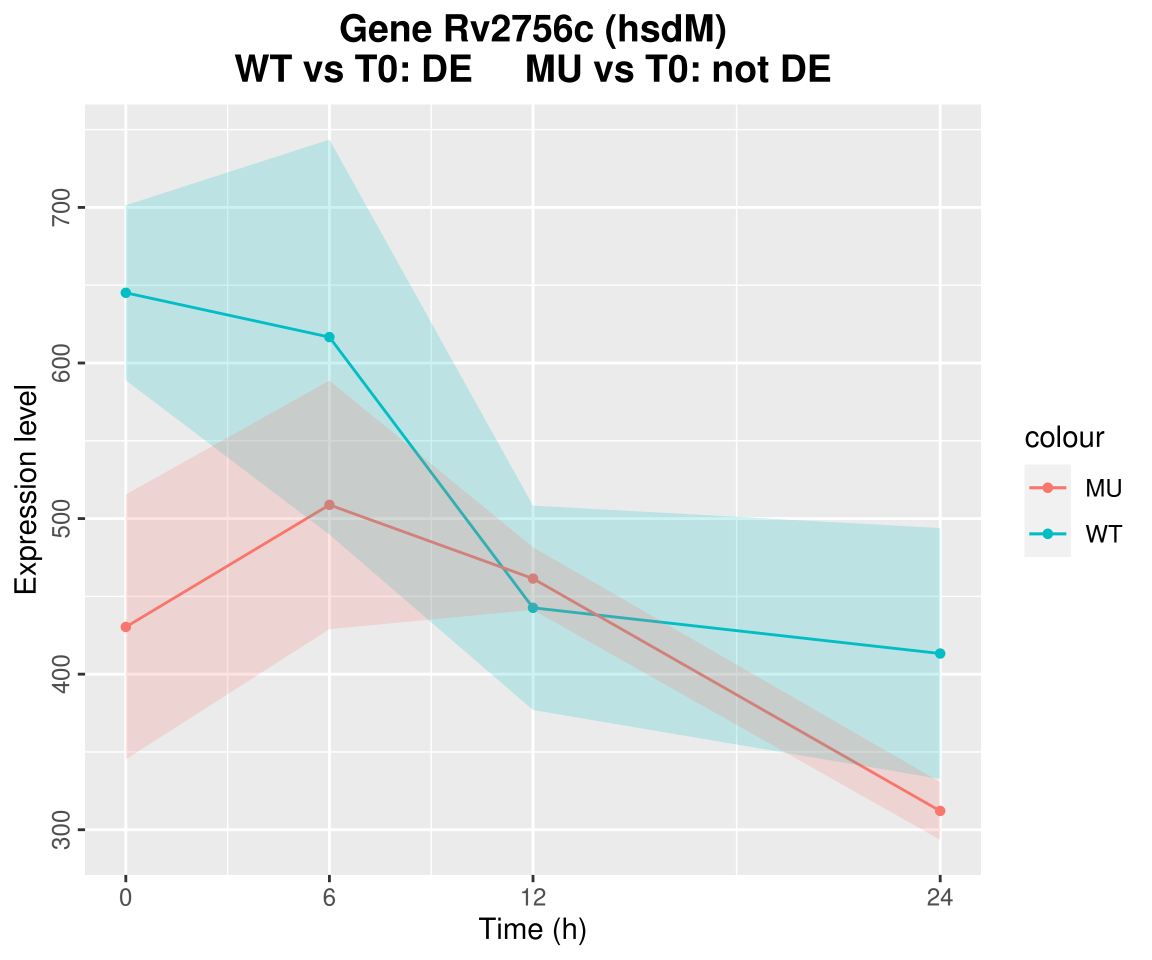
Gene Rv2756c (hsdM)
WT vs T0: DE   MU vs T0: not DE
Expression level
700
600
500
400
300
0
6
12
24
Time (h)
colour
MU
WT

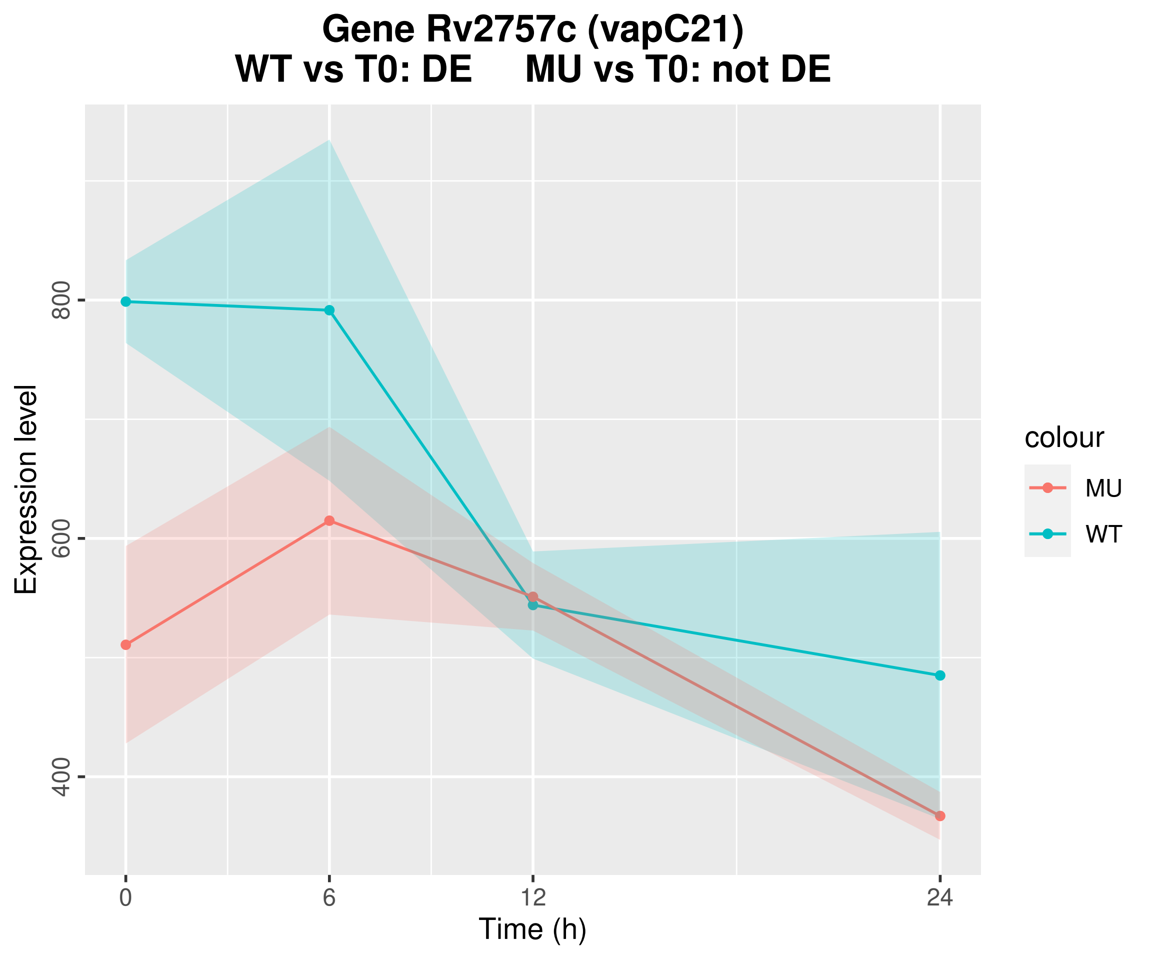

Gene Rv2757c (vapC21)
WT vs T0: DE MU vs T0: not DE
Expression level
800
600
400
0
6
12
24
Time (h)
colour
MU
WT

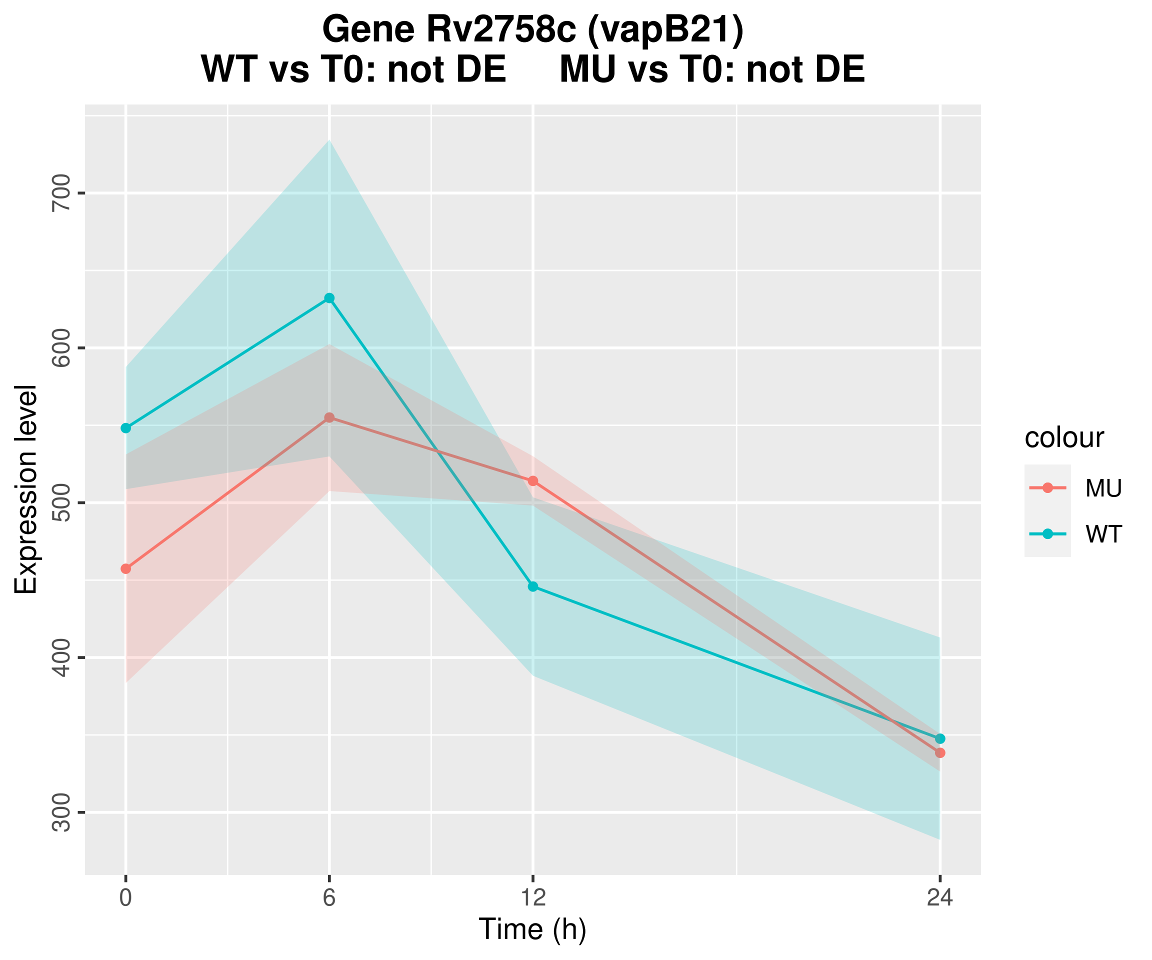

Gene Rv2758c (vapB21)
WT vs T0: not DE   MU vs T0: not DE
Expression level
300
400
500
600
700
0
6
12
24
Time (h)
colour
MU
WT

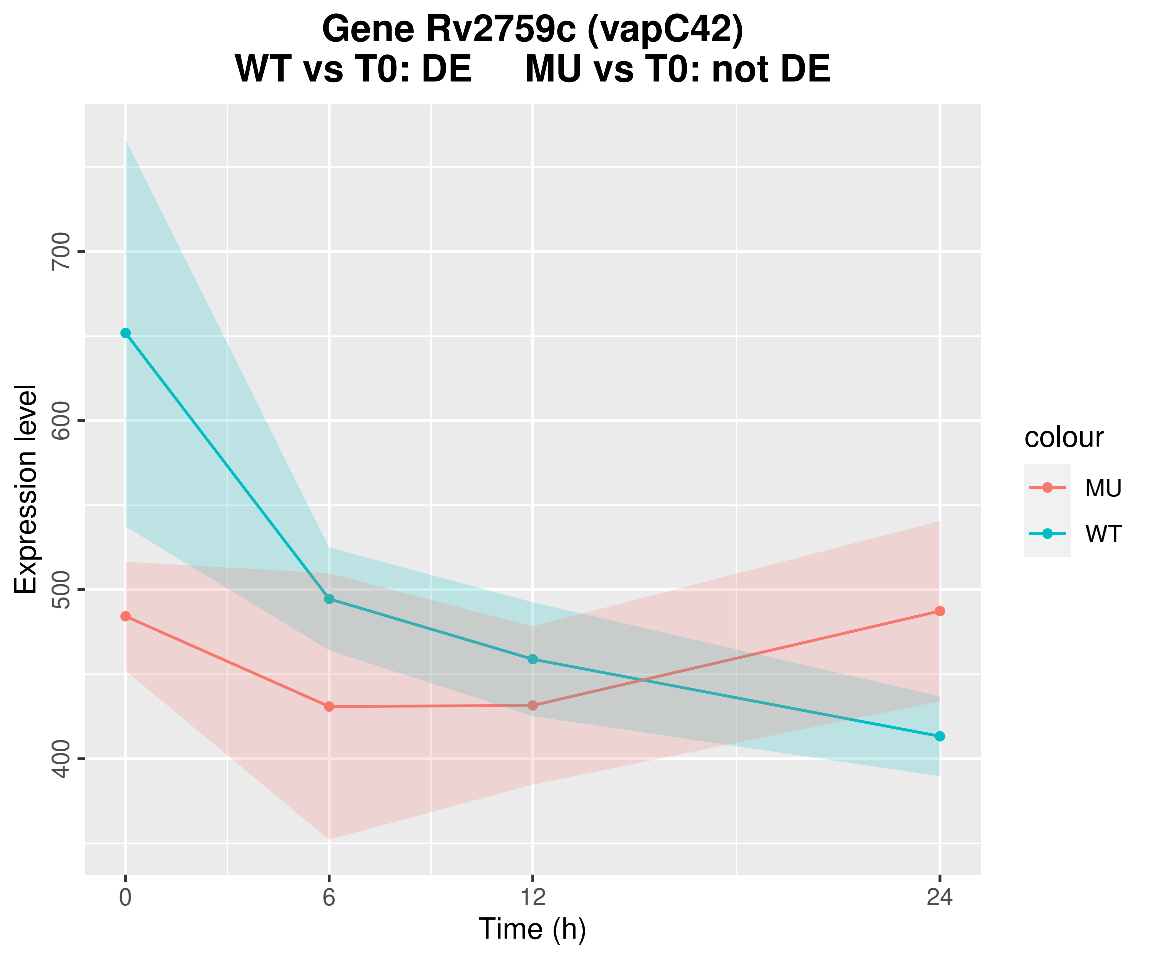
Gene Rv2759c (vapC42)
WT vs T0: DE   MU vs T0: not DE
Expression level
700
600
500
400
0
6
12
24
Time (h)
colour
MU
WT

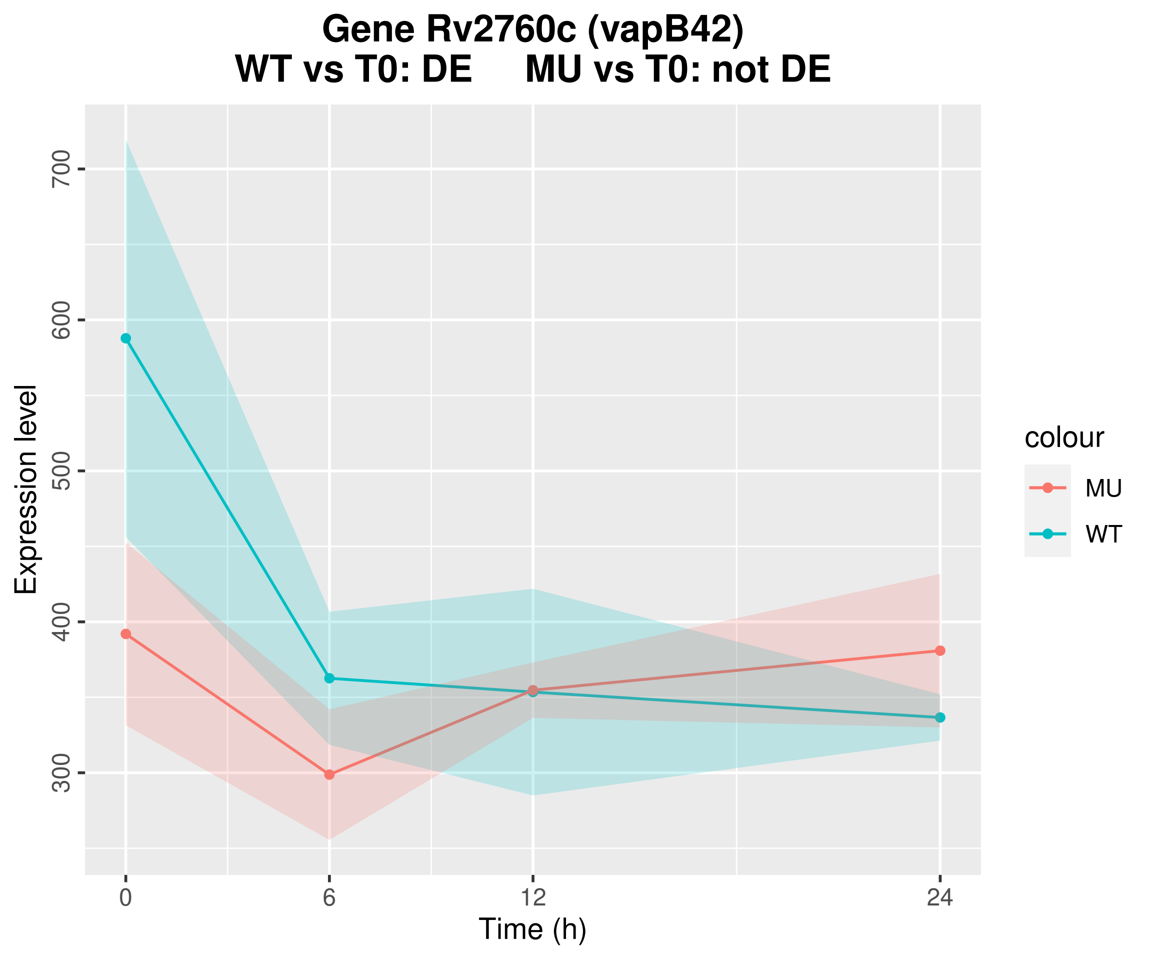

Gene Rv2760c (vapB42)
WT vs T0: DE MU vs T0: not DE
Expression level
700
600
500
400
300
0
6
12
24
Time (h)
colour
MU
WT

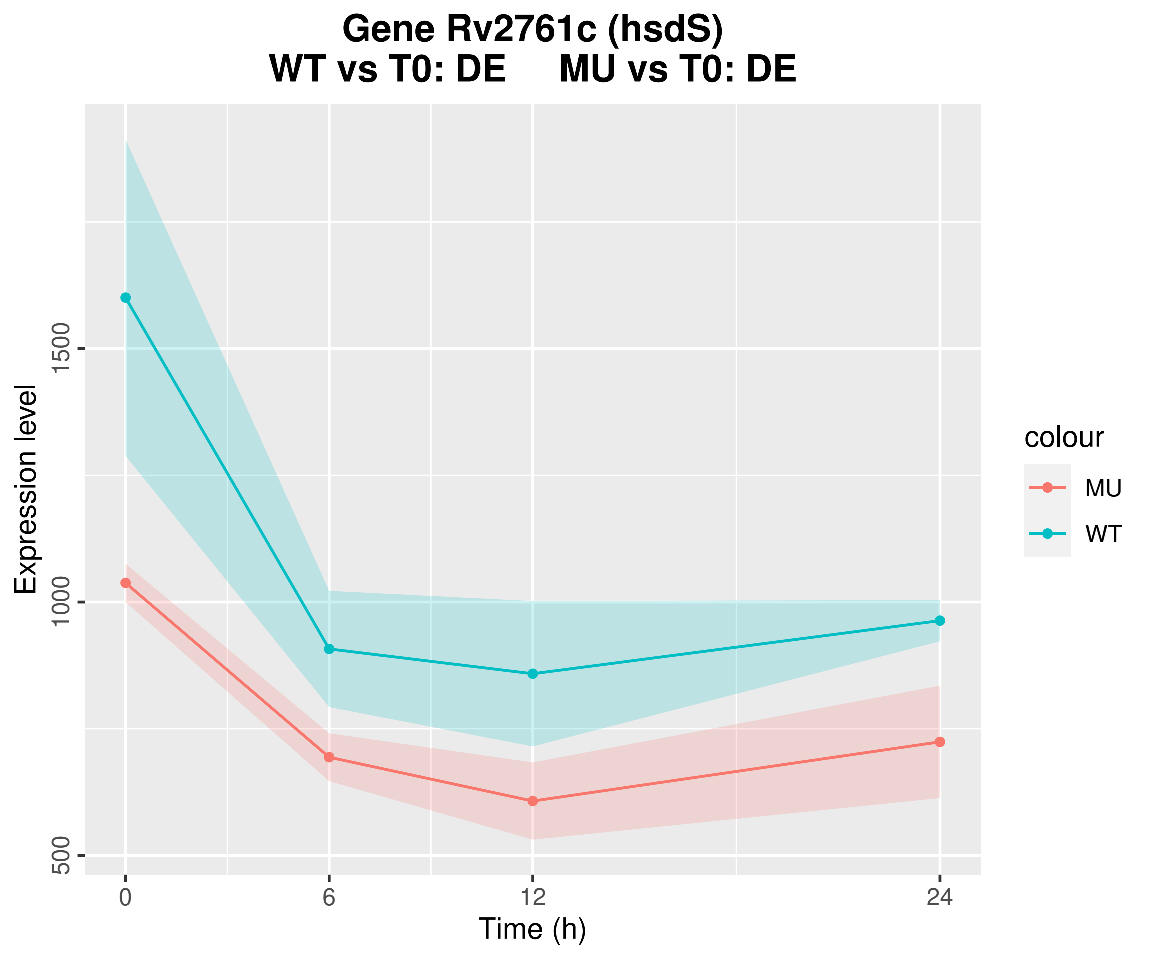
Gene Rv2761c (hsdS)
WT vs T0: DE MU vs T0: DE
Expression level
1500
1000
500
0
6
12
24
Time (h)
colour
MU
WT

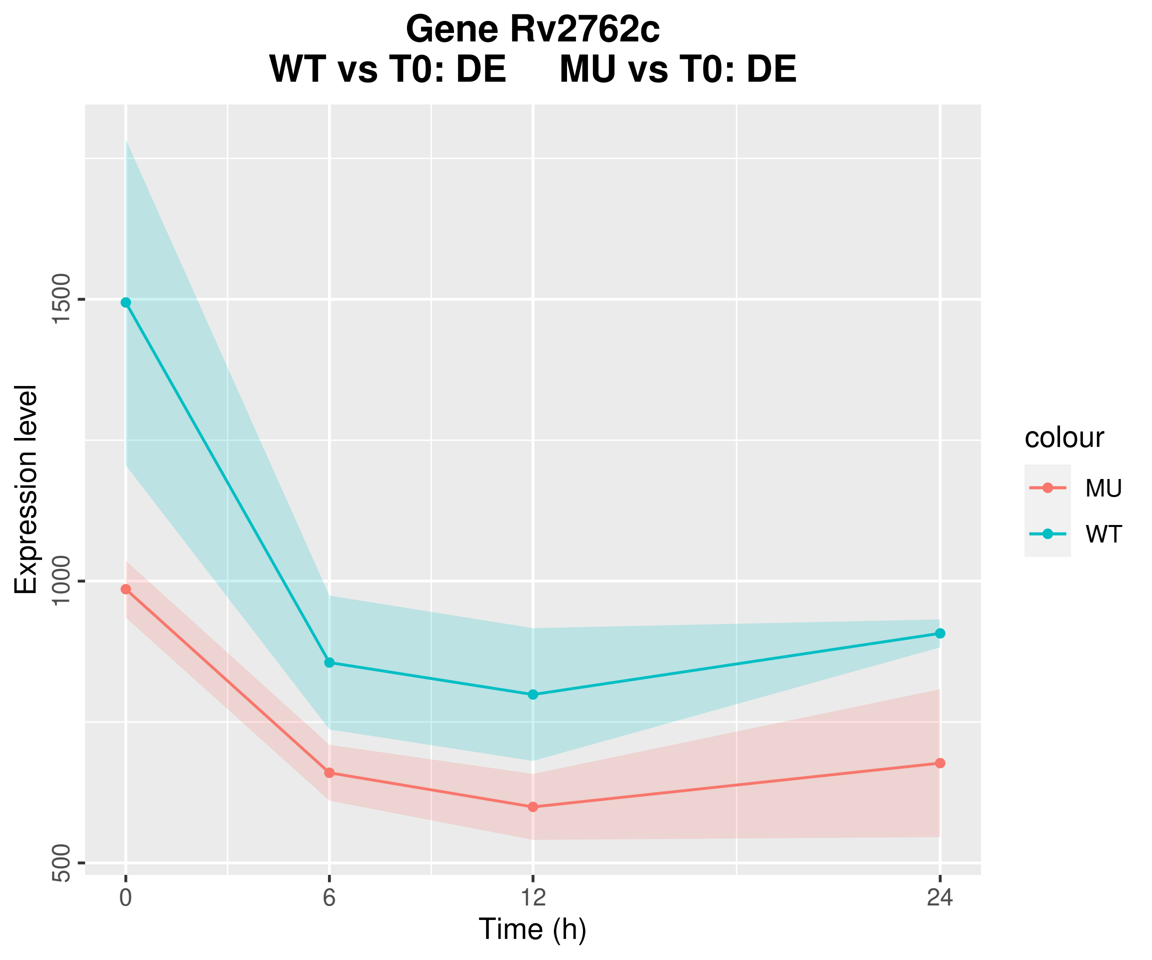
Gene Rv2762c
WT vs T0: DE   MU vs T0: DE
Expression level
1500
1000
500
0
6
12
24
Time (h)
colour
MU
WT

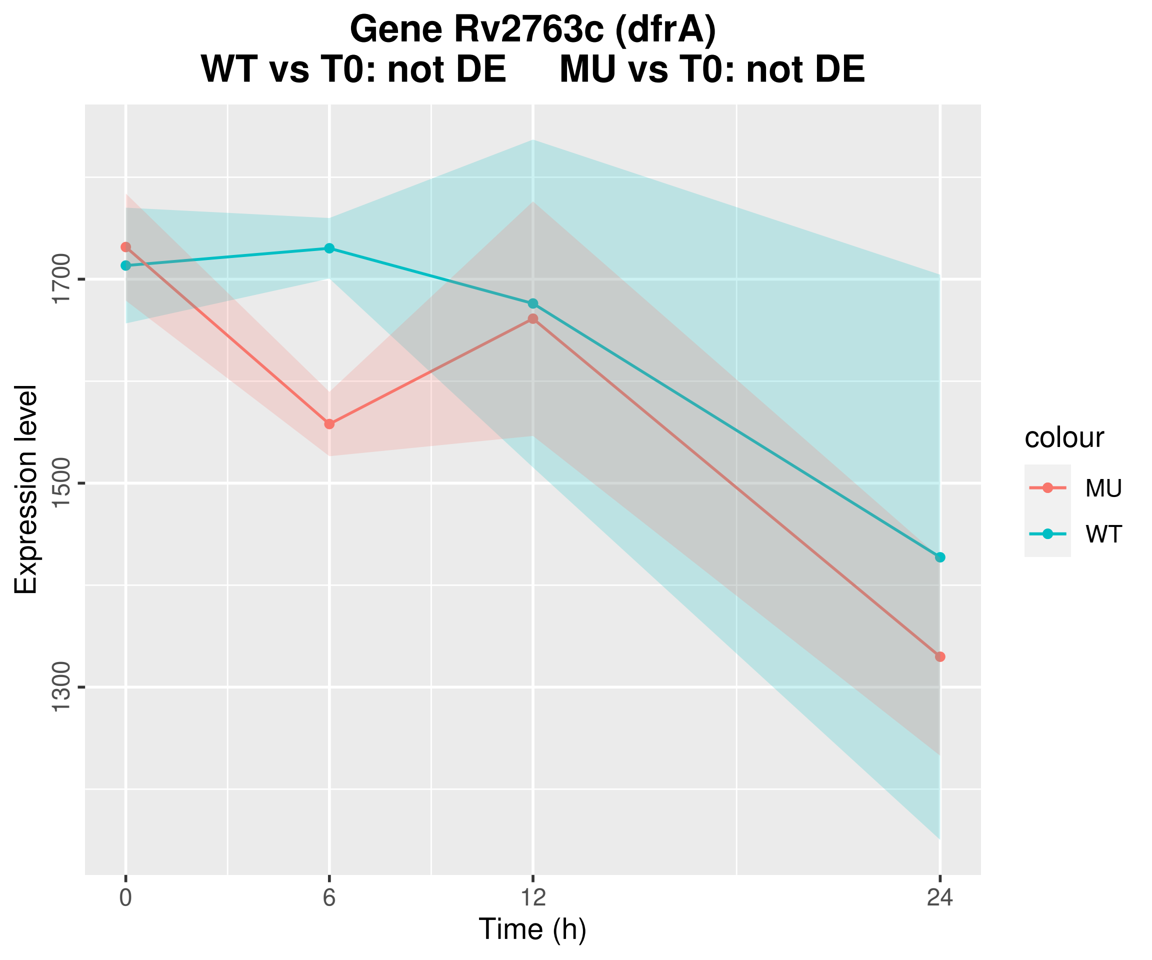

Gene Rv2763c (dfrA)
WT vs T0: not DE MU vs T0: not DE
Expression level
1700
1500
1300
0
6
12
24
Time (h)
colour
MU
WT

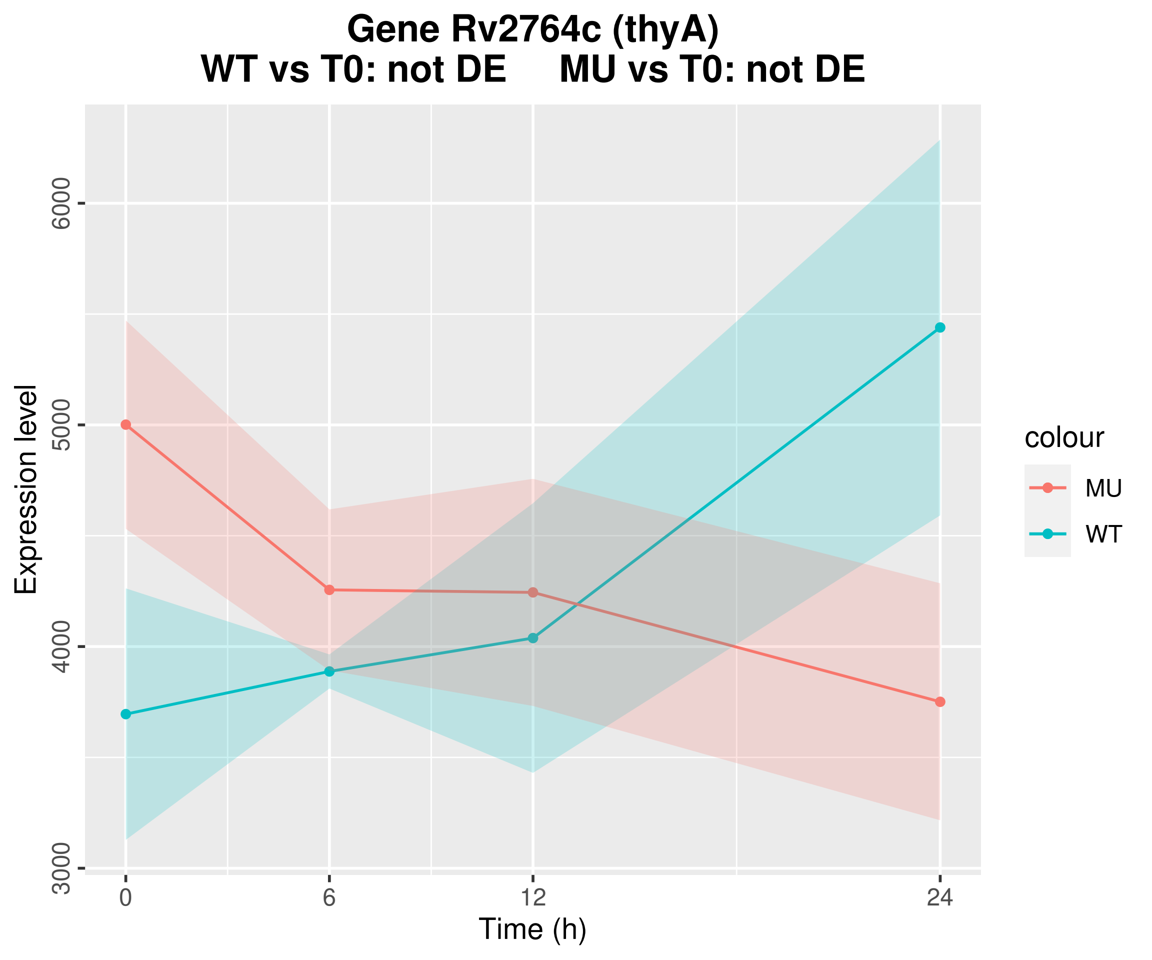

Gene Rv2764c (thyA)
WT vs T0: not DE MU vs T0: not DE
Expression level
6000
5000
4000
3000
0
6
12
24
Time (h)
colour
MU
WT

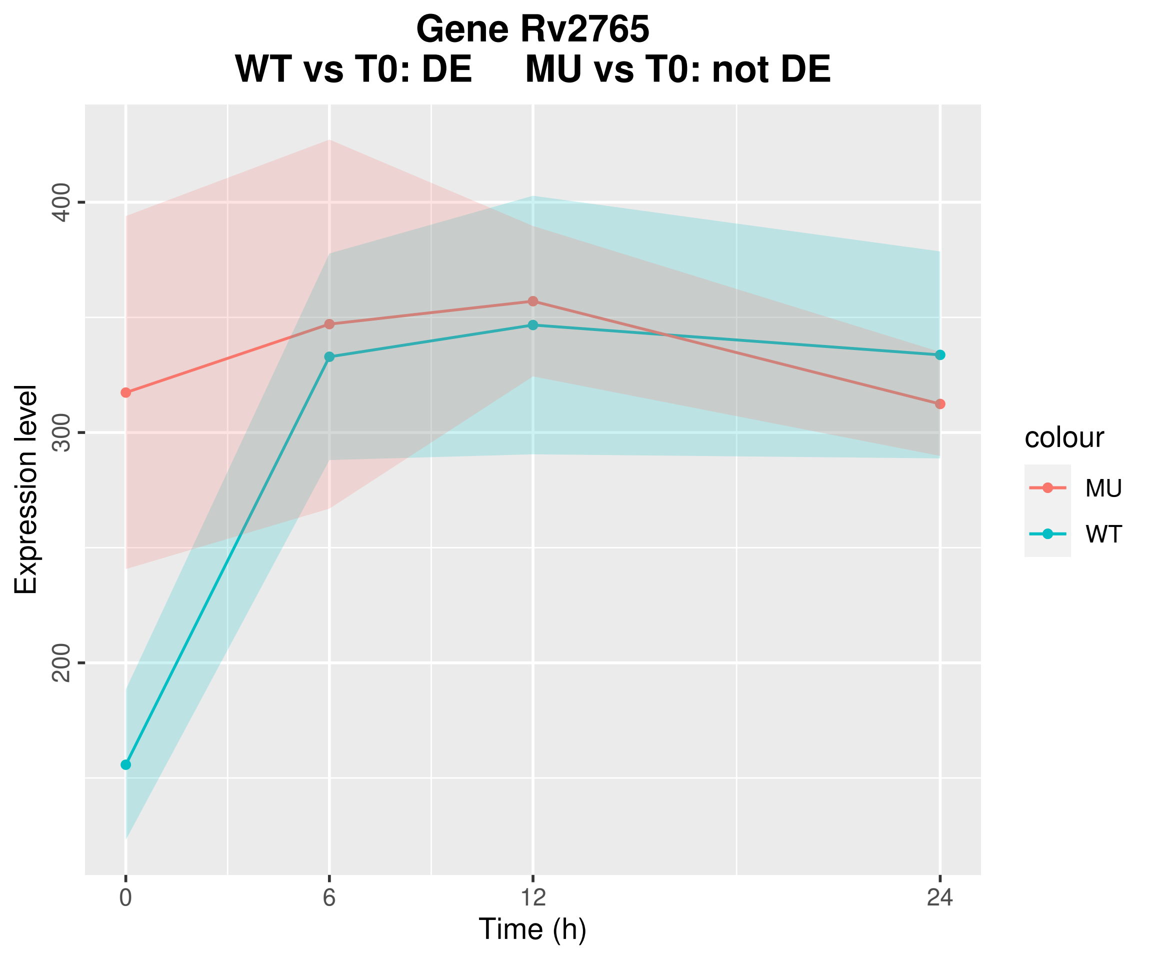

Gene Rv2765
WT vs T0: DE MU vs T0: not DE
Expression level
400
300
200
0
6
12
24
Time (h)
colour
MU
WT

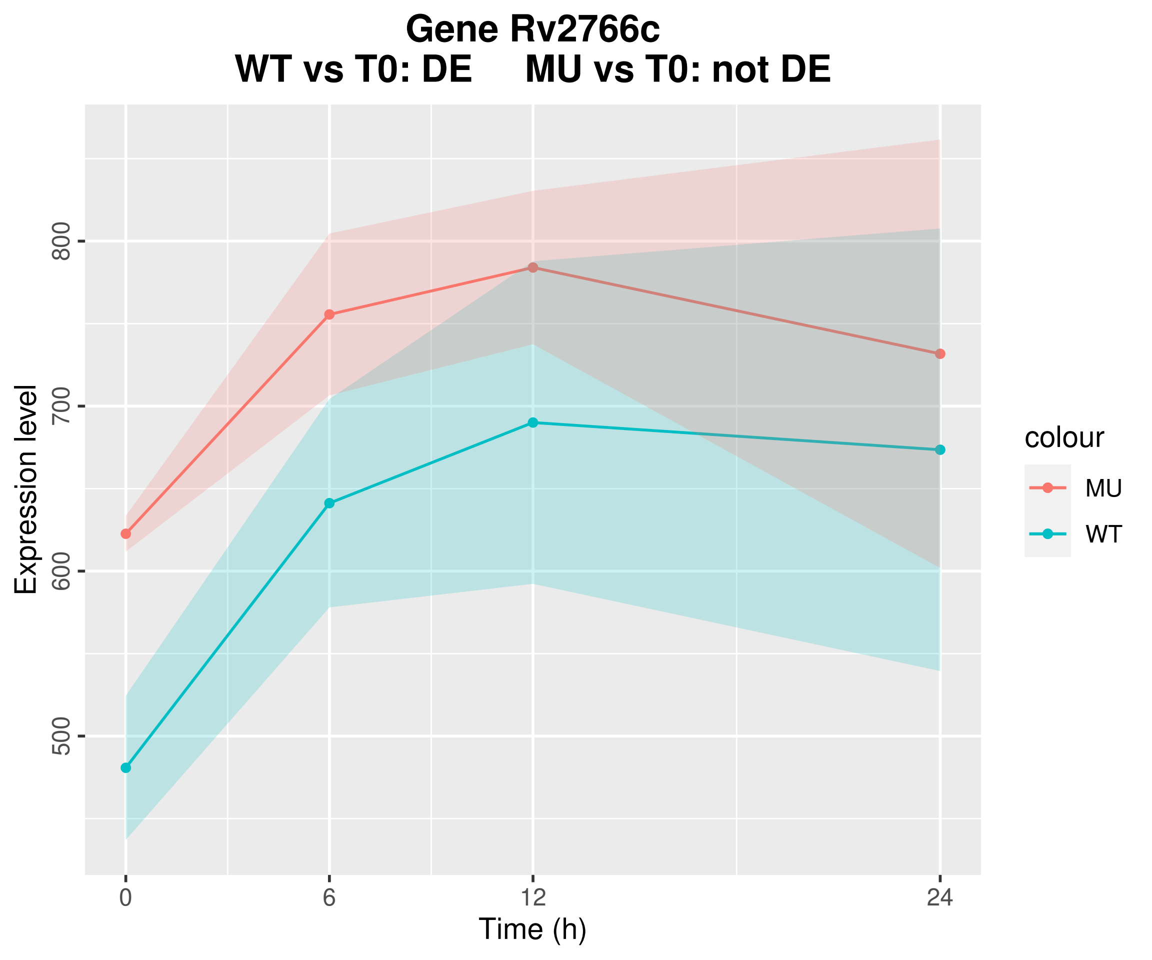

Gene Rv2766c
WT vs T0: DE   MU vs T0: not DE
Expression level
800
700
600
500
0
6
12
24
Time (h)
colour
MU
WT

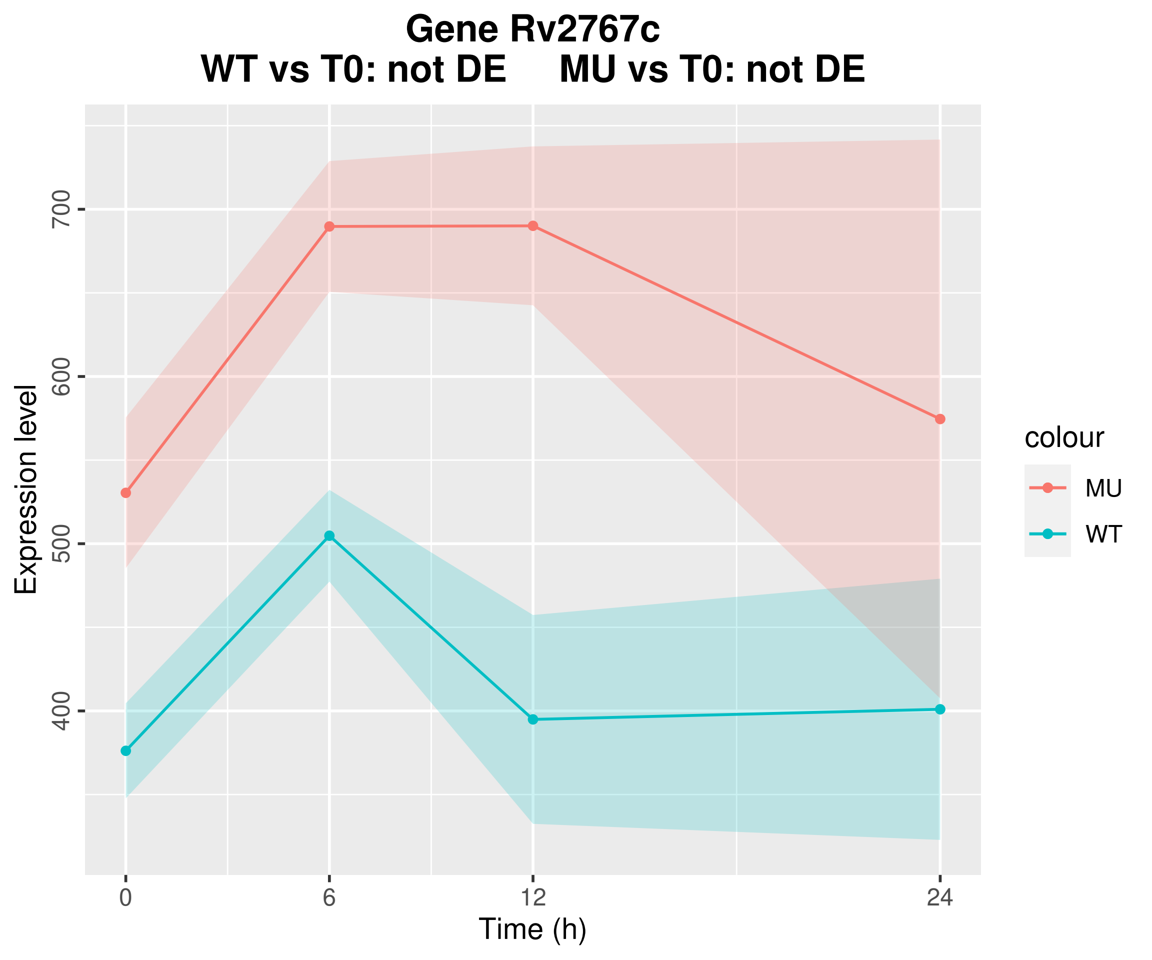

Gene Rv2767c
WT vs T0: not DE   MU vs T0: not DE
Expression level
400
500
600
700
0
6
12
24
Time (h)
colour
MU
WT

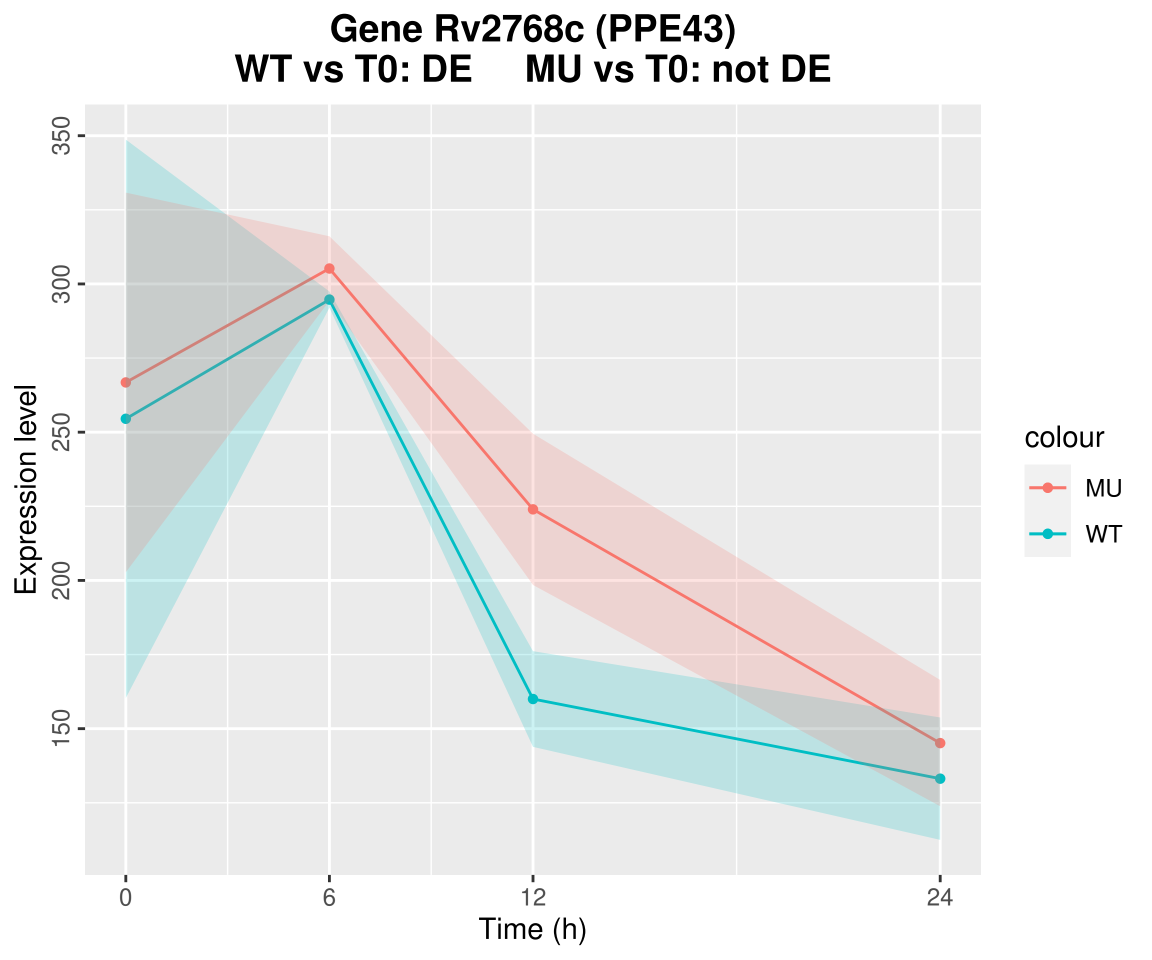
Gene Rv2768c (PPE43)
WT vs T0: DE   MU vs T0: not DE
Expression level
350
300
250
200
150
Time (h)
0
6
12
24
colour
MU
WT

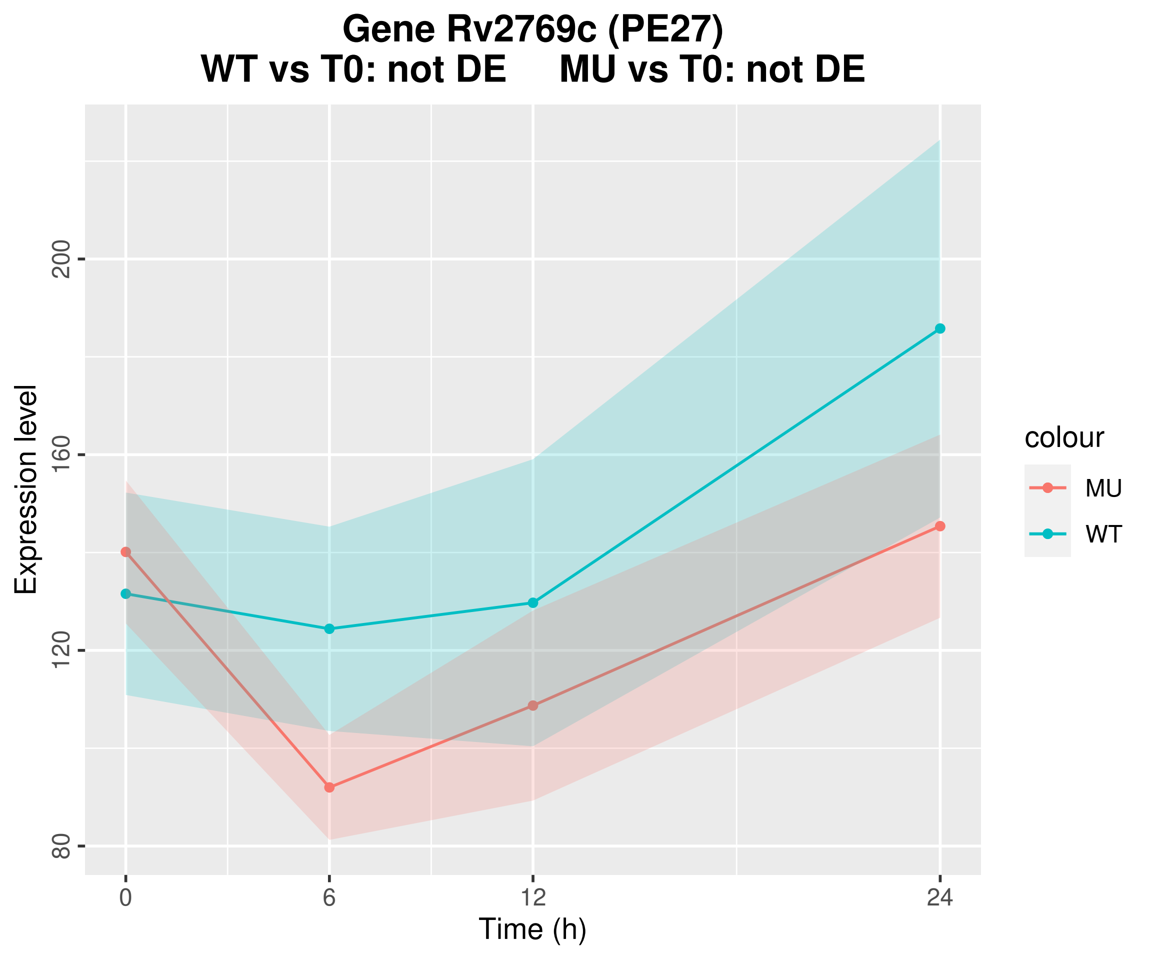
Gene Rv2769c (PE27)
WT vs T0: not DE   MU vs T0: not DE
Expression level
200
160
120
80
0
6
12
24
Time (h)
colour
MU
WT

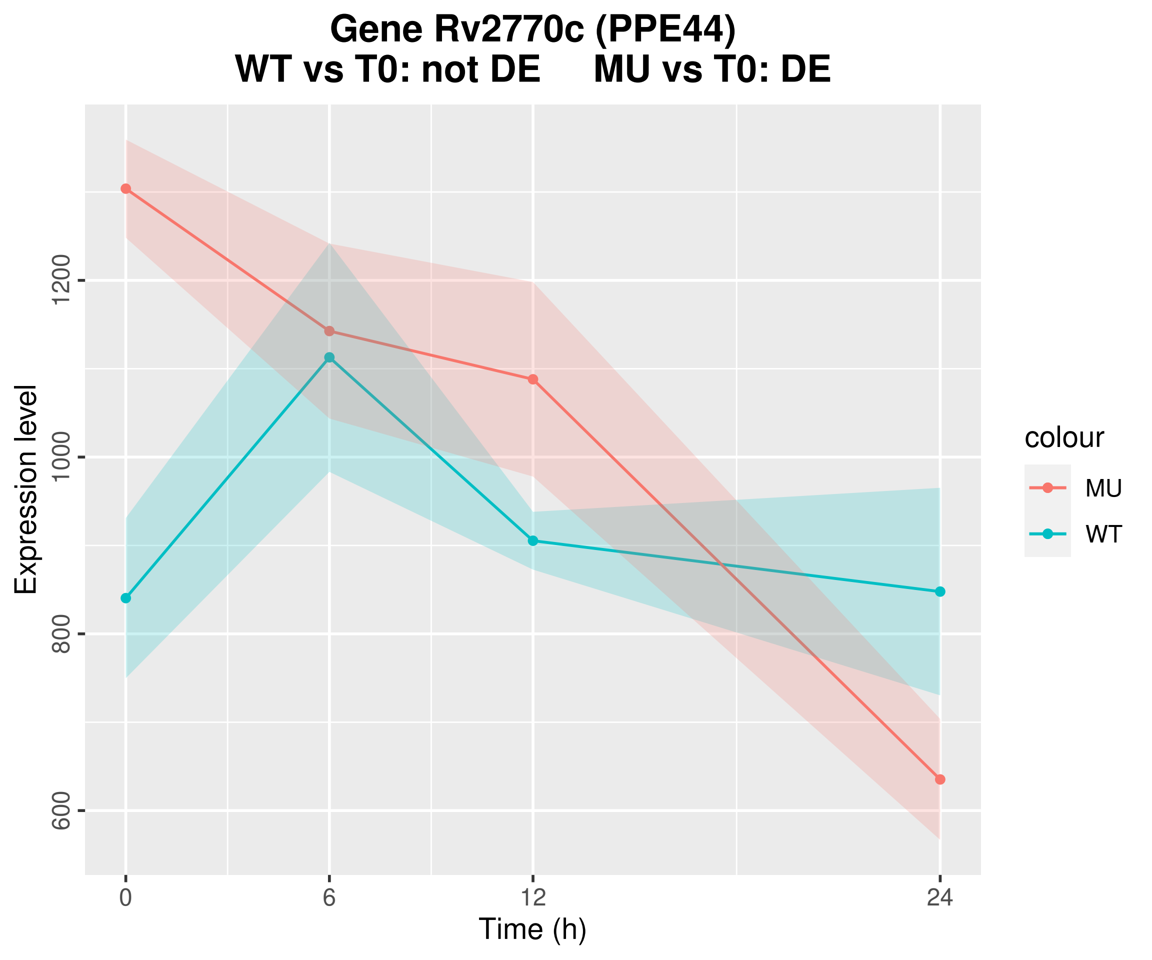

Gene Rv2770c (PPE44)
WT vs T0: not DE   MU vs T0: DE
Expression level
1200
1000
800
600
0
6
12
24
Time (h)
colour
MU
WT

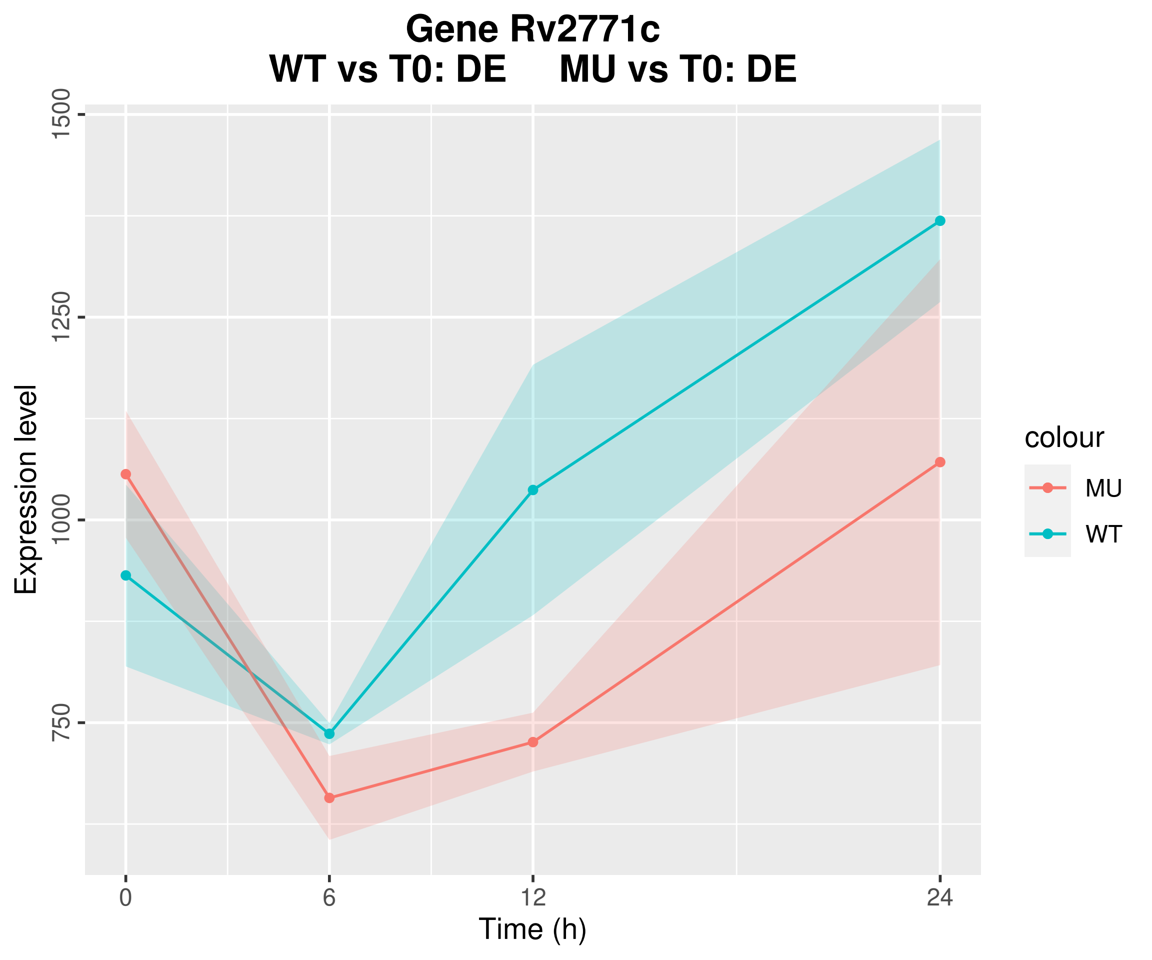
Gene Rv2771c
WT vs T0: DE   MU vs T0: DE
Expression level
1500
1250
1000
750
0
6
12
24
Time (h)
colour
MU
WT

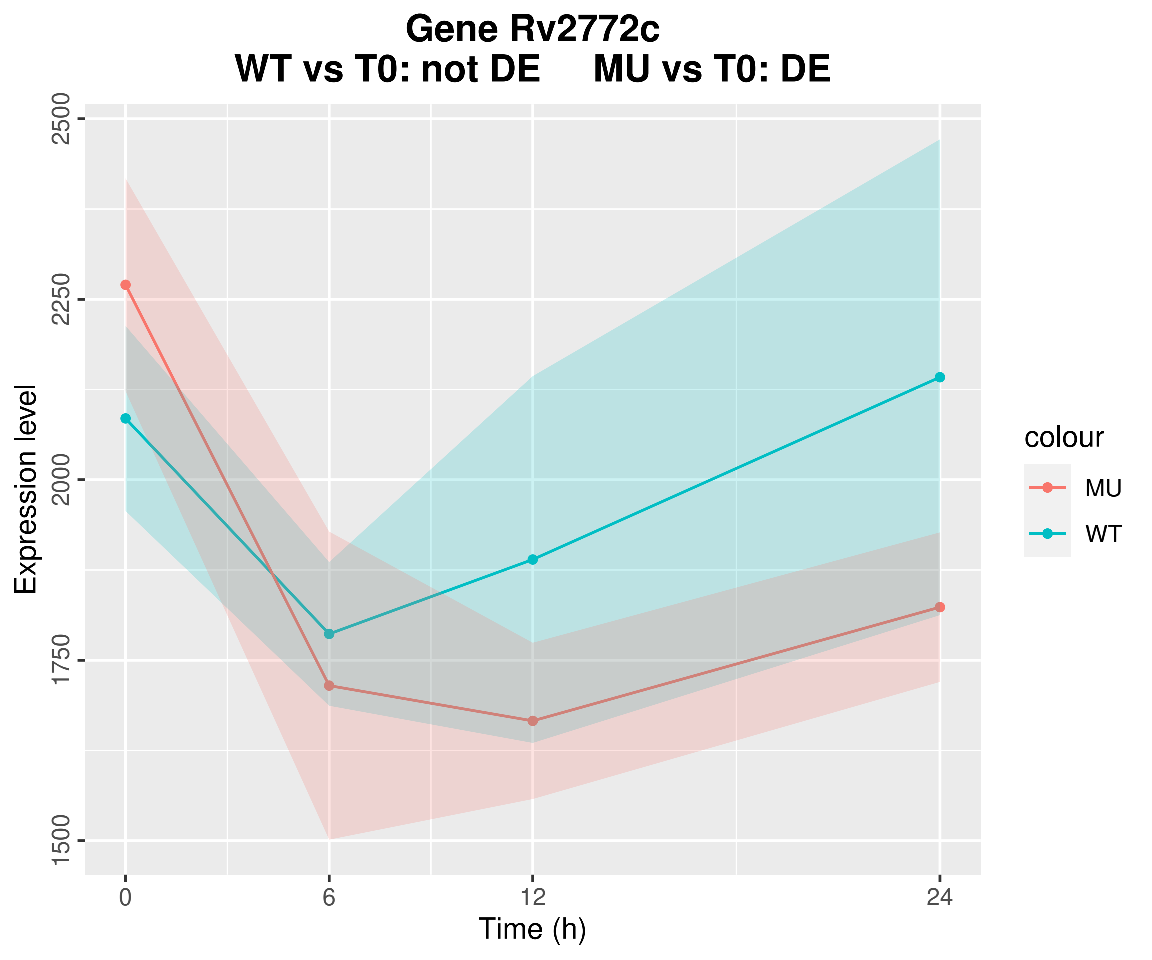
Gene Rv2772c
WT vs T0: not DE MU vs T0: DE
Expression level
2500
2250
2000
1750
1500
0
6
12
24
Time (h)
colour
MU
WT

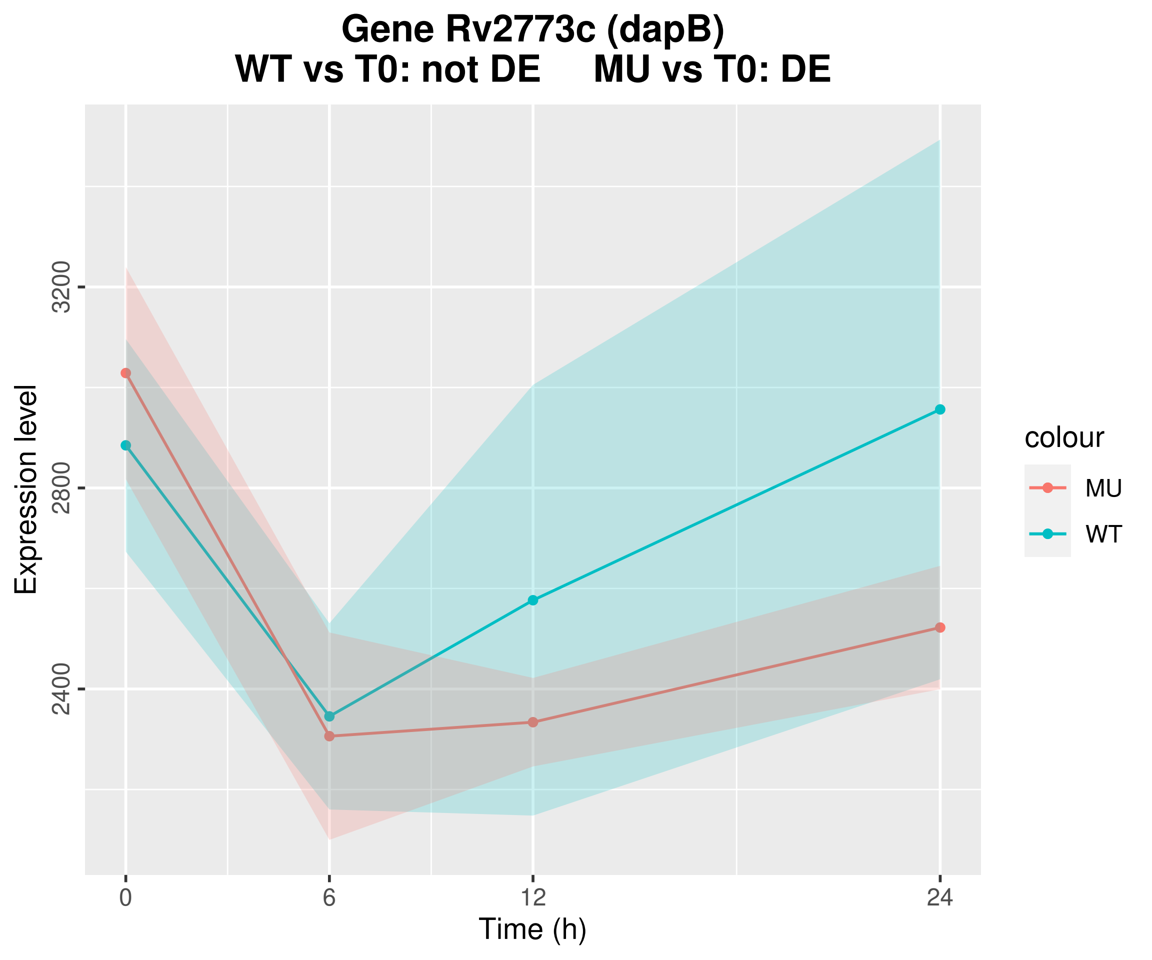

Gene Rv2773c (dapB)
WT vs T0: not DE   MU vs T0: DE
Expression level
3200
2800
2400
0
6
12
24
Time (h)
colour
MU
WT

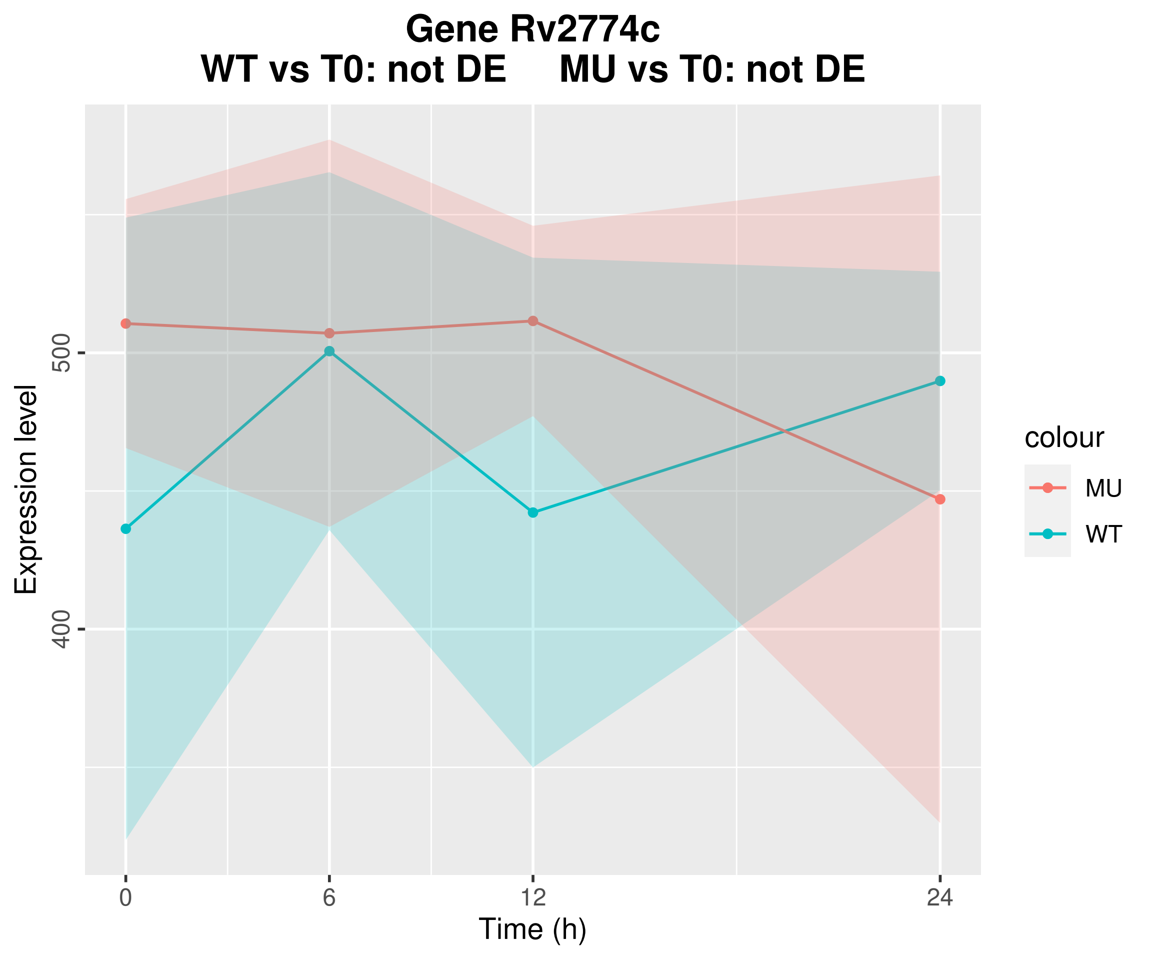
Gene Rv2774c
WT vs T0: not DE MU vs T0: not DE
Expression level
500
400
0
6
12
24
Time (h)
colour
MU
WT

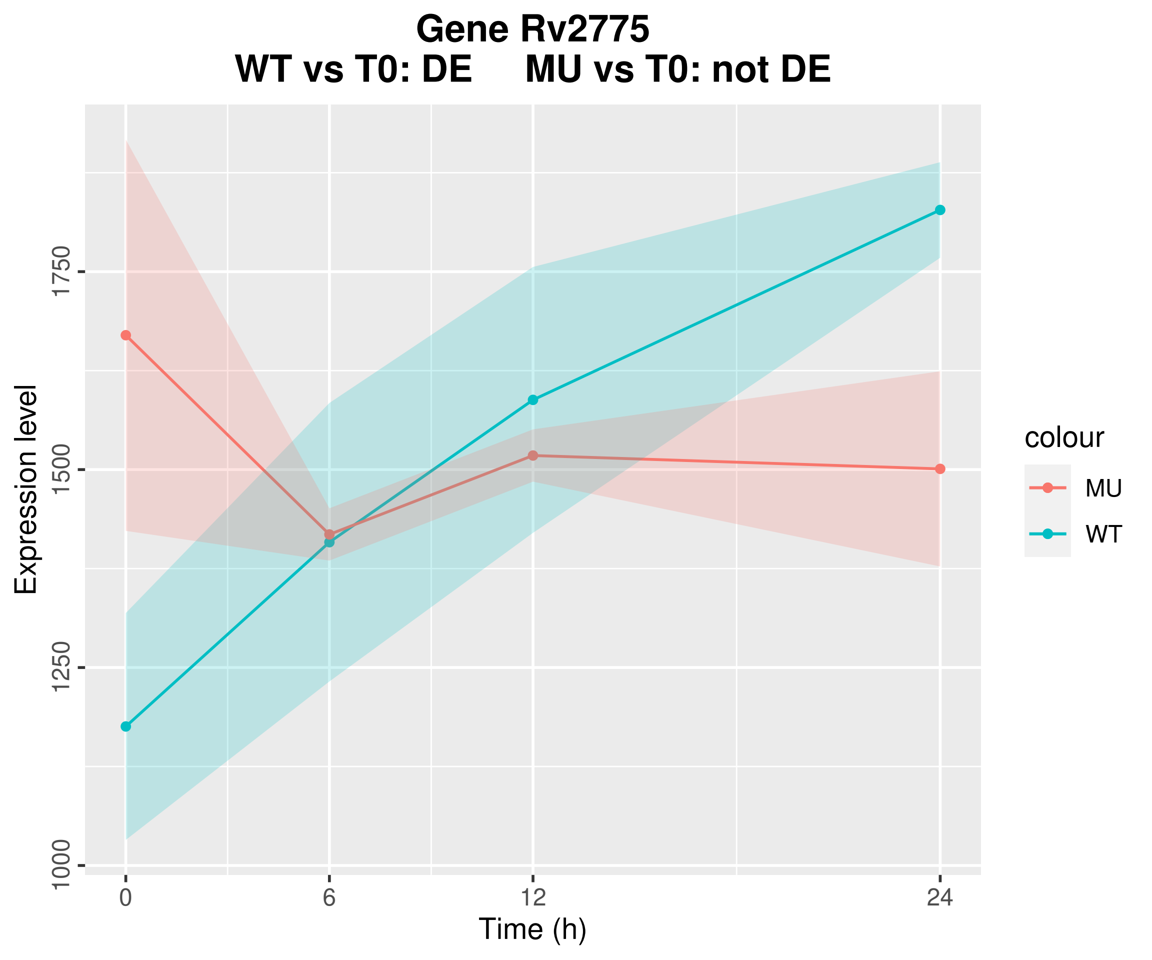
Gene Rv2775
WT vs T0: DE   MU vs T0: not DE
Expression level
1000
1250
1500
1750
0
6
12
24
Time (h)
colour
MU
WT

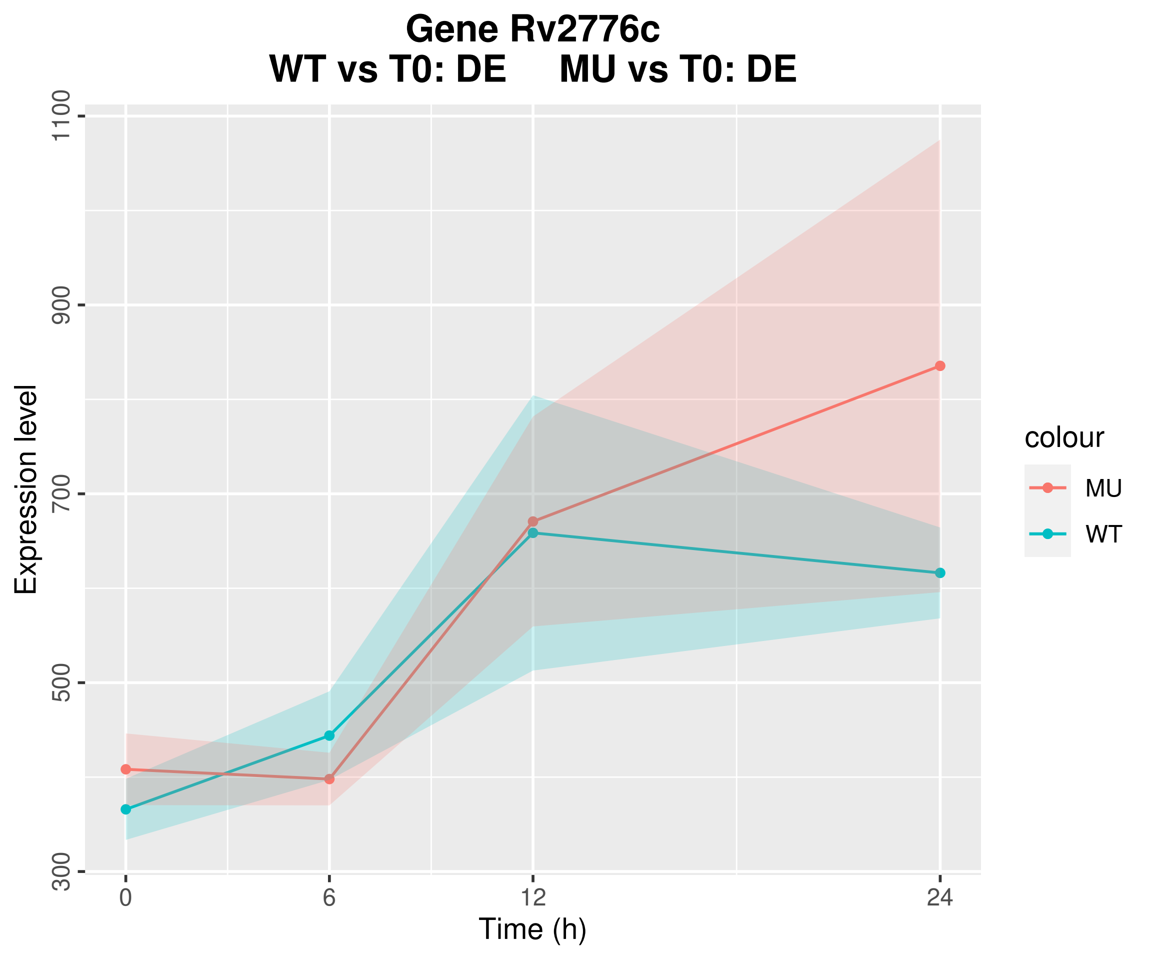

Gene Rv2776c
WT vs T0: DE   MU vs T0: DE
Expression level
1100
900
700
500
300
0
6
12
24
Time (h)
colour
MU
WT

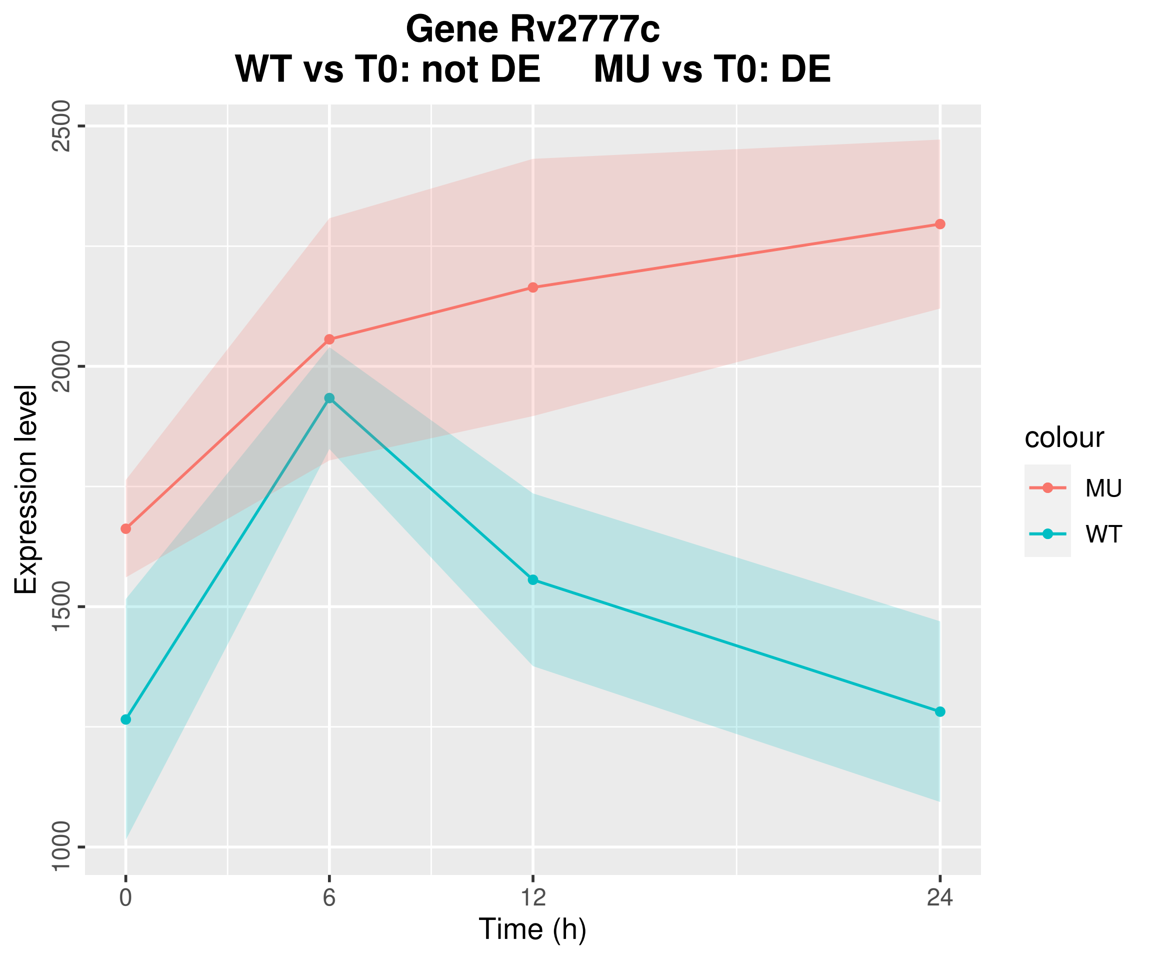

Gene Rv2777c
WT vs T0: not DE MU vs T0: DE
2500
2000
1500
1000
Expression level
0
6
12
24
Time (h)
colour
MU
WT

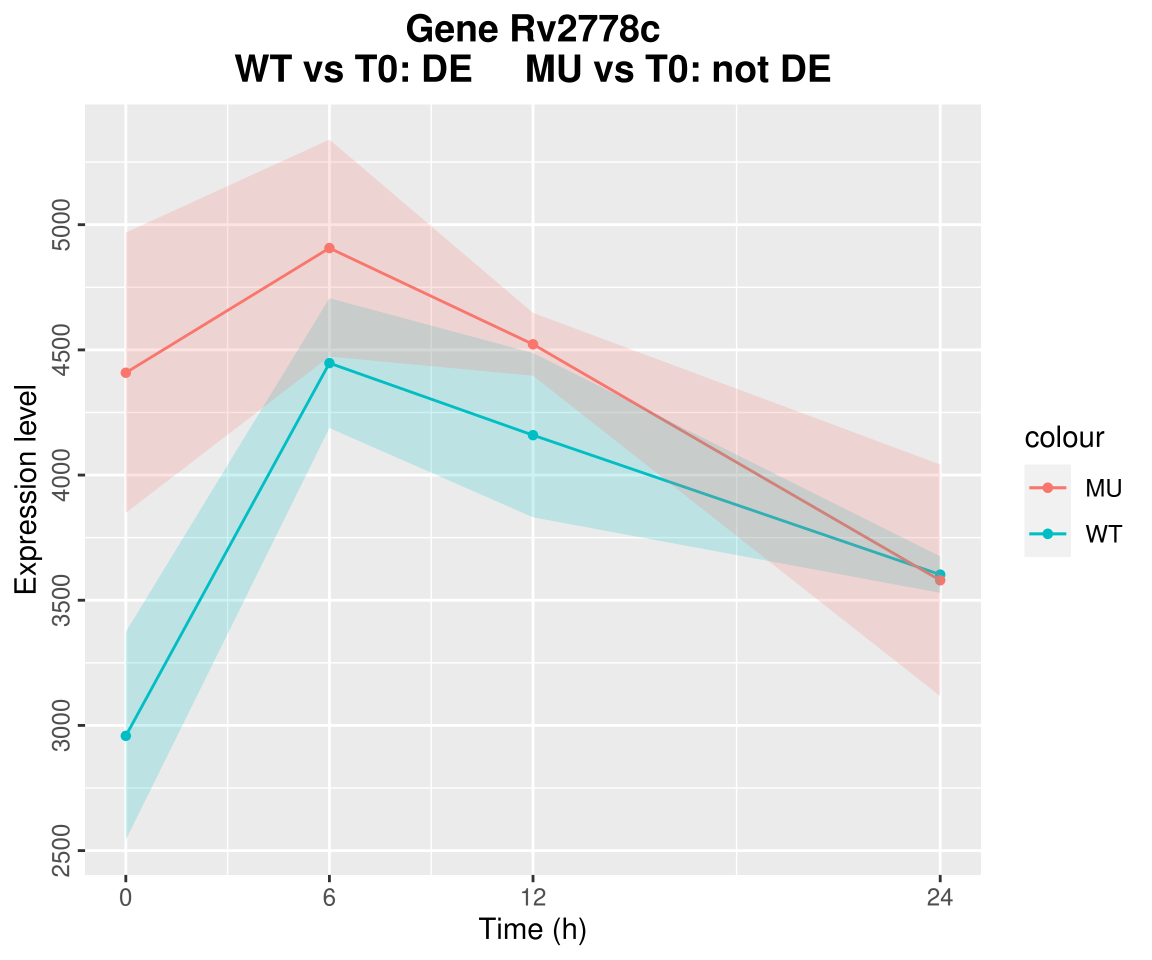
Gene Rv2778c
WT vs T0: DE   MU vs T0: not DE
Expression level
5000
4500
4000
3500
3000
2500
0
6
12
24
Time (h)
colour
MU
WT

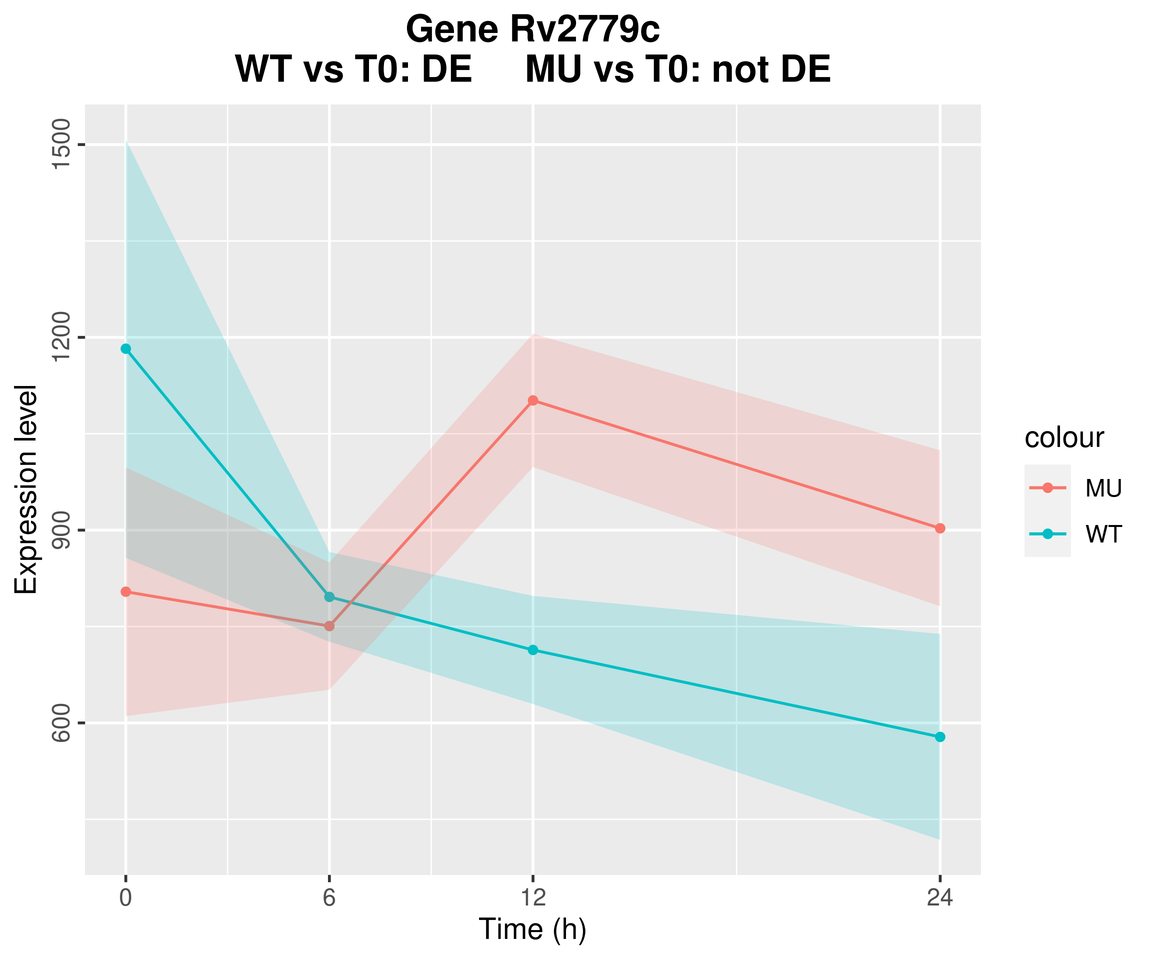
Gene Rv2779c
WT vs T0: DE   MU vs T0: not DE
Expression level
1500
1200
900
600
0
6
12
24
Time (h)
colour
MU
WT

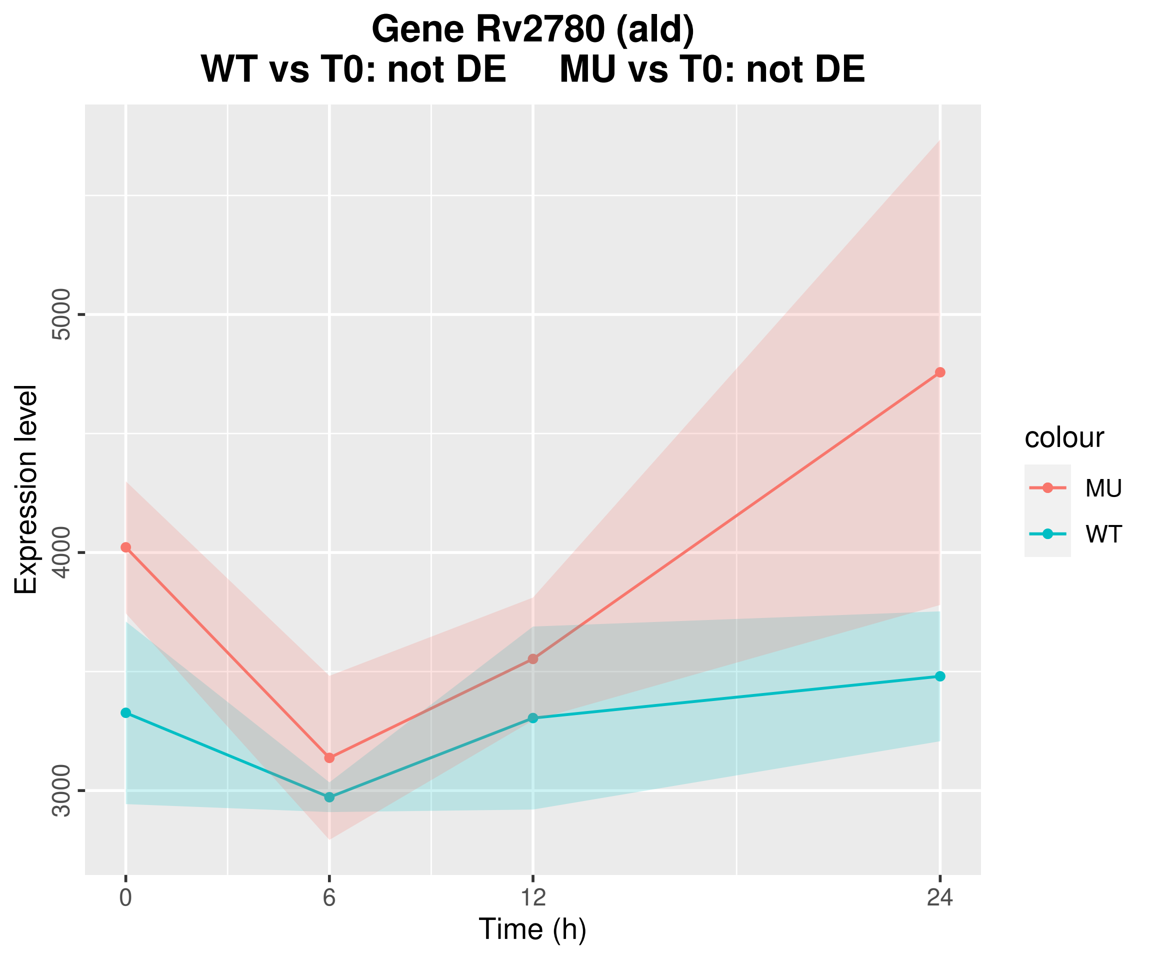
Gene Rv2780 (ald)
WT vs T0: not DE   MU vs T0: not DE
Expression level
5000
4000
3000
0
6
12
24
Time (h)
colour
MU
WT

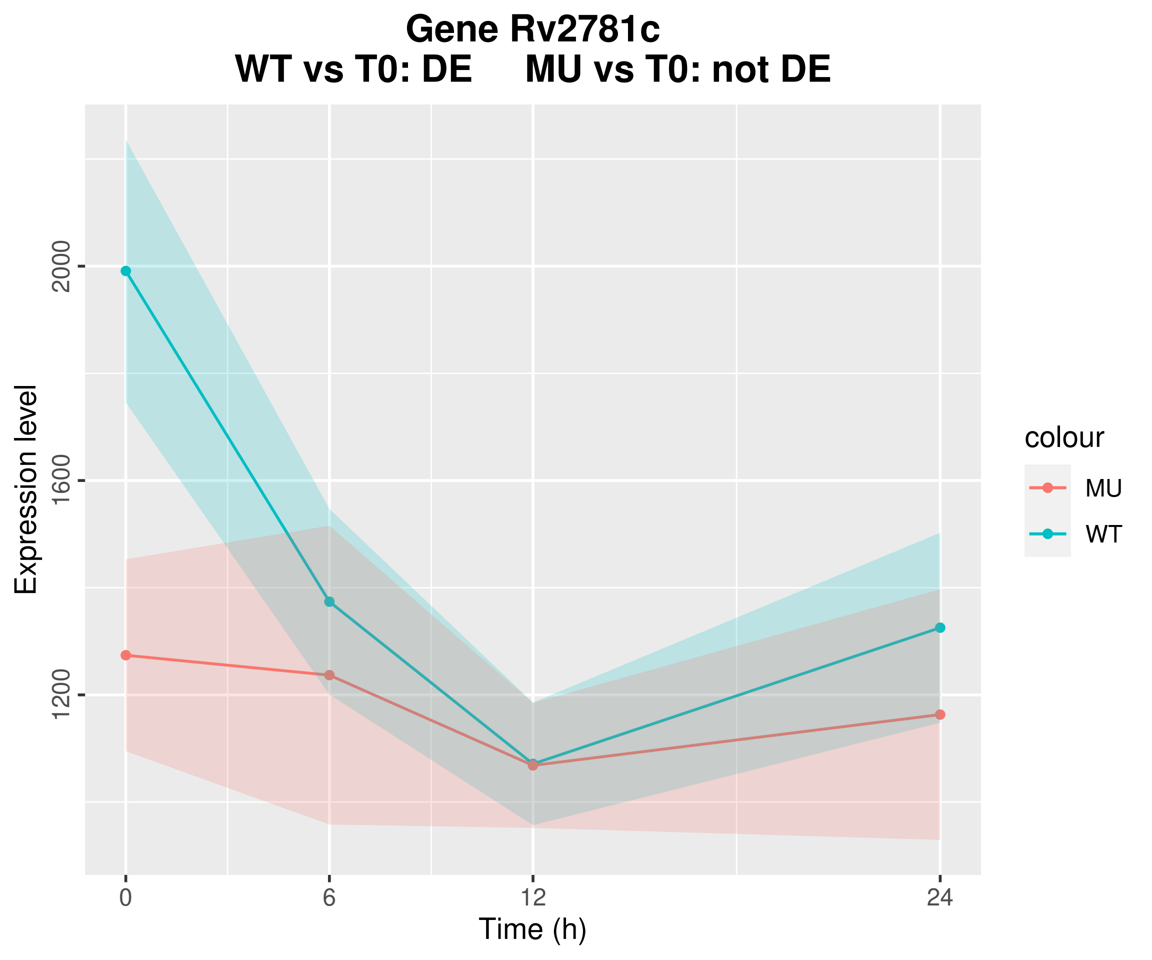

Gene Rv2781c
WT vs T0: DE MU vs T0: not DE
Expression level
2000
1600
1200
0
6
12
24
Time (h)
colour
MU
WT

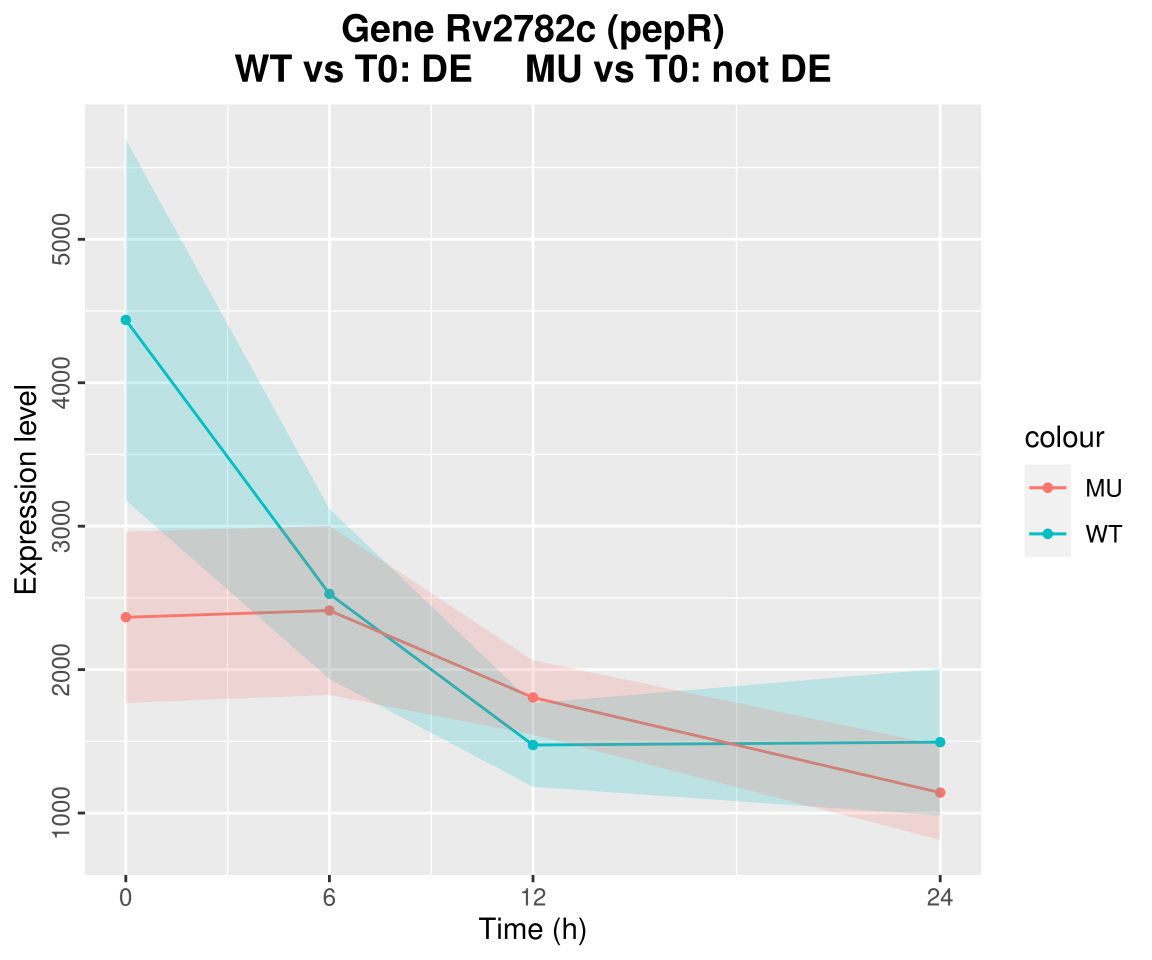
Gene Rv2782c (pepR)
WT vs T0: DE MU vs T0: not DE
Expression level
5000
4000
3000
2000
1000
0
6
12
24
Time (h)
colour
MU
WT

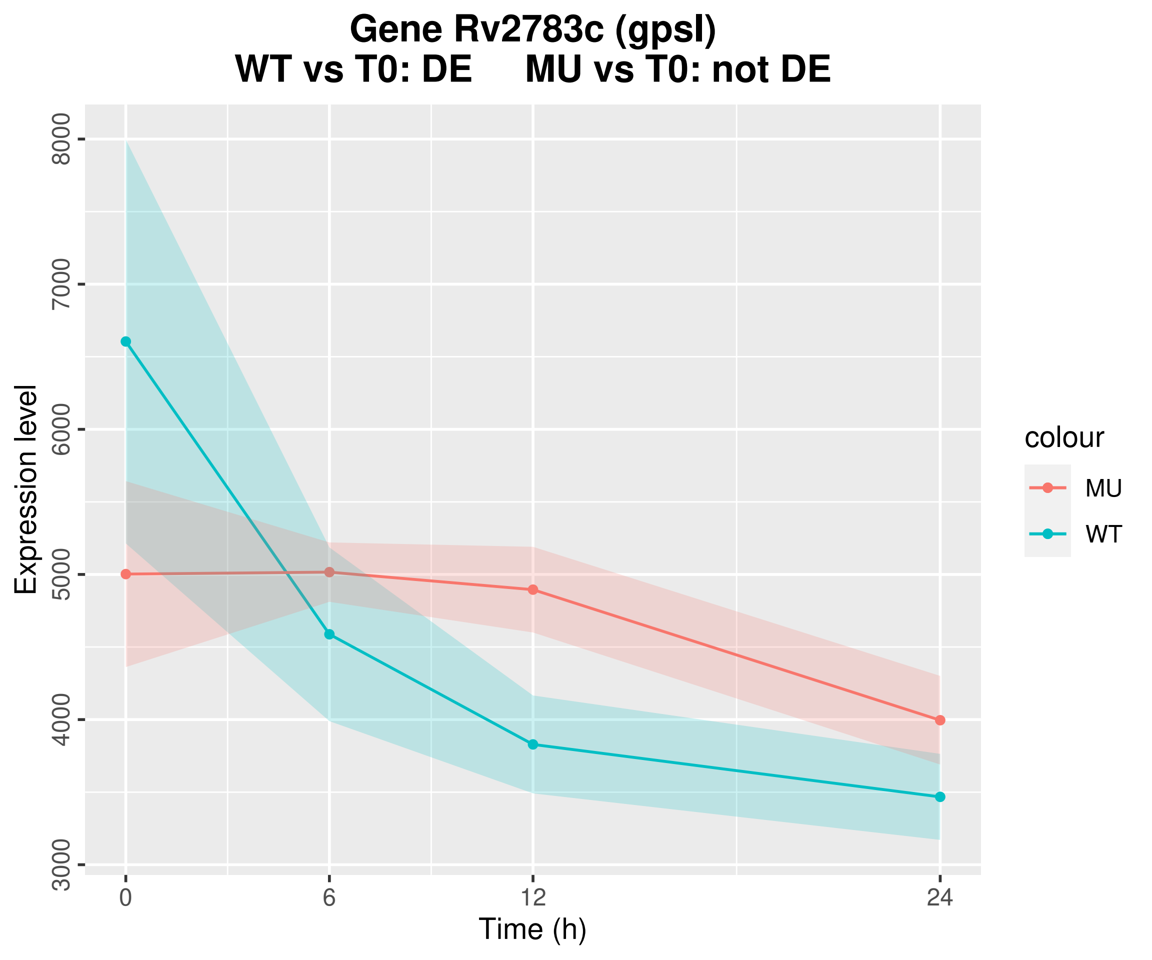

Gene Rv2783c (gpsI)
WT vs T0: DE   MU vs T0: not DE
Expression level
8000
7000
6000
5000
4000
3000
0
6
12
24
Time (h)
colour
MU
WT

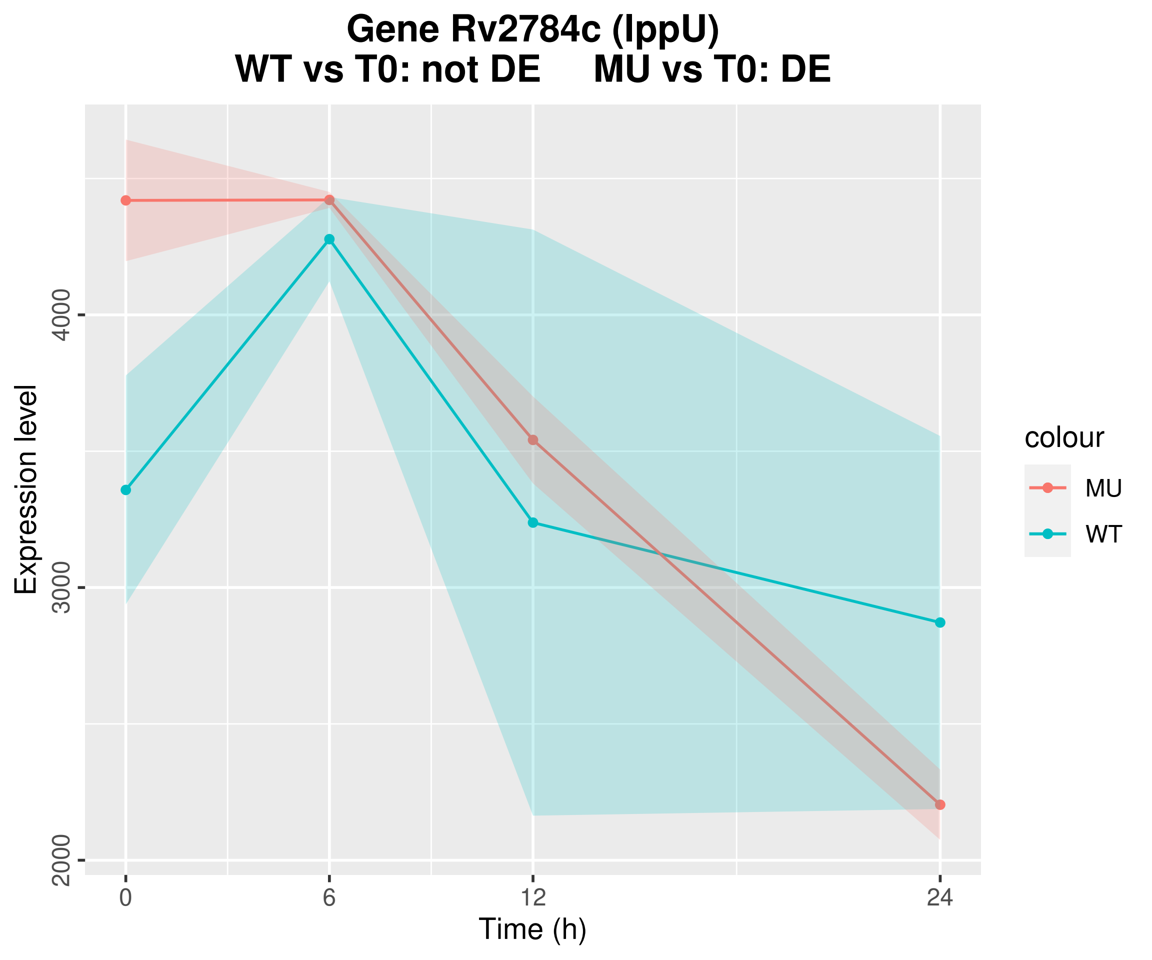
Gene Rv2784c (lppU)
WT vs T0: not DE   MU vs T0: DE
Expression level
4000
3000
2000
0
6
12
24
Time (h)
colour
MU
WT

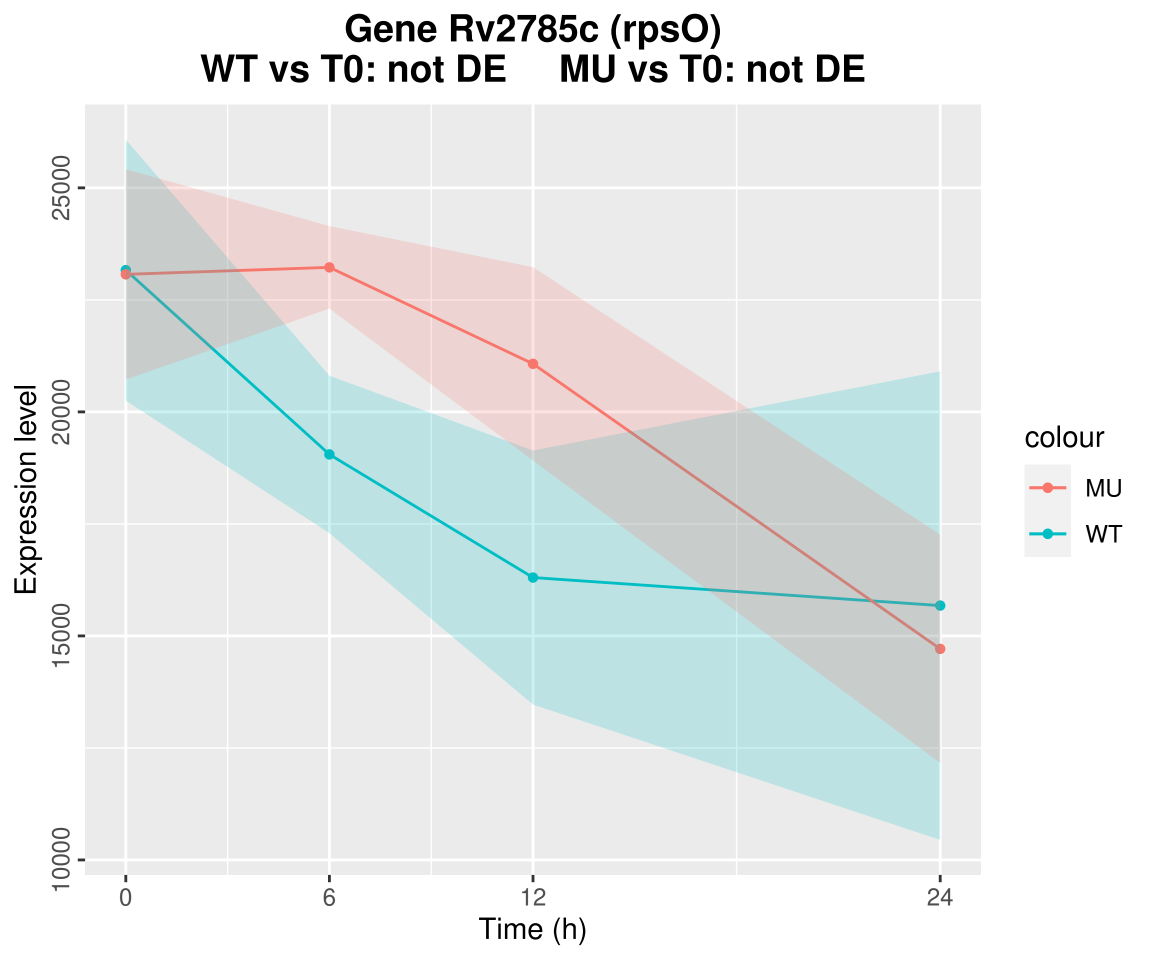

Gene Rv2785c (rpsO)
WT vs T0: not DE   MU vs T0: not DE
Expression level
25000
20000
15000
10000
0
6
12
24
Time (h)
colour
MU
WT

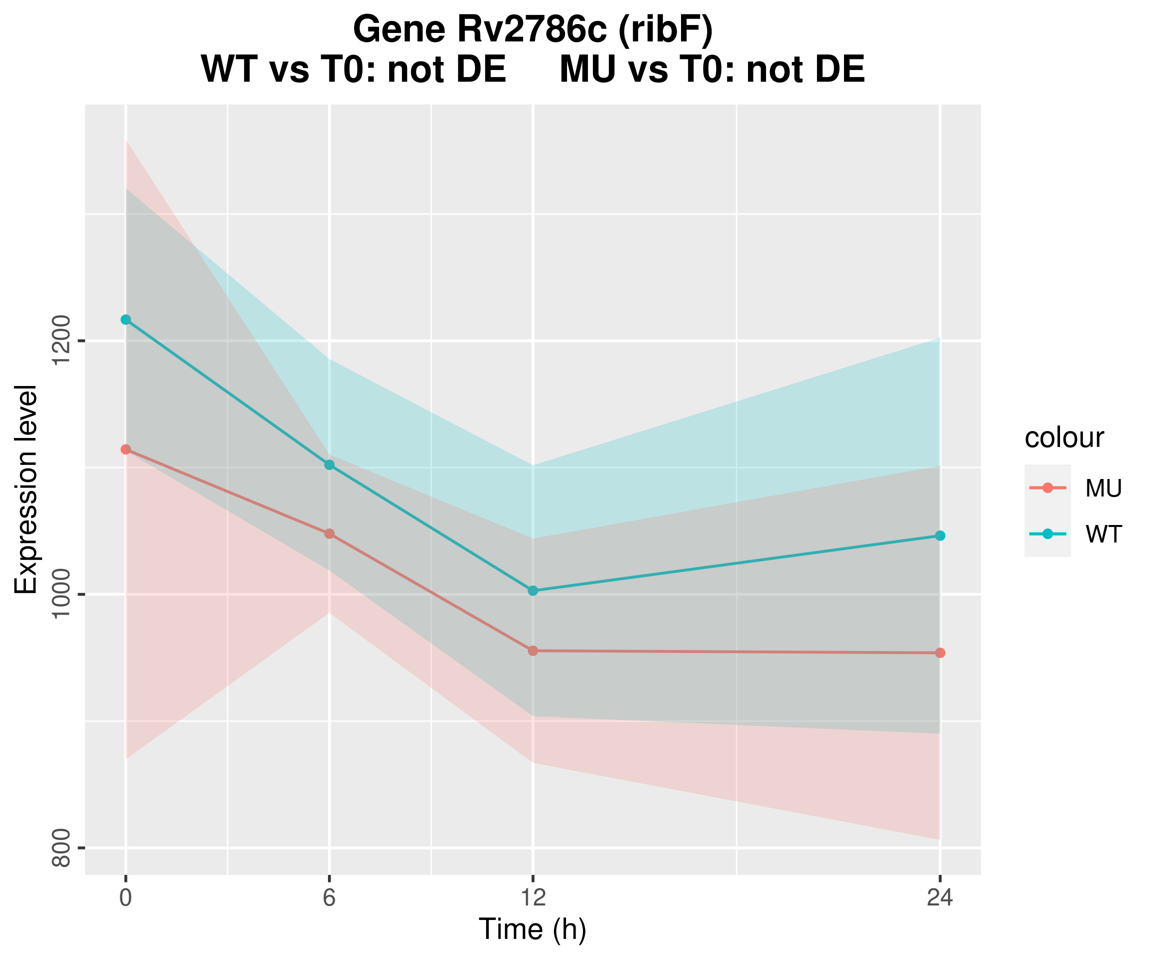

Gene Rv2786c (ribF)
WT vs T0: not DE   MU vs T0: not DE
Expression level
1200
1000
800
0
6
12
24
Time (h)
colour
MU
WT

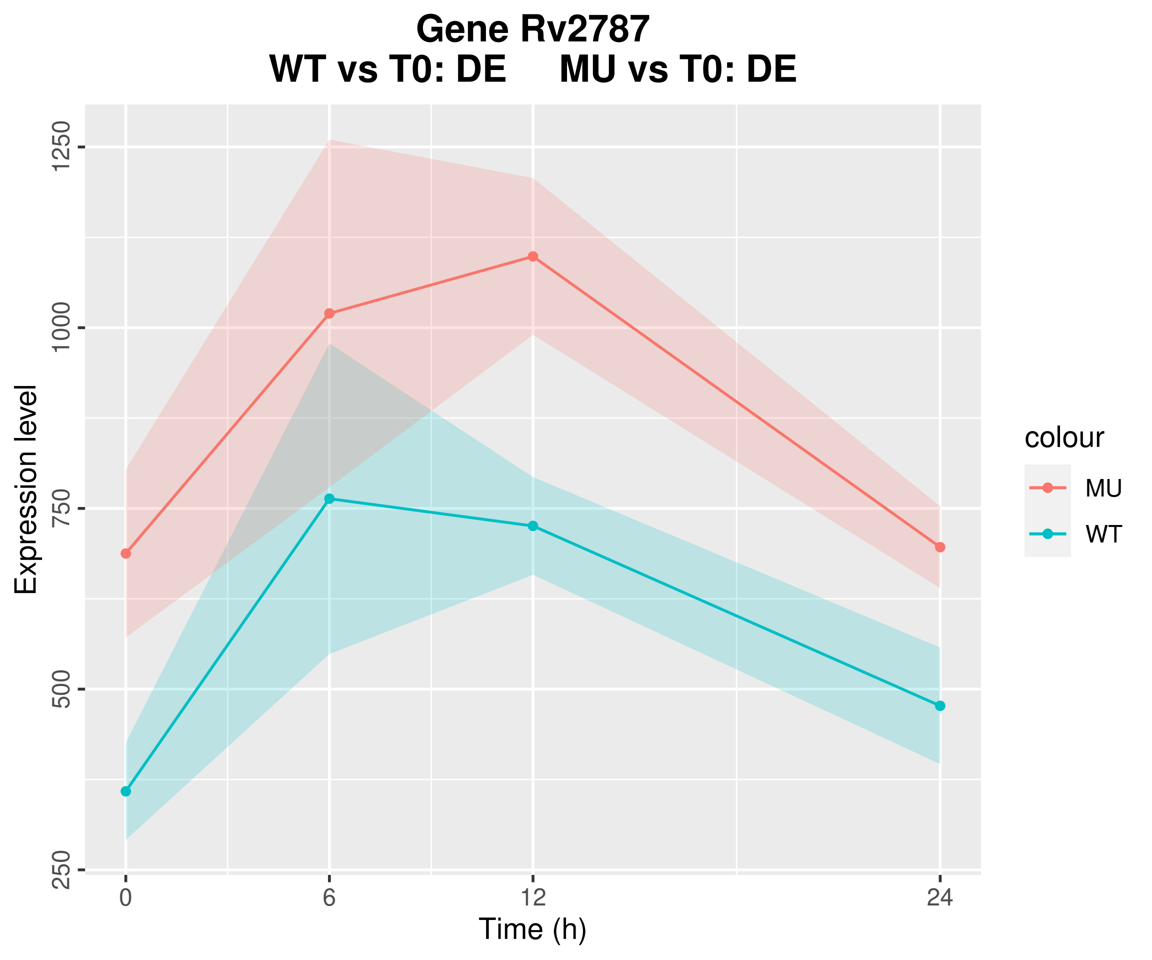
Gene Rv2787
WT vs T0: DE MU vs T0: DE
Expression level
1250
1000
750
500
250
0
6
12
24
Time (h)
colour
MU
WT

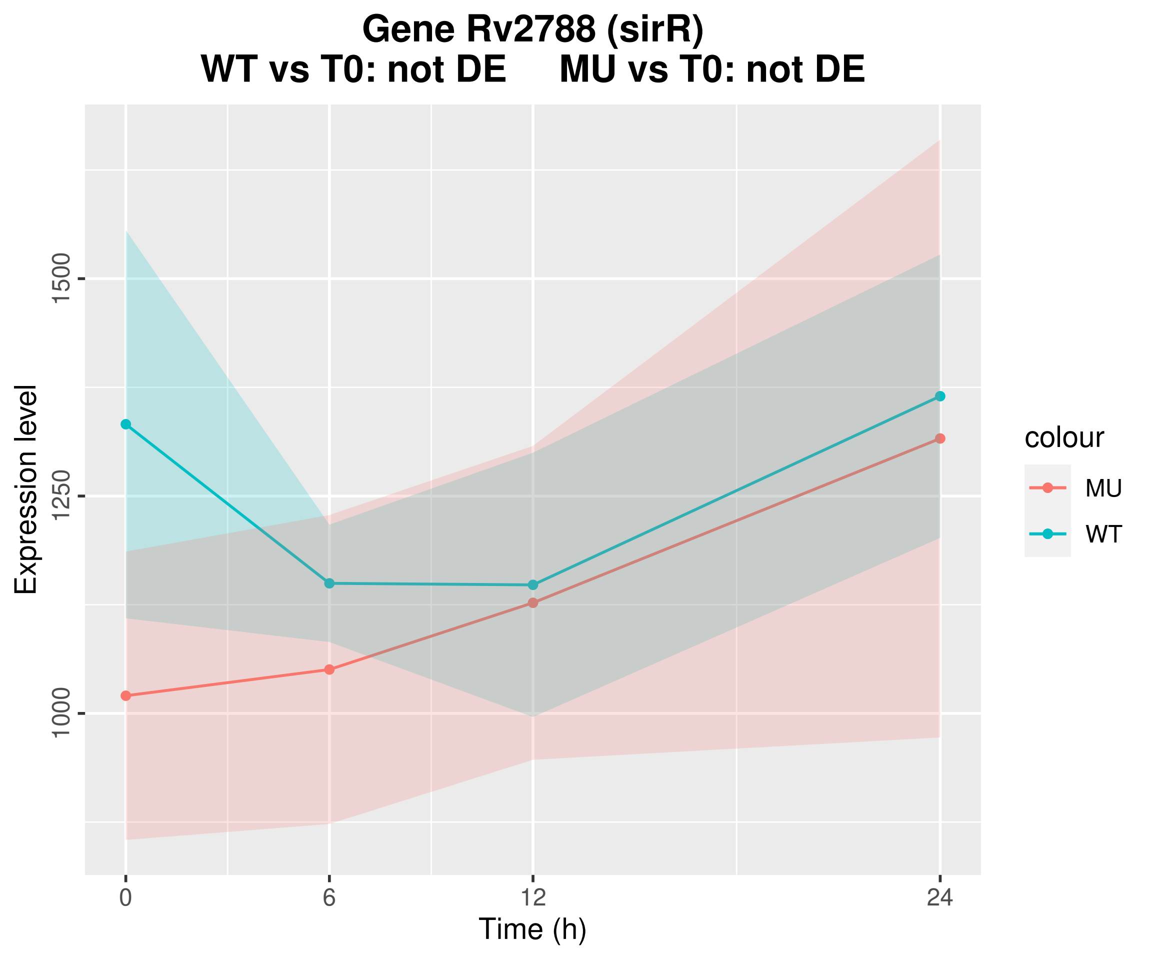
Gene Rv2788 (sirR)
WT vs T0: not DE   MU vs T0: not DE
Expression level
1500
1250
1000
0
6
12
24
Time (h)
colour
MU
WT

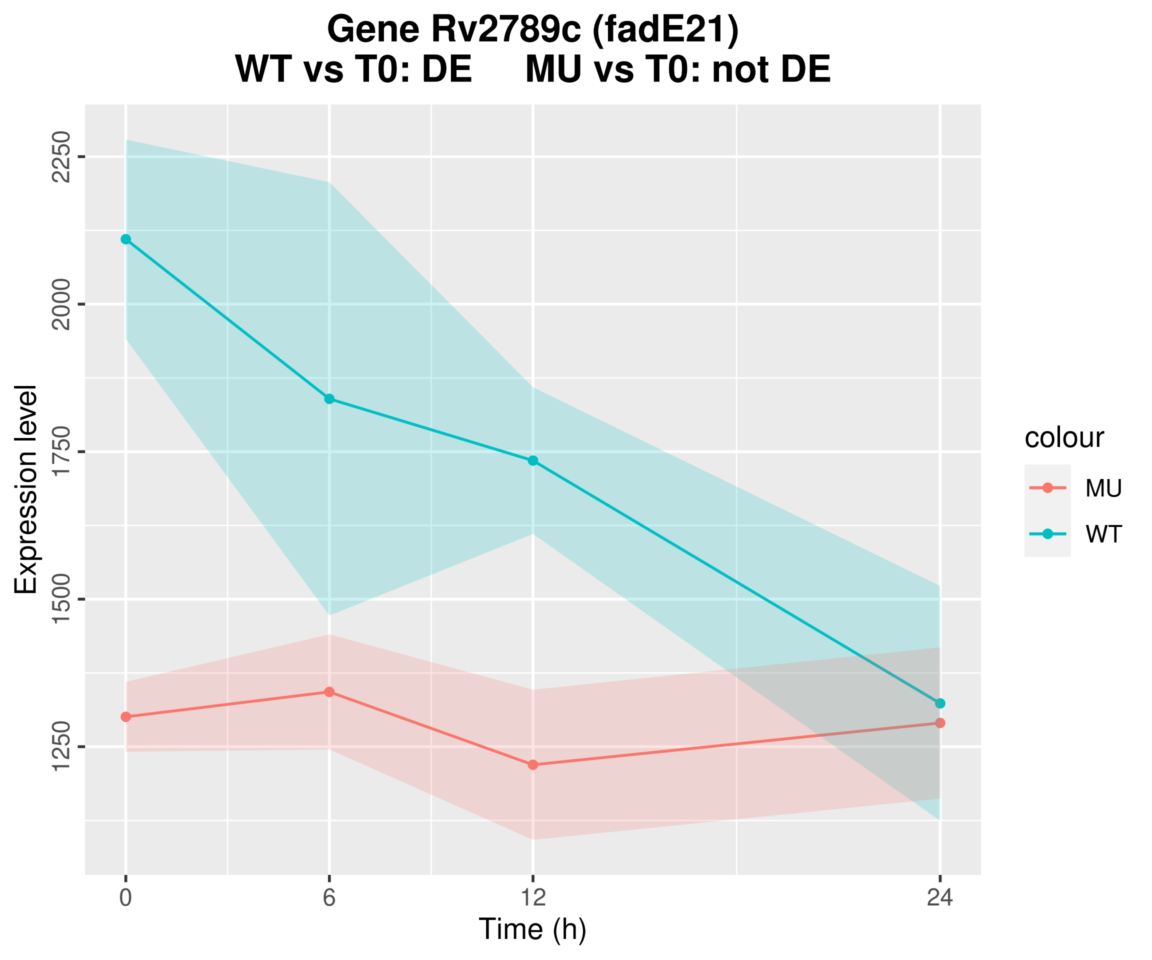
Gene Rv2789c (fadE21)
WT vs T0: DE   MU vs T0: not DE
Expression level
2250
2000
1750
1500
1250
0
6
12
24
Time (h)
colour
MU
WT

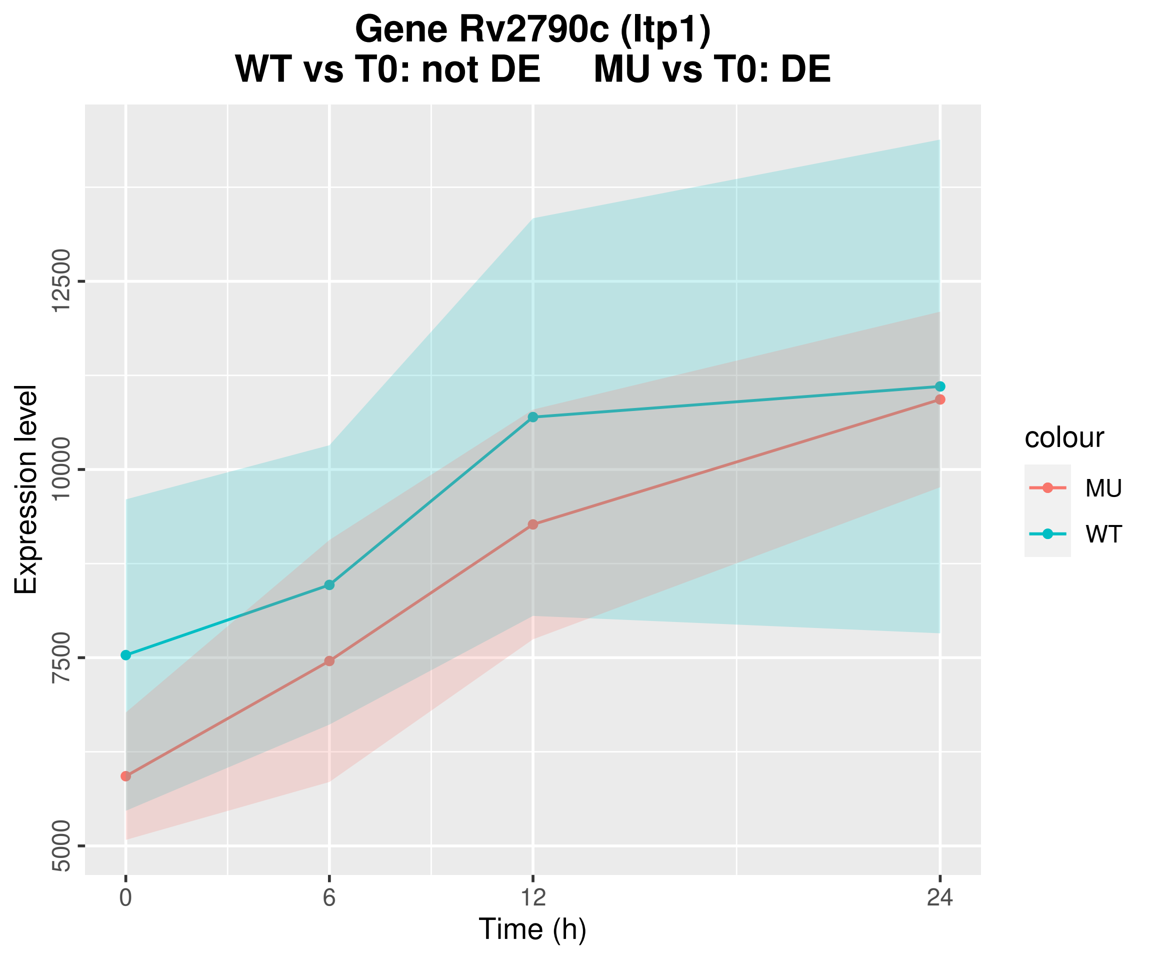

Gene Rv2790c (ltp1)
WT vs T0: not DE   MU vs T0: DE
Expression level
12500
10000
7500
5000
0
6
12
24
Time (h)
colour
MU
WT

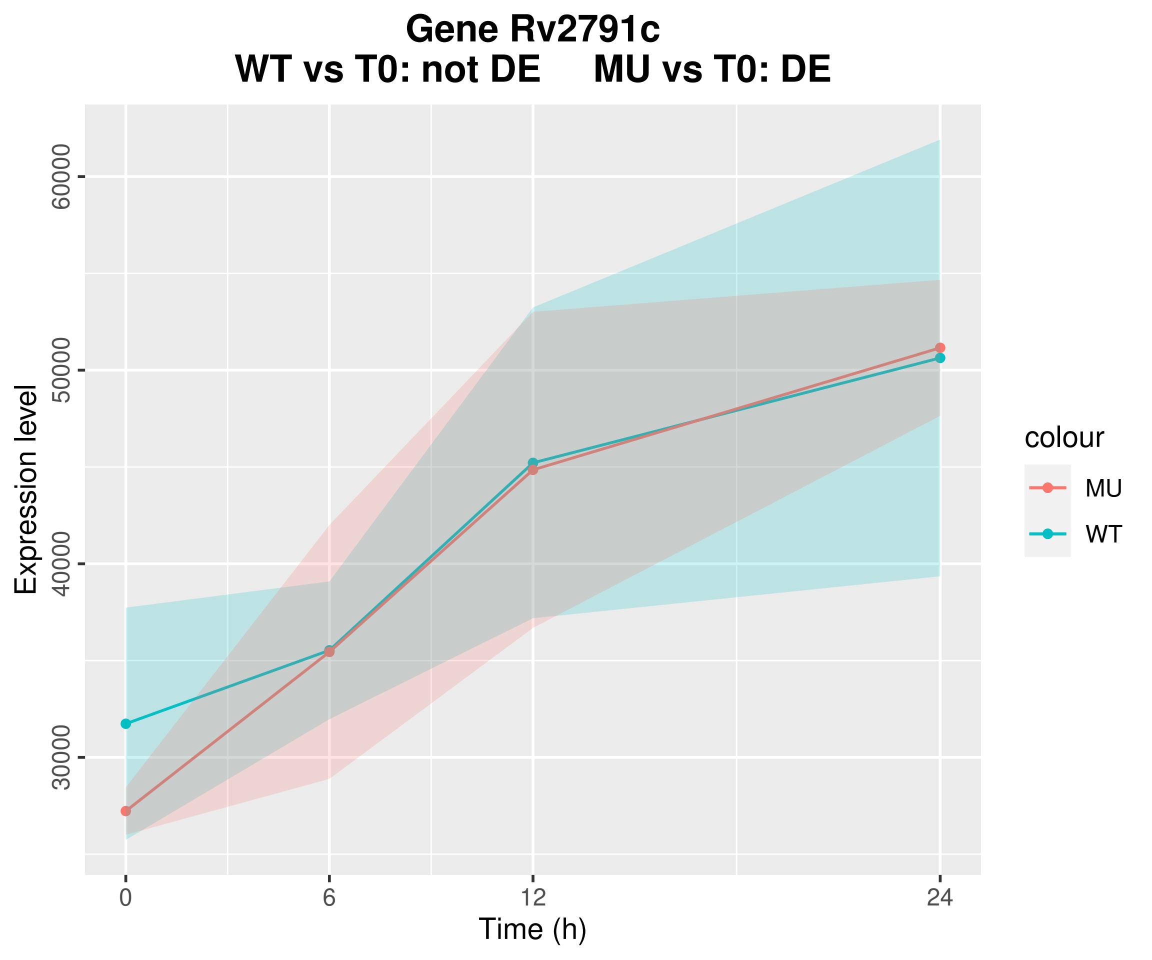

Gene Rv2791c
WT vs T0: not DE MU vs T0: DE
Expression level
60000
50000
40000
30000
0
6
12
24
Time (h)
colour
MU
WT

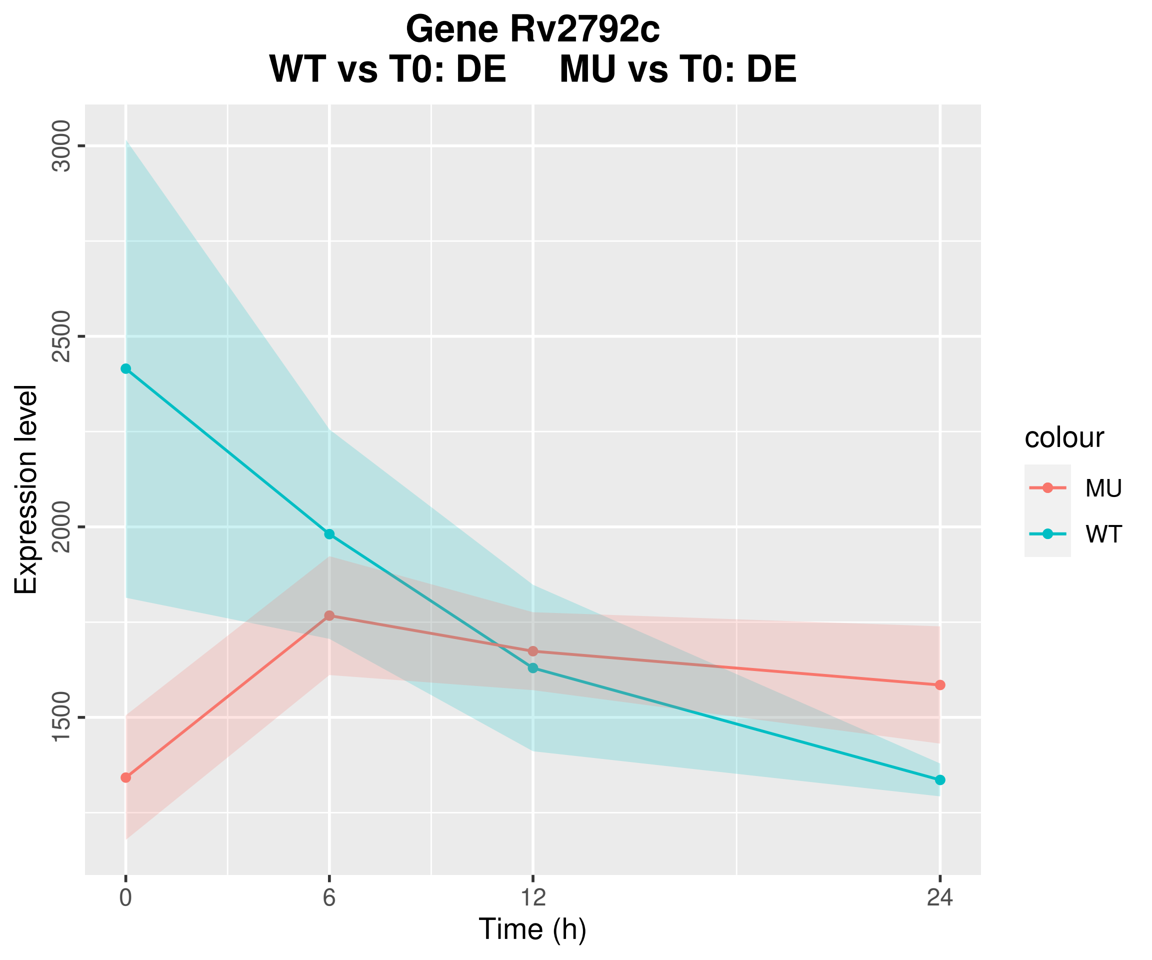
Gene Rv2792c
WT vs T0: DE MU vs T0: DE
Expression level
3000
2500
2000
1500
0
6
12
24
Time (h)
colour
MU
WT

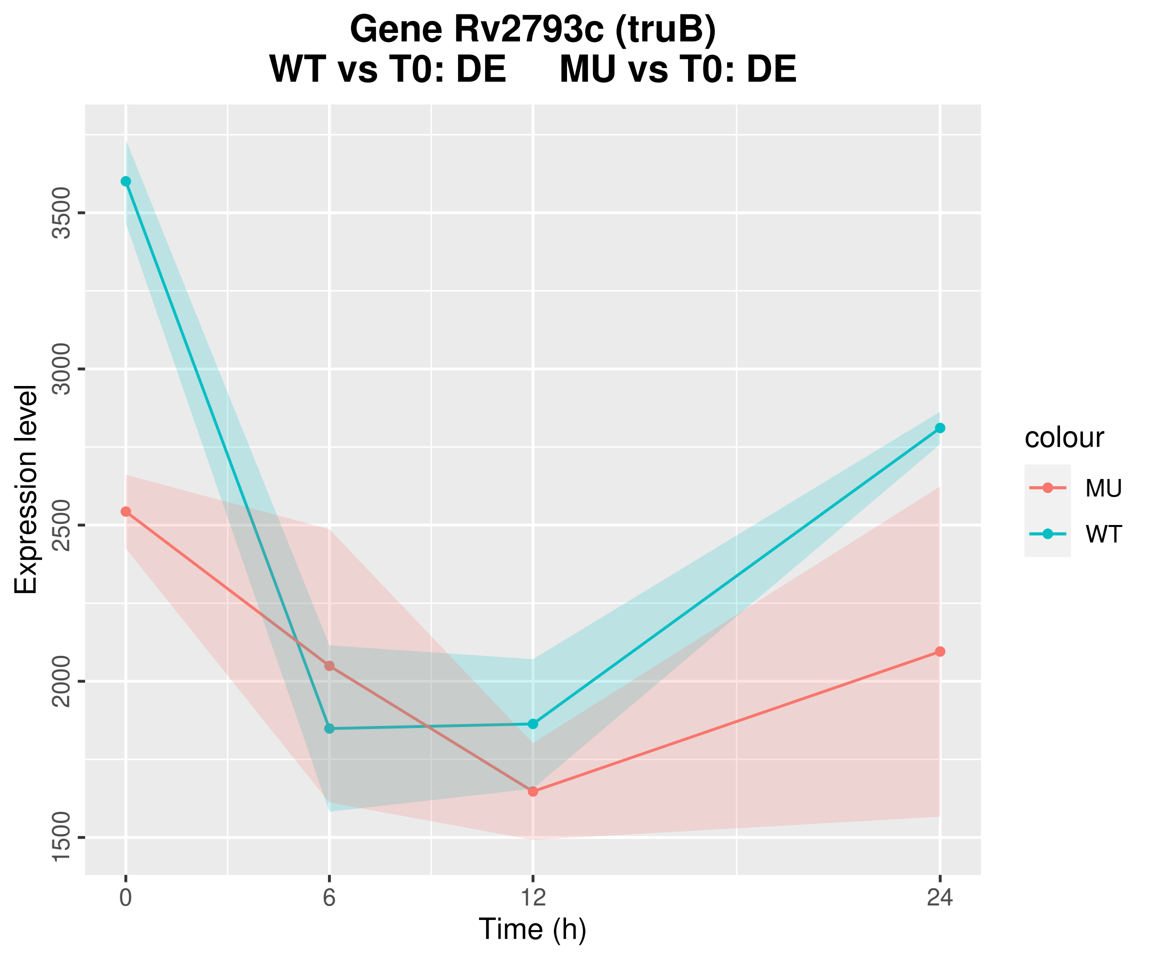
Gene Rv2793c (truB)
WT vs T0: DE MU vs T0: DE
Expression level
3500
3000
2500
2000
1500
0
6
12
24
Time (h)
colour
MU
WT

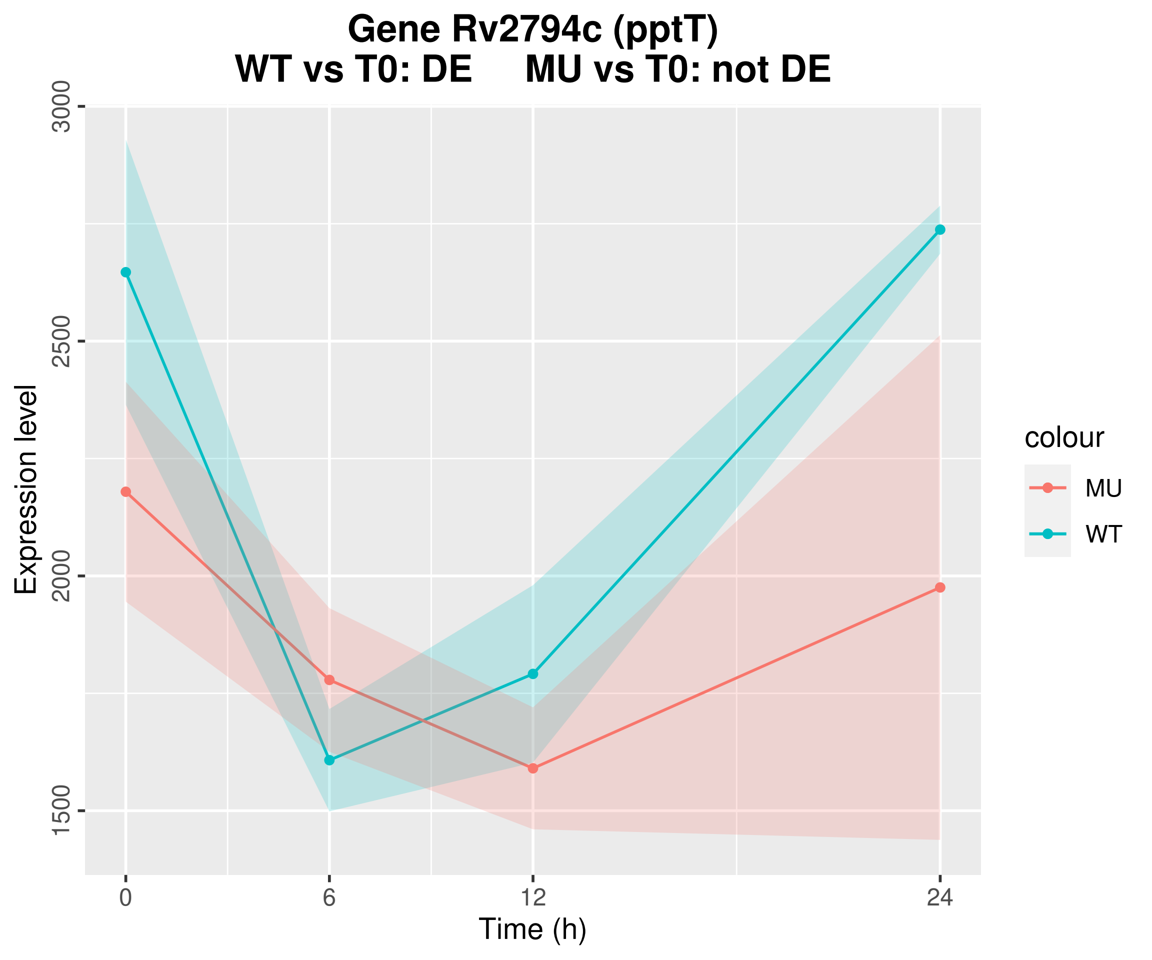
Gene Rv2794c (pptT)
WT vs T0: DE   MU vs T0: not DE
Expression level
3000
2500
2000
1500
0
6
12
24
Time (h)
colour
MU
WT

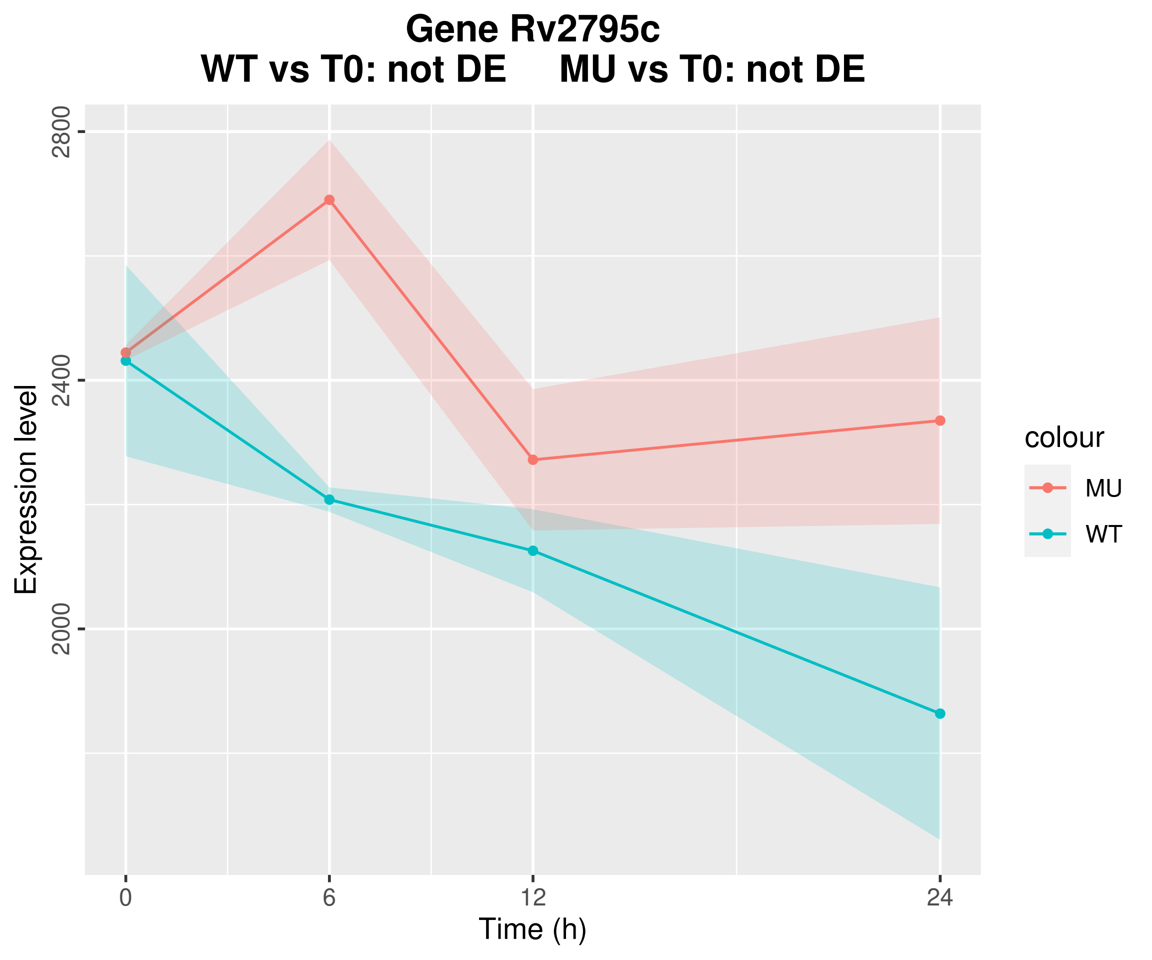

Gene Rv2795c
WT vs T0: not DE MU vs T0: not DE
2800
2400
2000
Expression level
0
6
12
24
Time (h)
colour
MU
WT

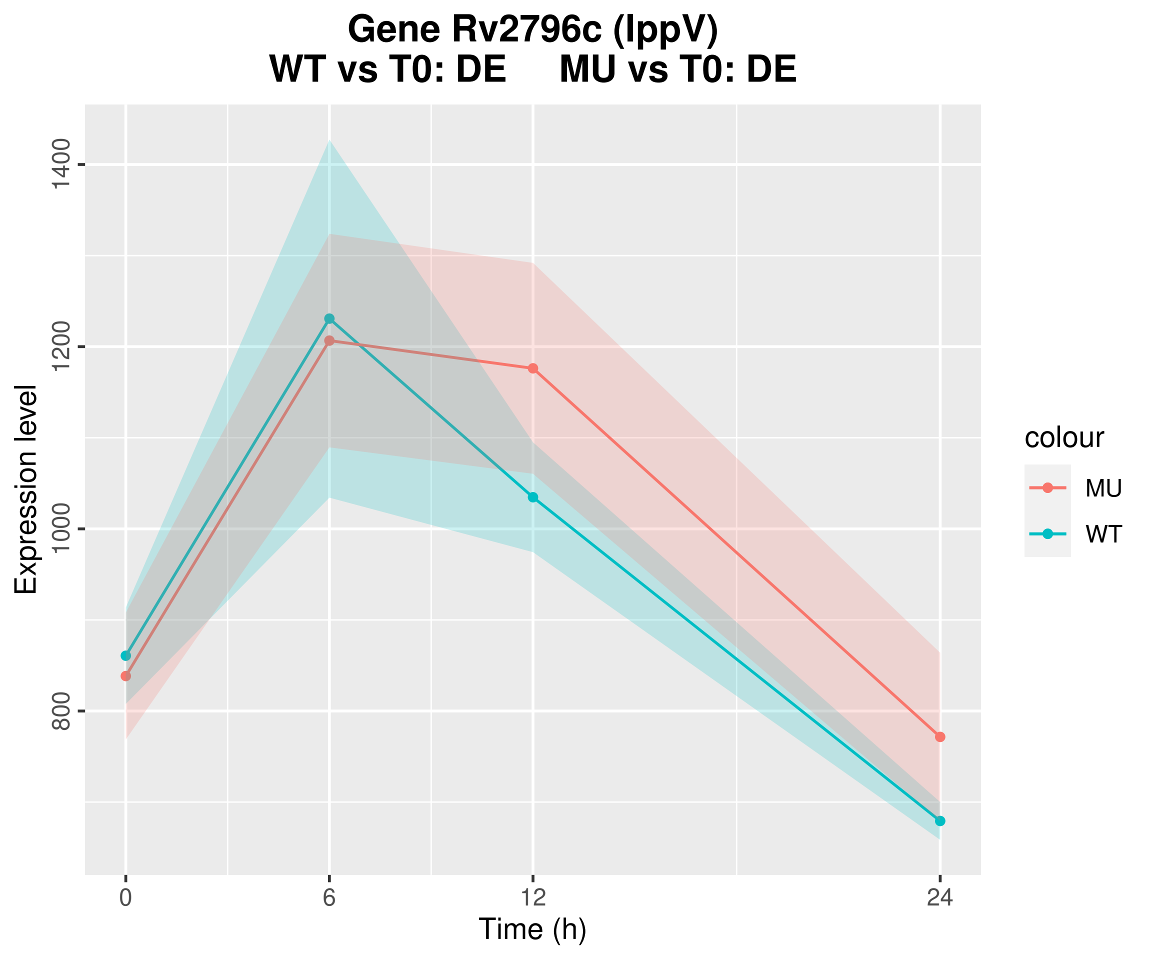
Gene Rv2796c (lppV)
WT vs T0: DE MU vs T0: DE
Expression level
1400
1200
1000
800
0
6
12
24
Time (h)
colour
MU
WT

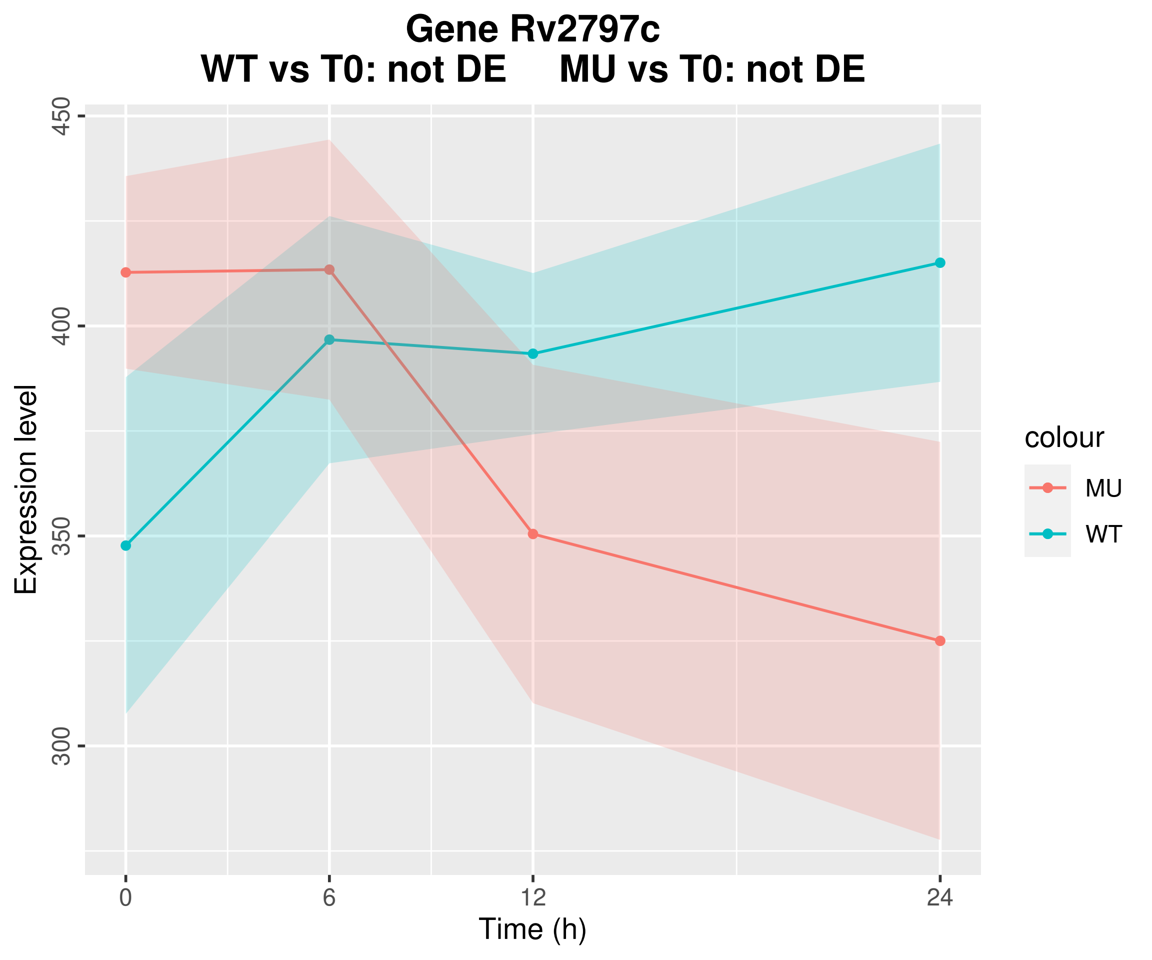

Gene Rv2797c
WT vs T0: not DE MU vs T0: not DE
Expression level
450
400
350
300
0
6
12
24
Time (h)
colour
MU
WT

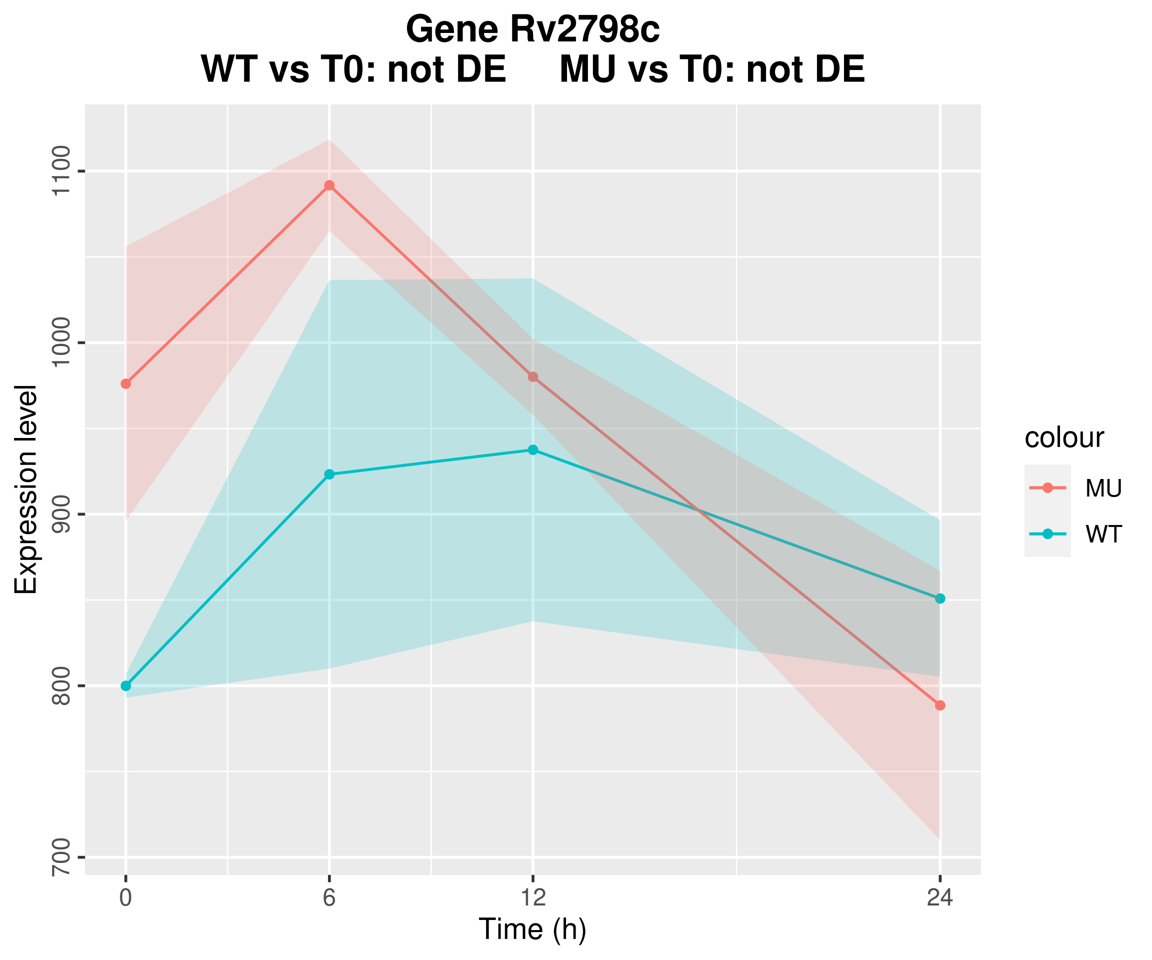

Gene Rv2798c
WT vs T0: not DE   MU vs T0: not DE
Expression level
1100
1000
900
800
700
0
6
12
24
Time (h)
colour
MU
WT

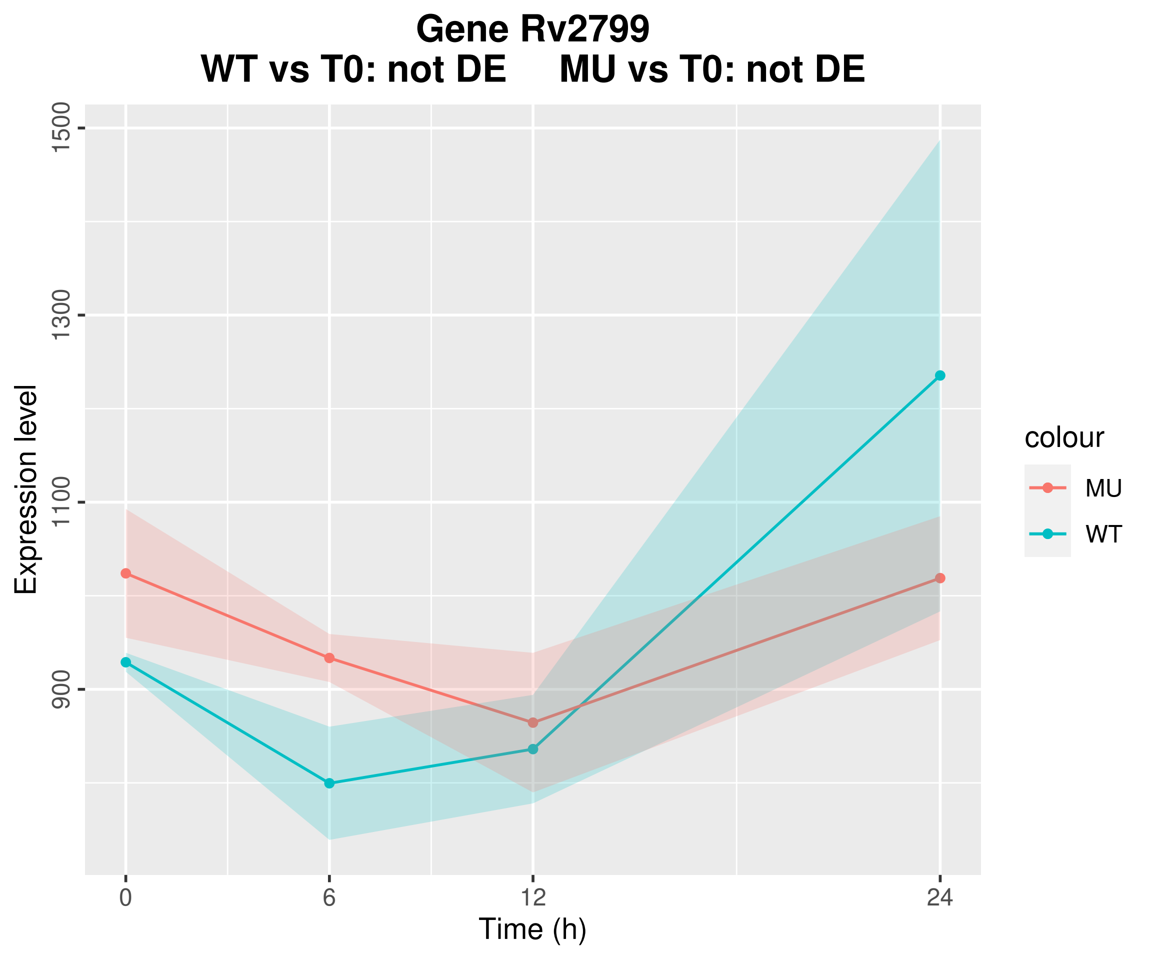
Gene Rv2799
WT vs T0: not DE   MU vs T0: not DE
Expression level
1500
1300
1100
900
0
6
12
24
Time (h)
colour
MU
WT

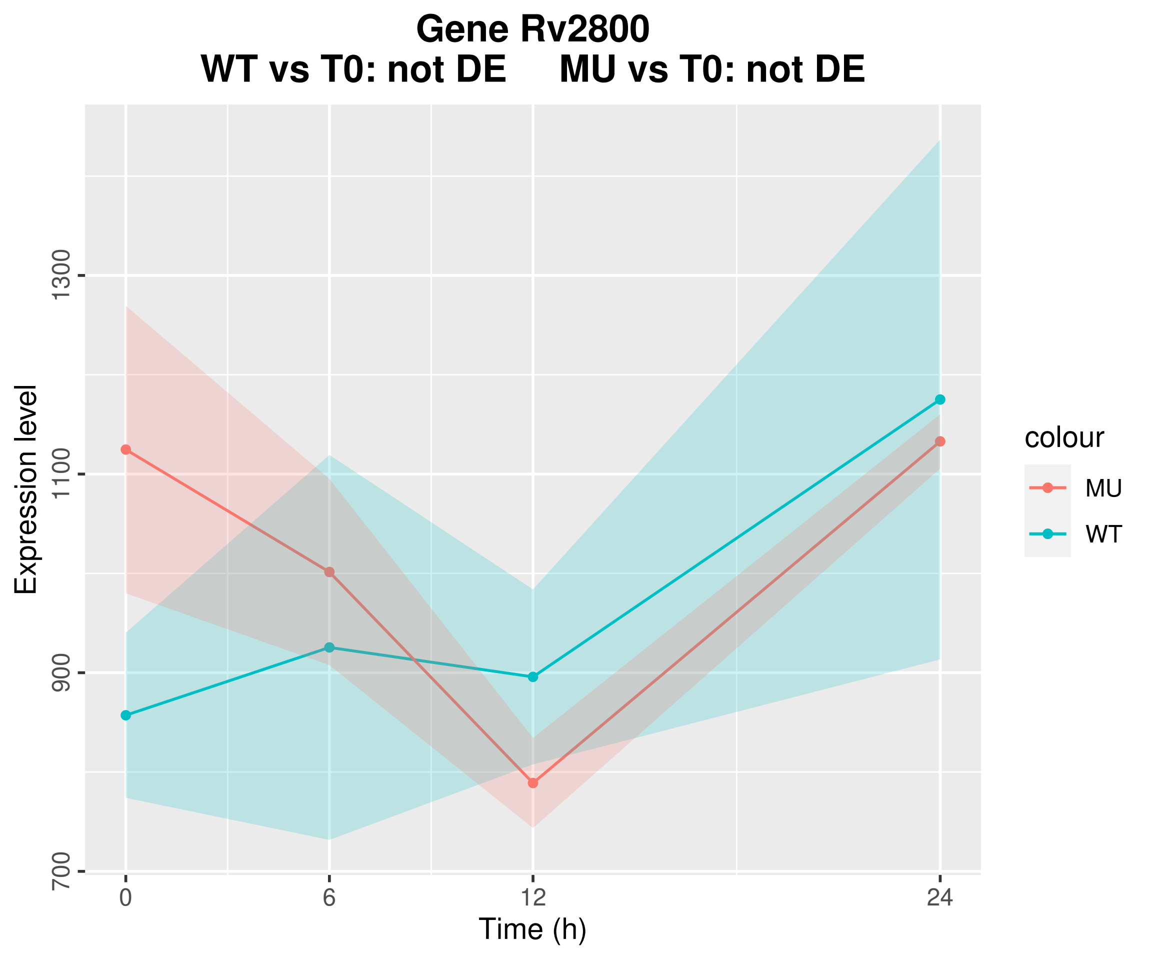
Gene Rv2800
WT vs T0: not DE   MU vs T0: not DE
Expression level
700
900
1100
1300
0
6
12
24
Time (h)
colour
MU
WT

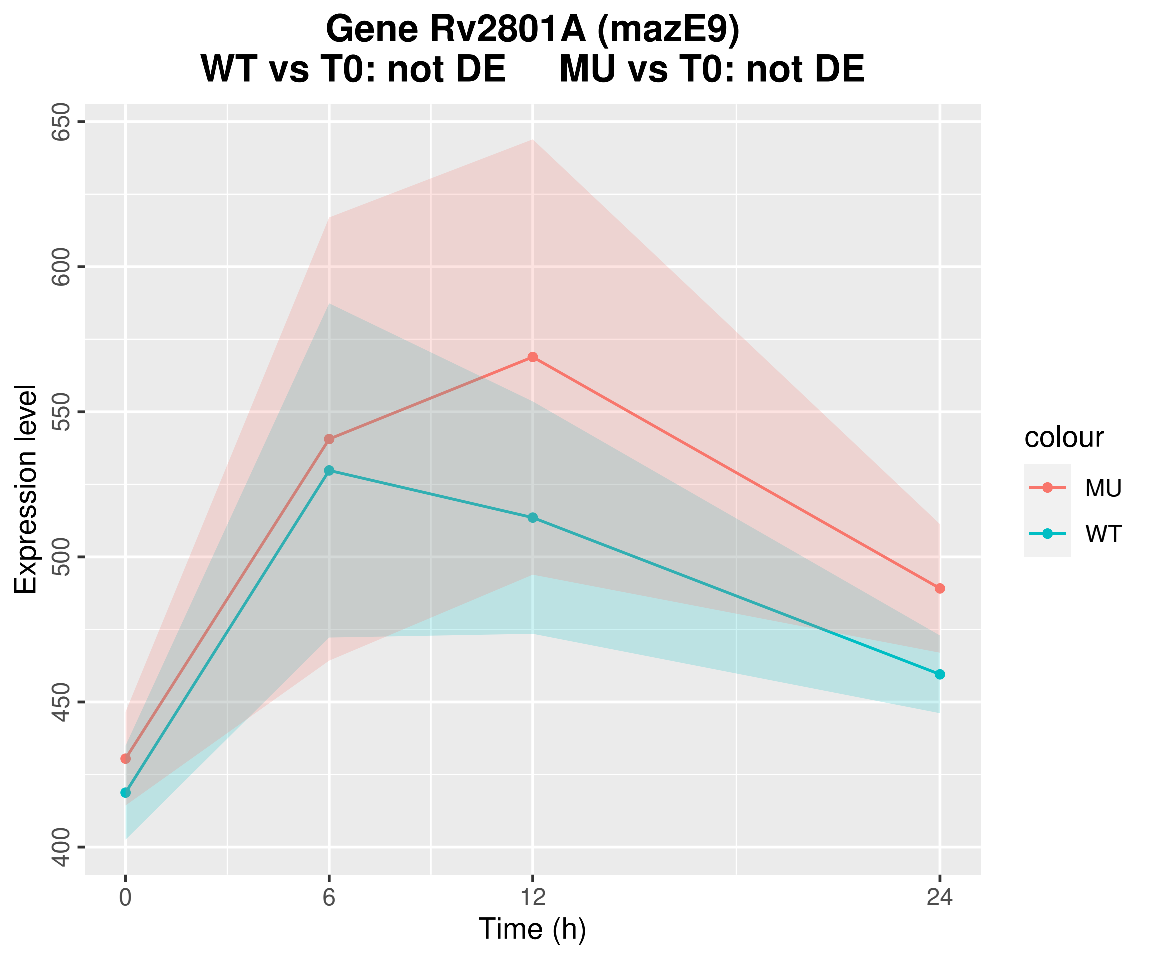

Gene Rv2801A (mazE9)
WT vs T0: not DE   MU vs T0: not DE
Expression level
650
600
550
500
450
400
0
6
12
24
Time (h)
colour
MU
WT

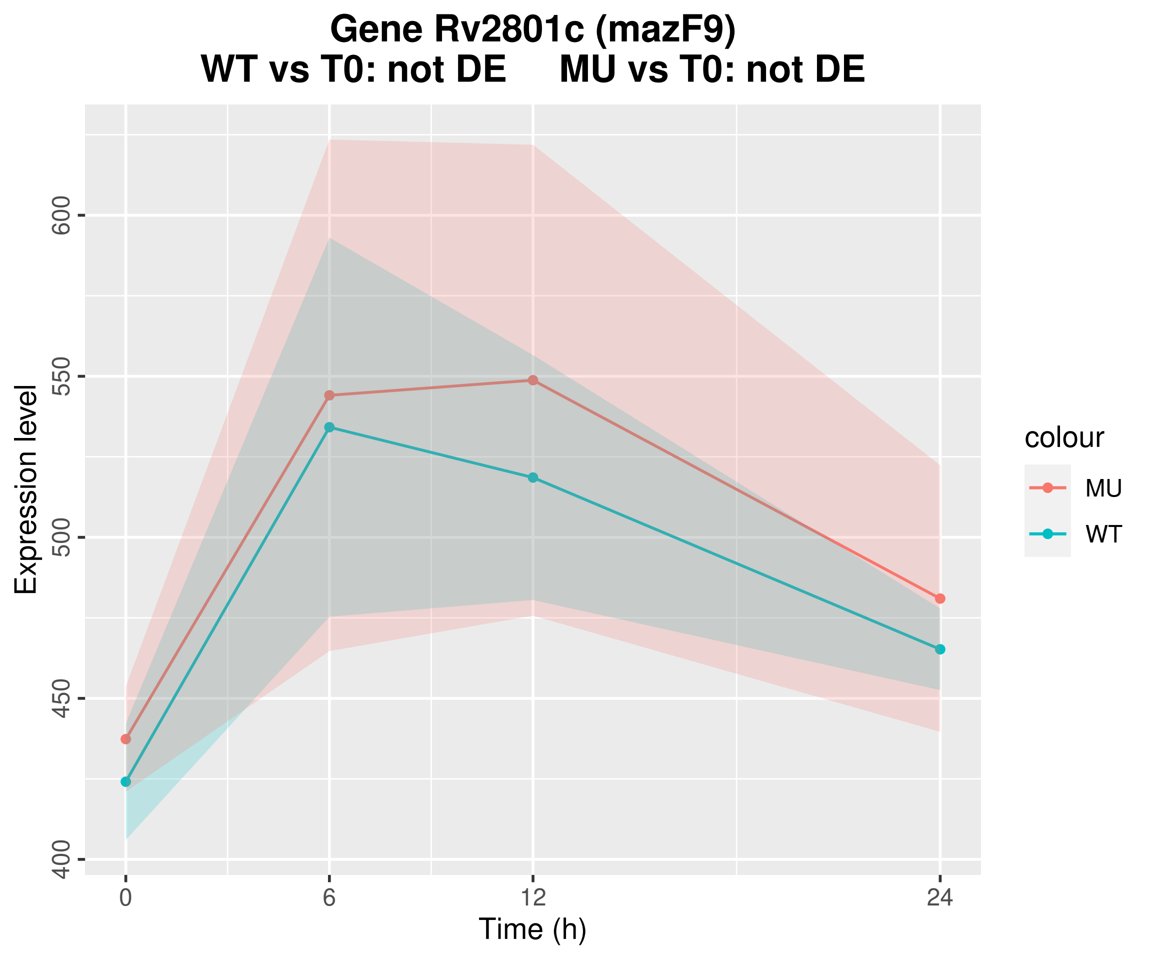

Gene Rv2801c (mazF9)
WT vs T0: not DE   MU vs T0: not DE
Expression level
600
550
500
450
400
0
6
12
24
Time (h)
colour
MU
WT

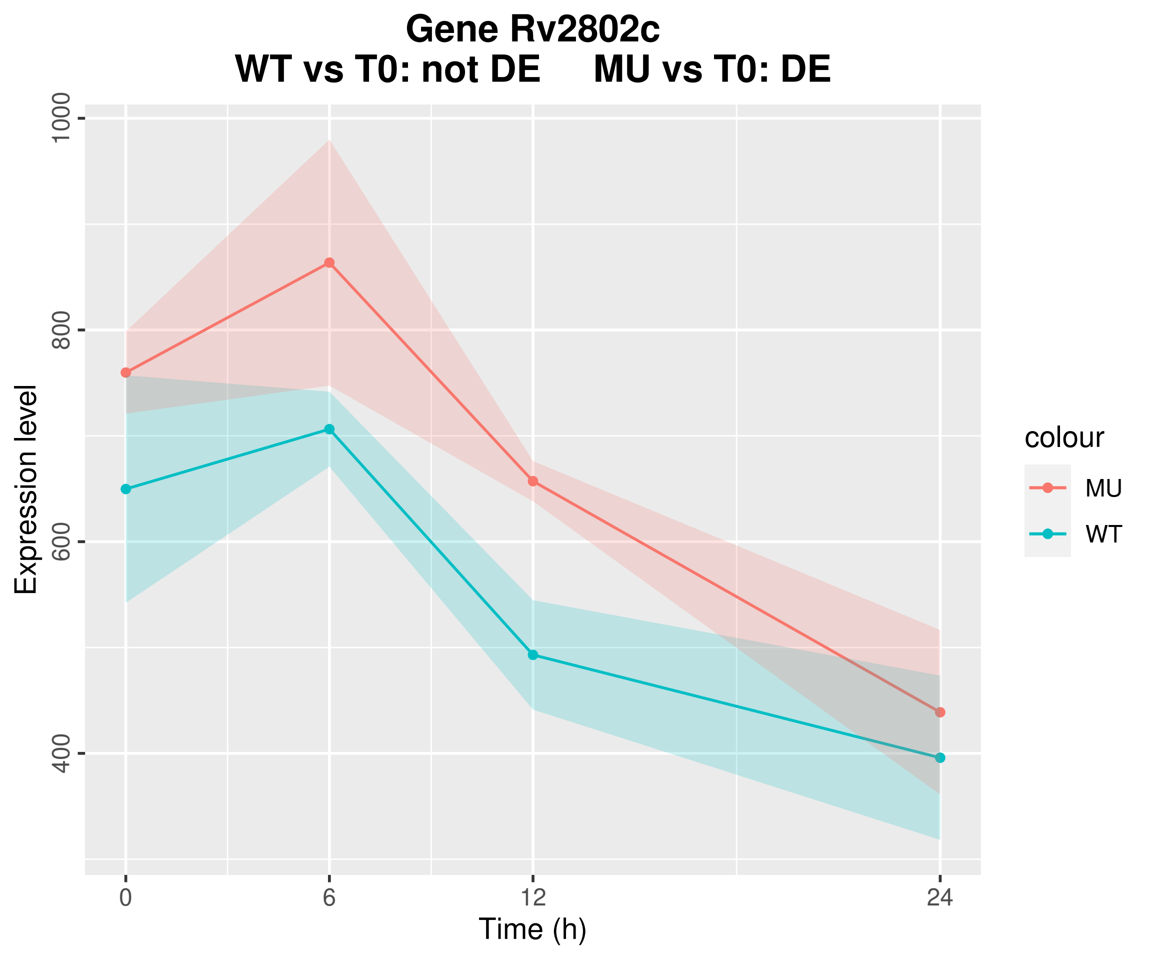

Gene Rv2802c
WT vs T0: not DE   MU vs T0: DE
Expression level
1000
800
600
400
0
6
12
24
Time (h)
colour
MU
WT

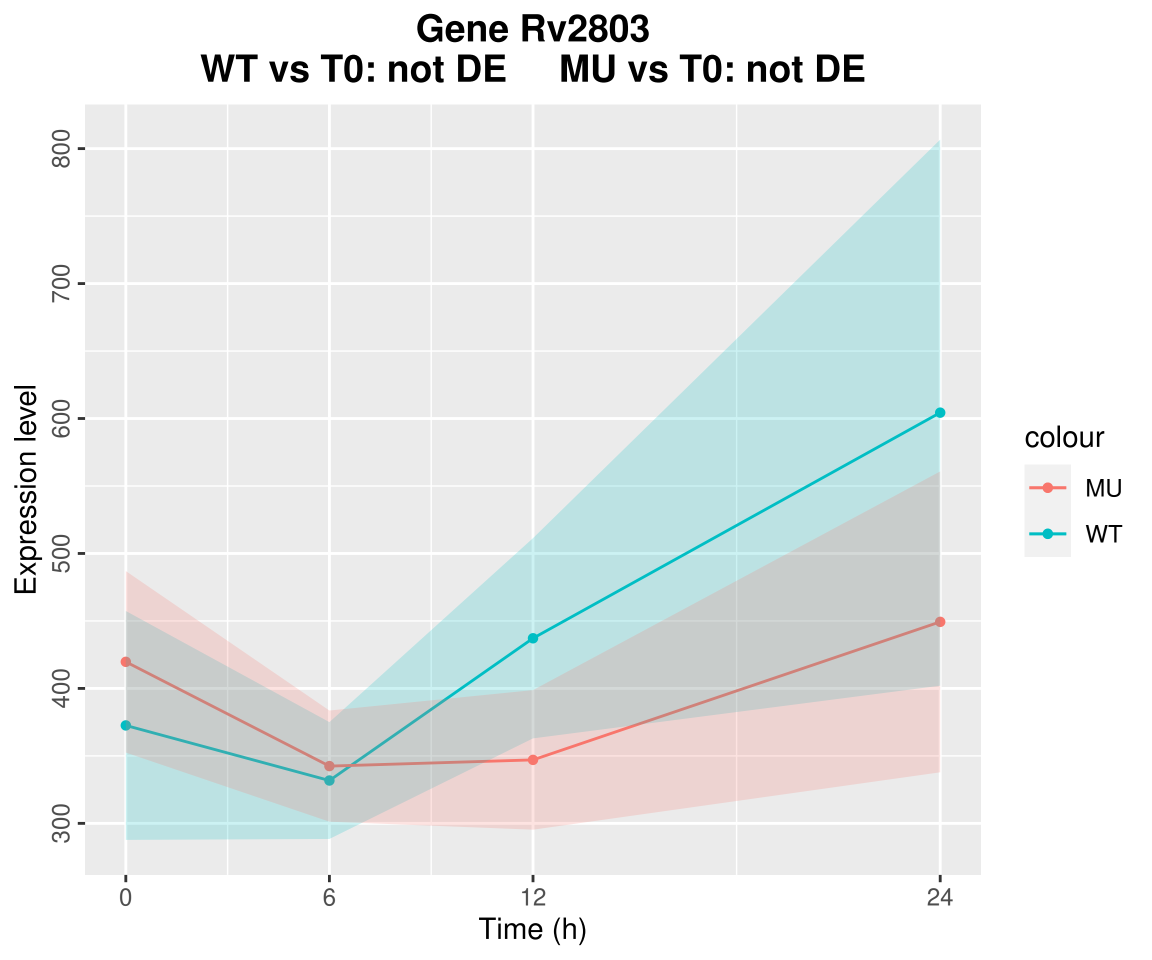

Gene Rv2803
WT vs T0: not DE MU vs T0: not DE
Expression level
800
700
600
500
400
300
0
6
12
24
Time (h)
colour
MU
WT

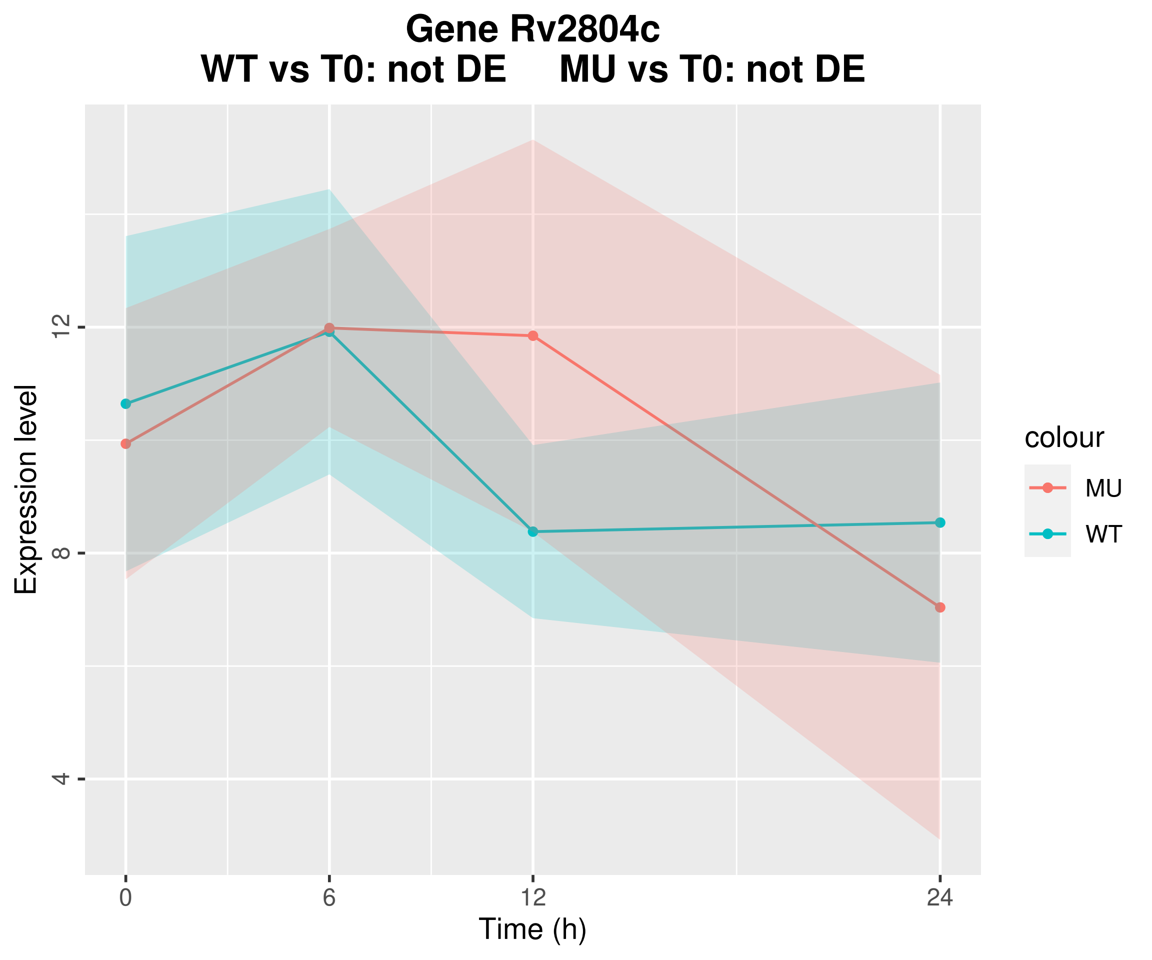
Gene Rv2804c
WT vs T0: not DE   MU vs T0: not DE
Expression level
12
8
4
0
6
12
24
Time (h)
colour
MU
WT

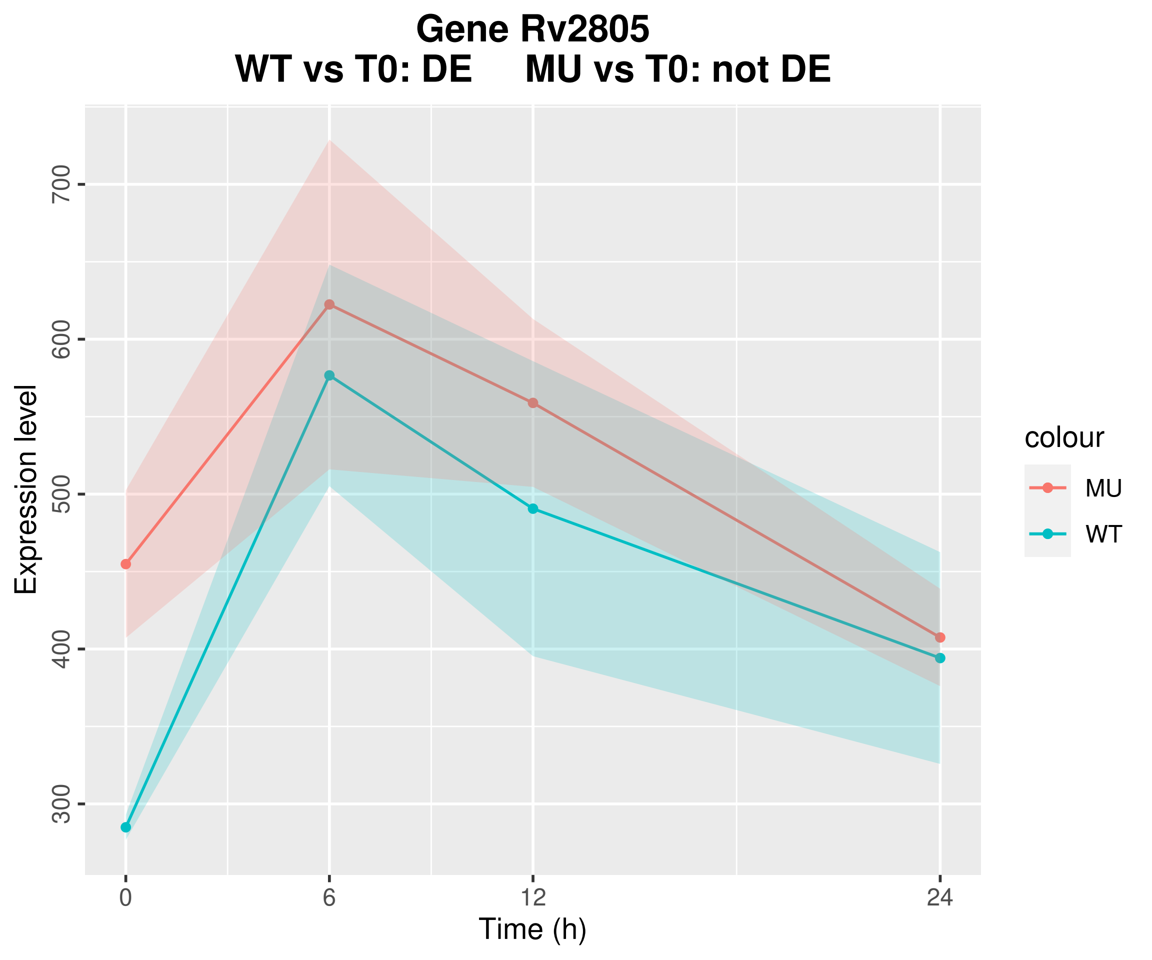

Gene Rv2805
WT vs T0: DE MU vs T0: not DE
Expression level
700
600
500
400
300
0
6
12
24
Time (h)
colour
MU
WT

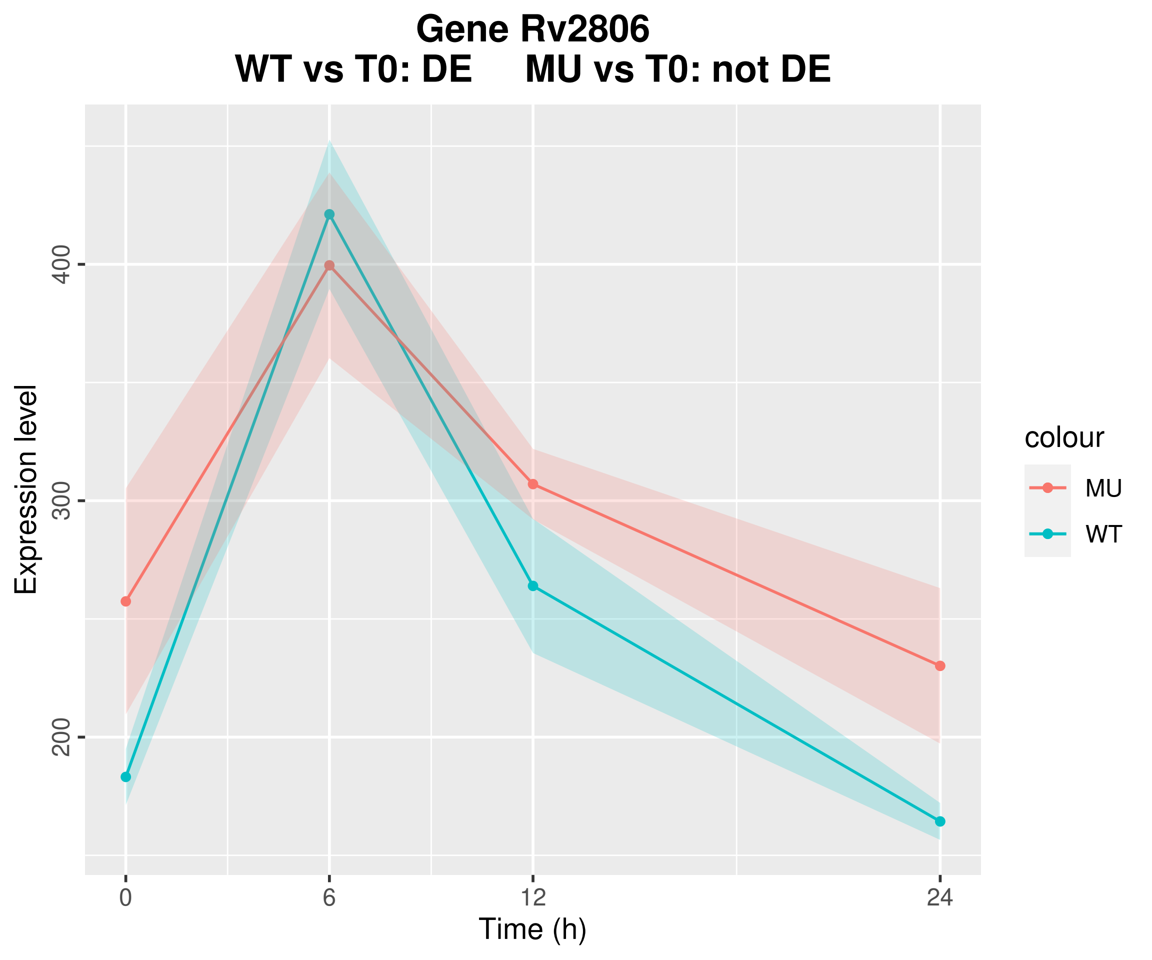

Gene Rv2806
WT vs T0: DE MU vs T0: not DE
Expression level
400
300
200
0
6
12
24
Time (h)
colour
MU
WT

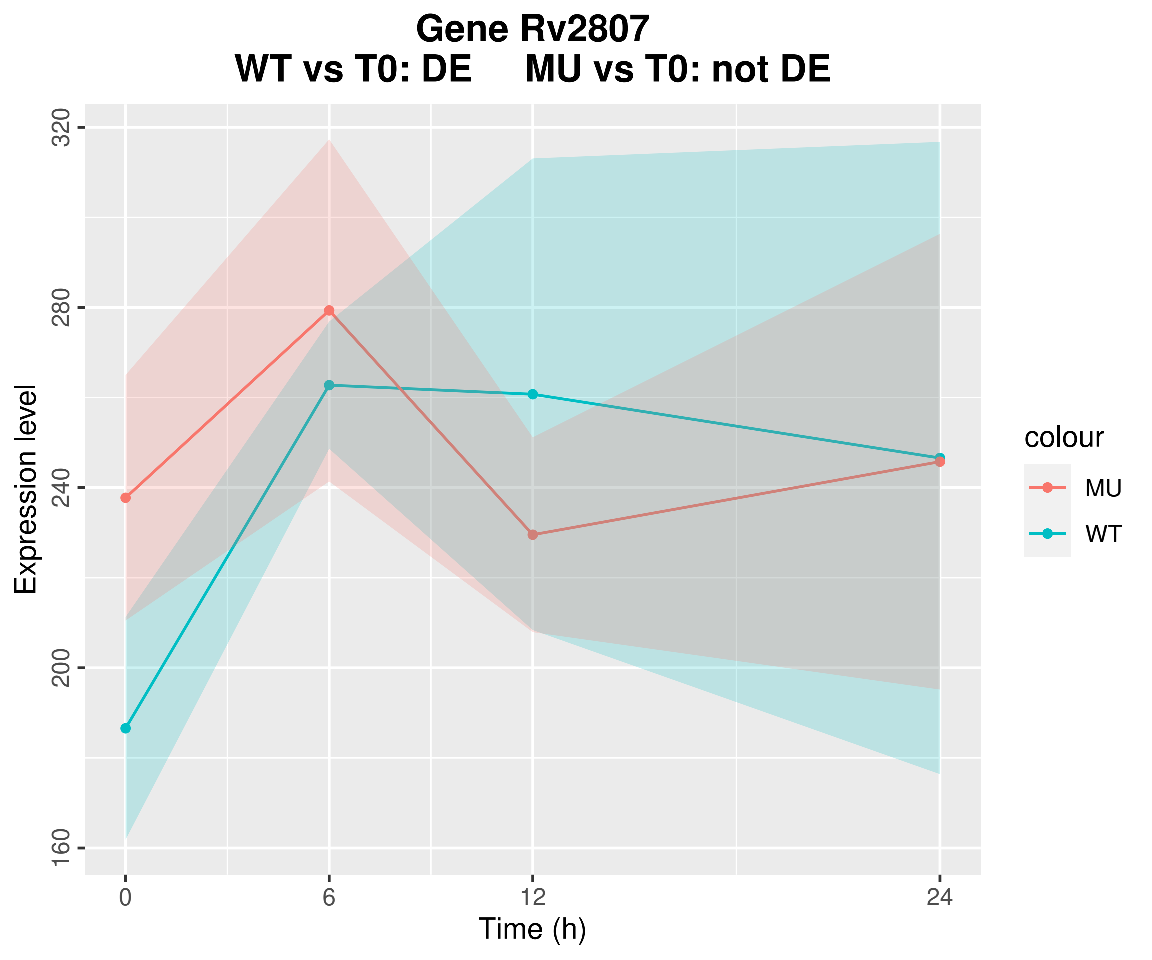

Gene Rv2807
WT vs T0: DE   MU vs T0: not DE
Expression level
320
280
240
200
160
0
6
12
24
Time (h)
colour
MU
WT

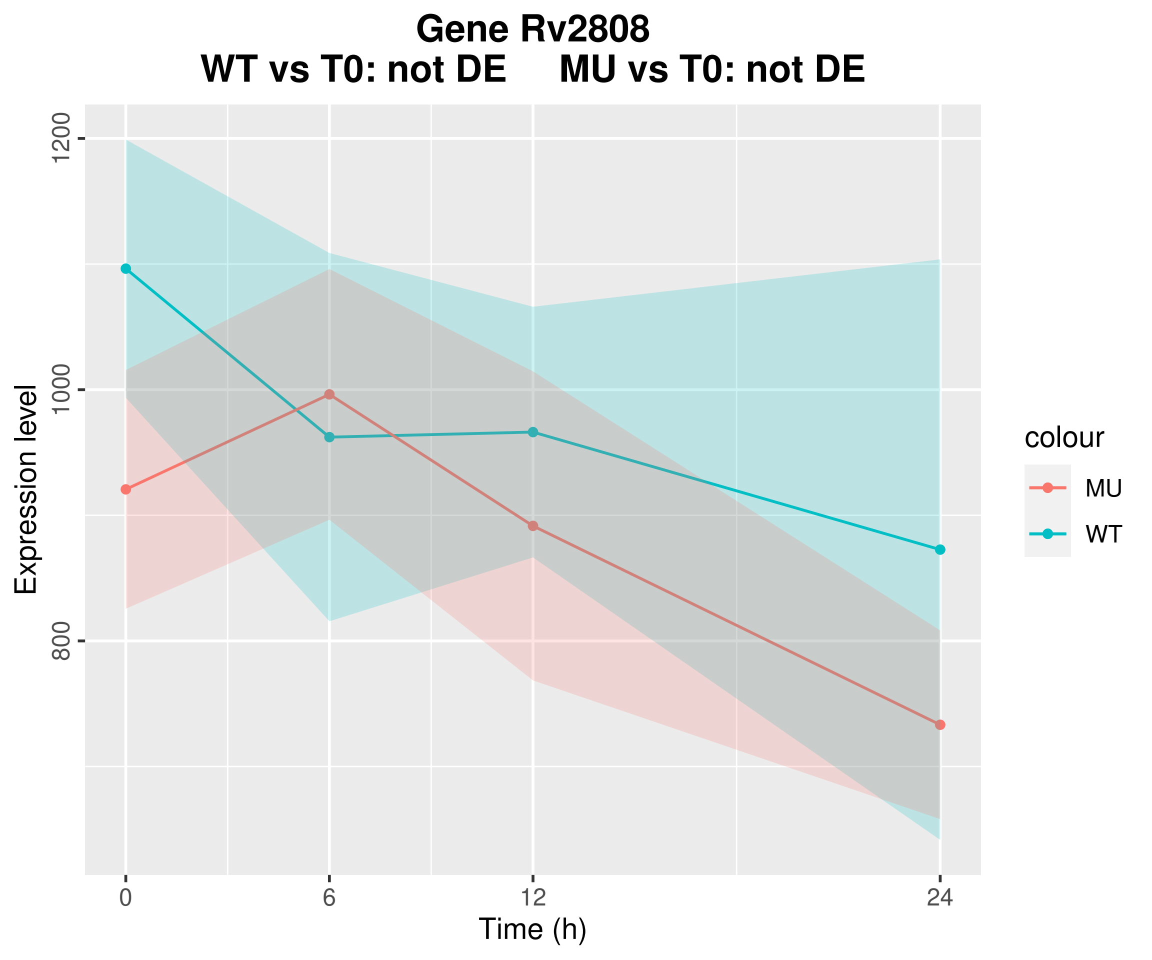

Gene Rv2808
WT vs T0: not DE   MU vs T0: not DE
1200
1000
800
Expression level
0
6
12
24
Time (h)
colour
MU
WT

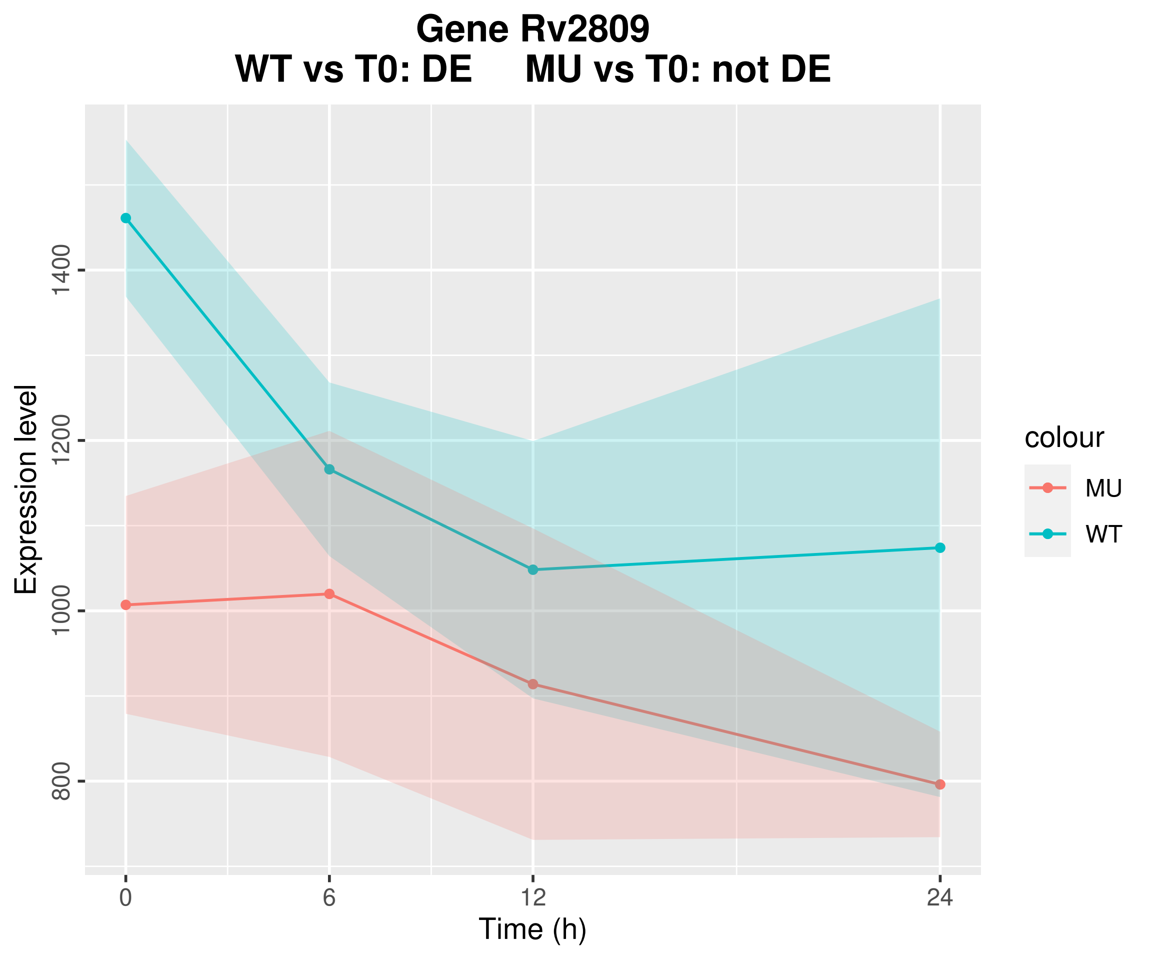
Gene Rv2809
WT vs T0: DE   MU vs T0: not DE
Expression level
1400
1200
1000
800
0
6
12
24
Time (h)
colour
MU
WT

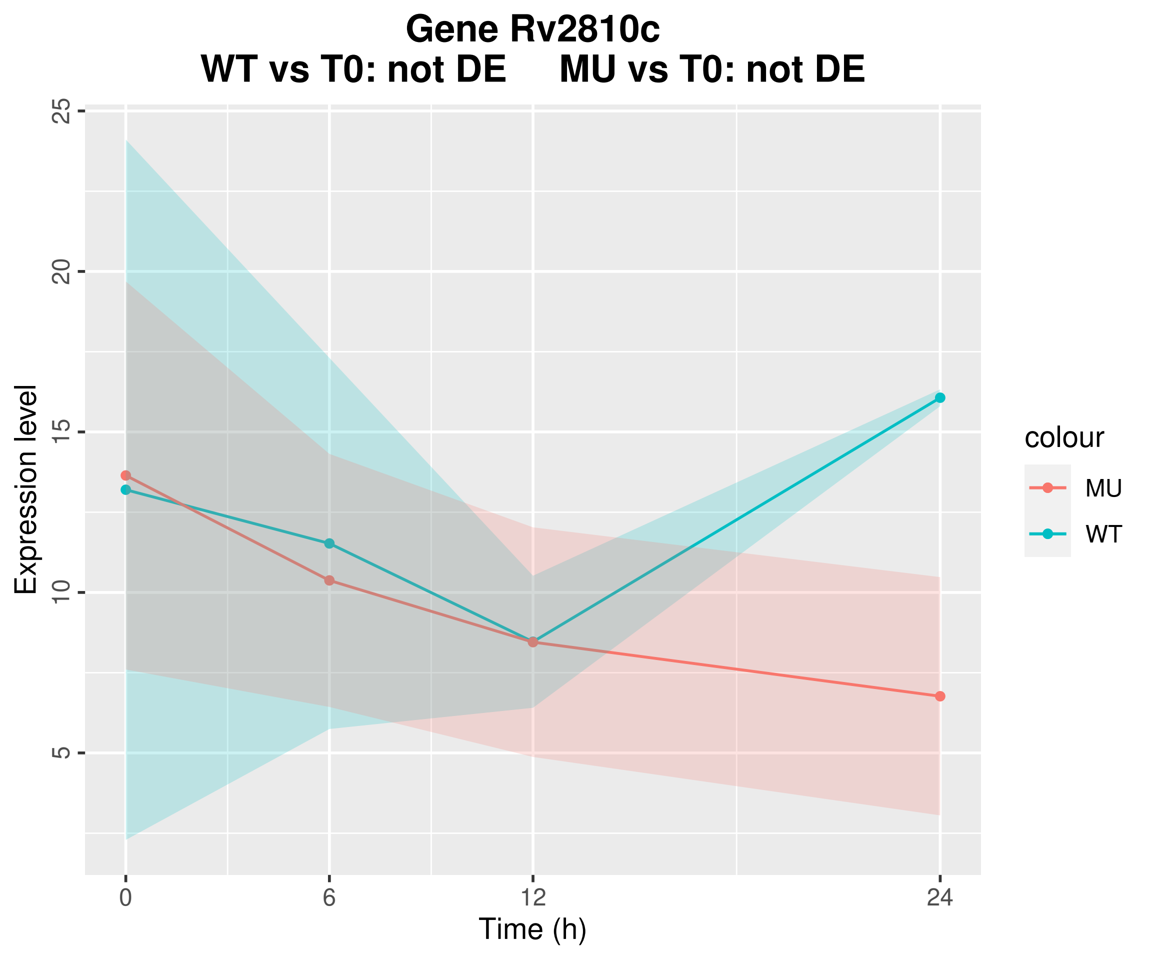

Gene Rv2810c
WT vs T0: not DE   MU vs T0: not DE
Expression level
Time (h)
25
20
15
10
5
0
6
12
24
colour
MU
WT

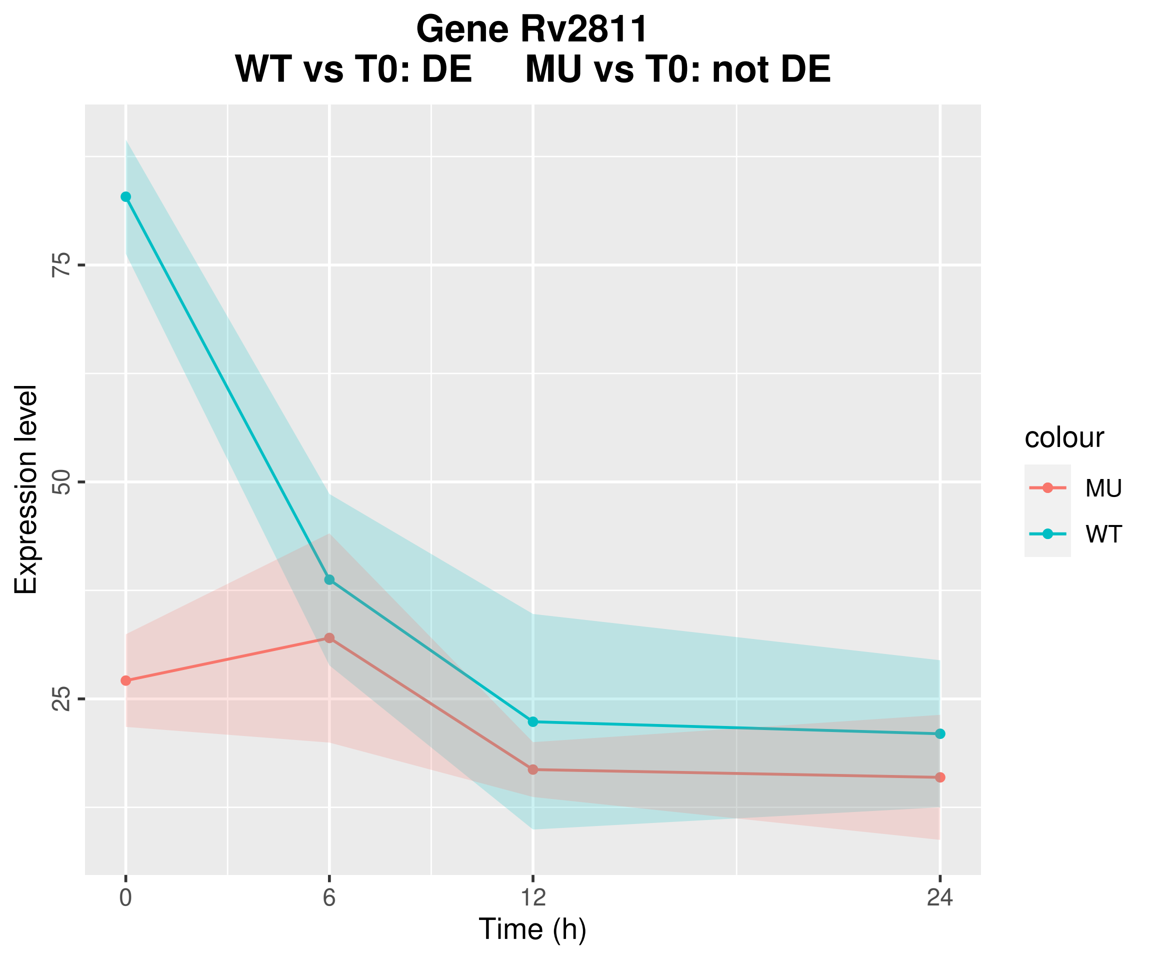

Gene Rv2811
WT vs T0: DE   MU vs T0: not DE
Expression level
75
50
25
0
6
12
24
Time (h)
colour
MU
WT

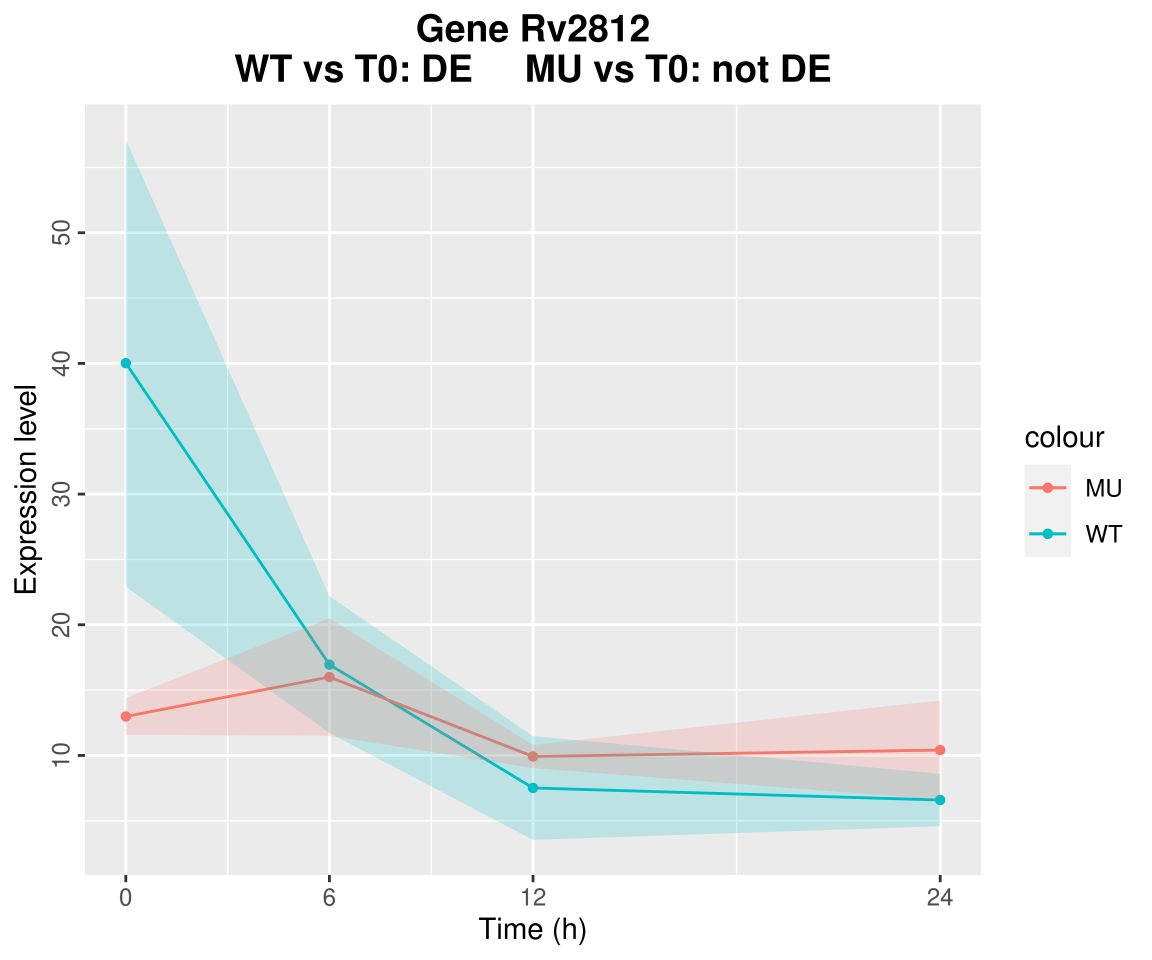

Gene Rv2812
WT vs T0: DE   MU vs T0: not DE
Expression level
50
40
30
20
10
0
6
12
24
Time (h)
colour
MU
WT

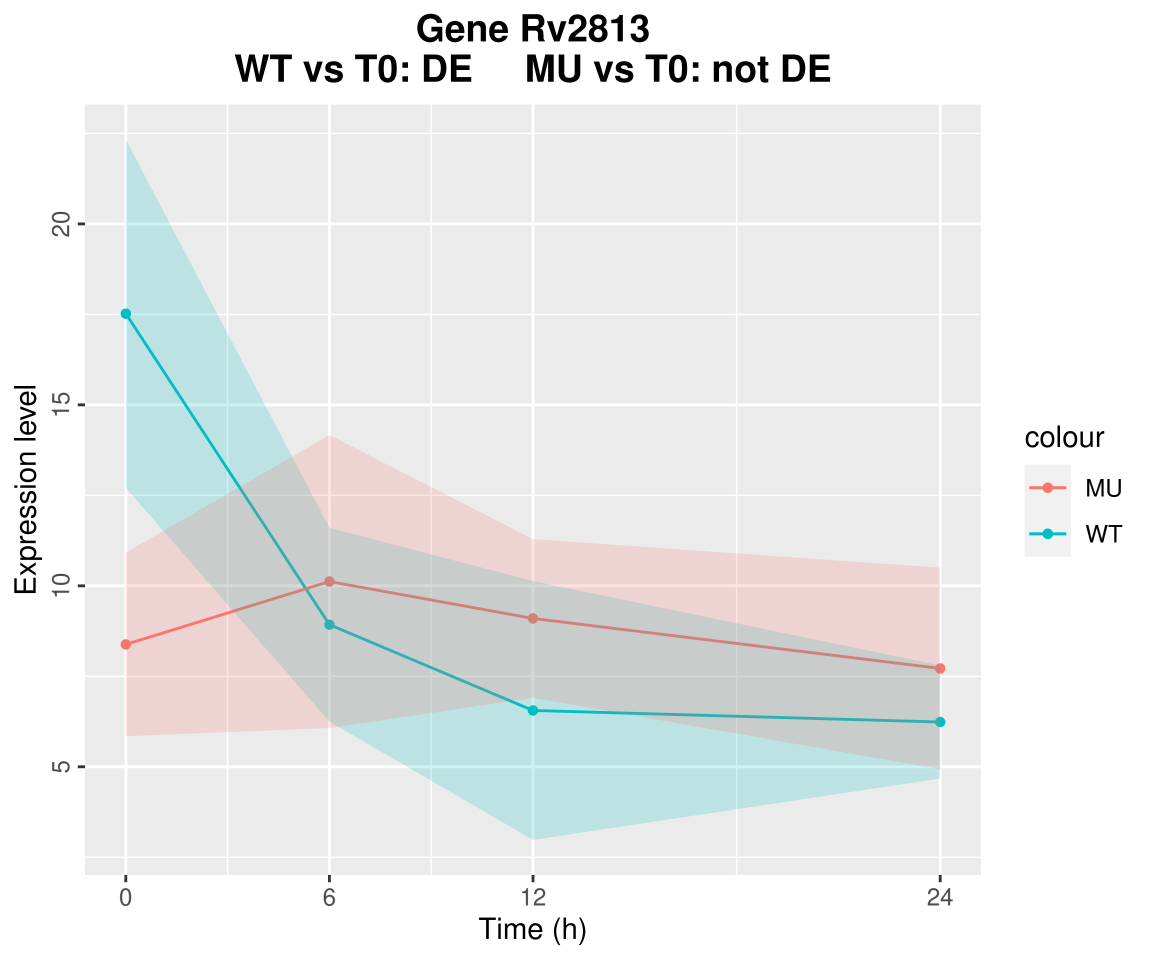
Gene Rv2813
WT vs T0: DE MU vs T0: not DE
Expression level
20
15
10
5
0
6
12
24
Time (h)
colour
MU
WT

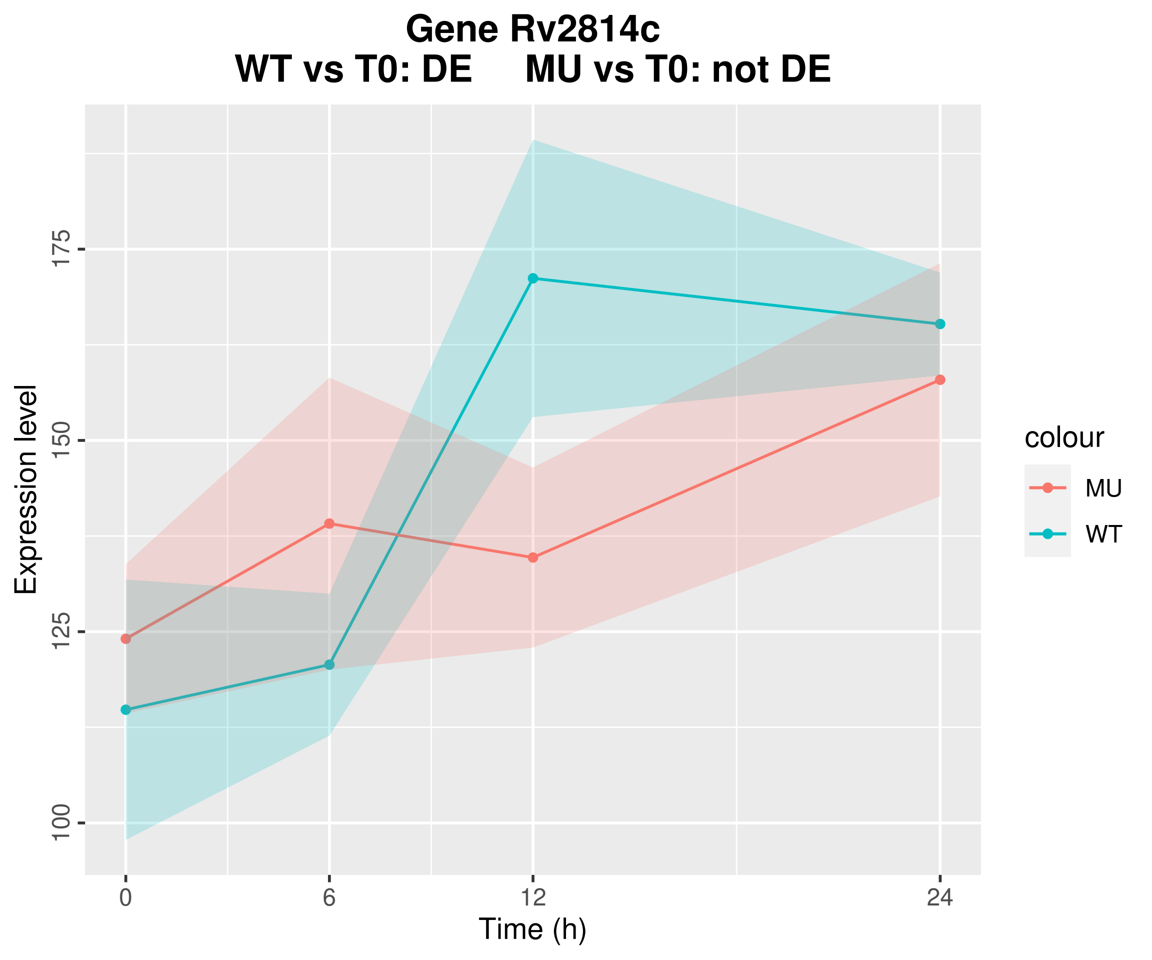

Gene Rv2814c
WT vs T0: DE   MU vs T0: not DE
Expression level
175
150
125
100
0
6
12
24
Time (h)
colour
MU
WT

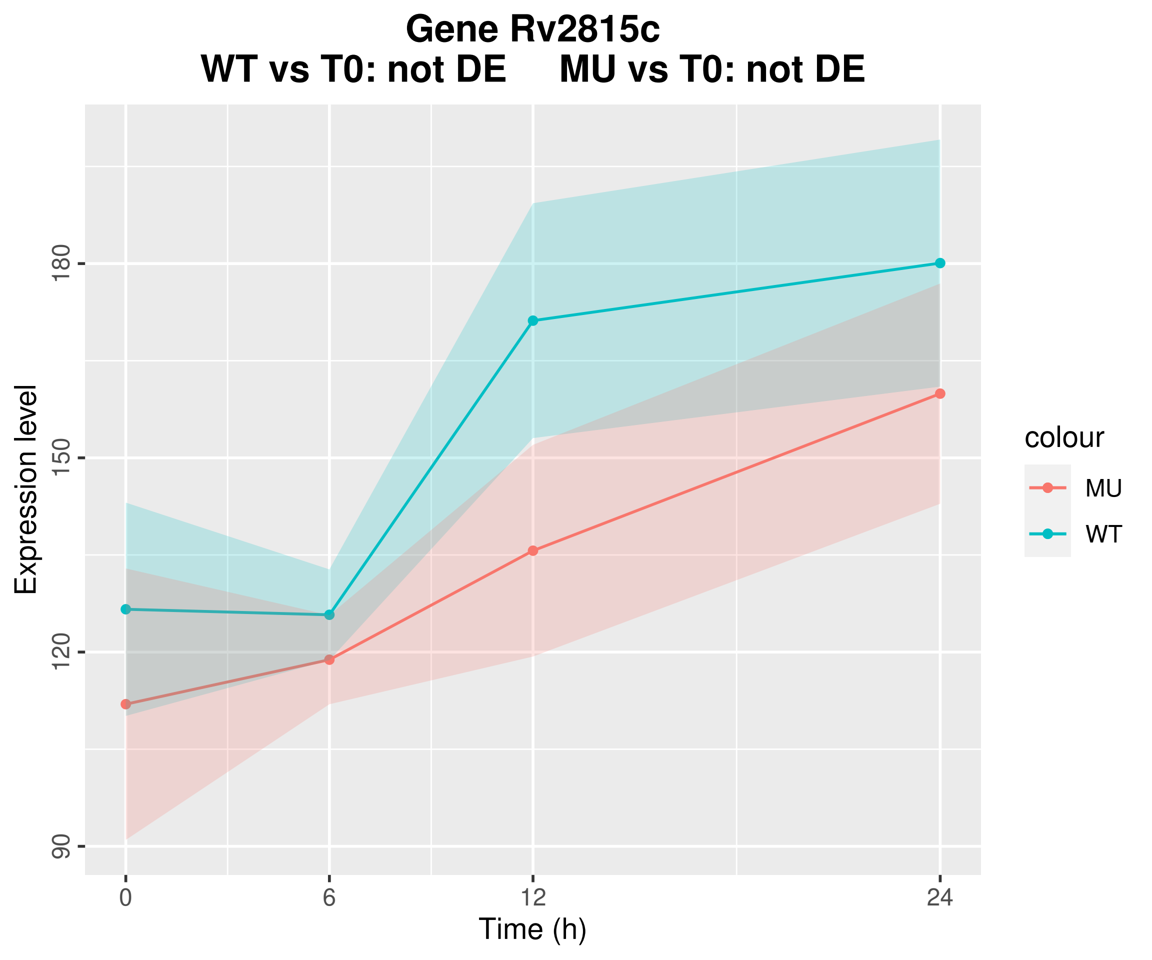
Gene Rv2815c
WT vs T0: not DE MU vs T0: not DE
Expression level
180
150
120
90
0
6
12
24
Time (h)
colour
MU
WT

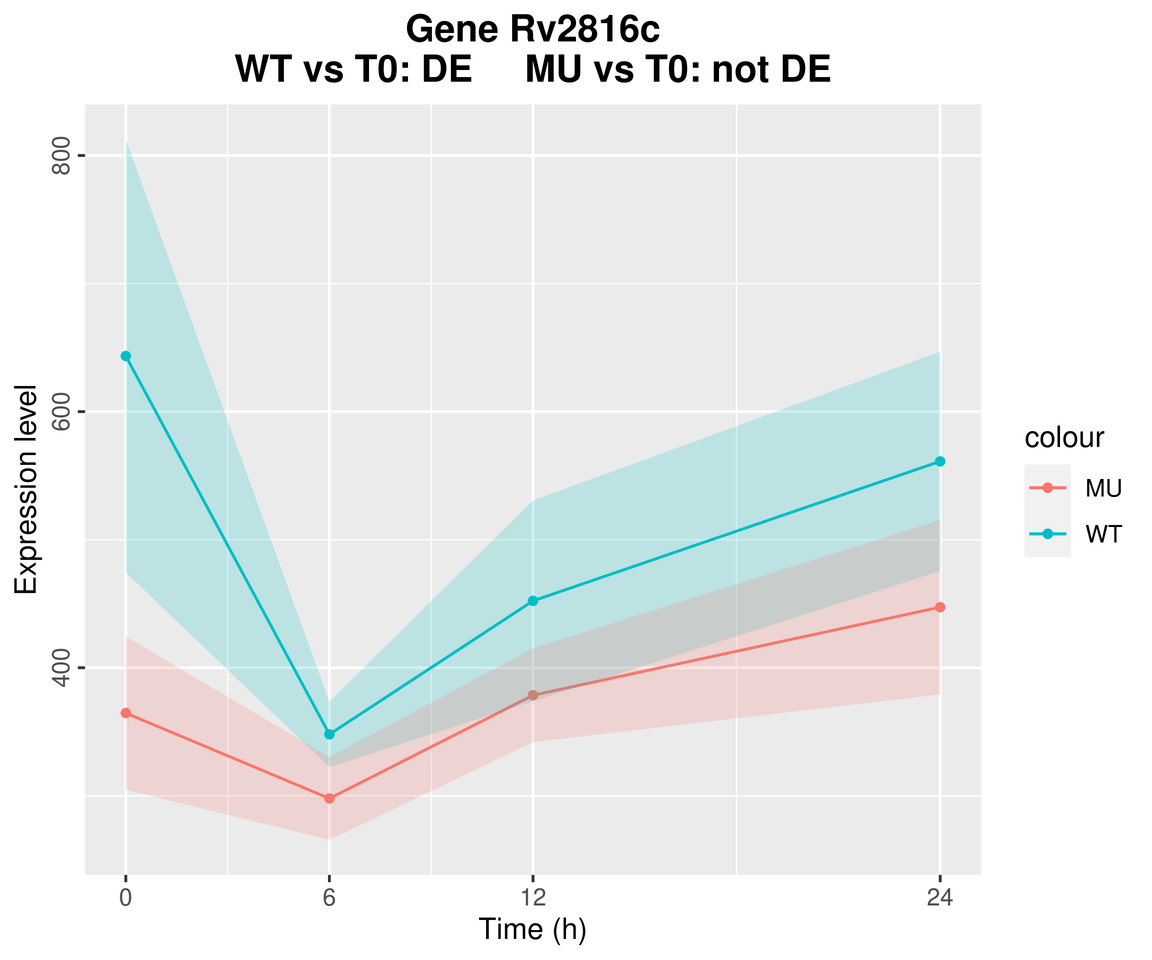

Gene Rv2816c
WT vs T0: DE MU vs T0: not DE
Expression level
800
600
400
0
6
12
24
Time (h)
colour
MU
WT

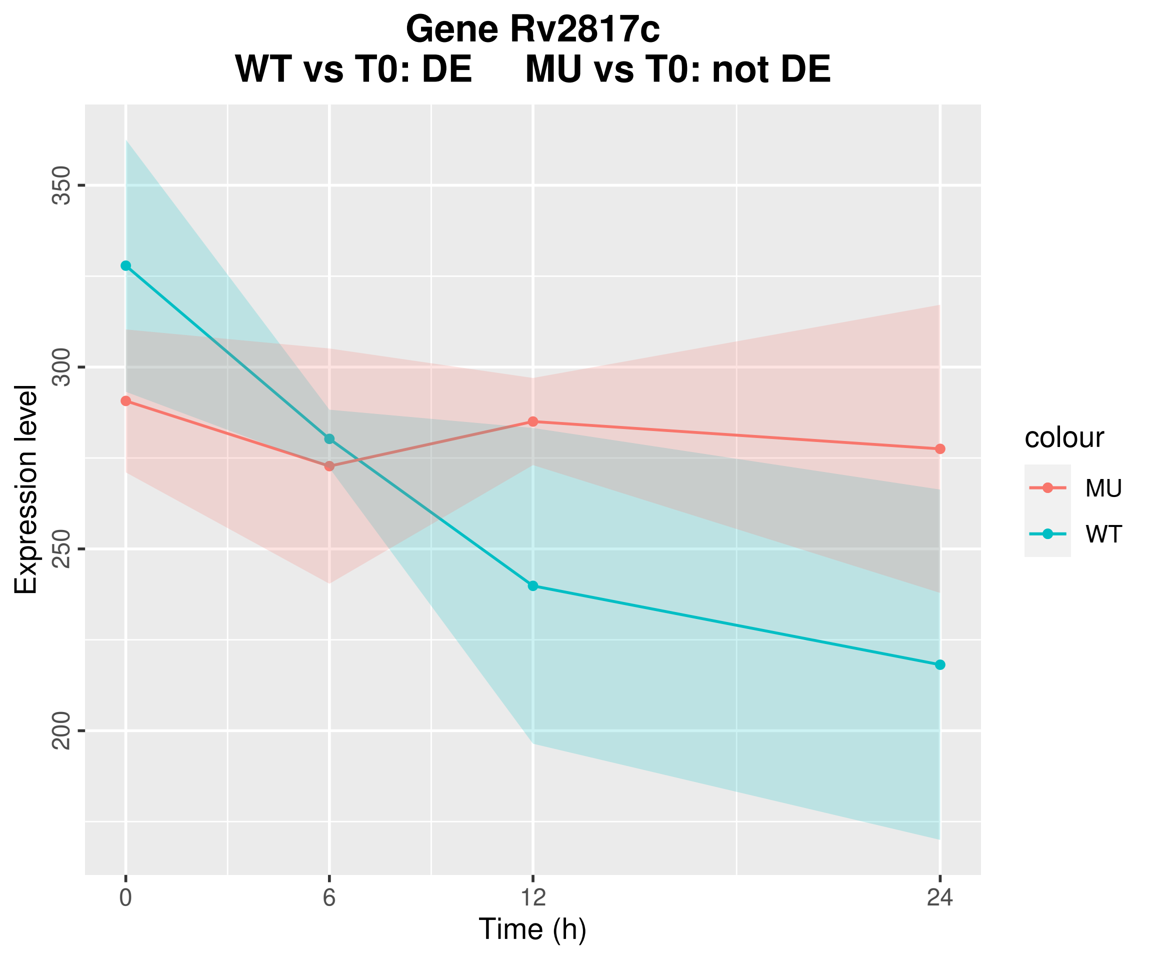
Gene Rv2817c
WT vs T0: DE MU vs T0: not DE
Expression level
350
300
250
200
0
6
12
24
Time (h)
colour
MU
WT

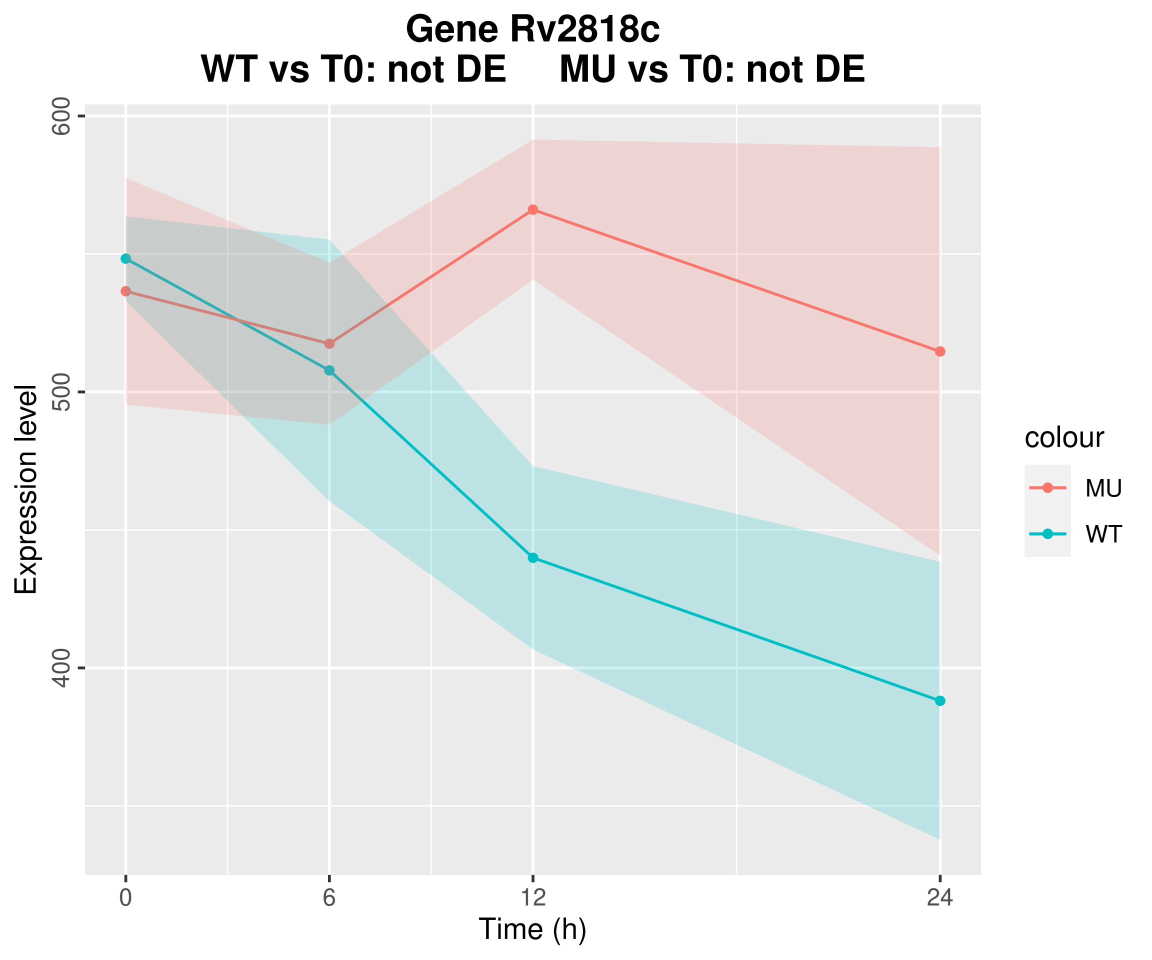

Gene Rv2818c
WT vs T0: not DE MU vs T0: not DE
600
500
400
Expression level
0
6
12
24
Time (h)
colour
MU
WT

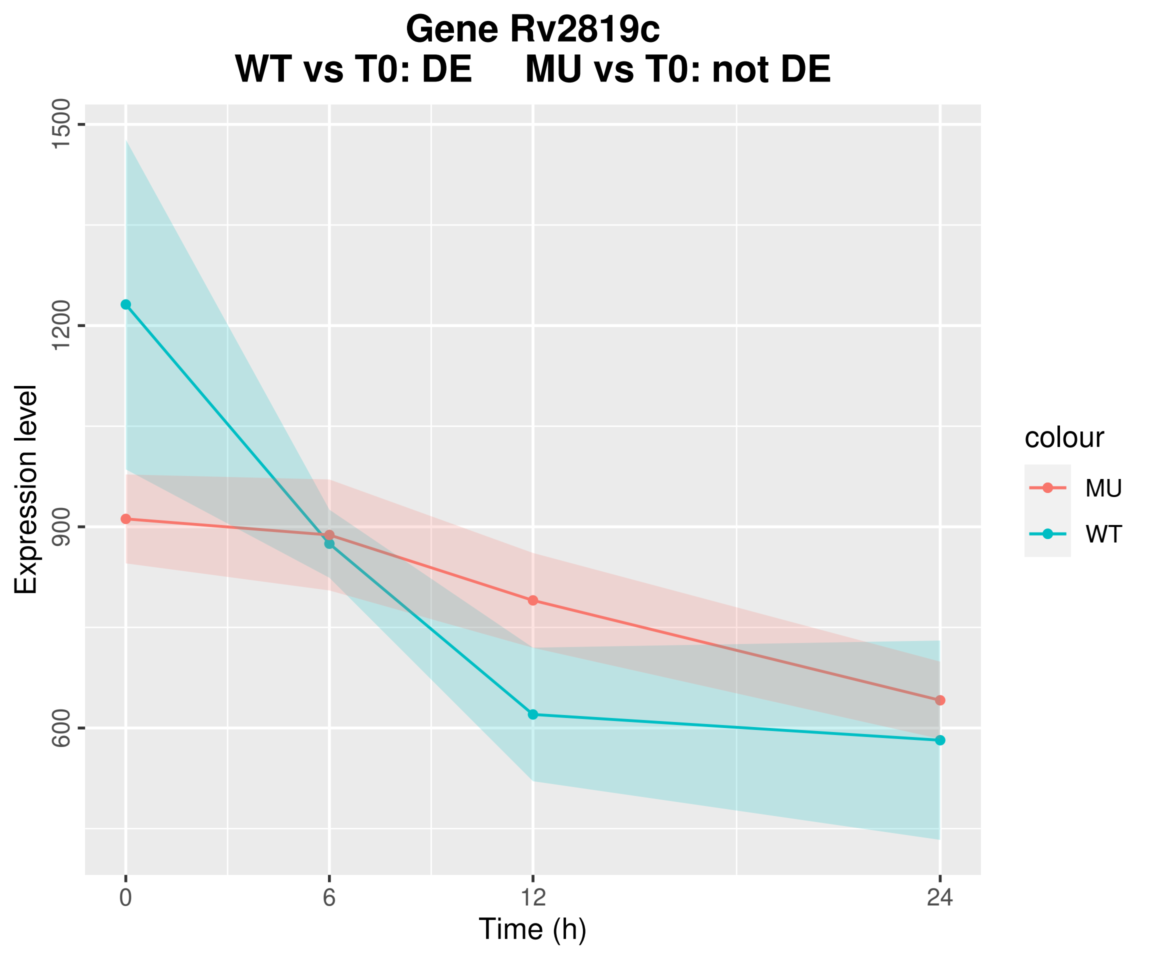
Gene Rv2819c
WT vs T0: DE   MU vs T0: not DE
Expression level
1500
1200
900
600
0
6
12
24
Time (h)
colour
MU
WT

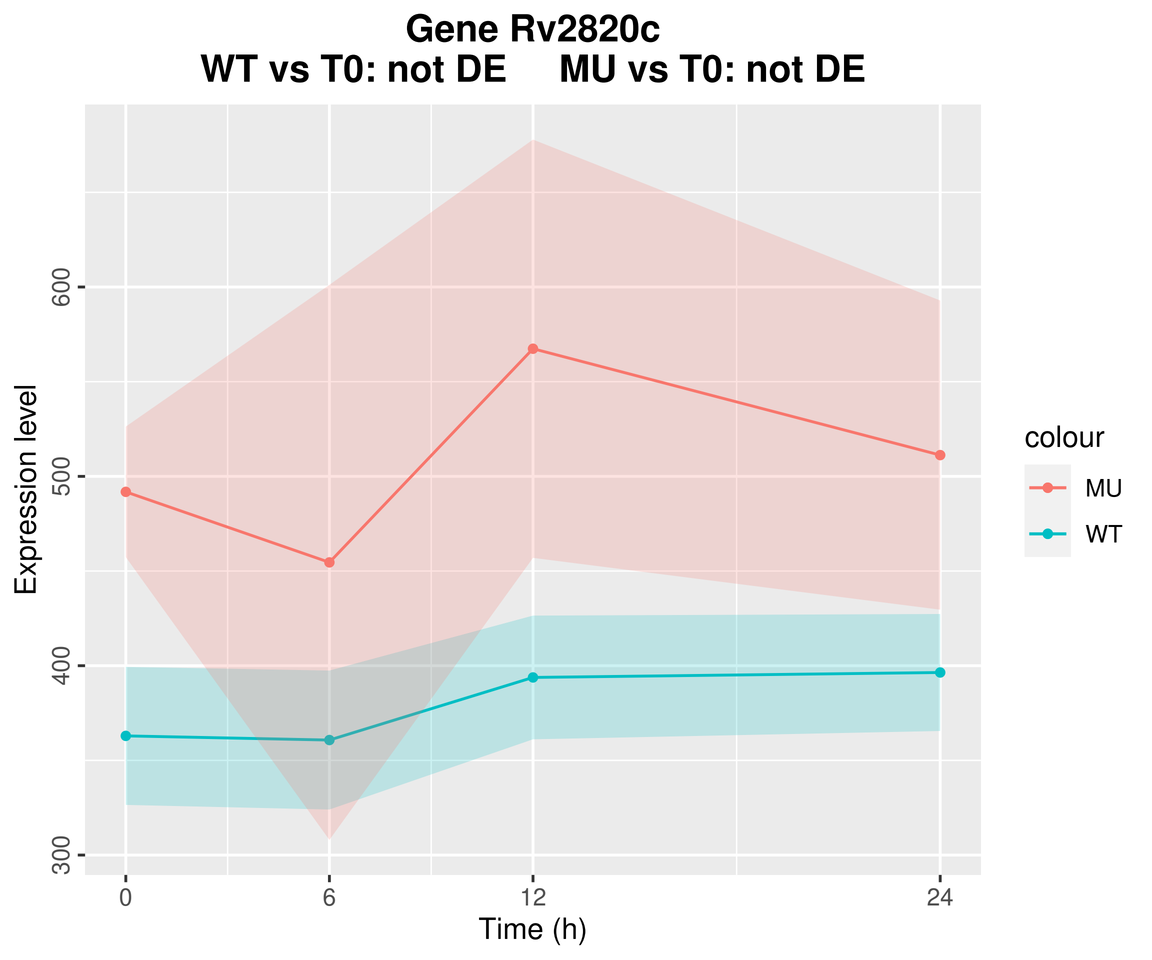
Gene Rv2820c
WT vs T0: not DE   MU vs T0: not DE
Expression level
600
500
400
300
0
6
12
24
Time (h)
colour
MU
WT

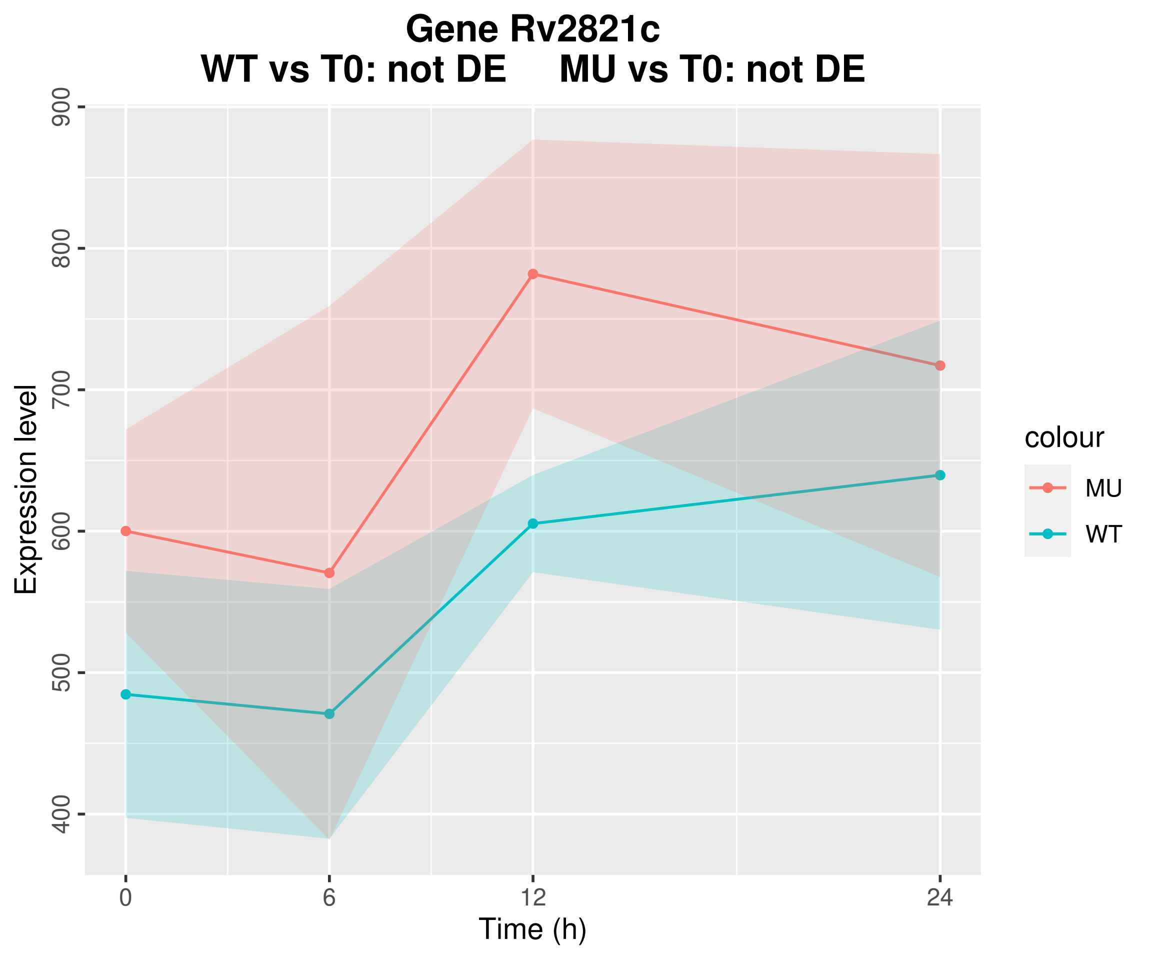
Gene Rv2821c
WT vs T0: not DE MU vs T0: not DE
Expression level
900
800
700
600
500
400
0
6
12
24
Time (h)
colour
MU
WT

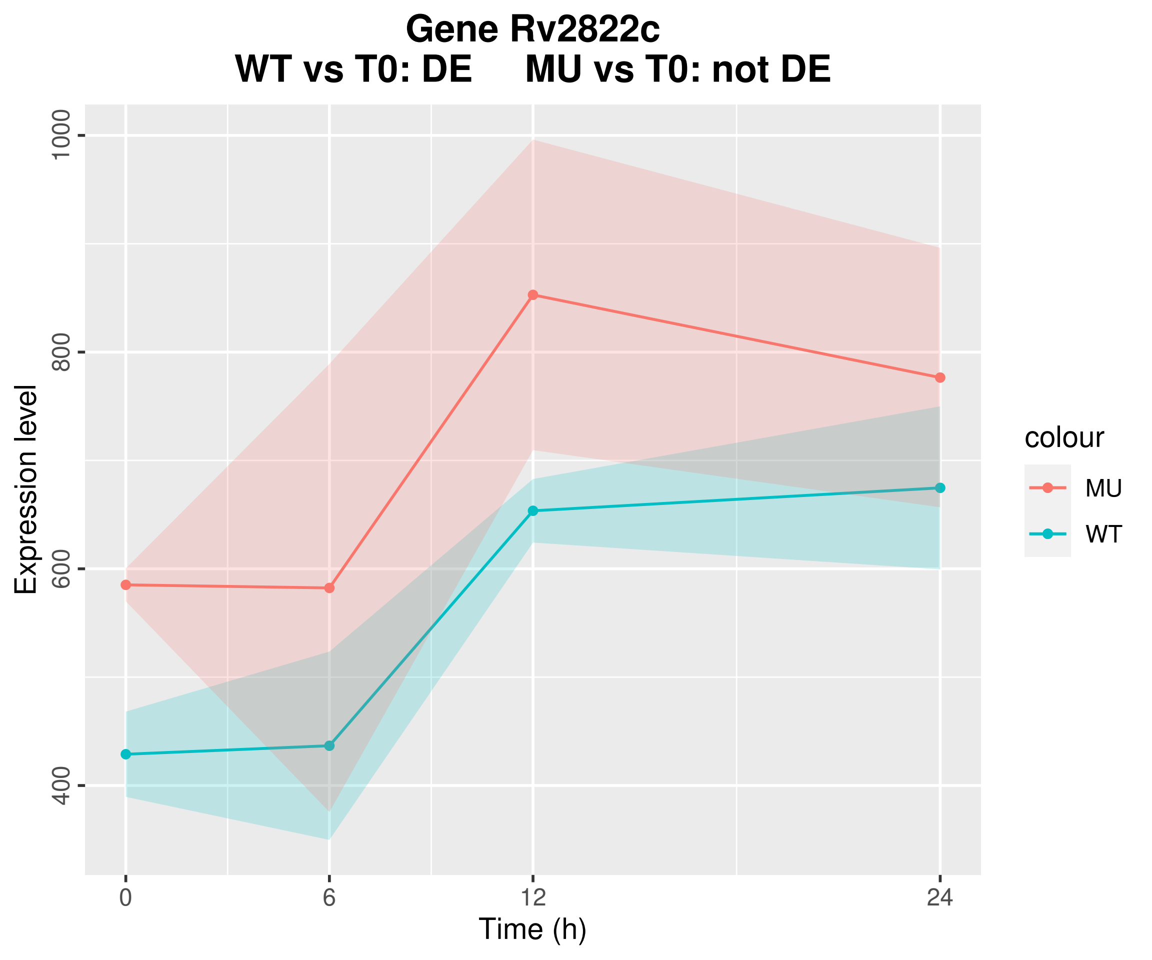

Gene Rv2822c
WT vs T0: DE MU vs T0: not DE
1000
800
600
400
Expression level
0
6
12
24
Time (h)
colour
MU
WT

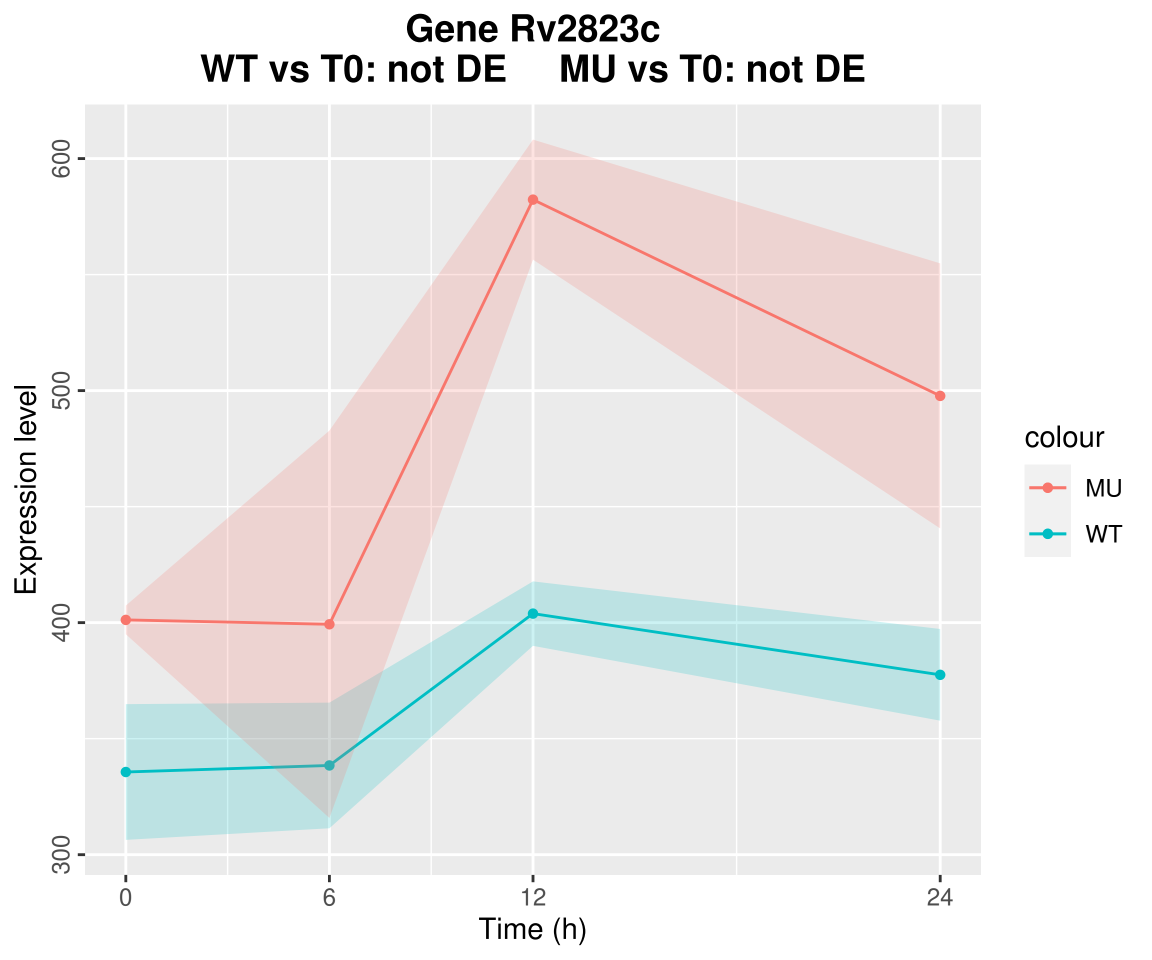
Gene Rv2823c
WT vs T0: not DE   MU vs T0: not DE
Expression level
600
500
400
300
Time (h)
0
6
12
24
colour
MU
WT

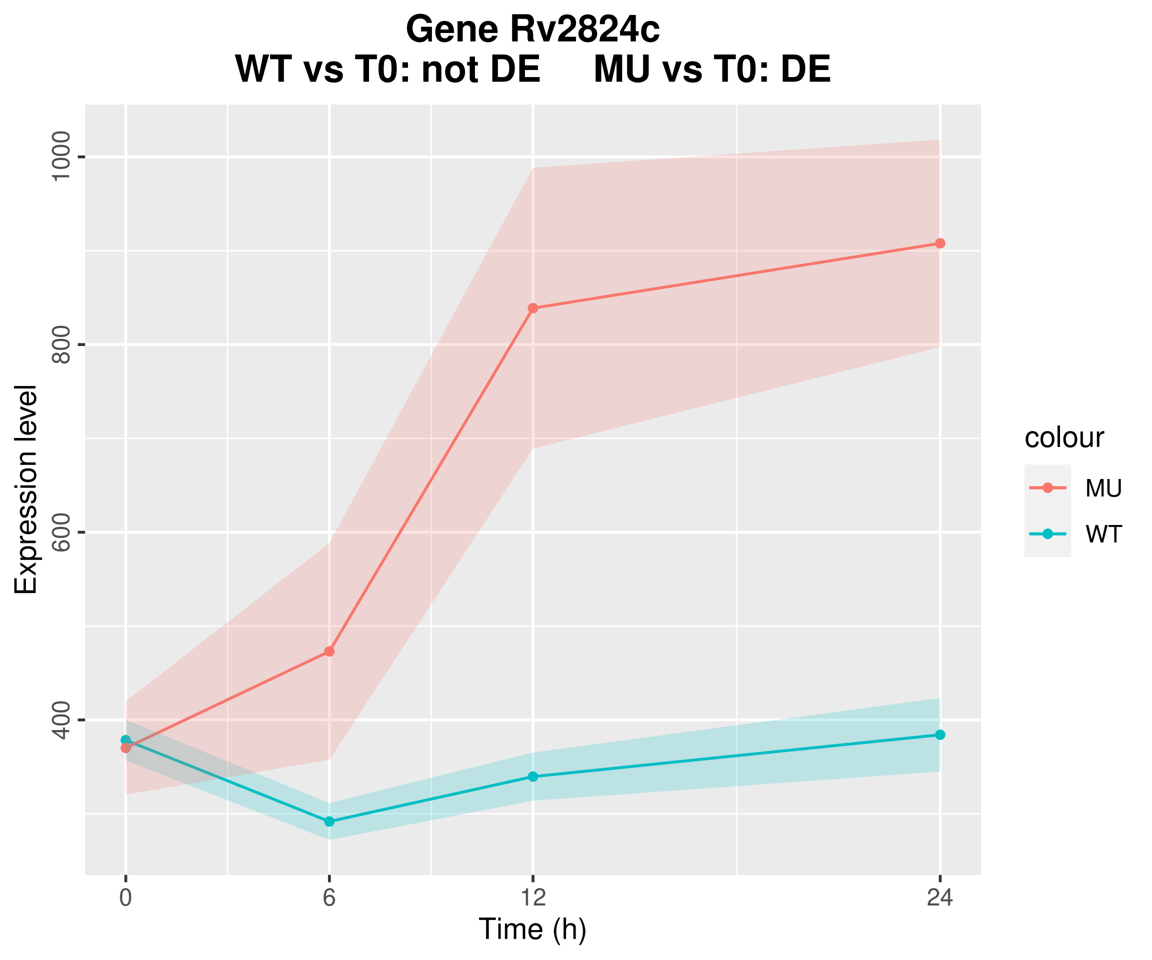

Gene Rv2824c
WT vs T0: not DE   MU vs T0: DE
Expression level
1000
800
600
400
0
6
12
24
Time (h)
colour
MU
WT

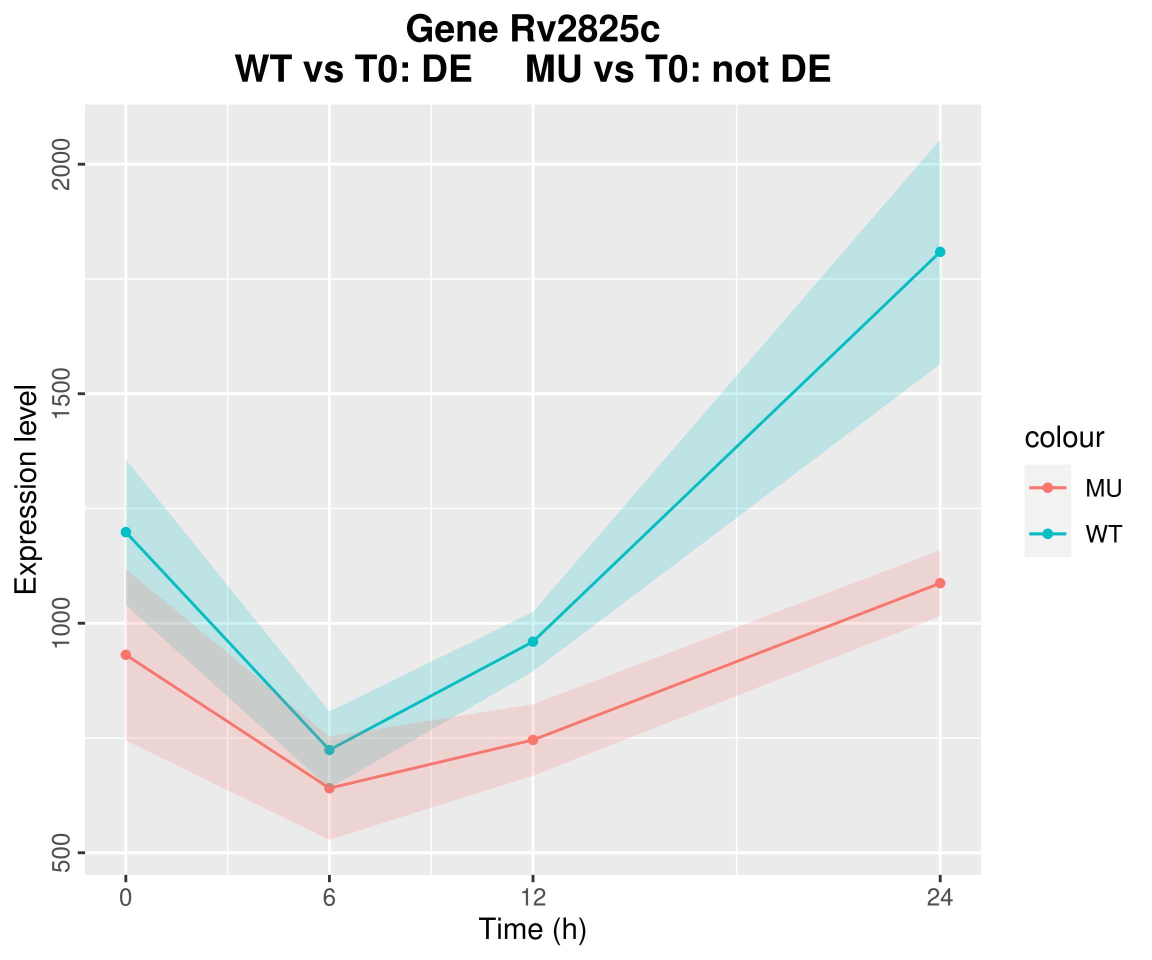
Gene Rv2825c
WT vs T0: DE MU vs T0: not DE
Expression level
2000
1500
1000
500
0
6
12
24
Time (h)
colour
MU
WT

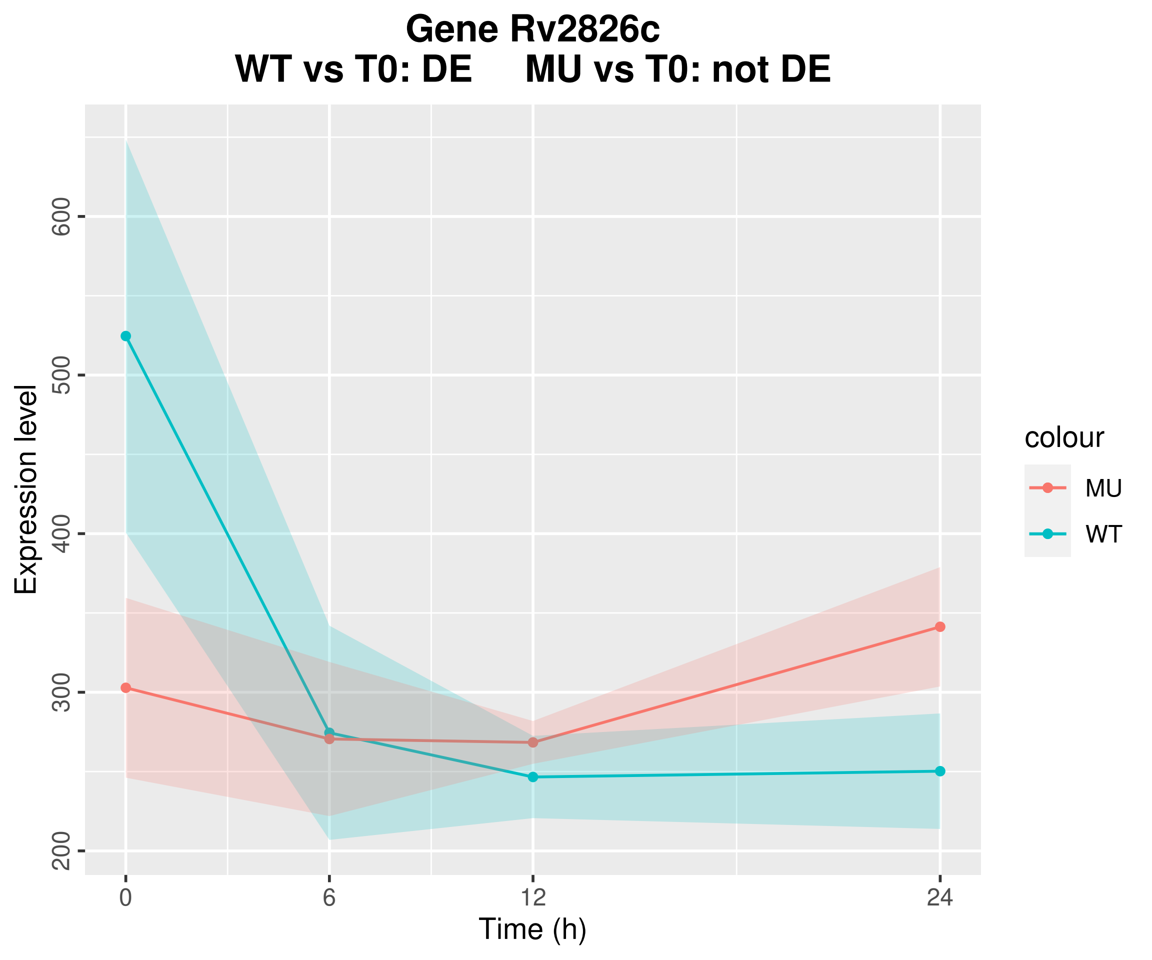

Gene Rv2826c
WT vs T0: DE MU vs T0: not DE
Expression level
200
300
400
500
600
0
6
12
24
Time (h)
colour
MU
WT

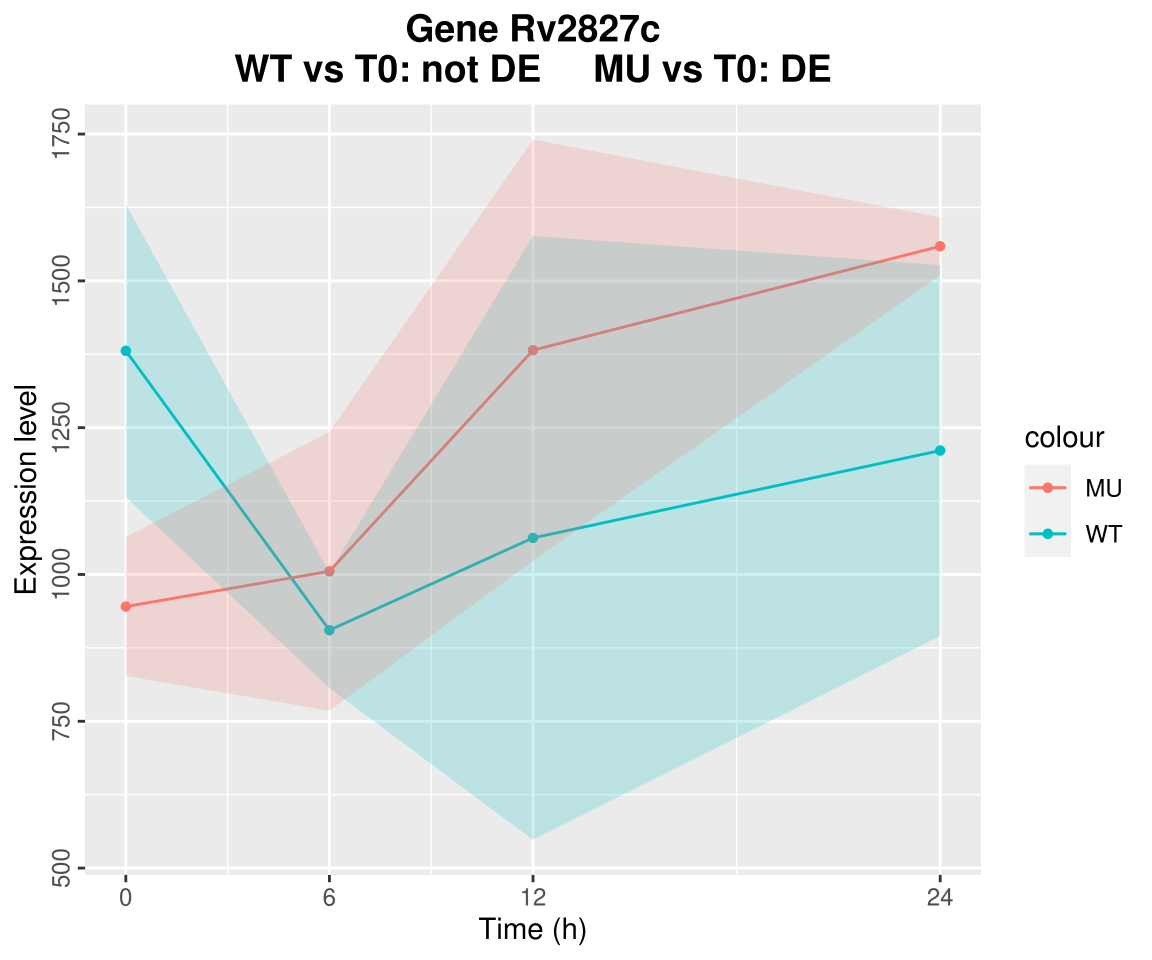

Gene Rv2827c
WT vs T0: not DE MU vs T0: DE
Expression level
1750
1500
1250
1000
750
500
0
6
12
24
Time (h)
colour
MU
WT

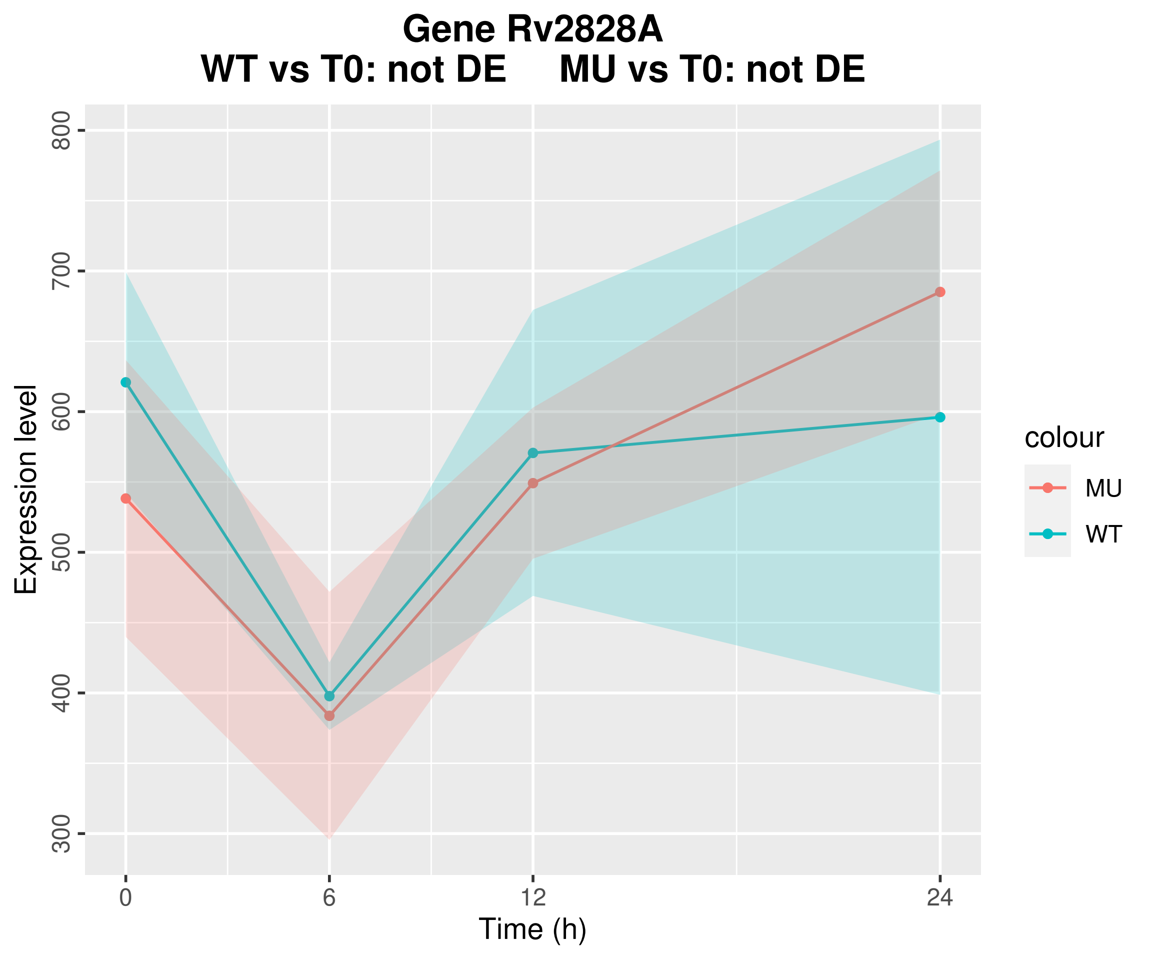
Gene Rv2828A
WT vs T0: not DE MU vs T0: not DE
Expression level
800
700
600
500
400
300
0
6
12
24
Time (h)
colour
MU
WT

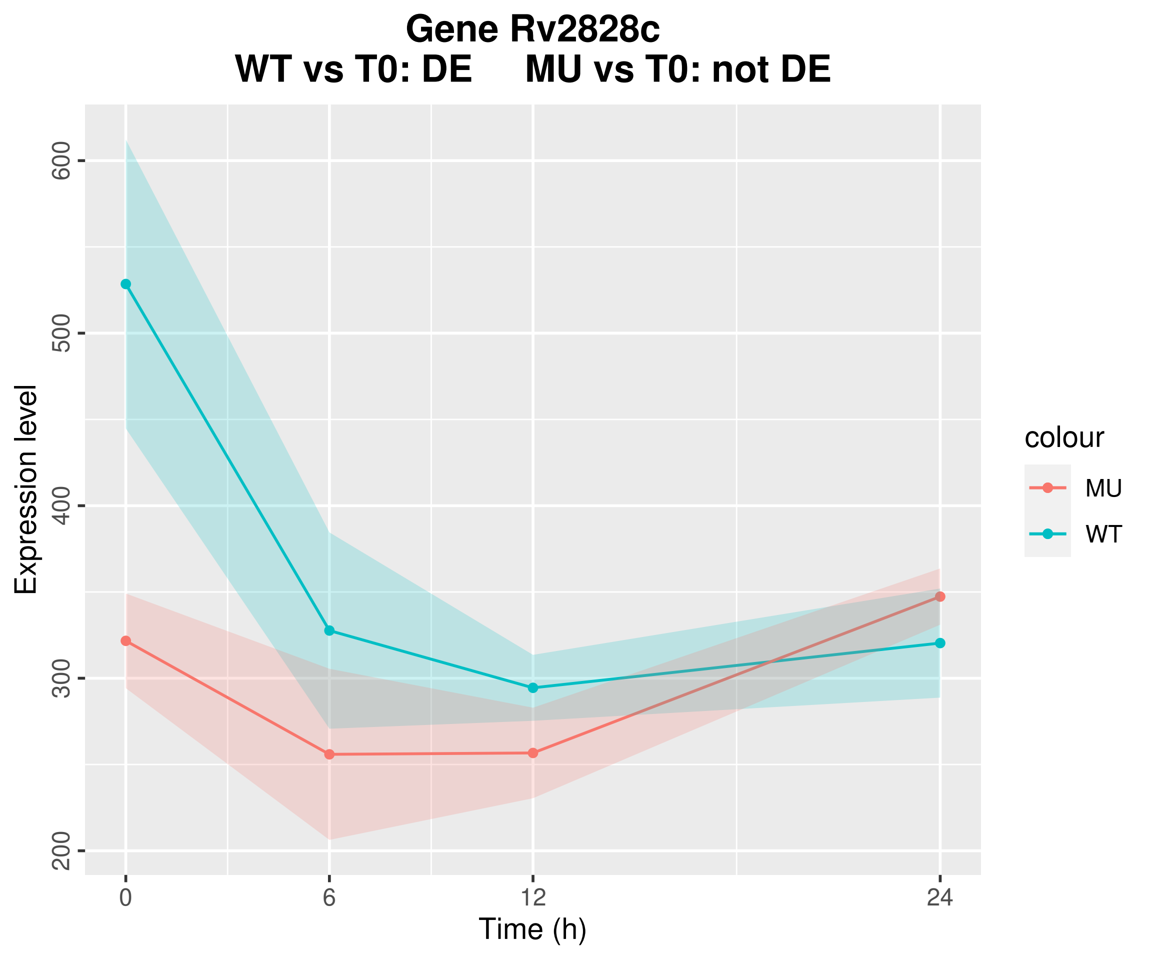
Gene Rv2828c
WT vs T0: DE MU vs T0: not DE
Expression level
600
500
400
300
200
0
6
12
24
Time (h)
colour
MU
WT

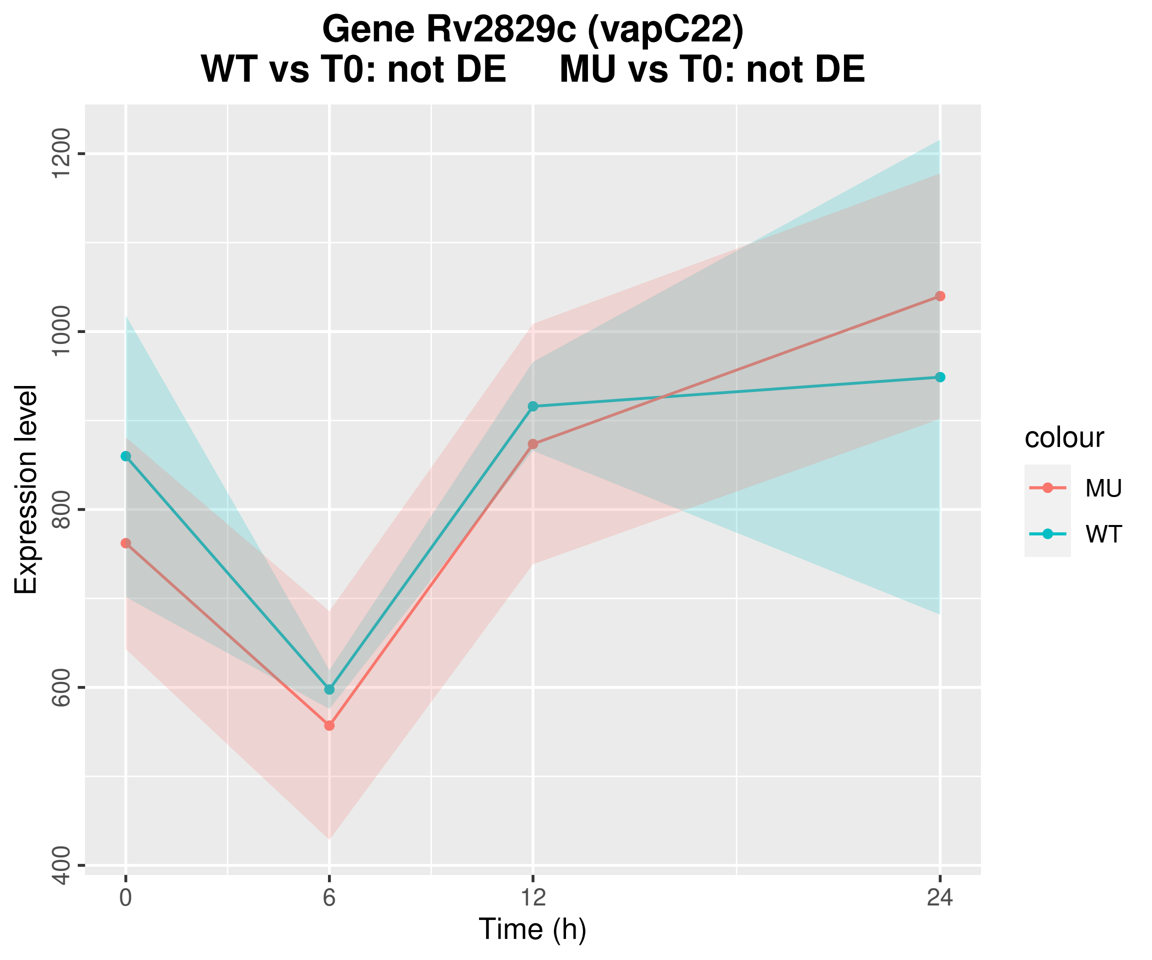

Gene Rv2829c (vapC22)
WT vs T0: not DE   MU vs T0: not DE
Expression level
1200
1000
800
600
400
0
6
12
24
Time (h)
colour
MU
WT

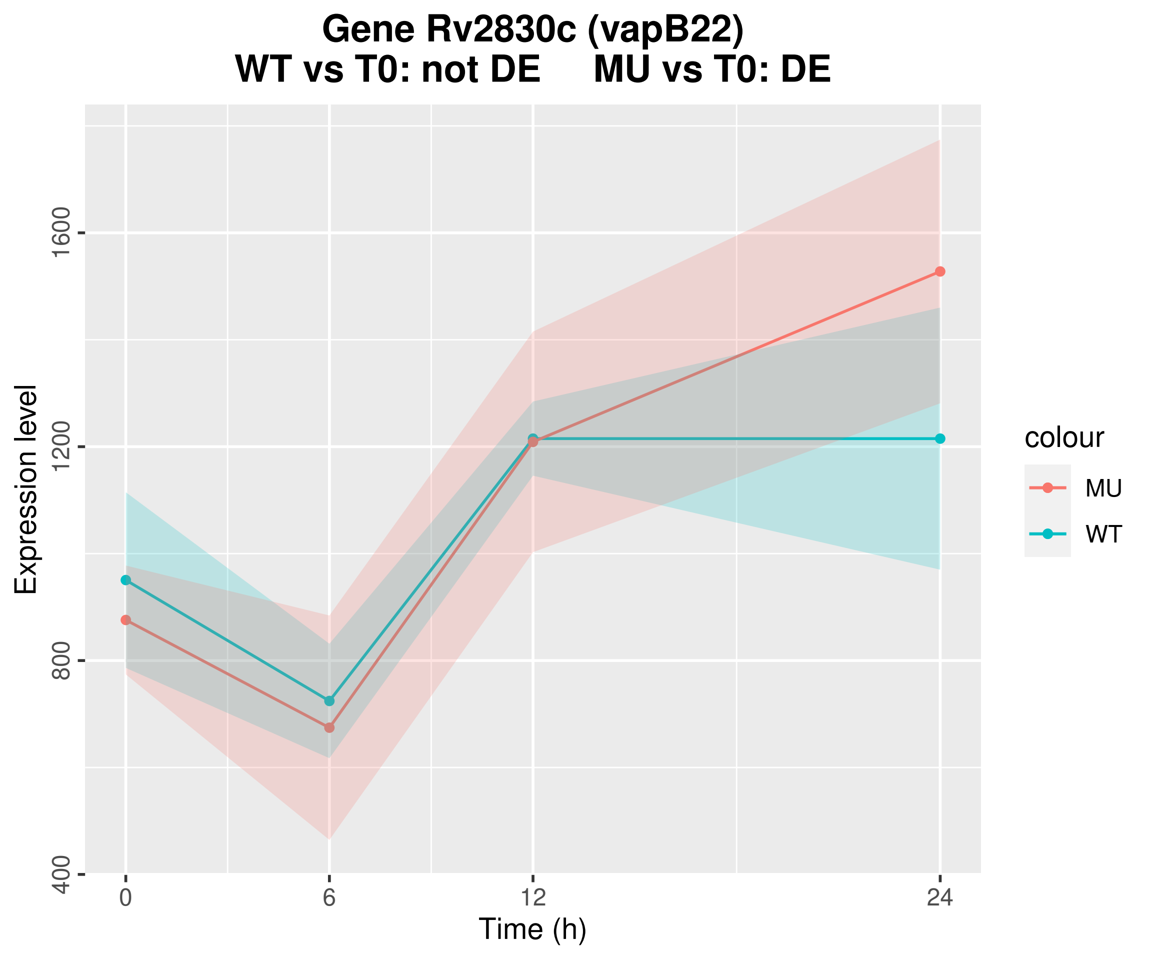

Gene Rv2830c (vapB22)
WT vs T0: not DE   MU vs T0: DE
Expression level
400
800
1200
1600
0
6
12
24
Time (h)
colour
MU
WT

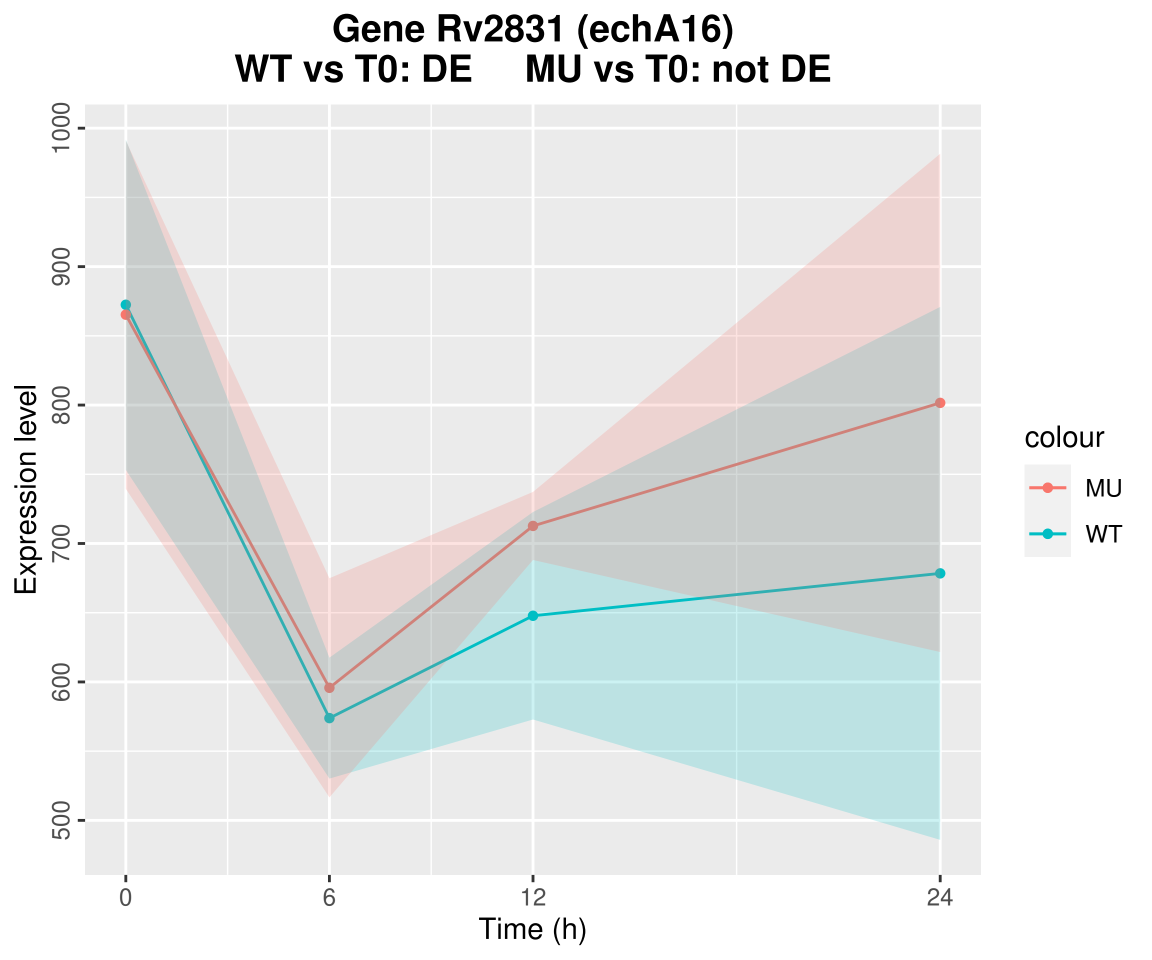

Gene Rv2831 (echA16)
WT vs T0: DE MU vs T0: not DE
Expression level
1000
900
800
700
600
500
0
6
12
24
Time (h)
colour
MU
WT

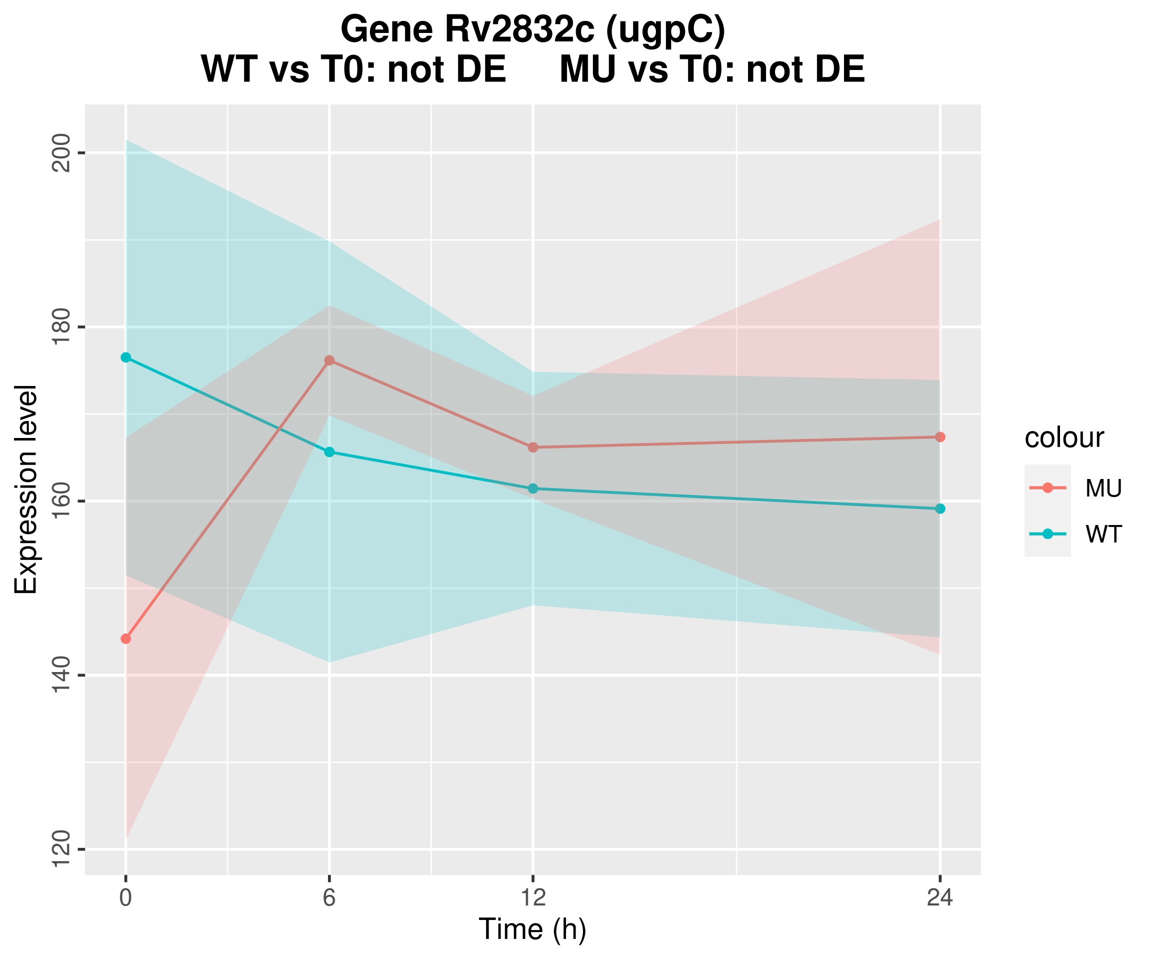

Gene Rv2832c (ugpC)
WT vs T0: not DE MU vs T0: not DE
Expression level
200
180
160
140
120
0
6
12
24
Time (h)
colour
MU
WT

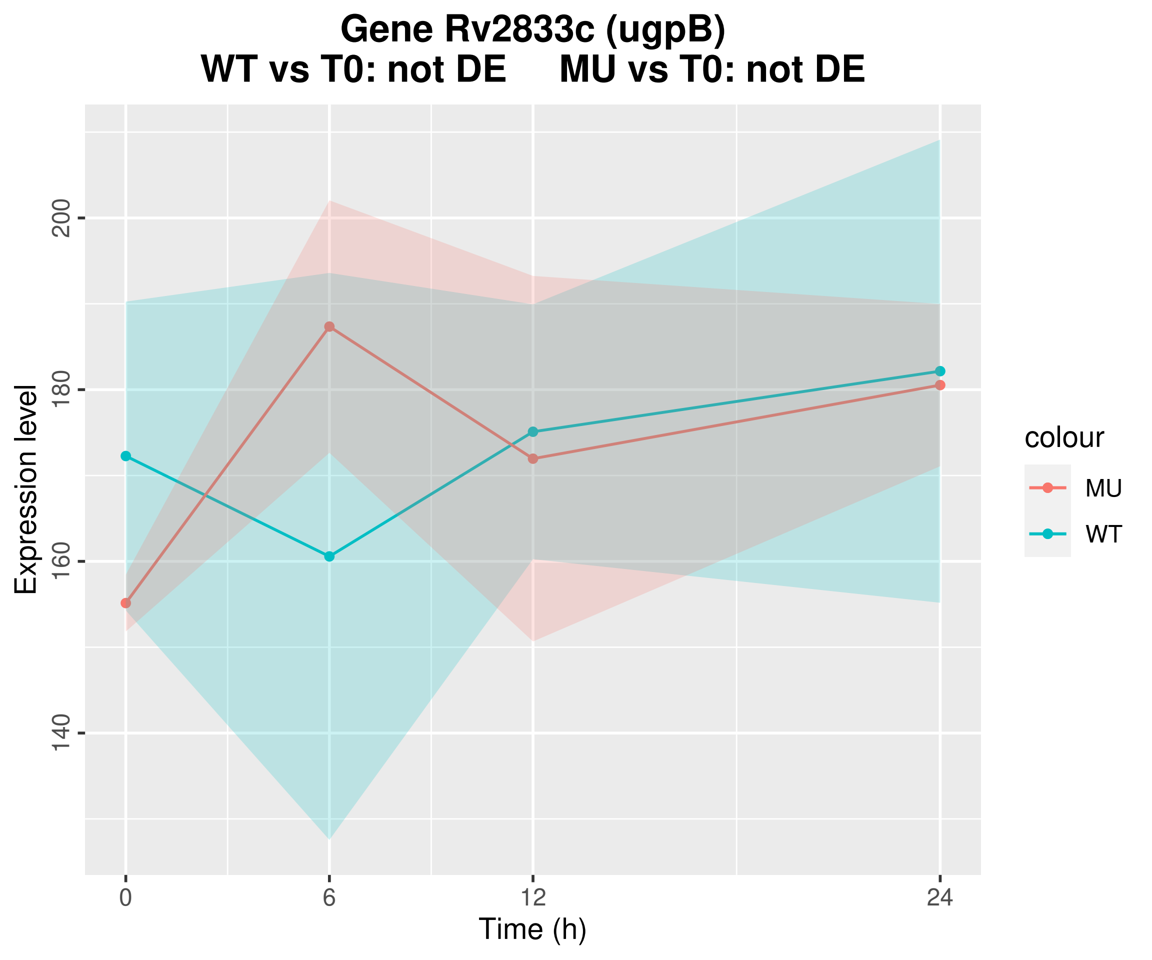
Gene Rv2833c (ugpB)
WT vs T0: not DE   MU vs T0: not DE
Expression level
200
180
160
140
0
6
12
24
Time (h)
colour
MU
WT

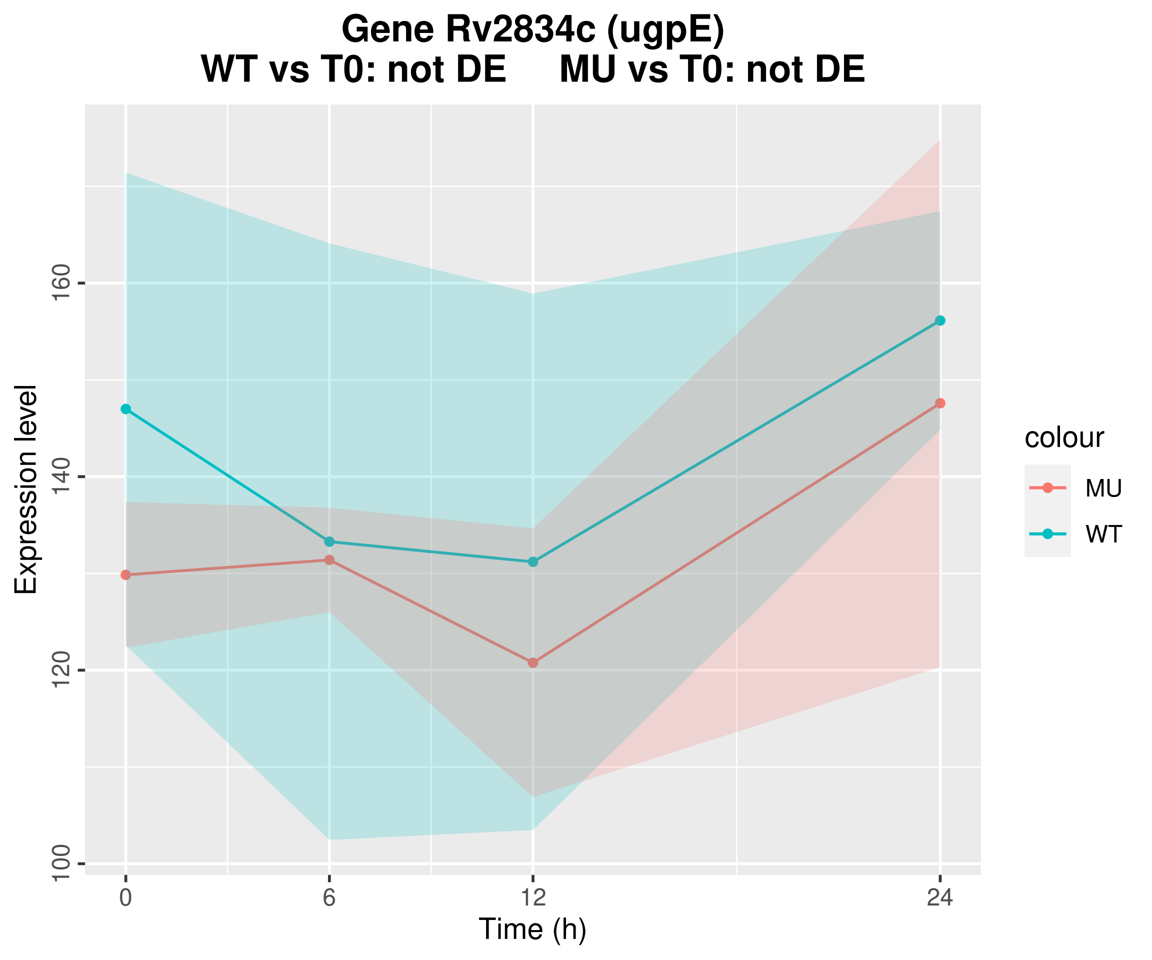

Gene Rv2834c (ugpE)
WT vs T0: not DE   MU vs T0: not DE
Expression level
160
140
120
100
0
6
12
24
Time (h)
colour
MU
WT

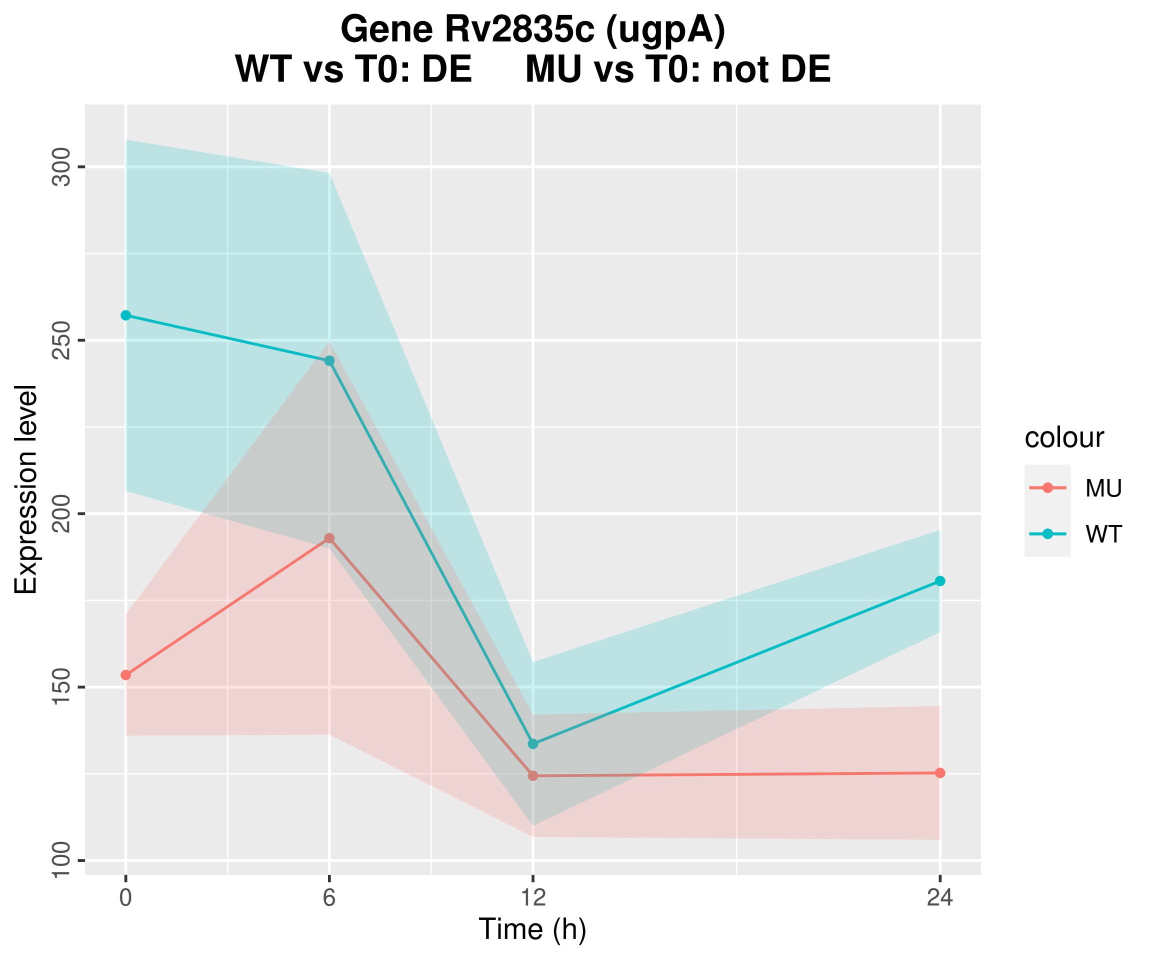

Gene Rv2835c (ugpA)
WT vs T0: DE   MU vs T0: not DE
Expression level
Time (h)
300
250
200
150
100
0
6
12
24
colour
MU
WT

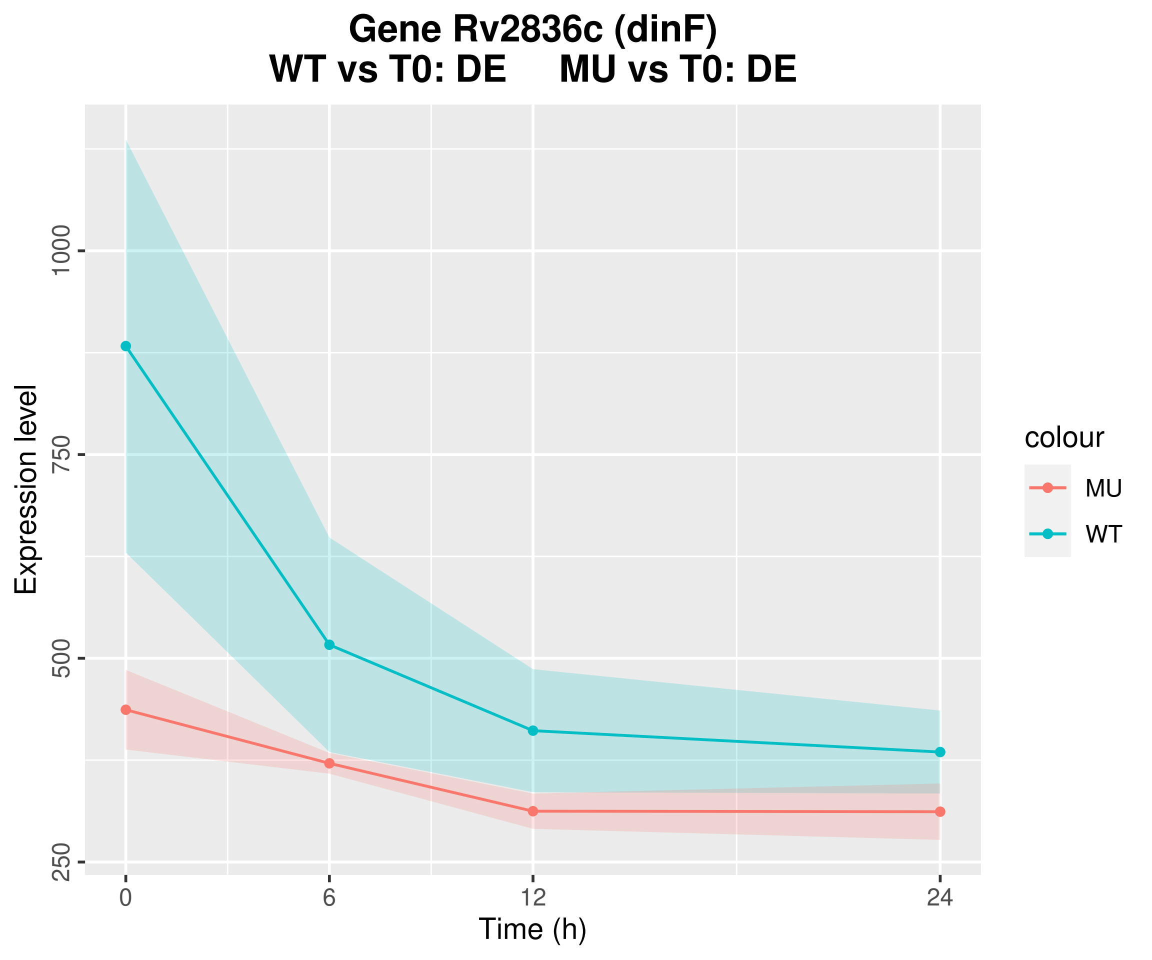

Gene Rv2836c (dinF)
WT vs T0: DE MU vs T0: DE
Expression level
1000
750
500
250
0
6
12
24
Time (h)
colour
MU
WT

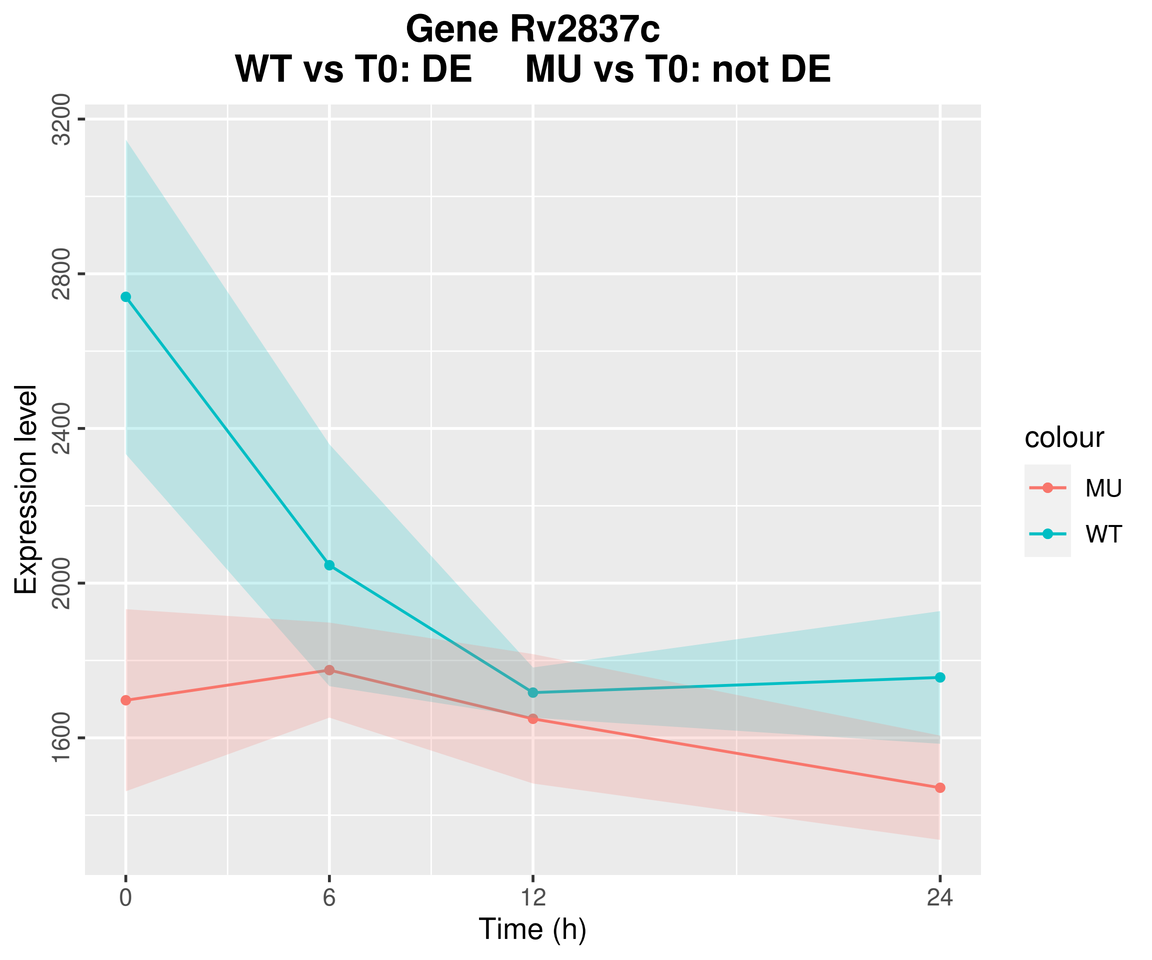

Gene Rv2837c
WT vs T0: DE MU vs T0: not DE
3200
2800
2400
2000
1600
Expression level
0
6
12
24
Time (h)
colour
MU
WT

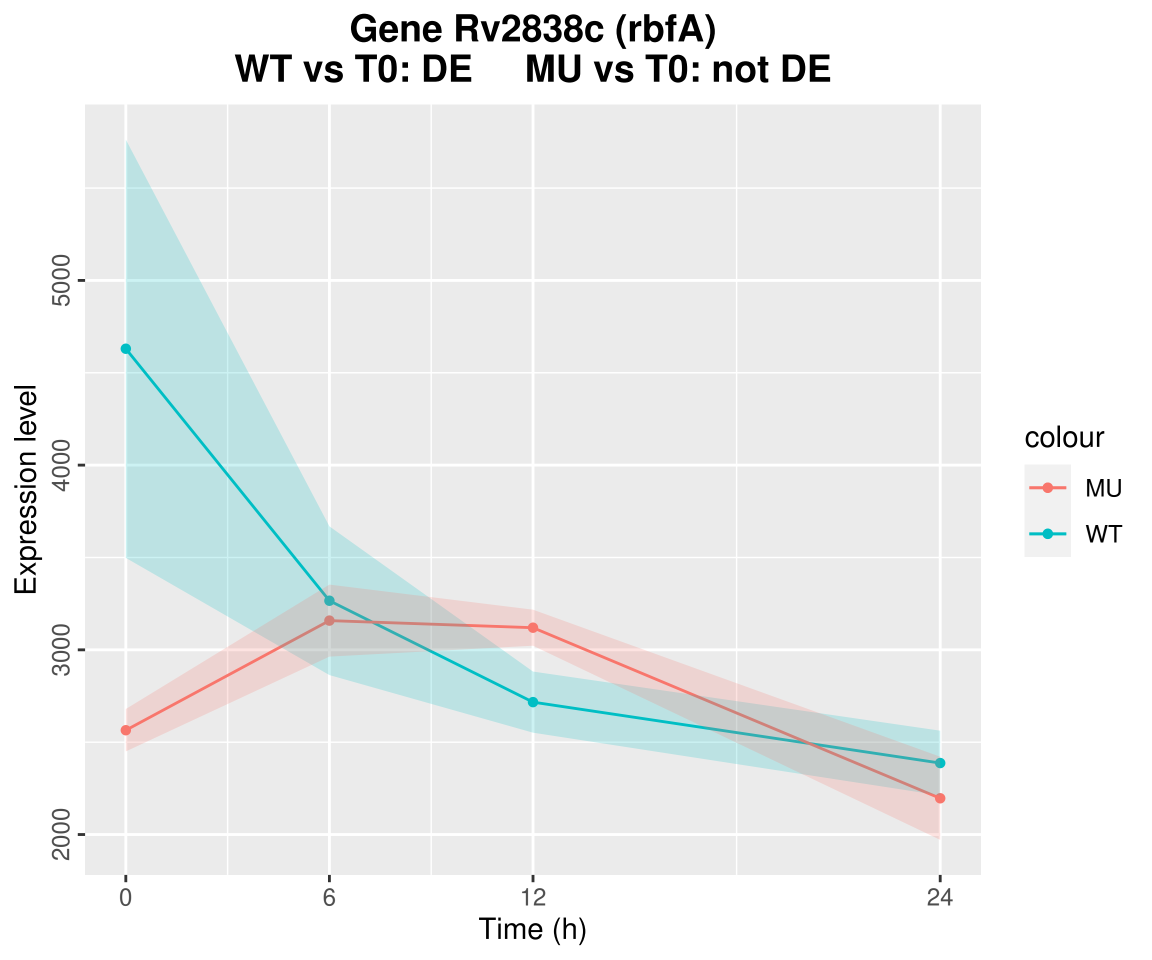

Gene Rv2838c (rbfA)
WT vs T0: DE MU vs T0: not DE
Expression level
5000
4000
3000
2000
0
6
12
24
Time (h)
colour
MU
WT

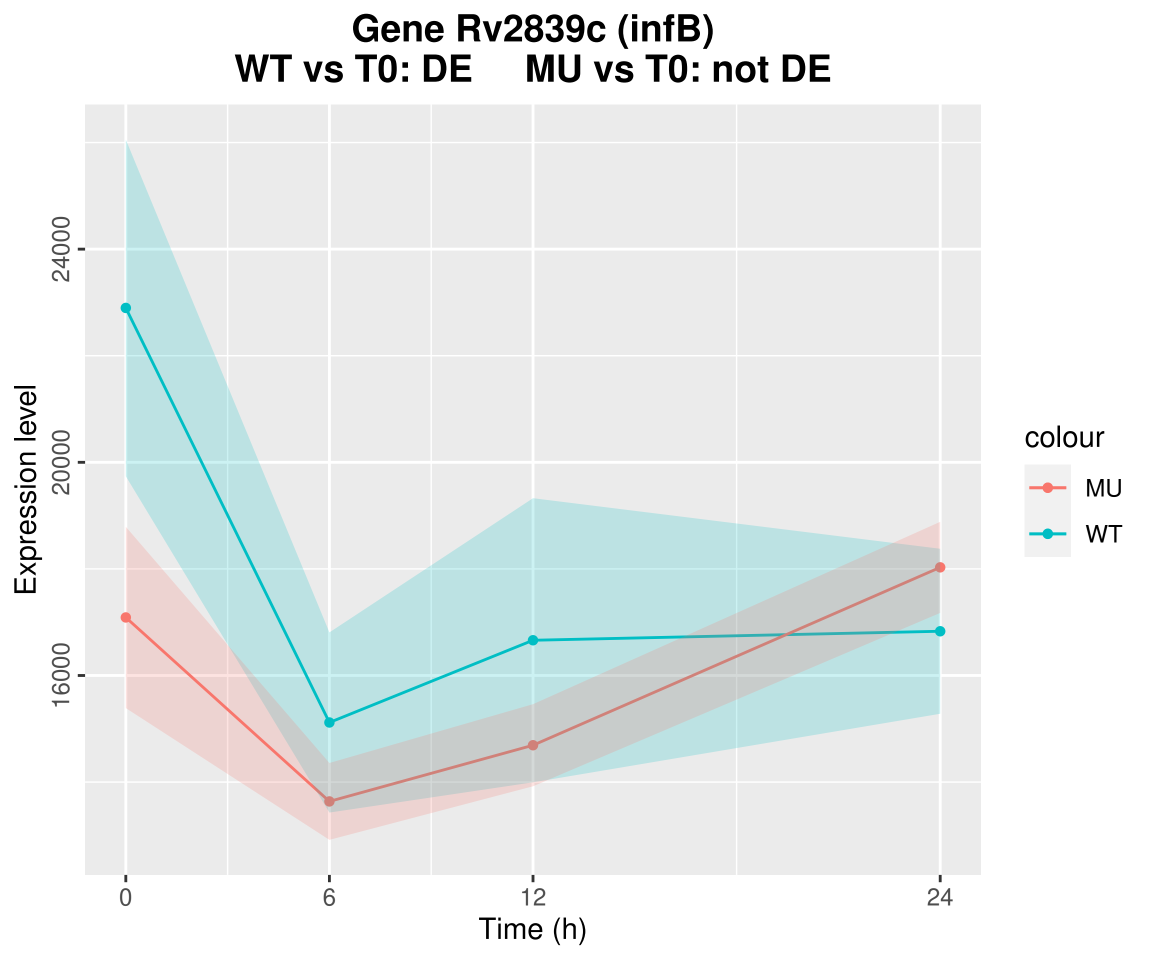

Gene Rv2839c (infB)
WT vs T0: DE MU vs T0: not DE
Expression level
24000
20000
16000
0
6
12
24
Time (h)
colour
MU
WT

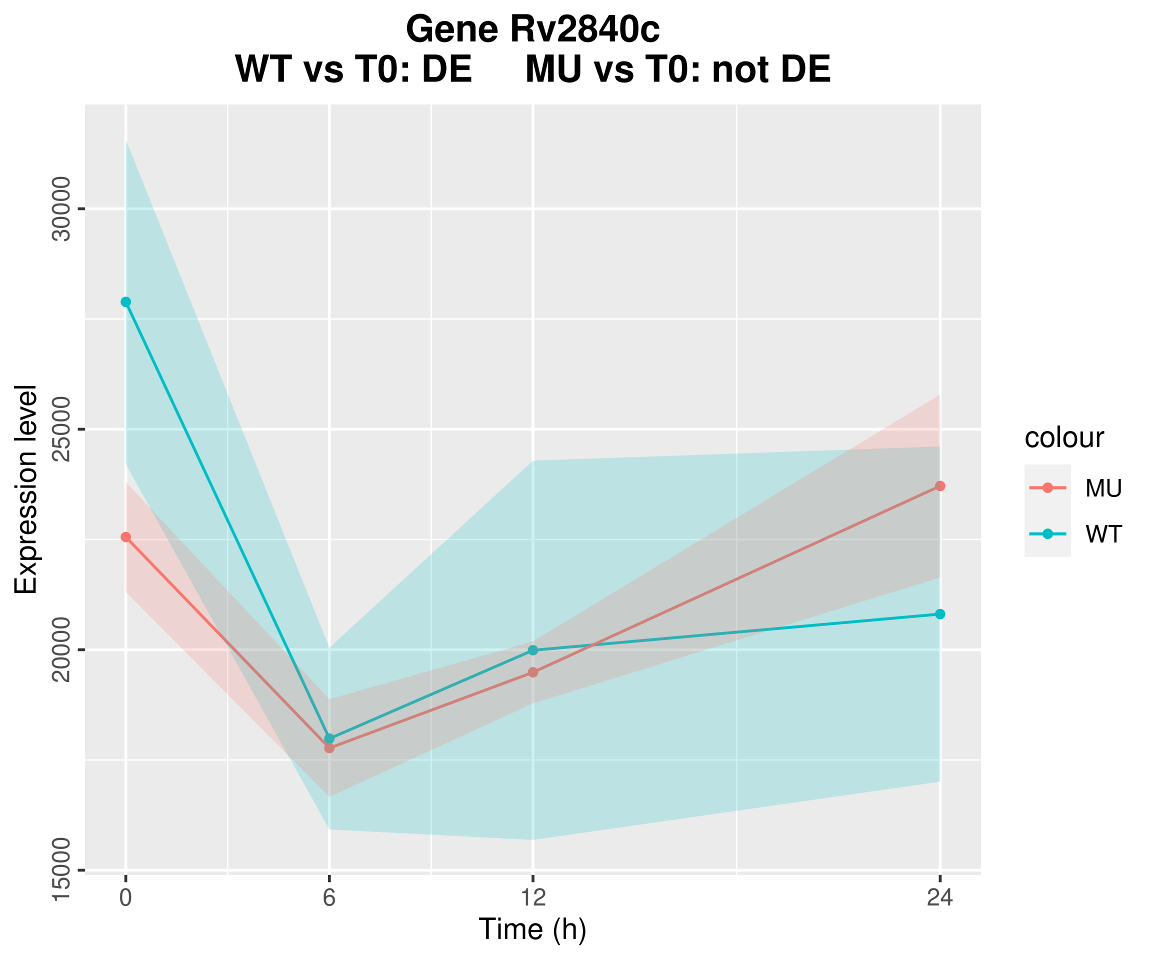

Gene Rv2840c
WT vs T0: DE   MU vs T0: not DE
Expression level
30000
25000
20000
15000
0
6
12
24
Time (h)
colour
MU
WT

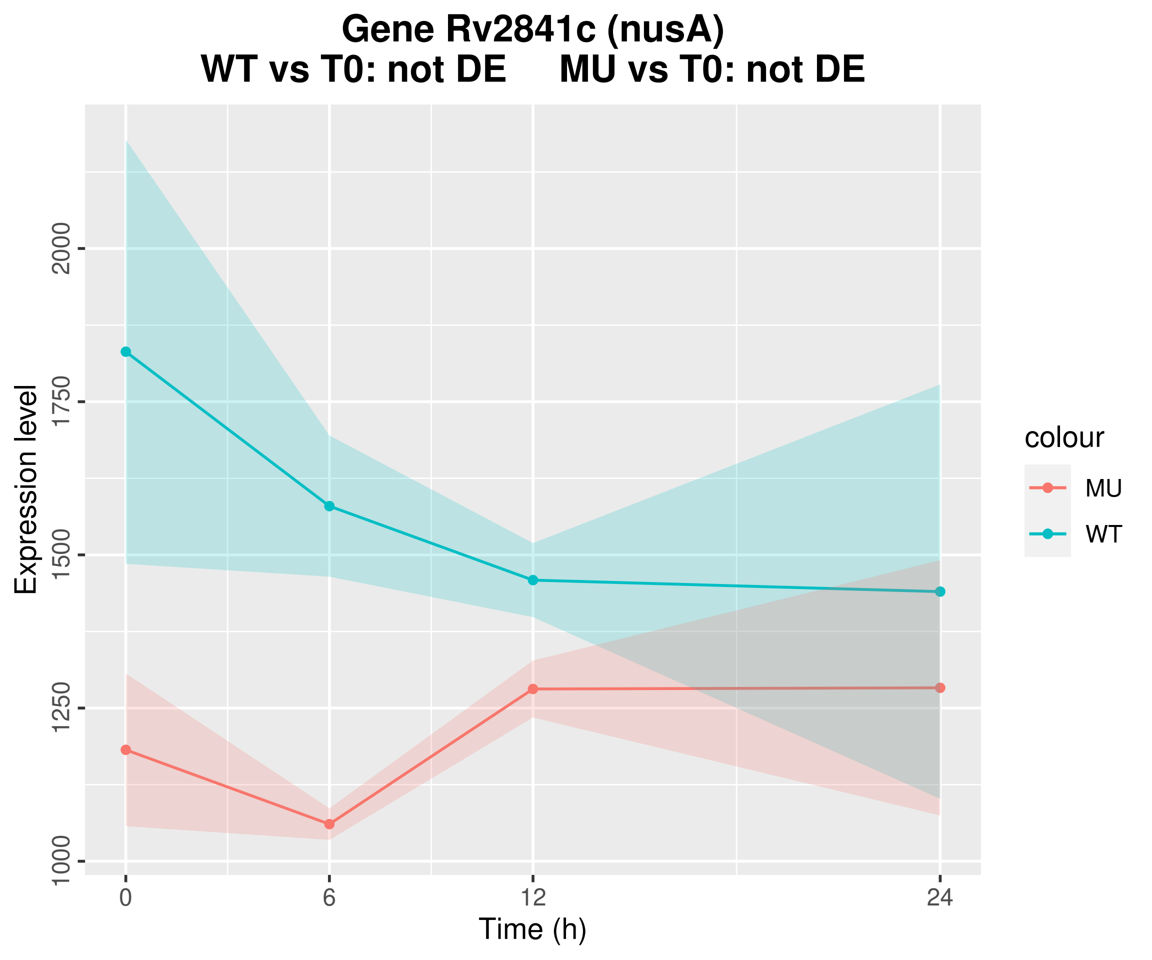

Gene Rv2841c (nusA)
WT vs T0: not DE MU vs T0: not DE
Expression level
2000
1750
1500
1250
1000
0
6
12
24
Time (h)
colour
MU
WT

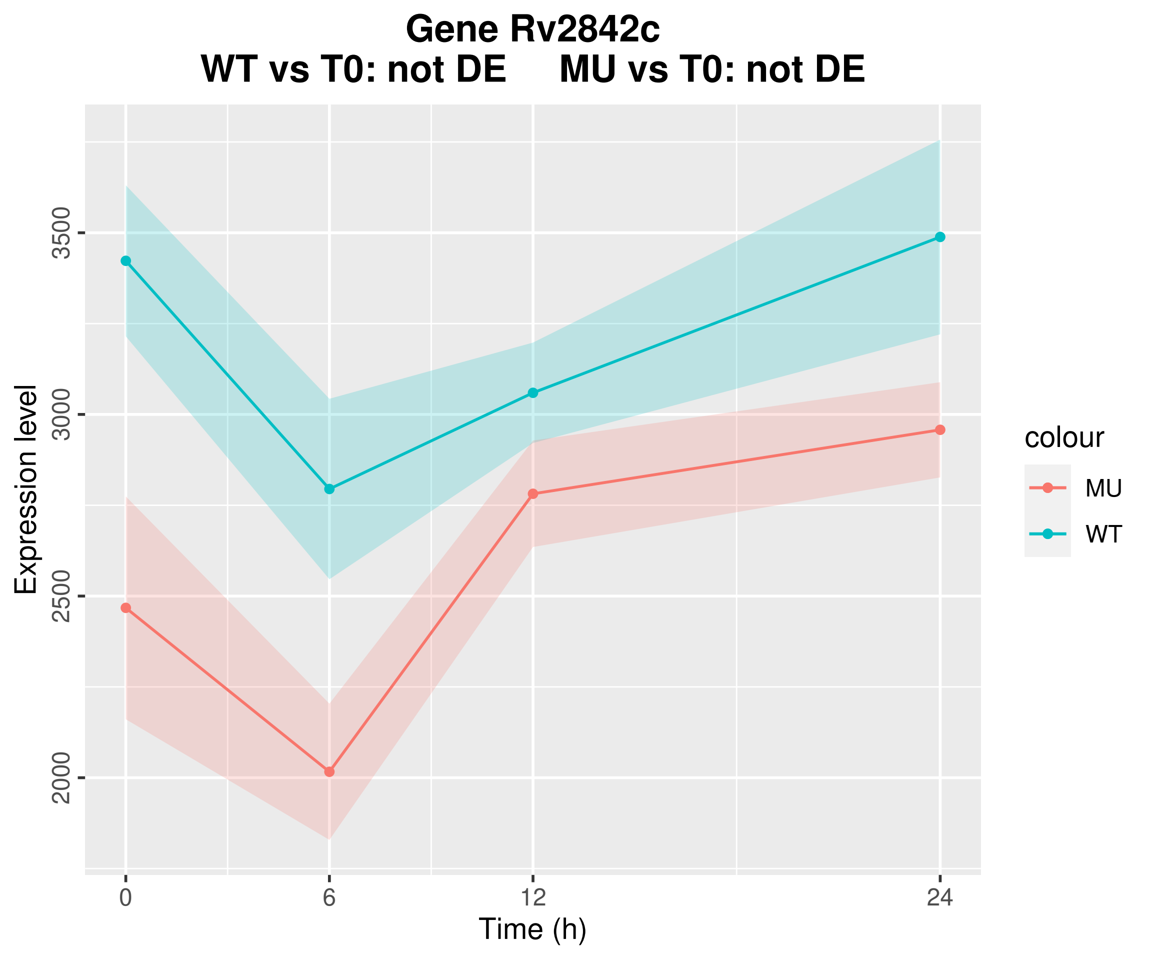
Gene Rv2842c
WT vs T0: not DE MU vs T0: not DE
Expression level
3500
3000
2500
2000
0
6
12
24
Time (h)
colour
MU
WT

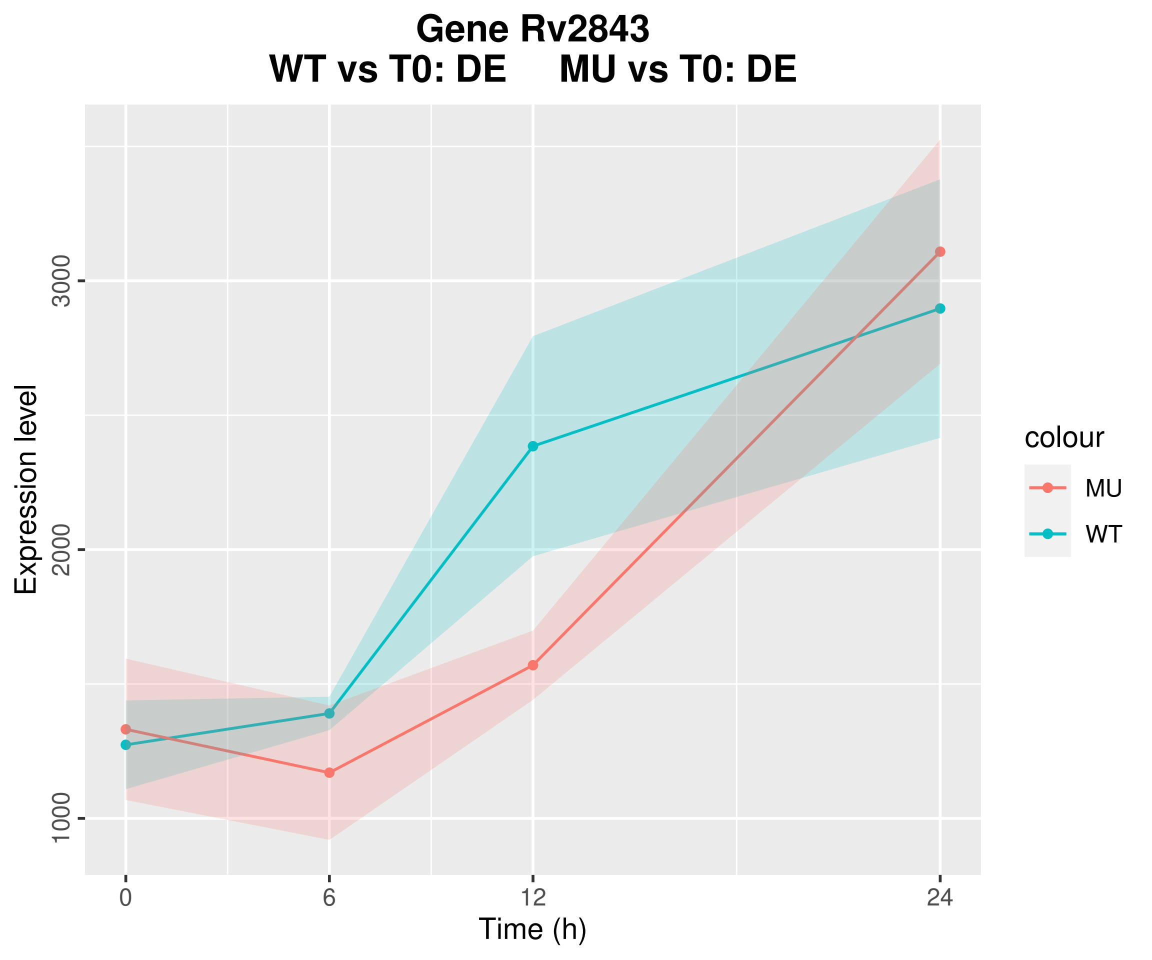
Gene Rv2843
WT vs T0: DE MU vs T0: DE
Expression level
3000
2000
1000
0
6
12
24
Time (h)
colour
MU
WT

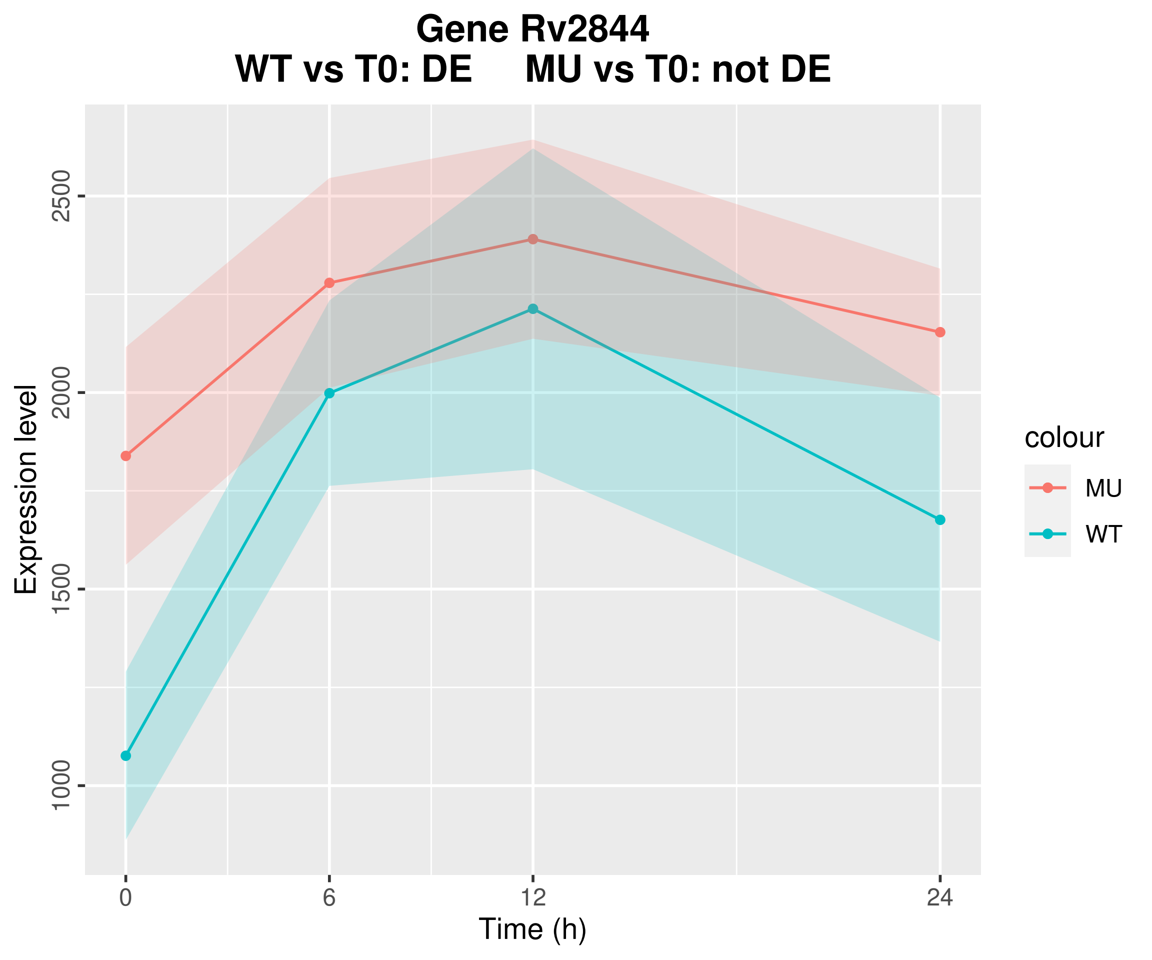

Gene Rv2844
WT vs T0: DE MU vs T0: not DE
Expression level
2500
2000
1500
1000
0
6
12
24
Time (h)
colour
MU
WT

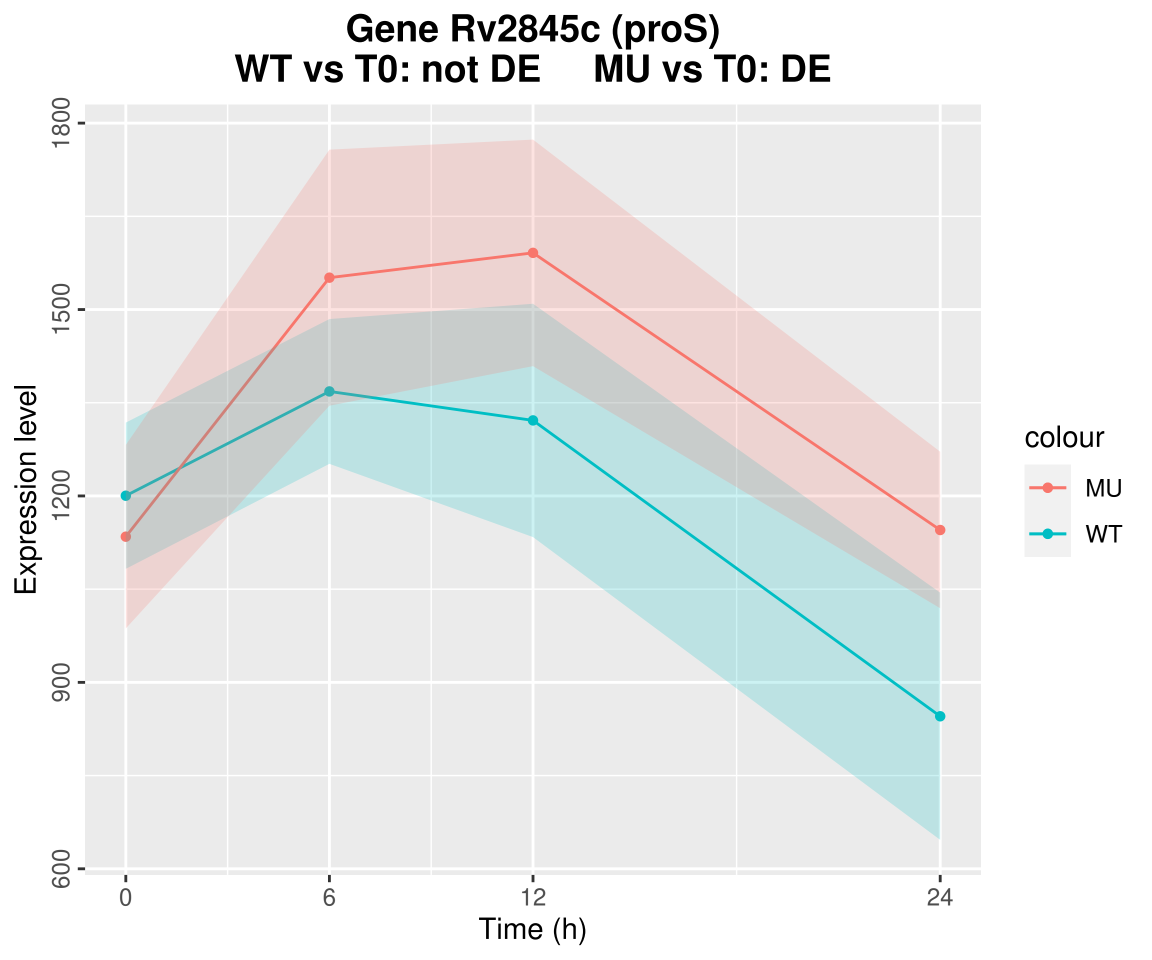
Gene Rv2845c (proS)
WT vs T0: not DE MU vs T0: DE
Expression level
1800
1500
1200
900
600
0
6
12
24
Time (h)
colour
MU
WT

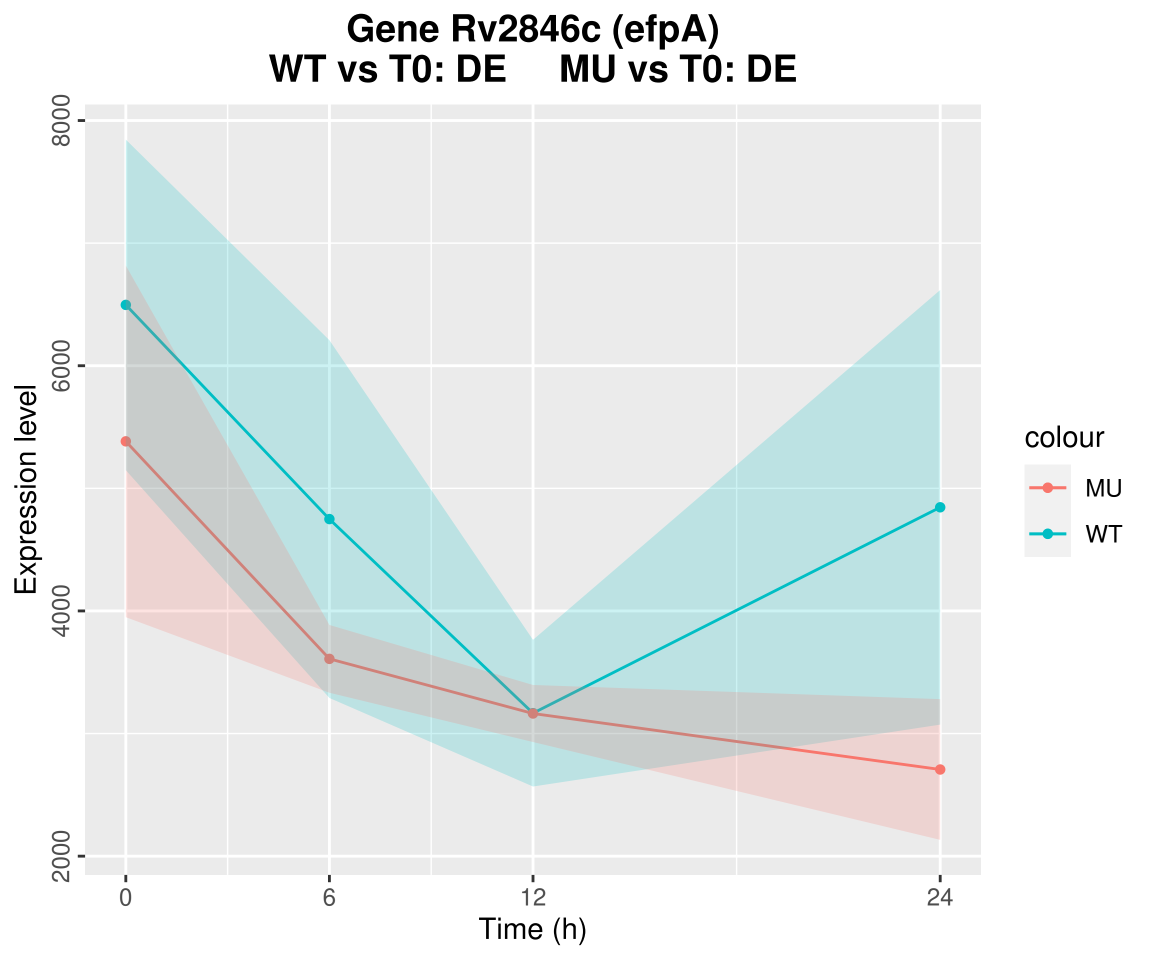
Gene Rv2846c (efpA)
WT vs T0: DE MU vs T0: DE
Expression level
8000
6000
4000
2000
0
6
12
24
Time (h)
colour
MU
WT

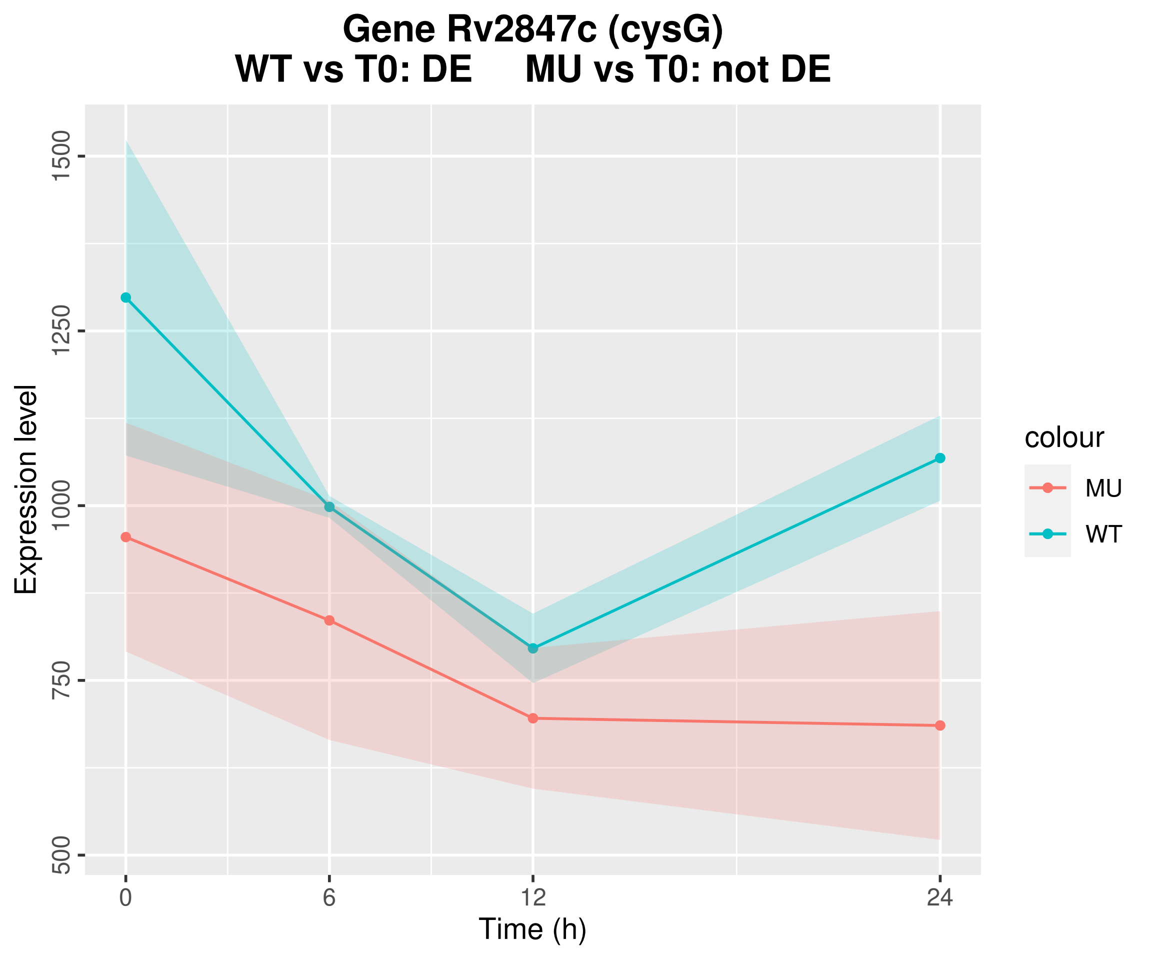

Gene Rv2847c (cysG)
WT vs T0: DE MU vs T0: not DE
1500
1250
1000
750
500
Expression level
0
6
12
24
Time (h)
colour
MU
WT

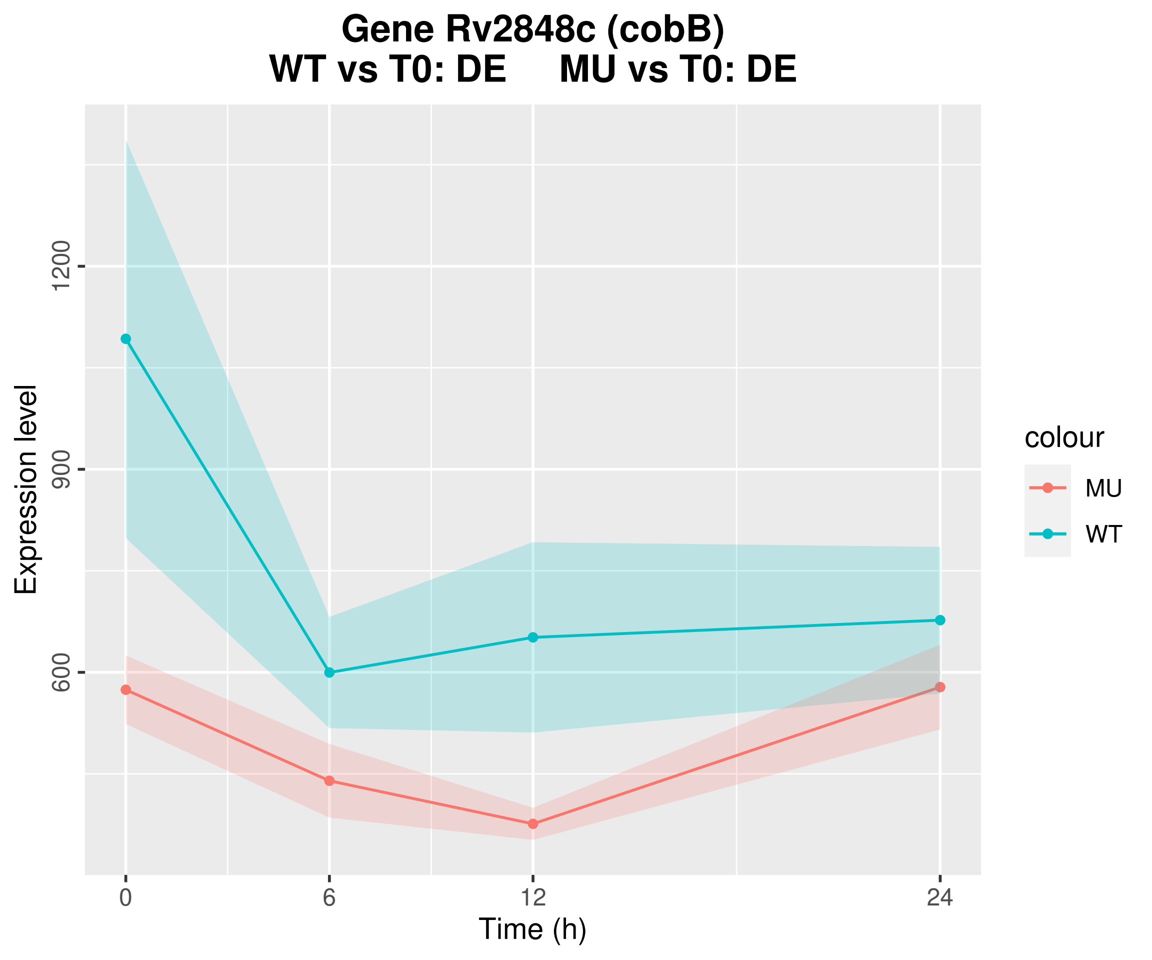
Gene Rv2848c (cobB)
WT vs T0: DE MU vs T0: DE
Expression level
1200
900
600
0
6
12
24
Time (h)
colour
MU
WT

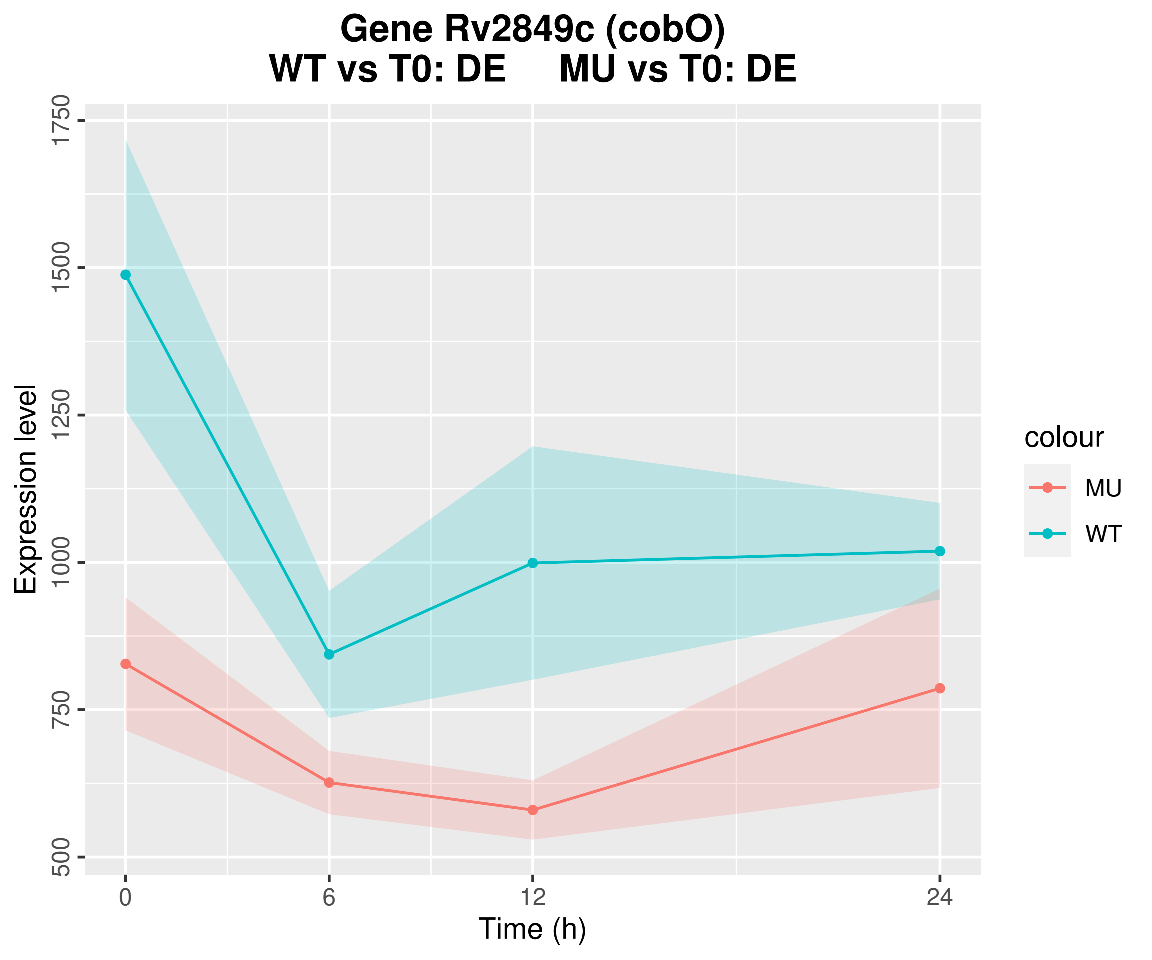
Gene Rv2849c (cobO)
WT vs T0: DE   MU vs T0: DE
Expression level
1750
1500
1250
1000
750
500
0
6
12
24
Time (h)
colour
MU
WT

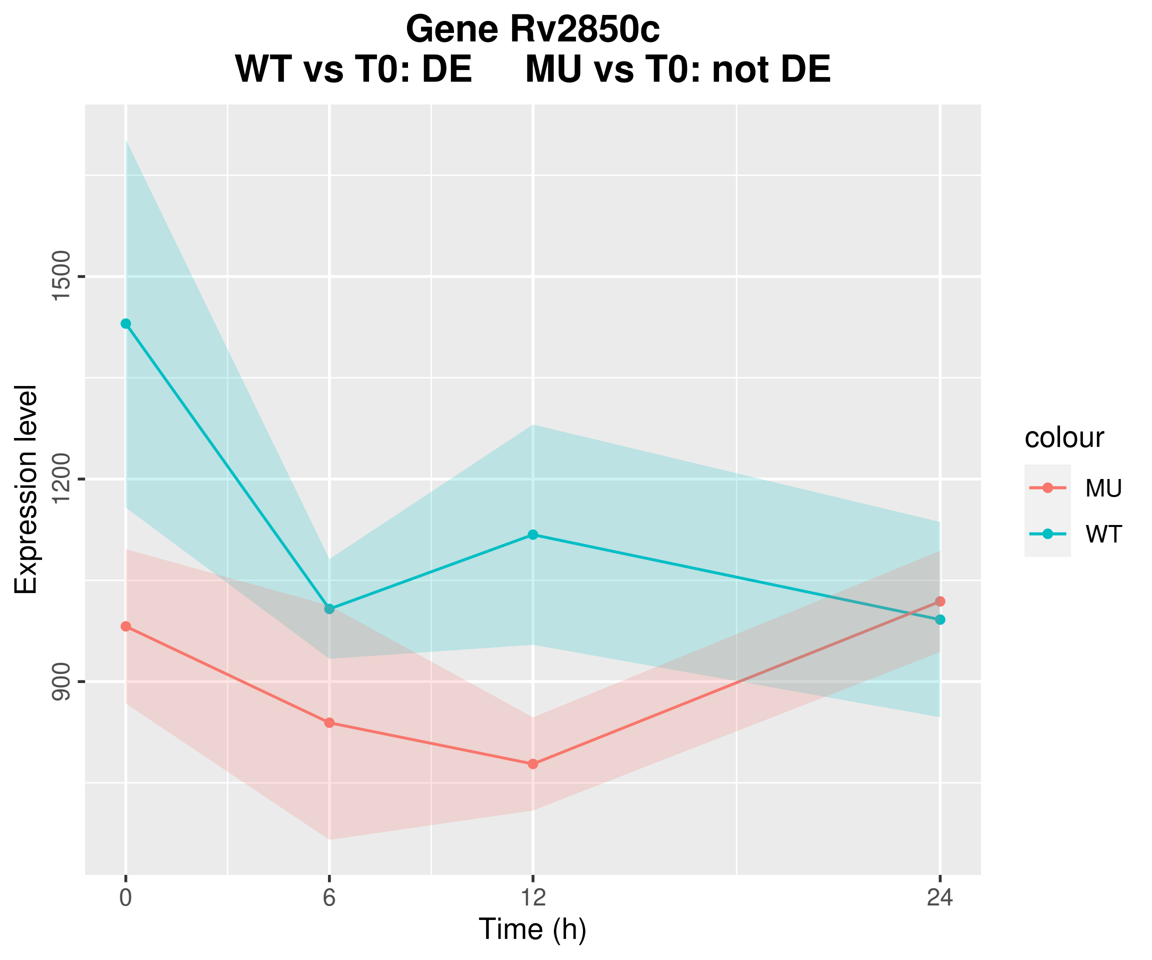

Gene Rv2850c
WT vs T0: DE MU vs T0: not DE
Expression level
1500
1200
900
0
6
12
24
Time (h)
colour
MU
WT

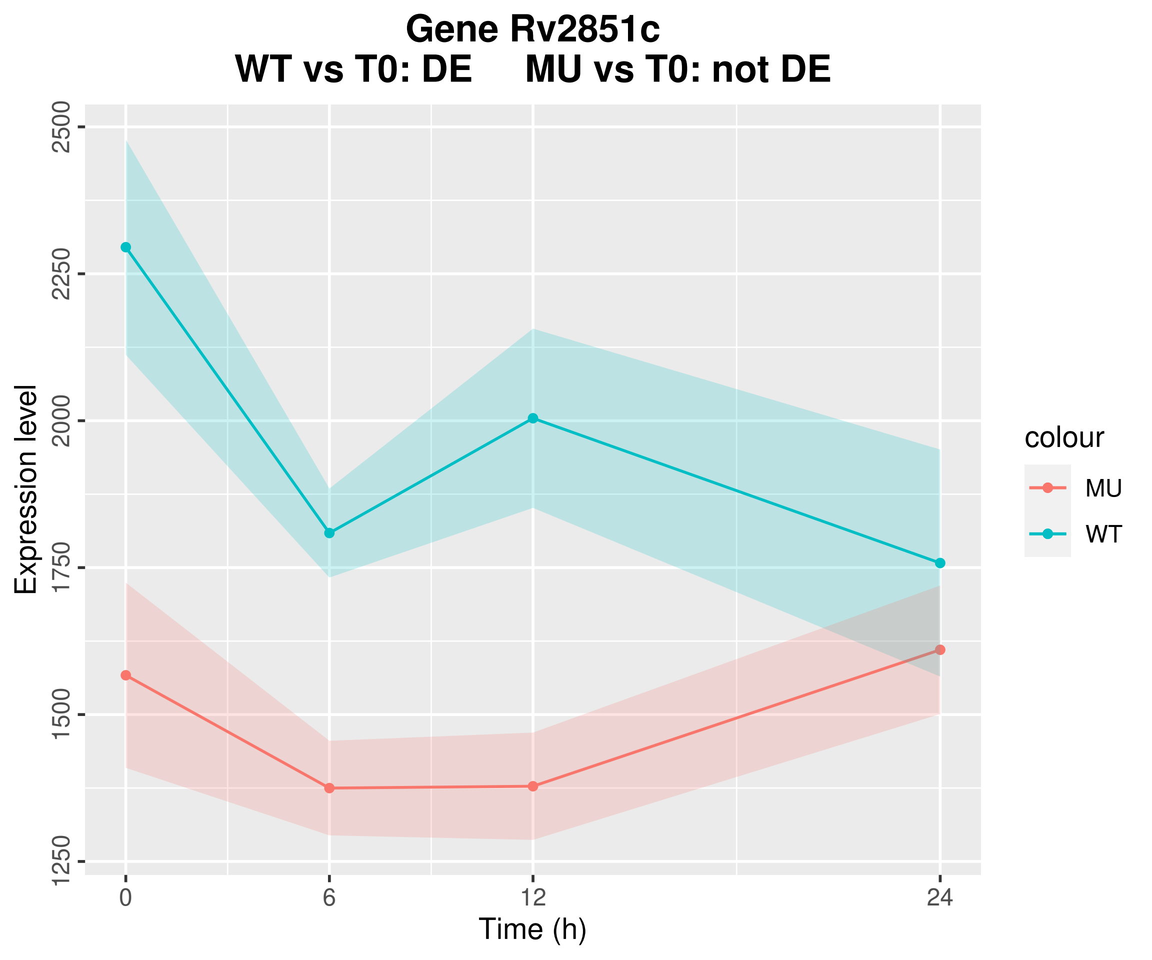

Gene Rv2851c
WT vs T0: DE MU vs T0: not DE
Expression level
2500
2250
2000
1750
1500
1250
0
6
12
24
Time (h)
colour
MU
WT

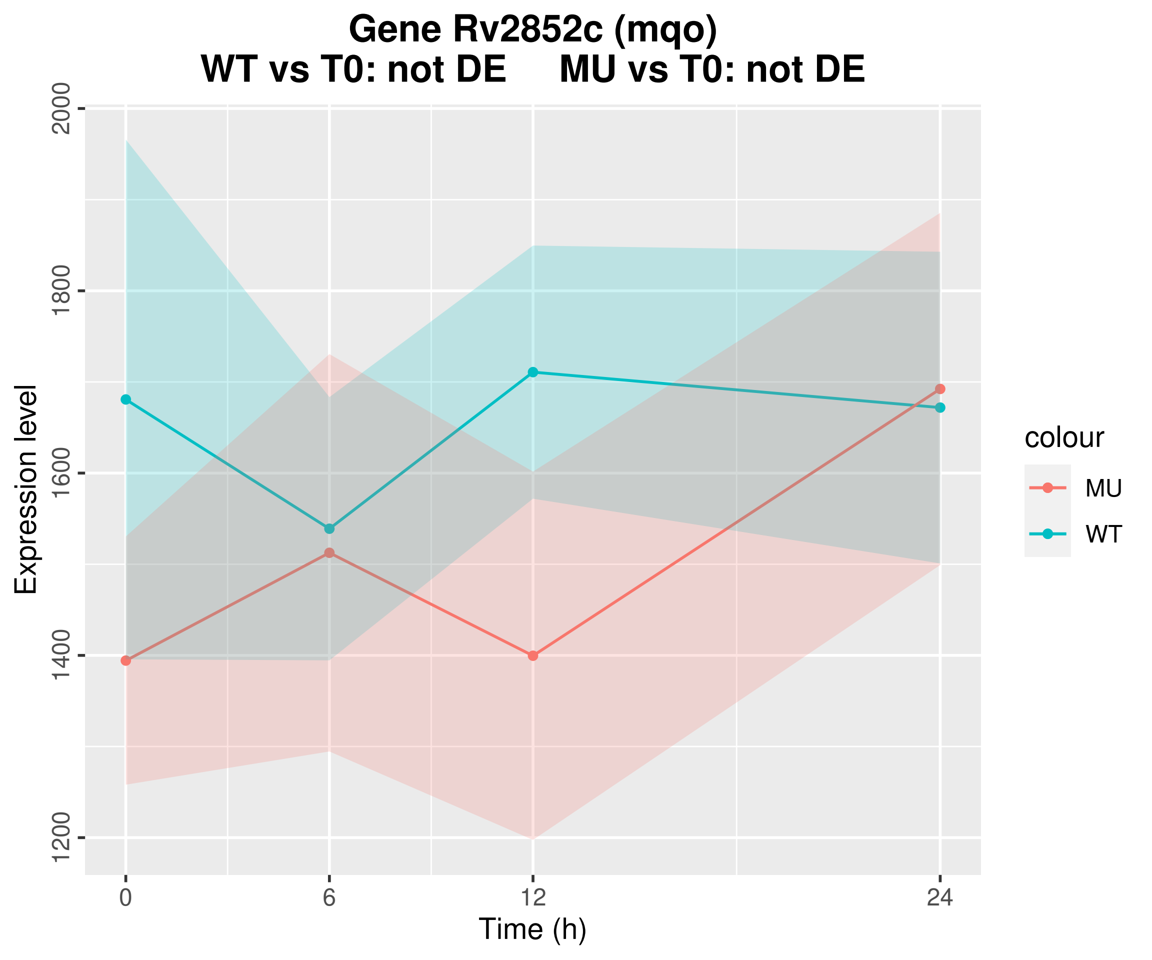
Gene Rv2852c (mqo)
WT vs T0: not DE   MU vs T0: not DE
Expression level
2000
1800
1600
1400
1200
0
6
12
24
Time (h)
colour
MU
WT

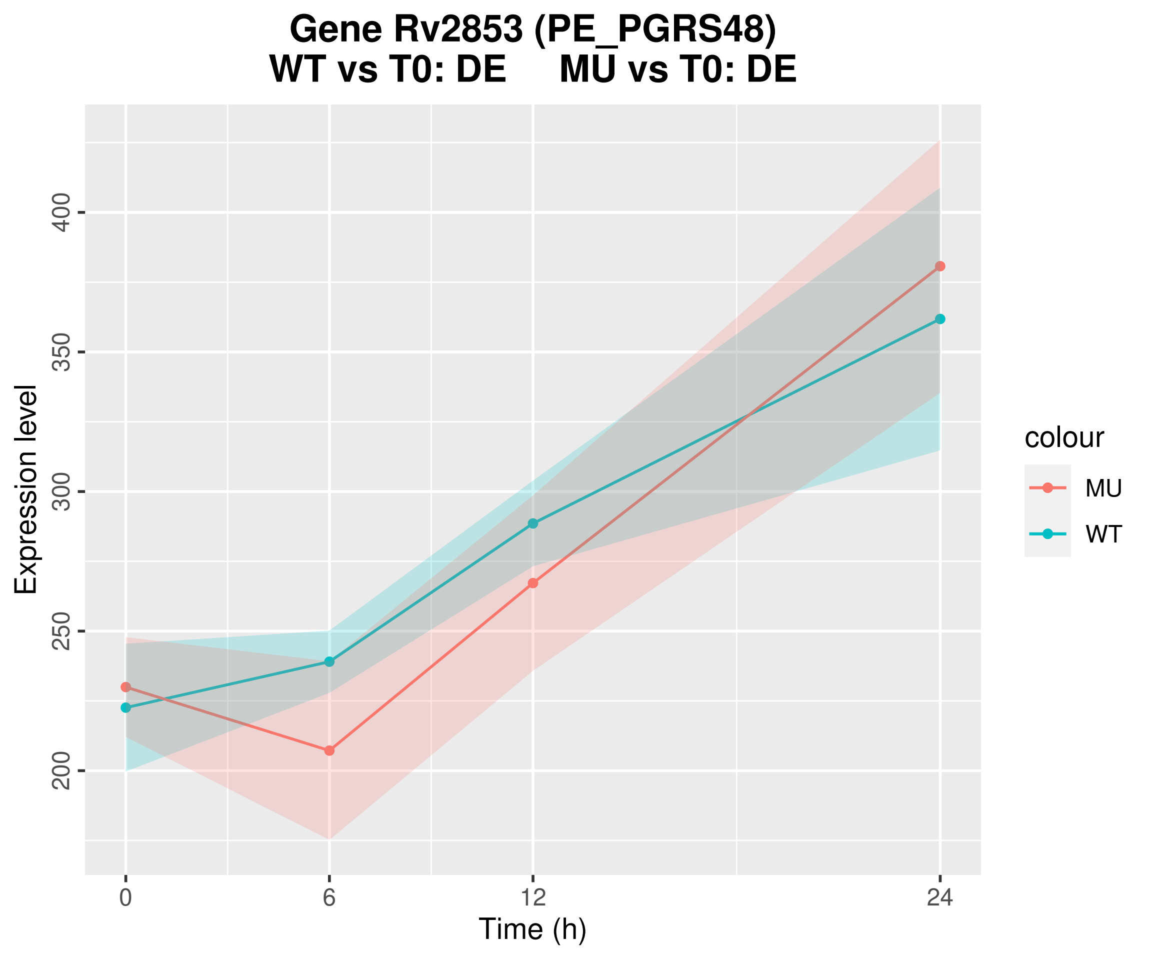

Gene Rv2853 (PE_PGRS48)
WT vs T0: DE MU vs T0: DE
Expression level
200
250
300
350
400
Time (h)
0
6
12
24
colour
MU
WT

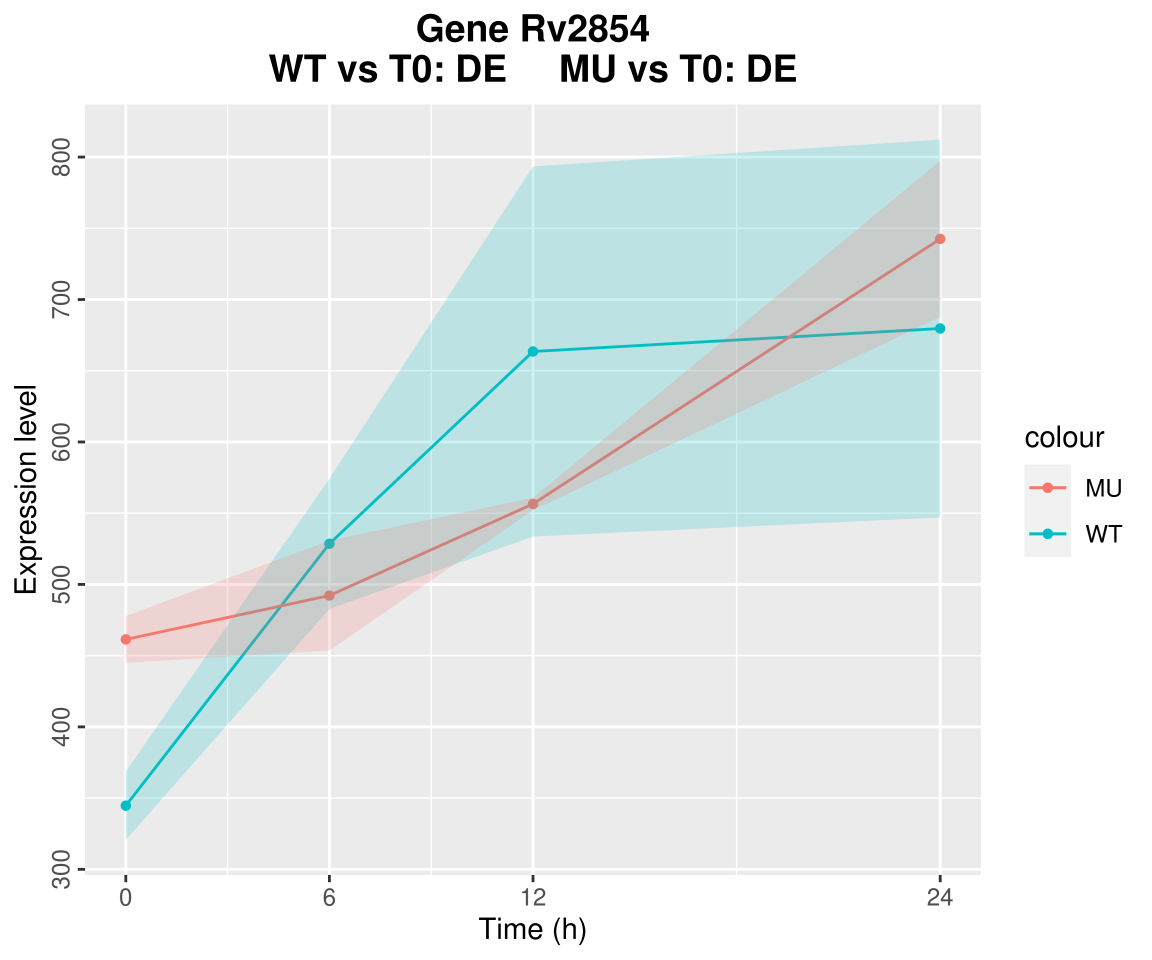

Gene Rv2854
WT vs T0: DE   MU vs T0: DE
Expression level
800
700
600
500
400
300
0
6
12
24
Time (h)
colour
MU
WT

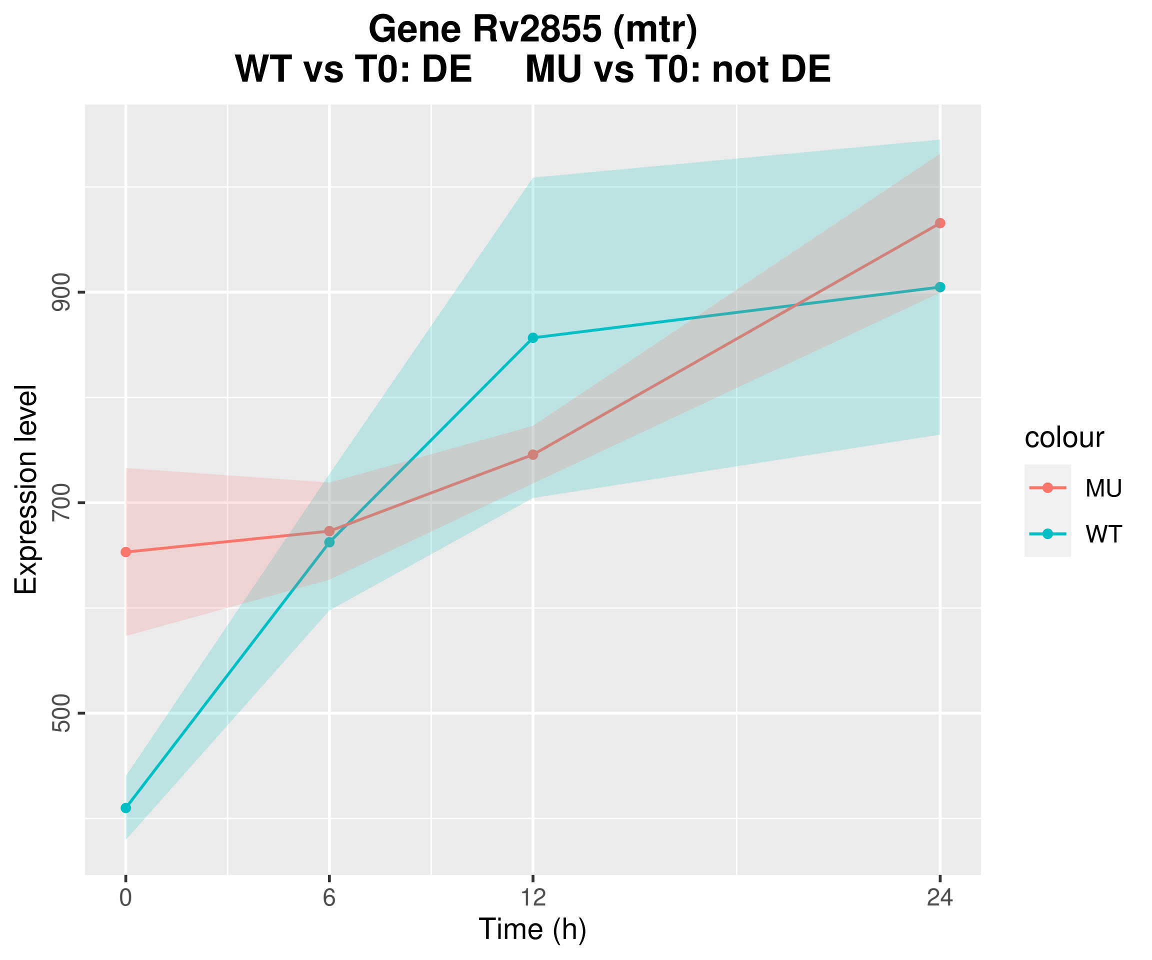
Gene Rv2855 (mtr)
WT vs T0: DE MU vs T0: not DE
Expression level
900
700
500
0
6
12
24
Time (h)
colour
MU
WT

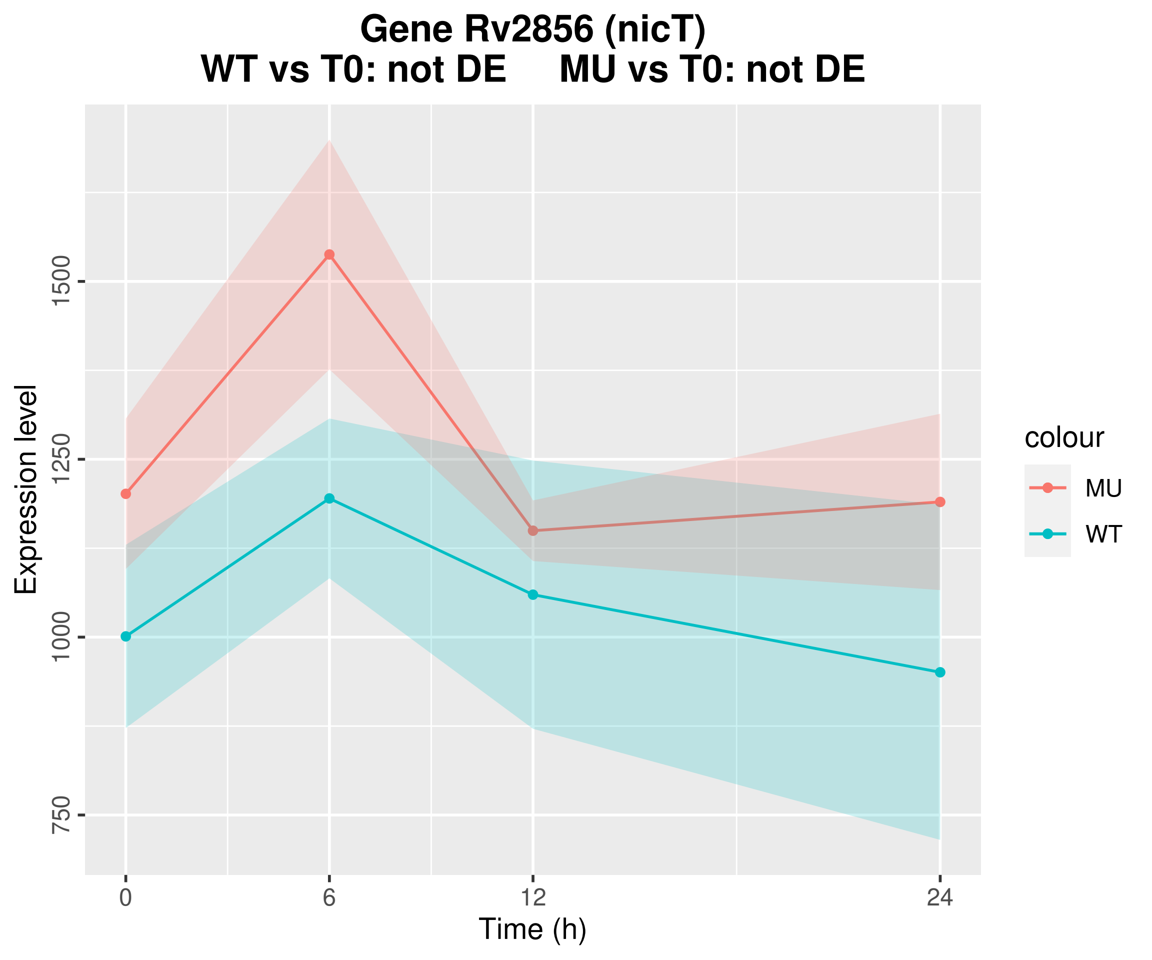
Gene Rv2856 (nicT)
WT vs T0: not DE   MU vs T0: not DE
Expression level
1500
1250
1000
750
0
6
12
24
Time (h)
colour
MU
WT

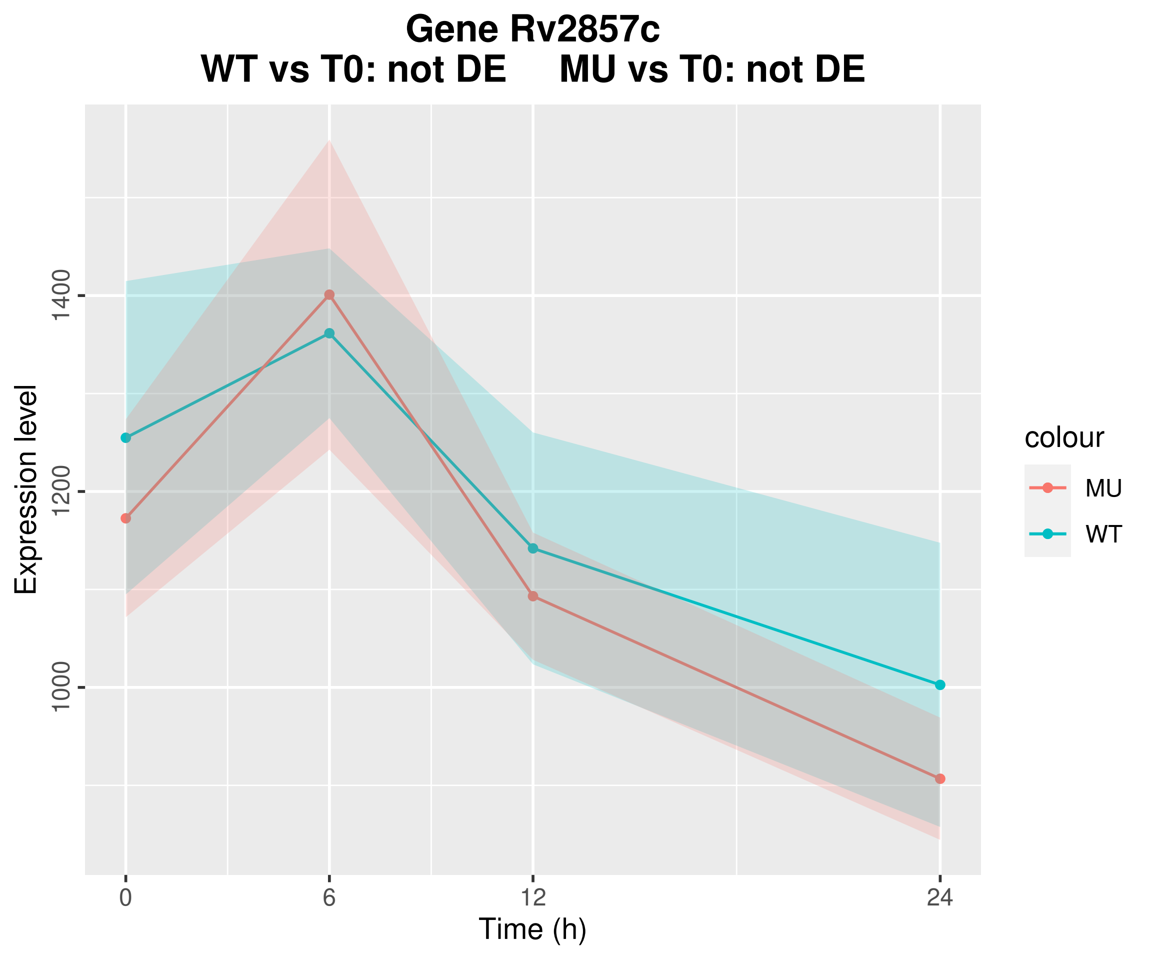
Gene Rv2857c
WT vs T0: not DE   MU vs T0: not DE
Expression level
1000
1200
1400
Time (h)
0
6
12
24
colour
MU
WT

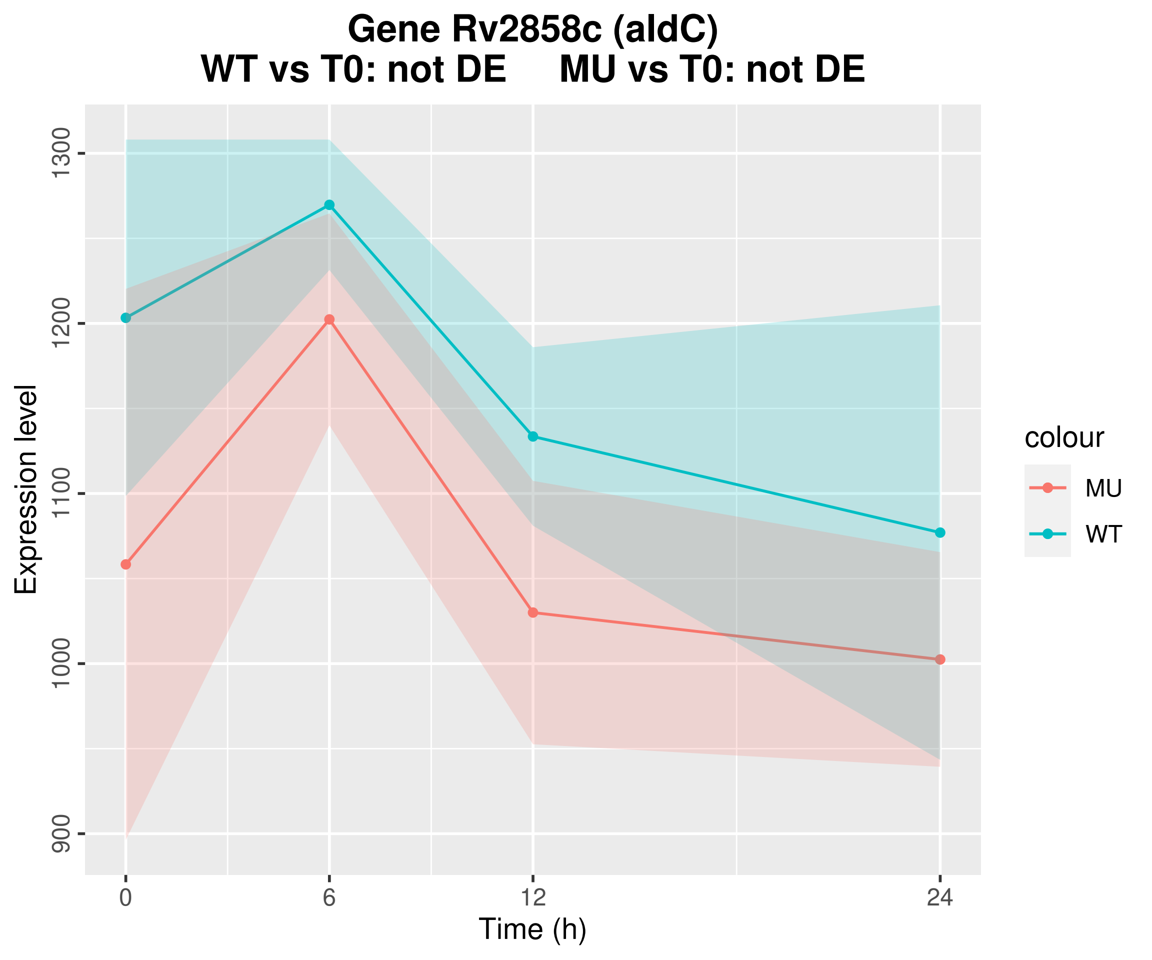
Gene Rv2858c (aldC)
WT vs T0: not DE   MU vs T0: not DE
Expression level
1300
1200
1100
1000
900
0
6
12
24
Time (h)
colour
MU
WT

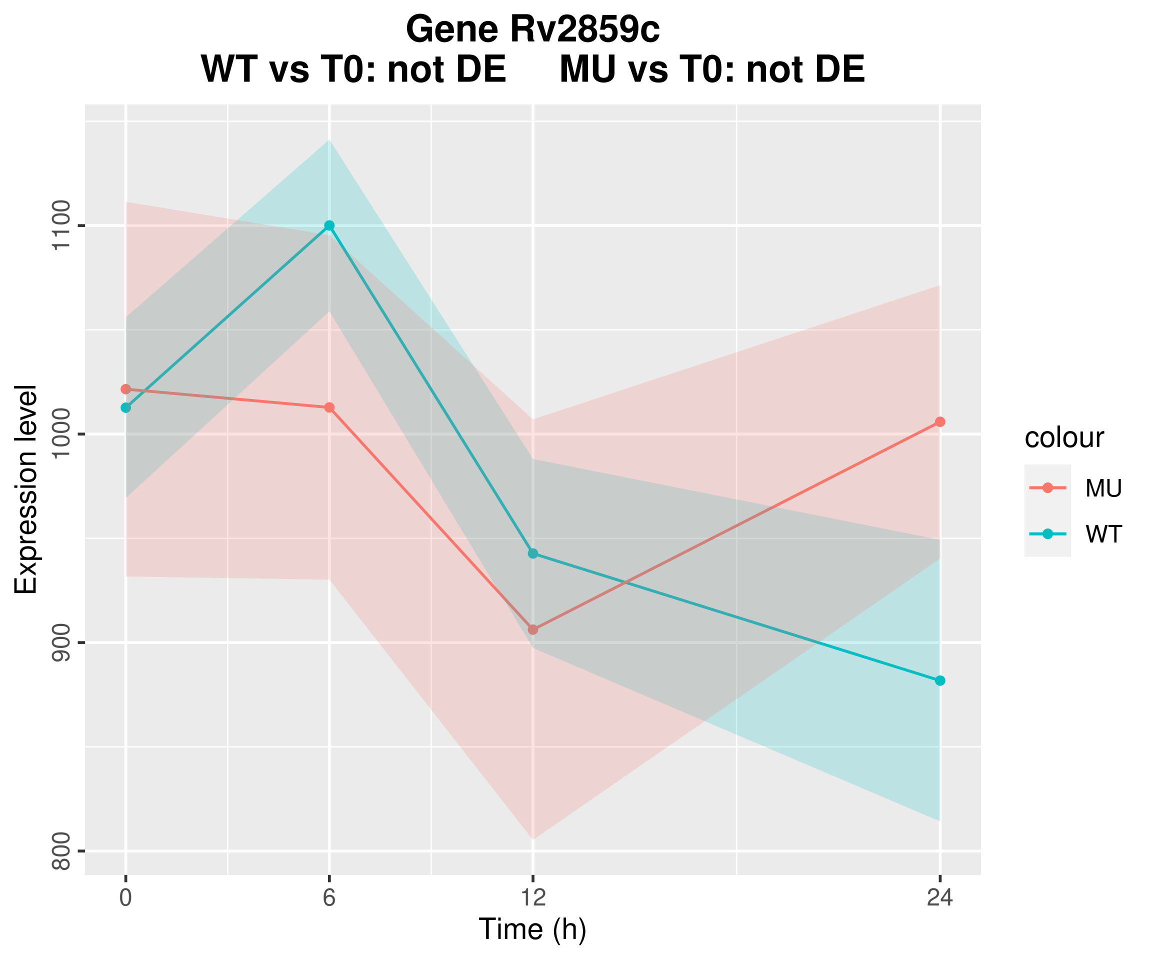
Gene Rv2859c
WT vs T0: not DE MU vs T0: not DE
Expression level
1100
1000
900
800
0
6
12
24
Time (h)
colour
MU
WT

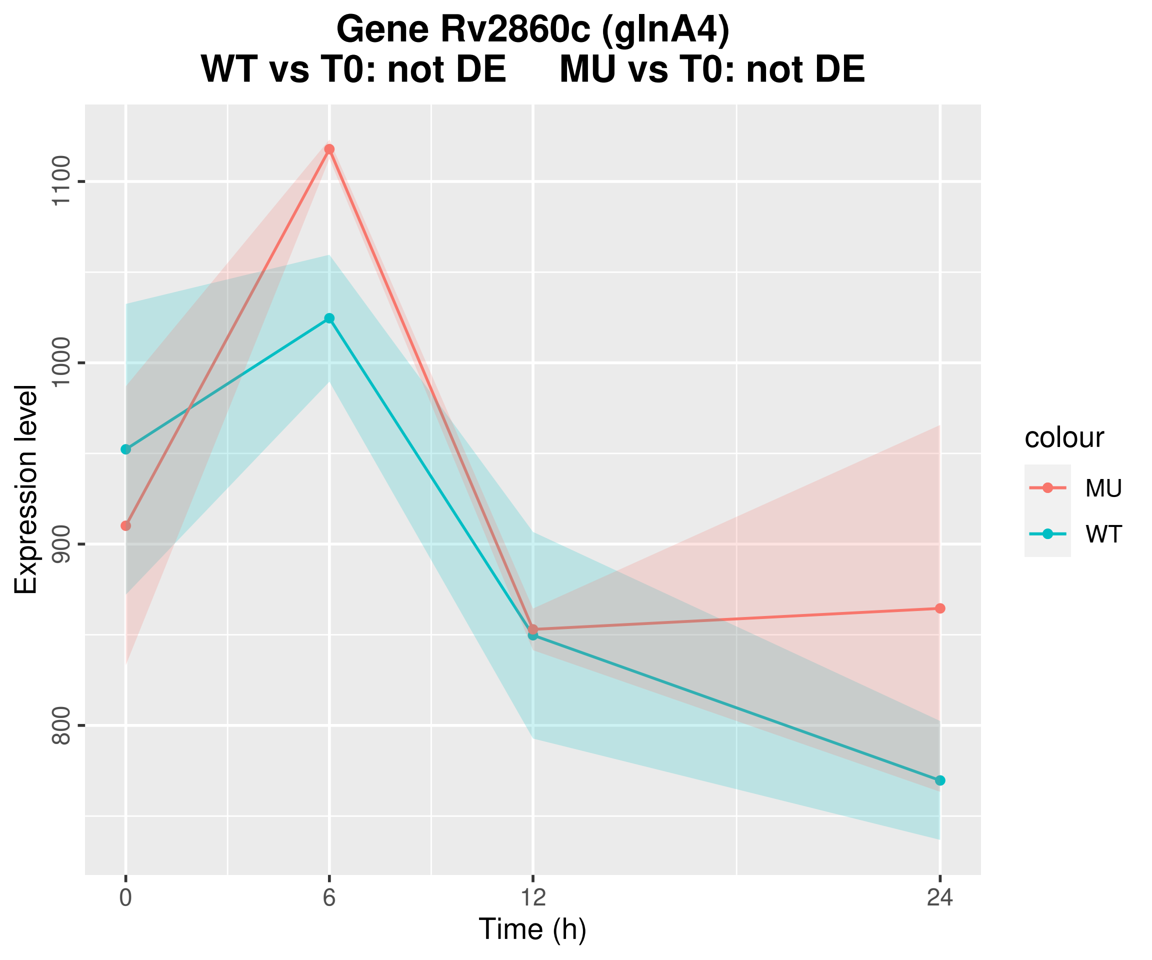
Gene Rv2860c (glnA4)
WT vs T0: not DE   MU vs T0: not DE
Expression level
1100
1000
900
800
0
6
12
24
Time (h)
colour
MU
WT

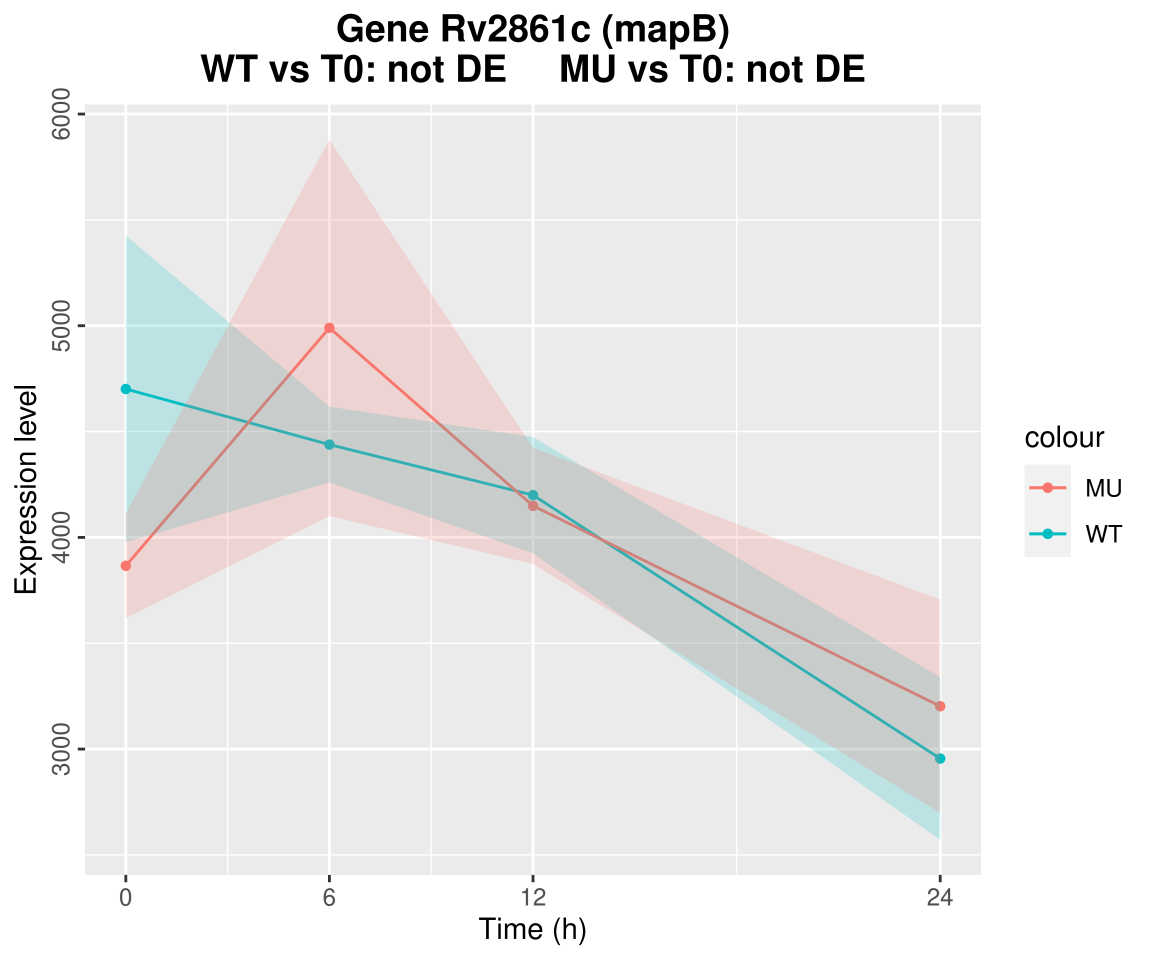
Gene Rv2861c (mapB)
WT vs T0: not DE   MU vs T0: not DE
6000
5000
4000
3000
Expression level
0
6
12
24
Time (h)
colour
MU
WT

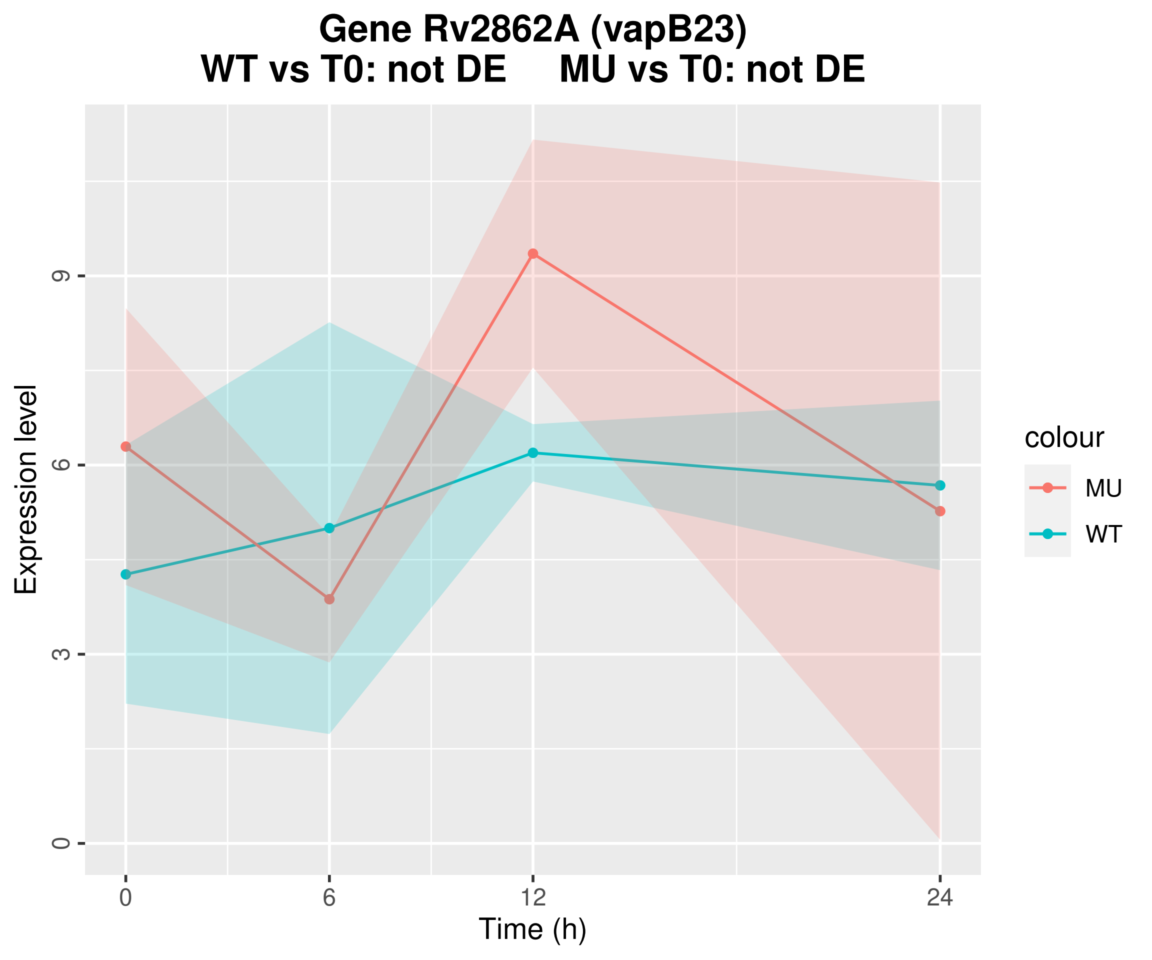
Gene Rv2862A (vapB23)
WT vs T0: not DE   MU vs T0: not DE
Expression level
9
6
3
0
0
6
12
24
Time (h)
colour
MU
WT

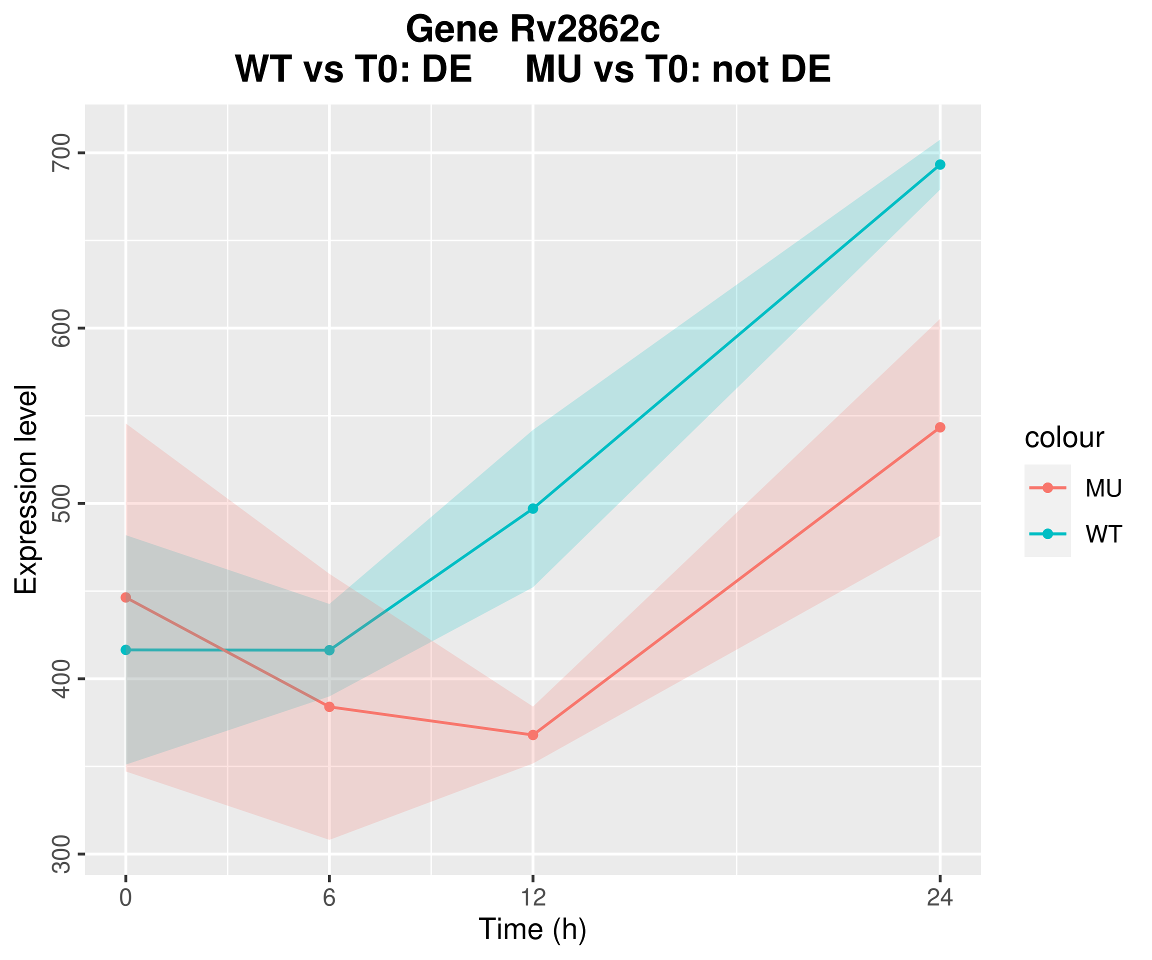
Gene Rv2862c
WT vs T0: DE   MU vs T0: not DE
700
600
500
400
300
Expression level
0
6
12
24
Time (h)
colour
MU
WT

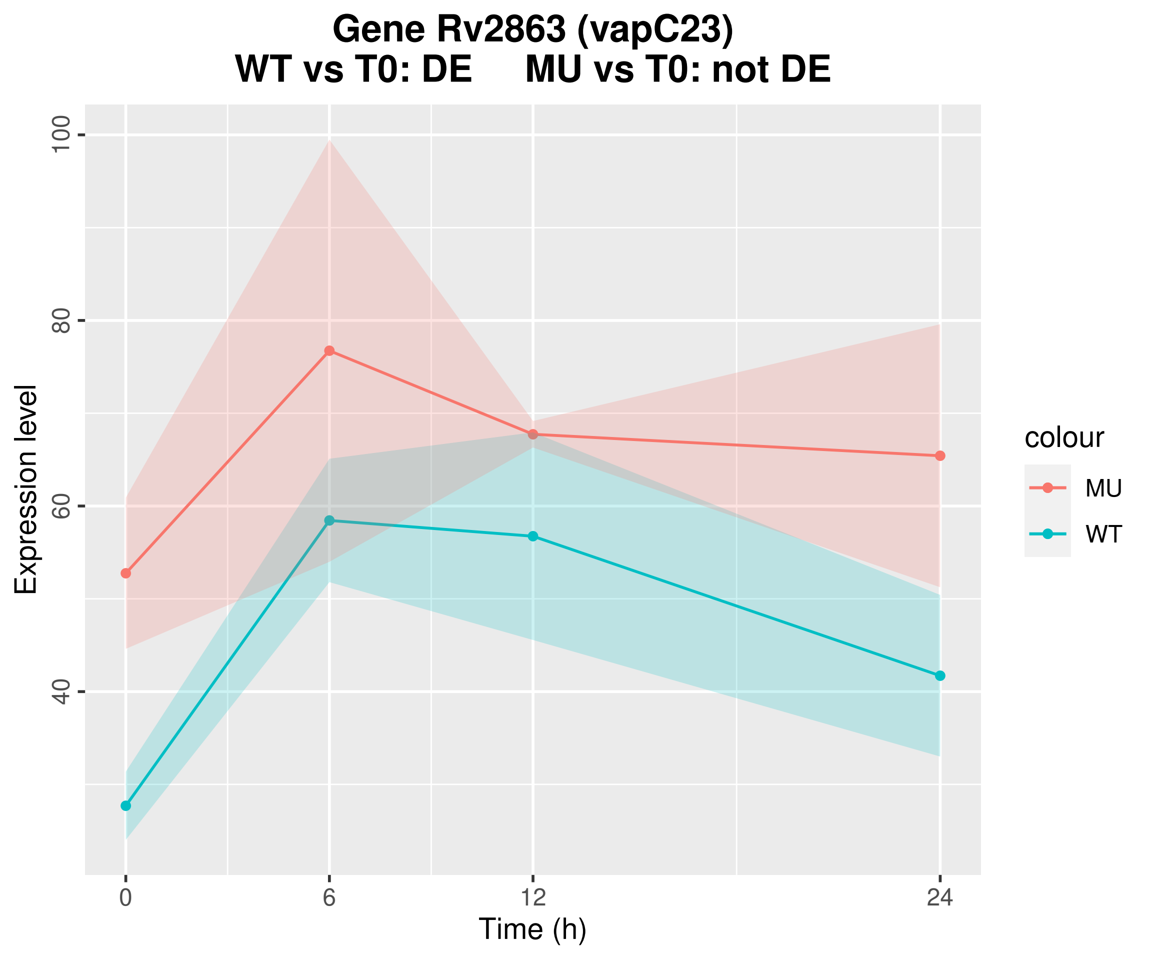

Gene Rv2863 (vapC23)
WT vs T0: DE   MU vs T0: not DE
Expression level
100
80
60
40
0
6
12
24
Time (h)
colour
MU
WT

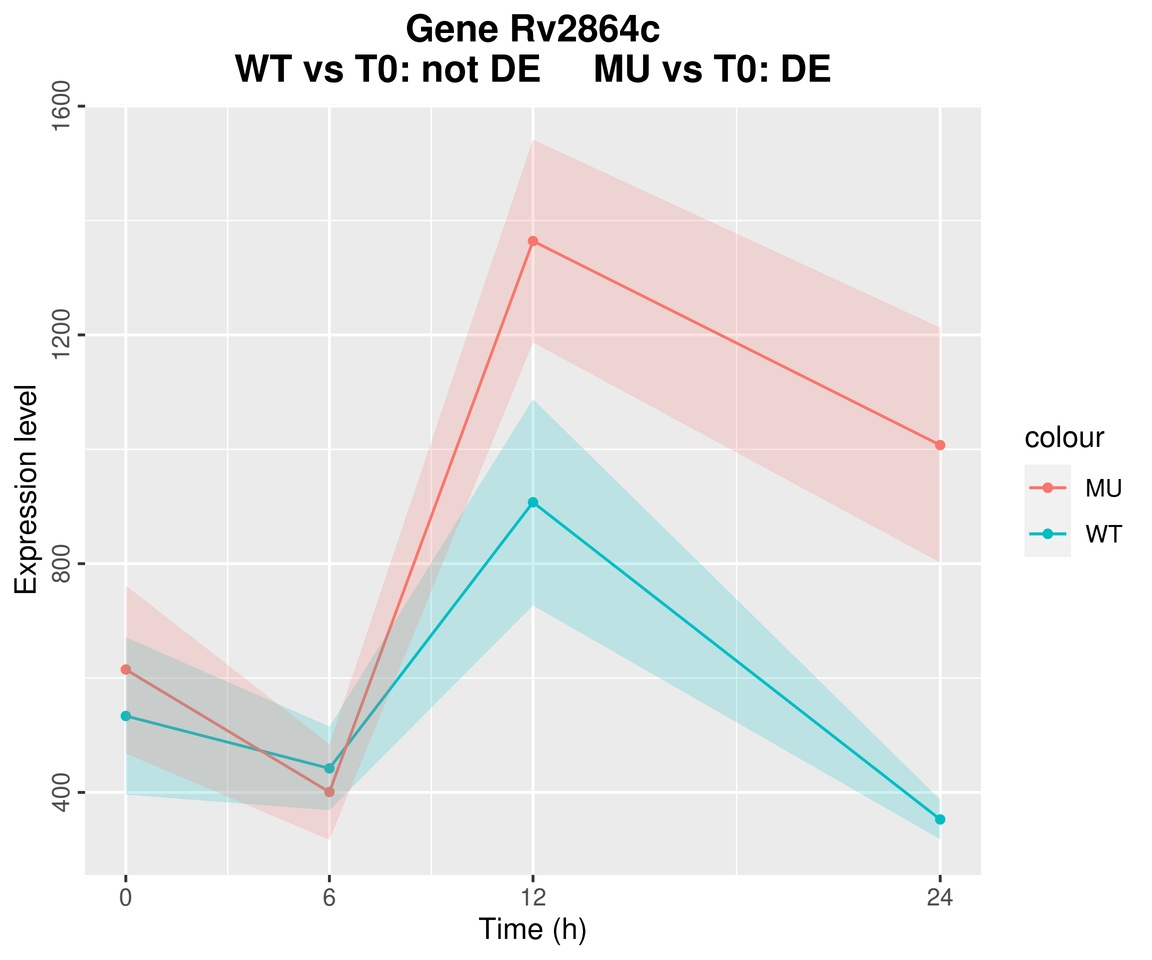

Gene Rv2864c
WT vs T0: not DE MU vs T0: DE
Expression level
1600
1200
800
400
0
6
12
24
Time (h)
colour
MU
WT

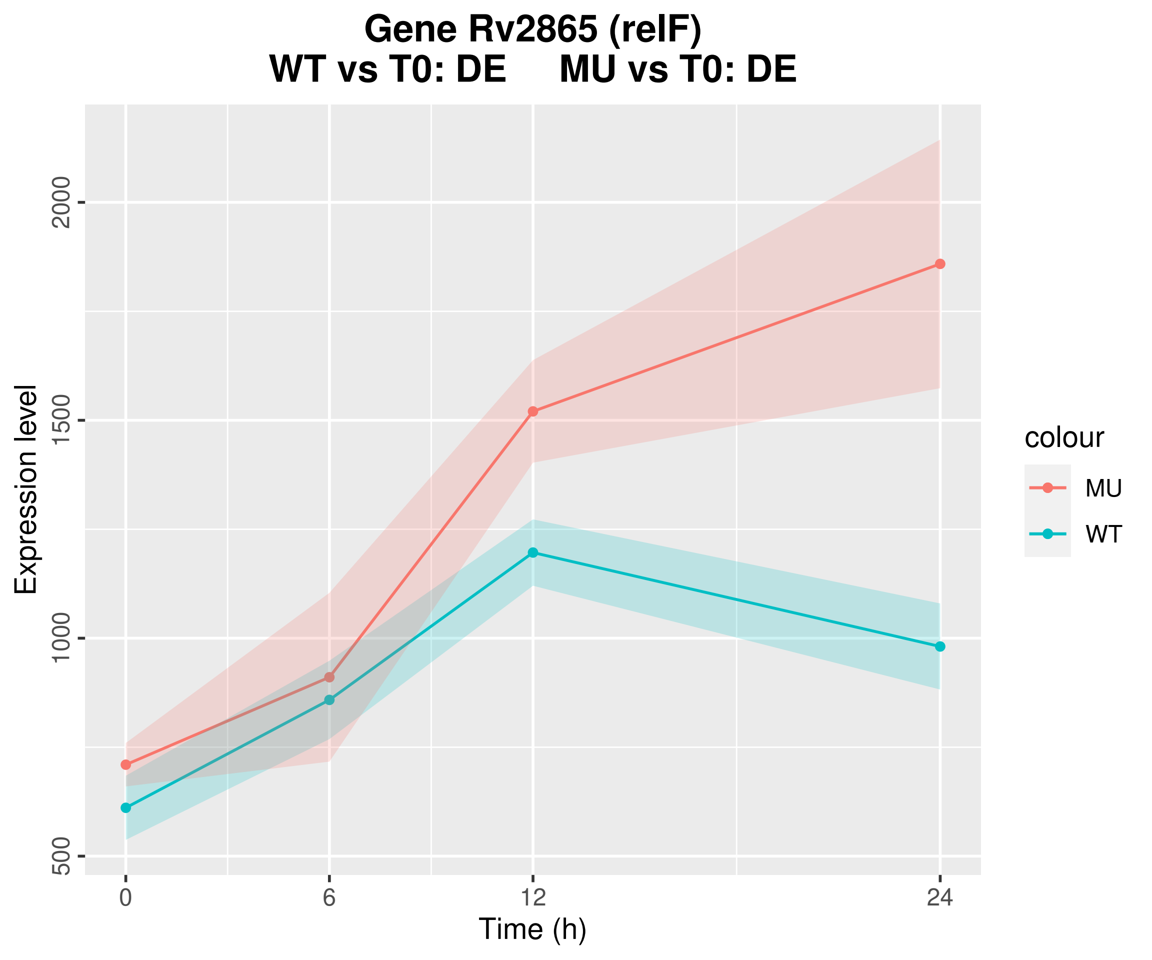

Gene Rv2865 (relF)
WT vs T0: DE   MU vs T0: DE
Expression level
2000
1500
1000
500
0
6
12
24
Time (h)
colour
MU
WT

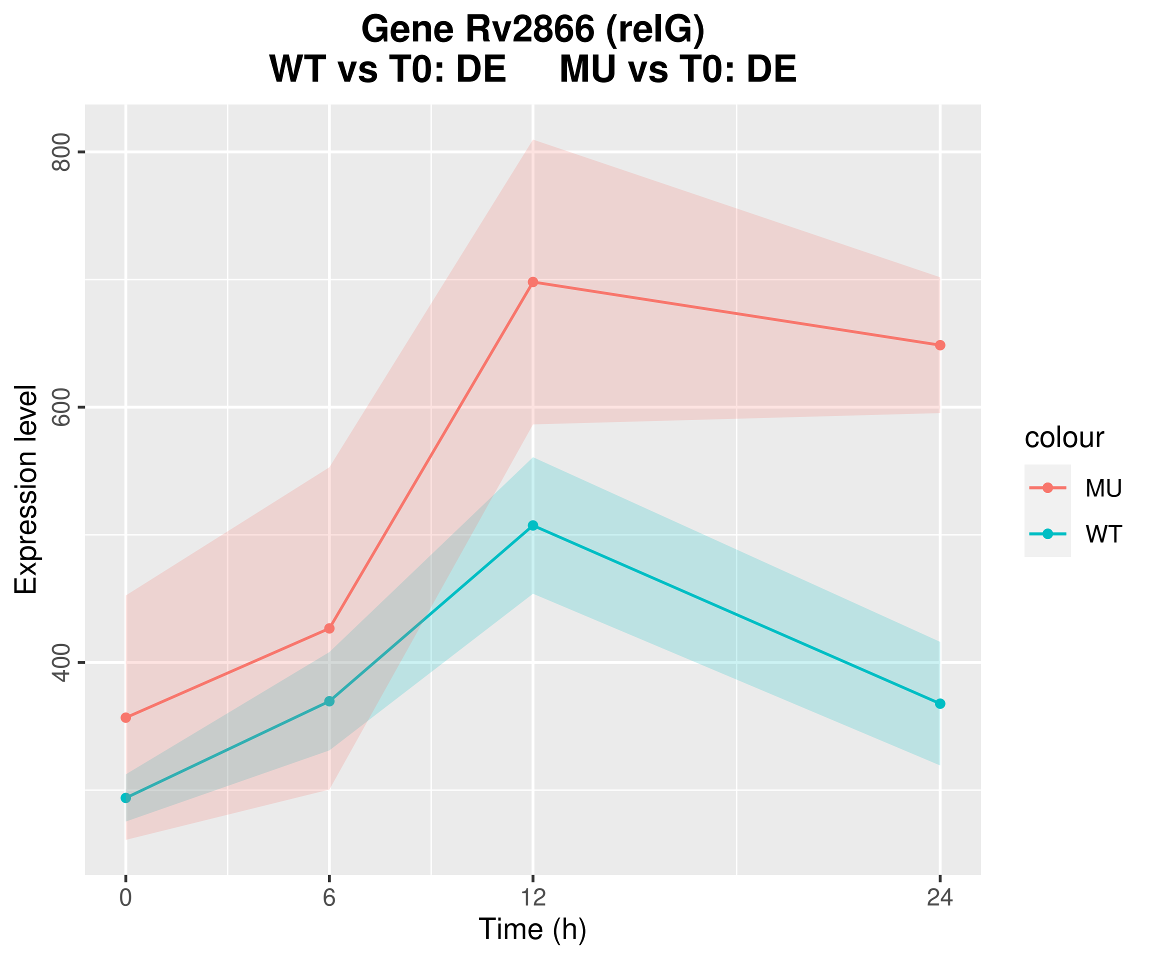

Gene Rv2866 (relG)
WT vs T0: DE MU vs T0: DE
800
600
400
Expression level
0
6
12
24
Time (h)
colour
MU
WT

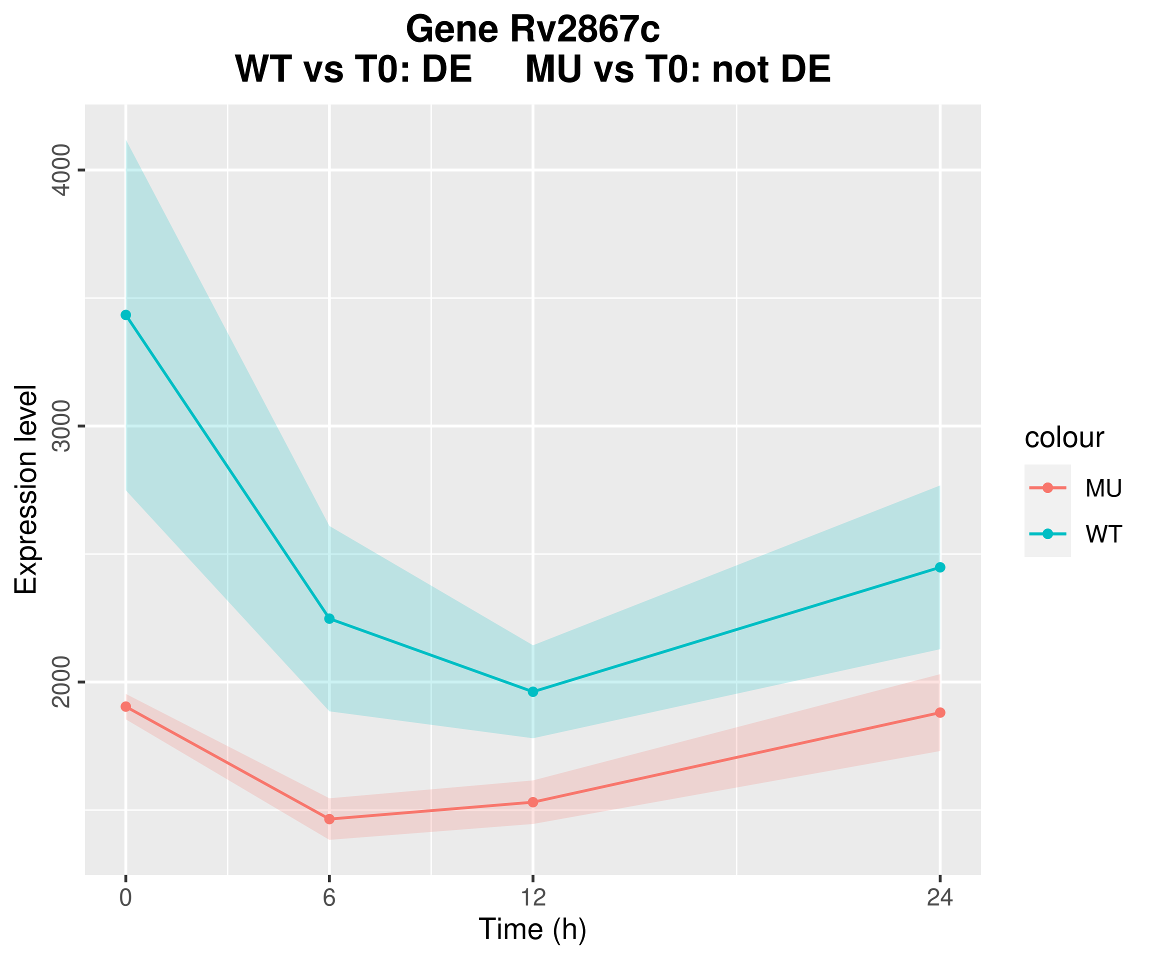
Gene Rv2867c
WT vs T0: DE MU vs T0: not DE
Expression level
4000
3000
2000
0
6
12
24
Time (h)
colour
MU
WT

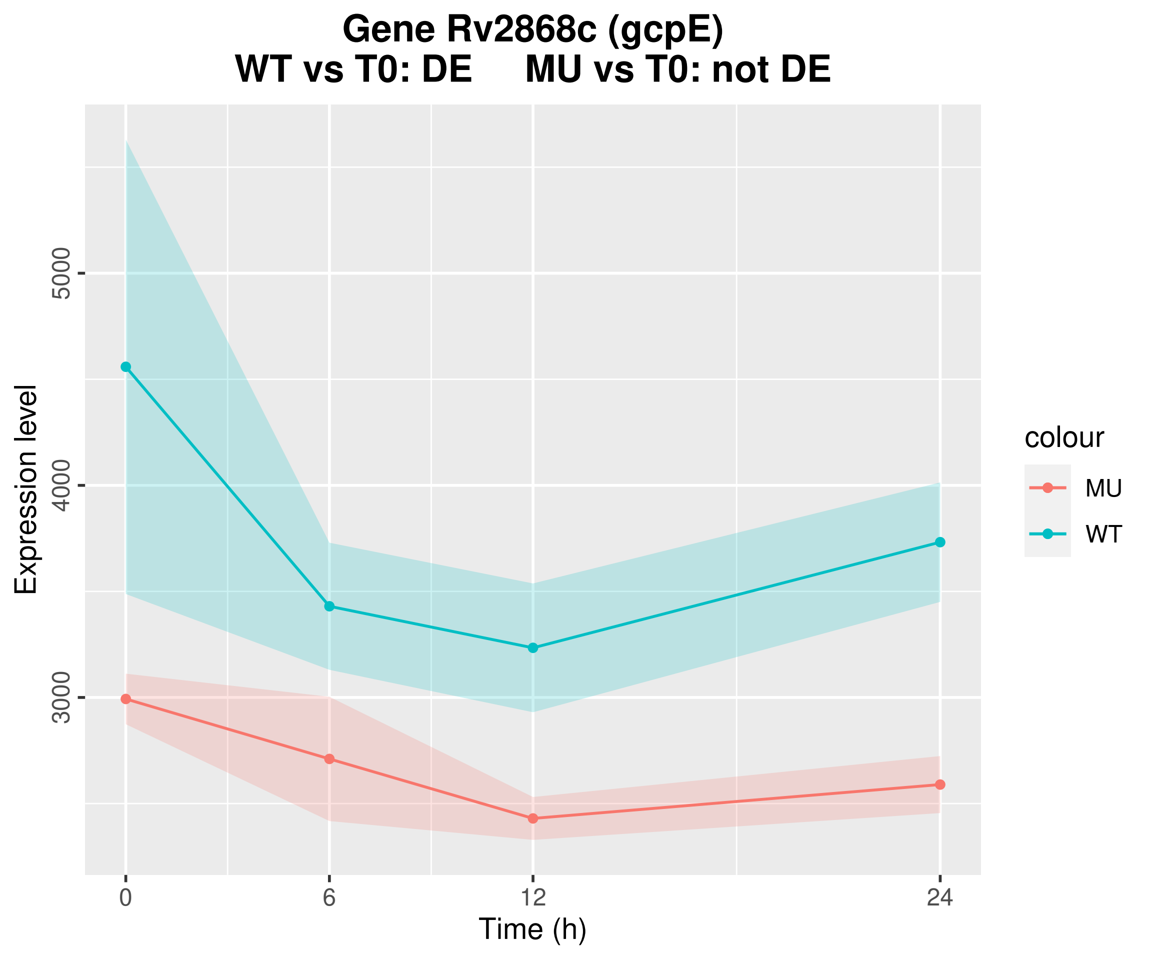

Gene Rv2868c (gcpE)
WT vs T0: DE MU vs T0: not DE
Expression level
5000
4000
3000
0
6
12
24
Time (h)
colour
MU
WT

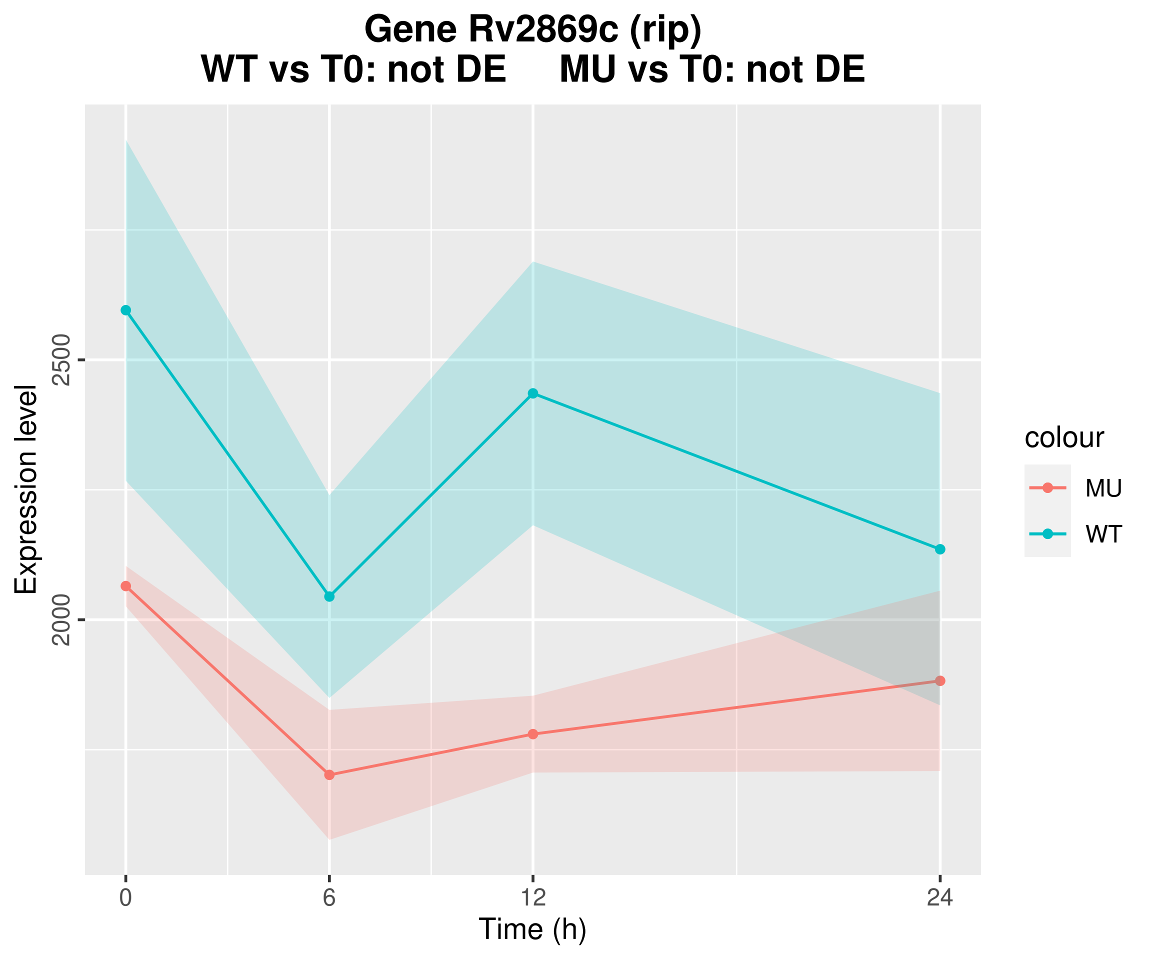

Gene Rv2869c (rip)
WT vs T0: not DE   MU vs T0: not DE
Expression level
2500
2000
0
6
12
24
Time (h)
colour
MU
WT

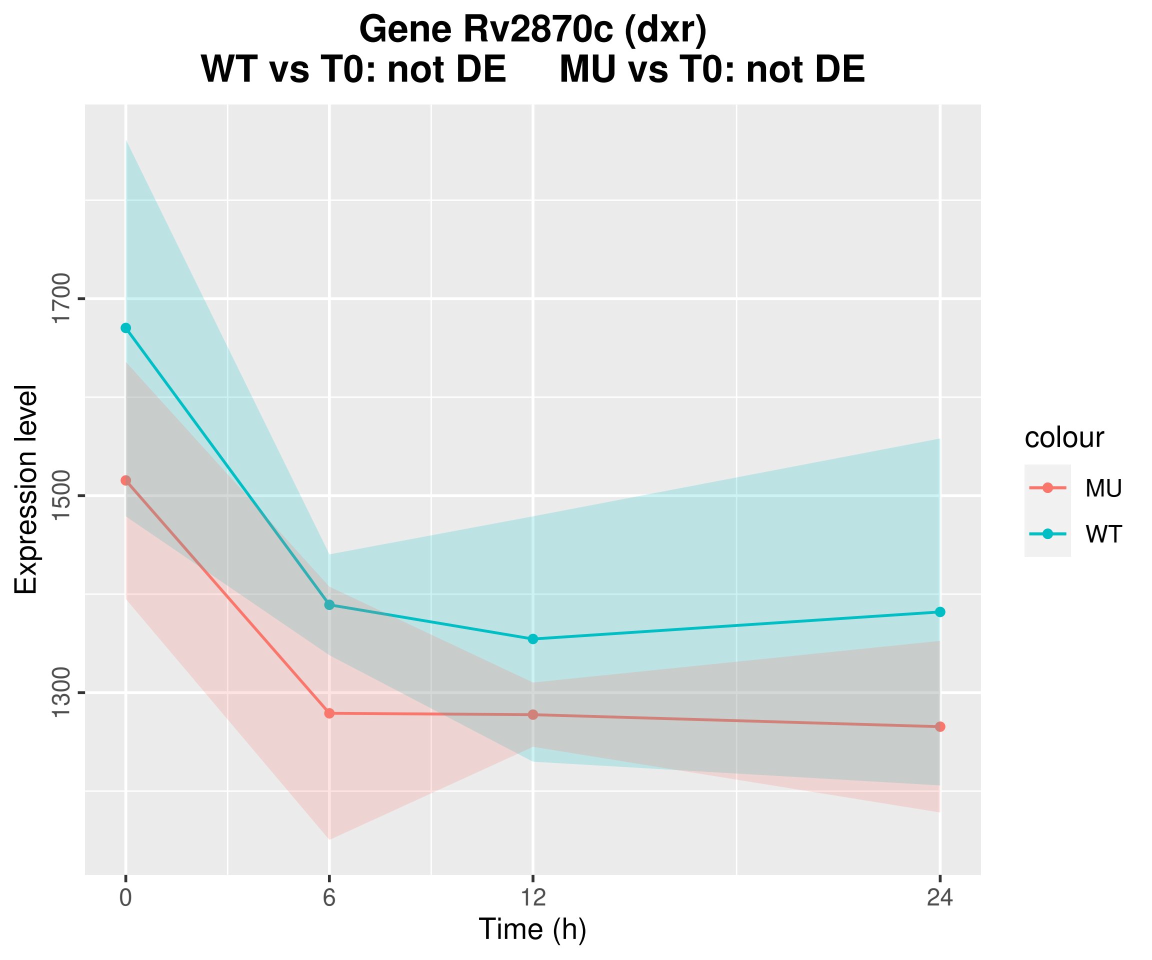

Gene Rv2870c (dxr)
WT vs T0: not DE   MU vs T0: not DE
Expression level
1300
1500
1700
Time (h)
0
6
12
24
colour
MU
WT

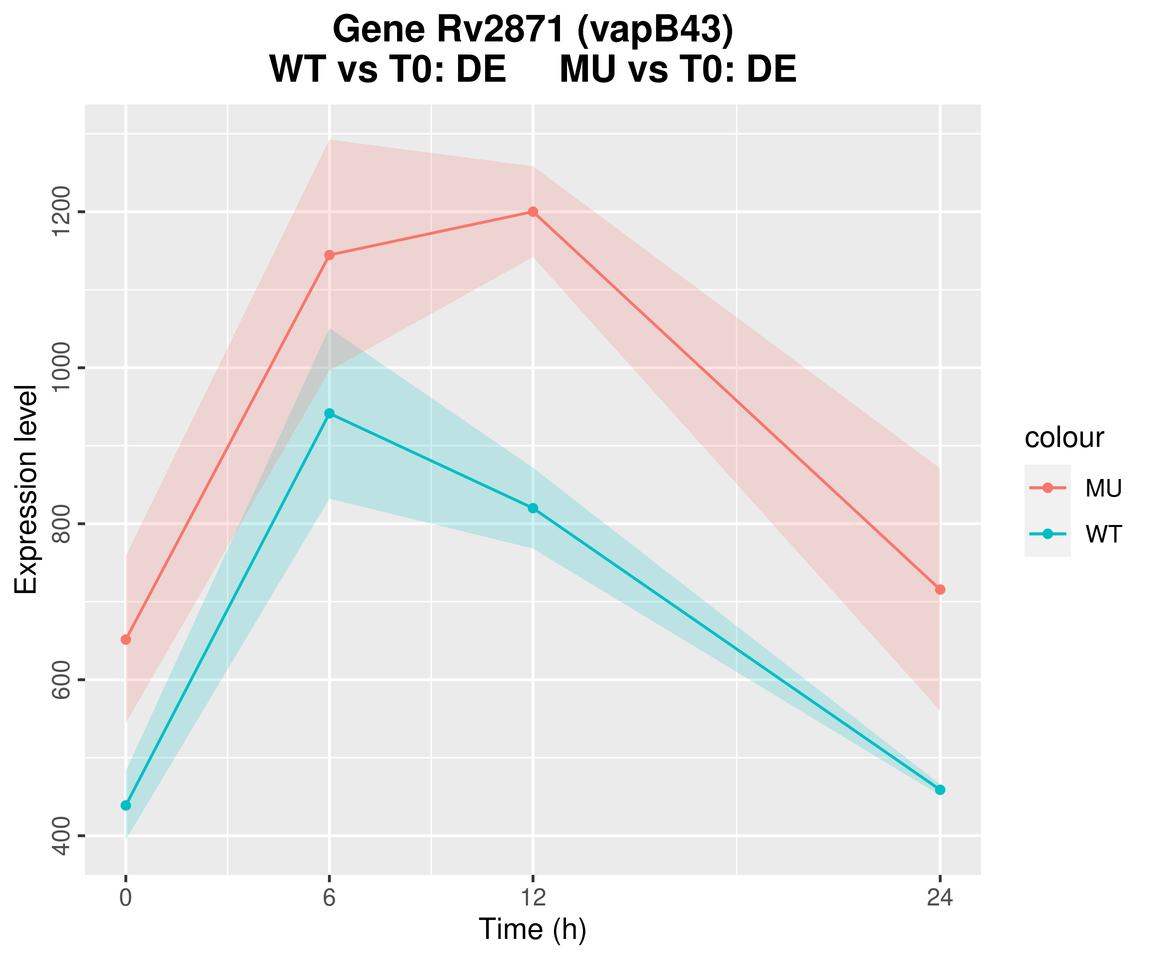

Gene Rv2871 (vapB43)
WT vs T0: DE   MU vs T0: DE
Expression level
400
600
800
1000
1200
0
6
12
24
Time (h)
colour
MU
WT

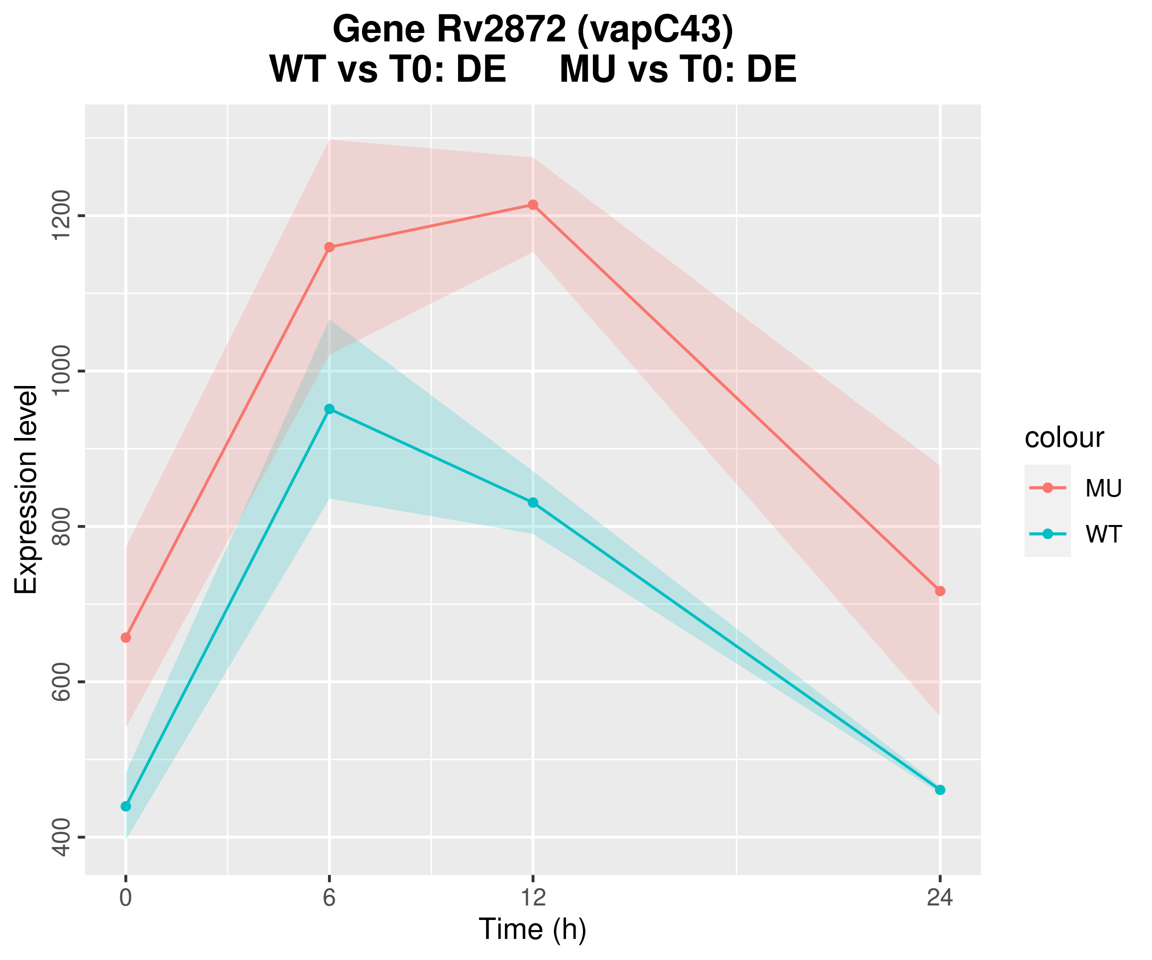
Gene Rv2872 (vapC43)
WT vs T0: DE MU vs T0: DE
Expression level
400
600
800
1000
1200
0
6
12
24
Time (h)
colour
MU
WT

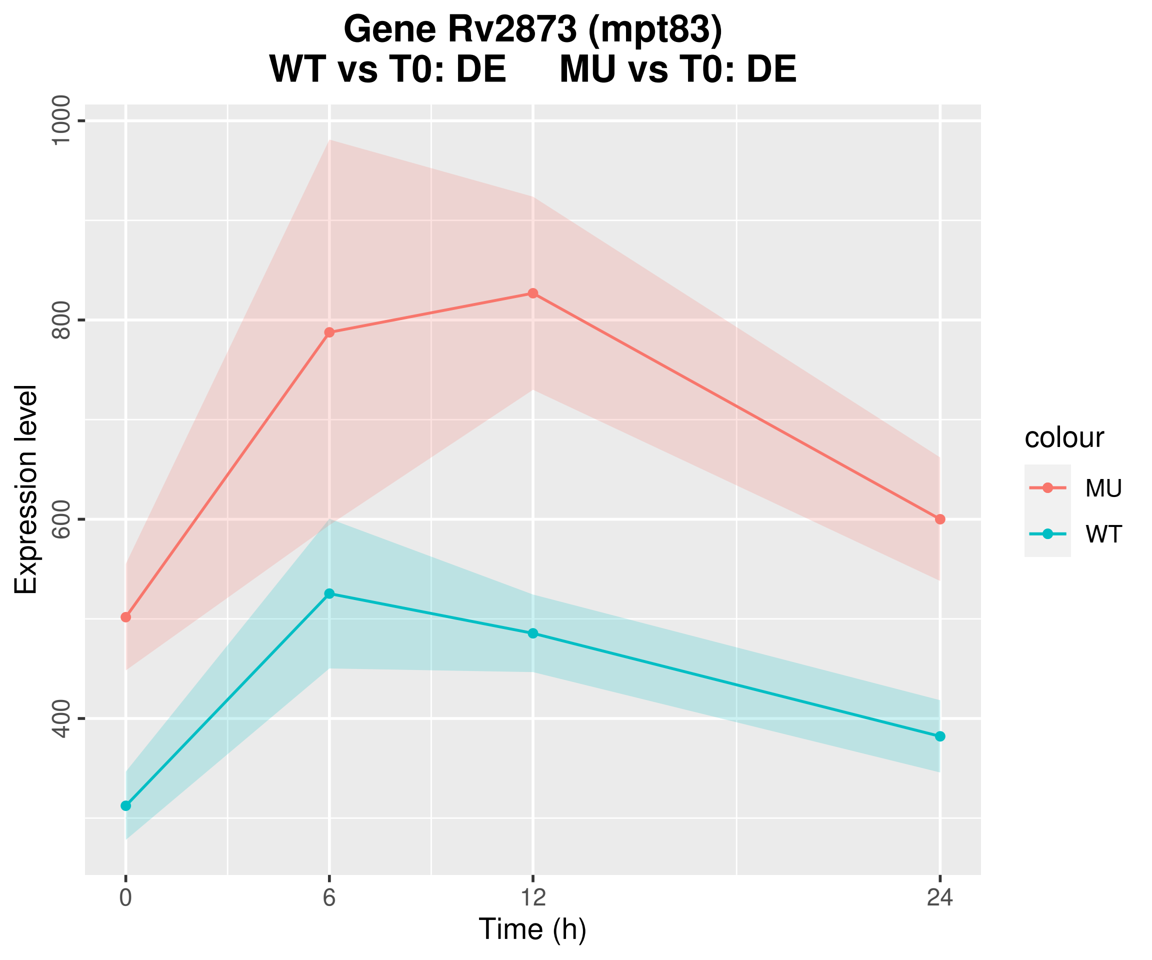
Gene Rv2873 (mpt83)
WT vs T0: DE   MU vs T0: DE
Expression level
1000
800
600
400
0
6
12
24
Time (h)
colour
MU
WT

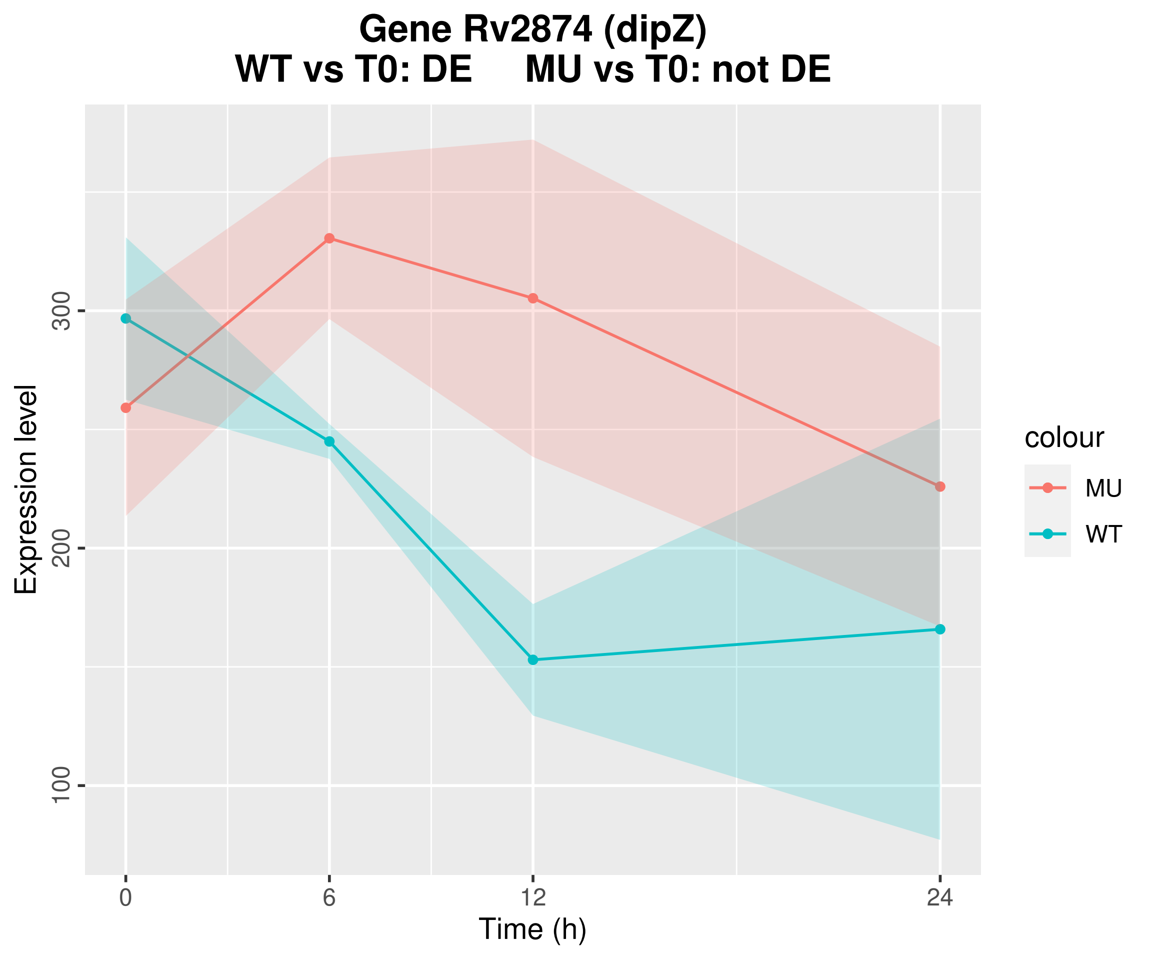

Gene Rv2874 (dipZ)
WT vs T0: DE MU vs T0: not DE
Expression level
300
200
100
0
6
12
24
Time (h)
colour
MU
WT

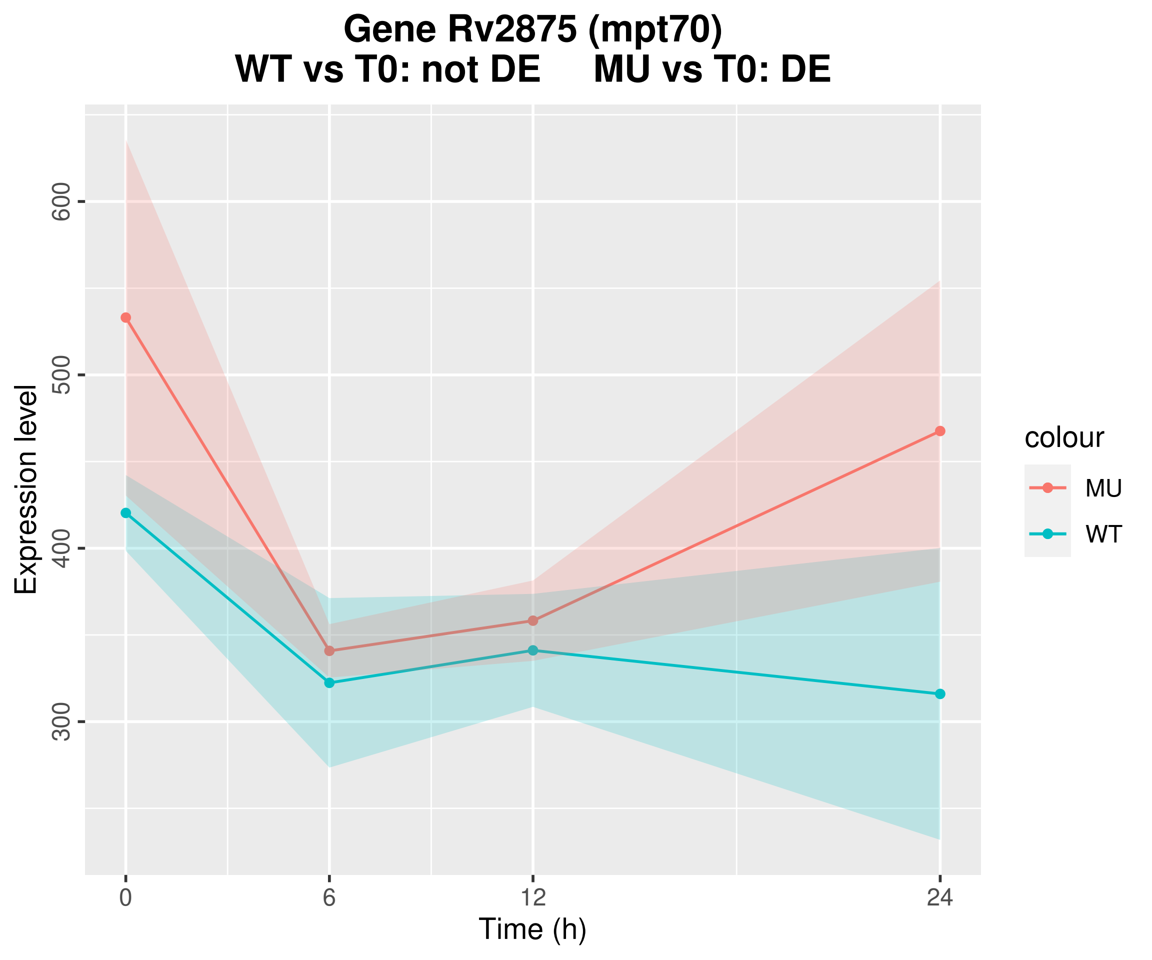

Gene Rv2875 (mpt70)
WT vs T0: not DE MU vs T0: DE
Expression level
600
500
400
300
0
6
12
24
Time (h)
colour
MU
WT

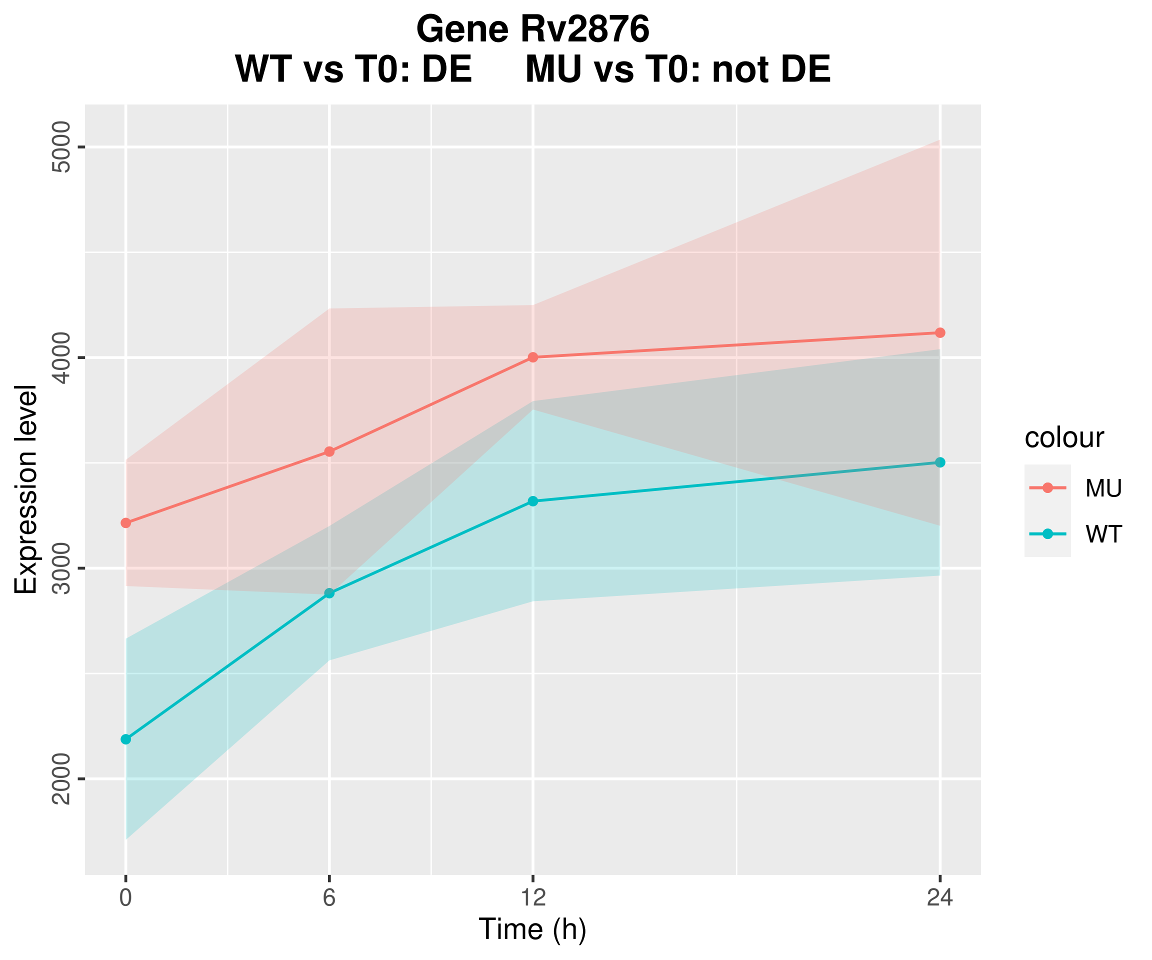
Gene Rv2876
WT vs T0: DE MU vs T0: not DE
Expression level
5000
4000
3000
2000
0
6
12
24
Time (h)
colour
MU
WT

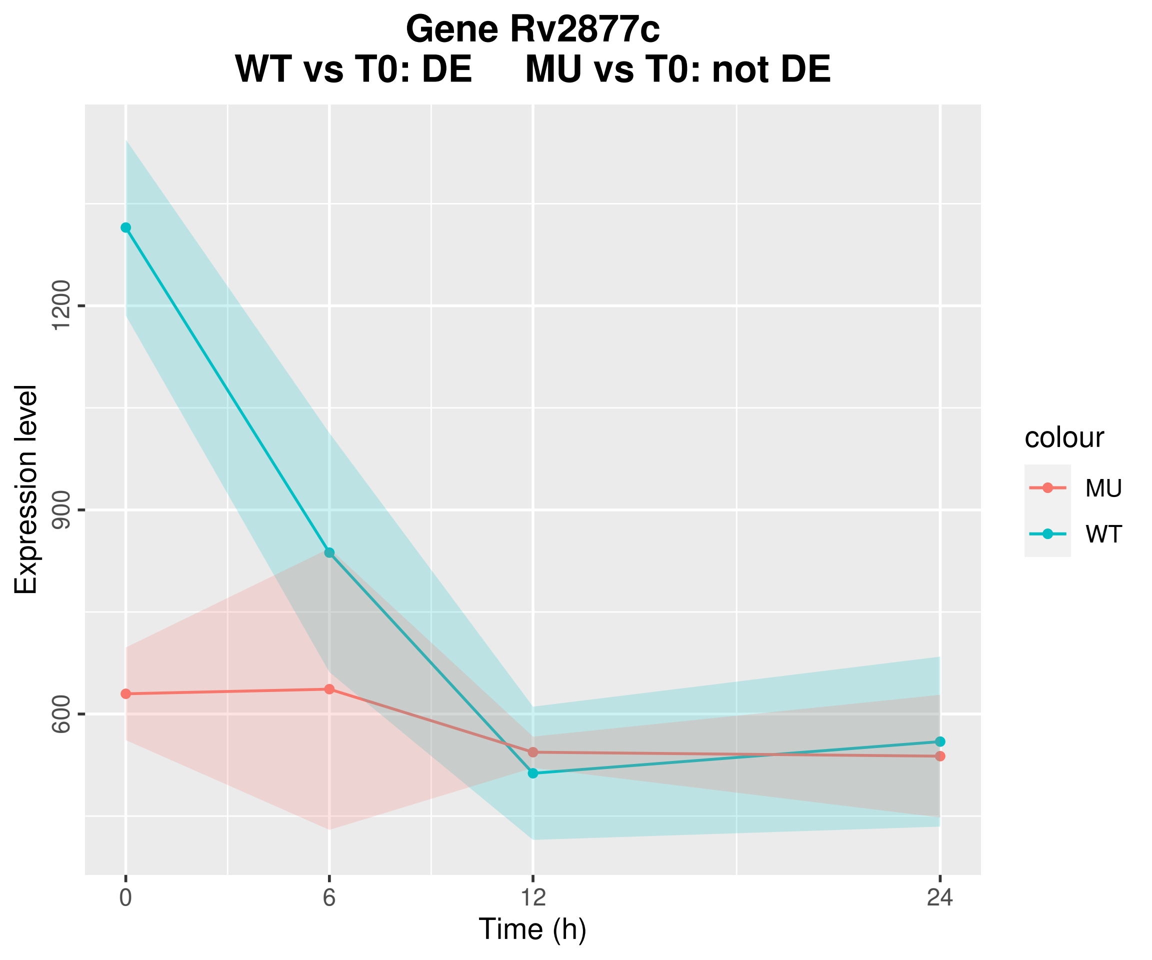

Gene Rv2877c
WT vs T0: DE MU vs T0: not DE
Expression level
1200
900
600
0
6
12
24
Time (h)
colour
MU
WT

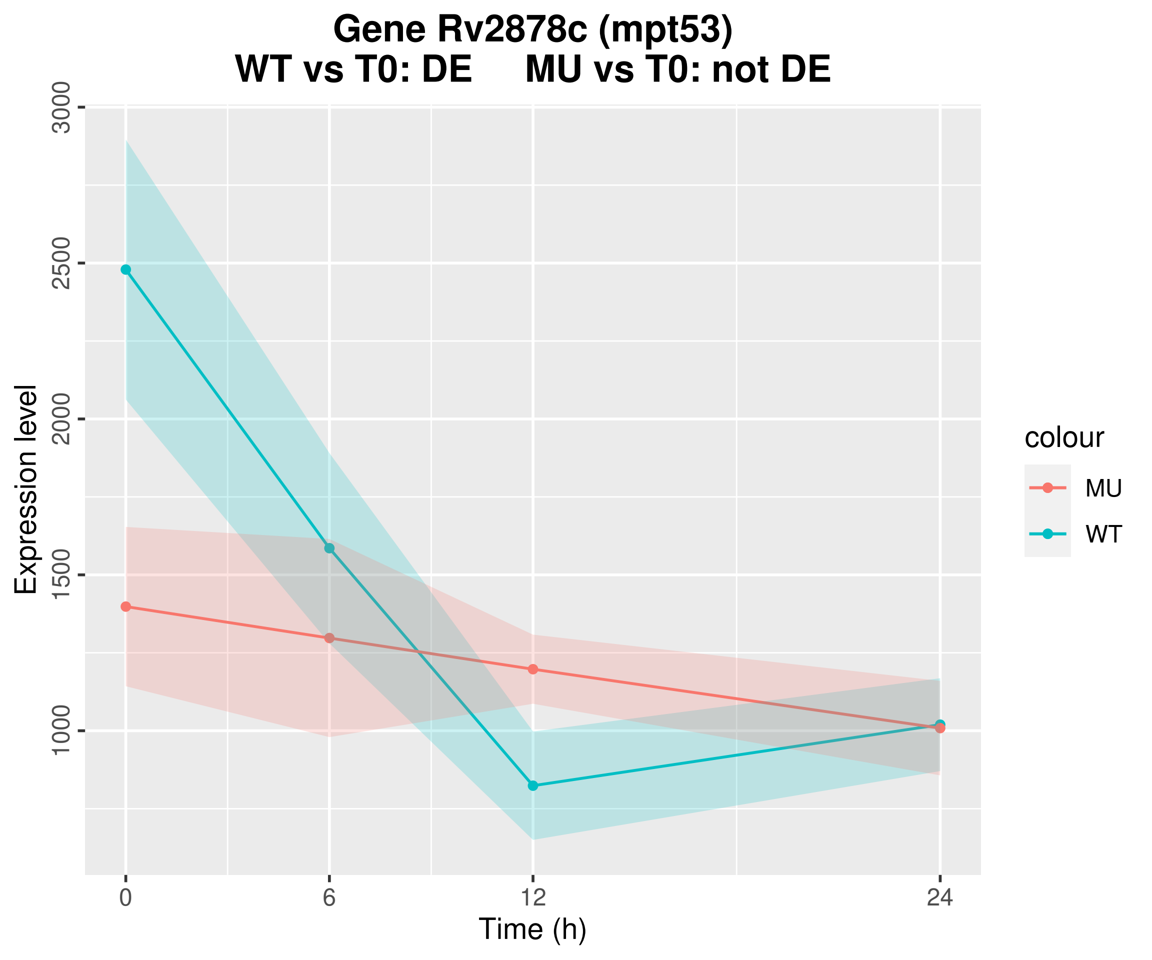

Gene Rv2878c (mpt53)
WT vs T0: DE   MU vs T0: not DE
Expression level
3000
2500
2000
1500
1000
0
6
12
24
Time (h)
colour
MU
WT

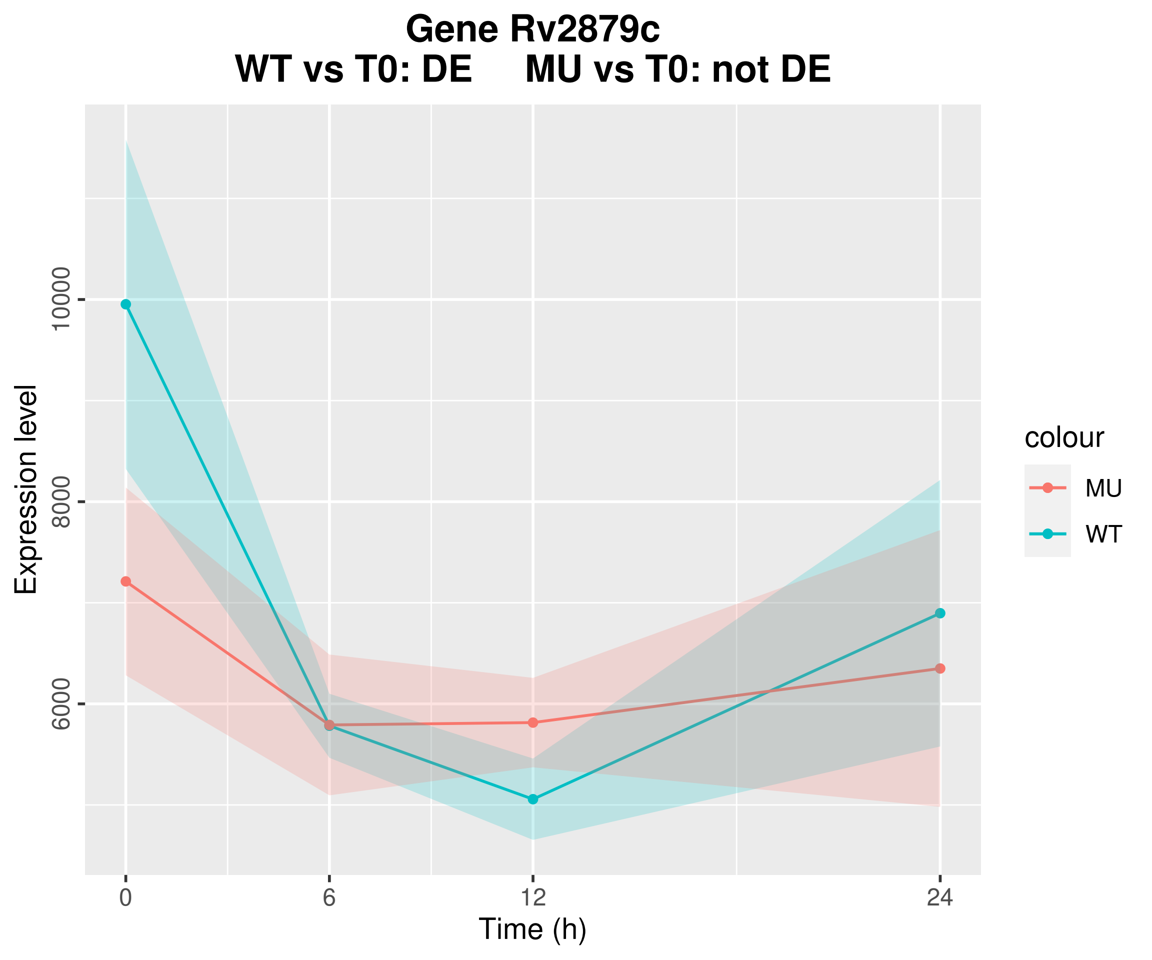

Gene Rv2879c
WT vs T0: DE   MU vs T0: not DE
Expression level
10000
8000
6000
0
6
12
24
Time (h)
colour
MU
WT

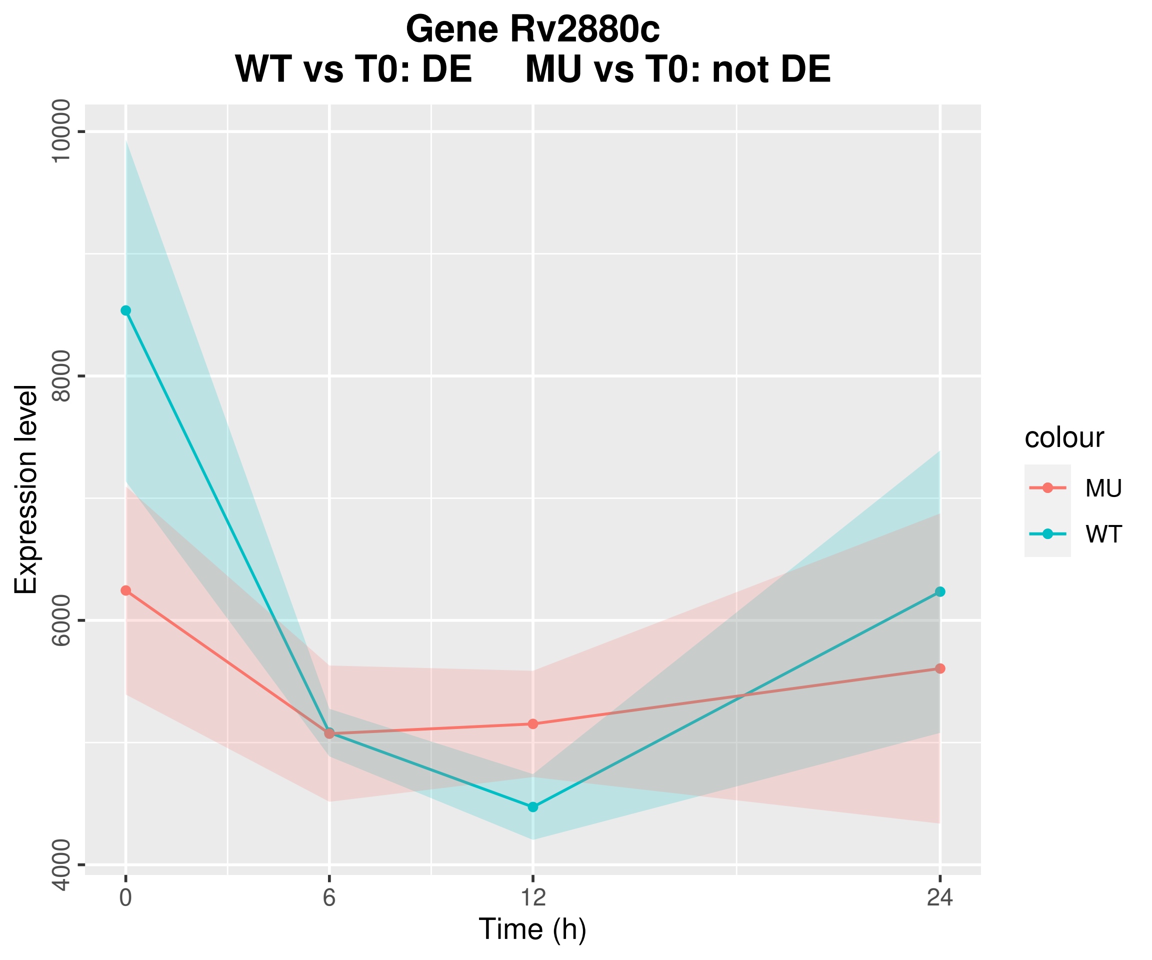
Gene Rv2880c
WT vs T0: DE MU vs T0: not DE
Expression level
10000
8000
6000
4000
0
6
12
24
Time (h)
colour
MU
WT

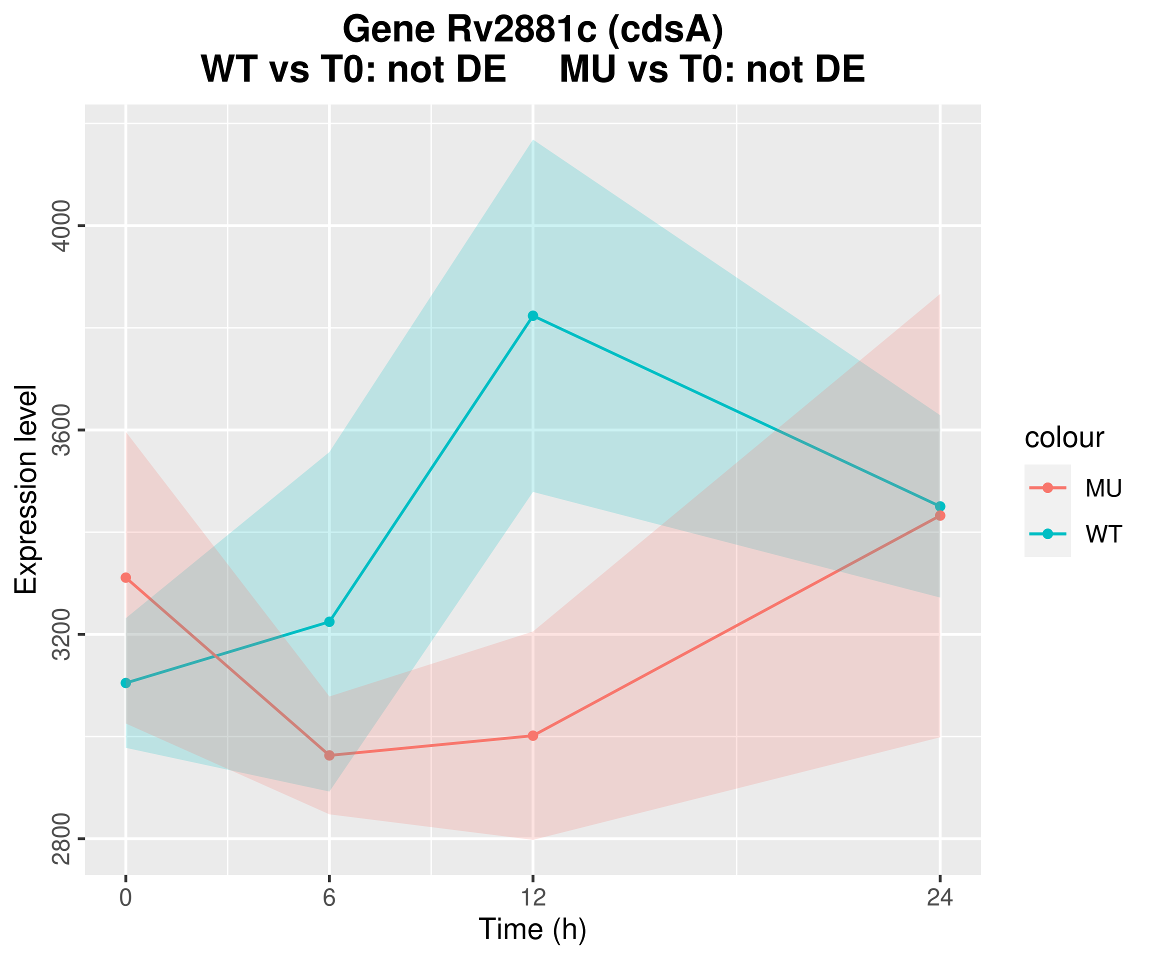
Gene Rv2881c (cdsA)
WT vs T0: not DE   MU vs T0: not DE
Expression level
4000
3600
3200
2800
0
6
12
24
Time (h)
colour
MU
WT

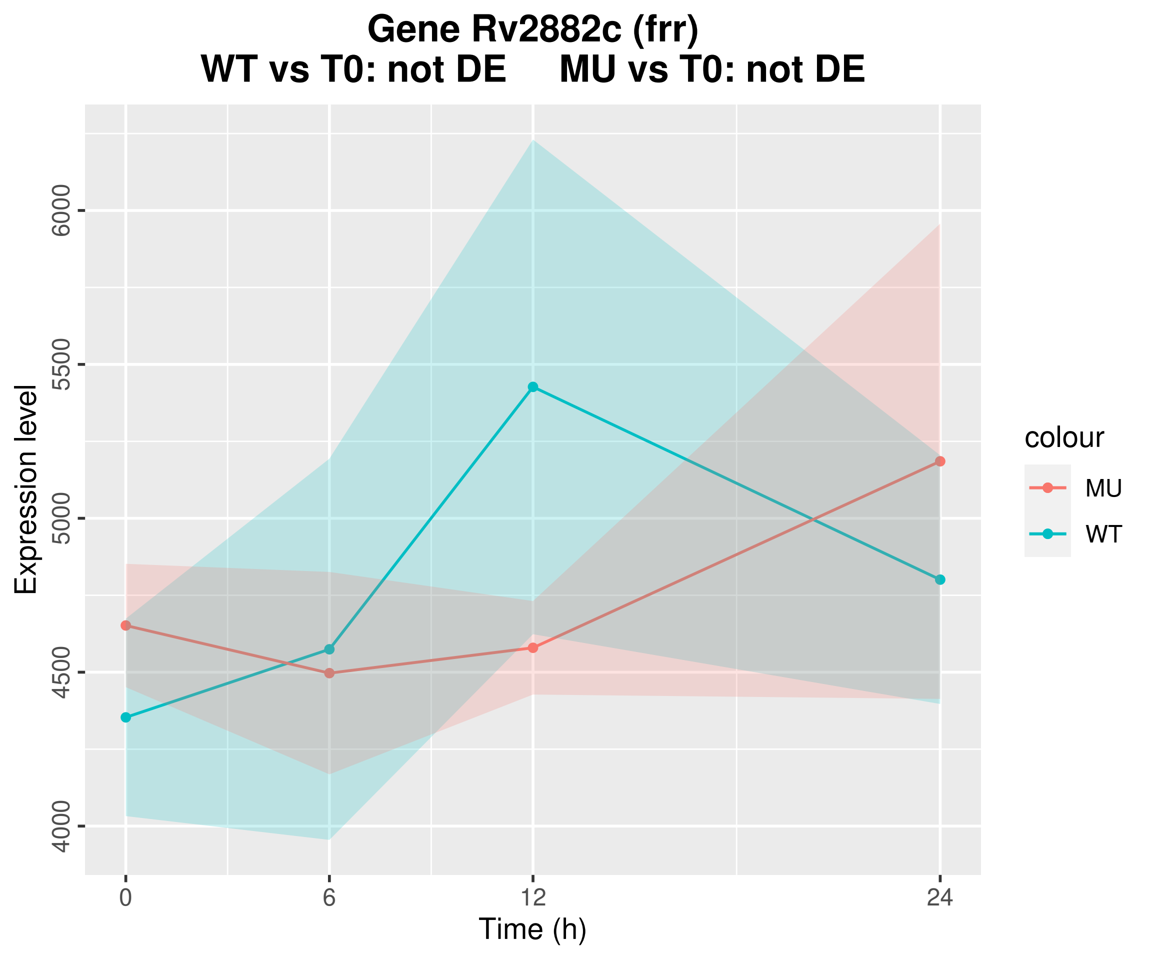
Gene Rv2882c (frr)
WT vs T0: not DE   MU vs T0: not DE
Expression level
6000
5500
5000
4500
4000
0
6
12
24
Time (h)
colour
MU
WT

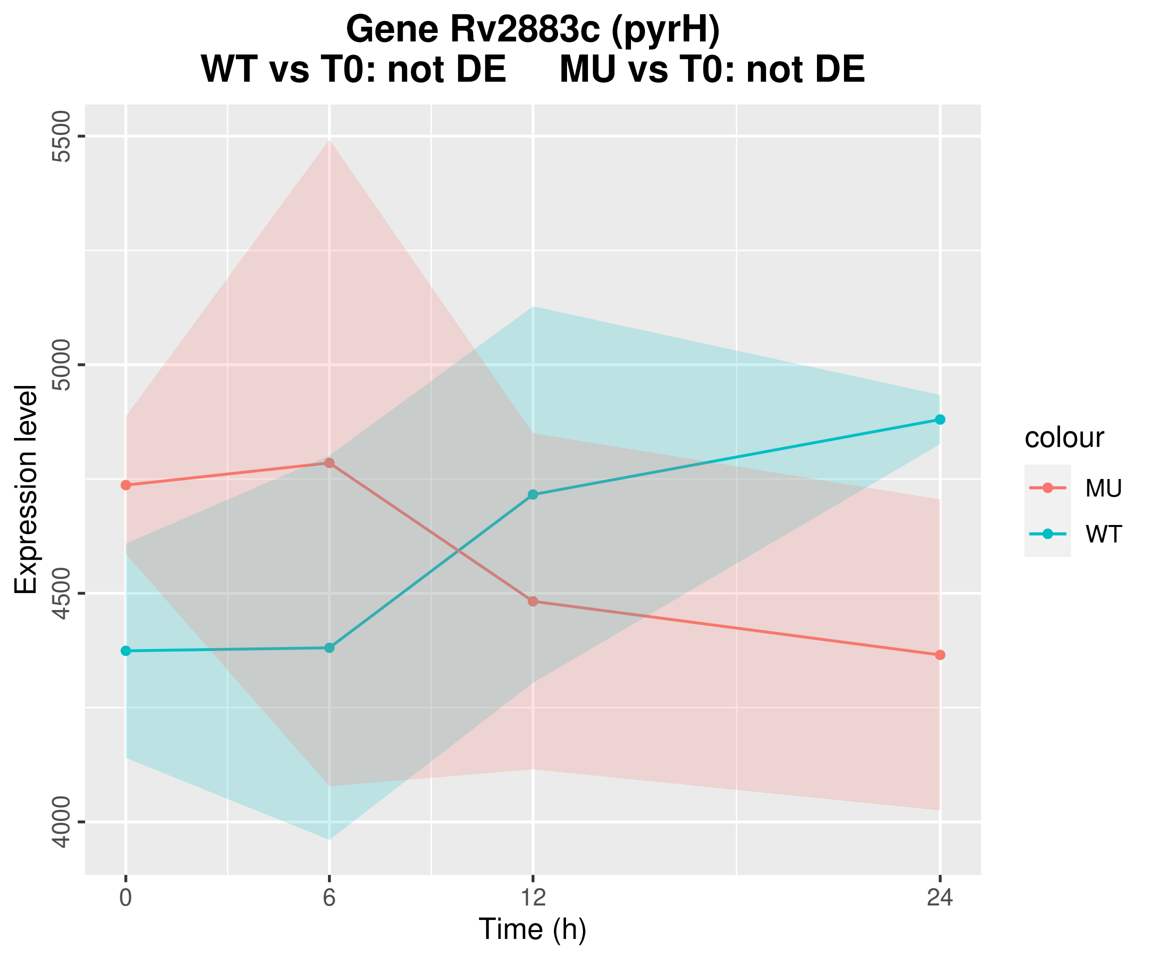
Gene Rv2883c (pyrH)
WT vs T0: not DE   MU vs T0: not DE
5500
5000
4500
4000
Expression level
0
6
12
24
Time (h)
colour
MU
WT

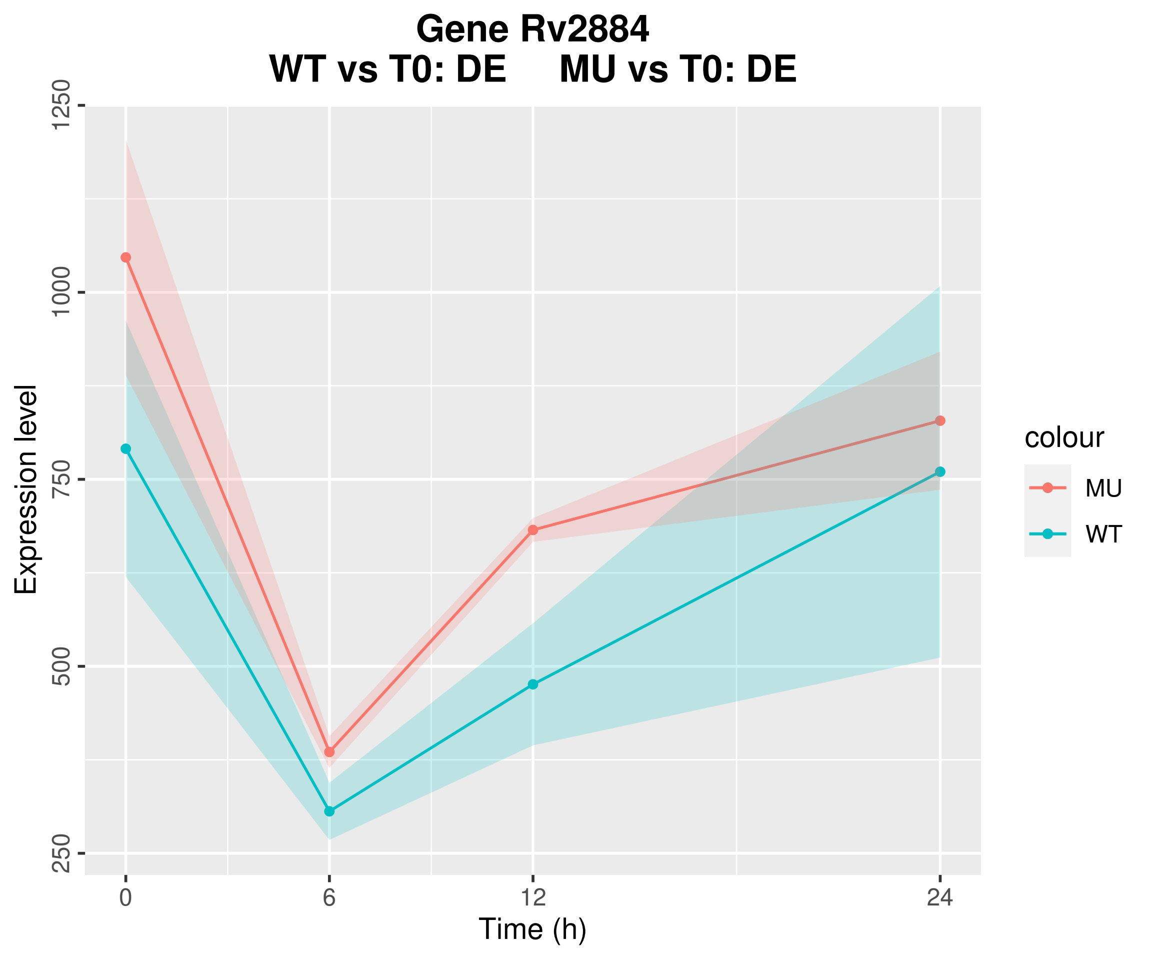

Gene Rv2884
WT vs T0: DE   MU vs T0: DE
Expression level
1250
1000
750
500
250
0
6
12
24
Time (h)
colour
MU
WT

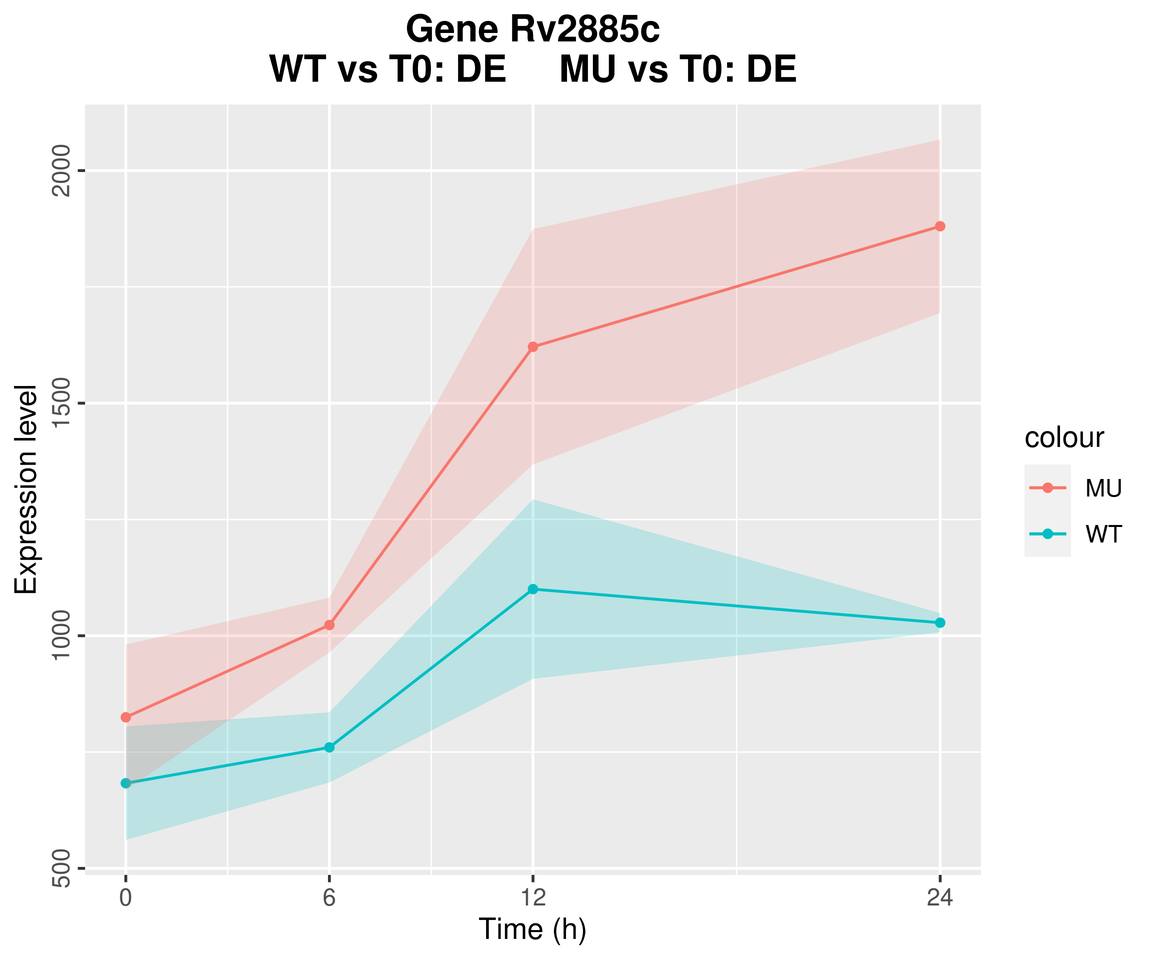
Gene Rv2885c
WT vs T0: DE MU vs T0: DE
Expression level
2000
1500
1000
500
0
6
12
24
Time (h)
colour
MU
WT

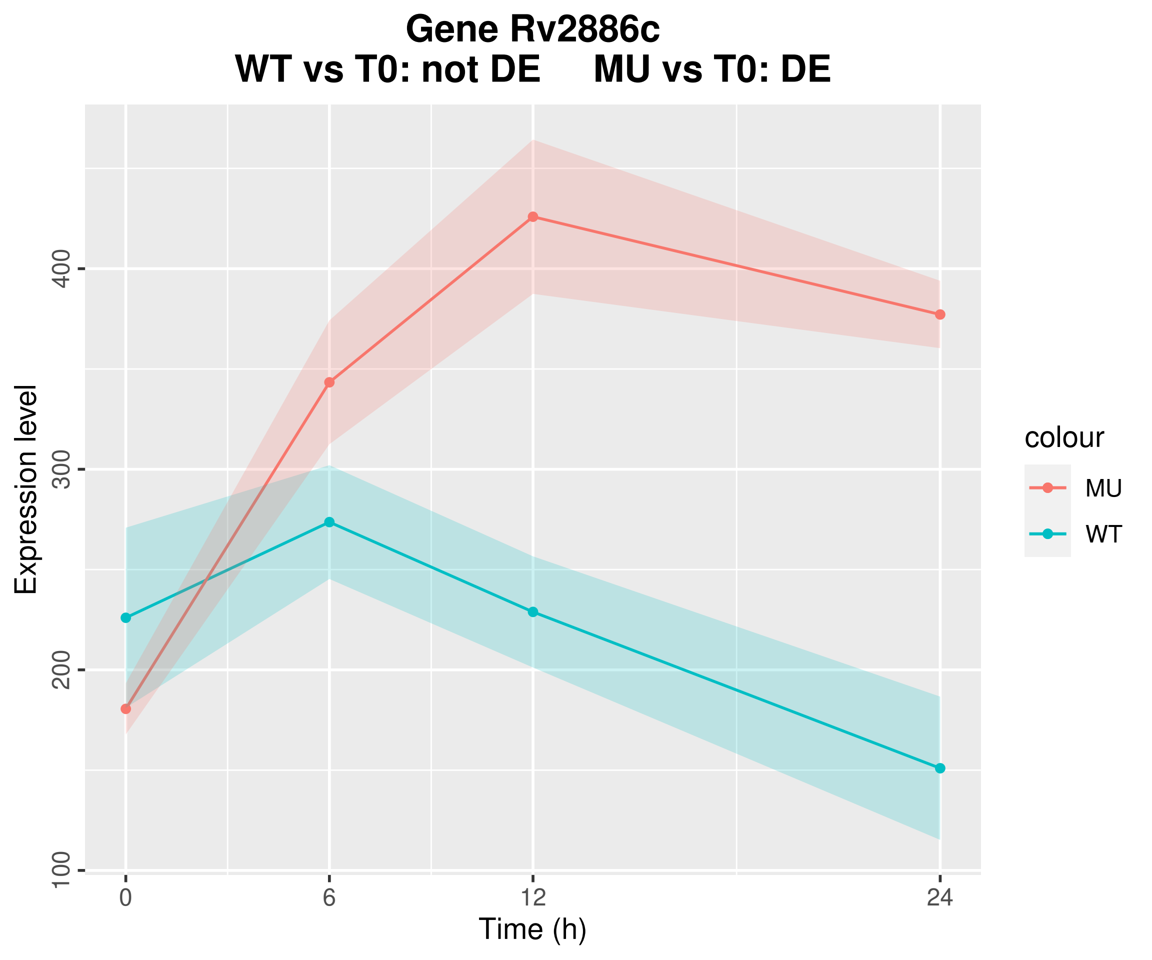

Gene Rv2886c
WT vs T0: not DE MU vs T0: DE
Expression level
100
200
300
400
0
6
12
24
Time (h)
colour
MU
WT

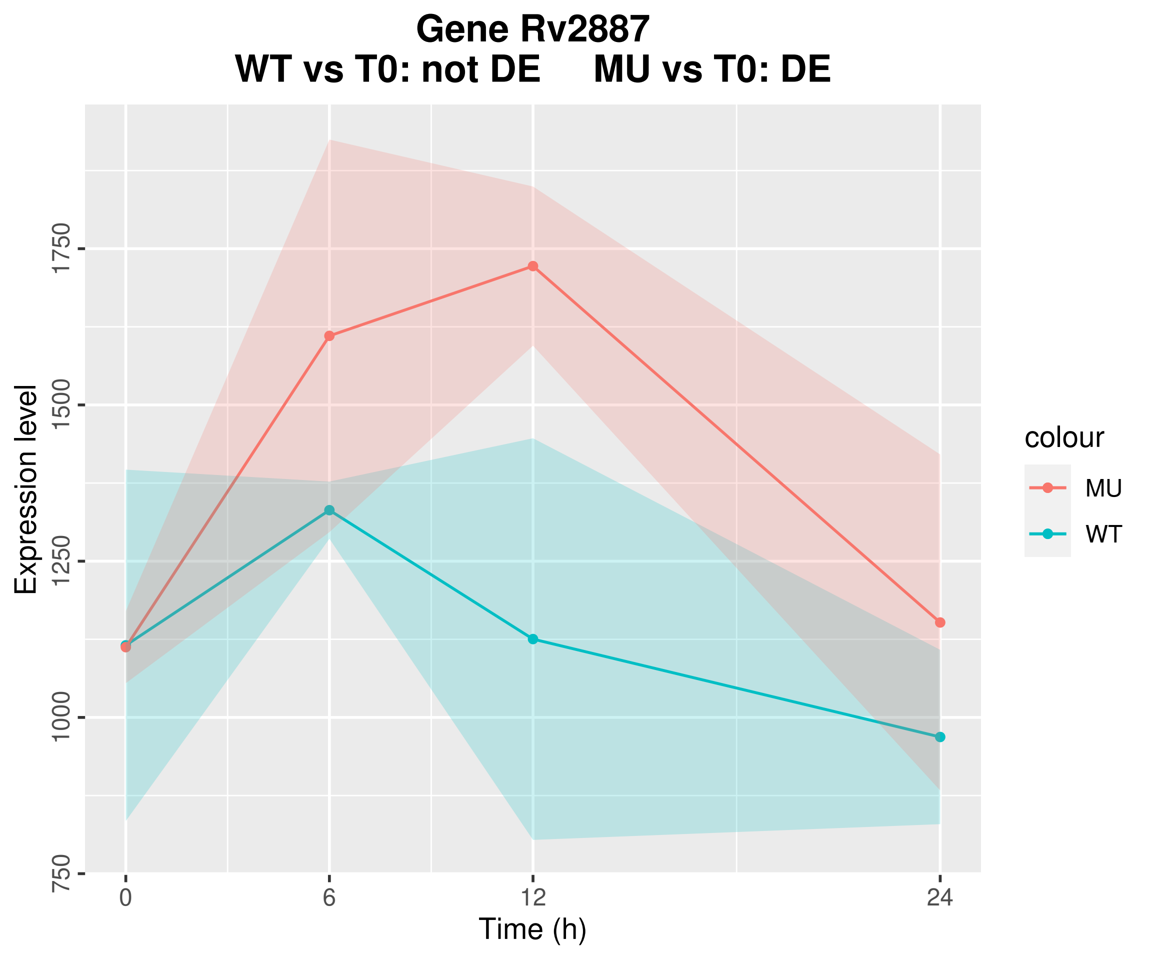

Gene Rv2887
WT vs T0: not DE MU vs T0: DE
Expression level
750
1000
1250
1500
1750
0
6
12
24
Time (h)
colour
MU
WT

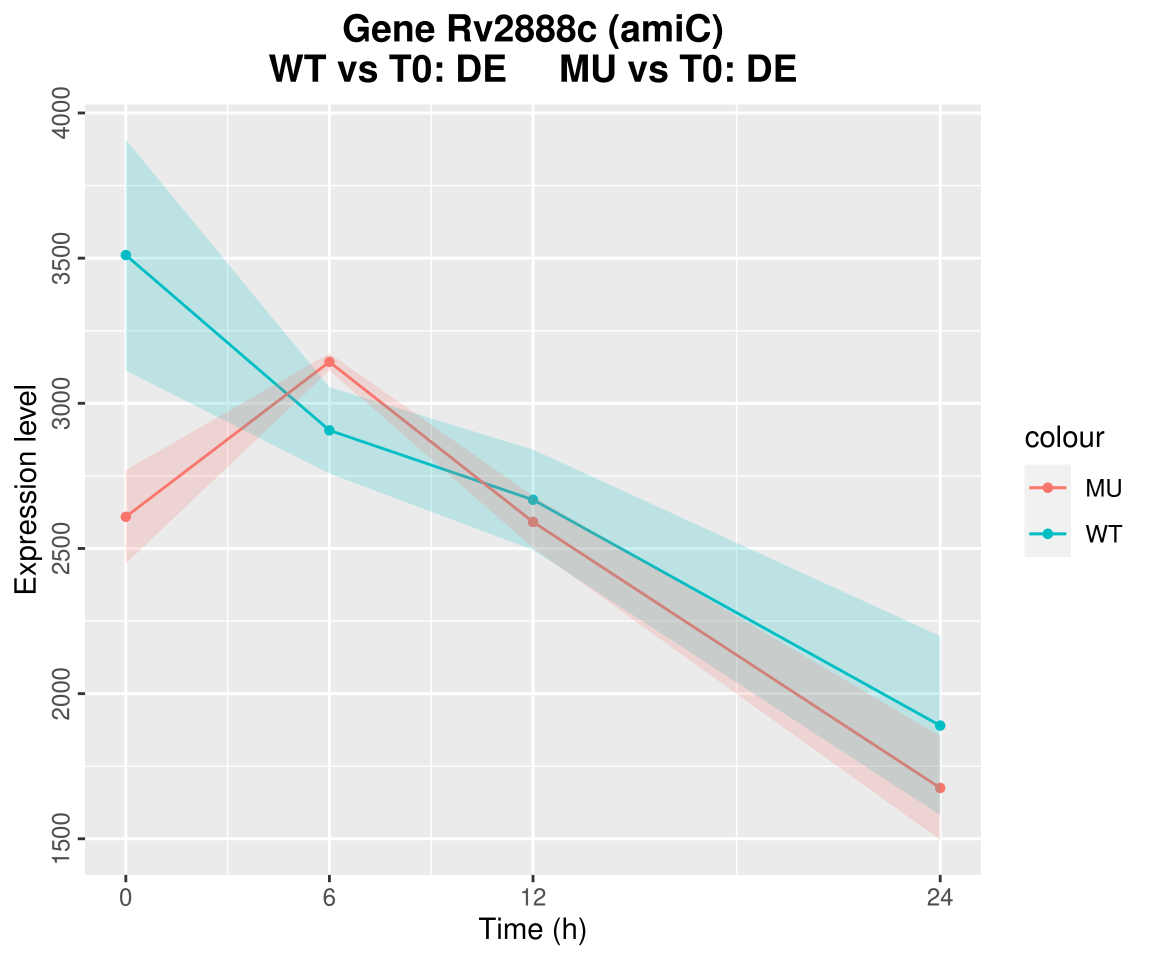
Gene Rv2888c (amiC)
WT vs T0: DE MU vs T0: DE
Expression level
4000
3500
3000
2500
2000
1500
0
6
12
24
Time (h)
colour
MU
WT

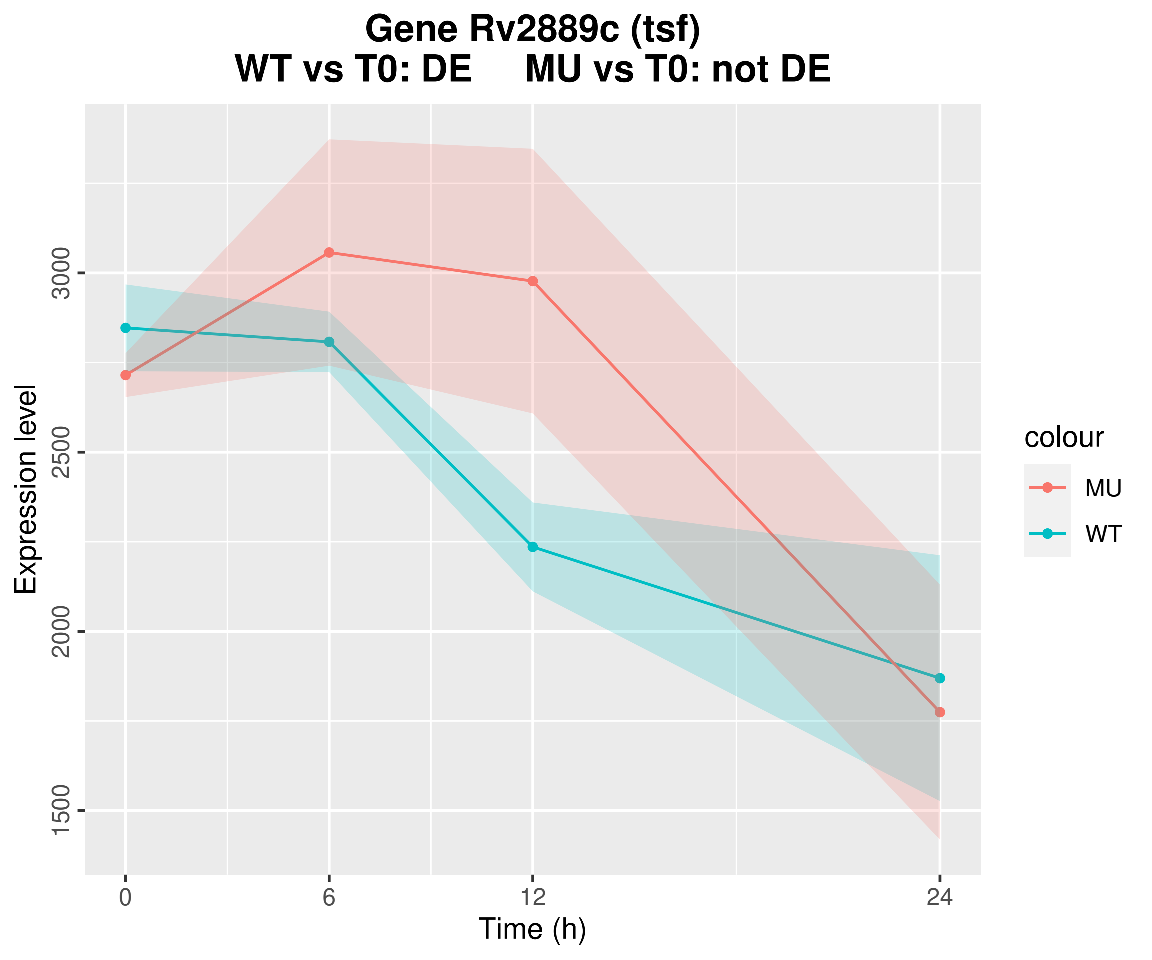

Gene Rv2889c (tsf)
WT vs T0: DE   MU vs T0: not DE
Expression level
3000
2500
2000
1500
0
6
12
24
Time (h)
colour
MU
WT

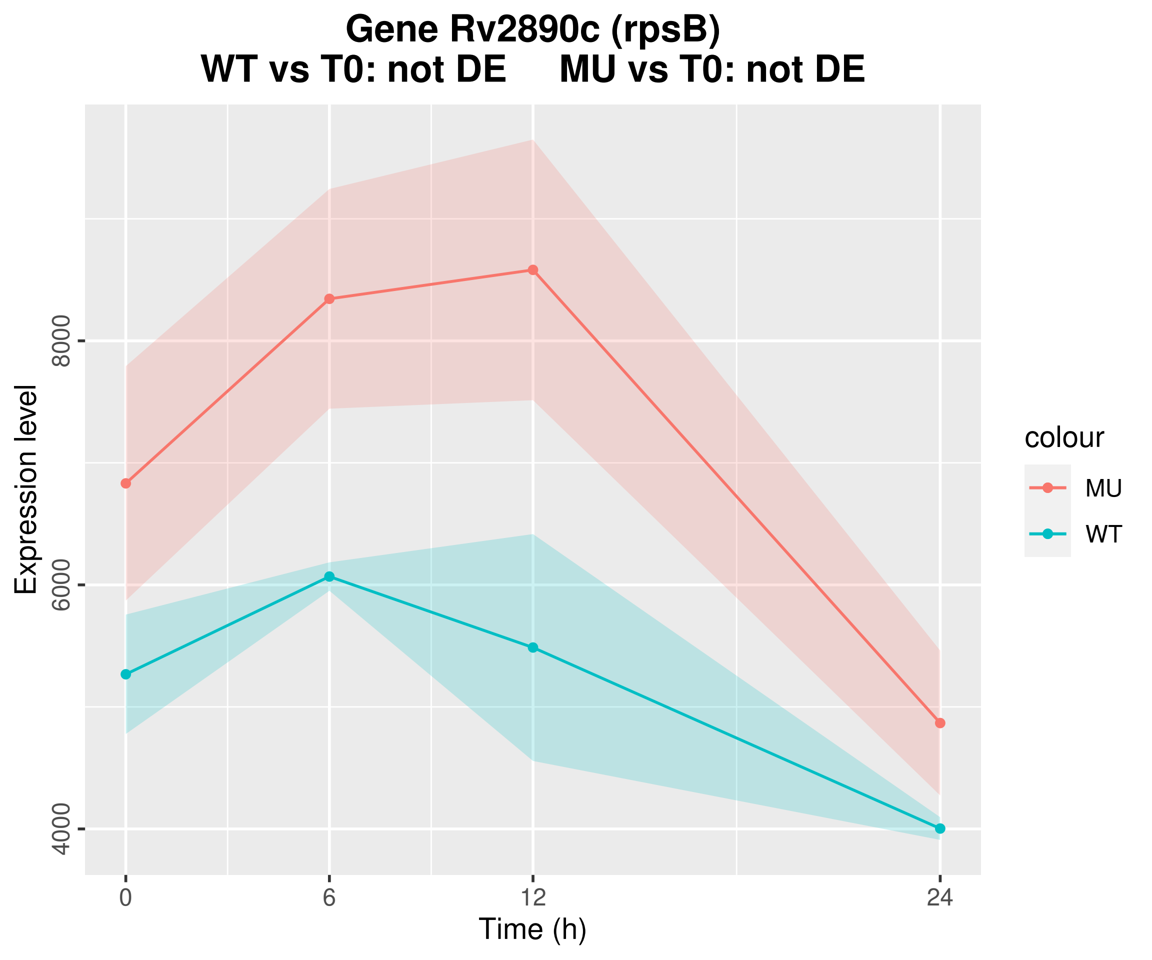

Gene Rv2890c (rpsB)
WT vs T0: not DE   MU vs T0: not DE
Expression level
8000
6000
4000
0
6
12
24
Time (h)
colour
MU
WT

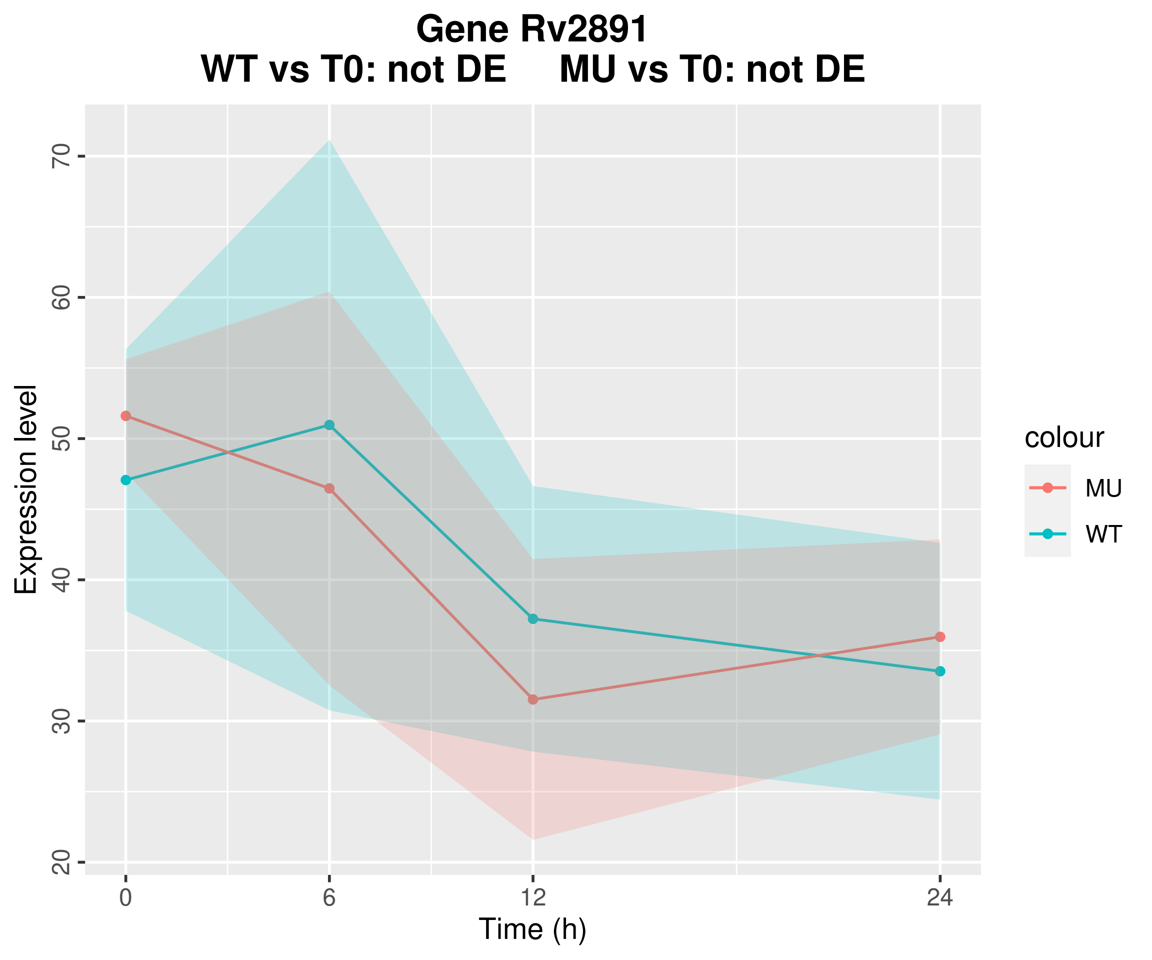
Gene Rv2891
WT vs T0: not DE   MU vs T0: not DE
Expression level
20
30
40
50
60
70
0
6
12
24
Time (h)
colour
MU
WT

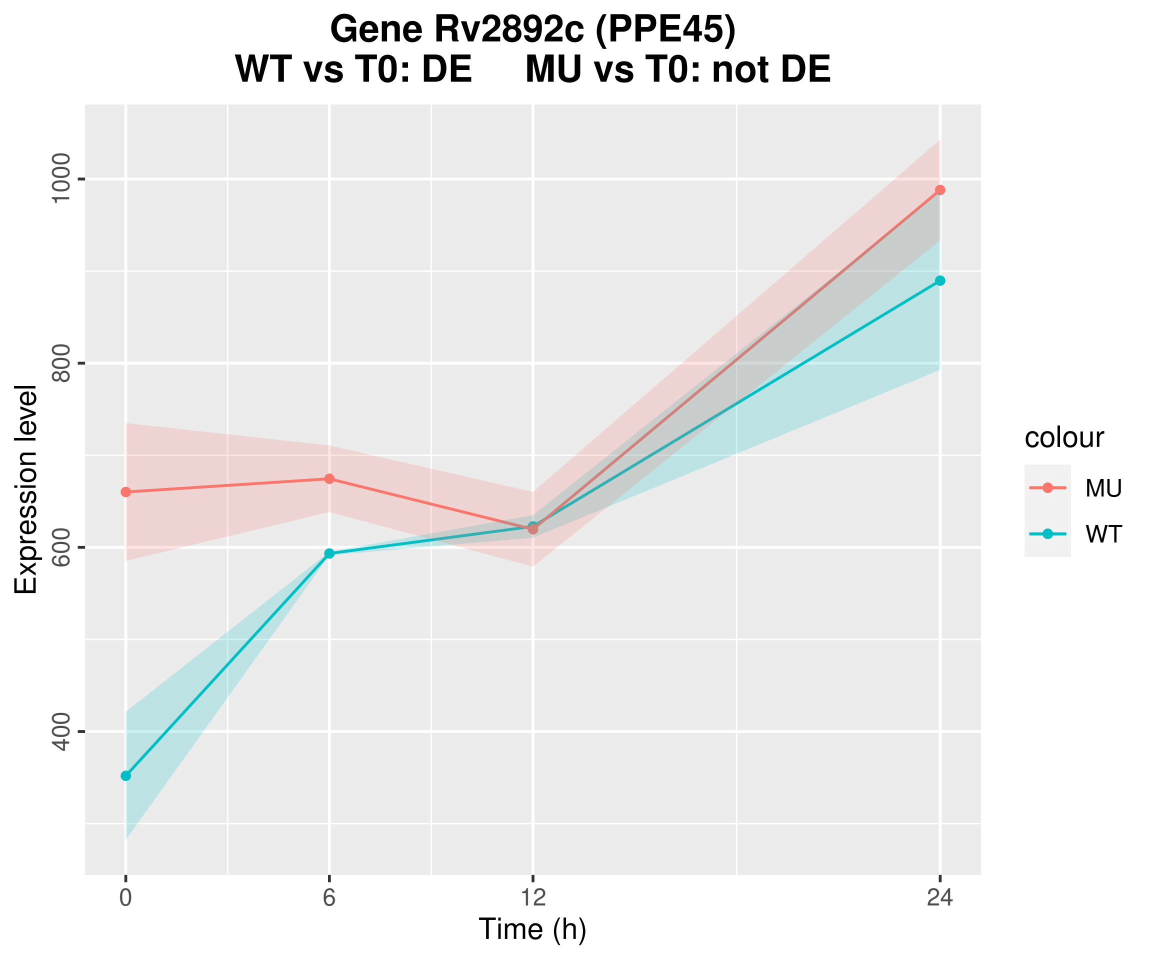
Gene Rv2892c (PPE45)
WT vs T0: DE   MU vs T0: not DE
Expression level
1000
800
600
400
0
6
12
24
Time (h)
colour
MU
WT

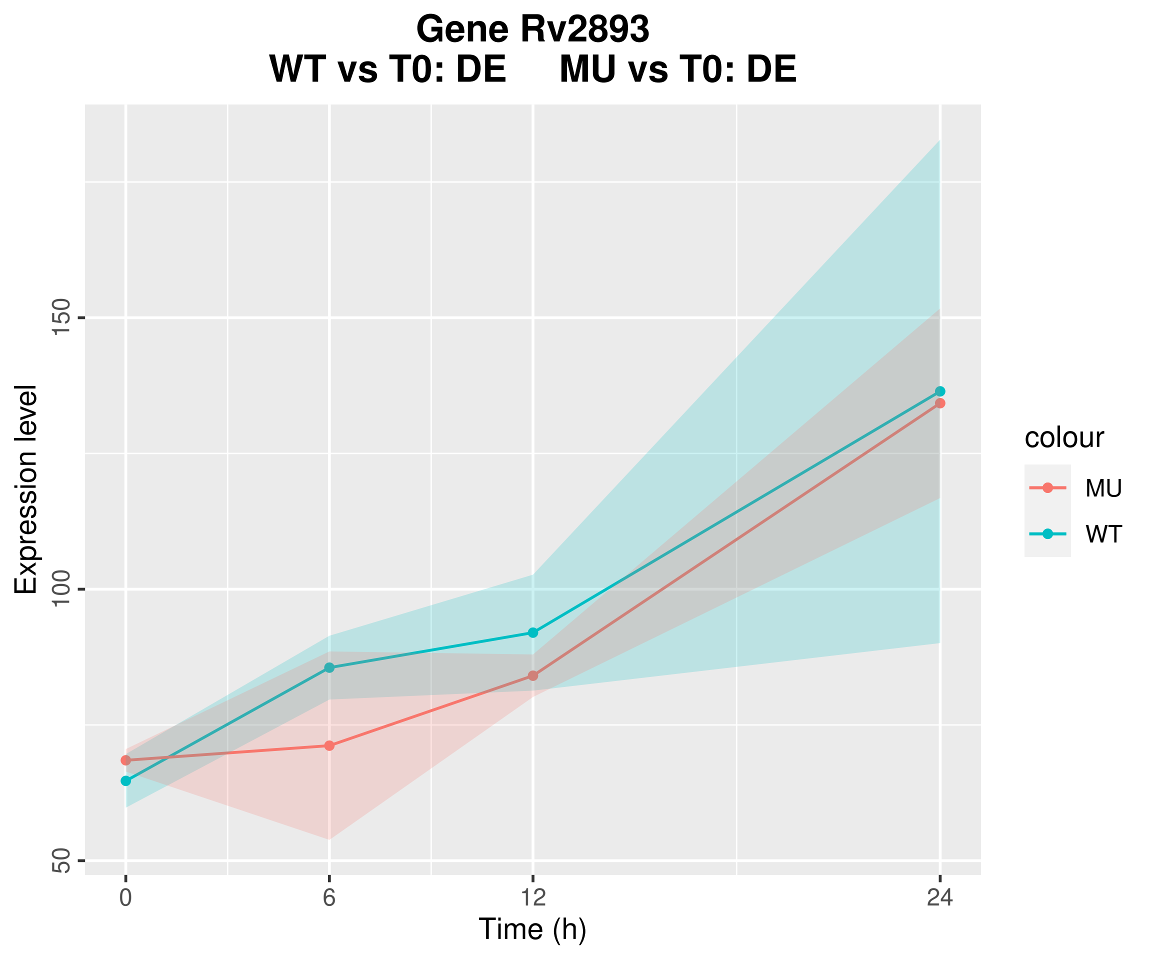

Gene Rv2893
WT vs T0: DE   MU vs T0: DE
Expression level
150
100
50
0
6
12
24
Time (h)
colour
MU
WT

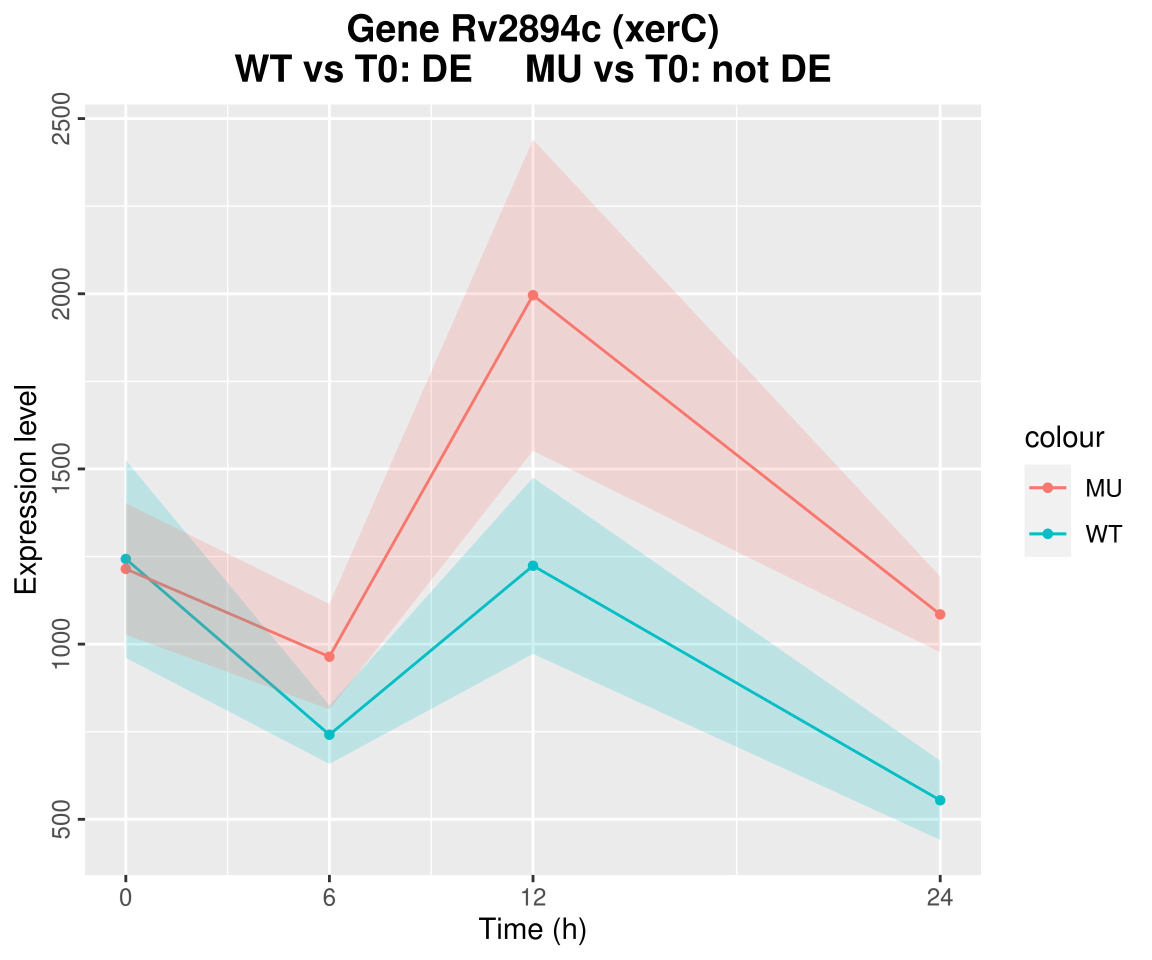
Gene Rv2894c (xerC)
WT vs T0: DE   MU vs T0: not DE
Expression level
2500
2000
1500
1000
500
0
6
12
24
Time (h)
colour
MU
WT

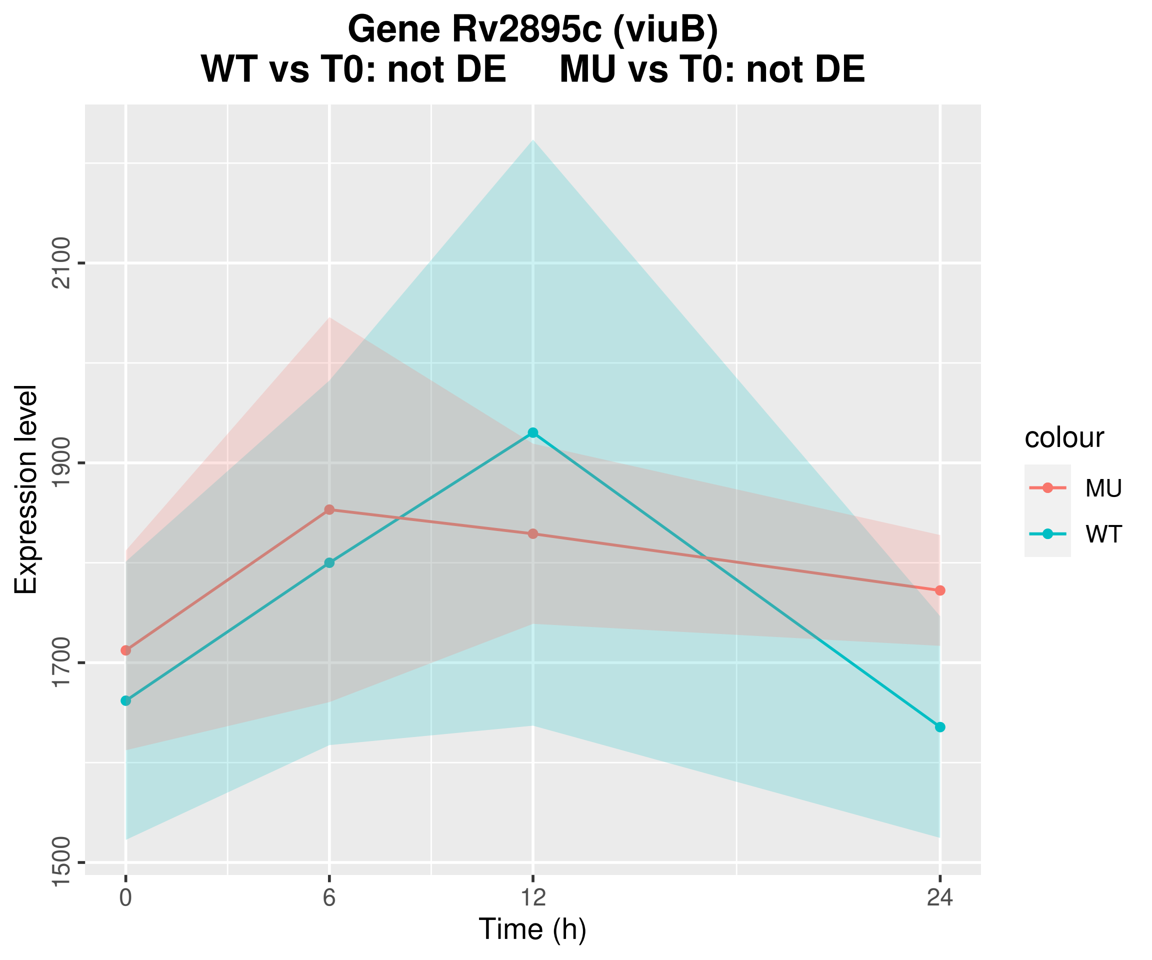
Gene Rv2895c (viuB)
WT vs T0: not DE   MU vs T0: not DE
Expression level
2100
1900
1700
1500
0
6
12
24
Time (h)
colour
MU
WT

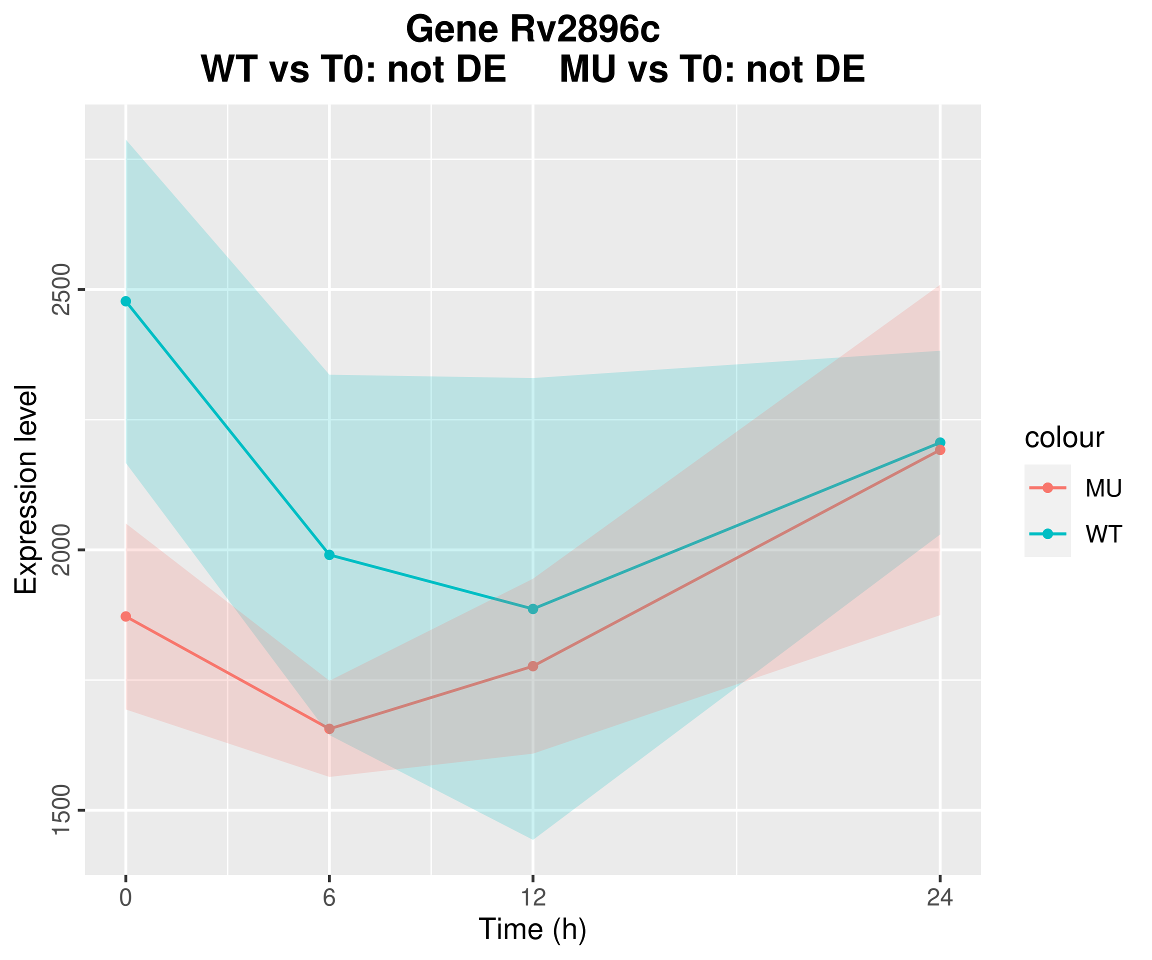

Gene Rv2896c
WT vs T0: not DE MU vs T0: not DE
Expression level
2500
2000
1500
0
6
12
24
Time (h)
colour
MU
WT

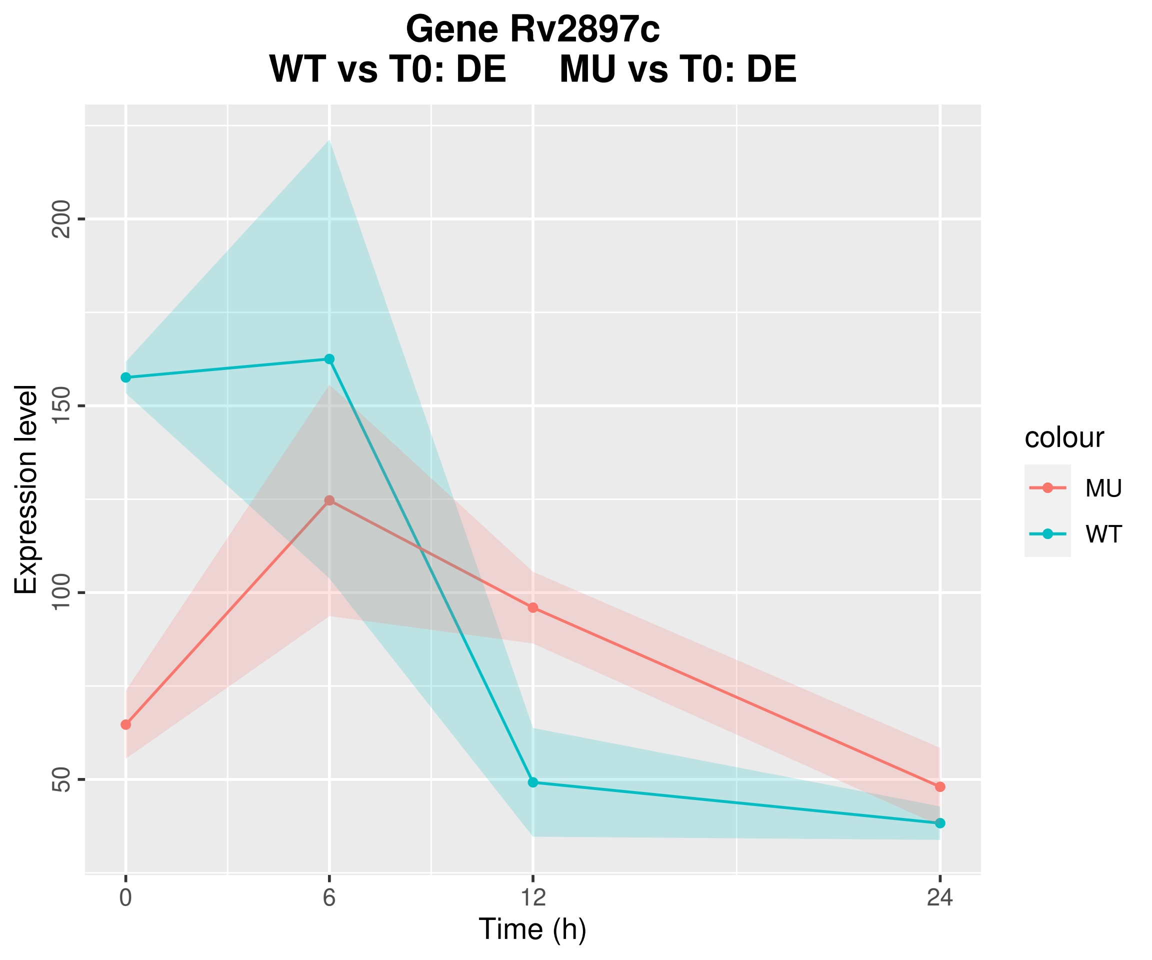

Gene Rv2897c
WT vs T0: DE MU vs T0: DE
Expression level
200
150
100
50
0
6
12
24
Time (h)
colour
MU
WT

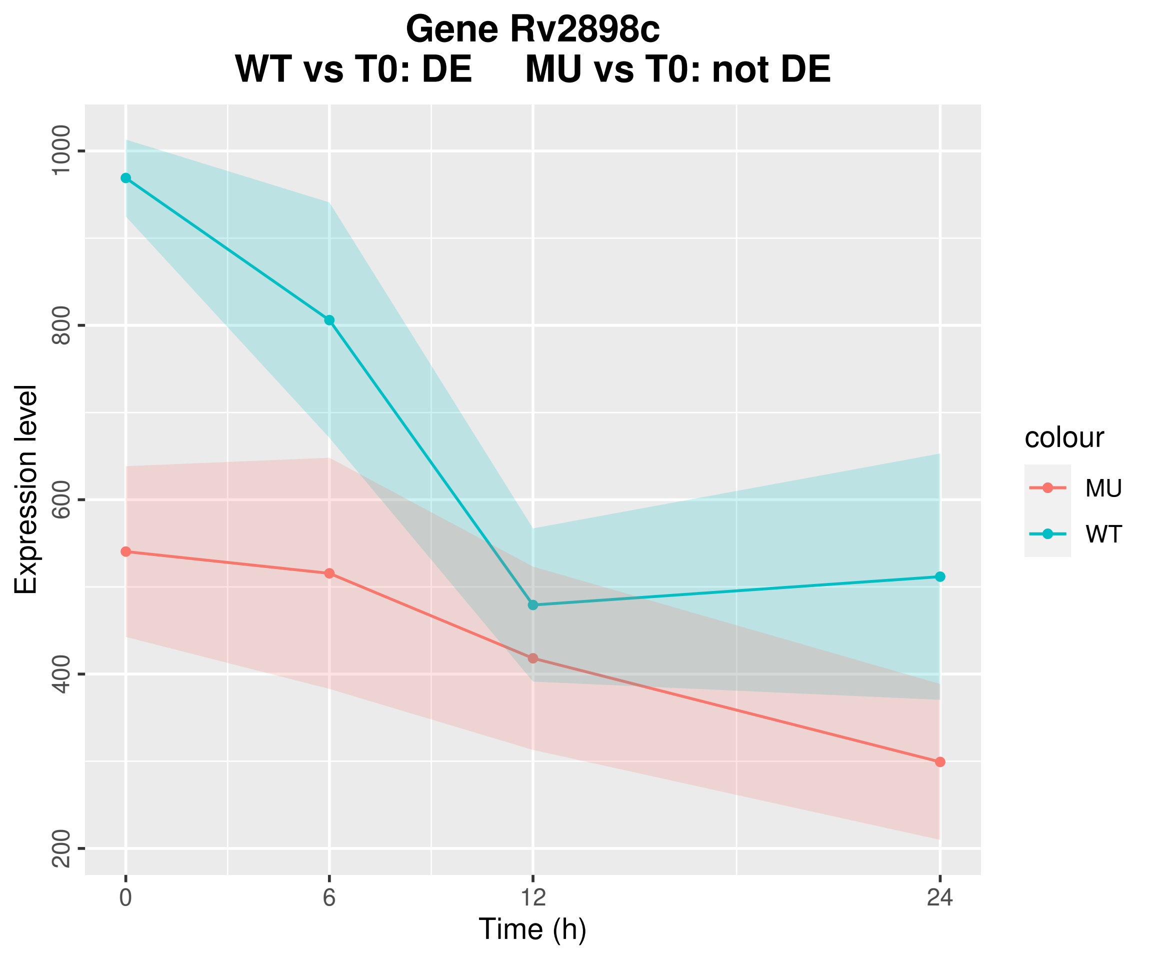

Gene Rv2898c
WT vs T0: DE MU vs T0: not DE
Expression level
1000
800
600
400
200
0
6
12
24
Time (h)
colour
MU
WT

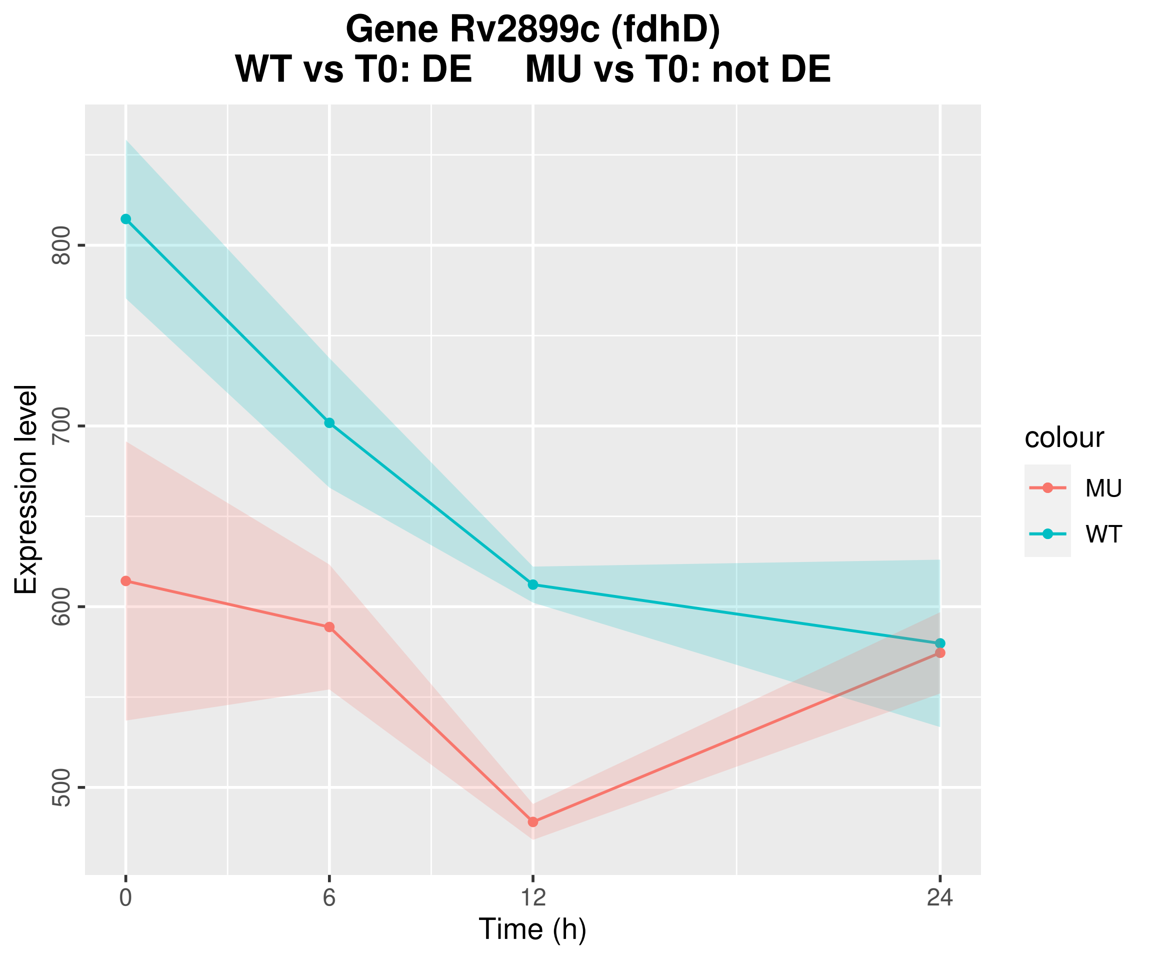

Gene Rv2899c (fdhD)
WT vs T0: DE   MU vs T0: not DE
Expression level
800
700
600
500
0
6
12
24
Time (h)
colour
MU
WT

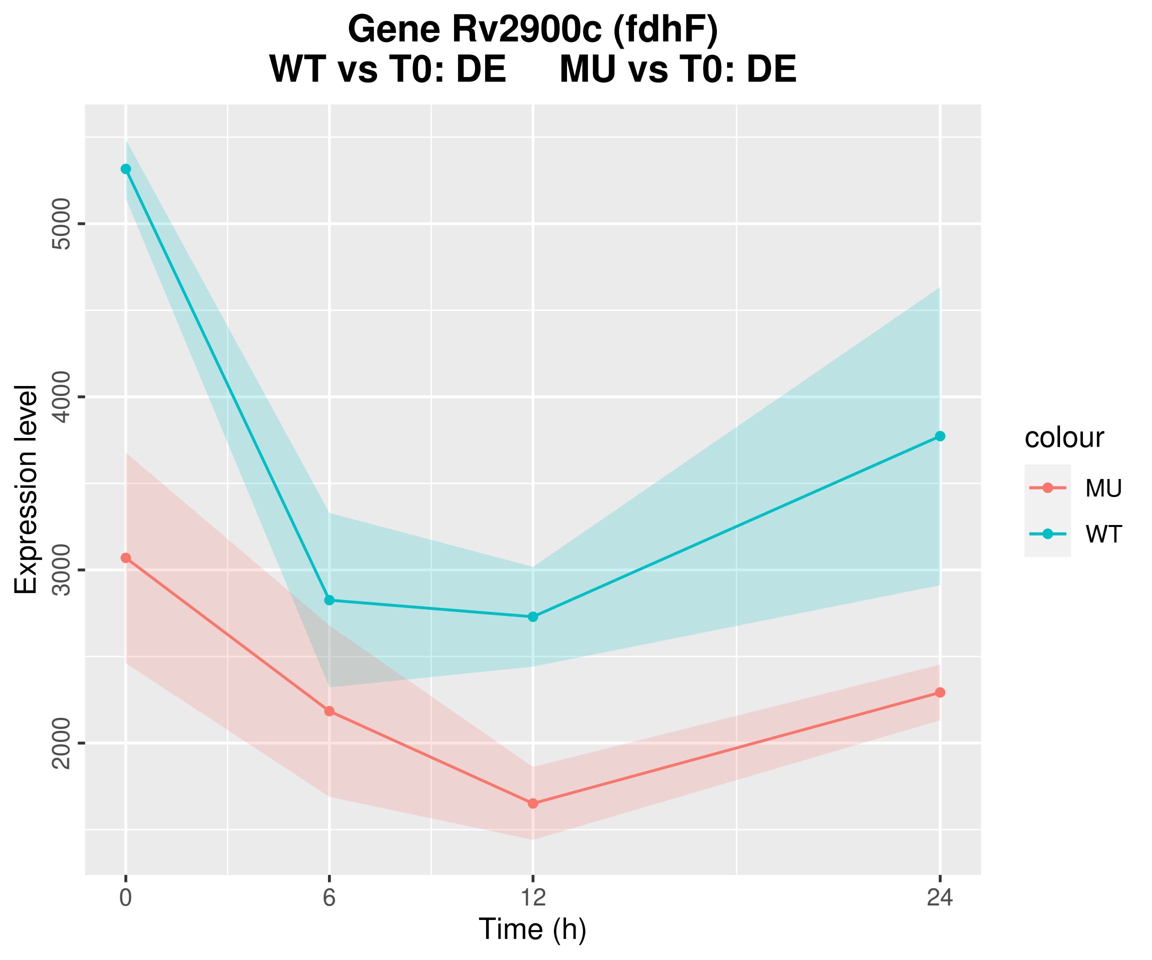
Gene Rv2900c (fdhF)
WT vs T0: DE MU vs T0: DE
Expression level
5000
4000
3000
2000
0
6
12
24
Time (h)
colour
MU
WT

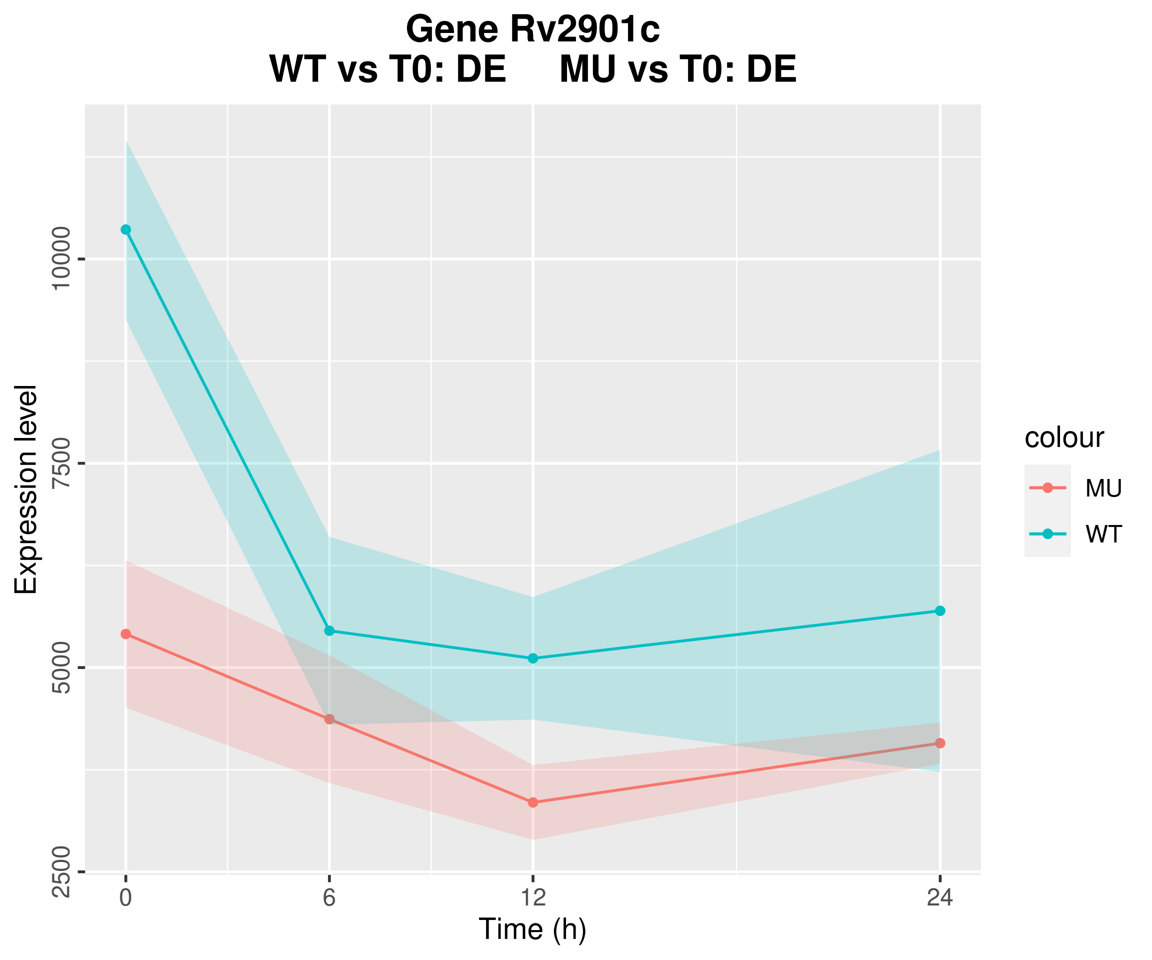
Gene Rv2901c
WT vs T0: DE   MU vs T0: DE
Expression level
10000
7500
5000
2500
0
6
12
24
Time (h)
colour
MU
WT

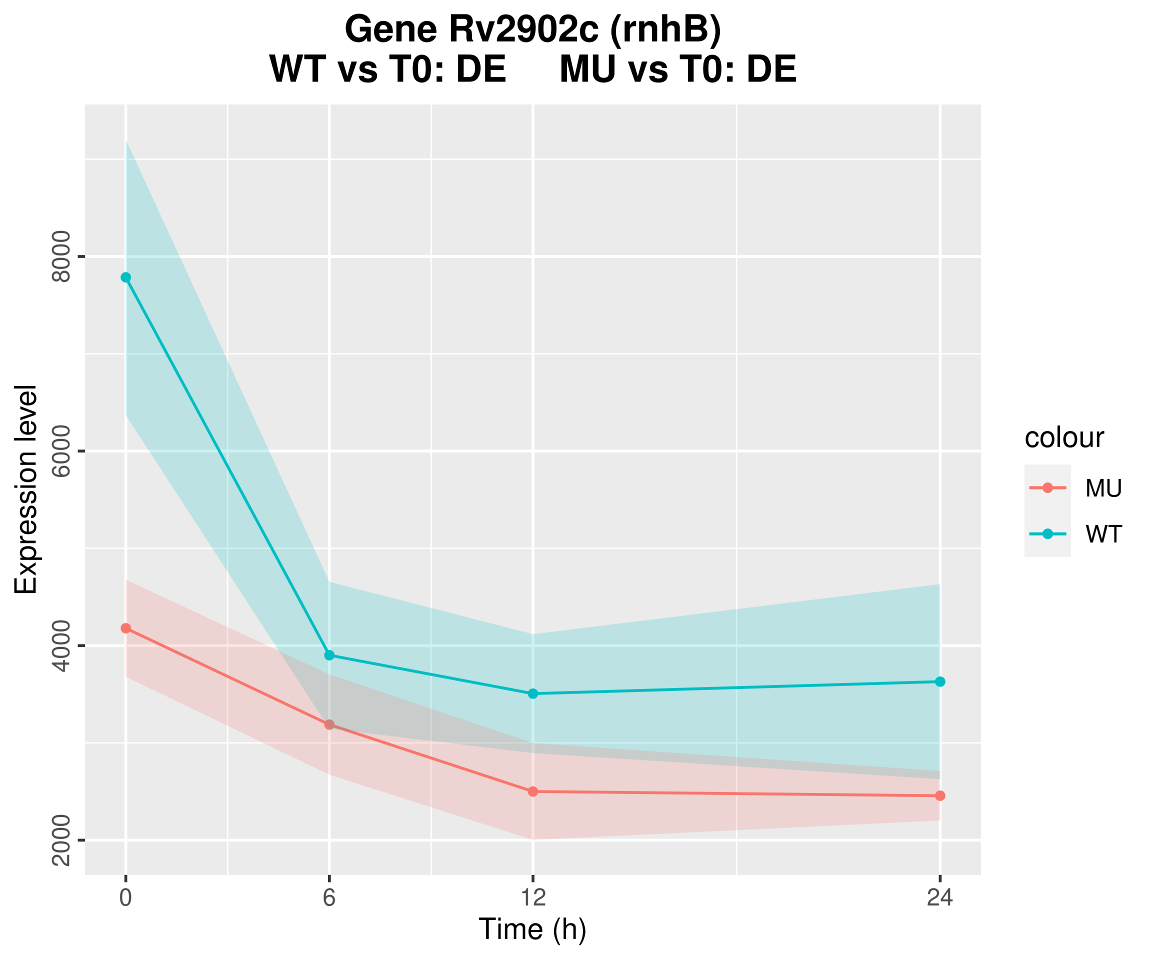

Gene Rv2902c (rnhB)
WT vs T0: DE   MU vs T0: DE
Expression level
8000
6000
4000
2000
0
6
12
24
Time (h)
colour
MU
WT

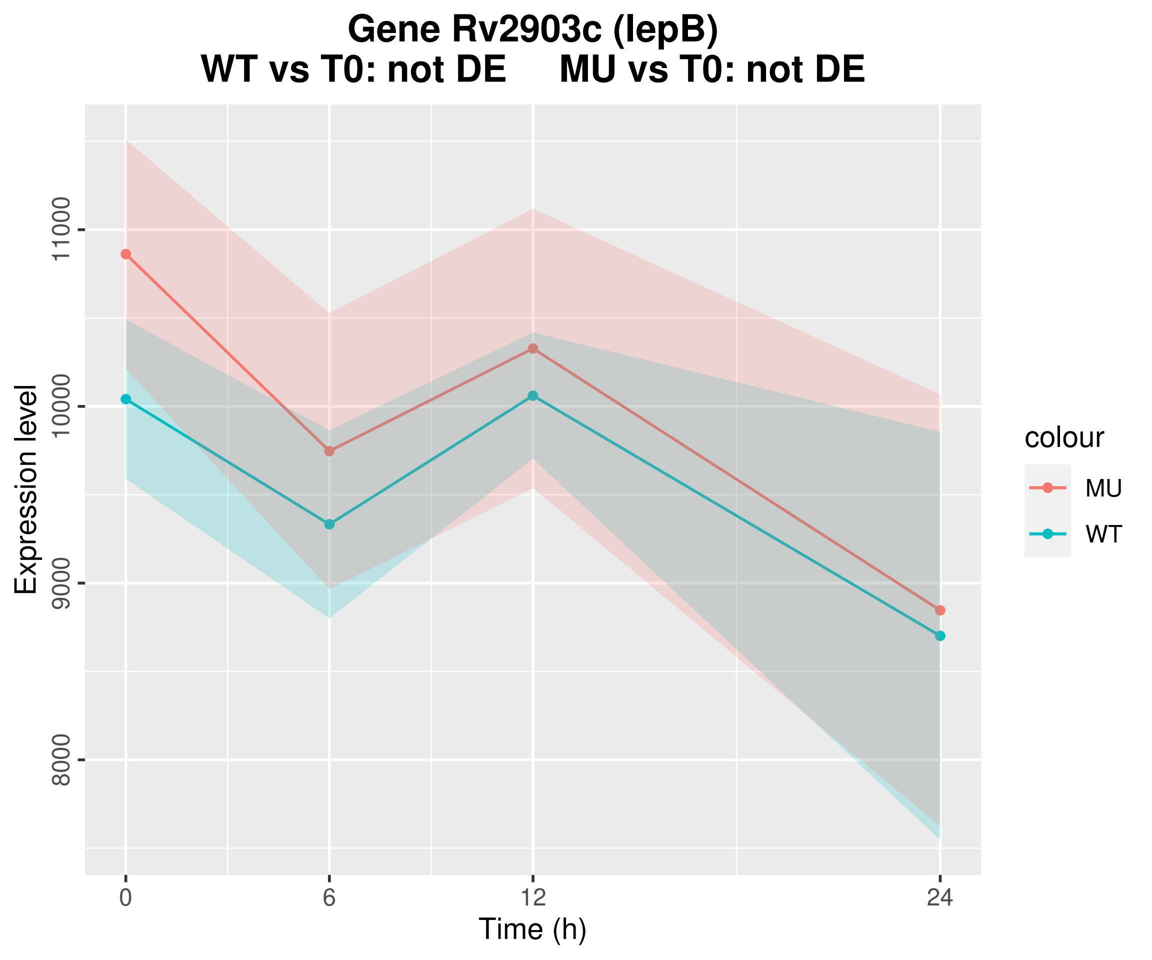

Gene Rv2903c (lepB)
WT vs T0: not DE   MU vs T0: not DE
Expression level
11000
10000
9000
8000
0
6
12
24
Time (h)
colour
MU
WT

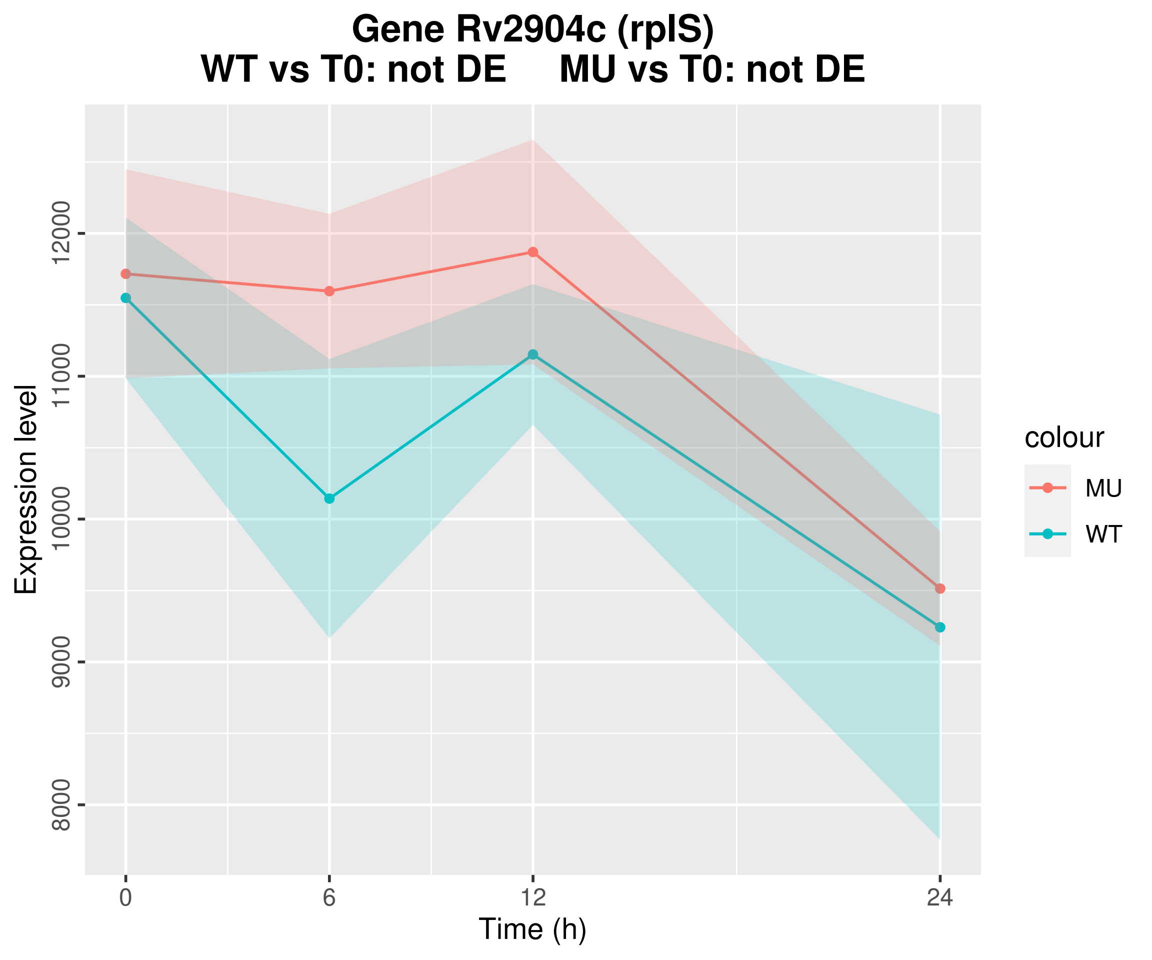

Gene Rv2904c (rplS)
WT vs T0: not DE MU vs T0: not DE
Expression level
12000
11000
10000
9000
8000
0
6
12
24
Time (h)
colour
MU
WT

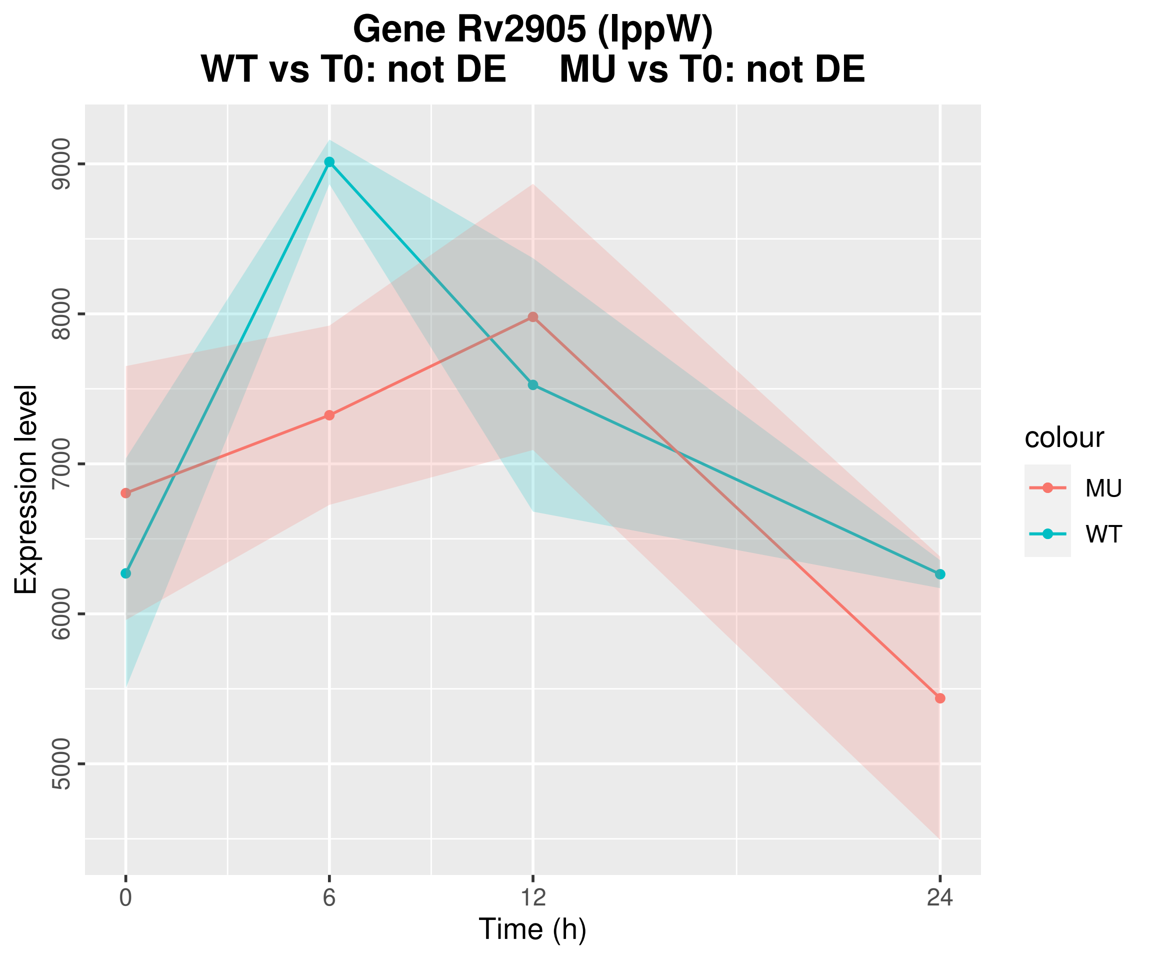
Gene Rv2905 (lppW)
WT vs T0: not DE MU vs T0: not DE
Expression level
9000
8000
7000
6000
5000
0
6
12
24
Time (h)
colour
MU
WT

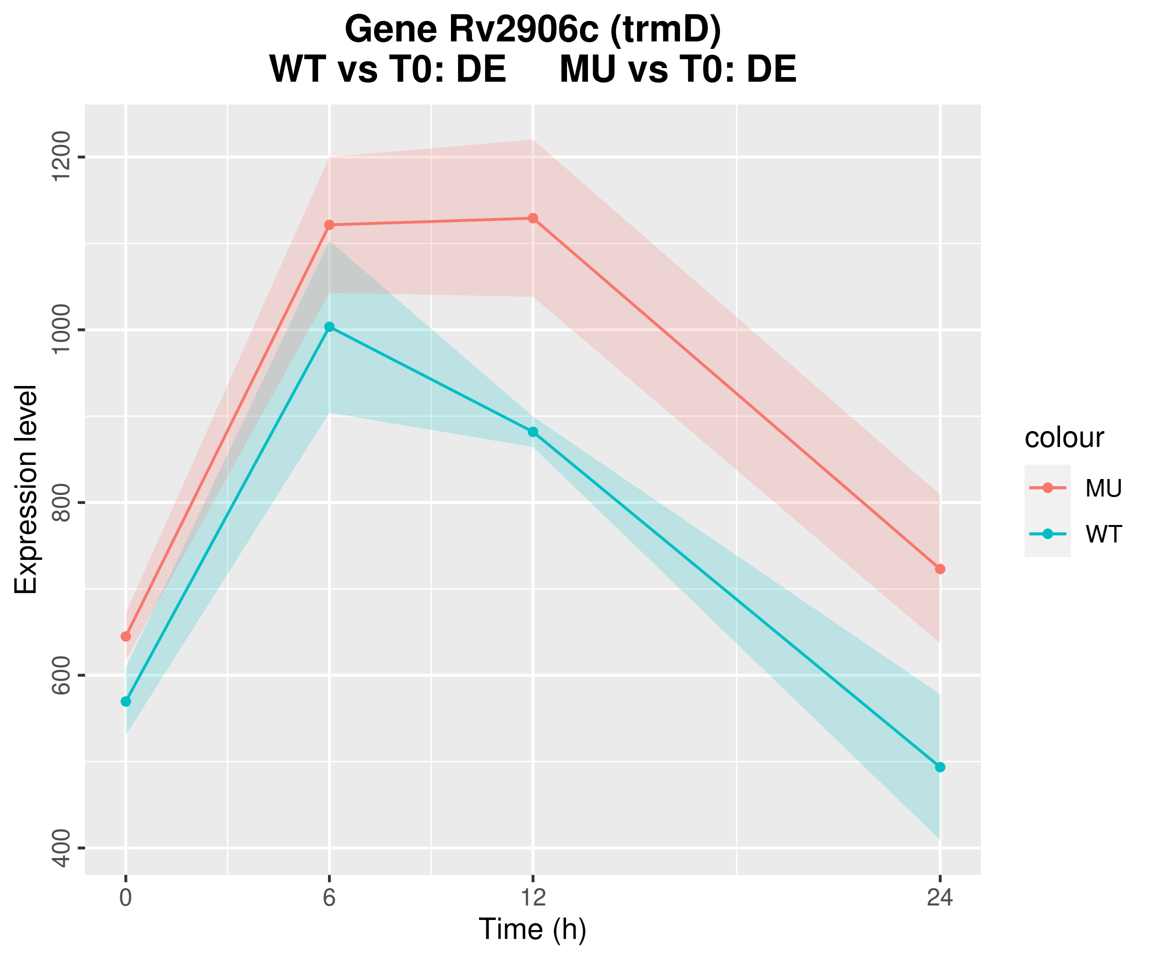

Gene Rv2906c (trmD)
WT vs T0: DE MU vs T0: DE
Expression level
1200
1000
800
600
400
0
6
12
24
Time (h)
colour
MU
WT

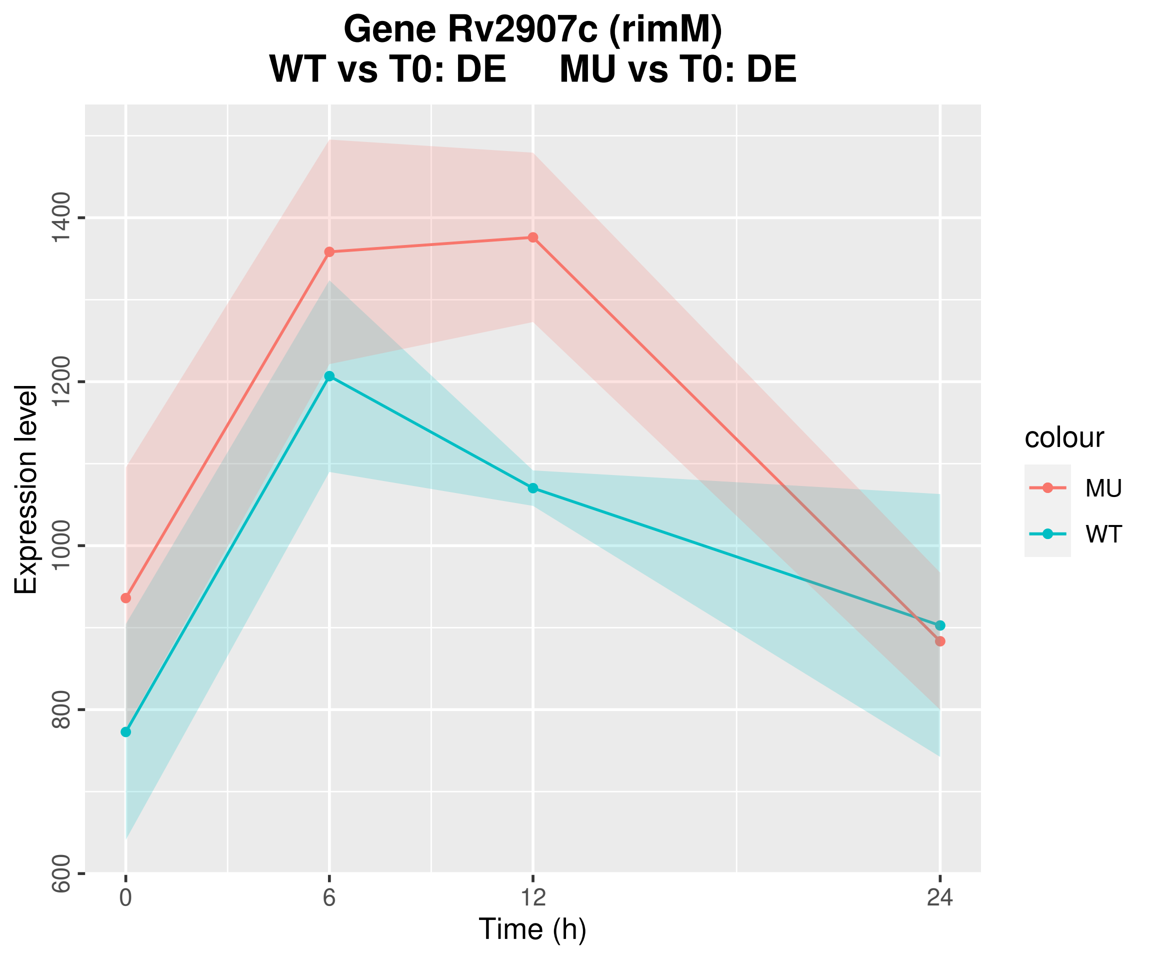

Gene Rv2907c (rimM)
WT vs T0: DE   MU vs T0: DE
Expression level
1400
1200
1000
800
600
0
6
12
24
Time (h)
colour
MU
WT

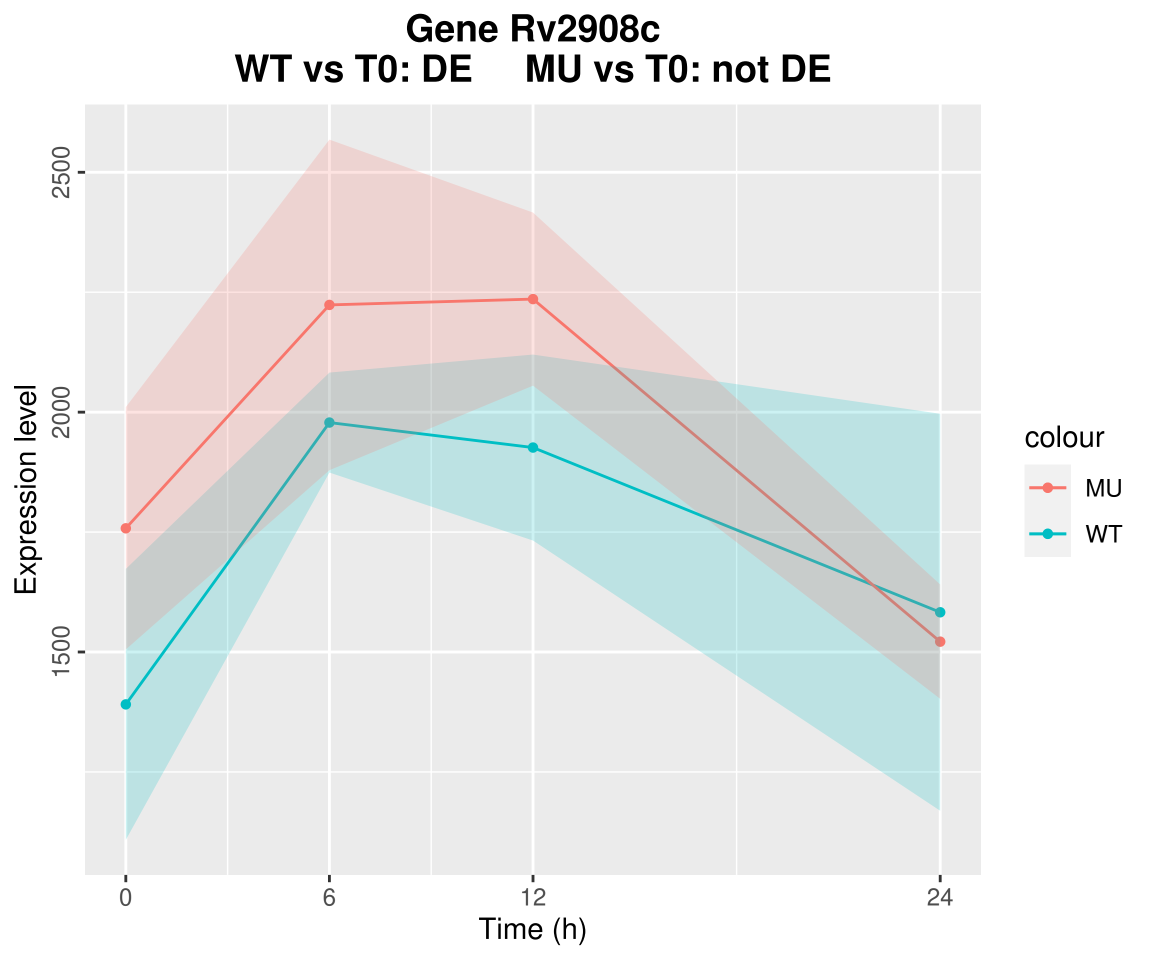

Gene Rv2908c
WT vs T0: DE   MU vs T0: not DE
2500
2000
1500
Expression level
0
6
12
24
Time (h)
colour
MU
WT

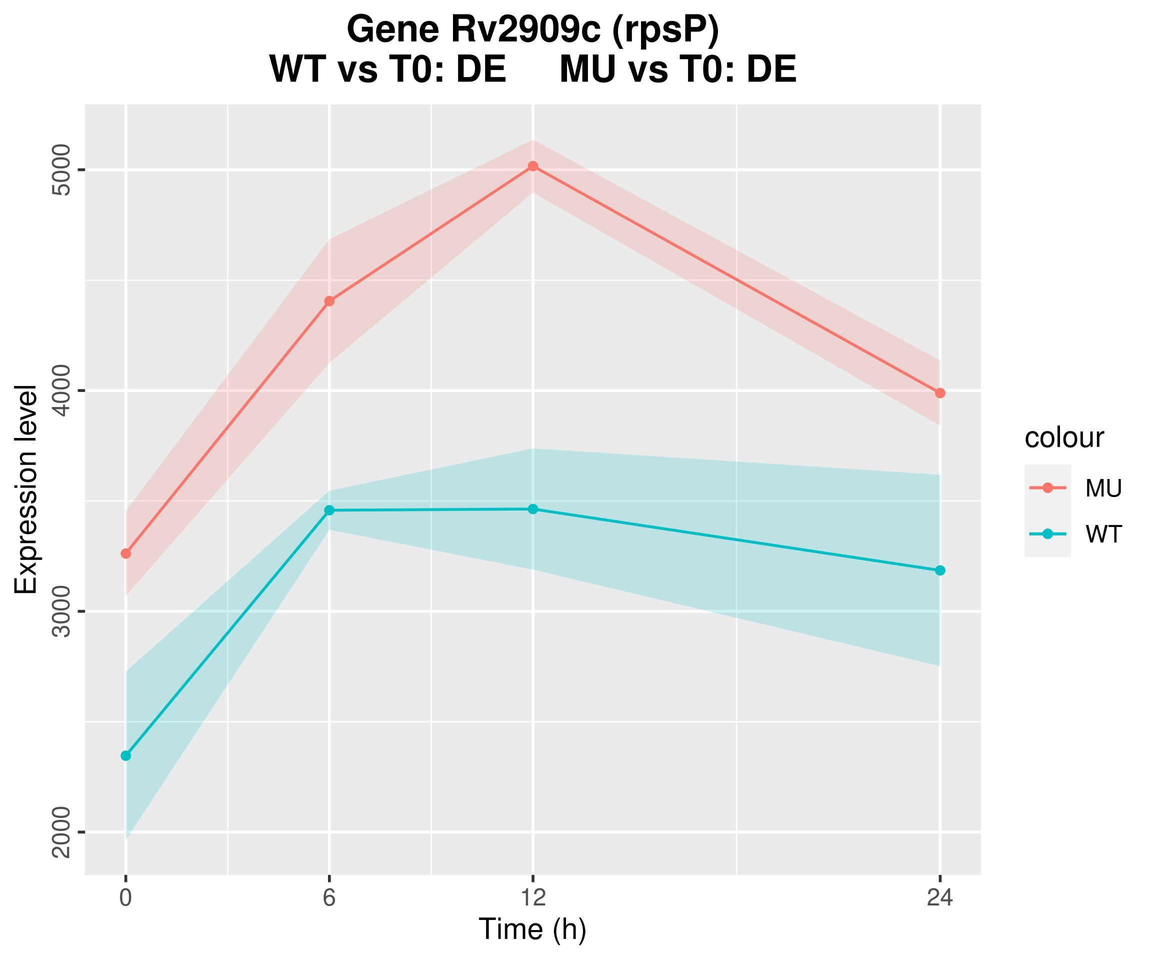
Gene Rv2909c (rpsP)
WT vs T0: DE MU vs T0: DE
Expression level
5000
4000
3000
2000
0
6
12
24
Time (h)
colour
MU
WT

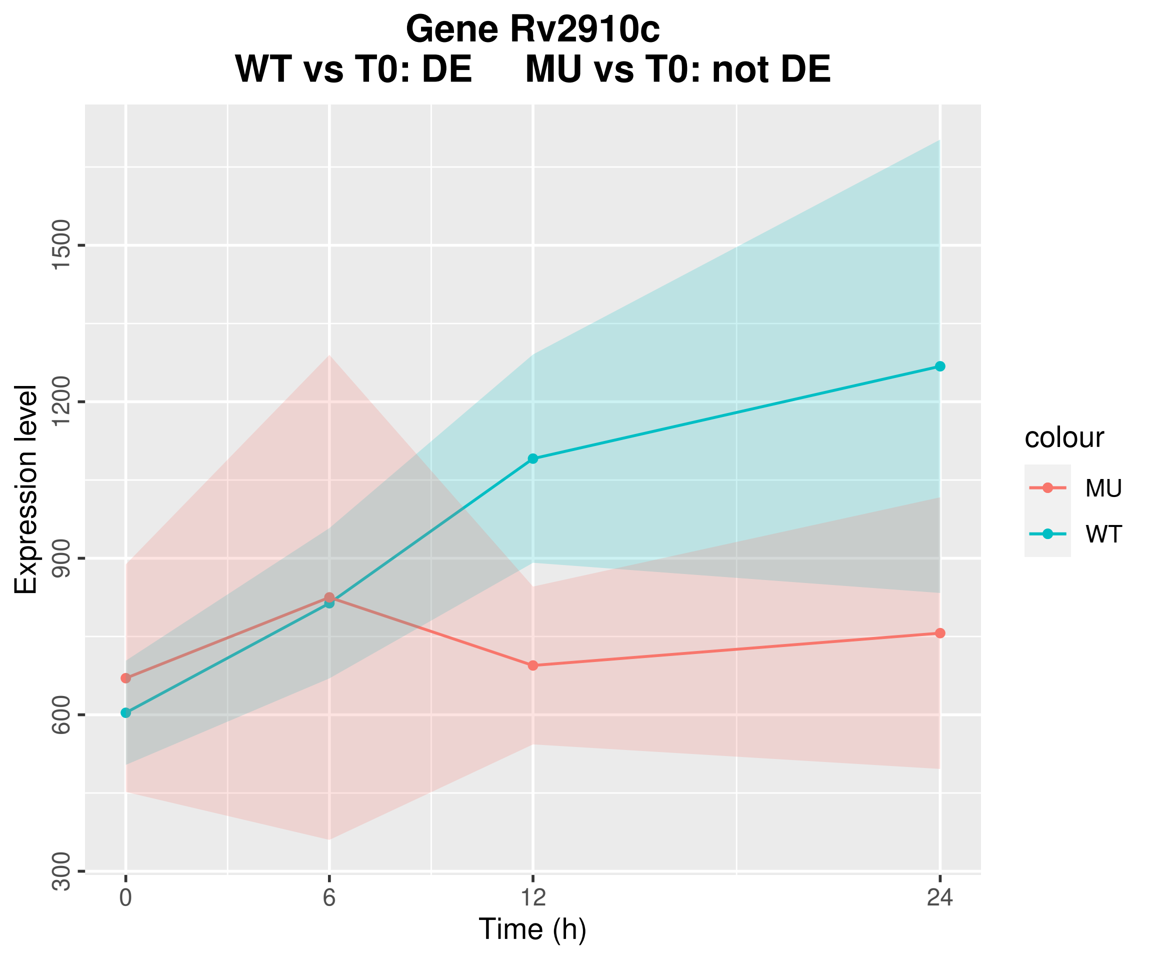
Gene Rv2910c
WT vs T0: DE MU vs T0: not DE
Expression level
1500
1200
900
600
300
0
6
12
24
Time (h)
colour
MU
WT

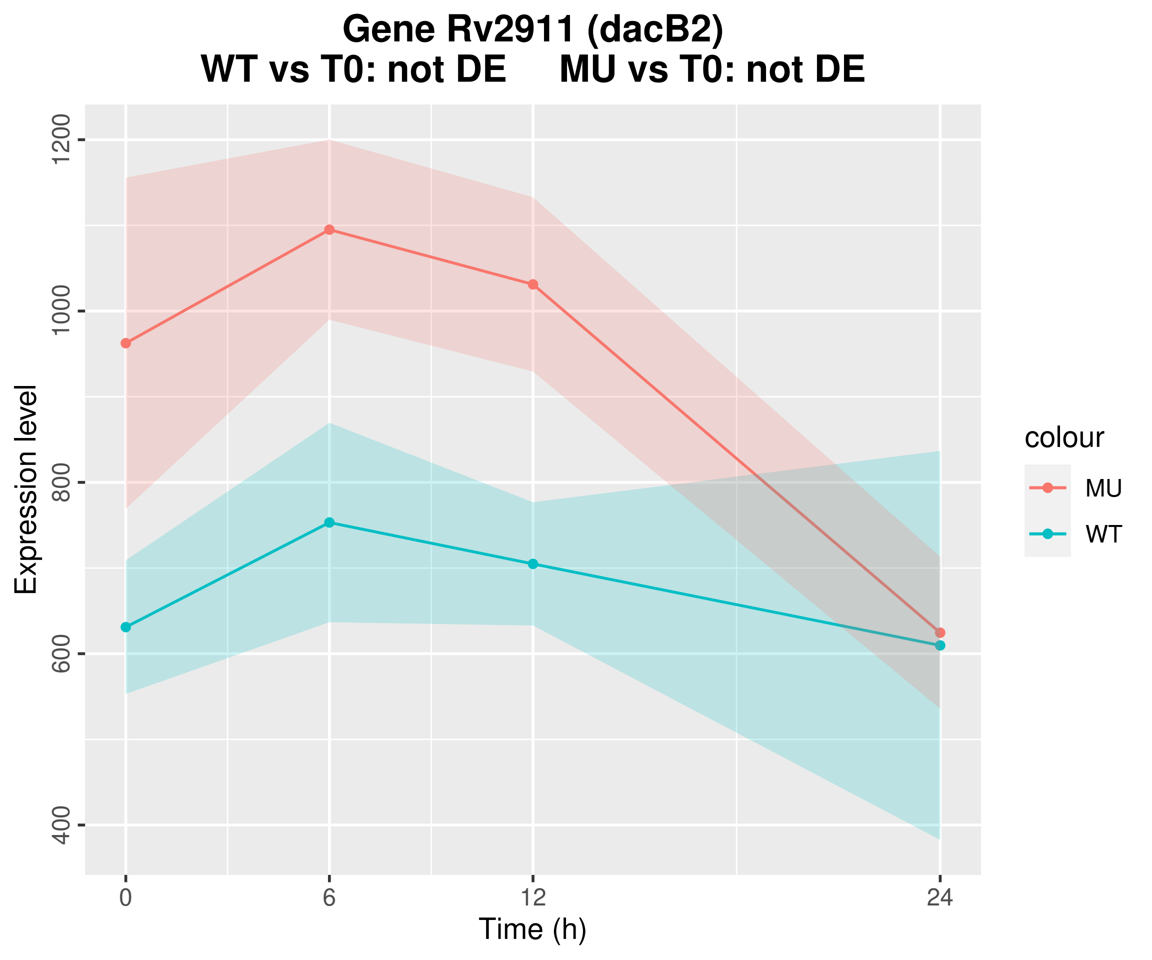
Gene Rv2911 (dacB2)
WT vs T0: not DE
MU vs T0: not DE
Expression level
1200
1000
800
600
400
0
6
12
24
Time (h)
colour
MU
WT

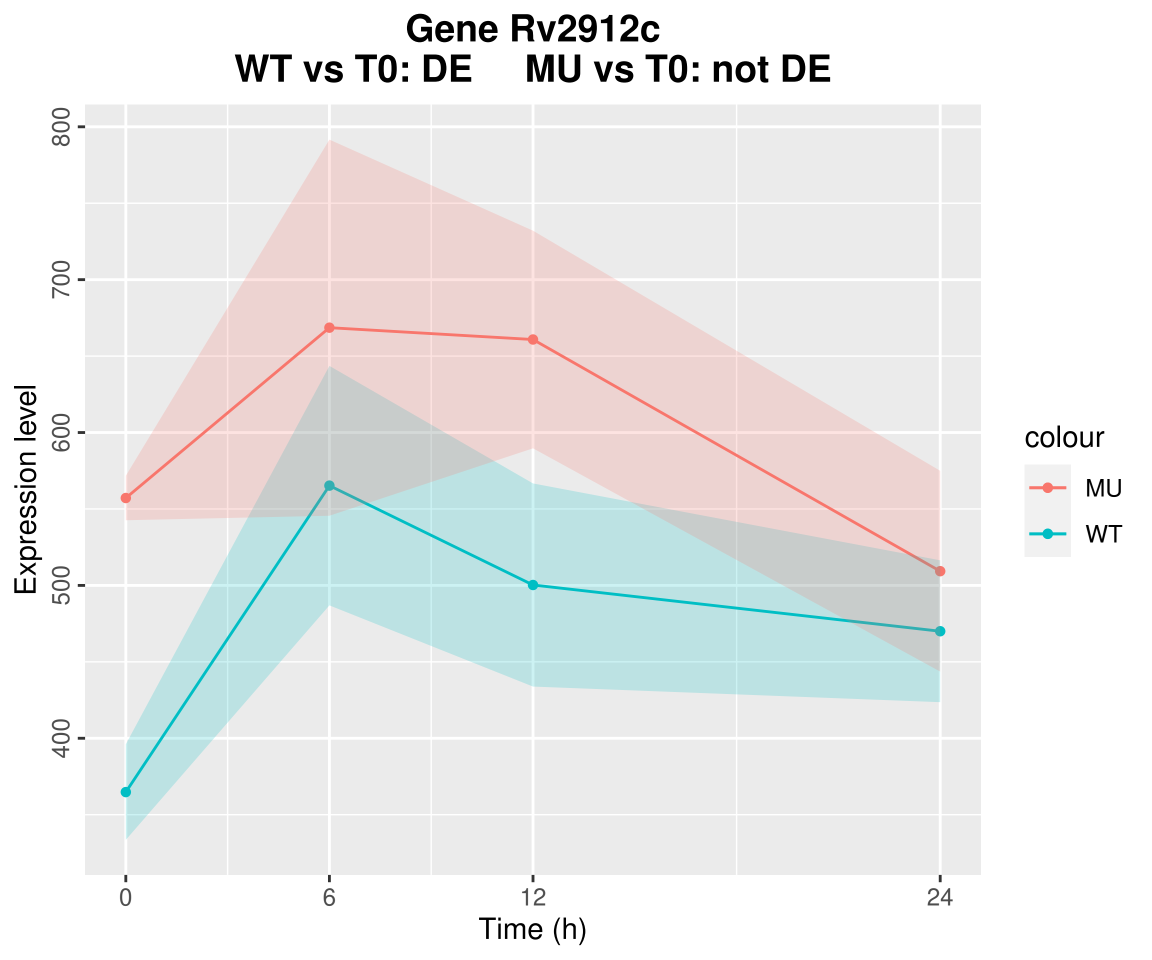
Gene Rv2912c
WT vs T0: DE MU vs T0: not DE
Expression level
800
700
600
500
400
0
6
12
24
Time (h)
colour
MU
WT

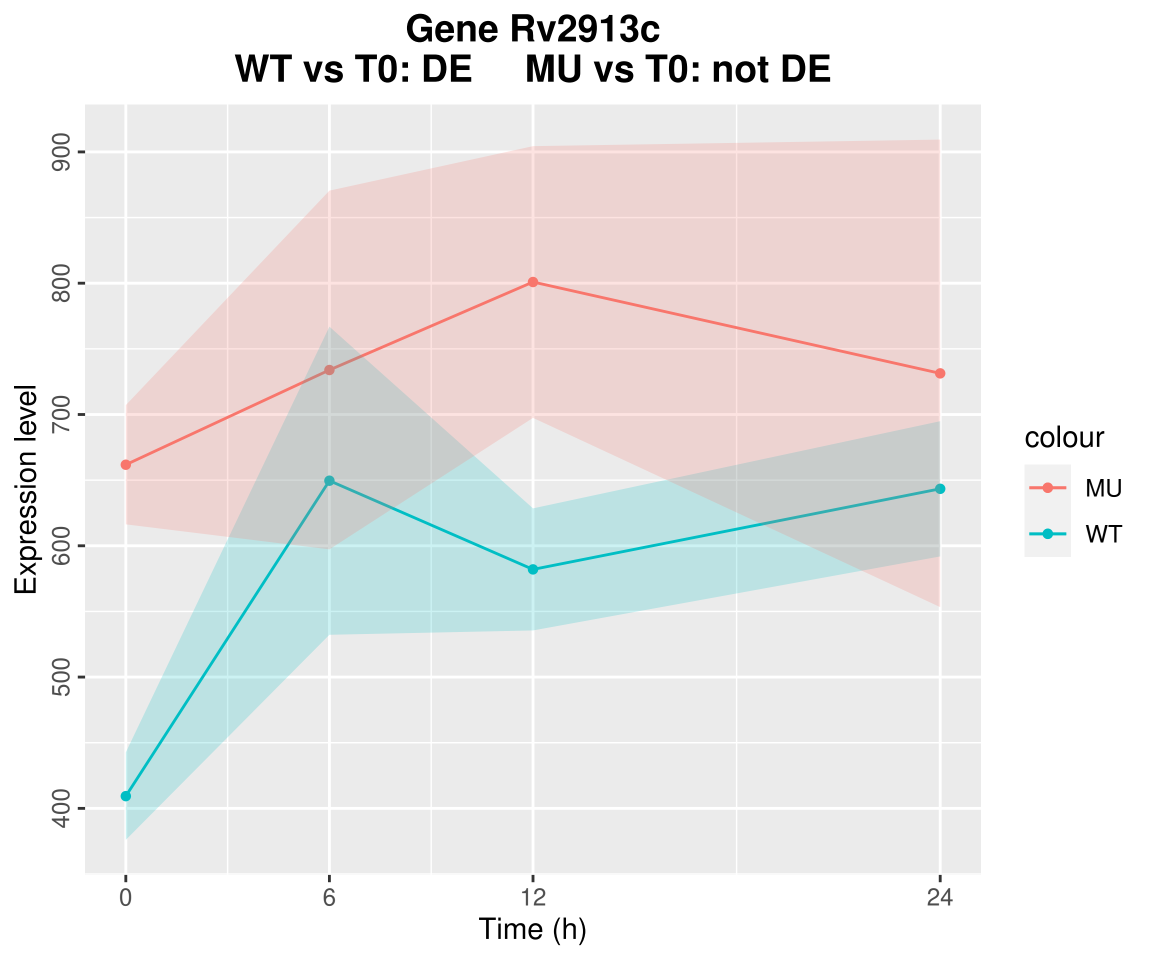
Gene Rv2913c
WT vs T0: DE   MU vs T0: not DE
Expression level
900
800
700
600
500
400
0
6
12
24
Time (h)
colour
MU
WT

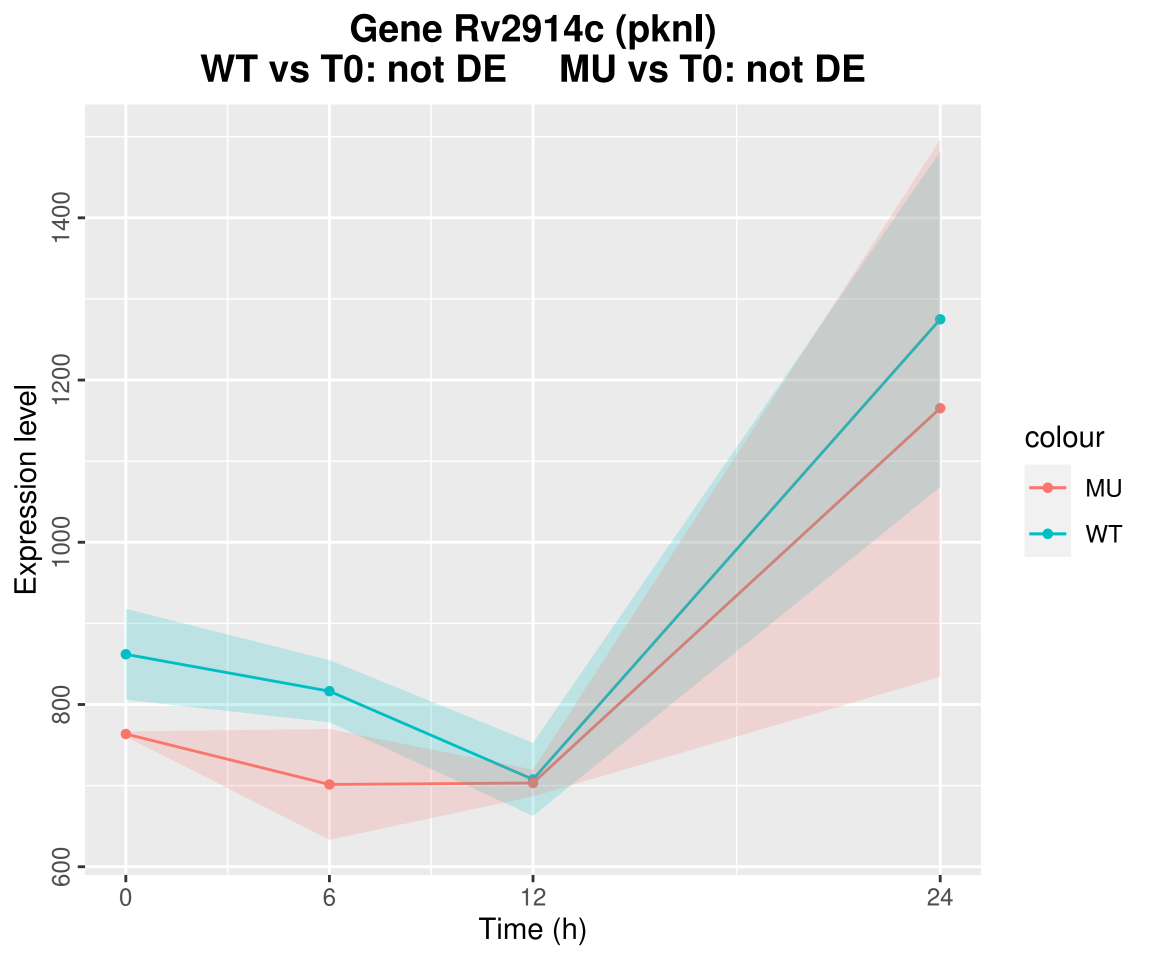
Gene Rv2914c (pknl)
WT vs T0: not DE   MU vs T0: not DE
Expression level
1400
1200
1000
800
600
0
6
12
24
Time (h)
colour
MU
WT

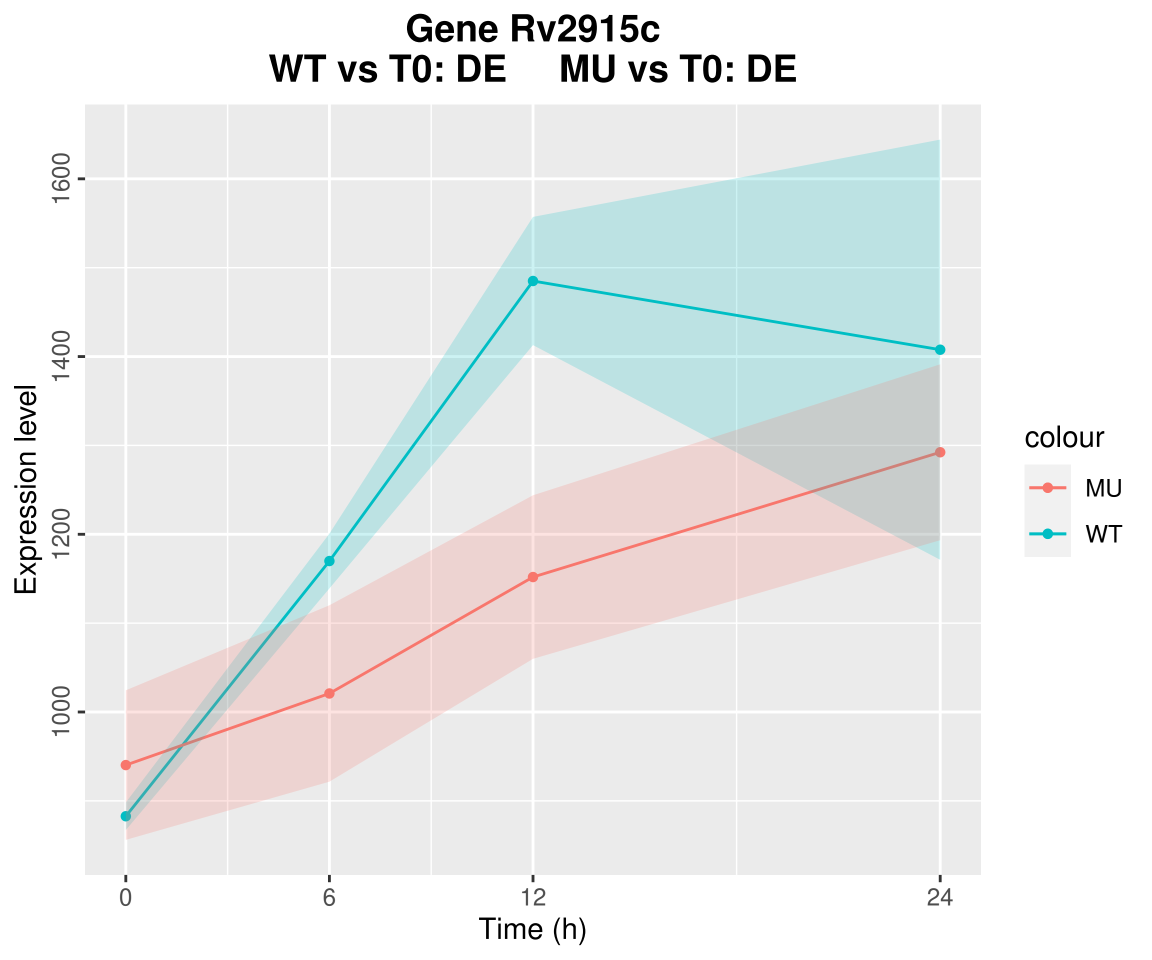

Gene Rv2915c
WT vs T0: DE MU vs T0: DE
Expression level
1000
1200
1400
1600
Time (h)
0
6
12
24
colour
MU
WT

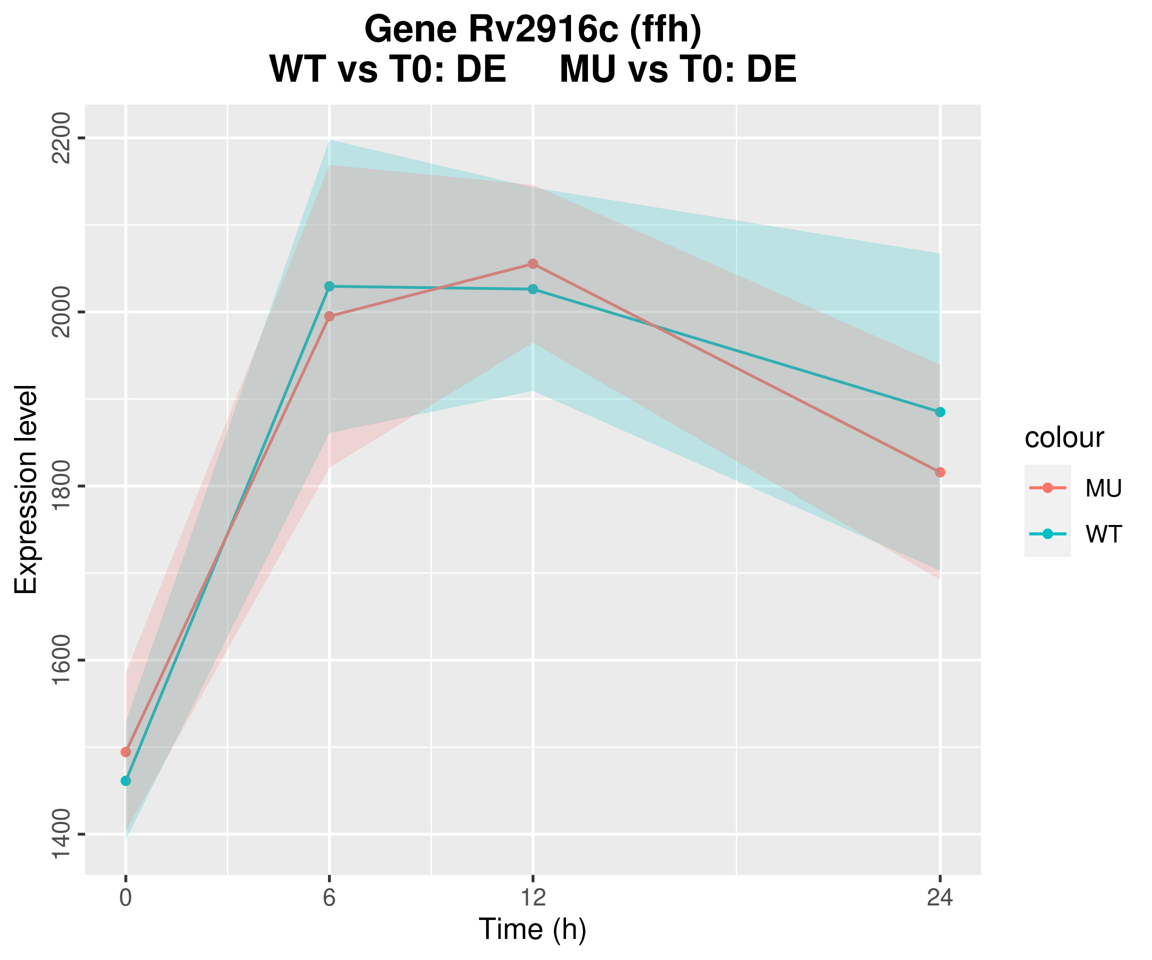
Gene Rv2916c (ffh)
WT vs T0: DE   MU vs T0: DE
Expression level
1400
1600
1800
2000
2200
0
6
12
24
Time (h)
colour
MU
WT

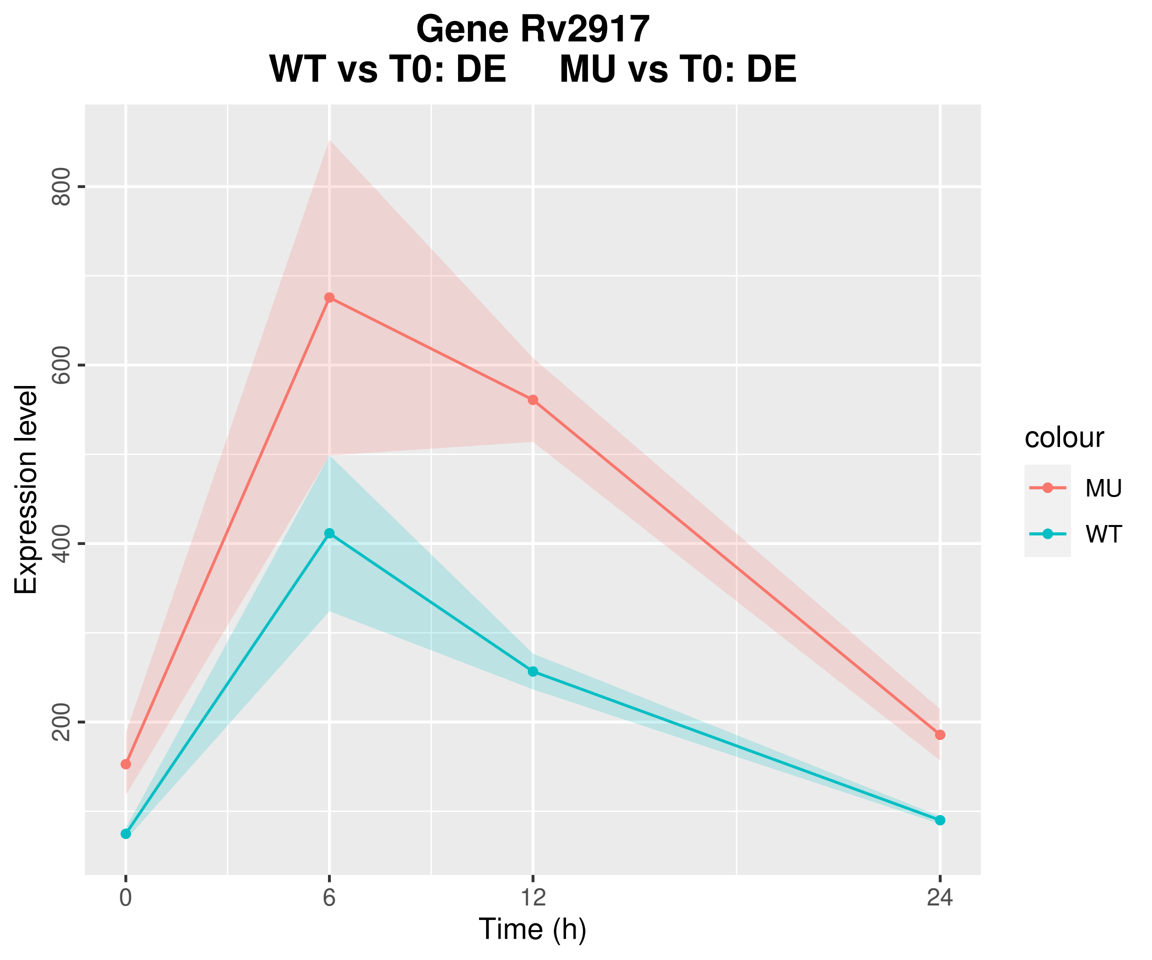

Gene Rv2917
WT vs T0: DE   MU vs T0: DE
Expression level
800
600
400
200
0
6
12
24
Time (h)
colour
MU
WT

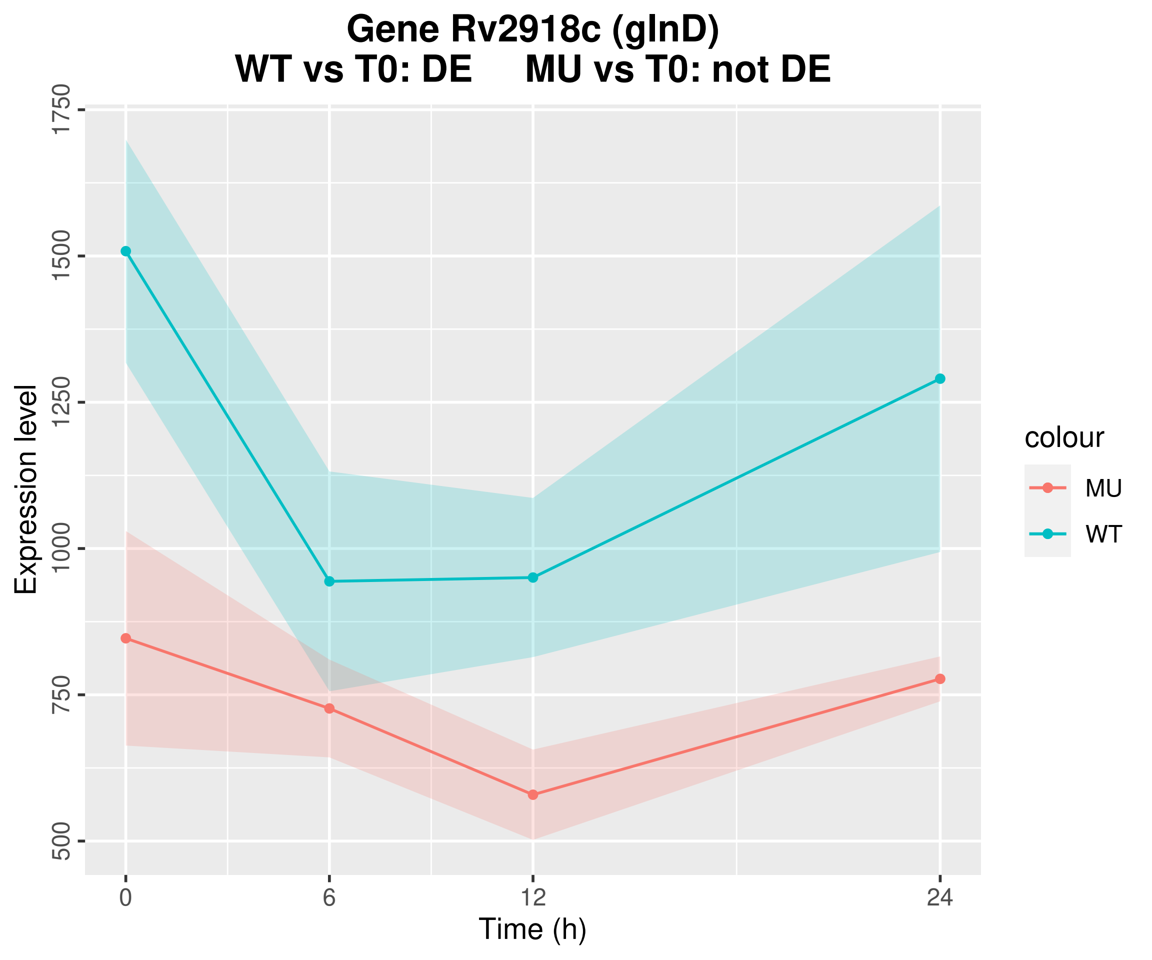
Gene Rv2918c (glnD)
WT vs T0: DE   MU vs T0: not DE
Expression level
1750
1500
1250
1000
750
500
0
6
12
24
Time (h)
colour
MU
WT

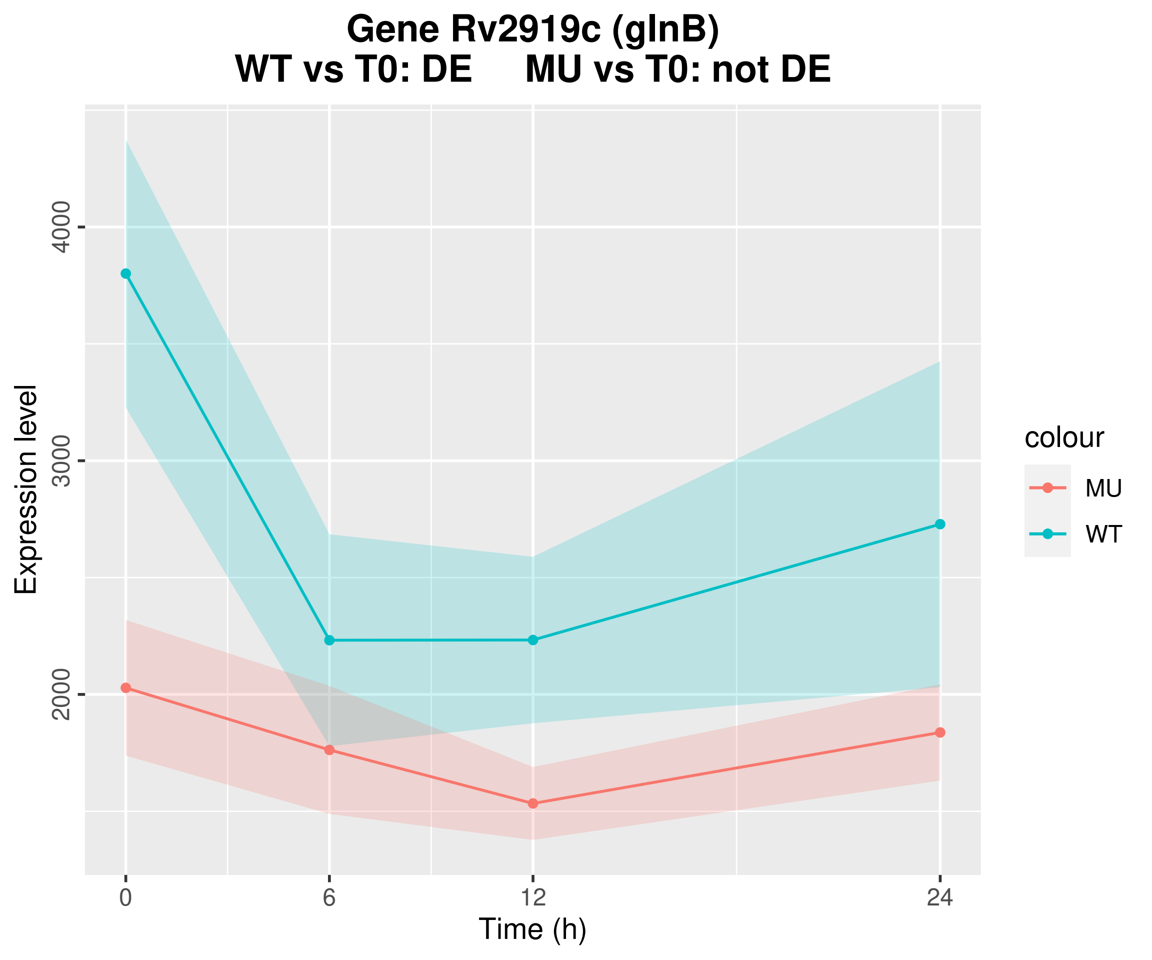

Gene Rv2919c (glnB)
WT vs T0: DE   MU vs T0: not DE
Expression level
4000
3000
2000
0
6
12
24
Time (h)
colour
MU
WT

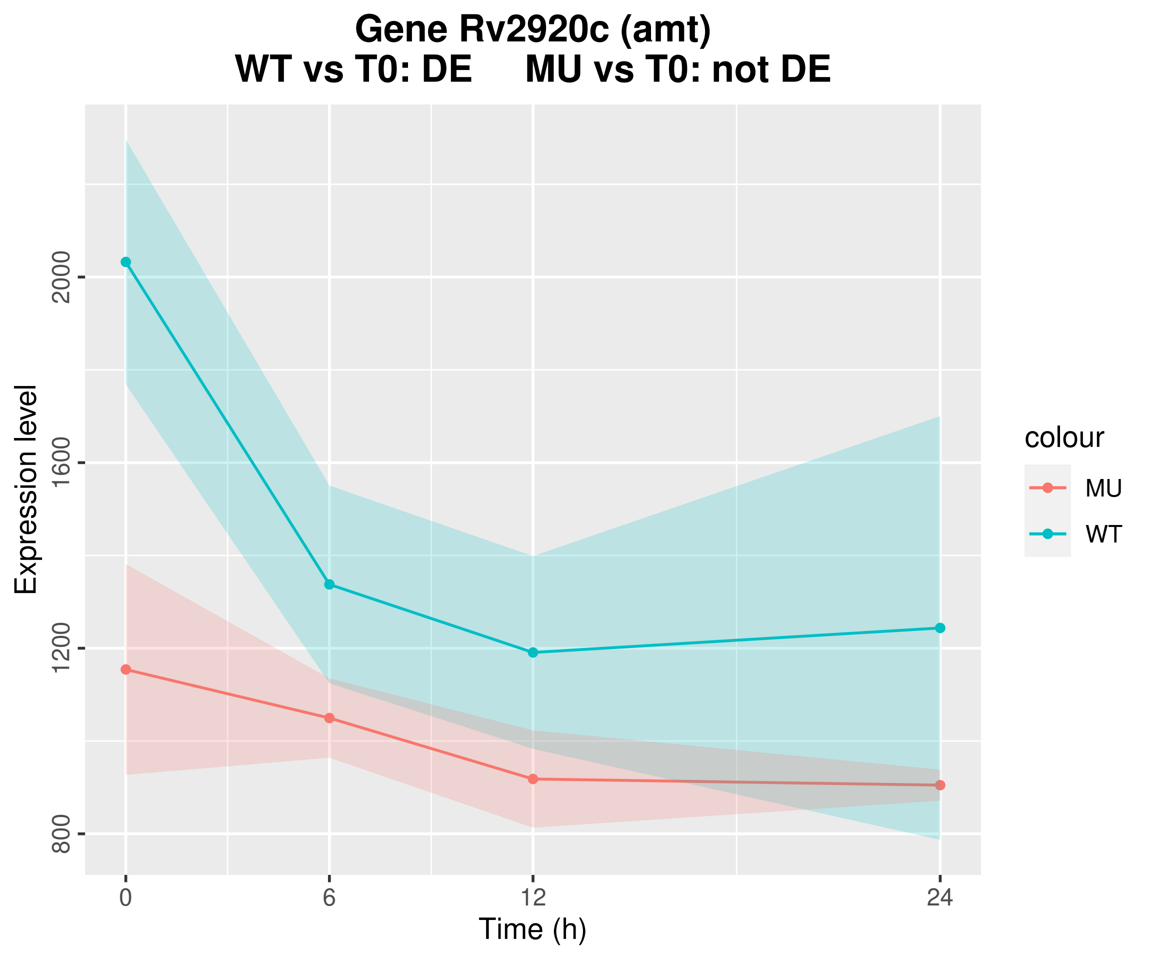

Gene Rv2920c (amt)
WT vs T0: DE   MU vs T0: not DE
Expression level
2000
1600
1200
800
0
6
12
24
Time (h)
colour
MU
WT

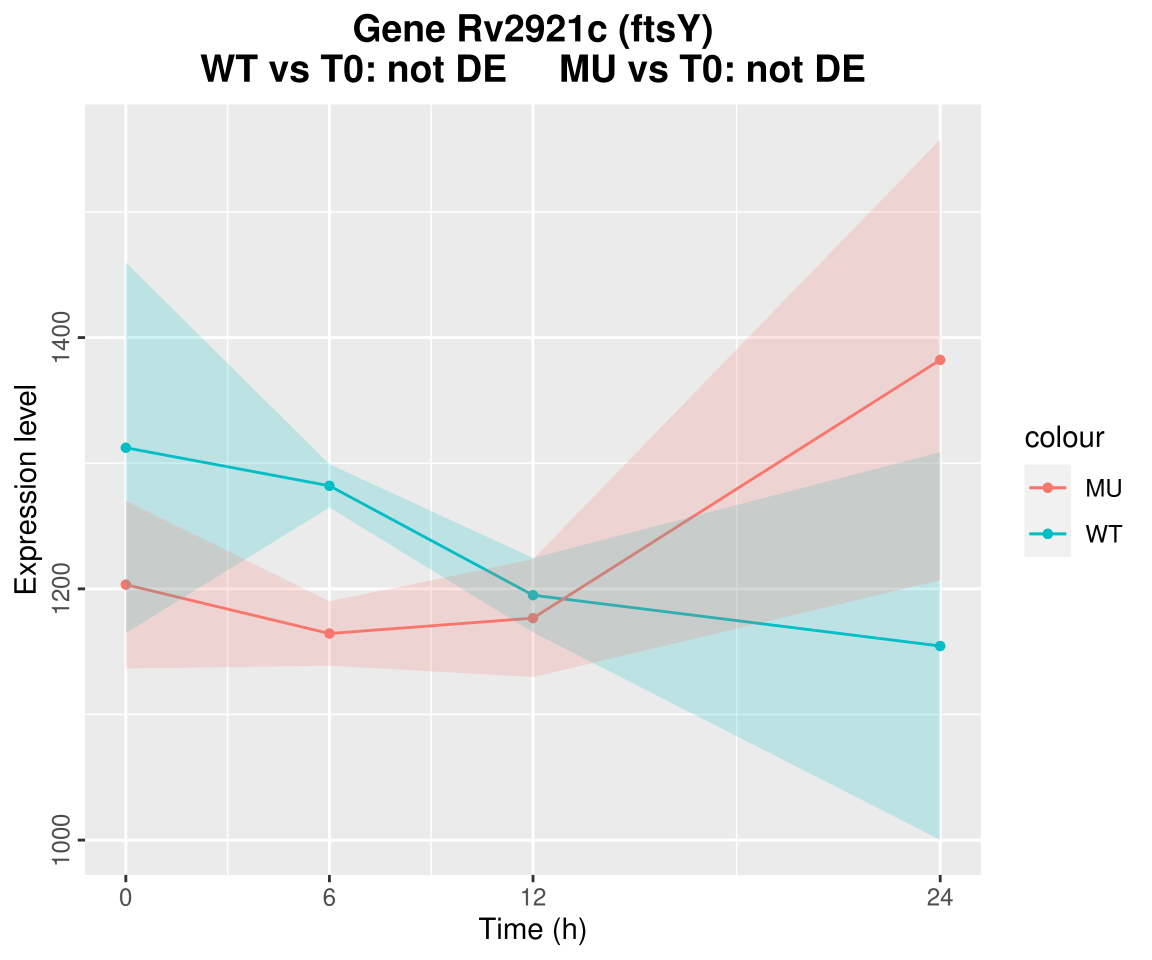
Gene Rv2921c (ftsY)
WT vs T0: not DE   MU vs T0: not DE
Expression level
1000
1200
1400
Time (h)
0
6
12
24
colour
MU
WT

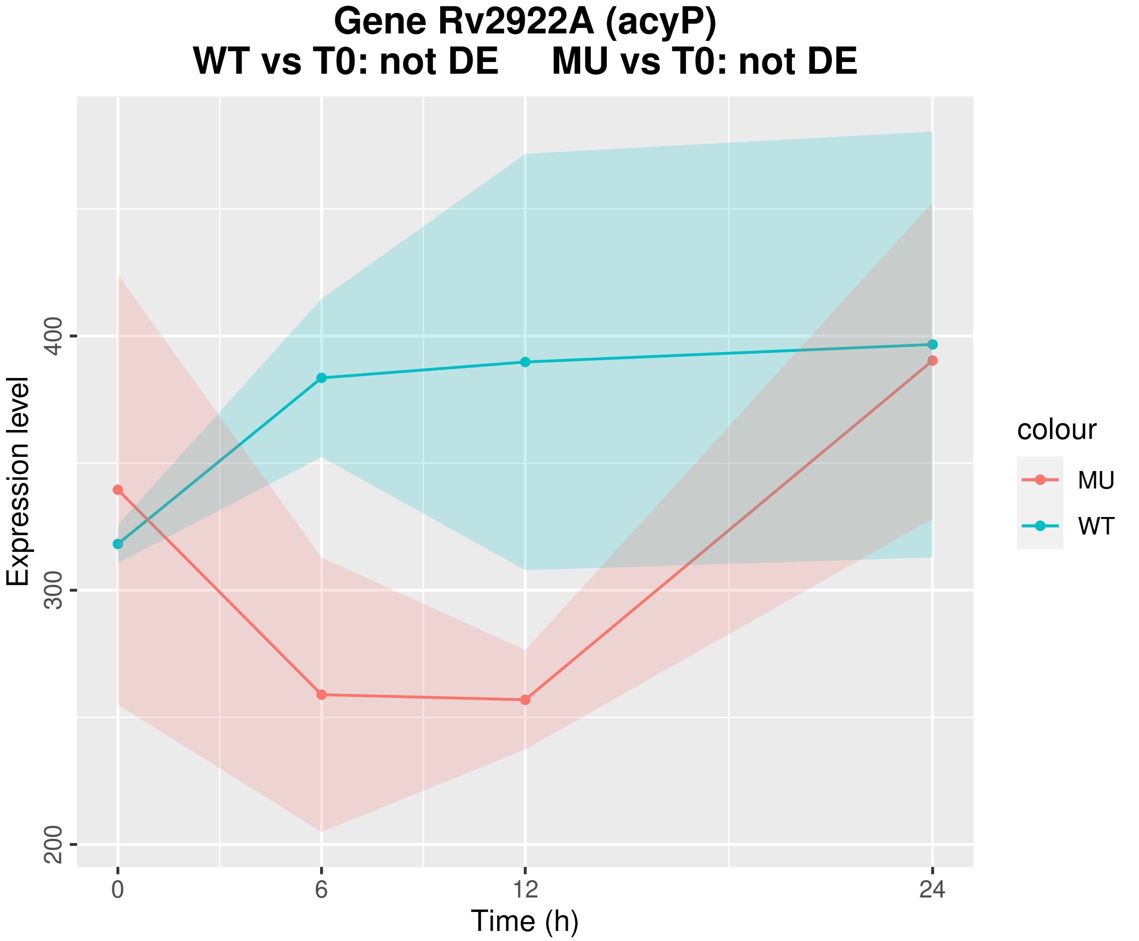

Gene Rv2922A (acyP)
WT vs T0: not DE   MU vs T0: not DE
Expression level
200
300
400
0
6
12
24
Time (h)
colour
MU
WT

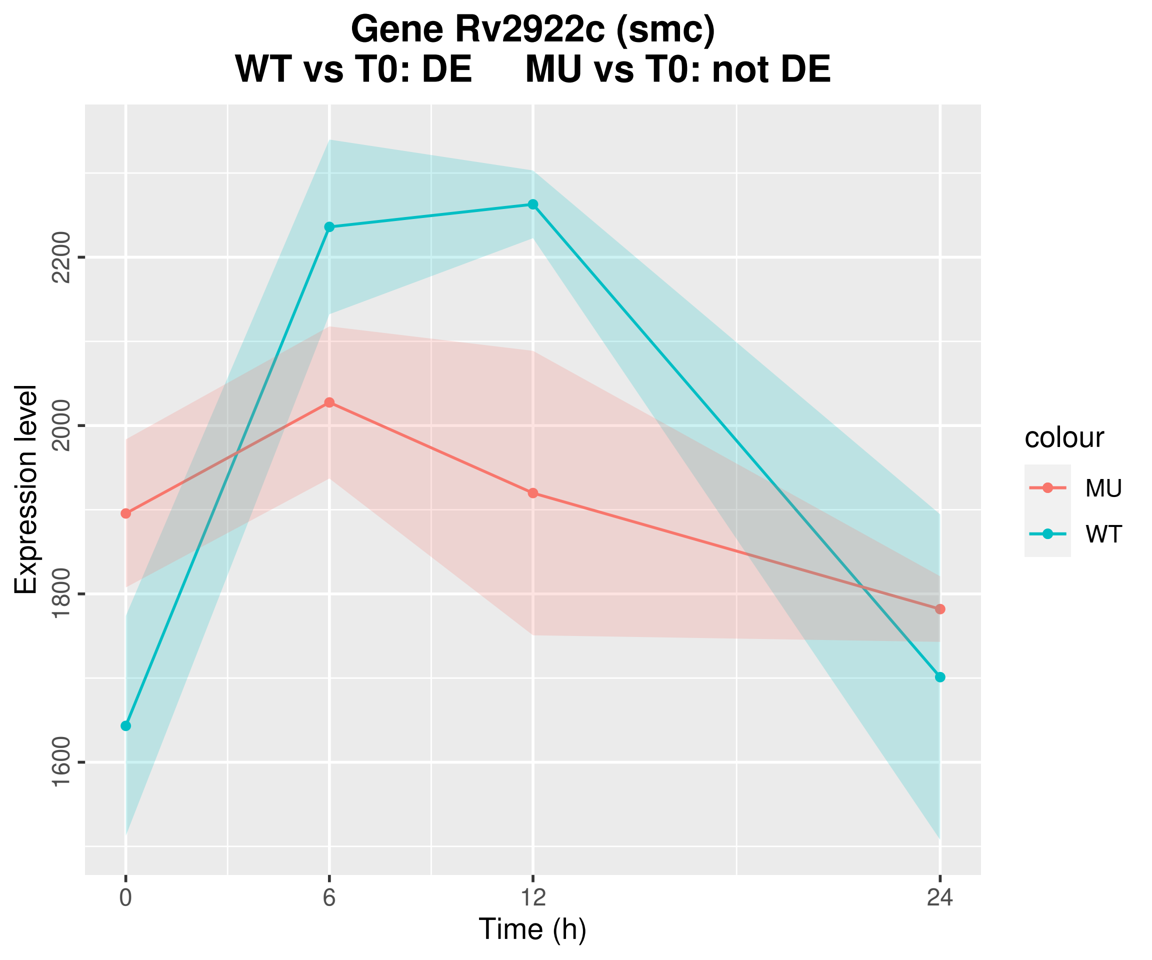

Gene Rv2922c (smc)
WT vs T0: DE MU vs T0: not DE
Expression level
1600
1800
2000
2200
0
6
12
24
Time (h)
colour
MU
WT

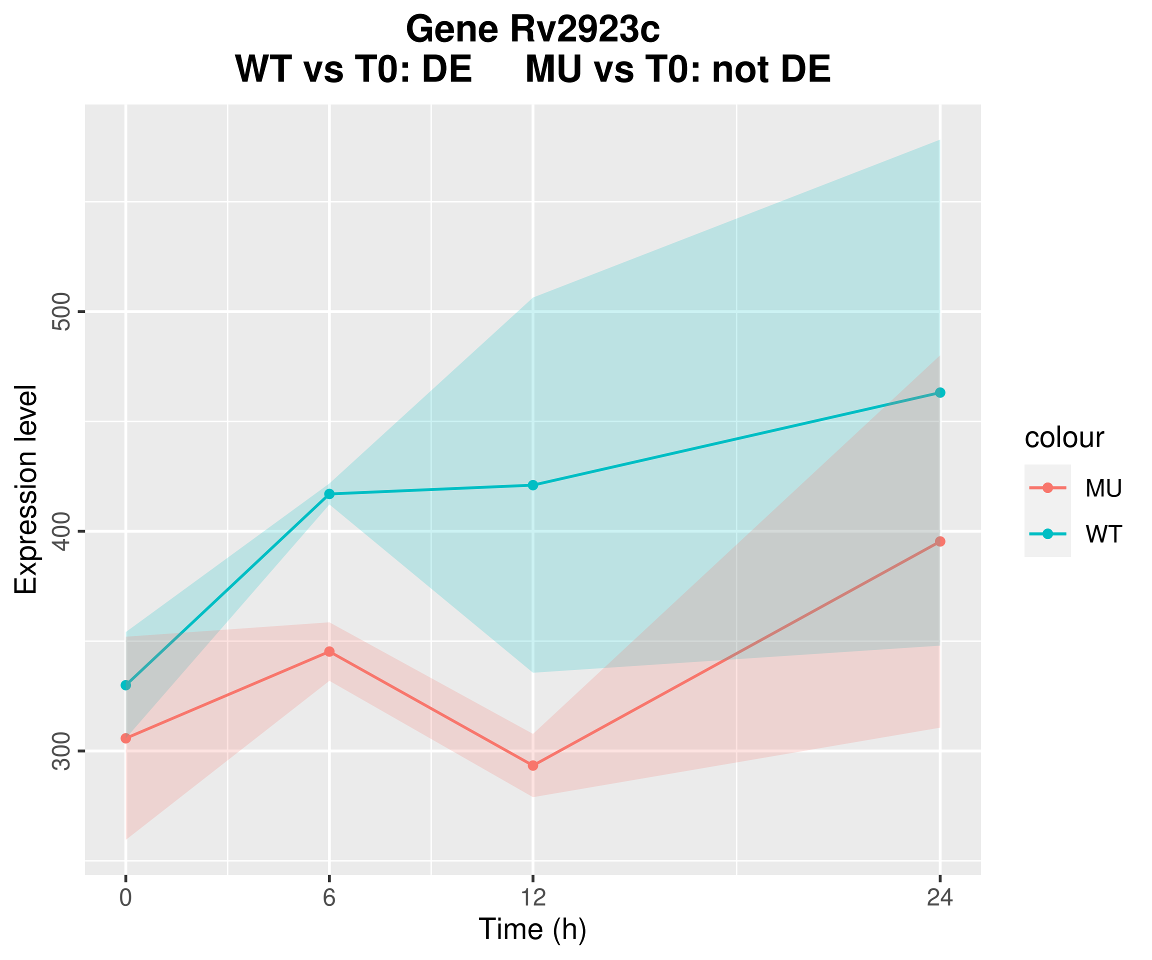
Gene Rv2923c
WT vs T0: DE   MU vs T0: not DE
Expression level
500
400
300
0
6
12
24
Time (h)
colour
MU
WT

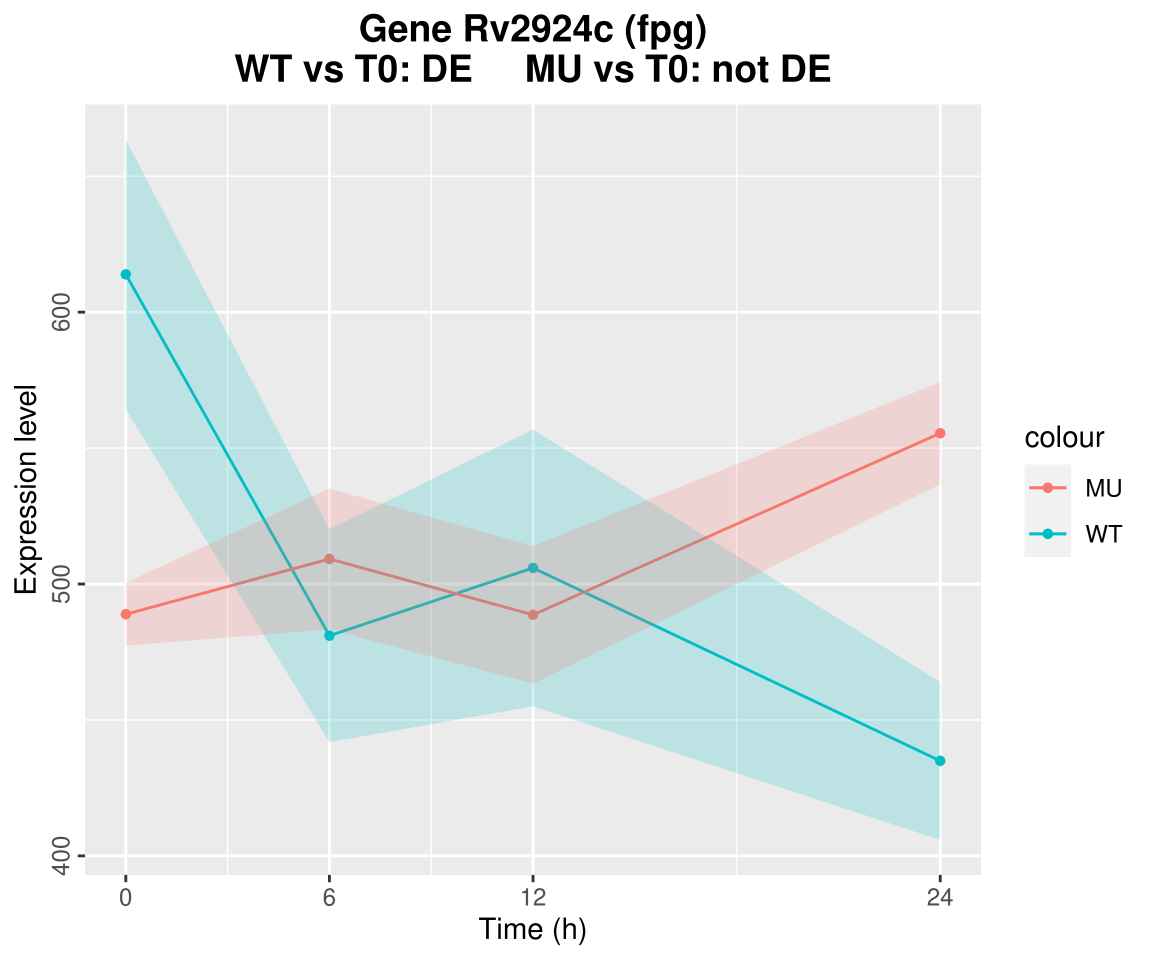
Gene Rv2924c (fpg)
WT vs T0: DE   MU vs T0: not DE
Expression level
600
500
400
0
6
12
24
Time (h)
colour
MU
WT

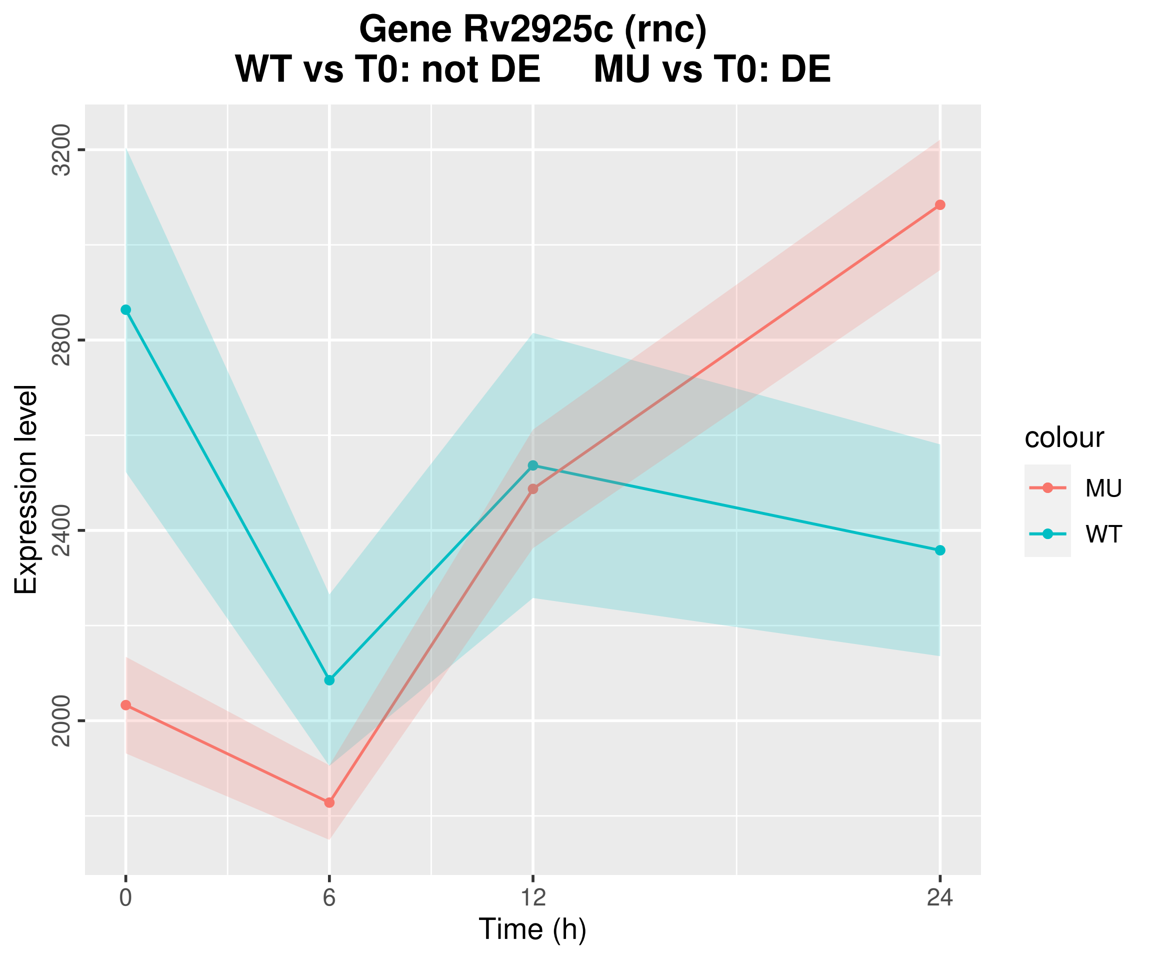

Gene Rv2925c (rnc)
WT vs T0: not DE   MU vs T0: DE
3200
2800
2400
2000
Expression level
0
6
12
24
Time (h)
colour
MU
WT

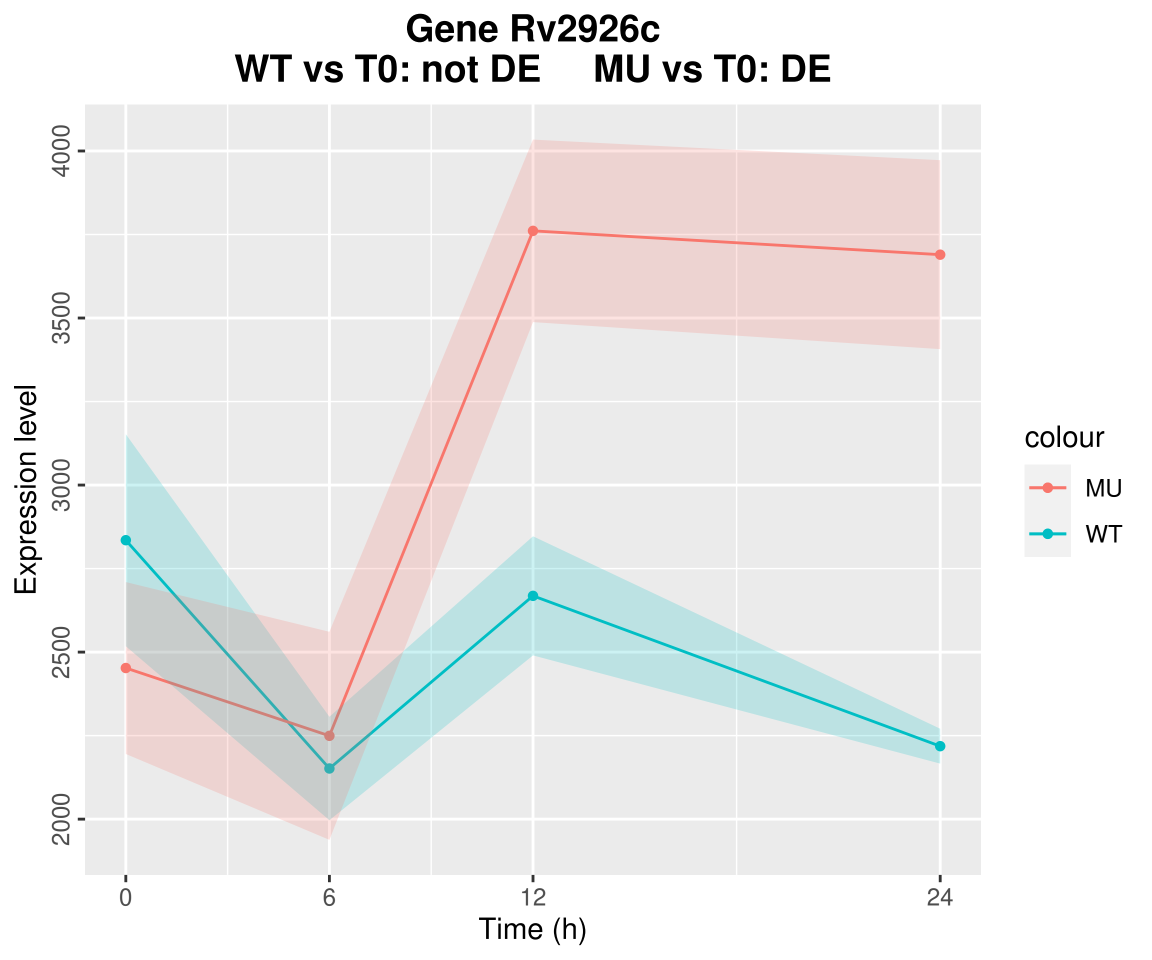
Gene Rv2926c
WT vs T0: not DE   MU vs T0: DE
Expression level
4000
3500
3000
2500
2000
0
6
12
24
Time (h)
colour
MU
WT

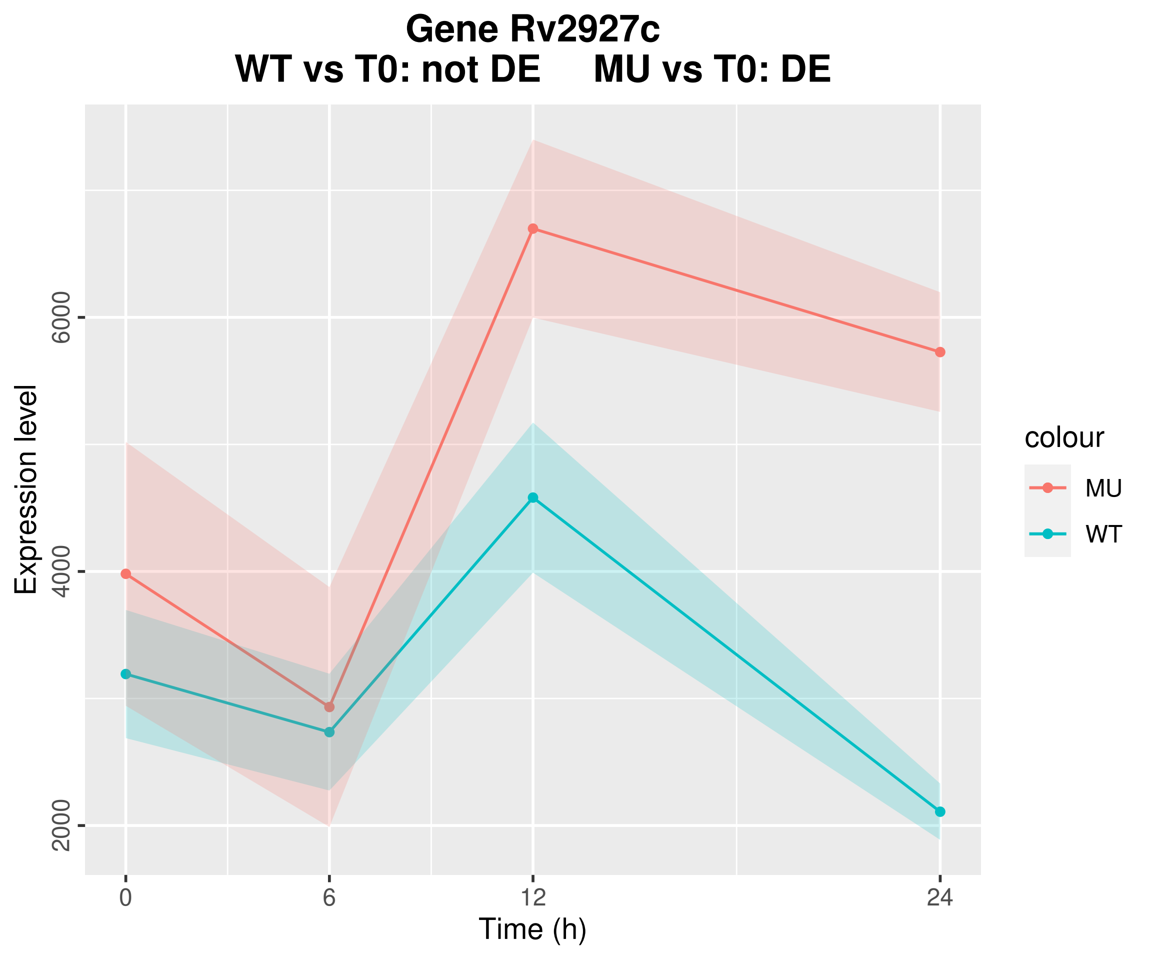
Gene Rv2927c
WT vs T0: not DE   MU vs T0: DE
Expression level
2000
4000
6000
0
6
12
24
Time (h)
colour
MU
WT

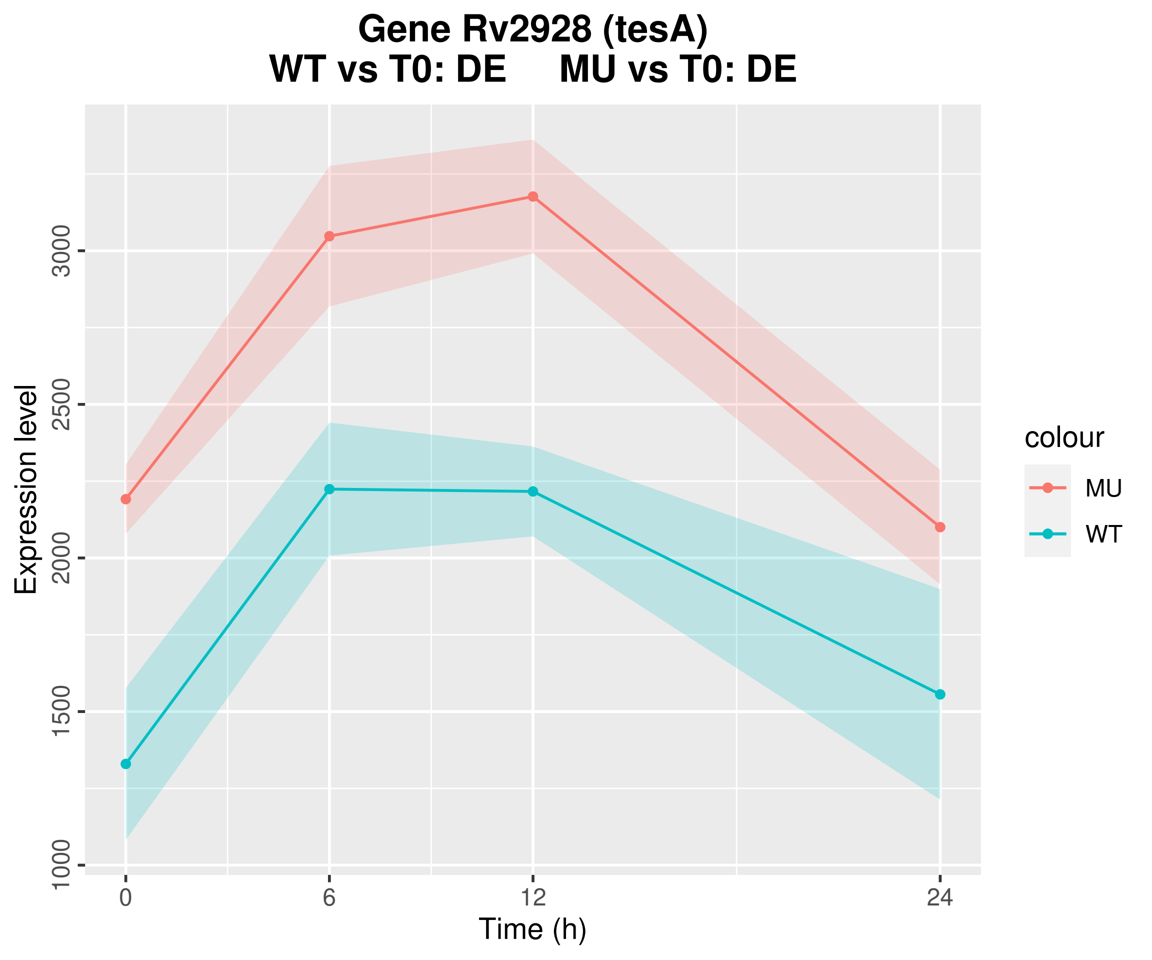

Gene Rv2928 (tesA)
WT vs T0: DE MU vs T0: DE
Expression level
1000
1500
2000
2500
3000
0
6
12
24
Time (h)
colour
MU
WT

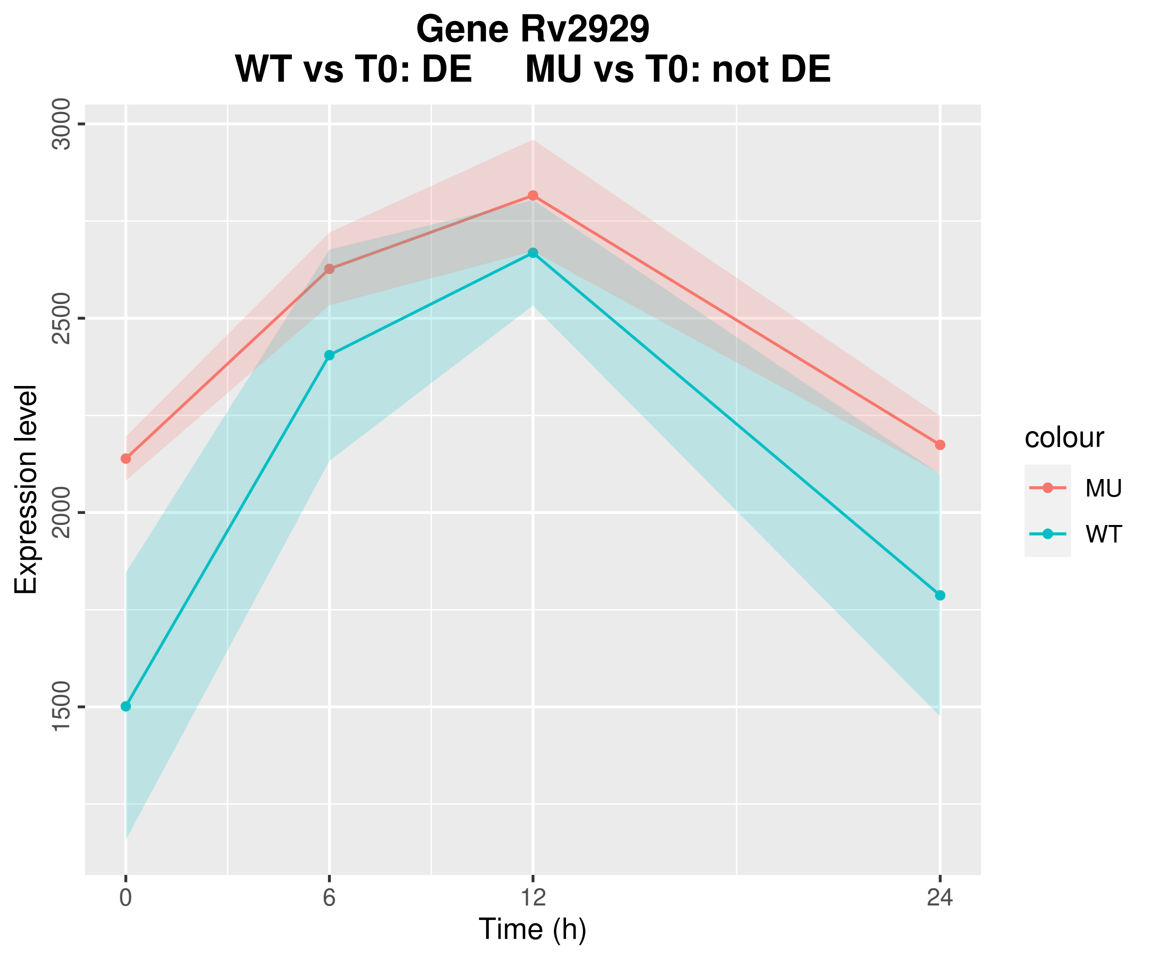

Gene Rv2929
WT vs T0: DE MU vs T0: not DE
3000
2500
2000
1500
Expression level
0
6
12
24
Time (h)
colour
MU
WT

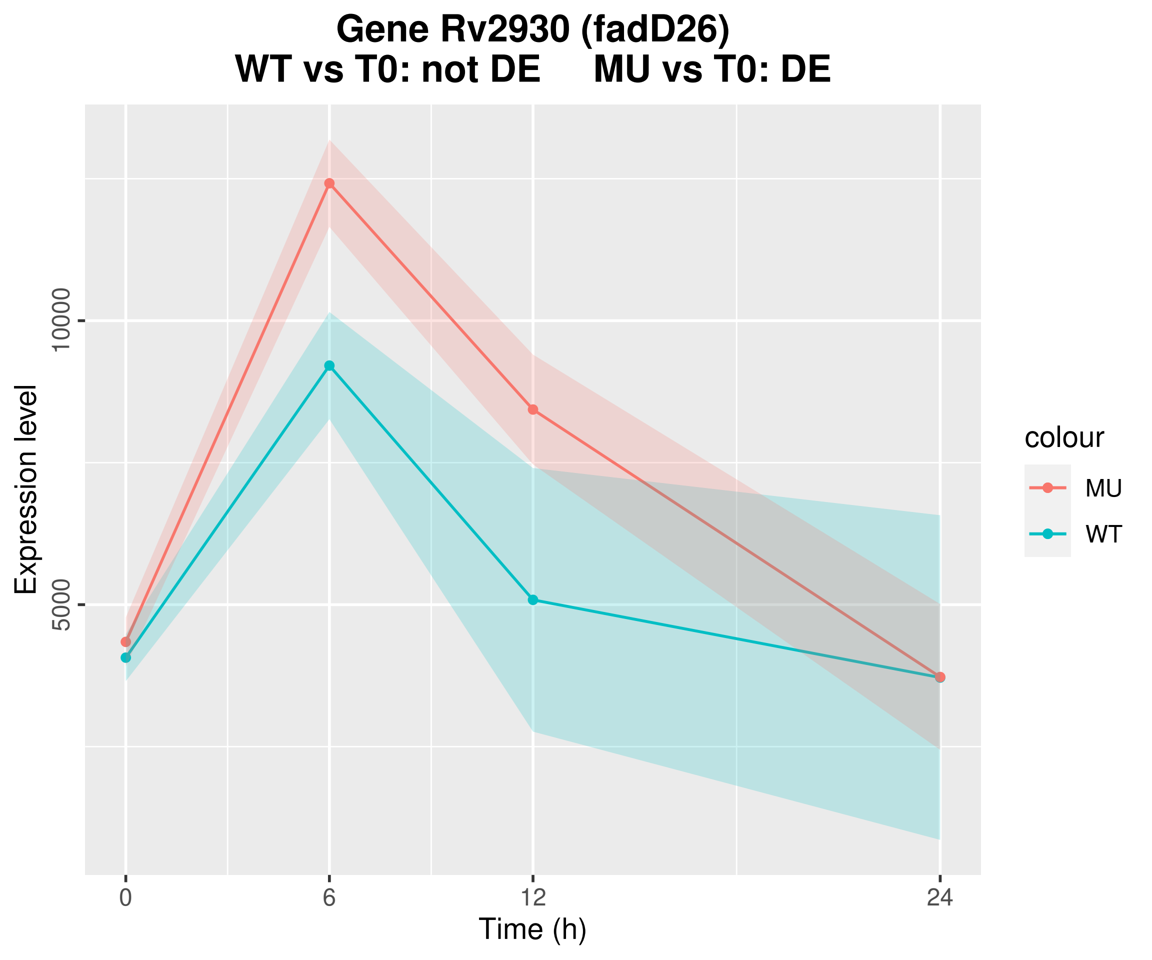

Gene Rv2930 (fadD26)
WT vs T0: not DE   MU vs T0: DE
Expression level
10000
5000
0
6
12
24
Time (h)
colour
MU
WT

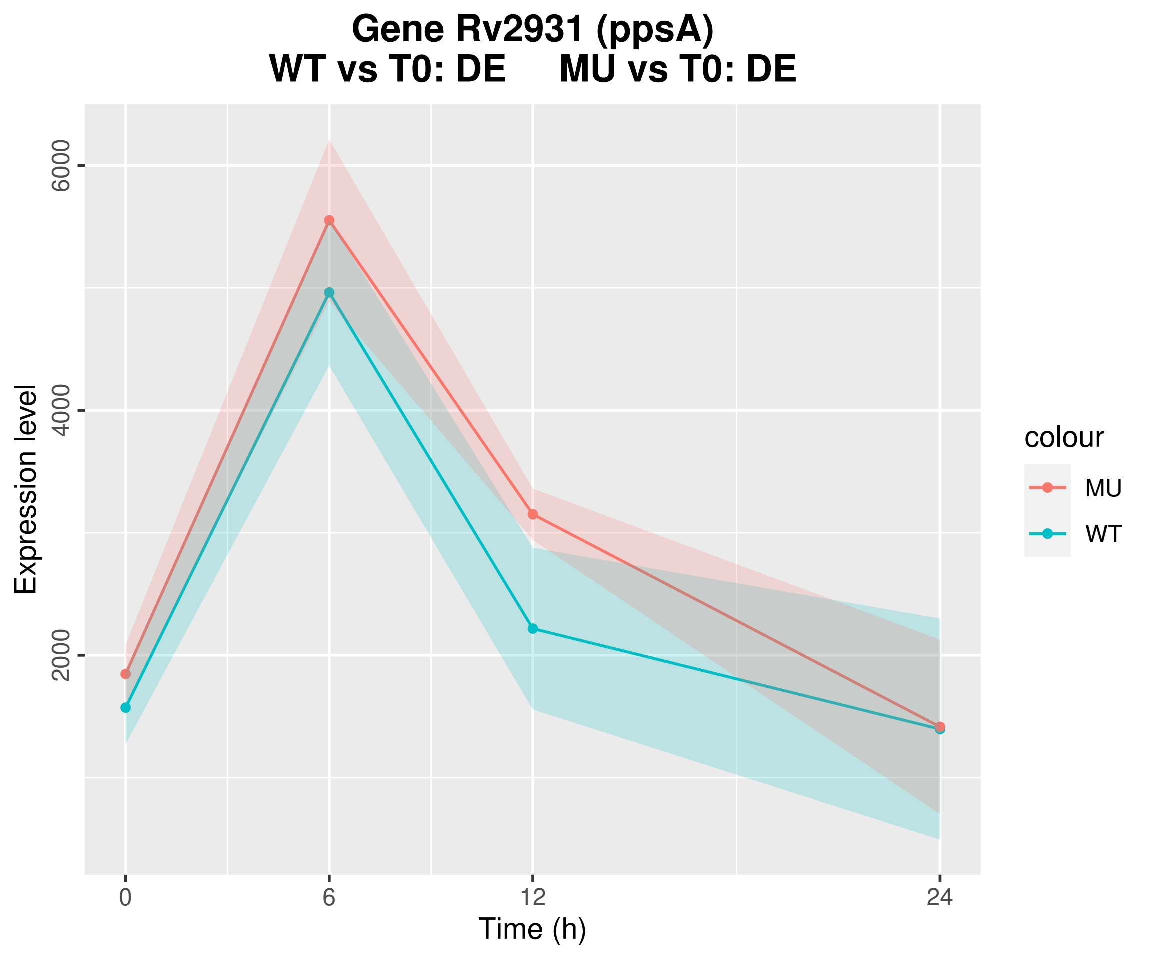
Gene Rv2931 (ppsA)
WT vs T0: DE MU vs T0: DE
Expression level
6000
4000
2000
0
6
12
24
Time (h)
colour
MU
WT

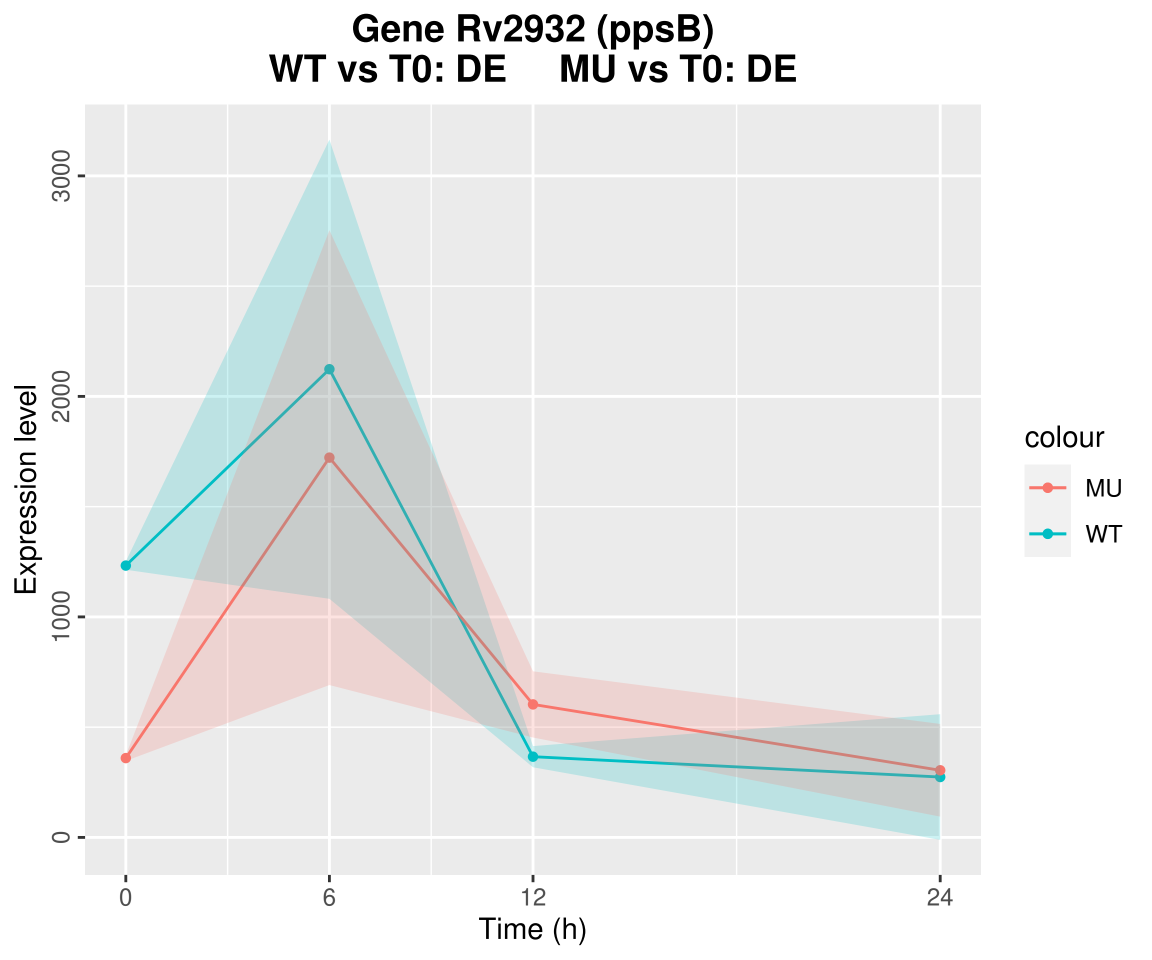

Gene Rv2932 (ppsB)
WT vs T0: DE MU vs T0: DE
3000
2000
1000
0
Expression level
0
6
12
24
Time (h)
colour
MU
WT

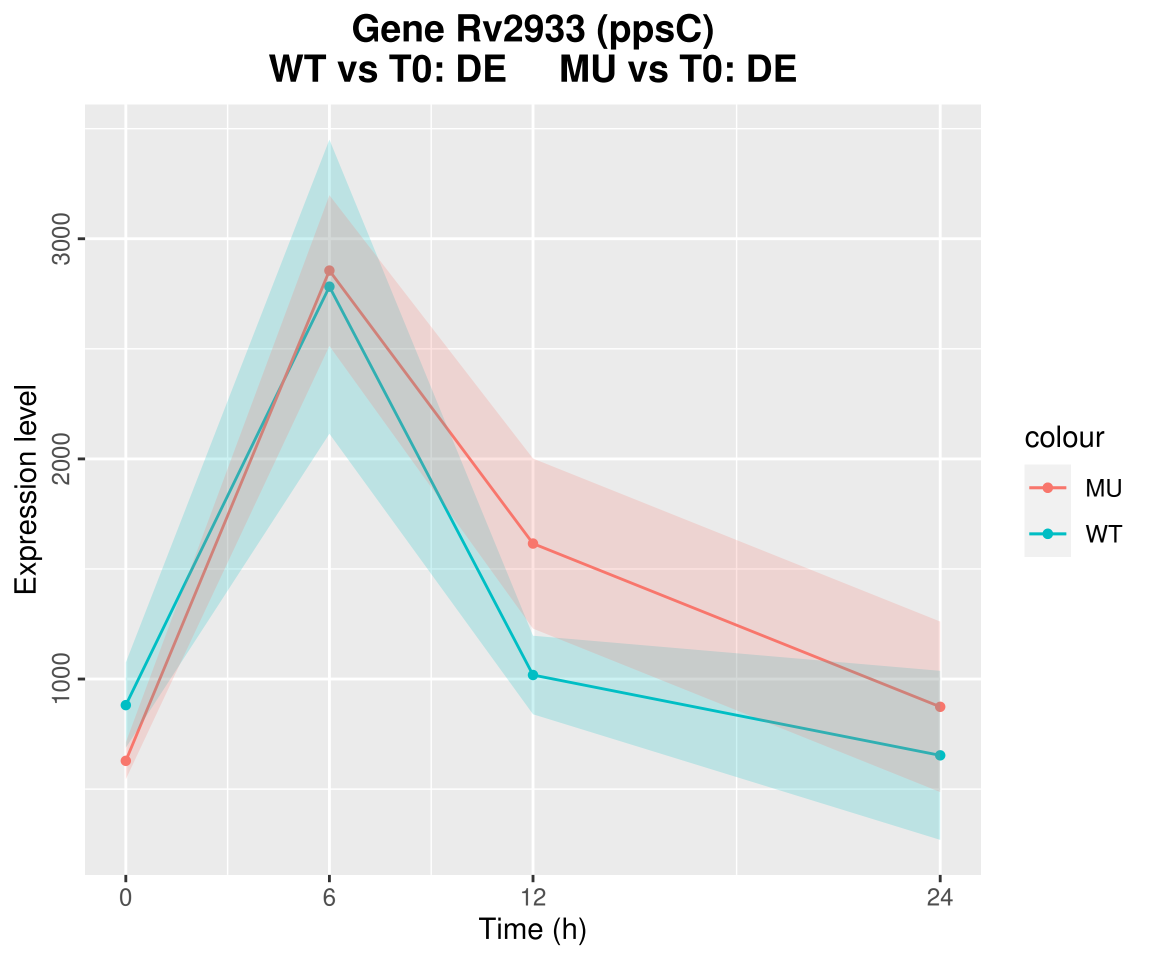
Gene Rv2933 (ppsC)
WT vs T0: DE   MU vs T0: DE
Expression level
3000
2000
1000
0
6
12
24
Time (h)
colour
MU
WT

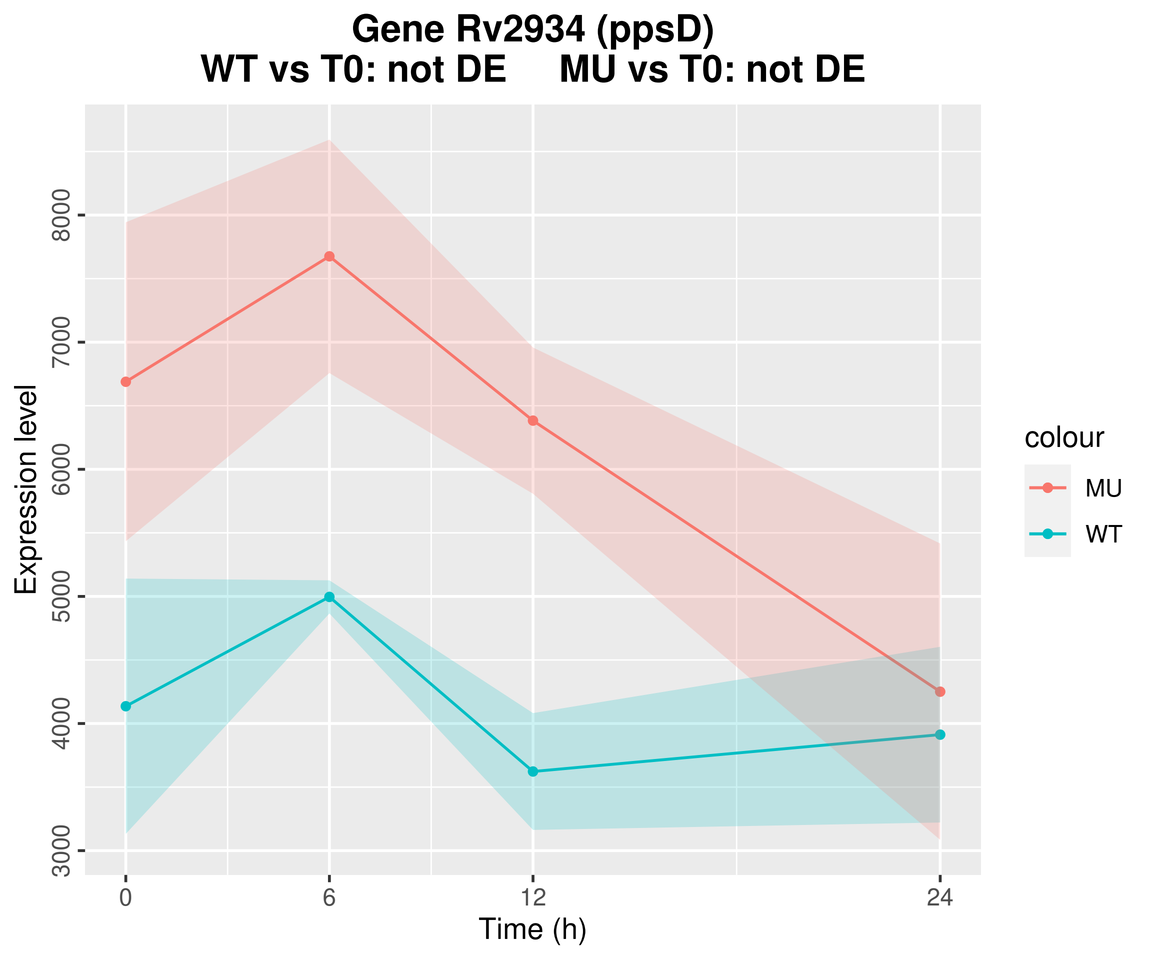
Gene Rv2934 (ppsD)
WT vs T0: not DE   MU vs T0: not DE
Expression level
3000
4000
5000
6000
7000
8000
0
6
12
24
Time (h)
colour
MU
WT

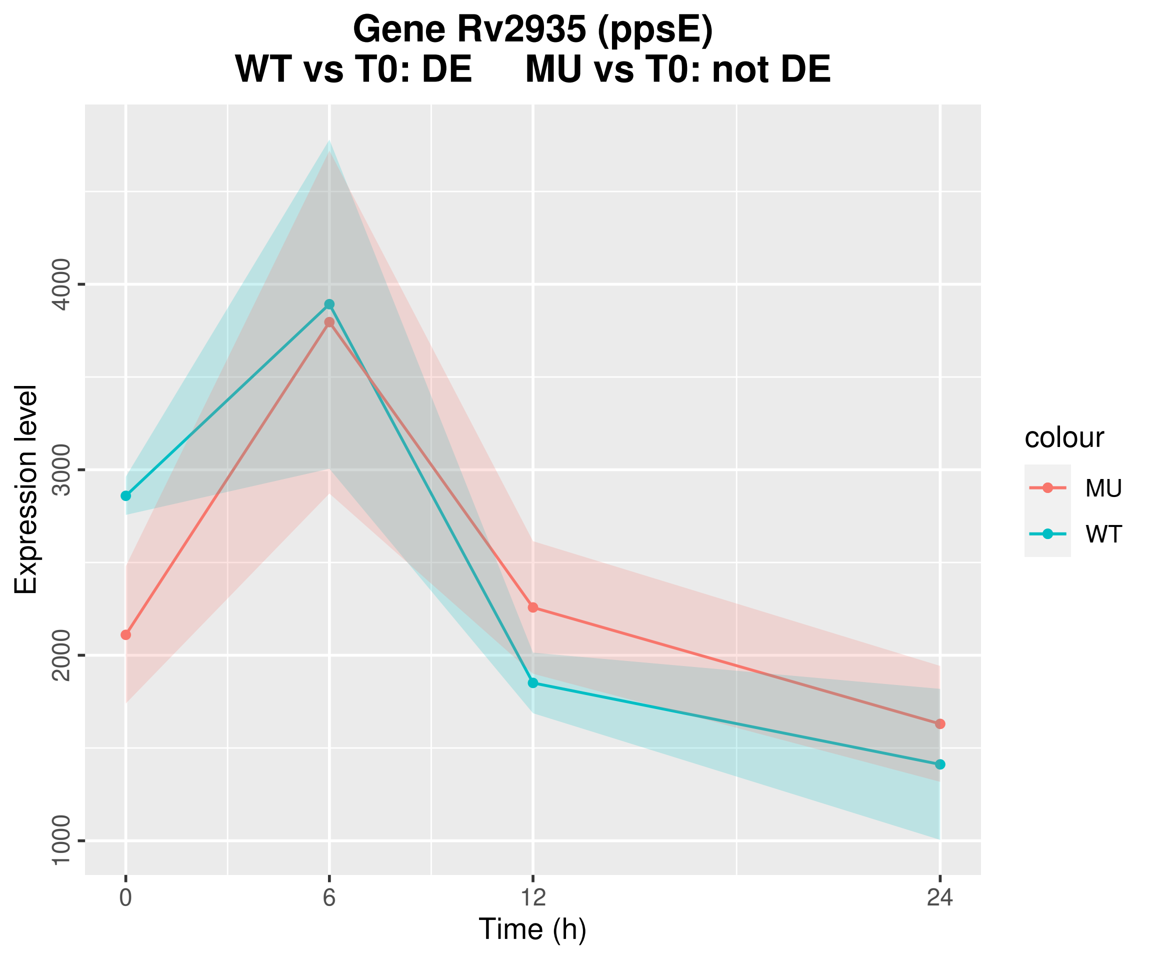
Gene Rv2935 (ppsE)
WT vs T0: DE MU vs T0: not DE
Expression level
1000
2000
3000
4000
Time (h)
0
6
12
24
colour
MU
WT

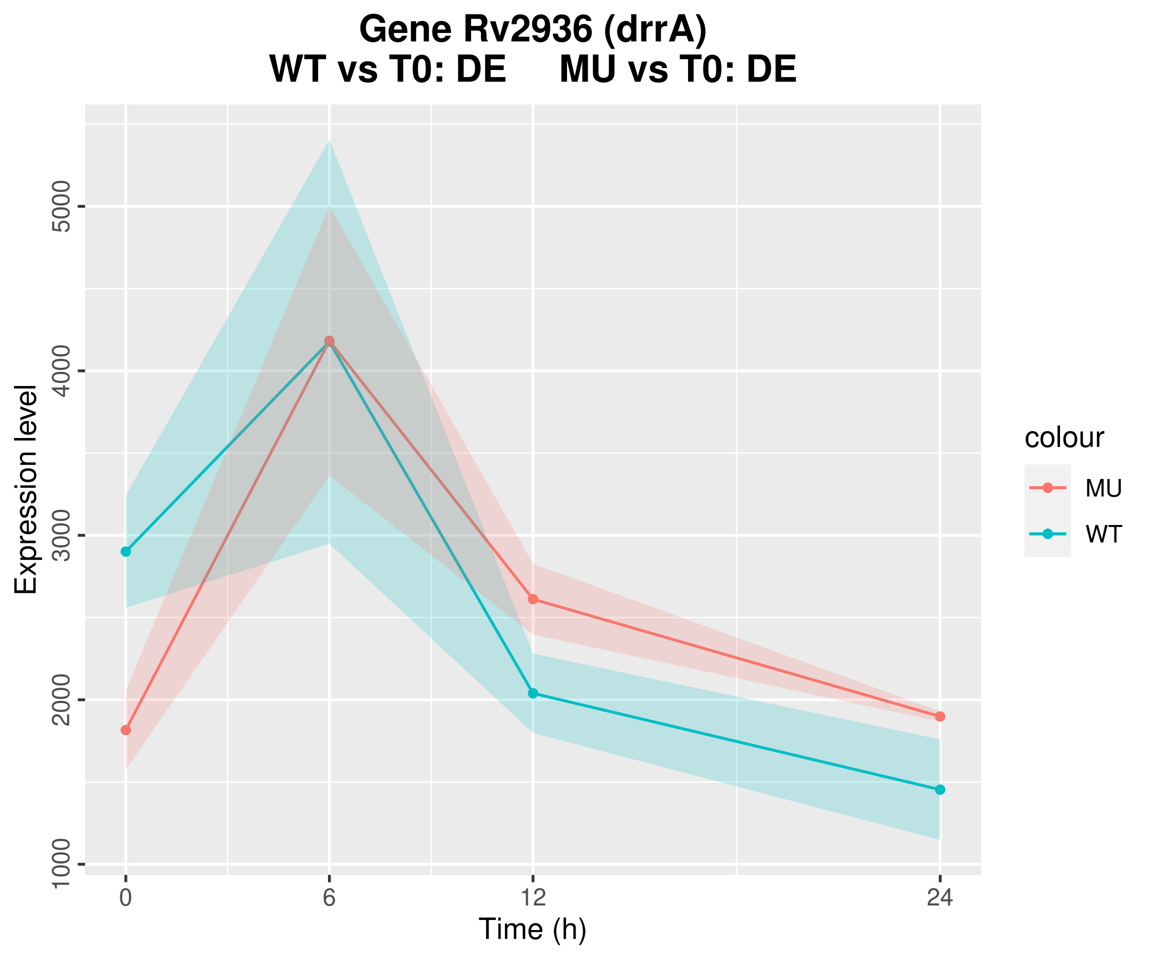

Gene Rv2936 (drrA)
WT vs T0: DE   MU vs T0: DE
Expression level
1000
2000
3000
4000
5000
0
6
12
24
Time (h)
colour
MU
WT

Gene Rv2937 (drrB)
WT vs T0: DE MU vs T0: DE

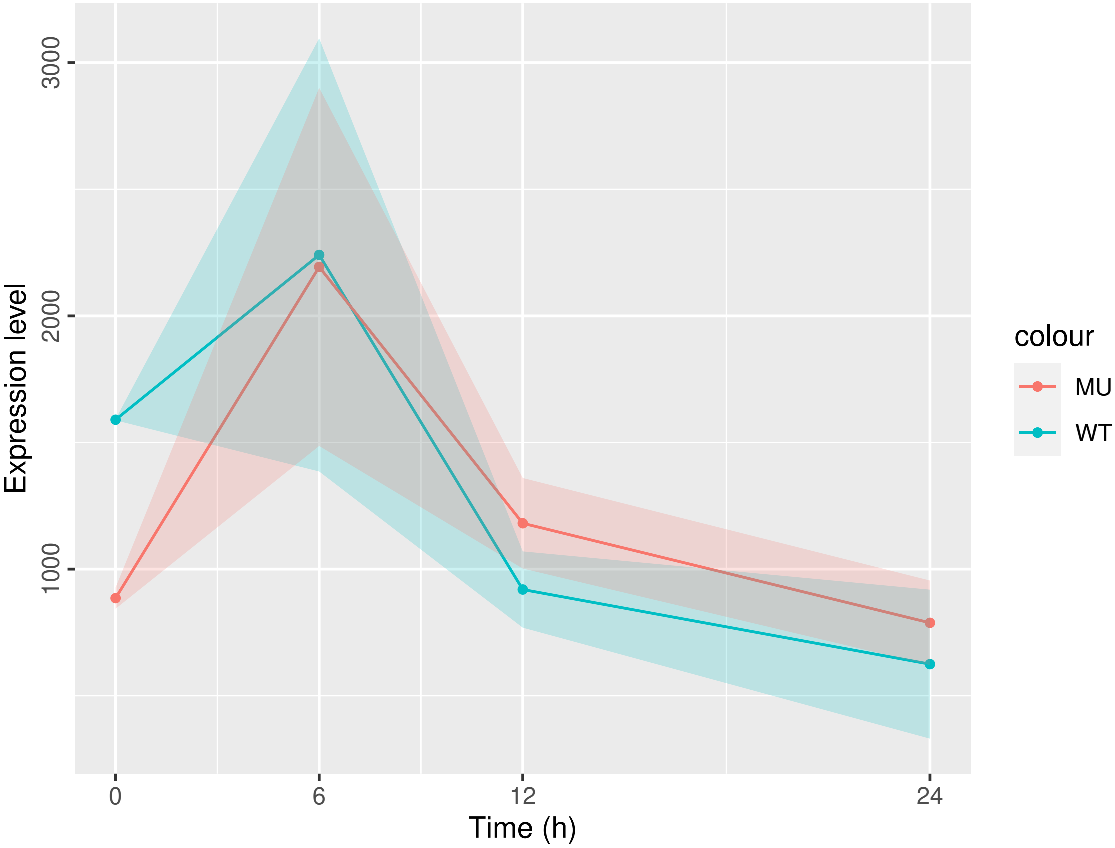

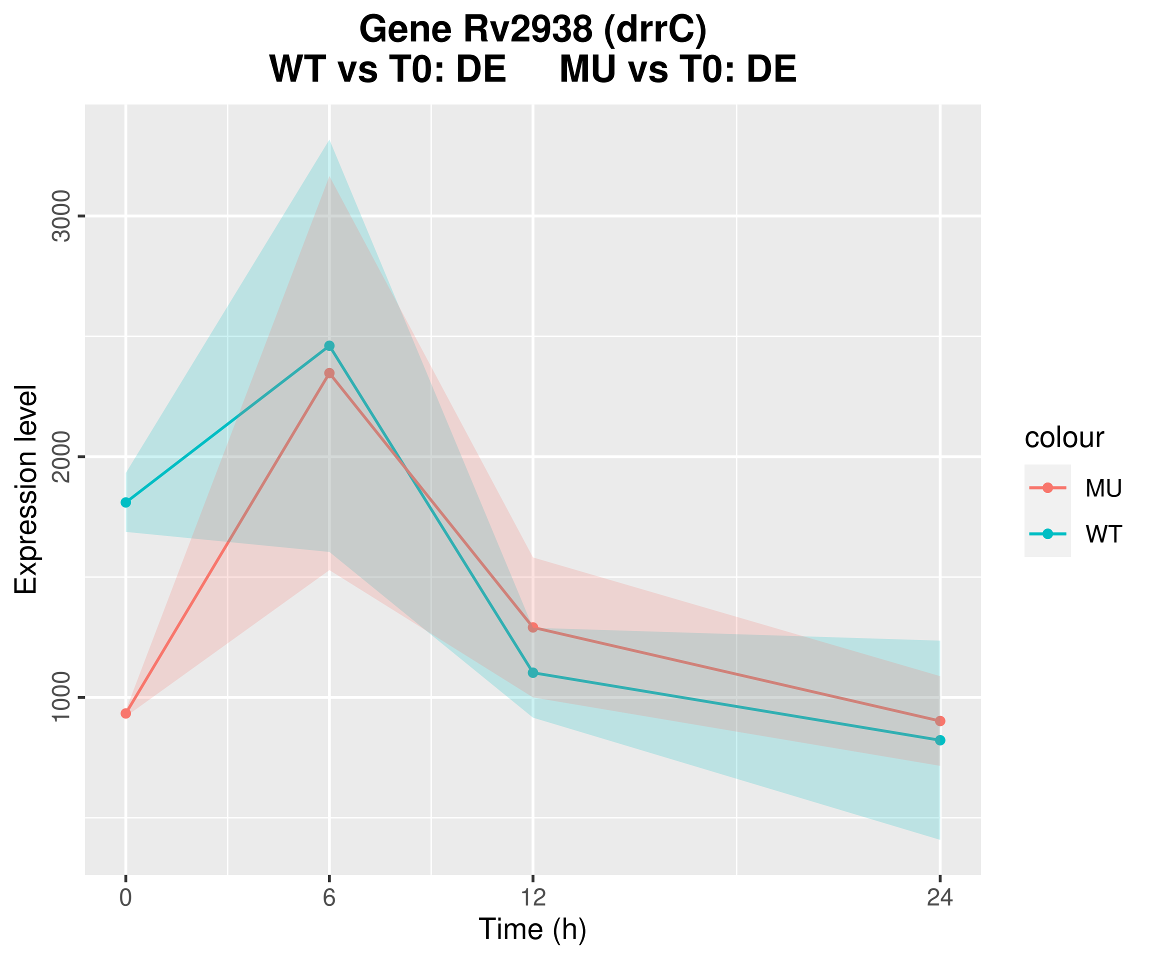
Gene Rv2938 (drrC)
WT vs T0: DE MU vs T0: DE
Expression level
3000
2000
1000
0
6
12
24
Time (h)
colour
MU
WT

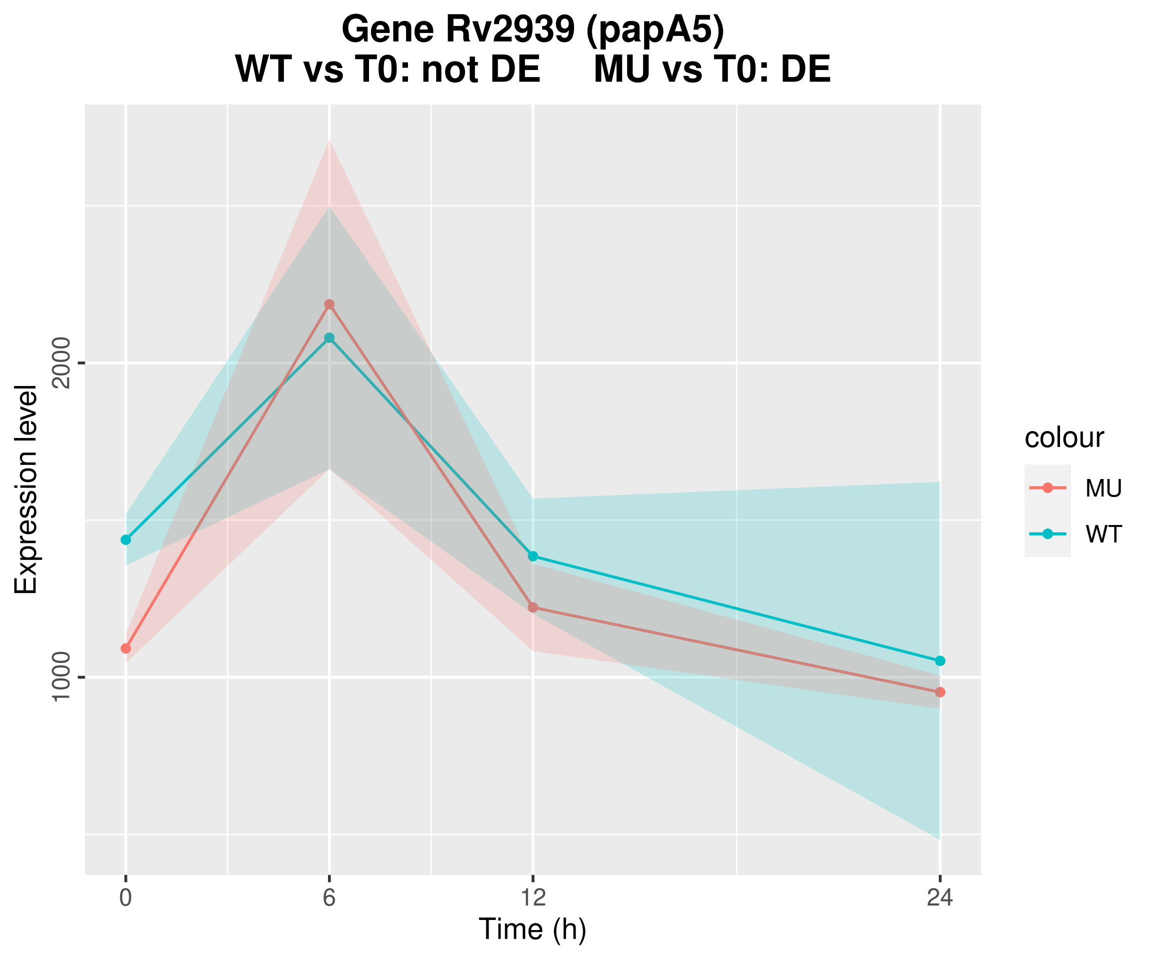
Gene Rv2939 (papA5)
WT vs T0: not DE MU vs T0: DE
Expression level
2000
1000
0
6
12
24
Time (h)
colour
MU
WT

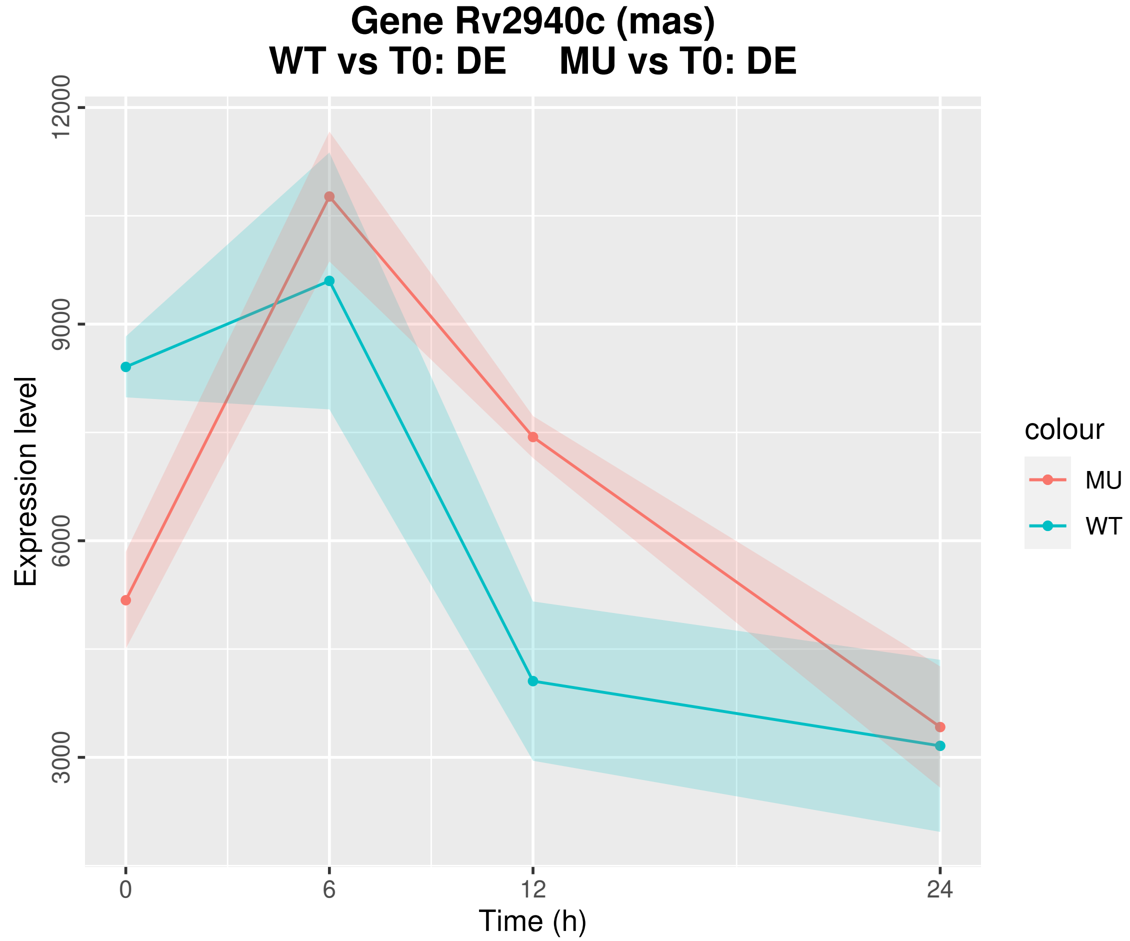
Gene Rv2940c (mas)
WT vs T0: DE MU vs T0: DE
Expression level
12000
9000
6000
3000
0
6
12
24
Time (h)
colour
MU
WT

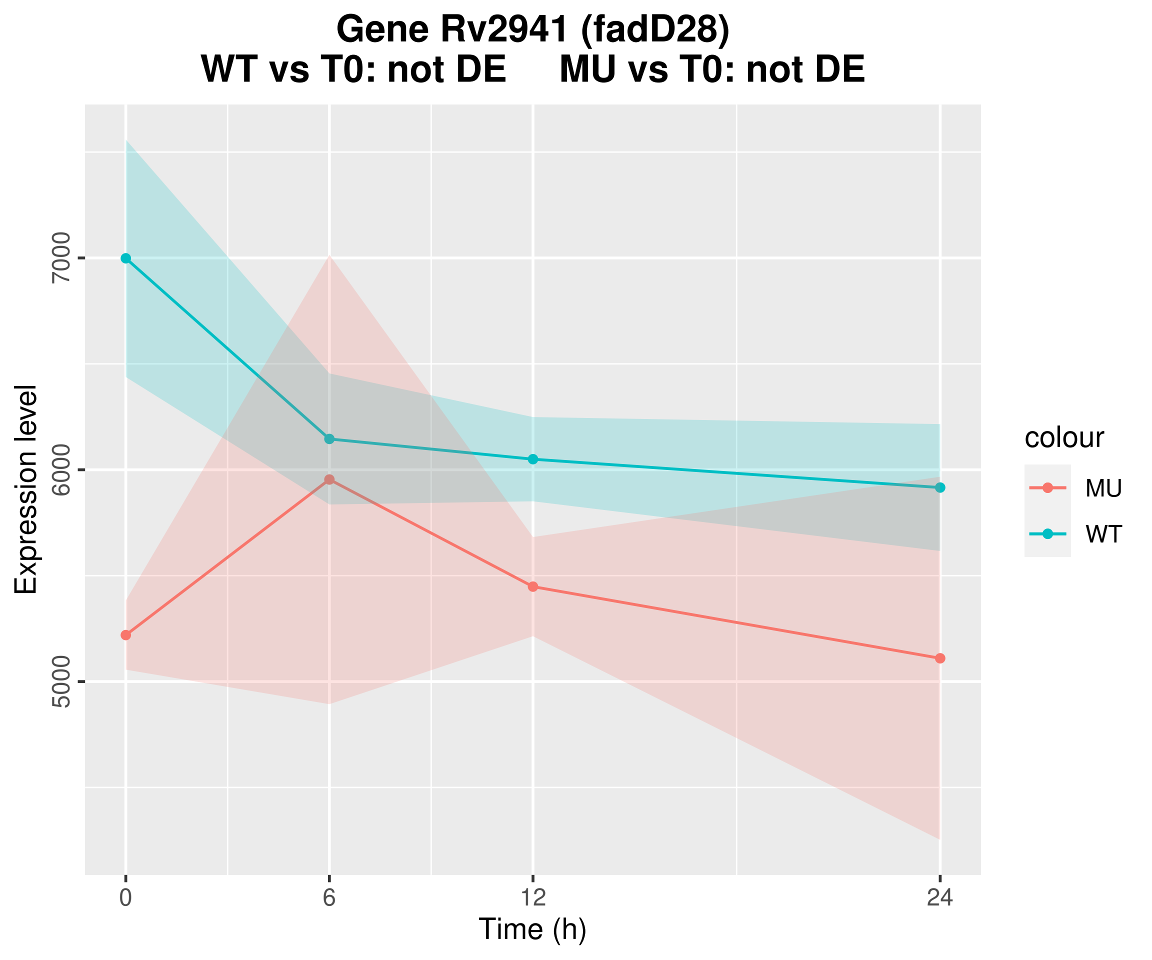
Gene Rv2941 (fadD28)
WT vs T0: not DE   MU vs T0: not DE
Expression level
7000
6000
5000
0
6
12
24
Time (h)
colour
MU
WT

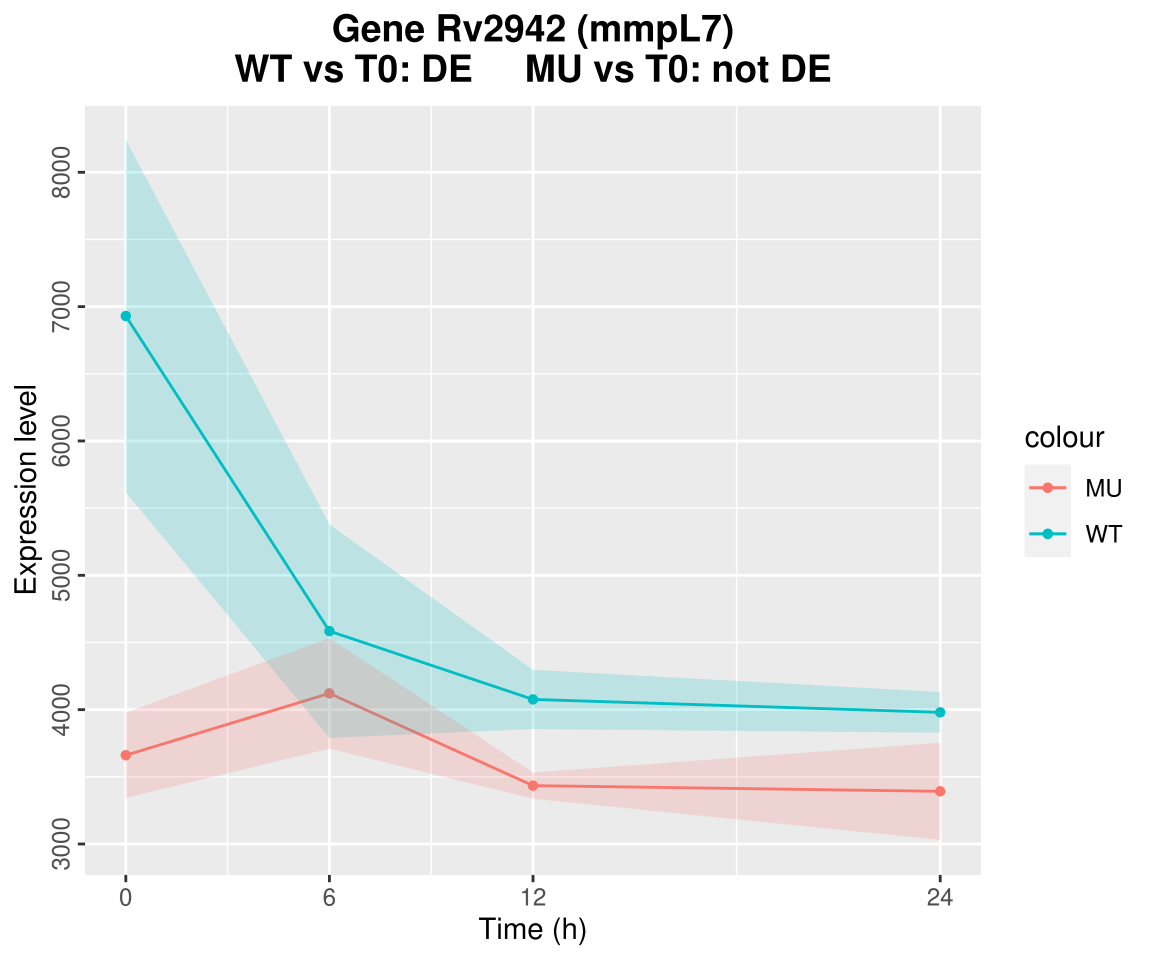
Gene Rv2942 (mmpL7)
WT vs T0: DE   MU vs T0: not DE
Expression level
8000
7000
6000
5000
4000
3000
0
6
12
24
Time (h)
colour
MU
WT

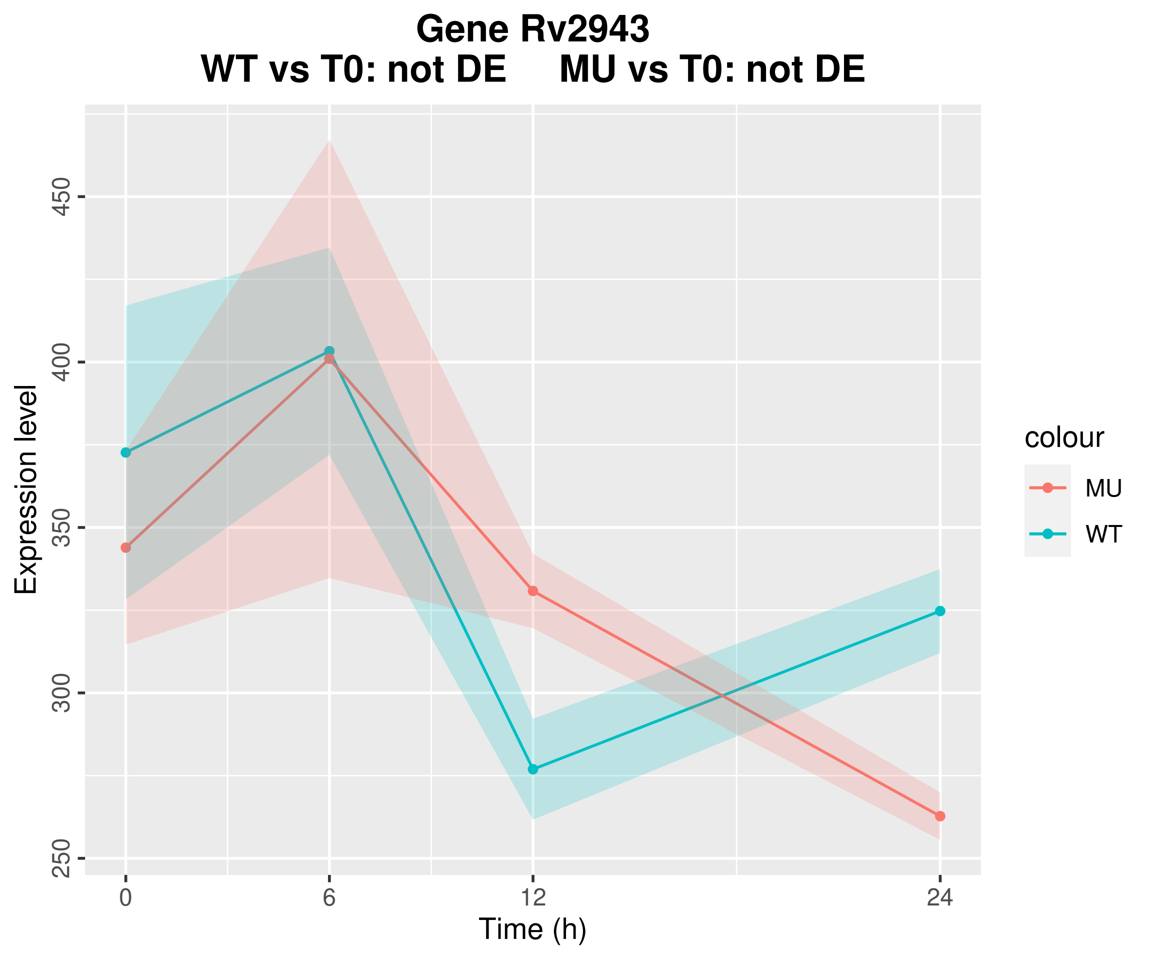

Gene Rv2943
WT vs T0: not DE   MU vs T0: not DE
Expression level
450
400
350
300
250
0
6
12
24
Time (h)
colour
MU
WT

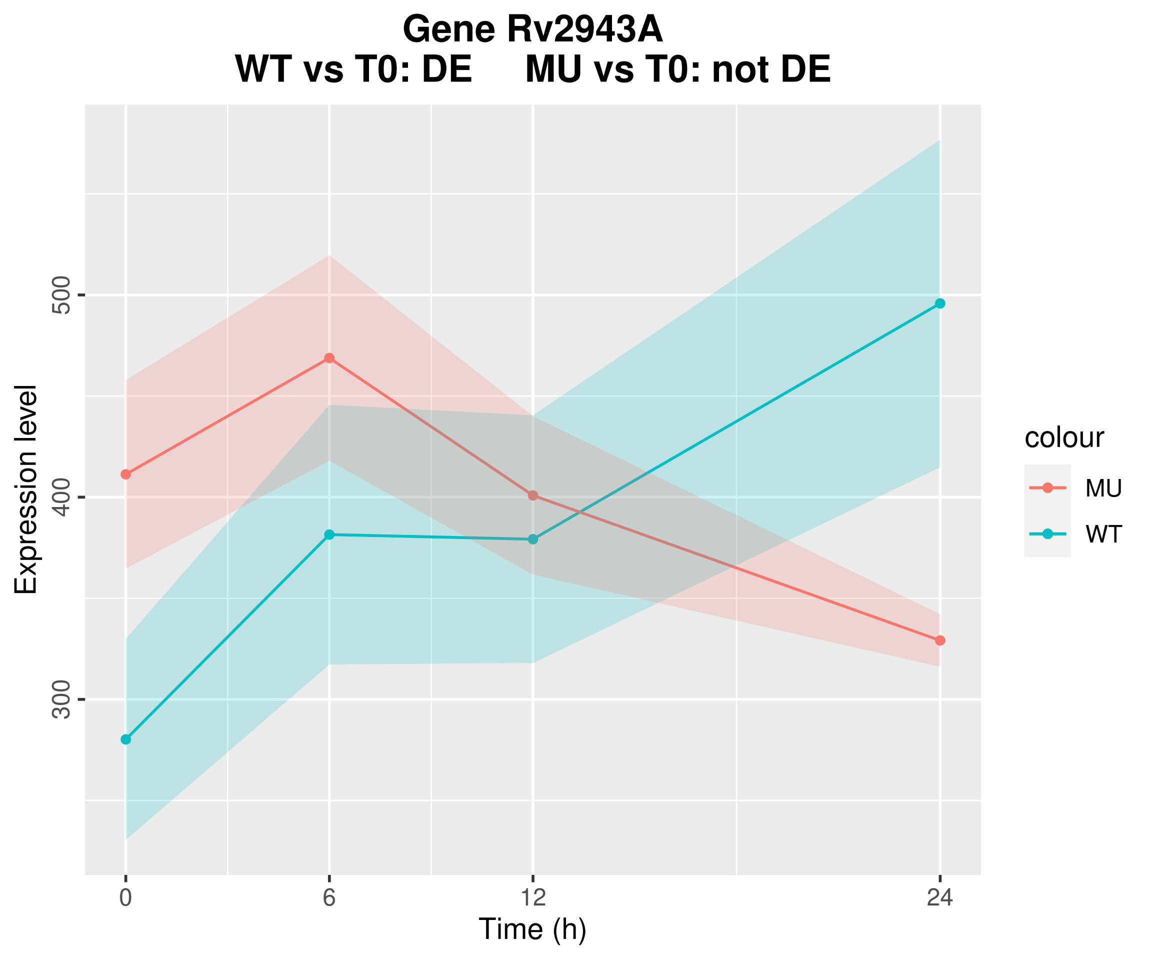
Gene Rv2943A
WT vs T0: DE   MU vs T0: not DE
Expression level
300
400
500
0
6
12
24
Time (h)
colour
MU
WT

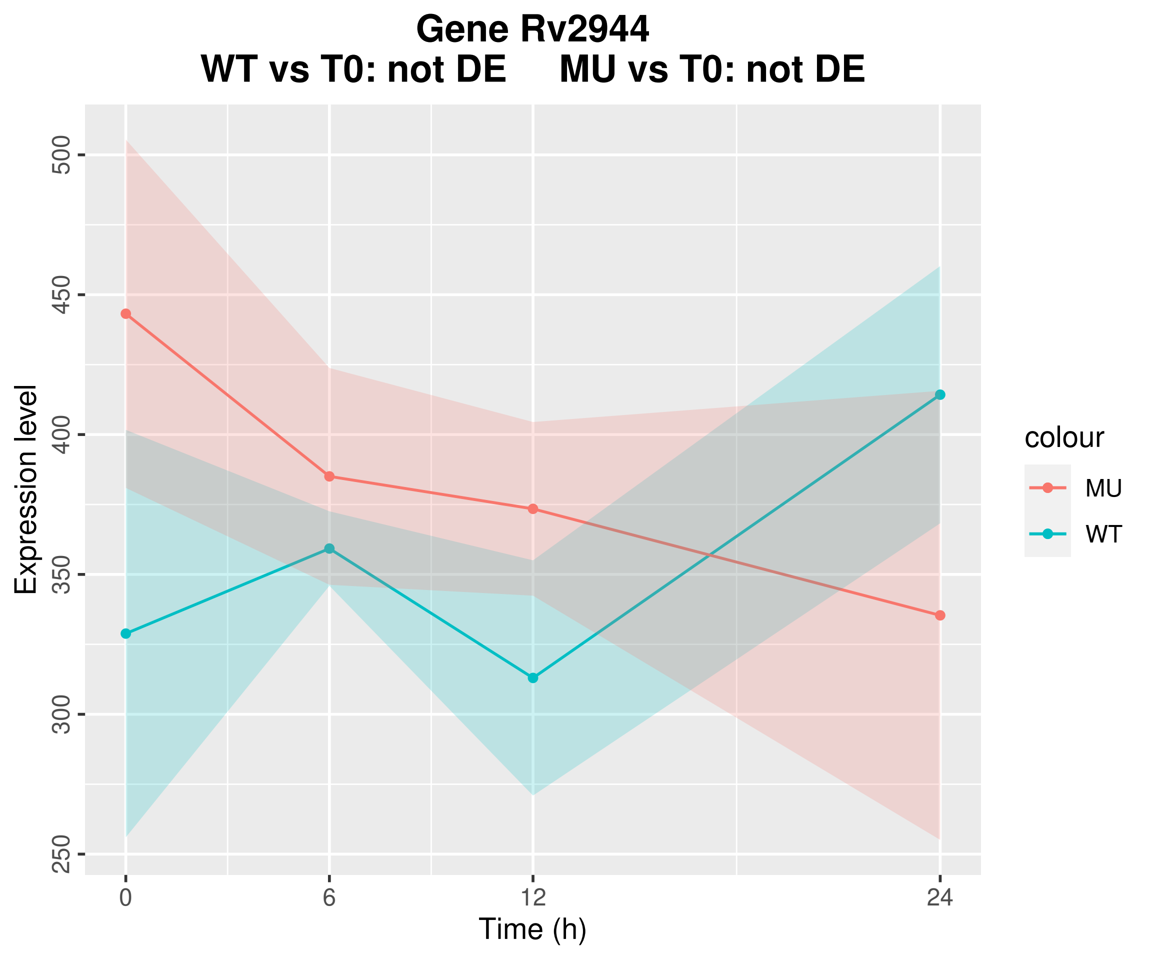

Gene Rv2944
WT vs T0: not DE MU vs T0: not DE
Expression level
500
450
400
350
300
250
0
6
12
24
Time (h)
colour
MU
WT

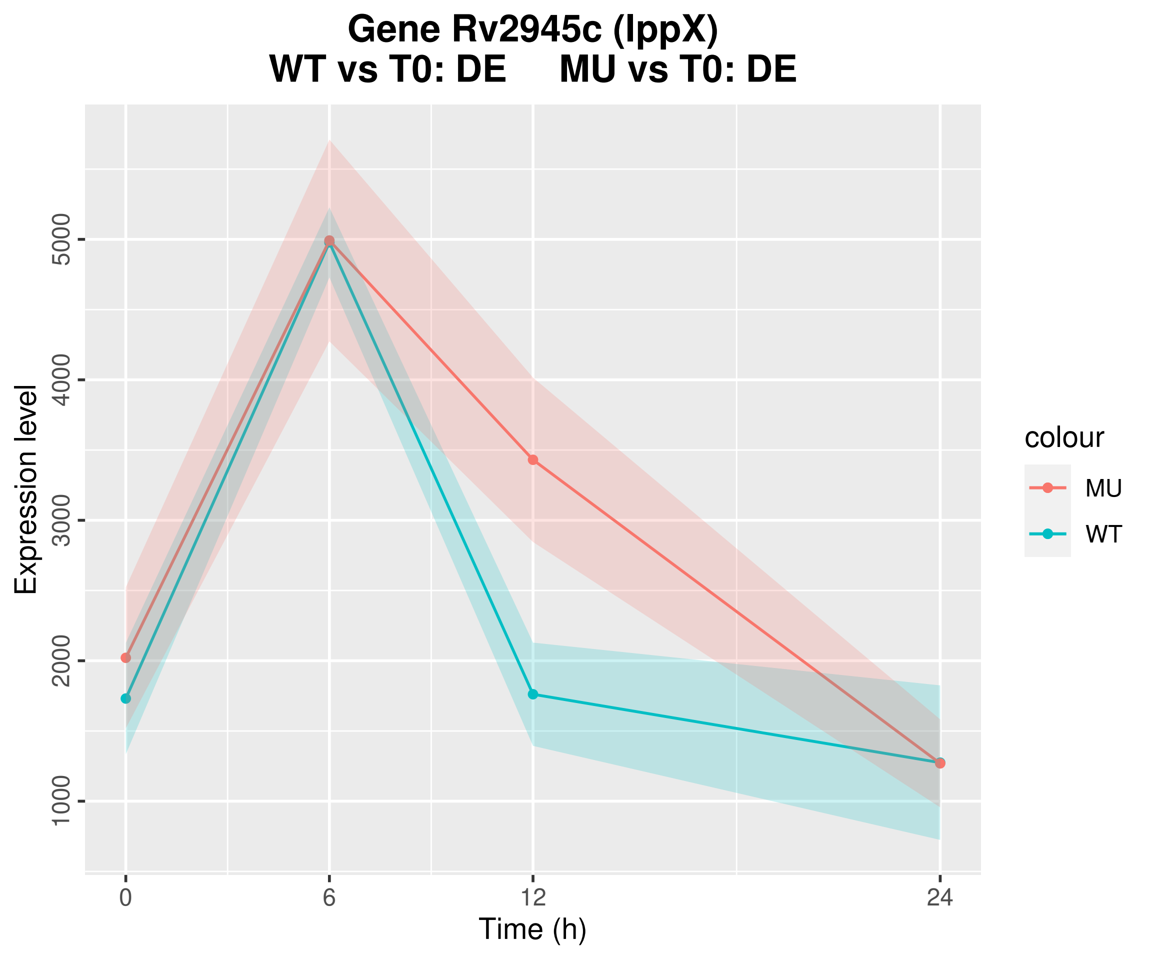
Gene Rv2945c (lppX)
WT vs T0: DE MU vs T0: DE
Expression level
5000
4000
3000
2000
1000
0
6
12
24
Time (h)
colour
MU
WT

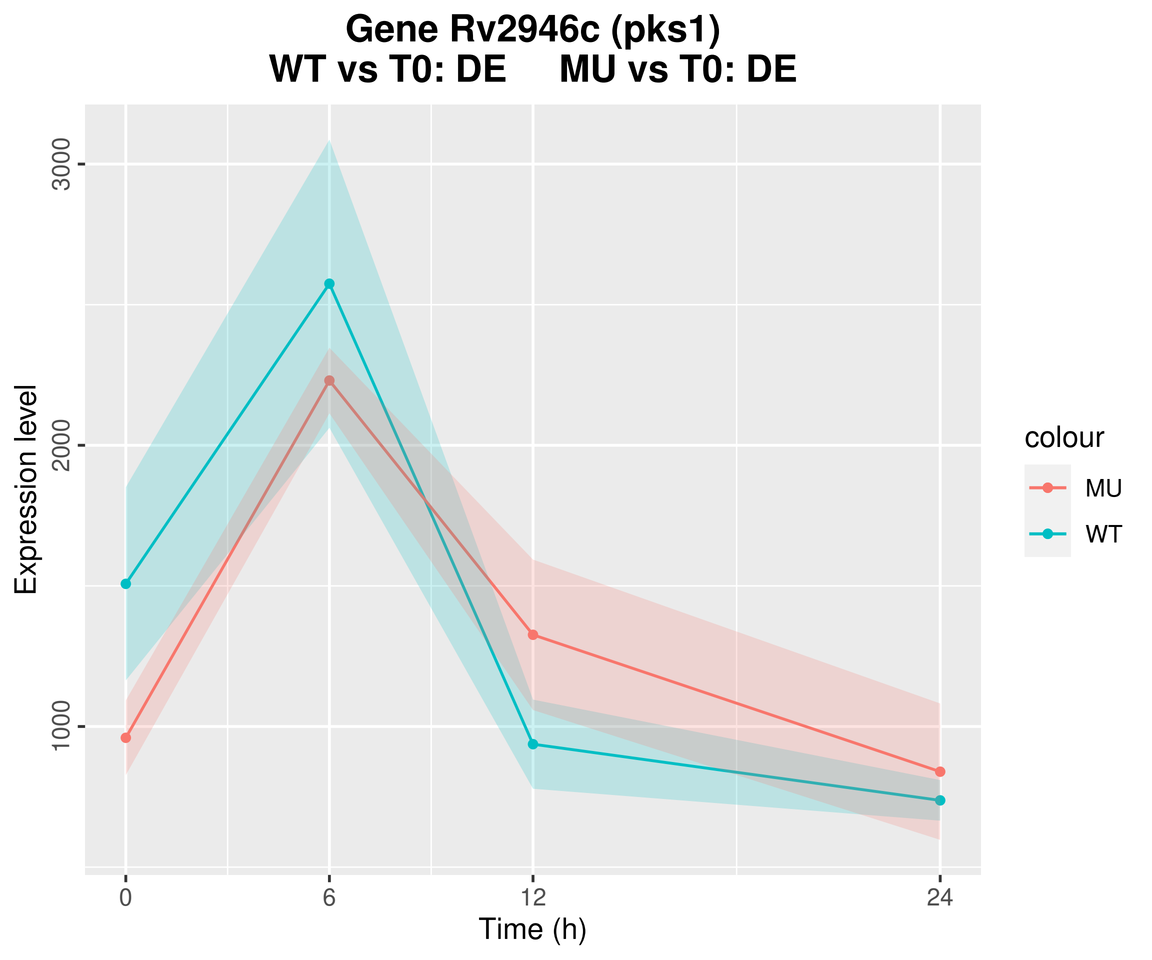
Gene Rv2946c (pks1)
WT vs T0: DE MU vs T0: DE
Expression level
3000
2000
1000
0
6
12
24
Time (h)
colour
MU
WT

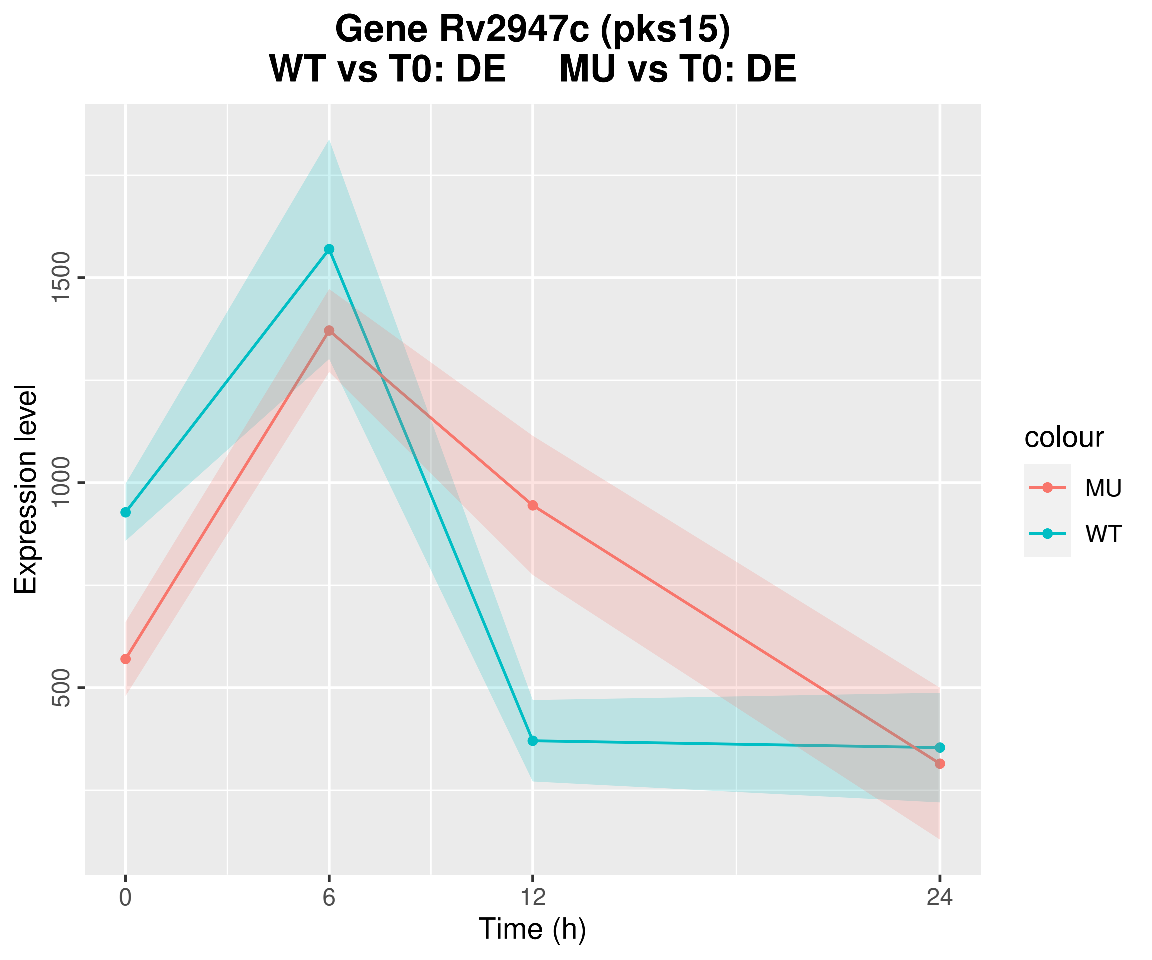

Gene Rv2947c (pks15)
WT vs T0: DE MU vs T0: DE
Expression level
1500
1000
500
0
6
12
24
Time (h)
colour
MU
WT

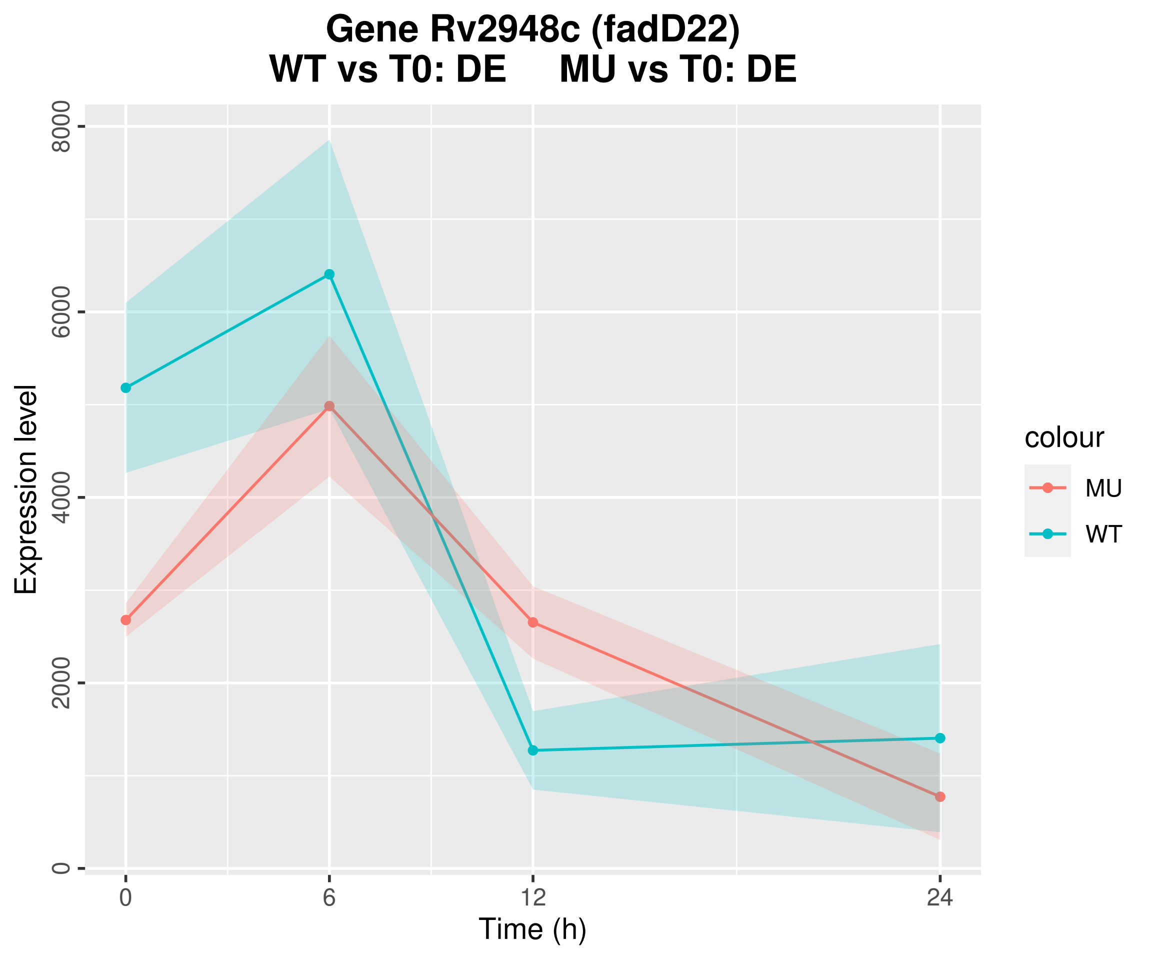

Gene Rv2948c (fadD22)
WT vs T0: DE   MU vs T0: DE
Expression level
8000
6000
4000
2000
0
0
6
12
24
Time (h)
colour
MU
WT

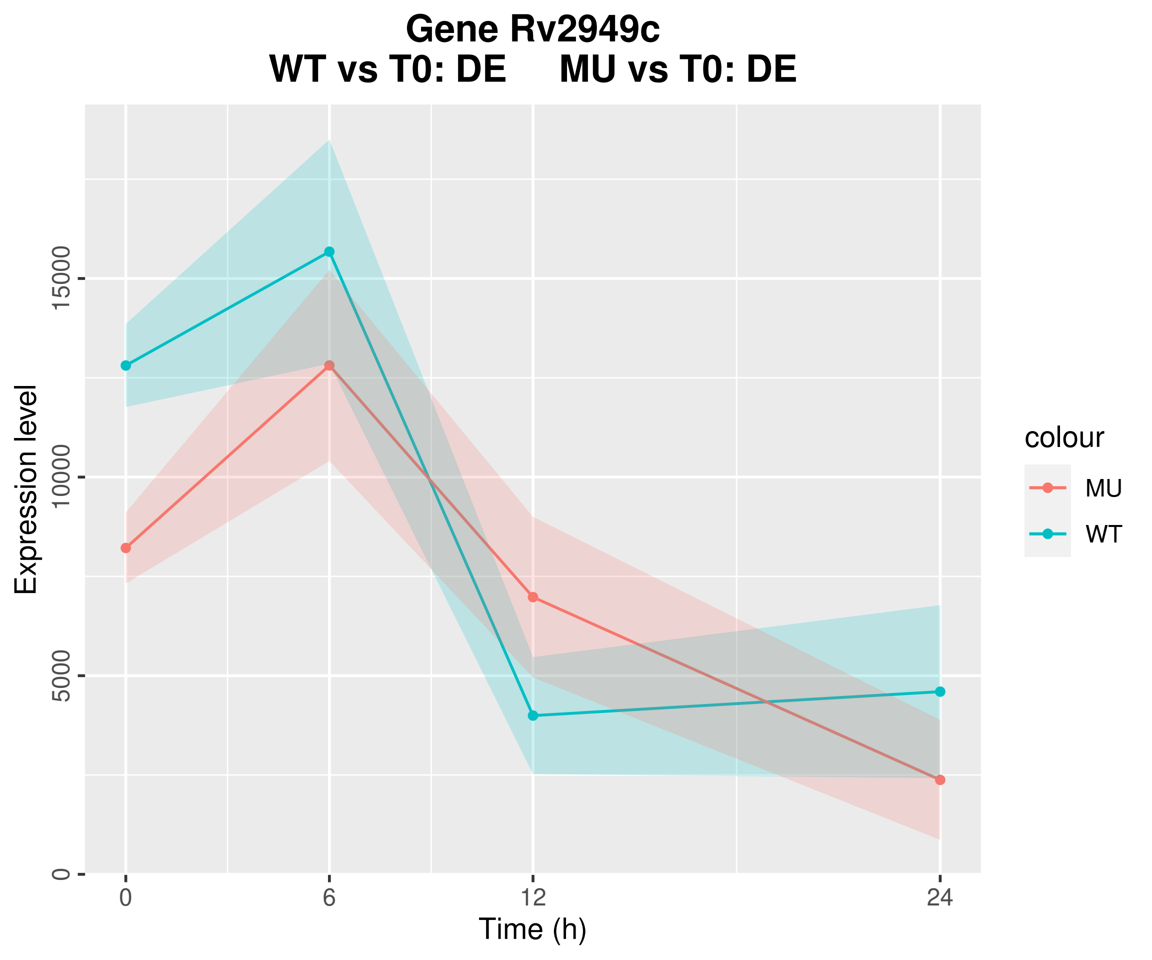
Gene Rv2949c
WT vs T0: DE   MU vs T0: DE
Expression level
0
5000
10000
15000
0
6
12
24
Time (h)
colour
MU
WT

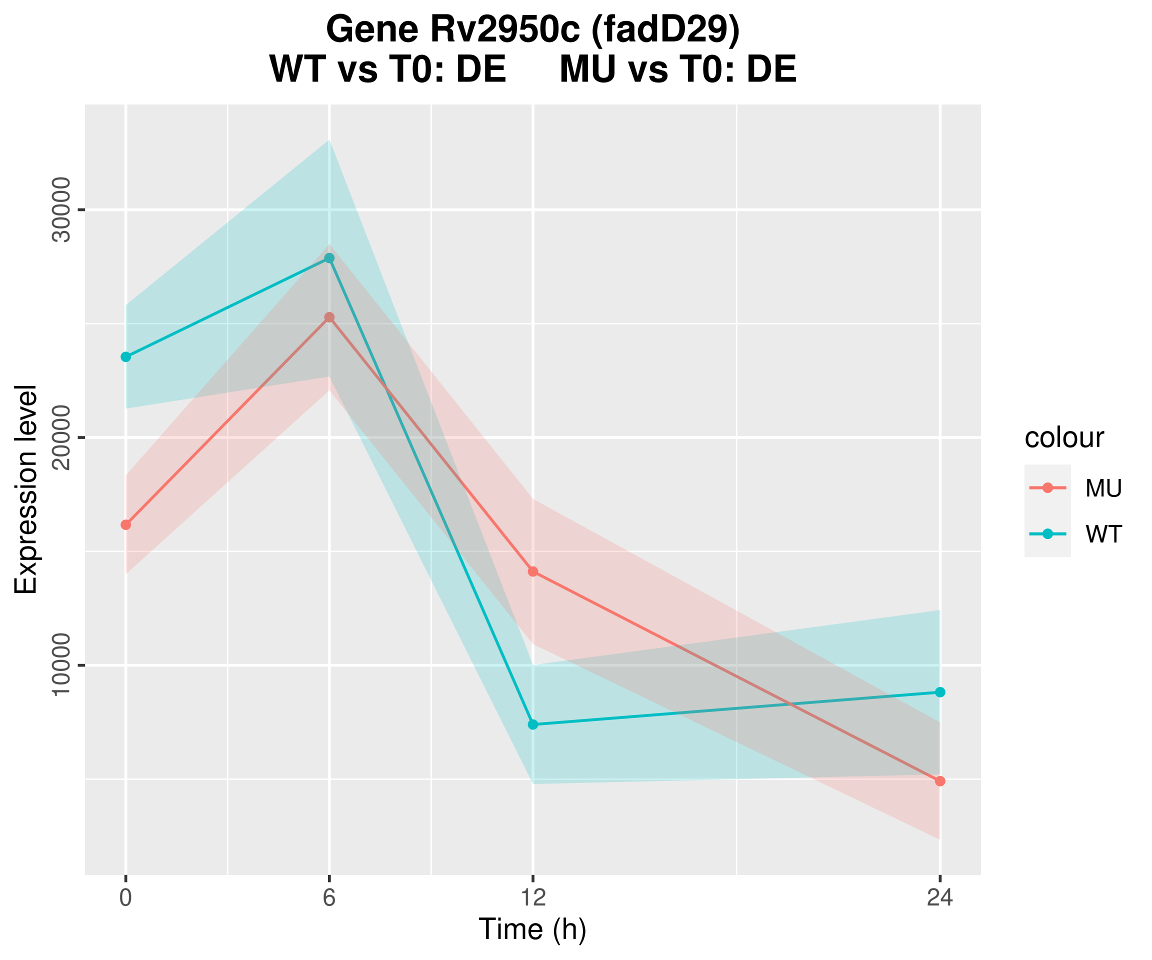

Gene Rv2950c (fadD29)
WT vs T0: DE MU vs T0: DE
30000
20000
10000
Expression level
0
6
12
24
Time (h)
colour
MU
WT

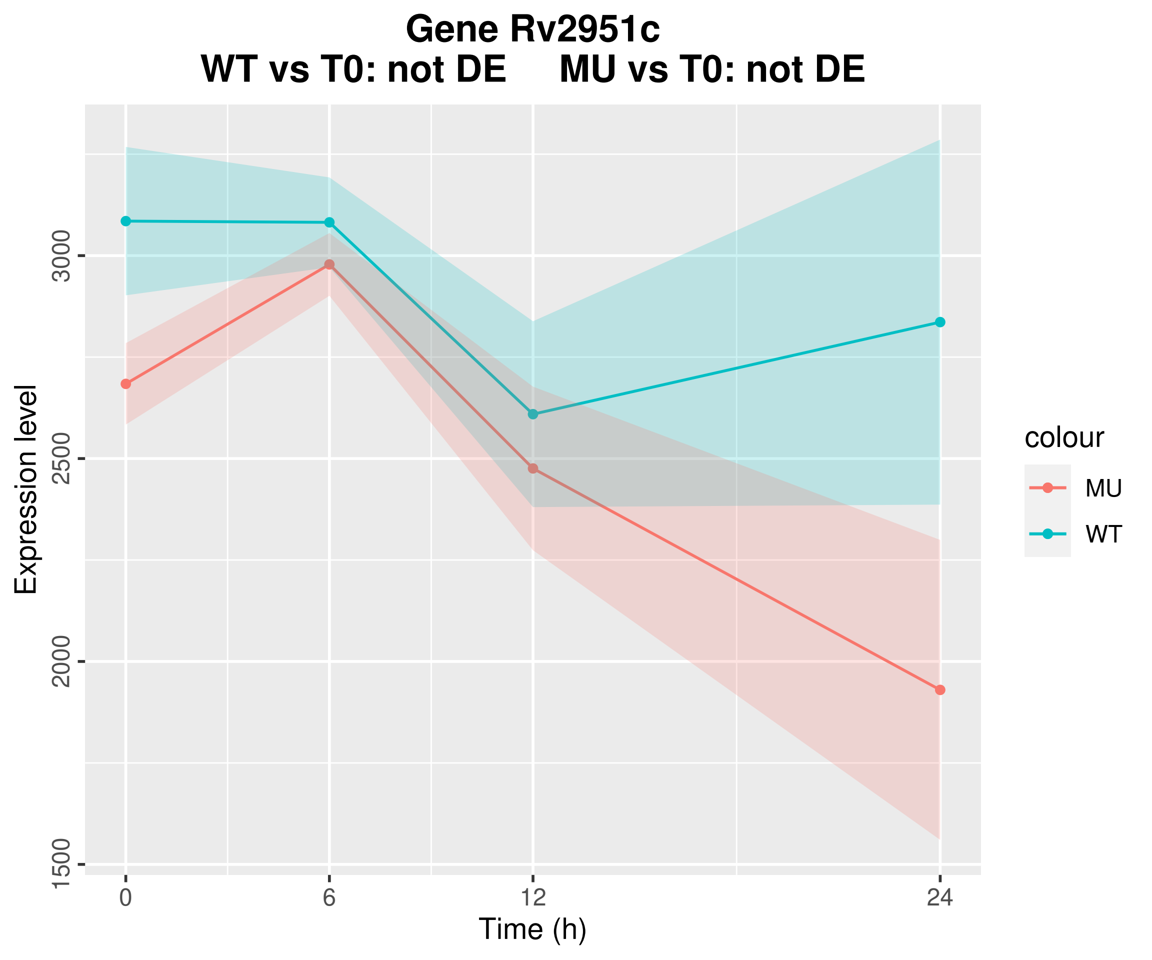
Gene Rv2951c
WT vs T0: not DE MU vs T0: not DE
Expression level
3000
2500
2000
1500
0
6
12
24
Time (h)
colour
MU
WT

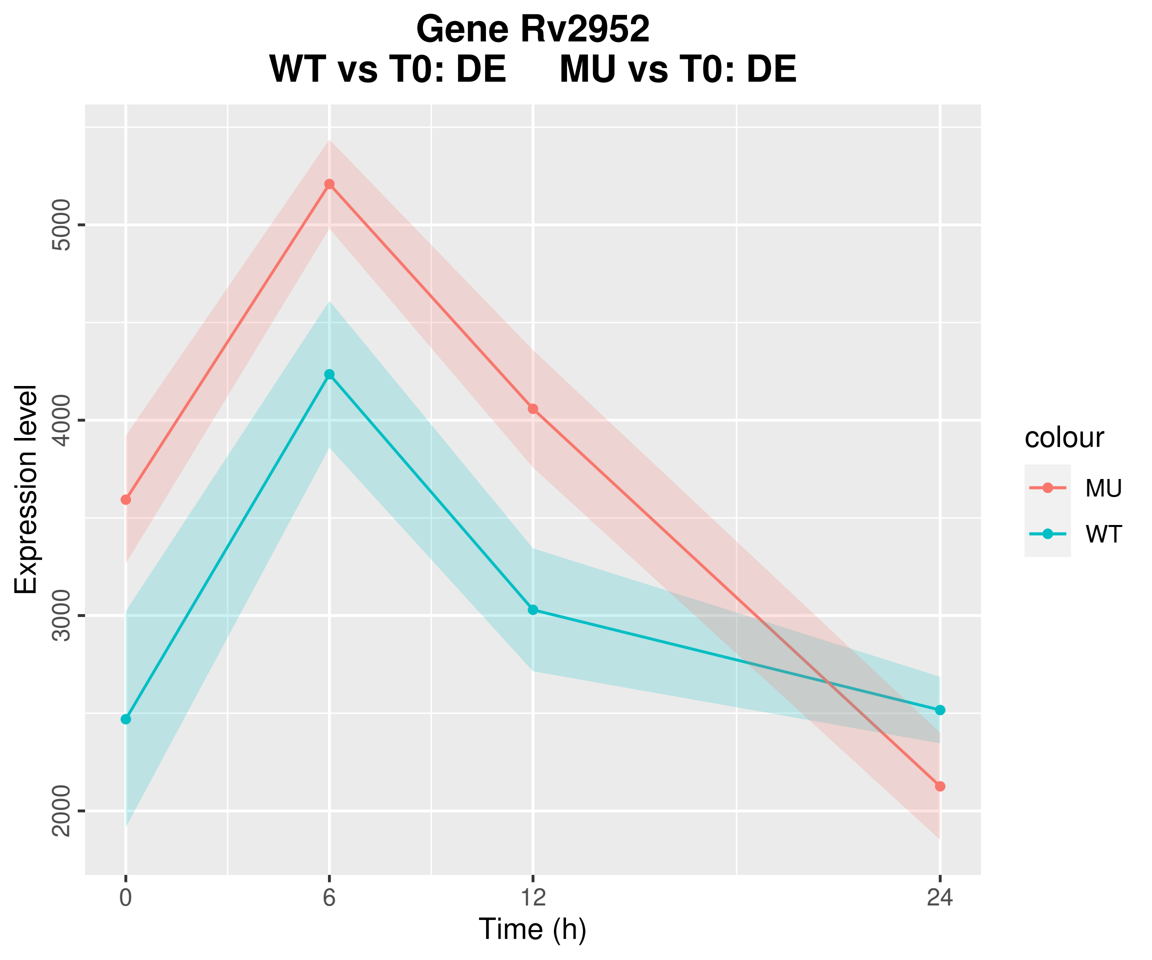
Gene Rv2952
WT vs T0: DE   MU vs T0: DE
Expression level
5000
4000
3000
2000
0
6
12
24
Time (h)
colour
MU
WT

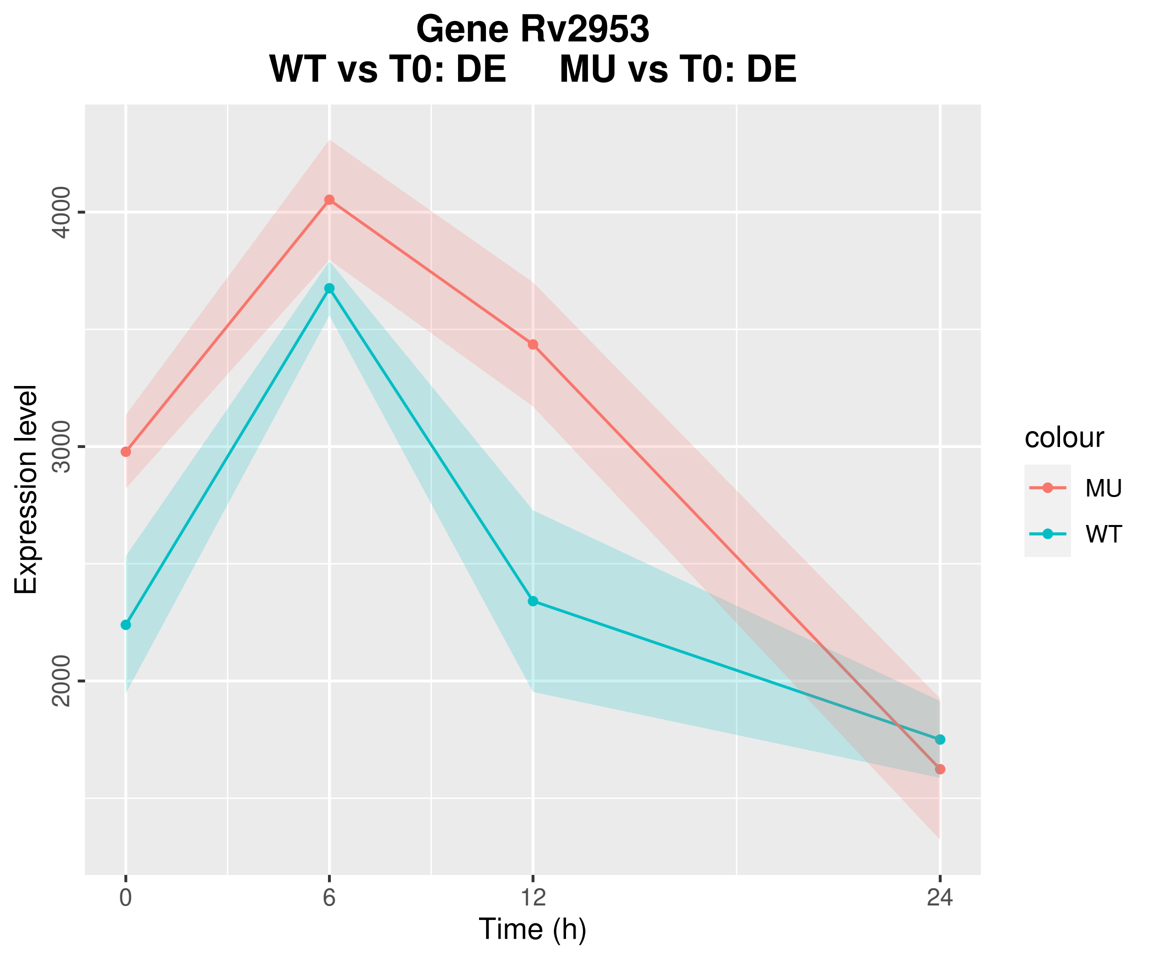
Gene Rv2953
WT vs T0: DE   MU vs T0: DE
Expression level
4000
3000
2000
0
6
12
24
Time (h)
colour
MU
WT

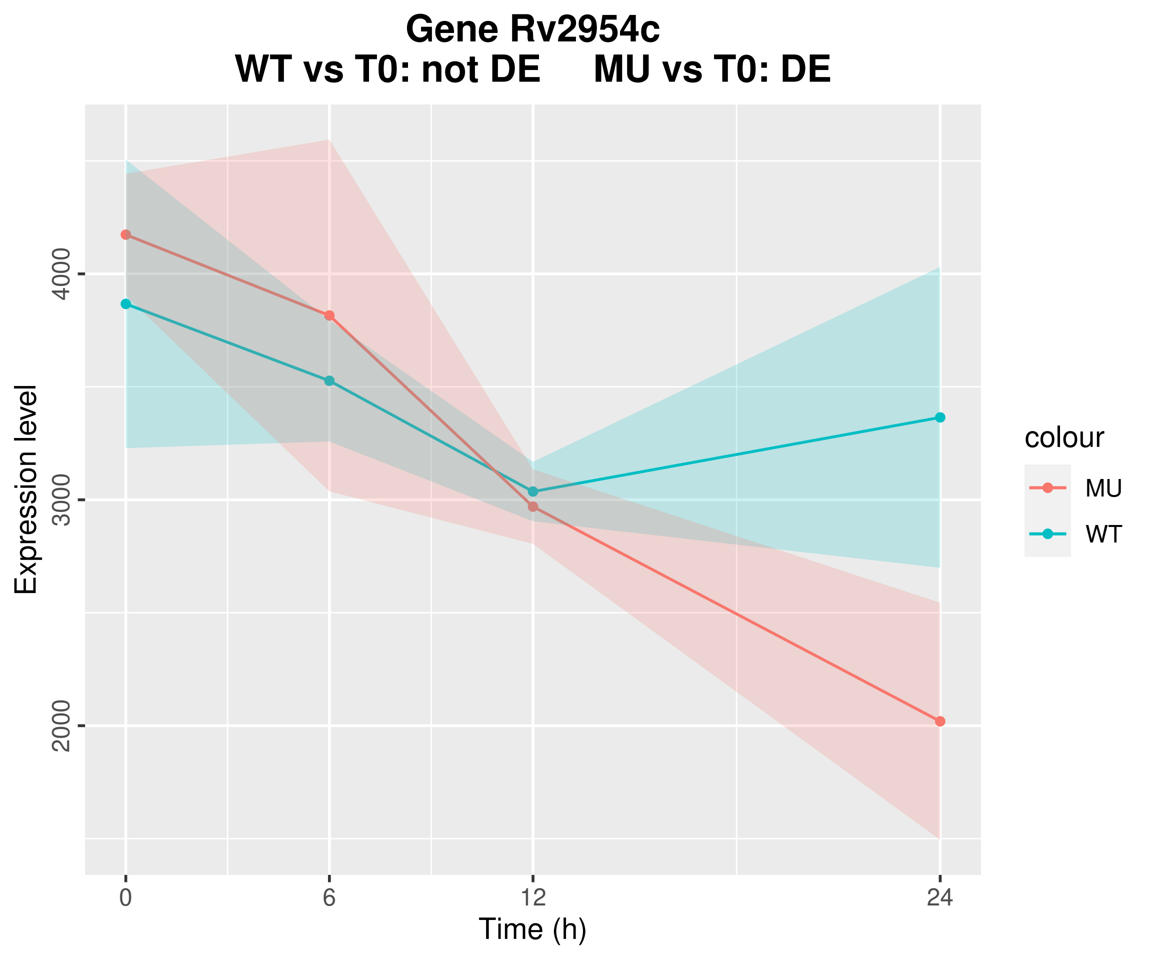

Gene Rv2954c
WT vs T0: not DE   MU vs T0: DE
Expression level
4000
3000
2000
0
6
12
24
Time (h)
colour
MU
WT

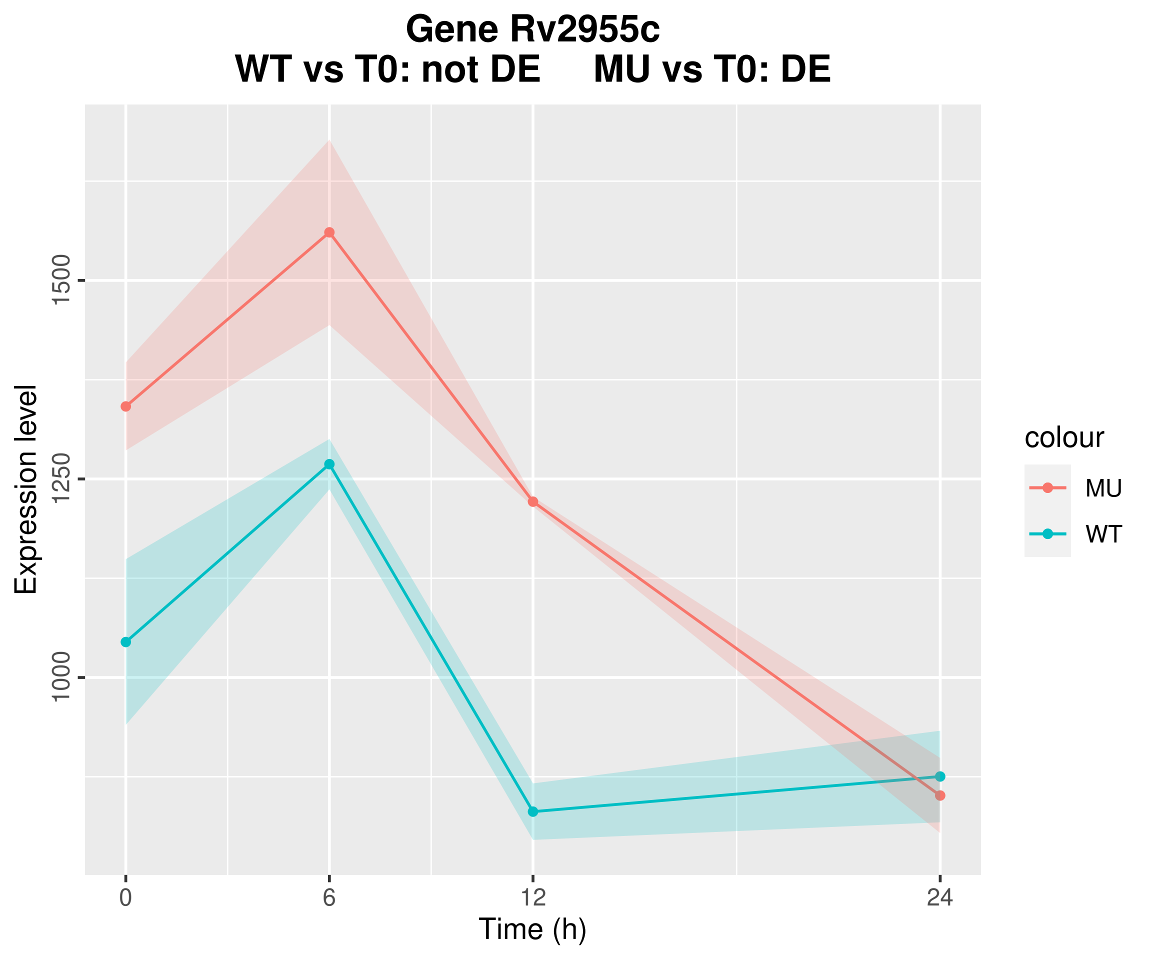

Gene Rv2955c
WT vs T0: not DE MU vs T0: DE
Expression level
1500
1250
1000
0
6
12
24
Time (h)
colour
MU
WT

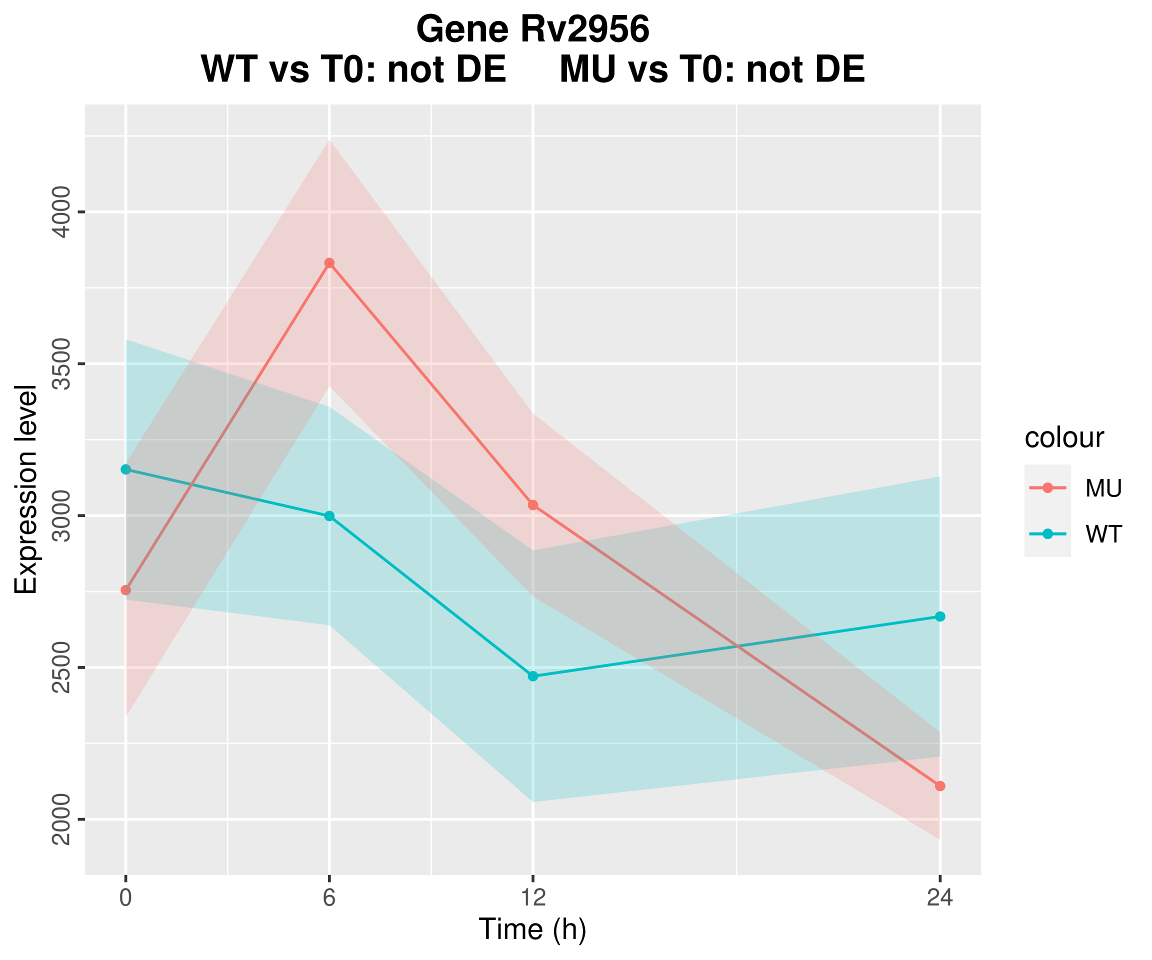
Gene Rv2956
WT vs T0: not DE MU vs T0: not DE
Expression level
4000
3500
3000
2500
2000
0
6
12
24
Time (h)
colour
MU
WT

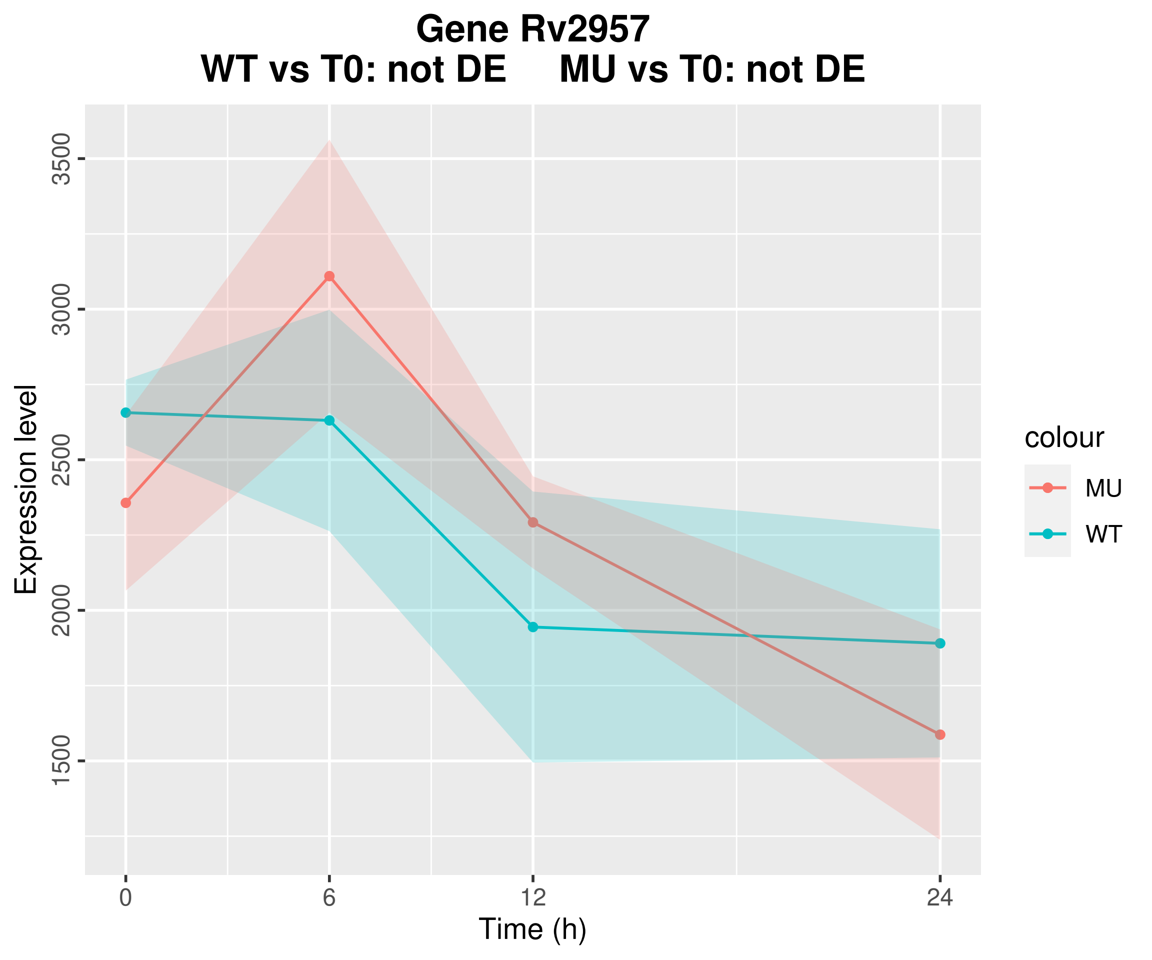

Gene Rv2957
WT vs T0: not DE MU vs T0: not DE
Expression level
3500
3000
2500
2000
1500
0
6
12
24
Time (h)
colour
MU
WT

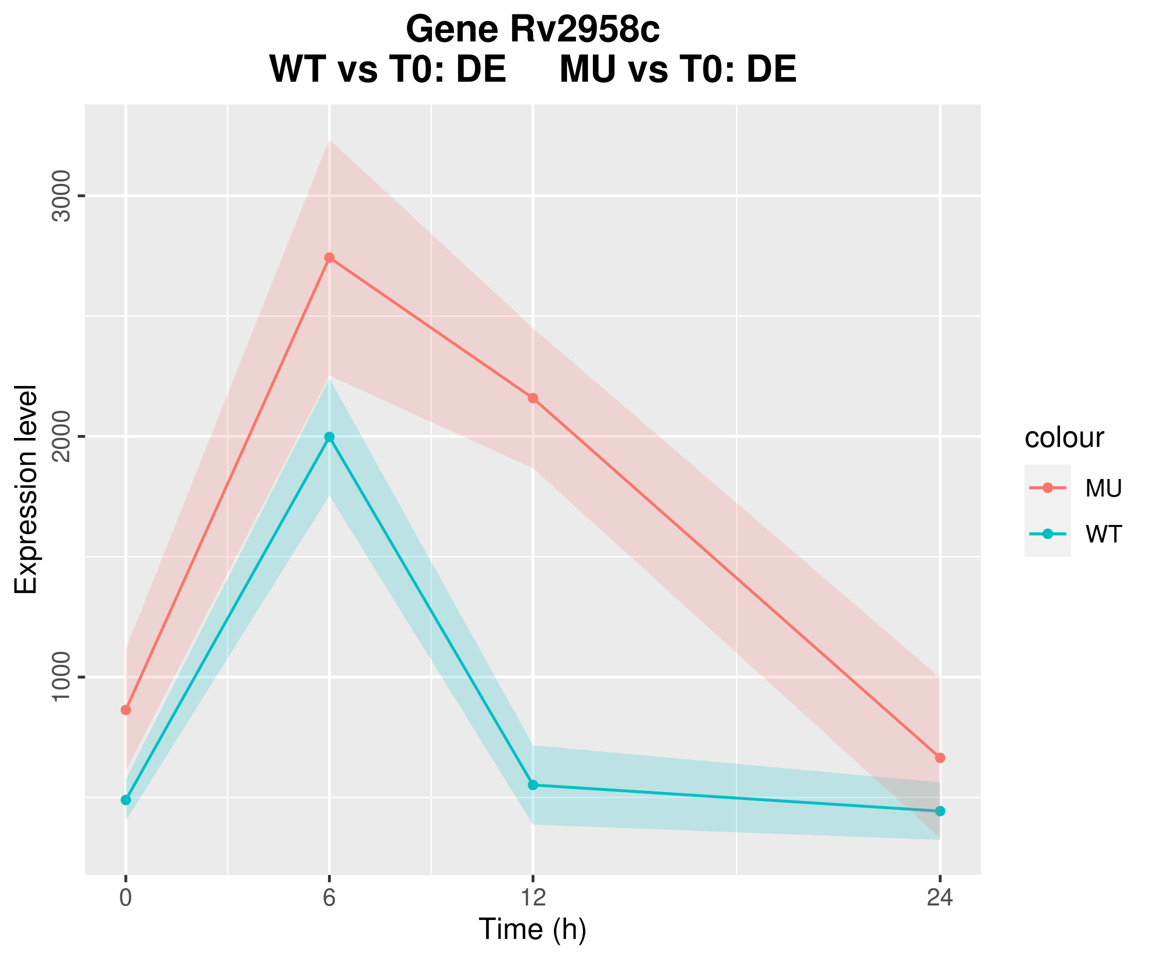

Gene Rv2958c
WT vs T0: DE   MU vs T0: DE
Expression level
3000
2000
1000
0
6
12
24
Time (h)
colour
MU
WT

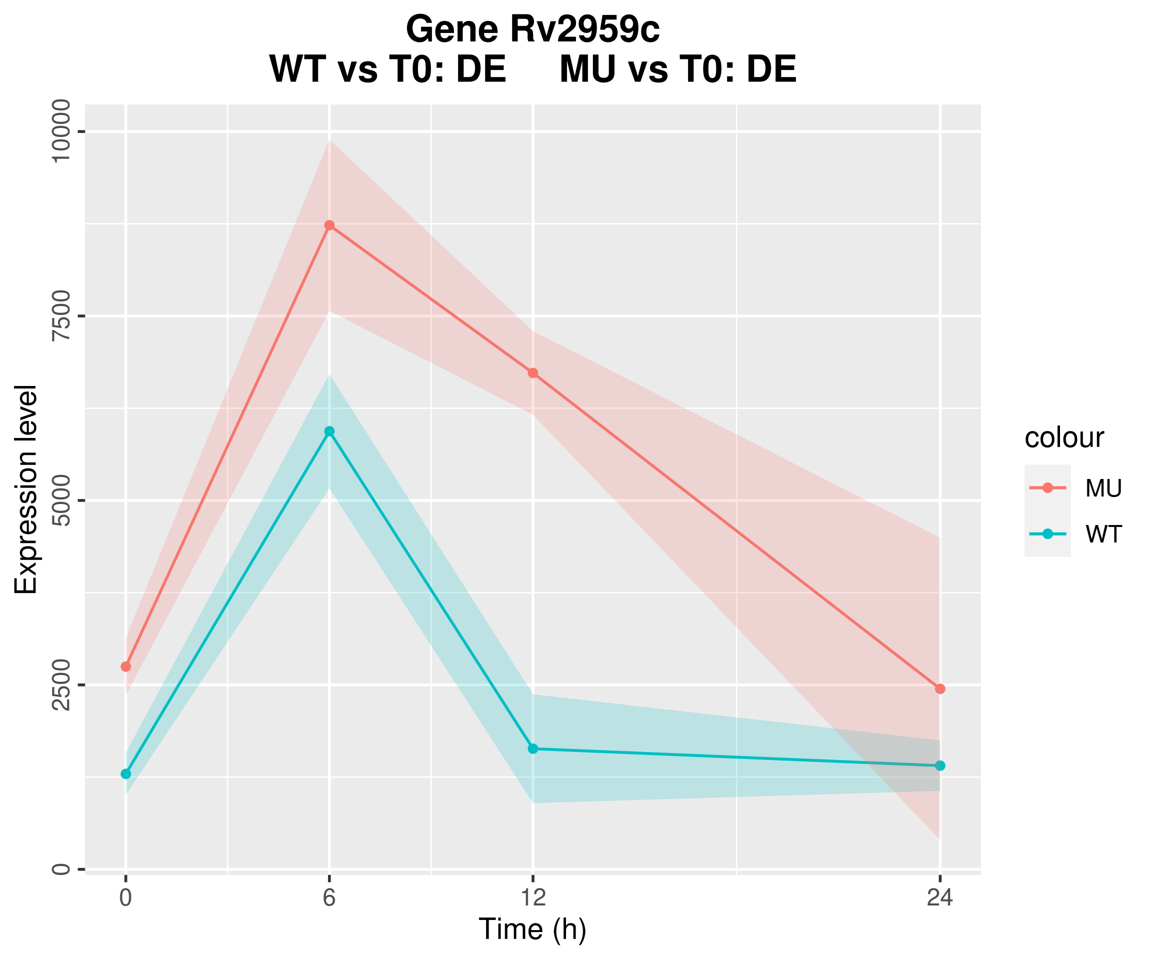
Gene Rv2959c
WT vs T0: DE MU vs T0: DE
Expression level
10000
7500
5000
2500
0
0
6
12
24
Time (h)
colour
MU
WT

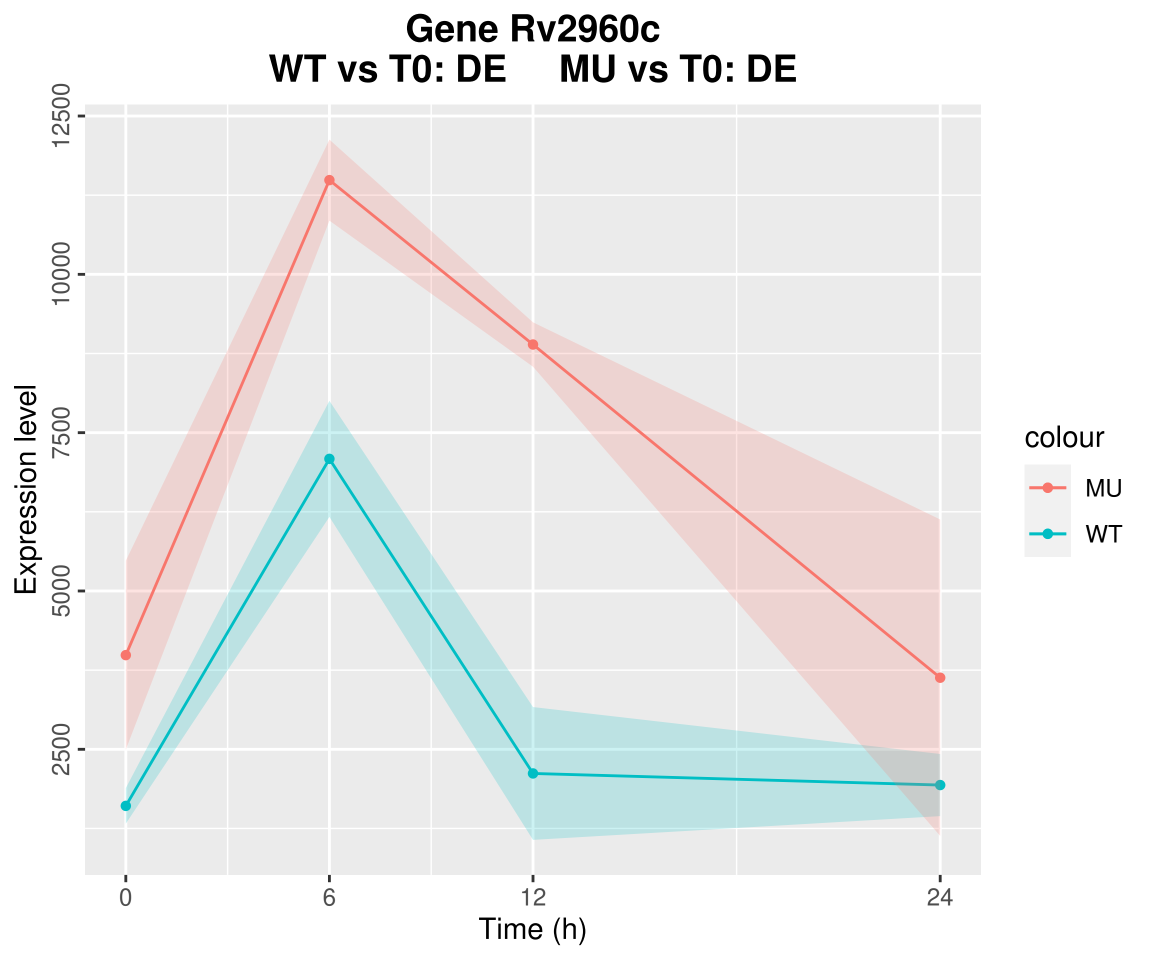
Gene Rv2960c
WT vs T0: DE MU vs T0: DE
Expression level
12500
10000
7500
5000
2500
0
6
12
24
Time (h)
colour
MU
WT

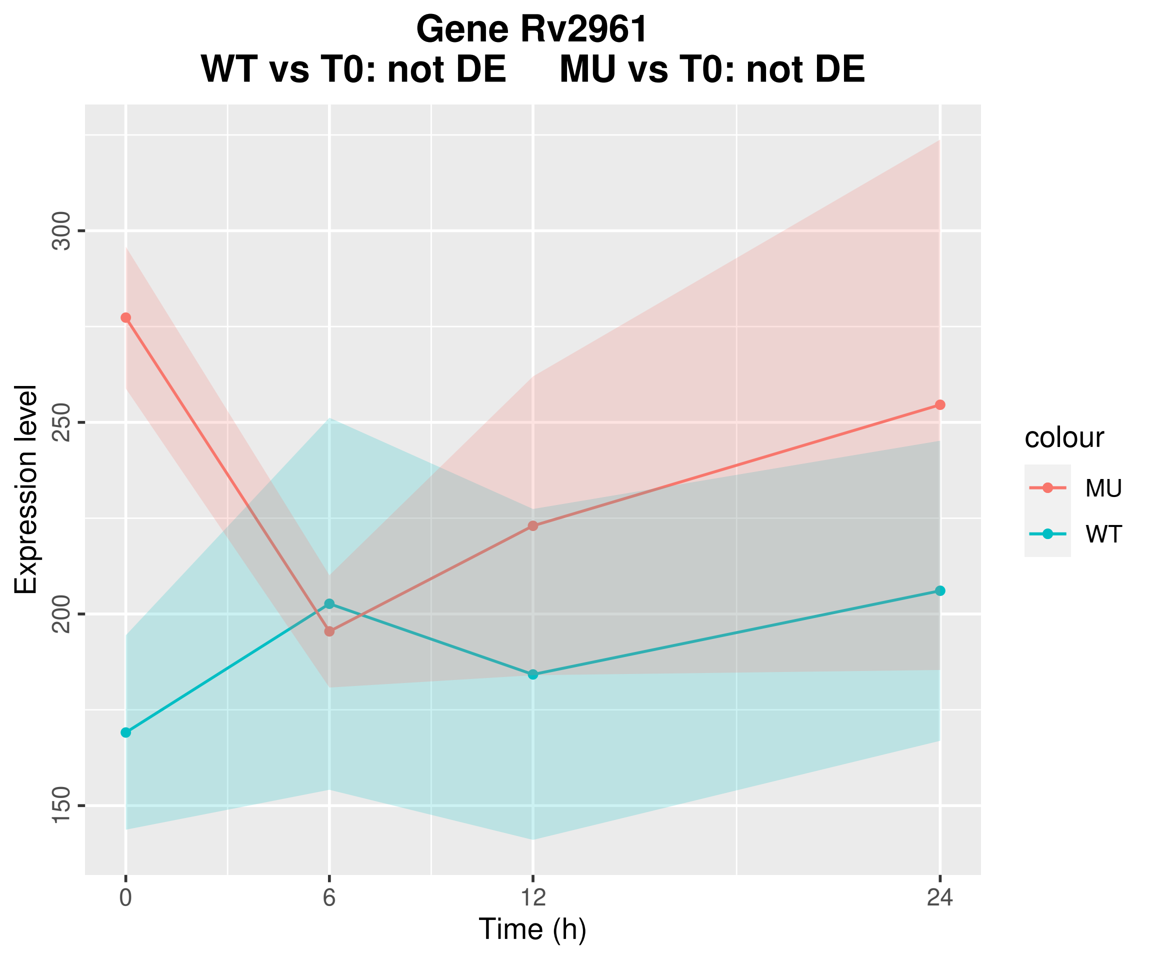

Gene Rv2961
WT vs T0: not DE   MU vs T0: not DE
Expression level
300
250
200
150
0
6
12
24
Time (h)
colour
MU
WT

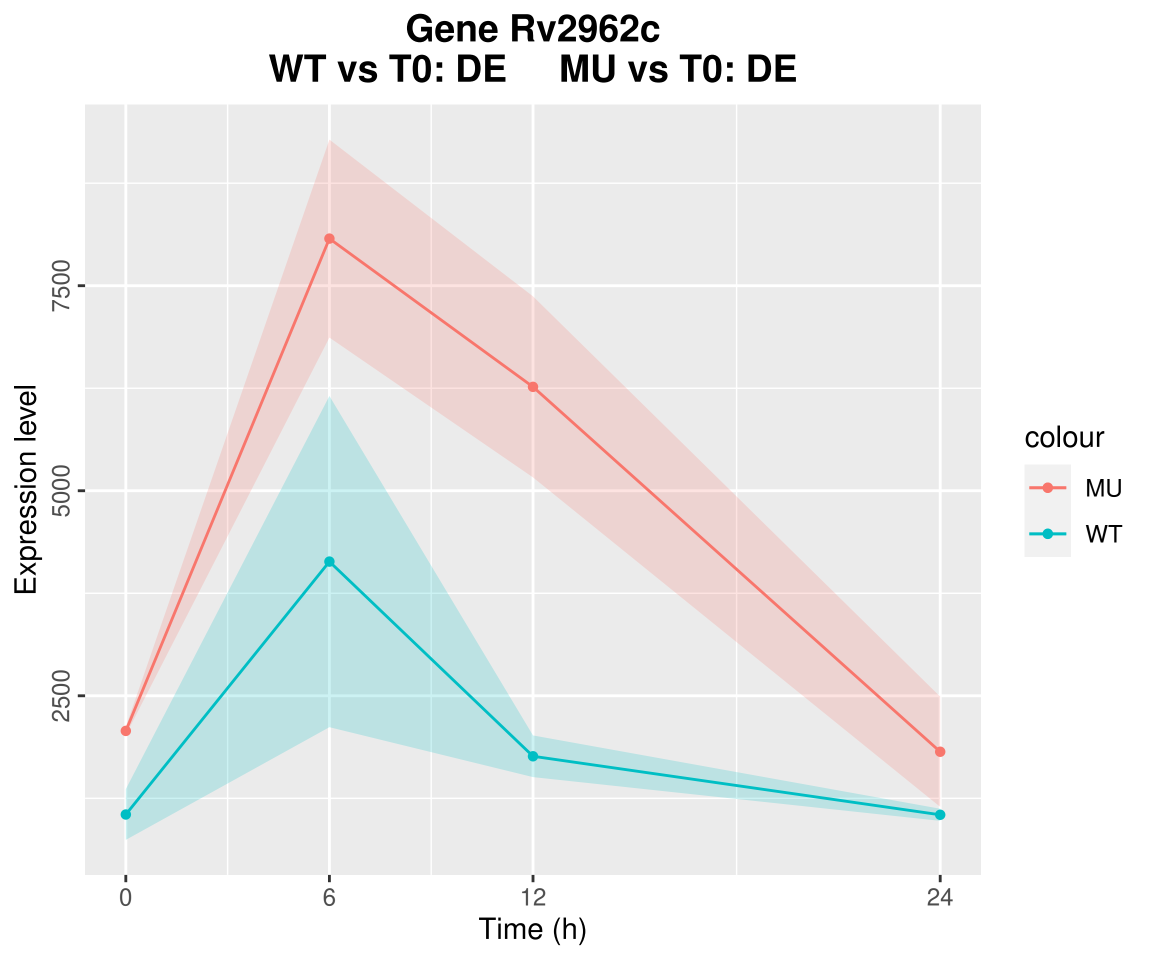
Gene Rv2962c
WT vs T0: DE MU vs T0: DE
Expression level
7500
5000
2500
0
6
12
24
Time (h)
colour
MU
WT

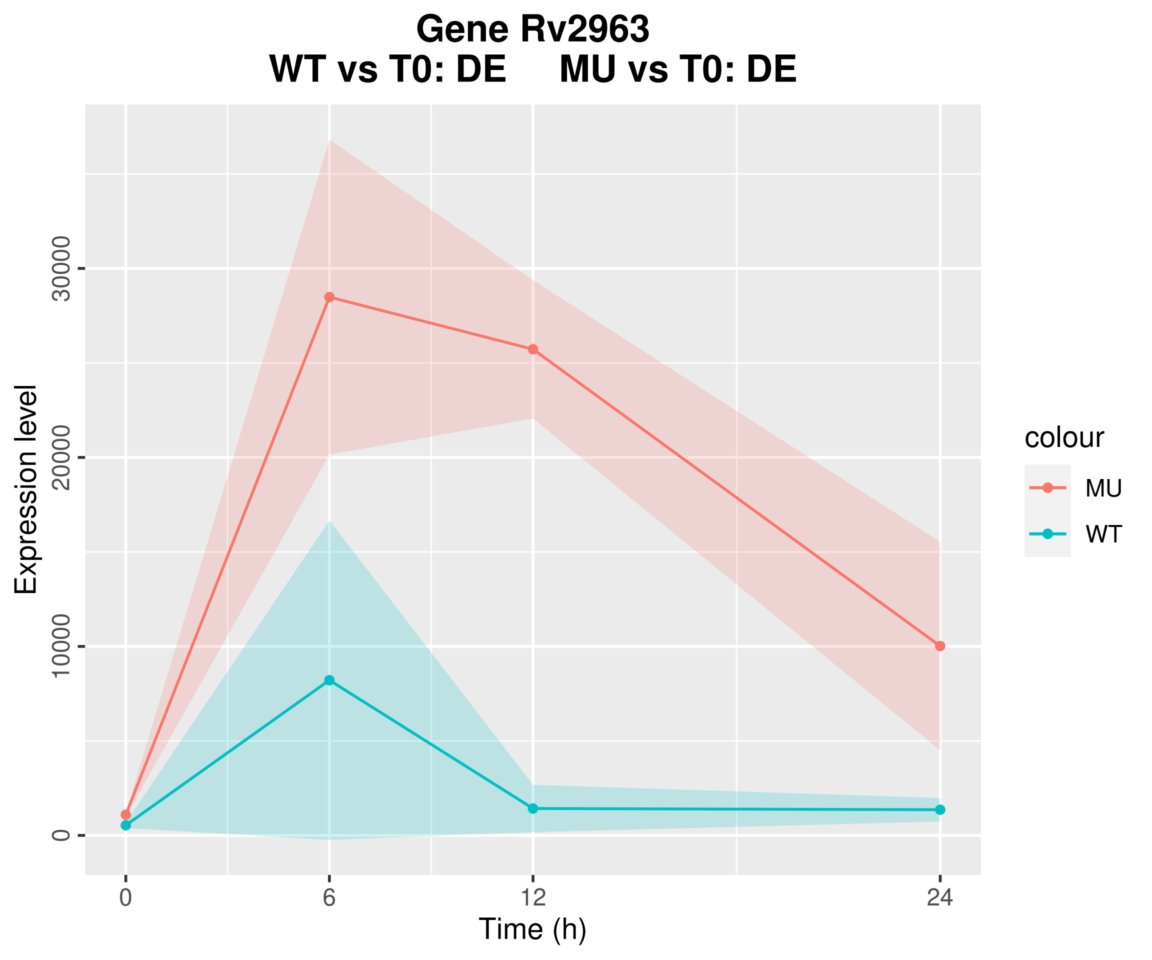
Gene Rv2963
WT vs T0: DE MU vs T0: DE
Expression level
30000
20000
10000
0
0
6
12
24
Time (h)
colour
MU
WT

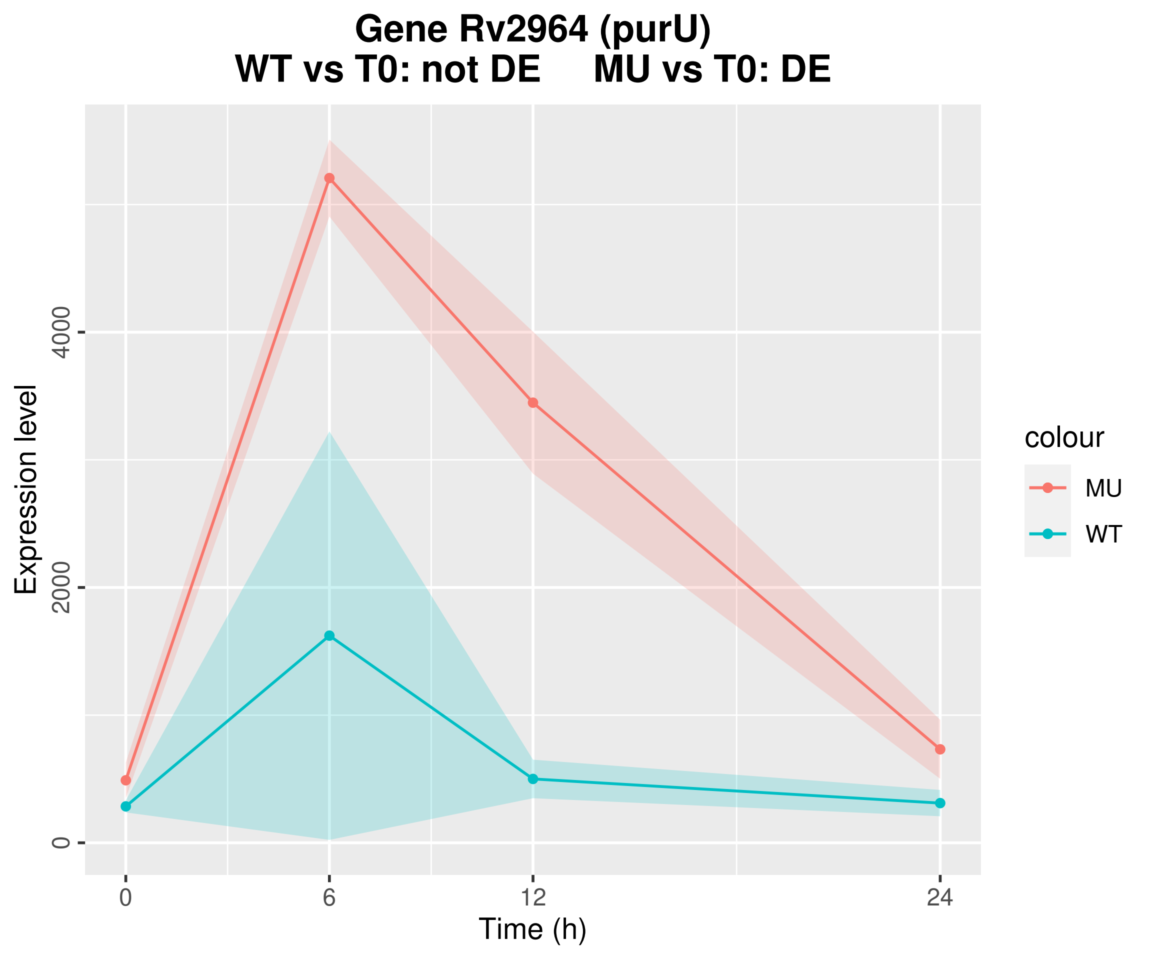
Gene Rv2964 (purU)
WT vs T0: not DE   MU vs T0: DE
Expression level
0
2000
4000
0
6
12
24
Time (h)
colour
MU
WT

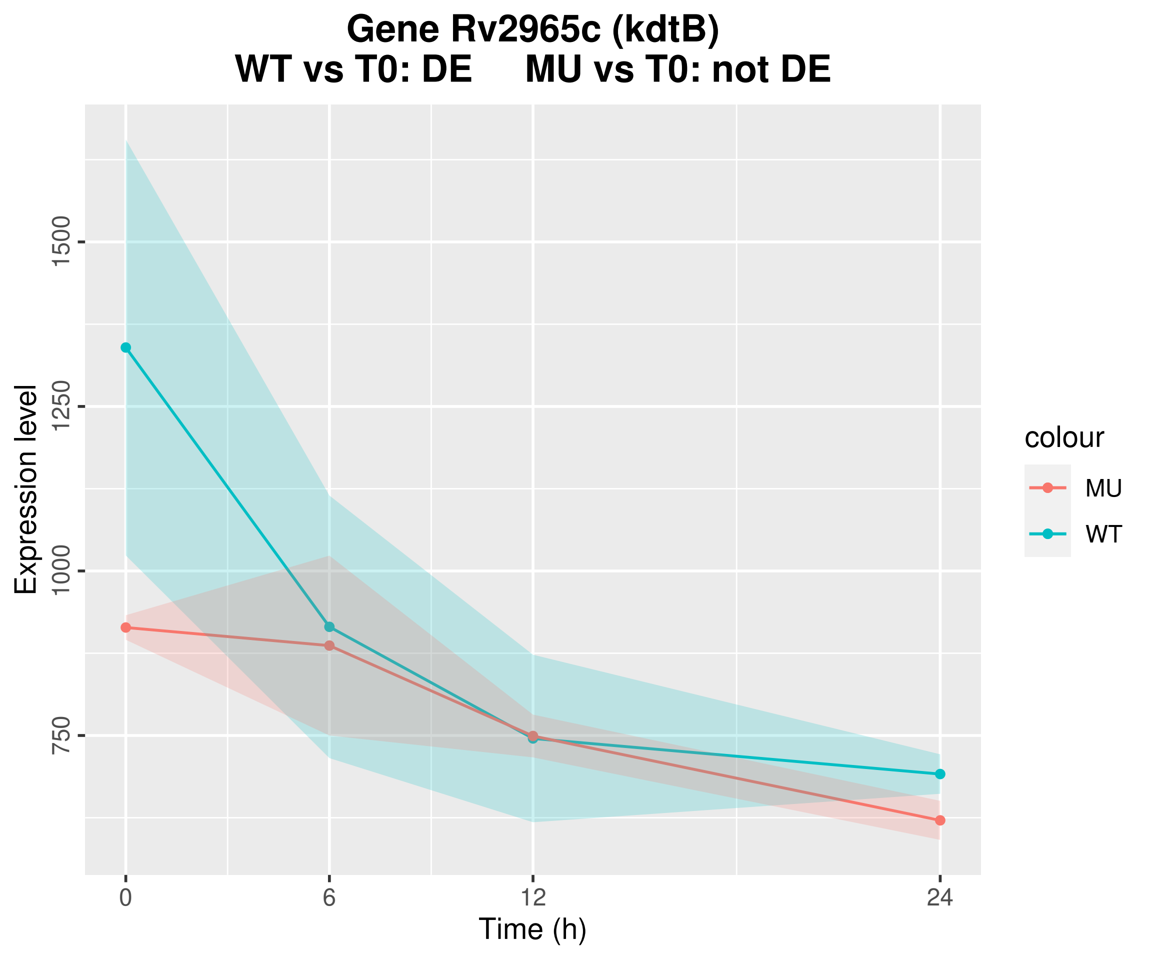
Gene Rv2965c (kdtB)
WT vs T0: DE   MU vs T0: not DE
Expression level
1500
1250
1000
750
0
6
12
24
Time (h)
colour
MU
WT

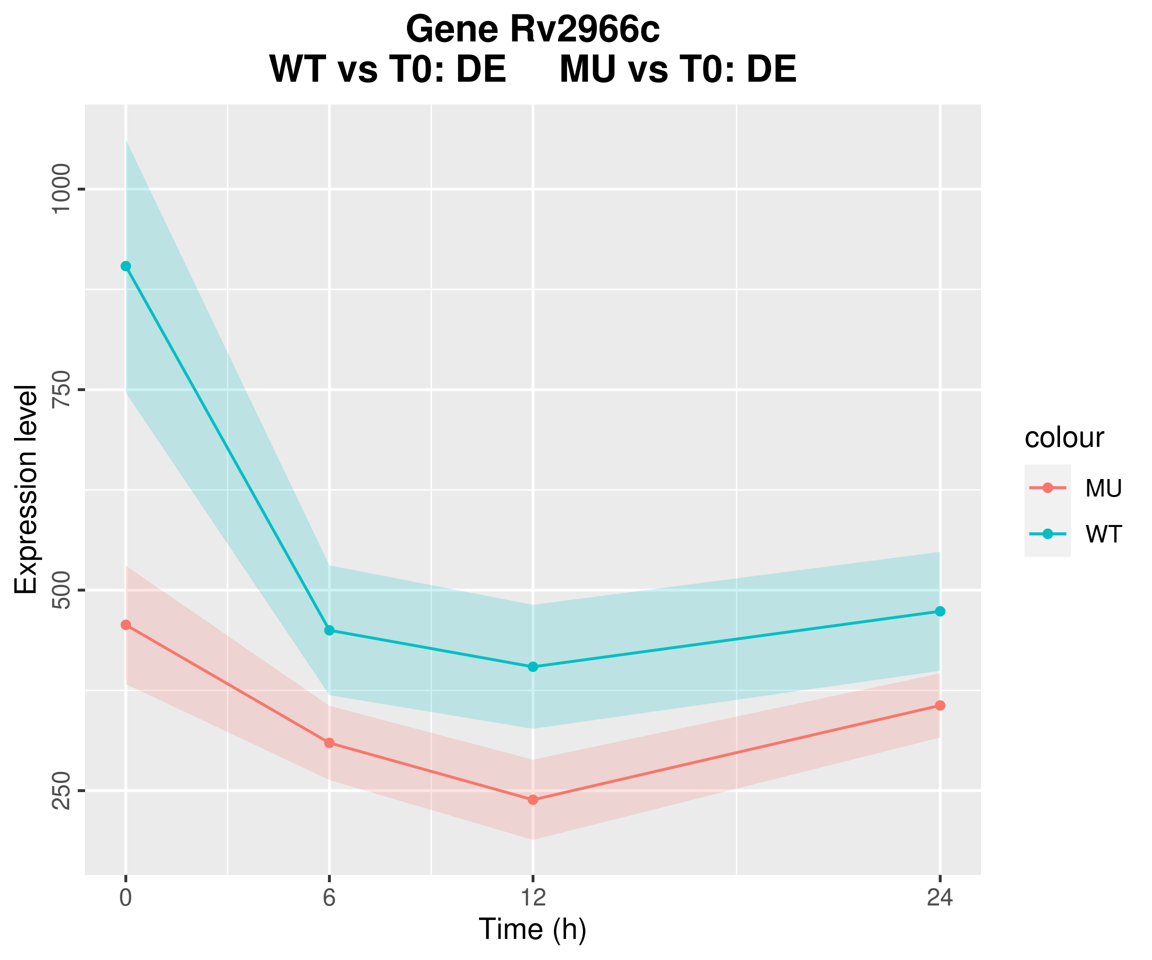

Gene Rv2966c
WT vs T0: DE MU vs T0: DE
Expression level
1000
750
500
250
0
6
12
24
Time (h)
colour
MU
WT

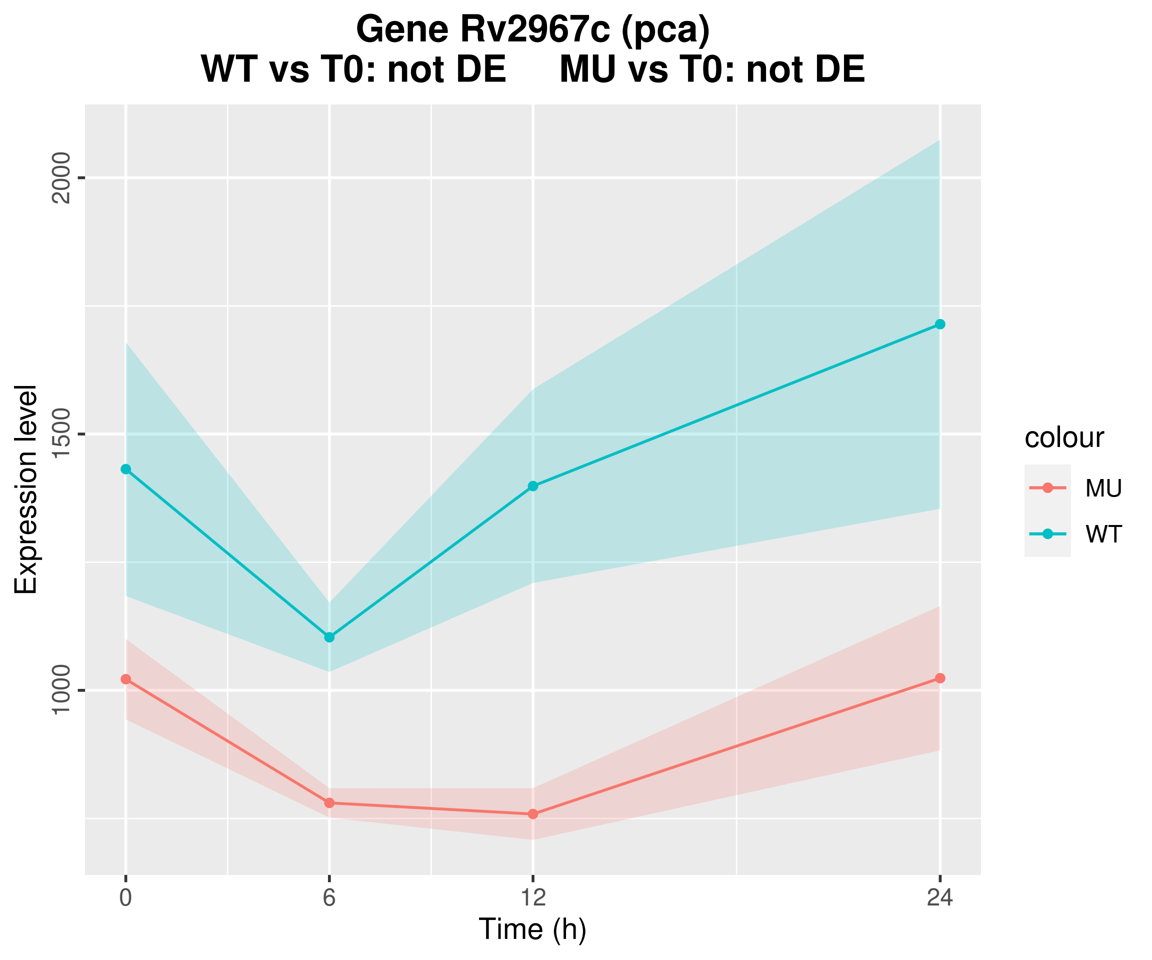

Gene Rv2967c (pca)
WT vs T0: not DE   MU vs T0: not DE
Expression level
2000
1500
1000
0
6
12
24
Time (h)
colour
MU
WT

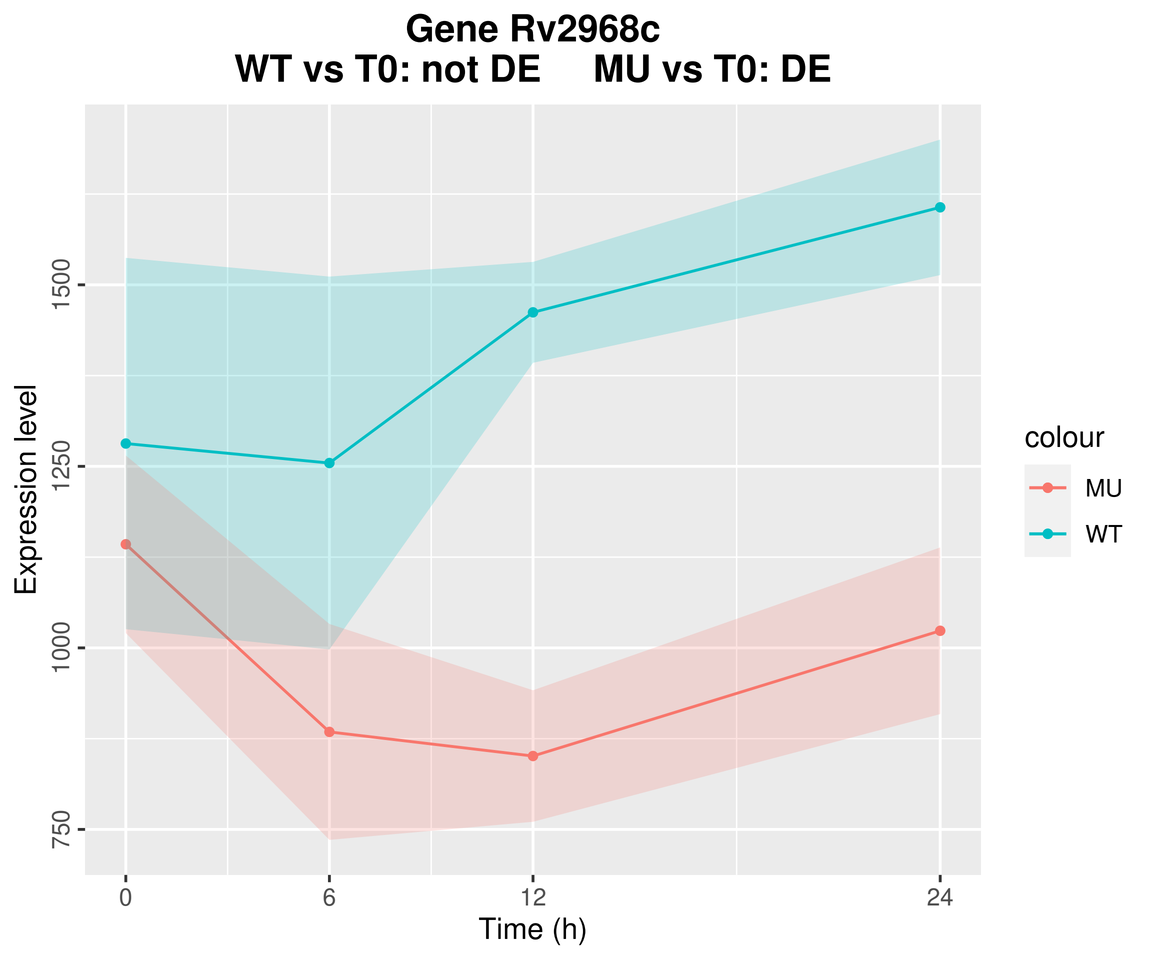

Gene Rv2968c
WT vs T0: not DE   MU vs T0: DE
Expression level
1500
1250
1000
750
0
6
12
24
Time (h)
colour
MU
WT

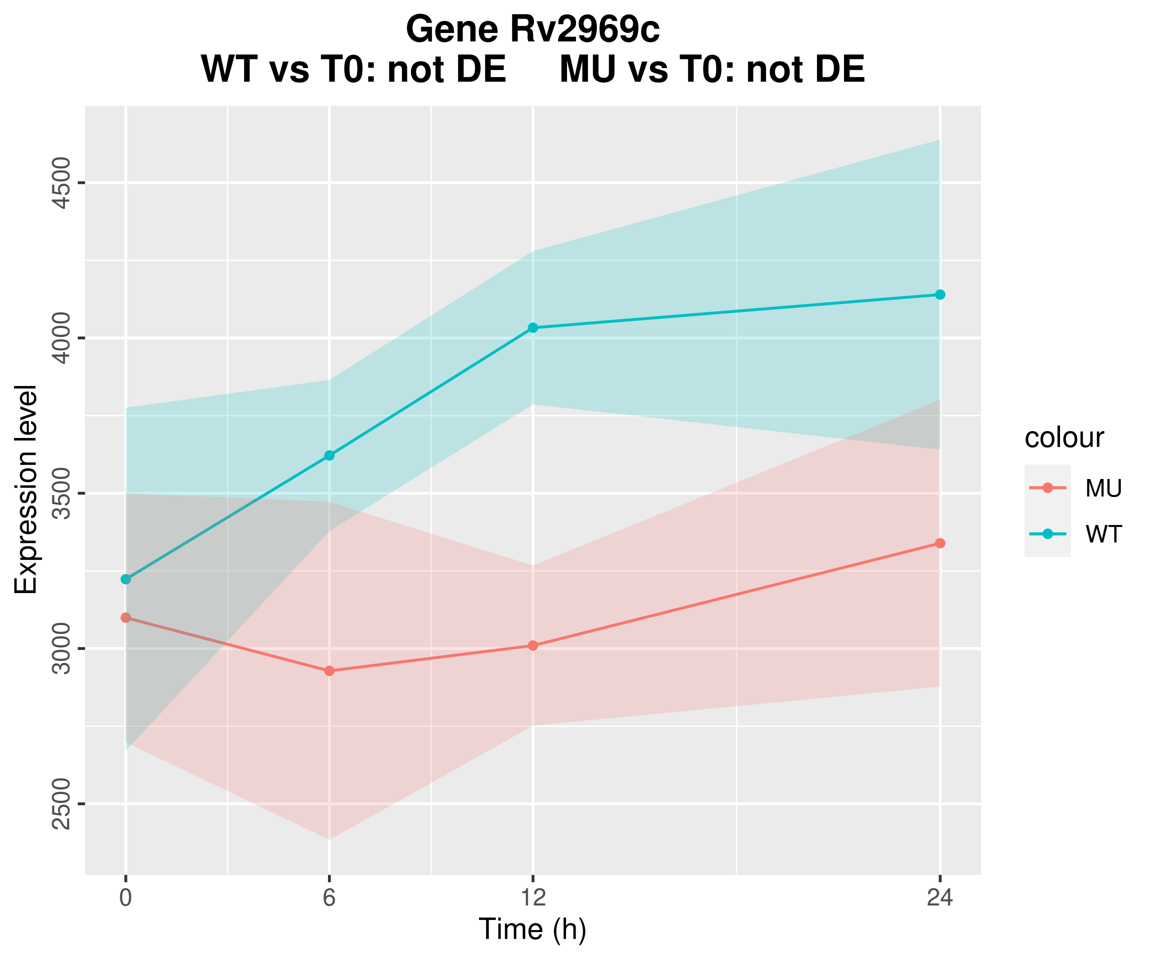
Gene Rv2969c
WT vs T0: not DE   MU vs T0: not DE
Expression level
4500
4000
3500
3000
2500
0
6
12
24
Time (h)
colour
MU
WT

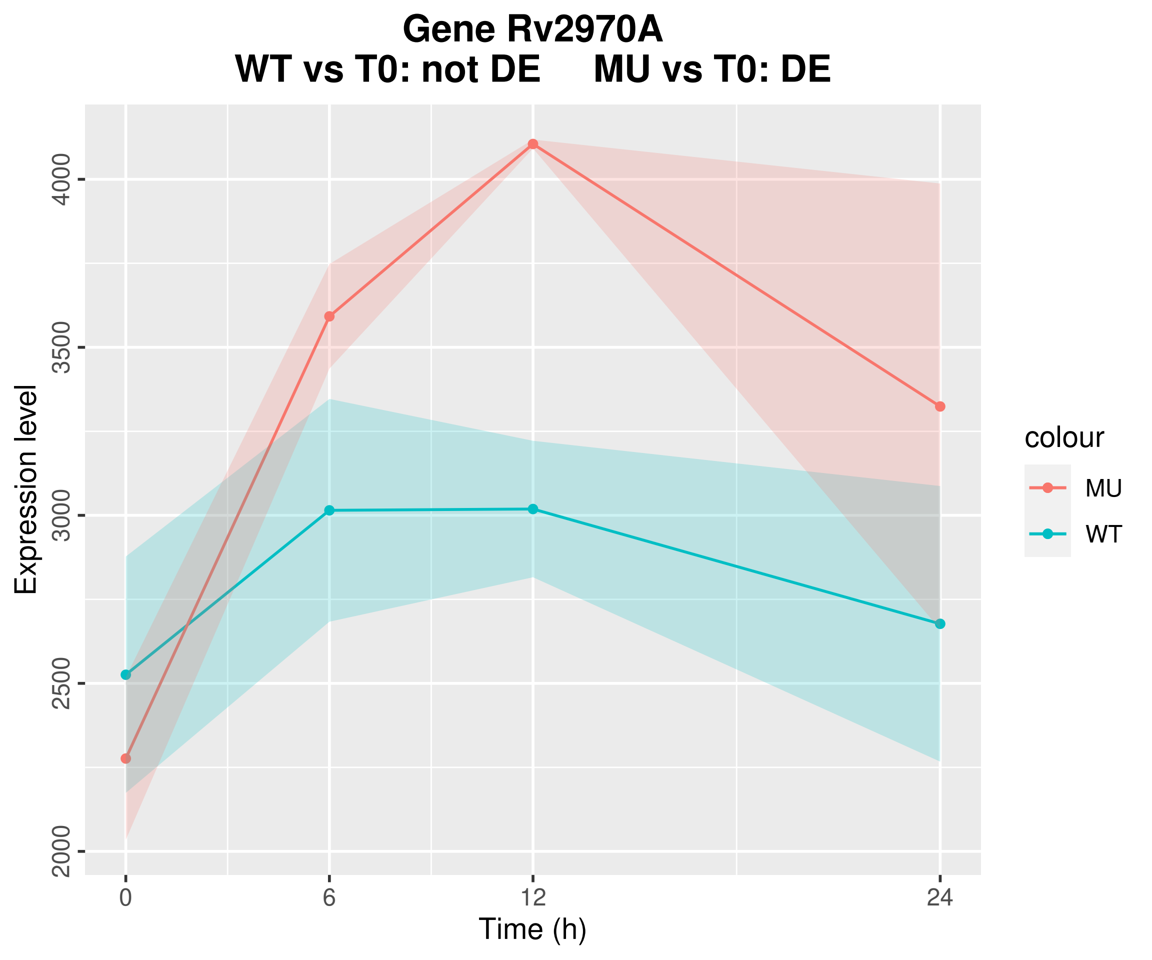
Gene Rv2970A
WT vs T0: not DE MU vs T0: DE
Expression level
4000
3500
3000
2500
2000
0
6
12
24
Time (h)
colour
MU
WT

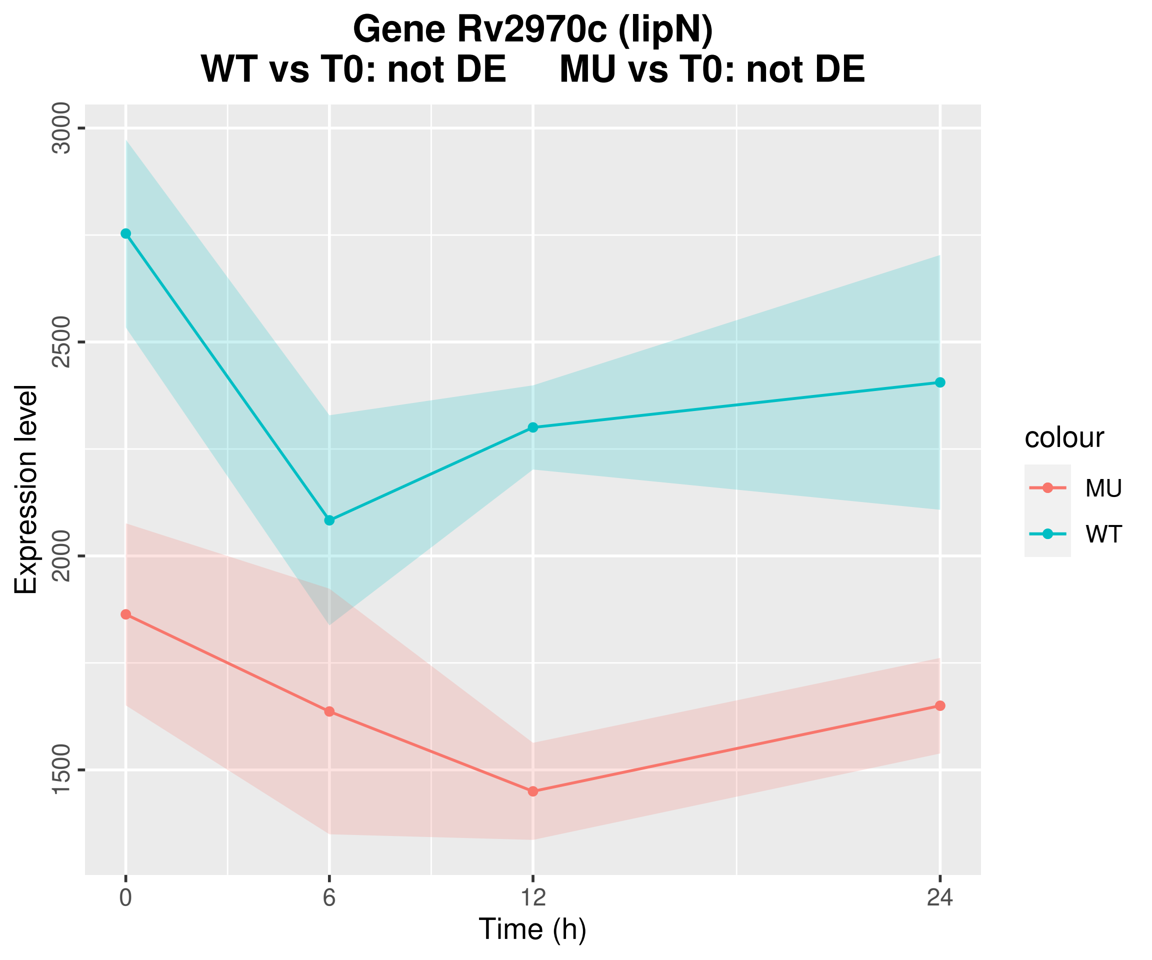

Gene Rv2970c (lipN)
WT vs T0: not DE   MU vs T0: not DE
Expression level
3000
2500
2000
1500
0
6
12
24
Time (h)
colour
MU
WT

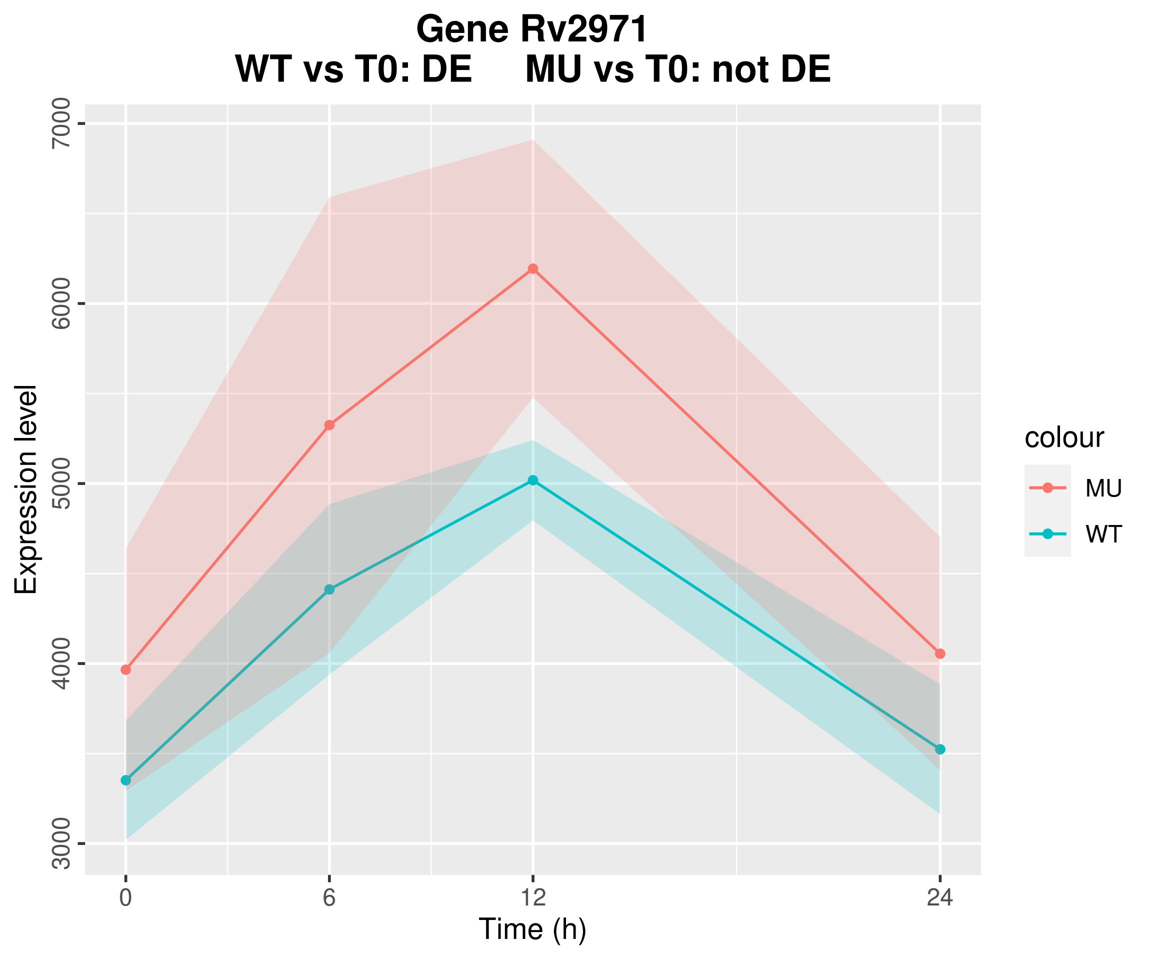
Gene Rv2971
WT vs T0: DE   MU vs T0: not DE
Expression level
7000
6000
5000
4000
3000
0
6
12
24
Time (h)
colour
MU
WT

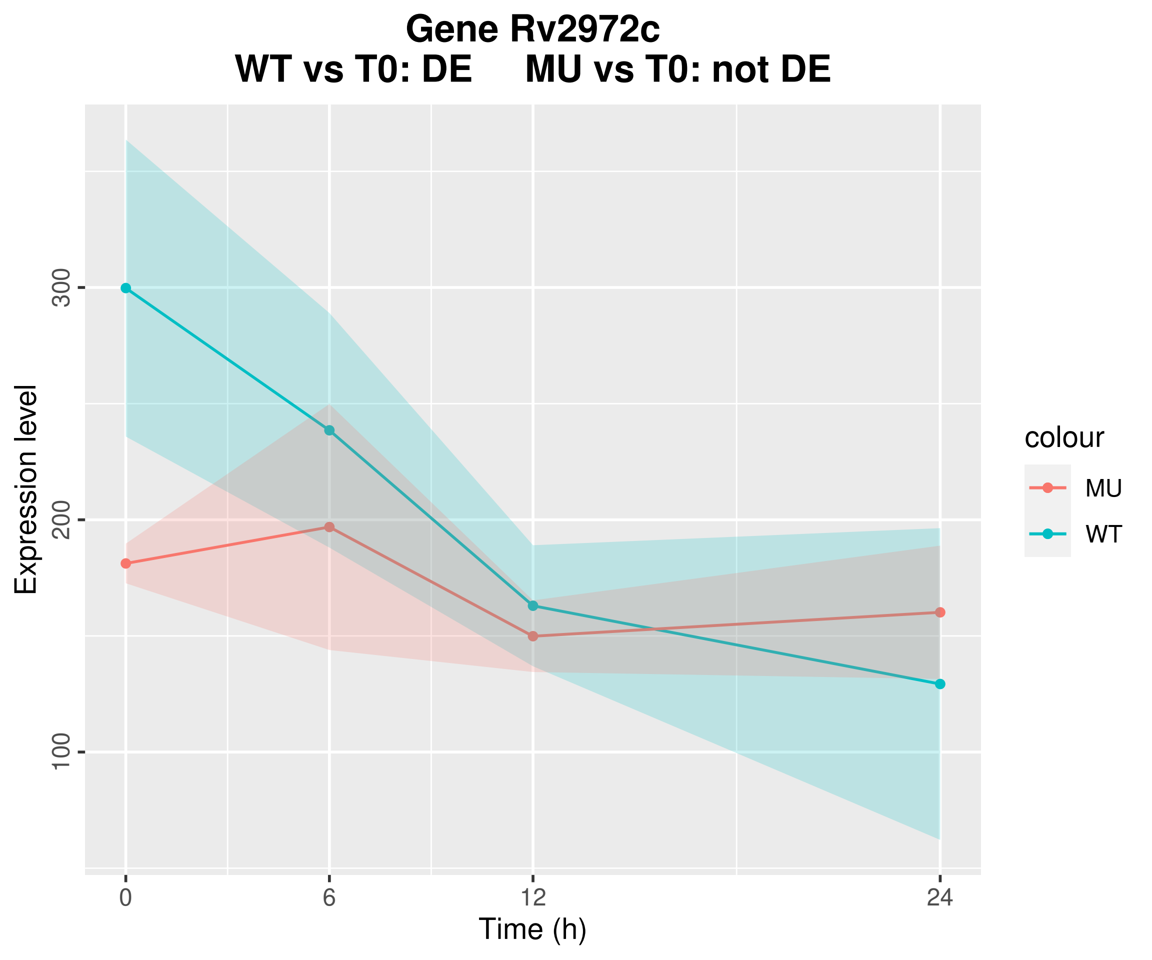

Gene Rv2972c
WT vs T0: DE   MU vs T0: not DE
Expression level
300
200
100
0
6
12
24
Time (h)
colour
MU
WT

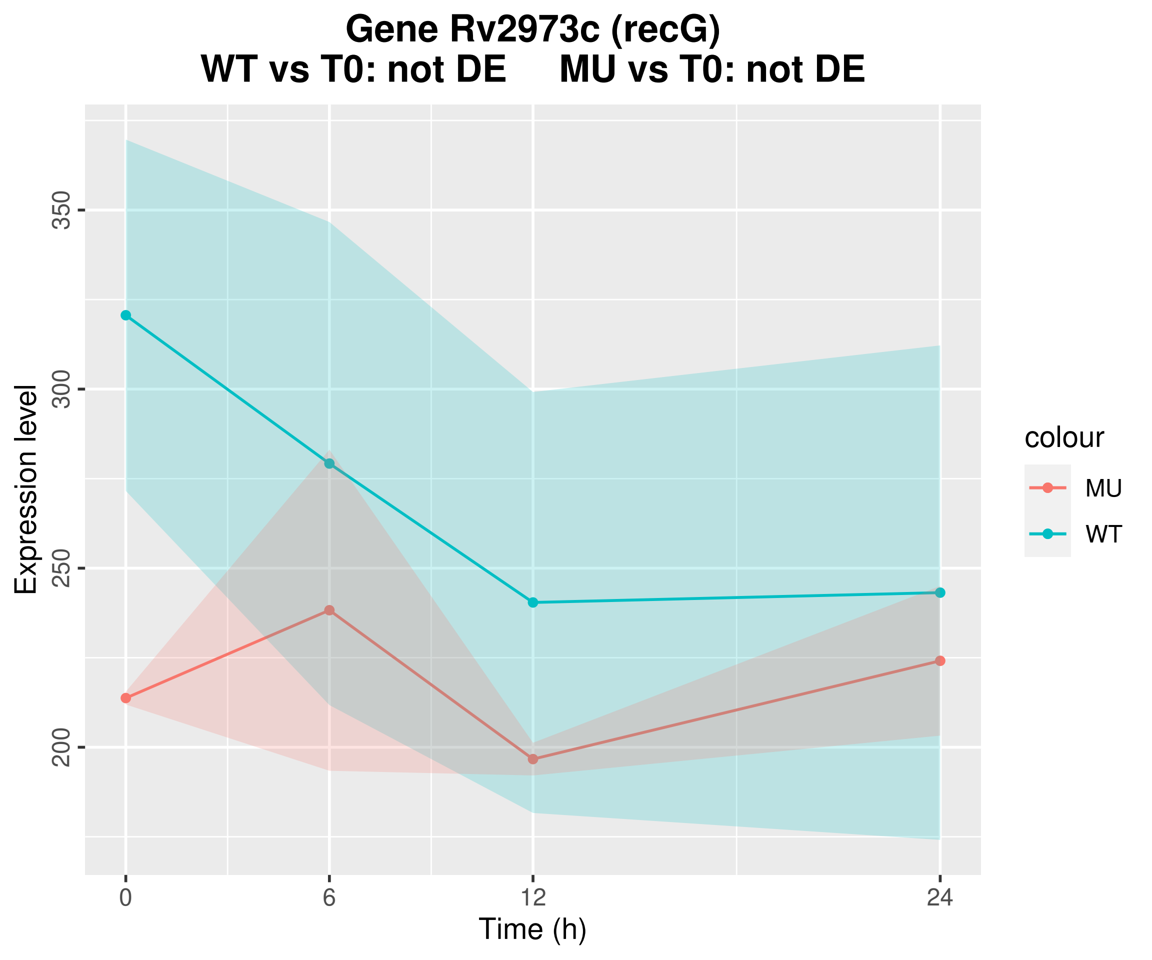
Gene Rv2973c (recG)
WT vs T0: not DE   MU vs T0: not DE
Expression level
350
300
250
200
0
6
12
24
Time (h)
colour
MU
WT

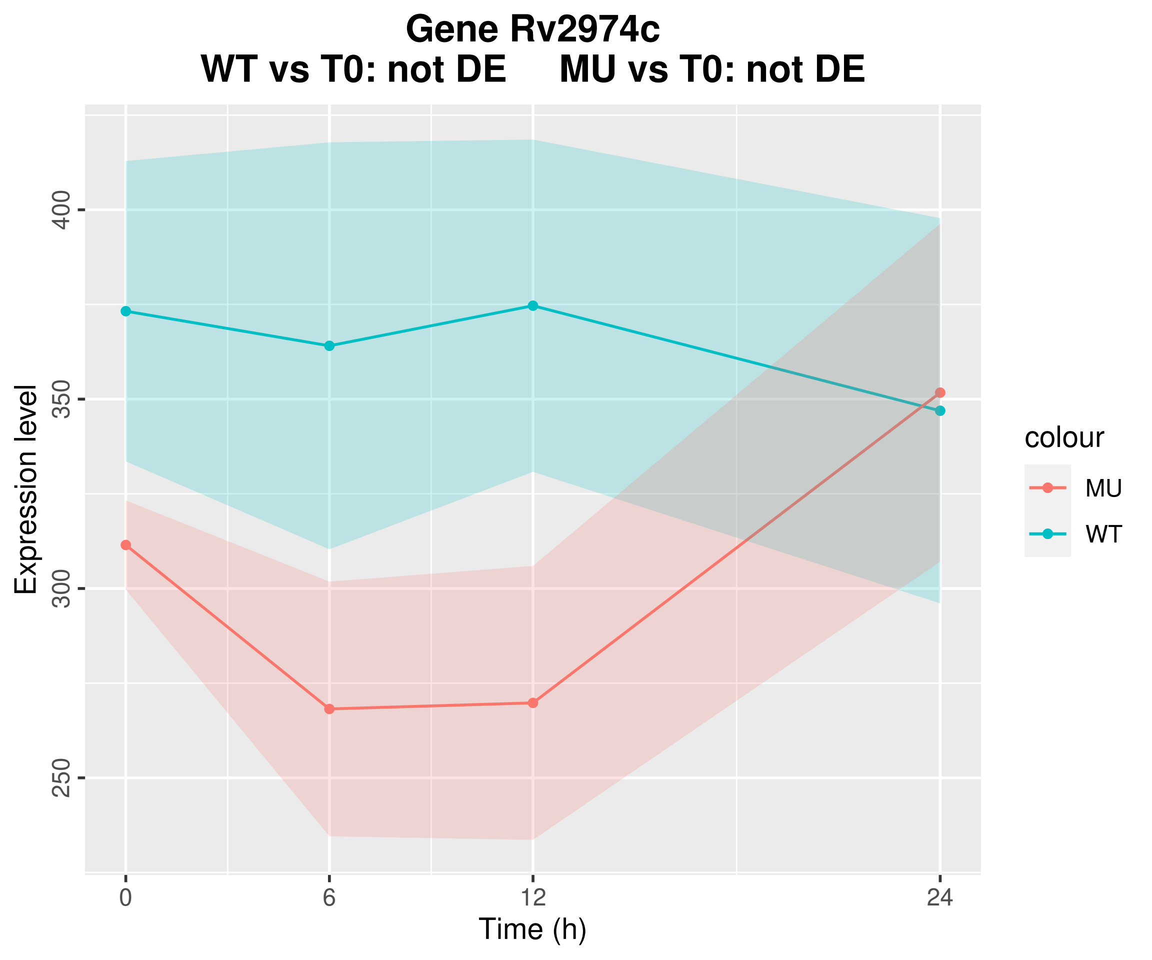

Gene Rv2974c
WT vs T0: not DE   MU vs T0: not DE
Expression level
400
350
300
250
0
6
12
24
Time (h)
colour
MU
WT

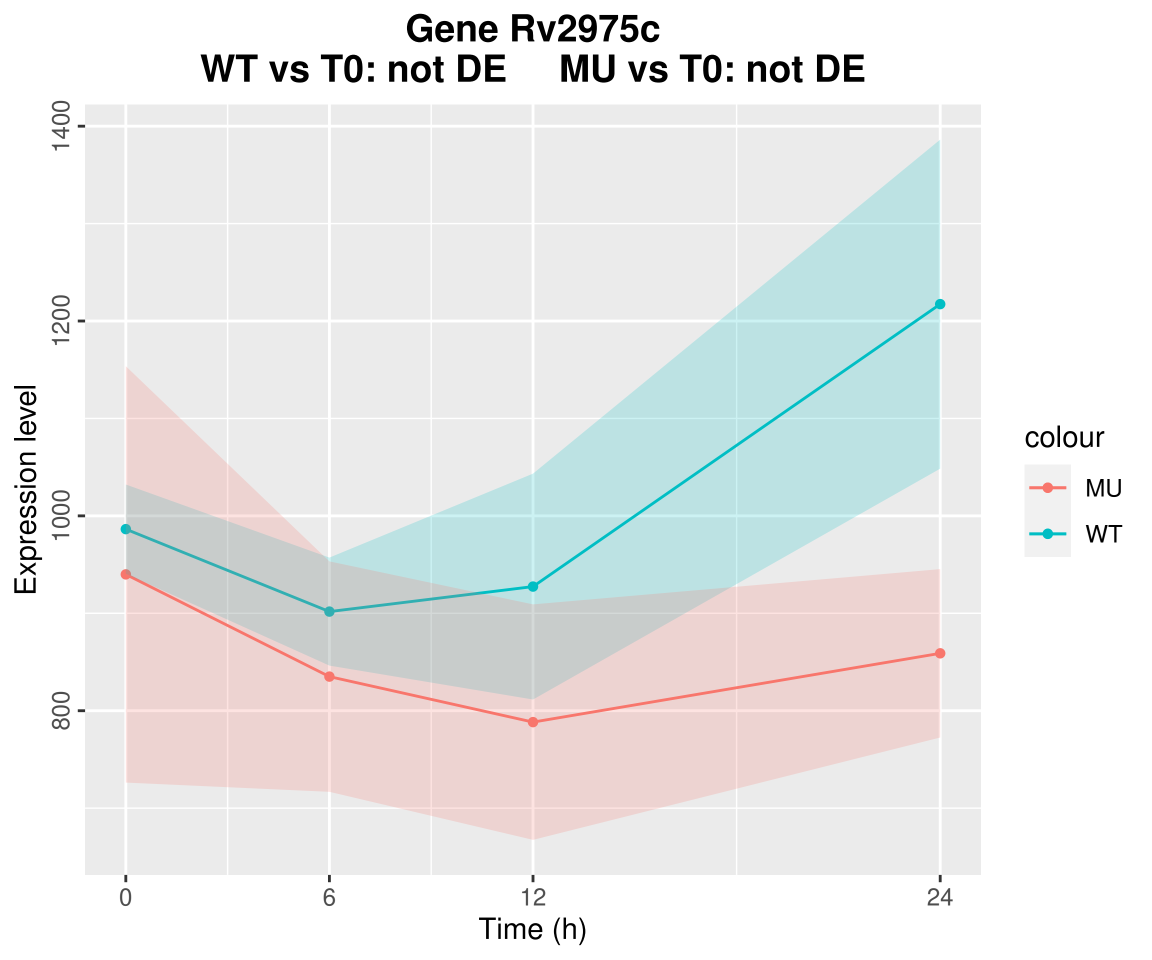
Gene Rv2975c
WT vs T0: not DE MU vs T0: not DE
Expression level
1400
1200
1000
800
0
6
12
24
Time (h)
colour
MU
WT

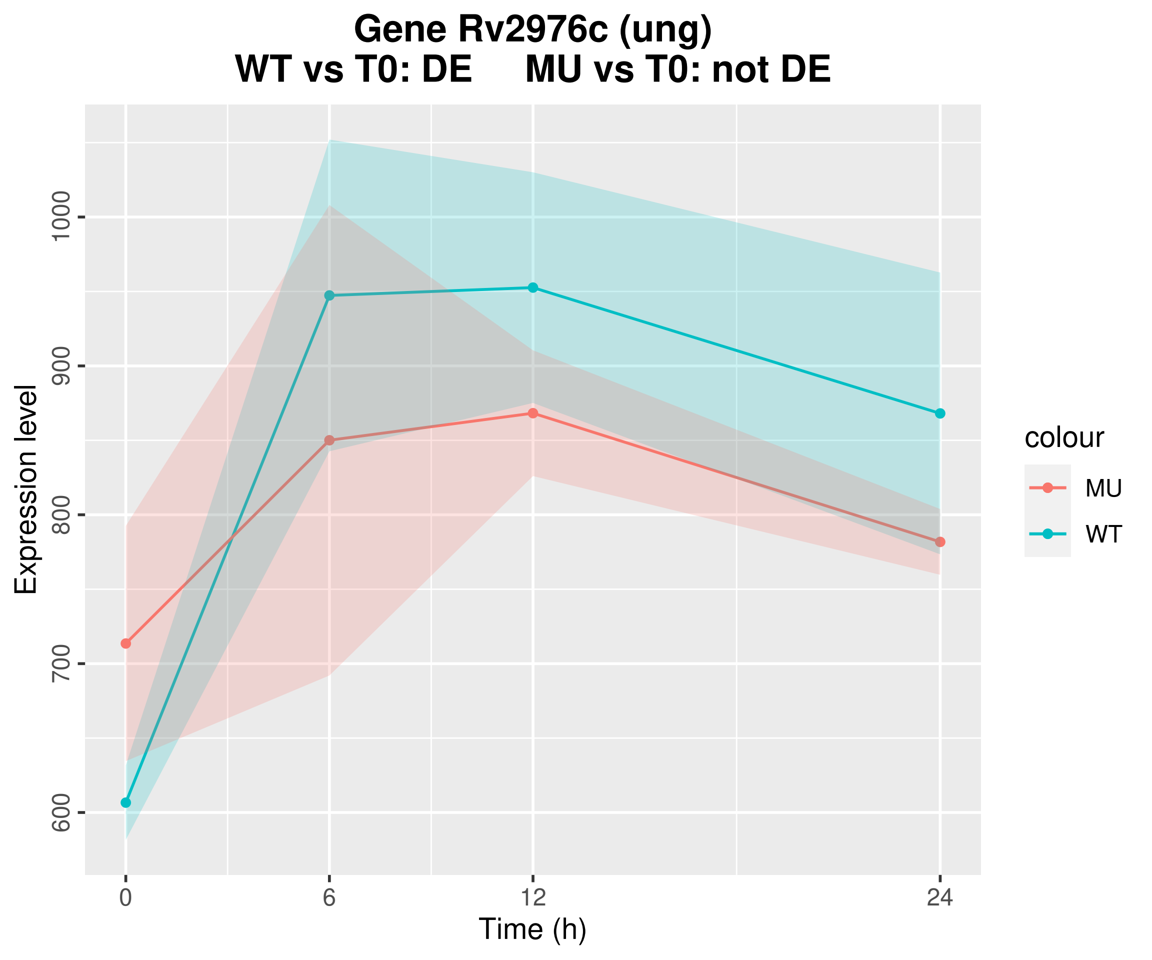

Gene Rv2976c (ung)
WT vs T0: DE   MU vs T0: not DE
Expression level
1000
900
800
700
600
0
6
12
24
Time (h)
colour
MU
WT

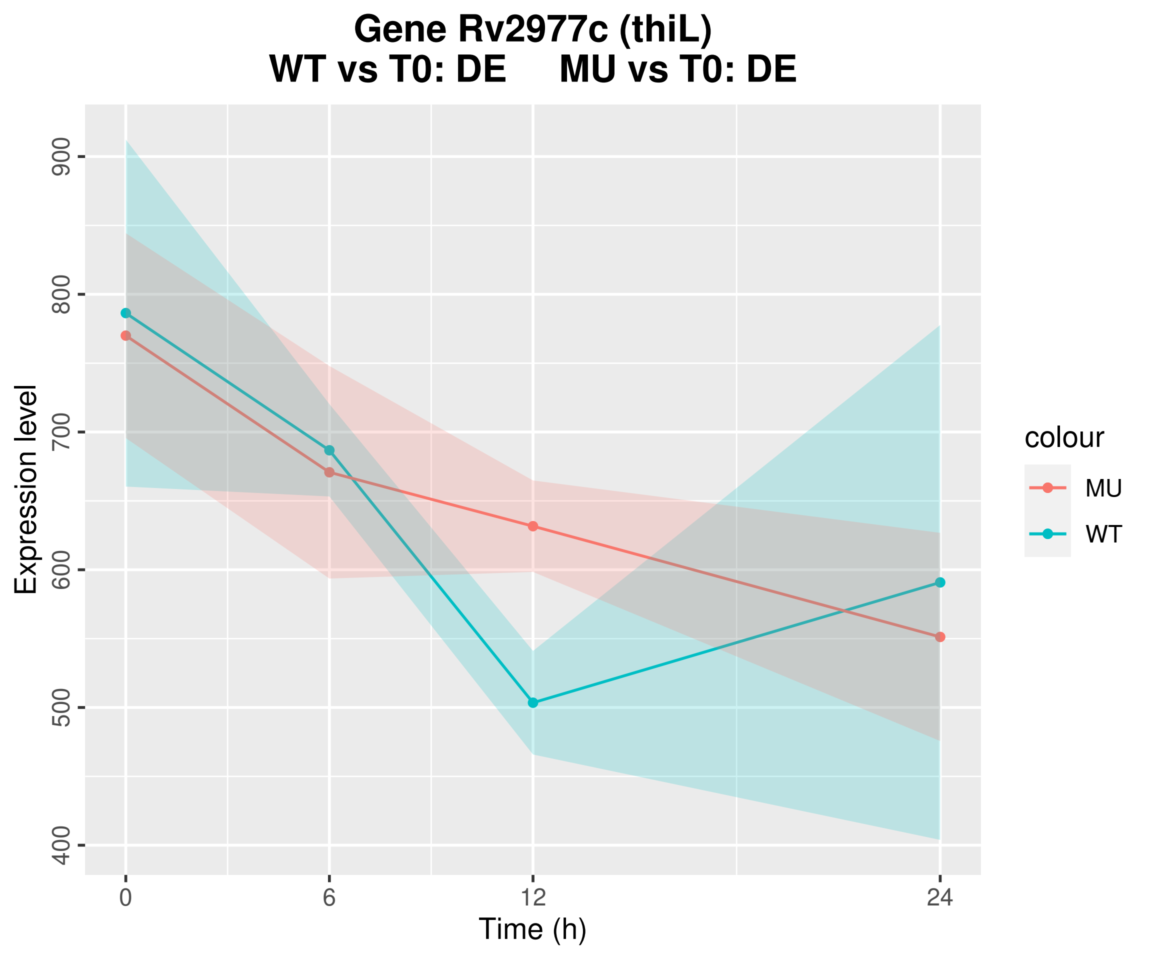
Gene Rv2977c (thiL)
WT vs T0: DE MU vs T0: DE
Expression level
900
800
700
600
500
400
0
6
12
24
Time (h)
colour
MU
WT

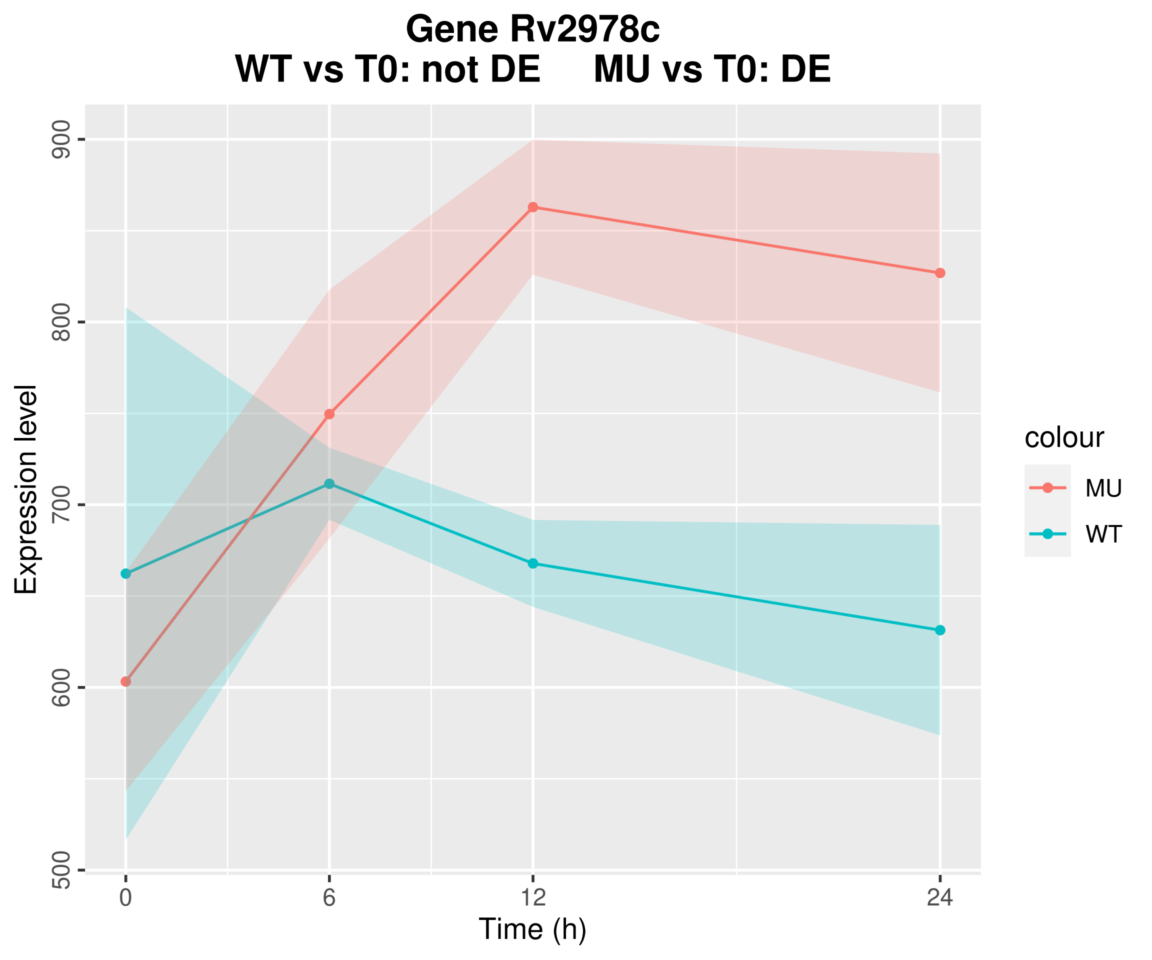
Gene Rv2978c
WT vs T0: not DE   MU vs T0: DE
Expression level
900
800
700
600
500
0
6
12
24
Time (h)
colour
MU
WT

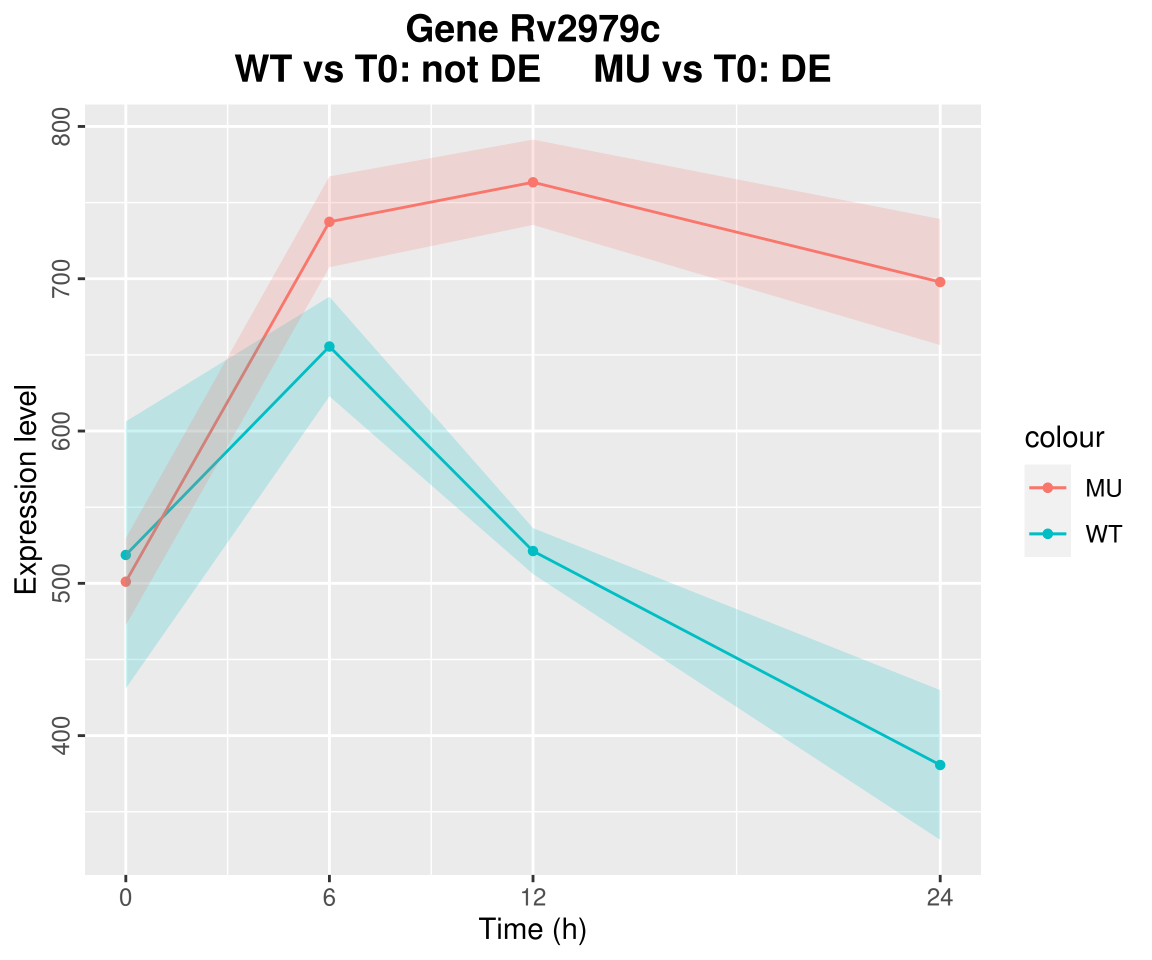

Gene Rv2979c
WT vs T0: not DE   MU vs T0: DE
800
700
600
500
400
Expression level
0
6
12
24
Time (h)
colour
MU
WT

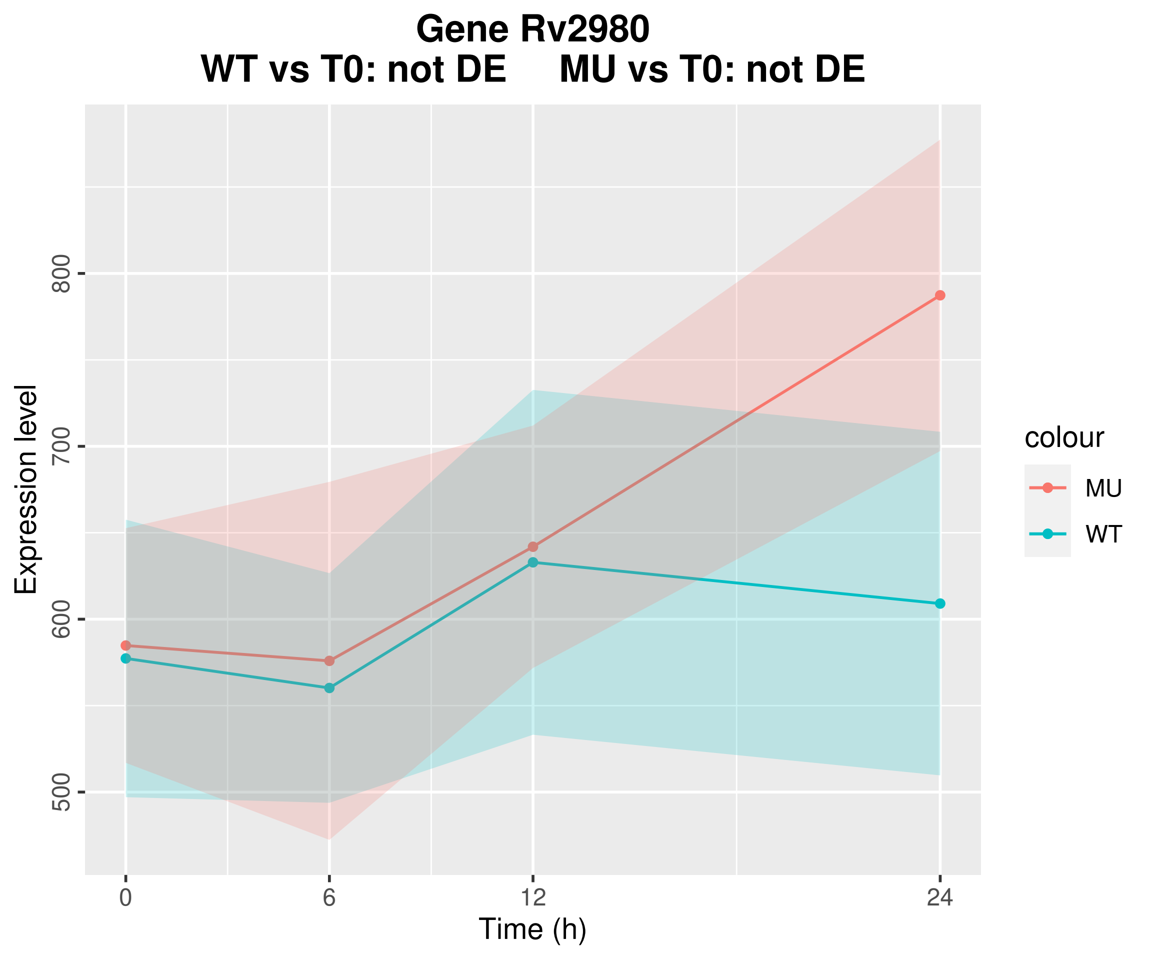

Gene Rv2980
WT vs T0: not DE   MU vs T0: not DE
Expression level
500
600
700
800
0
6
12
24
Time (h)
colour
MU
WT

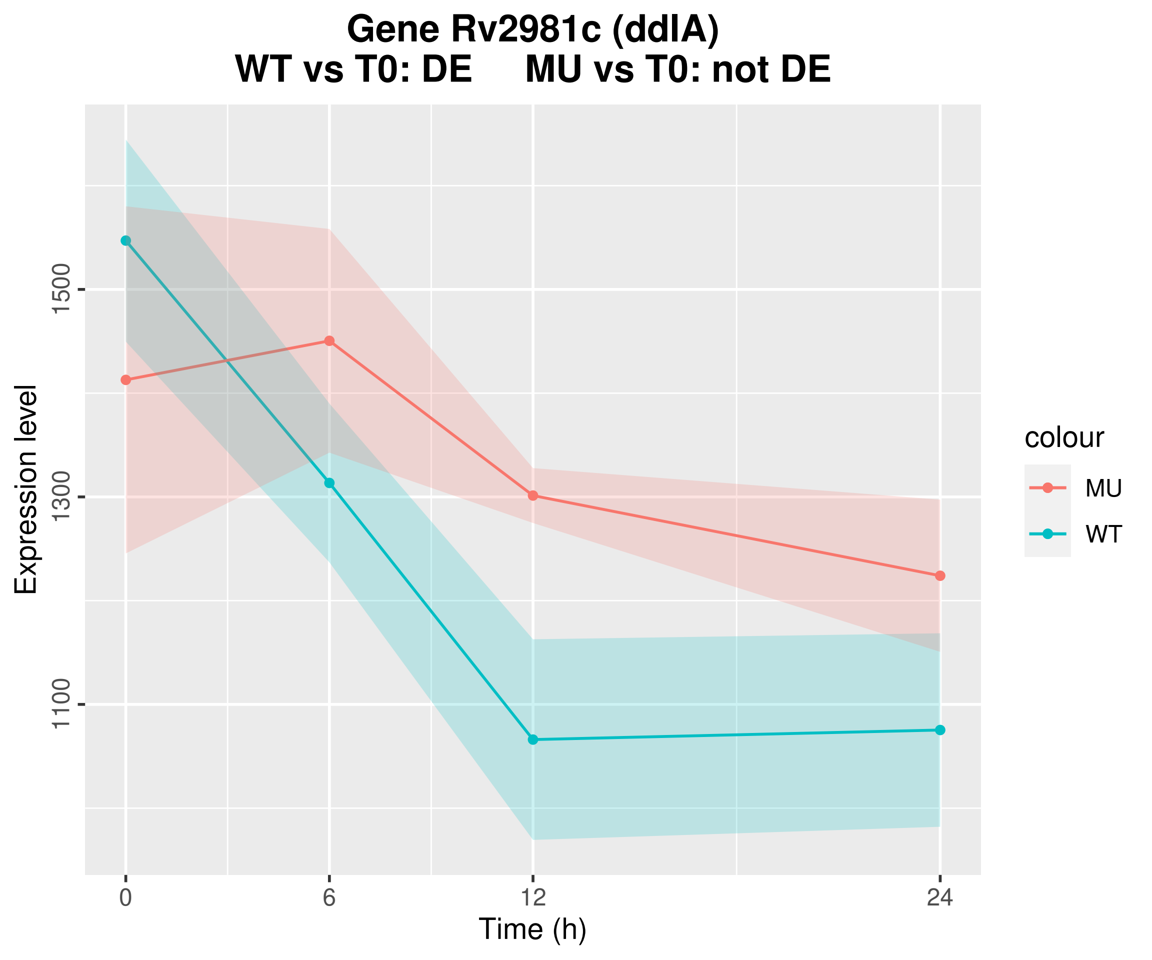

Gene Rv2981c (ddlA)
WT vs T0: DE   MU vs T0: not DE
Expression level
1500
1300
1100
0
6
12
24
Time (h)
colour
MU
WT

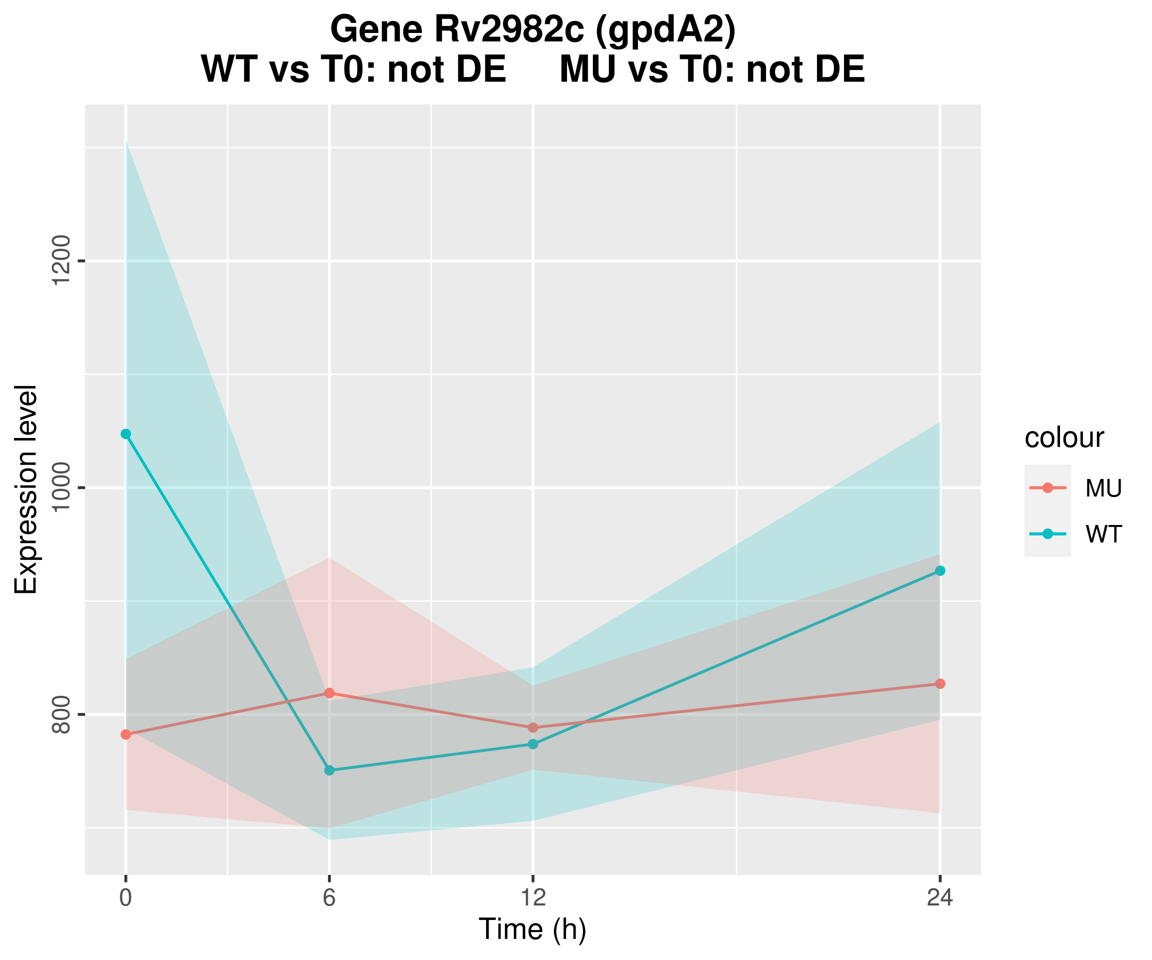
Gene Rv2982c (gpdA2)
WT vs T0: not DE   MU vs T0: not DE
Expression level
1200
1000
800
0
6
12
24
Time (h)
colour
MU
WT

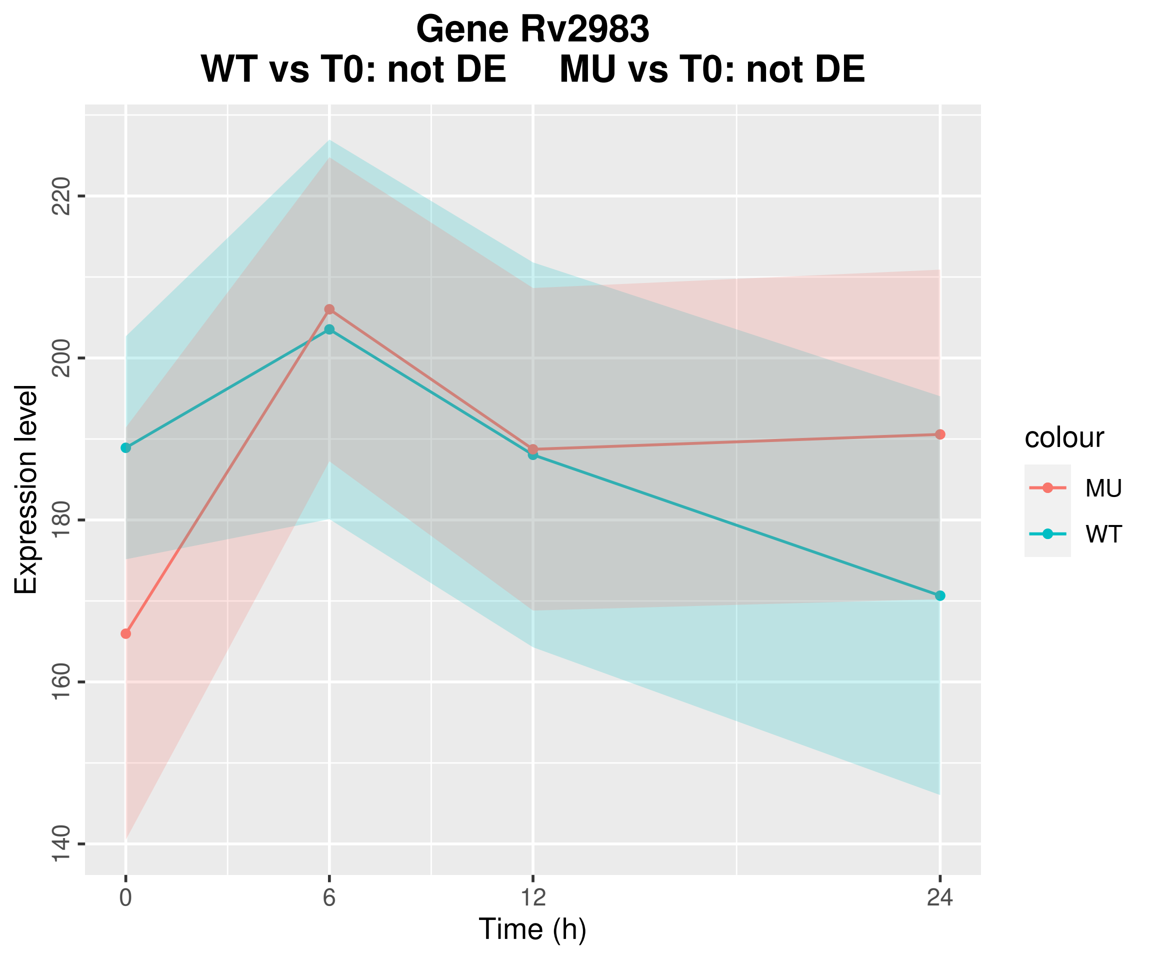

Gene Rv2983
WT vs T0: not DE MU vs T0: not DE
Expression level
220
200
180
160
140
0
6
12
24
Time (h)
colour
MU
WT

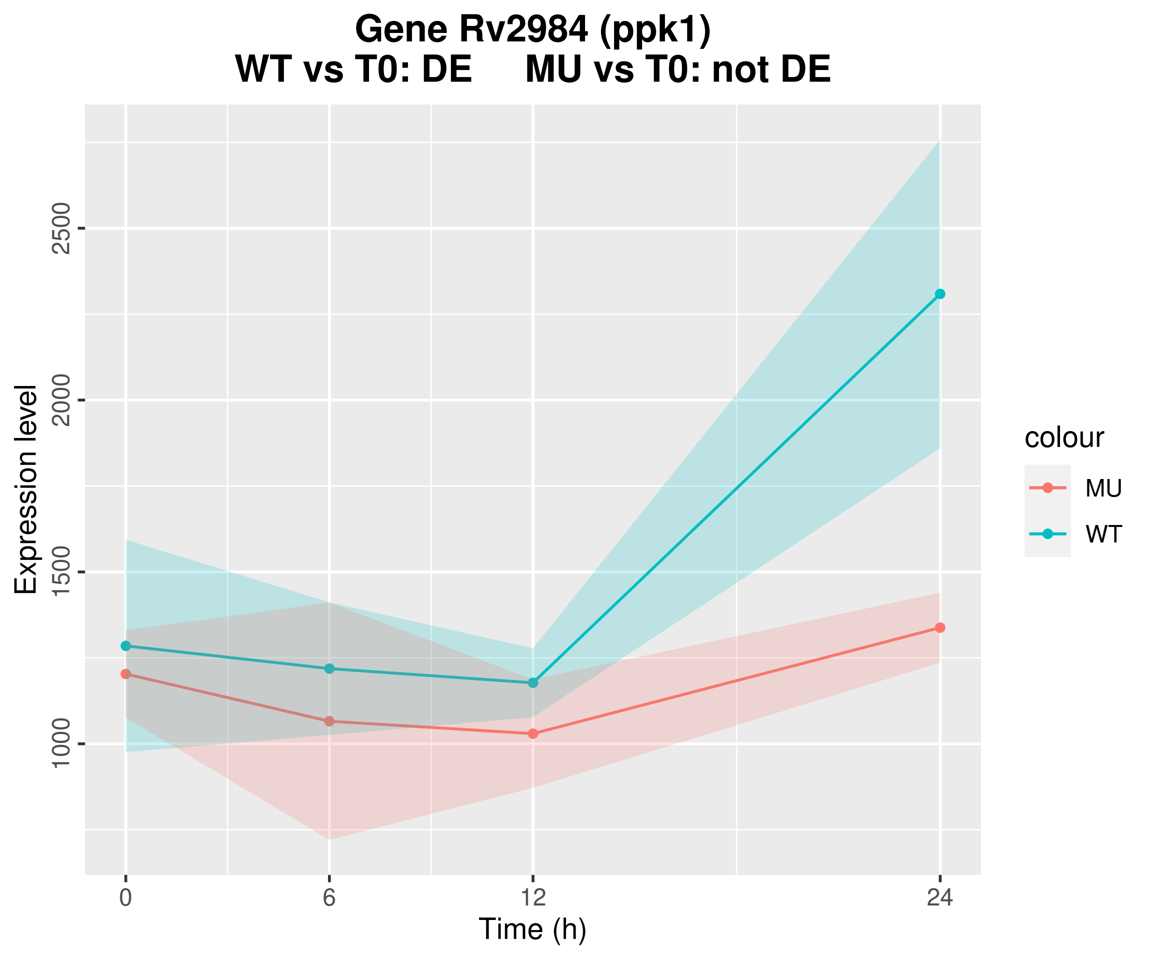

Gene Rv2984 (ppk1)
WT vs T0: DE   MU vs T0: not DE
Expression level
2500
2000
1500
1000
0
6
12
24
Time (h)
colour
MU
WT

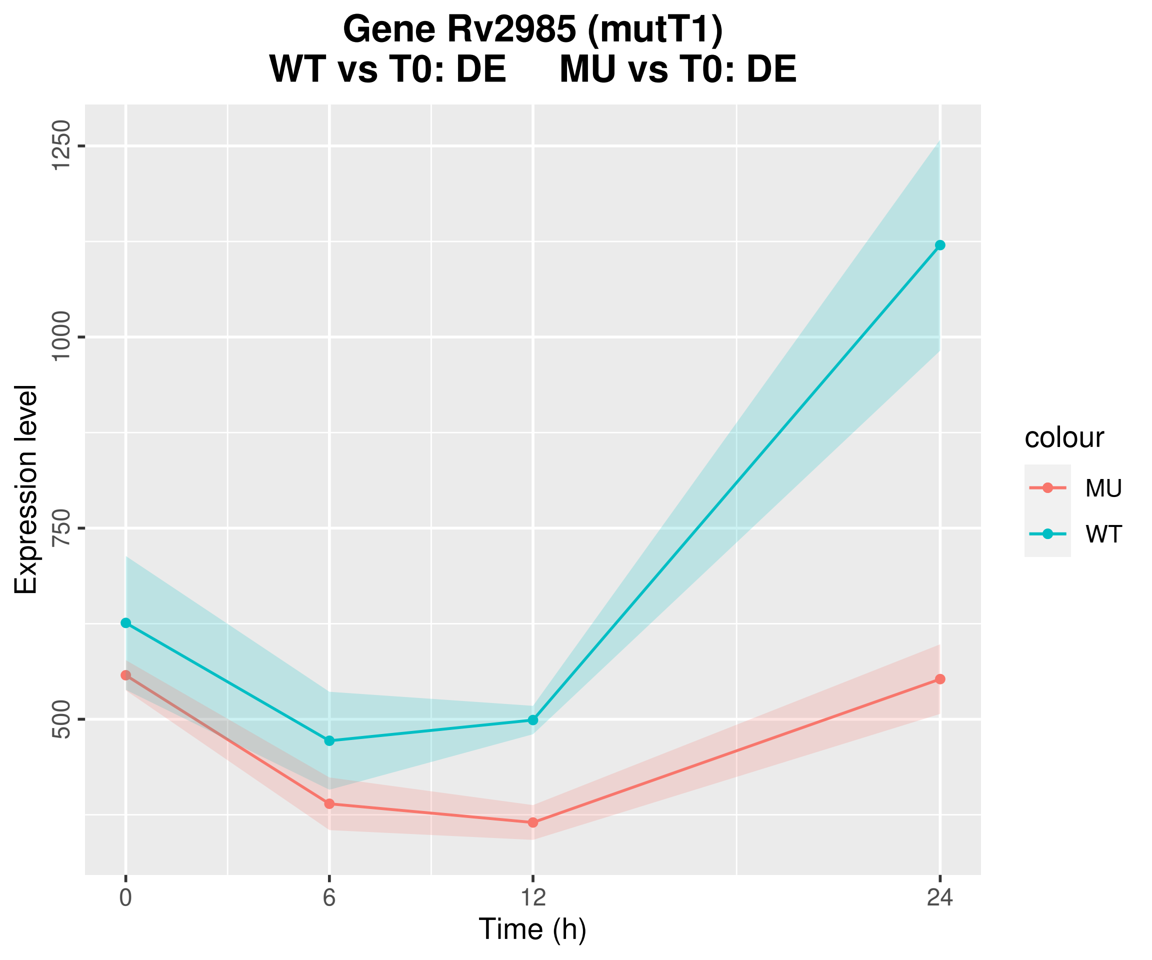
Gene Rv2985 (mutT1)
WT vs T0: DE   MU vs T0: DE
Expression level
1250
1000
750
500
0
6
12
24
Time (h)
colour
MU
WT

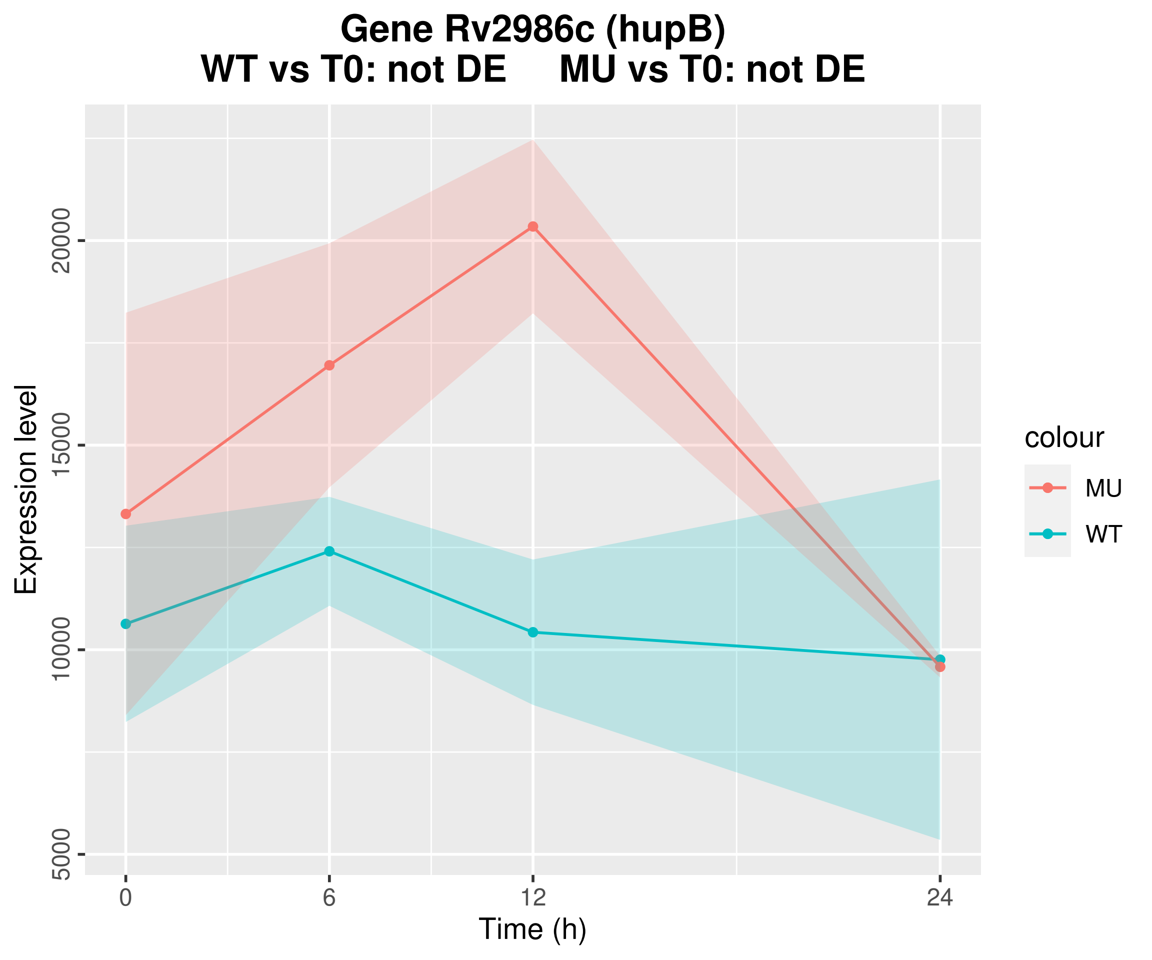

Gene Rv2986c (hupB)
WT vs T0: not DE MU vs T0: not DE
Expression level
20000
15000
10000
5000
0
6
12
24
Time (h)
colour
MU
WT

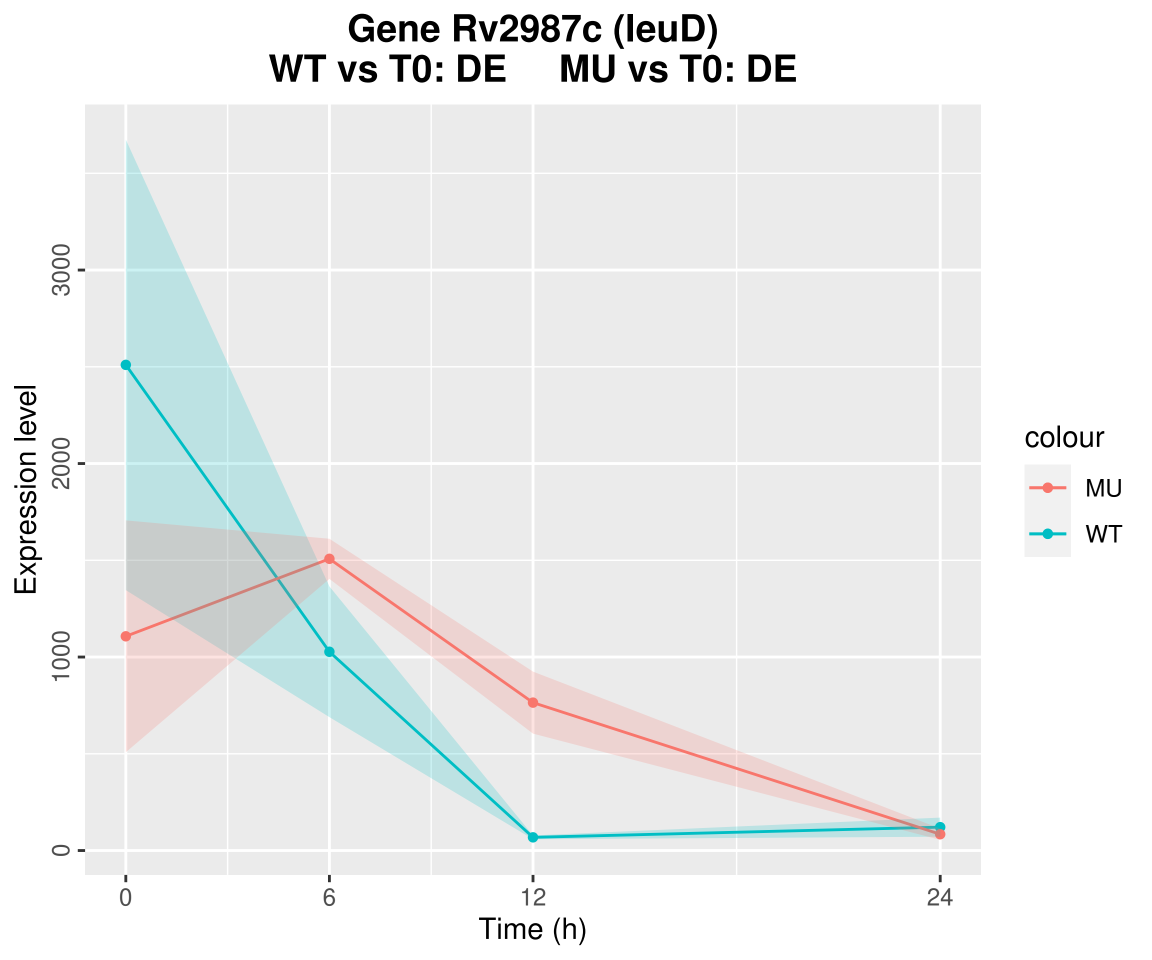
Gene Rv2987c (leuD)
WT vs T0: DE   MU vs T0: DE
Expression level
3000
2000
1000
0
0
6
12
24
Time (h)
colour
MU
WT

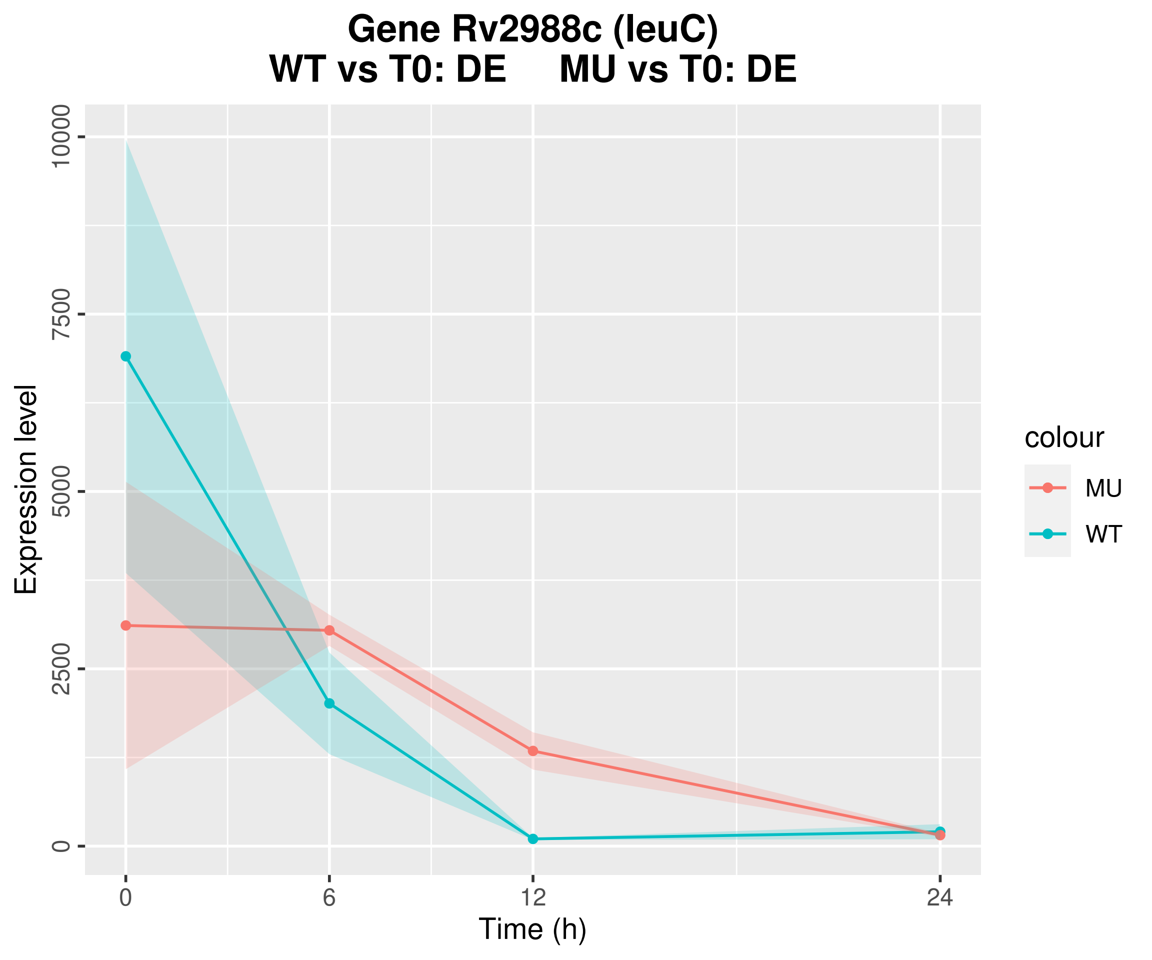
Gene Rv2988c (leuC)
WT vs T0: DE MU vs T0: DE
Expression level
10000
7500
5000
2500
0
0
6
12
24
Time (h)
colour
MU
WT

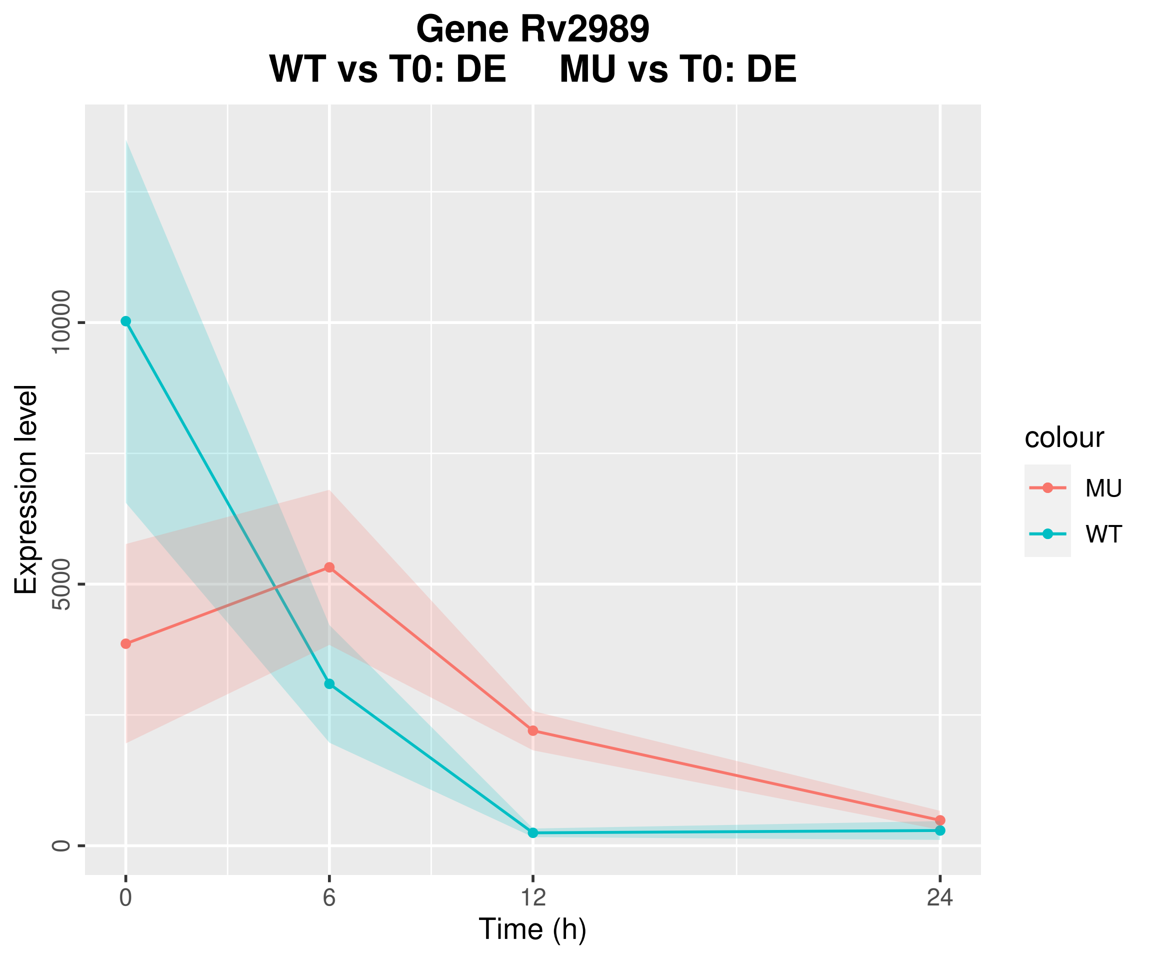
Gene Rv2989
WT vs T0: DE MU vs T0: DE
Expression level
10000
5000
0
0
6
12
24
Time (h)
colour
MU
WT

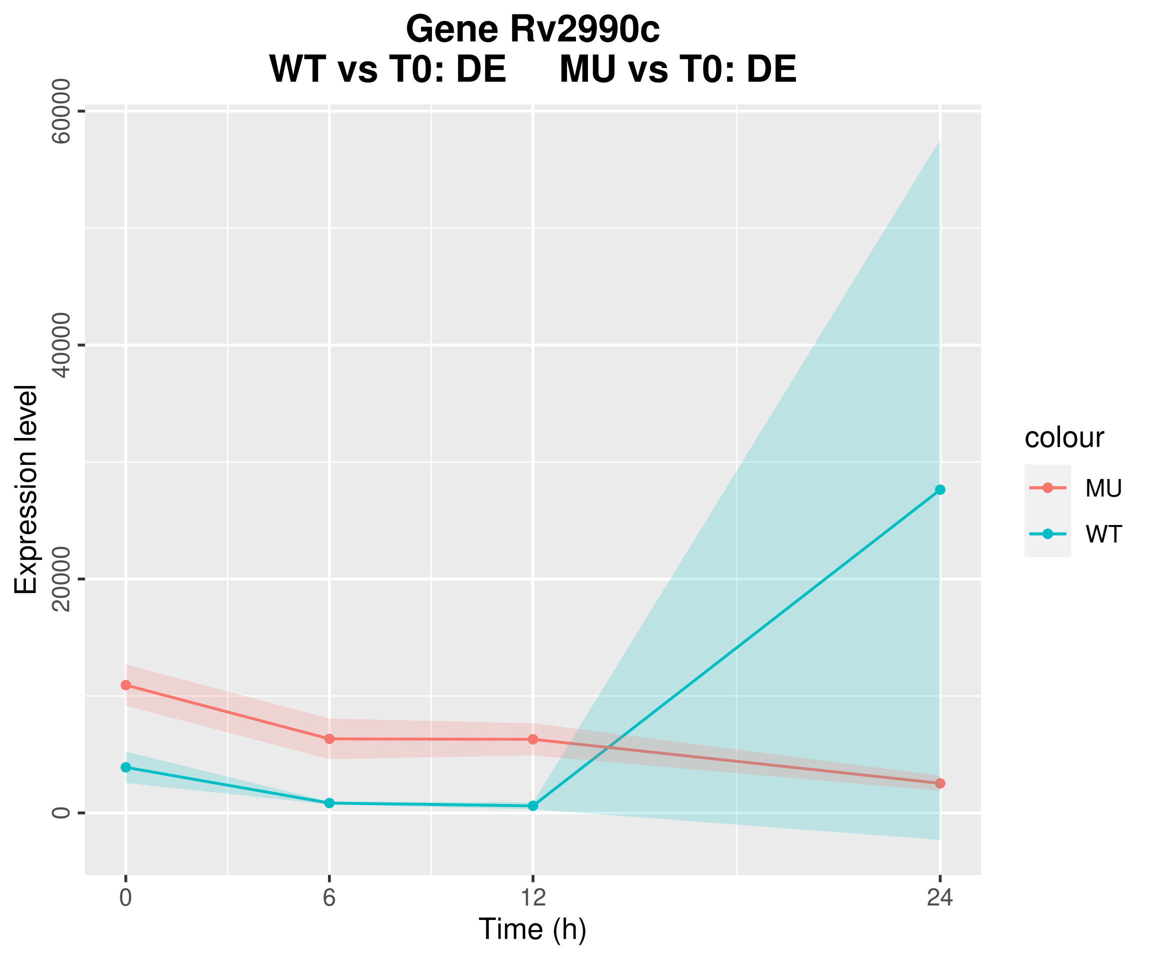

Gene Rv2990c
WT vs T0: DE   MU vs T0: DE
Expression level
60000
40000
20000
0
0
6
12
24
Time (h)
colour
MU
WT

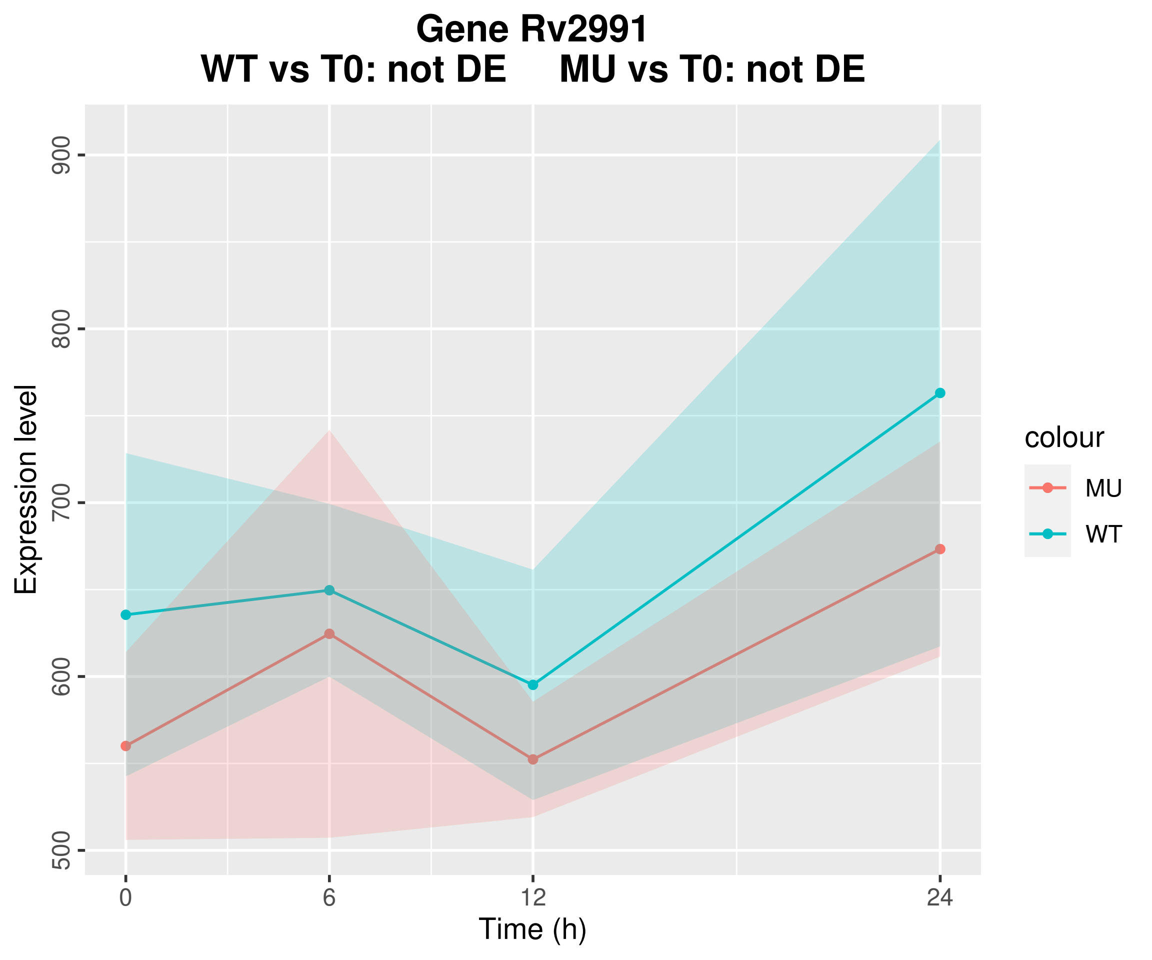

Gene Rv2991
WT vs T0: not DE   MU vs T0: not DE
Expression level
900
800
700
600
500
0
6
12
24
Time (h)
colour
MU
WT

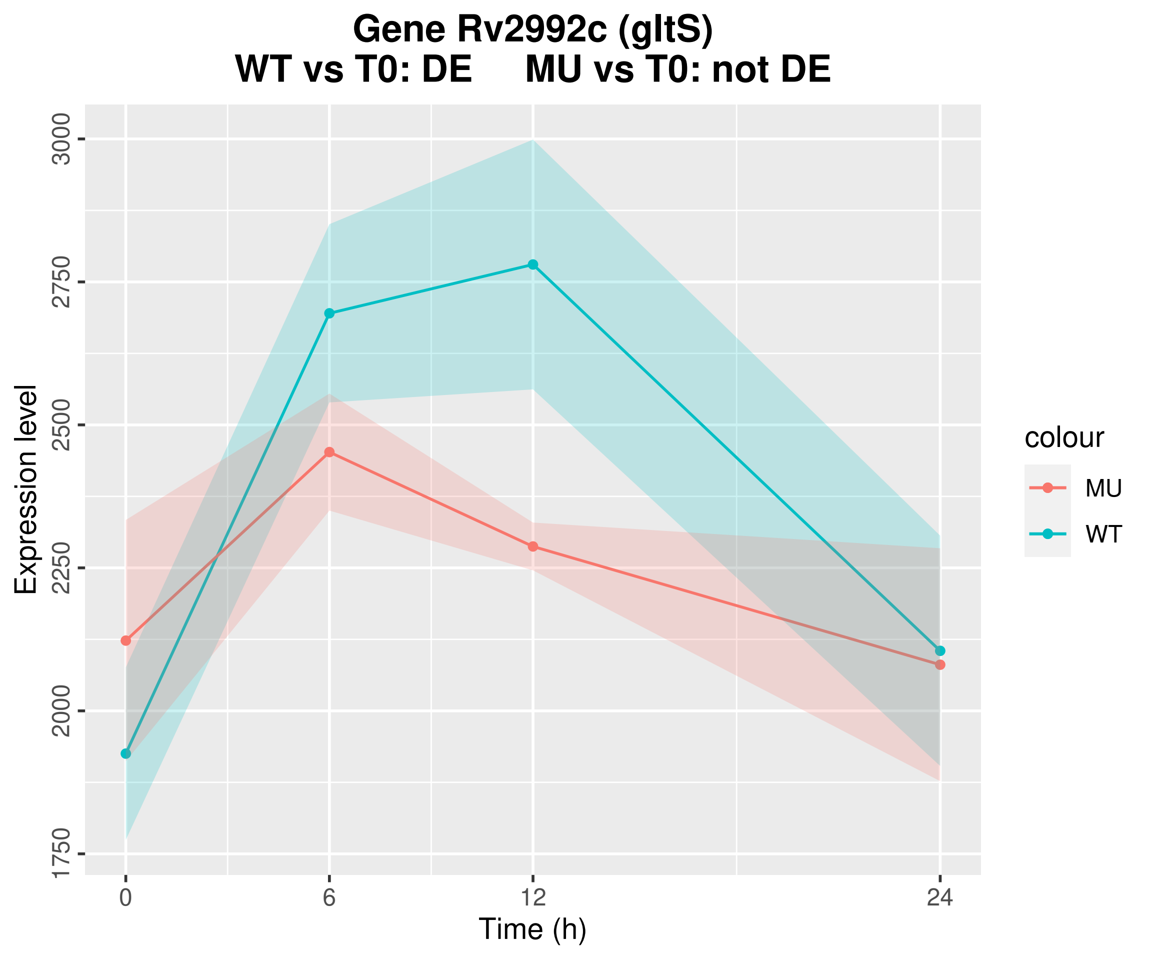

Gene Rv2992c (gltS)
WT vs T0: DE   MU vs T0: not DE
Expression level
3000
2750
2500
2250
2000
1750
0
6
12
24
Time (h)
colour
MU
WT

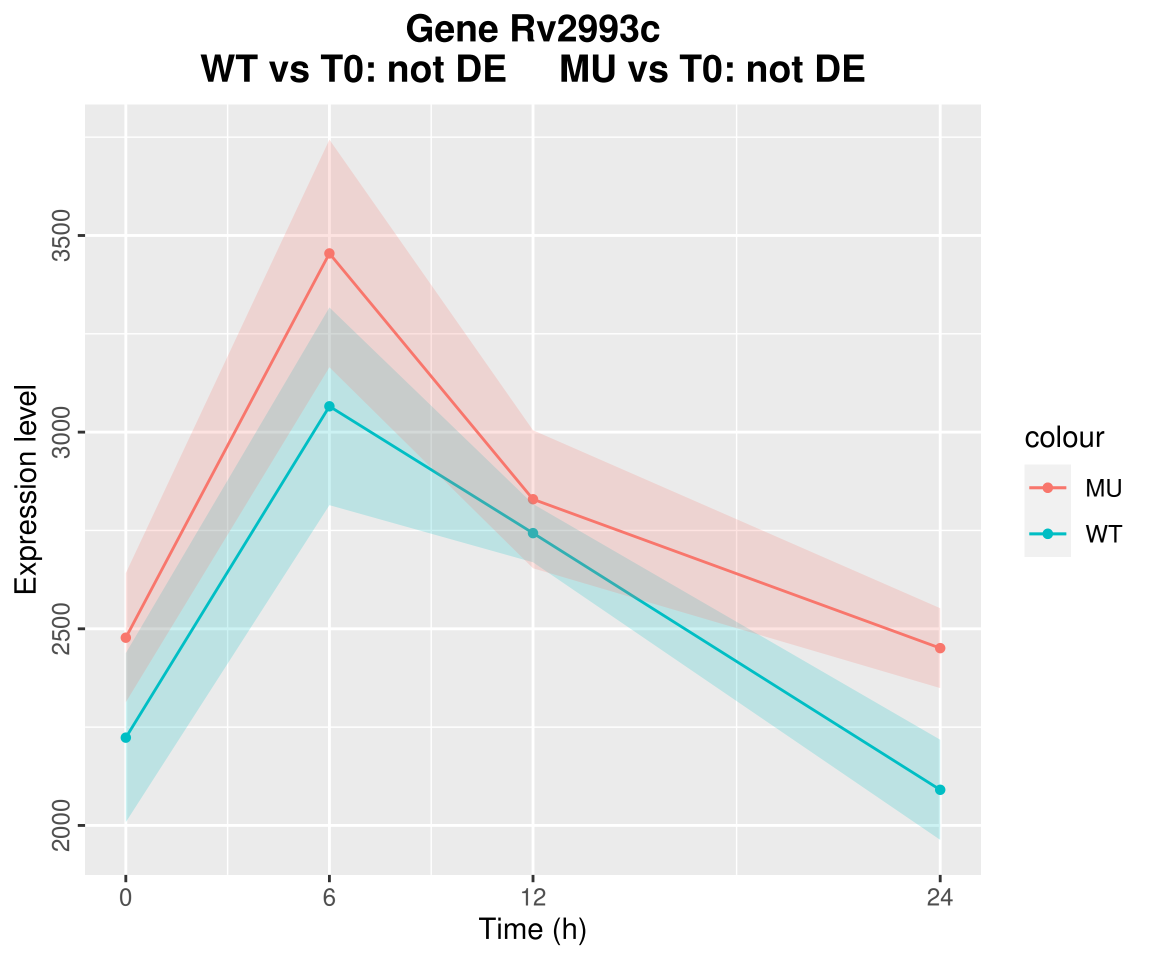
Gene Rv2993c
WT vs T0: not DE   MU vs T0: not DE
Expression level
3500
3000
2500
2000
0
6
12
24
Time (h)
colour
MU
WT

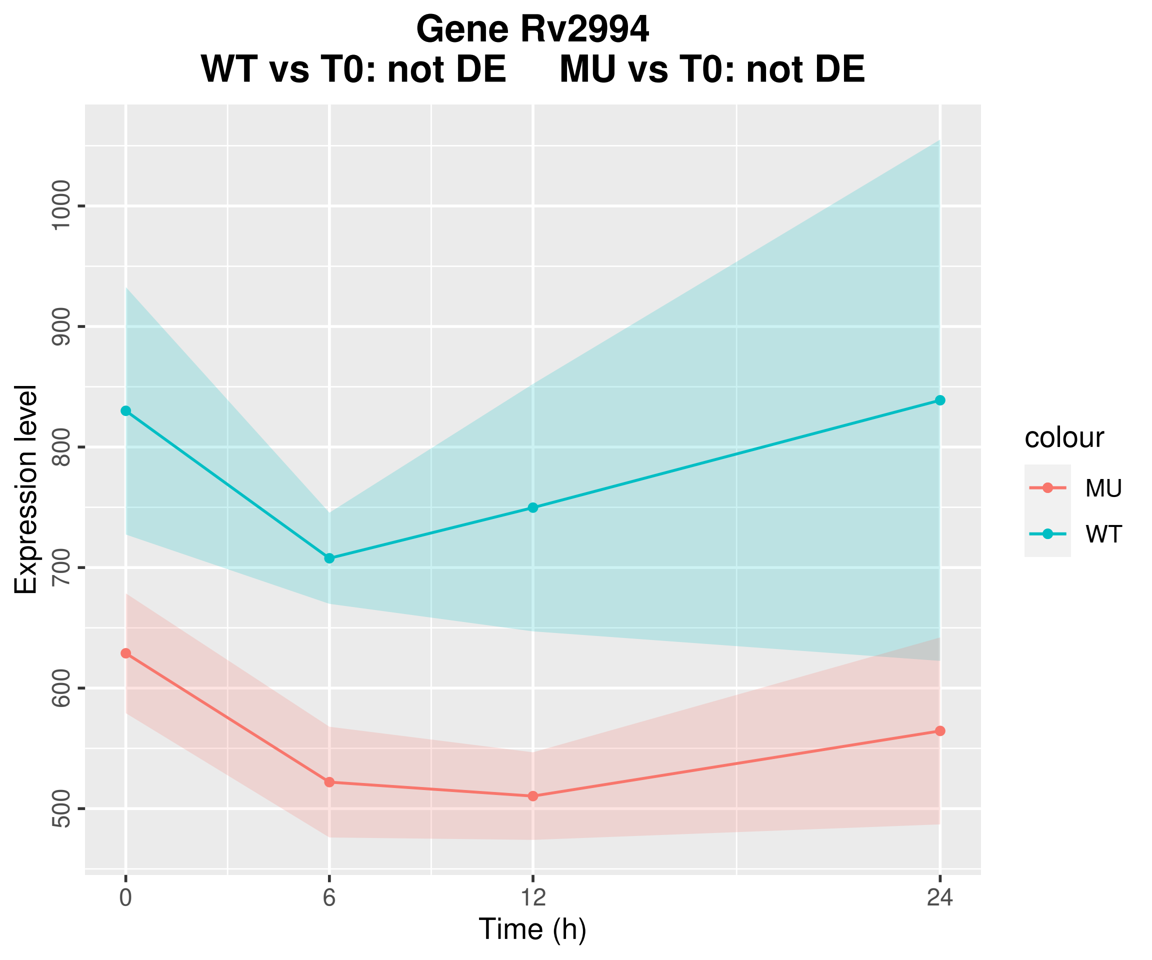
Gene Rv2994
WT vs T0: not DE MU vs T0: not DE
Expression level
1000
900
800
700
600
500
0
6
12
24
Time (h)
colour
MU
WT

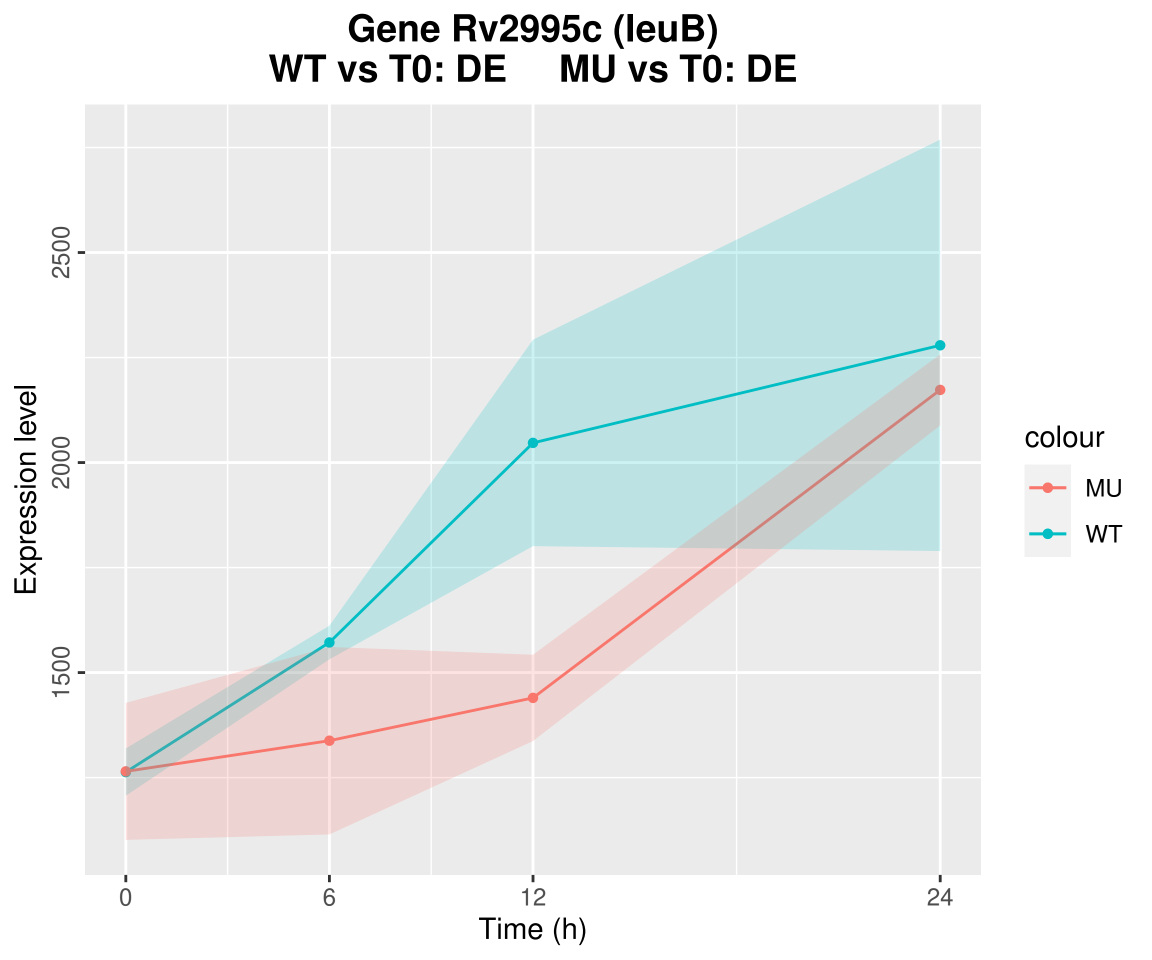
Gene Rv2995c (leuB)
WT vs T0: DE   MU vs T0: DE
Expression level
2500
2000
1500
0
6
12
24
Time (h)
colour
MU
WT

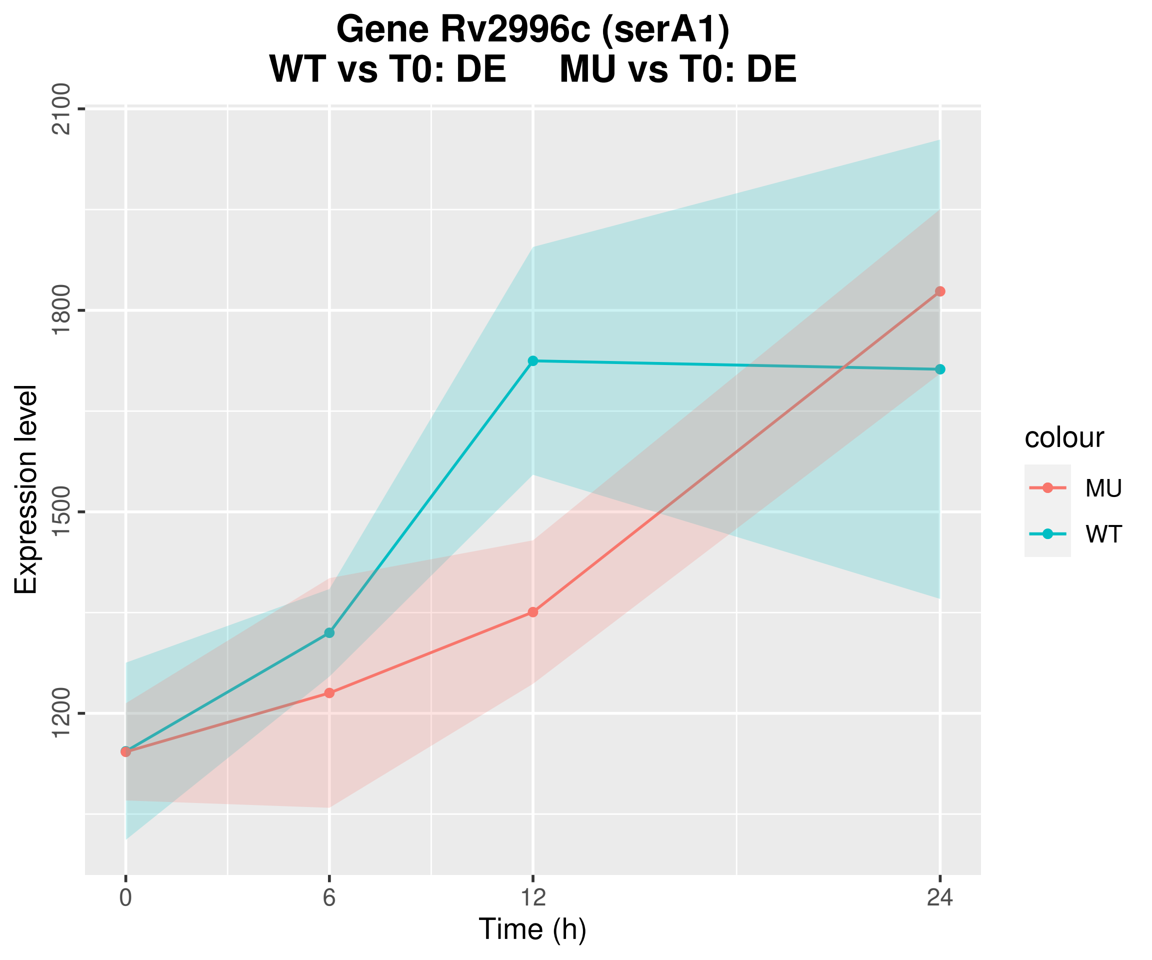
Gene Rv2996c (serA1)
WT vs T0: DE   MU vs T0: DE
Expression level
2100
1800
1500
1200
0
6
12
24
Time (h)
colour
MU
WT

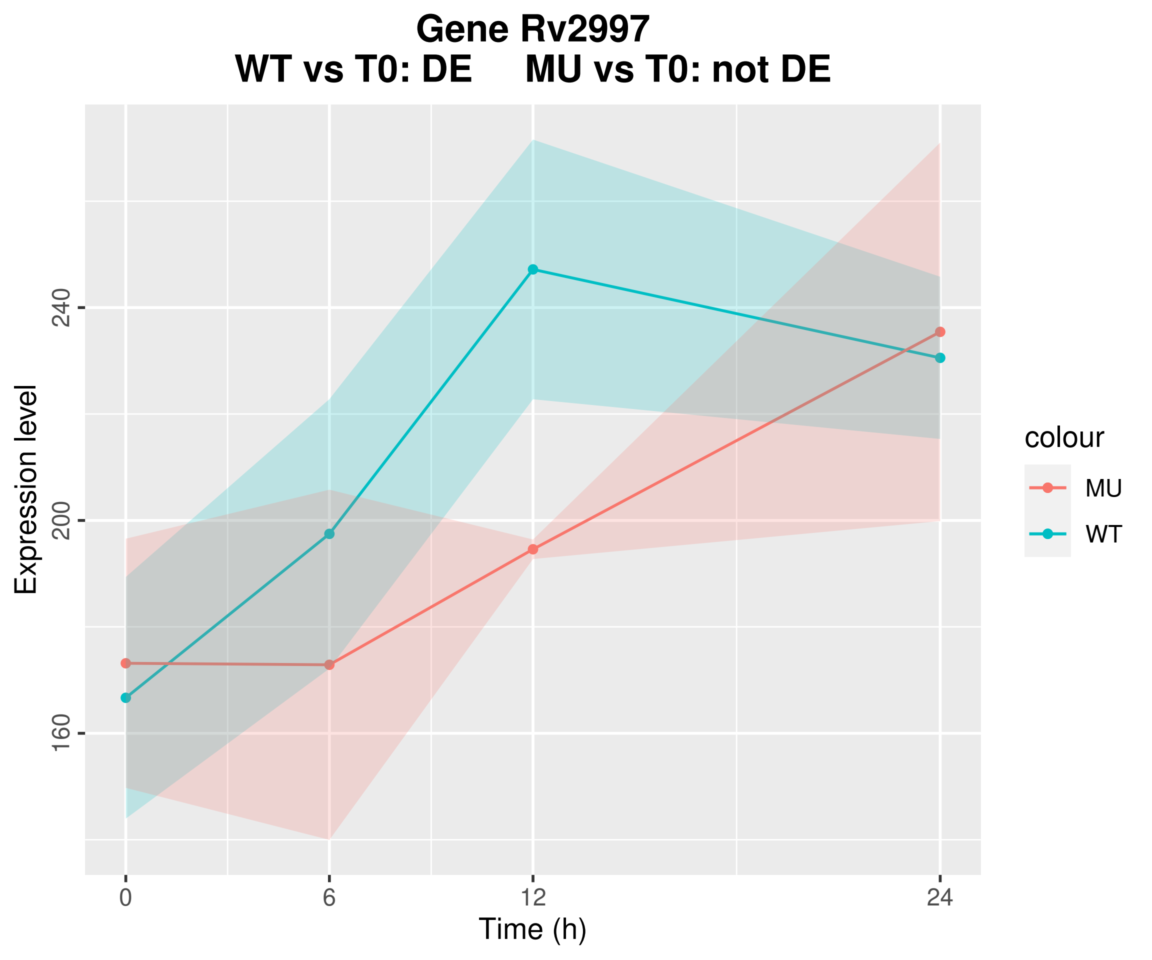
Gene Rv2997
WT vs T0: DE   MU vs T0: not DE
Expression level
240
200
160
0
6
12
24
Time (h)
colour
MU
WT

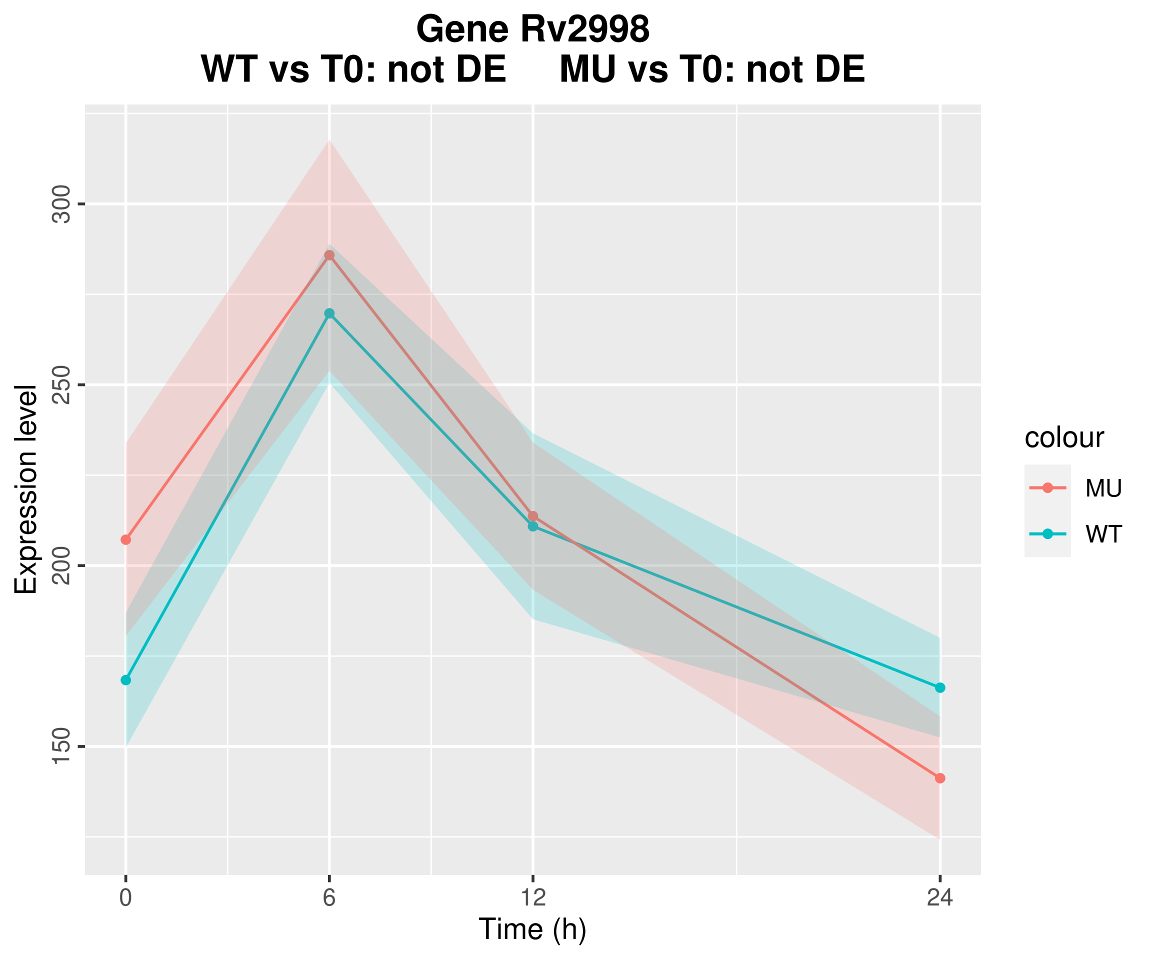
Gene Rv2998
WT vs T0: not DE MU vs T0: not DE
Expression level
300
250
200
150
0
6
12
24
Time (h)
colour
MU
WT

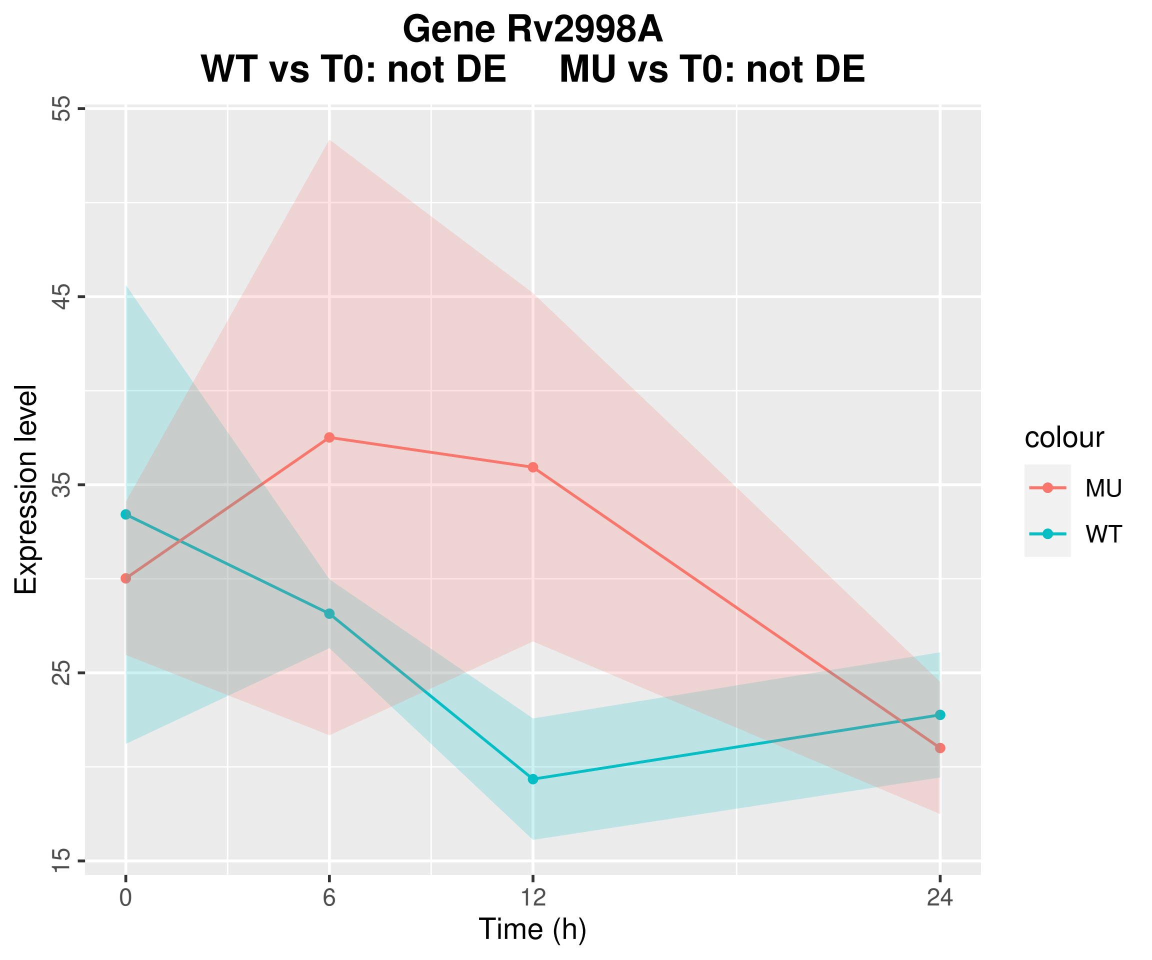
Gene Rv2998A
WT vs T0: not DE   MU vs T0: not DE
Expression level
55
45
35
25
15
0
6
12
24
Time (h)
colour
MU
WT

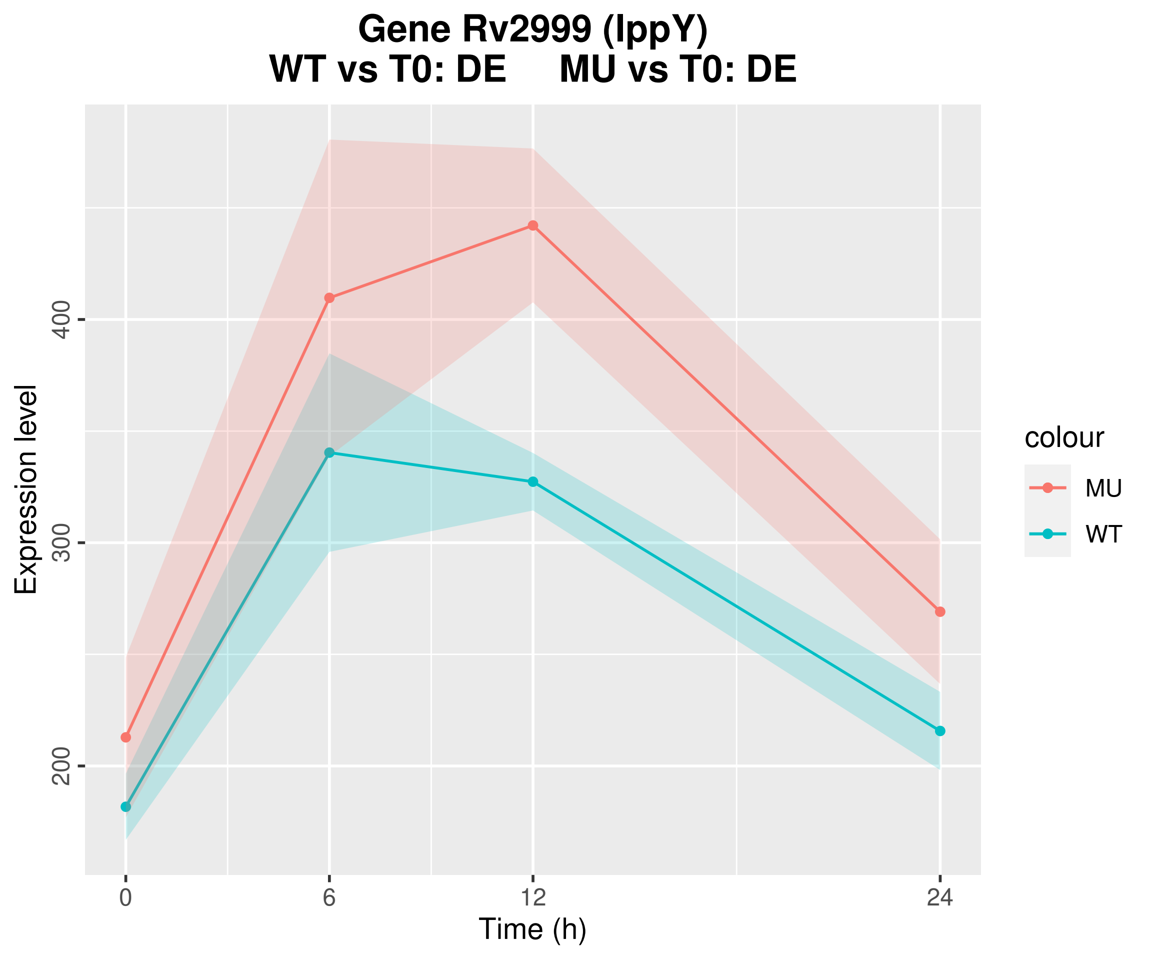

Gene Rv2999 (lppY)
WT vs T0: DE   MU vs T0: DE
Expression level
200
300
400
0
6
12
24
Time (h)
colour
MU
WT

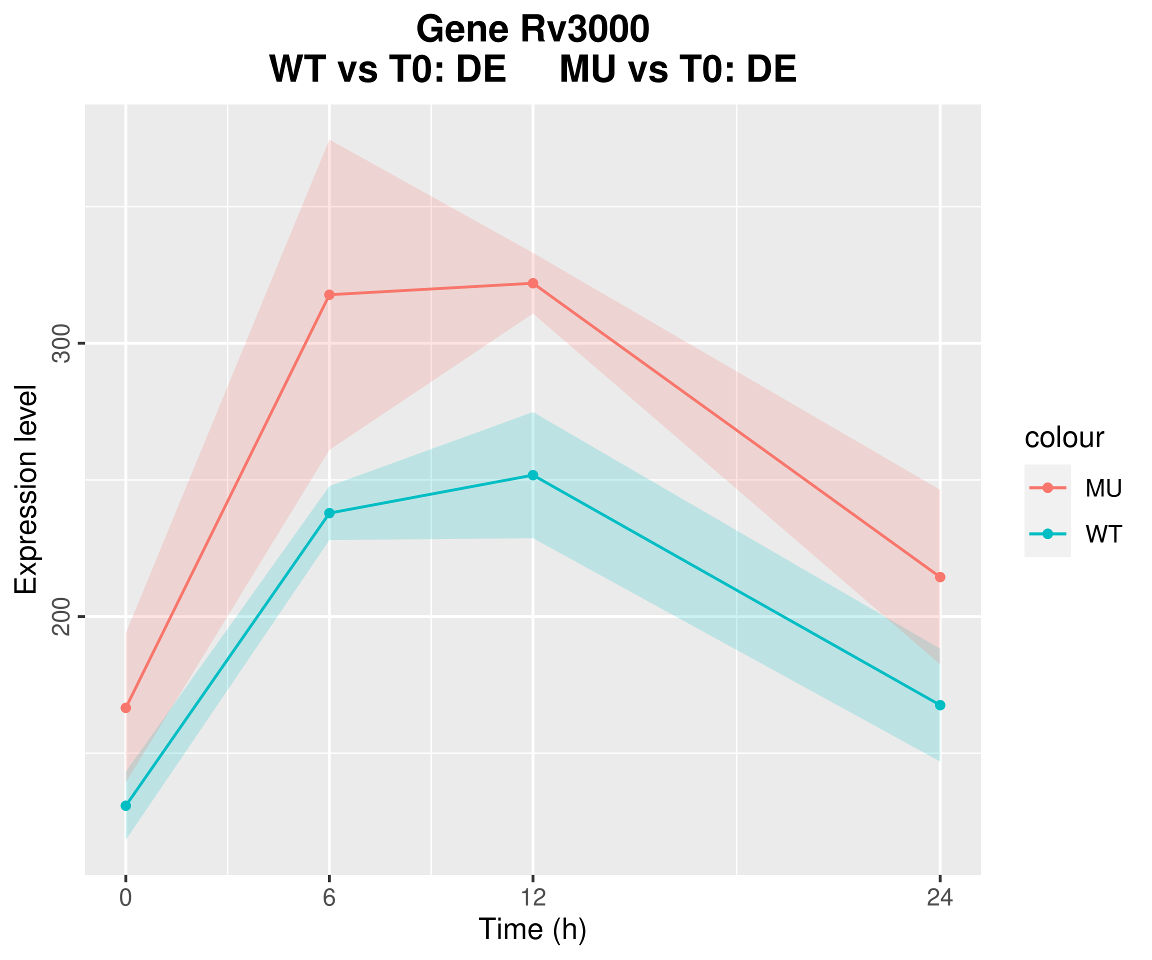
Gene Rv3000
WT vs T0: DE MU vs T0: DE
Expression level
300
200
0
6
12
24
Time (h)
colour
MU
WT

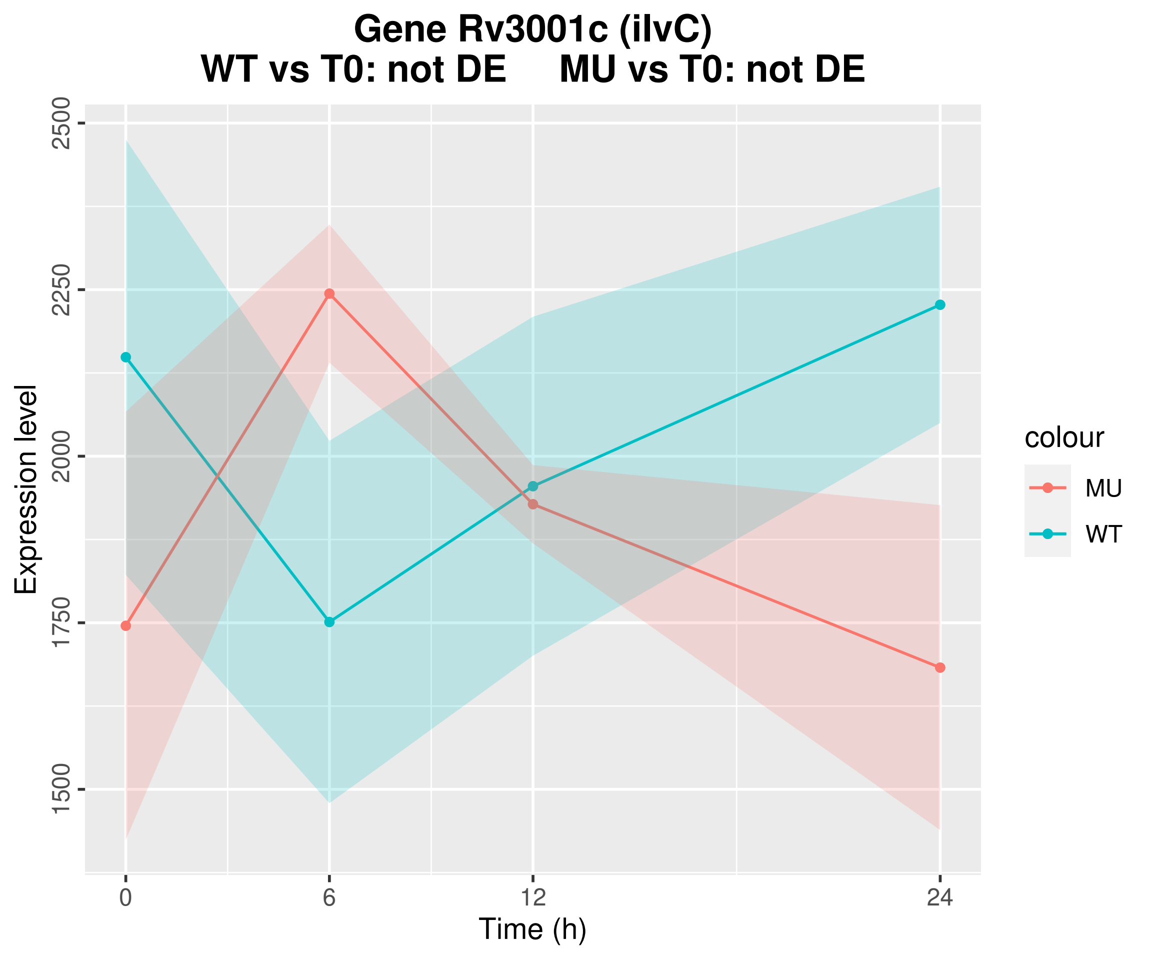
Gene Rv3001c (ilvC)
WT vs T0: not DE MU vs T0: not DE
Expression level
2500
2250
2000
1750
1500
0
6
12
24
Time (h)
colour
MU
WT

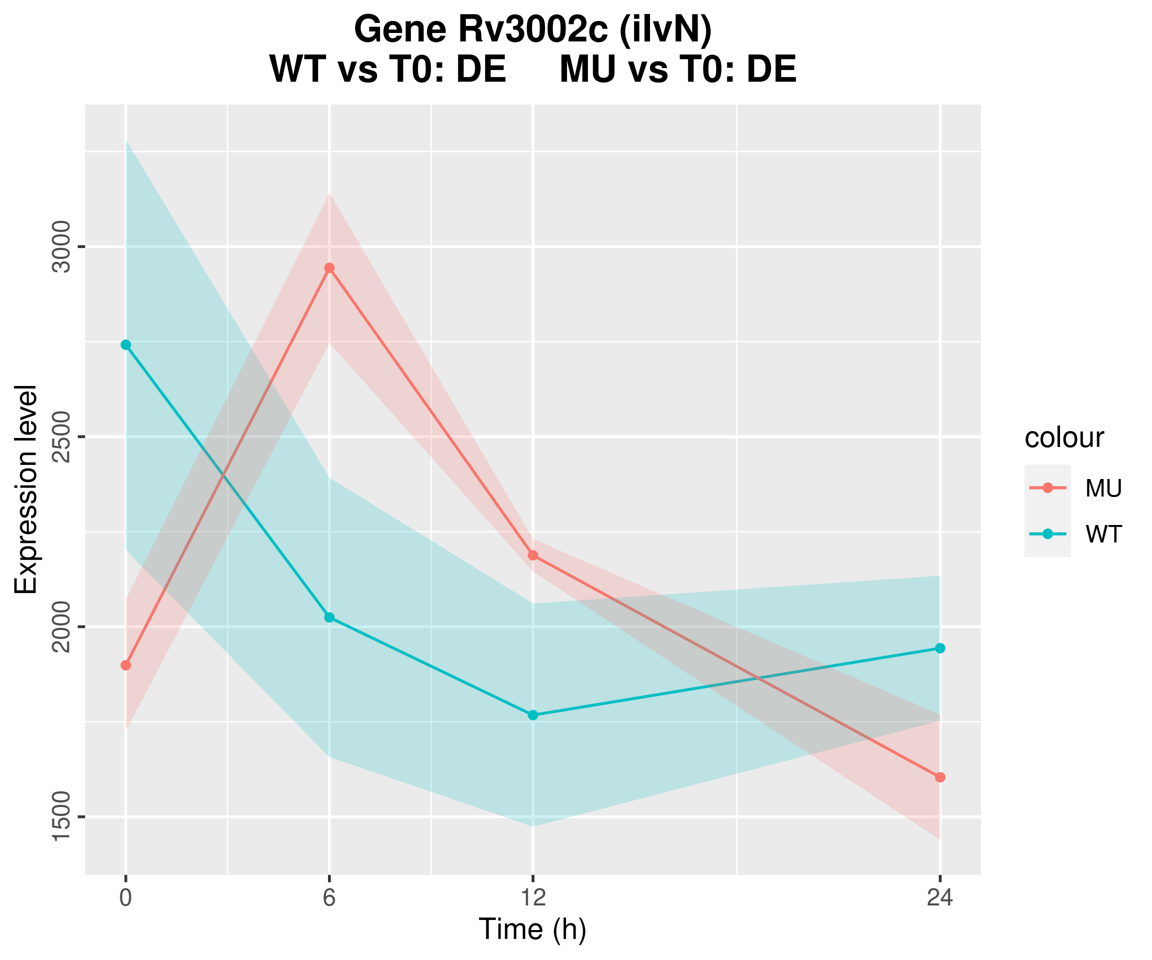

Gene Rv3002c (ilvN)
WT vs T0: DE MU vs T0: DE
Expression level
3000
2500
2000
1500
0
6
12
24
Time (h)
colour
MU
WT

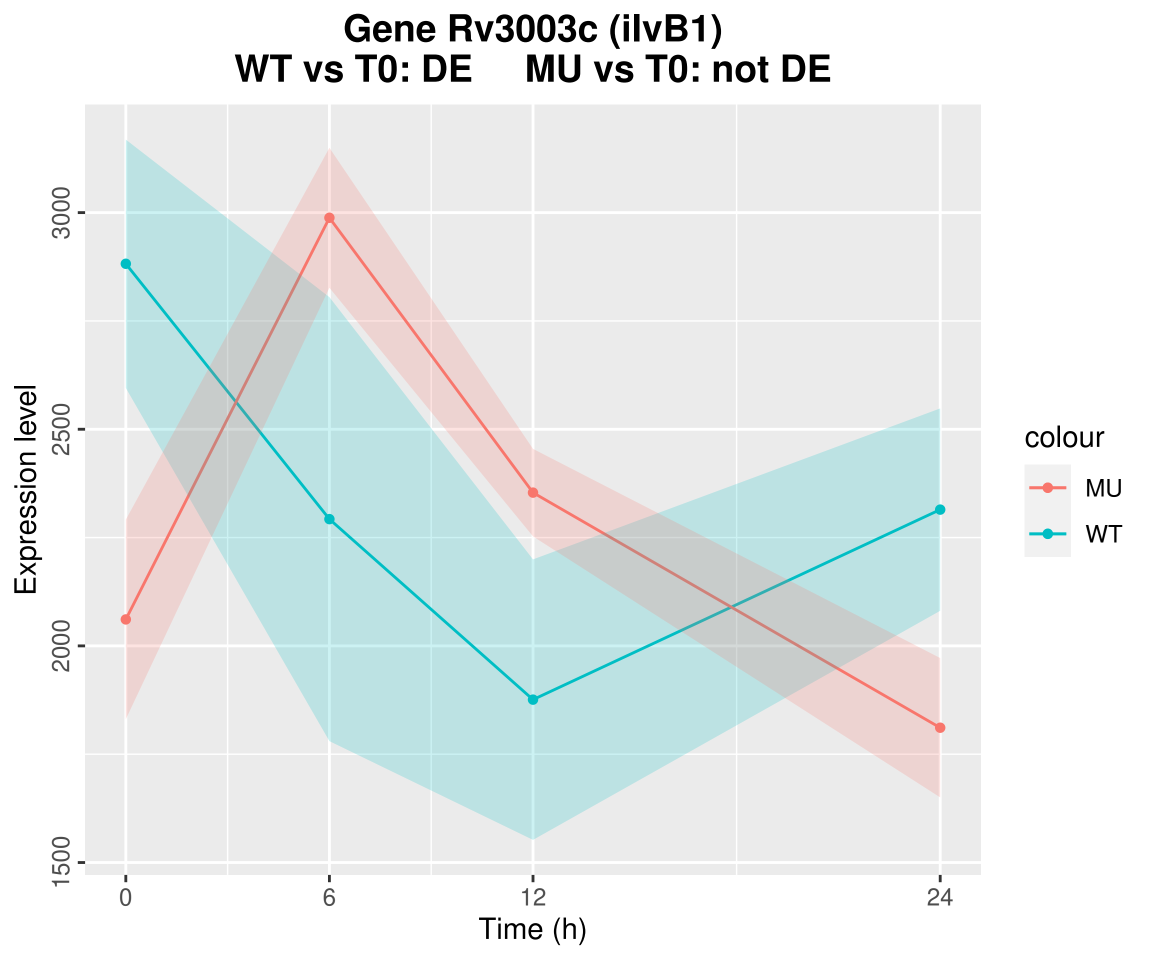

Gene Rv3003c (ilvB1)
WT vs T0: DE MU vs T0: not DE
Expression level
1500
2000
2500
3000
0
6
12
24
Time (h)
colour
MU
WT

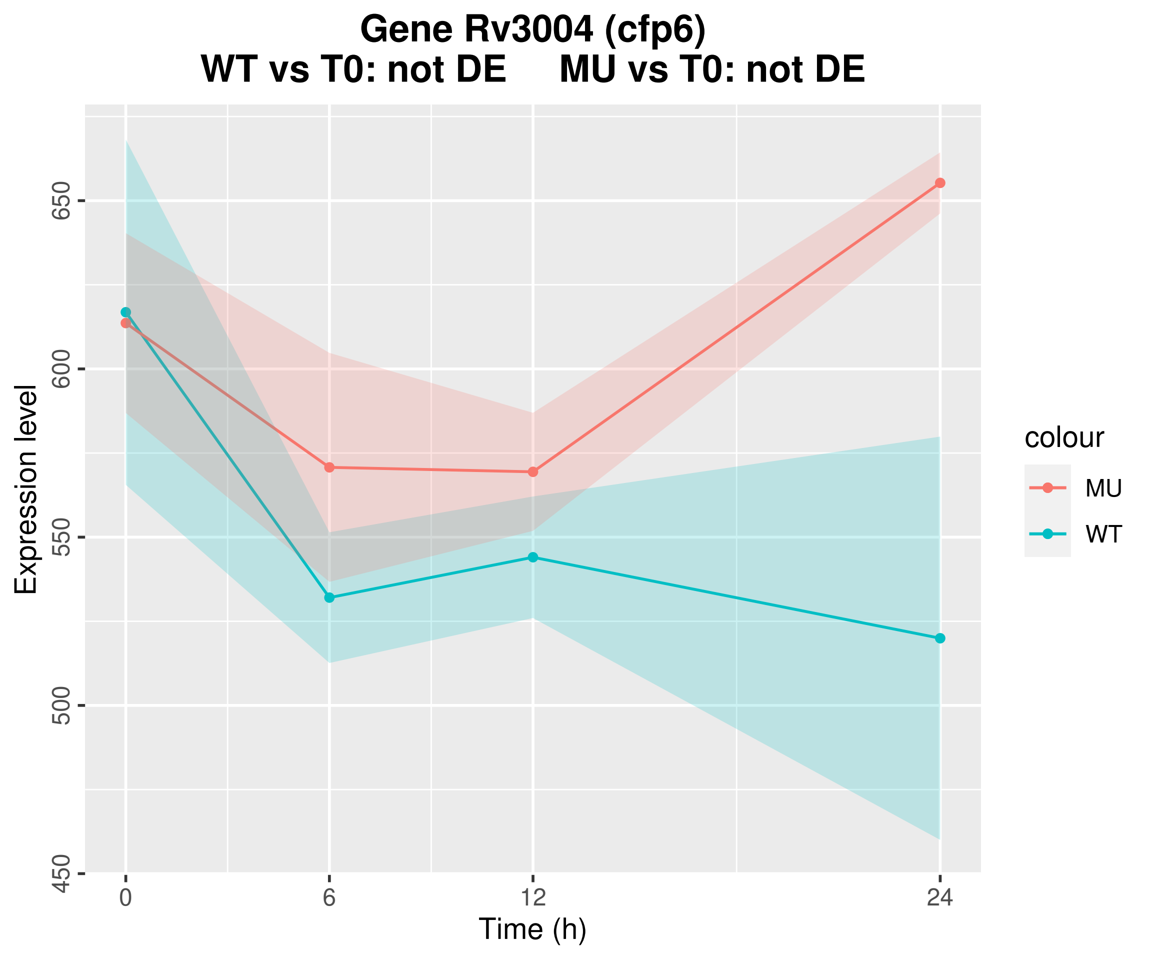

Gene Rv3004 (cfp6)
WT vs T0: not DE MU vs T0: not DE
Expression level
650
600
550
500
450
0
6
12
24
Time (h)
colour
MU
WT

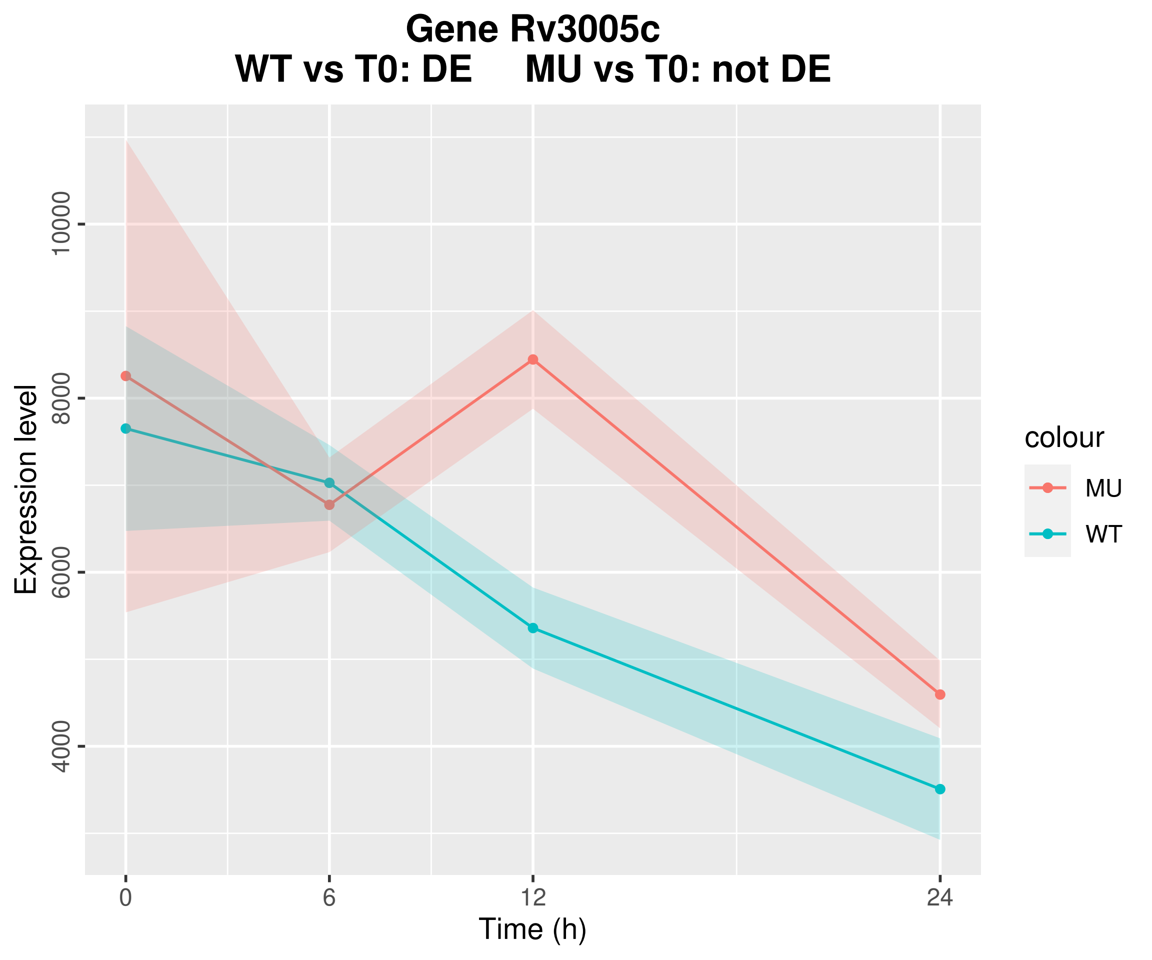
Gene Rv3005c
WT vs T0: DE   MU vs T0: not DE
Expression level
10000
8000
6000
4000
0
6
12
24
Time (h)
colour
MU
WT

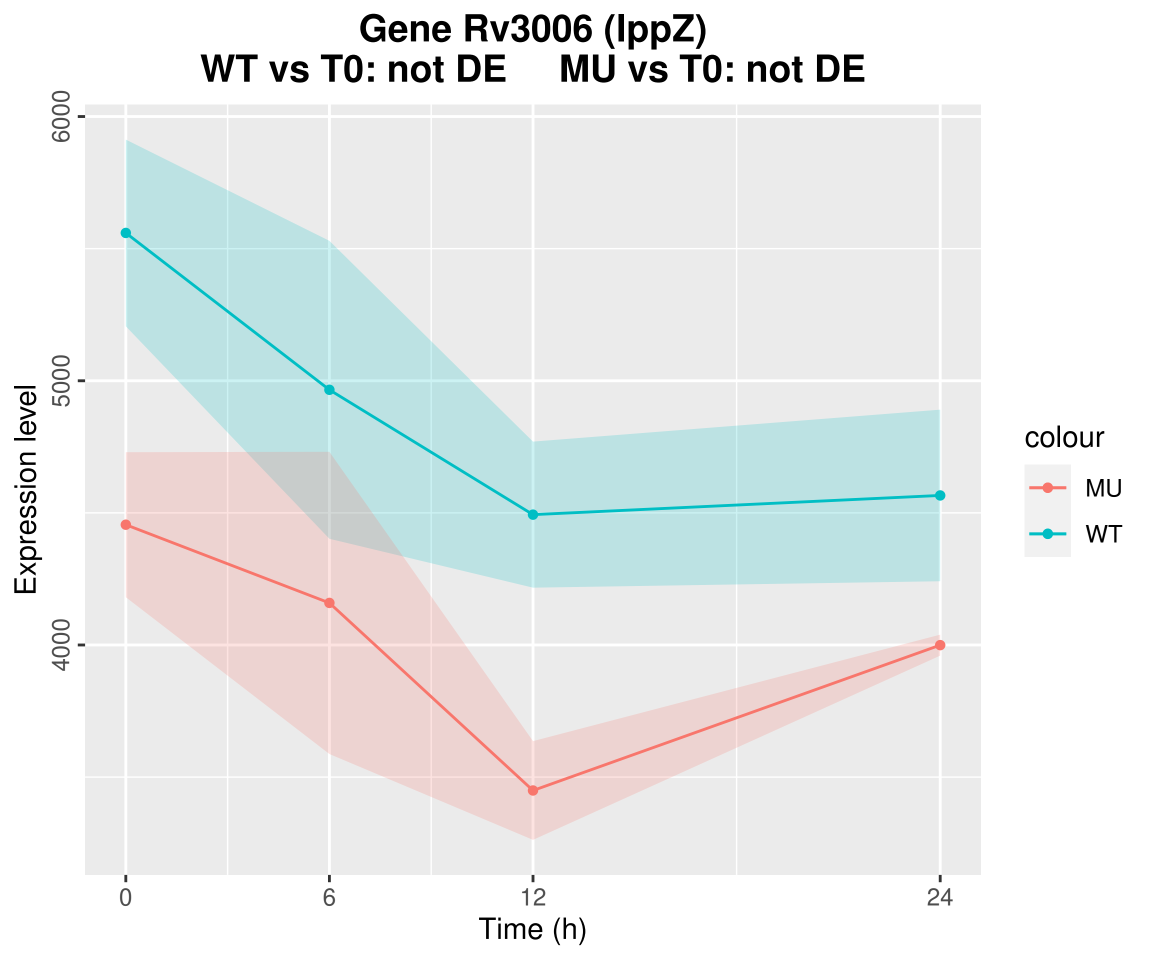

Gene Rv3006 (lppZ)
WT vs T0: not DE MU vs T0: not DE
6000
5000
4000
Expression level
0
6
12
24
Time (h)
colour
MU
WT

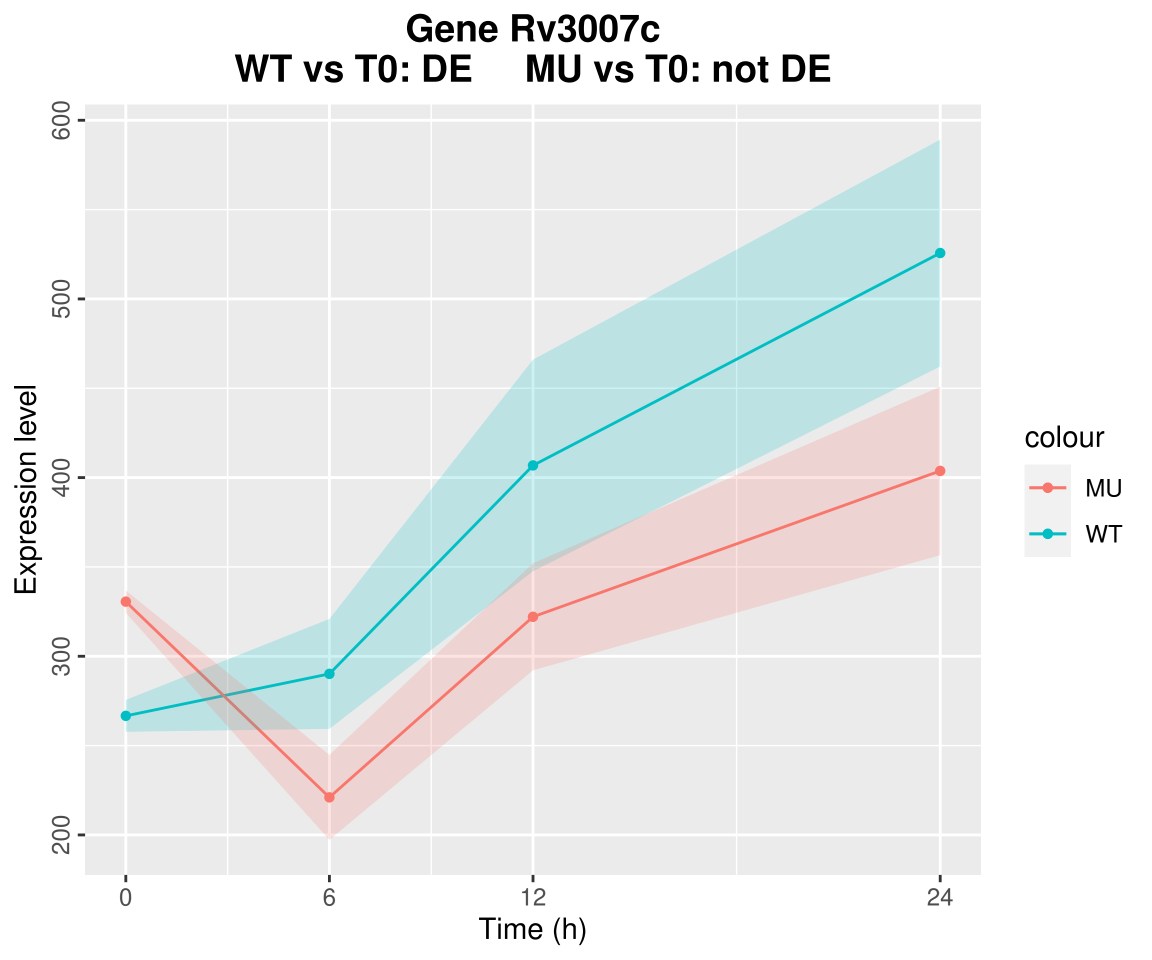

Gene Rv3007c
WT vs T0: DE   MU vs T0: not DE
Expression level
600
500
400
300
200
0
6
12
24
Time (h)
colour
MU
WT

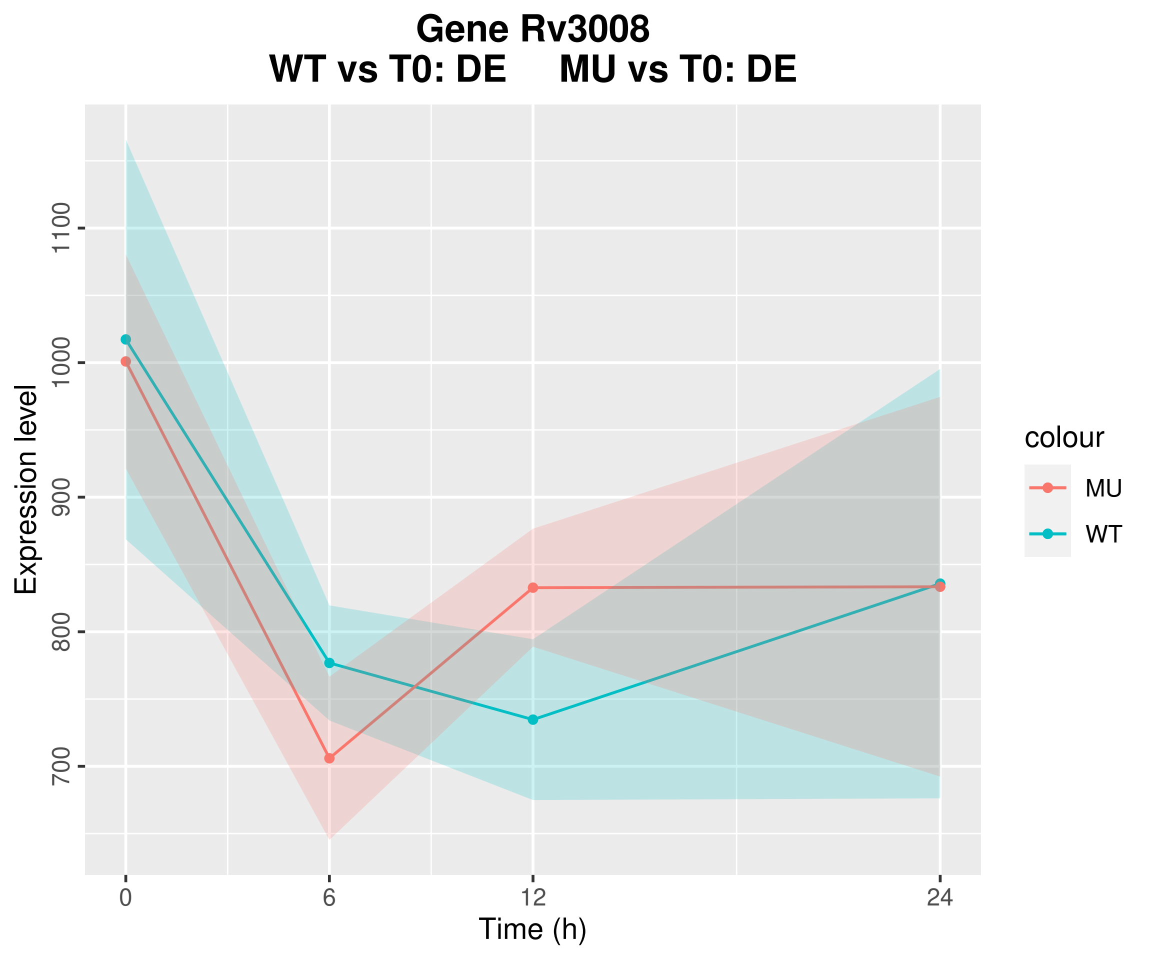
Gene Rv3008
WT vs T0: DE MU vs T0: DE
Expression level
1100
1000
900
800
700
0
6
12
24
Time (h)
colour
MU
WT

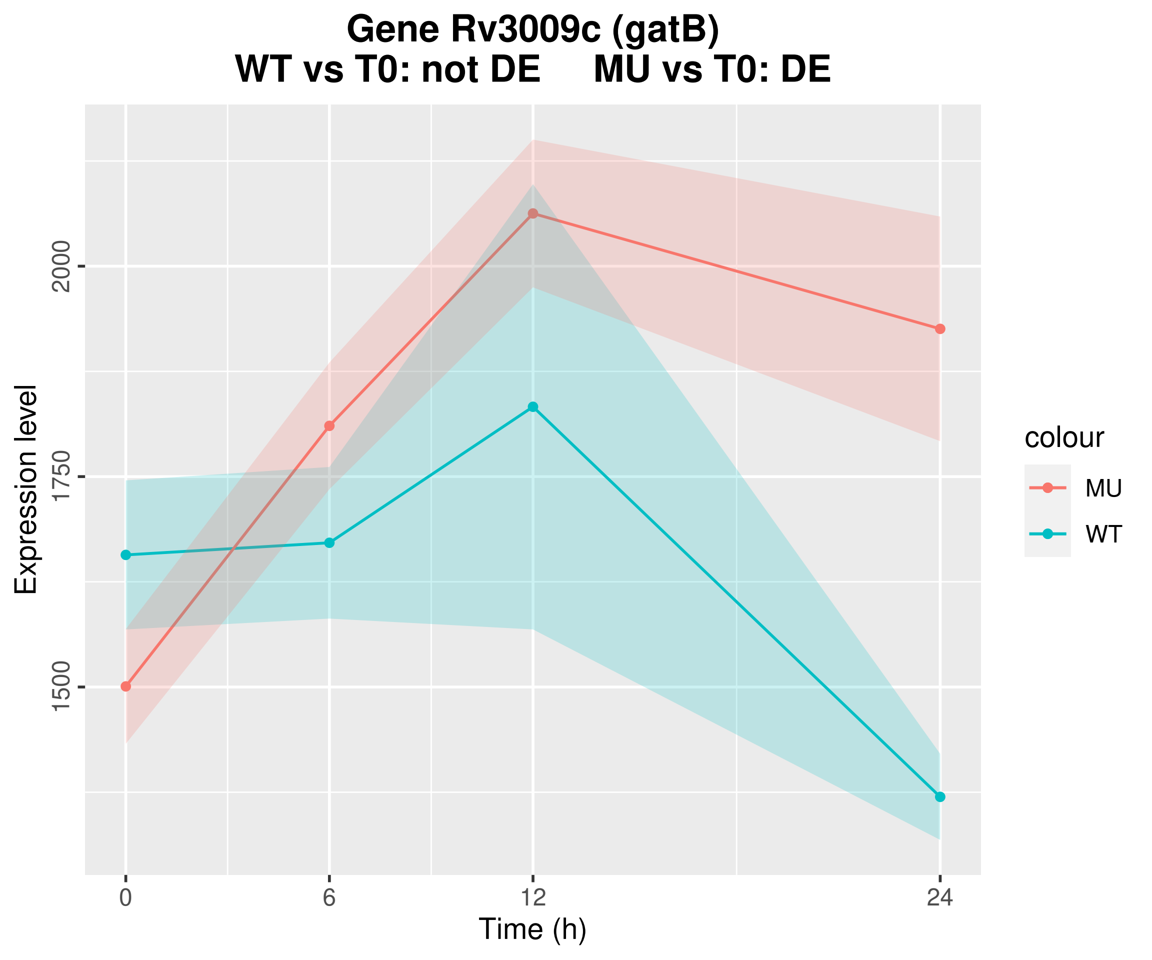
Gene Rv3009c (gatB)
WT vs T0: not DE   MU vs T0: DE
Expression level
2000
1750
1500
0
6
12
24
Time (h)
colour
MU
WT

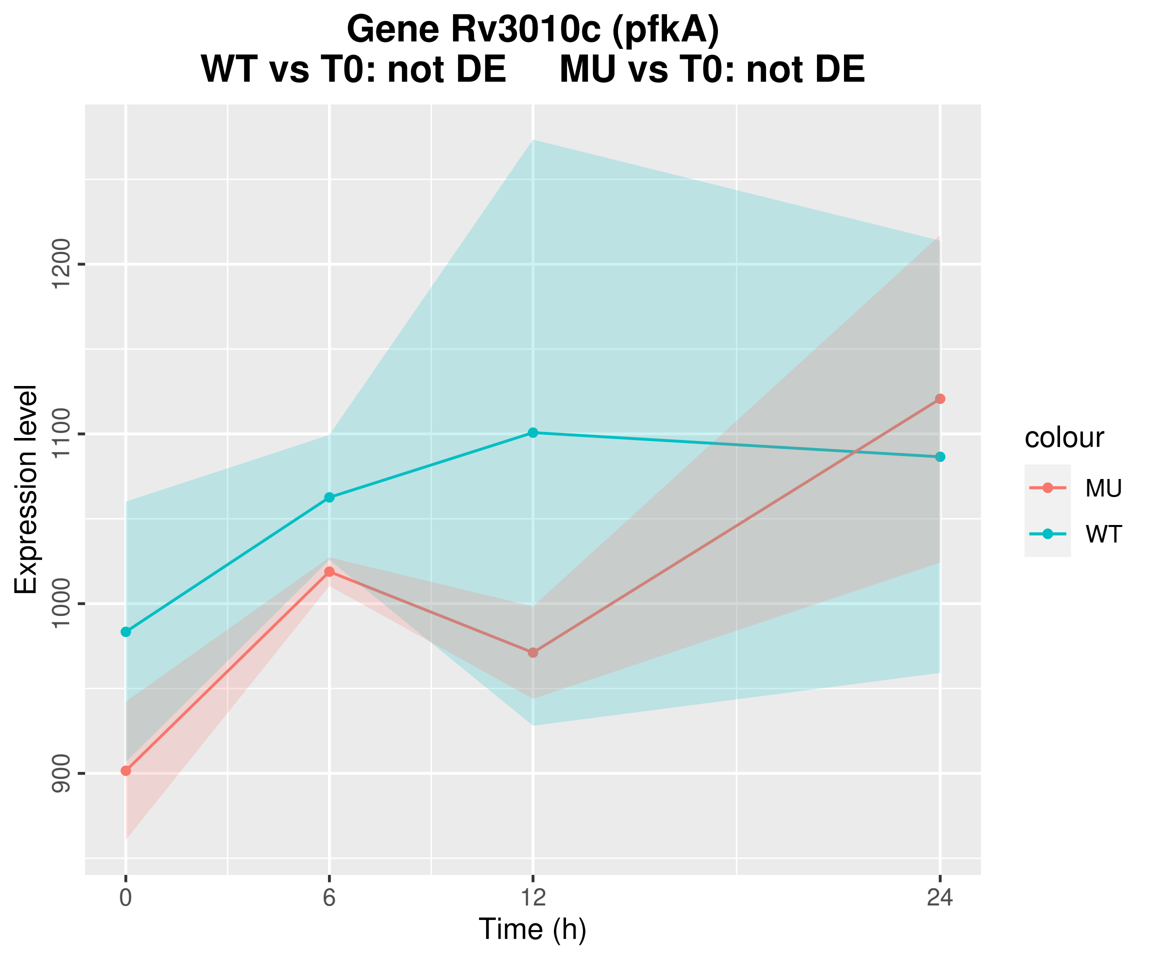
Gene Rv3010c (pfkA)
WT vs T0: not DE   MU vs T0: not DE
Expression level
900
1000
1100
1200
0
6
12
24
Time (h)
colour
MU
WT

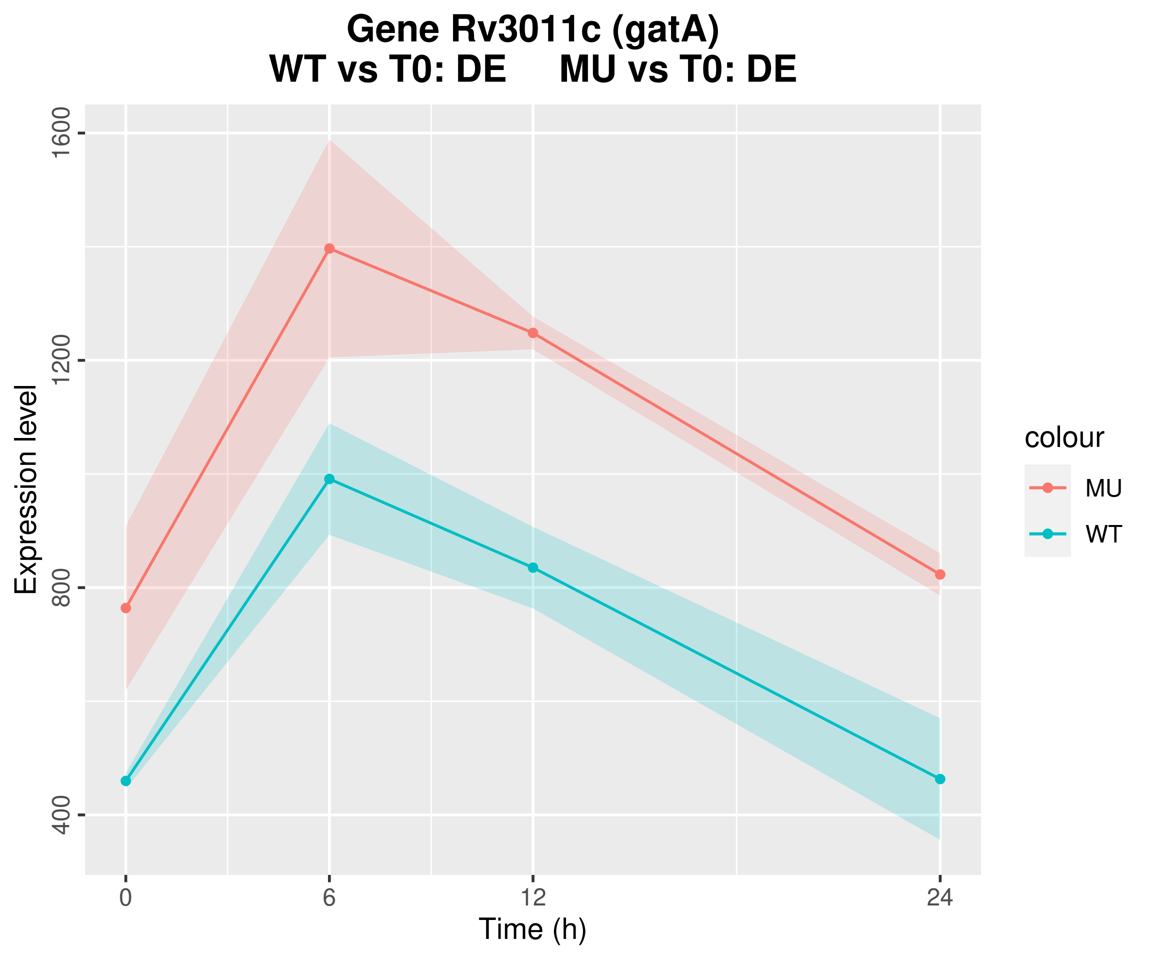
Gene Rv3011c (gatA)
WT vs T0: DE   MU vs T0: DE
Expression level
1600
1200
800
400
0
6
12
24
Time (h)
colour
MU
WT

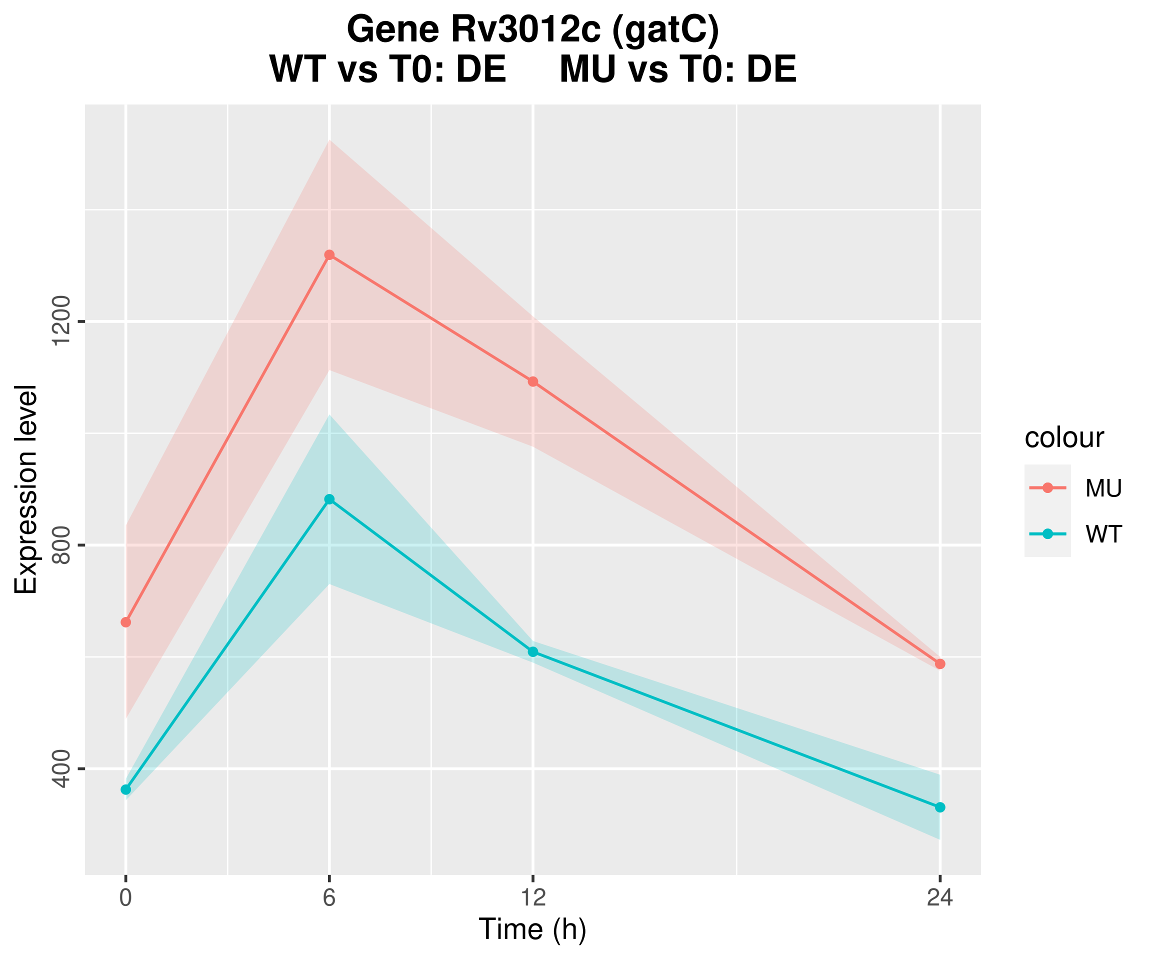
Gene Rv3012c (gatC)
WT vs T0: DE   MU vs T0: DE
Expression level
1200
800
400
0
6
12
24
Time (h)
colour
MU
WT

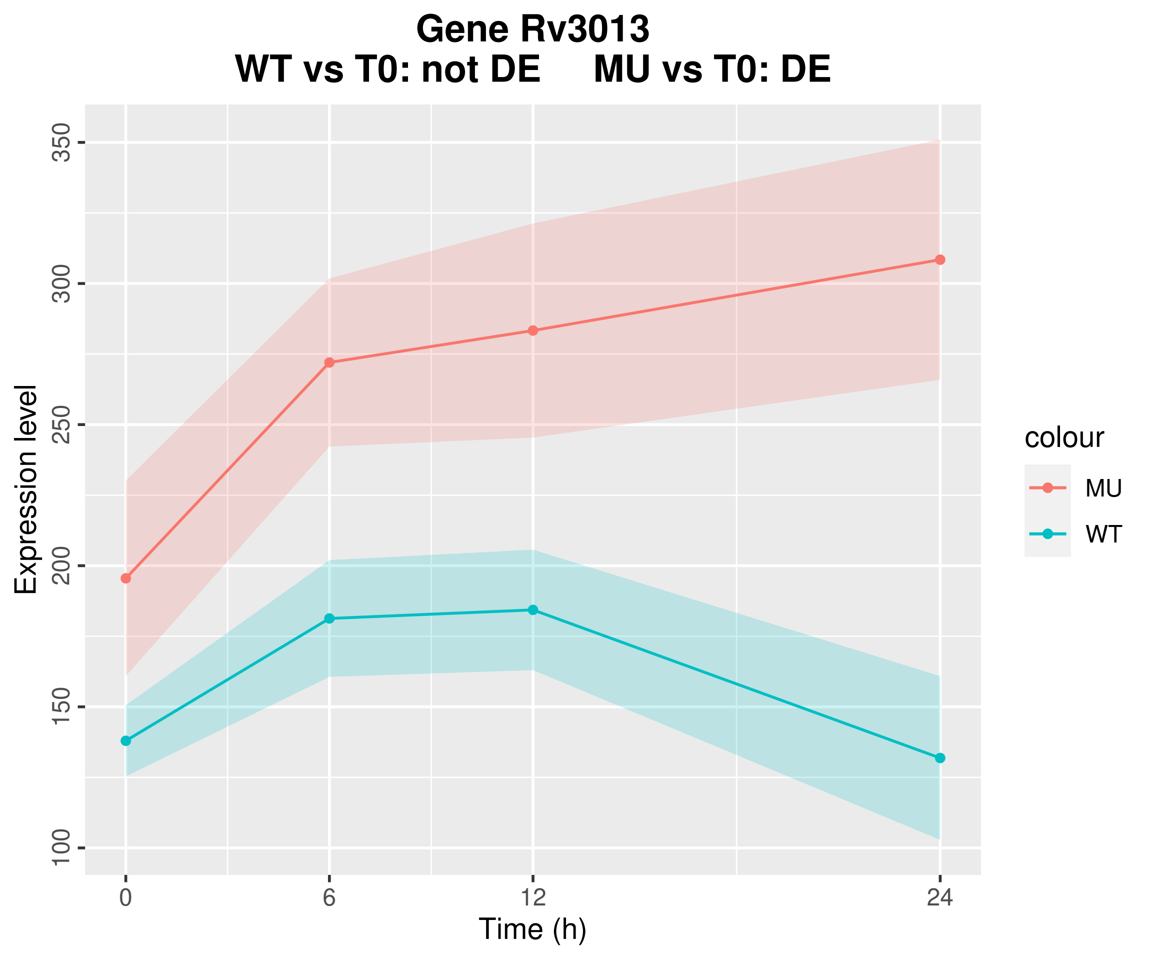

Gene Rv3013
WT vs T0: not DE   MU vs T0: DE
Expression level
350
300
250
200
150
100
0
6
12
24
Time (h)
colour
MU
WT

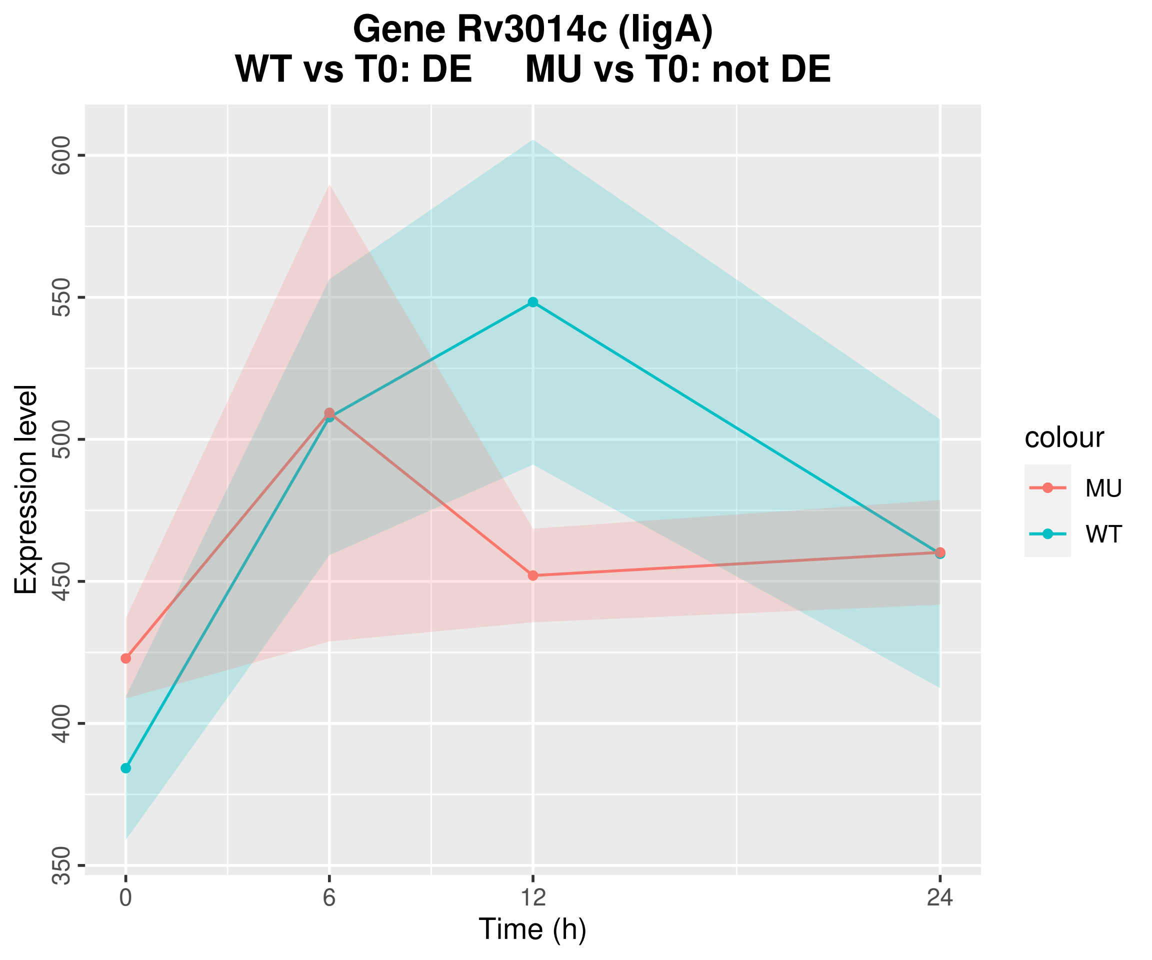
Gene Rv3014c (ligA)
WT vs T0: DE MU vs T0: not DE
Expression level
600
550
500
450
400
350
0
6
12
24
Time (h)
colour
MU
WT

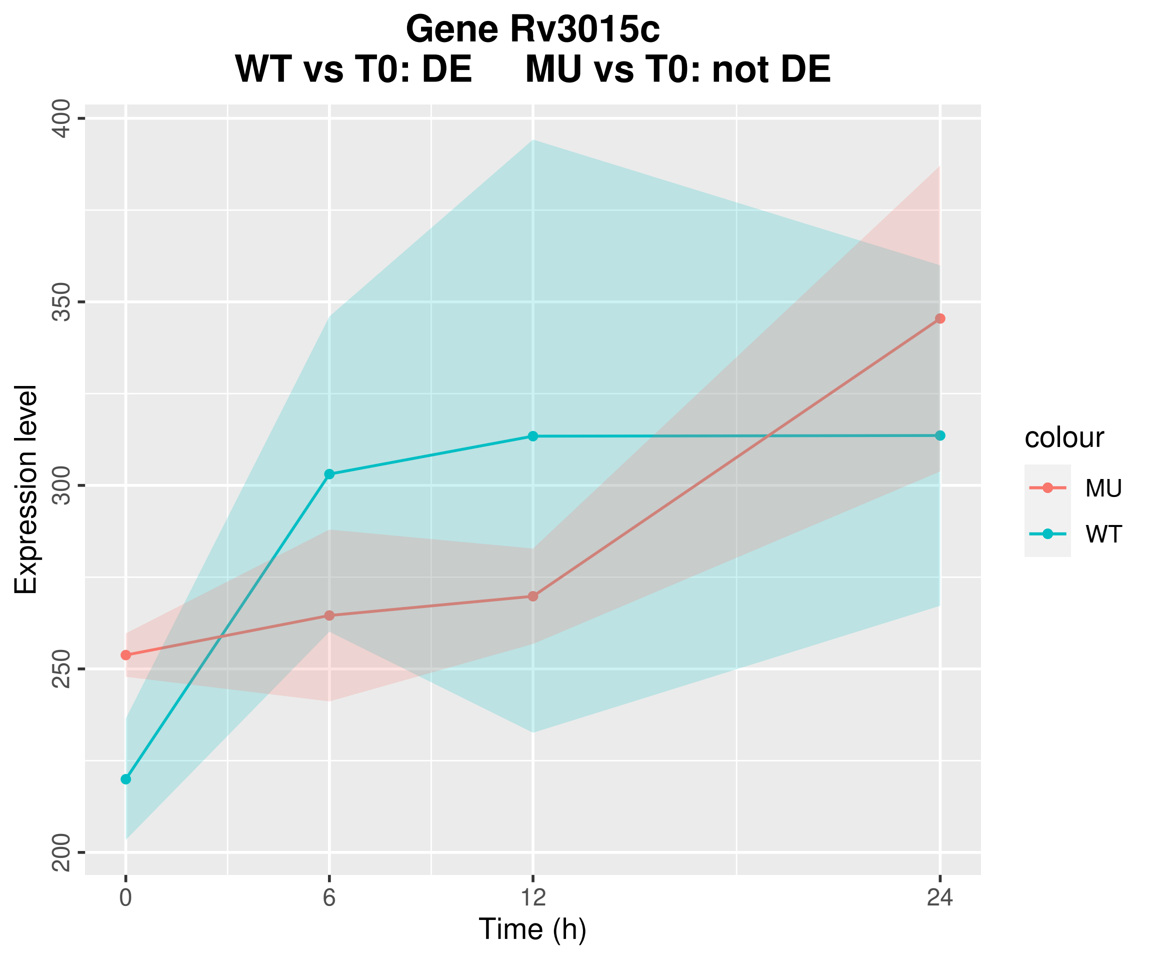

Gene Rv3015c
WT vs T0: DE   MU vs T0: not DE
Expression level
400
350
300
250
200
0
6
12
24
Time (h)
colour
MU
WT

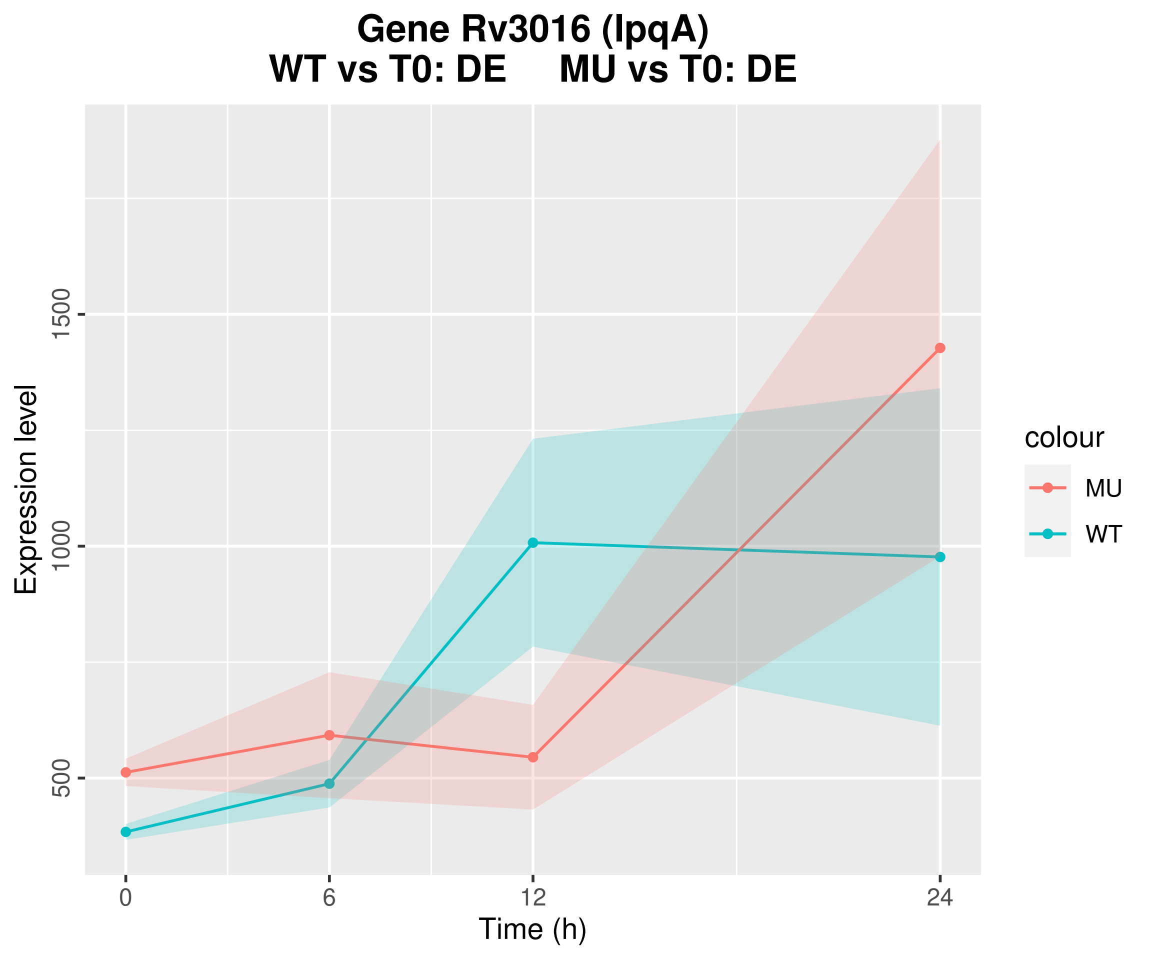

Gene Rv3016 (lpqA)
WT vs T0: DE   MU vs T0: DE
Expression level
1500
1000
500
0
6
12
24
Time (h)
colour
MU
WT

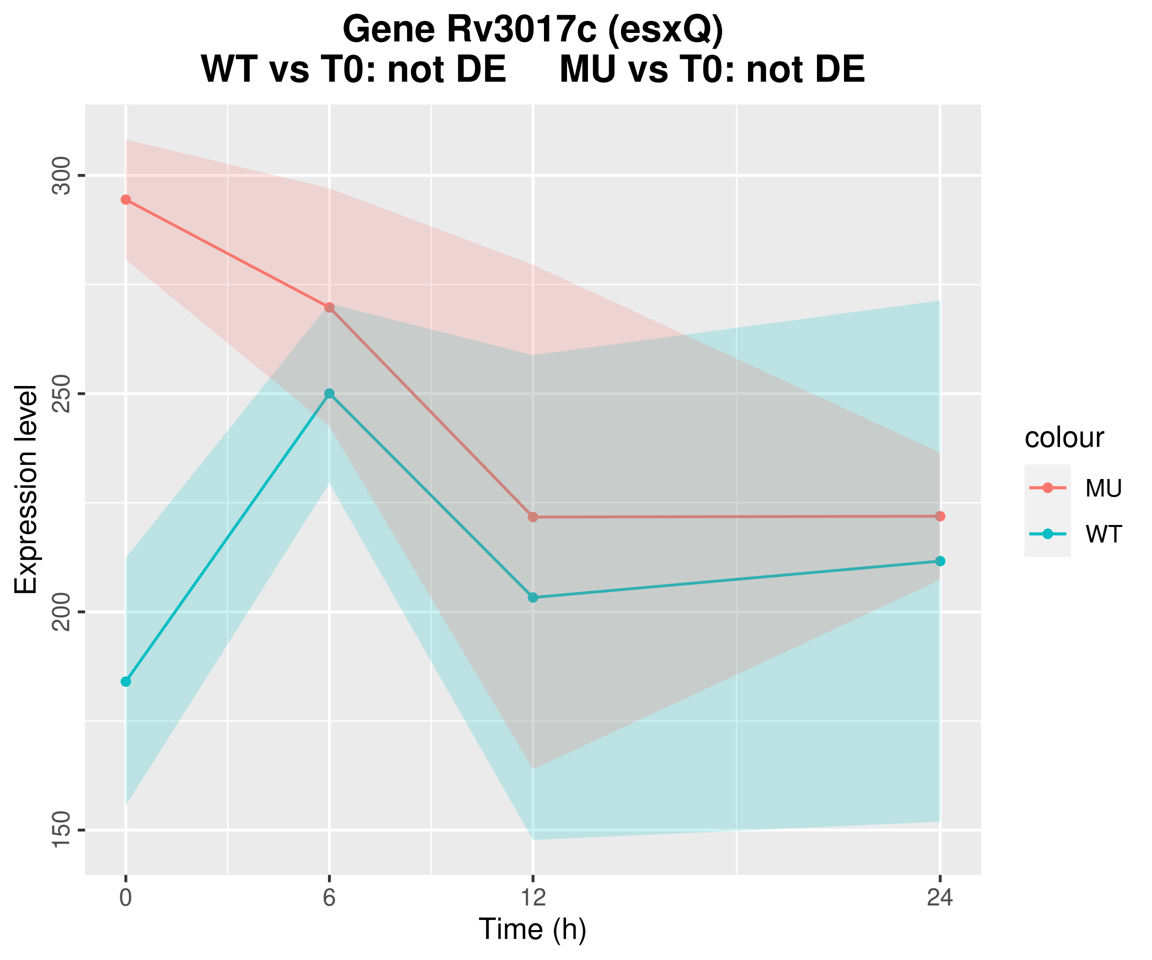
Gene Rv3017c (esxQ)
WT vs T0: not DE   MU vs T0: not DE
Expression level
300
250
200
150
0
6
12
24
Time (h)
colour
MU
WT

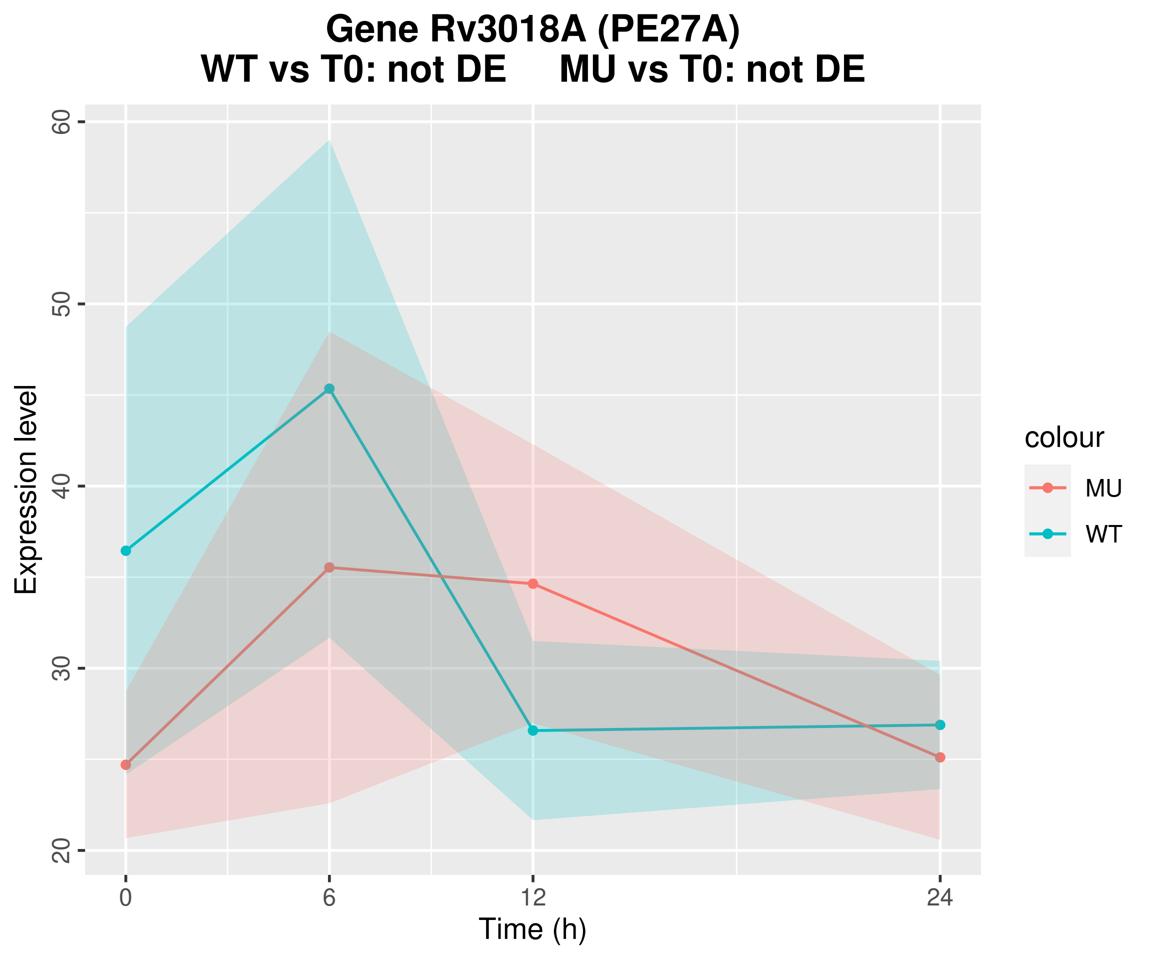
Gene Rv3018A (PE27A)
WT vs T0: not DE MU vs T0: not DE
Expression level
20
30
40
50
60
0
6
12
24
Time (h)
colour
MU
WT

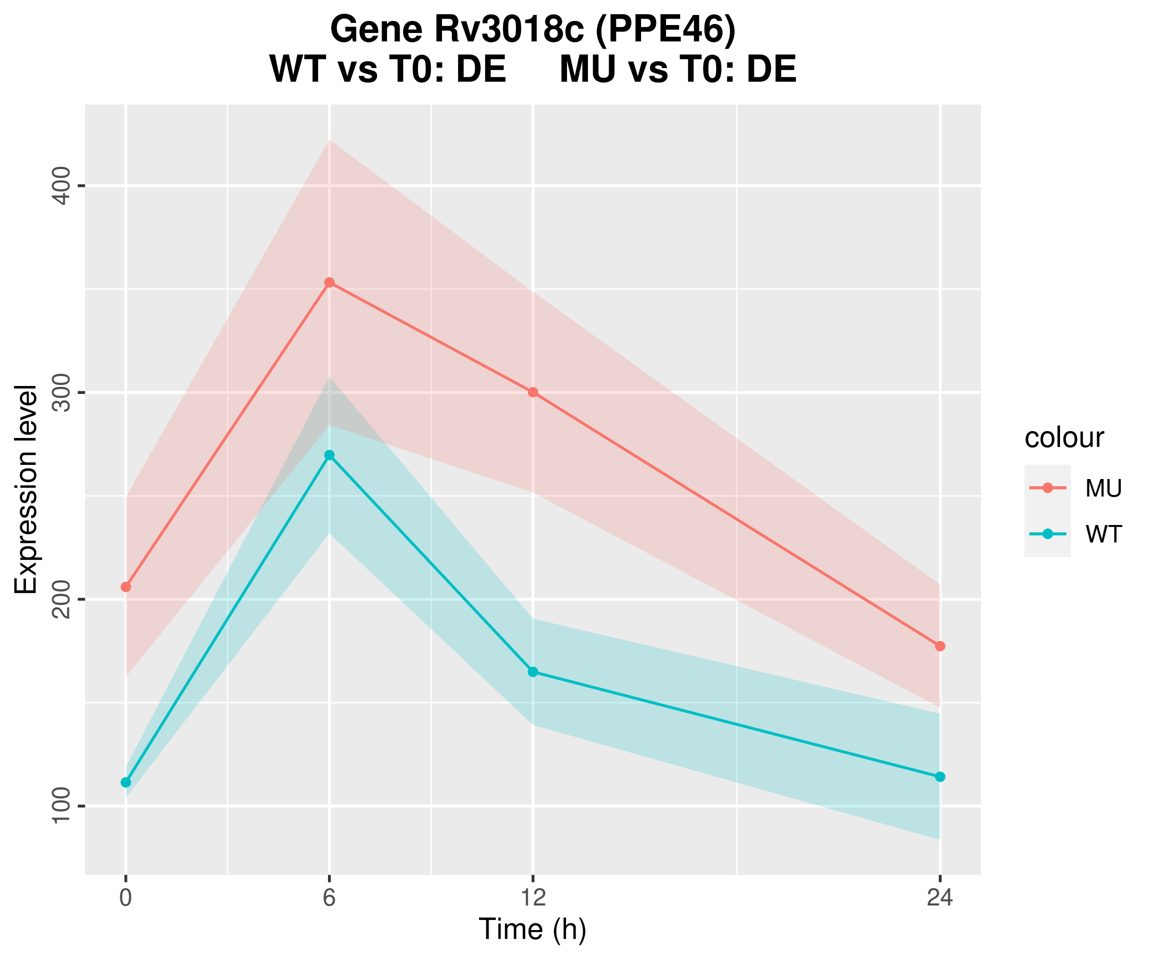
Gene Rv3018c (PPE46)
WT vs T0: DE   MU vs T0: DE
Expression level
400
300
200
100
0
6
12
24
Time (h)
colour
MU
WT

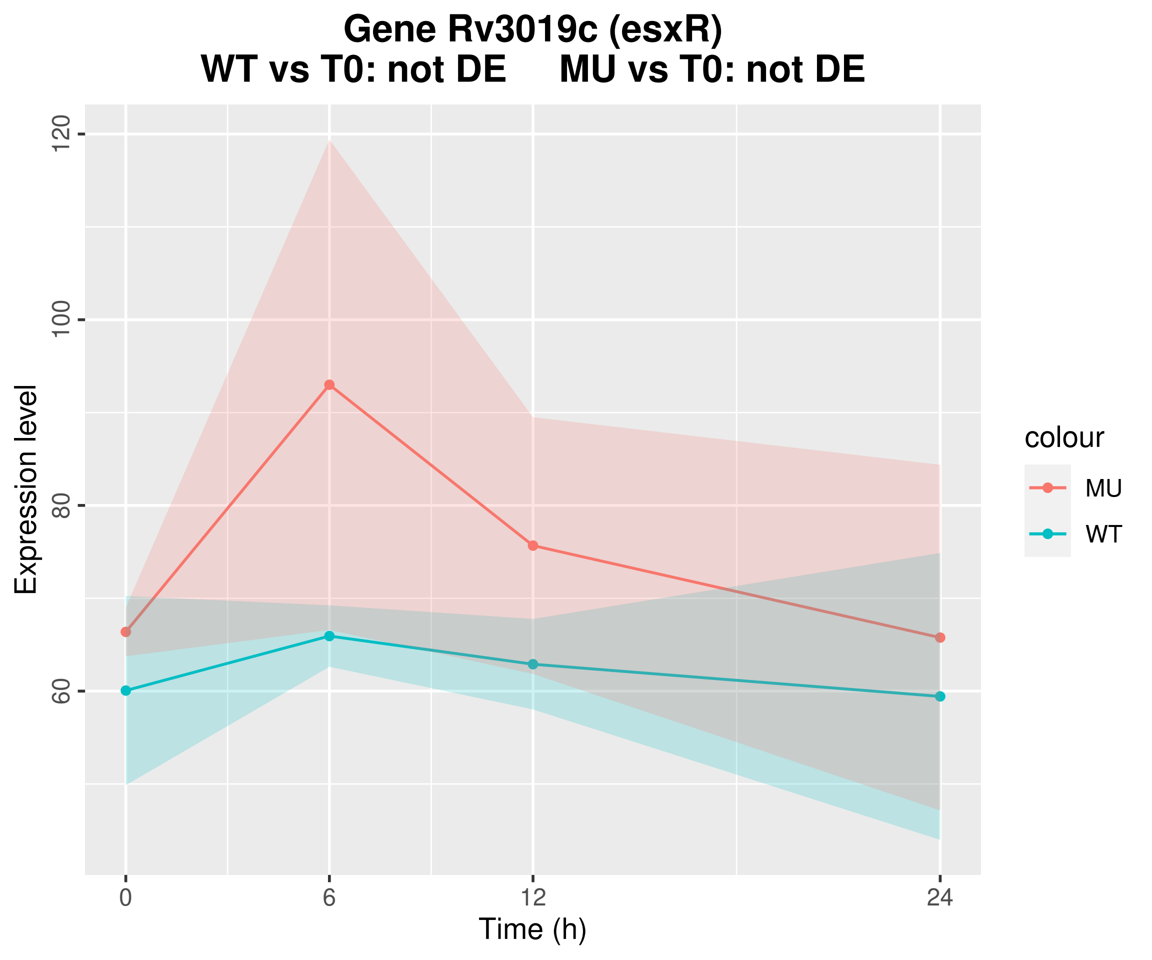
Gene Rv3019c (esxR)
WT vs T0: not DE   MU vs T0: not DE
Expression level
120
100
80
60
0
6
12
24
Time (h)
colour
MU
WT

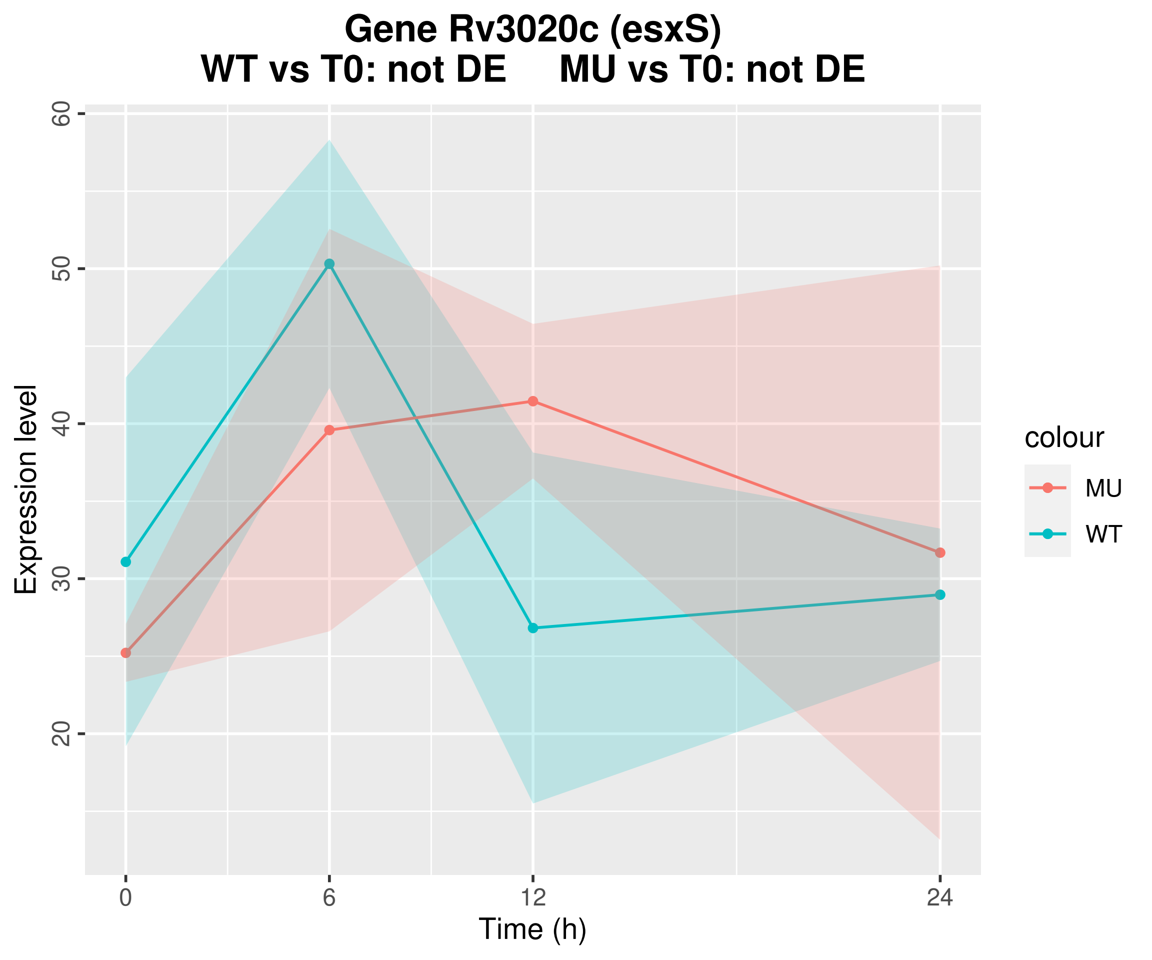

Gene Rv3020c (esxS)
WT vs T0: not DE   MU vs T0: not DE
Expression level
60
50
40
30
20
0
6
12
24
Time (h)
colour
MU
WT

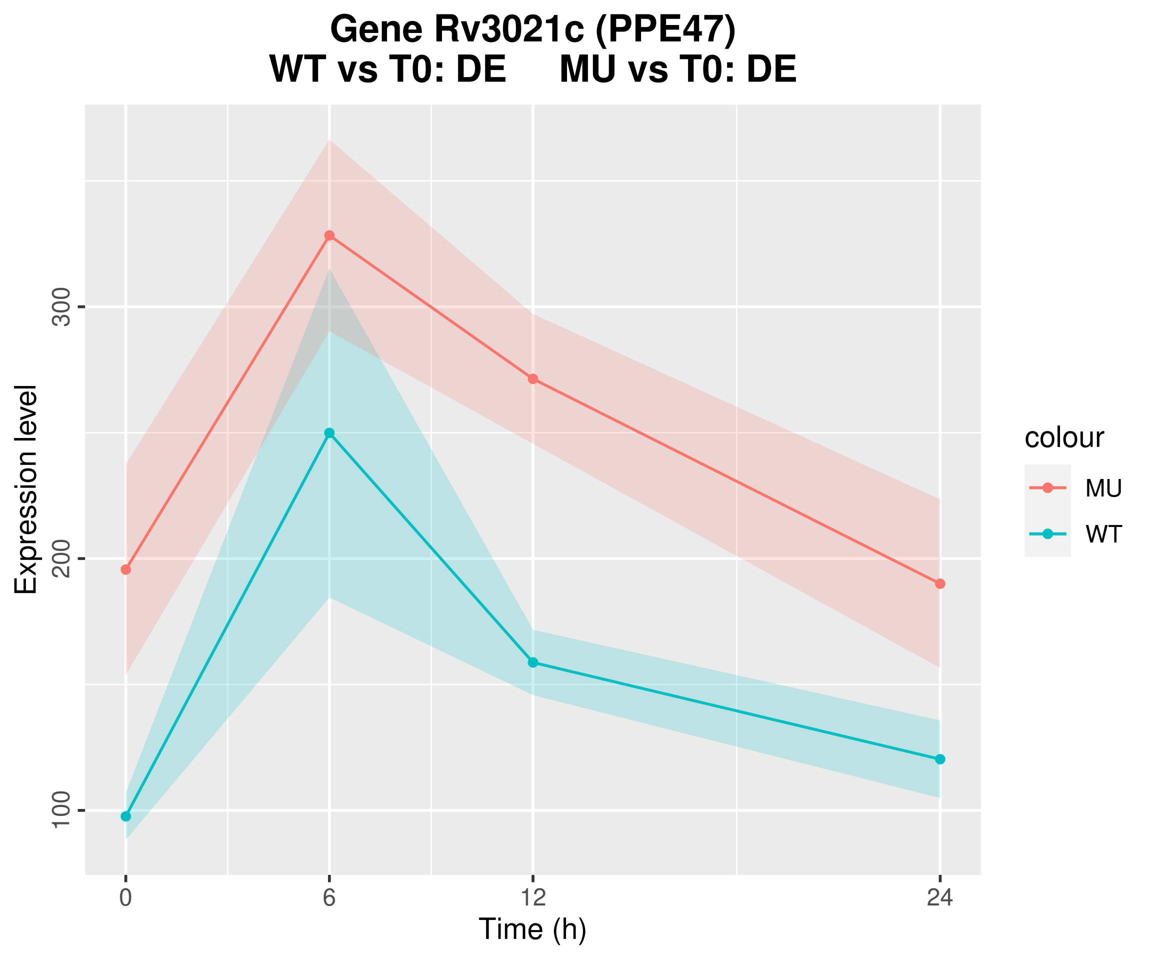

Gene Rv3021c (PPE47)
WT vs T0: DE   MU vs T0: DE
Expression level
300
200
100
0
6
12
24
Time (h)
colour
MU
WT

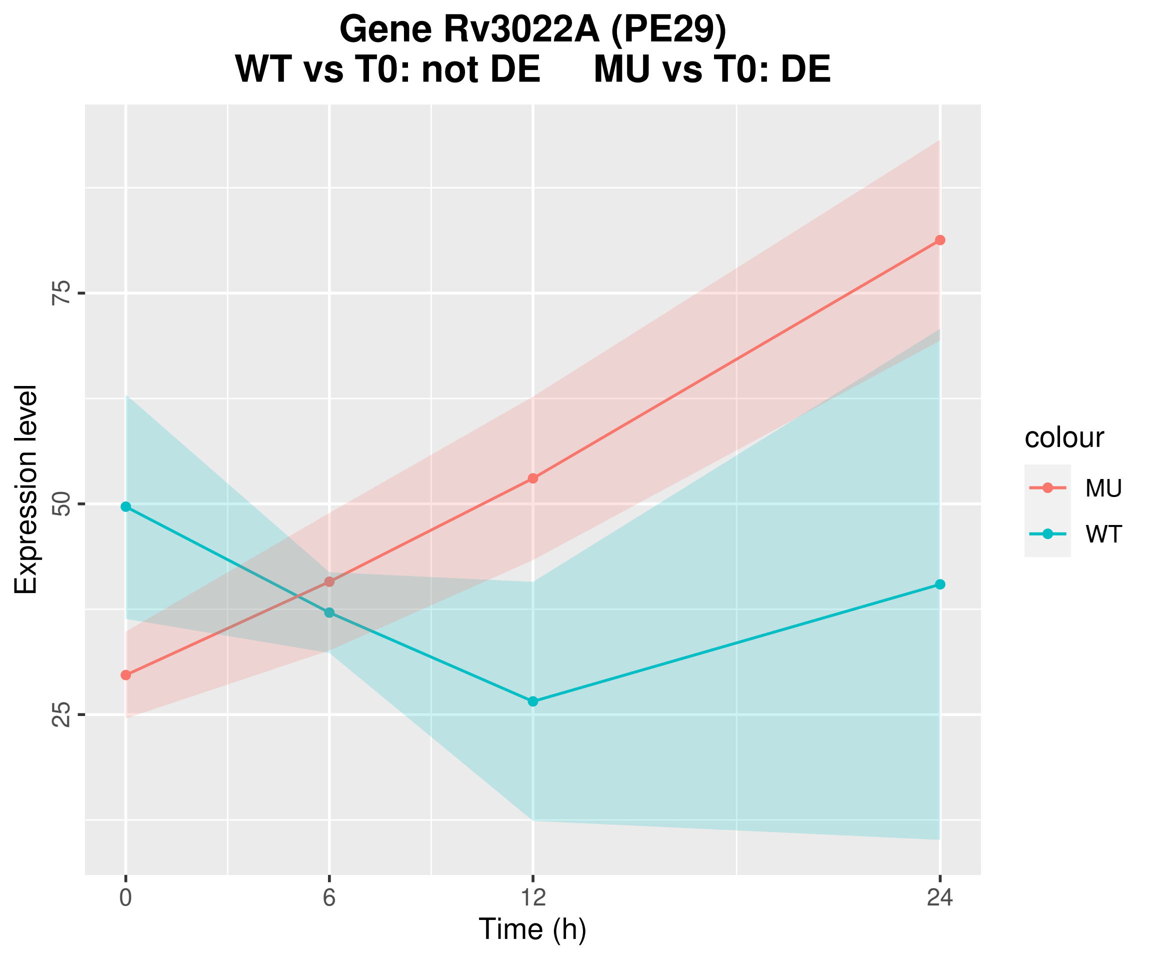
Gene Rv3022A (PE29)
WT vs T0: not DE   MU vs T0: DE
Expression level
75
50
25
0
6
12
24
Time (h)
colour
MU
WT

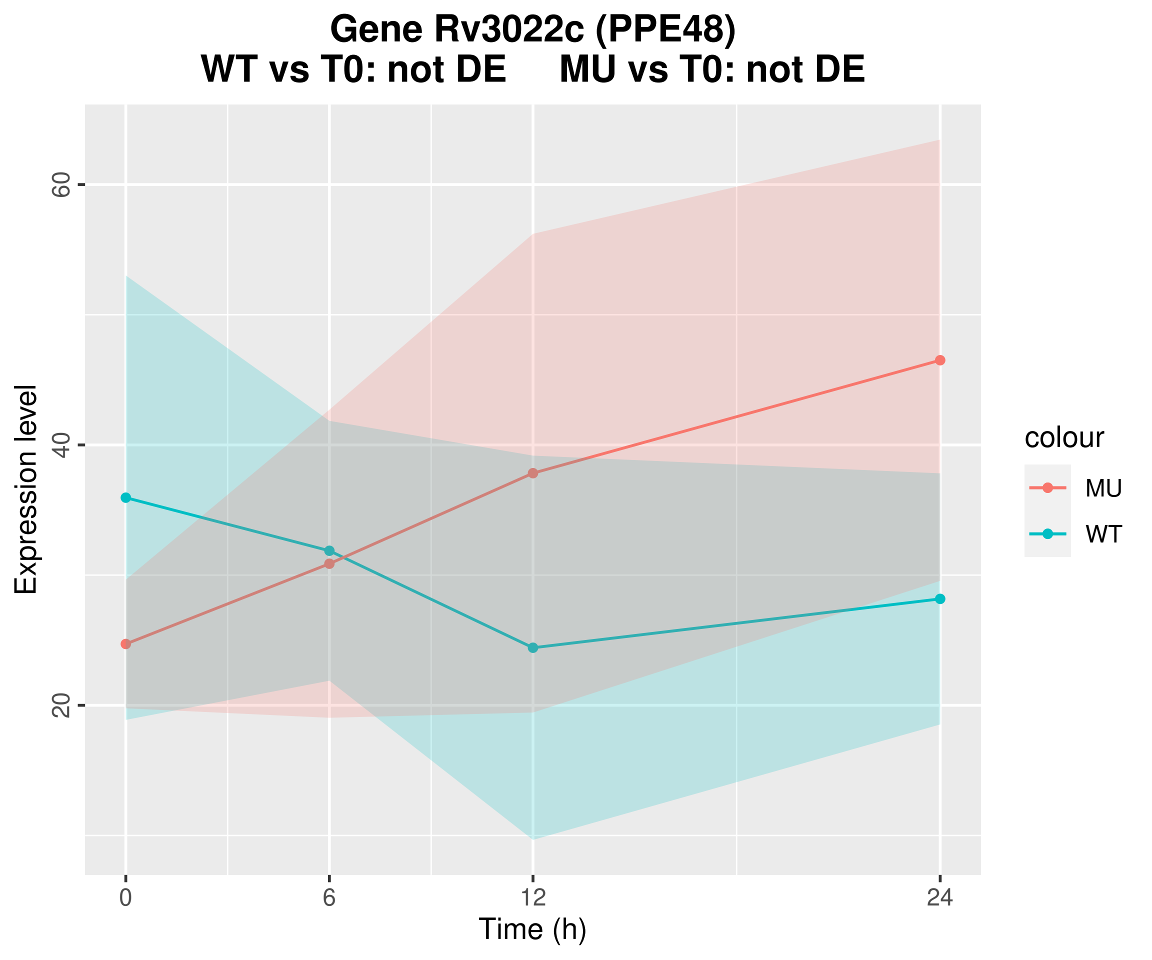

Gene Rv3022c (PPE48)
WT vs T0: not DE MU vs T0: not DE
Expression level
60
40
20
0
6
12
24
Time (h)
colour
MU
WT

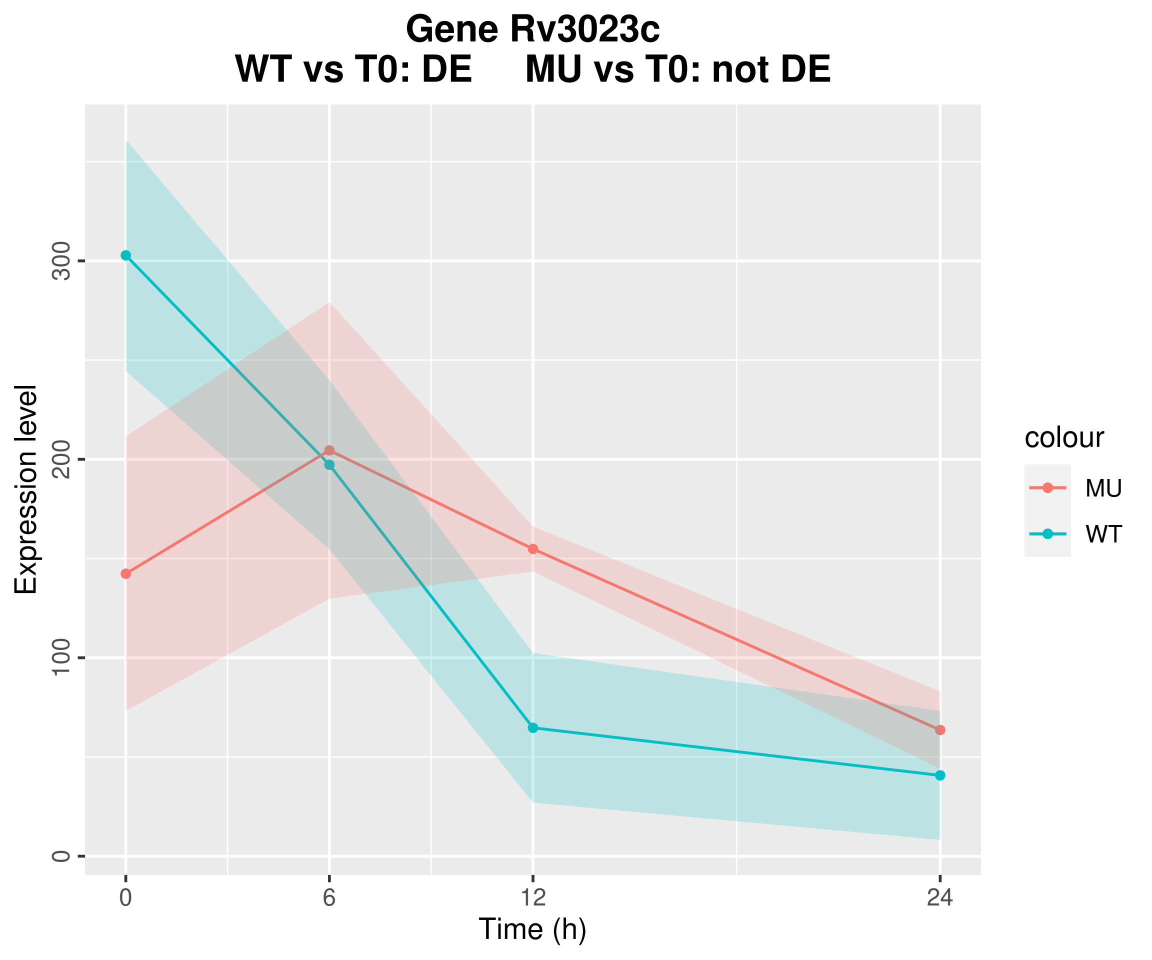

Gene Rv3023c
WT vs T0: DE MU vs T0: not DE
Expression level
300
200
100
0
0
6
12
24
Time (h)
colour
MU
WT

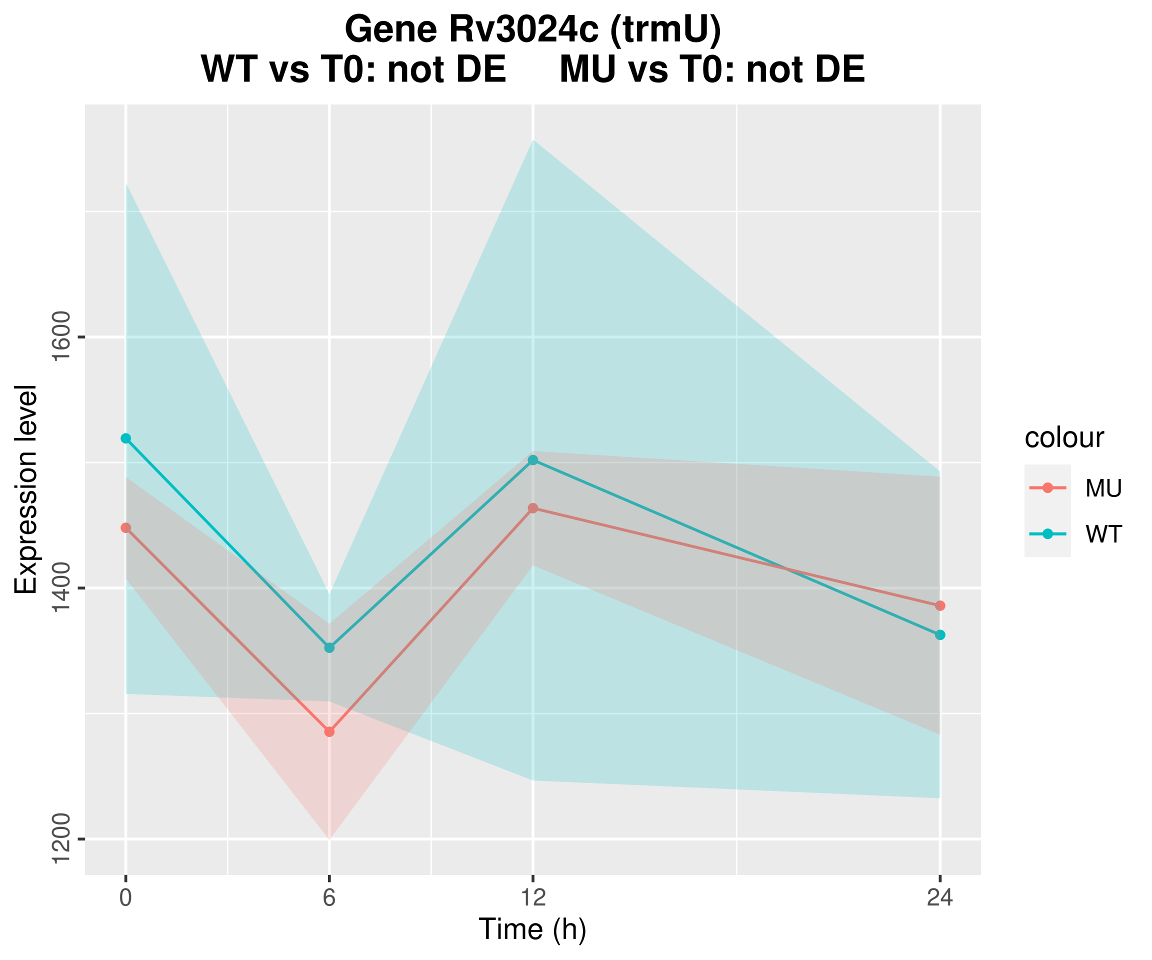
Gene Rv3024c (trmU)
WT vs T0: not DE   MU vs T0: not DE
Expression level
1200
1400
1600
0
6
12
24
Time (h)
colour
MU
WT

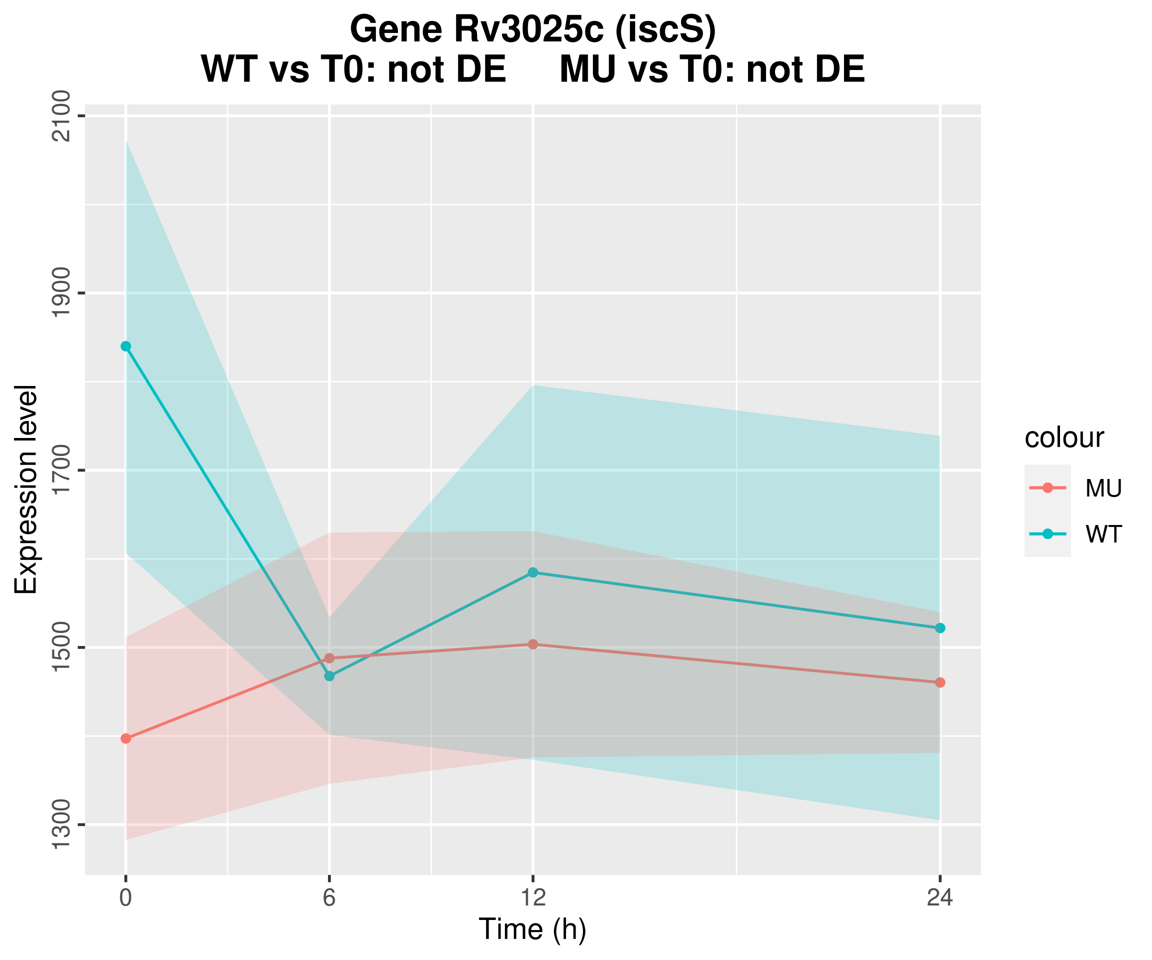
Gene Rv3025c (iscS)
WT vs T0: not DE   MU vs T0: not DE
Expression level
2100
1900
1700
1500
1300
0
6
12
24
Time (h)
colour
MU
WT

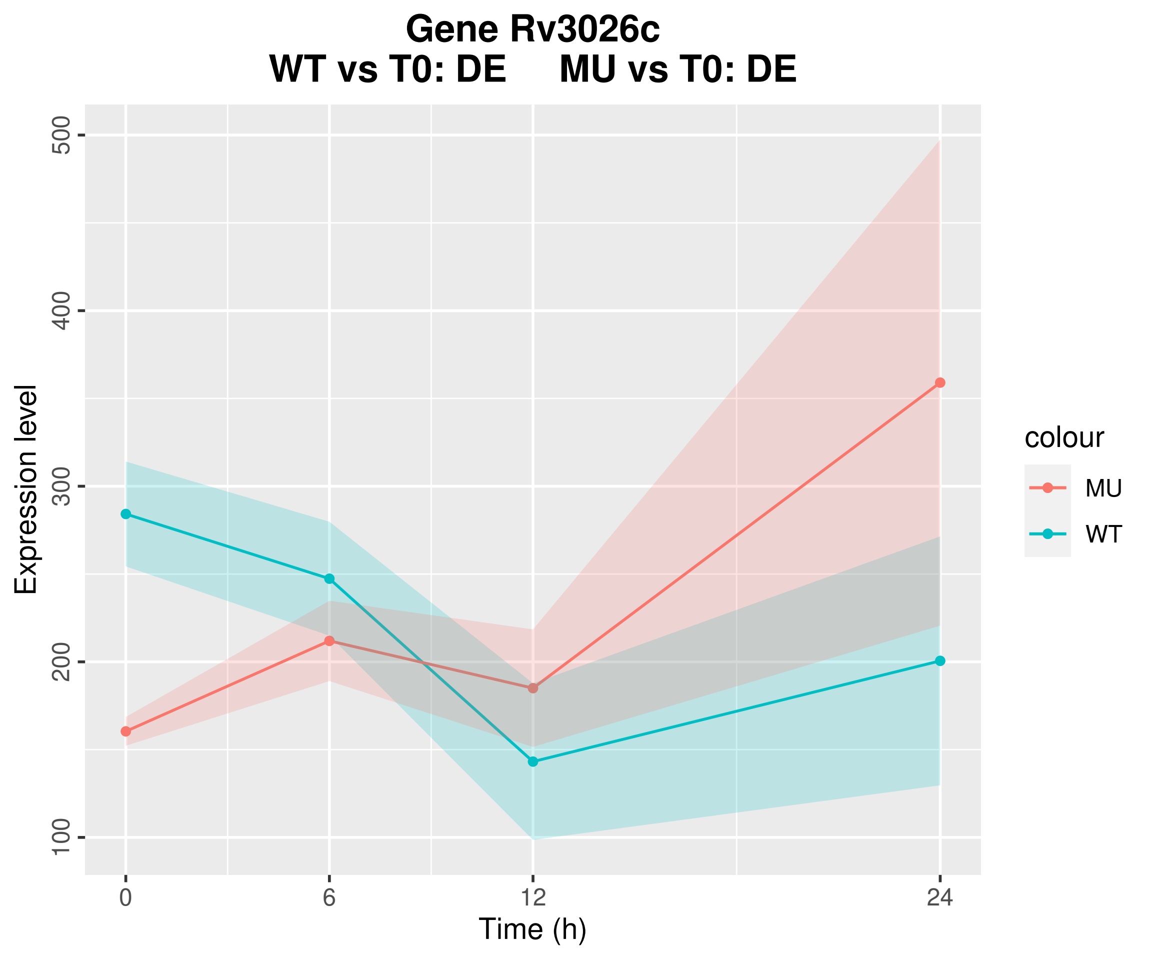
Gene Rv3026c
WT vs T0: DE MU vs T0: DE
Expression level
500
400
300
200
100
0
6
12
24
Time (h)
colour
MU
WT

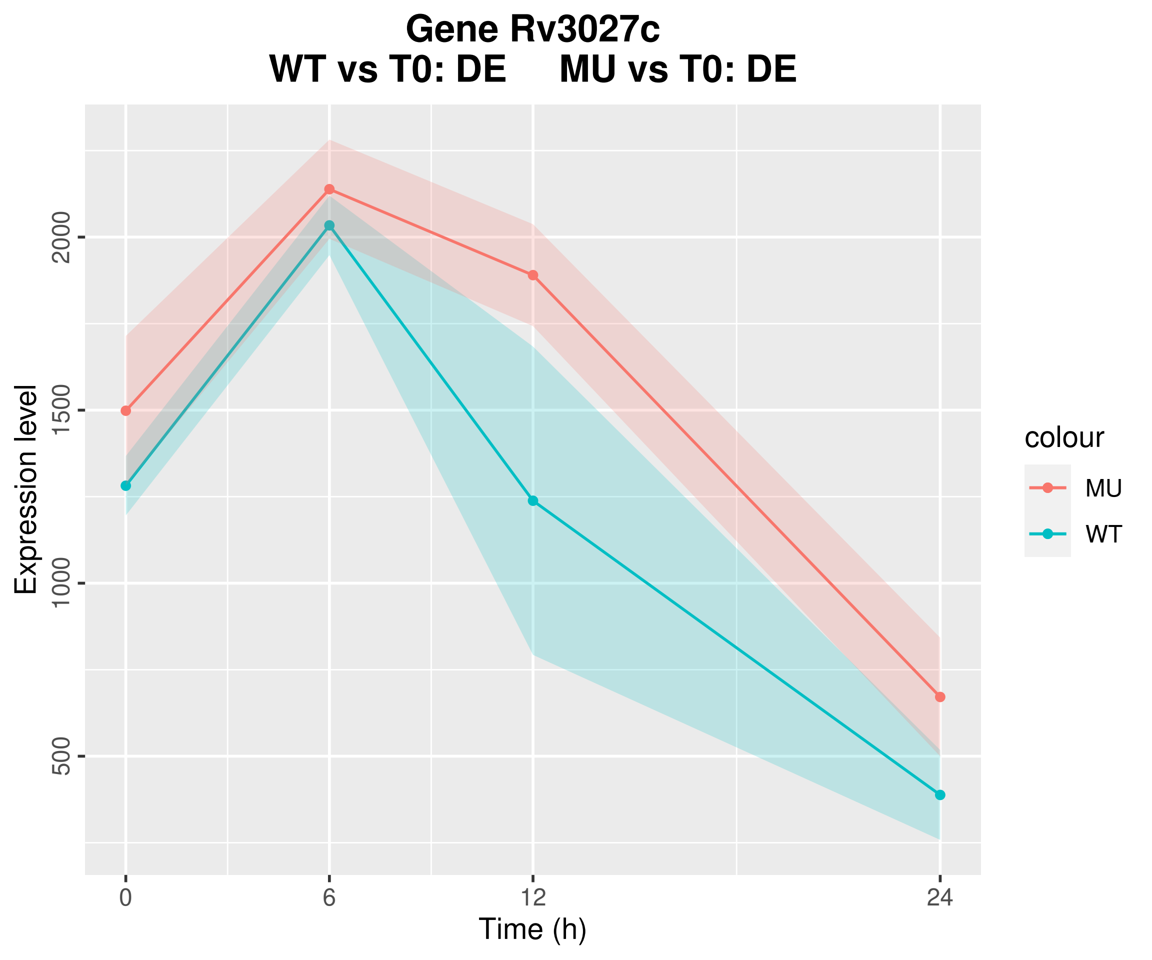

Gene Rv3027c
WT vs T0: DE   MU vs T0: DE
2000
1500
1000
500
Expression level
0
6
12
24
Time (h)
colour
MU
WT

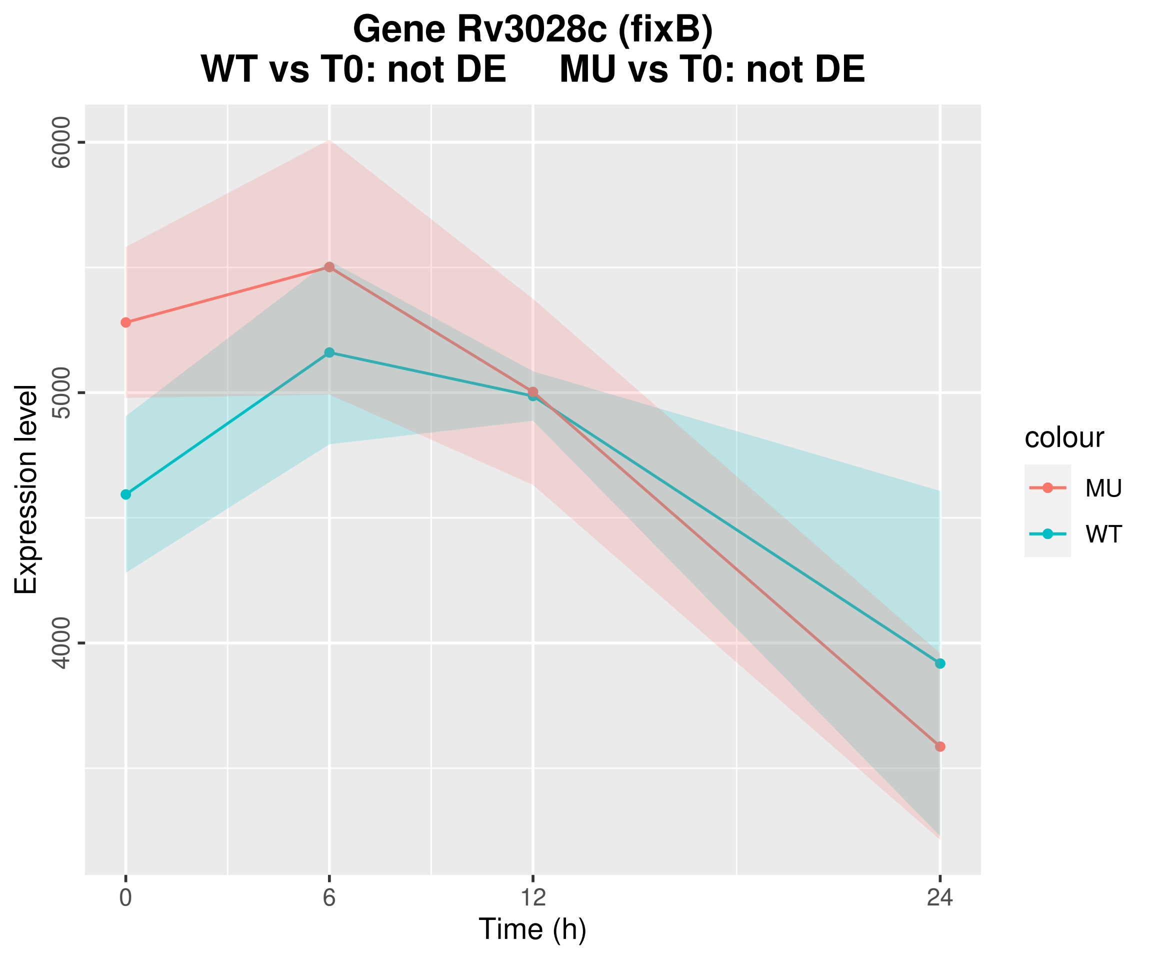

Gene Rv3028c (fixB)
WT vs T0: not DE MU vs T0: not DE
Expression level
6000
5000
4000
0
6
12
24
Time (h)
colour
MU
WT

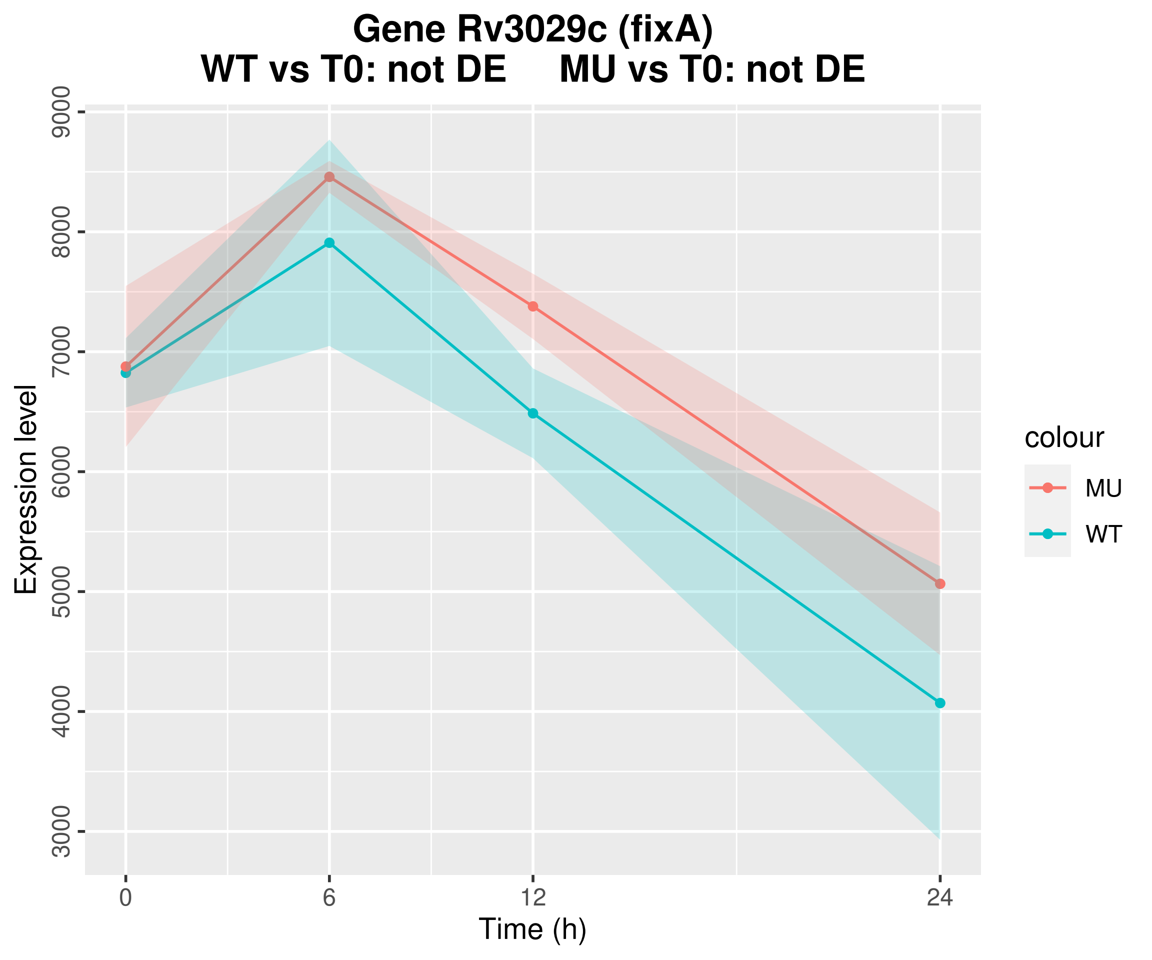

Gene Rv3029c (fixA)
WT vs T0: not DE   MU vs T0: not DE
Expression level
9000
8000
7000
6000
5000
4000
3000
0
6
12
24
Time (h)
colour
MU
WT

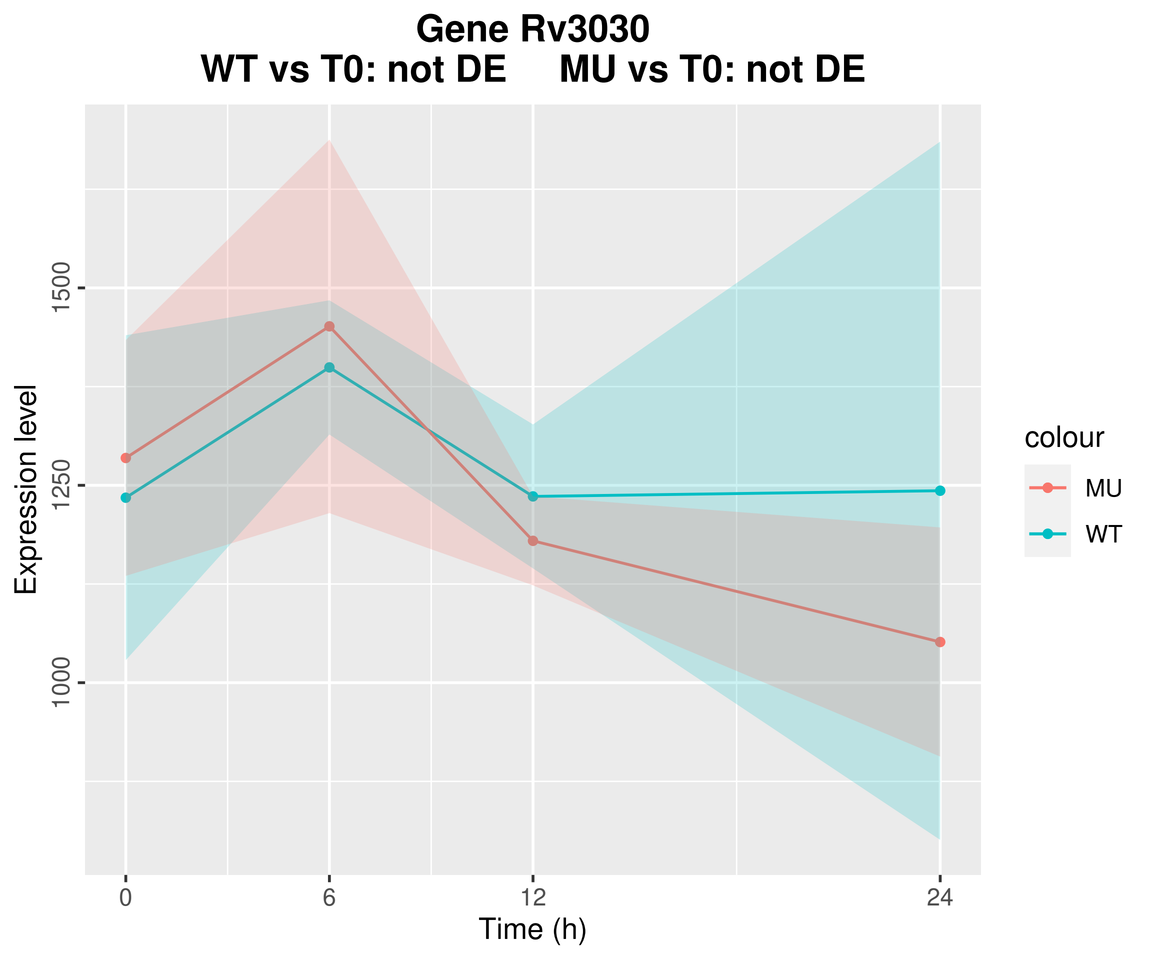
Gene Rv3030
WT vs T0: not DE   MU vs T0: not DE
Expression level
1500
1250
1000
0
6
12
24
Time (h)
colour
MU
WT

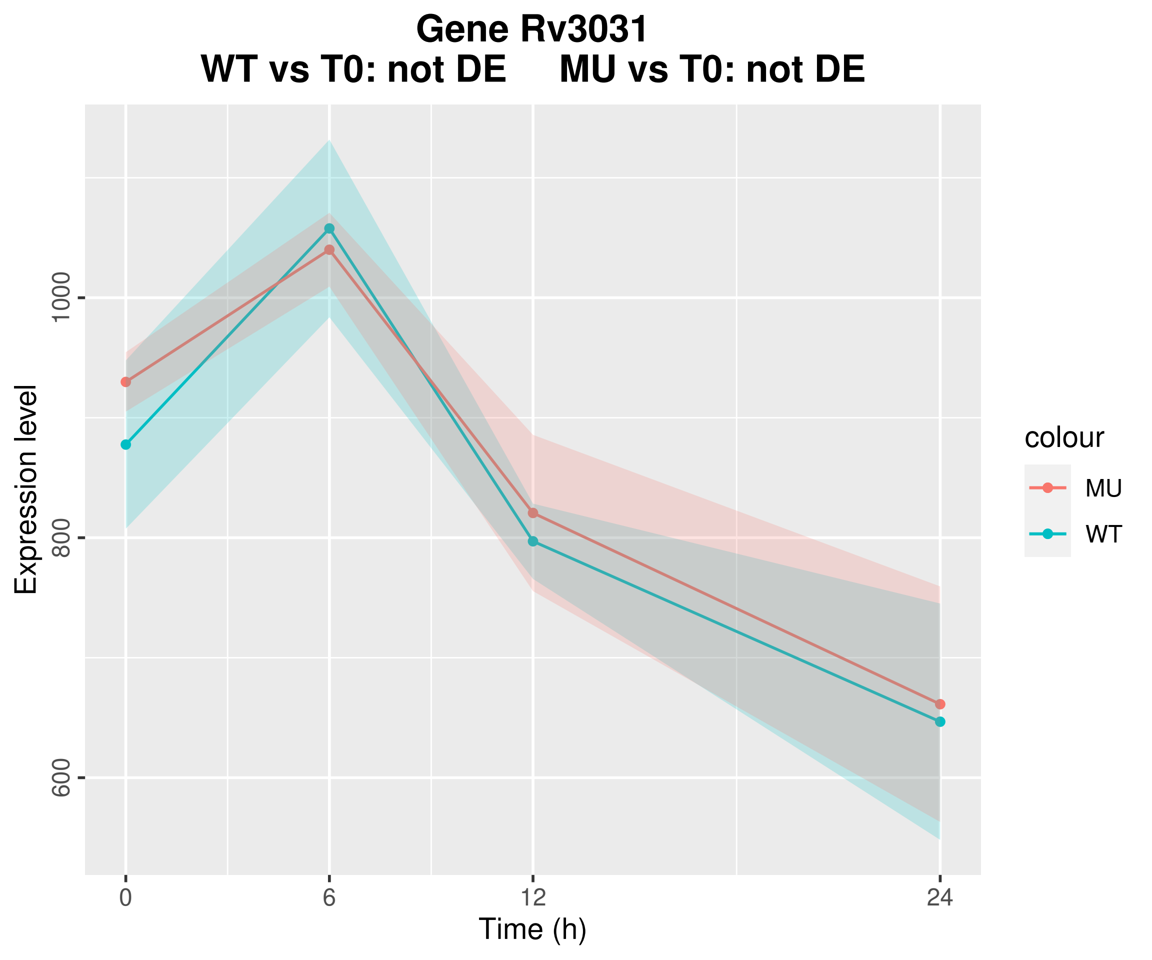

Gene Rv3031
WT vs T0: not DE   MU vs T0: not DE
1000
800
600
Expression level
0
6
12
24
Time (h)
colour
MU
WT

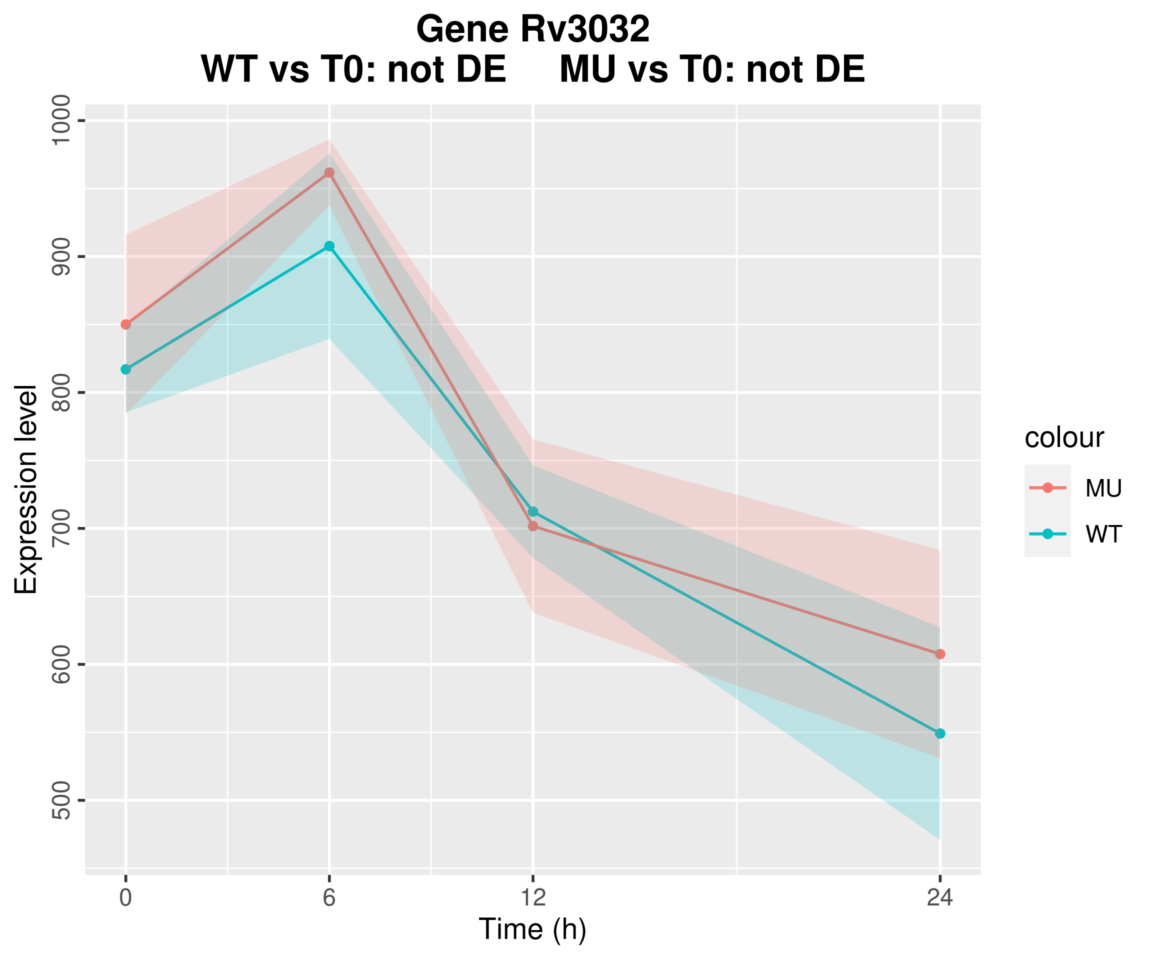

Gene Rv3032
WT vs T0: not DE   MU vs T0: not DE
1000
900
800
700
600
500
Expression level
0
6
12
24
Time (h)
colour
MU
WT

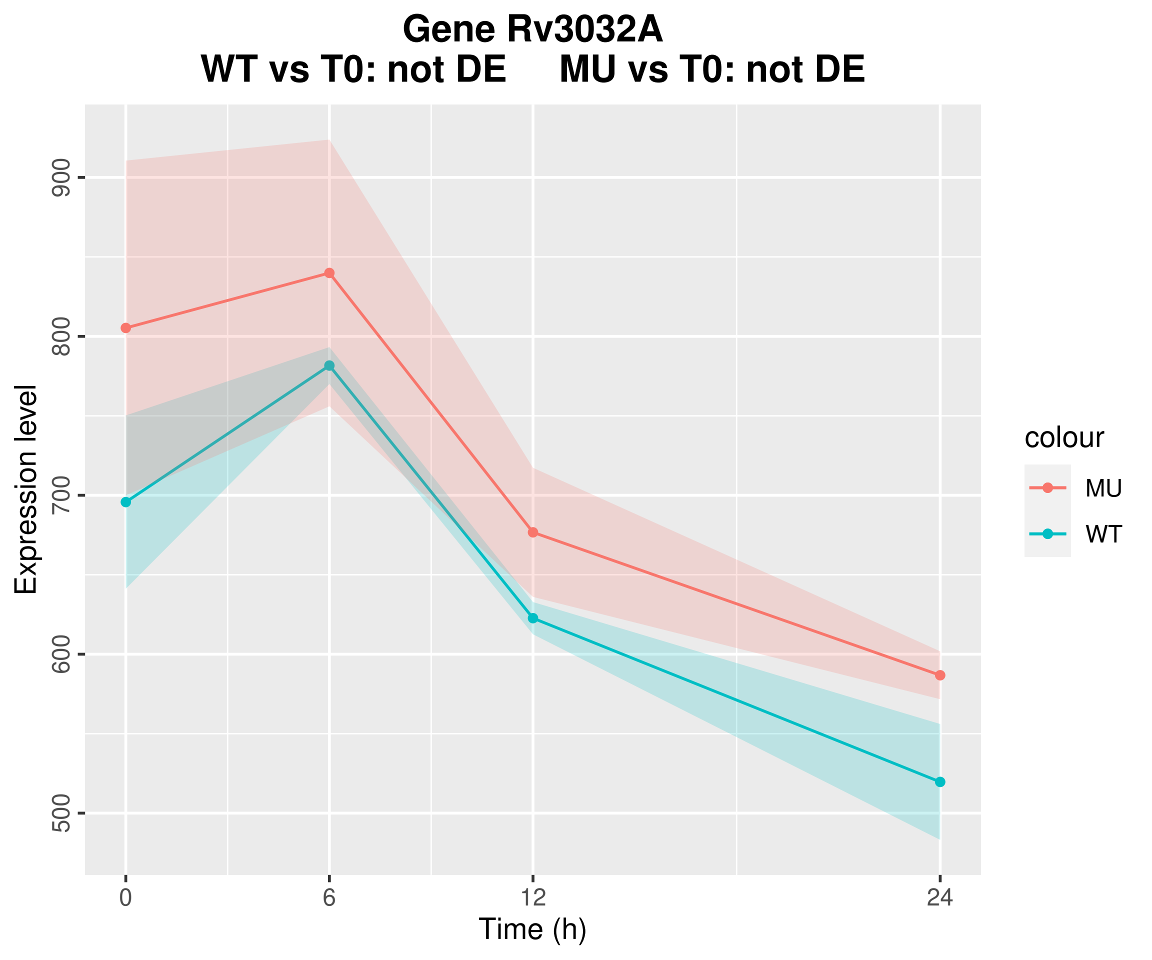
Gene Rv3032A
WT vs T0: not DE   MU vs T0: not DE
Expression level
900
800
700
600
500
0
6
12
24
Time (h)
colour
MU
WT

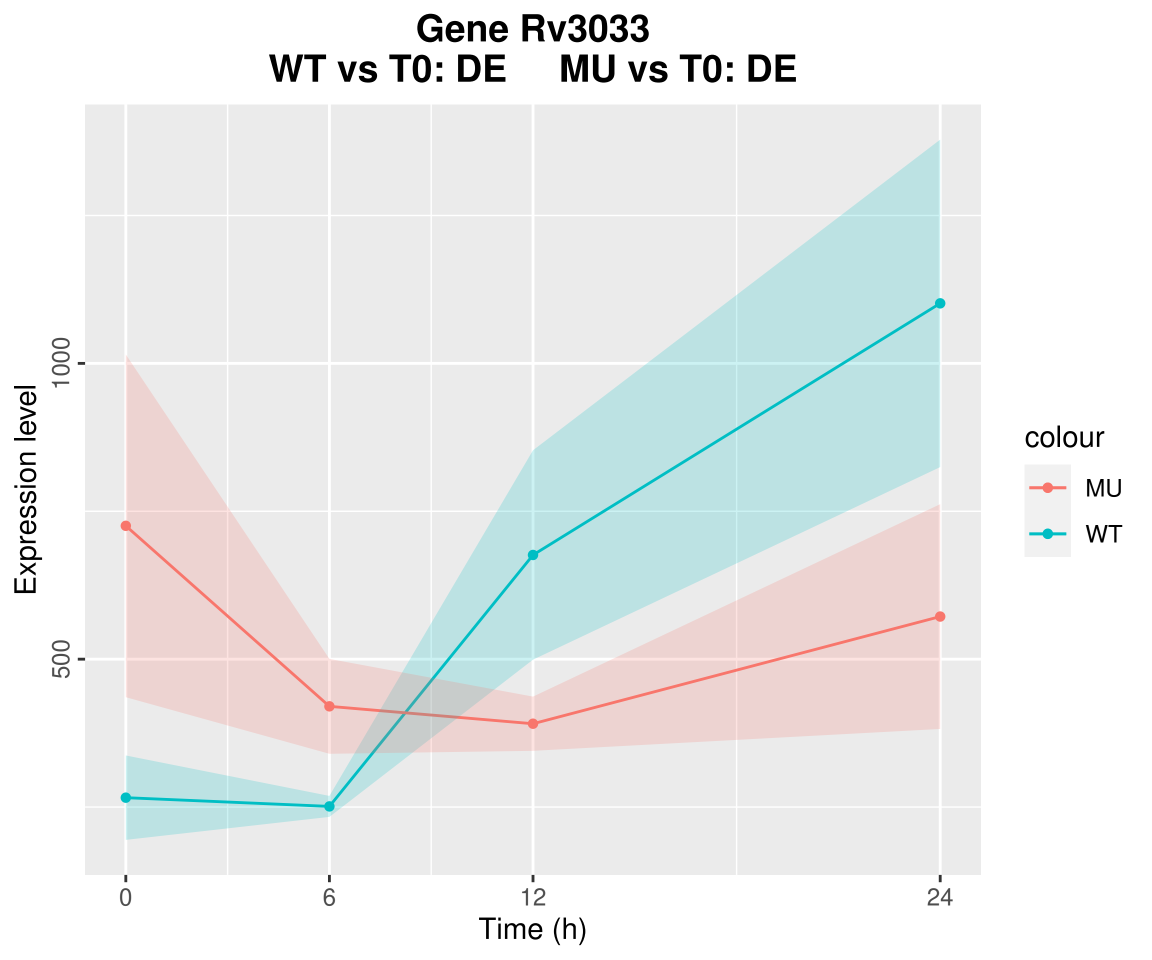

Gene Rv3033
WT vs T0: DE MU vs T0: DE
Expression level
1000
500
0
6
12
24
Time (h)
colour
MU
WT

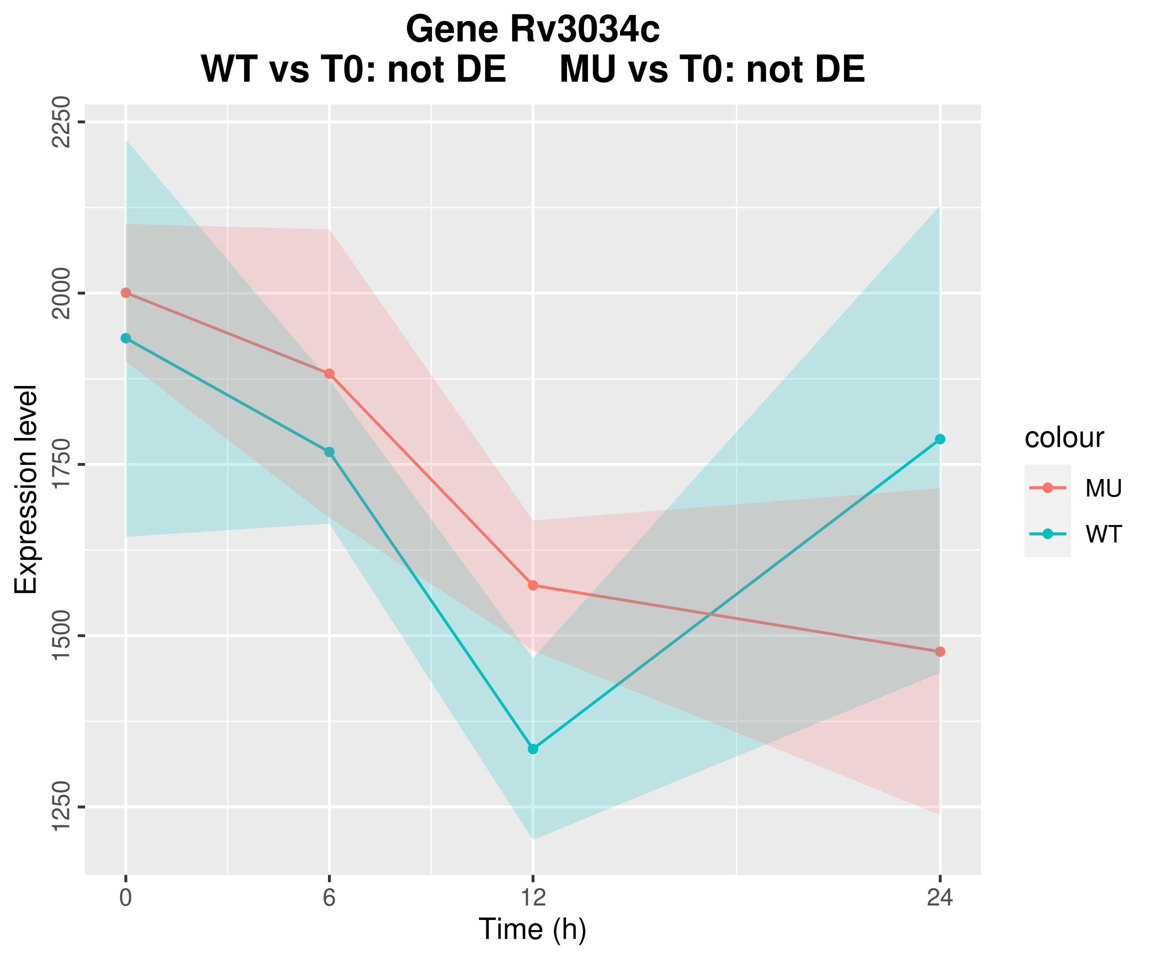

Gene Rv3034c
WT vs T0: not DE   MU vs T0: not DE
Expression level
2250
2000
1750
1500
1250
0
6
12
24
Time (h)
colour
MU
WT

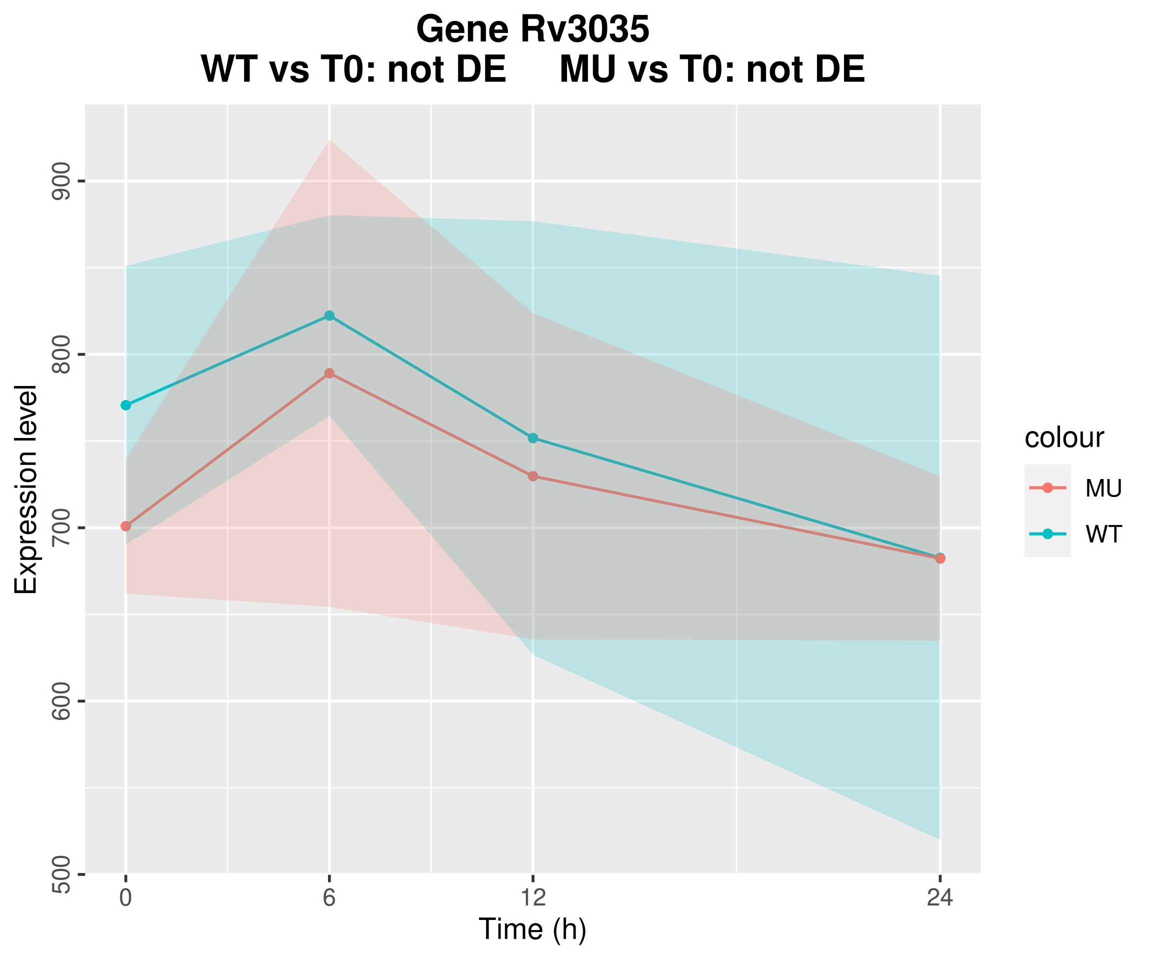
Gene Rv3035
WT vs T0: not DE MU vs T0: not DE
Expression level
900
800
700
600
500
0
6
12
24
Time (h)
colour
MU
WT

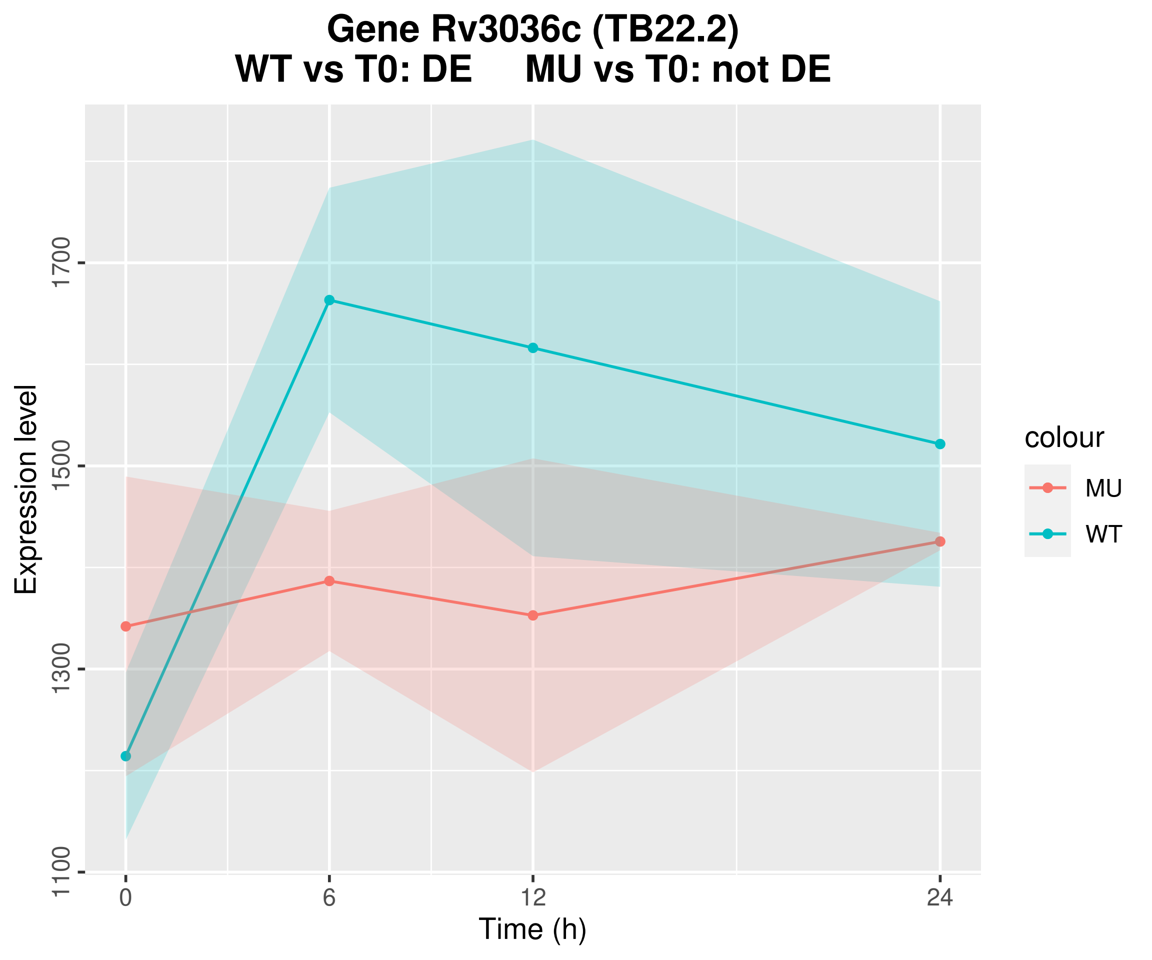
Gene Rv3036c (TB22.2)
WT vs T0: DE   MU vs T0: not DE
Expression level
1100
1300
1500
1700
0
6
12
24
Time (h)
colour
MU
WT

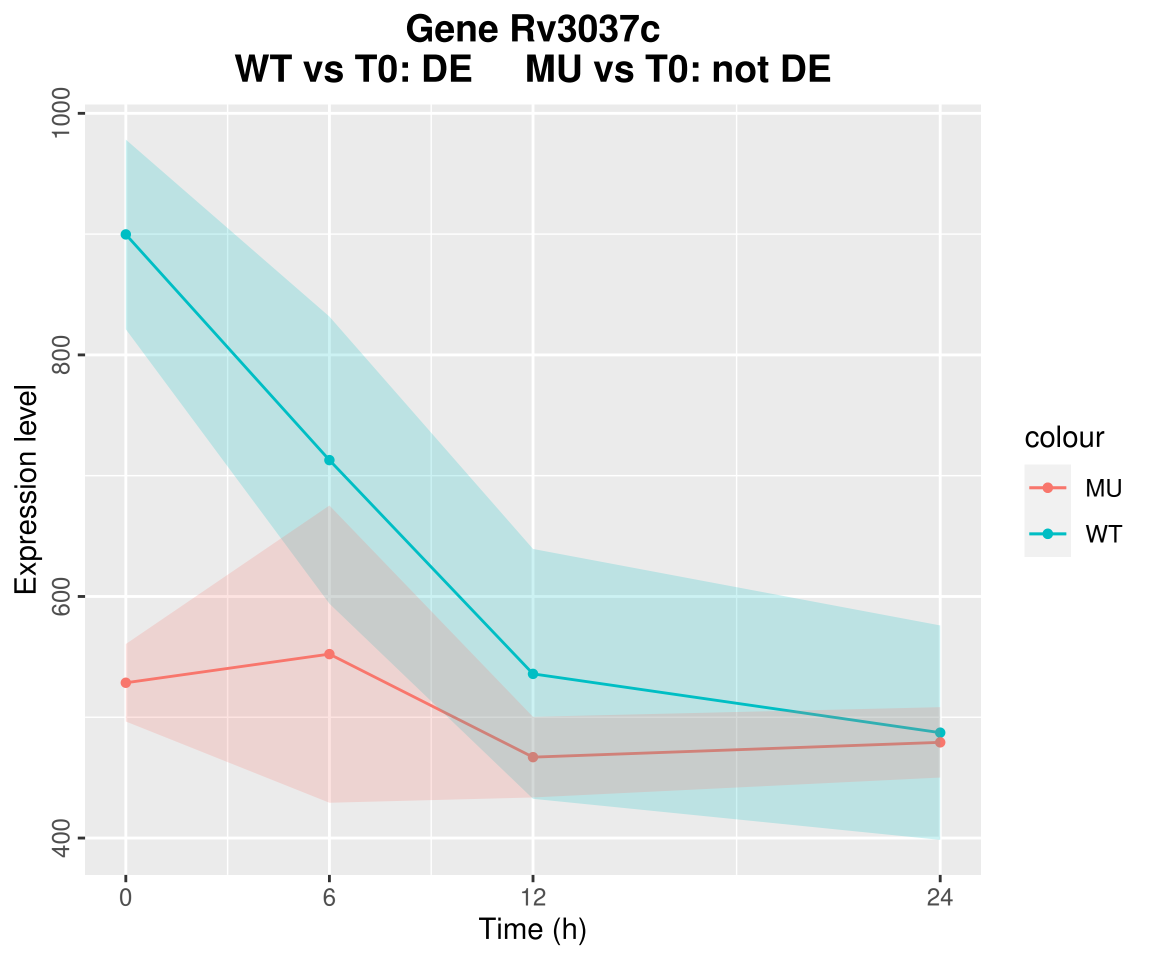
Gene Rv3037c
WT vs T0: DE   MU vs T0: not DE
Expression level
1000
800
600
400
0
6
12
24
Time (h)
colour
MU
WT

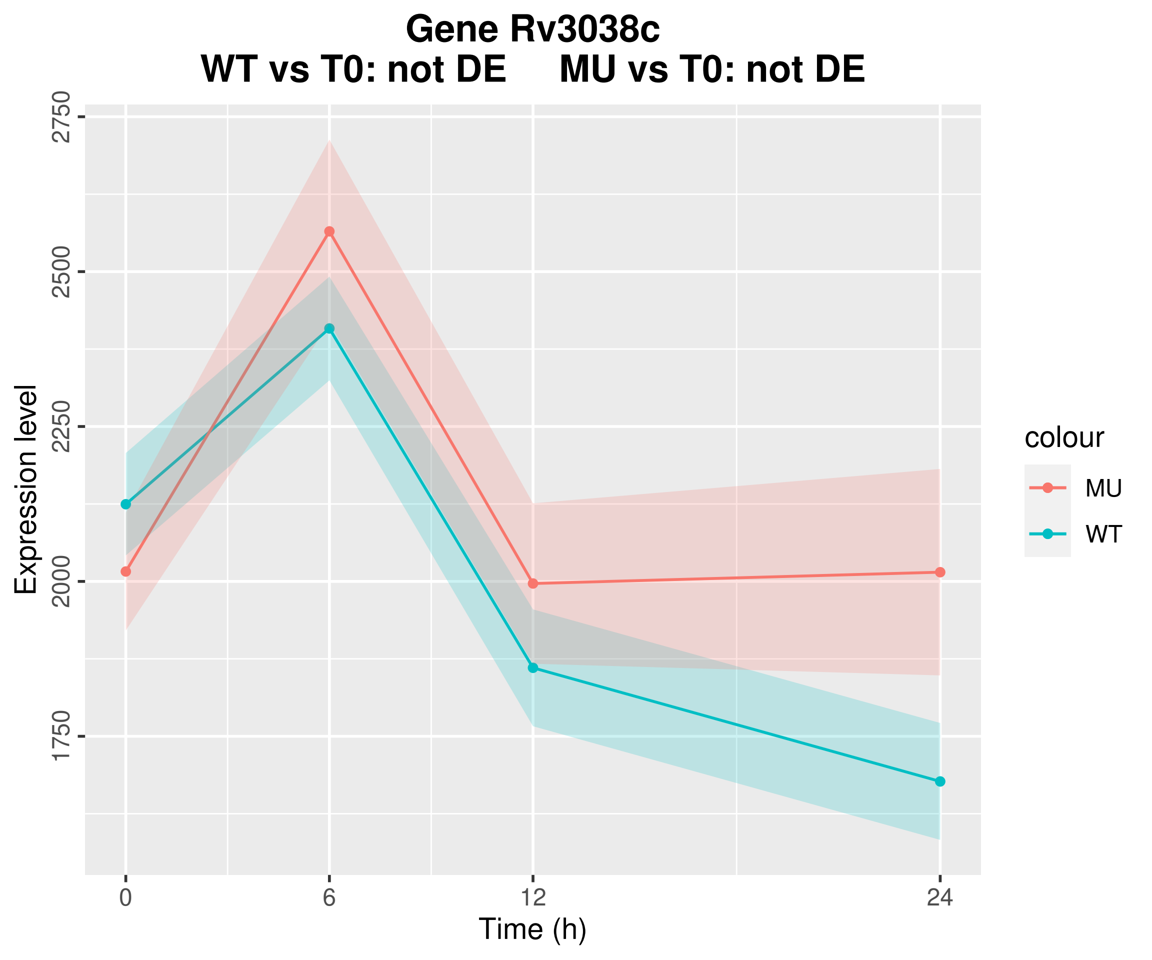

Gene Rv3038c
WT vs T0: not DE   MU vs T0: not DE
Expression level
2750
2500
2250
2000
1750
0
6
12
24
Time (h)
colour
MU
WT

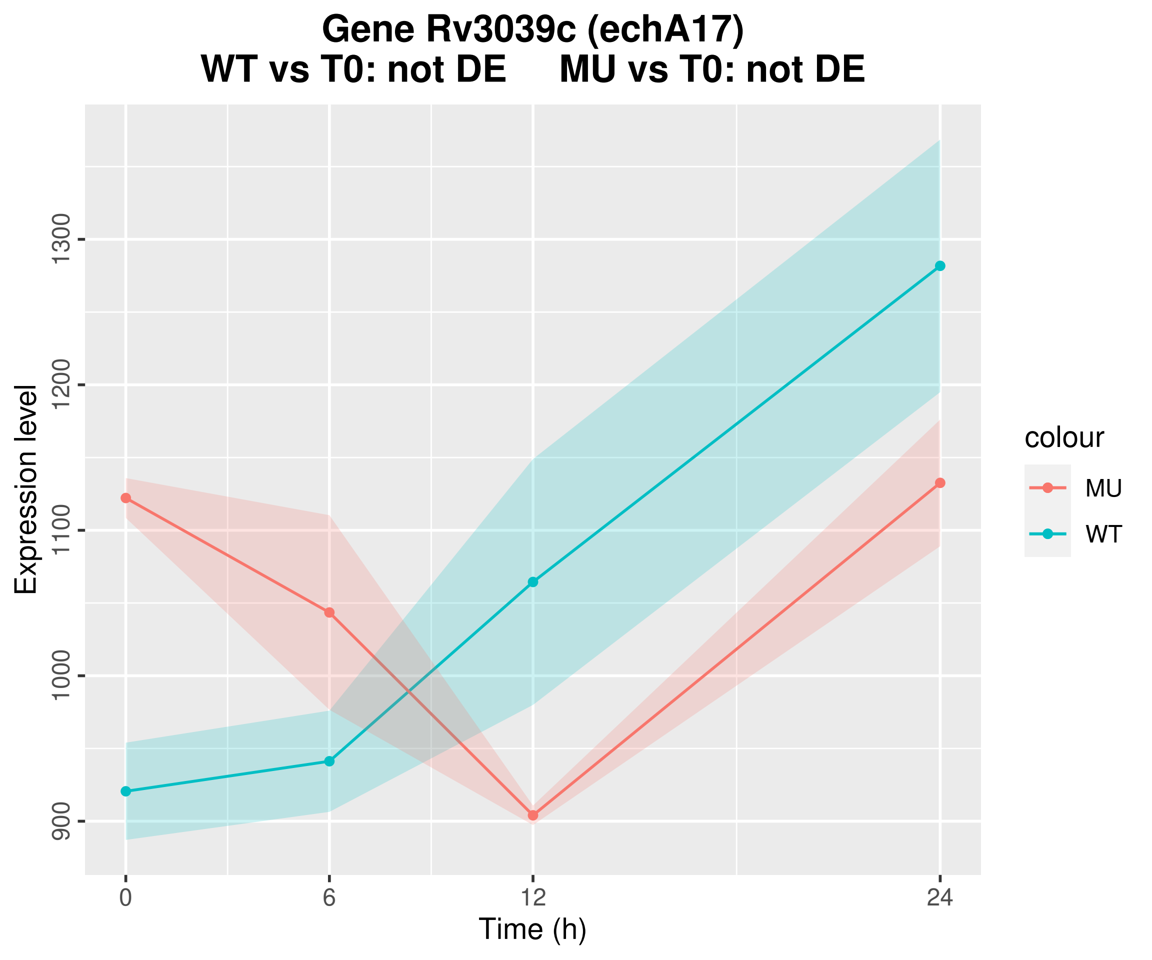
Gene Rv3039c (echA17)
WT vs T0: not DE   MU vs T0: not DE
Expression level
1300
1200
1100
1000
900
0
6
12
24
Time (h)
colour
MU
WT

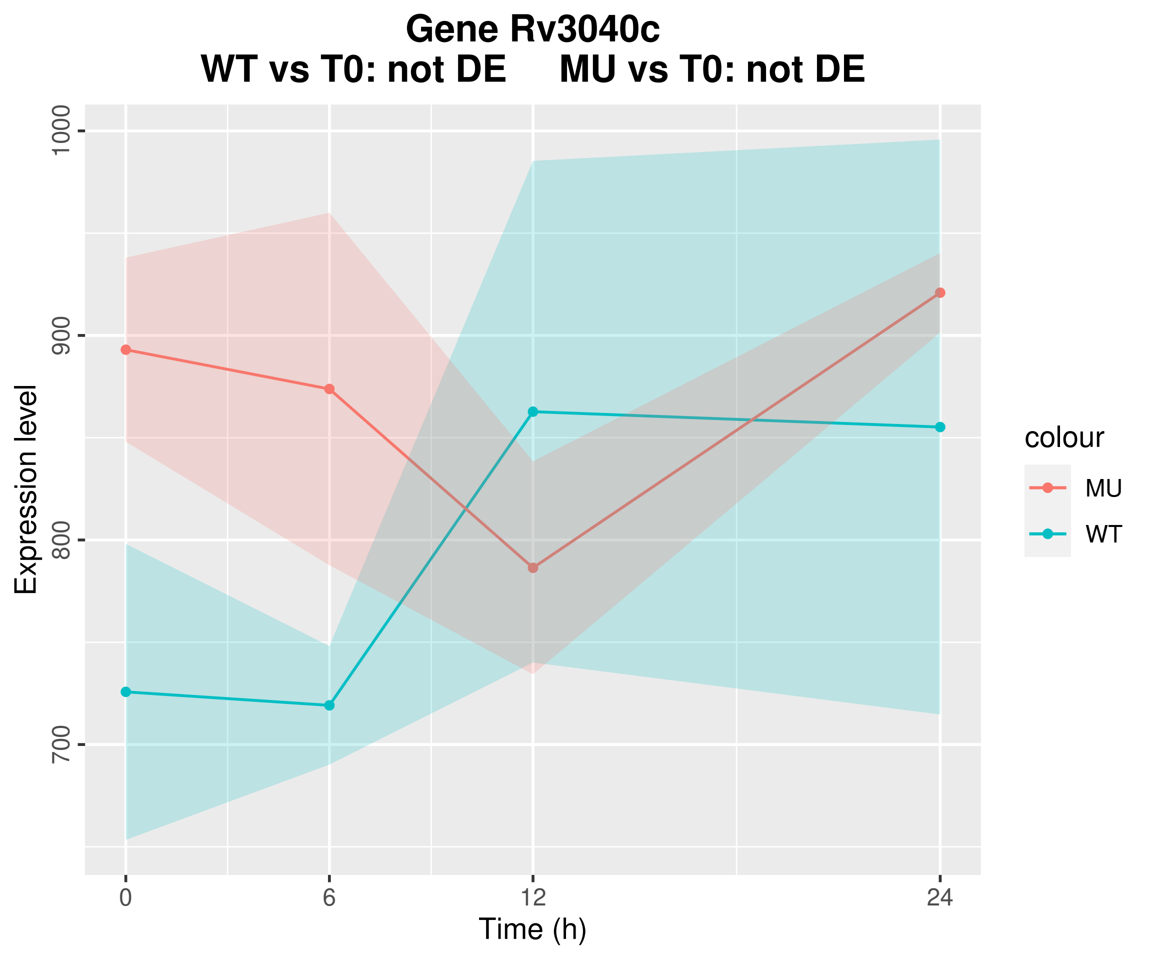
Gene Rv3040c
WT vs T0: not DE MU vs T0: not DE
1000
900
800
700
Expression level
0
6
12
24
Time (h)
colour
MU
WT

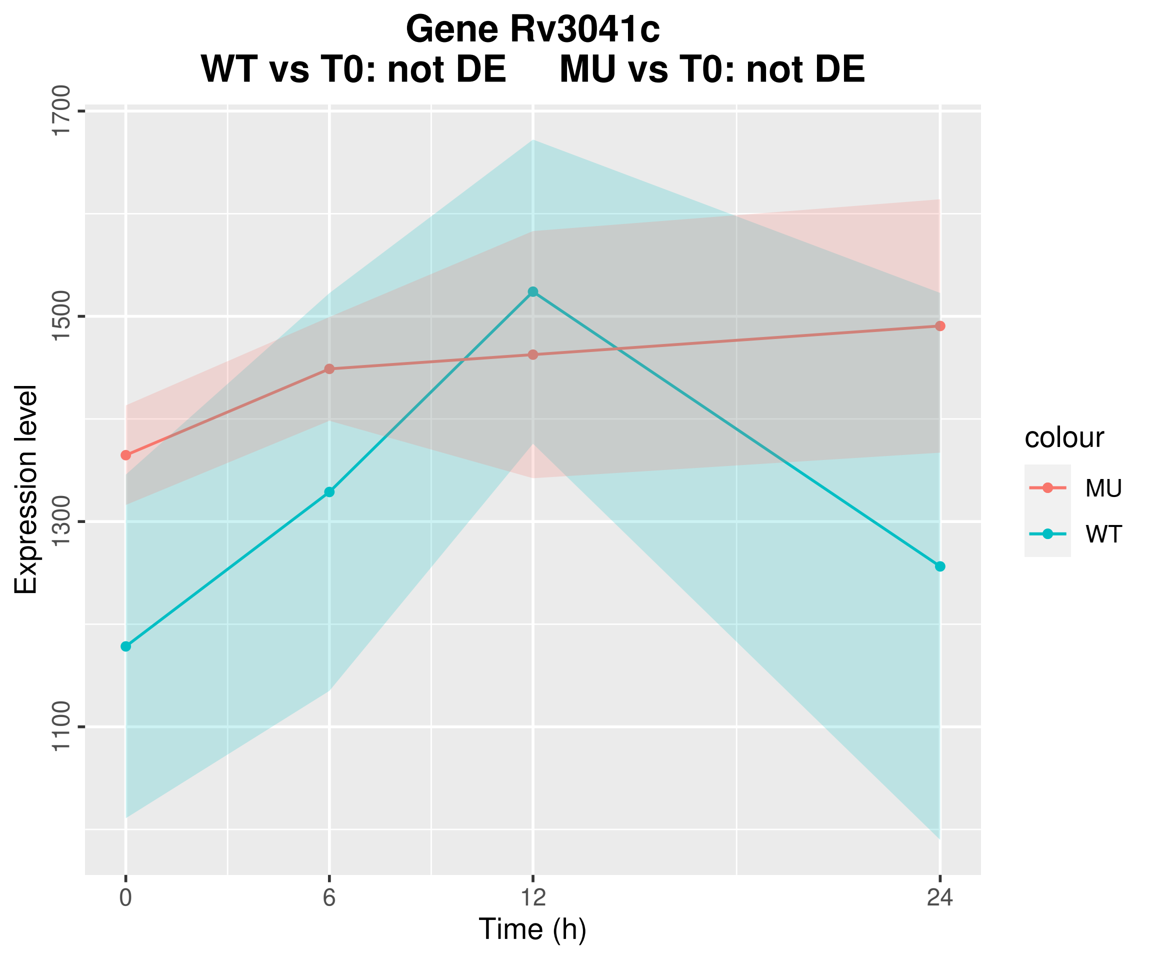
Gene Rv3041c
WT vs T0: not DE MU vs T0: not DE
1700
1500
1300
1100
Expression level
0
6
12
24
Time (h)
colour
MU
WT

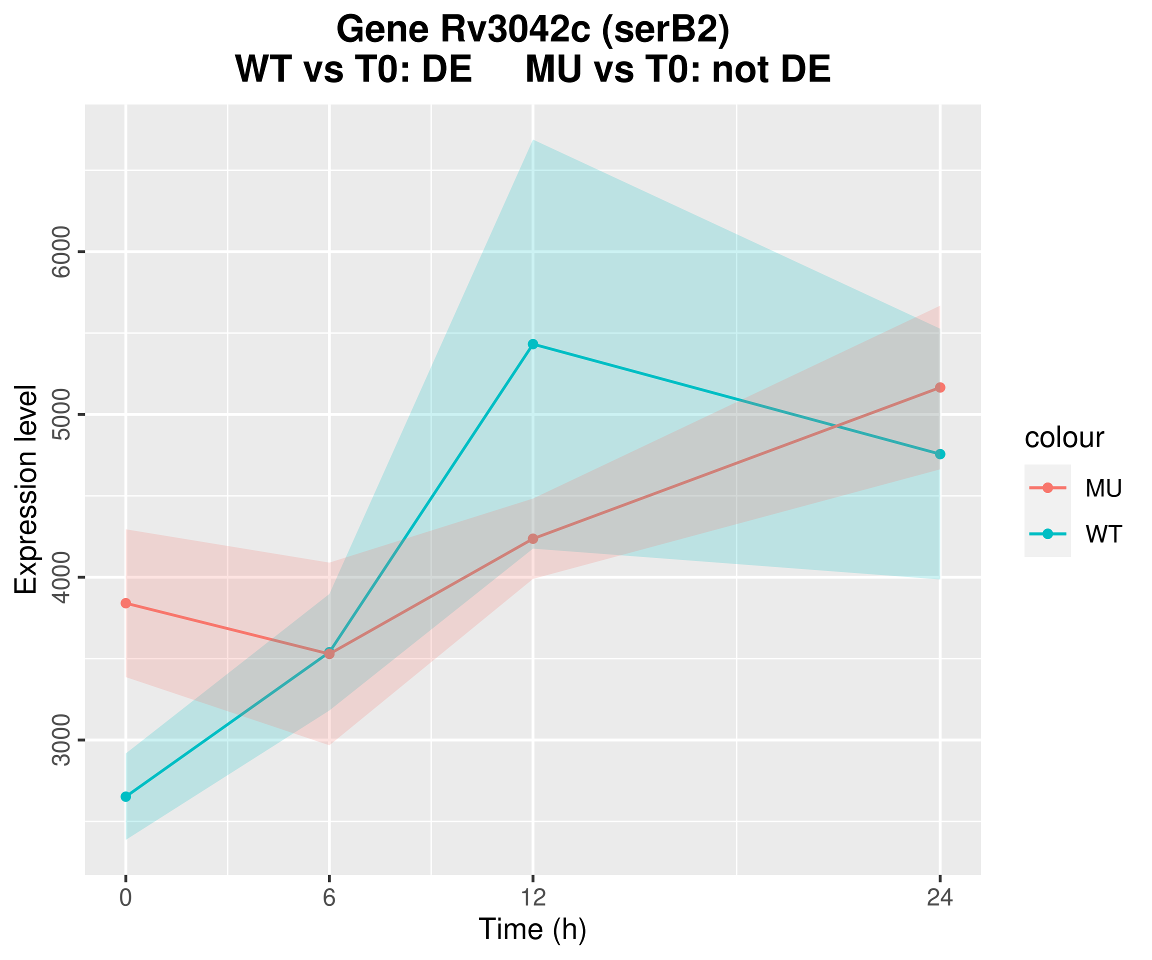

Gene Rv3042c (serB2)
WT vs T0: DE   MU vs T0: not DE
Expression level
6000
5000
4000
3000
0
6
12
24
Time (h)
colour
MU
WT

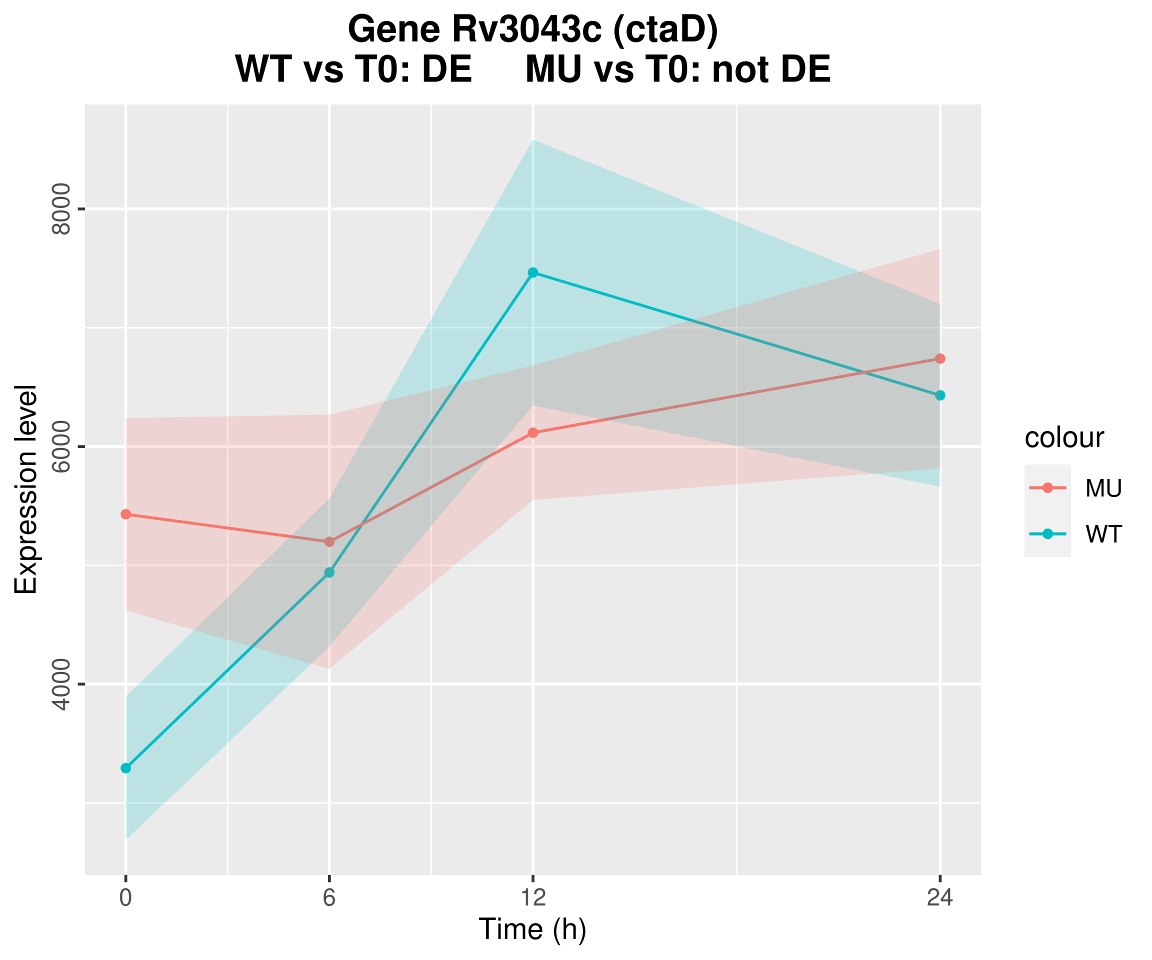

Gene Rv3043c (ctaD)
WT vs T0: DE   MU vs T0: not DE
Expression level
8000
6000
4000
0
6
12
24
Time (h)
colour
MU
WT

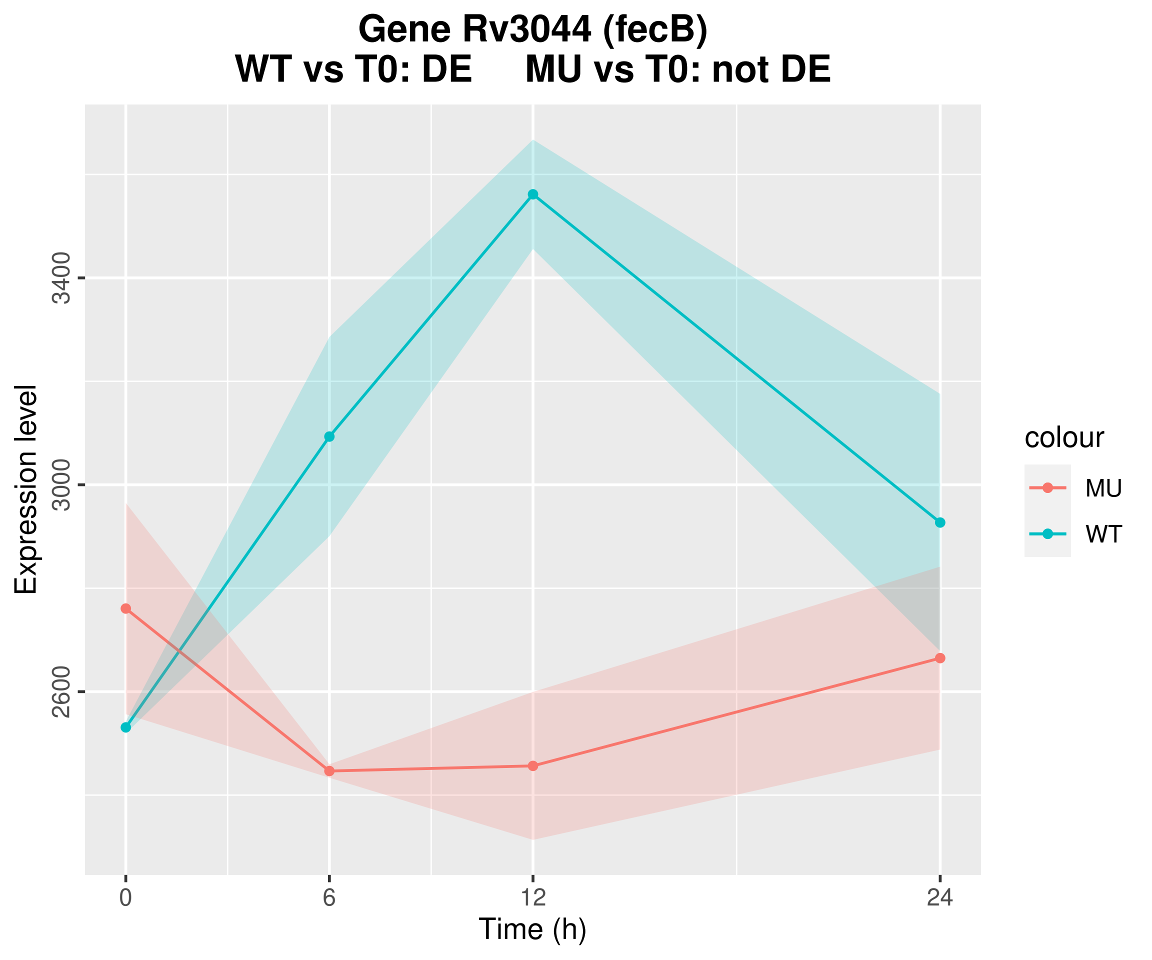
Gene Rv3044 (fecB)
WT vs T0: DE   MU vs T0: not DE
Expression level
3400
3000
2600
0
6
12
24
Time (h)
colour
MU
WT

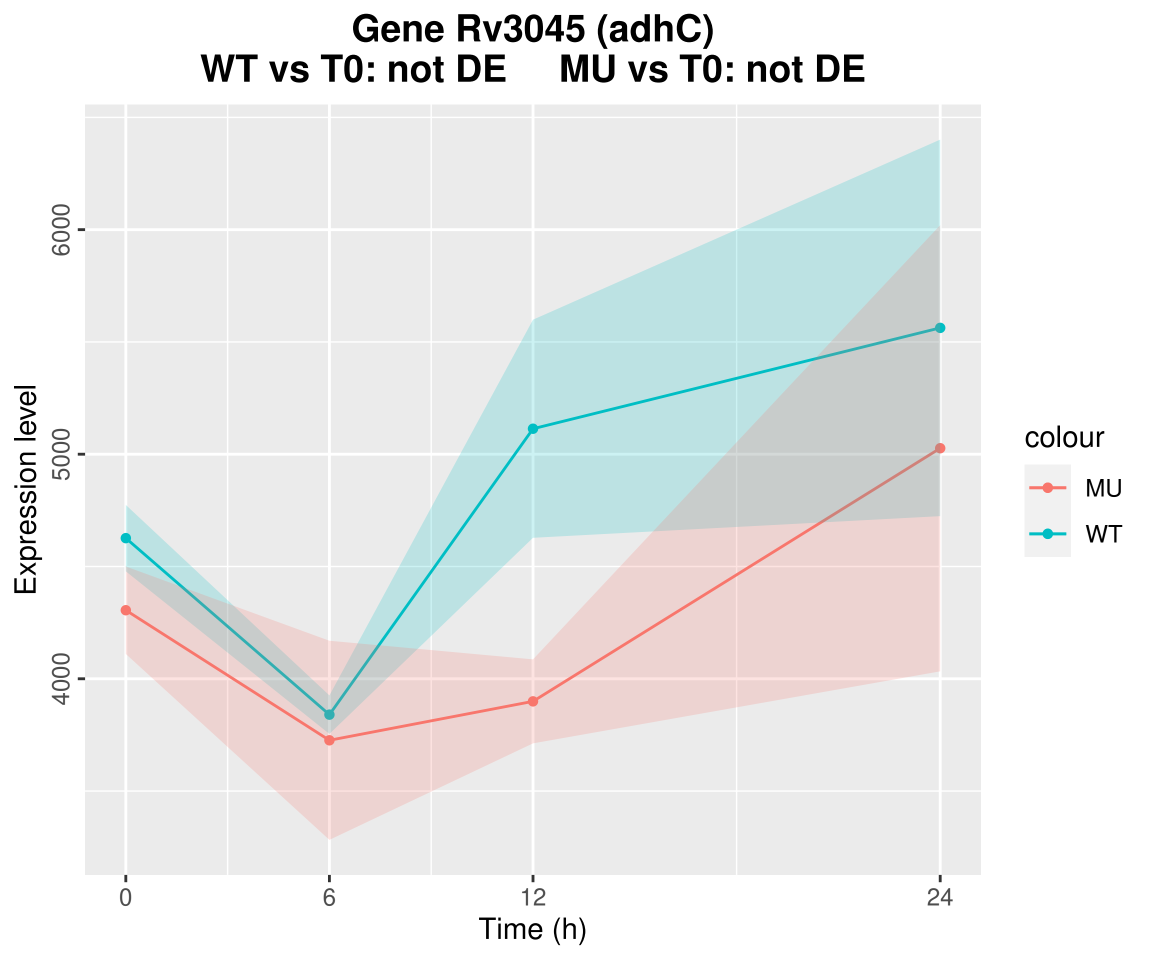

Gene Rv3045 (adhC)
WT vs T0: not DE MU vs T0: not DE
Expression level
6000
5000
4000
0
6
12
24
Time (h)
colour
MU
WT

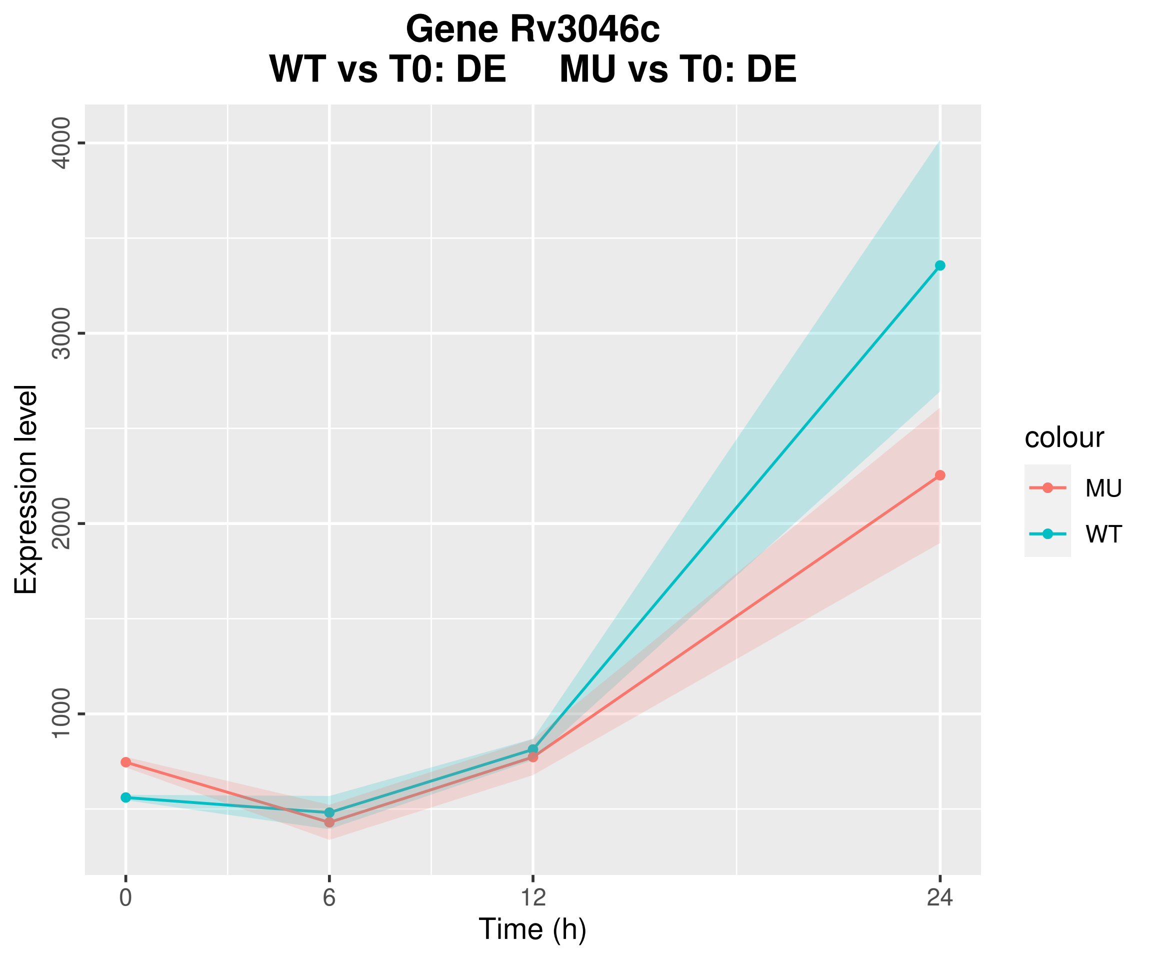

Gene Rv3046c
WT vs T0: DE   MU vs T0: DE
Expression level
4000
3000
2000
1000
0
6
12
24
Time (h)
colour
MU
WT

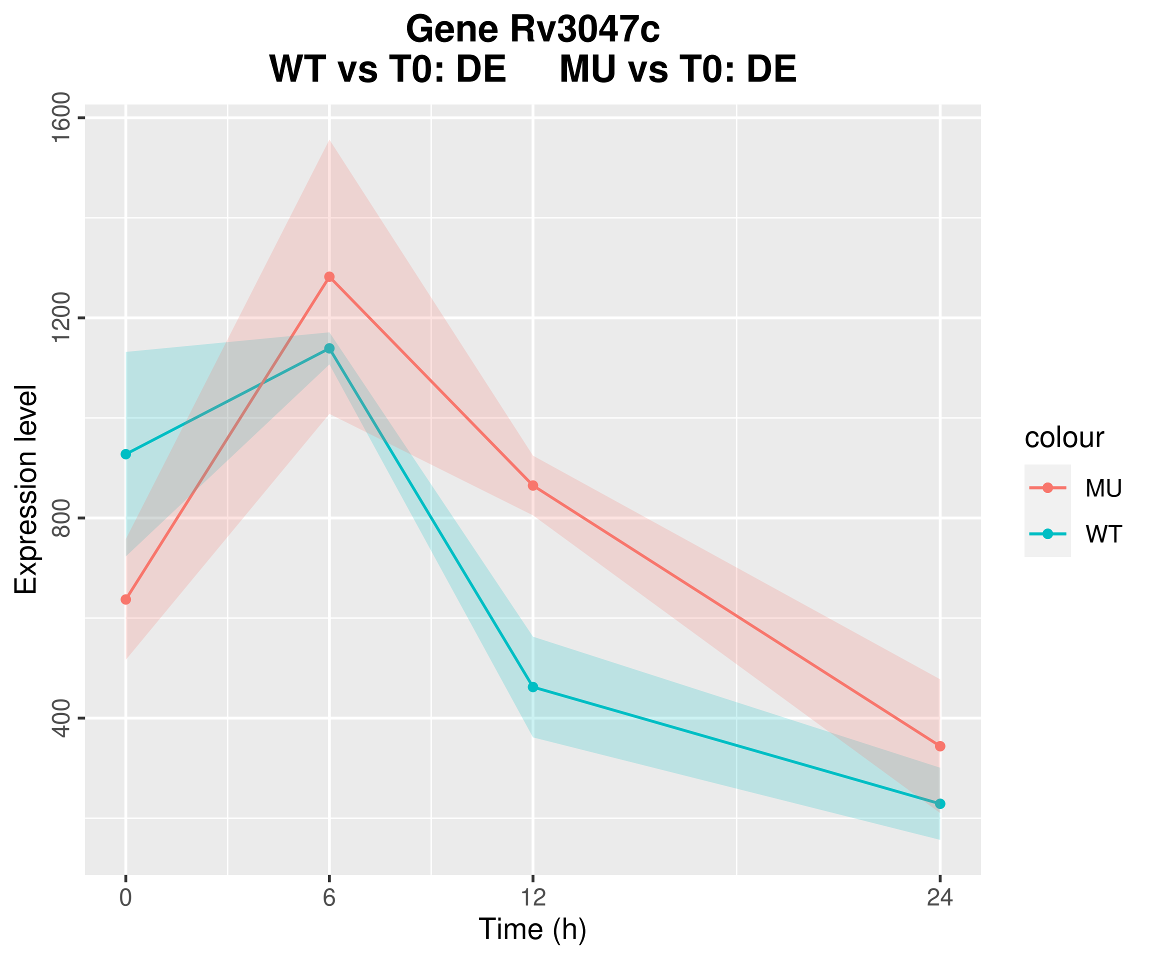

Gene Rv3047c
WT vs T0: DE   MU vs T0: DE
1600
1200
800
400
Expression level
0
6
12
24
Time (h)
colour
MU
WT

Gene Rv3048c (nrdF2)
WT vs T0: DE   MU vs T0: not DE

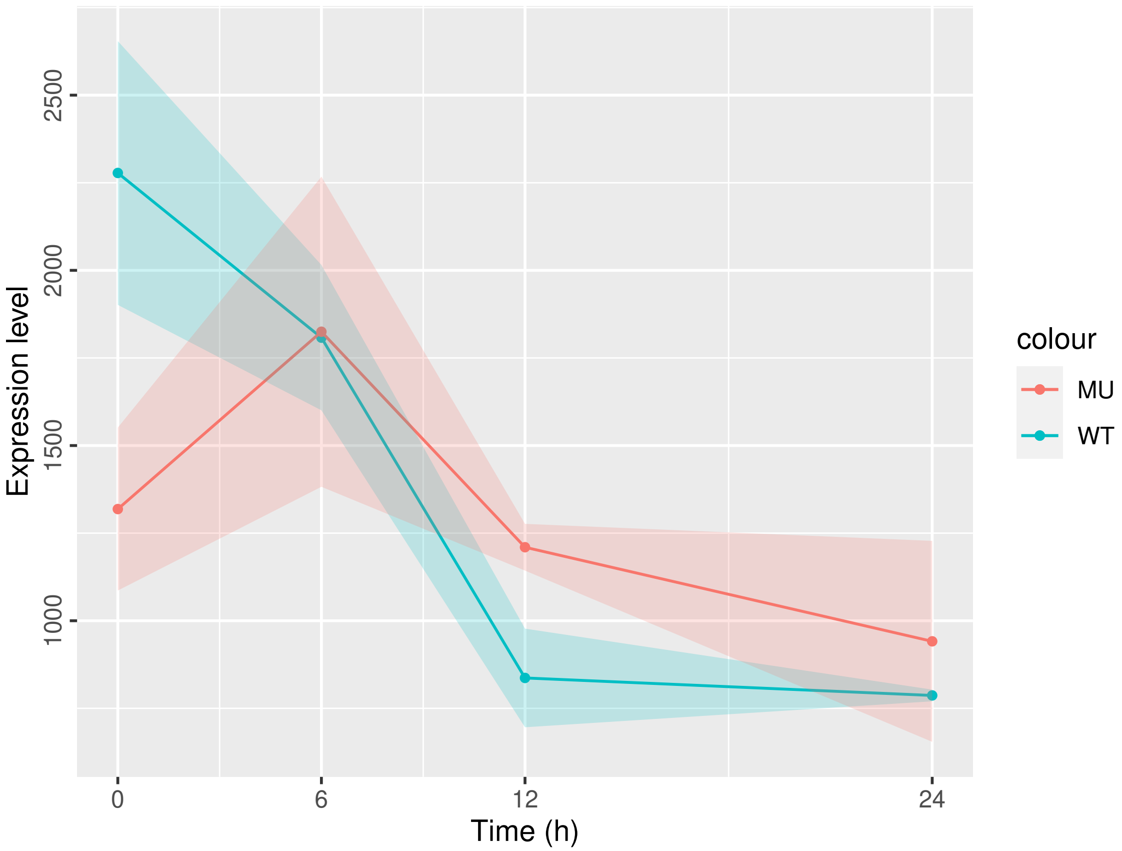

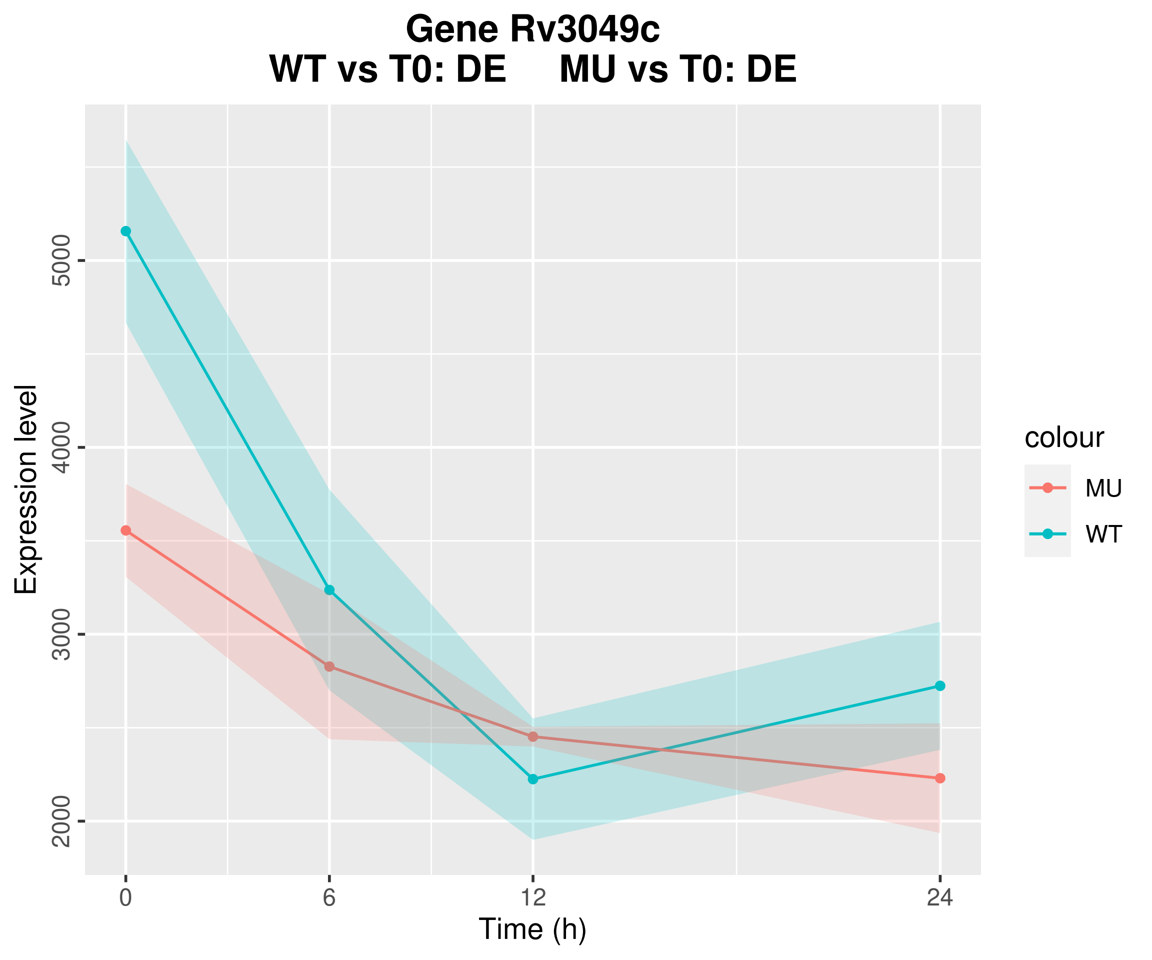

Gene Rv3049c
WT vs T0: DE   MU vs T0: DE
Expression level
5000
4000
3000
2000
0
6
12
24
Time (h)
colour
MU
WT

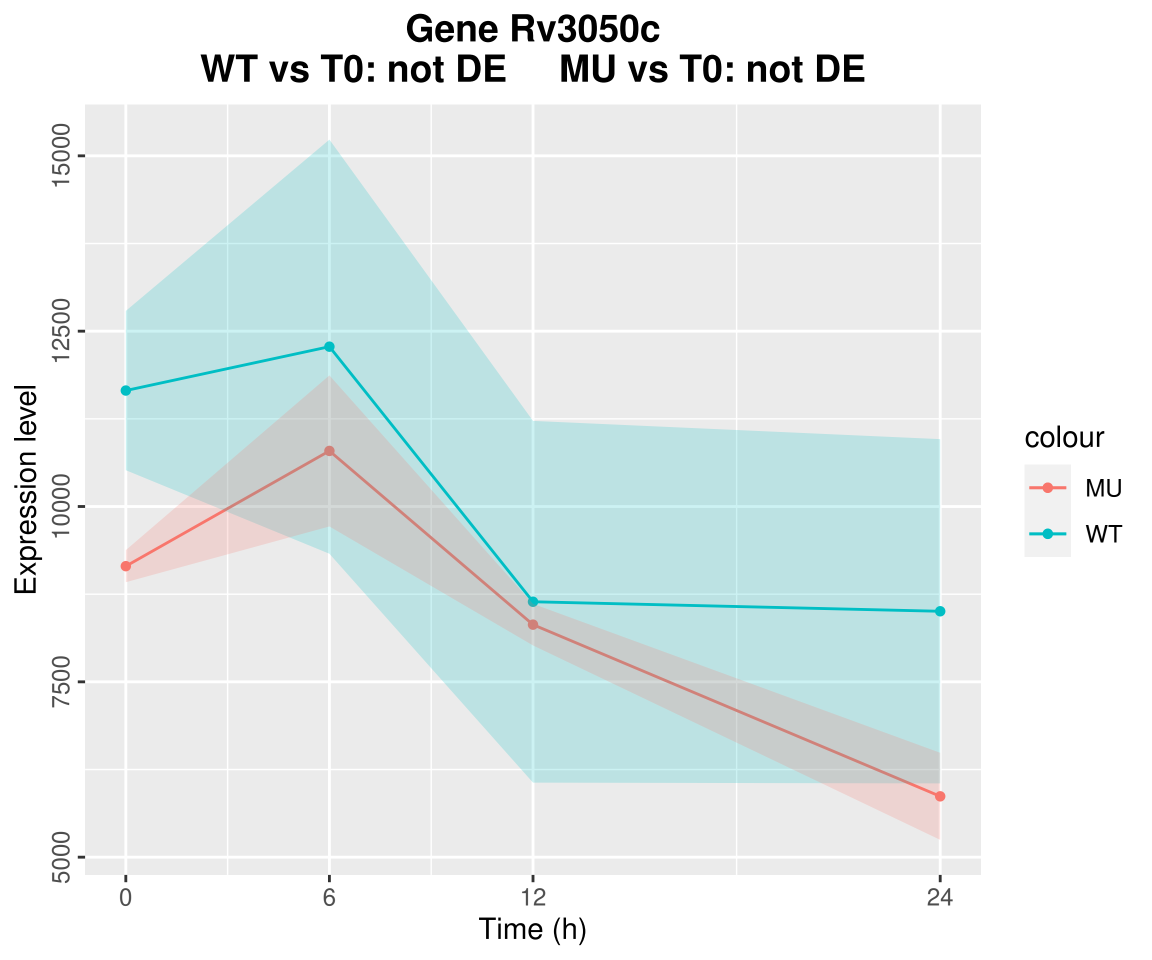

Gene Rv3050c
WT vs T0: not DE MU vs T0: not DE
Expression level
15000
12500
10000
7500
5000
0
6
12
24
Time (h)
colour
MU
WT

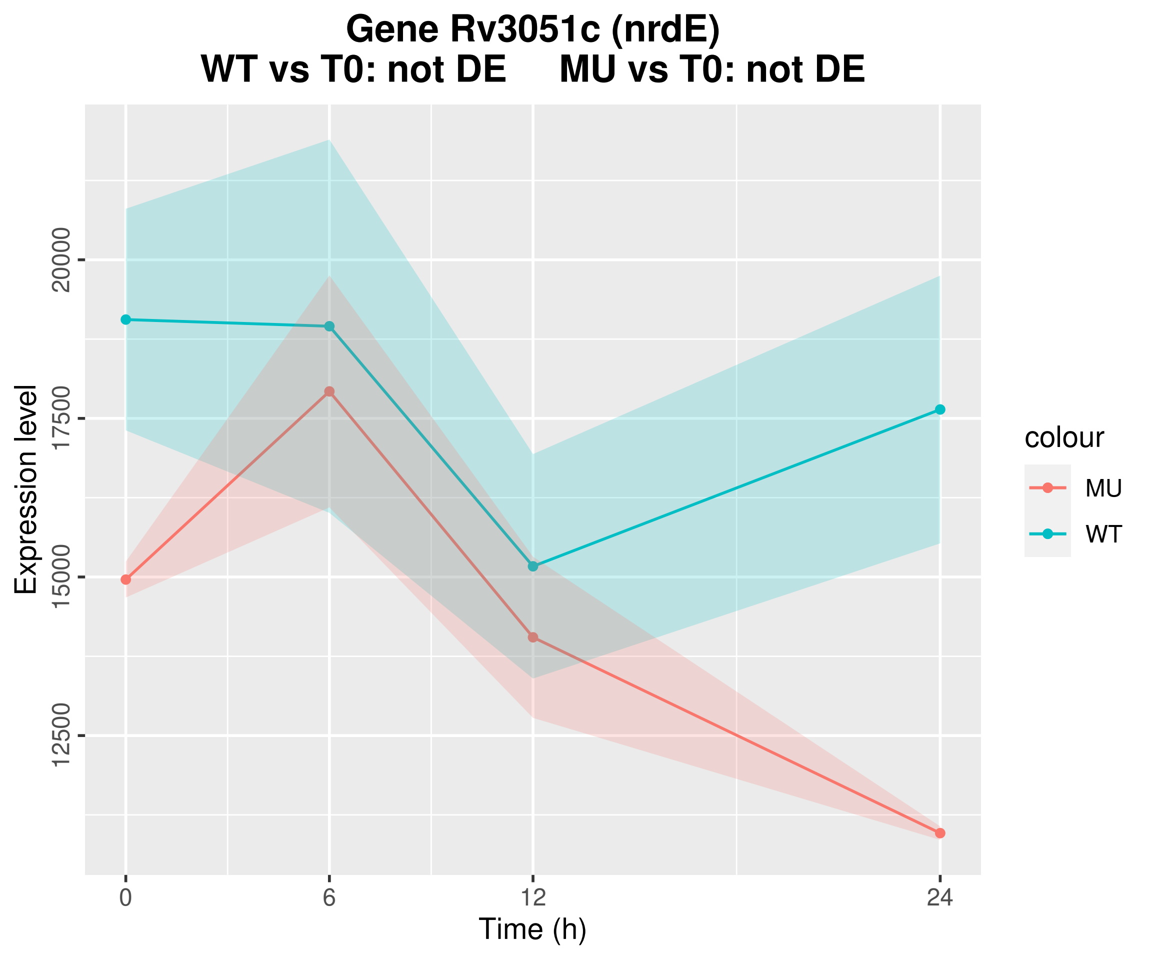
Gene Rv3051c (nrdE)
WT vs T0: not DE MU vs T0: not DE
Expression level
20000
17500
15000
12500
0
6
12
24
Time (h)
colour
MU
WT

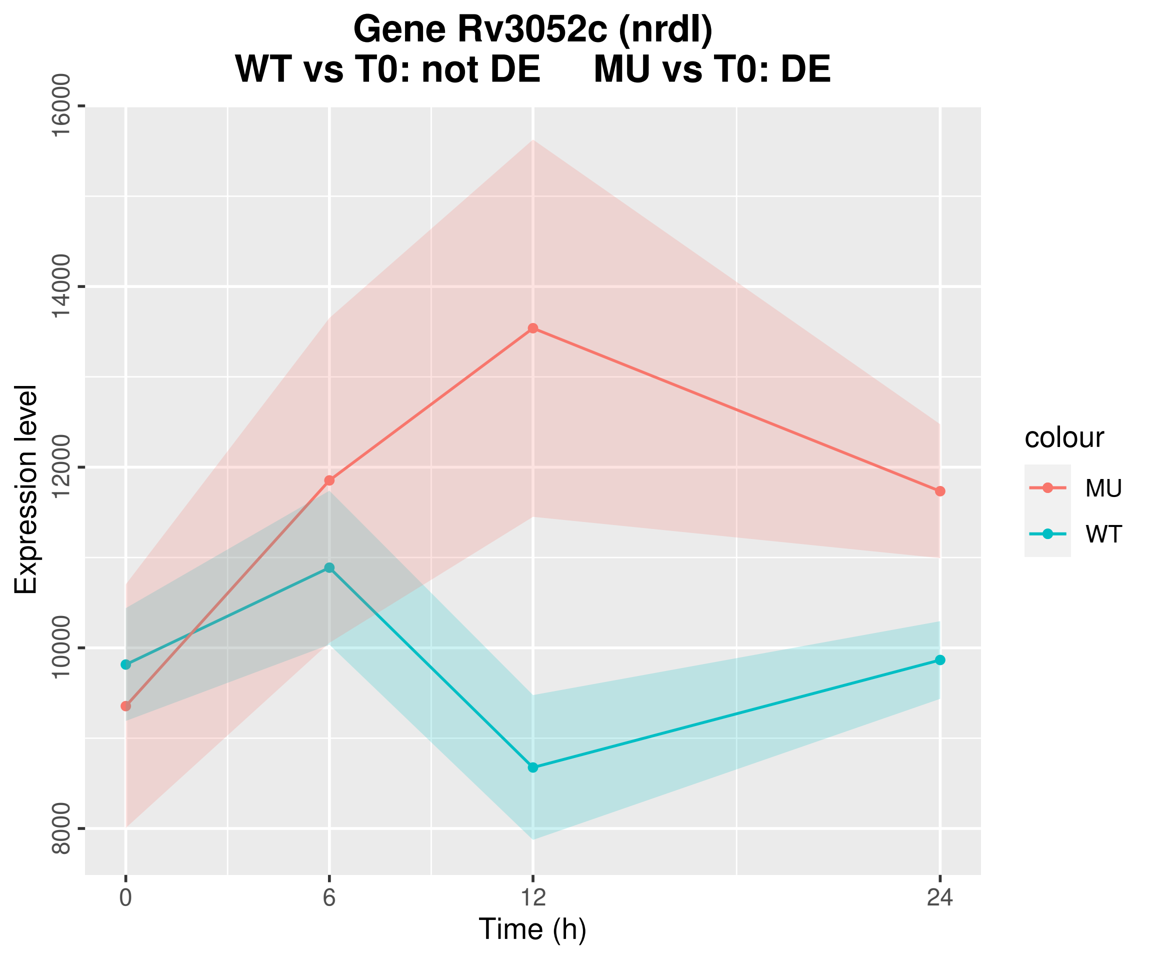
Gene Rv3052c (nrdI)
WT vs T0: not DE MU vs T0: DE
16000
14000
12000
10000
8000
Expression level
0
6
12
24
Time (h)
colour
MU
WT

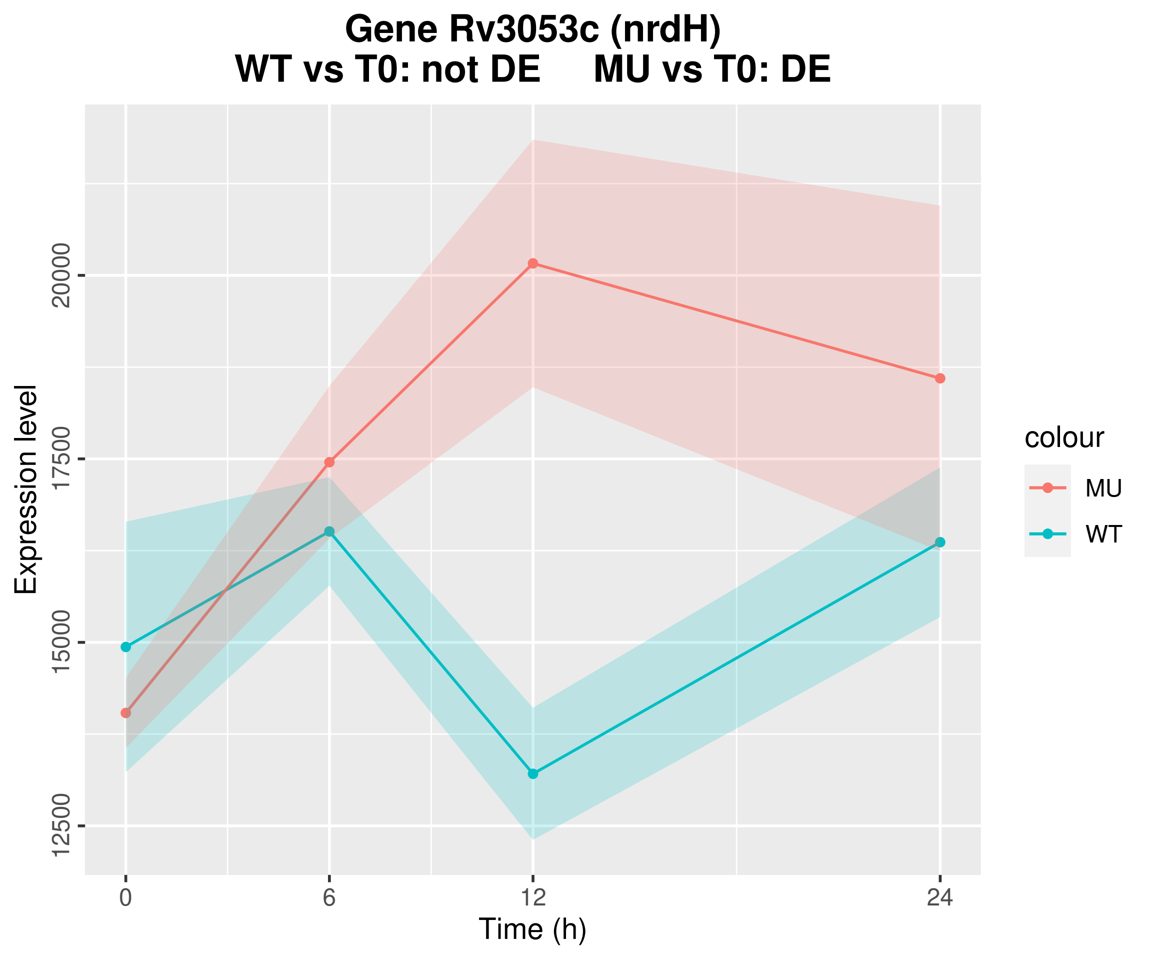
Gene Rv3053c (nrdH)
WT vs T0: not DE MU vs T0: DE
Expression level
20000
17500
15000
12500
0
6
12
24
Time (h)
colour
MU
WT

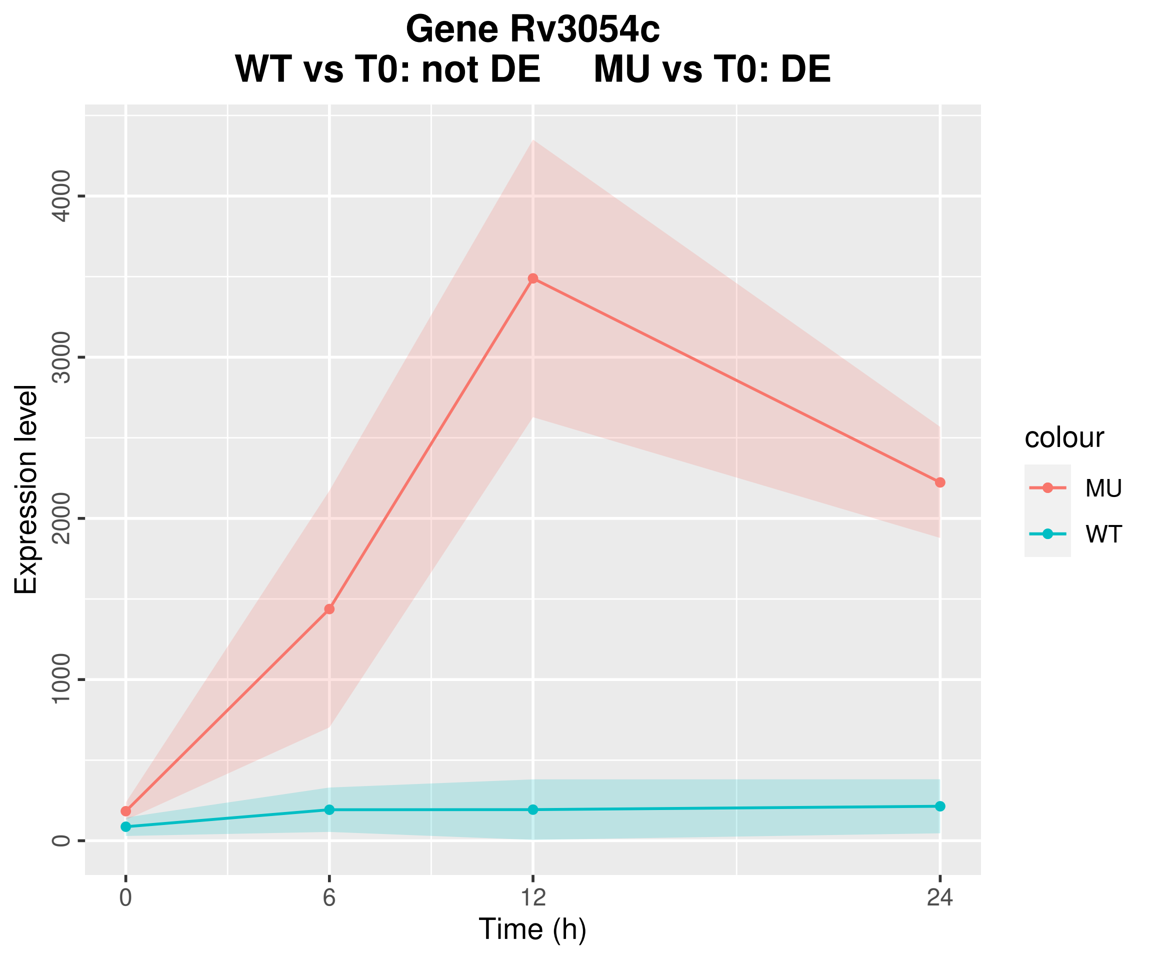
Gene Rv3054c
WT vs T0: not DE   MU vs T0: DE
Expression level
4000
3000
2000
1000
0
0
6
12
24
Time (h)
colour
MU
WT

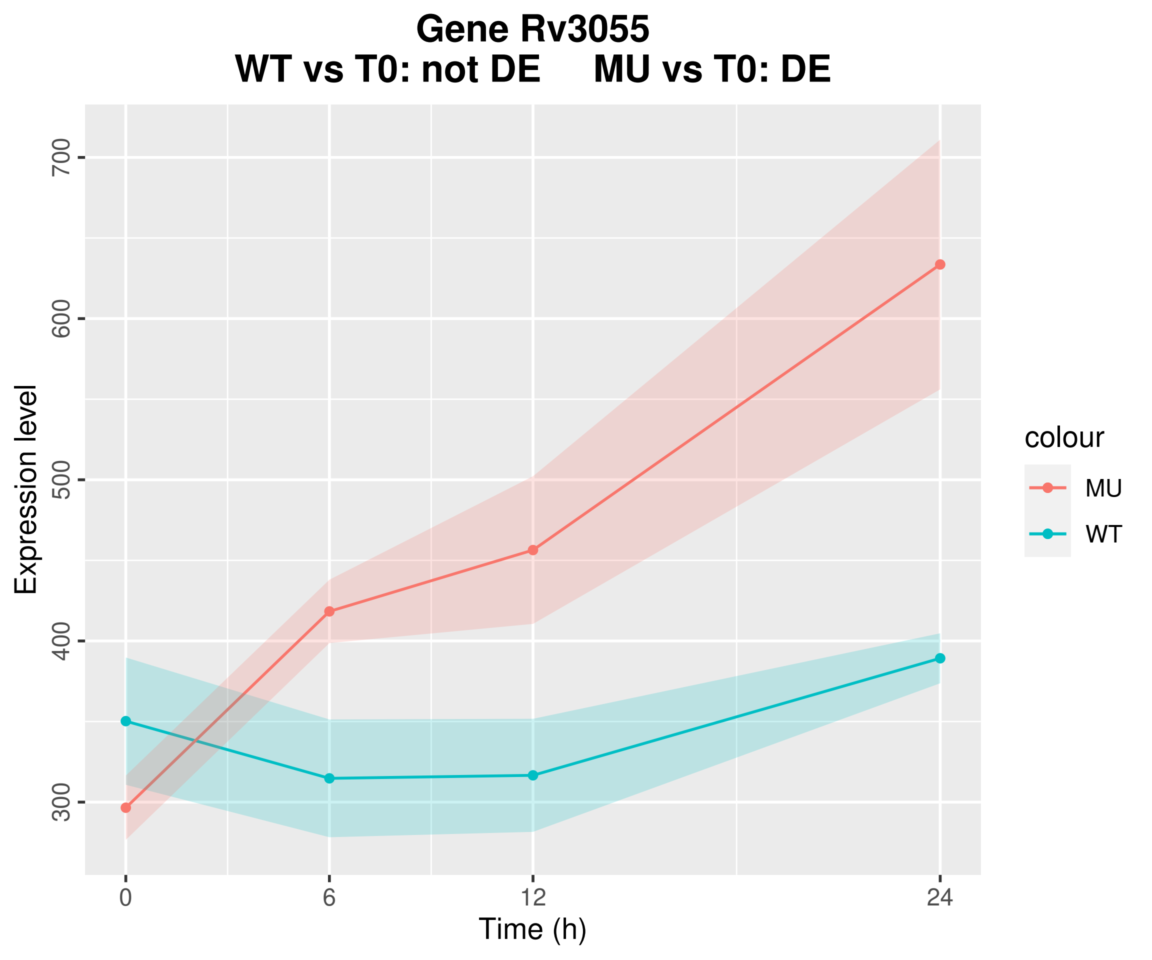
Gene Rv3055
WT vs T0: not DE MU vs T0: DE
Expression level
700
600
500
400
300
0
6
12
24
Time (h)
colour
MU
WT

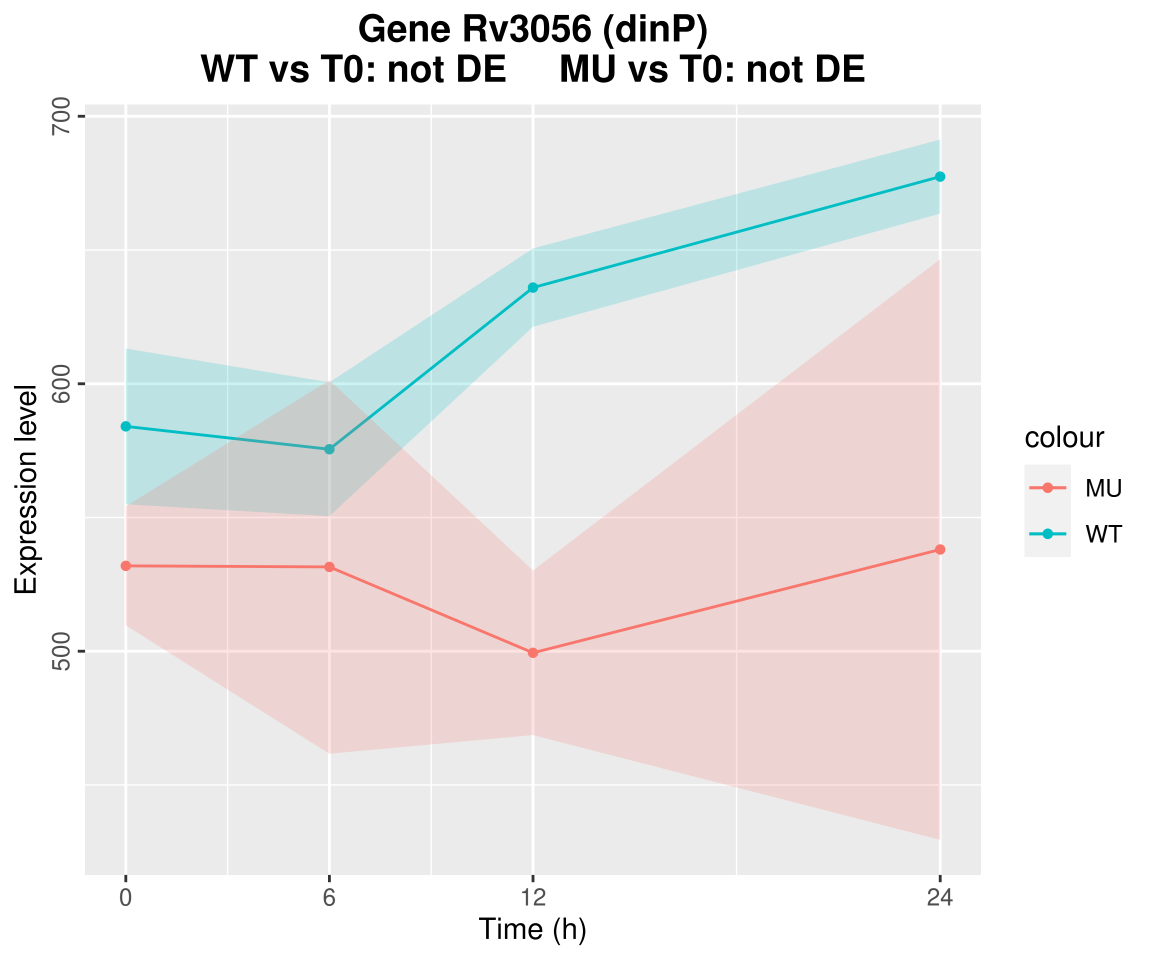

Gene Rv3056 (dinP)
WT vs T0: not DE   MU vs T0: not DE
700
600
500
Expression level
0
6
12
24
Time (h)
colour
MU
WT

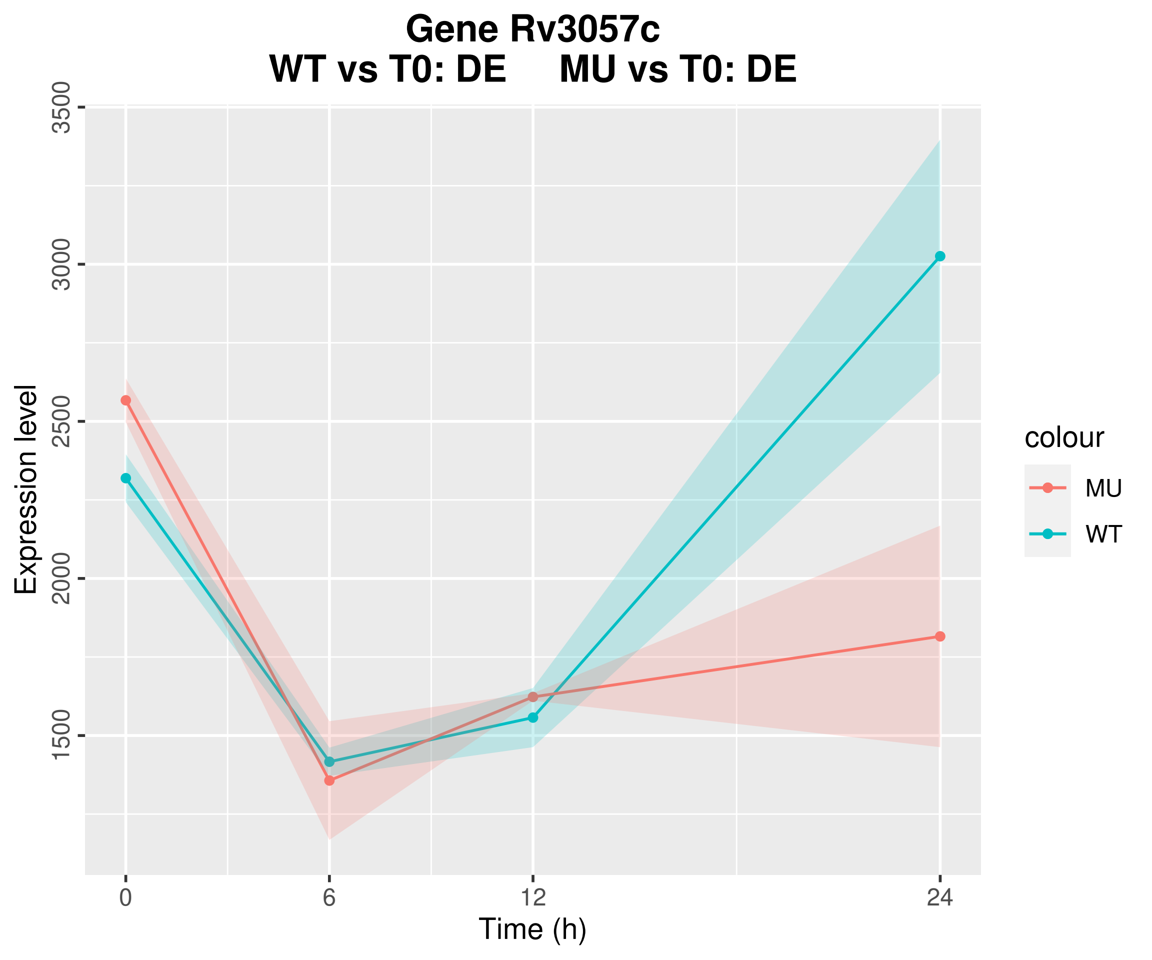

Gene Rv3057c
WT vs T0: DE   MU vs T0: DE
3500
3000
2500
2000
1500
Expression level
0
6
12
24
Time (h)
colour
MU
WT

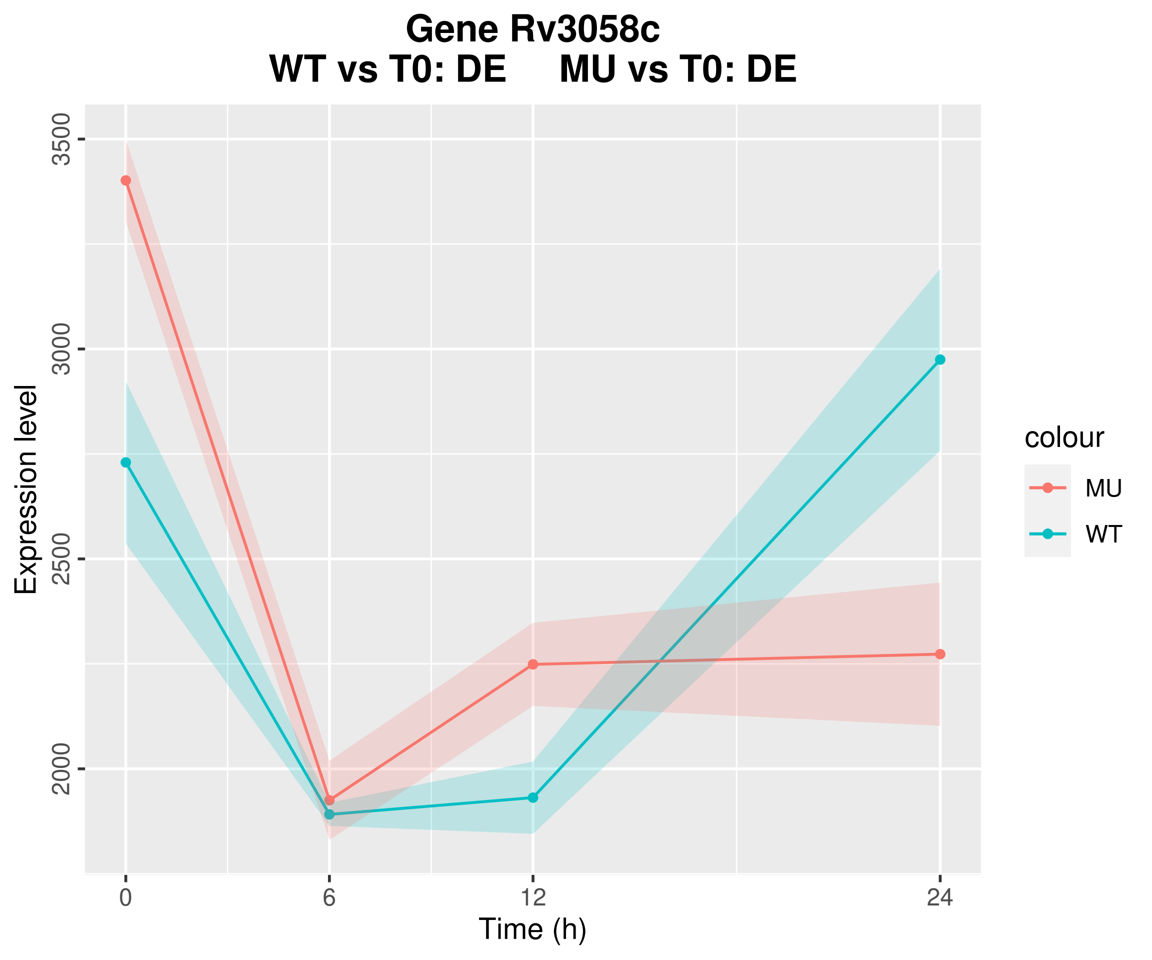

Gene Rv3058c
WT vs T0: DE MU vs T0: DE
Expression level
3500
3000
2500
2000
0
6
12
24
Time (h)
colour
MU
WT

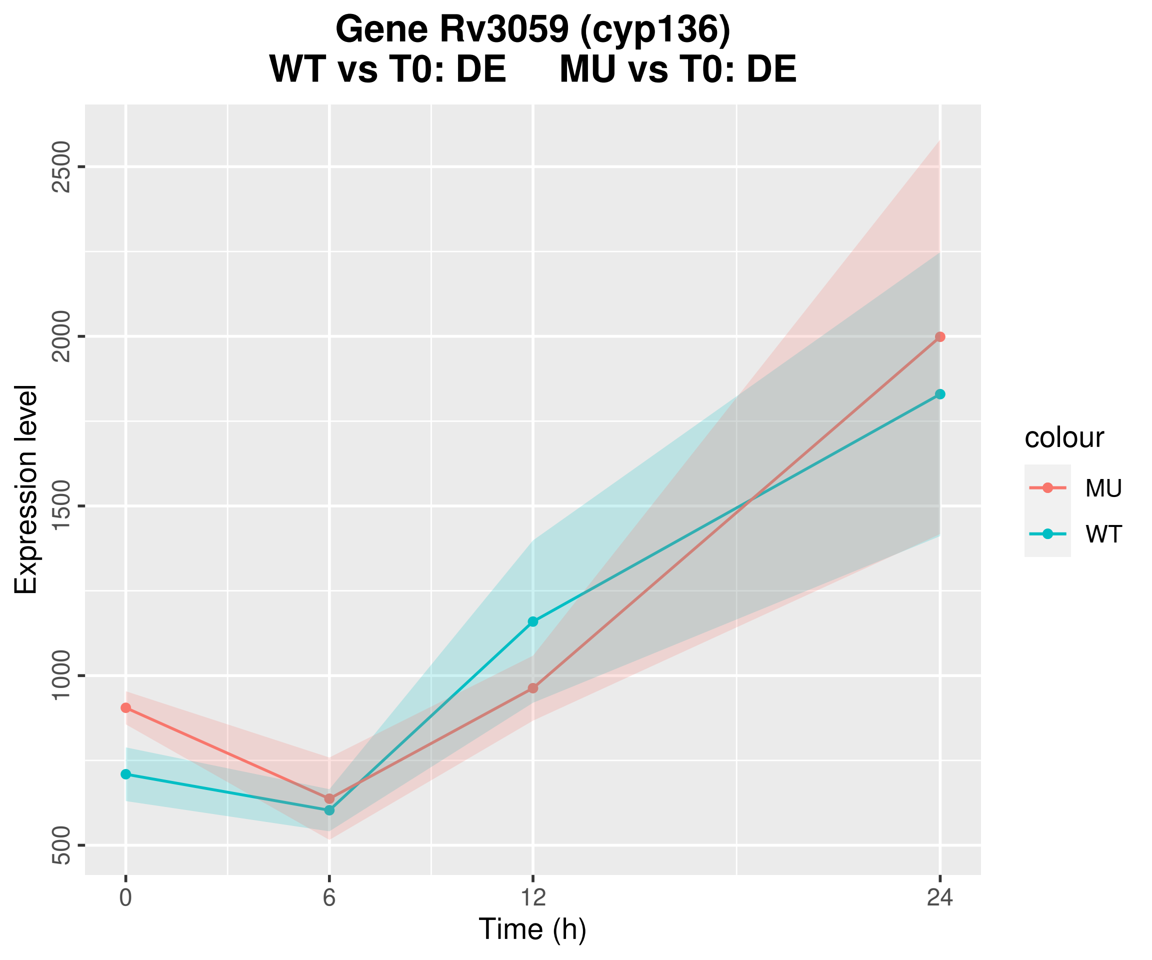
Gene Rv3059 (cyp136)
WT vs T0: DE   MU vs T0: DE
Expression level
2500
2000
1500
1000
500
0
6
12
24
Time (h)
colour
MU
WT

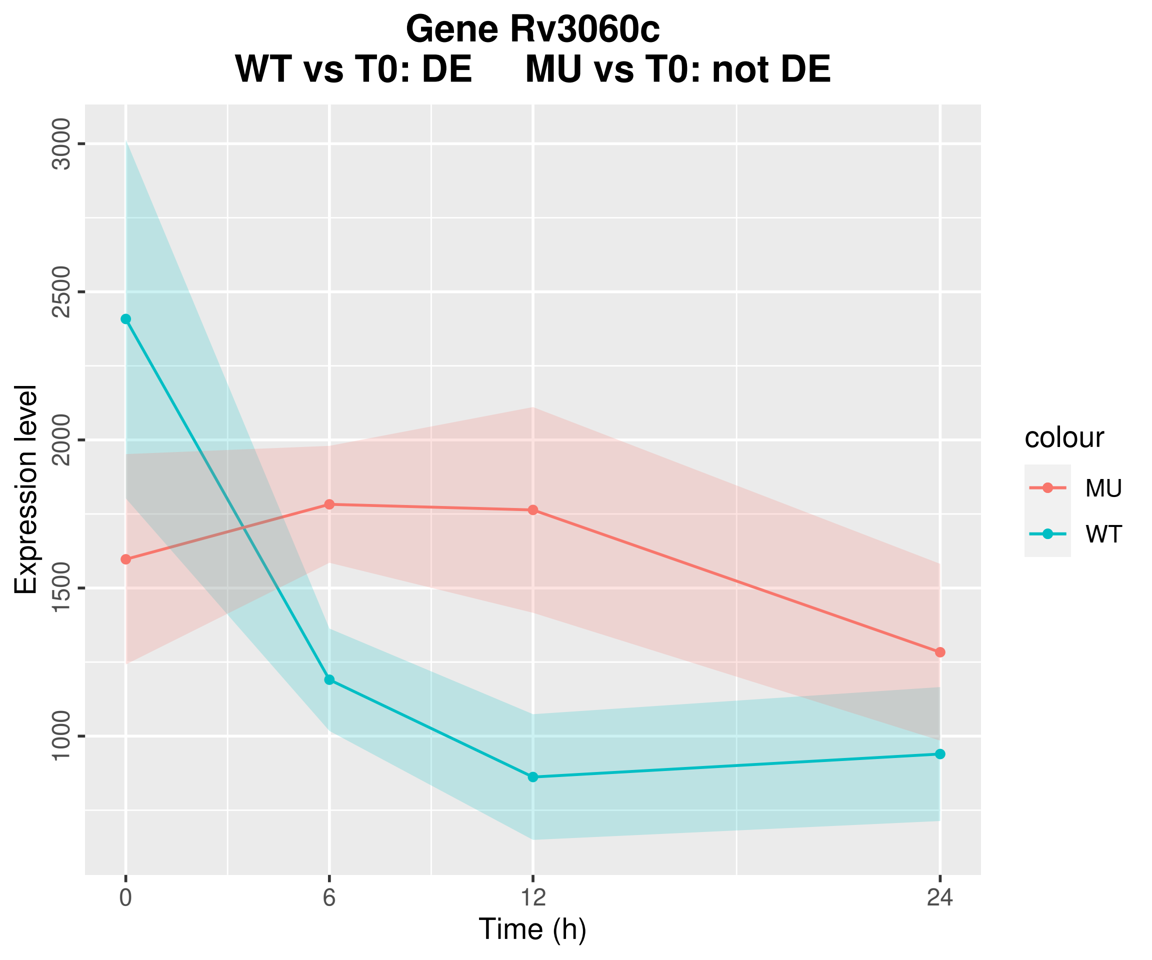

Gene Rv3060c
WT vs T0: DE MU vs T0: not DE
3000
2500
2000
1500
1000
Expression level
0
6
12
24
Time (h)
colour
MU
WT

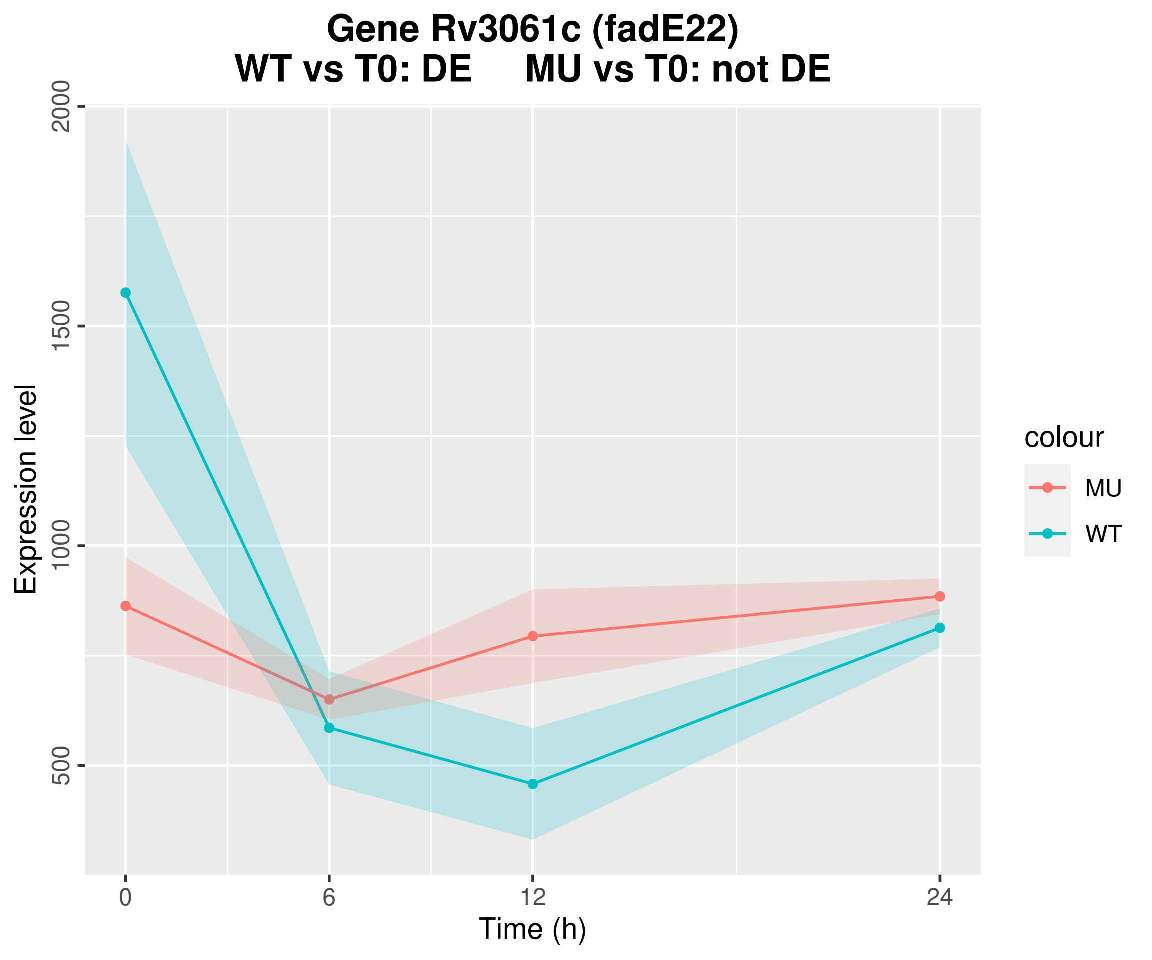
Gene Rv3061c (fadE22)
WT vs T0: DE   MU vs T0: not DE
Expression level
2000
1500
1000
500
0
6
12
24
Time (h)
colour
MU
WT

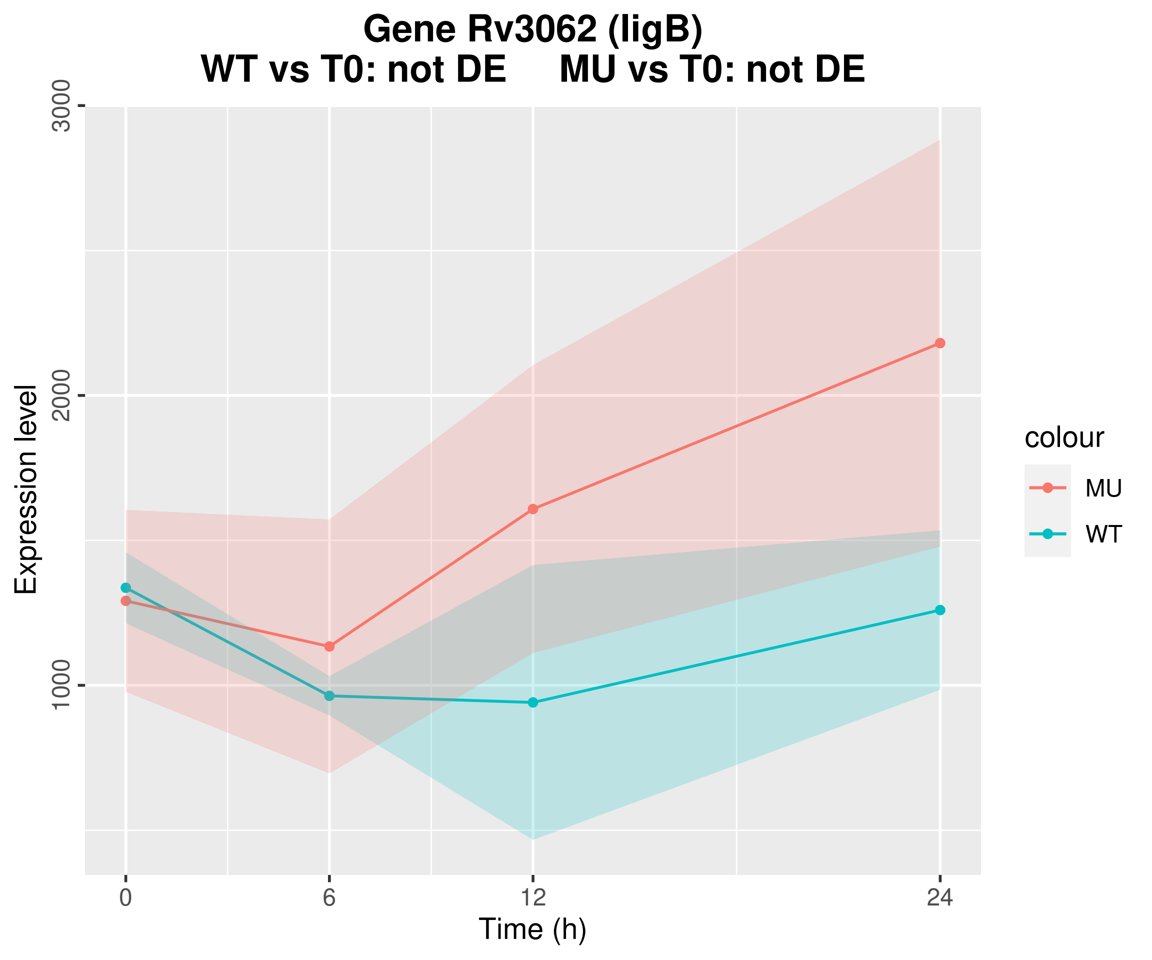

Gene Rv3062 (ligB)
WT vs T0: not DE MU vs T0: not DE
3000
2000
1000
Expression level
0
6
12
24
Time (h)
colour
MU
WT

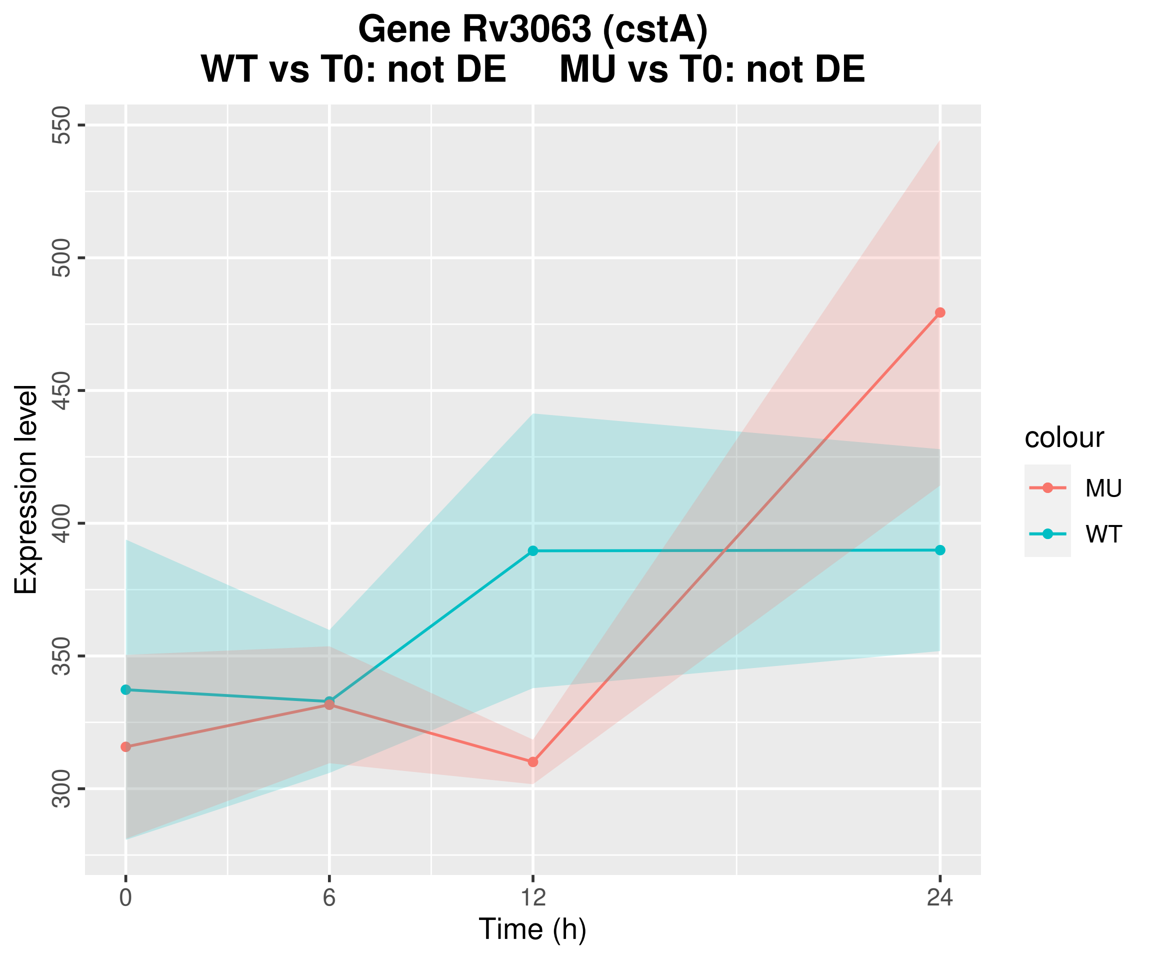
Gene Rv3063 (cstA)
WT vs T0: not DE   MU vs T0: not DE
Expression level
550
500
450
400
350
300
0
6
12
24
Time (h)
colour
MU
WT

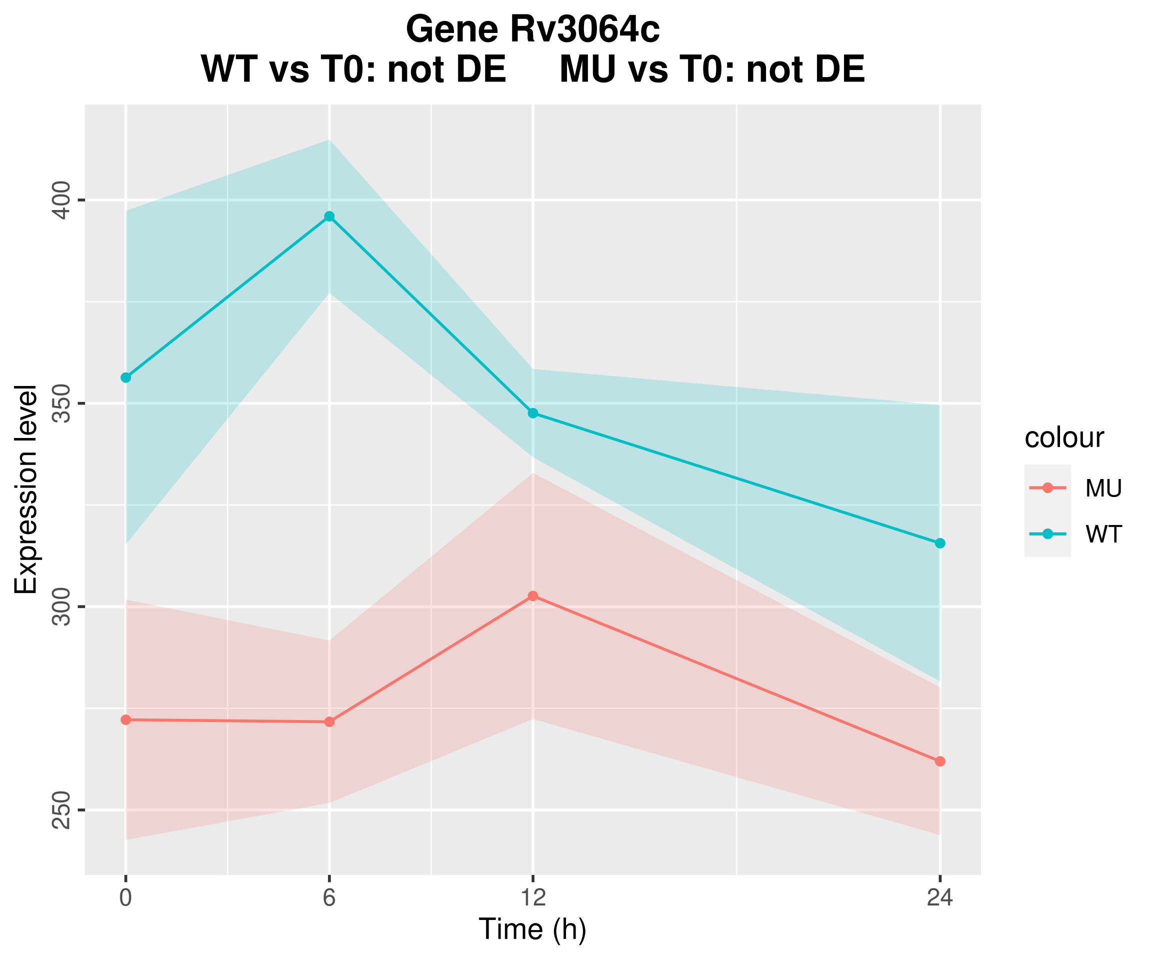
Gene Rv3064c
WT vs T0: not DE MU vs T0: not DE
400
350
300
250
Expression level
0
6
12
24
Time (h)
colour
MU
WT

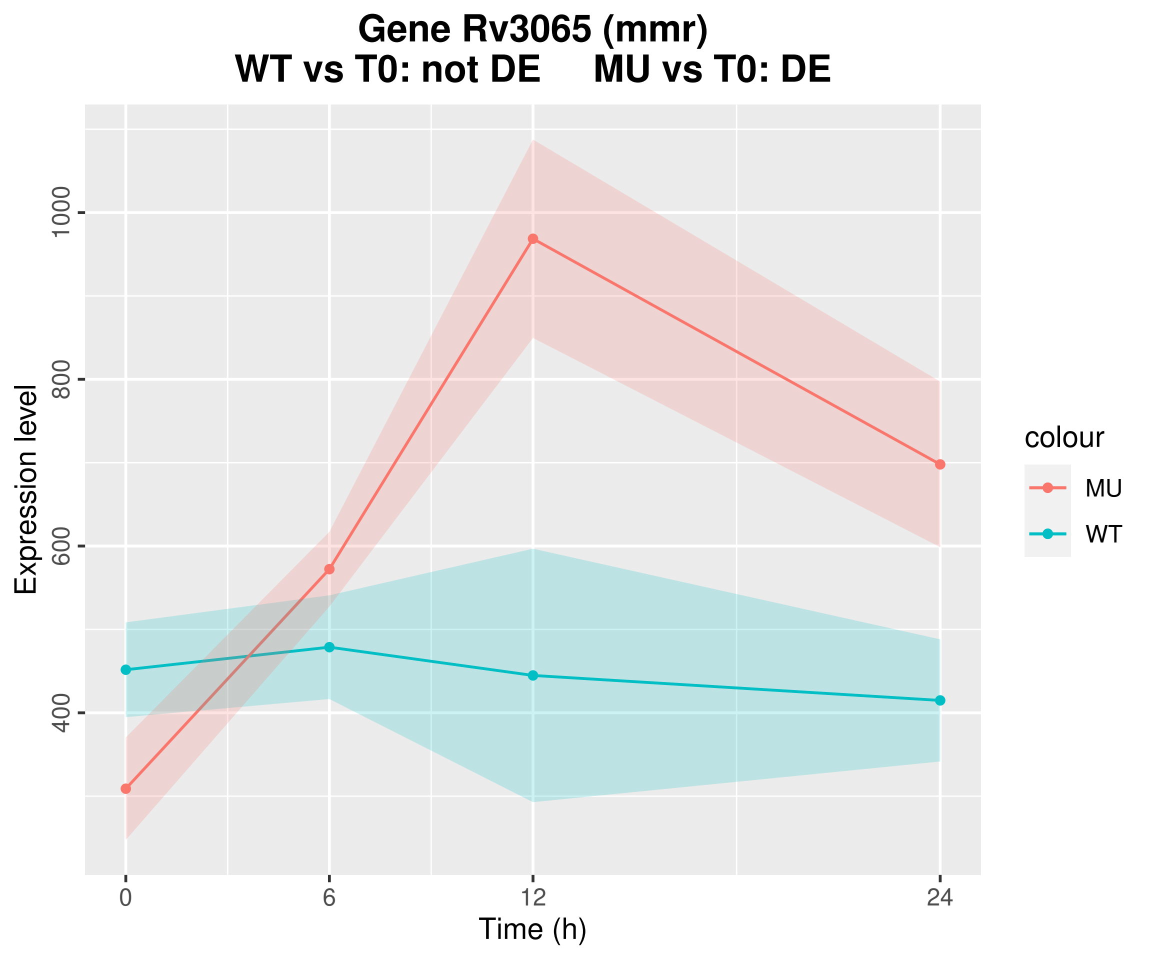
Gene Rv3065 (mmr)
WT vs T0: not DE MU vs T0: DE
Expression level
1000
800
600
400
0
6
12
24
Time (h)
colour
MU
WT

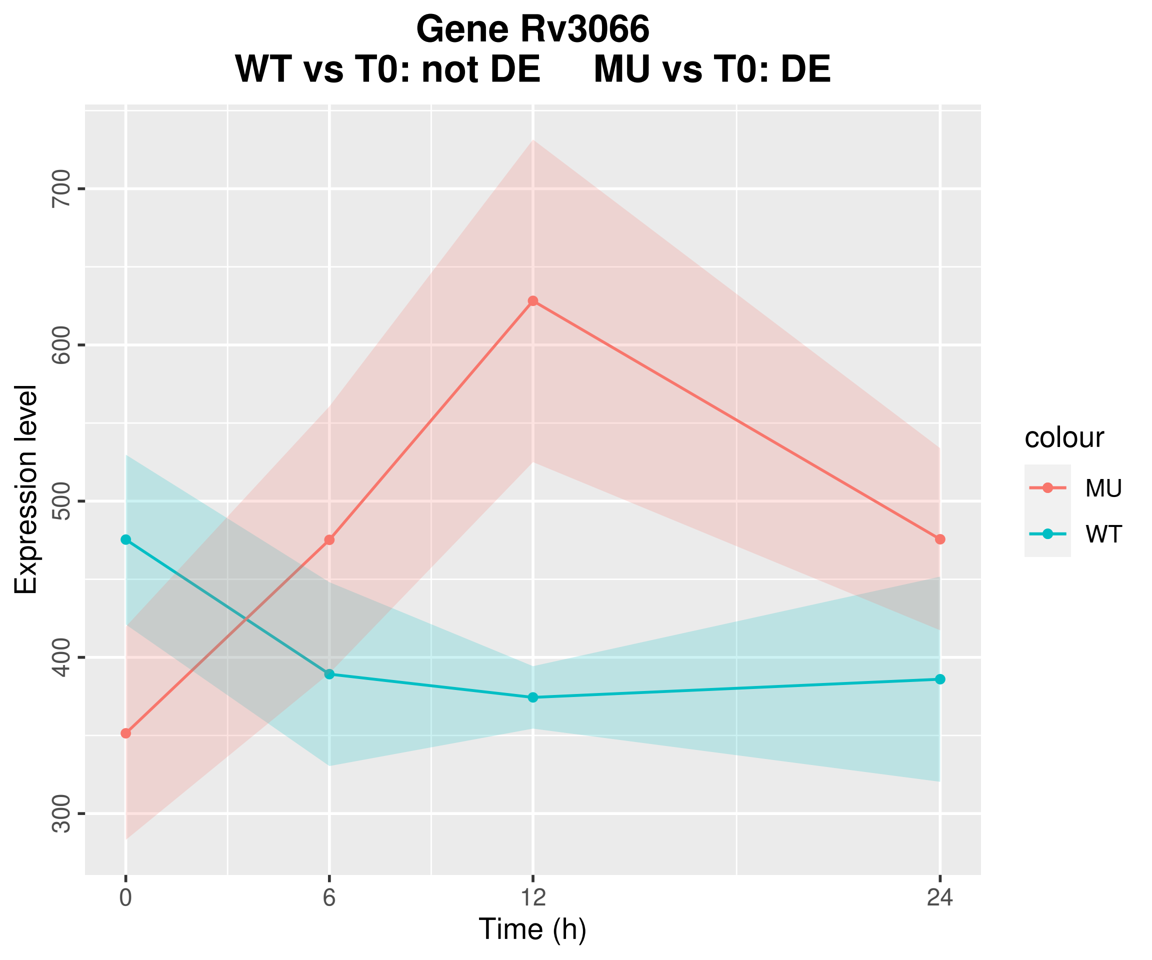
Gene Rv3066
WT vs T0: not DE MU vs T0: DE
Expression level
700
600
500
400
300
0
6
12
24
Time (h)
colour
MU
WT

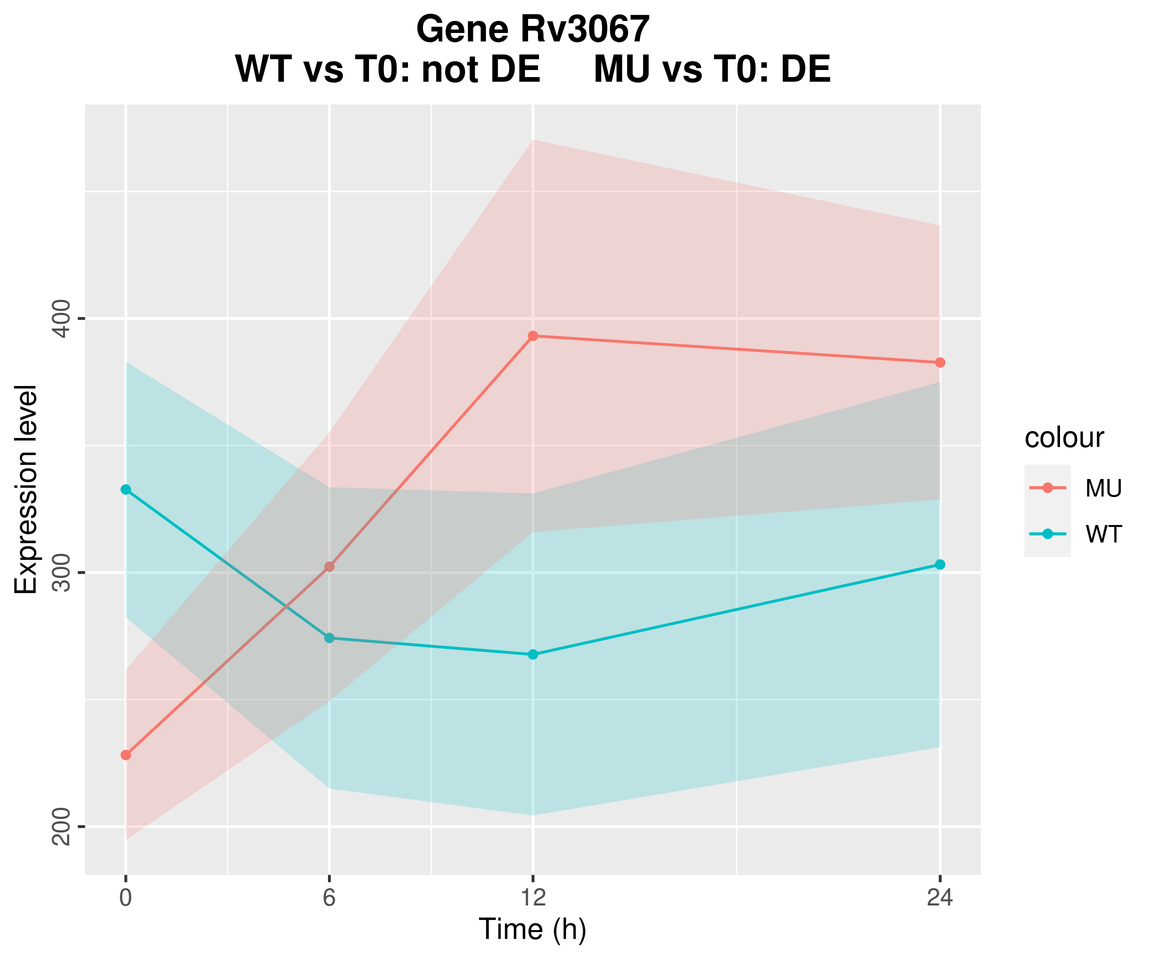
Gene Rv3067
WT vs T0: not DE MU vs T0: DE
Expression level
400
300
200
0
6
12
24
Time (h)
colour
MU
WT

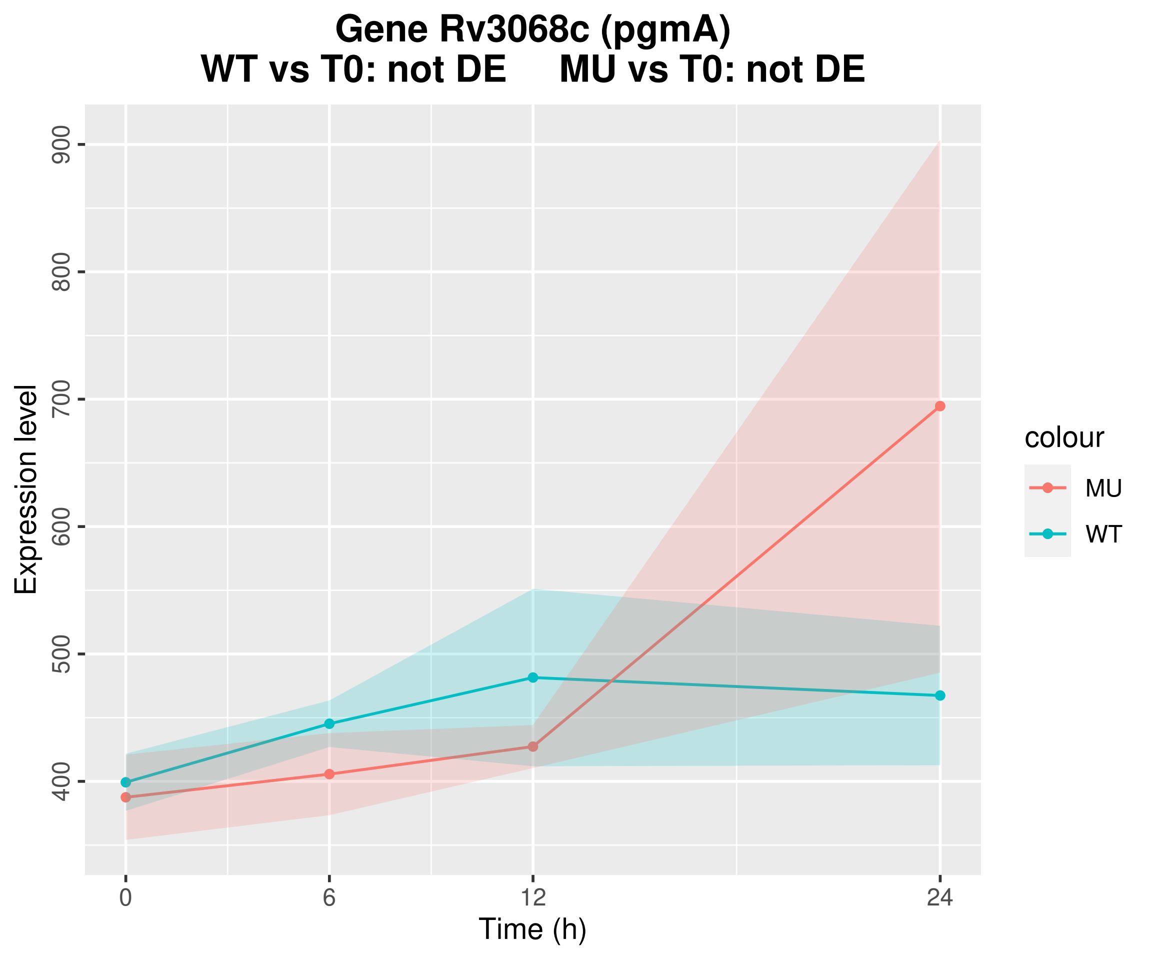

Gene Rv3068c (pgmA)
WT vs T0: not DE MU vs T0: not DE
Expression level
900
800
700
600
500
400
0
6
12
24
Time (h)
colour
MU
WT

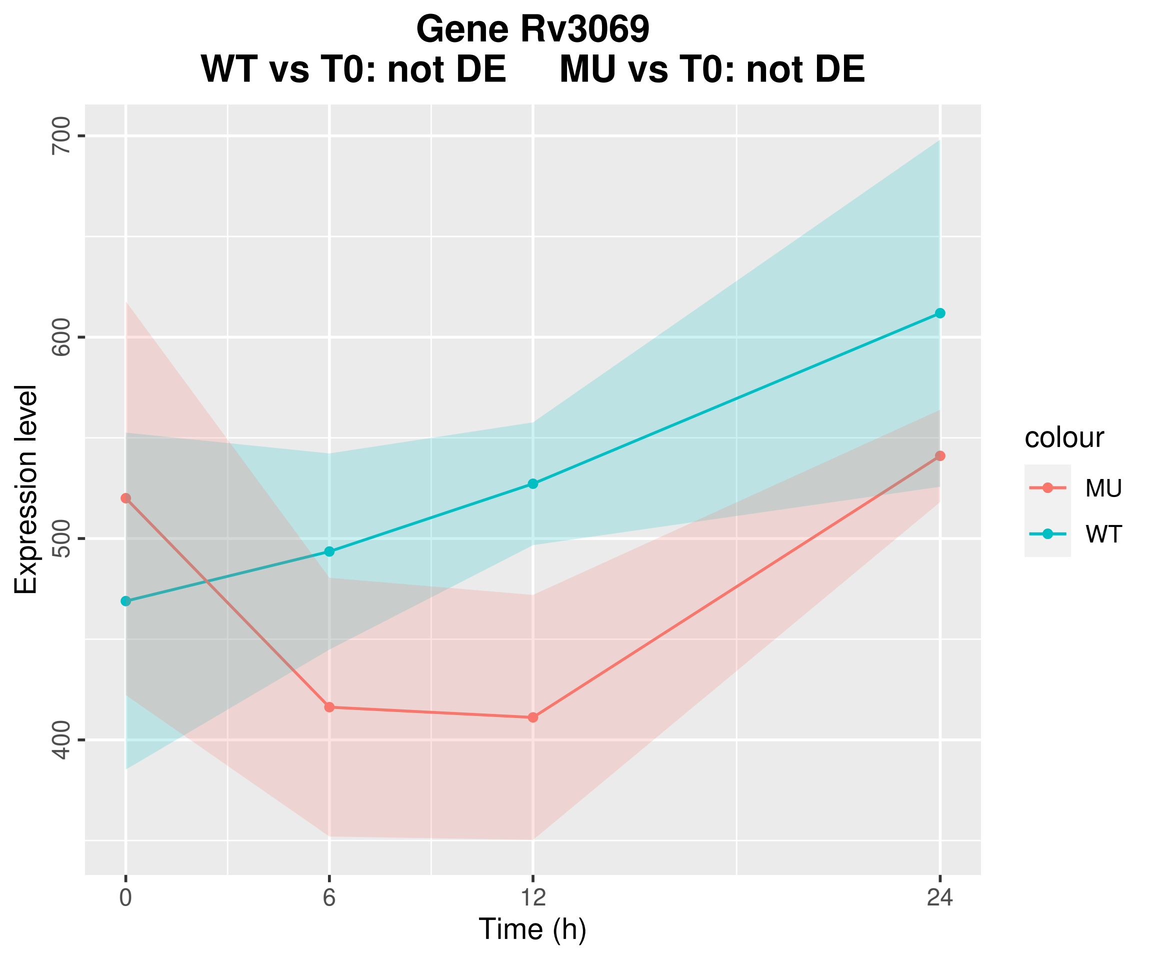
Gene Rv3069
WT vs T0: not DE   MU vs T0: not DE
Expression level
700
600
500
400
0
6
12
24
Time (h)
colour
MU
WT

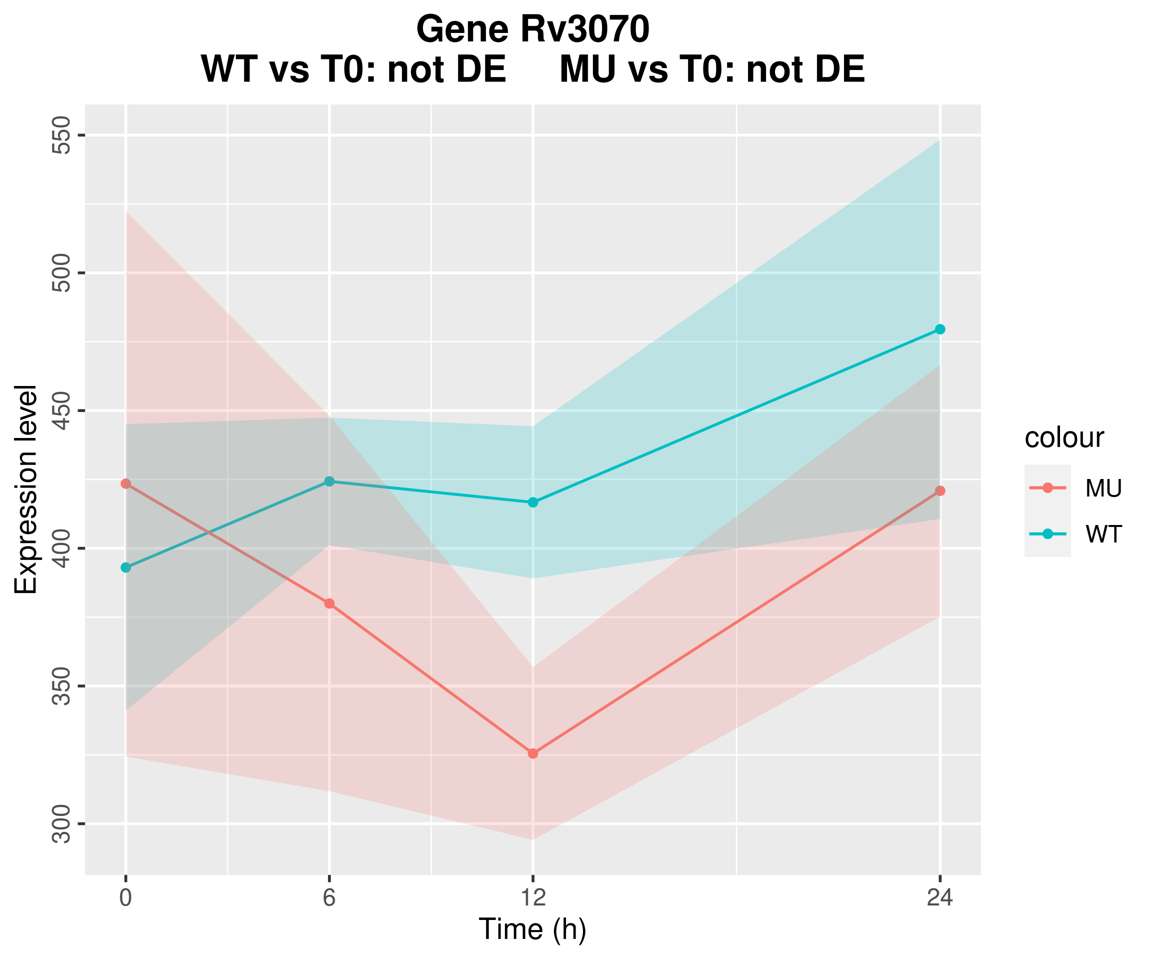
Gene Rv3070
WT vs T0: not DE MU vs T0: not DE
Expression level
550
500
450
400
350
300
Time (h)
0
6
12
24
colour
MU
WT

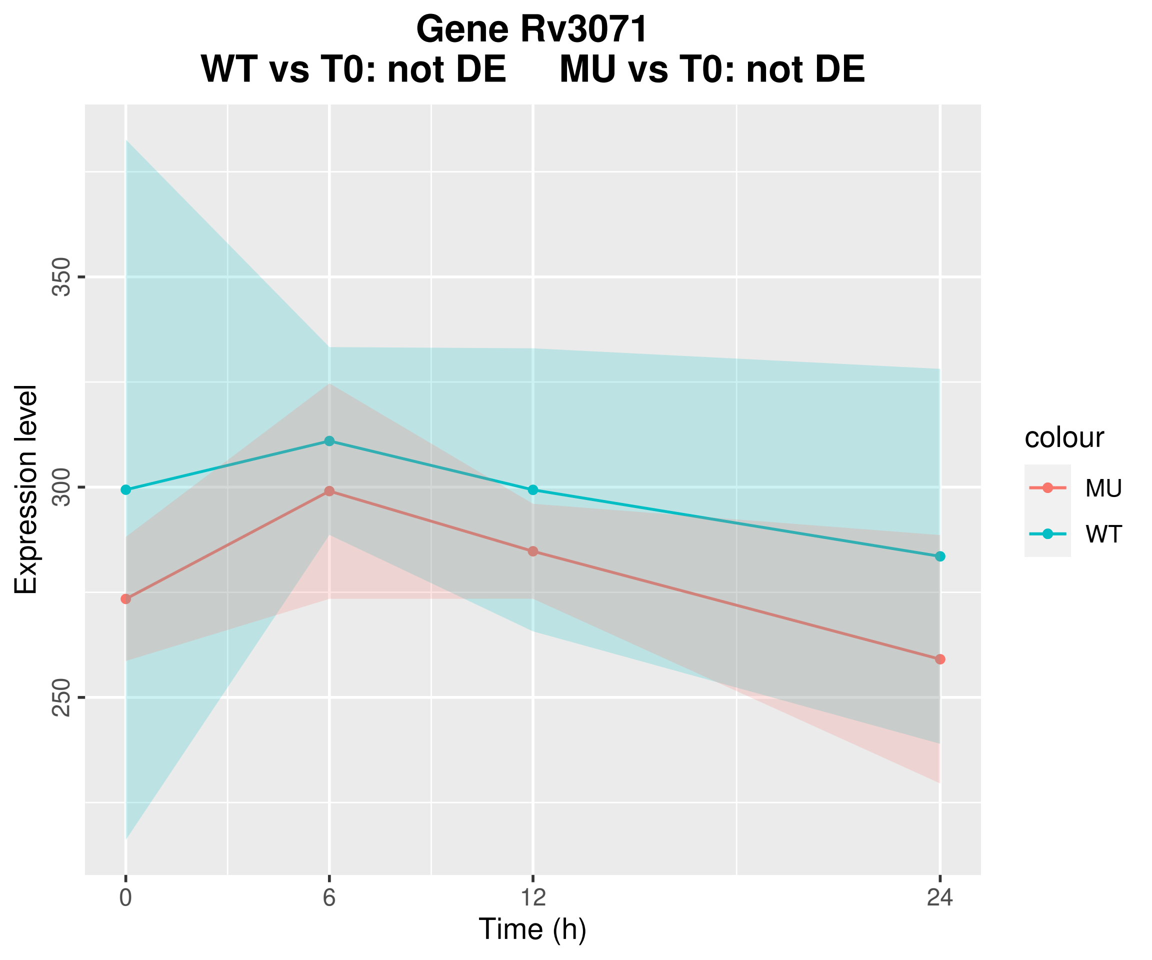
Gene Rv3071
WT vs T0: not DE   MU vs T0: not DE
Expression level
350
300
250
0
6
12
24
Time (h)
colour
MU
WT

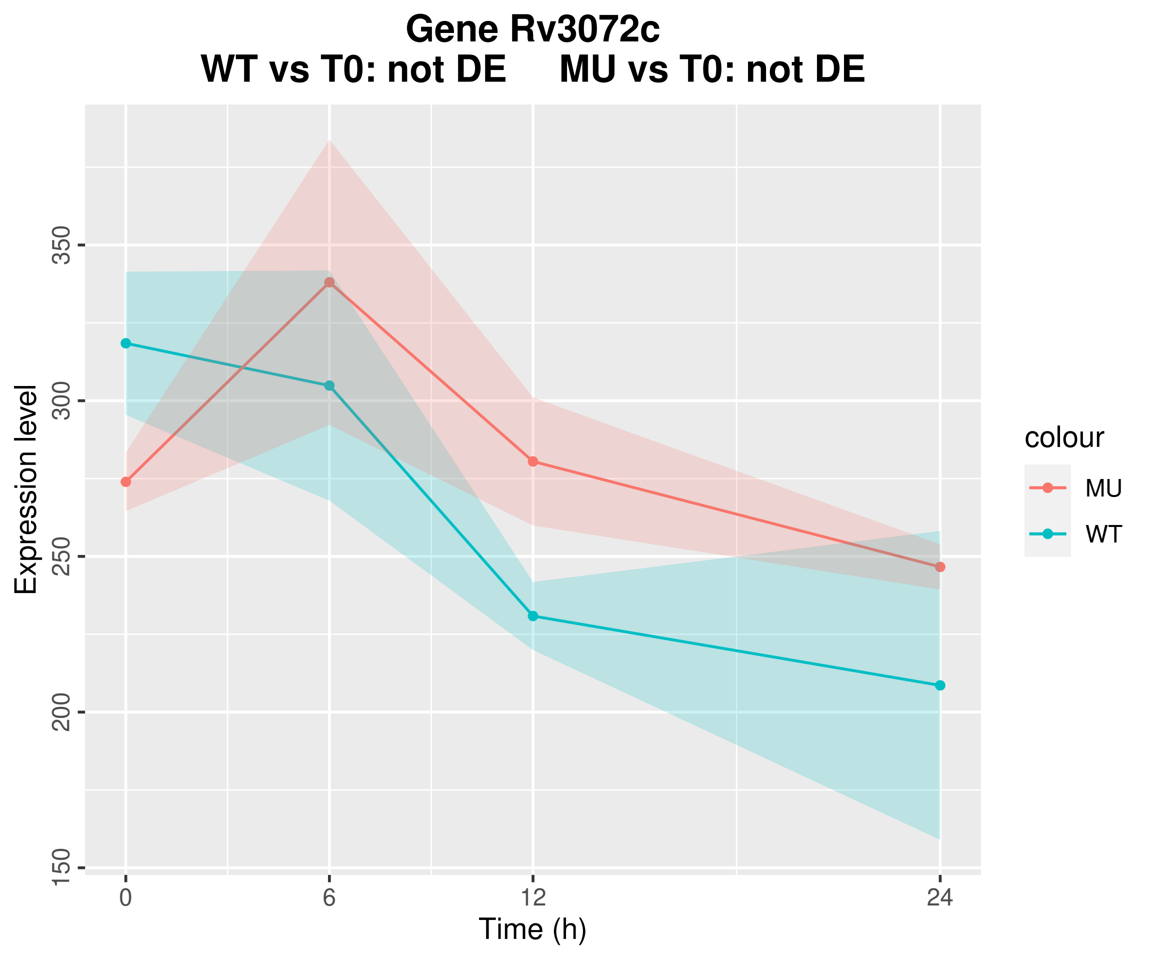
Gene Rv3072c
WT vs T0: not DE   MU vs T0: not DE
Expression level
350
300
250
200
150
0
6
12
24
Time (h)
colour
MU
WT

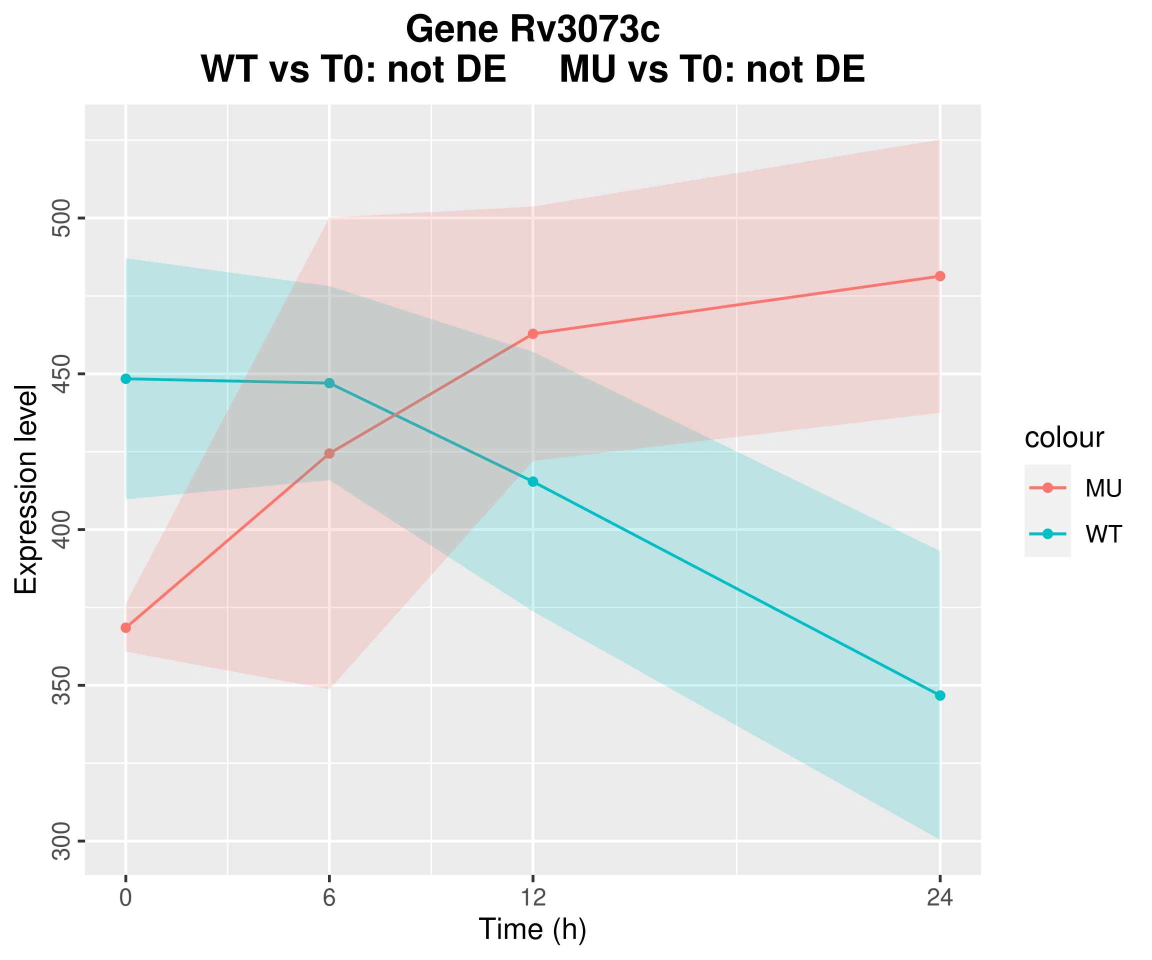

Gene Rv3073c
WT vs T0: not DE   MU vs T0: not DE
Expression level
300
350
400
450
500
0
6
12
24
Time (h)
colour
MU
WT

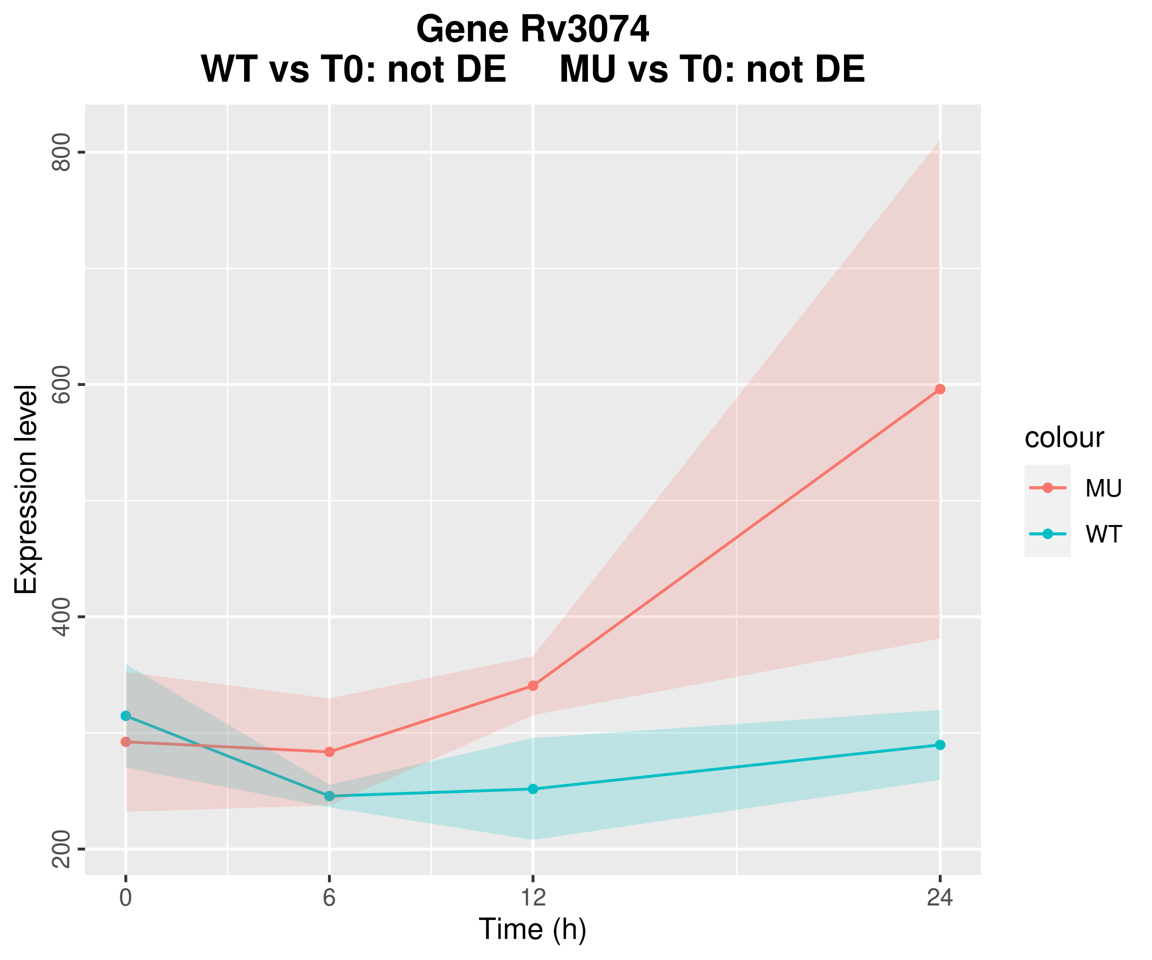
Gene Rv3074
WT vs T0: not DE MU vs T0: not DE
Expression level
200
400
600
800
0
6
12
24
Time (h)
colour
MU
WT

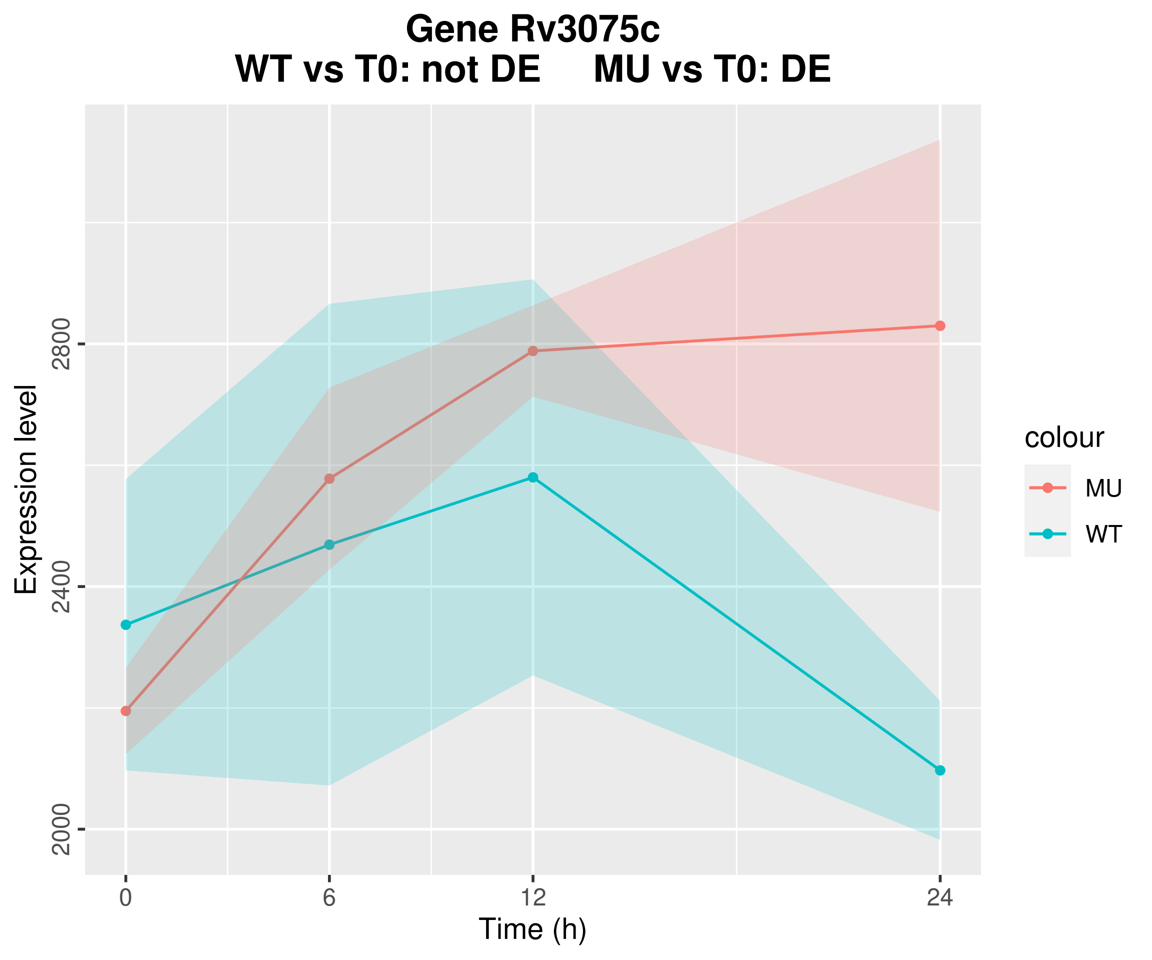
Gene Rv3075c
WT vs T0: not DE
MU vs T0: DE
Expression level
2000
2400
2800
0
6
12
24
Time (h)
colour
MU
WT

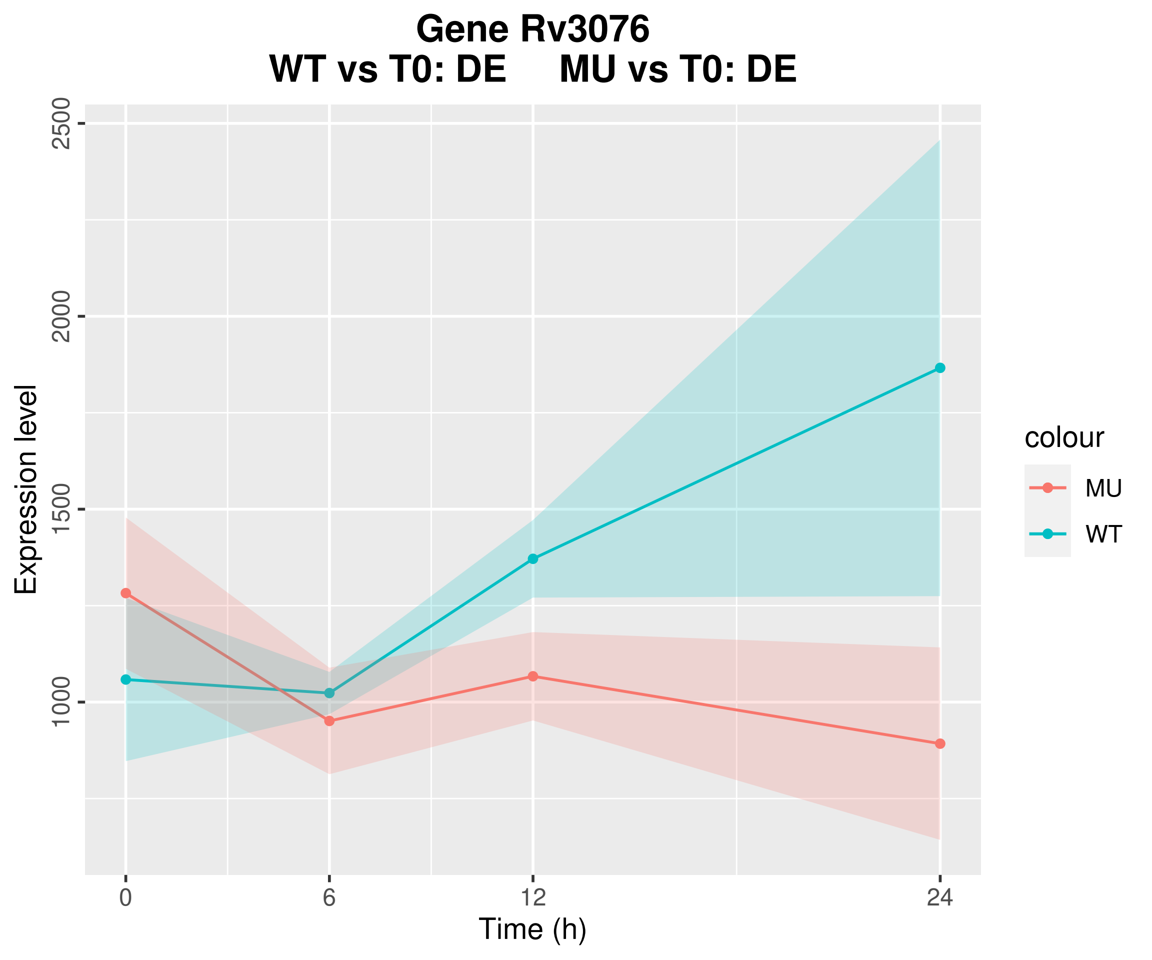
Gene Rv3076
WT vs T0: DE MU vs T0: DE
Expression level
2500
2000
1500
1000
0
6
12
24
Time (h)
colour
MU
WT

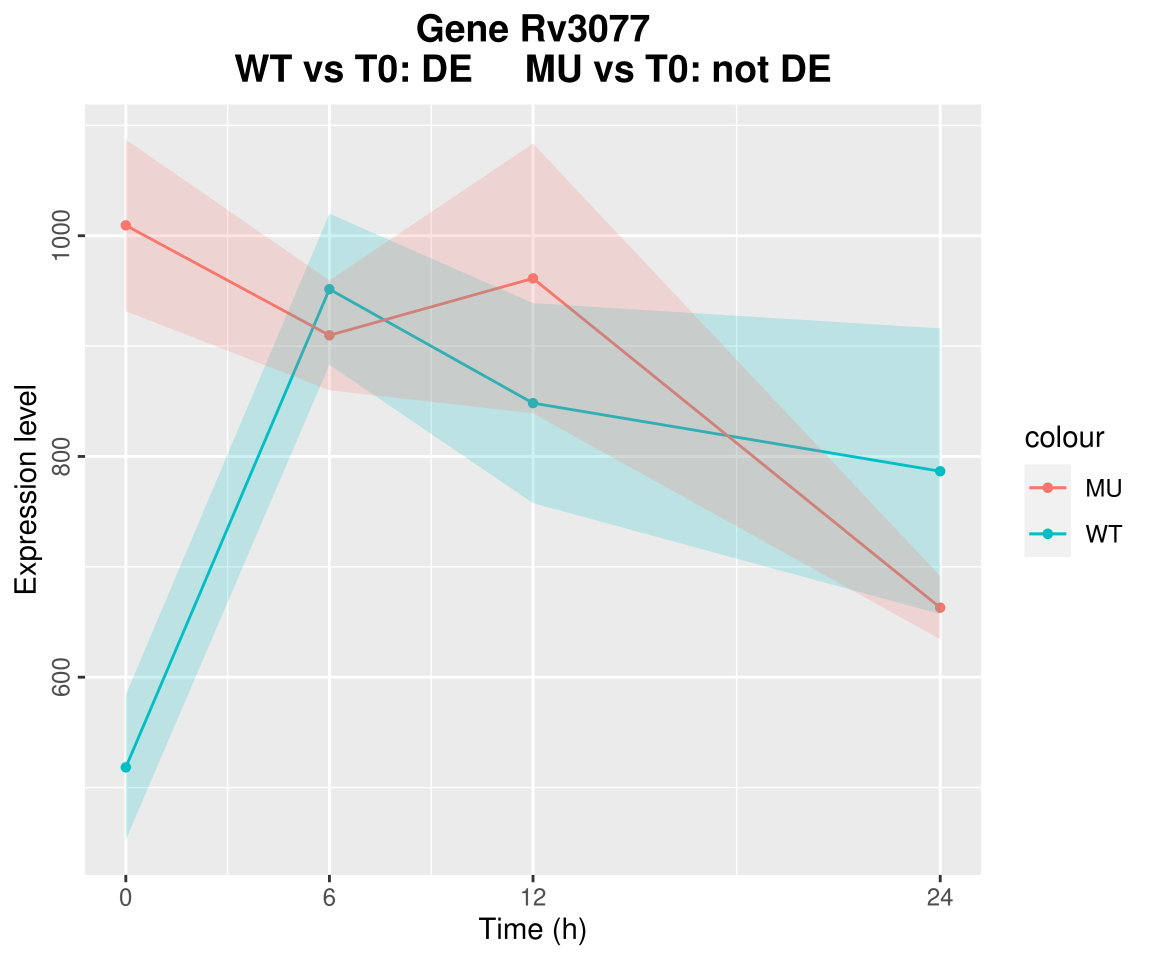
Gene Rv3077
WT vs T0: DE   MU vs T0: not DE
Expression level
1000
800
600
0
6
12
24
Time (h)
colour
MU
WT

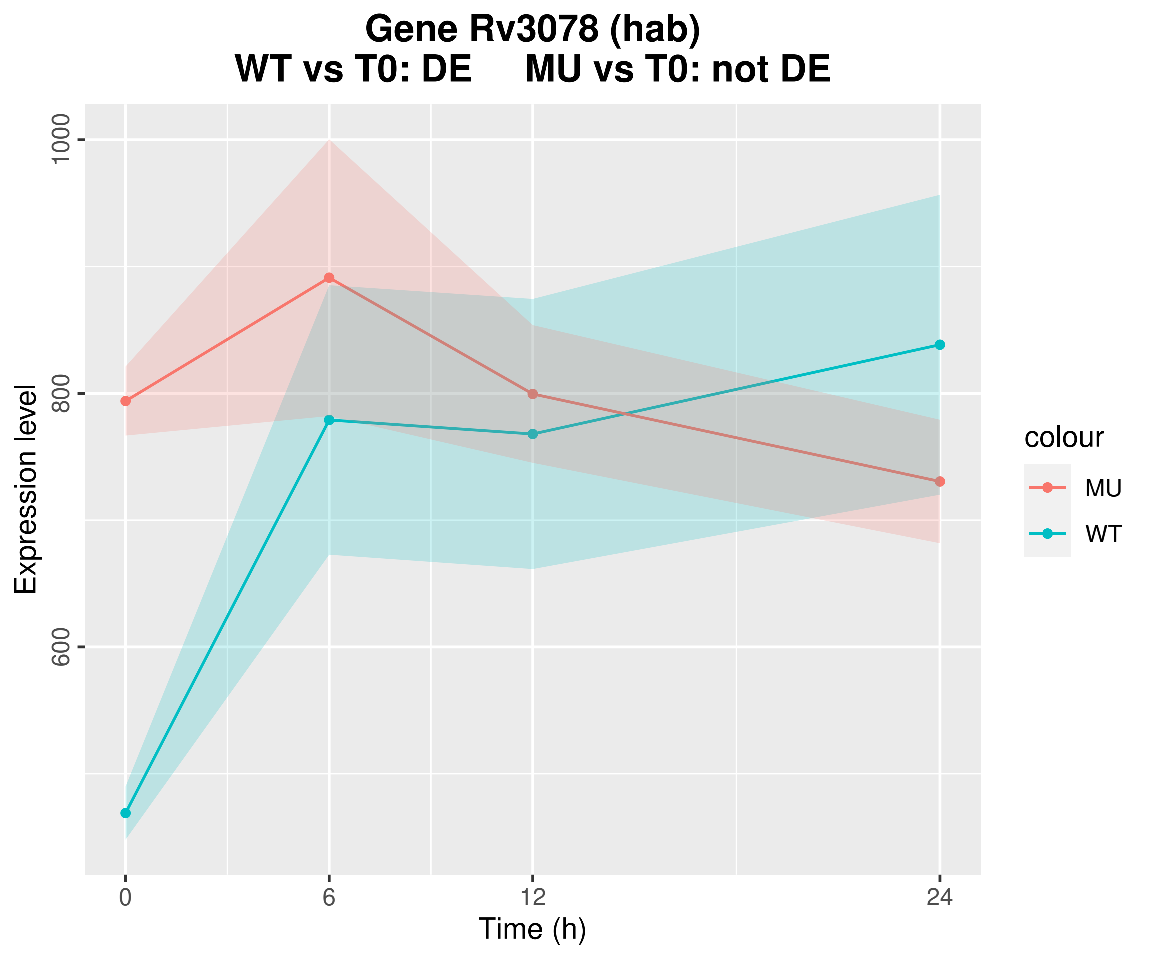
Gene Rv3078 (hab)
WT vs T0: DE MU vs T0: not DE
Expression level
1000
800
600
0
6
12
24
Time (h)
colour
MU
WT

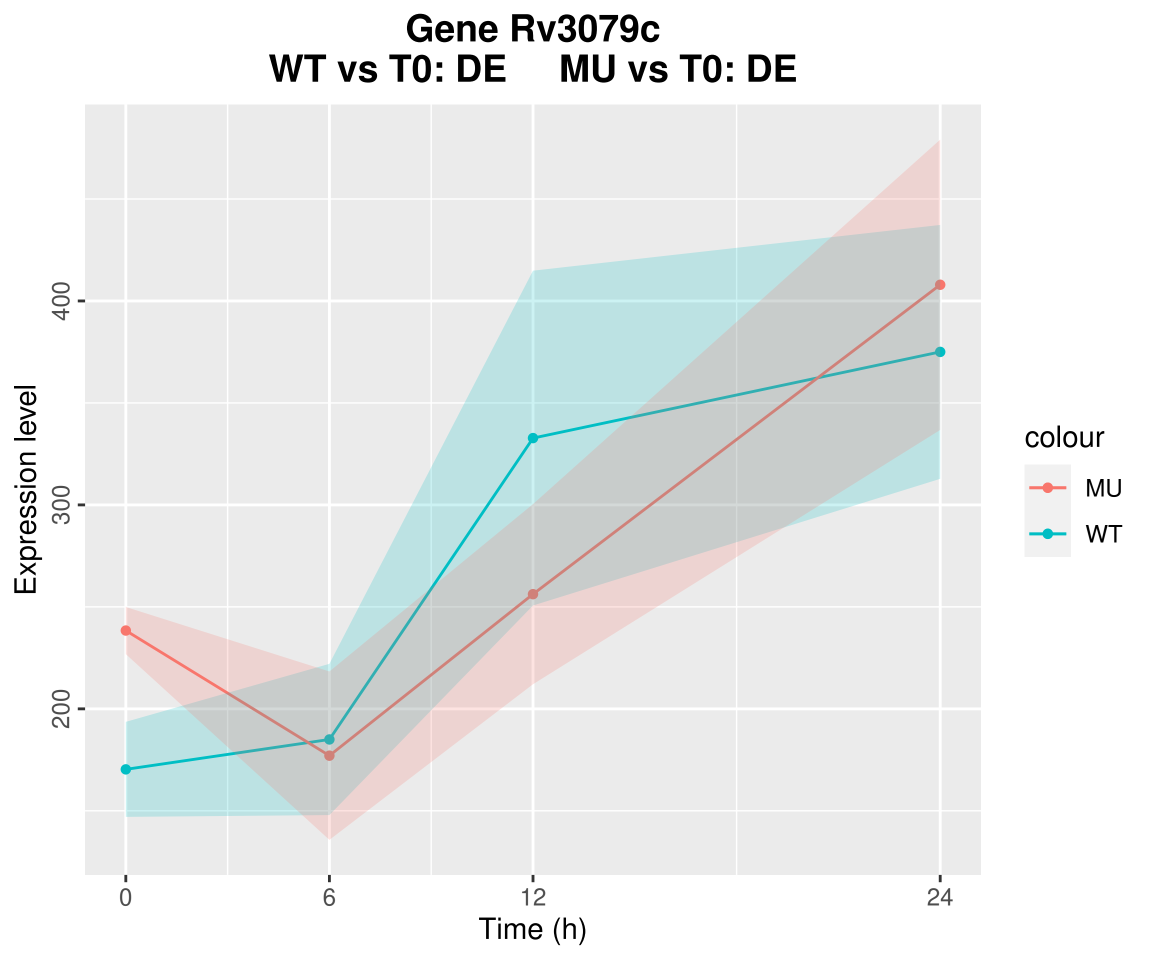

Gene Rv3079c
WT vs T0: DE MU vs T0: DE
Expression level
400
300
200
0
6
12
24
Time (h)
colour
MU
WT

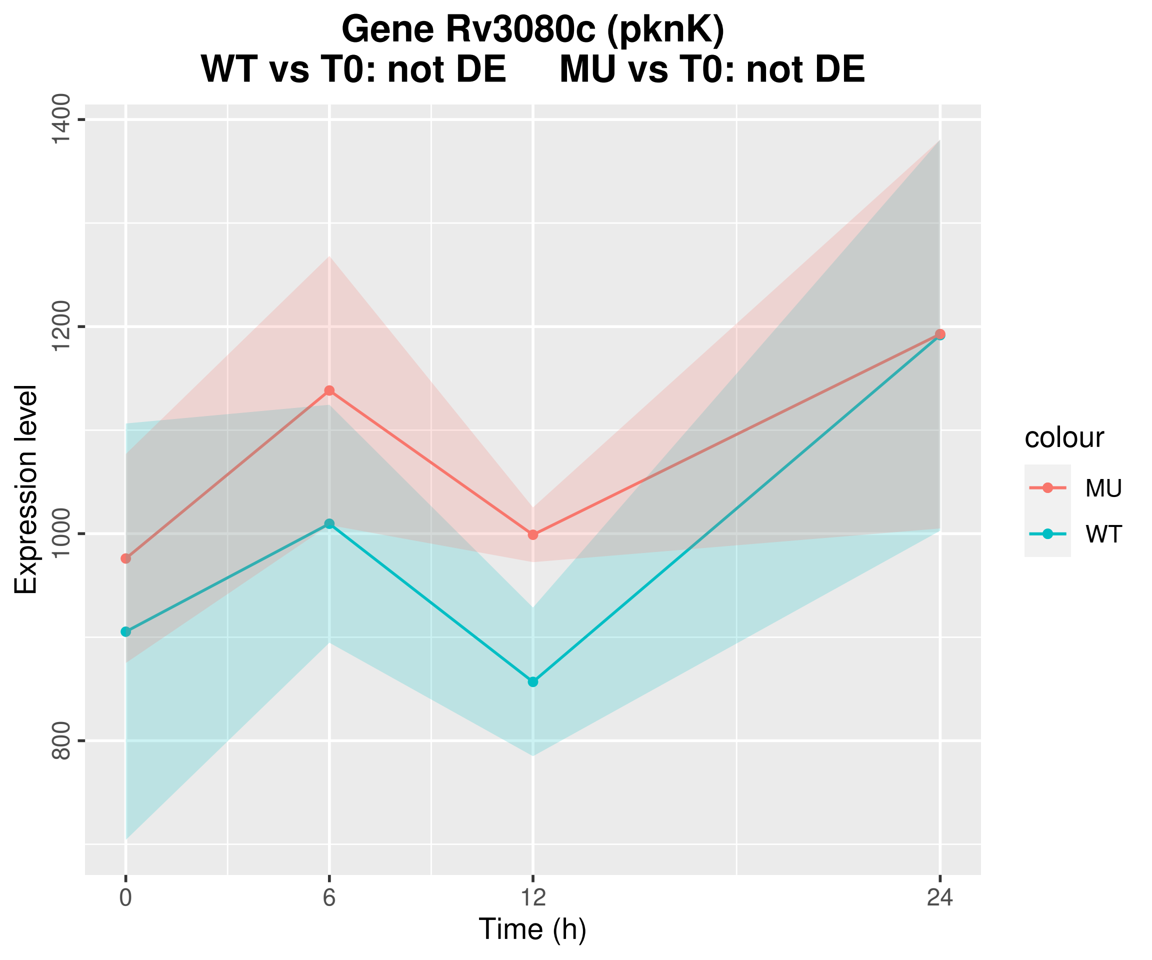
Gene Rv3080c (pknK)
WT vs T0: not DE   MU vs T0: not DE
Expression level
1400
1200
1000
800
0
6
12
24
Time (h)
colour
MU
WT

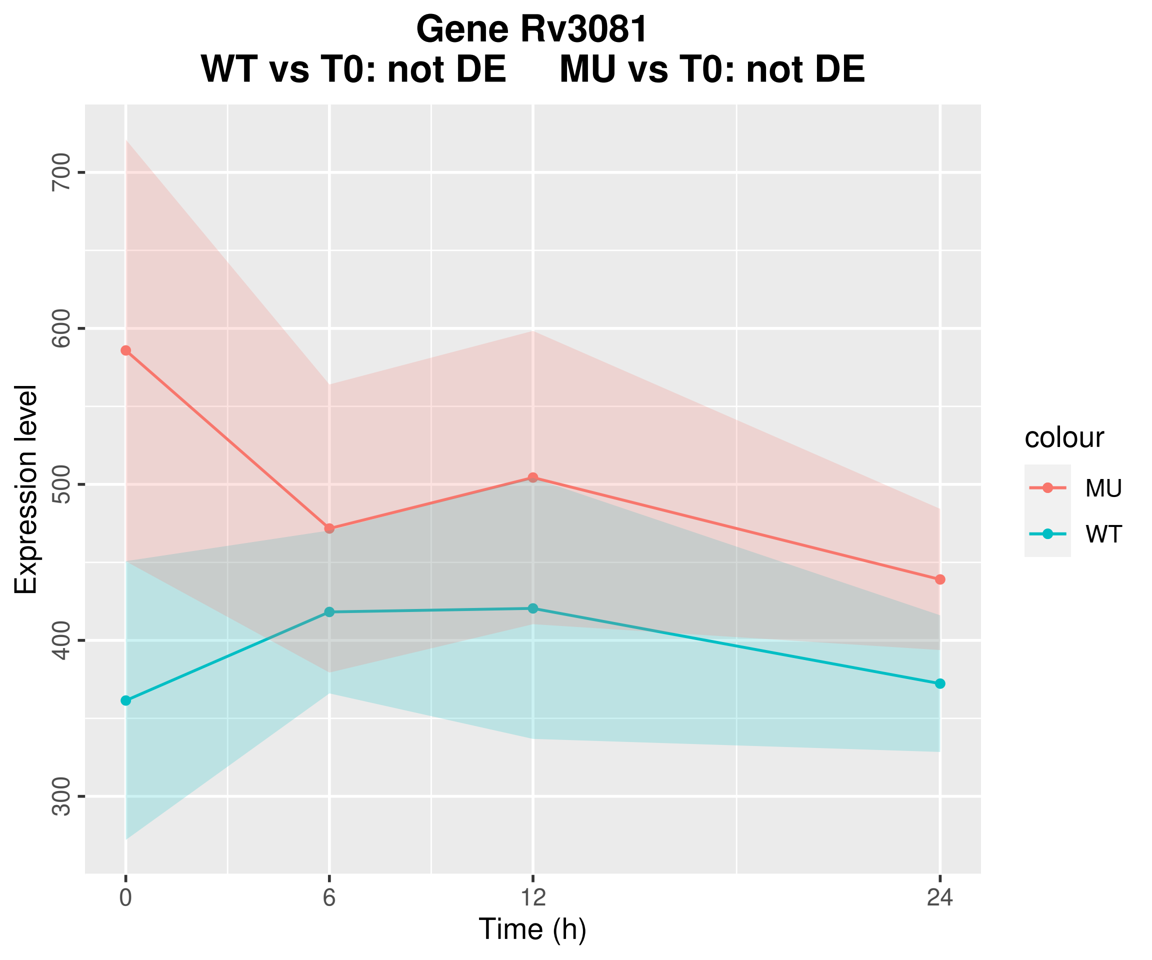
Gene Rv3081
WT vs T0: not DE MU vs T0: not DE
700
600
500
400
300
Expression level
0
6
12
24
Time (h)
colour
MU
WT

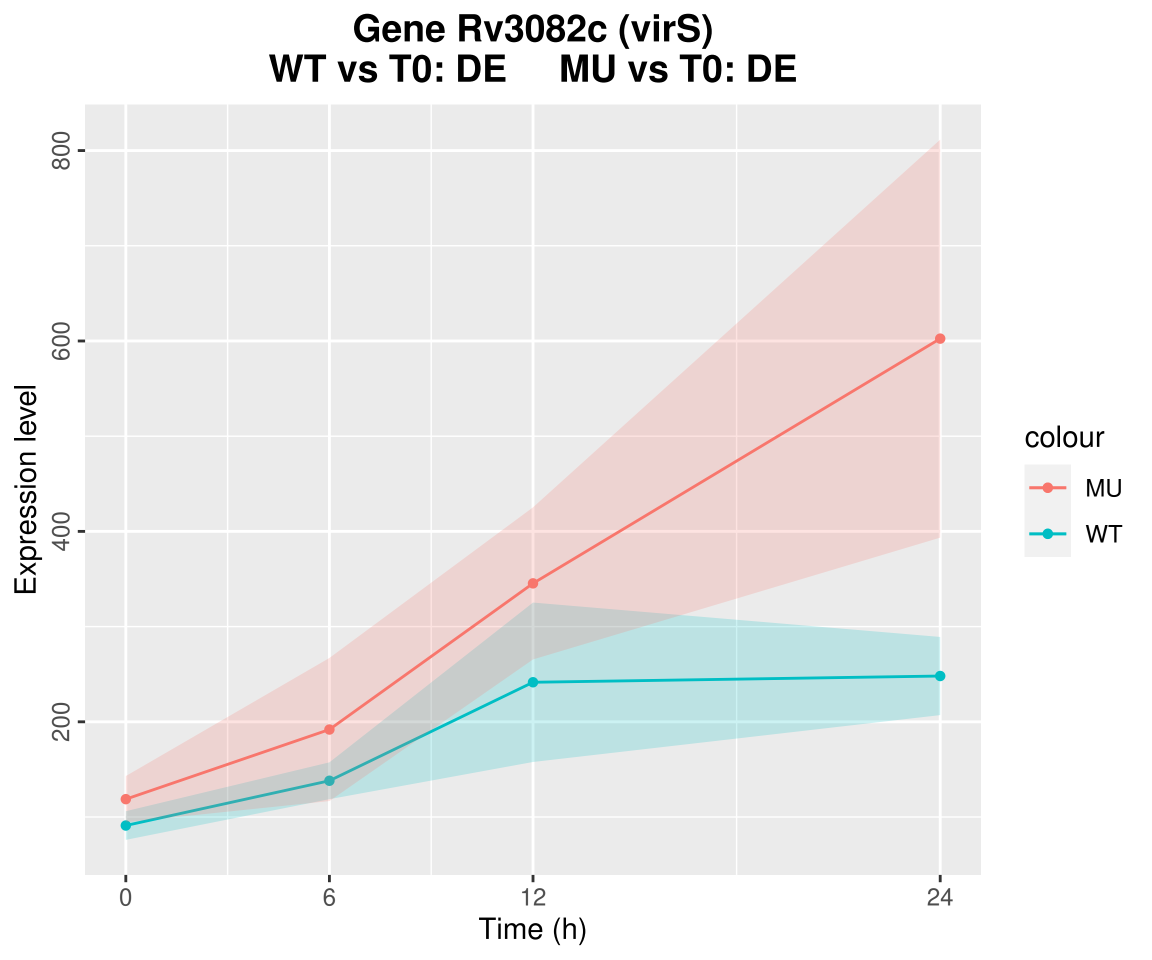
Gene Rv3082c (virS)
WT vs T0: DE   MU vs T0: DE
Expression level
800
600
400
200
0
6
12
24
Time (h)
colour
MU
WT

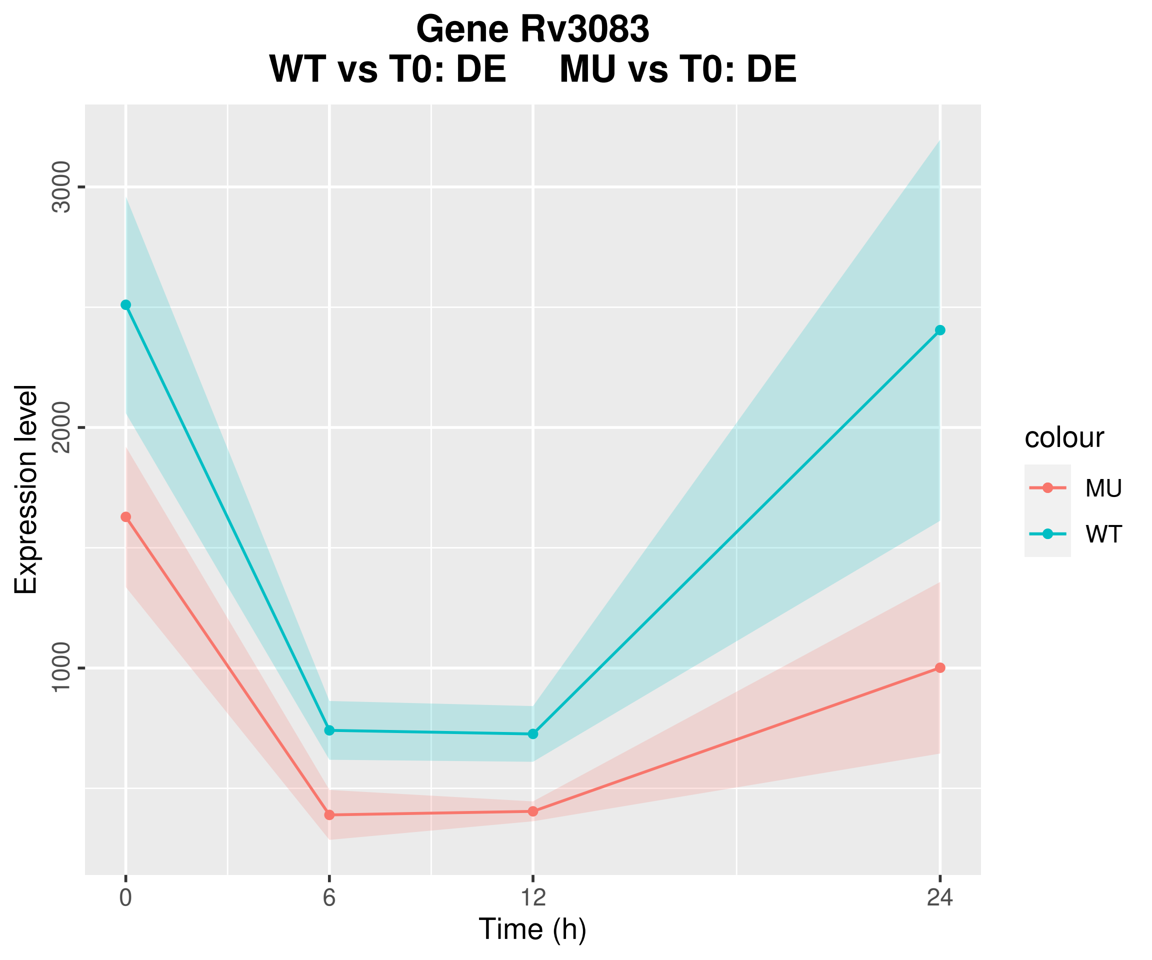
Gene Rv3083
WT vs T0: DE MU vs T0: DE
Expression level
3000
2000
1000
0
6
12
24
Time (h)
colour
MU
WT

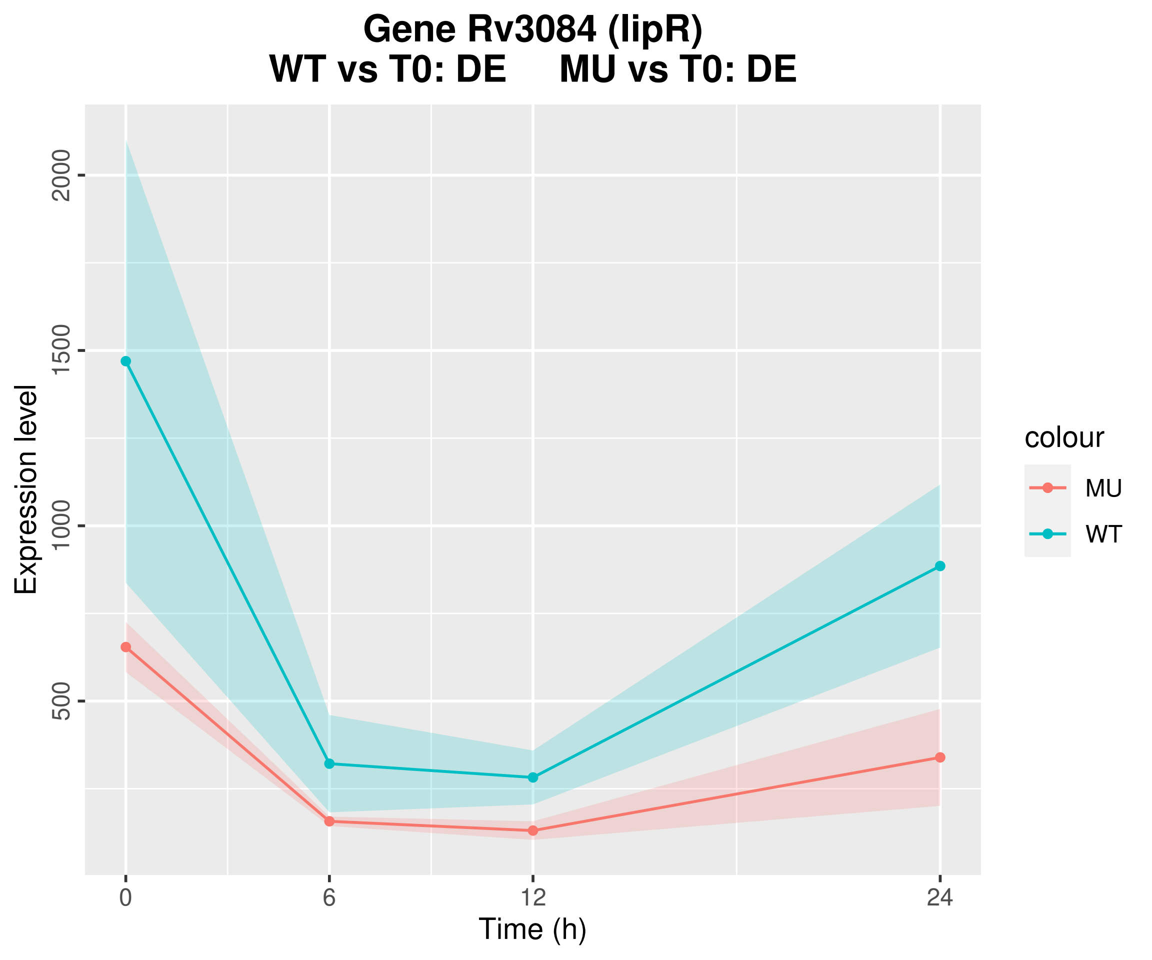
Gene Rv3084 (lipR)
WT vs T0: DE   MU vs T0: DE
Expression level
2000
1500
1000
500
0
6
12
24
Time (h)
colour
MU
WT

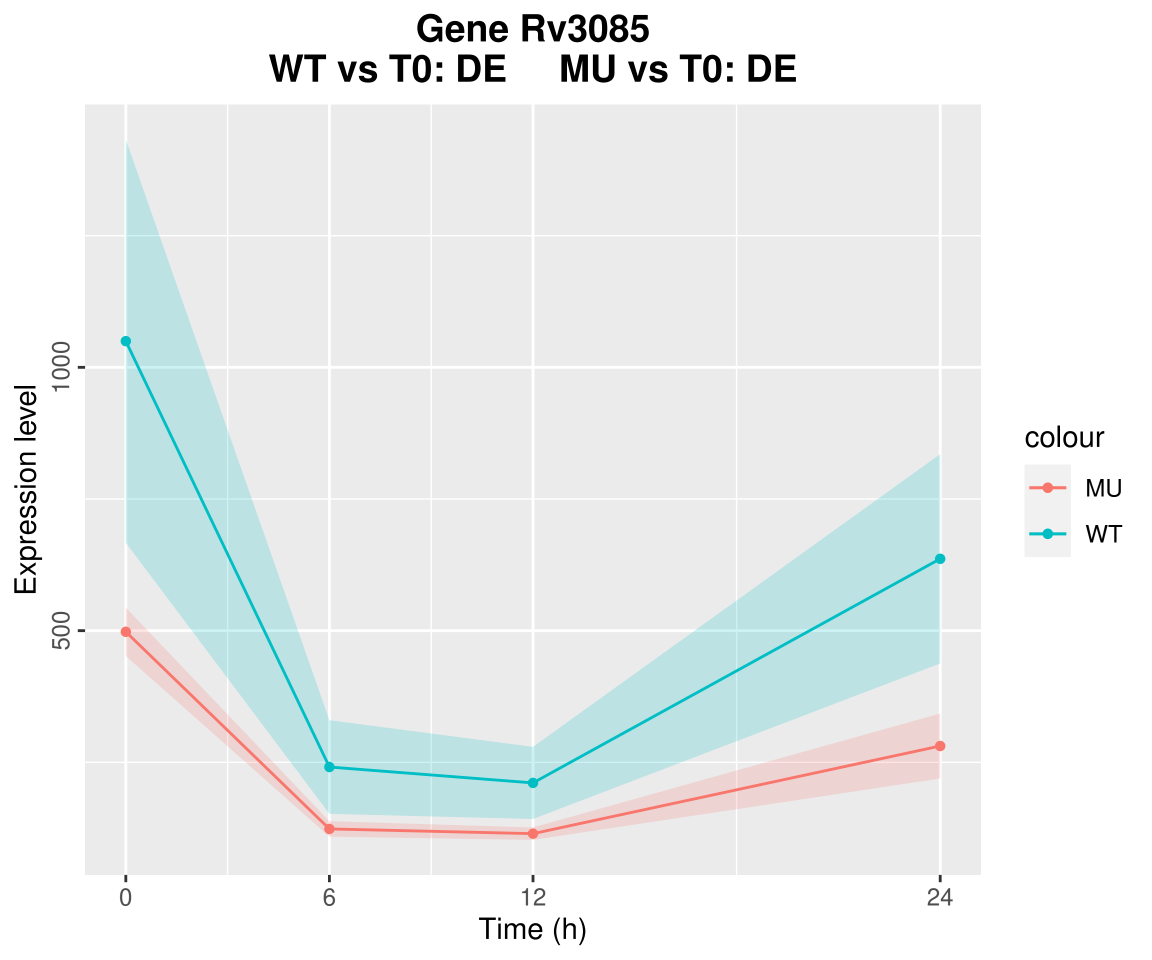
Gene Rv3085
WT vs T0: DE   MU vs T0: DE
Expression level
1000
500
0
6
12
24
Time (h)
colour
MU
WT

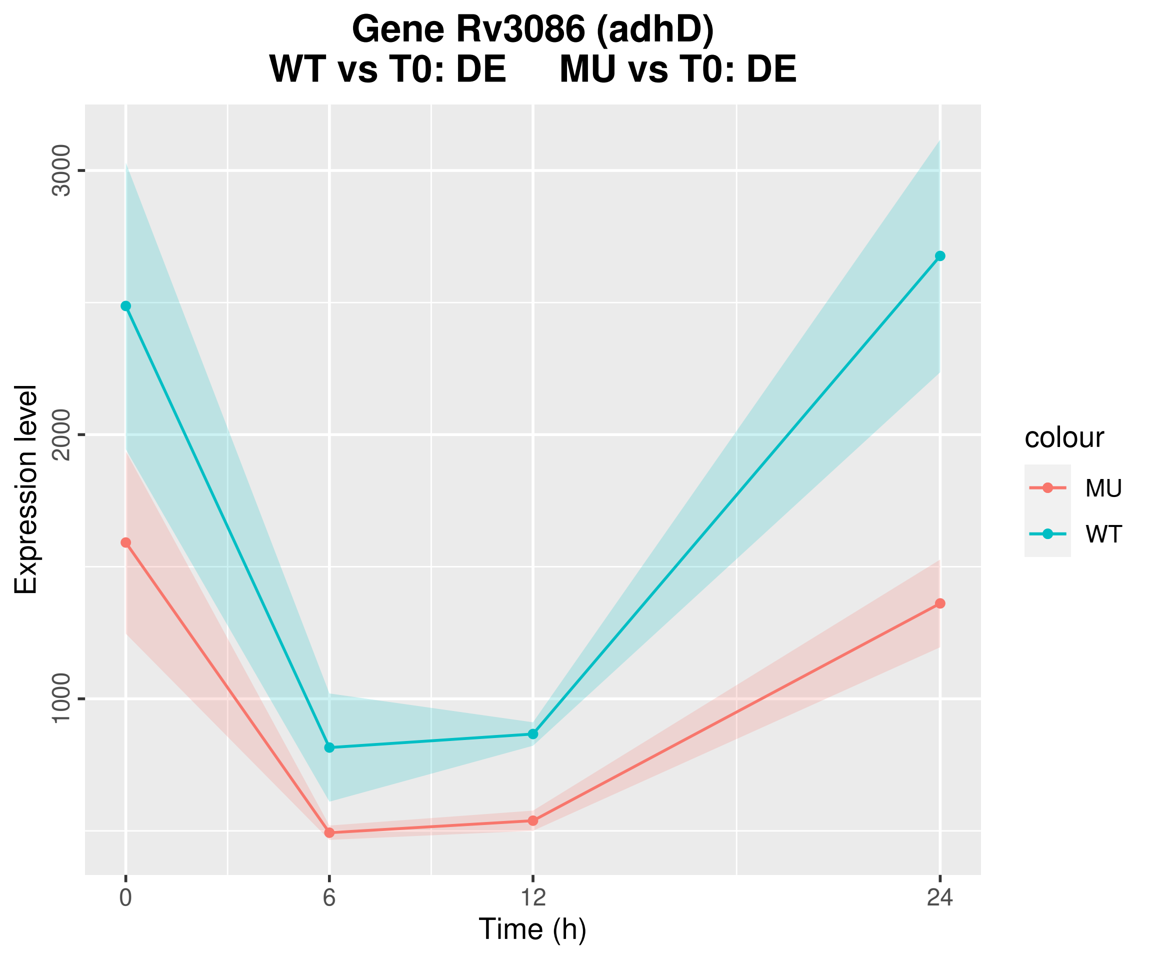
Gene Rv3086 (adhD)
WT vs T0: DE   MU vs T0: DE
Expression level
3000
2000
1000
0
6
12
24
Time (h)
colour
MU
WT

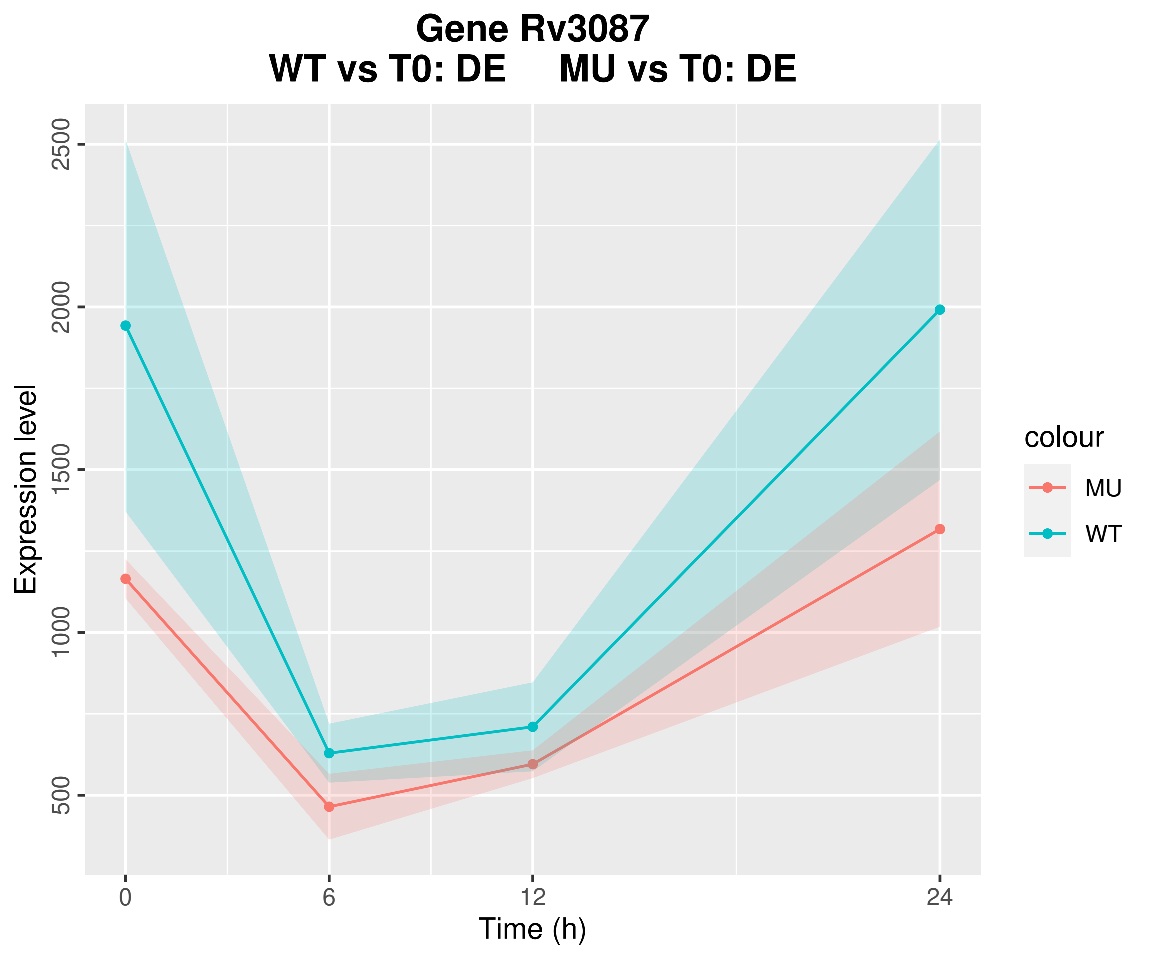
Gene Rv3087
WT vs T0: DE   MU vs T0: DE
Expression level
2500
2000
1500
1000
500
0
6
12
24
Time (h)
colour
MU
WT

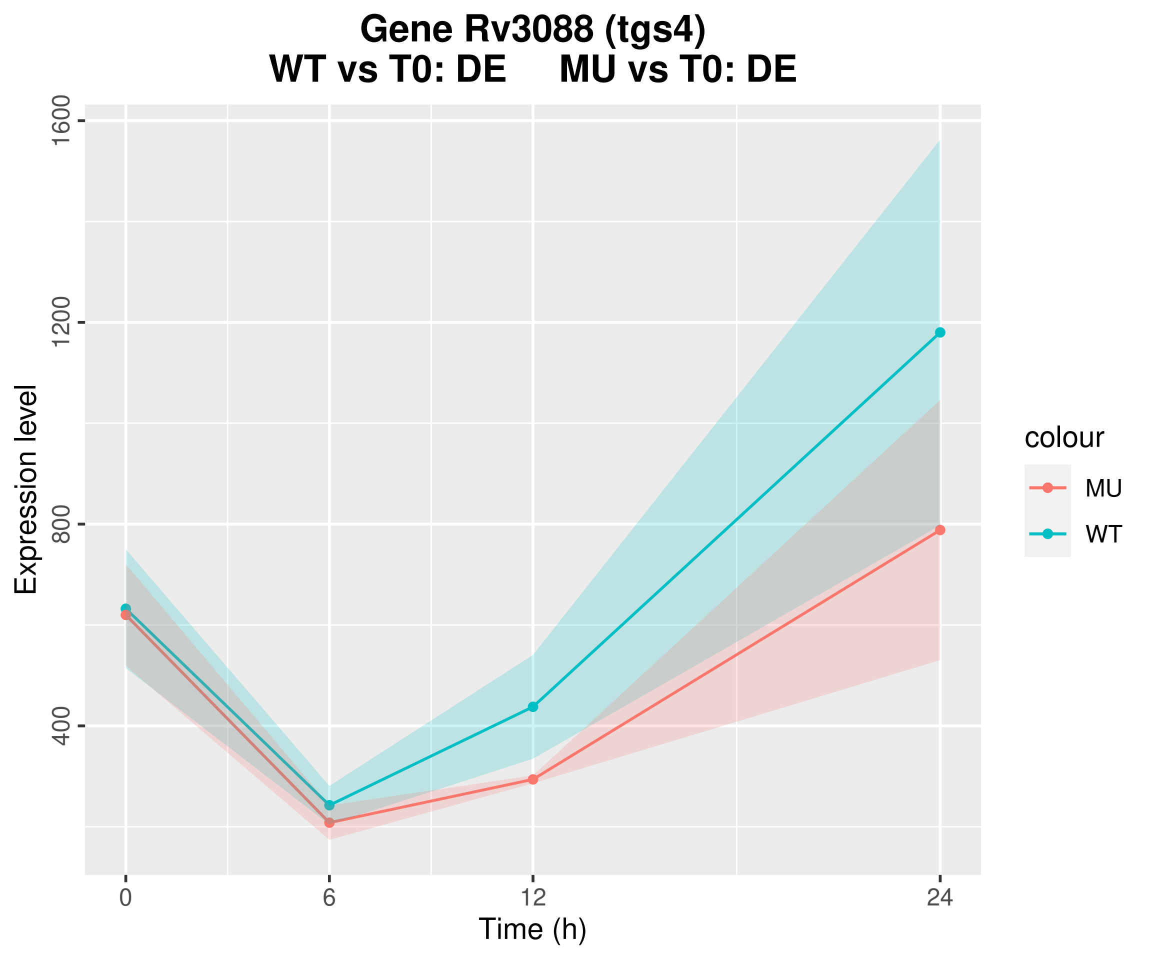
Gene Rv3088 (tgs4)
WT vs T0: DE MU vs T0: DE
1600
1200
800
400
Expression level
0
6
12
24
Time (h)
colour
MU
WT

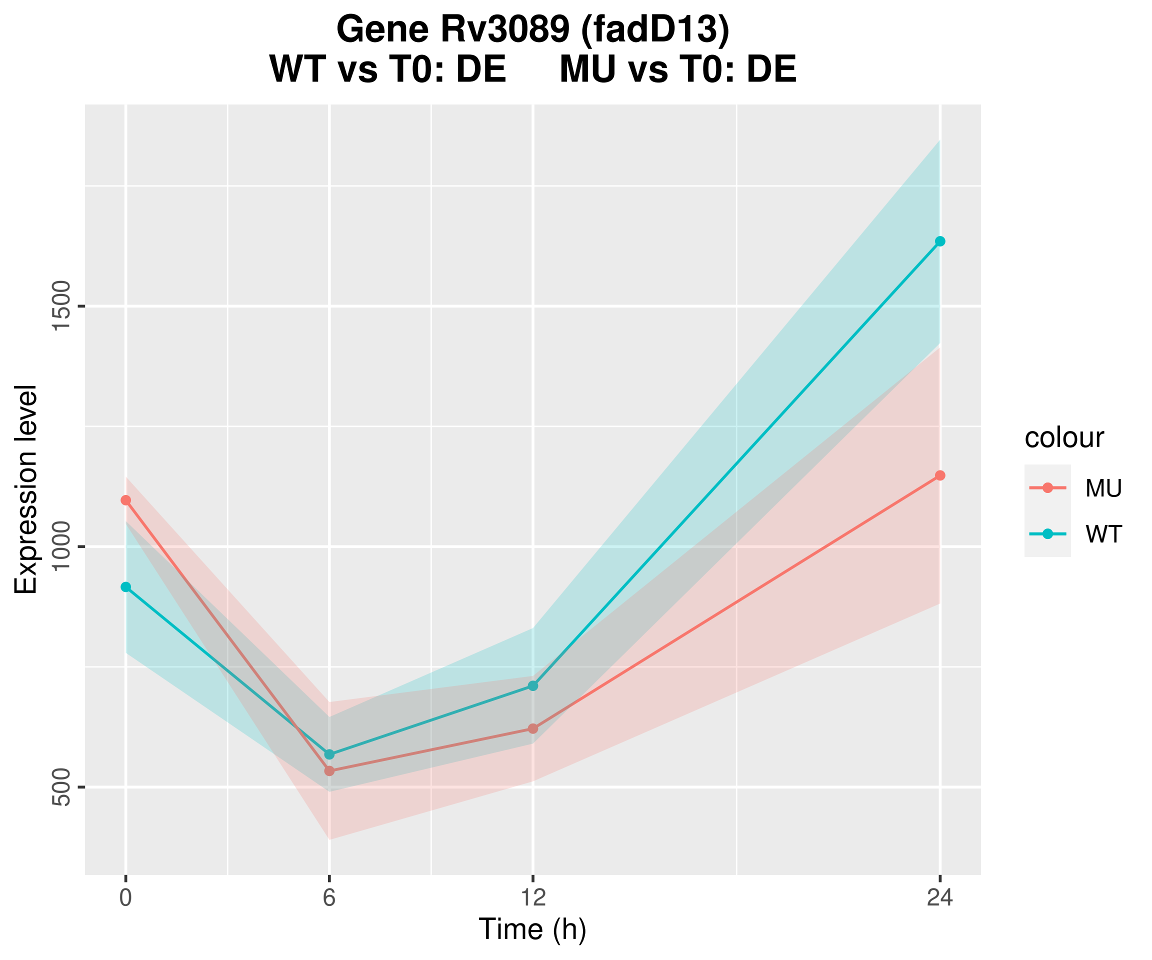

Gene Rv3089 (fadD13)
WT vs T0: DE   MU vs T0: DE
Expression level
500
1000
1500
0
6
12
24
Time (h)
colour
MU
WT

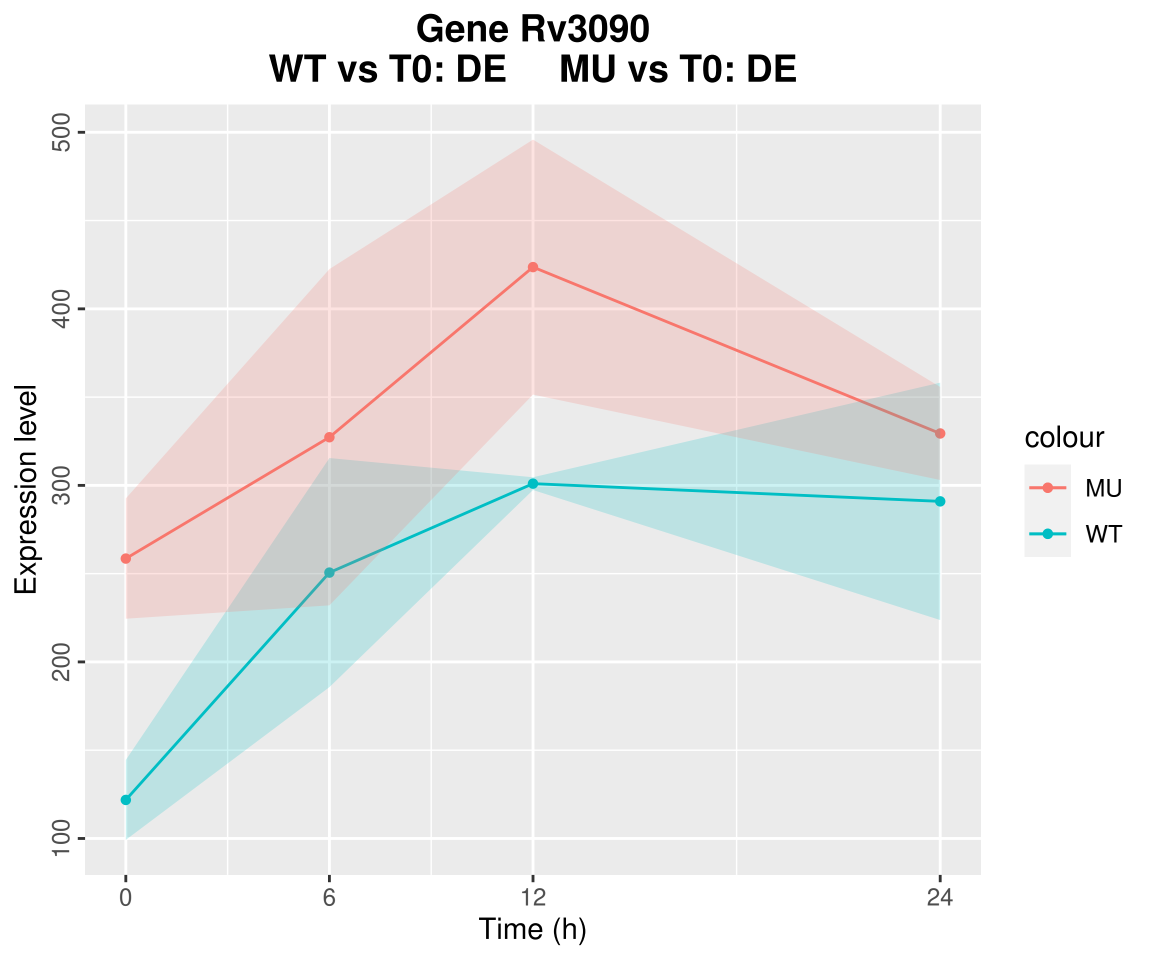

Gene Rv3090
WT vs T0: DE   MU vs T0: DE
Expression level
500
400
300
200
100
0
6
12
24
Time (h)
colour
MU
WT

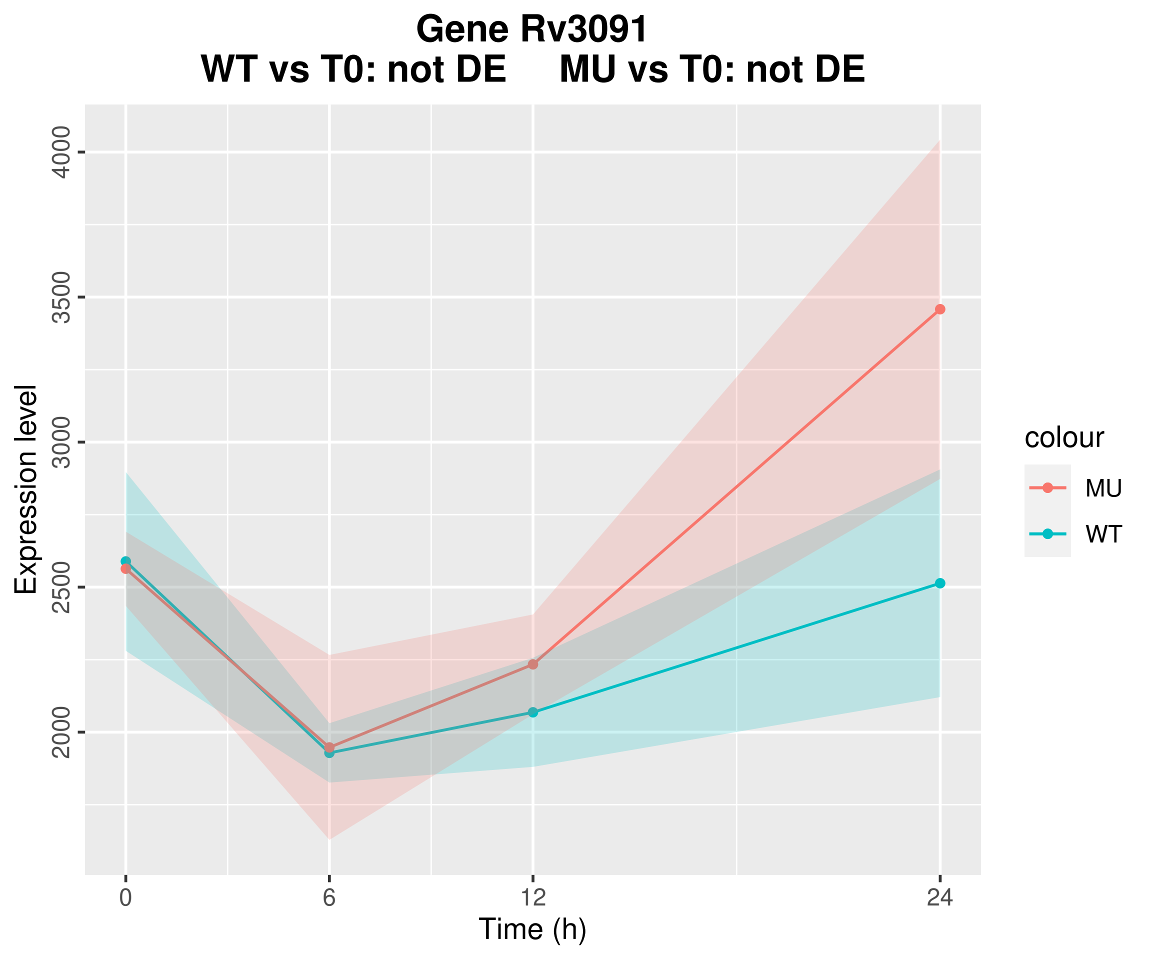

Gene Rv3091
WT vs T0: not DE   MU vs T0: not DE
Expression level
4000
3500
3000
2500
2000
0
6
12
24
Time (h)
colour
MU
WT

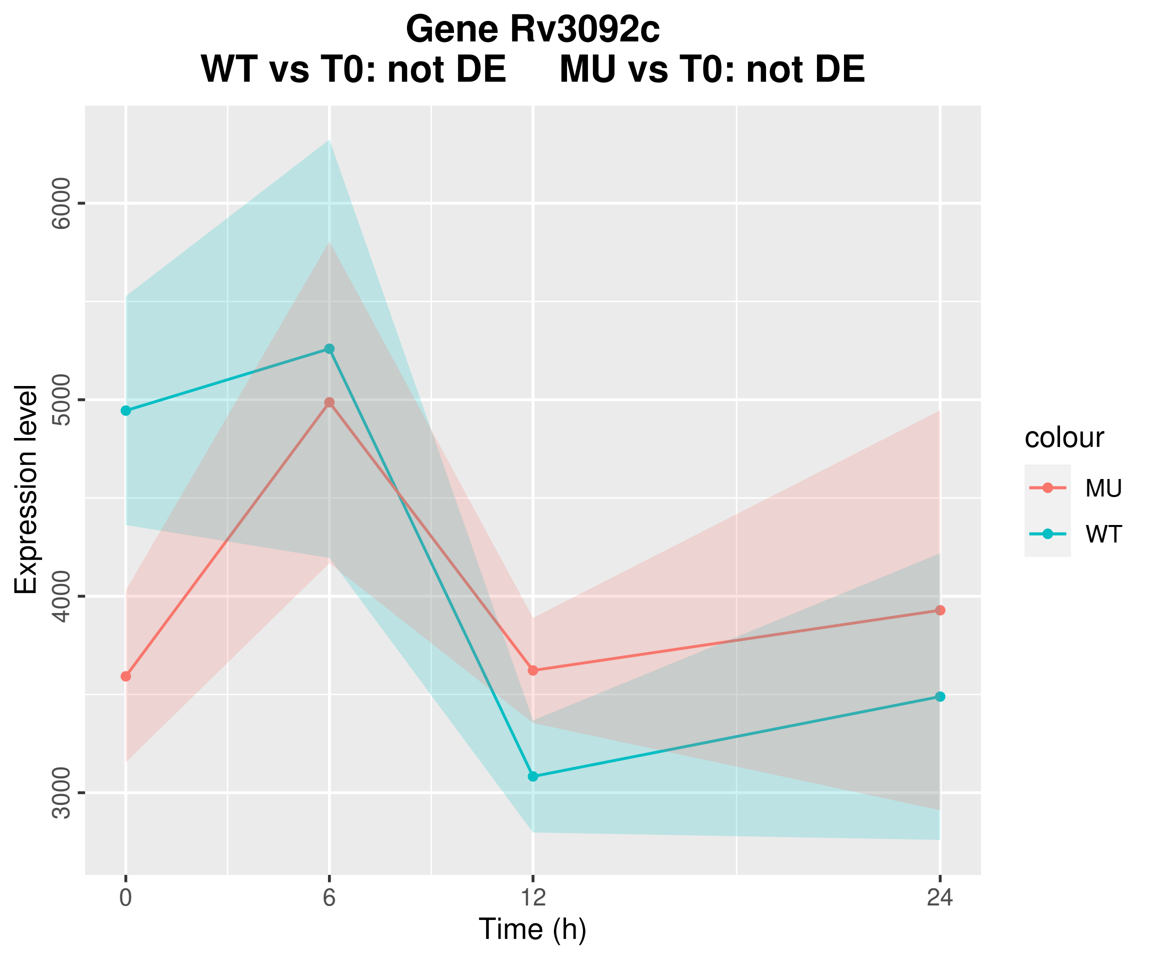
Gene Rv3092c
WT vs T0: not DE   MU vs T0: not DE
Expression level
6000
5000
4000
3000
0
6
12
24
Time (h)
colour
MU
WT

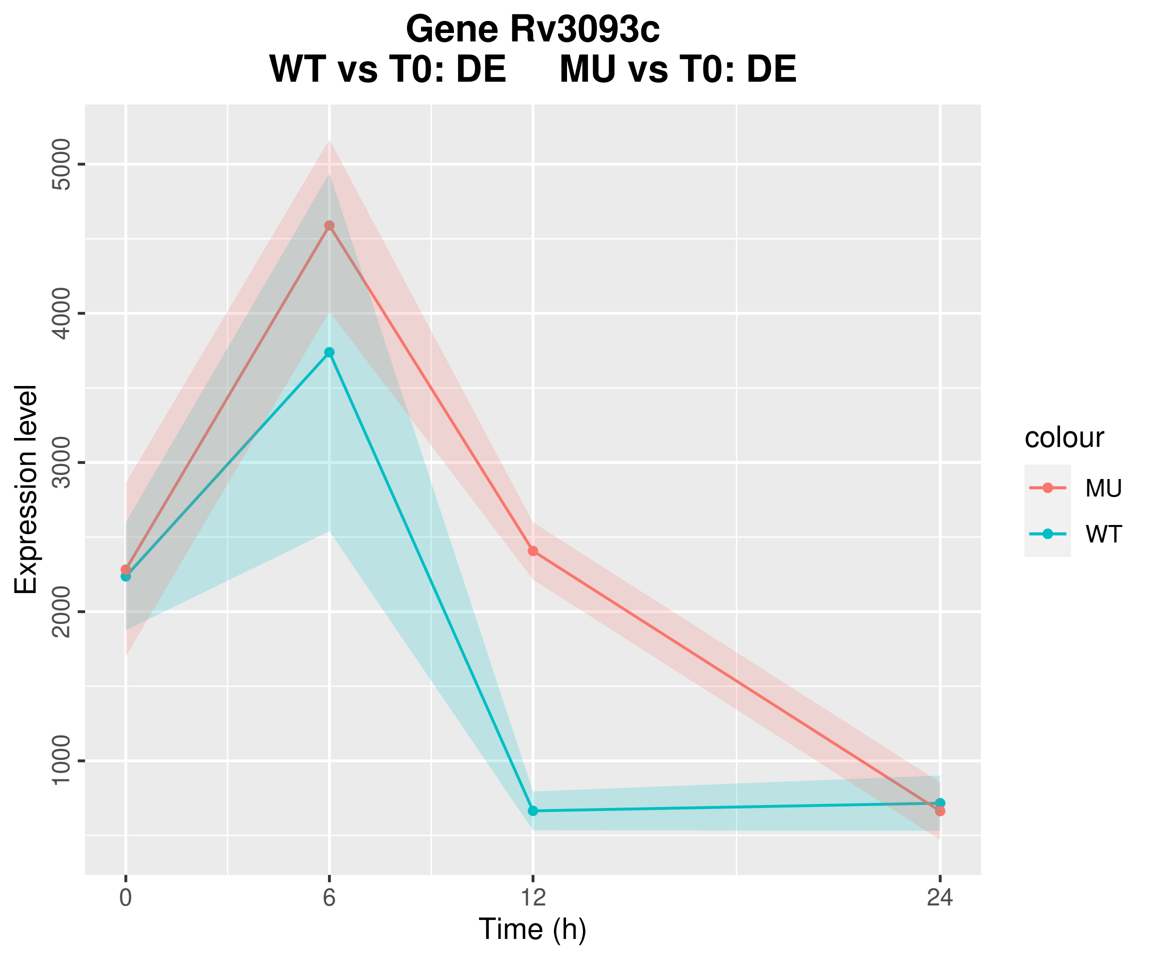

Gene Rv3093c
WT vs T0: DE MU vs T0: DE
Expression level
5000
4000
3000
2000
1000
0
6
12
24
Time (h)
colour
MU
WT

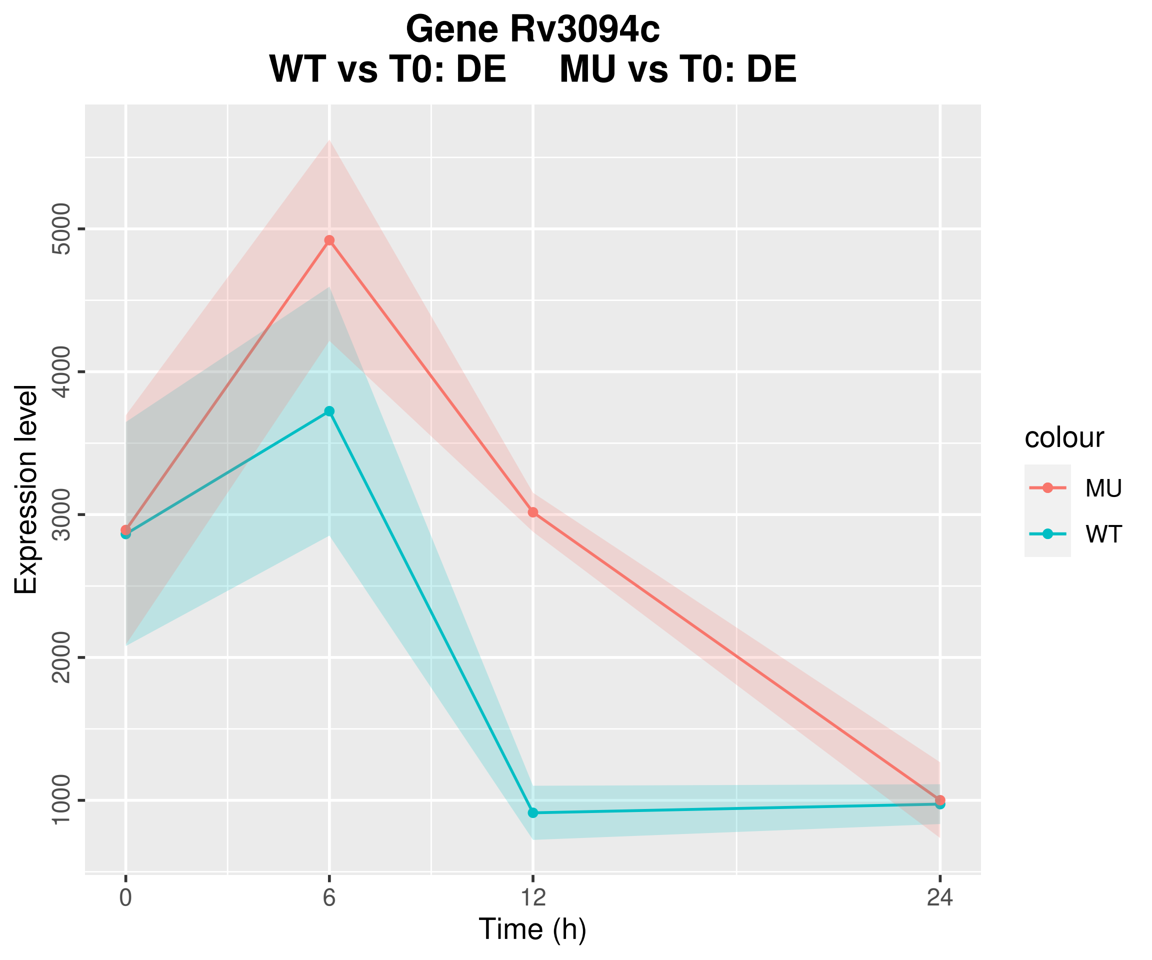
Gene Rv3094c
WT vs T0: DE MU vs T0: DE
Expression level
5000
4000
3000
2000
1000
0
6
12
24
Time (h)
colour
MU
WT

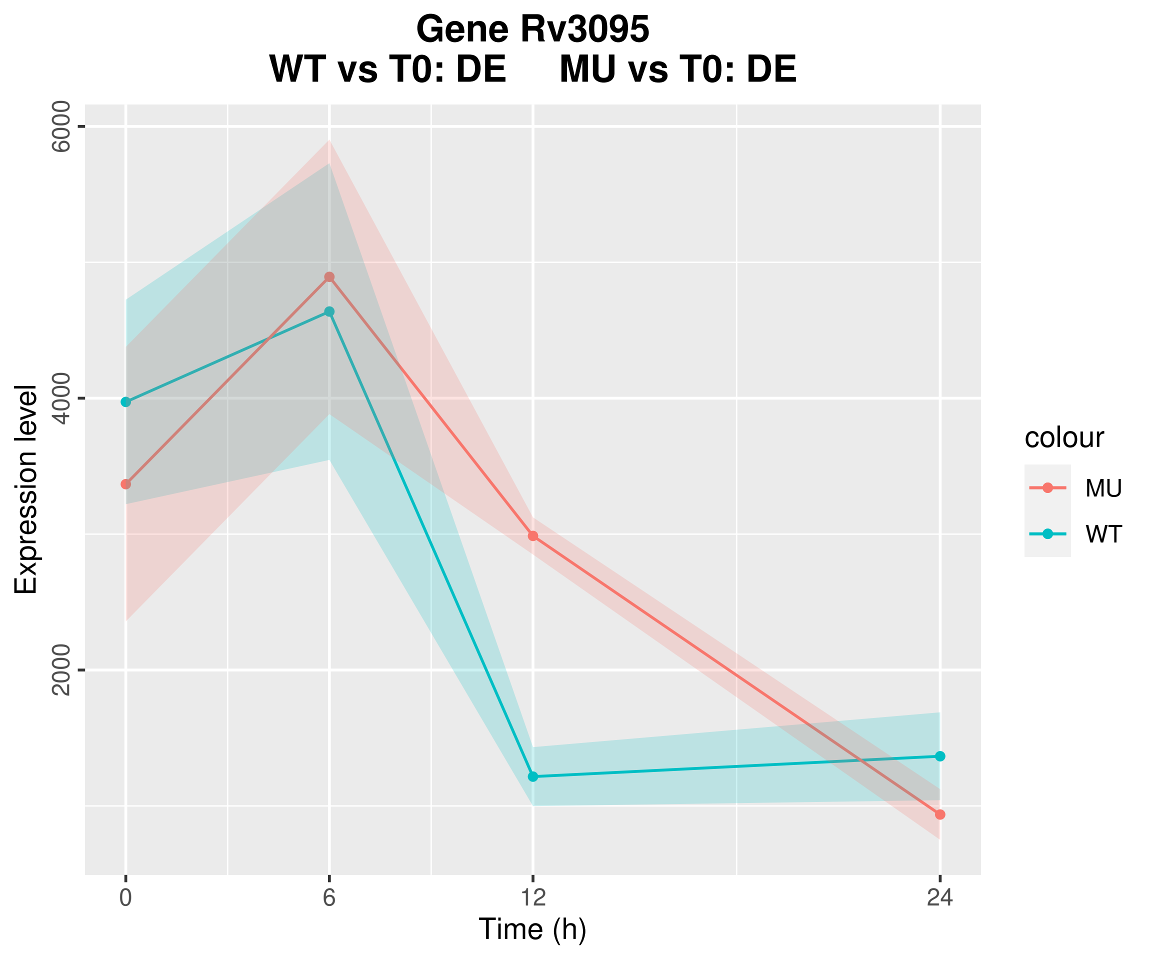
Gene Rv3095
WT vs T0: DE   MU vs T0: DE
Expression level
6000
4000
2000
0
6
12
24
Time (h)
colour
MU
WT

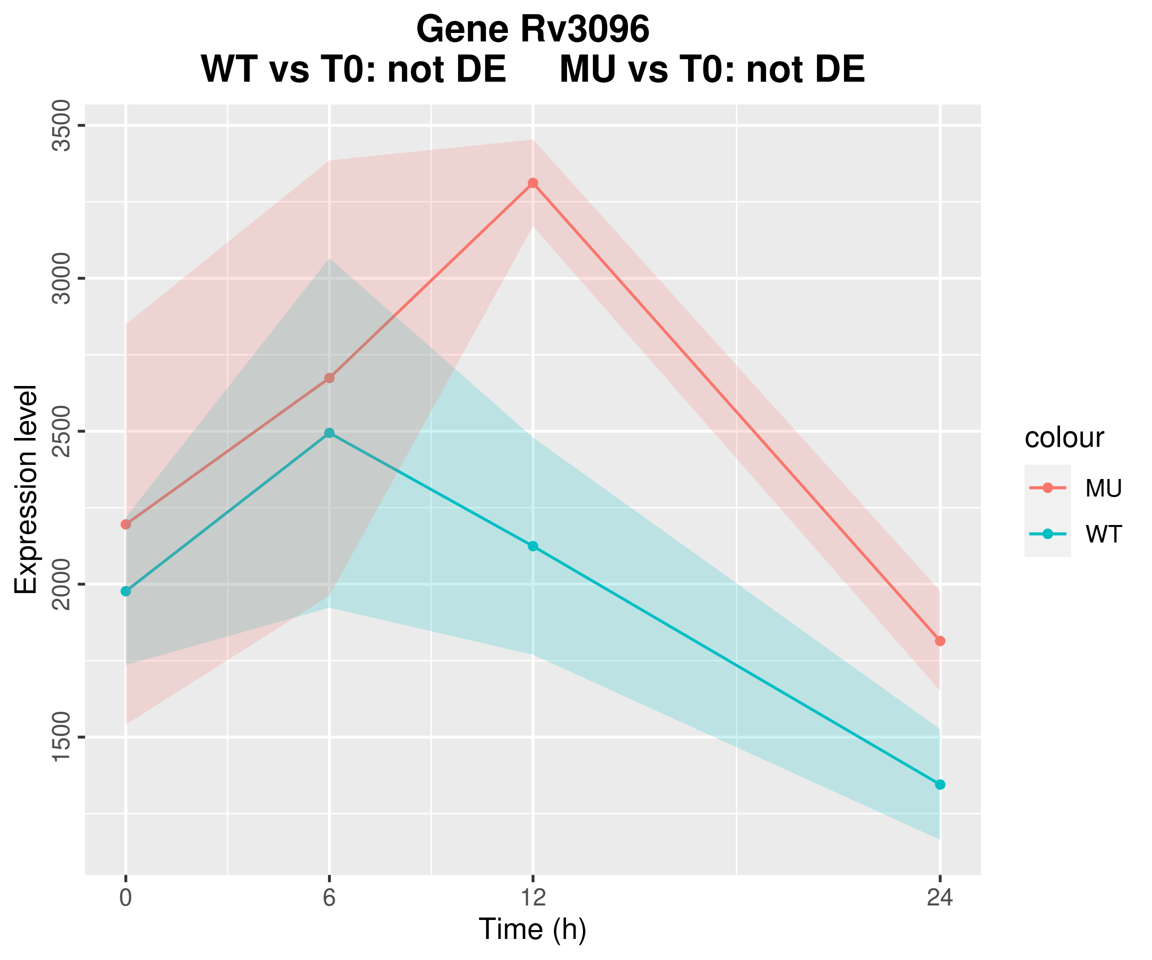
Gene Rv3096
WT vs T0: not DE MU vs T0: not DE
Expression level
3500
3000
2500
2000
1500
0
6
12
24
Time (h)
colour
MU
WT

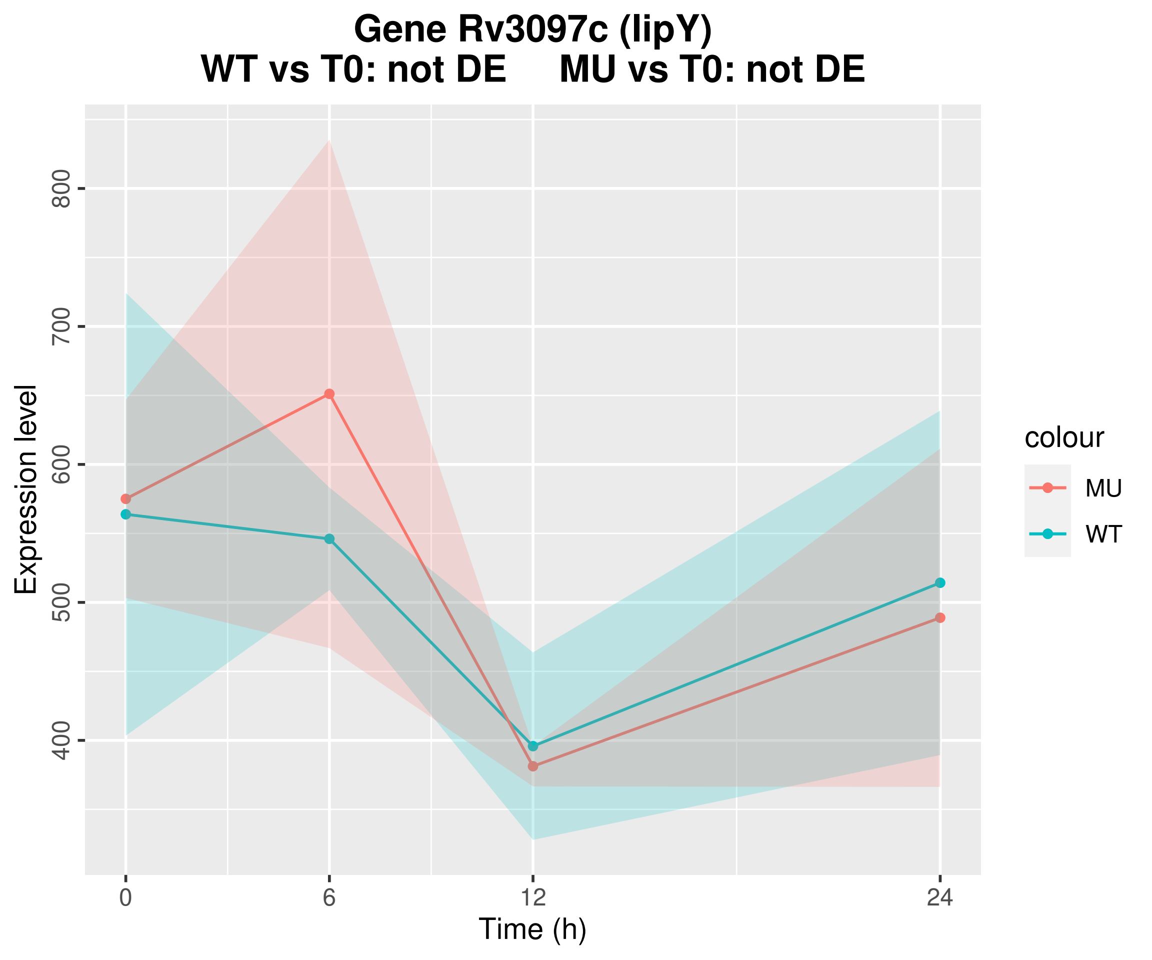
Gene Rv3097c (lipY)
WT vs T0: not DE   MU vs T0: not DE
Expression level
800
700
600
500
400
0
6
12
24
Time (h)
colour
MU
WT

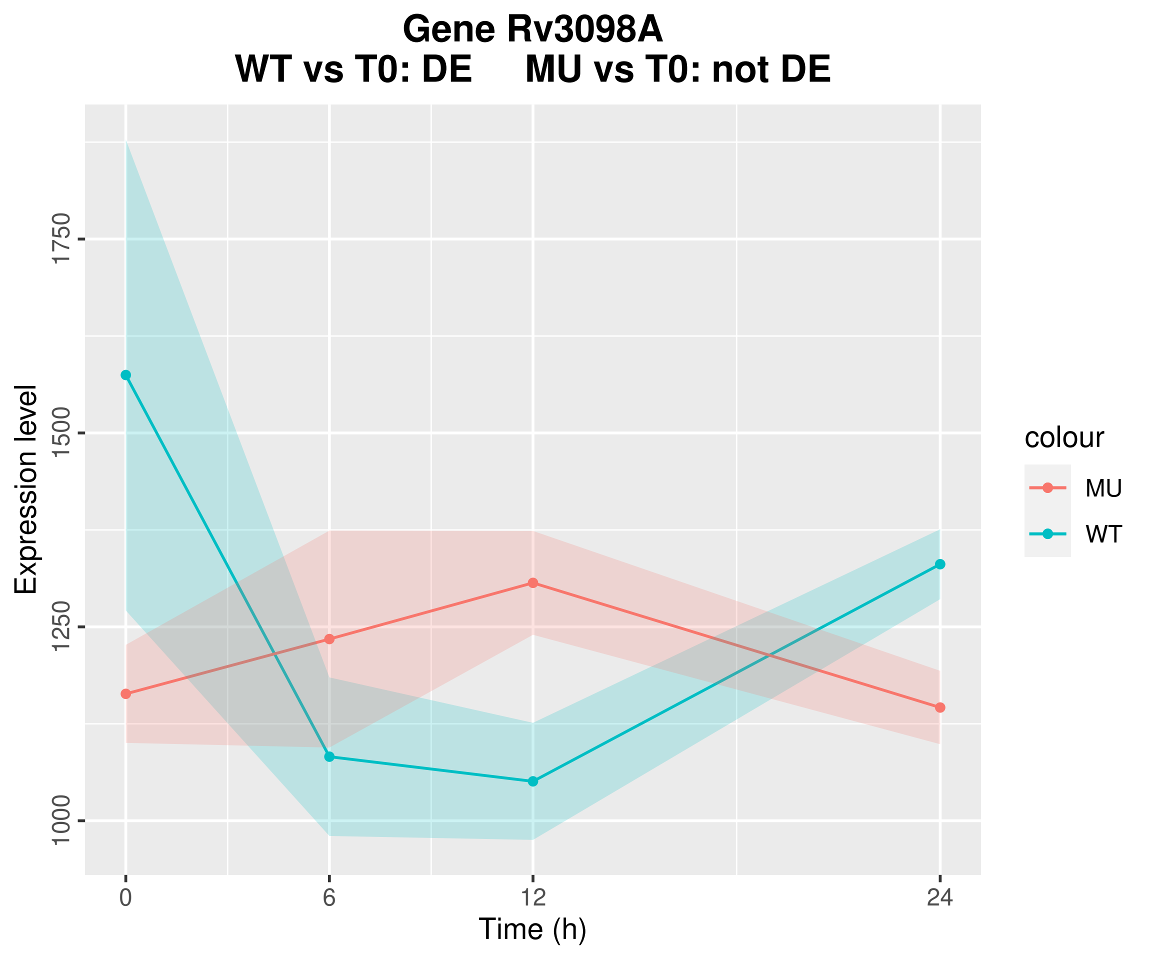
Gene Rv3098A
WT vs T0: DE   MU vs T0: not DE
Expression level
1000
1250
1500
1750
0
6
12
24
Time (h)
colour
MU
WT

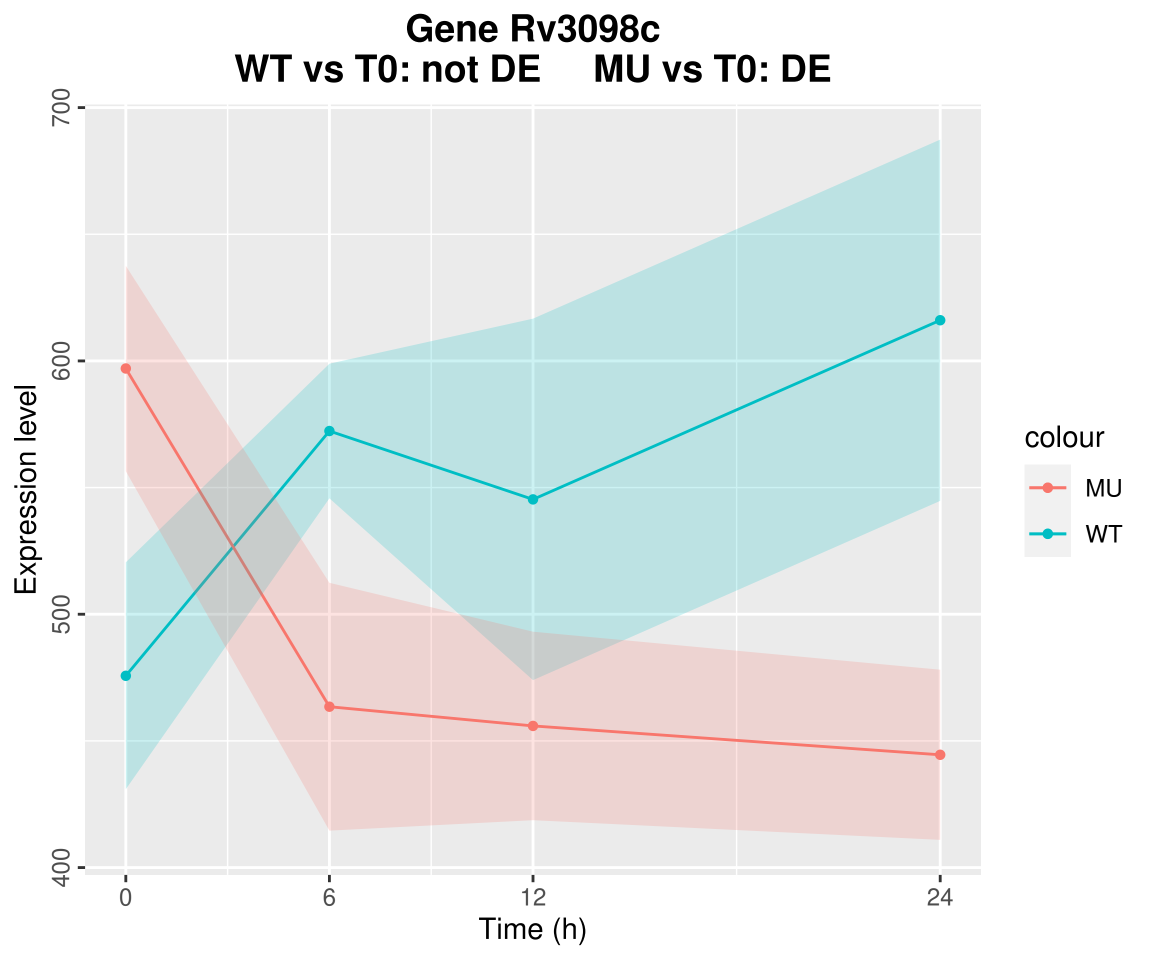
Gene Rv3098c
WT vs T0: not DE   MU vs T0: DE
700
600
500
400
Expression level
0
6
12
24
Time (h)
colour
MU
WT

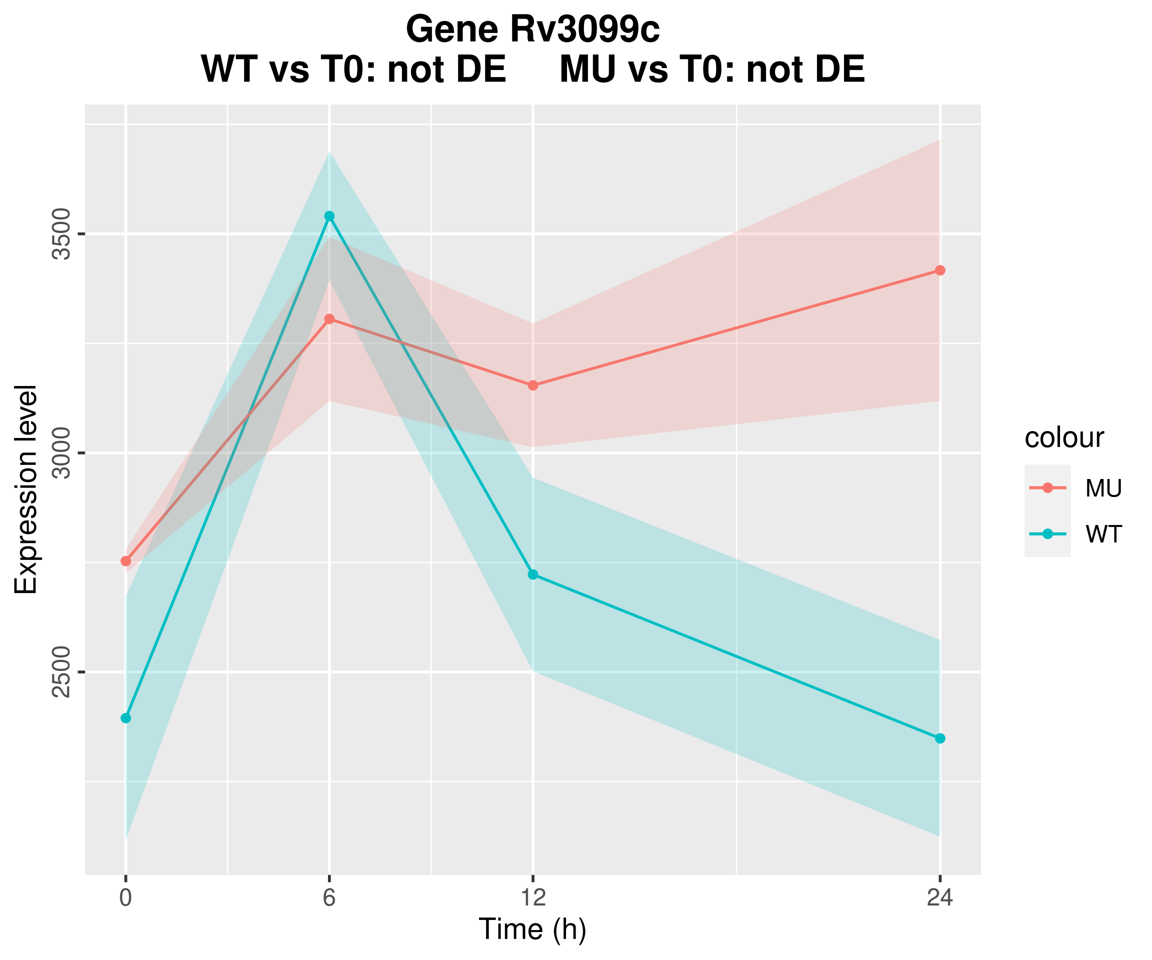
Gene Rv3099c
WT vs T0: not DE   MU vs T0: not DE
Expression level
3500
3000
2500
0
6
12
24
Time (h)
colour
MU
WT

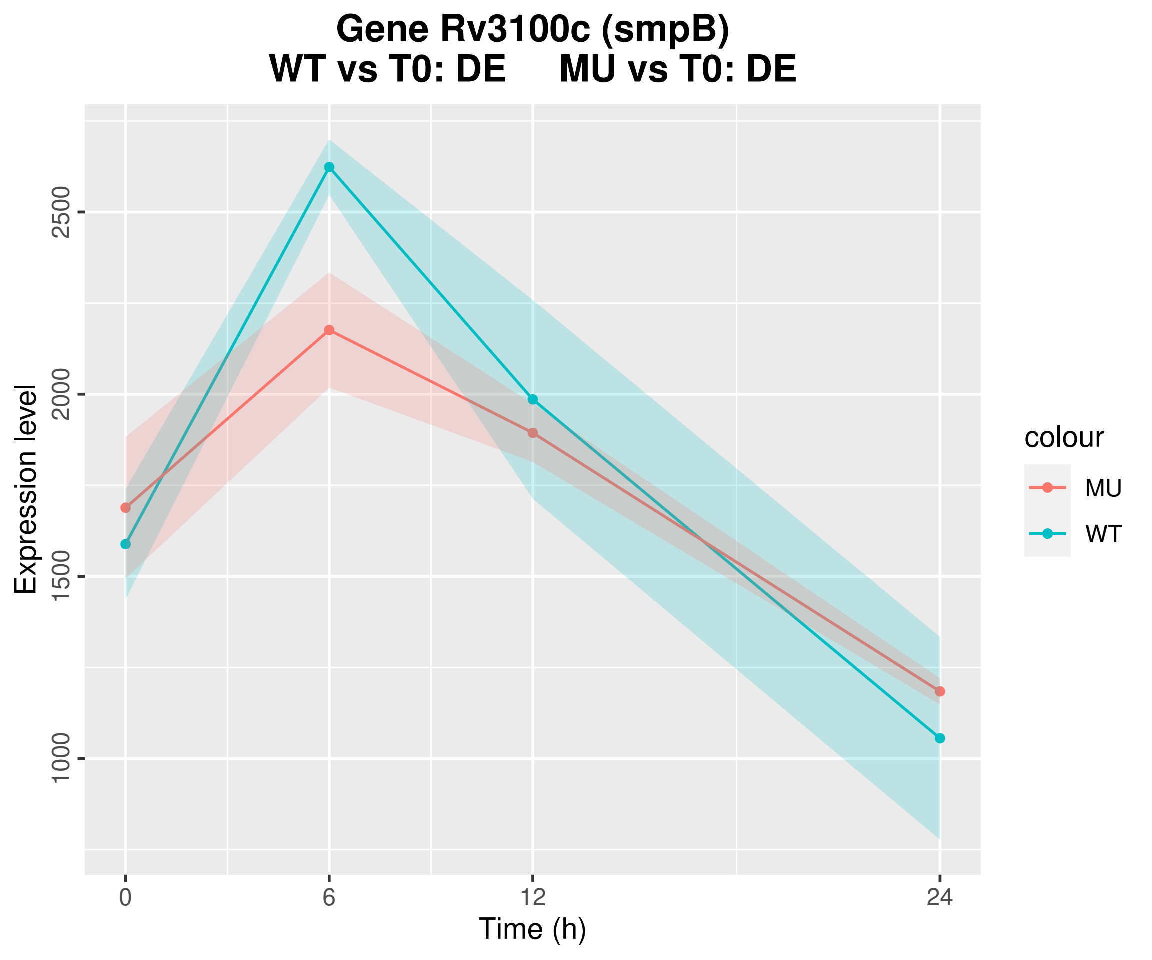
Gene Rv3100c (smpB)
WT vs T0: DE MU vs T0: DE
Expression level
2500
2000
1500
1000
0
6
12
24
Time (h)
colour
MU
WT

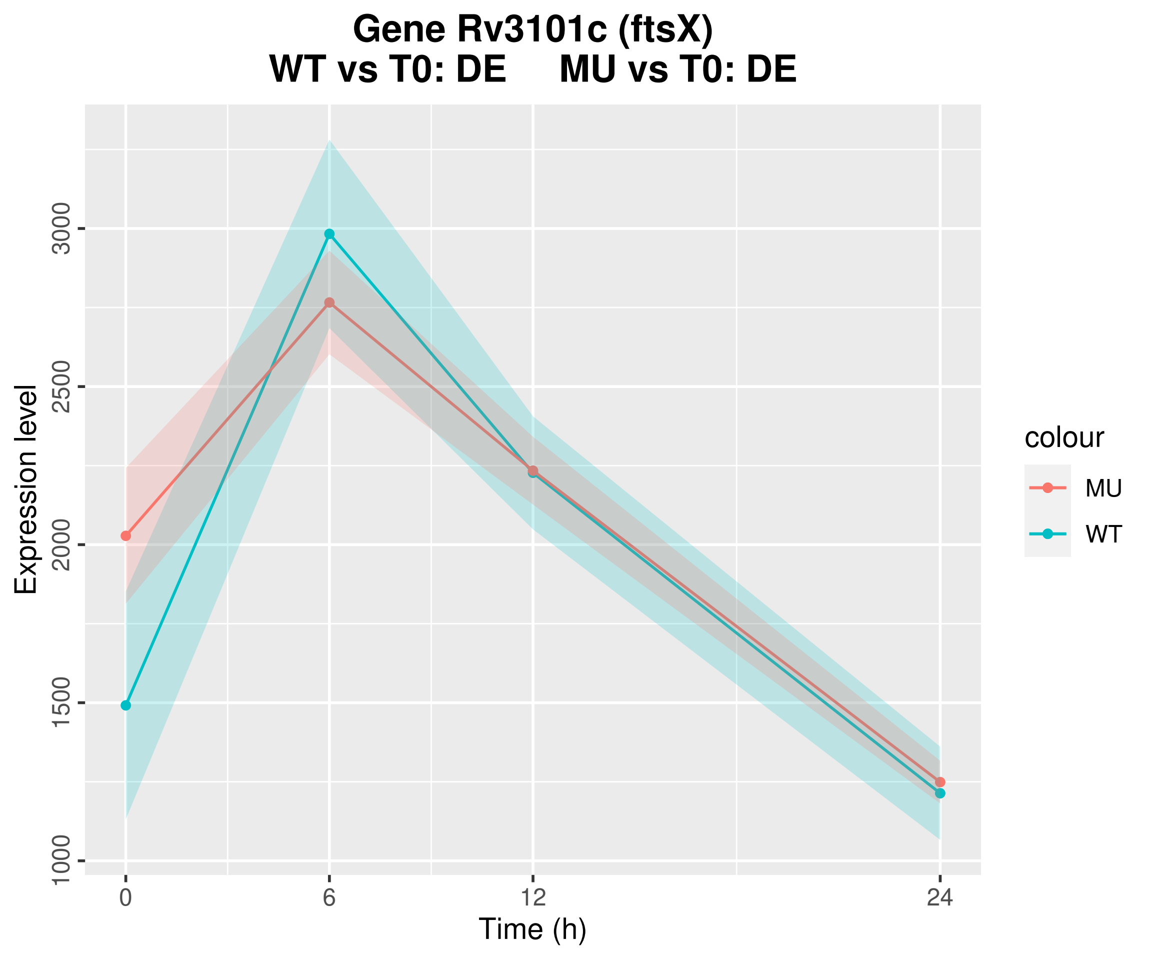
Gene Rv3101c (ftsX)
WT vs T0: DE MU vs T0: DE
Expression level
3000
2500
2000
1500
1000
0
6
12
24
Time (h)
colour
MU
WT

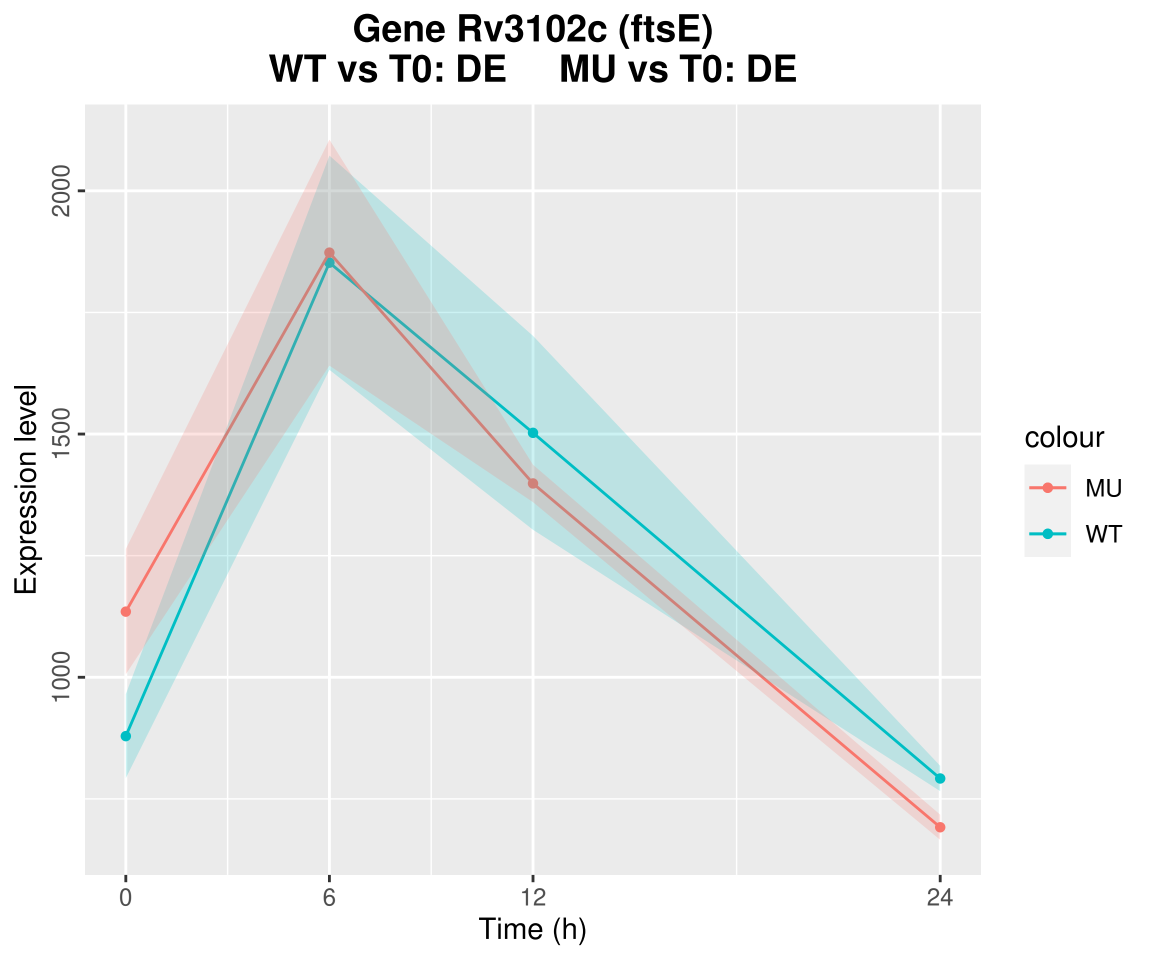
Gene Rv3102c (ftsE)
WT vs T0: DE MU vs T0: DE
Expression level
2000
1500
1000
0
6
12
24
Time (h)
colour
MU
WT

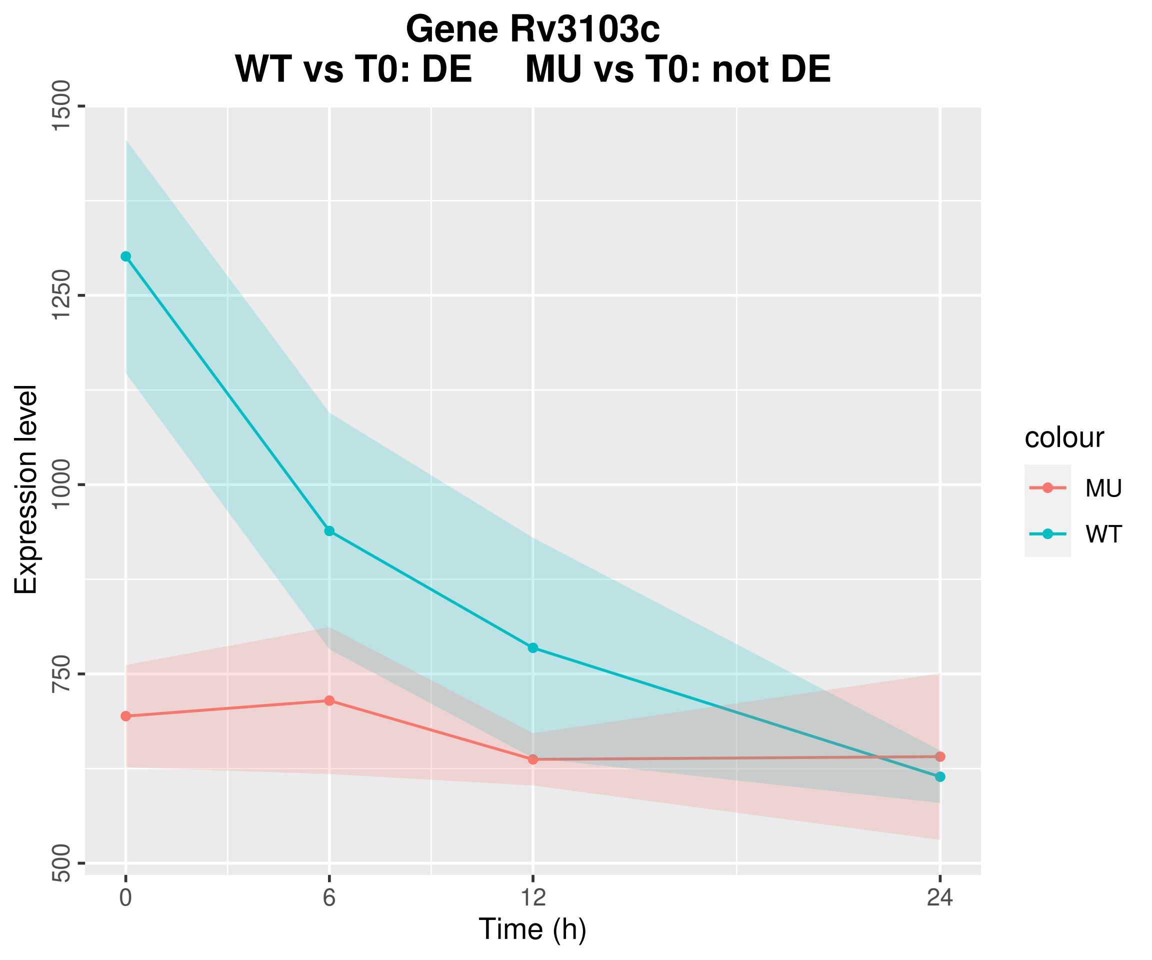

Gene Rv3103c
WT vs T0: DE MU vs T0: not DE
1500
1250
1000
750
500
Expression level
0
6
12
24
Time (h)
colour
MU
WT

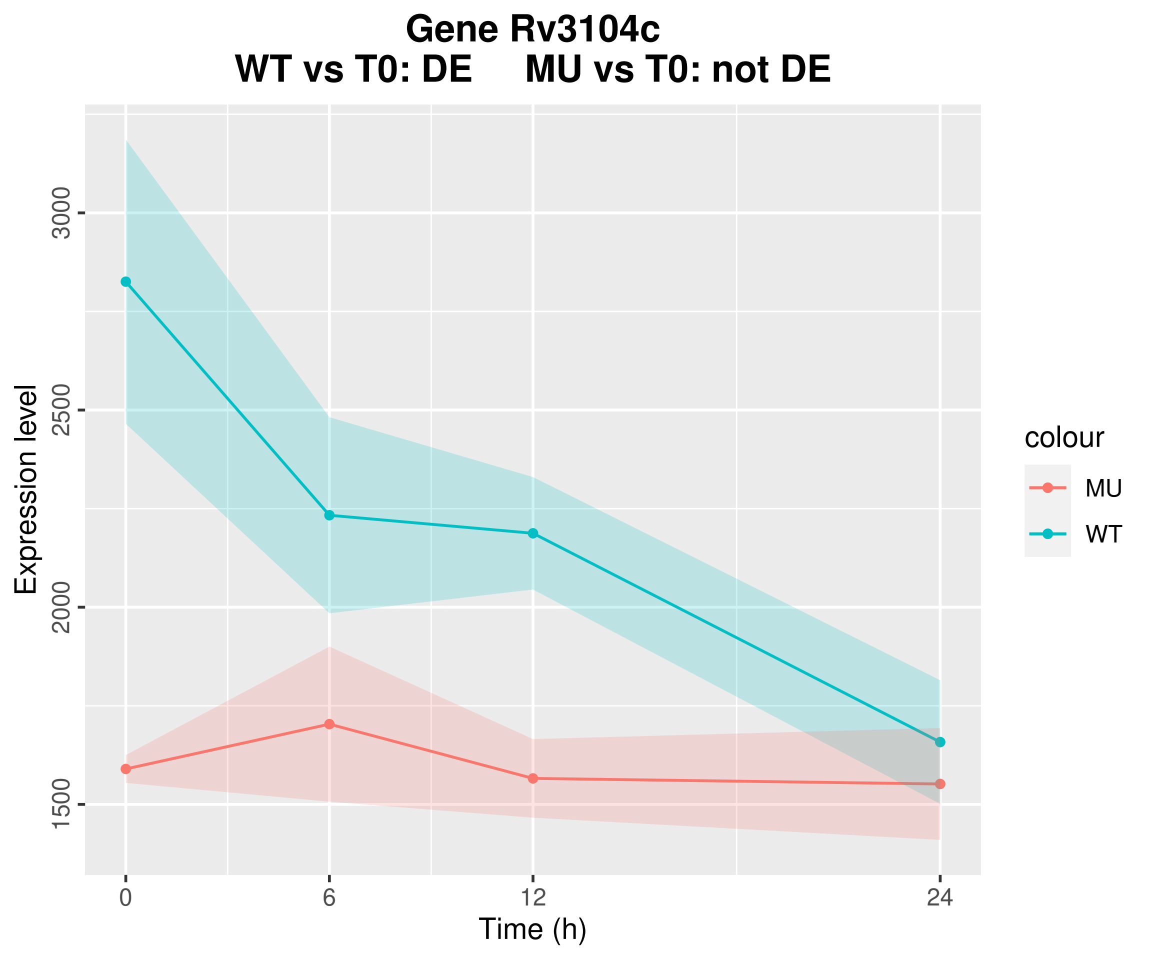
Gene Rv3104c
WT vs T0: DE MU vs T0: not DE
Expression level
3000
2500
2000
1500
0
6
12
24
Time (h)
colour
MU
WT

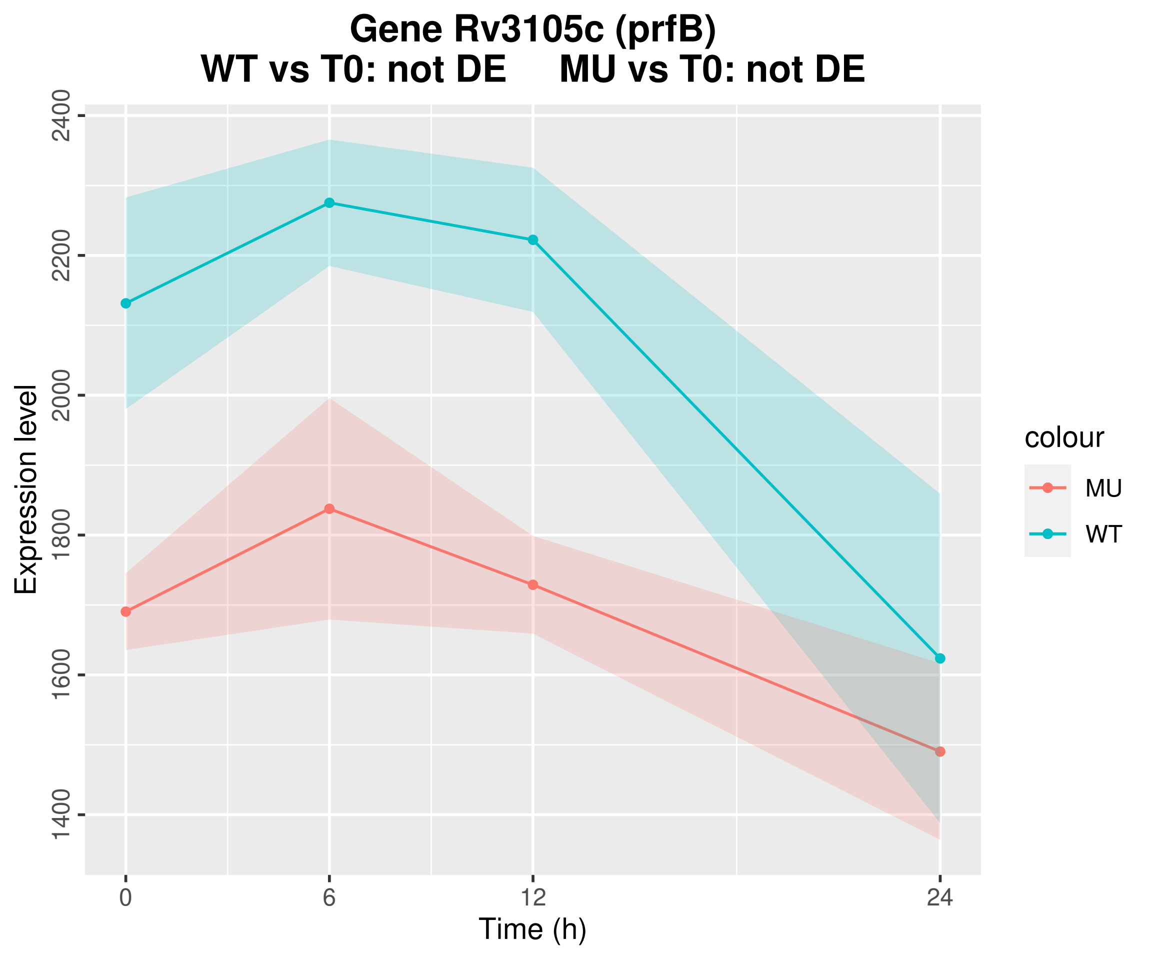
Gene Rv3105c (prfB)
WT vs T0: not DE MU vs T0: not DE
Expression level
2400
2200
2000
1800
1600
1400
0
6
12
24
Time (h)
colour
MU
WT

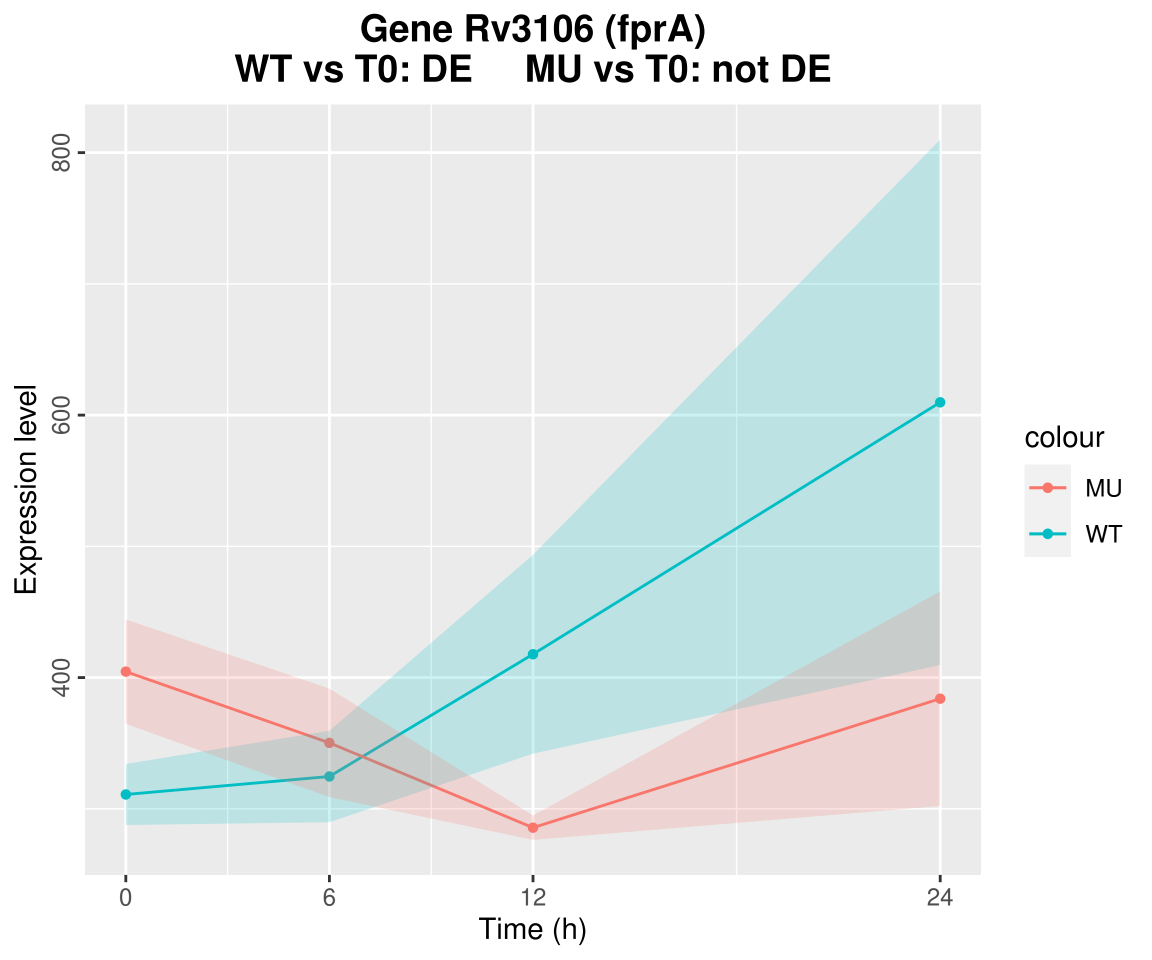
Gene Rv3106 (fprA)
WT vs T0: DE   MU vs T0: not DE
Expression level
800
600
400
Time (h)
0
6
12
24
colour
MU
WT

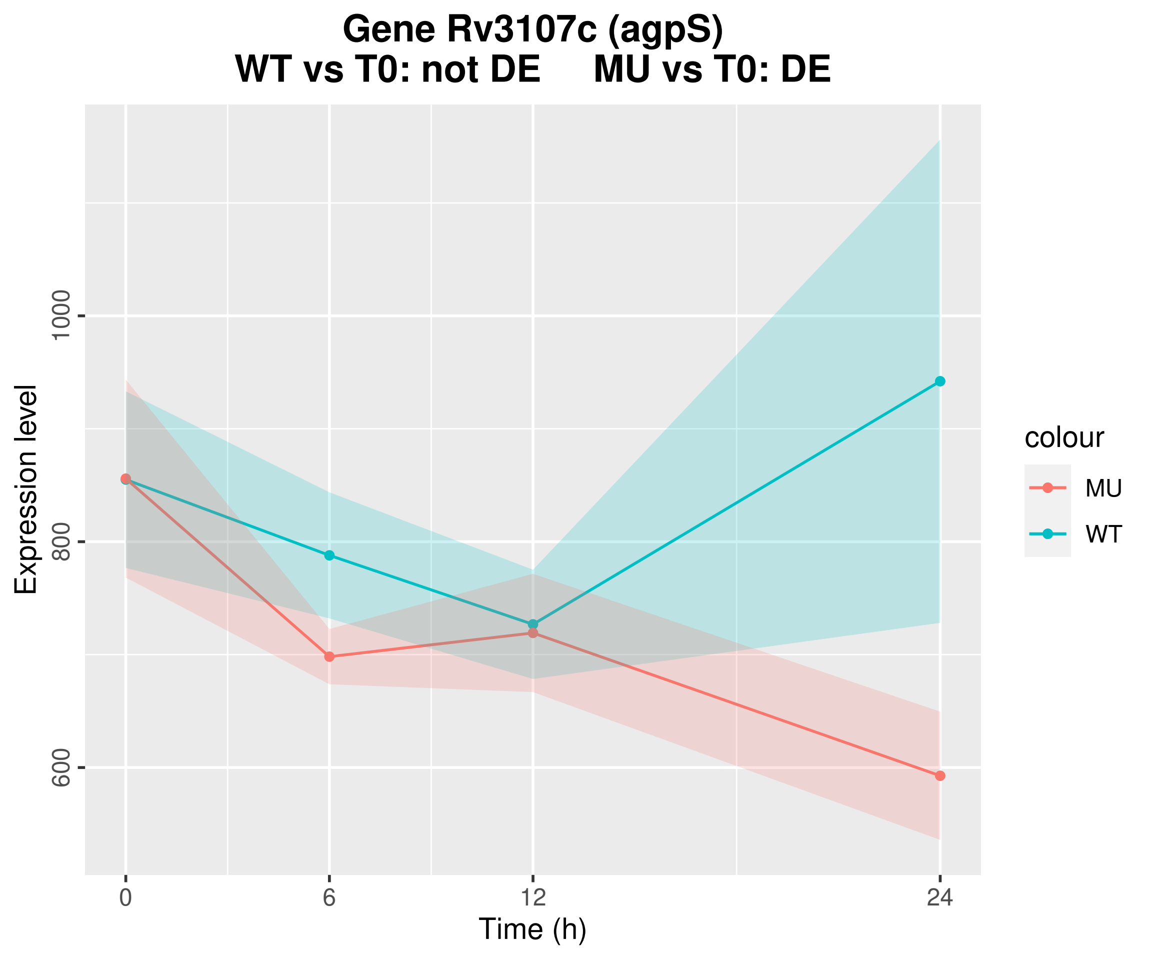
Gene Rv3107c (agpS)
WT vs T0: not DE   MU vs T0: DE
Expression level
1000
800
600
0
6
12
24
Time (h)
colour
MU
WT

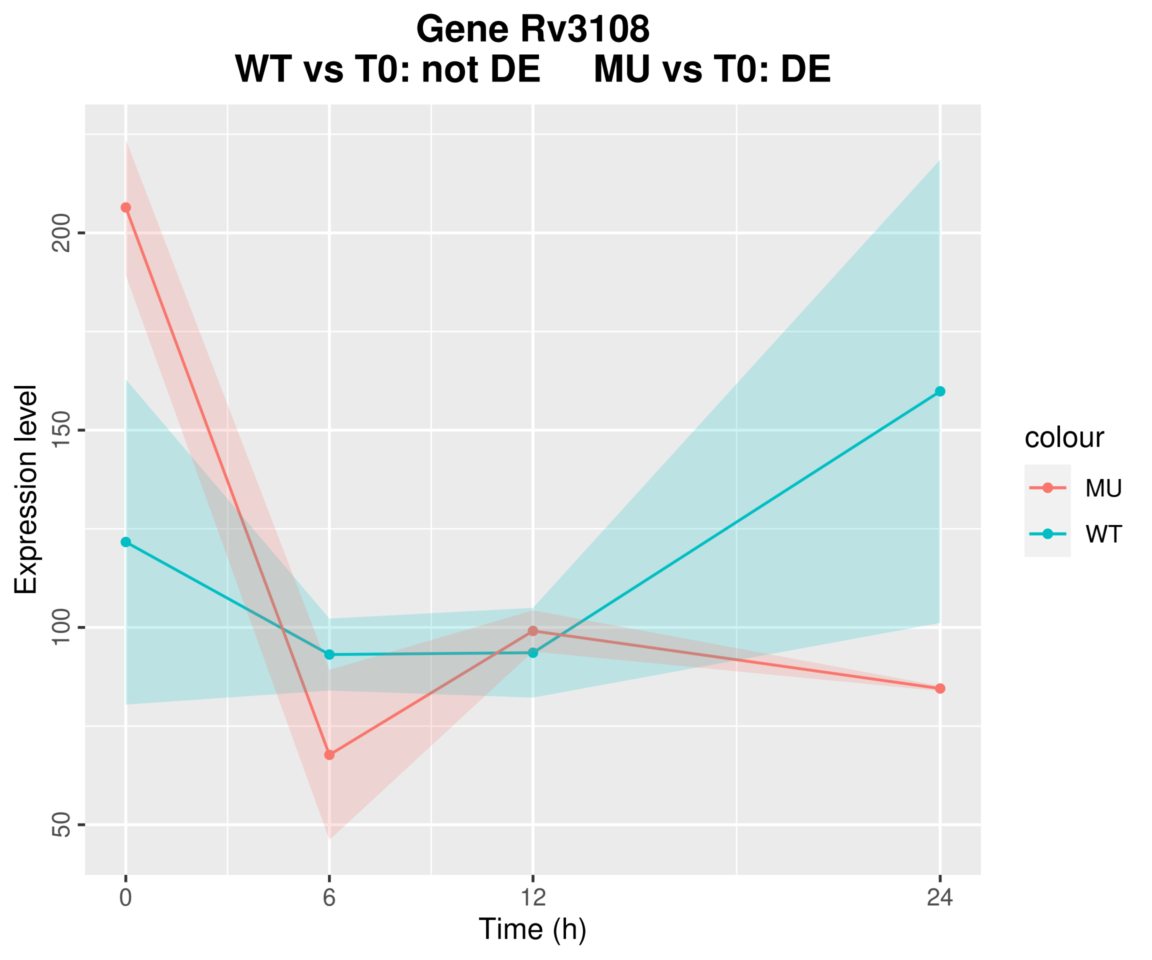

Gene Rv3108
WT vs T0: not DE   MU vs T0: DE
Expression level
200
150
100
50
0
6
12
24
Time (h)
colour
MU
WT

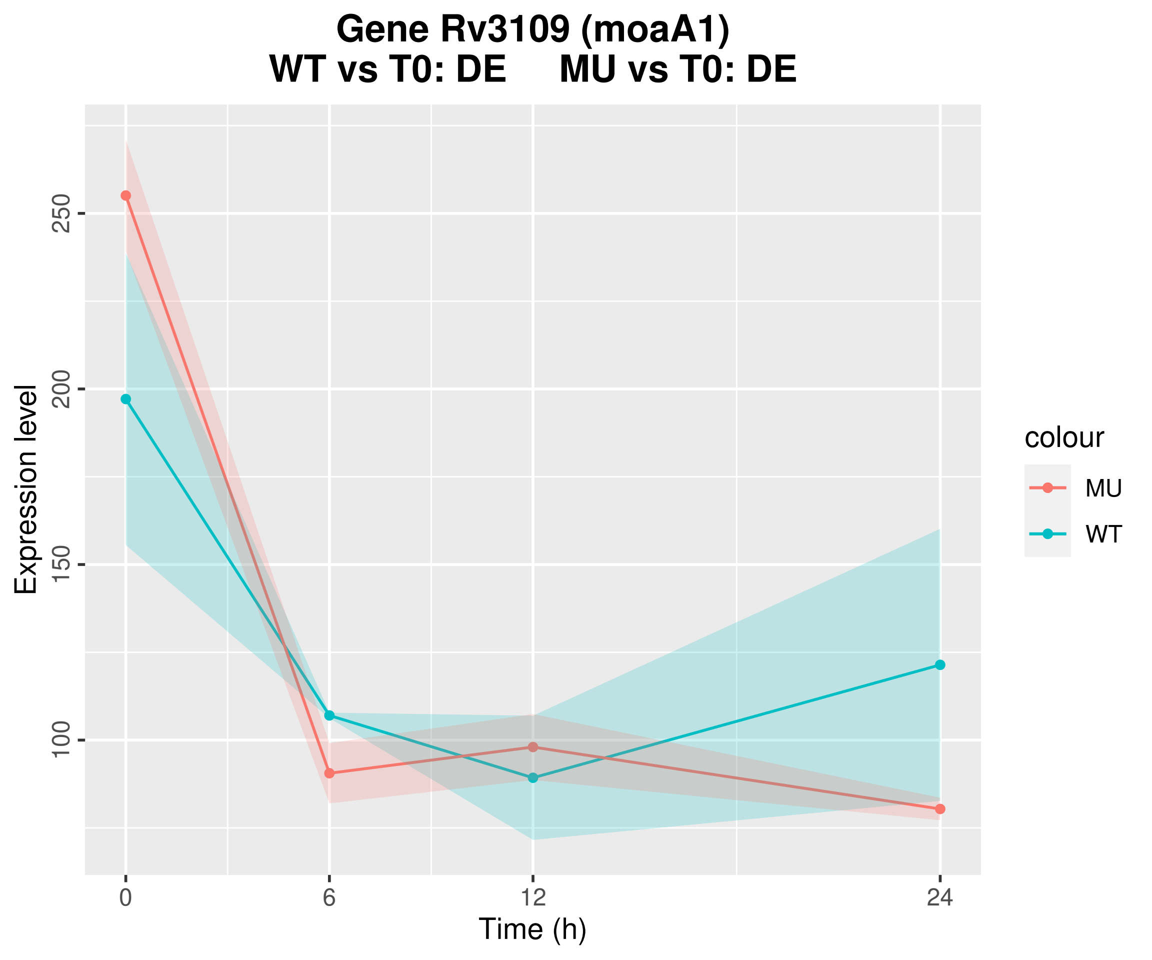

Gene Rv3109 (moaA1)
WT vs T0: DE MU vs T0: DE
Expression level
250
200
150
100
0
6
12
24
Time (h)
colour
MU
WT

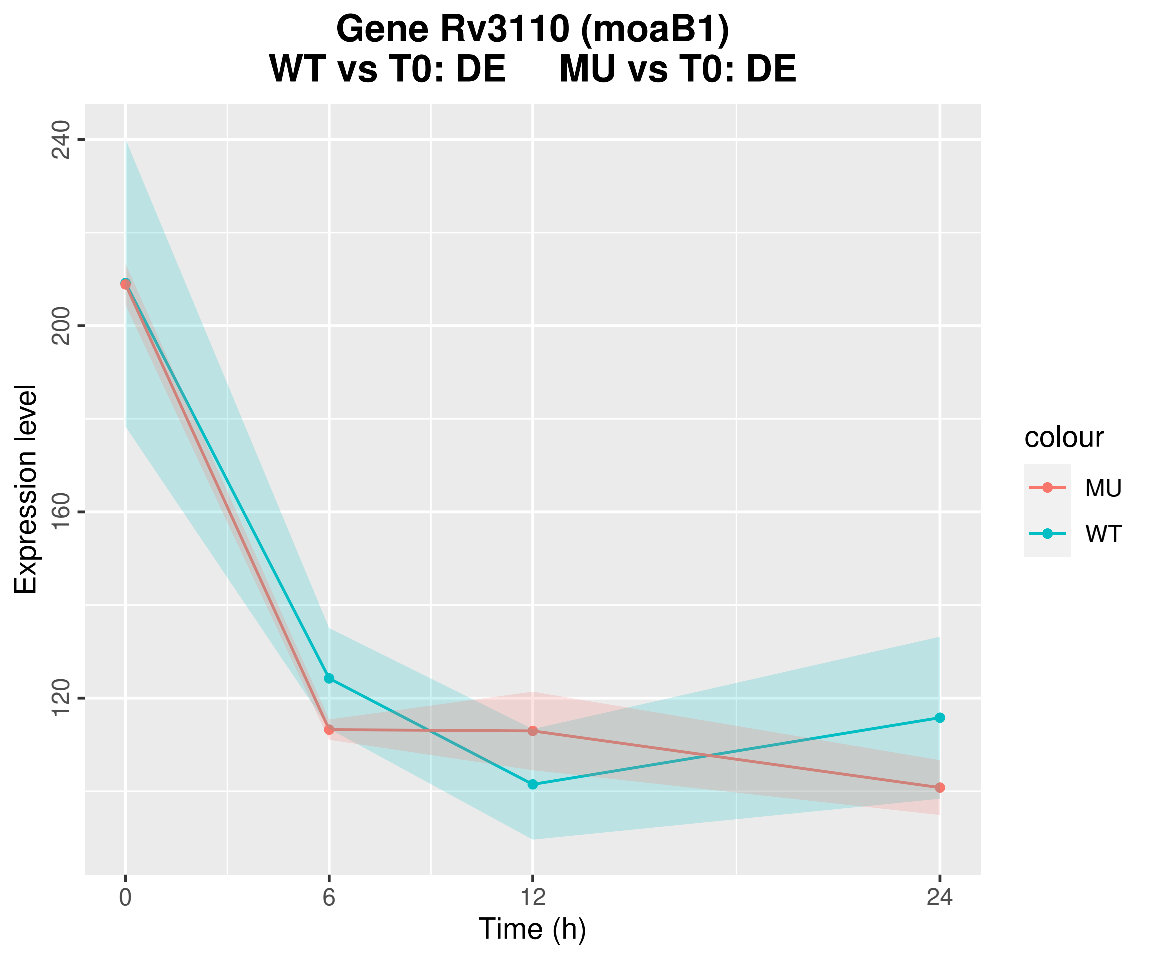
Gene Rv3110 (moaB1)
WT vs T0: DE MU vs T0: DE
Expression level
240
200
160
120
0
6
12
24
Time (h)
colour
MU
WT

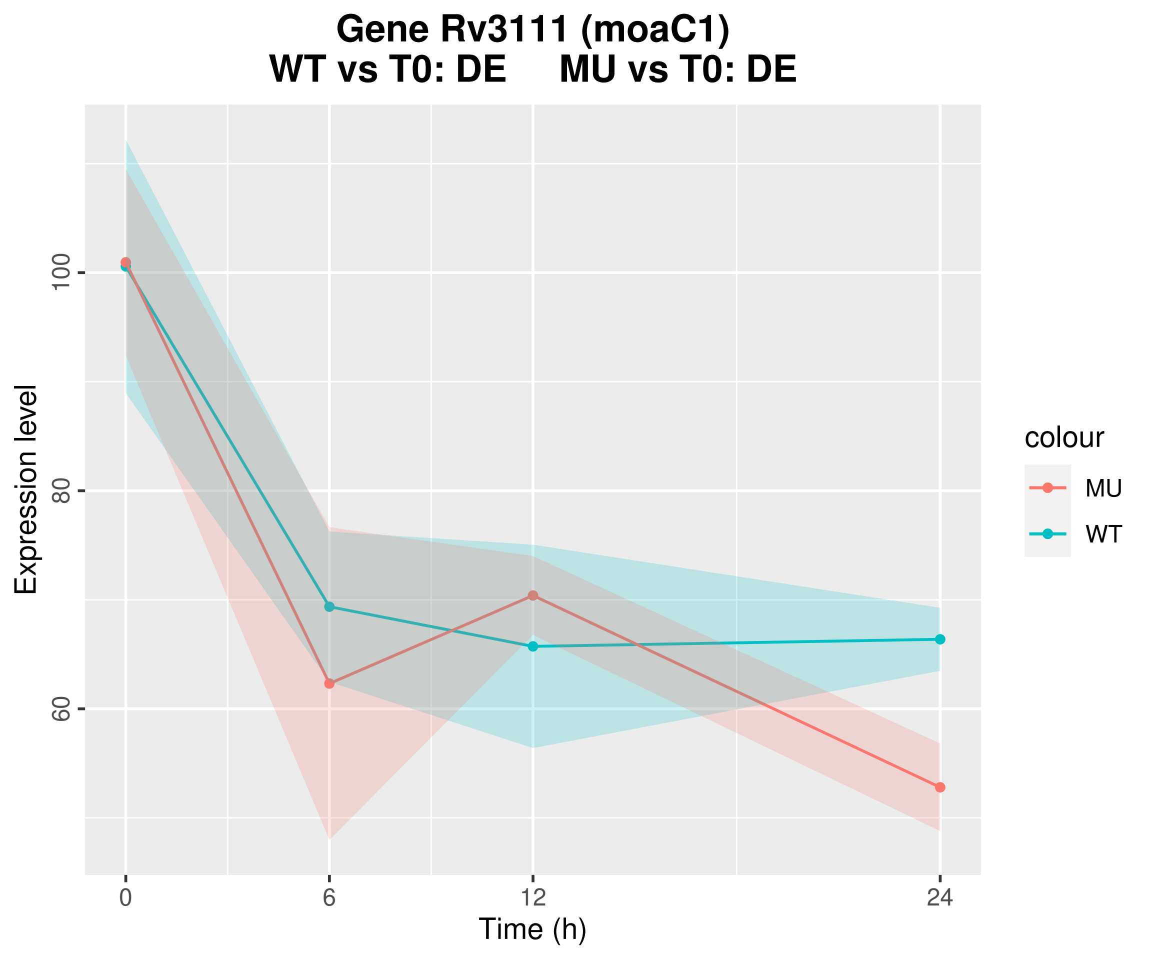
Gene Rv3111 (moaC1)
WT vs T0: DE MU vs T0: DE
Expression level
100
80
60
0
6
12
24
Time (h)
colour
MU
WT

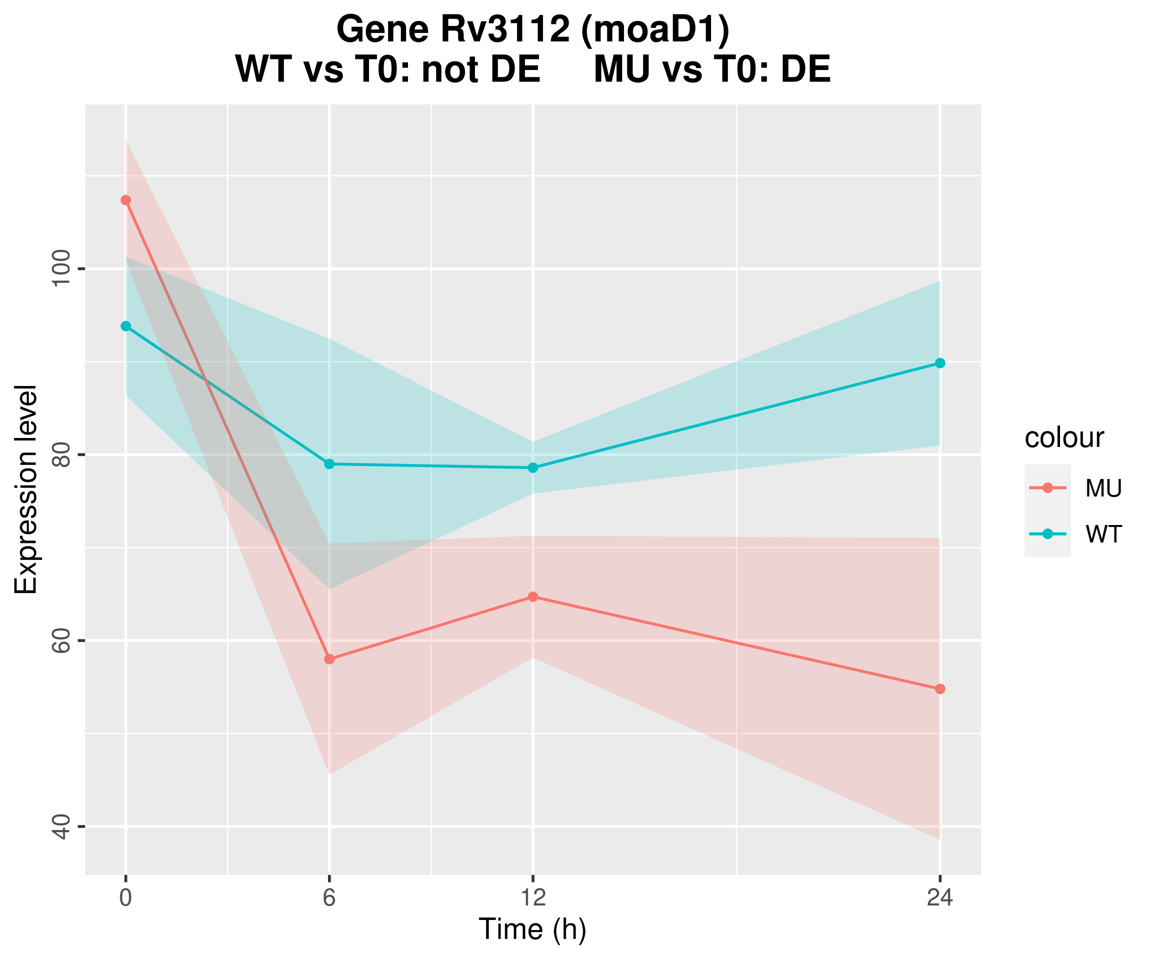
Gene Rv3112 (moaD1)
WT vs T0: not DE MU vs T0: DE
Expression level
100
80
60
40
0
6
12
24
Time (h)
colour
MU
WT

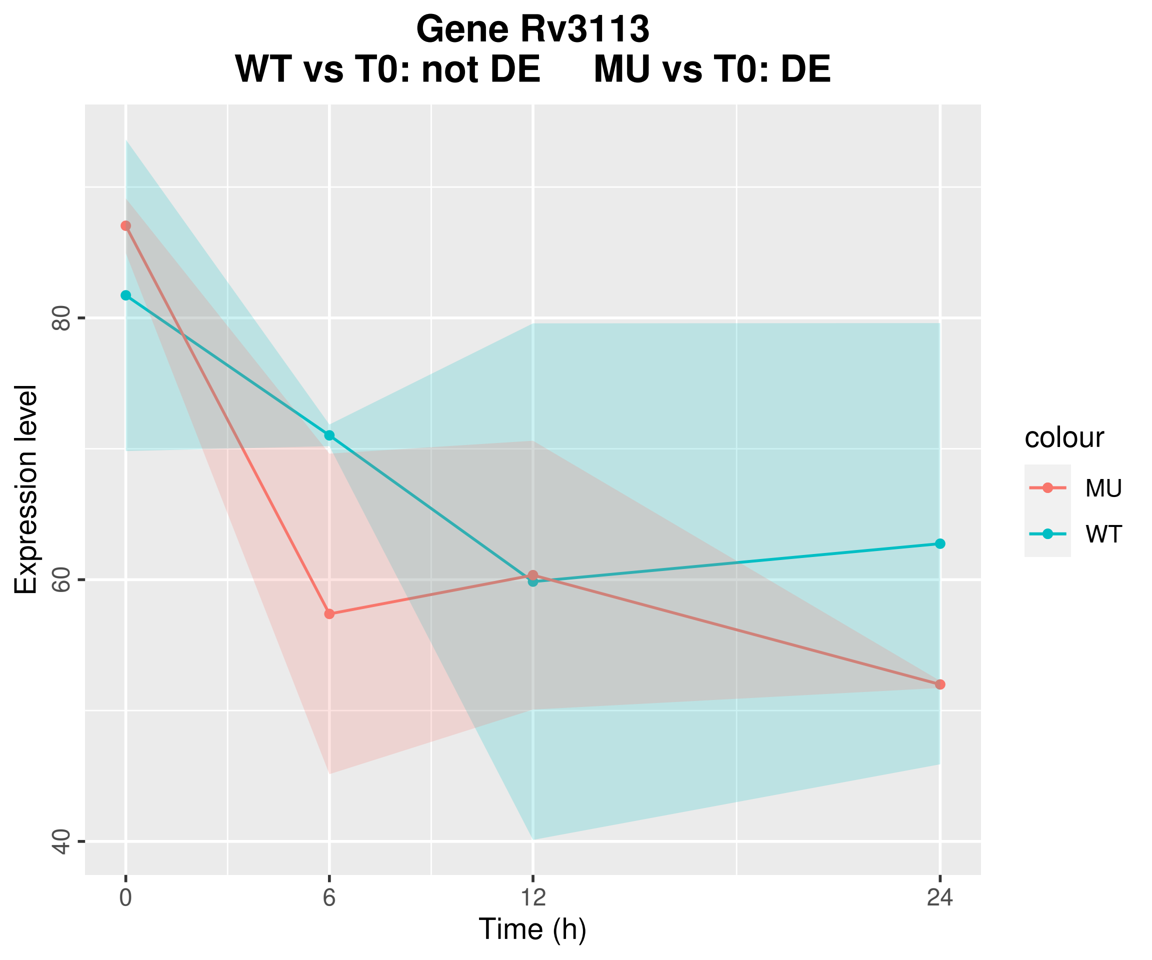

Gene Rv3113
WT vs T0: not DE MU vs T0: DE
Expression level
80
60
40
0
6
12
24
Time (h)
colour
MU
WT

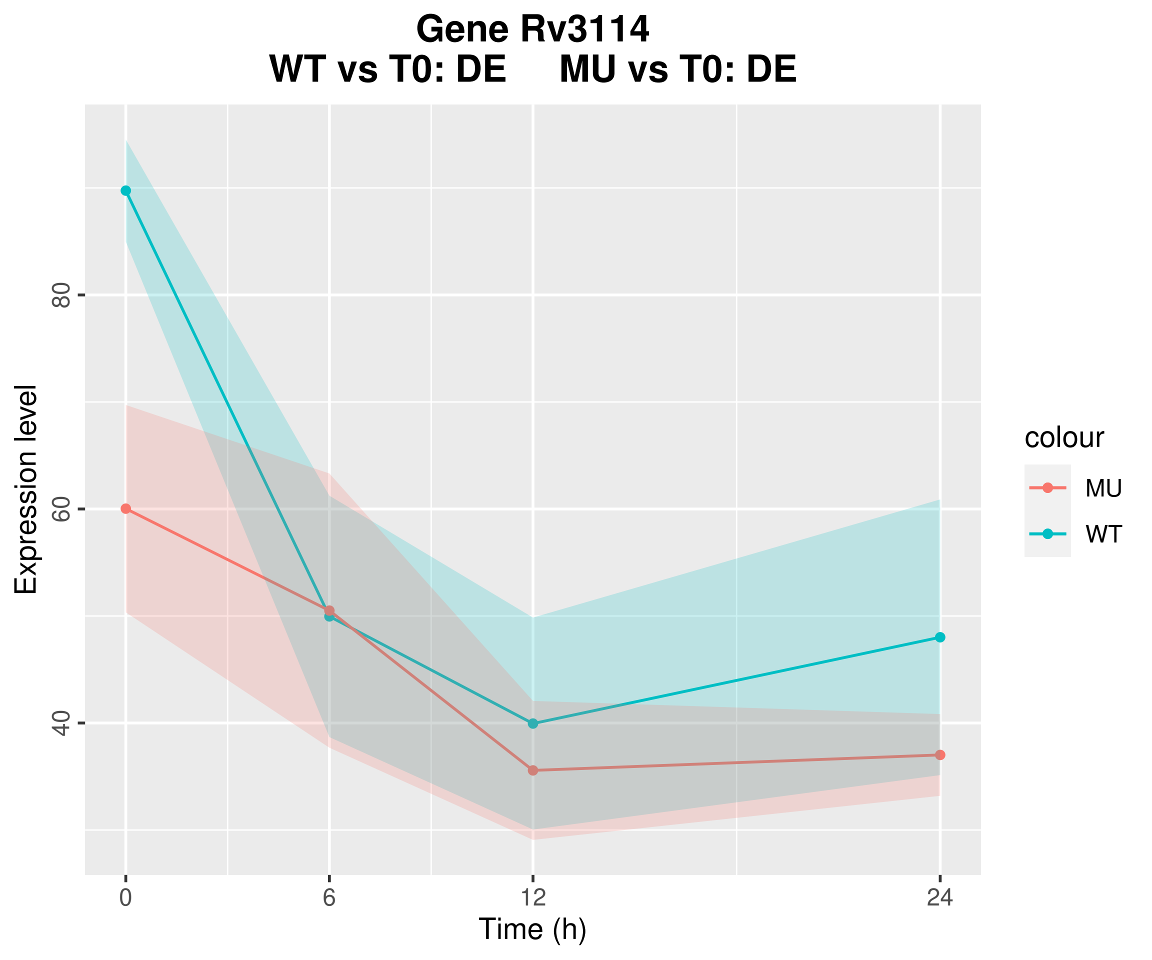
Gene Rv3114
WT vs T0: DE   MU vs T0: DE
Expression level
80
60
40
0
6
12
24
Time (h)
colour
MU
WT

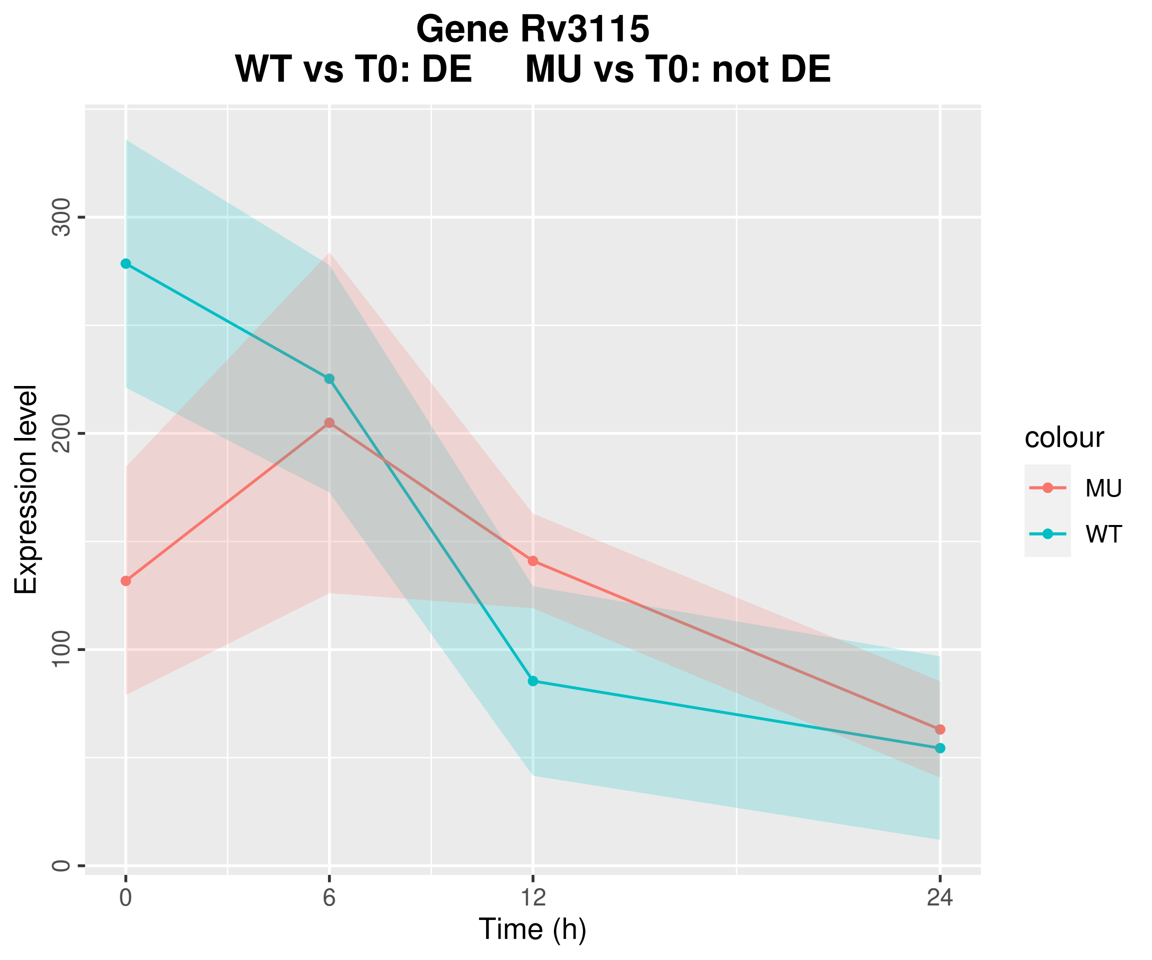

Gene Rv3115
WT vs T0: DE MU vs T0: not DE
Expression level
0
100
200
300
Time (h)
0
6
12
24
colour
MU
WT

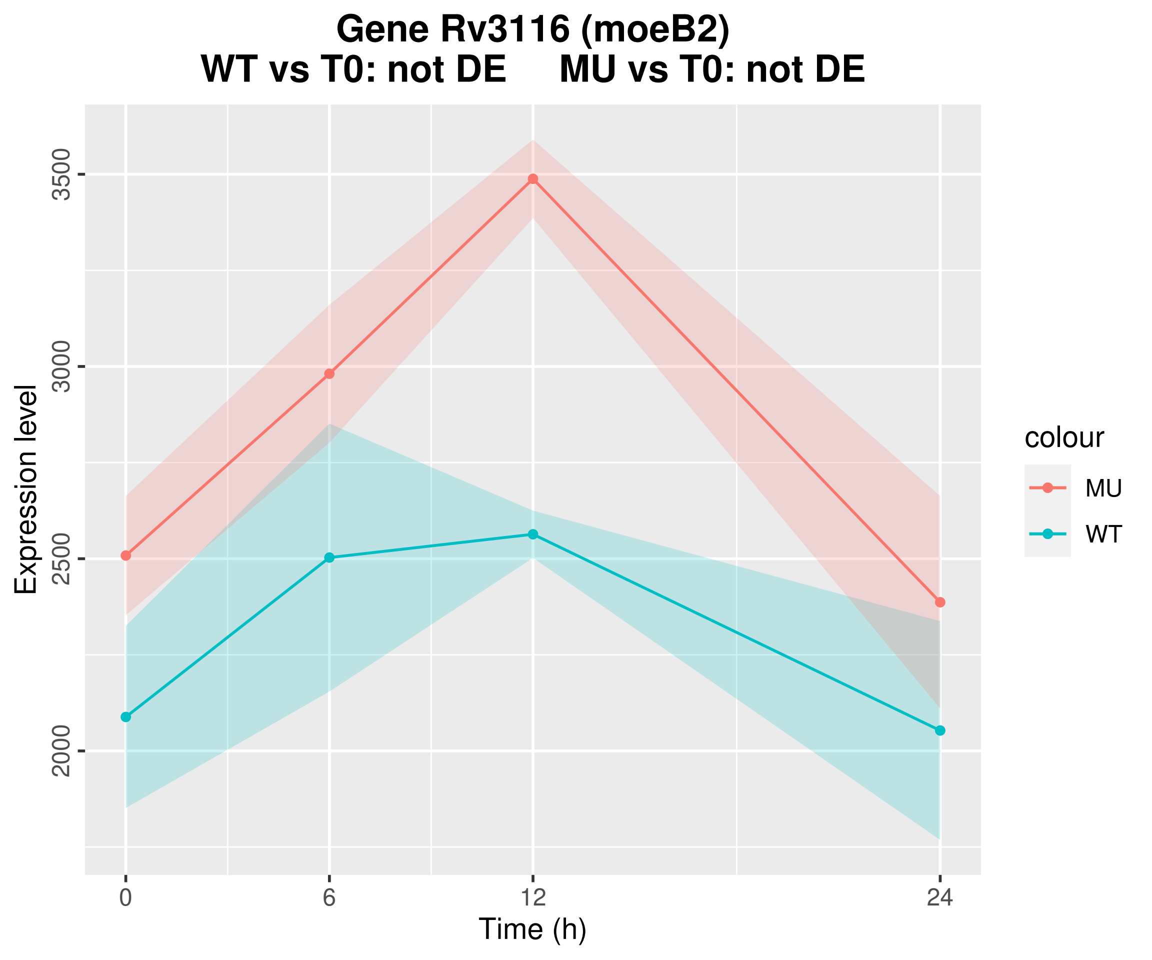
Gene Rv3116 (moeB2)
WT vs T0: not DE   MU vs T0: not DE
Expression level
3500
3000
2500
2000
0
6
12
24
Time (h)
colour
MU
WT

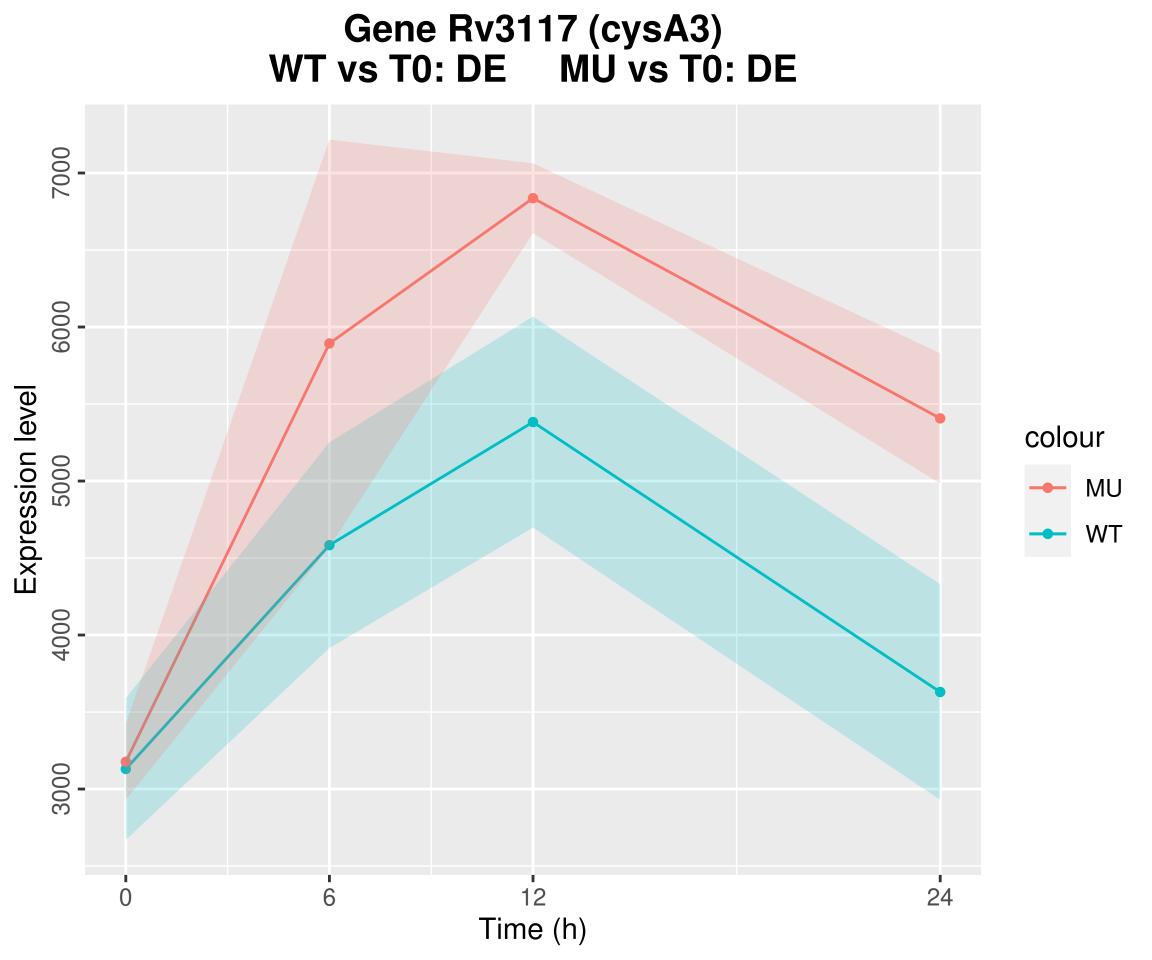
Gene Rv3117 (cysA3)
WT vs T0: DE MU vs T0: DE
7000
6000
5000
4000
3000
Expression level
0
6
12
24
Time (h)
colour
MU
WT

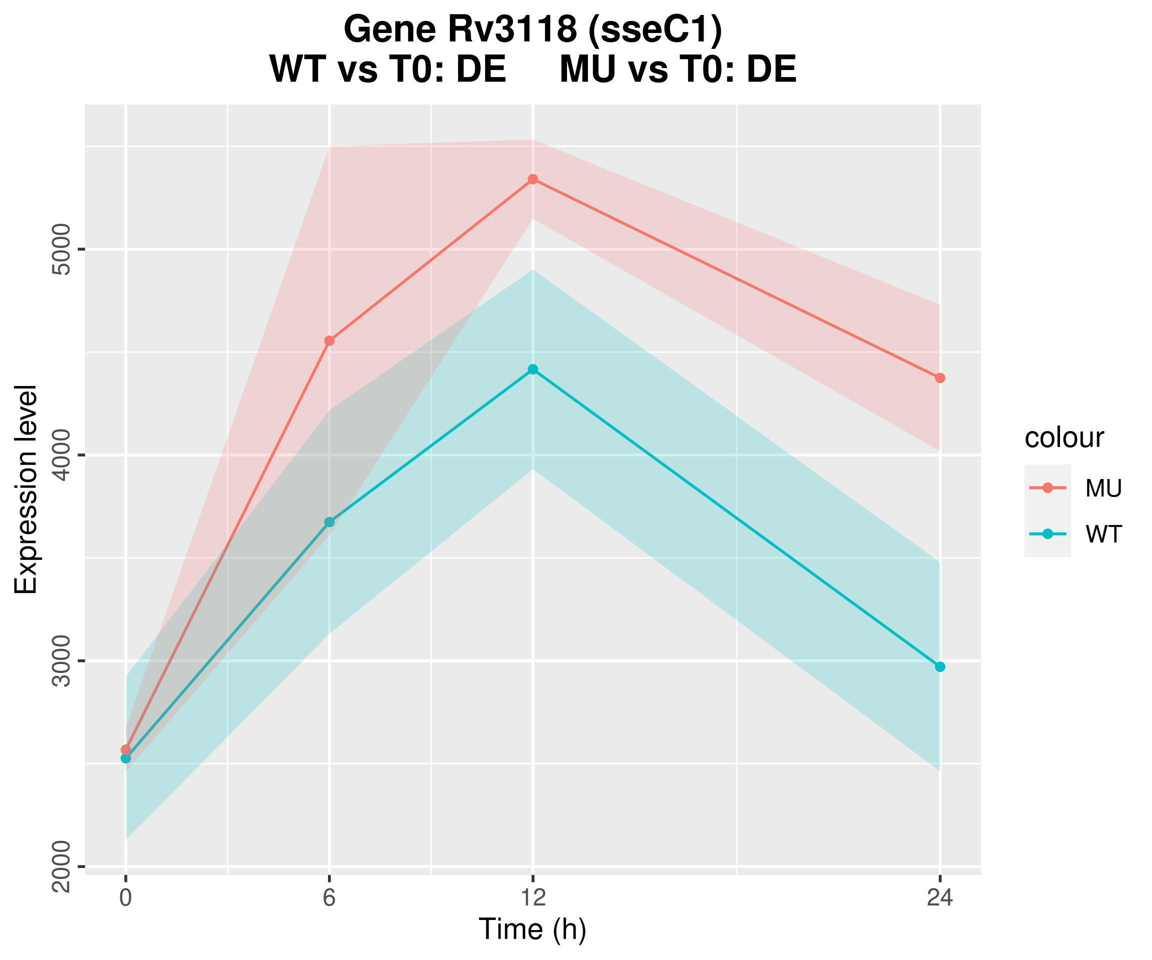

Gene Rv3118 (sseC1)
WT vs T0: DE MU vs T0: DE
Expression level
5000
4000
3000
2000
0
6
12
24
Time (h)
colour
MU
WT

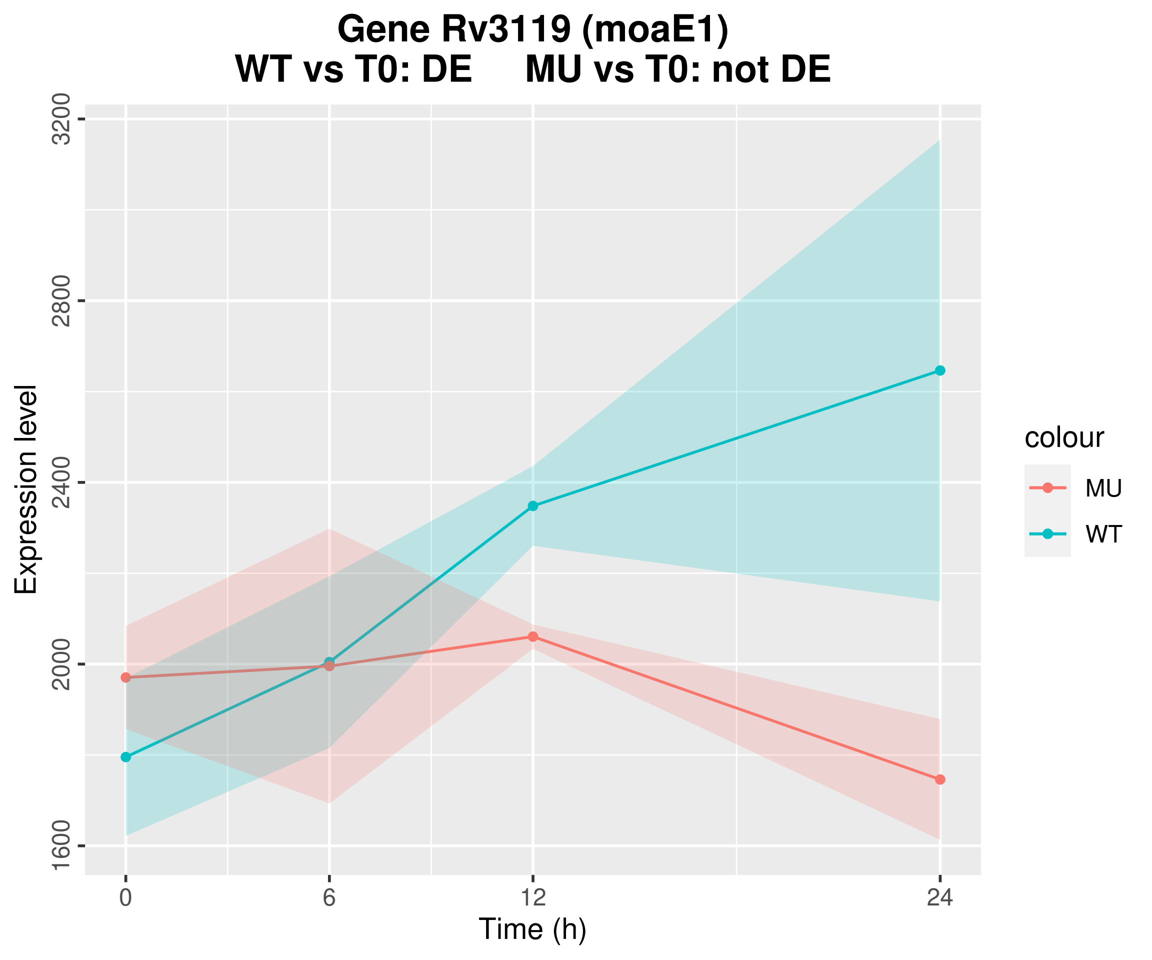

Gene Rv3119 (moaE1)
WT vs T0: DE   MU vs T0: not DE
Expression level
3200
2800
2400
2000
1600
0
6
12
24
Time (h)
colour
MU
WT

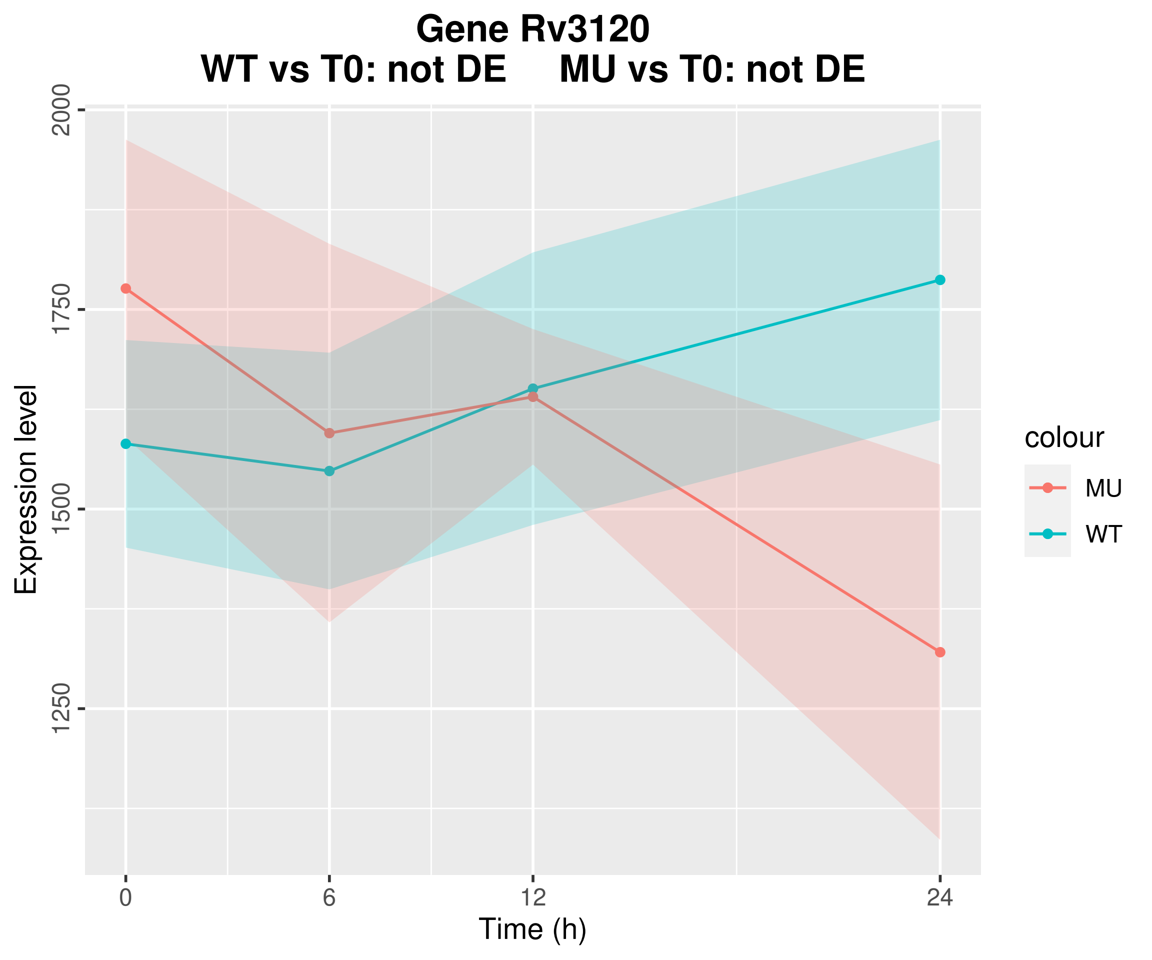
Gene Rv3120
WT vs T0: not DE MU vs T0: not DE
2000
1750
1500
1250
Expression level
0
6
12
24
Time (h)
colour
MU
WT

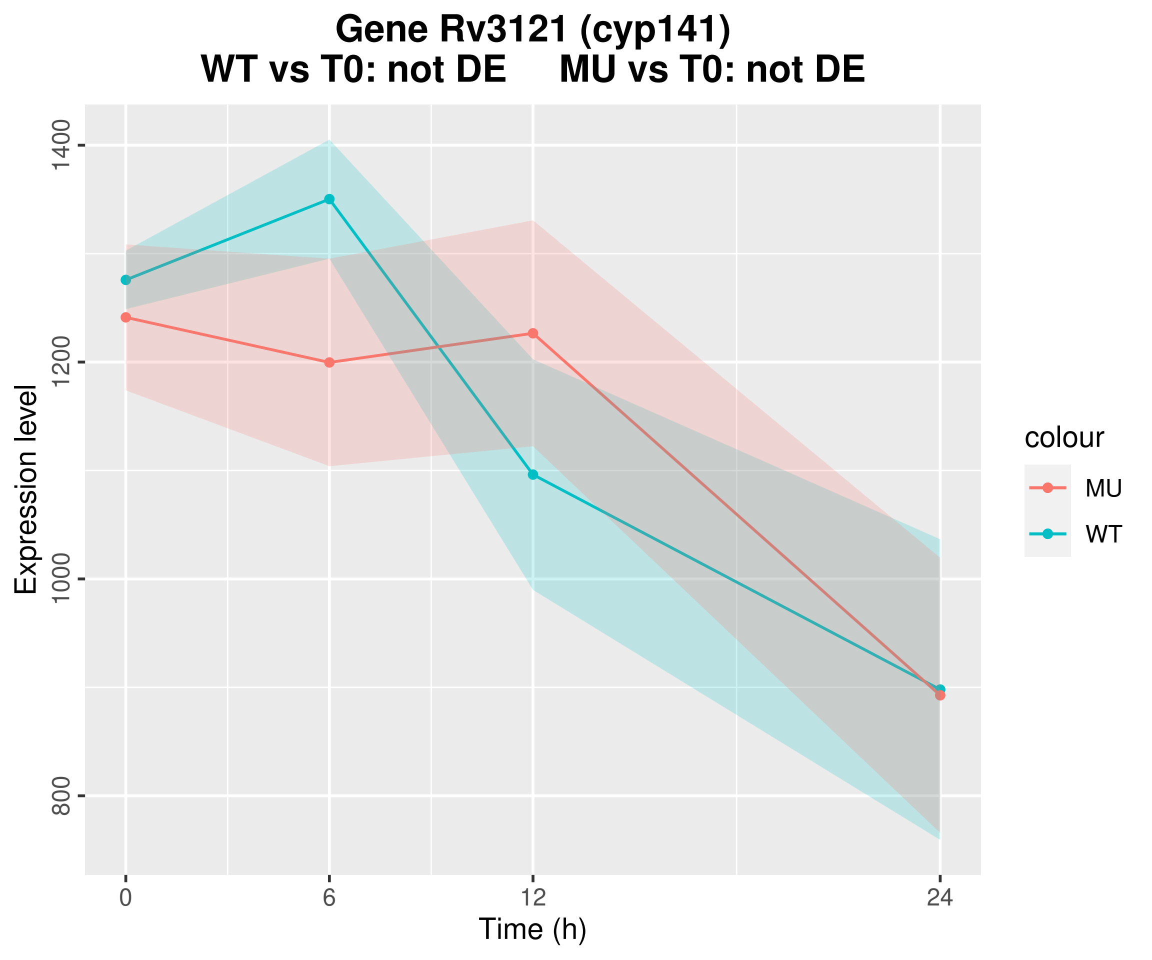

Gene Rv3121 (cyp141)
WT vs T0: not DE   MU vs T0: not DE
1400
1200
1000
800
Expression level
0
6
12
24
Time (h)
colour
MU
WT

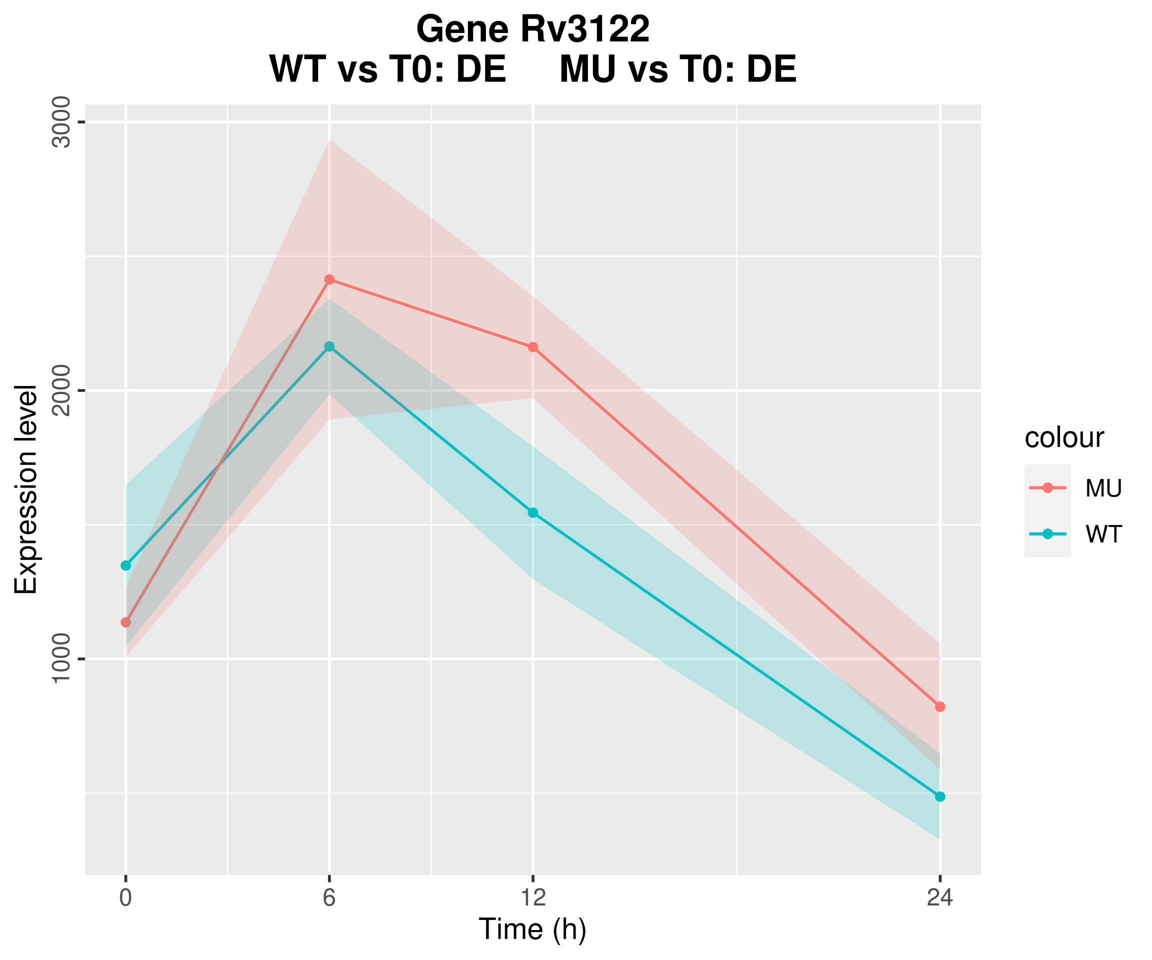
Gene Rv3122
WT vs T0: DE MU vs T0: DE
3000
2000
1000
Expression level
0
6
12
24
Time (h)
colour
MU
WT

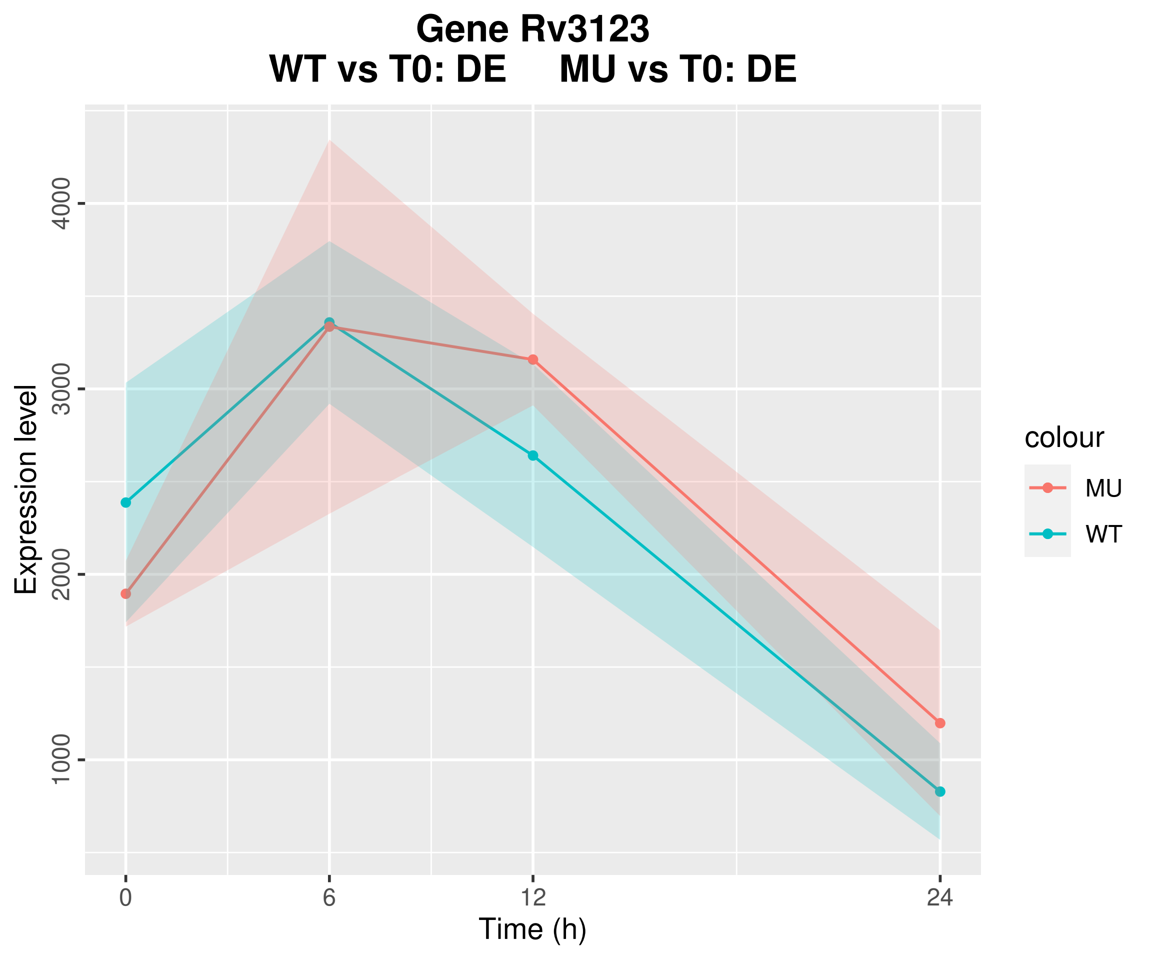
Gene Rv3123
WT vs T0: DE MU vs T0: DE
Expression level
4000
3000
2000
1000
0
6
12
24
Time (h)
colour
MU
WT

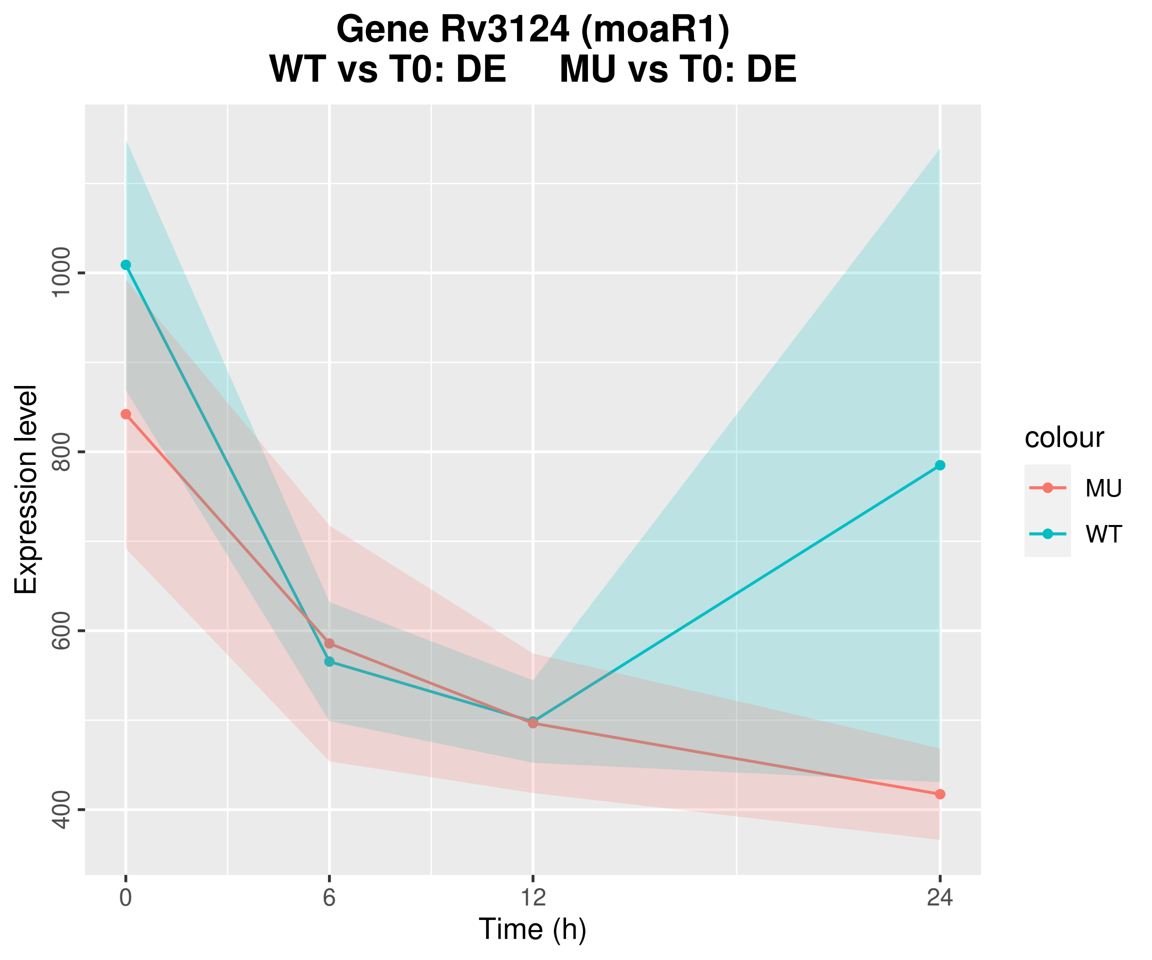
Gene Rv3124 (moaR1)
WT vs T0: DE   MU vs T0: DE
Expression level
1000
800
600
400
0
6
12
24
Time (h)
colour
MU
WT

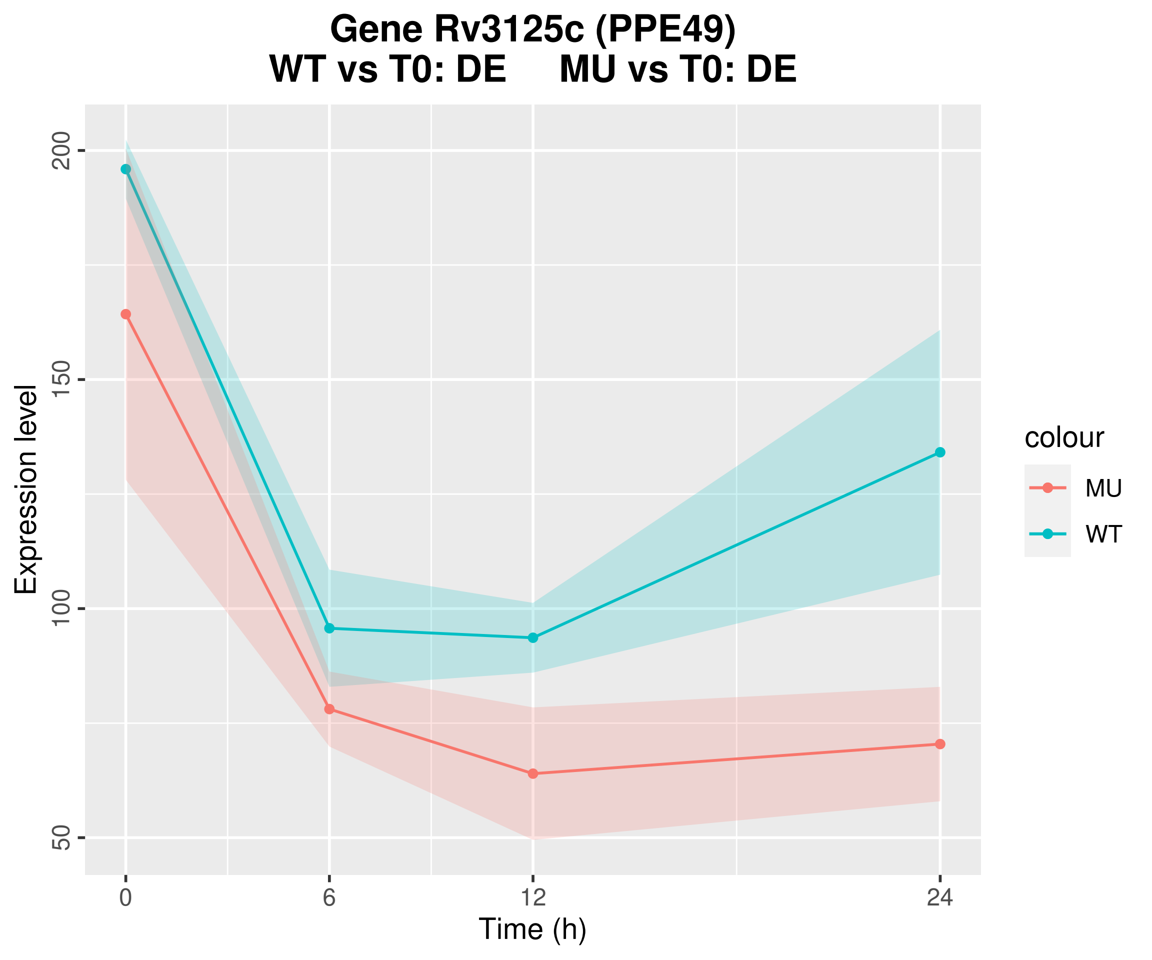

Gene Rv3125c (PPE49)
WT vs T0: DE MU vs T0: DE
Expression level
200
150
100
50
0
6
12
24
Time (h)
colour
MU
WT

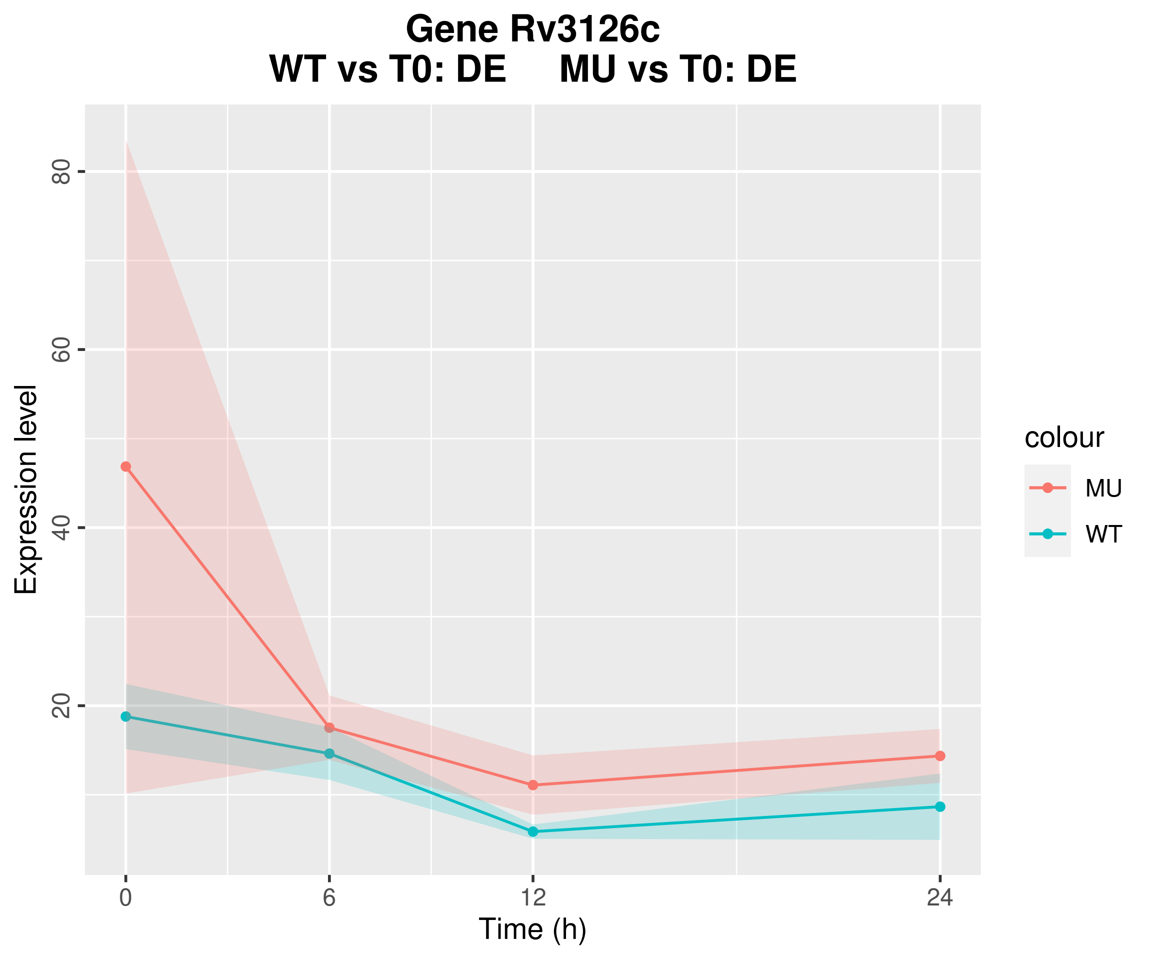
Gene Rv3126c
WT vs T0: DE MU vs T0: DE
Expression level
80
60
40
20
0
6
12
24
Time (h)
colour
MU
WT

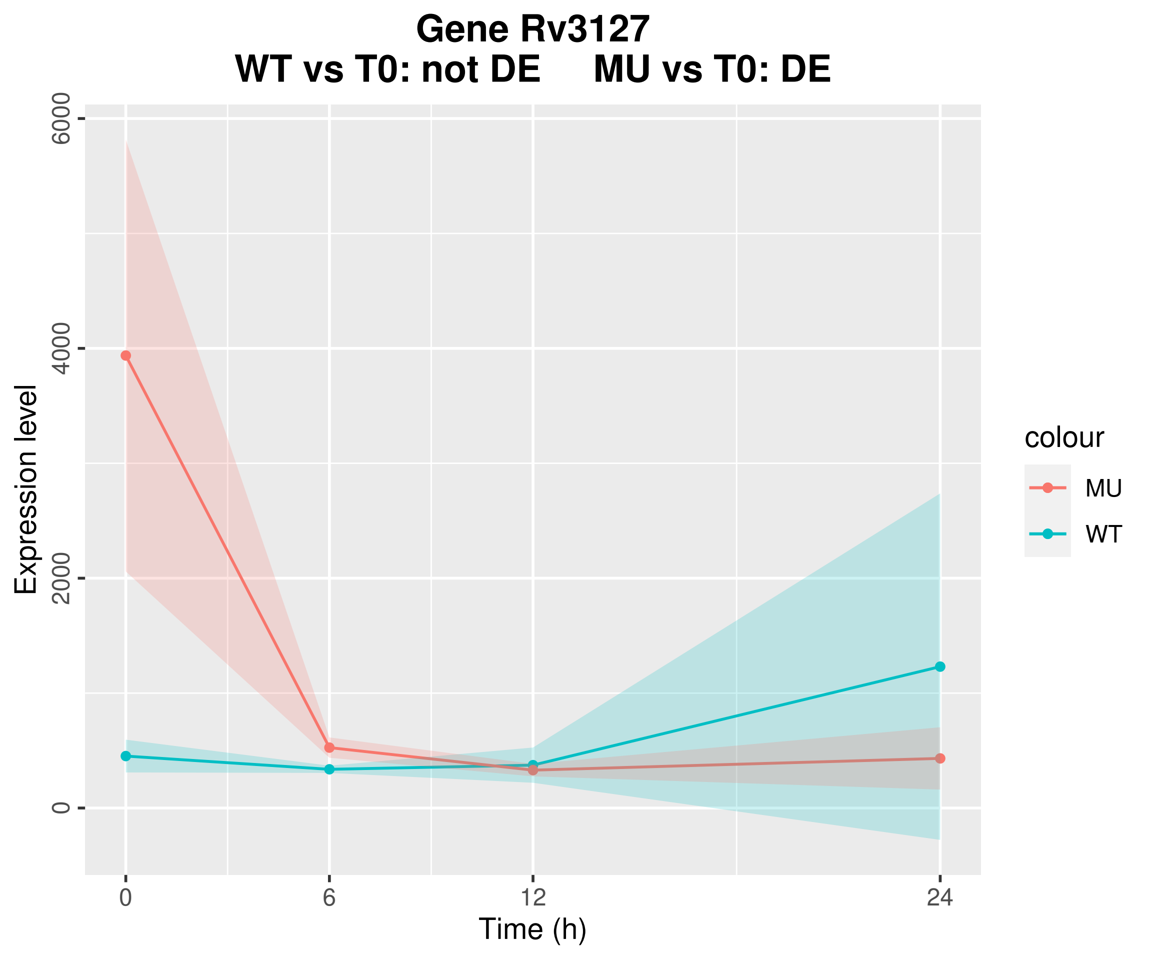
Gene Rv3127
WT vs T0: not DE   MU vs T0: DE
Expression level
6000
4000
2000
0
0
6
12
24
Time (h)
colour
MU
WT

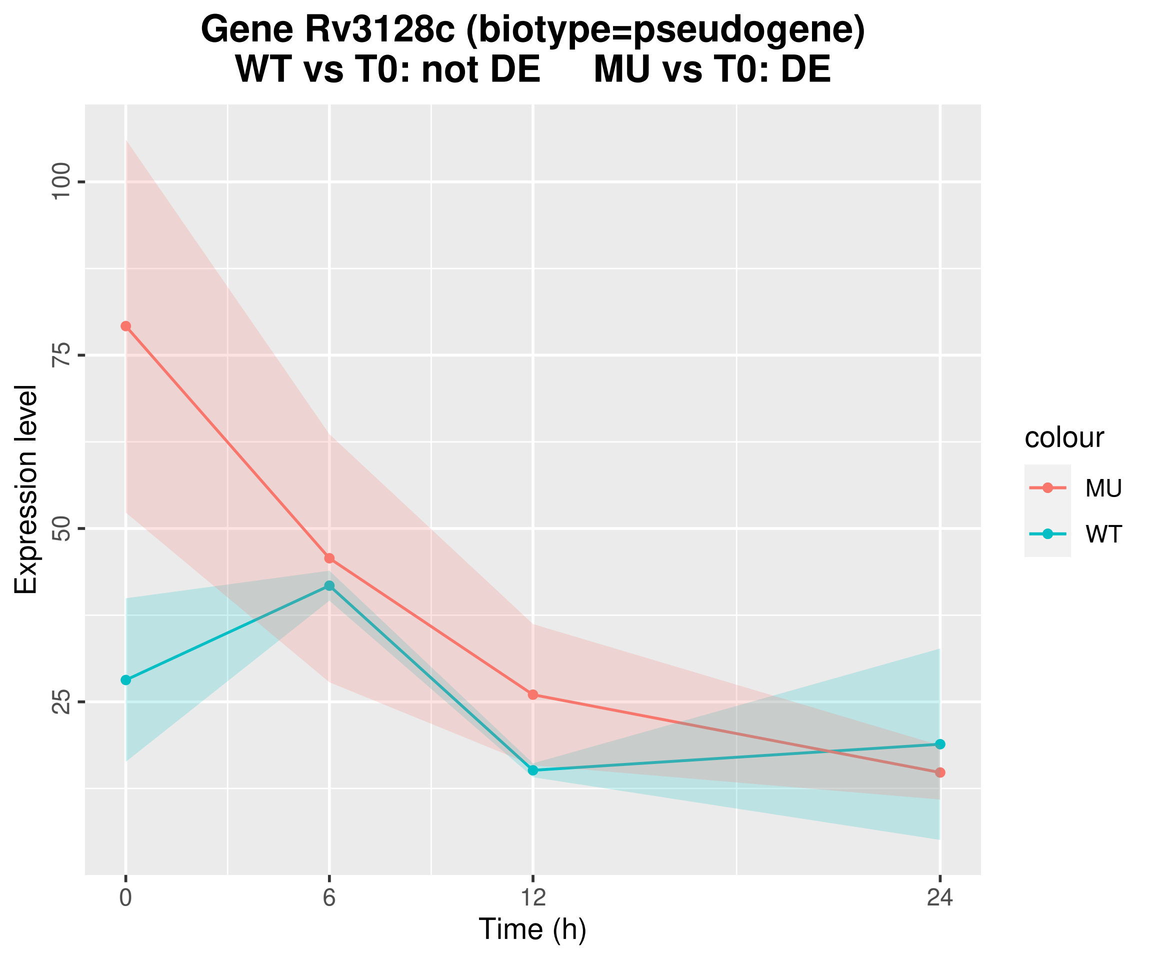

Gene Rv3128c (biotype=pseudogene)
WT vs T0: not DE   MU vs T0: DE
Expression level
100
75
50
25
0
6
12
24
Time (h)
colour
MU
WT

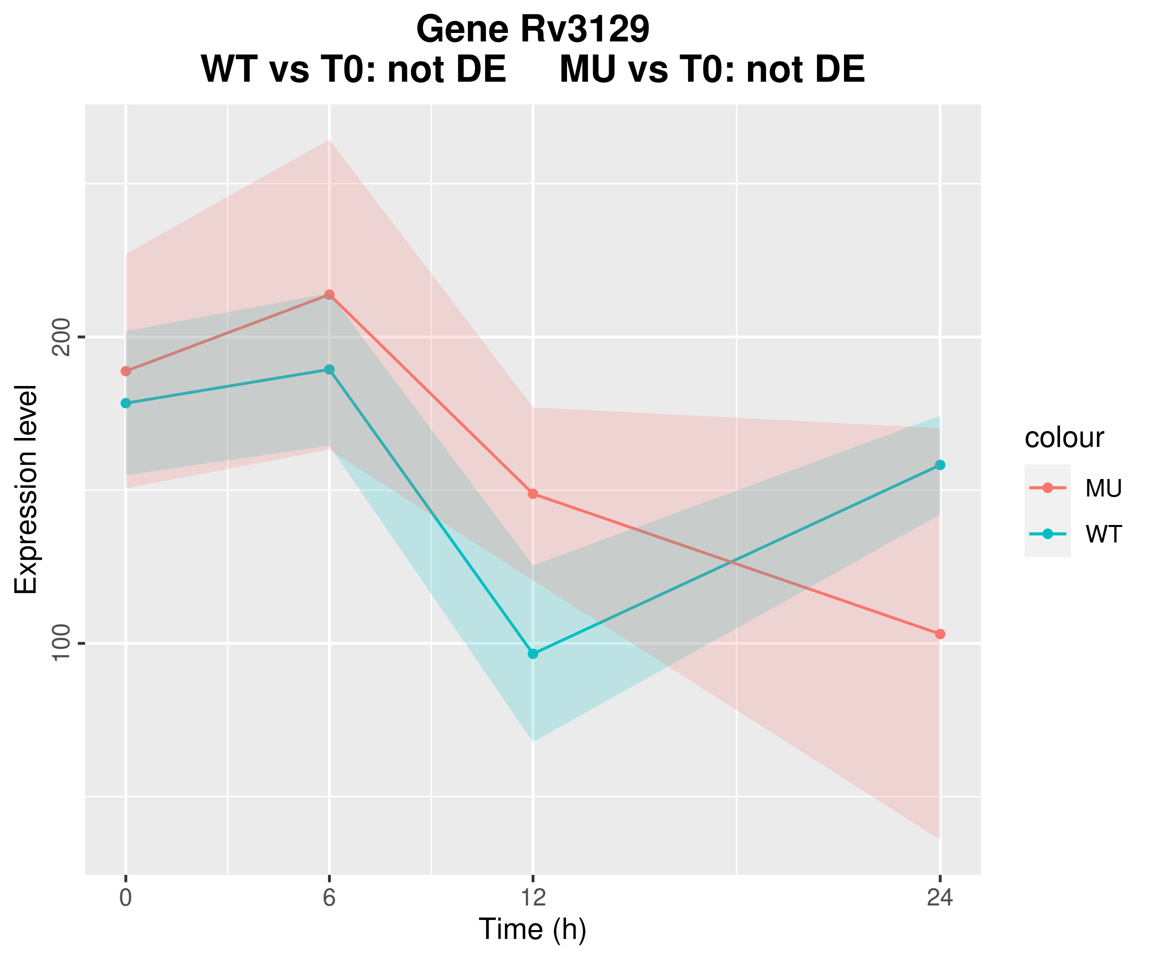
Gene Rv3129
WT vs T0: not DE   MU vs T0: not DE
Expression level
200
100
0
6
12
24
Time (h)
colour
MU
WT

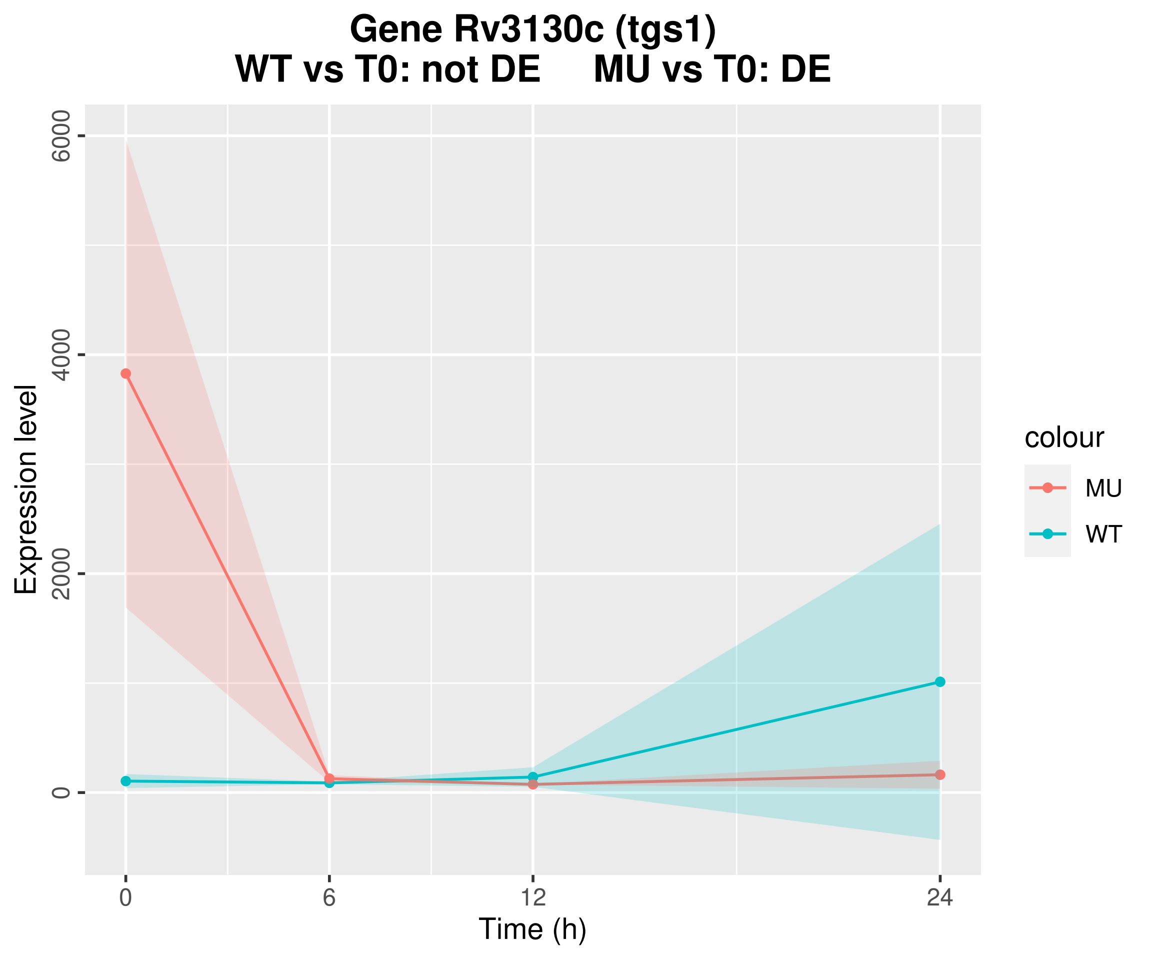

Gene Rv3130c (tgs1)
WT vs T0: not DE   MU vs T0: DE
Expression level
6000
4000
2000
0
0
6
12
24
Time (h)
colour
MU
WT

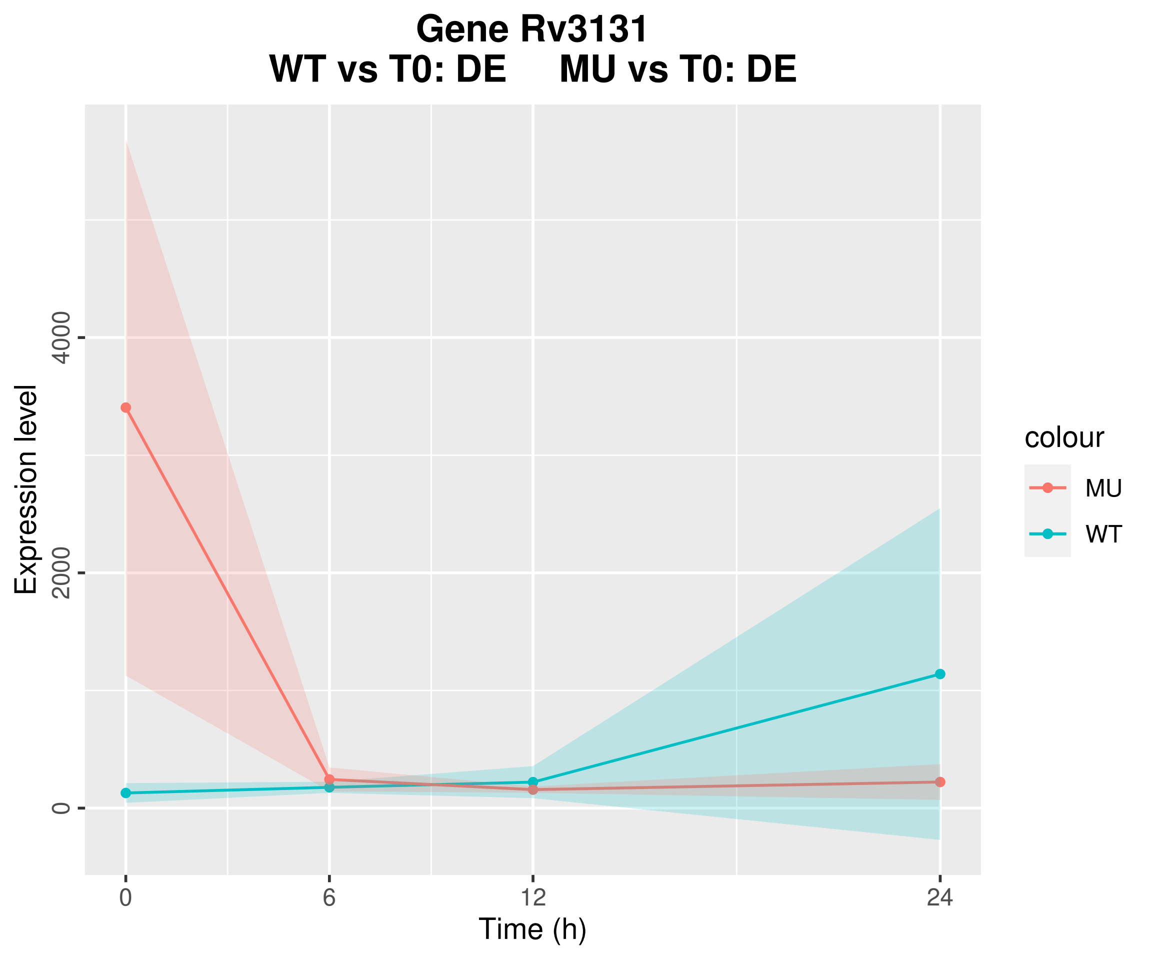
Gene Rv3131
WT vs T0: DE MU vs T0: DE
Expression level
4000
2000
0
0
6
12
24
Time (h)
colour
MU
WT

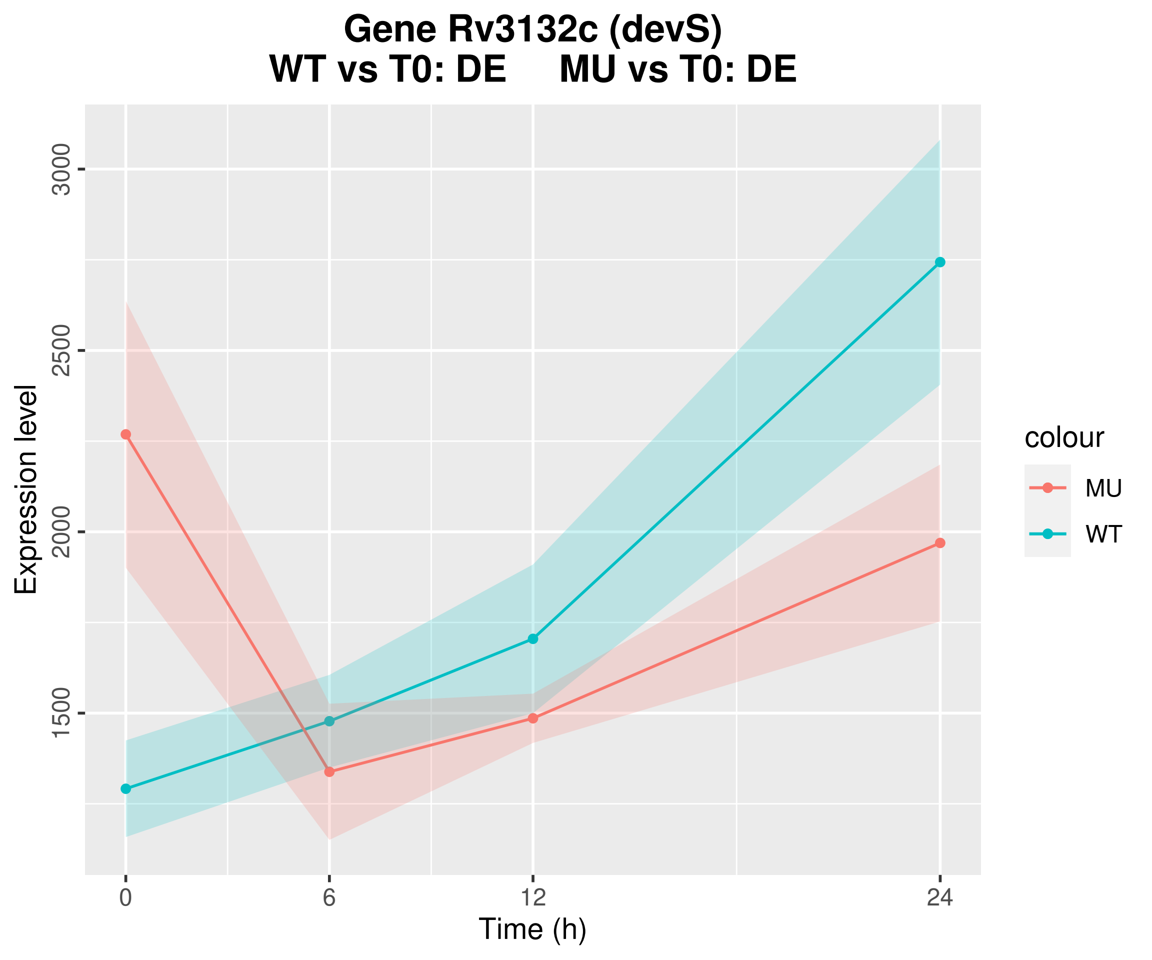

Gene Rv3132c (devS)
WT vs T0: DE MU vs T0: DE
Expression level
3000
2500
2000
1500
0
6
12
24
Time (h)
colour
MU
WT

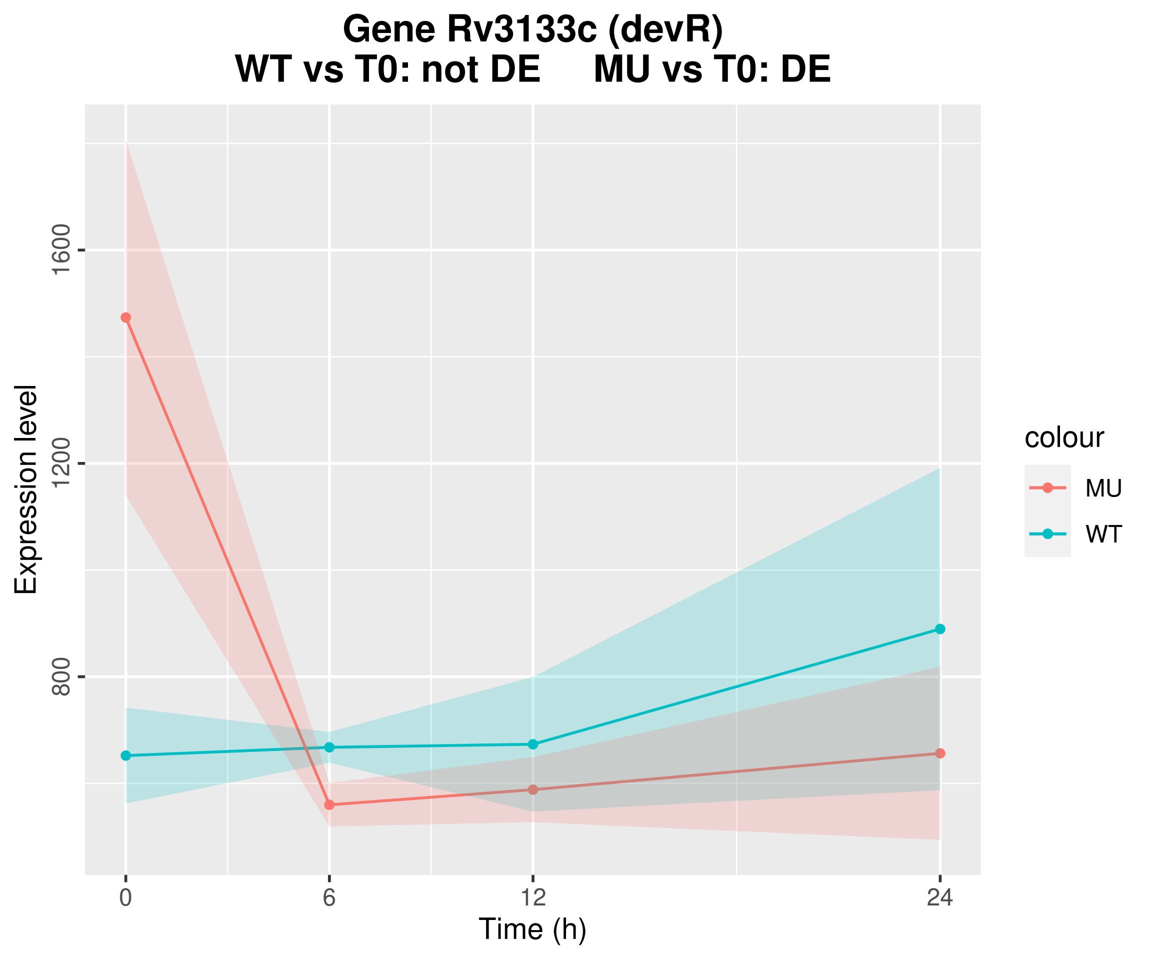
Gene Rv3133c (devR)
WT vs T0: not DE MU vs T0: DE
Expression level
1600
1200
800
0
6
12
24
Time (h)
colour
MU
WT

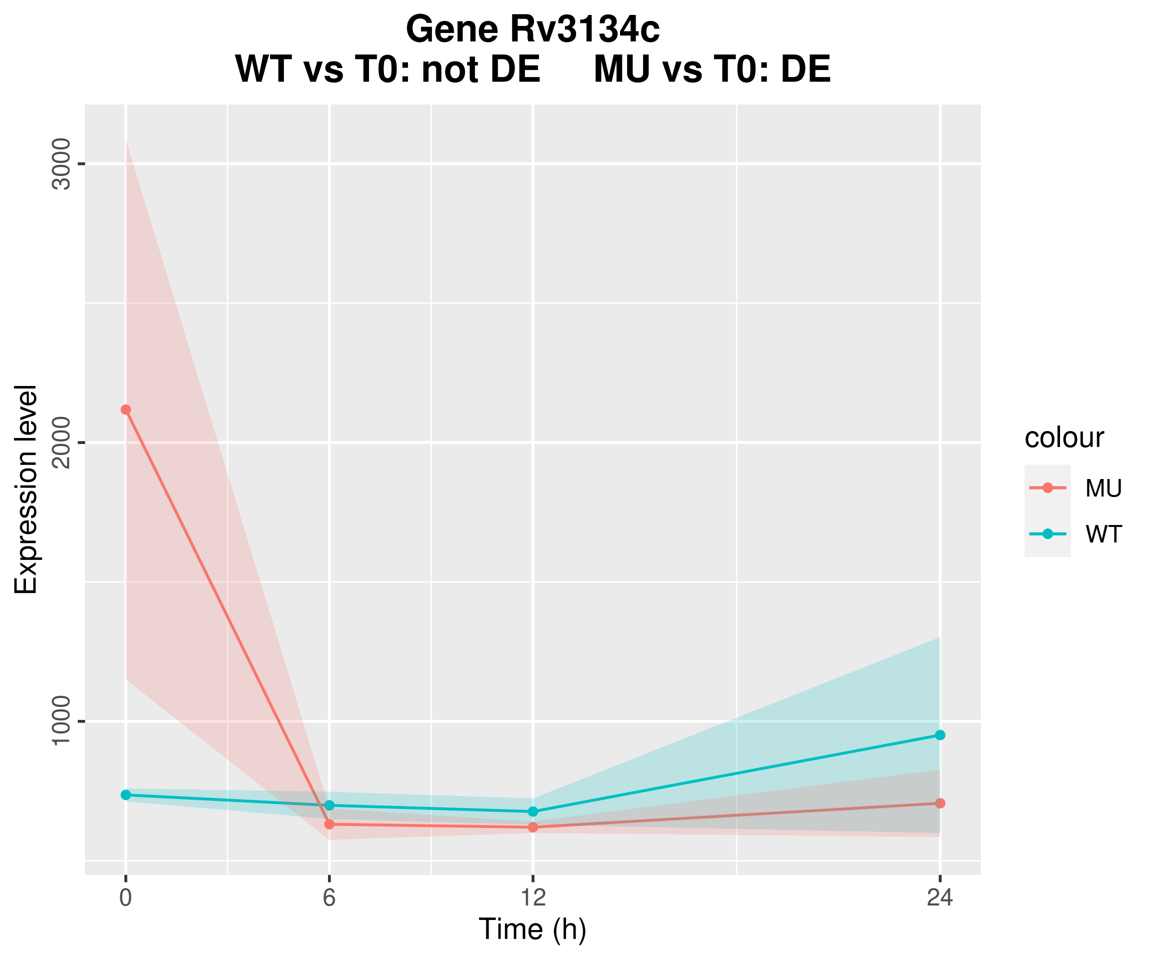

Gene Rv3134c
WT vs T0: not DE MU vs T0: DE
Expression level
3000
2000
1000
0
6
12
24
Time (h)
colour
MU
WT

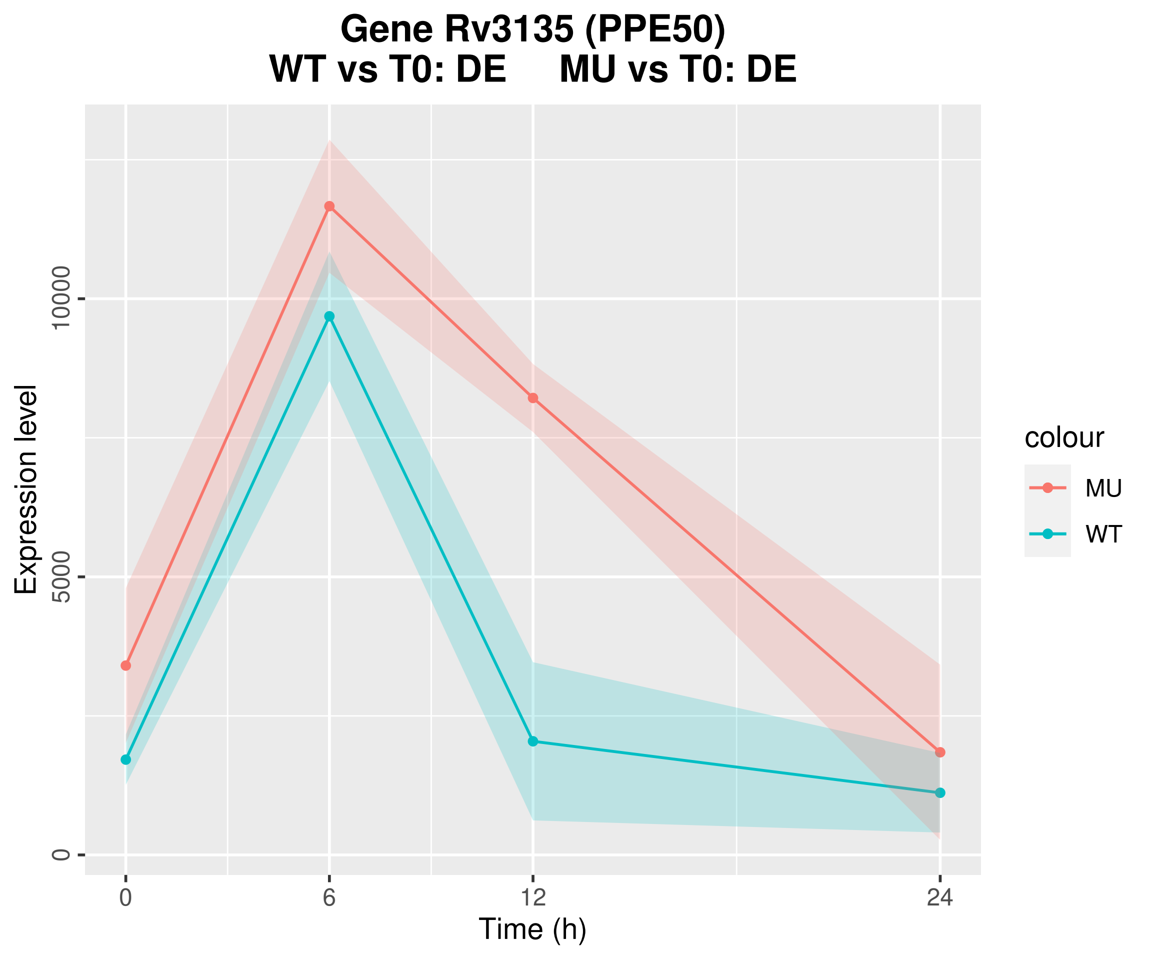

Gene Rv3135 (PPE50)
WT vs T0: DE MU vs T0: DE
Expression level
10000
5000
0
0
6
12
24
Time (h)
colour
MU
WT

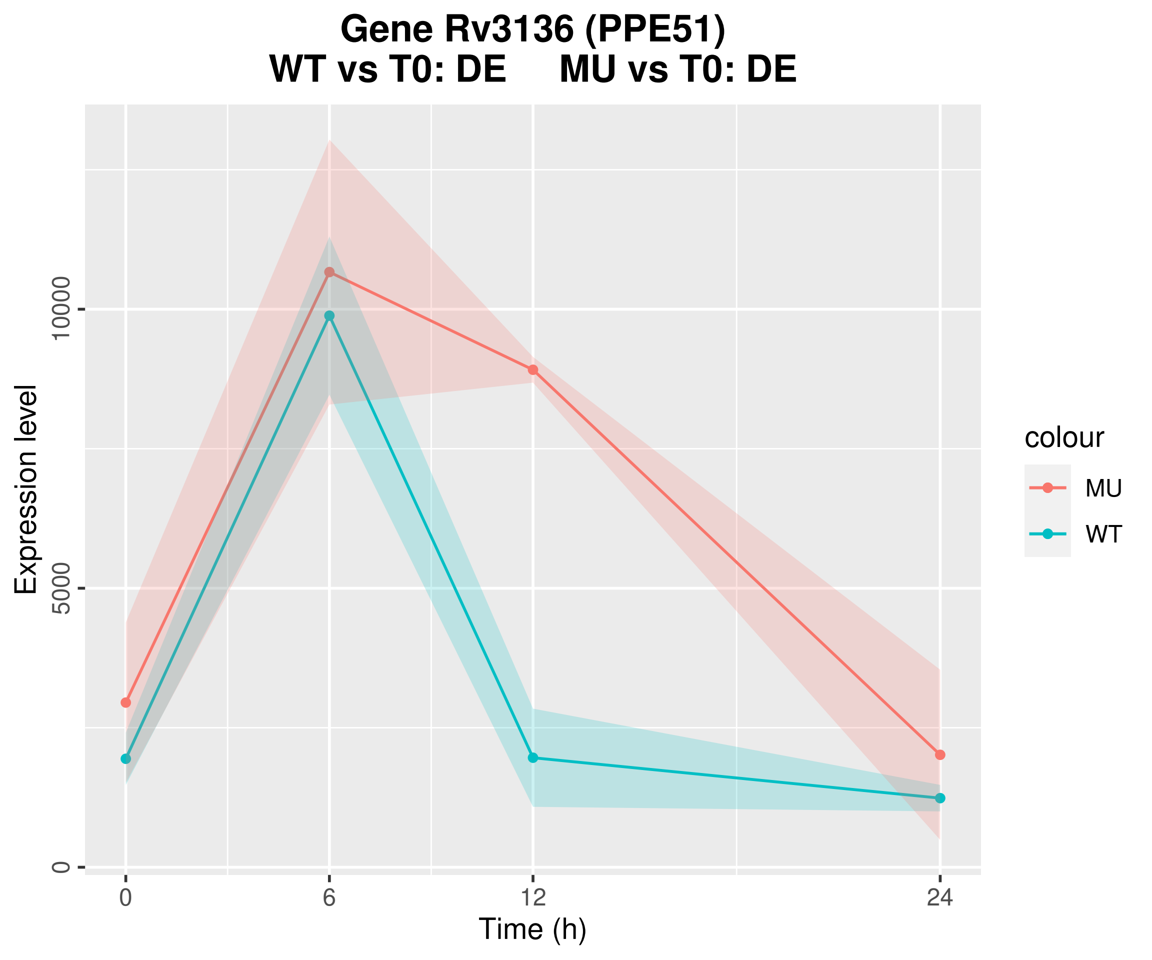
Gene Rv3136 (PPE51)
WT vs T0: DE MU vs T0: DE
Expression level
10000
5000
0
0
6
12
24
Time (h)
colour
MU
WT

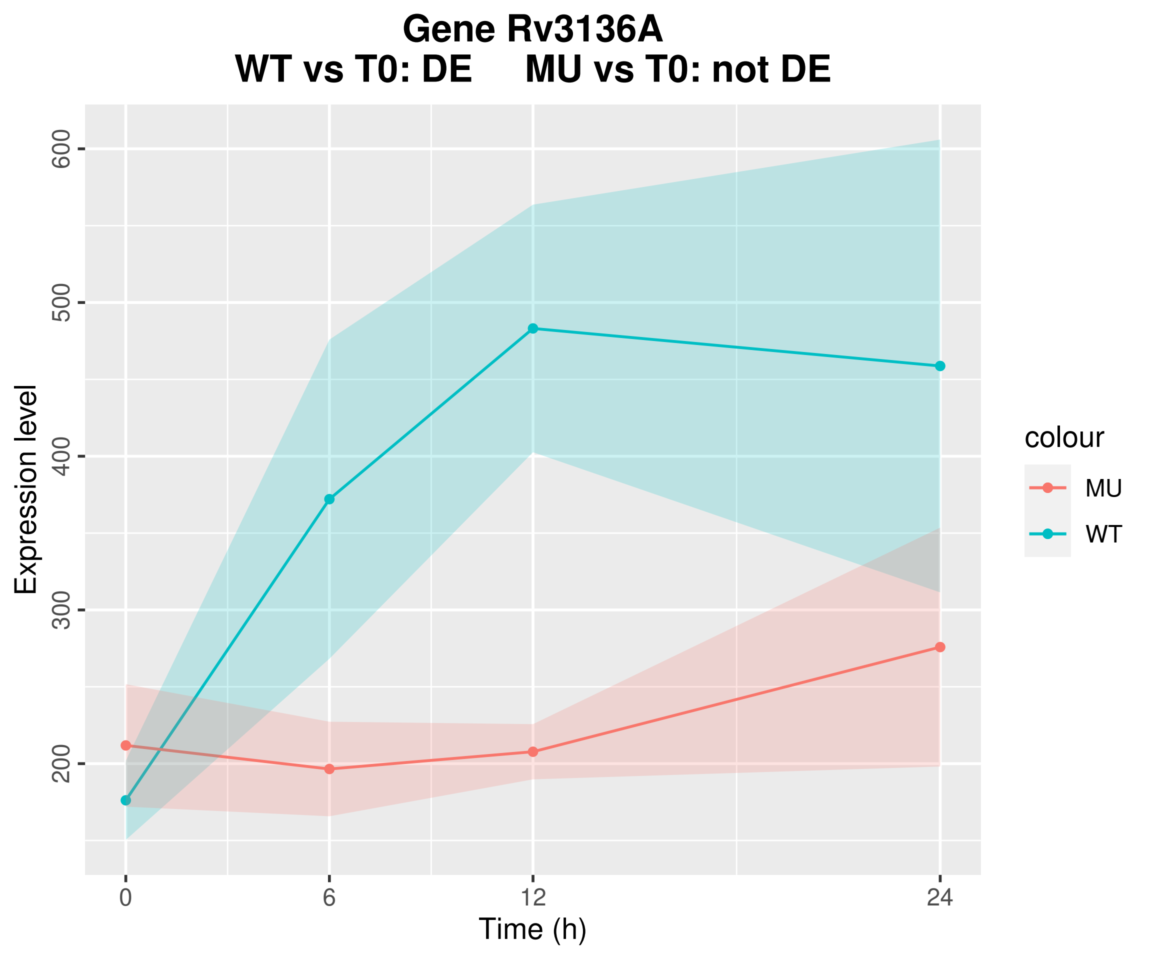
Gene Rv3136A
WT vs T0: DE   MU vs T0: not DE
Expression level
200
300
400
500
600
Time (h)
0
6
12
24
colour
MU
WT

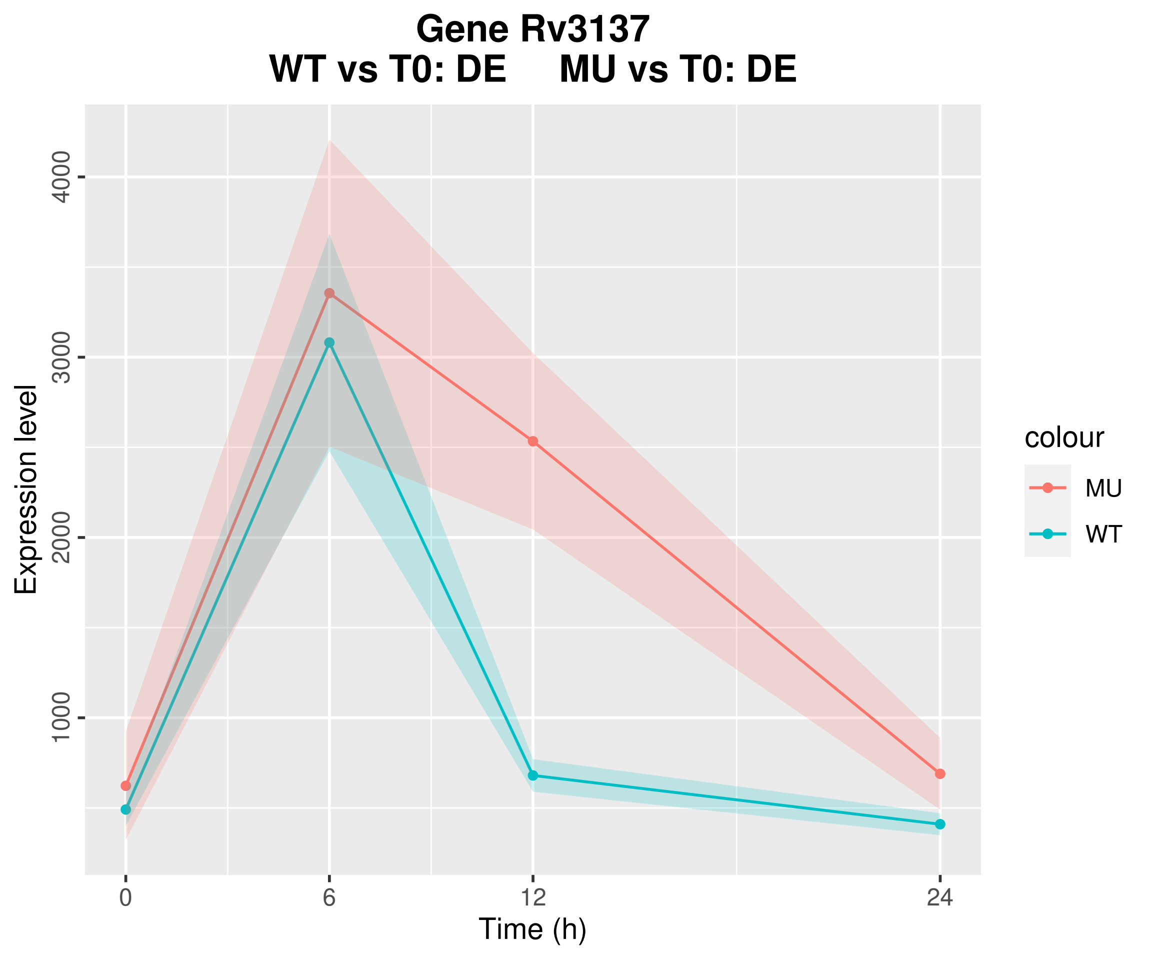

Gene Rv3137
WT vs T0: DE   MU vs T0: DE
Expression level
1000
2000
3000
4000
0
6
12
24
Time (h)
colour
MU
WT

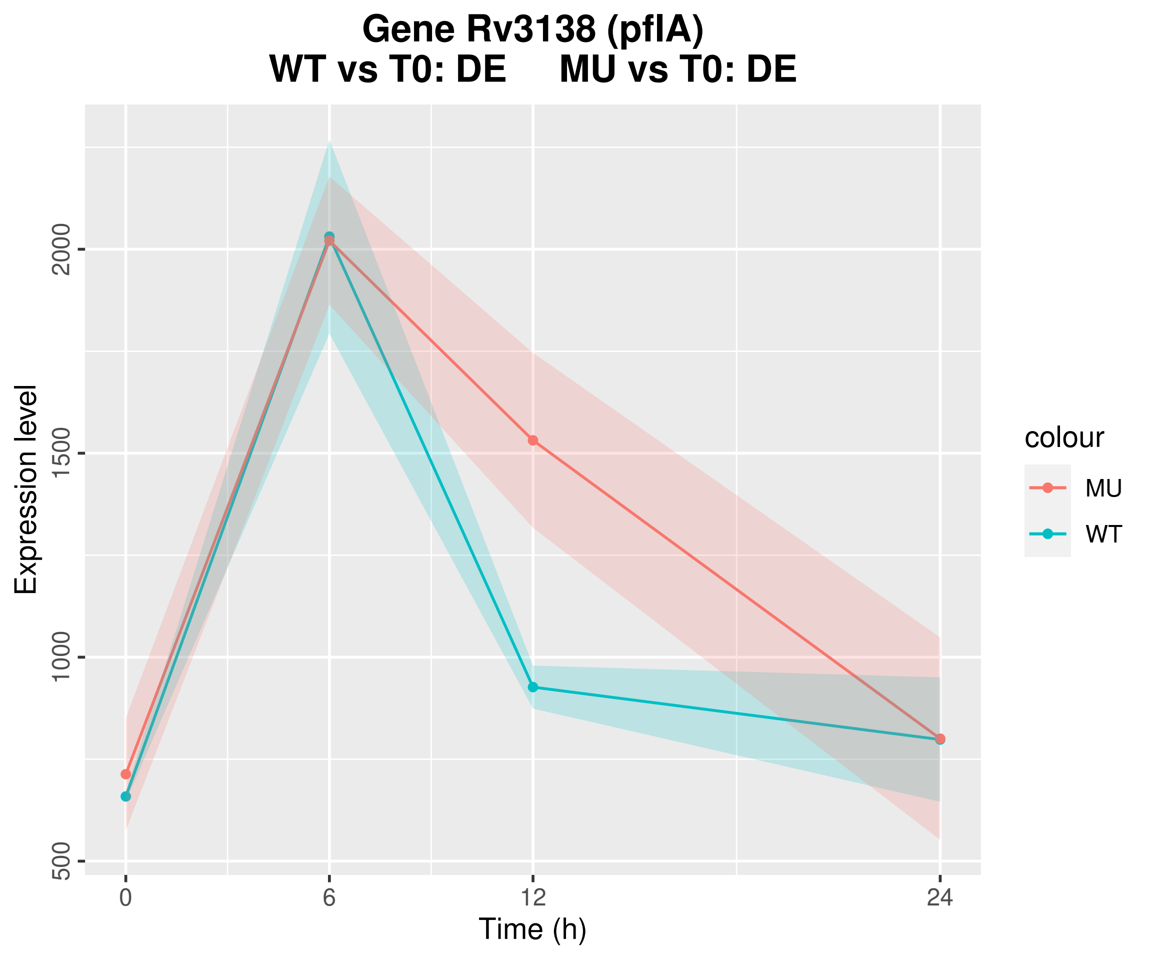

Gene Rv3138 (pflA)
WT vs T0: DE   MU vs T0: DE
Expression level
2000
1500
1000
500
0
6
12
24
Time (h)
colour
MU
WT

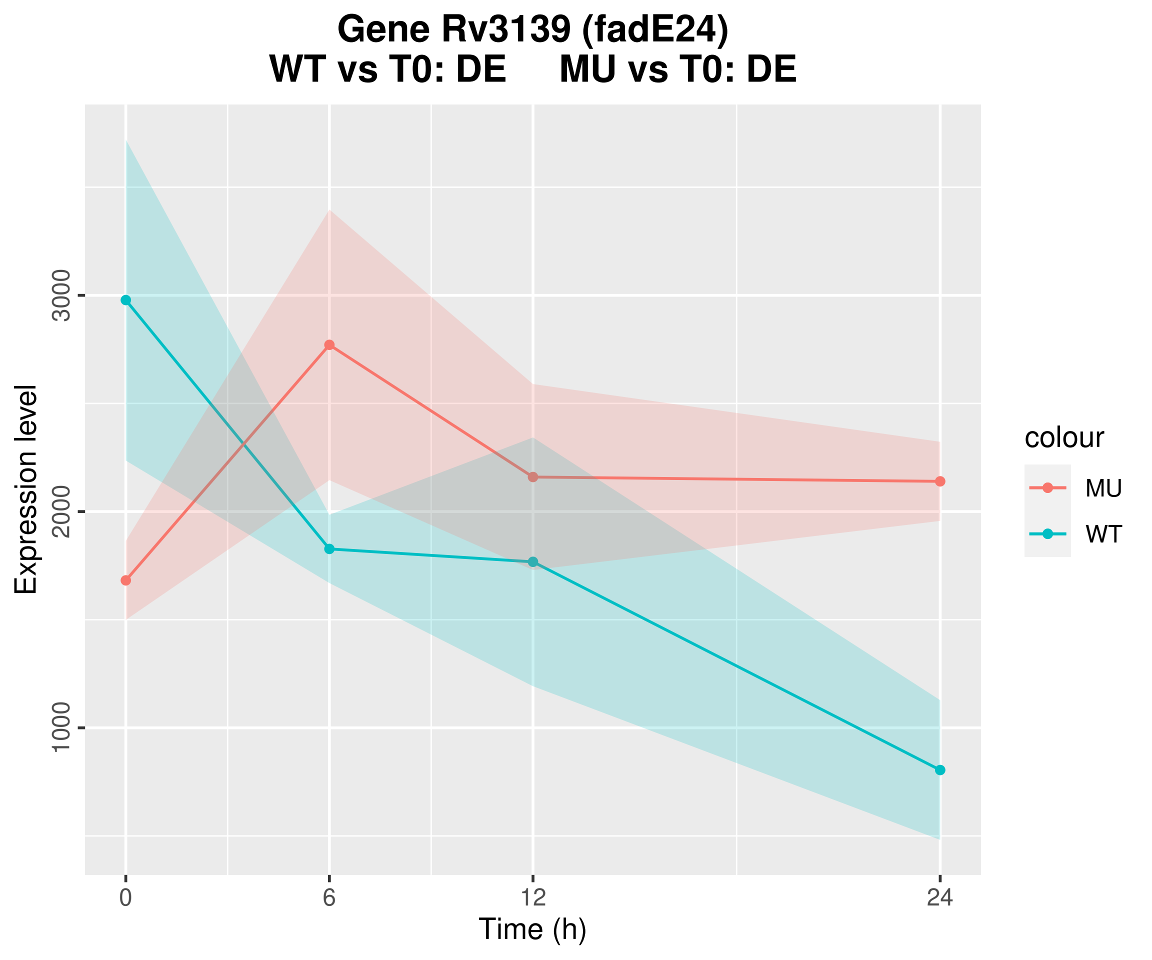
Gene Rv3139 (fadE24)
WT vs T0: DE   MU vs T0: DE
Expression level
3000
2000
1000
0
6
12
24
Time (h)
colour
MU
WT

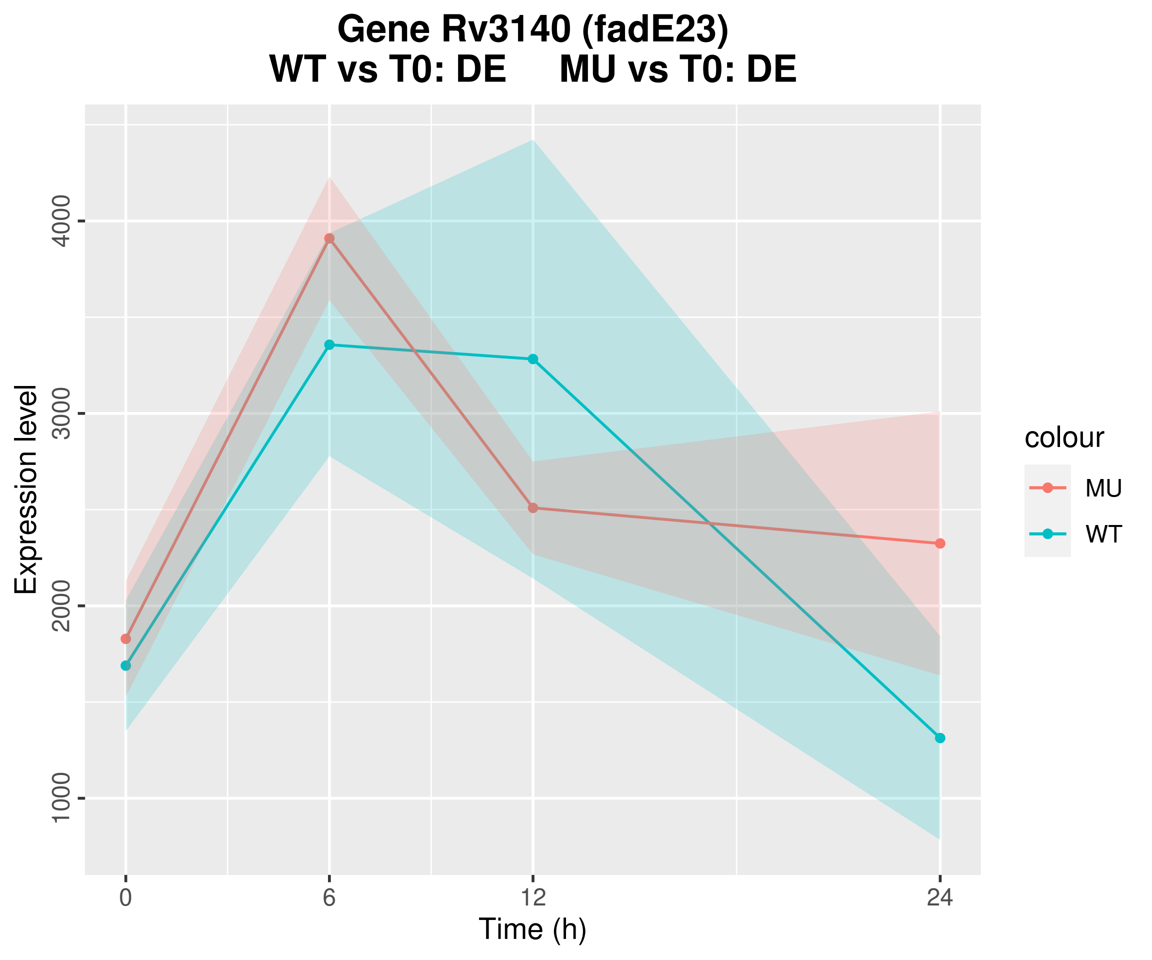

Gene Rv3140 (fadE23)
WT vs T0: DE MU vs T0: DE
Expression level
1000
2000
3000
4000
0
6
12
24
Time (h)
colour
MU
WT

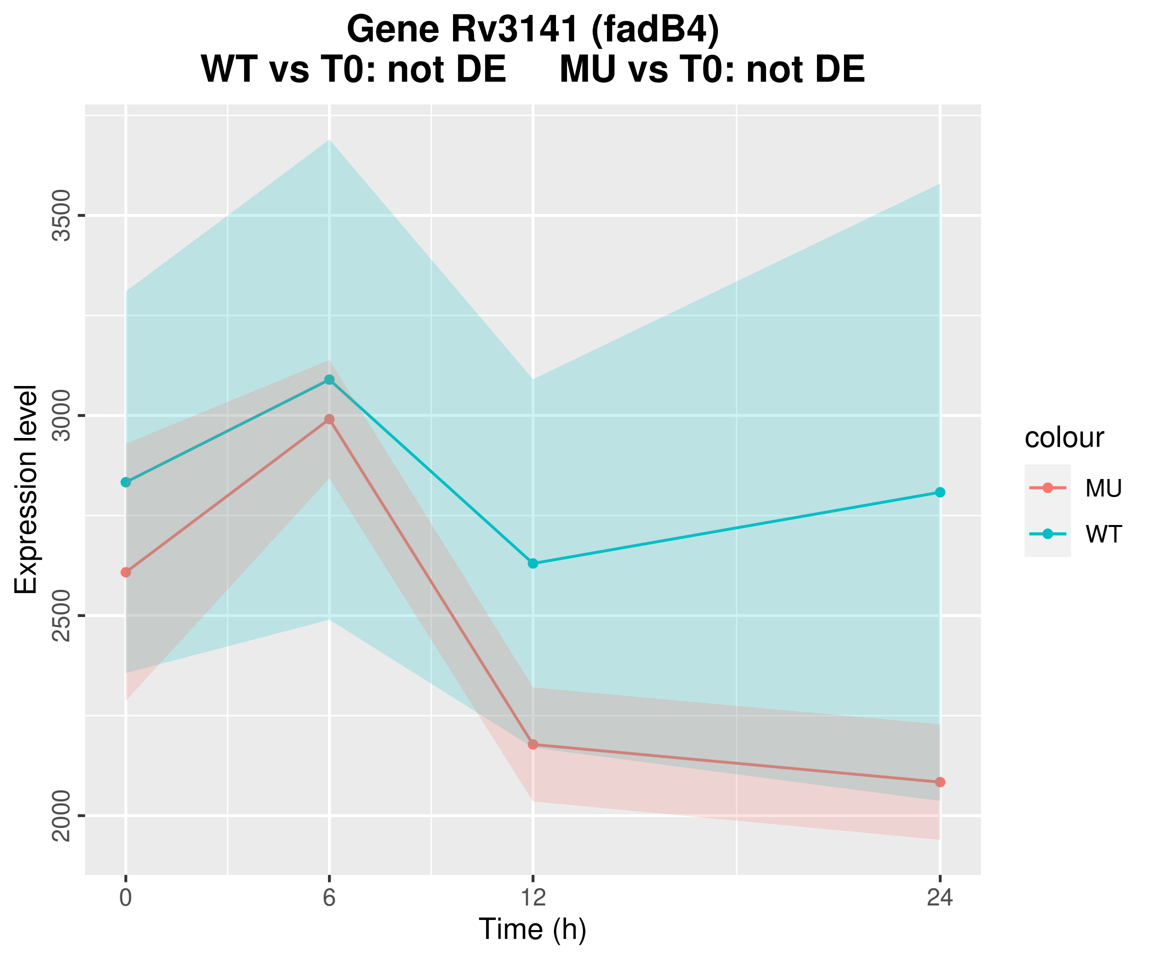
Gene Rv3141 (fadB4)
WT vs T0: not DE   MU vs T0: not DE
Expression level
3500
3000
2500
2000
0
6
12
24
Time (h)
colour
MU
WT

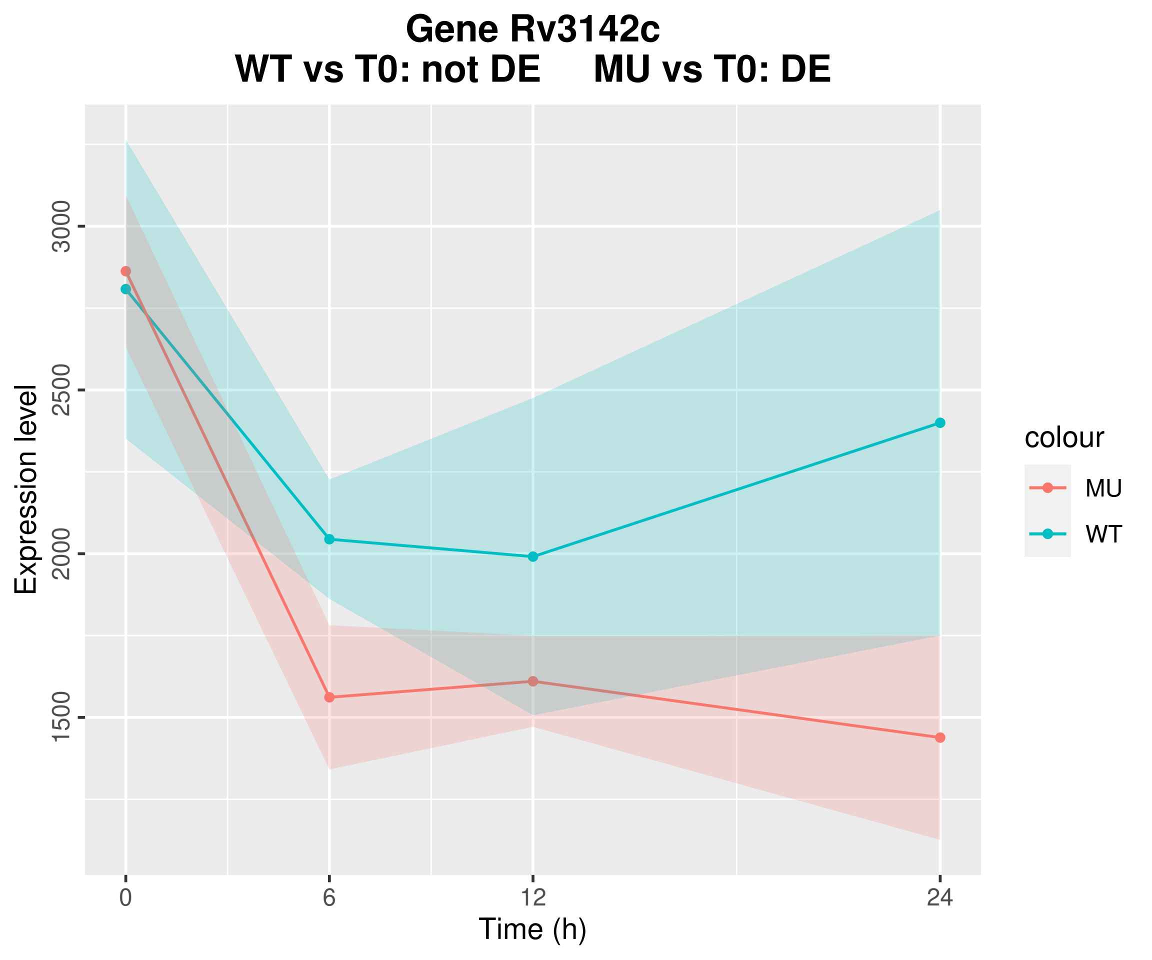
Gene Rv3142c
WT vs T0: not DE MU vs T0: DE
Expression level
3000
2500
2000
1500
0
6
12
24
Time (h)
colour
MU
WT

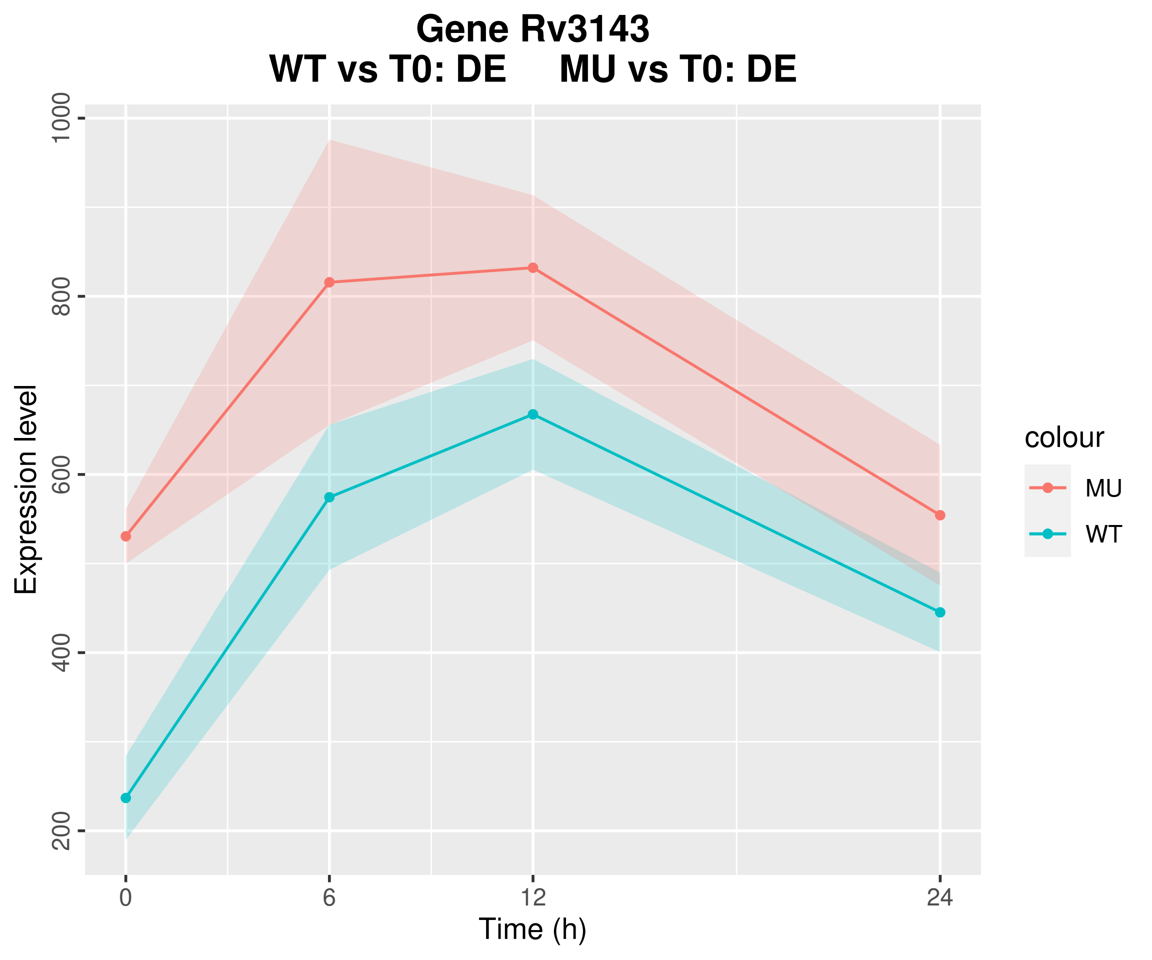
Gene Rv3143
WT vs T0: DE MU vs T0: DE
Expression level
1000
800
600
400
200
0
6
12
24
Time (h)
colour
MU
WT

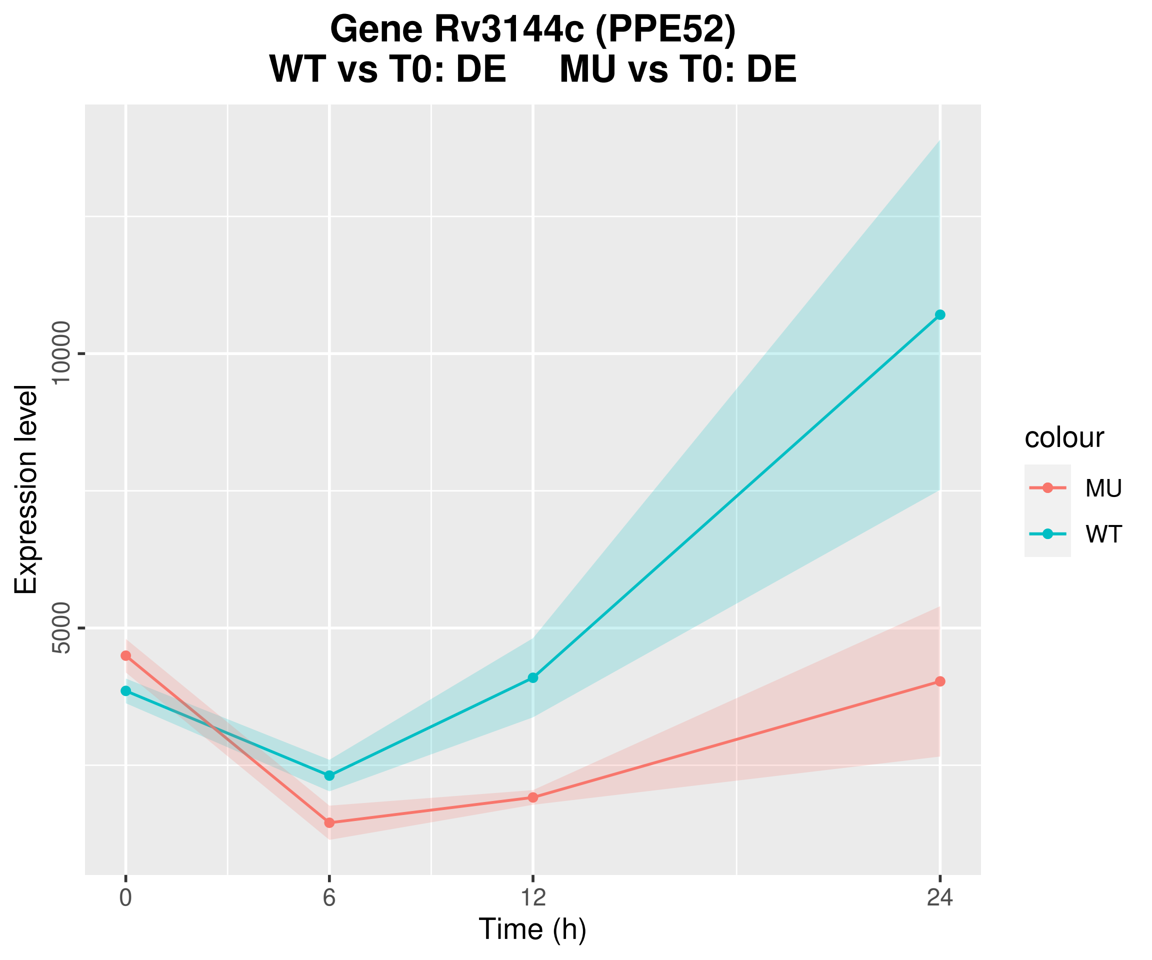

Gene Rv3144c (PPE52)
WT vs T0: DE MU vs T0: DE
Expression level
10000
5000
0
6
12
24
Time (h)
colour
MU
WT

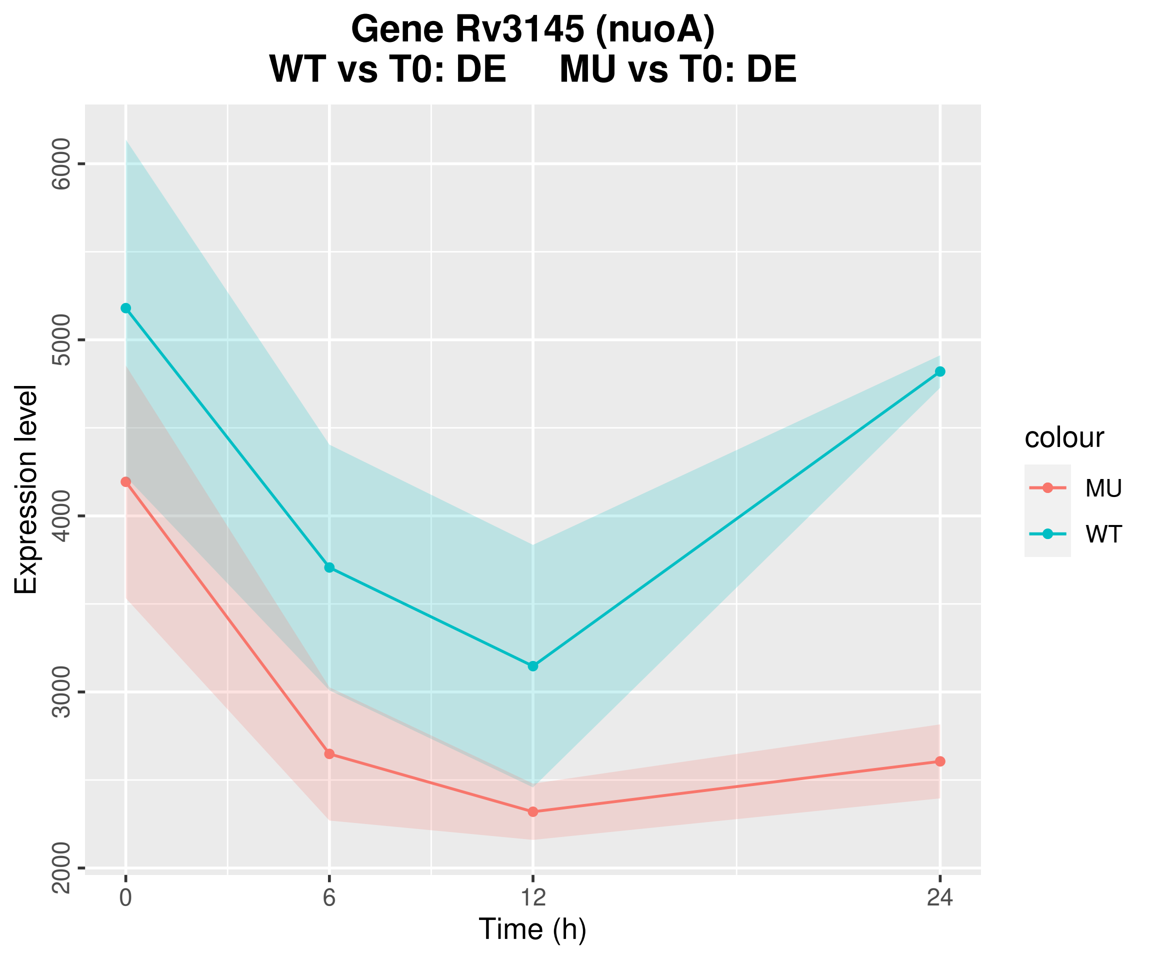
Gene Rv3145 (nuoA)
WT vs T0: DE MU vs T0: DE
Expression level
6000
5000
4000
3000
2000
0
6
12
24
Time (h)
colour
MU
WT

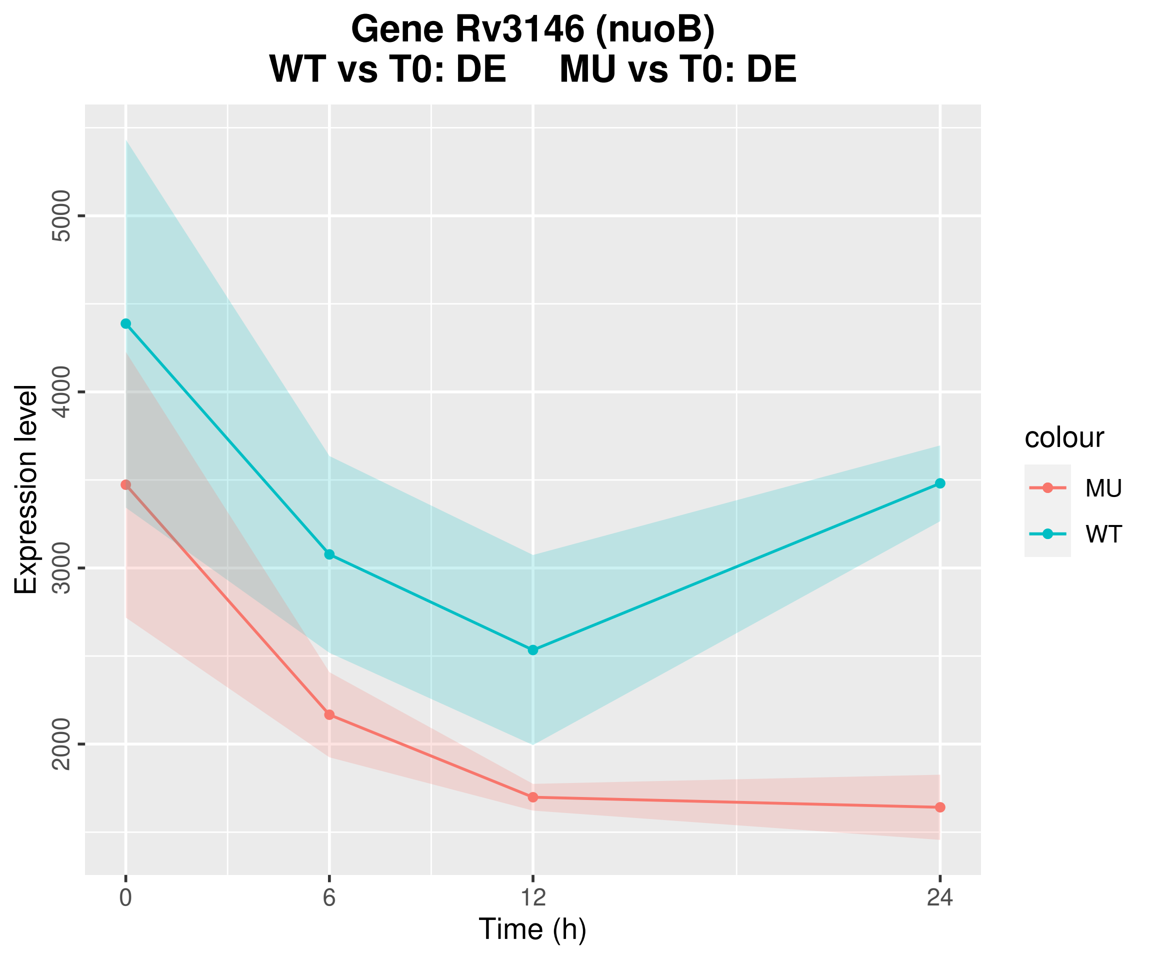

Gene Rv3146 (nuoB)
WT vs T0: DE MU vs T0: DE
Expression level
5000
4000
3000
2000
0
6
12
24
Time (h)
colour
MU
WT

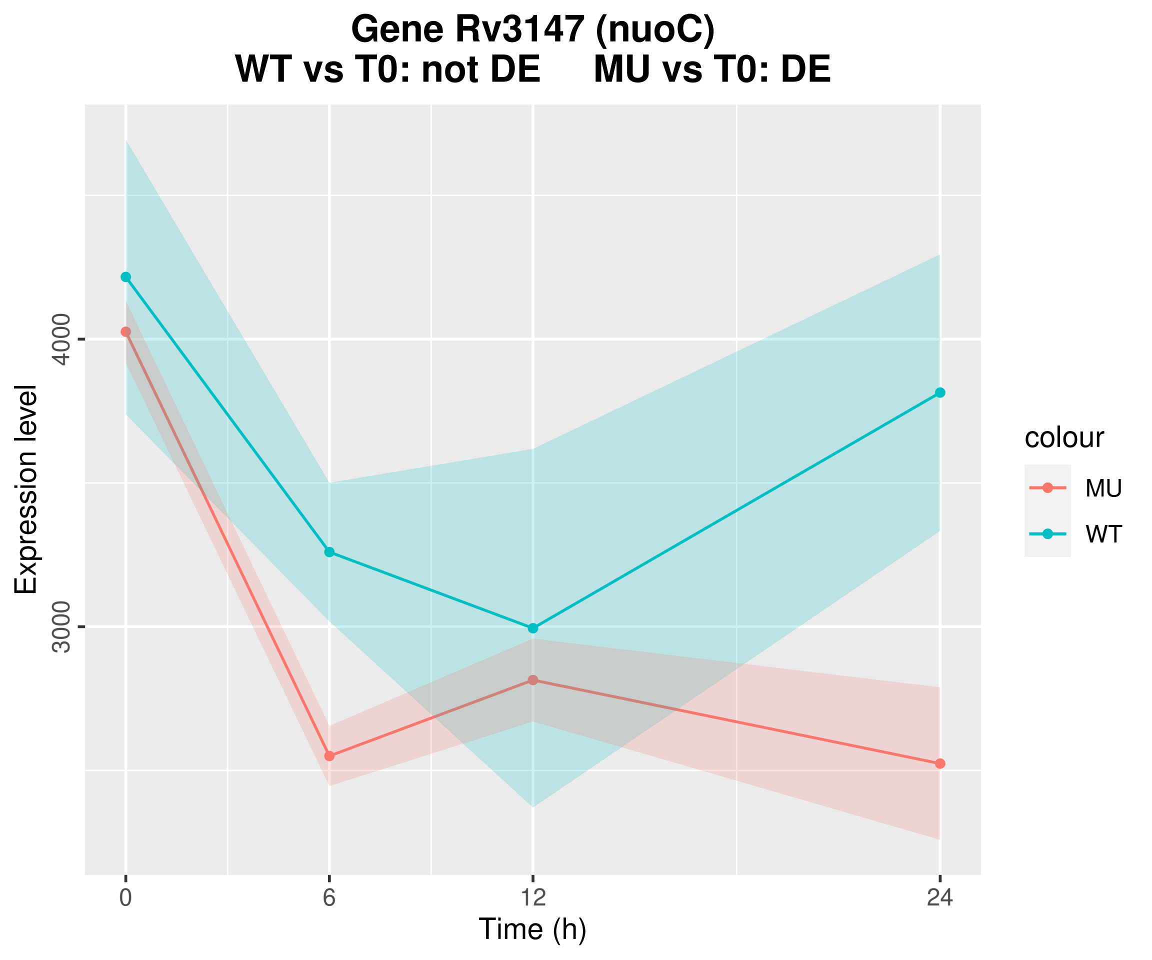
Gene Rv3147 (nuoC)
WT vs T0: not DE MU vs T0: DE
Expression level
4000
3000
0
6
12
24
Time (h)
colour
MU
WT

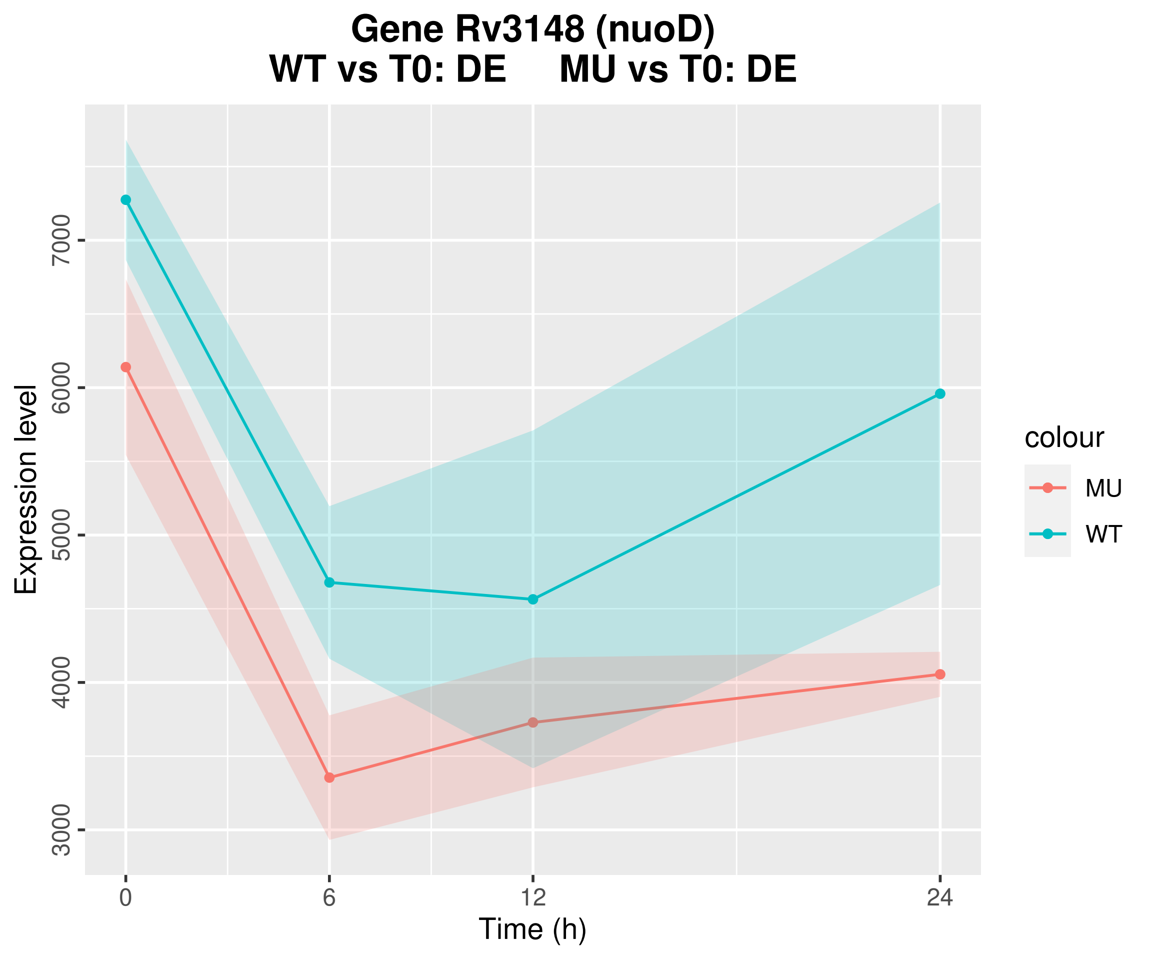

Gene Rv3148 (nuoD)
WT vs T0: DE   MU vs T0: DE
Expression level
3000
4000
5000
6000
7000
0
6
12
24
Time (h)
colour
MU
WT

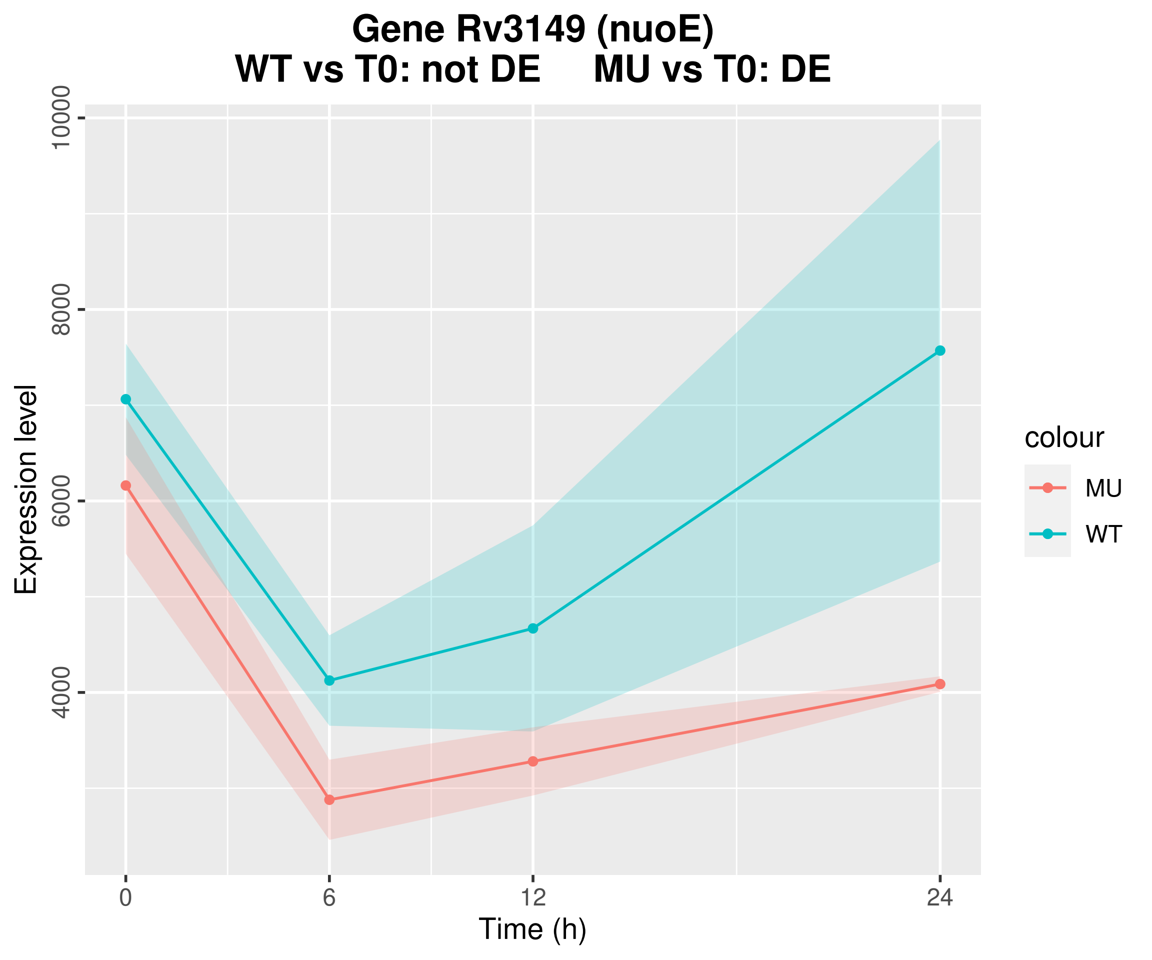
Gene Rv3149 (nuoE)
WT vs T0: not DE MU vs T0: DE
Expression level
10000
8000
6000
4000
0
6
12
24
Time (h)
colour
MU
WT

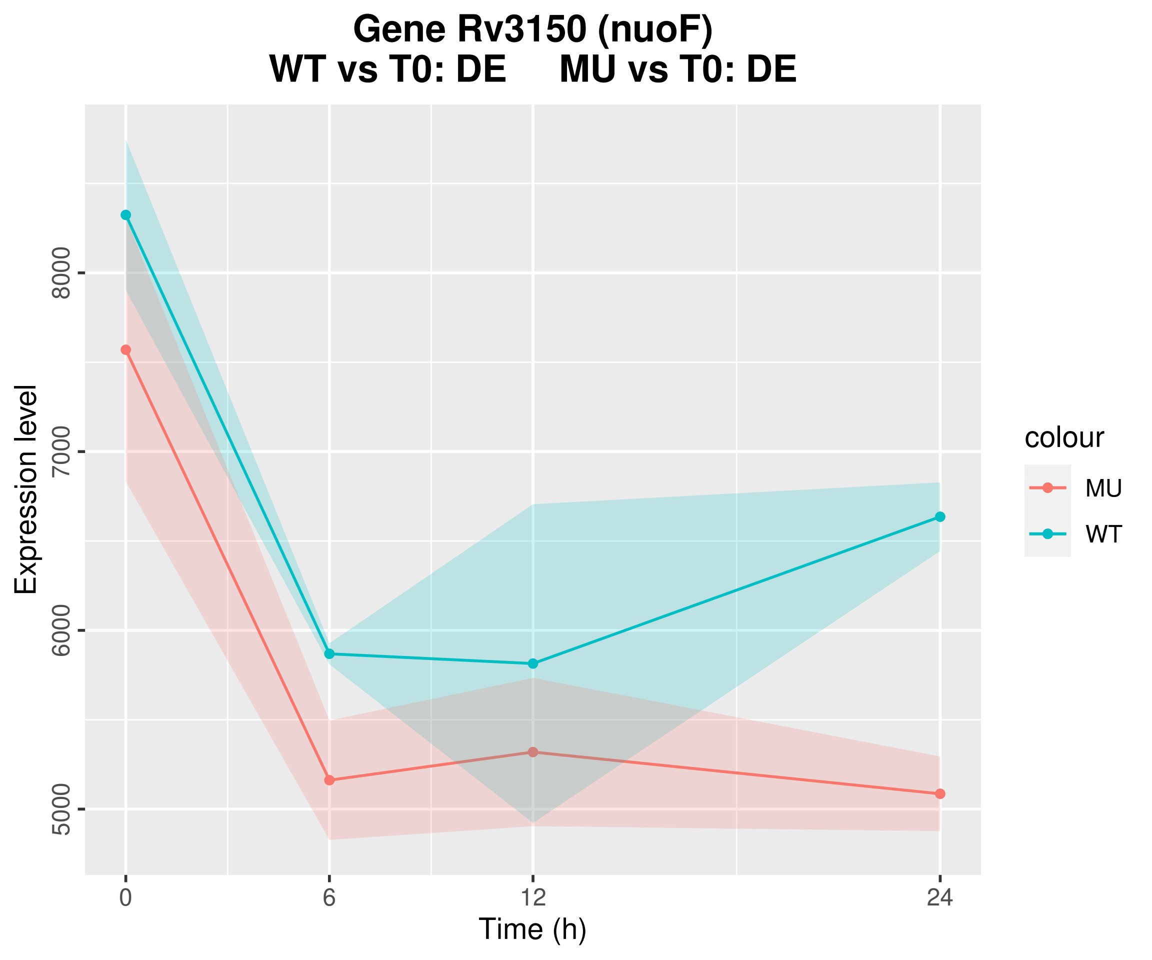

Gene Rv3150 (nuoF)
WT vs T0: DE MU vs T0: DE
Expression level
5000
6000
7000
8000
0
6
12
24
Time (h)
colour
MU
WT

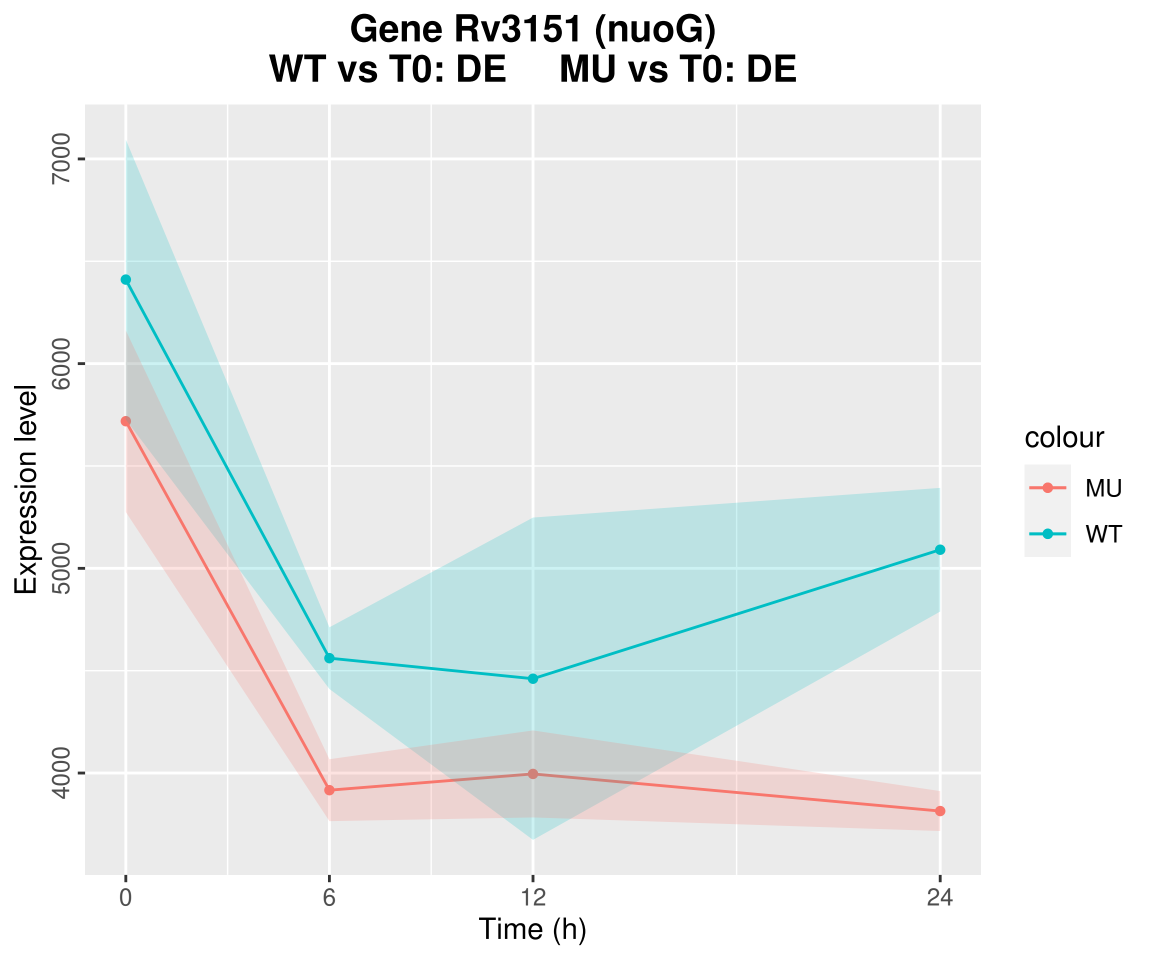

Gene Rv3151 (nuoG)
WT vs T0: DE MU vs T0: DE
Expression level
7000
6000
5000
4000
0
6
12
24
Time (h)
colour
MU
WT

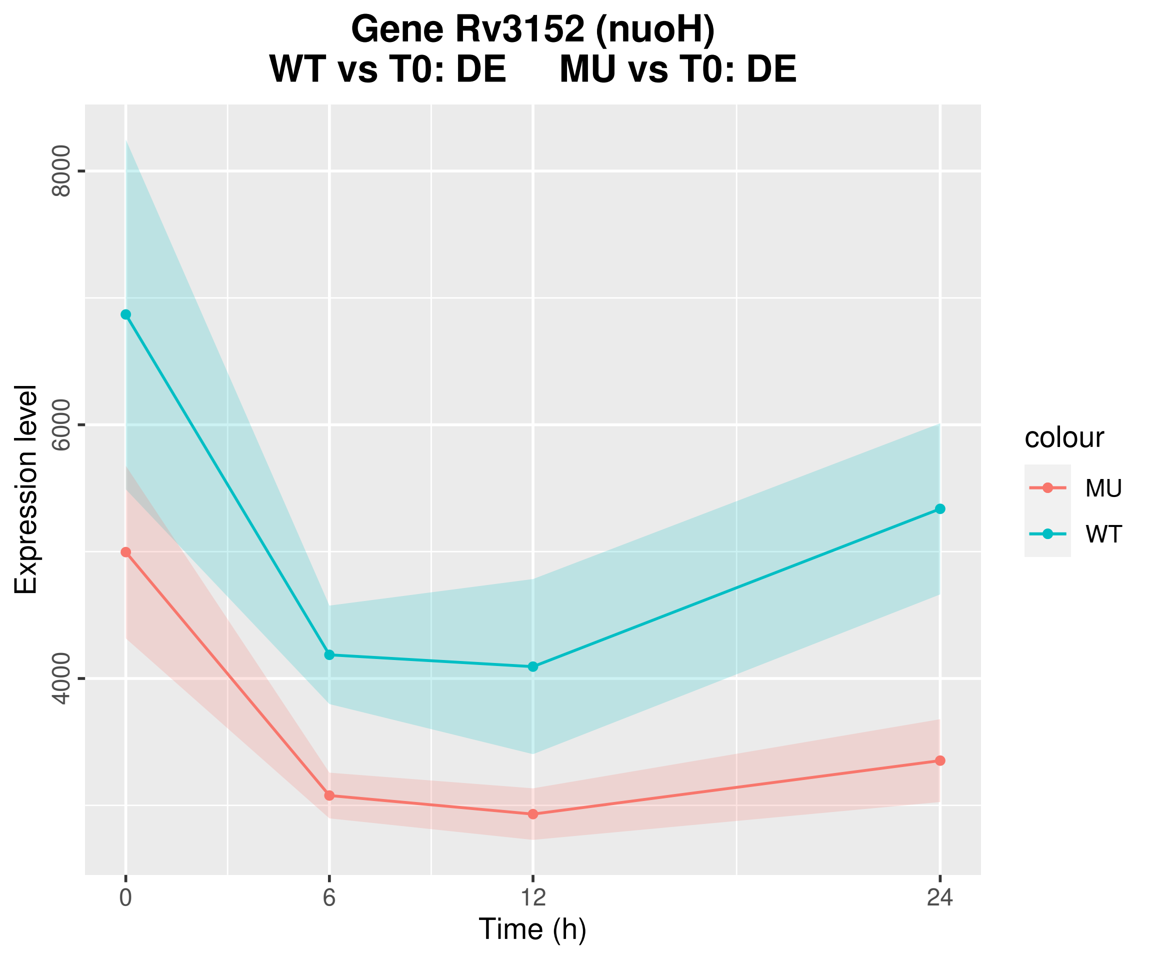
Gene Rv3152 (nuoH)
WT vs T0: DE MU vs T0: DE
Expression level
8000
6000
4000
Time (h)
0
6
12
24
colour
MU
WT

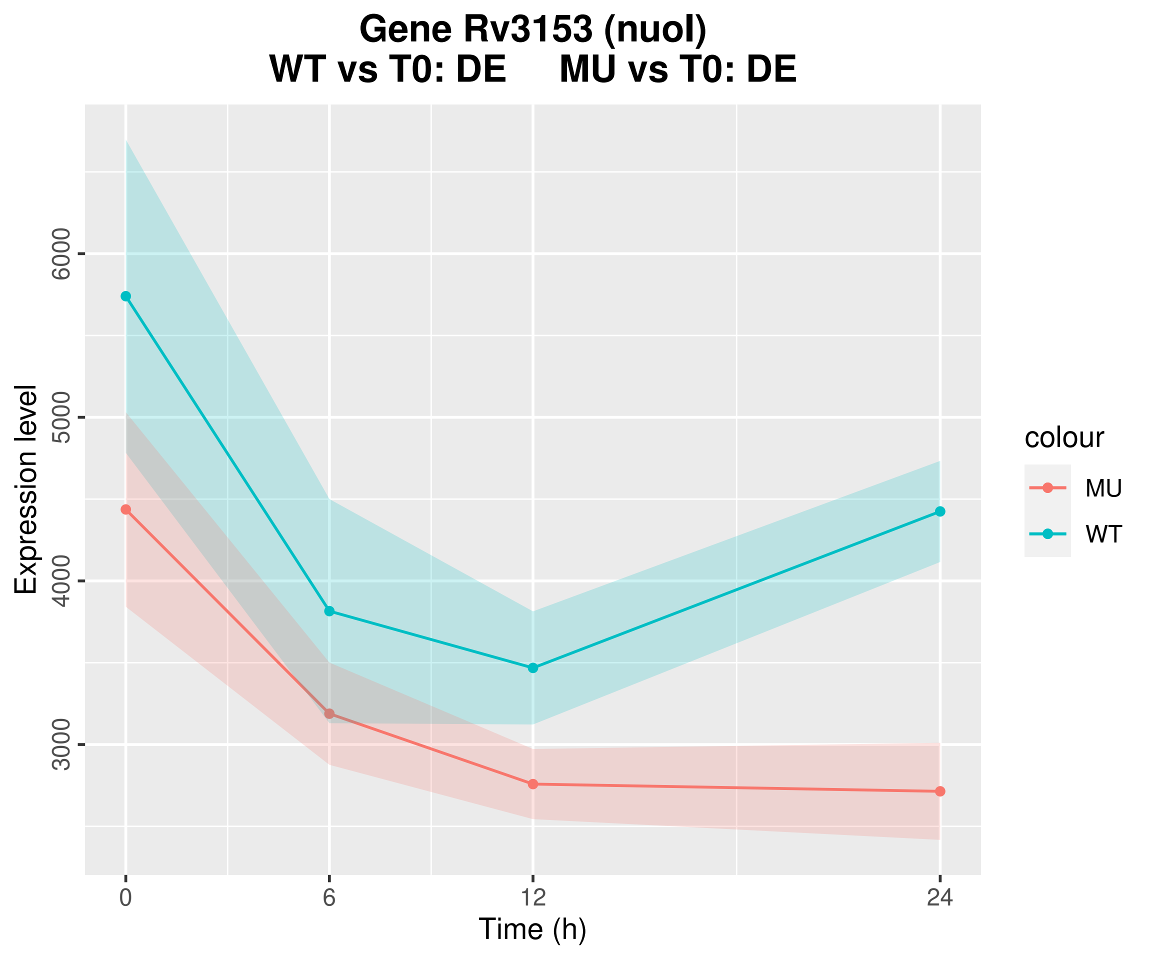

Gene Rv3153 (nuoI)
WT vs T0: DE MU vs T0: DE
Expression level
6000
5000
4000
3000
0
6
12
24
Time (h)
colour
MU
WT

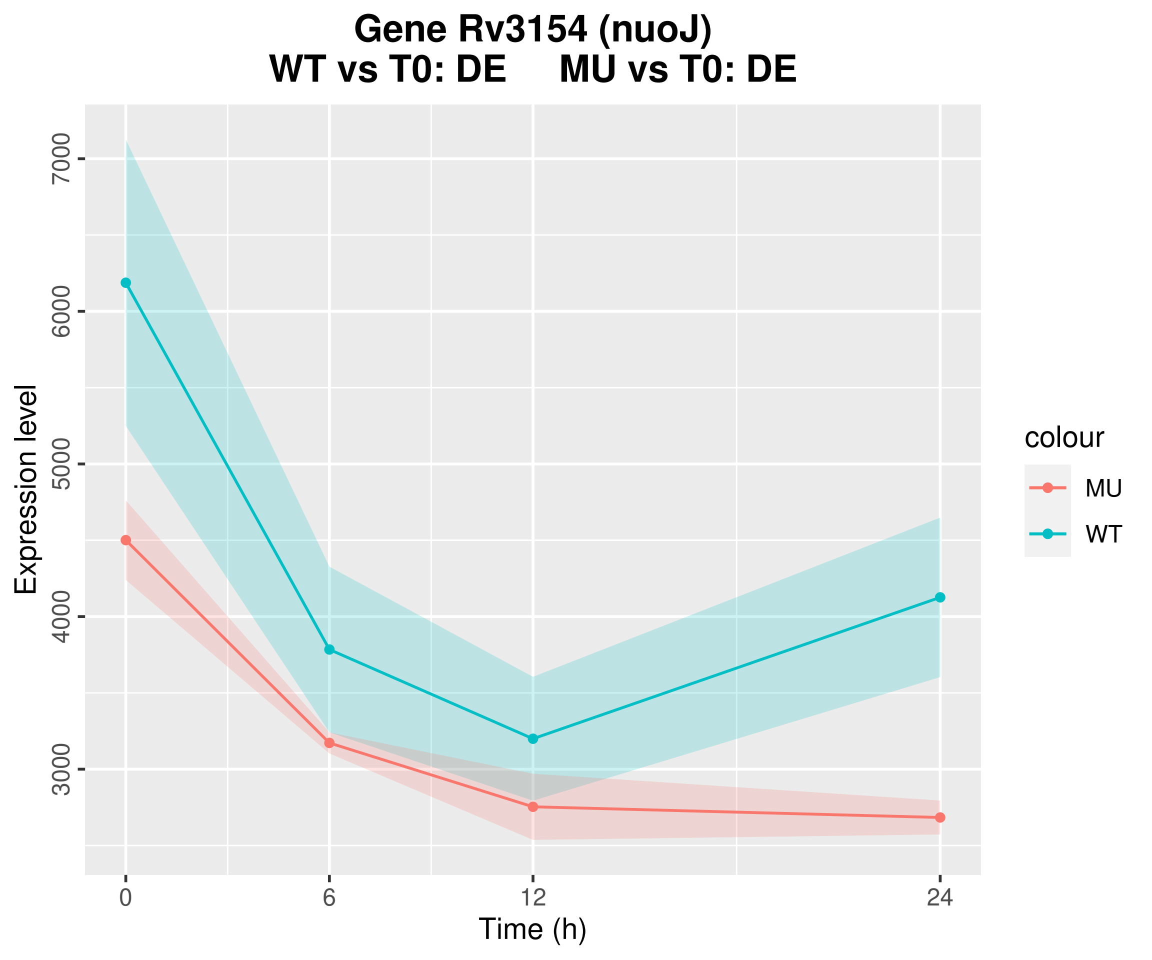
Gene Rv3154 (nuoJ)
WT vs T0: DE MU vs T0: DE
Expression level
7000
6000
5000
4000
3000
0
6
12
24
Time (h)
colour
MU
WT

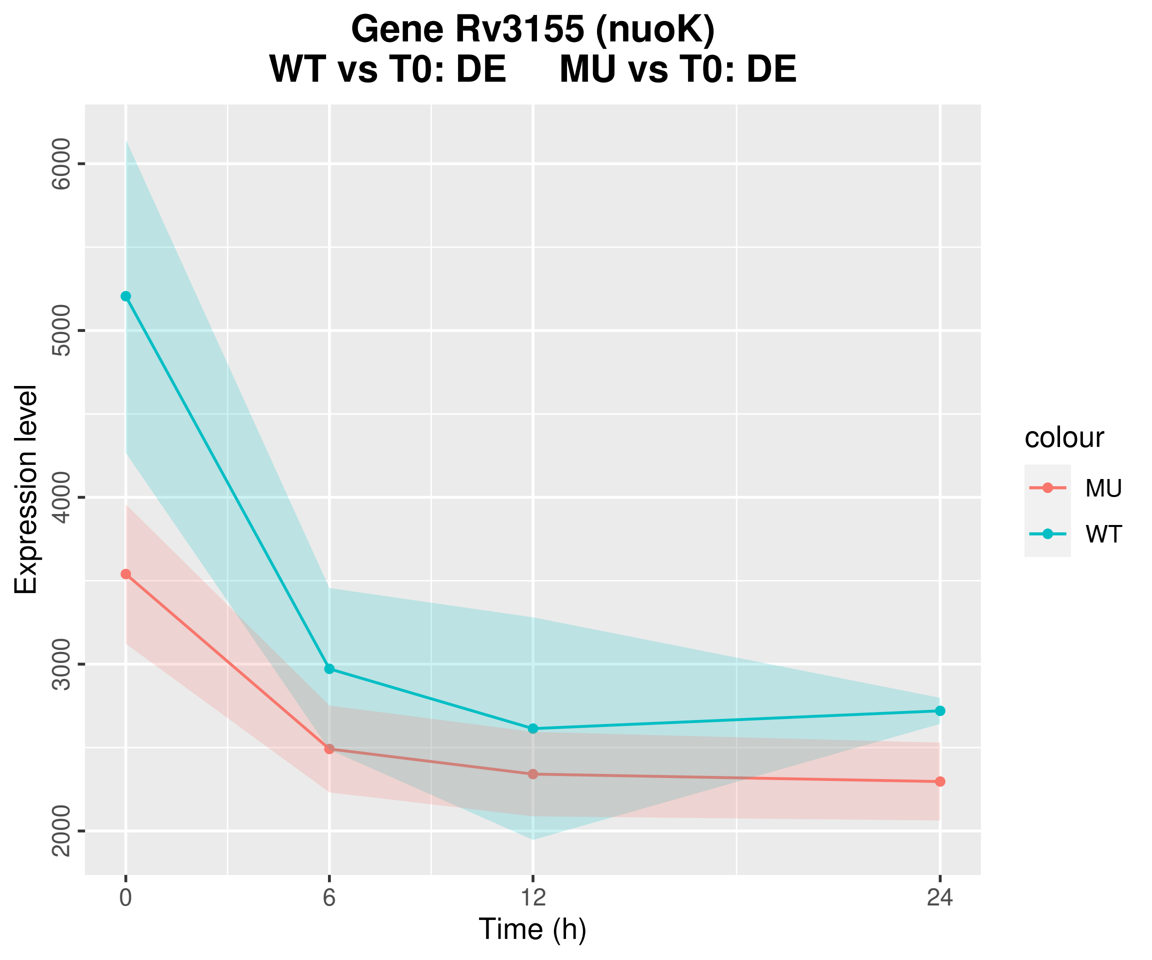
Gene Rv3155 (nuoK)
WT vs T0: DE MU vs T0: DE
Expression level
6000
5000
4000
3000
2000
0
6
12
24
Time (h)
colour
MU
WT

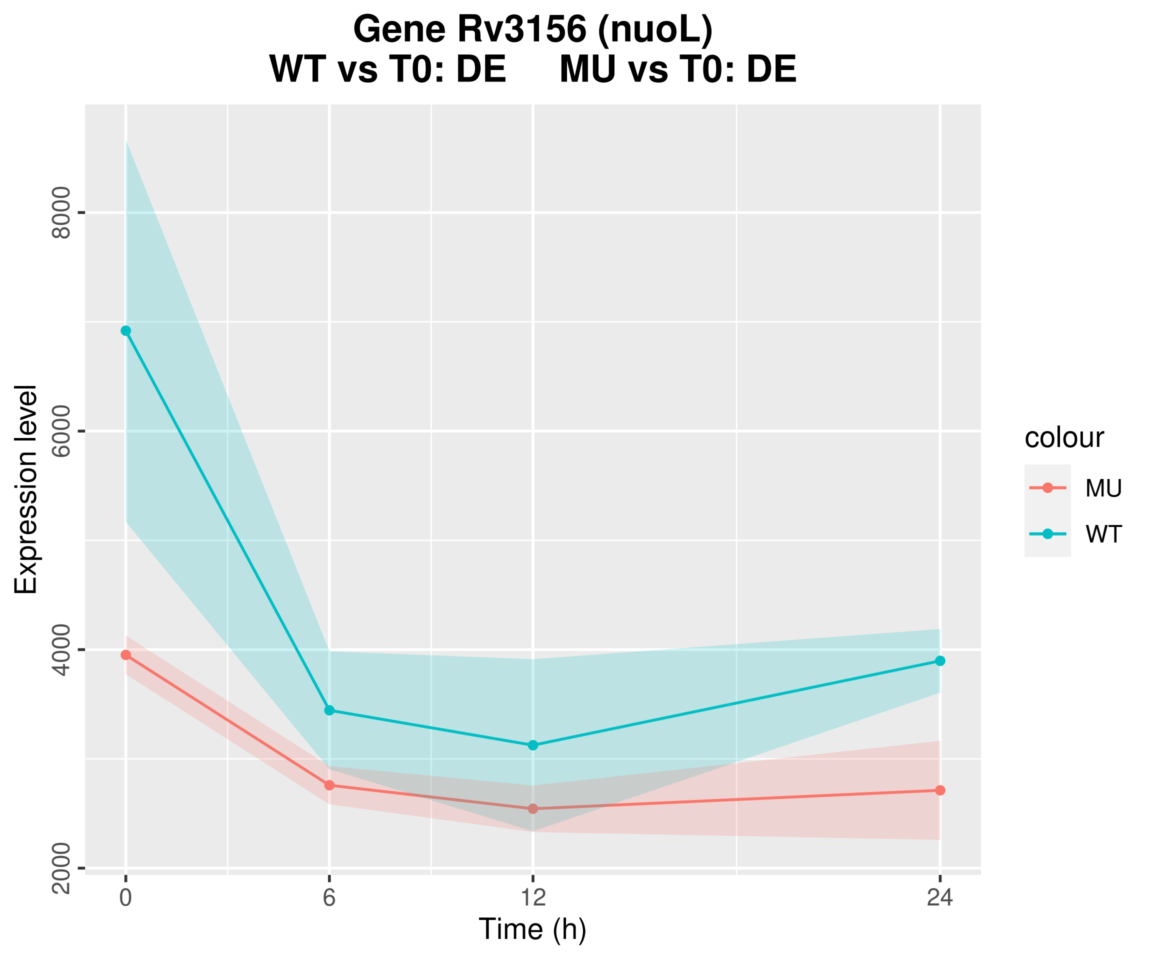

Gene Rv3156 (nuoL)
WT vs T0: DE MU vs T0: DE
Expression level
8000
6000
4000
2000
0
6
12
24
Time (h)
colour
MU
WT

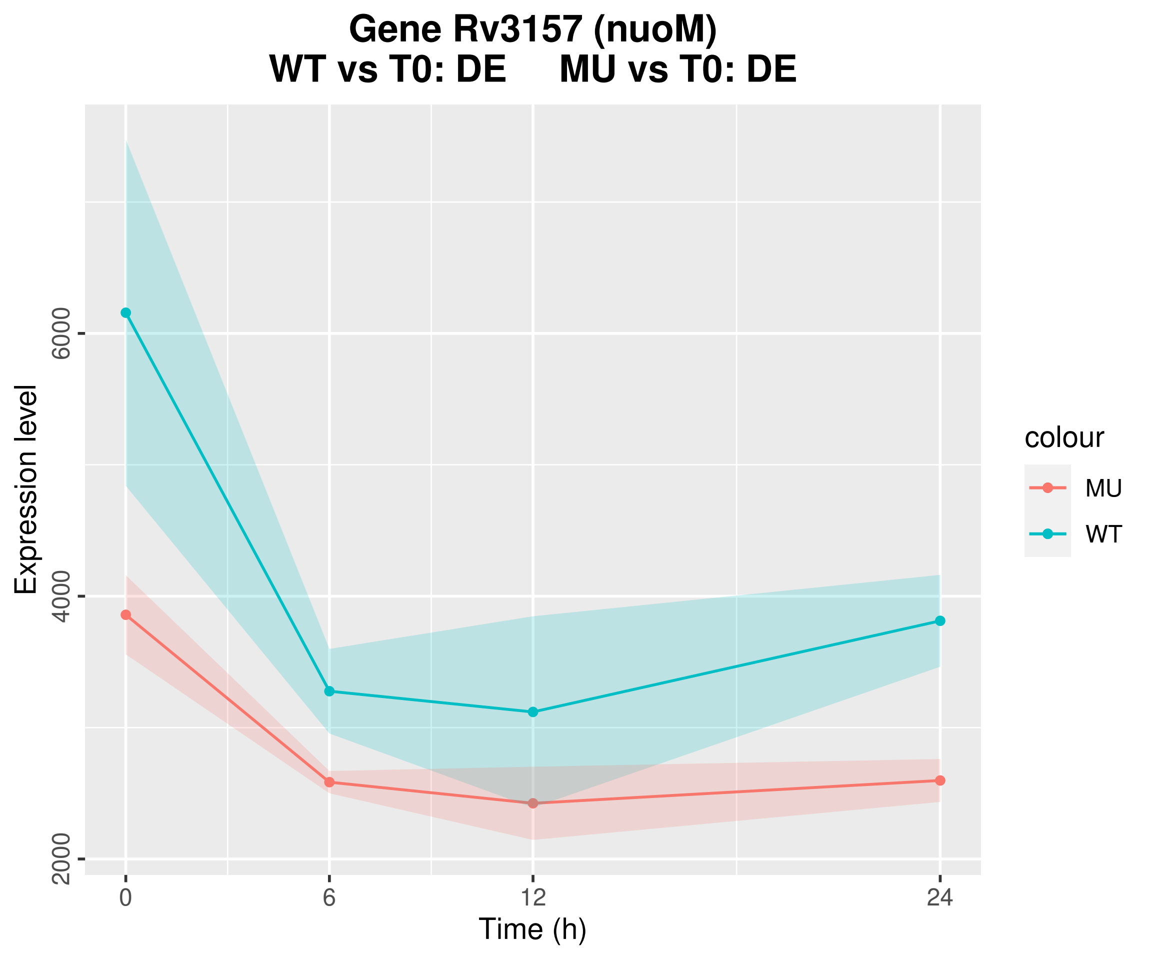
Gene Rv3157 (nuoM)
WT vs T0: DE   MU vs T0: DE
Expression level
2000
4000
6000
0
6
12
24
Time (h)
colour
MU
WT

Gene Rv3158 (nuoN)
WT vs T0: DE   MU vs T0: DE

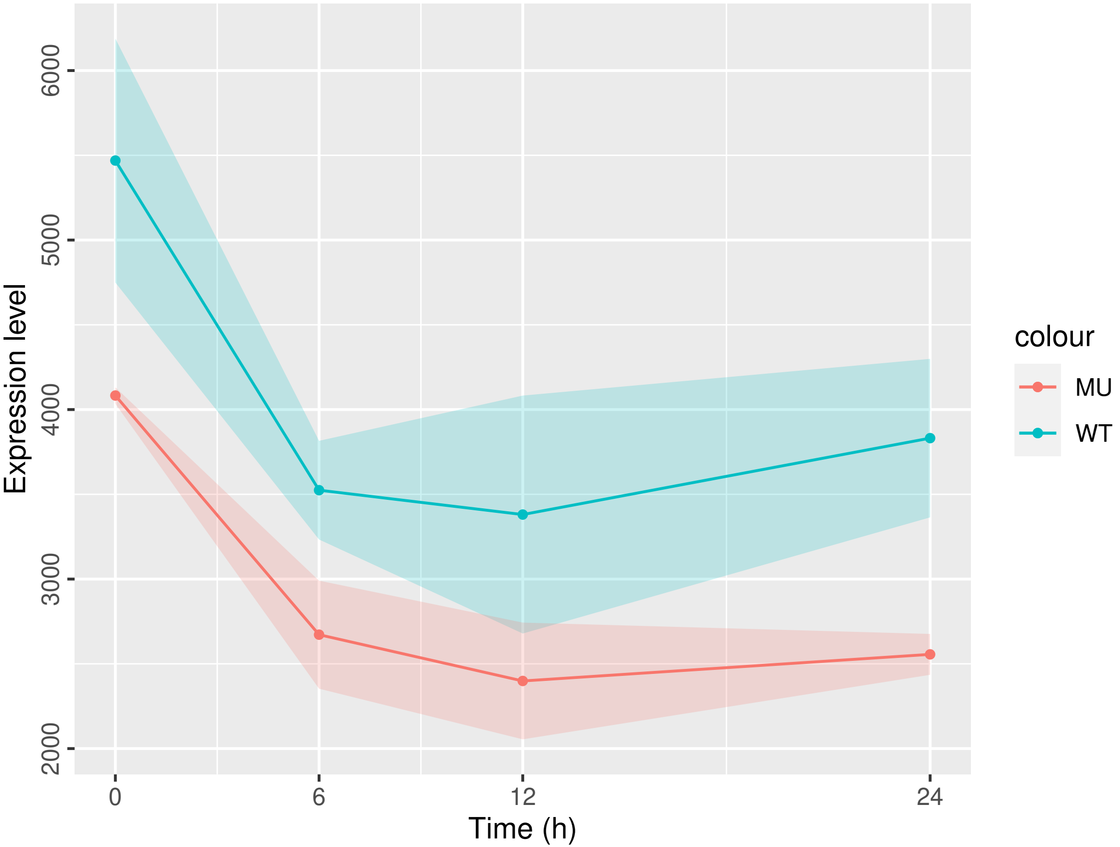

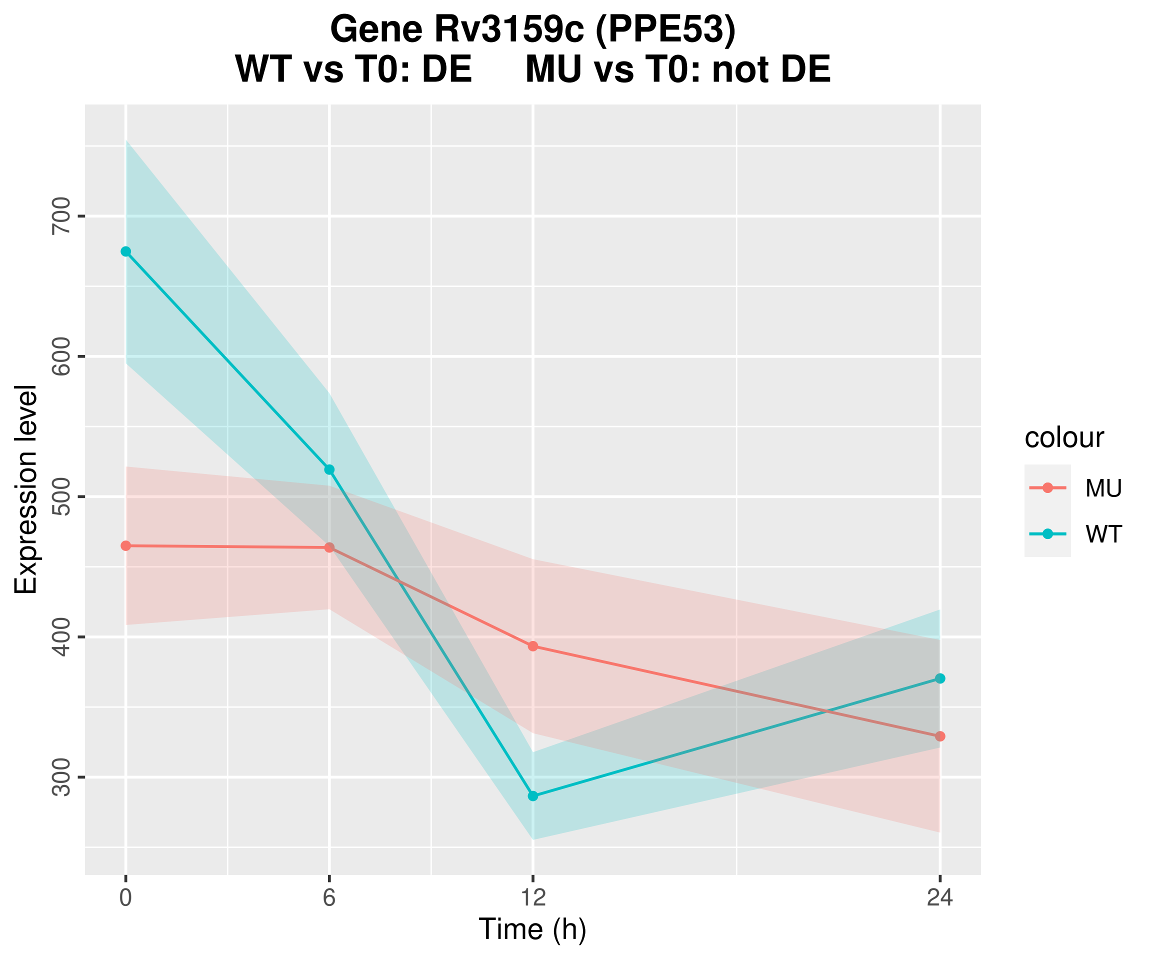

Gene Rv3159c (PPE53)
WT vs T0: DE   MU vs T0: not DE
Expression level
700
600
500
400
300
0
6
12
24
Time (h)
colour
MU
WT

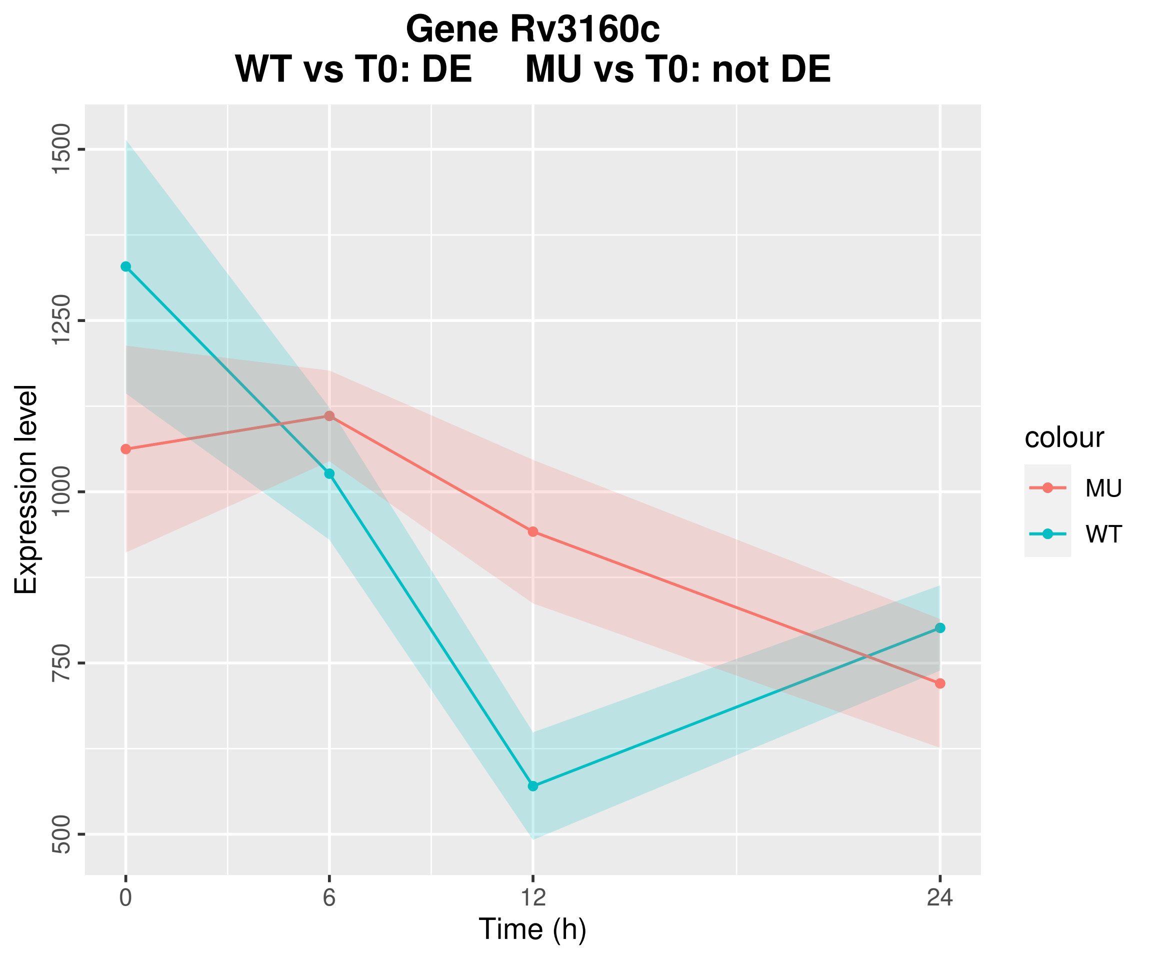

Gene Rv3160c
WT vs T0: DE MU vs T0: not DE
1500
1250
1000
750
500
Expression level
0
6
12
24
Time (h)
colour
MU
WT

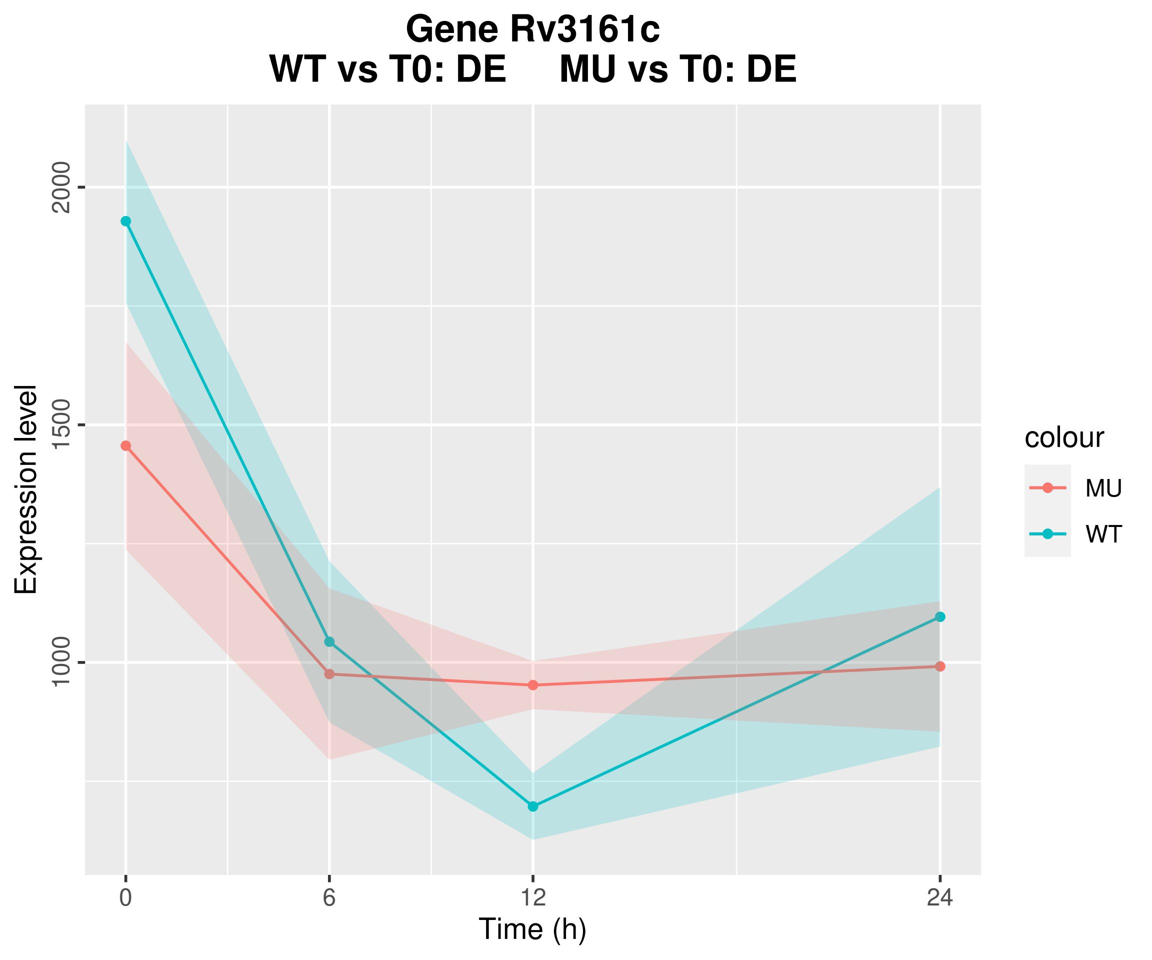
Gene Rv3161c
WT vs T0: DE MU vs T0: DE
2000
1500
1000
Expression level
0
6
12
24
Time (h)
colour
MU
WT

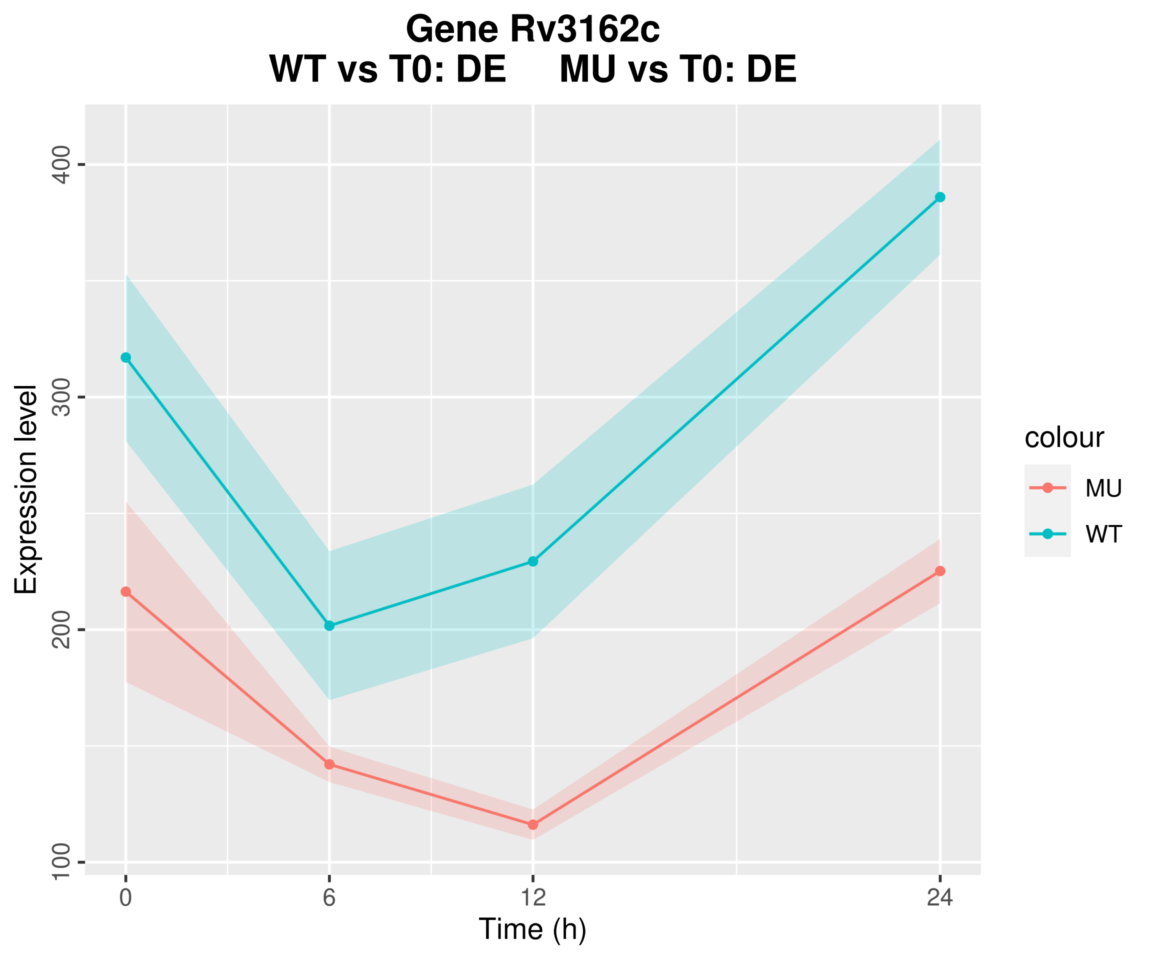

Gene Rv3162c
WT vs T0: DE   MU vs T0: DE
Expression level
400
300
200
100
0
6
12
24
Time (h)
colour
MU
WT

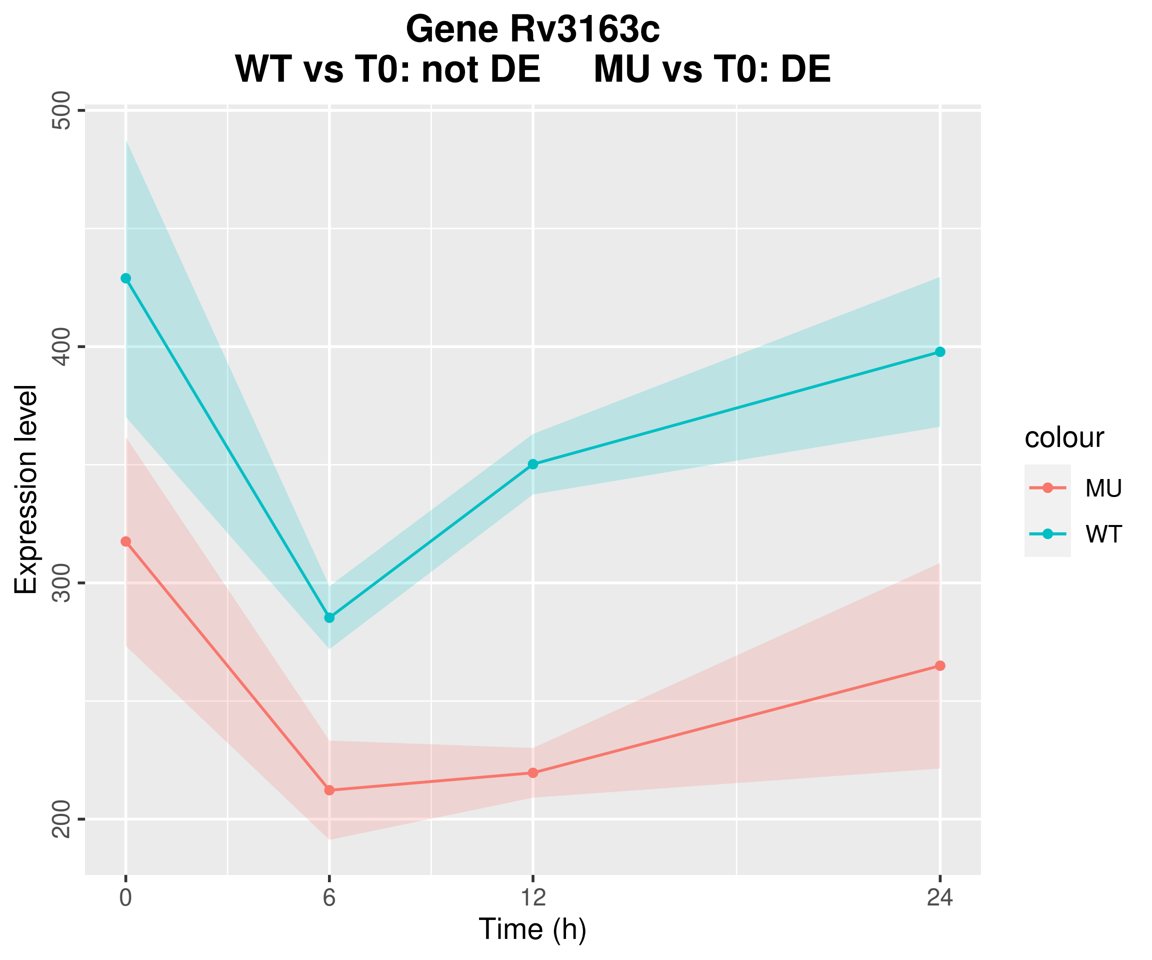
Gene Rv3163c
WT vs T0: not DE   MU vs T0: DE
Expression level
500
400
300
200
0
6
12
24
Time (h)
colour
MU
WT

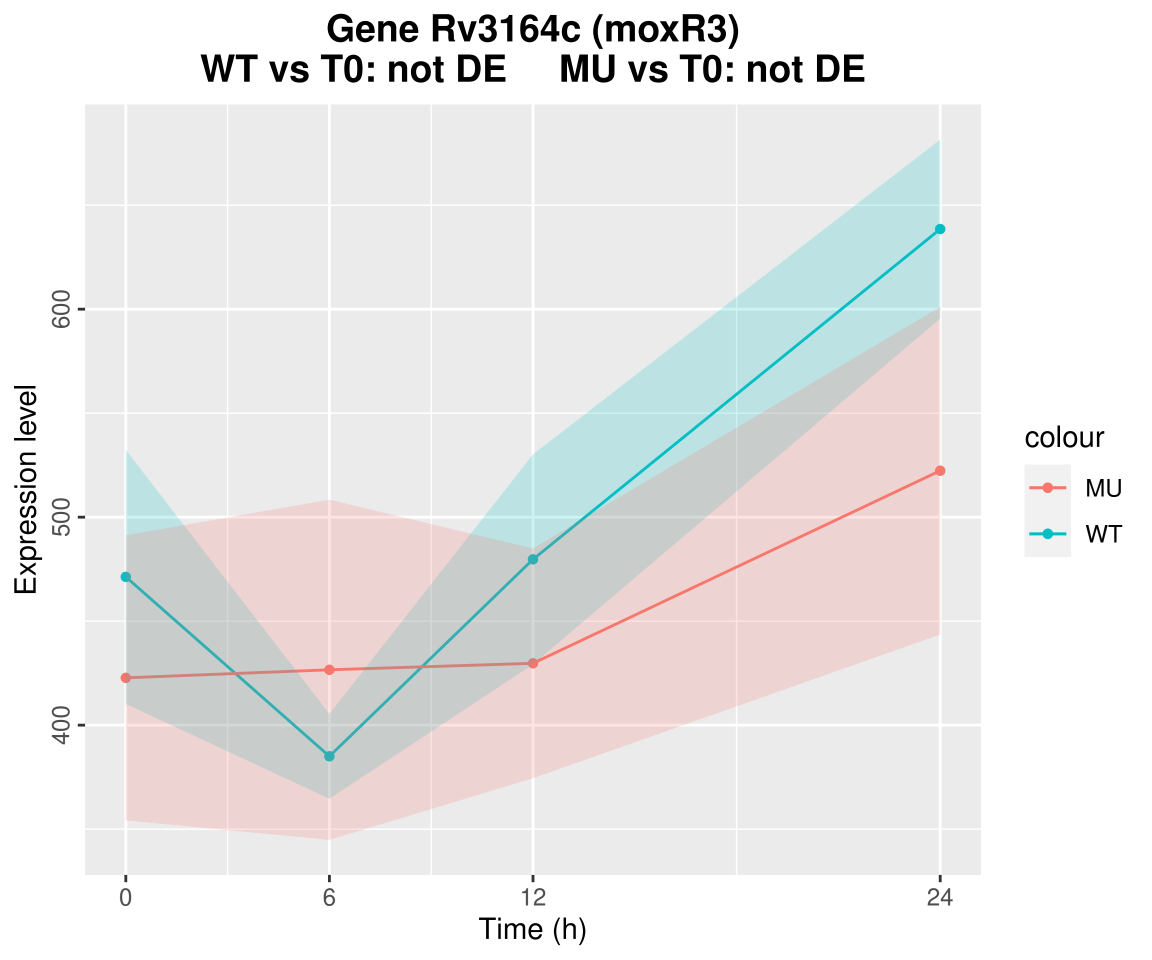

Gene Rv3164c (moxR3)
WT vs T0: not DE MU vs T0: not DE
Expression level
400
500
600
0
6
12
24
Time (h)
colour
MU
WT

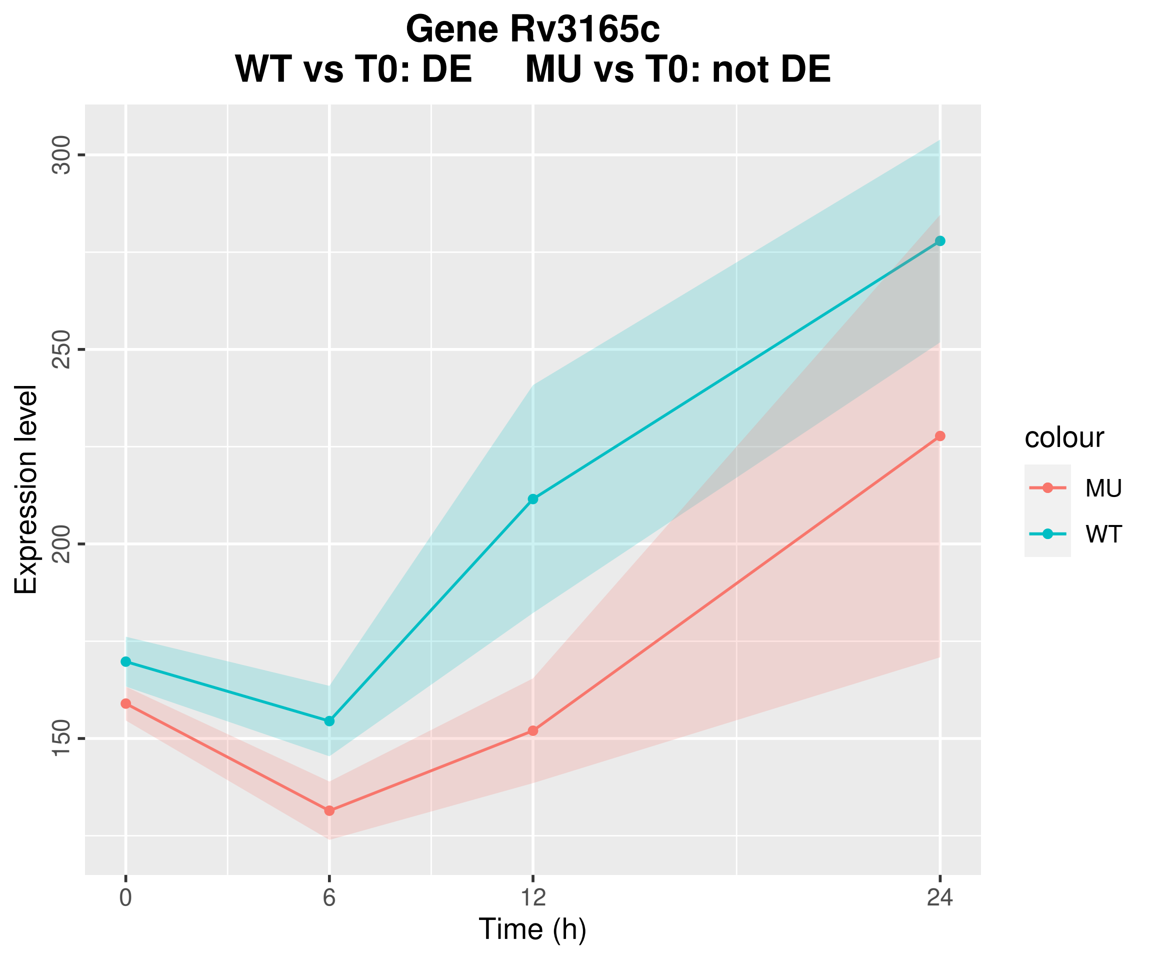

Gene Rv3165c
WT vs T0: DE MU vs T0: not DE
Expression level
300
250
200
150
0
6
12
24
Time (h)
colour
MU
WT

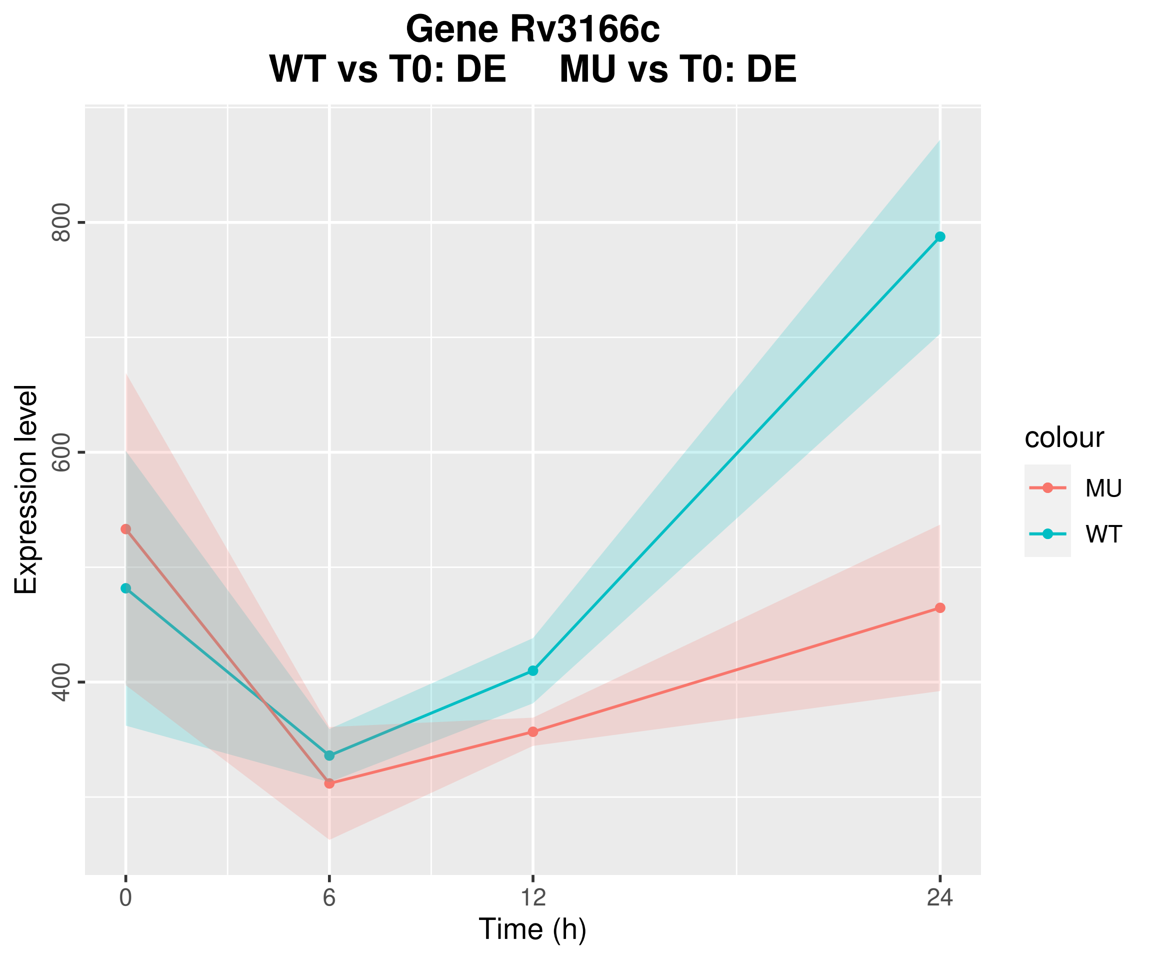
Gene Rv3166c
WT vs T0: DE MU vs T0: DE
Expression level
800
600
400
0
6
12
24
Time (h)
colour
MU
WT

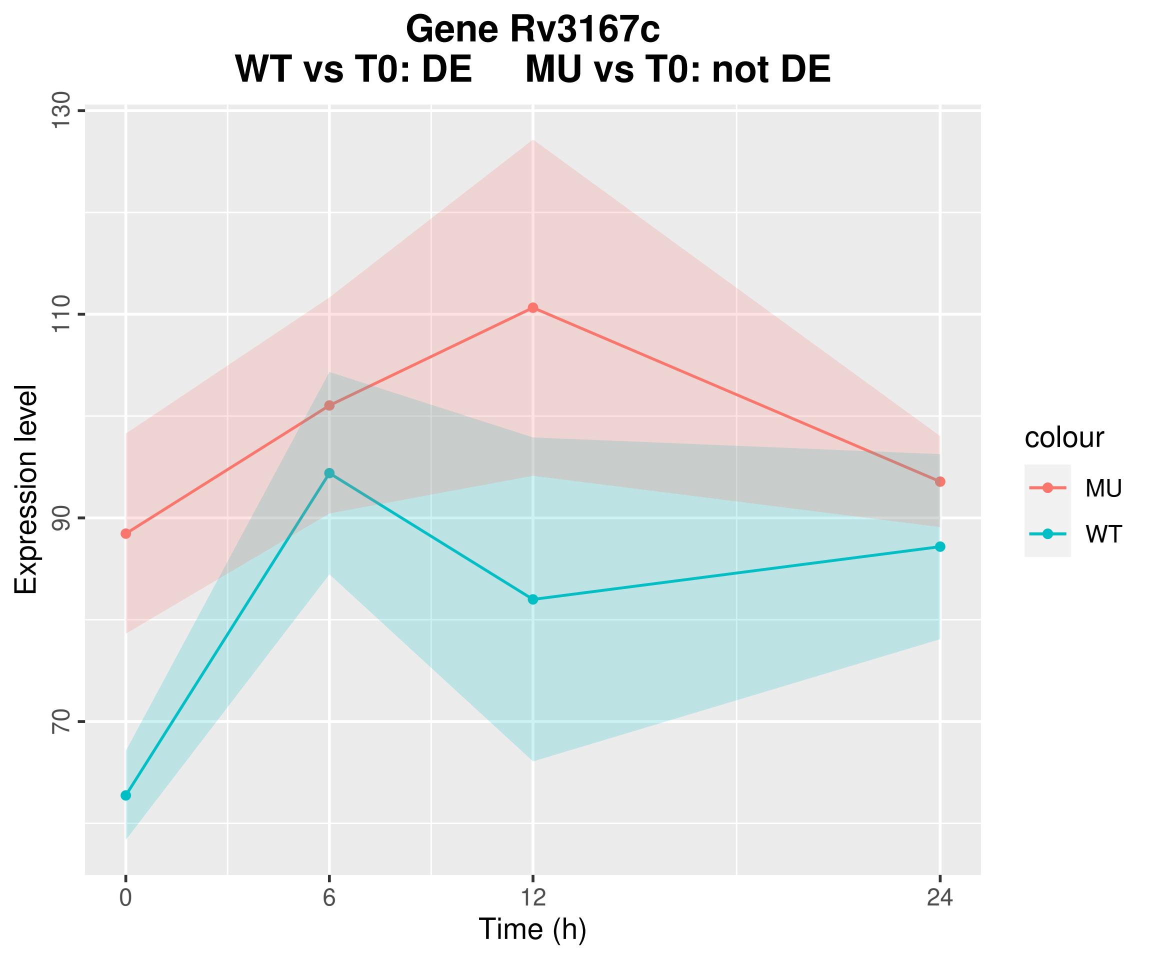
Gene Rv3167c
WT vs T0: DE   MU vs T0: not DE
Expression level
130
110
90
70
0
6
12
24
Time (h)
colour
MU
WT

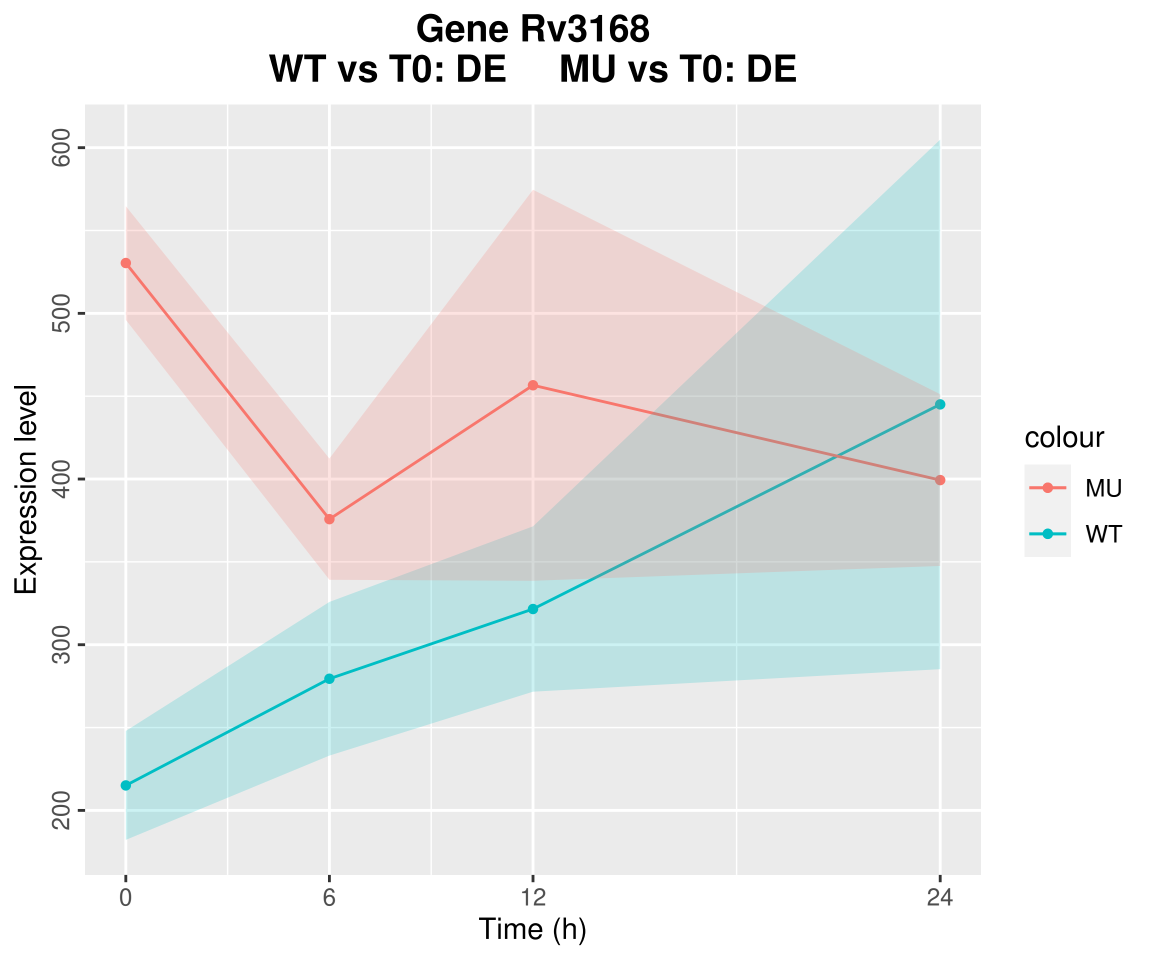
Gene Rv3168
WT vs T0: DE MU vs T0: DE
Expression level
600
500
400
300
200
0
6
12
24
Time (h)
colour
MU
WT

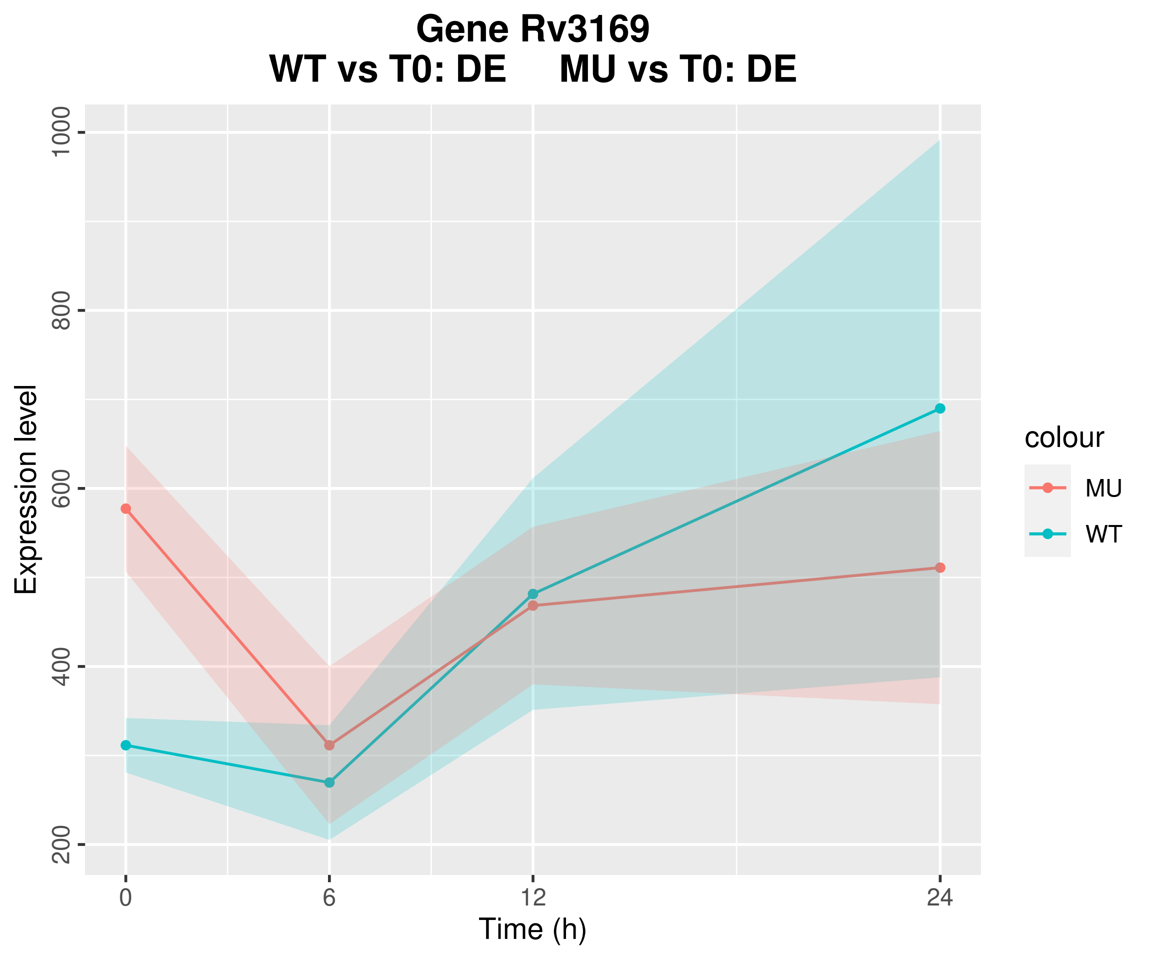

Gene Rv3169
WT vs T0: DE   MU vs T0: DE
Expression level
1000
800
600
400
200
0
6
12
24
Time (h)
colour
MU
WT

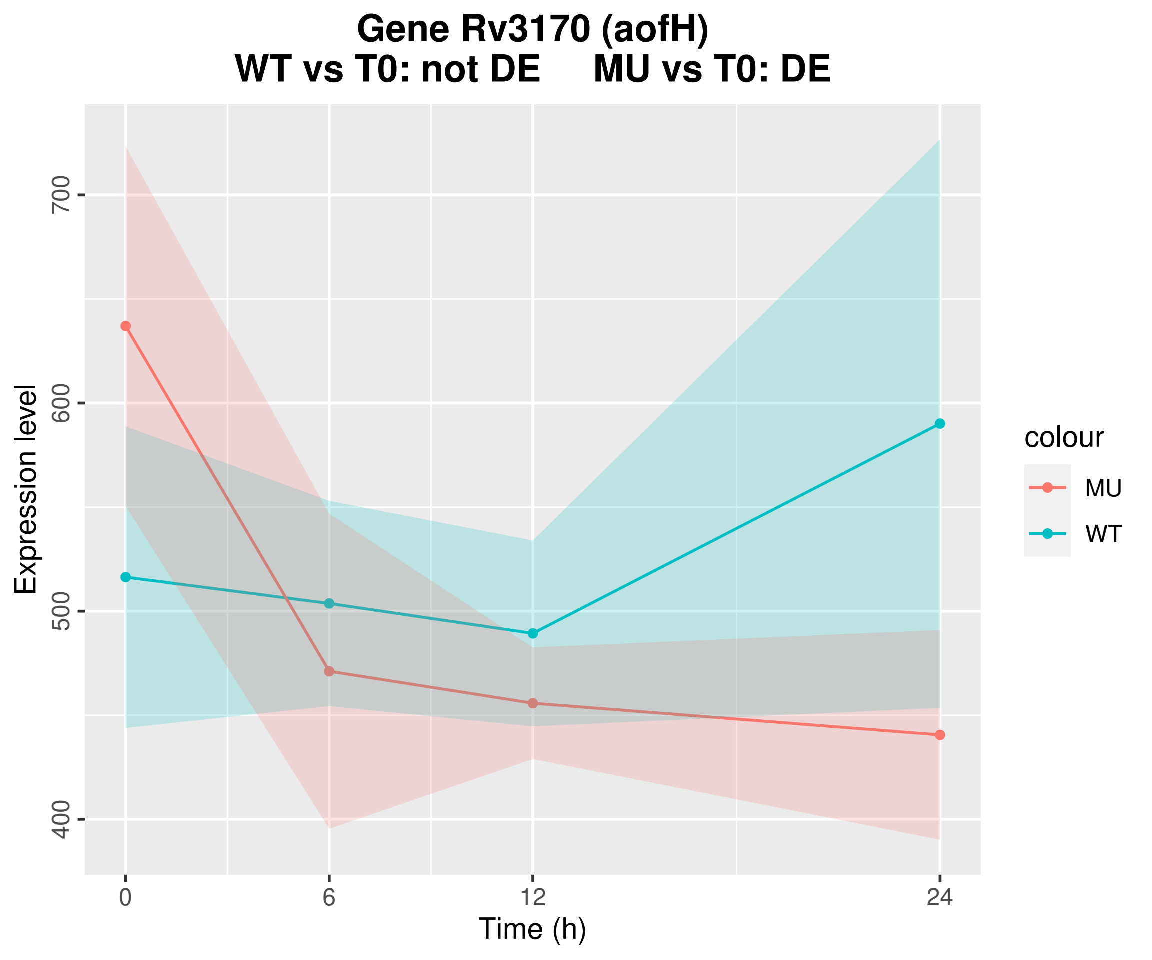

Gene Rv3170 (aofH)
WT vs T0: not DE   MU vs T0: DE
Expression level
700
600
500
400
0
6
12
24
Time (h)
colour
MU
WT

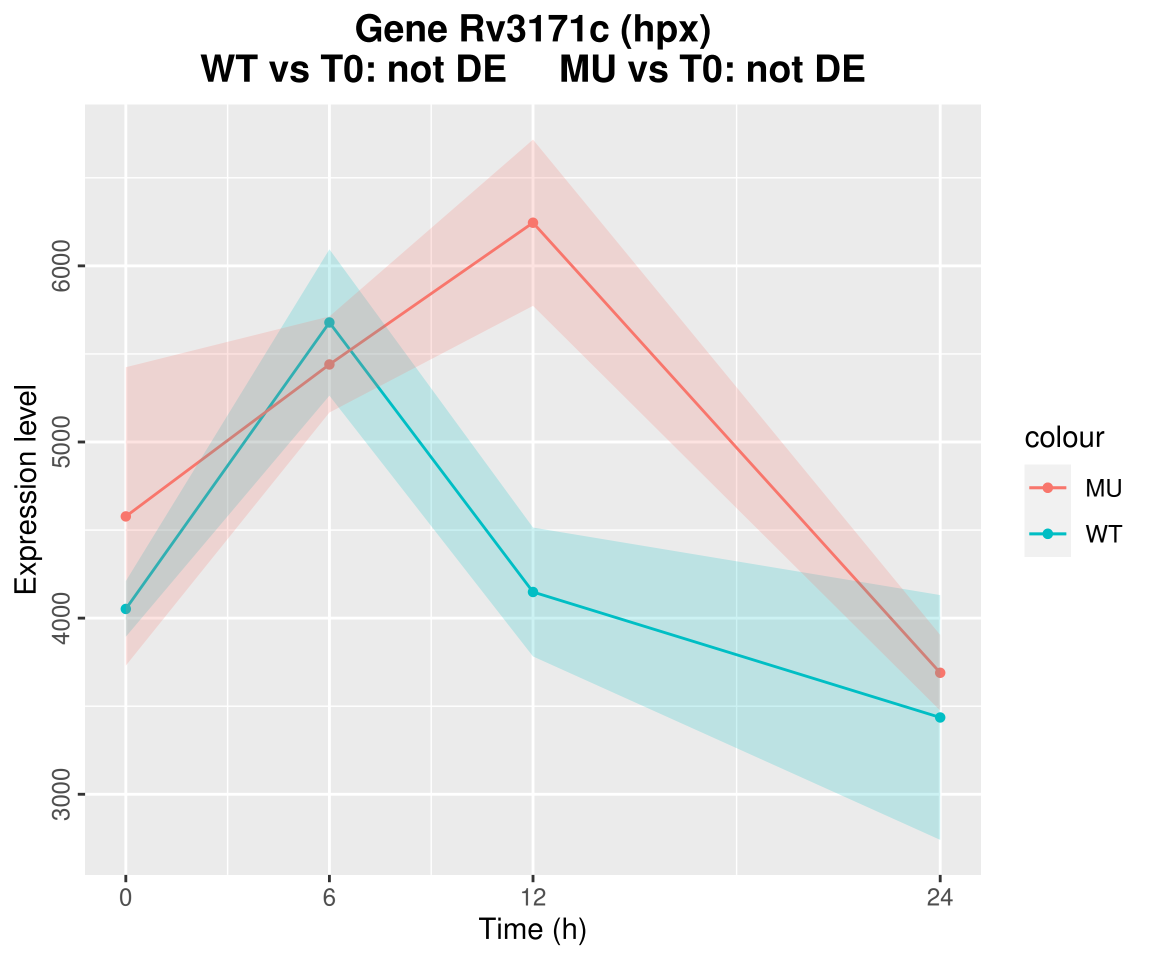
Gene Rv3171c (hpx)
WT vs T0: not DE MU vs T0: not DE
Expression level
3000
4000
5000
6000
0
6
12
24
Time (h)
colour
MU
WT

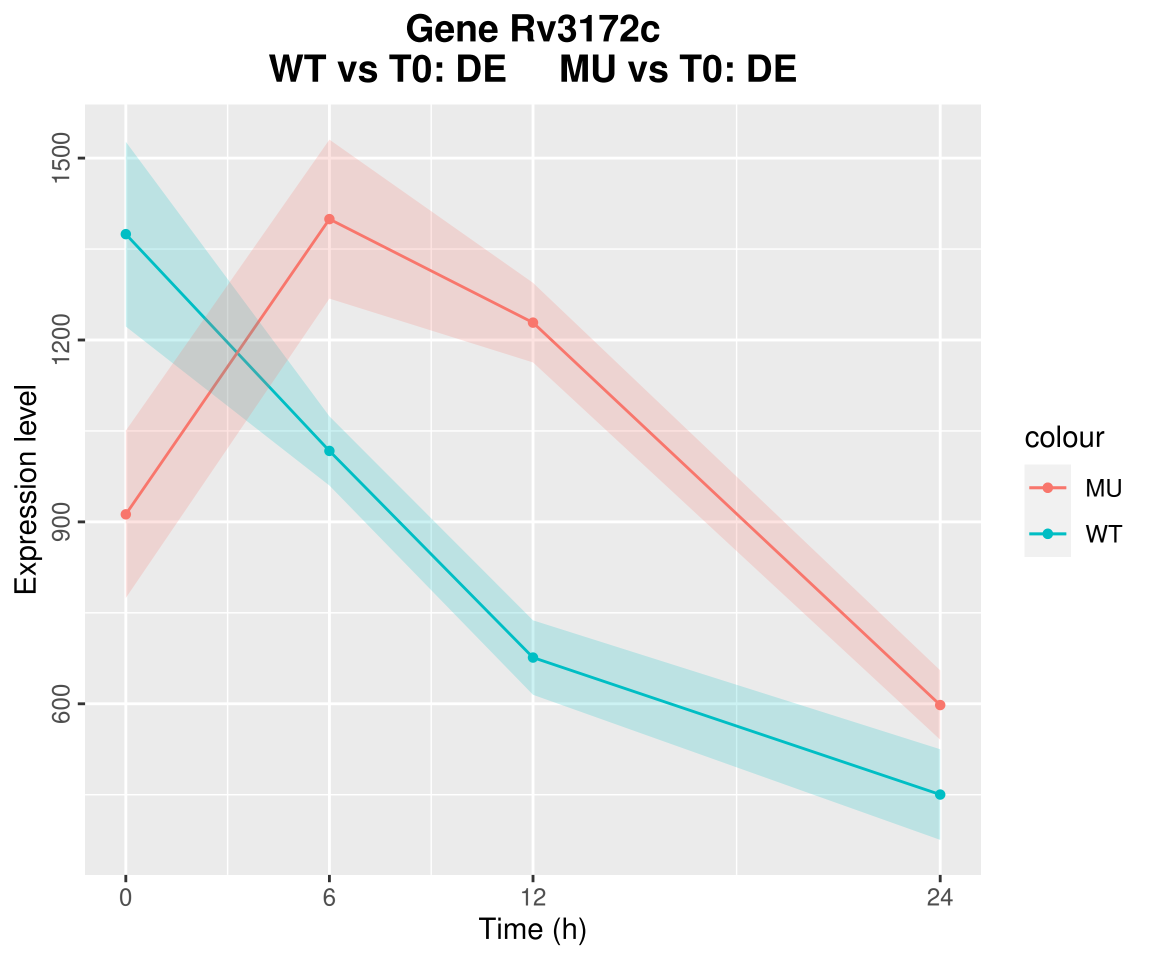

Gene Rv3172c
WT vs T0: DE   MU vs T0: DE
Expression level
1500
1200
900
600
0
6
12
24
Time (h)
colour
MU
WT

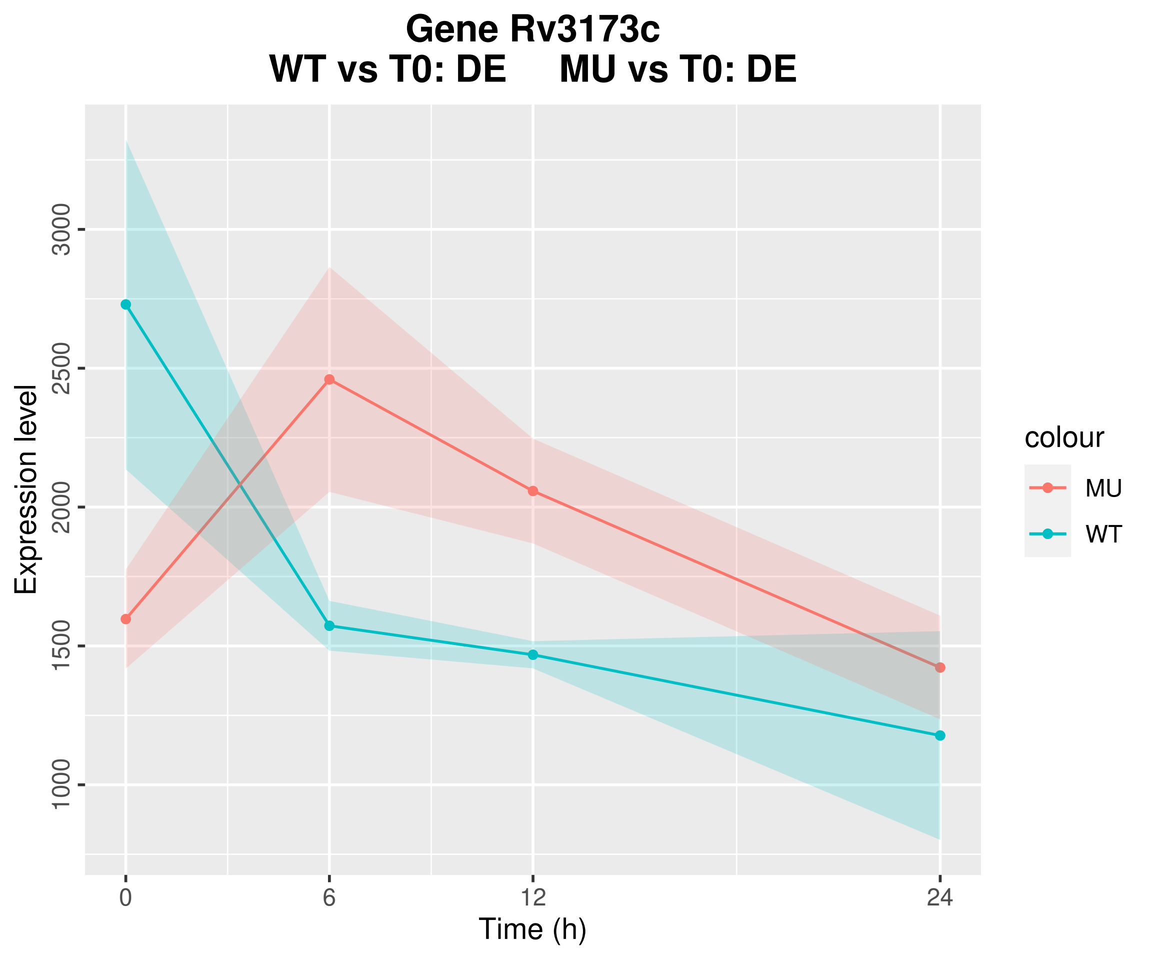
Gene Rv3173c
WT vs T0: DE MU vs T0: DE
Expression level
3000
2500
2000
1500
1000
0
6
12
24
Time (h)
colour
MU
WT

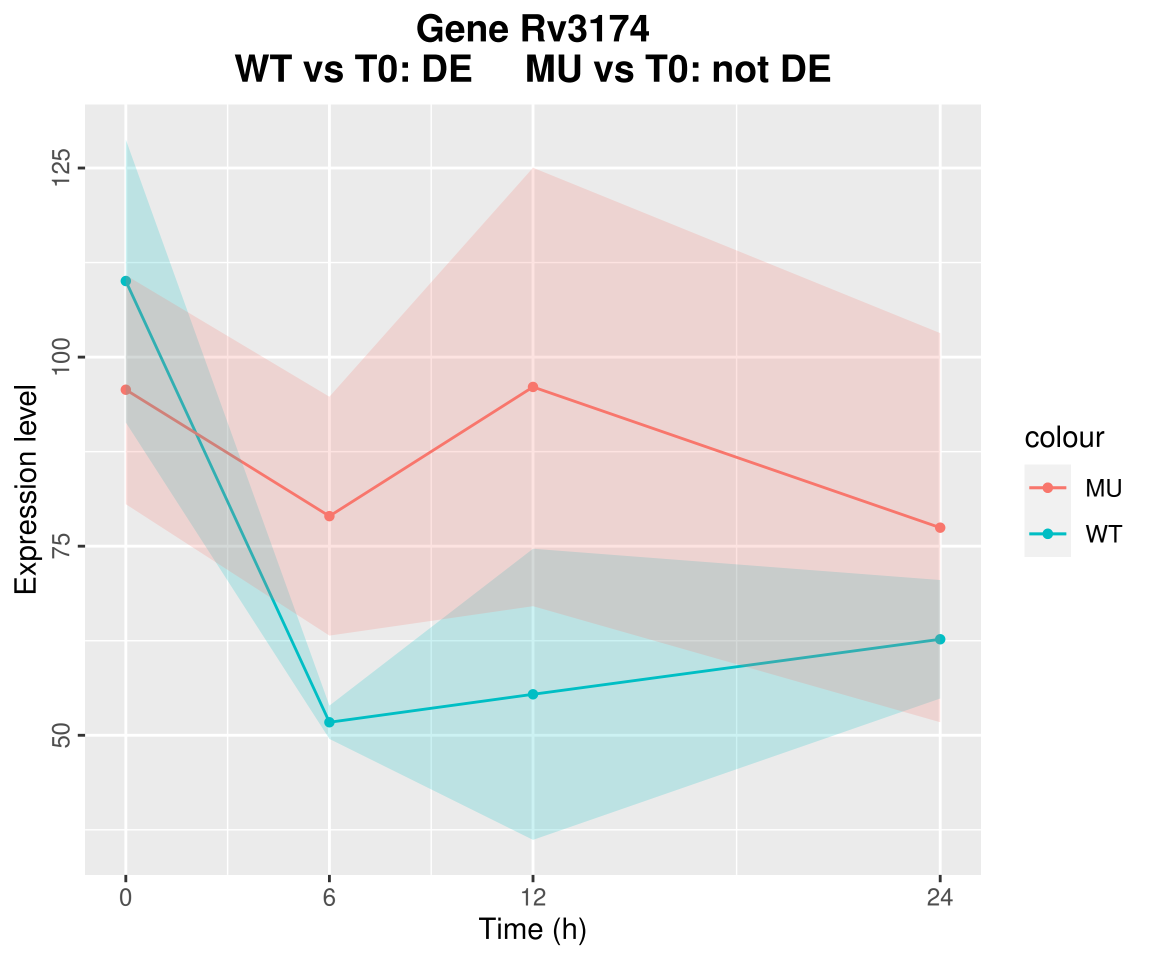
Gene Rv3174
WT vs T0: DE   MU vs T0: not DE
Expression level
125
100
75
50
0
6
12
24
Time (h)
colour
MU
WT

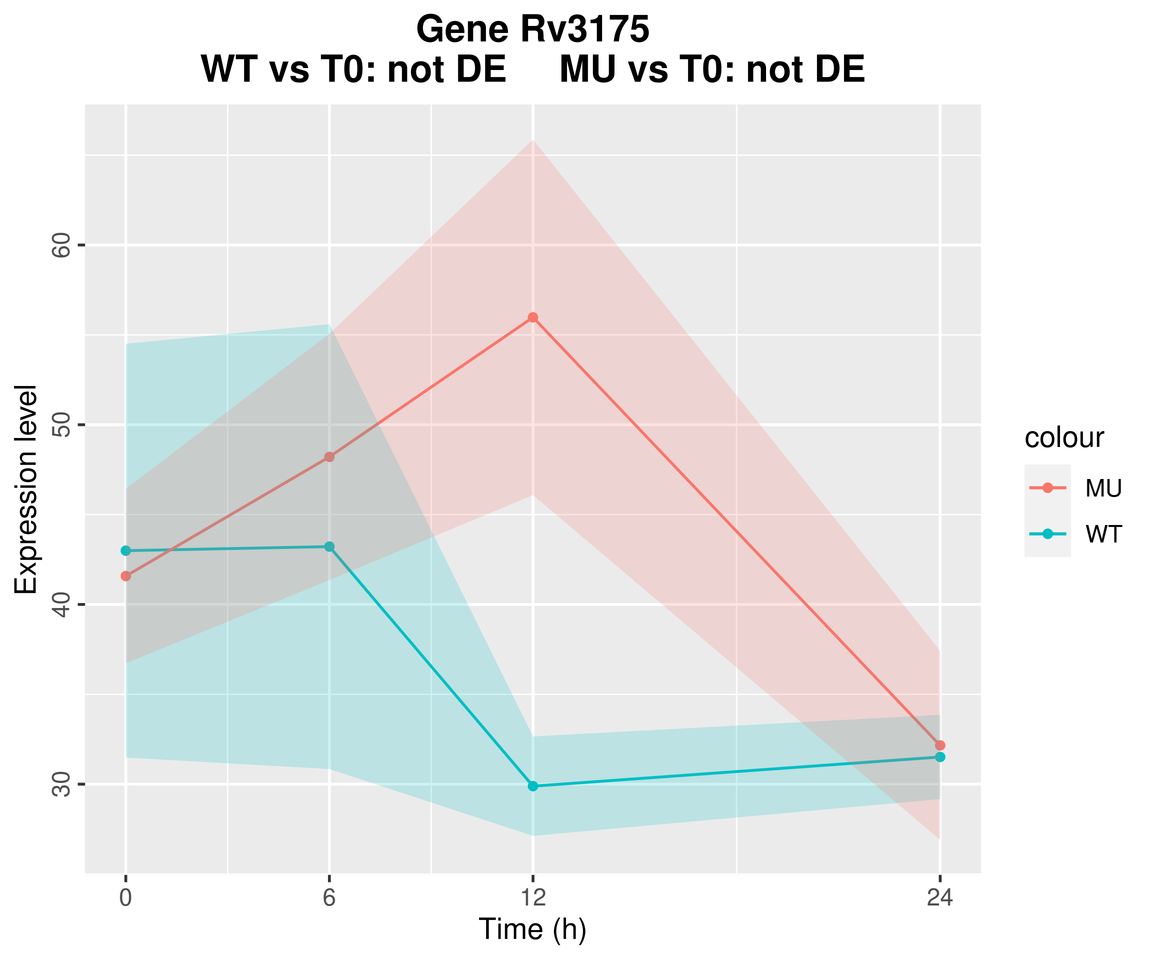

Gene Rv3175
WT vs T0: not DE MU vs T0: not DE
Expression level
30
40
50
60
0
6
12
24
Time (h)
colour
MU
WT

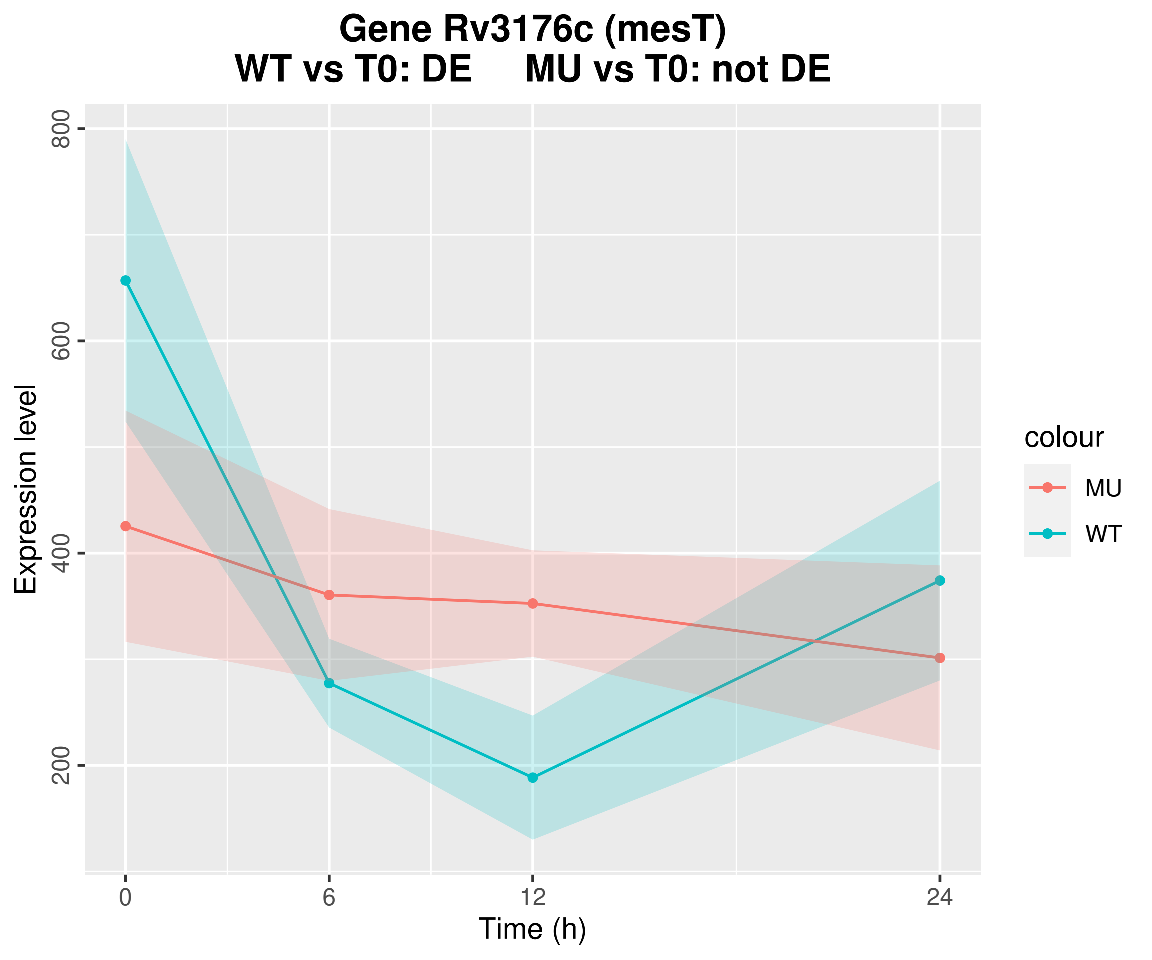
Gene Rv3176c (mesT)
WT vs T0: DE   MU vs T0: not DE
Expression level
800
600
400
200
0
6
12
24
Time (h)
colour
MU
WT

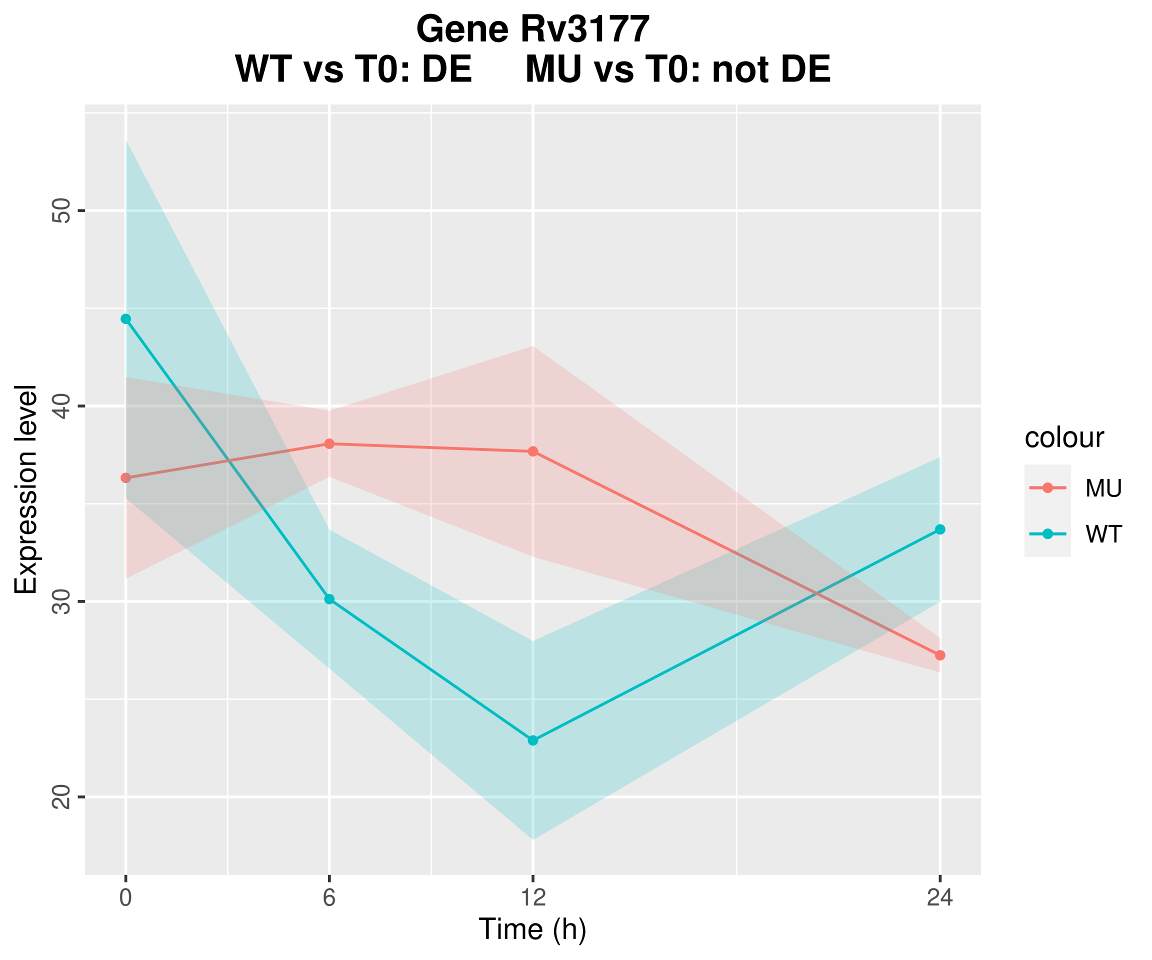

Gene Rv3177
WT vs T0: DE MU vs T0: not DE
Expression level
50
40
30
20
0
6
12
24
Time (h)
colour
MU
WT

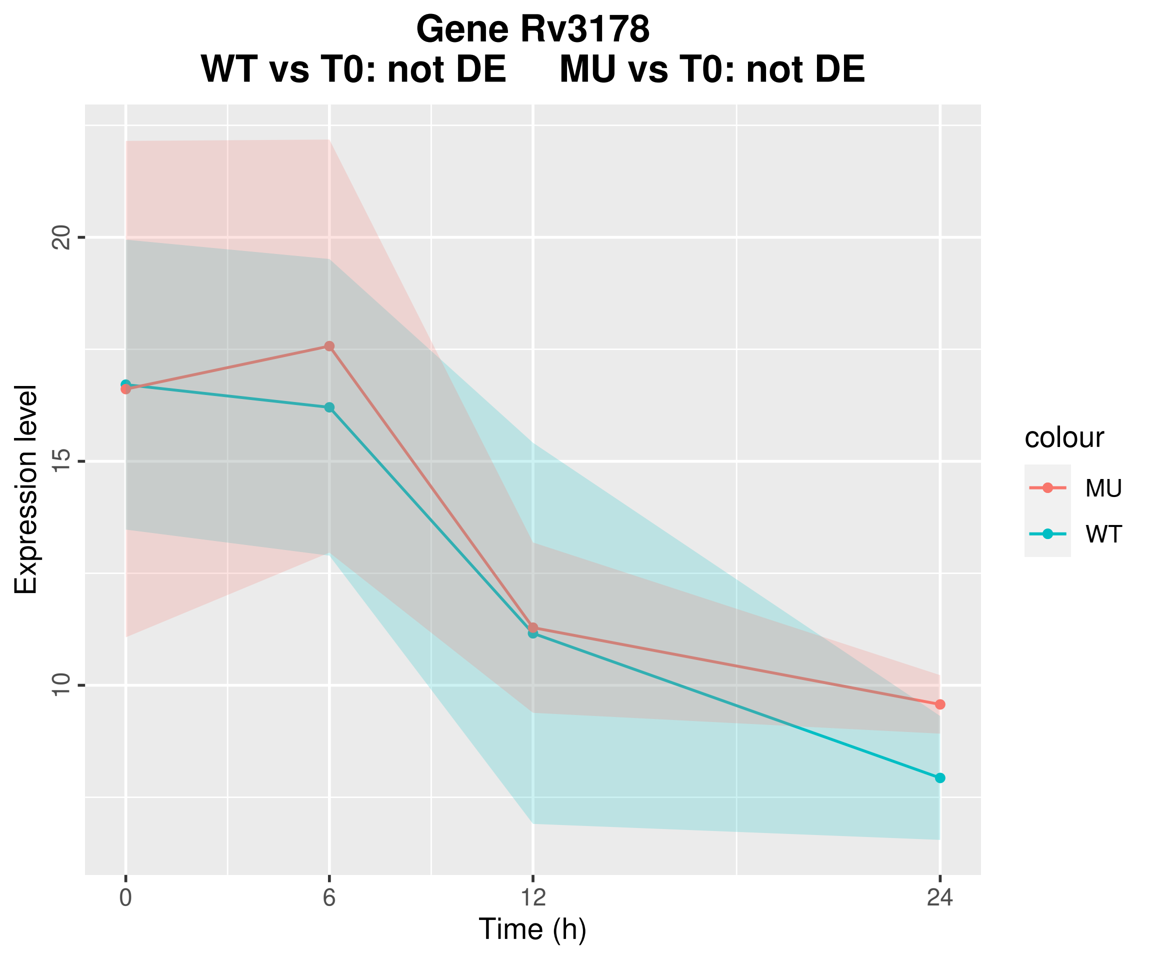
Gene Rv3178
WT vs T0: not DE   MU vs T0: not DE
Expression level
20
15
10
0
6
12
24
Time (h)
colour
MU
WT

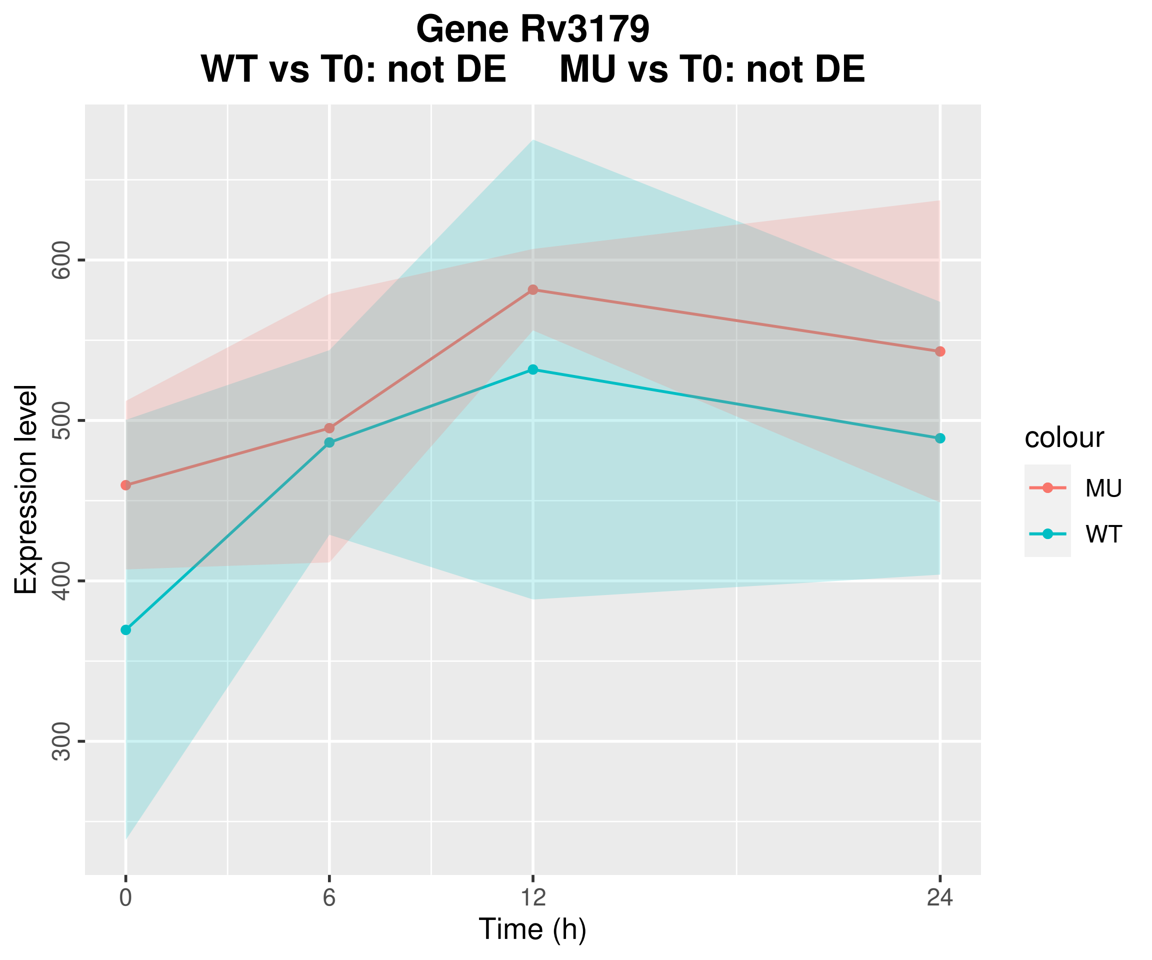

Gene Rv3179
WT vs T0: not DE MU vs T0: not DE
Expression level
300
400
500
600
0
6
12
24
Time (h)
colour
MU
WT

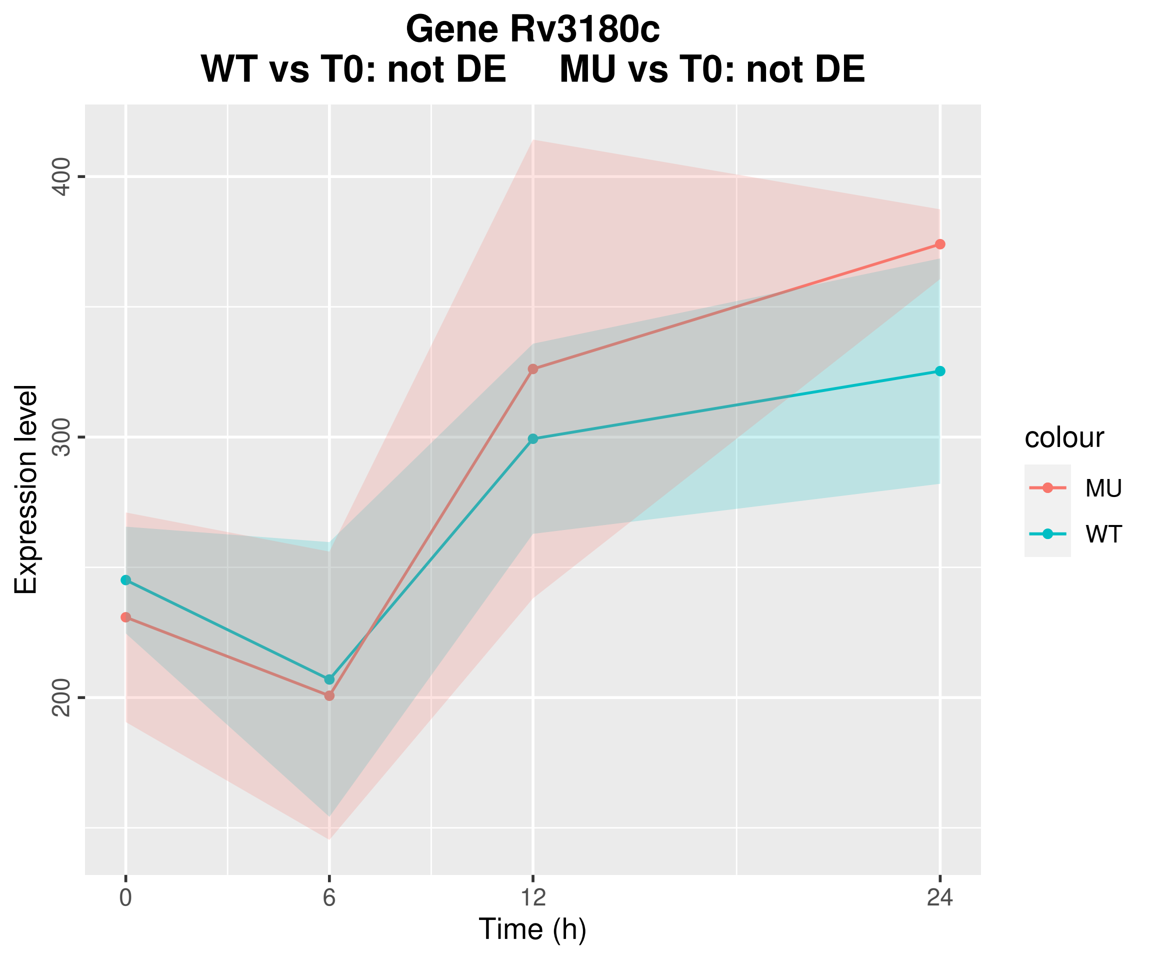
Gene Rv3180c
WT vs T0: not DE   MU vs T0: not DE
Expression level
400
300
200
0
6
12
24
Time (h)
colour
MU
WT

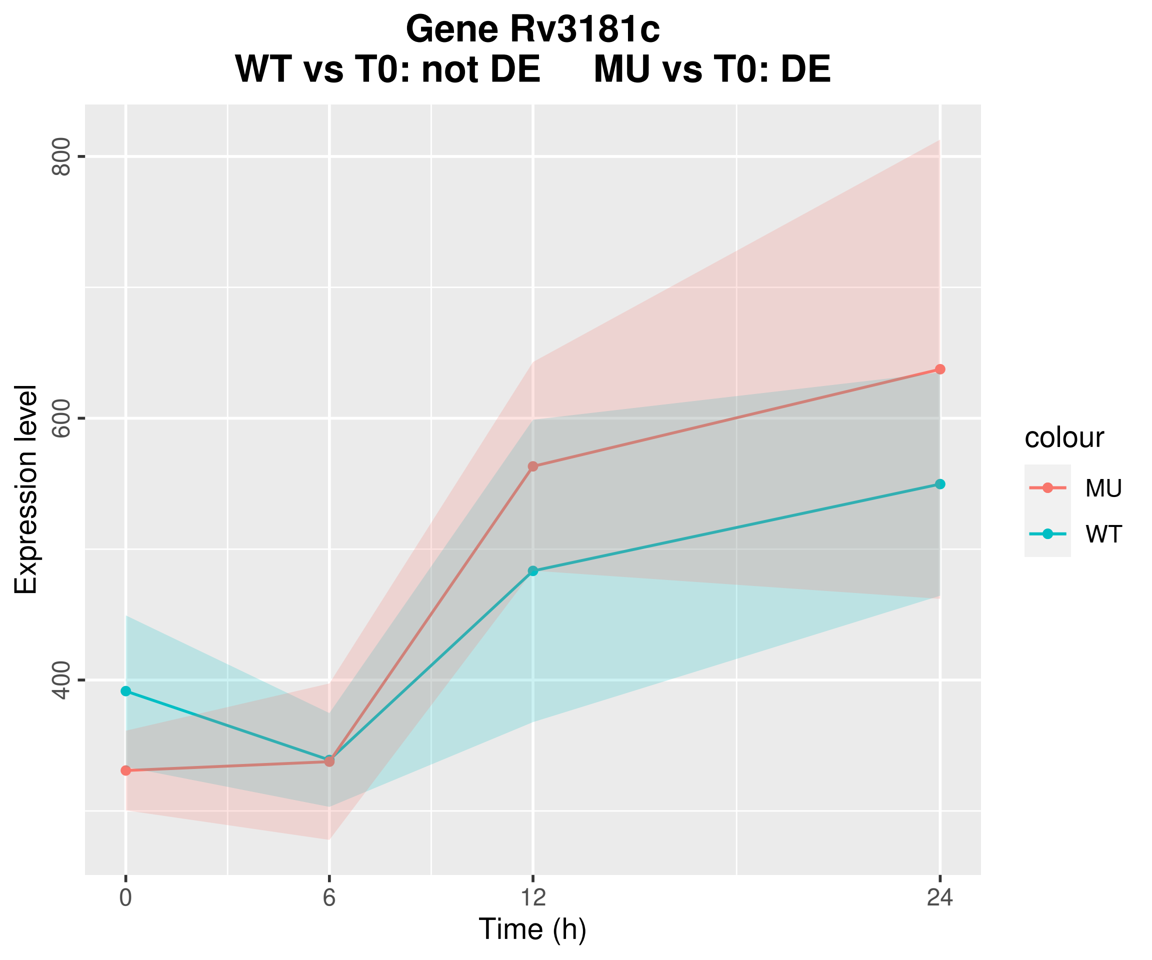
Gene Rv3181c
WT vs T0: not DE MU vs T0: DE
Expression level
800
600
400
0
6
12
24
Time (h)
colour
MU
WT

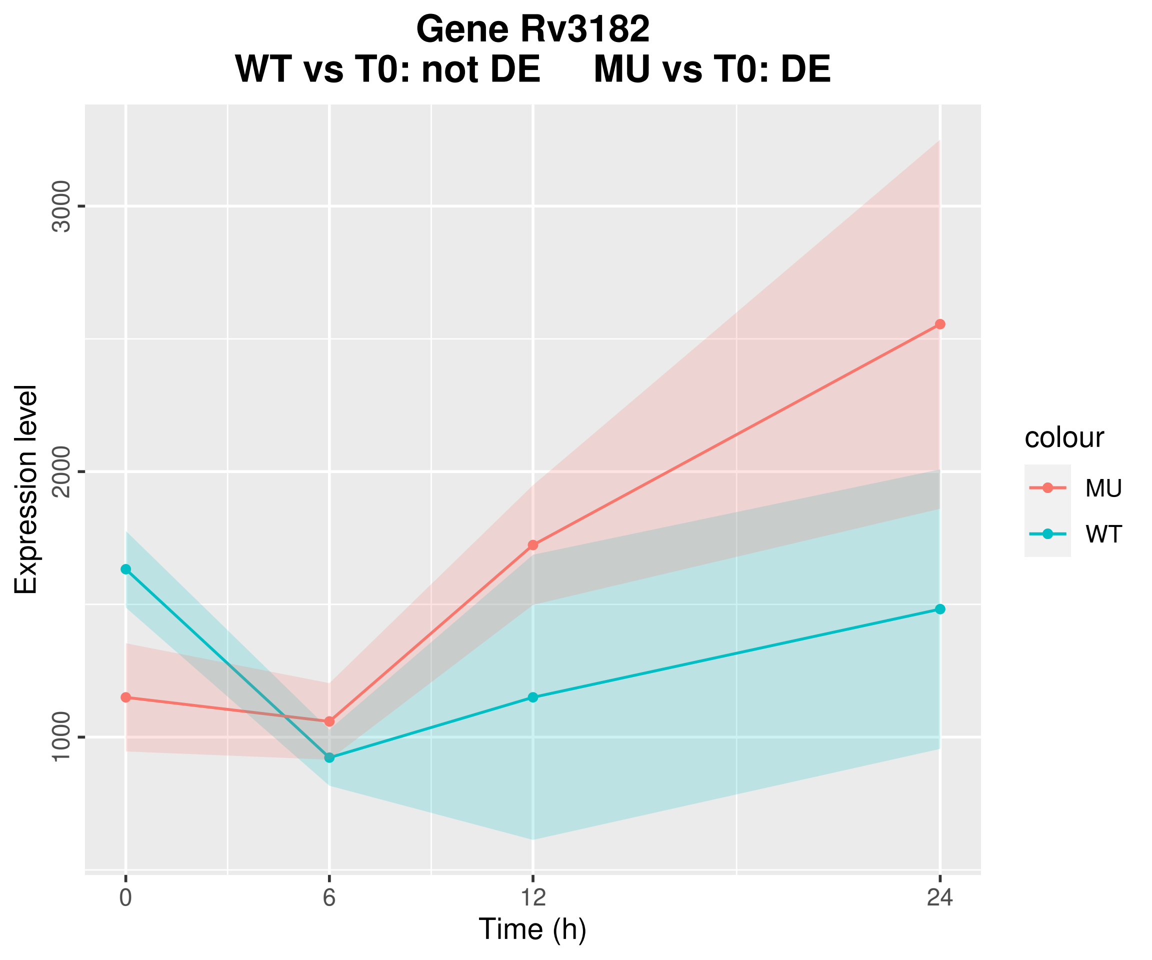

Gene Rv3182
WT vs T0: not DE   MU vs T0: DE
Expression level
3000
2000
1000
0
6
12
24
Time (h)
colour
MU
WT

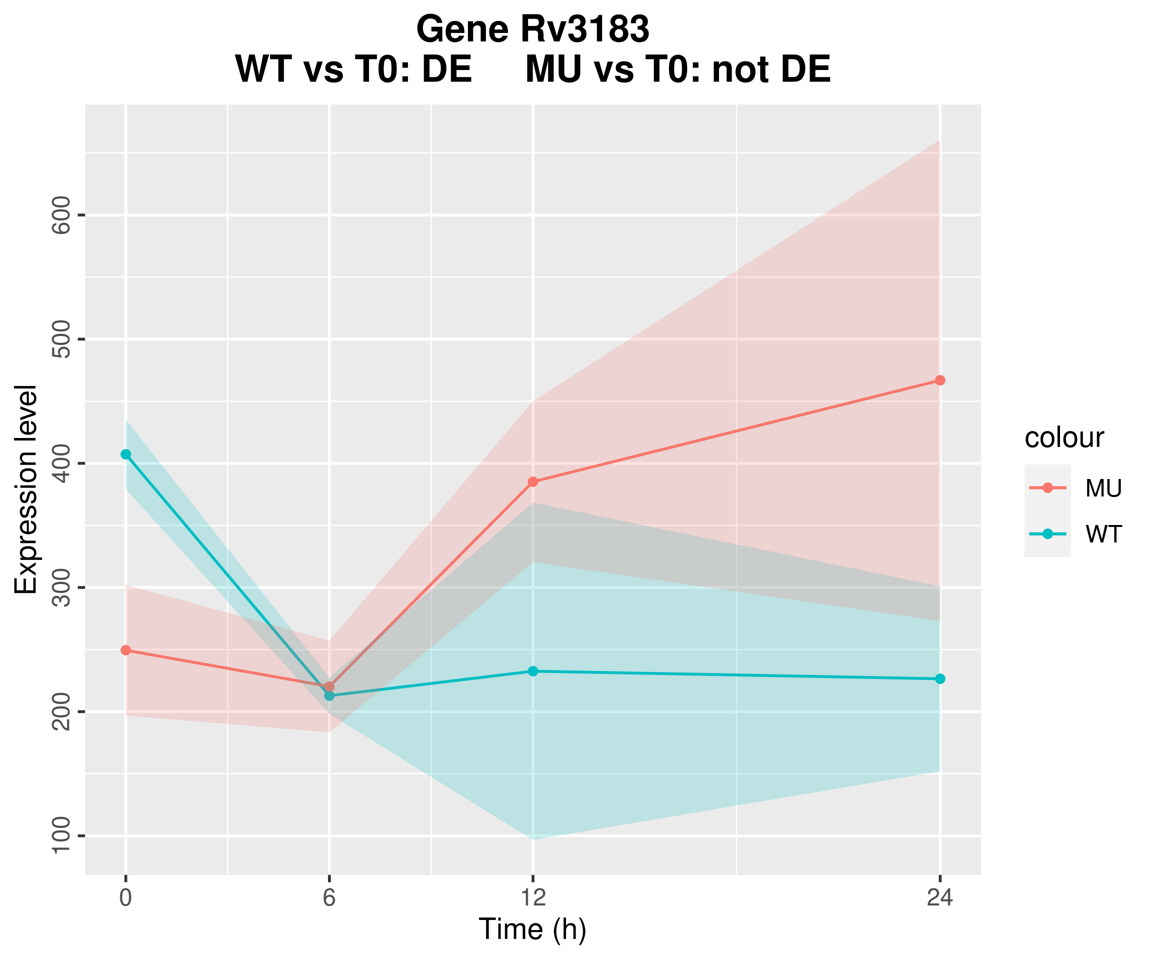

Gene Rv3183
WT vs T0: DE   MU vs T0: not DE
Expression level
100
200
300
400
500
600
0
6
12
24
Time (h)
colour
MU
WT

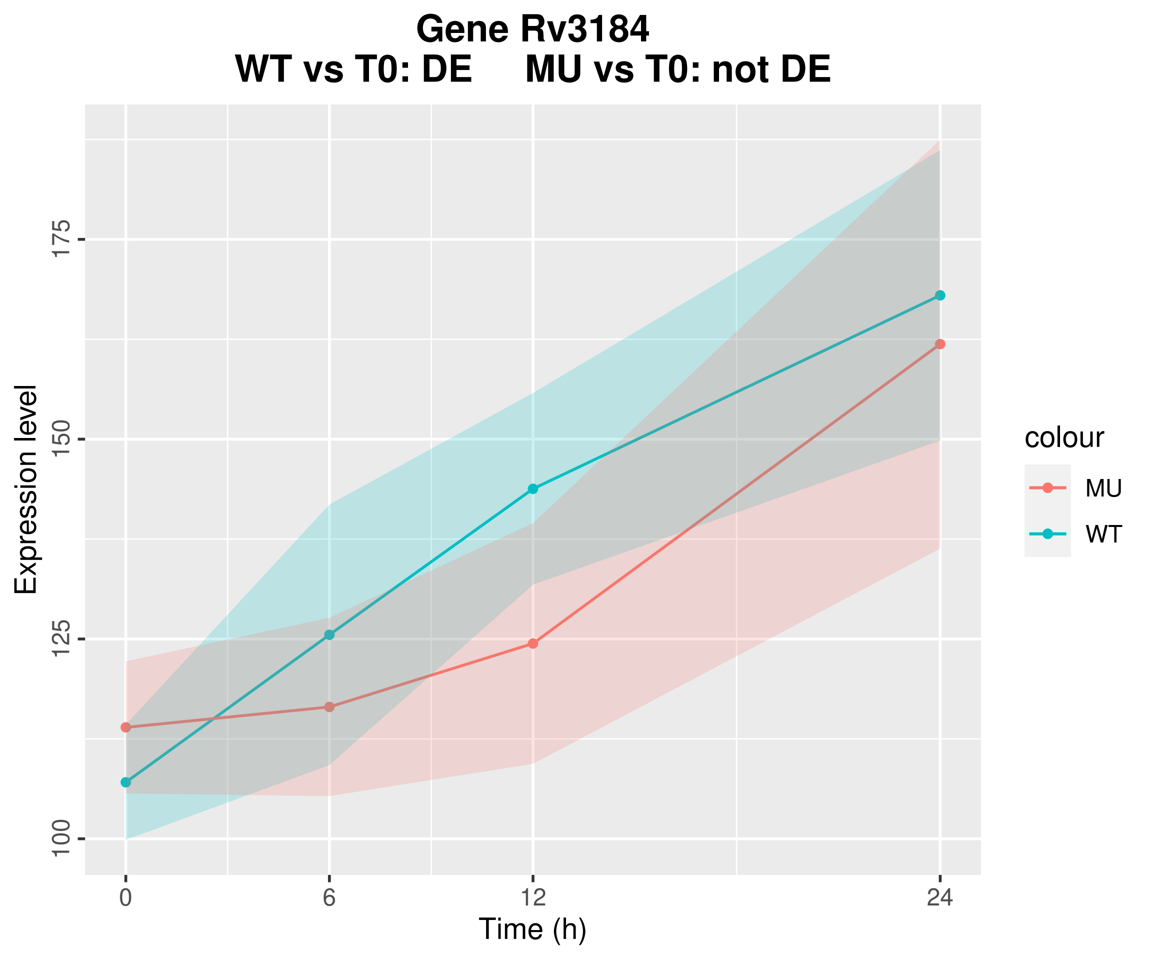
Gene Rv3184
WT vs T0: DE MU vs T0: not DE
Expression level
100
125
150
175
0
6
12
24
Time (h)
colour
MU
WT

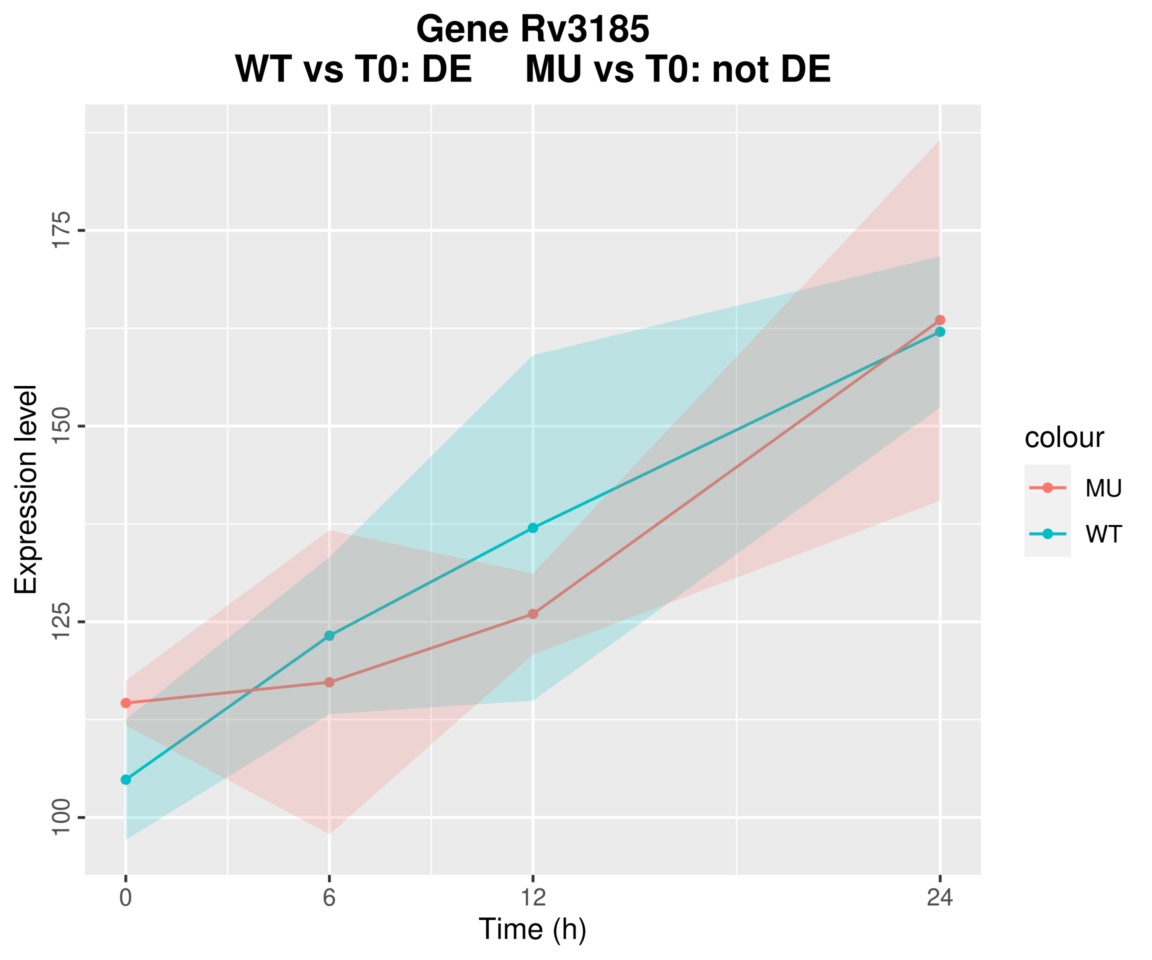
Gene Rv3185
WT vs T0: DE   MU vs T0: not DE
Expression level
175
150
125
100
0
6
12
24
Time (h)
colour
MU
WT

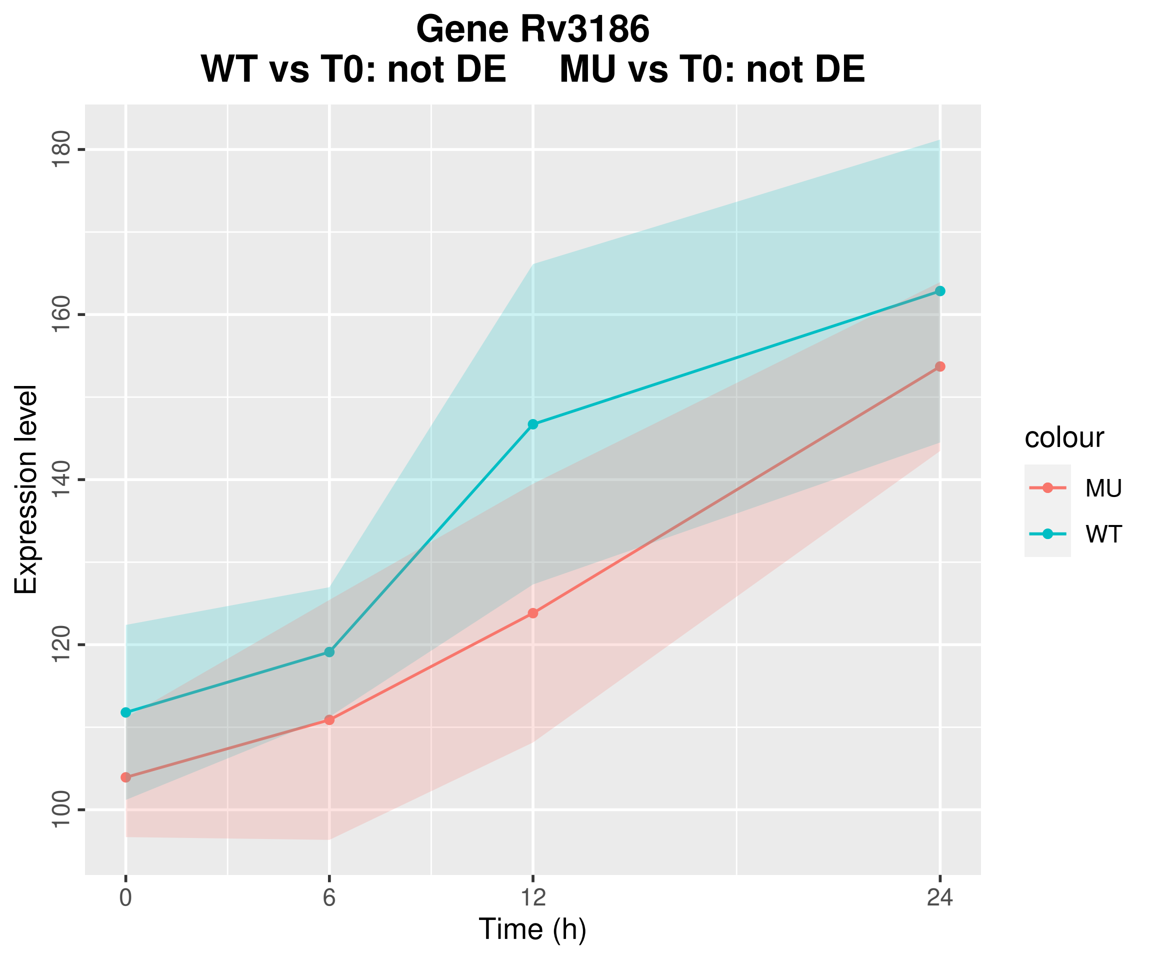
Gene Rv3186
WT vs T0: not DE MU vs T0: not DE
Expression level
180
160
140
120
100
0
6
12
24
Time (h)
colour
MU
WT

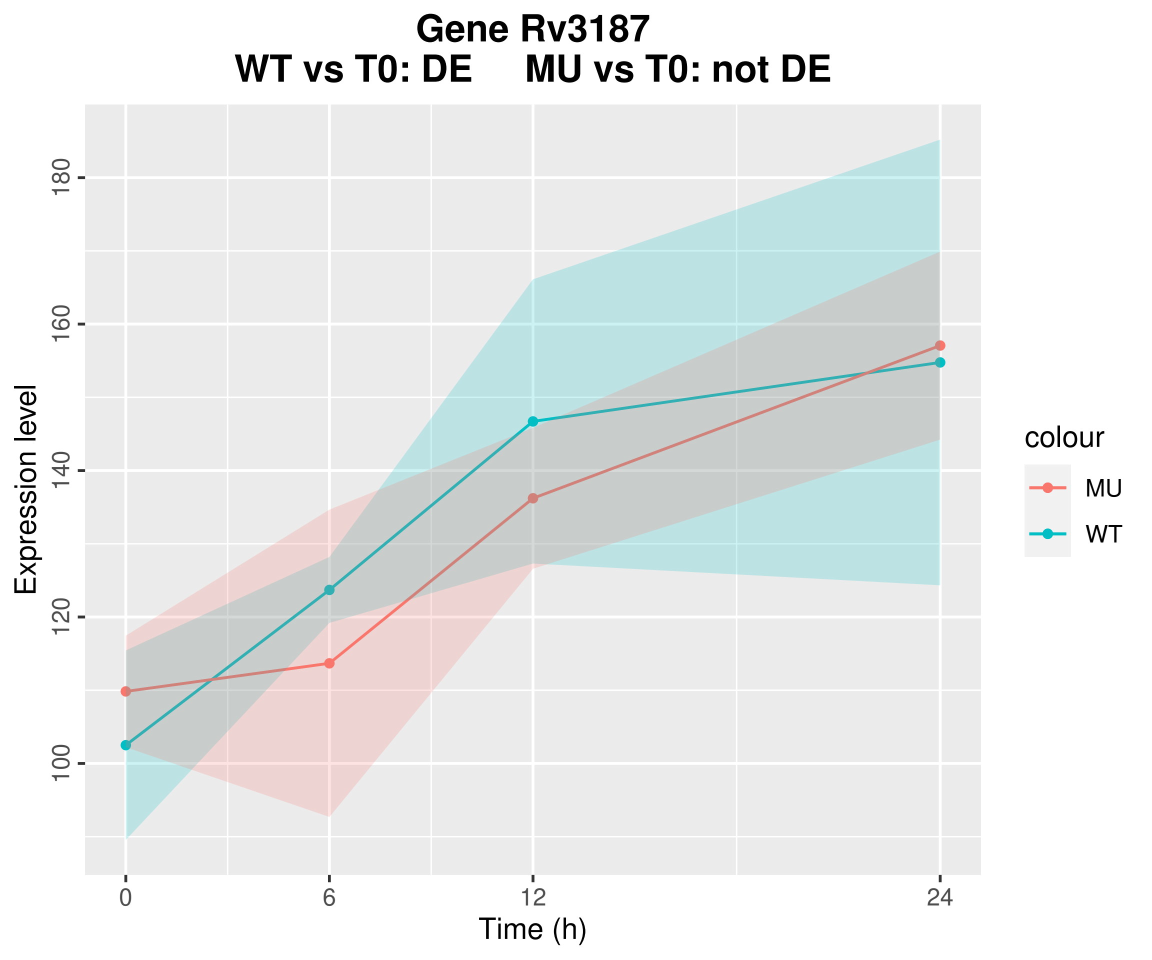

Gene Rv3187
WT vs T0: DE MU vs T0: not DE
Expression level
180
160
140
120
100
0
6
12
24
Time (h)
colour
MU
WT

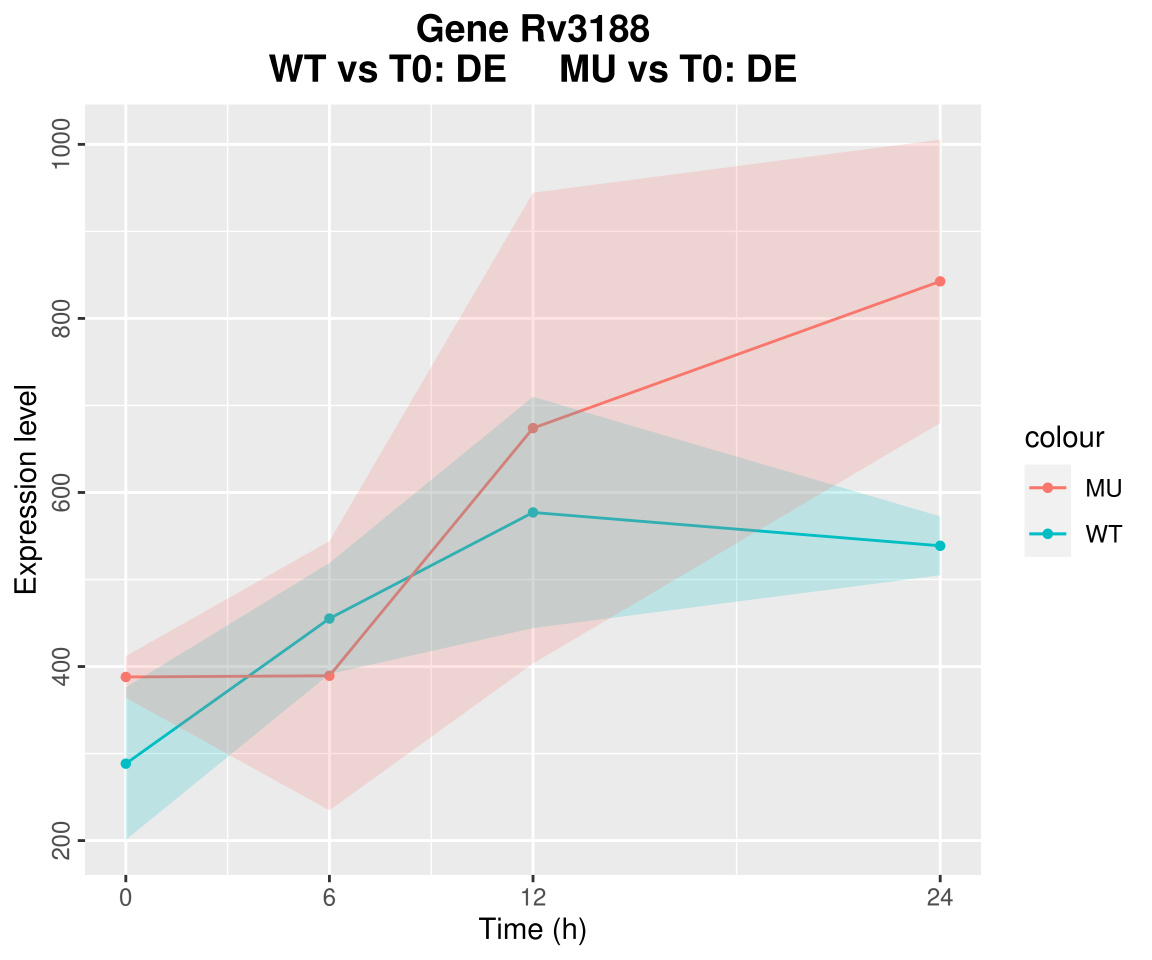

Gene Rv3188
WT vs T0: DE MU vs T0: DE
Expression level
Time (h)
200
400
600
800
1000
0
6
12
24
colour
MU
WT

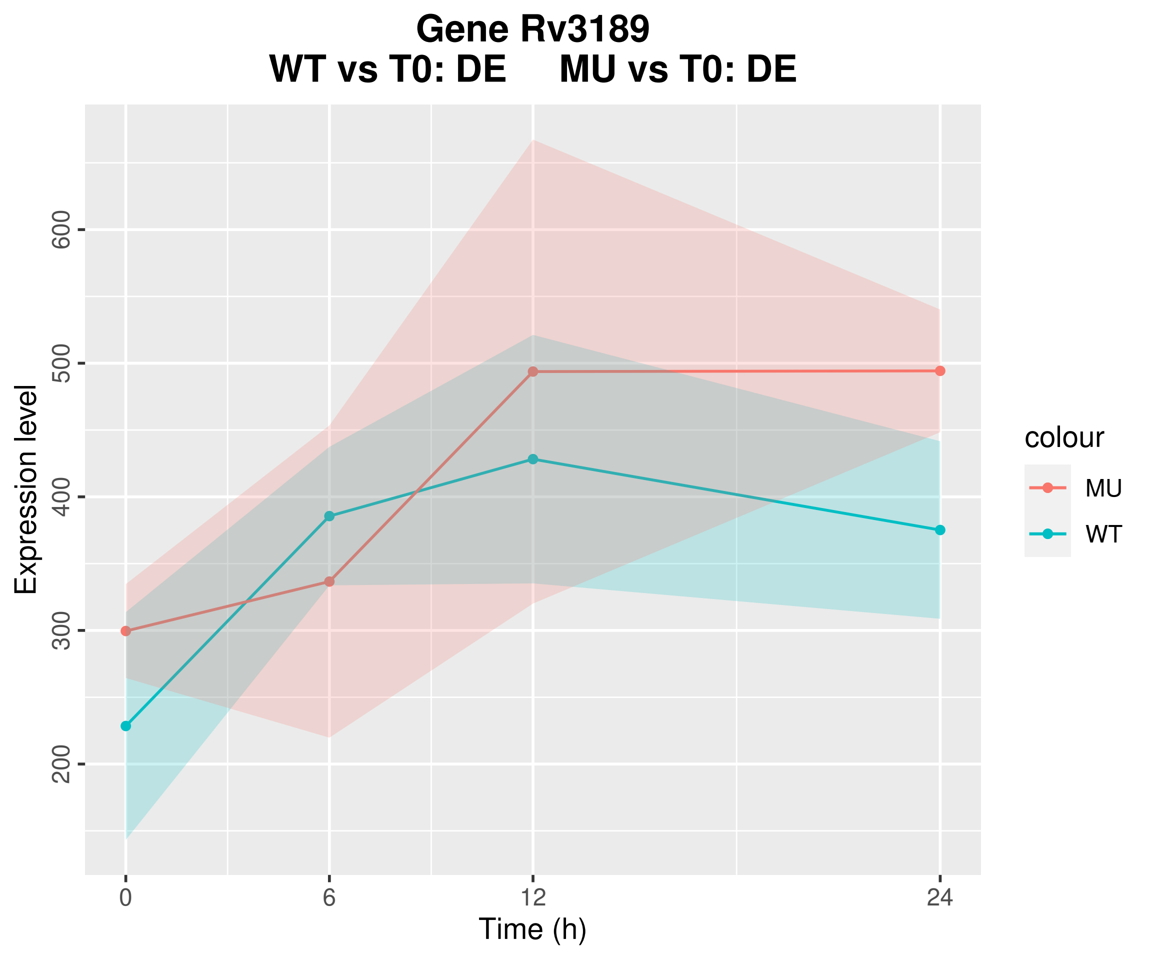

Gene Rv3189
WT vs T0: DE   MU vs T0: DE
Expression level
600
500
400
300
200
0
6
12
24
Time (h)
colour
MU
WT

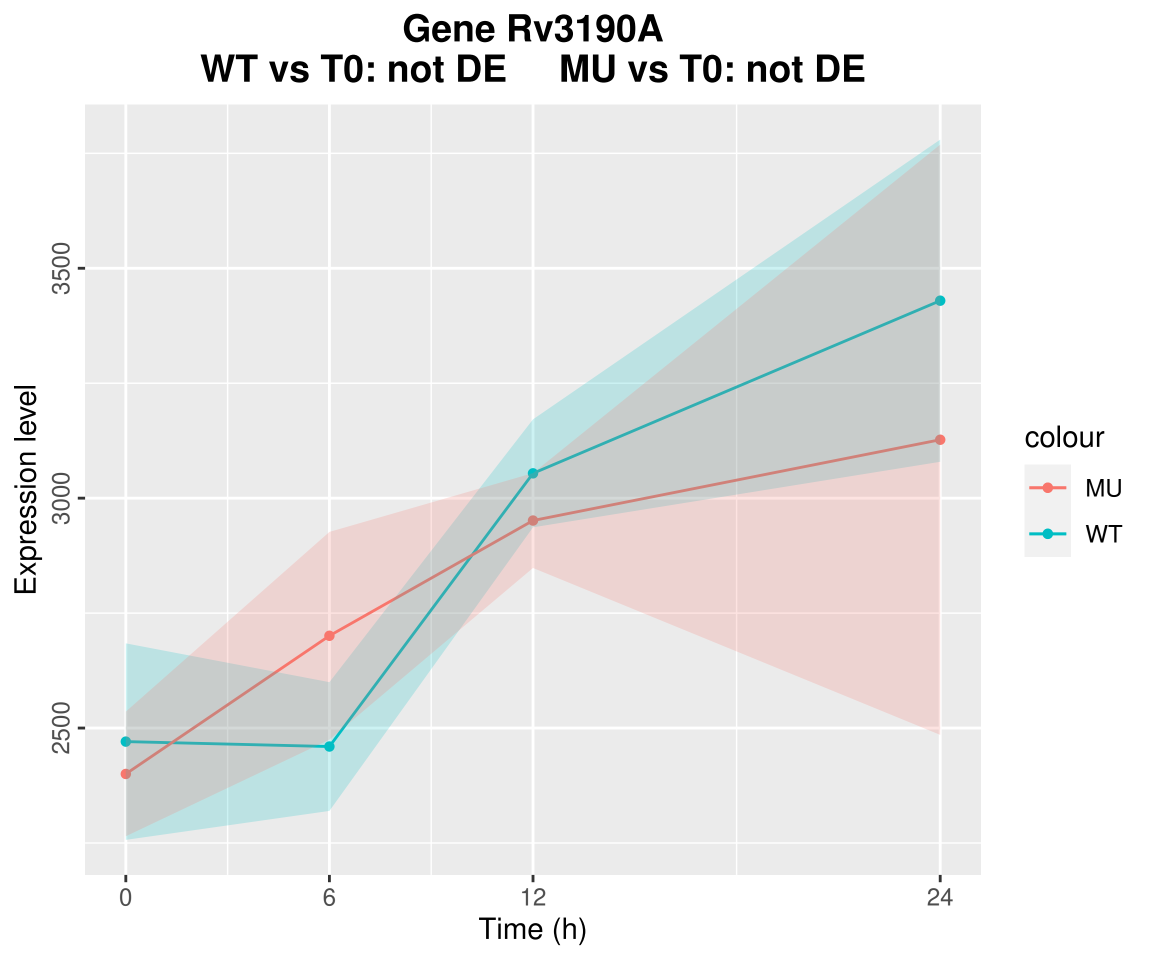

Gene Rv3190A
WT vs T0: not DE   MU vs T0: not DE
Expression level
3500
3000
2500
0
6
12
24
Time (h)
colour
MU
WT

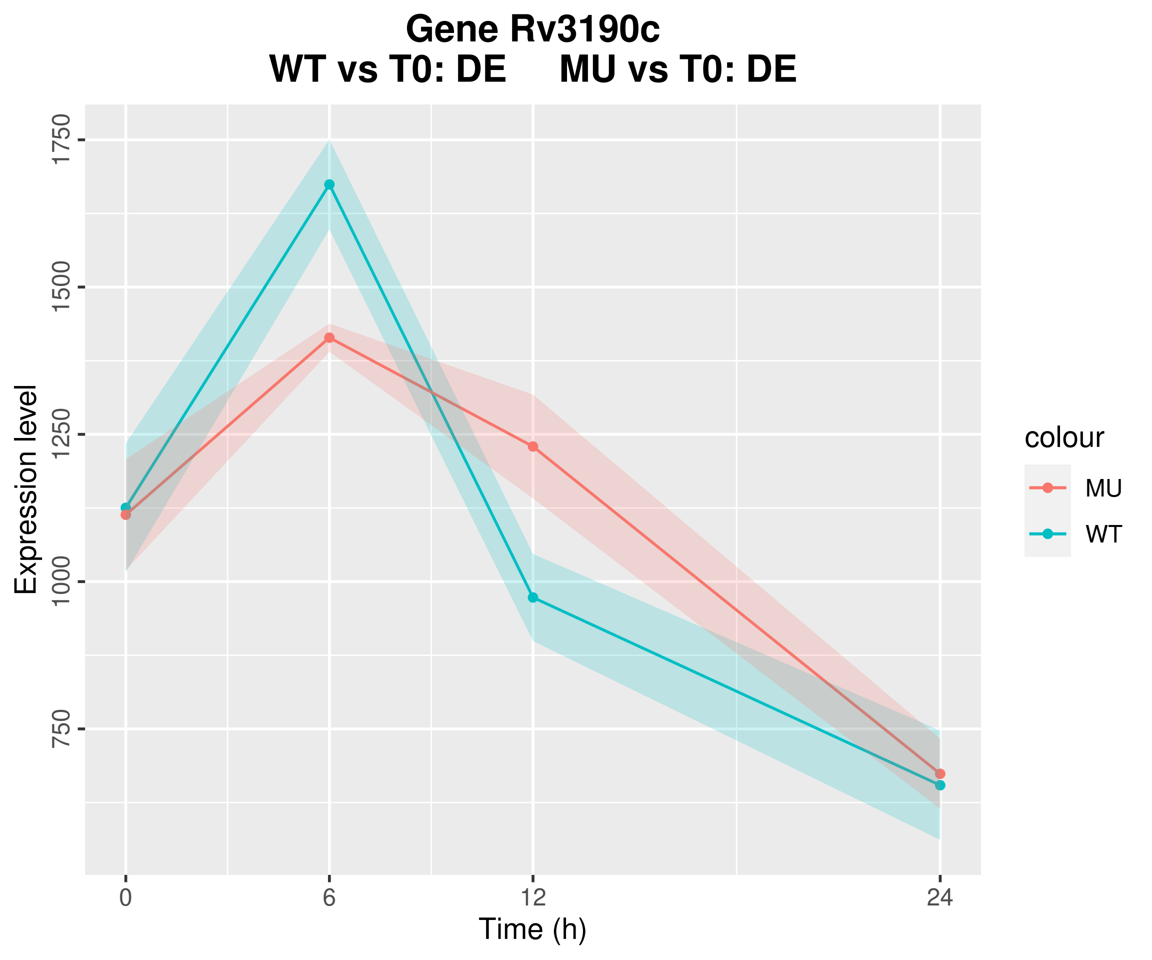

Gene Rv3190c
WT vs T0: DE   MU vs T0: DE
Expression level
1750
1500
1250
1000
750
0
6
12
24
Time (h)
colour
MU
WT

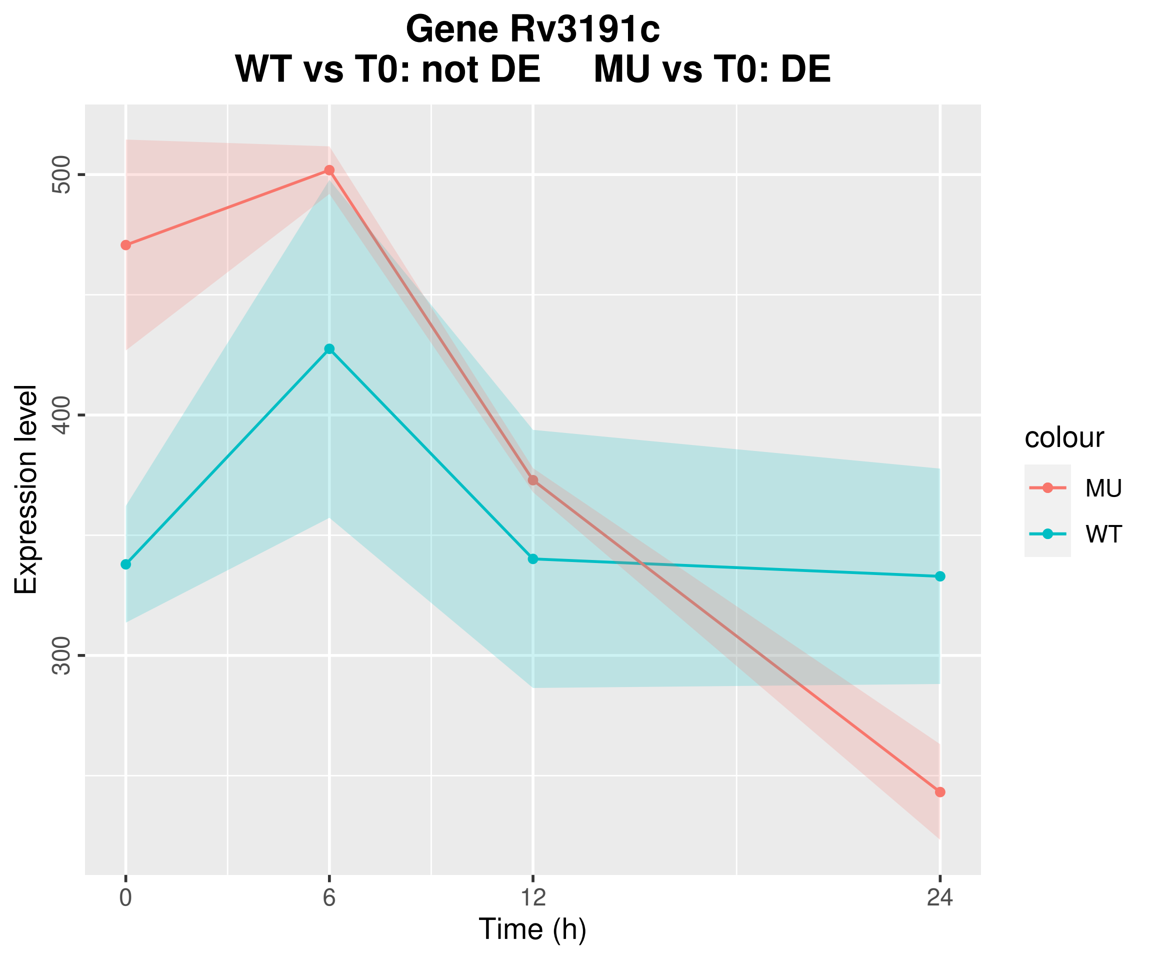

Gene Rv3191c
WT vs T0: not DE MU vs T0: DE
Expression level
500
400
300
0
6
12
24
Time (h)
colour
MU
WT

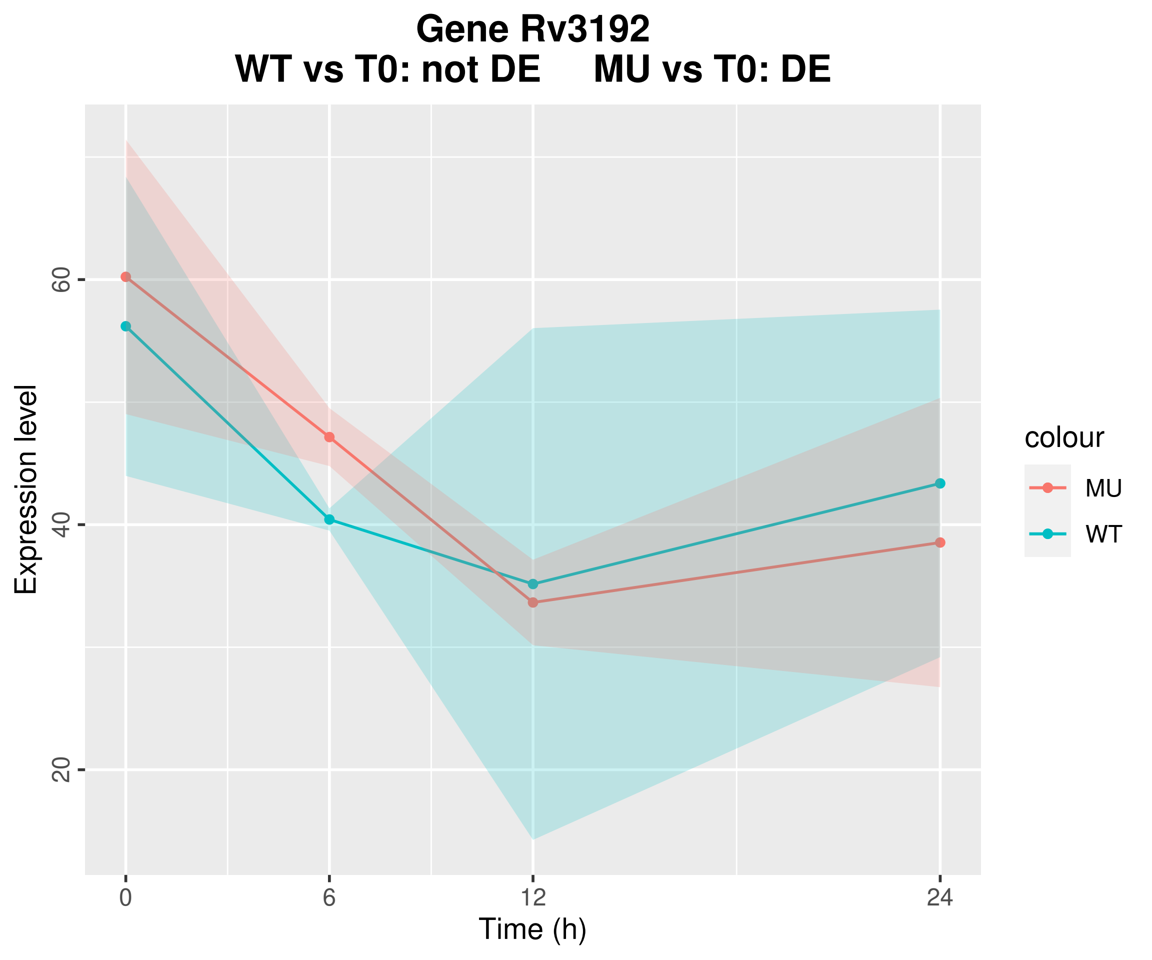

Gene Rv3192
WT vs T0: not DE MU vs T0: DE
Expression level
60
40
20
0
6
12
24
Time (h)
colour
MU
WT

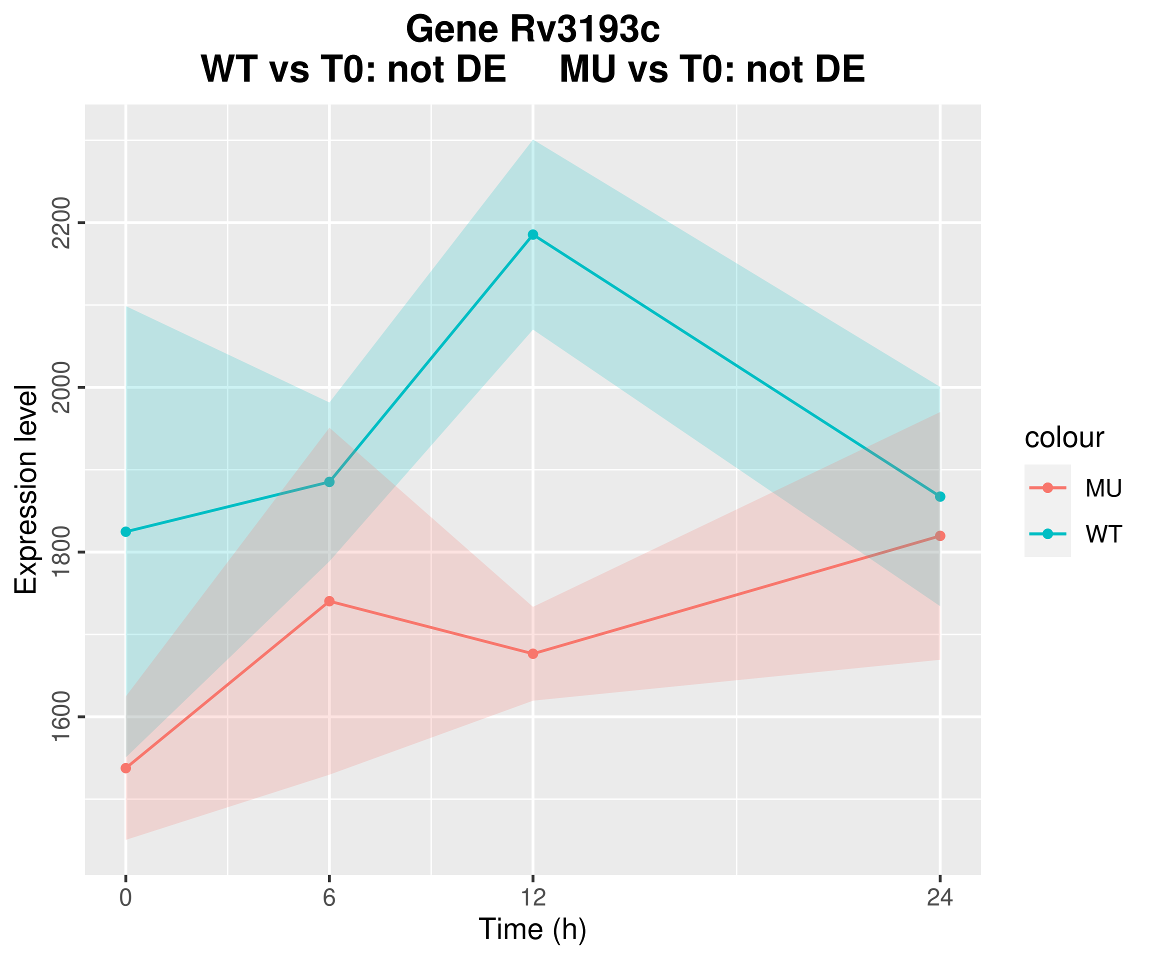
Gene Rv3193c
WT vs T0: not DE MU vs T0: not DE
Expression level
2200
2000
1800
1600
0
6
12
24
Time (h)
colour
MU
WT

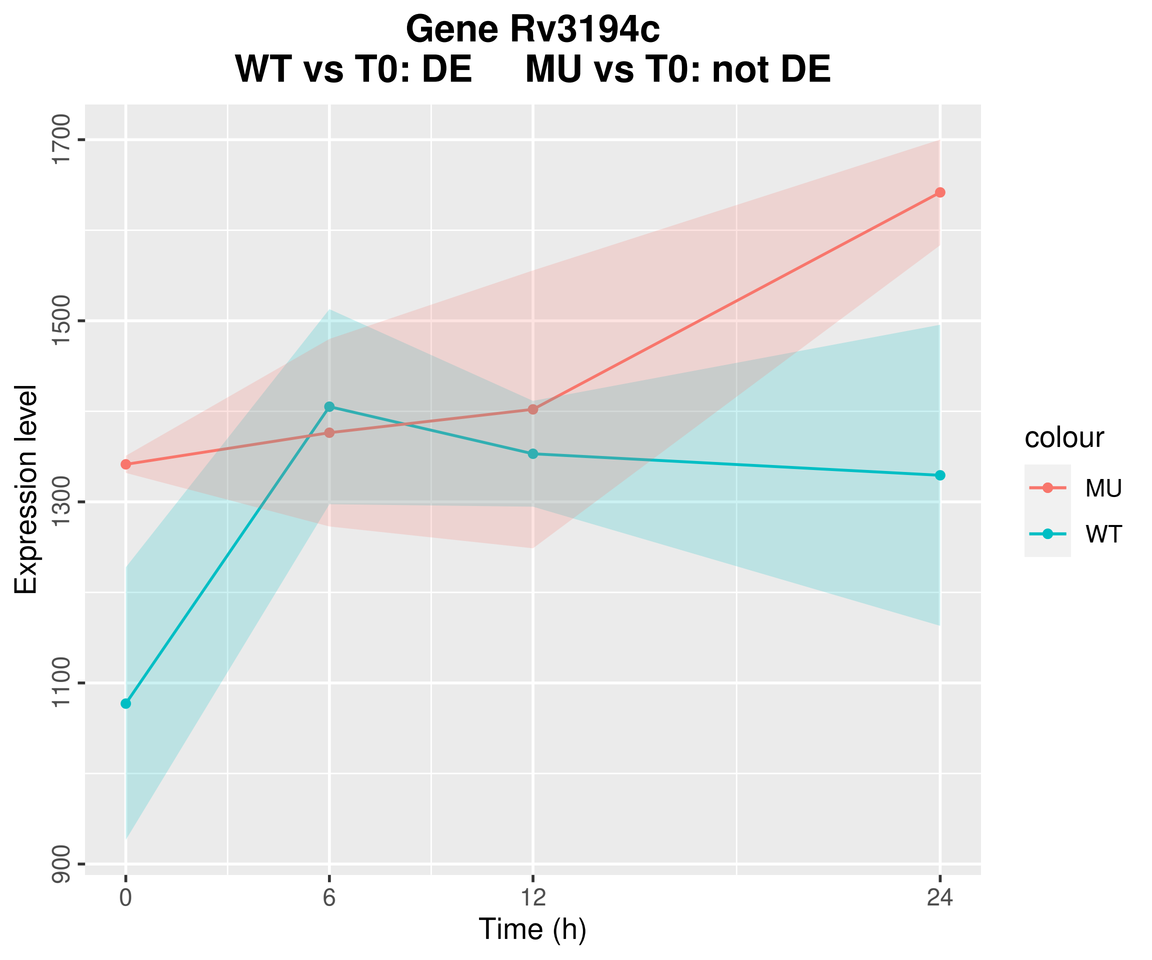

Gene Rv3194c
WT vs T0: DE   MU vs T0: not DE
Expression level
1700
1500
1300
1100
900
0
6
12
24
Time (h)
colour
MU
WT

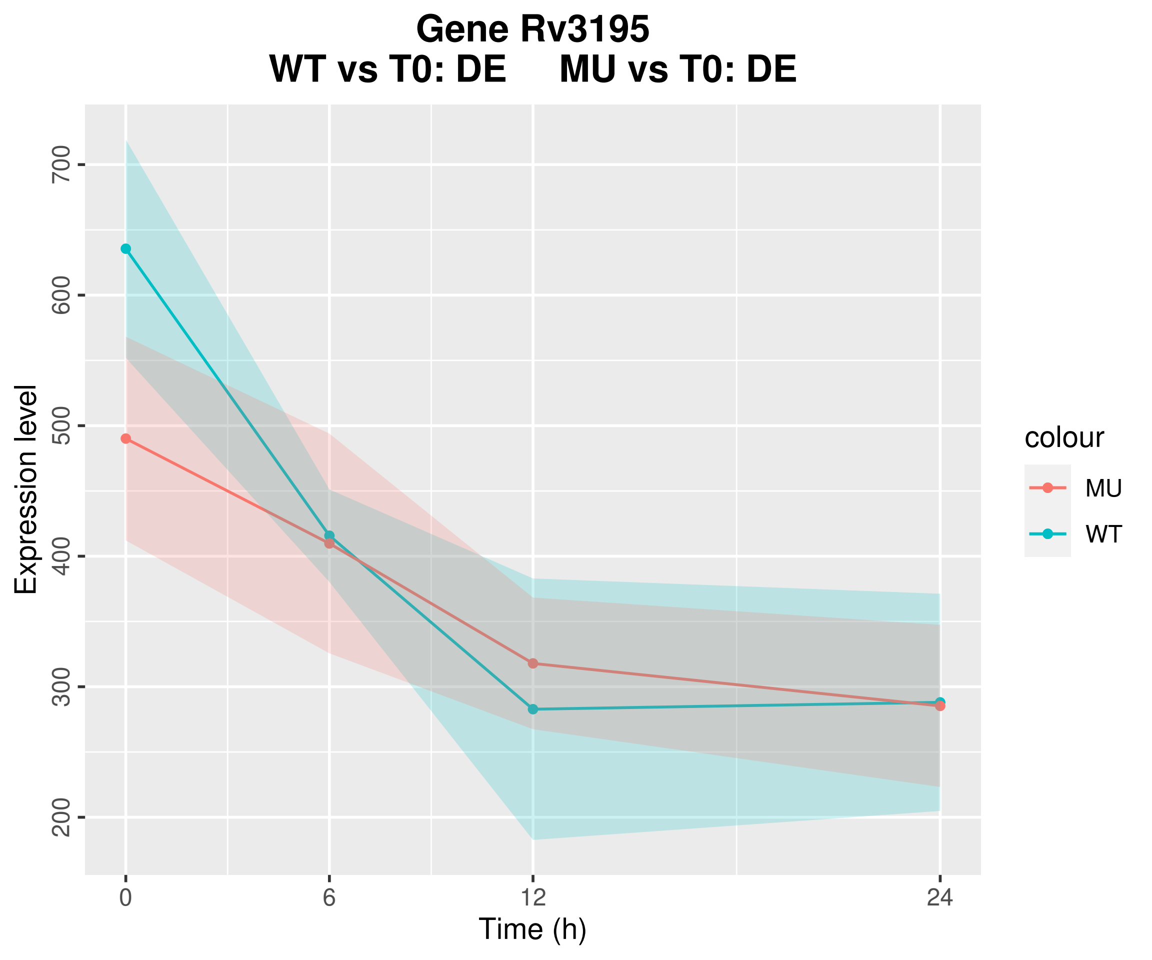

Gene Rv3195
WT vs T0: DE   MU vs T0: DE
Expression level
700
600
500
400
300
200
0
6
12
24
Time (h)
colour
MU
WT

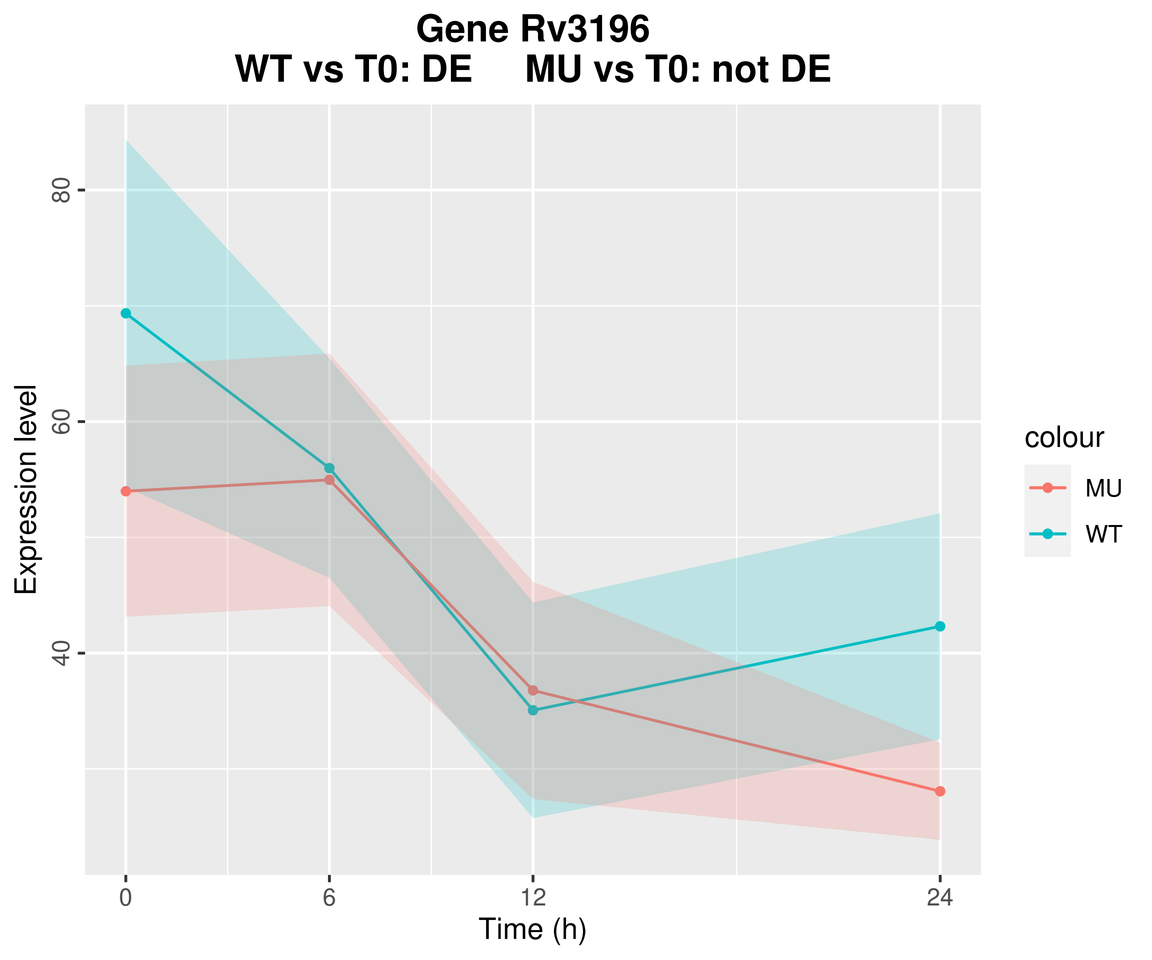
Gene Rv3196
WT vs T0: DE   MU vs T0: not DE
Expression level
80
60
40
0
6
12
24
Time (h)
colour
MU
WT

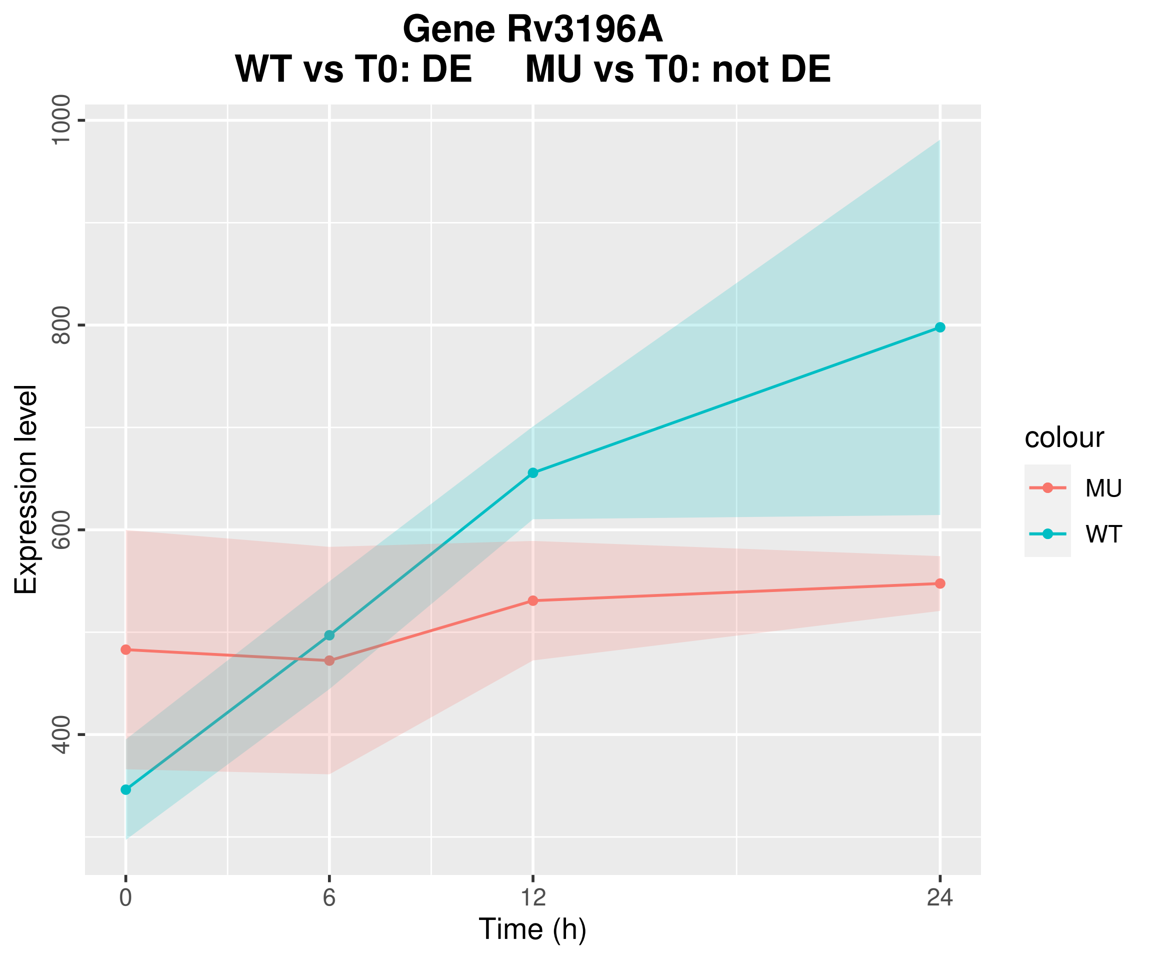

Gene Rv3196A
WT vs T0: DE   MU vs T0: not DE
Expression level
1000
800
600
400
0
6
12
24
Time (h)
colour
MU
WT

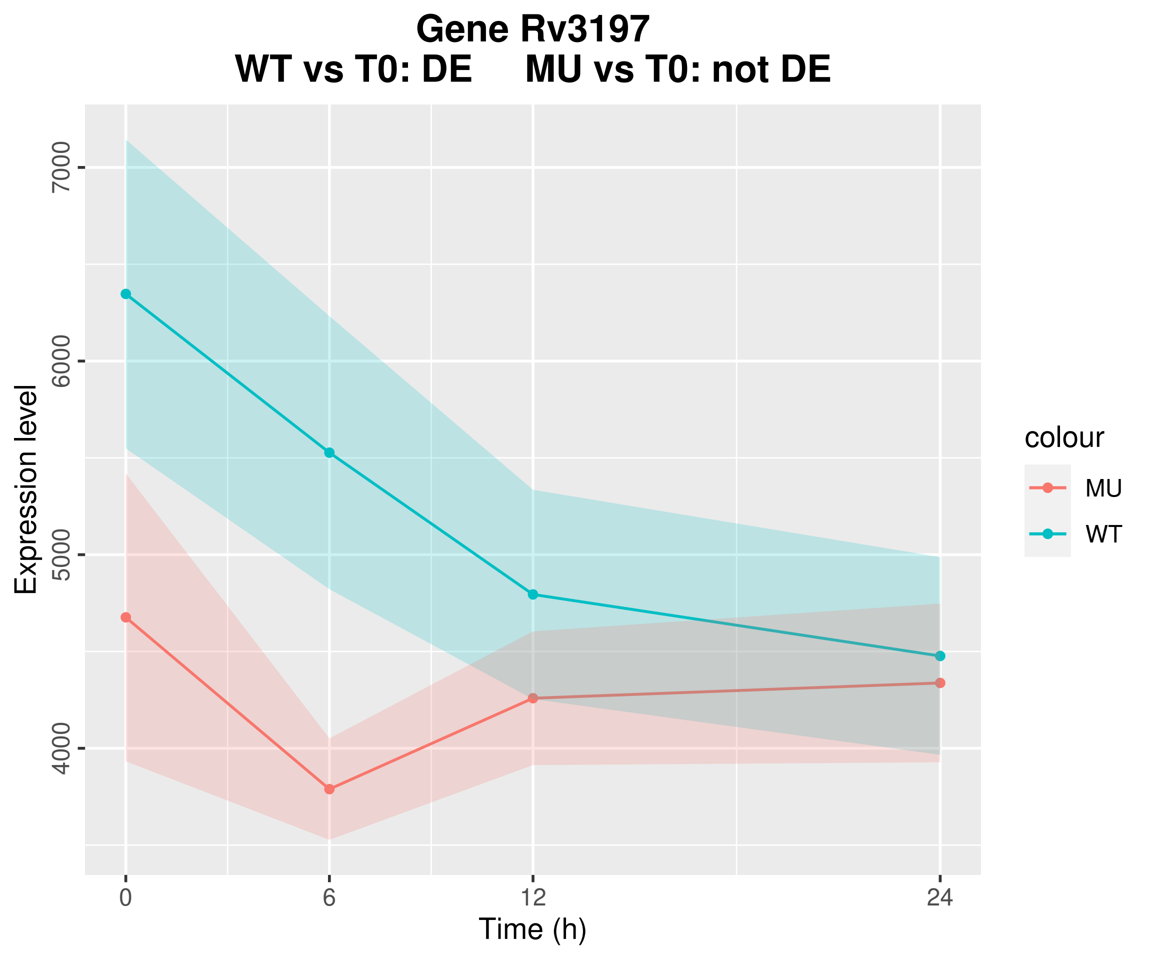

Gene Rv3197
WT vs T0: DE   MU vs T0: not DE
Expression level
7000
6000
5000
4000
0
6
12
24
Time (h)
colour
MU
WT

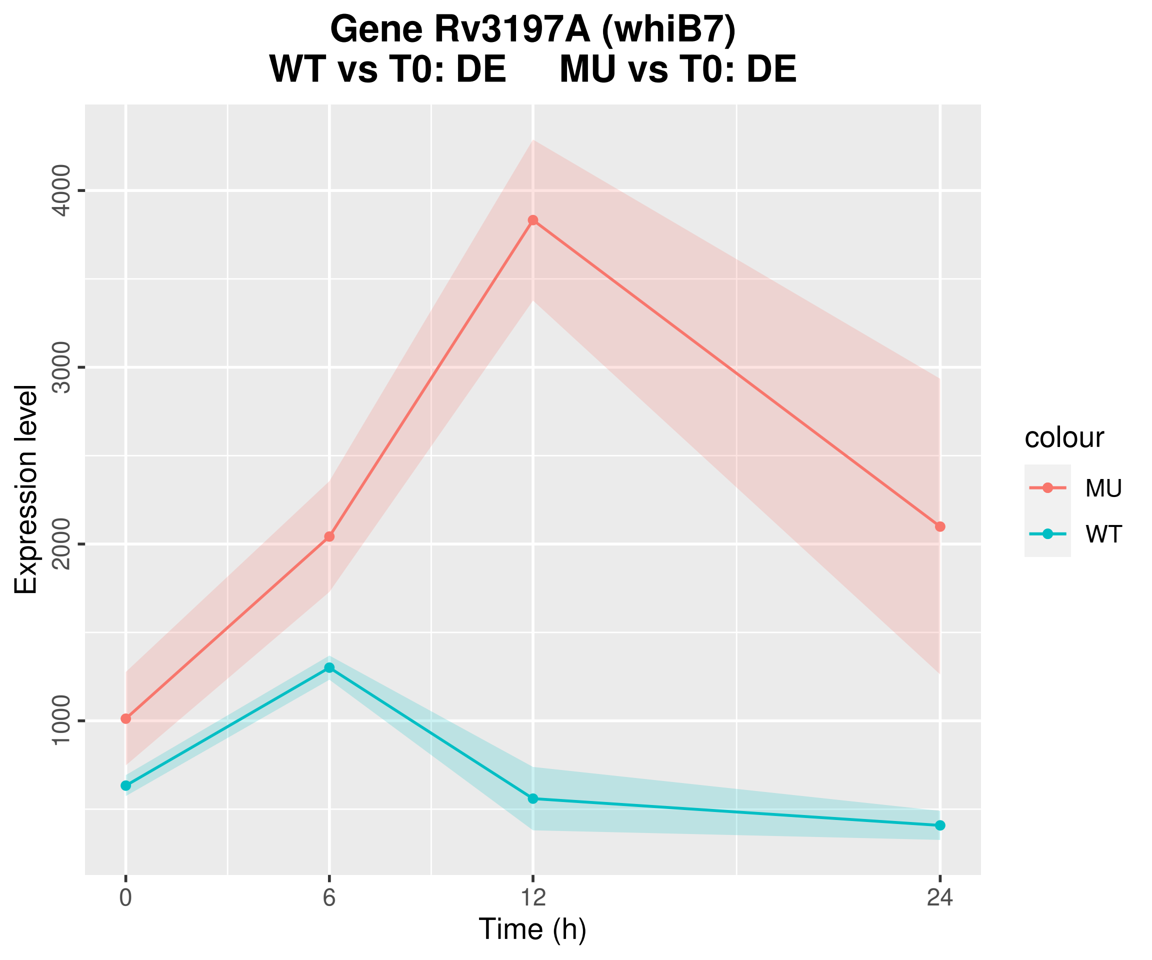
Gene Rv3197A (whiB7)
WT vs T0: DE MU vs T0: DE
Expression level
4000
3000
2000
1000
0
6
12
24
Time (h)
colour
MU
WT

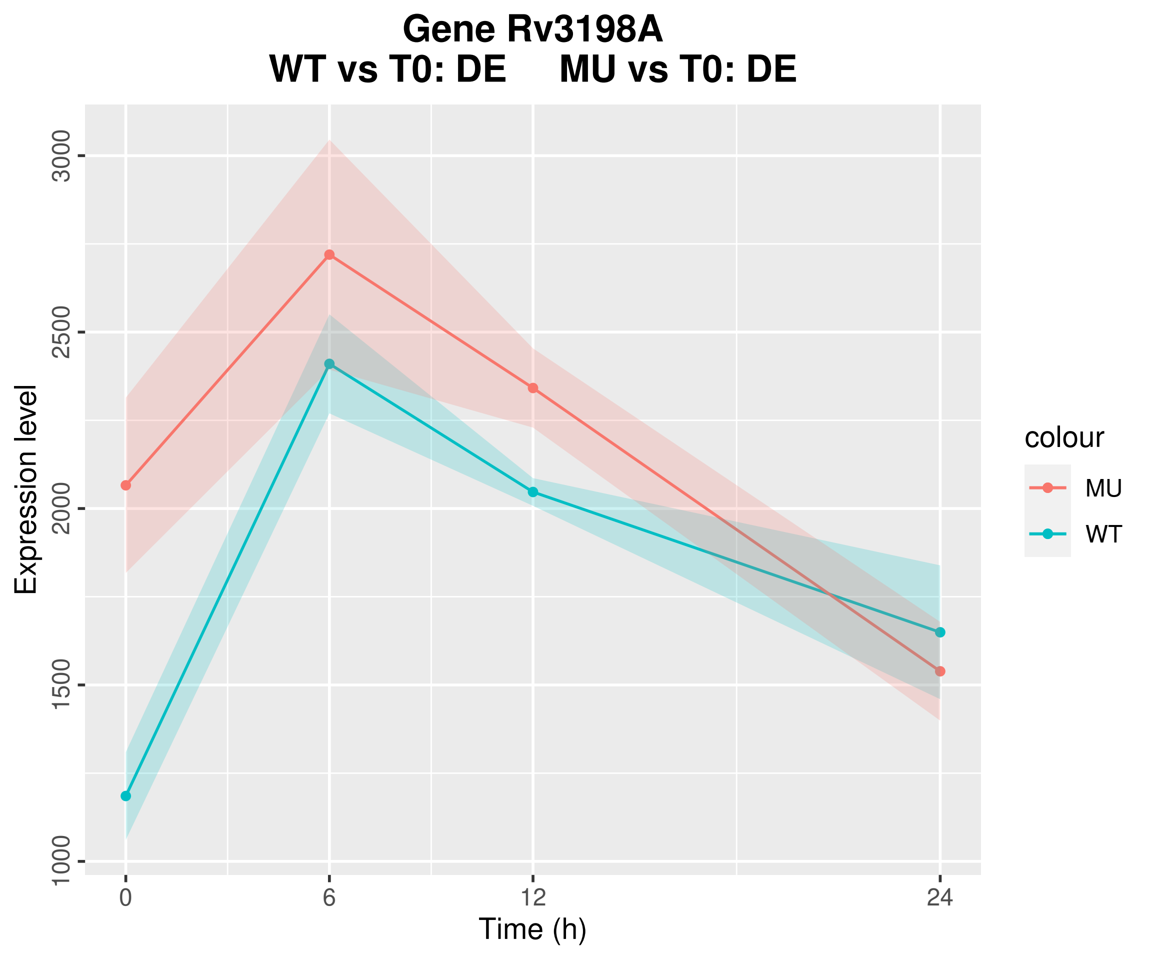

Gene Rv3198A
WT vs T0: DE   MU vs T0: DE
Expression level
3000
2500
2000
1500
1000
Time (h)
0
6
12
24
colour
MU
WT

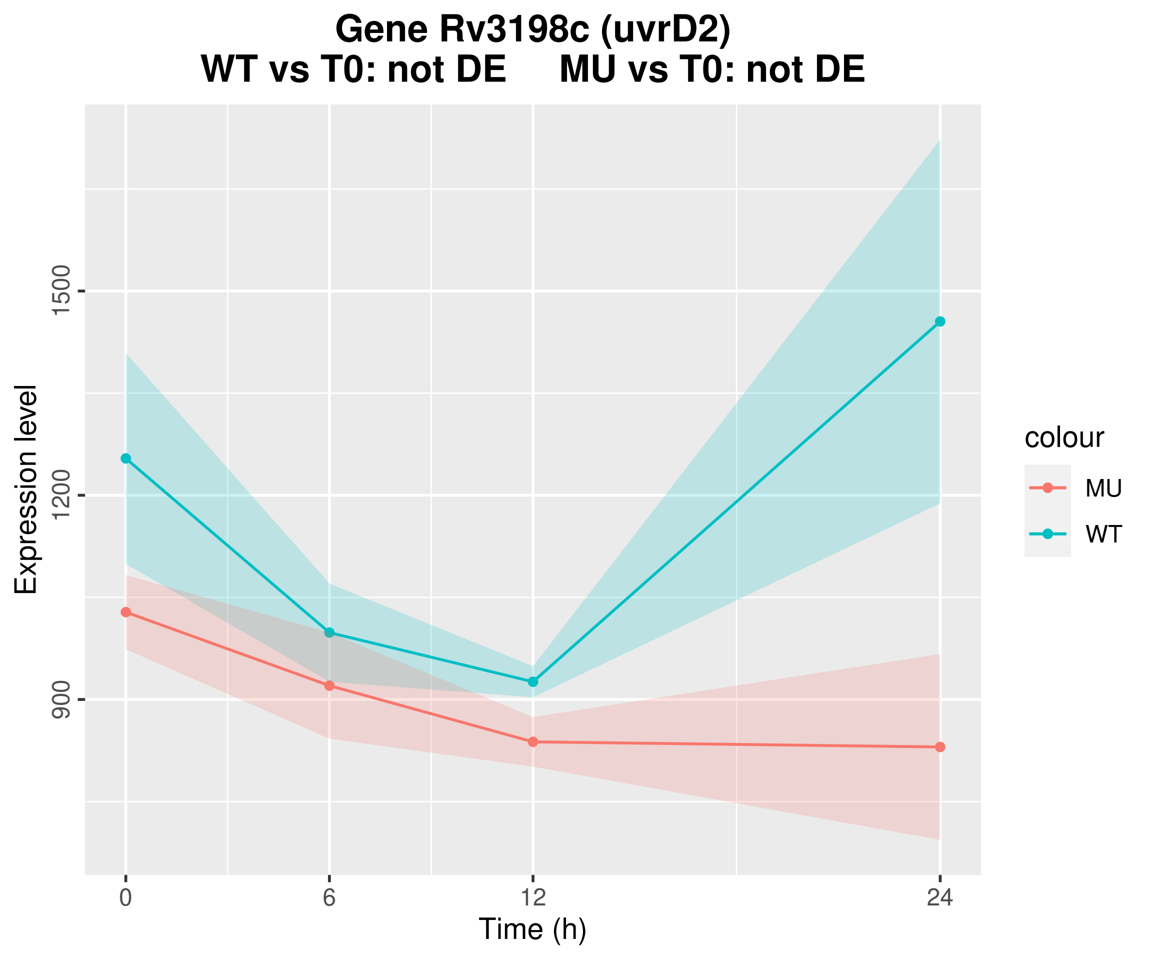

Gene Rv3198c (uvrD2)
WT vs T0: not DE   MU vs T0: not DE
Expression level
1500
1200
900
0
6
12
24
Time (h)
colour
MU
WT

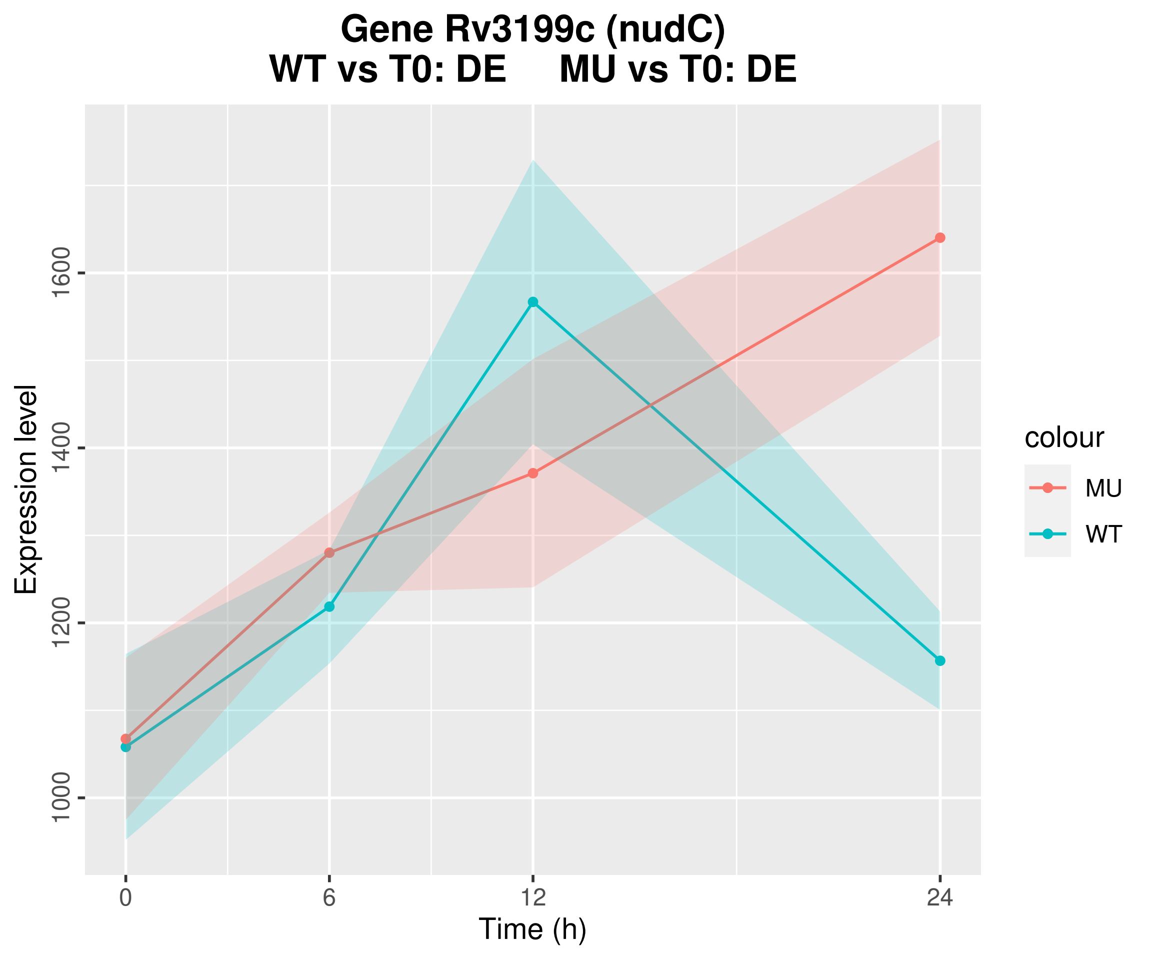
Gene Rv3199c (nudC)
WT vs T0: DE MU vs T0: DE
Expression level
1000
1200
1400
1600
0
6
12
24
Time (h)
colour
MU
WT

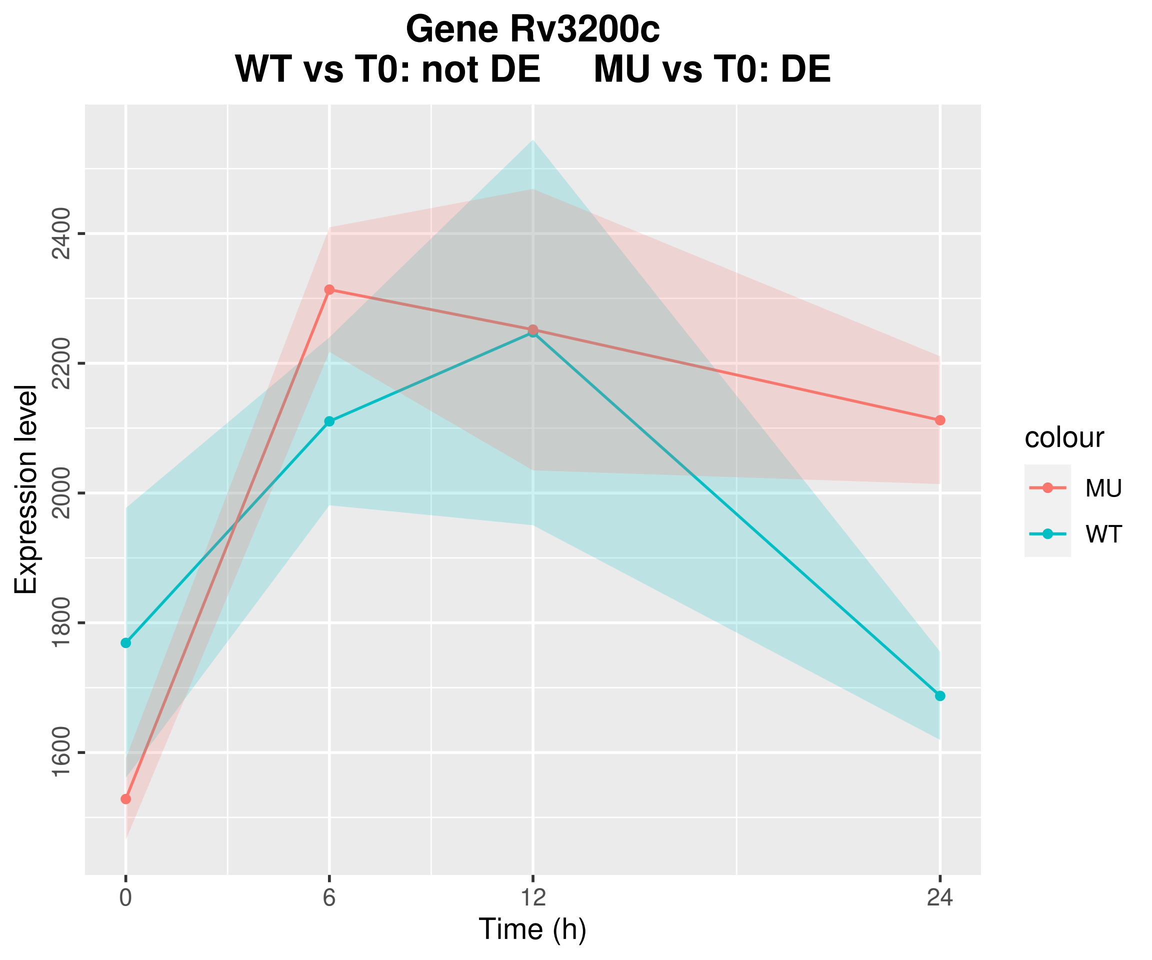
Gene Rv3200c
WT vs T0: not DE MU vs T0: DE
Expression level
2400
2200
2000
1800
1600
0
6
12
24
Time (h)
colour
MU
WT

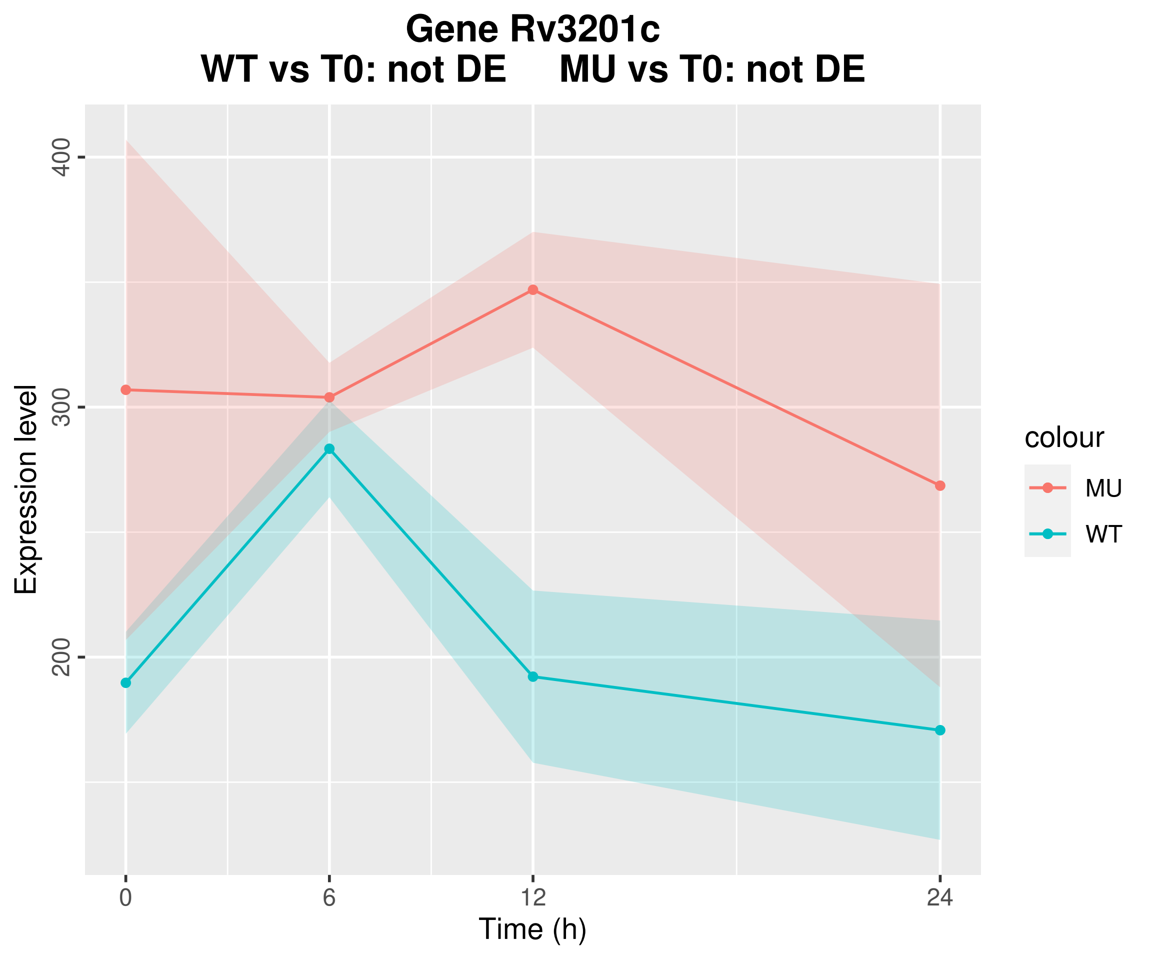

Gene Rv3201c
WT vs T0: not DE MU vs T0: not DE
Expression level
400
300
200
0
6
12
24
Time (h)
colour
MU
WT

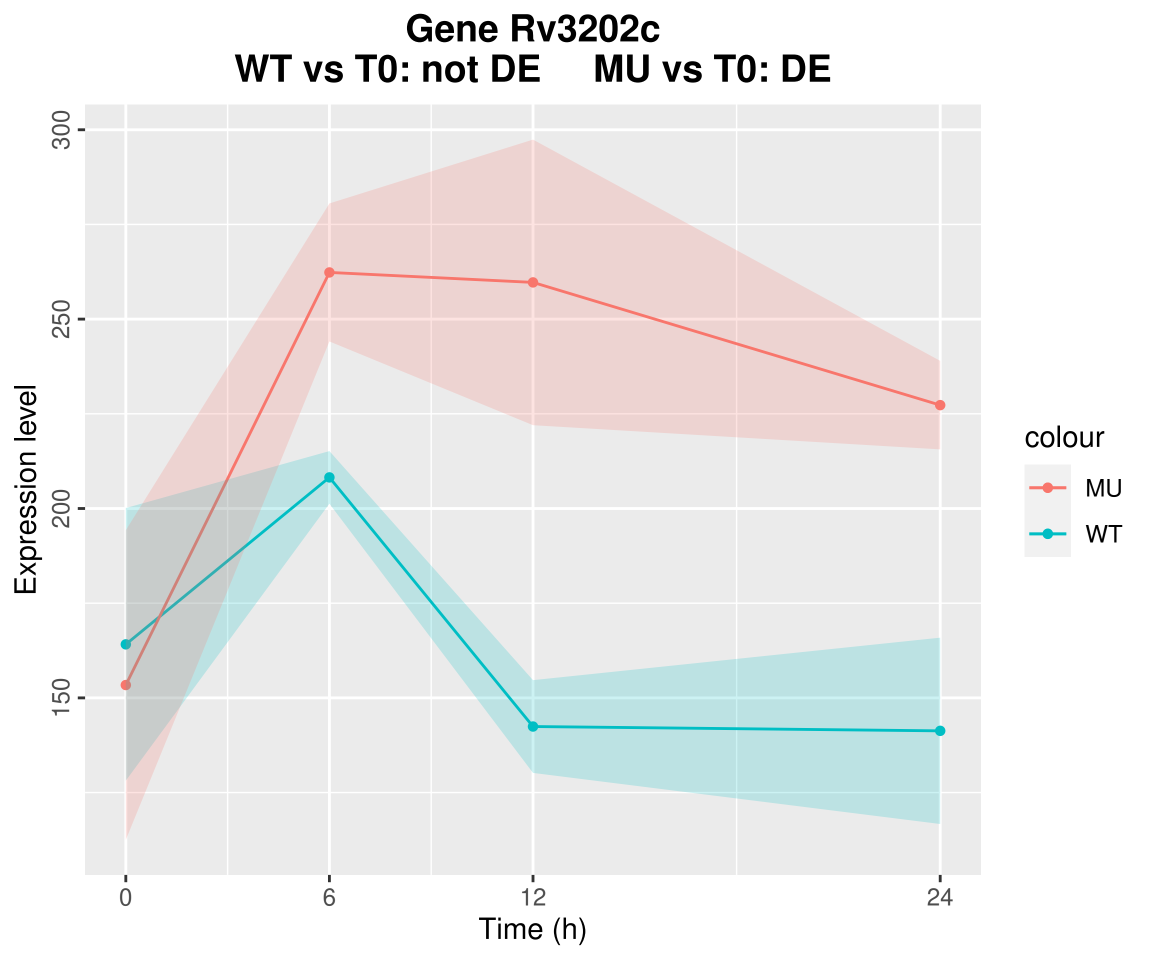
Gene Rv3202c
WT vs T0: not DE MU vs T0: DE
Expression level
300
250
200
150
0
6
12
24
Time (h)
colour
MU
WT

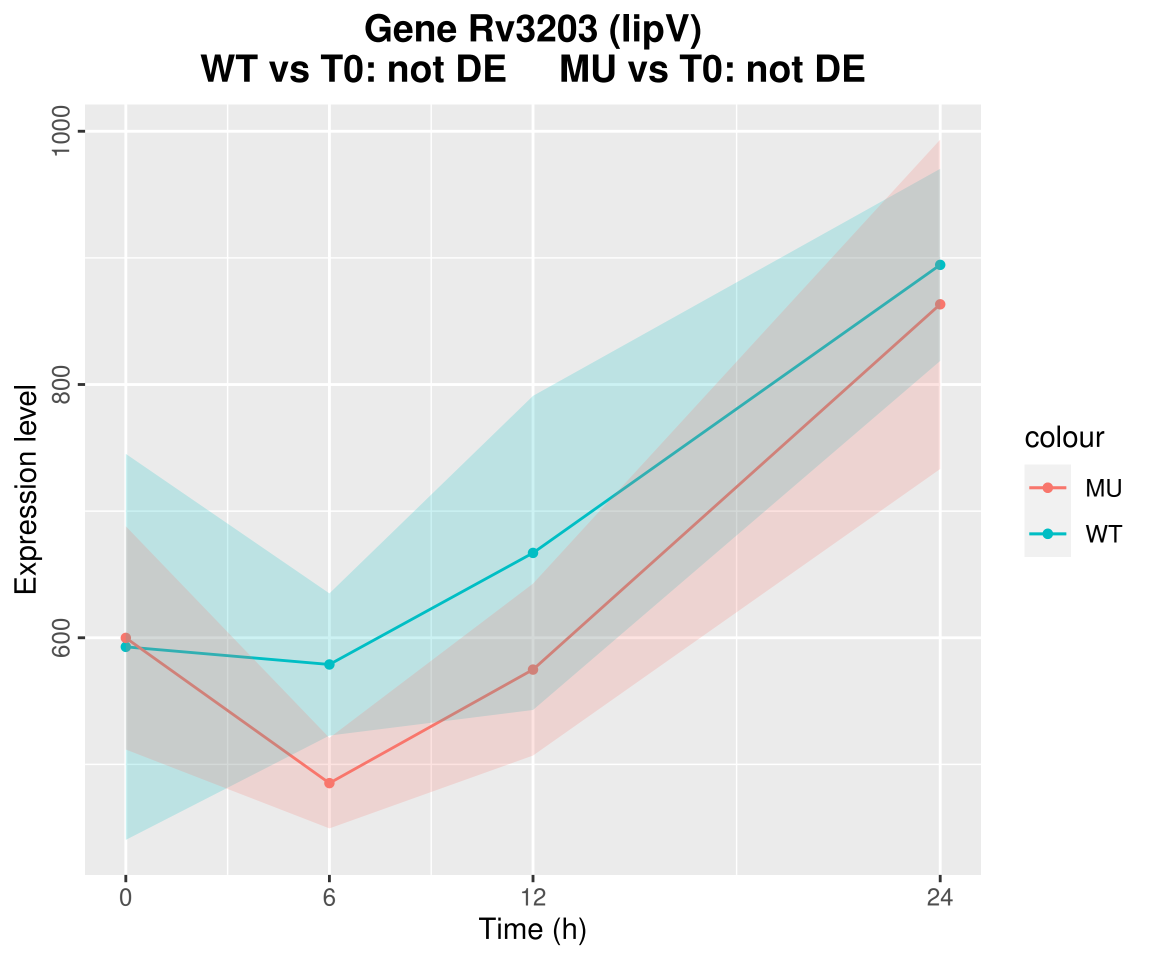

Gene Rv3203 (lipV)
WT vs T0: not DE
MU vs T0: not DE
1000
800
600
Expression level
0
6
12
24
Time (h)
colour
MU
WT

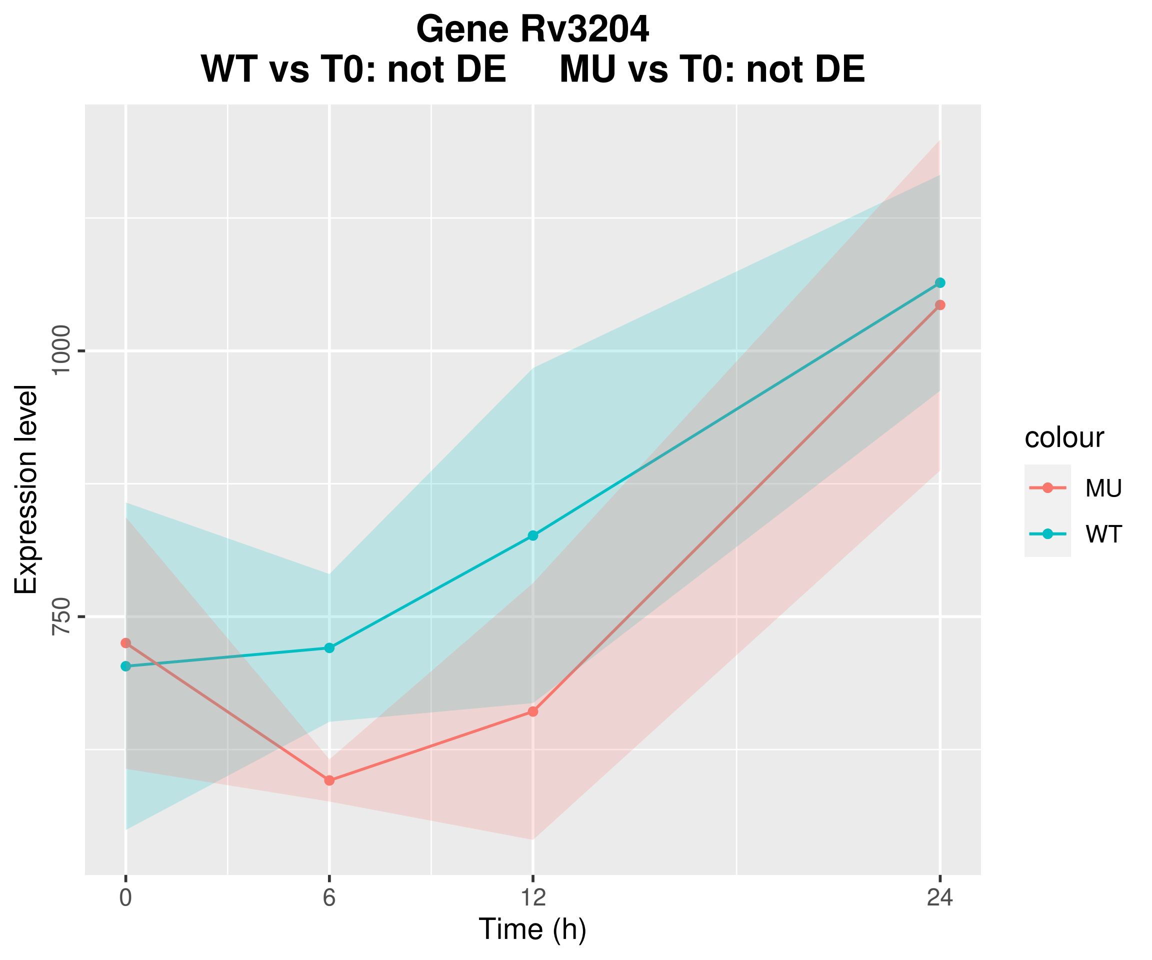

Gene Rv3204
WT vs T0: not DE MU vs T0: not DE
Expression level
1000
750
0
6
12
24
Time (h)
colour
MU
WT

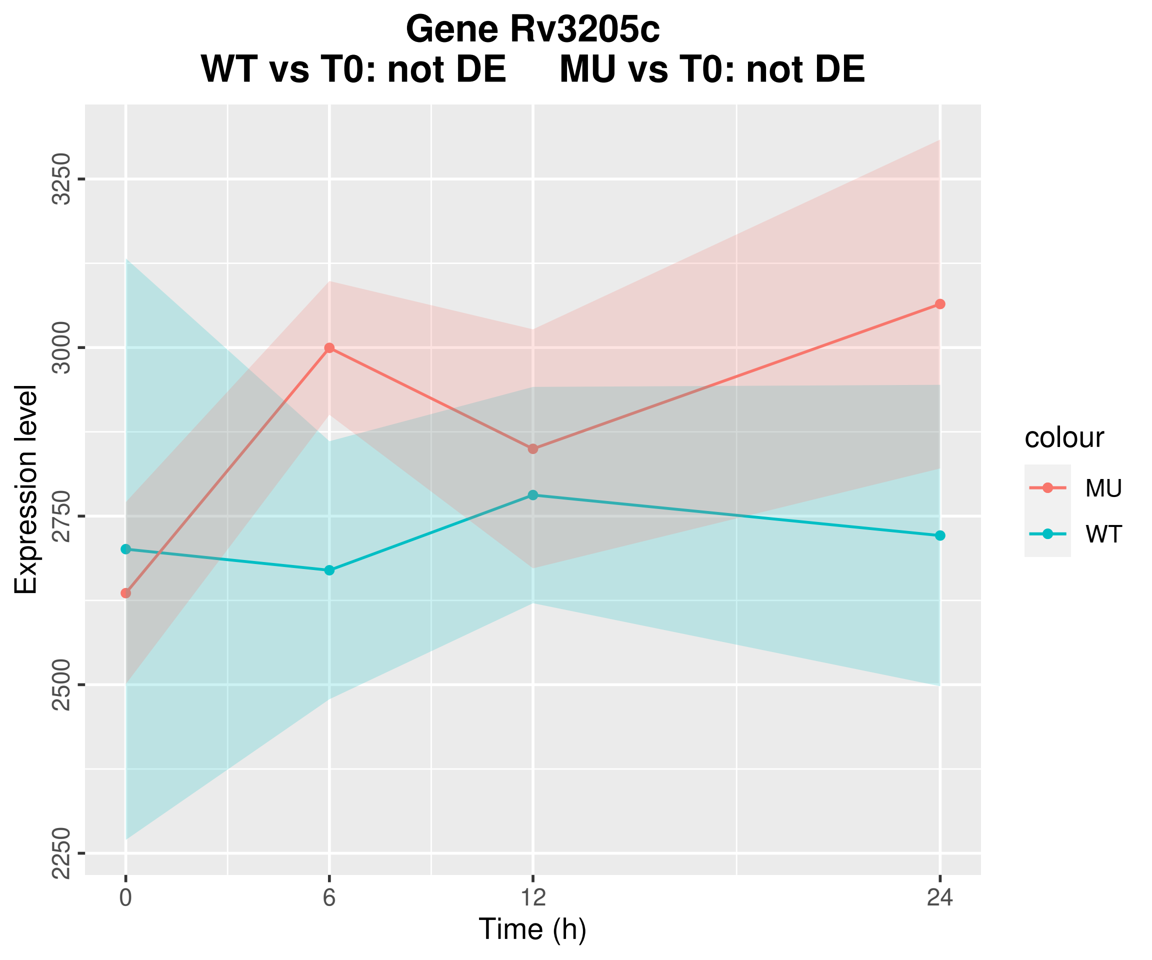

Gene Rv3205c
WT vs T0: not DE   MU vs T0: not DE
Expression level
3250
3000
2750
2500
2250
0
6
12
24
Time (h)
colour
MU
WT

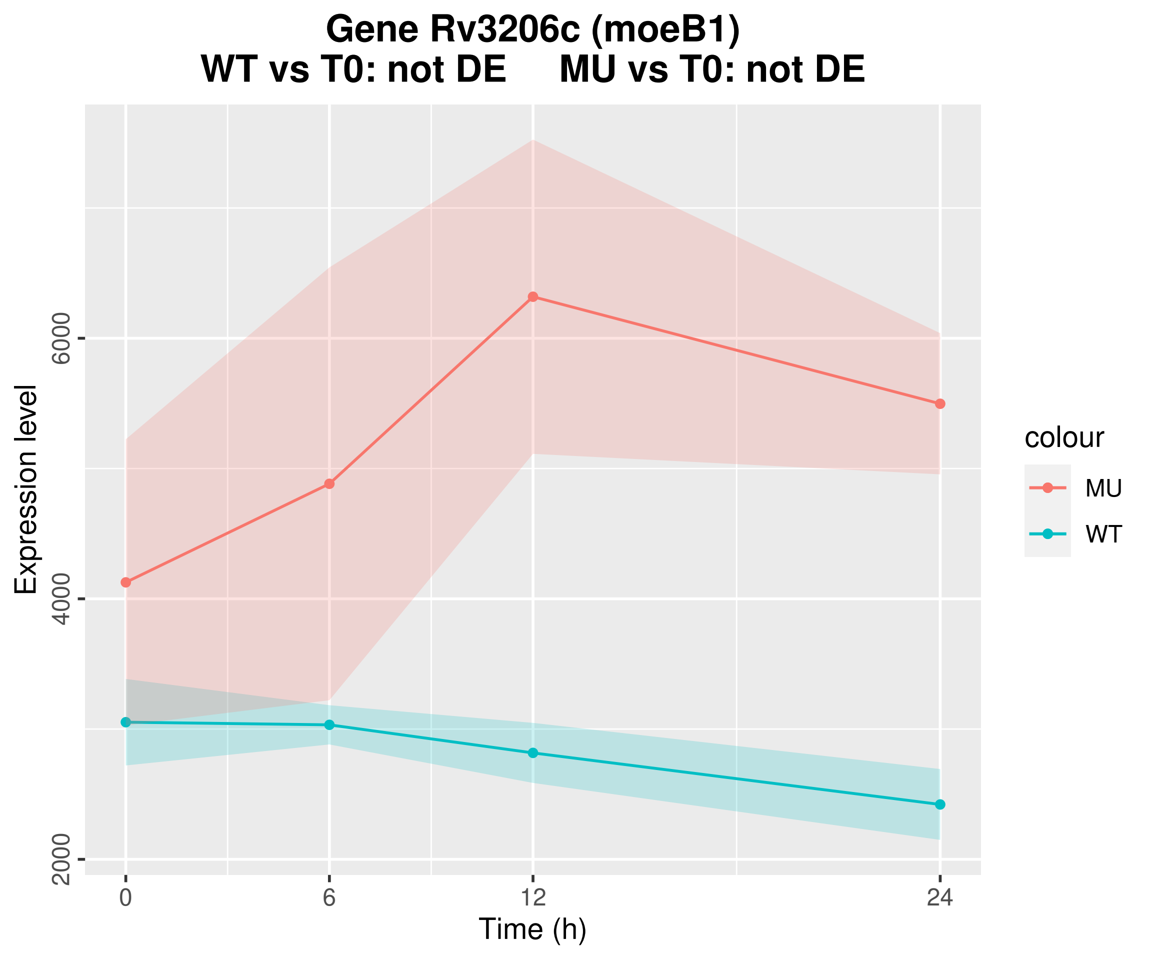
Gene Rv3206c (moeB1)
WT vs T0: not DE MU vs T0: not DE
Expression level
6000
4000
2000
0
6
12
24
Time (h)
colour
MU
WT

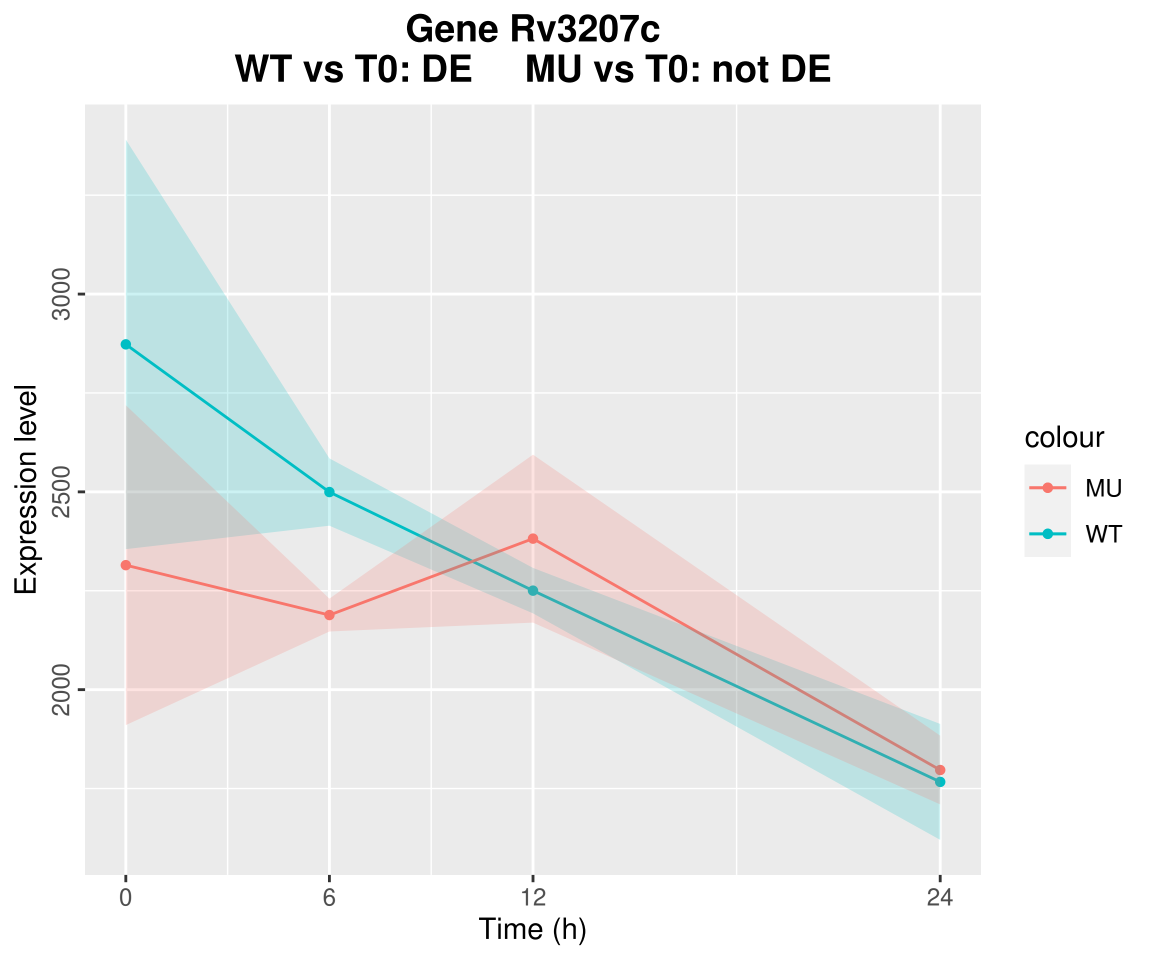
Gene Rv3207c
WT vs T0: DE   MU vs T0: not DE
Expression level
3000
2500
2000
Time (h)
0
6
12
24
colour
MU
WT

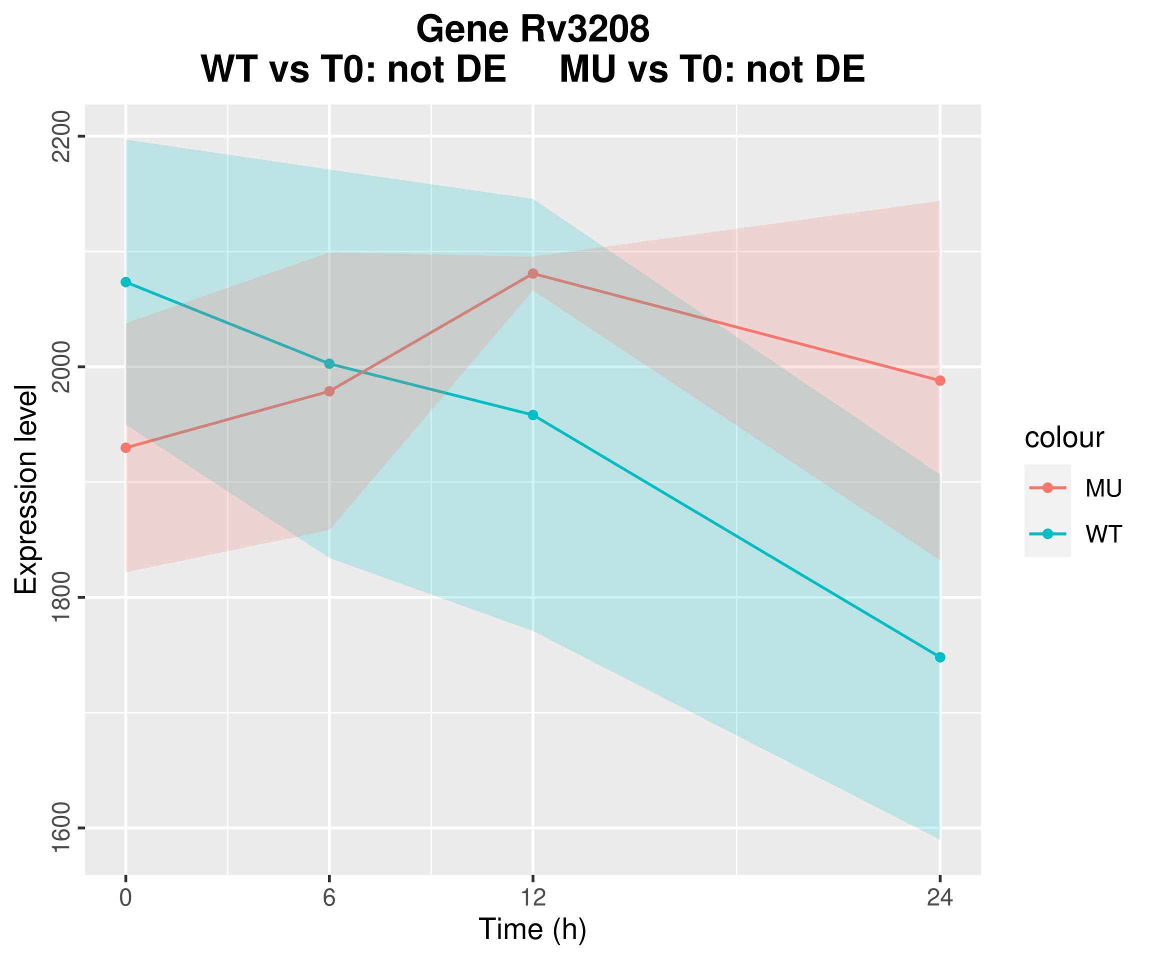

Gene Rv3208
WT vs T0: not DE MU vs T0: not DE
Expression level
2200
2000
1800
1600
0
6
12
24
Time (h)
colour
MU
WT

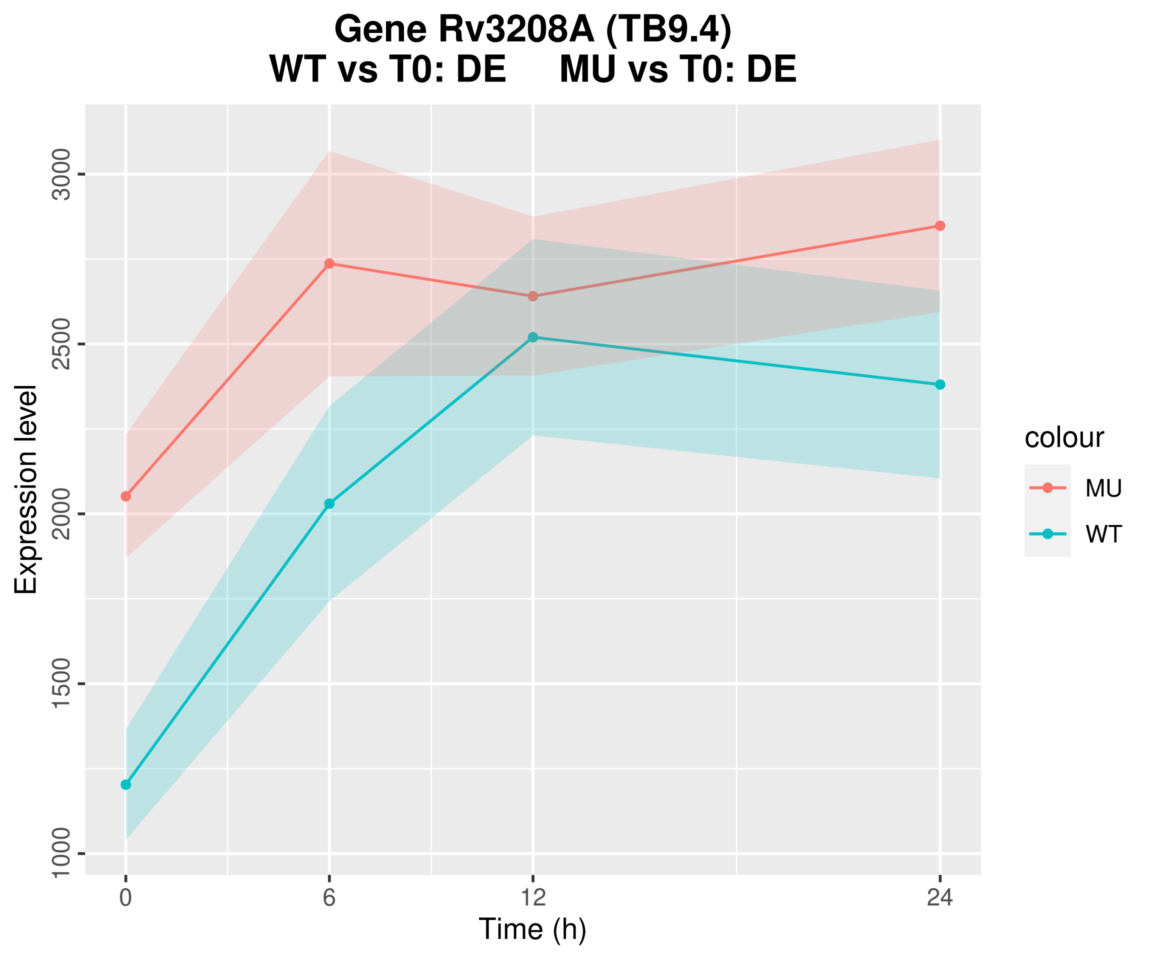
Gene Rv3208A (TB9.4)
WT vs T0: DE   MU vs T0: DE
Expression level
3000
2500
2000
1500
1000
0
6
12
24
Time (h)
colour
MU
WT

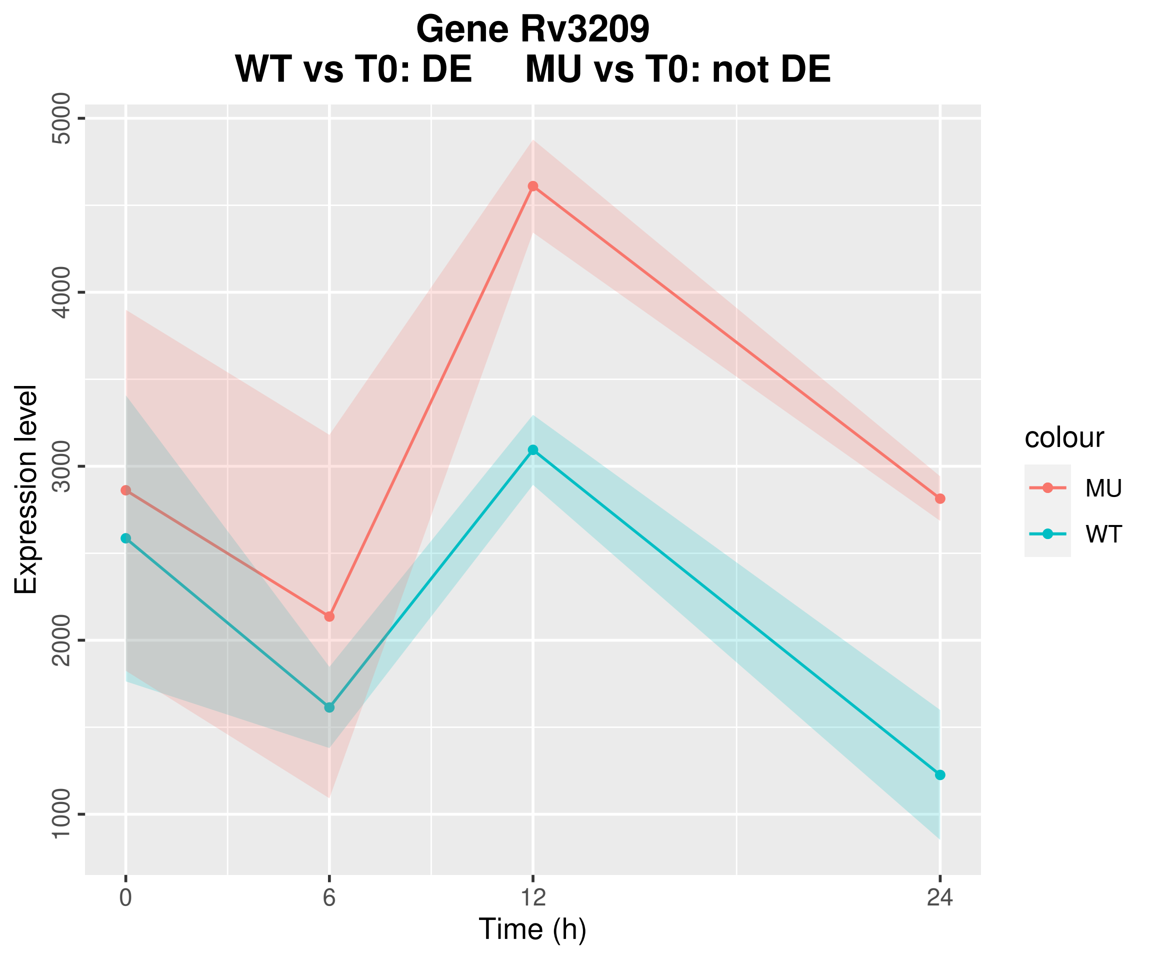
Gene Rv3209
WT vs T0: DE   MU vs T0: not DE
Expression level
5000
4000
3000
2000
1000
0
6
12
24
Time (h)
colour
MU
WT

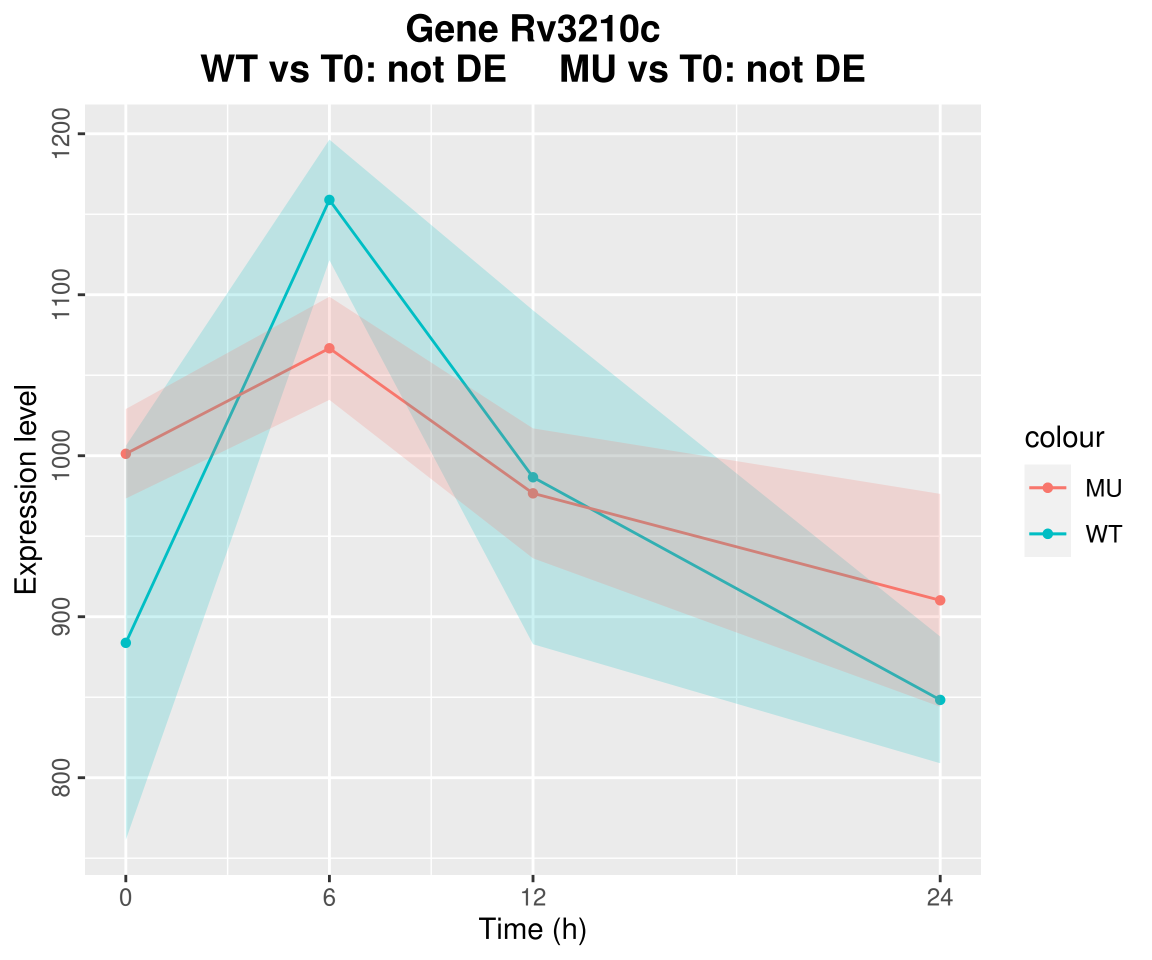

Gene Rv3210c
WT vs T0: not DE   MU vs T0: not DE
Expression level
1200
1100
1000
900
800
0
6
12
24
Time (h)
colour
MU
WT

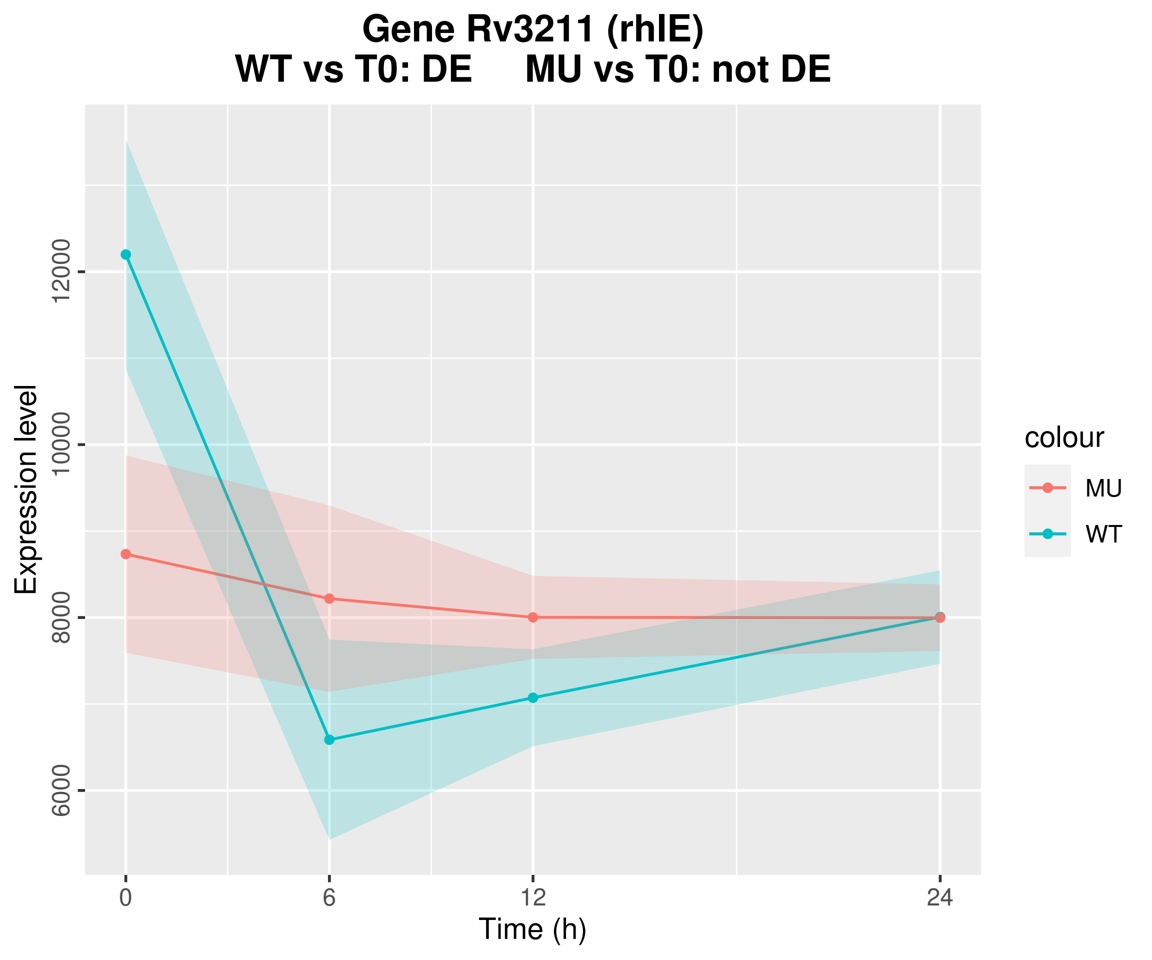

Gene Rv3211 (rhlE)
WT vs T0: DE MU vs T0: not DE
Expression level
12000
10000
8000
6000
0
6
12
24
Time (h)
colour
MU
WT

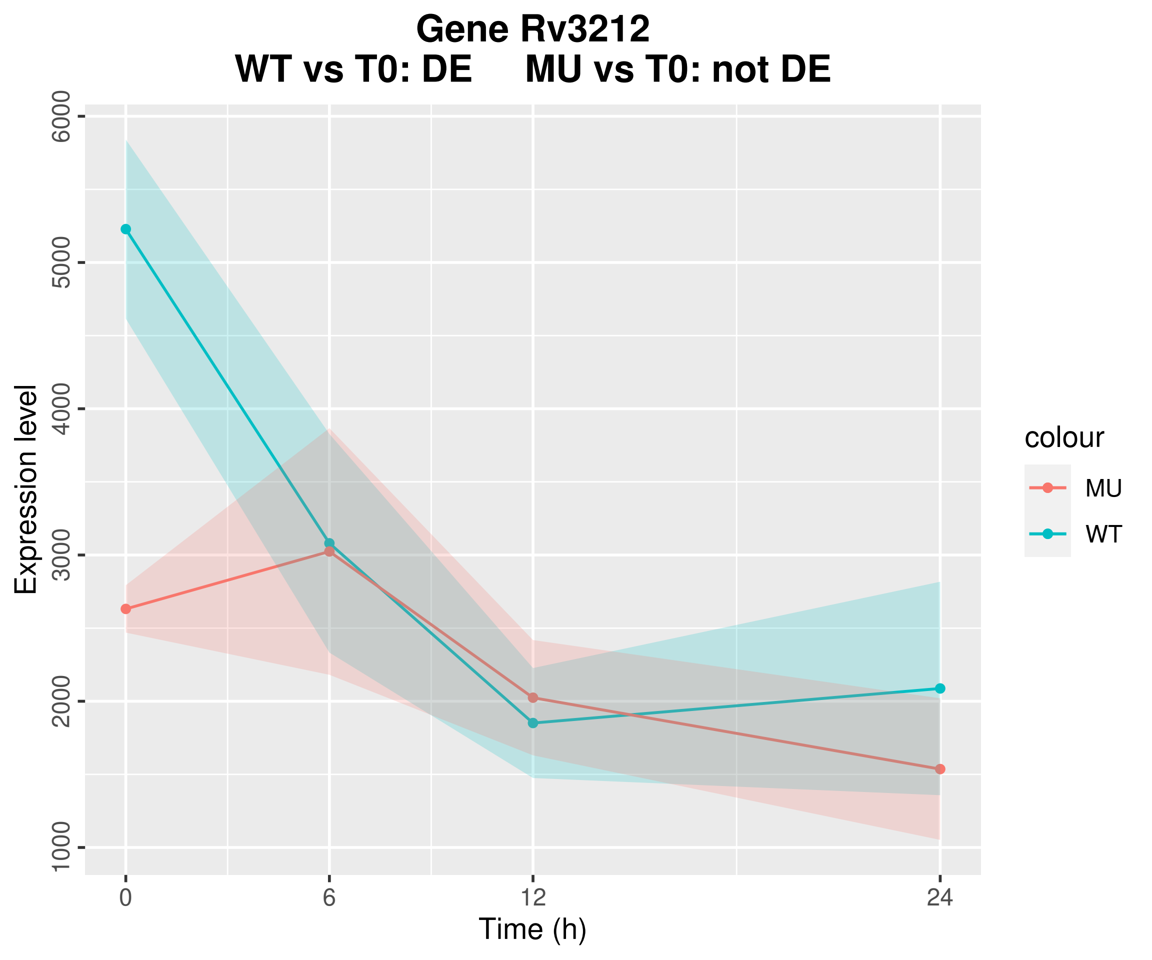
Gene Rv3212
WT vs T0: DE   MU vs T0: not DE
Expression level
6000
5000
4000
3000
2000
1000
0
6
12
24
Time (h)
colour
MU
WT

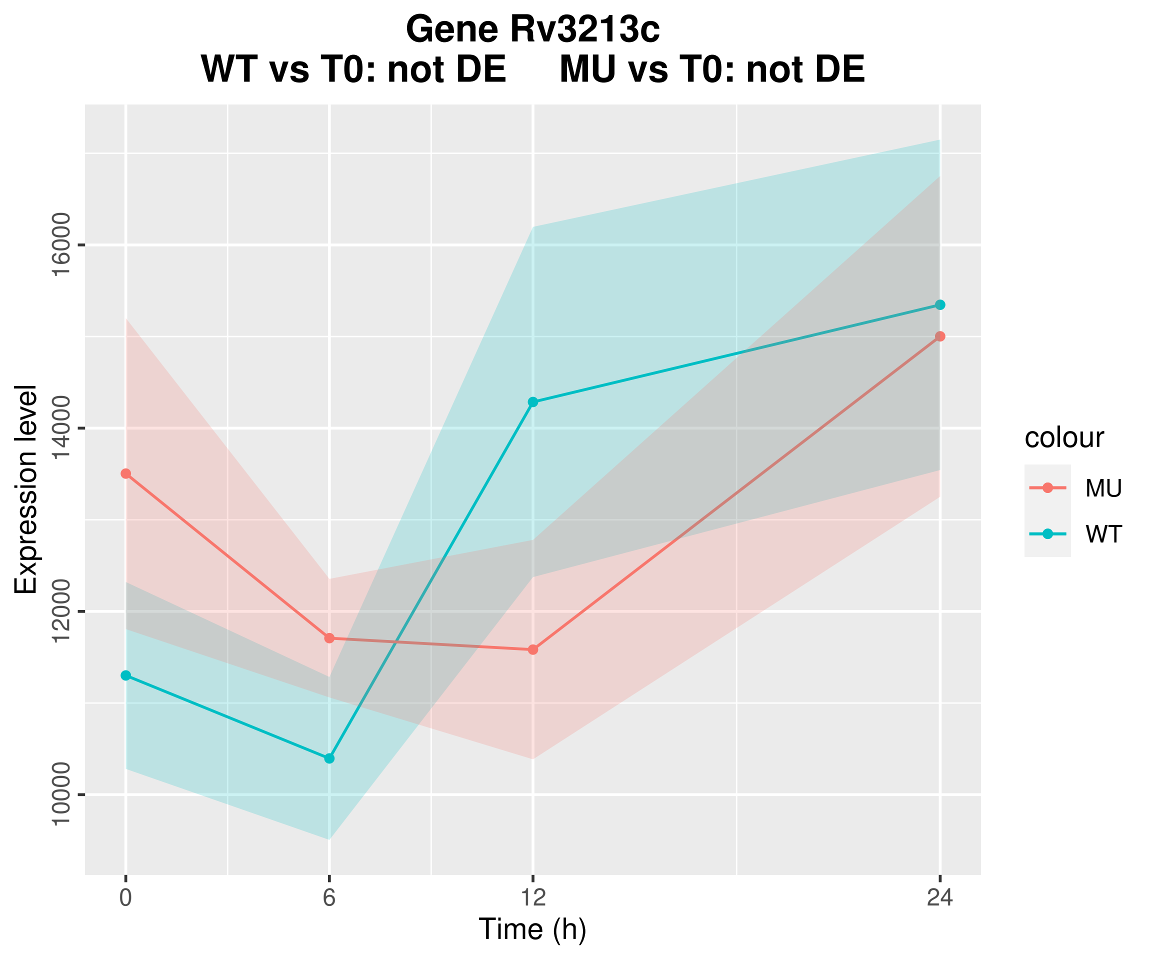
Gene Rv3213c
WT vs T0: not DE   MU vs T0: not DE
Expression level
10000
12000
14000
16000
0
6
12
24
Time (h)
colour
MU
WT

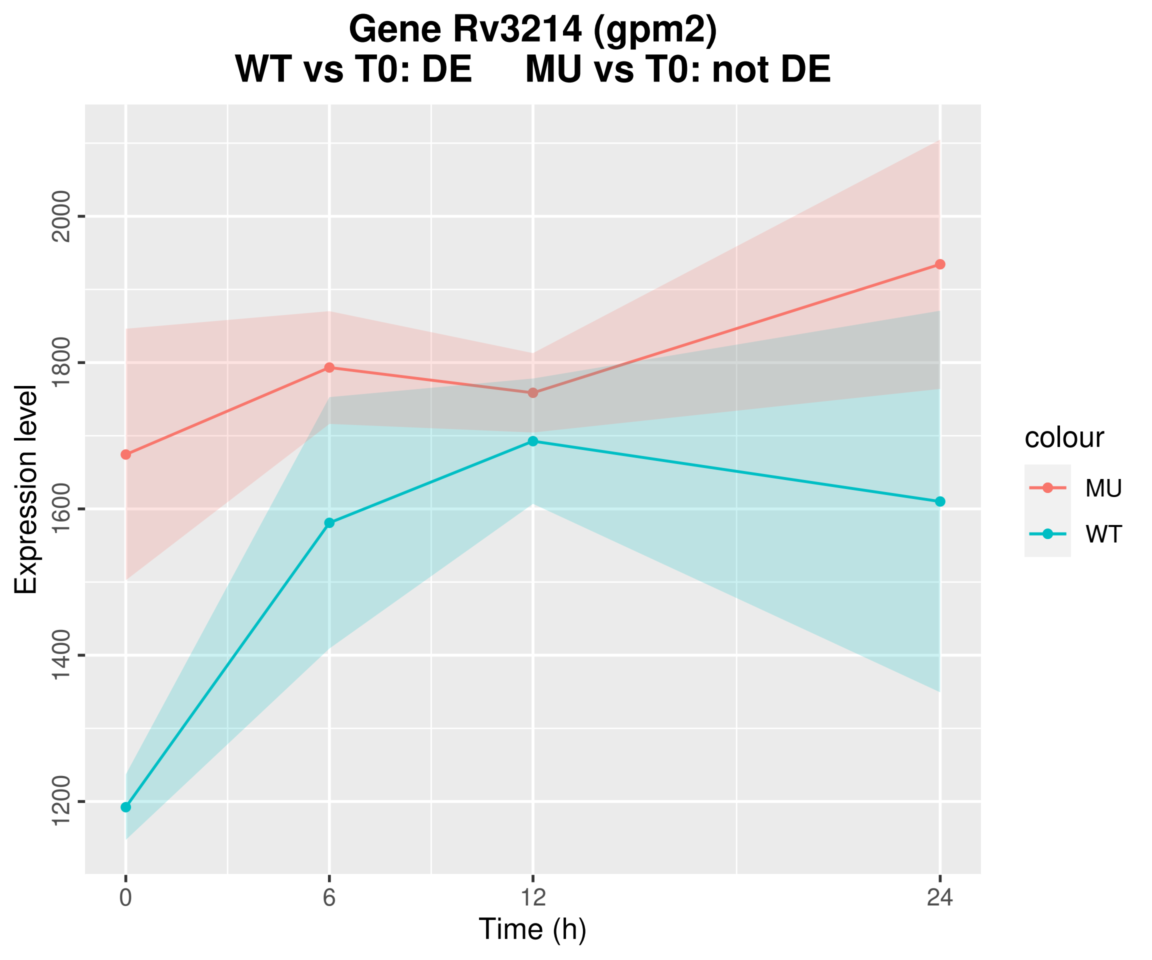
Gene Rv3214 (gpm2)
WT vs T0: DE   MU vs T0: not DE
Expression level
2000
1800
1600
1400
1200
0
6
12
24
Time (h)
colour
MU
WT

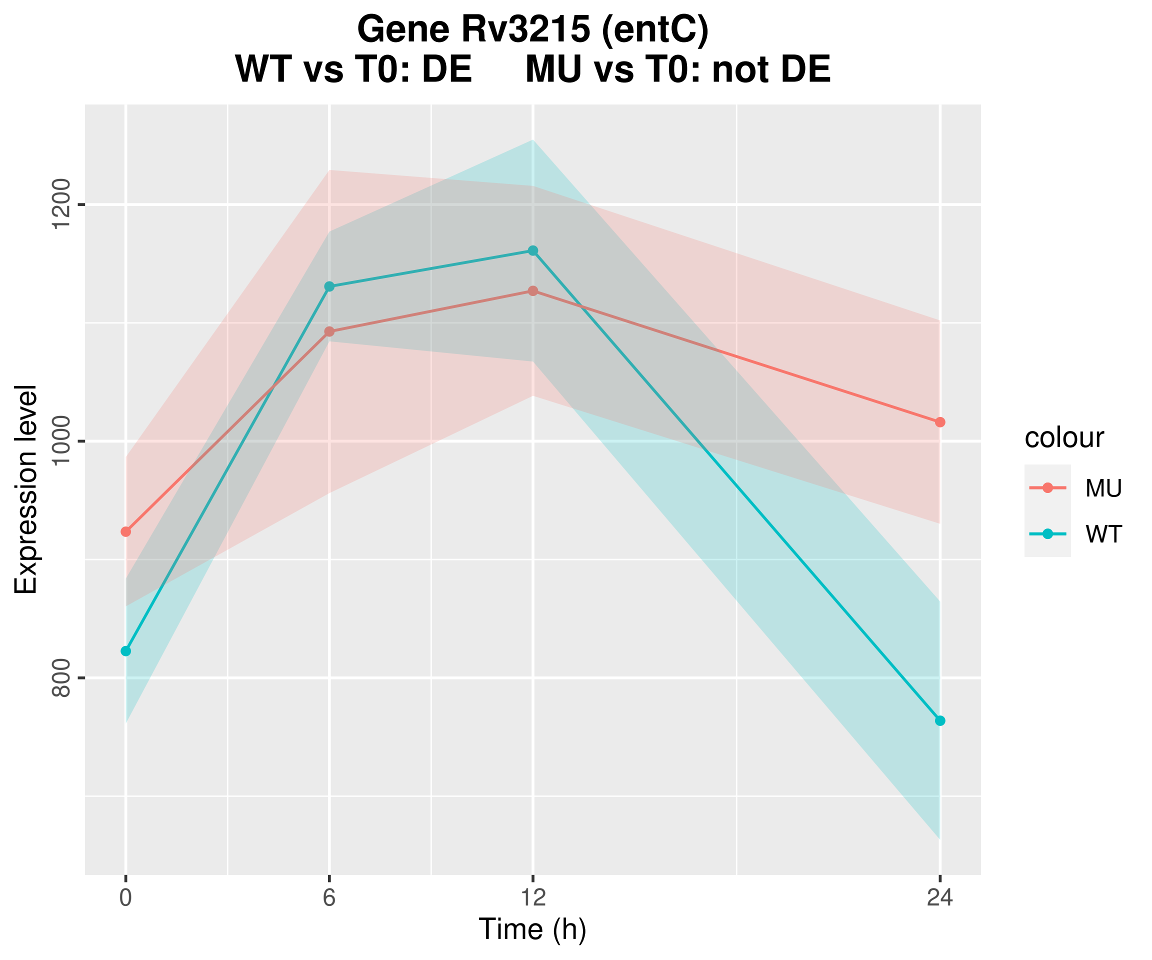

Gene Rv3215 (entC)
WT vs T0: DE MU vs T0: not DE
Expression level
1200
1000
800
0
6
12
24
Time (h)
colour
MU
WT

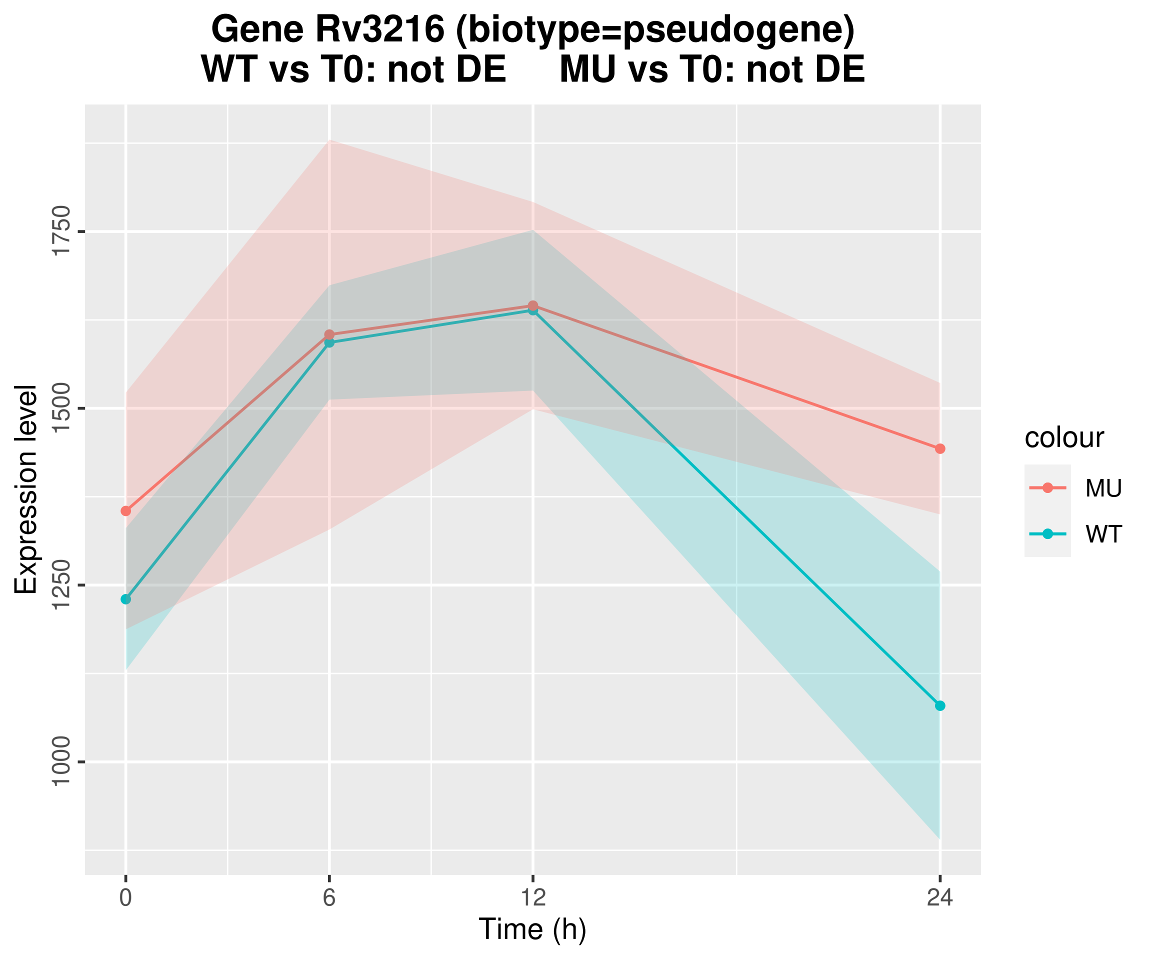
Gene Rv3216 (biotype=pseudogene)
WT vs T0: not DE MU vs T0: not DE
Expression level
1000
1250
1500
1750
0
6
12
24
Time (h)
colour
MU
WT

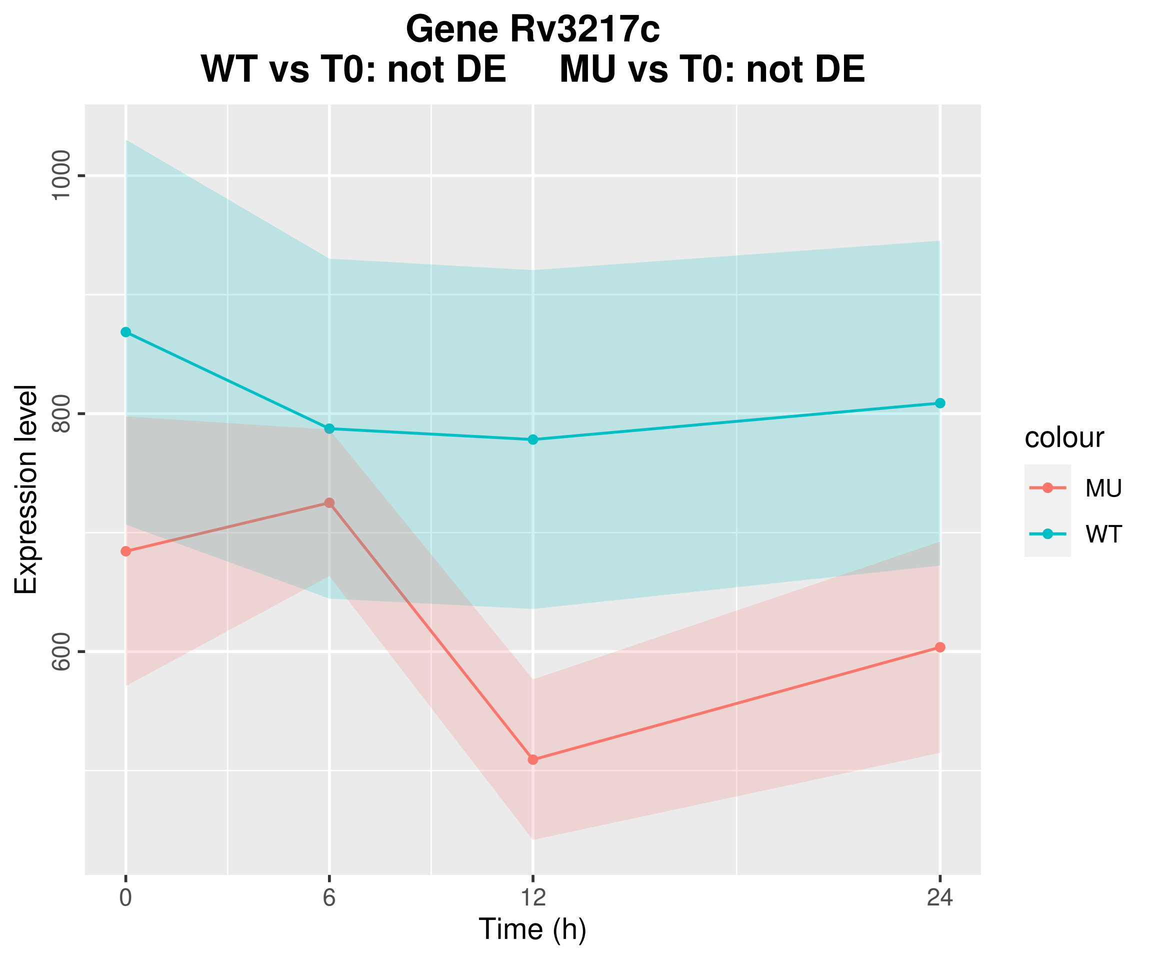

Gene Rv3217c
WT vs T0: not DE MU vs T0: not DE
Expression level
1000
800
600
0
6
12
24
Time (h)
colour
MU
WT

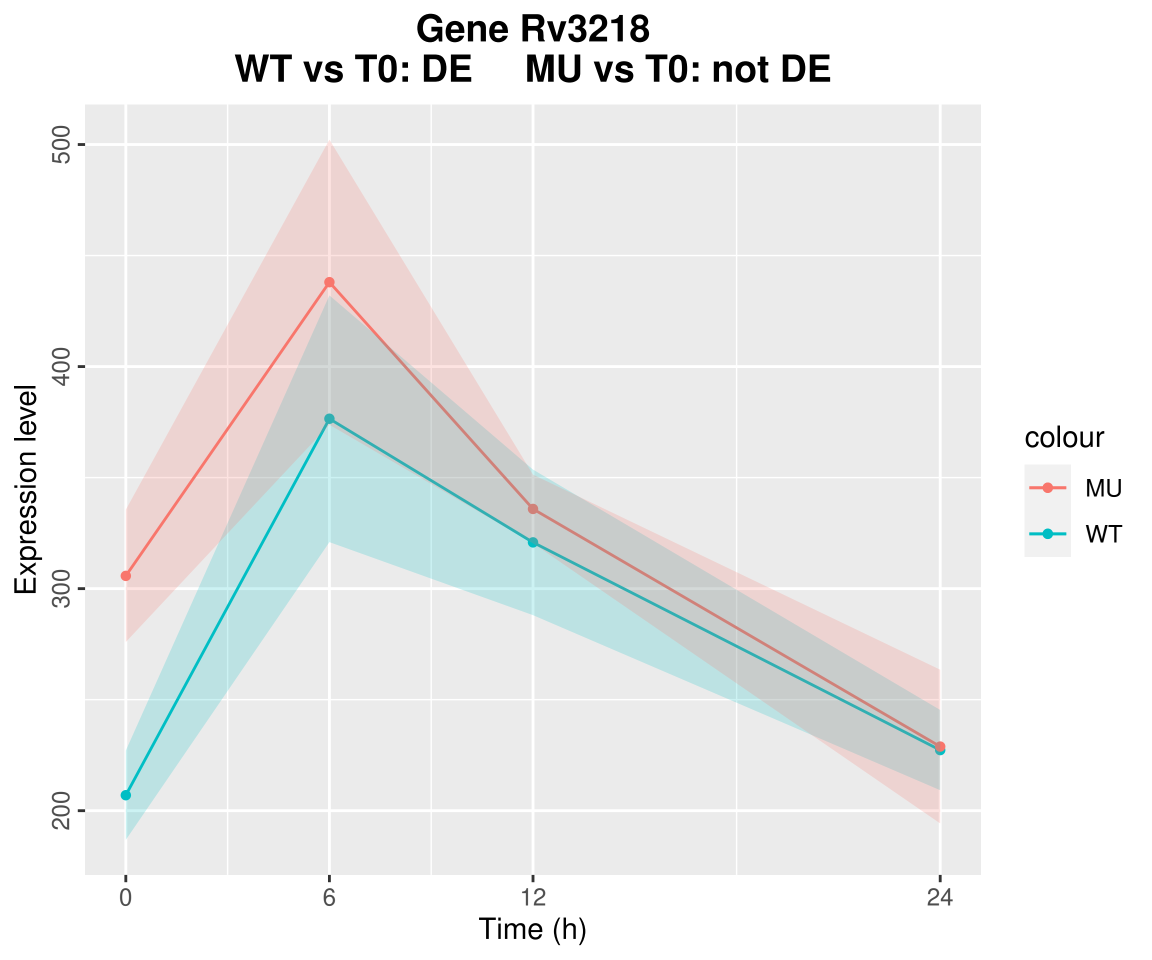
Gene Rv3218
WT vs T0: DE MU vs T0: not DE
Expression level
500
400
300
200
0
6
12
24
Time (h)
colour
MU
WT

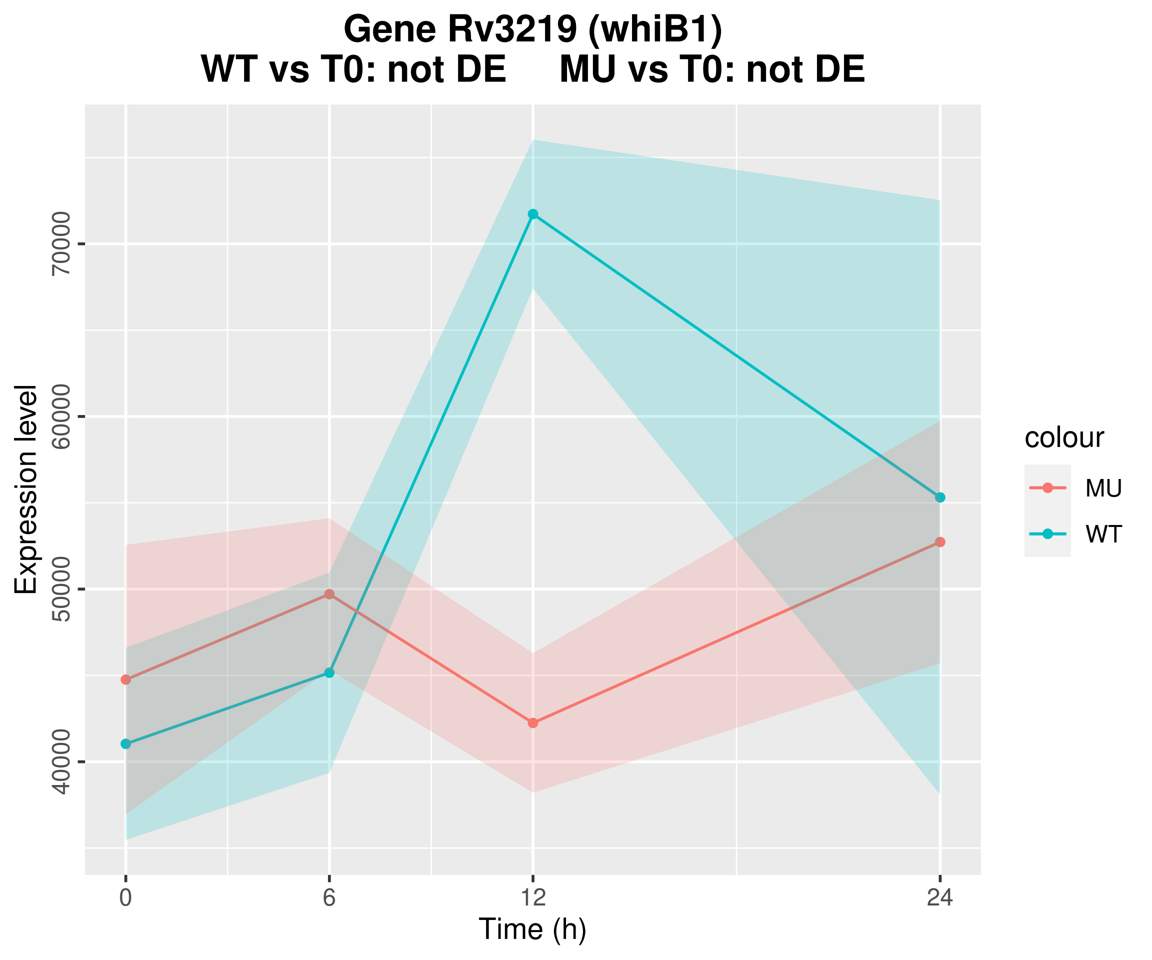
Gene Rv3219 (whiB1)
WT vs T0: not DE   MU vs T0: not DE
Expression level
70000
60000
50000
40000
0
6
12
24
Time (h)
colour
MU
WT

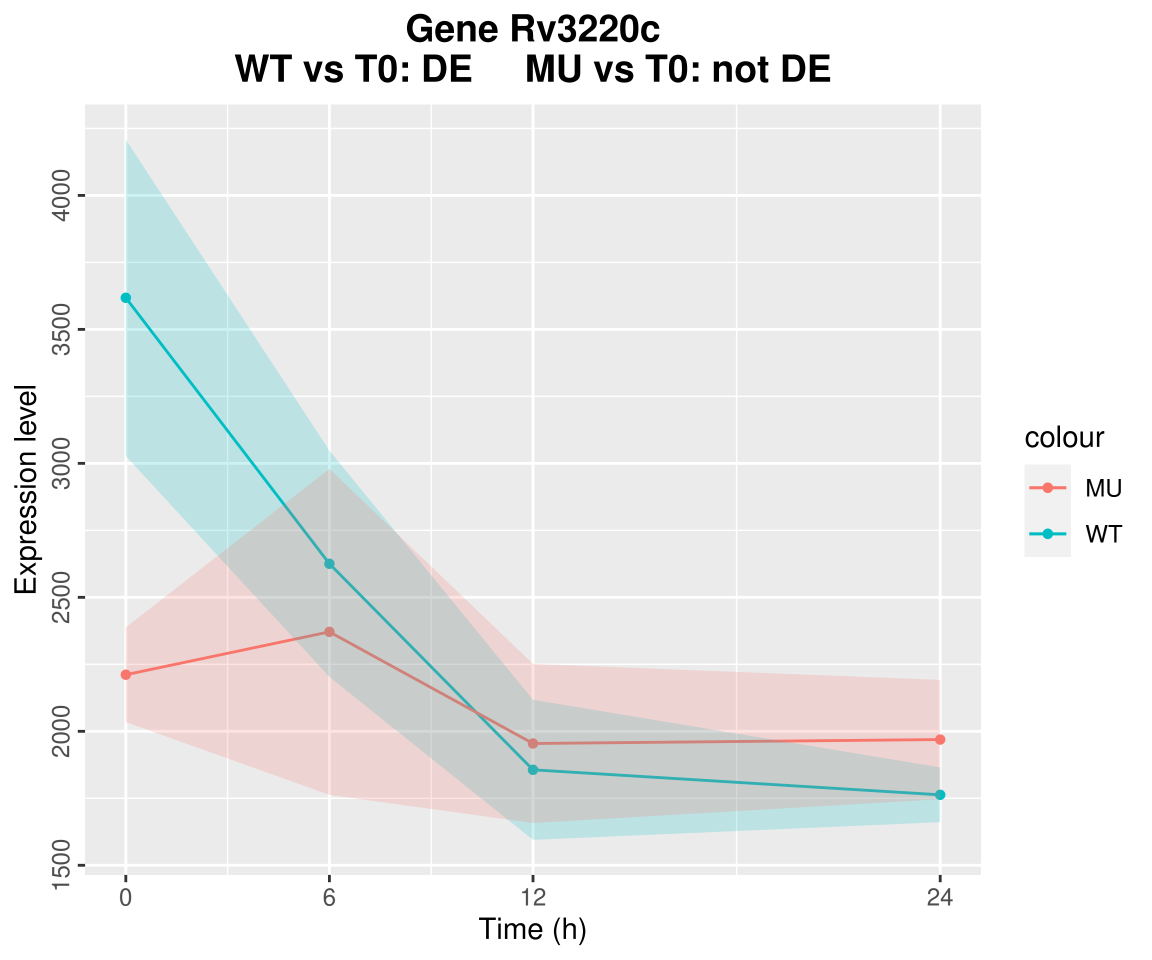

Gene Rv3220c
WT vs T0: DE   MU vs T0: not DE
Expression level
4000
3500
3000
2500
2000
1500
0
6
12
24
Time (h)
colour
MU
WT

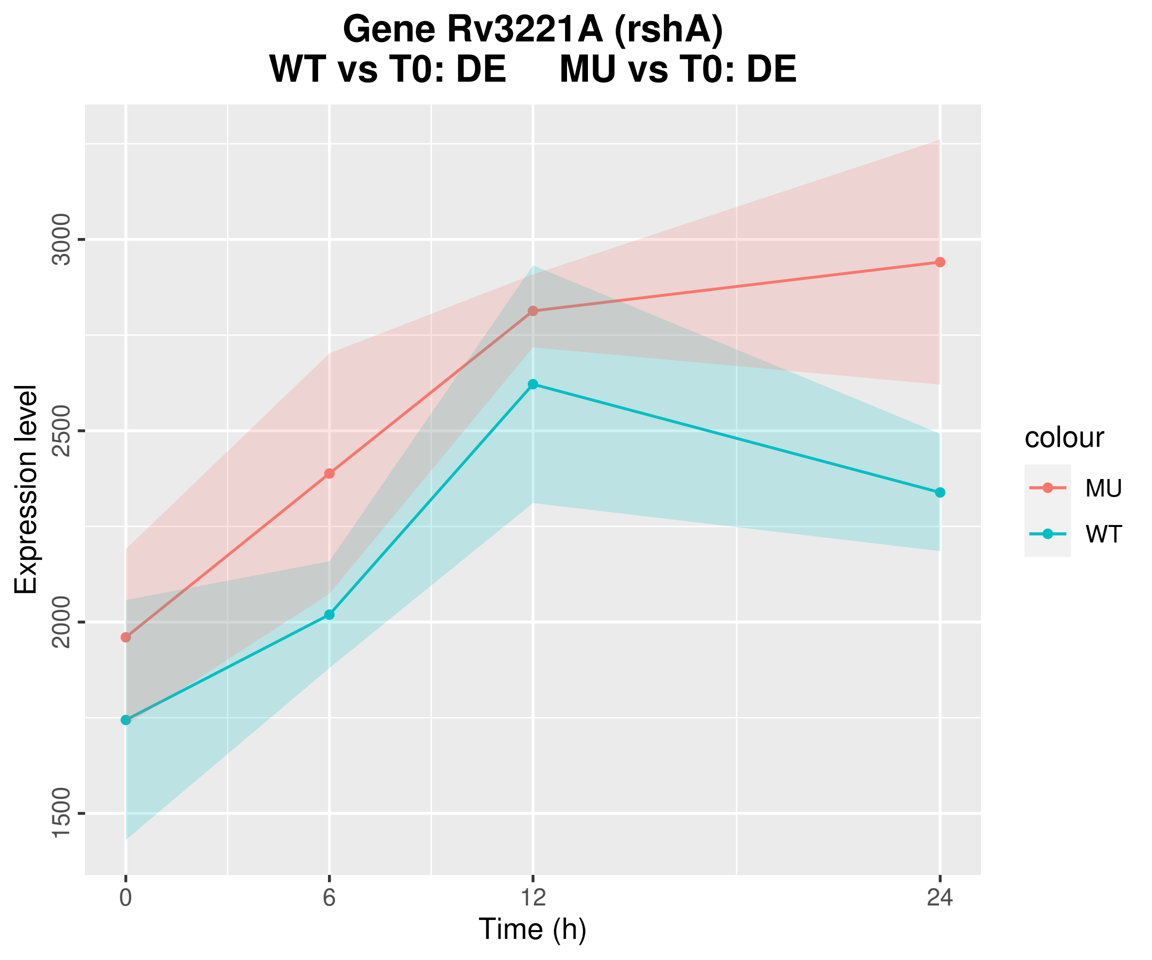
Gene Rv3221A (rshA)
WT vs T0: DE MU vs T0: DE
Expression level
3000
2500
2000
1500
0
6
12
24
Time (h)
colour
MU
WT

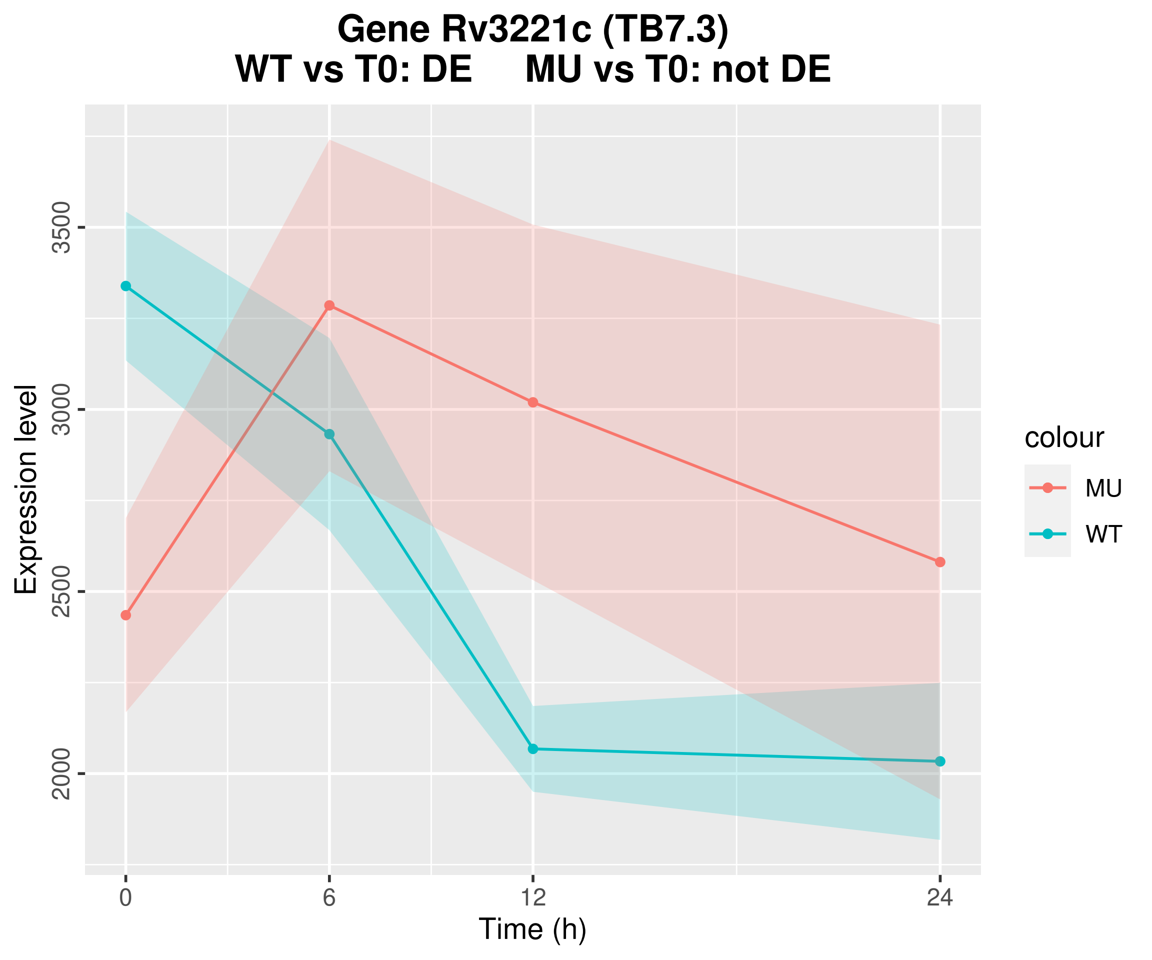
Gene Rv3221c (TB7.3)
WT vs T0: DE   MU vs T0: not DE
Expression level
3500
3000
2500
2000
0
6
12
24
Time (h)
colour
MU
WT

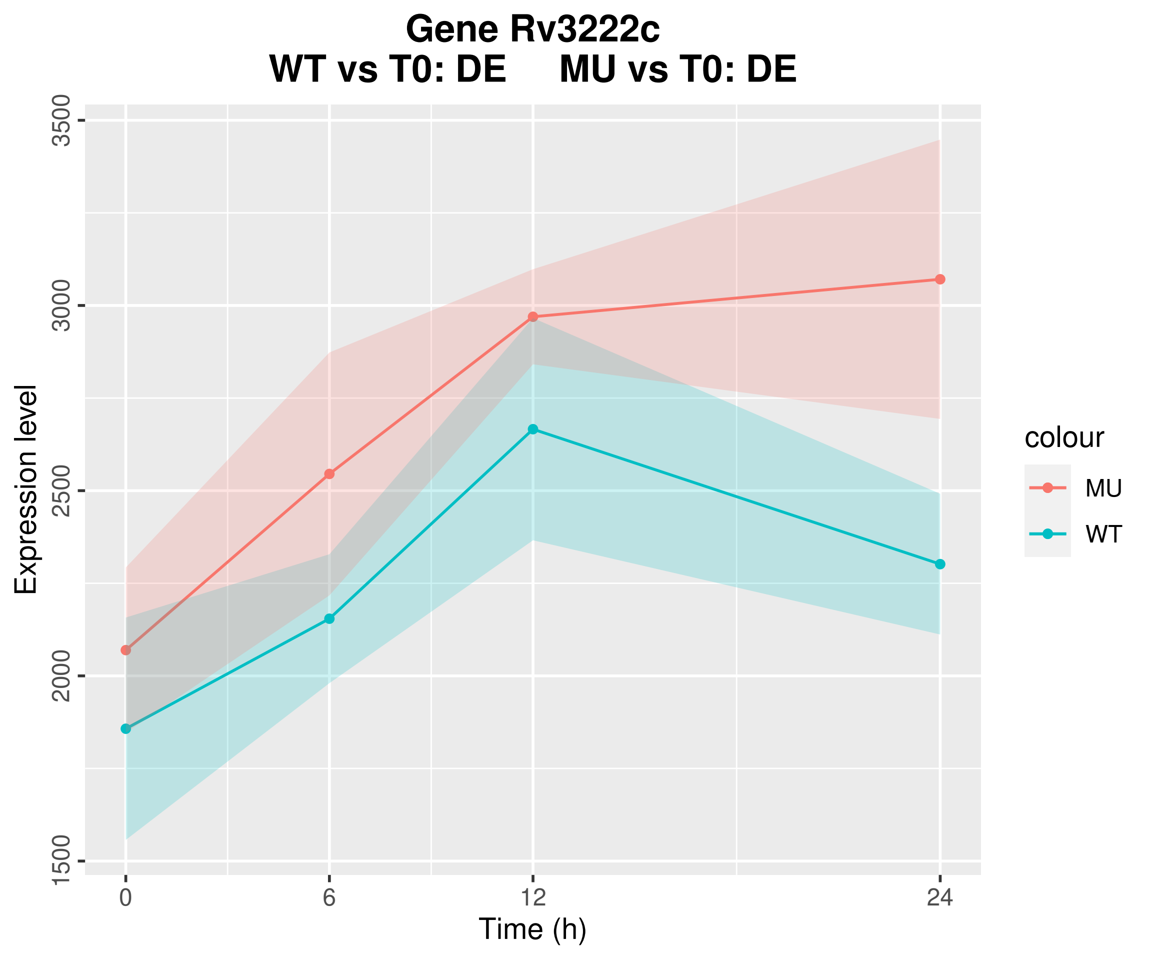
Gene Rv3222c
WT vs T0: DE MU vs T0: DE
Expression level
3500
3000
2500
2000
1500
0
6
12
24
Time (h)
colour
MU
WT

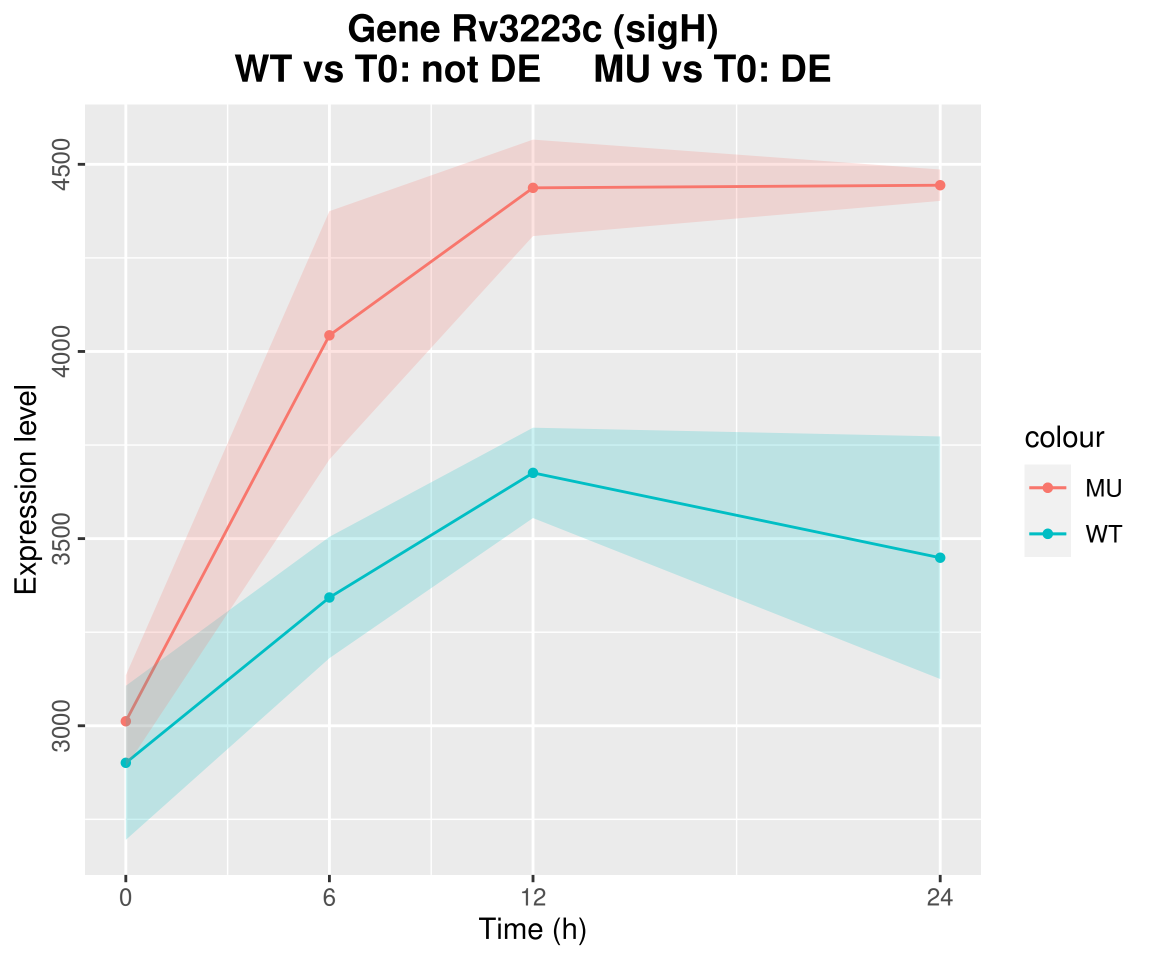
Gene Rv3223c (sigH)
WT vs T0: not DE   MU vs T0: DE
Expression level
4500
4000
3500
3000
0
6
12
24
Time (h)
colour
MU
WT

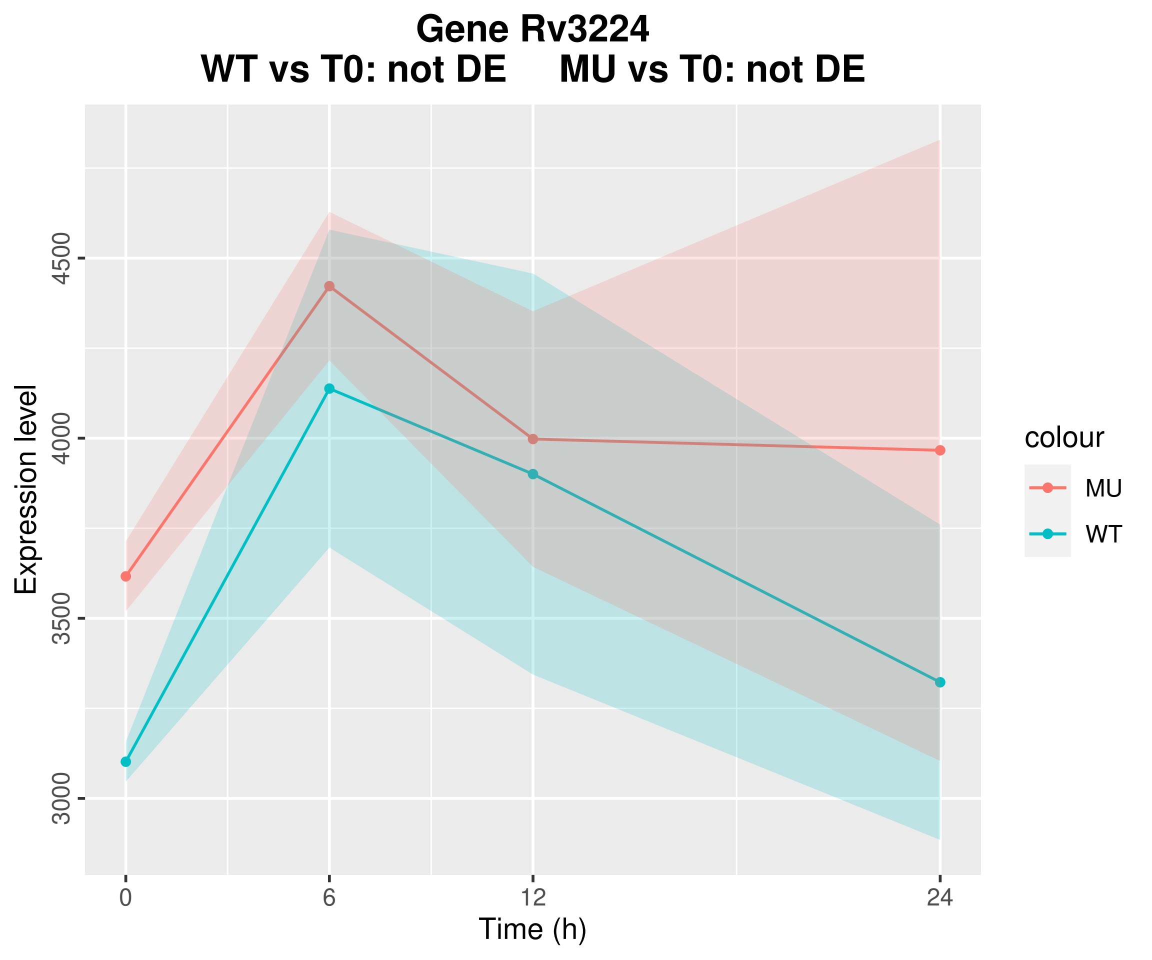

Gene Rv3224
WT vs T0: not DE   MU vs T0: not DE
Expression level
4500
4000
3500
3000
0
6
12
24
Time (h)
colour
MU
WT

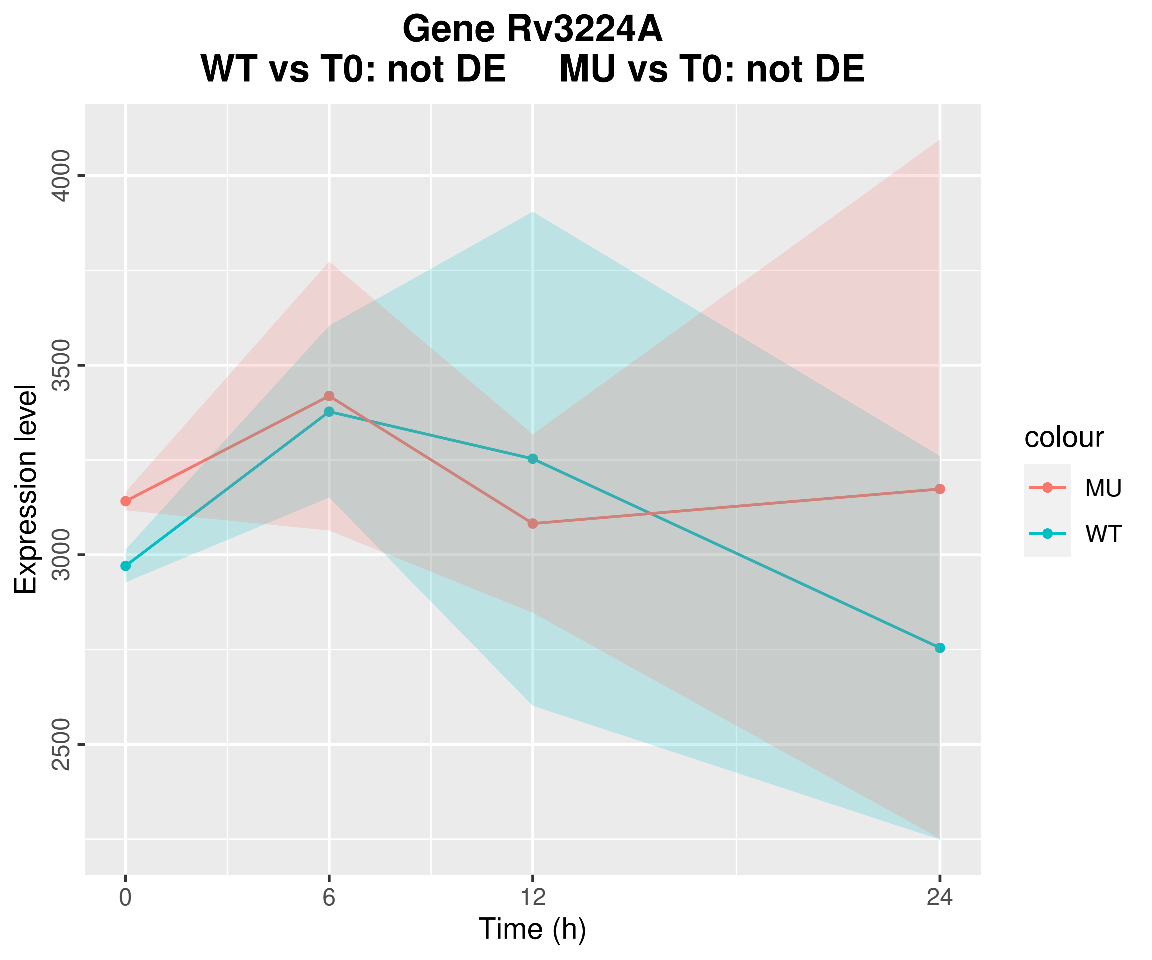
Gene Rv3224A
WT vs T0: not DE   MU vs T0: not DE
Expression level
4000
3500
3000
2500
0
6
12
24
Time (h)
colour
MU
WT

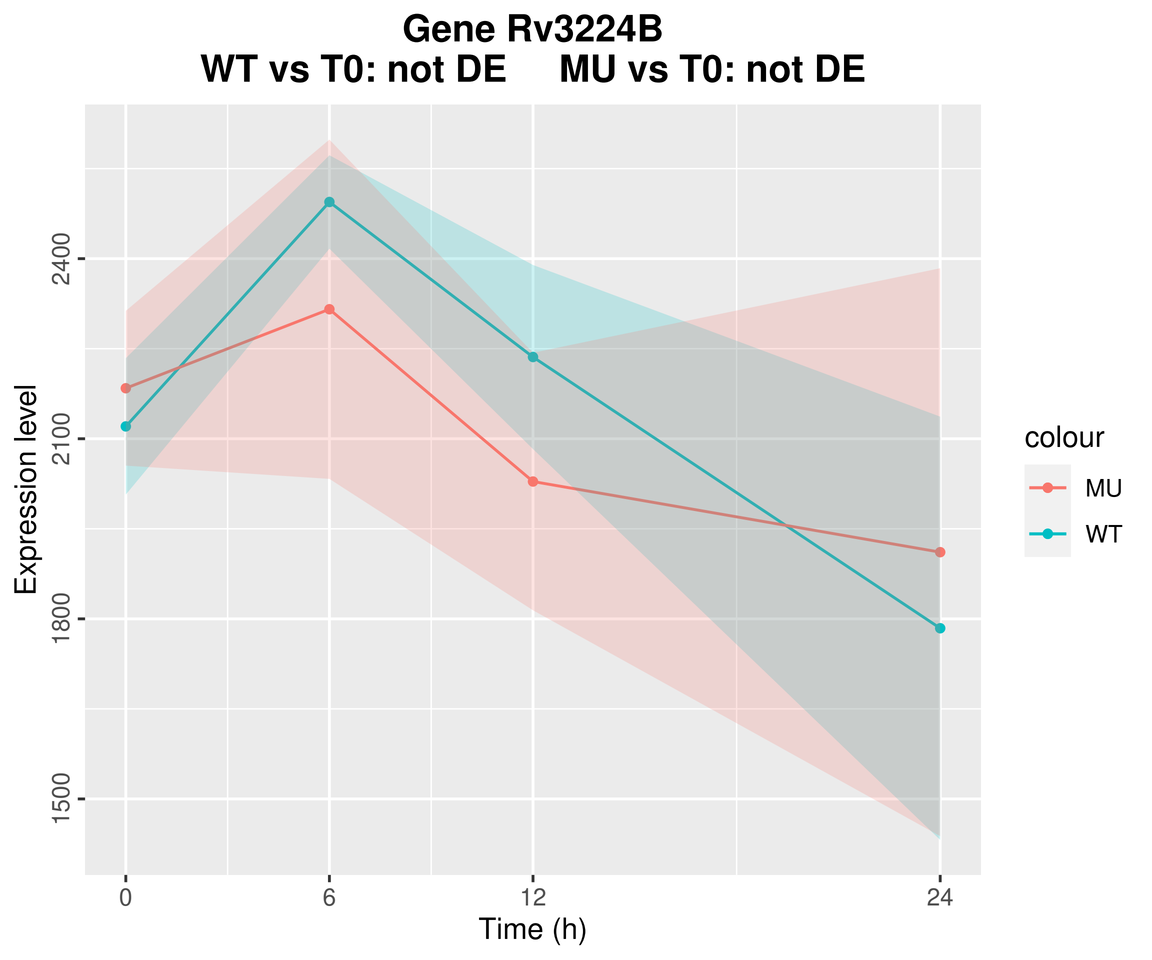
Gene Rv3224B
WT vs T0: not DE   MU vs T0: not DE
Expression level
2400
2100
1800
1500
0
6
12
24
Time (h)
colour
MU
WT

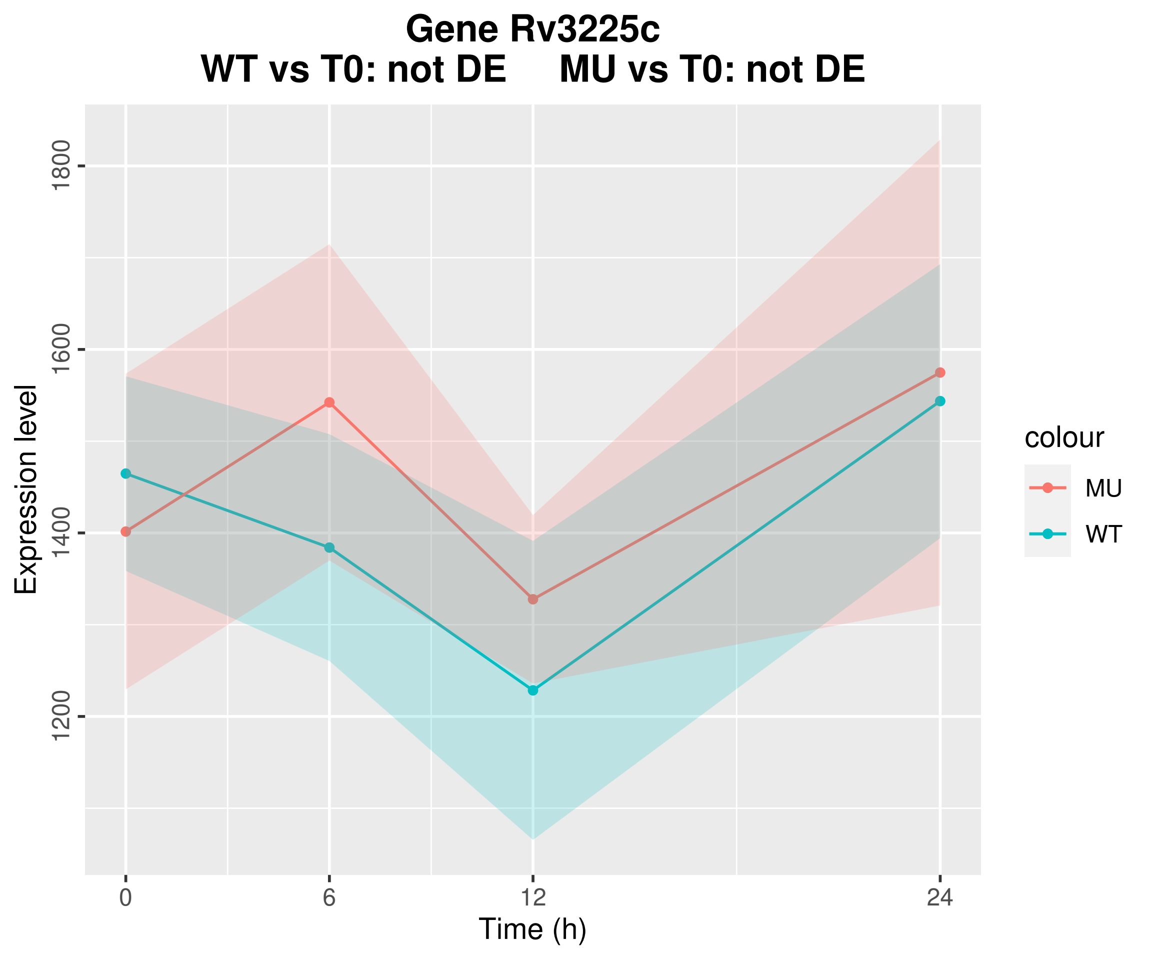

Gene Rv3225c
WT vs T0: not DE MU vs T0: not DE
1800
1600
1400
1200
Expression level
0
6
12
24
Time (h)
colour
MU
WT

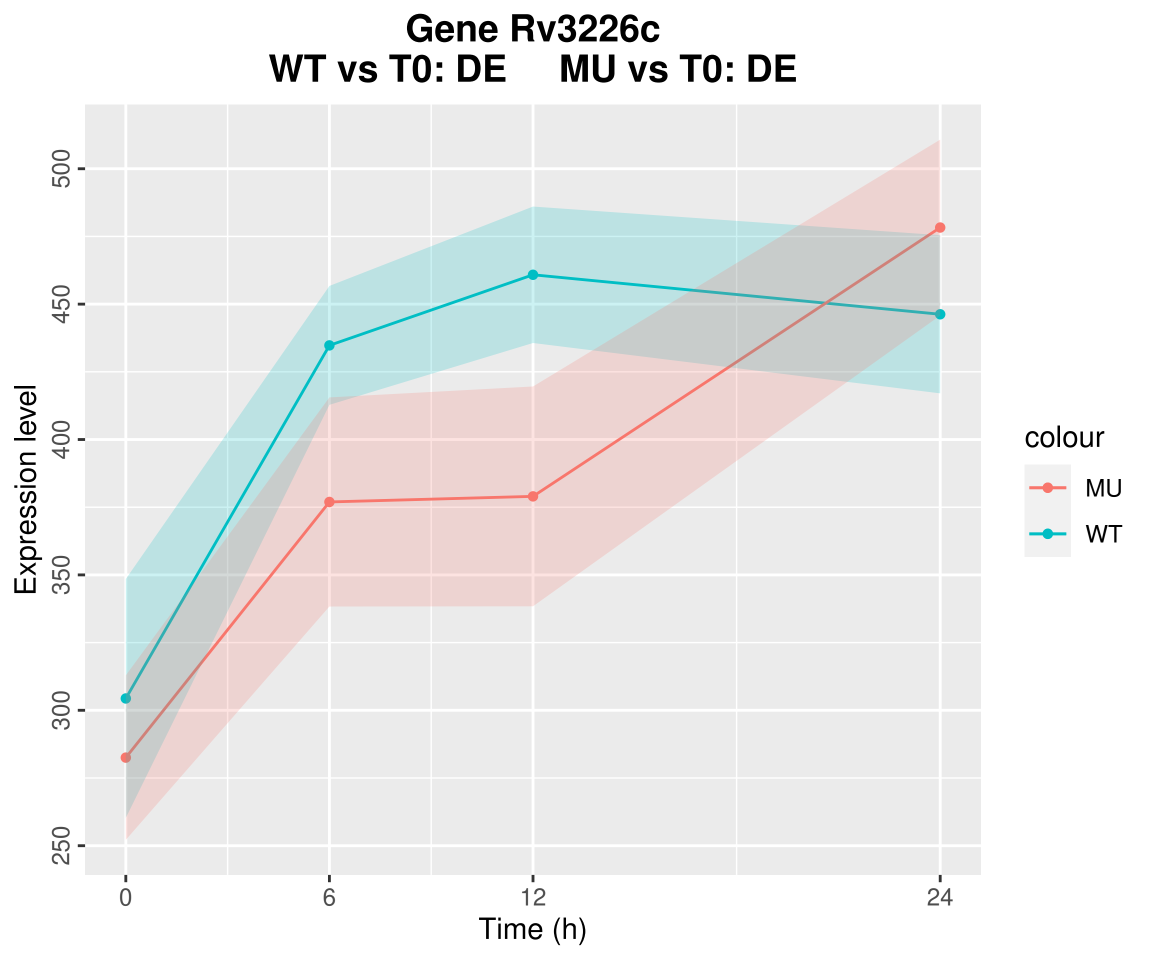

Gene Rv3226c
WT vs T0: DE MU vs T0: DE
Expression level
250
300
350
400
450
500
0
6
12
24
Time (h)
colour
MU
WT

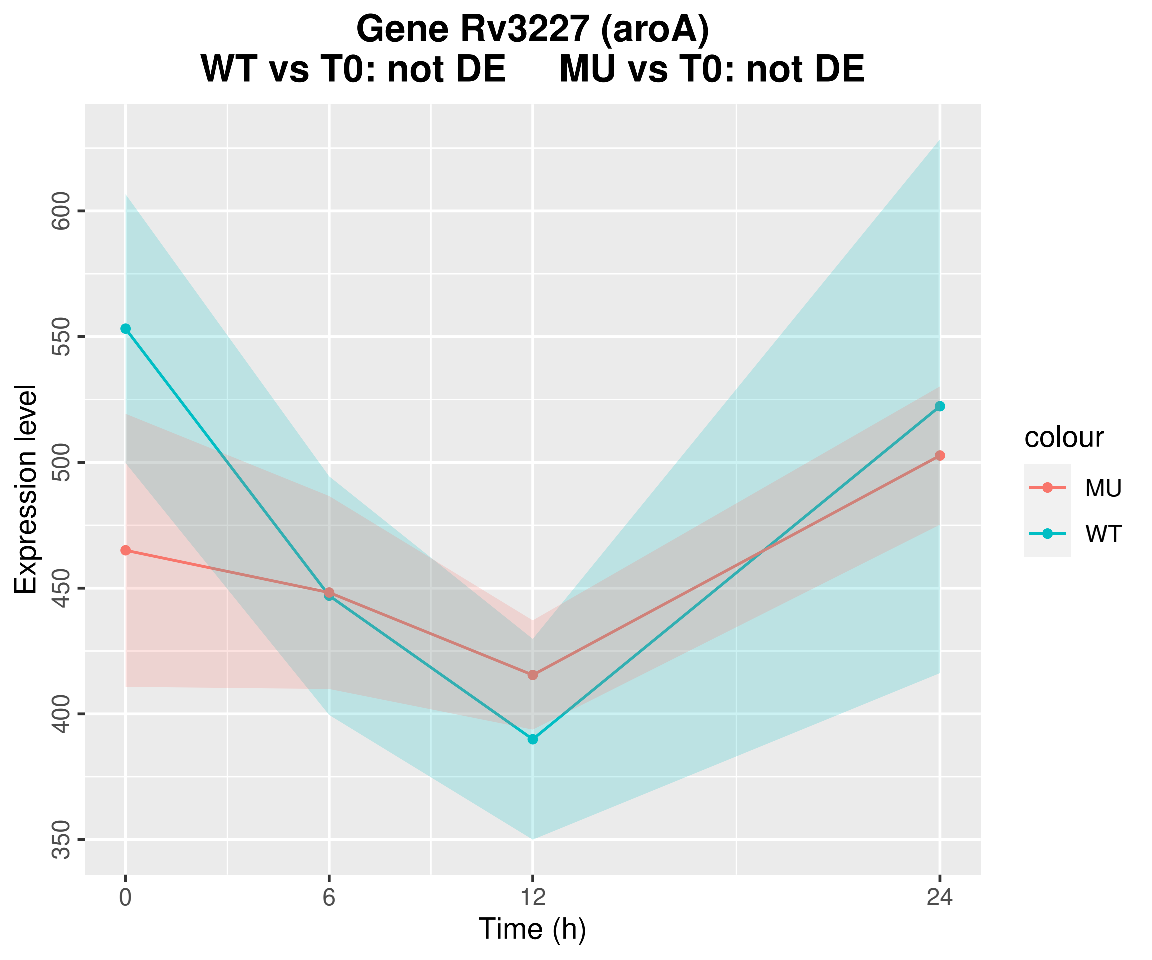
Gene Rv3227 (aroA)
WT vs T0: not DE   MU vs T0: not DE
Expression level
350
400
450
500
550
600
0
6
12
24
Time (h)
colour
MU
WT

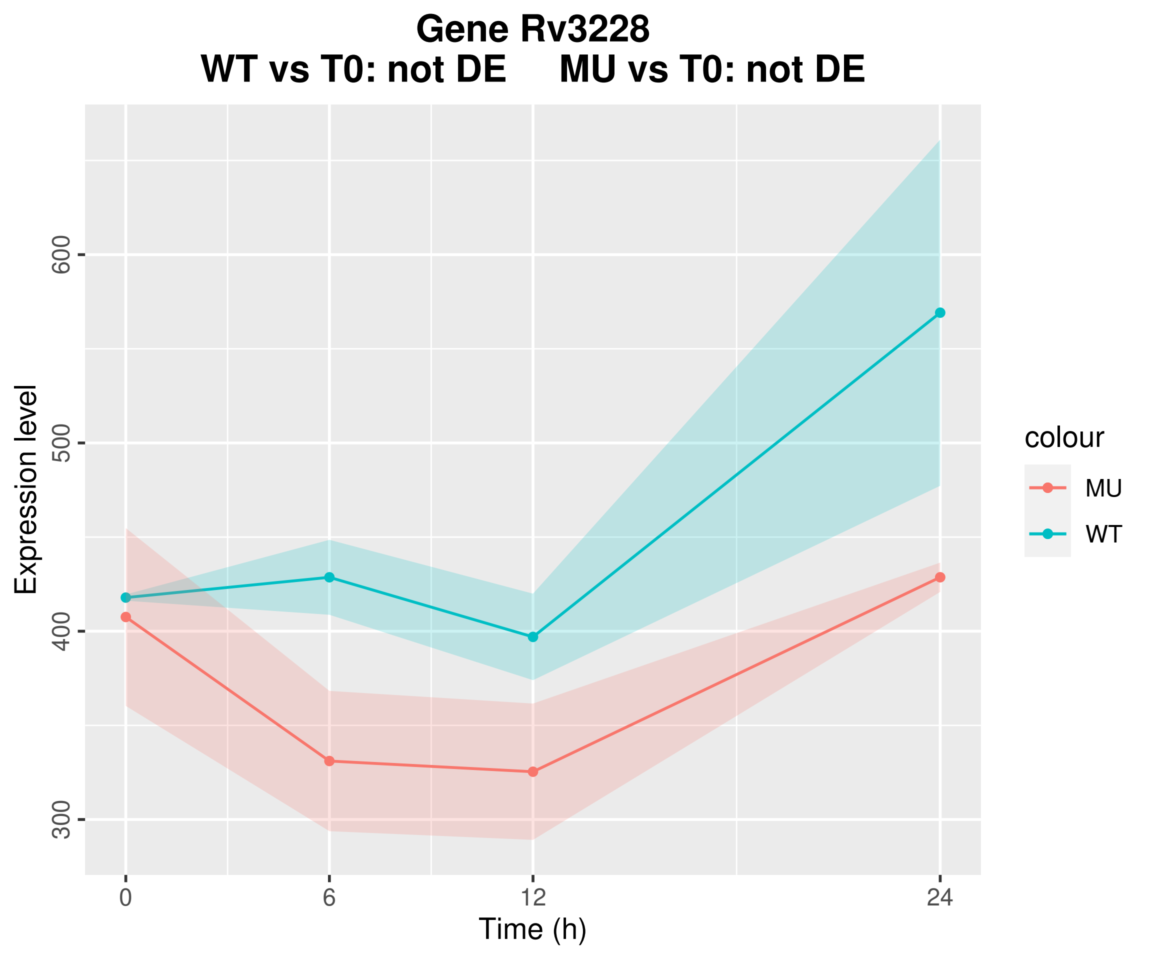
Gene Rv3228
WT vs T0: not DE   MU vs T0: not DE
Expression level
600
500
400
300
0
6
12
24
Time (h)
colour
MU
WT

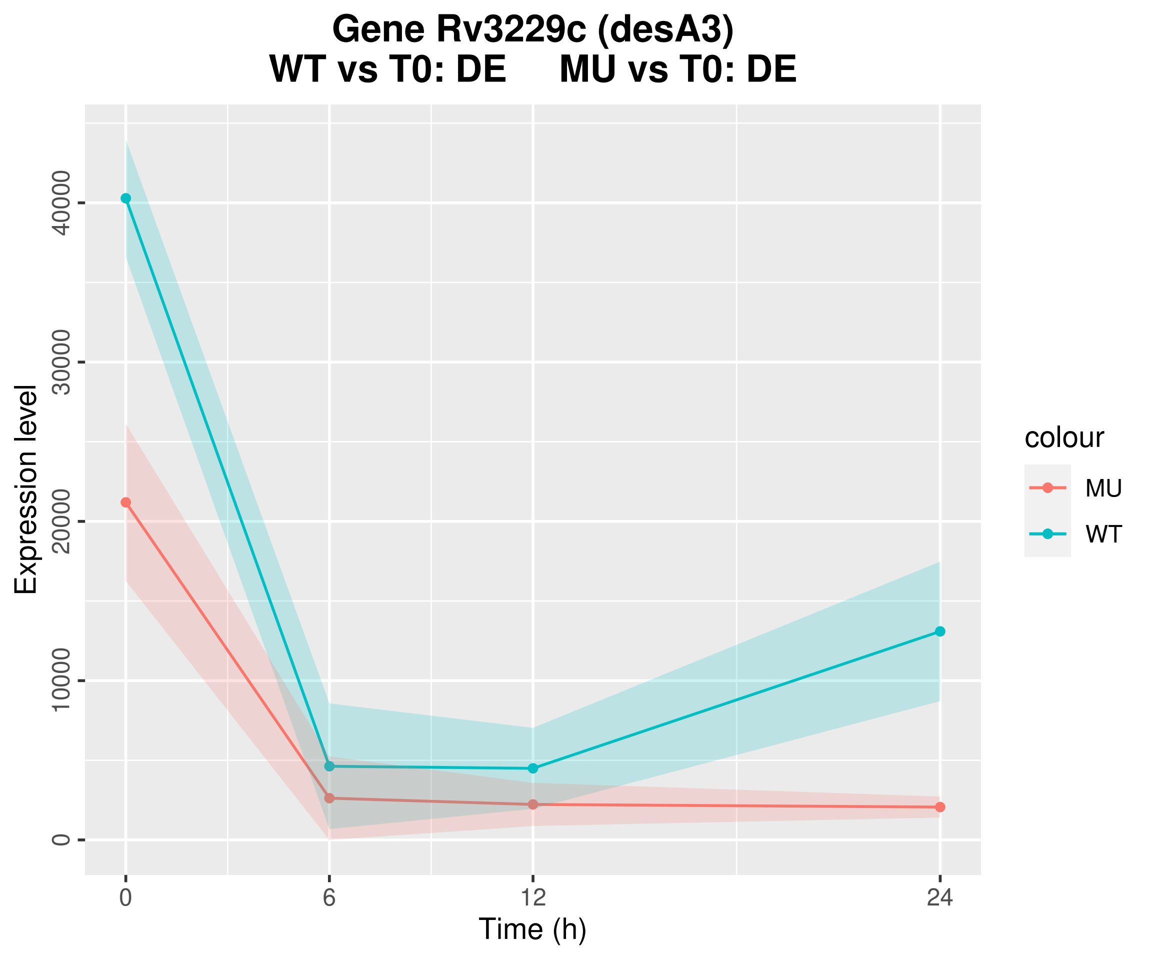
Gene Rv3229c (desA3)
WT vs T0: DE   MU vs T0: DE
Expression level
40000
30000
20000
10000
0
0
6
12
24
Time (h)
colour
MU
WT

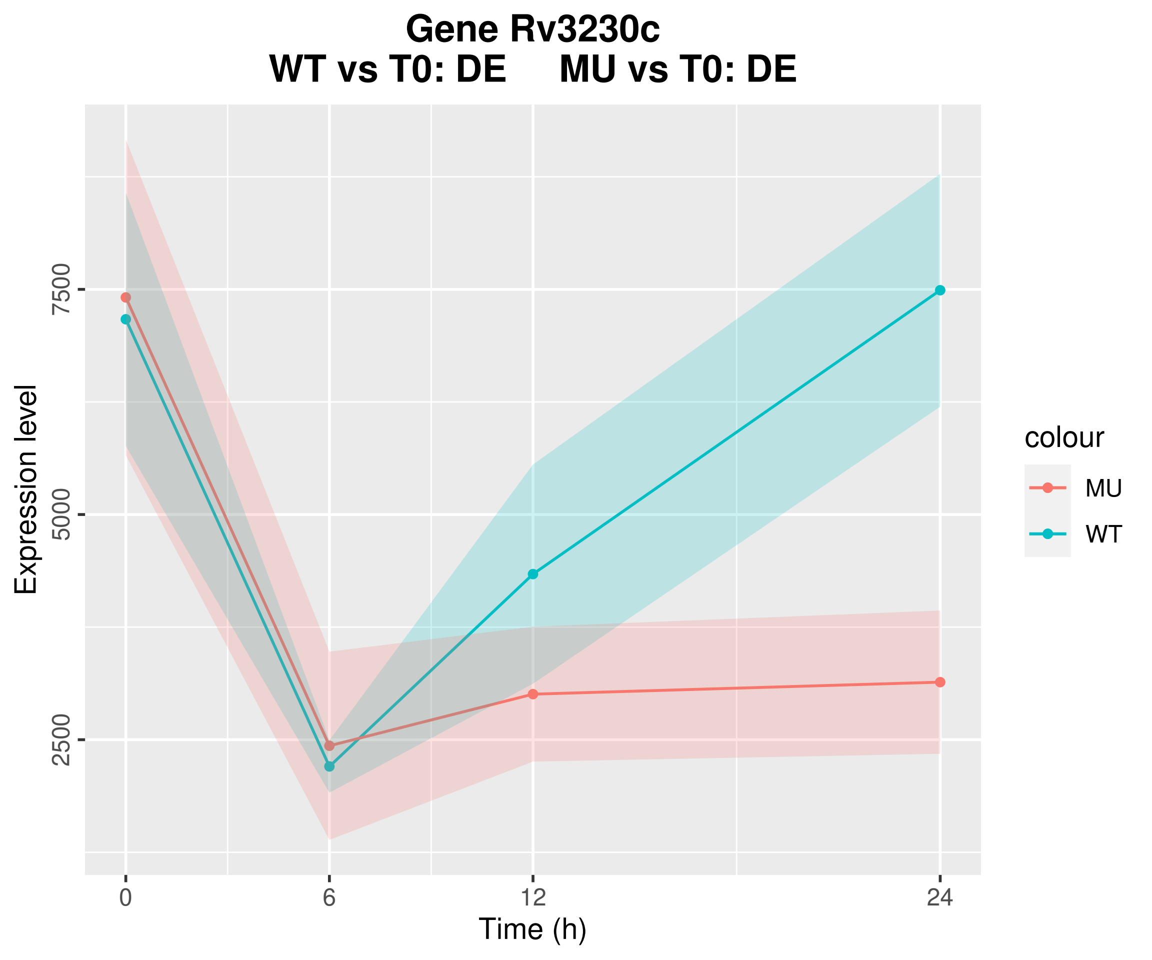

Gene Rv3230c
WT vs T0: DE   MU vs T0: DE
Expression level
7500
5000
2500
0
6
12
24
Time (h)
colour
MU
WT

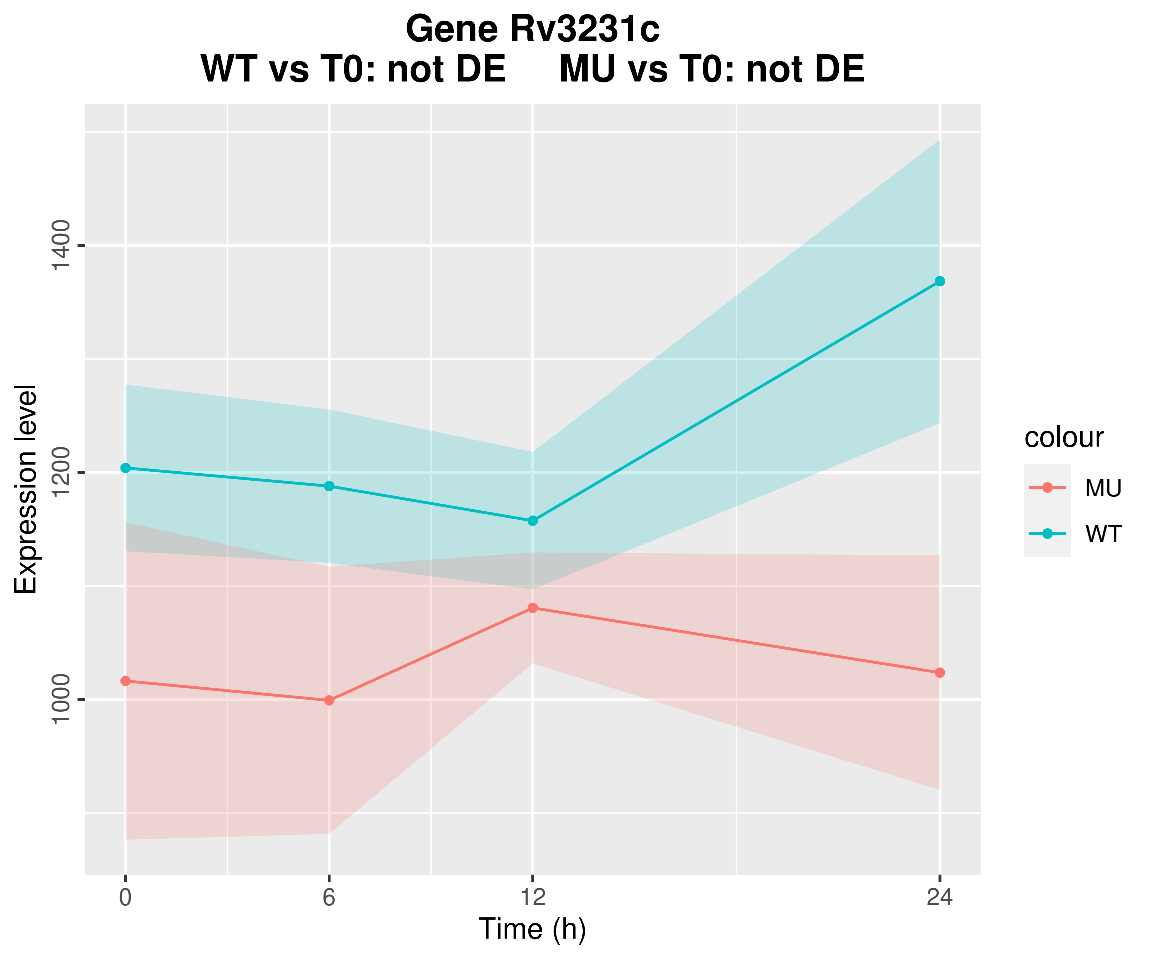
Gene Rv3231c
WT vs T0: not DE   MU vs T0: not DE
Expression level
1400
1200
1000
0
6
12
24
Time (h)
colour
MU
WT

Gene Rv3232c (ppk2)
WT vs T0: not DE   MU vs T0: not DE

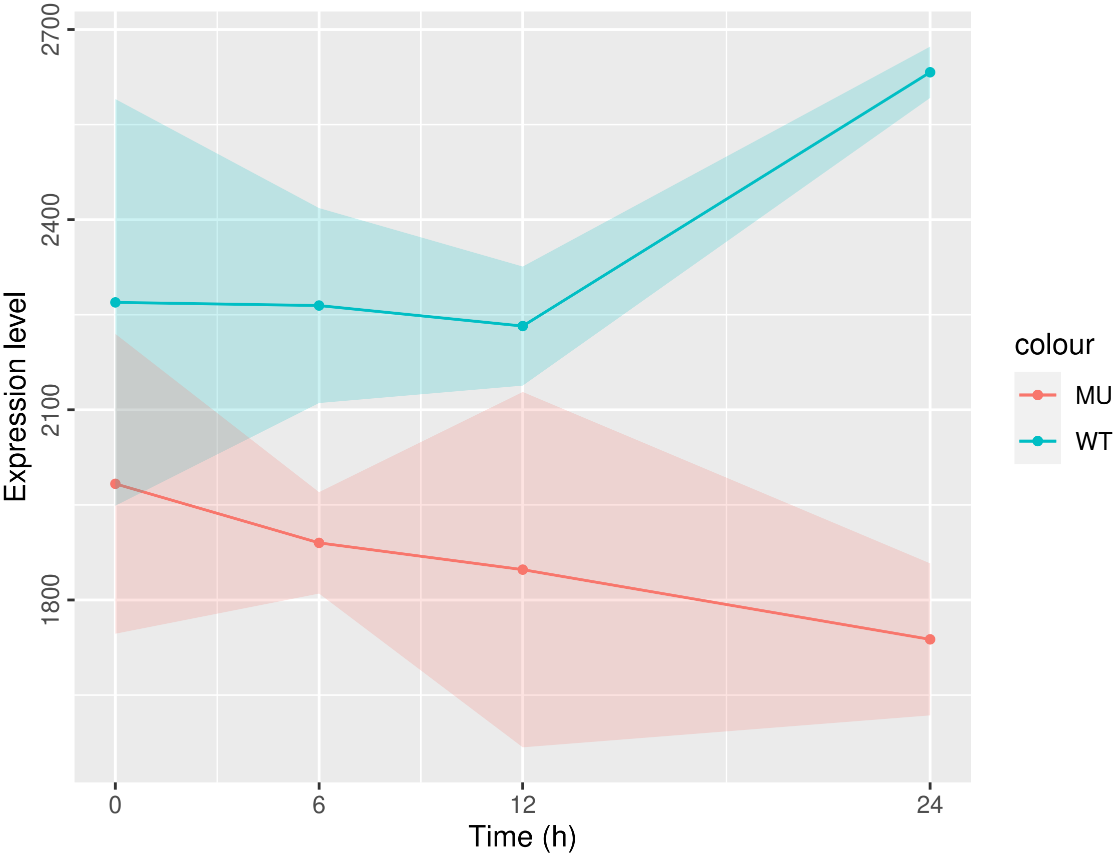

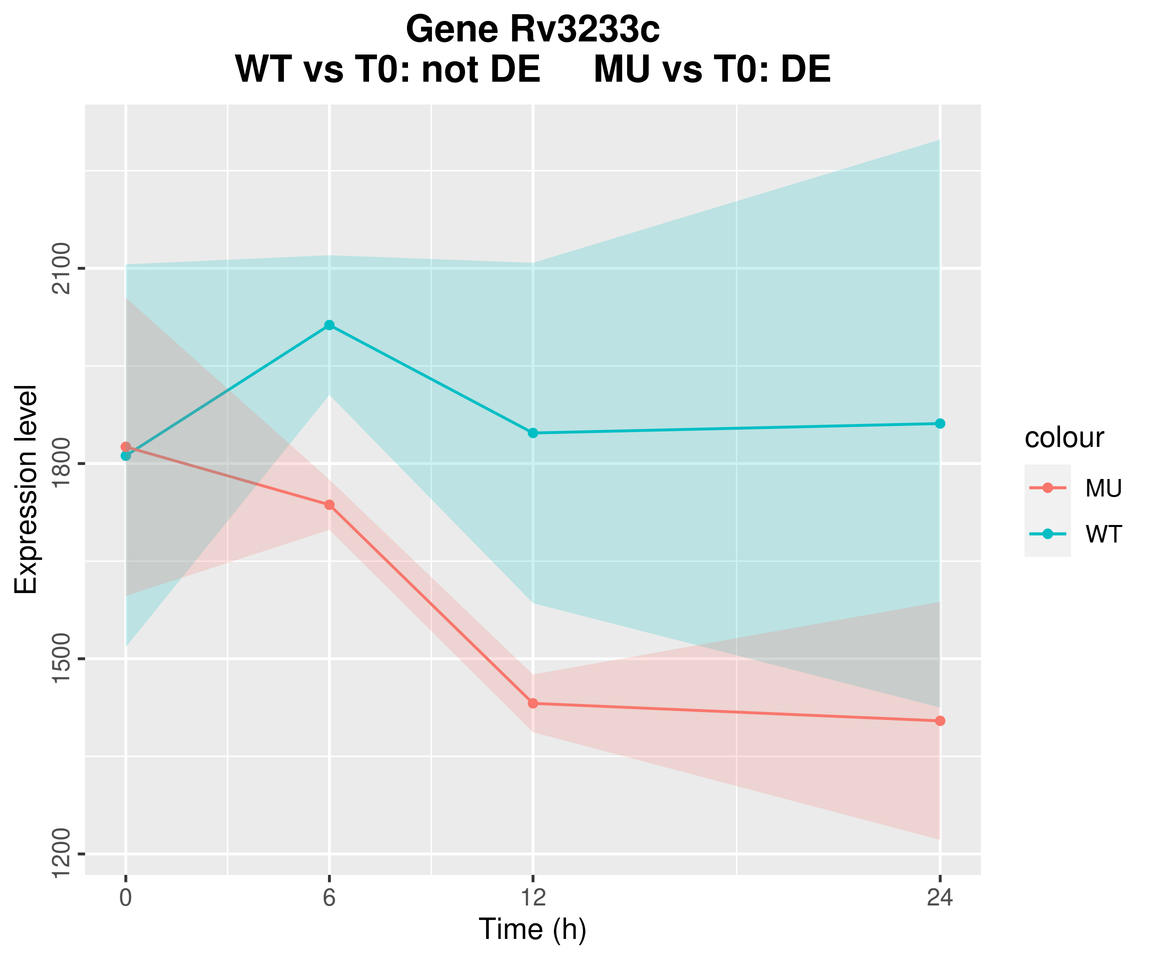
Gene Rv3233c
WT vs T0: not DE   MU vs T0: DE
Expression level
2100
1800
1500
1200
0
6
12
24
Time (h)
colour
MU
WT

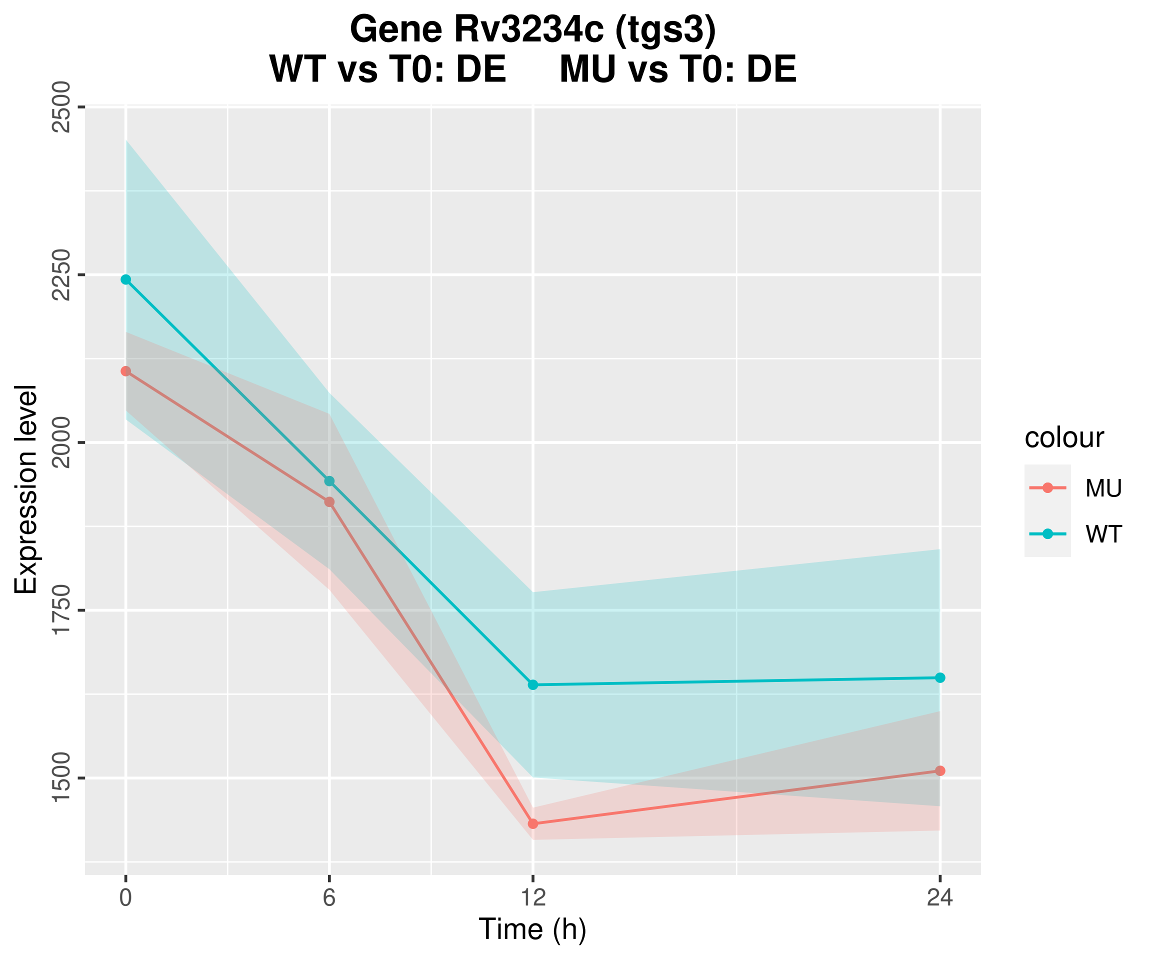
Gene Rv3234c (tgs3)
WT vs T0: DE   MU vs T0: DE
2500
2250
2000
1750
1500
Expression level
0
6
12
24
Time (h)
colour
MU
WT

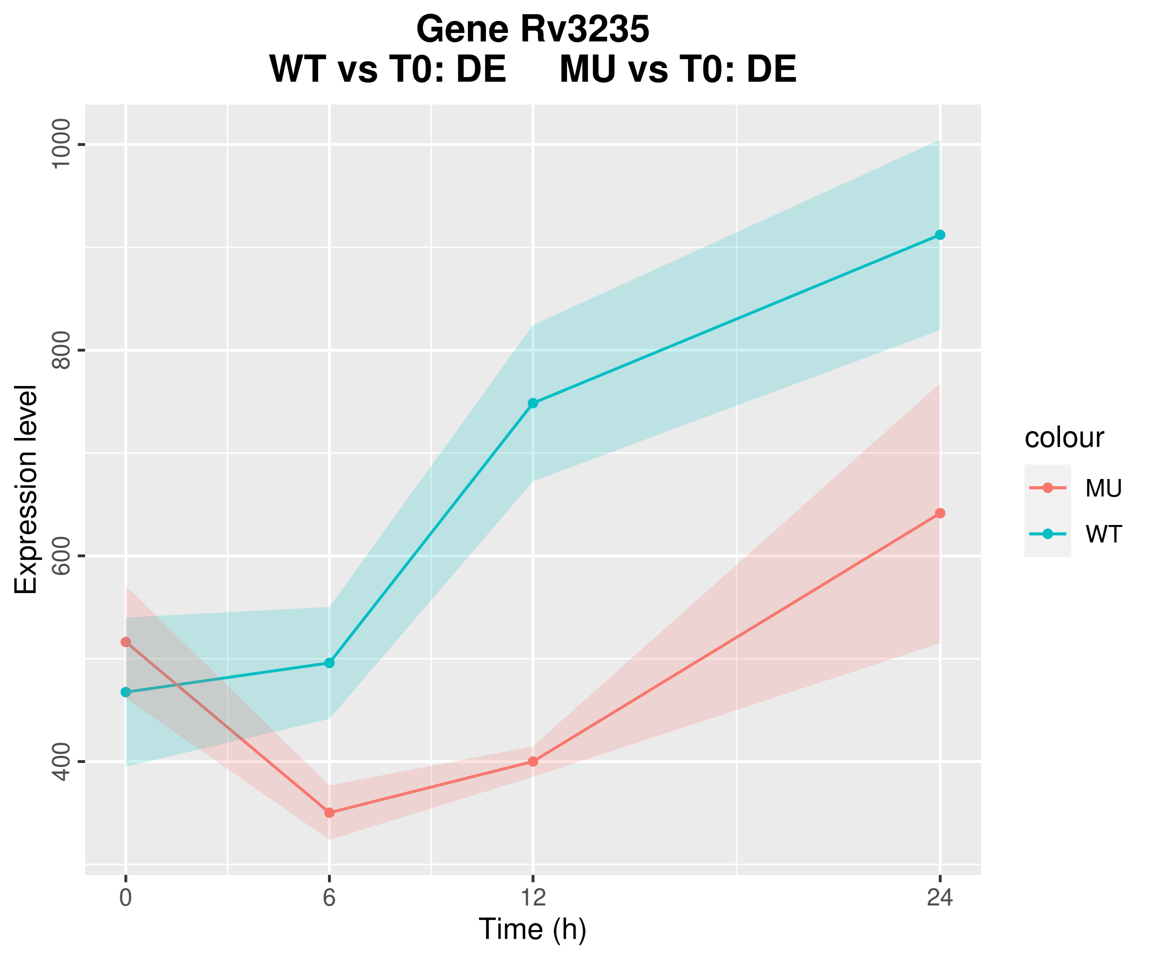

Gene Rv3235
WT vs T0: DE   MU vs T0: DE
1000
800
600
400
Expression level
0
6
12
24
Time (h)
colour
MU
WT

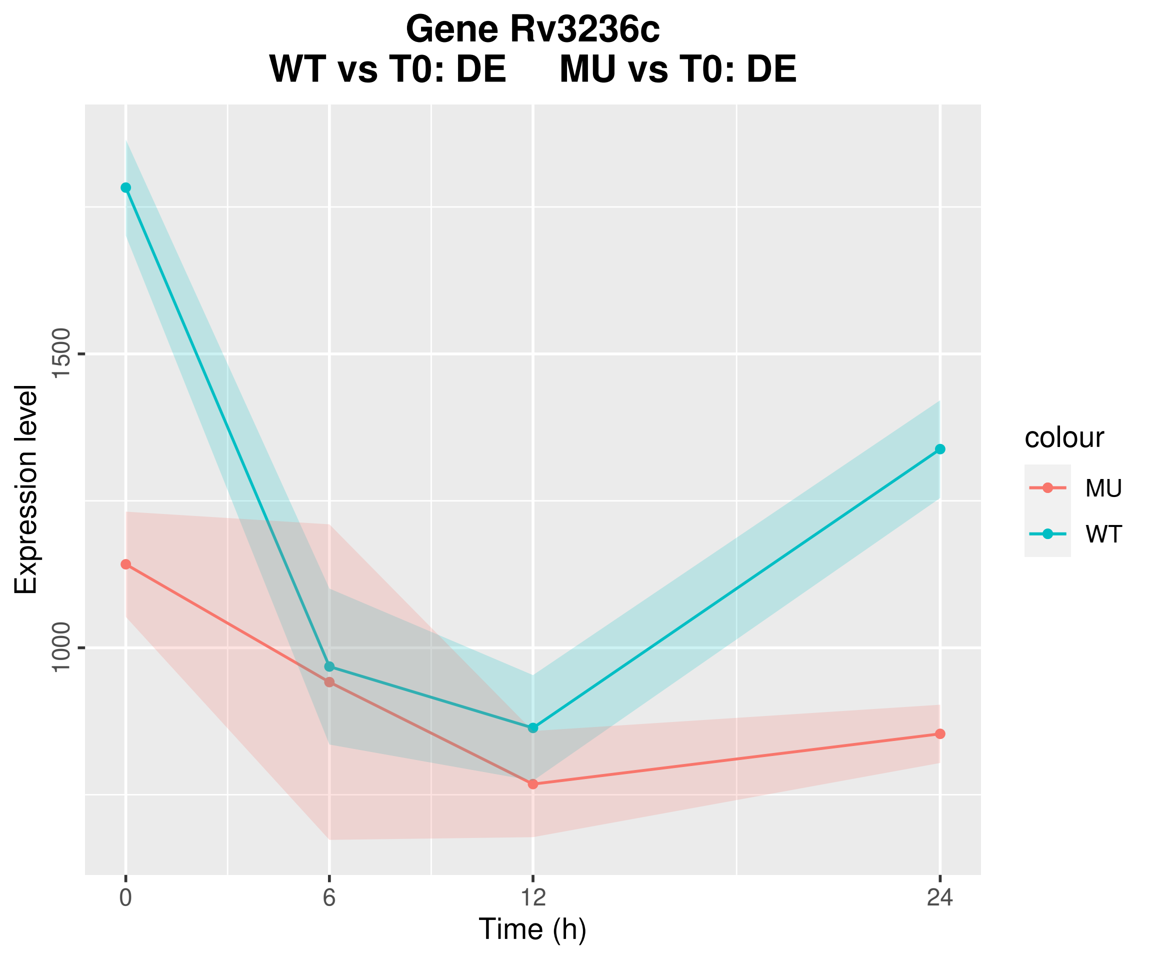
Gene Rv3236c
WT vs T0: DE   MU vs T0: DE
Expression level
1500
1000
0
6
12
24
Time (h)
colour
MU
WT

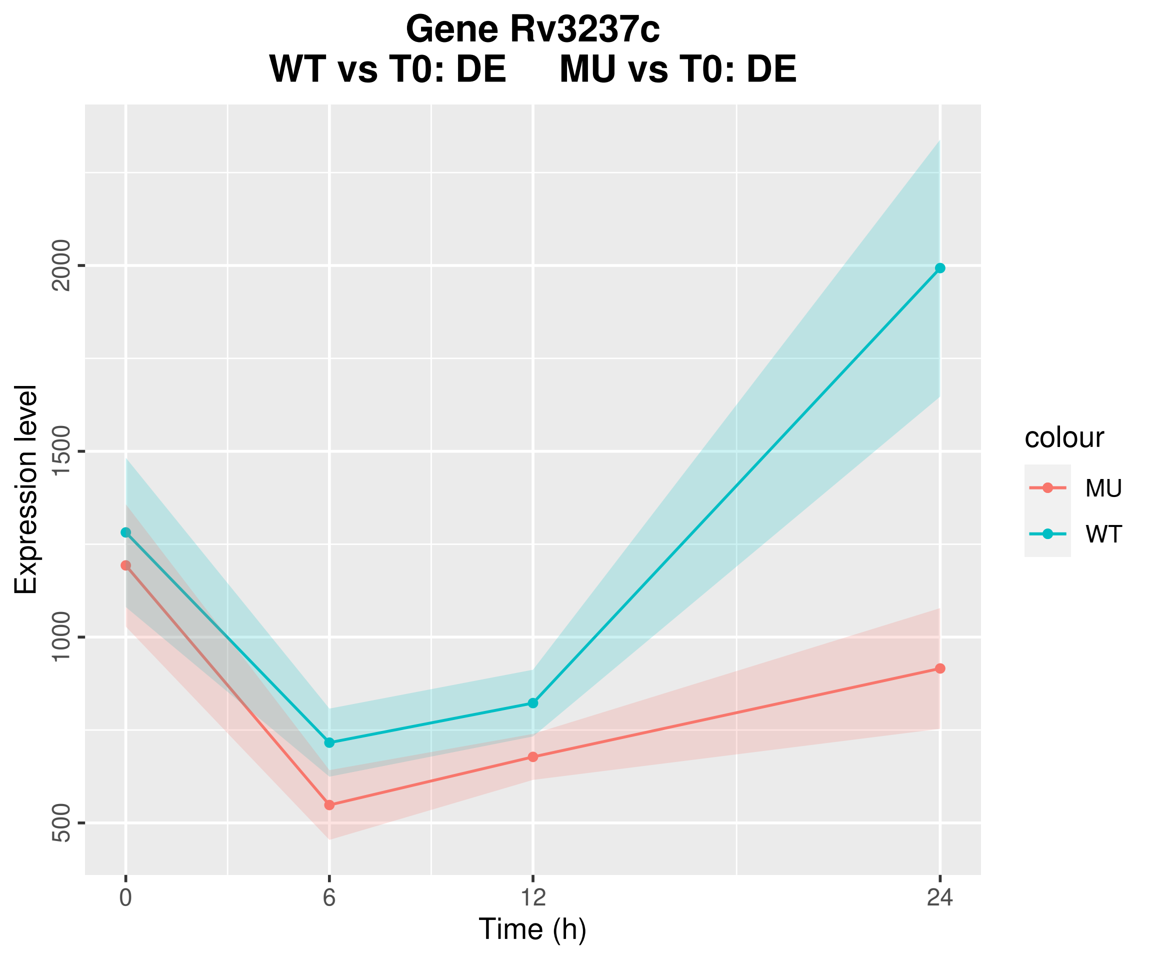
Gene Rv3237c
WT vs T0: DE MU vs T0: DE
Expression level
500
1000
1500
2000
0
6
12
24
Time (h)
colour
MU
WT

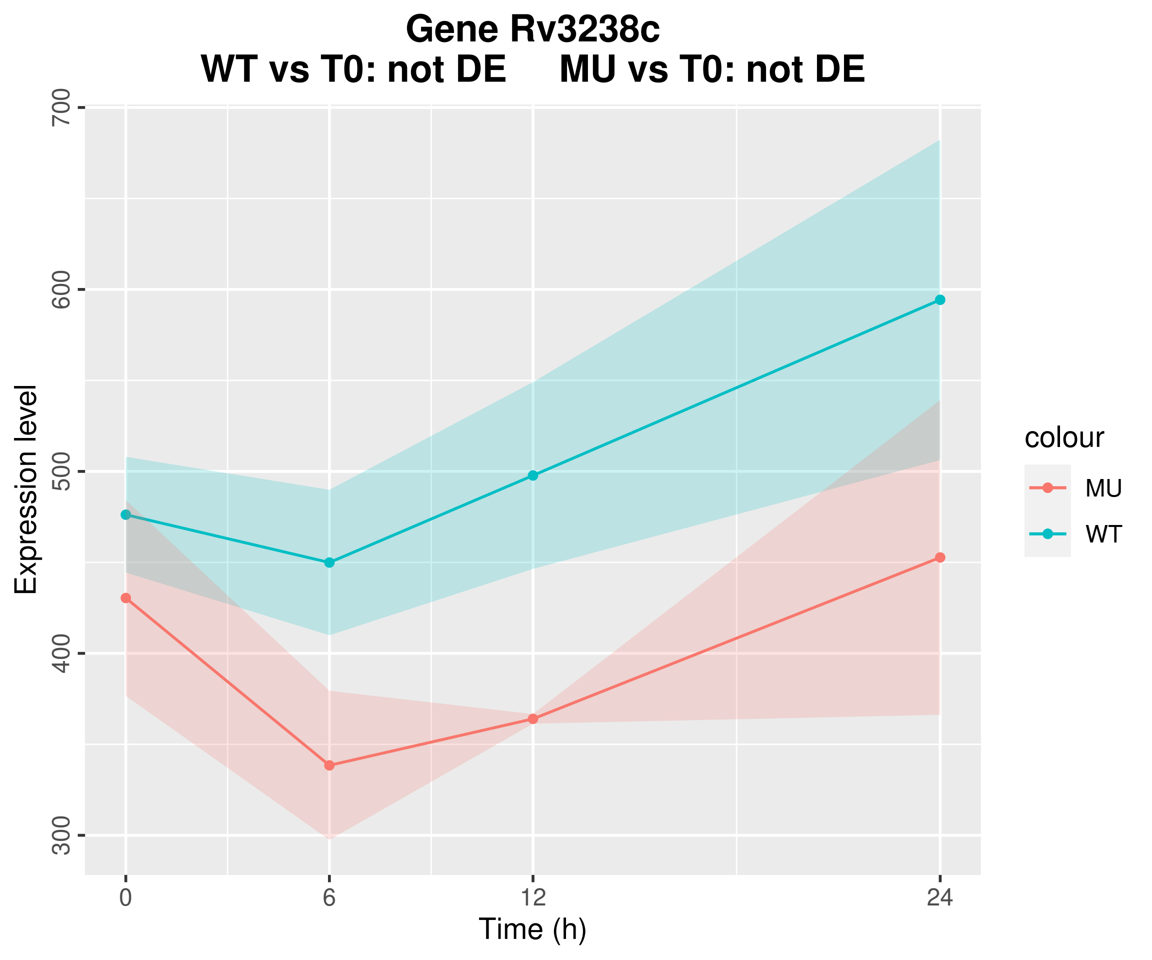

Gene Rv3238c
WT vs T0: not DE   MU vs T0: not DE
700
600
500
400
300
Expression level
0
6
12
24
Time (h)
colour
MU
WT

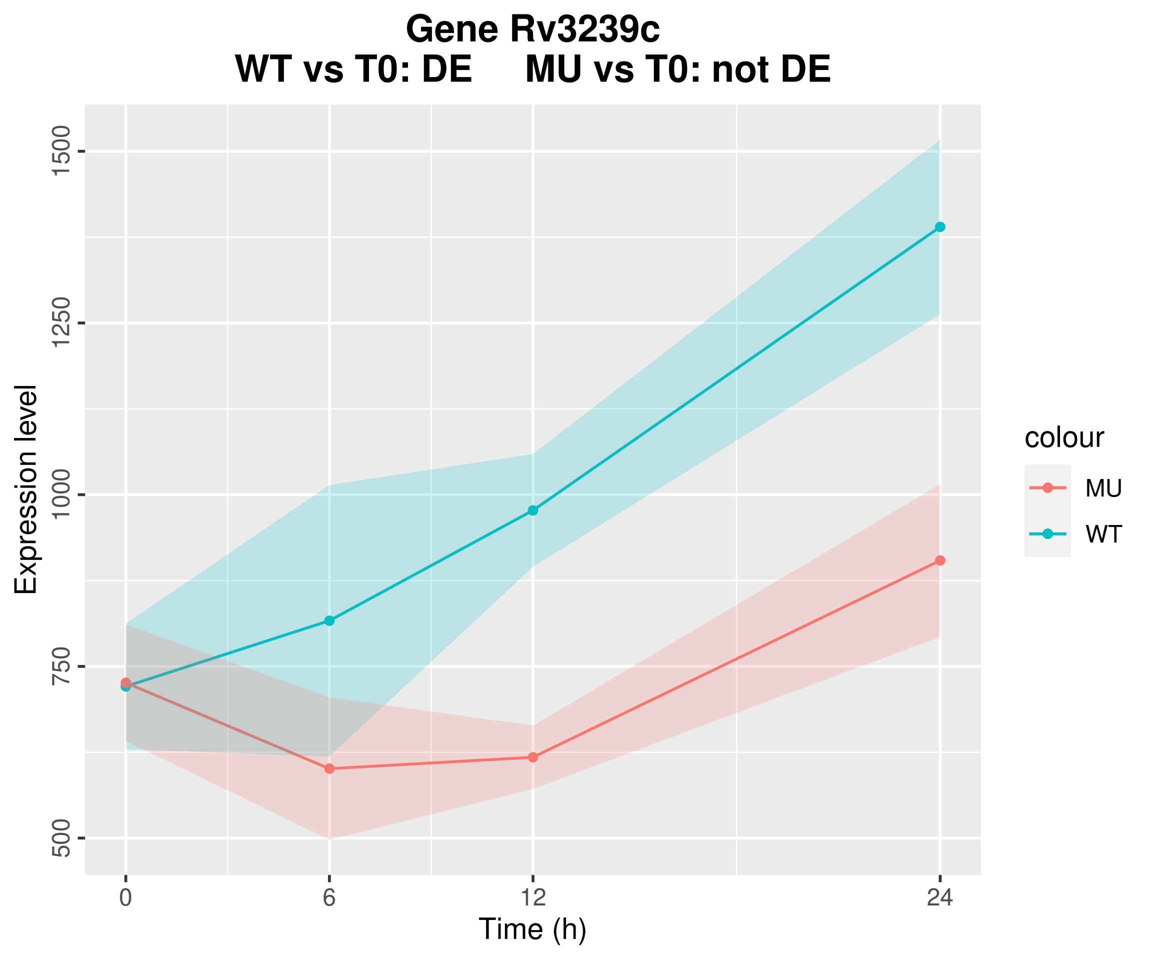

Gene Rv3239c
WT vs T0: DE MU vs T0: not DE
Expression level
1500
1250
1000
750
500
0
6
12
24
Time (h)
colour
MU
WT

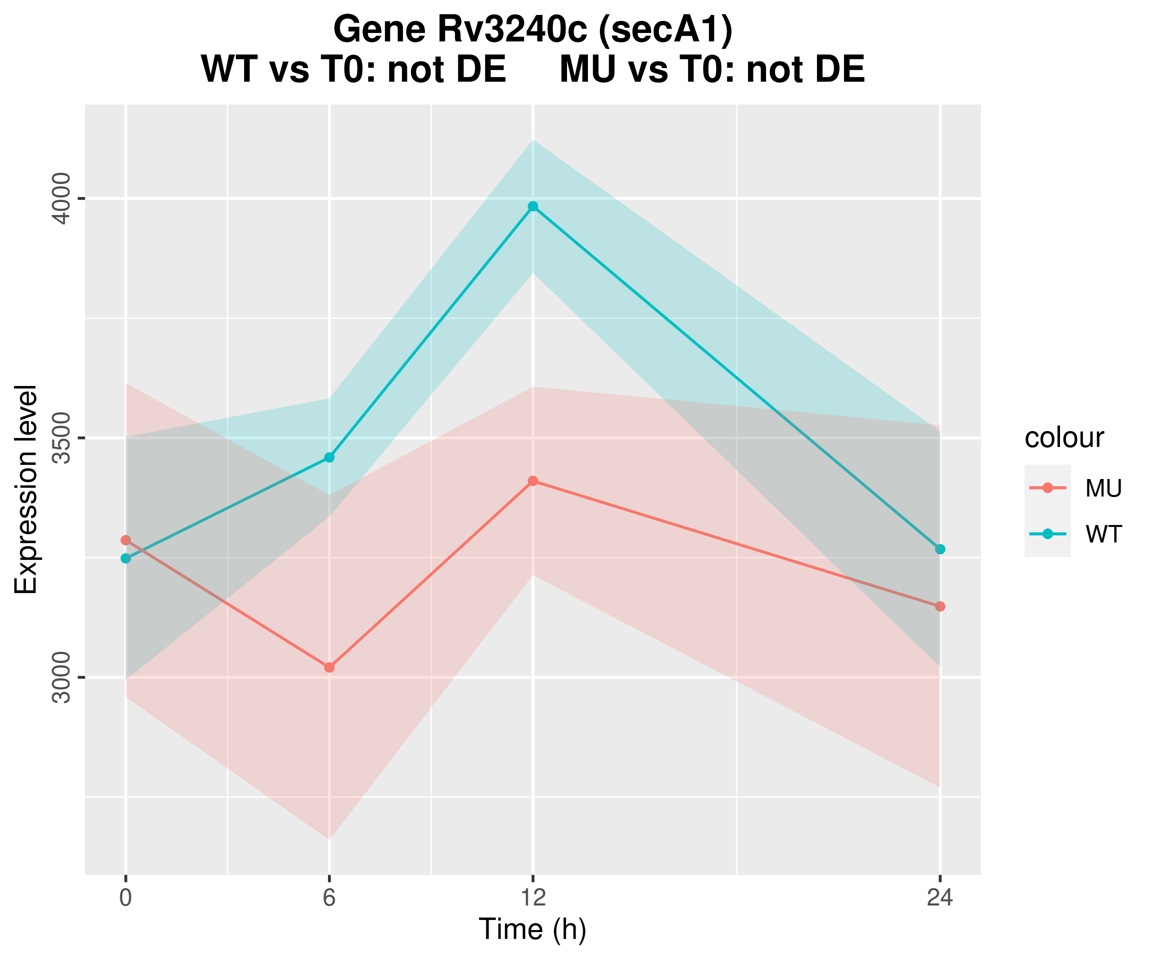

Gene Rv3240c (secA1)
WT vs T0: not DE   MU vs T0: not DE
Expression level
4000
3500
3000
0
6
12
24
Time (h)
colour
MU
WT

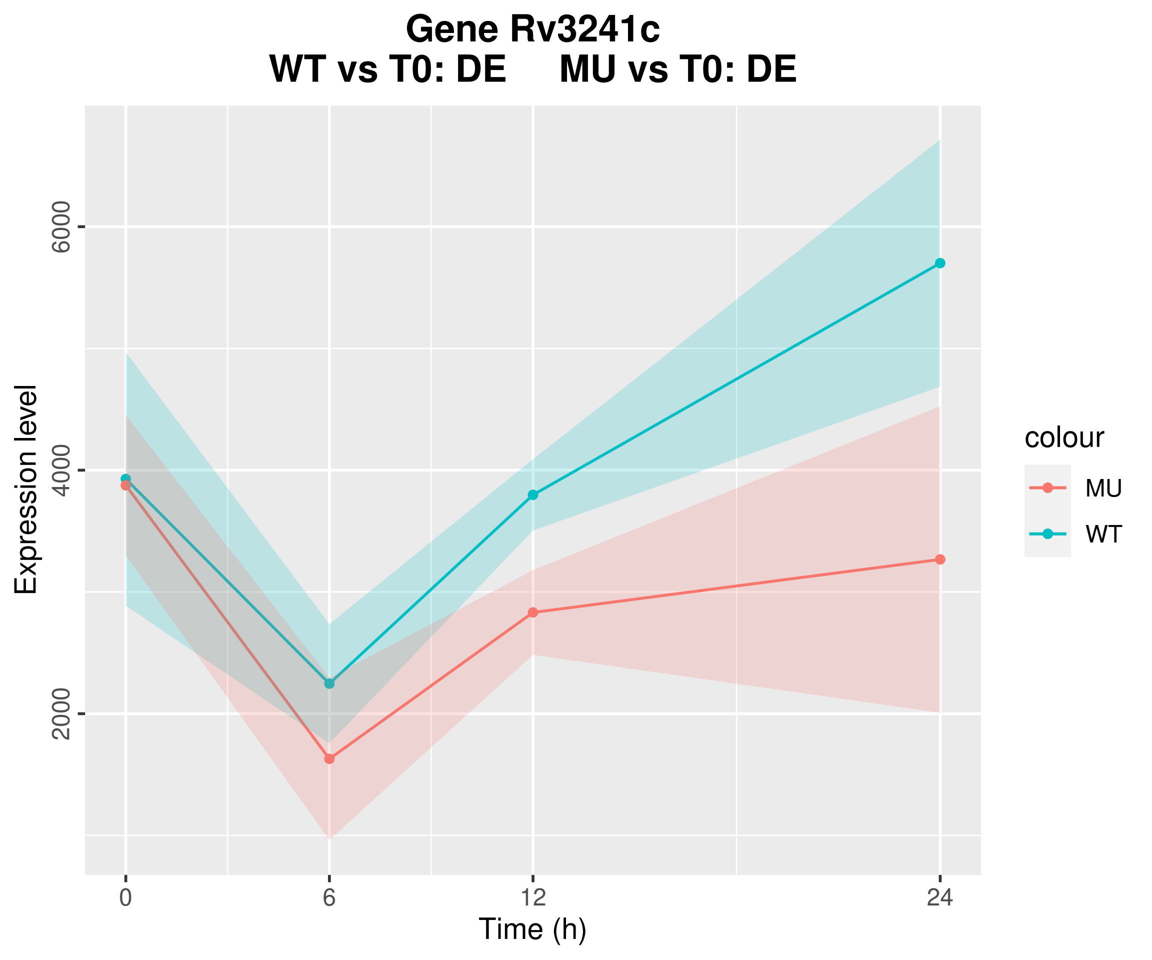
Gene Rv3241c
WT vs T0: DE MU vs T0: DE
Expression level
6000
4000
2000
0
6
12
24
Time (h)
colour
MU
WT

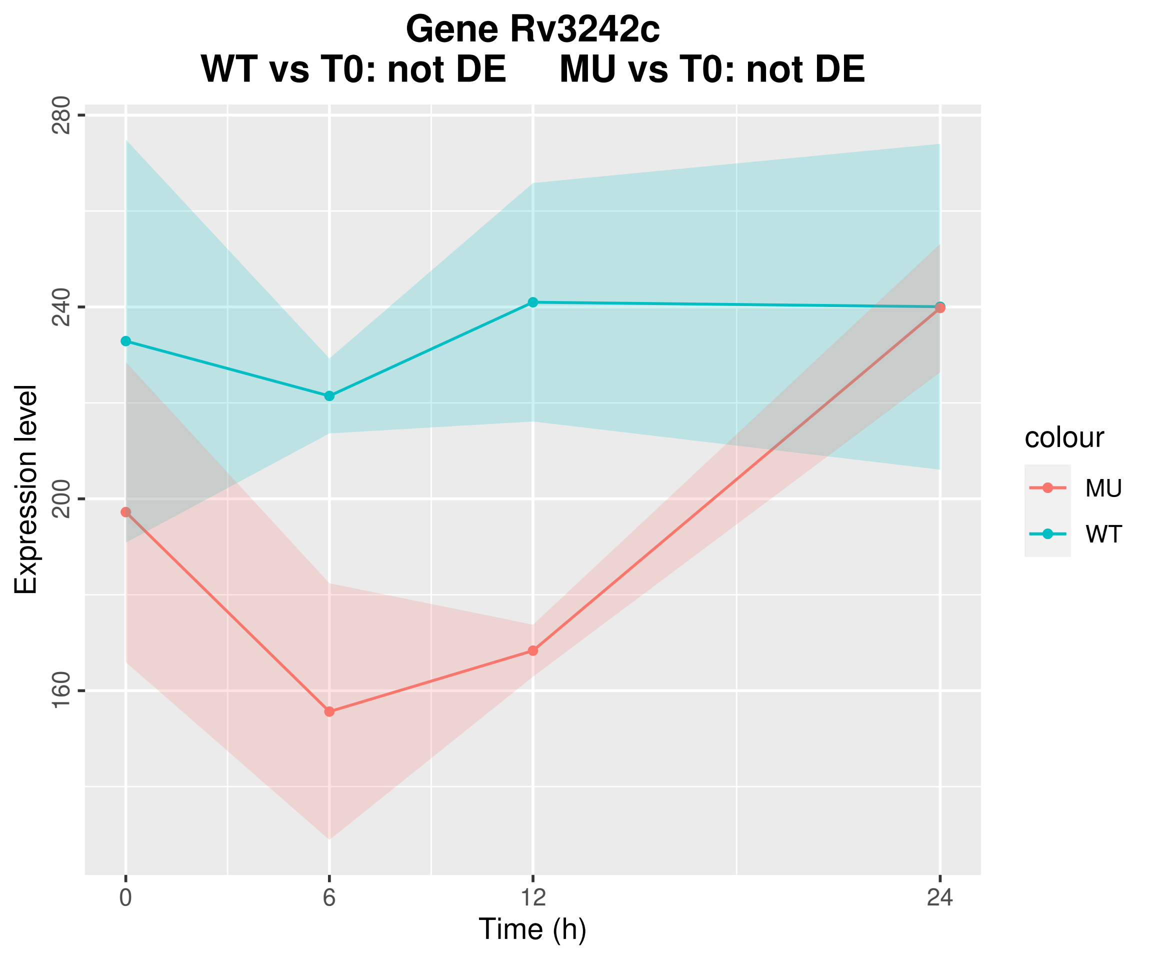
Gene Rv3242c
WT vs T0: not DE MU vs T0: not DE
280
240
200
160
Expression level
0
6
12
24
Time (h)
colour
MU
WT

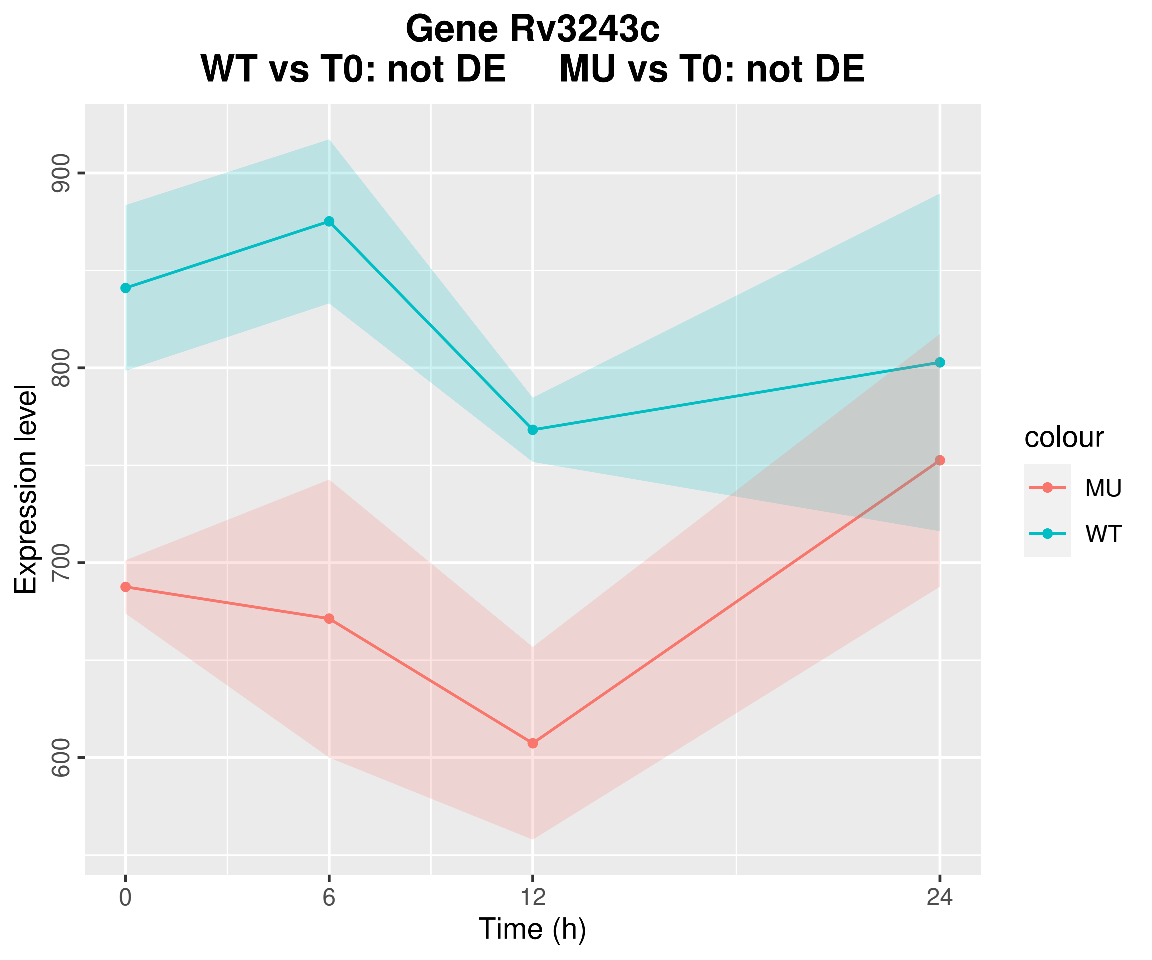

Gene Rv3243c
WT vs T0: not DE   MU vs T0: not DE
Expression level
900
800
700
600
0
6
12
24
Time (h)
colour
MU
WT

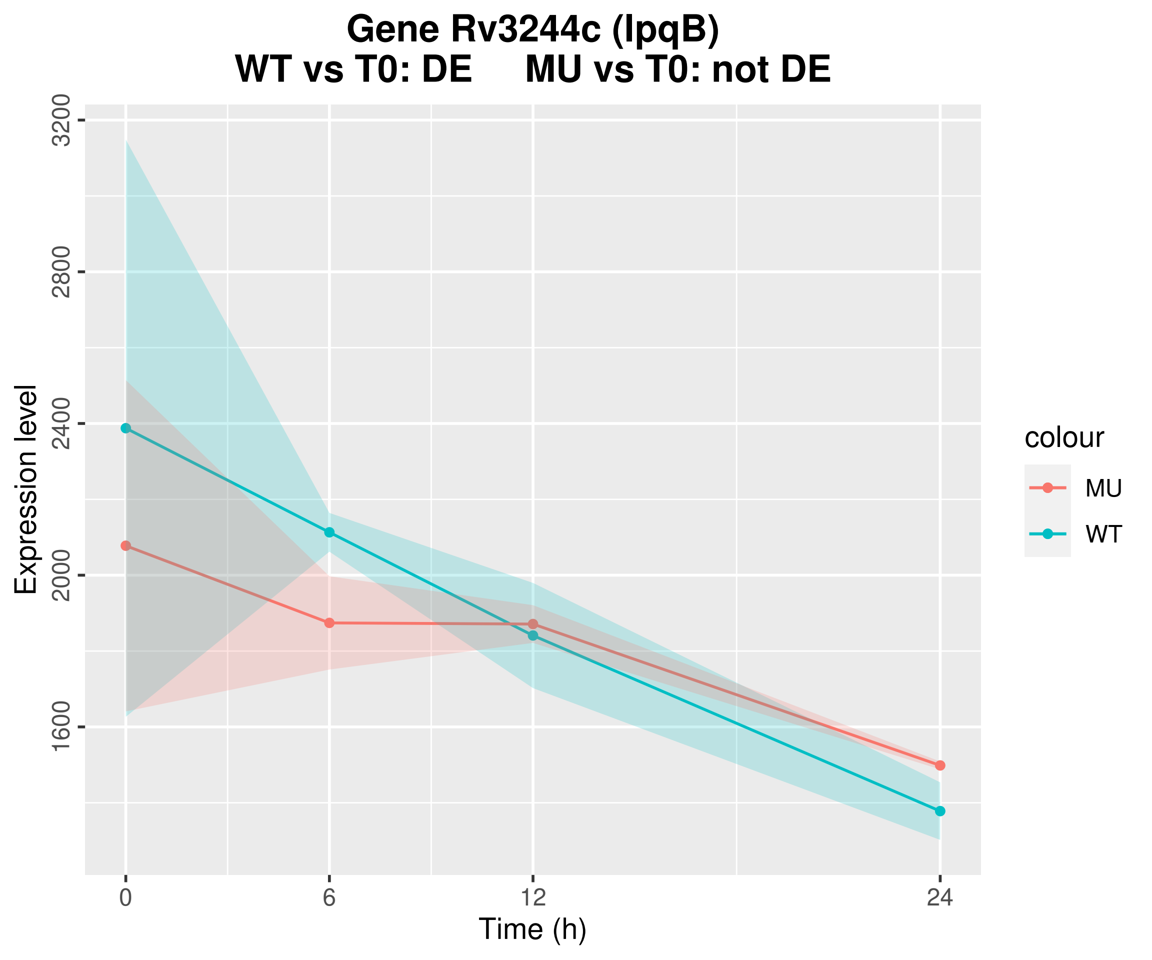

Gene Rv3244c (lpqB)
WT vs T0: DE MU vs T0: not DE
3200
2800
2400
2000
1600
Expression level
0
6
12
24
Time (h)
colour
MU
WT

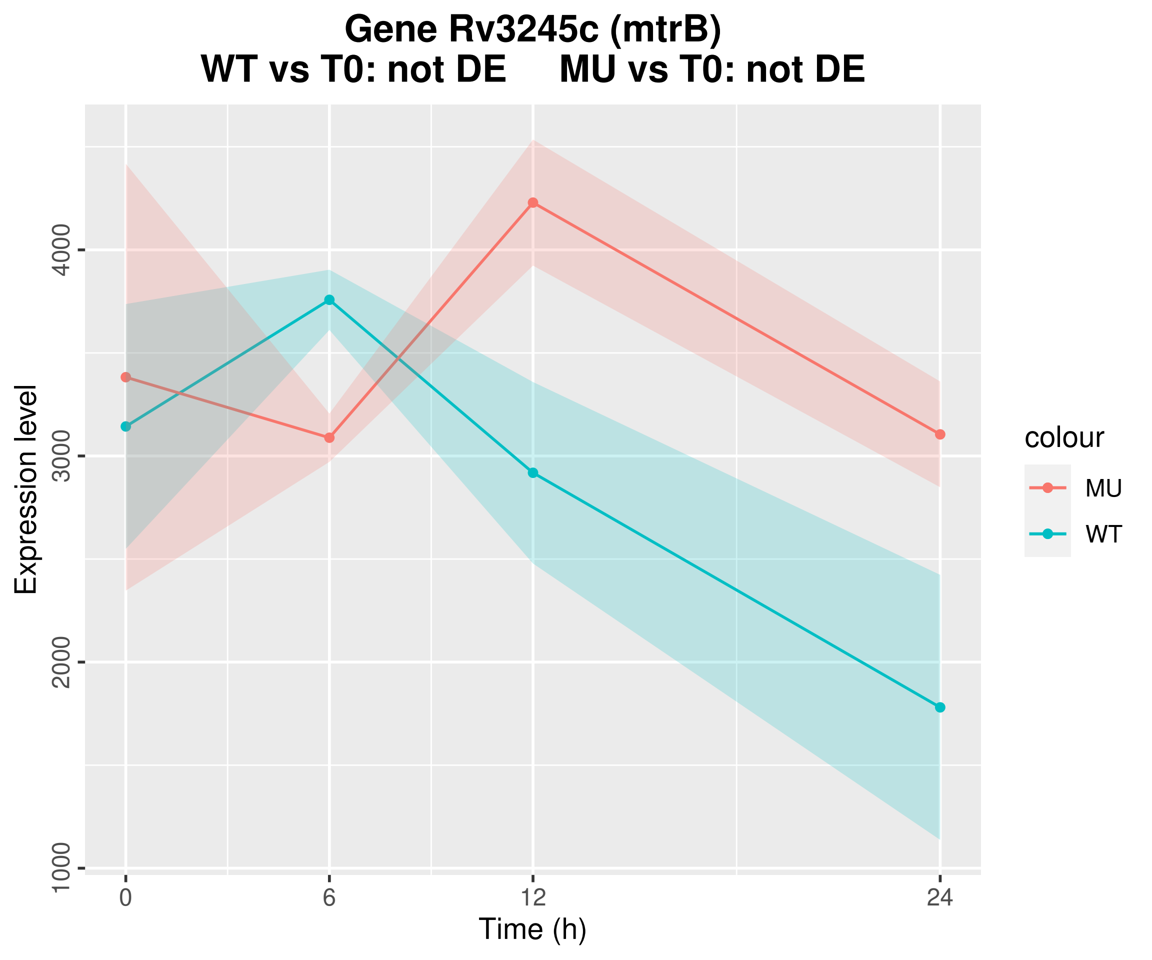
Gene Rv3245c (mtrB)
WT vs T0: not DE   MU vs T0: not DE
Expression level
1000
2000
3000
4000
0
6
12
24
Time (h)
colour
MU
WT

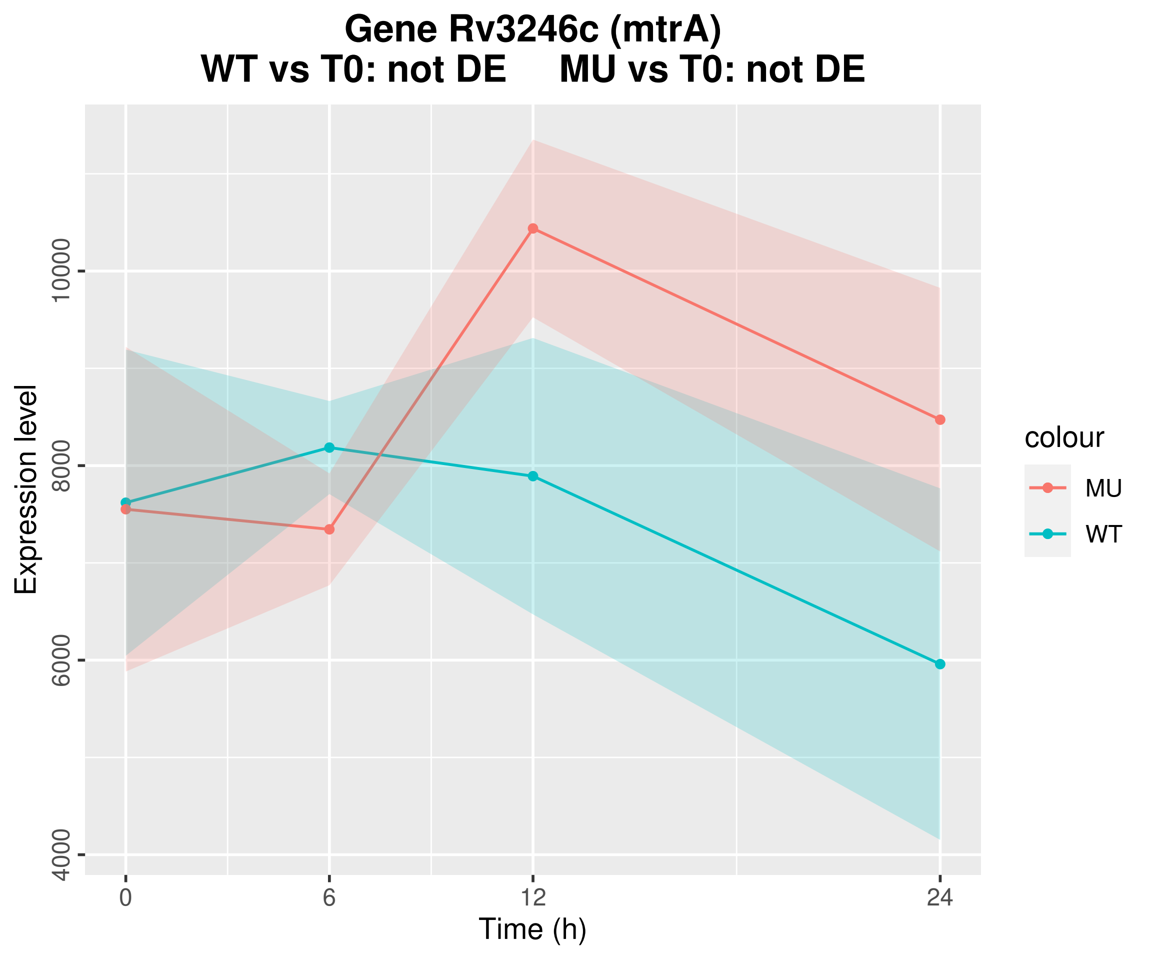

Gene Rv3246c (mtrA)
WT vs T0: not DE MU vs T0: not DE
Expression level
4000
6000
8000
10000
0
6
12
24
Time (h)
colour
MU
WT

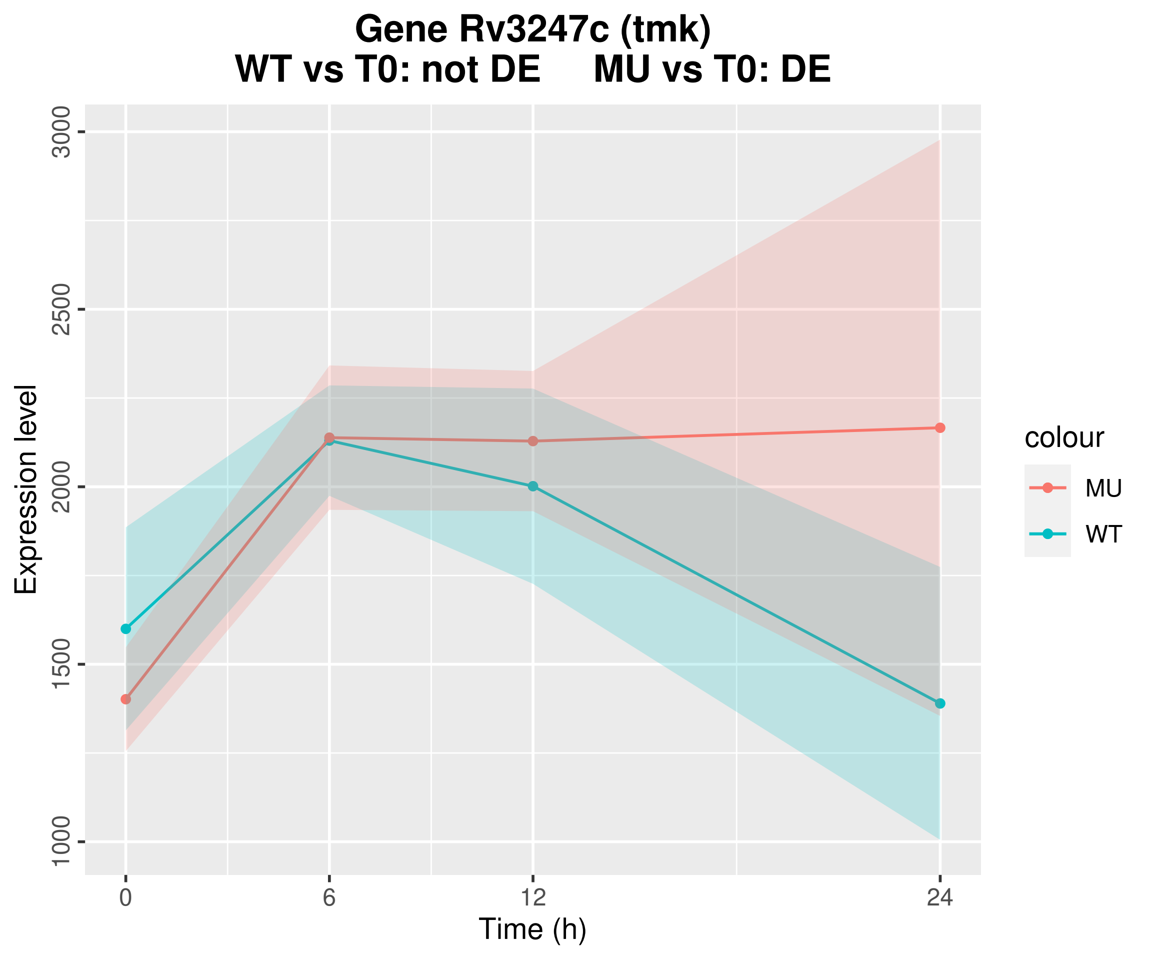

Gene Rv3247c (tmk)
WT vs T0: not DE MU vs T0: DE
Expression level
3000
2500
2000
1500
1000
0
6
12
24
Time (h)
colour
MU
WT

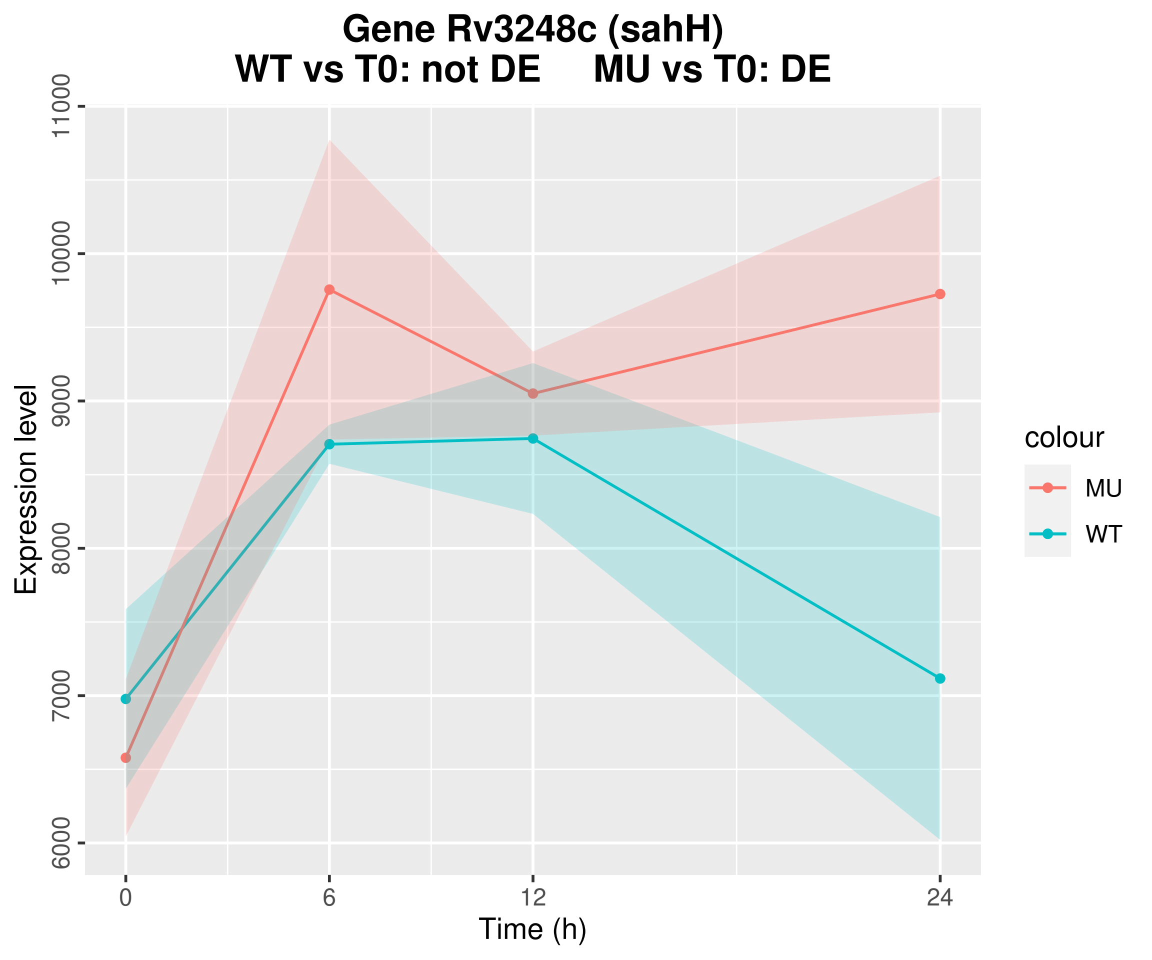

Gene Rv3248c (sahH)
WT vs T0: not DE   MU vs T0: DE
Expression level
11000
10000
9000
8000
7000
6000
0
6
12
24
Time (h)
colour
MU
WT

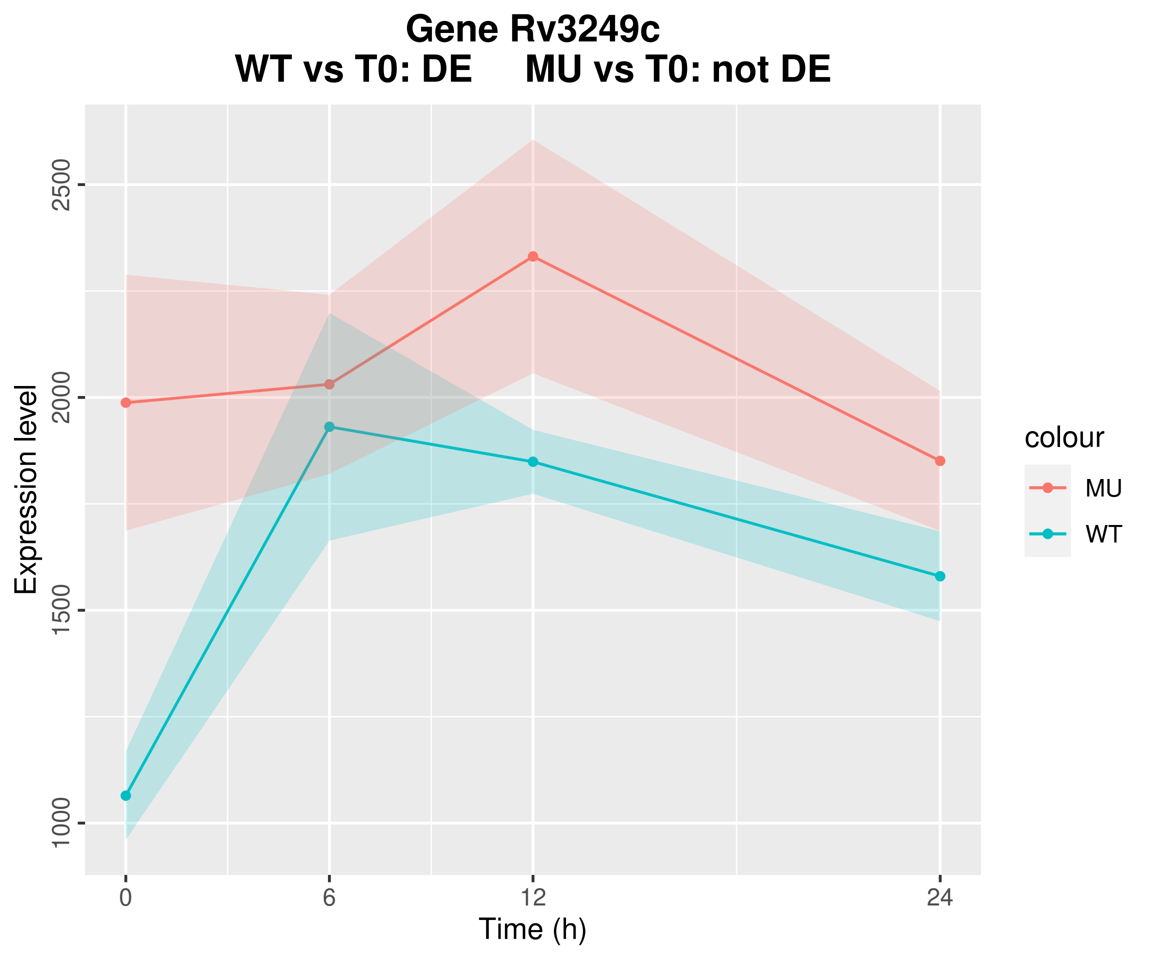
Gene Rv3249c
WT vs T0: DE MU vs T0: not DE
Expression level
2500
2000
1500
1000
0
6
12
24
Time (h)
colour
MU
WT

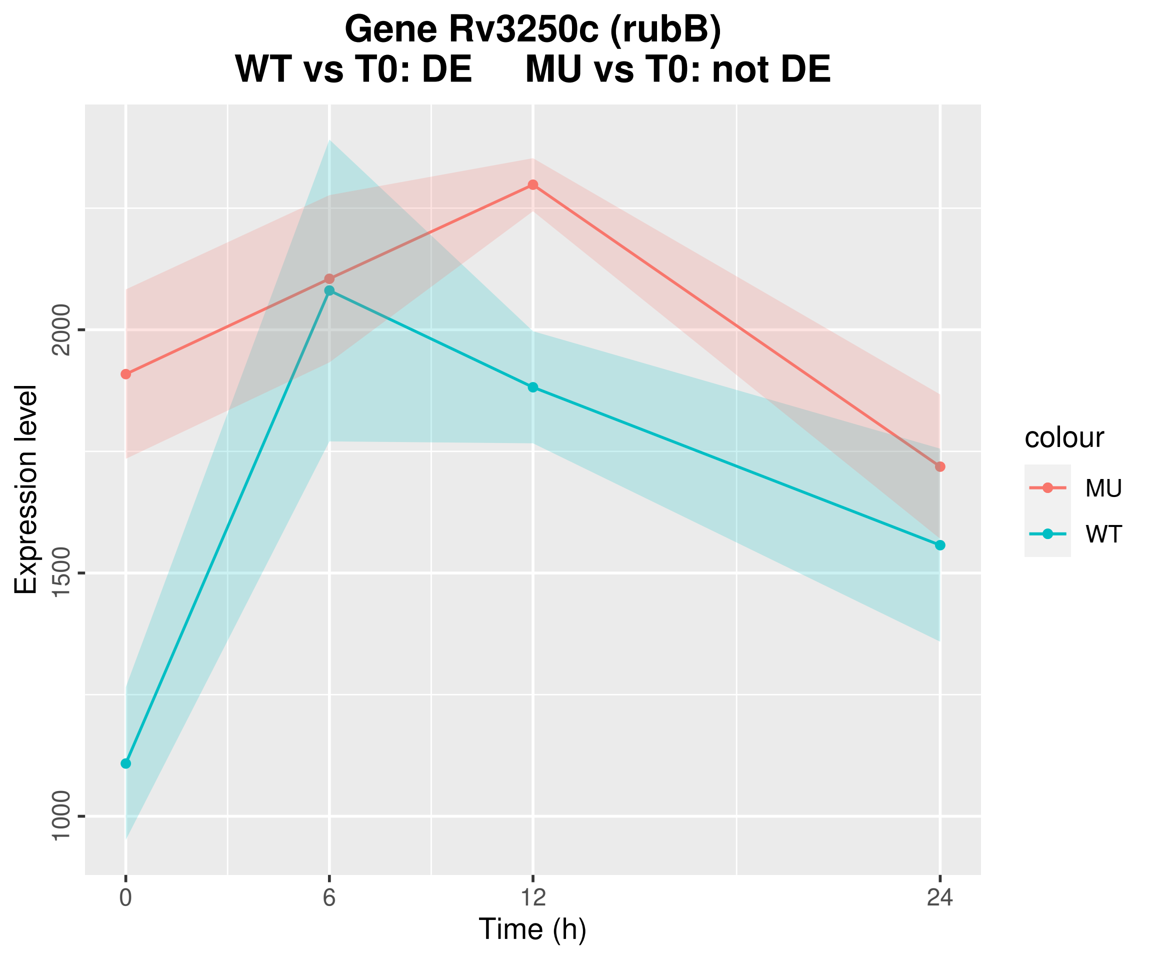

Gene Rv3250c (rubB)
WT vs T0: DE   MU vs T0: not DE
Expression level
2000
1500
1000
0
6
12
24
Time (h)
colour
MU
WT

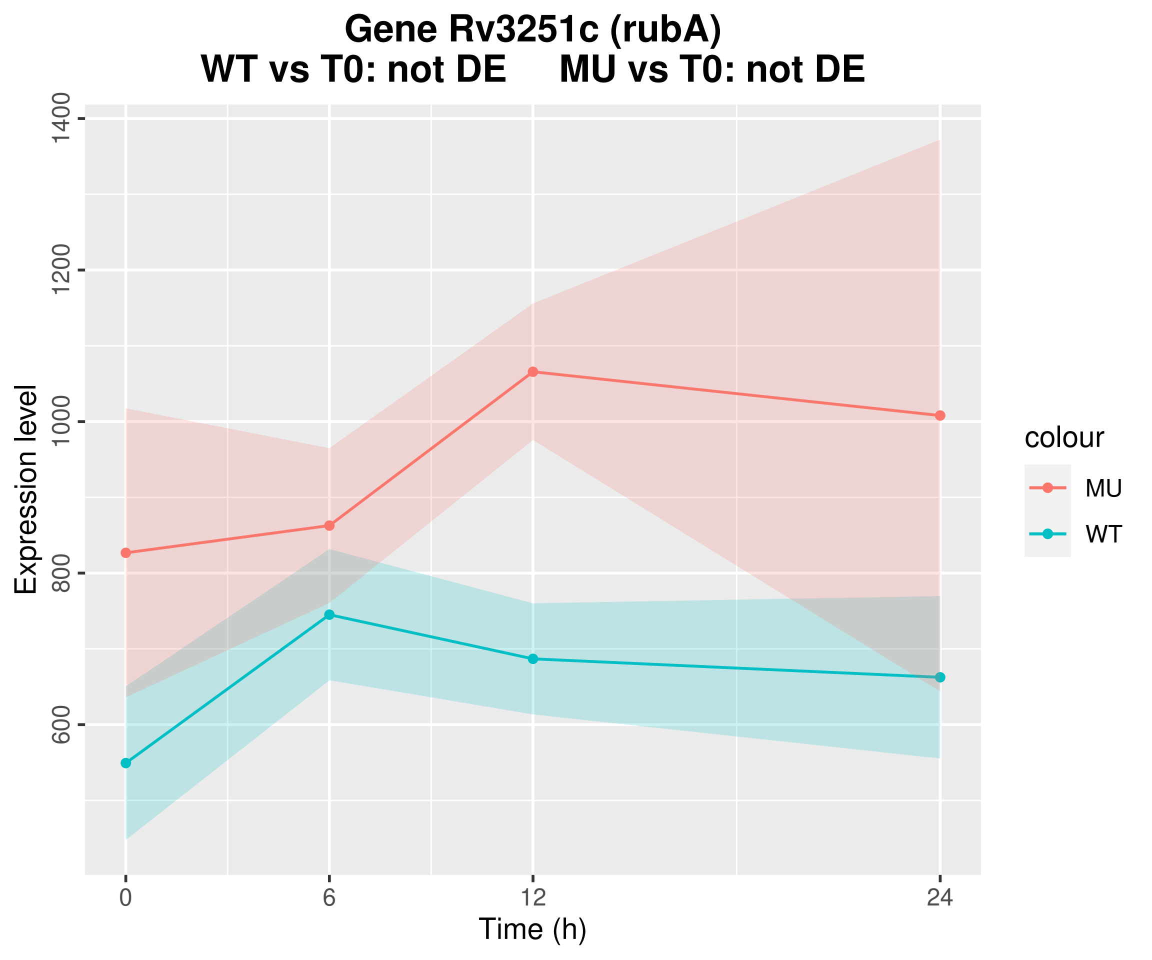

Gene Rv3251c (rubA)
WT vs T0: not DE MU vs T0: not DE
Expression level
1400
1200
1000
800
600
0
6
12
24
Time (h)
colour
MU
WT

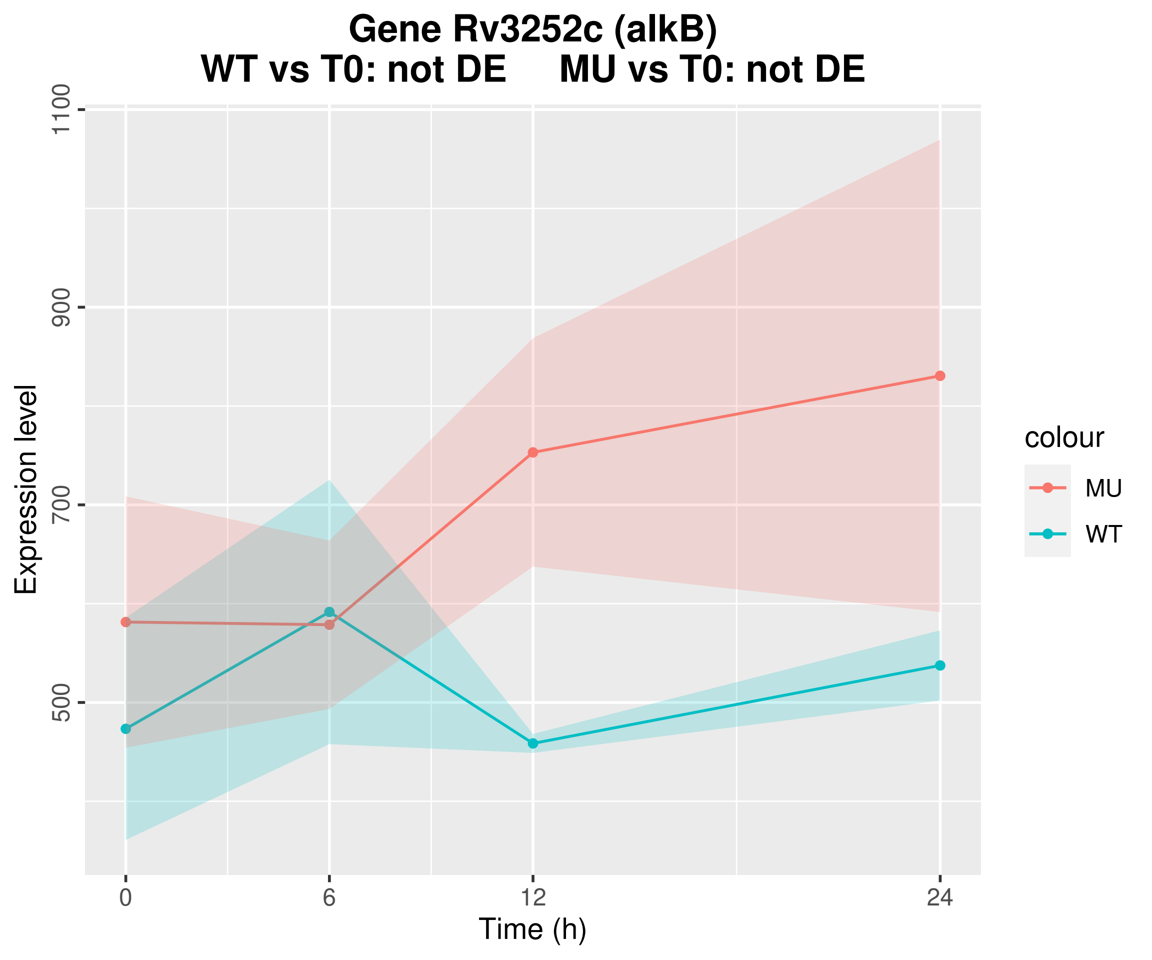
Gene Rv3252c (alkB)
WT vs T0: not DE   MU vs T0: not DE
Expression level
Time (h)
1100
900
700
500
0
6
12
24
colour
MU
WT

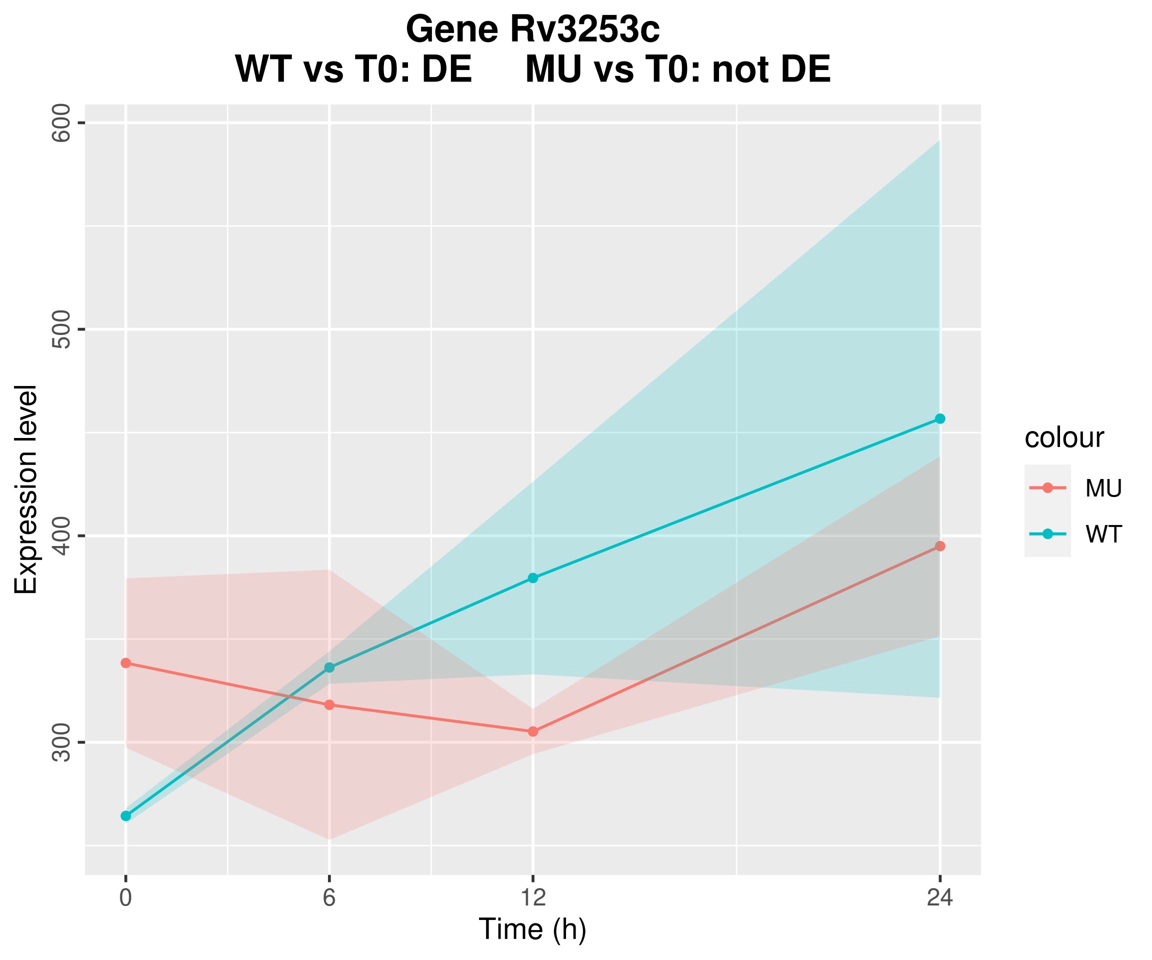
Gene Rv3253c
WT vs T0: DE   MU vs T0: not DE
Expression level
600
500
400
300
0
6
12
24
Time (h)
colour
MU
WT

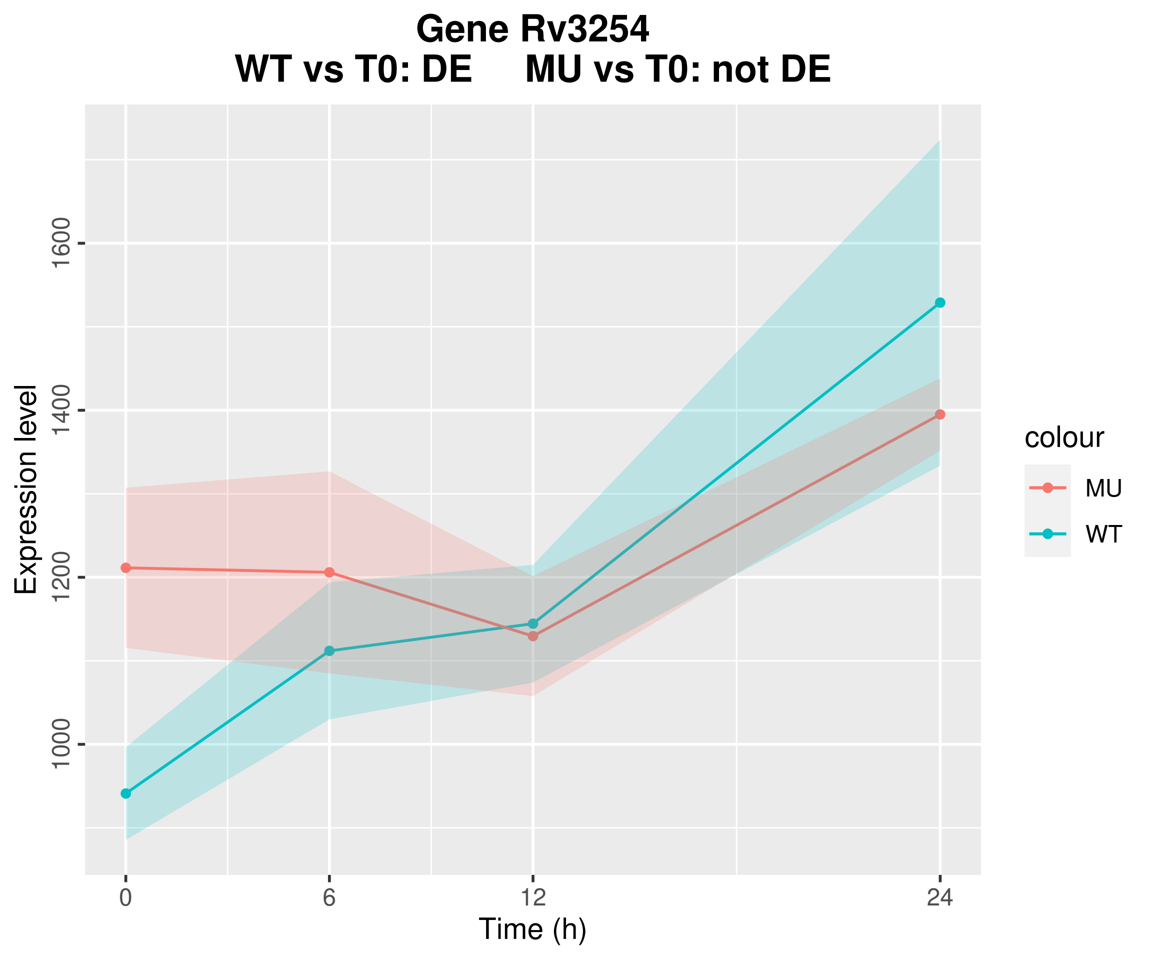

Gene Rv3254
WT vs T0: DE   MU vs T0: not DE
Expression level
1000
1200
1400
1600
0
6
12
24
Time (h)
colour
MU
WT

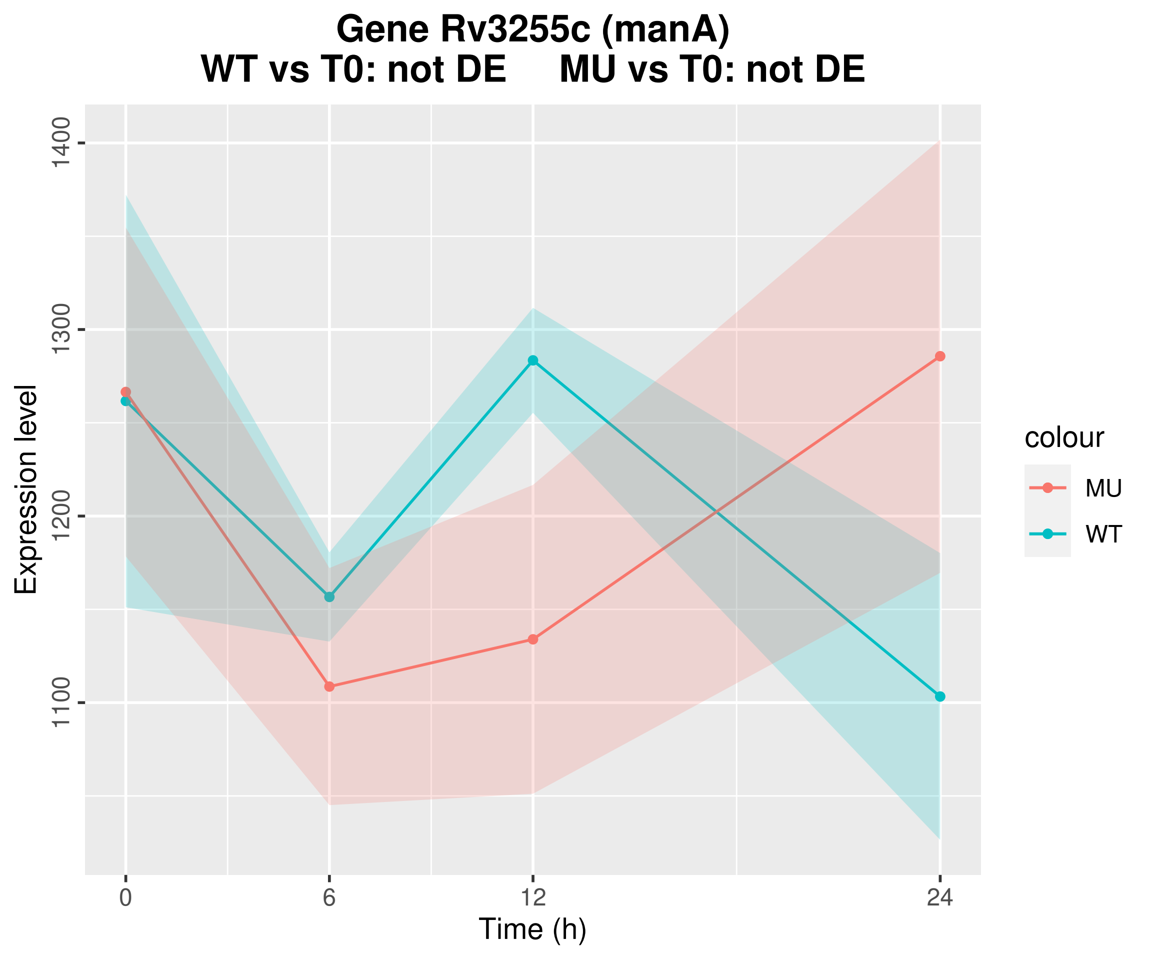

Gene Rv3255c (manA)
WT vs T0: not DE   MU vs T0: not DE
Expression level
1100
1200
1300
1400
0
6
12
24
Time (h)
colour
MU
WT

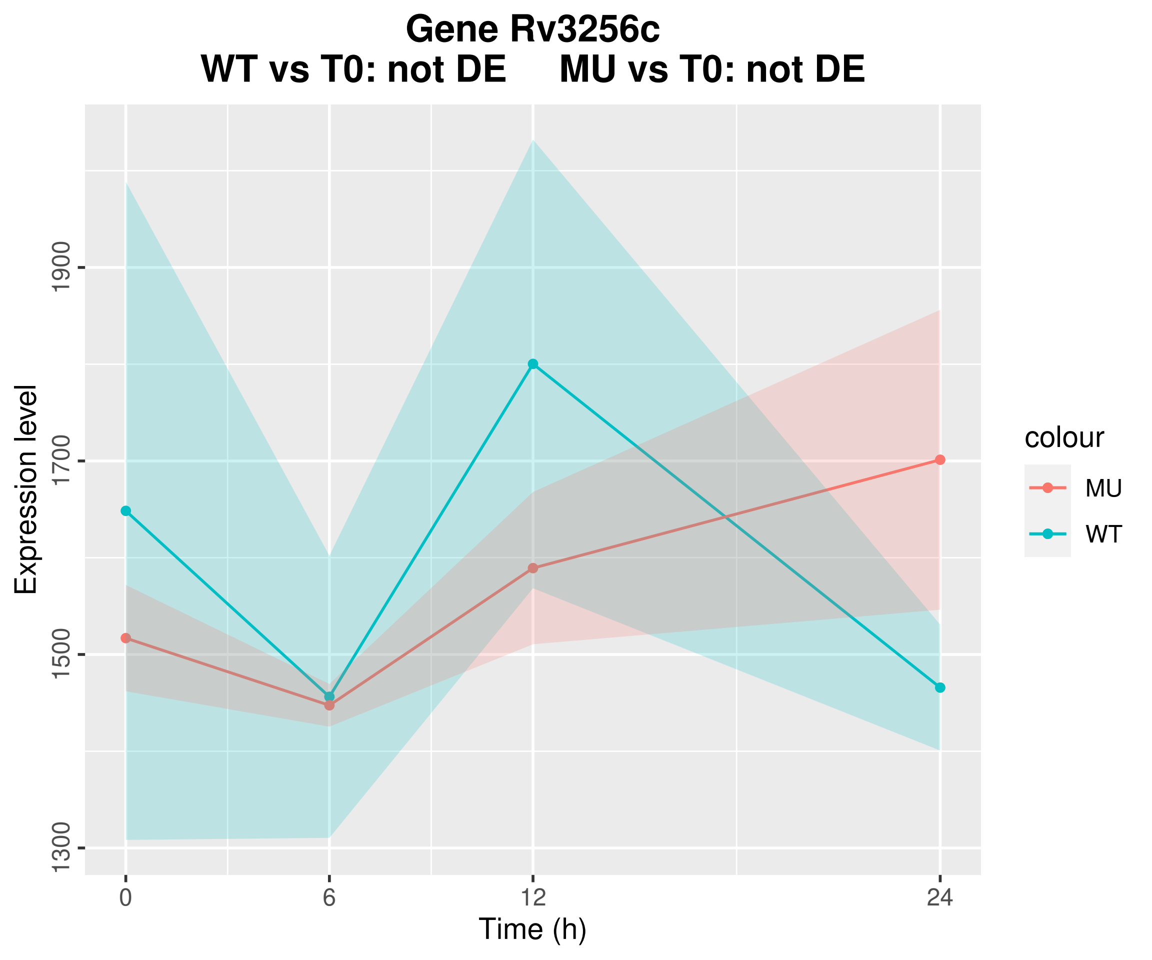

Gene Rv3256c
WT vs T0: not DE MU vs T0: not DE
Expression level
1300
1500
1700
1900
0
6
12
24
Time (h)
colour
MU
WT

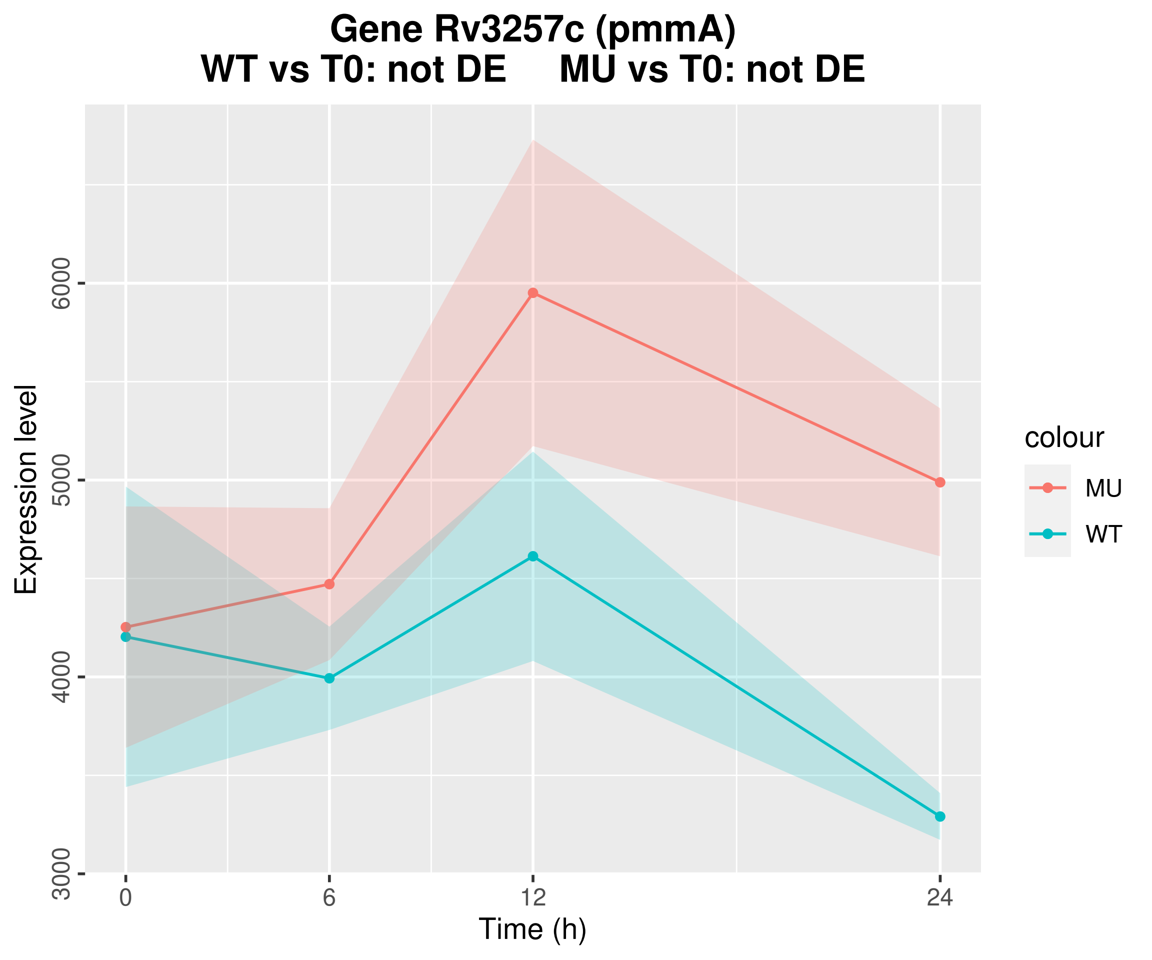
Gene Rv3257c (pmmA)
WT vs T0: not DE MU vs T0: not DE
Expression level
3000
4000
5000
6000
0
6
12
24
Time (h)
colour
MU
WT

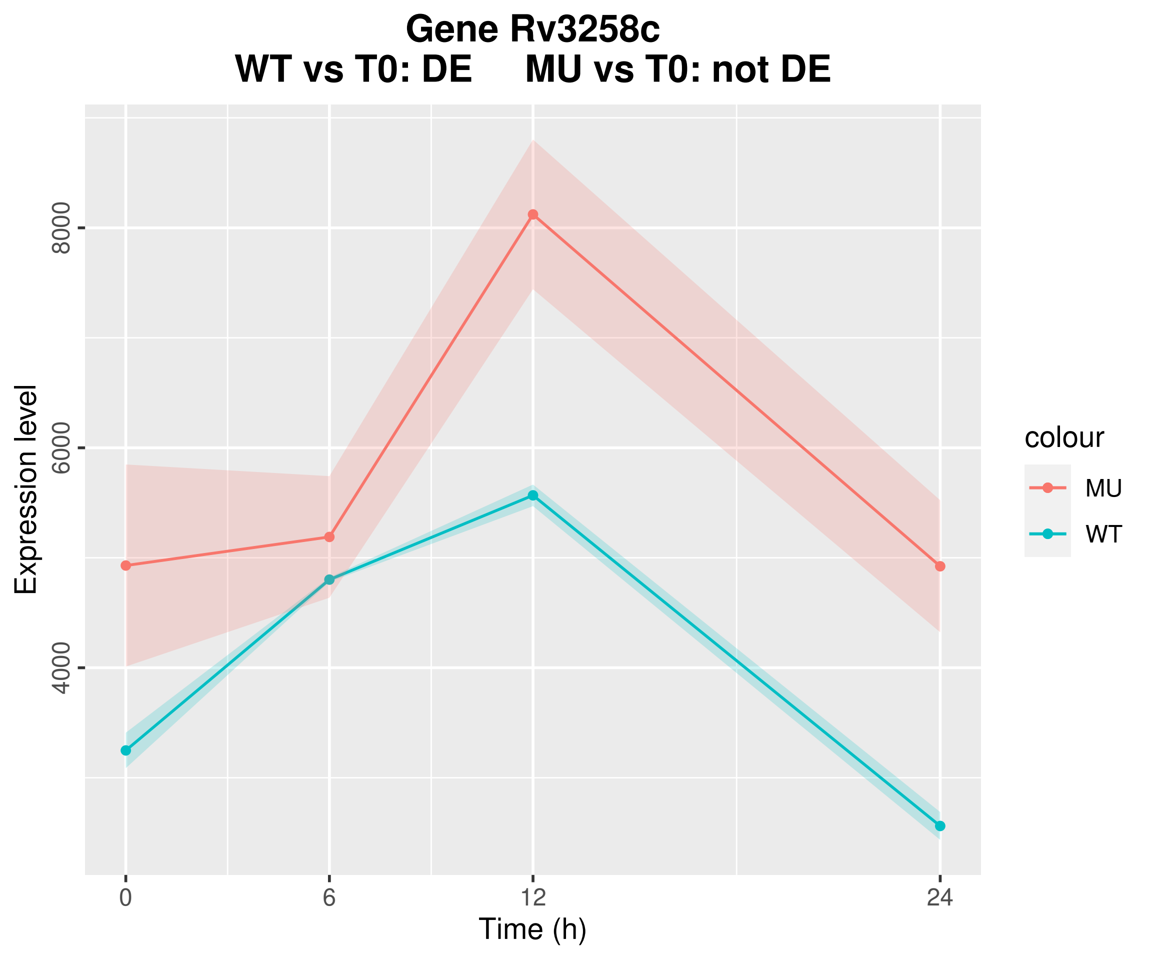
Gene Rv3258c
WT vs T0: DE   MU vs T0: not DE
Expression level
8000
6000
4000
0
6
12
24
Time (h)
colour
MU
WT

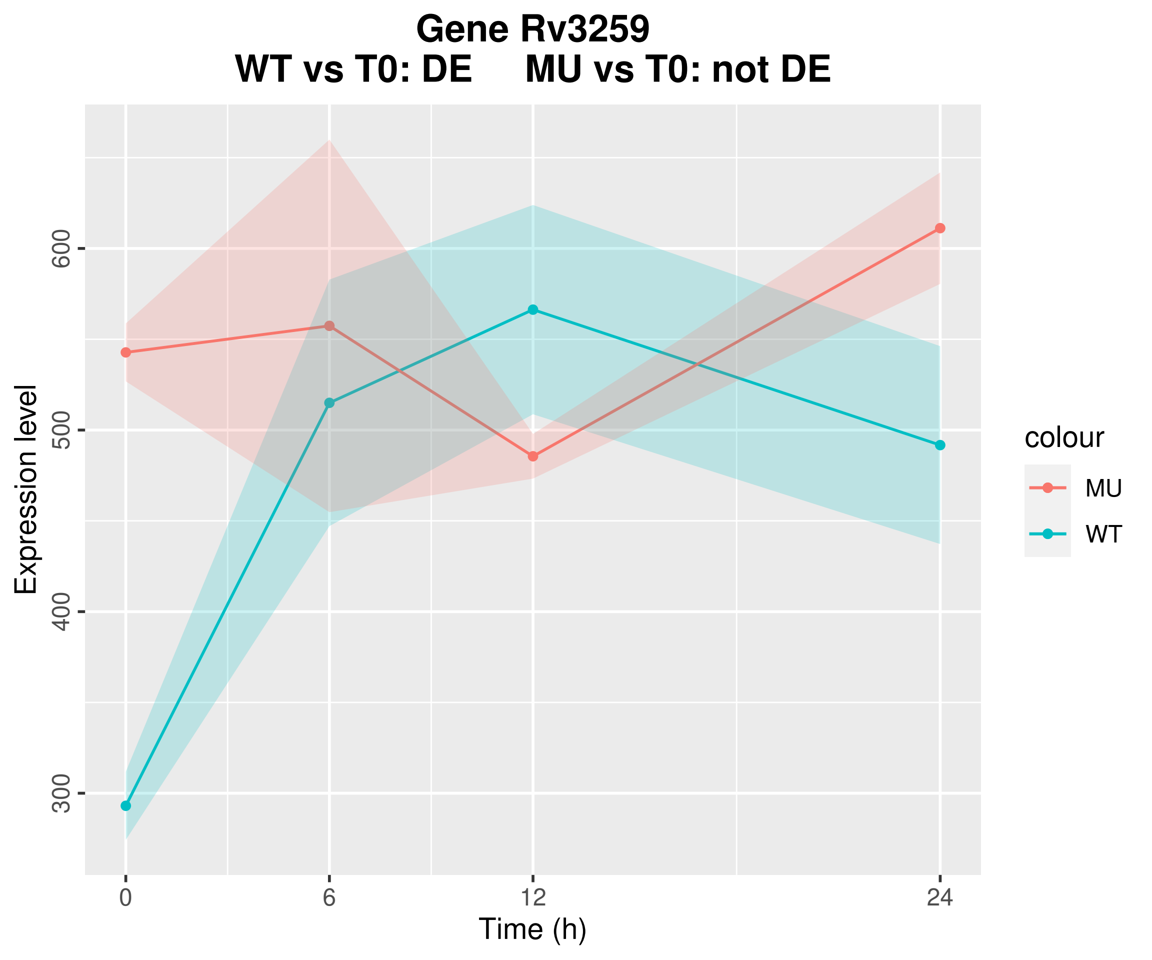
Gene Rv3259
WT vs T0: DE MU vs T0: not DE
Expression level
600
500
400
300
0
6
12
24
Time (h)
colour
MU
WT

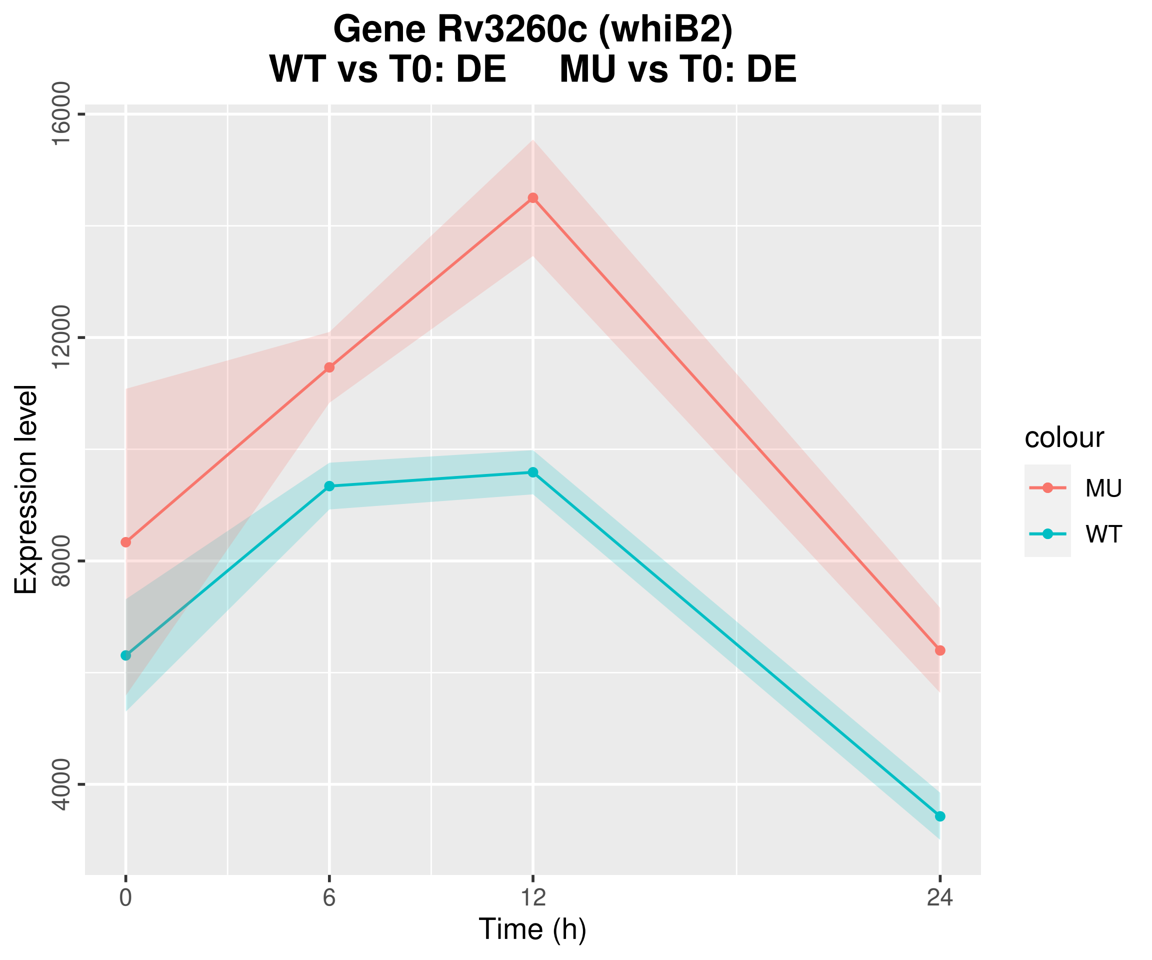

Gene Rv3260c (whiB2)
WT vs T0: DE MU vs T0: DE
Expression level
16000
12000
8000
4000
0
6
12
24
Time (h)
colour
MU
WT

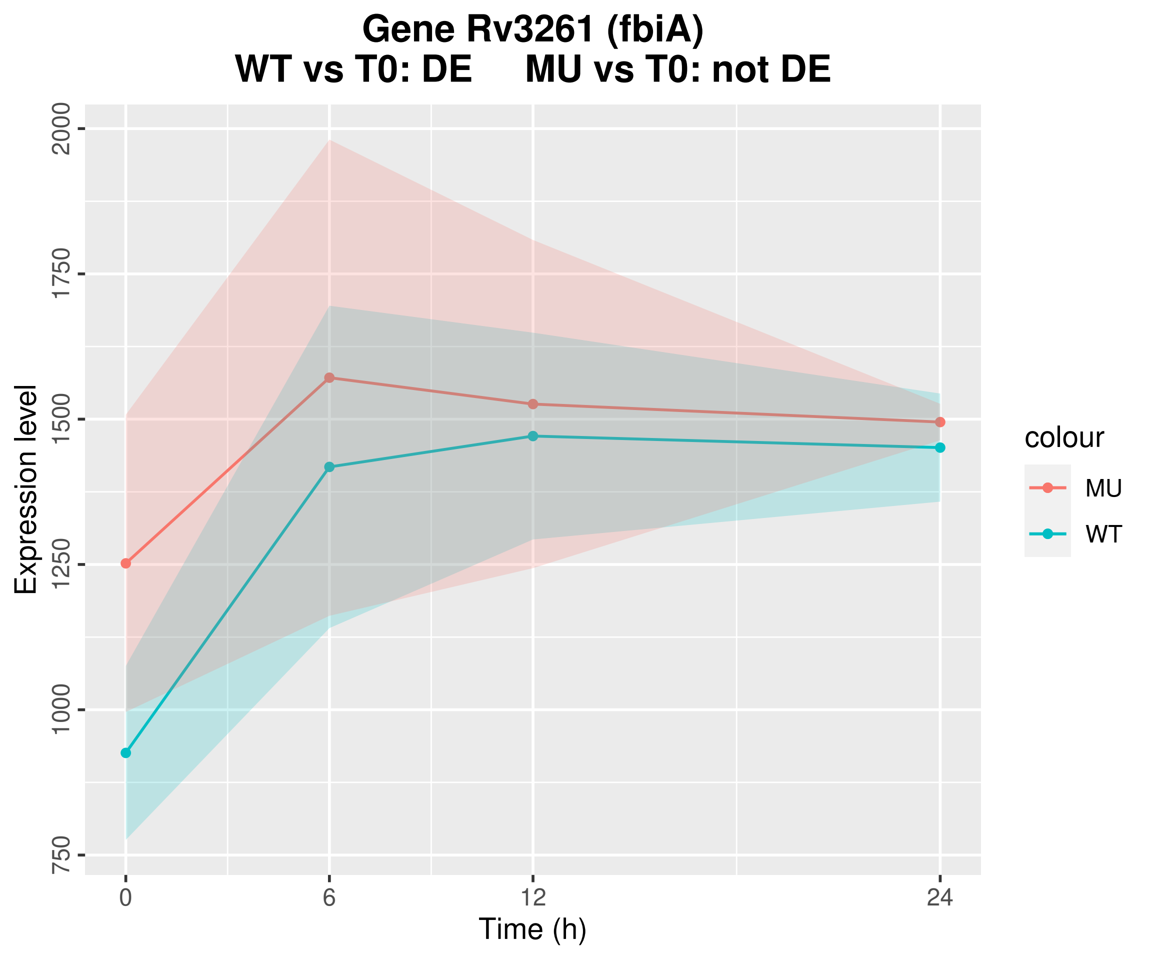

Gene Rv3261 (fbiA)
WT vs T0: DE MU vs T0: not DE
Expression level
2000
1750
1500
1250
1000
750
0
6
12
24
Time (h)
colour
MU
WT

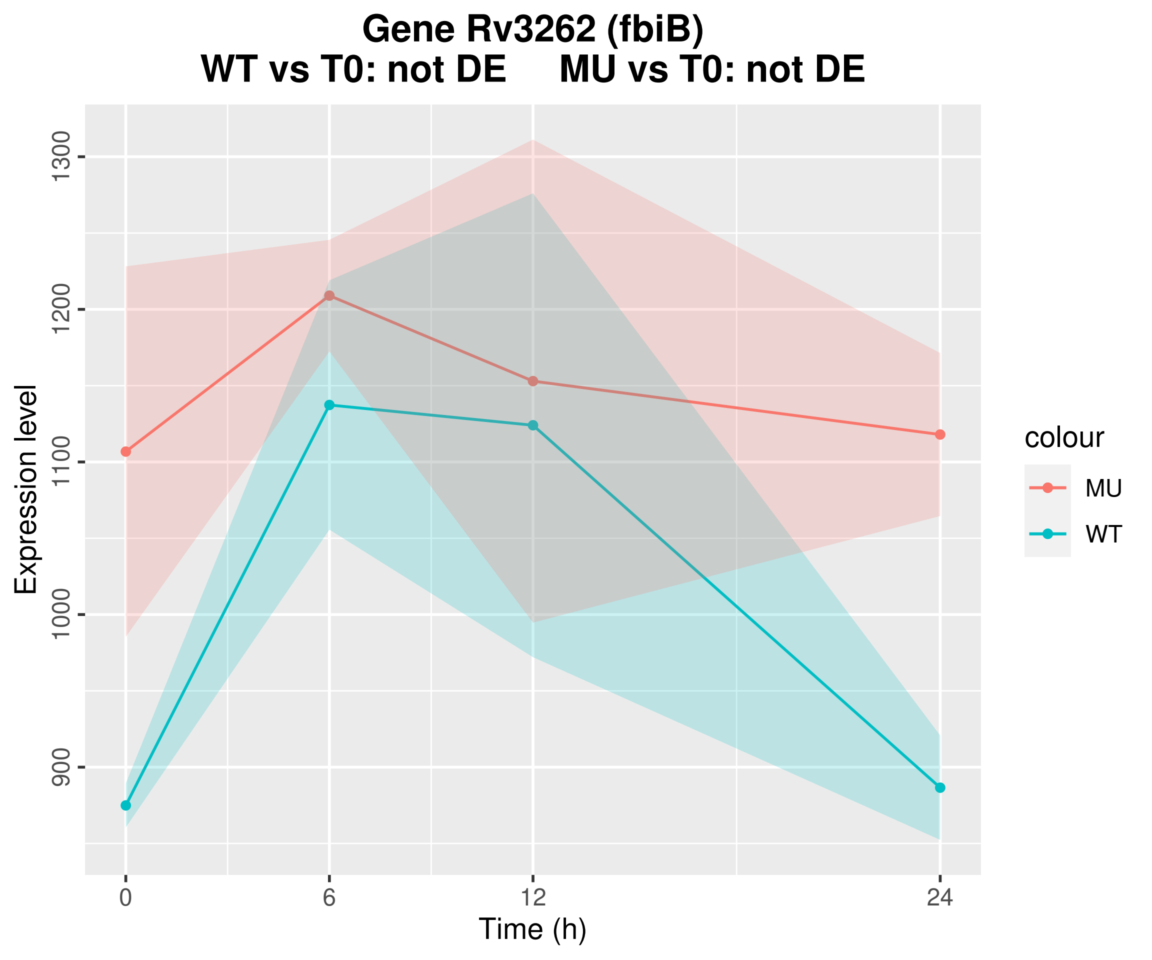

Gene Rv3262 (fbiB)
WT vs T0: not DE   MU vs T0: not DE
Expression level
1300
1200
1100
1000
900
0
6
12
24
Time (h)
colour
MU
WT

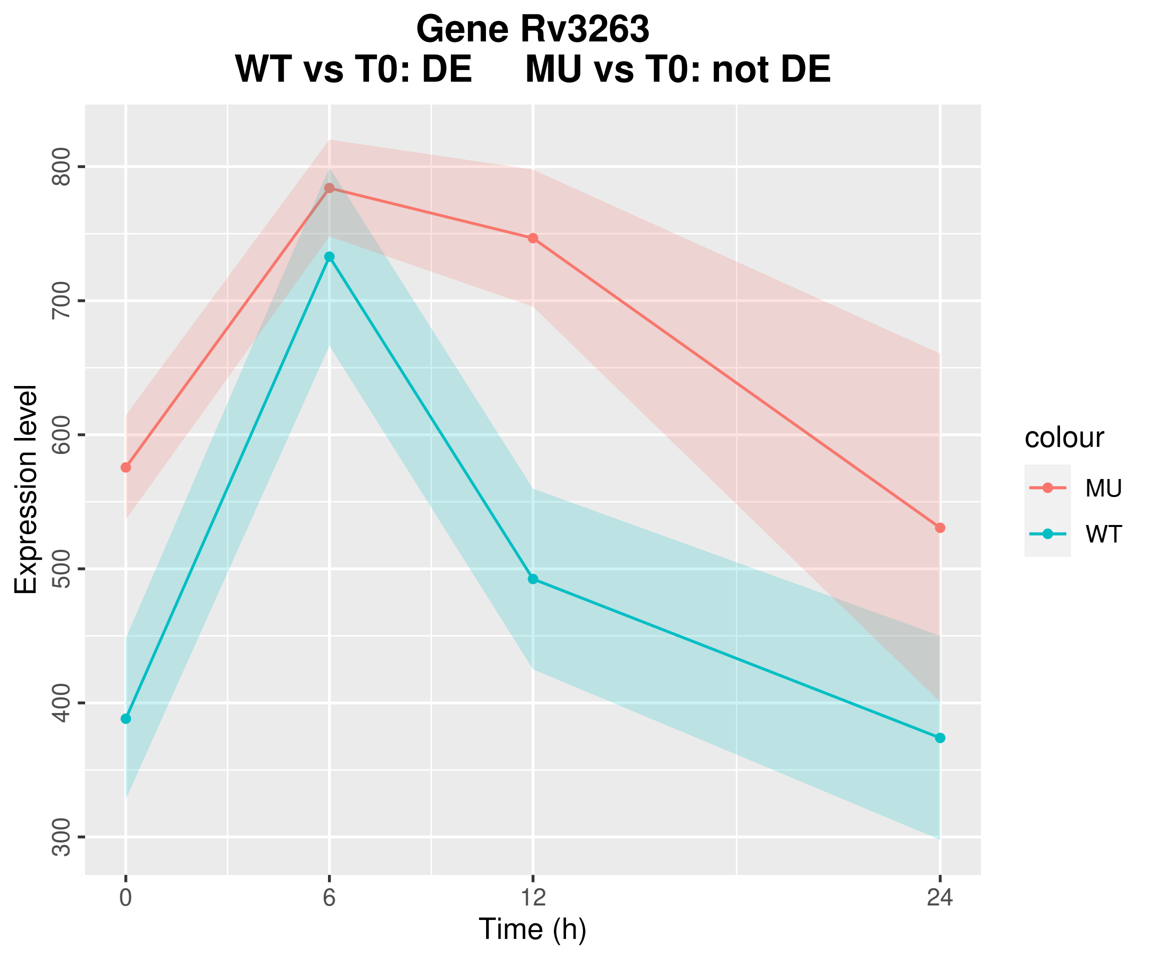
Gene Rv3263
WT vs T0: DE   MU vs T0: not DE
Expression level
800
700
600
500
400
300
0
6
12
24
Time (h)
colour
MU
WT

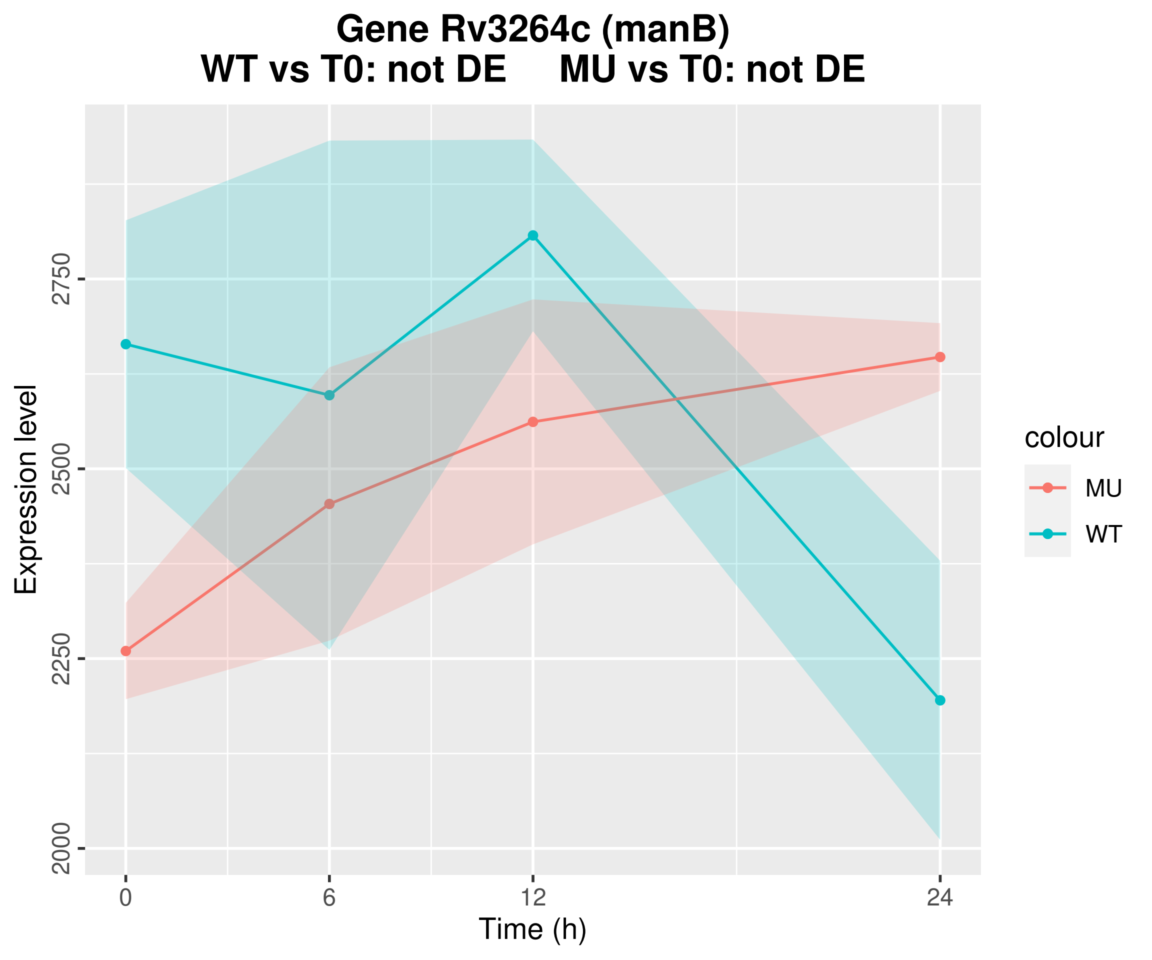
Gene Rv3264c (manB)
WT vs T0: not DE   MU vs T0: not DE
Expression level
2000
2250
2500
2750
0
6
12
24
Time (h)
colour
MU
WT

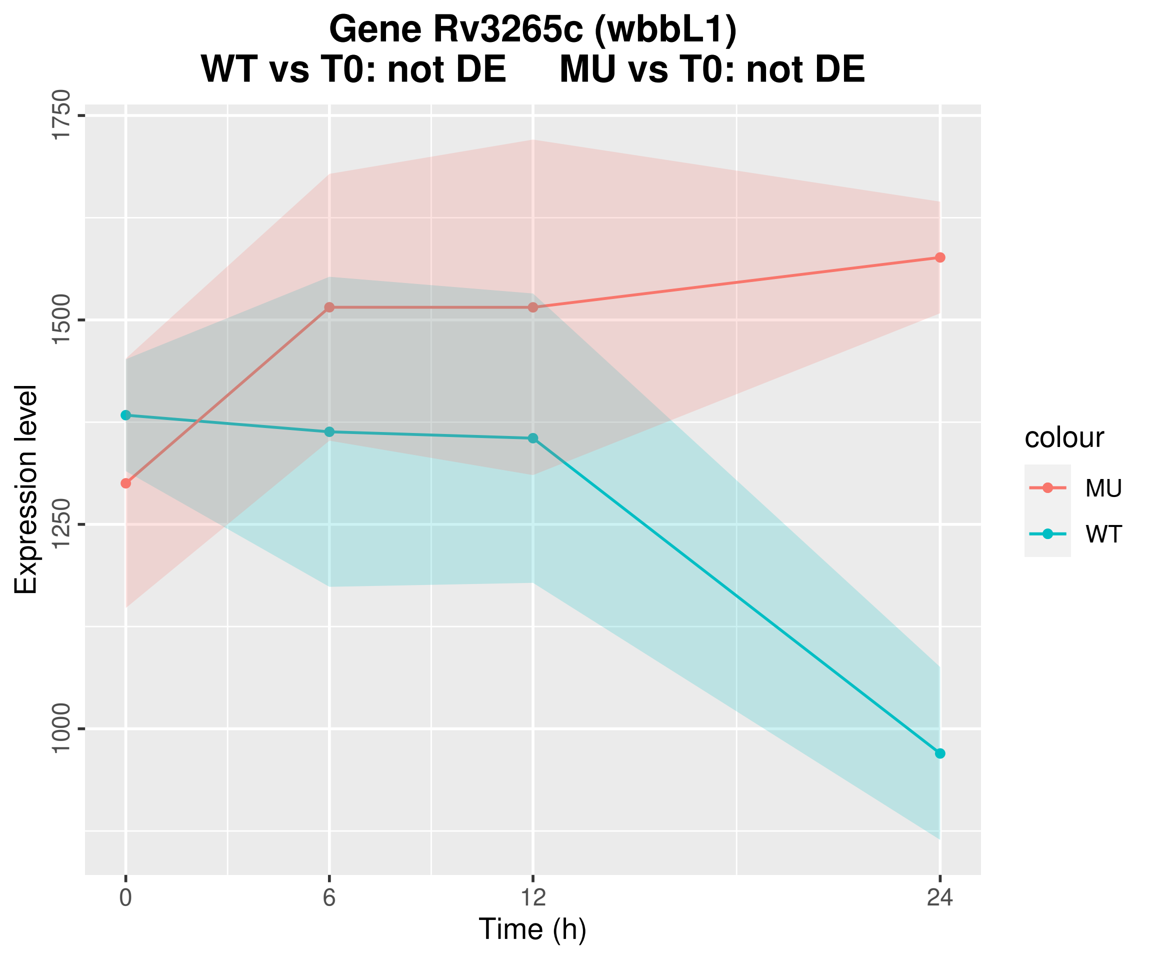

Gene Rv3265c (wbbL1)
WT vs T0: not DE MU vs T0: not DE
Expression level
1750
1500
1250
1000
0
6
12
24
Time (h)
colour
MU
WT

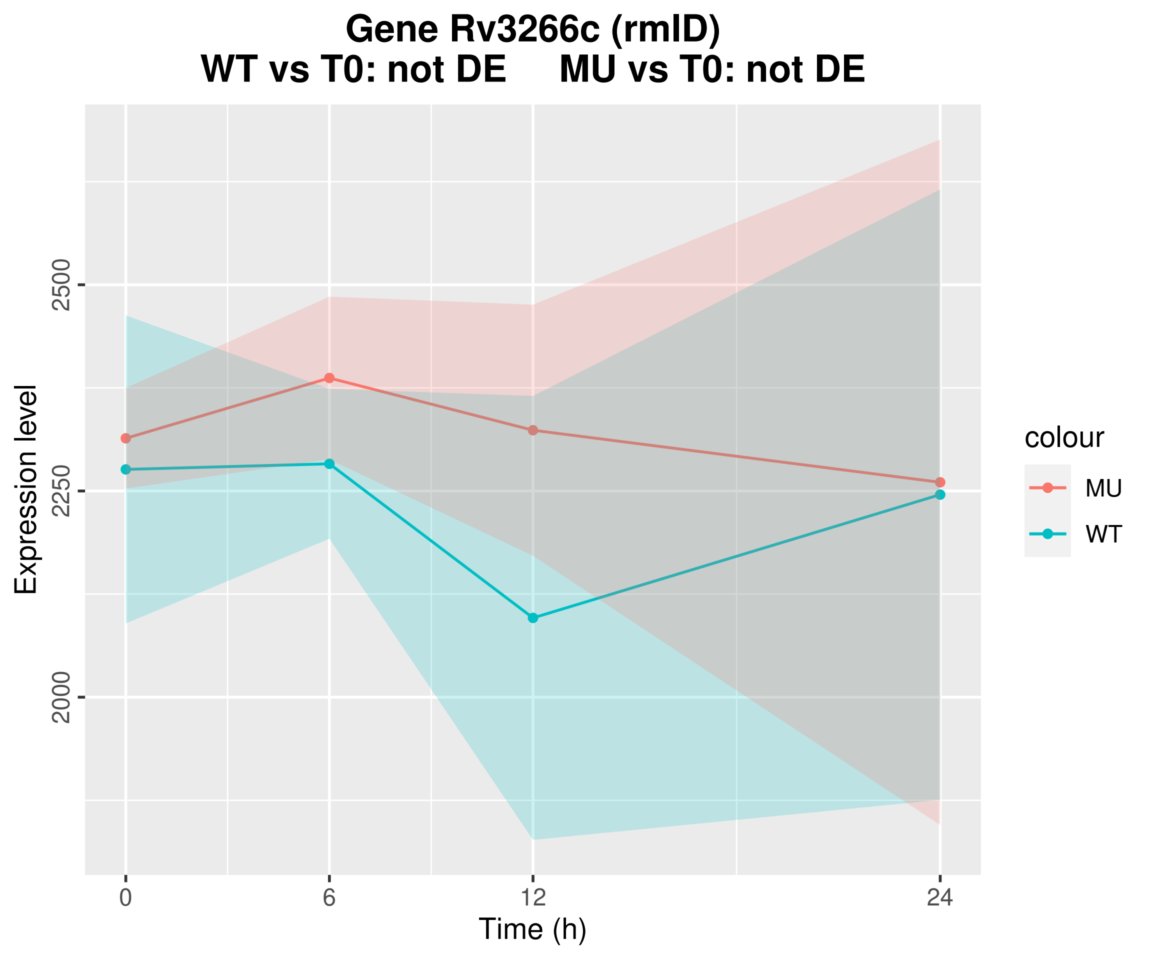

Gene Rv3266c (rmlD)
WT vs T0: not DE   MU vs T0: not DE
Expression level
2000
2250
2500
0
6
12
24
Time (h)
colour
MU
WT

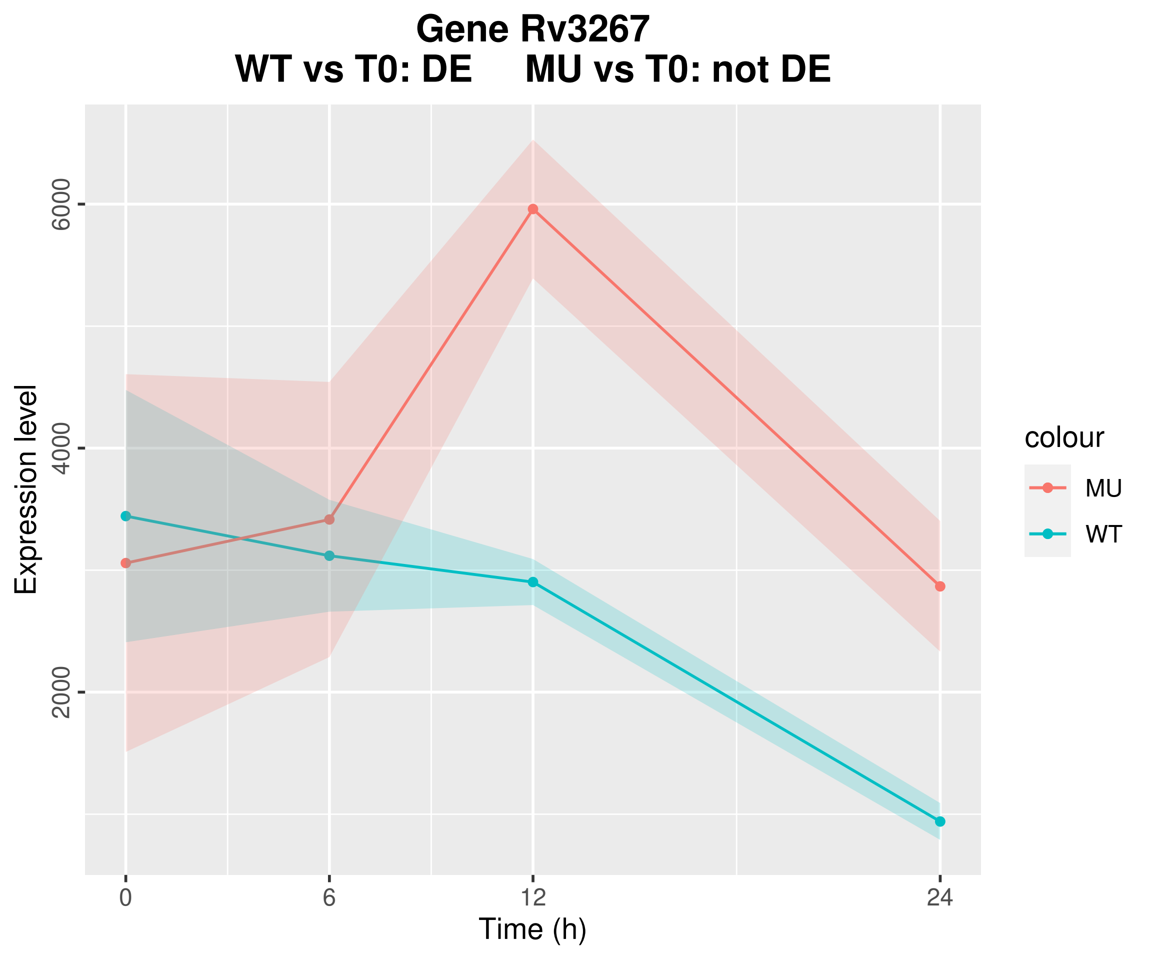
Gene Rv3267
WT vs T0: DE   MU vs T0: not DE
Expression level
6000
4000
2000
0
6
12
24
Time (h)
colour
MU
WT

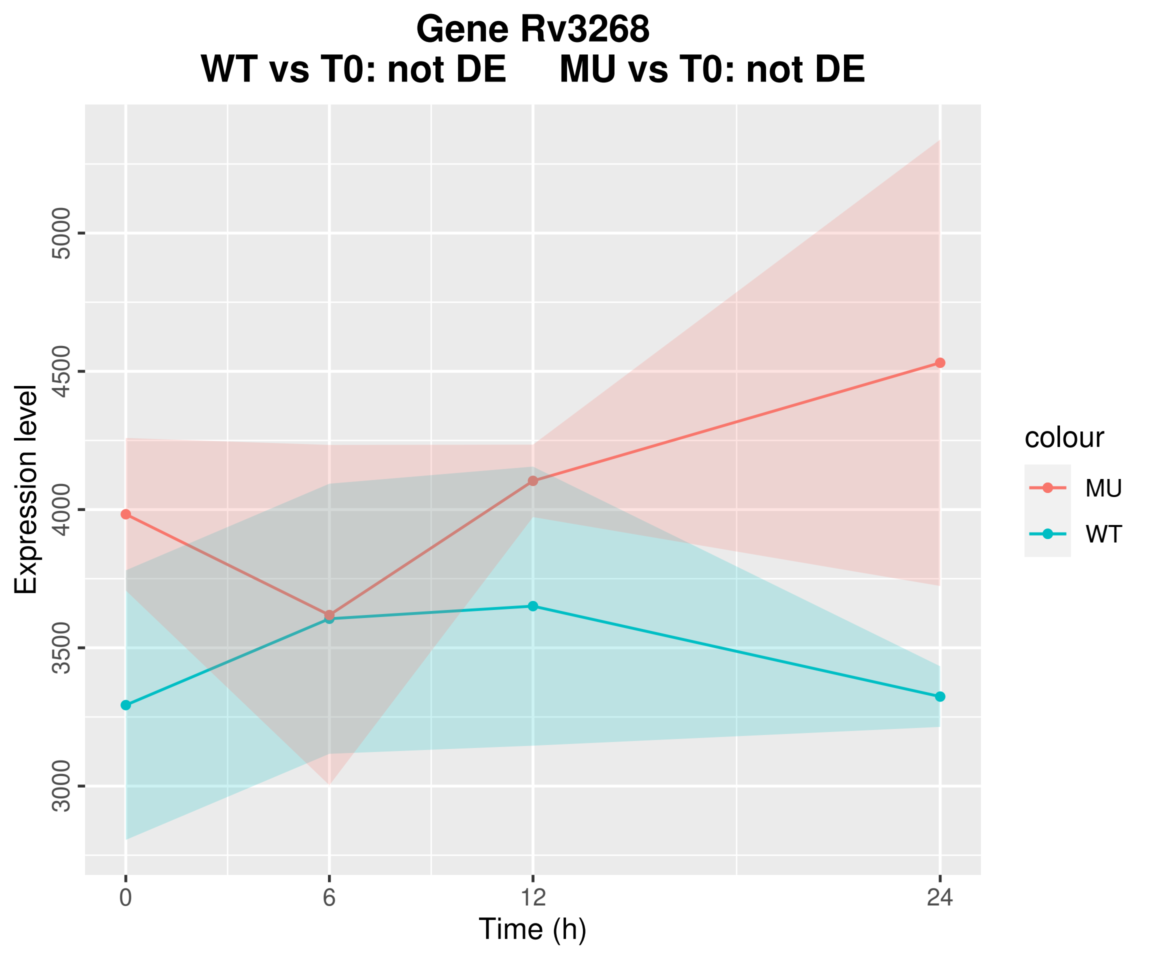

Gene Rv3268
WT vs T0: not DE MU vs T0: not DE
Expression level
3000
3500
4000
4500
5000
0
6
12
24
Time (h)
colour
MU
WT

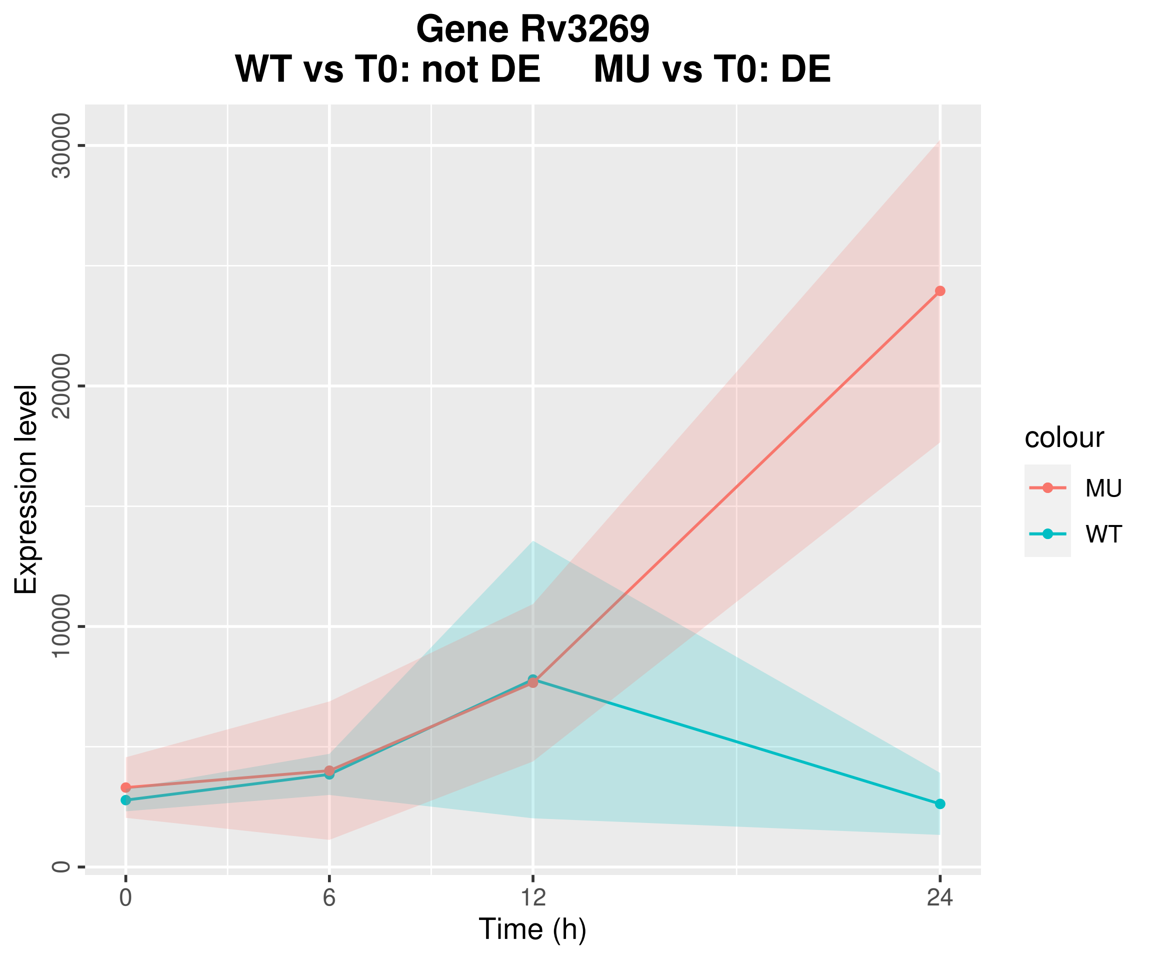

Gene Rv3269
WT vs T0: not DE MU vs T0: DE
Expression level
30000
20000
10000
0
0
6
12
24
Time (h)
colour
MU
WT

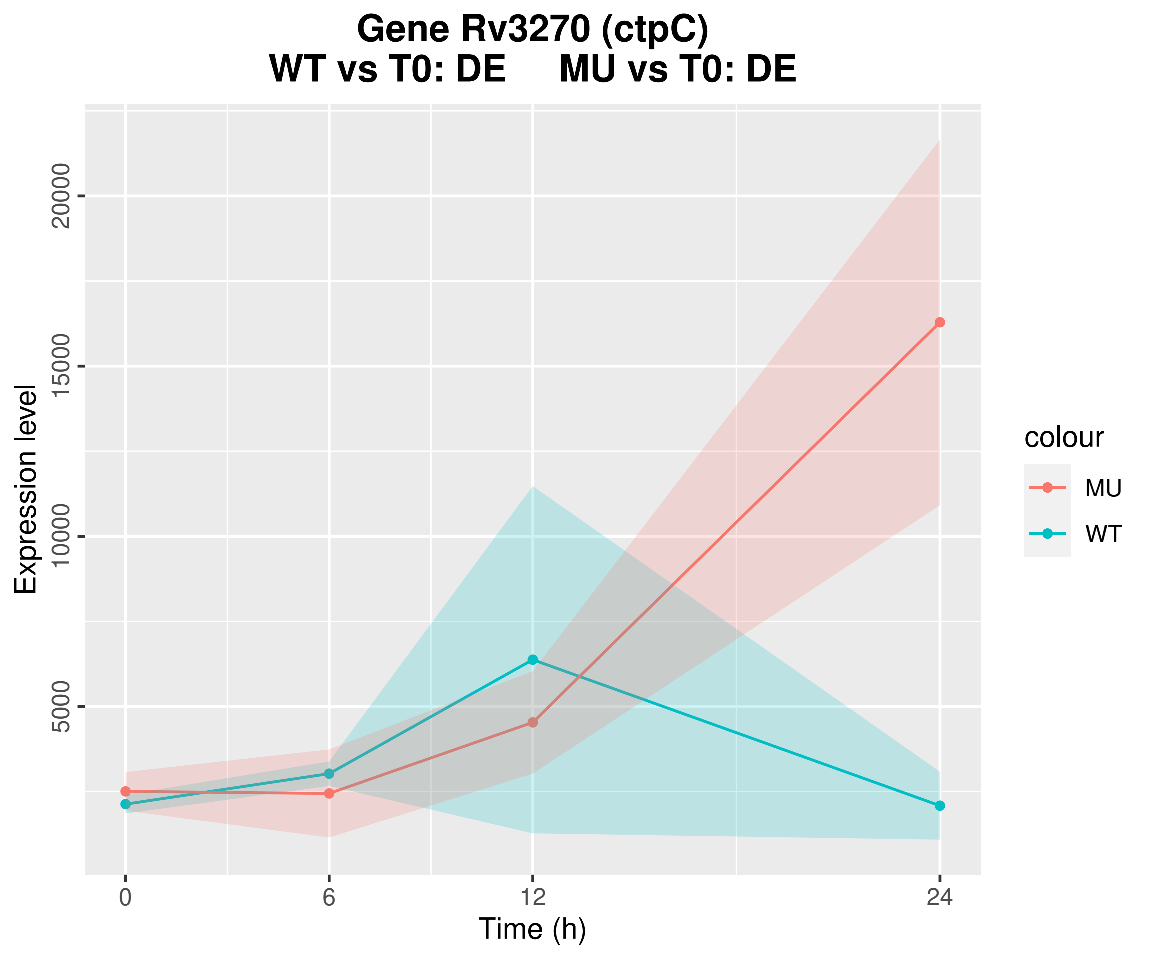

Gene Rv3270 (ctpC)
WT vs T0: DE MU vs T0: DE
Expression level
20000
15000
10000
5000
0
6
12
24
Time (h)
colour
MU
WT

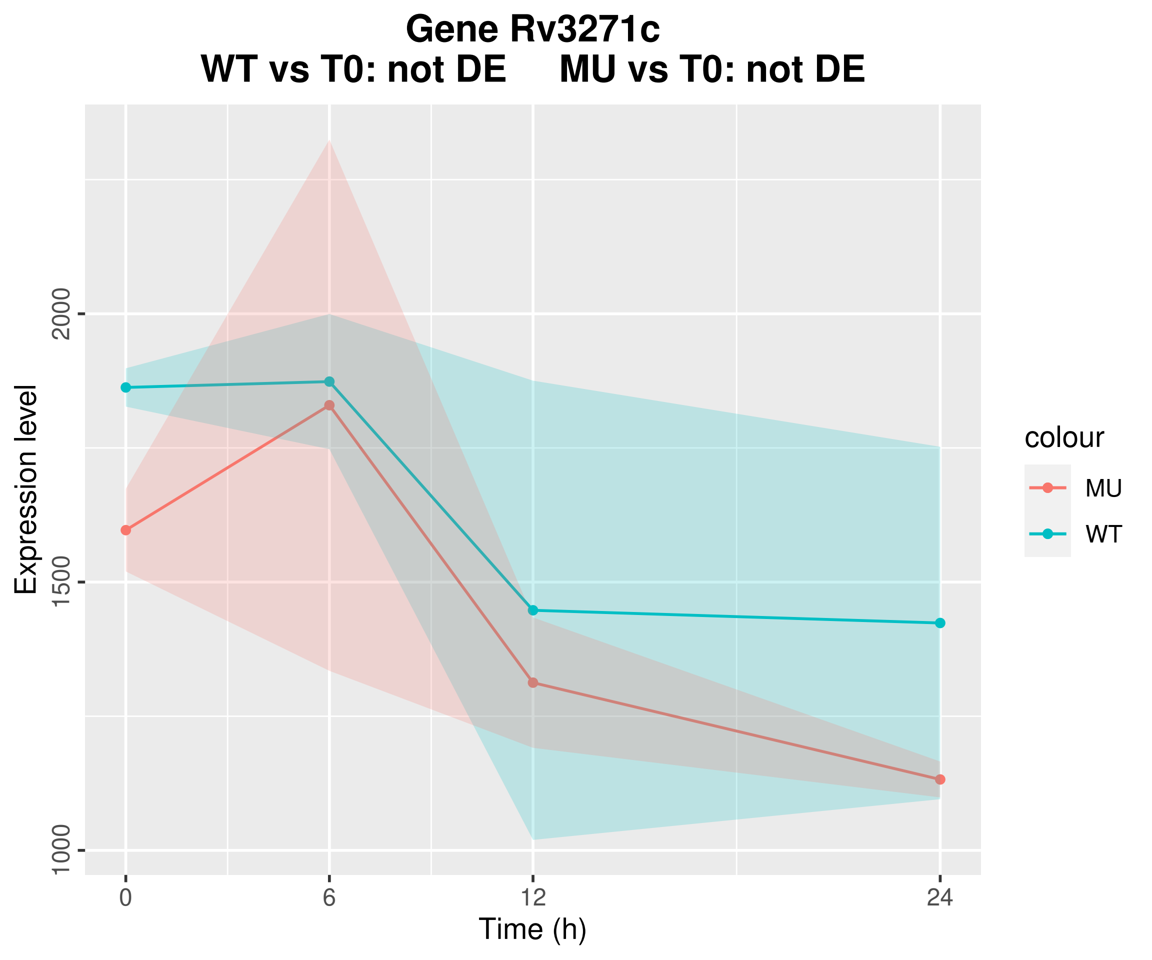

Gene Rv3271c
WT vs T0: not DE   MU vs T0: not DE
Expression level
1000
1500
2000
0
6
12
24
Time (h)
colour
MU
WT

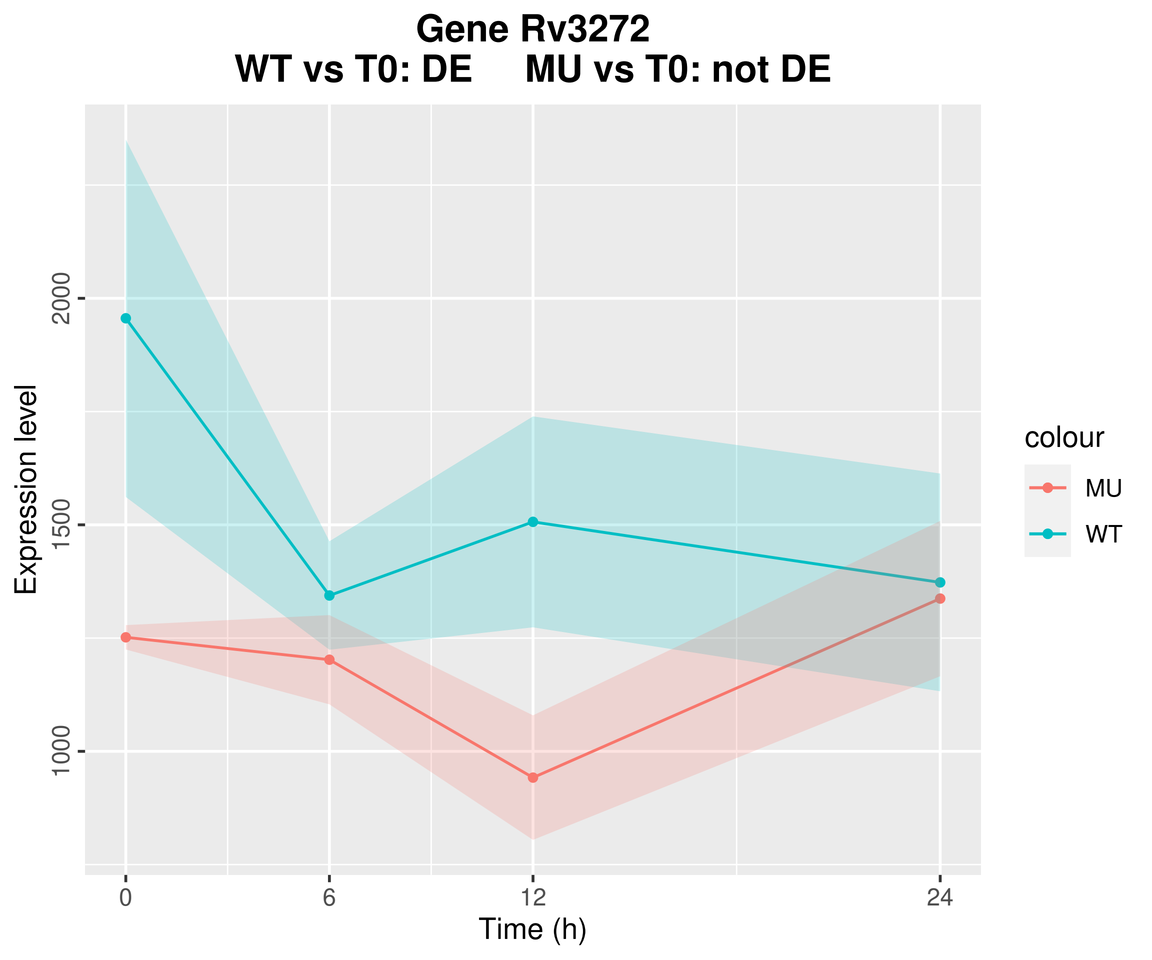
Gene Rv3272
WT vs T0: DE   MU vs T0: not DE
Expression level
2000
1500
1000
0
6
12
24
Time (h)
colour
MU
WT

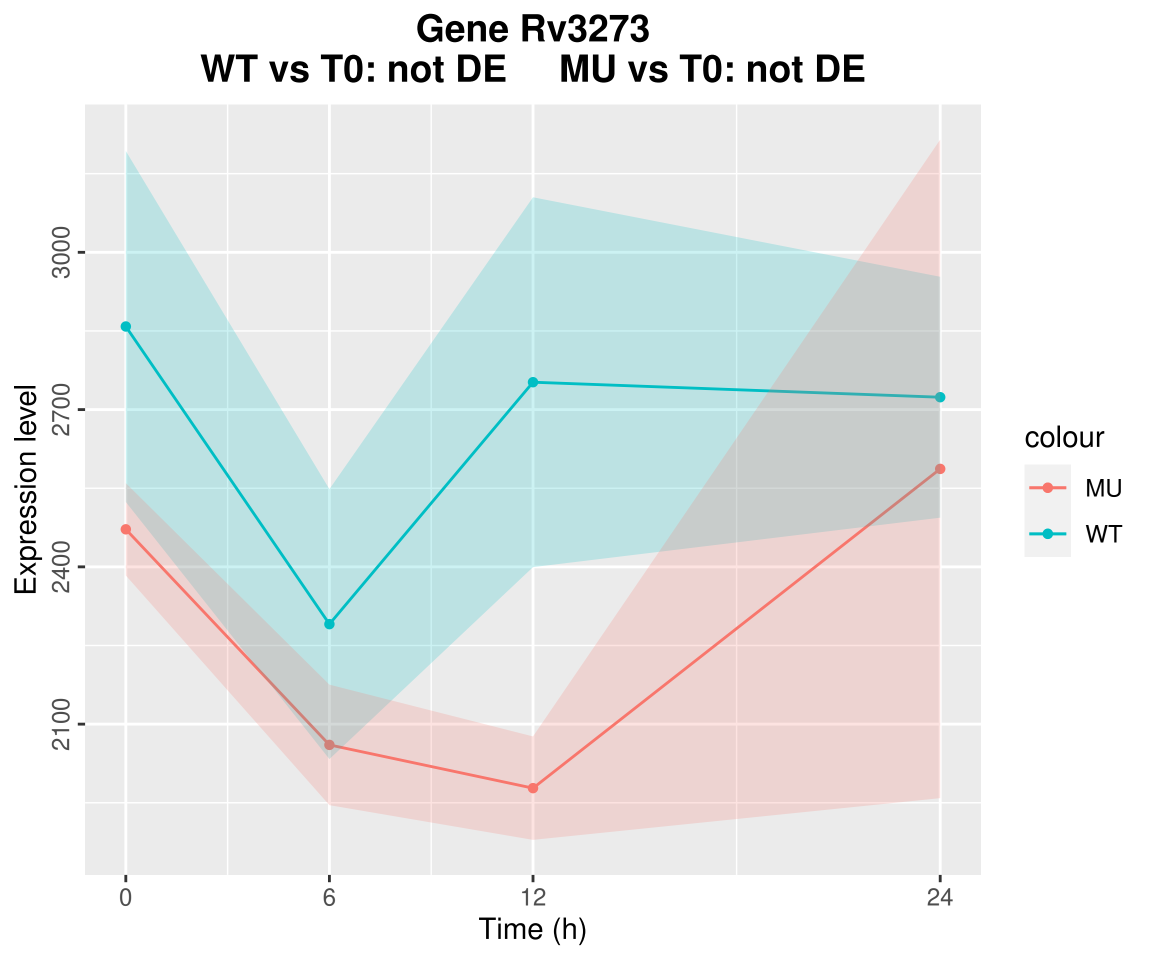

Gene Rv3273
WT vs T0: not DE MU vs T0: not DE
Expression level
3000
2700
2400
2100
0
6
12
24
Time (h)
colour
MU
WT

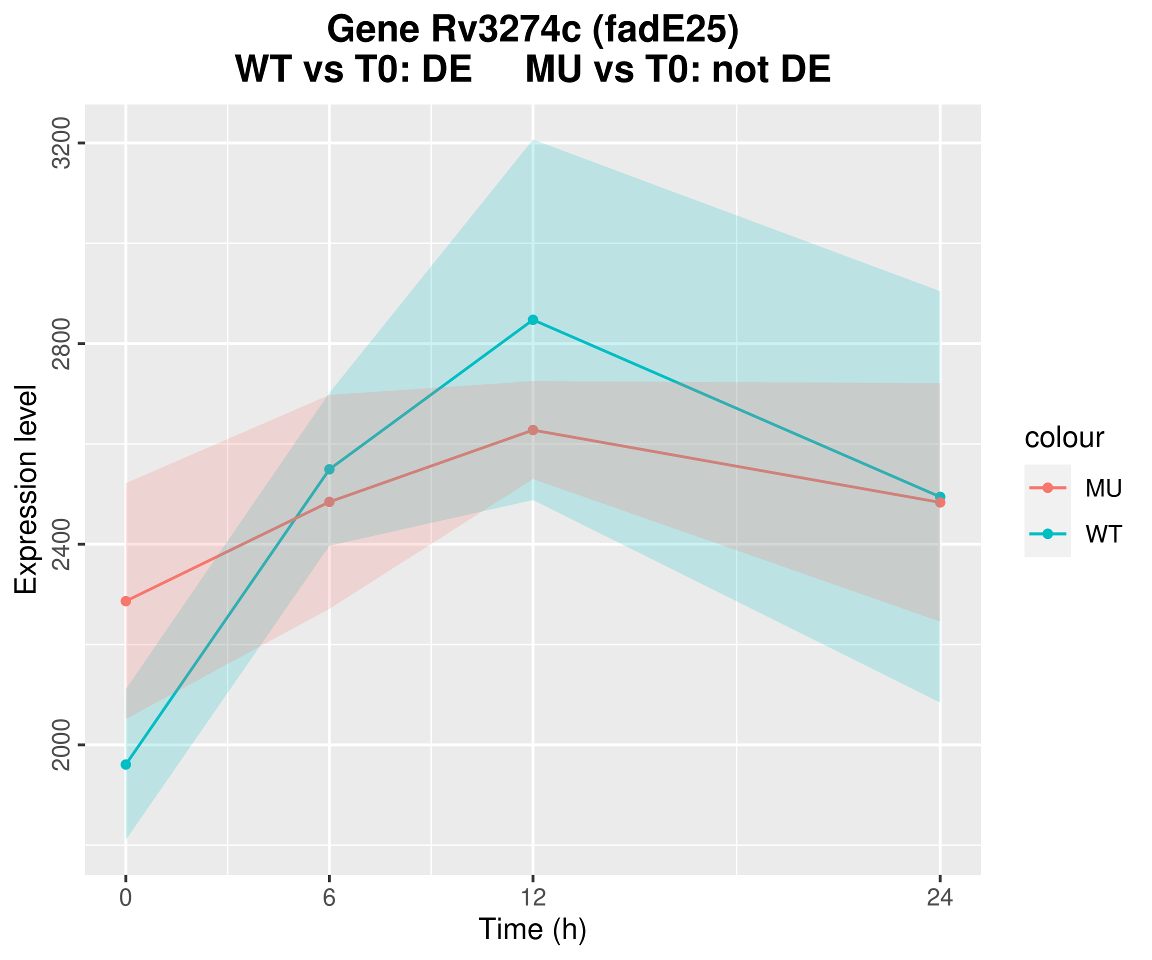
Gene Rv3274c (fadE25)
WT vs T0: DE   MU vs T0: not DE
Expression level
3200
2800
2400
2000
0
6
12
24
Time (h)
colour
MU
WT

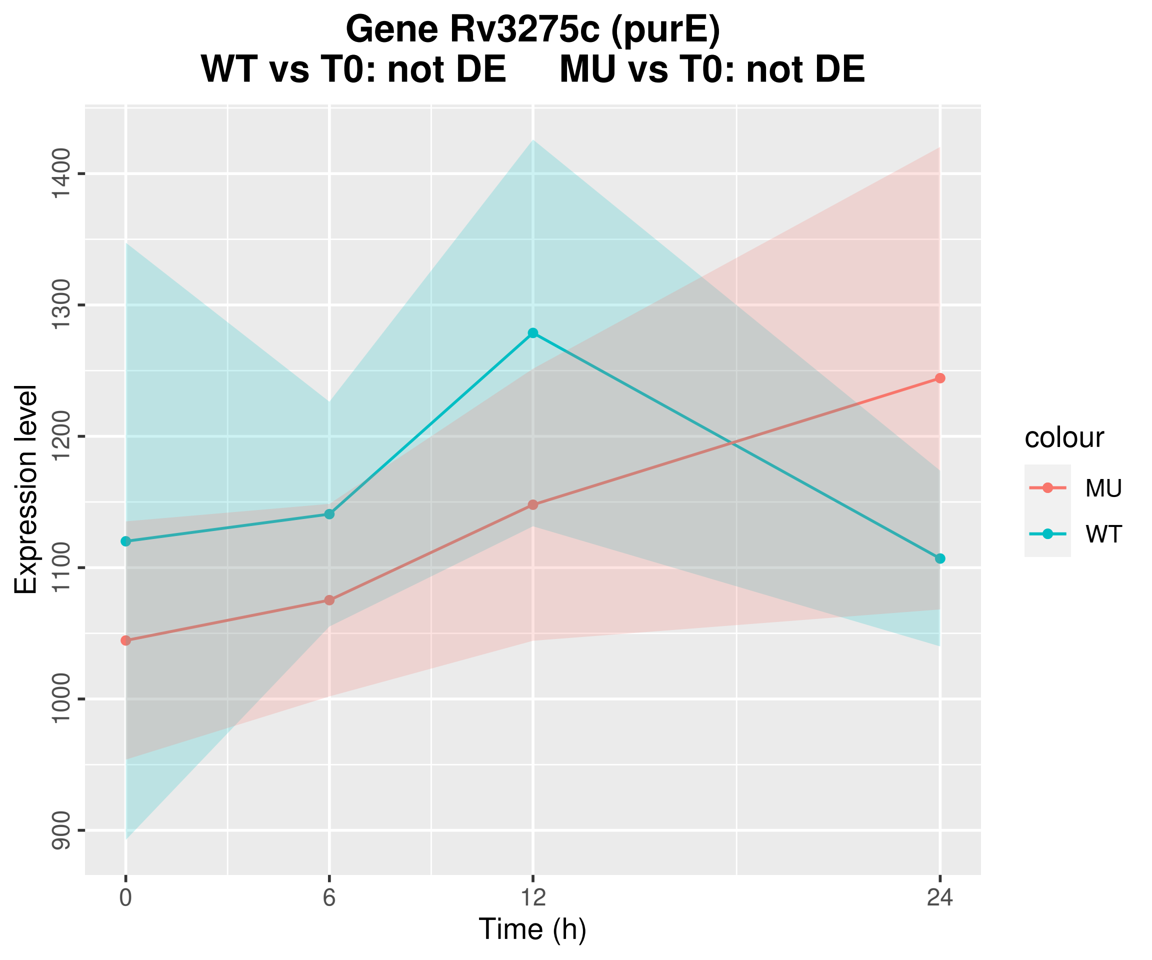
Gene Rv3275c (purE)
WT vs T0: not DE   MU vs T0: not DE
Expression level
1400
1300
1200
1100
1000
900
0
6
12
24
Time (h)
colour
MU
WT

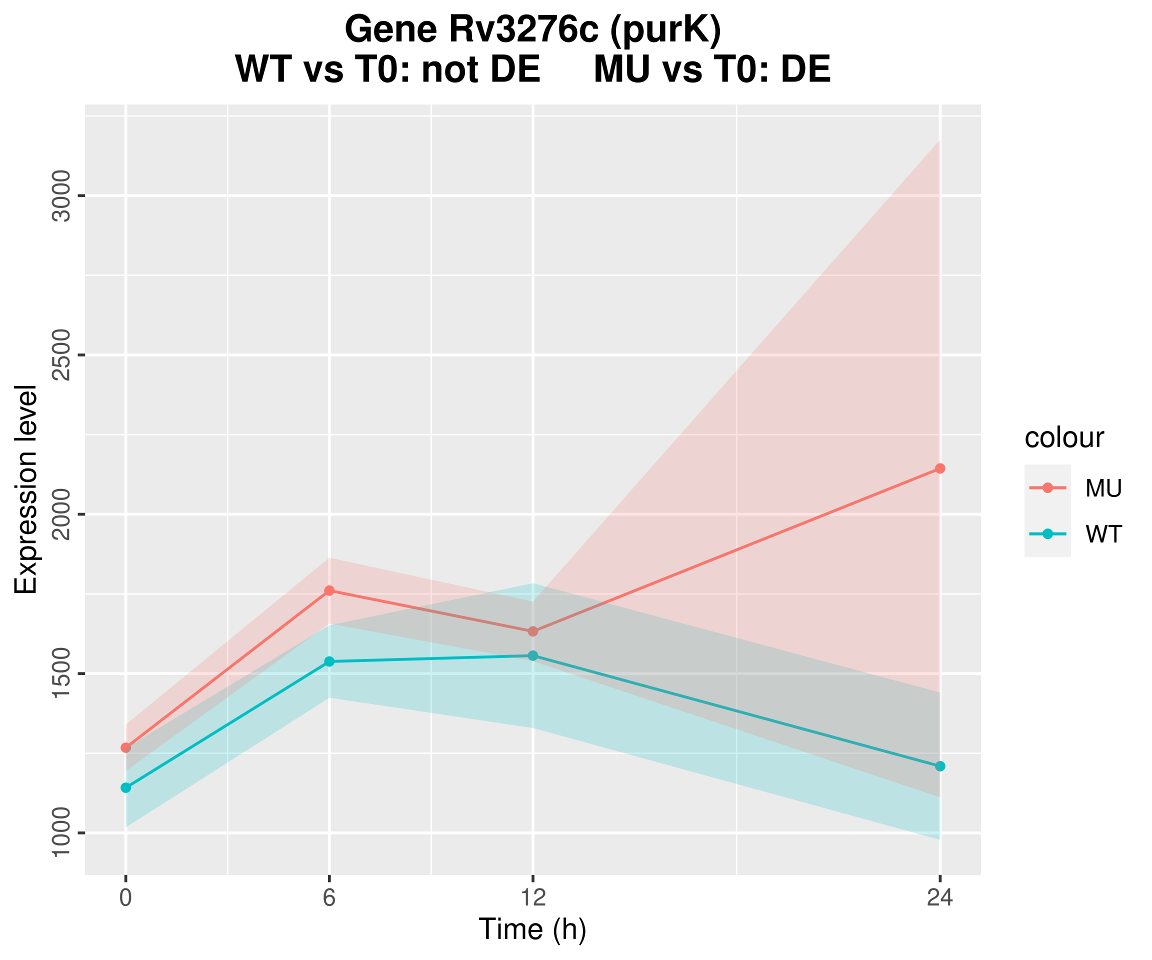
Gene Rv3276c (purK)
WT vs T0: not DE MU vs T0: DE
Expression level
1000
1500
2000
2500
3000
0
6
12
24
Time (h)
colour
MU
WT

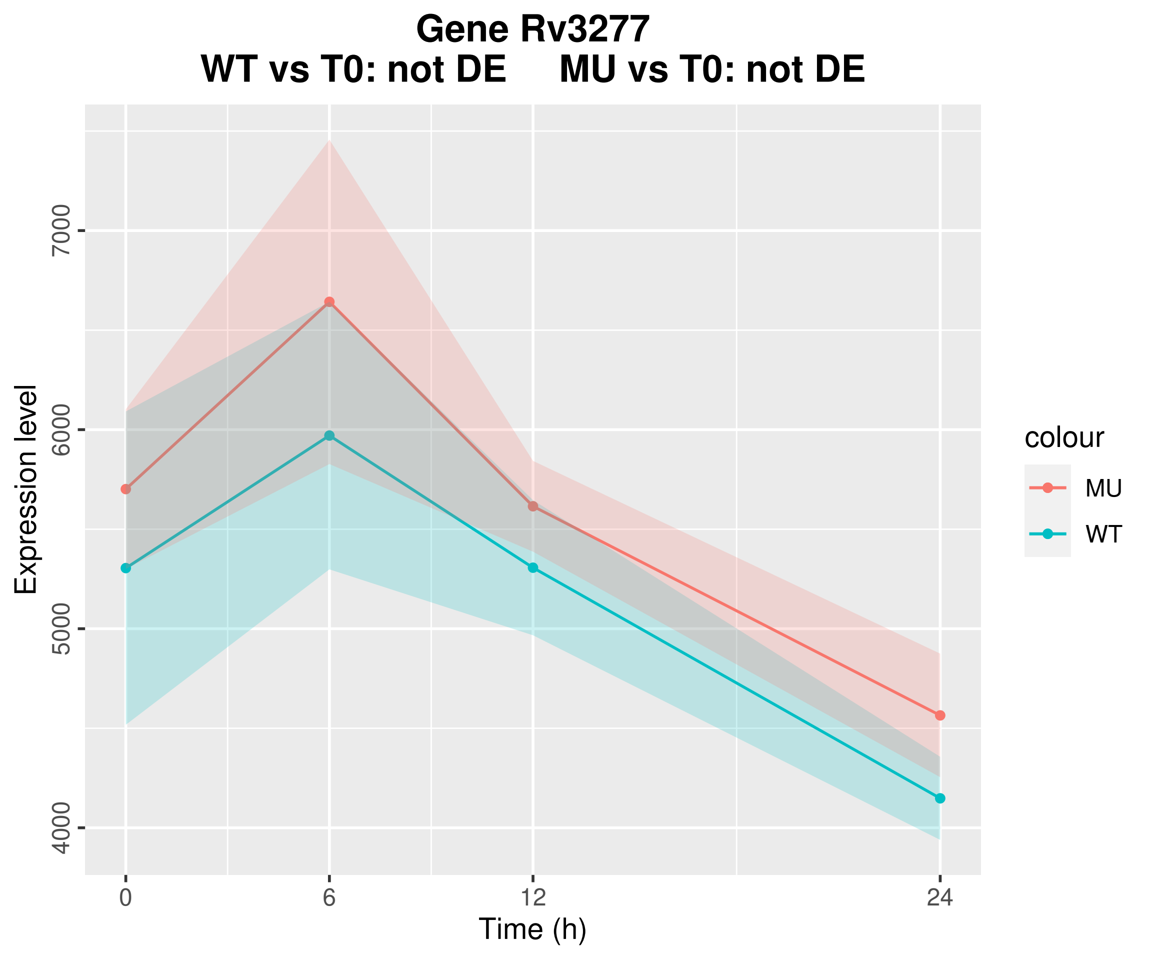

Gene Rv3277
WT vs T0: not DE   MU vs T0: not DE
Expression level
7000
6000
5000
4000
0
6
12
24
Time (h)
colour
MU
WT

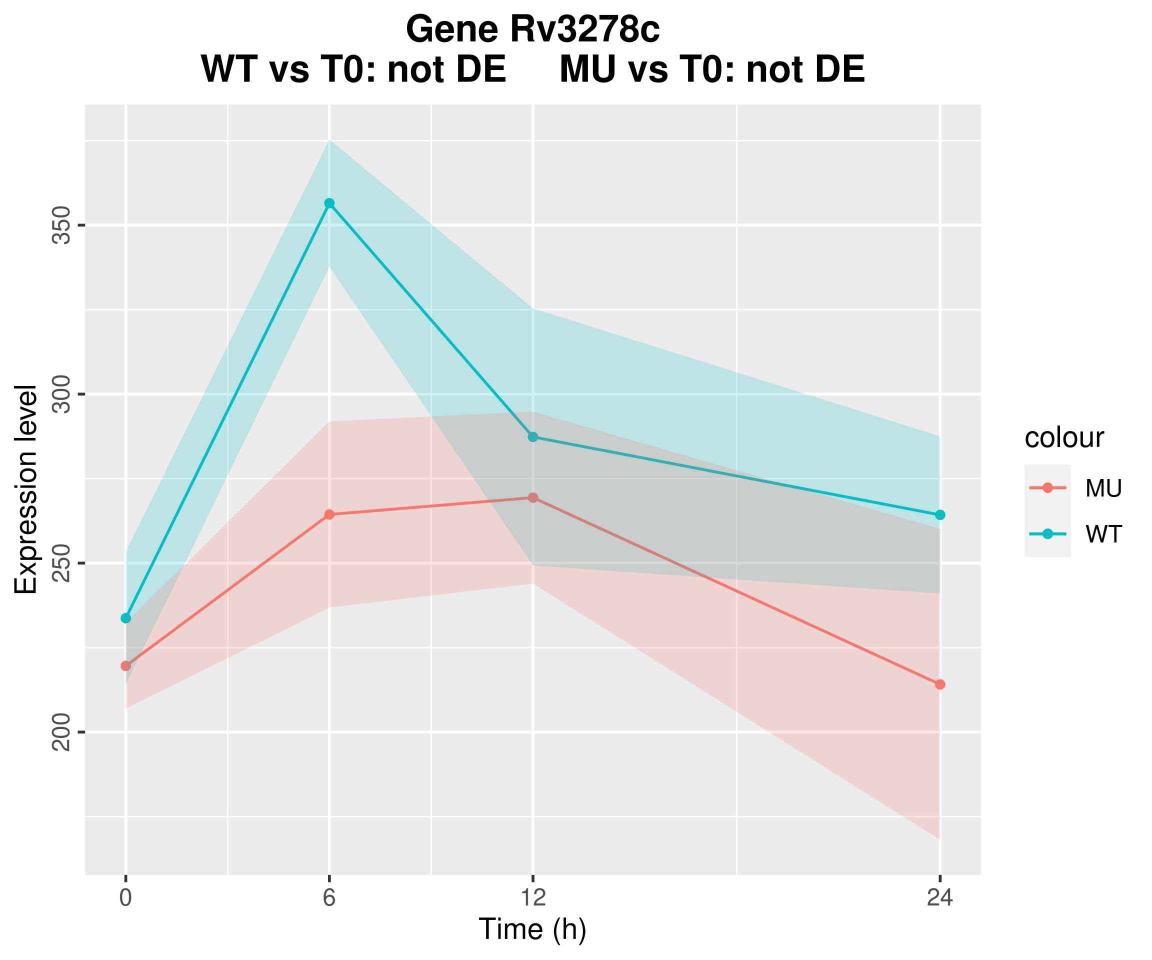

Gene Rv3278c
WT vs T0: not DE   MU vs T0: not DE
Expression level
350
300
250
200
0
6
12
24
Time (h)
colour
MU
WT

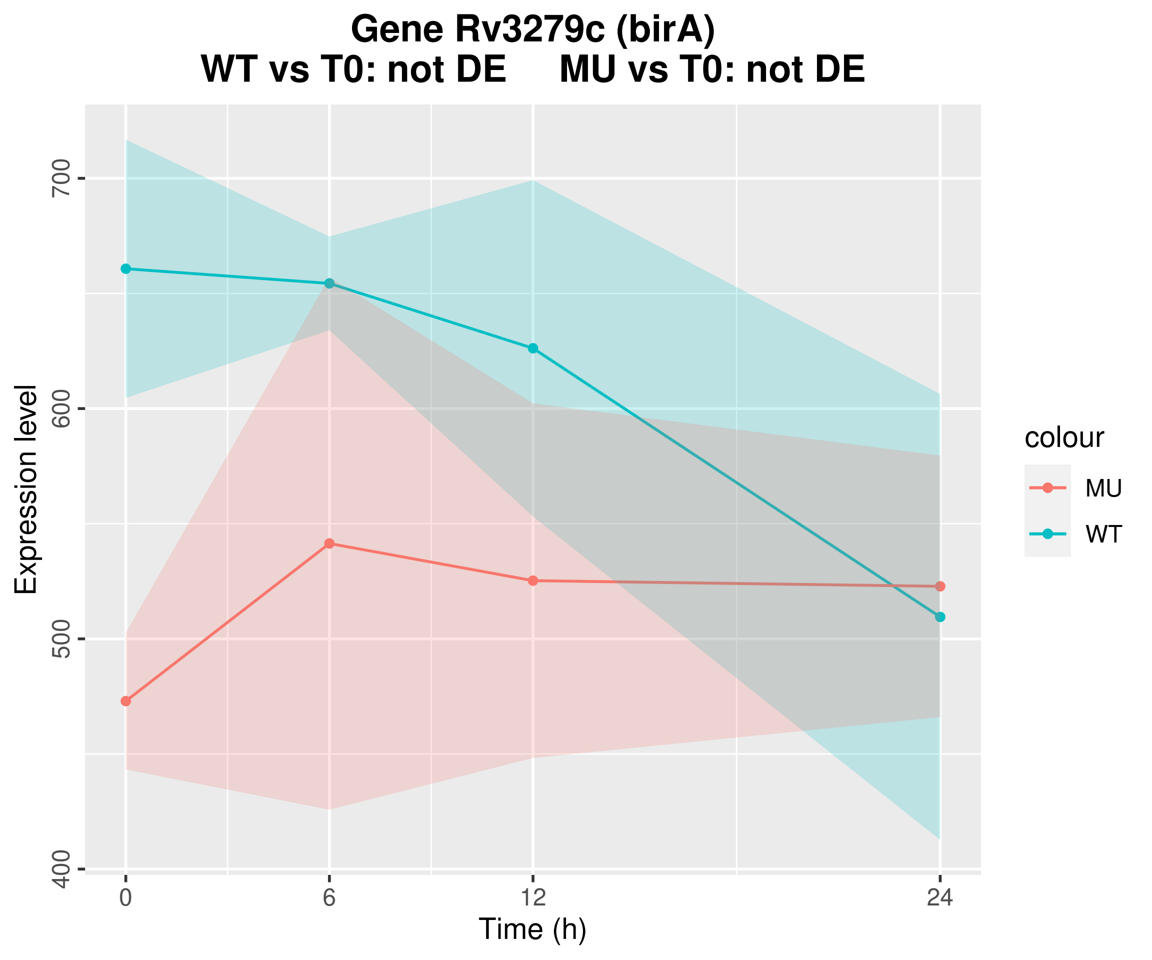

Gene Rv3279c (birA)
WT vs T0: not DE MU vs T0: not DE
Expression level
700
600
500
400
0
6
12
24
Time (h)
colour
MU
WT

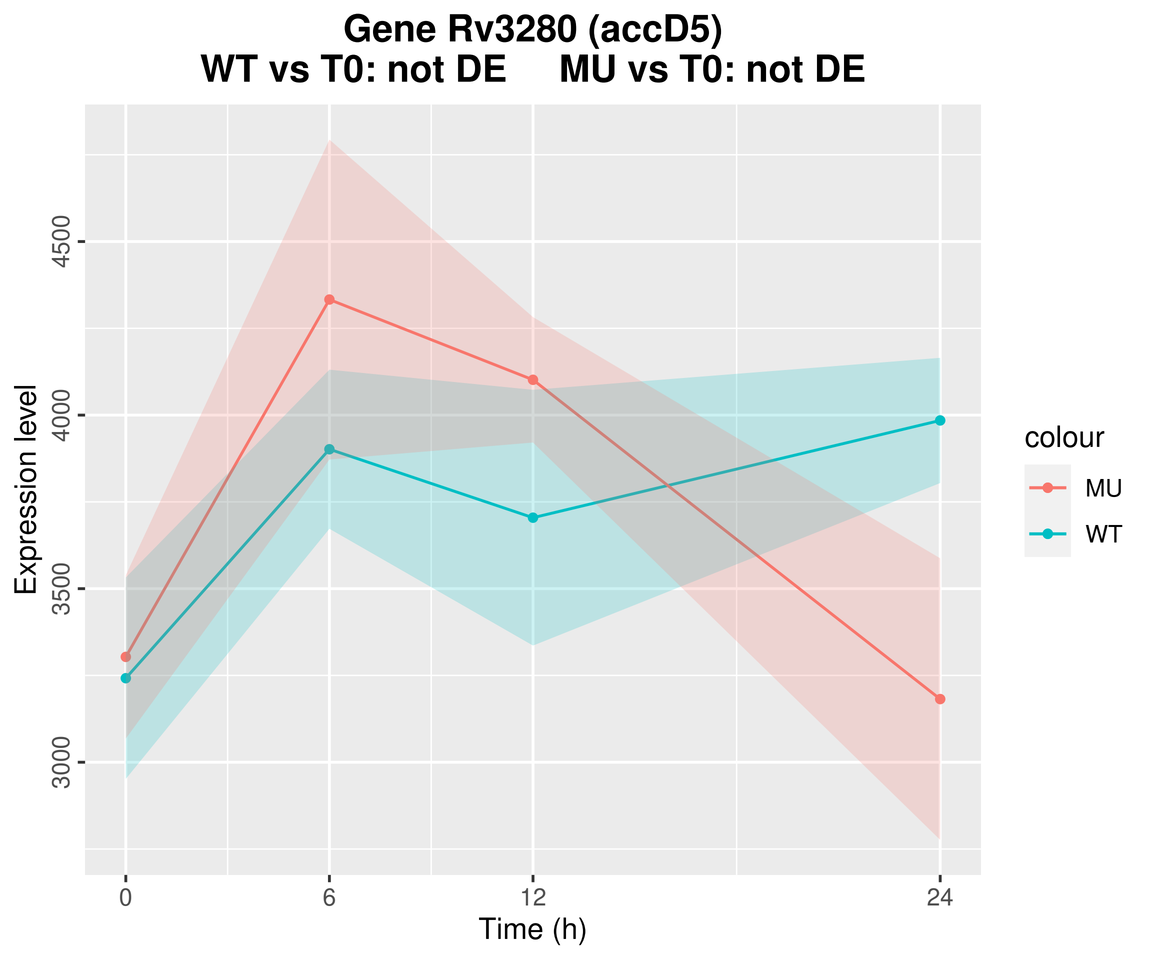

Gene Rv3280 (accD5)
WT vs T0: not DE MU vs T0: not DE
Expression level
4500
4000
3500
3000
0
6
12
24
Time (h)
colour
MU
WT

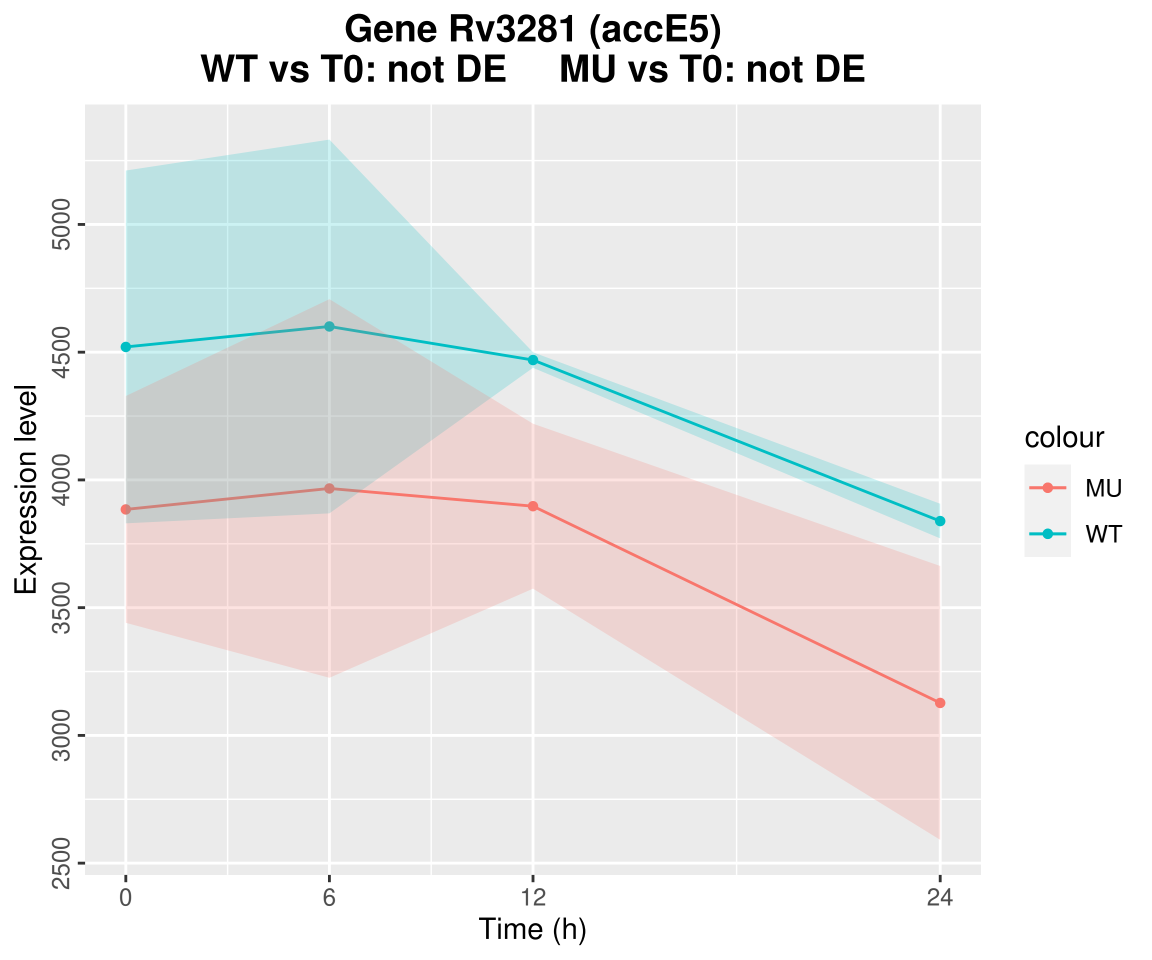
Gene Rv3281 (accE5)
WT vs T0: not DE   MU vs T0: not DE
Expression level
2500
3000
3500
4000
4500
5000
0
6
12
24
Time (h)
colour
MU
WT

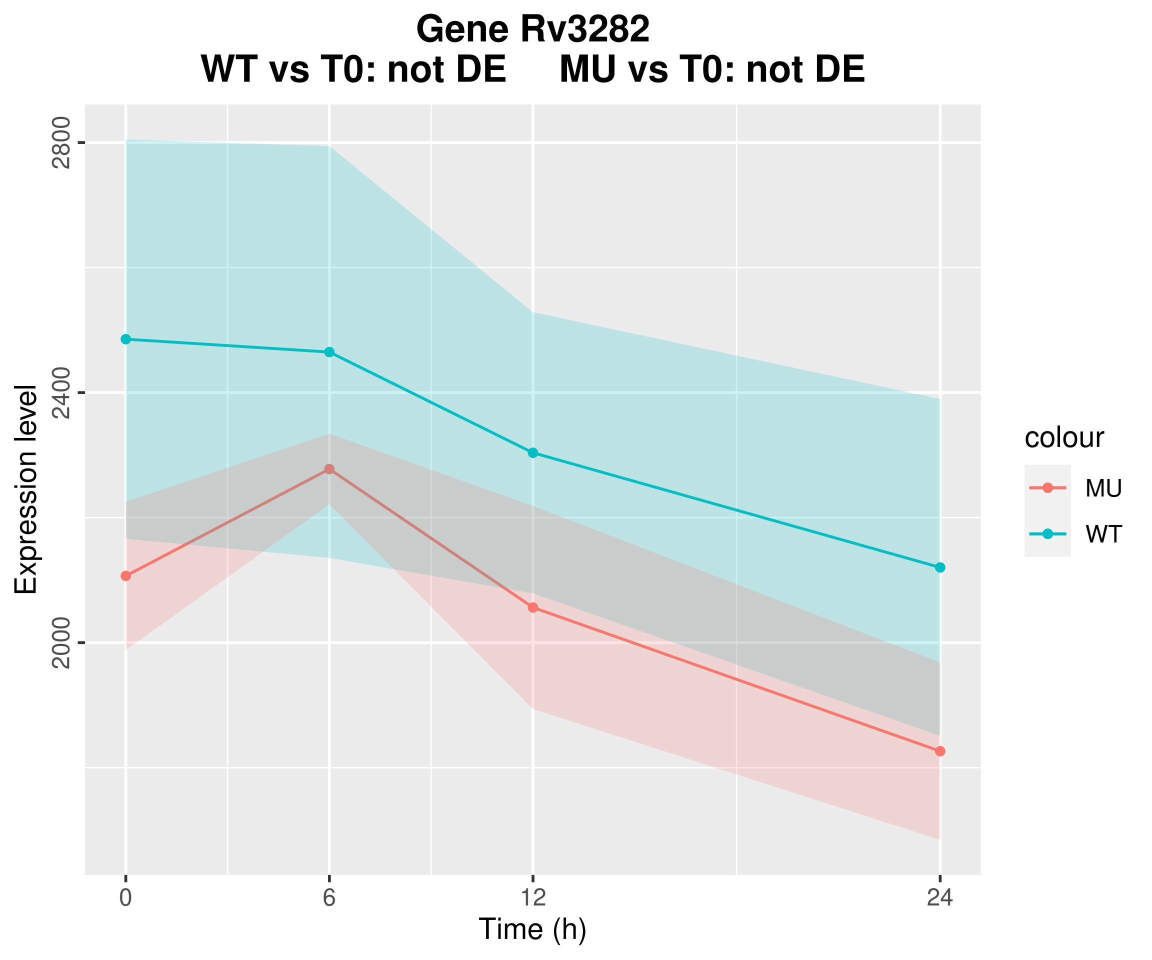

Gene Rv3282
WT vs T0: not DE   MU vs T0: not DE
Expression level
2800
2400
2000
0
6
12
24
Time (h)
colour
MU
WT

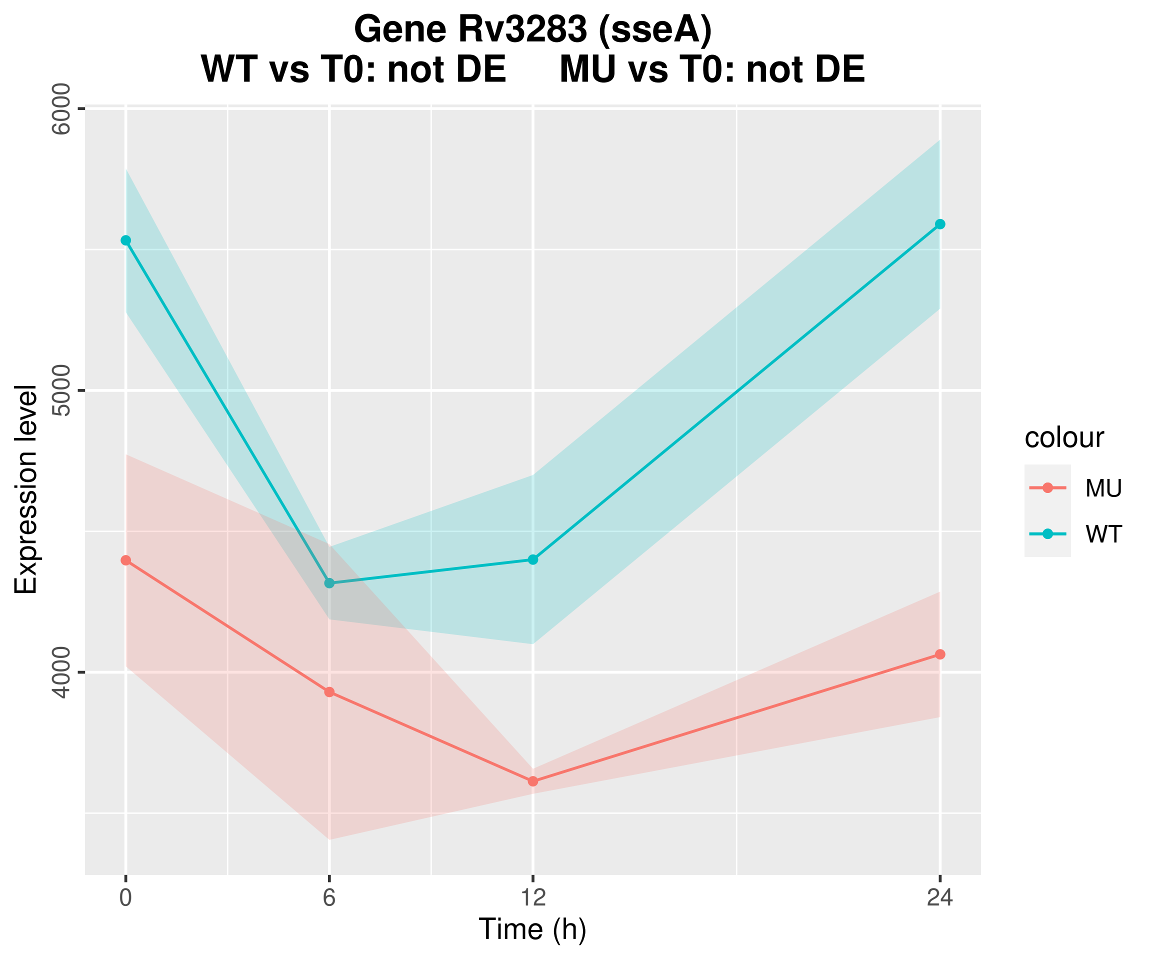
Gene Rv3283 (sseA)
WT vs T0: not DE   MU vs T0: not DE
6000
5000
4000
Expression level
0
6
12
24
Time (h)
colour
MU
WT

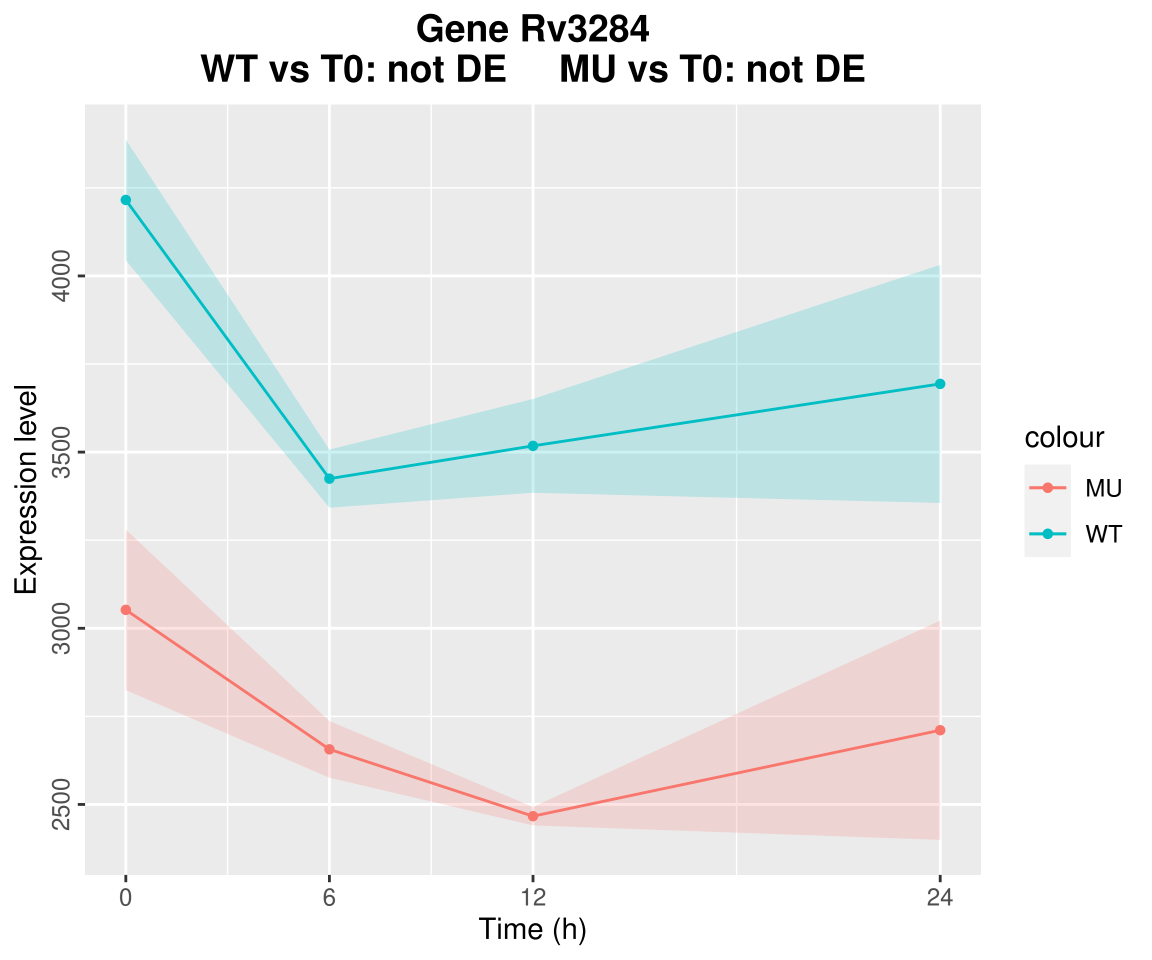
Gene Rv3284
WT vs T0: not DE   MU vs T0: not DE
Expression level
2500
3000
3500
4000
0
6
12
24
Time (h)
colour
MU
WT

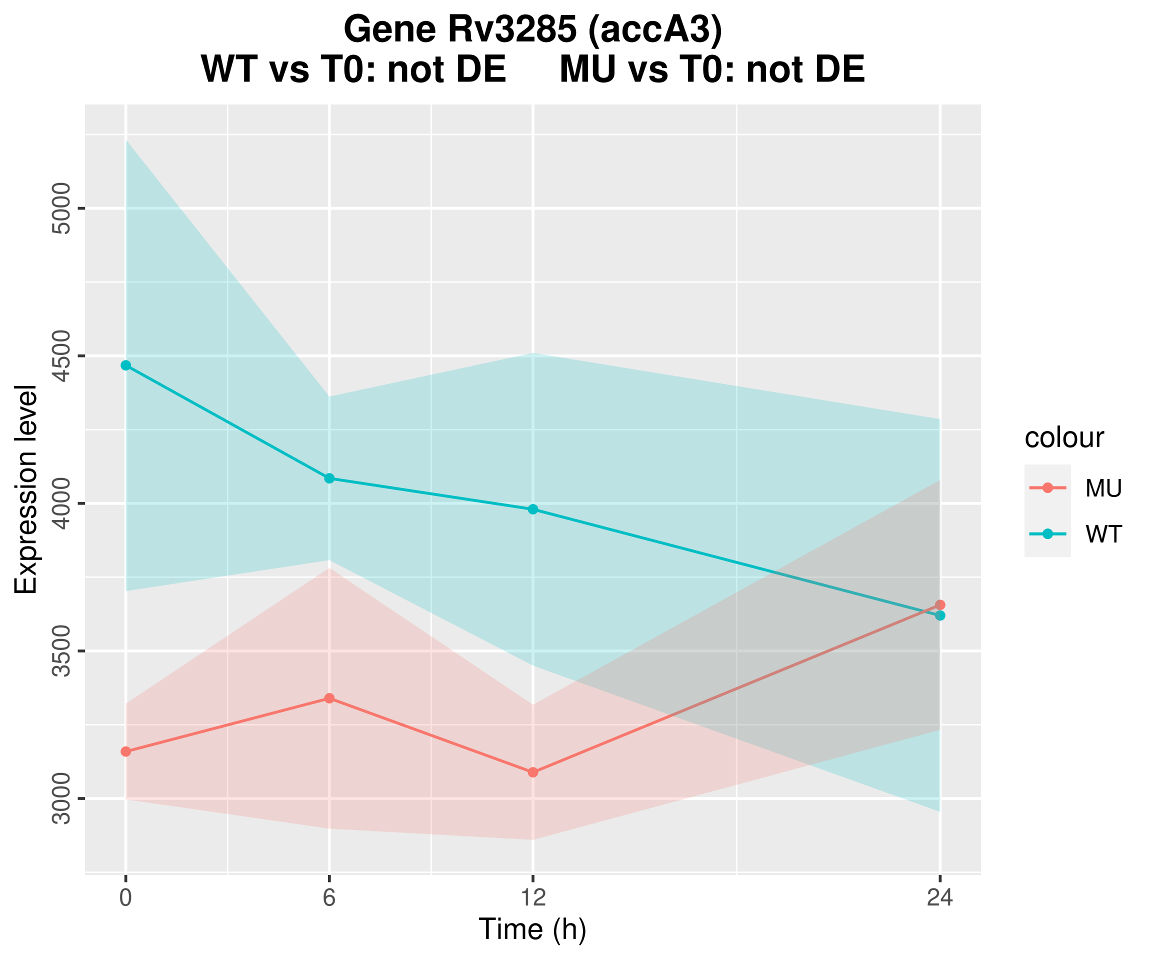
Gene Rv3285 (accA3)
WT vs T0: not DE   MU vs T0: not DE
Expression level
5000
4500
4000
3500
3000
0
6
12
24
Time (h)
colour
MU
WT

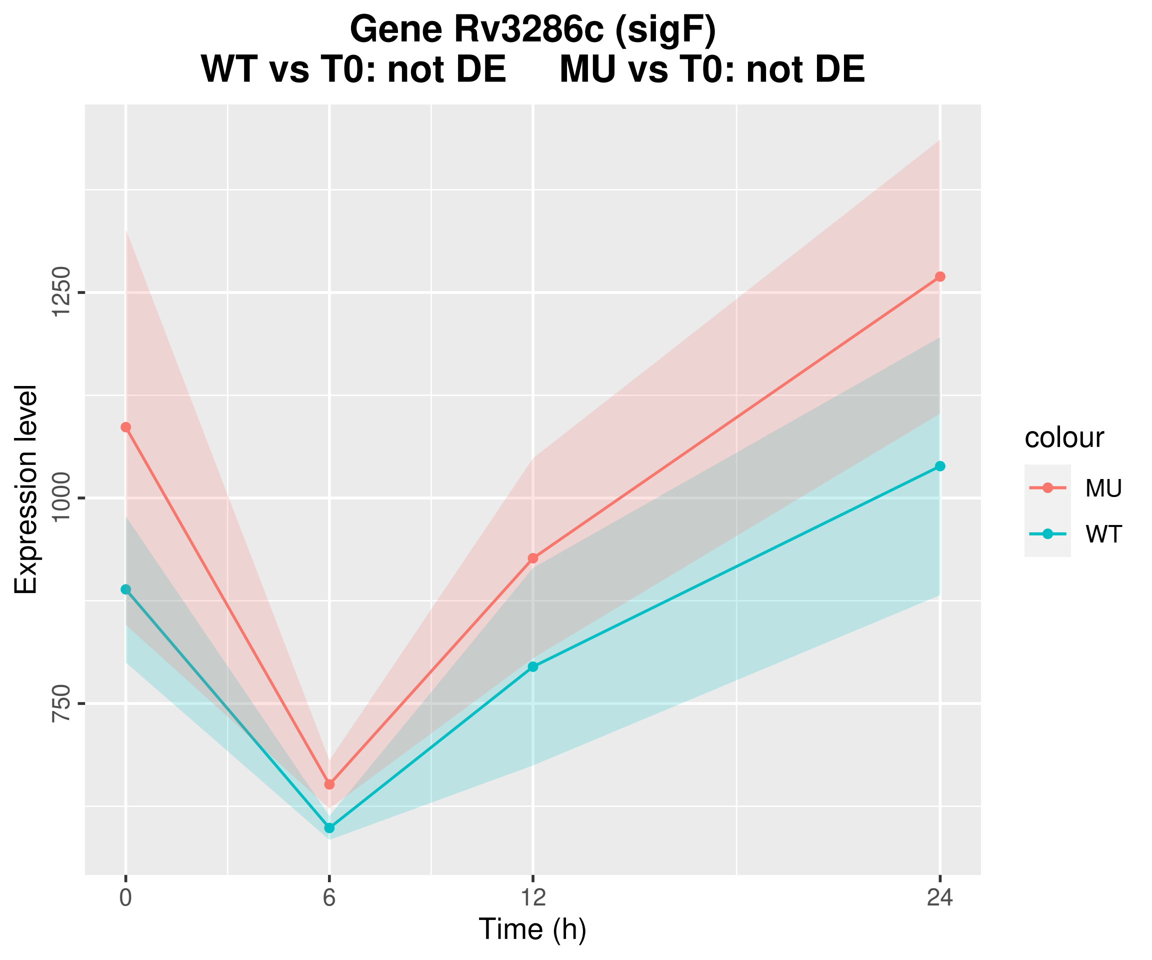

Gene Rv3286c (sigF)
WT vs T0: not DE MU vs T0: not DE
Expression level
1250
1000
750
0
6
12
24
Time (h)
colour
MU
WT

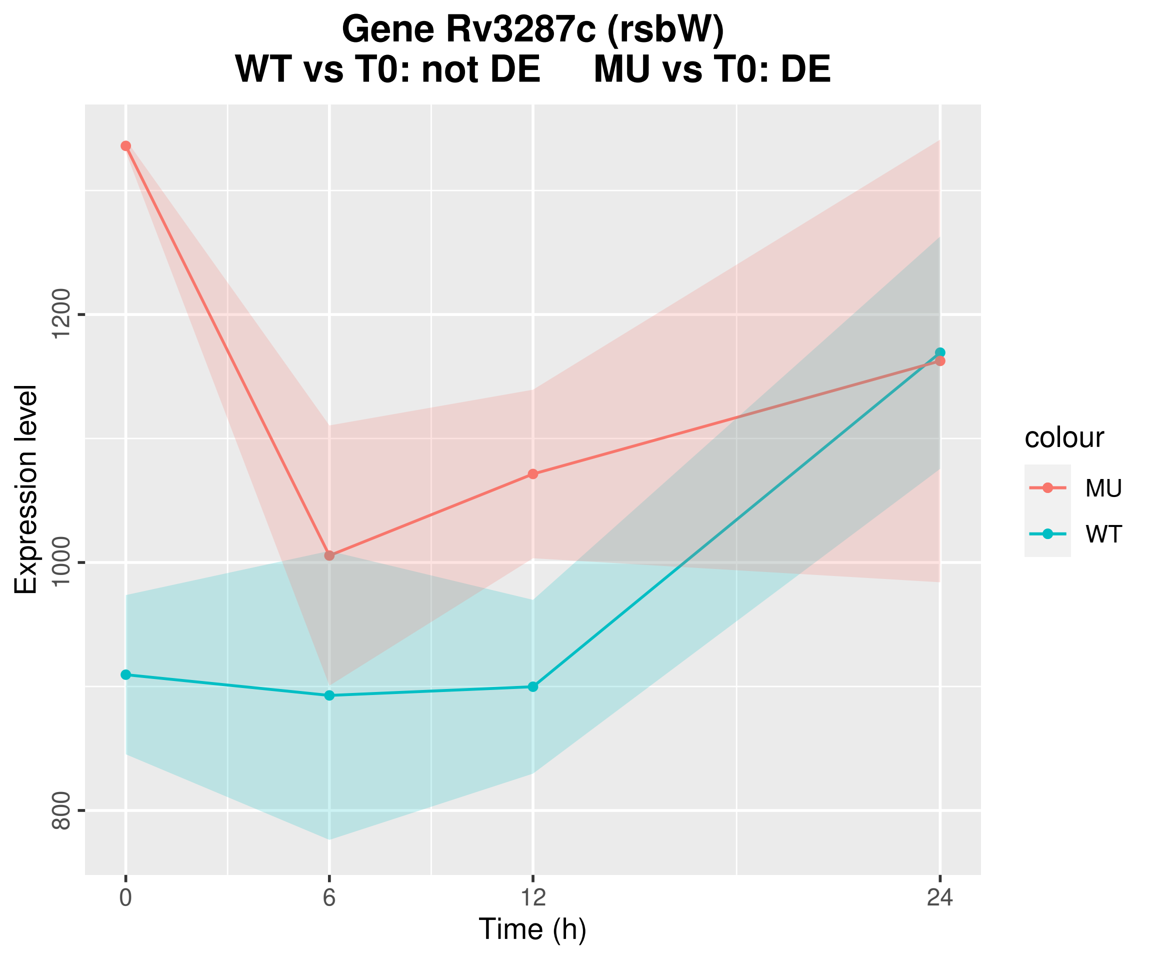

Gene Rv3287c (rsbW)
WT vs T0: not DE   MU vs T0: DE
Expression level
1200
1000
800
0
6
12
24
Time (h)
colour
MU
WT

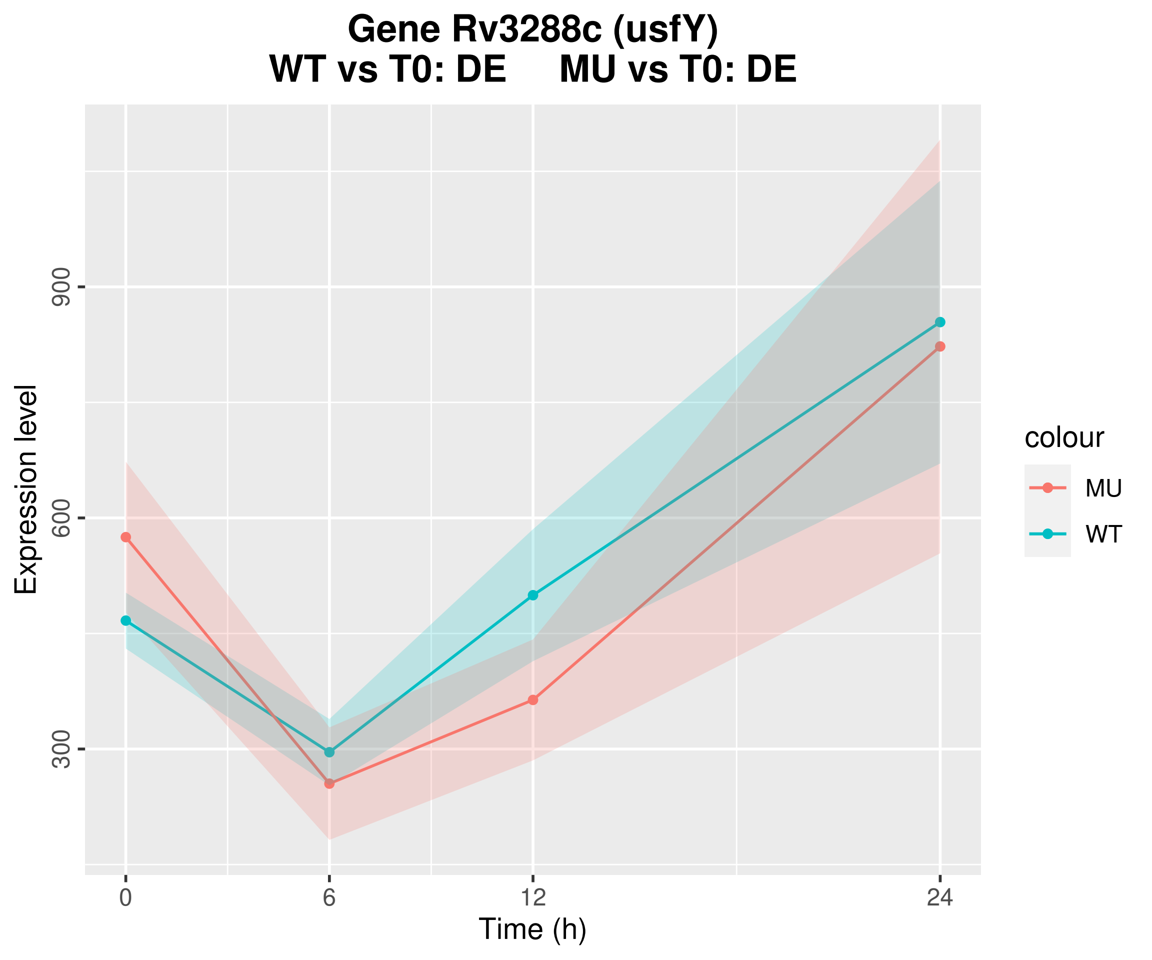
Gene Rv3288c (usfY)
WT vs T0: DE MU vs T0: DE
Expression level
900
600
300
0
6
12
24
Time (h)
colour
MU
WT

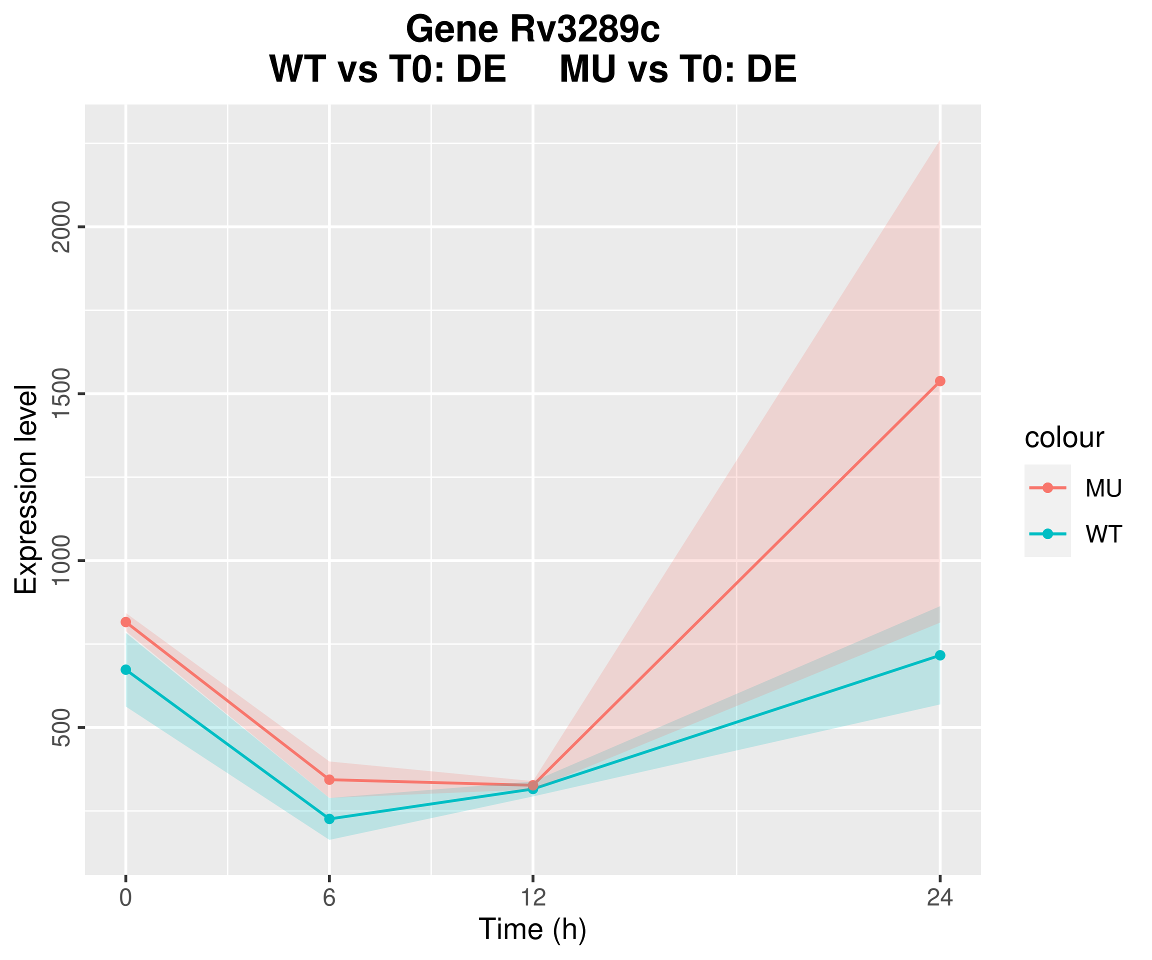

Gene Rv3289c
WT vs T0: DE   MU vs T0: DE
Expression level
2000
1500
1000
500
0
6
12
24
Time (h)
colour
MU
WT

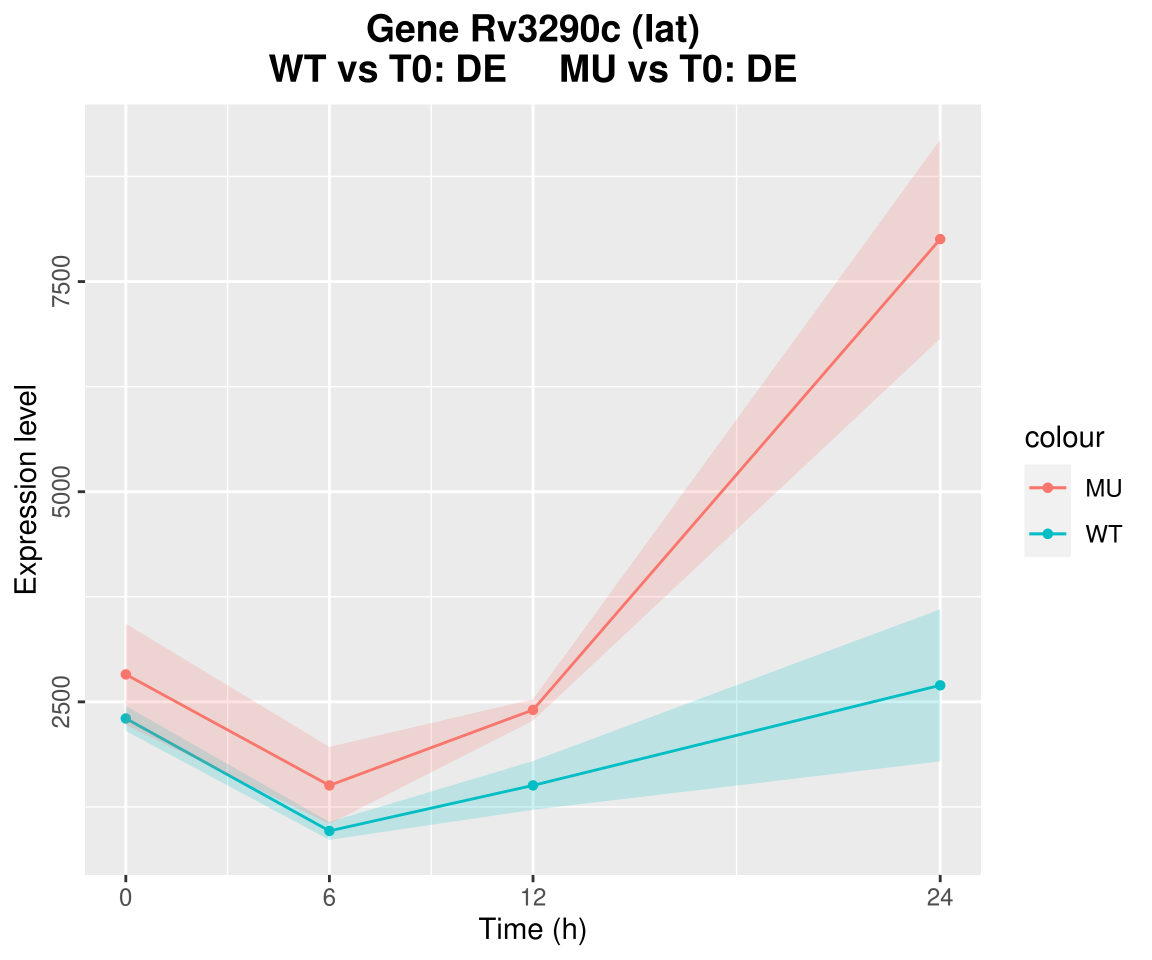
Gene Rv3290c (lat)
WT vs T0: DE MU vs T0: DE
Expression level
7500
5000
2500
0
6
12
24
Time (h)
colour
MU
WT

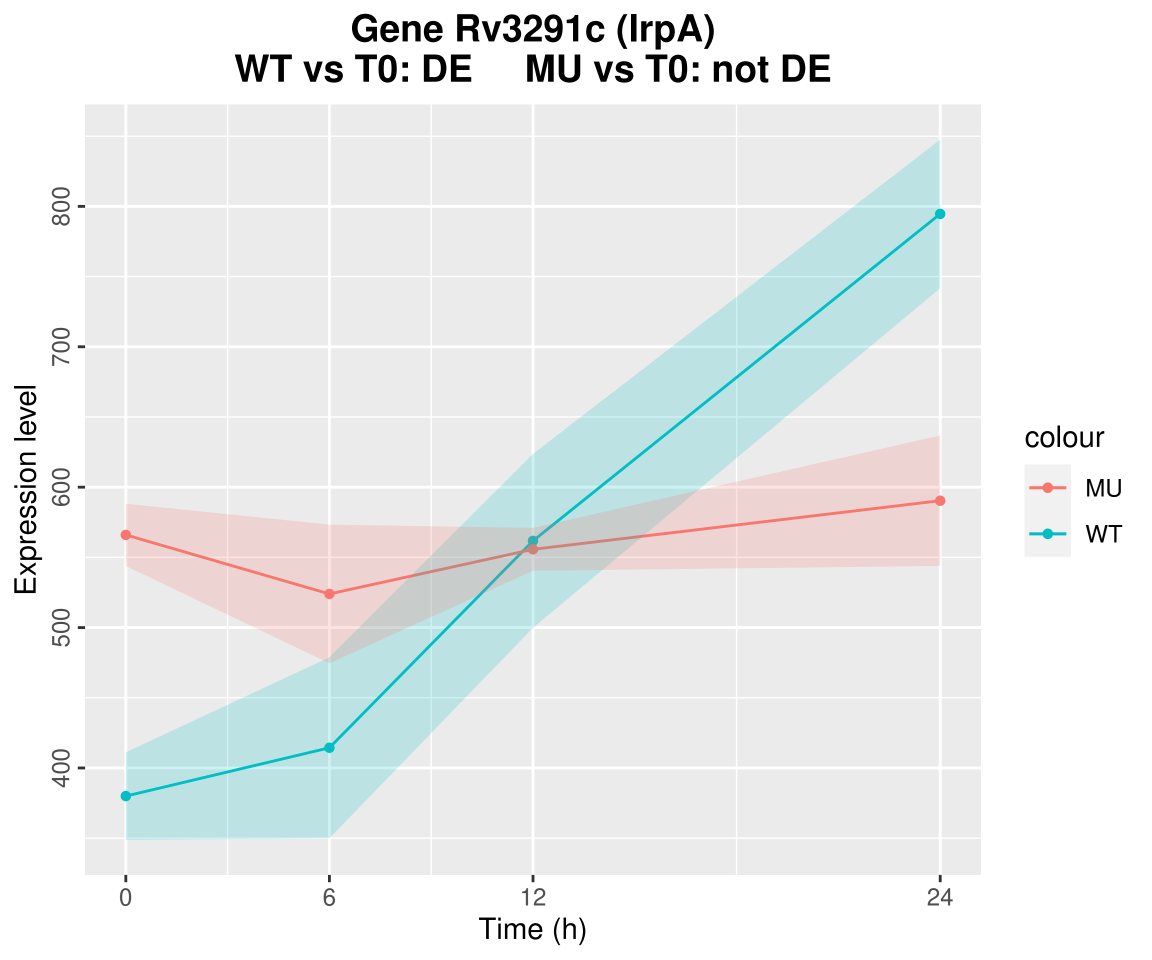
Gene Rv3291c (lrpA)
WT vs T0: DE   MU vs T0: not DE
Expression level
800
700
600
500
400
0
6
12
24
Time (h)
colour
MU
WT

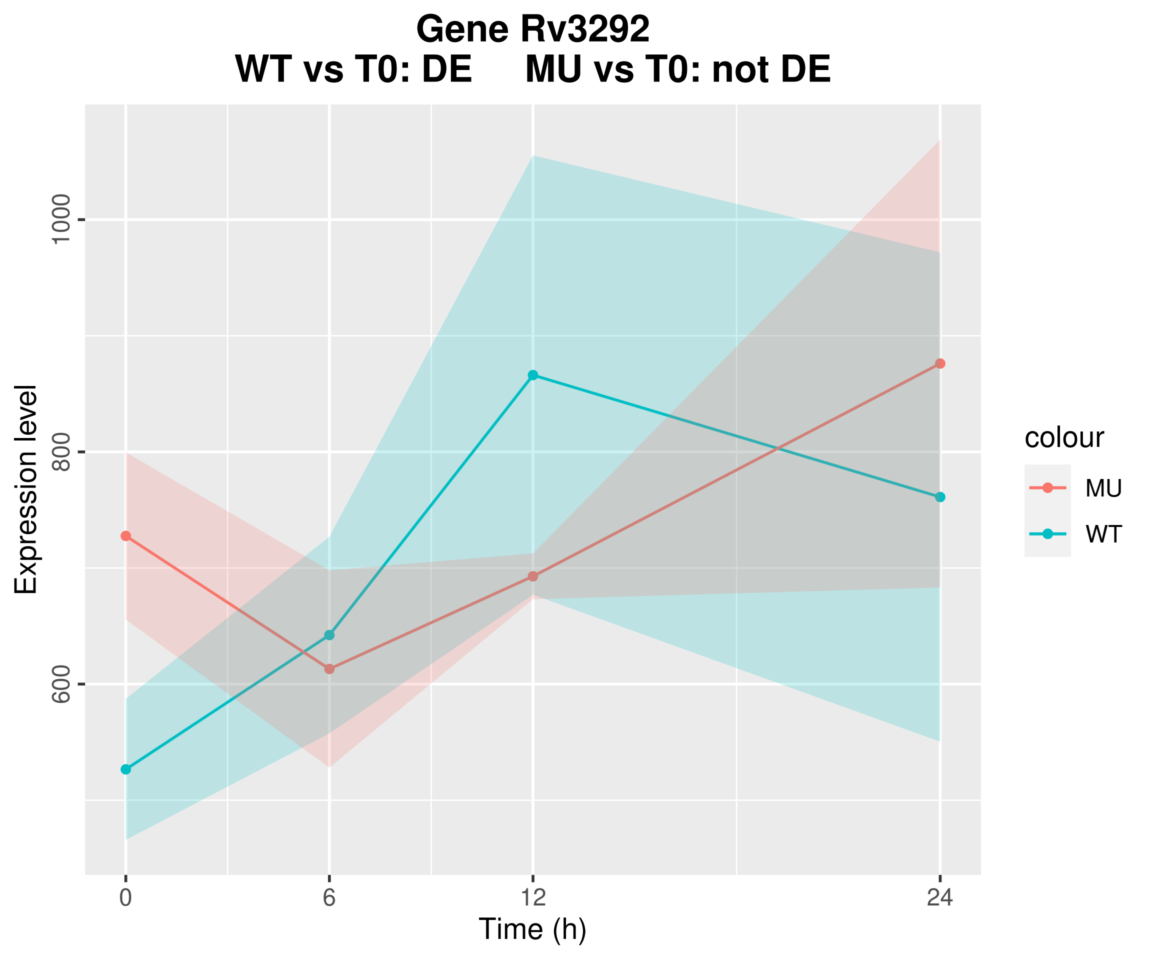
Gene Rv3292
WT vs T0: DE MU vs T0: not DE
Expression level
1000
800
600
Time (h)
0
6
12
24
colour
MU
WT

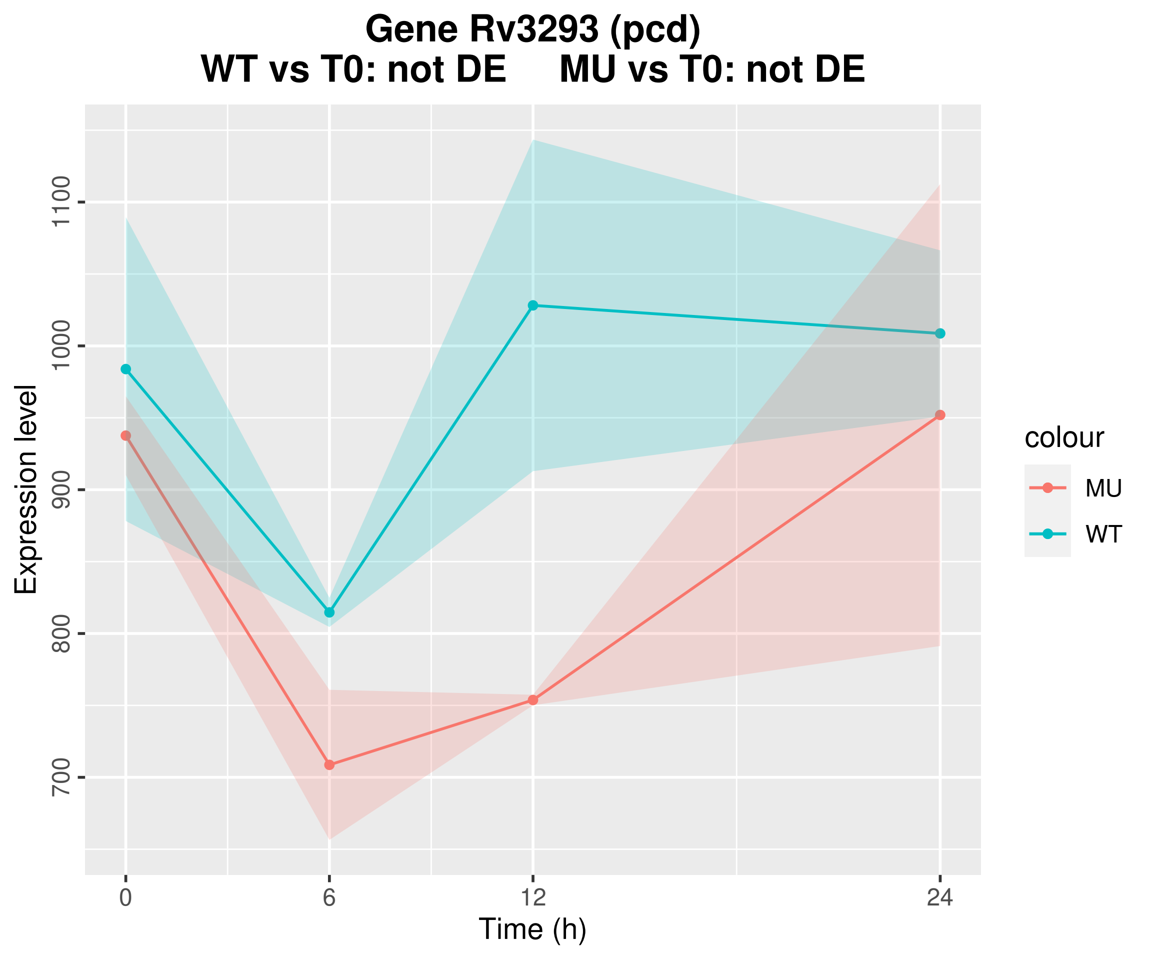

Gene Rv3293 (pcd)
WT vs T0: not DE   MU vs T0: not DE
Expression level
1100
1000
900
800
700
0
6
12
24
Time (h)
colour
MU
WT

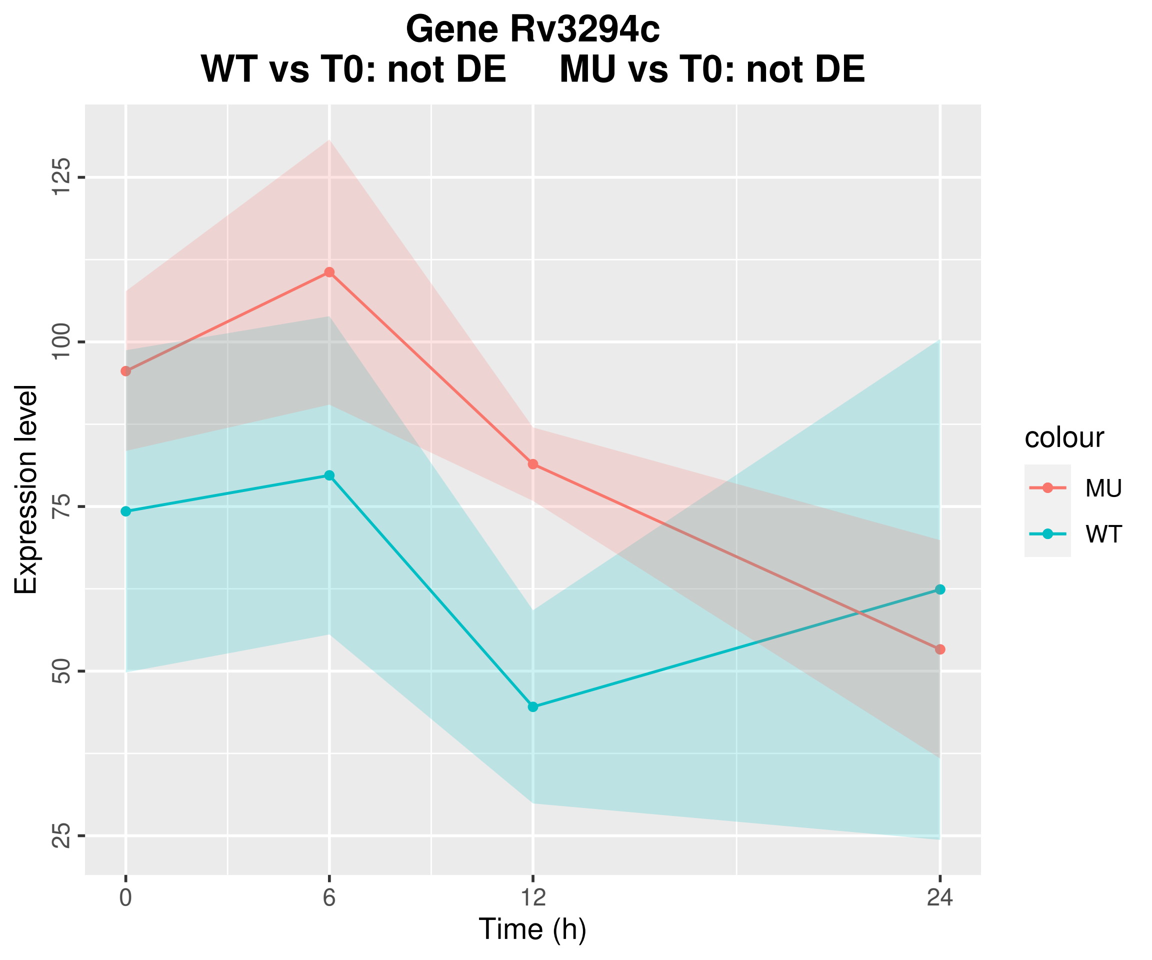
Gene Rv3294c
WT vs T0: not DE MU vs T0: not DE
Expression level
125
100
75
50
25
0
6
12
24
Time (h)
colour
MU
WT

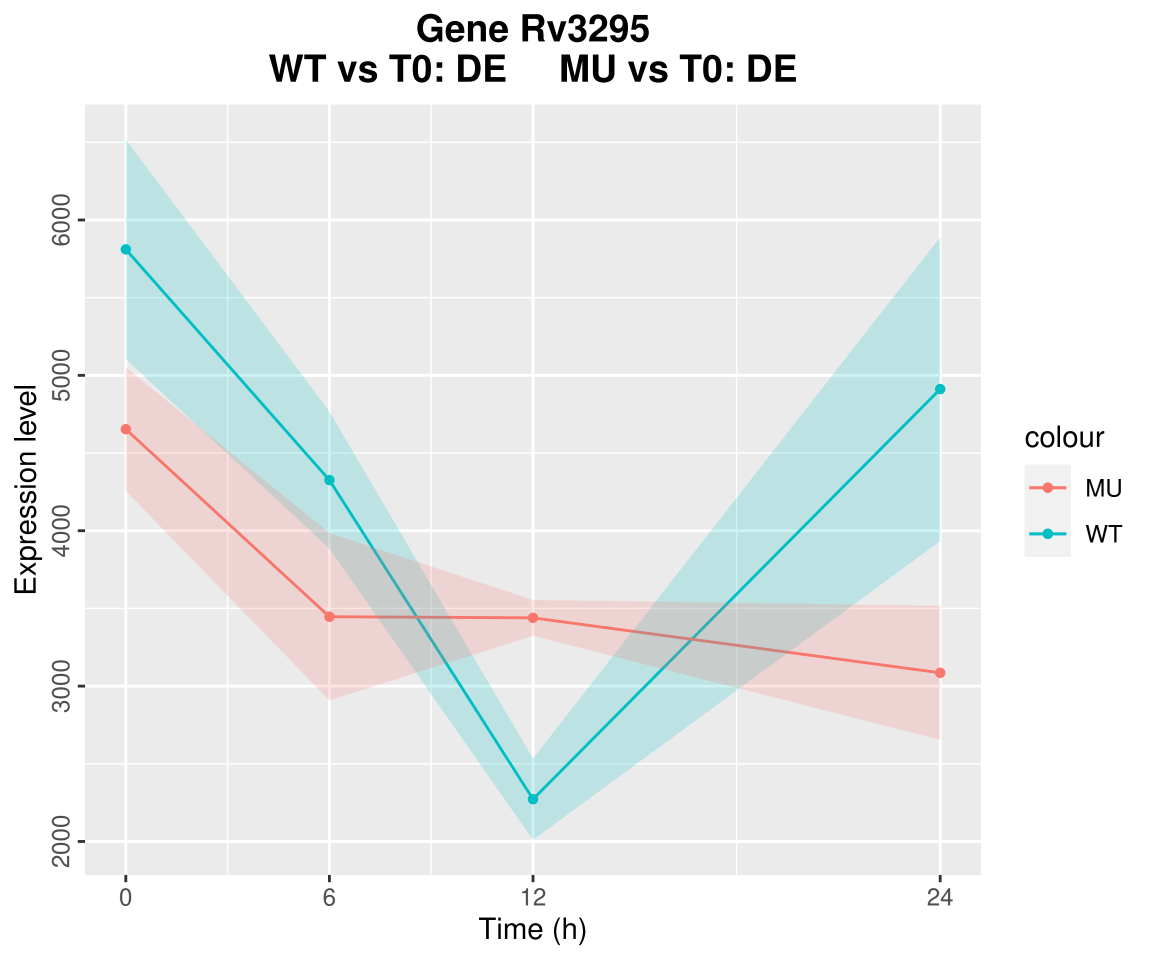

Gene Rv3295
WT vs T0: DE MU vs T0: DE
Expression level
6000
5000
4000
3000
2000
0
6
12
24
Time (h)
colour
MU
WT

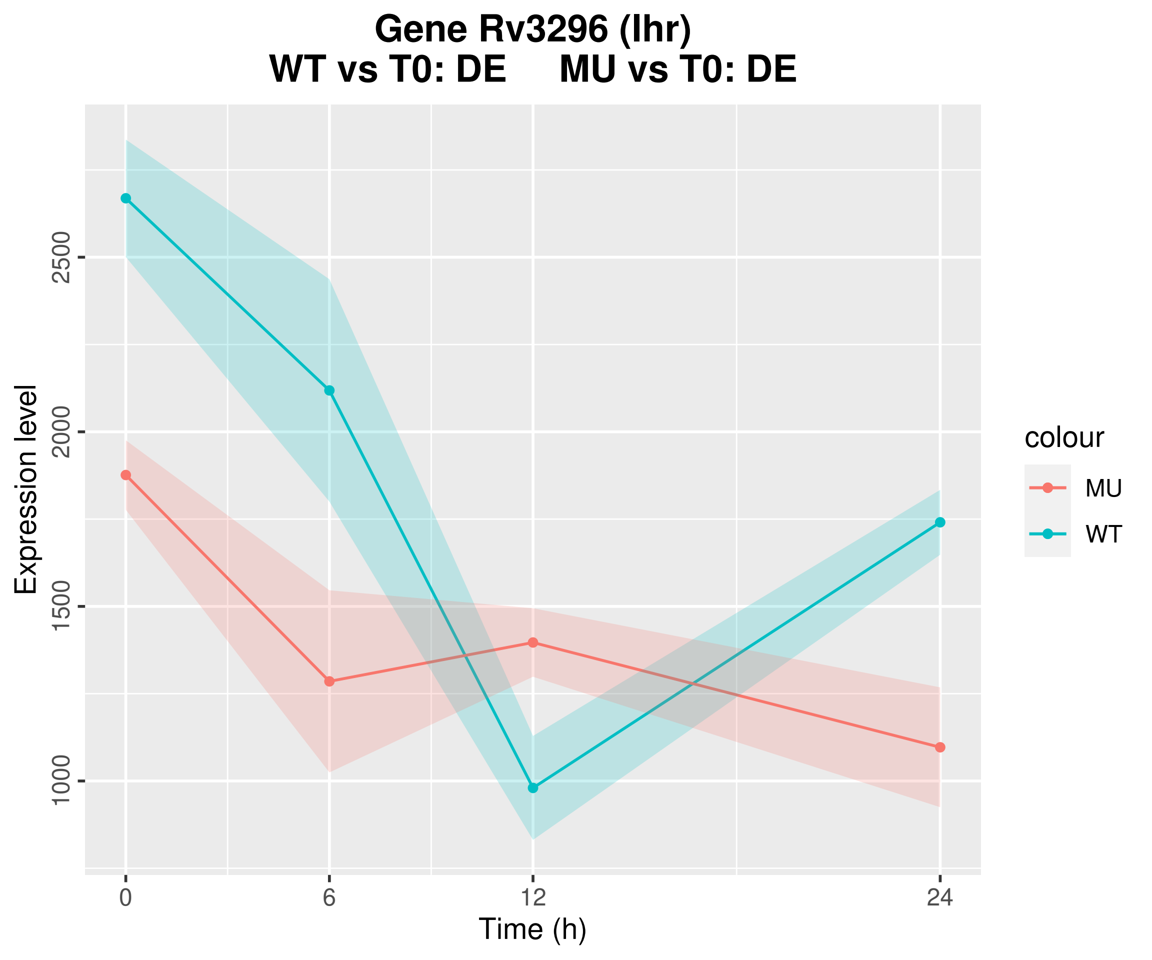

Gene Rv3296 (lhr)
WT vs T0: DE   MU vs T0: DE
Expression level
2500
2000
1500
1000
0
6
12
24
Time (h)
colour
MU
WT

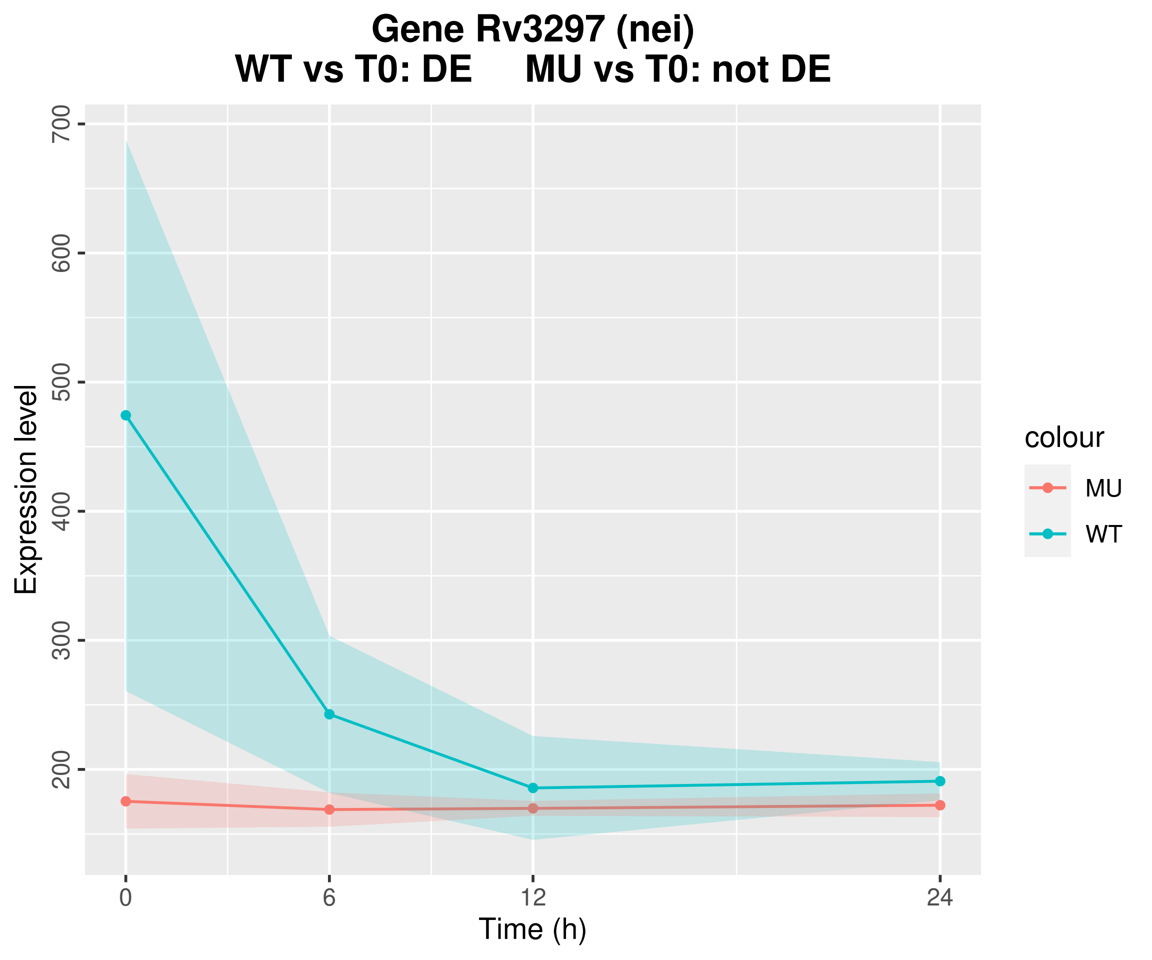
Gene Rv3297 (nei)
WT vs T0: DE MU vs T0: not DE
Expression level
700
600
500
400
300
200
0
6
12
24
Time (h)
colour
MU
WT

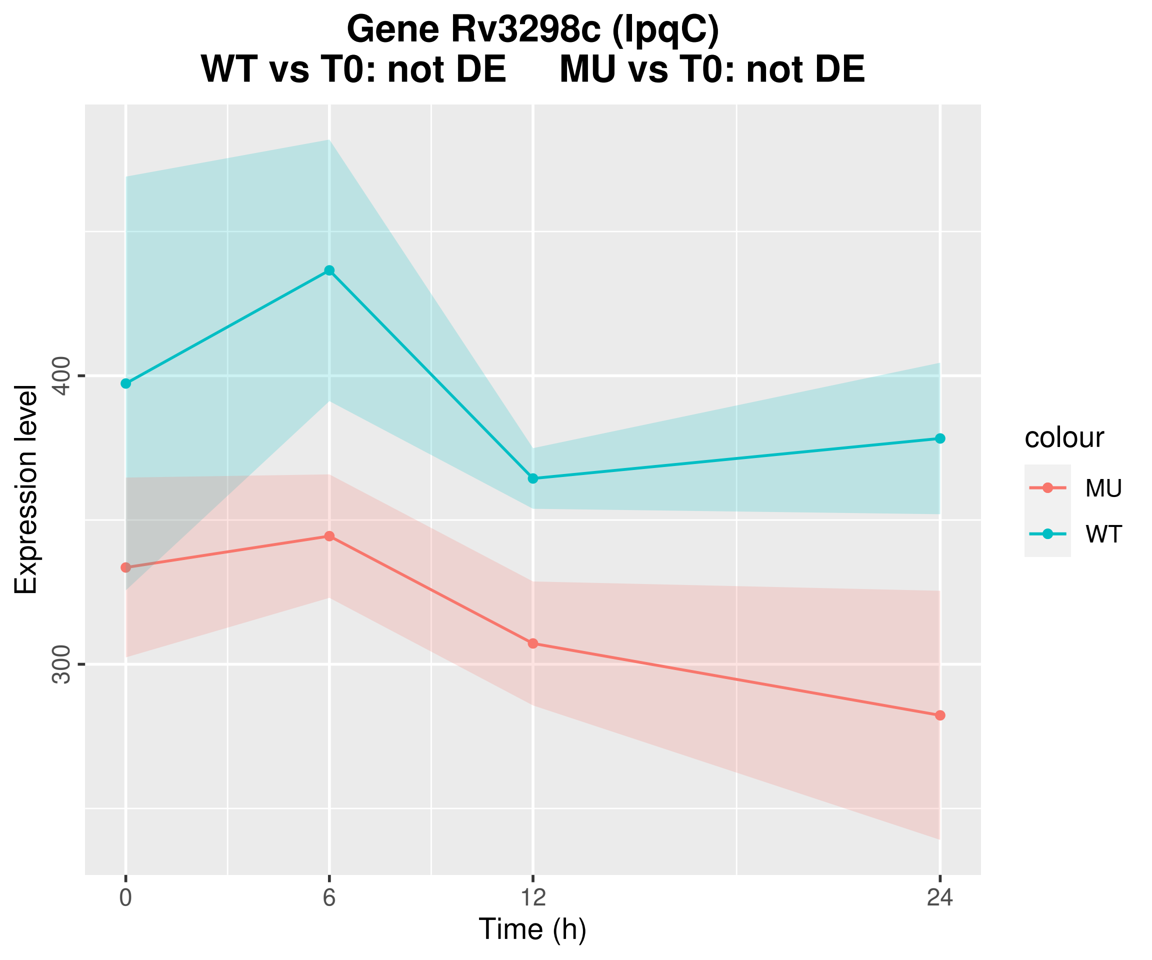

Gene Rv3298c (lpqC)
WT vs T0: not DE MU vs T0: not DE
Expression level
400
300
0
6
12
24
Time (h)
colour
MU
WT

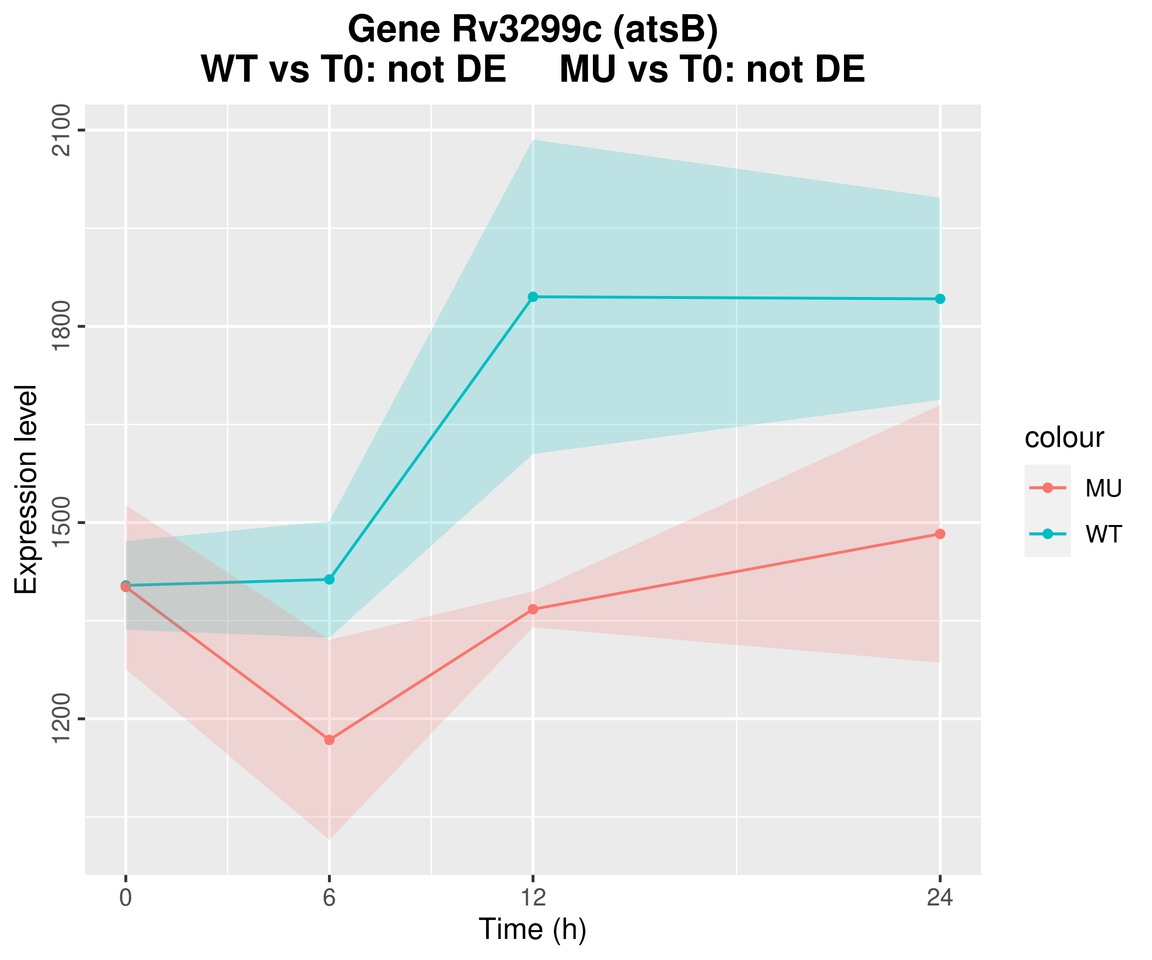

Gene Rv3299c (atsB)
WT vs T0: not DE MU vs T0: not DE
Expression level
2100
1800
1500
1200
0
6
12
24
Time (h)
colour
MU
WT

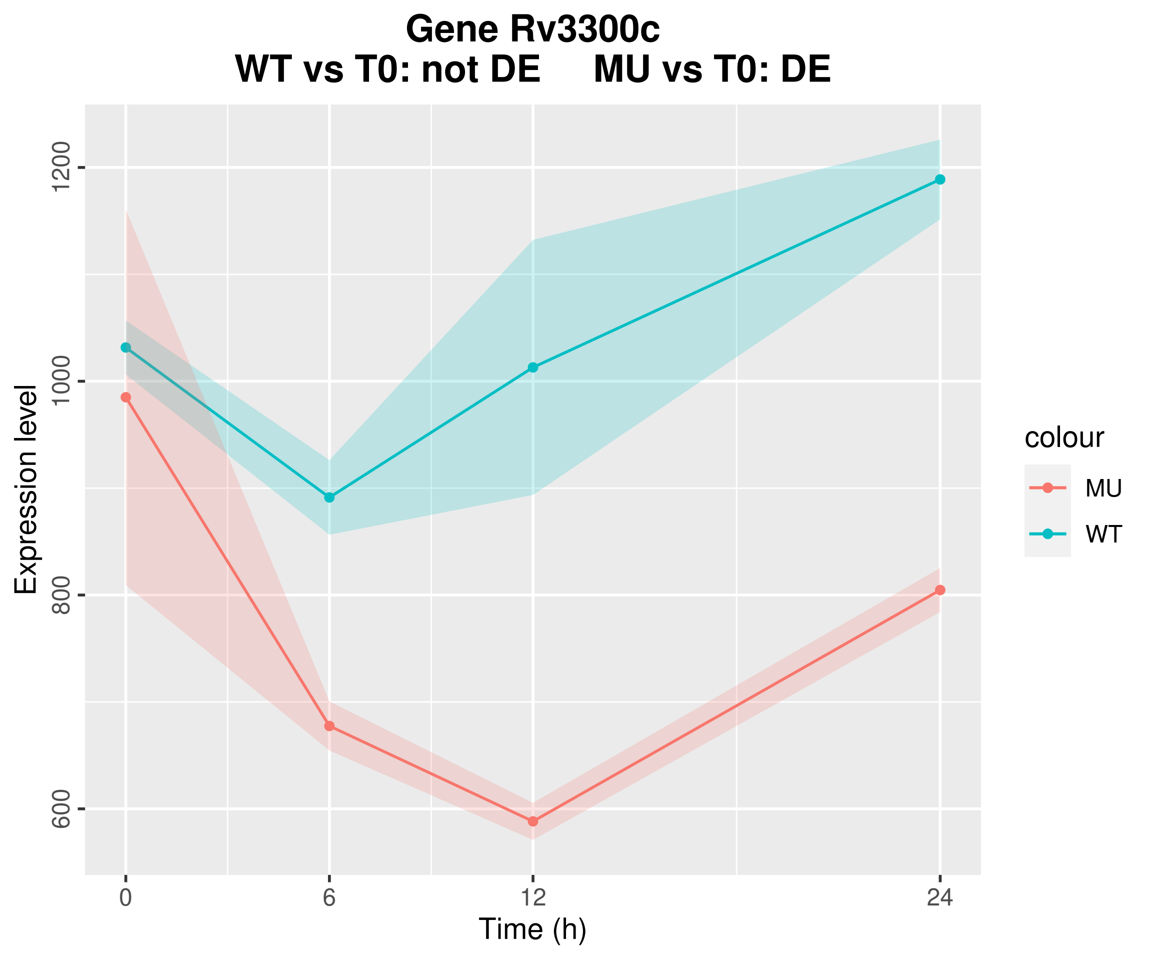
Gene Rv3300c
WT vs T0: not DE MU vs T0: DE
Expression level
1200
1000
800
600
0
6
12
24
Time (h)
colour
MU
WT

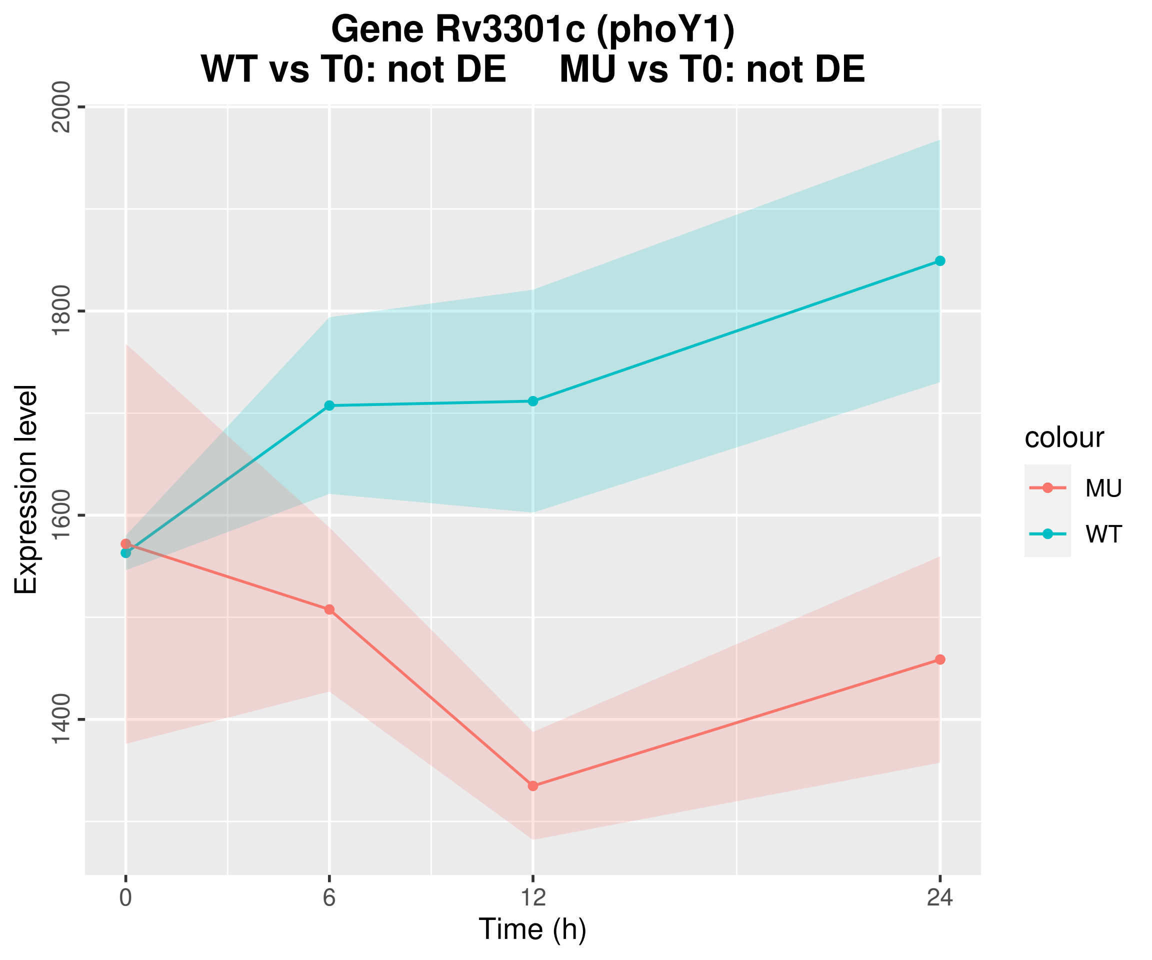

Gene Rv3301c (phoY1)
WT vs T0: not DE   MU vs T0: not DE
Expression level
2000
1800
1600
1400
0
6
12
24
Time (h)
colour
MU
WT

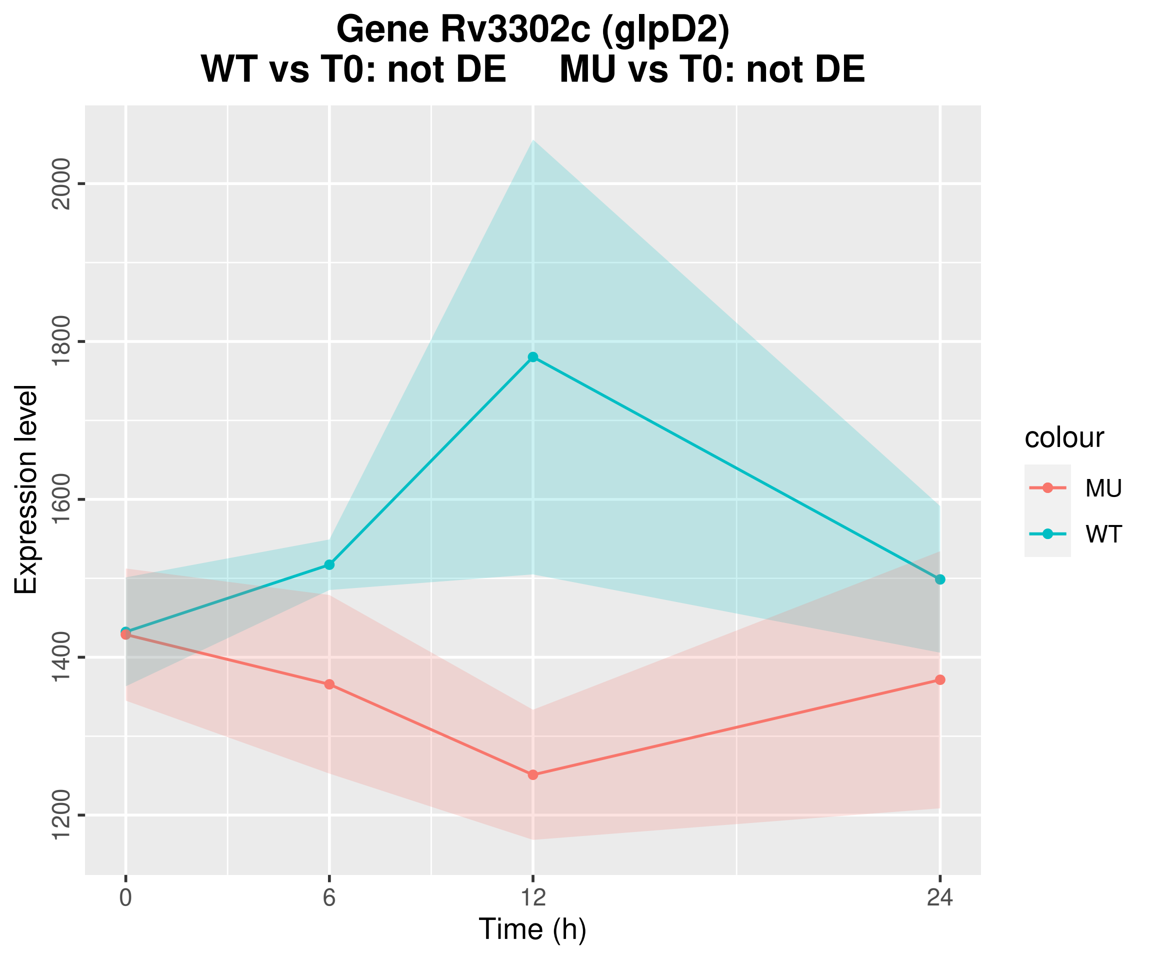

Gene Rv3302c (glpD2)
WT vs T0: not DE   MU vs T0: not DE
Expression level
2000
1800
1600
1400
1200
0
6
12
24
Time (h)
colour
MU
WT

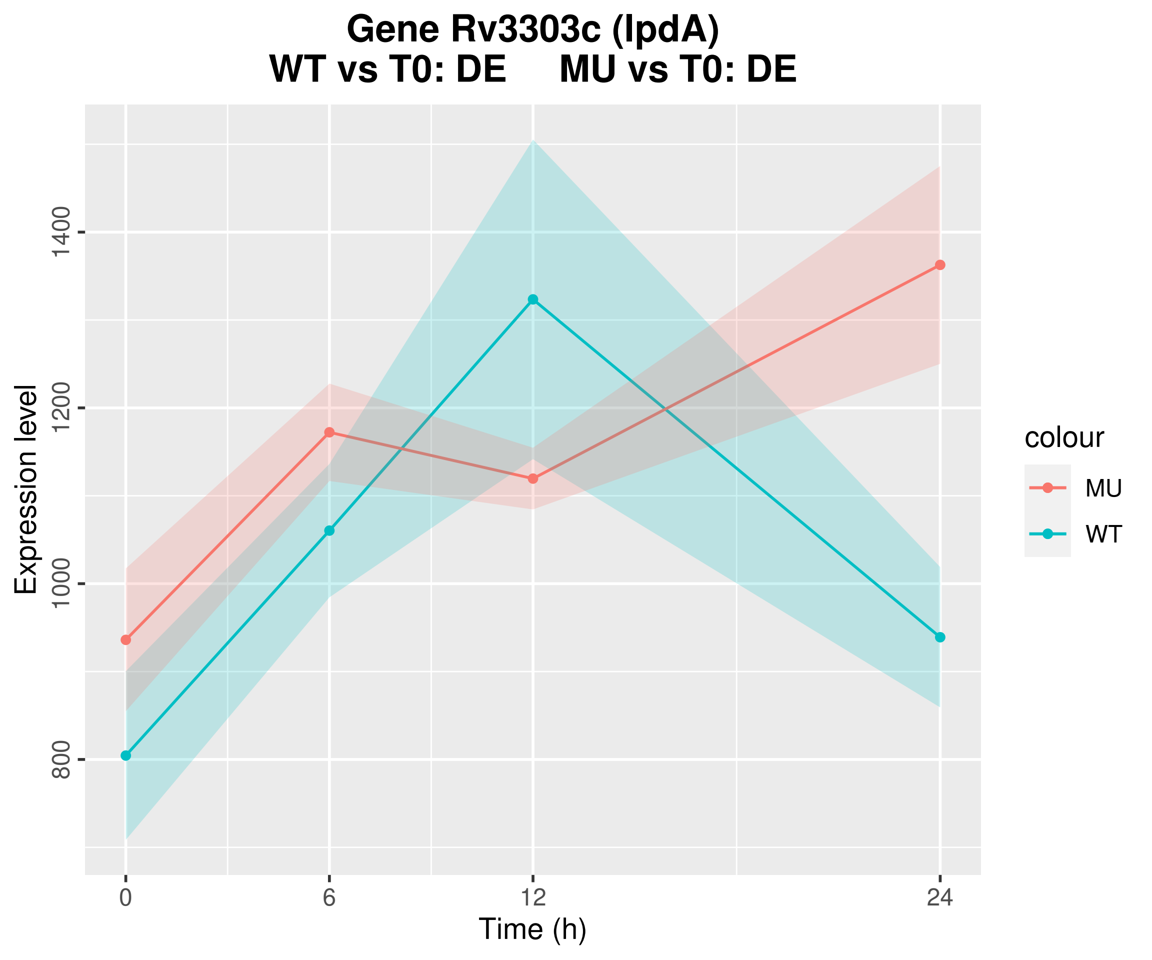

Gene Rv3303c (lpdA)
WT vs T0: DE MU vs T0: DE
Expression level
1400
1200
1000
800
0
6
12
24
Time (h)
colour
MU
WT

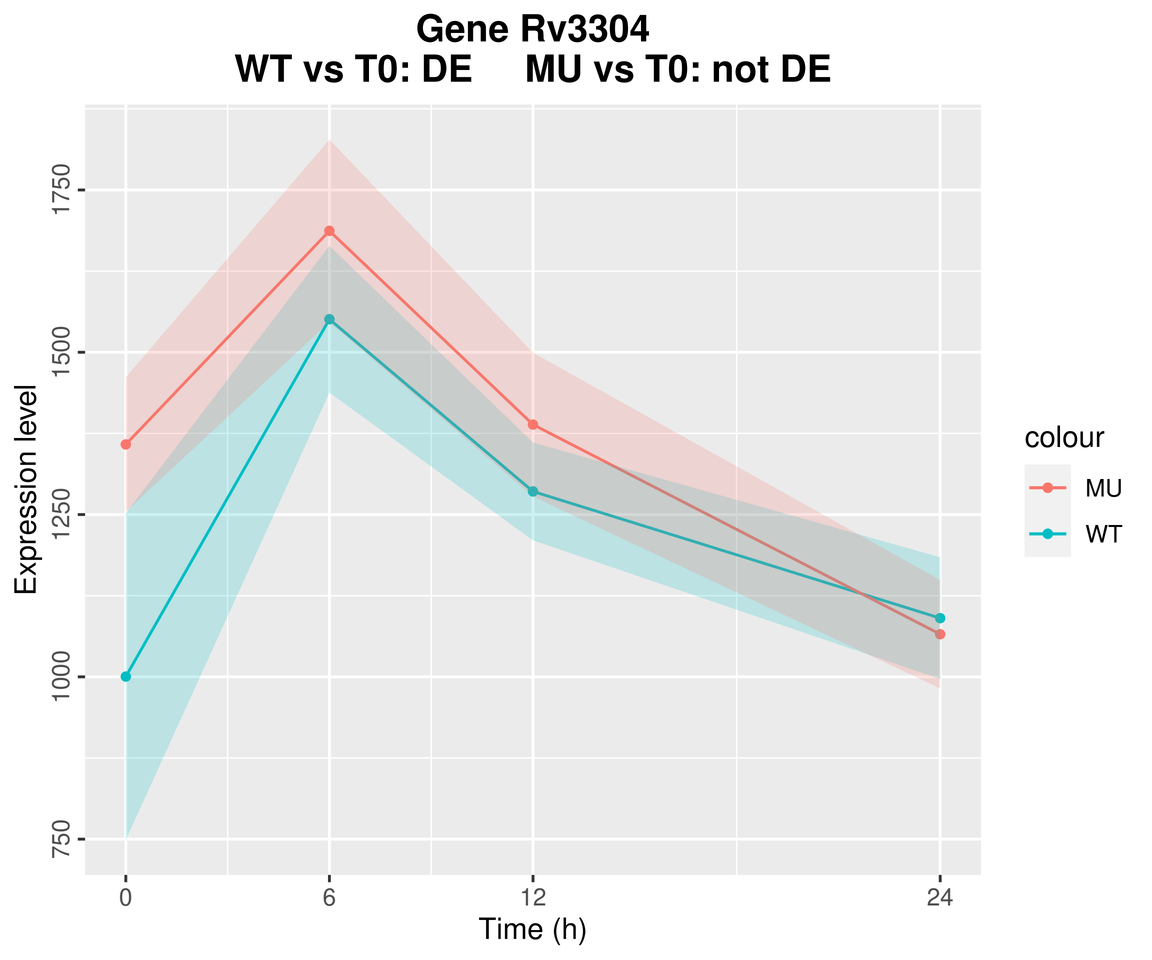

Gene Rv3304
WT vs T0: DE   MU vs T0: not DE
Expression level
1750
1500
1250
1000
750
0
6
12
24
Time (h)
colour
MU
WT

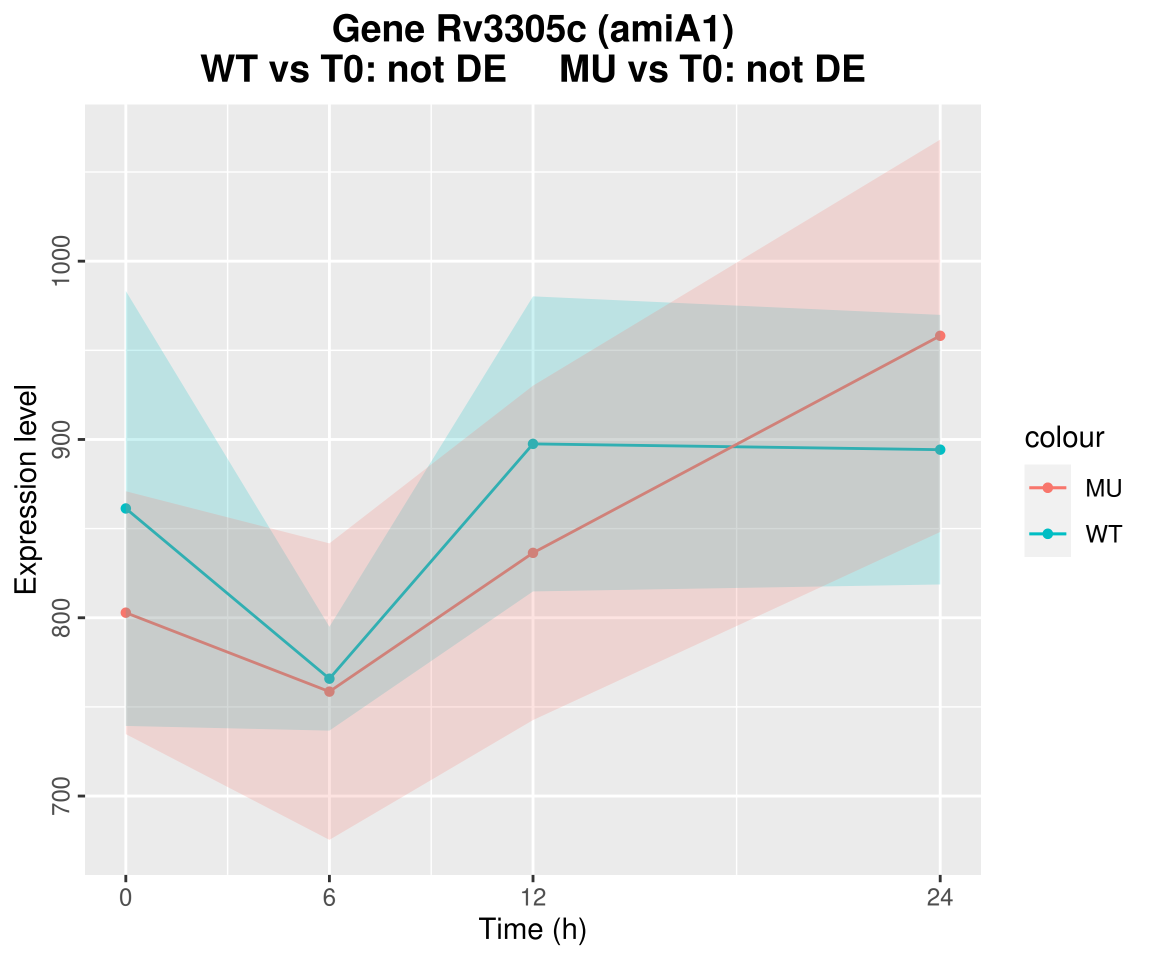
Gene Rv3305c (amiA1)
WT vs T0: not DE   MU vs T0: not DE
Expression level
1000
900
800
700
0
6
12
24
Time (h)
colour
MU
WT

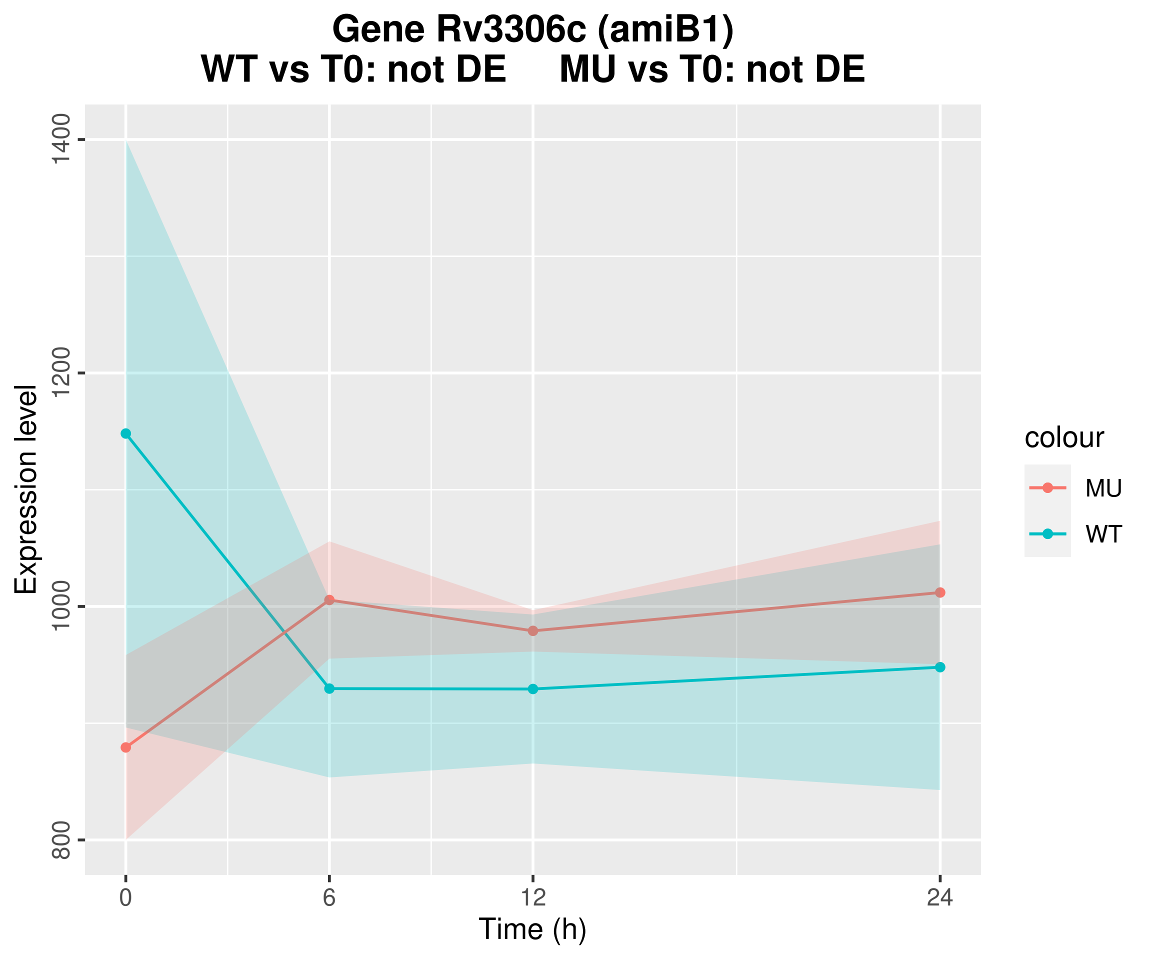

Gene Rv3306c (amiB1)
WT vs T0: not DE MU vs T0: not DE
Expression level
1400
1200
1000
800
0
6
12
24
Time (h)
colour
MU
WT

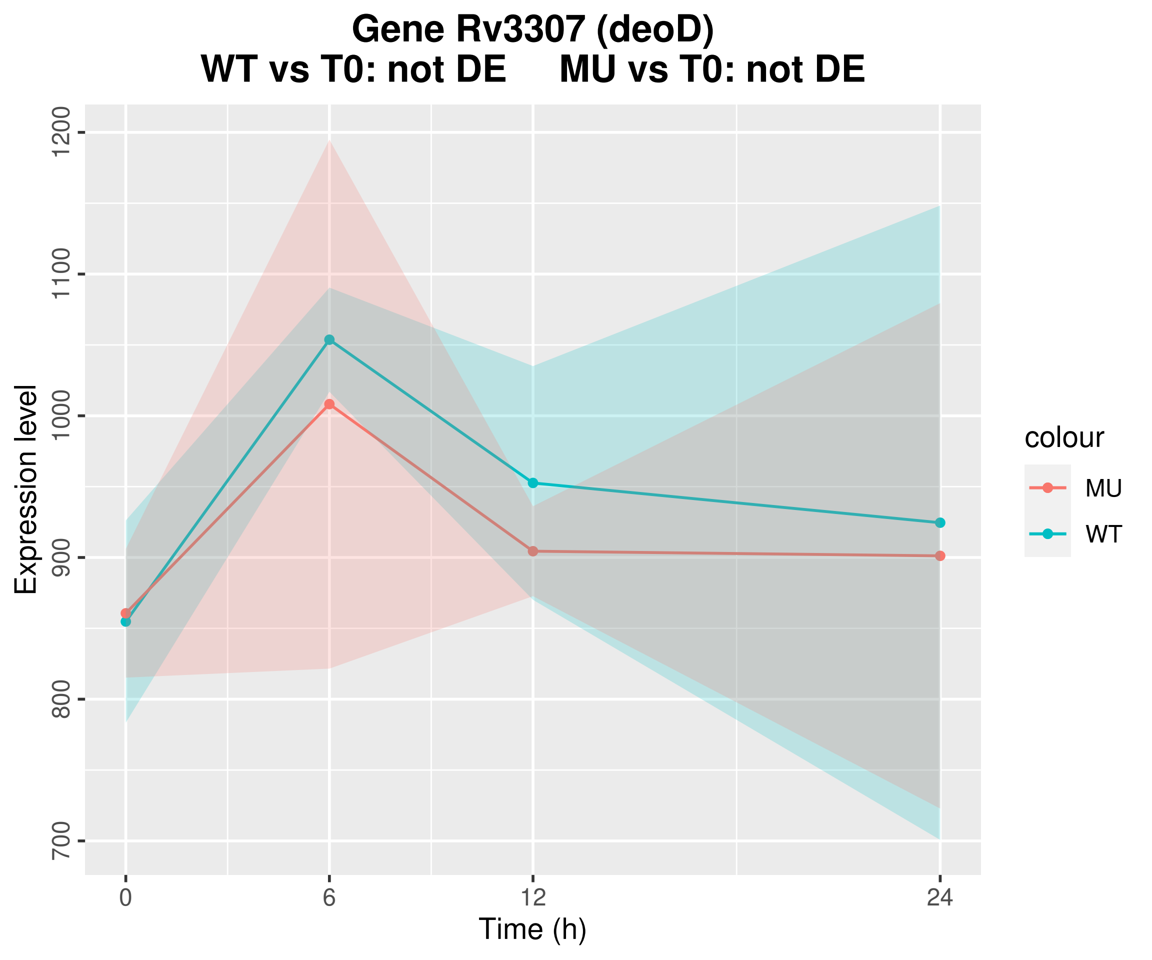
Gene Rv3307 (deoD)
WT vs T0: not DE   MU vs T0: not DE
Expression level
1200
1100
1000
900
800
700
0
6
12
24
Time (h)
colour
MU
WT

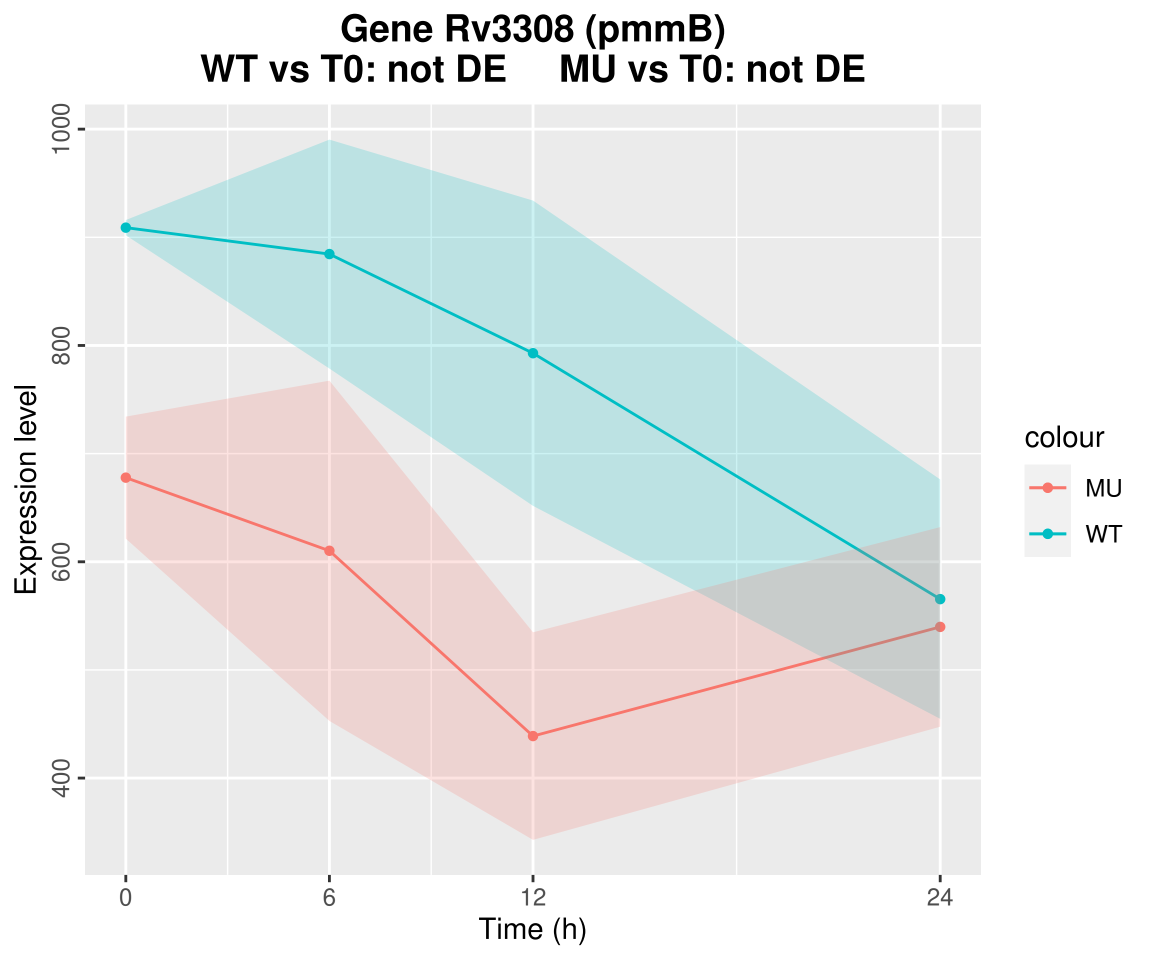
Gene Rv3308 (pmmB)
WT vs T0: not DE   MU vs T0: not DE
Expression level
1000
800
600
400
0
6
12
24
Time (h)
colour
MU
WT

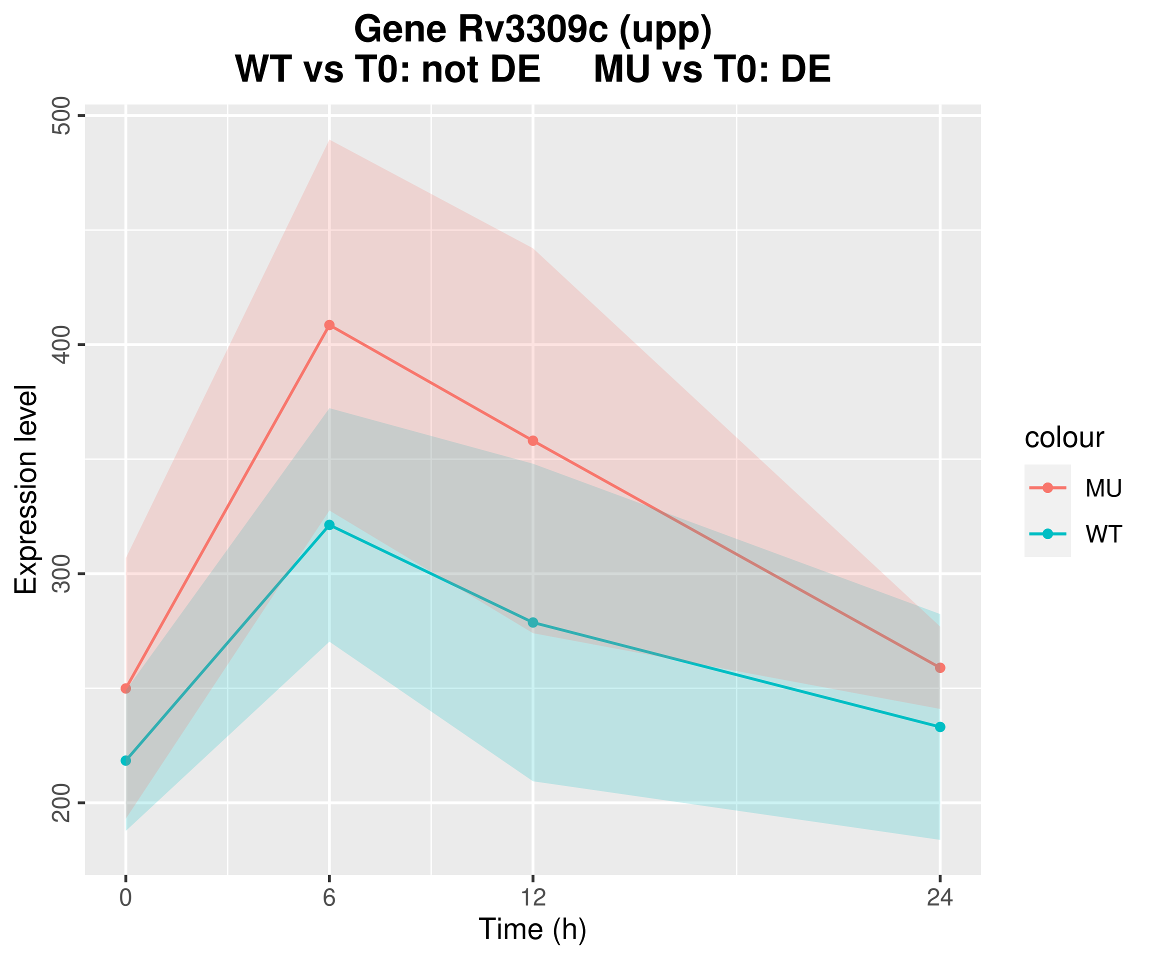
Gene Rv3309c (upp)
WT vs T0: not DE   MU vs T0: DE
500
400
300
200
Expression level
0
6
12
24
Time (h)
colour
MU
WT

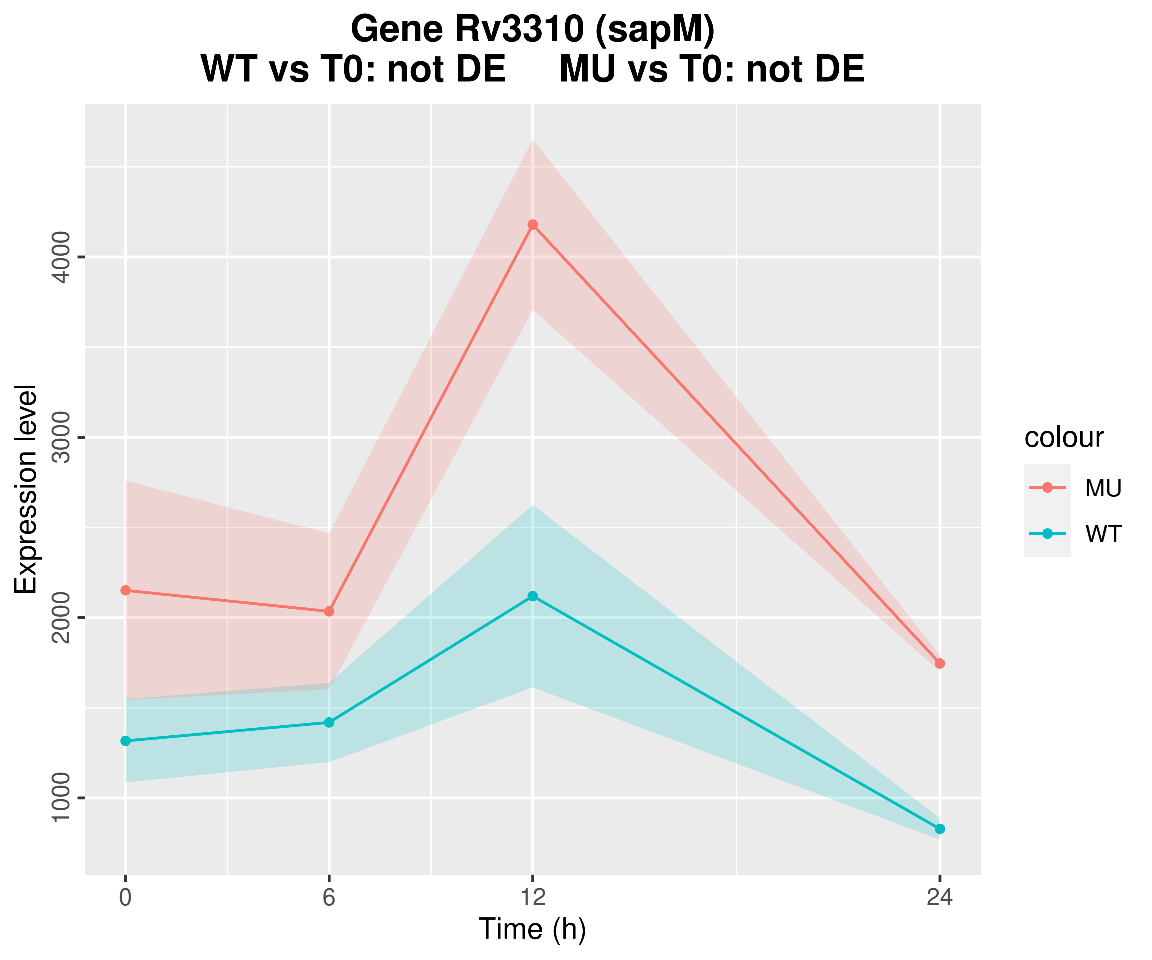

Gene Rv3310 (sapM)
WT vs T0: not DE   MU vs T0: not DE
Expression level
4000
3000
2000
1000
0
6
12
24
Time (h)
colour
MU
WT

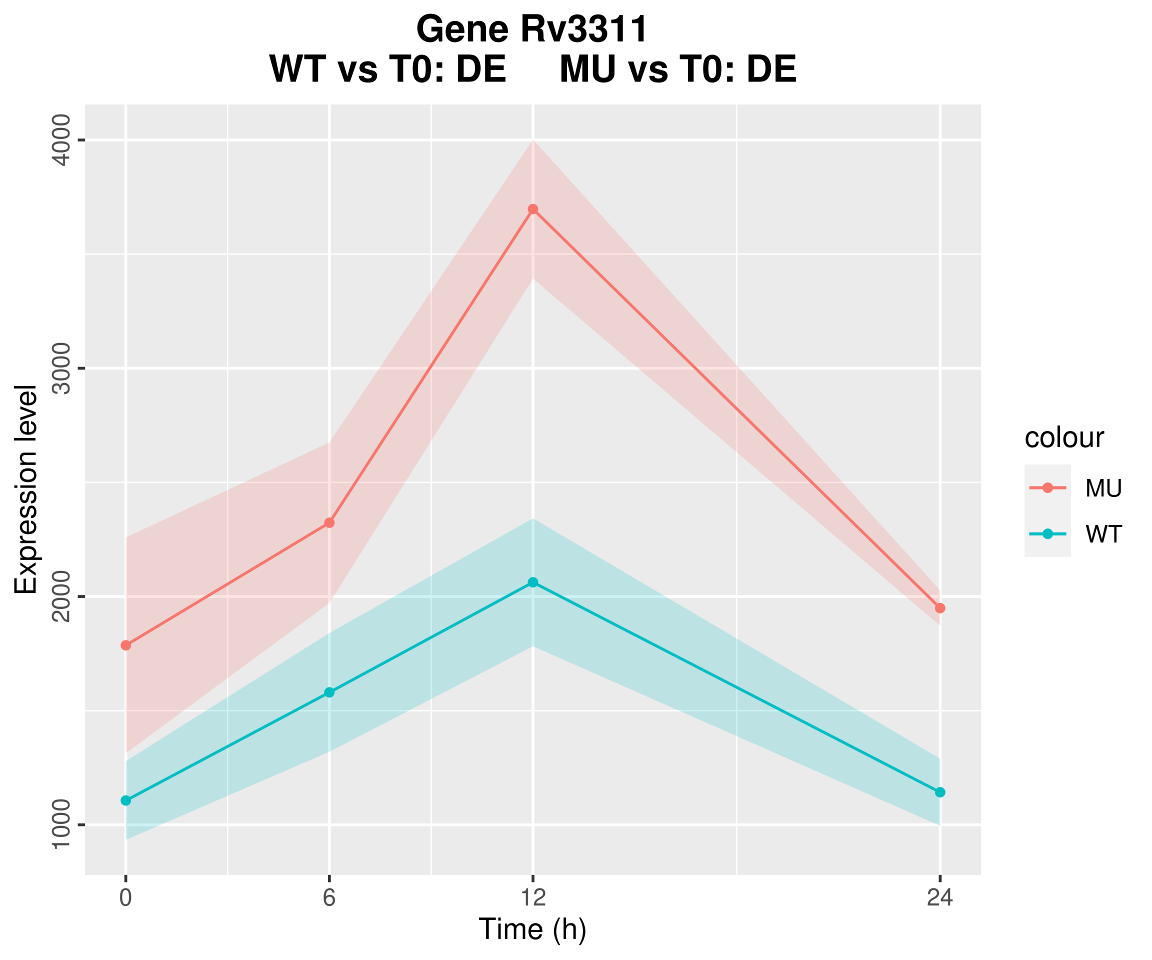
Gene Rv3311
WT vs T0: DE   MU vs T0: DE
4000
3000
2000
1000
Expression level
0
6
12
24
Time (h)
colour
MU
WT

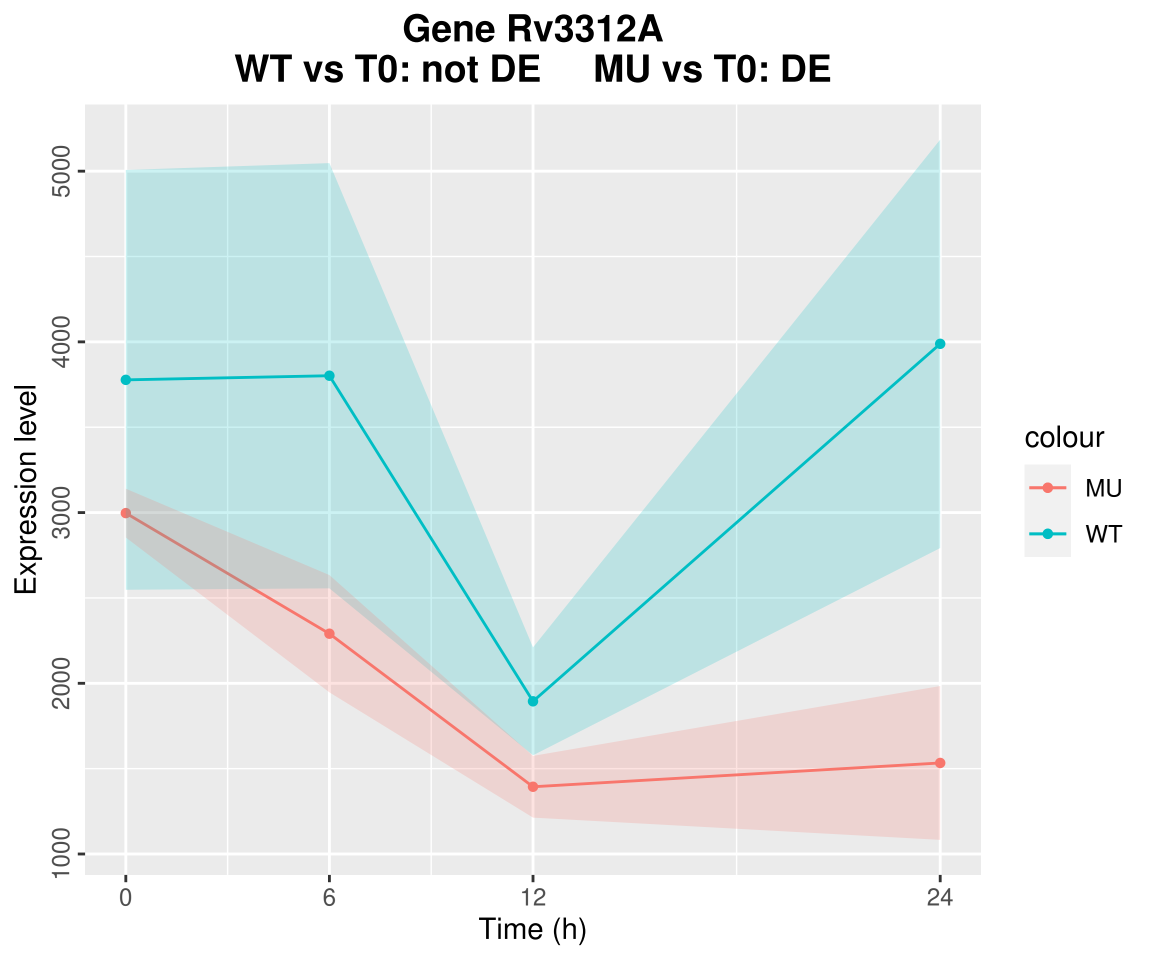

Gene Rv3312A
WT vs T0: not DE   MU vs T0: DE
Expression level
5000
4000
3000
2000
1000
0
6
12
24
Time (h)
colour
MU
WT

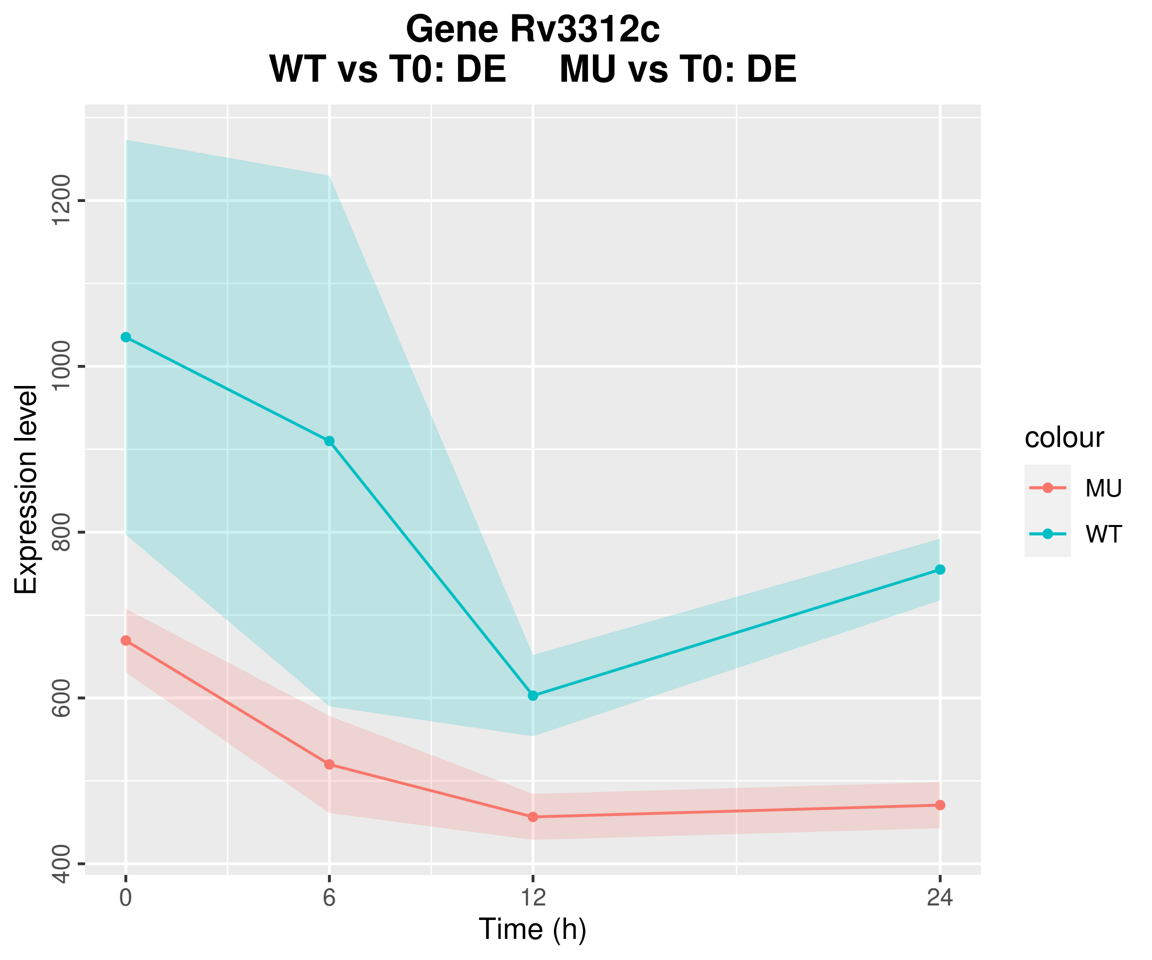

Gene Rv3312c
WT vs T0: DE MU vs T0: DE
Expression level
1200
1000
800
600
400
0
6
12
24
Time (h)
colour
MU
WT

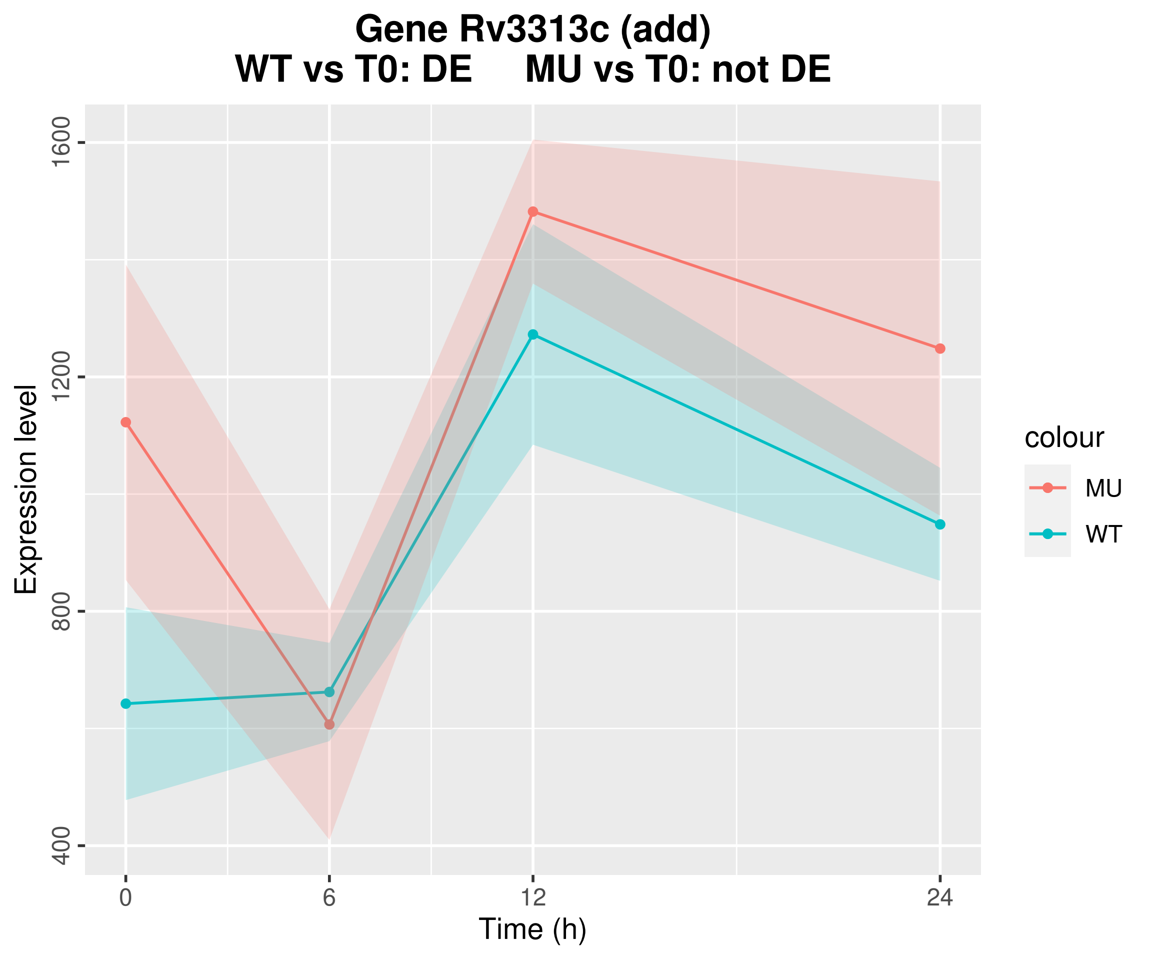

Gene Rv3313c (add)
WT vs T0: DE   MU vs T0: not DE
Expression level
1600
1200
800
400
0
6
12
24
Time (h)
colour
MU
WT

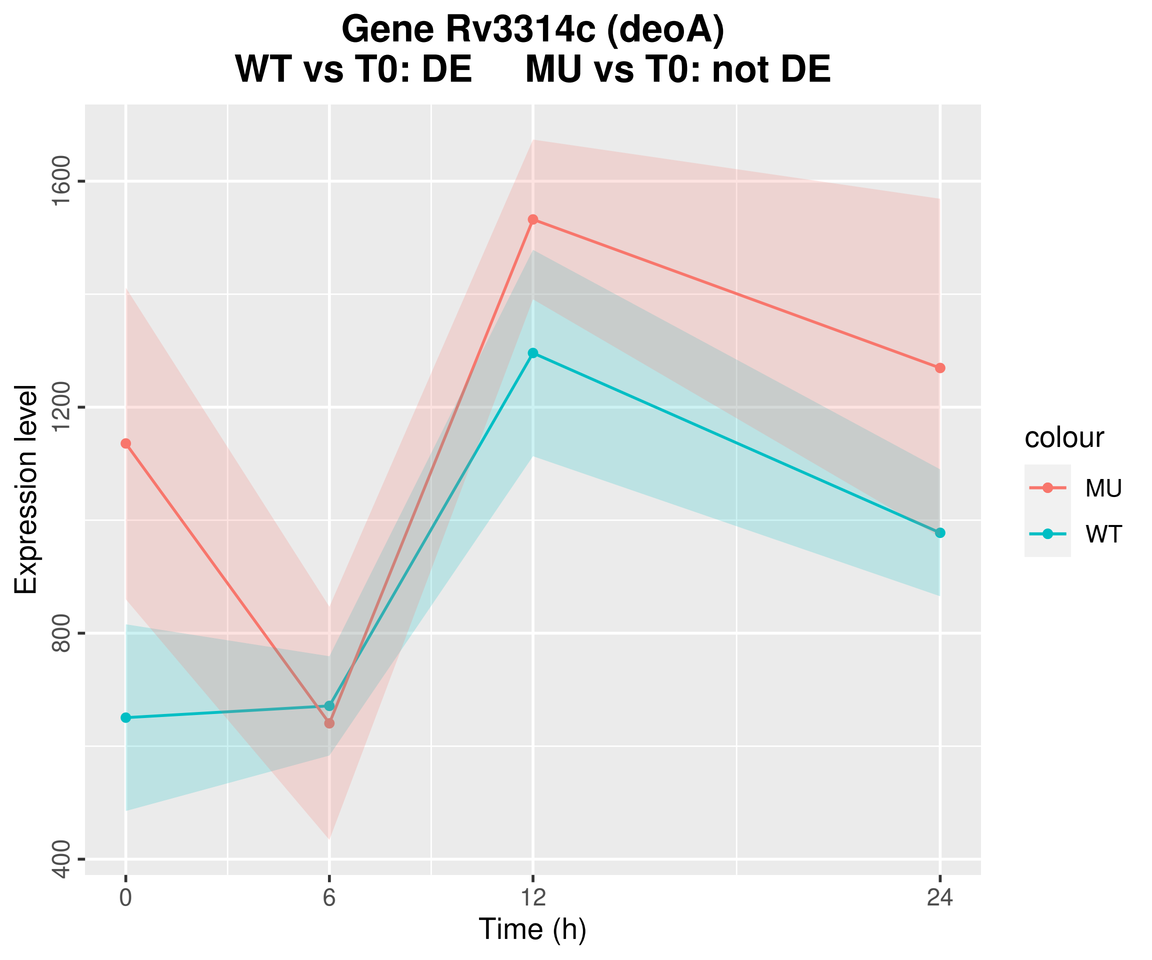

Gene Rv3314c (deoA)
WT vs T0: DE   MU vs T0: not DE
Expression level
1600
1200
800
400
0
6
12
24
Time (h)
colour
MU
WT

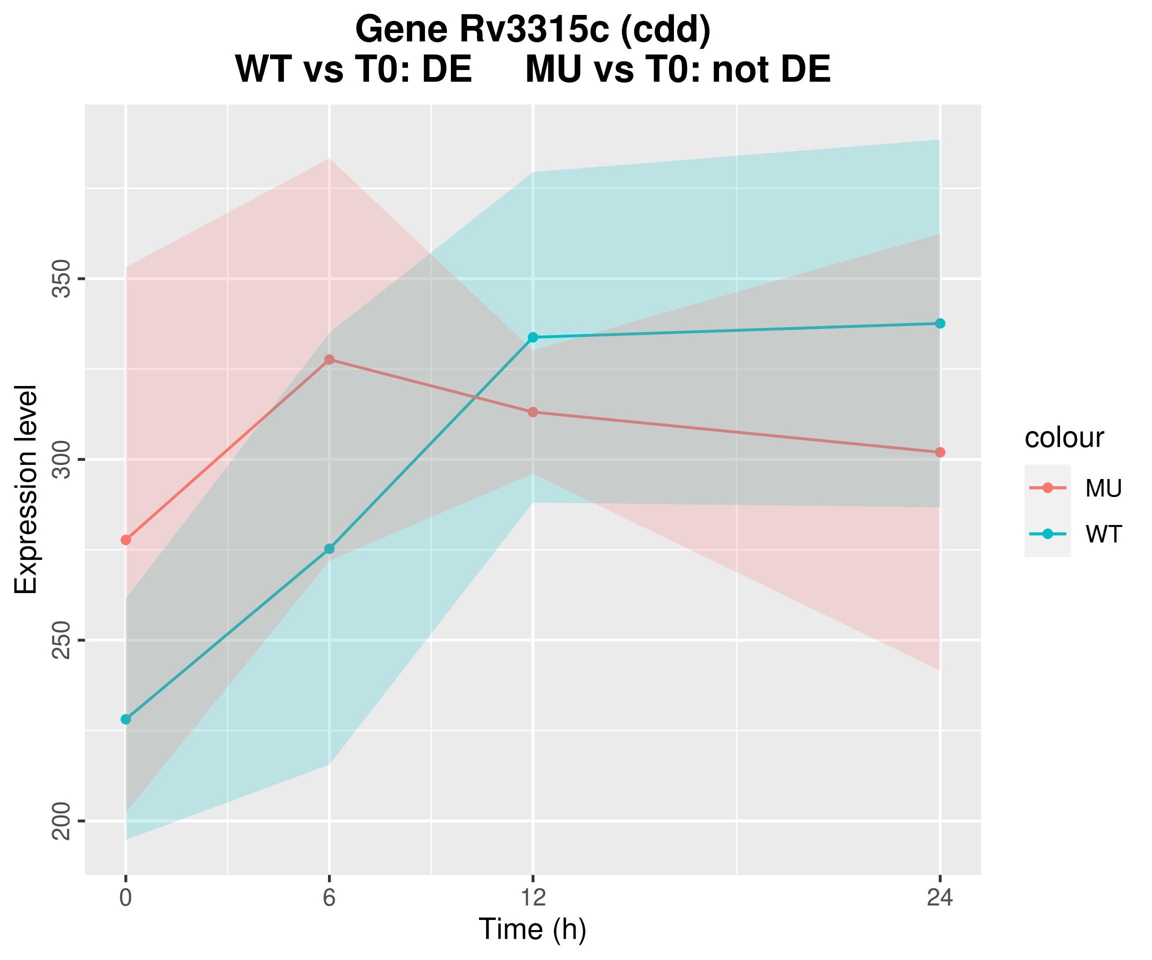

Gene Rv3315c (cdd)
WT vs T0: DE MU vs T0: not DE
Expression level
350
300
250
200
0
6
12
24
Time (h)
colour
MU
WT

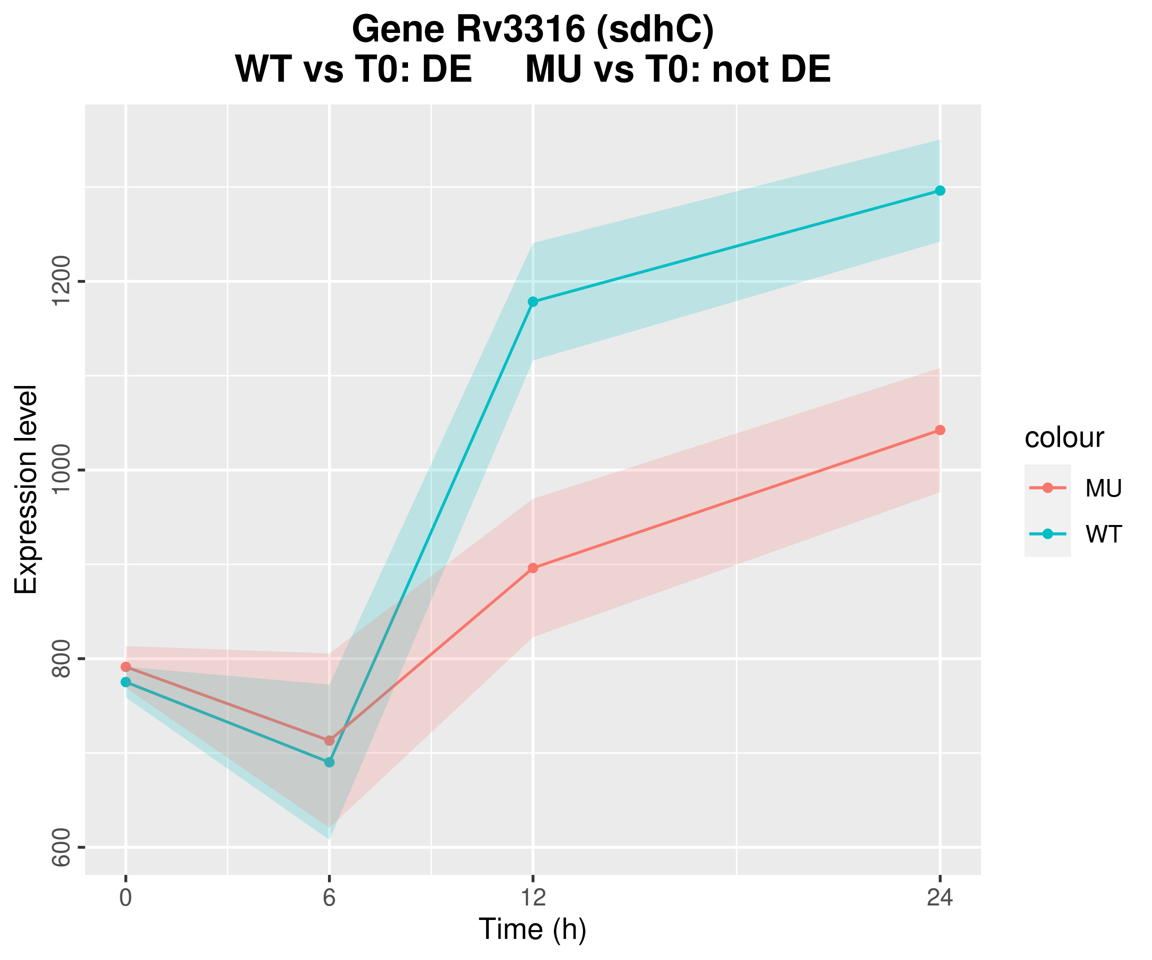
Gene Rv3316 (sdhC)
WT vs T0: DE MU vs T0: not DE
Expression level
1200
1000
800
600
0
6
12
24
Time (h)
colour
MU
WT

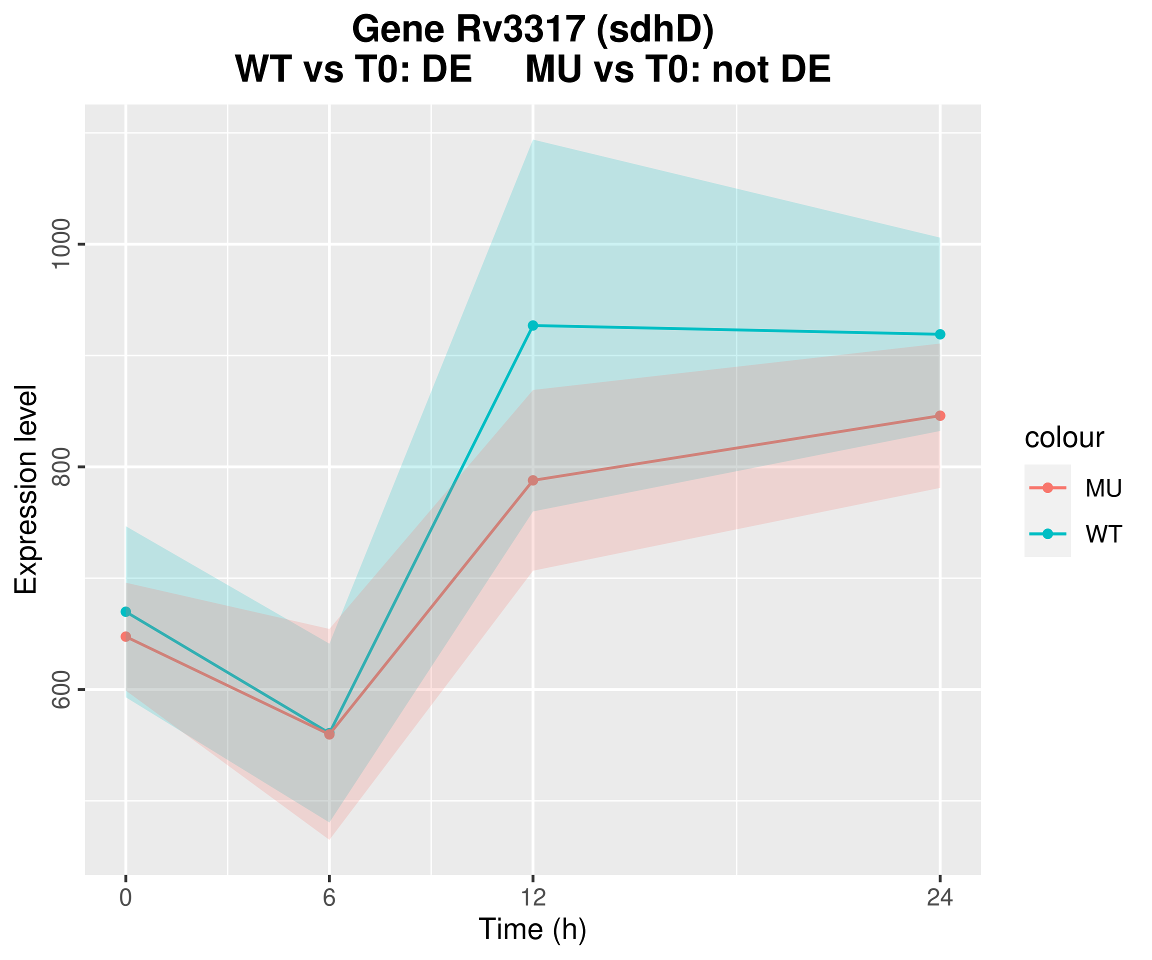

Gene Rv3317 (sdhD)
WT vs T0: DE   MU vs T0: not DE
Expression level
1000
800
600
0
6
12
24
Time (h)
colour
MU
WT

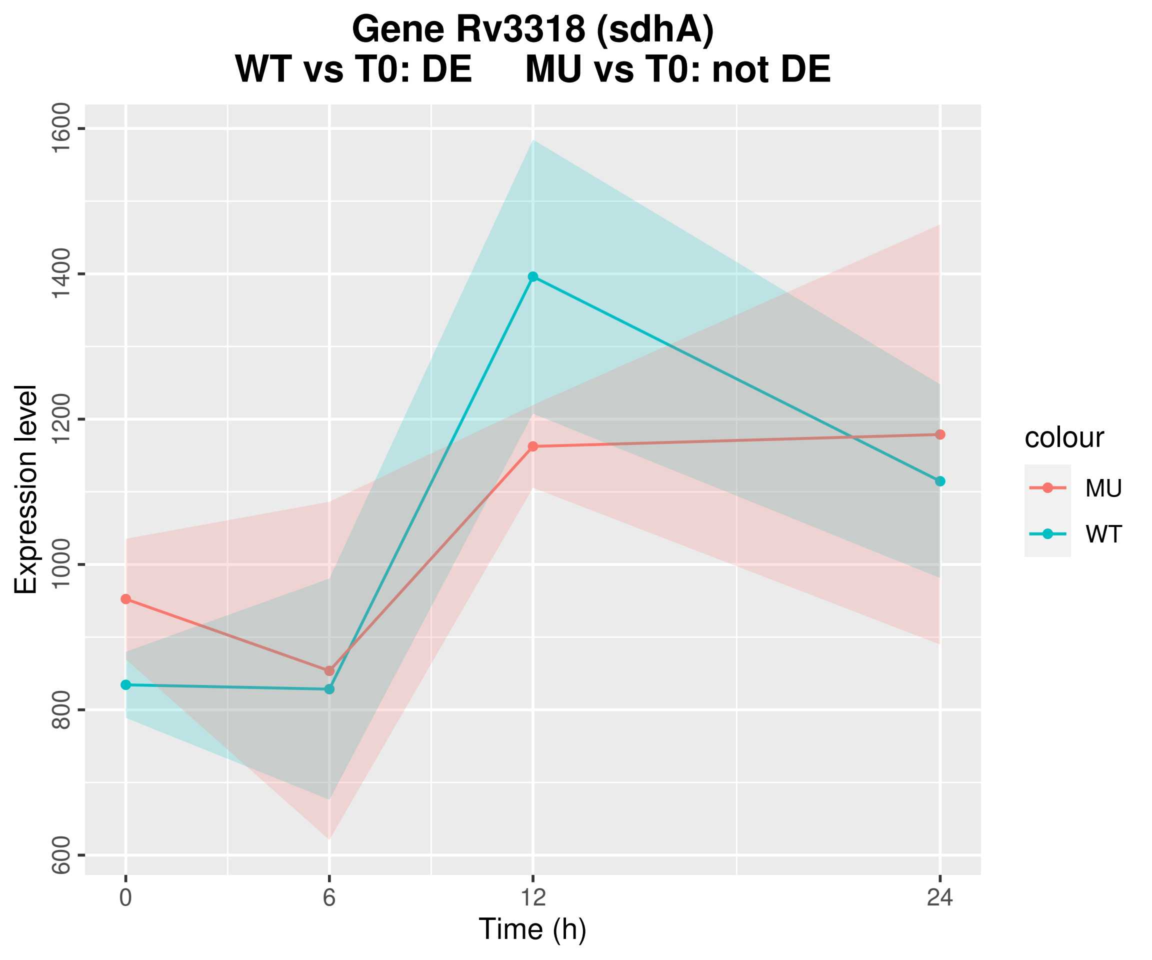
Gene Rv3318 (sdhA)
WT vs T0: DE   MU vs T0: not DE
Expression level
1600
1400
1200
1000
800
600
0
6
12
24
Time (h)
colour
MU
WT

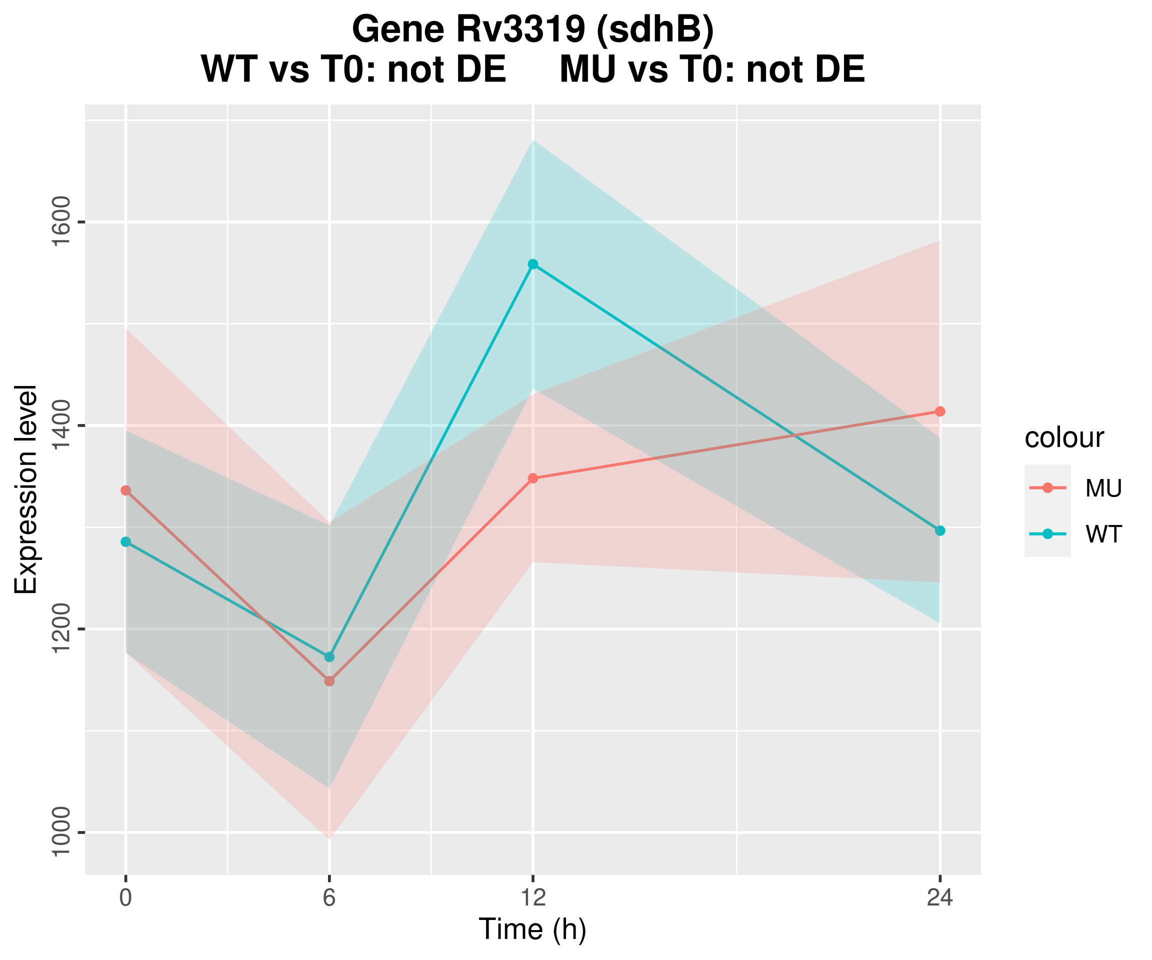

Gene Rv3319 (sdhB)
WT vs T0: not DE MU vs T0: not DE
Expression level
1000
1200
1400
1600
0
6
12
24
Time (h)
colour
MU
WT

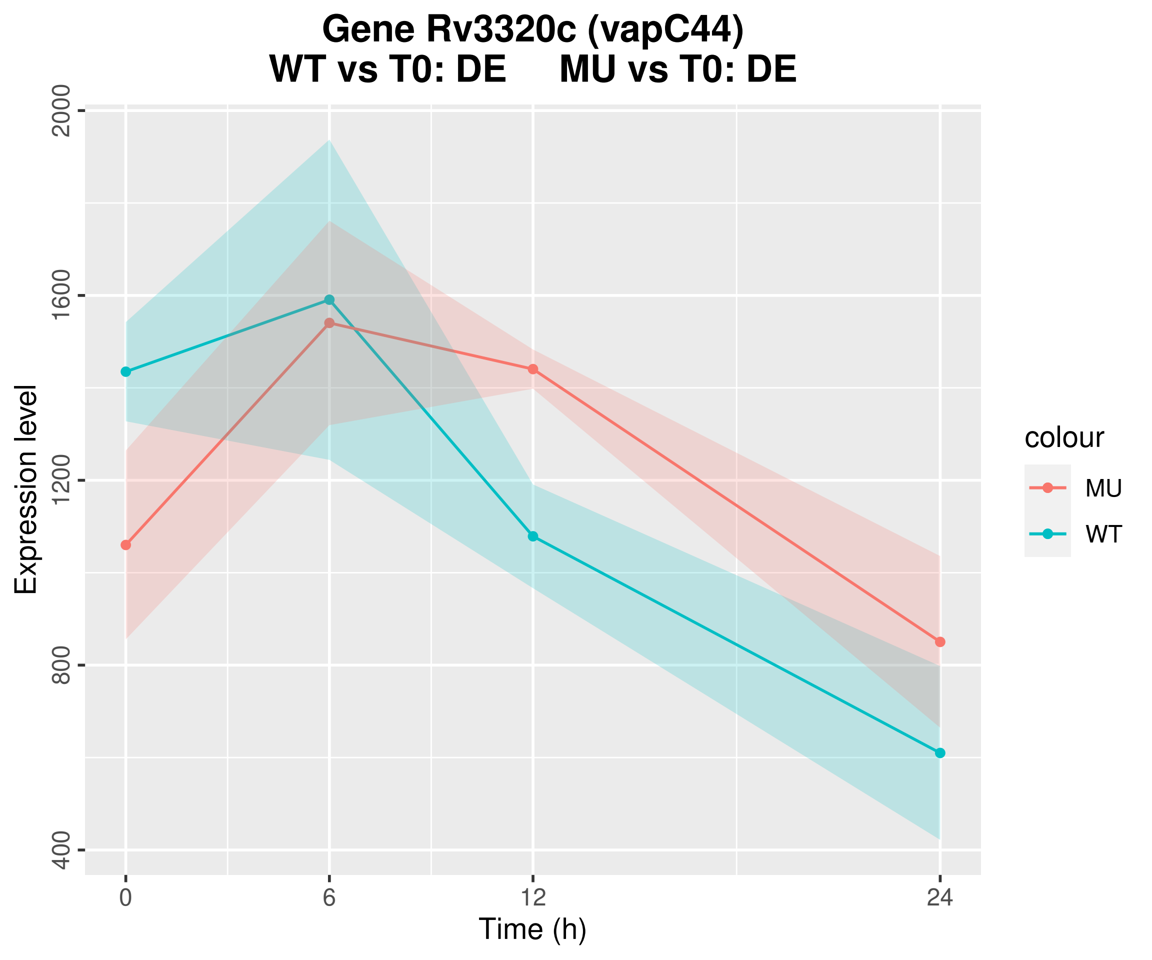

Gene Rv3320c (vapC44)
WT vs T0: DE MU vs T0: DE
Expression level
2000
1600
1200
800
400
0
6
12
24
Time (h)
colour
MU
WT

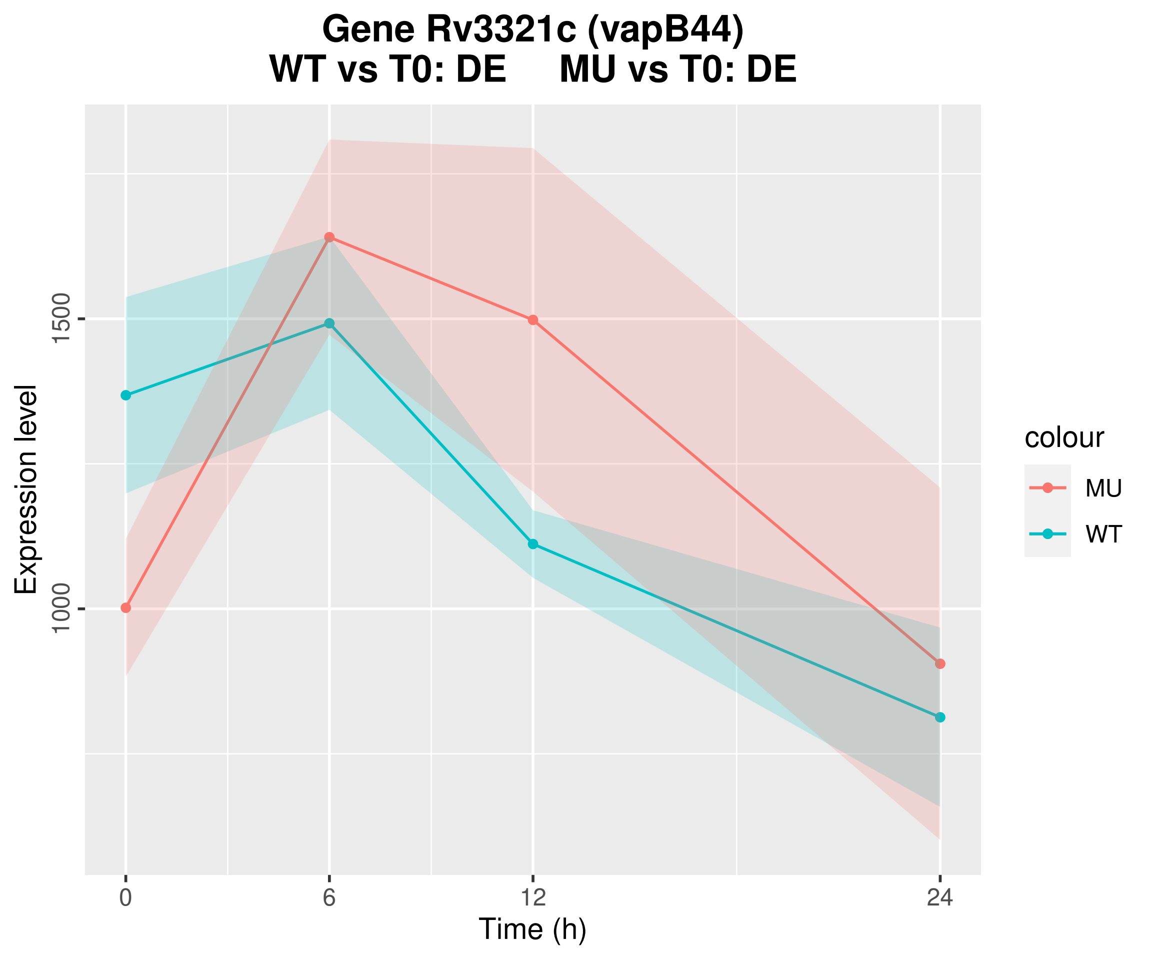

Gene Rv3321c (vapB44)
WT vs T0: DE   MU vs T0: DE
Expression level
1500
1000
0
6
12
24
Time (h)
colour
MU
WT

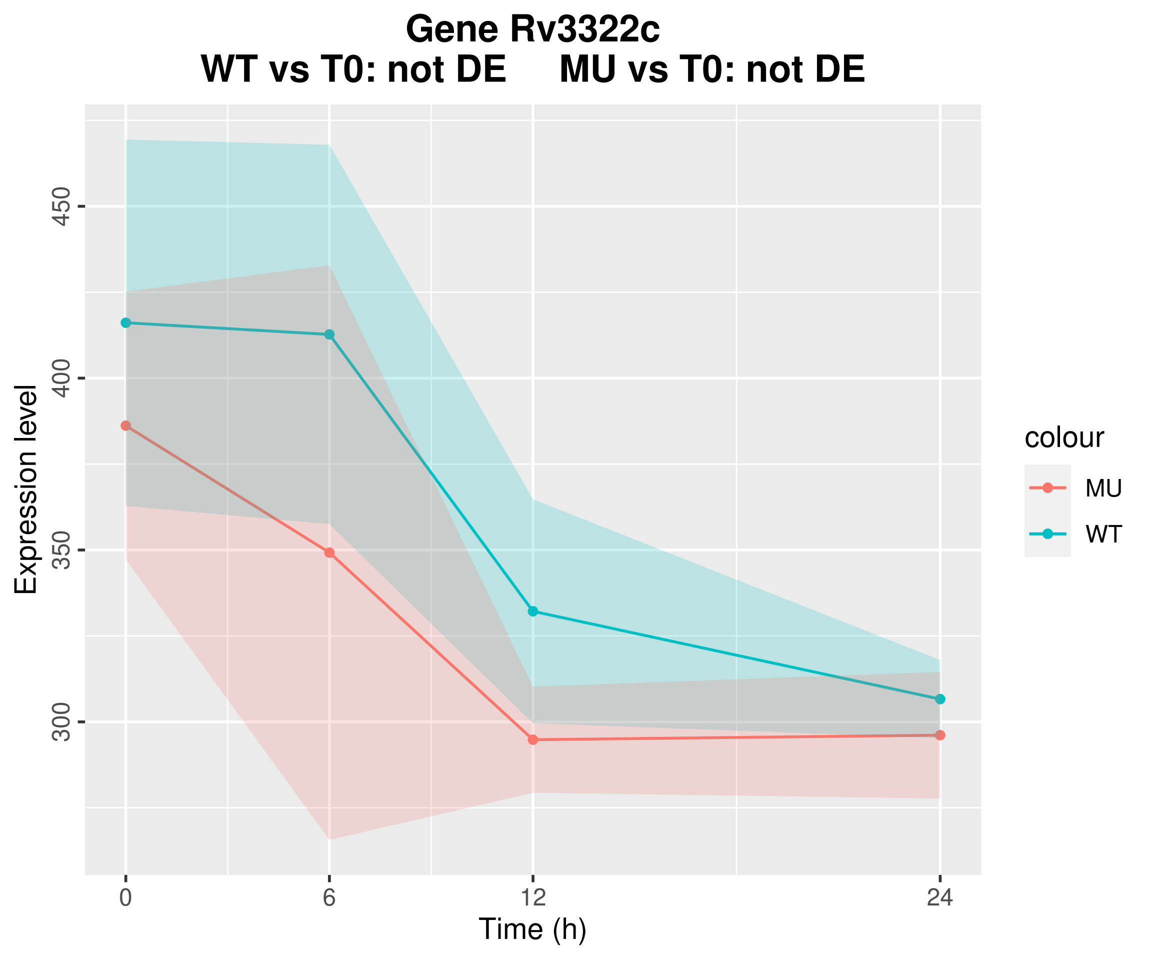

Gene Rv3322c
WT vs T0: not DE   MU vs T0: not DE
Expression level
450
400
350
300
0
6
12
24
Time (h)
colour
MU
WT

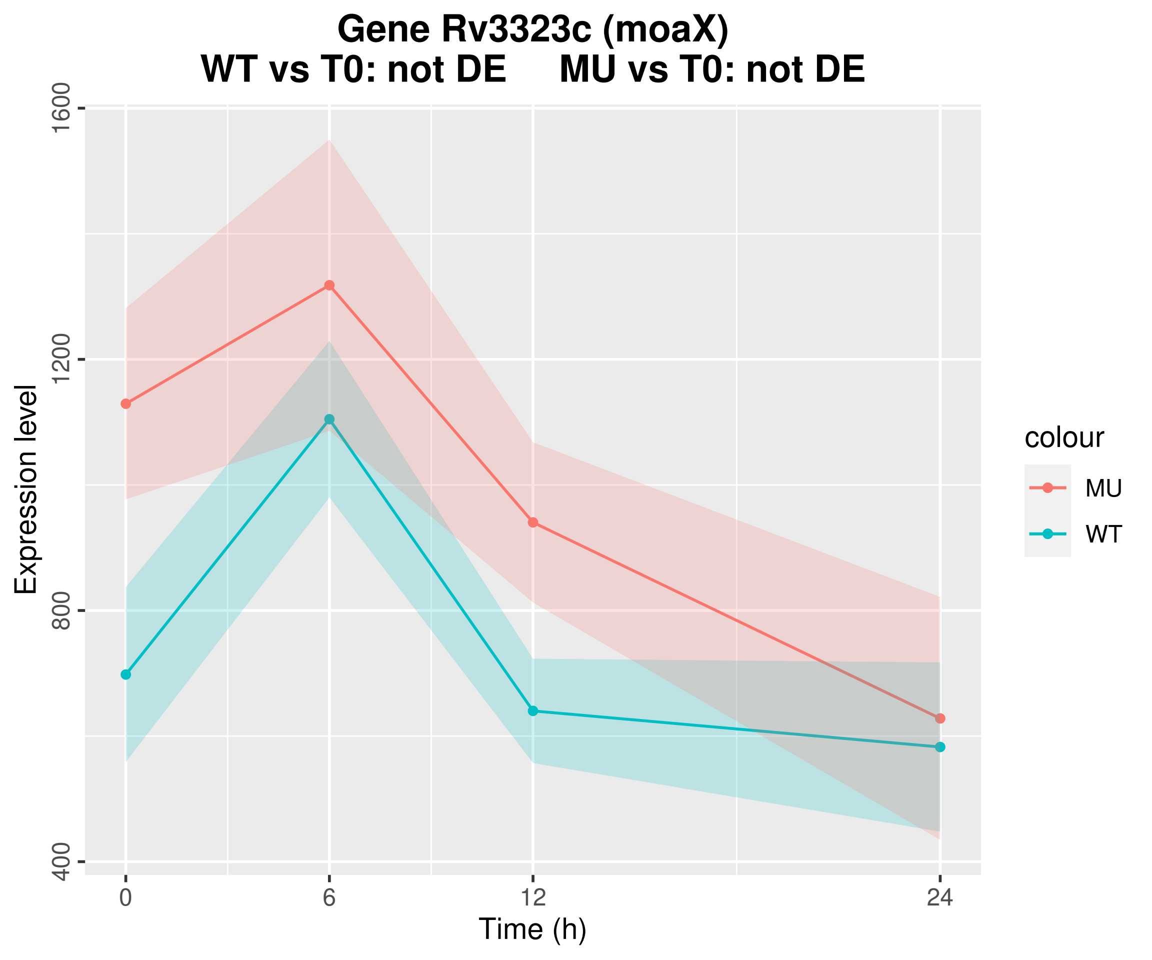

Gene Rv3323c (moaX)
WT vs T0: not DE MU vs T0: not DE
1600
1200
800
400
Expression level
0
6
12
24
Time (h)
colour
MU
WT

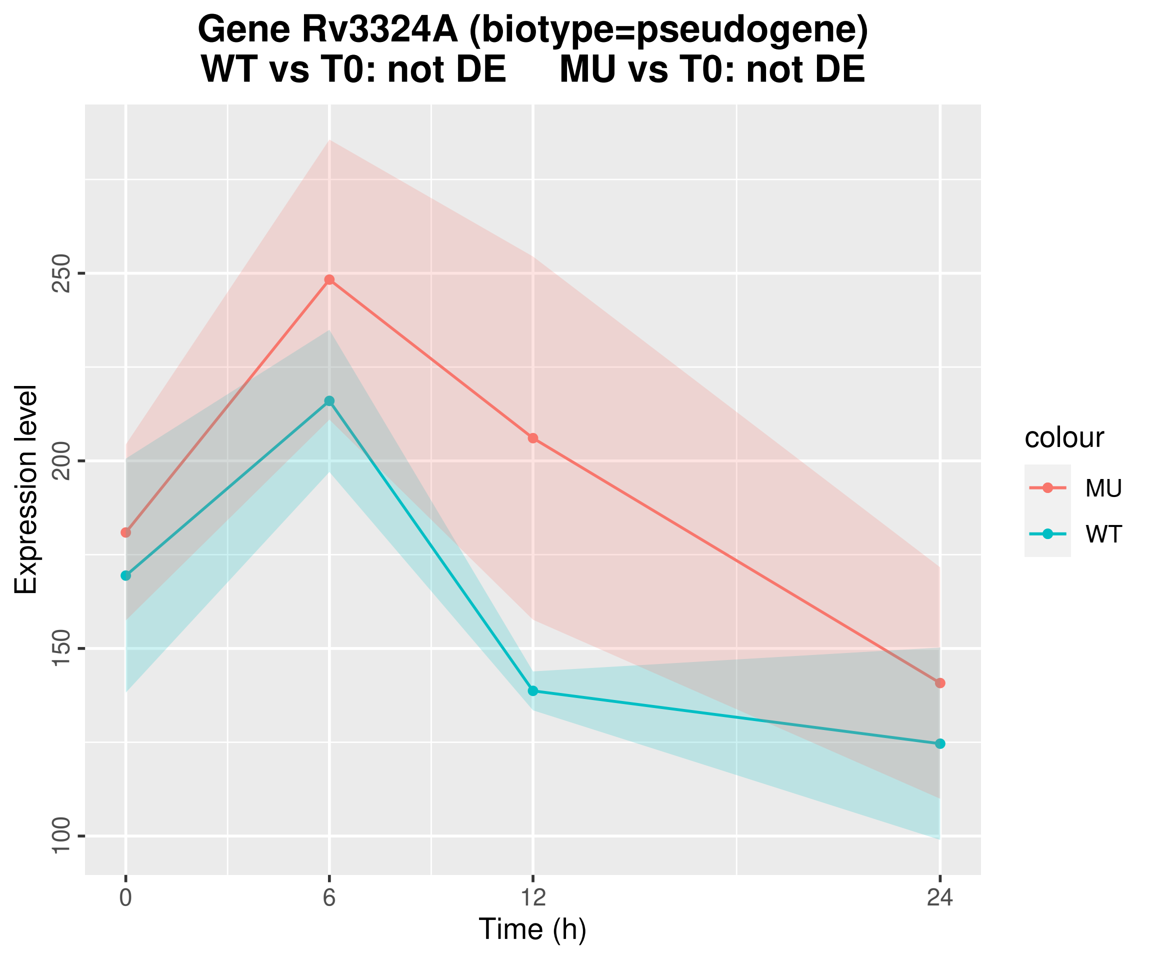
Gene Rv3324A (biotype=pseudogene)
WT vs T0: not DE   MU vs T0: not DE
Expression level
100
150
200
250
0
6
12
24
Time (h)
colour
MU
WT

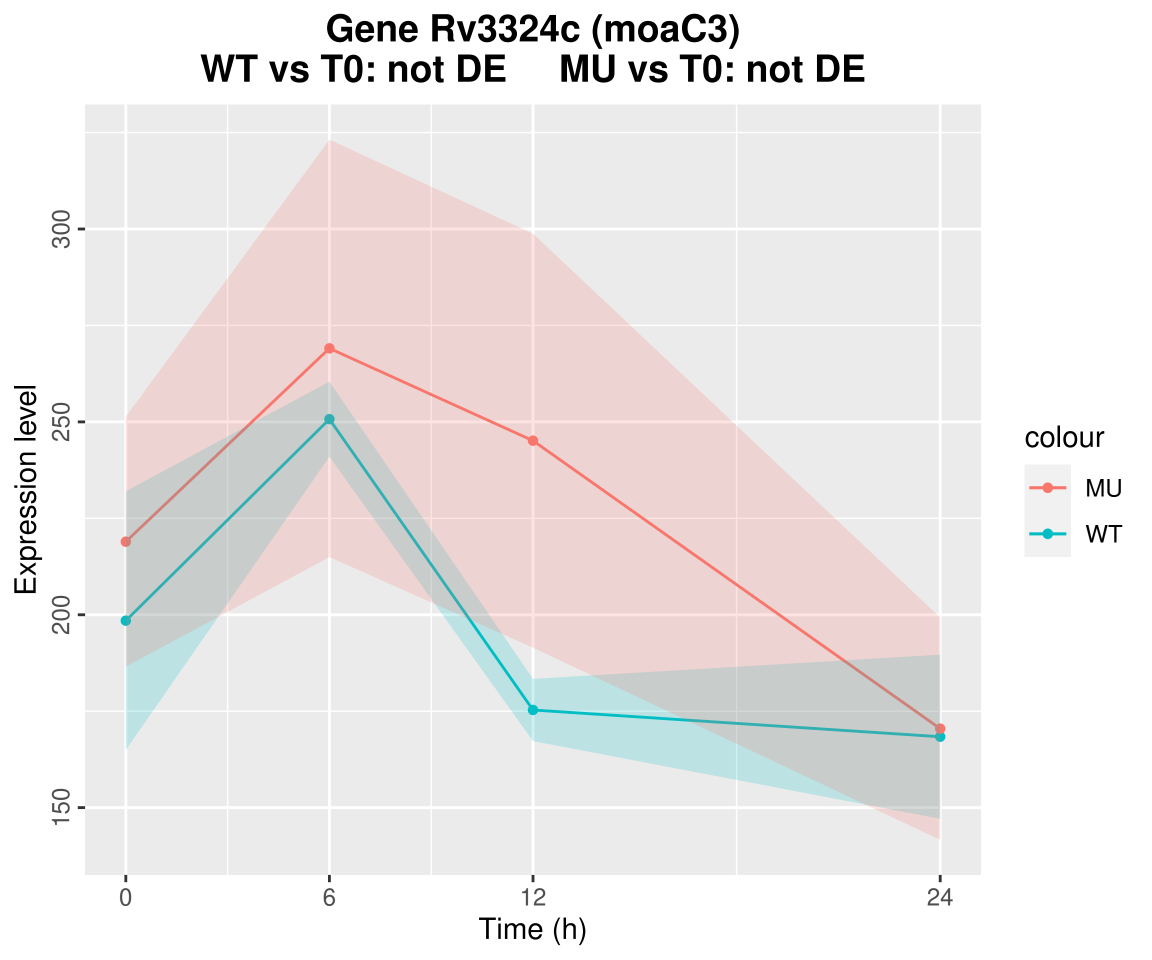

Gene Rv3324c (moaC3)
WT vs T0: not DE   MU vs T0: not DE
Expression level
300
250
200
150
0
6
12
24
Time (h)
colour
MU
WT

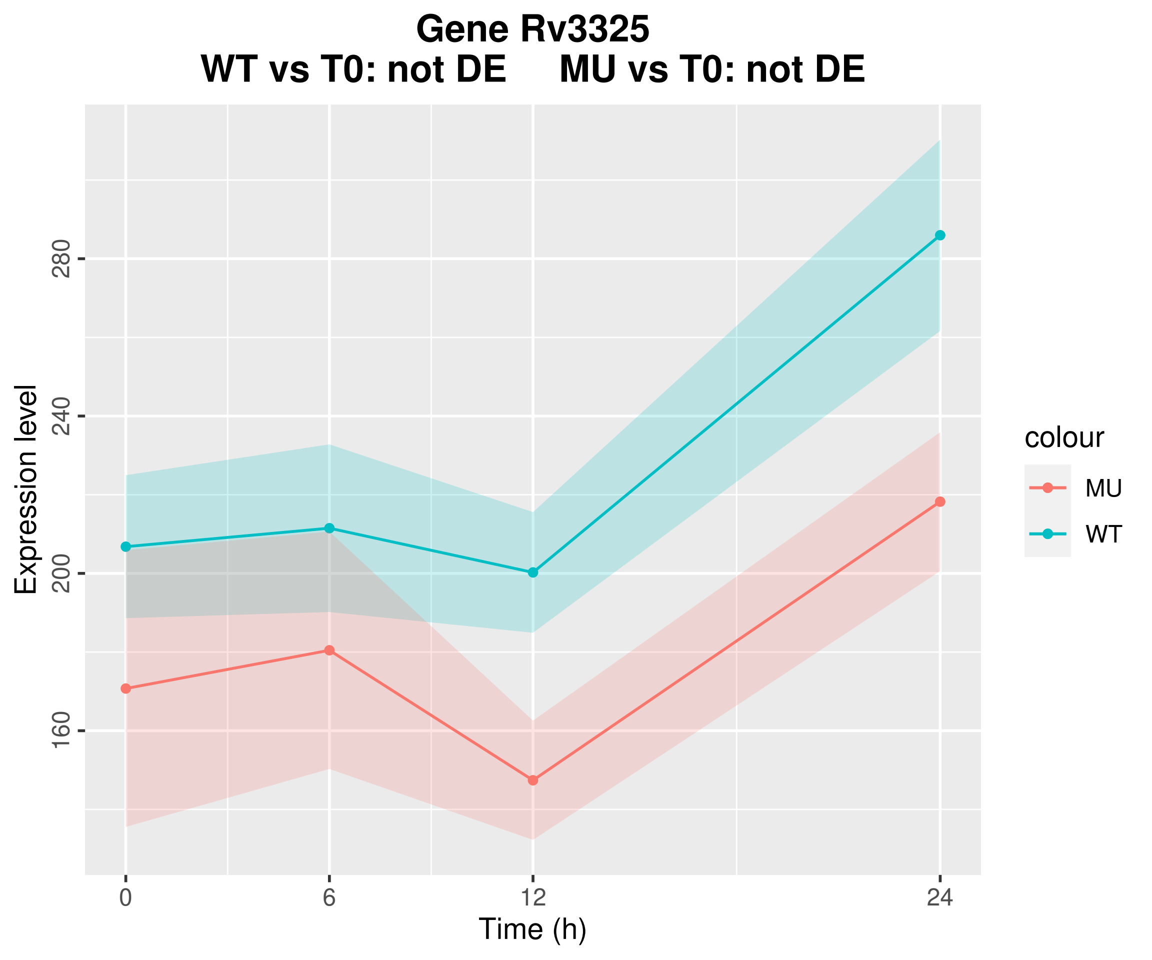

Gene Rv3325
WT vs T0: not DE   MU vs T0: not DE
Expression level
160
200
240
280
0
6
12
24
Time (h)
colour
MU
WT

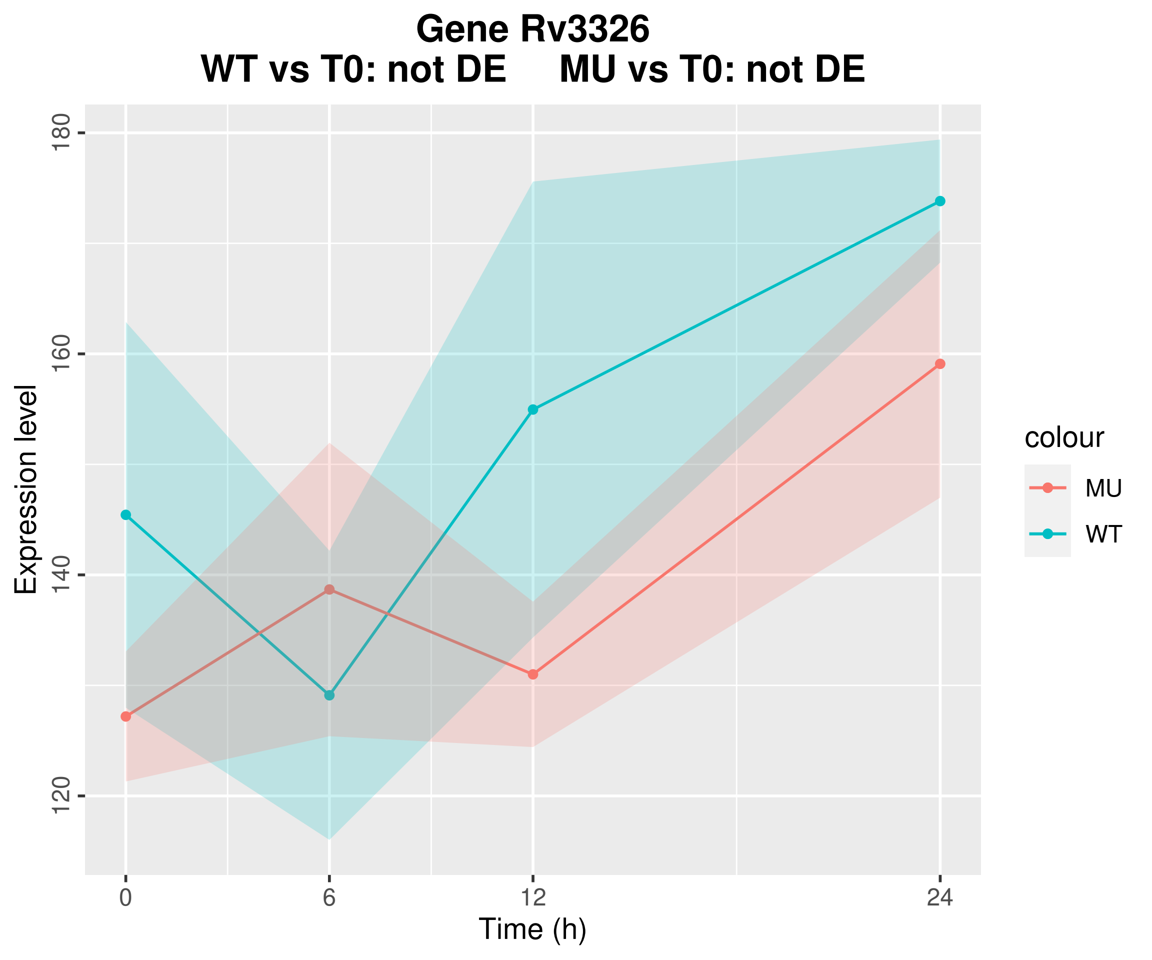
Gene Rv3326
WT vs T0: not DE   MU vs T0: not DE
Expression level
180
160
140
120
0
6
12
24
Time (h)
colour
MU
WT

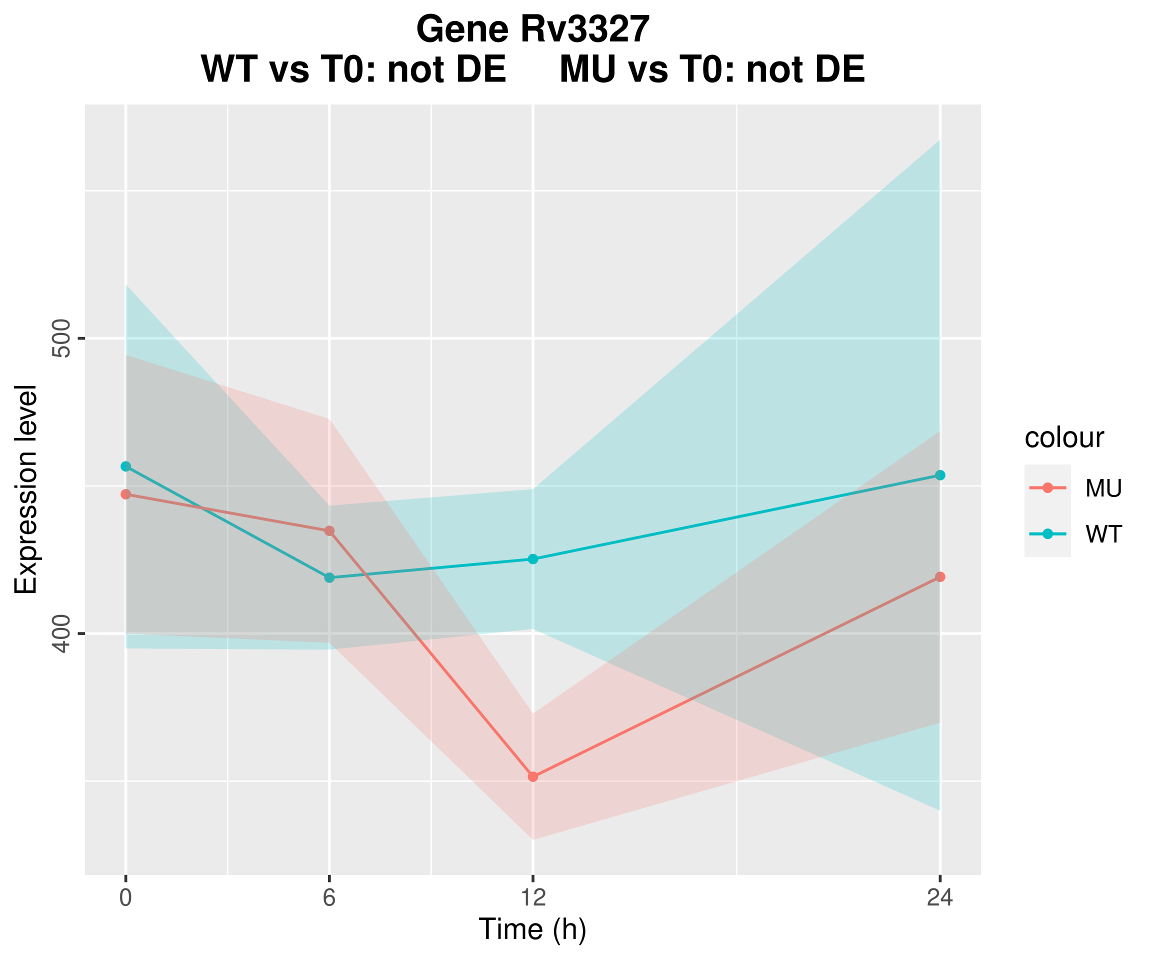
Gene Rv3327
WT vs T0: not DE MU vs T0: not DE
Expression level
500
400
0
6
12
24
Time (h)
colour
MU
WT

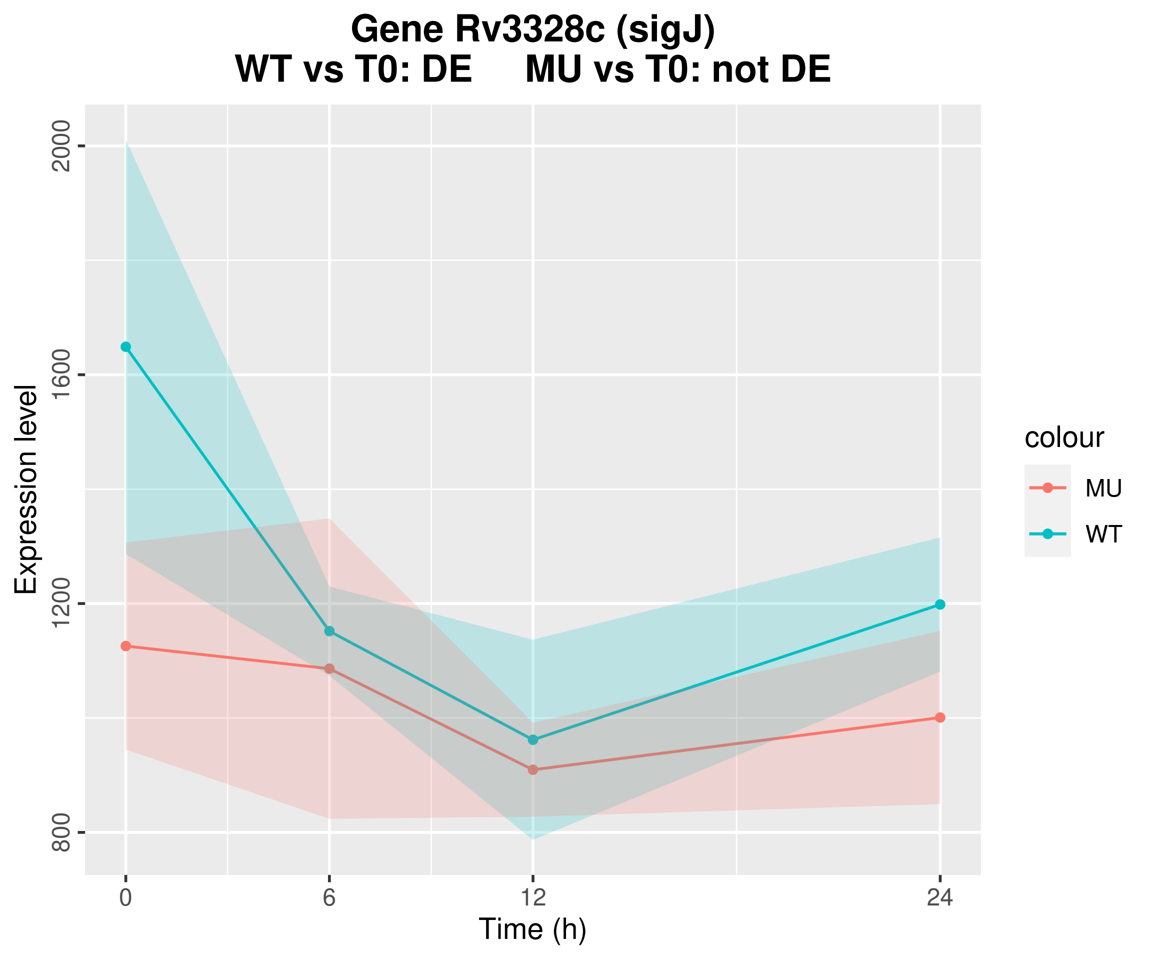

Gene Rv3328c (sigJ)
WT vs T0: DE MU vs T0: not DE
Expression level
2000
1600
1200
800
0
6
12
24
Time (h)
colour
MU
WT

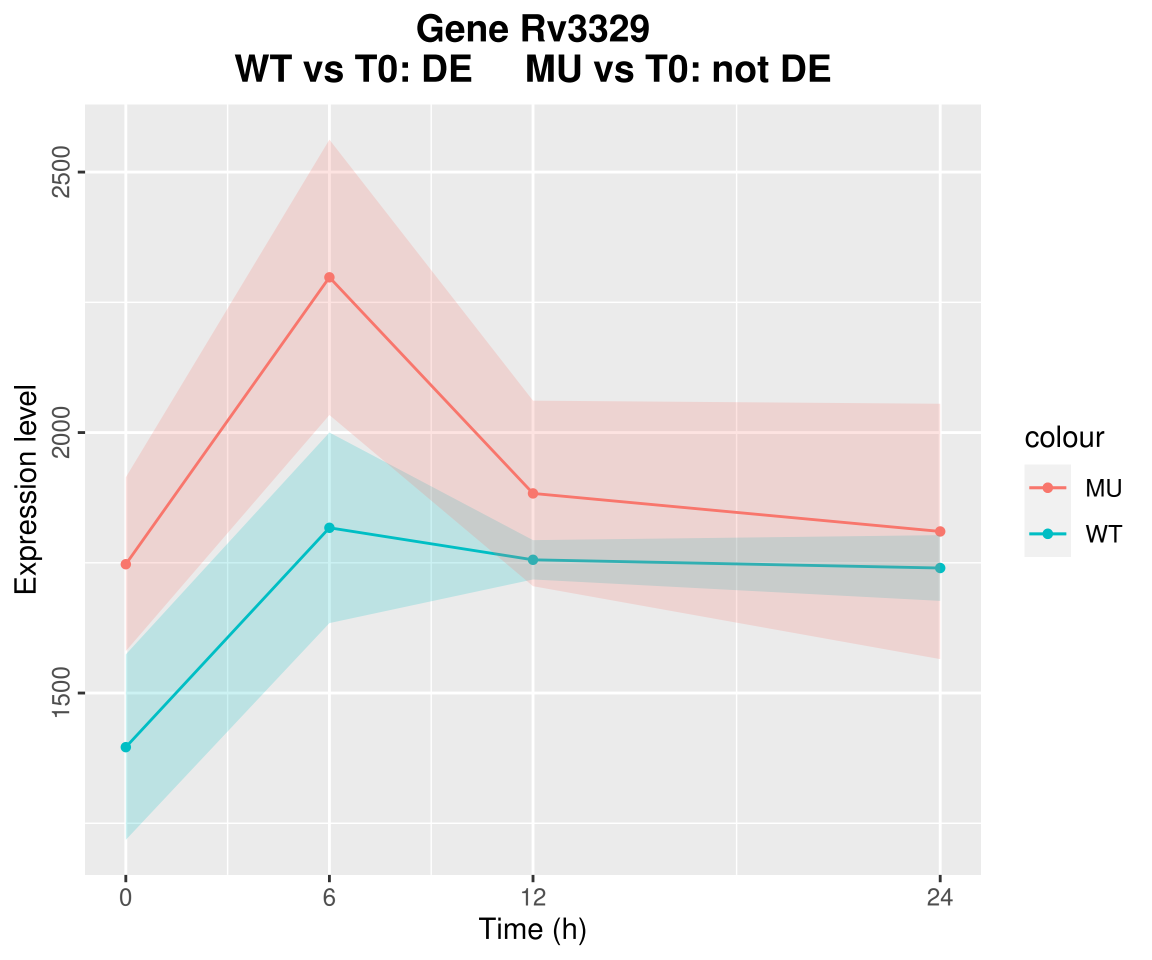
Gene Rv3329
WT vs T0: DE MU vs T0: not DE
Expression level
2500
2000
1500
0
6
12
24
Time (h)
colour
MU
WT

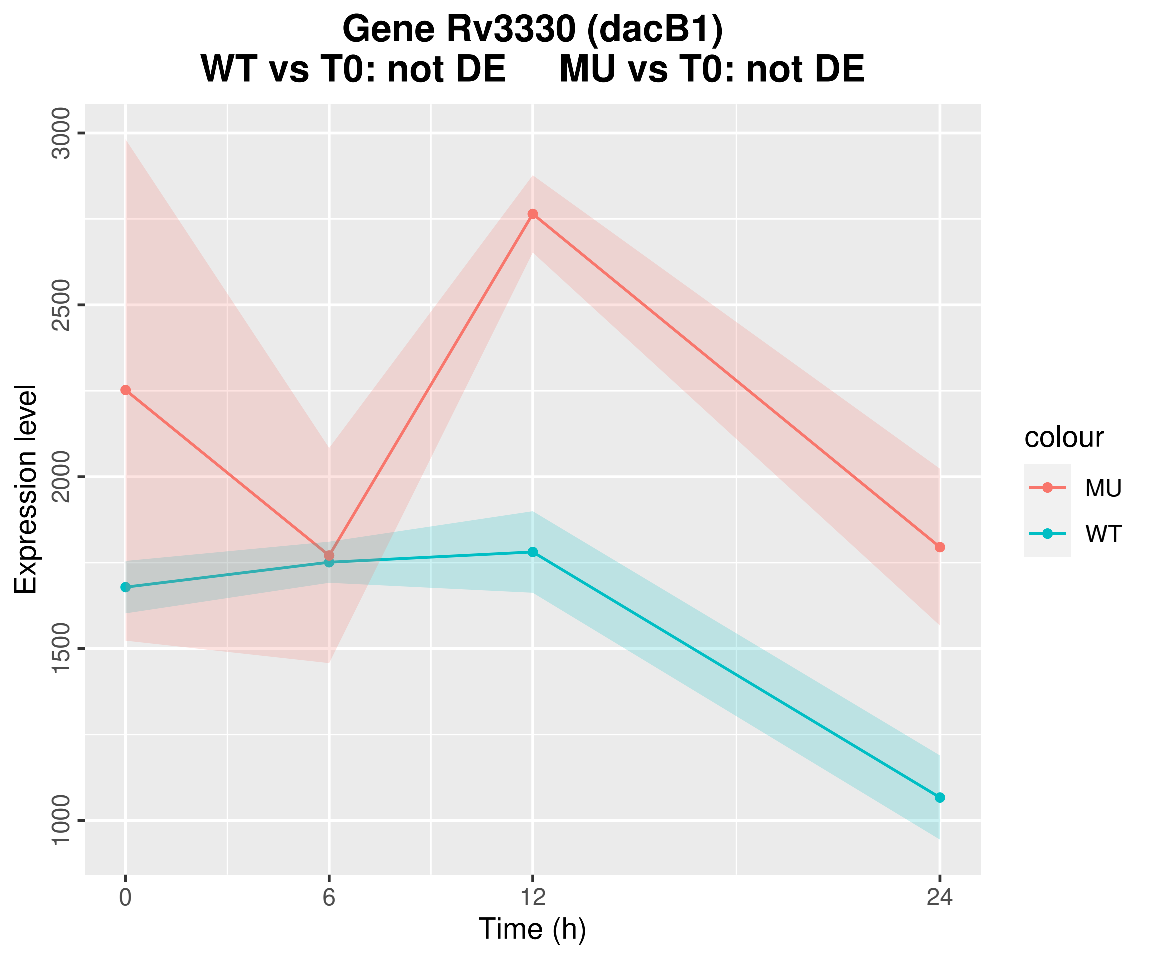
Gene Rv3330 (dacB1)
WT vs T0: not DE   MU vs T0: not DE
Expression level
3000
2500
2000
1500
1000
0
6
12
24
Time (h)
colour
MU
WT

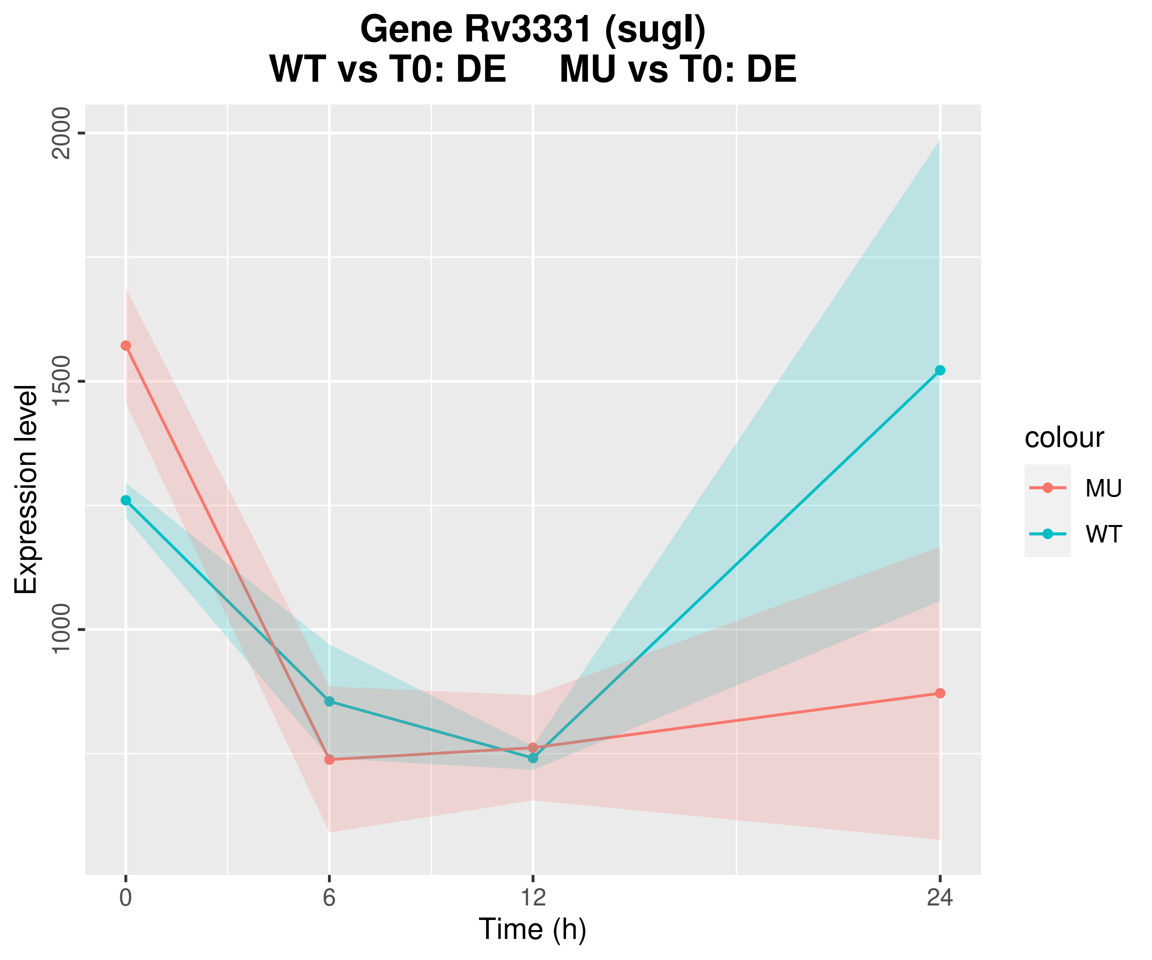
Gene Rv3331 (sugI)
WT vs T0: DE   MU vs T0: DE
Expression level
2000
1500
1000
0
6
12
24
Time (h)
colour
MU
WT

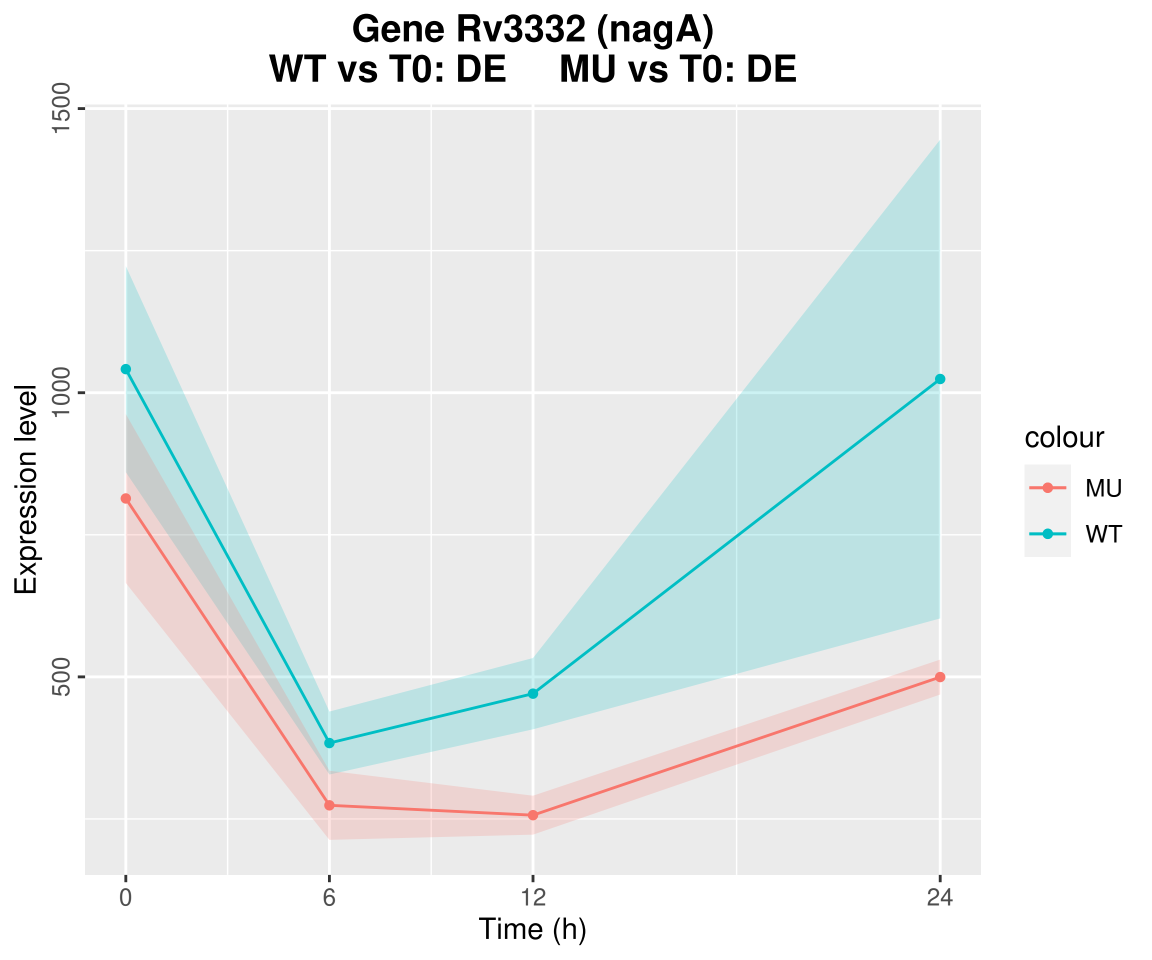
Gene Rv3332 (nagA)
WT vs T0: DE   MU vs T0: DE
1500
1000
500
Expression level
0
6
12
24
Time (h)
colour
MU
WT

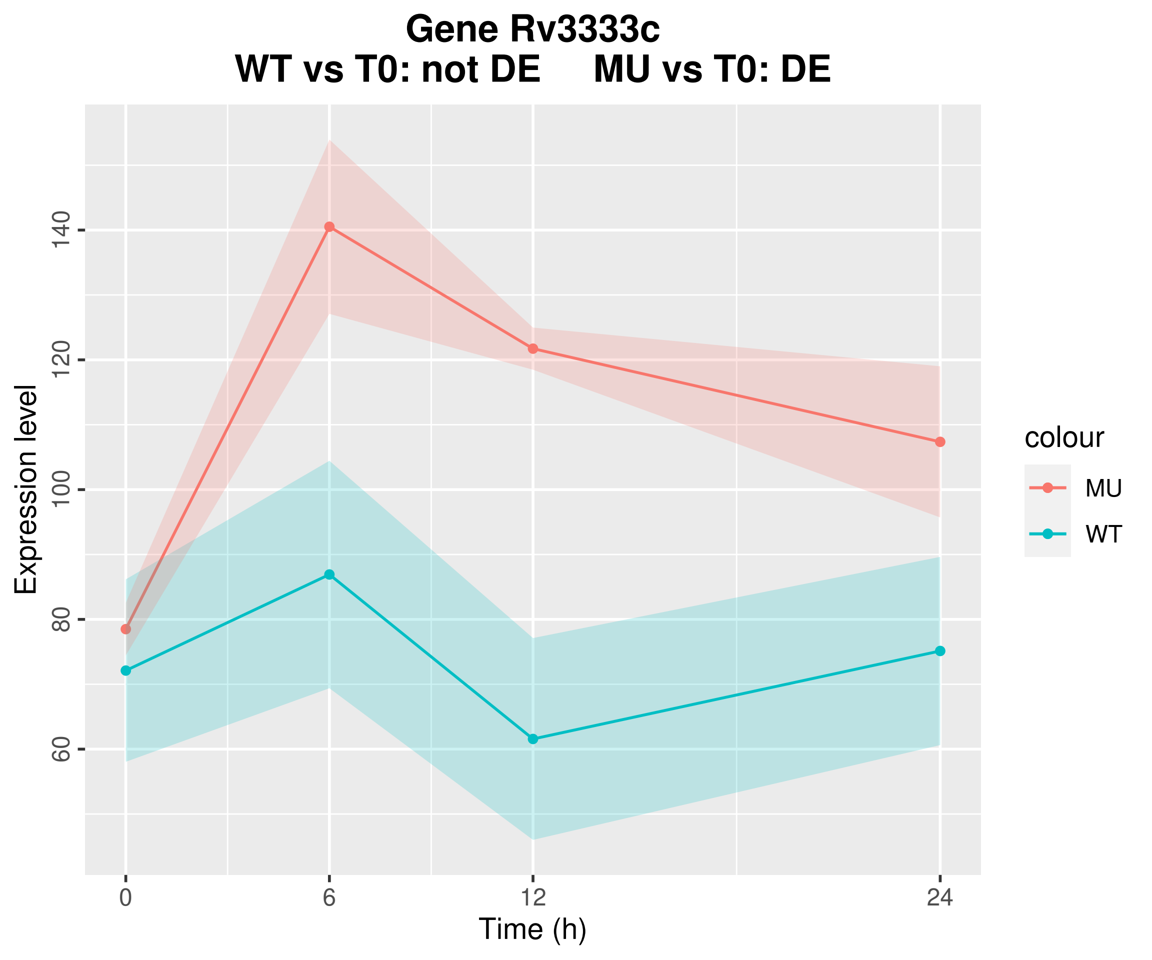
Gene Rv3333c
WT vs T0: not DE MU vs T0: DE
Expression level
60
80
100
120
140
0
6
12
24
Time (h)
colour
MU
WT

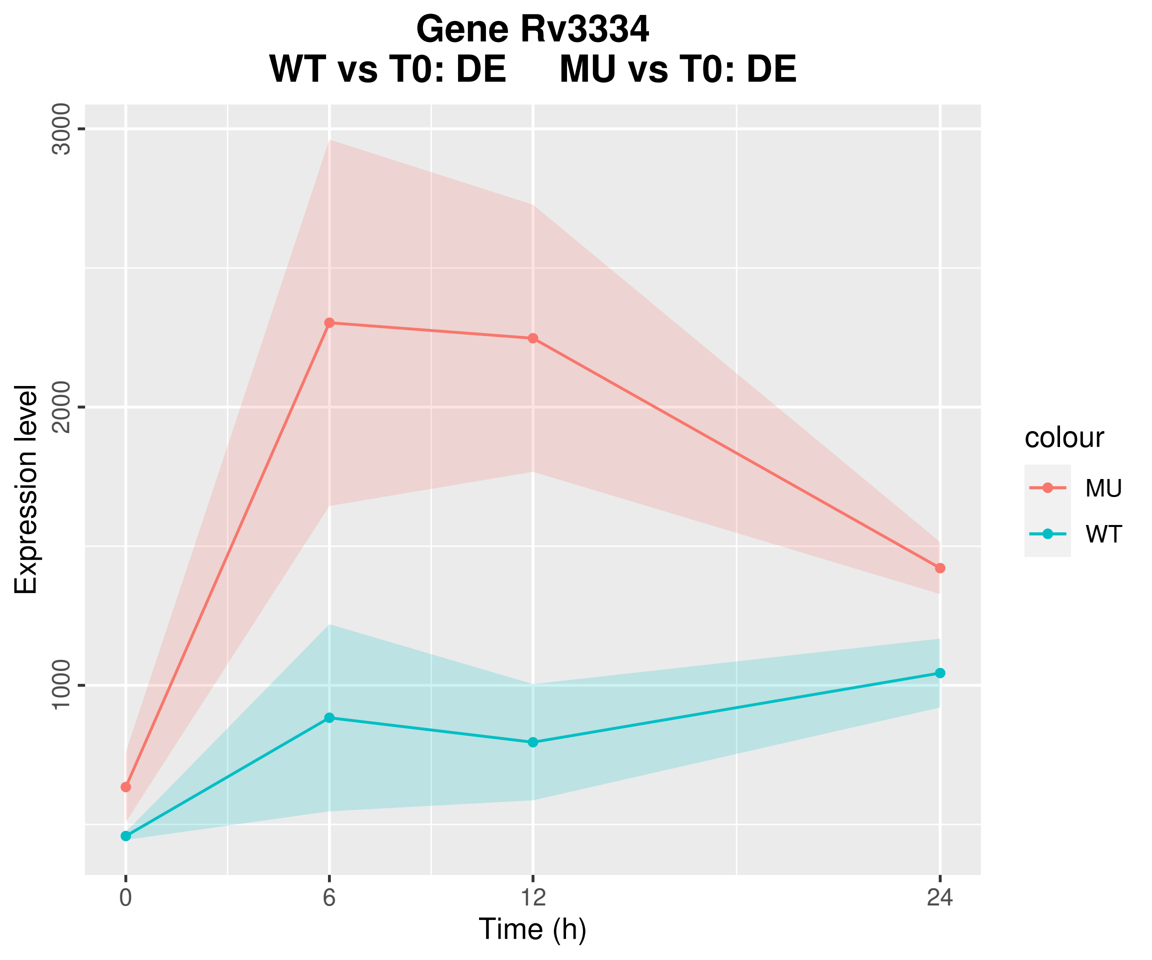
Gene Rv3334
WT vs T0: DE MU vs T0: DE
Expression level
3000
2000
1000
0
6
12
24
Time (h)
colour
MU
WT

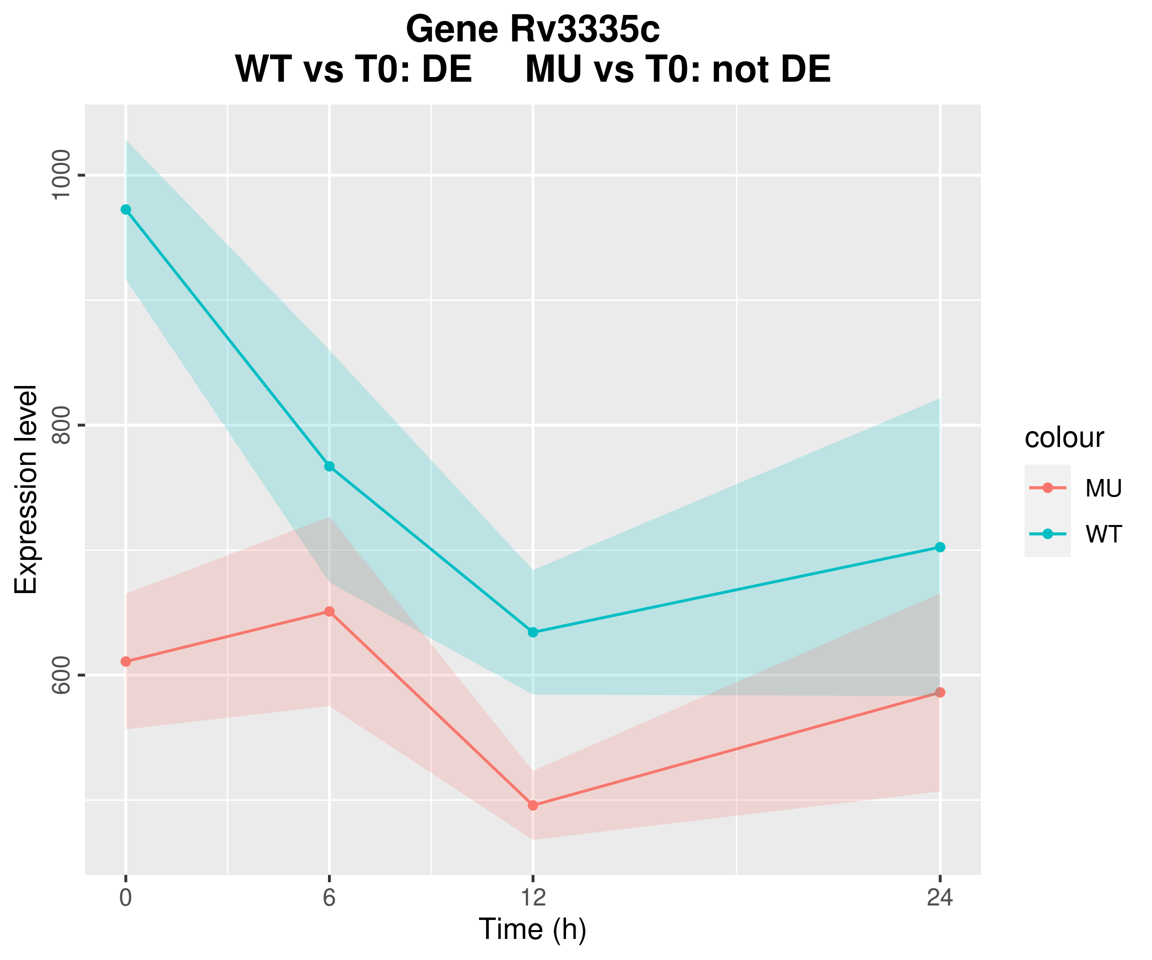
Gene Rv3335c
WT vs T0: DE MU vs T0: not DE
Expression level
1000
800
600
0
6
12
24
Time (h)
colour
MU
WT

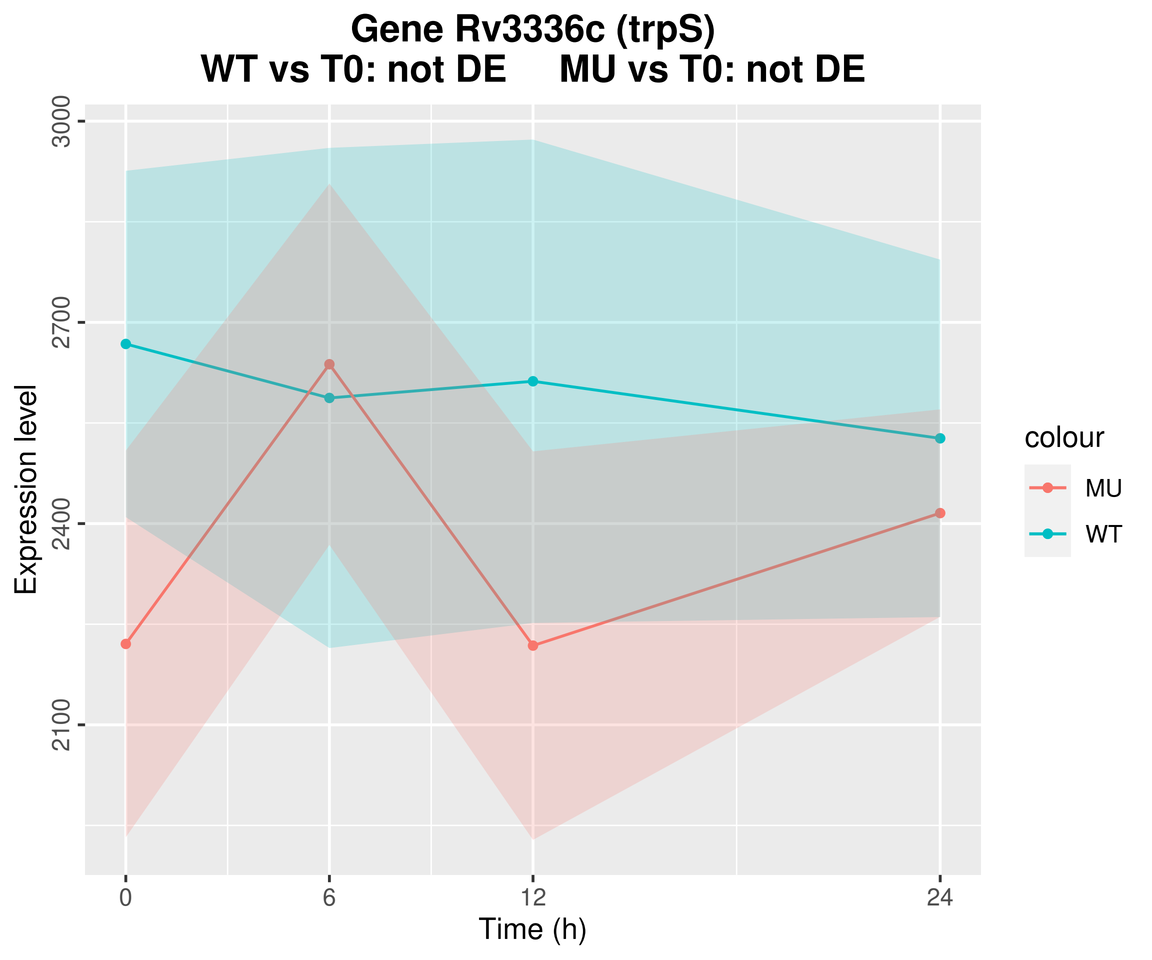

Gene Rv3336c (trpS)
WT vs T0: not DE MU vs T0: not DE
Expression level
3000
2700
2400
2100
0
6
12
24
Time (h)
colour
MU
WT

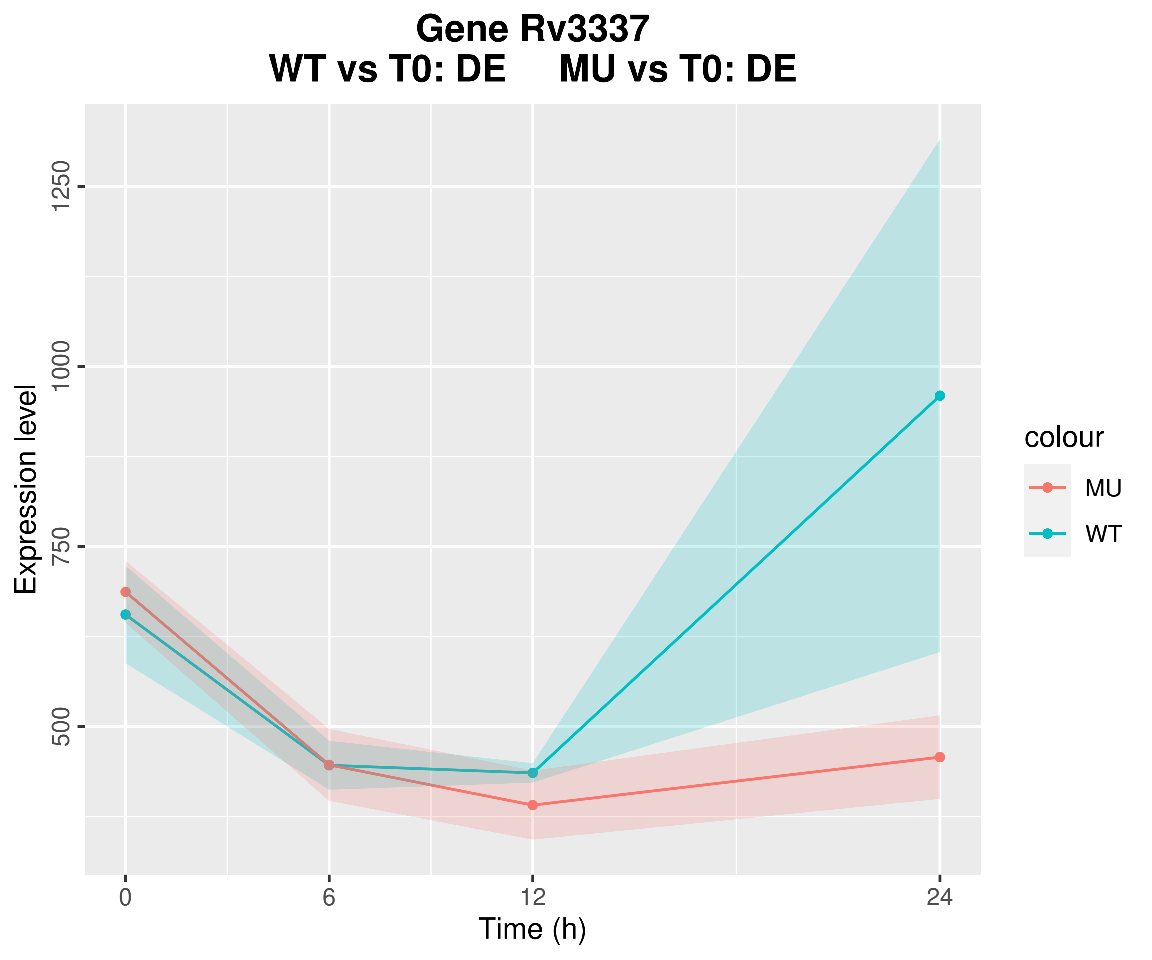

Gene Rv3337
WT vs T0: DE   MU vs T0: DE
Expression level
1250
1000
750
500
0
6
12
24
Time (h)
colour
MU
WT

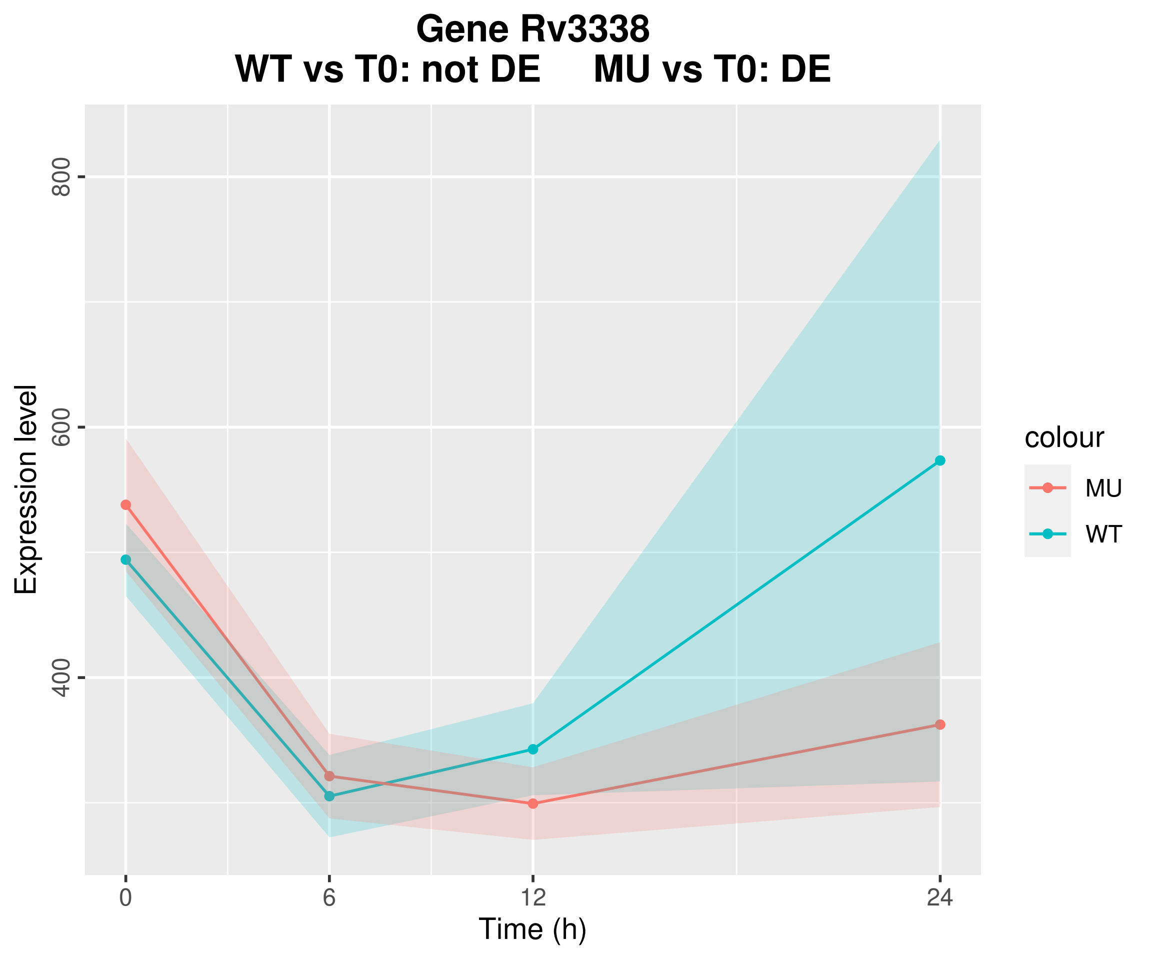

Gene Rv3338
WT vs T0: not DE   MU vs T0: DE
Expression level
800
600
400
0
6
12
24
Time (h)
colour
MU
WT

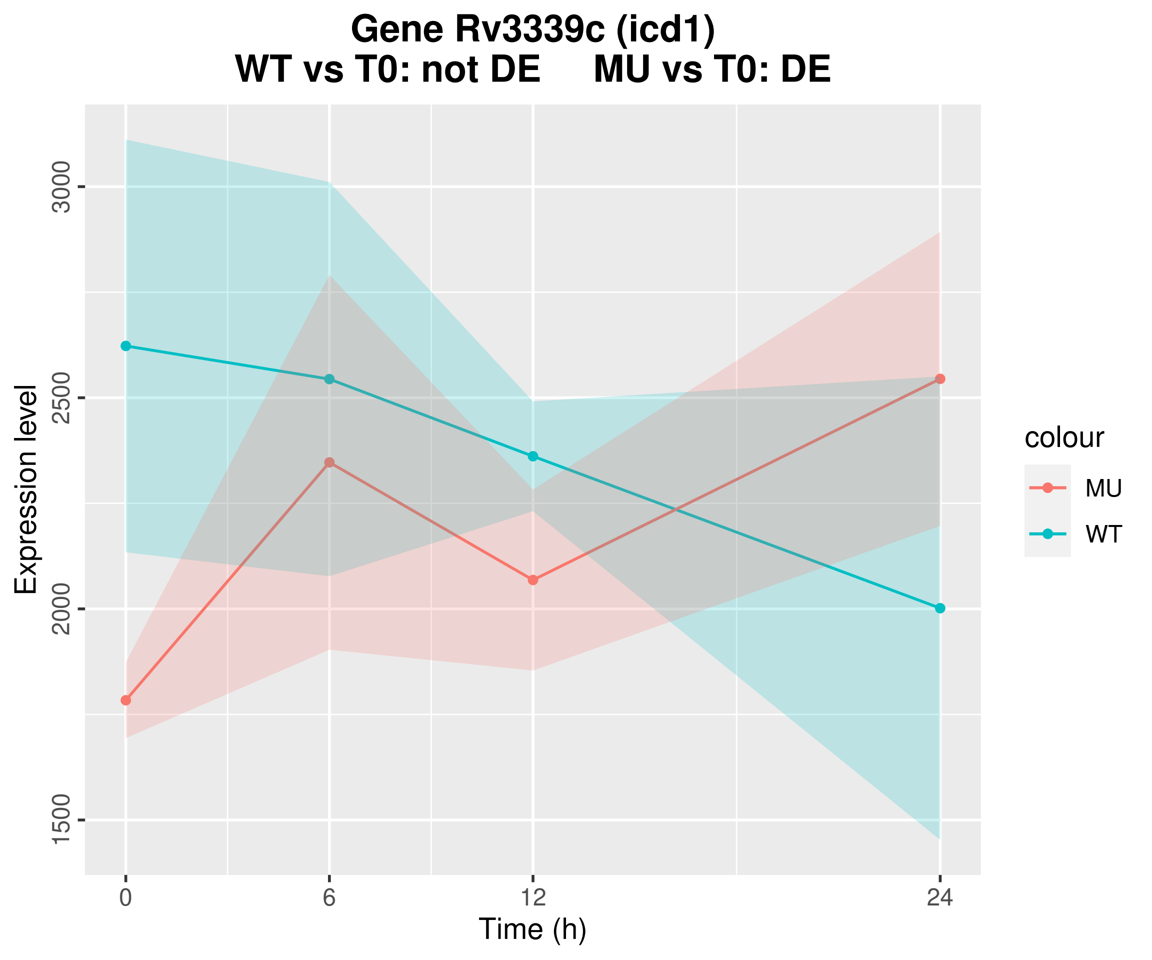
Gene Rv3339c (icd1)
WT vs T0: not DE   MU vs T0: DE
Expression level
3000
2500
2000
1500
0
6
12
24
Time (h)
colour
MU
WT

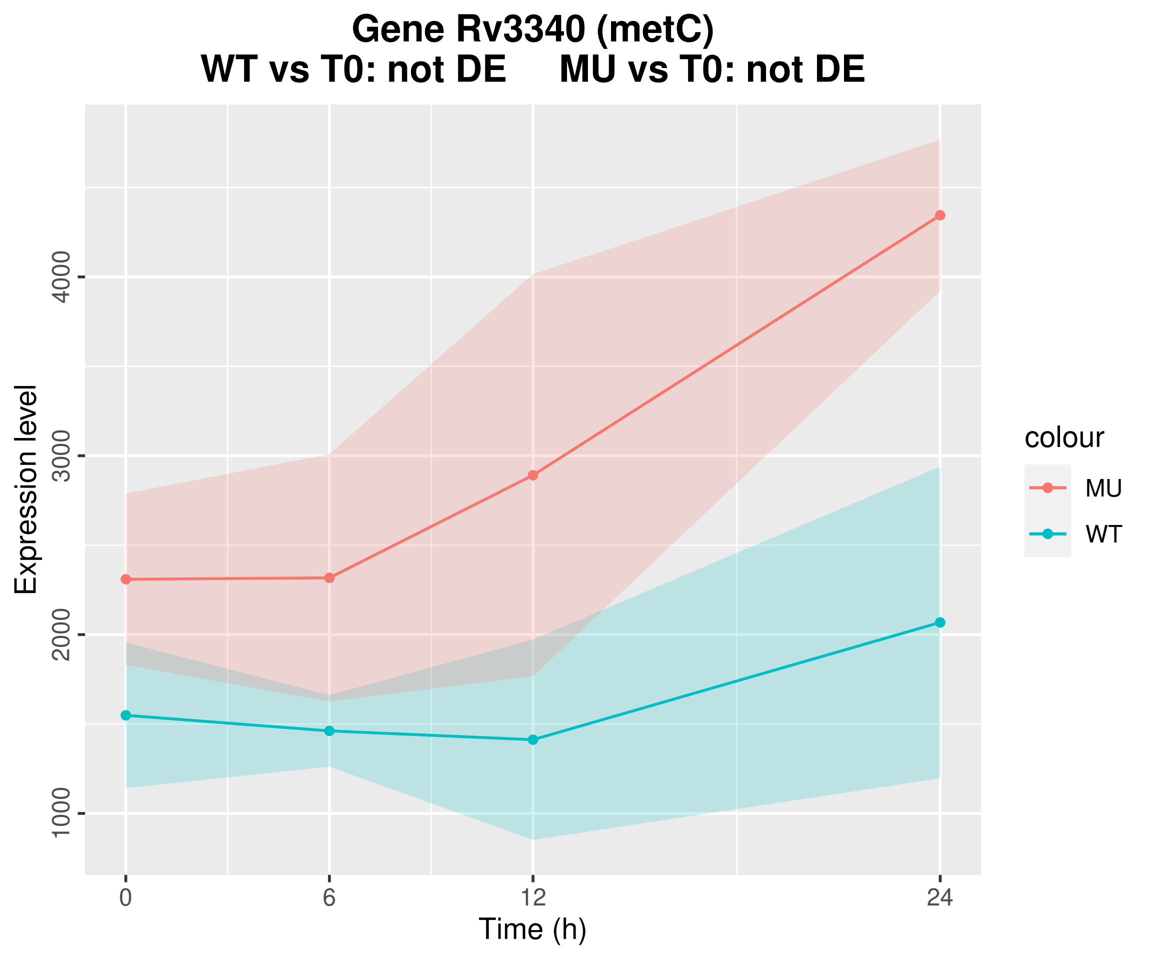
Gene Rv3340 (metC)
WT vs T0: not DE   MU vs T0: not DE
Expression level
1000
2000
3000
4000
0
6
12
24
Time (h)
colour
MU
WT

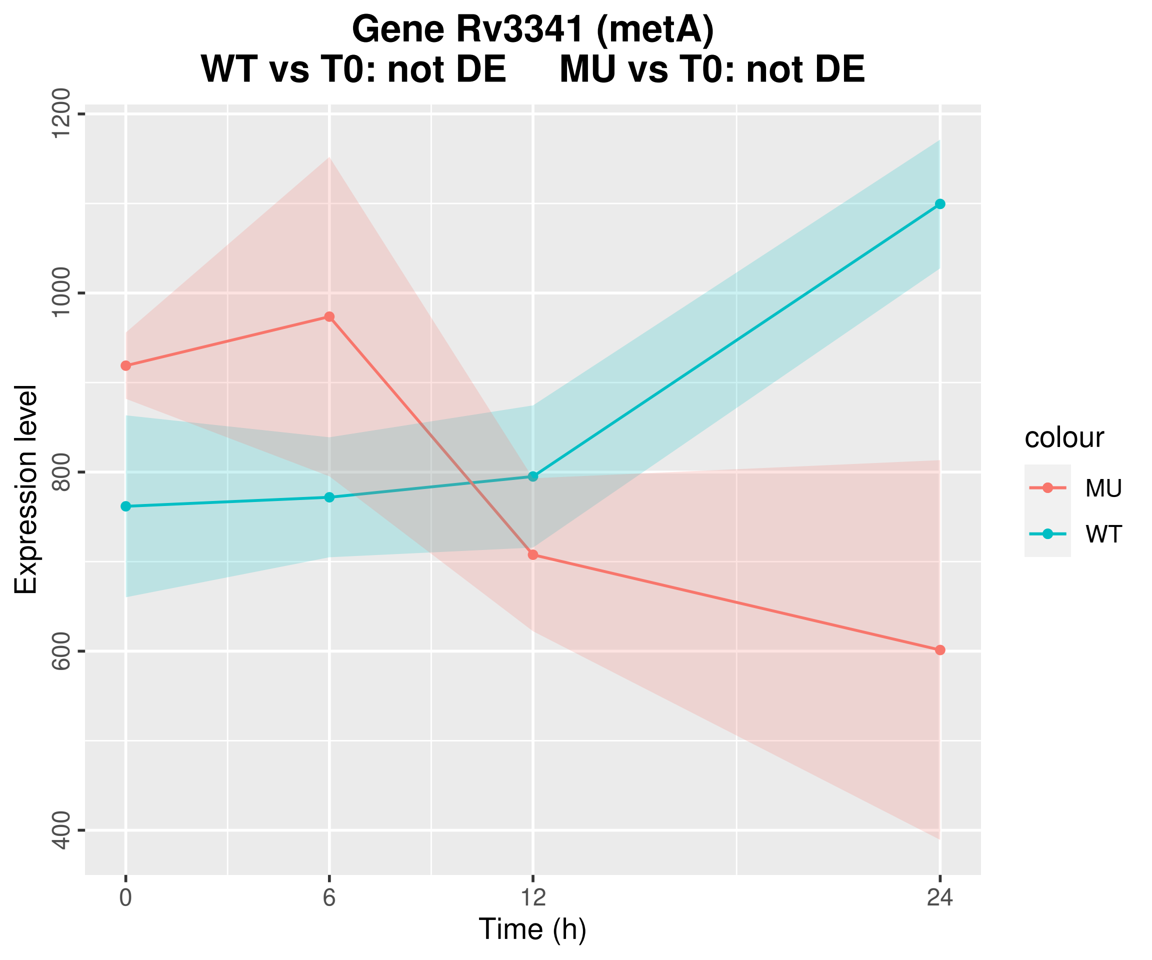
Gene Rv3341 (metA)
WT vs T0: not DE MU vs T0: not DE
Expression level
1200
1000
800
600
400
0
6
12
24
Time (h)
colour
MU
WT

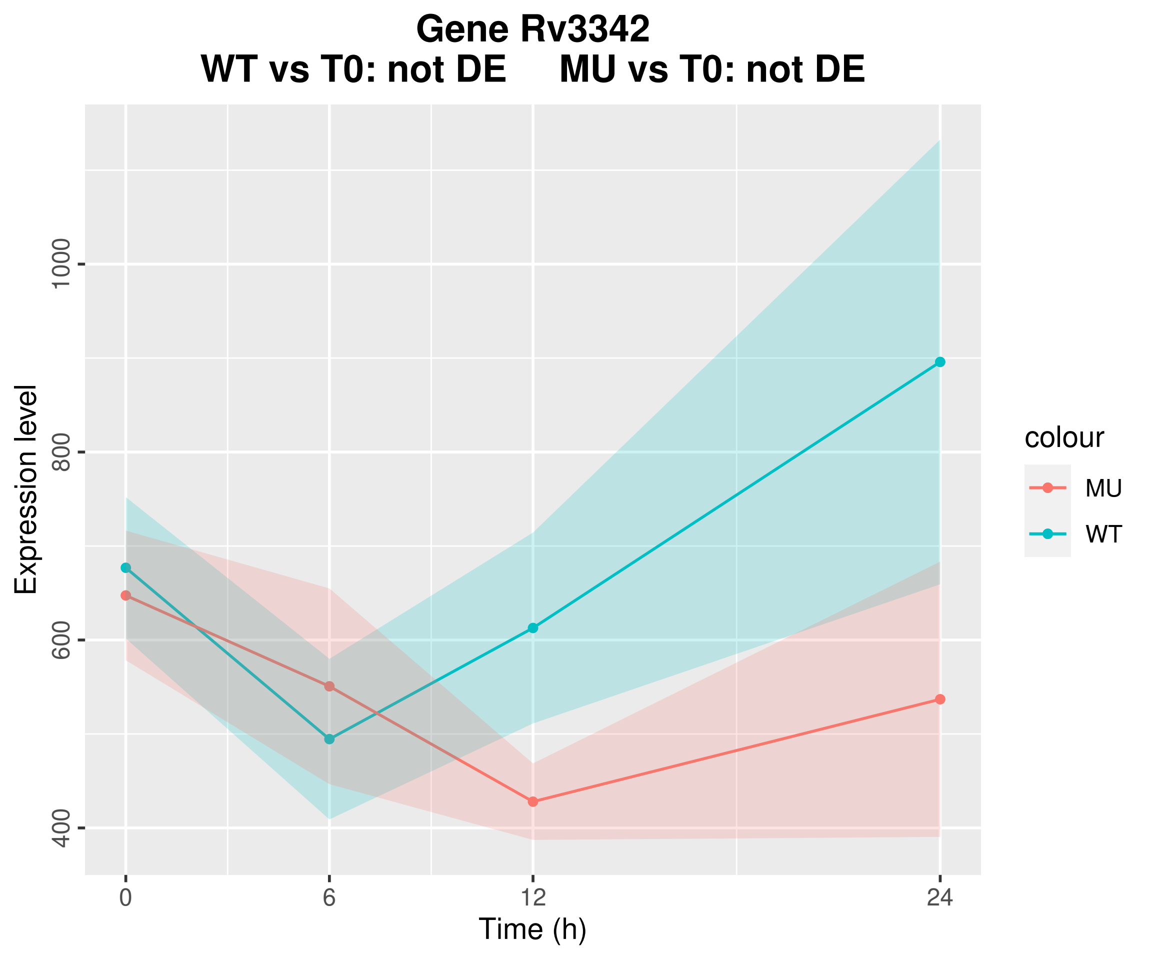
Gene Rv3342
WT vs T0: not DE MU vs T0: not DE
Expression level
400
600
800
1000
0
6
12
24
Time (h)
colour
MU
WT

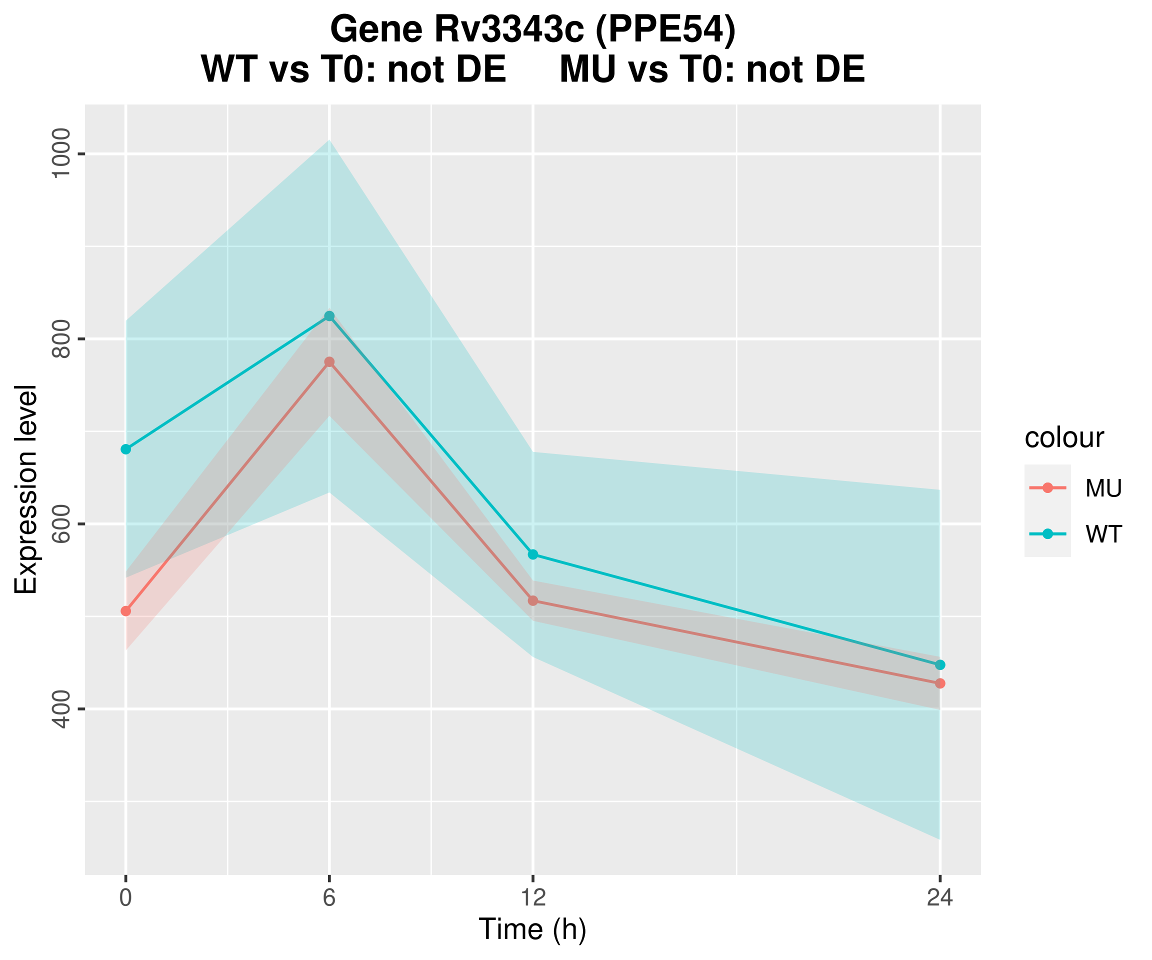
Gene Rv3343c (PPE54)
WT vs T0: not DE   MU vs T0: not DE
1000
800
600
400
Expression level
0
6
12
24
Time (h)
colour
MU
WT

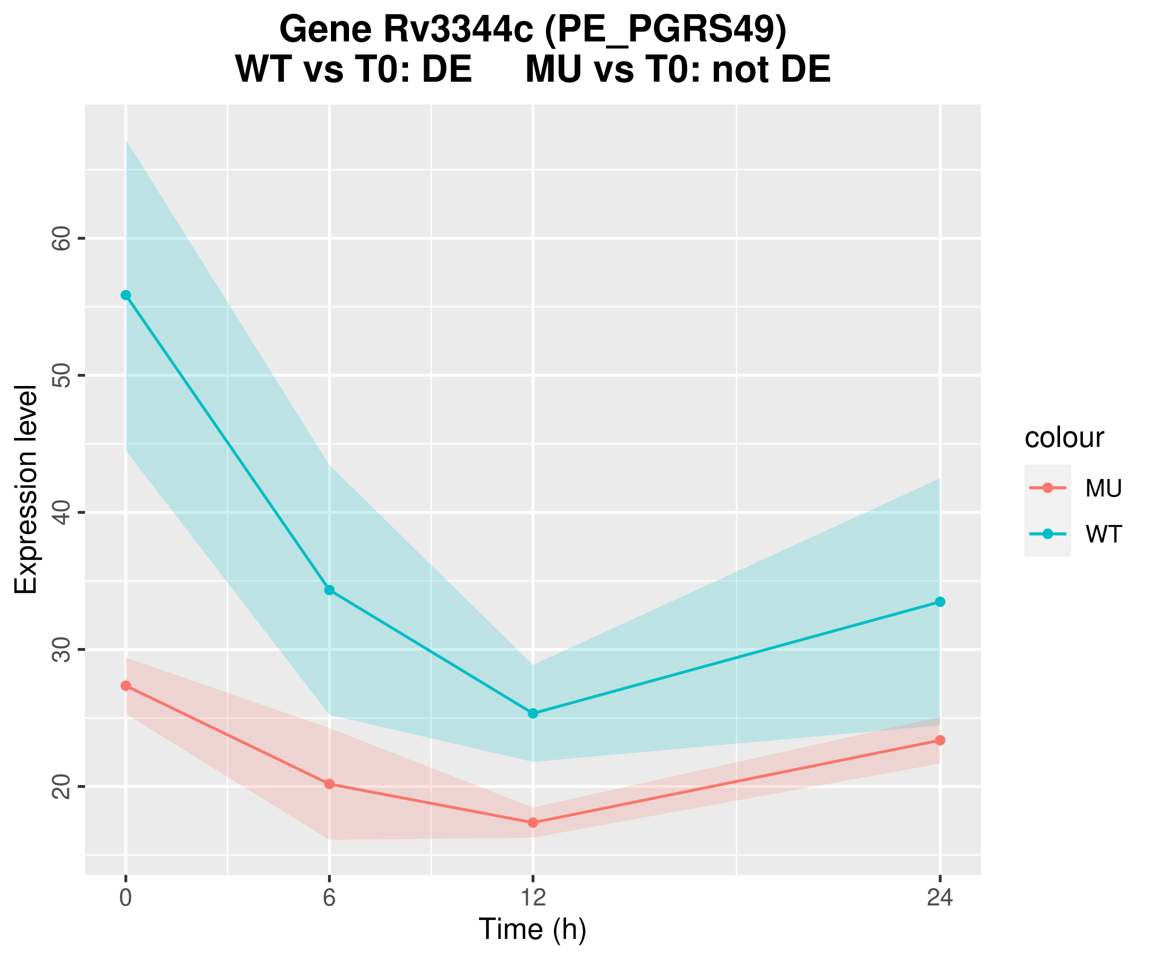
Gene Rv3344c (PE_PGRS49)
WT vs T0: DE   MU vs T0: not DE
Expression level
60
50
40
30
20
0
6
12
24
Time (h)
colour
MU
WT

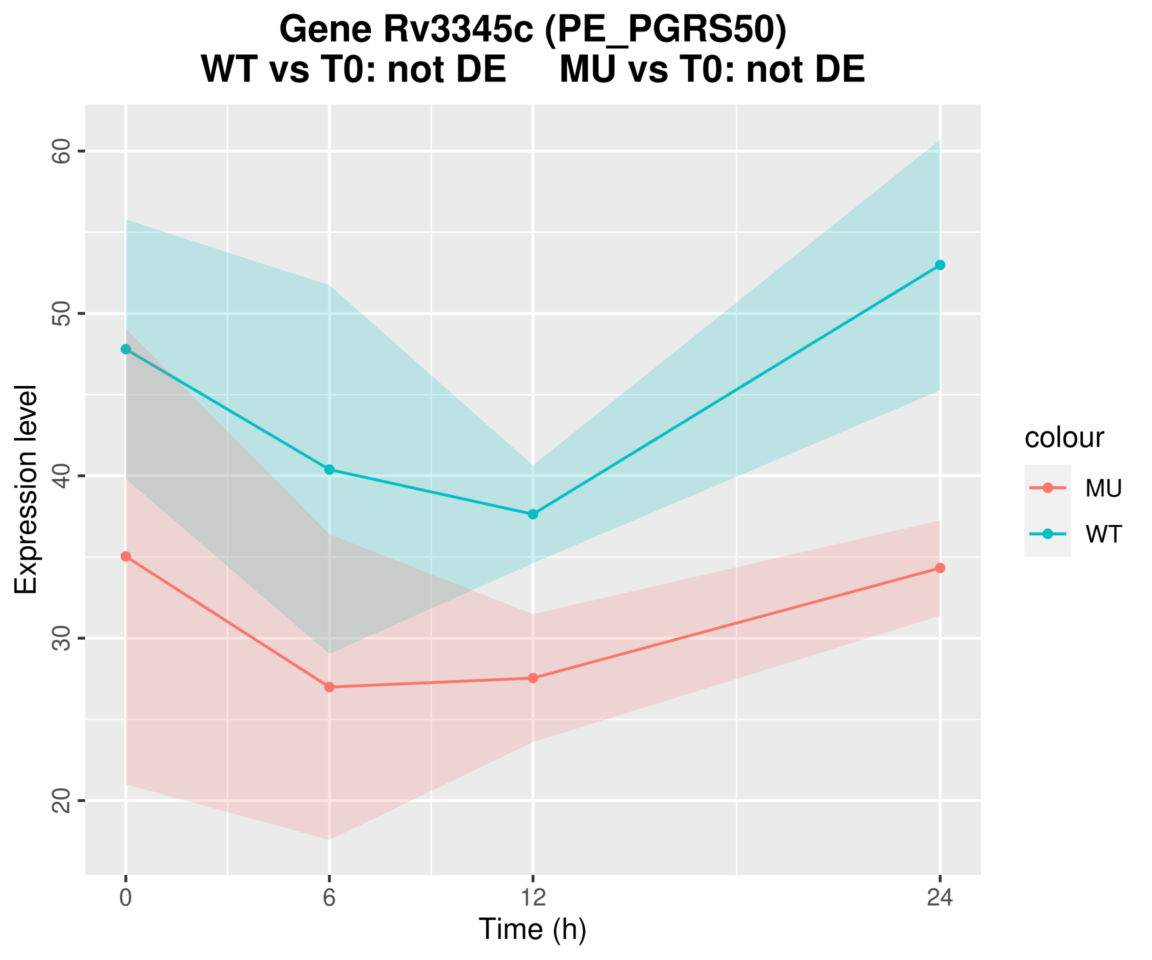
Gene Rv3345c (PE_PGRS50)
WT vs T0: not DE MU vs T0: not DE
Expression level
60
50
40
30
20
0
6
12
24
Time (h)
colour
MU
WT

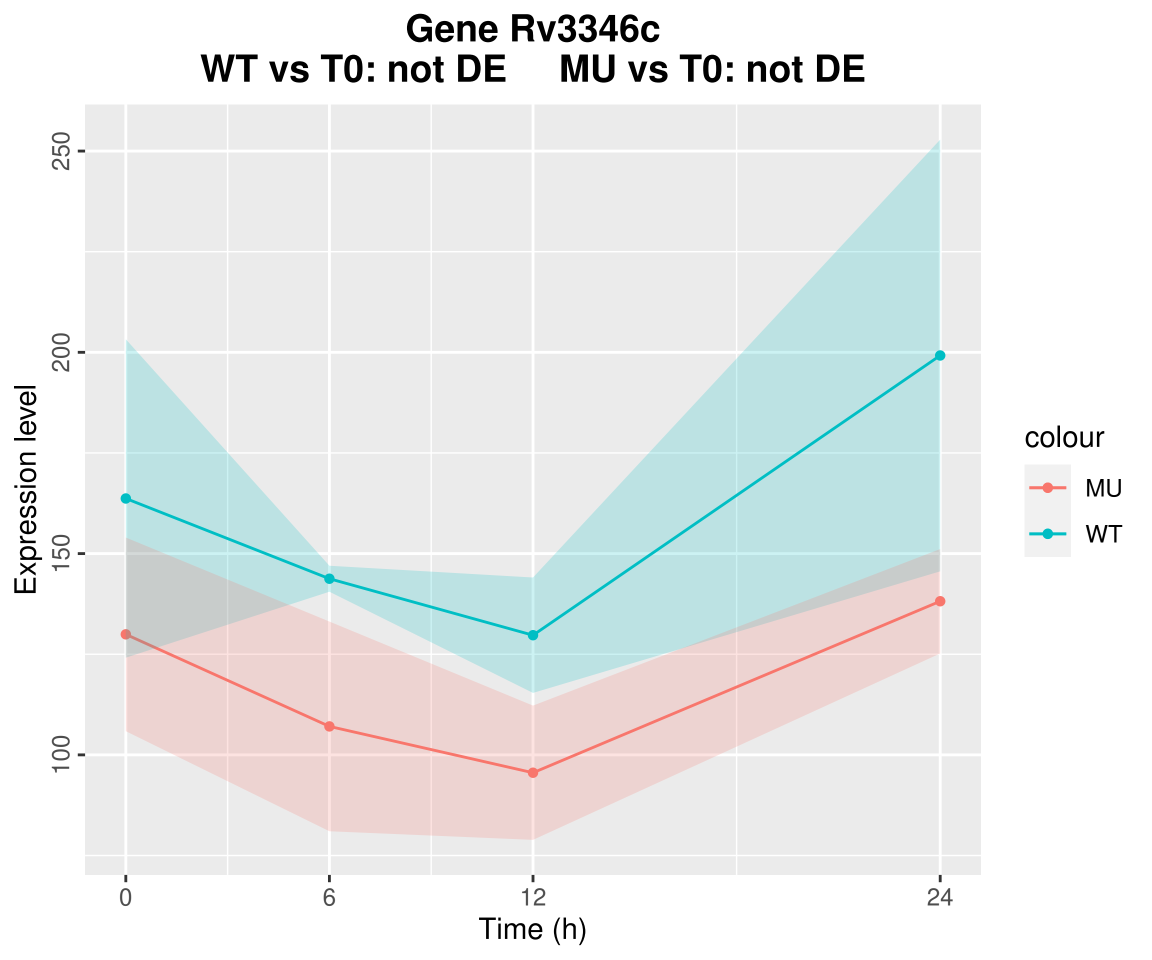
Gene Rv3346c
WT vs T0: not DE MU vs T0: not DE
Expression level
250
200
150
100
0
6
12
24
Time (h)
colour
MU
WT

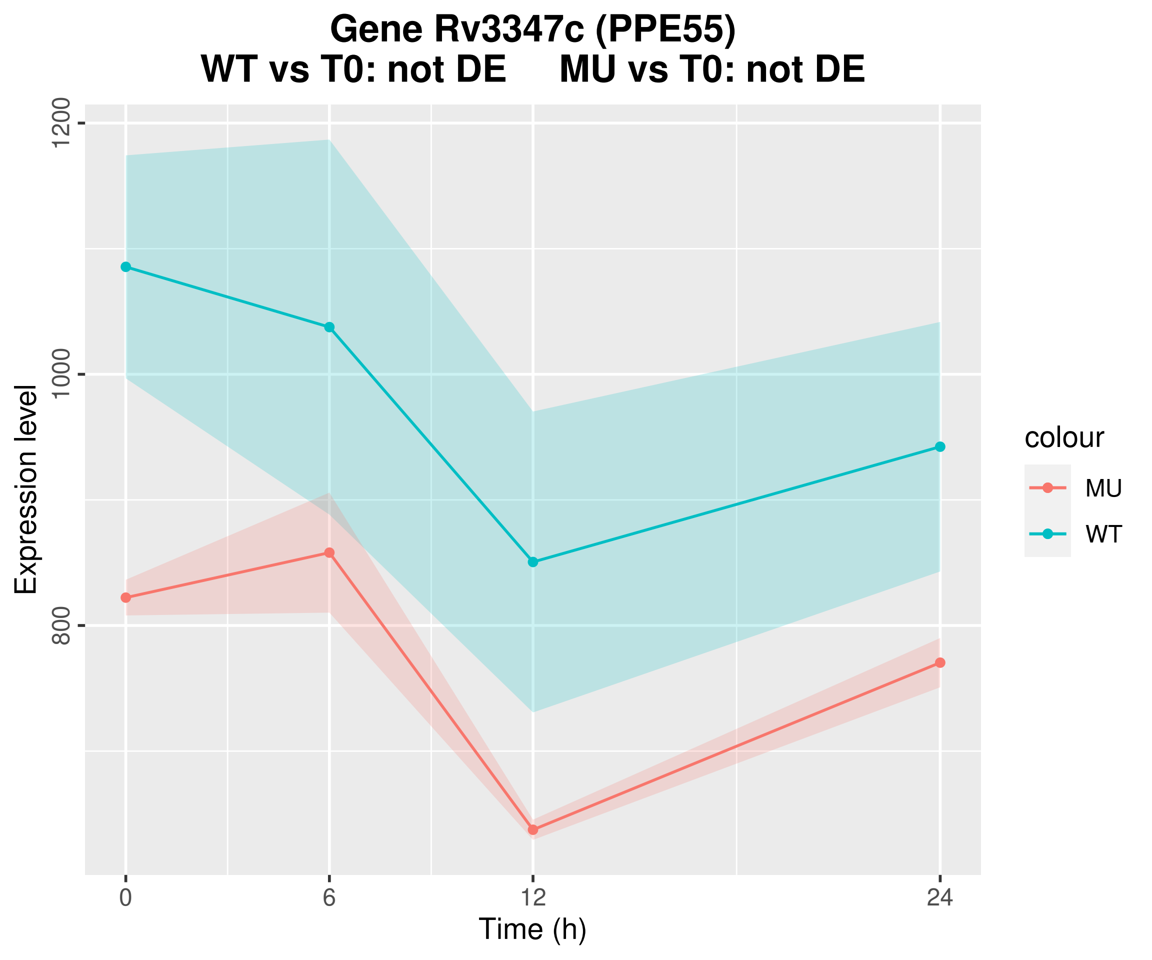

Gene Rv3347c (PPE55)
WT vs T0: not DE   MU vs T0: not DE
1200
1000
800
Expression level
0
6
12
24
Time (h)
colour
MU
WT

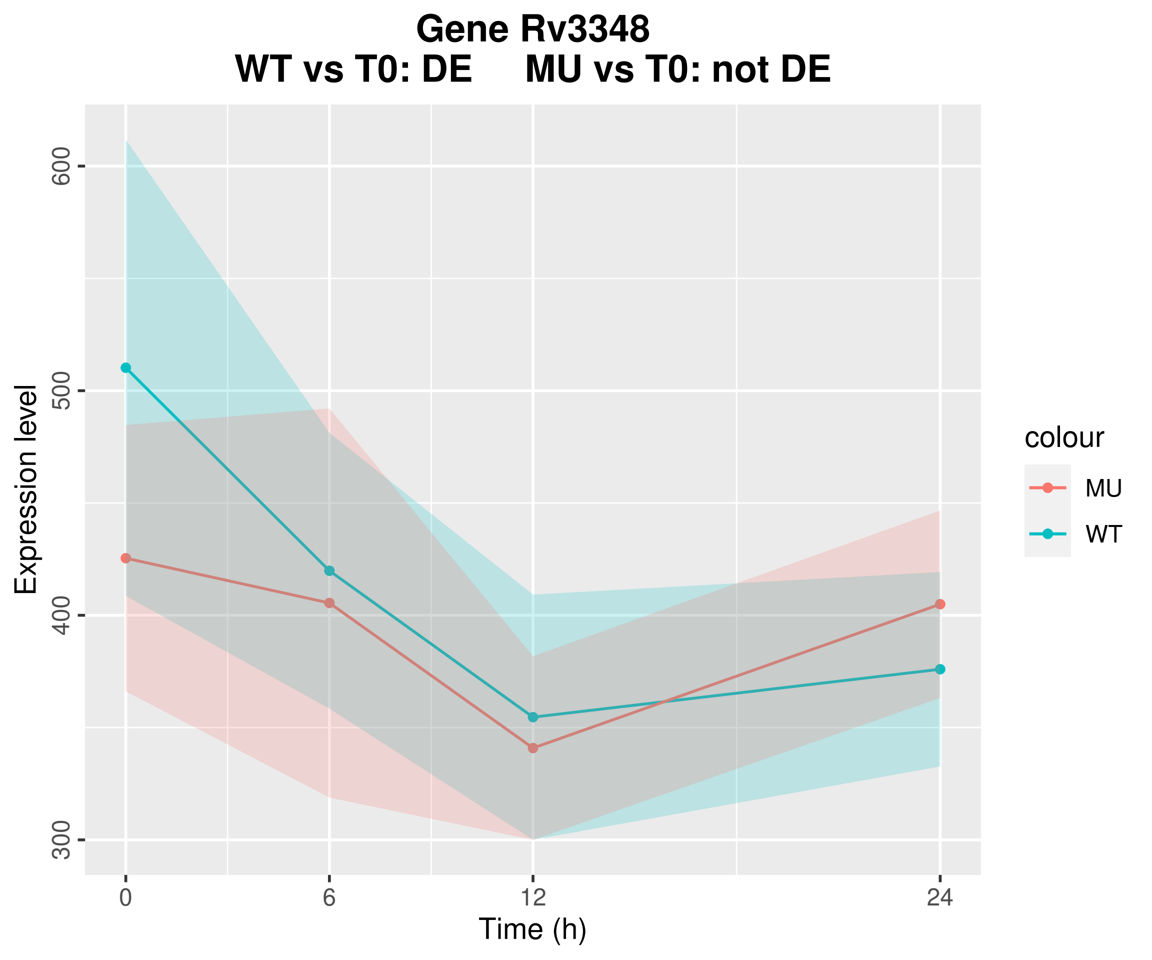
Gene Rv3348
WT vs T0: DE   MU vs T0: not DE
600
500
400
300
Expression level
0
6
12
24
Time (h)
colour
MU
WT

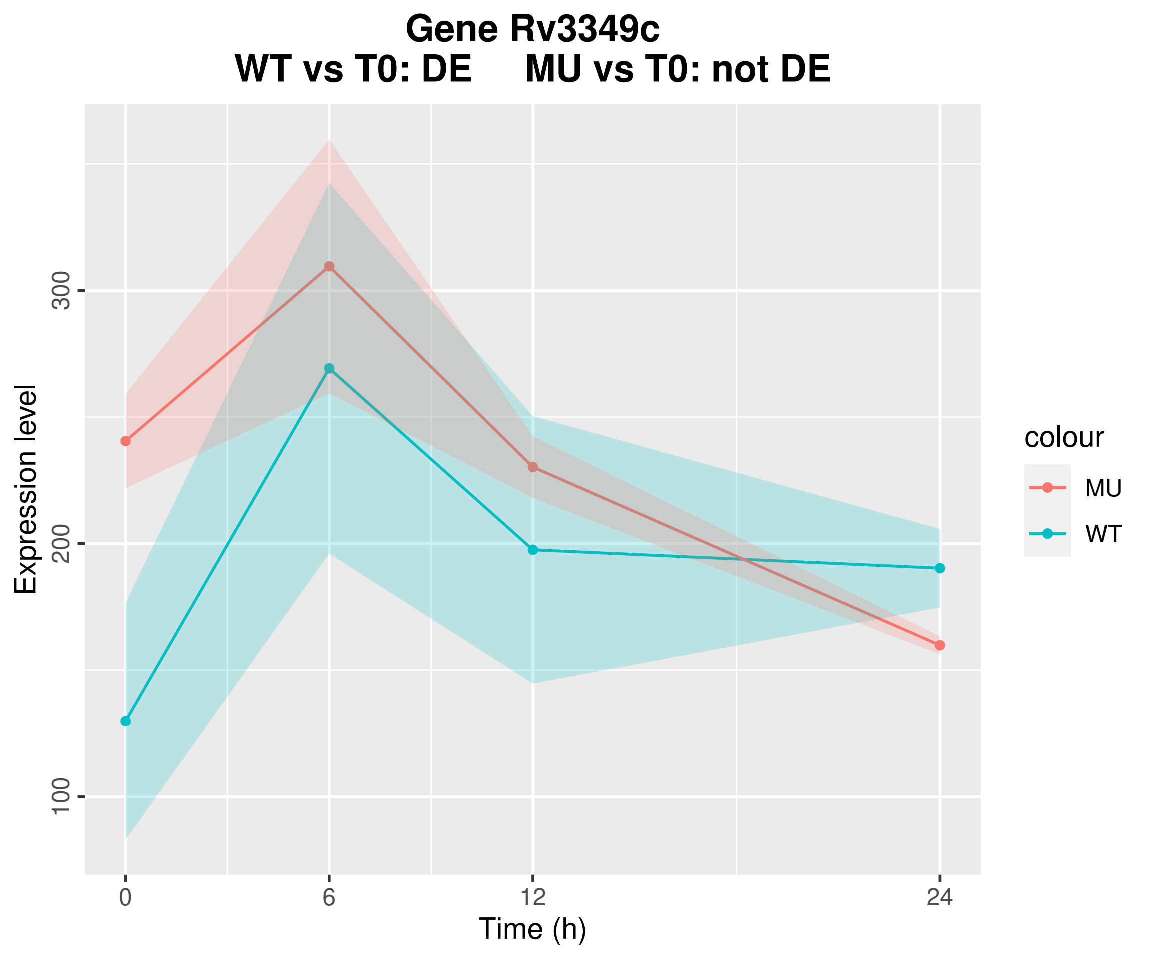
Gene Rv3349c
WT vs T0: DE MU vs T0: not DE
Expression level
300
200
100
0
6
12
24
Time (h)
colour
MU
WT

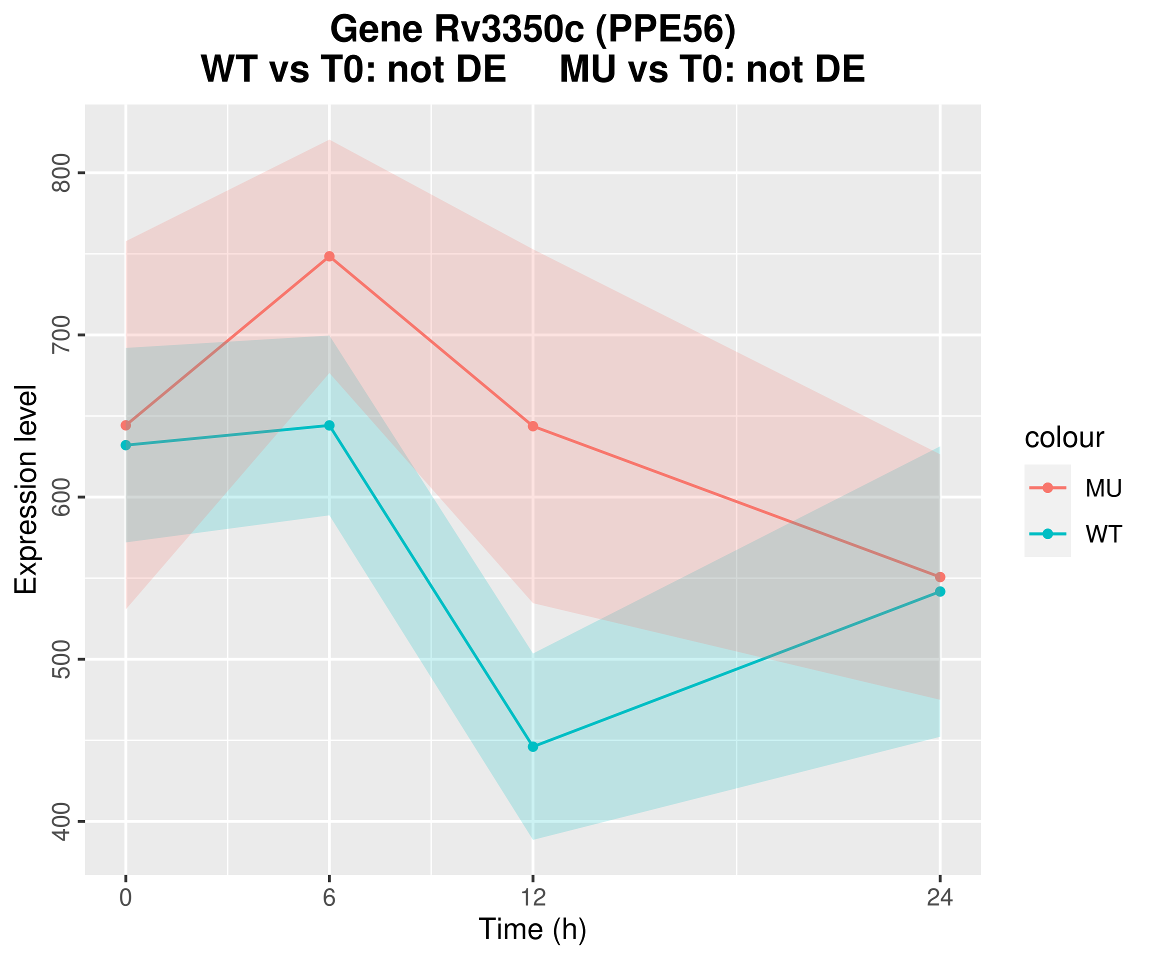
Gene Rv3350c (PPE56)
WT vs T0: not DE MU vs T0: not DE
Expression level
800
700
600
500
400
0
6
12
24
Time (h)
colour
MU
WT

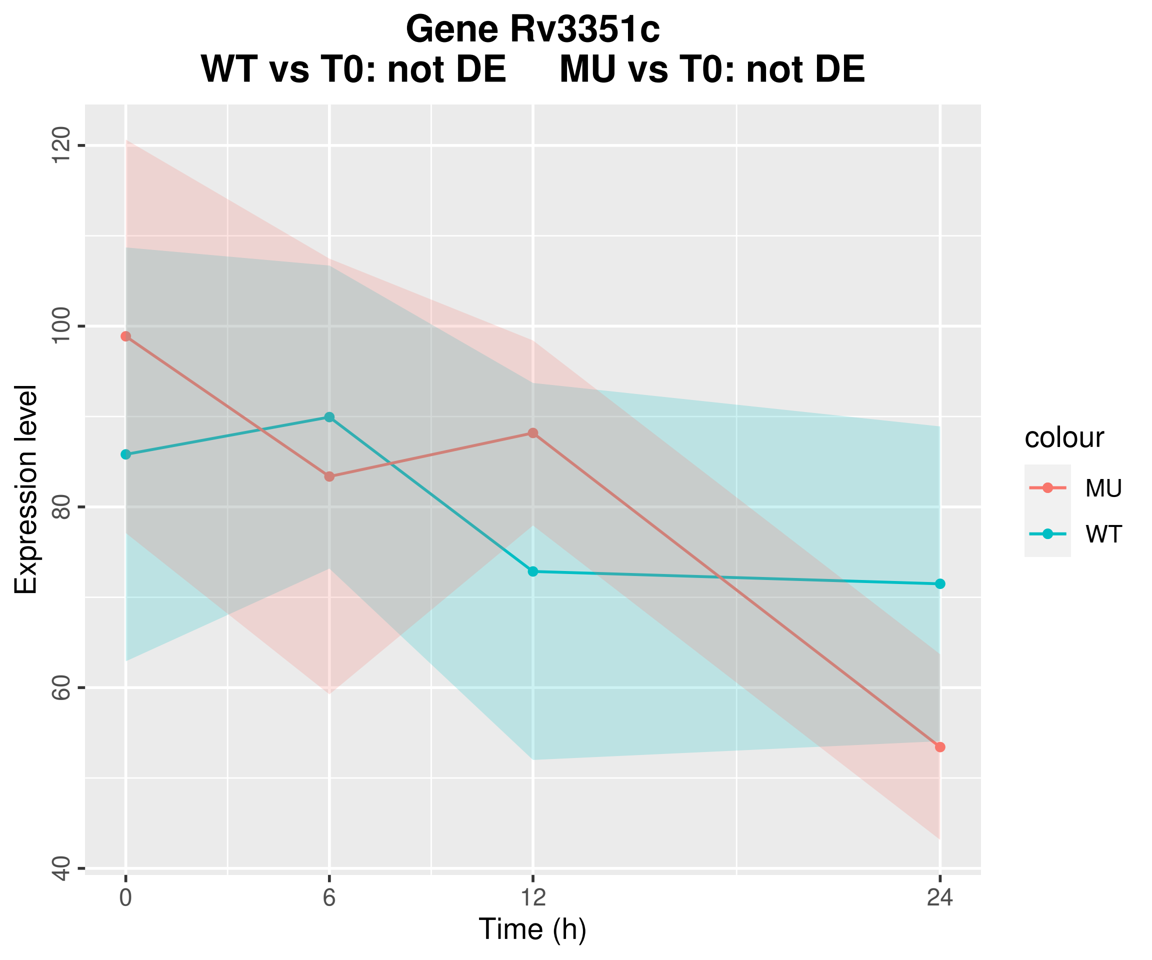

Gene Rv3351c
WT vs T0: not DE MU vs T0: not DE
Expression level
Time (h)
colour
MU
WT
120
100
80
60
40
0
6
12
24

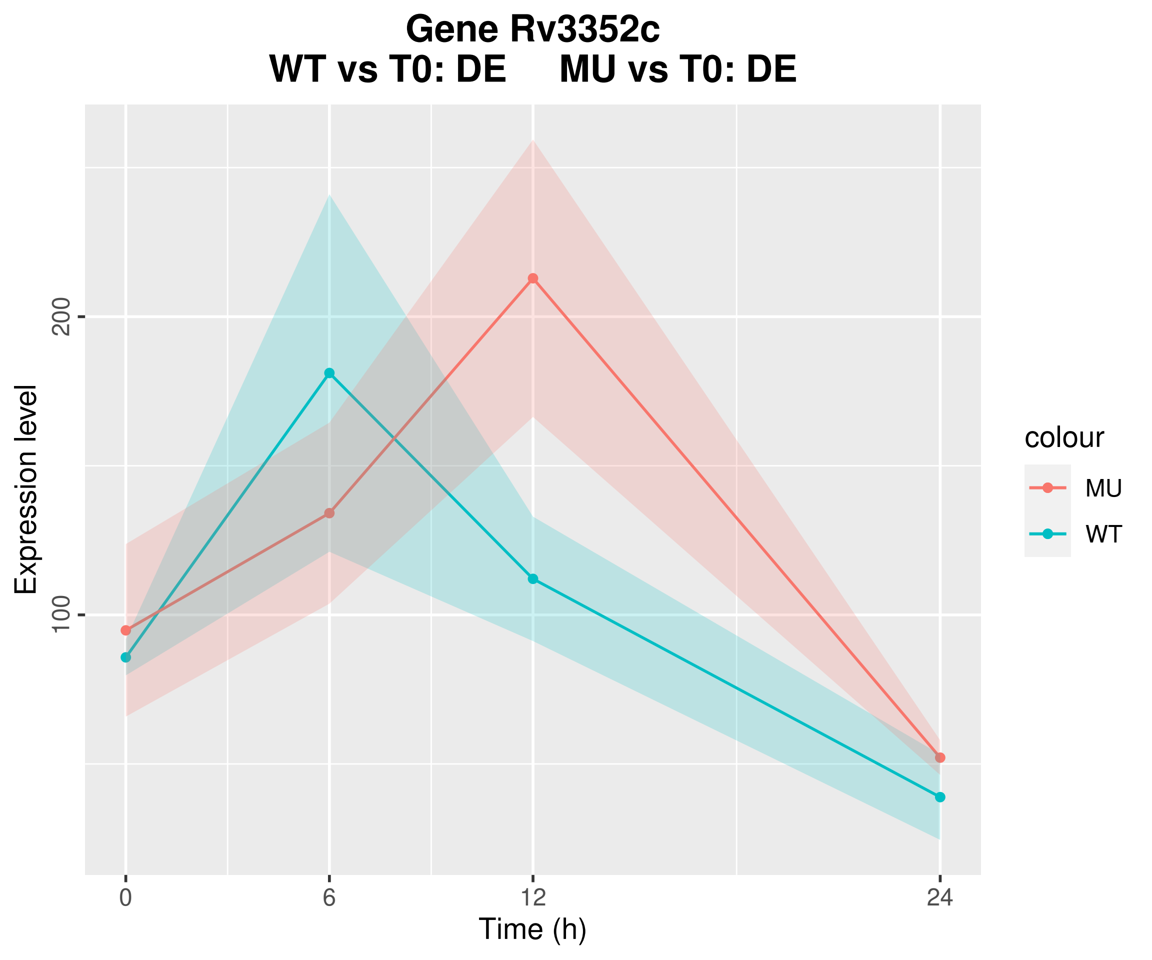
Gene Rv3352c
WT vs T0: DE   MU vs T0: DE
Expression level
200
100
0
6
12
24
Time (h)
colour
MU
WT

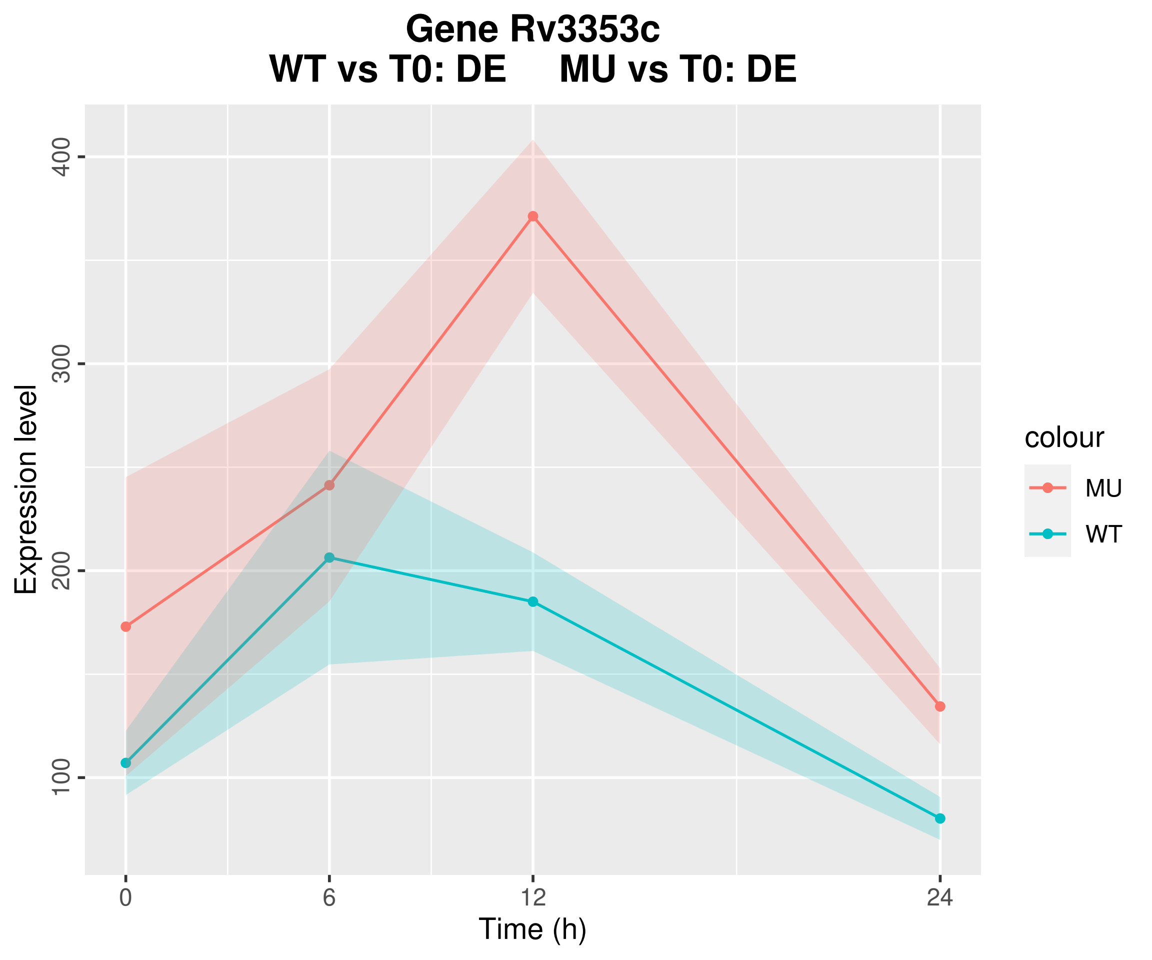
Gene Rv3353c
WT vs T0: DE   MU vs T0: DE
Expression level
400
300
200
100
0
6
12
24
Time (h)
colour
MU
WT

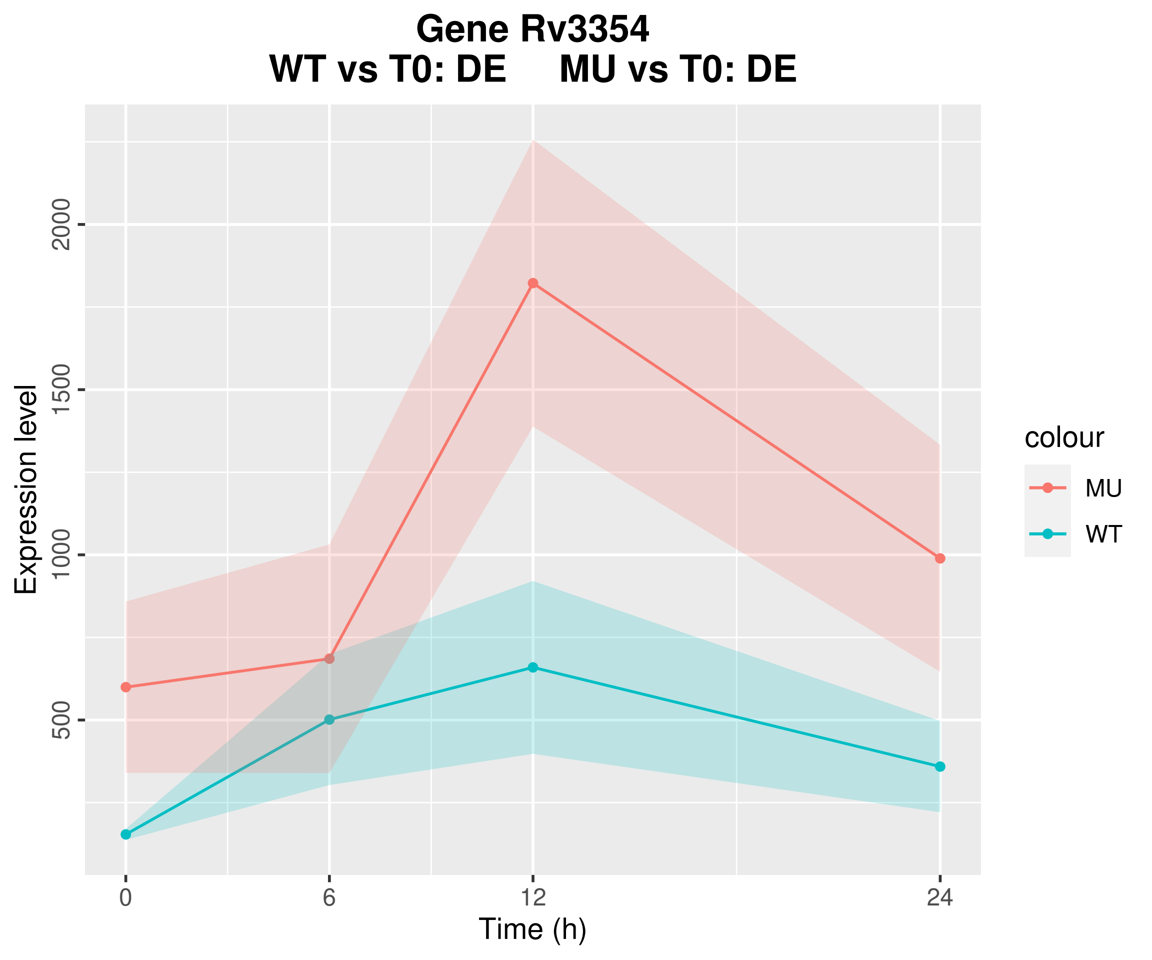
Gene Rv3354
WT vs T0: DE MU vs T0: DE
Expression level
2000
1500
1000
500
0
6
12
24
Time (h)
colour
MU
WT

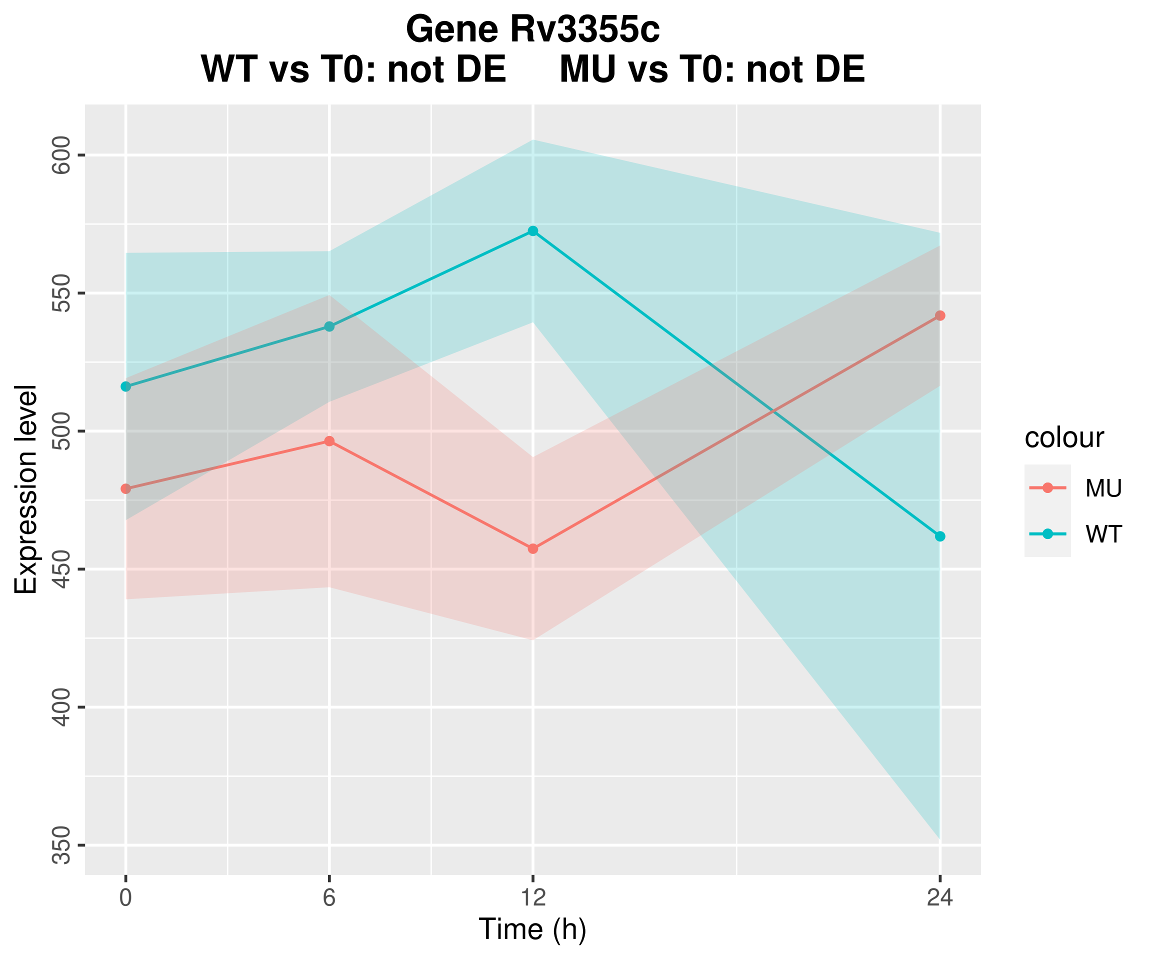

Gene Rv3355c
WT vs T0: not DE   MU vs T0: not DE
Expression level
600
550
500
450
400
350
0
6
12
24
Time (h)
colour
MU
WT

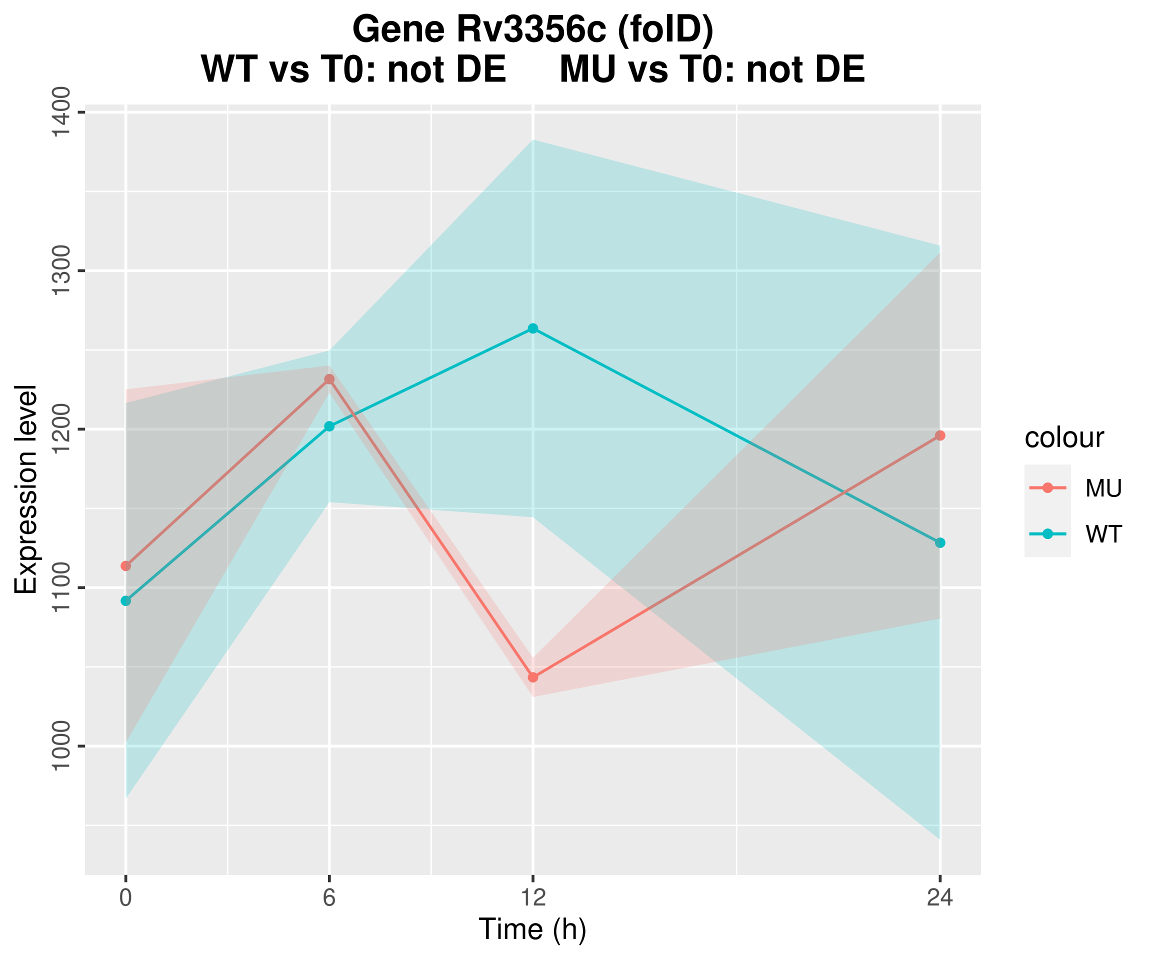
Gene Rv3356c (folD)
WT vs T0: not DE MU vs T0: not DE
1400
1300
1200
1100
1000
Expression level
0
6
12
24
Time (h)
colour
MU
WT

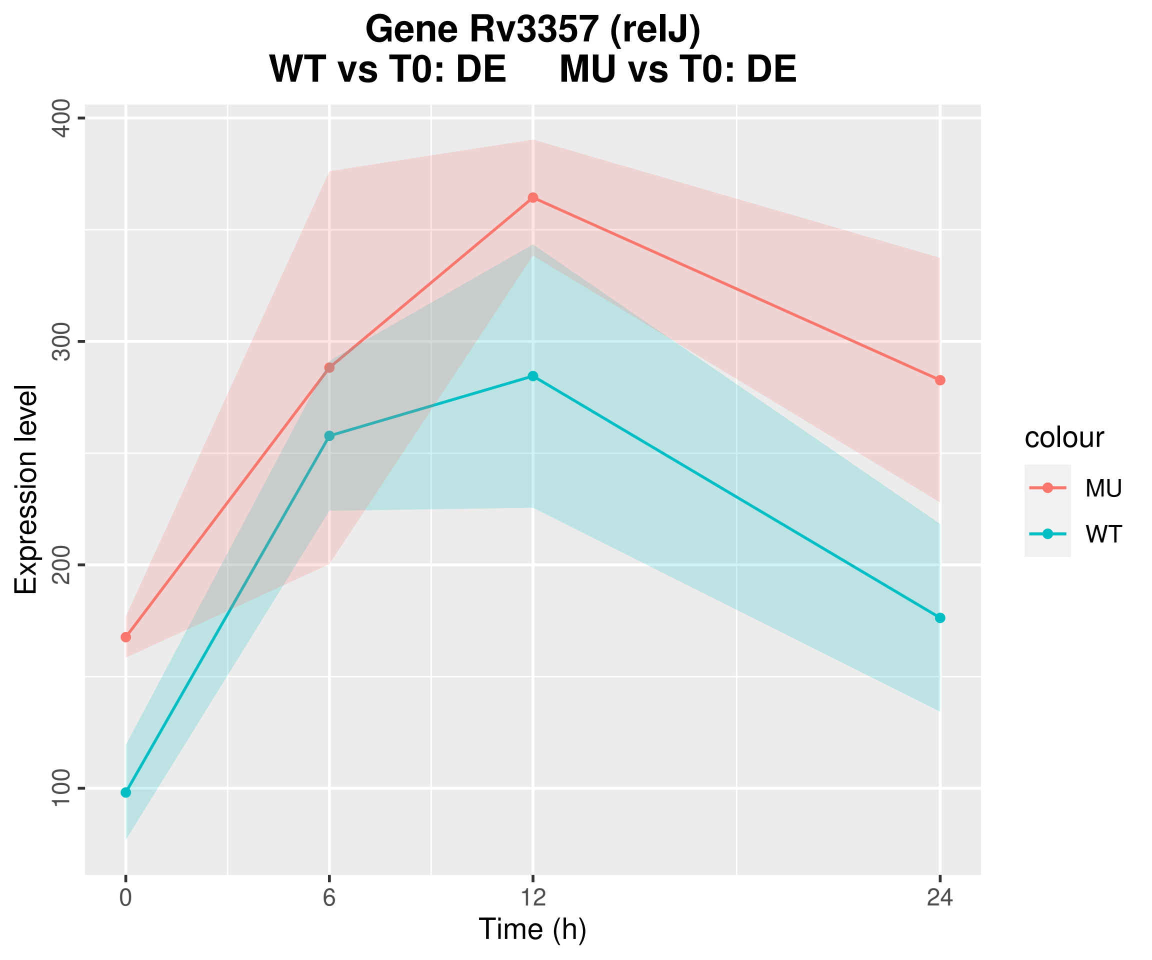

Gene Rv3357 (relJ)
WT vs T0: DE MU vs T0: DE
Expression level
400
300
200
100
0
6
12
24
Time (h)
colour
MU
WT

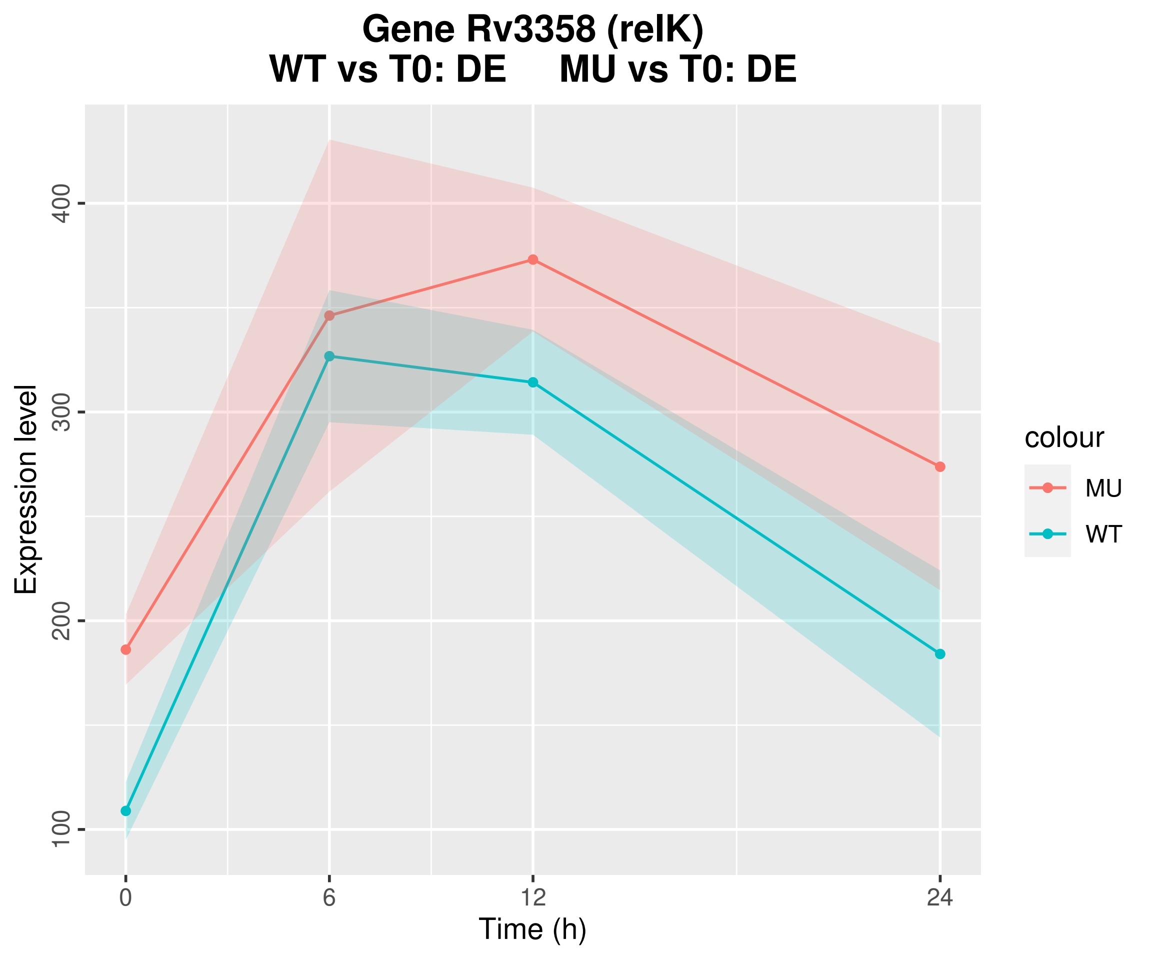

Gene Rv3358 (relK)
WT vs T0: DE MU vs T0: DE
Expression level
100
200
300
400
0
6
12
24
Time (h)
colour
MU
WT

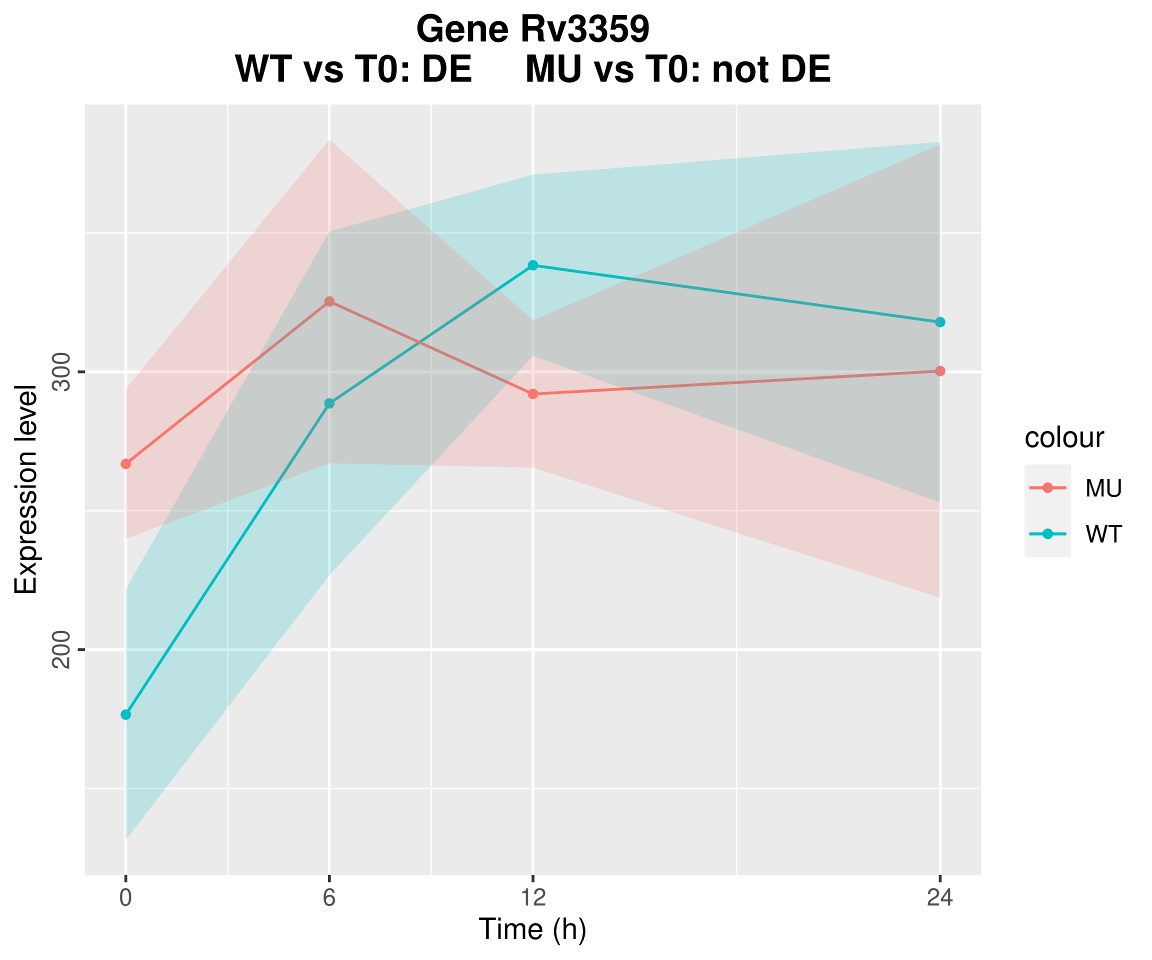

Gene Rv3359
WT vs T0: DE   MU vs T0: not DE
Expression level
300
200
0
6
12
24
Time (h)
colour
MU
WT

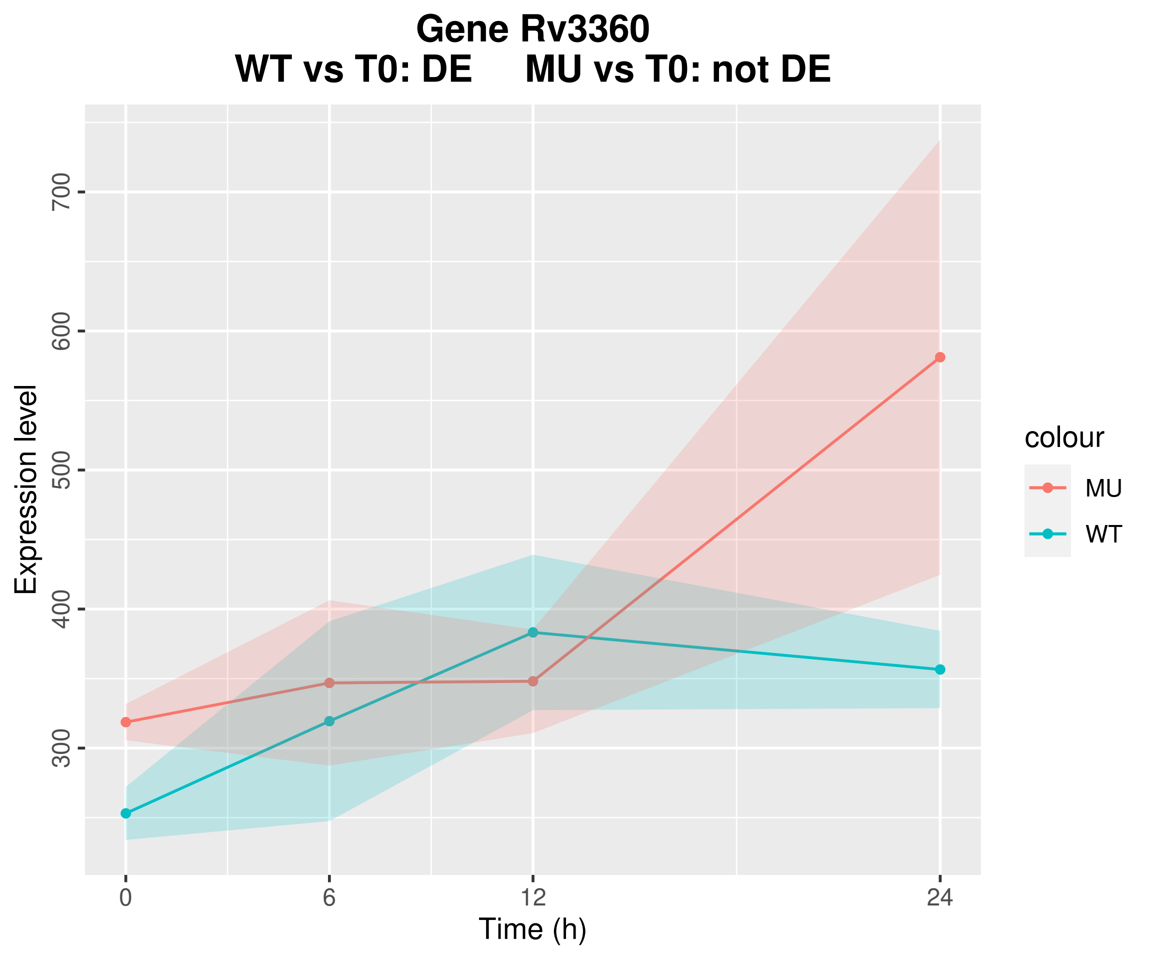

Gene Rv3360
WT vs T0: DE   MU vs T0: not DE
Expression level
700
600
500
400
300
0
6
12
24
Time (h)
colour
MU
WT

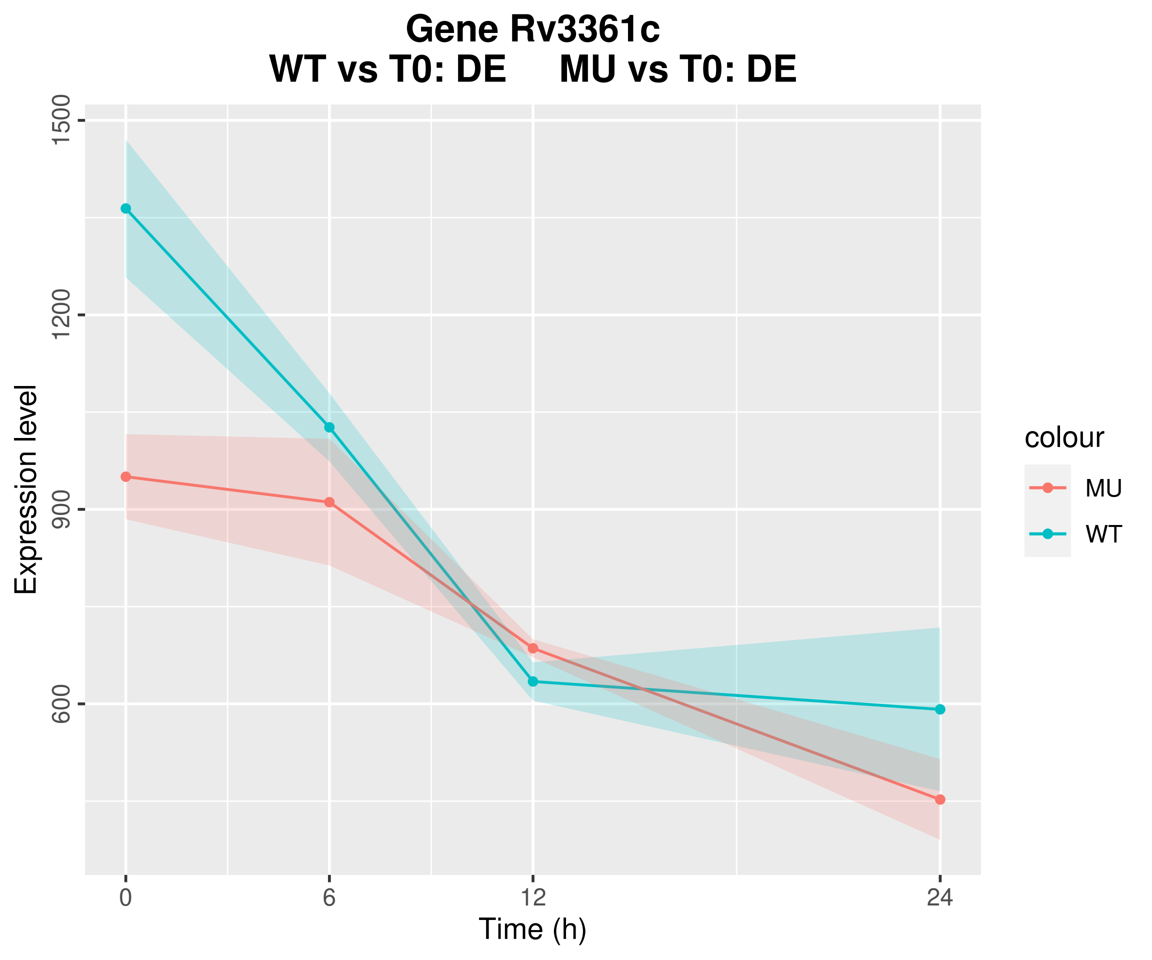

Gene Rv3361c
WT vs T0: DE   MU vs T0: DE
1500
1200
900
600
Expression level
0
6
12
24
Time (h)
colour
MU
WT

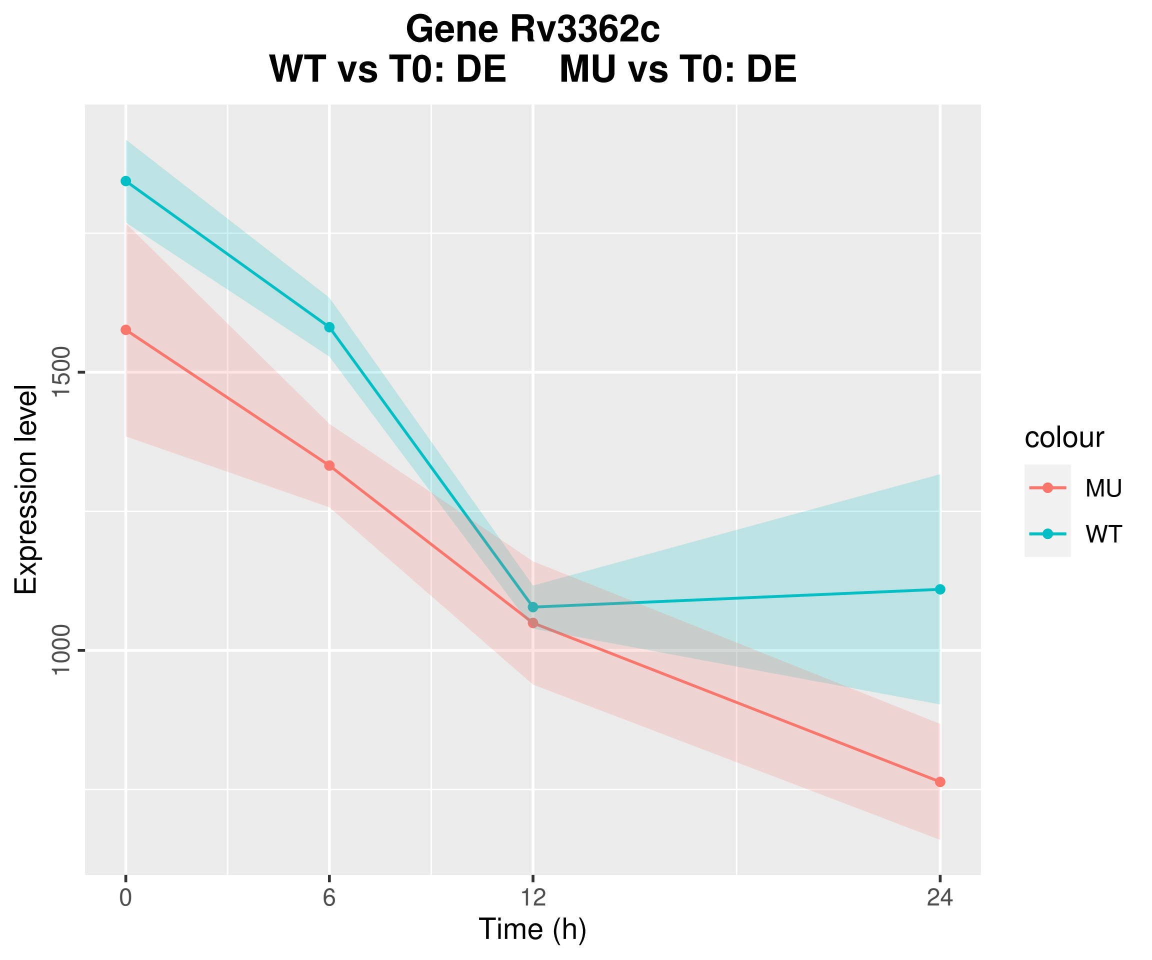

Gene Rv3362c
WT vs T0: DE   MU vs T0: DE
Expression level
1500
1000
0
6
12
24
Time (h)
colour
MU
WT

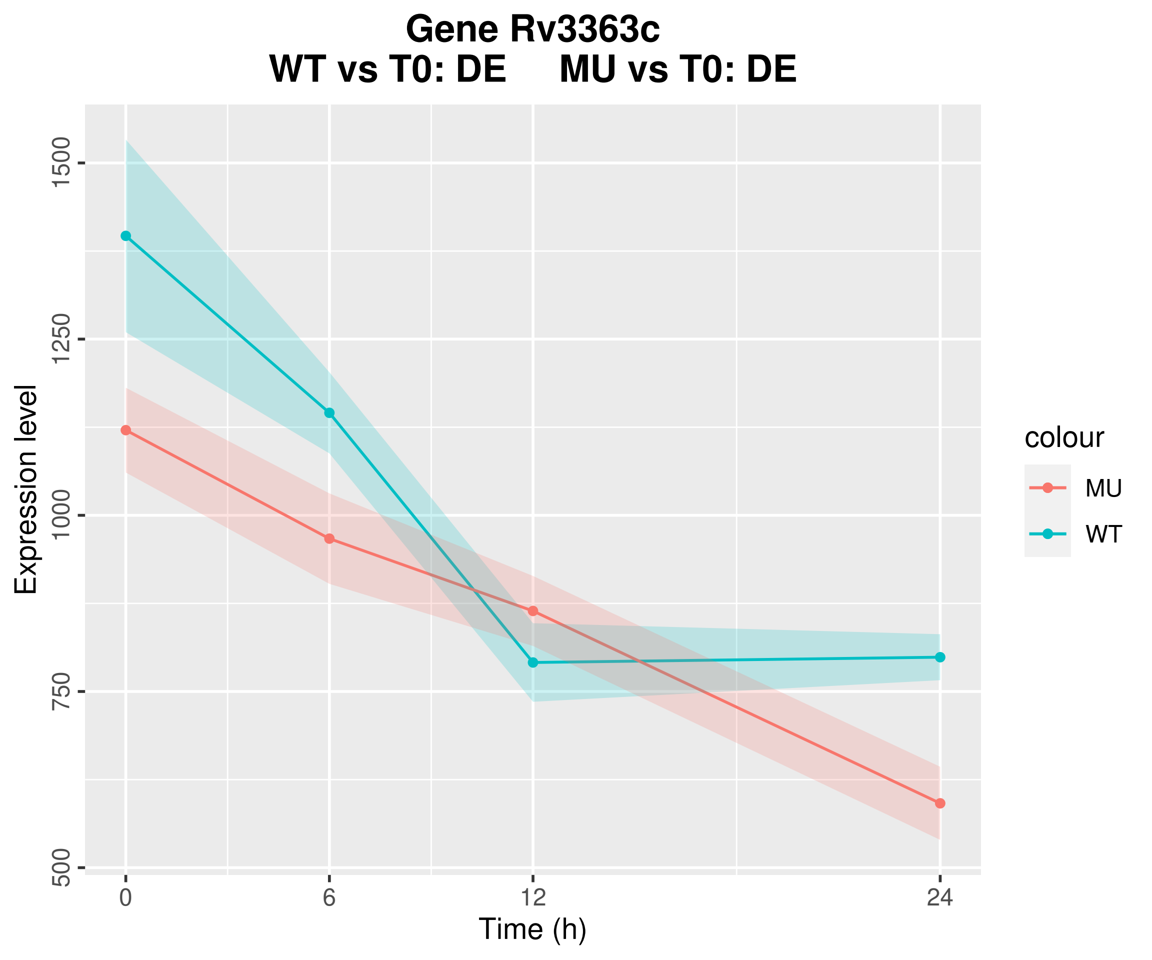
Gene Rv3363c
WT vs T0: DE MU vs T0: DE
Expression level
1500
1250
1000
750
500
0
6
12
24
Time (h)
colour
MU
WT

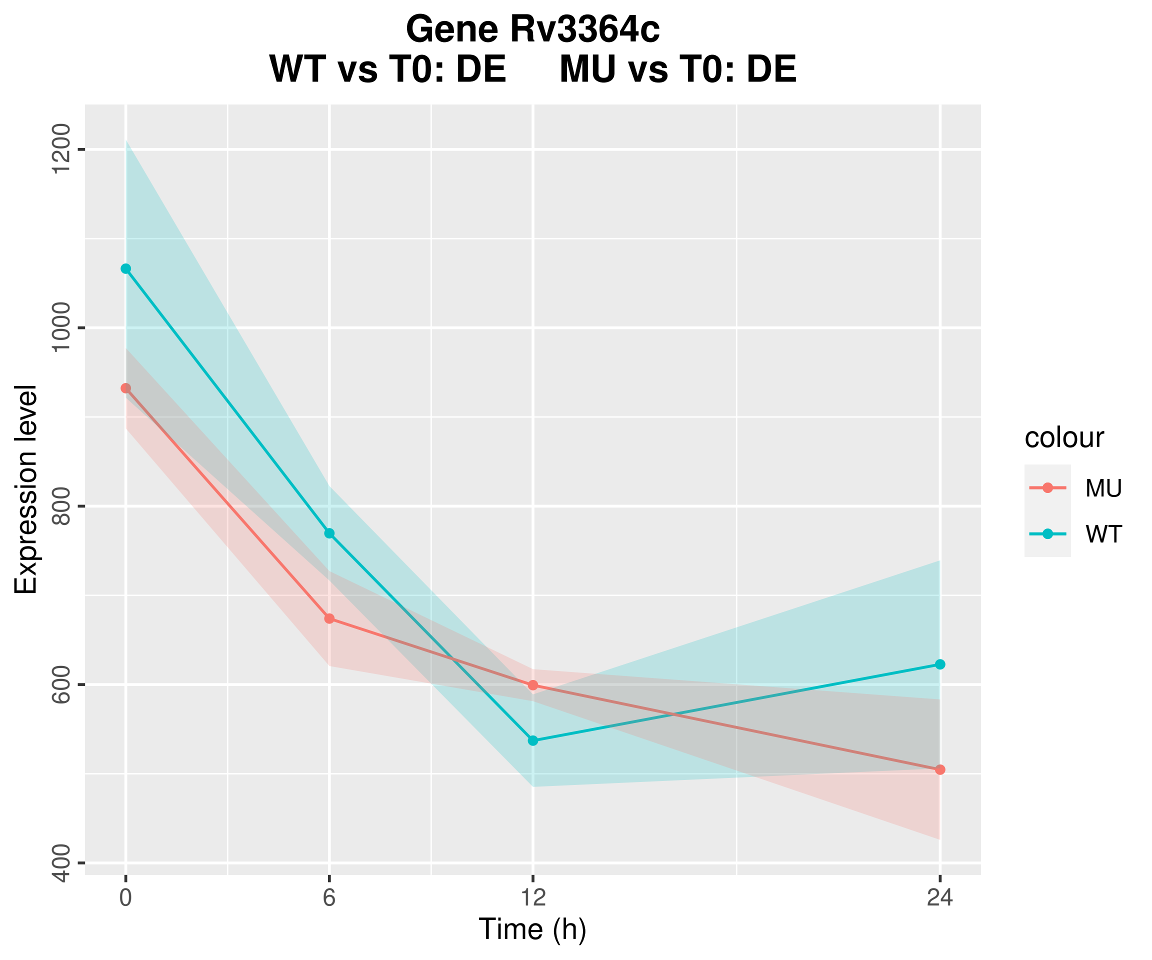

Gene Rv3364c
WT vs T0: DE   MU vs T0: DE
Expression level
1200
1000
800
600
400
0
6
12
24
Time (h)
colour
MU
WT

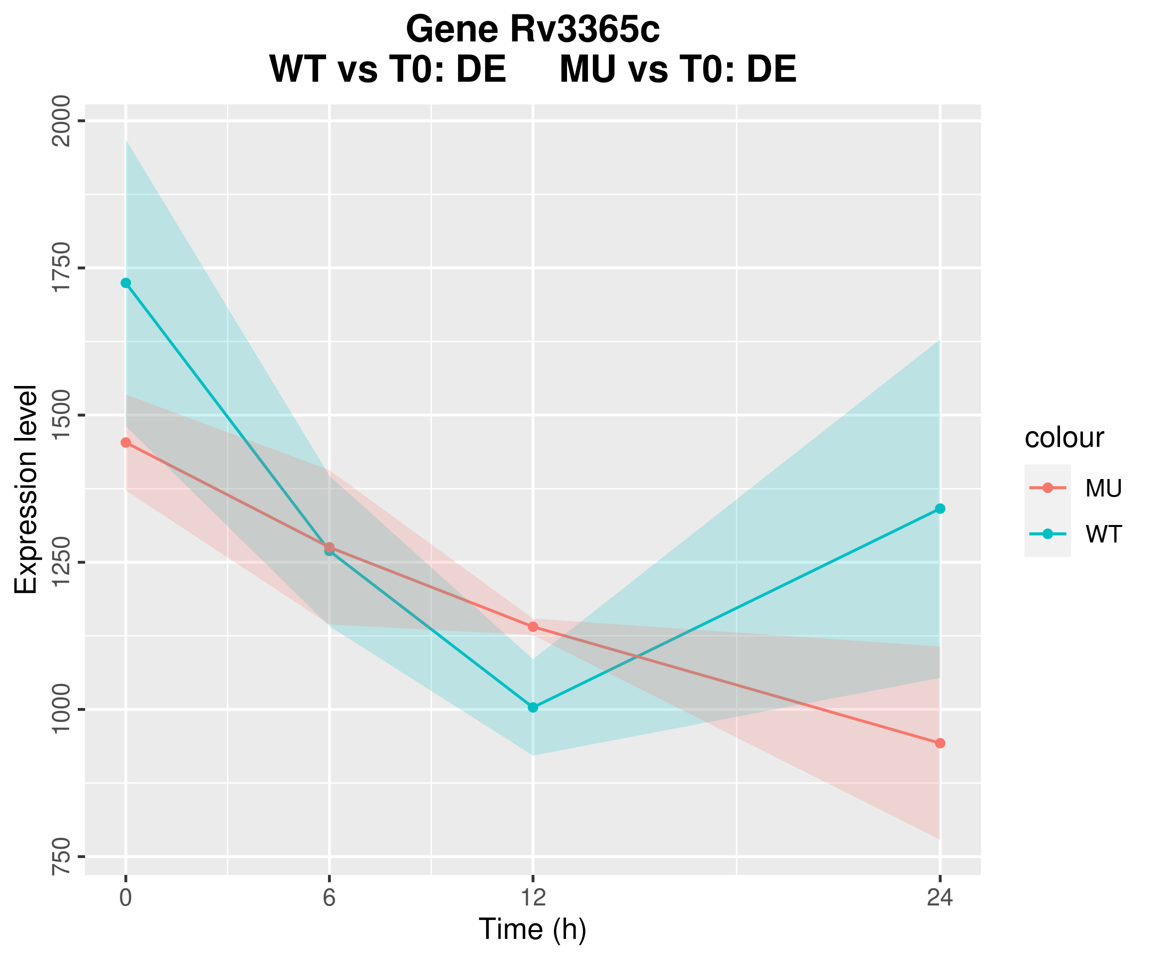

Gene Rv3365c
WT vs T0: DE   MU vs T0: DE
Expression level
2000
1750
1500
1250
1000
750
0
6
12
24
Time (h)
colour
MU
WT

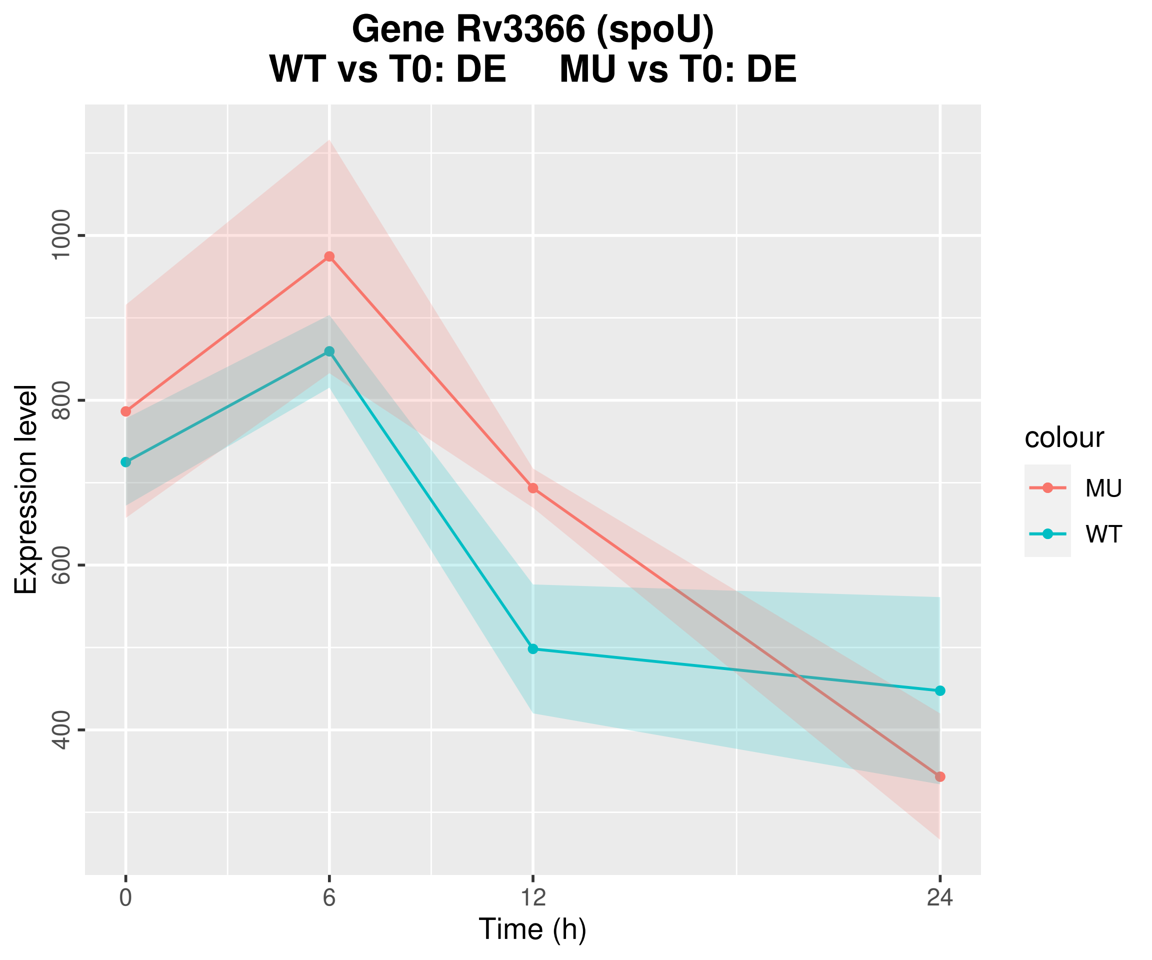
Gene Rv3366 (spoU)
WT vs T0: DE MU vs T0: DE
Expression level
1000
800
600
400
0
6
12
24
Time (h)
colour
MU
WT

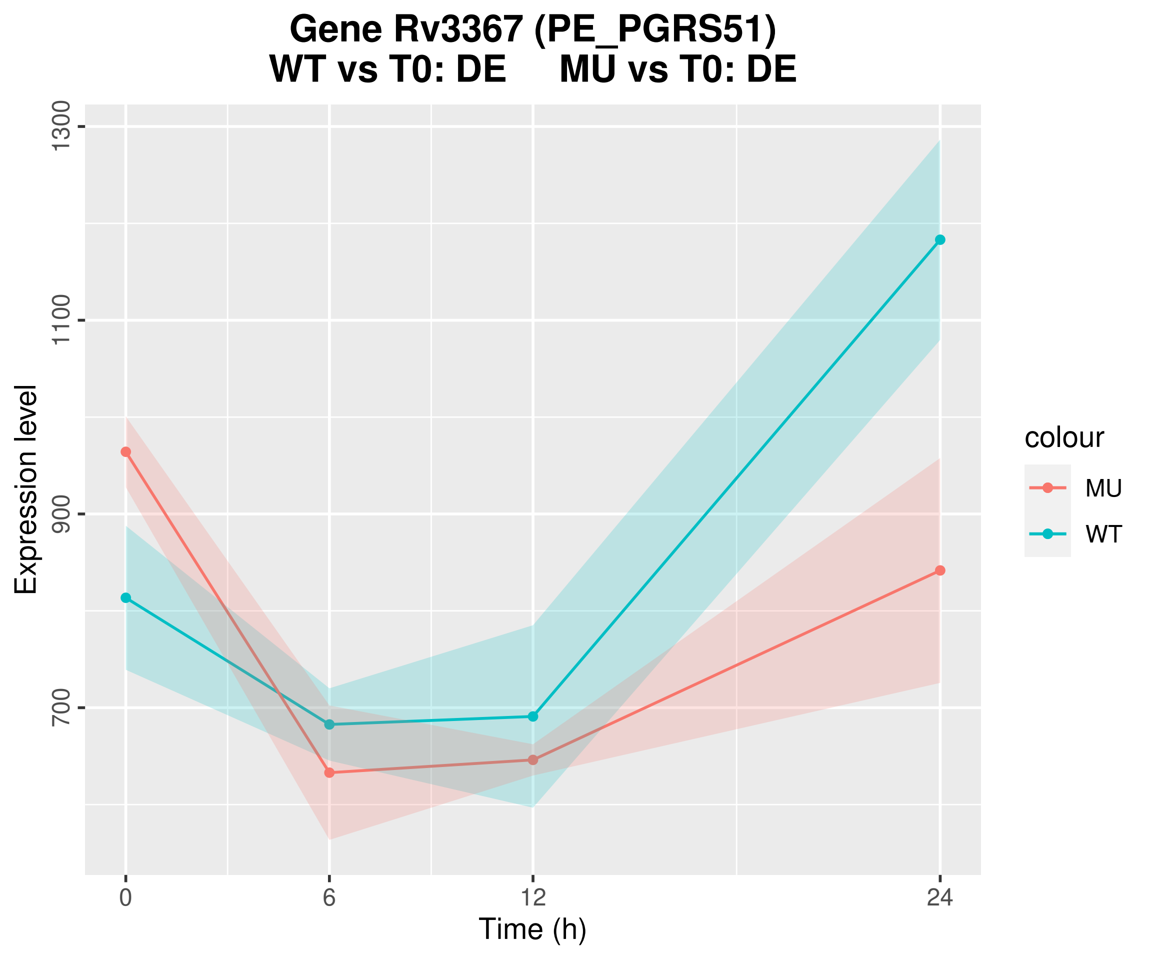
Gene Rv3367 (PE_PGRS51)
WT vs T0: DE   MU vs T0: DE
1300
1100
900
700
Expression level
0
6
12
24
Time (h)
colour
MU
WT

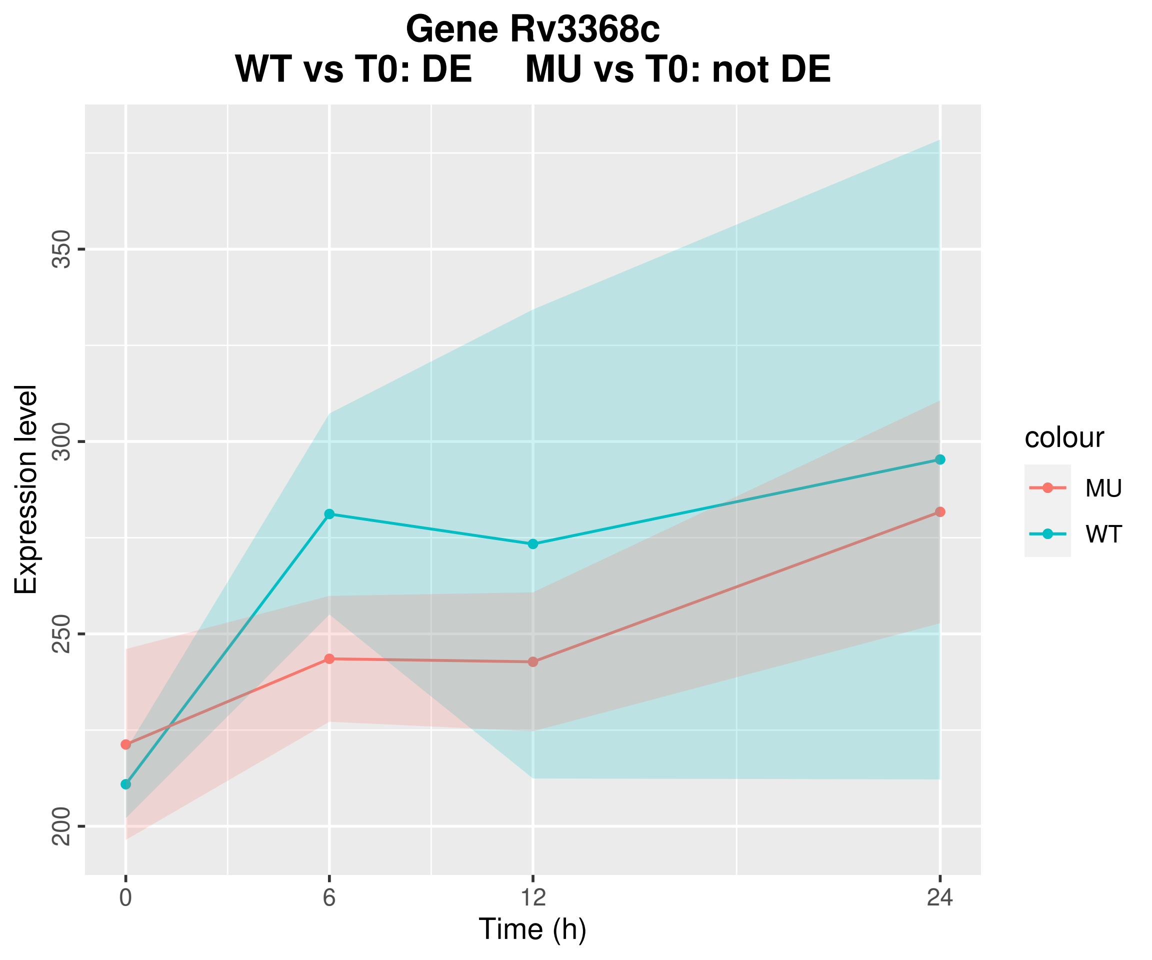
Gene Rv3368c
WT vs T0: DE MU vs T0: not DE
Expression level
200
250
300
350
0
6
12
24
Time (h)
colour
MU
WT

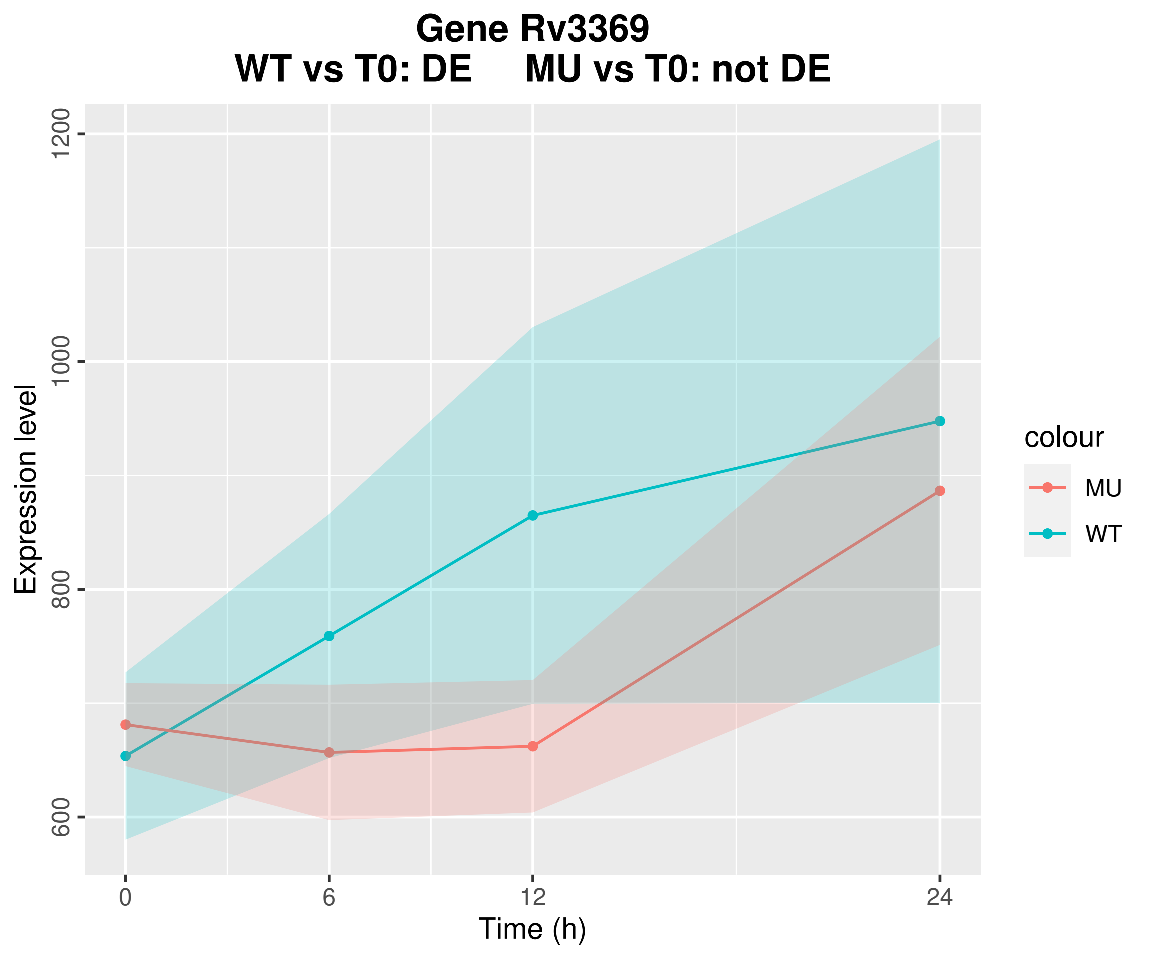
Gene Rv3369
WT vs T0: DE   MU vs T0: not DE
Expression level
1200
1000
800
600
0
6
12
24
Time (h)
colour
MU
WT

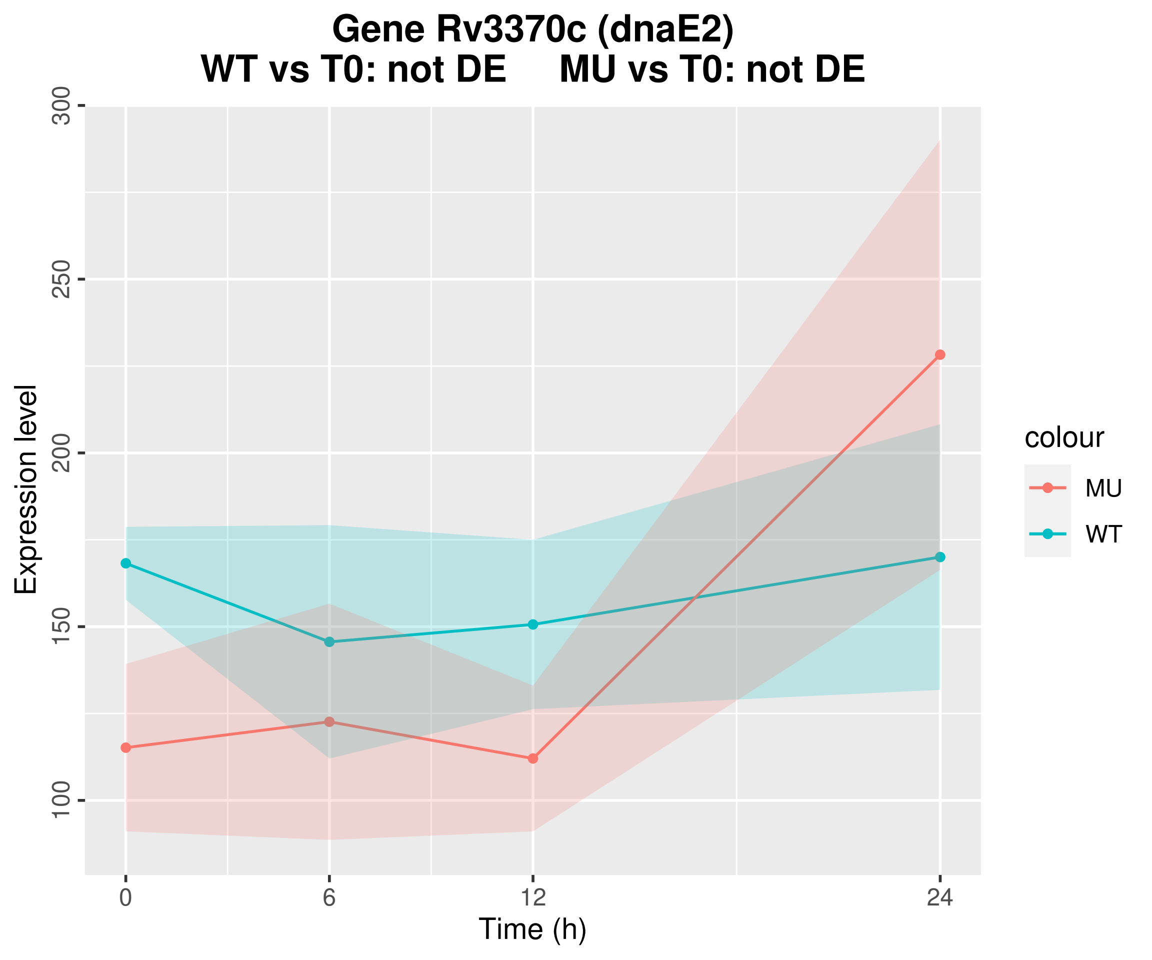

Gene Rv3370c (dnaE2)
WT vs T0: not DE   MU vs T0: not DE
Expression level
Time (h)
300
250
200
150
100
0
6
12
24
colour
MU
WT

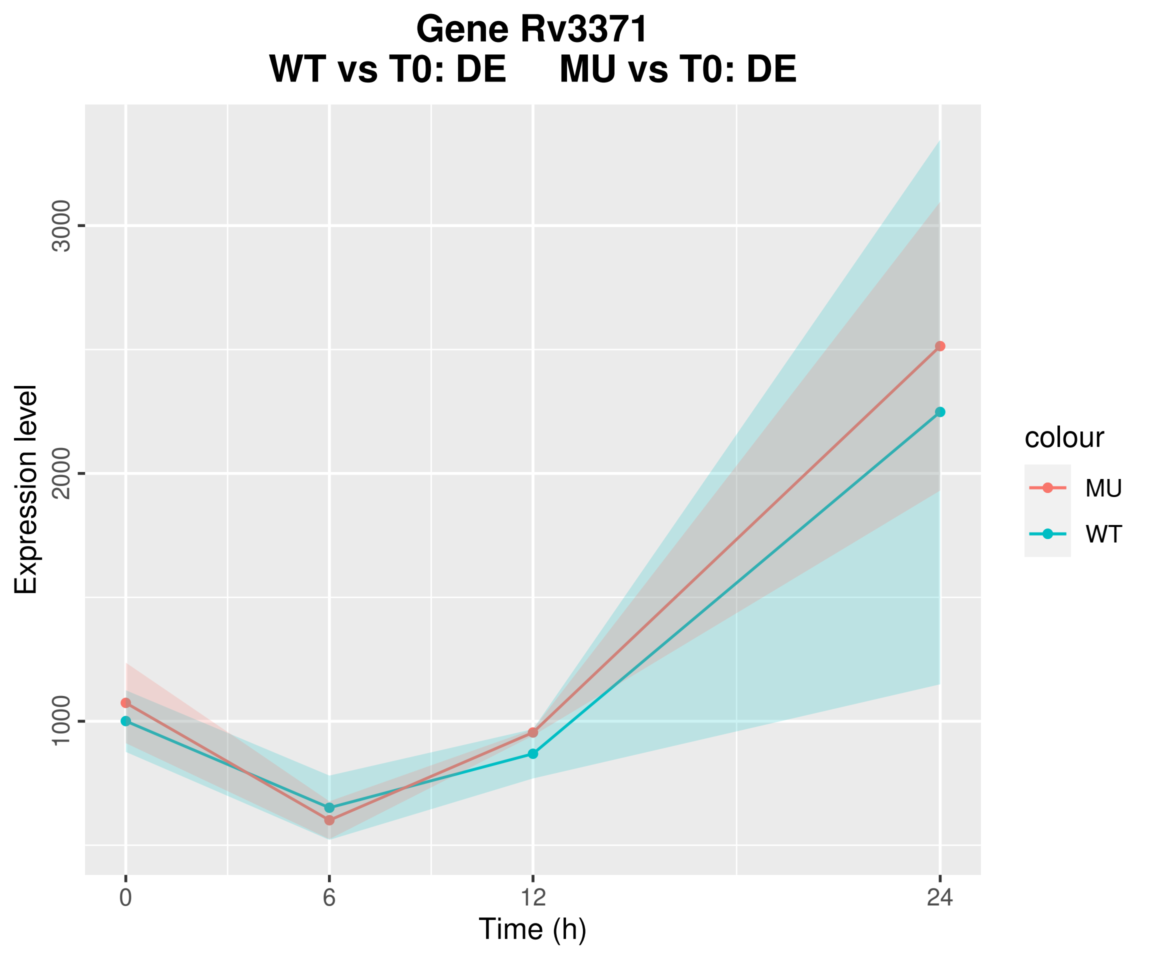

Gene Rv3371
WT vs T0: DE   MU vs T0: DE
Expression level
3000
2000
1000
0
6
12
24
Time (h)
colour
MU
WT

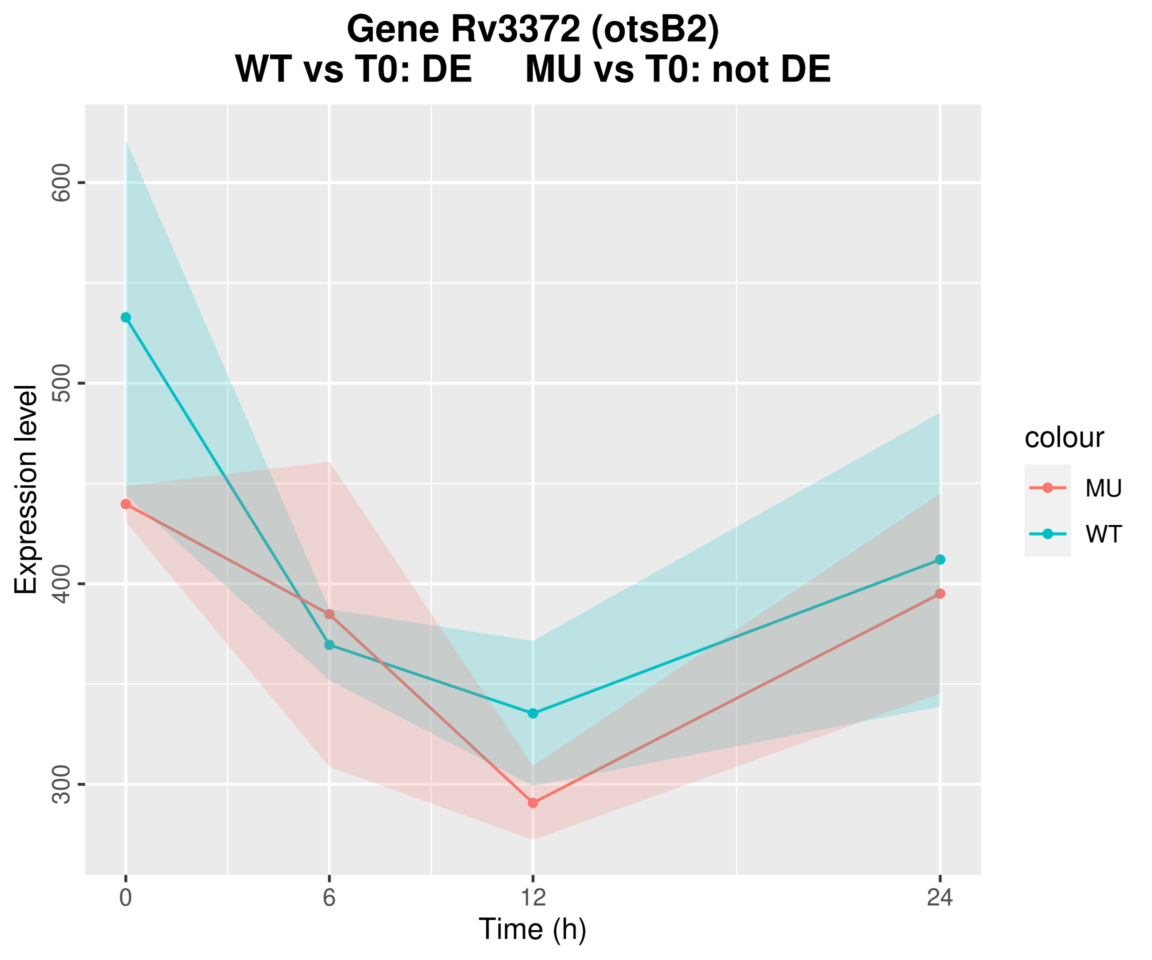
Gene Rv3372 (otsB2)
WT vs T0: DE   MU vs T0: not DE
Expression level
600
500
400
300
0
6
12
24
Time (h)
colour
MU
WT

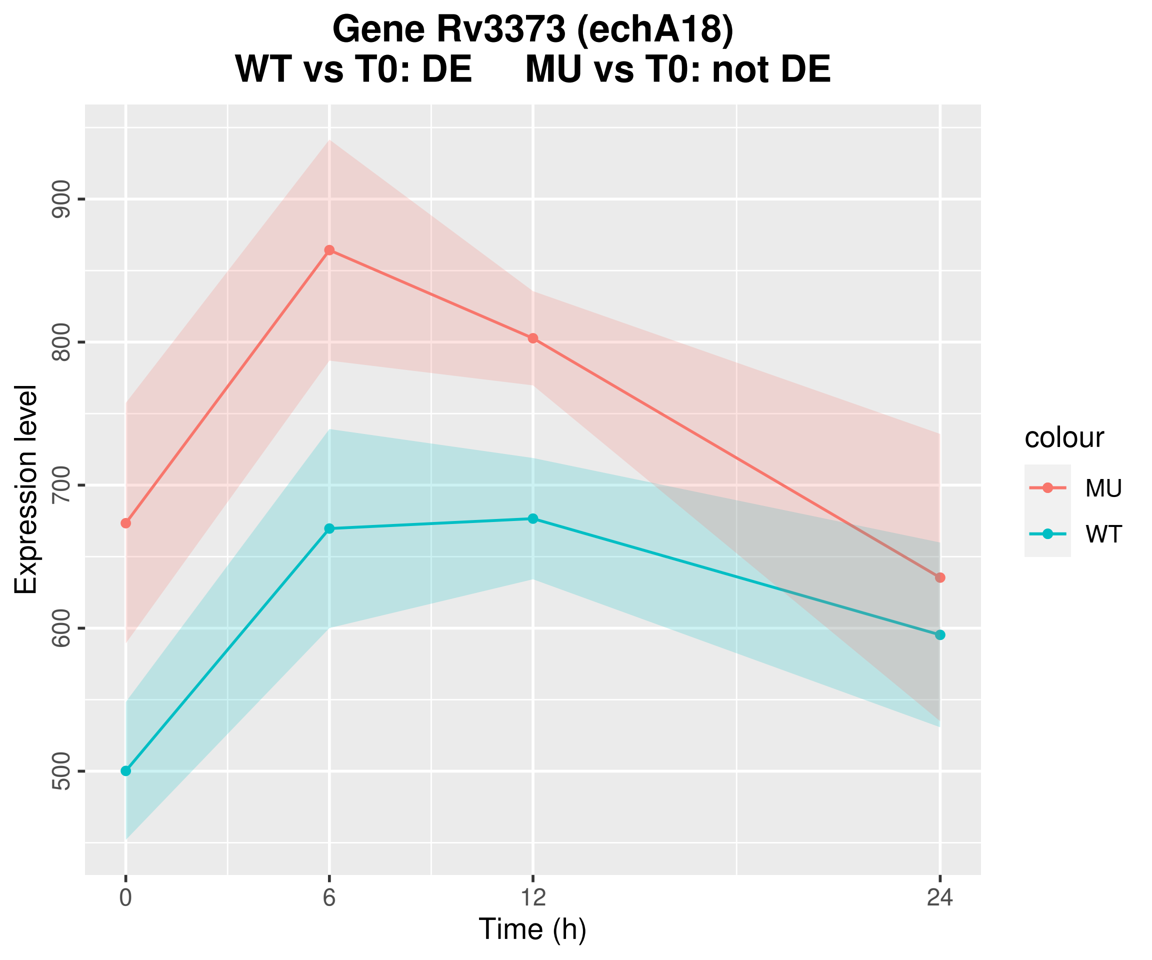

Gene Rv3373 (echA18)
WT vs T0: DE   MU vs T0: not DE
Expression level
900
800
700
600
500
0
6
12
24
Time (h)
colour
MU
WT

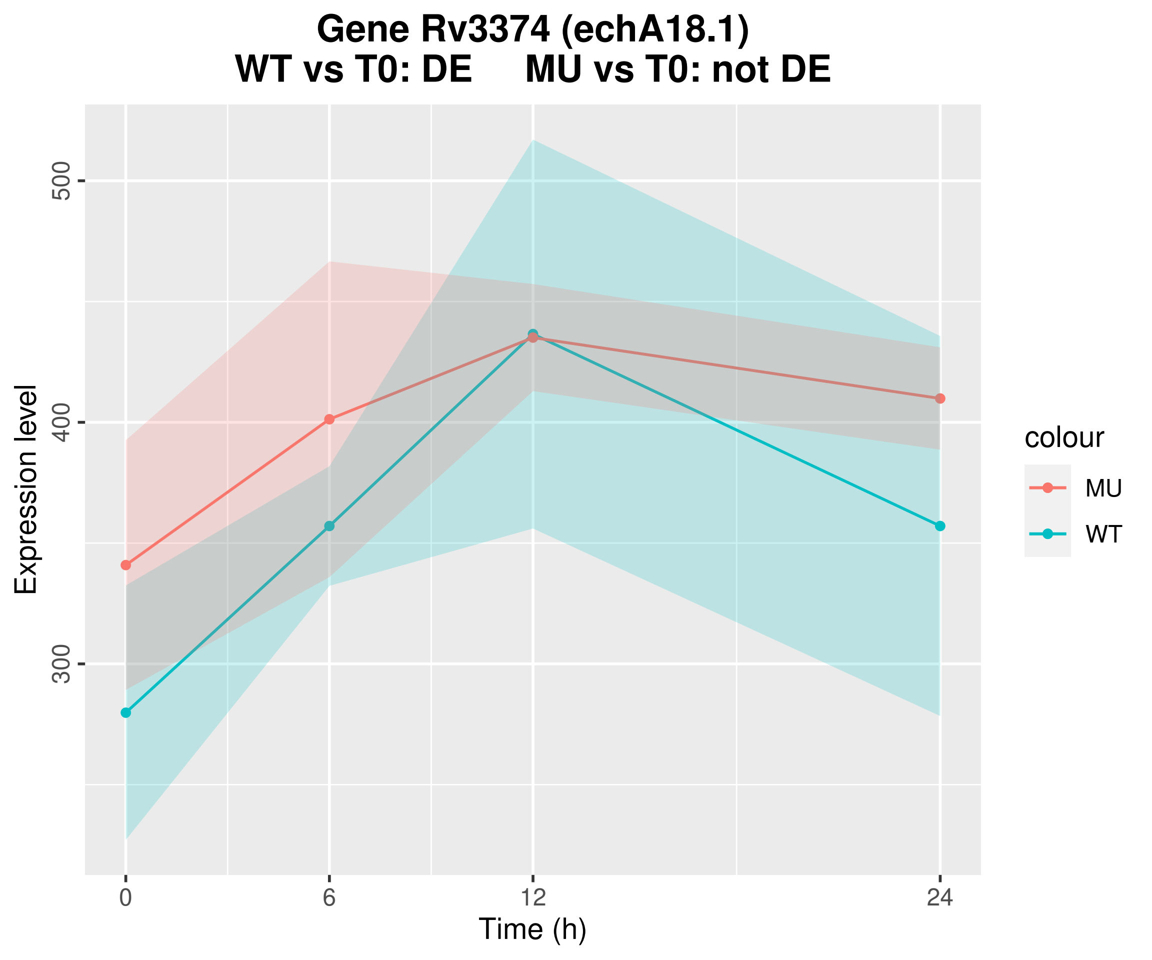

Gene Rv3374 (echA18.1)
WT vs T0: DE   MU vs T0: not DE
500
400
300
Expression level
0
6
12
24
Time (h)
colour
MU
WT

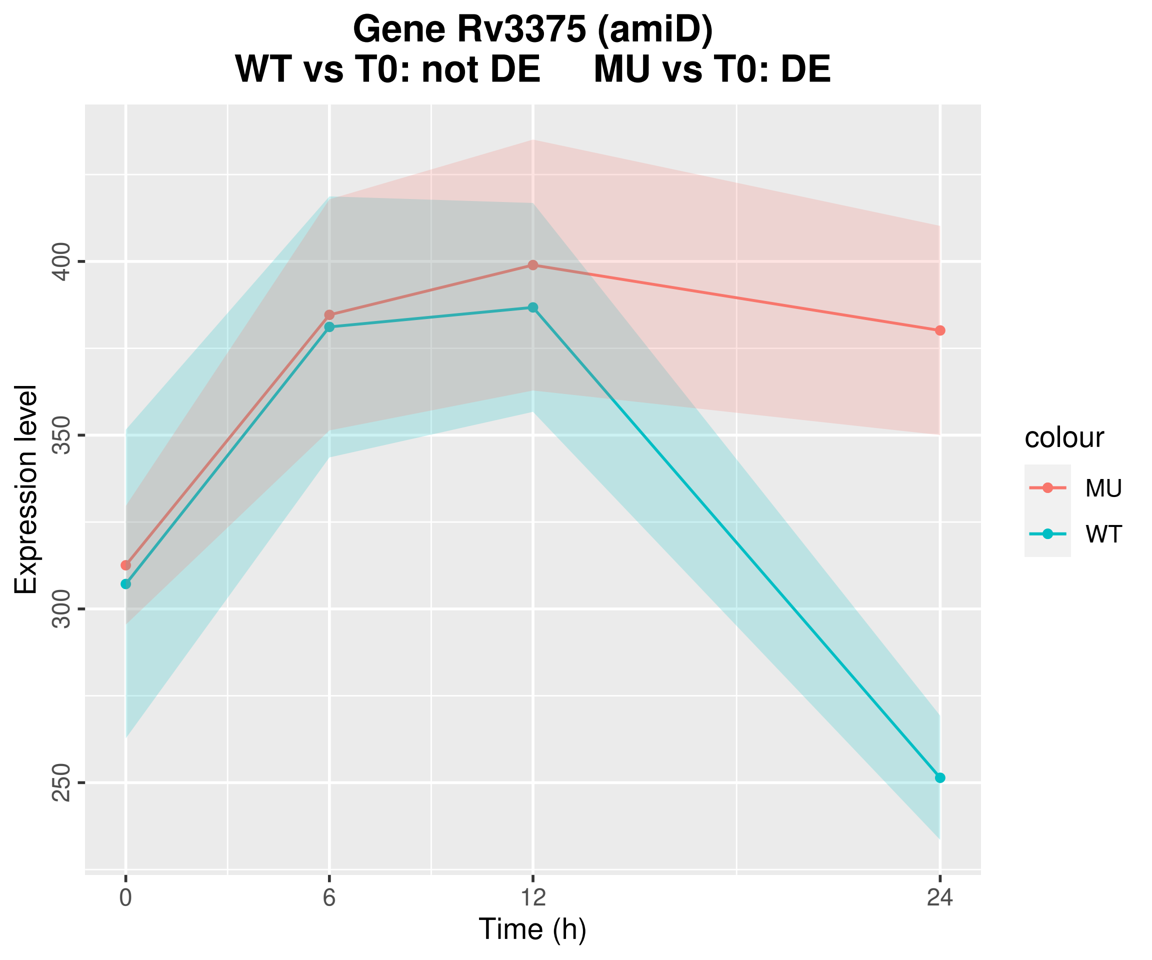
Gene Rv3375 (amiD)
WT vs T0: not DE   MU vs T0: DE
Expression level
400
350
300
250
0
6
12
24
Time (h)
colour
MU
WT

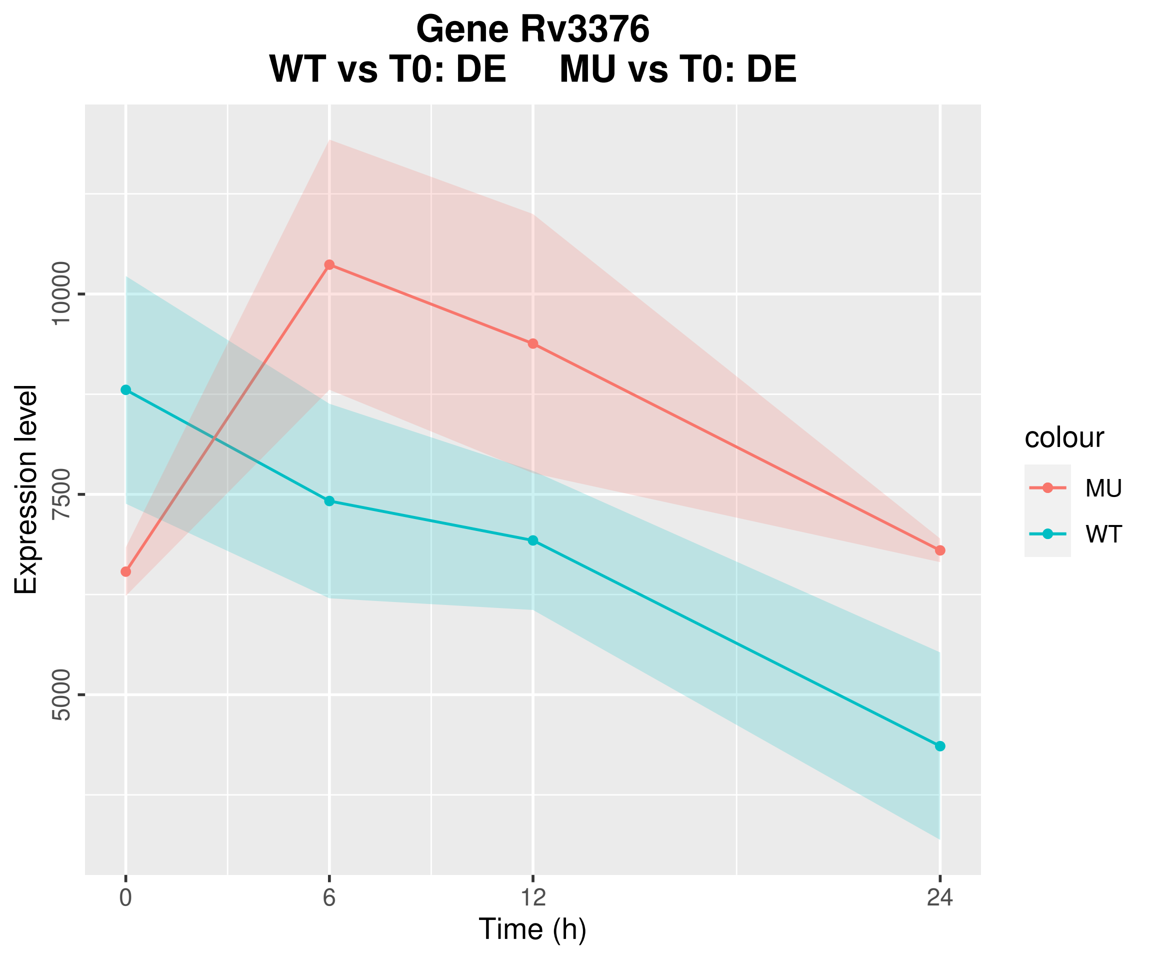
Gene Rv3376
WT vs T0: DE   MU vs T0: DE
Expression level
10000
7500
5000
0
6
12
24
Time (h)
colour
MU
WT

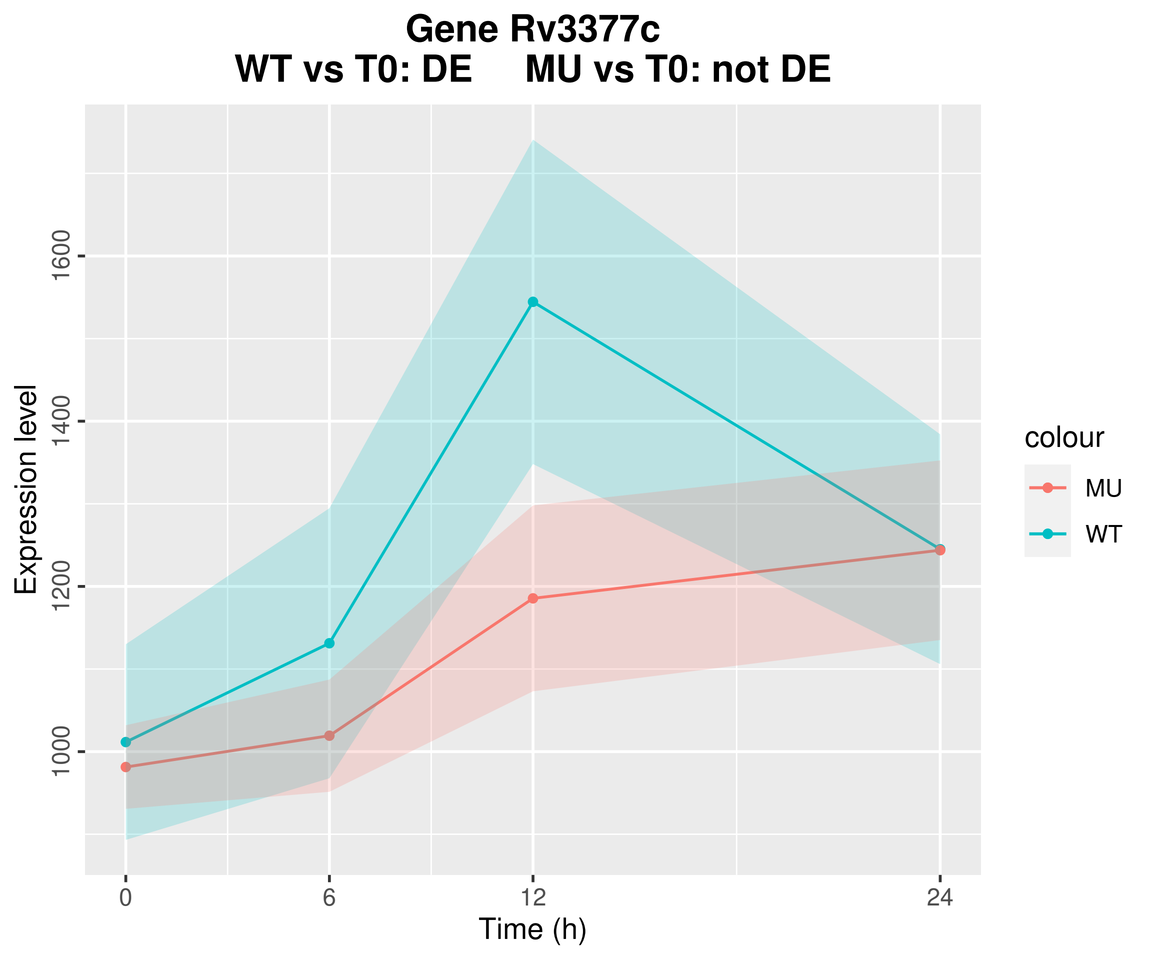

Gene Rv3377c
WT vs T0: DE   MU vs T0: not DE
Expression level
1000
1200
1400
1600
0
6
12
24
Time (h)
colour
MU
WT

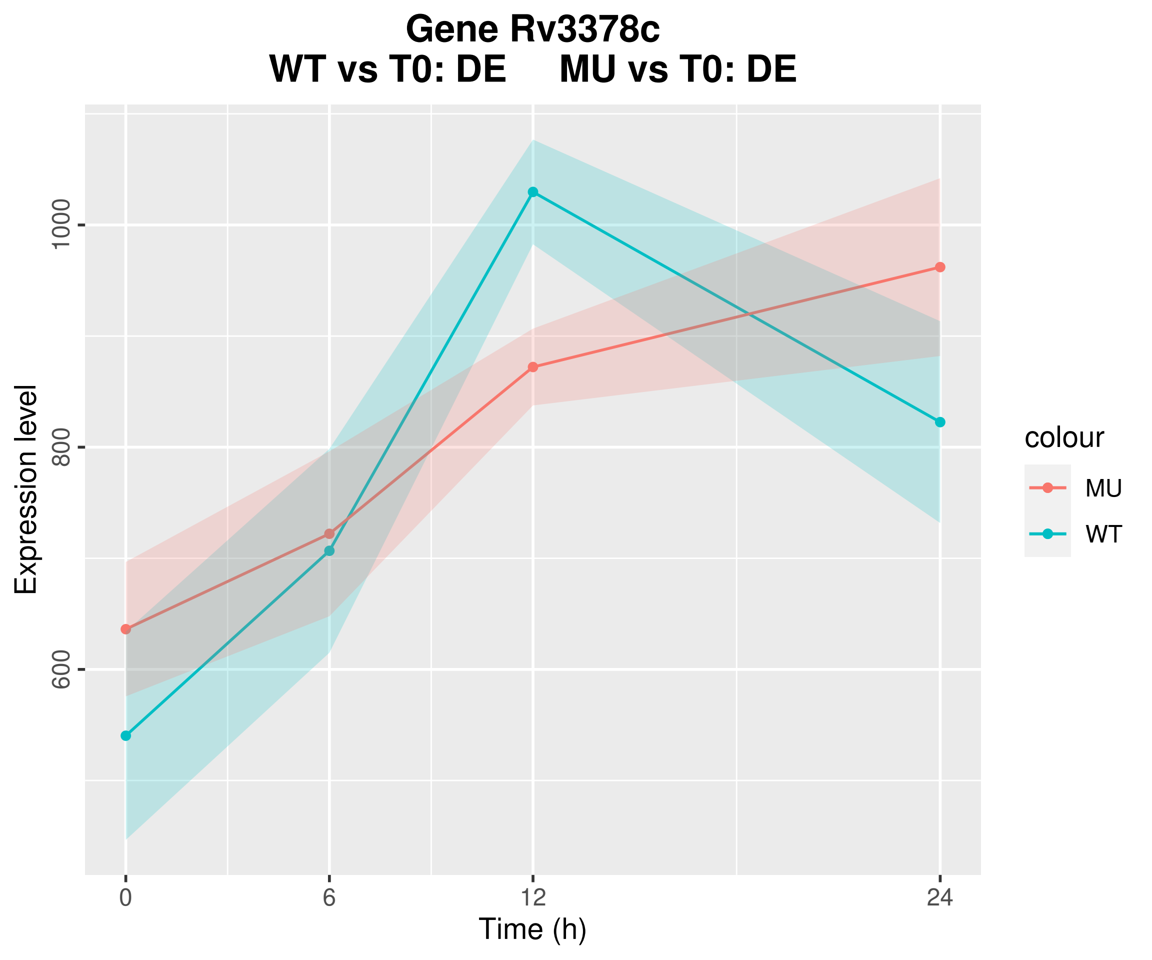
Gene Rv3378c
WT vs T0: DE   MU vs T0: DE
Expression level
1000
800
600
0
6
12
24
Time (h)
colour
MU
WT

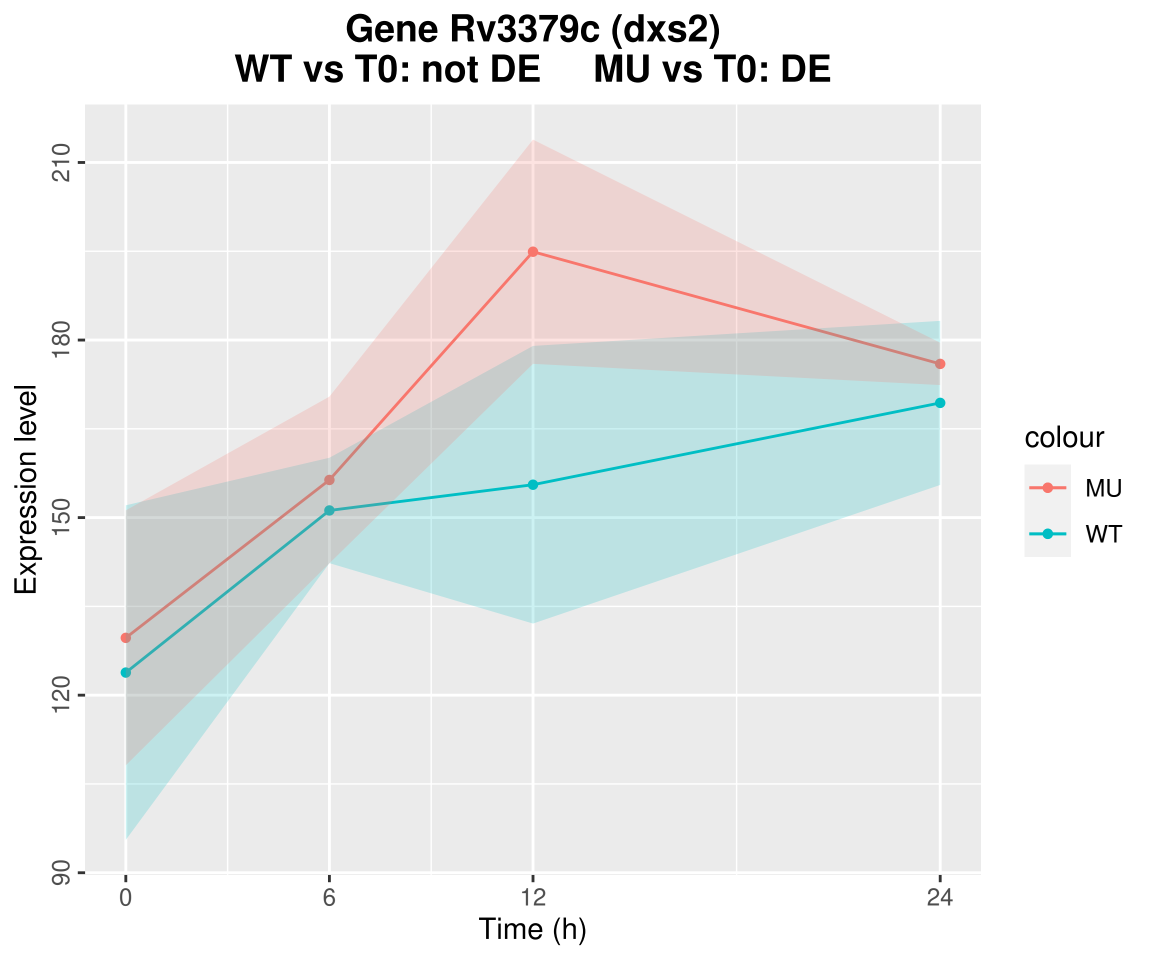

Gene Rv3379c (dxs2)
WT vs T0: not DE MU vs T0: DE
Expression level
210
180
150
120
90
0
6
12
24
Time (h)
colour
MU
WT

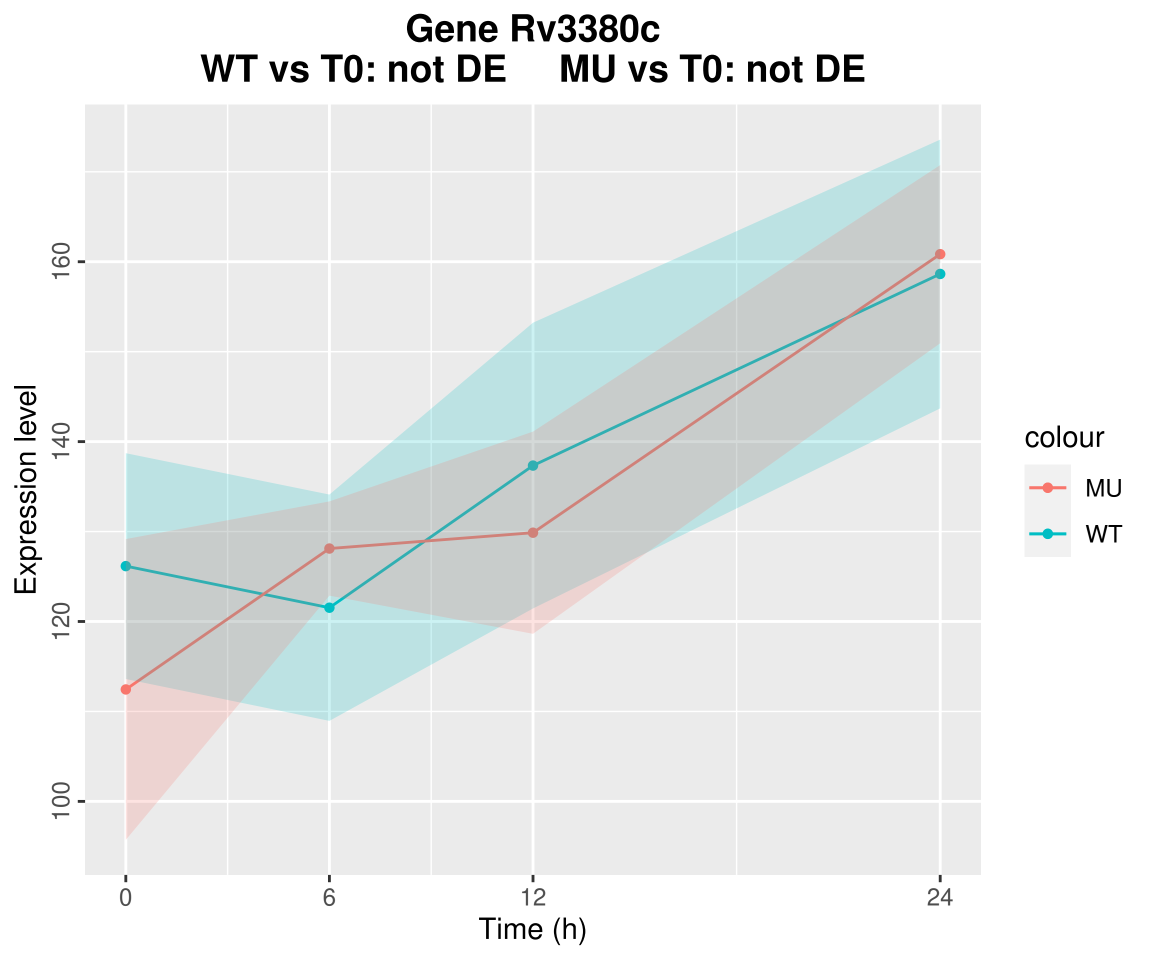

Gene Rv3380c
WT vs T0: not DE MU vs T0: not DE
Expression level
100
120
140
160
0
6
12
24
Time (h)
colour
MU
WT

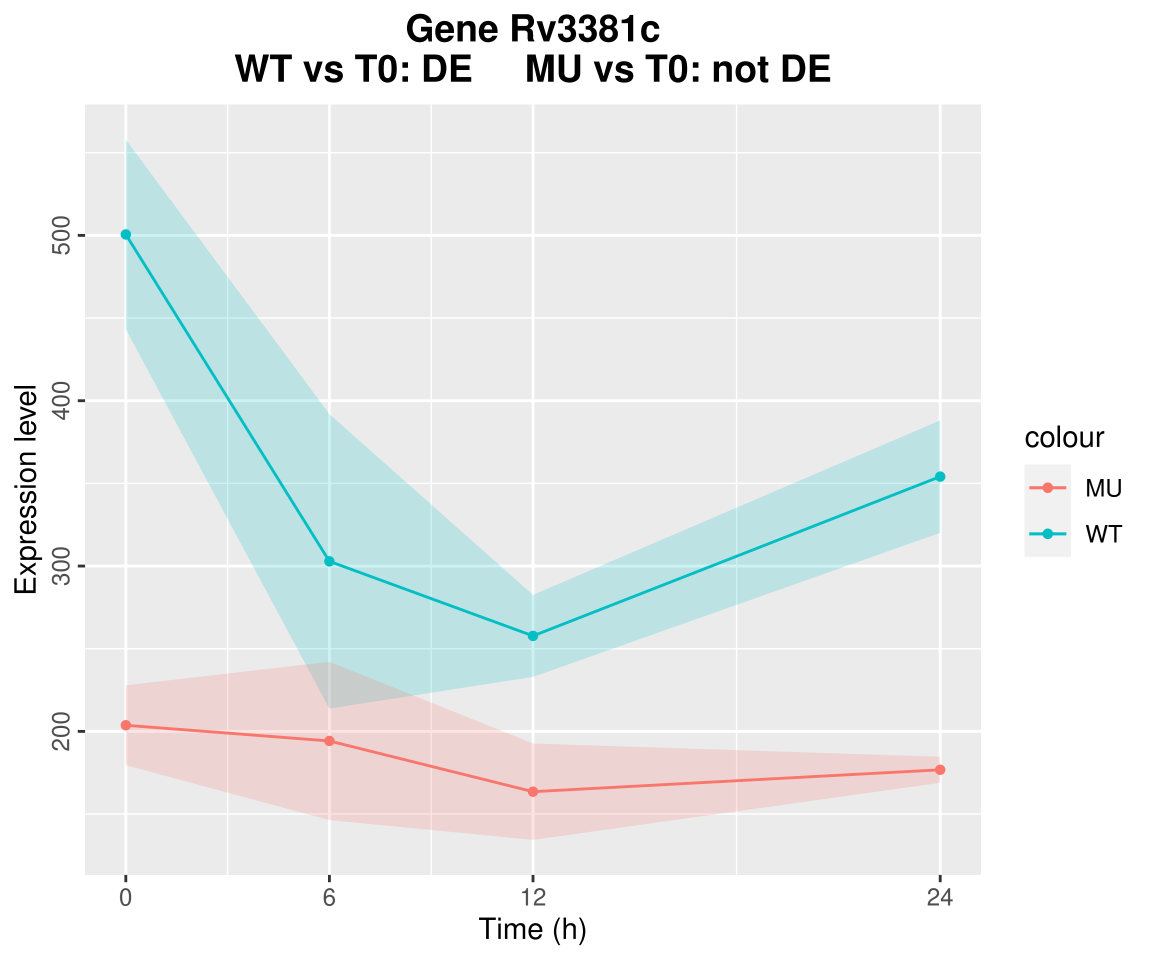
Gene Rv3381c
WT vs T0: DE MU vs T0: not DE
Expression level
500
400
300
200
0
6
12
24
Time (h)
colour
MU
WT

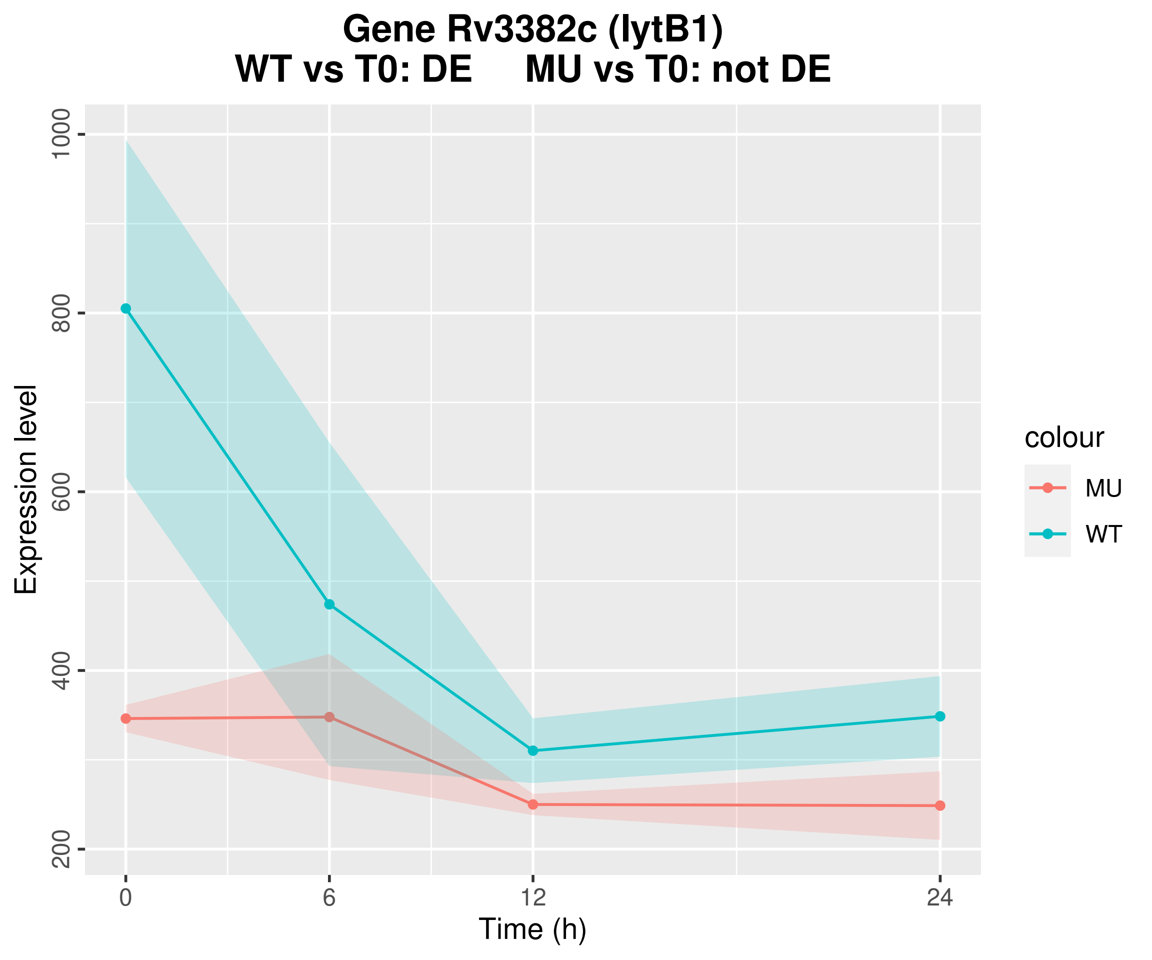

Gene Rv3382c (lytB1)
WT vs T0: DE MU vs T0: not DE
Expression level
1000
800
600
400
200
0
6
12
24
Time (h)
colour
MU
WT

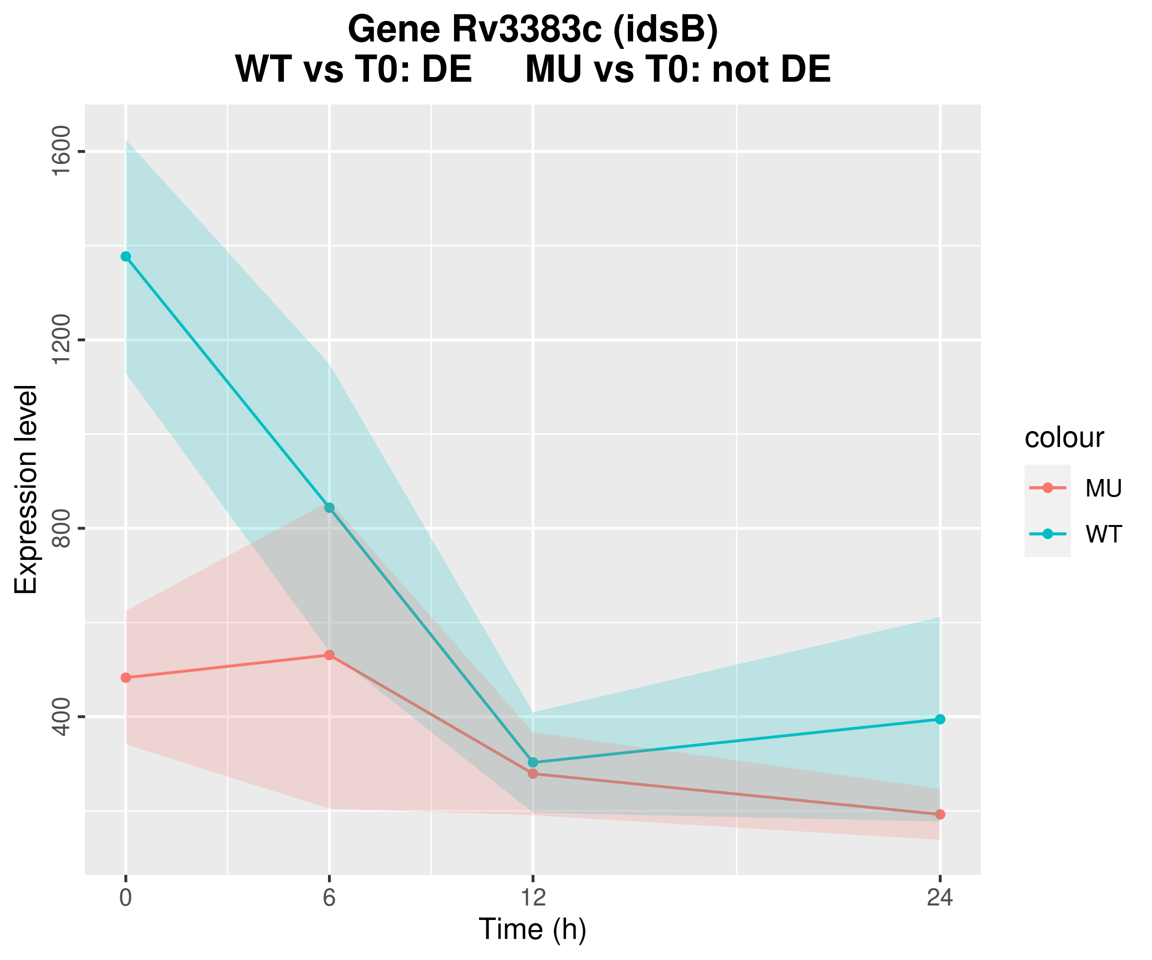

Gene Rv3383c (idsB)
WT vs T0: DE   MU vs T0: not DE
1600
1200
800
400
0
6
12
24
Time (h)
Expression level
colour
MU
WT

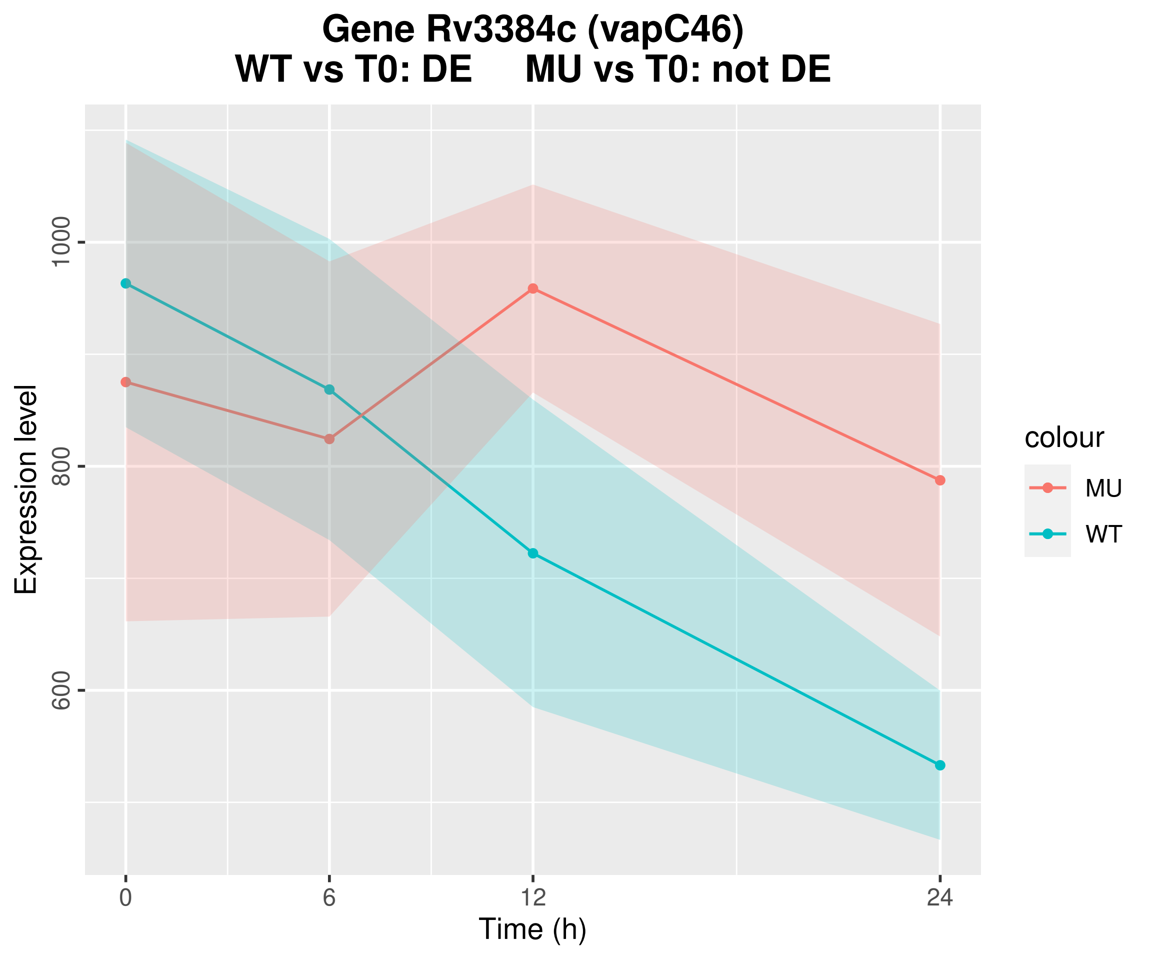

Gene Rv3384c (vapC46)
WT vs T0: DE   MU vs T0: not DE
Expression level
1000
800
600
0
6
12
24
Time (h)
colour
MU
WT

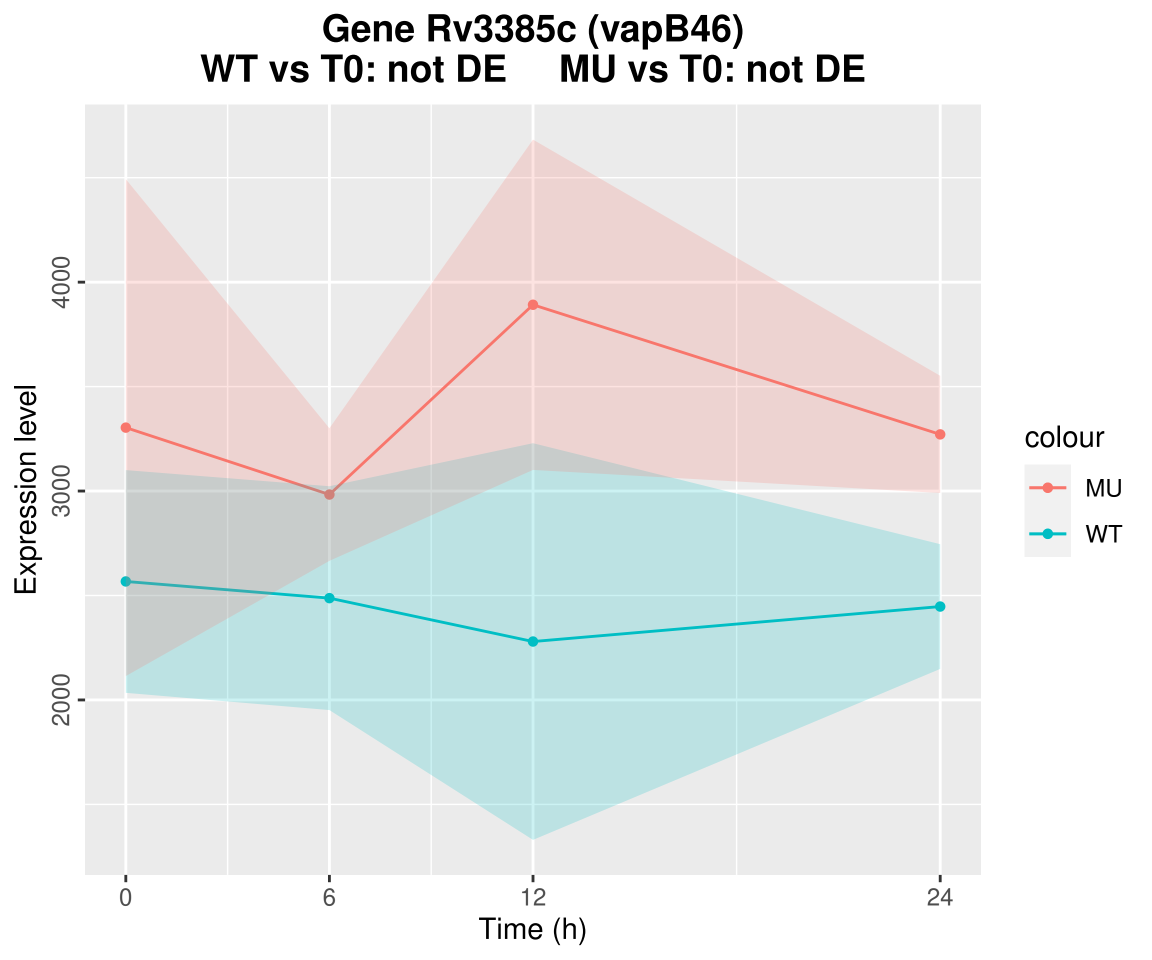
Gene Rv3385c (vapB46)
WT vs T0: not DE MU vs T0: not DE
Expression level
4000
3000
2000
0
6
12
24
Time (h)
colour
MU
WT

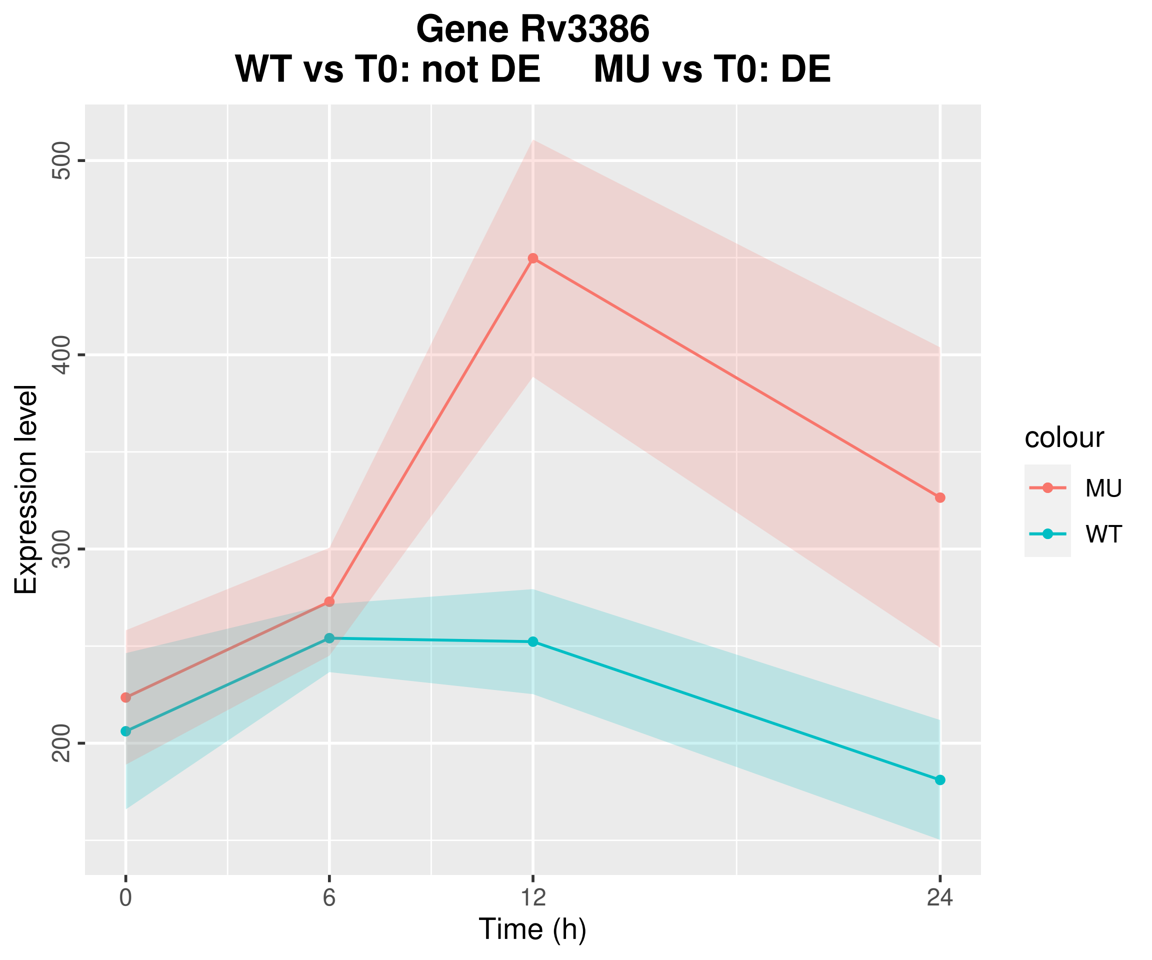
Gene Rv3386
WT vs T0: not DE MU vs T0: DE
Expression level
500
400
300
200
0
6
12
24
Time (h)
colour
MU
WT

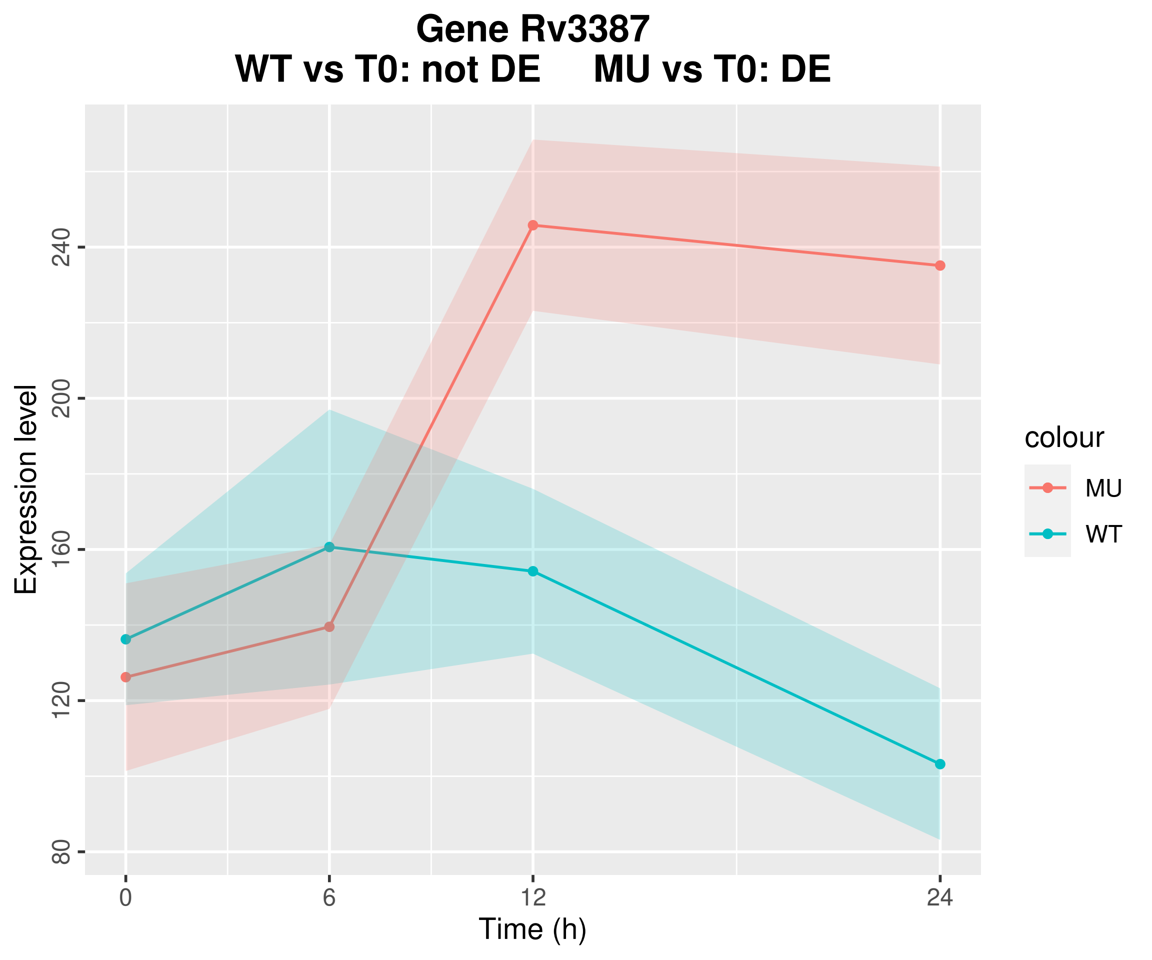
Gene Rv3387
WT vs T0: not DE   MU vs T0: DE
Expression level
240
200
160
120
80
0
6
12
24
Time (h)
colour
MU
WT

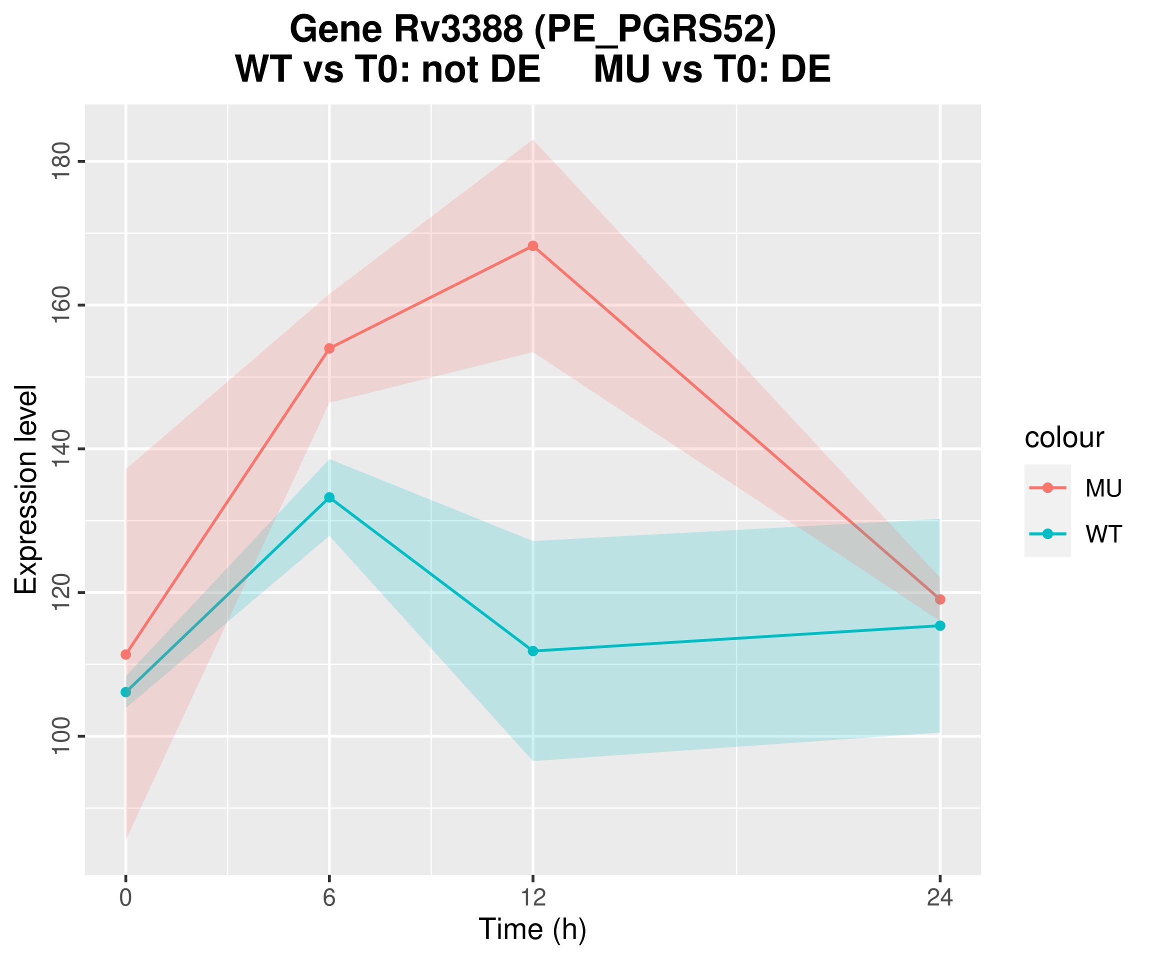

Gene Rv3388 (PE_PGRS52)
WT vs T0: not DE MU vs T0: DE
Expression level
180
160
140
120
100
0
6
12
24
Time (h)
colour
MU
WT

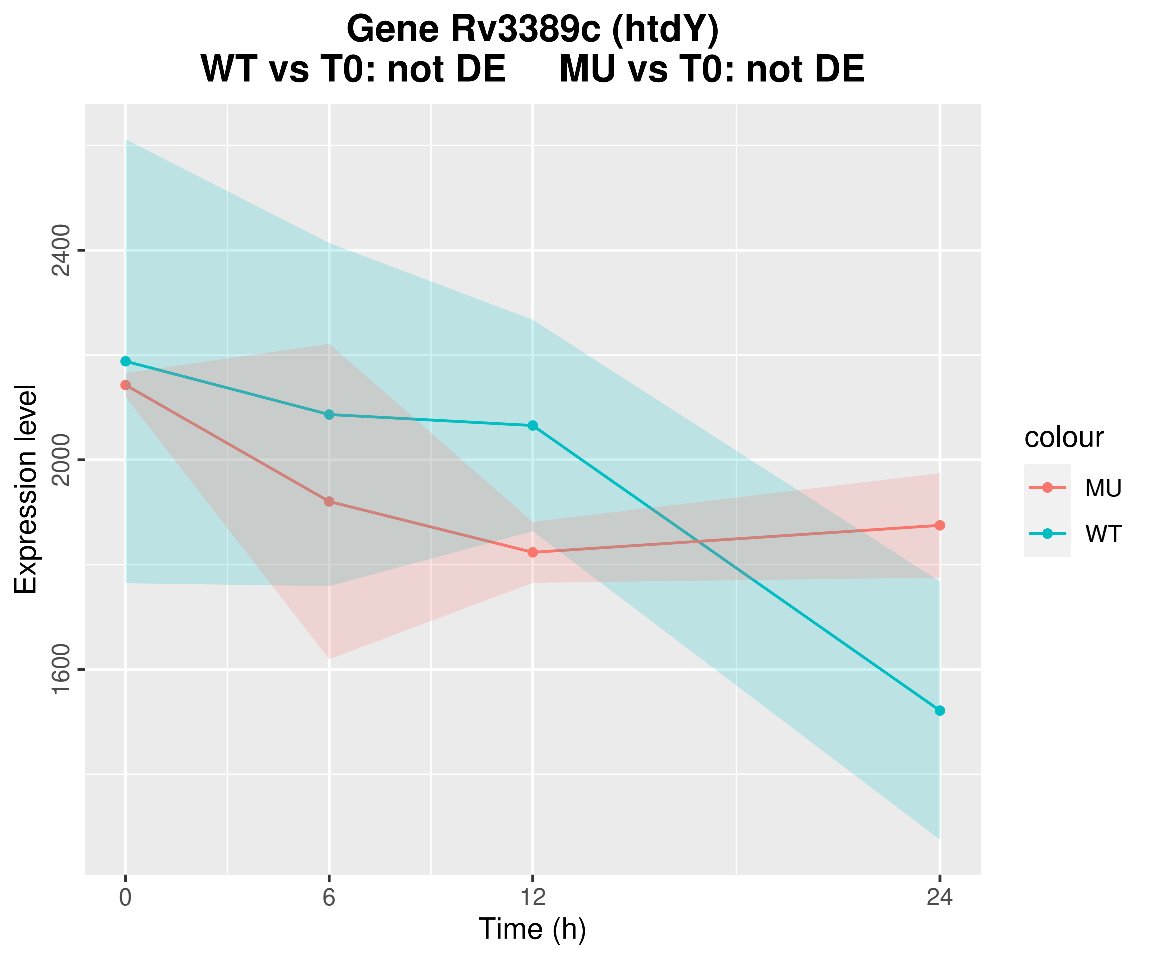

Gene Rv3389c (htdY)
WT vs T0: not DE MU vs T0: not DE
2400
2000
1600
Expression level
0
6
12
24
Time (h)
colour
MU
WT

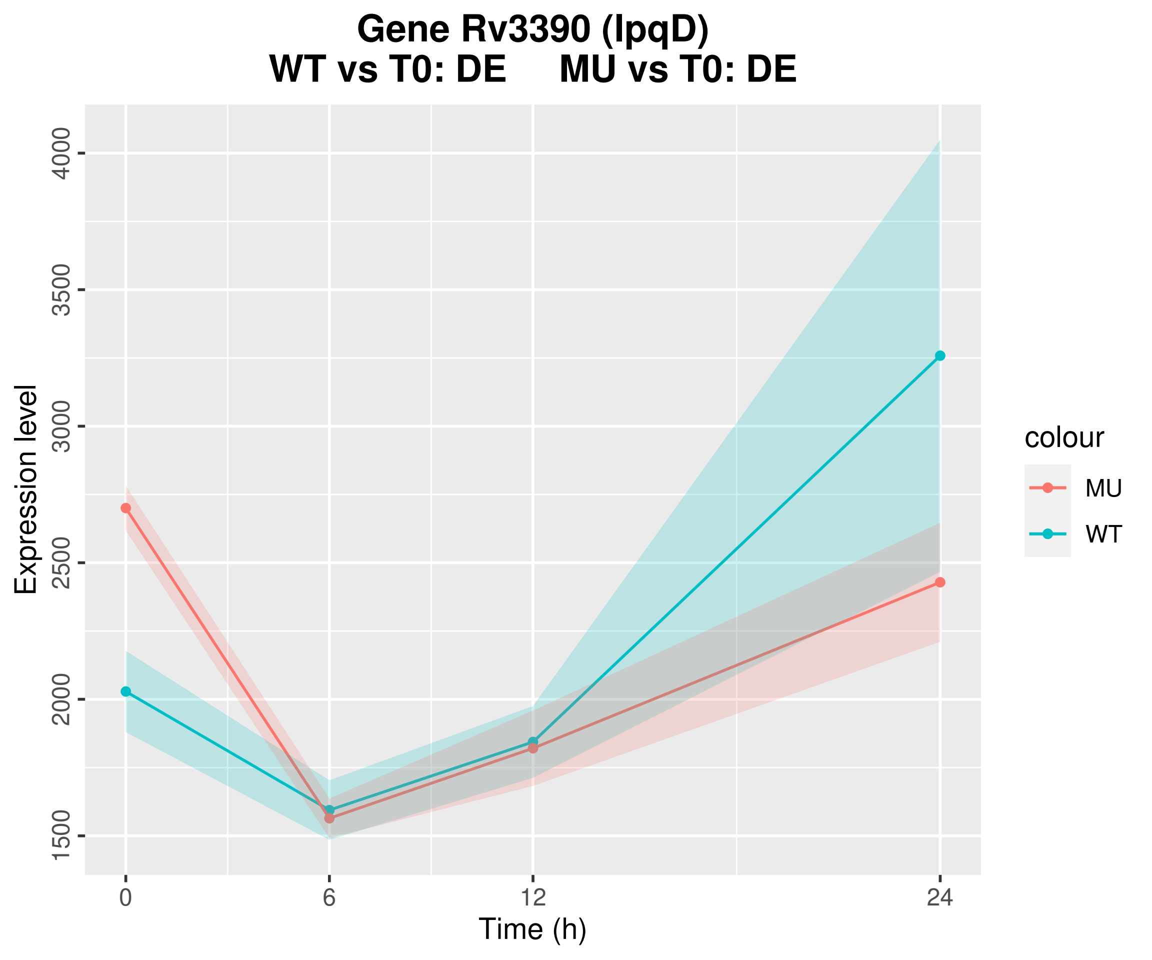

Gene Rv3390 (lpqD)
WT vs T0: DE MU vs T0: DE
Expression level
4000
3500
3000
2500
2000
1500
0
6
12
24
Time (h)
colour
MU
WT

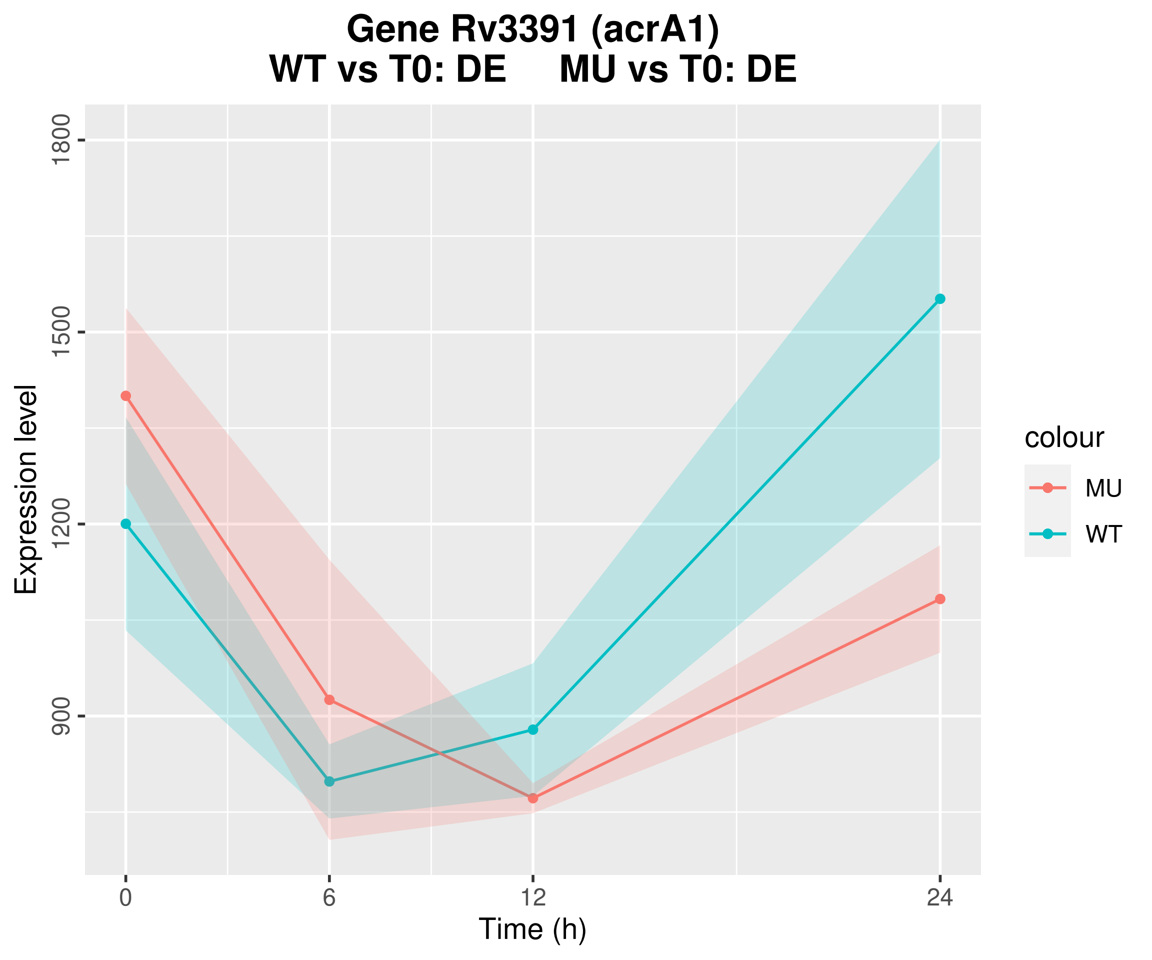

Gene Rv3391 (acrA1)
WT vs T0: DE MU vs T0: DE
Expression level
1800
1500
1200
900
0
6
12
24
Time (h)
colour
MU
WT

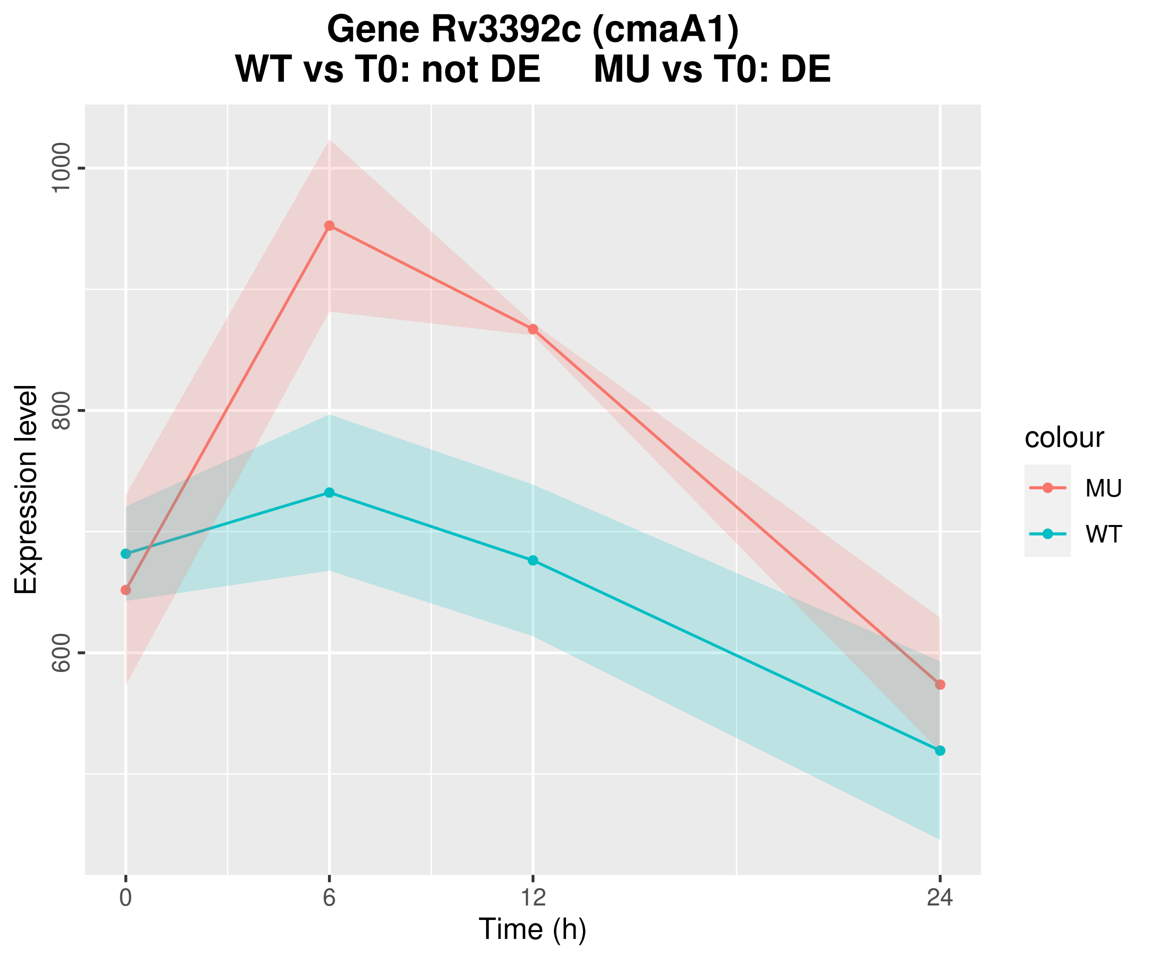

Gene Rv3392c (cmaA1)
WT vs T0: not DE MU vs T0: DE
Expression level
1000
800
600
0
6
12
24
Time (h)
colour
MU
WT

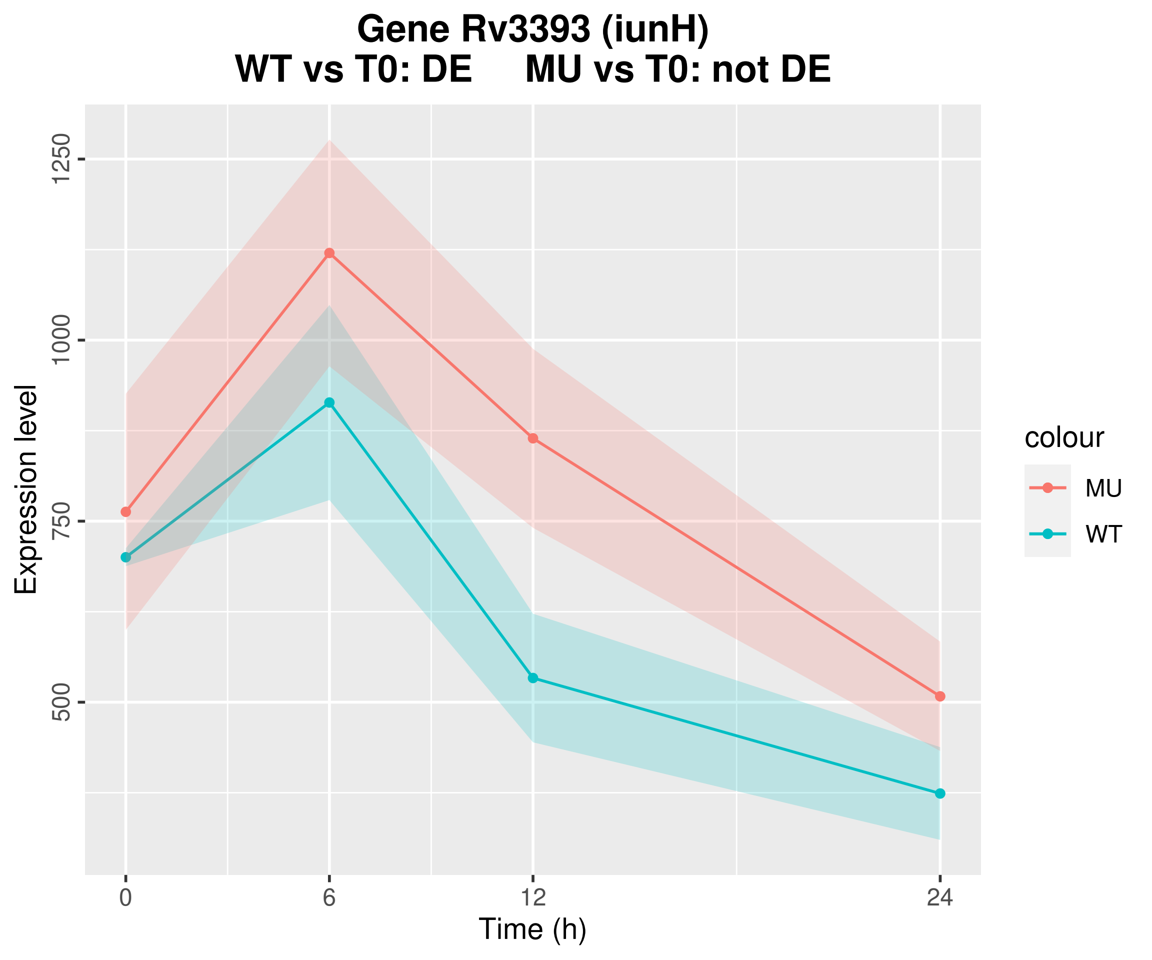
Gene Rv3393 (iunH)
WT vs T0: DE   MU vs T0: not DE
1250
1000
750
500
Expression level
0
6
12
24
Time (h)
colour
MU
WT

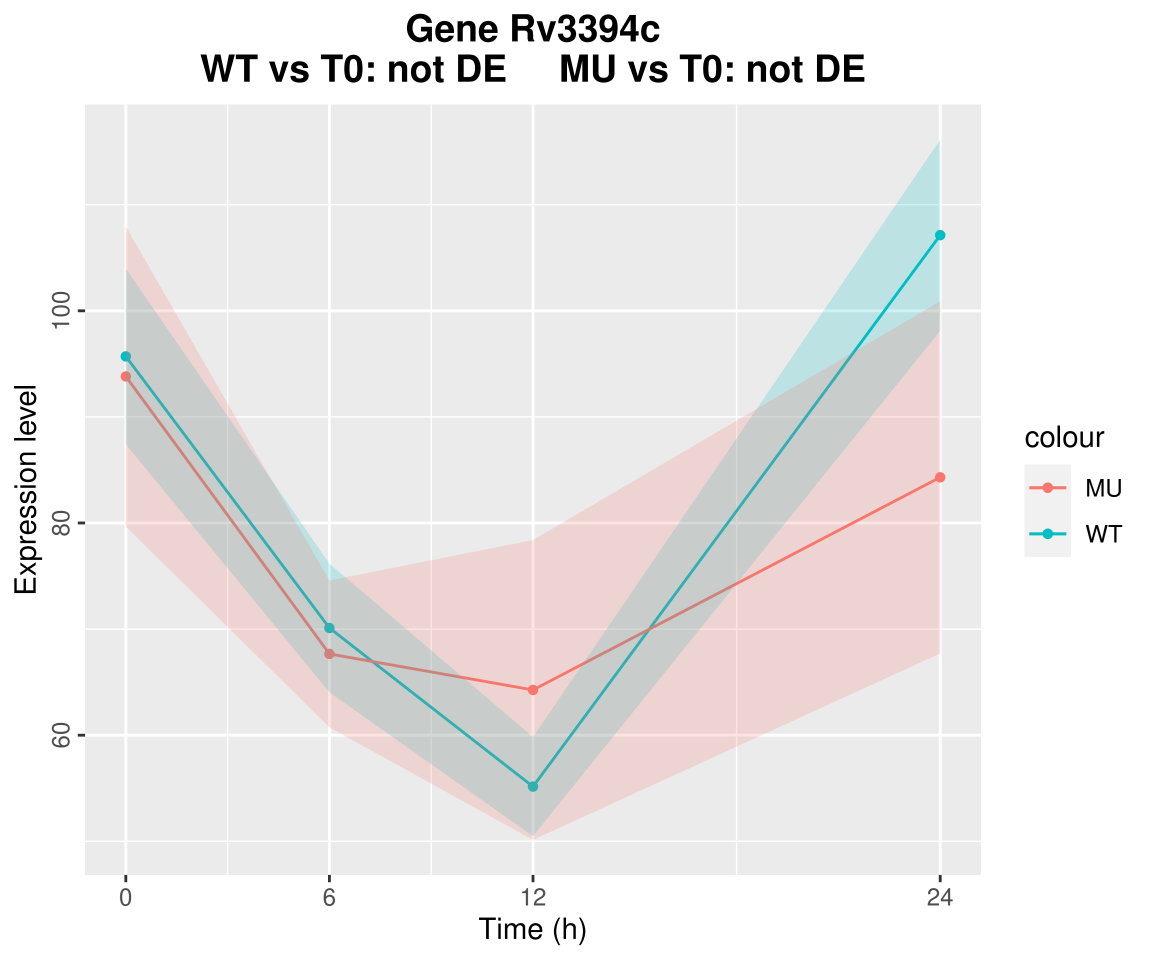
Gene Rv3394c
WT vs T0: not DE   MU vs T0: not DE
Expression level
100
80
60
0
6
12
24
Time (h)
colour
MU
WT

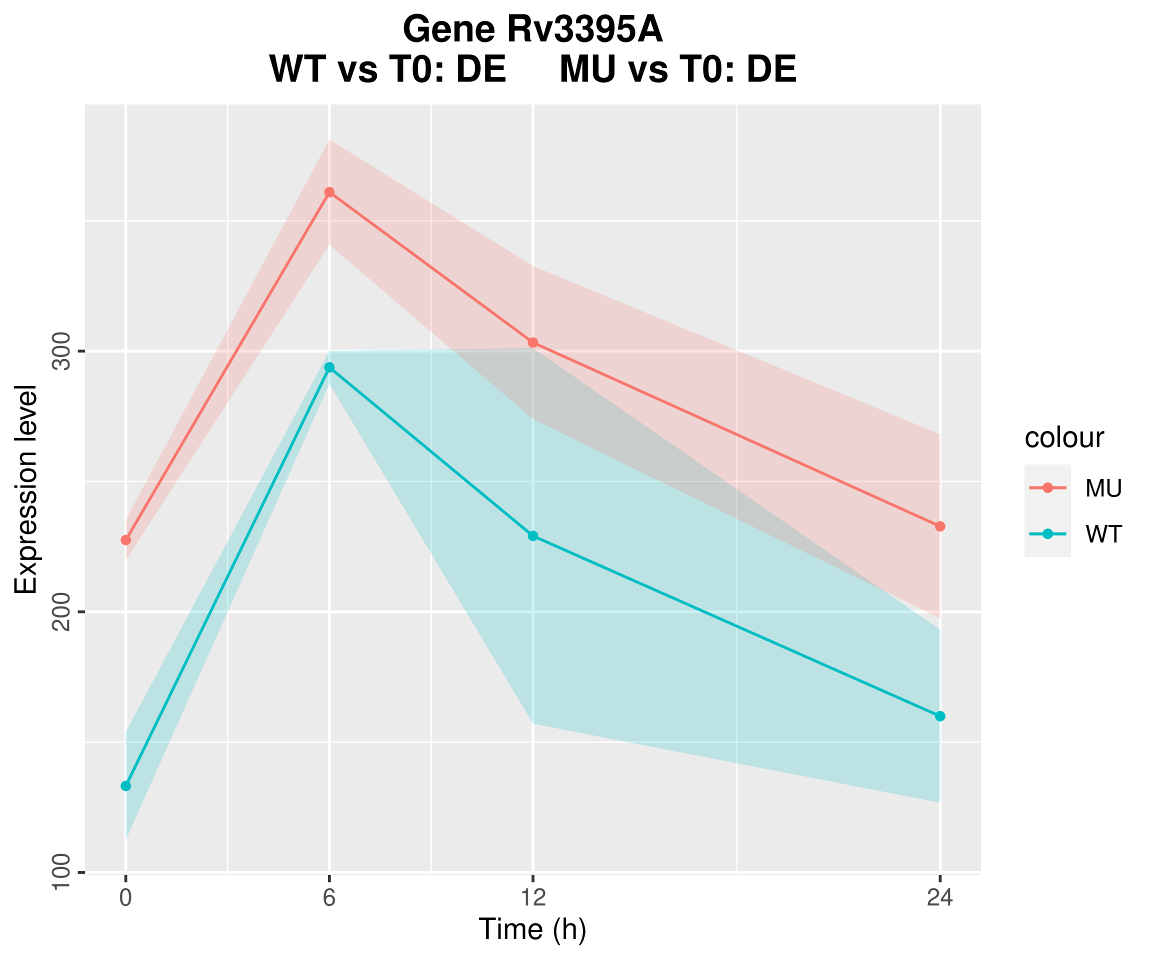
Gene Rv3395A
WT vs T0: DE MU vs T0: DE
Expression level
300
200
100
0
6
12
24
Time (h)
colour
MU
WT

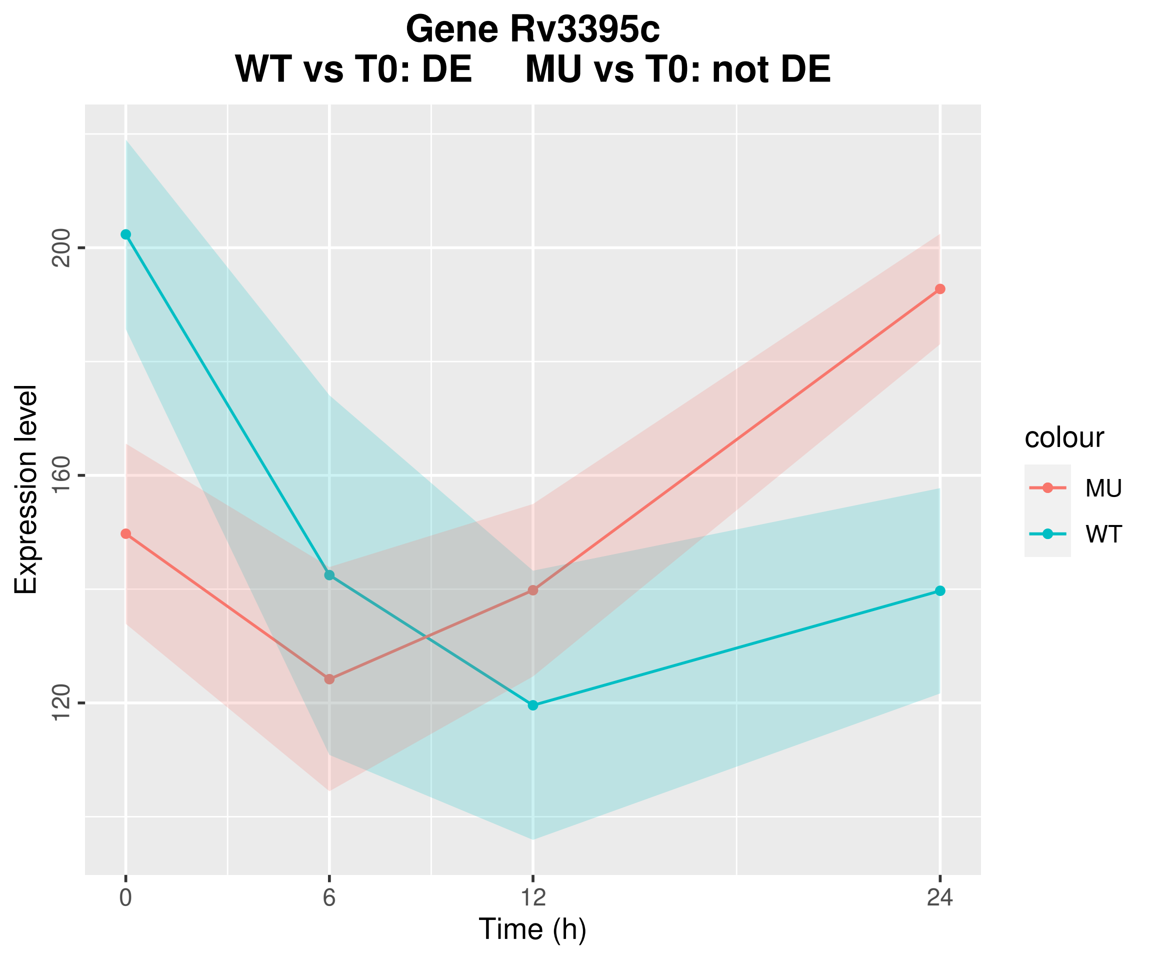

Gene Rv3395c
WT vs T0: DE MU vs T0: not DE
Expression level
200
160
120
0
6
12
24
Time (h)
colour
MU
WT

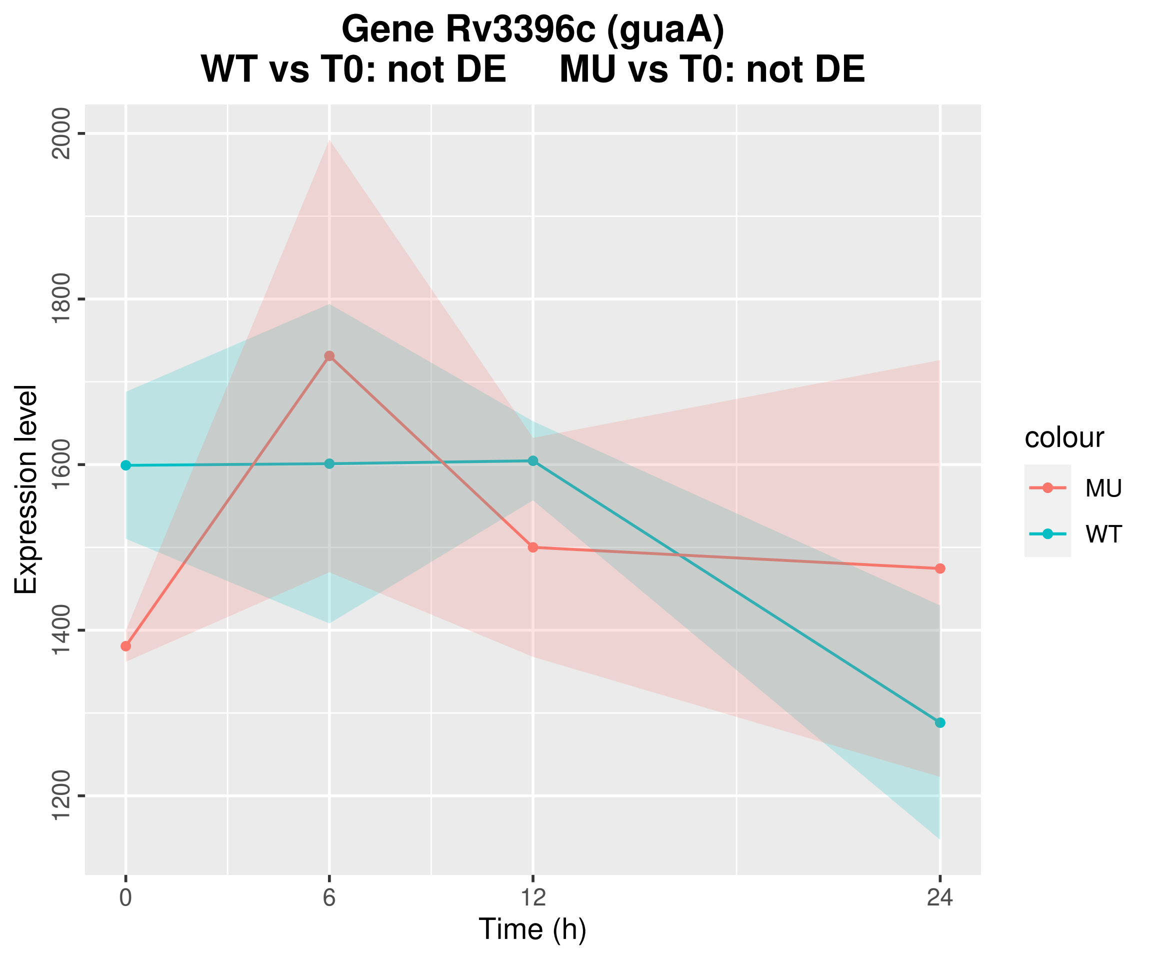
Gene Rv3396c (guaA)
WT vs T0: not DE   MU vs T0: not DE
Expression level
2000
1800
1600
1400
1200
0
6
12
24
Time (h)
colour
MU
WT

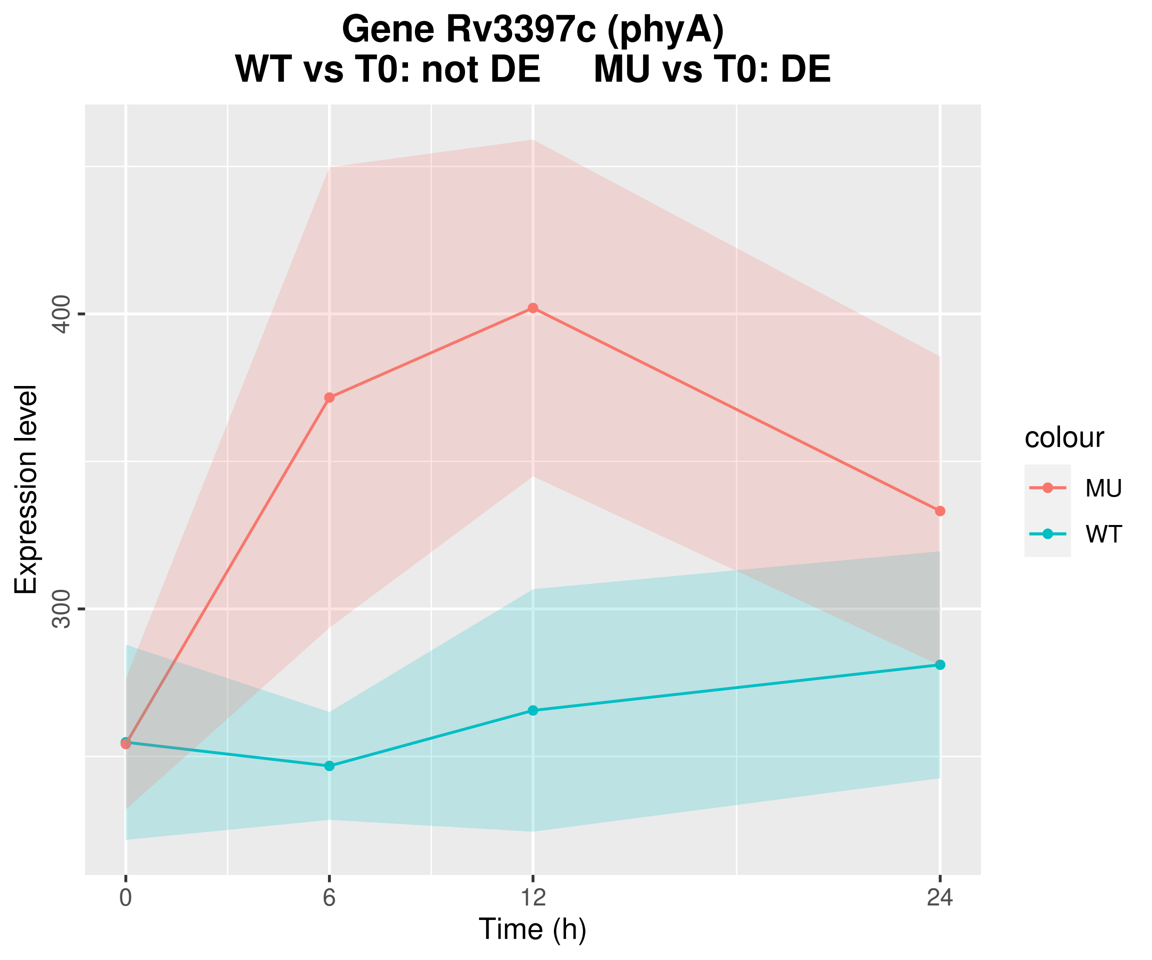
Gene Rv3397c (phyA)
WT vs T0: not DE MU vs T0: DE
Expression level
400
300
0
6
12
24
Time (h)
colour
MU
WT

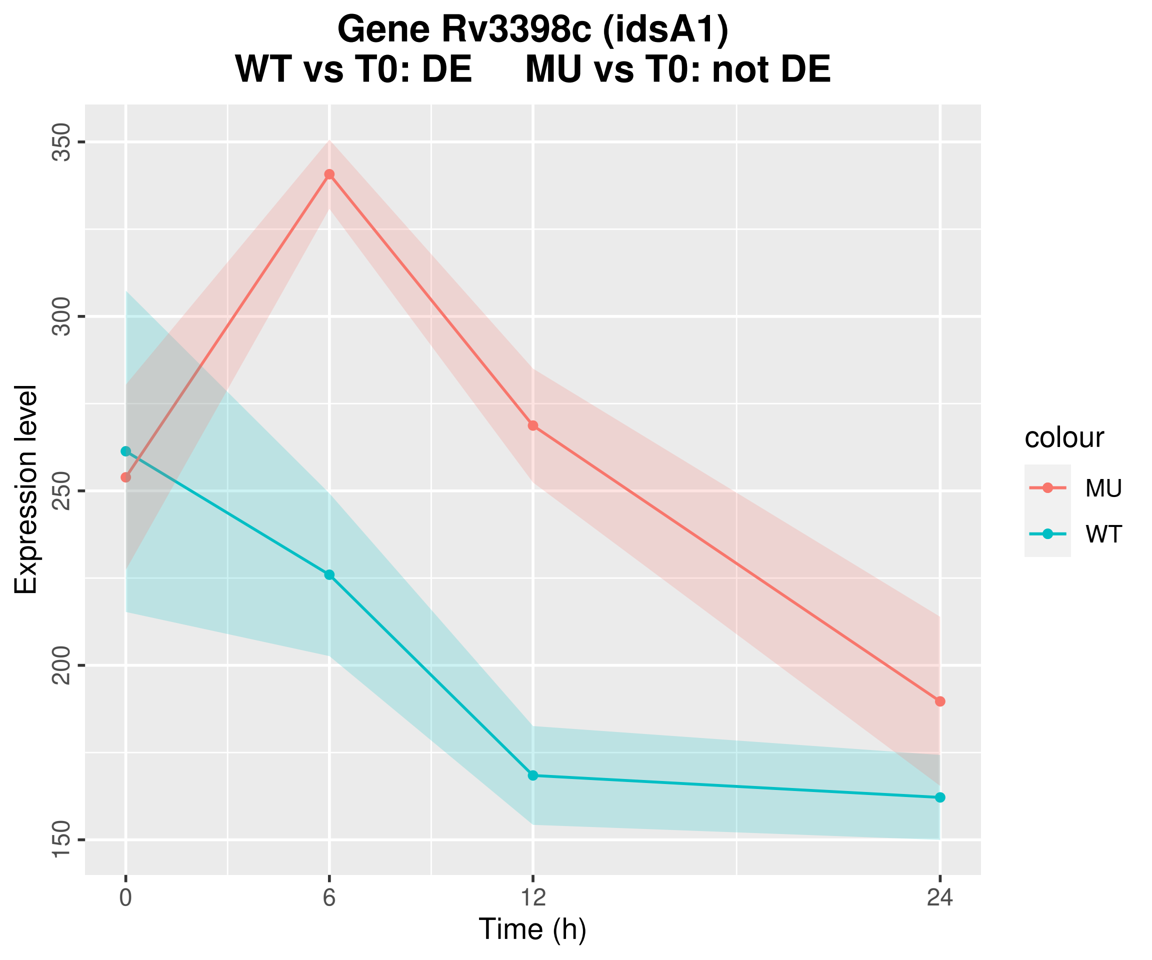

Gene Rv3398c (idsA1)
WT vs T0: DE   MU vs T0: not DE
Expression level
350
300
250
200
150
0
6
12
24
Time (h)
colour
MU
WT

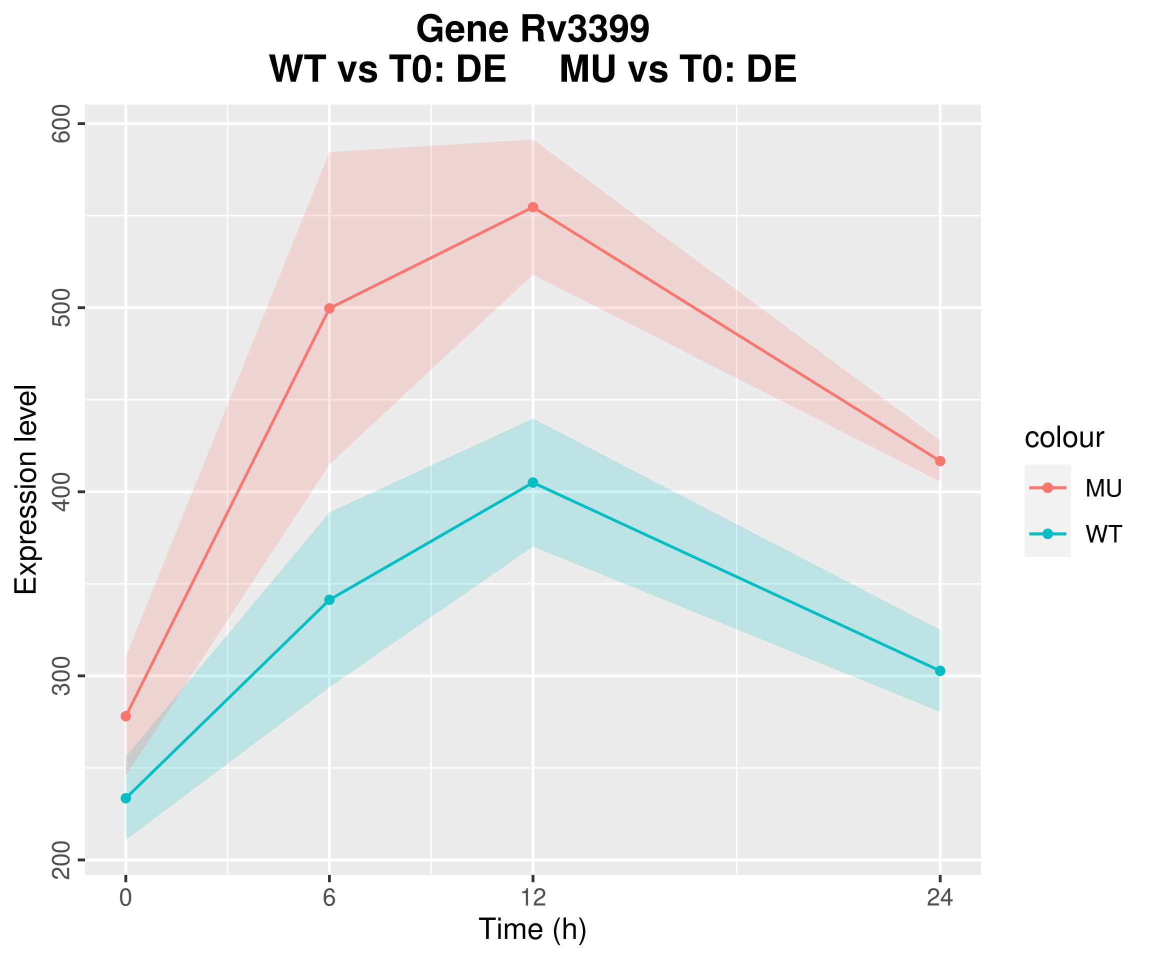
Gene Rv3399
WT vs T0: DE   MU vs T0: DE
Expression level
600
500
400
300
200
0
6
12
24
Time (h)
colour
MU
WT

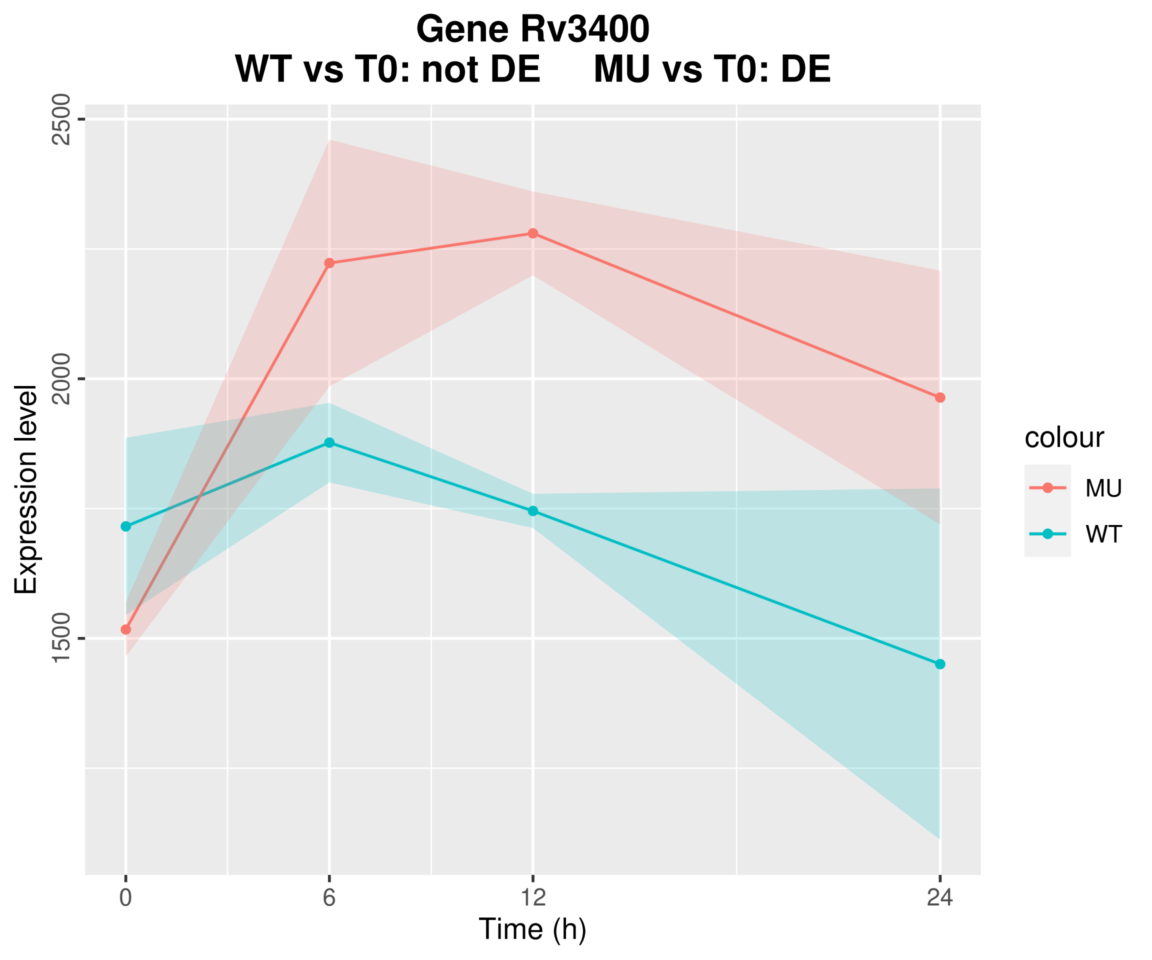

Gene Rv3400
WT vs T0: not DE   MU vs T0: DE
2500
2000
1500
Expression level
0
6
12
24
Time (h)
colour
MU
WT

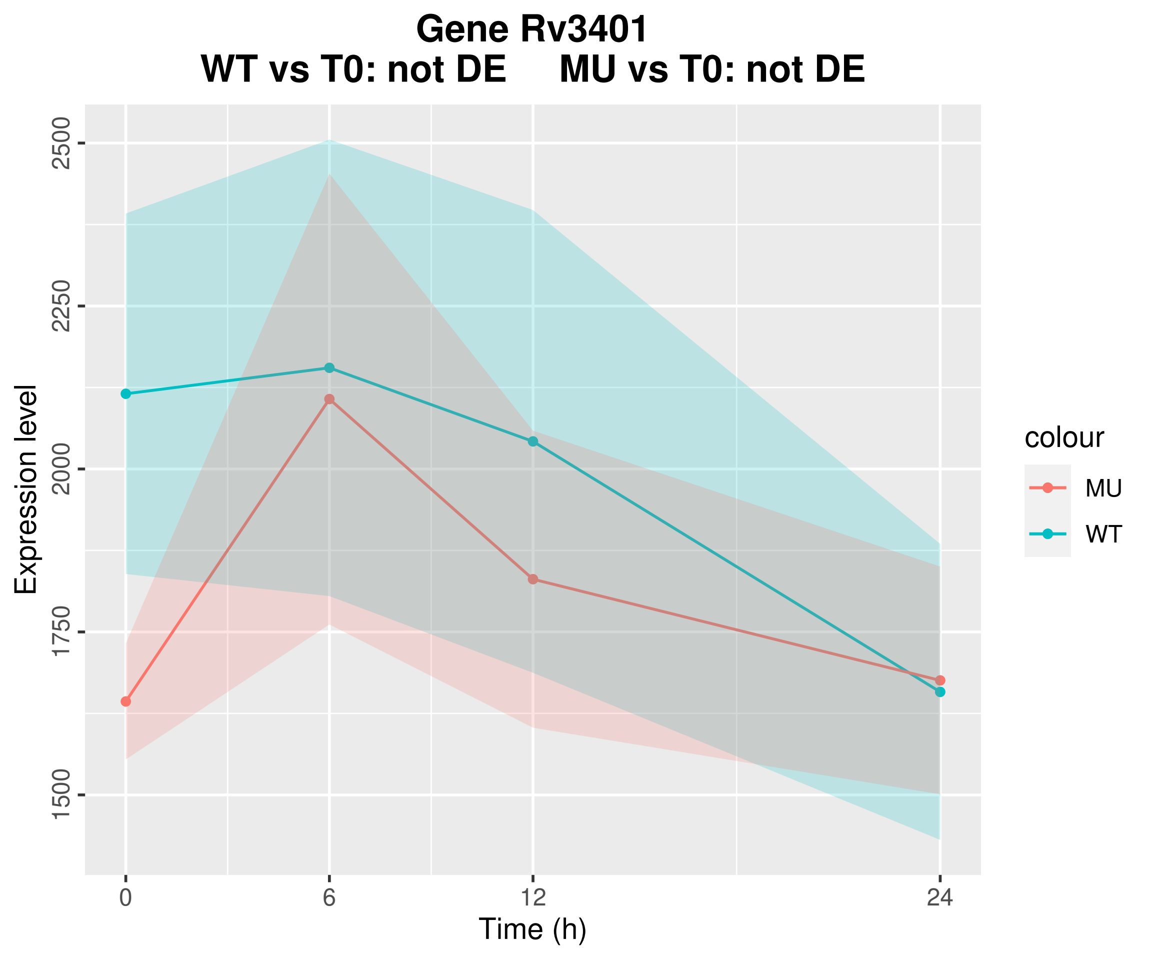

Gene Rv3401
WT vs T0: not DE MU vs T0: not DE
Expression level
2500
2250
2000
1750
1500
Time (h)
0
6
12
24
colour
MU
WT

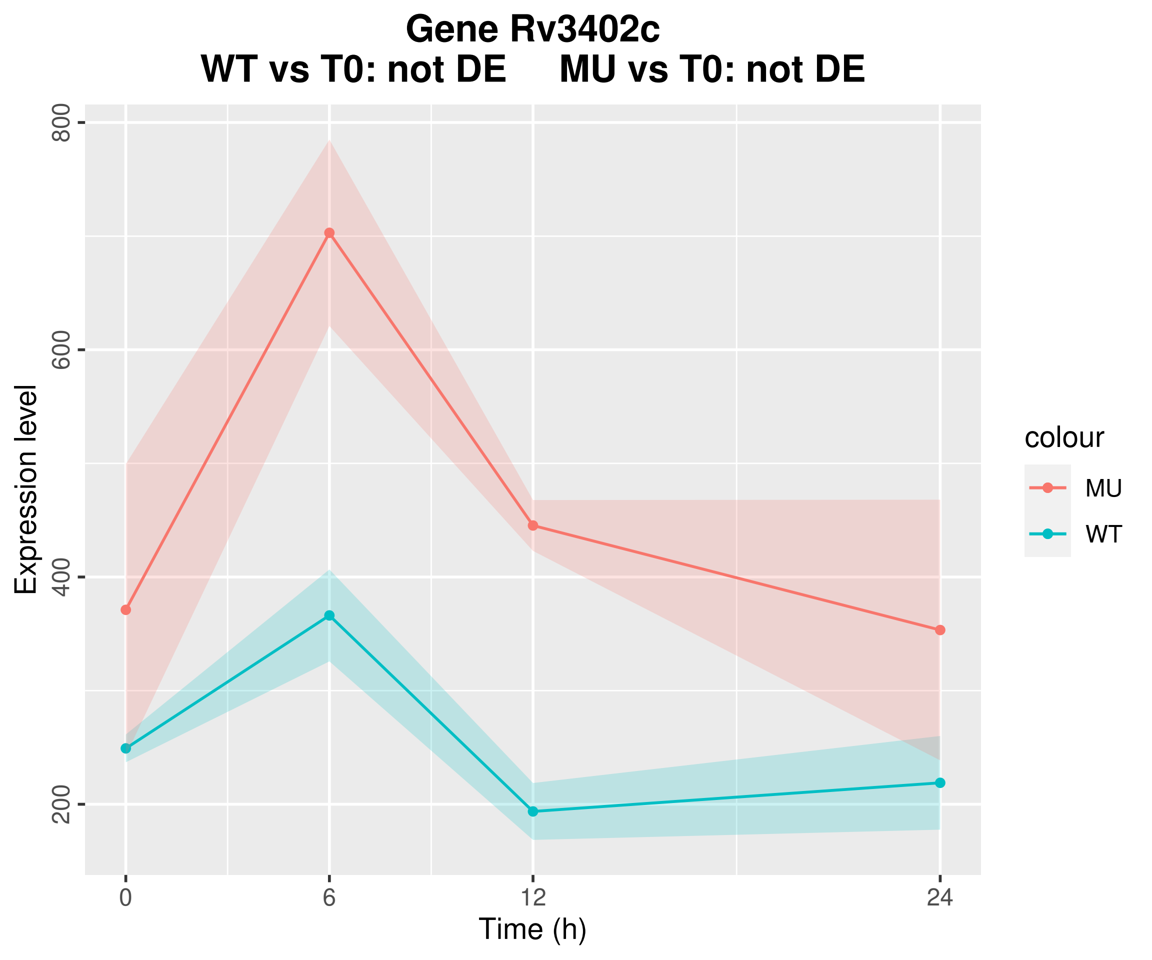

Gene Rv3402c
WT vs T0: not DE MU vs T0: not DE
Expression level
800
600
400
200
0
6
12
24
Time (h)
colour
MU
WT

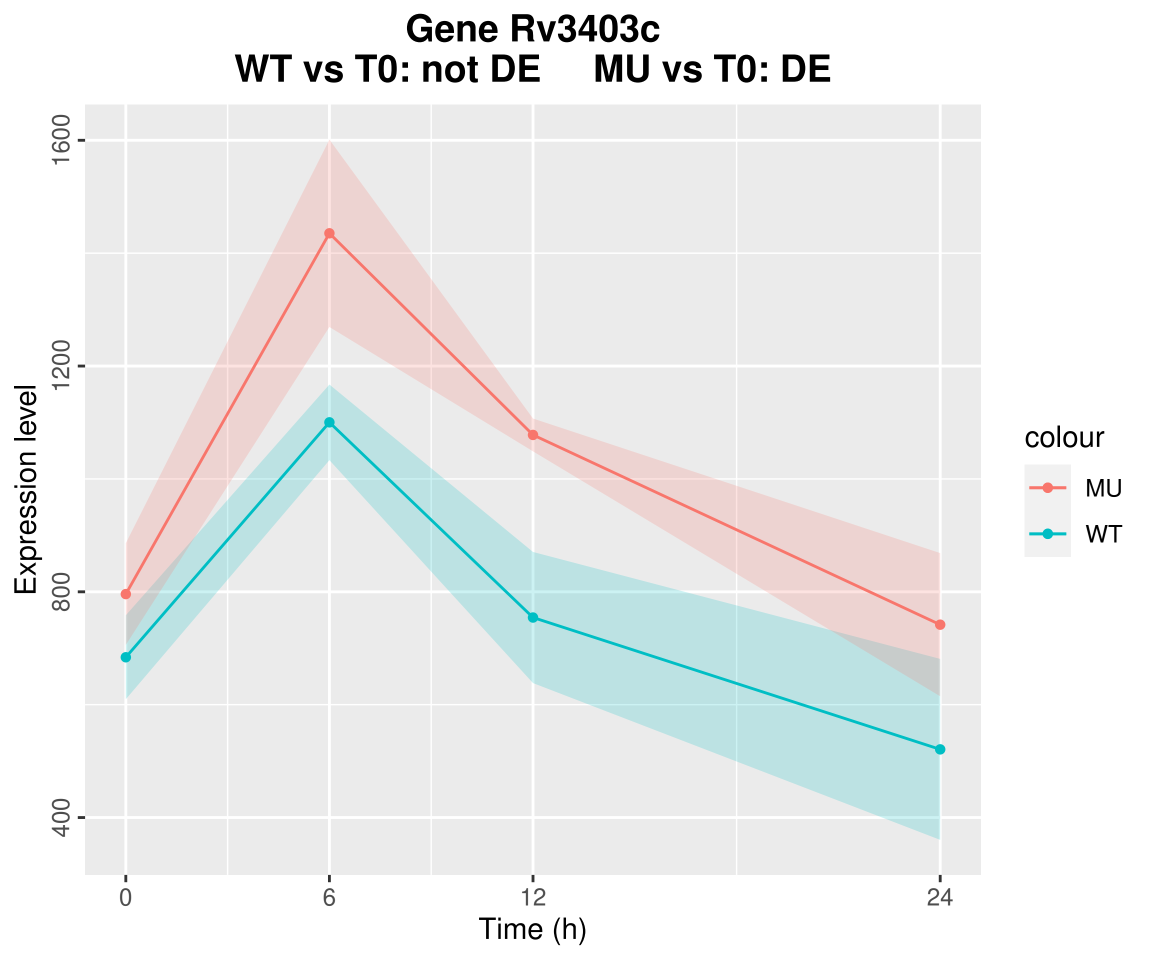

Gene Rv3403c
WT vs T0: not DE   MU vs T0: DE
Expression level
1600
1200
800
400
0
6
12
24
Time (h)
colour
MU
WT

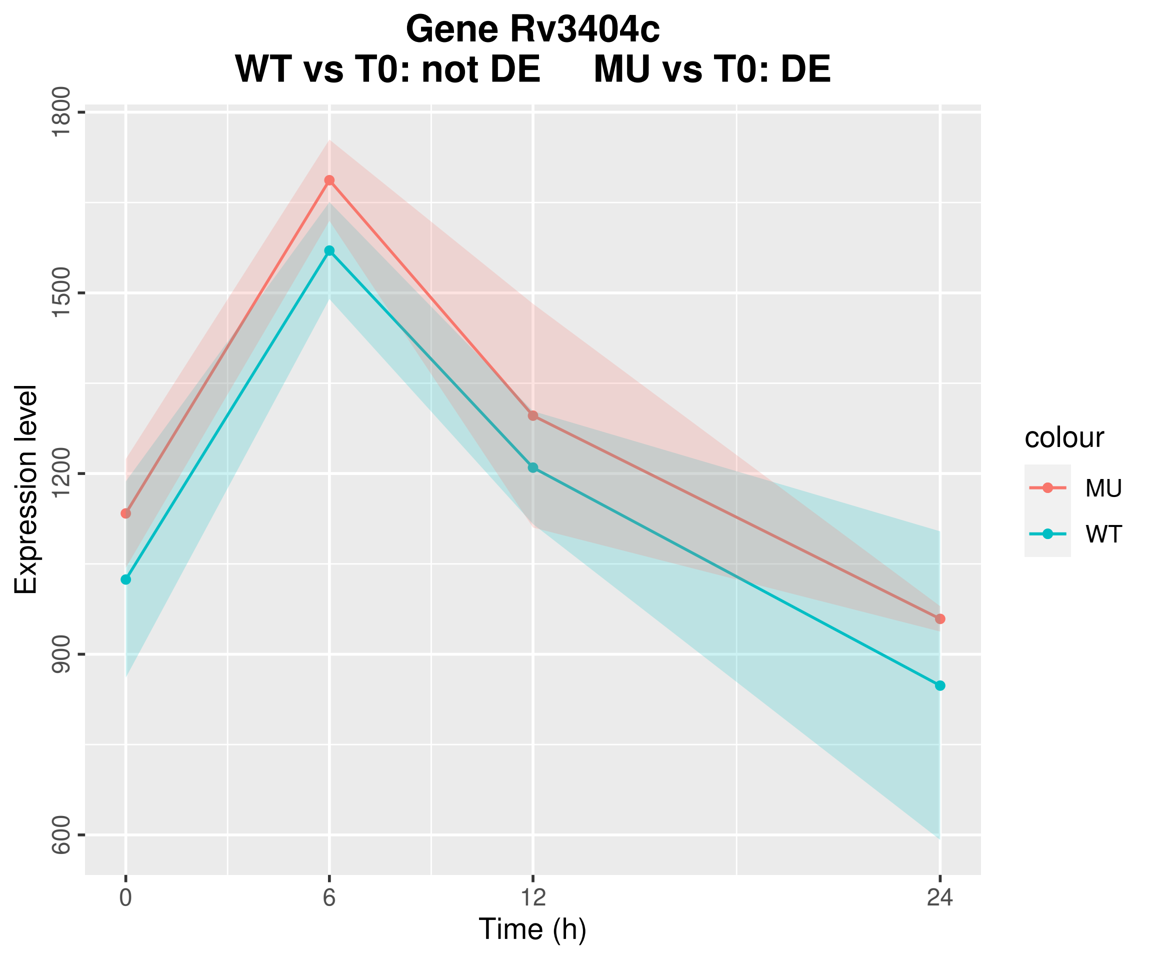

Gene Rv3404c
WT vs T0: not DE MU vs T0: DE
1800
1500
1200
900
600
Expression level
0
6
12
24
Time (h)
colour
MU
WT

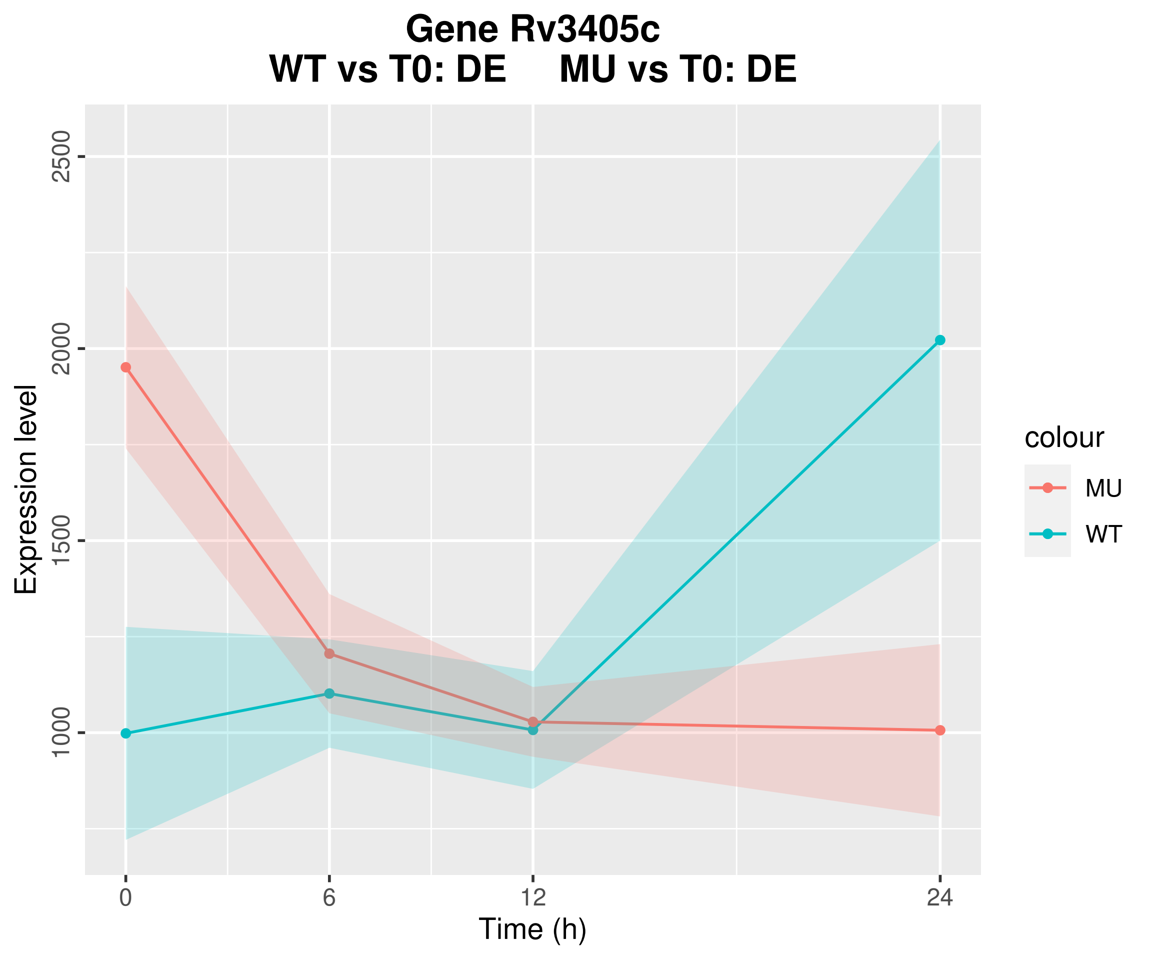

Gene Rv3405c
WT vs T0: DE   MU vs T0: DE
Expression level
2500
2000
1500
1000
0
6
12
24
Time (h)
colour
MU
WT

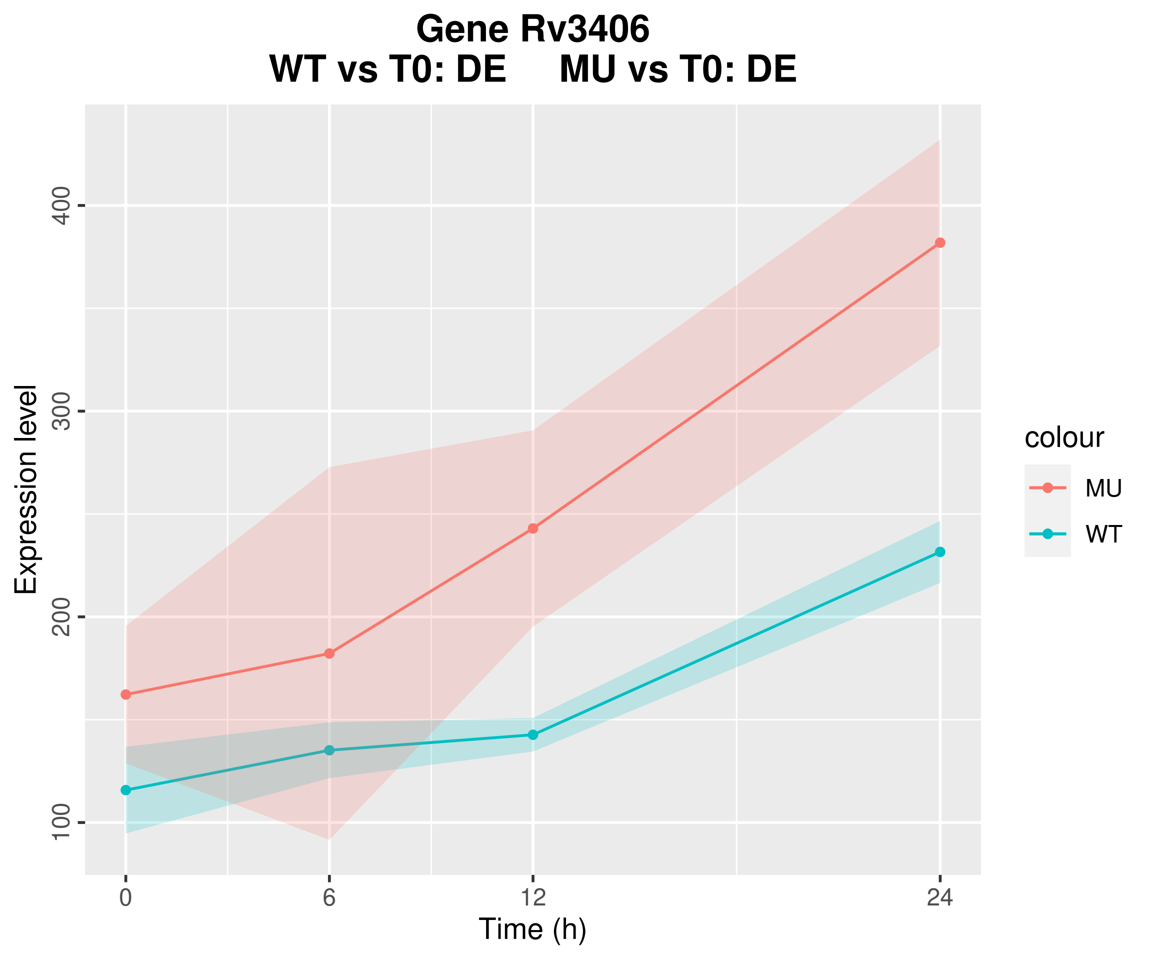
Gene Rv3406
WT vs T0: DE   MU vs T0: DE
Expression level
400
300
200
100
0
6
12
24
Time (h)
colour
MU
WT

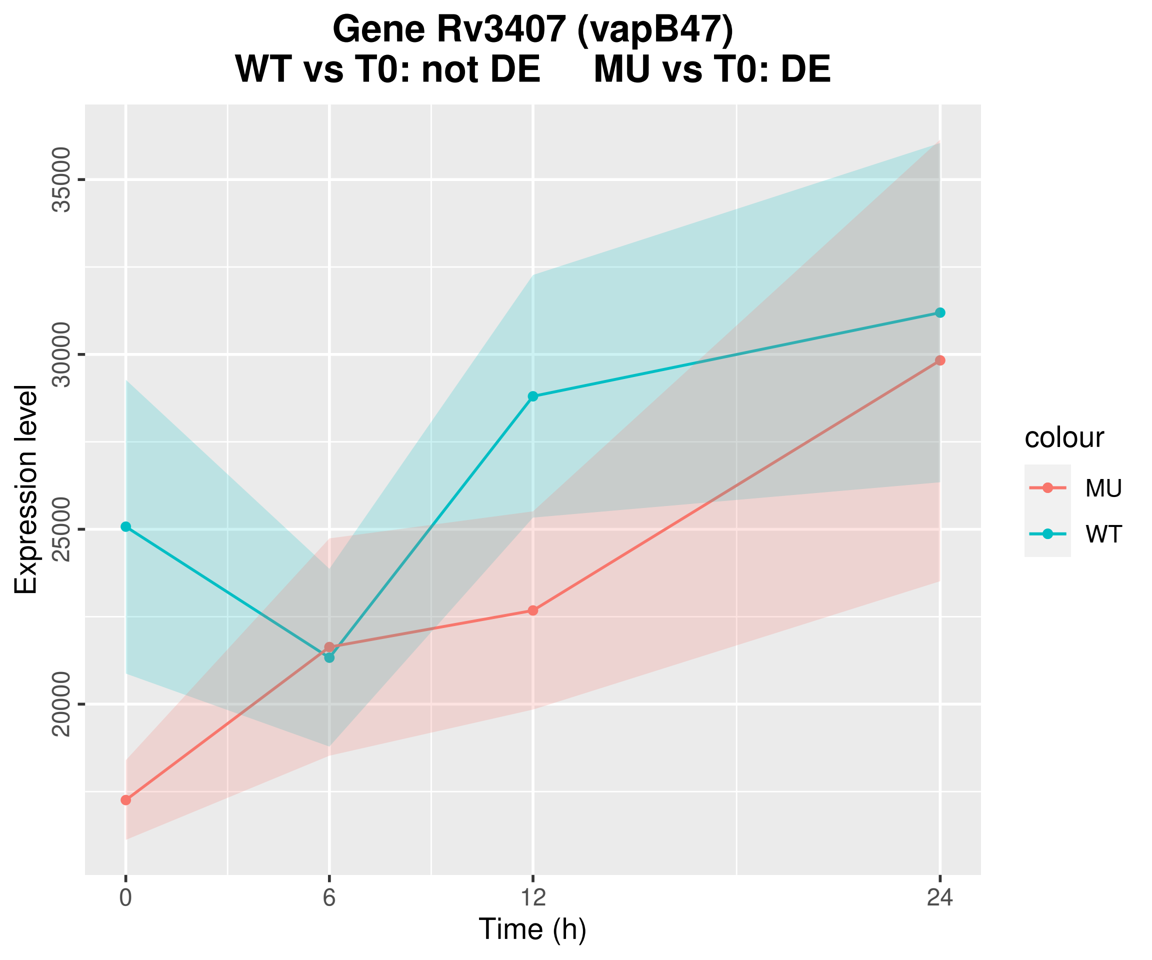

Gene Rv3407 (vapB47)
WT vs T0: not DE MU vs T0: DE
Expression level
35000
30000
25000
20000
0
6
12
24
Time (h)
colour
MU
WT

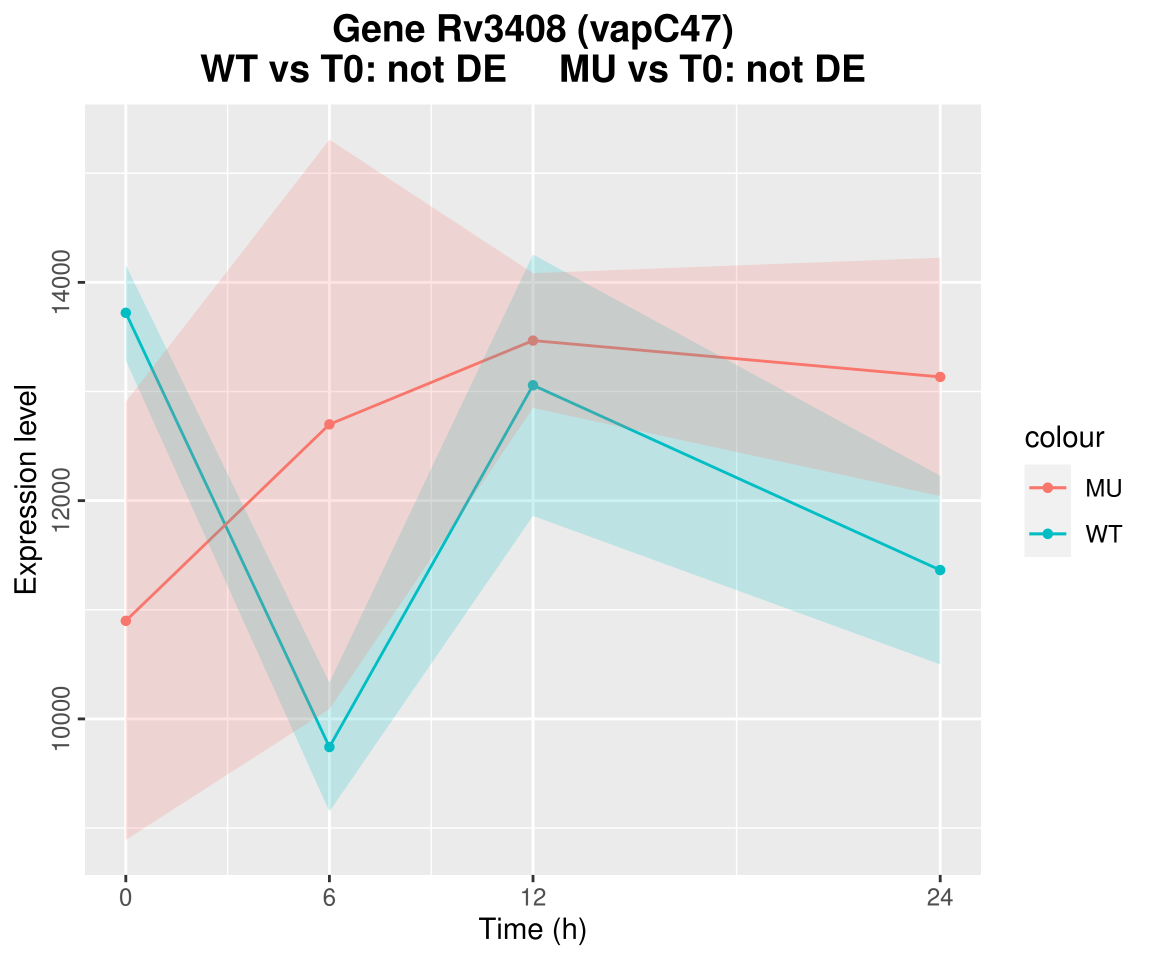

Gene Rv3408 (vapC47)
WT vs T0: not DE   MU vs T0: not DE
Expression level
14000
12000
10000
0
6
12
24
Time (h)
colour
MU
WT

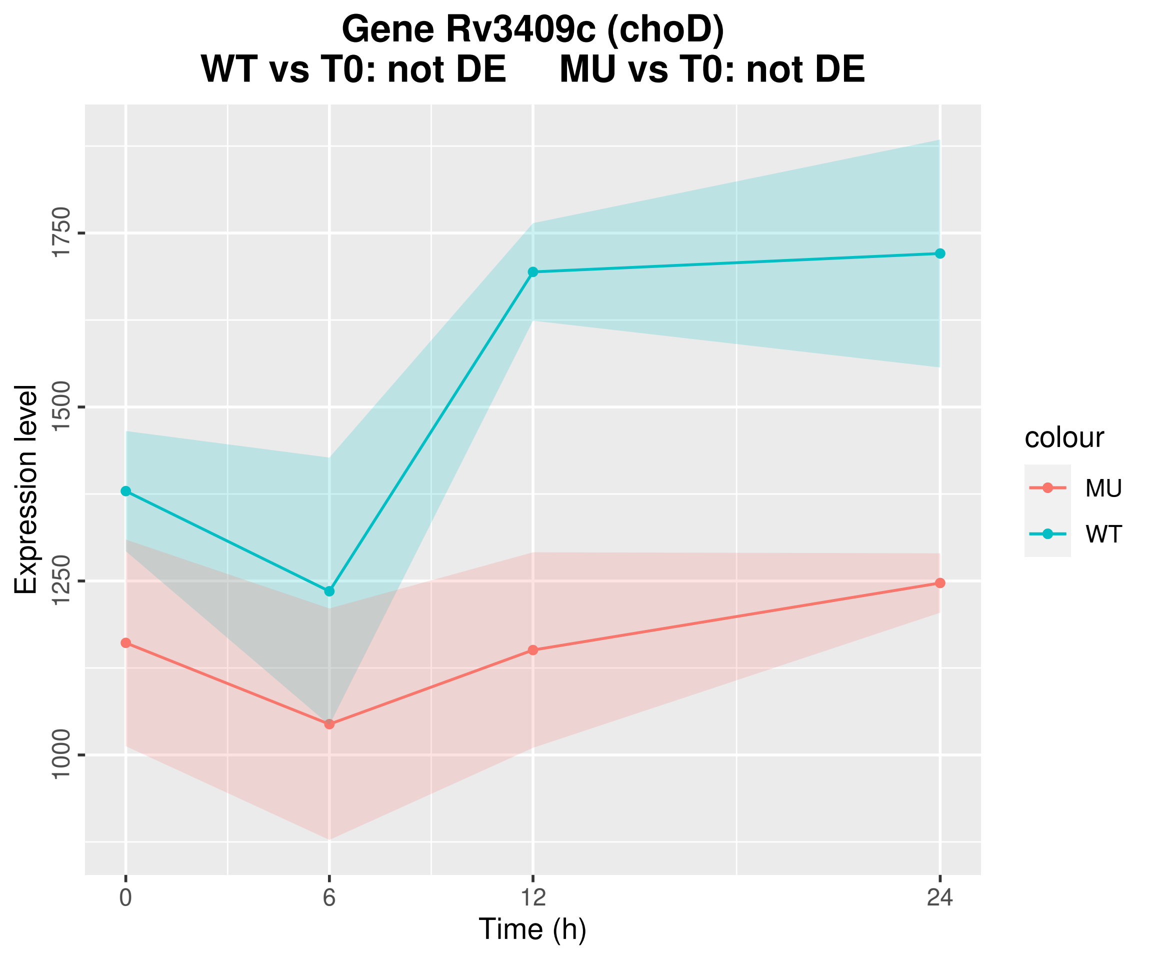
Gene Rv3409c (choD)
WT vs T0: not DE   MU vs T0: not DE
Expression level
1000
1250
1500
1750
0
6
12
24
Time (h)
colour
MU
WT

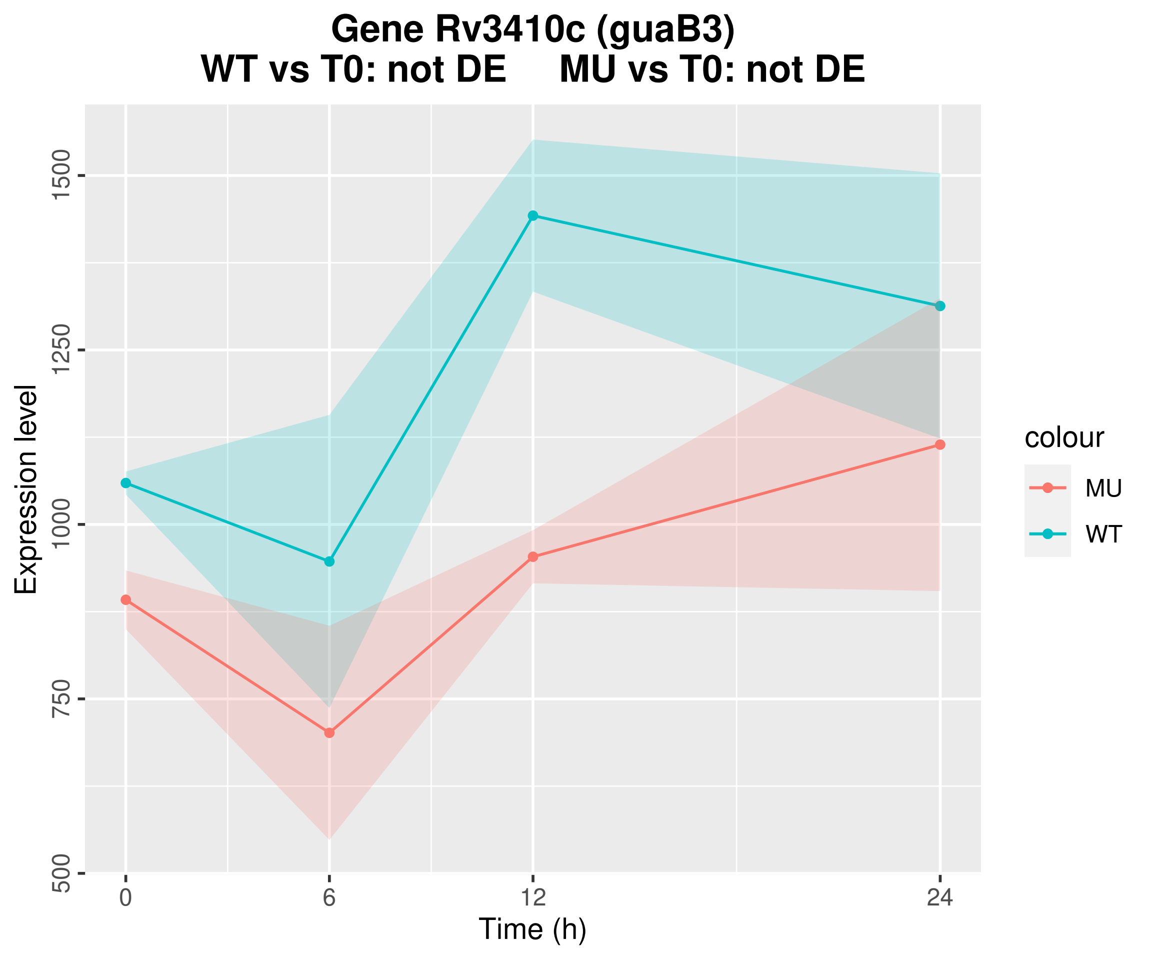

Gene Rv3410c (guaB3)
WT vs T0: not DE   MU vs T0: not DE
Expression level
1500
1250
1000
750
500
0
6
12
24
Time (h)
colour
MU
WT

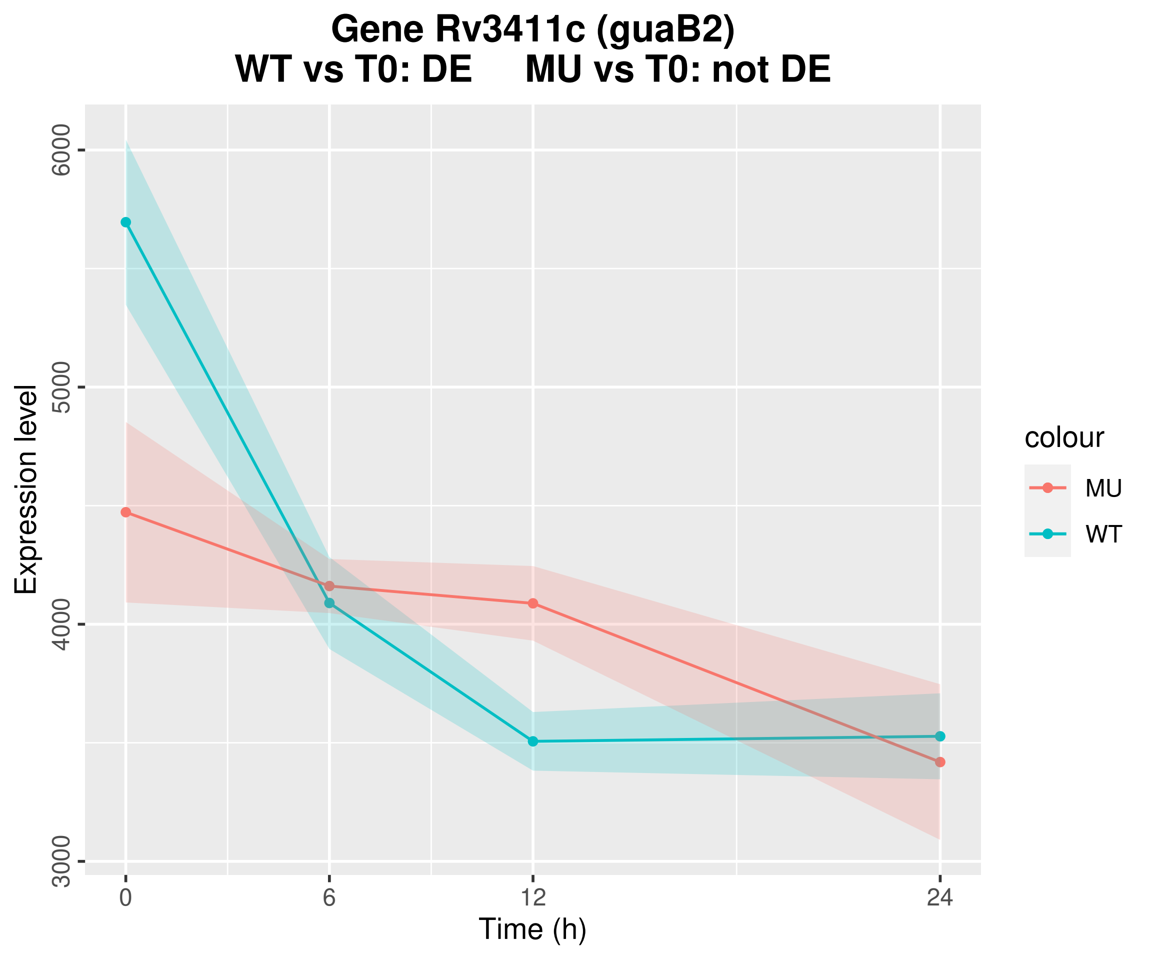
Gene Rv3411c (guaB2)
WT vs T0: DE   MU vs T0: not DE
Expression level
6000
5000
4000
3000
0
6
12
24
Time (h)
colour
MU
WT

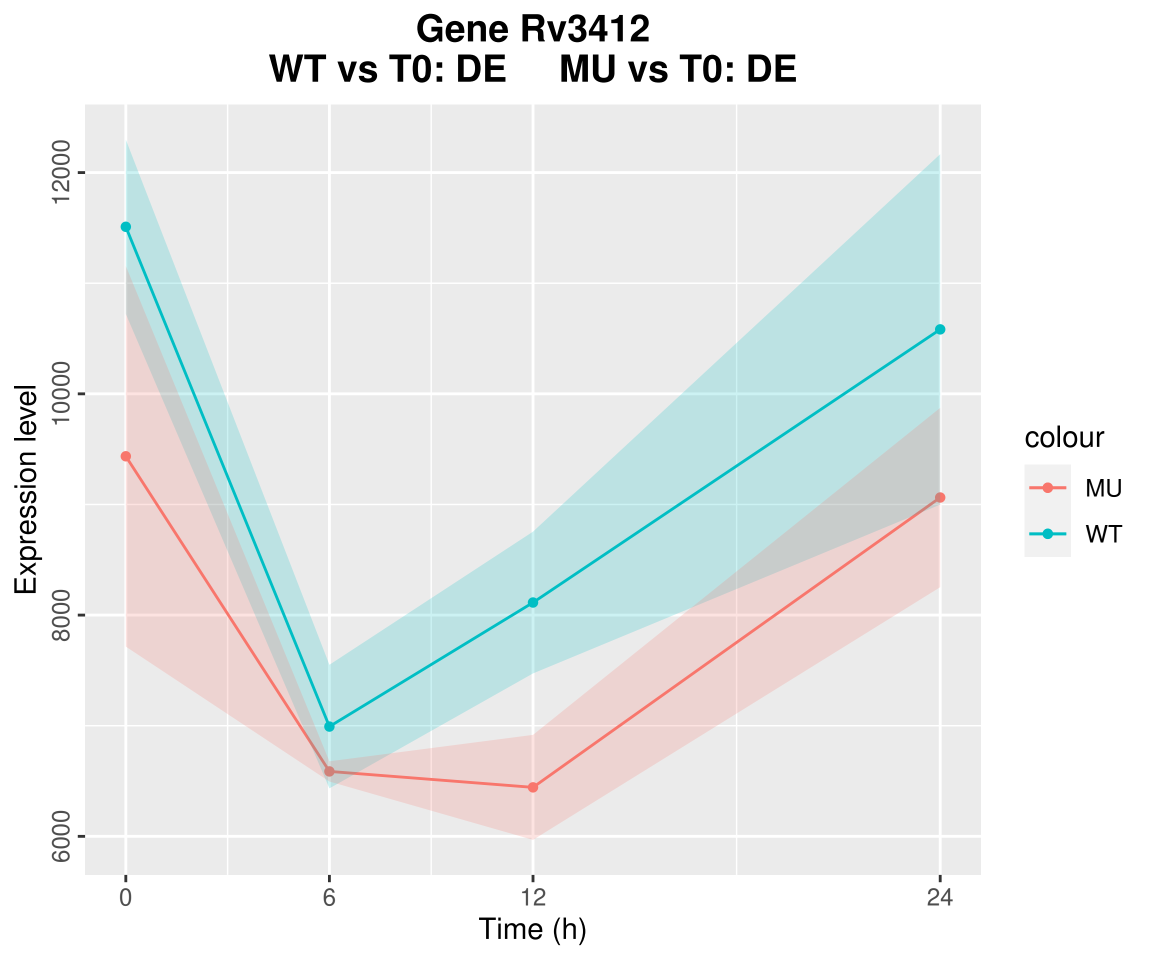
Gene Rv3412
WT vs T0: DE MU vs T0: DE
Expression level
12000
10000
8000
6000
0
6
12
24
Time (h)
colour
MU
WT

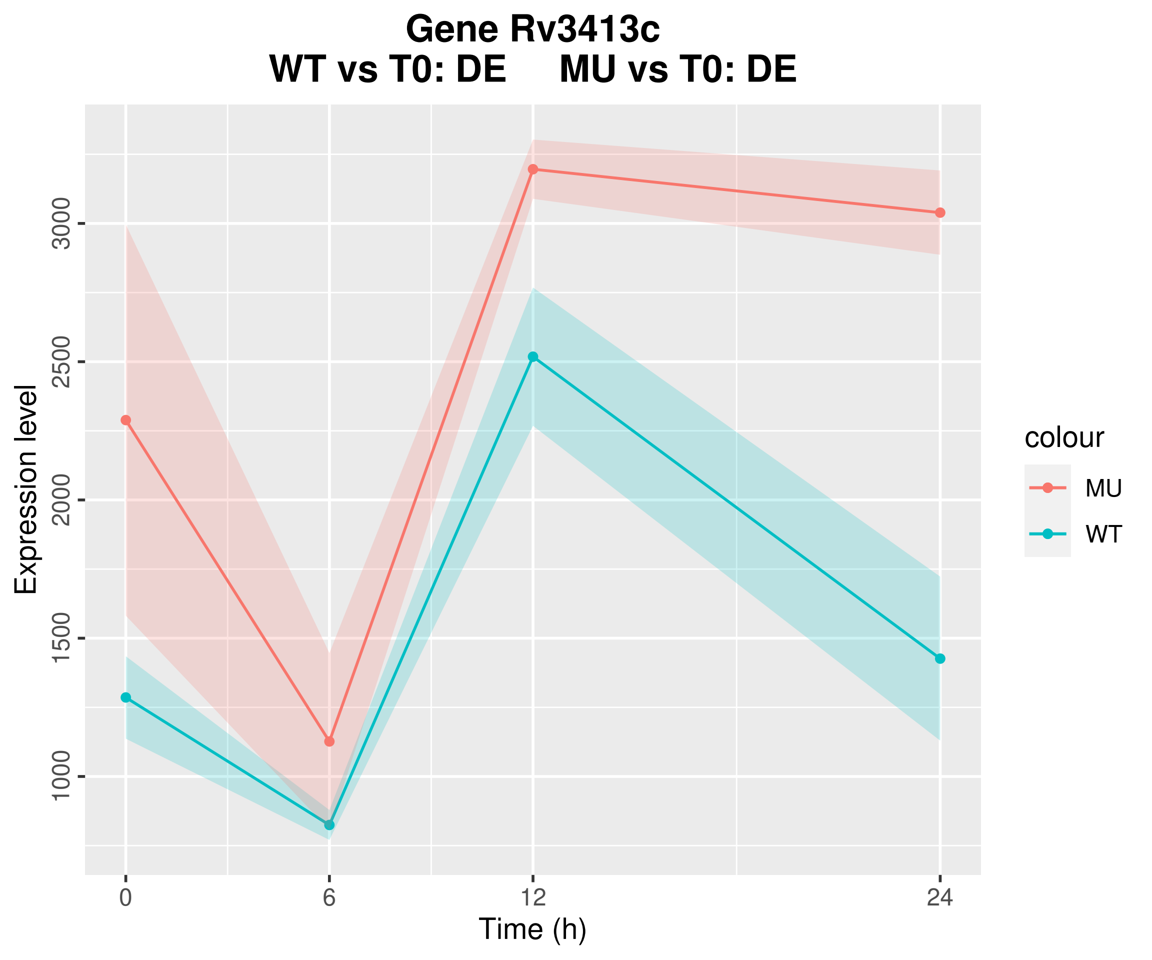
Gene Rv3413c
WT vs T0: DE MU vs T0: DE
Expression level
3000
2500
2000
1500
1000
0
6
12
24
Time (h)
colour
MU
WT

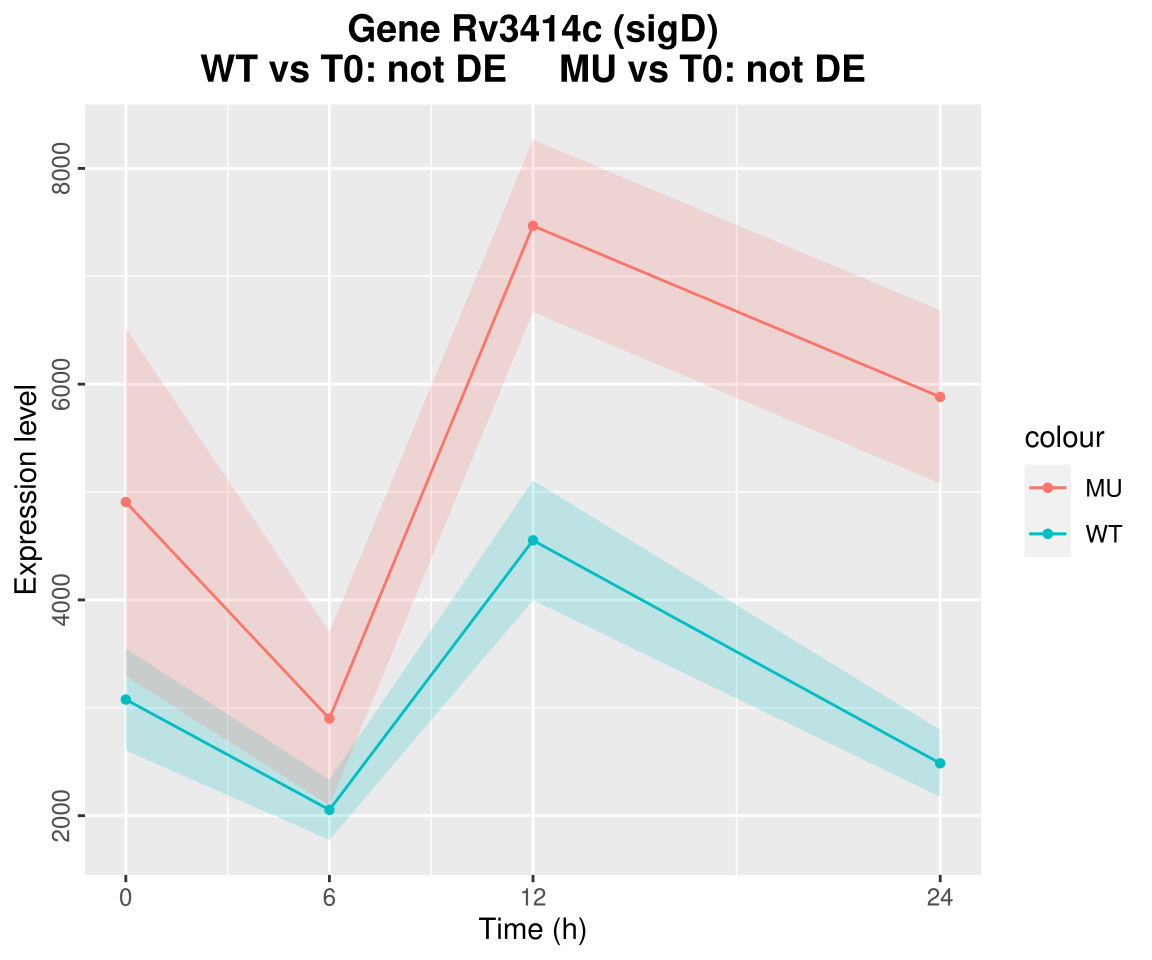
Gene Rv3414c (sigD)
WT vs T0: not DE MU vs T0: not DE
8000
6000
4000
2000
Expression level
0
6
12
24
Time (h)
colour
MU
WT

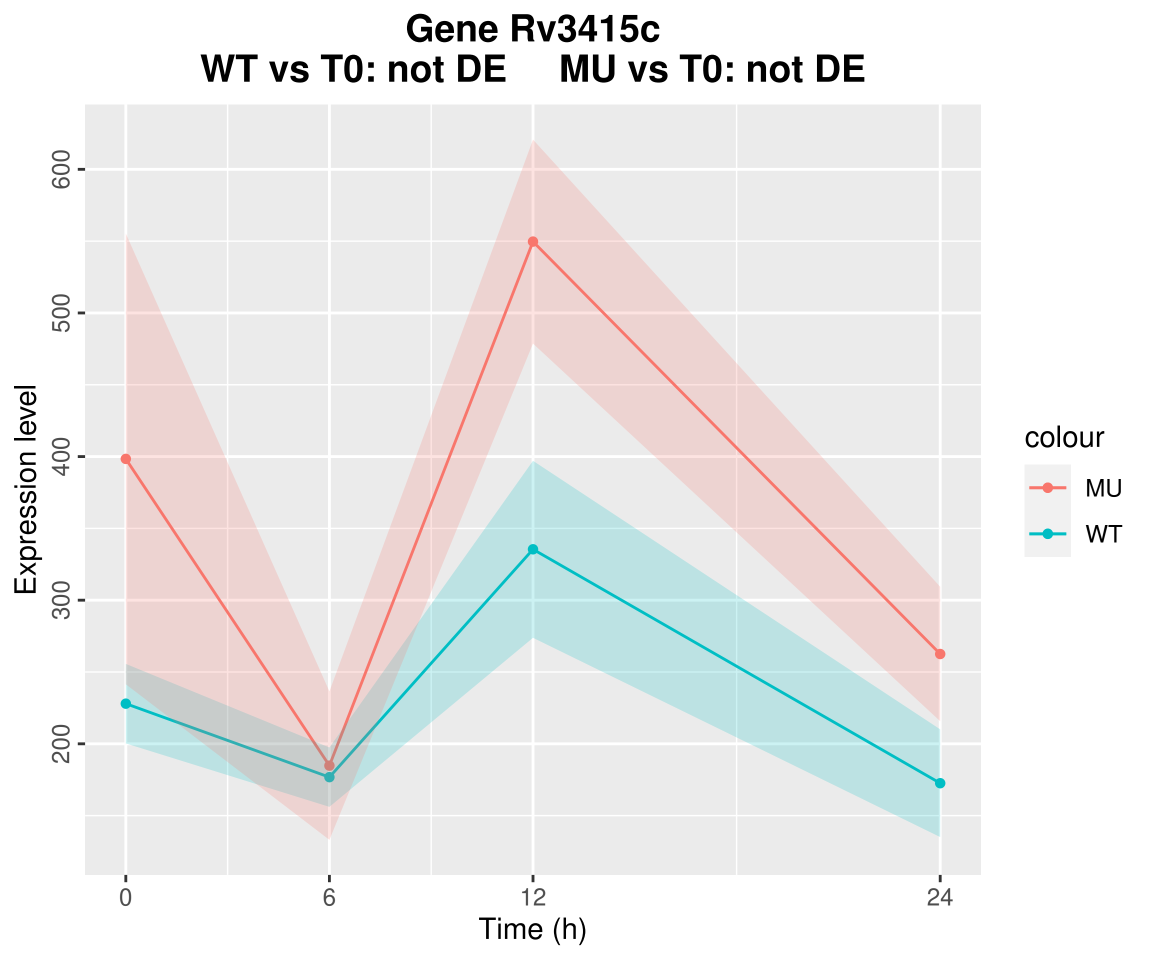
Gene Rv3415c
WT vs T0: not DE   MU vs T0: not DE
600
500
400
300
200
Expression level
0
6
12
24
Time (h)
colour
MU
WT

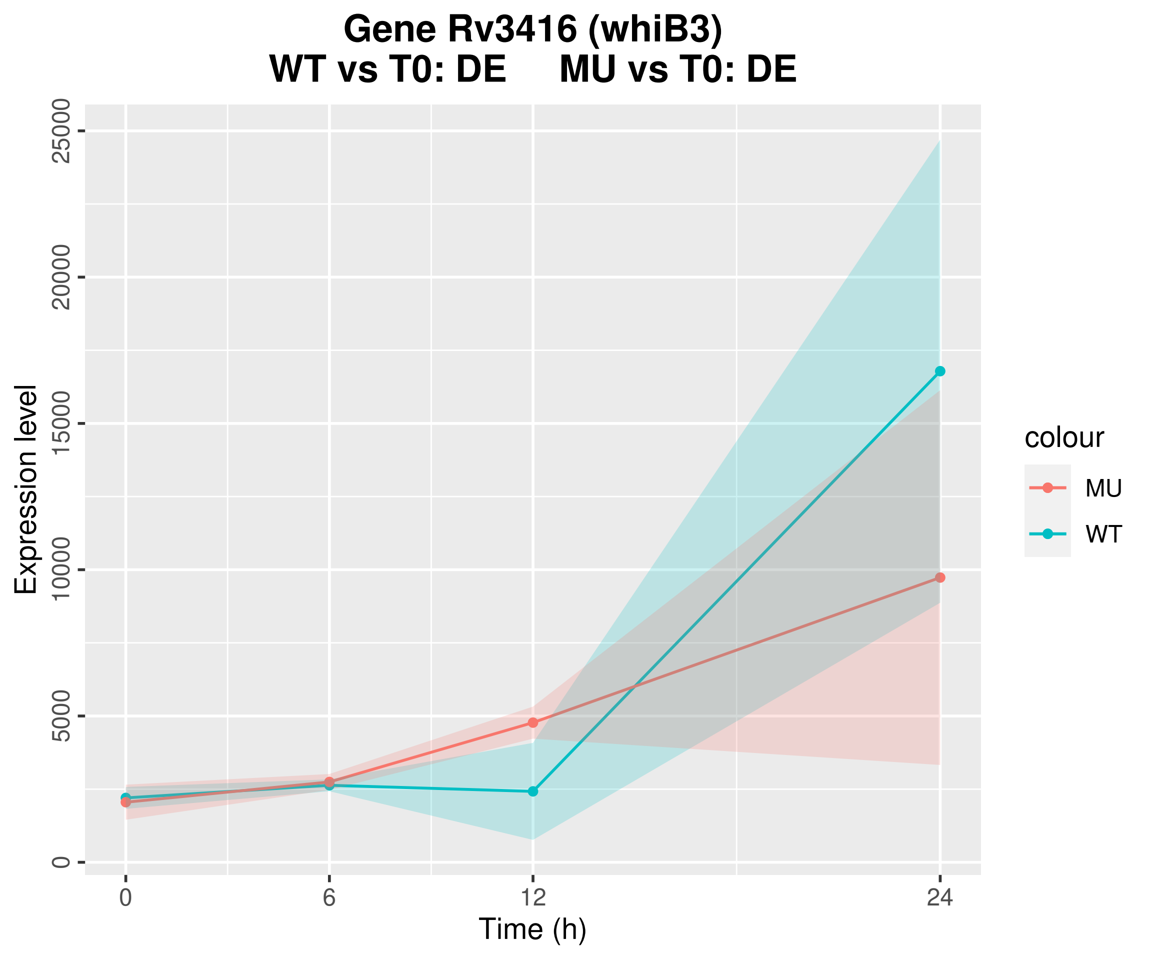
Gene Rv3416 (whiB3)
WT vs T0: DE   MU vs T0: DE
Expression level
0
5000
10000
15000
20000
25000
0
6
12
24
Time (h)
colour
MU
WT

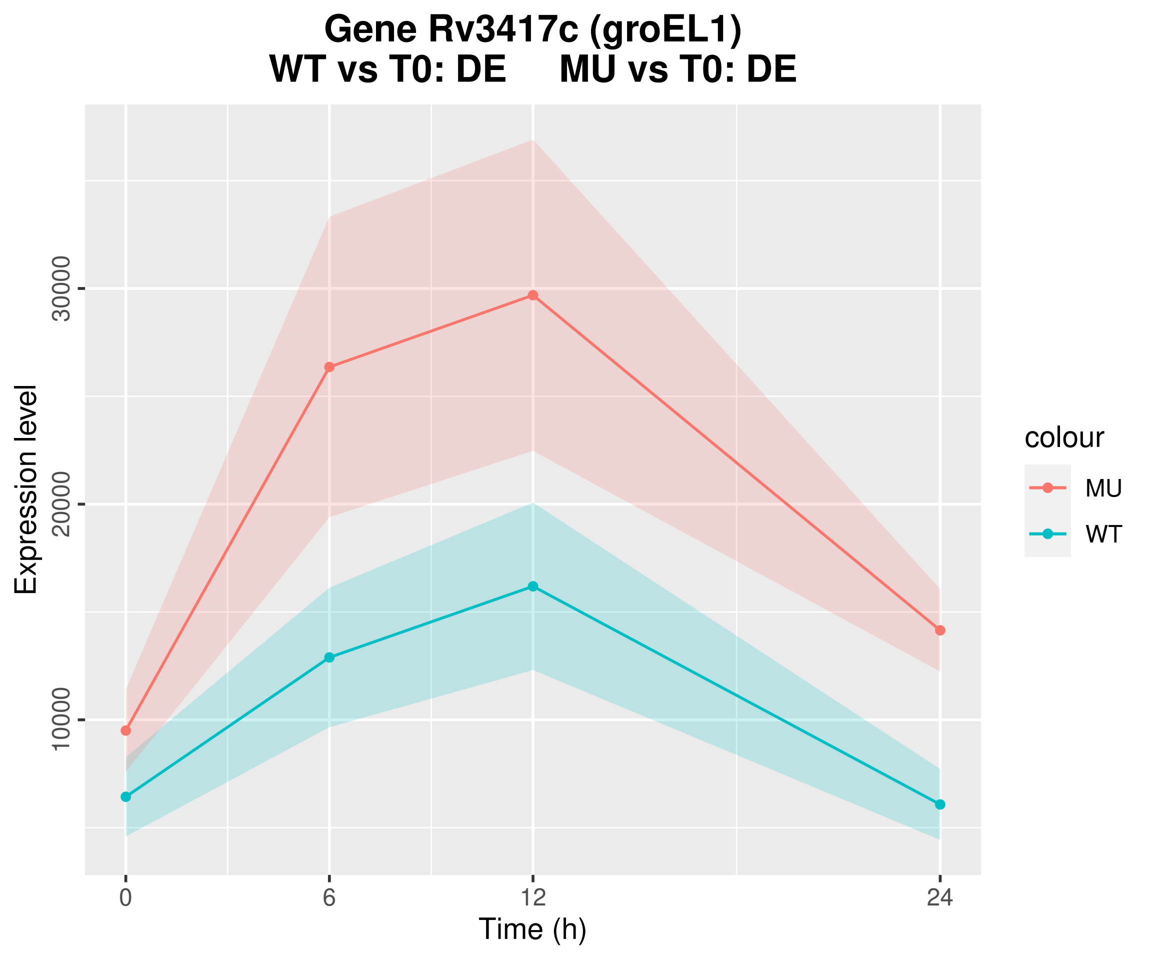

Gene Rv3417c (groEL1)
WT vs T0: DE   MU vs T0: DE
Expression level
30000
20000
10000
0
6
12
24
Time (h)
colour
MU
WT

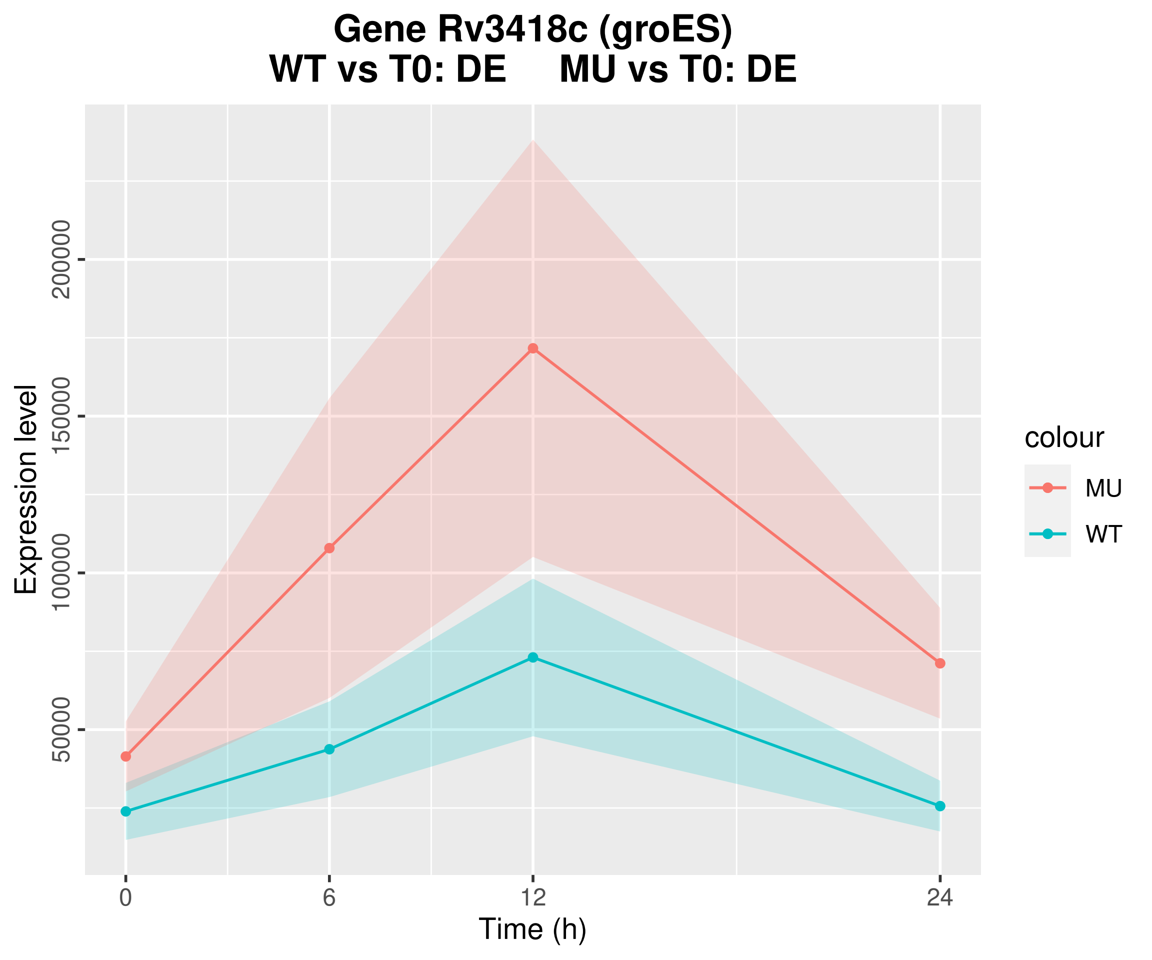

Gene Rv3418c (groES)
WT vs T0: DE MU vs T0: DE
Expression level
200000
150000
100000
50000
0
6
12
24
Time (h)
colour
MU
WT

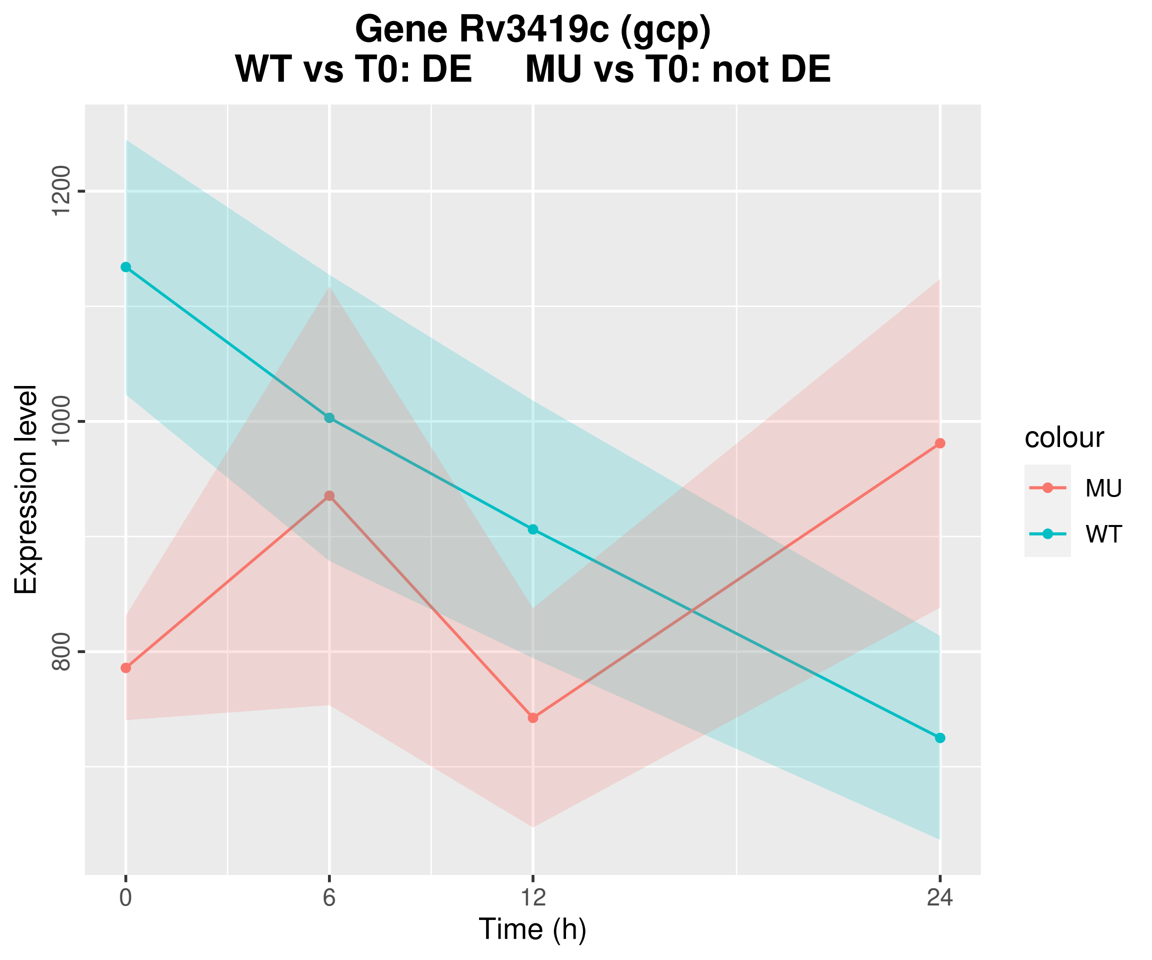

Gene Rv3419c (gcp)
WT vs T0: DE MU vs T0: not DE
Expression level
1200
1000
800
0
6
12
24
Time (h)
colour
MU
WT

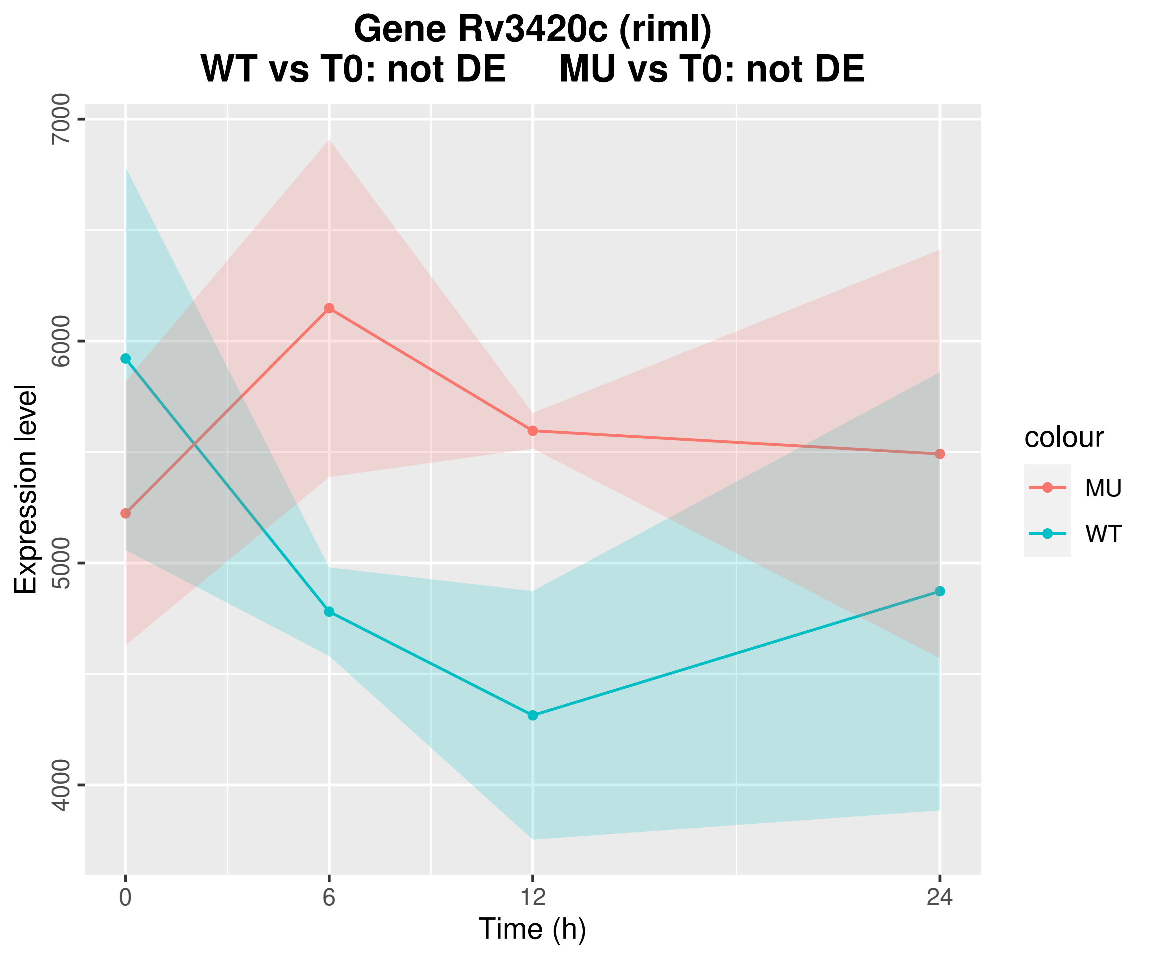
Gene Rv3420c (rimI)
WT vs T0: not DE   MU vs T0: not DE
7000
6000
5000
4000
Expression level
0
6
12
24
Time (h)
colour
MU
WT

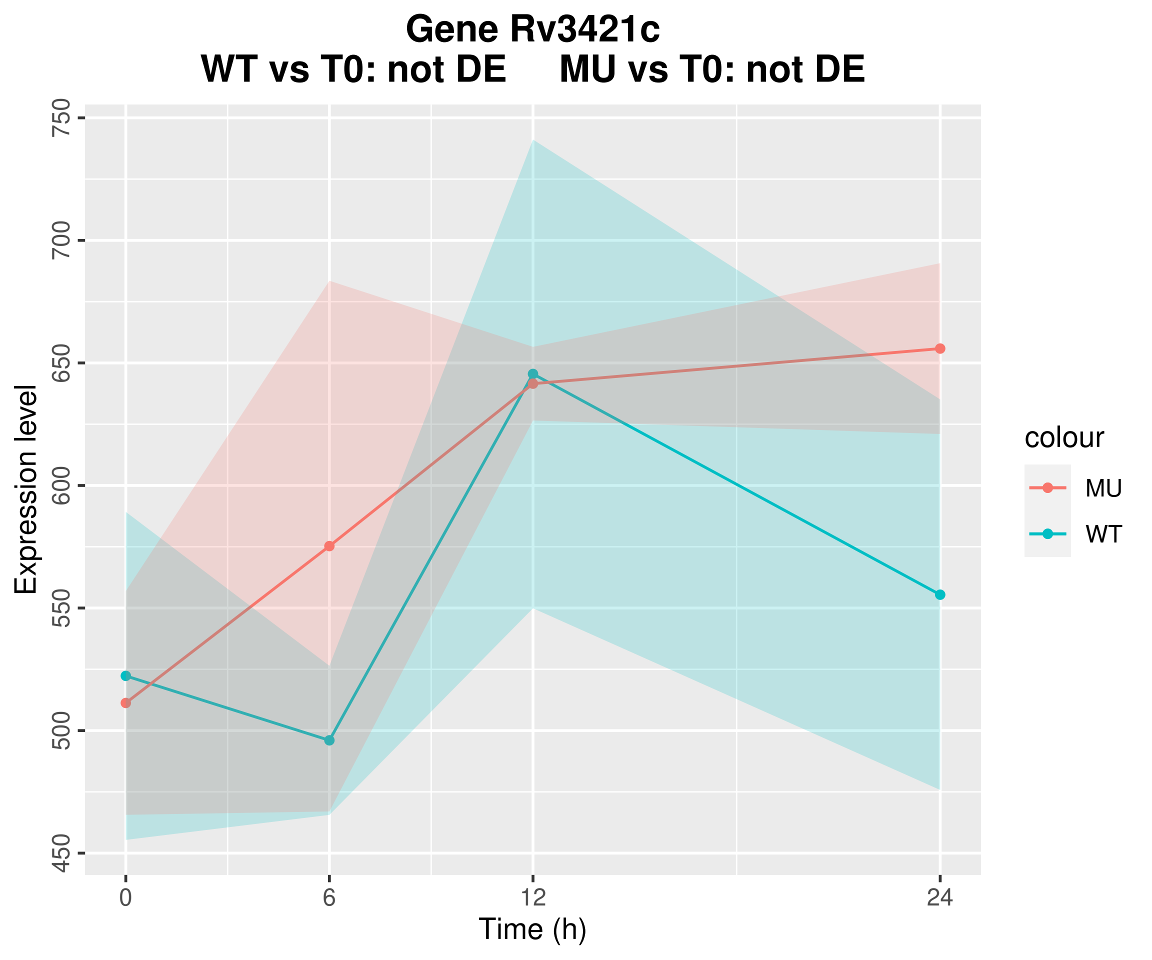
Gene Rv3421c
WT vs T0: not DE MU vs T0: not DE
Expression level
750
700
650
600
550
500
450
0
6
12
24
Time (h)
colour
MU
WT

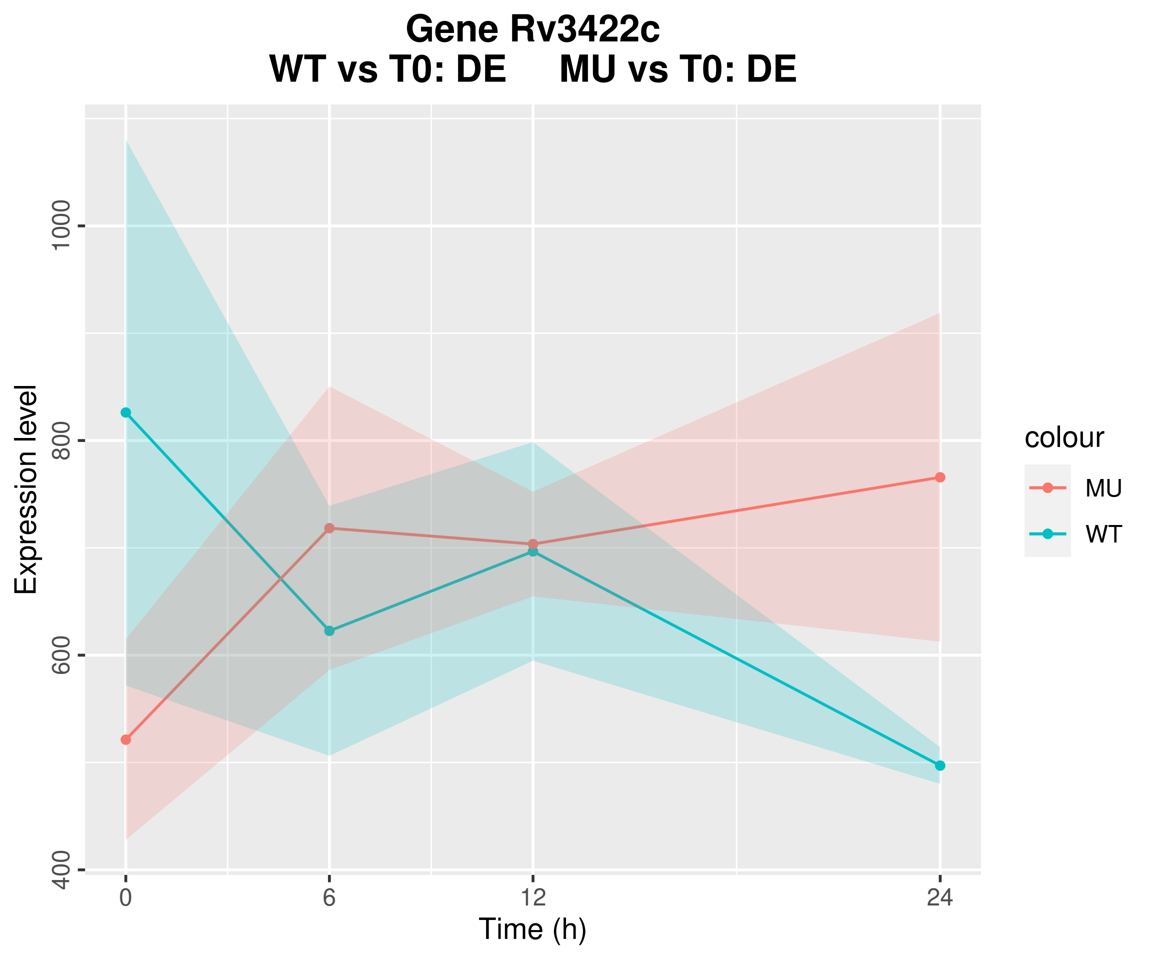
Gene Rv3422c
WT vs T0: DE MU vs T0: DE
Expression level
1000
800
600
400
0
6
12
24
Time (h)
colour
MU
WT

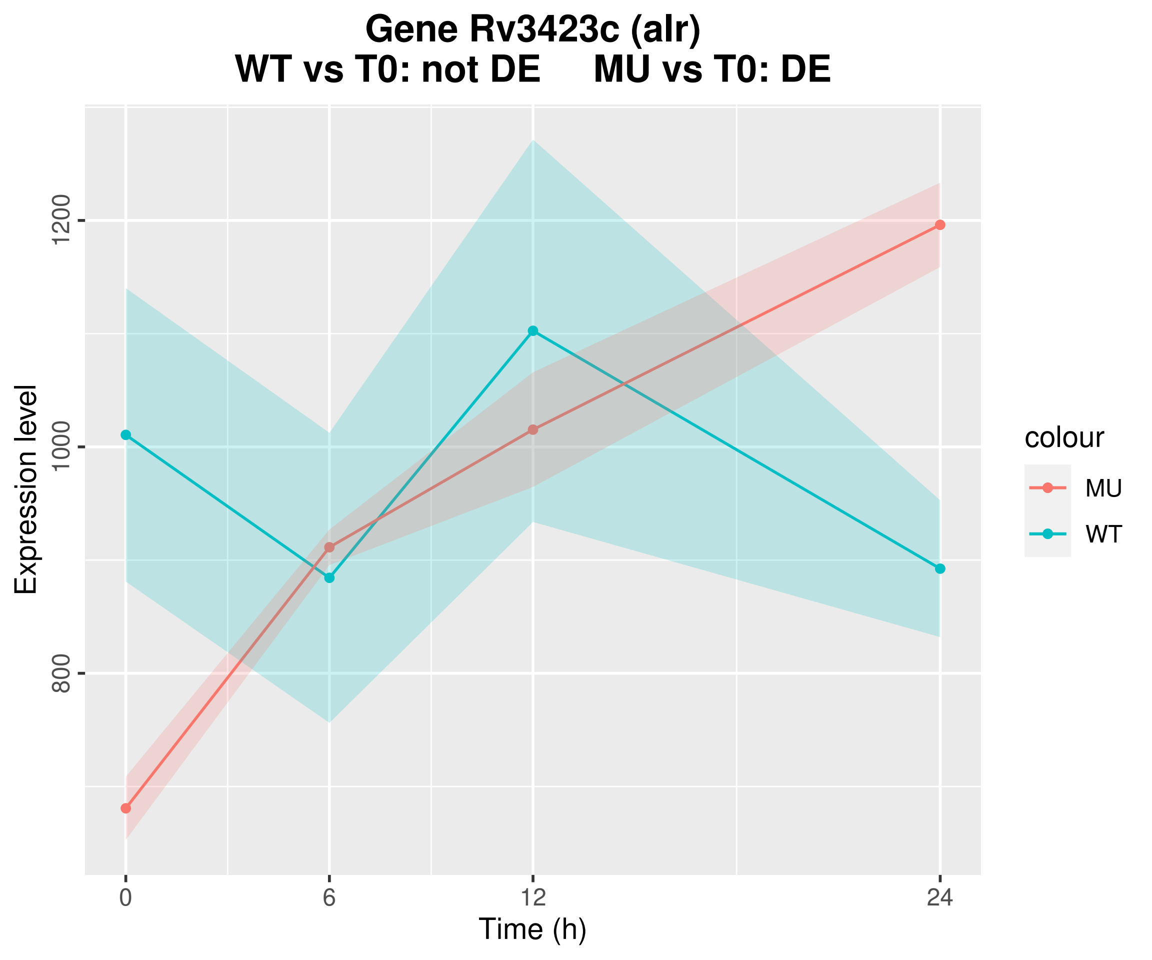

Gene Rv3423c (alr)
WT vs T0: not DE   MU vs T0: DE
Expression level
1200
1000
800
0
6
12
24
Time (h)
colour
MU
WT

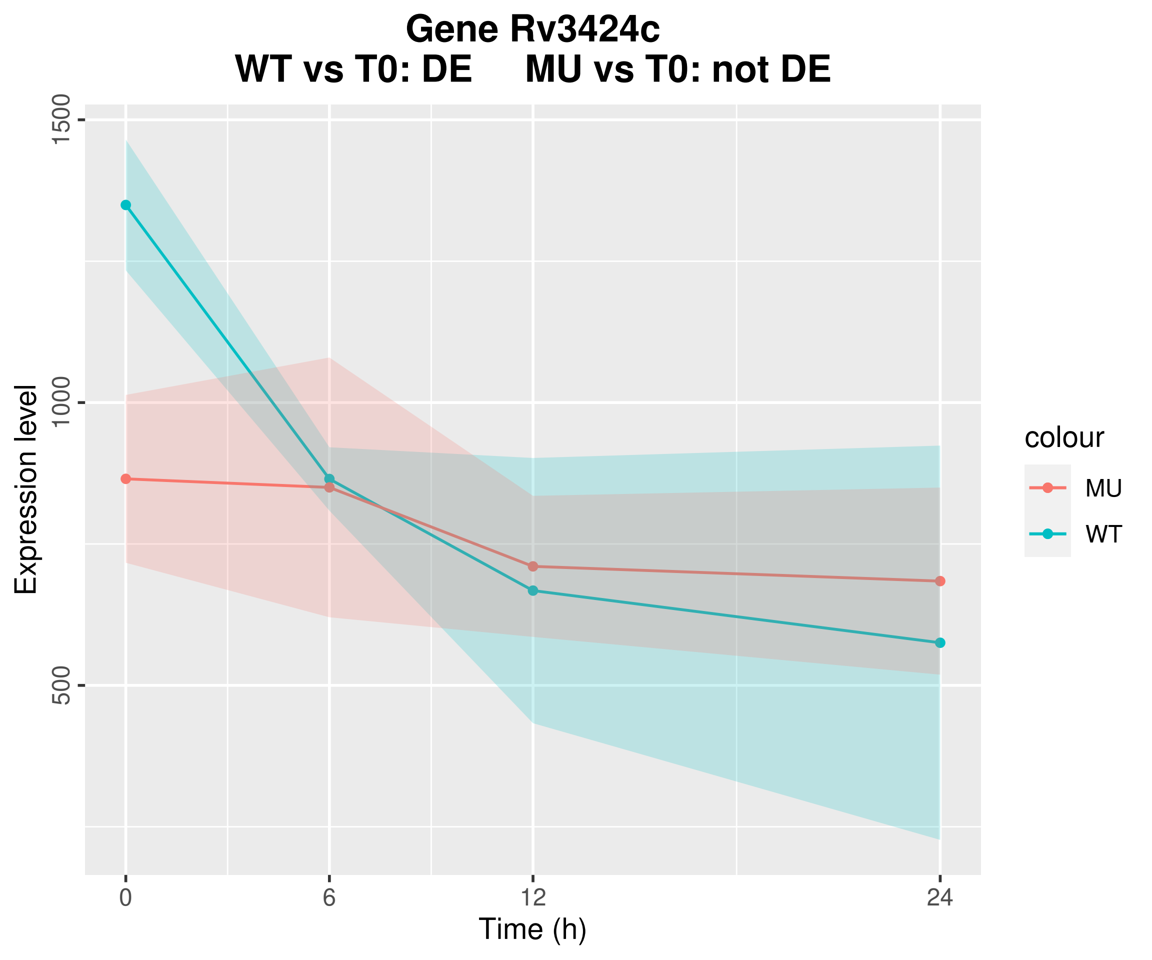

Gene Rv3424c
WT vs T0: DE   MU vs T0: not DE
1500
1000
500
Expression level
0
6
12
24
Time (h)
colour
MU
WT

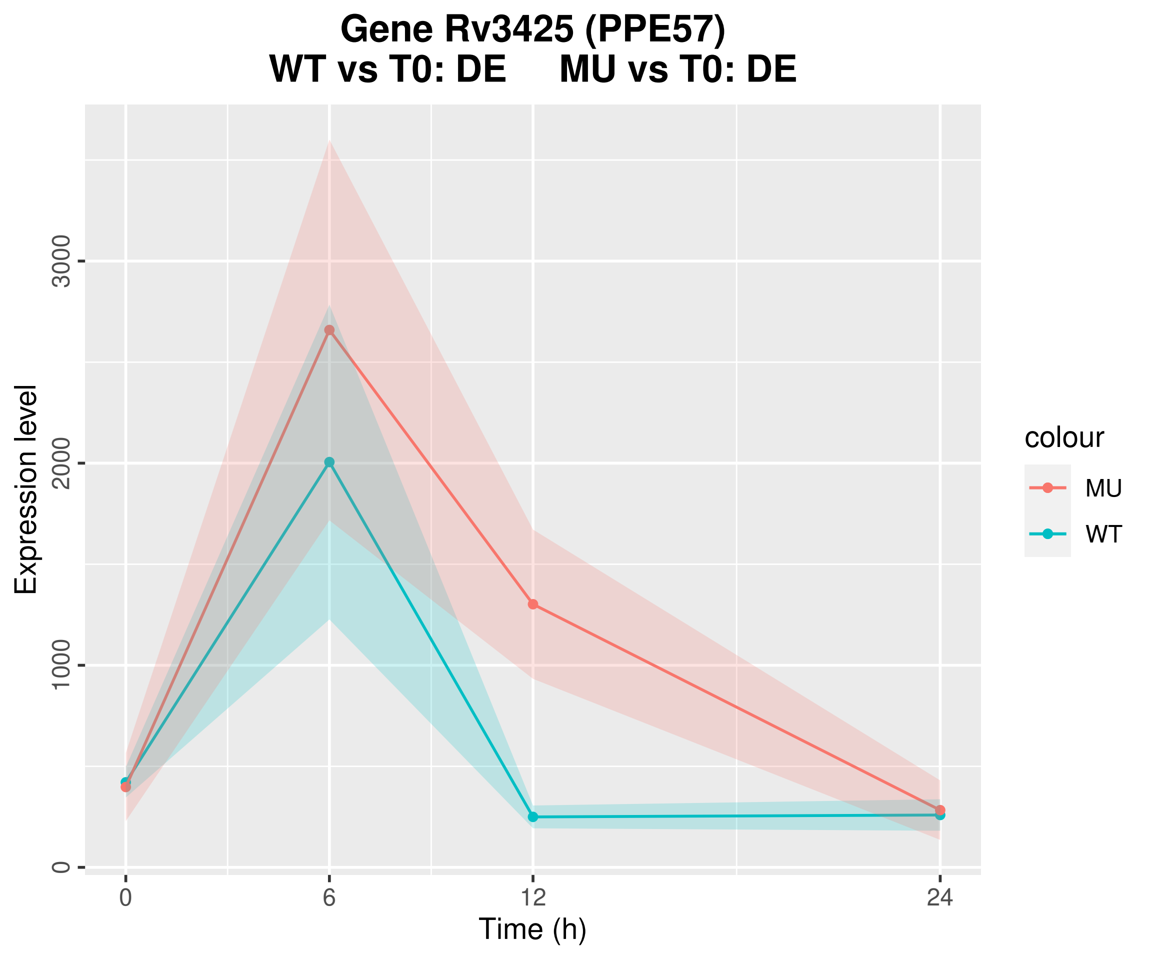
Gene Rv3425 (PPE57)
WT vs T0: DE   MU vs T0: DE
Expression level
3000
2000
1000
0
0
6
12
24
Time (h)
colour
MU
WT

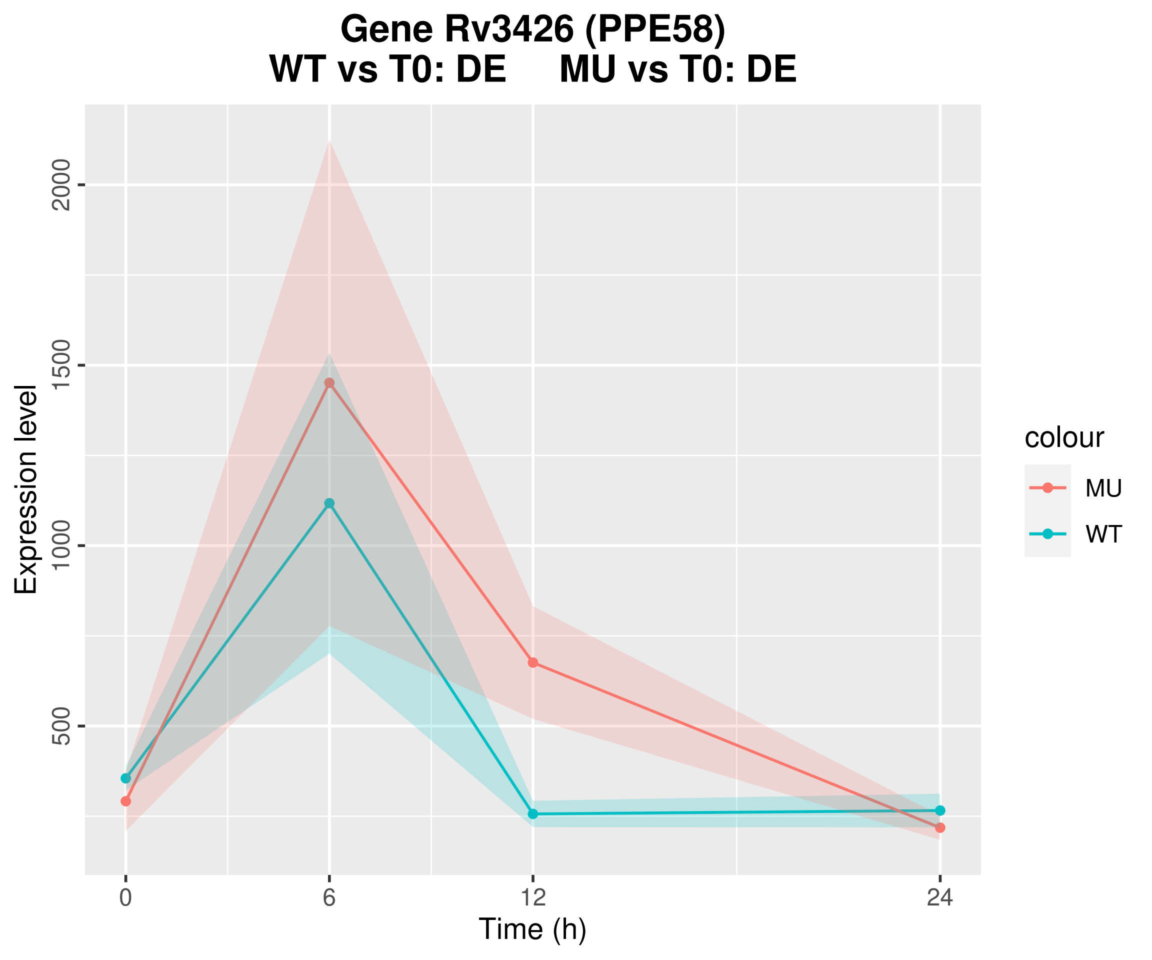

Gene Rv3426 (PPE58)
WT vs T0: DE MU vs T0: DE
Expression level
2000
1500
1000
500
0
6
12
24
Time (h)
colour
MU
WT

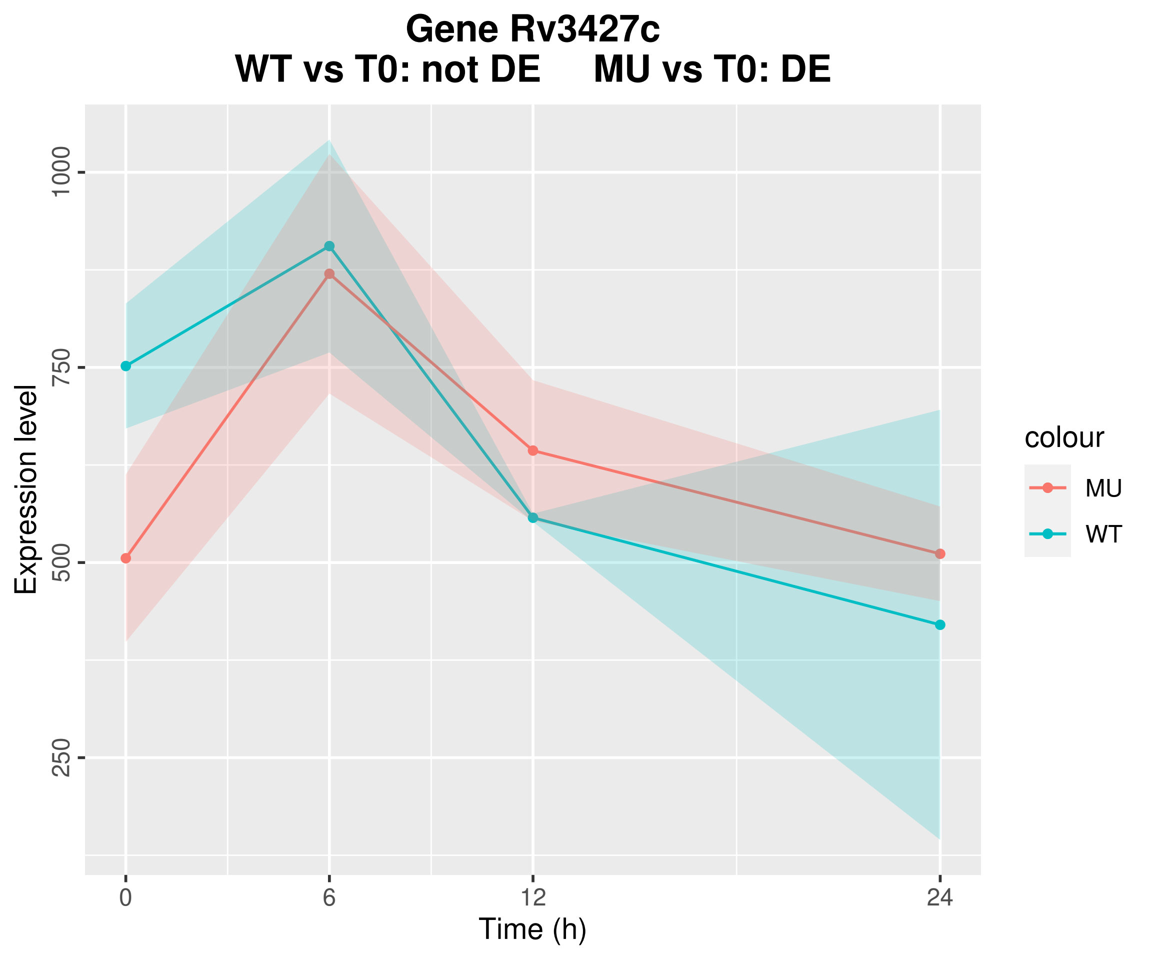

Gene Rv3427c
WT vs T0: not DE   MU vs T0: DE
Expression level
1000
750
500
250
0
6
12
24
Time (h)
colour
MU
WT

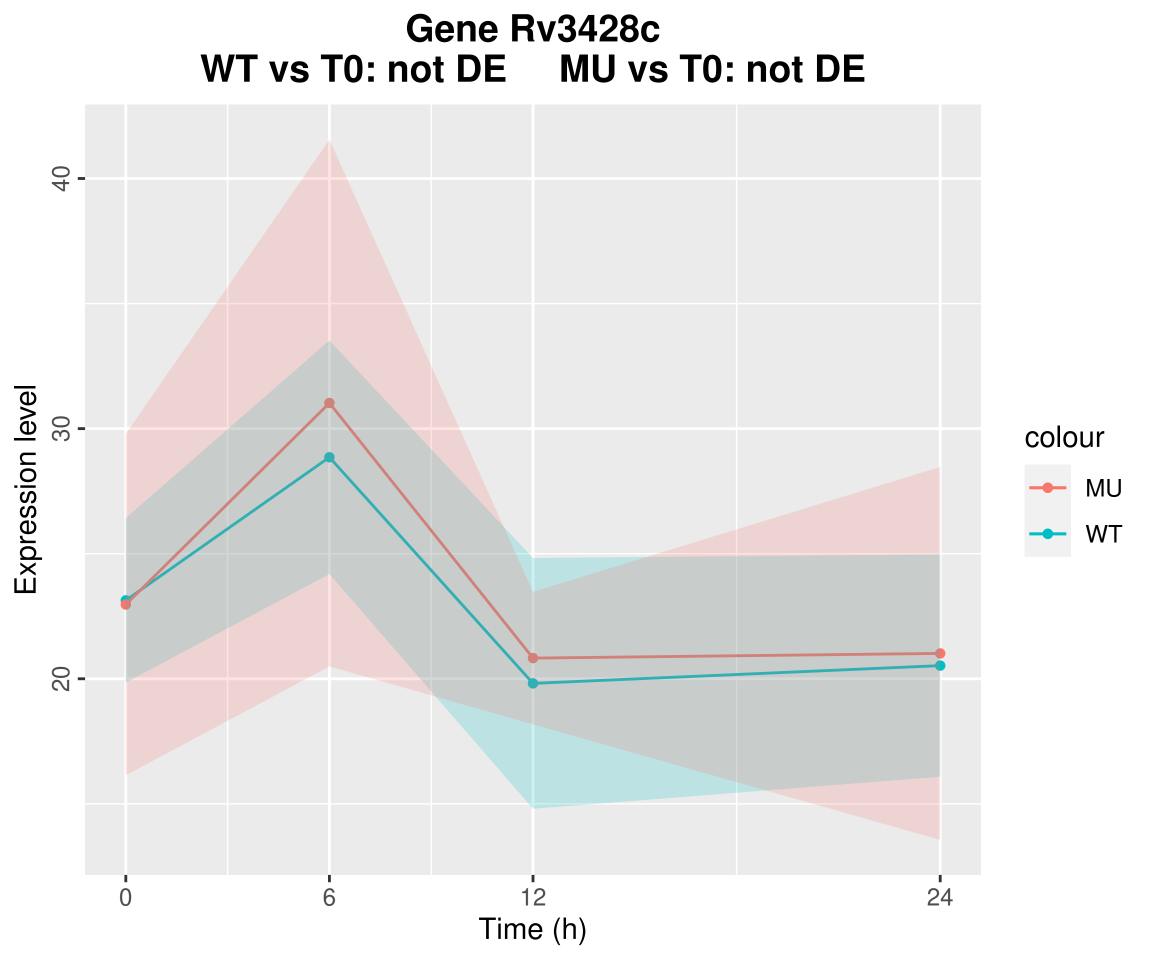
Gene Rv3428c
WT vs T0: not DE MU vs T0: not DE
Expression level
40
30
20
0
6
12
24
Time (h)
colour
MU
WT

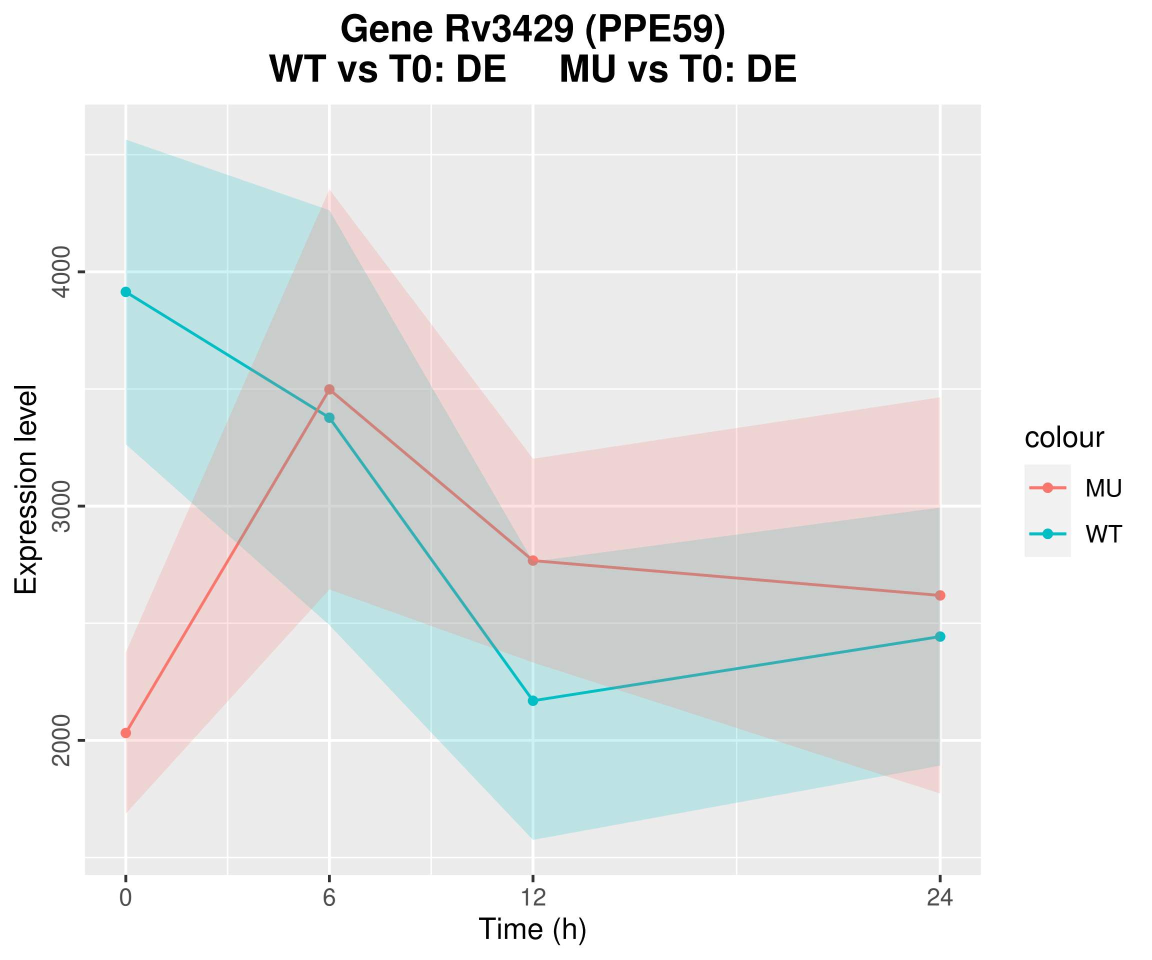
Gene Rv3429 (PPE59)
WT vs T0: DE   MU vs T0: DE
Expression level
4000
3000
2000
0
6
12
24
Time (h)
colour
MU
WT

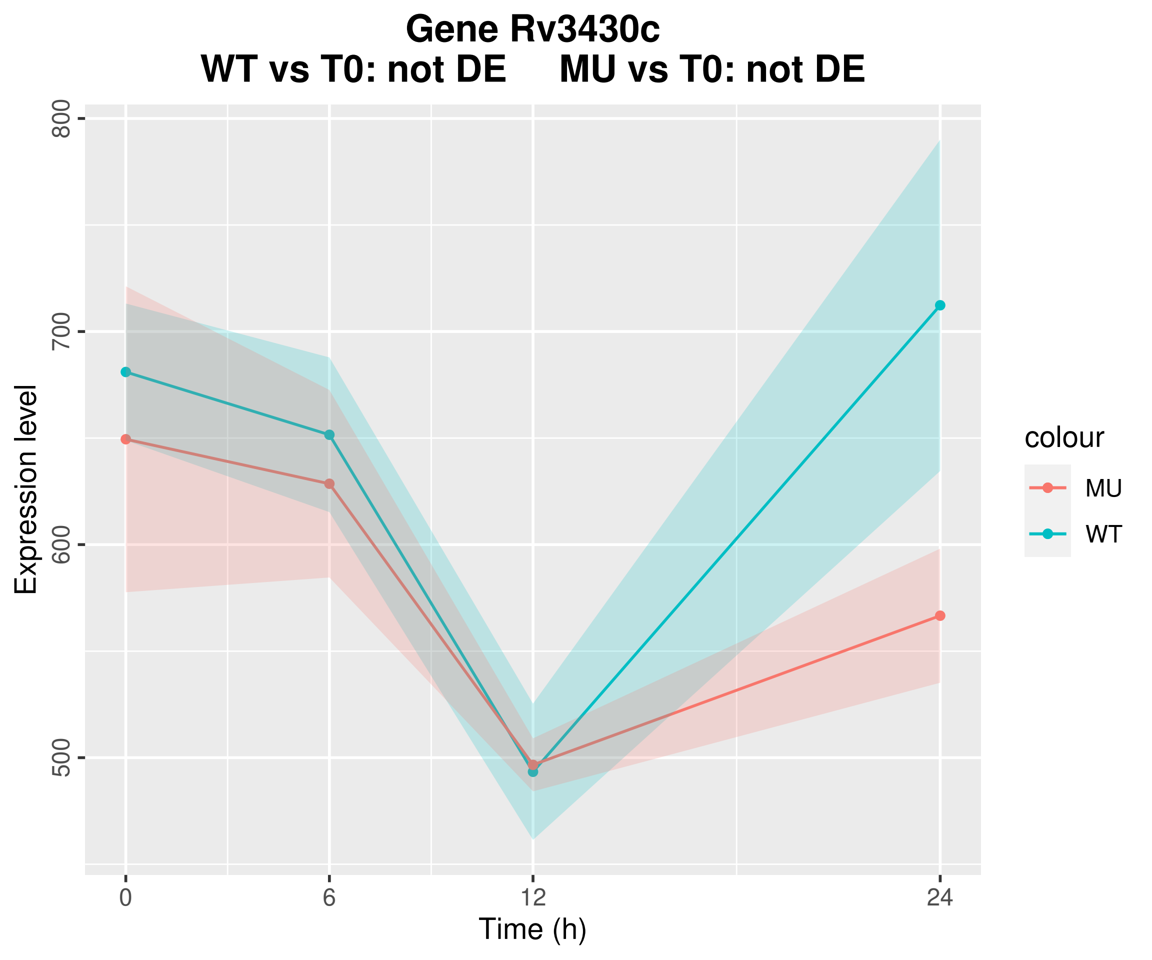
Gene Rv3430c
WT vs T0: not DE
MU vs T0: not DE
Expression level
800
700
600
500
0
6
12
24
Time (h)
colour
MU
WT

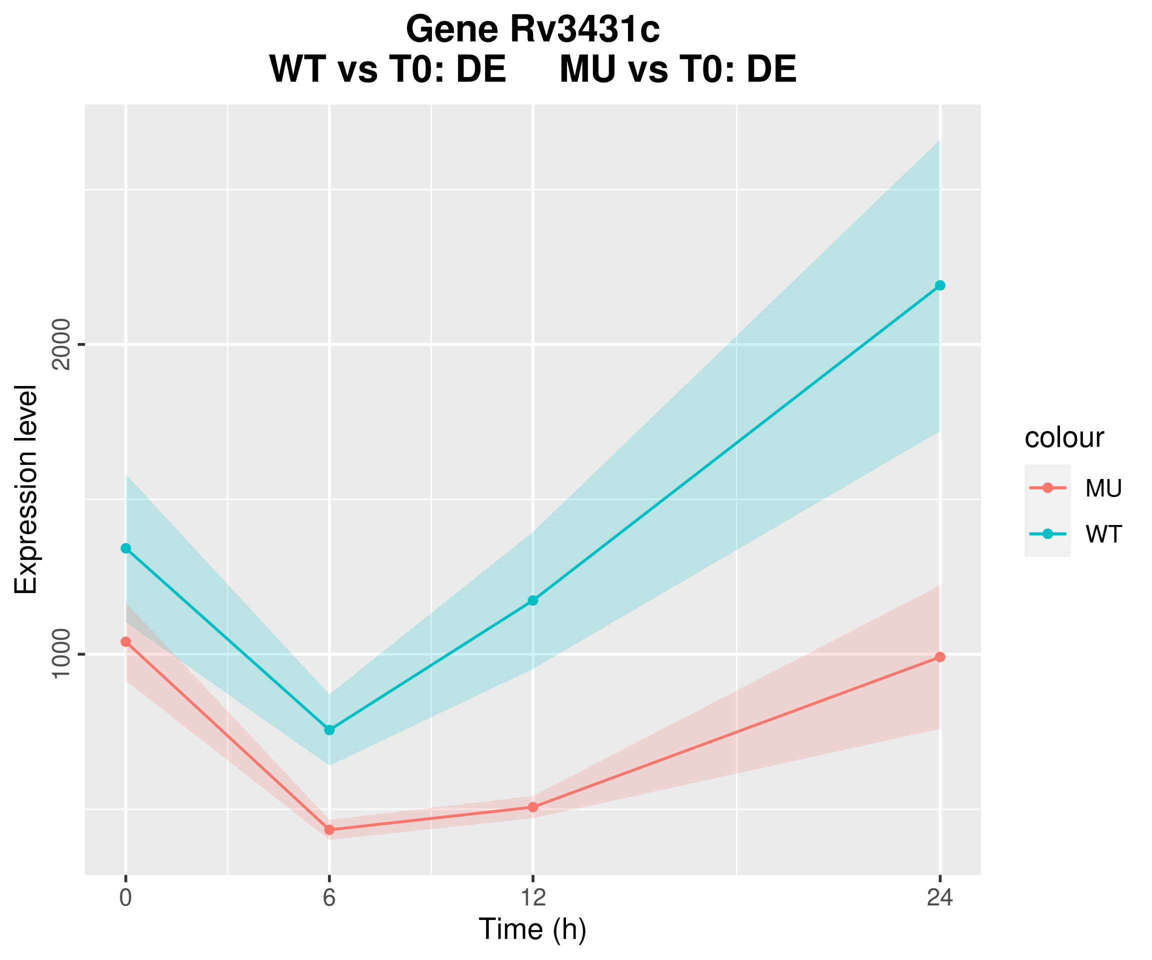
Gene Rv3431c
WT vs T0: DE MU vs T0: DE
Expression level
2000
1000
0
6
12
24
Time (h)
colour
MU
WT

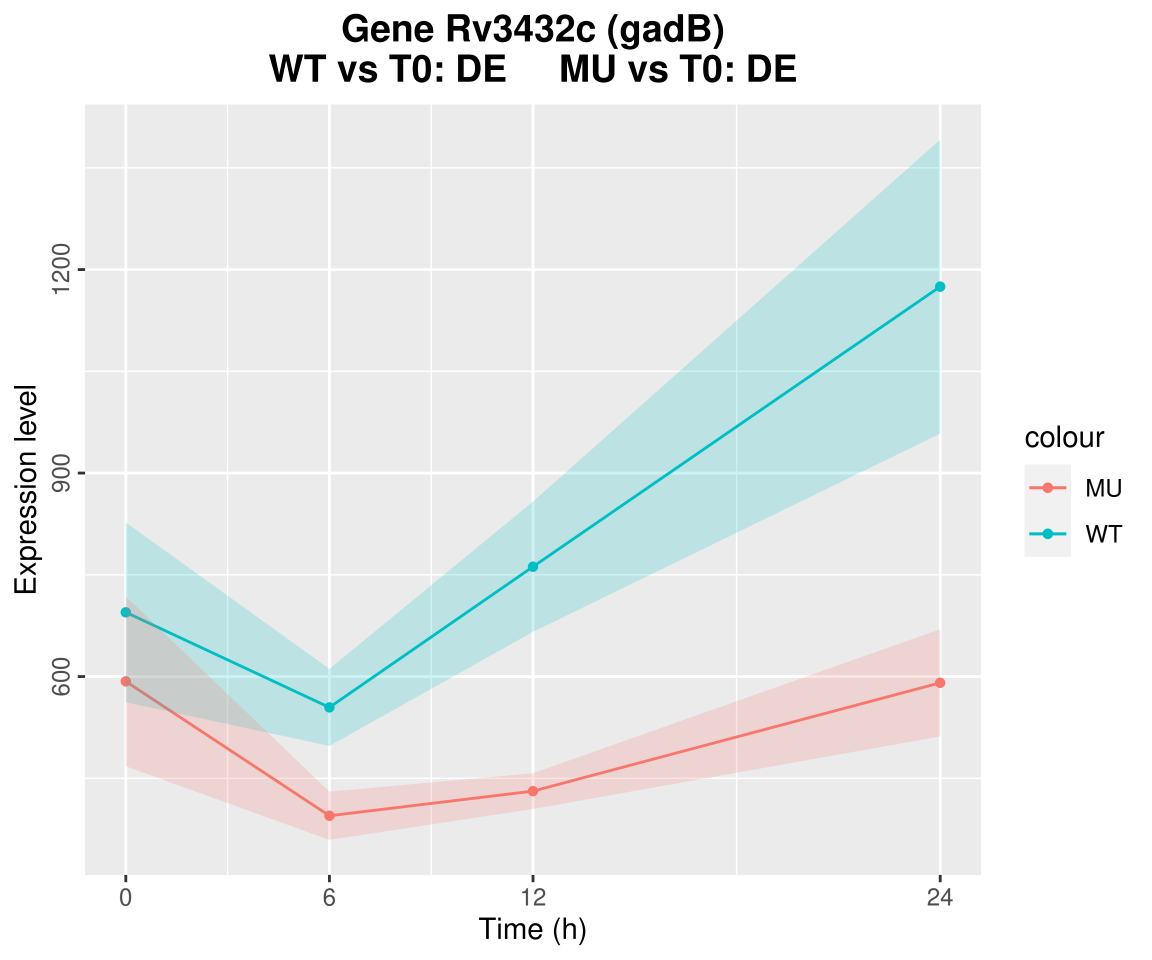
Gene Rv3432c (gadB)
WT vs T0: DE   MU vs T0: DE
Expression level
1200
900
600
0
6
12
24
Time (h)
colour
MU
WT

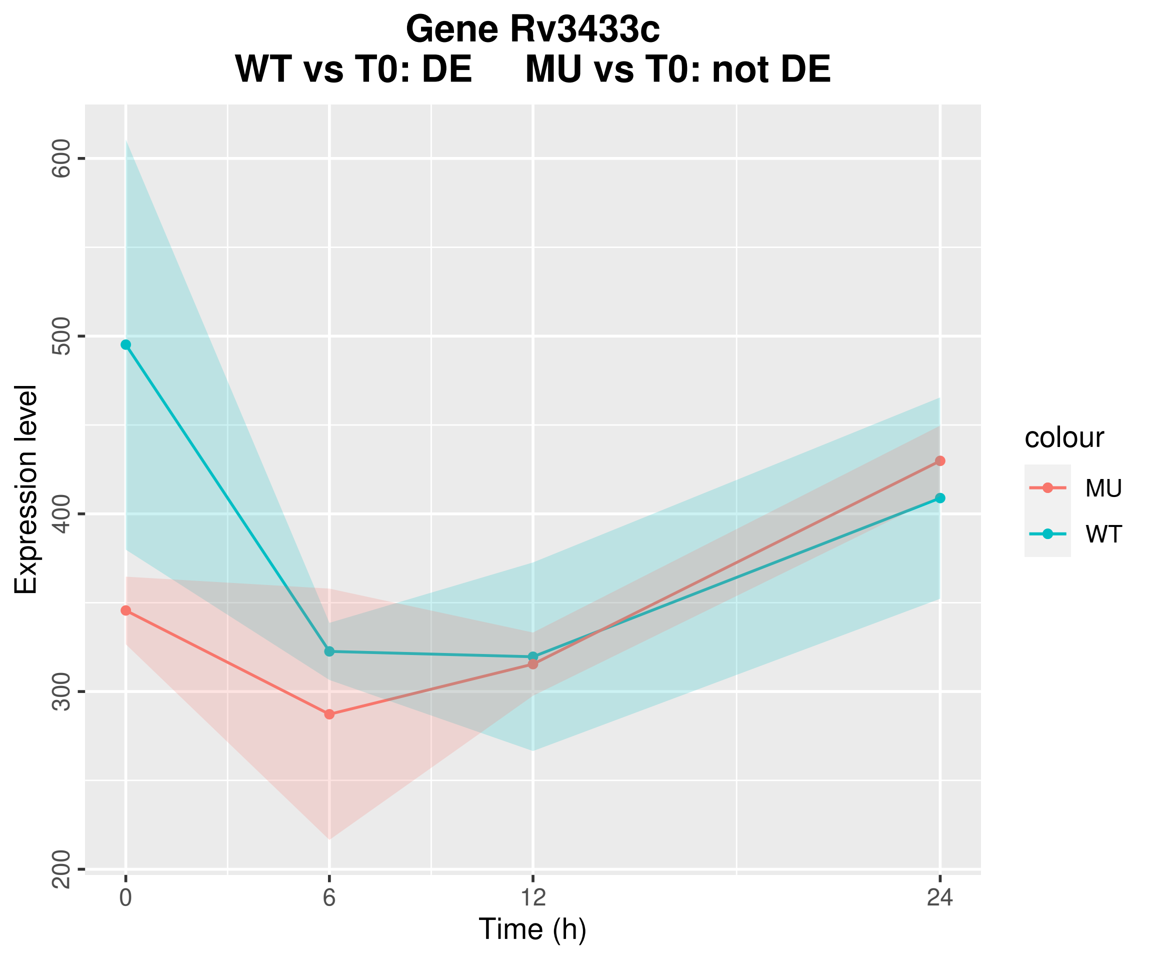
Gene Rv3433c
WT vs T0: DE MU vs T0: not DE
600
500
400
300
200
Expression level
0
6
12
24
Time (h)
colour
MU
WT

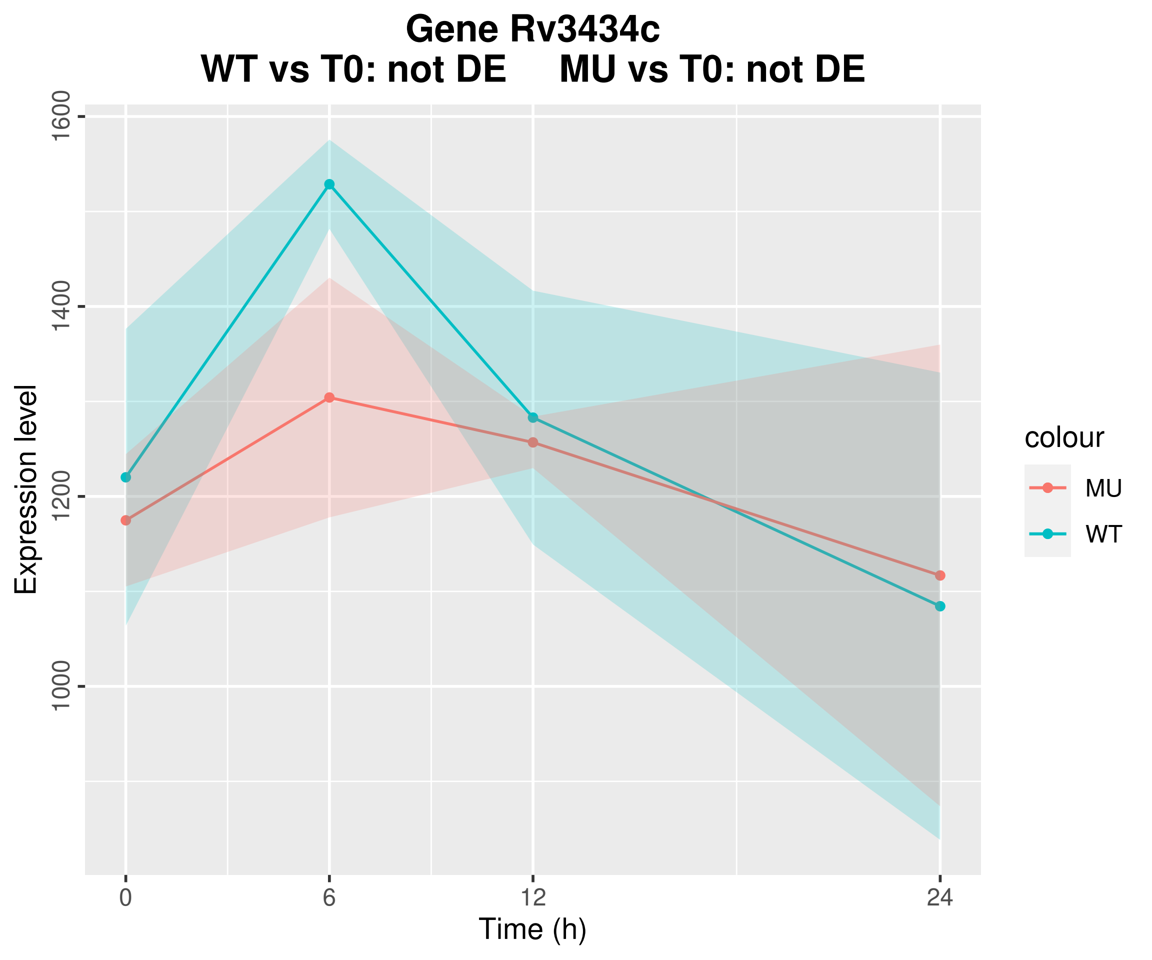

Gene Rv3434c
WT vs T0: not DE   MU vs T0: not DE
Expression level
1000
1200
1400
1600
Time (h)
0
6
12
24
colour
MU
WT

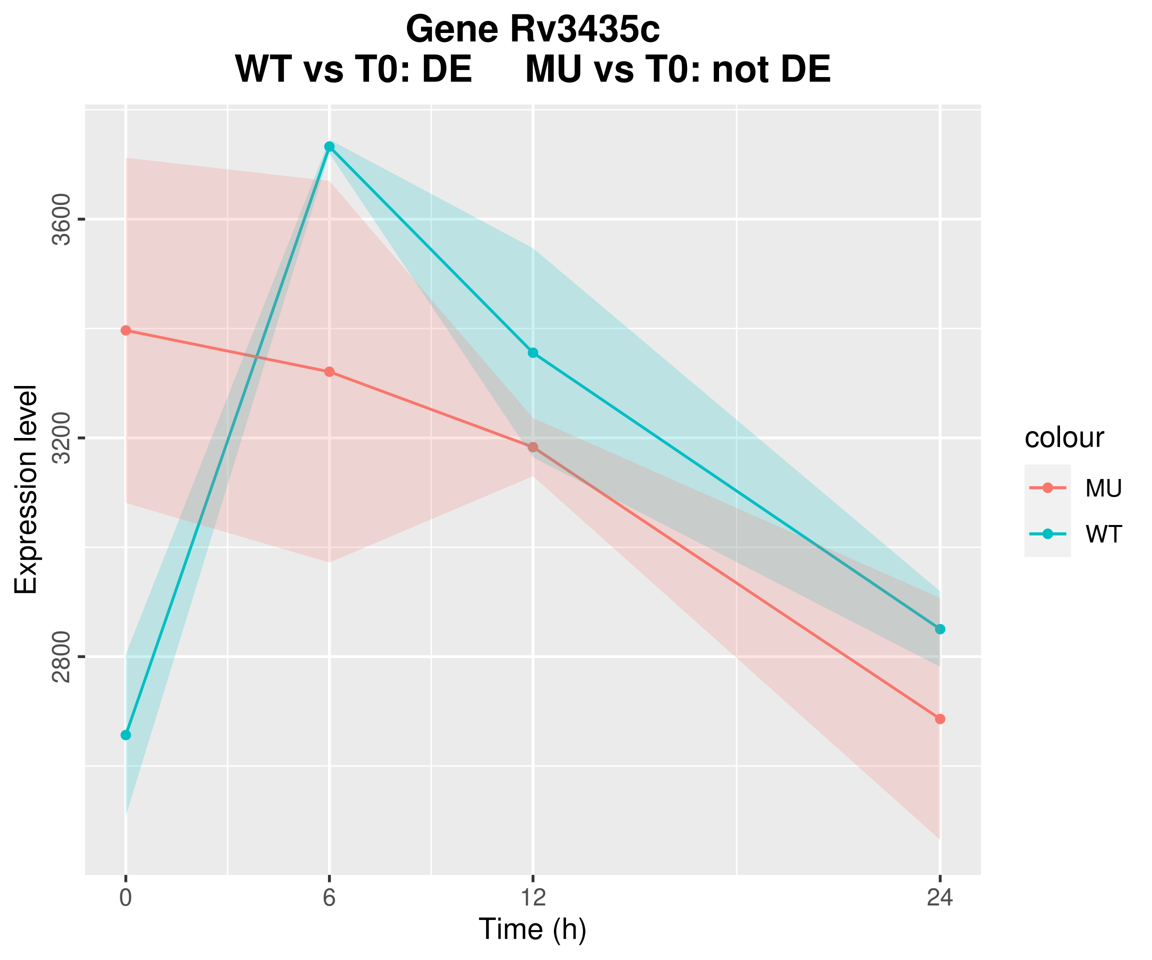
Gene Rv3435c
WT vs T0: DE   MU vs T0: not DE
3600
3200
2800
Expression level
0
6
12
24
Time (h)
colour
MU
WT

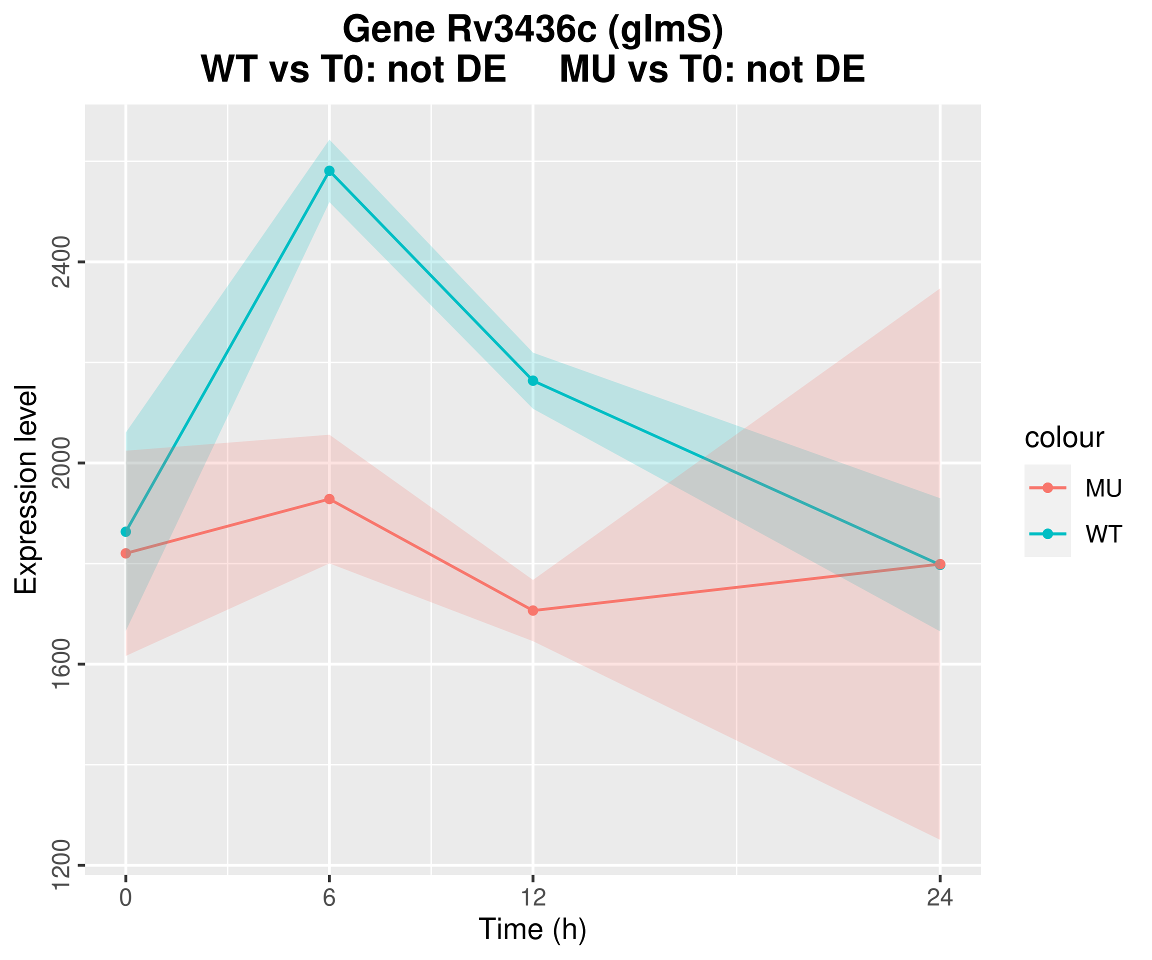

Gene Rv3436c (glmS)
WT vs T0: not DE   MU vs T0: not DE
Expression level
2400
2000
1600
1200
0
6
12
24
Time (h)
colour
MU
WT

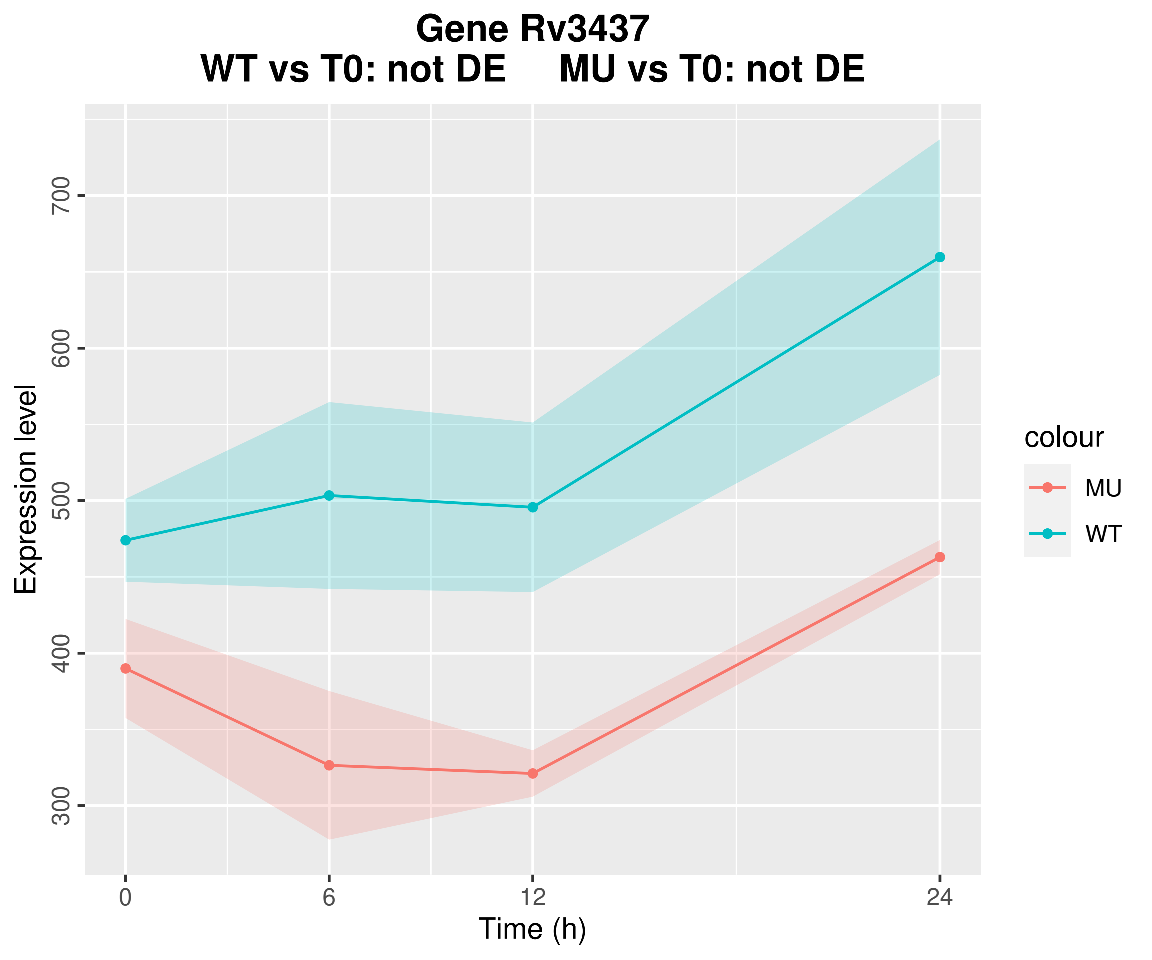

Gene Rv3437
WT vs T0: not DE MU vs T0: not DE
Expression level
300
400
500
600
700
0
6
12
24
Time (h)
colour
MU
WT

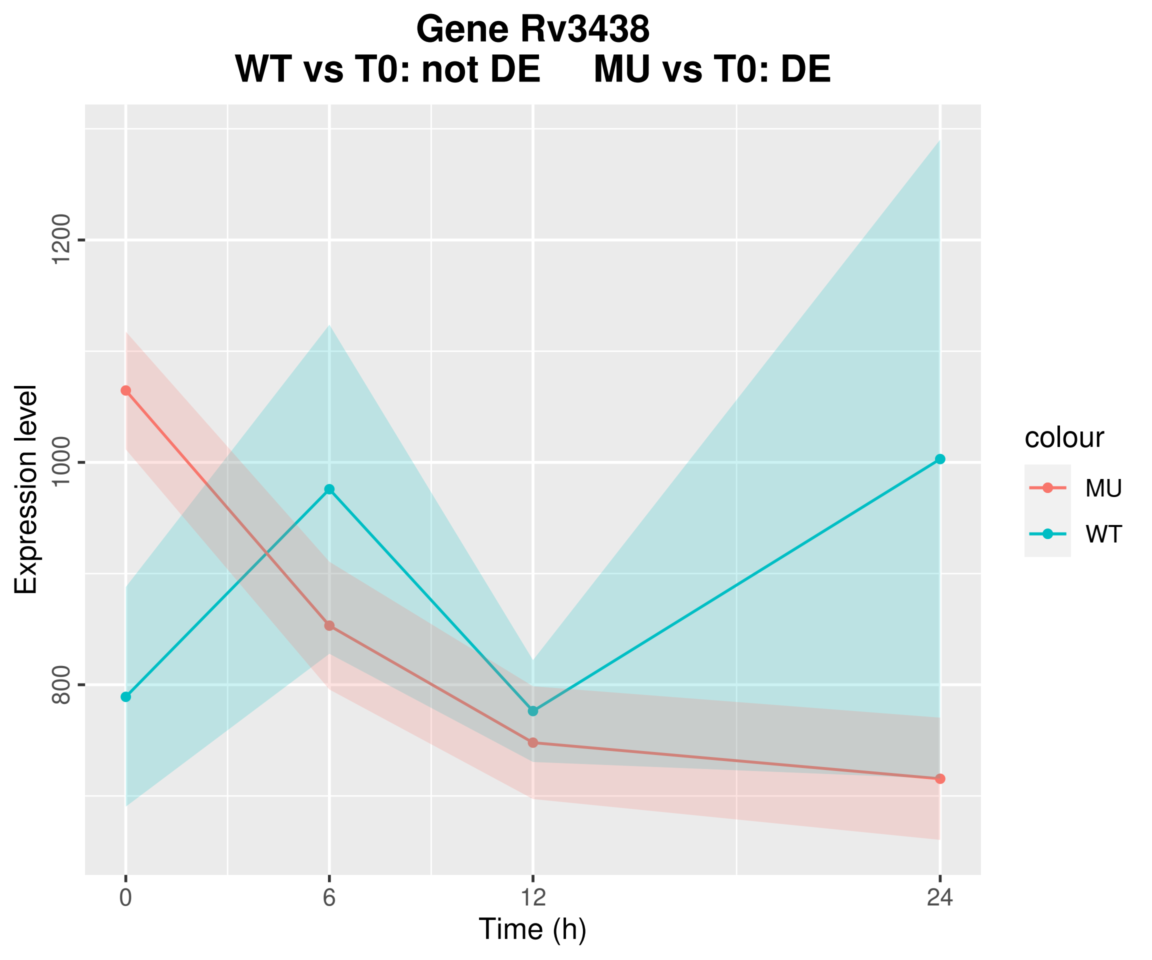
Gene Rv3438
WT vs T0: not DE MU vs T0: DE
Expression level
1200
1000
800
0
6
12
24
Time (h)
colour
MU
WT

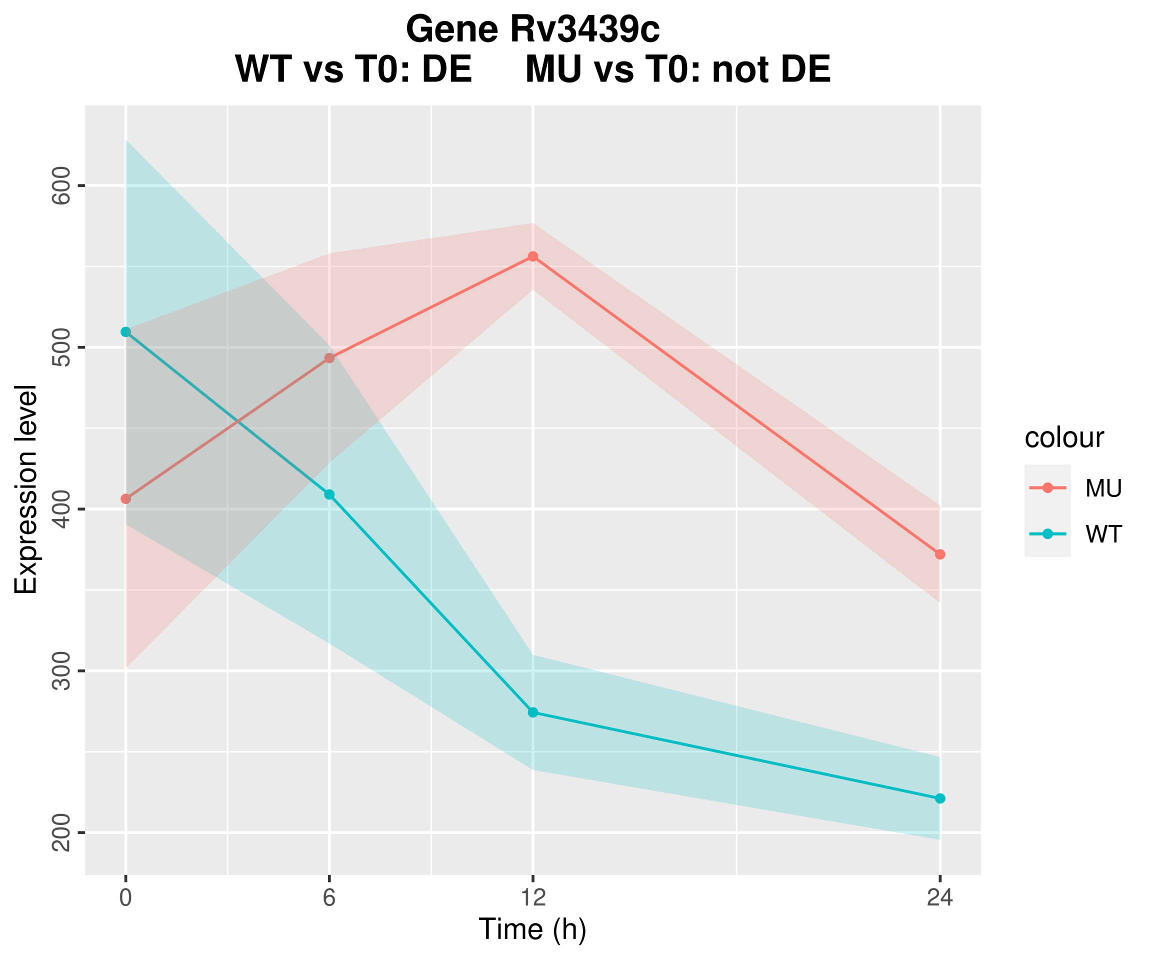
Gene Rv3439c
WT vs T0: DE MU vs T0: not DE
Expression level
600
500
400
300
200
0
6
12
24
Time (h)
colour
MU
WT

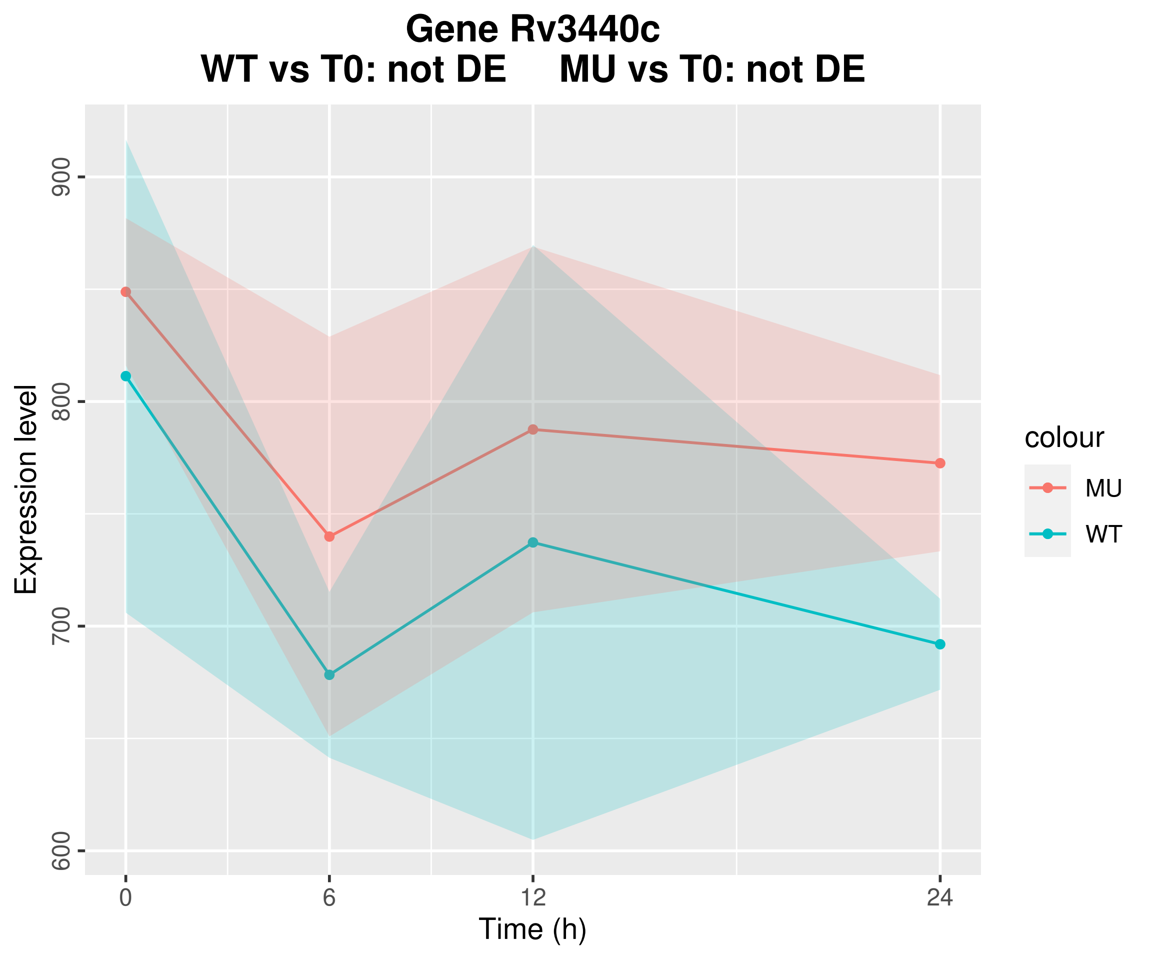

Gene Rv3440c
WT vs T0: not DE   MU vs T0: not DE
Expression level
600
700
800
900
0
6
12
24
Time (h)
colour
MU
WT

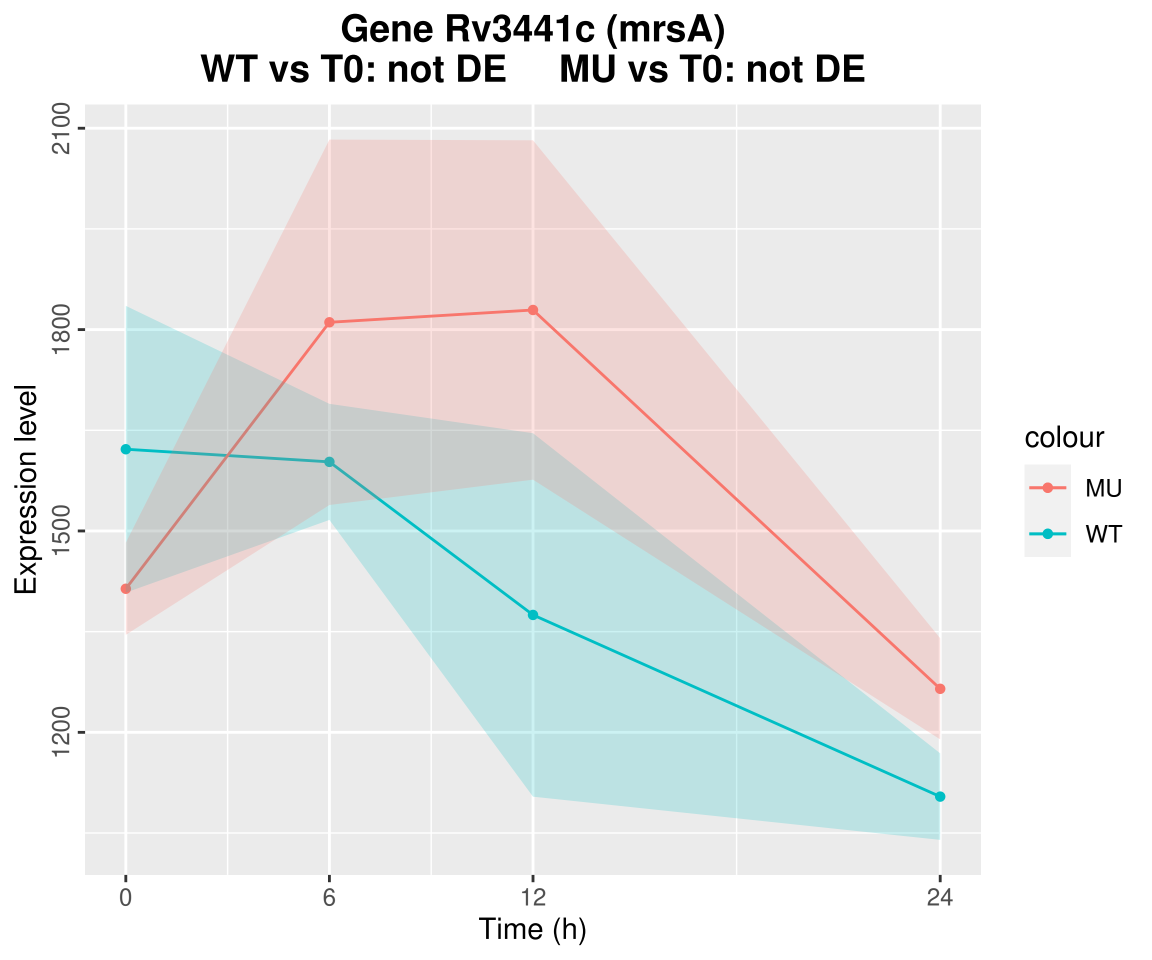

Gene Rv3441c (mrsA)
WT vs T0: not DE MU vs T0: not DE
Expression level
2100
1800
1500
1200
0
6
12
24
Time (h)
colour
MU
WT

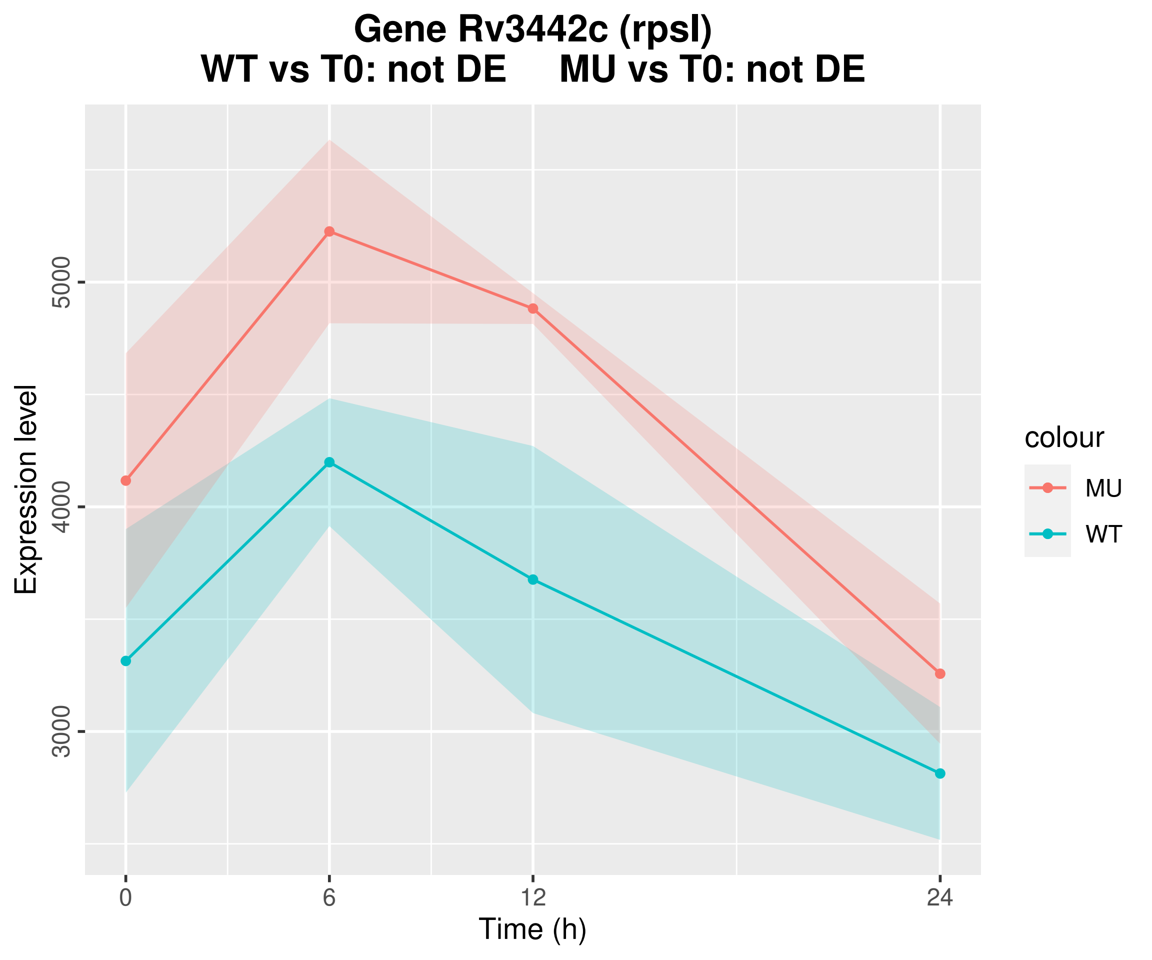
Gene Rv3442c (rpsI)
WT vs T0: not DE   MU vs T0: not DE
Expression level
5000
4000
3000
0
6
12
24
Time (h)
colour
MU
WT

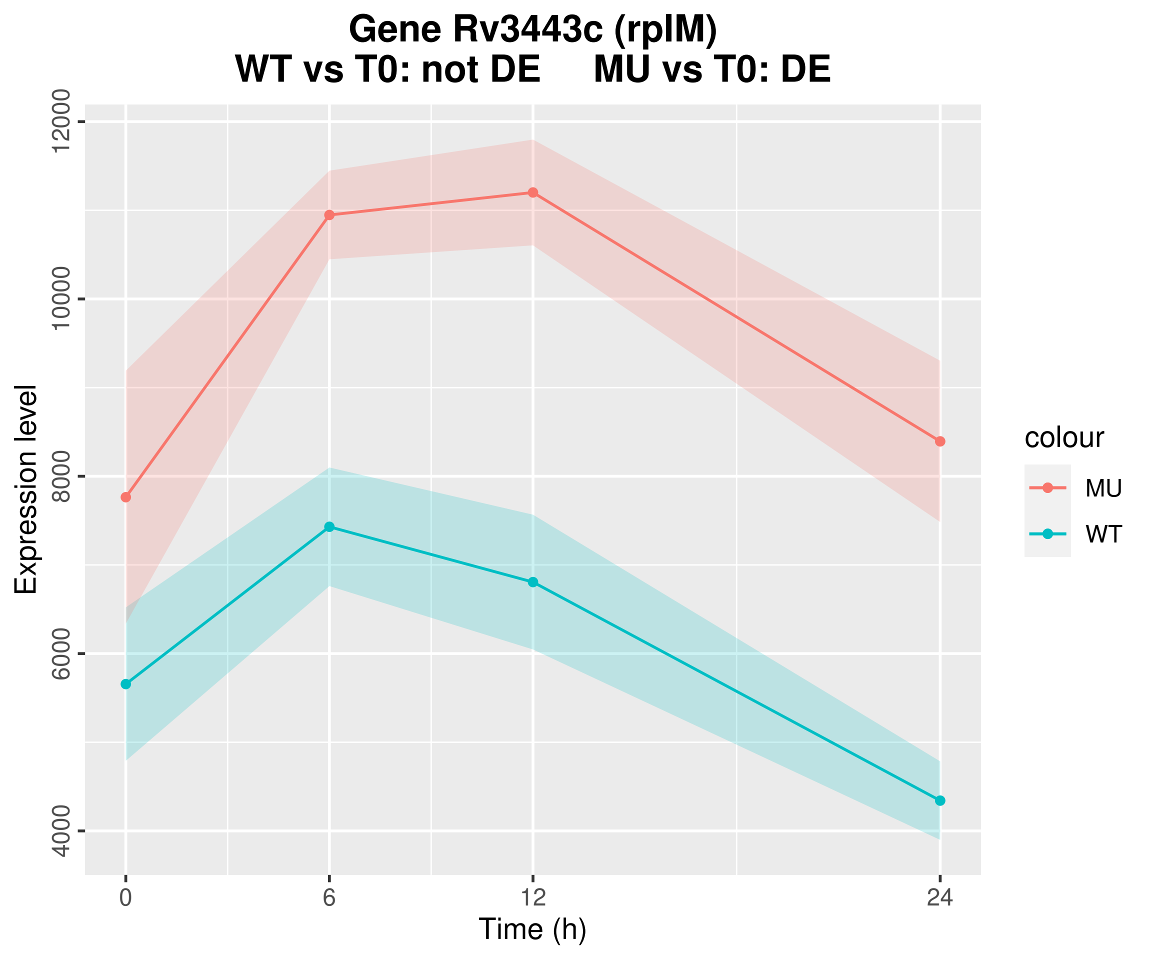
Gene Rv3443c (rplM)
WT vs T0: not DE   MU vs T0: DE
Expression level
4000
6000
8000
10000
12000
0
6
12
24
Time (h)
colour
MU
WT

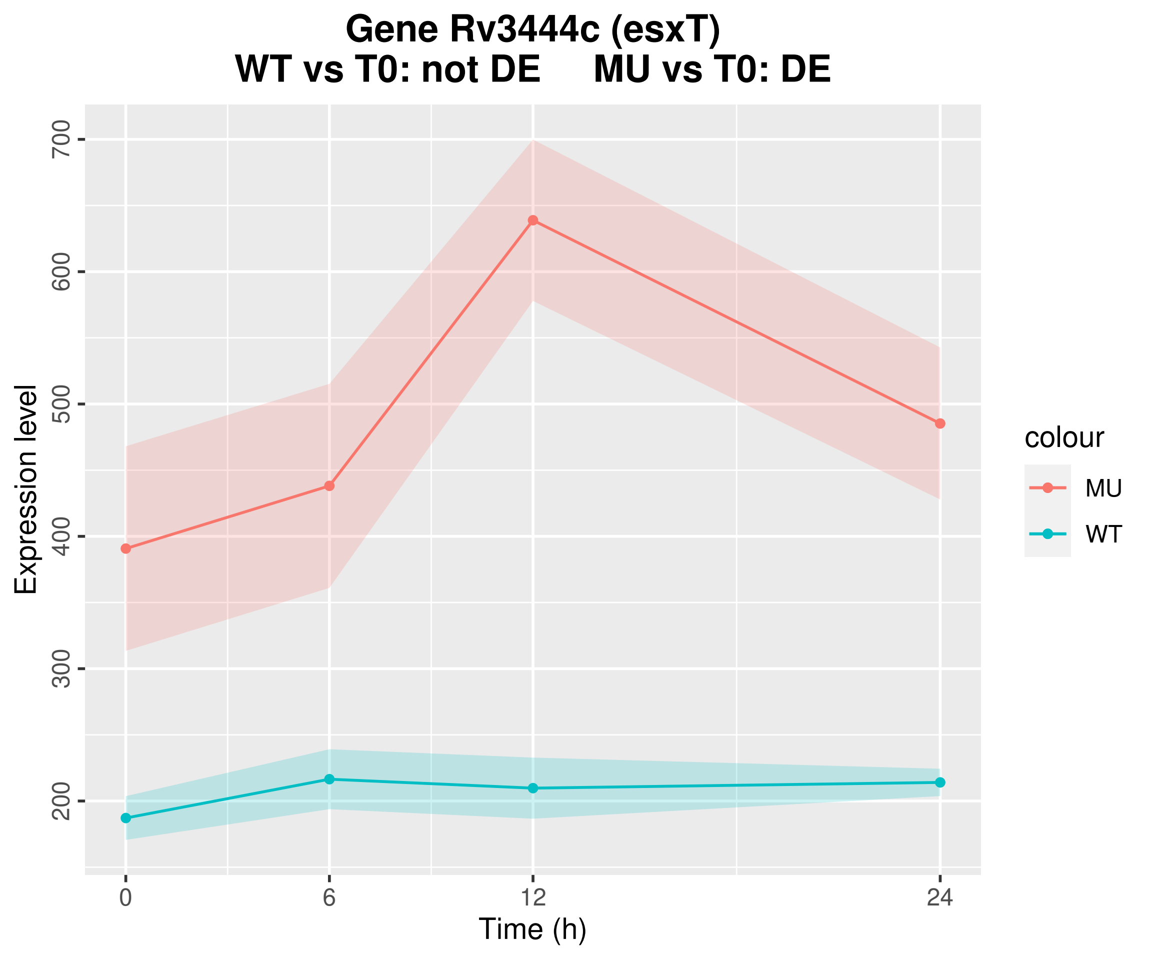

Gene Rv3444c (esxT)
WT vs T0: not DE   MU vs T0: DE
Expression level
700
600
500
400
300
200
0
6
12
24
Time (h)
colour
MU
WT

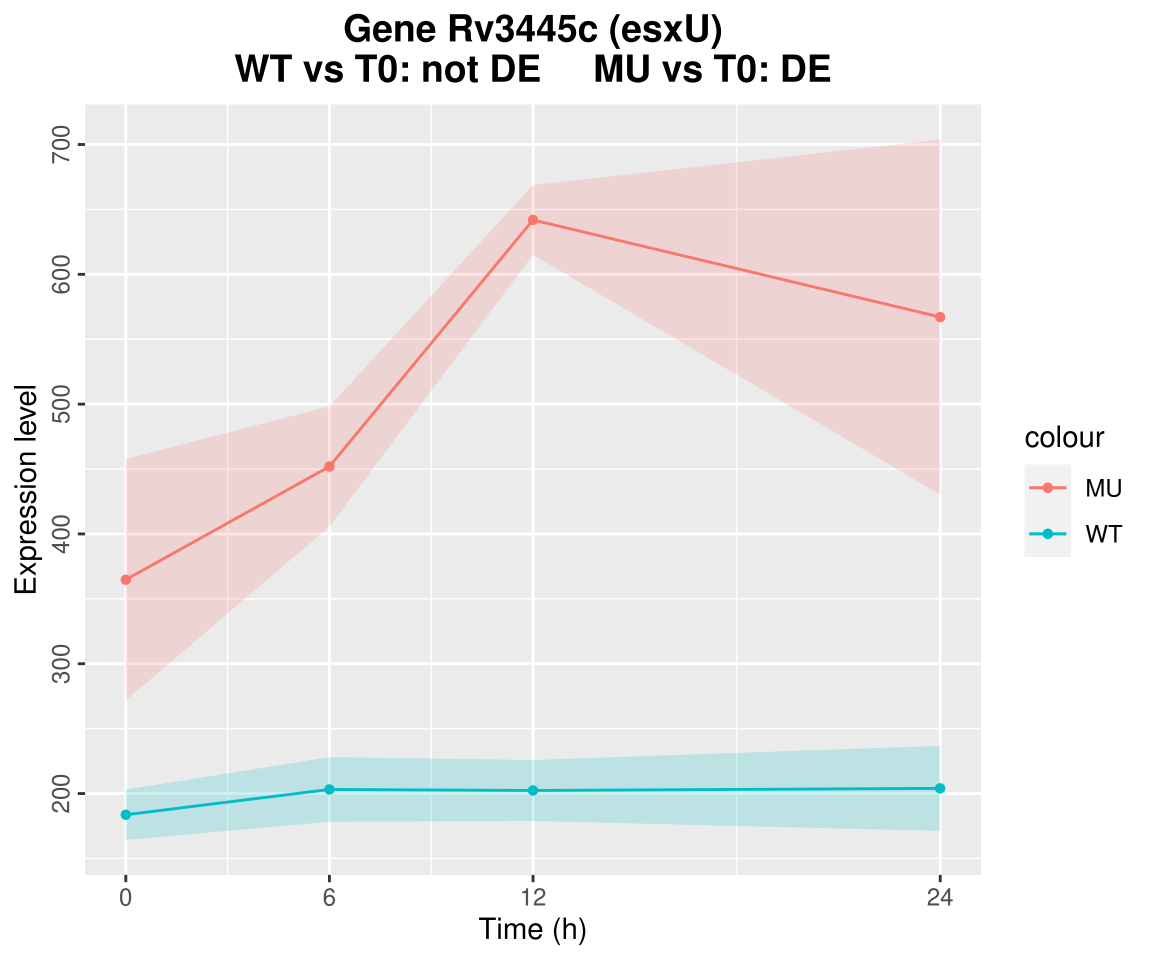
Gene Rv3445c (esxU)
WT vs T0: not DE   MU vs T0: DE
Expression level
700
600
500
400
300
200
0
6
12
24
Time (h)
colour
MU
WT

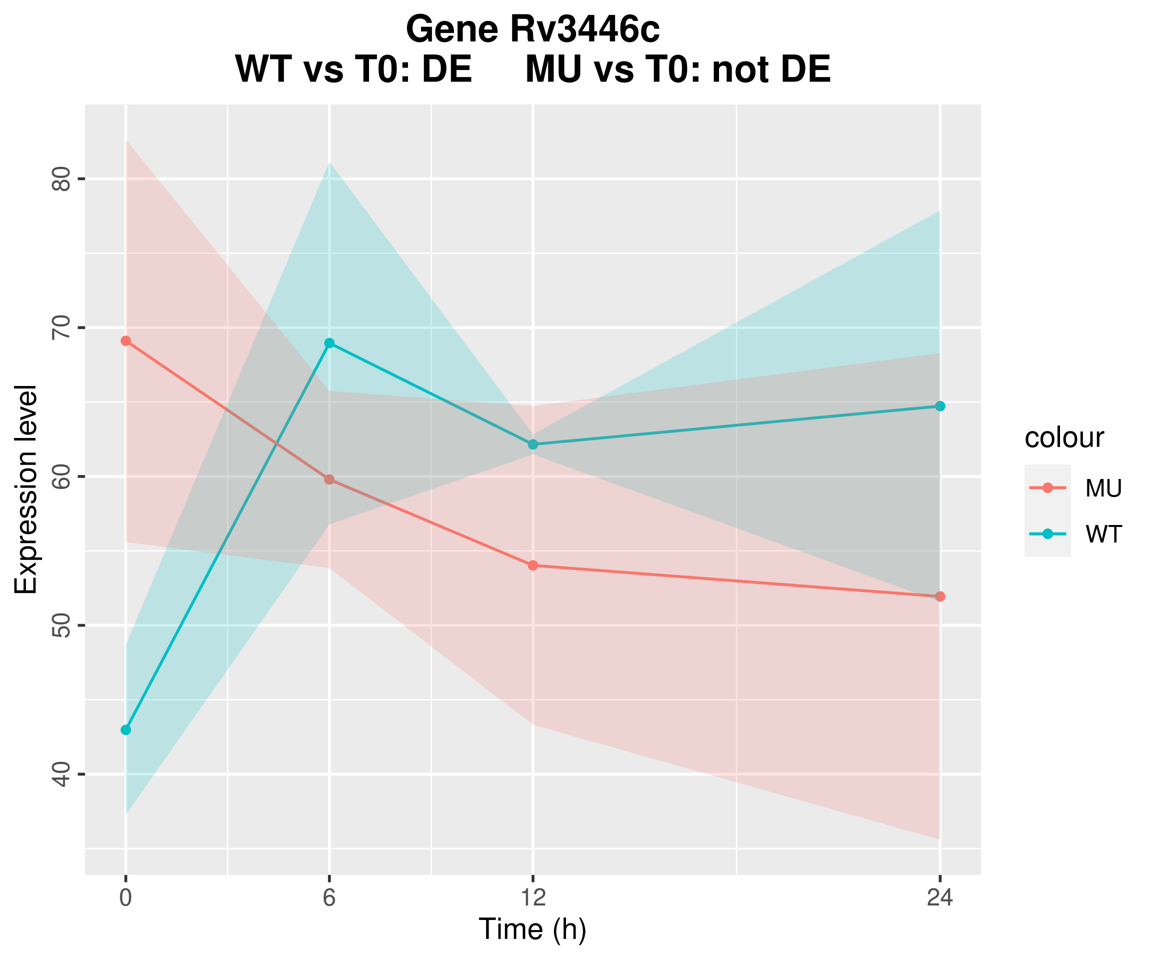
Gene Rv3446c
WT vs T0: DE   MU vs T0: not DE
Expression level
80
70
60
50
40
0
6
12
24
Time (h)
colour
MU
WT

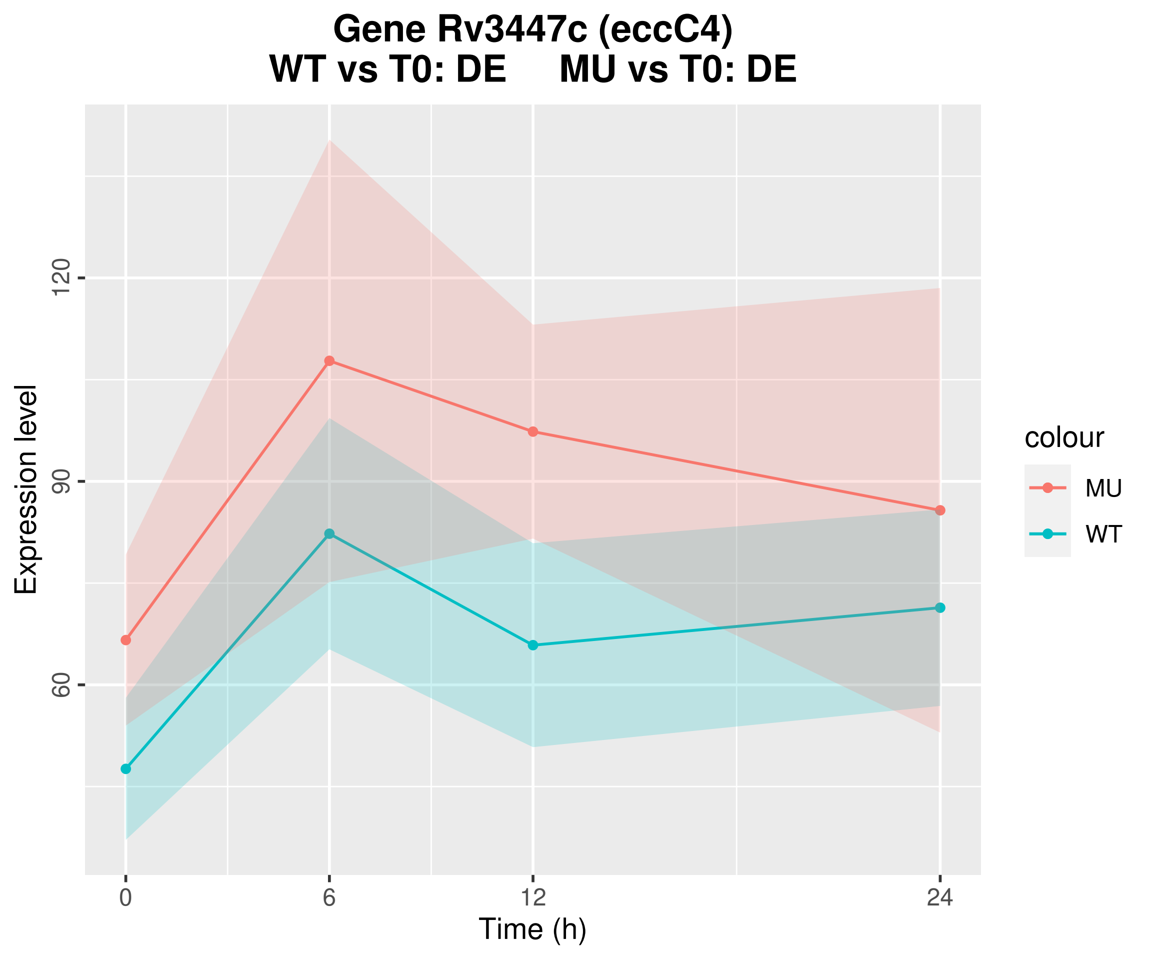

Gene Rv3447c (eccC4)
WT vs T0: DE   MU vs T0: DE
Expression level
120
90
60
0
6
12
24
Time (h)
colour
MU
WT

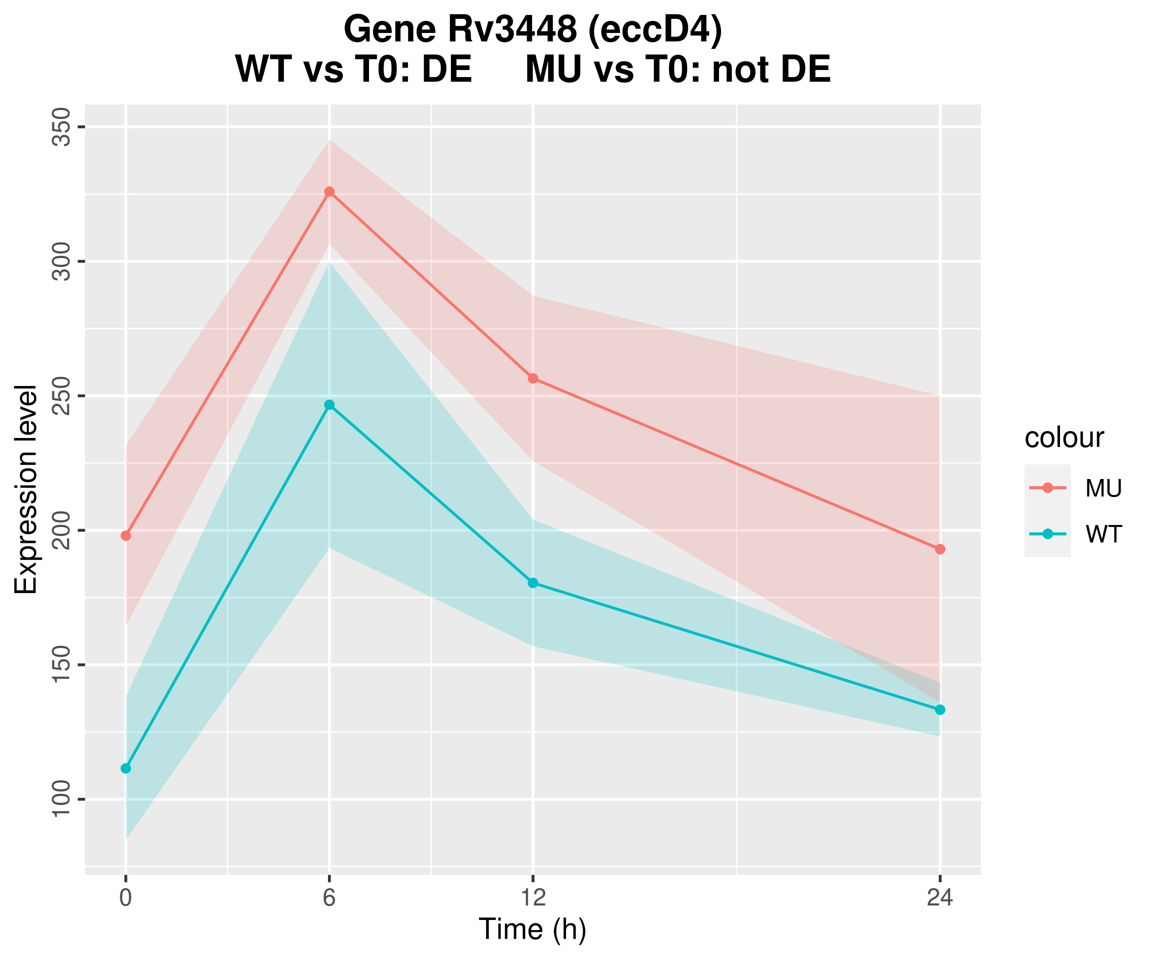
Gene Rv3448 (eccD4)
WT vs T0: DE   MU vs T0: not DE
Expression level
350
300
250
200
150
100
0
6
12
24
Time (h)
colour
MU
WT

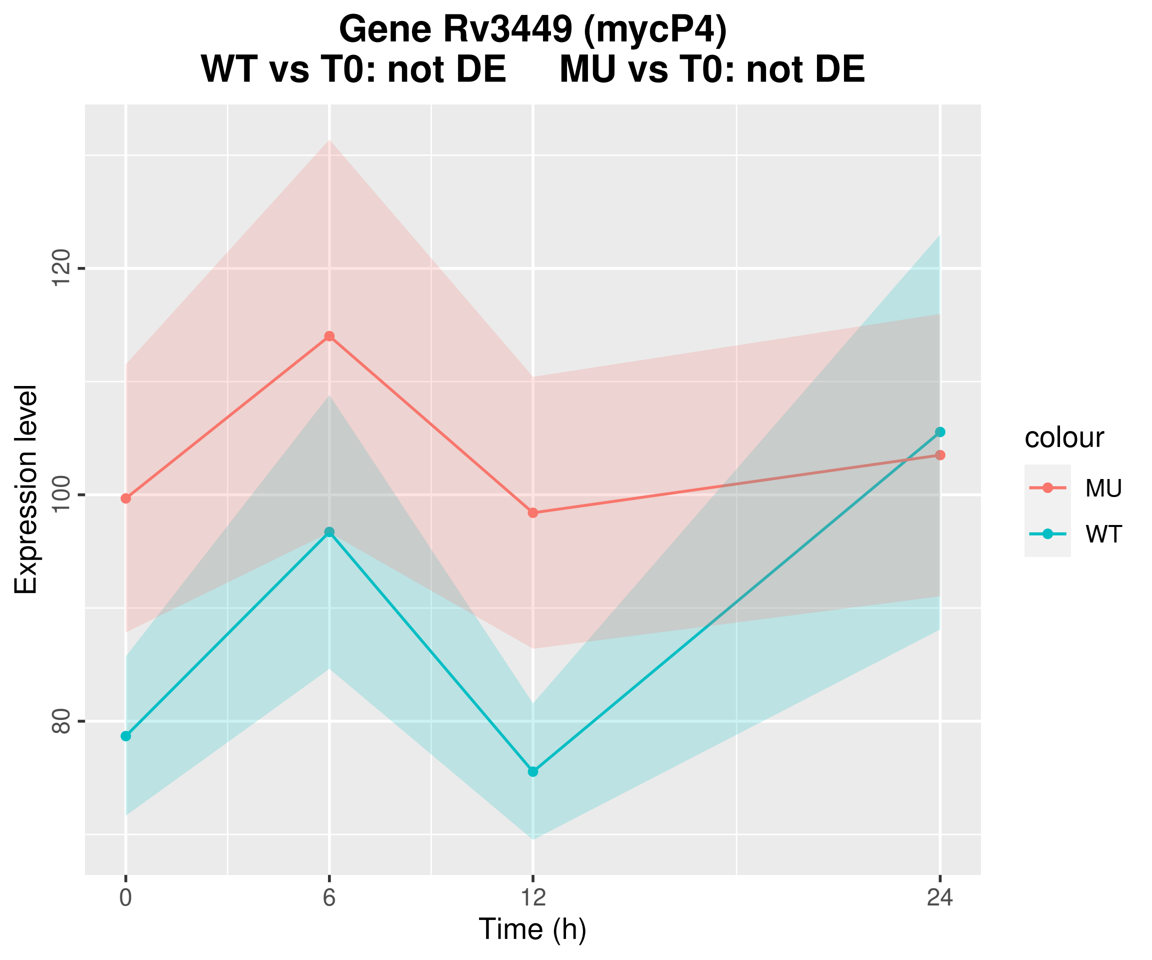
Gene Rv3449 (mycP4)
WT vs T0: not DE   MU vs T0: not DE
Expression level
120
100
80
0
6
12
24
Time (h)
colour
MU
WT

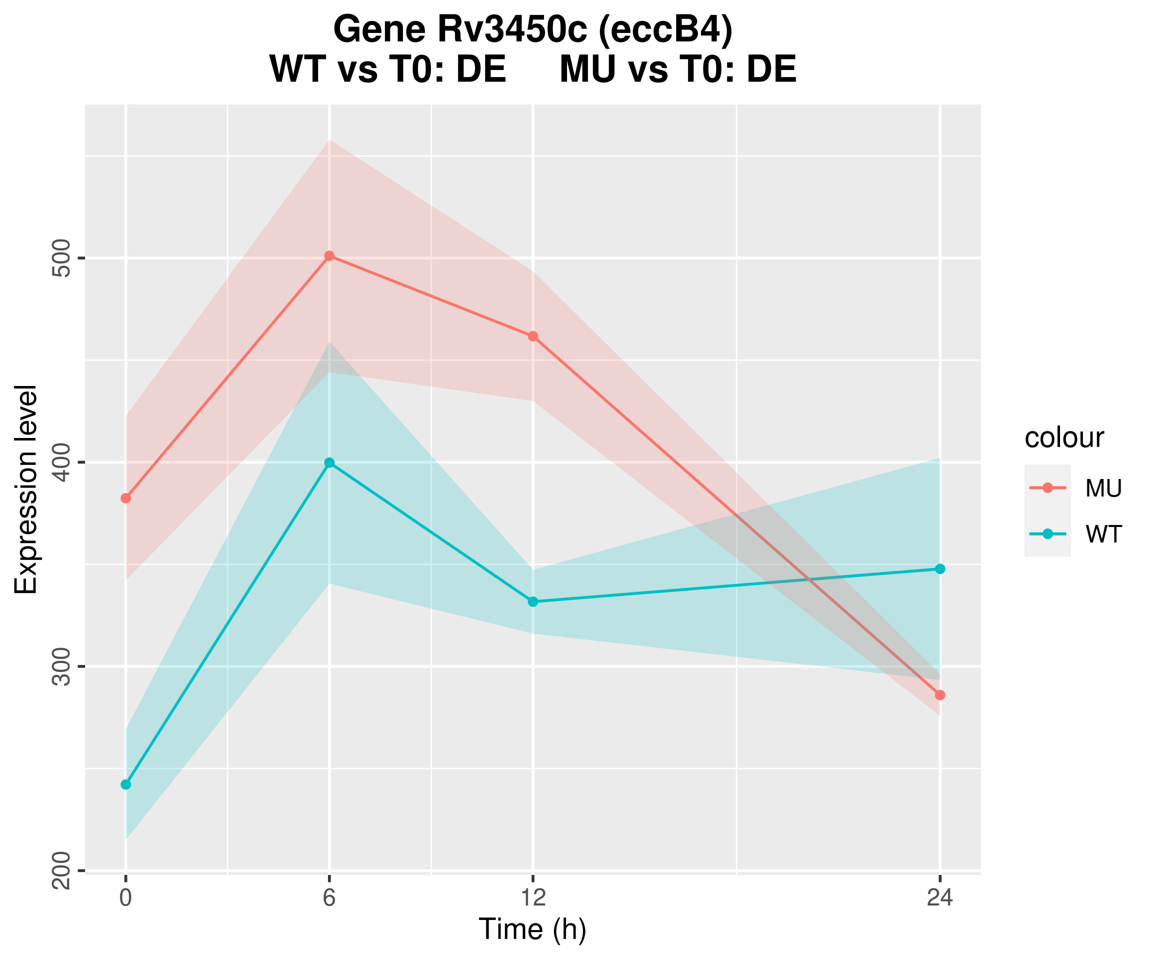

Gene Rv3450c (eccB4)
WT vs T0: DE MU vs T0: DE
Expression level
500
400
300
200
0
6
12
24
Time (h)
colour
MU
WT

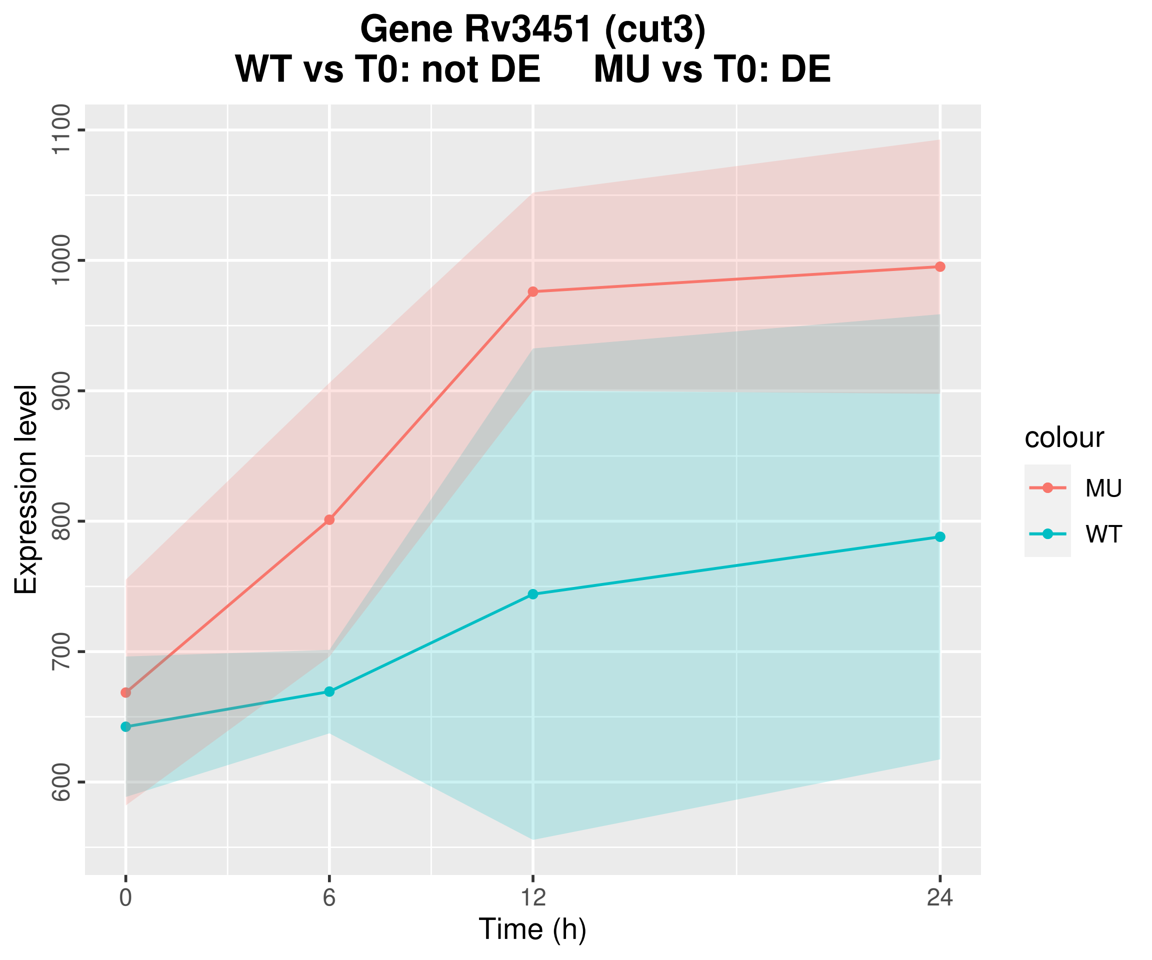
Gene Rv3451 (cut3)
WT vs T0: not DE   MU vs T0: DE
Expression level
1100
1000
900
800
700
600
0
6
12
24
Time (h)
colour
MU
WT

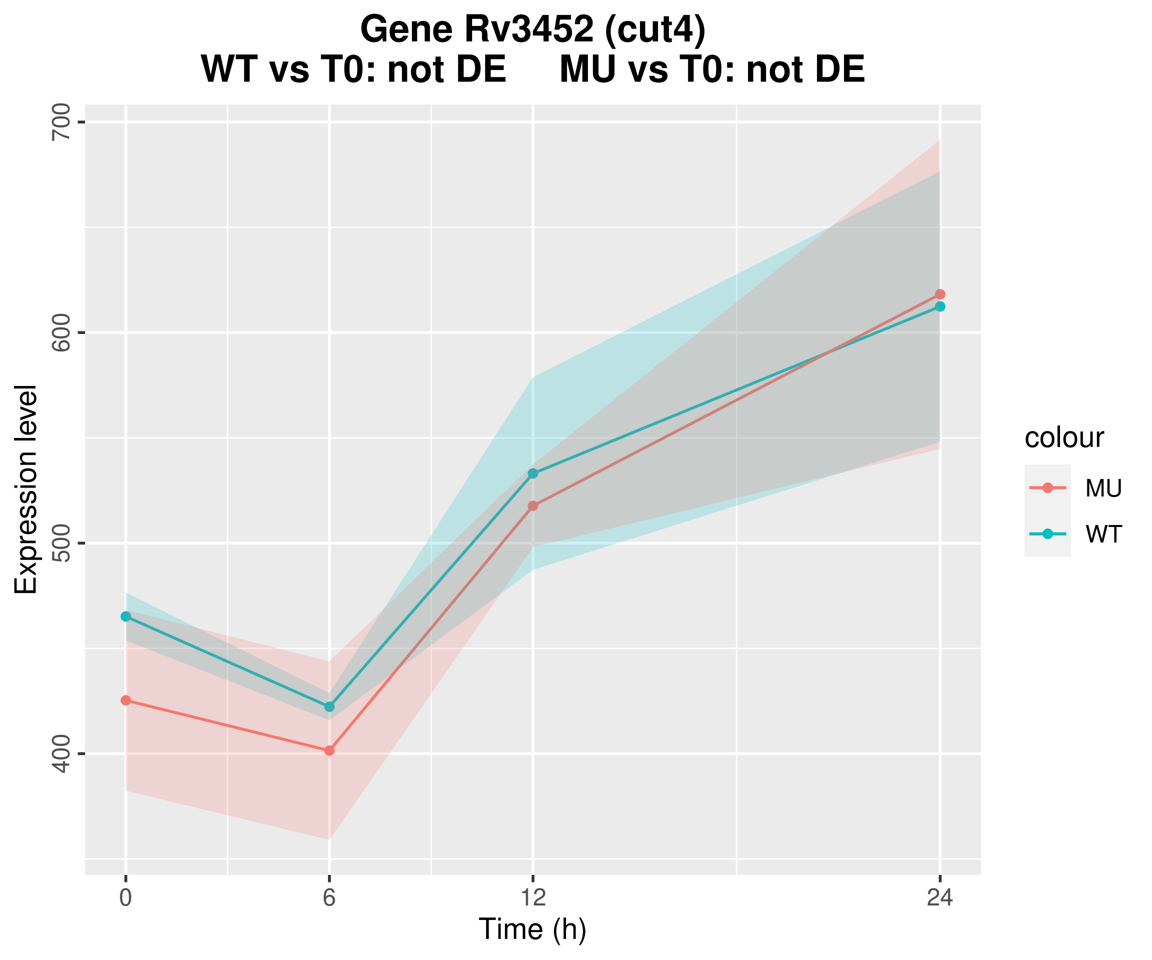

Gene Rv3452 (cut4)
WT vs T0: not DE MU vs T0: not DE
Expression level
700
600
500
400
0
6
12
24
Time (h)
colour
MU
WT

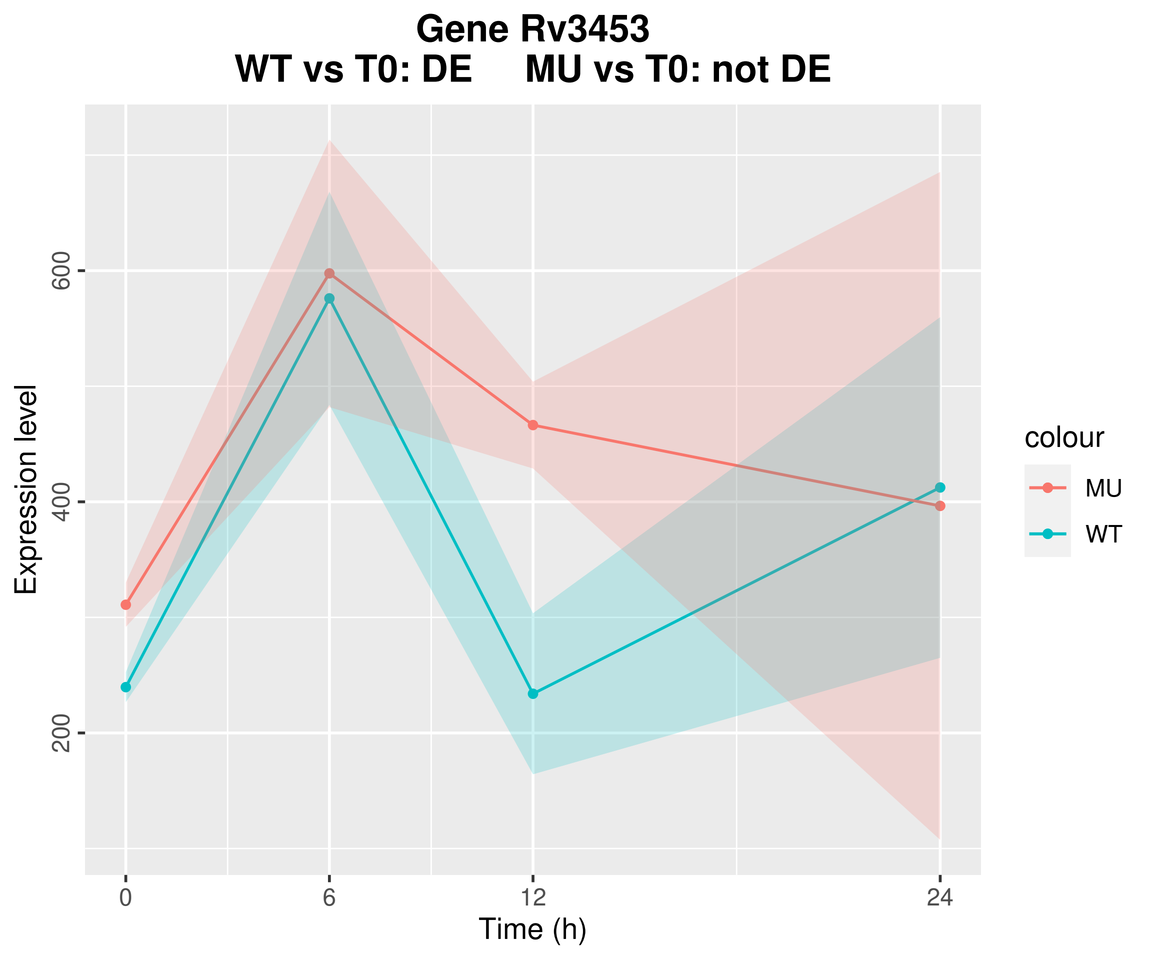
Gene Rv3453
WT vs T0: DE   MU vs T0: not DE
Expression level
600
400
200
0
6
12
24
Time (h)
colour
MU
WT

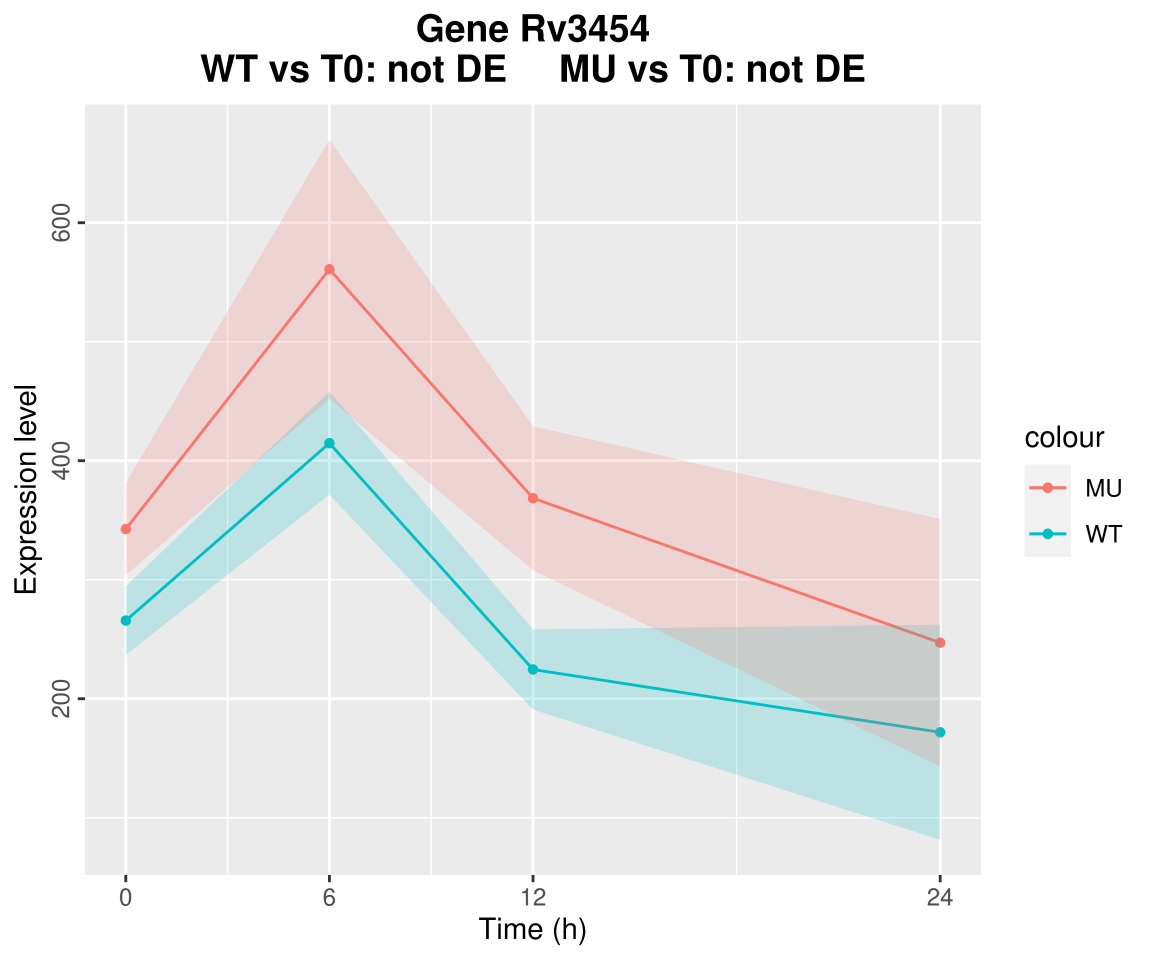
Gene Rv3454
WT vs T0: not DE
MU vs T0: not DE
600
400
200
Expression level
0
6
12
24
Time (h)
colour
MU
WT

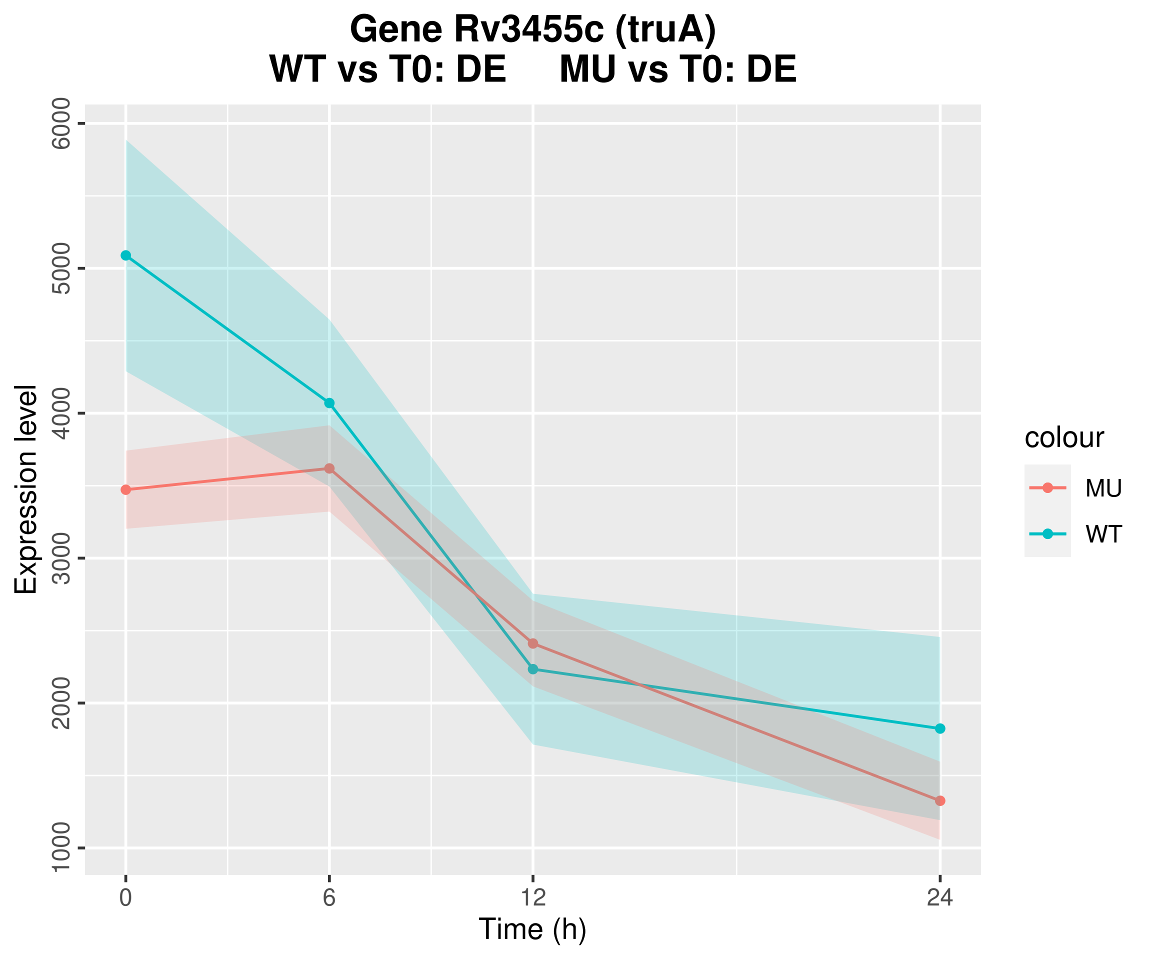
Gene Rv3455c (truA)
WT vs T0: DE MU vs T0: DE
Expression level
1000
2000
3000
4000
5000
6000
0
6
12
24
Time (h)
colour
MU
WT

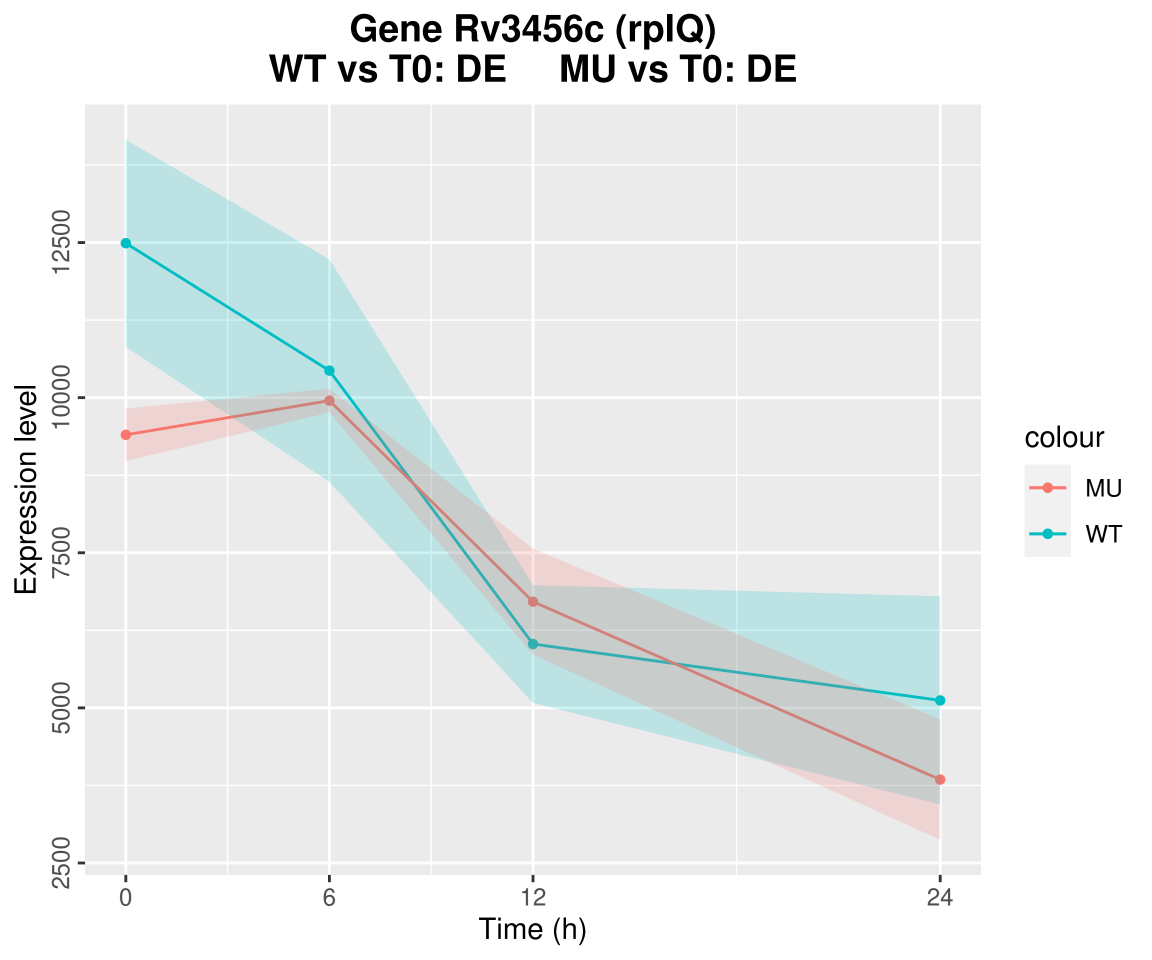
Gene Rv3456c (rplQ)
WT vs T0: DE MU vs T0: DE
Expression level
12500
10000
7500
5000
2500
0
6
12
24
Time (h)
colour
MU
WT

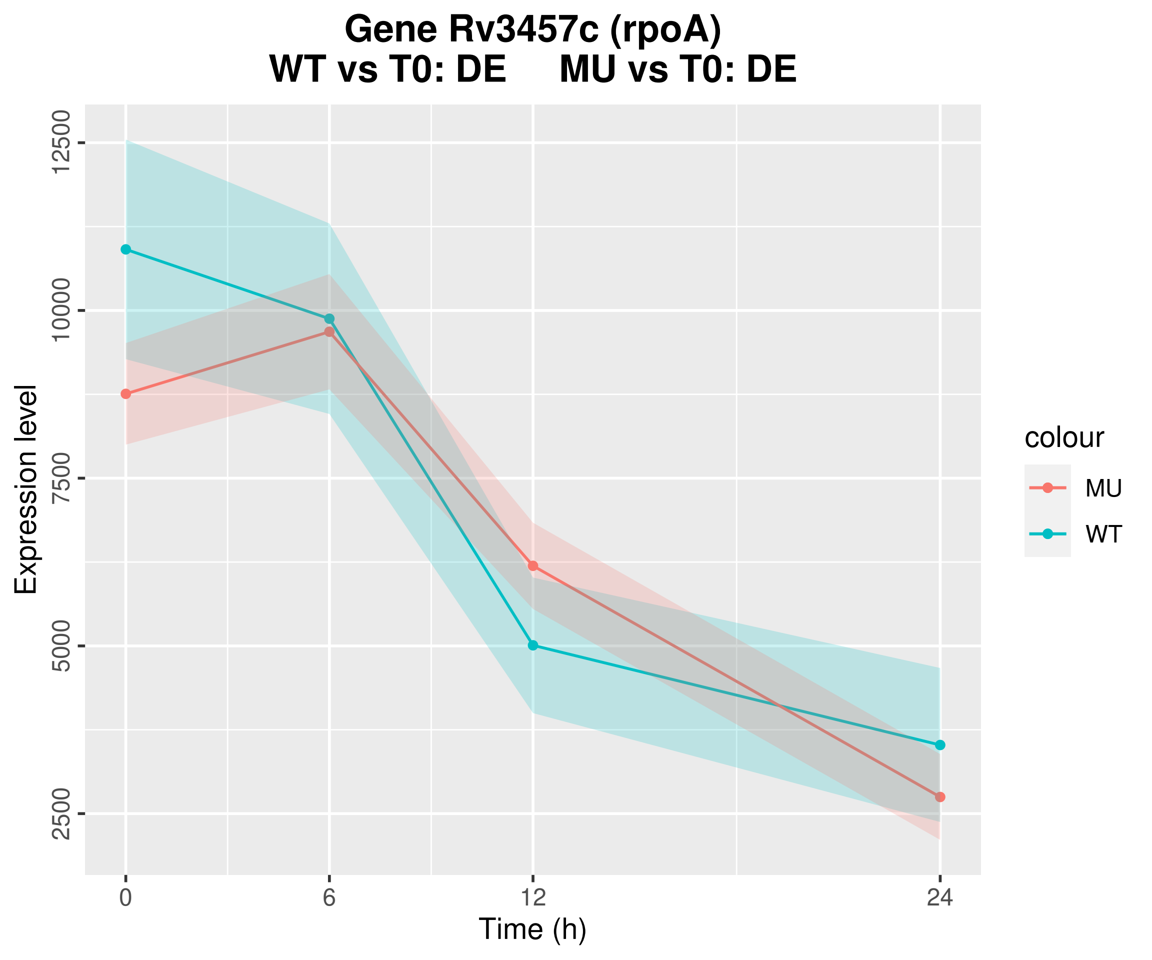

Gene Rv3457c (rpoA)
WT vs T0: DE MU vs T0: DE
Expression level
12500
10000
7500
5000
2500
0
6
12
24
Time (h)
colour
MU
WT

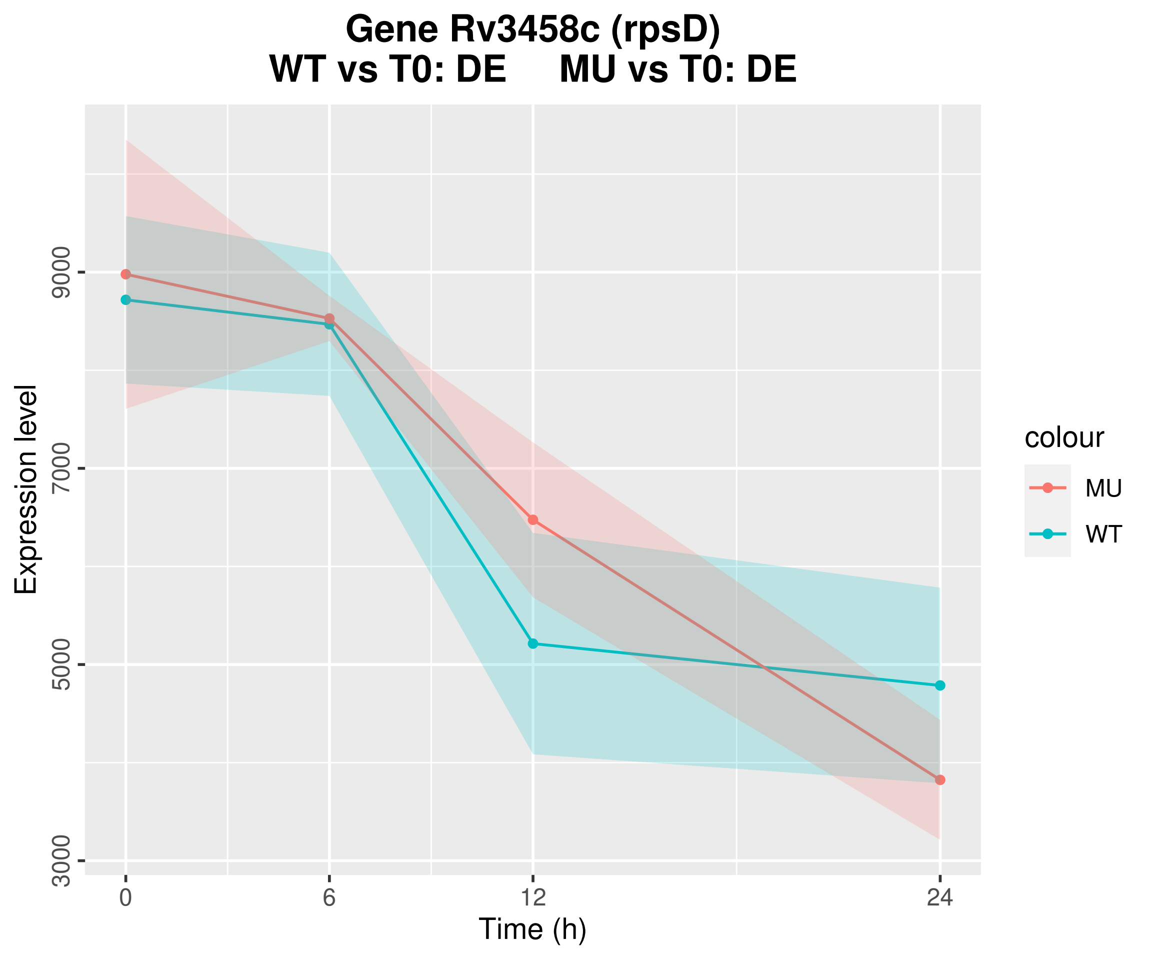

Gene Rv3458c (rpsD)
WT vs T0: DE MU vs T0: DE
Expression level
9000
7000
5000
3000
0
6
12
24
Time (h)
colour
MU
WT

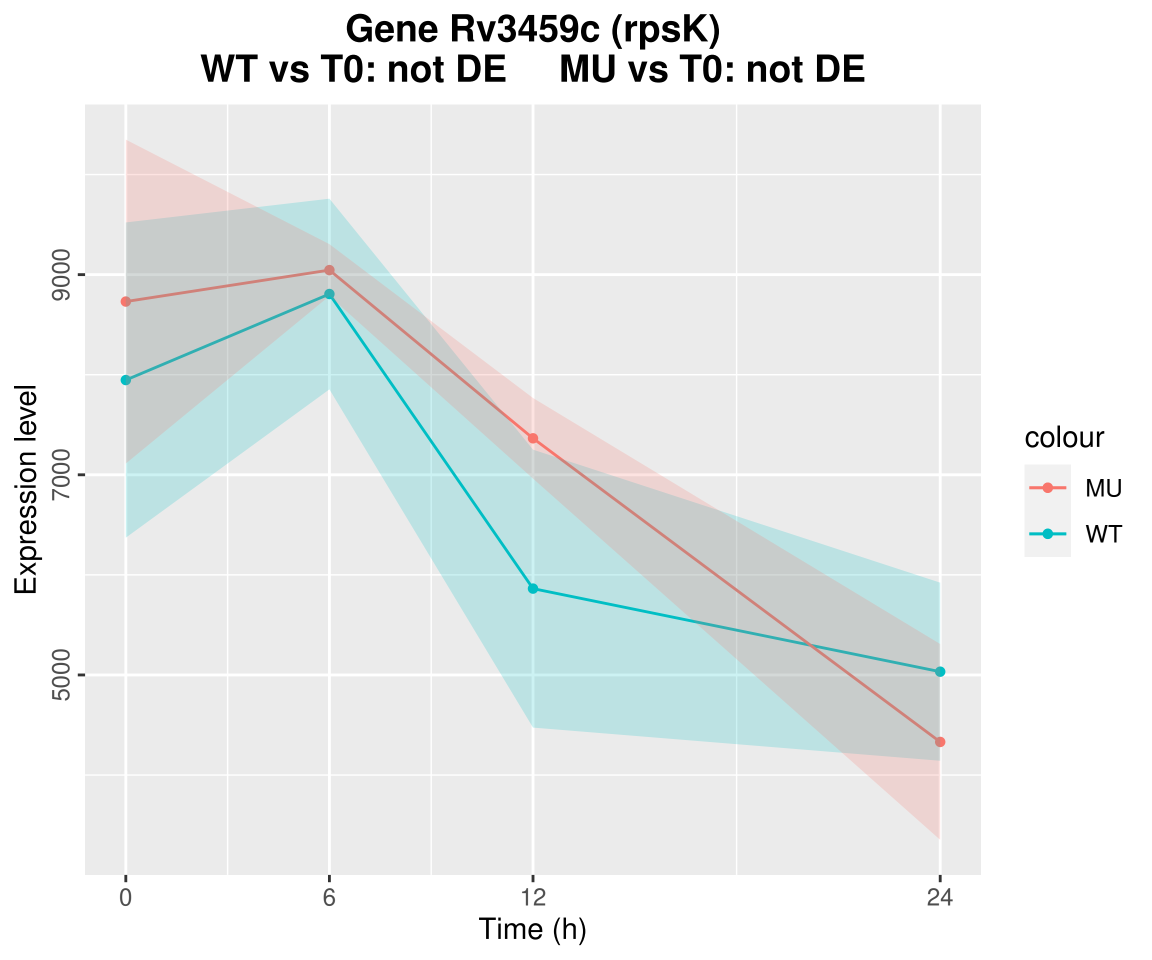

Gene Rv3459c (rpsK)
WT vs T0: not DE   MU vs T0: not DE
Expression level
9000
7000
5000
0
6
12
24
Time (h)
colour
MU
WT

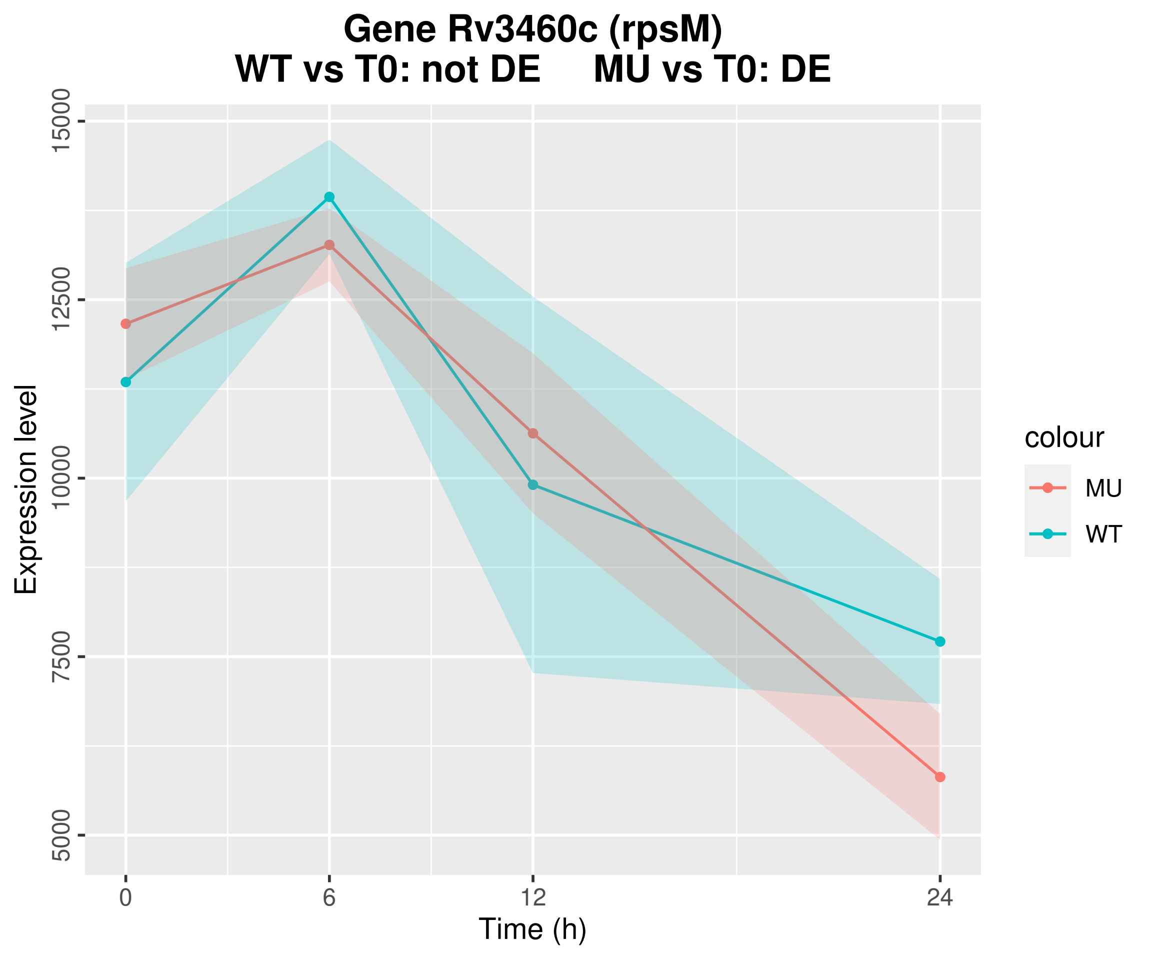
Gene Rv3460c (rpsM)
WT vs T0: not DE   MU vs T0: DE
15000
12500
10000
7500
5000
Expression level
0
6
12
24
Time (h)
colour
MU
WT

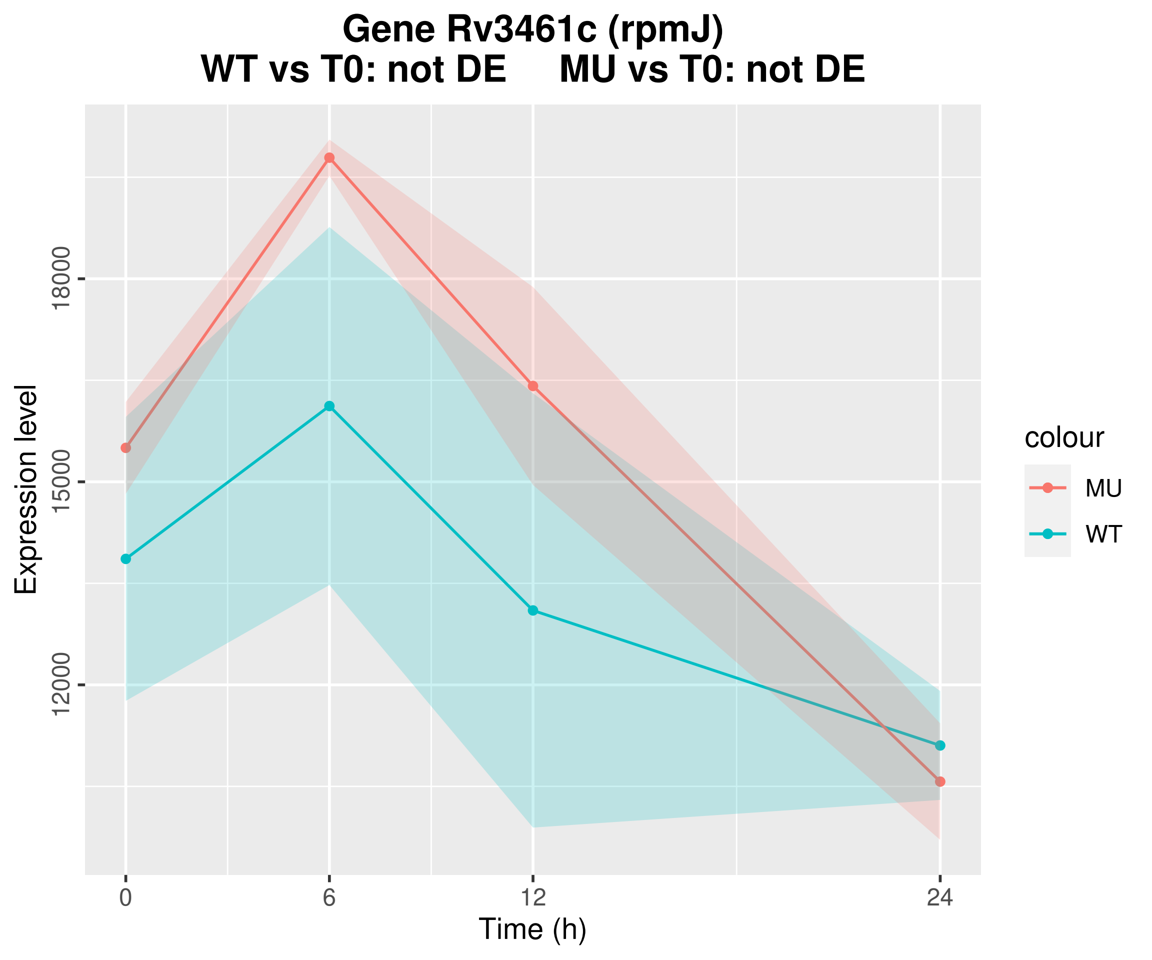

Gene Rv3461c (rpmJ)
WT vs T0: not DE   MU vs T0: not DE
Expression level
18000
15000
12000
0
6
12
24
Time (h)
colour
MU
WT

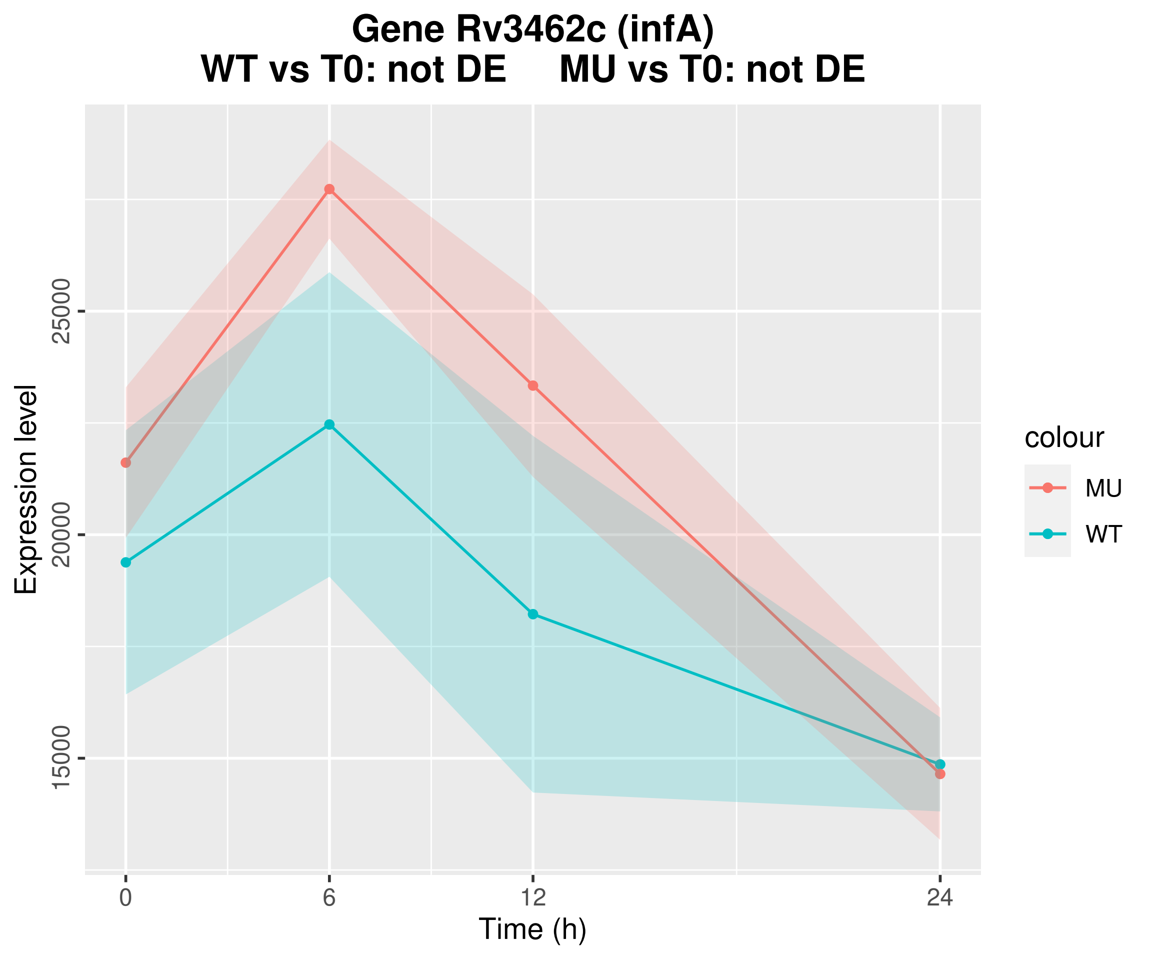
Gene Rv3462c (infA)
WT vs T0: not DE   MU vs T0: not DE
Expression level
25000
20000
15000
0
6
12
24
Time (h)
colour
MU
WT

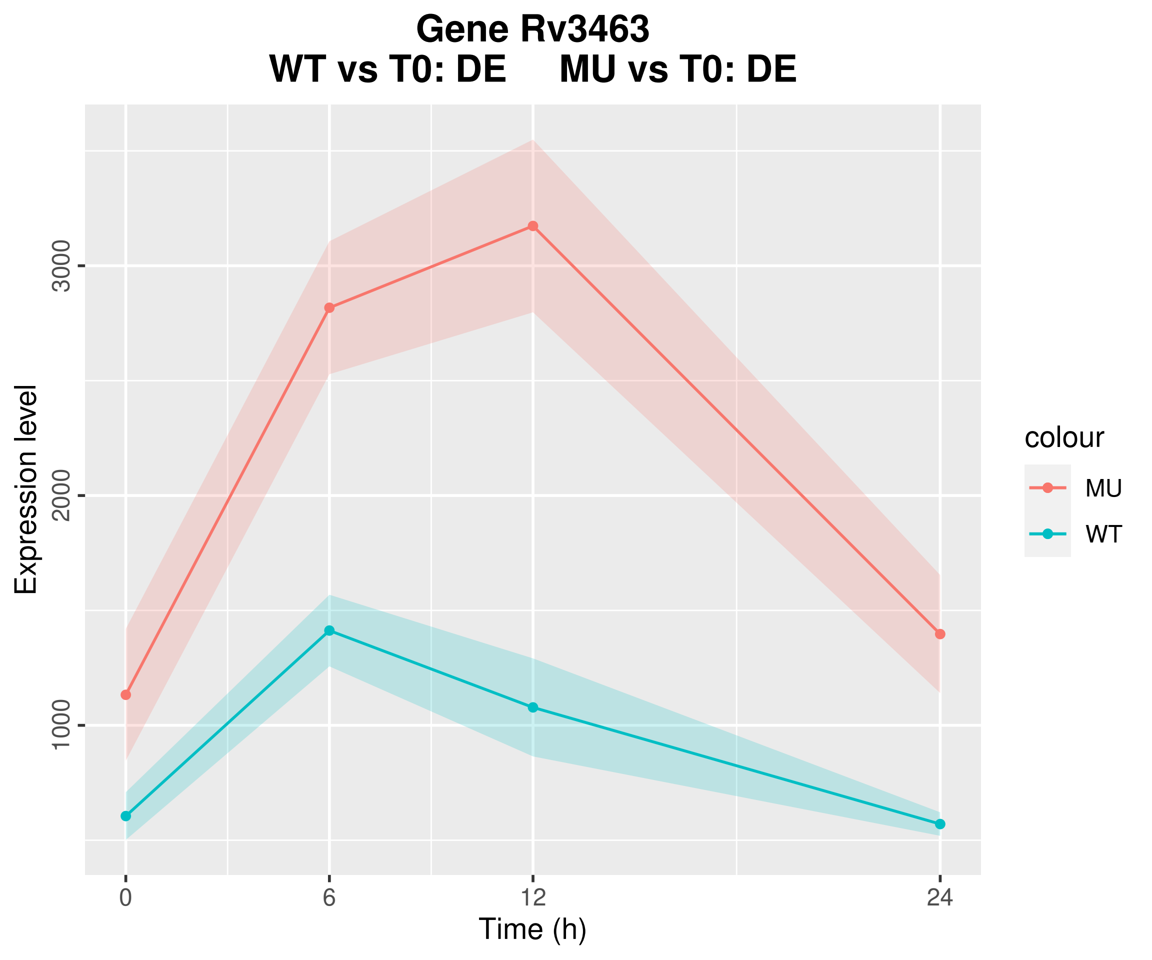
Gene Rv3463
WT vs T0: DE   MU vs T0: DE
Expression level
3000
2000
1000
0
6
12
24
Time (h)
colour
MU
WT

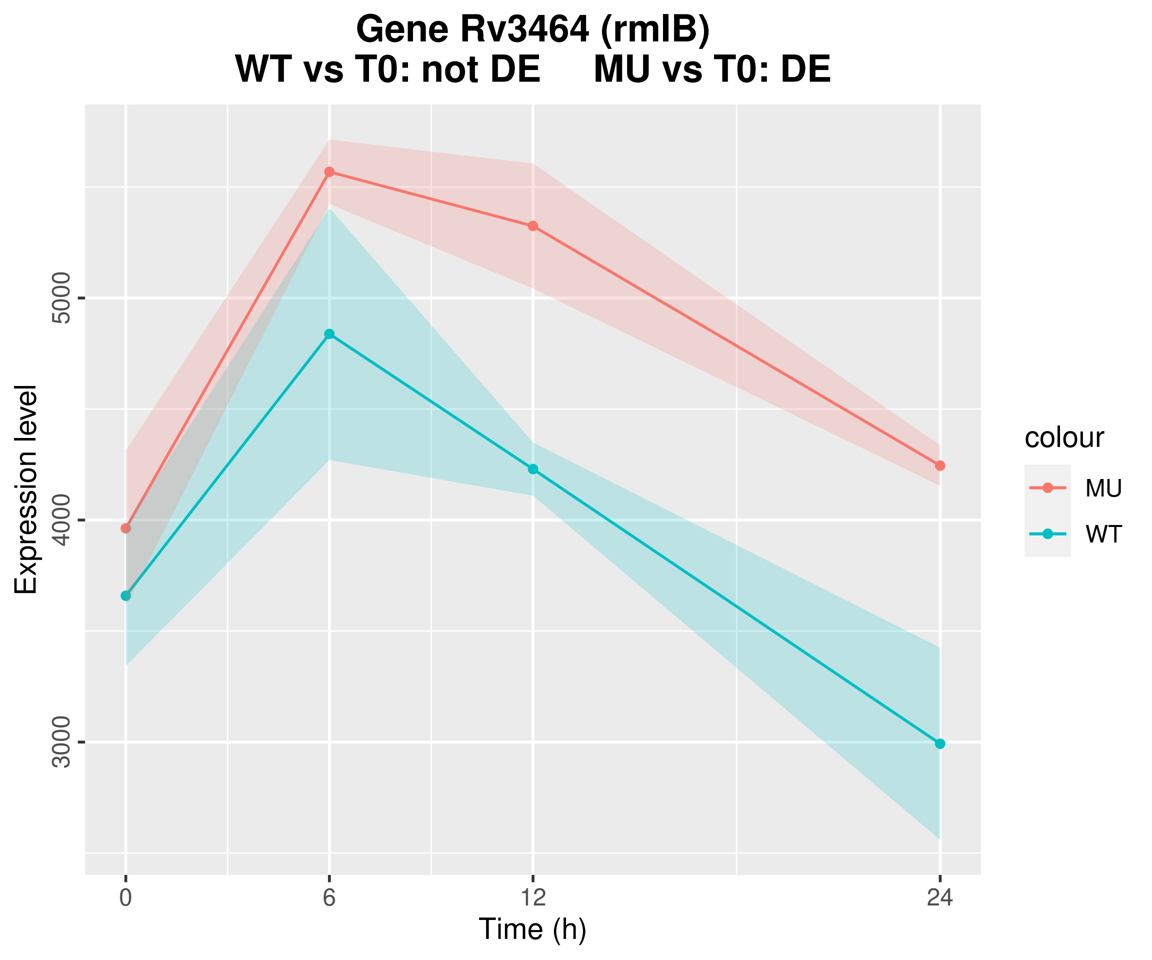

Gene Rv3464 (rmlB)
WT vs T0: not DE MU vs T0: DE
Expression level
5000
4000
3000
0
6
12
24
Time (h)
colour
MU
WT

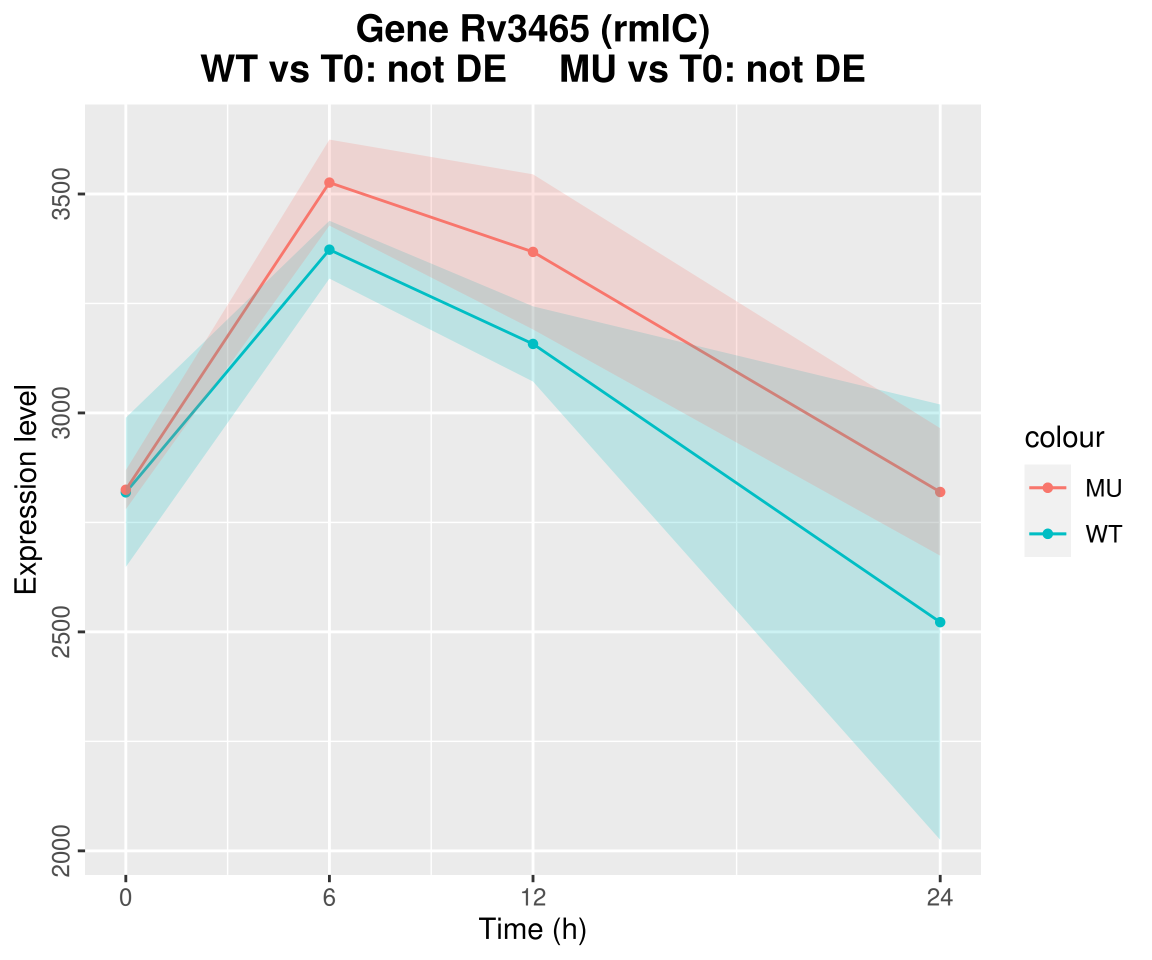

Gene Rv3465 (rmlC)
WT vs T0: not DE
MU vs T0: not DE
Expression level
3500
3000
2500
2000
0
6
12
24
Time (h)
colour
MU
WT

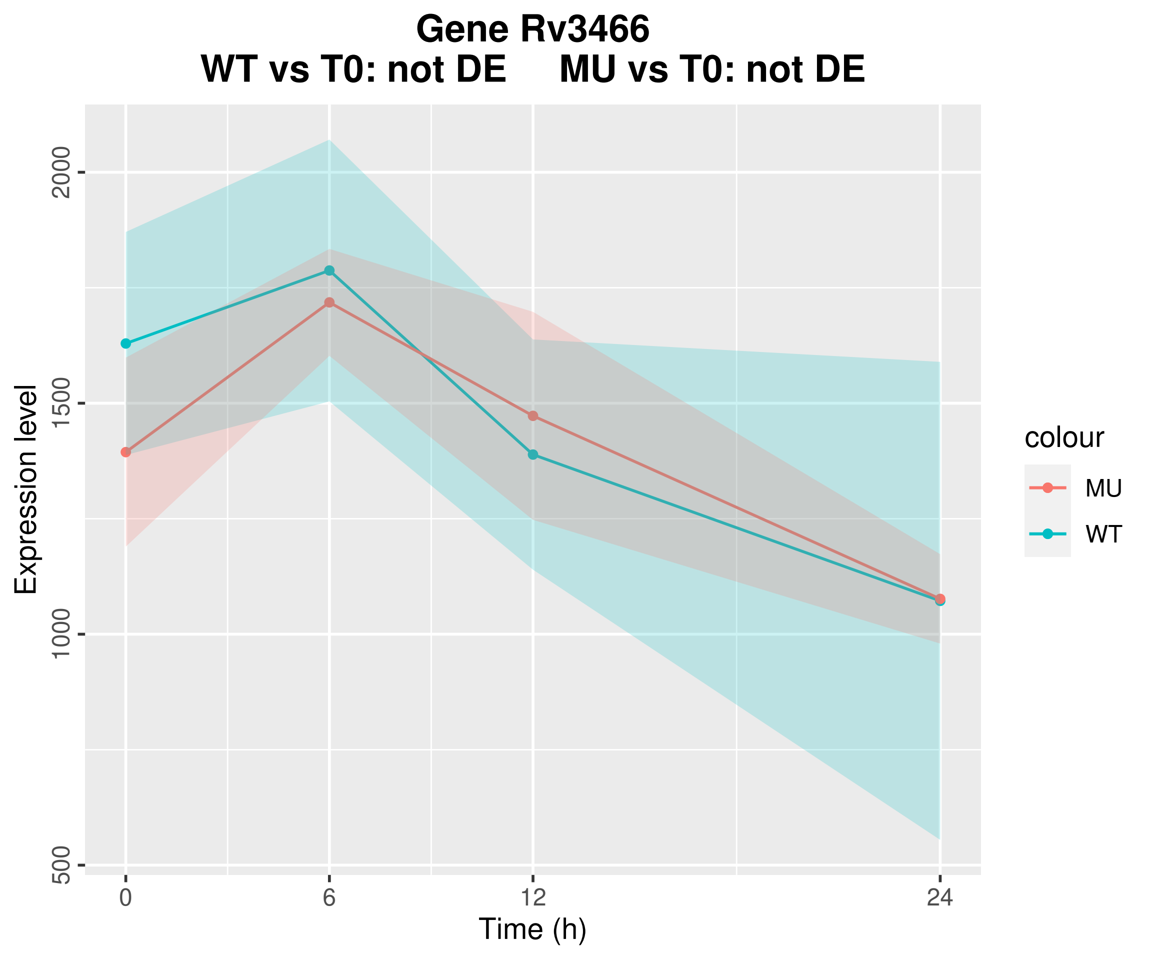
Gene Rv3466
WT vs T0: not DE MU vs T0: not DE
Expression level
2000
1500
1000
500
0
6
12
24
Time (h)
colour
MU
WT

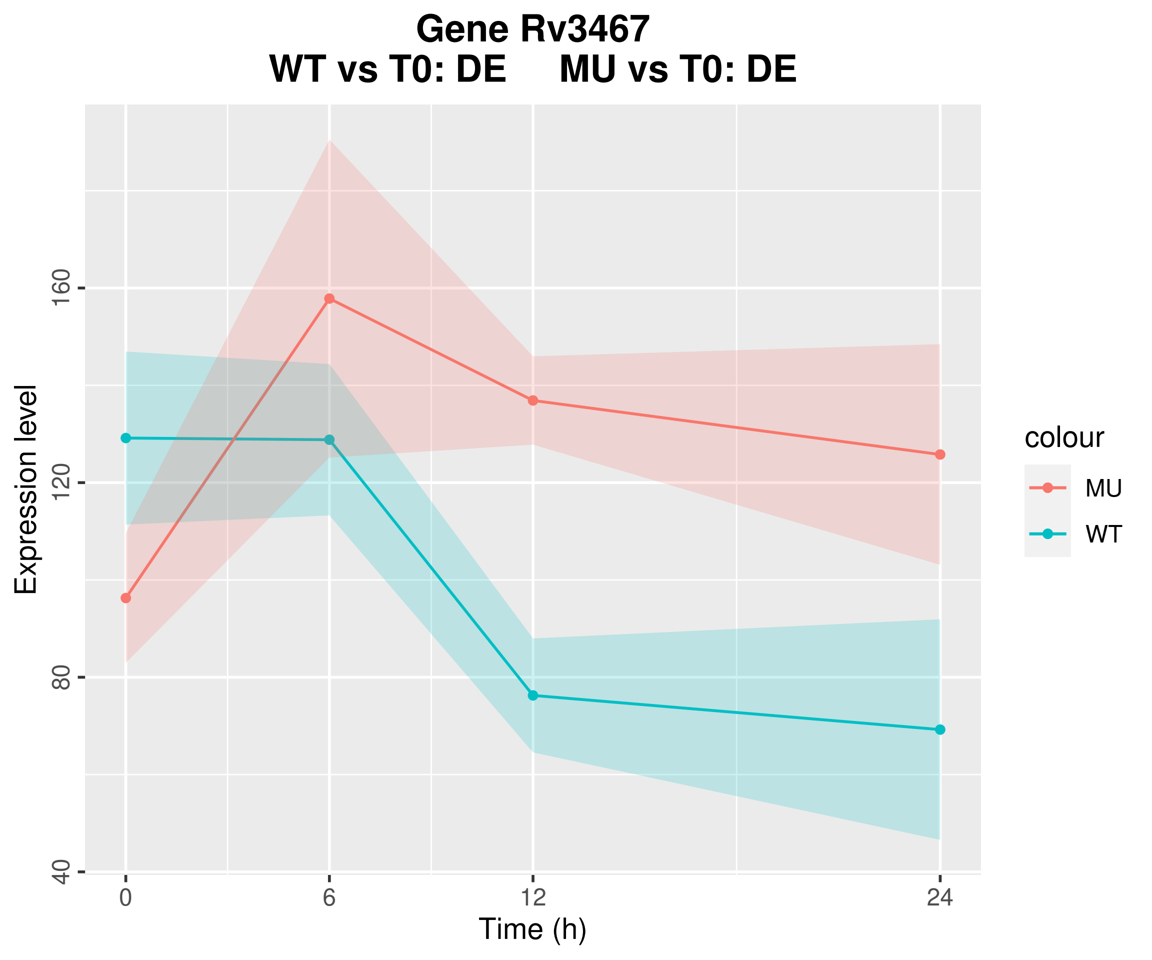
Gene Rv3467
WT vs T0: DE   MU vs T0: DE
Expression level
160
120
80
40
0
6
12
24
Time (h)
colour
MU
WT

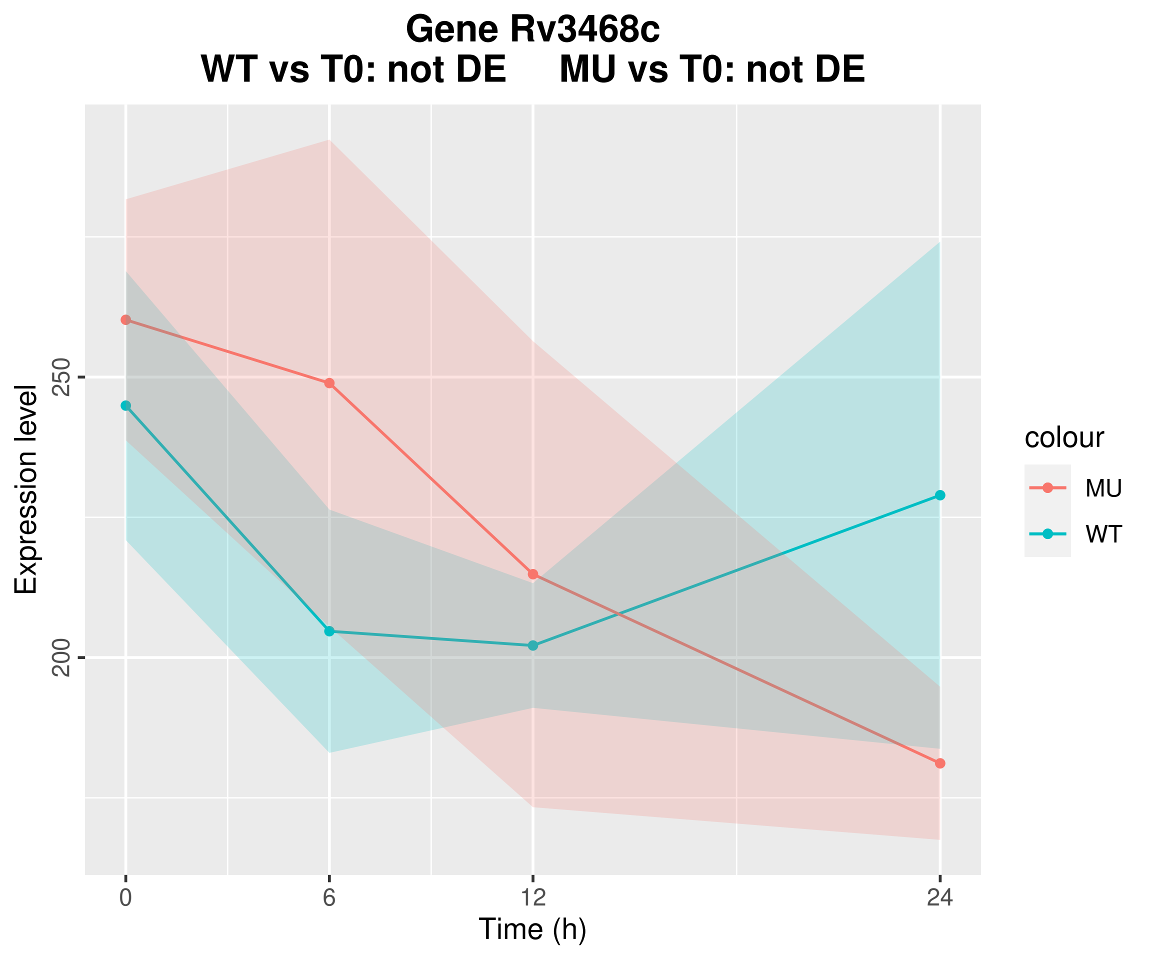

Gene Rv3468c
WT vs T0: not DE MU vs T0: not DE
Expression level
250
200
0
6
12
24
Time (h)
colour
MU
WT

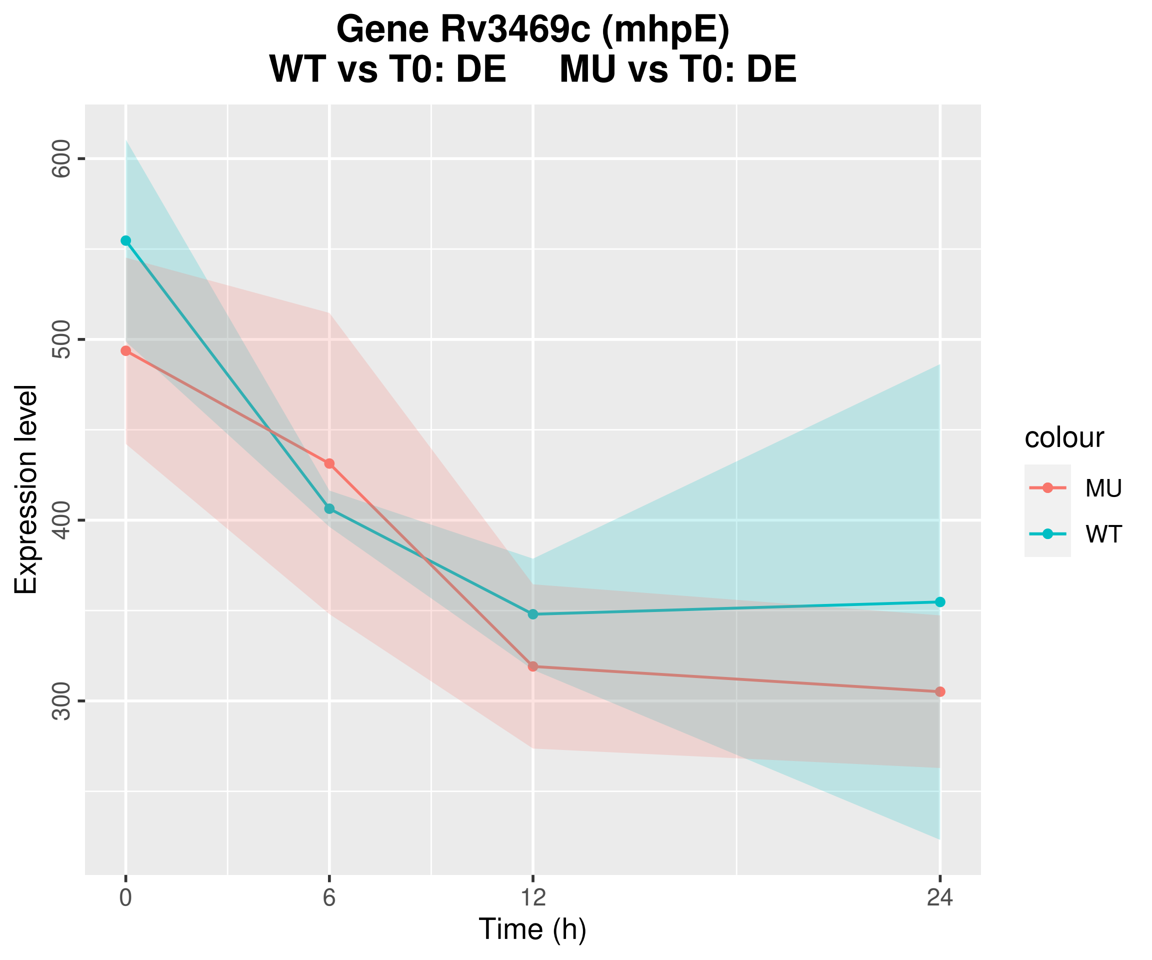

Gene Rv3469c (mhpE)
WT vs T0: DE MU vs T0: DE
Expression level
600
500
400
300
0
6
12
24
Time (h)
colour
MU
WT

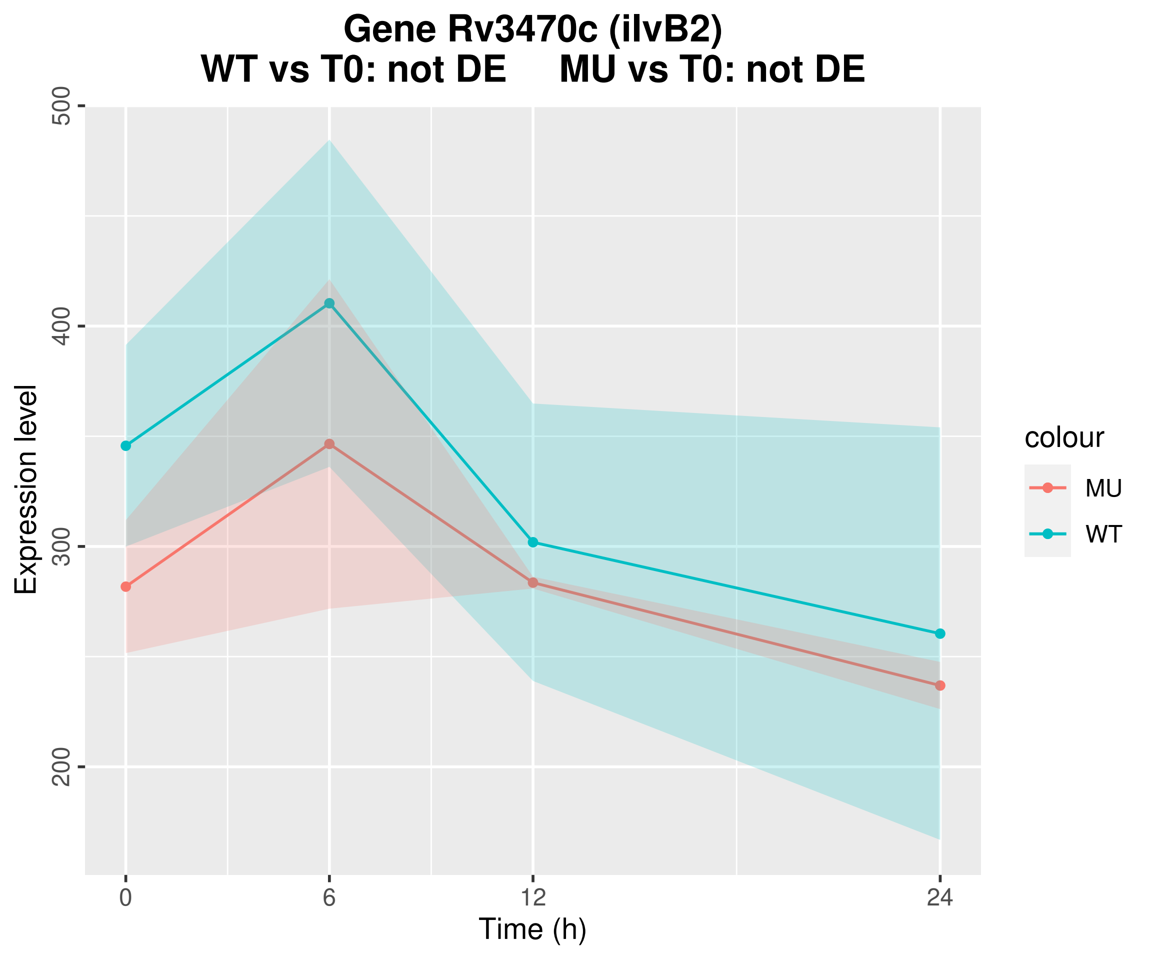
Gene Rv3470c (ilvB2)
WT vs T0: not DE MU vs T0: not DE
Expression level
500
400
300
200
0
6
12
24
Time (h)
colour
MU
WT

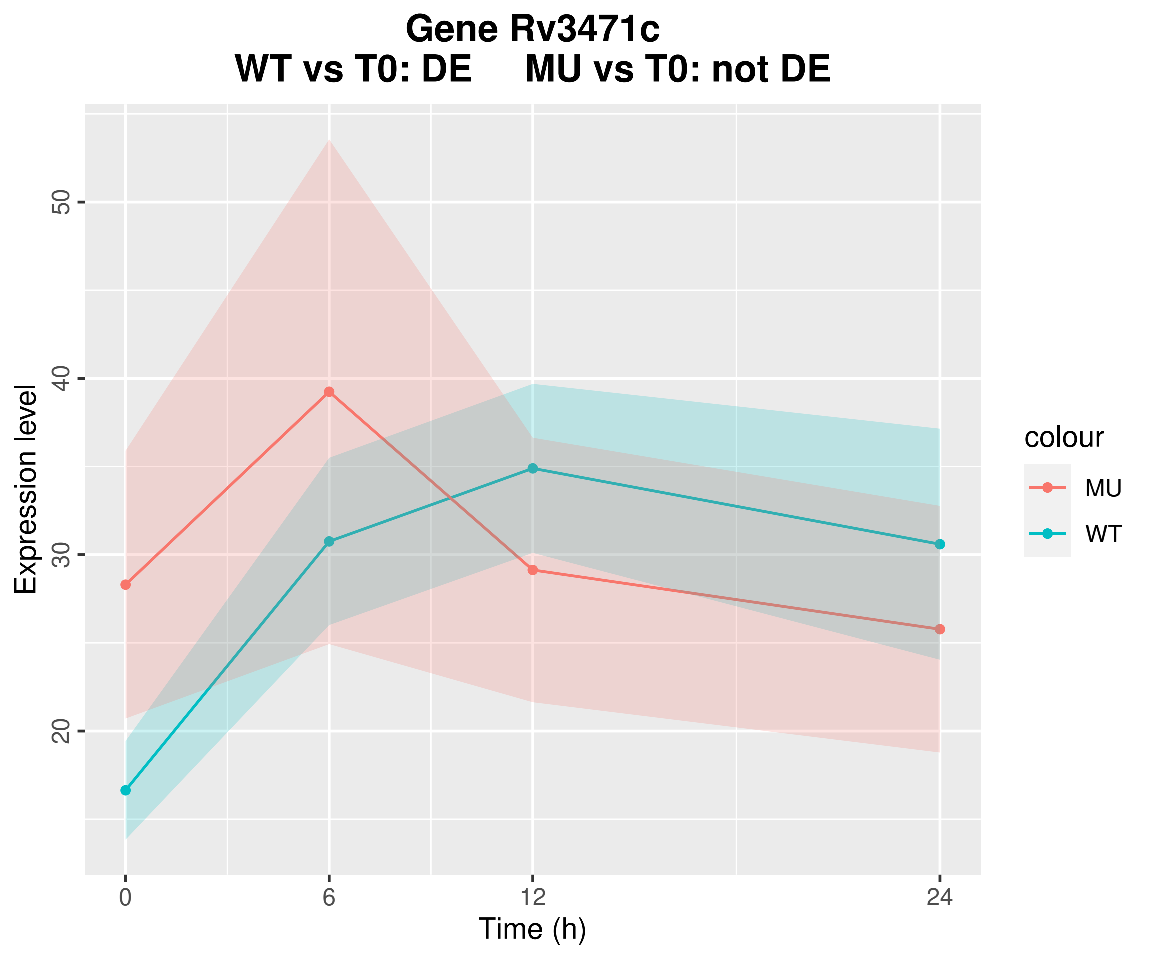

Gene Rv3471c
WT vs T0: DE MU vs T0: not DE
Expression level
50
40
30
20
0
6
12
24
Time (h)
colour
MU
WT

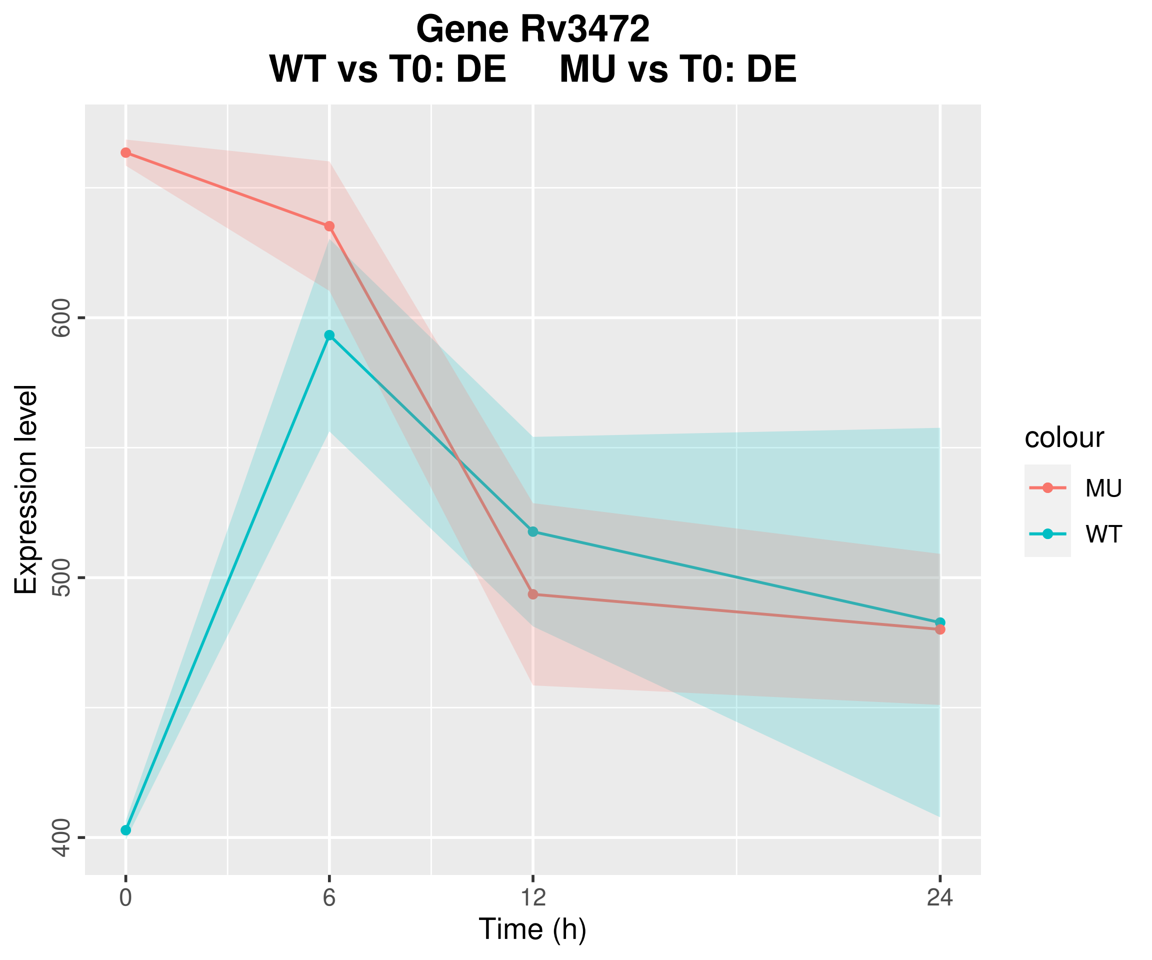

Gene Rv3472
WT vs T0: DE MU vs T0: DE
Expression level
600
500
400
0
6
12
24
Time (h)
colour
MU
WT

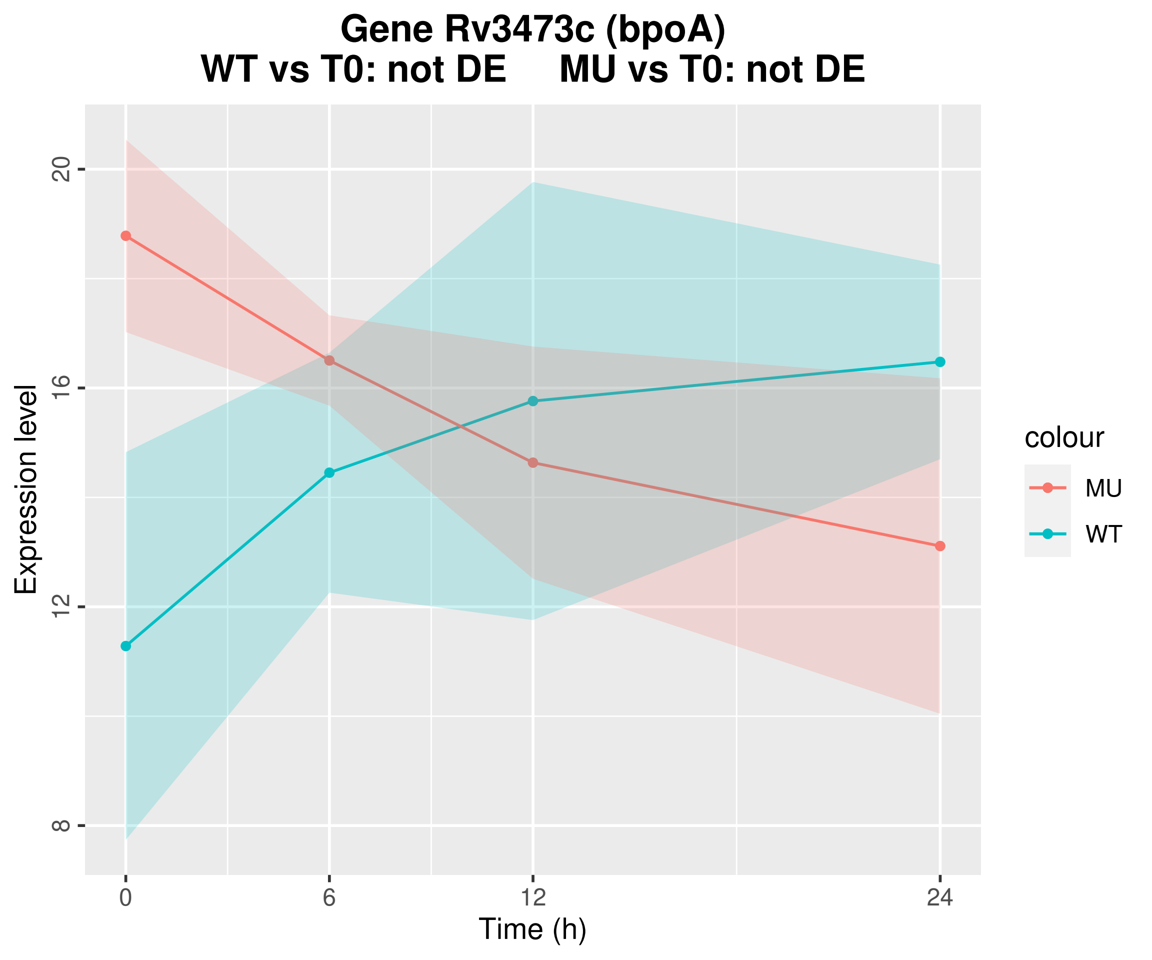
Gene Rv3473c (bpoA)
WT vs T0: not DE   MU vs T0: not DE
Expression level
20
16
12
8
0
6
12
24
Time (h)
colour
MU
WT

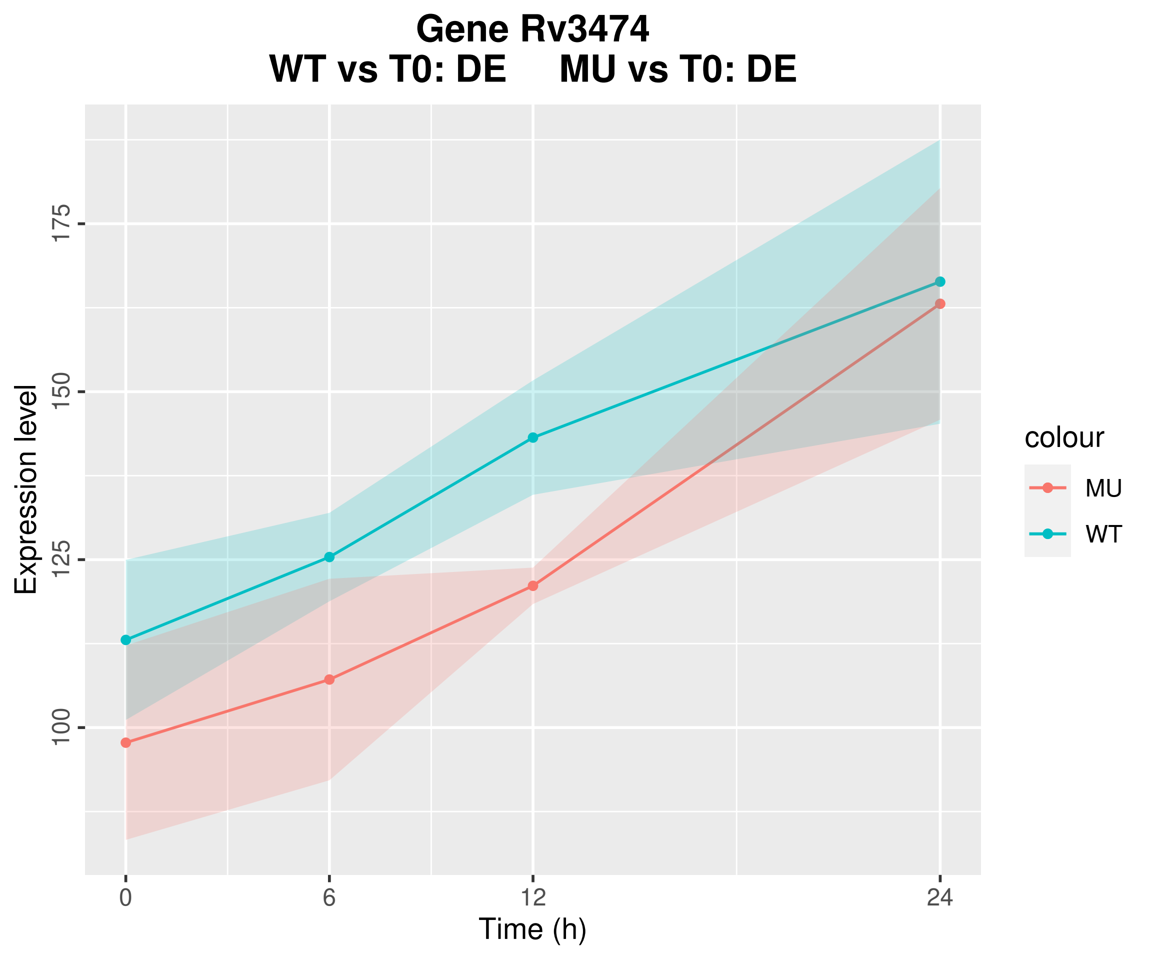

Gene Rv3474
WT vs T0: DE   MU vs T0: DE
175
150
125
100
Expression level
0
6
12
24
Time (h)
colour
MU
WT

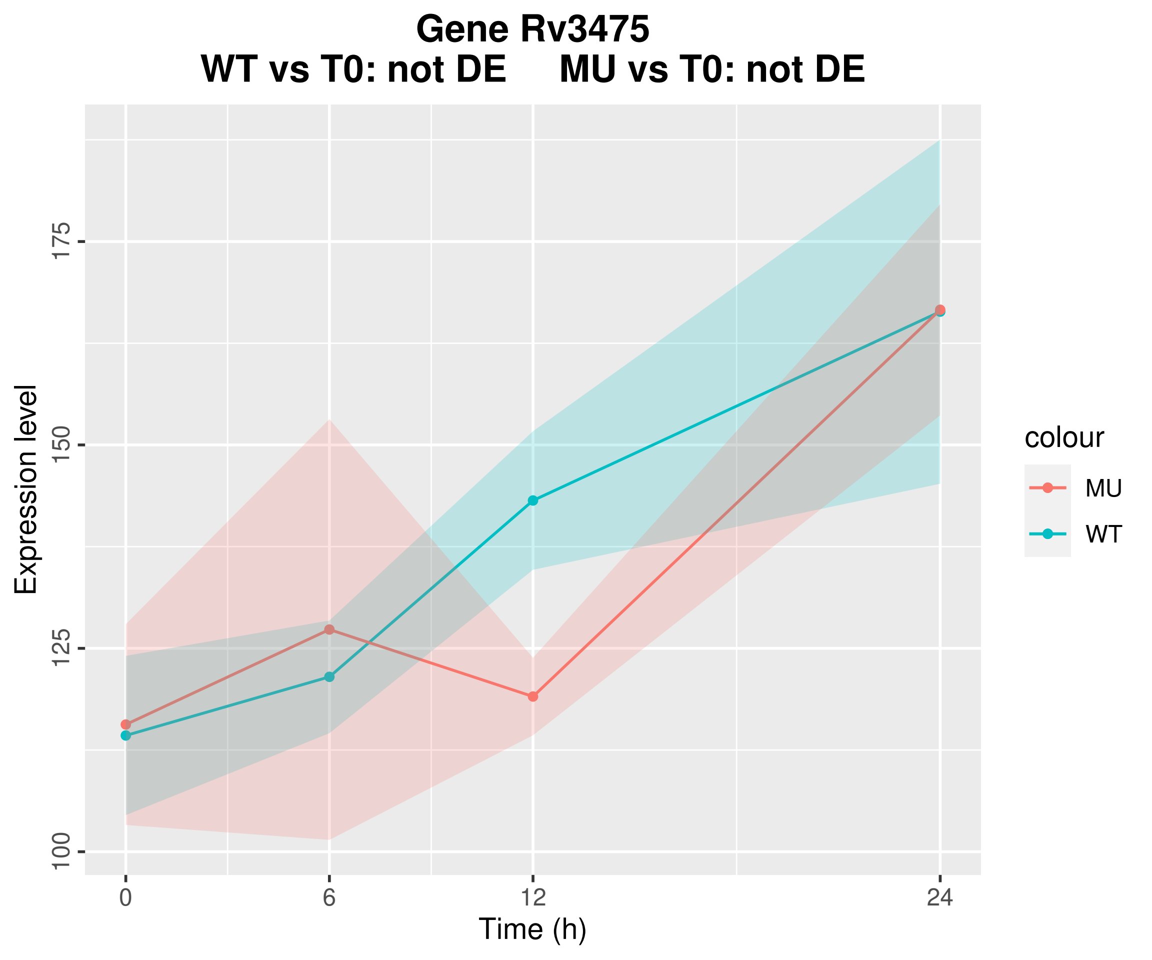
Gene Rv3475
WT vs T0: not DE MU vs T0: not DE
Expression level
100
125
150
175
0
6
12
24
Time (h)
colour
MU
WT

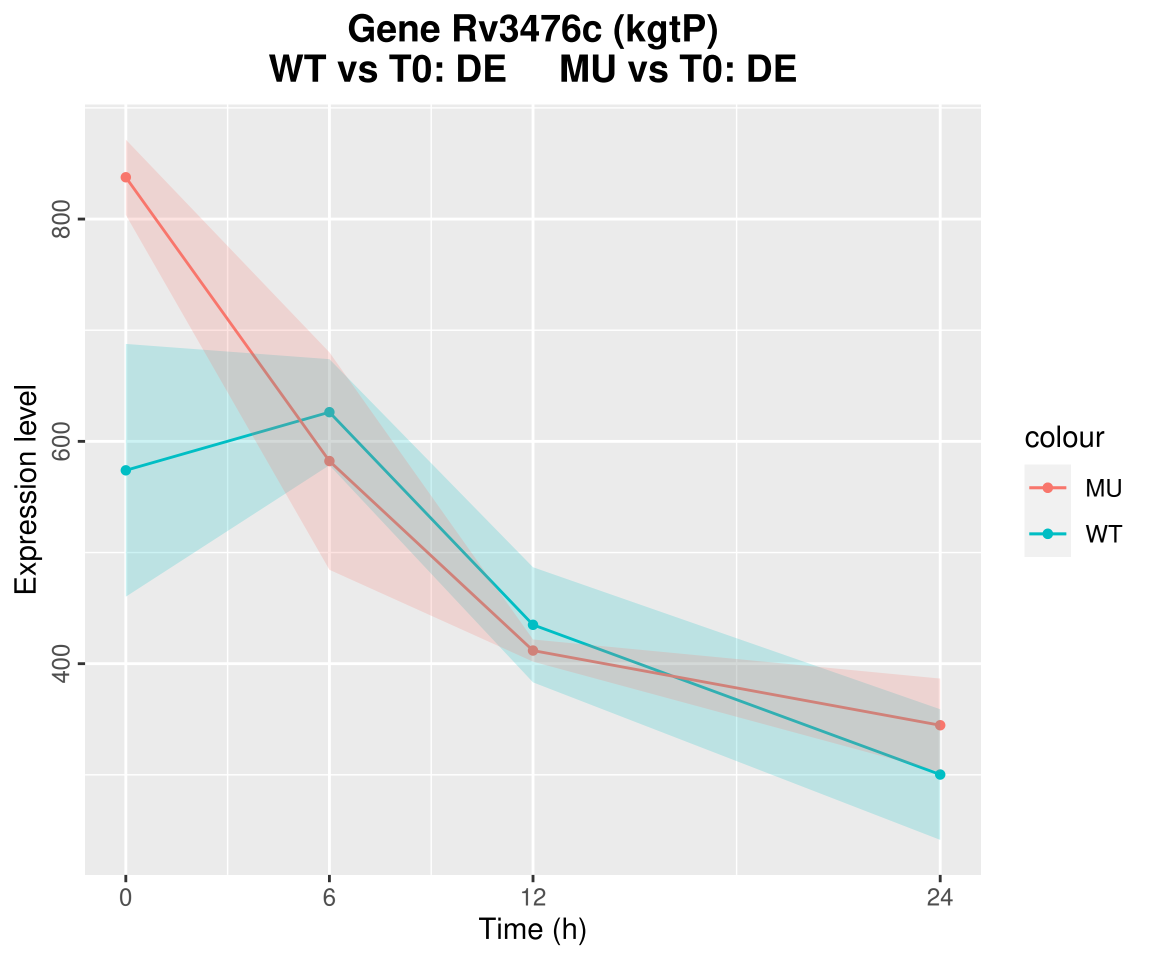

Gene Rv3476c (kgtP)
WT vs T0: DE   MU vs T0: DE
Expression level
800
600
400
0
6
12
24
Time (h)
colour
MU
WT

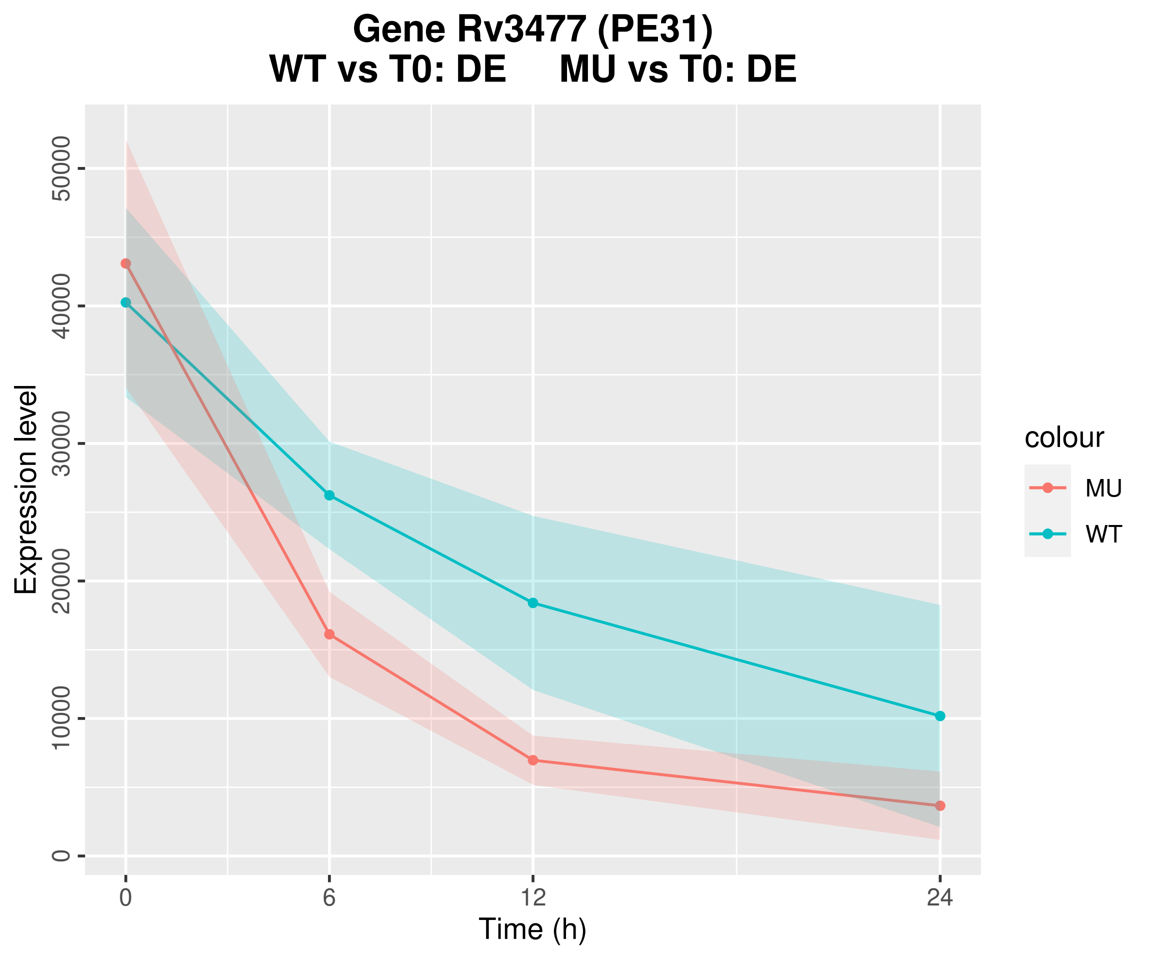

Gene Rv3477 (PE31)
WT vs T0: DE   MU vs T0: DE
Expression level
50000
40000
30000
20000
10000
0
0
6
12
24
Time (h)
colour
MU
WT

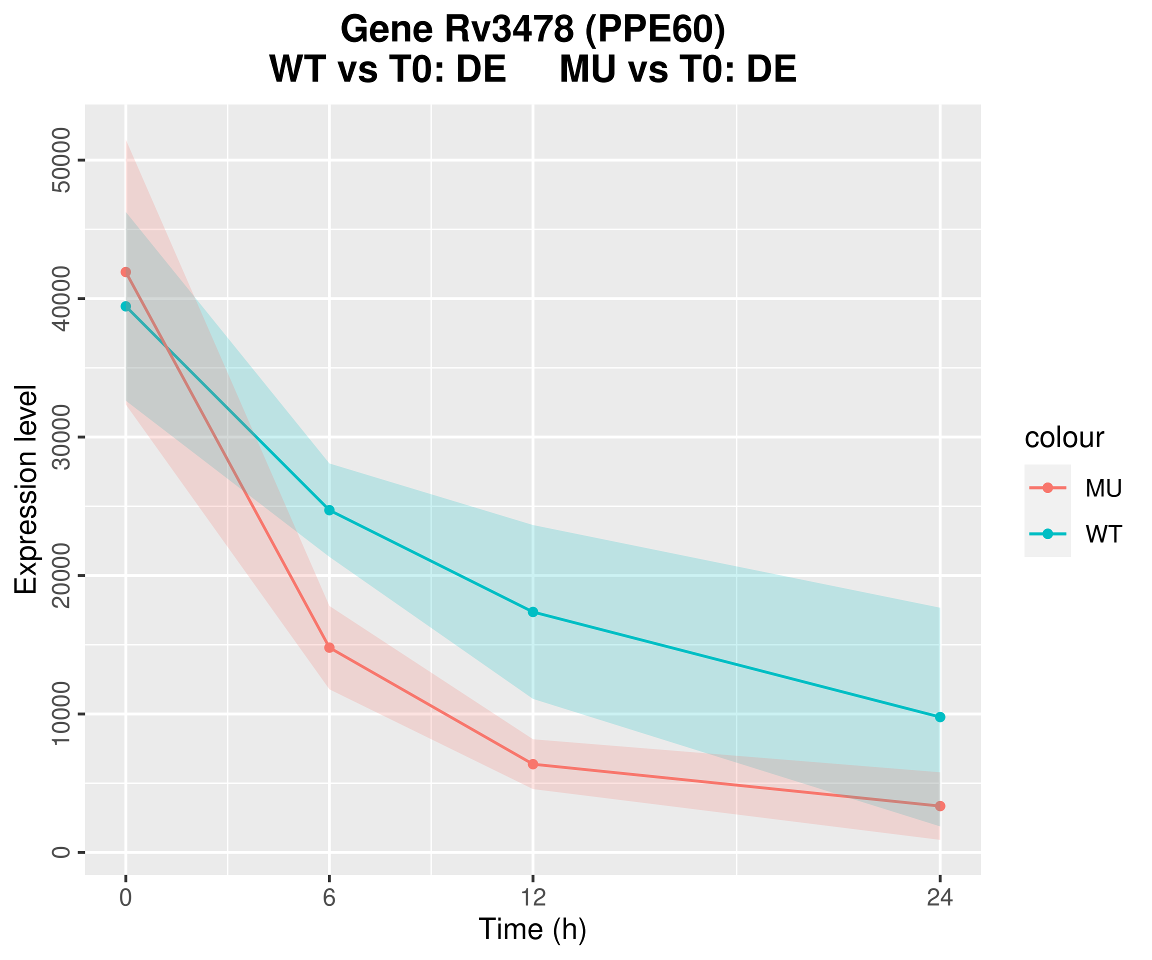

Gene Rv3478 (PPE60)
WT vs T0: DE   MU vs T0: DE
Expression level
0
10000
20000
30000
40000
50000
0
6
12
24
Time (h)
colour
MU
WT

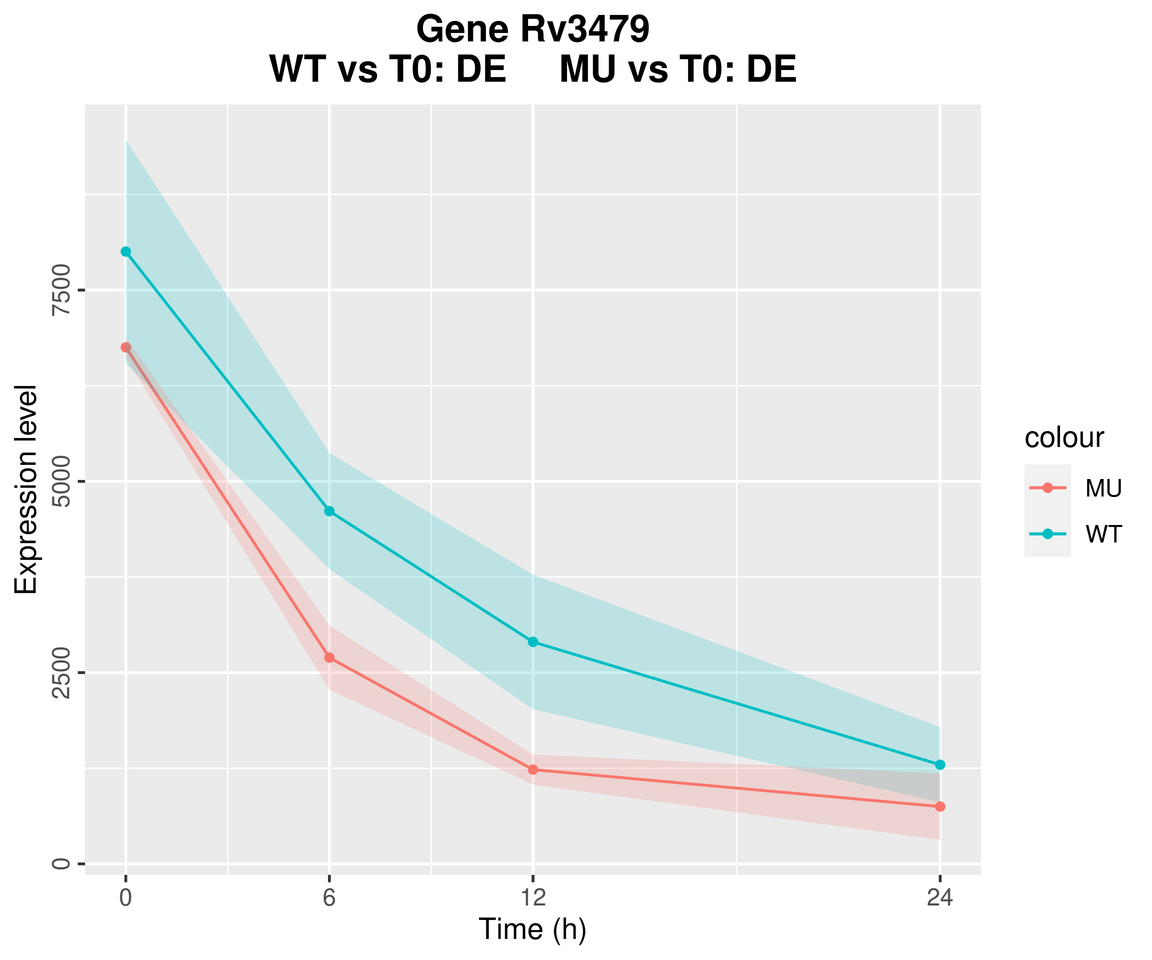
Gene Rv3479
WT vs T0: DE MU vs T0: DE
Expression level
7500
5000
2500
0
0
6
12
24
Time (h)
colour
MU
WT

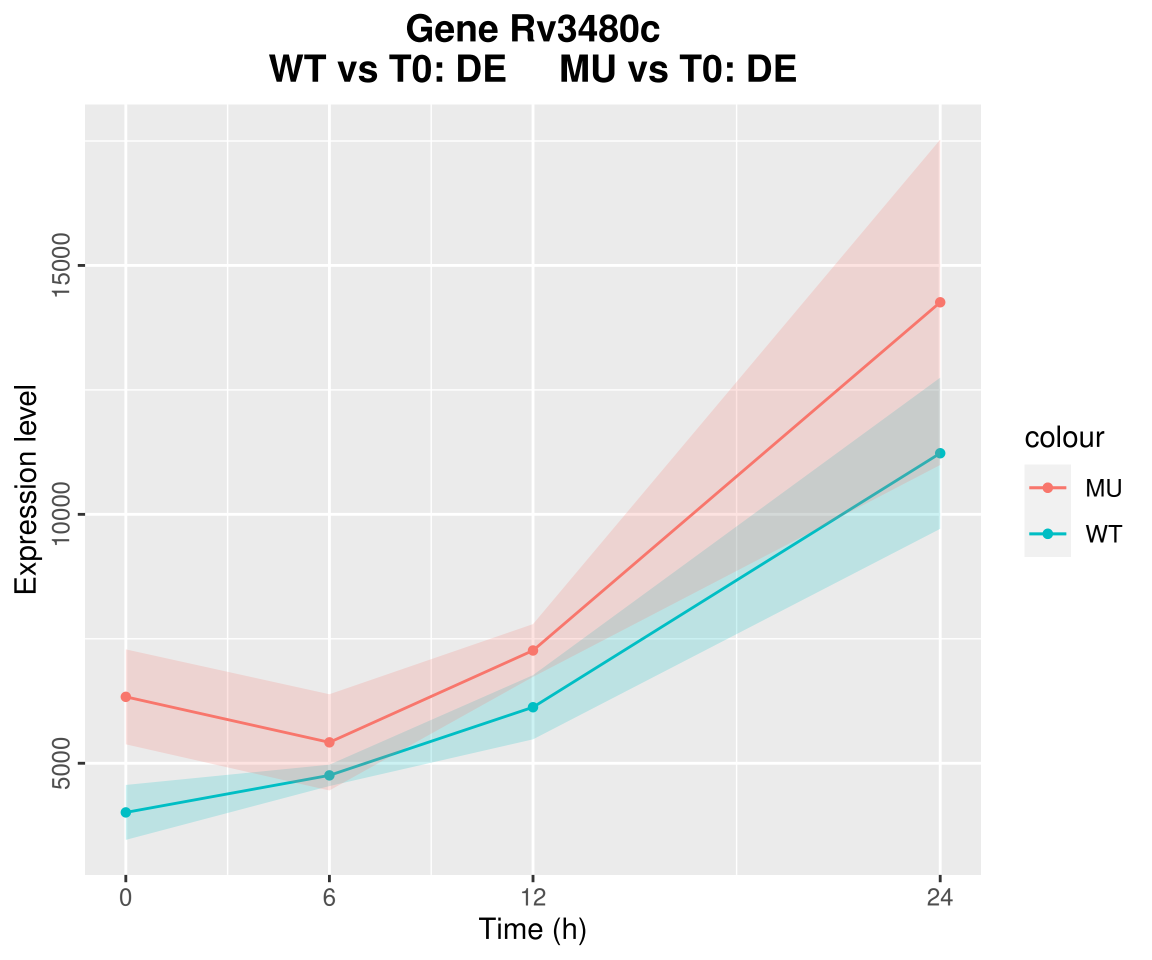

Gene Rv3480c
WT vs T0: DE MU vs T0: DE
Expression level
15000
10000
5000
0
6
12
24
Time (h)
colour
MU
WT

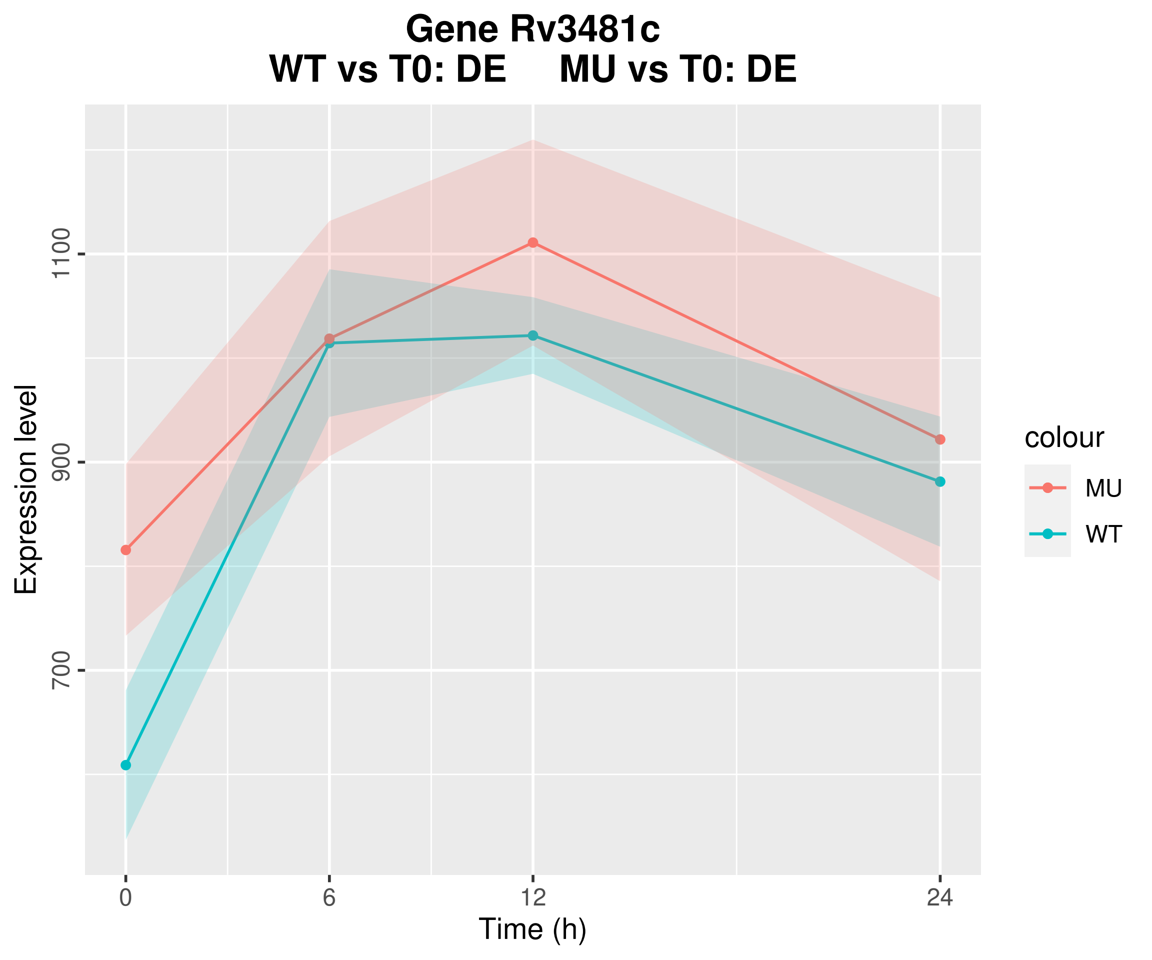

Gene Rv3481c
WT vs T0: DE MU vs T0: DE
Expression level
700
900
1100
Time (h)
0
6
12
24
colour
MU
WT

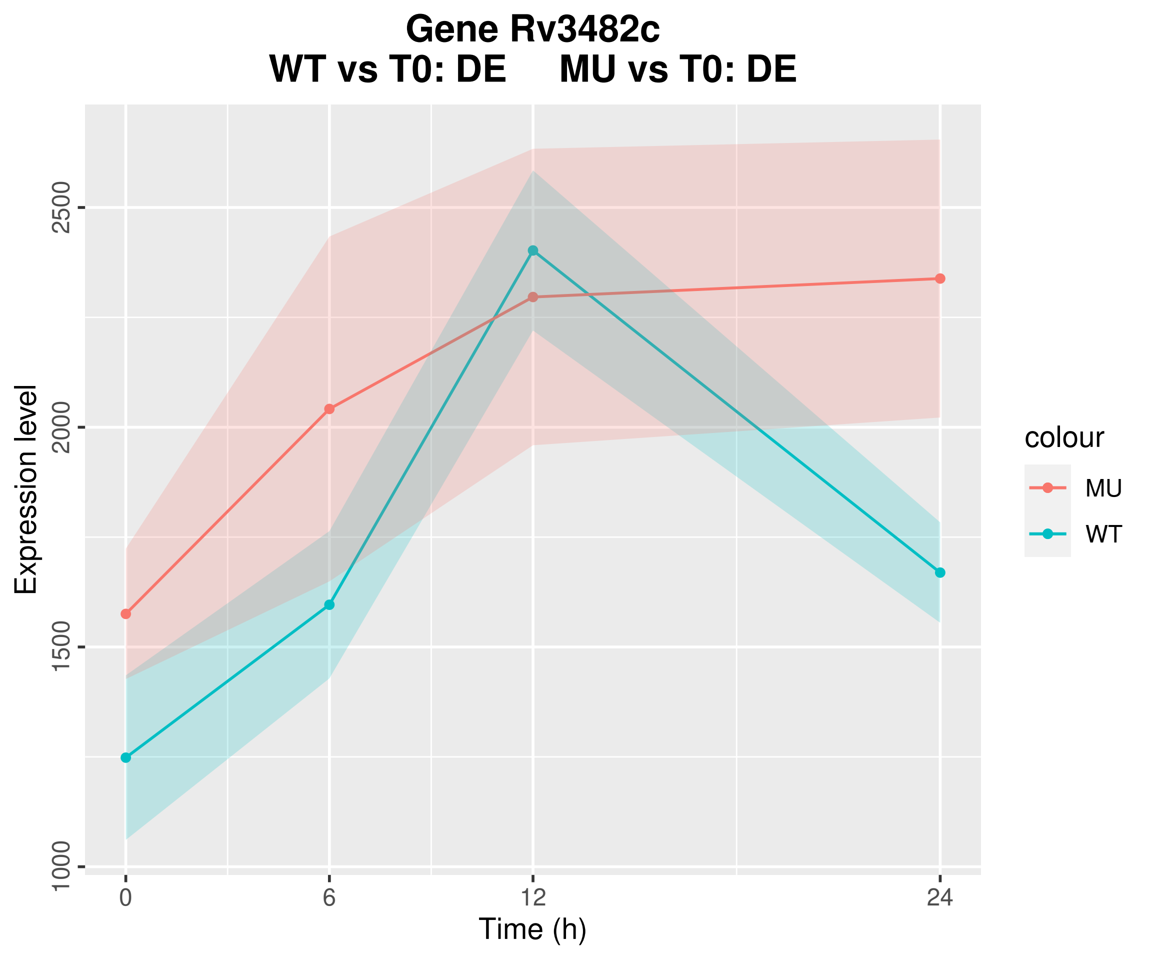

Gene Rv3482c
WT vs T0: DE   MU vs T0: DE
Expression level
2500
2000
1500
1000
0
6
12
24
Time (h)
colour
MU
WT

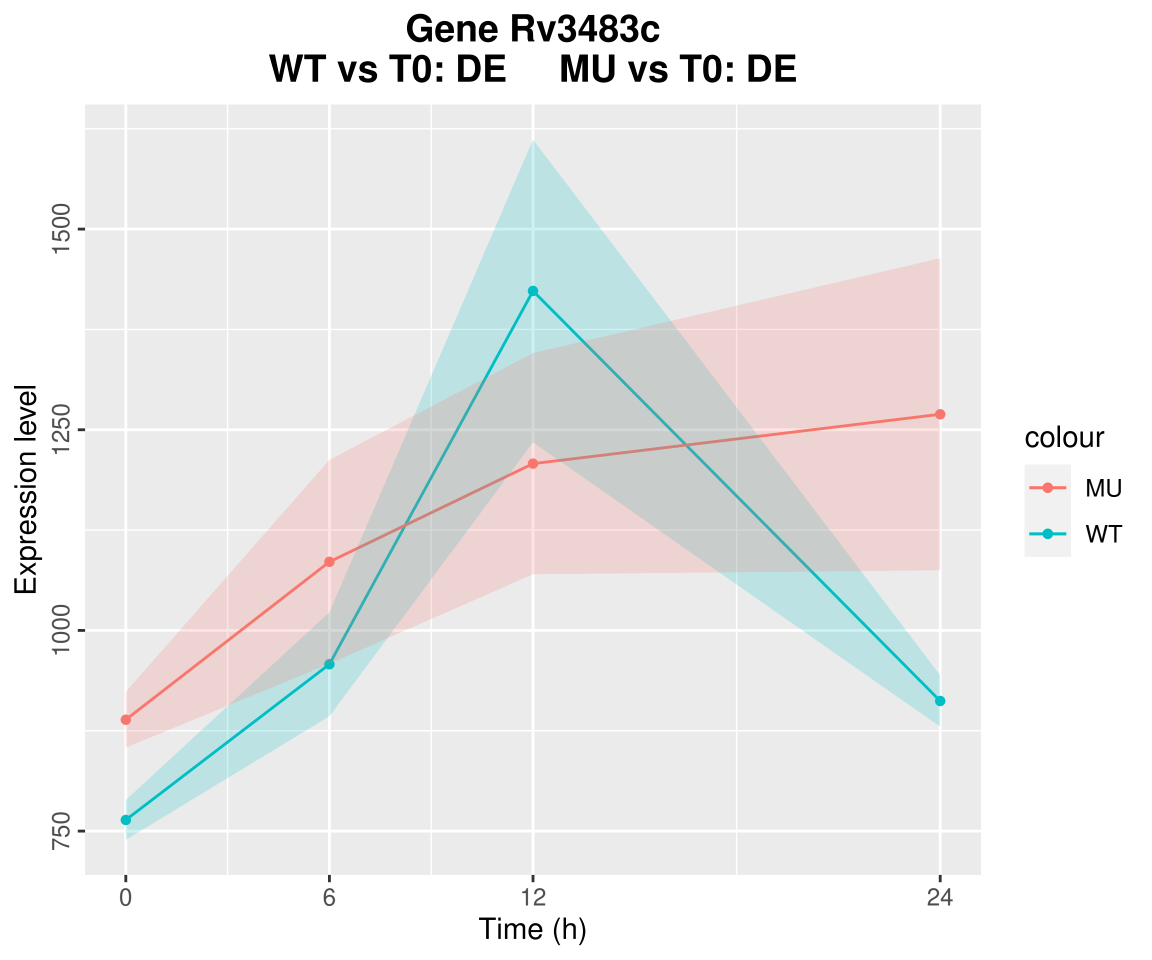
Gene Rv3483c
WT vs T0: DE MU vs T0: DE
Expression level
Time (h)
750
1000
1250
1500
0
6
12
24
colour
MU
WT

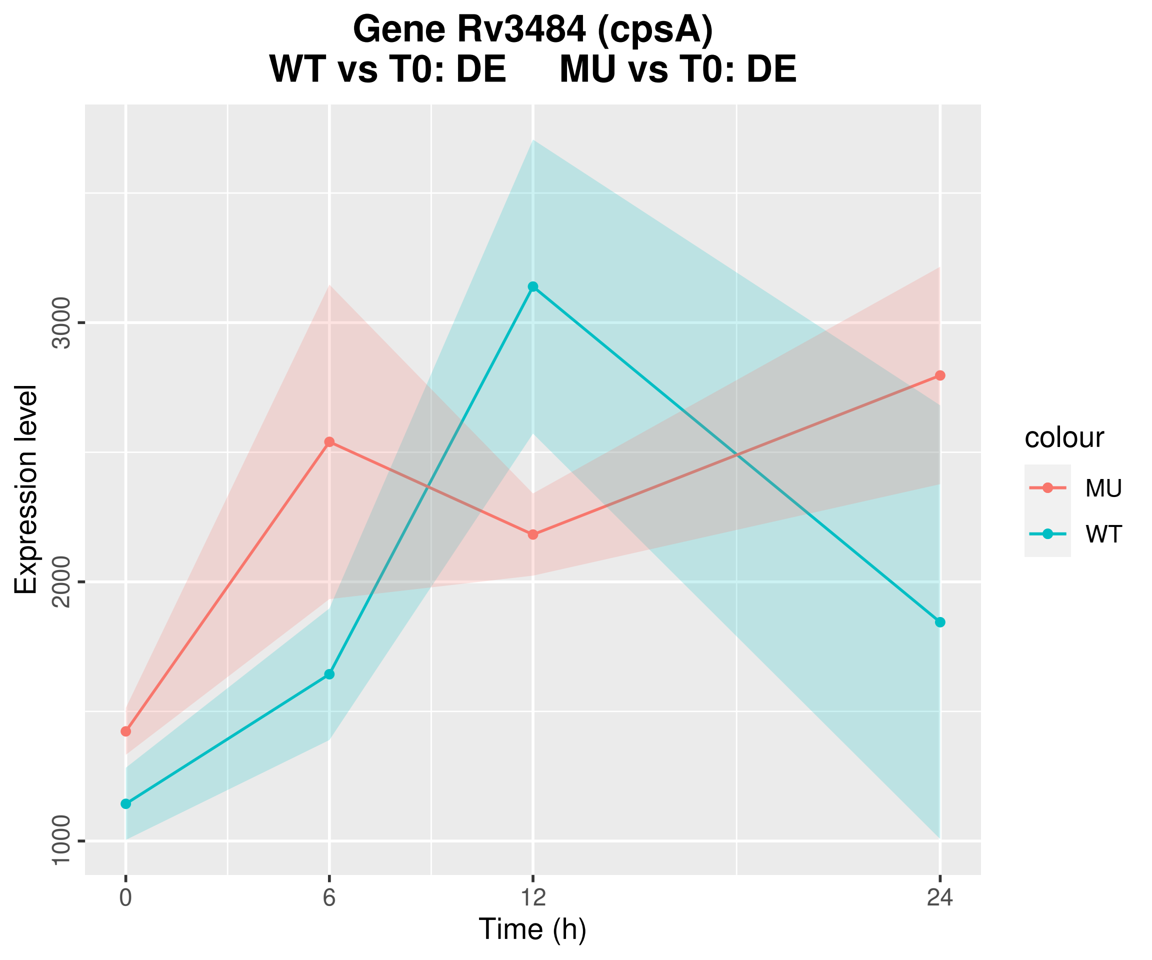

Gene Rv3484 (cpsA)
WT vs T0: DE MU vs T0: DE
Expression level
3000
2000
1000
0
6
12
24
Time (h)
colour
MU
WT

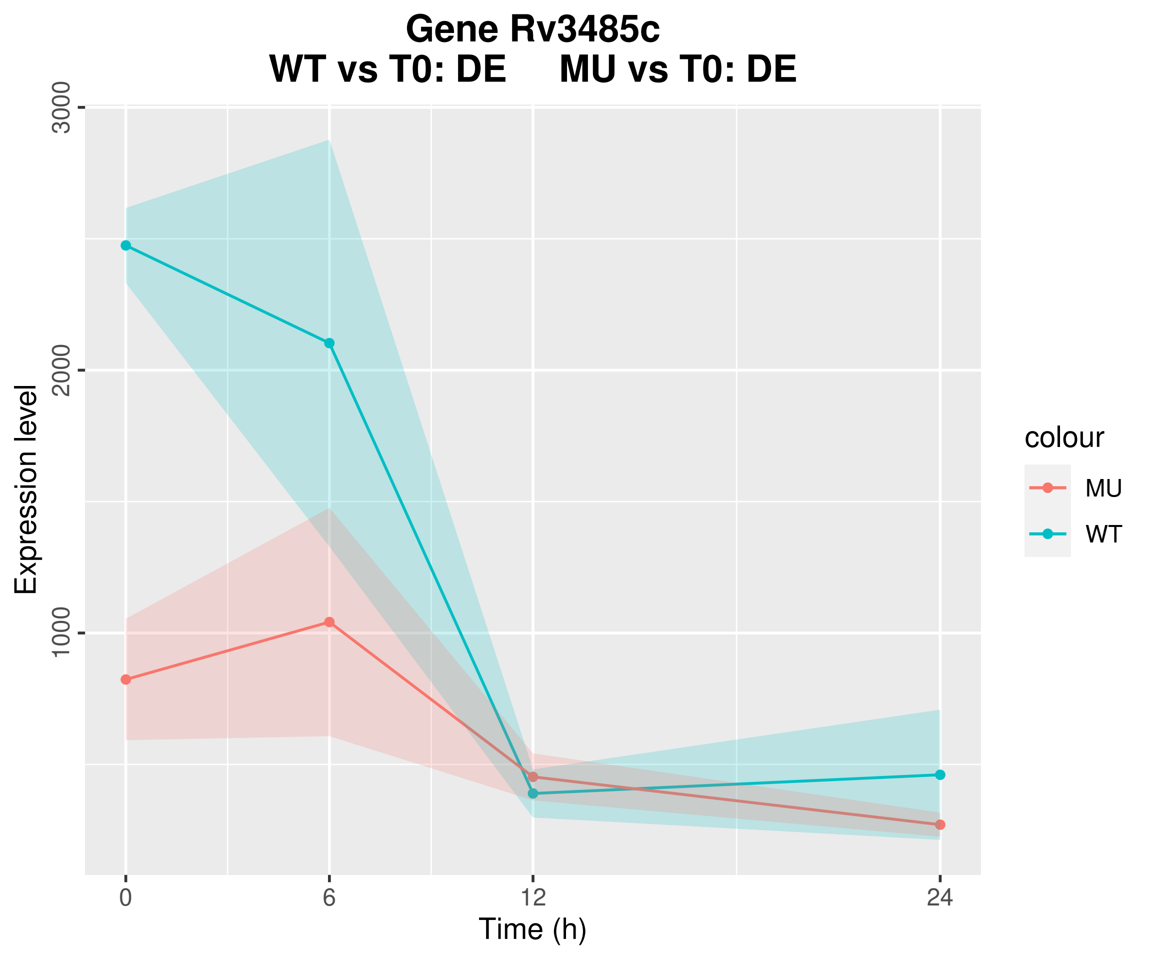
Gene Rv3485c
WT vs T0: DE   MU vs T0: DE
3000
2000
1000
Expression level
0
6
12
24
Time (h)
colour
MU
WT

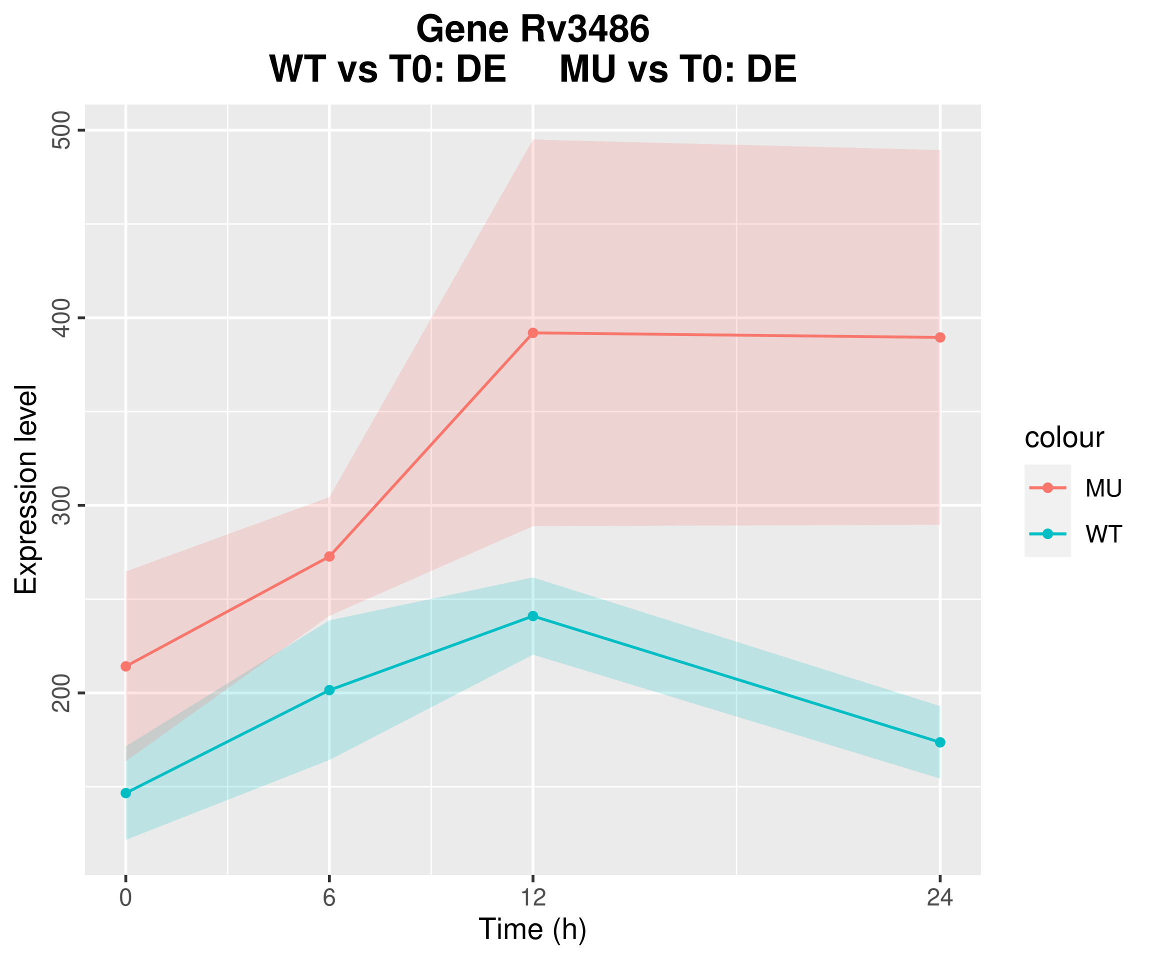
Gene Rv3486
WT vs T0: DE MU vs T0: DE
Expression level
500
400
300
200
0
6
12
24
Time (h)
colour
MU
WT

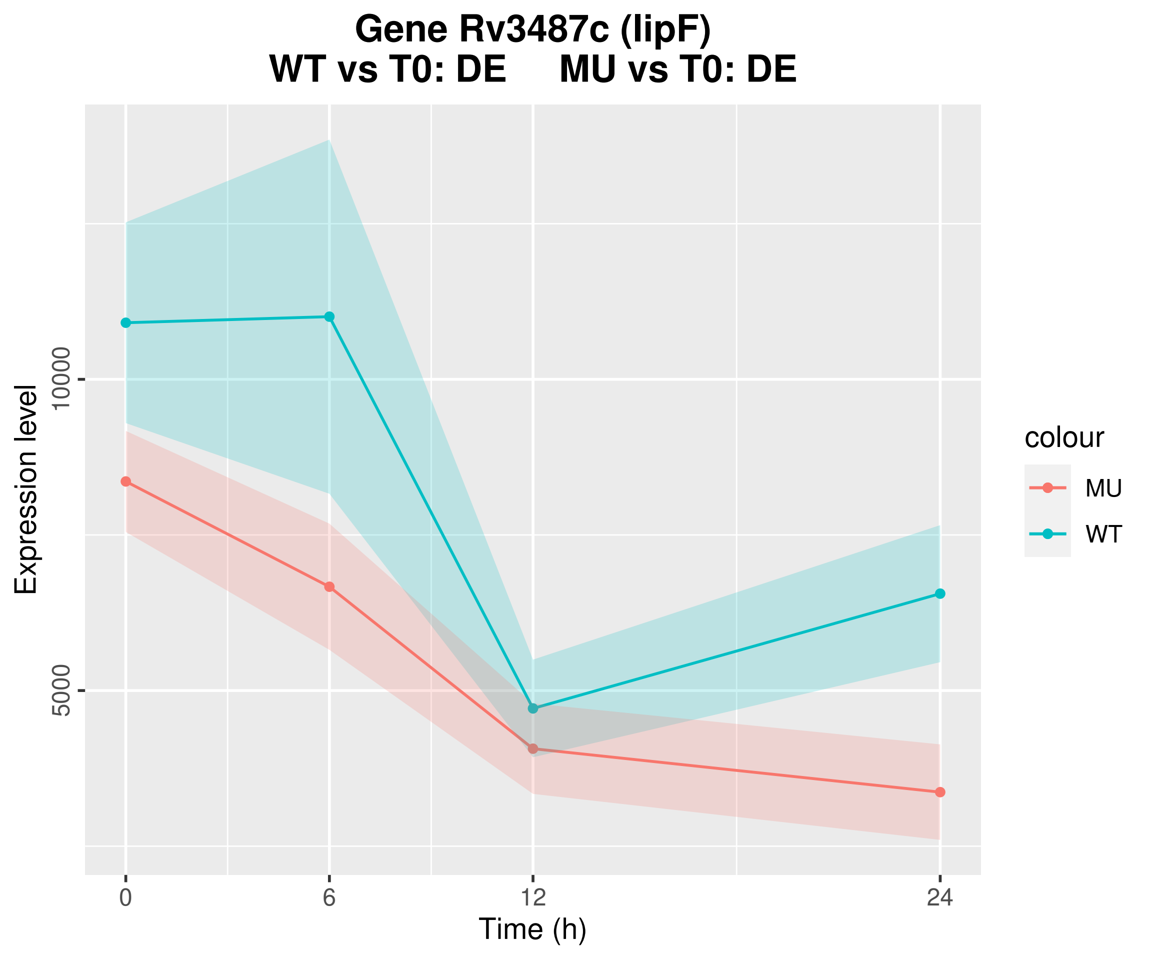
Gene Rv3487c (lipF)
WT vs T0: DE MU vs T0: DE
Expression level
10000
5000
0
6
12
24
Time (h)
colour
MU
WT

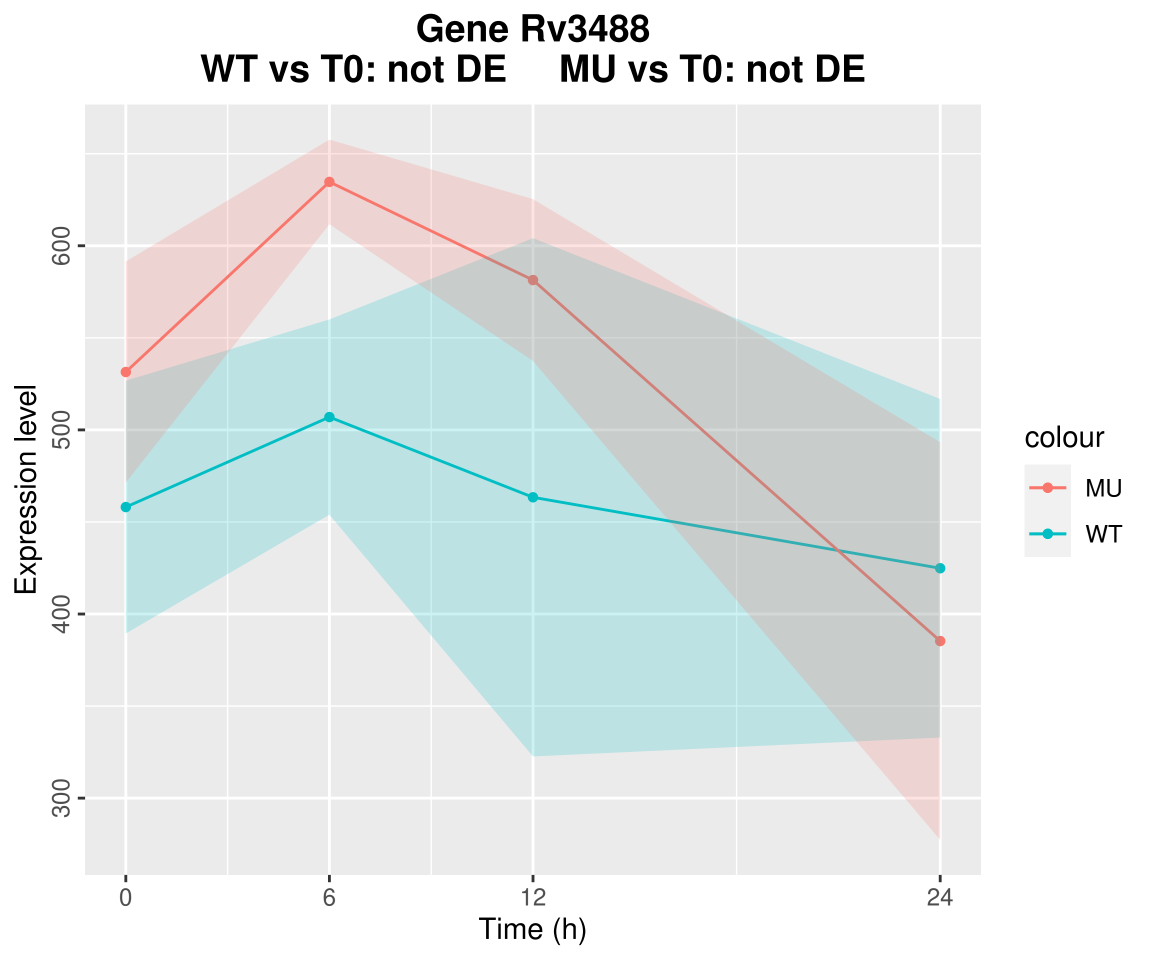

Gene Rv3488
WT vs T0: not DE MU vs T0: not DE
Expression level
600
500
400
300
0
6
12
24
Time (h)
colour
MU
WT

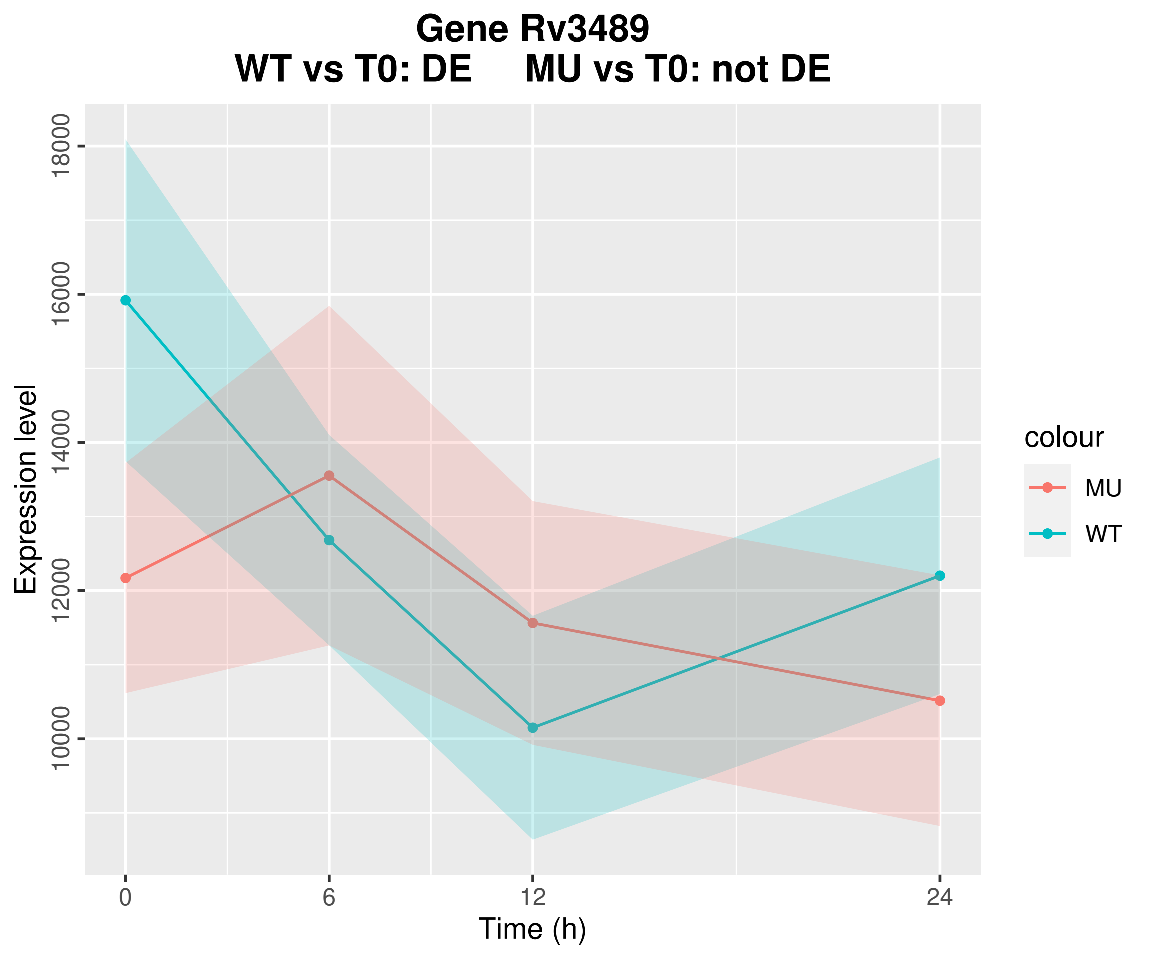
Gene Rv3489
WT vs T0: DE   MU vs T0: not DE
Expression level
18000
16000
14000
12000
10000
Time (h)
0
6
12
24
colour
MU
WT

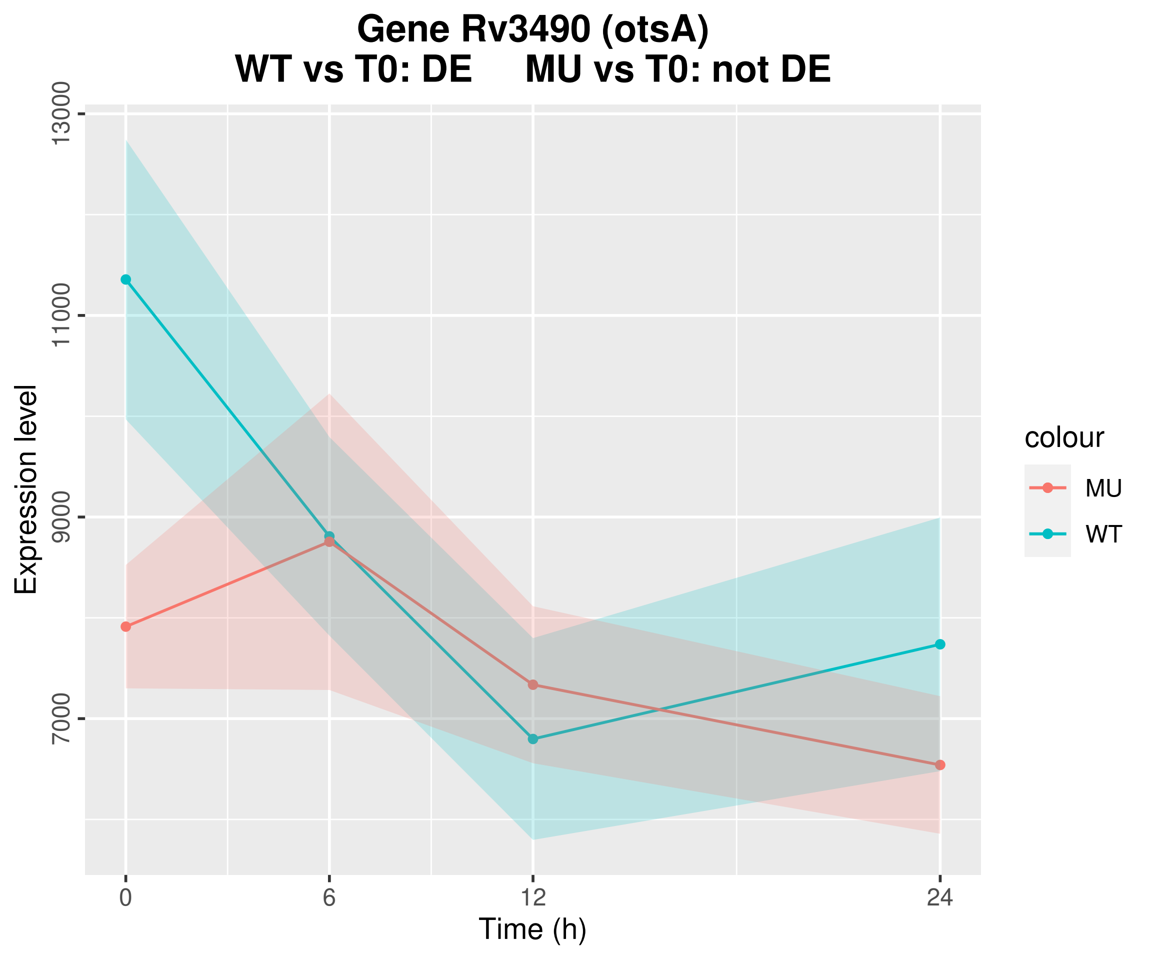
Gene Rv3490 (otsA)
WT vs T0: DE   MU vs T0: not DE
Expression level
13000
11000
9000
7000
0
6
12
24
Time (h)
colour
MU
WT

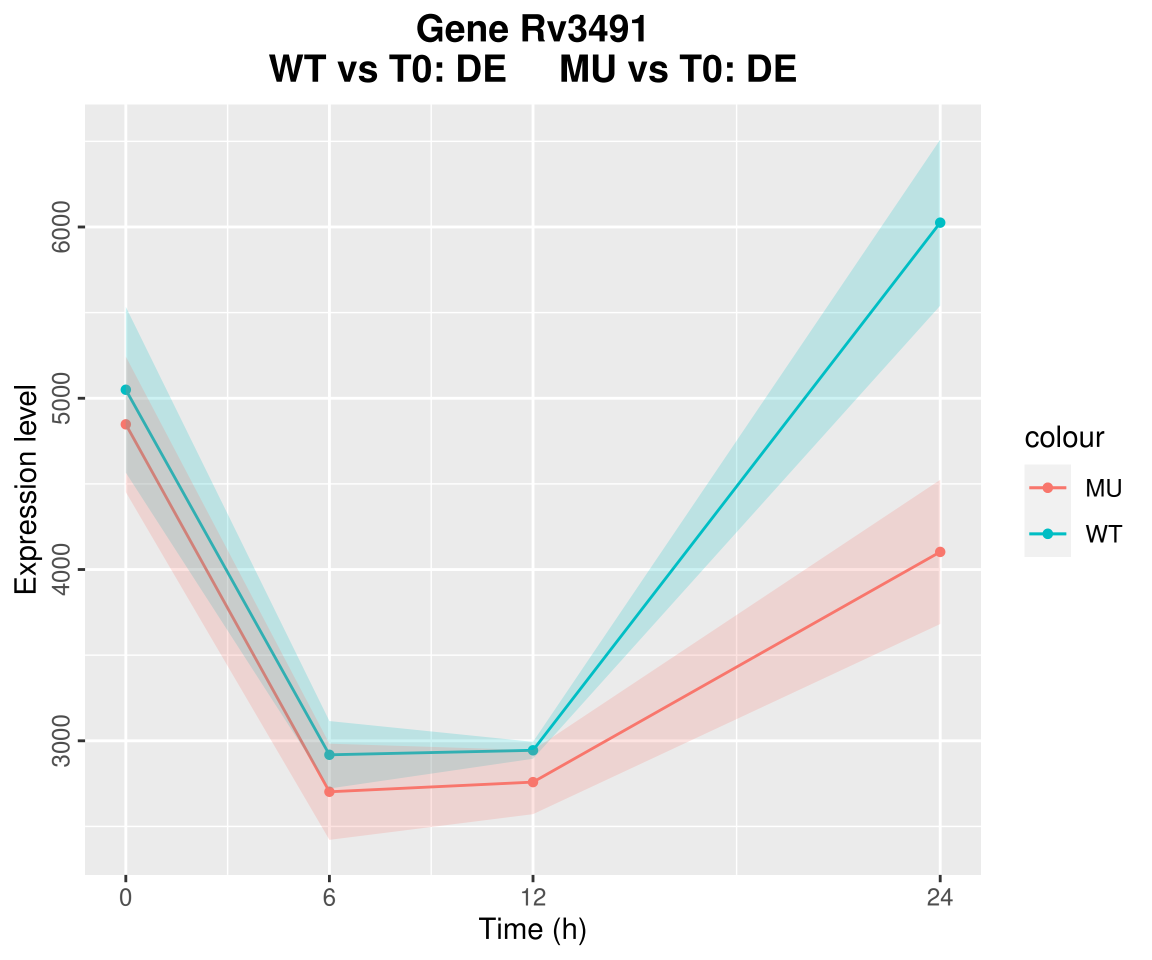

Gene Rv3491
WT vs T0: DE MU vs T0: DE
6000
5000
4000
3000
Expression level
0
6
12
24
Time (h)
colour
MU
WT

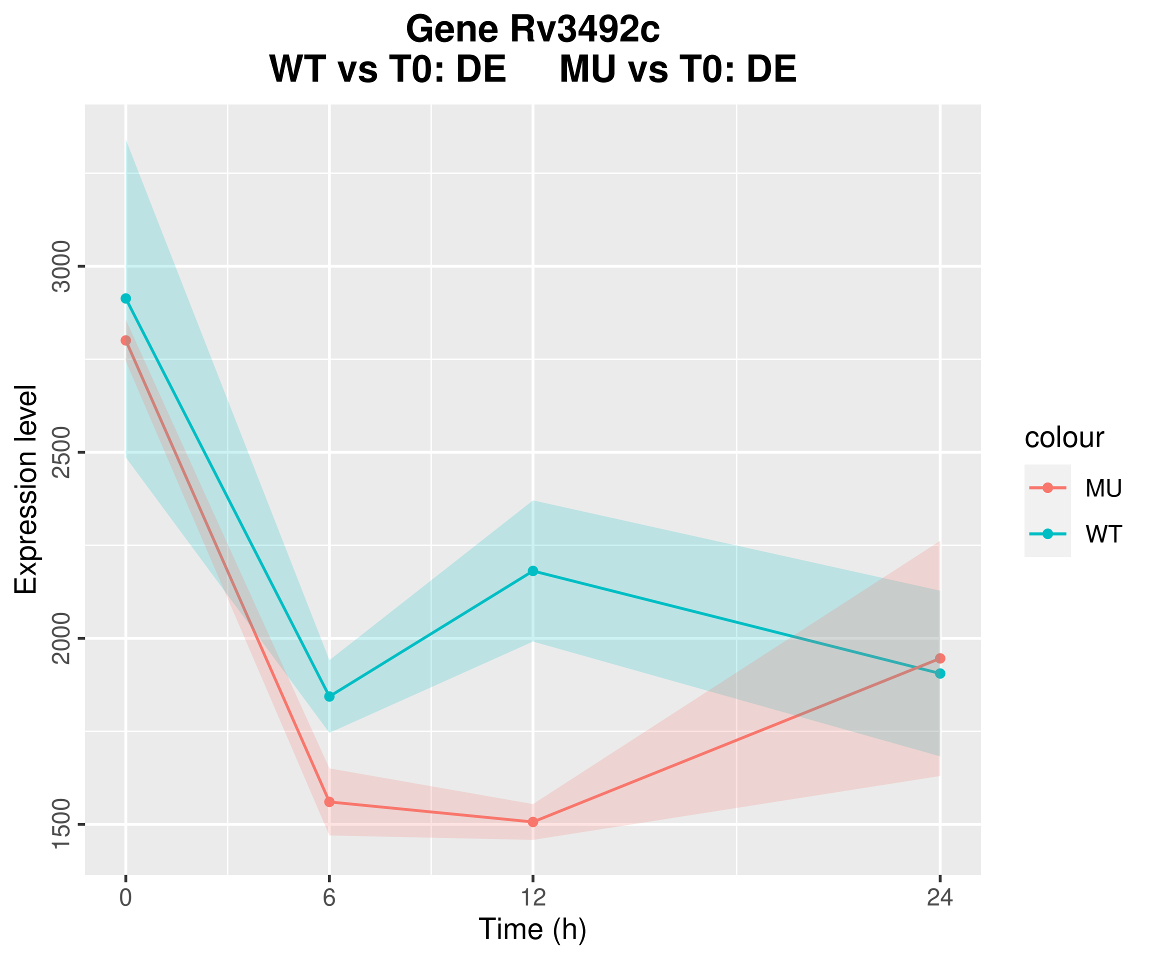
Gene Rv3492c
WT vs T0: DE   MU vs T0: DE
Expression level
3000
2500
2000
1500
0
6
12
24
Time (h)
colour
MU
WT

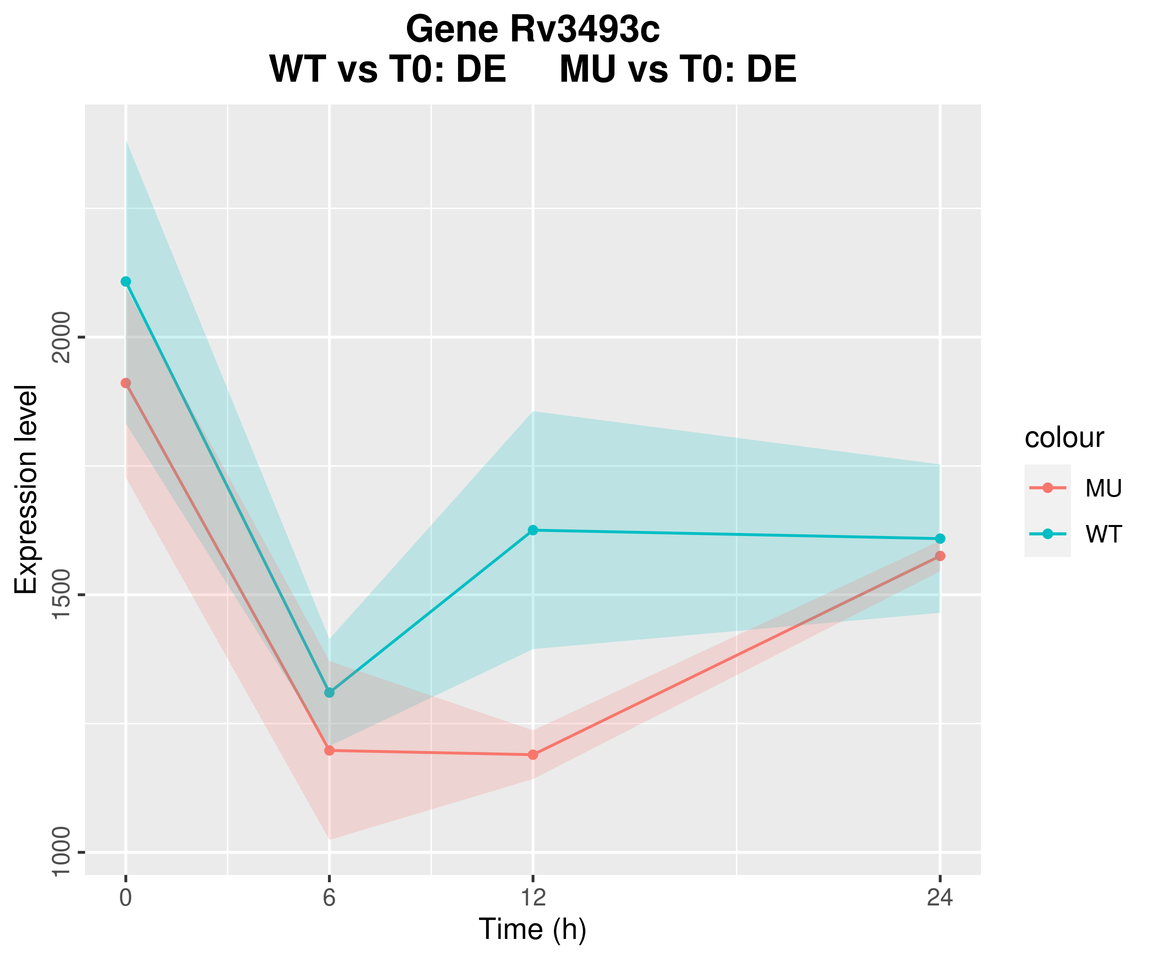

Gene Rv3493c
WT vs T0: DE   MU vs T0: DE
Expression level
2000
1500
1000
0
6
12
24
Time (h)
colour
MU
WT

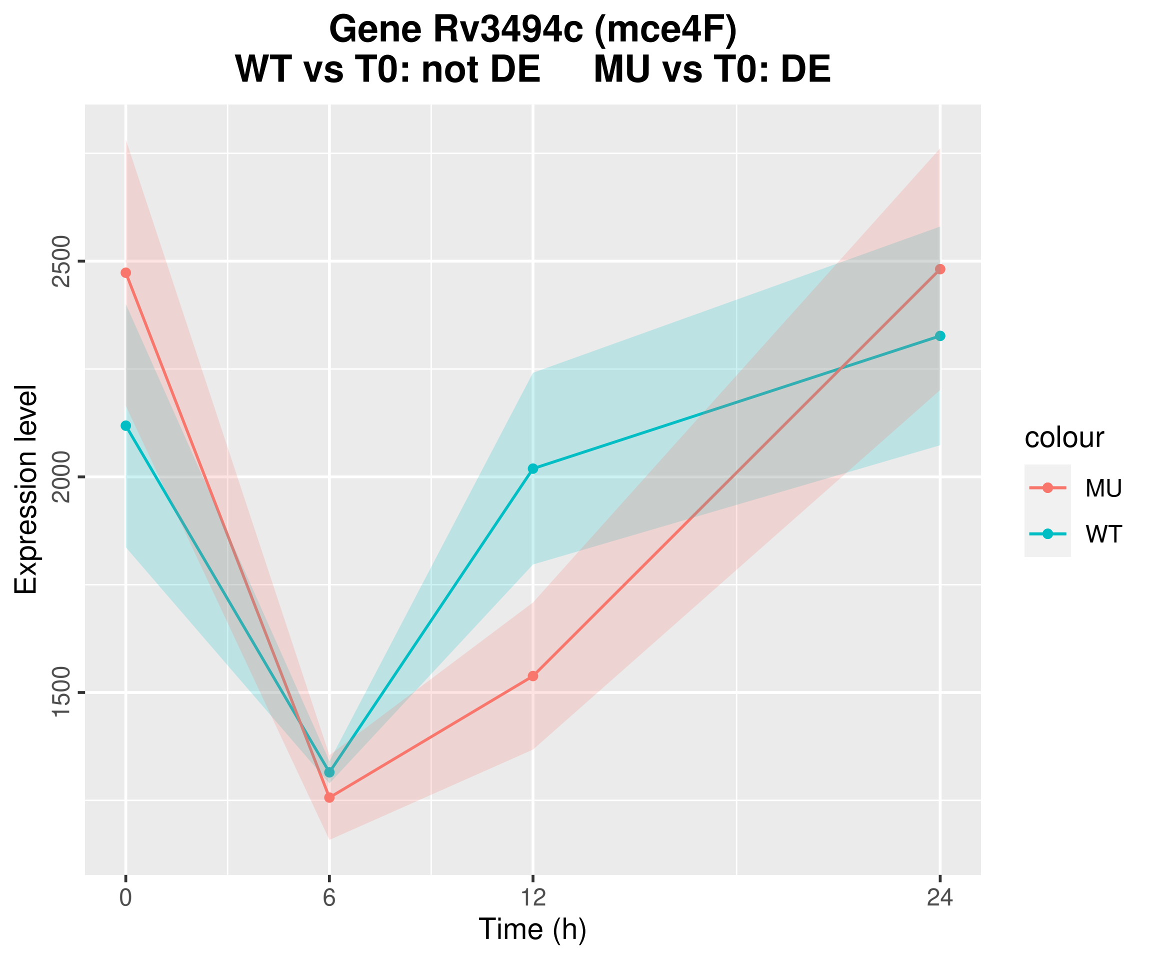
Gene Rv3494c (mce4F)
WT vs T0: not DE   MU vs T0: DE
Expression level
2500
2000
1500
0
6
12
24
Time (h)
colour
MU
WT

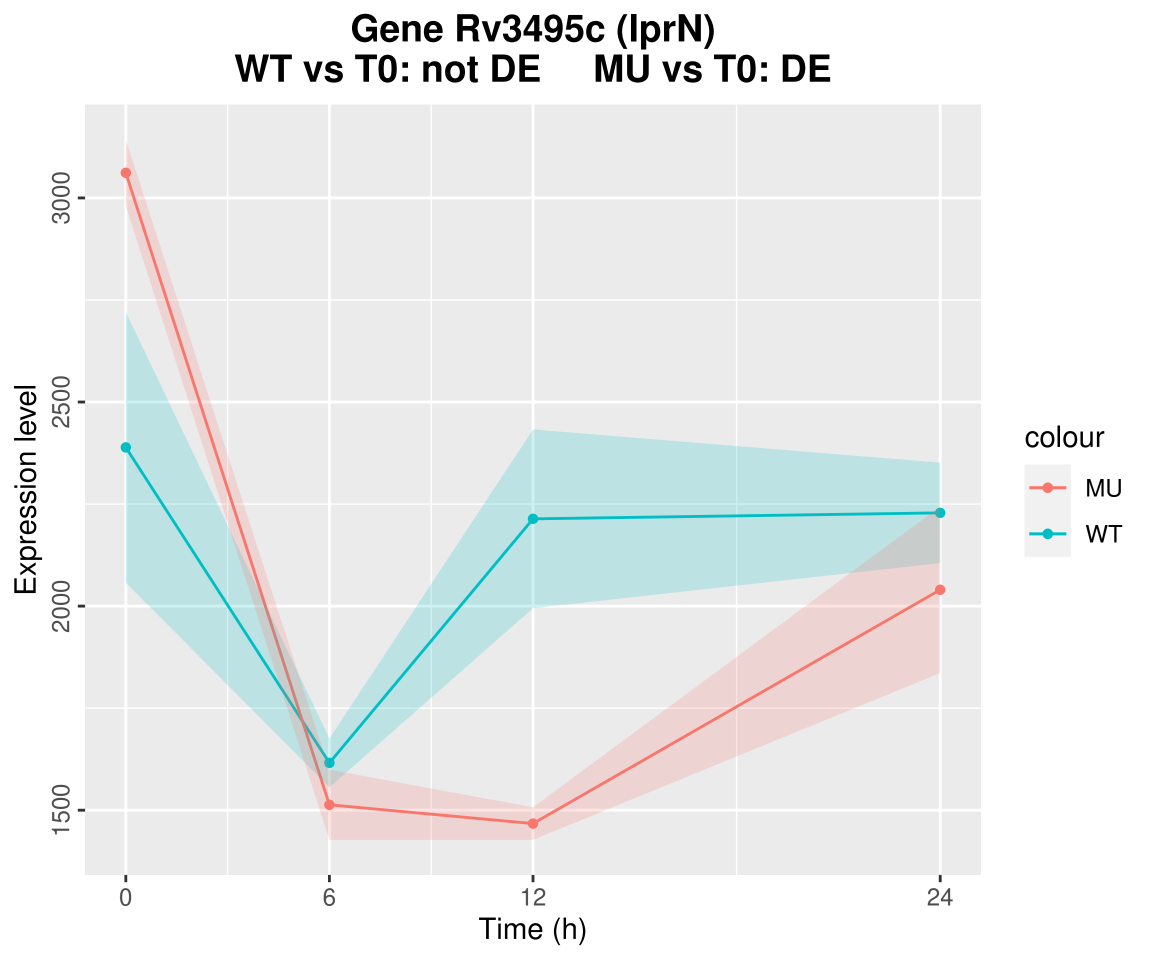
Gene Rv3495c (lprN)
WT vs T0: not DE   MU vs T0: DE
Expression level
3000
2500
2000
1500
0
6
12
24
Time (h)
colour
MU
WT

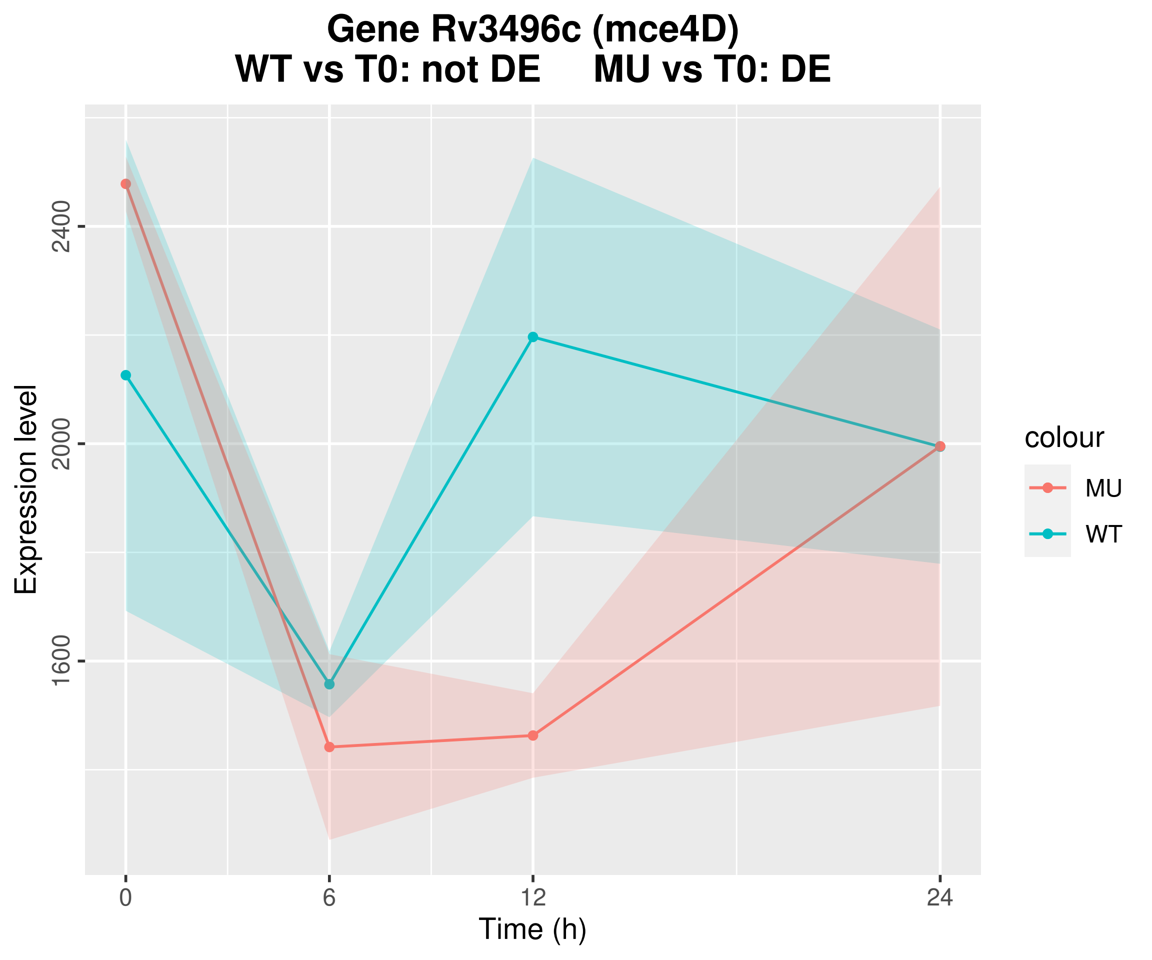

Gene Rv3496c (mce4D)
WT vs T0: not DE   MU vs T0: DE
Expression level
2400
2000
1600
0
6
12
24
Time (h)
colour
MU
WT

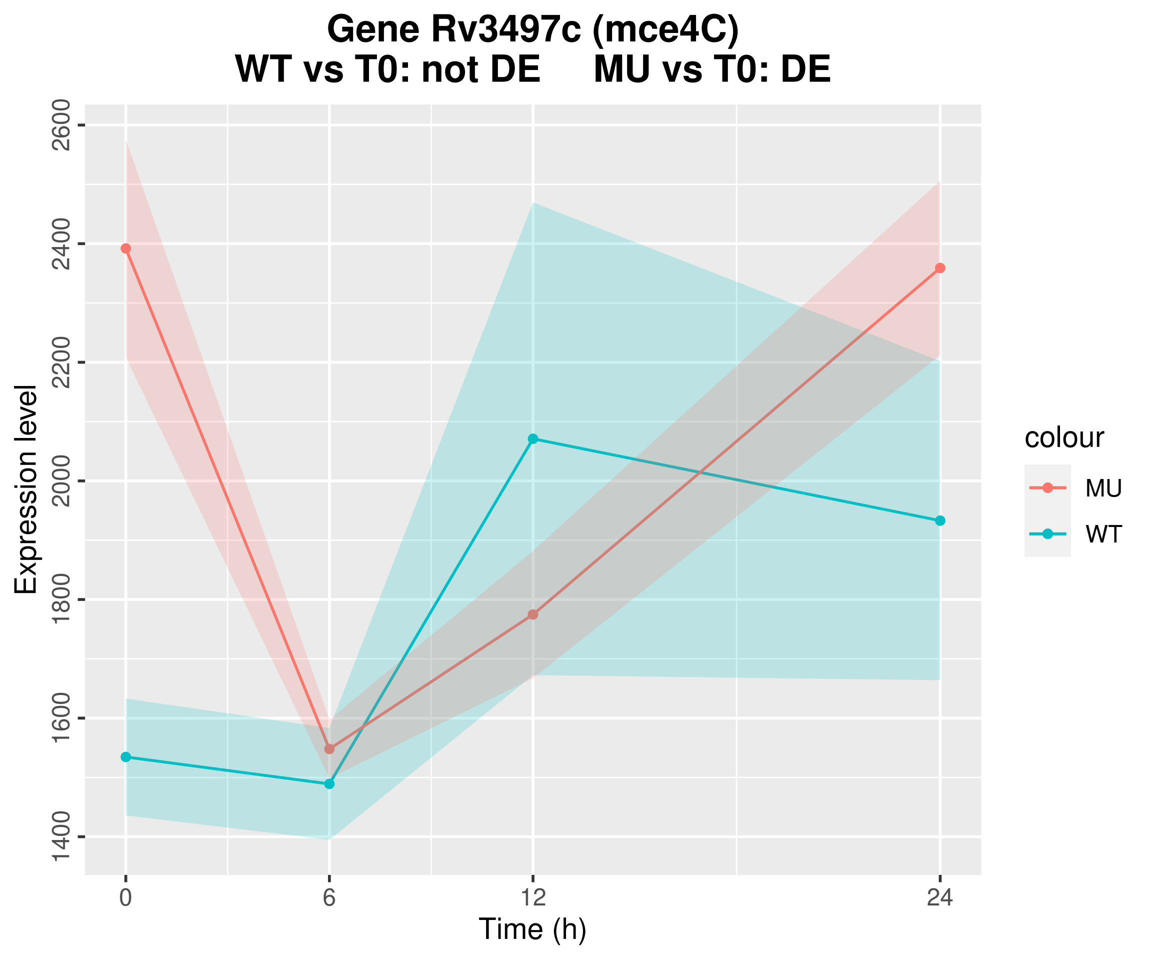

Gene Rv3497c (mce4C)
WT vs T0: not DE   MU vs T0: DE
Expression level
2600
2400
2200
2000
1800
1600
1400
0
6
12
24
Time (h)
colour
MU
WT

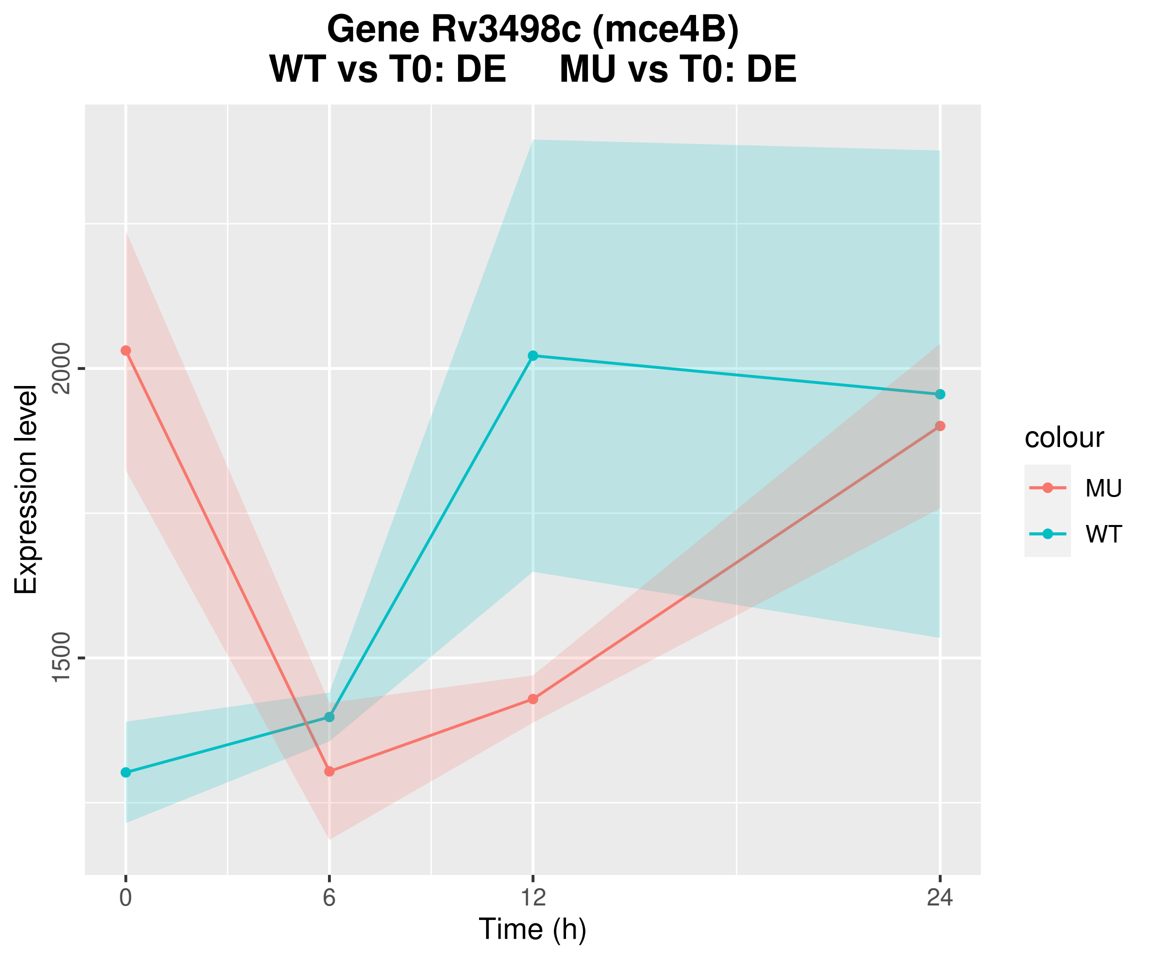
Gene Rv3498c (mce4B)
WT vs T0: DE   MU vs T0: DE
Expression level
2000
1500
0
6
12
24
Time (h)
colour
MU
WT

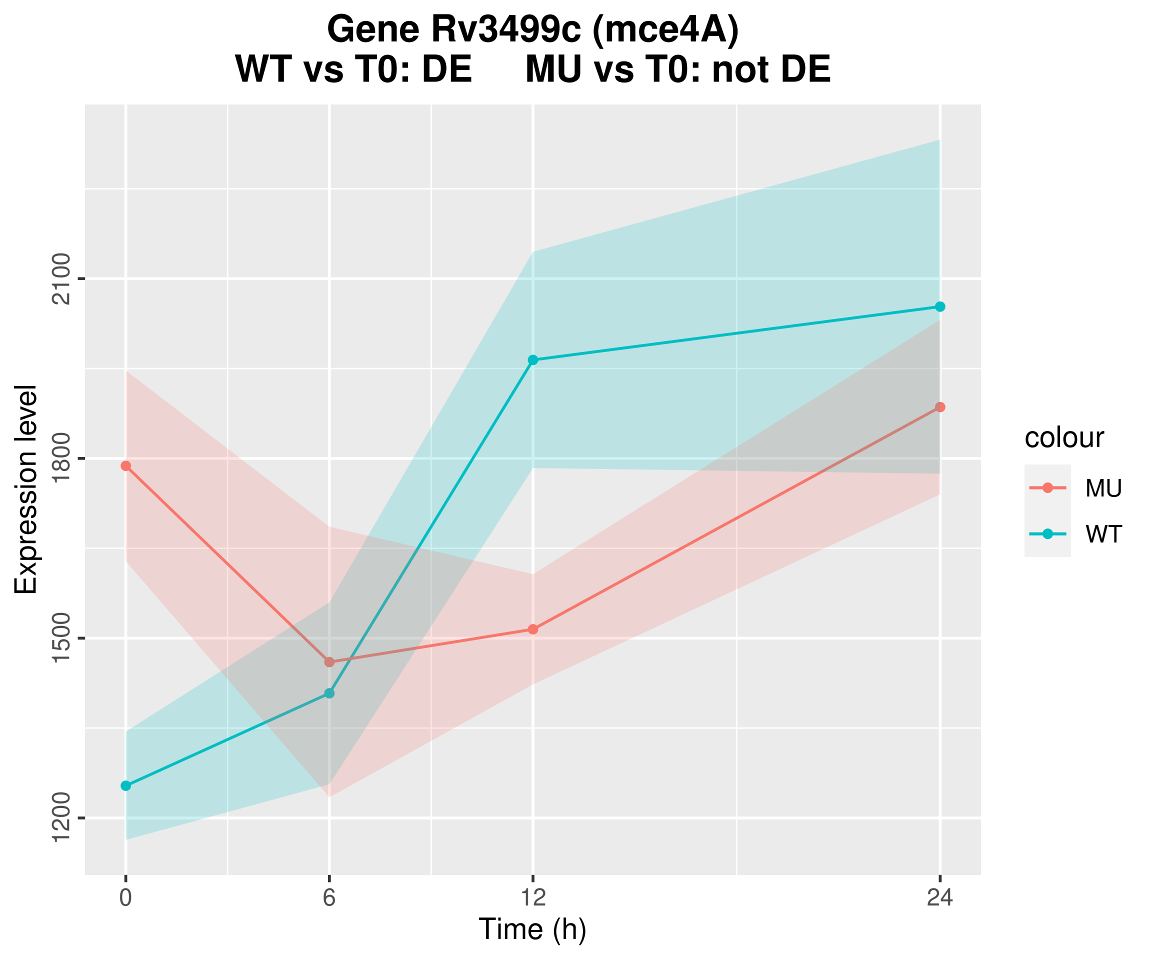
Gene Rv3499c (mce4A)
WT vs T0: DE   MU vs T0: not DE
Expression level
2100
1800
1500
1200
0
6
12
24
Time (h)
colour
MU
WT

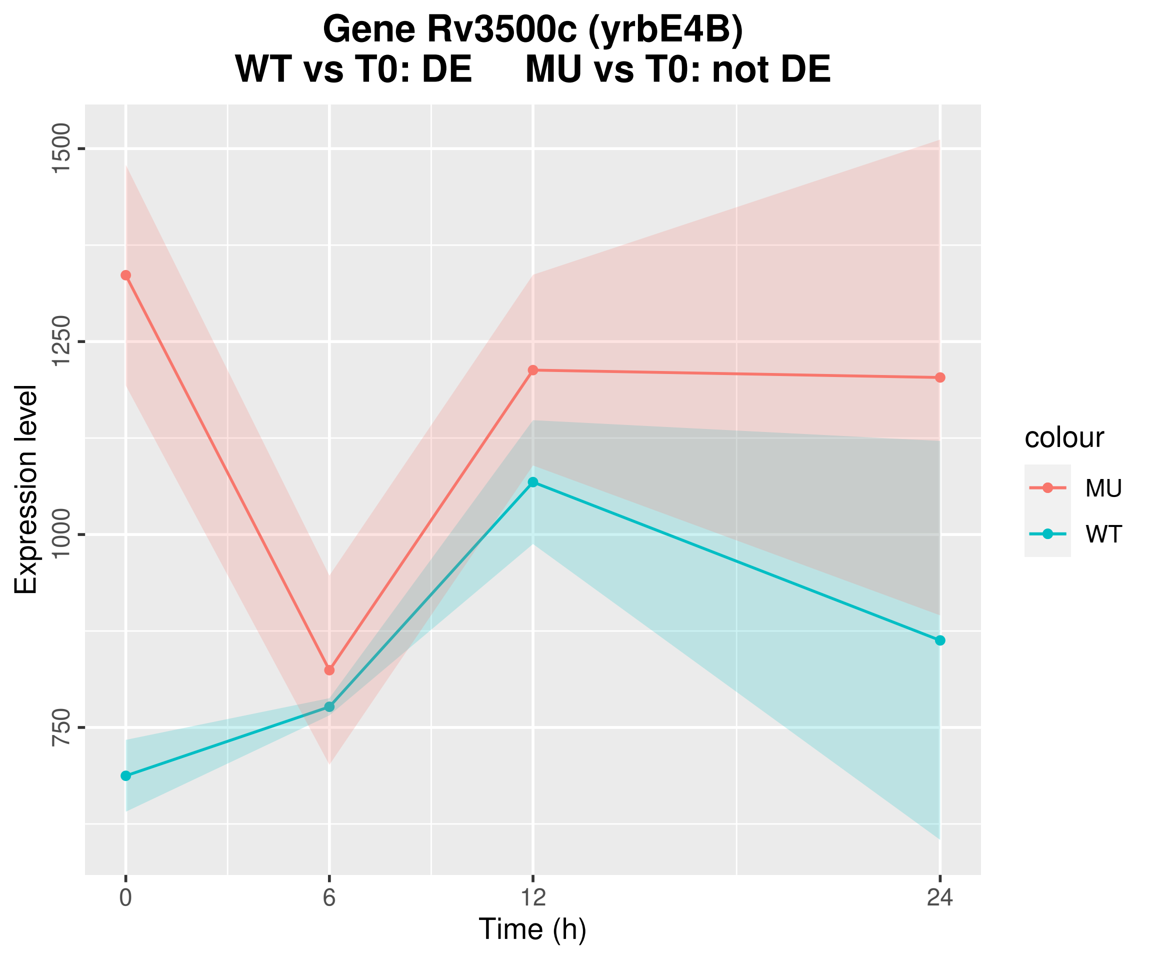

Gene Rv3500c (yrbE4B)
WT vs T0: DE MU vs T0: not DE
1500
1250
1000
750
Expression level
0
6
12
24
Time (h)
colour
MU
WT

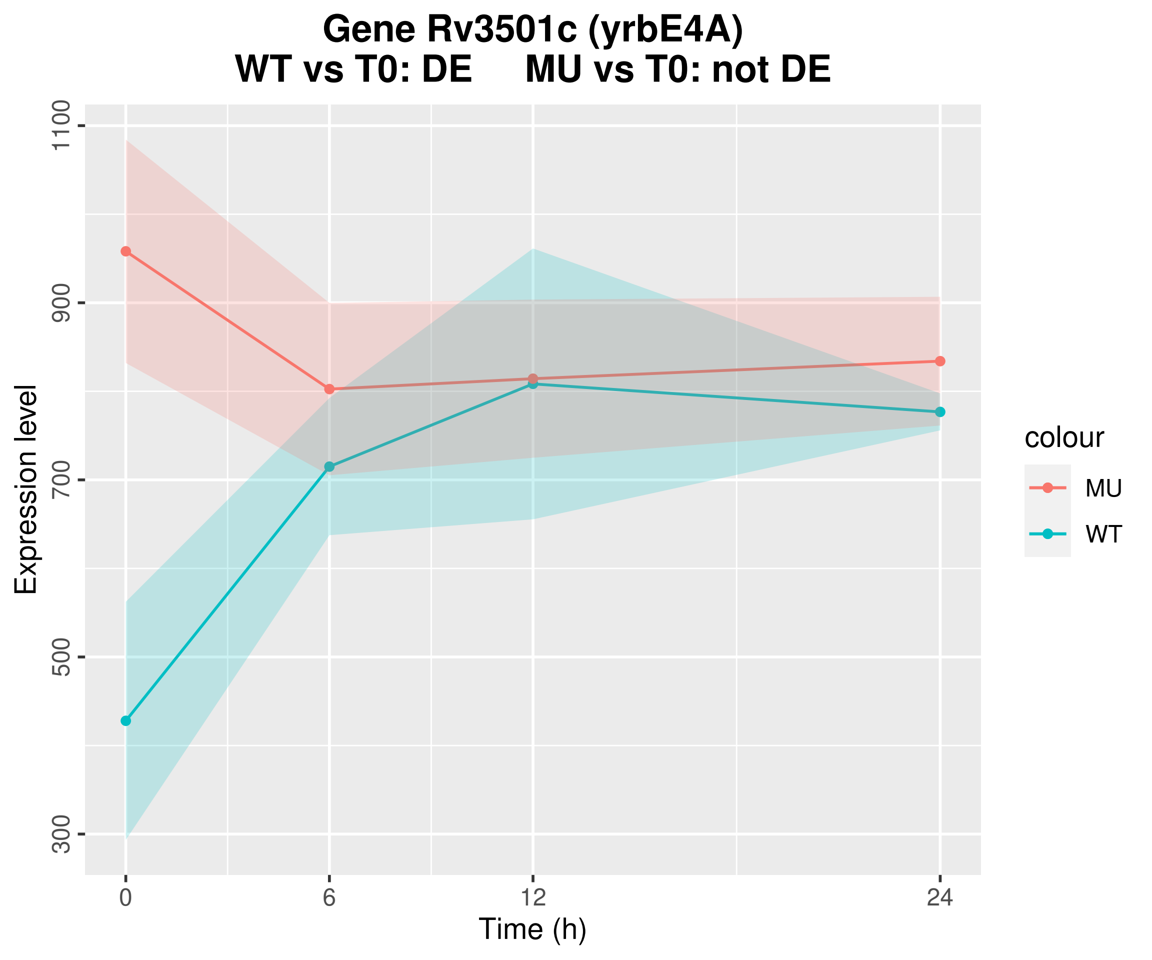

Gene Rv3501c (yrbE4A)
WT vs T0: DE MU vs T0: not DE
Expression level
1100
900
700
500
300
0
6
12
24
Time (h)
colour
MU
WT

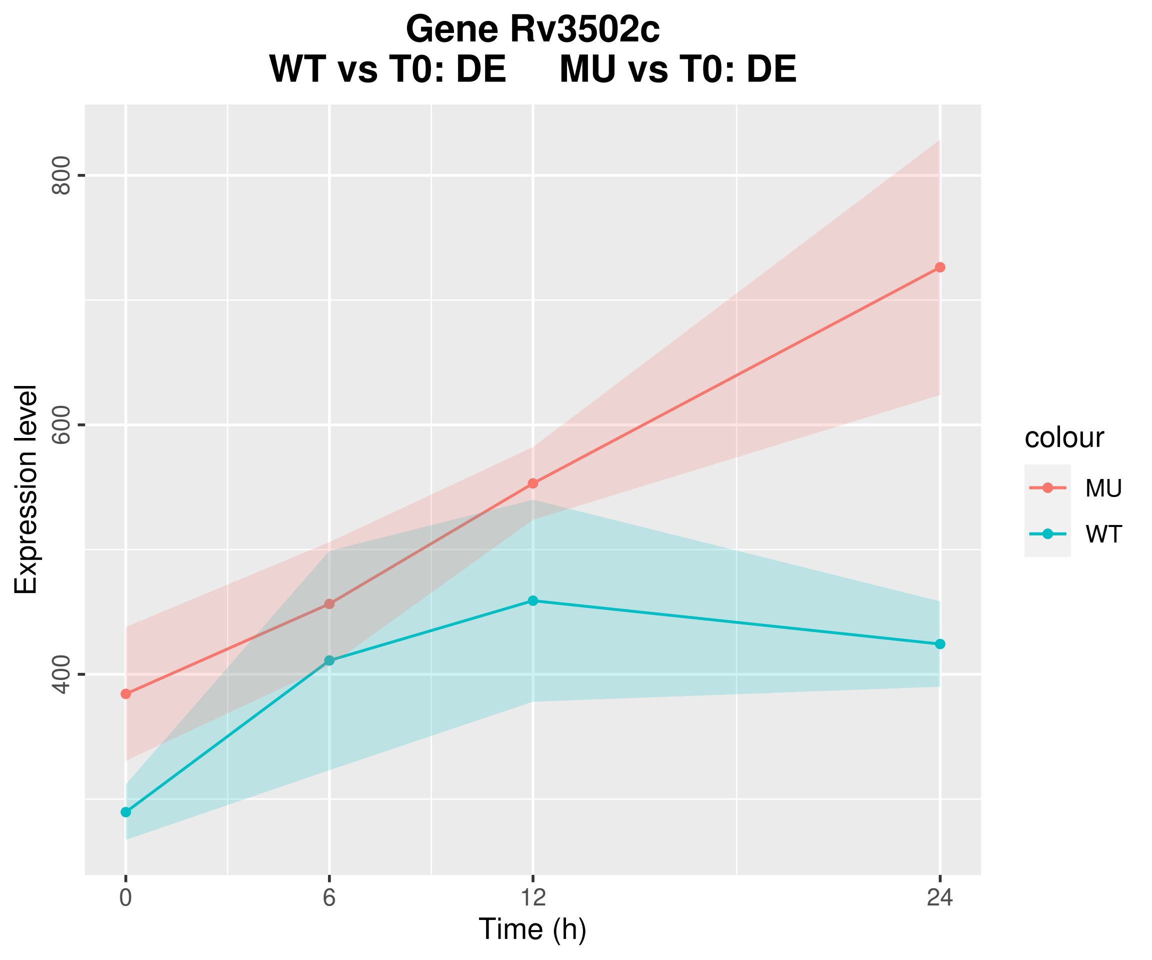
Gene Rv3502c
WT vs T0: DE MU vs T0: DE
Expression level
800
600
400
0
6
12
24
Time (h)
colour
MU
WT

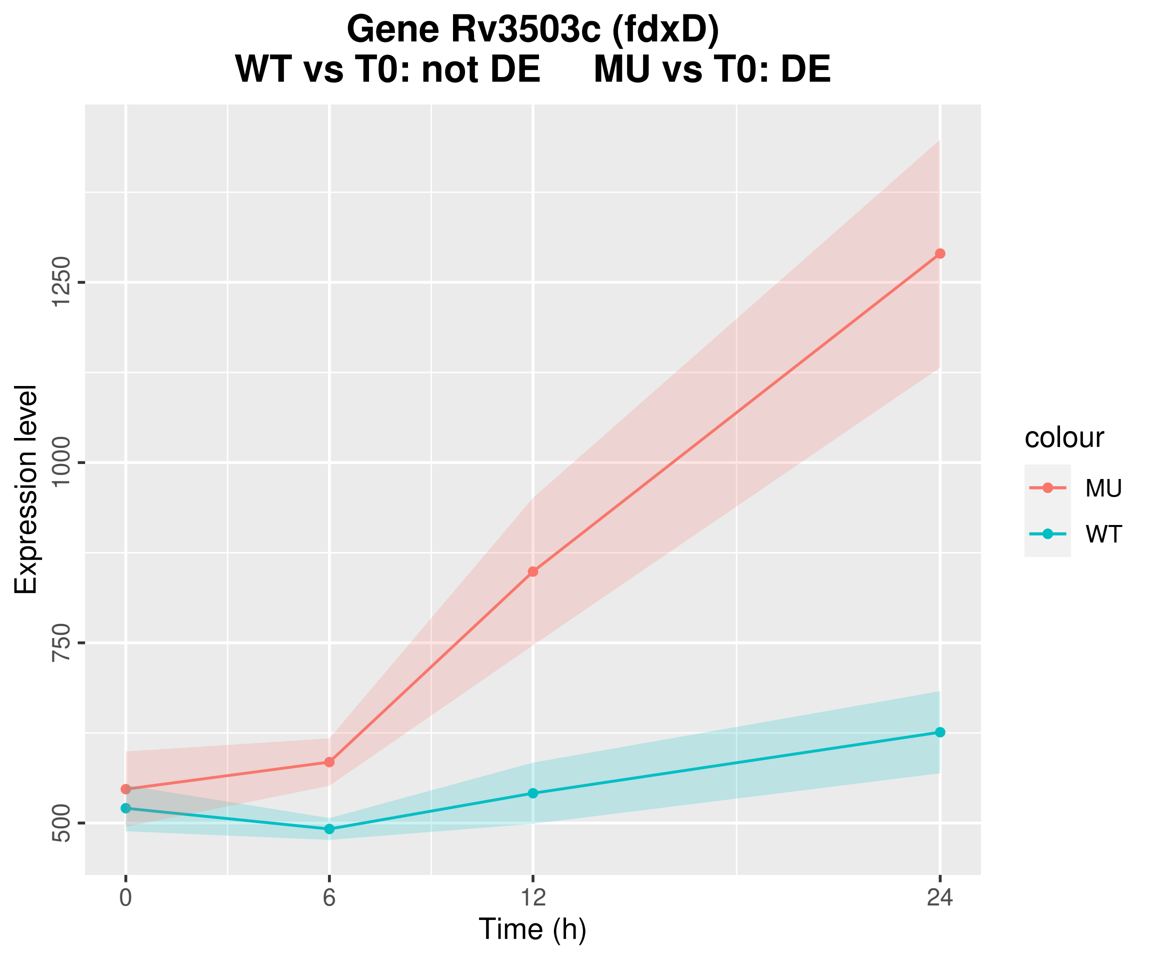
Gene Rv3503c (fdxD)
WT vs T0: not DE MU vs T0: DE
Expression level
1250
1000
750
500
0
6
12
24
Time (h)
colour
MU
WT

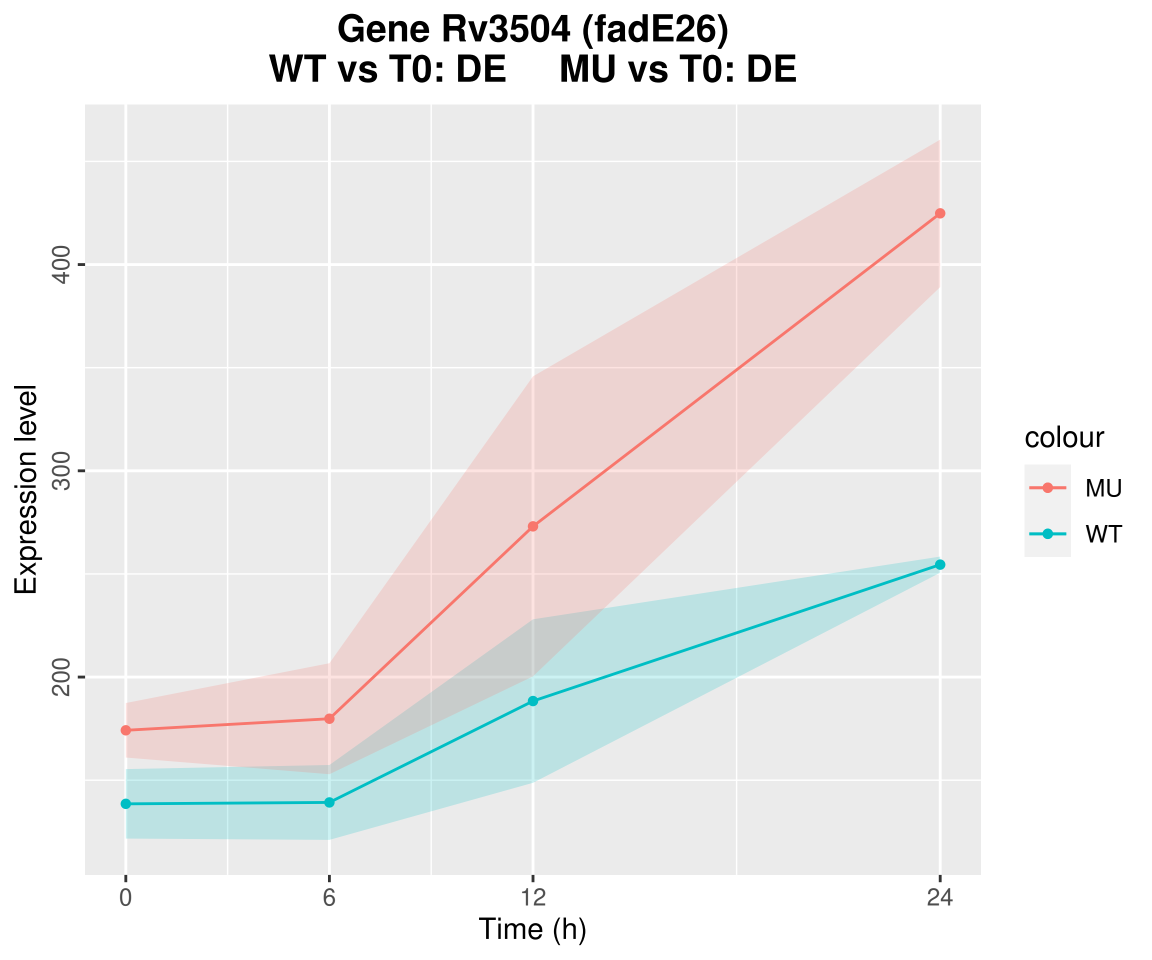
Gene Rv3504 (fadE26)
WT vs T0: DE MU vs T0: DE
Expression level
400
300
200
0
6
12
24
Time (h)
colour
MU
WT

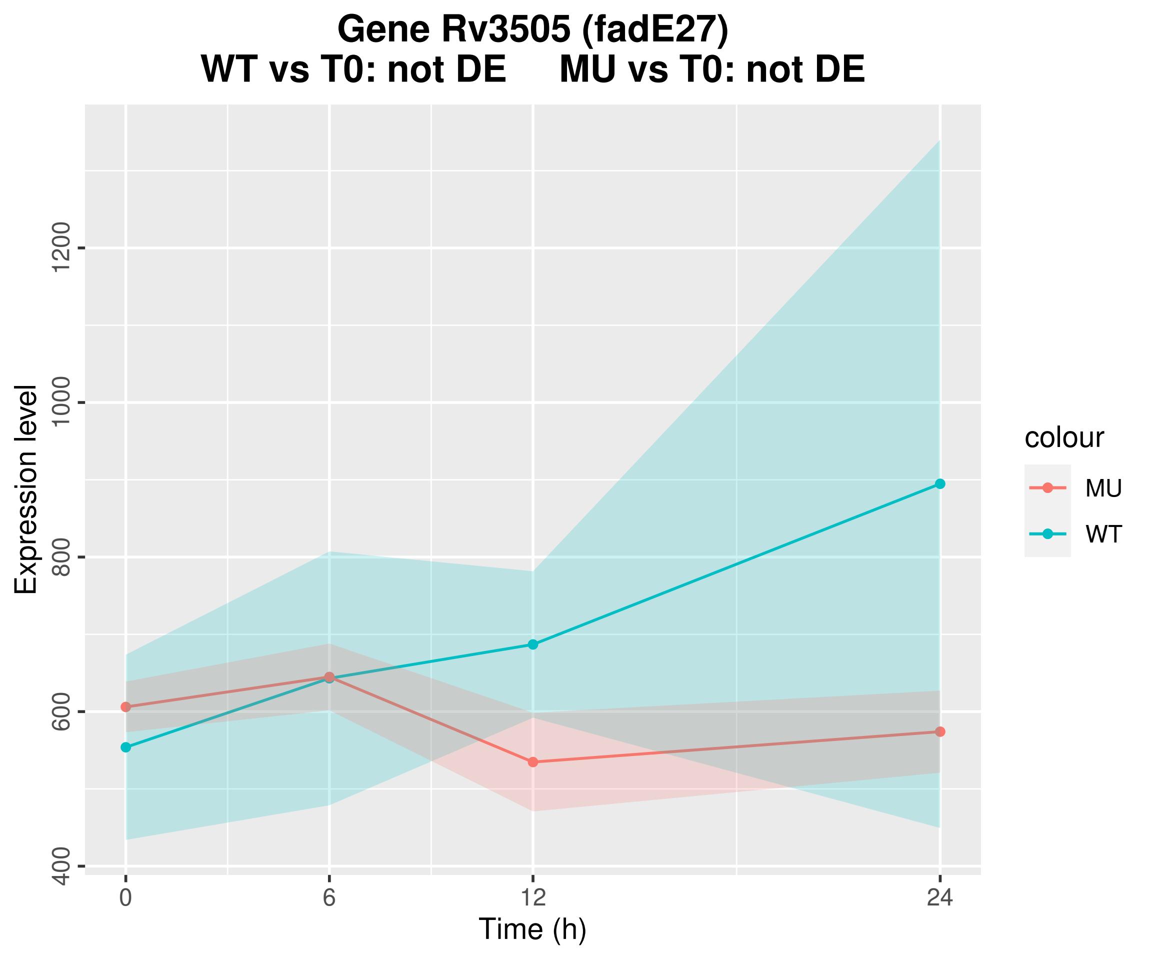
Gene Rv3505 (fadE27)
WT vs T0: not DE   MU vs T0: not DE
Expression level
1200
1000
800
600
400
0
6
12
24
Time (h)
colour
MU
WT

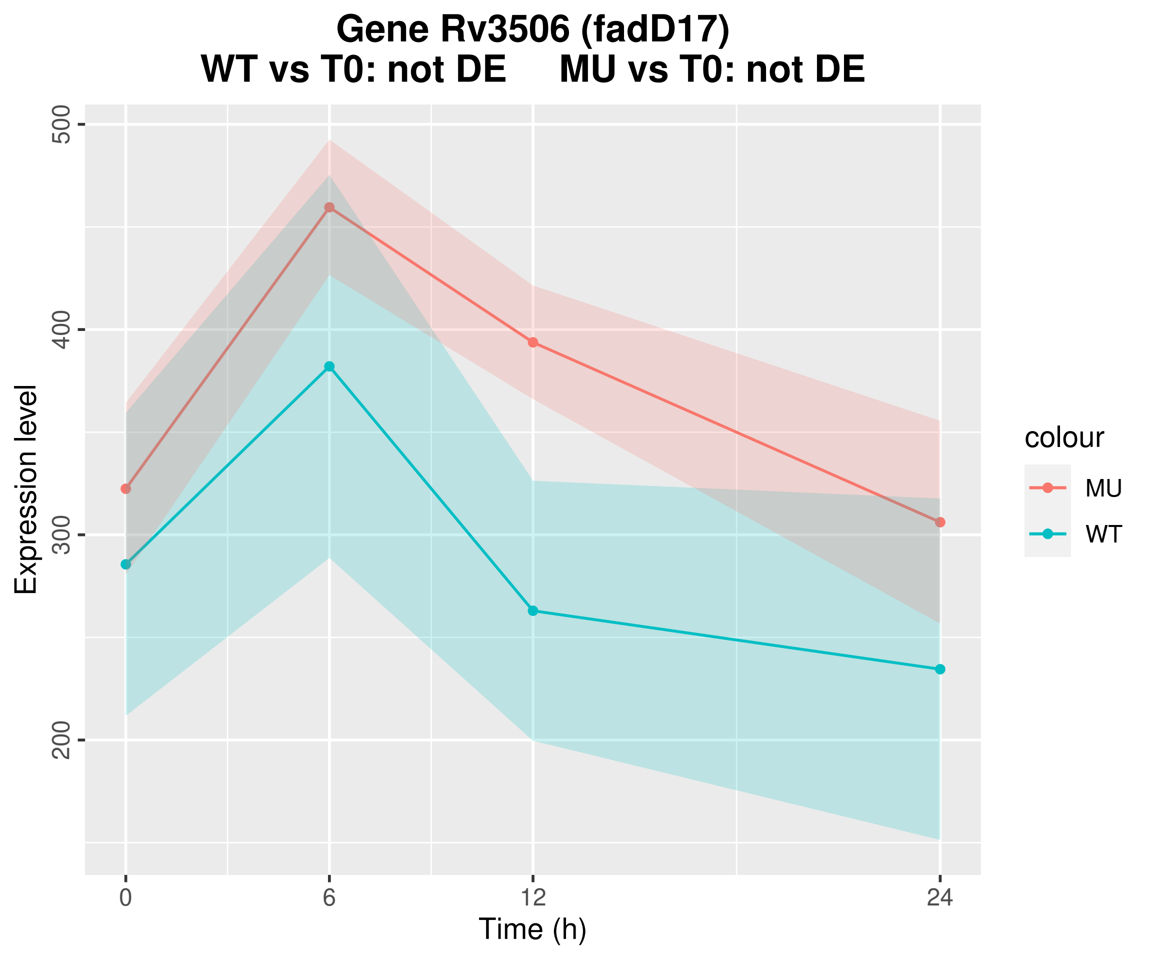

Gene Rv3506 (fadD17)
WT vs T0: not DE MU vs T0: not DE
Expression level
500
400
300
200
0
6
12
24
Time (h)
colour
MU
WT

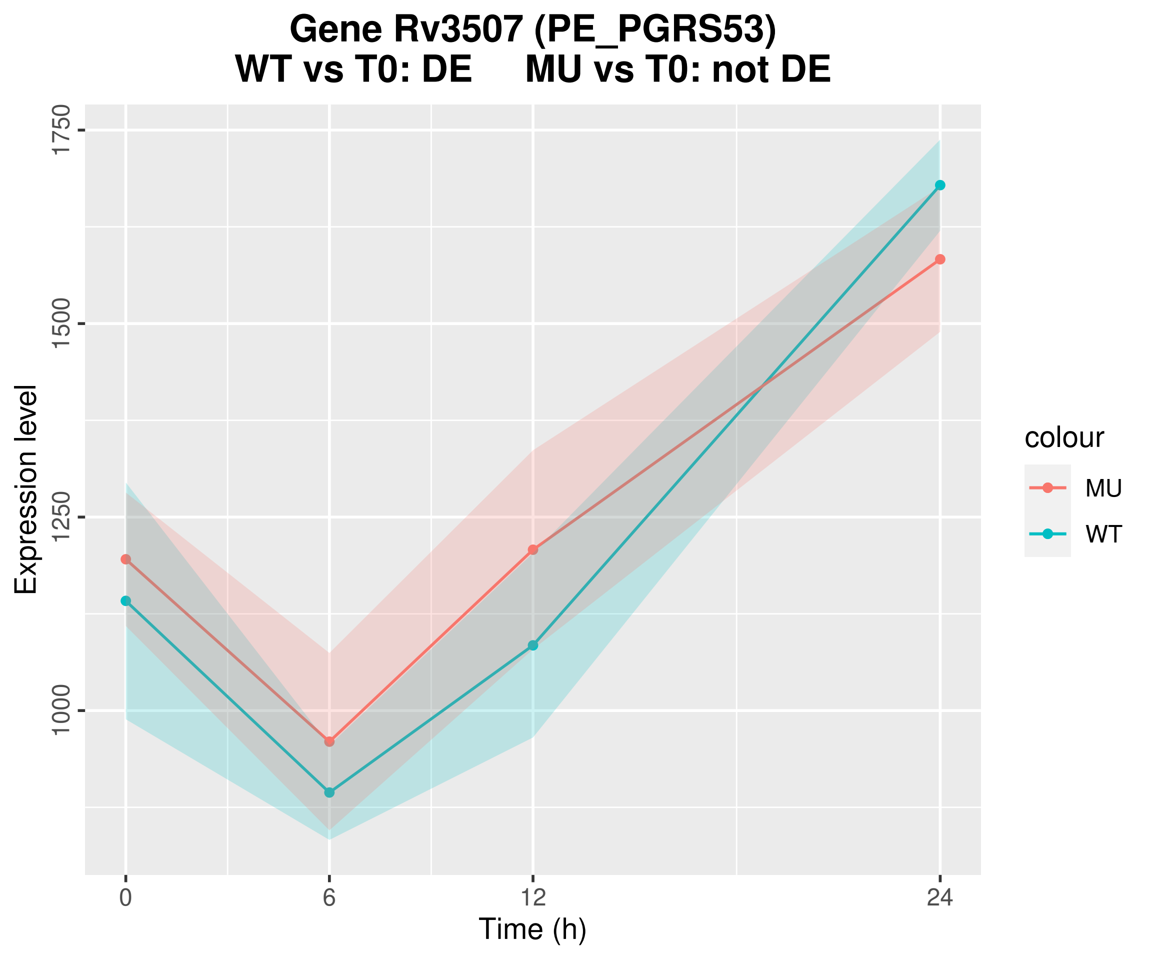
Gene Rv3507 (PE_PGRS53)
WT vs T0: DE MU vs T0: not DE
Expression level
1750
1500
1250
1000
0
6
12
24
Time (h)
colour
MU
WT

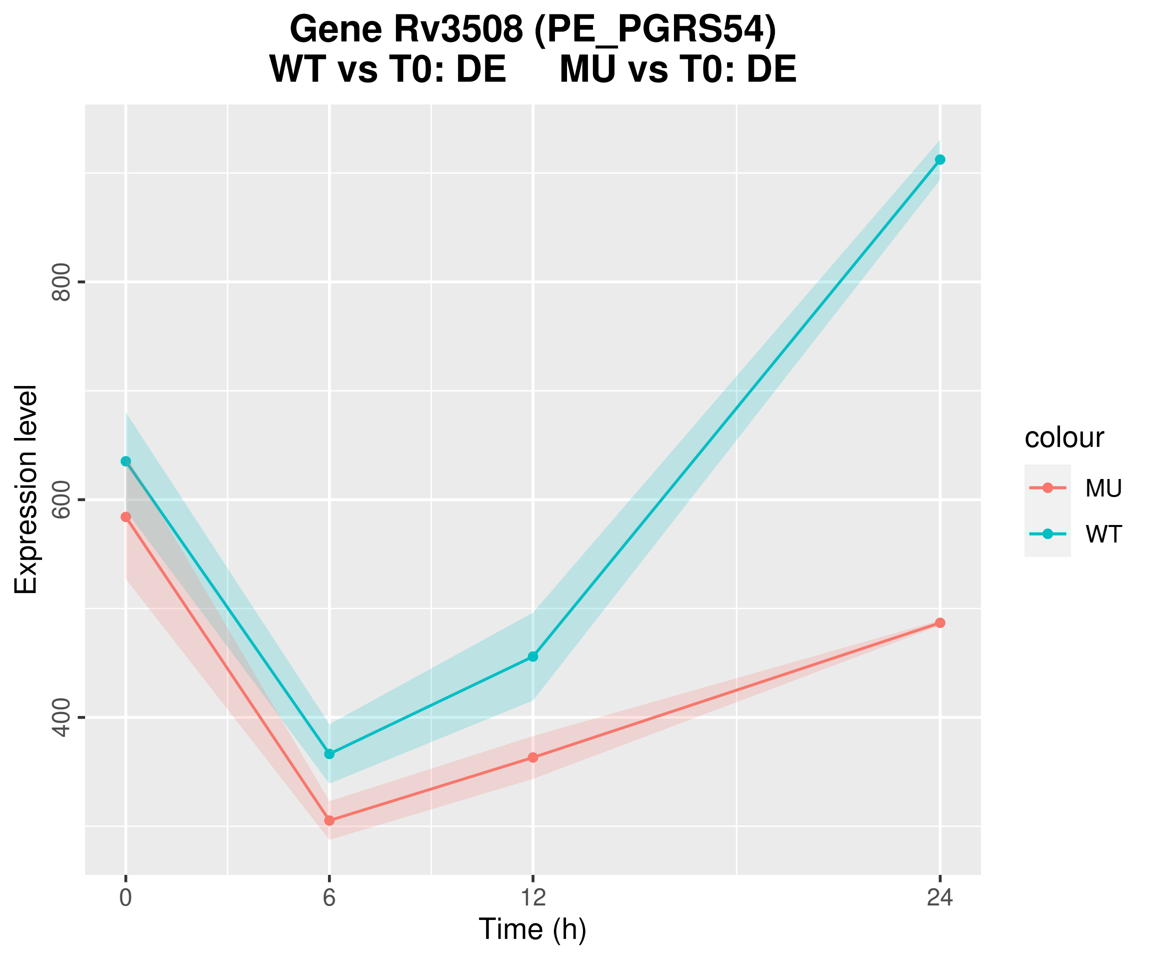
Gene Rv3508 (PE_PGRS54)
WT vs T0: DE MU vs T0: DE
Expression level
800
600
400
0
6
12
24
Time (h)
colour
MU
WT

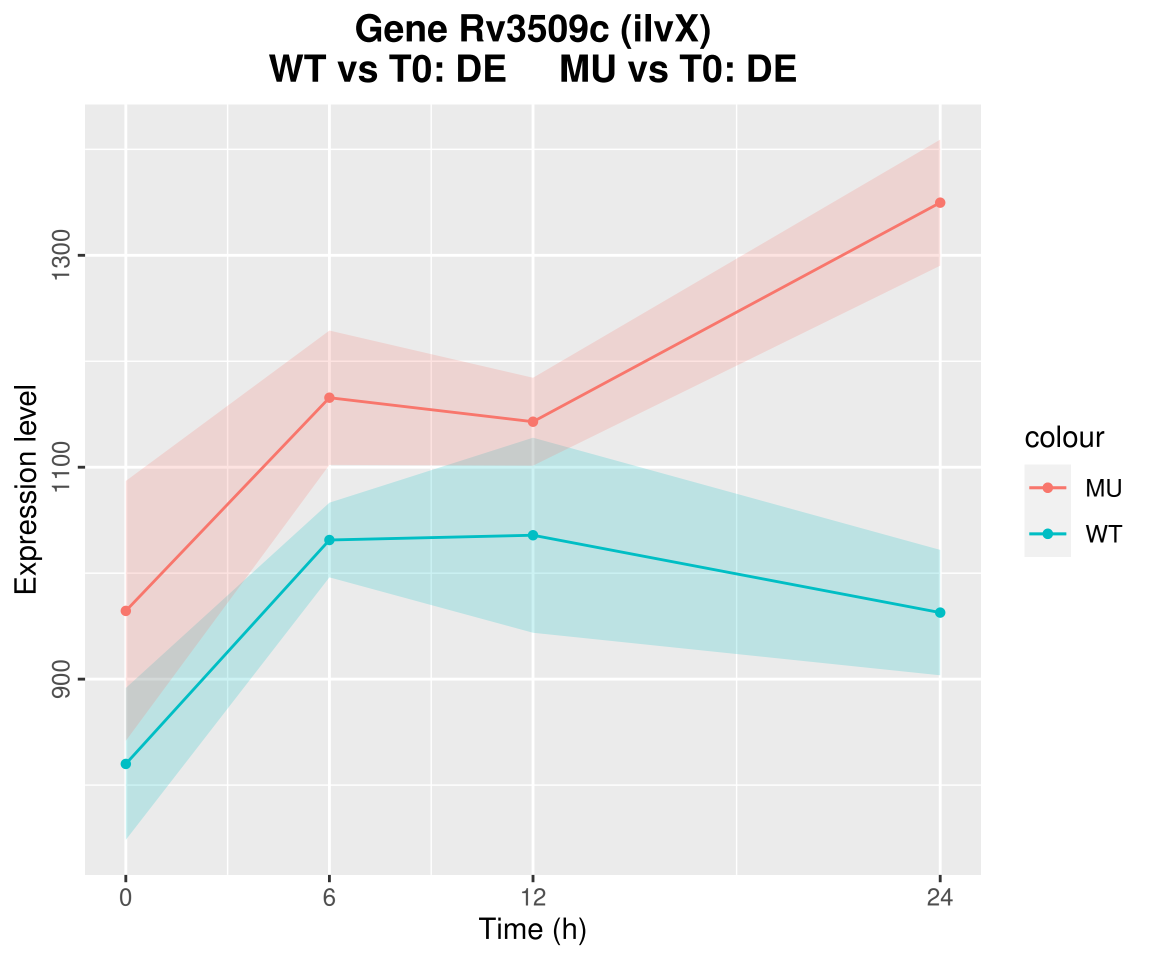

Gene Rv3509c (ilvX)
WT vs T0: DE MU vs T0: DE
Expression level
1300
1100
900
0
6
12
24
Time (h)
colour
MU
WT

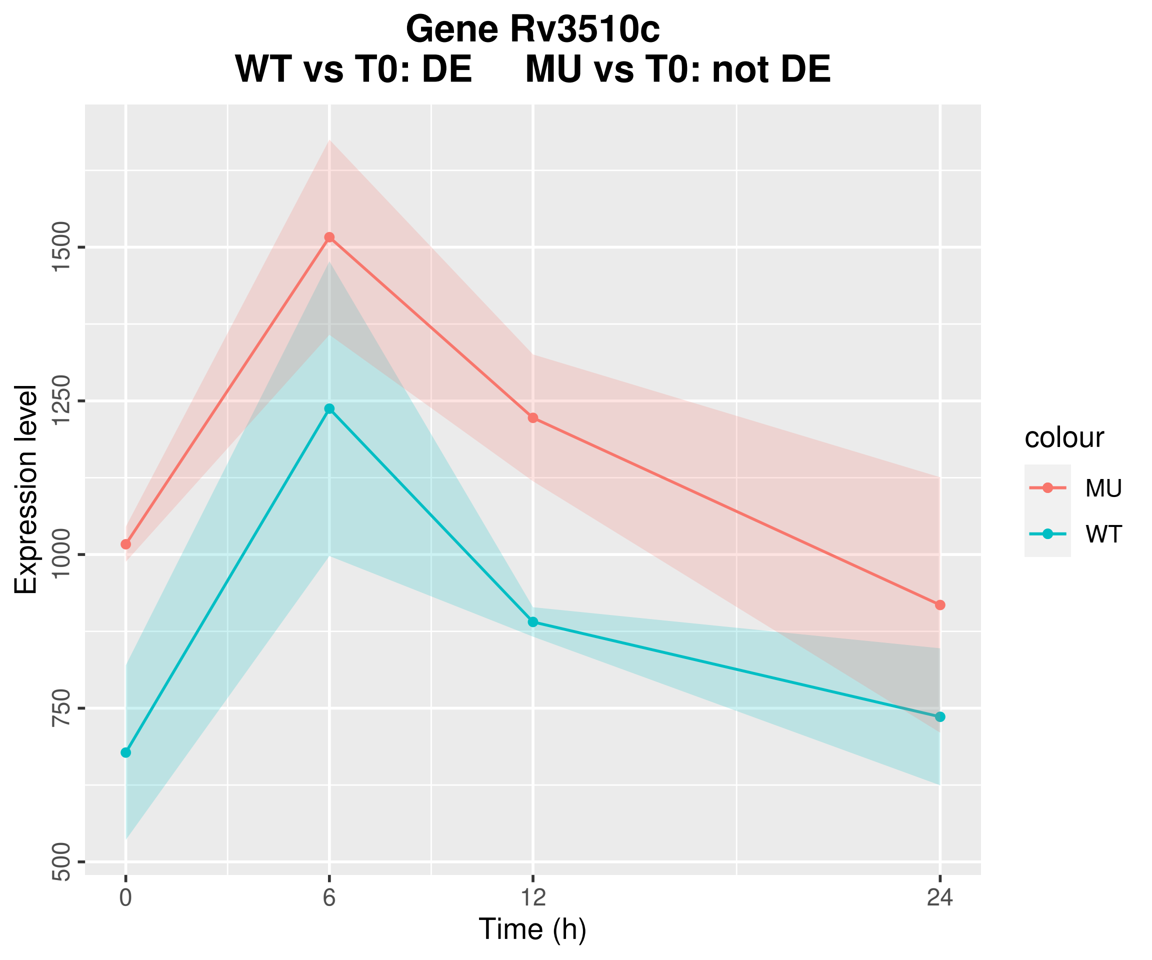
Gene Rv3510c
WT vs T0: DE MU vs T0: not DE
Expression level
1500
1250
1000
750
500
0
6
12
24
Time (h)
colour
MU
WT

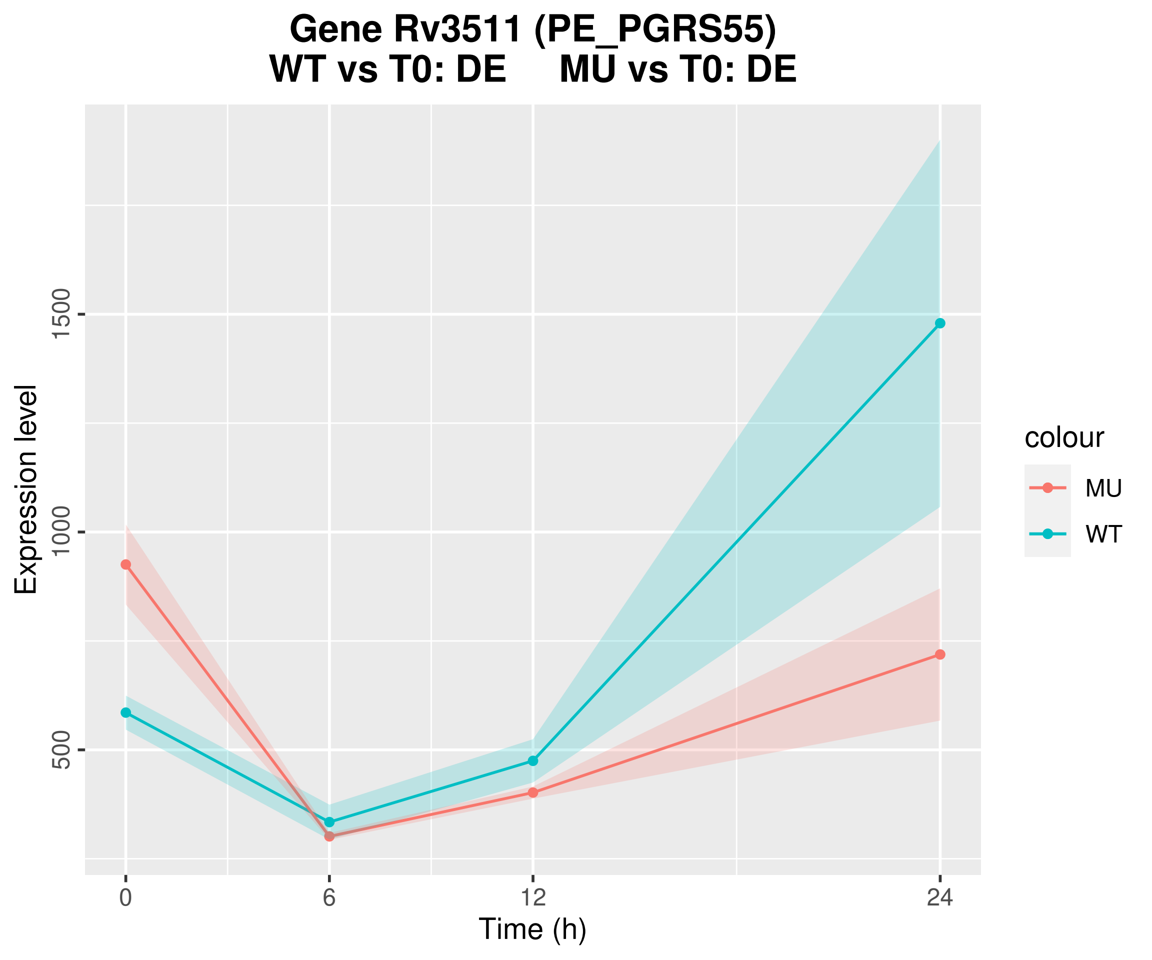

Gene Rv3511 (PE_PGRS55)
WT vs T0: DE   MU vs T0: DE
Expression level
1500
1000
500
0
6
12
24
Time (h)
colour
MU
WT

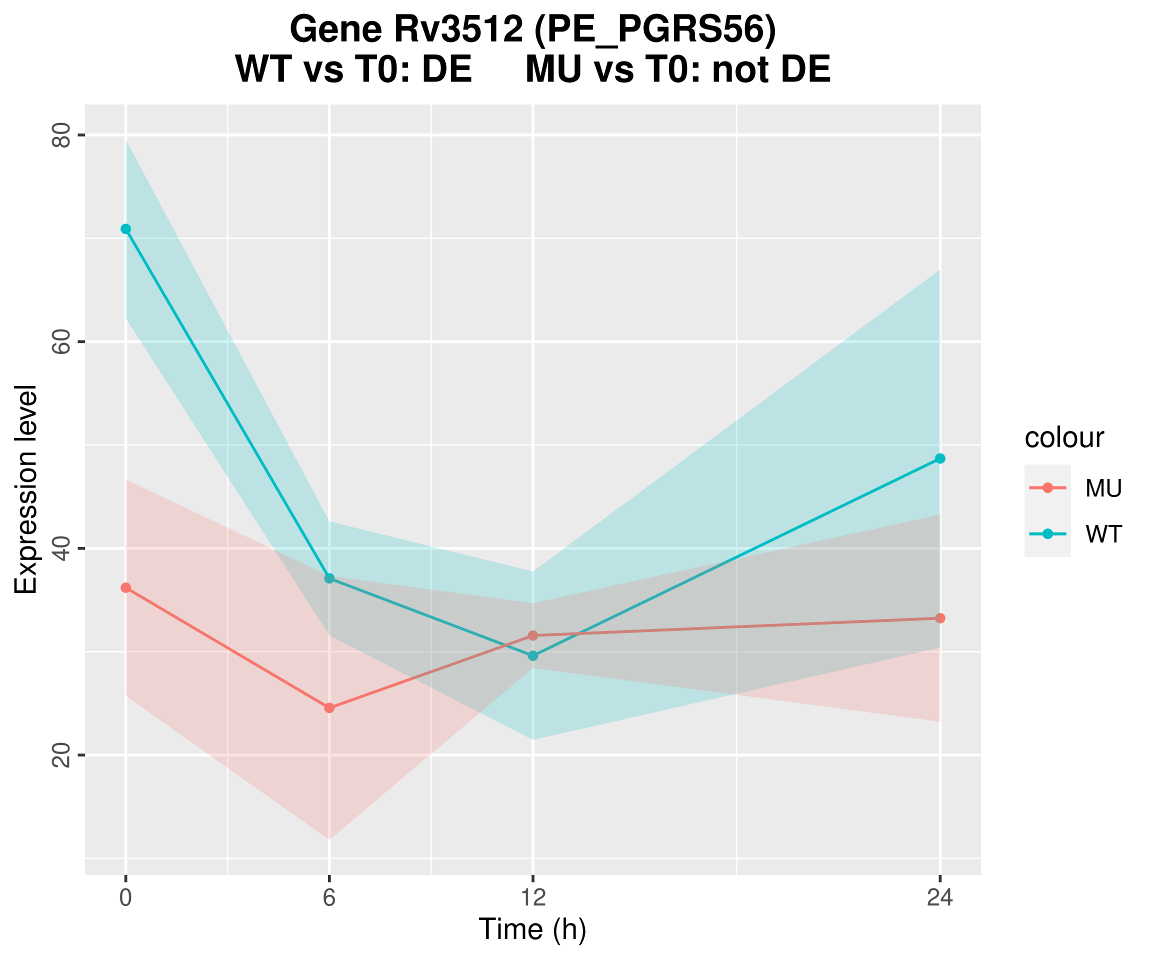
Gene Rv3512 (PE_PGRS56)
WT vs T0: DE   MU vs T0: not DE
Expression level
80
60
40
20
0
6
12
24
Time (h)
colour
MU
WT

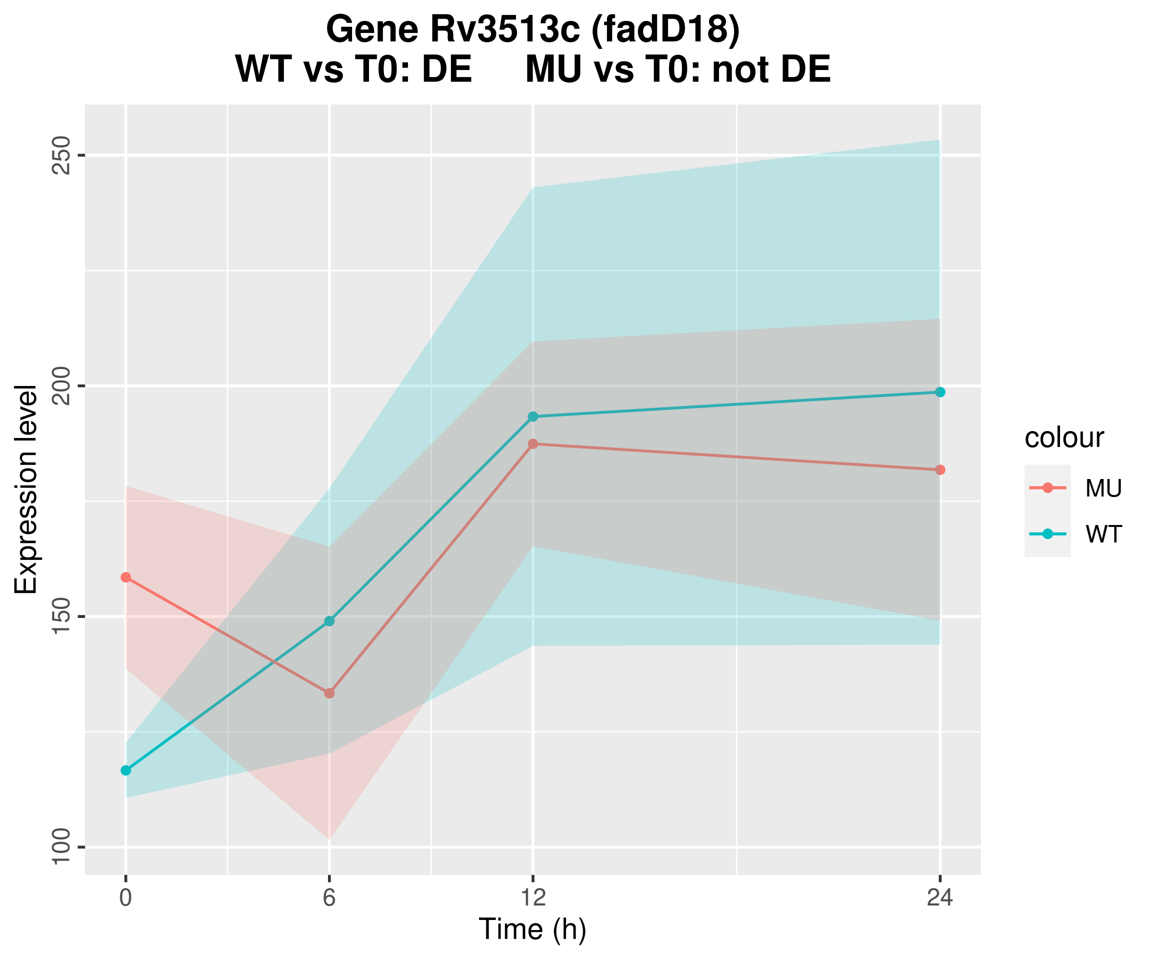
Gene Rv3513c (fadD18)
WT vs T0: DE   MU vs T0: not DE
Expression level
250
200
150
100
0
6
12
24
Time (h)
colour
MU
WT

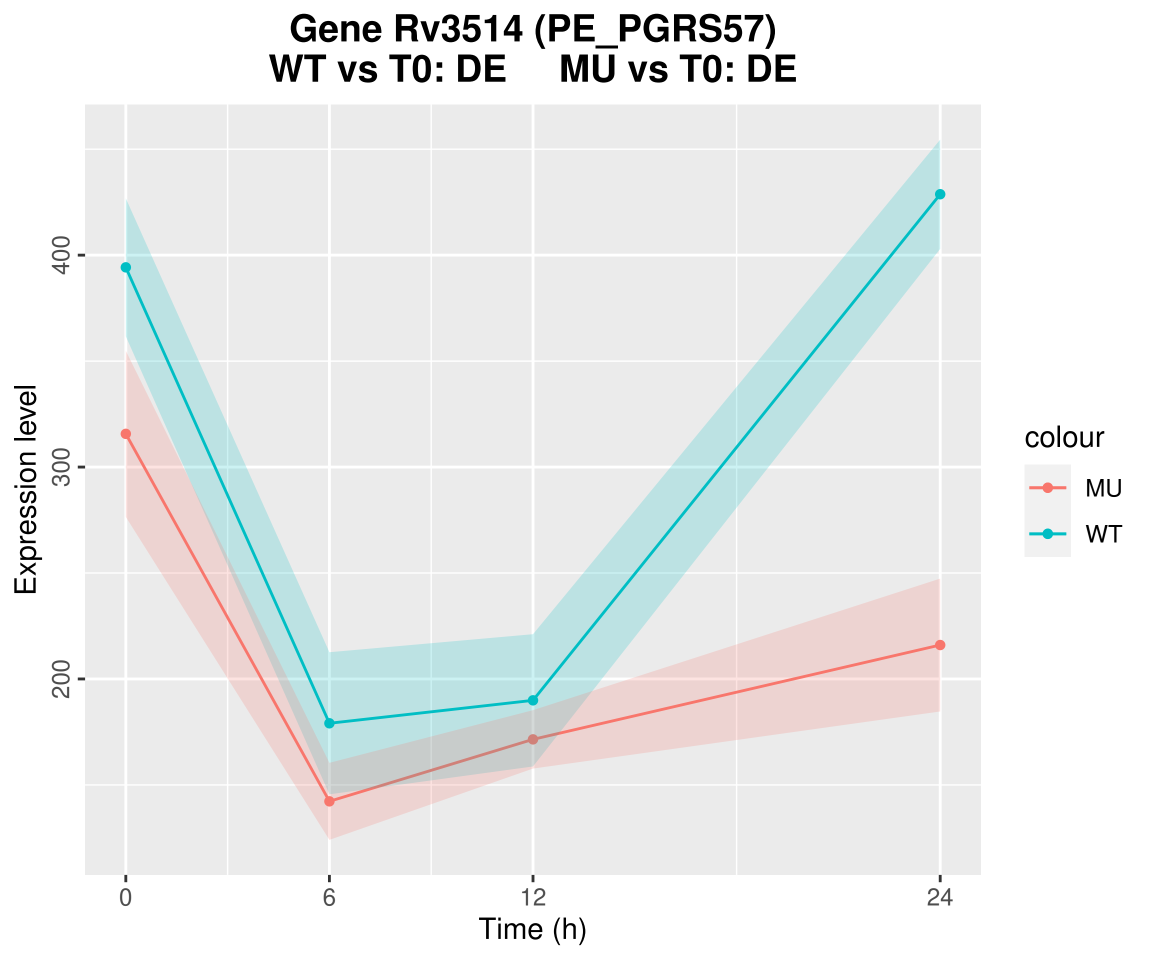
Gene Rv3514 (PE_PGRS57)
WT vs T0: DE MU vs T0: DE
Expression level
400
300
200
0
6
12
24
Time (h)
colour
MU
WT

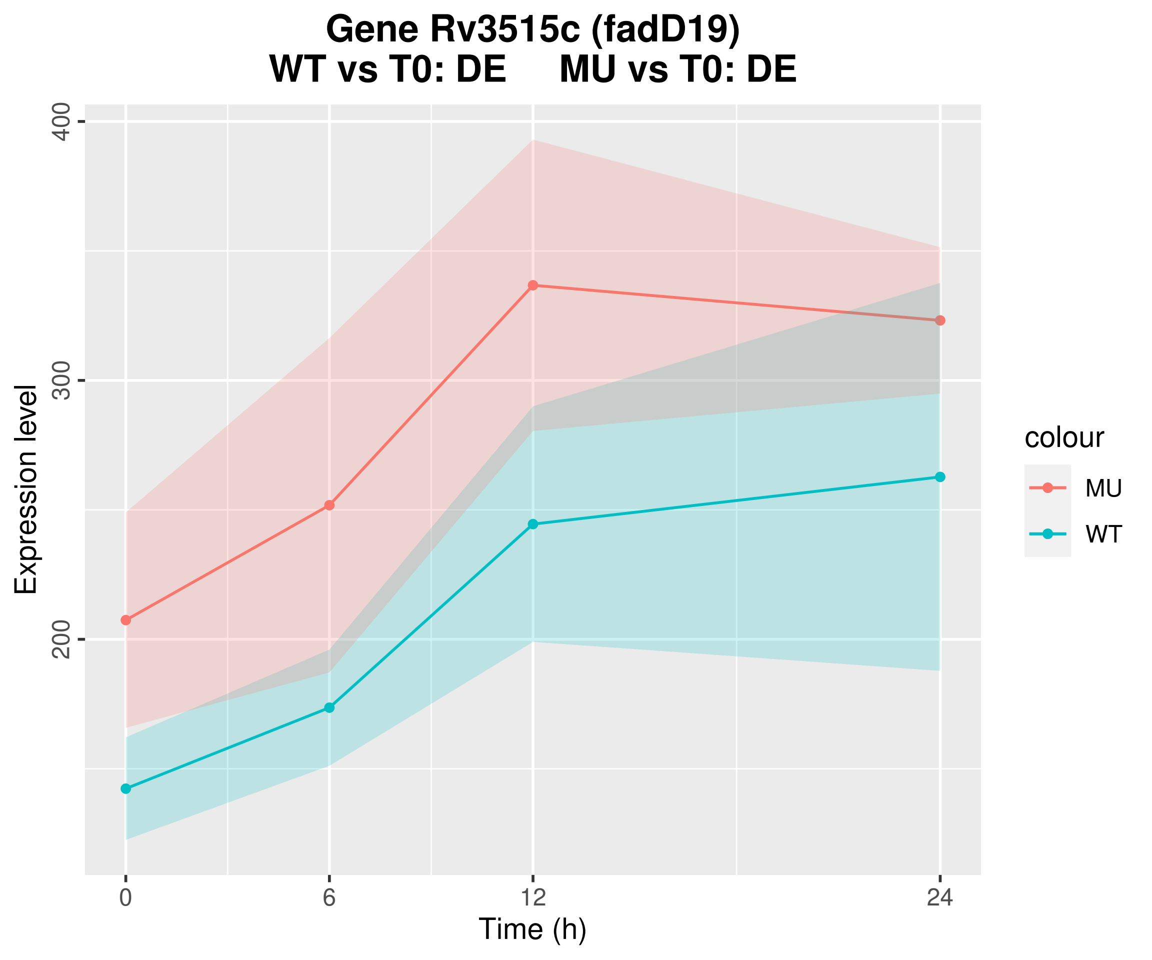

Gene Rv3515c (fadD19)
WT vs T0: DE MU vs T0: DE
400
300
200
Expression level
0
6
12
24
Time (h)
colour
MU
WT

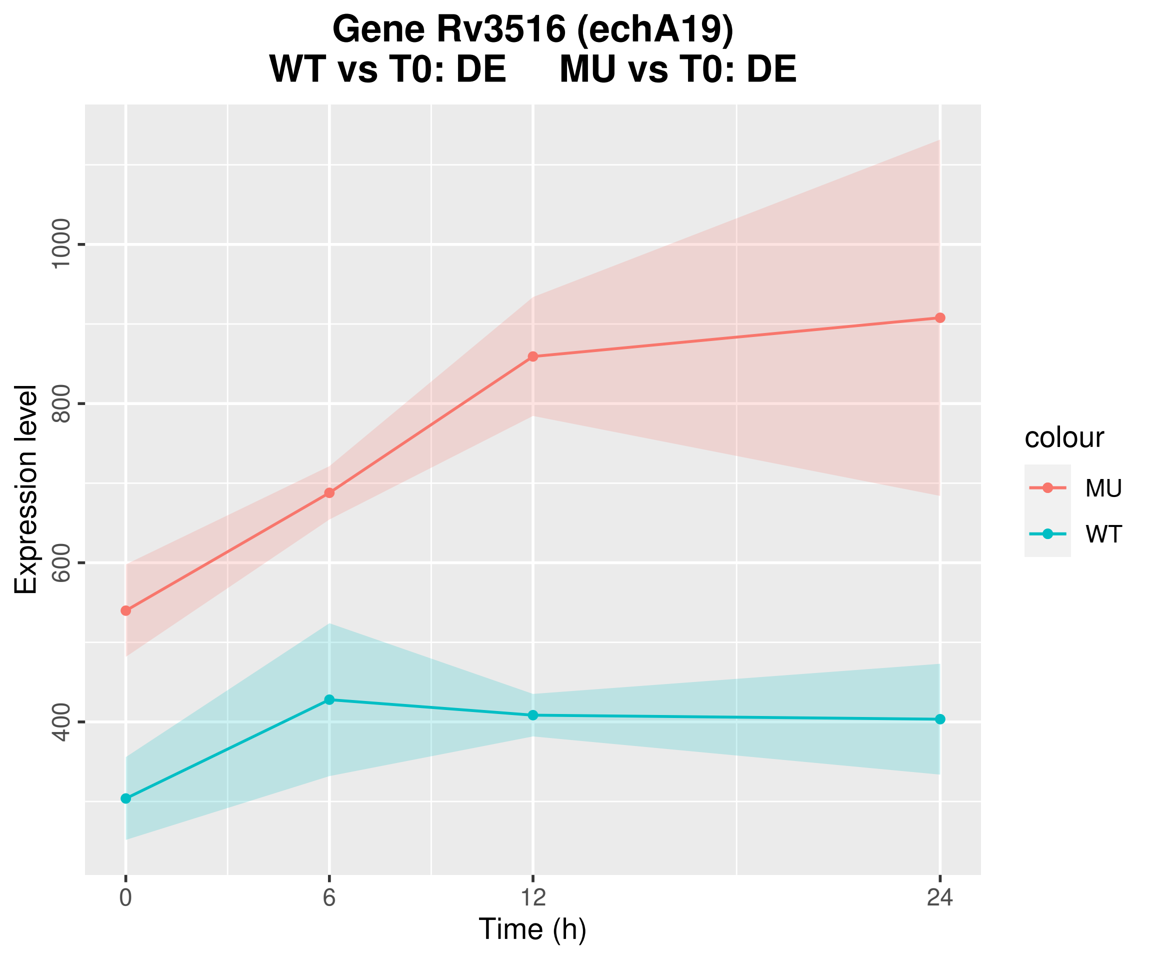

Gene Rv3516 (echA19)
WT vs T0: DE   MU vs T0: DE
Expression level
1000
800
600
400
0
6
12
24
Time (h)
colour
MU
WT

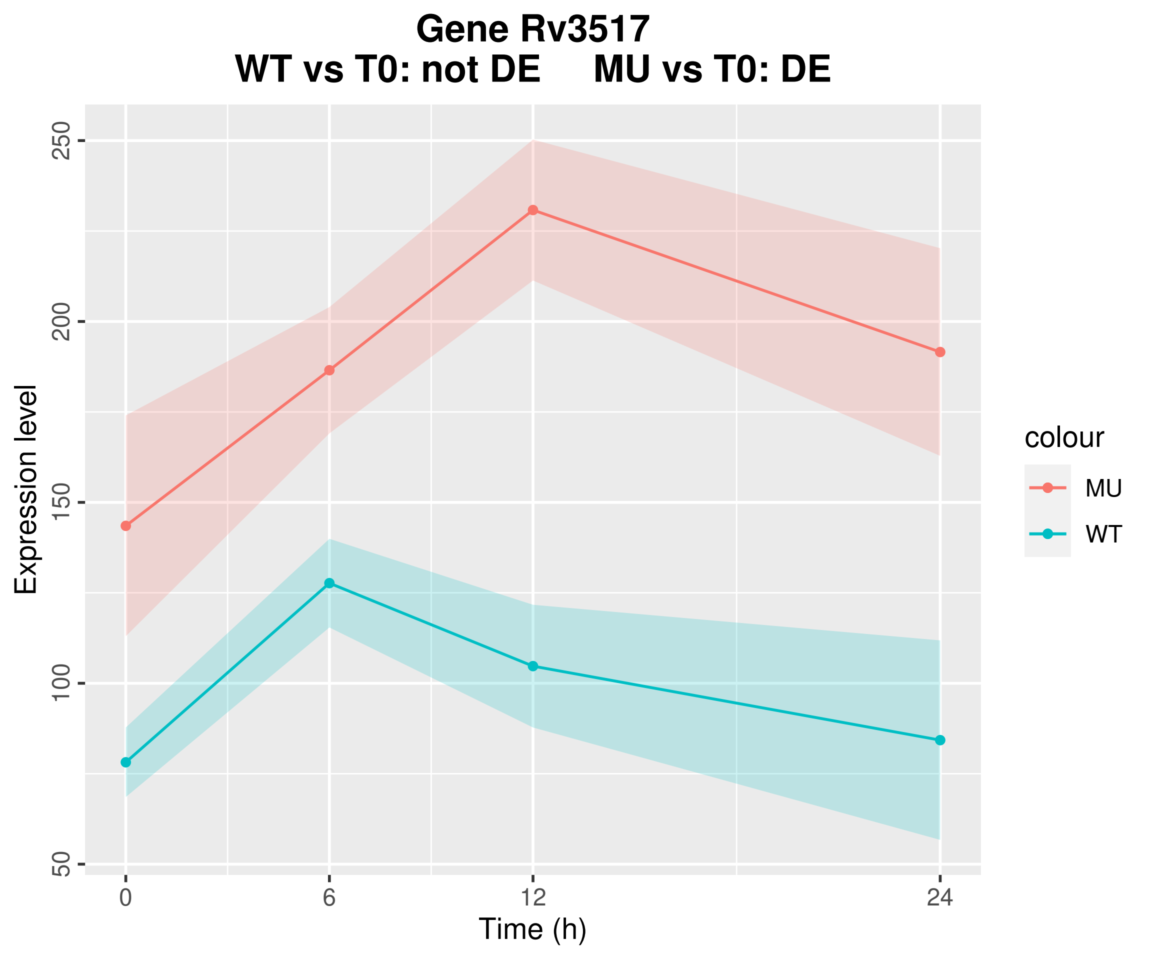
Gene Rv3517
WT vs T0: not DE   MU vs T0: DE
Expression level
250
200
150
100
50
0
6
12
24
Time (h)
colour
MU
WT

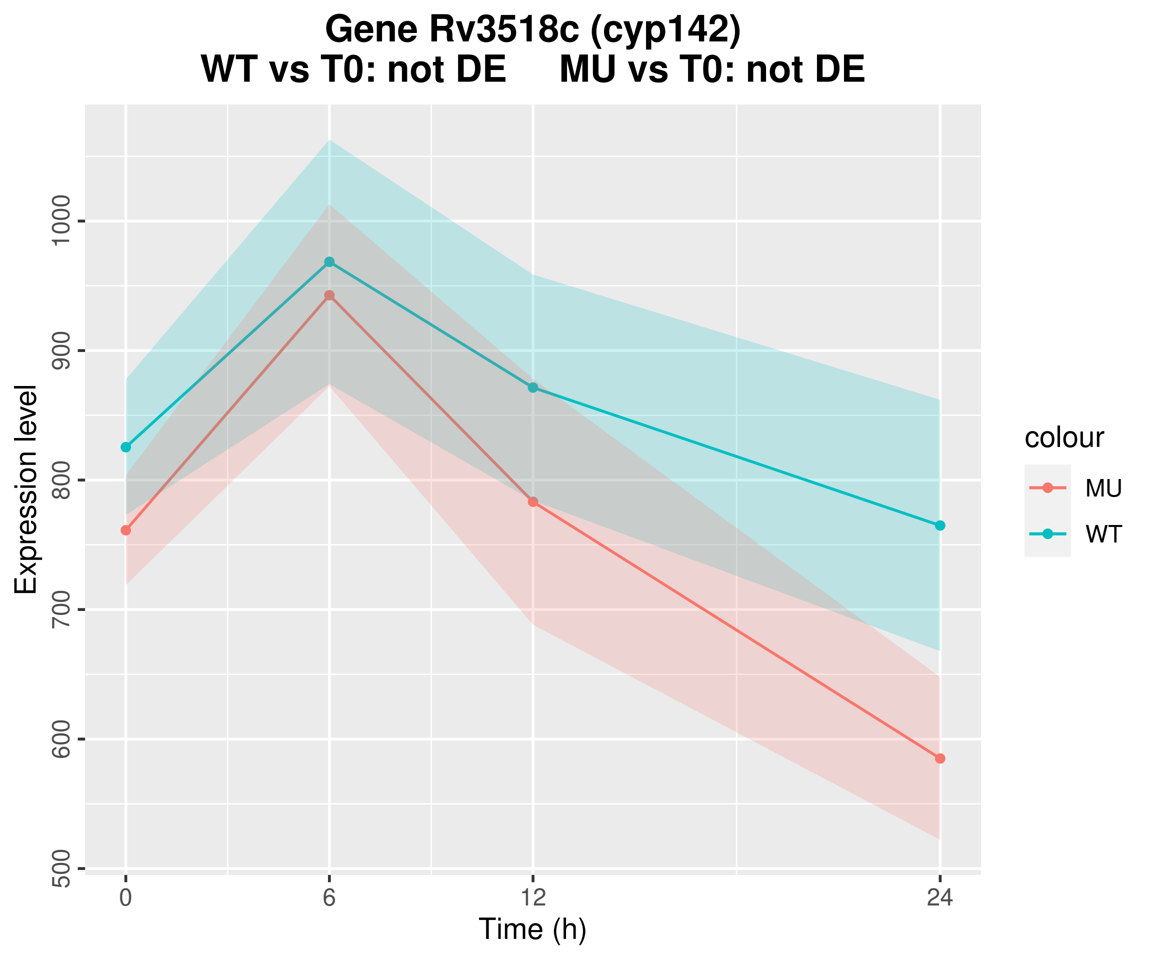

Gene Rv3518c (cyp142)
WT vs T0: not DE MU vs T0: not DE
Expression level
1000
900
800
700
600
500
0
6
12
24
Time (h)
colour
MU
WT

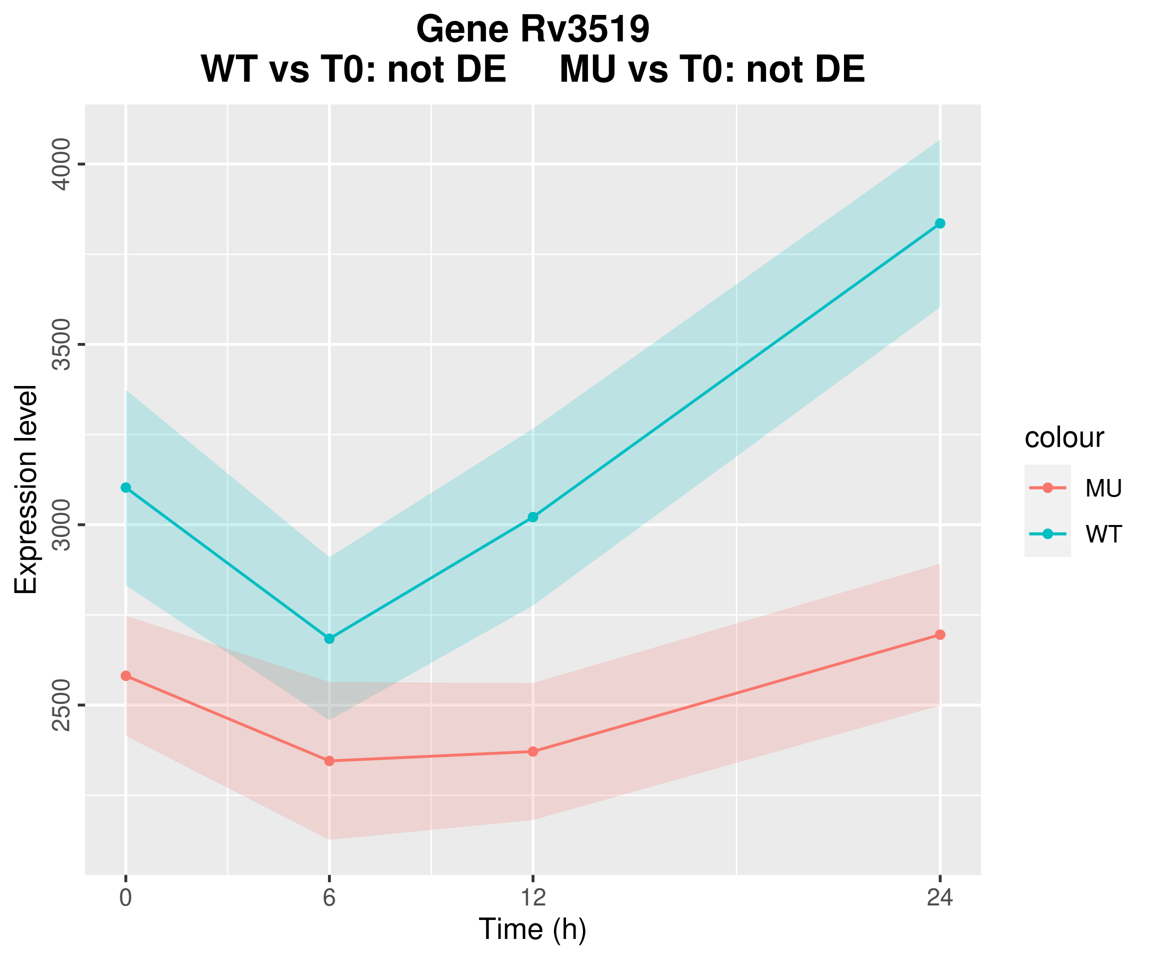
Gene Rv3519
WT vs T0: not DE   MU vs T0: not DE
Expression level
4000
3500
3000
2500
Time (h)
0
6
12
24
colour
MU
WT

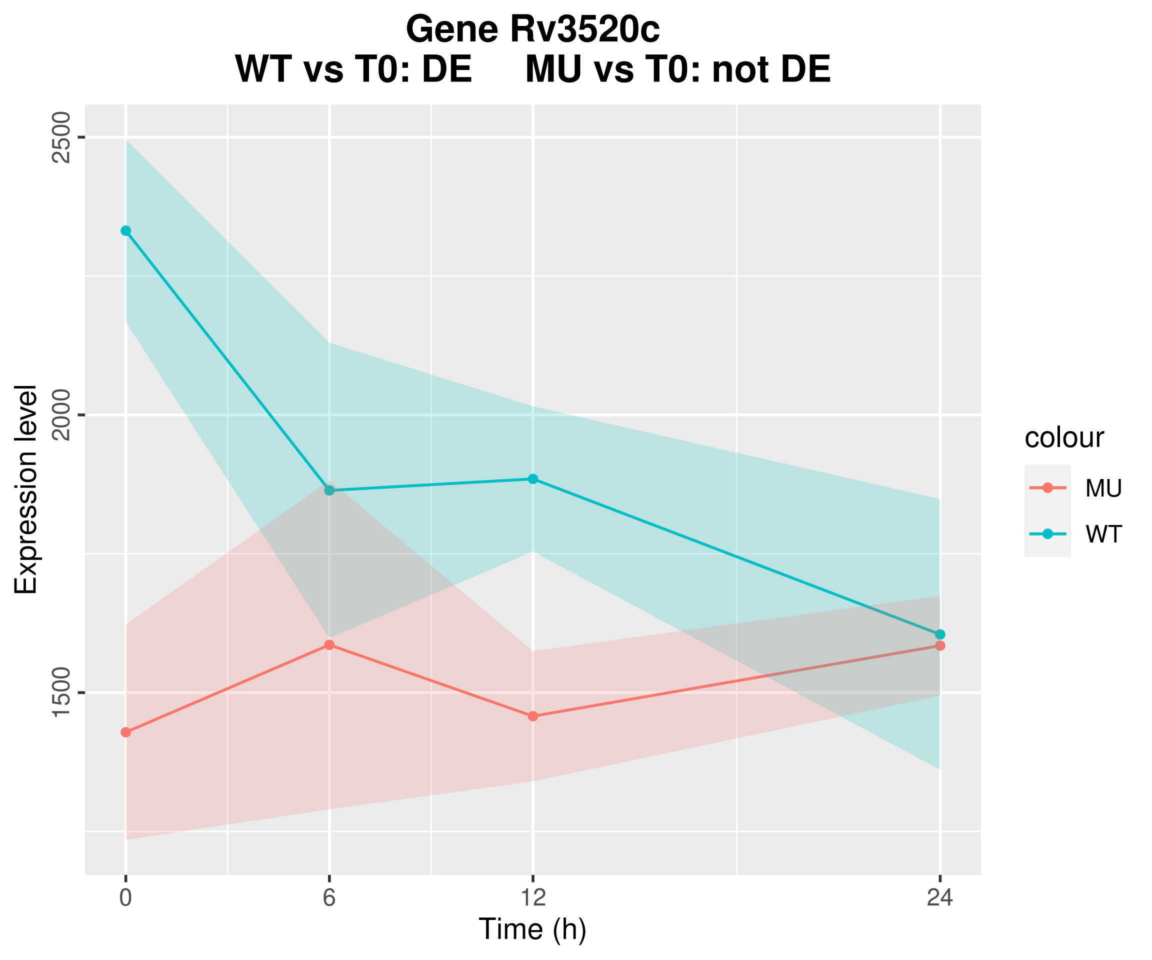
Gene Rv3520c
WT vs T0: DE MU vs T0: not DE
Expression level
2500
2000
1500
0
6
12
24
Time (h)
colour
MU
WT

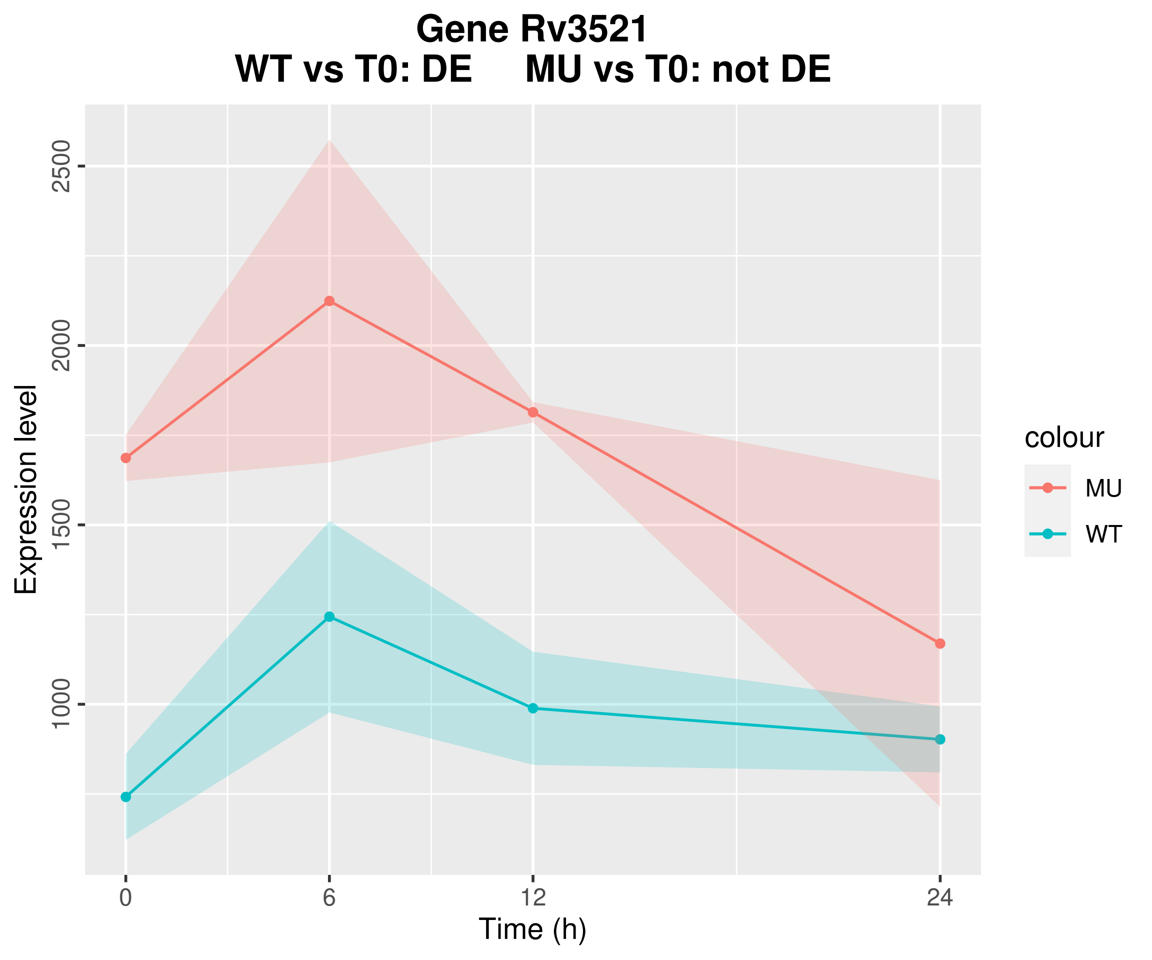
Gene Rv3521
WT vs T0: DE   MU vs T0: not DE
2500
2000
1500
1000
Expression level
0
6
12
24
Time (h)
colour
MU
WT

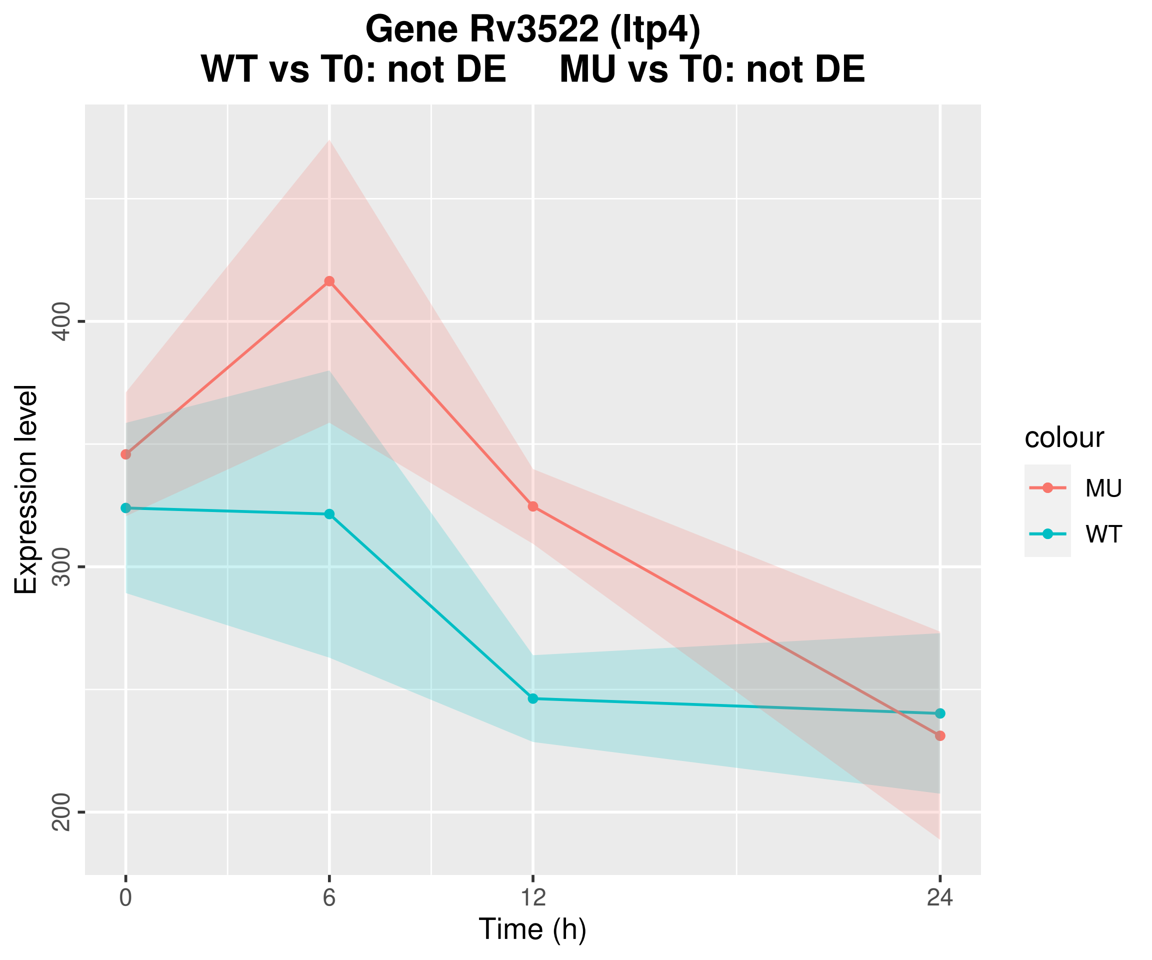

Gene Rv3522 (ltp4)
WT vs T0: not DE   MU vs T0: not DE
Expression level
400
300
200
0
6
12
24
Time (h)
colour
MU
WT

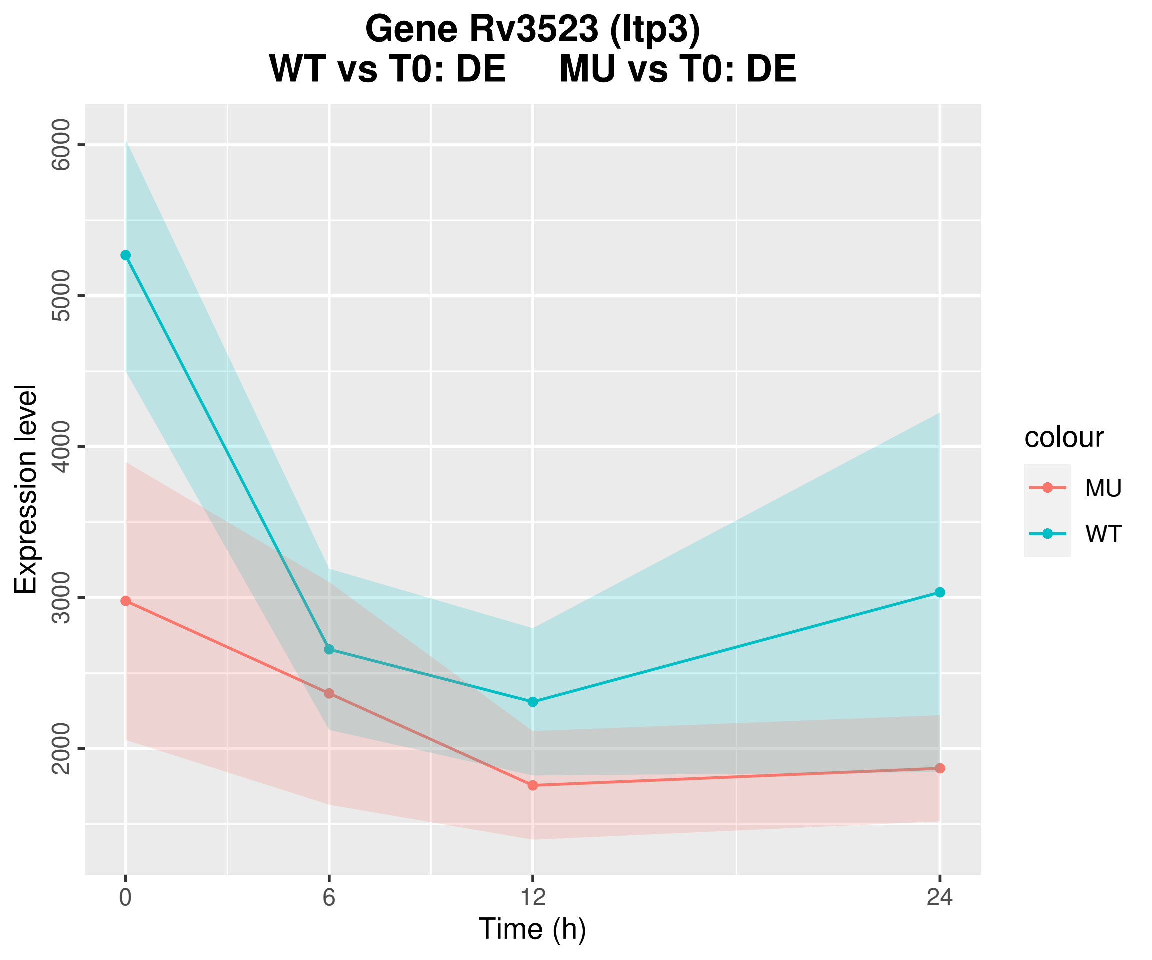
Gene Rv3523 (ltp3)
WT vs T0: DE MU vs T0: DE
Expression level
6000
5000
4000
3000
2000
0
6
12
24
Time (h)
colour
MU
WT

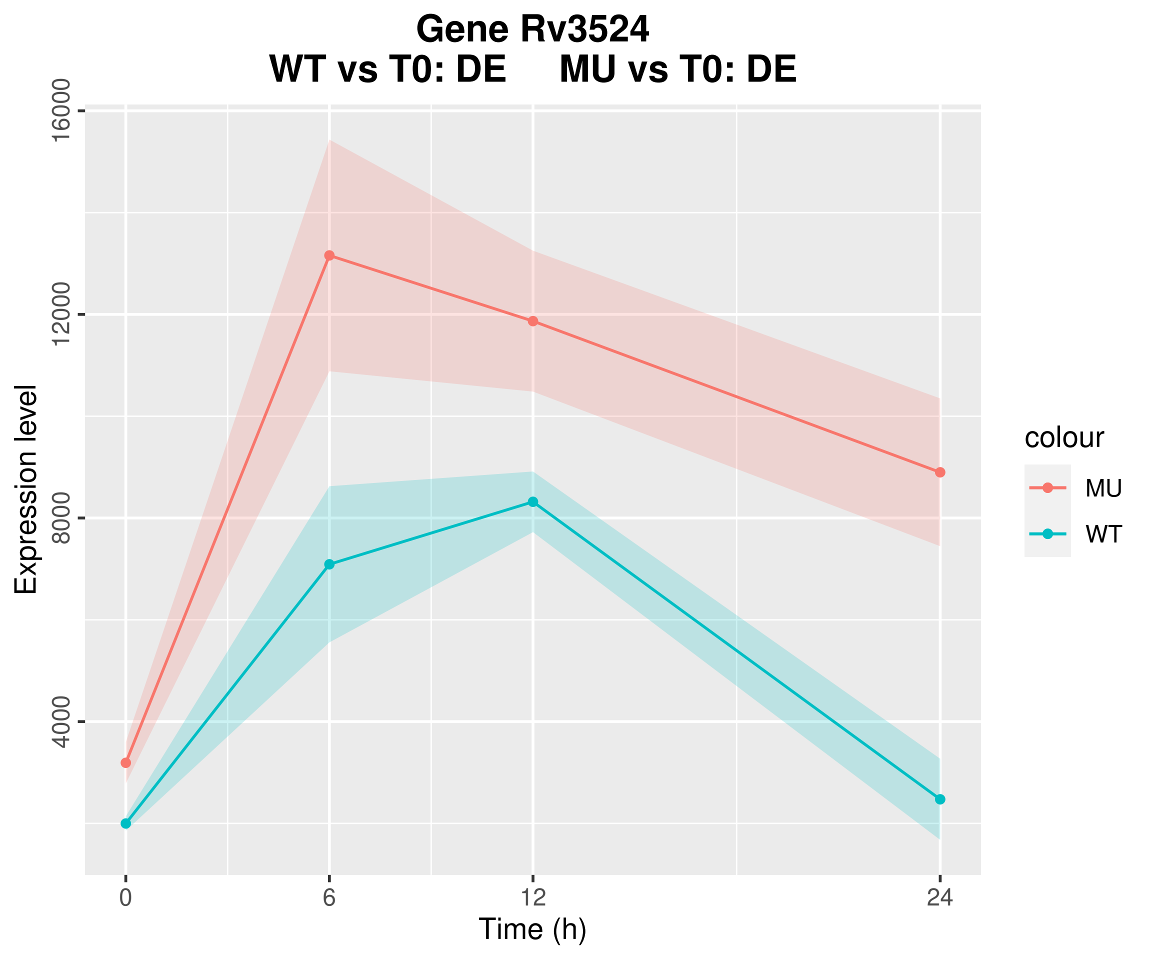

Gene Rv3524
WT vs T0: DE MU vs T0: DE
Expression level
16000
12000
8000
4000
0
6
12
24
Time (h)
colour
MU
WT

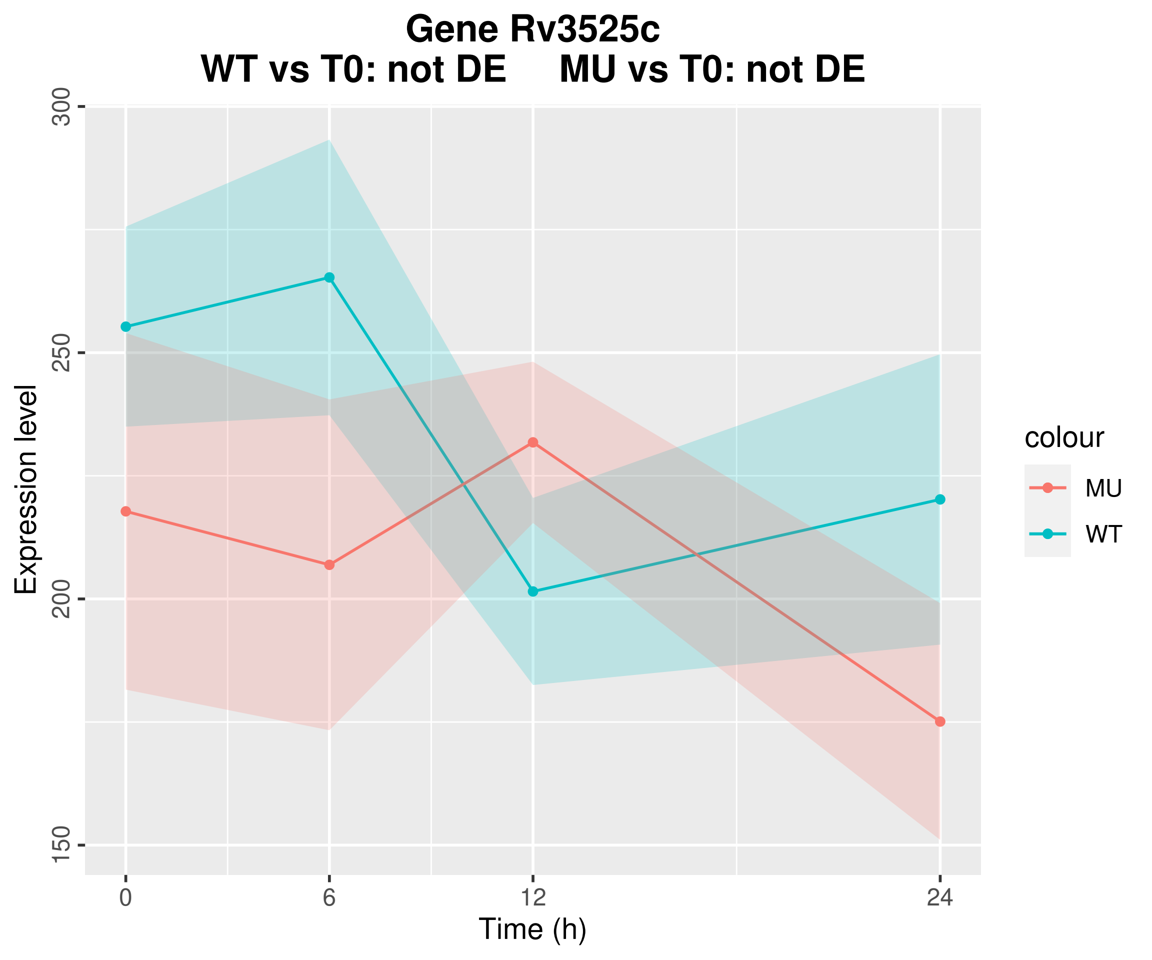
Gene Rv3525c
WT vs T0: not DE MU vs T0: not DE
Expression level
300
250
200
150
0
6
12
24
Time (h)
colour
MU
WT

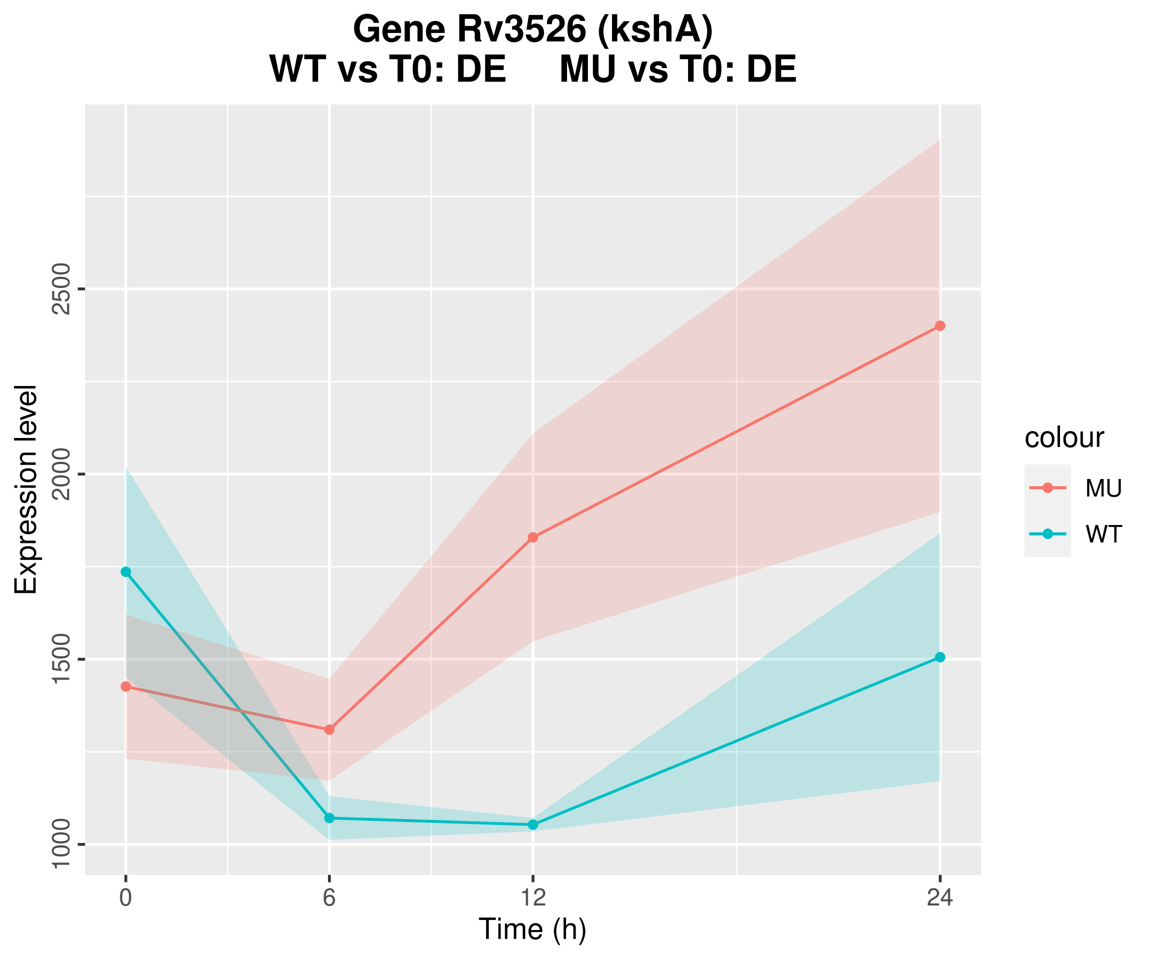

Gene Rv3526 (kshA)
WT vs T0: DE MU vs T0: DE
Expression level
2500
2000
1500
1000
0
6
12
24
Time (h)
colour
MU
WT

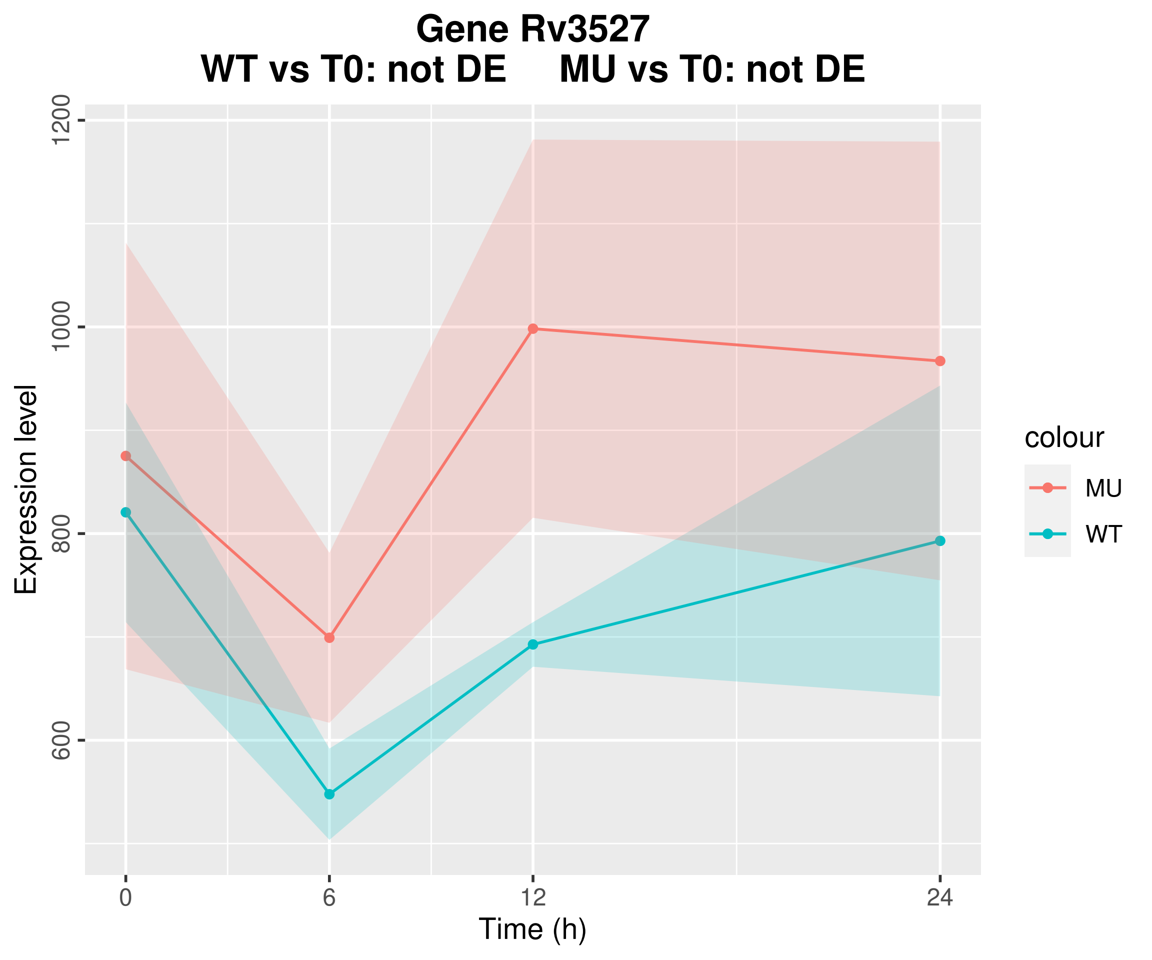

Gene Rv3527
WT vs T0: not DE MU vs T0: not DE
Expression level
1200
1000
800
600
0
6
12
24
Time (h)
colour
MU
WT

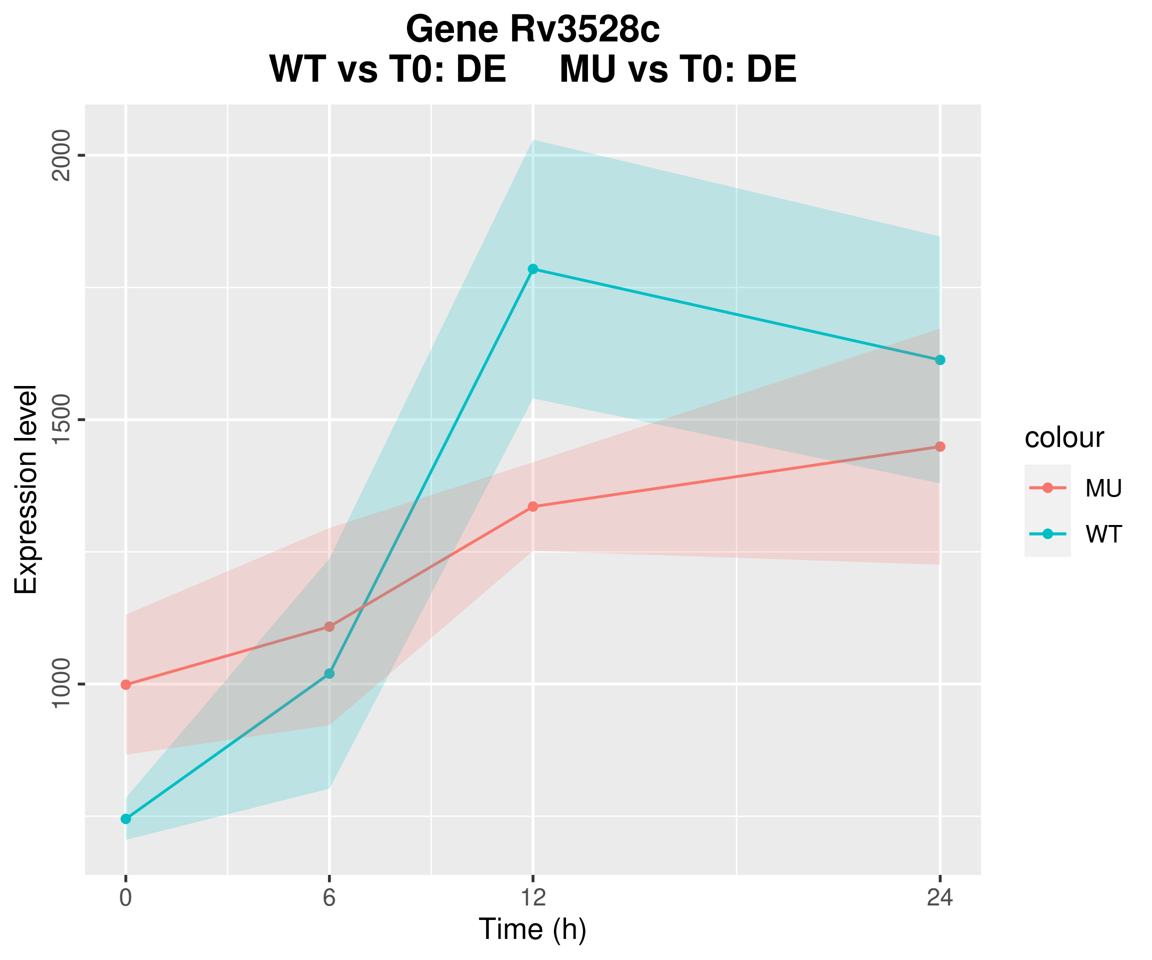

Gene Rv3528c
WT vs T0: DE MU vs T0: DE
Expression level
2000
1500
1000
0
6
12
24
Time (h)
colour
MU
WT

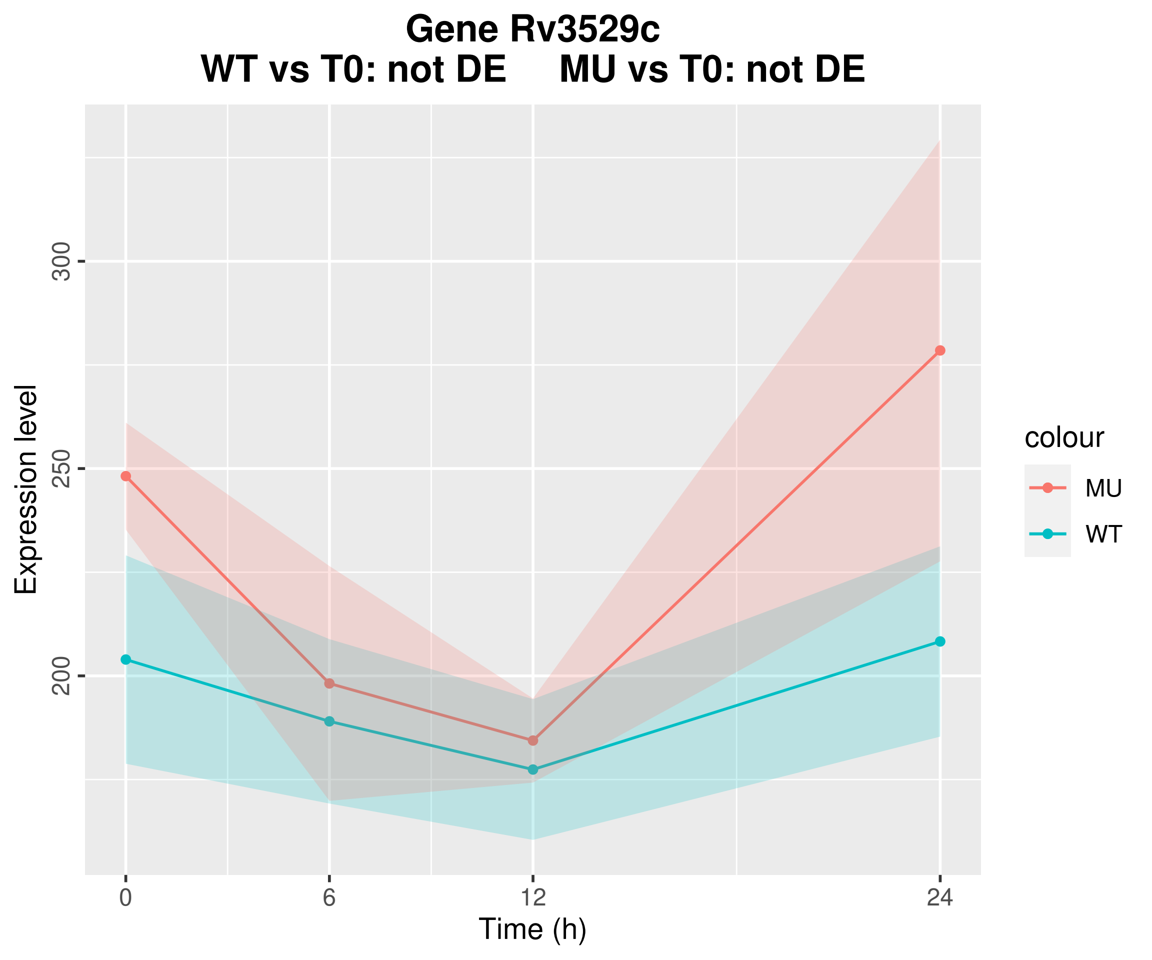
Gene Rv3529c
WT vs T0: not DE MU vs T0: not DE
Expression level
300
250
200
0
6
12
24
Time (h)
colour
MU
WT

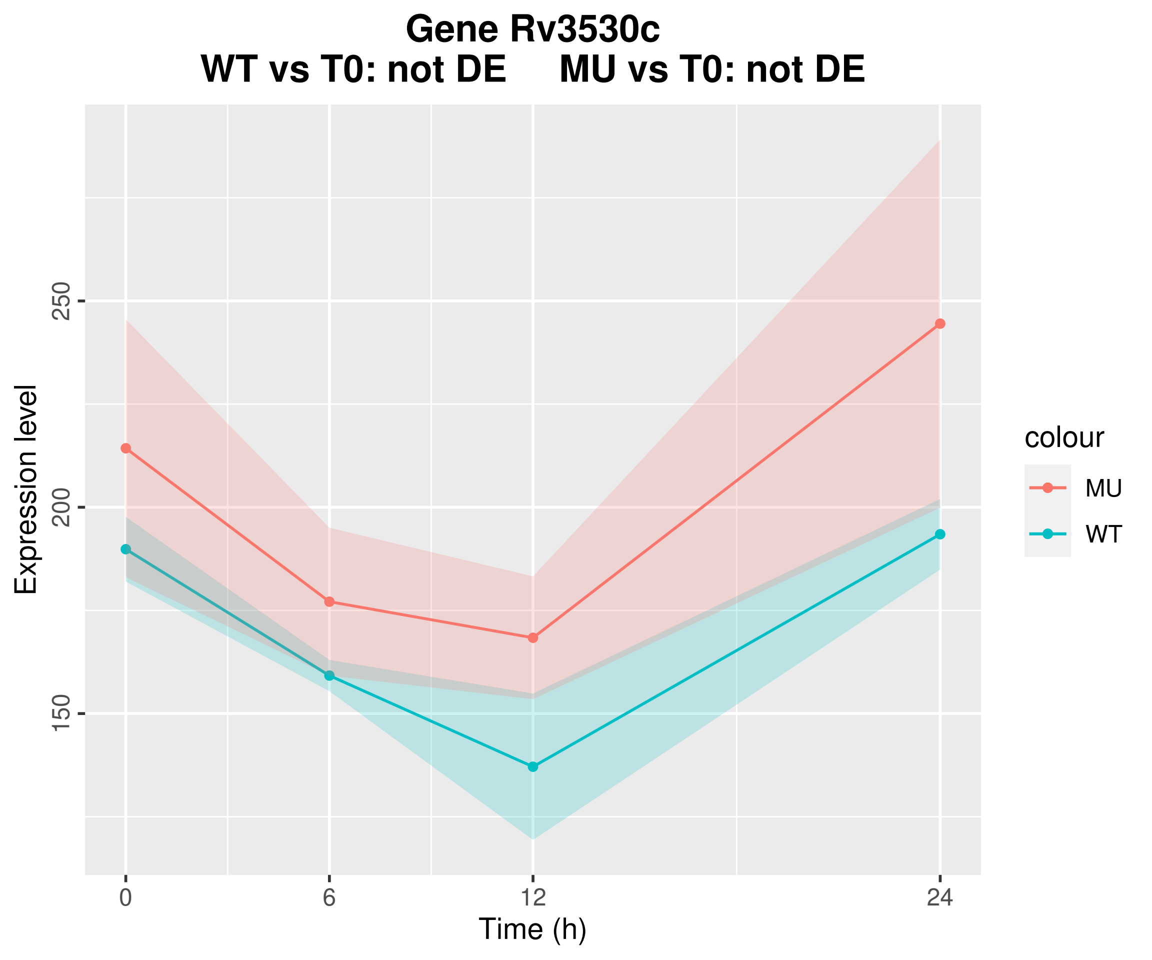

Gene Rv3530c
WT vs T0: not DE MU vs T0: not DE
Expression level
250
200
150
0
6
12
24
Time (h)
colour
MU
WT

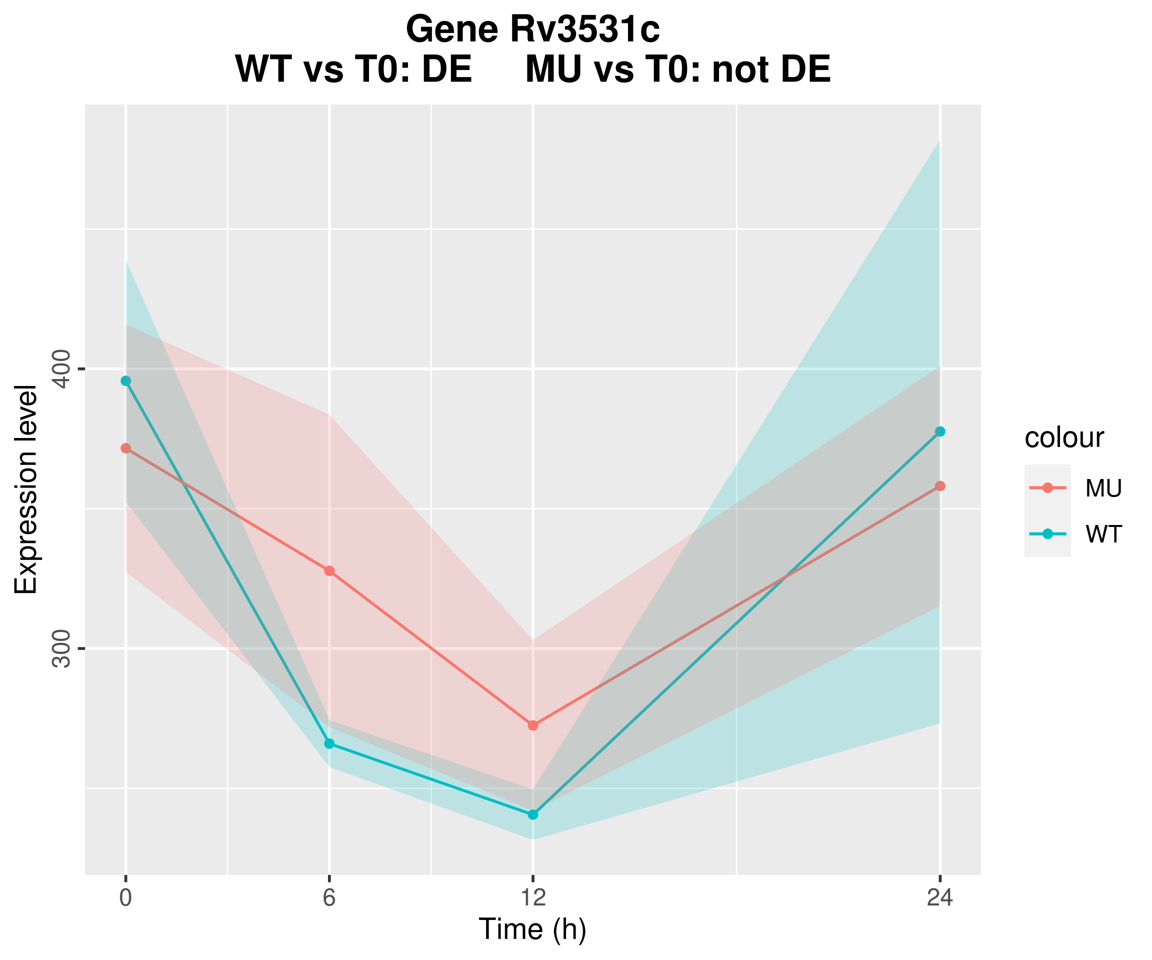
Gene Rv3531c
WT vs T0: DE   MU vs T0: not DE
Expression level
400
300
0
6
12
24
Time (h)
colour
MU
WT

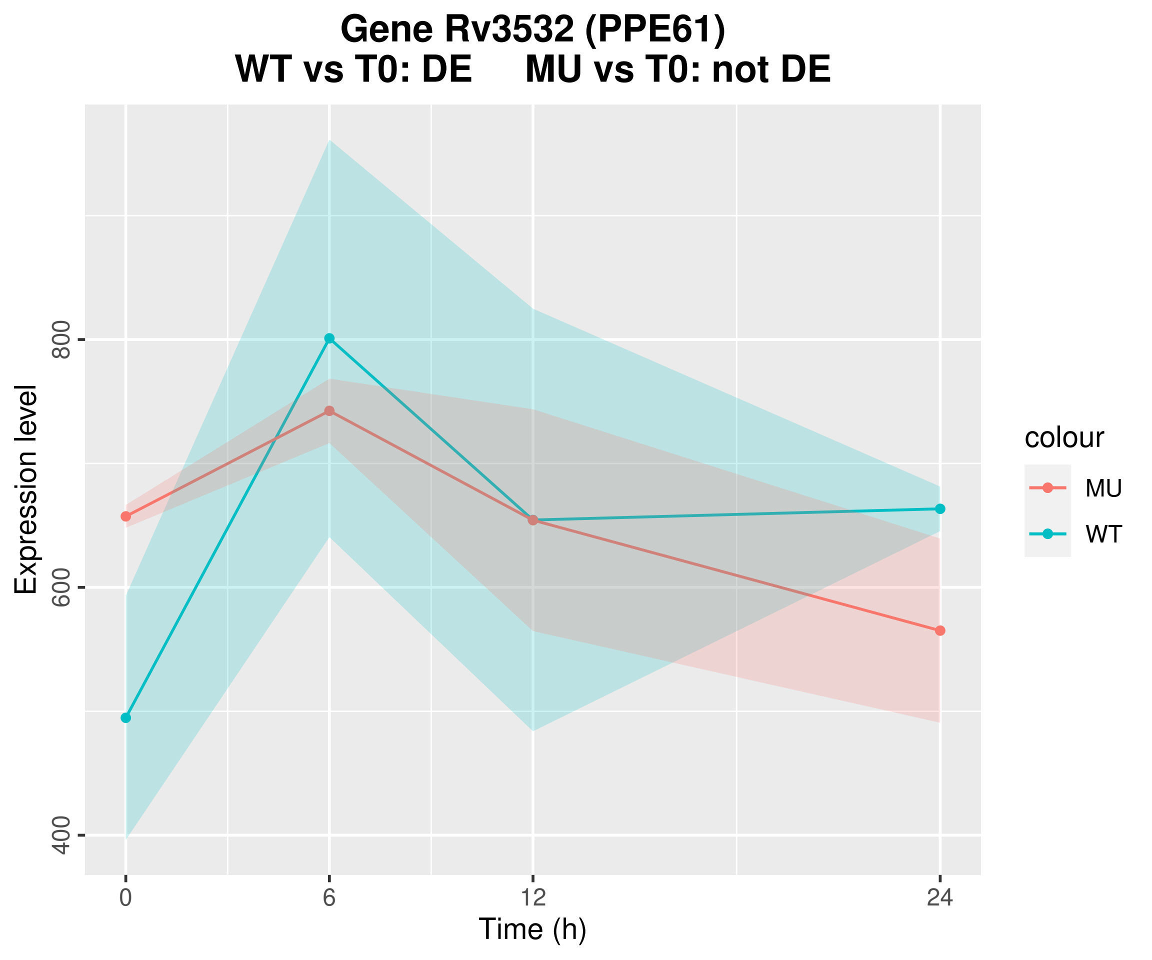

Gene Rv3532 (PPE61)
WT vs T0: DE   MU vs T0: not DE
Expression level
800
600
400
0
6
12
24
Time (h)
colour
MU
WT

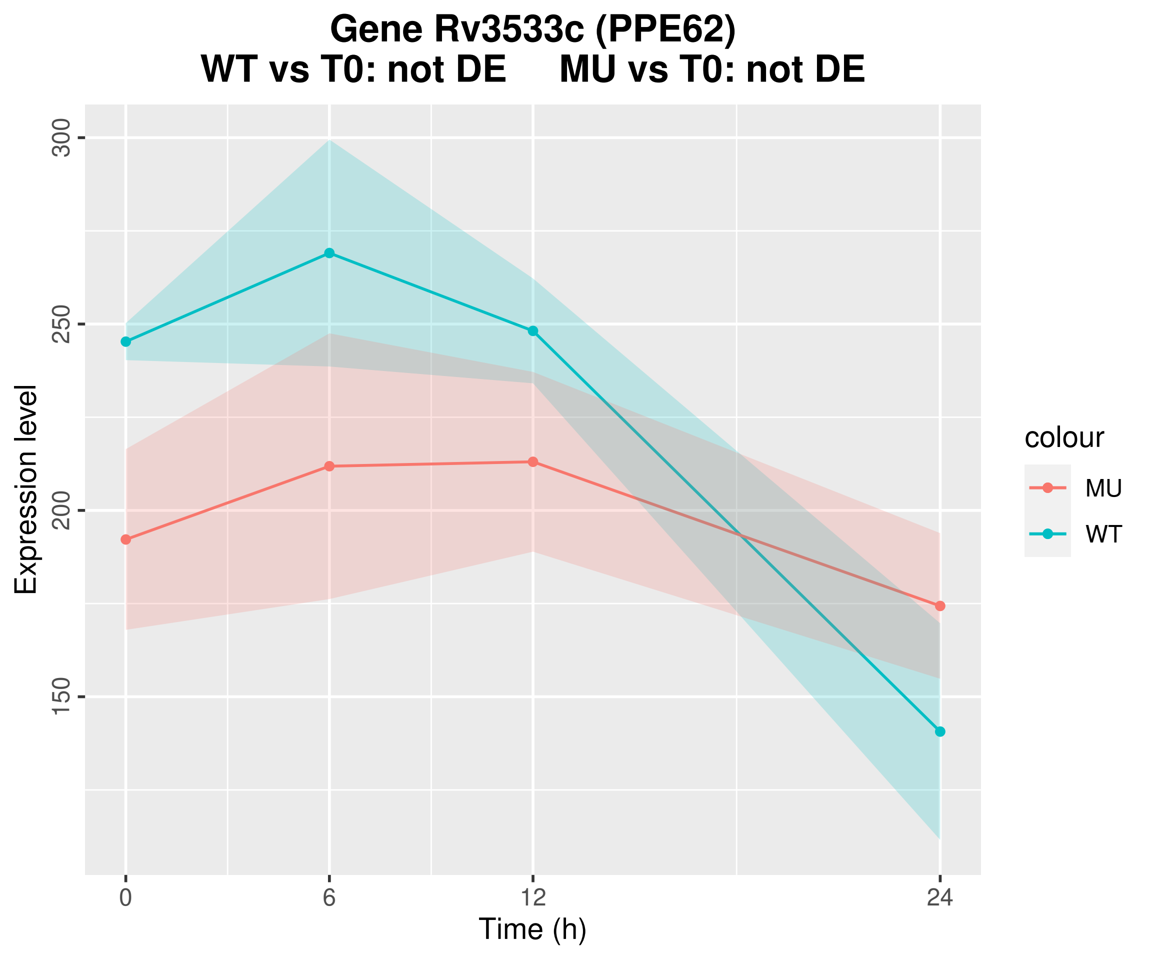
Gene Rv3533c (PPE62)
WT vs T0: not DE   MU vs T0: not DE
300
250
200
150
Expression level
0
6
12
24
Time (h)
colour
MU
WT

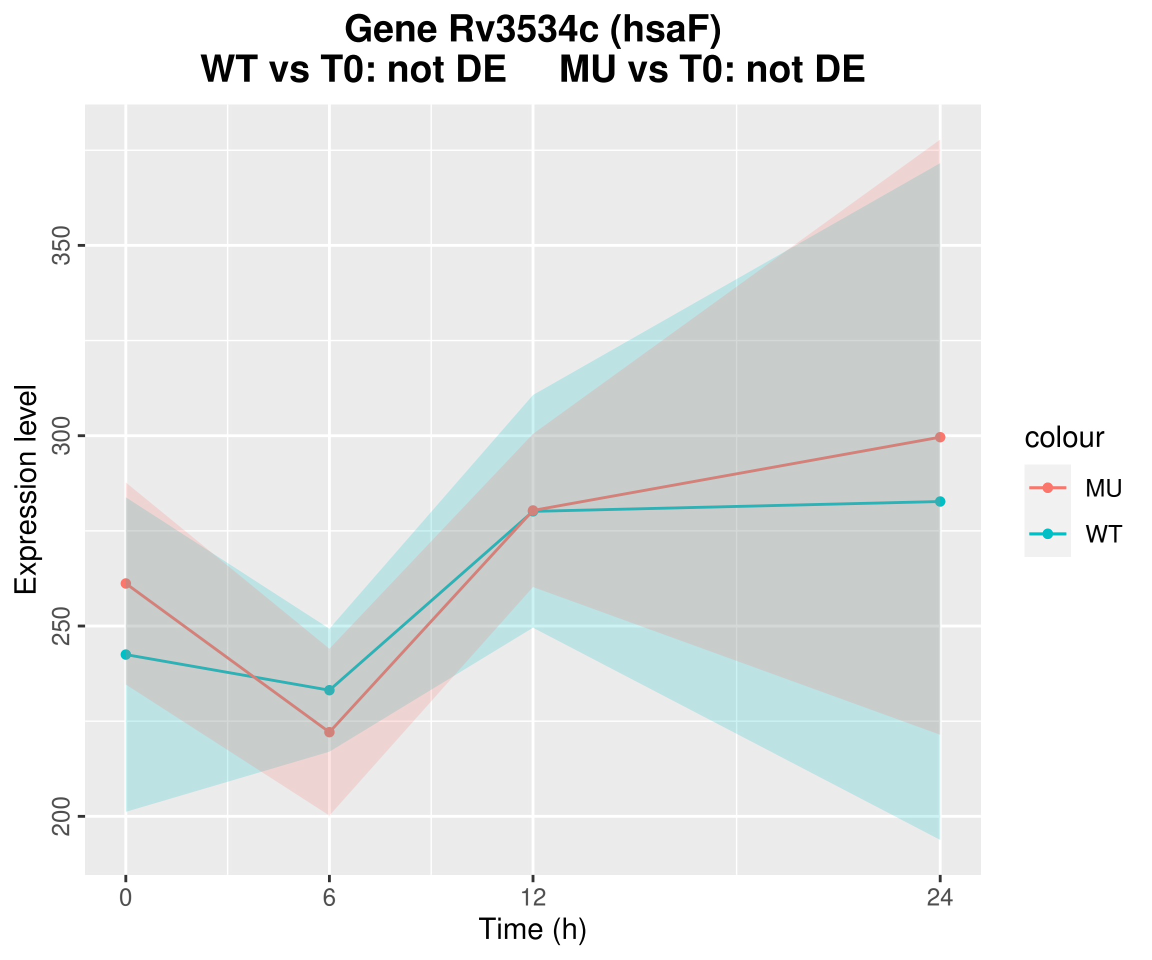

Gene Rv3534c (hsaF)
WT vs T0: not DE MU vs T0: not DE
Expression level
350
300
250
200
0
6
12
24
Time (h)
colour
MU
WT

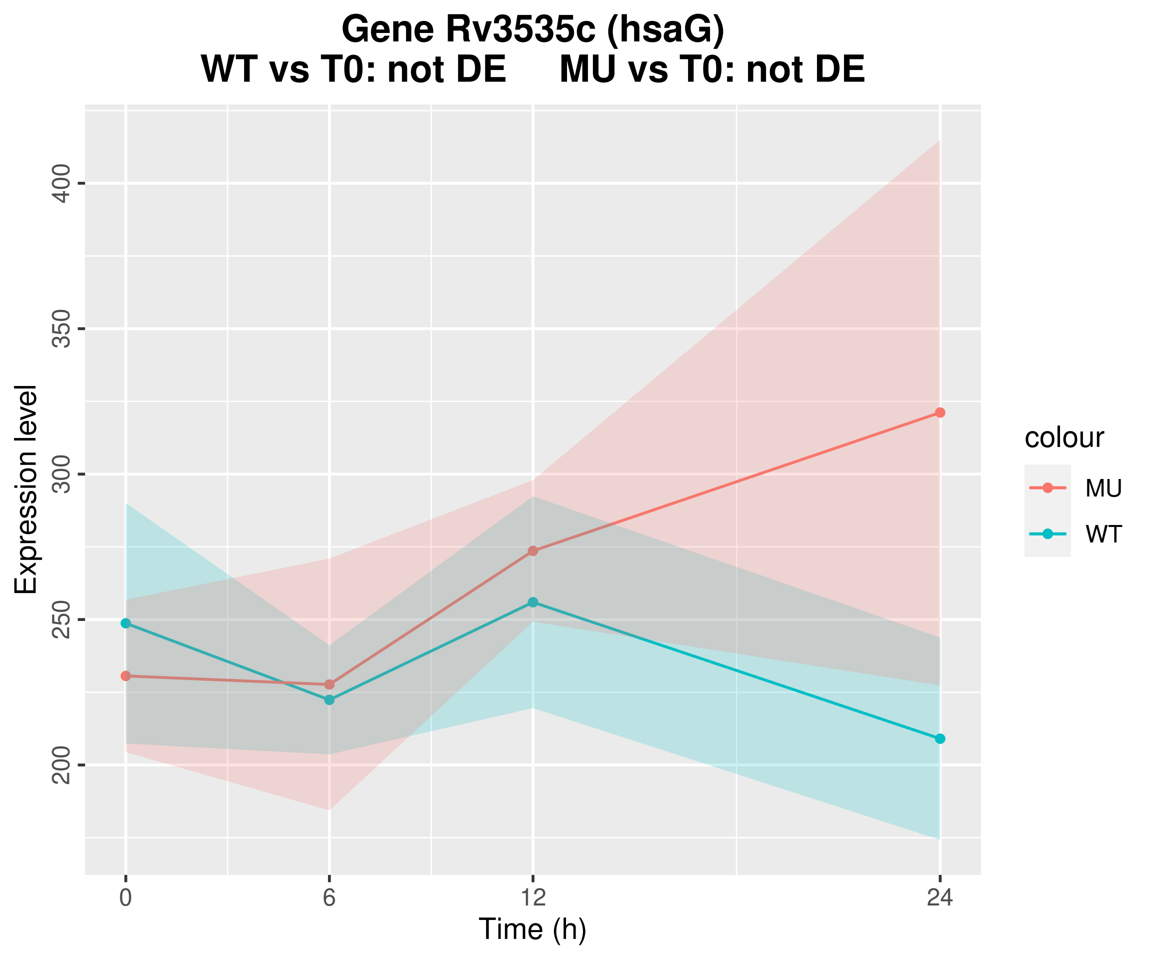
Gene Rv3535c (hsaG)
WT vs T0: not DE
MU vs T0: not DE
Expression level
400
350
300
250
200
Time (h)
0
6
12
24
colour
MU
WT

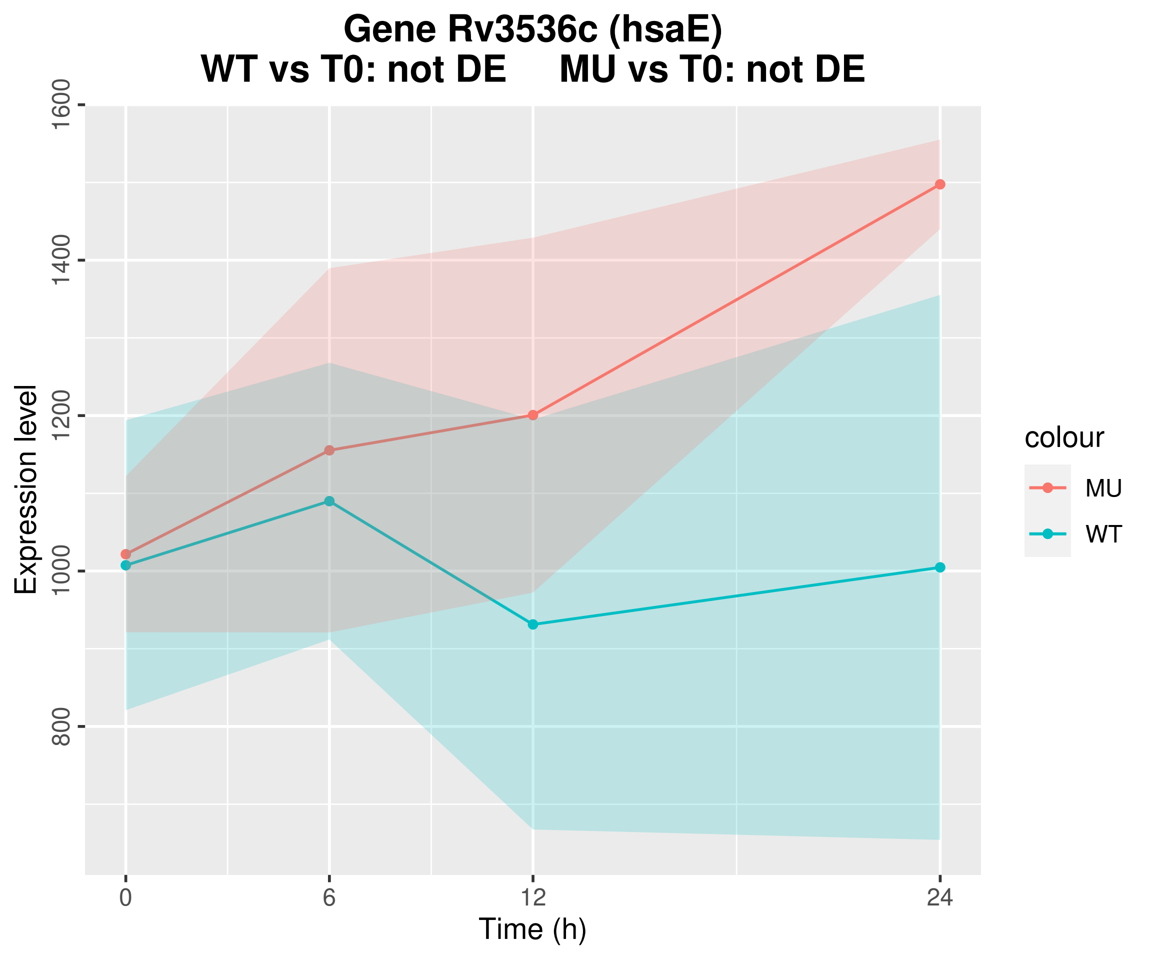

Gene Rv3536c (hsaE)
WT vs T0: not DE   MU vs T0: not DE
Expression level
1600
1400
1200
1000
800
0
6
12
24
Time (h)
colour
MU
WT

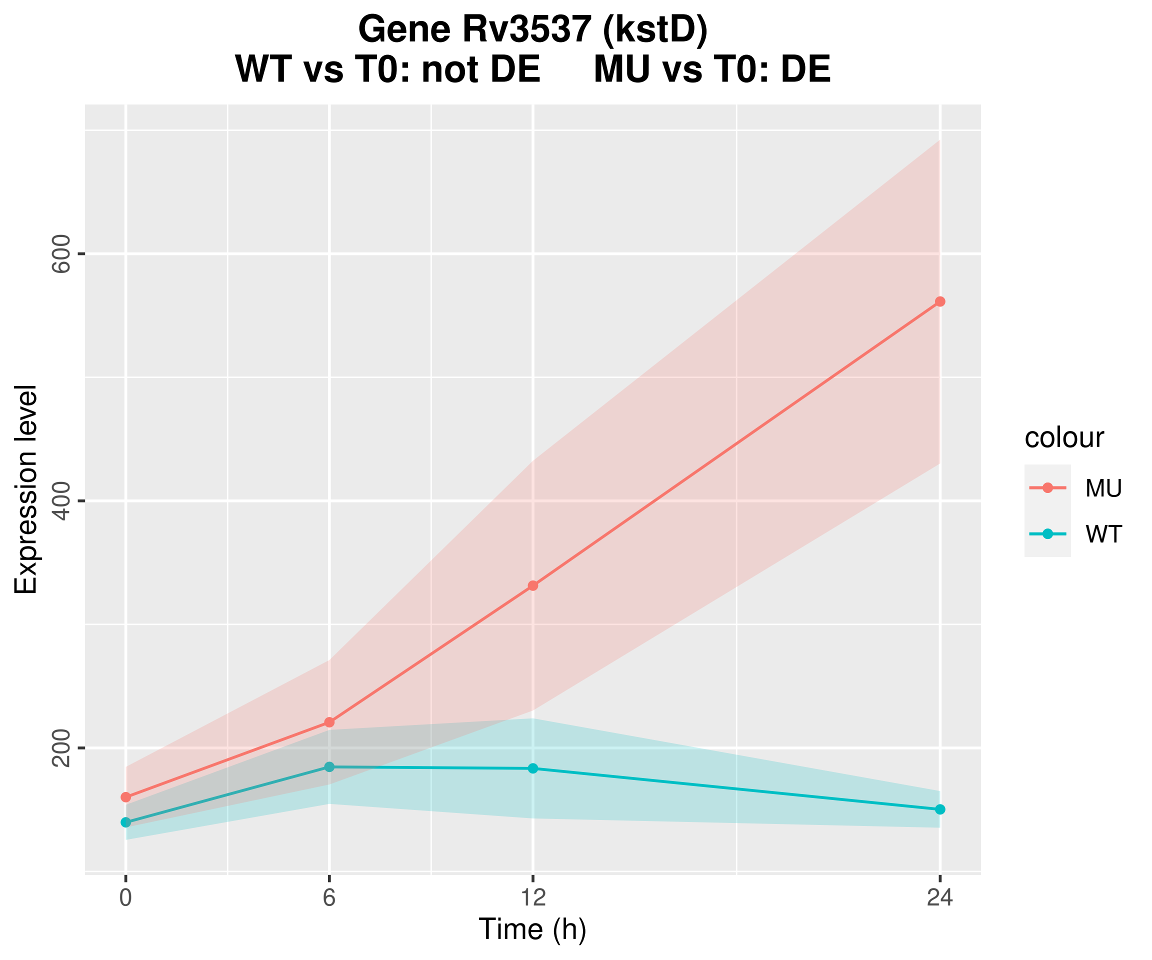

Gene Rv3537 (kstD)
WT vs T0: not DE MU vs T0: DE
Expression level
600
400
200
0
6
12
24
Time (h)
colour
MU
WT

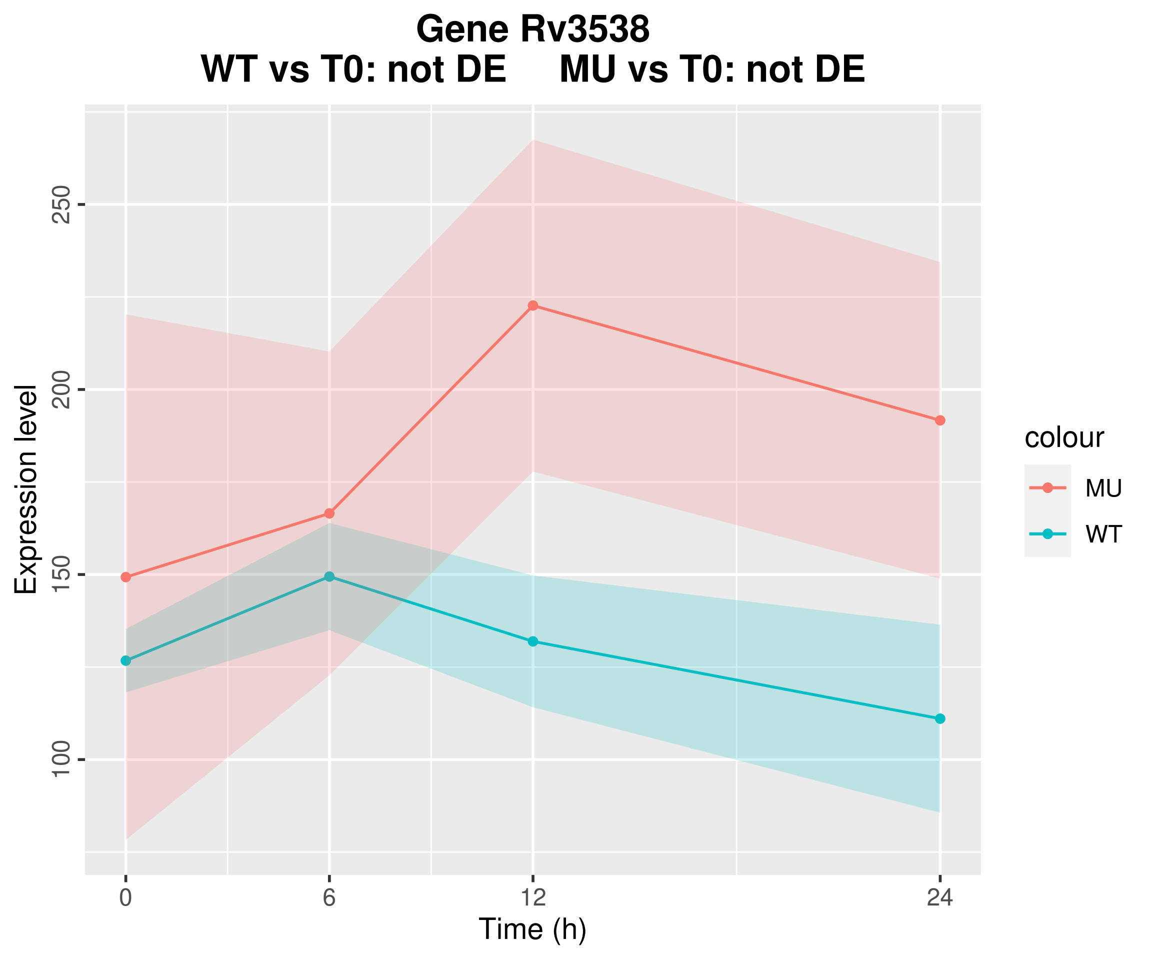
Gene Rv3538
WT vs T0: not DE   MU vs T0: not DE
Expression level
250
200
150
100
0
6
12
24
Time (h)
colour
MU
WT

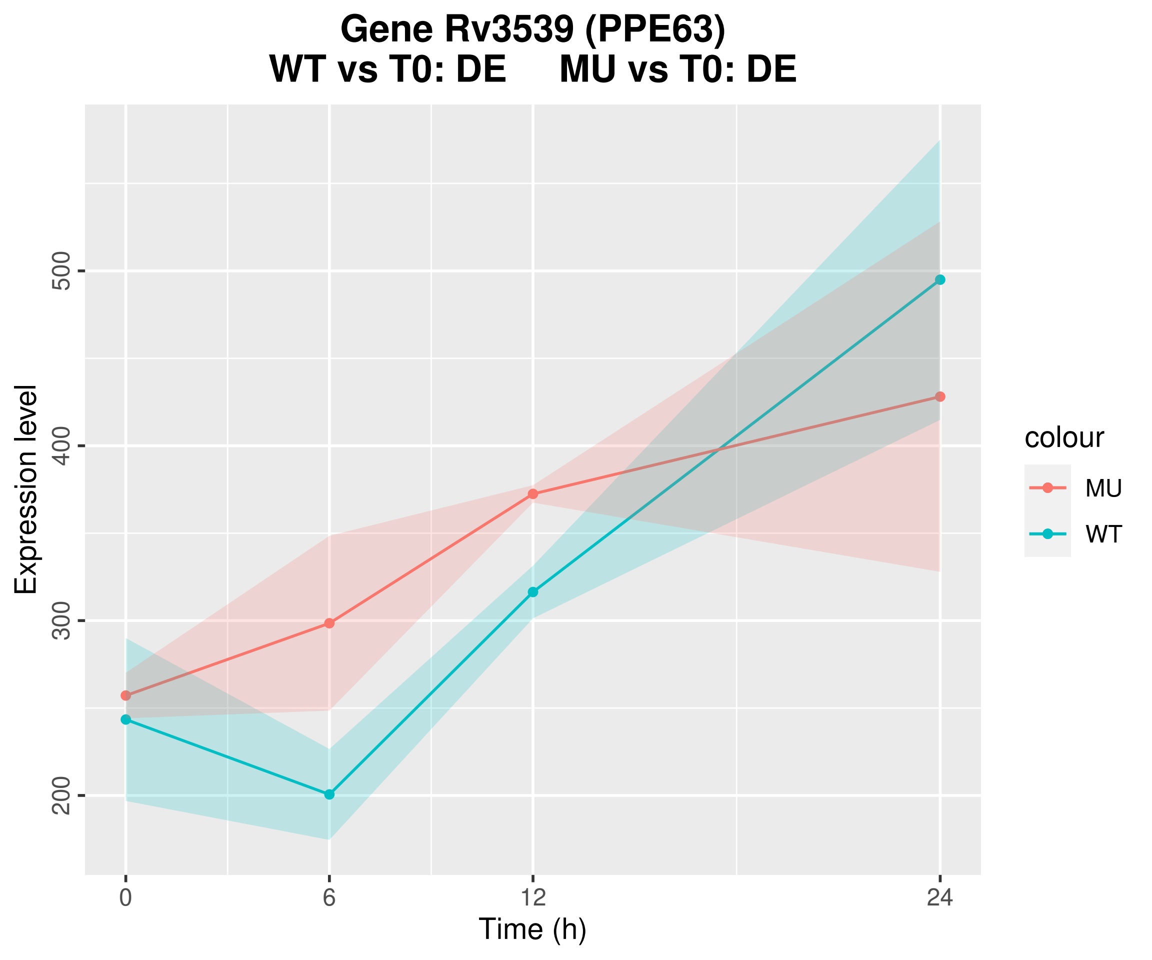

Gene Rv3539 (PPE63)
WT vs T0: DE MU vs T0: DE
Expression level
200
300
400
500
0
6
12
24
Time (h)
colour
MU
WT

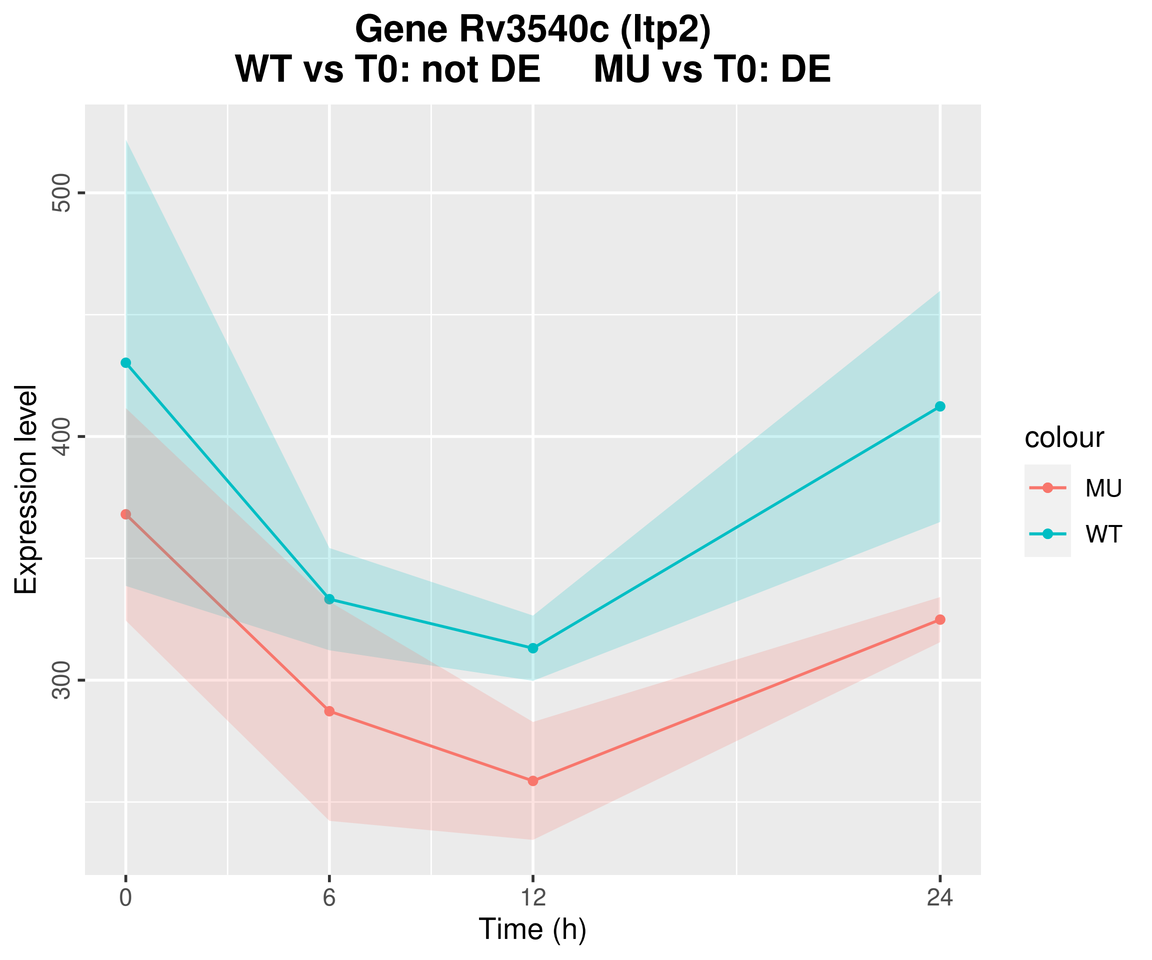
Gene Rv3540c (ltp2)
WT vs T0: not DE   MU vs T0: DE
500
400
300
Expression level
0
6
12
24
Time (h)
colour
MU
WT

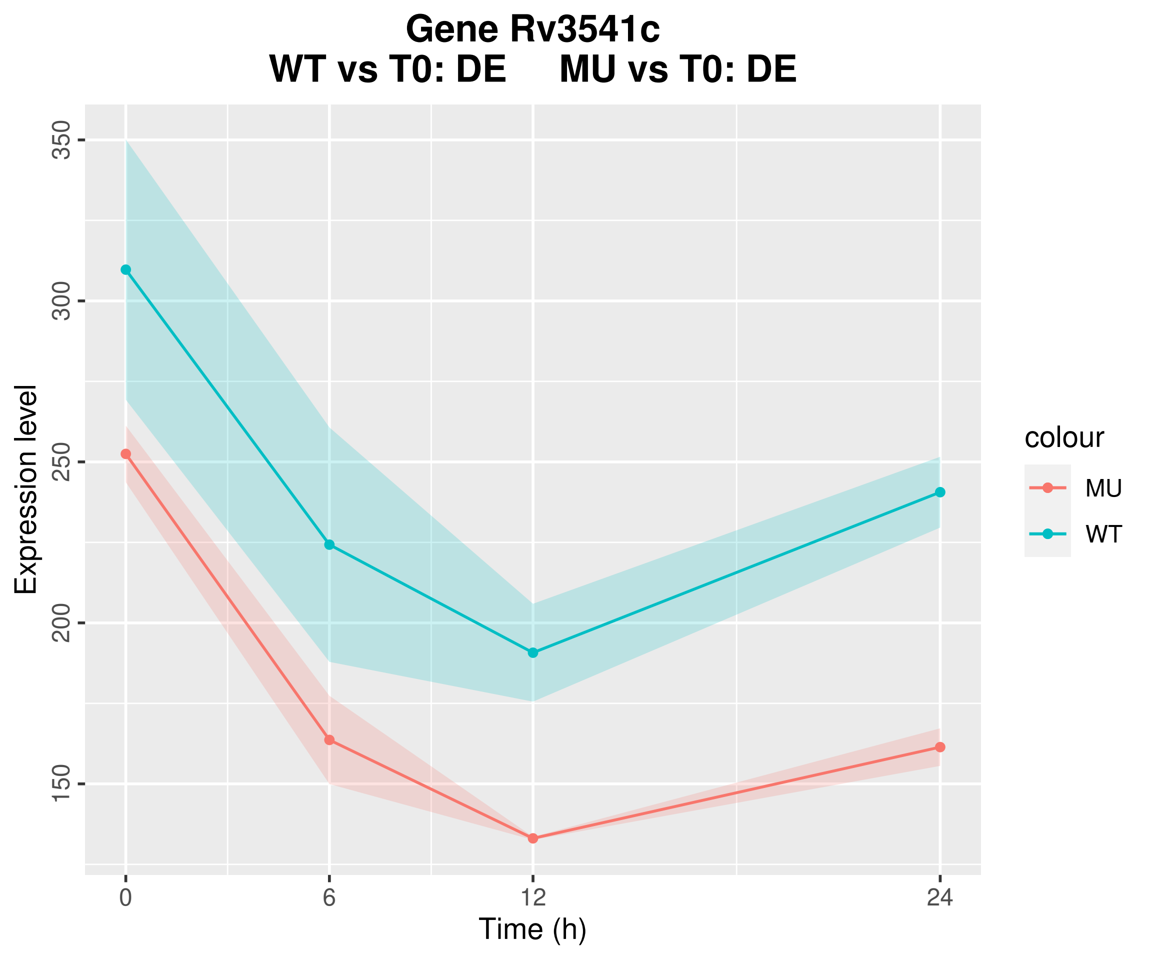
Gene Rv3541c
WT vs T0: DE   MU vs T0: DE
Expression level
350
300
250
200
150
0
6
12
24
Time (h)
colour
MU
WT

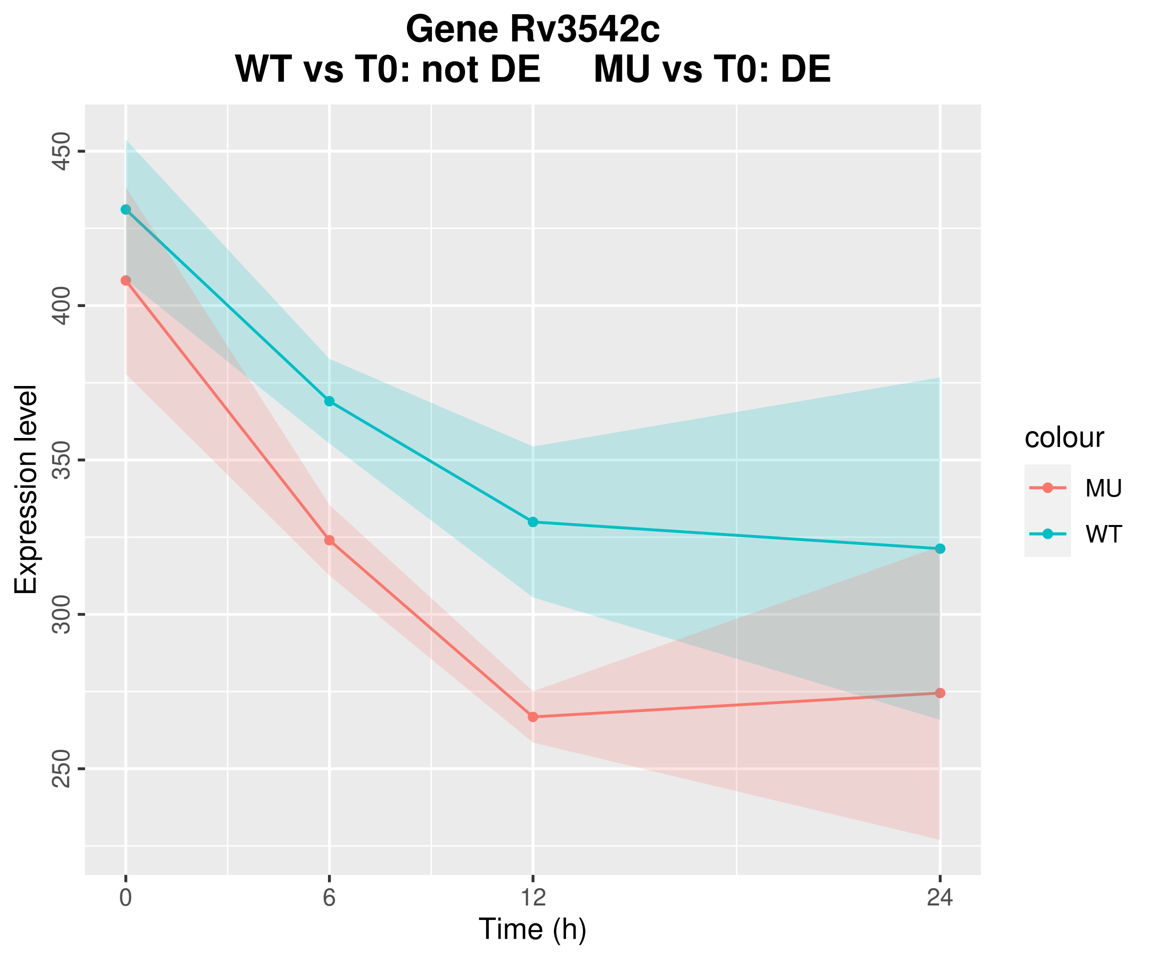
Gene Rv3542c
WT vs T0: not DE MU vs T0: DE
Expression level
450
400
350
300
250
0
6
12
24
Time (h)
colour
MU
WT

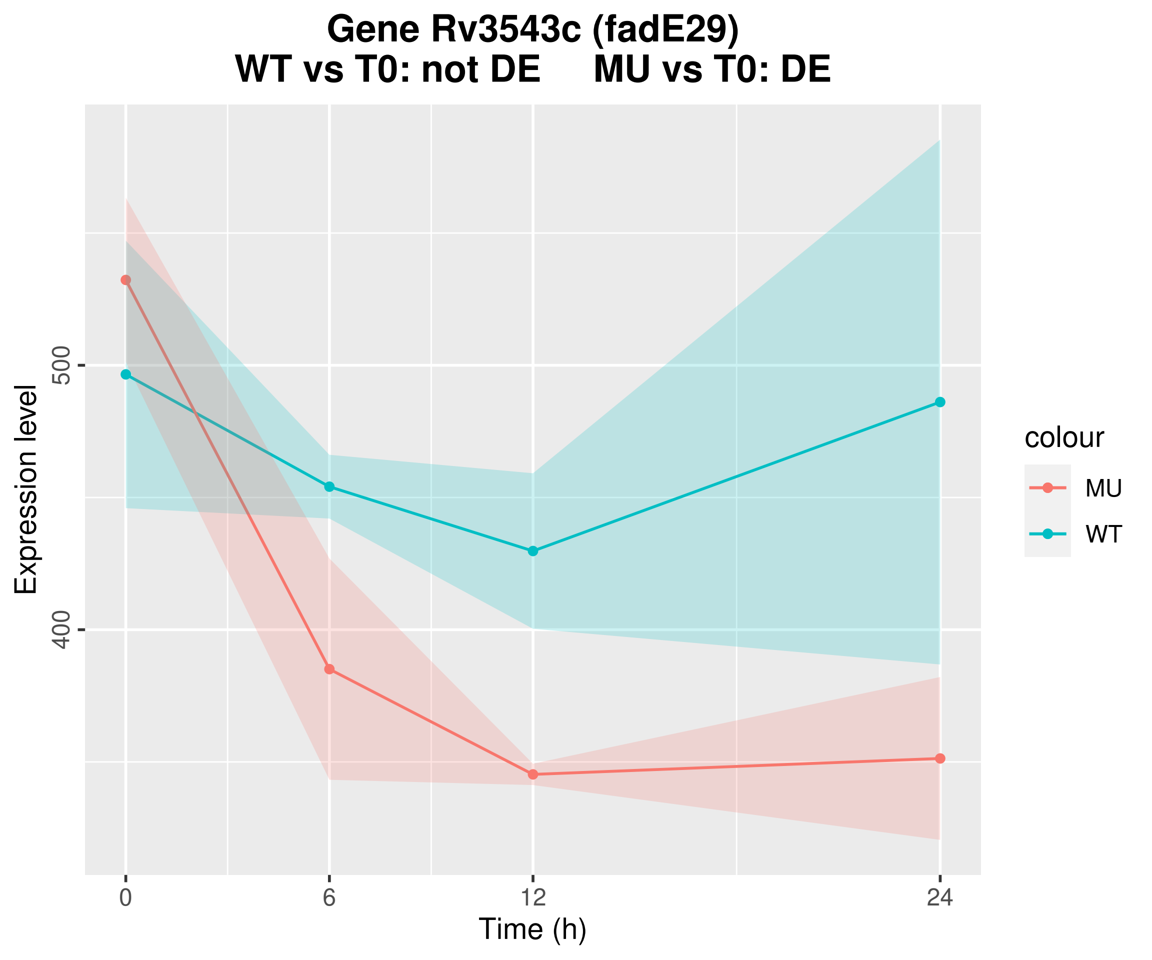

Gene Rv3543c (fadE29)
WT vs T0: not DE MU vs T0: DE
Expression level
500
400
0
6
12
24
Time (h)
colour
MU
WT

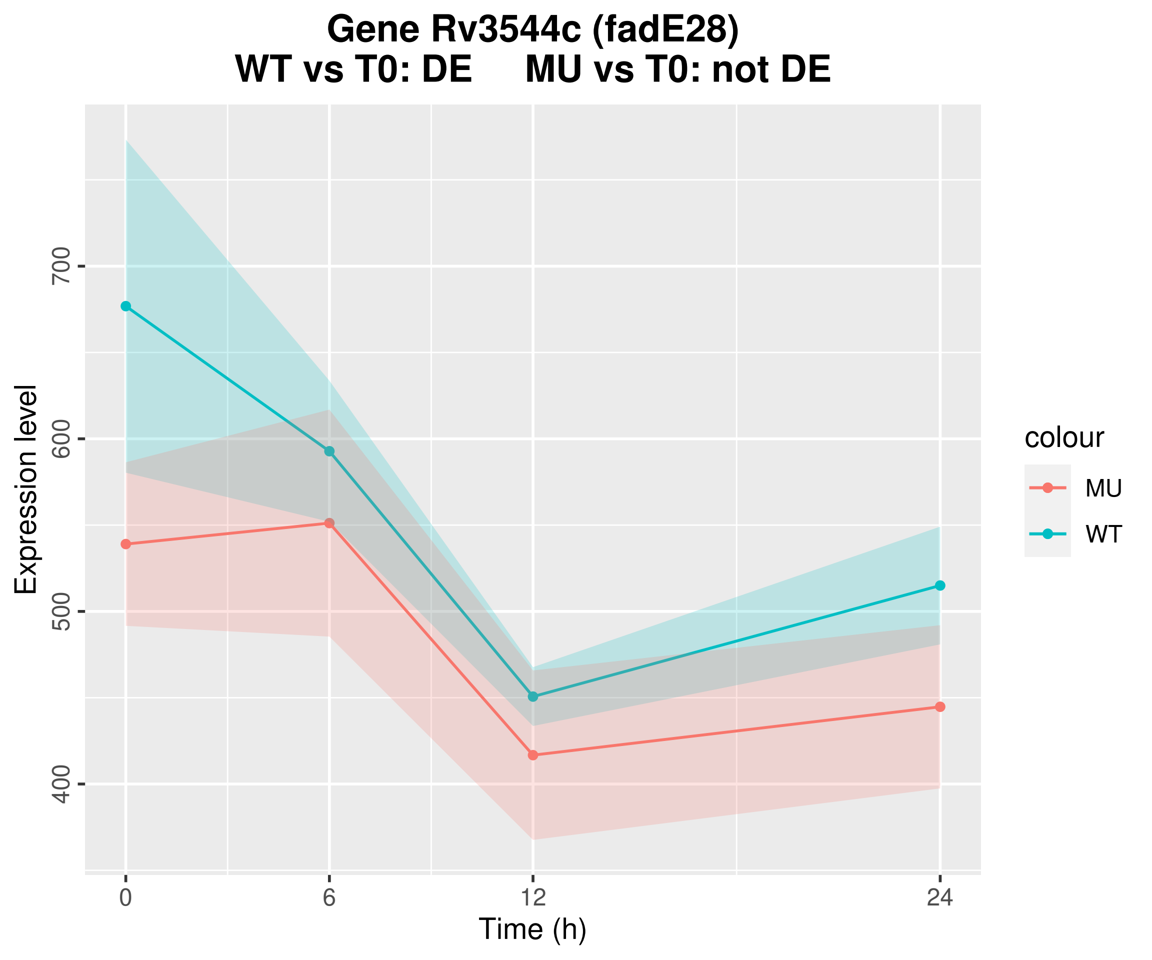

Gene Rv3544c (fadE28)
WT vs T0: DE MU vs T0: not DE
Expression level
700
600
500
400
0
6
12
24
Time (h)
colour
MU
WT

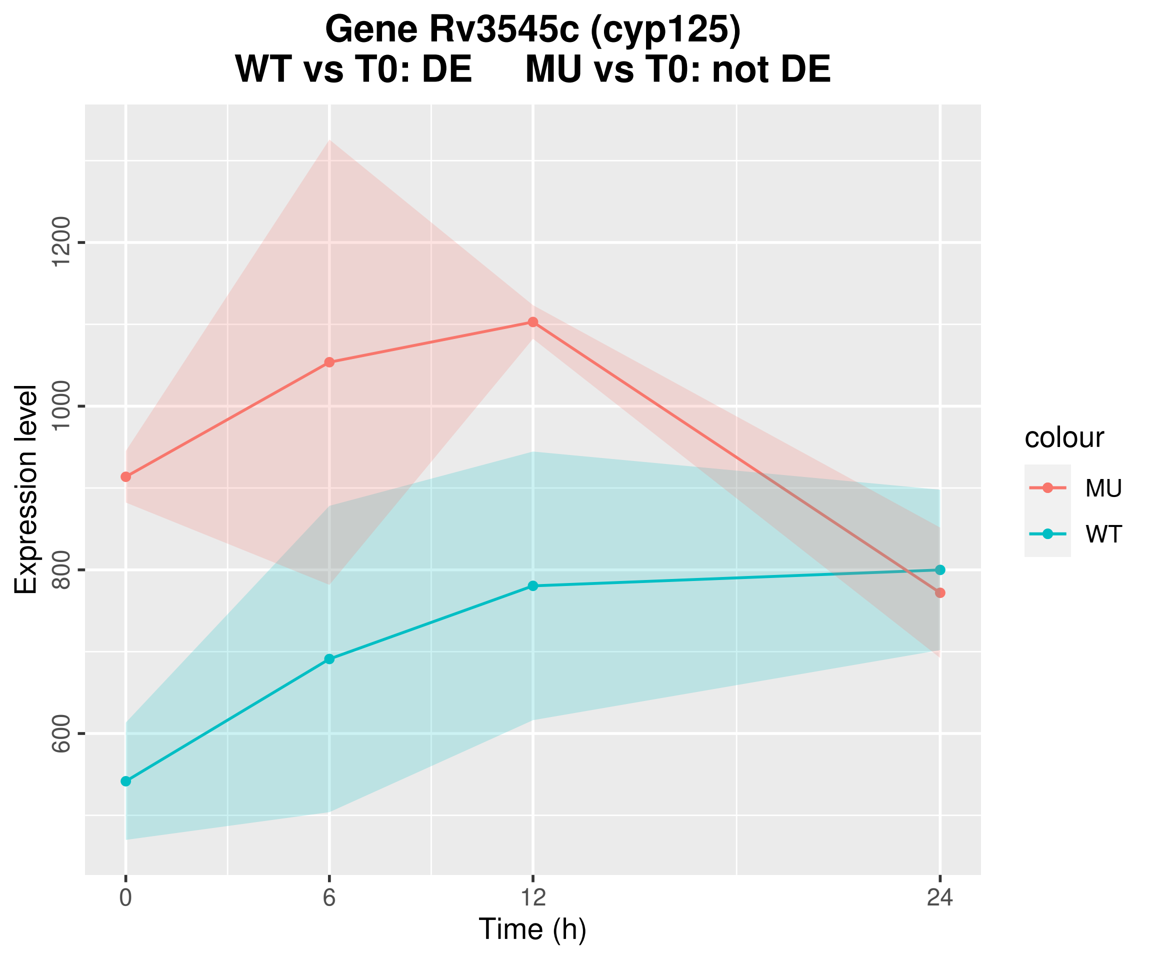

Gene Rv3545c (cyp125)
WT vs T0: DE MU vs T0: not DE
Expression level
1200
1000
800
600
0
6
12
24
Time (h)
colour
MU
WT

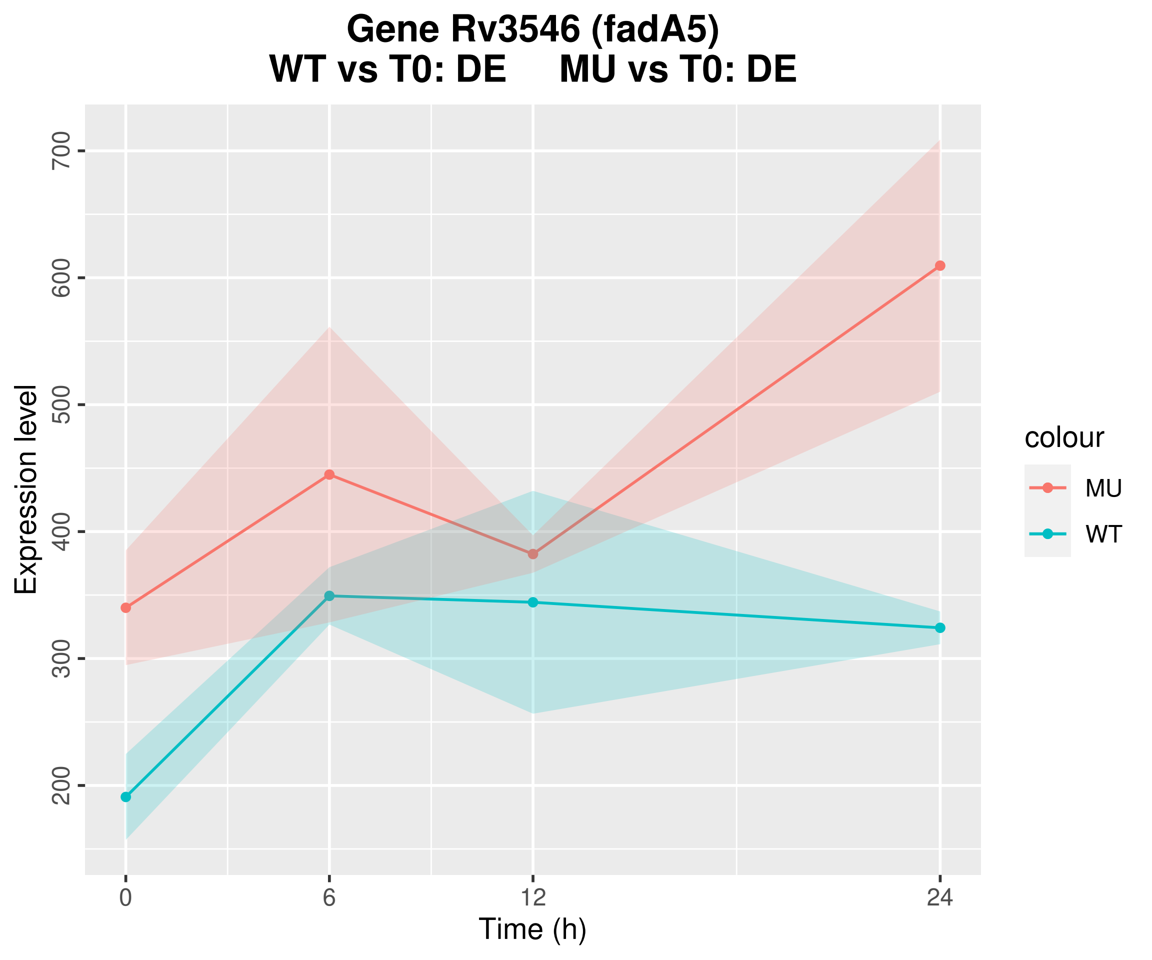

Gene Rv3546 (fadA5)
WT vs T0: DE MU vs T0: DE
Expression level
700
600
500
400
300
200
0
6
12
24
Time (h)
colour
MU
WT

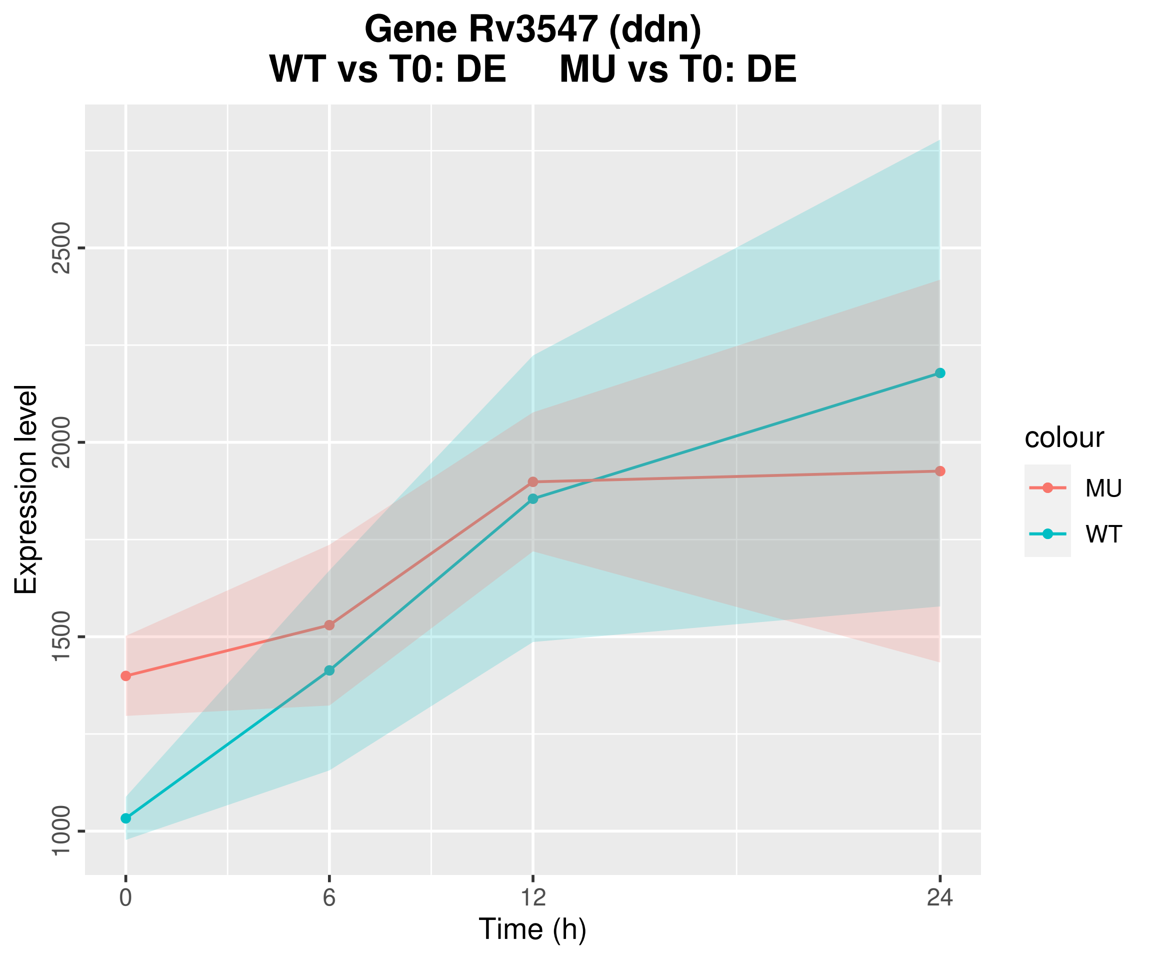
Gene Rv3547 (ddn)
WT vs T0: DE   MU vs T0: DE
Expression level
2500
2000
1500
1000
0
6
12
24
Time (h)
colour
MU
WT

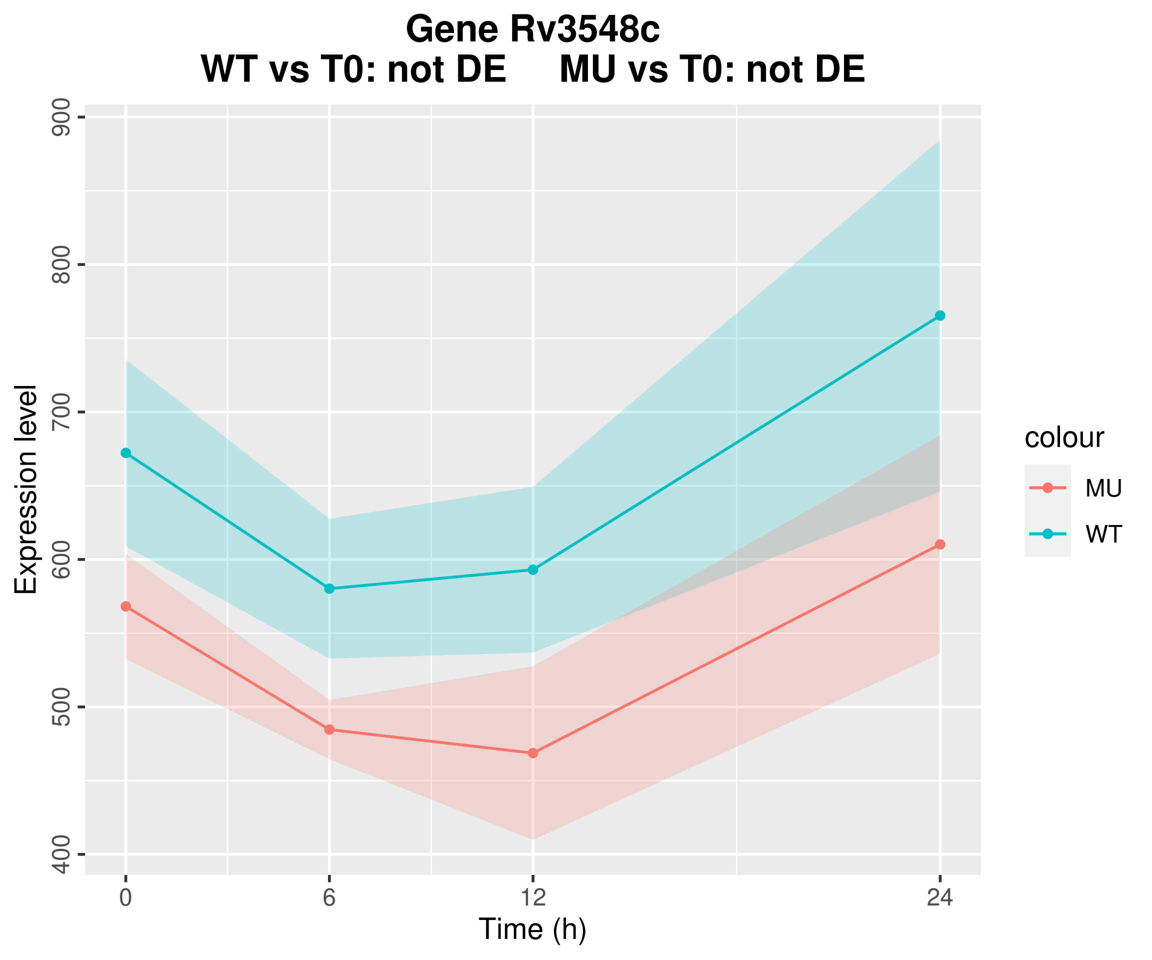

Gene Rv3548c
WT vs T0: not DE MU vs T0: not DE
Expression level
900
800
700
600
500
400
0
6
12
24
Time (h)
colour
MU
WT

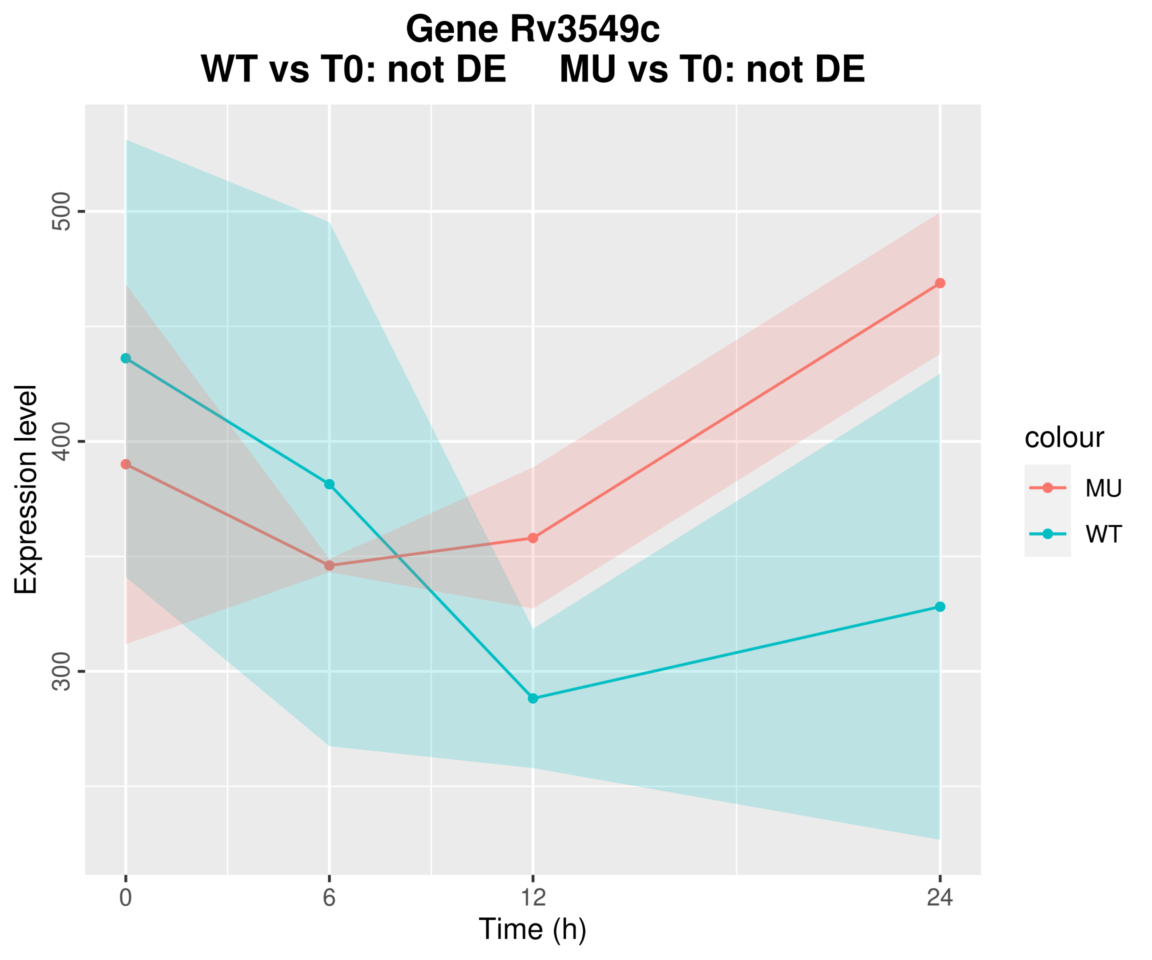
Gene Rv3549c
WT vs T0: not DE   MU vs T0: not DE
Expression level
500
400
300
0
6
12
24
Time (h)
colour
MU
WT

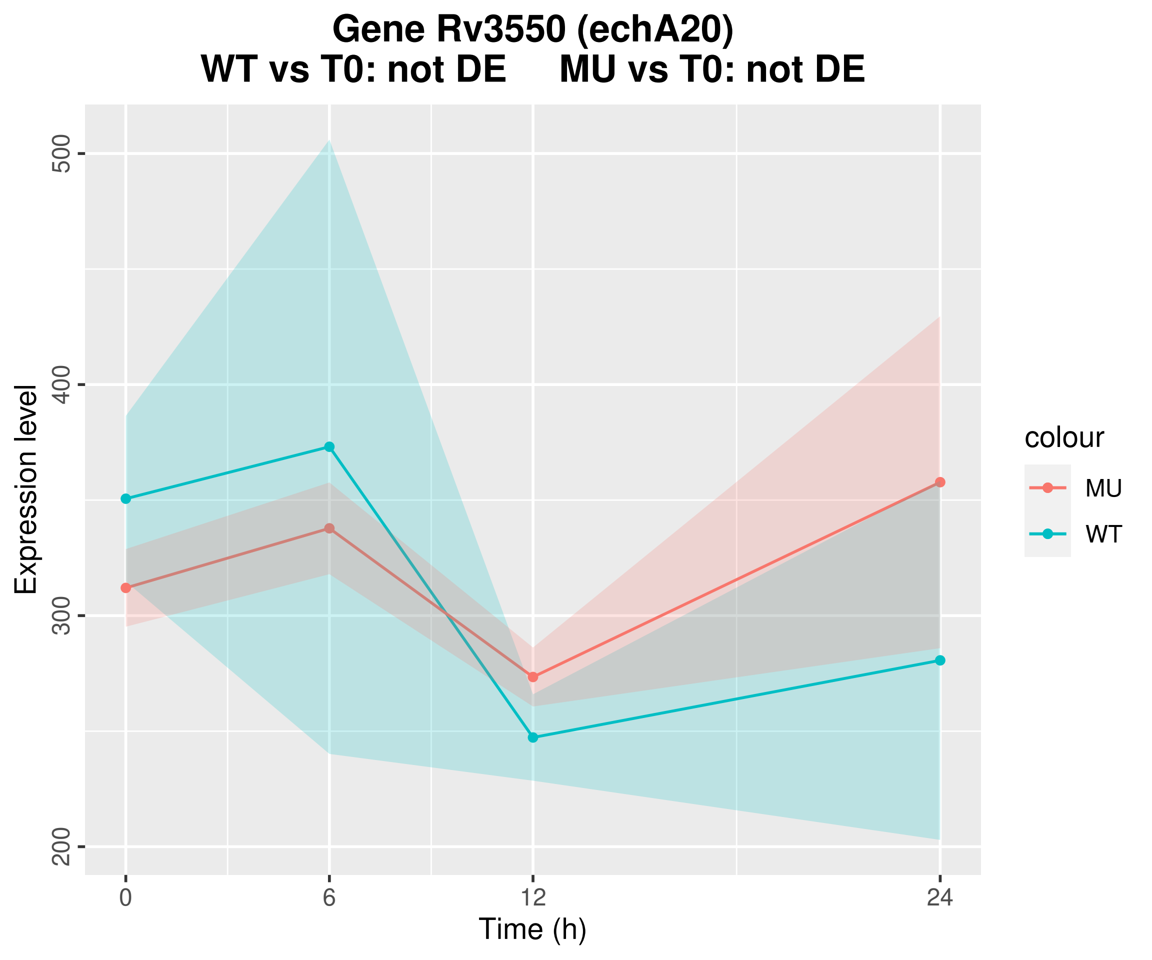

Gene Rv3550 (echA20)
WT vs T0: not DE MU vs T0: not DE
Expression level
500
400
300
200
0
6
12
24
Time (h)
colour
MU
WT

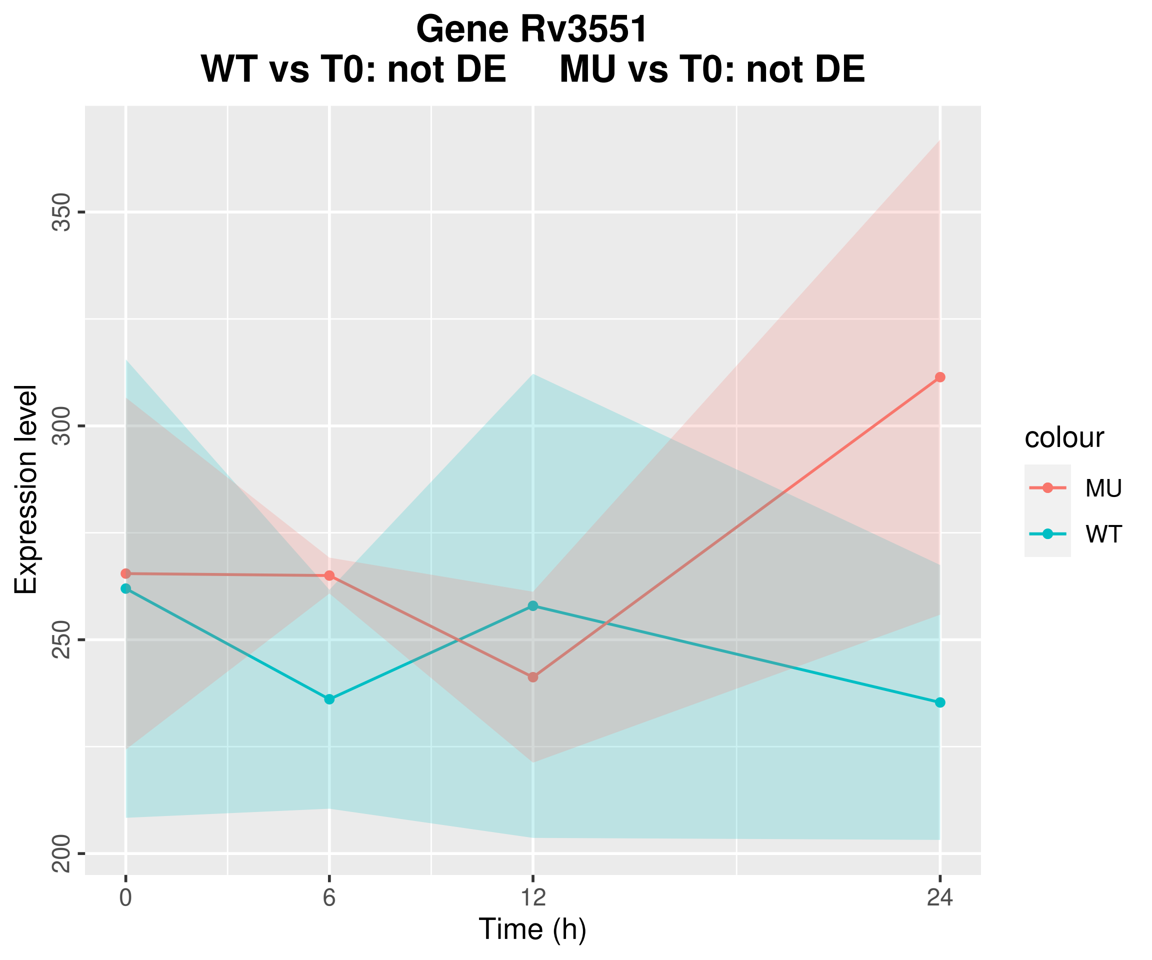

Gene Rv3551
WT vs T0: not DE   MU vs T0: not DE
Expression level
350
300
250
200
0
6
12
24
Time (h)
colour
MU
WT

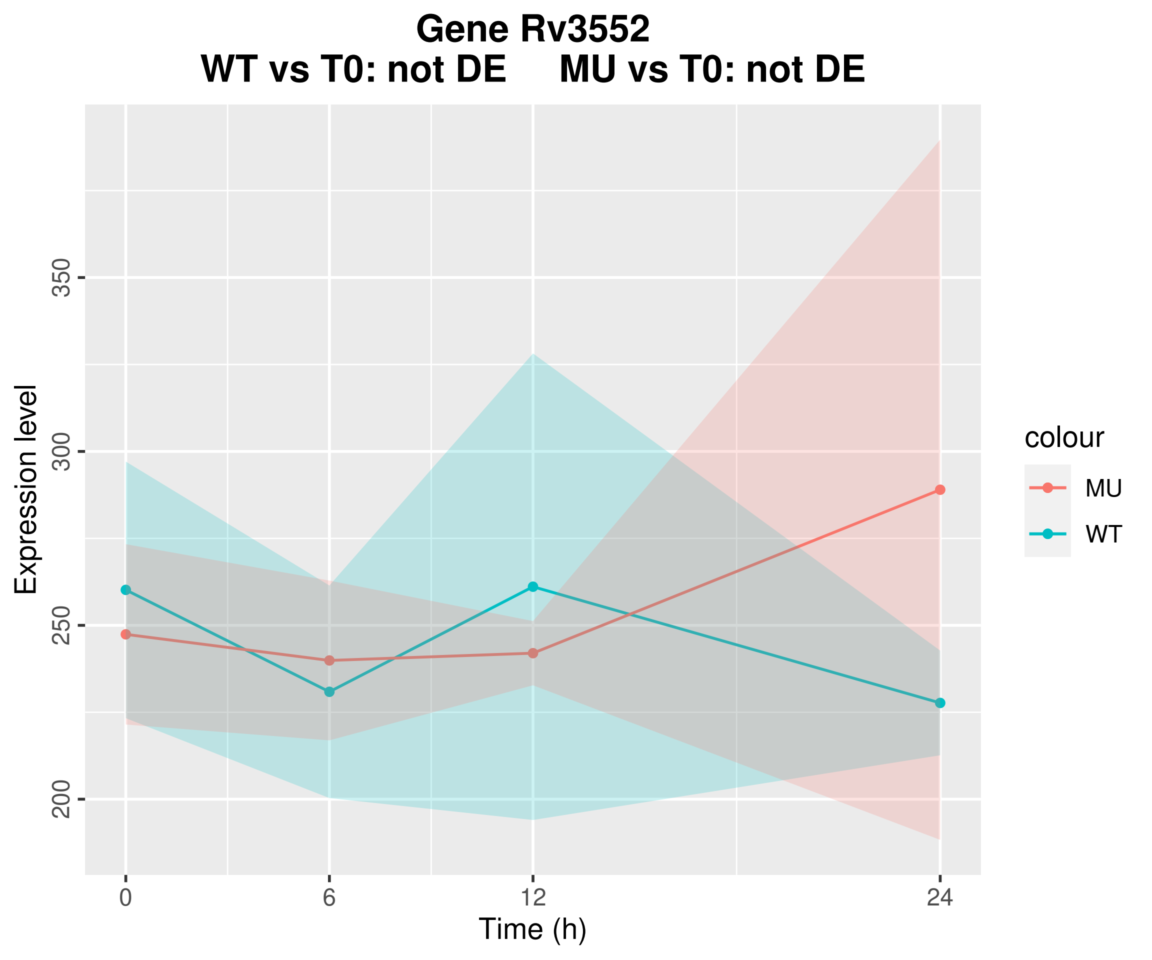
Gene Rv3552
WT vs T0: not DE   MU vs T0: not DE
Expression level
200
250
300
350
Time (h)
0
6
12
24
colour
MU
WT

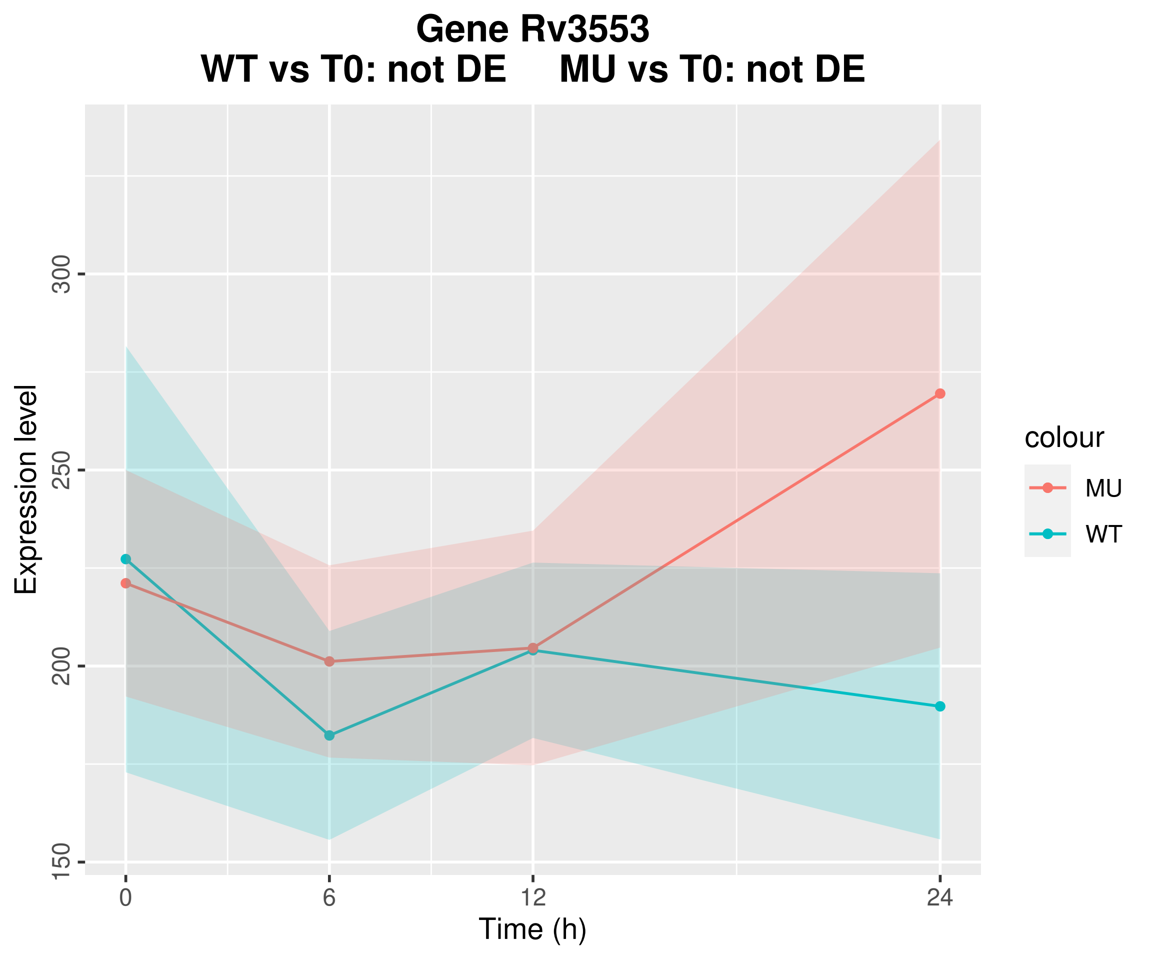

Gene Rv3553
WT vs T0: not DE MU vs T0: not DE
Expression level
300
250
200
150
0
6
12
24
Time (h)
colour
MU
WT

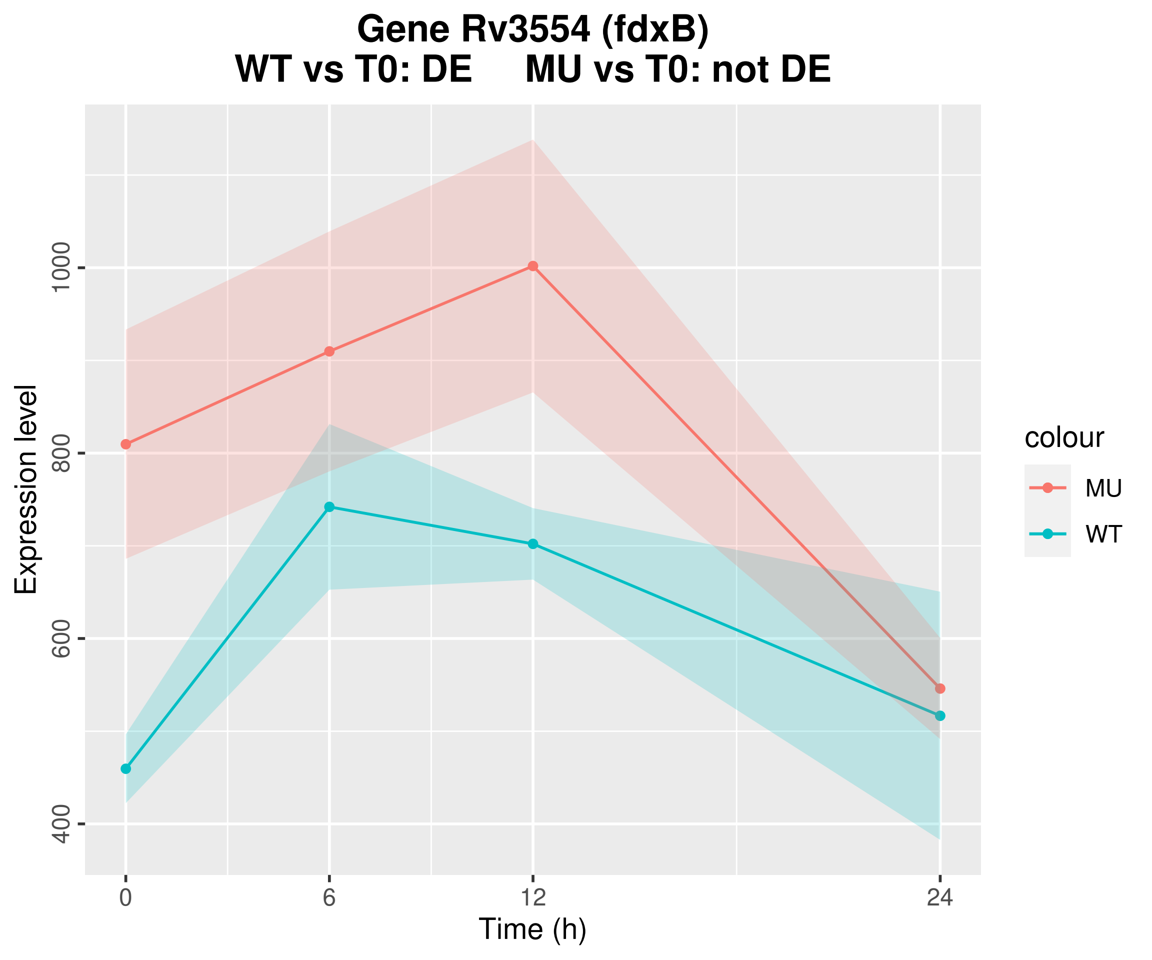

Gene Rv3554 (fdxB)
WT vs T0: DE   MU vs T0: not DE
Expression level
1000
800
600
400
0
6
12
24
Time (h)
colour
MU
WT

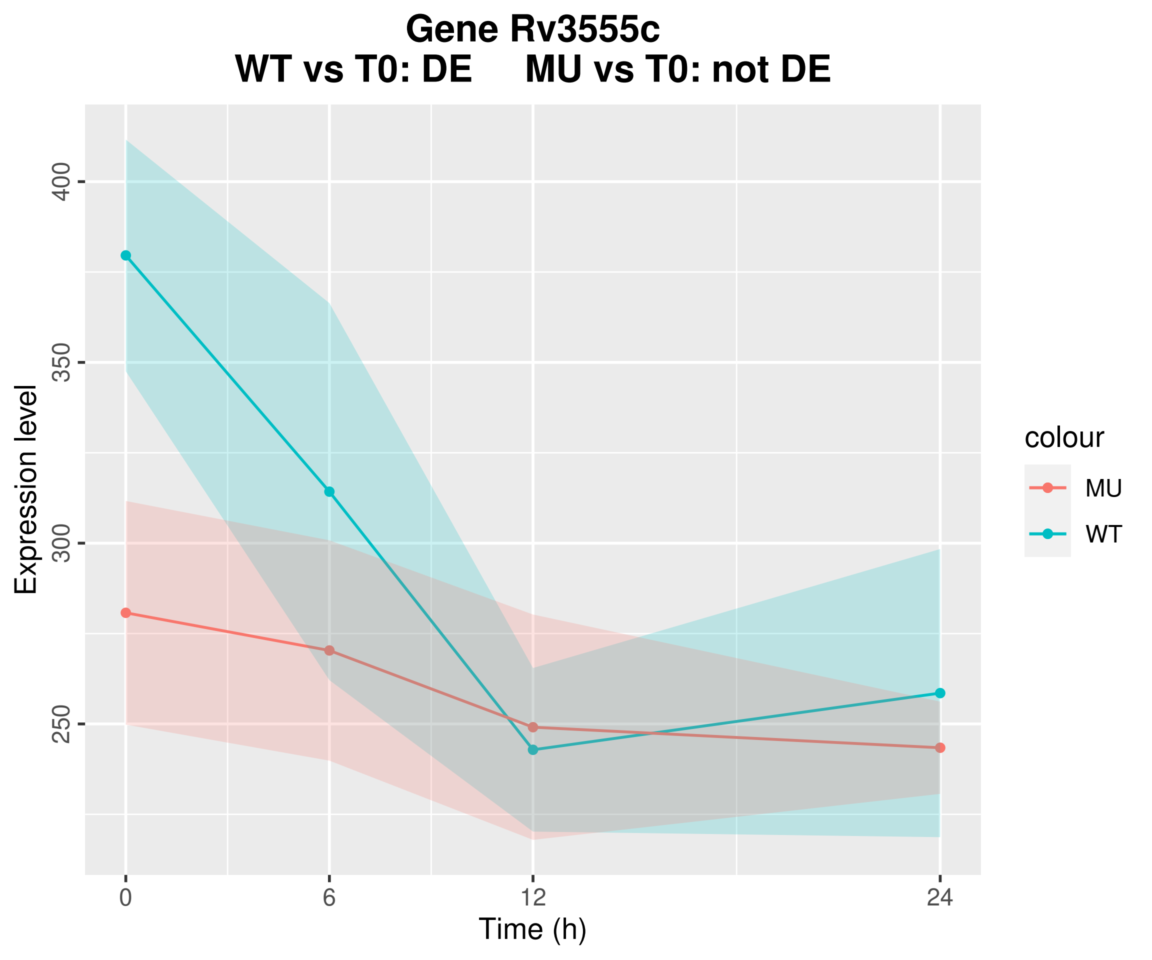

Gene Rv3555c
WT vs T0: DE   MU vs T0: not DE
Expression level
400
350
300
250
0
6
12
24
Time (h)
colour
MU
WT

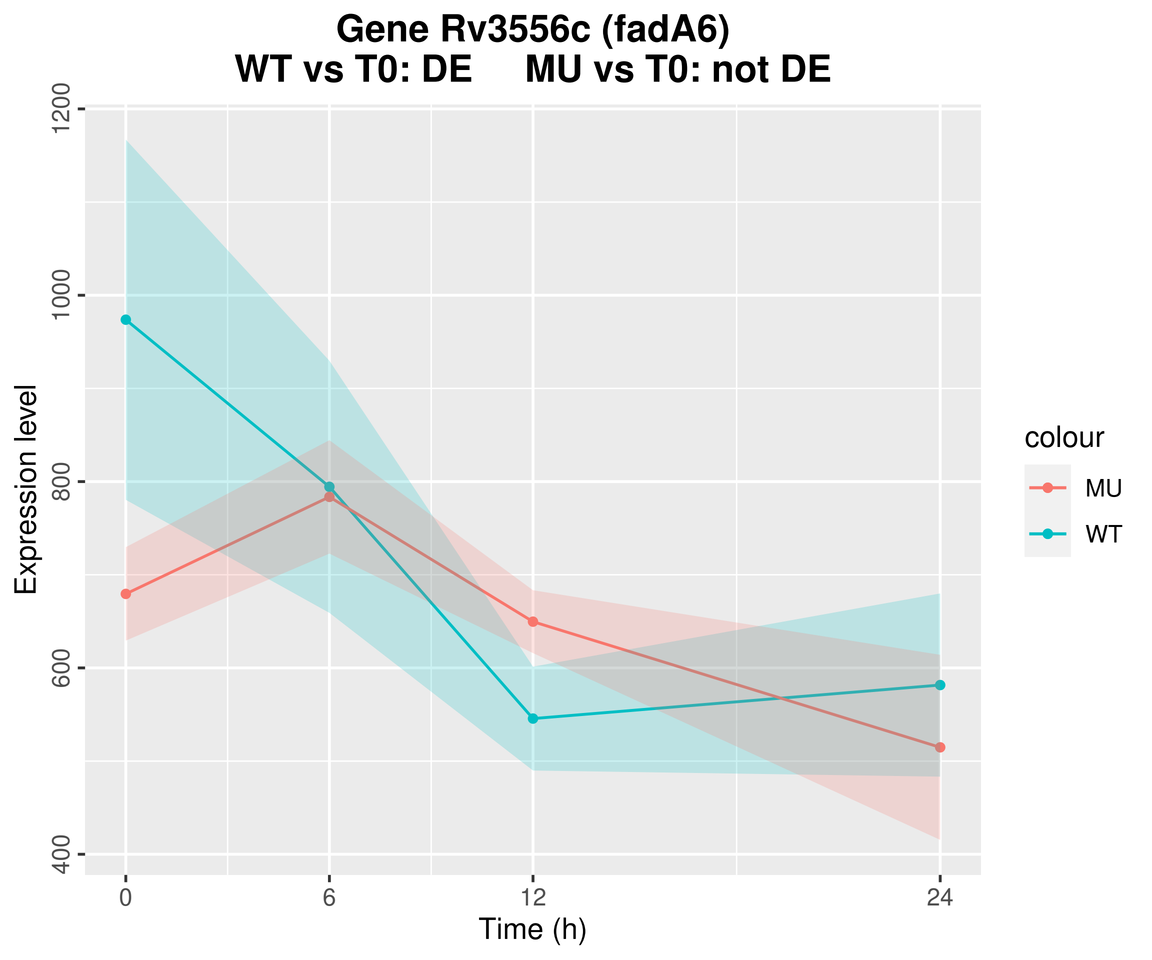

Gene Rv3556c (fadA6)
WT vs T0: DE   MU vs T0: not DE
Expression level
1200
1000
800
600
400
0
6
12
24
Time (h)
colour
MU
WT

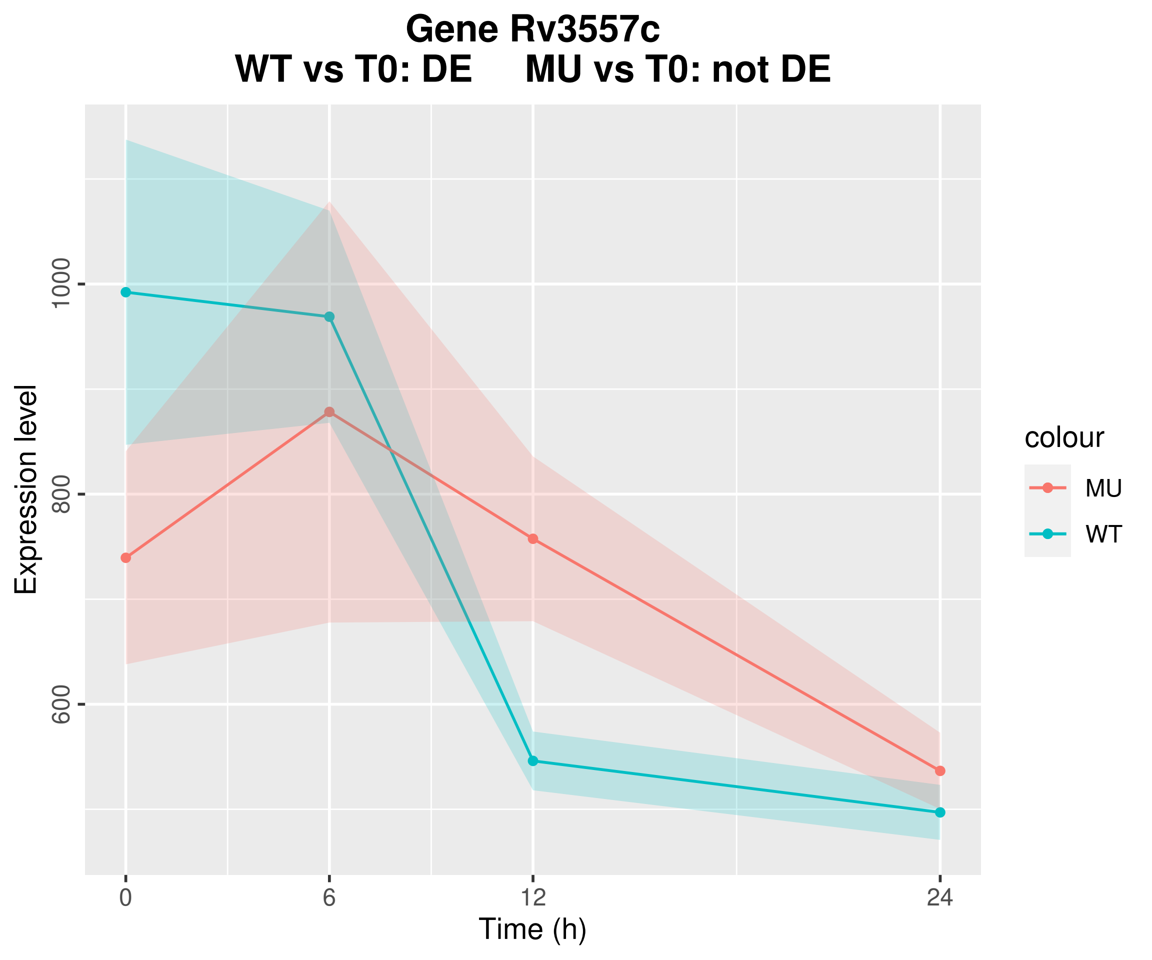
Gene Rv3557c
WT vs T0: DE   MU vs T0: not DE
Expression level
1000
800
600
0
6
12
24
Time (h)
colour
MU
WT

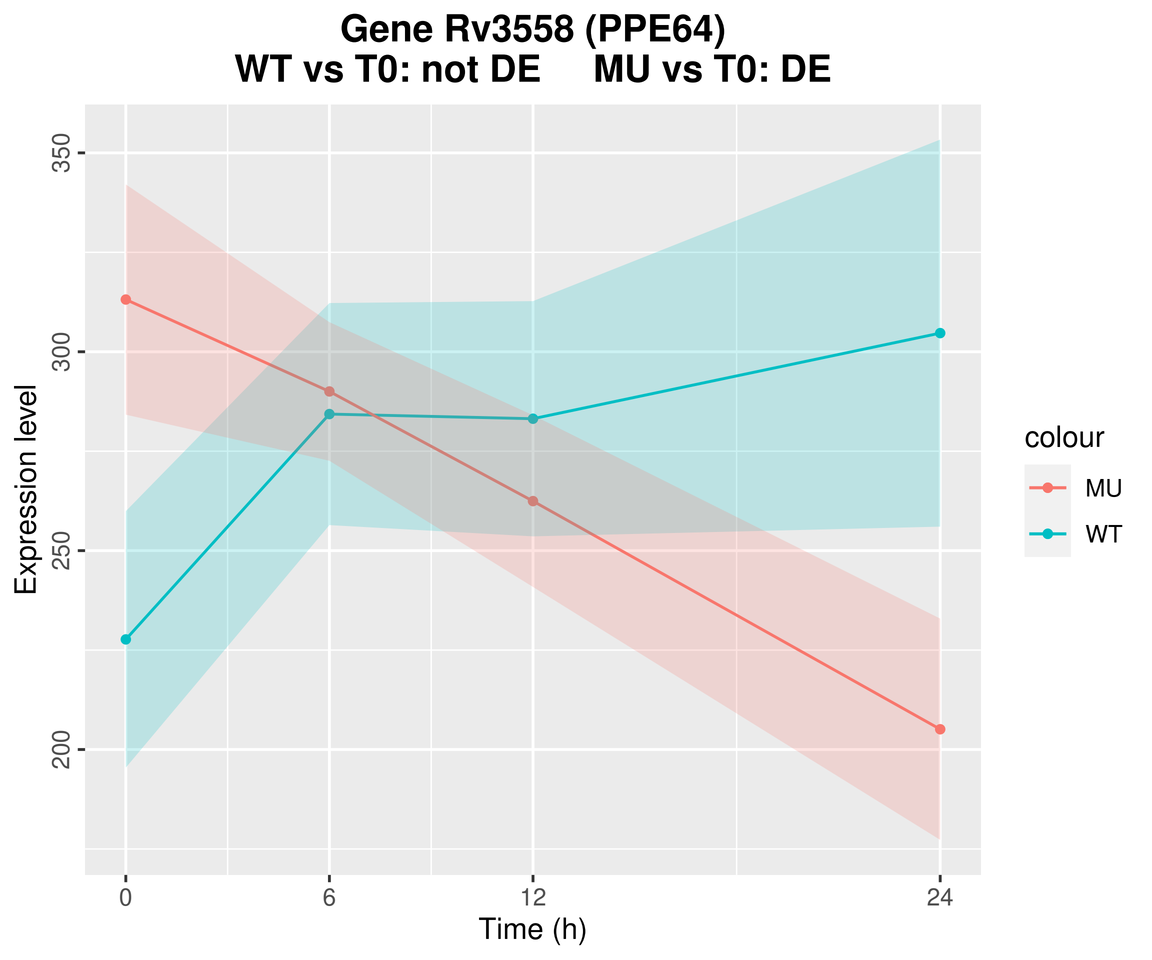

Gene Rv3558 (PPE64)
WT vs T0: not DE MU vs T0: DE
Expression level
350
300
250
200
0
6
12
24
Time (h)
colour
MU
WT

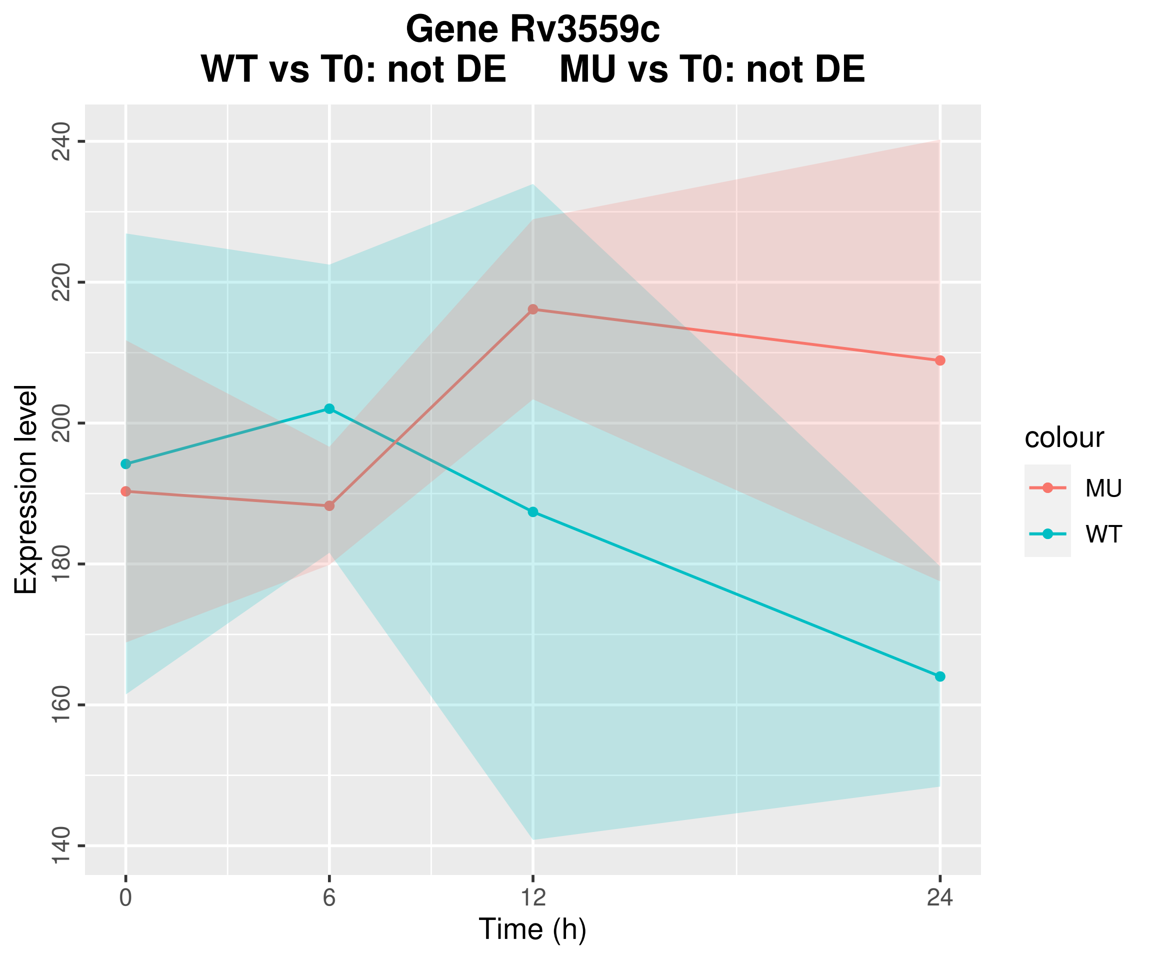
Gene Rv3559c
WT vs T0: not DE   MU vs T0: not DE
Expression level
240
220
200
180
160
140
0
6
12
24
Time (h)
colour
MU
WT

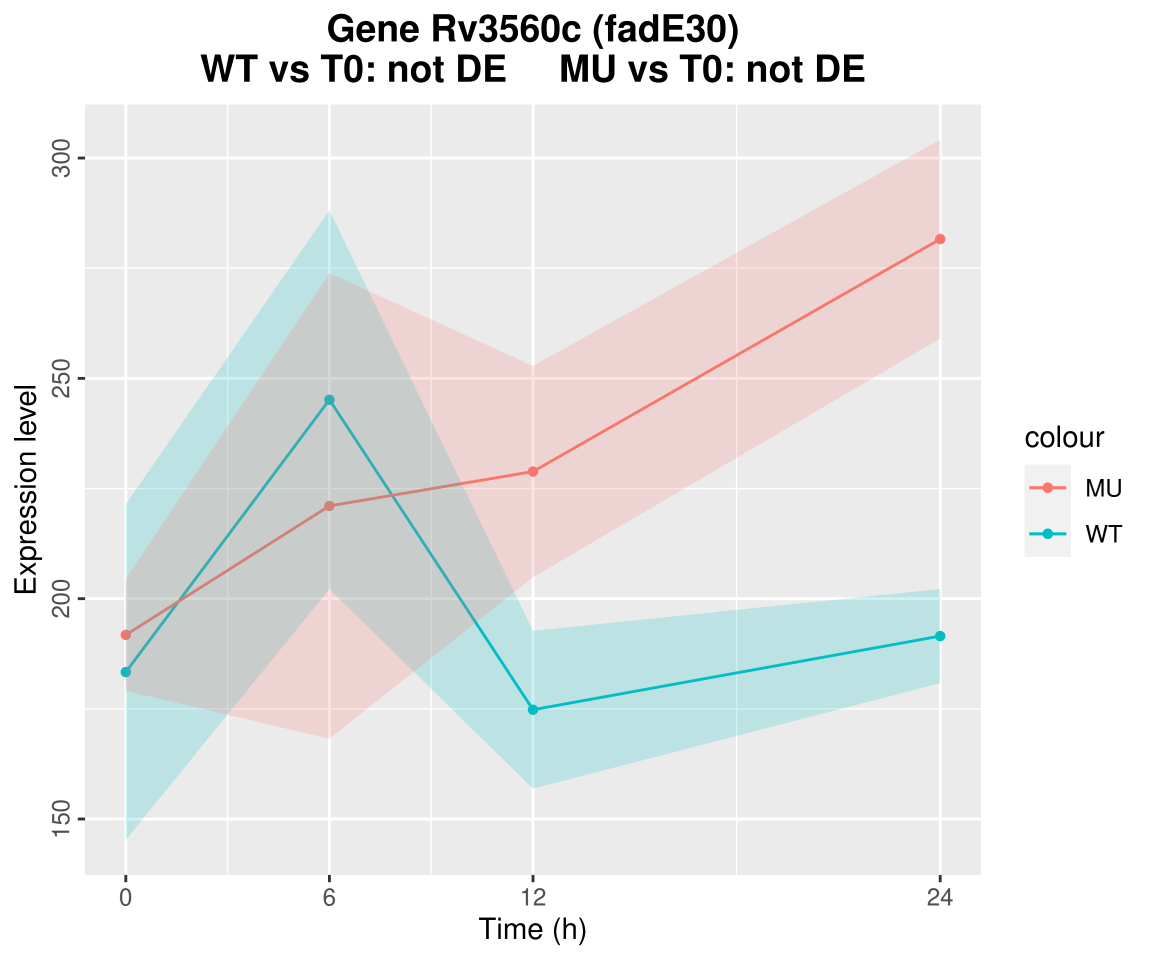
Gene Rv3560c (fadE30)
WT vs T0: not DE MU vs T0: not DE
Expression level
300
250
200
150
0
6
12
24
Time (h)
colour
MU
WT

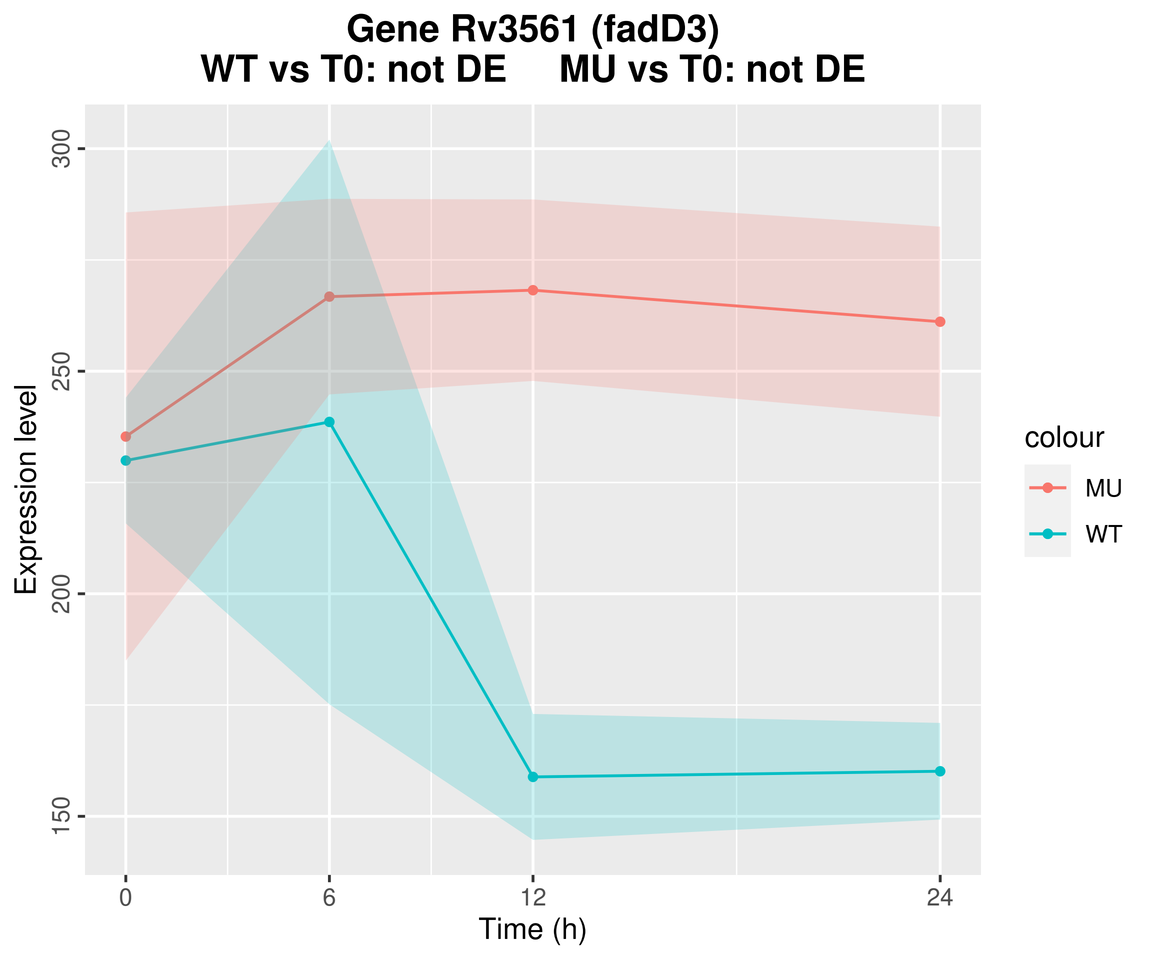
Gene Rv3561 (fadD3)
WT vs T0: not DE MU vs T0: not DE
Expression level
300
250
200
150
0
6
12
24
Time (h)
colour
MU
WT

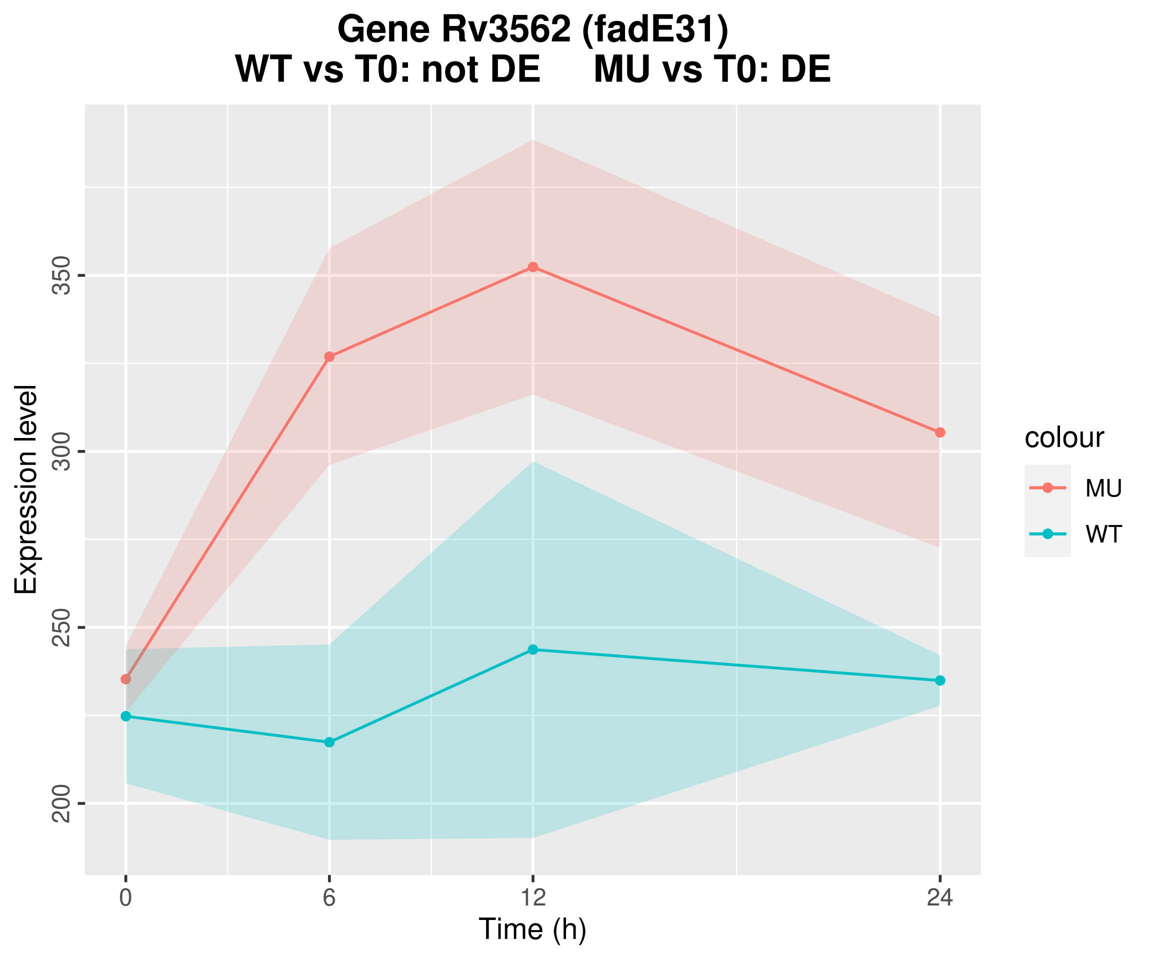

Gene Rv3562 (fadE31)
WT vs T0: not DE MU vs T0: DE
Expression level
200
250
300
350
0
6
12
24
Time (h)
colour
MU
WT

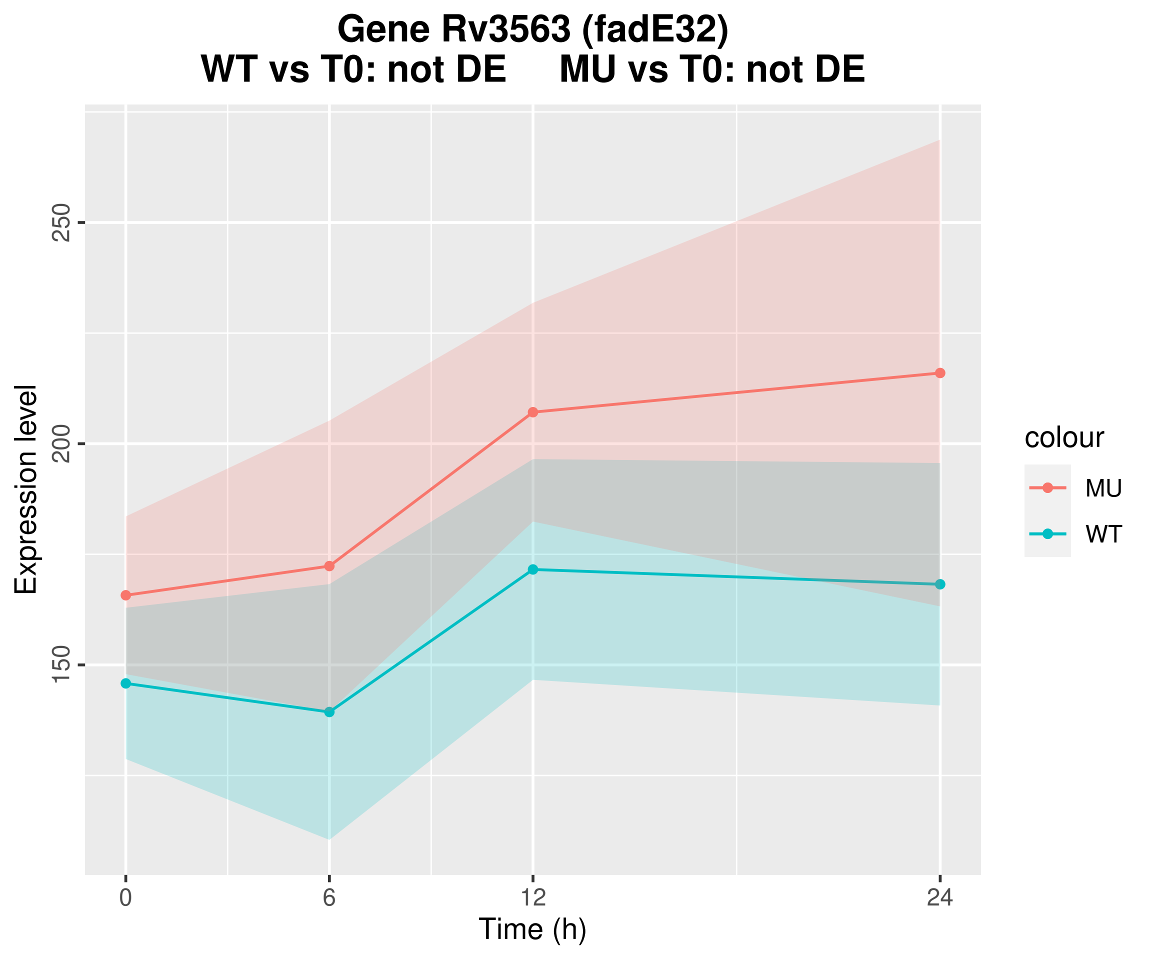
Gene Rv3563 (fadE32)
WT vs T0: not DE   MU vs T0: not DE
Expression level
250
200
150
0
6
12
24
Time (h)
colour
MU
WT

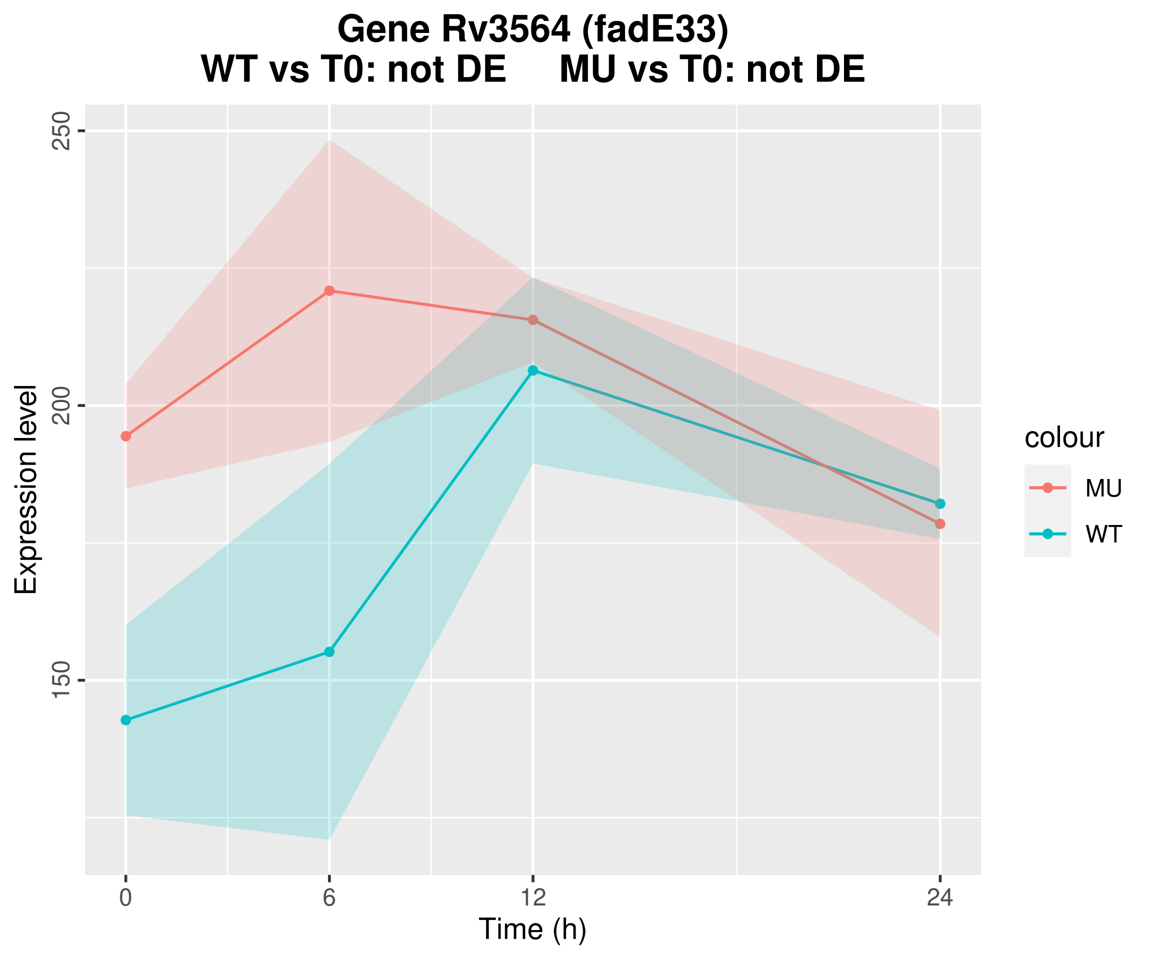
Gene Rv3564 (fadE33)
WT vs T0: not DE   MU vs T0: not DE
Expression level
250
200
150
0
6
12
24
Time (h)
colour
MU
WT

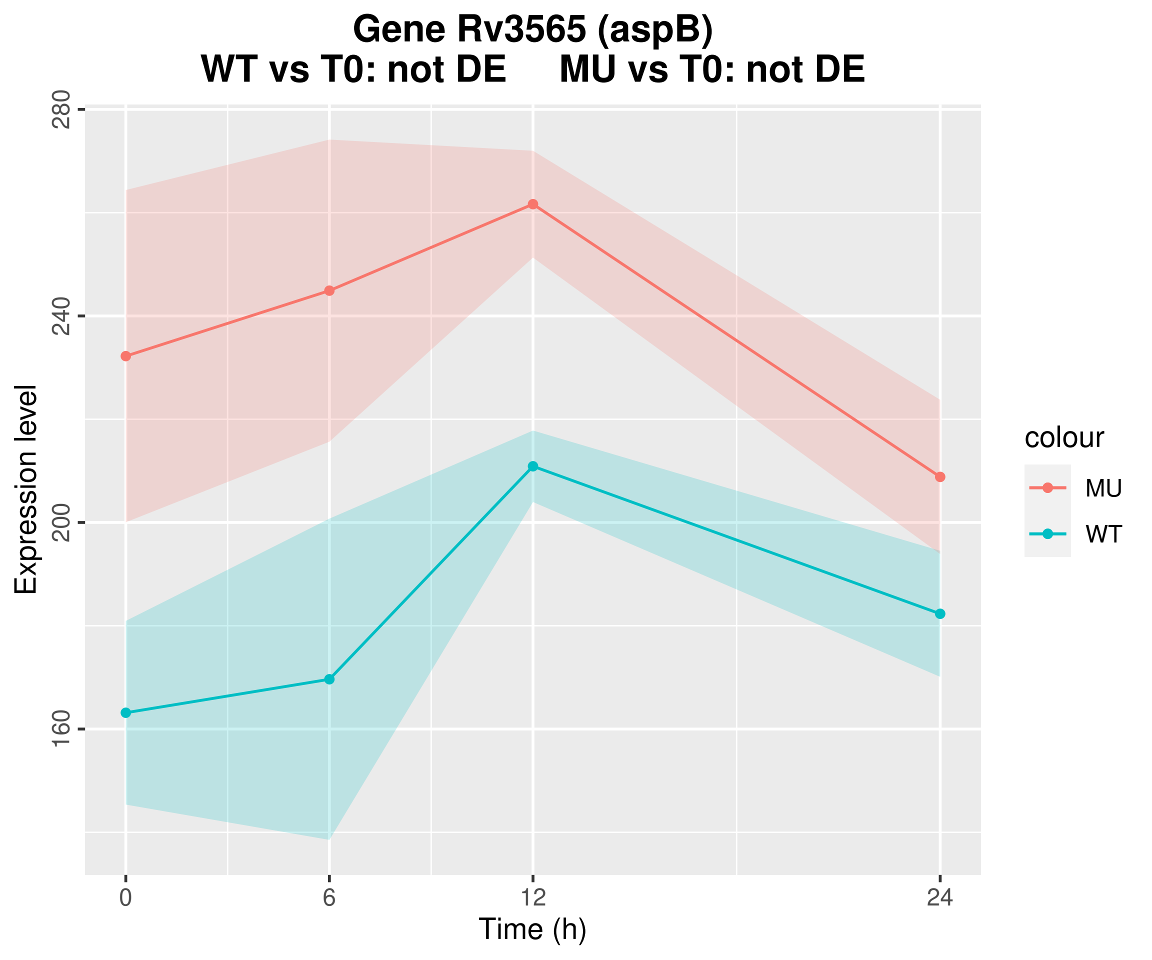

Gene Rv3565 (aspB)
WT vs T0: not DE MU vs T0: not DE
280
240
200
160
Expression level
0
6
12
24
Time (h)
colour
MU
WT

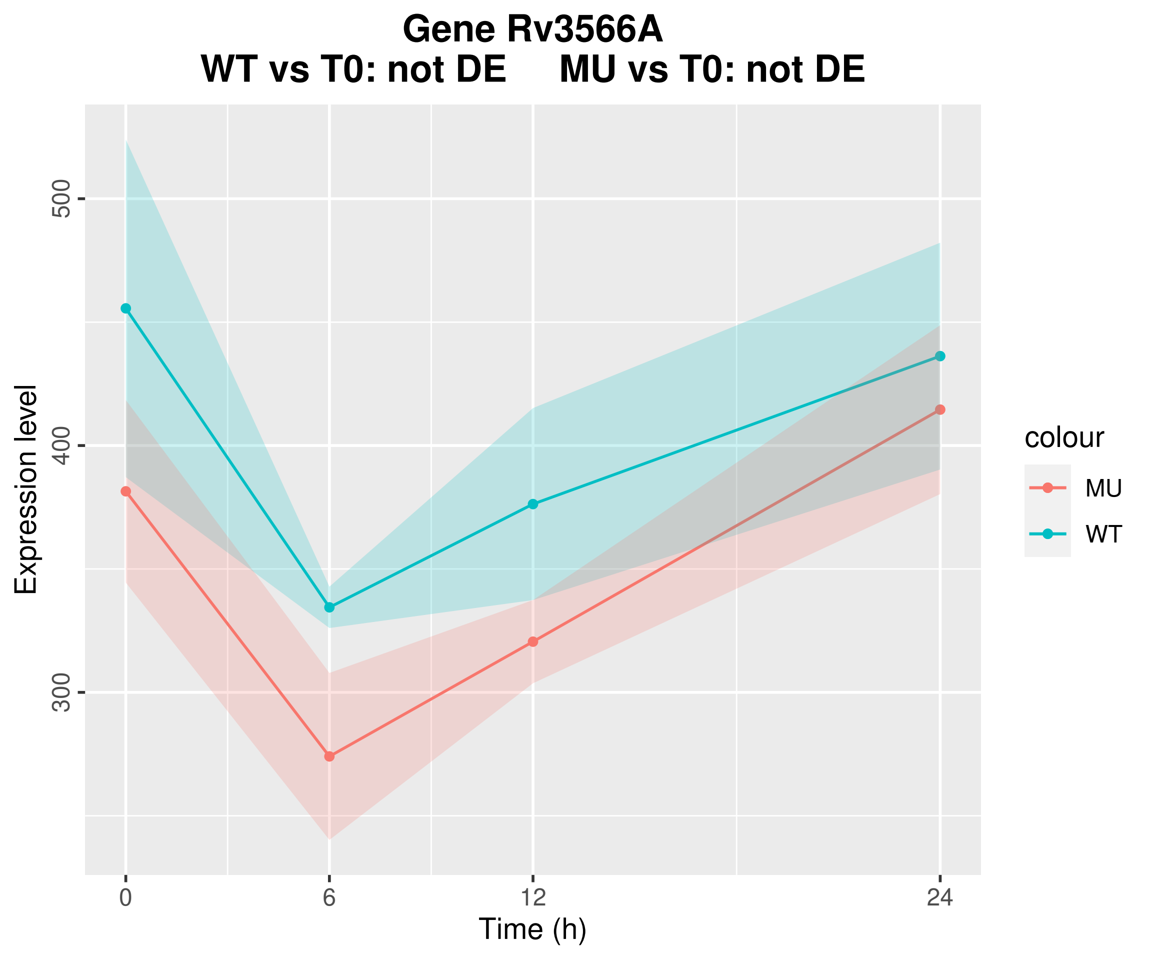

Gene Rv3566A
WT vs T0: not DE MU vs T0: not DE
Expression level
500
400
300
0
6
12
24
Time (h)
colour
MU
WT

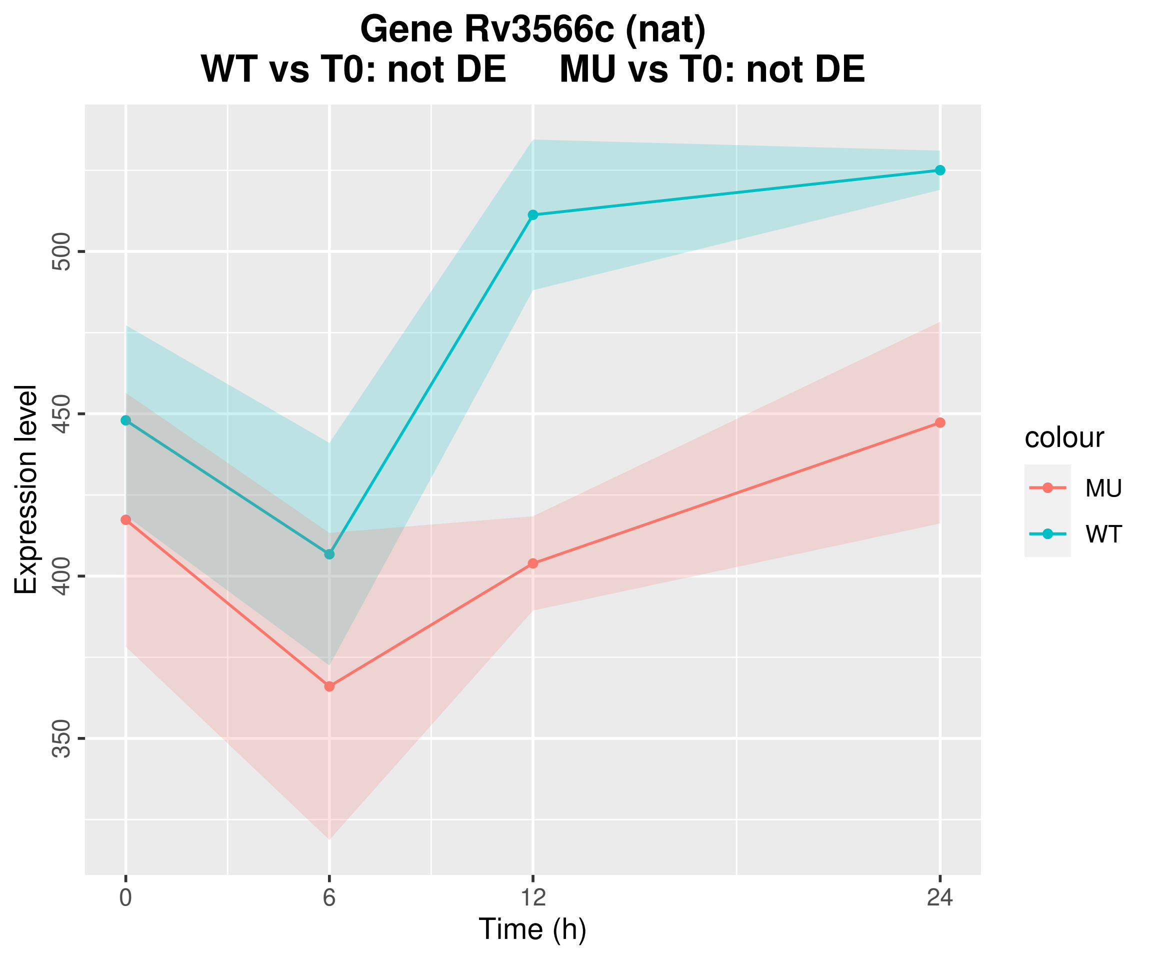
Gene Rv3566c (nat)
WT vs T0: not DE   MU vs T0: not DE
Expression level
350
400
450
500
0
6
12
24
Time (h)
colour
MU
WT

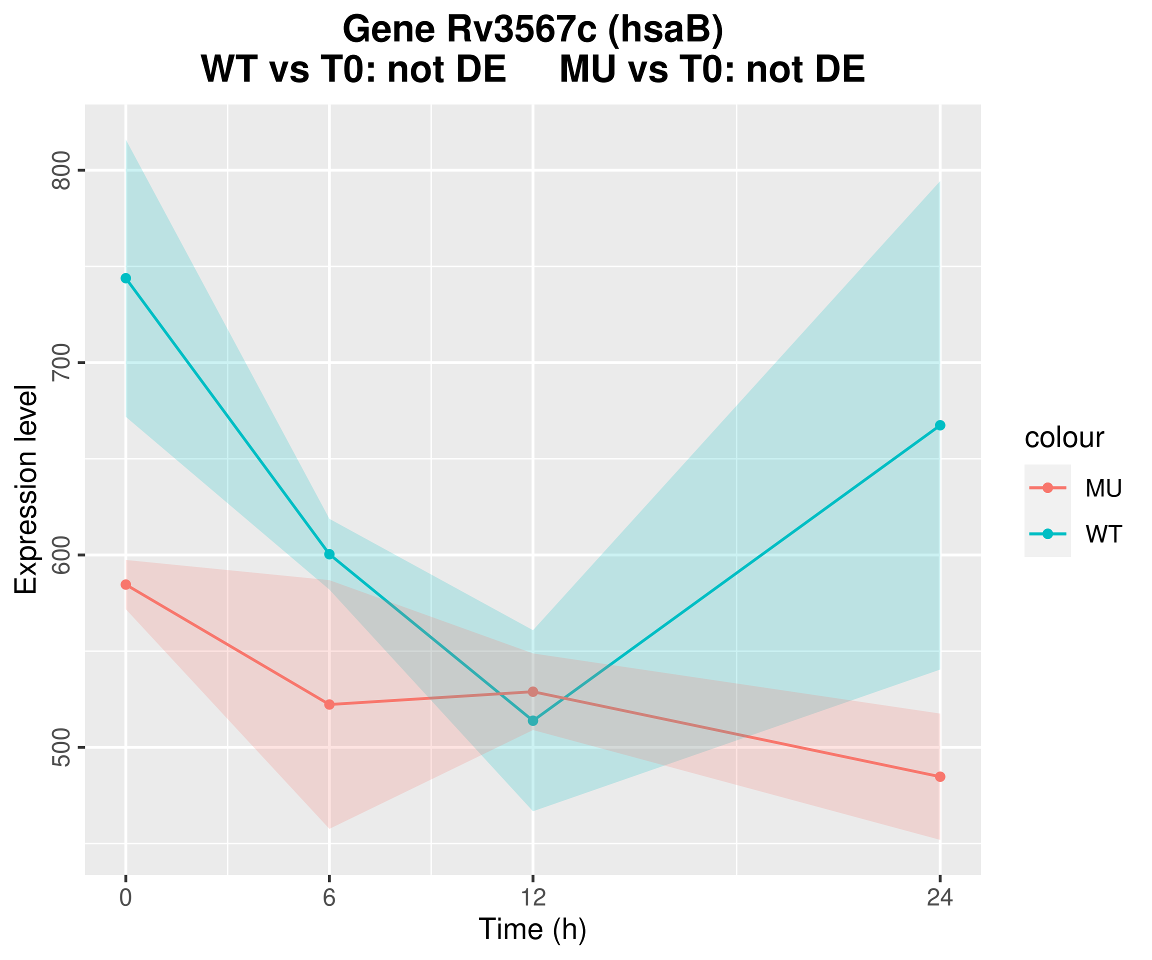

Gene Rv3567c (hsaB)
WT vs T0: not DE   MU vs T0: not DE
800
700
600
500
Expression level
0
6
12
24
Time (h)
colour
MU
WT

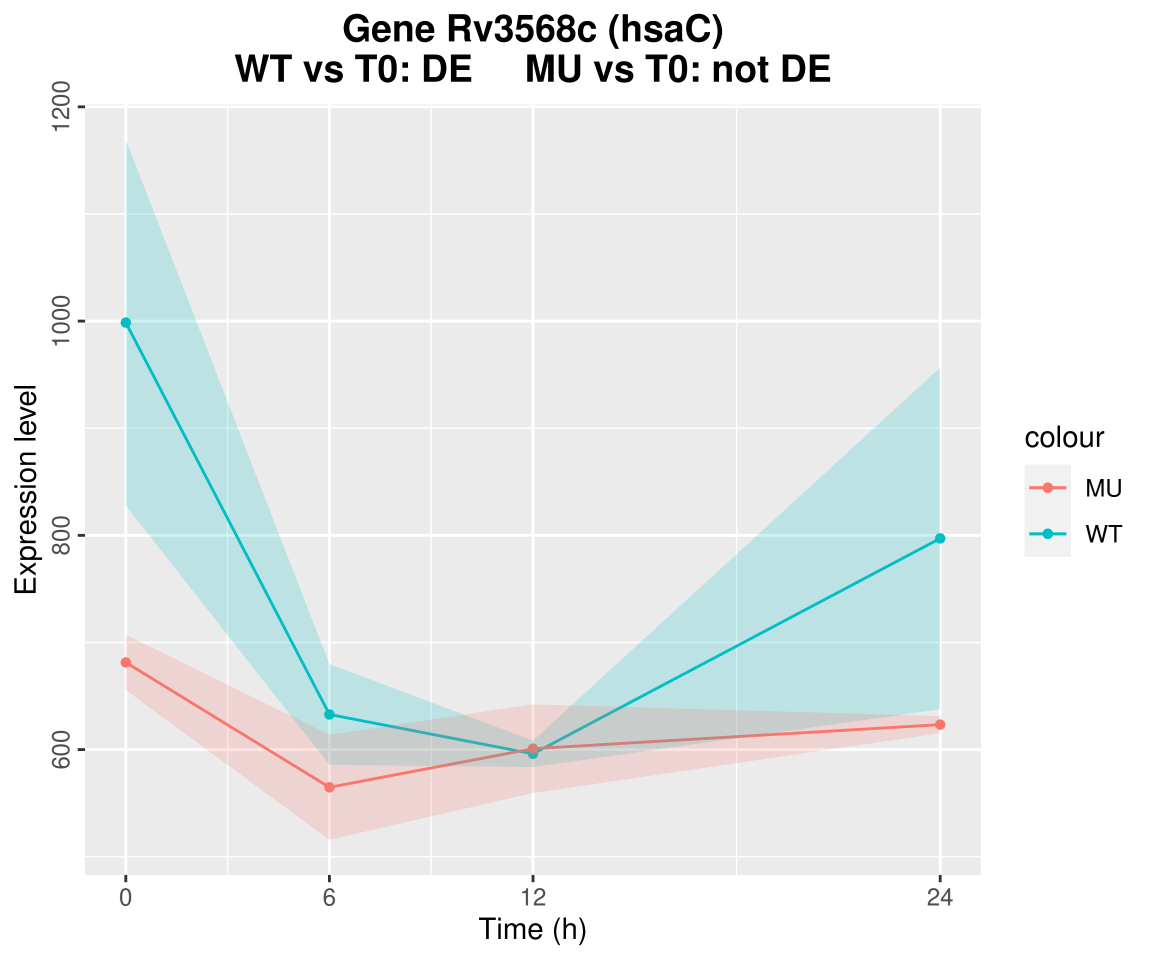
Gene Rv3568c (hsaC)
WT vs T0: DE MU vs T0: not DE
Expression level
1200
1000
800
600
0
6
12
24
Time (h)
colour
MU
WT

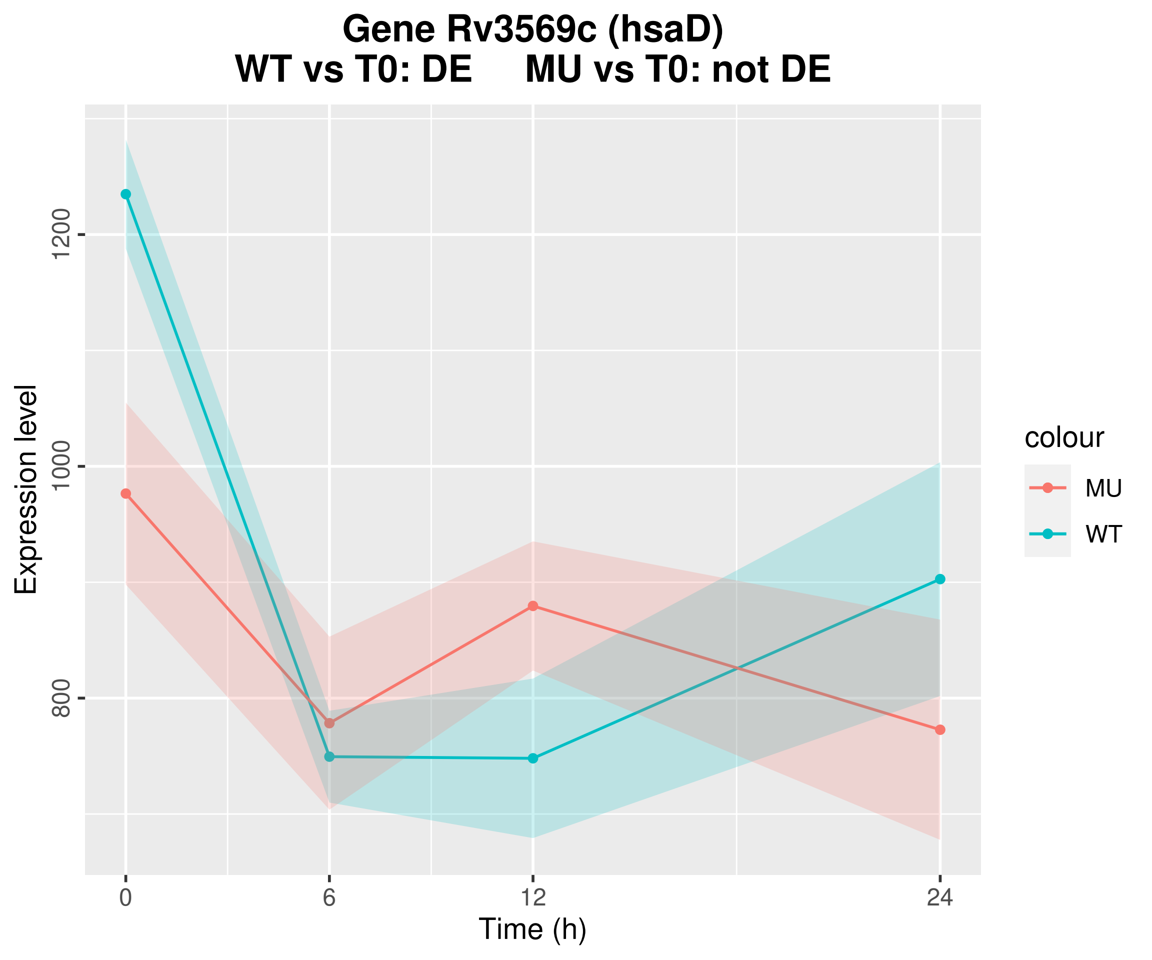

Gene Rv3569c (hsaD)
WT vs T0: DE MU vs T0: not DE
Expression level
1200
1000
800
0
6
12
24
Time (h)
colour
MU
WT

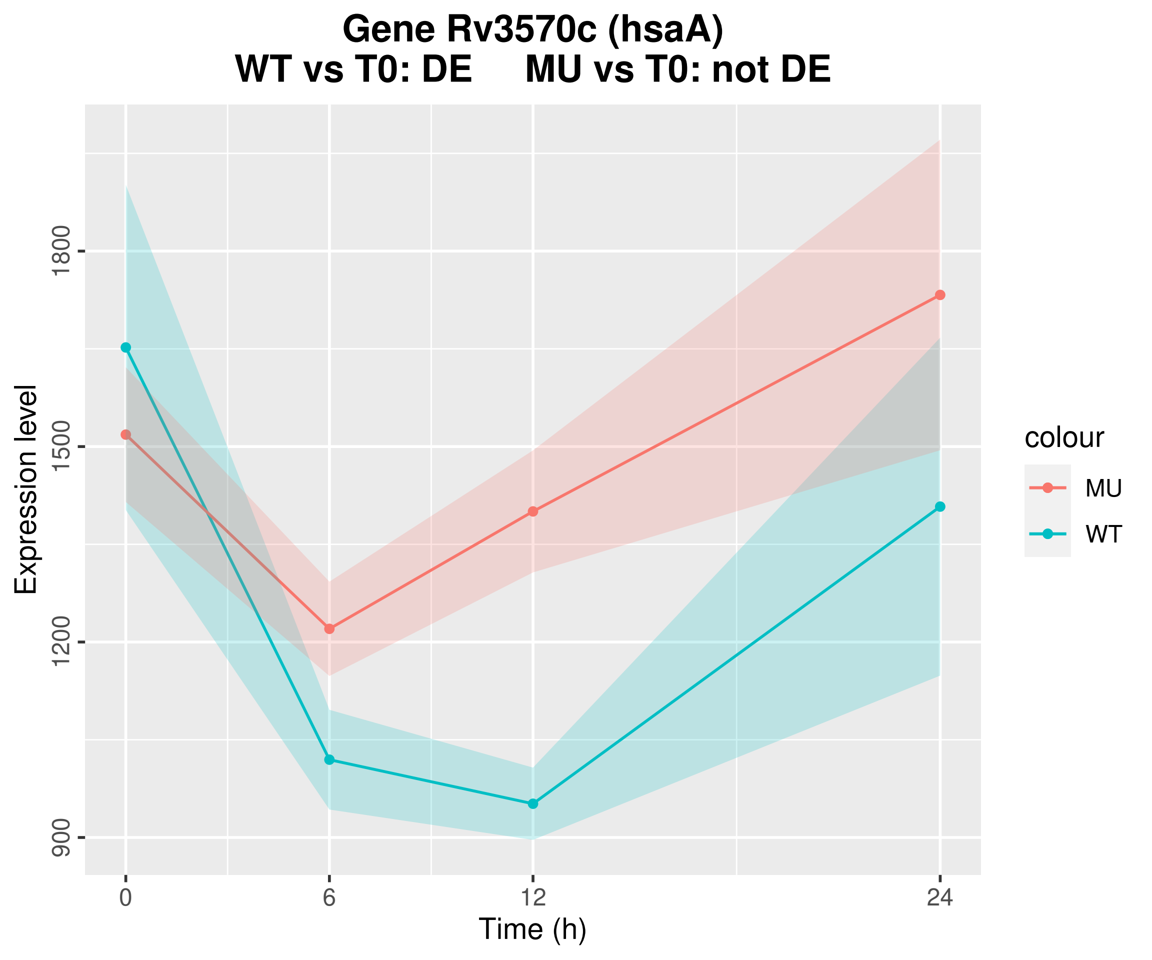

Gene Rv3570c (hsaA)
WT vs T0: DE   MU vs T0: not DE
Expression level
1800
1500
1200
900
0
6
12
24
Time (h)
colour
MU
WT

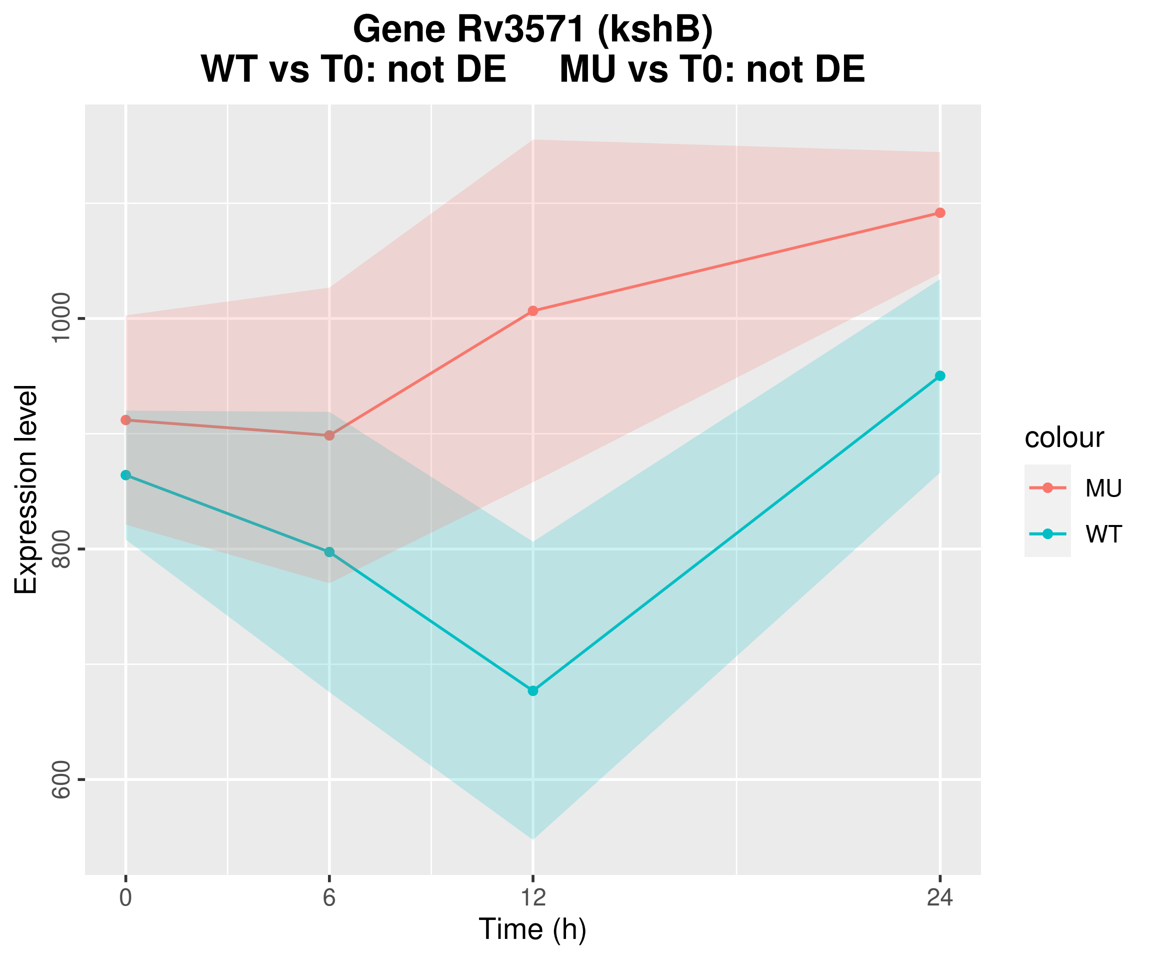

Gene Rv3571 (kshB)
WT vs T0: not DE   MU vs T0: not DE
Expression level
1000
800
600
0
6
12
24
Time (h)
colour
MU
WT

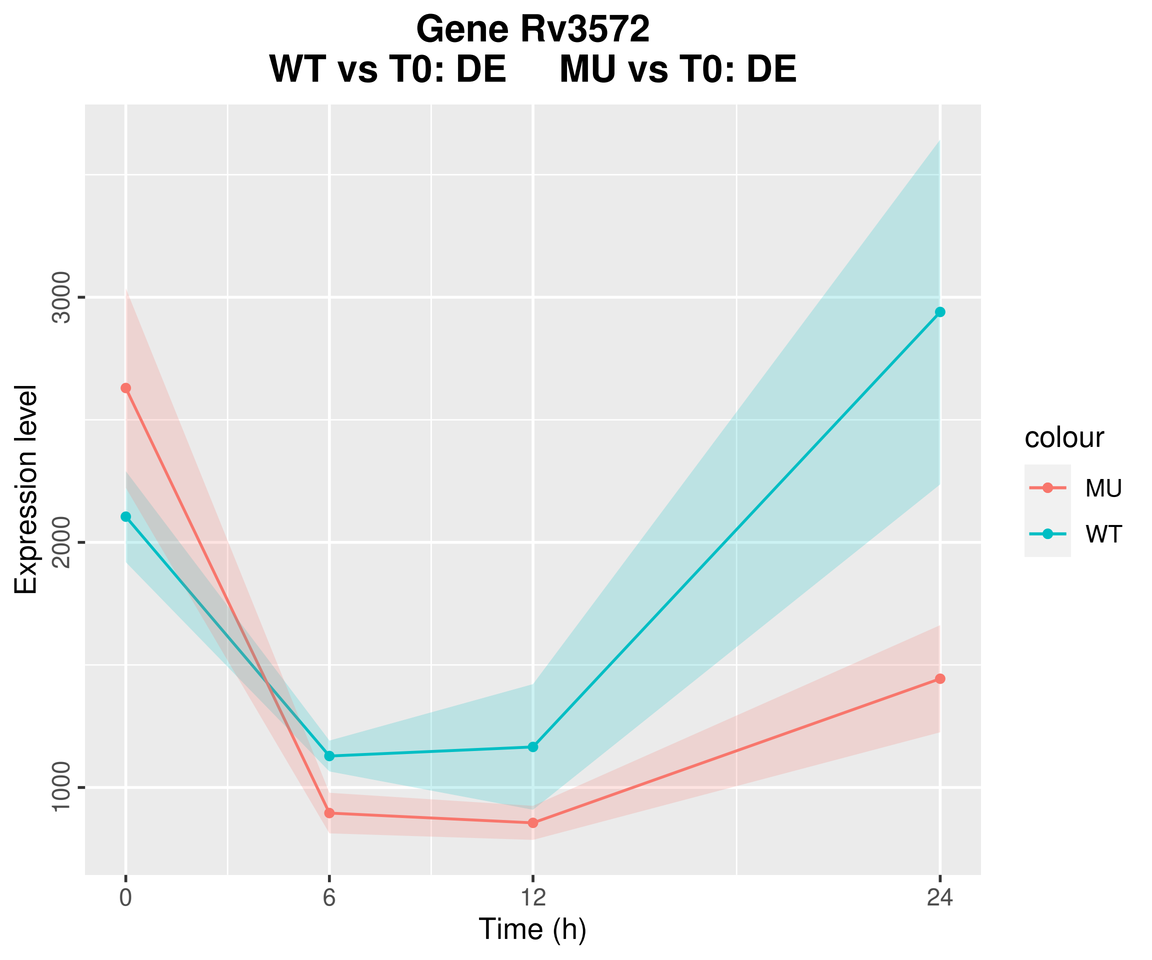
Gene Rv3572
WT vs T0: DE   MU vs T0: DE
Expression level
3000
2000
1000
0
6
12
24
Time (h)
colour
MU
WT

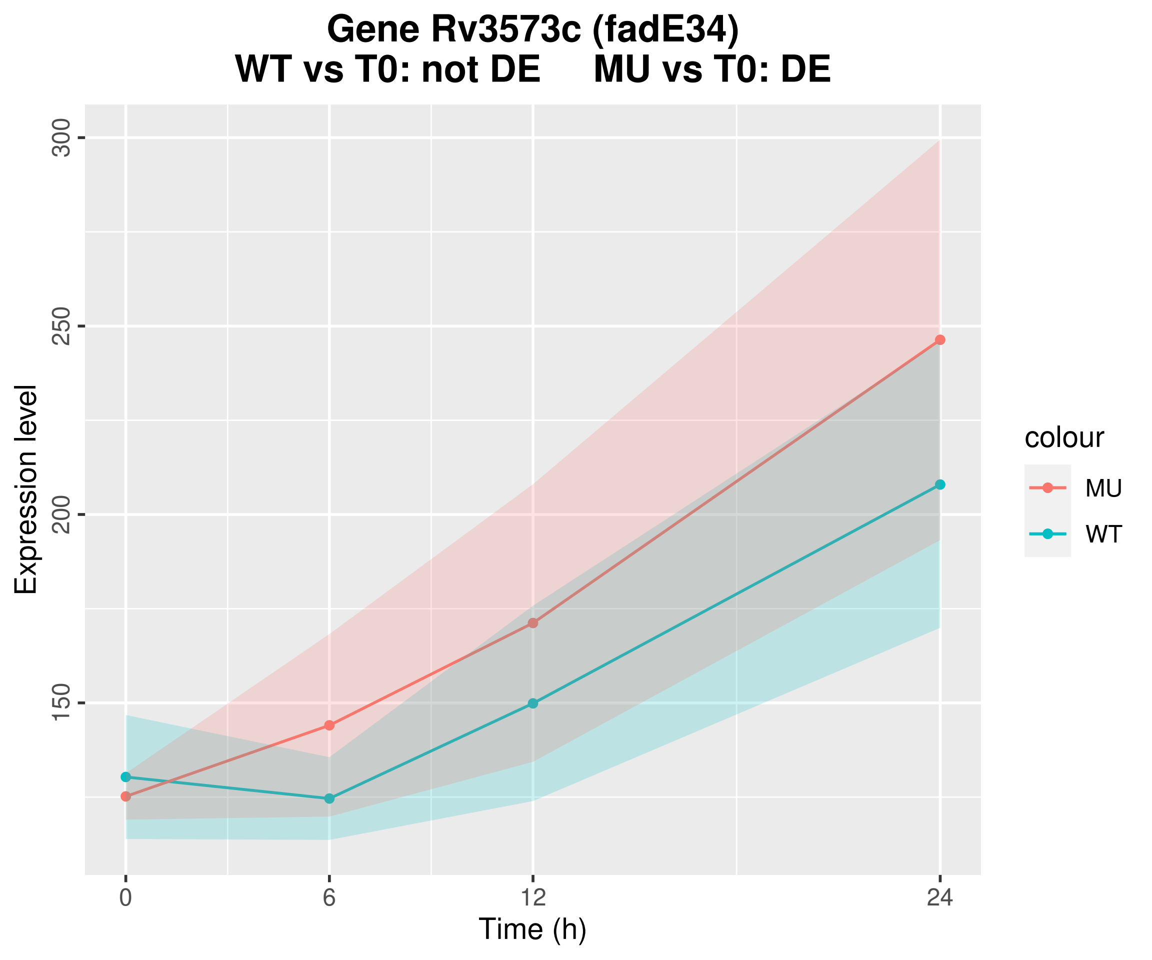
Gene Rv3573c (fadE34)
WT vs T0: not DE   MU vs T0: DE
Expression level
300
250
200
150
0
6
12
24
Time (h)
colour
MU
WT

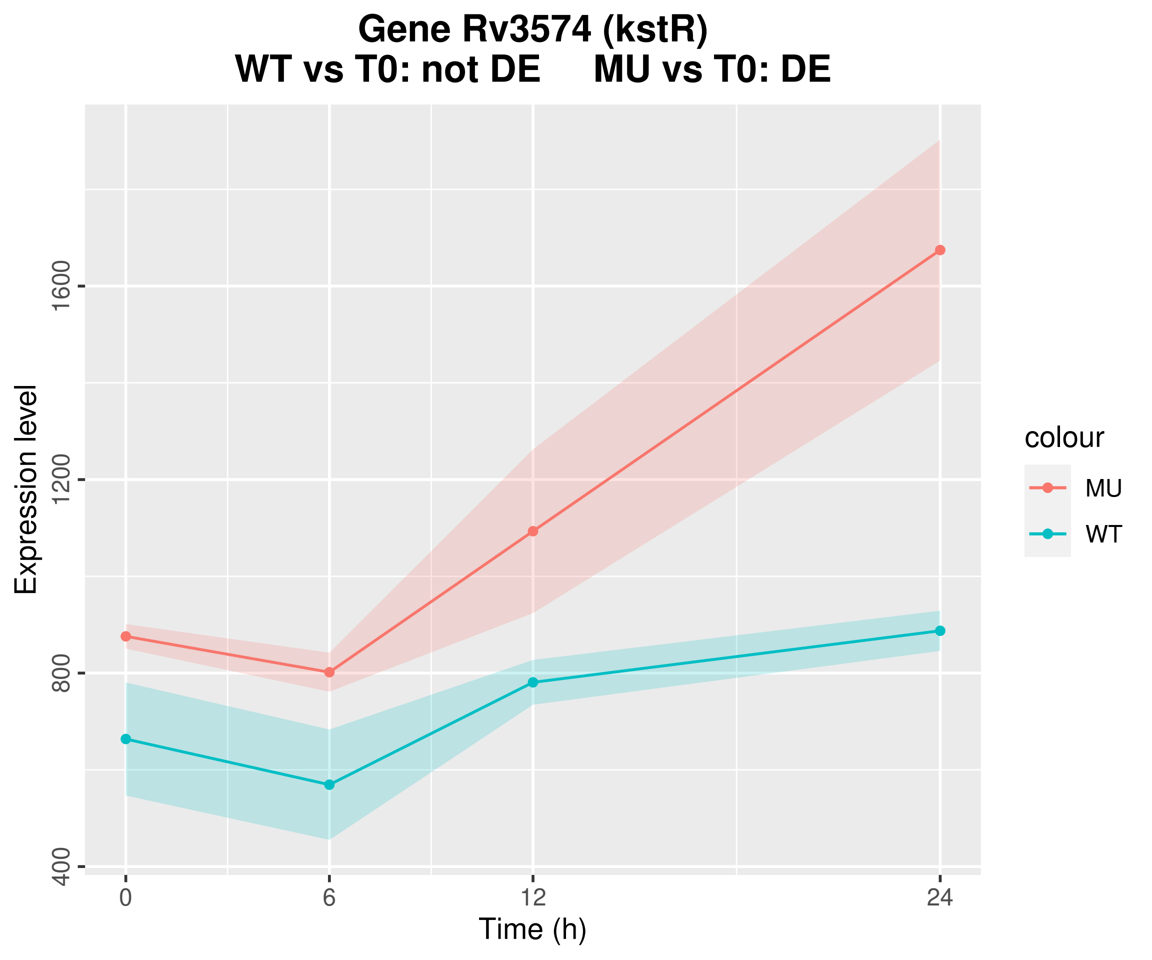

Gene Rv3574 (kstR)
WT vs T0: not DE MU vs T0: DE
Expression level
1600
1200
800
400
0
6
12
24
Time (h)
colour
MU
WT

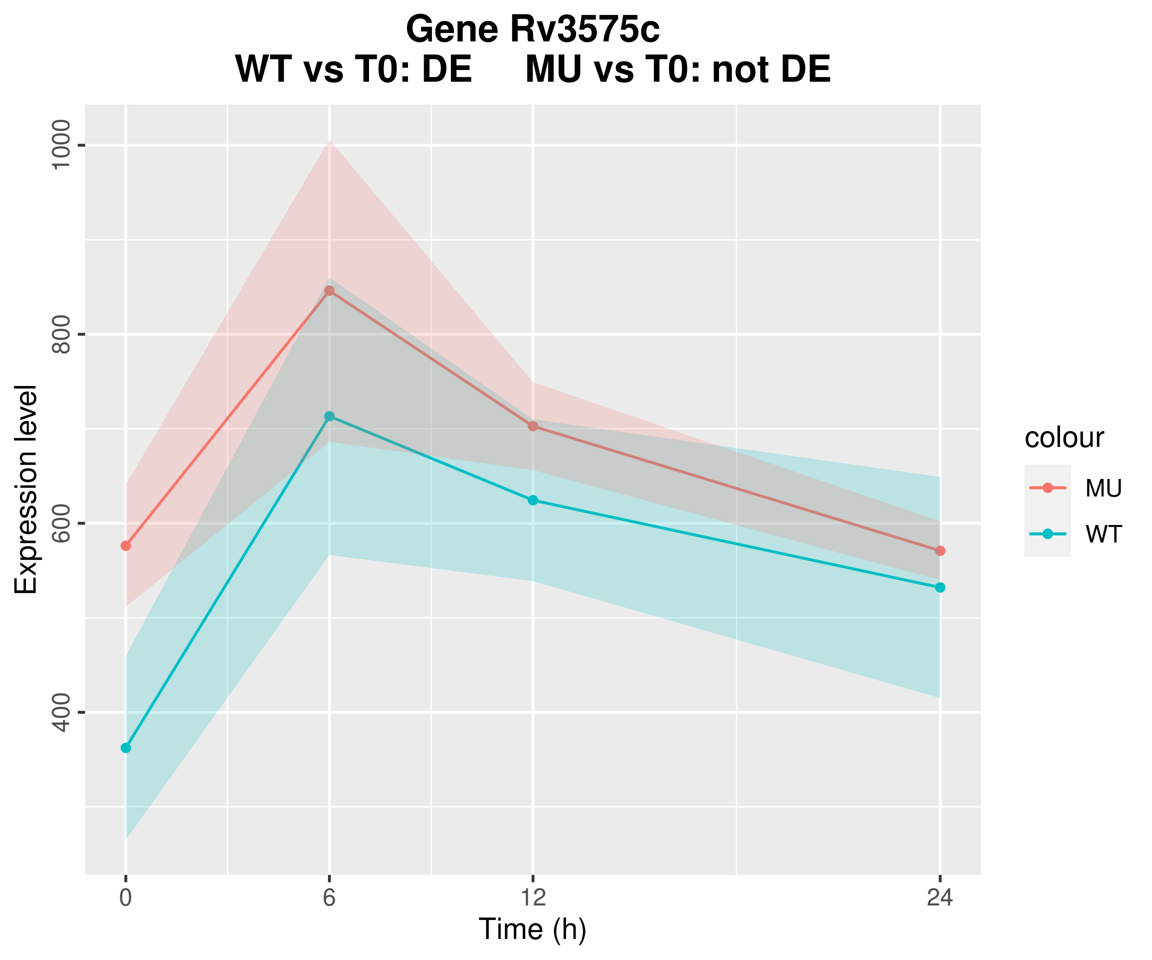

Gene Rv3575c
WT vs T0: DE MU vs T0: not DE
Expression level
1000
800
600
400
0
6
12
24
Time (h)
colour
MU
WT

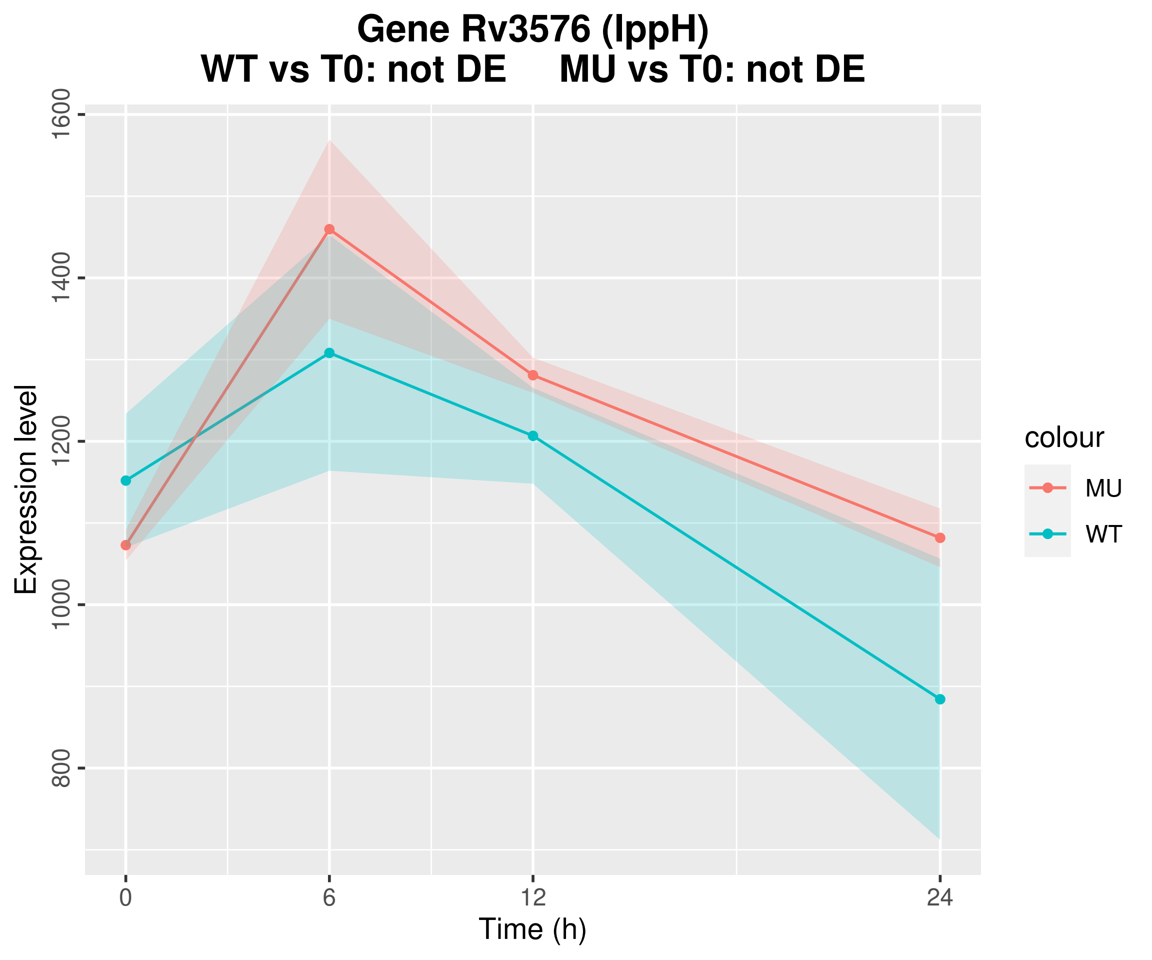

Gene Rv3576 (lppH)
WT vs T0: not DE MU vs T0: not DE
Expression level
1600
1400
1200
1000
800
0
6
12
24
Time (h)
colour
MU
WT

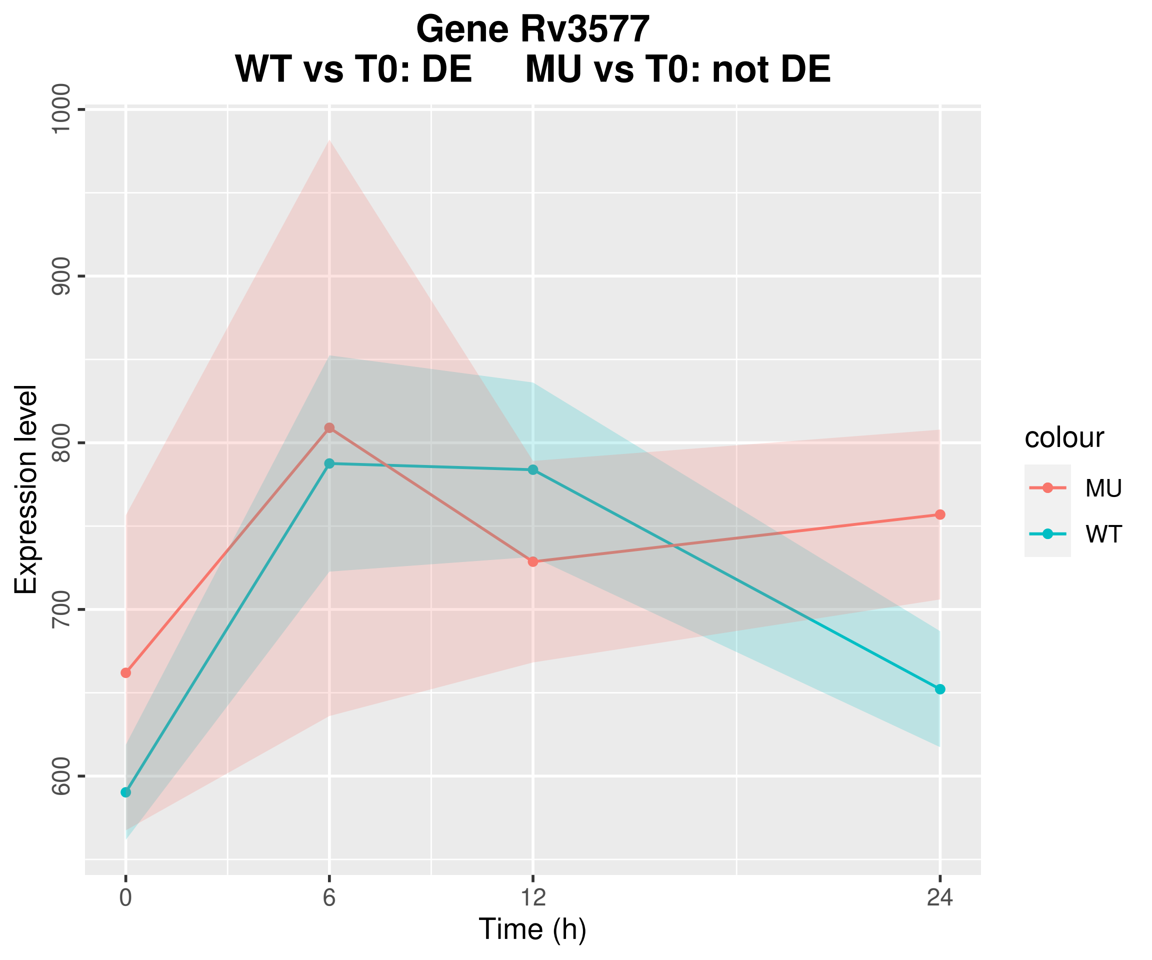
Gene Rv3577
WT vs T0: DE MU vs T0: not DE
1000
900
800
700
600
Expression level
0
6
12
24
Time (h)
colour
MU
WT

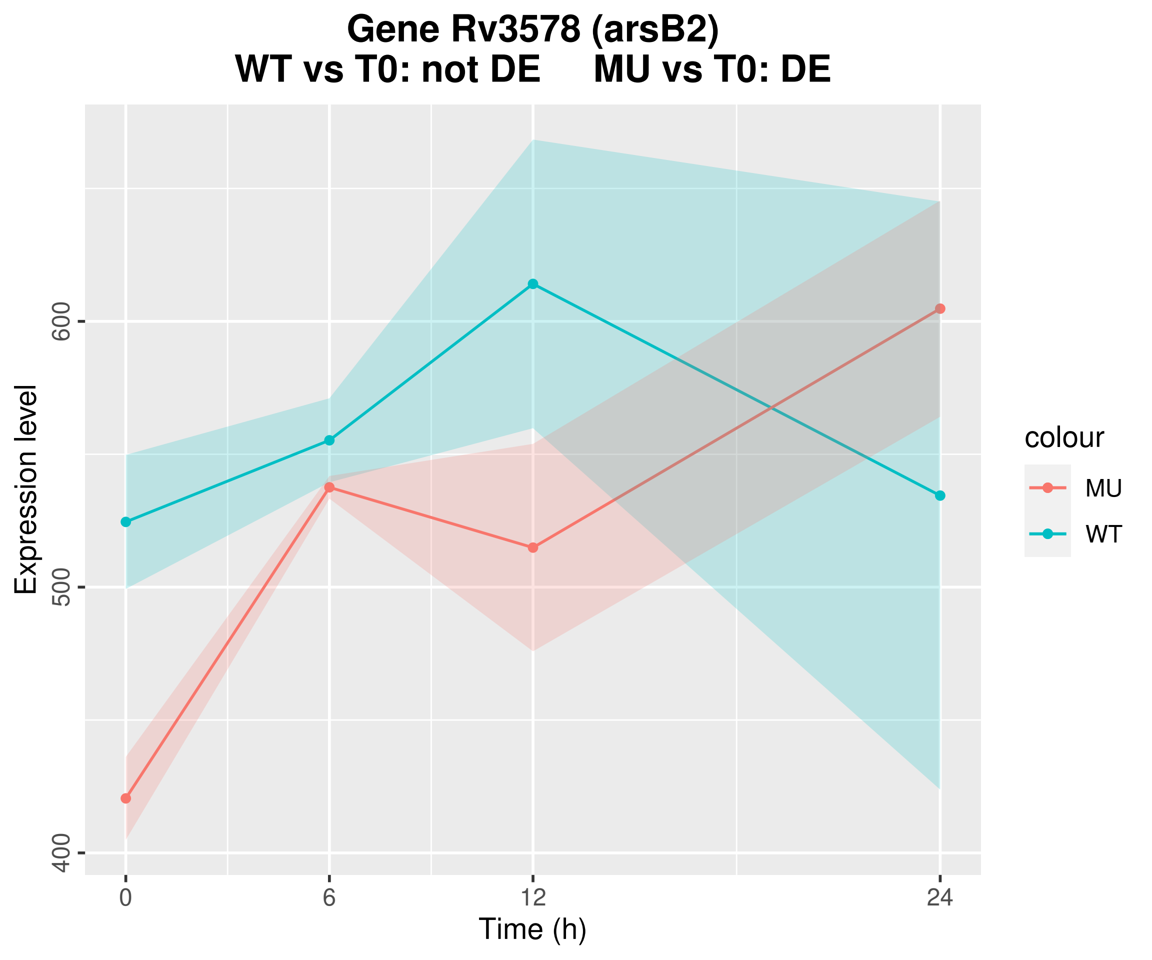
Gene Rv3578 (arsB2)
WT vs T0: not DE   MU vs T0: DE
Expression level
600
500
400
0
6
12
24
Time (h)
colour
MU
WT

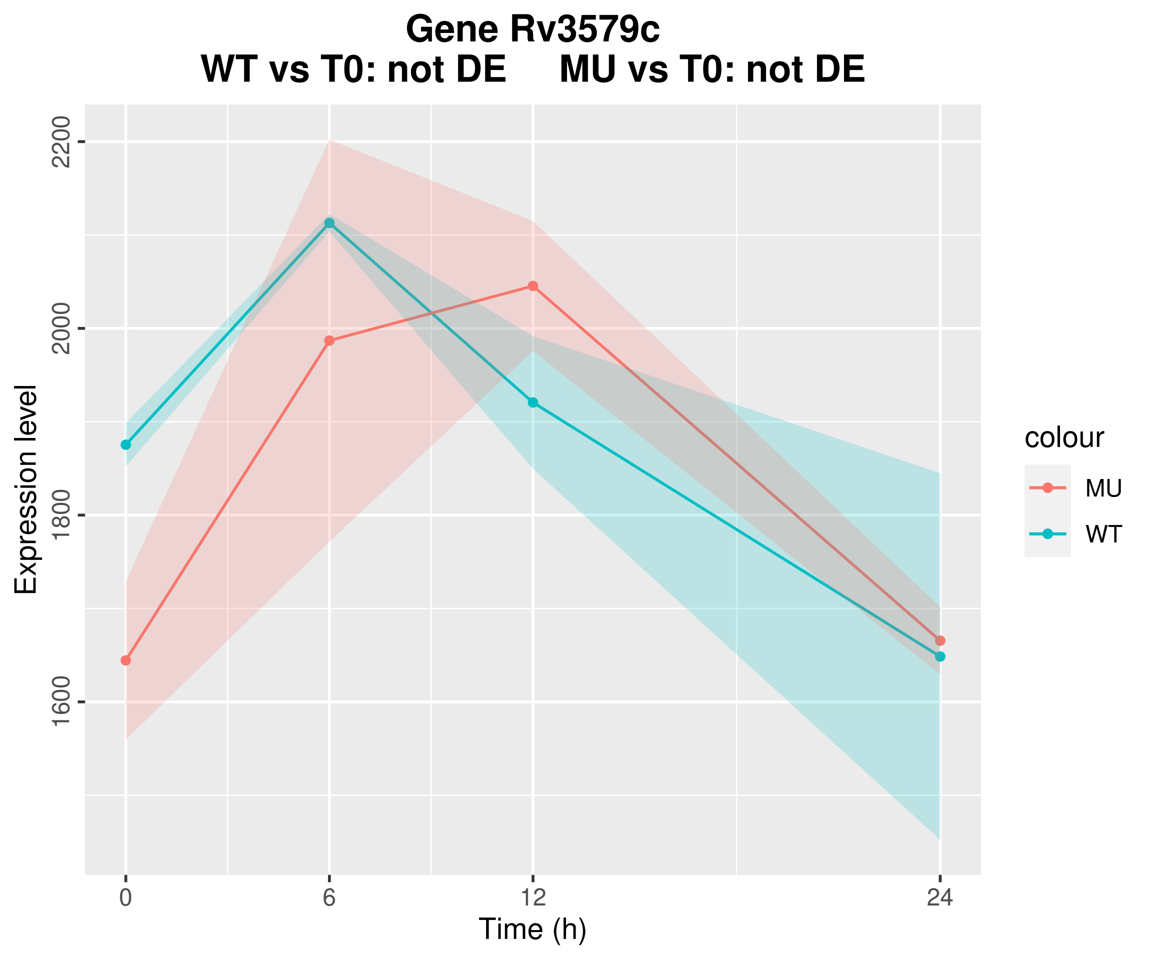

Gene Rv3579c
WT vs T0: not DE MU vs T0: not DE
Expression level
2200
2000
1800
1600
0
6
12
24
Time (h)
colour
MU
WT

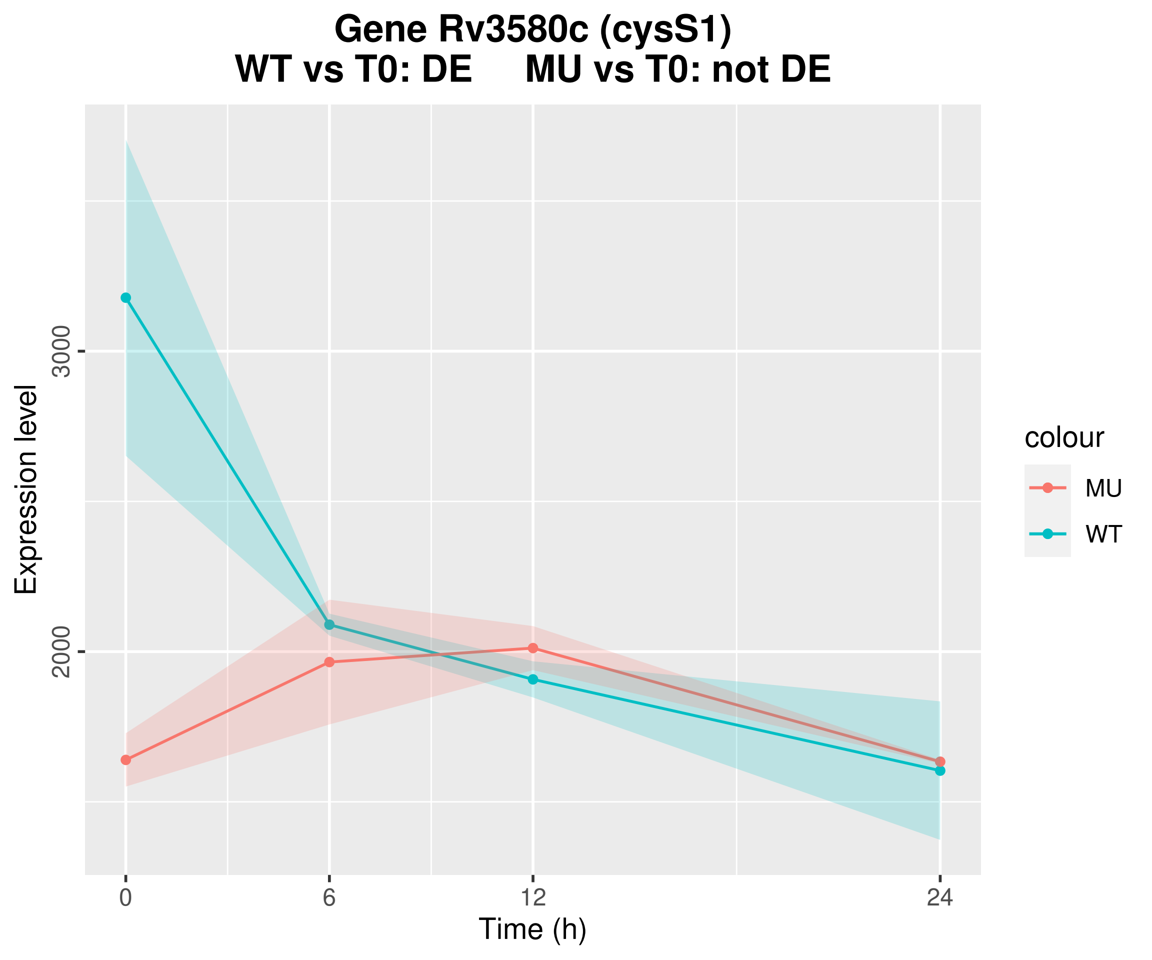

Gene Rv3580c (cysS1)
WT vs T0: DE MU vs T0: not DE
3000
2000
Expression level
0
6
12
24
Time (h)
colour
MU
WT

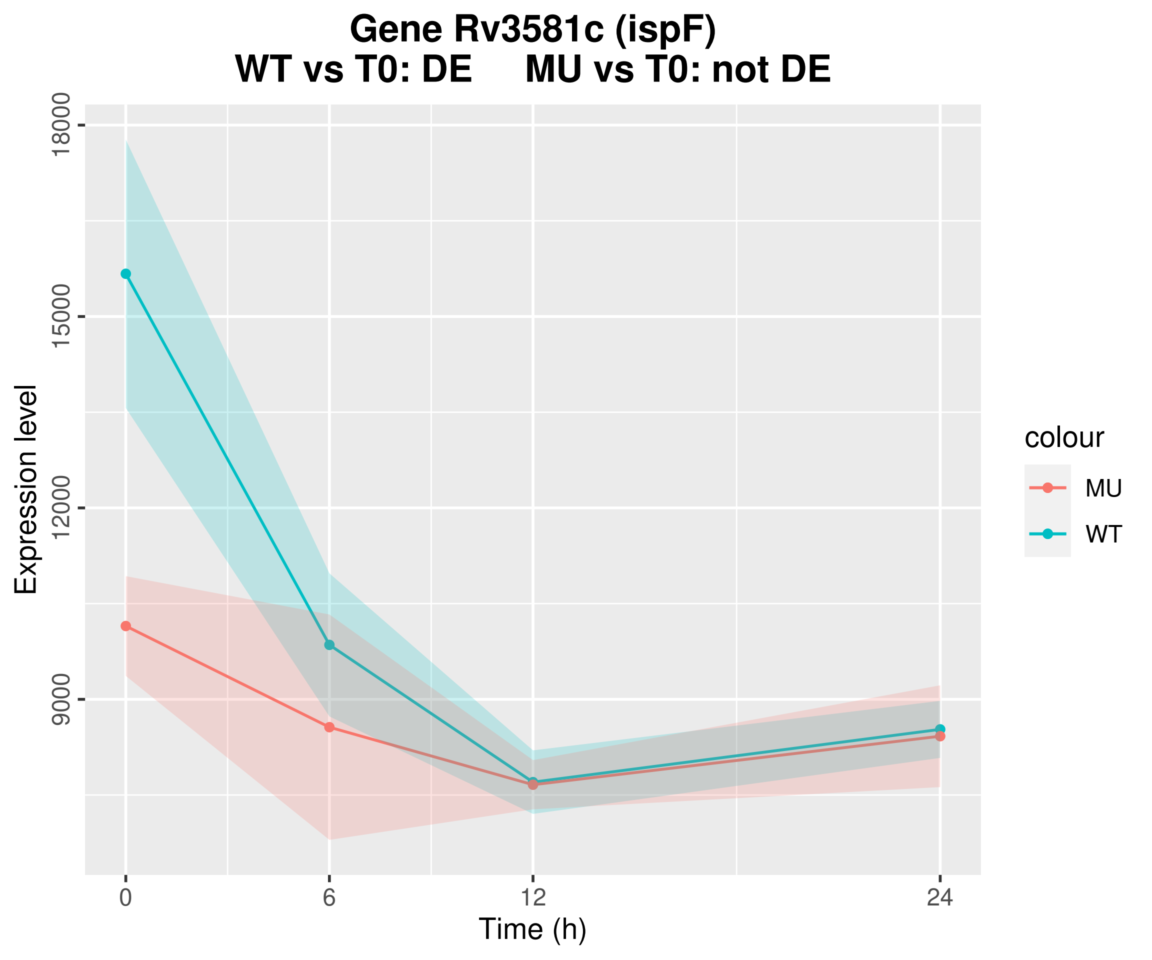

Gene Rv3581c (ispF)
WT vs T0: DE   MU vs T0: not DE
18000
15000
12000
9000
Expression level
0
6
12
24
Time (h)
colour
MU
WT

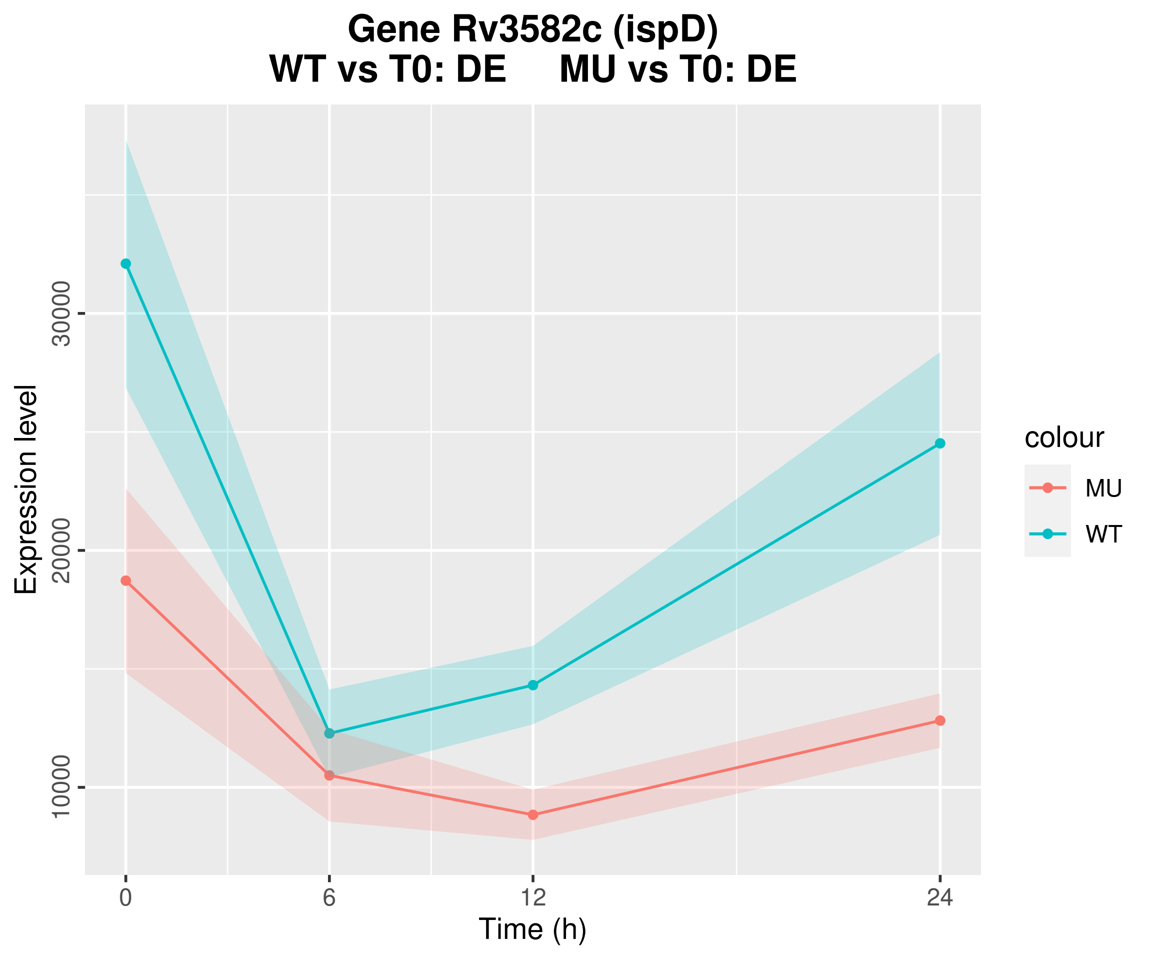
Gene Rv3582c (ispD)
WT vs T0: DE MU vs T0: DE
Expression level
30000
20000
10000
0
6
12
24
Time (h)
colour
MU
WT

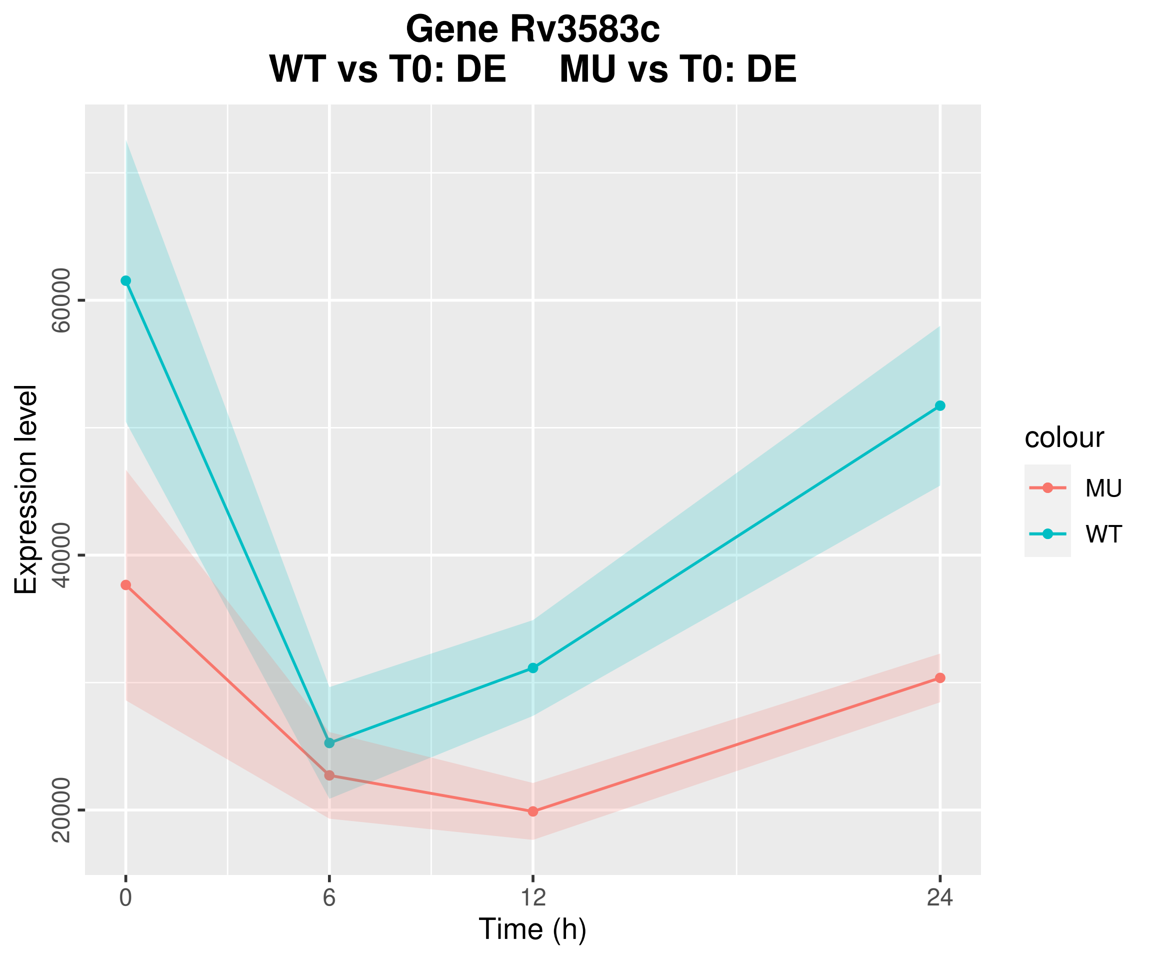
Gene Rv3583c
WT vs T0: DE   MU vs T0: DE
Expression level
60000
40000
20000
0
6
12
24
Time (h)
colour
MU
WT

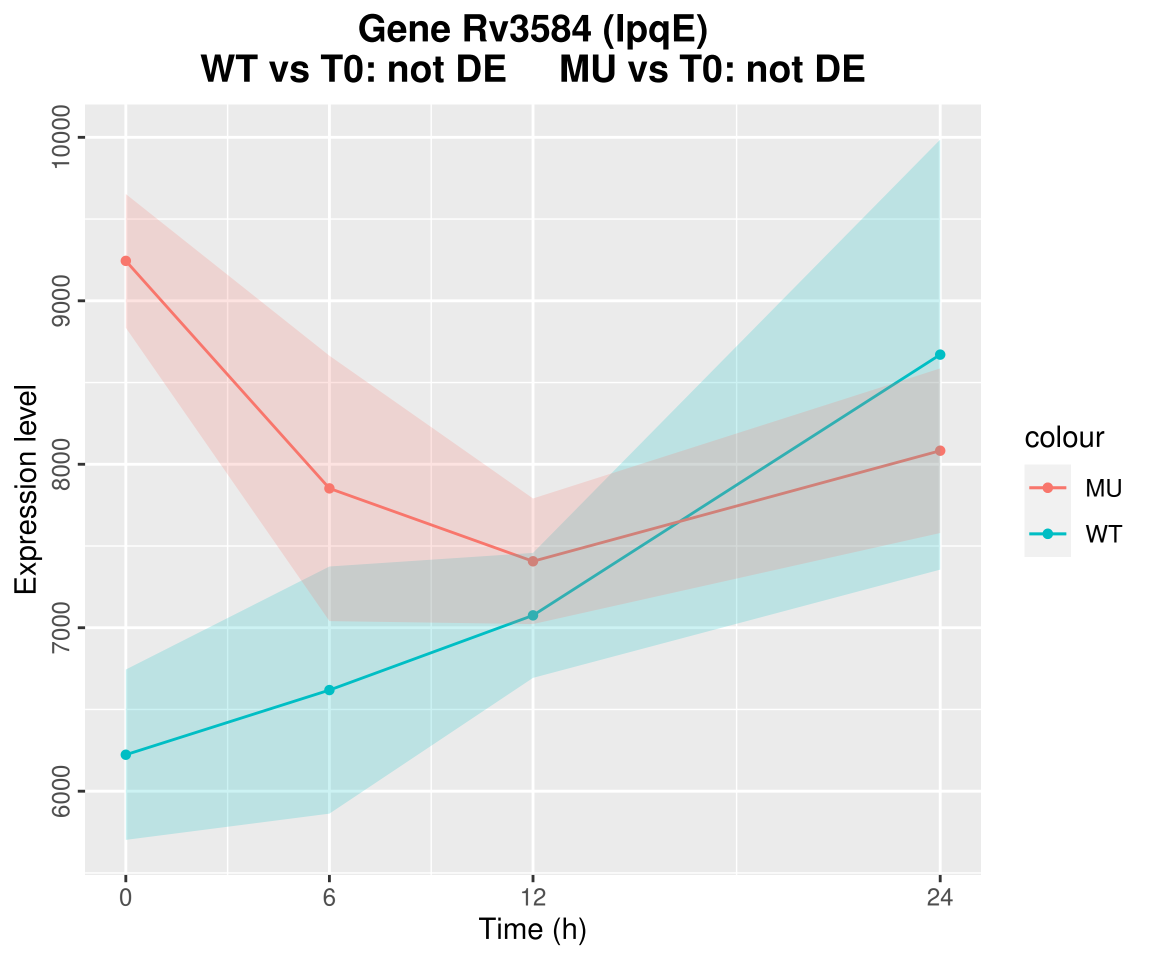

Gene Rv3584 (lpqE)
WT vs T0: not DE   MU vs T0: not DE
Expression level
10000
9000
8000
7000
6000
Time (h)
0
6
12
24
colour
MU
WT

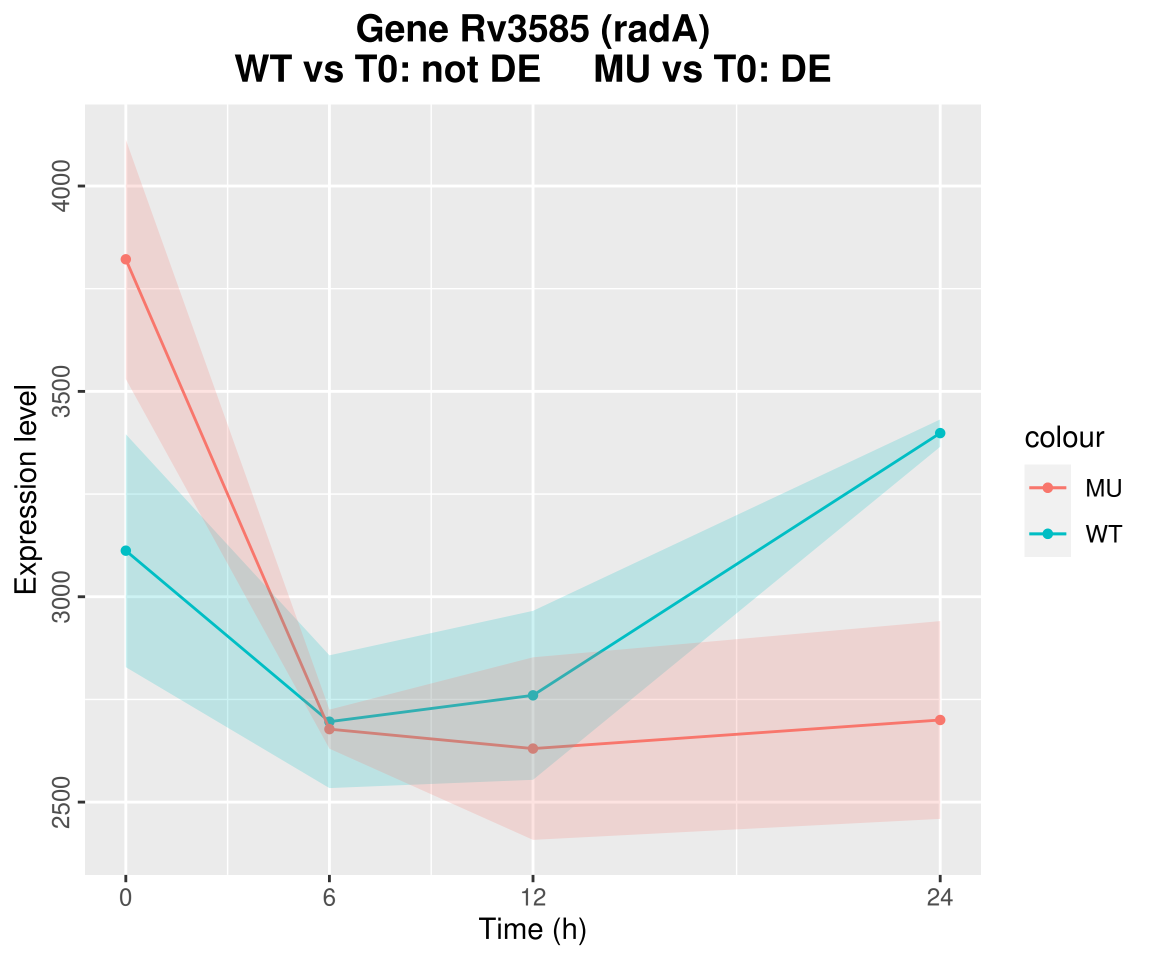

Gene Rv3585 (radA)
WT vs T0: not DE   MU vs T0: DE
Expression level
4000
3500
3000
2500
0
6
12
24
Time (h)
colour
MU
WT

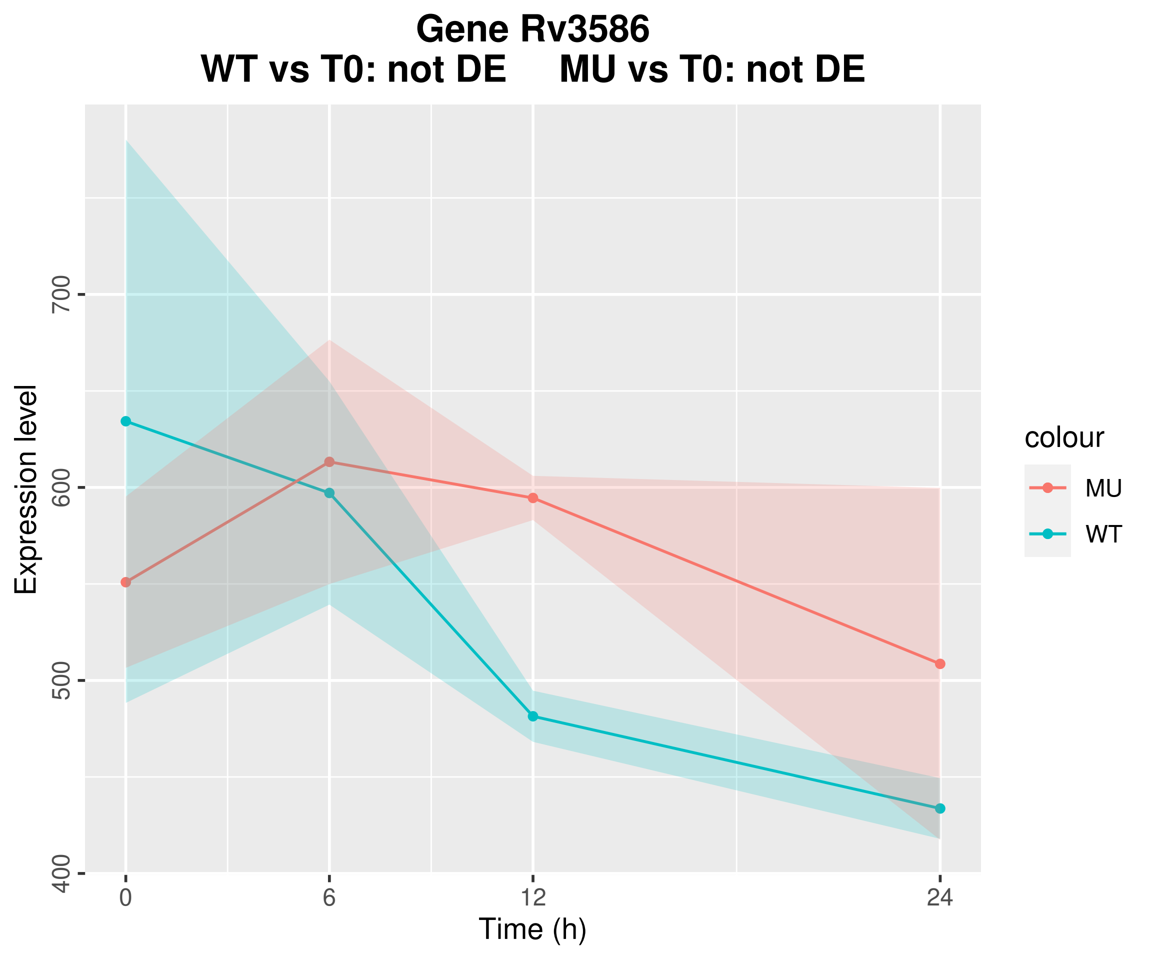

Gene Rv3586
WT vs T0: not DE   MU vs T0: not DE
Expression level
700
600
500
400
0
6
12
24
Time (h)
colour
MU
WT

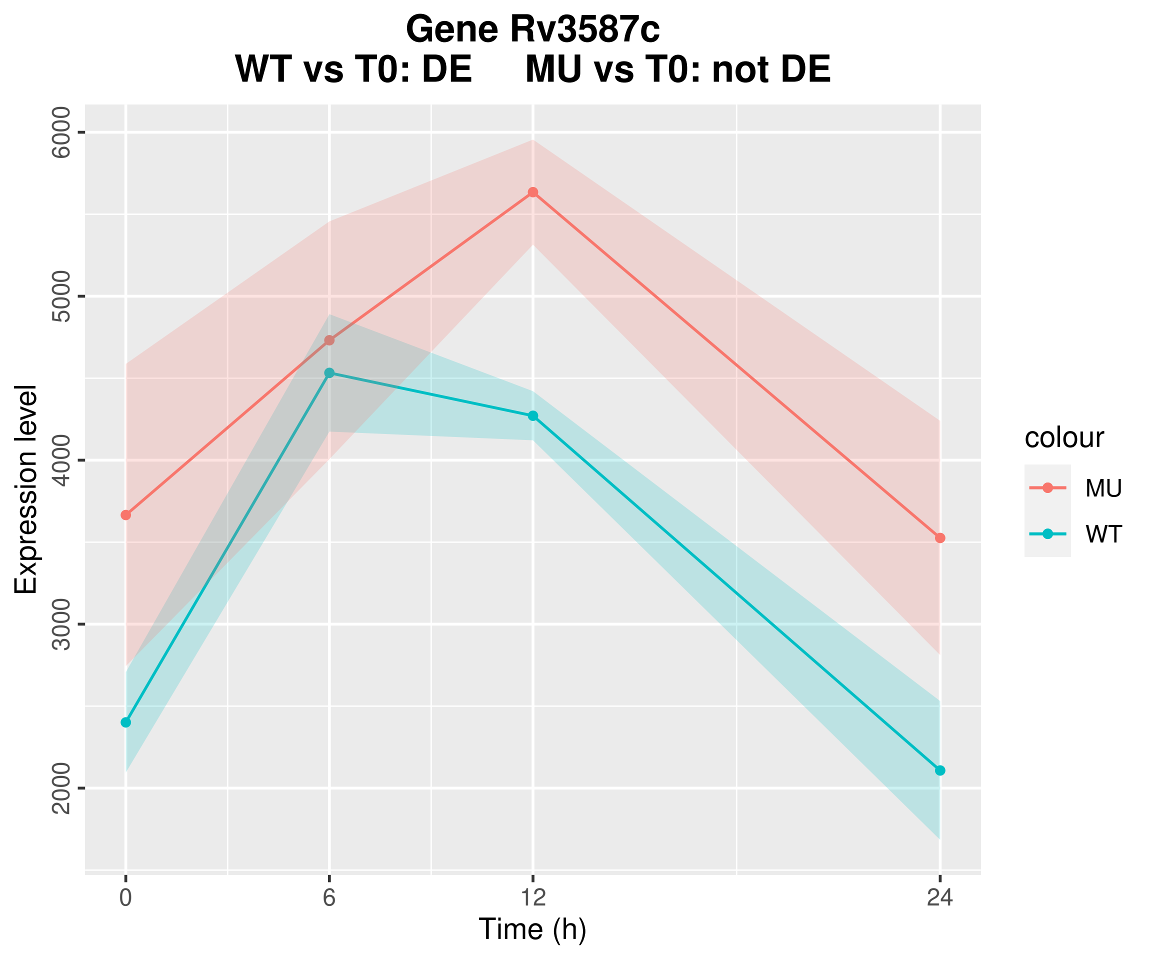
Gene Rv3587c
WT vs T0: DE   MU vs T0: not DE
Expression level
6000
5000
4000
3000
2000
0
6
12
24
Time (h)
colour
MU
WT

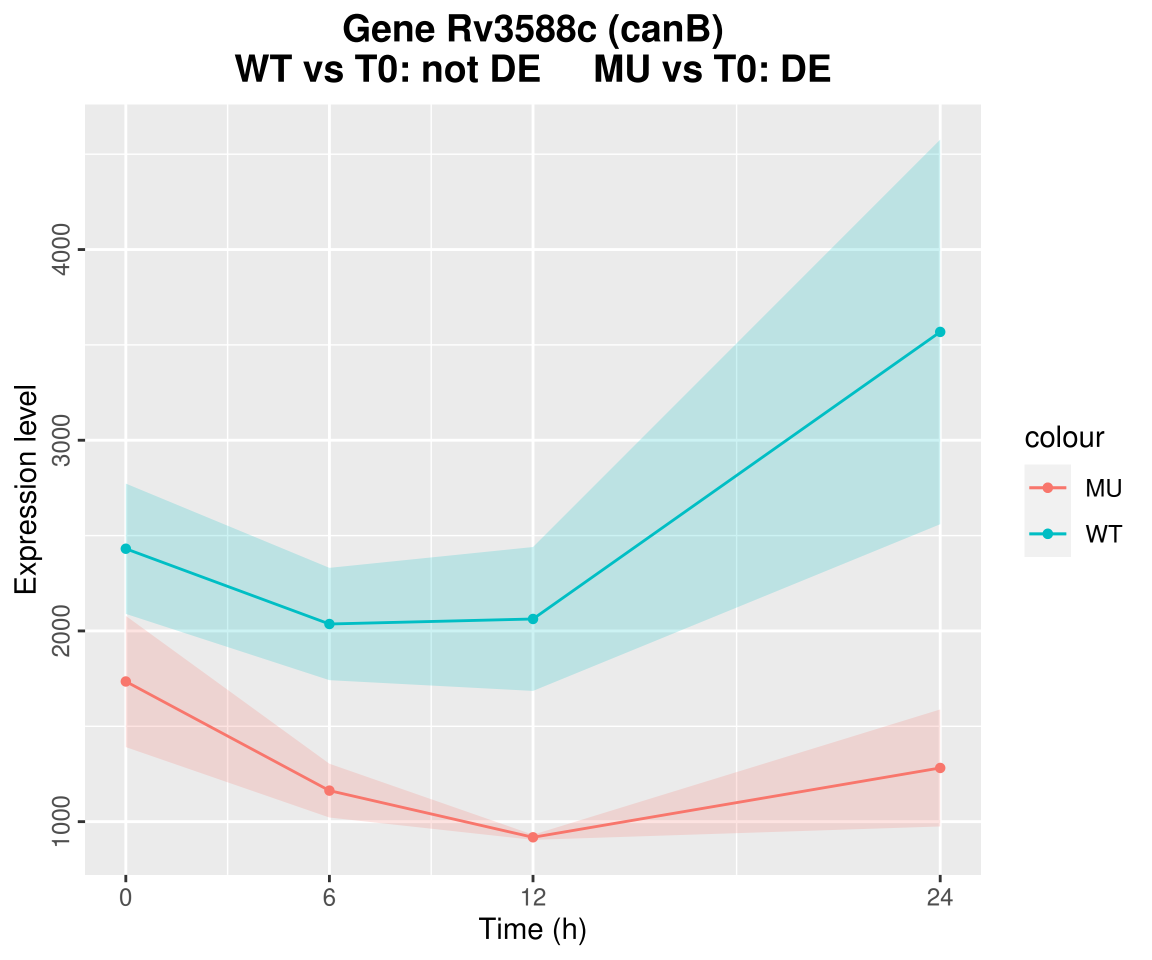
Gene Rv3588c (canB)
WT vs T0: not DE MU vs T0: DE
Expression level
1000
2000
3000
4000
0
6
12
24
Time (h)
colour
MU
WT

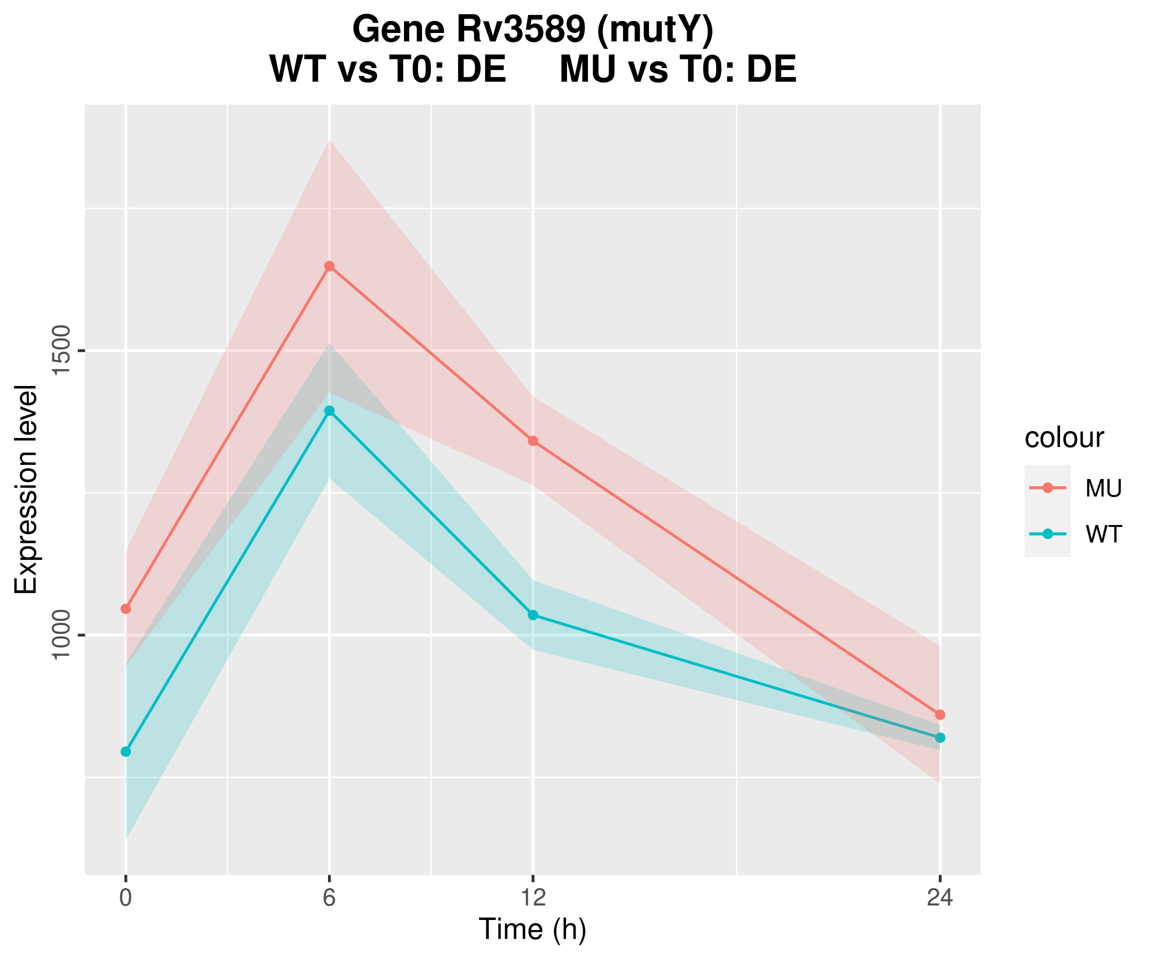

Gene Rv3589 (mutY)
WT vs T0: DE   MU vs T0: DE
Expression level
1500
1000
0
6
12
24
Time (h)
colour
MU
WT

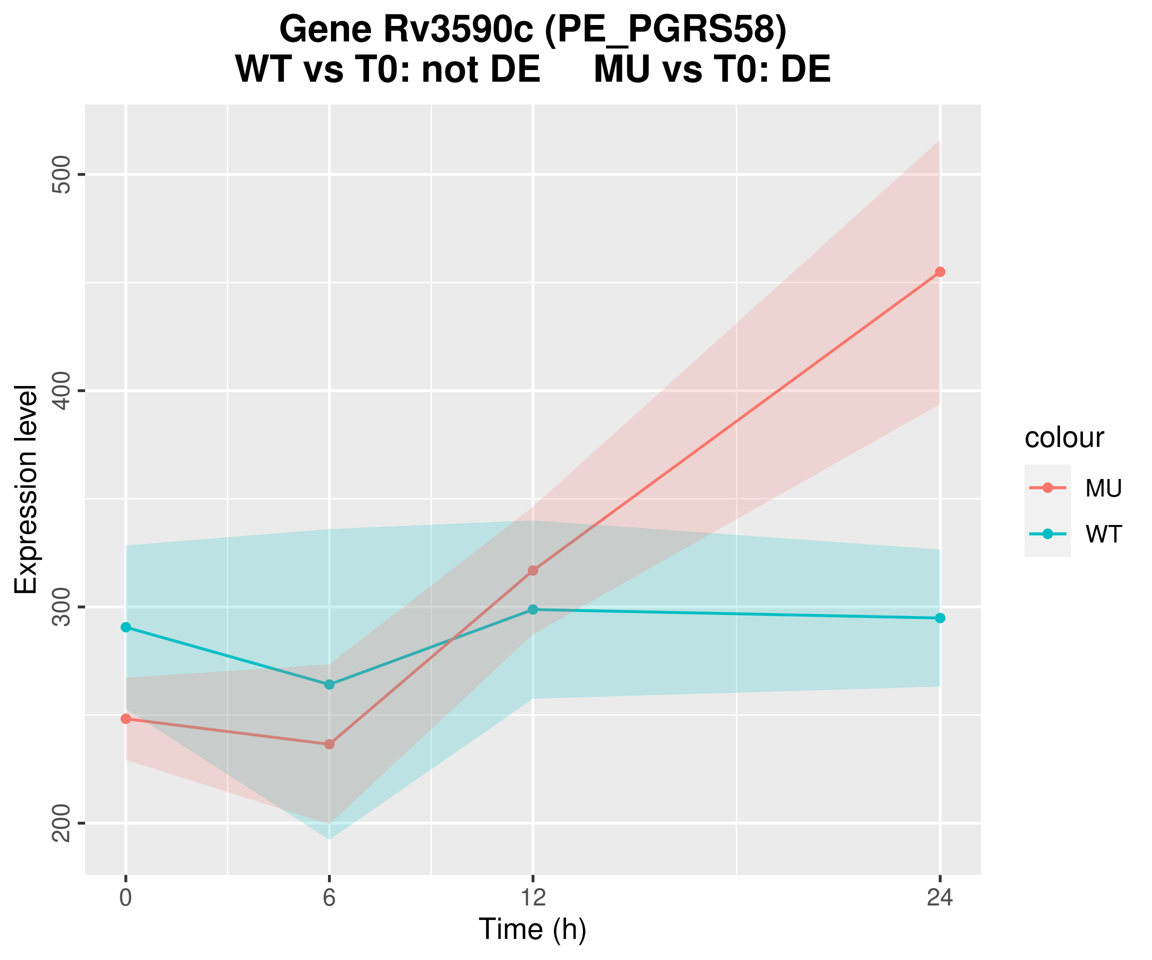
Gene Rv3590c (PE_PGRS58)
WT vs T0: not DE   MU vs T0: DE
Expression level
500
400
300
200
0
6
12
24
Time (h)
colour
MU
WT

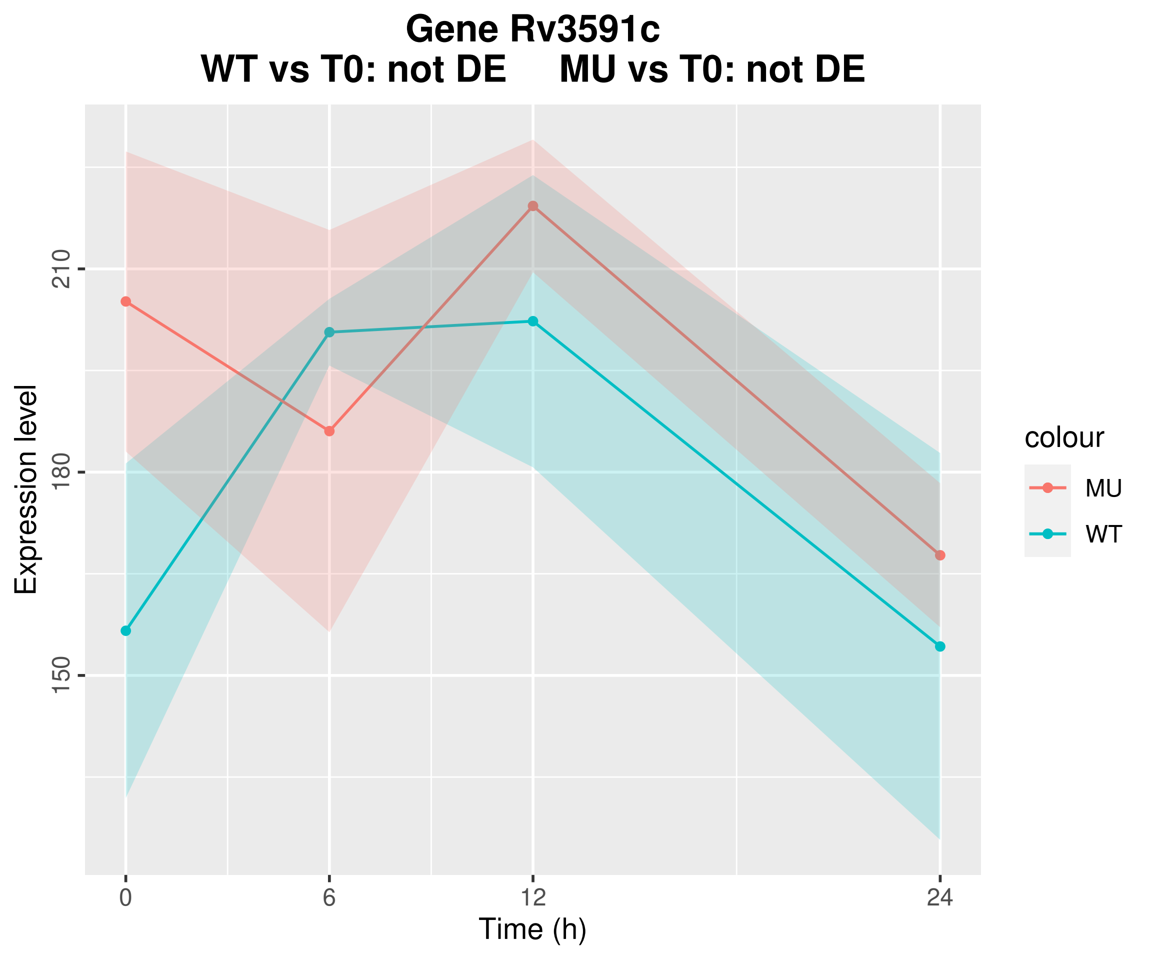
Gene Rv3591c
WT vs T0: not DE   MU vs T0: not DE
Expression level
210
180
150
0
6
12
24
Time (h)
colour
MU
WT

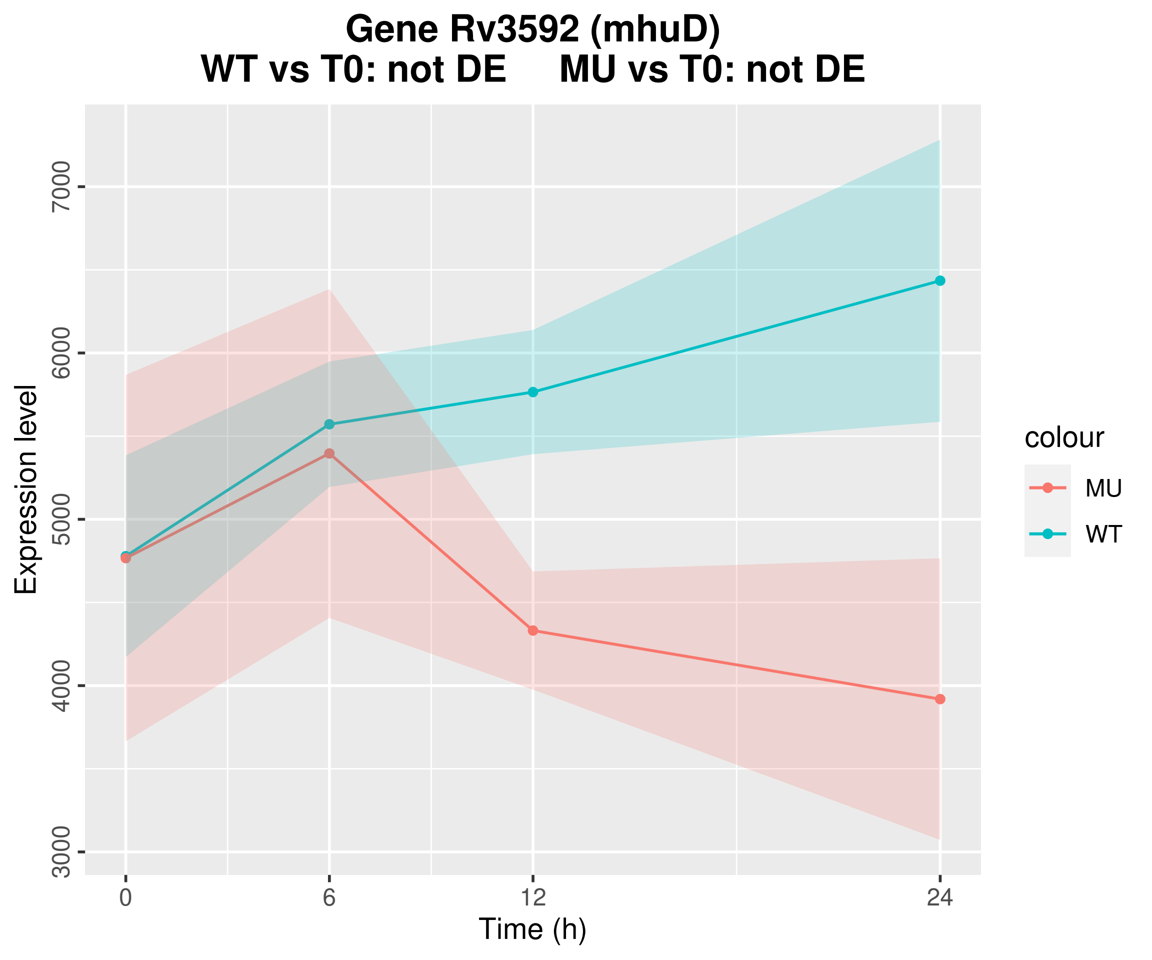

Gene Rv3592 (mhuD)
WT vs T0: not DE   MU vs T0: not DE
Expression level
Time (h)
7000
6000
5000
4000
3000
0
6
12
24
colour
MU
WT

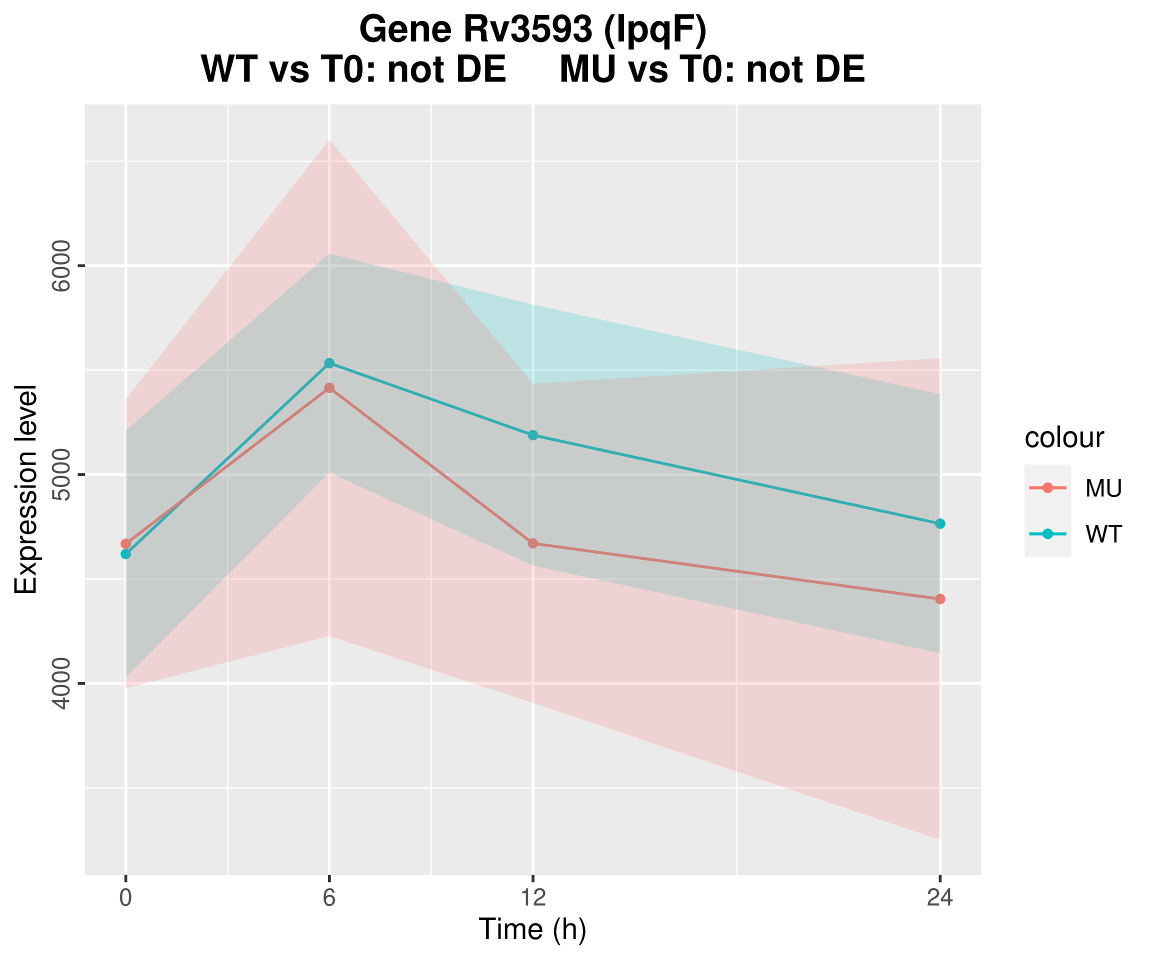
Gene Rv3593 (lpqF)
WT vs T0: not DE   MU vs T0: not DE
Expression level
6000
5000
4000
0
6
12
24
Time (h)
colour
MU
WT

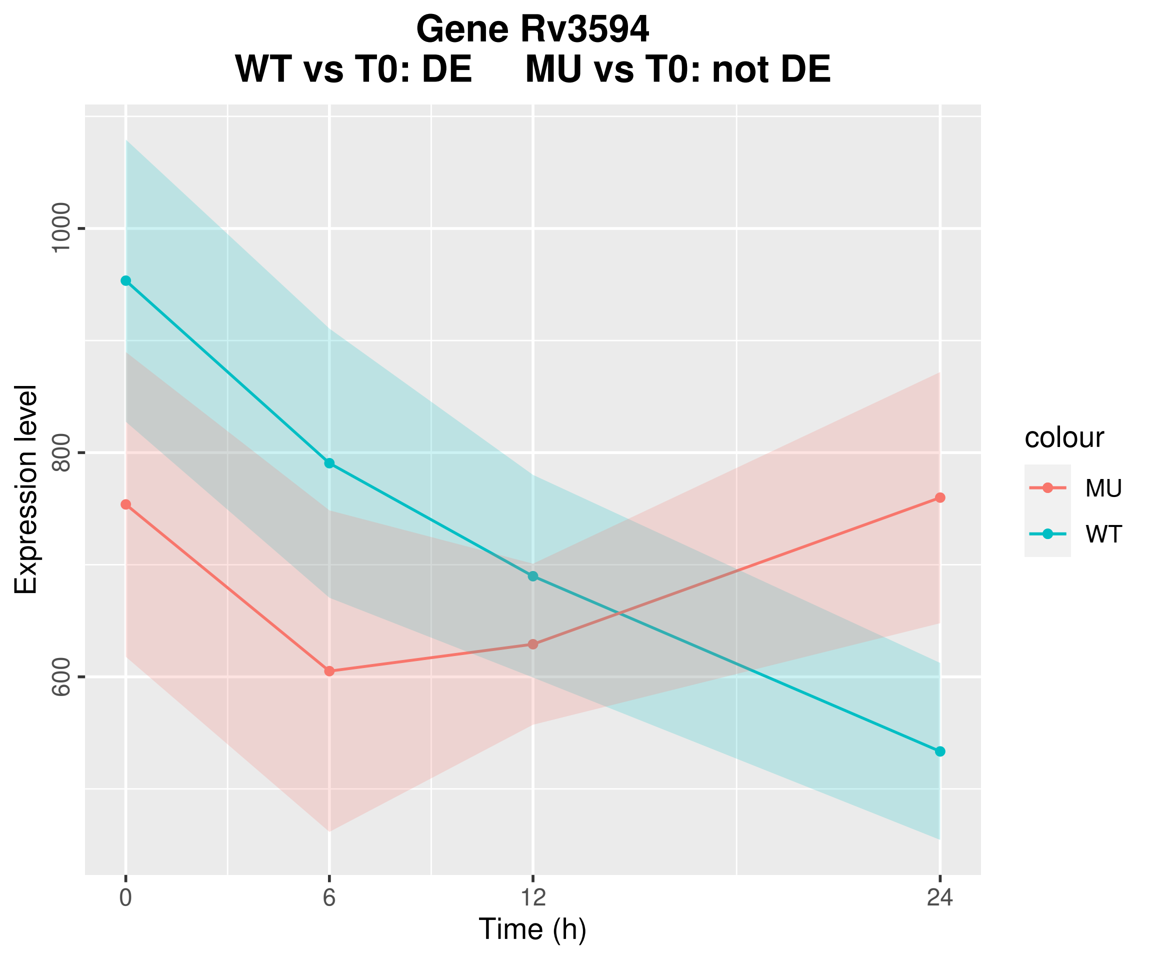
Gene Rv3594
WT vs T0: DE   MU vs T0: not DE
Expression level
1000
800
600
0
6
12
24
Time (h)
colour
MU
WT

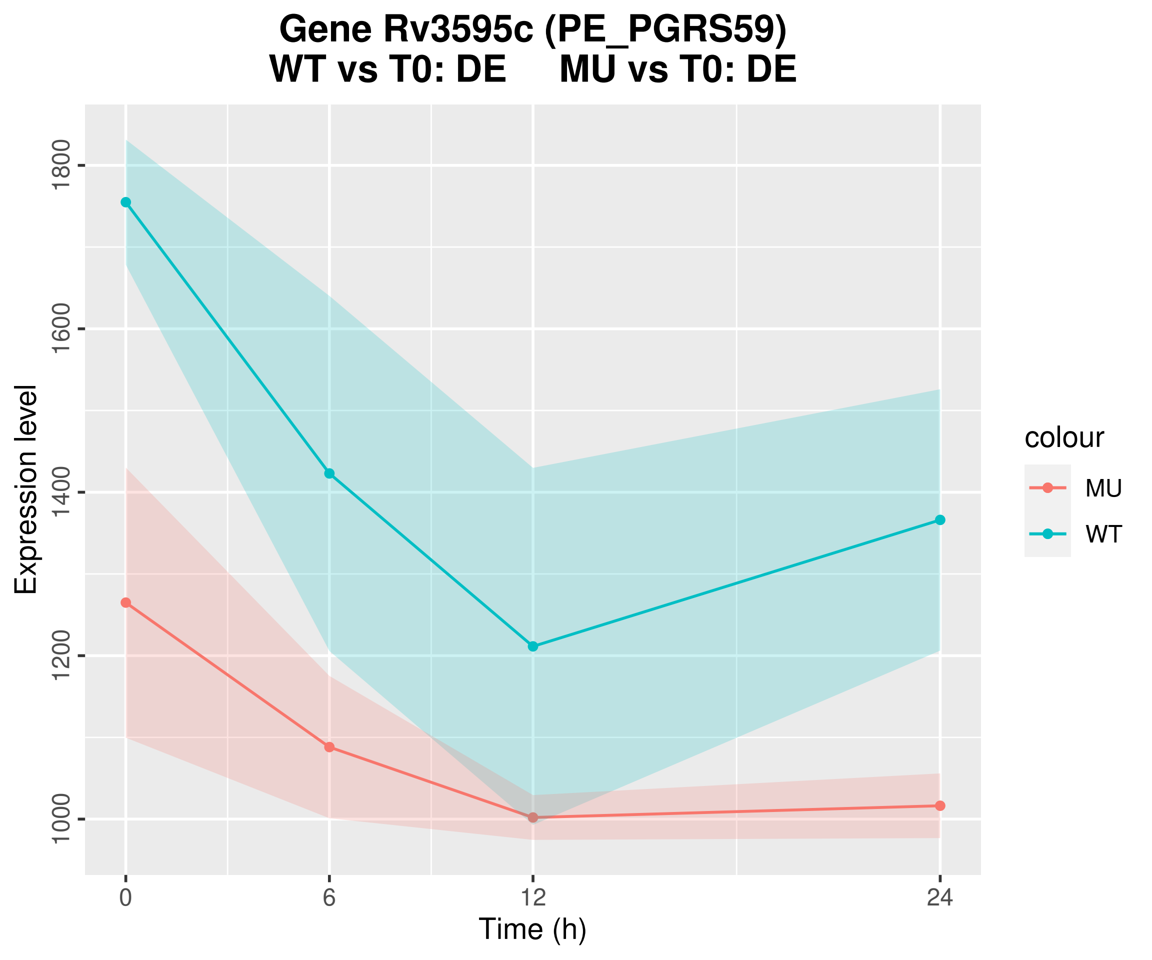

Gene Rv3595c (PE_PGRS59)
WT vs T0: DE MU vs T0: DE
Expression level
1800
1600
1400
1200
1000
0
6
12
24
Time (h)
colour
MU
WT

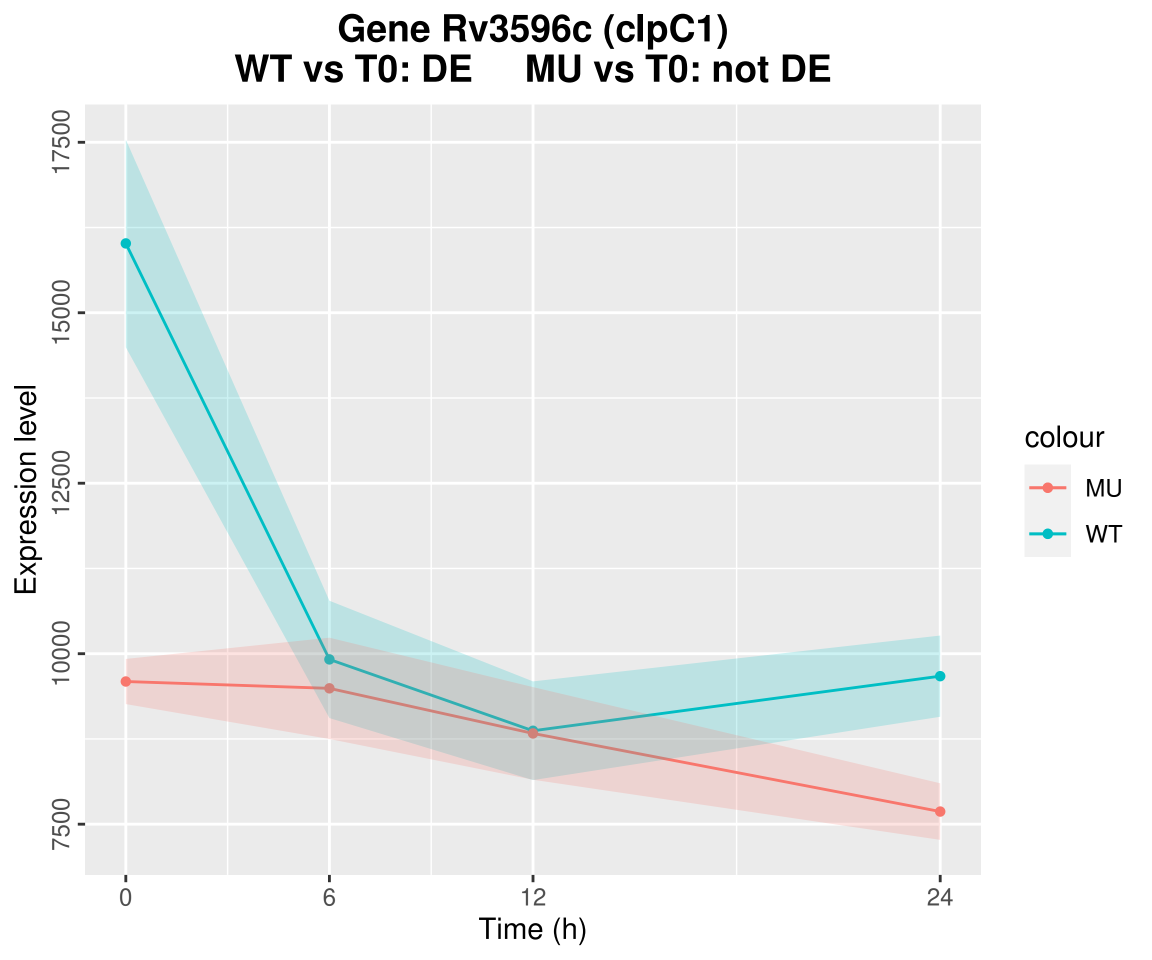
Gene Rv3596c (clpC1)
WT vs T0: DE MU vs T0: not DE
Expression level
17500
15000
12500
10000
7500
0
6
12
24
Time (h)
colour
MU
WT

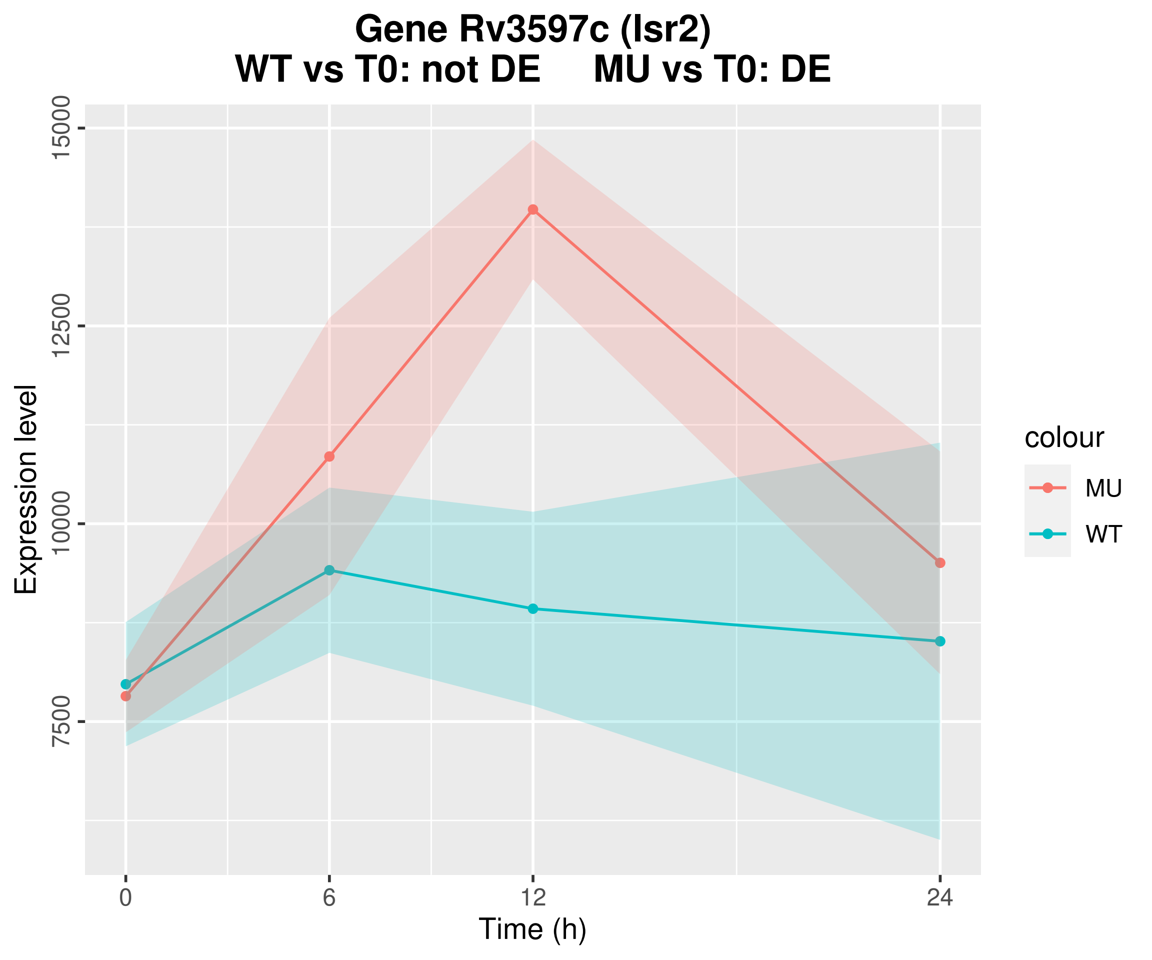
Gene Rv3597c (lsr2)
WT vs T0: not DE   MU vs T0: DE
Expression level
15000
12500
10000
7500
0
6
12
24
Time (h)
colour
MU
WT

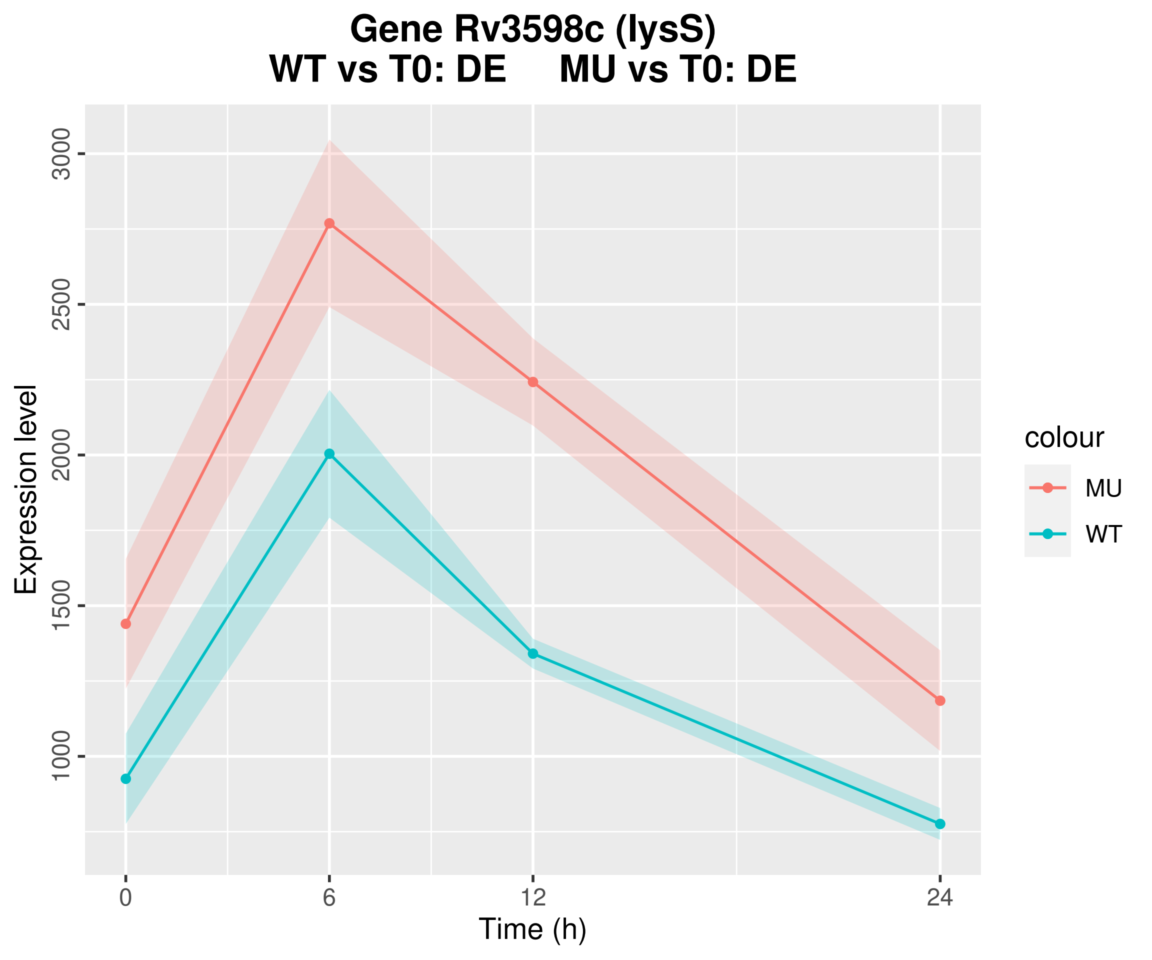
Gene Rv3598c (lysS)
WT vs T0: DE MU vs T0: DE
Expression level
3000
2500
2000
1500
1000
0
6
12
24
Time (h)
colour
MU
WT

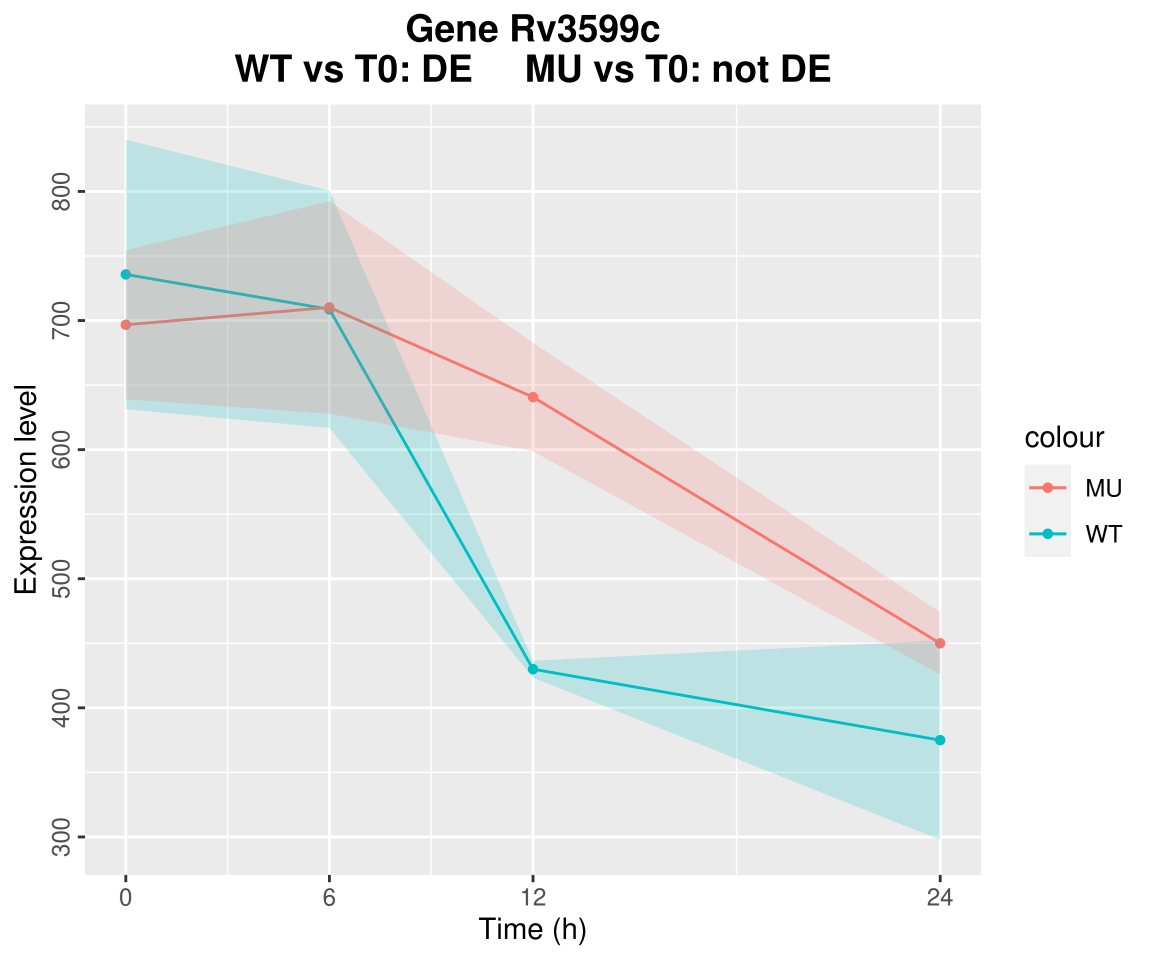

Gene Rv3599c
WT vs T0: DE MU vs T0: not DE
Expression level
800
700
600
500
400
300
0
6
12
24
Time (h)
colour
MU
WT

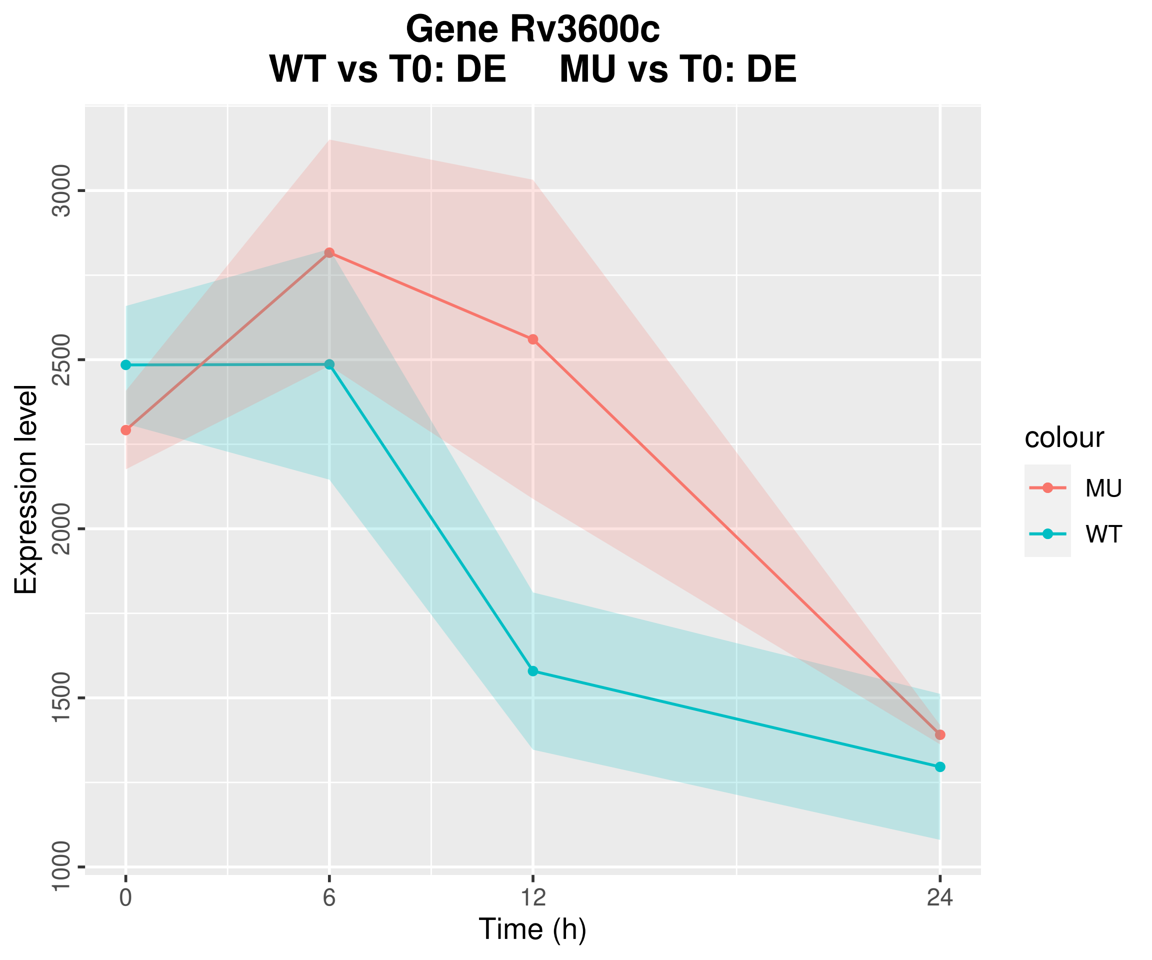

Gene Rv3600c
WT vs T0: DE   MU vs T0: DE
Expression level
3000
2500
2000
1500
1000
0
6
12
24
Time (h)
colour
MU
WT

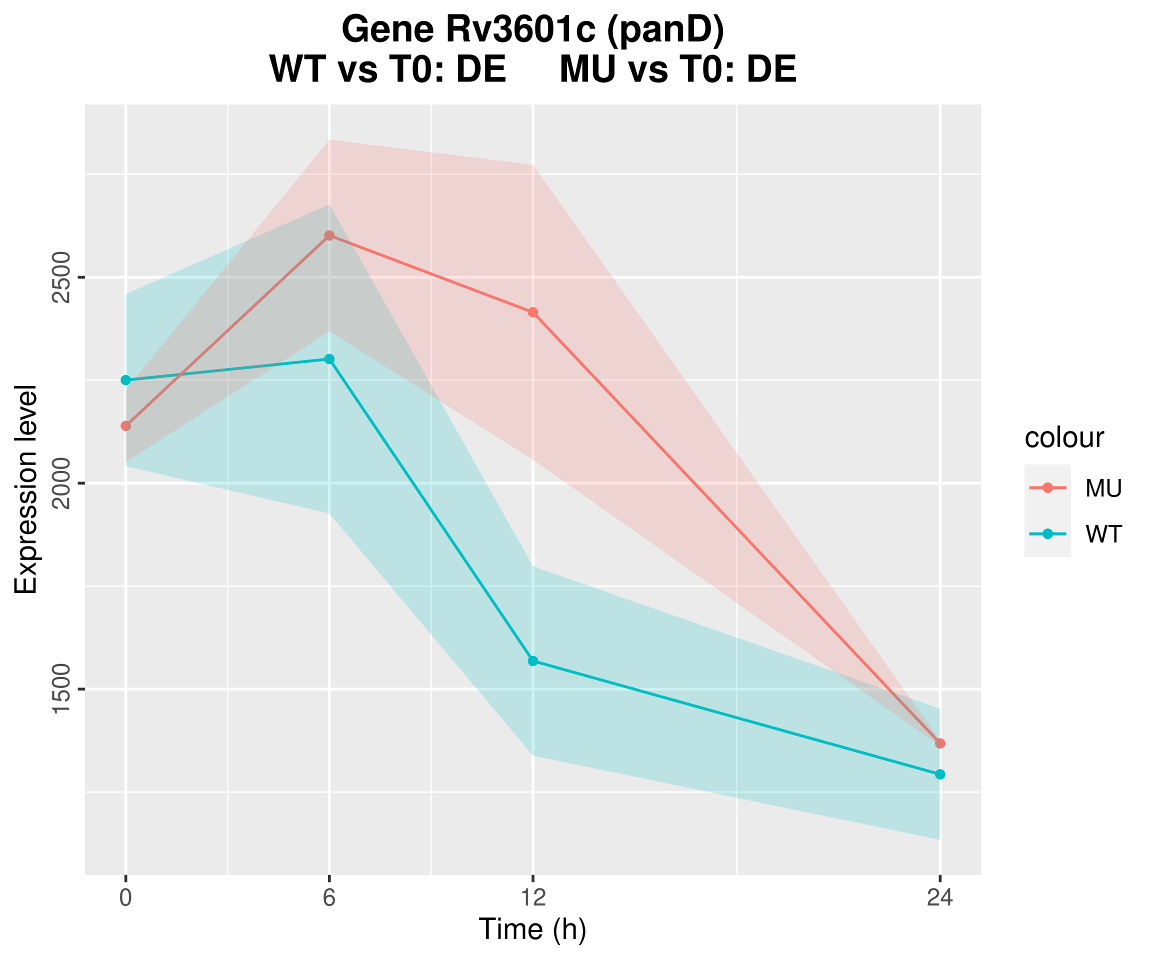

Gene Rv3601c (panD)
WT vs T0: DE MU vs T0: DE
Expression level
2500
2000
1500
0
6
12
24
Time (h)
colour
MU
WT

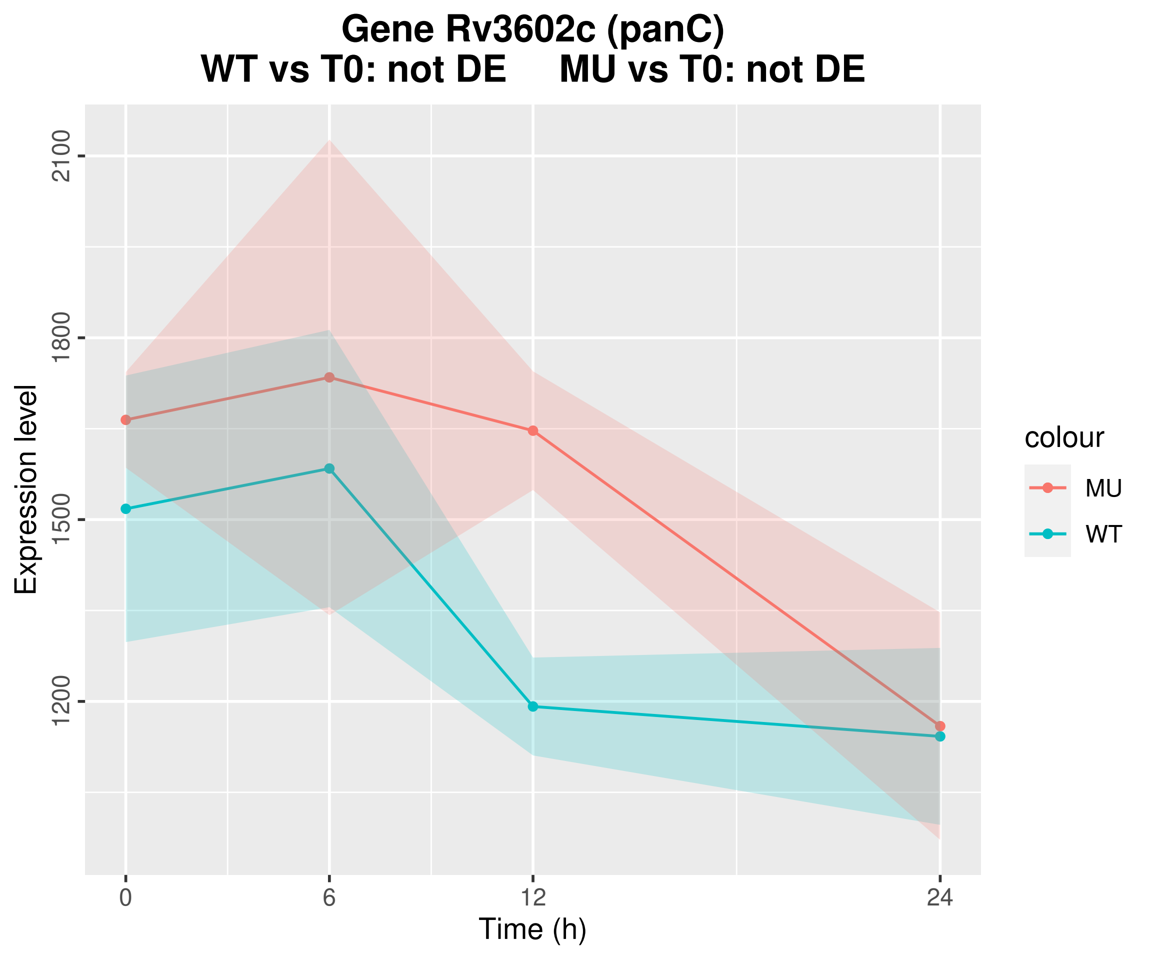

Gene Rv3602c (panC)
WT vs T0: not DE MU vs T0: not DE
2100
1800
1500
1200
Expression level
0
6
12
24
Time (h)
colour
MU
WT

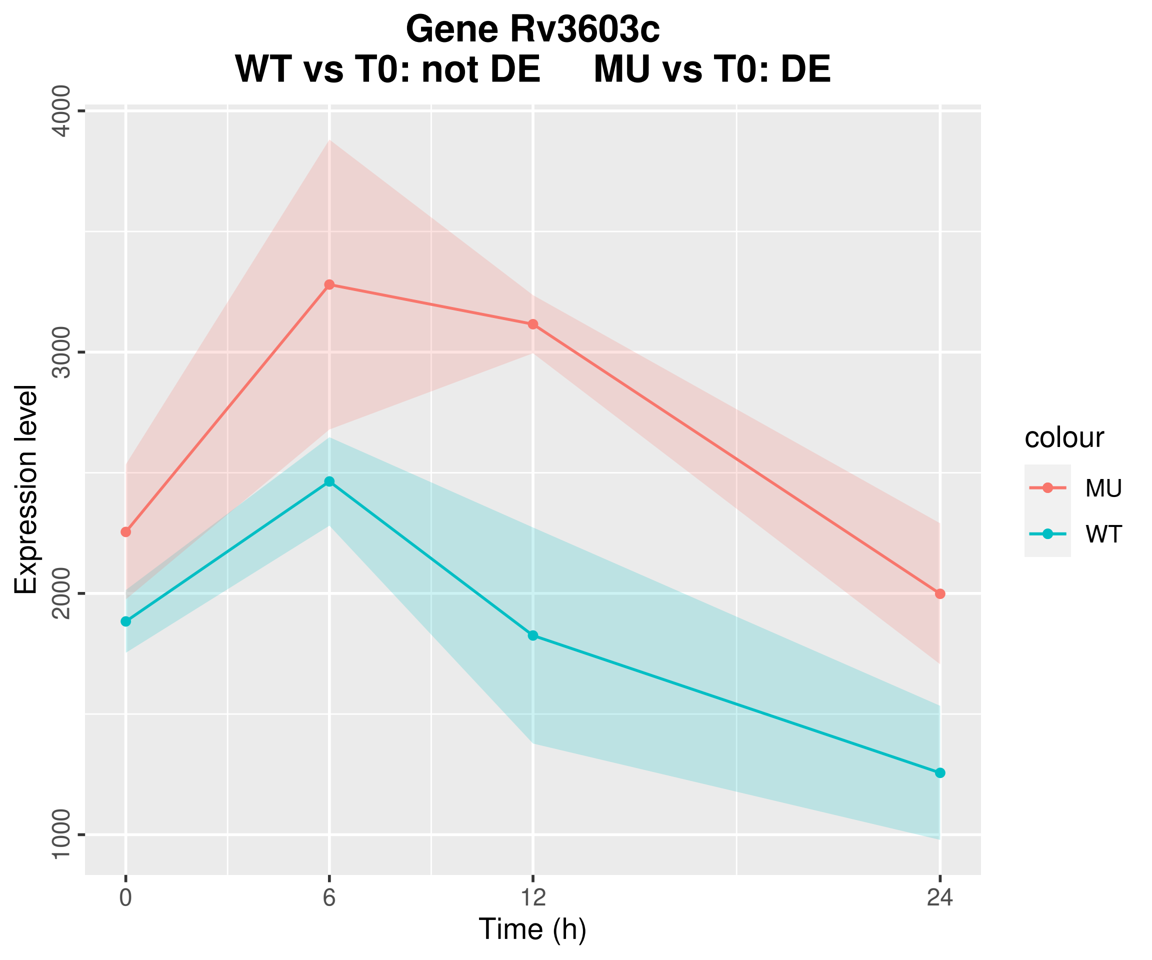
Gene Rv3603c
WT vs T0: not DE MU vs T0: DE
Expression level
4000
3000
2000
1000
0
6
12
24
Time (h)
colour
MU
WT

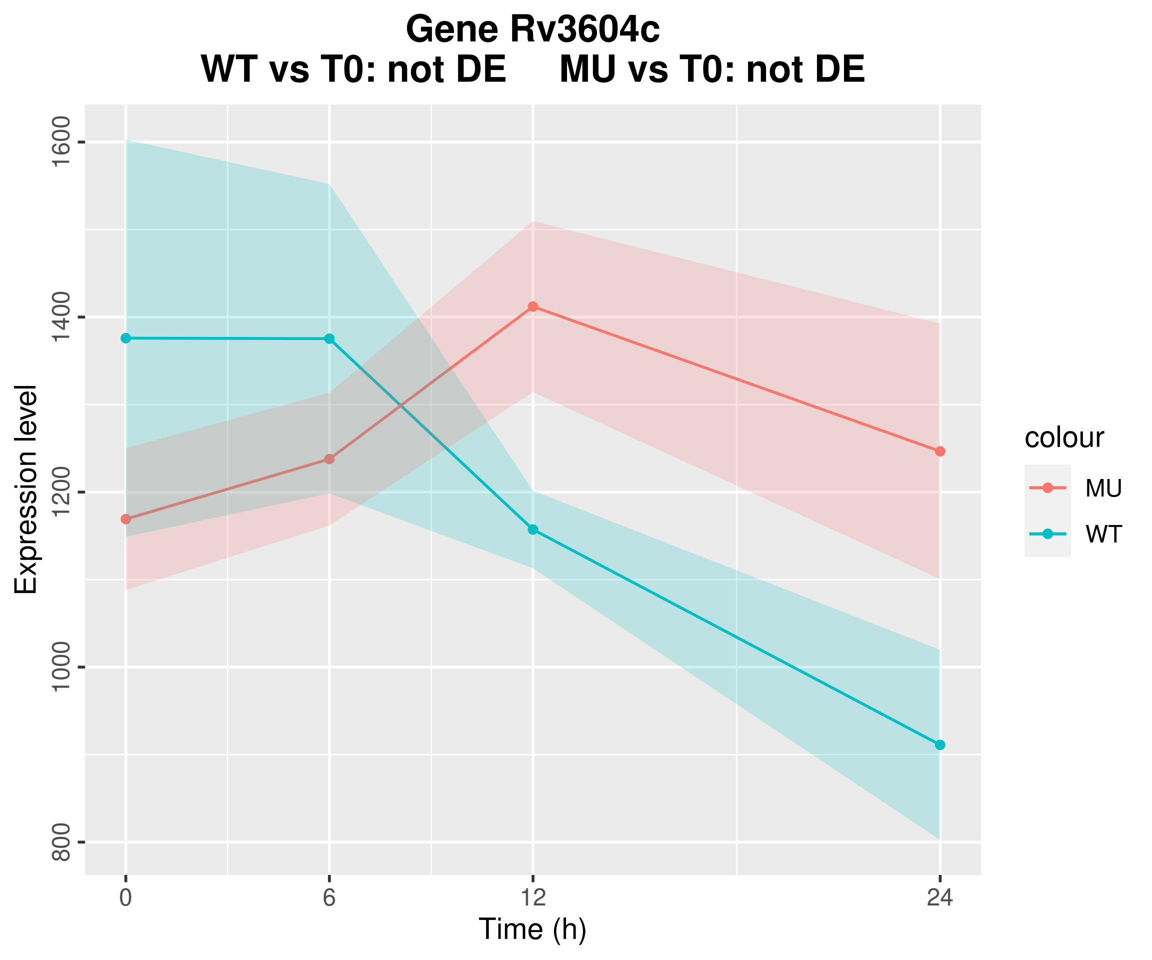
Gene Rv3604c
WT vs T0: not DE   MU vs T0: not DE
Expression level
1600
1400
1200
1000
800
0
6
12
24
Time (h)
colour
MU
WT

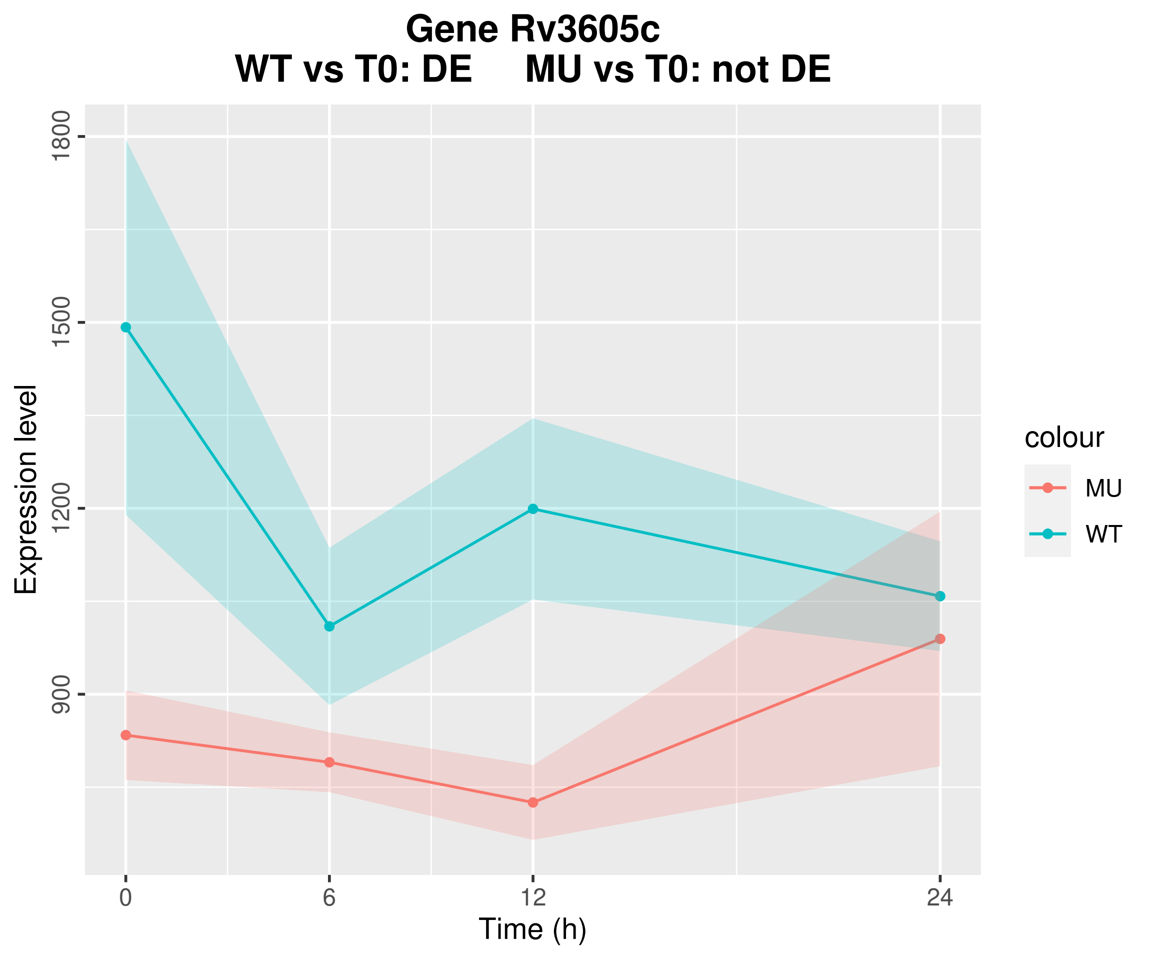

Gene Rv3605c
WT vs T0: DE   MU vs T0: not DE
Expression level
1800
1500
1200
900
0
6
12
24
Time (h)
colour
MU
WT

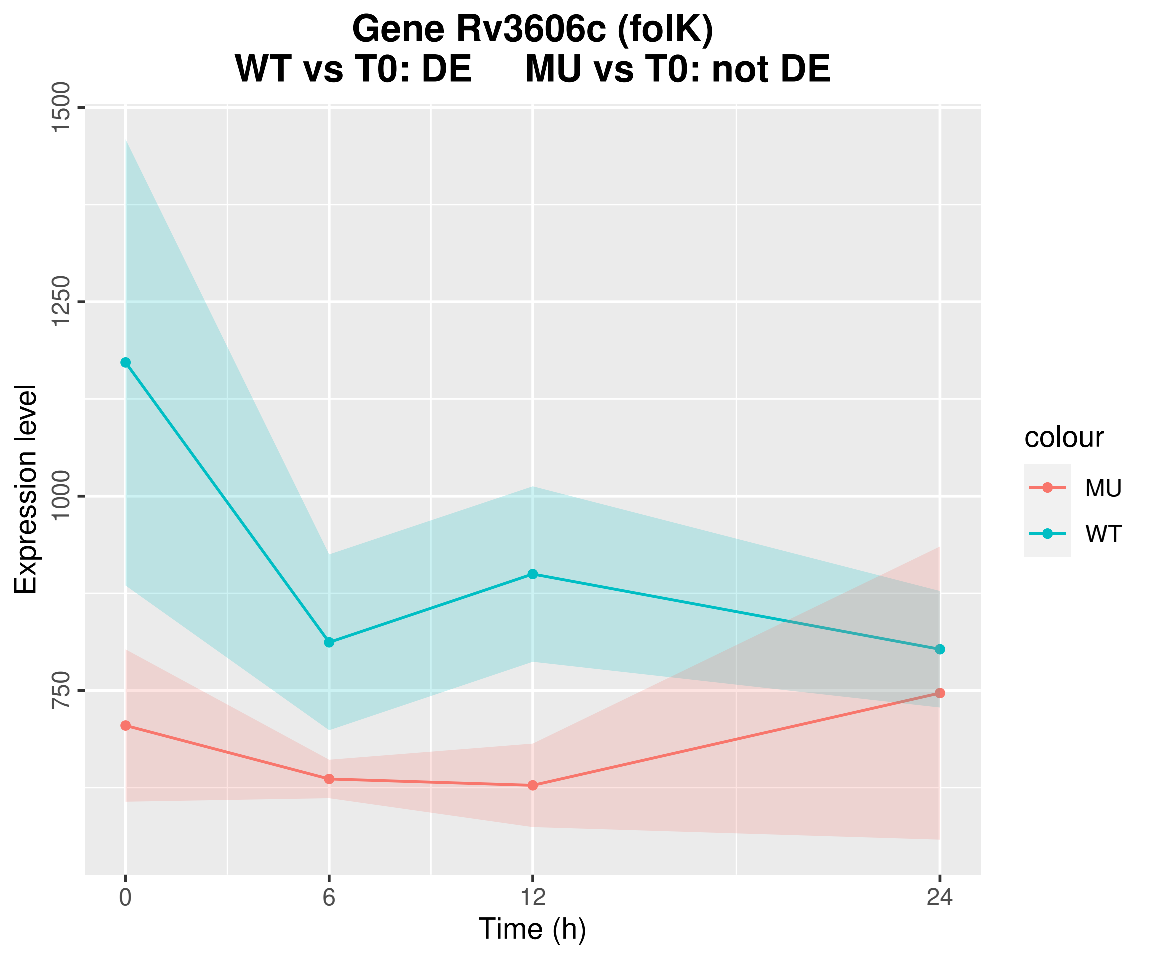
Gene Rv3606c (folK)
WT vs T0: DE MU vs T0: not DE
1500
1250
1000
750
Expression level
0
6
12
24
Time (h)
colour
MU
WT

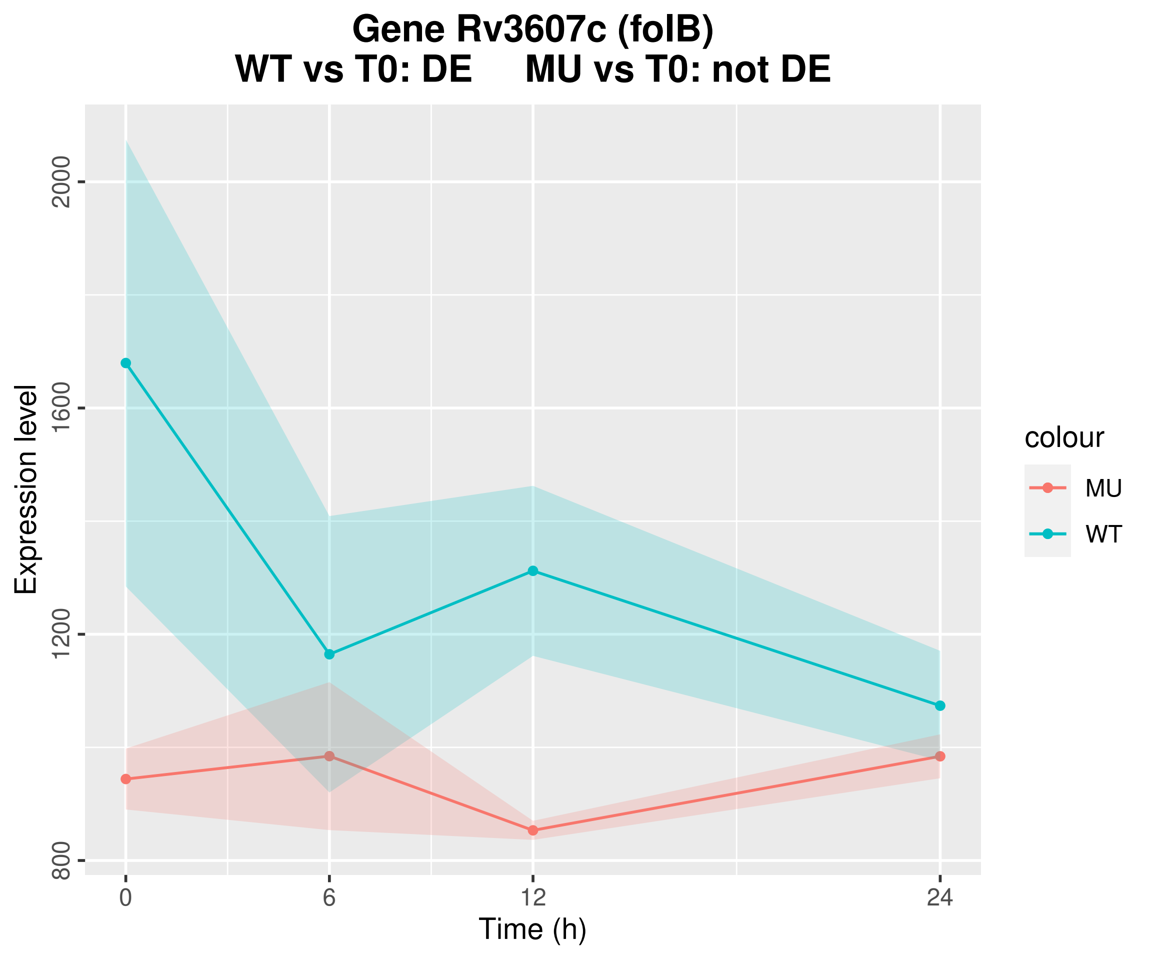

Gene Rv3607c (folB)
WT vs T0: DE MU vs T0: not DE
2000
1600
1200
800
Expression level
0
6
12
24
Time (h)
colour
MU
WT

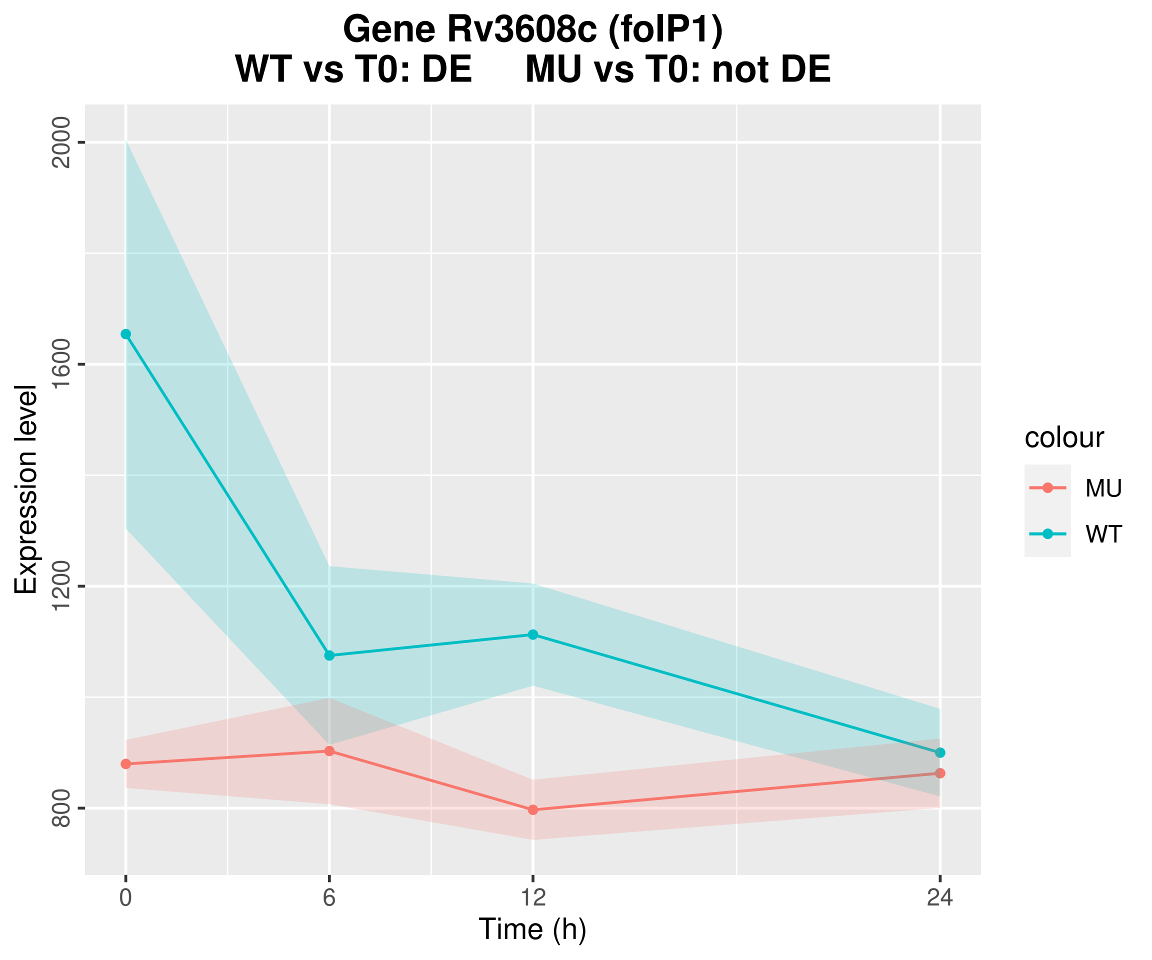

Gene Rv3608c (folP1)
WT vs T0: DE MU vs T0: not DE
Expression level
2000
1600
1200
800
0
6
12
24
Time (h)
colour
MU
WT

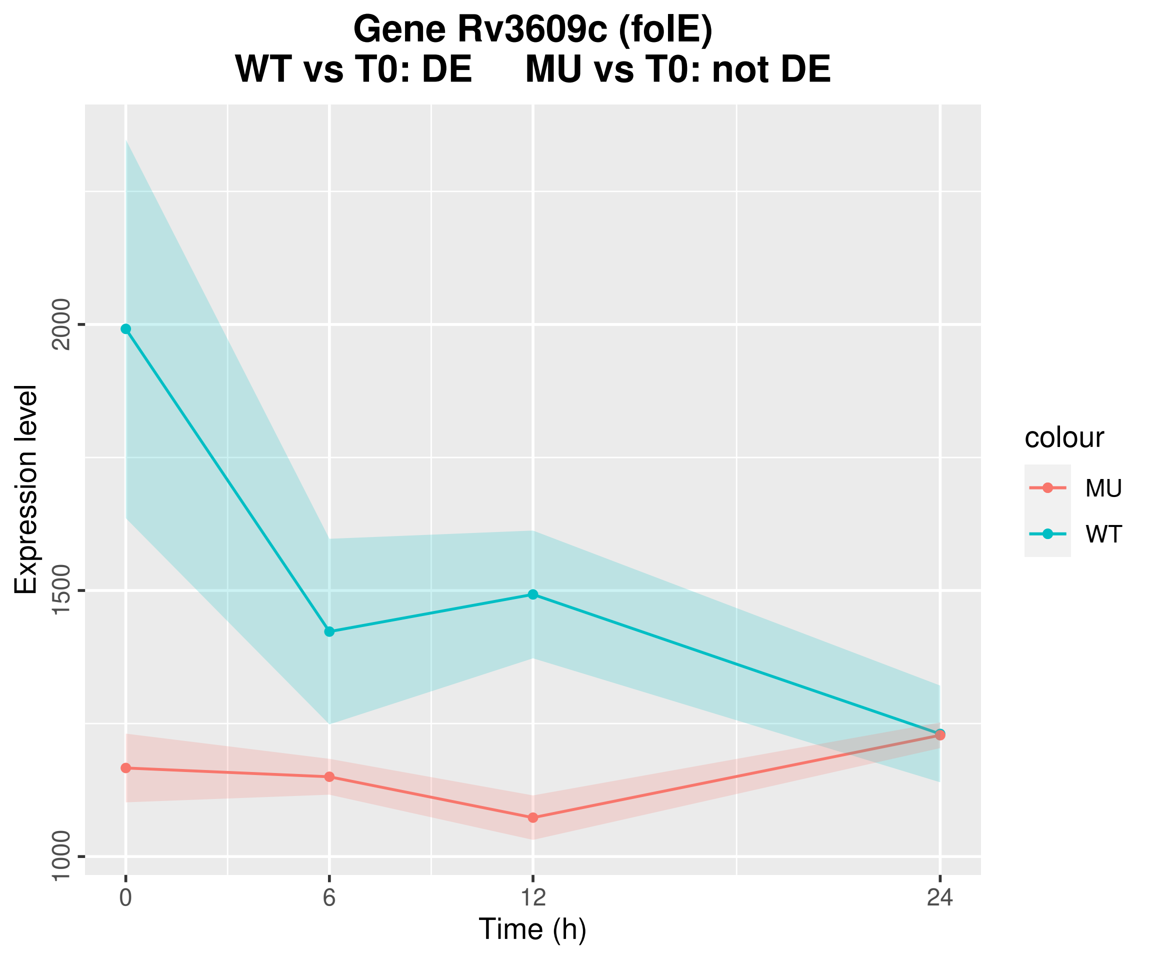
Gene Rv3609c (folE)
WT vs T0: DE MU vs T0: not DE
Expression level
2000
1500
1000
0
6
12
24
Time (h)
colour
MU
WT

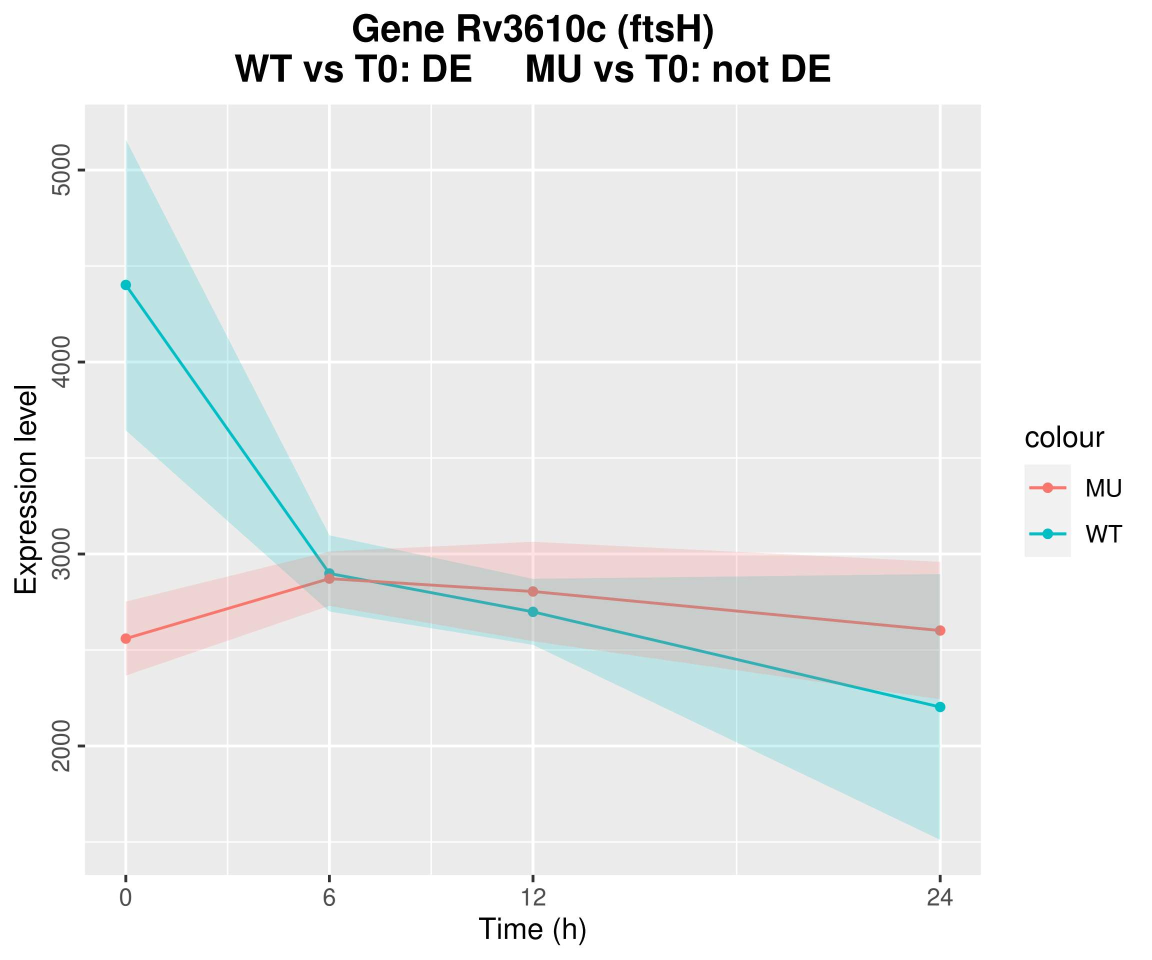
Gene Rv3610c (ftsH)
WT vs T0: DE MU vs T0: not DE
Expression level
5000
4000
3000
2000
0
6
12
24
Time (h)
colour
MU
WT

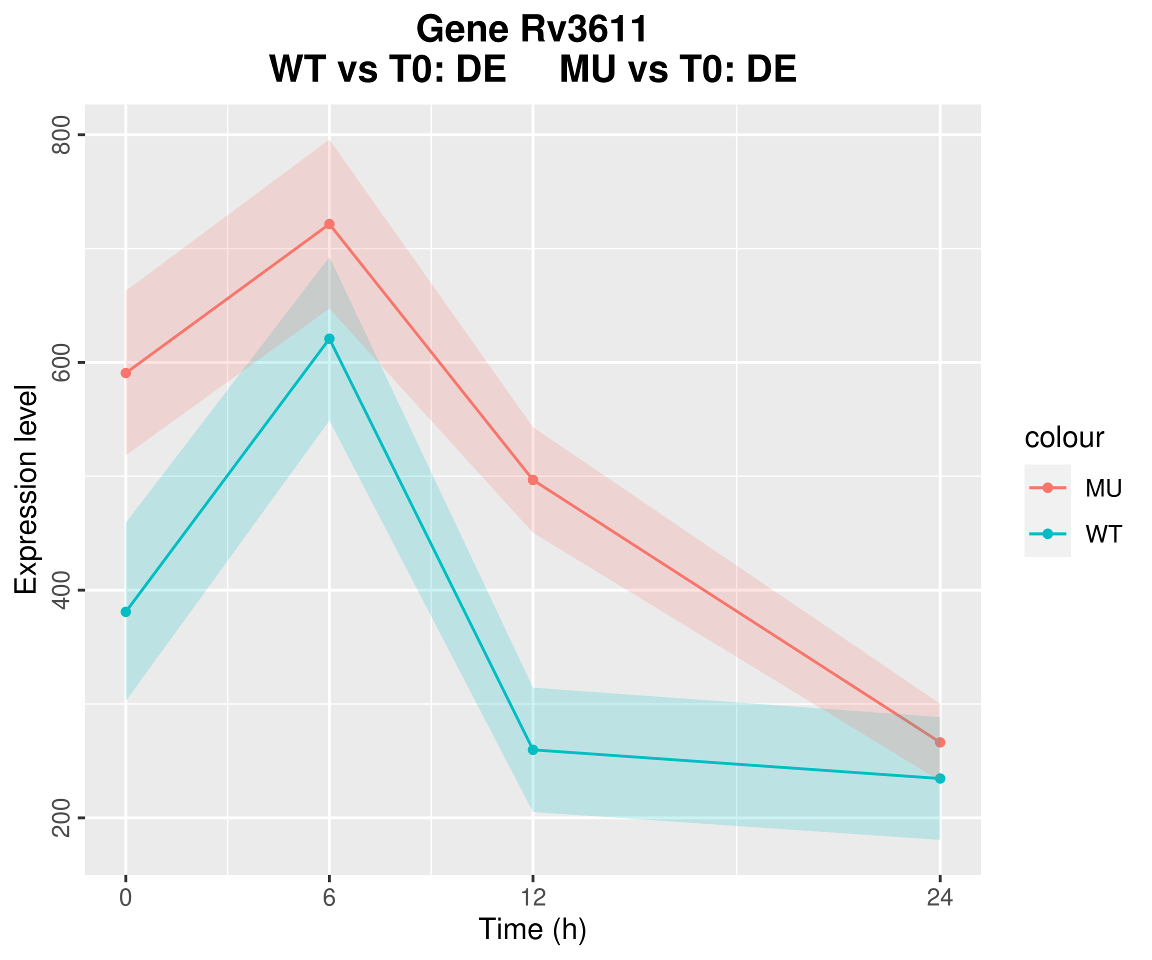

Gene Rv3611
WT vs T0: DE MU vs T0: DE
800
600
400
200
Expression level
0
6
12
24
Time (h)
colour
MU
WT

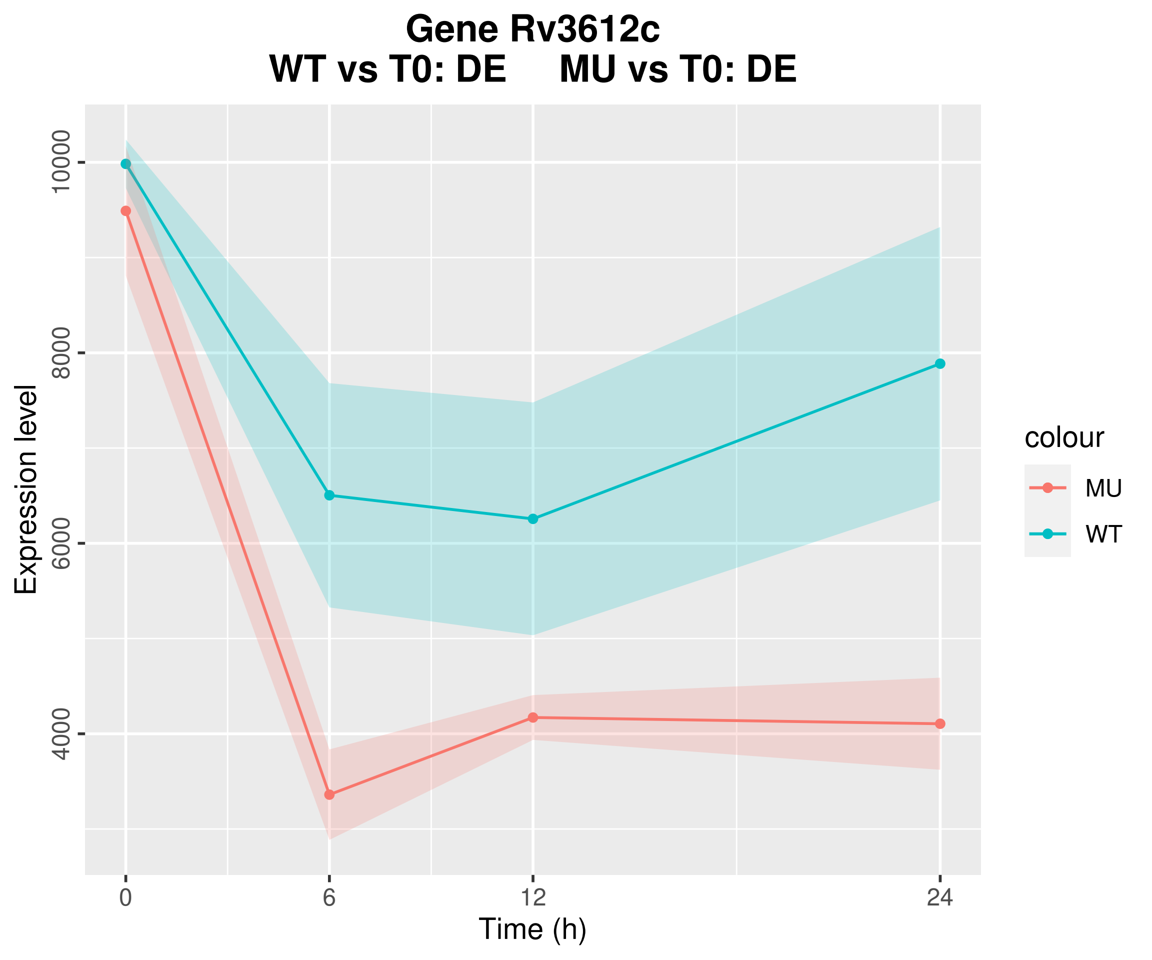
Gene Rv3612c
WT vs T0: DE MU vs T0: DE
Expression level
10000
8000
6000
4000
0
6
12
24
Time (h)
colour
MU
WT

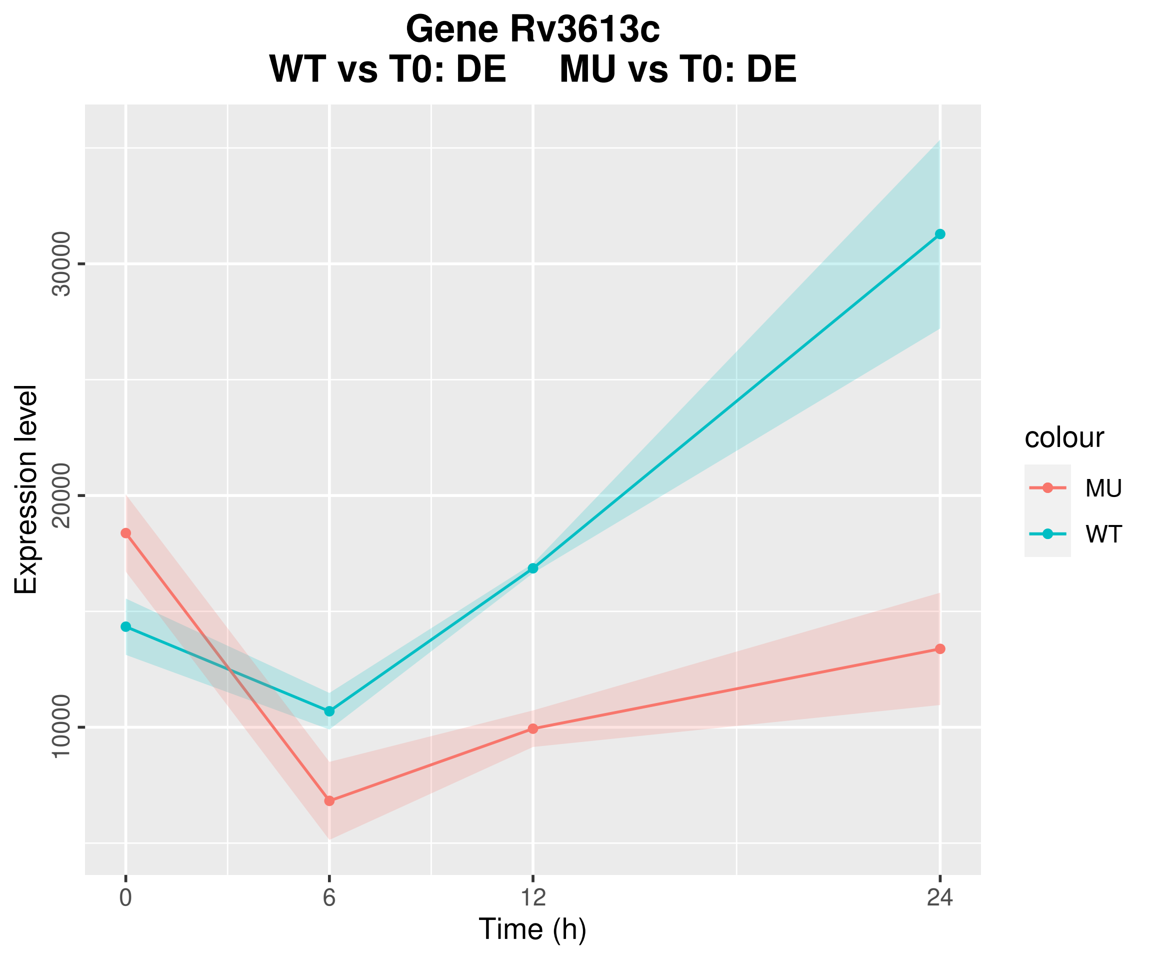
Gene Rv3613c
WT vs T0: DE MU vs T0: DE
Expression level
30000
20000
10000
0
6
12
24
Time (h)
colour
MU
WT

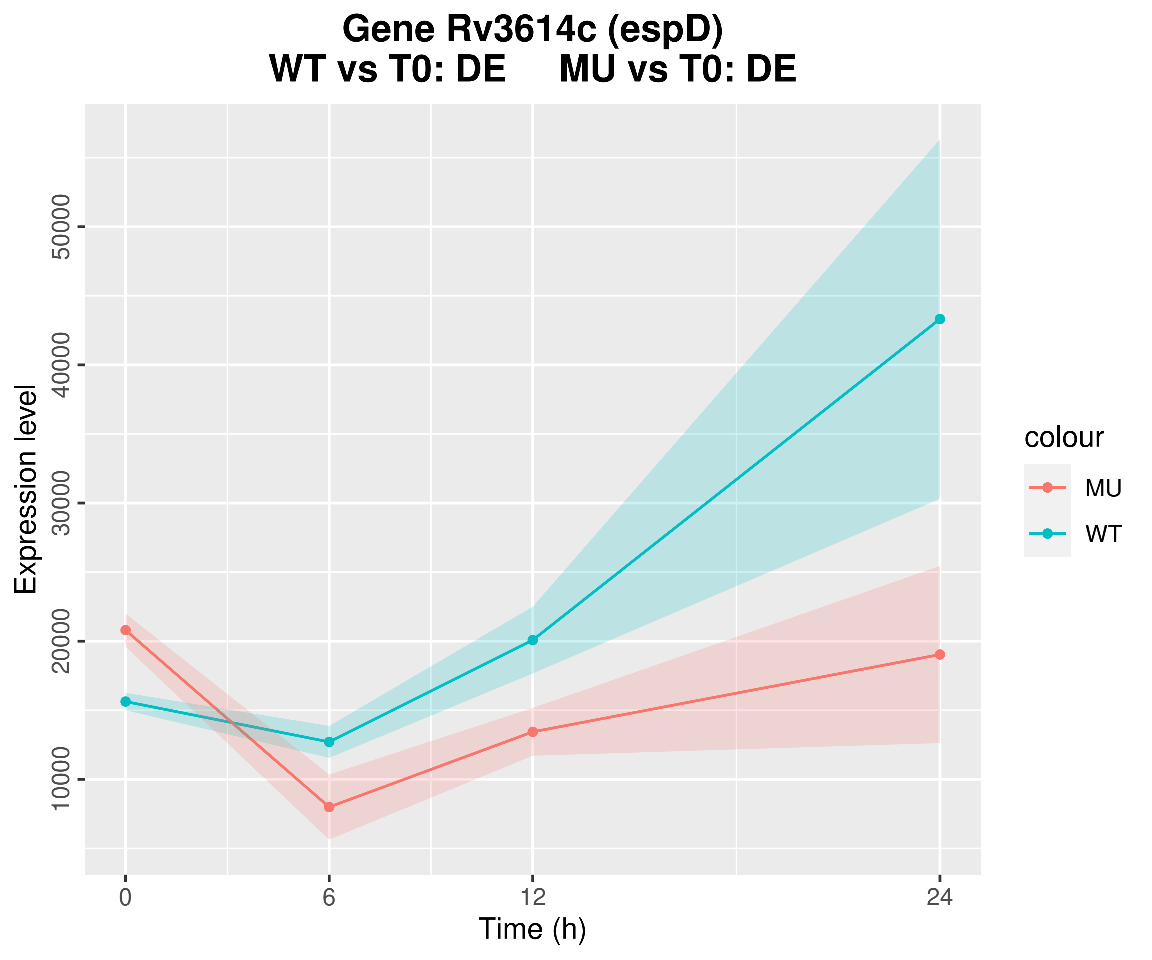

Gene Rv3614c (espD)
WT vs T0: DE   MU vs T0: DE
Expression level
50000
40000
30000
20000
10000
0
6
12
24
Time (h)
colour
MU
WT

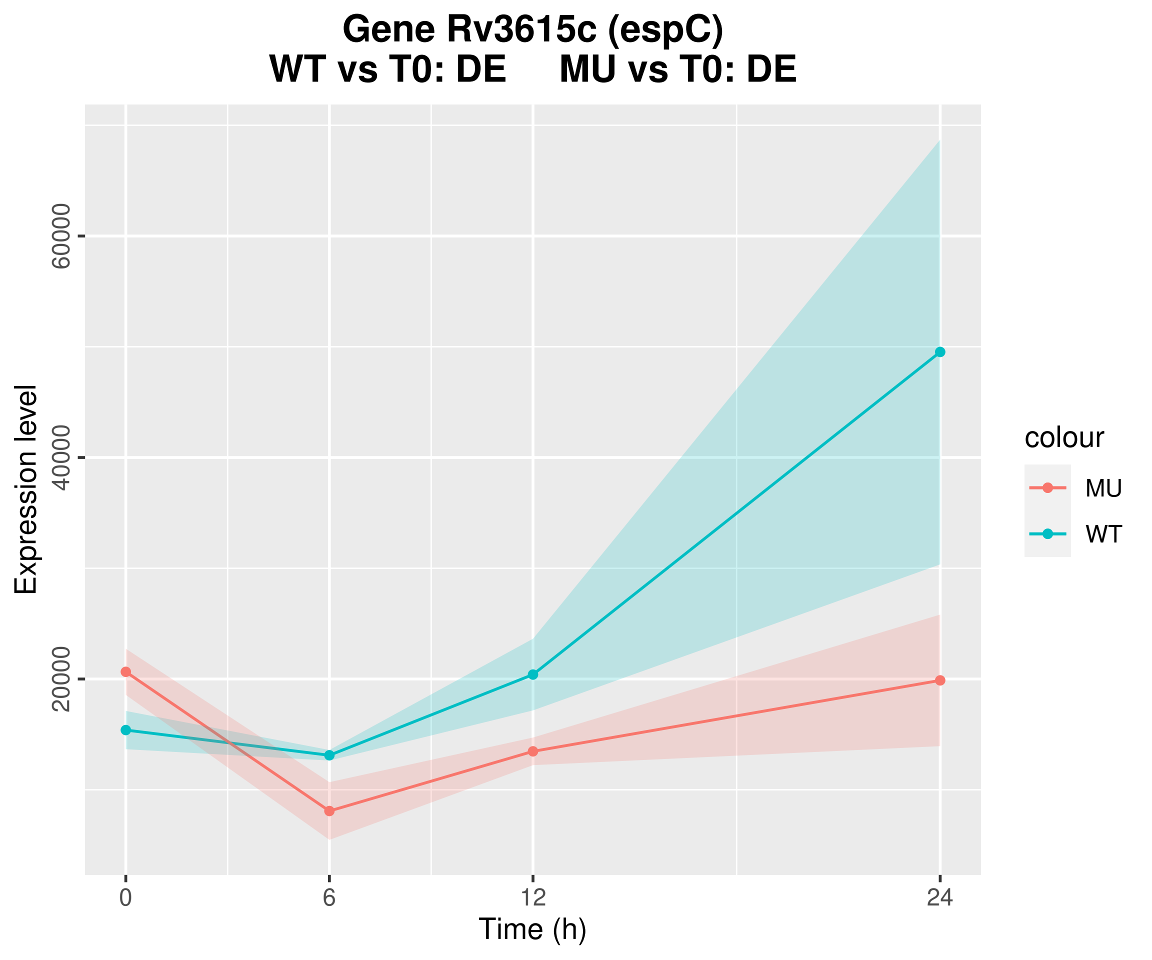
Gene Rv3615c (espC)
WT vs T0: DE   MU vs T0: DE
Expression level
60000
40000
20000
0
6
12
24
Time (h)
colour
MU
WT

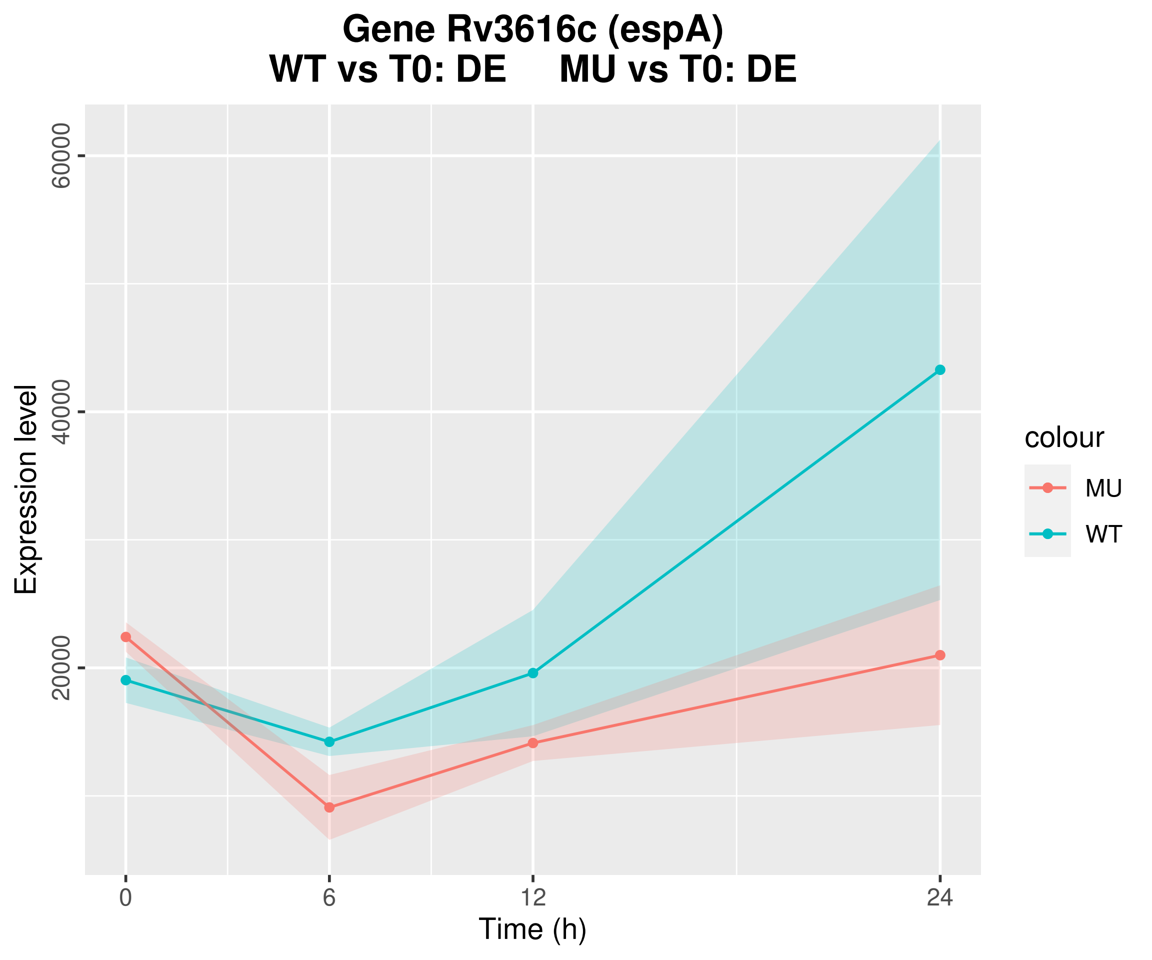

Gene Rv3616c (espA)
WT vs T0: DE MU vs T0: DE
Expression level
60000
40000
20000
0
6
12
24
Time (h)
colour
MU
WT

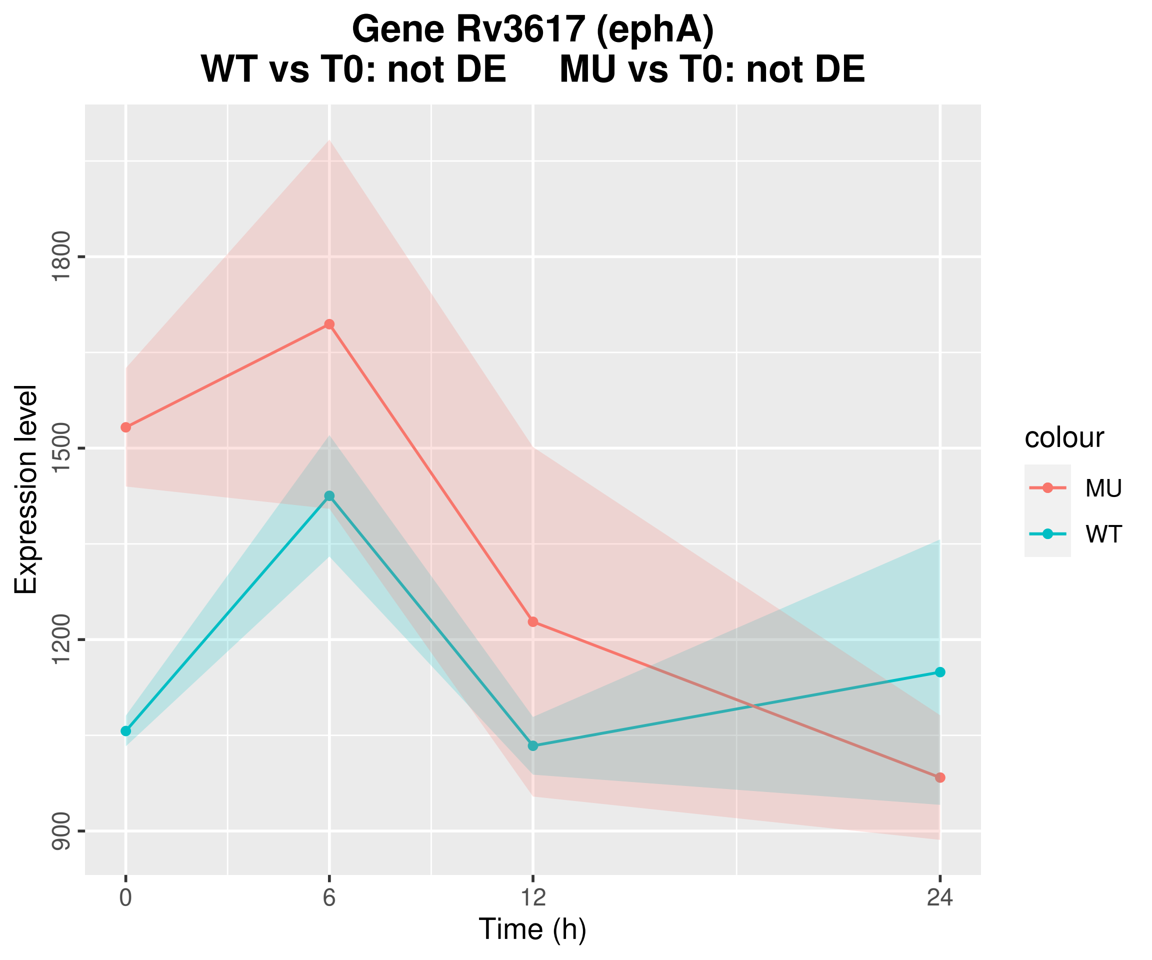
Gene Rv3617 (ephA)
WT vs T0: not DE   MU vs T0: not DE
Expression level
1800
1500
1200
900
0
6
12
24
Time (h)
colour
MU
WT

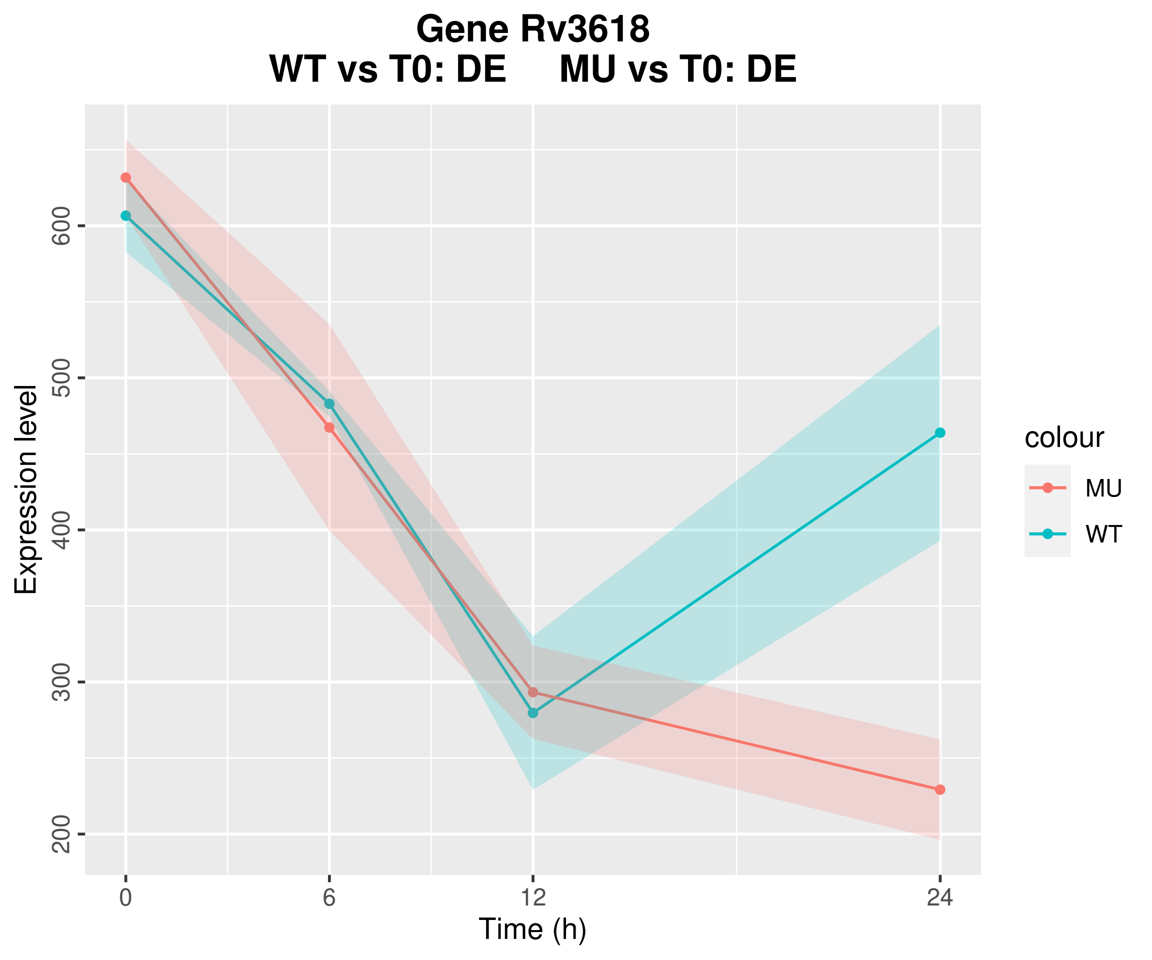

Gene Rv3618
WT vs T0: DE   MU vs T0: DE
Expression level
600
500
400
300
200
0
6
12
24
Time (h)
colour
MU
WT

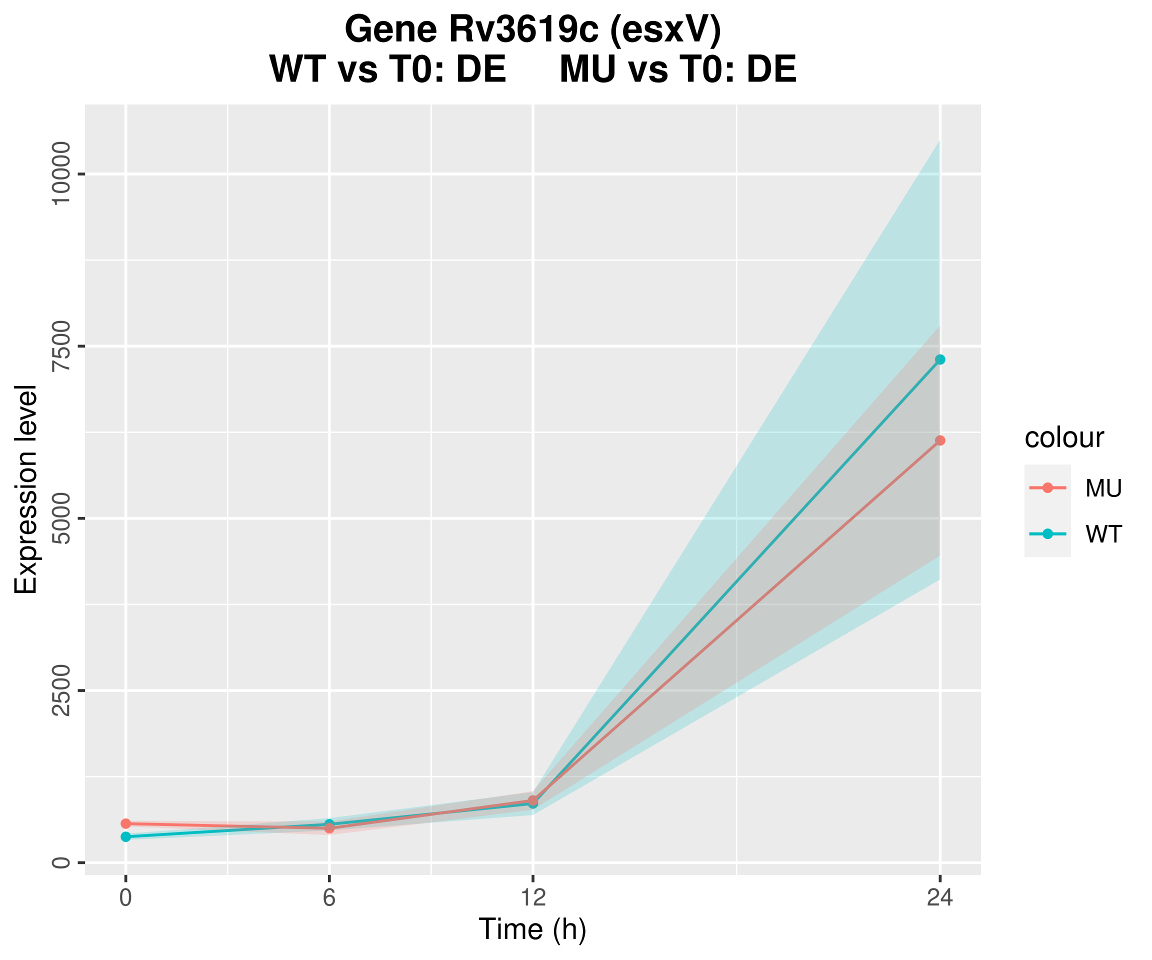
Gene Rv3619c (esxV)
WT vs T0: DE MU vs T0: DE
Expression level
10000
7500
5000
2500
0
0
6
12
24
Time (h)
colour
MU
WT

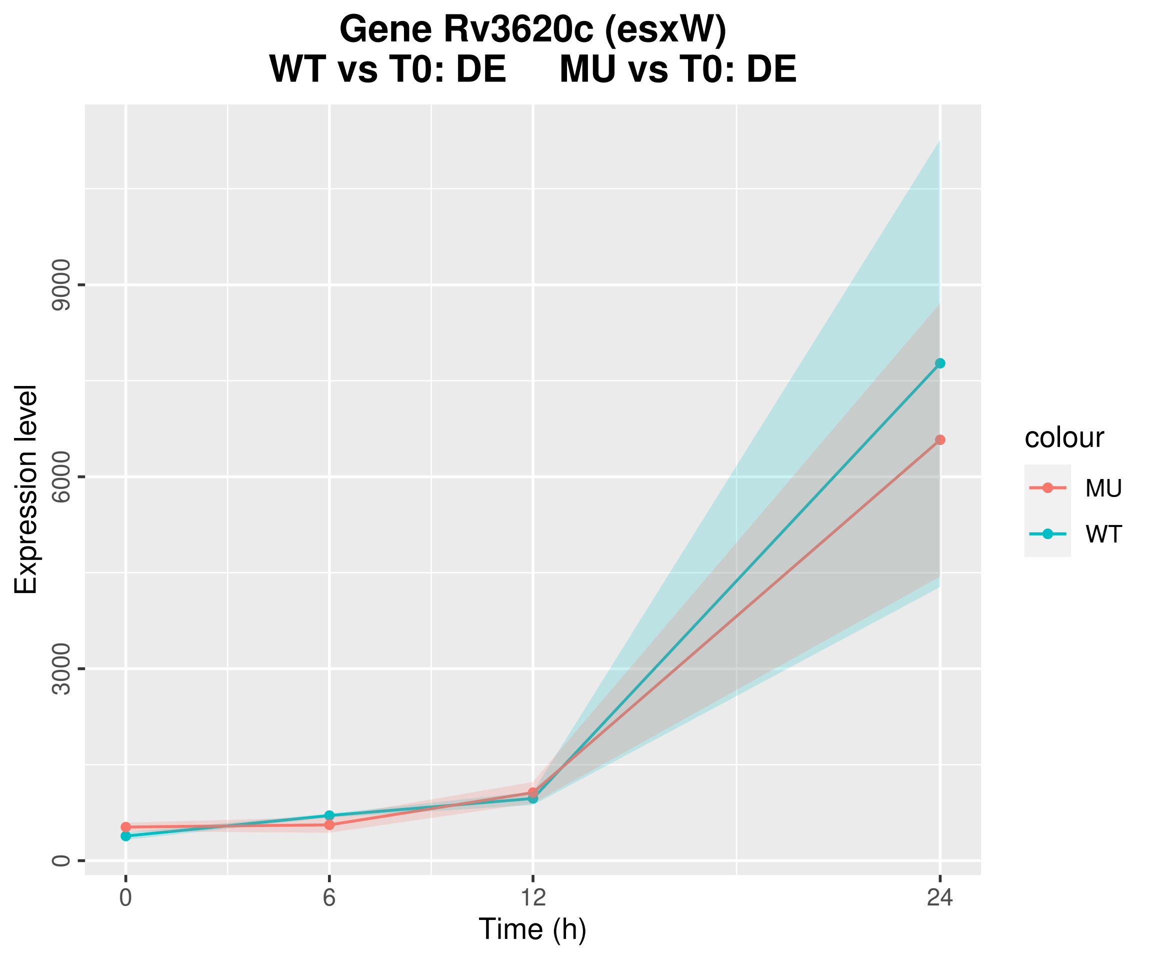
Gene Rv3620c (esxW)
WT vs T0: DE   MU vs T0: DE
Expression level
9000
6000
3000
0
0
6
12
24
Time (h)
colour
MU
WT

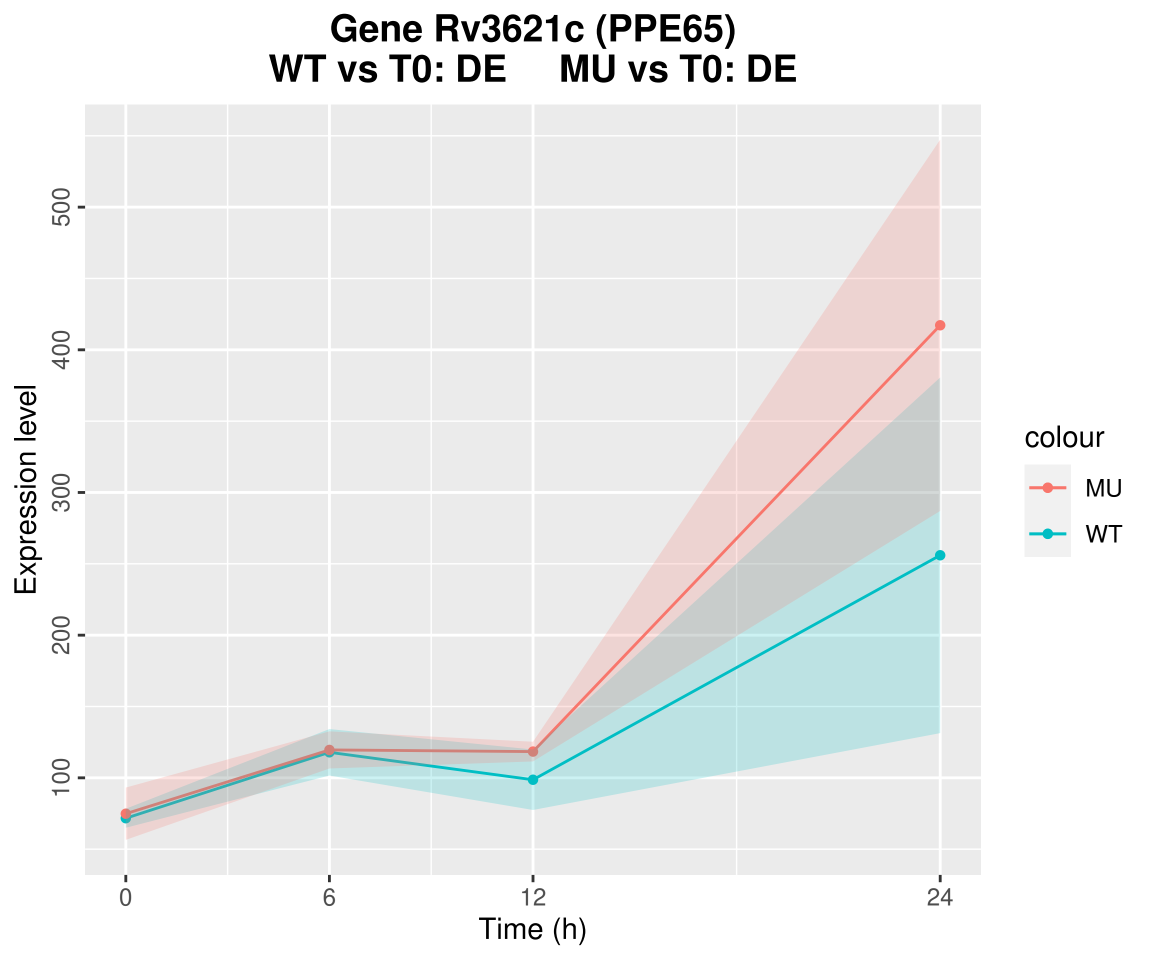

Gene Rv3621c (PPE65)
WT vs T0: DE MU vs T0: DE
Expression level
500
400
300
200
100
0
6
12
24
Time (h)
colour
MU
WT

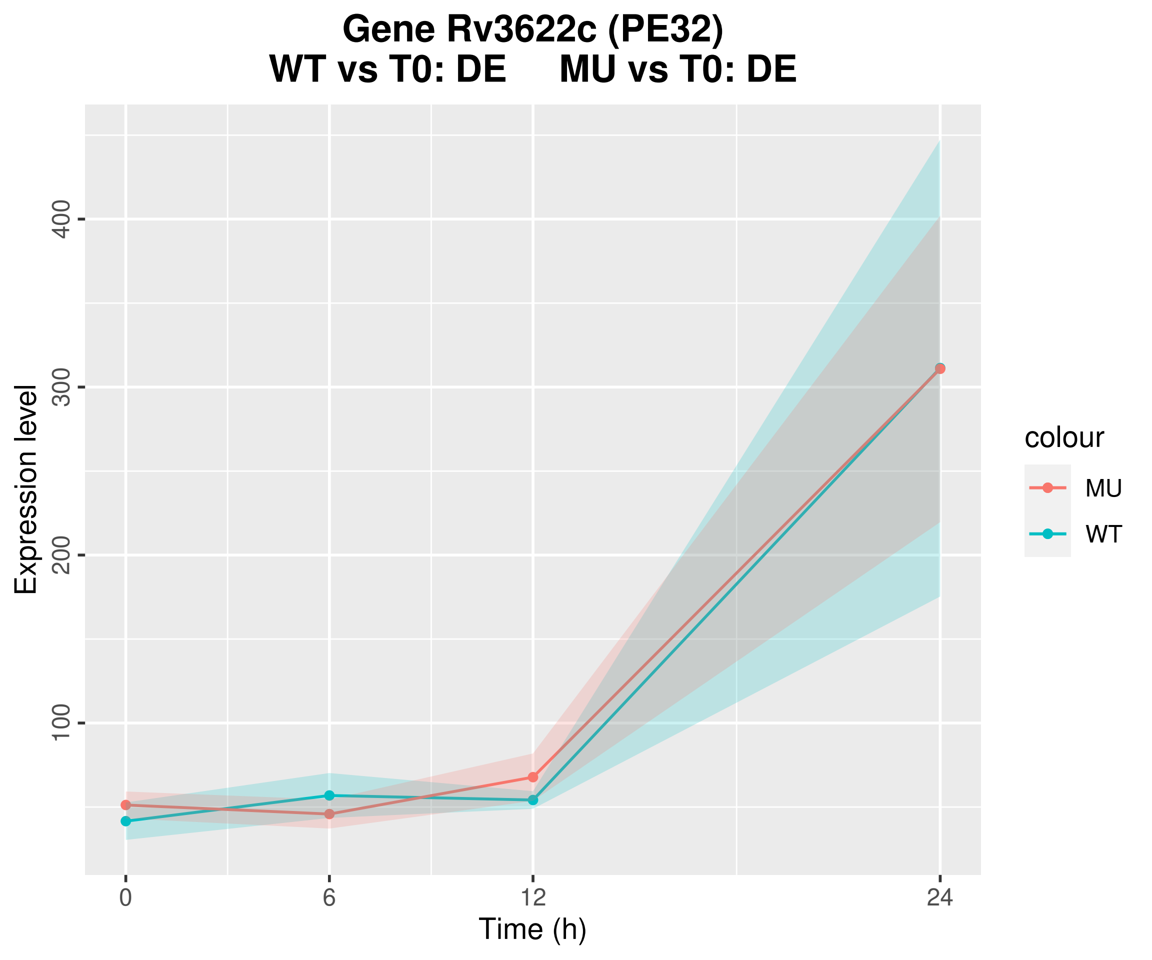

Gene Rv3622c (PE32)
WT vs T0: DE   MU vs T0: DE
Expression level
400
300
200
100
0
6
12
24
Time (h)
colour
MU
WT

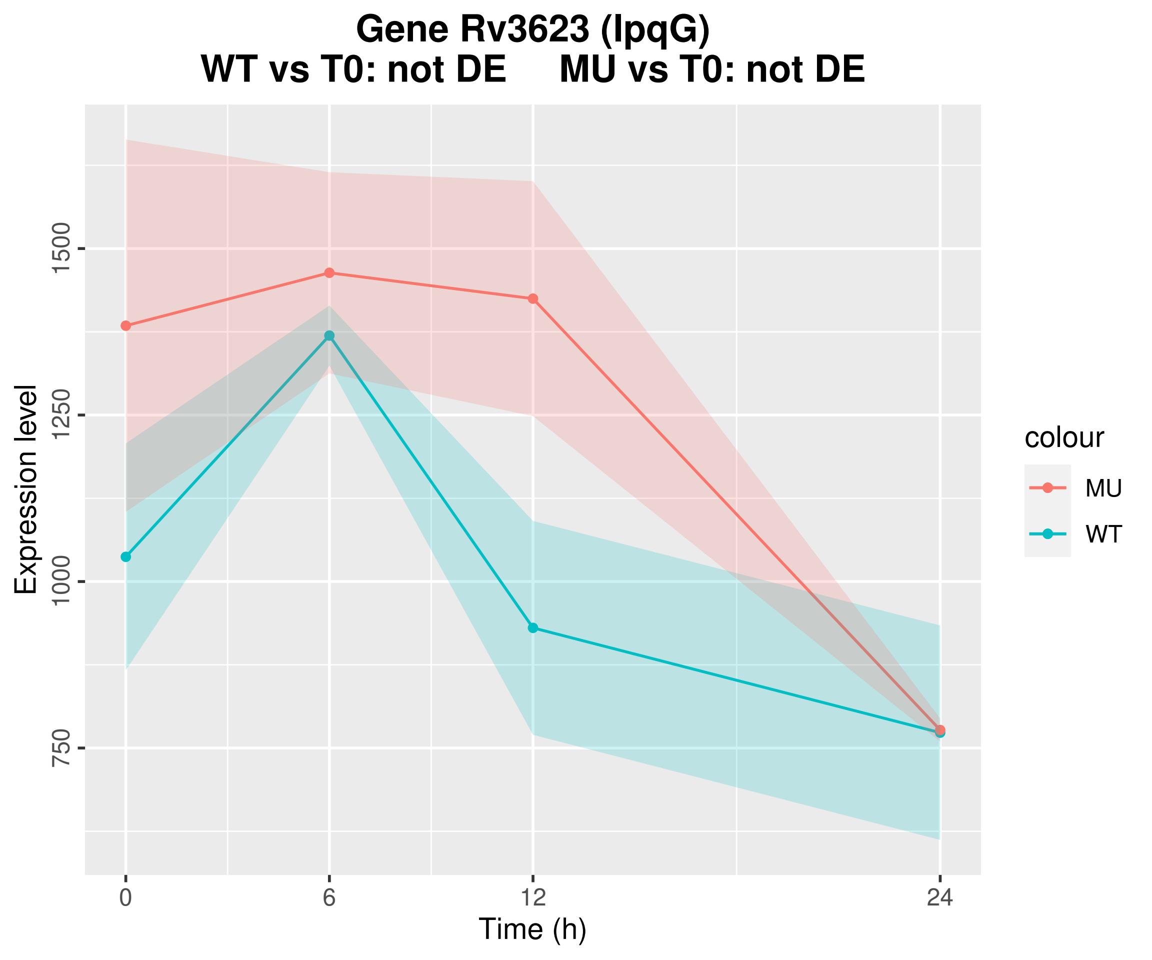

Gene Rv3623 (lpqG)
WT vs T0: not DE   MU vs T0: not DE
Expression level
1500
1250
1000
750
0
6
12
24
Time (h)
colour
MU
WT

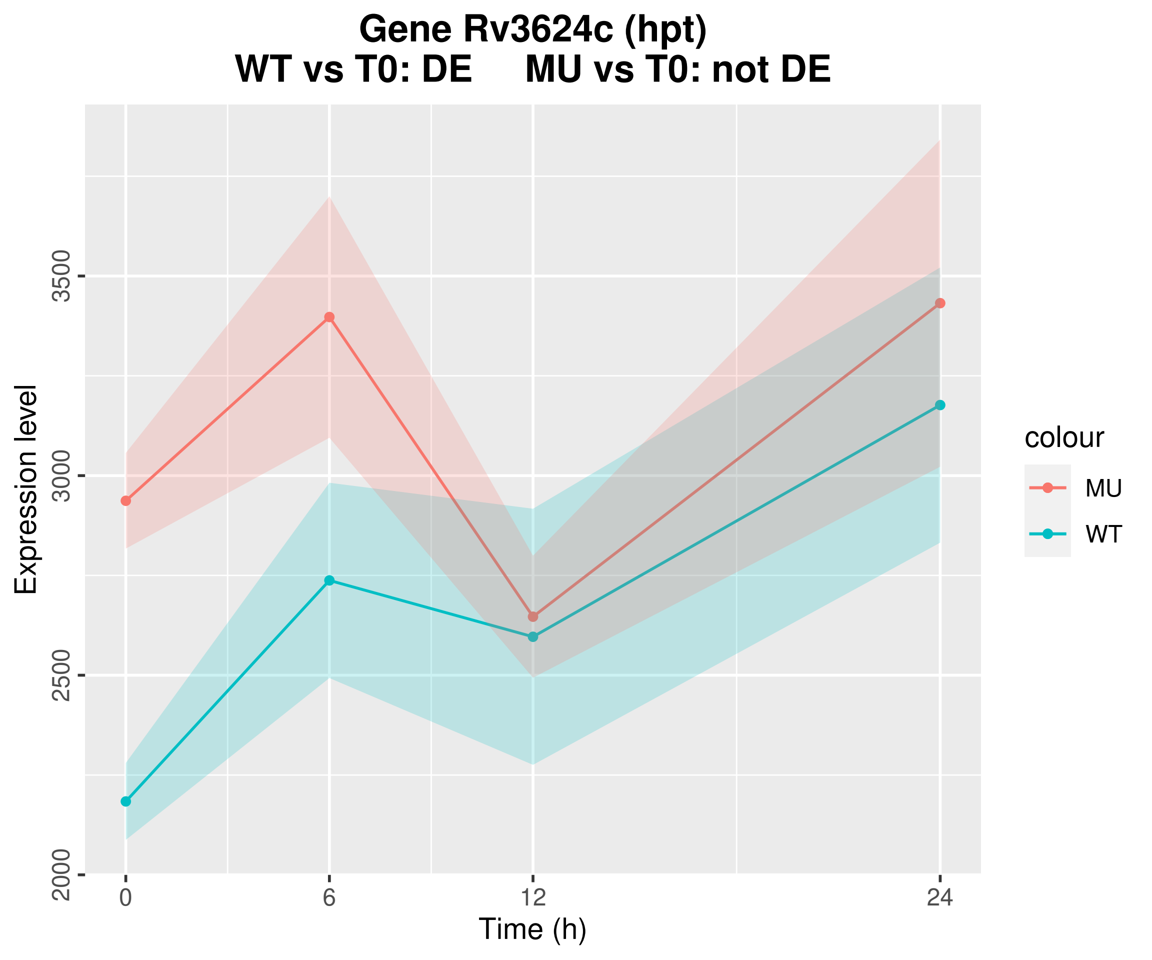
Gene Rv3624c (hpt)
WT vs T0: DE   MU vs T0: not DE
Expression level
2000
2500
3000
3500
0
6
12
24
Time (h)
colour
MU
WT

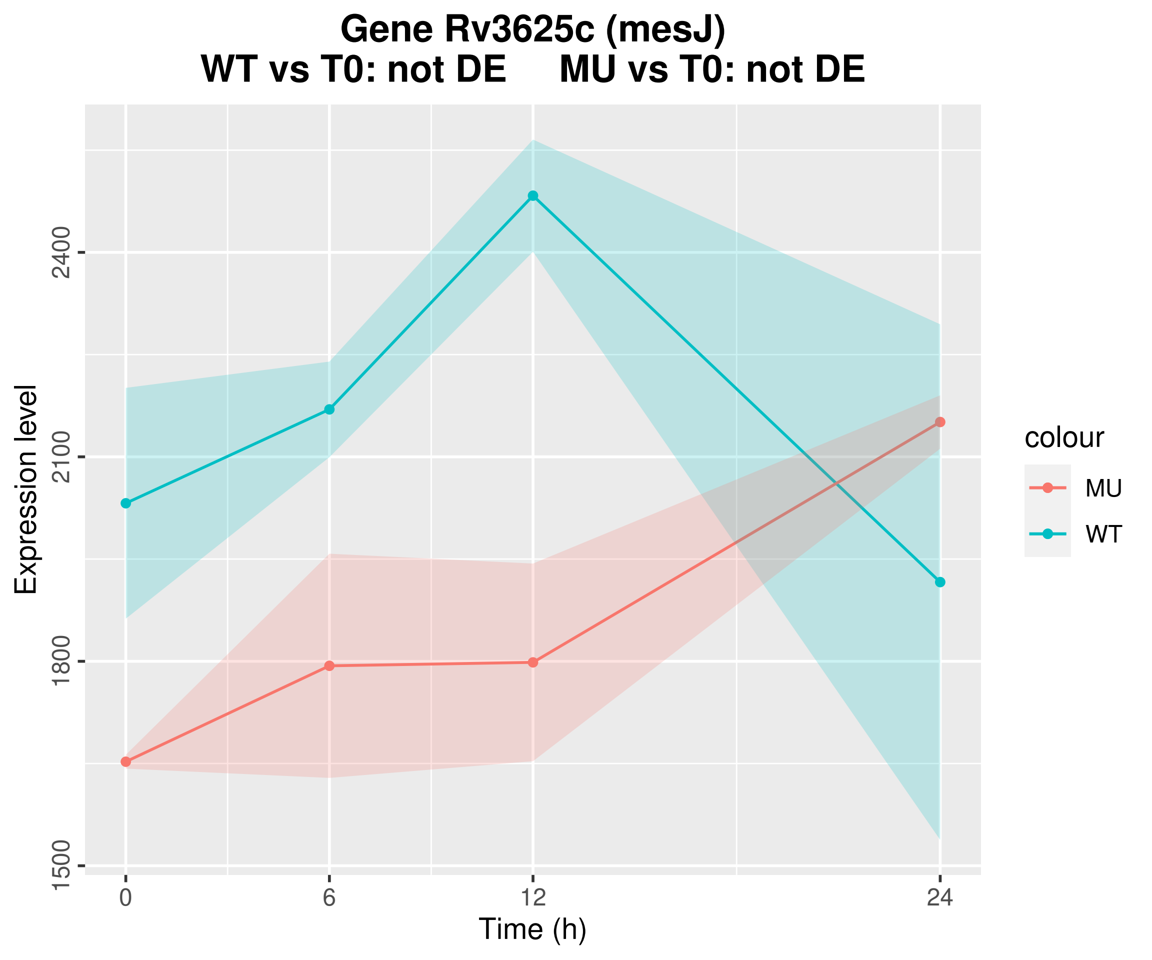
Gene Rv3625c (mesJ)
WT vs T0: not DE   MU vs T0: not DE
Expression level
2400
2100
1800
1500
0
6
12
24
Time (h)
colour
MU
WT

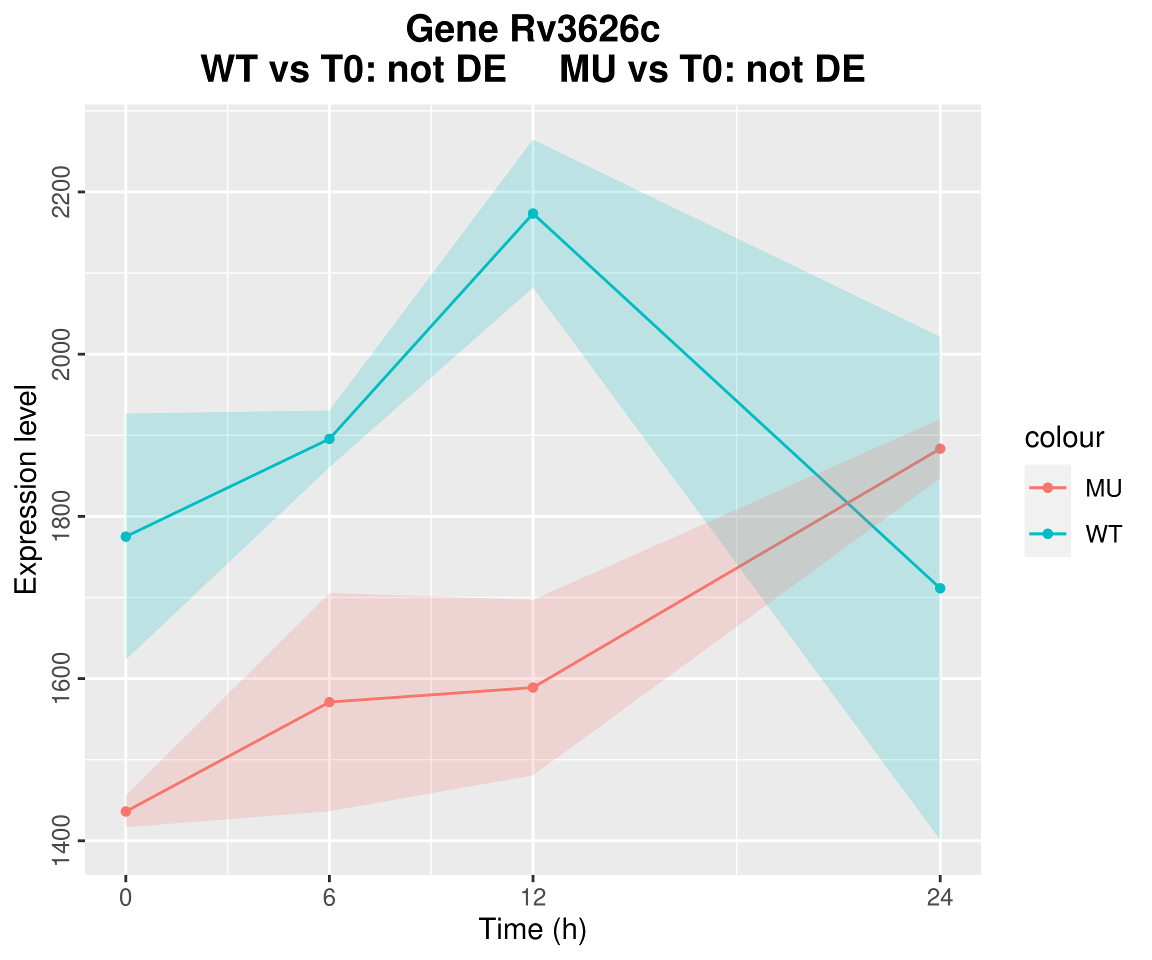
Gene Rv3626c
WT vs T0: not DE   MU vs T0: not DE
Expression level
2200
2000
1800
1600
1400
0
6
12
24
Time (h)
colour
MU
WT

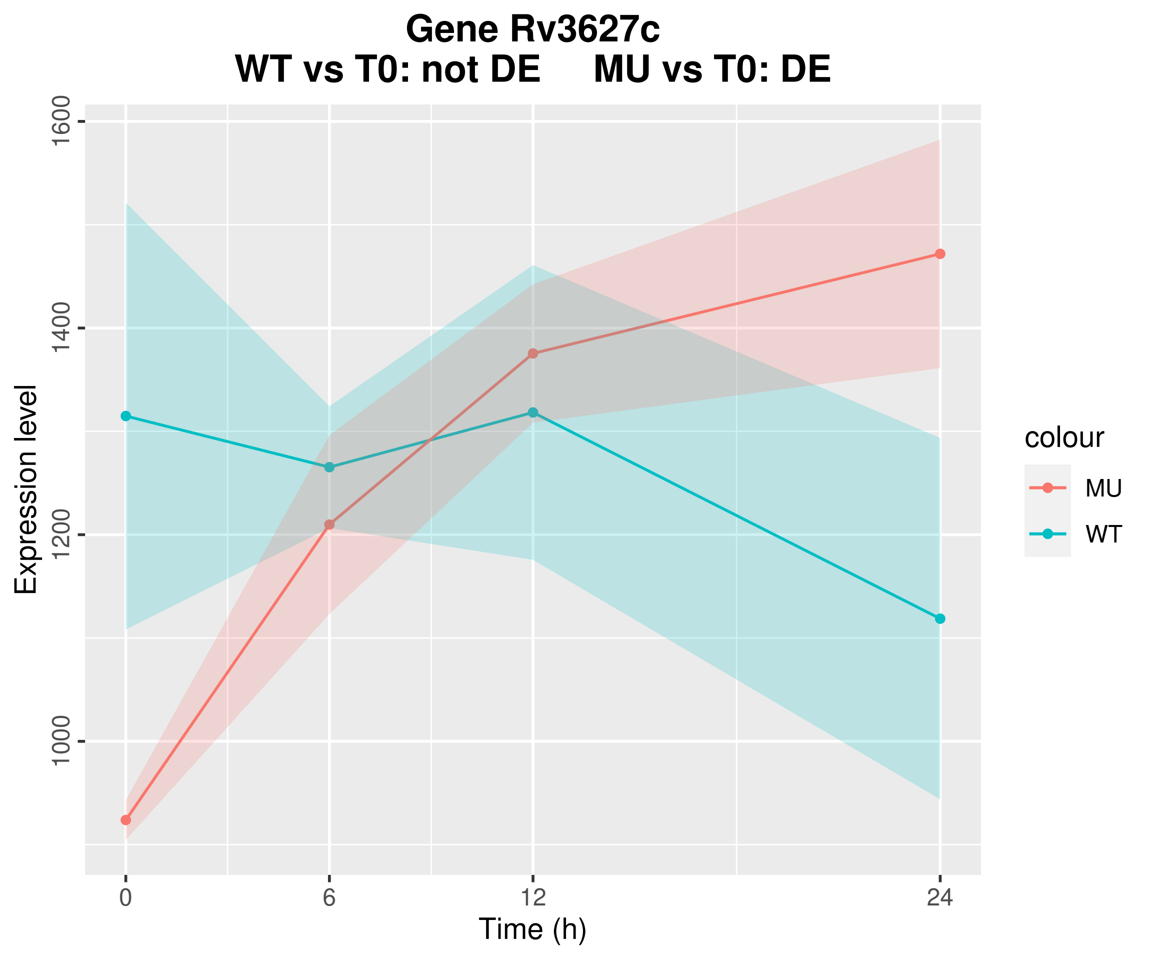
Gene Rv3627c
WT vs T0: not DE   MU vs T0: DE
Expression level
1600
1400
1200
1000
0
6
12
24
Time (h)
colour
MU
WT

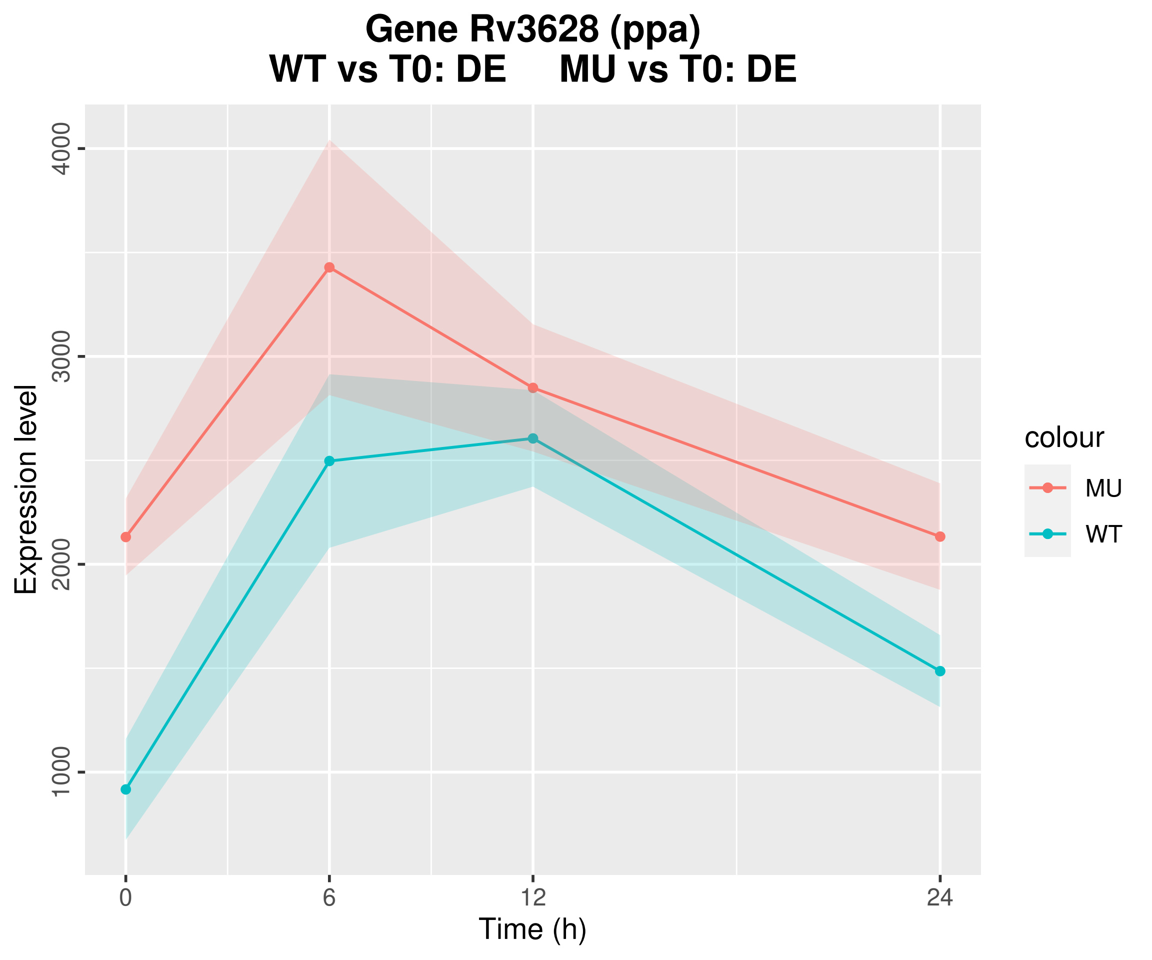

Gene Rv3628 (ppa)
WT vs T0: DE MU vs T0: DE
Expression level
4000
3000
2000
1000
0
6
12
24
Time (h)
colour
MU
WT

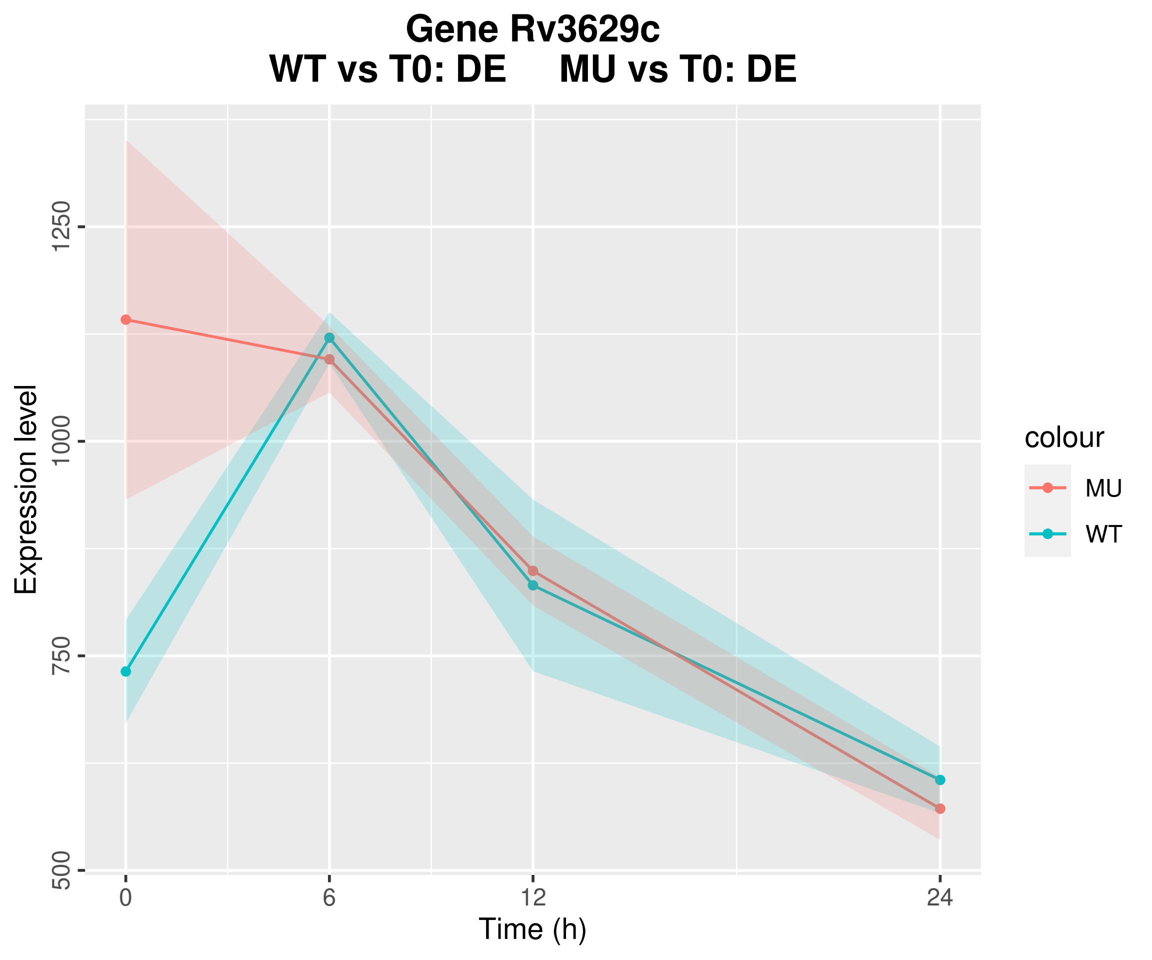
Gene Rv3629c
WT vs T0: DE   MU vs T0: DE
Expression level
1250
1000
750
500
0
6
12
24
Time (h)
colour
MU
WT

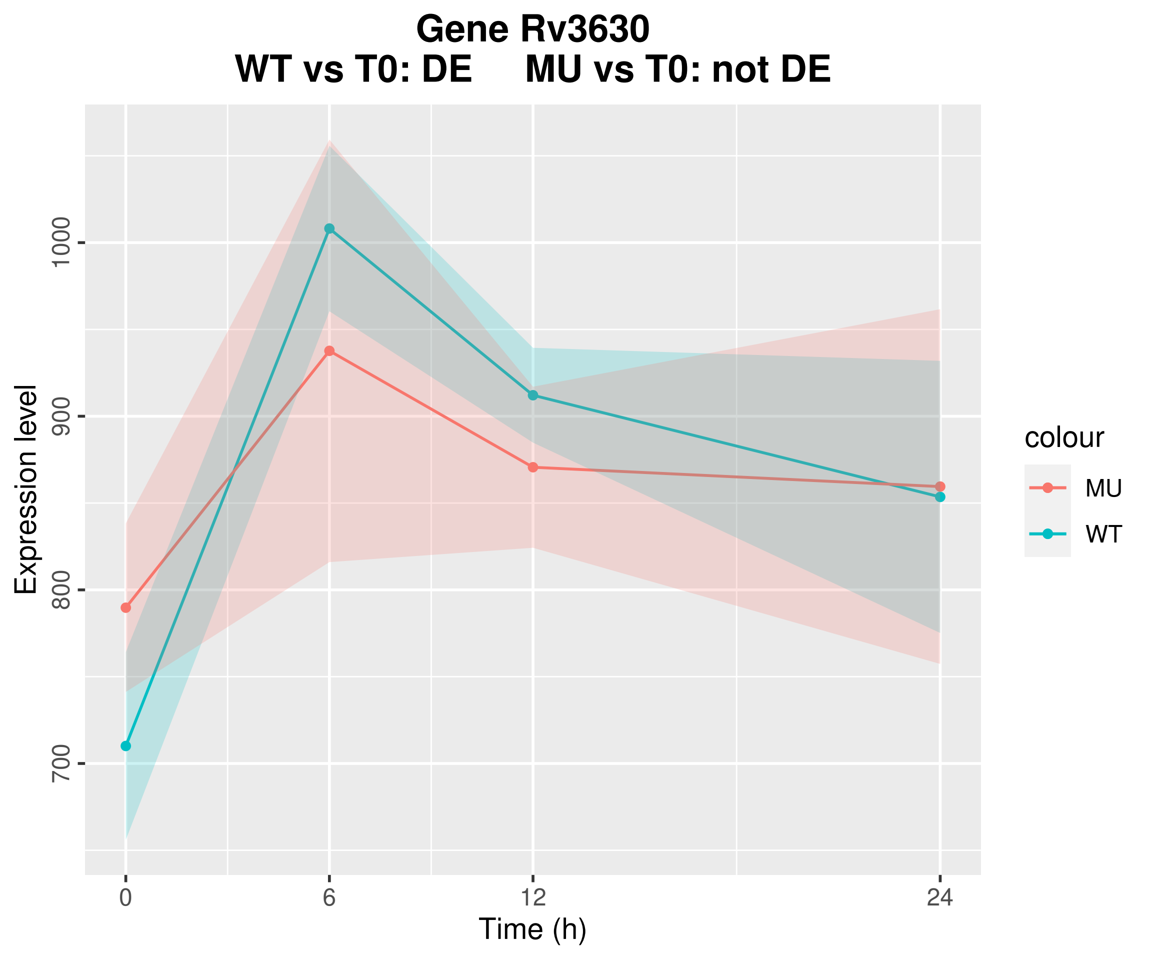
Gene Rv3630
WT vs T0: DE   MU vs T0: not DE
Expression level
1000
900
800
700
0
6
12
24
Time (h)
colour
MU
WT

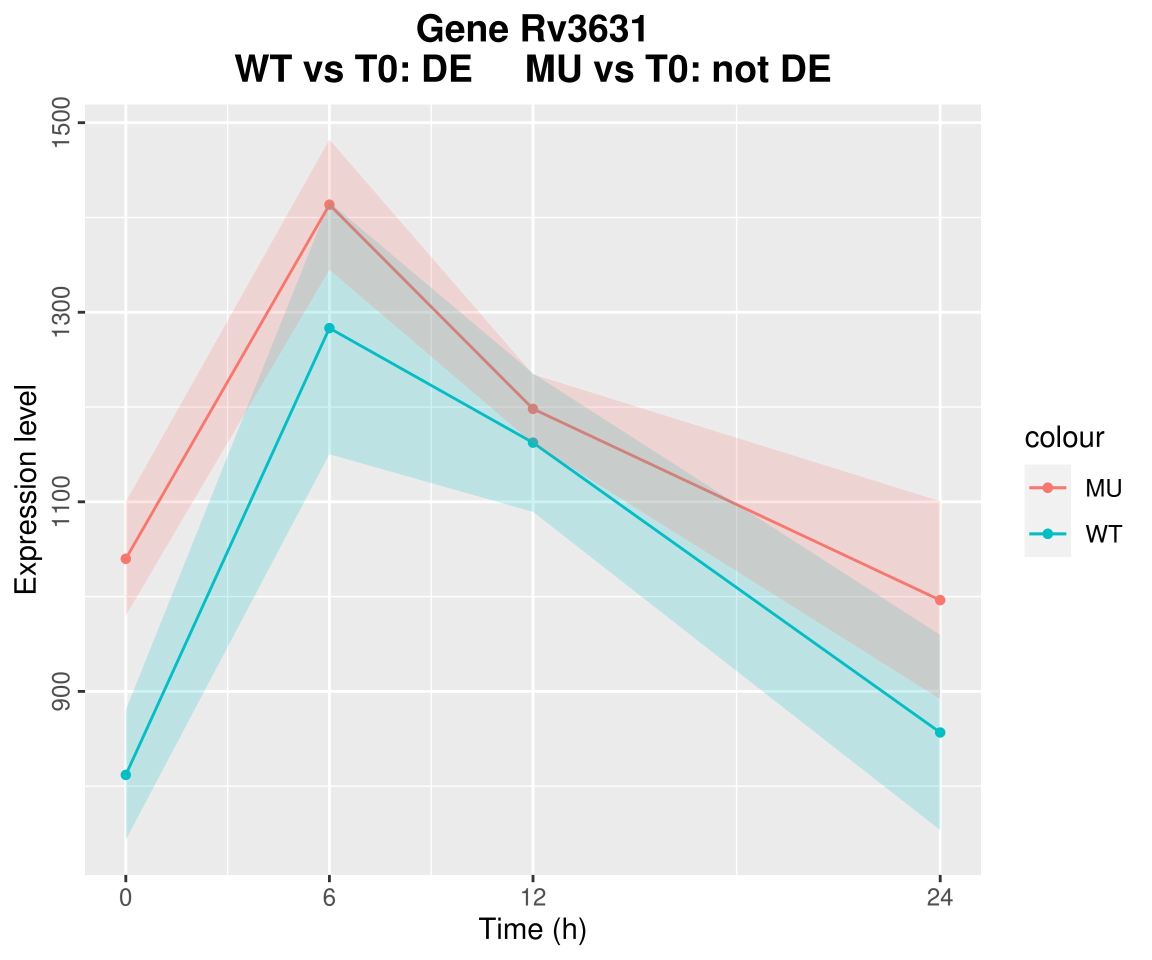
Gene Rv3631
WT vs T0: DE   MU vs T0: not DE
Expression level
1500
1300
1100
900
0
6
12
24
Time (h)
colour
MU
WT

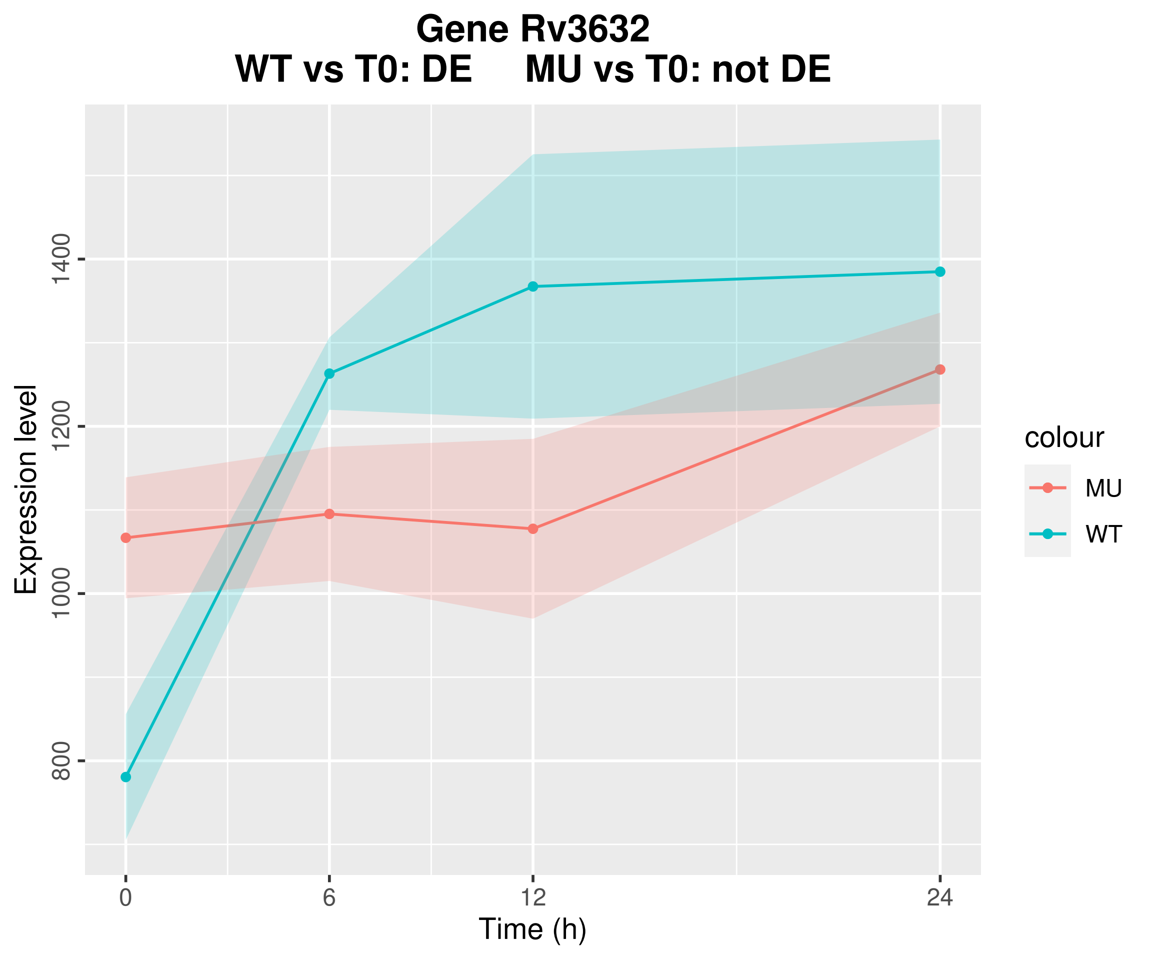

Gene Rv3632
WT vs T0: DE   MU vs T0: not DE
Expression level
800
1000
1200
1400
0
6
12
24
Time (h)
colour
MU
WT

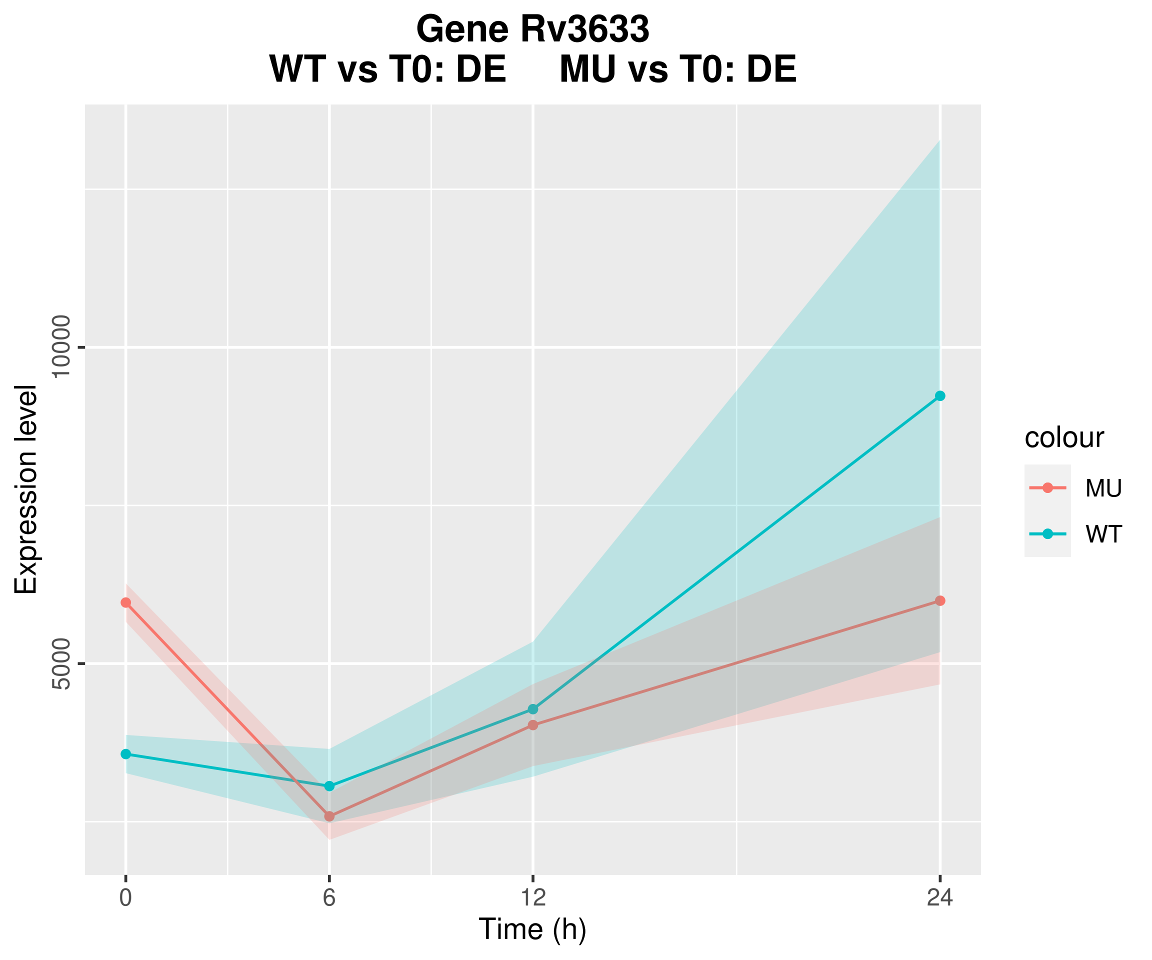

Gene Rv3633
WT vs T0: DE MU vs T0: DE
Expression level
10000
5000
0
6
12
24
Time (h)
colour
MU
WT

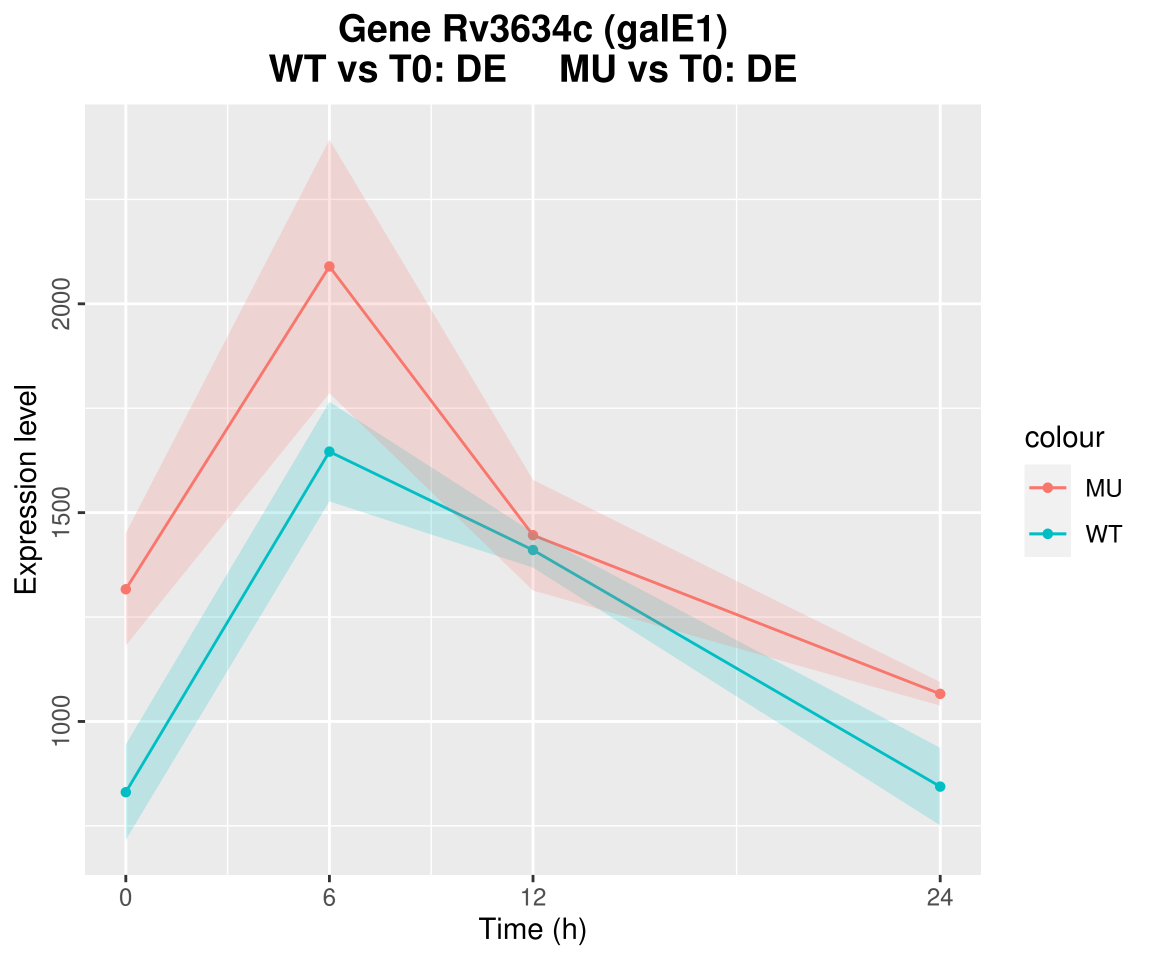

Gene Rv3634c (galE1)
WT vs T0: DE MU vs T0: DE
2000
1500
1000
Expression level
0
6
12
24
Time (h)
colour
MU
WT

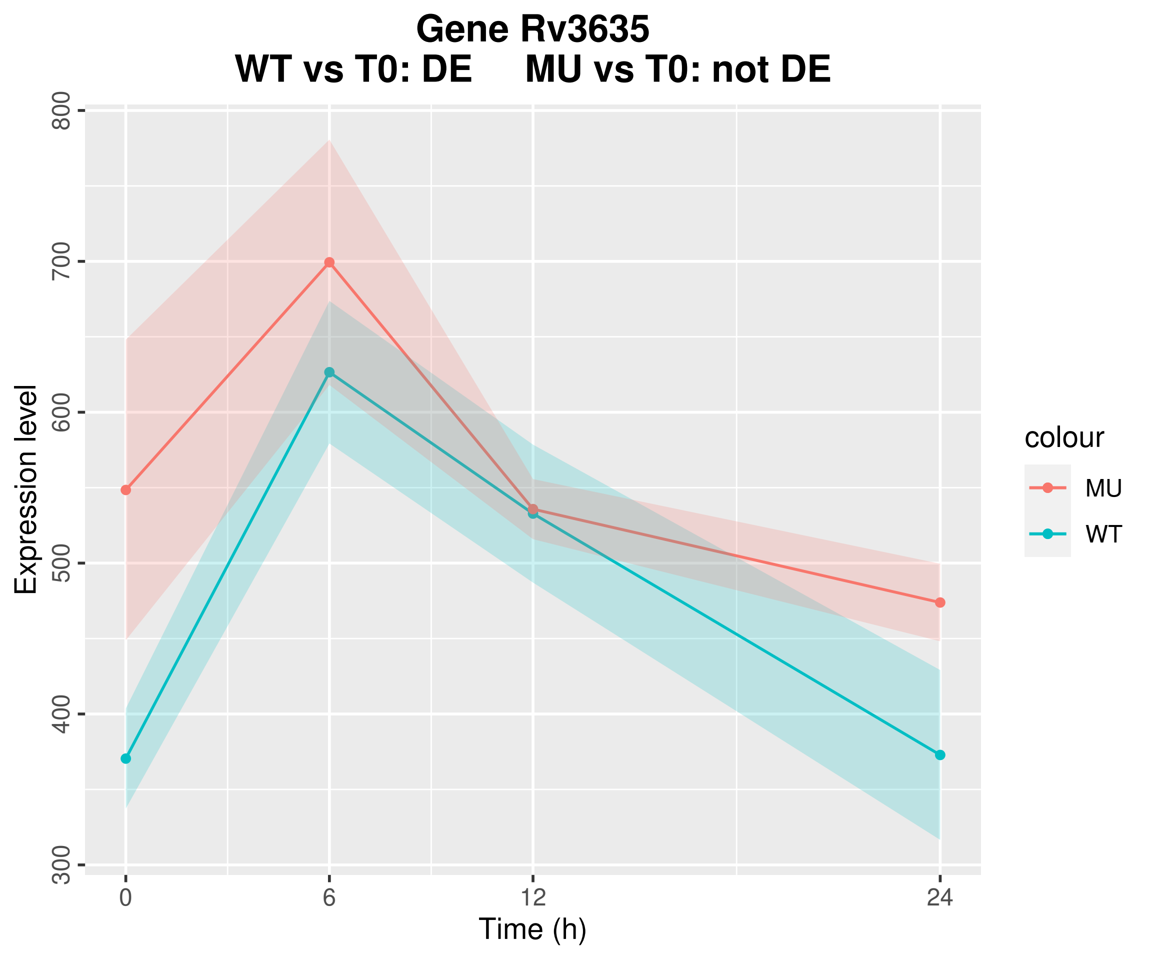

Gene Rv3635
WT vs T0: DE   MU vs T0: not DE
Expression level
800
700
600
500
400
300
0
6
12
24
Time (h)
colour
MU
WT

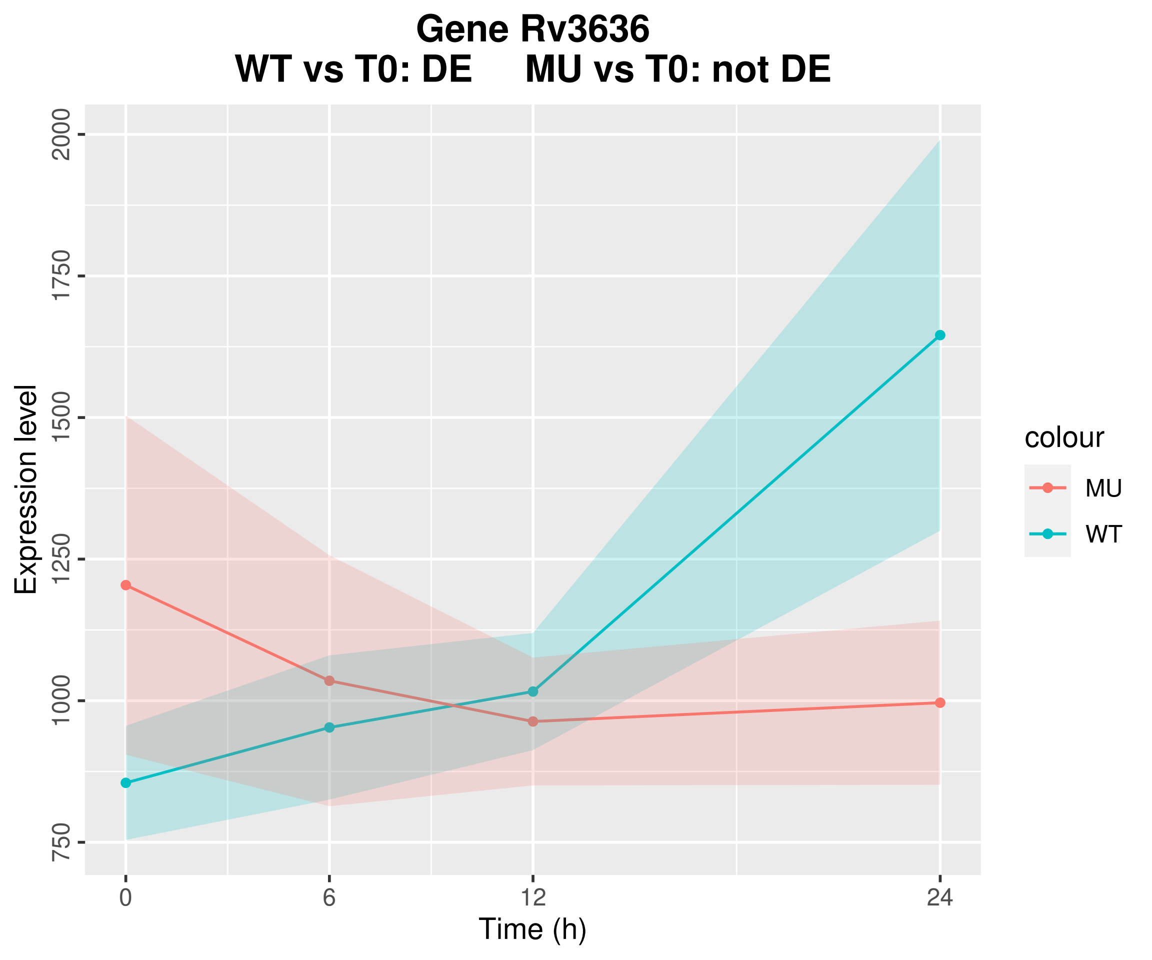
Gene Rv3636
WT vs T0: DE   MU vs T0: not DE
Expression level
2000
1750
1500
1250
1000
750
0
6
12
24
Time (h)
colour
MU
WT

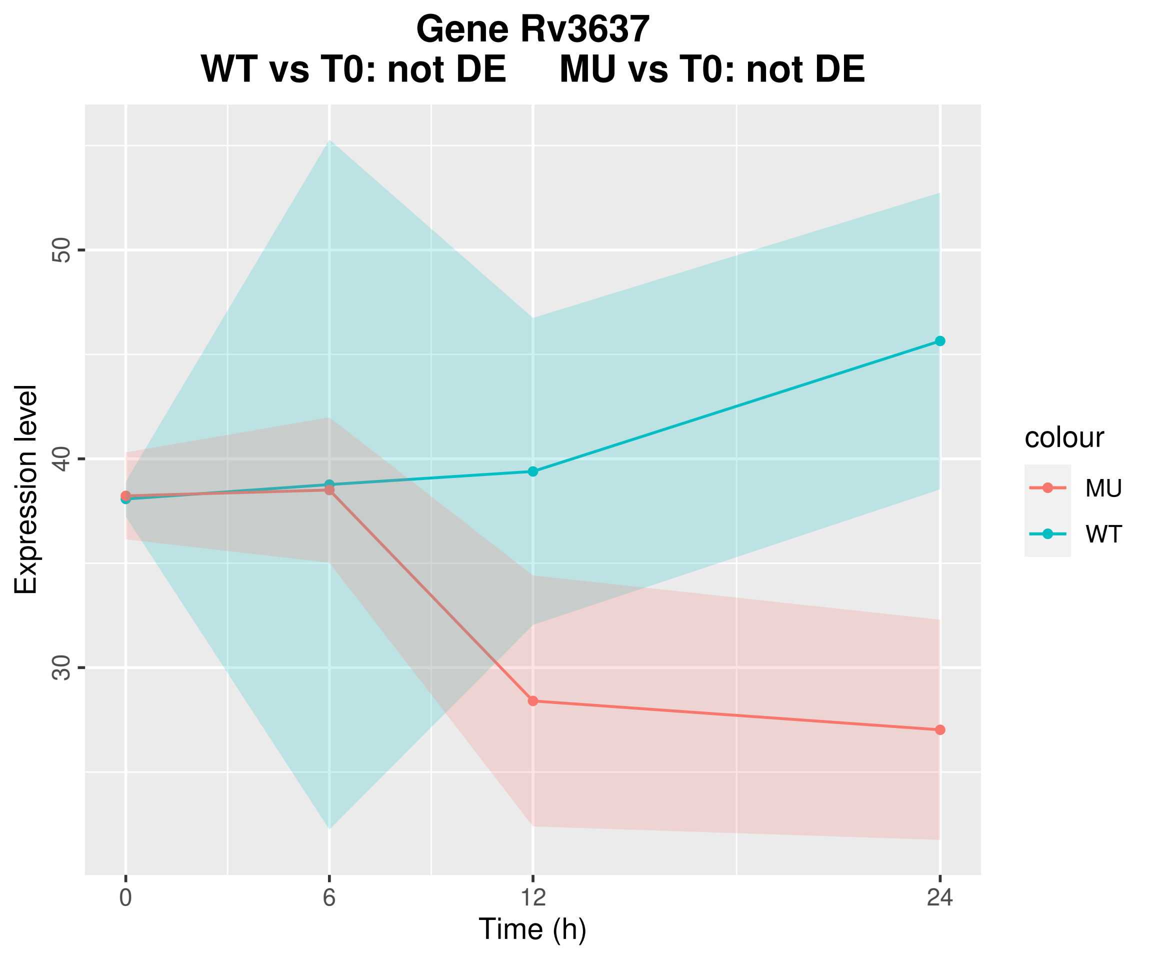
Gene Rv3637
WT vs T0: not DE   MU vs T0: not DE
Expression level
50
40
30
0
6
12
24
Time (h)
colour
MU
WT

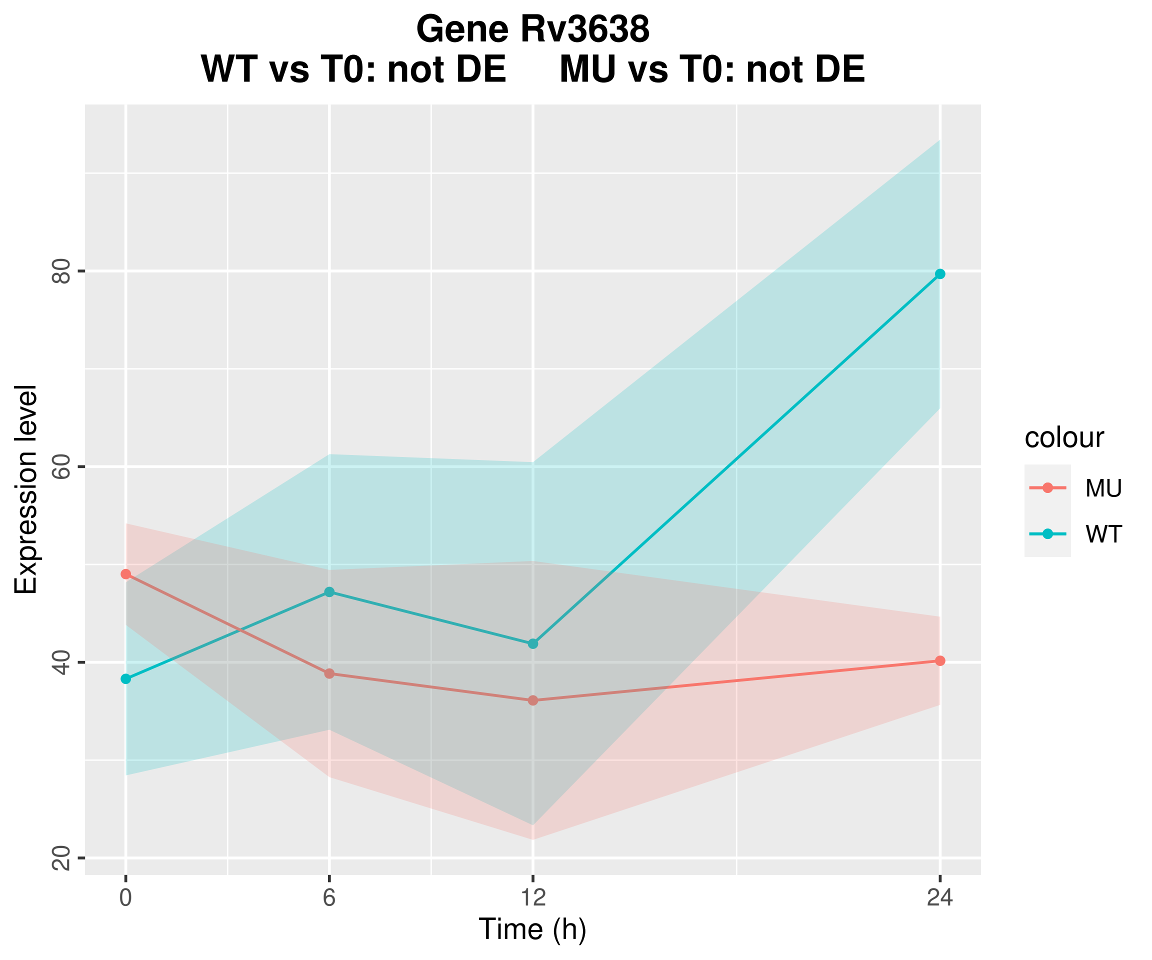

Gene Rv3638
WT vs T0: not DE   MU vs T0: not DE
Expression level
80
60
40
20
0
6
12
24
Time (h)
colour
MU
WT

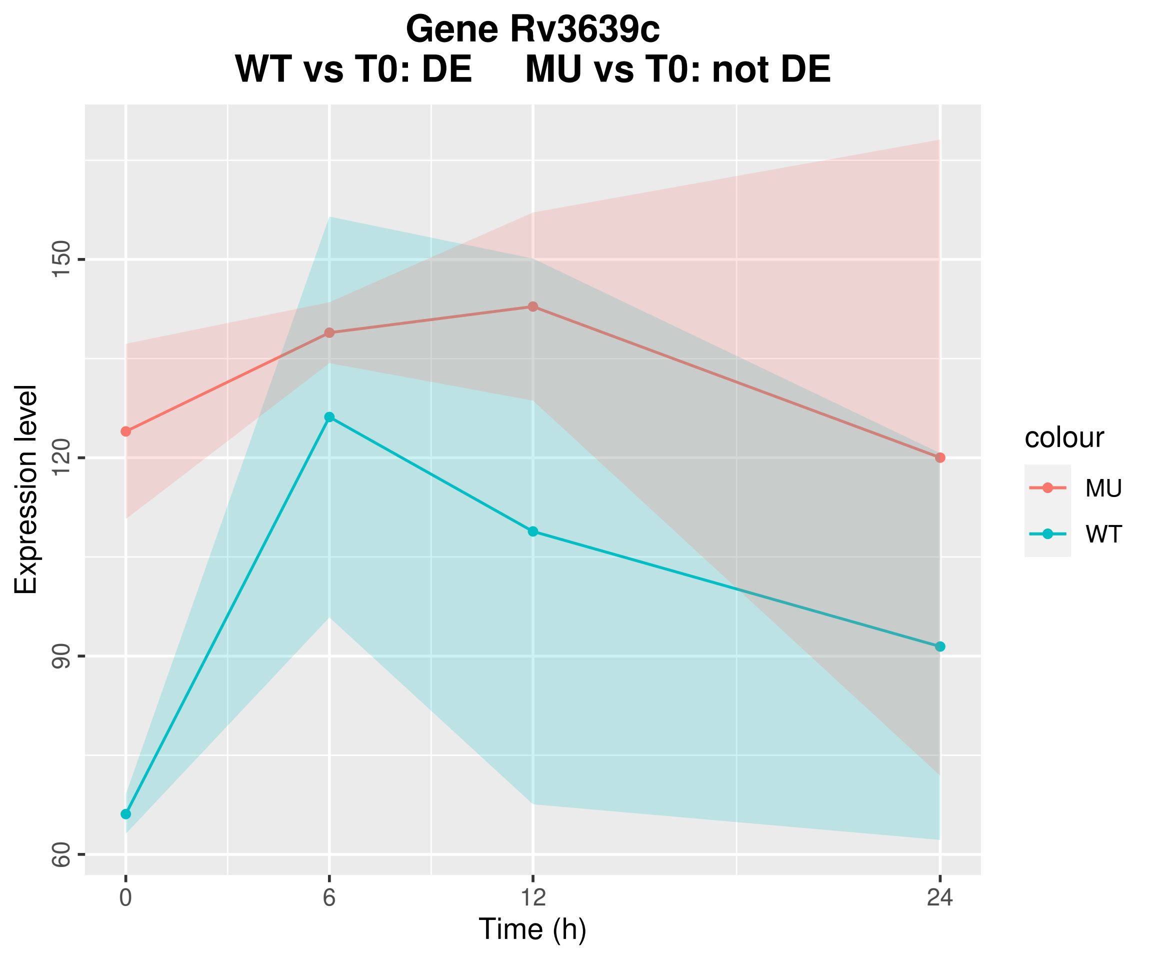

Gene Rv3639c
WT vs T0: DE   MU vs T0: not DE
Expression level
150
120
90
60
0
6
12
24
Time (h)
colour
MU
WT

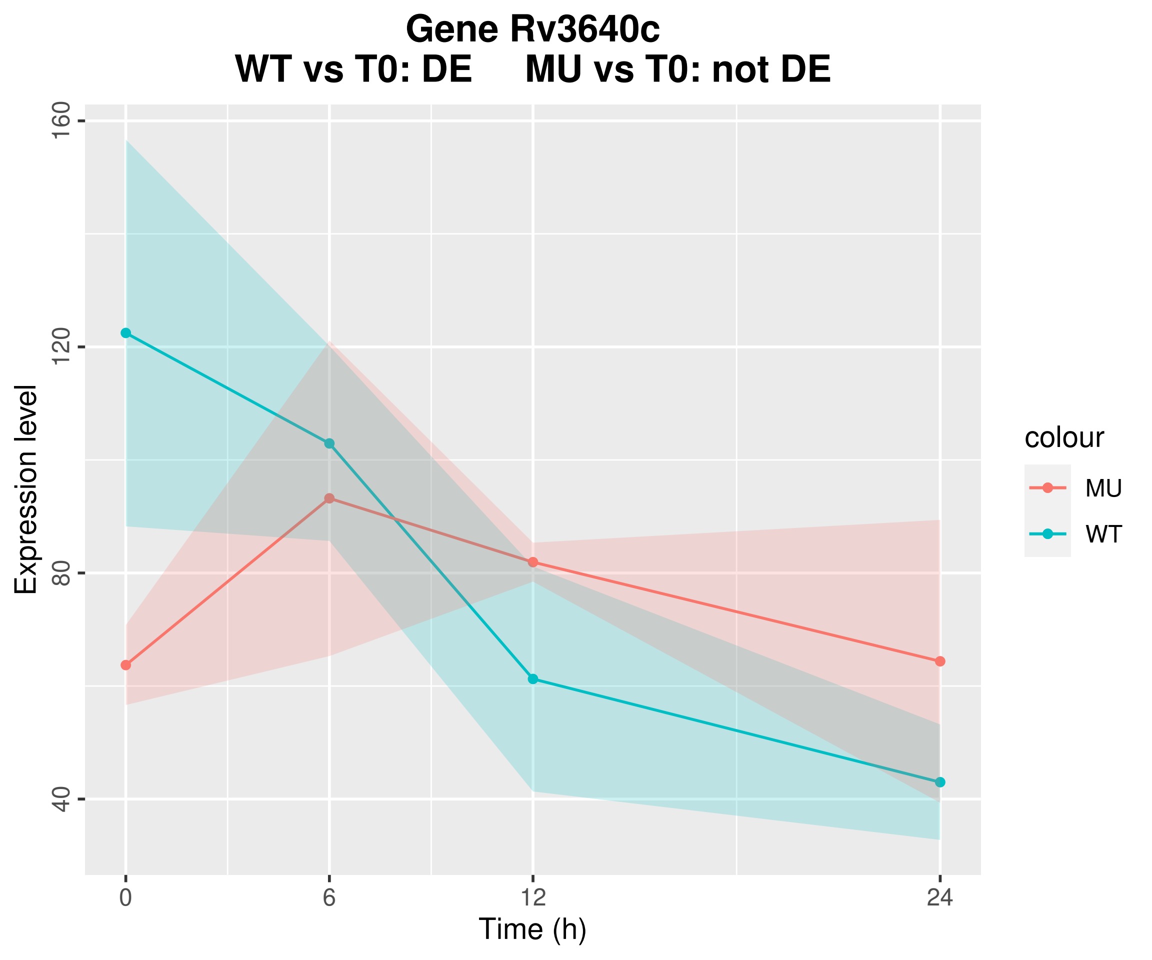

Gene Rv3640c
WT vs T0: DE   MU vs T0: not DE
160
120
80
40
Expression level
0
6
12
24
Time (h)
colour
MU
WT

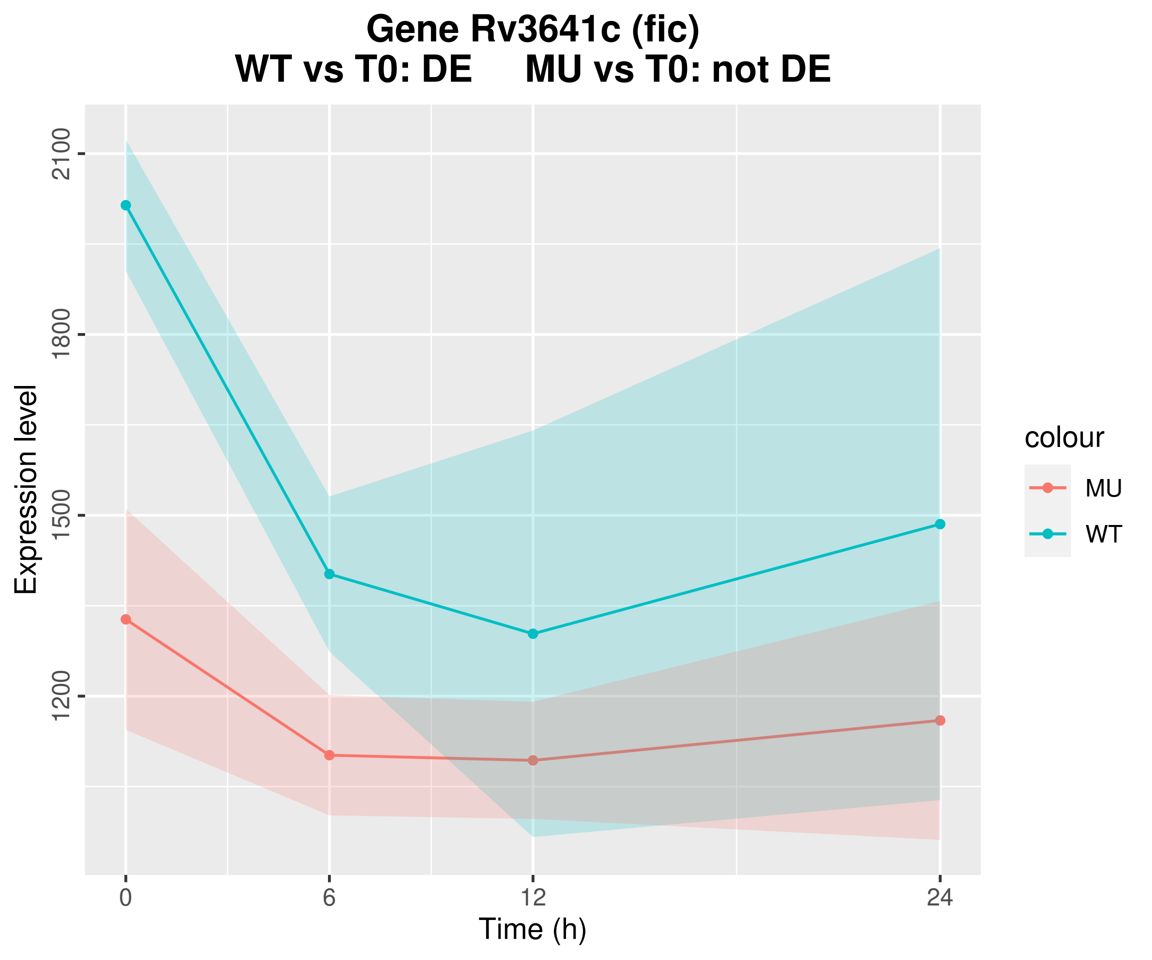

Gene Rv3641c (fic)
WT vs T0: DE   MU vs T0: not DE
Expression level
2100
1800
1500
1200
0
6
12
24
Time (h)
colour
MU
WT

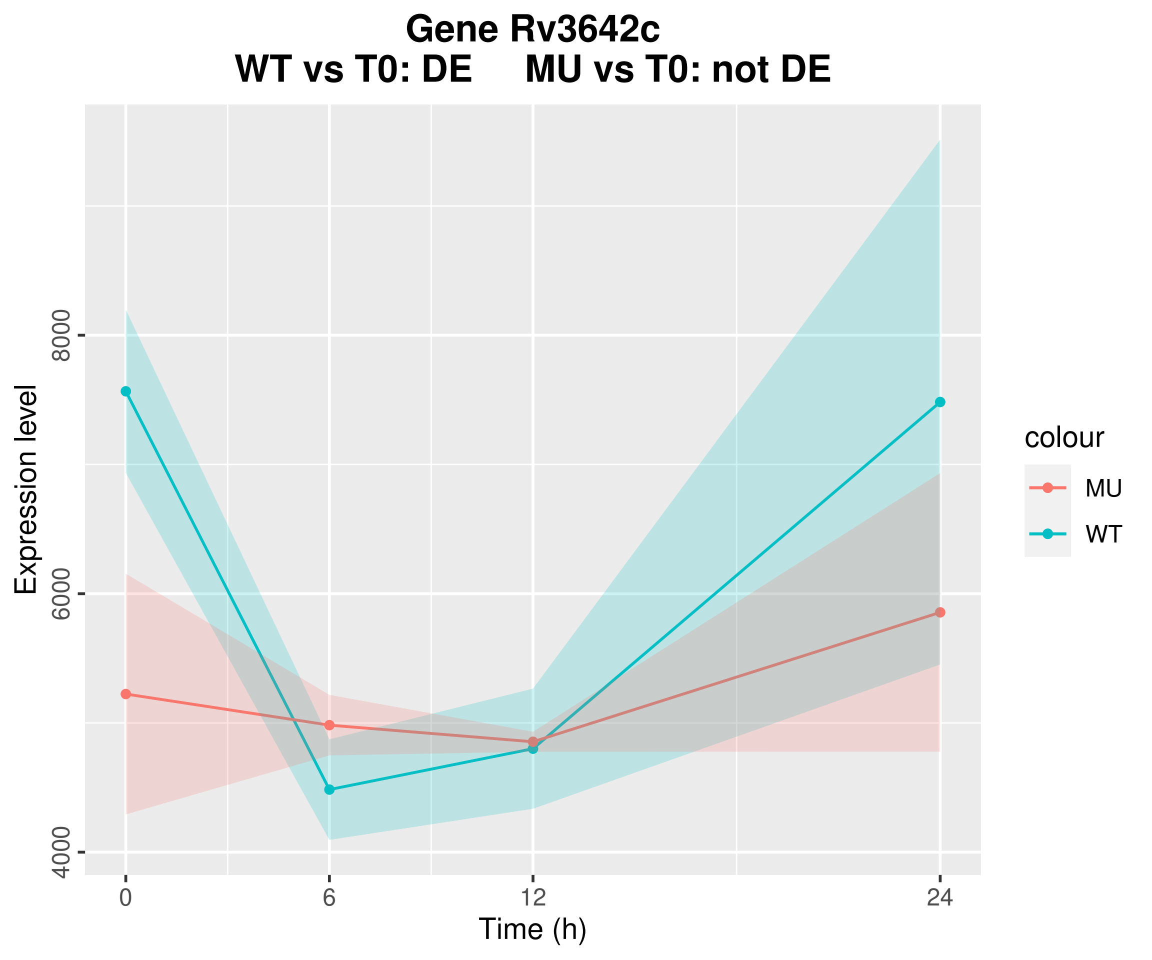
Gene Rv3642c
WT vs T0: DE
MU vs T0: not DE
Expression level
8000
6000
4000
0
6
12
24
Time (h)
colour
MU
WT

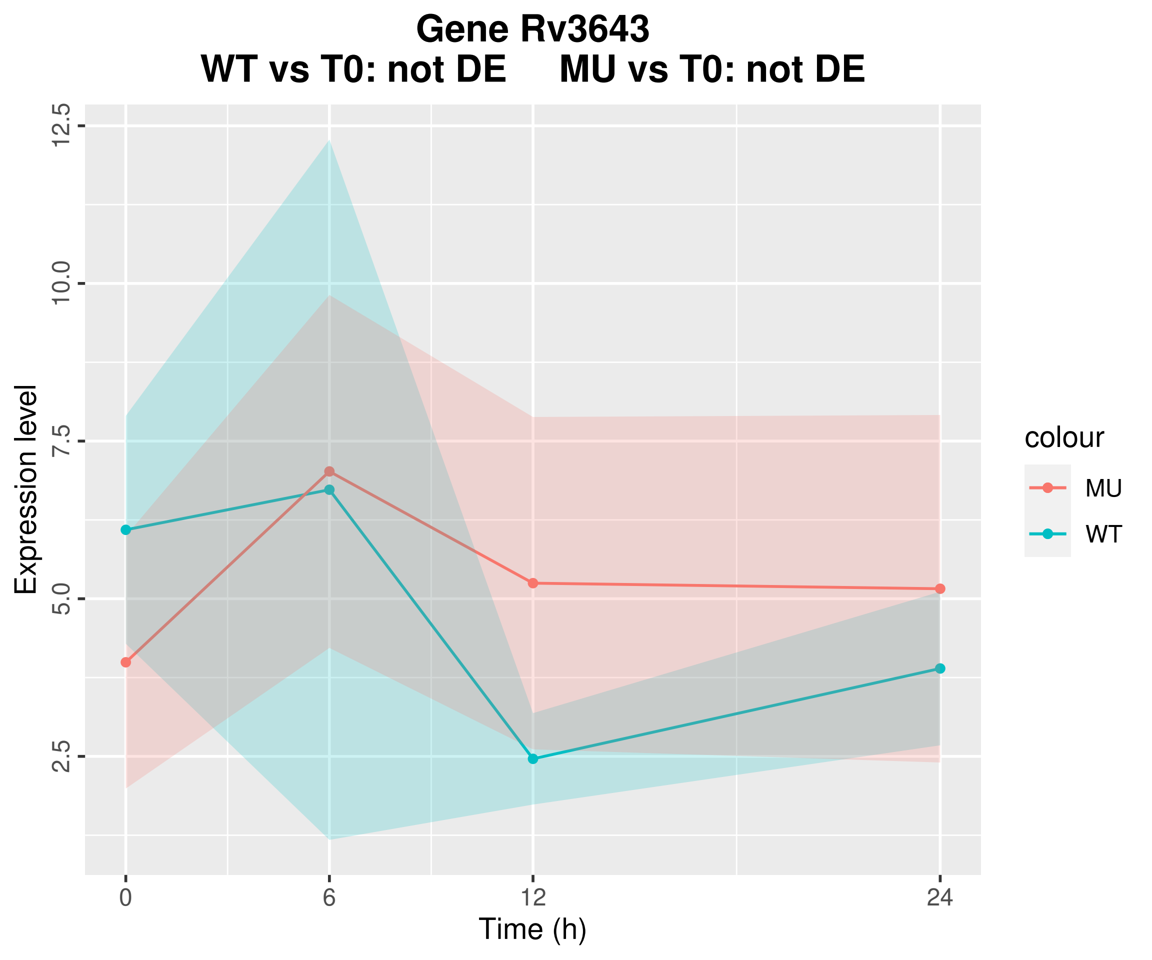
Gene Rv3643
WT vs T0: not DE MU vs T0: not DE
Expression level
12.5
10.0
7.5
5.0
2.5
0
6
12
24
Time (h)
colour
MU
WT

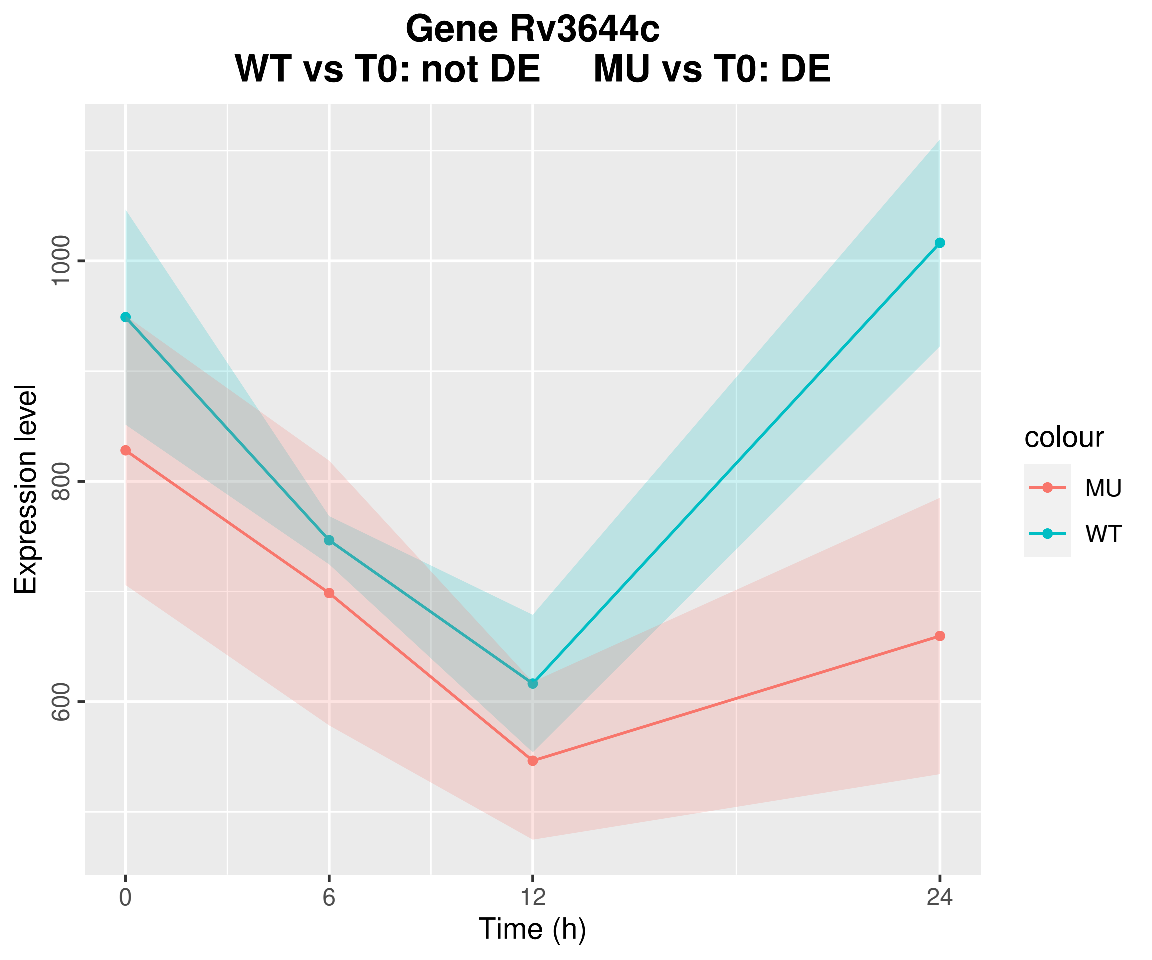

Gene Rv3644c
WT vs T0: not DE   MU vs T0: DE
Expression level
1000
800
600
0
6
12
24
Time (h)
colour
MU
WT

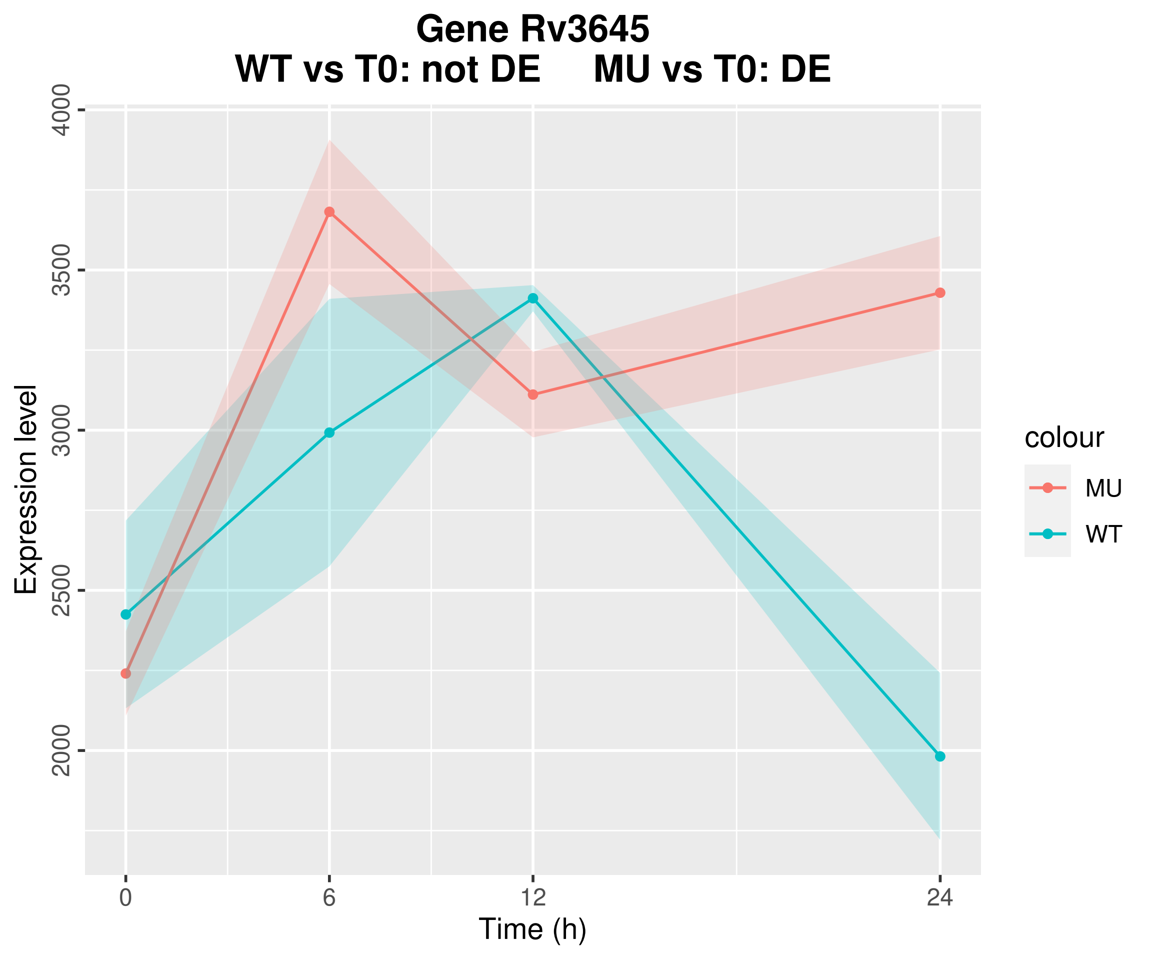
Gene Rv3645
WT vs T0: not DE MU vs T0: DE
Expression level
4000
3500
3000
2500
2000
0
6
12
24
Time (h)
colour
MU
WT

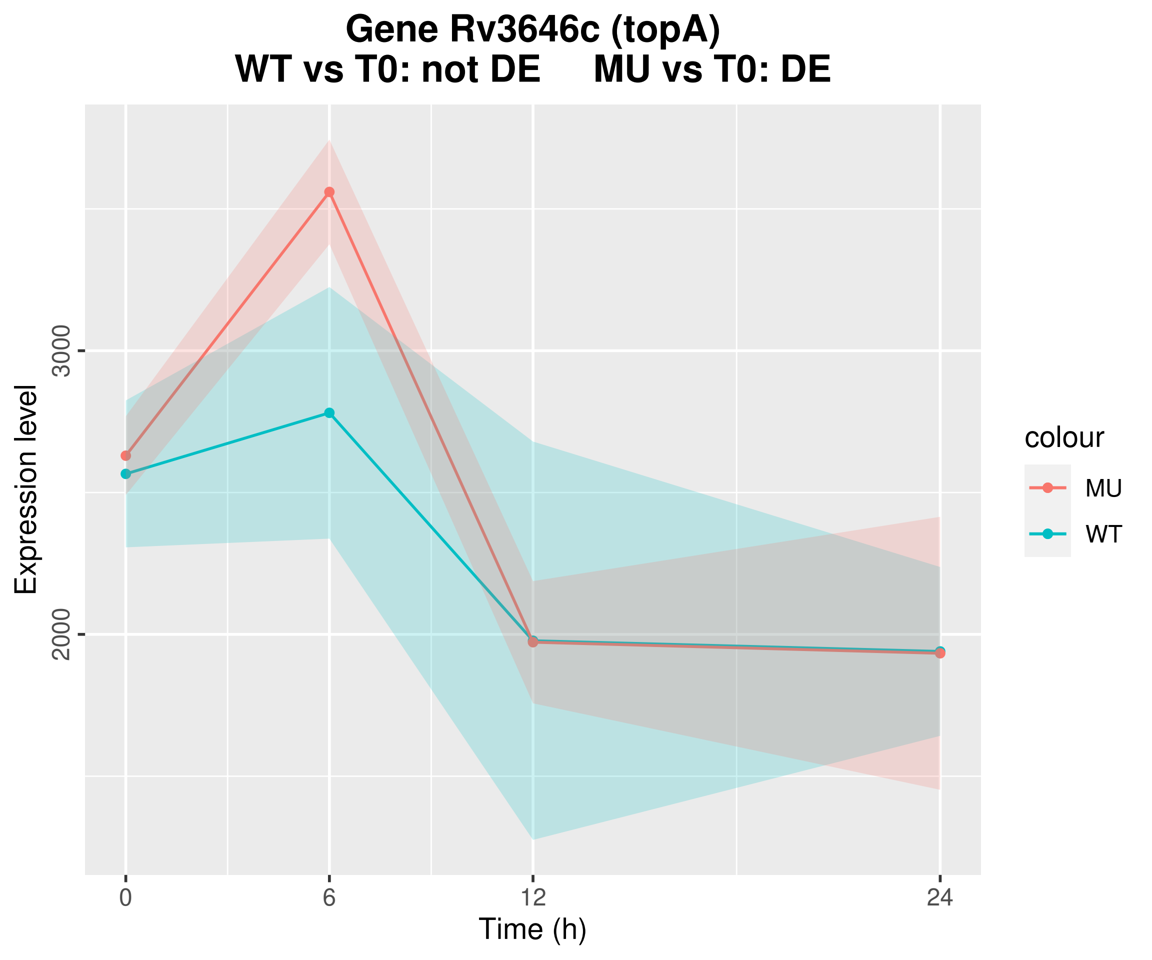

Gene Rv3646c (topA)
WT vs T0: not DE   MU vs T0: DE
Expression level
3000
2000
0
6
12
24
Time (h)
colour
MU
WT

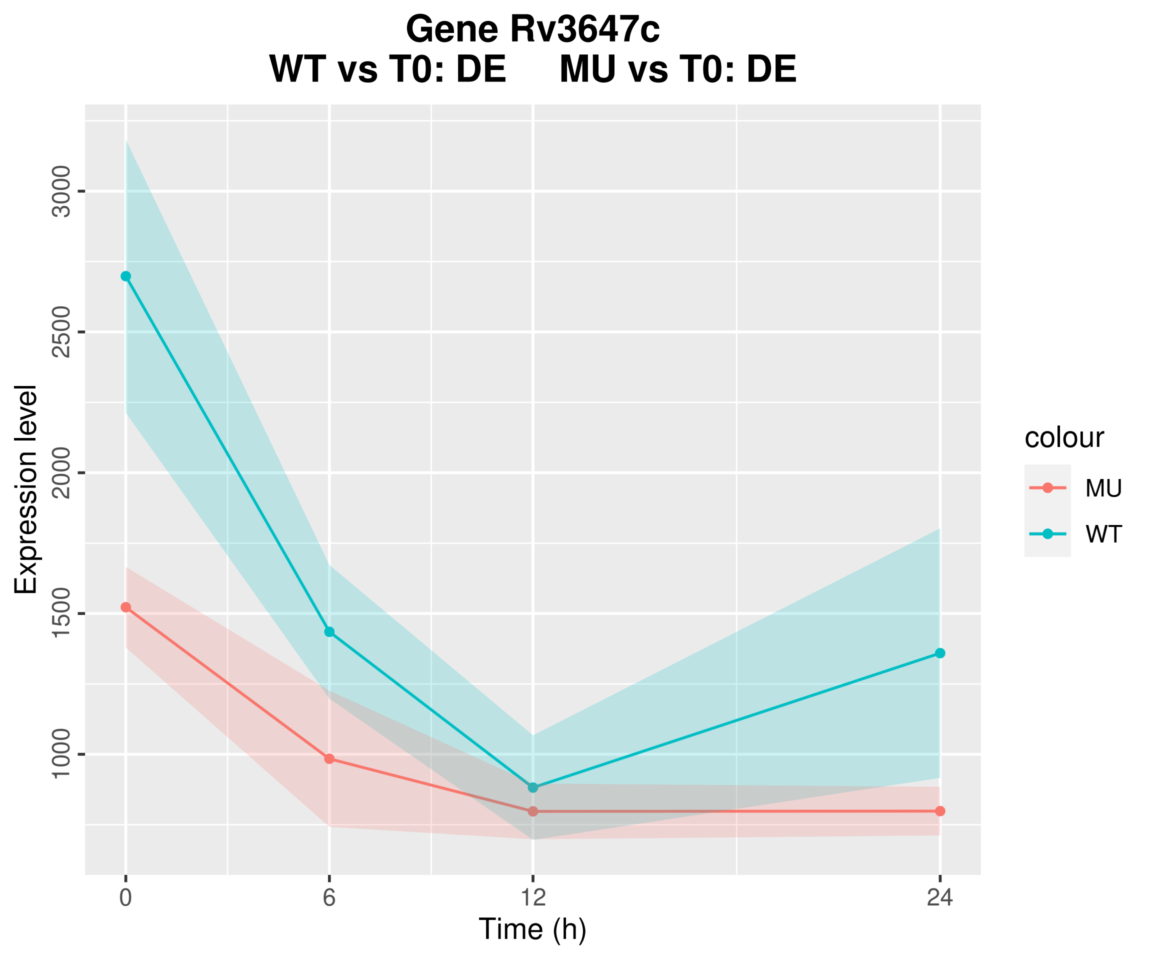

Gene Rv3647c
WT vs T0: DE   MU vs T0: DE
Expression level
3000
2500
2000
1500
1000
0
6
12
24
Time (h)
colour
MU
WT

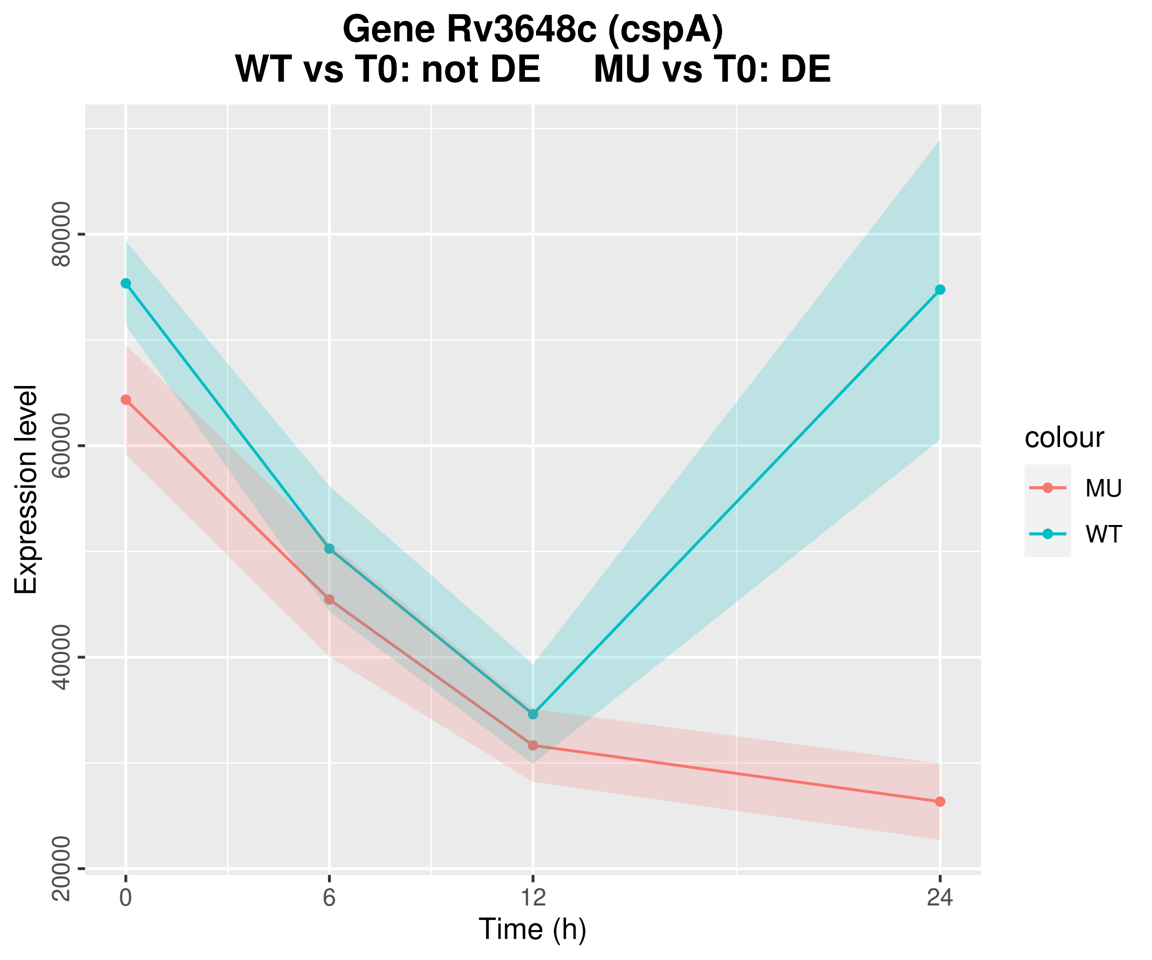

Gene Rv3648c (cspA)
WT vs T0: not DE MU vs T0: DE
Expression level
80000
60000
40000
20000
0
6
12
24
Time (h)
colour
MU
WT

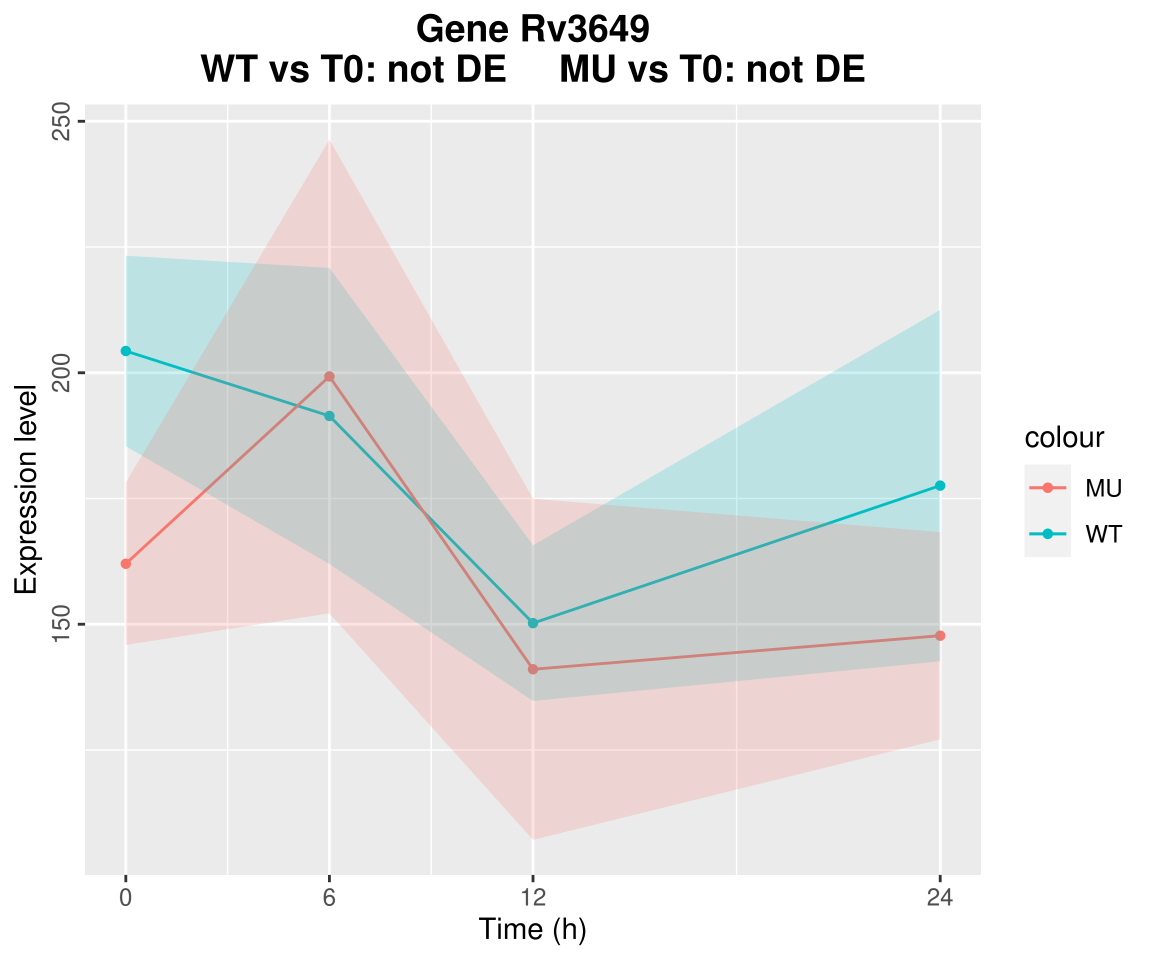
Gene Rv3649
WT vs T0: not DE   MU vs T0: not DE
250
200
150
Expression level
0
6
12
24
Time (h)
colour
MU
WT

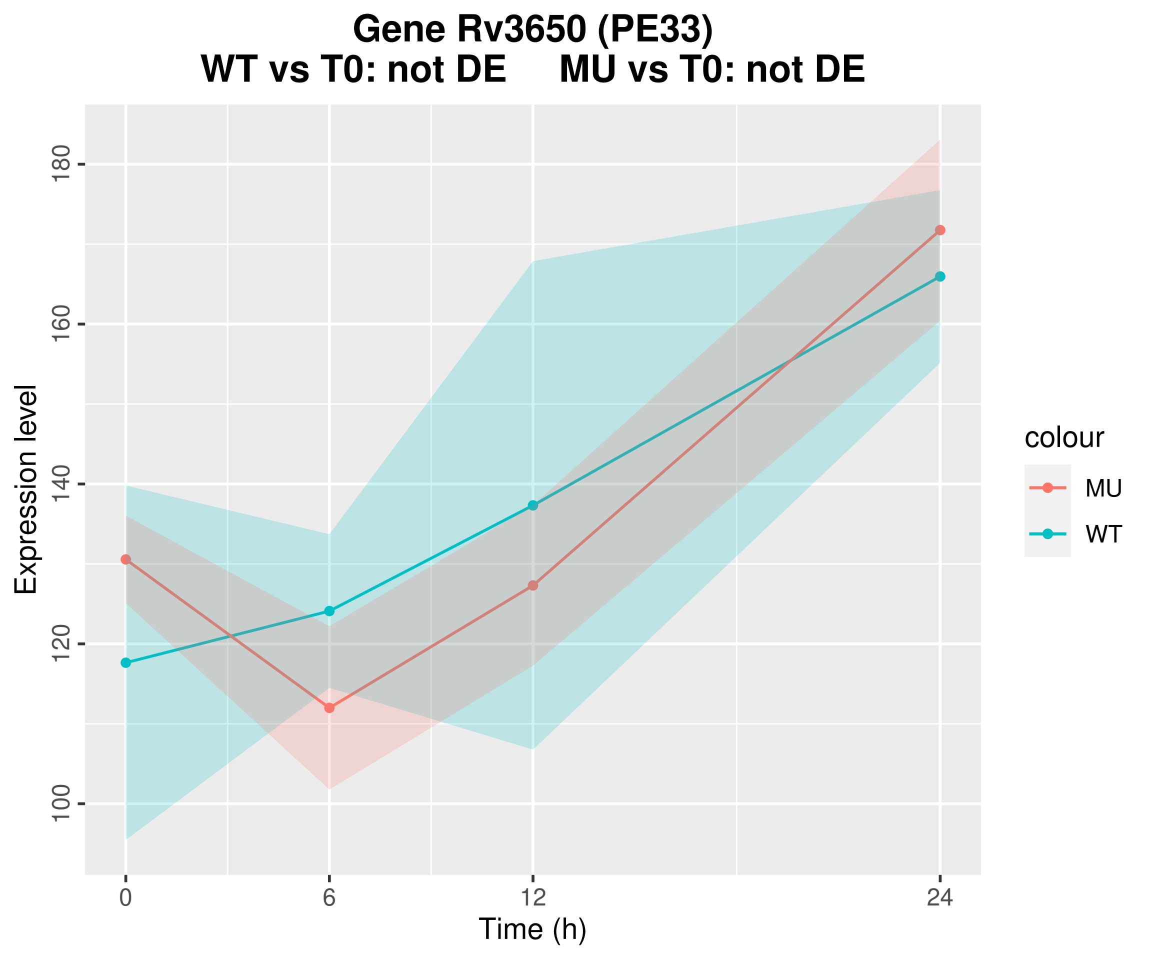
Gene Rv3650 (PE33)
WT vs T0: not DE   MU vs T0: not DE
Expression level
180
160
140
120
100
0
6
12
24
Time (h)
colour
MU
WT

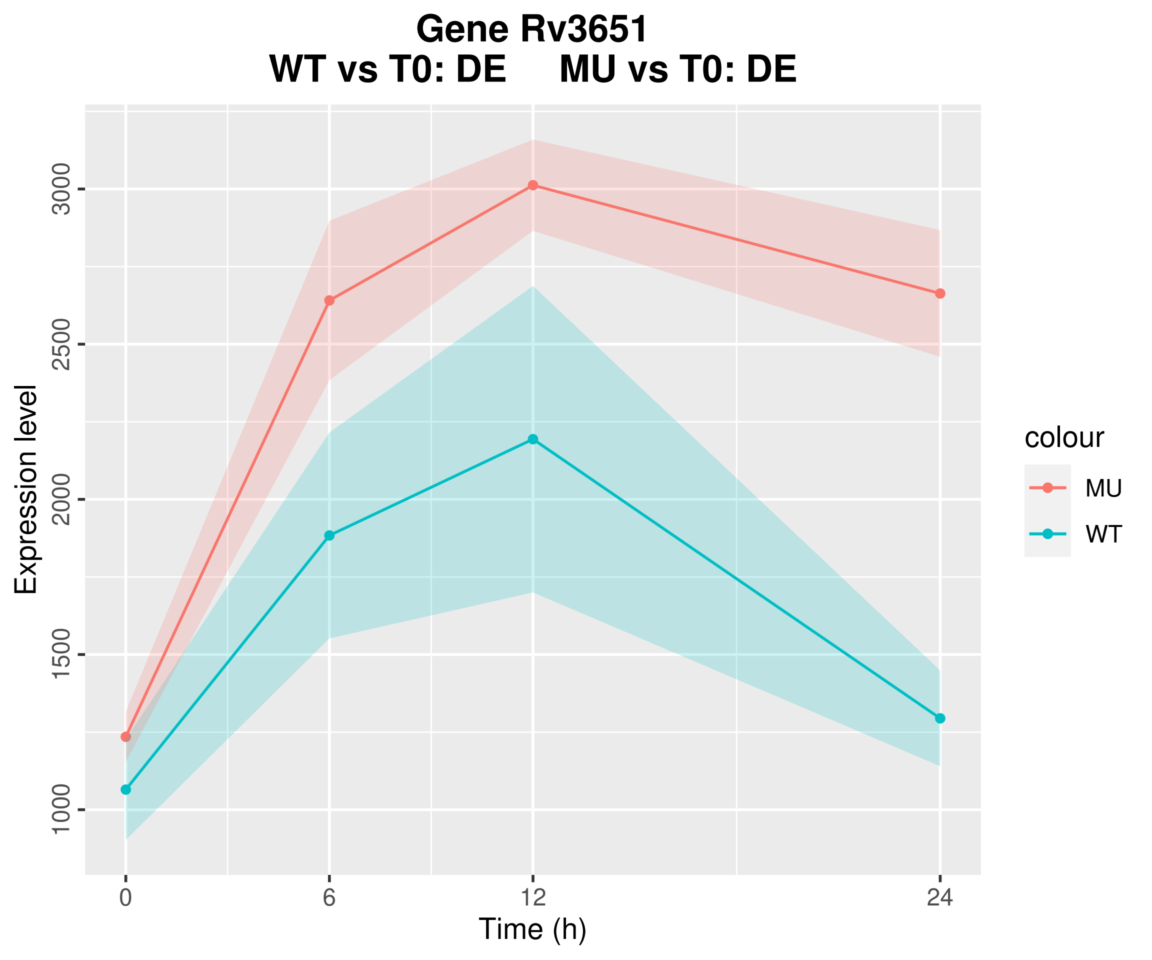

Gene Rv3651
WT vs T0: DE MU vs T0: DE
Expression level
3000
2500
2000
1500
1000
0
6
12
24
Time (h)
colour
MU
WT

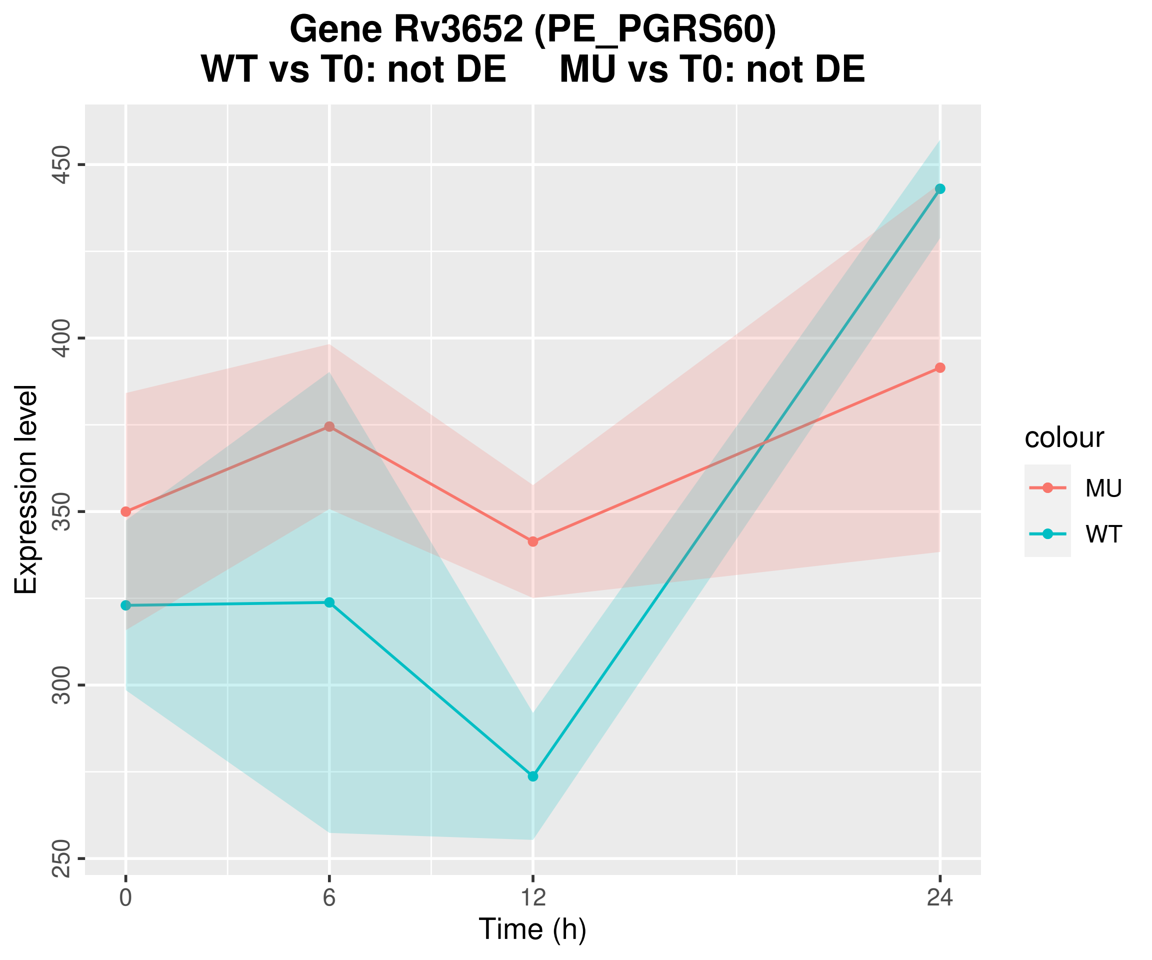

Gene Rv3652 (PE_PGRS60)
WT vs T0: not DE MU vs T0: not DE
Expression level
450
400
350
300
250
0
6
12
24
Time (h)
colour
MU
WT

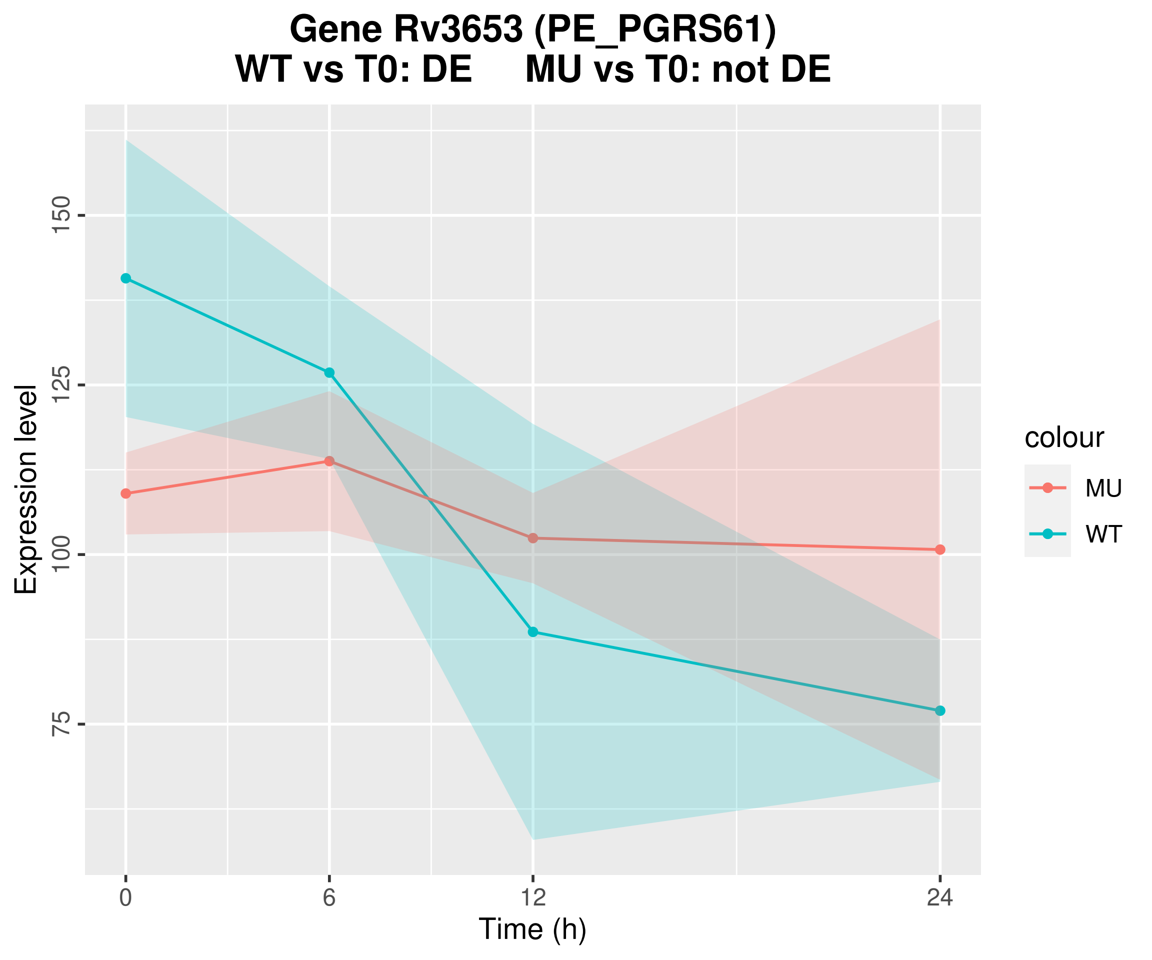
Gene Rv3653 (PE_PGRS61)
WT vs T0: DE   MU vs T0: not DE
Expression level
150
125
100
75
0
6
12
24
Time (h)
colour
MU
WT

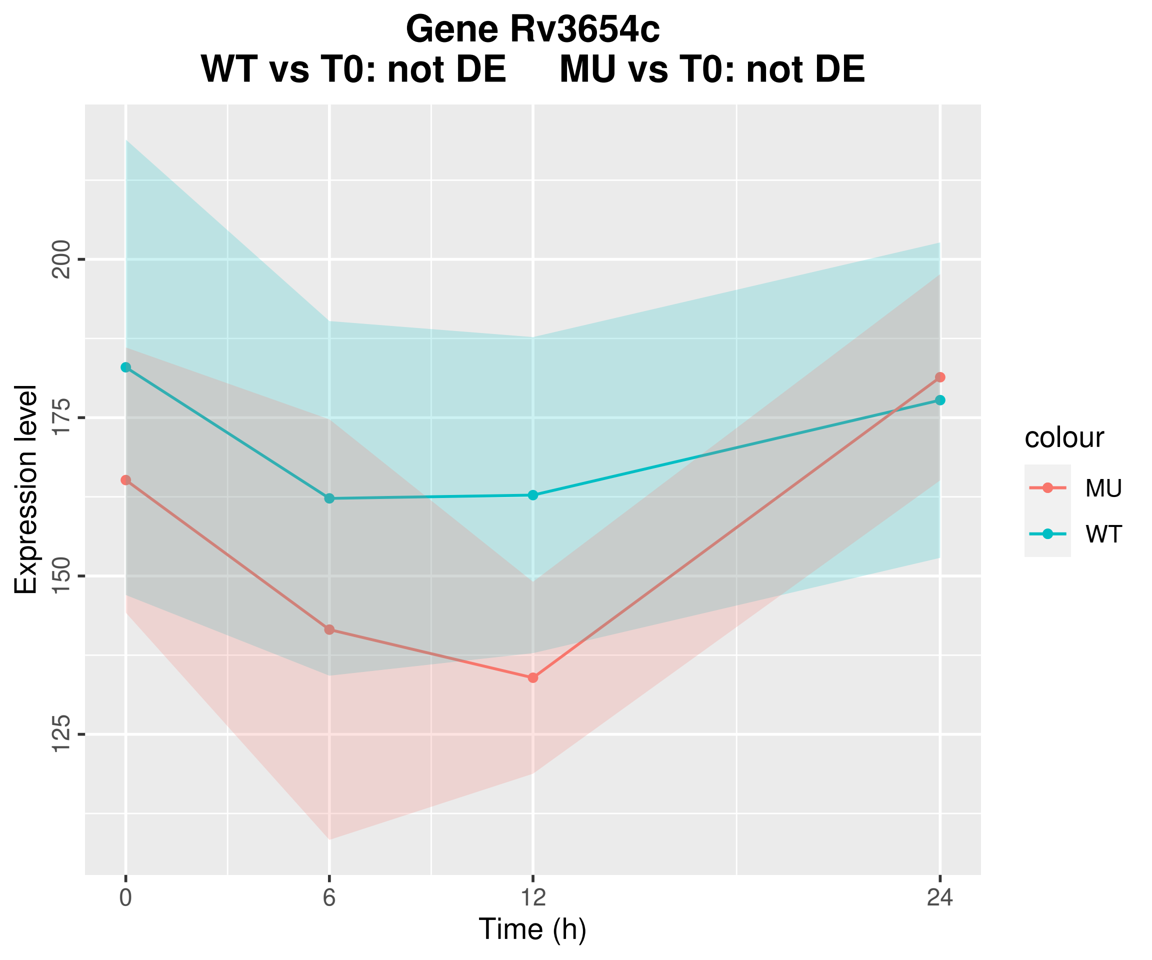
Gene Rv3654c
WT vs T0: not DE   MU vs T0: not DE
Expression level
200
175
150
125
0
6
12
24
Time (h)
colour
MU
WT

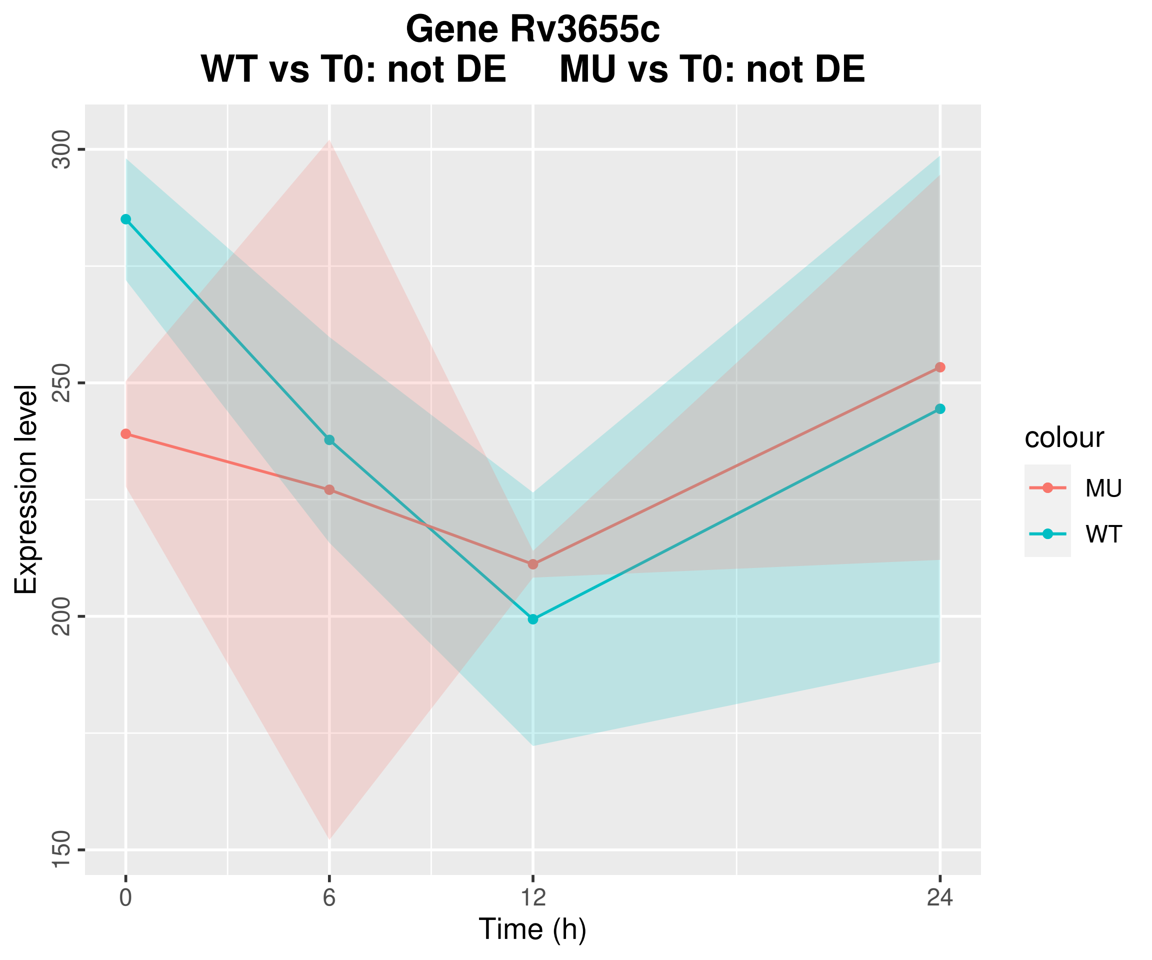

Gene Rv3655c
WT vs T0: not DE   MU vs T0: not DE
Expression level
300
250
200
150
0
6
12
24
Time (h)
colour
MU
WT

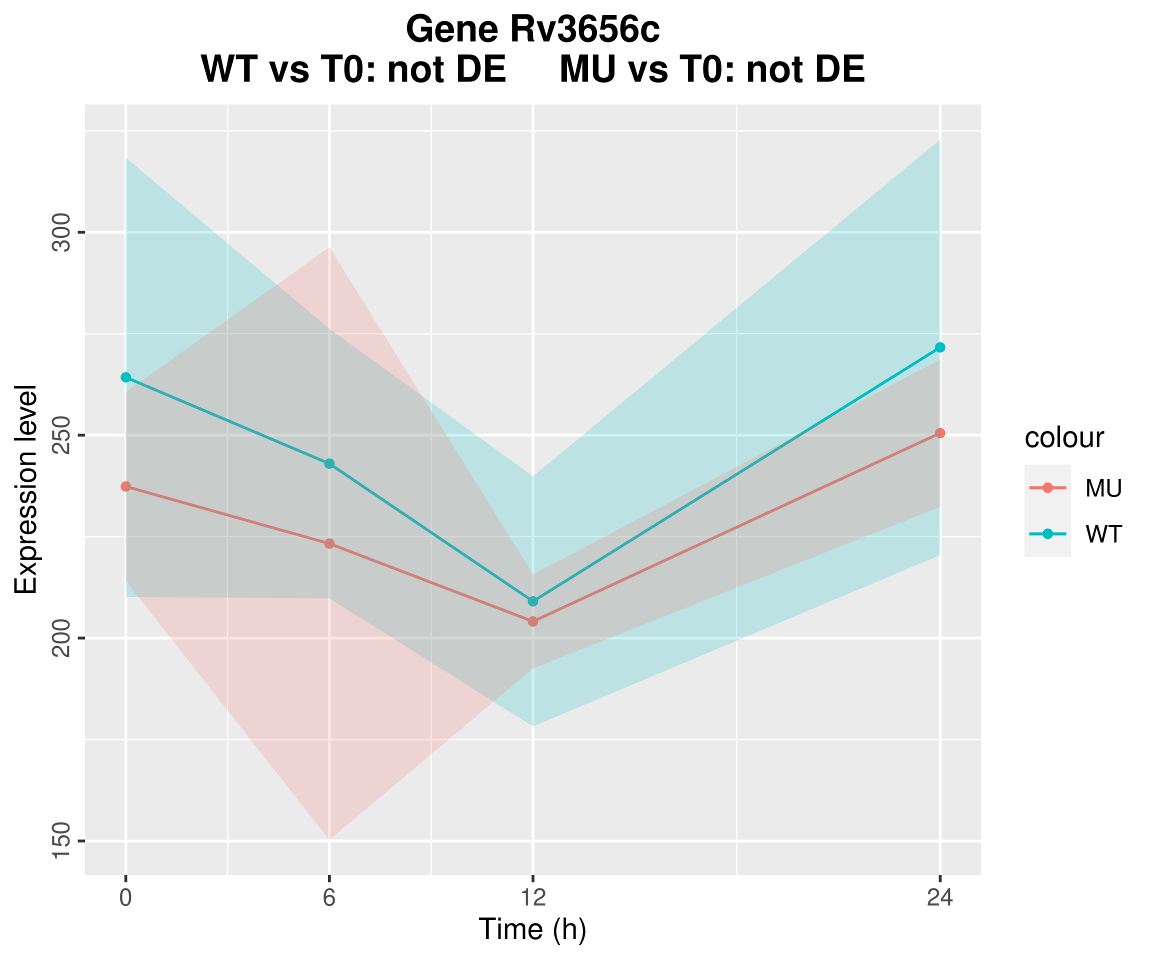

Gene Rv3656c
WT vs T0: not DE MU vs T0: not DE
Expression level
300
250
200
150
0
6
12
24
Time (h)
colour
MU
WT

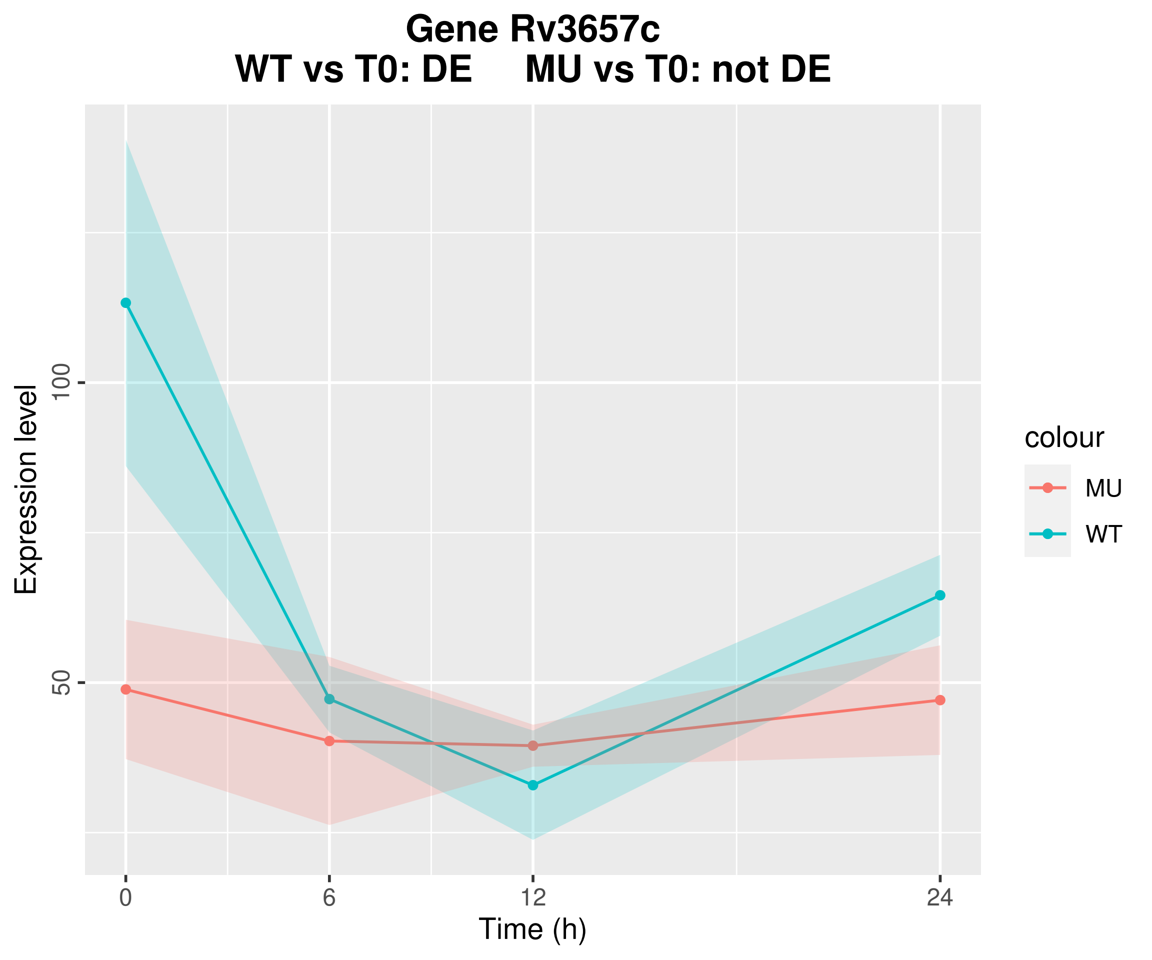
Gene Rv3657c
WT vs T0: DE   MU vs T0: not DE
Expression level
100
50
0
6
12
24
Time (h)
colour
MU
WT

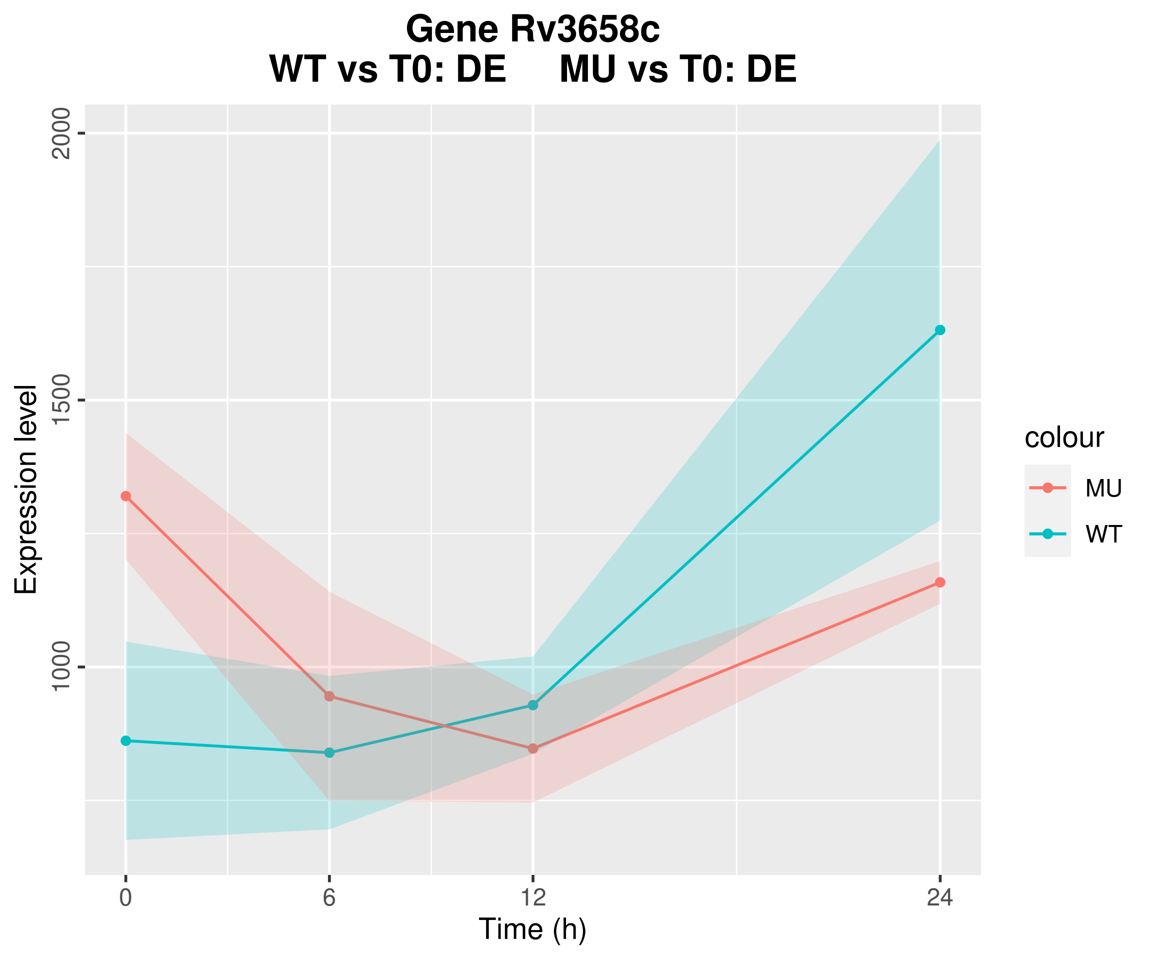
Gene Rv3658c
WT vs T0: DE MU vs T0: DE
2000
1500
1000
Expression level
0
6
12
24
Time (h)
colour
MU
WT

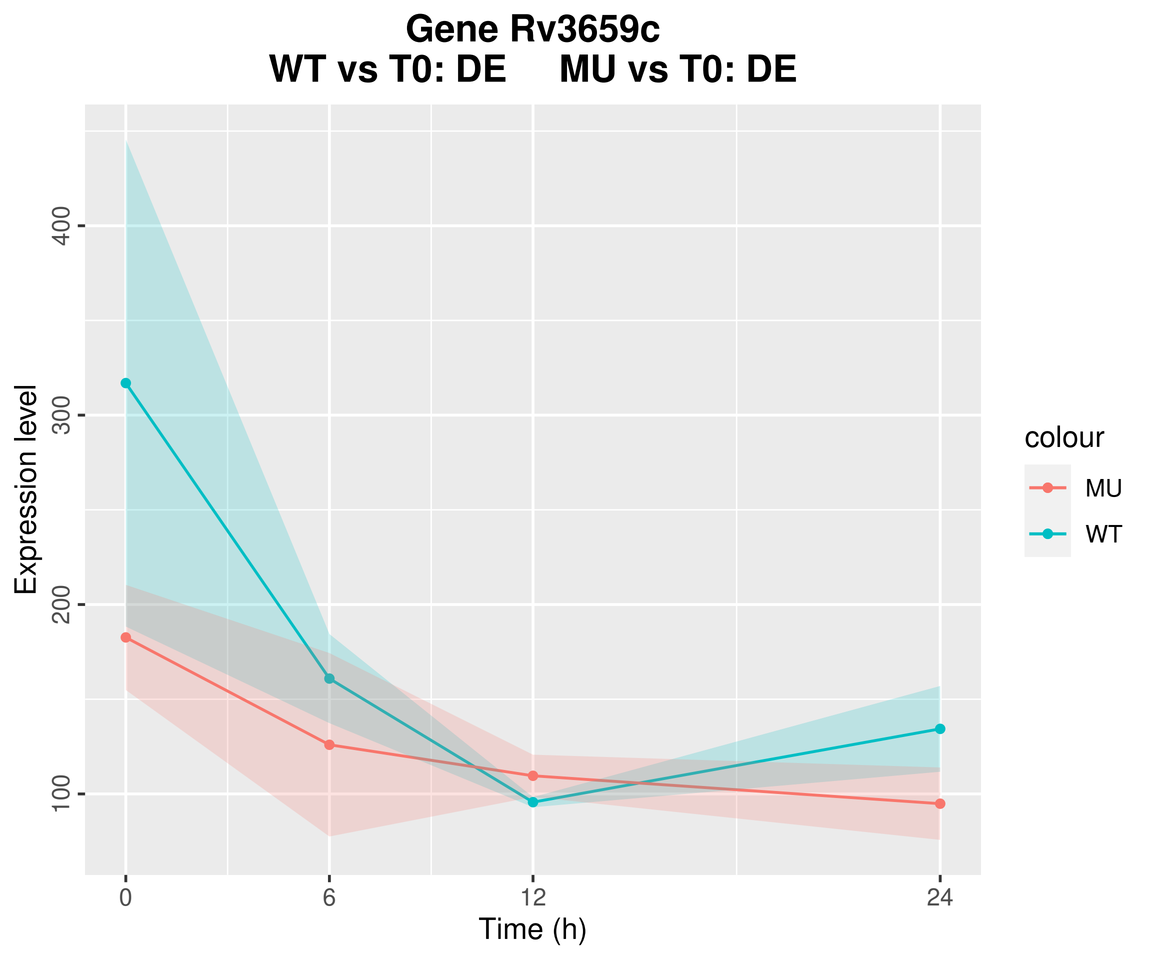

Gene Rv3659c
WT vs T0: DE   MU vs T0: DE
Expression level
400
300
200
100
0
6
12
24
Time (h)
colour
MU
WT

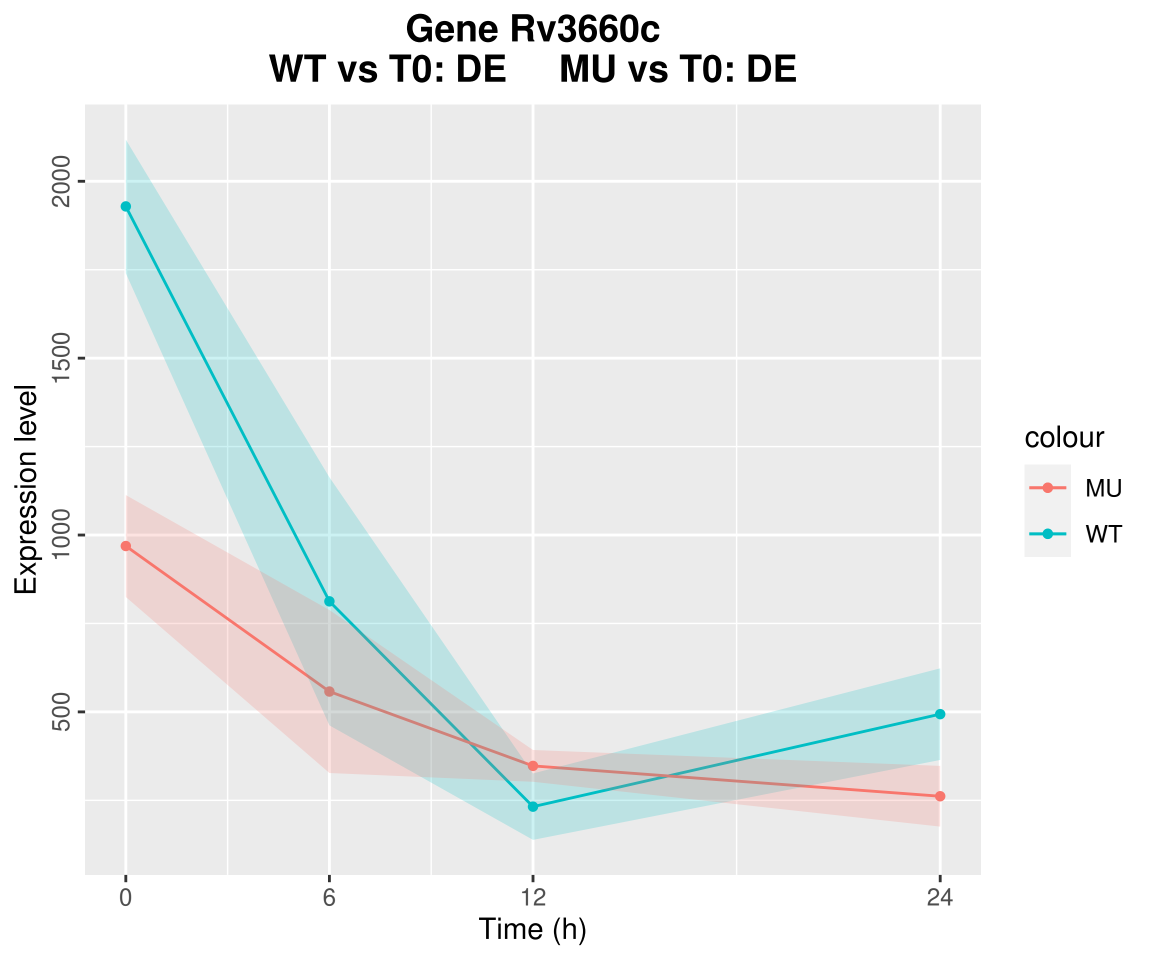
Gene Rv3660c
WT vs T0: DE MU vs T0: DE
Expression level
2000
1500
1000
500
0
6
12
24
Time (h)
colour
MU
WT

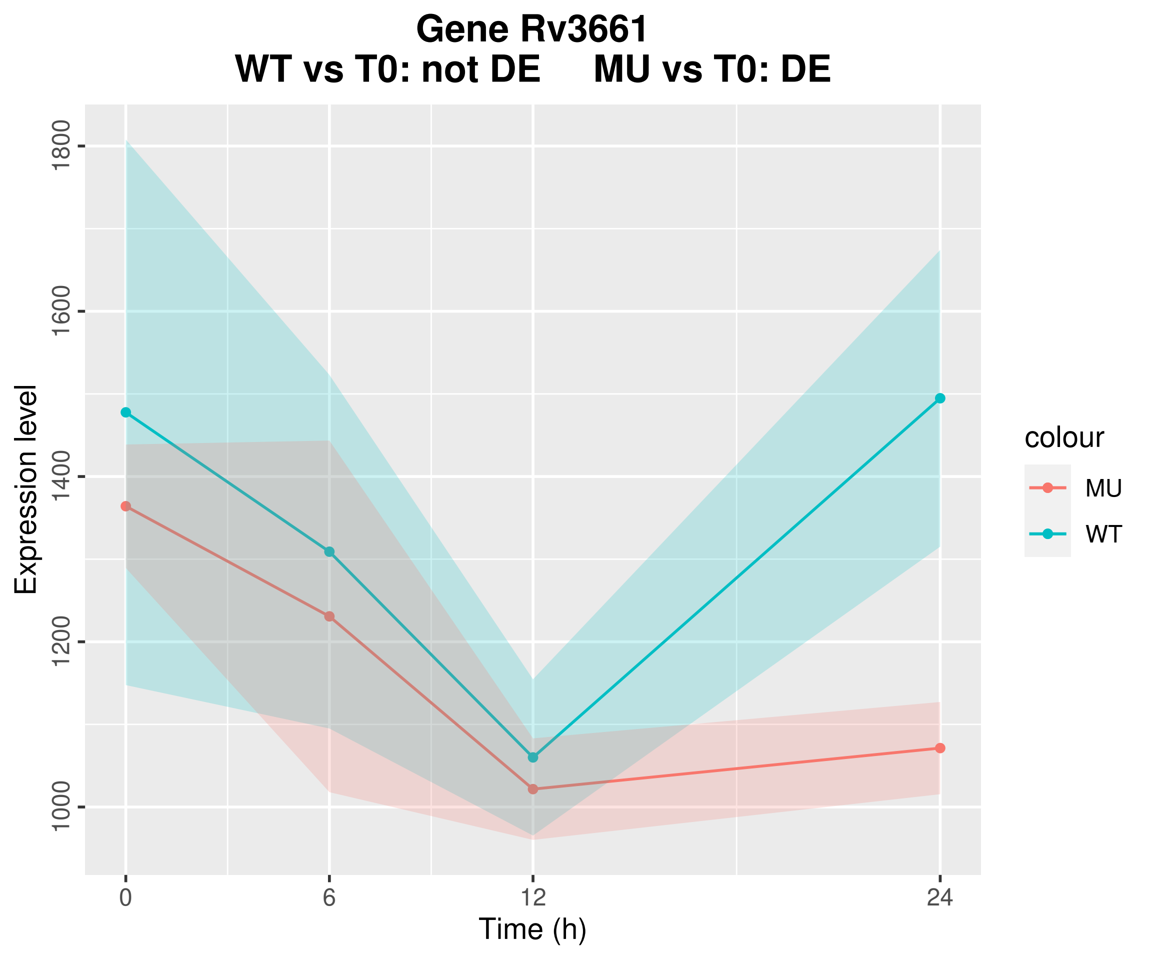

Gene Rv3661
WT vs T0: not DE MU vs T0: DE
Expression level
Time (h)
1000
1200
1400
1600
1800
0
6
12
24
colour
MU
WT

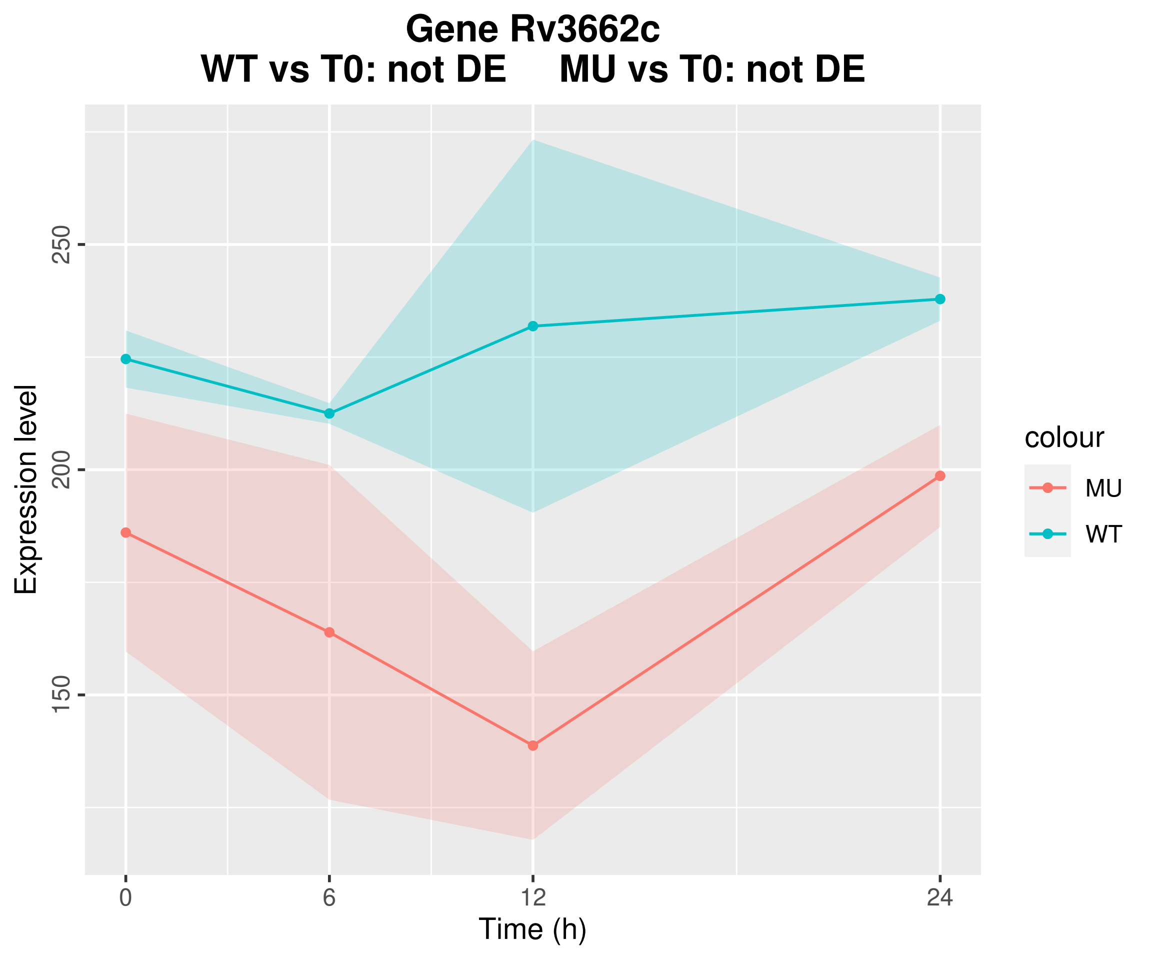
Gene Rv3662c
WT vs T0: not DE   MU vs T0: not DE
Expression level
250
200
150
Time (h)
0
6
12
24
colour
MU
WT

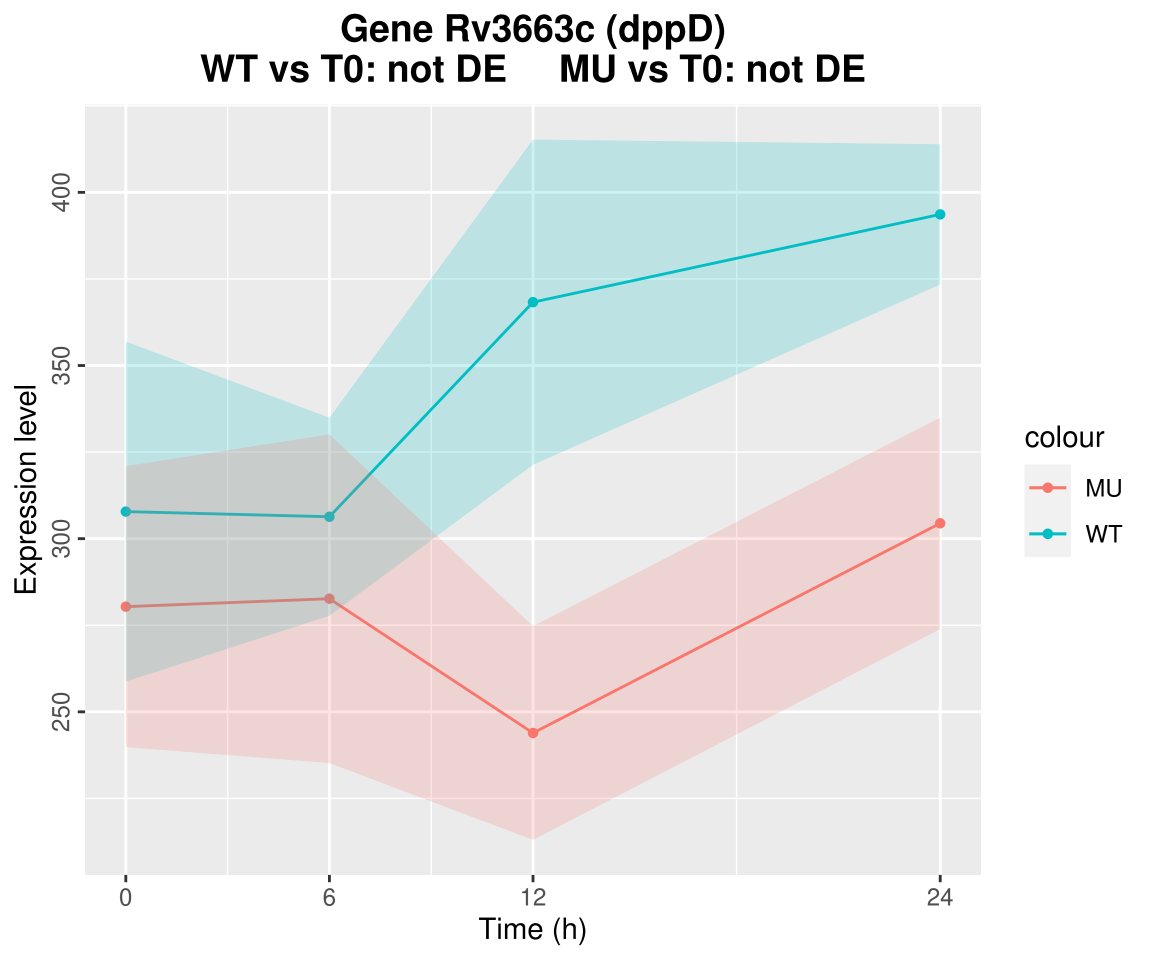

Gene Rv3663c (dppD)
WT vs T0: not DE   MU vs T0: not DE
Expression level
400
350
300
250
0
6
12
24
Time (h)
colour
MU
WT

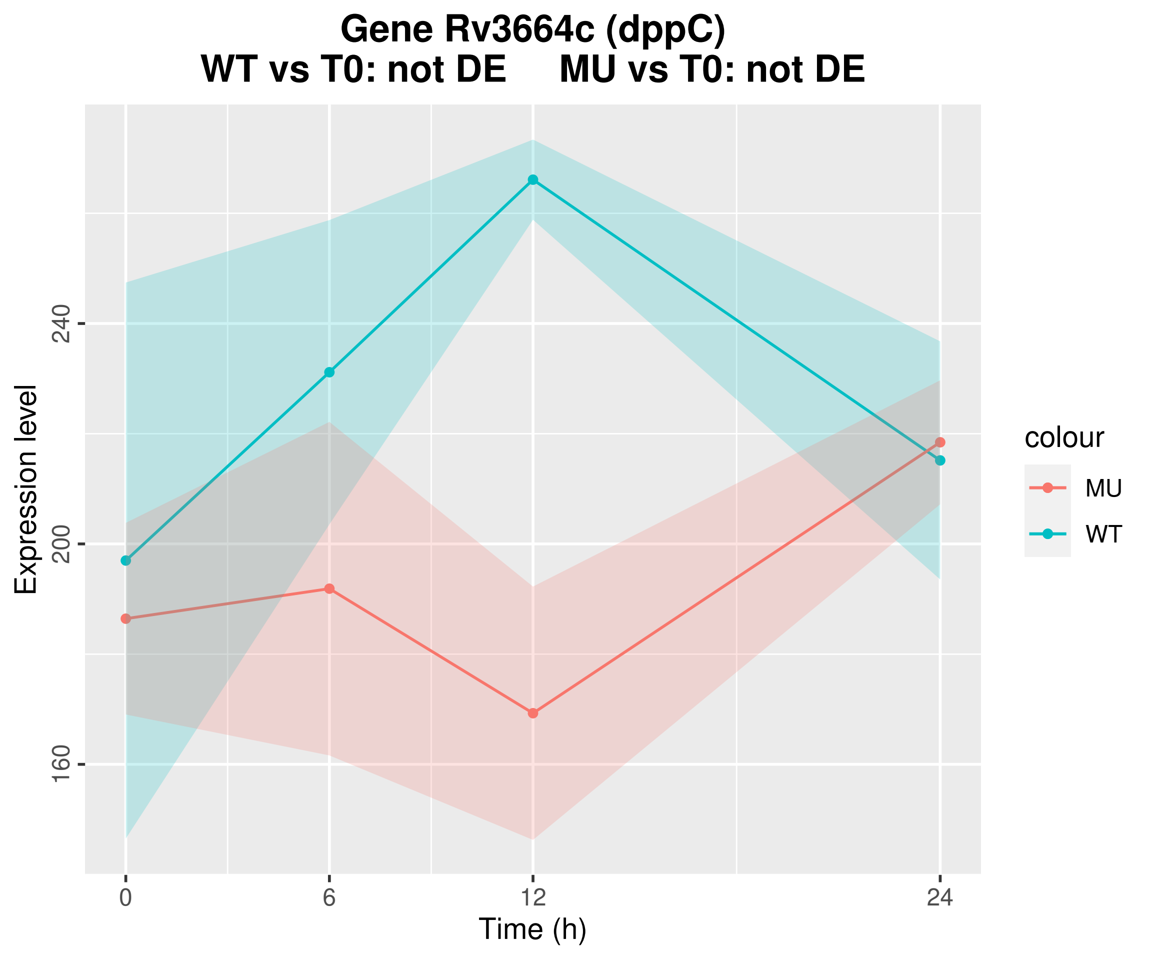

Gene Rv3664c (dppC)
WT vs T0: not DE   MU vs T0: not DE
Expression level
240
200
160
0
6
12
24
Time (h)
colour
MU
WT

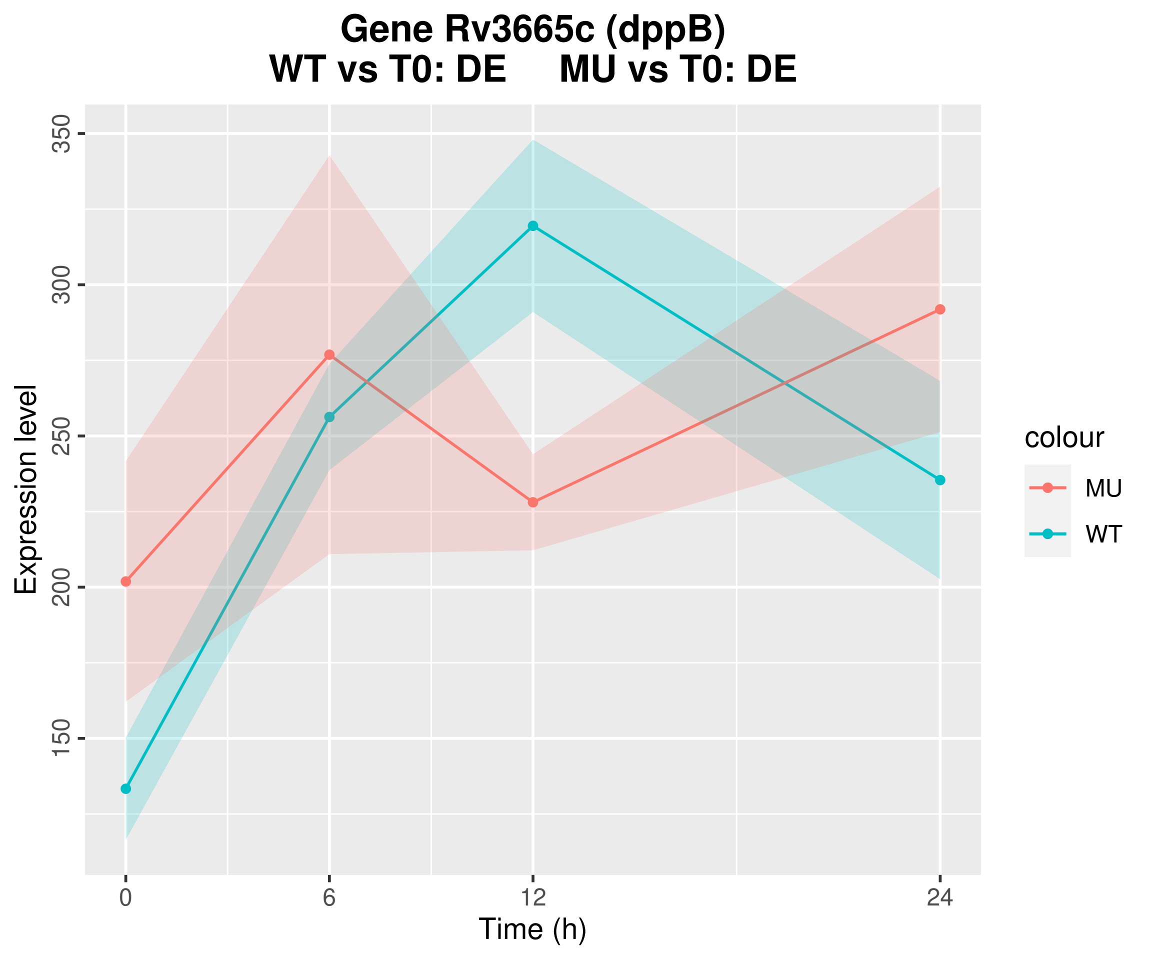
Gene Rv3665c (dppB)
WT vs T0: DE   MU vs T0: DE
Expression level
350
300
250
200
150
0
6
12
24
Time (h)
colour
MU
WT

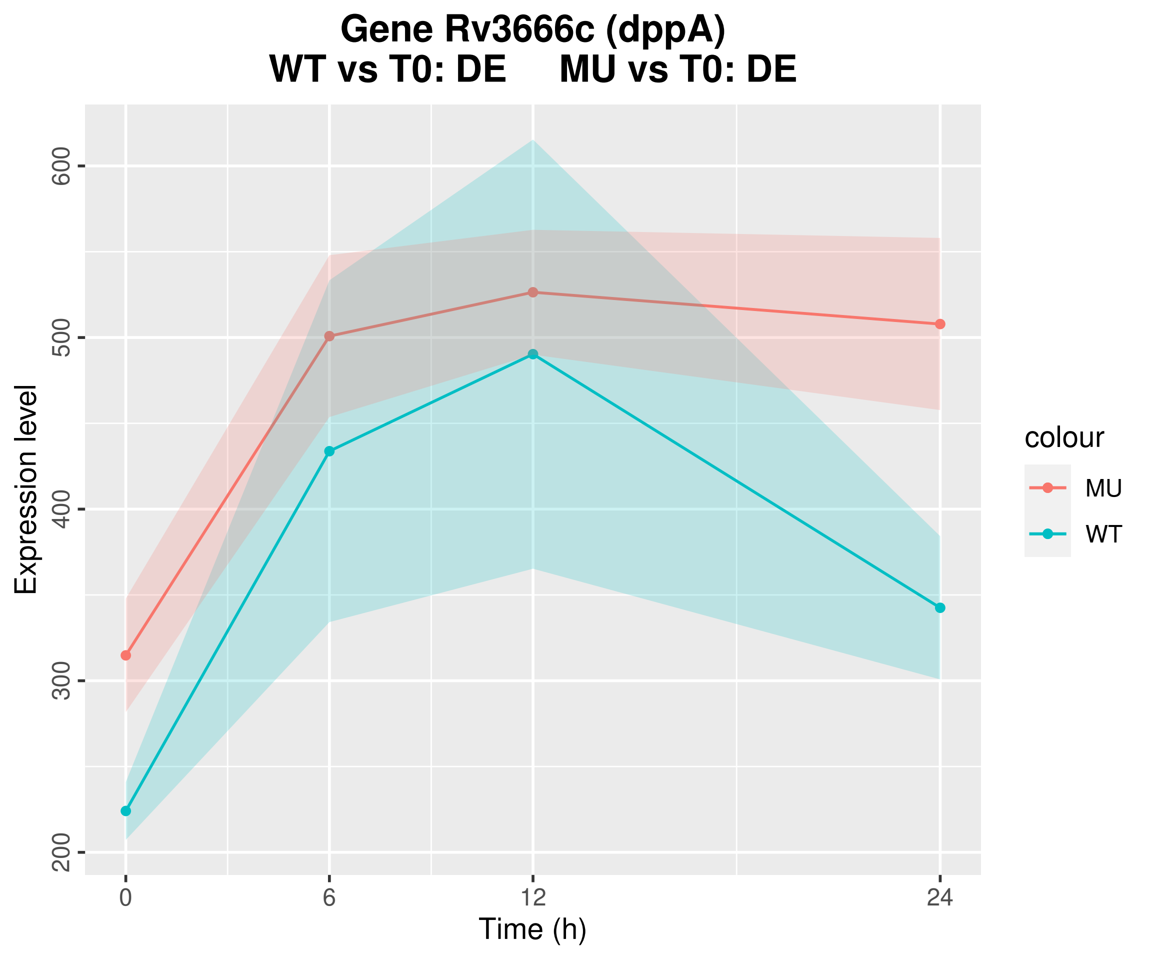

Gene Rv3666c (dppA)
WT vs T0: DE   MU vs T0: DE
Expression level
200
300
400
500
600
0
6
12
24
Time (h)
colour
MU
WT

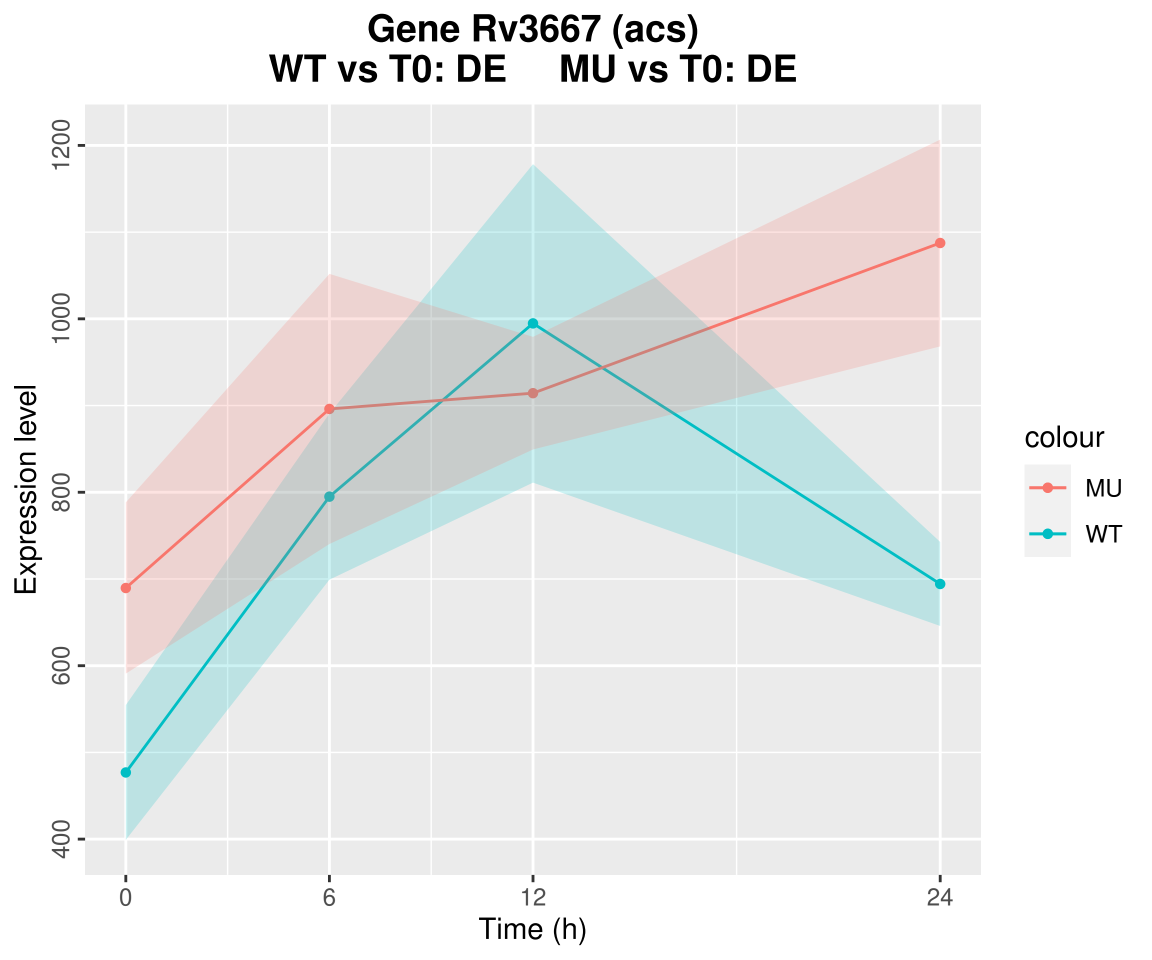
Gene Rv3667 (acs)
WT vs T0: DE   MU vs T0: DE
Expression level
1200
1000
800
600
400
0
6
12
24
Time (h)
colour
MU
WT

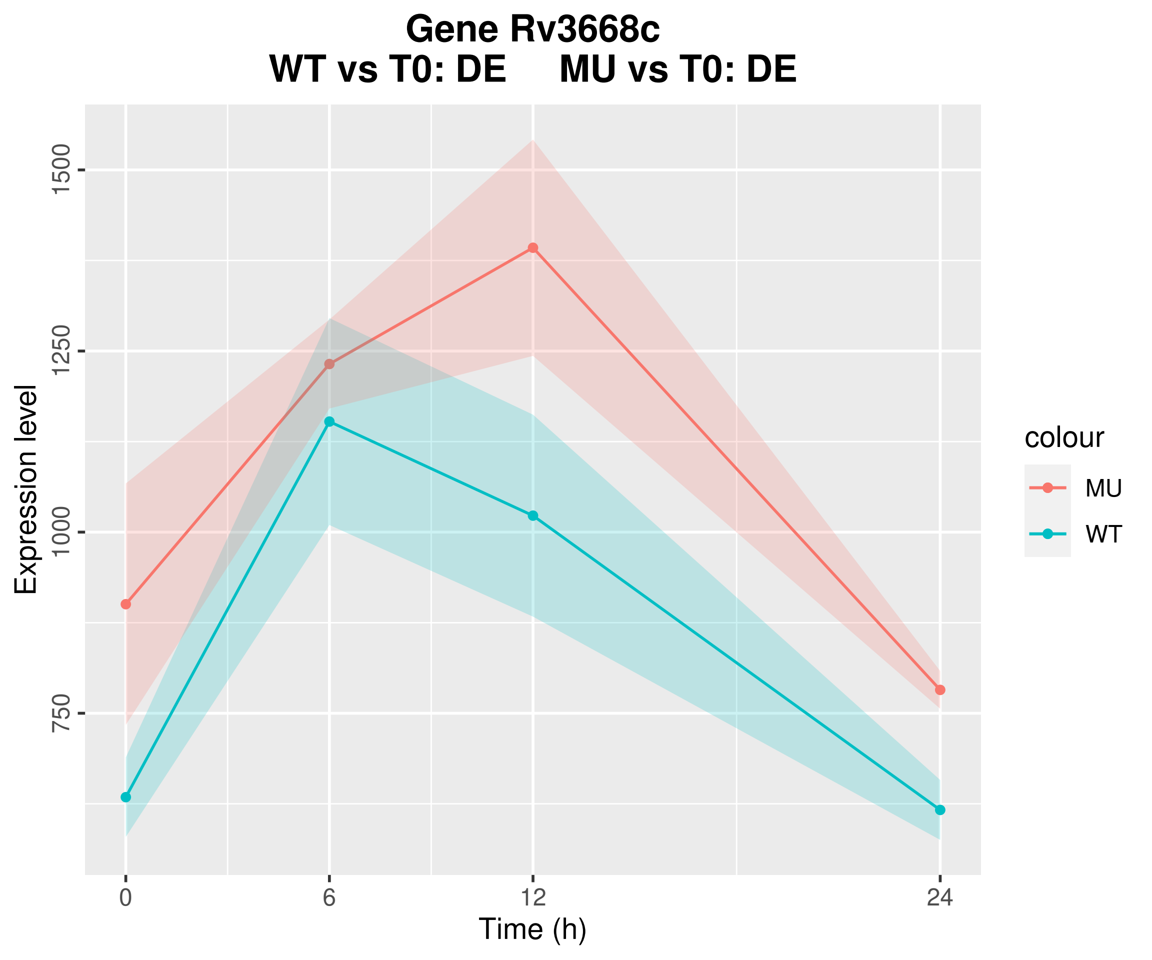
Gene Rv3668c
WT vs T0: DE MU vs T0: DE
Expression level
1500
1250
1000
750
0
6
12
24
Time (h)
colour
MU
WT

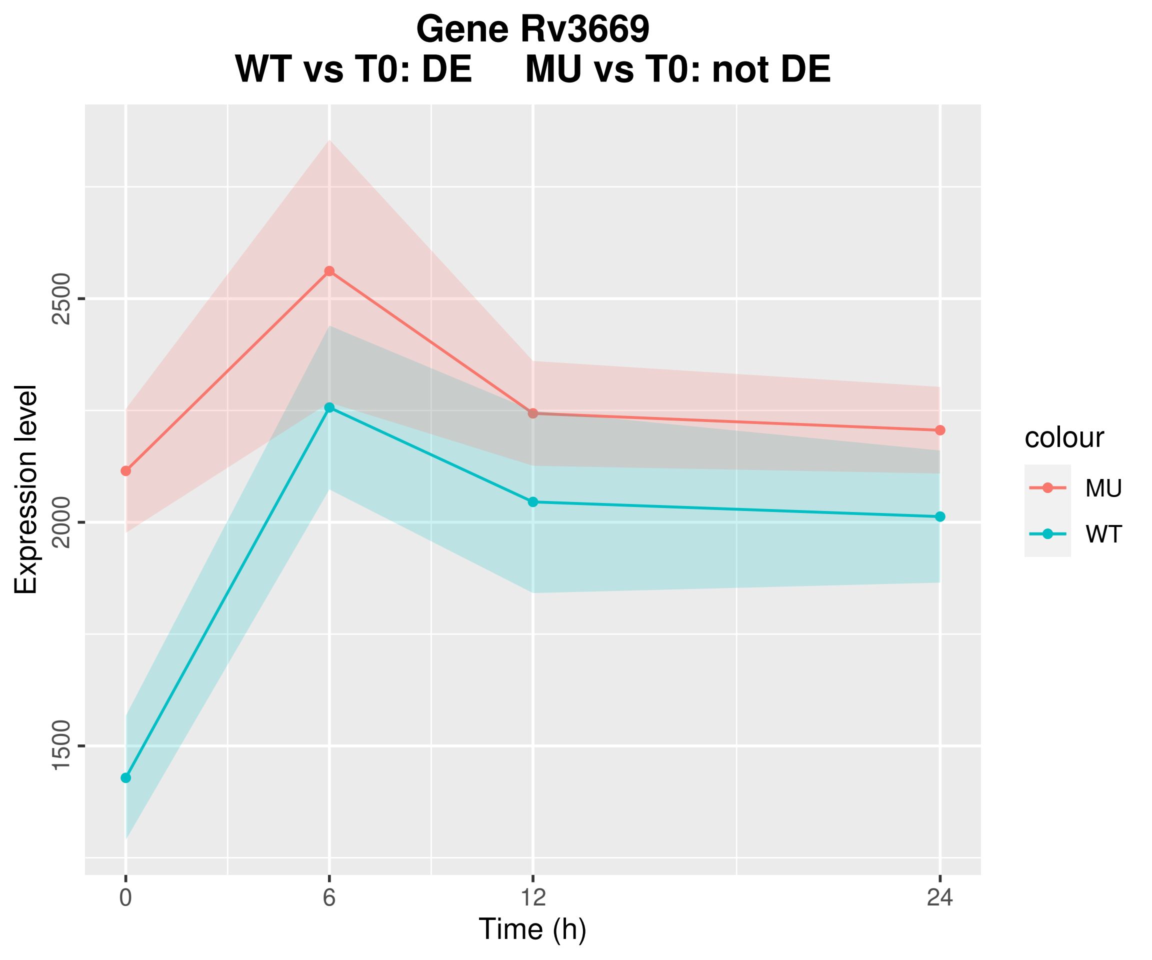
Gene Rv3669
WT vs T0: DE MU vs T0: not DE
Expression level
2500
2000
1500
0
6
12
24
Time (h)
colour
MU
WT

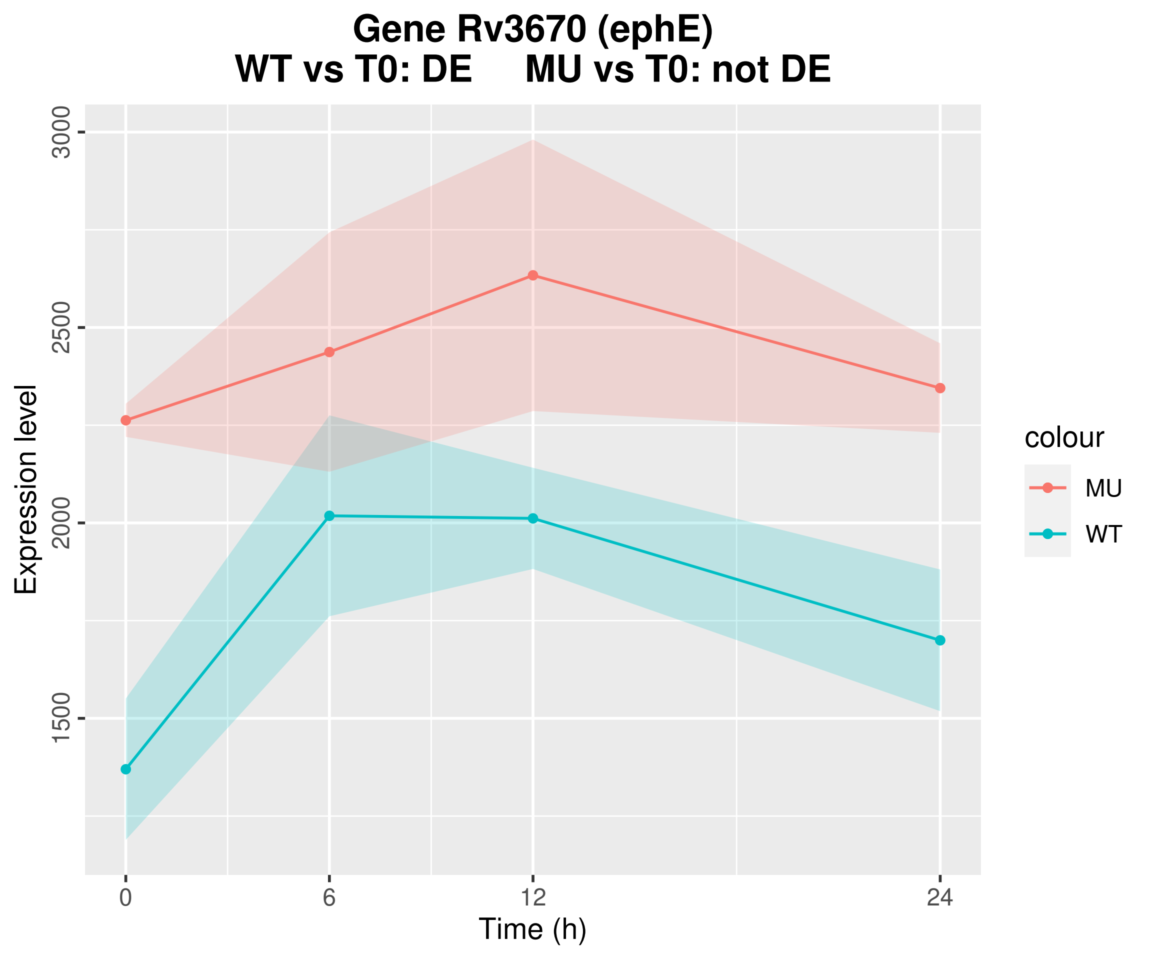

Gene Rv3670 (ephE)
WT vs T0: DE   MU vs T0: not DE
Expression level
3000
2500
2000
1500
0
6
12
24
Time (h)
colour
MU
WT

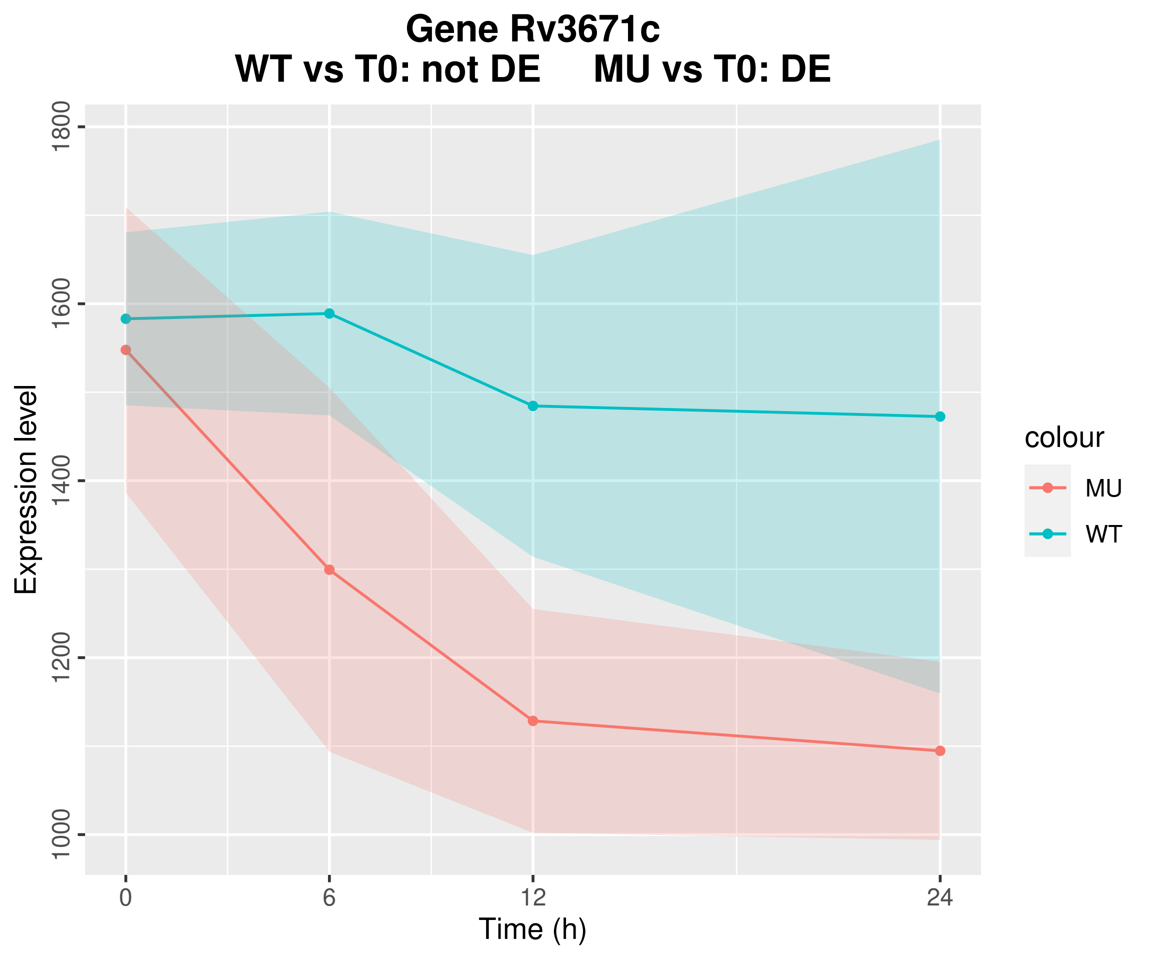

Gene Rv3671c
WT vs T0: not DE   MU vs T0: DE
Expression level
1800
1600
1400
1200
1000
0
6
12
24
Time (h)
colour
MU
WT

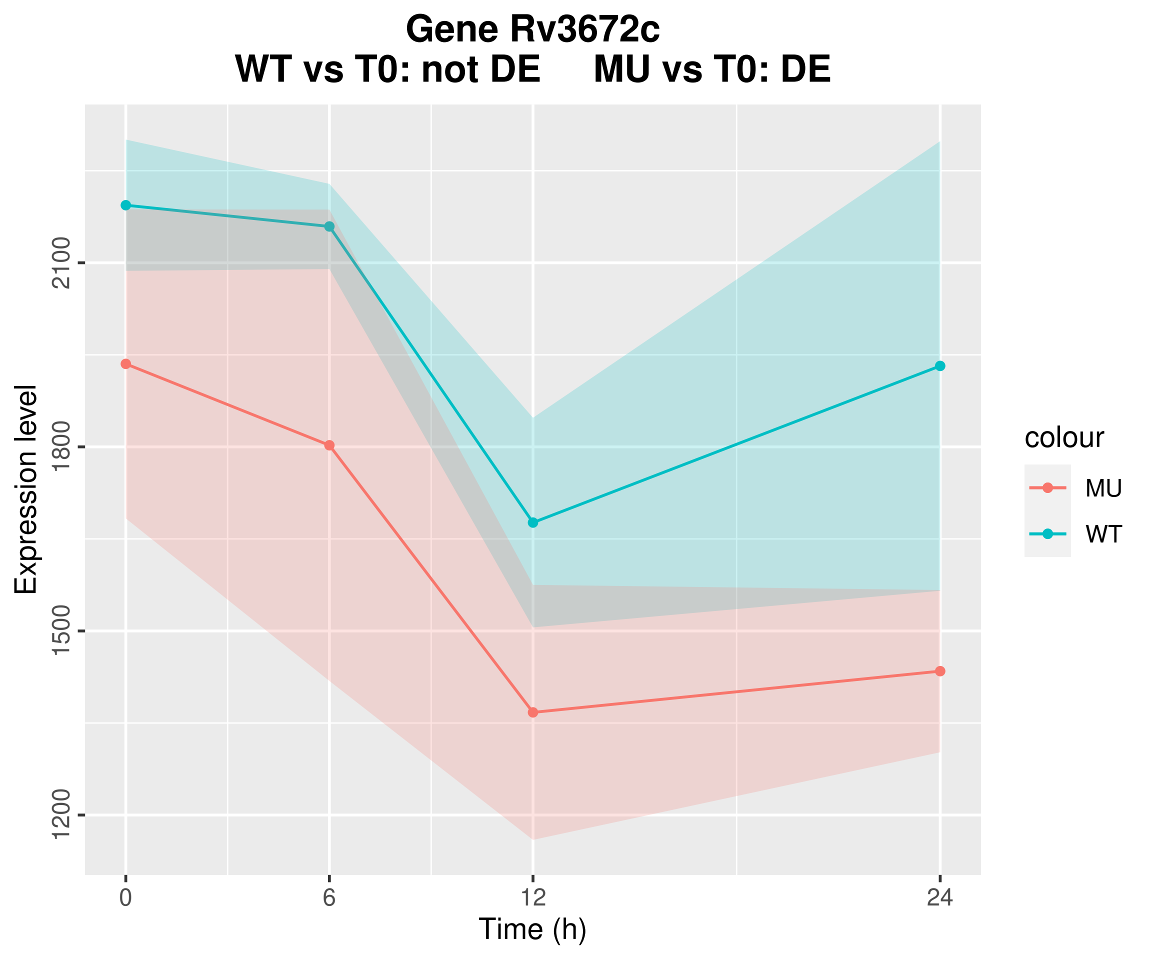

Gene Rv3672c
WT vs T0: not DE MU vs T0: DE
Expression level
2100
1800
1500
1200
0
6
12
24
Time (h)
colour
MU
WT

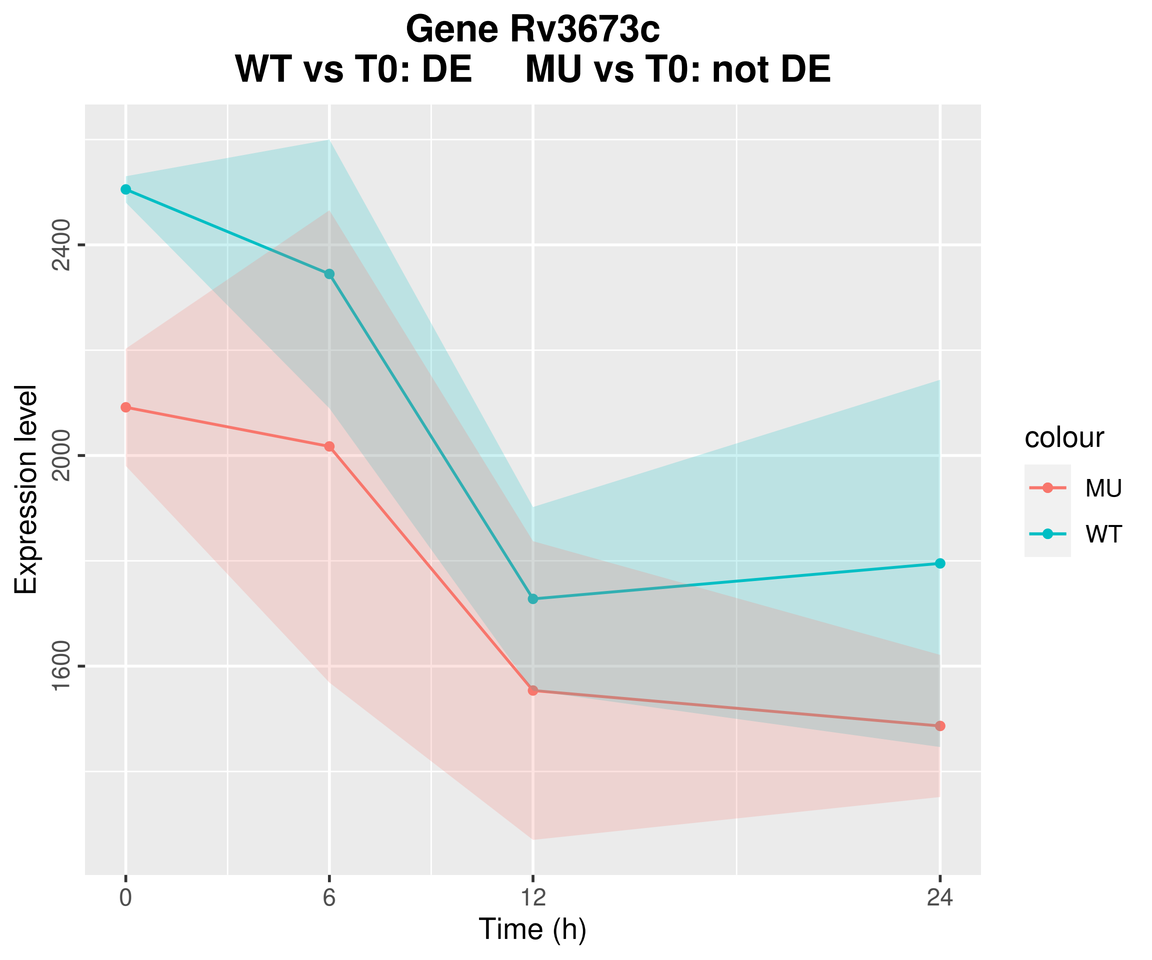

Gene Rv3673c
WT vs T0: DE   MU vs T0: not DE
Expression level
2400
2000
1600
0
6
12
24
Time (h)
colour
MU
WT

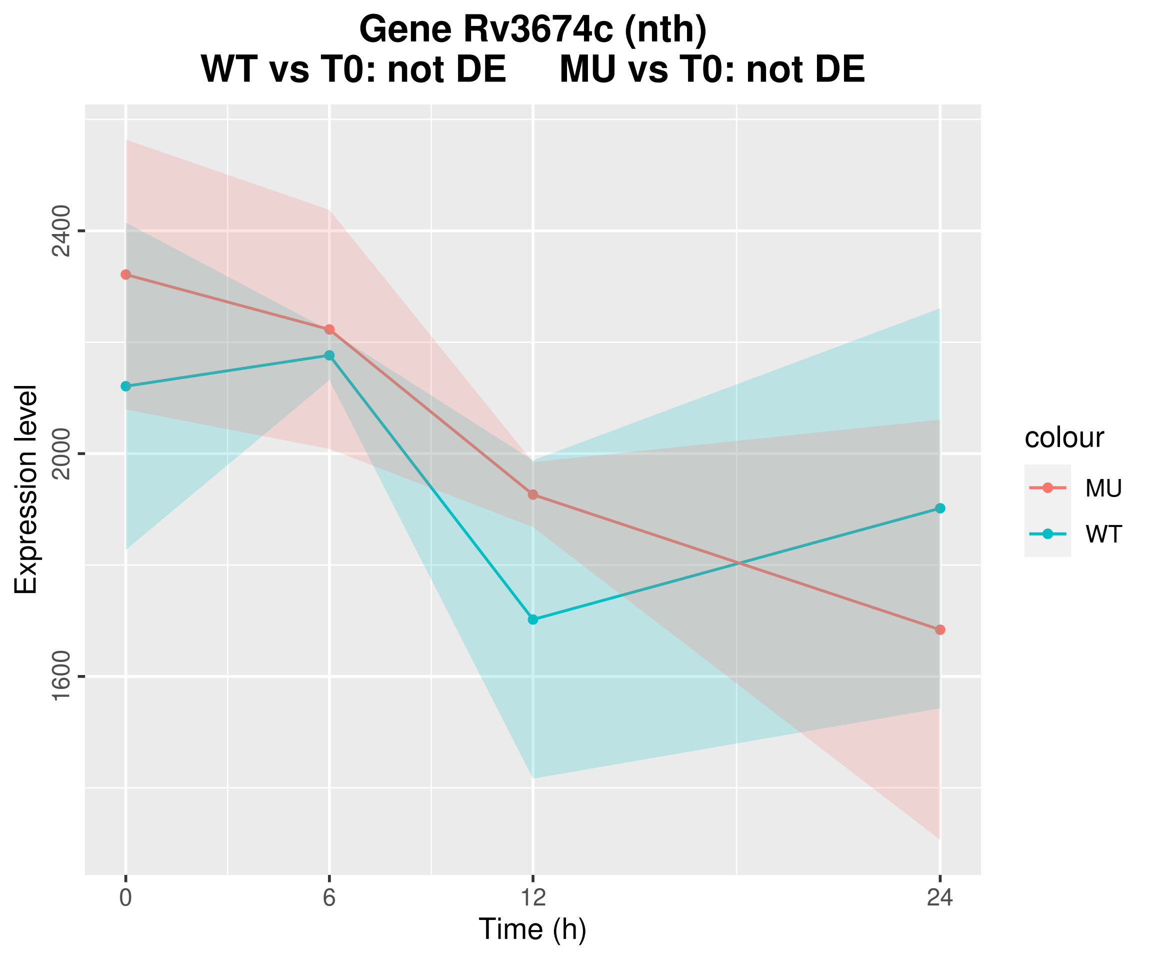

Gene Rv3674c (nth)
WT vs T0: not DE MU vs T0: not DE
Expression level
2400
2000
1600
0
6
12
24
Time (h)
colour
MU
WT

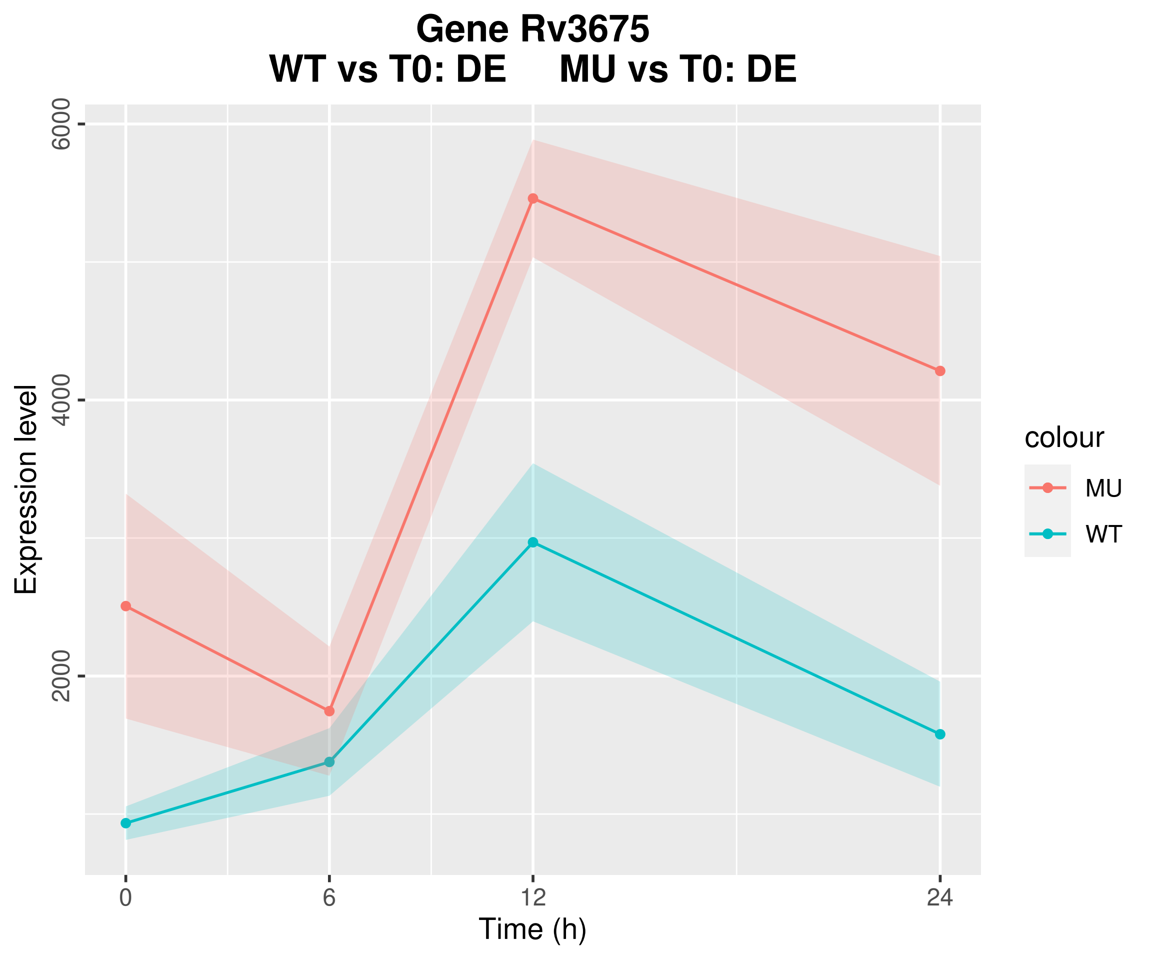
Gene Rv3675
WT vs T0: DE MU vs T0: DE
Expression level
6000
4000
2000
0
6
12
24
Time (h)
colour
MU
WT

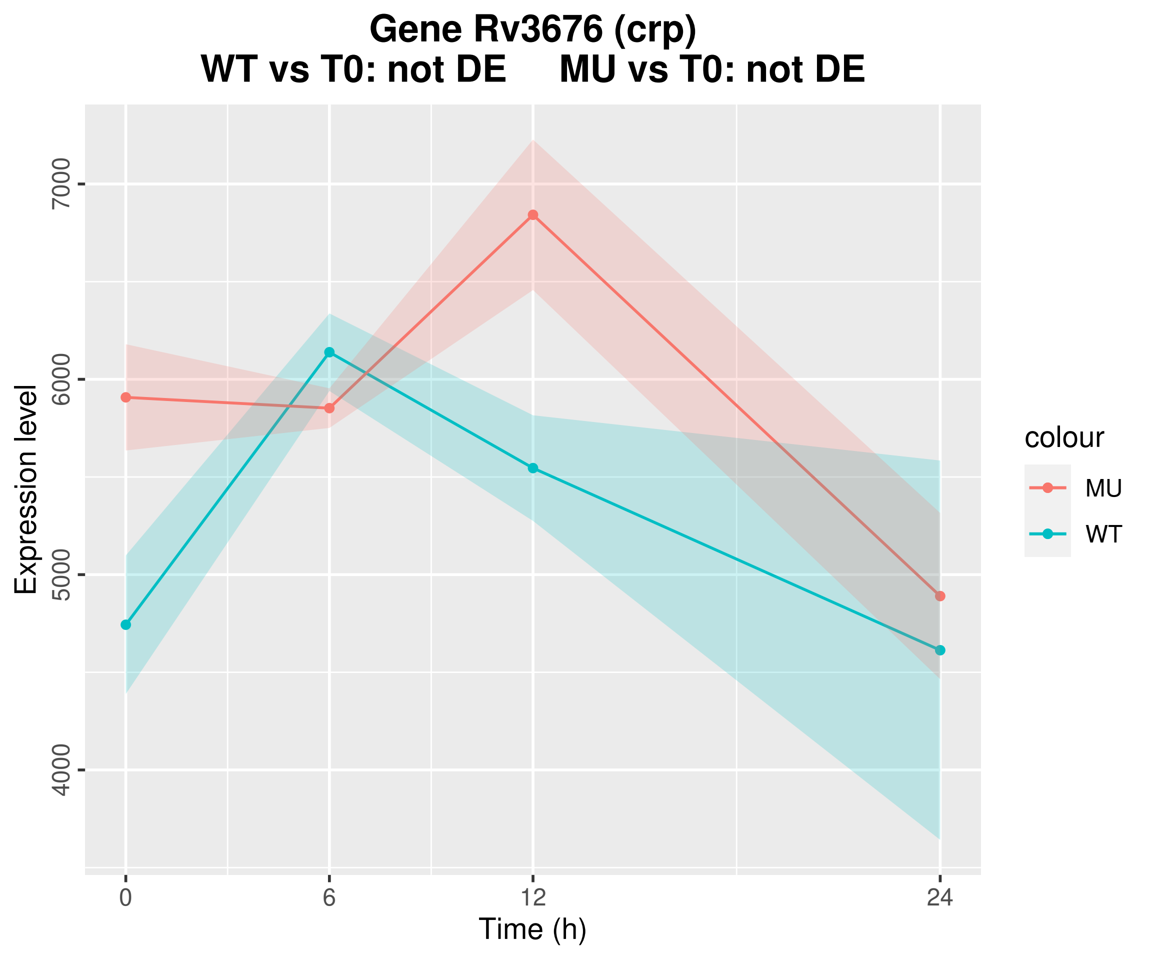
Gene Rv3676 (crp)
WT vs T0: not DE MU vs T0: not DE
Expression level
7000
6000
5000
4000
0
6
12
24
Time (h)
colour
MU
WT

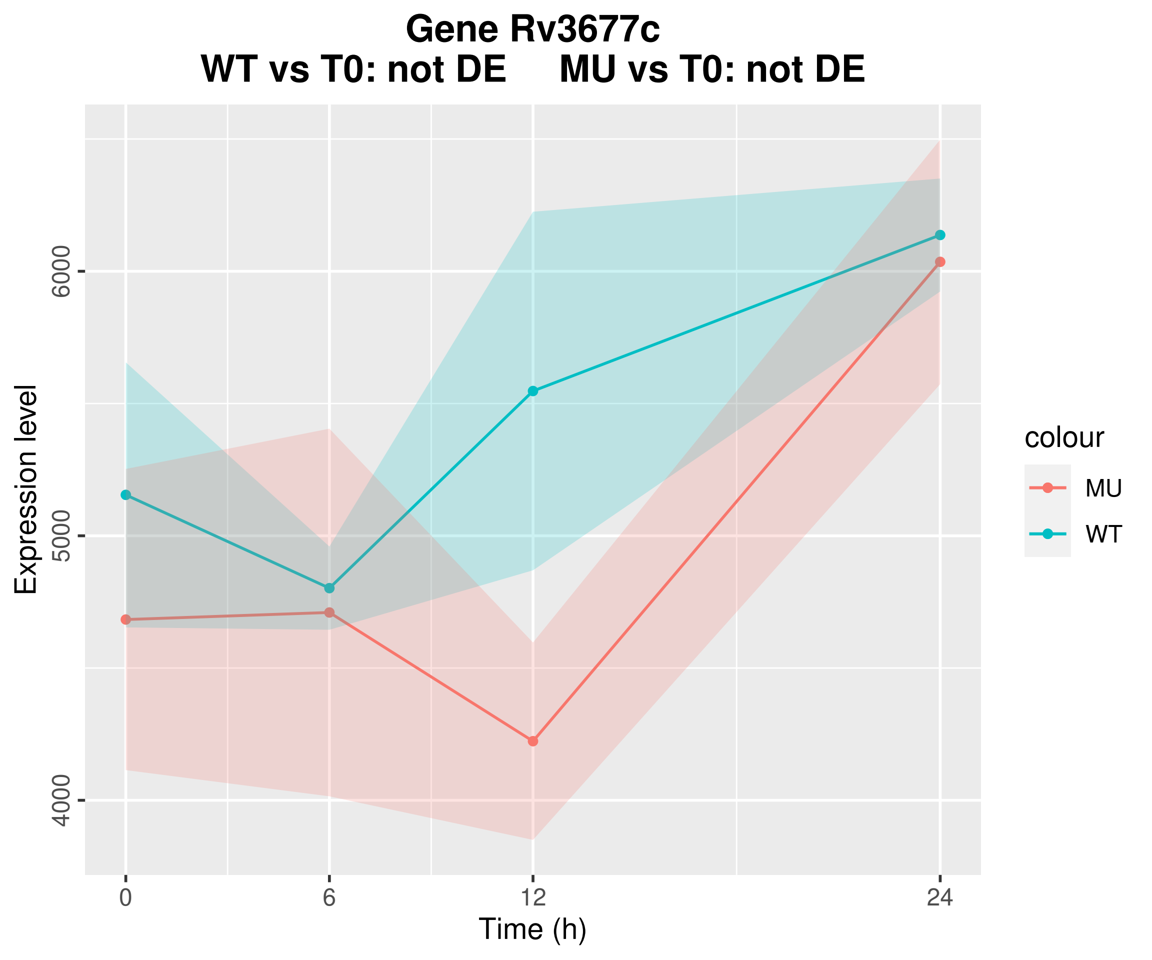

Gene Rv3677c
WT vs T0: not DE MU vs T0: not DE
Expression level
4000
5000
6000
0
6
12
24
Time (h)
colour
MU
WT

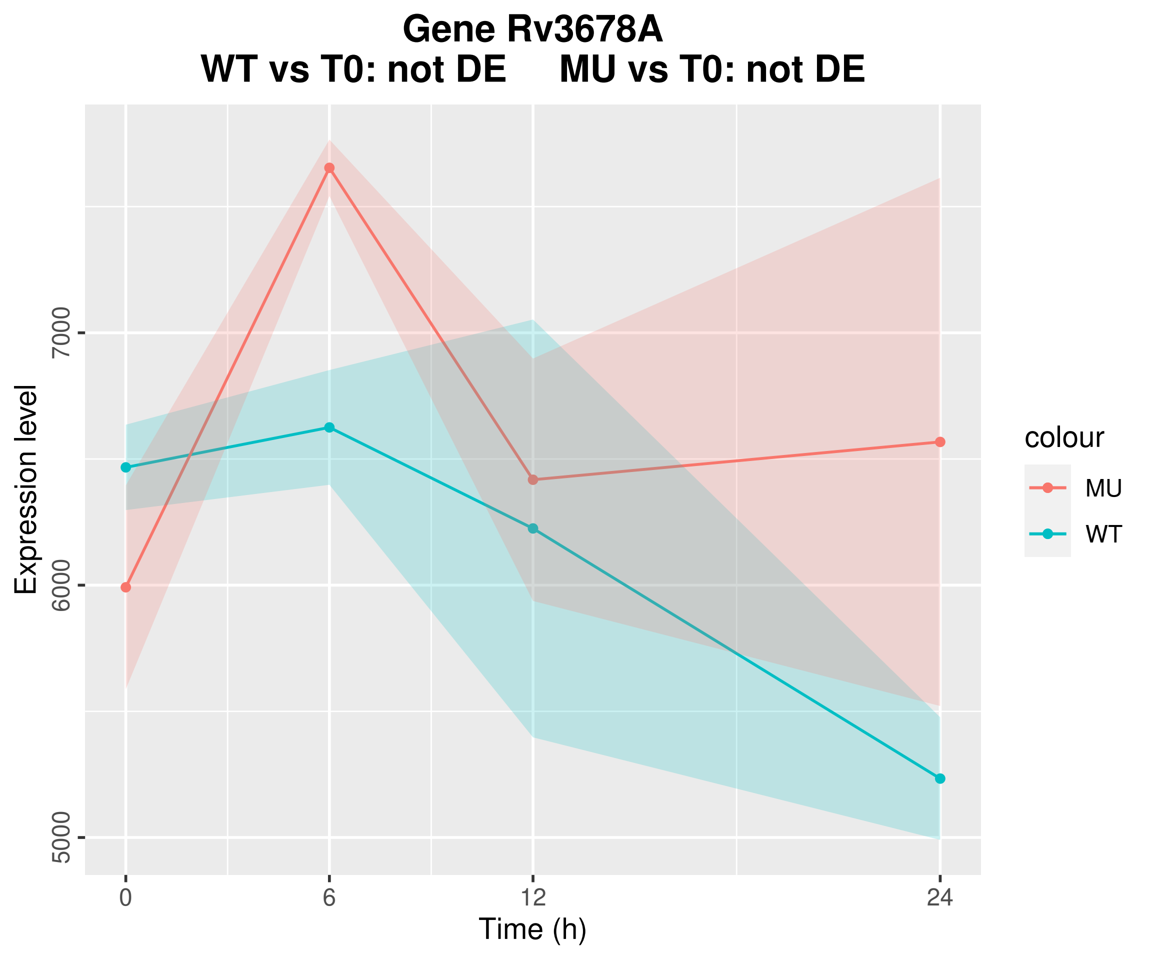
Gene Rv3678A
WT vs T0: not DE MU vs T0: not DE
Expression level
5000
6000
7000
0
6
12
24
Time (h)
colour
MU
WT

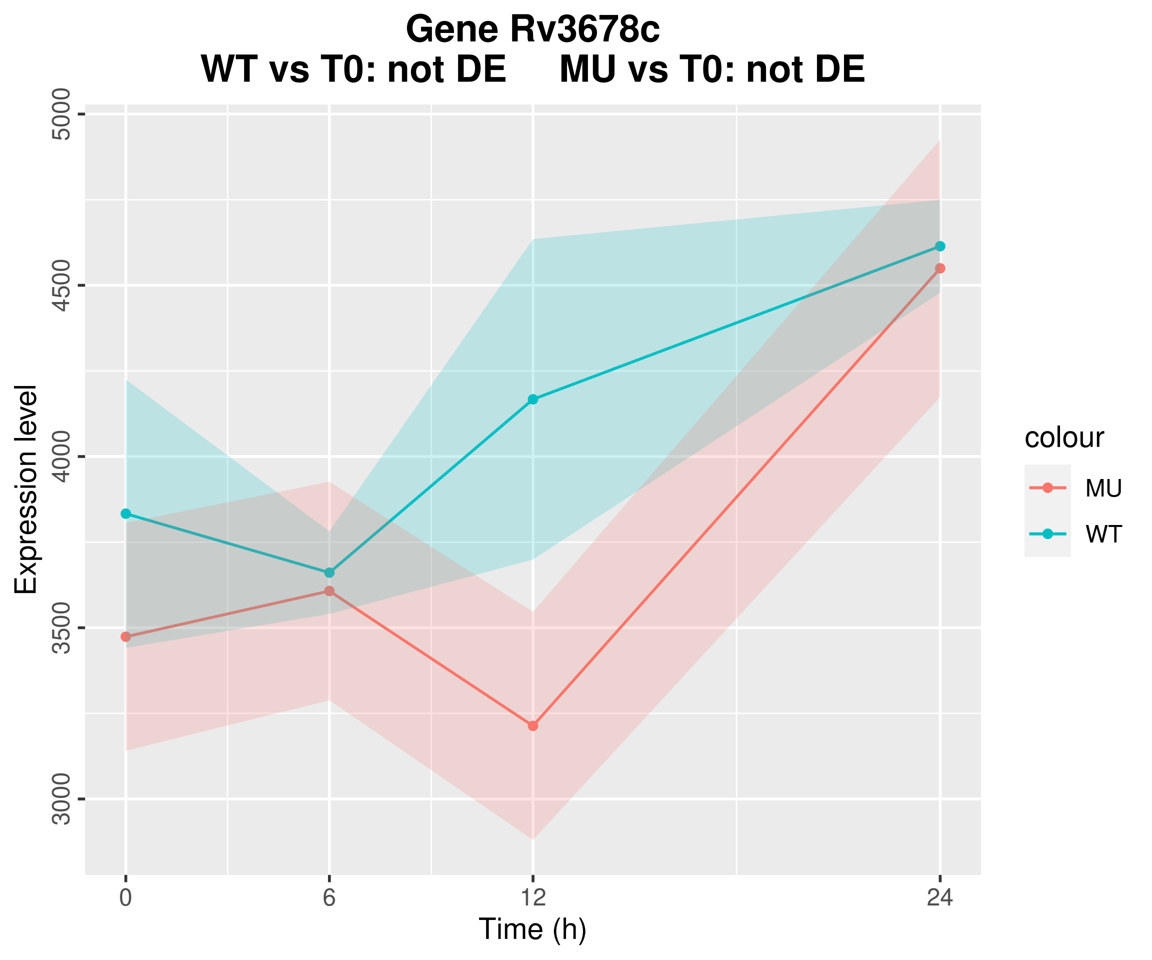

Gene Rv3678c
WT vs T0: not DE   MU vs T0: not DE
Expression level
5000
4500
4000
3500
3000
0
6
12
24
Time (h)
colour
MU
WT

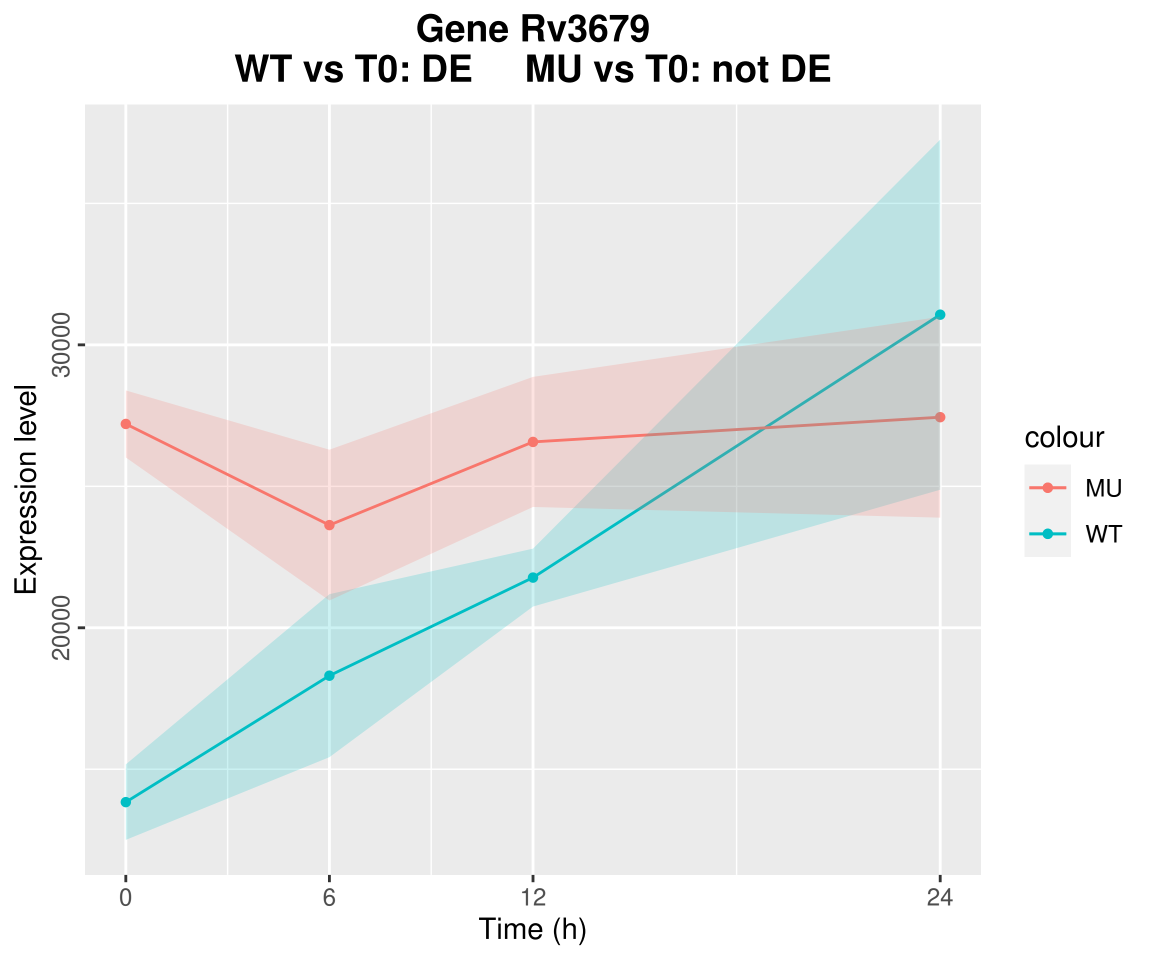
Gene Rv3679
WT vs T0: DE MU vs T0: not DE
Expression level
30000
20000
0
6
12
24
Time (h)
colour
MU
WT

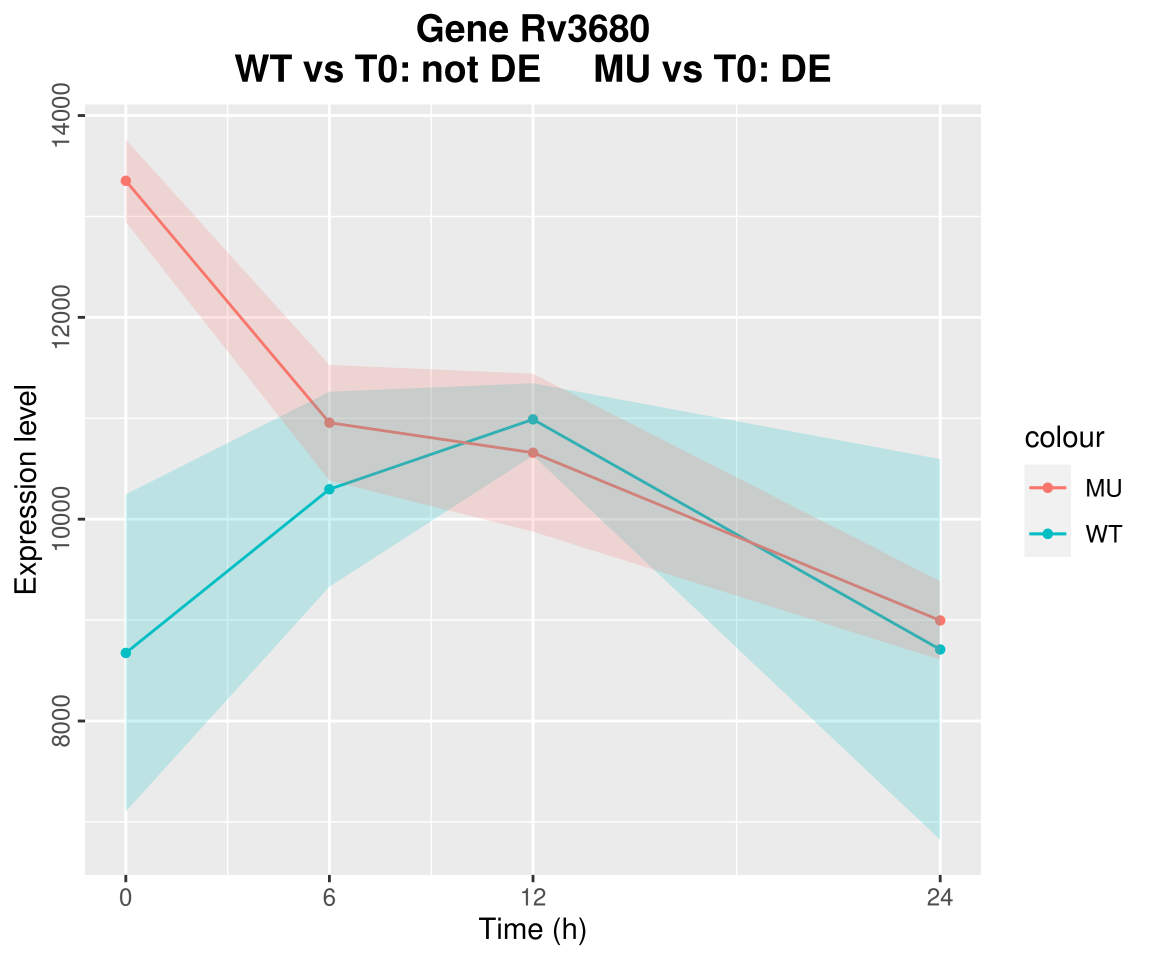
Gene Rv3680
WT vs T0: not DE MU vs T0: DE
Expression level
14000
12000
10000
8000
0
6
12
24
Time (h)
colour
MU
WT

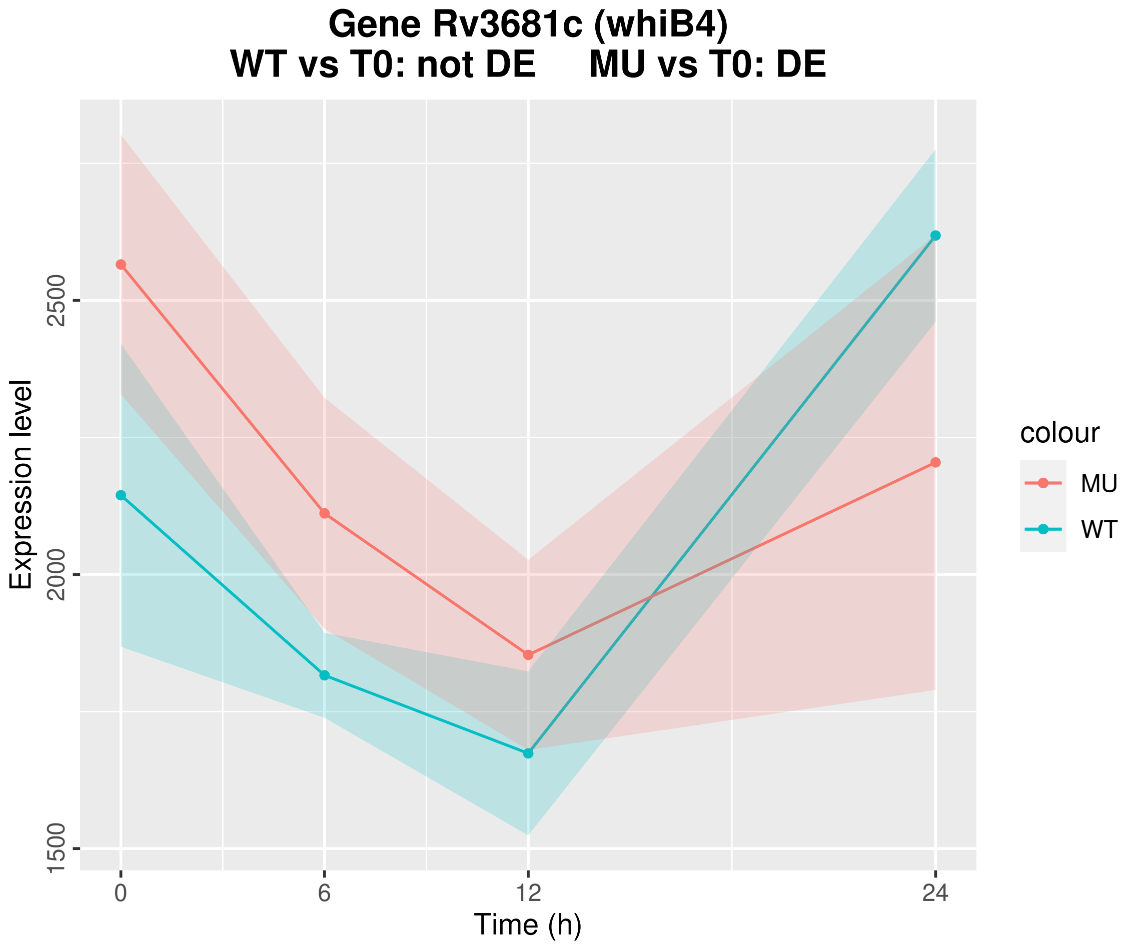
Gene Rv3681c (whiB4)
WT vs T0: not DE   MU vs T0: DE
Expression level
2500
2000
1500
0
6
12
24
Time (h)
colour
MU
WT

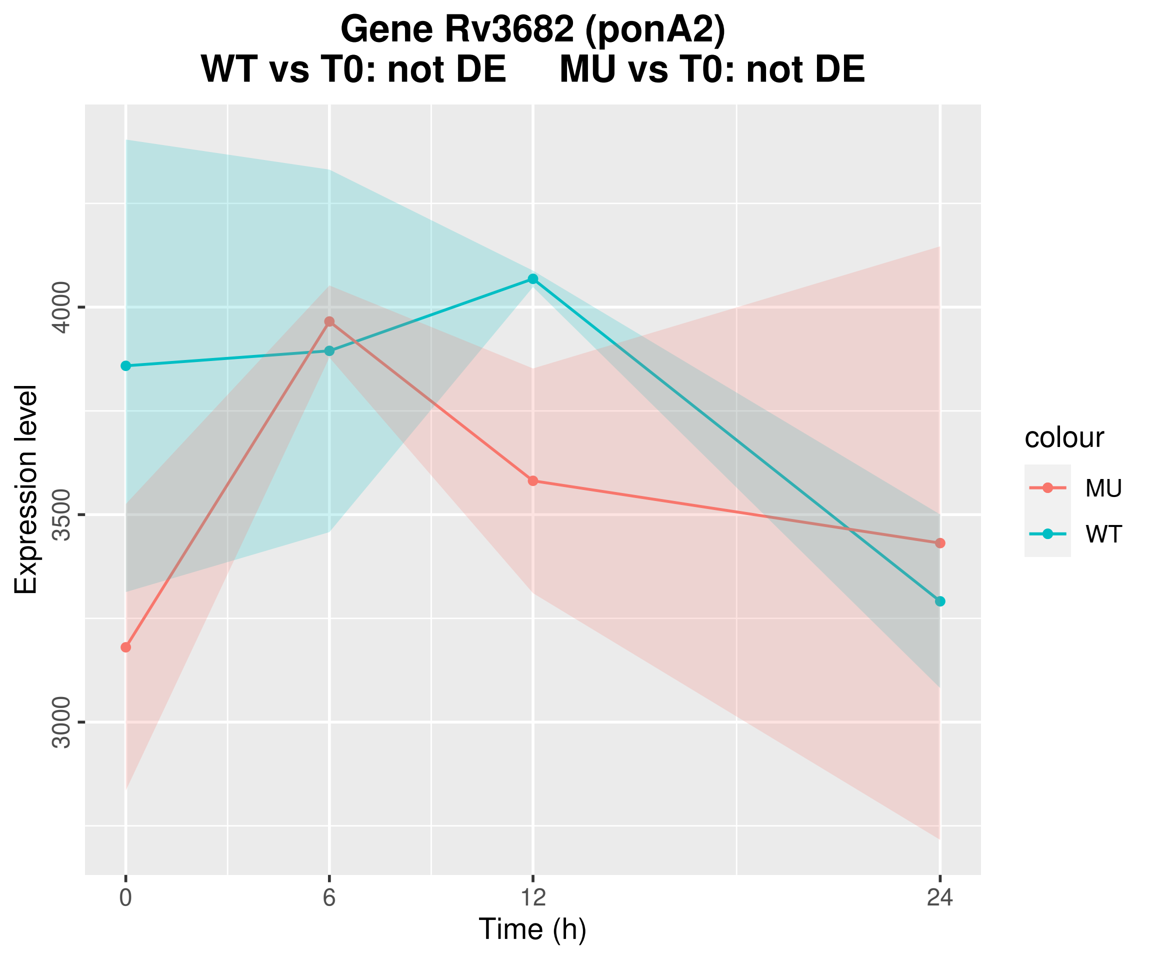
Gene Rv3682 (ponA2)
WT vs T0: not DE   MU vs T0: not DE
Expression level
3000
3500
4000
0
6
12
24
Time (h)
colour
MU
WT

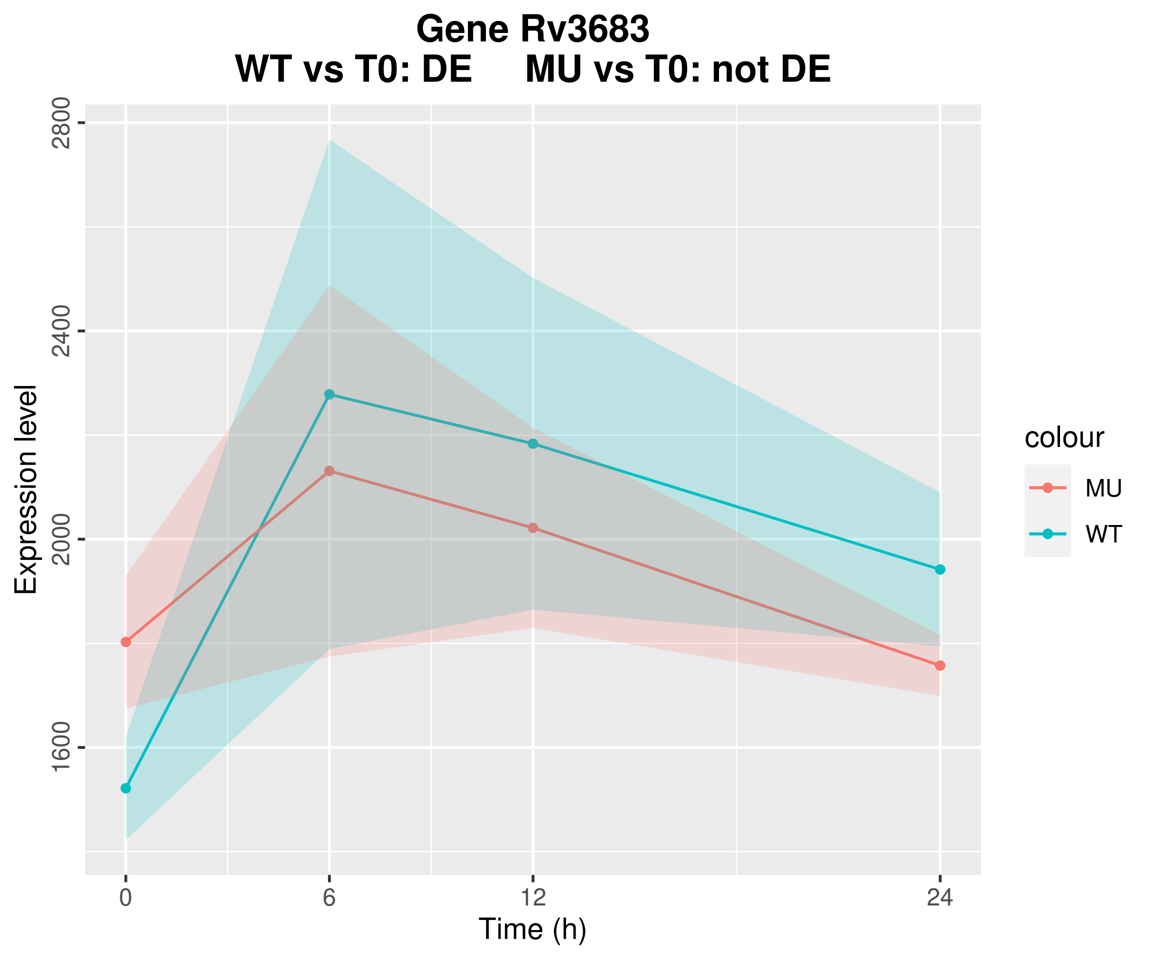
Gene Rv3683
WT vs T0: DE MU vs T0: not DE
Expression level
2800
2400
2000
1600
0
6
12
24
Time (h)
colour
MU
WT

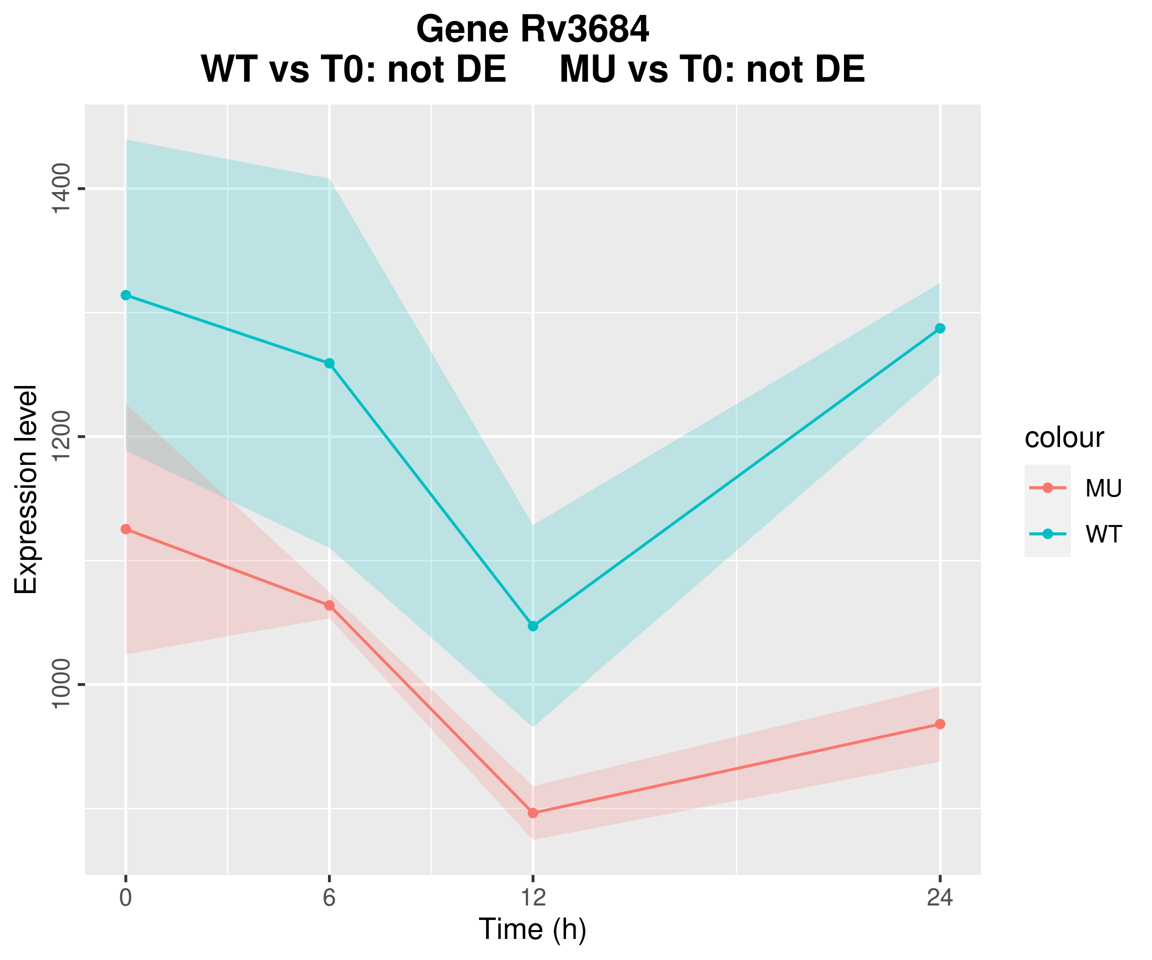

Gene Rv3684
WT vs T0: not DE MU vs T0: not DE
Expression level
1400
1200
1000
Time (h)
0
6
12
24
colour
MU
WT

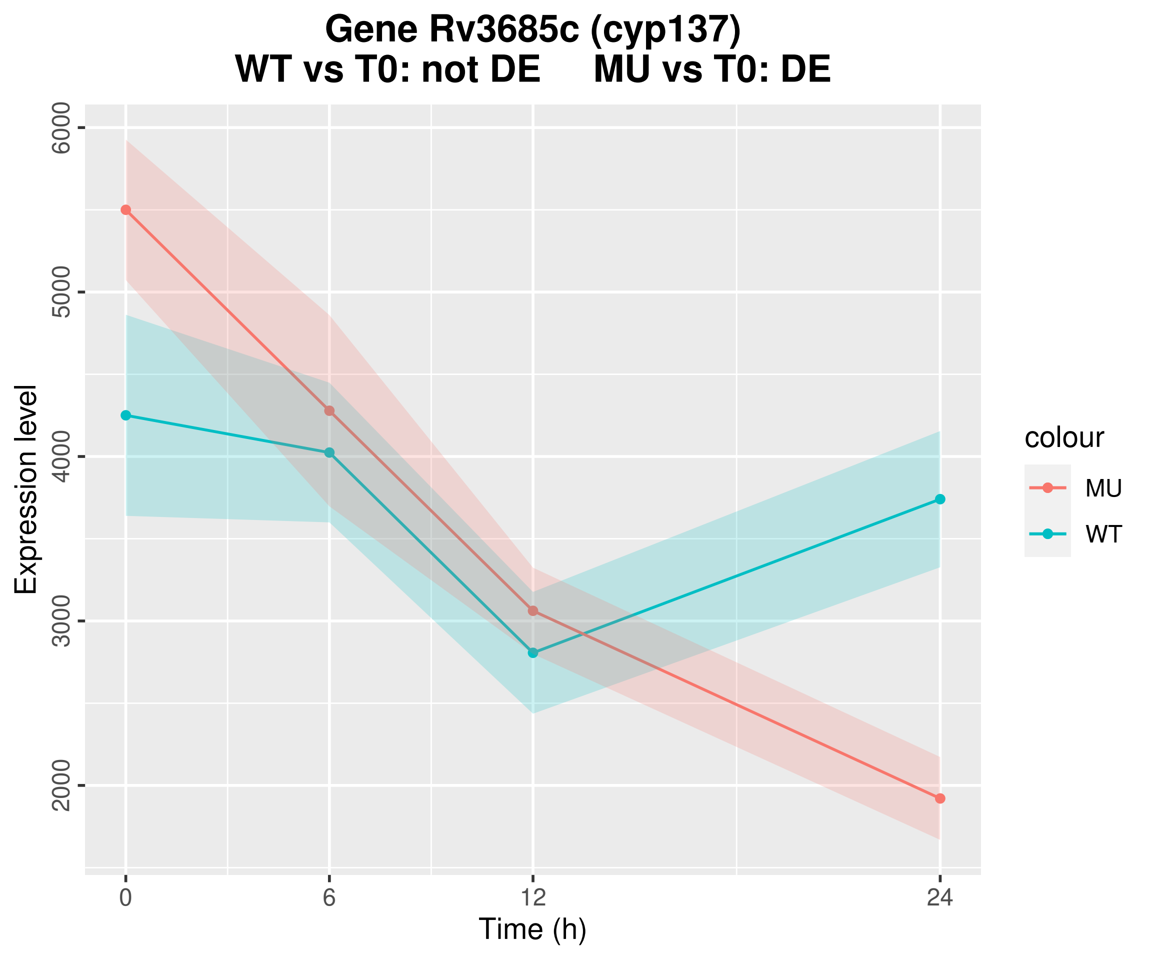

Gene Rv3685c (cyp137)
WT vs T0: not DE   MU vs T0: DE
Expression level
6000
5000
4000
3000
2000
0
6
12
24
Time (h)
colour
MU
WT

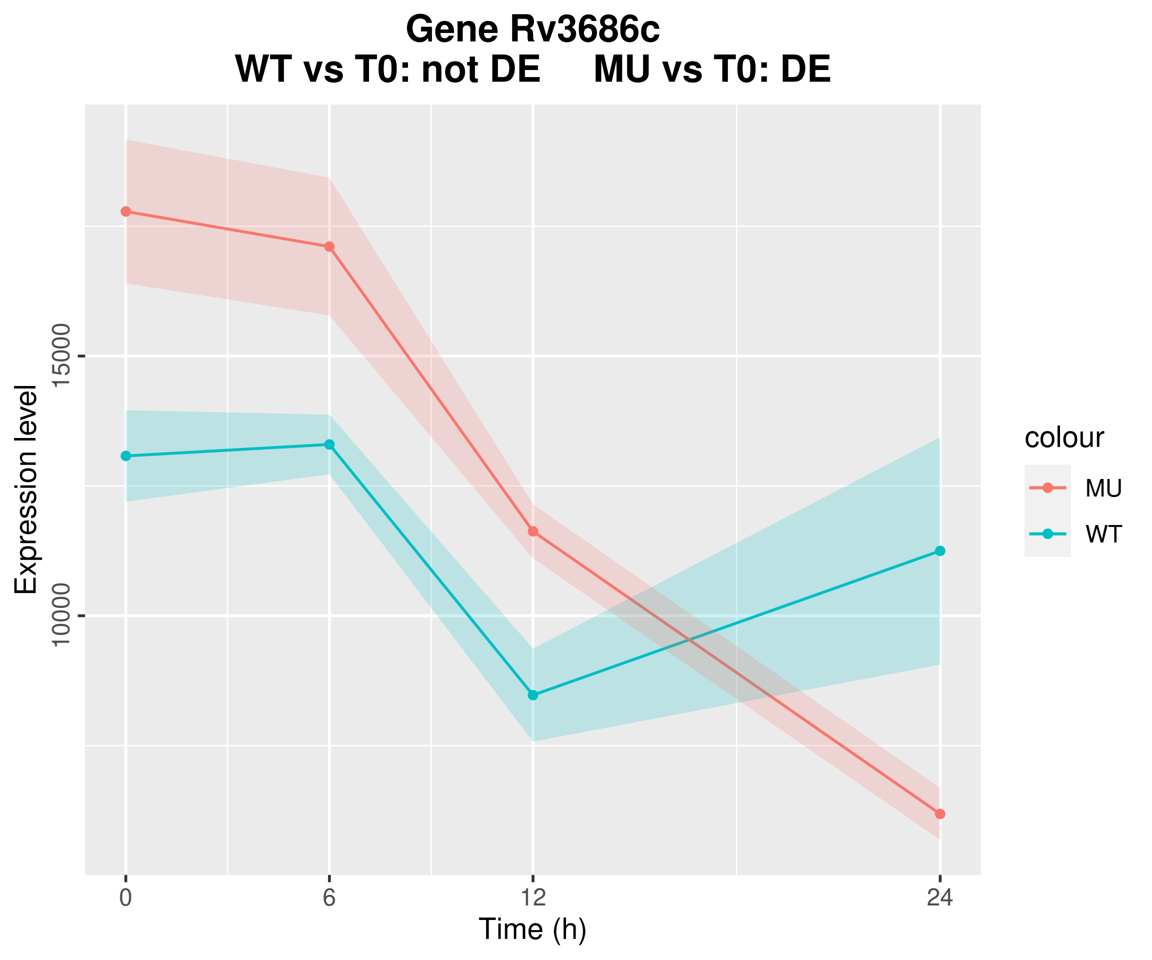
Gene Rv3686c
WT vs T0: not DE MU vs T0: DE
Expression level
15000
10000
0
6
12
24
Time (h)
colour
MU
WT

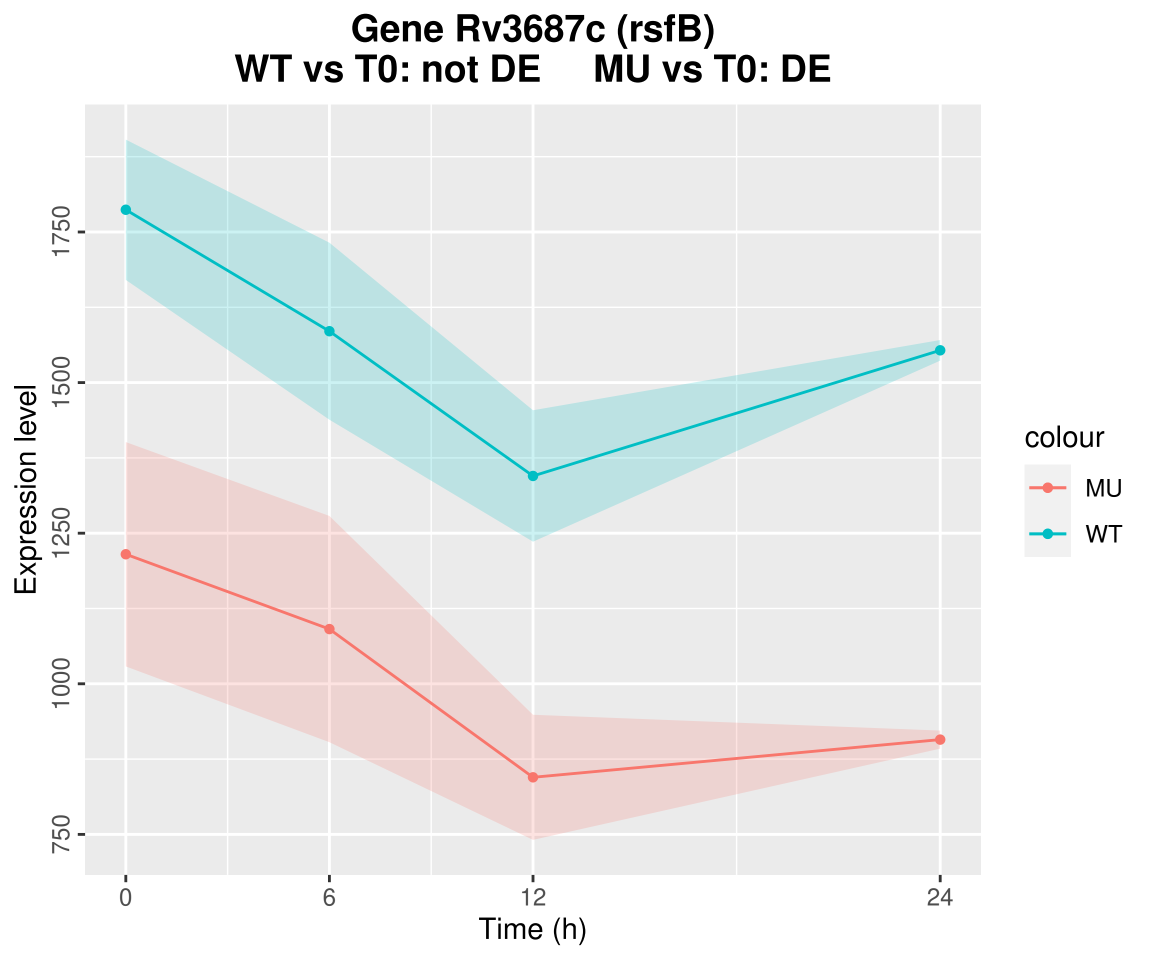

Gene Rv3687c (rsfB)
WT vs T0: not DE MU vs T0: DE
Expression level
750
1000
1250
1500
1750
0
6
12
24
Time (h)
colour
MU
WT

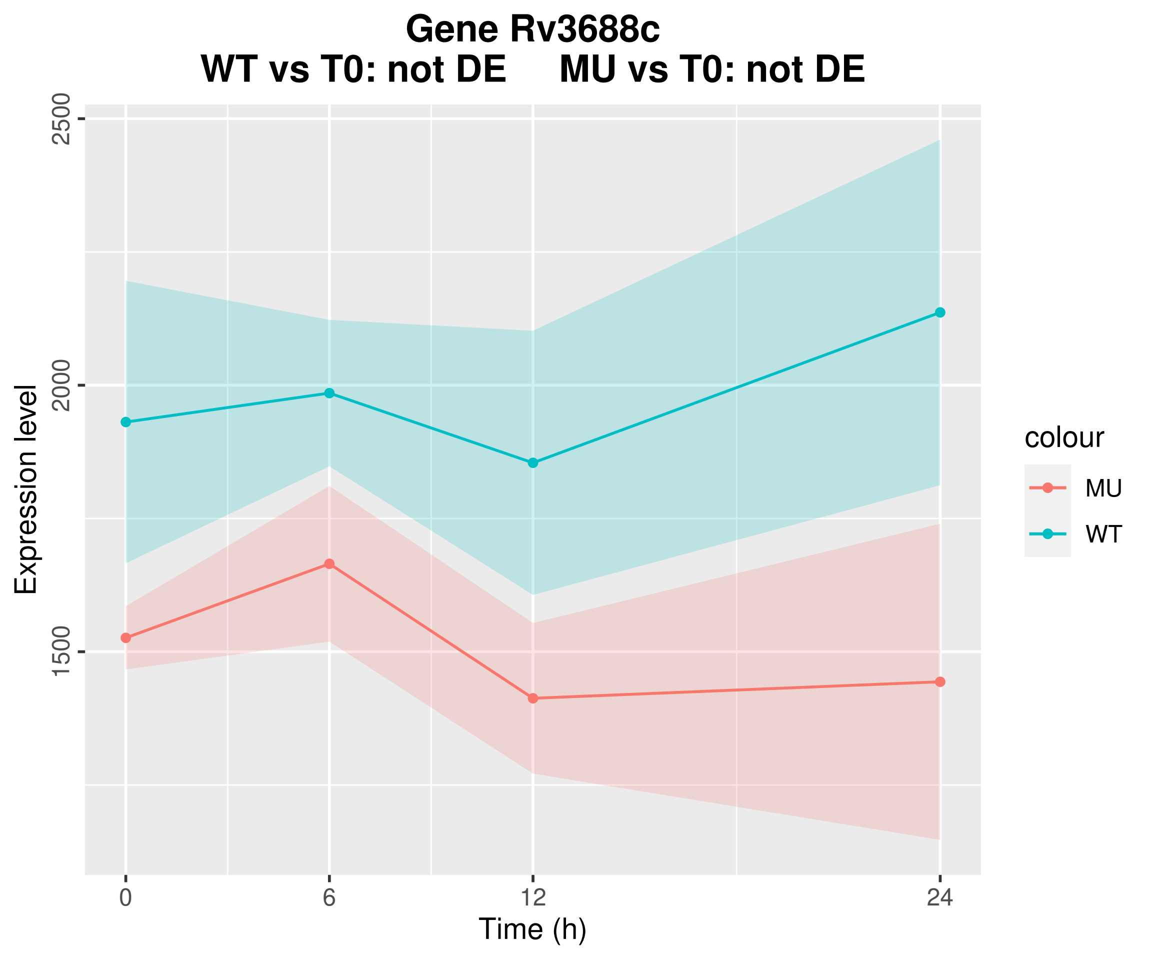

Gene Rv3688c
WT vs T0: not DE MU vs T0: not DE
2500
2000
1500
Expression level
0
6
12
24
Time (h)
colour
MU
WT

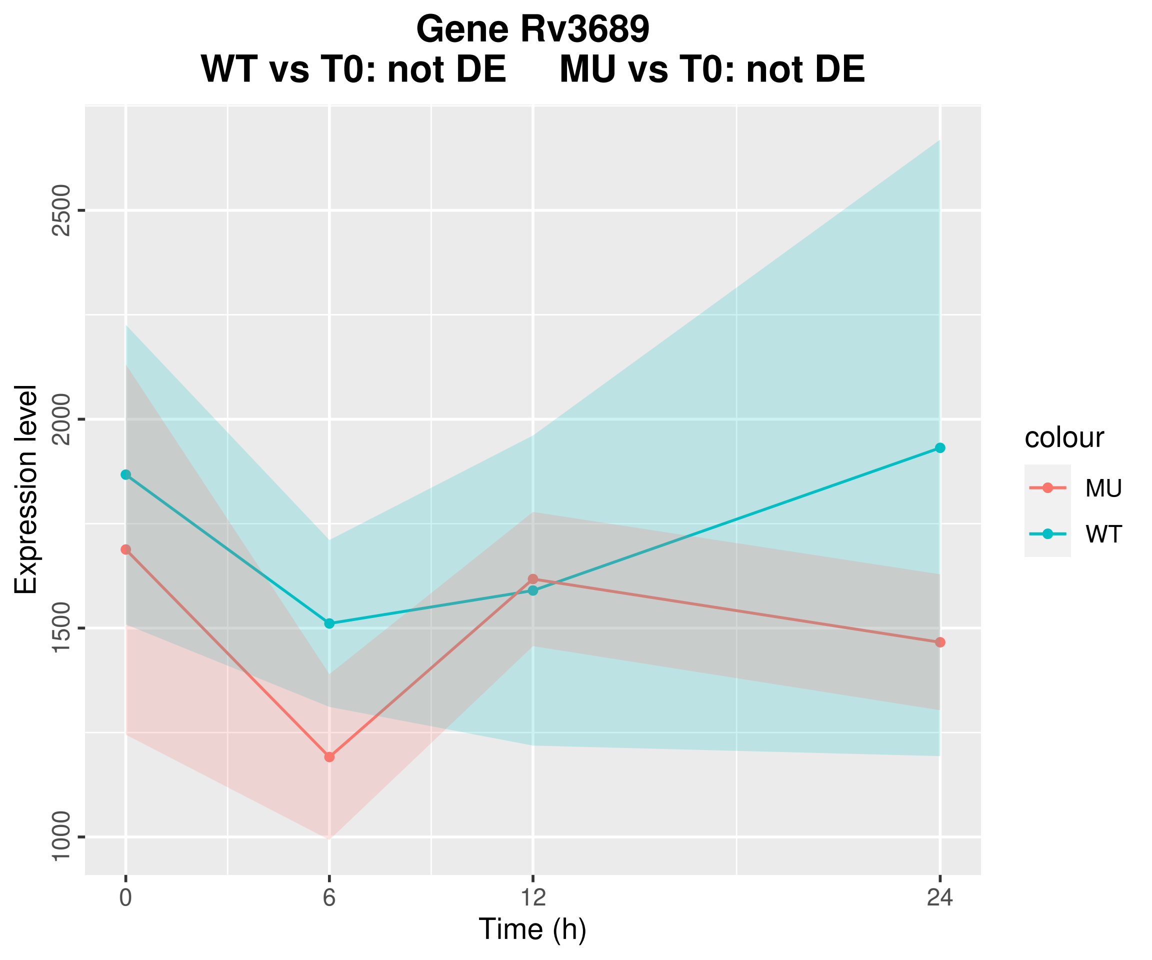

Gene Rv3689
WT vs T0: not DE   MU vs T0: not DE
Expression level
2500
2000
1500
1000
0
6
12
24
Time (h)
colour
MU
WT

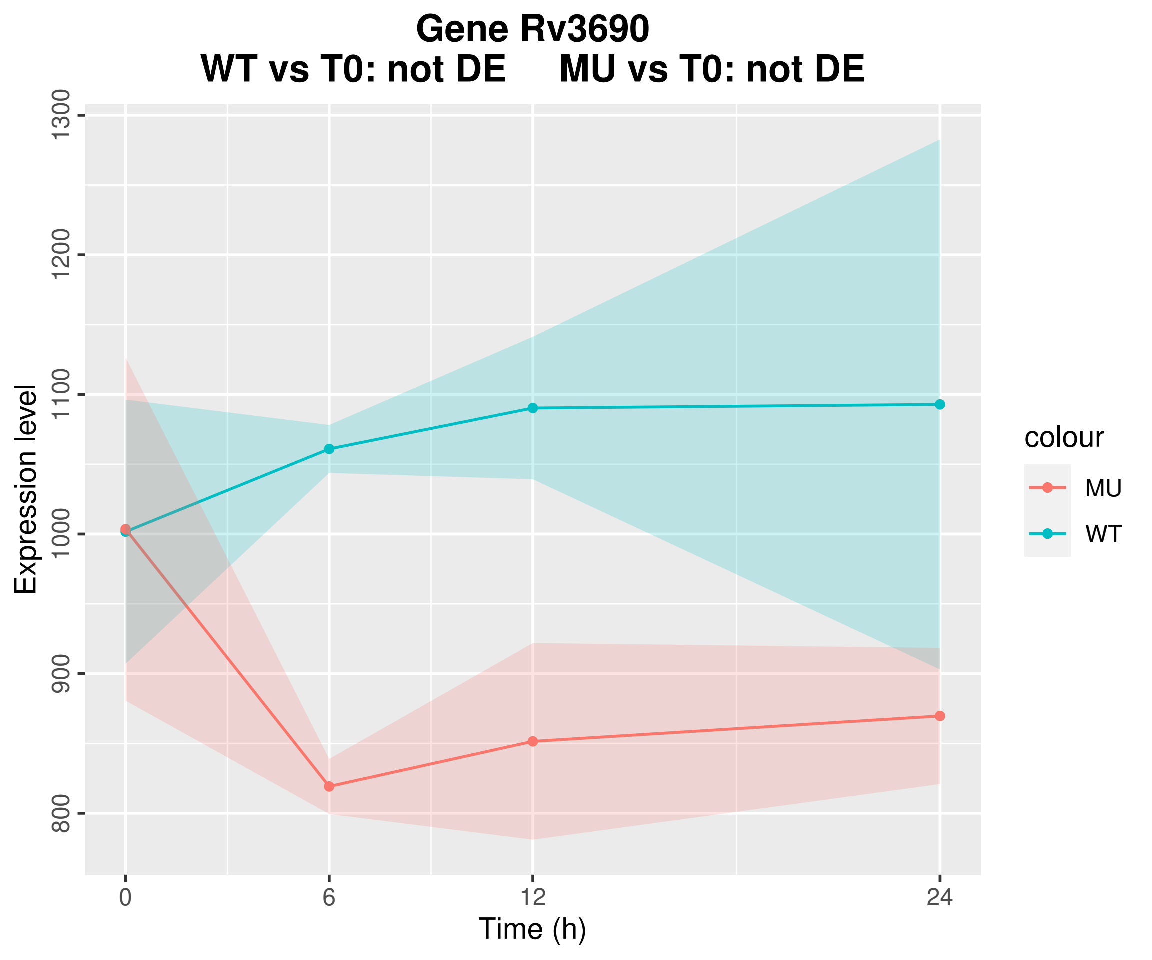
Gene Rv3690
WT vs T0: not DE MU vs T0: not DE
Expression level
1300
1200
1100
1000
900
800
0
6
12
24
Time (h)
colour
MU
WT

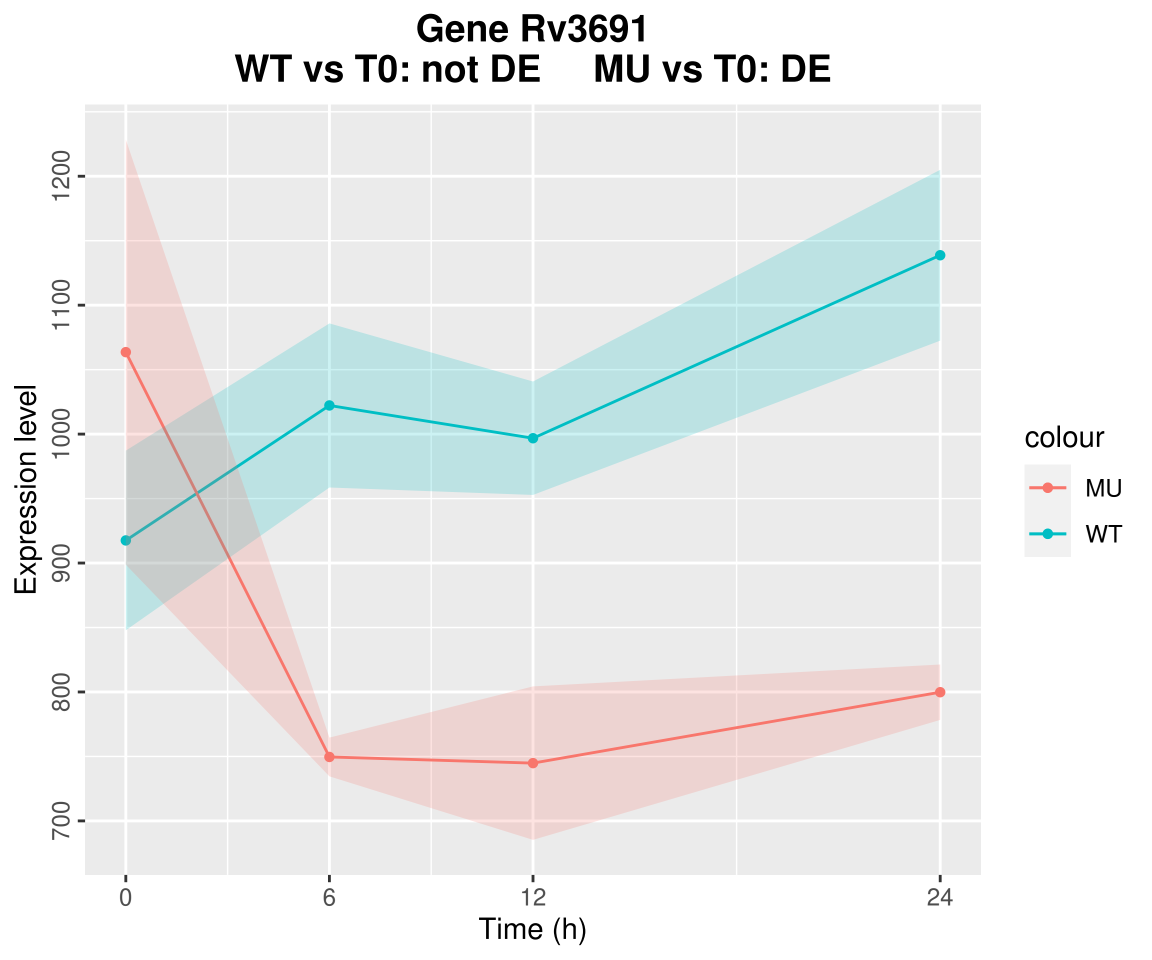
Gene Rv3691
WT vs T0: not DE   MU vs T0: DE
Expression level
700
800
900
1000
1100
1200
0
6
12
24
Time (h)
colour
MU
WT

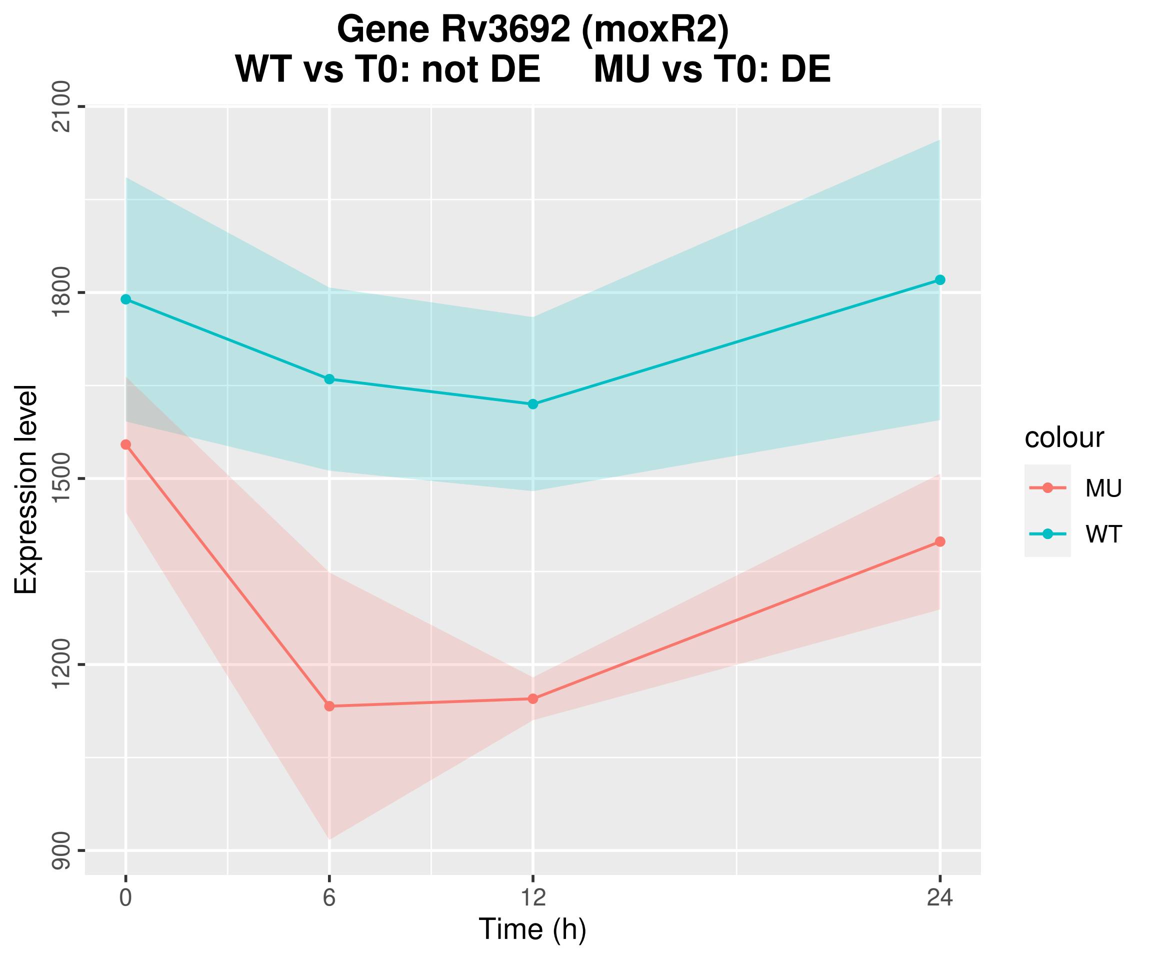

Gene Rv3692 (moxR2)
WT vs T0: not DE   MU vs T0: DE
Expression level
2100
1800
1500
1200
900
0
6
12
24
Time (h)
colour
MU
WT

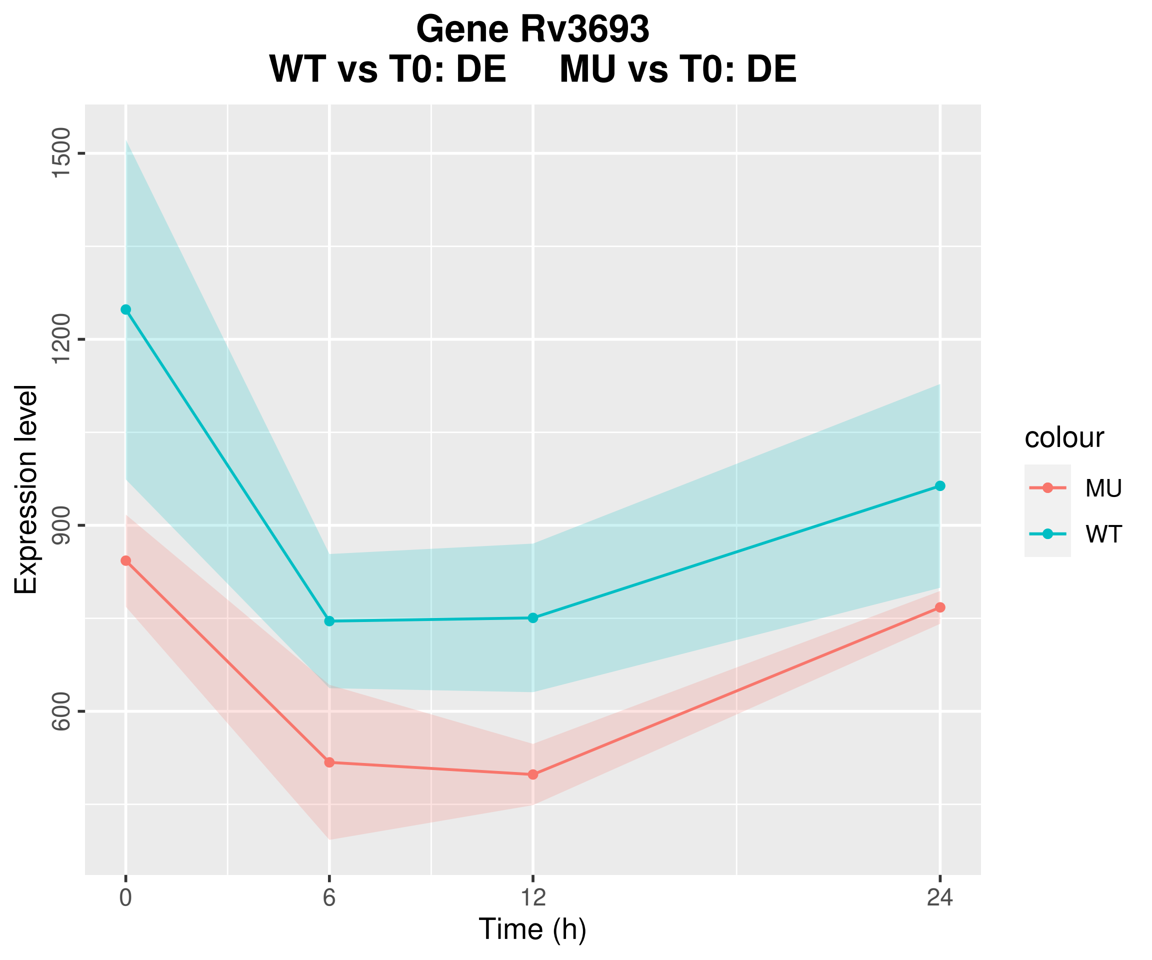

Gene Rv3693
WT vs T0: DE   MU vs T0: DE
Expression level
1500
1200
900
600
0
6
12
24
Time (h)
colour
MU
WT

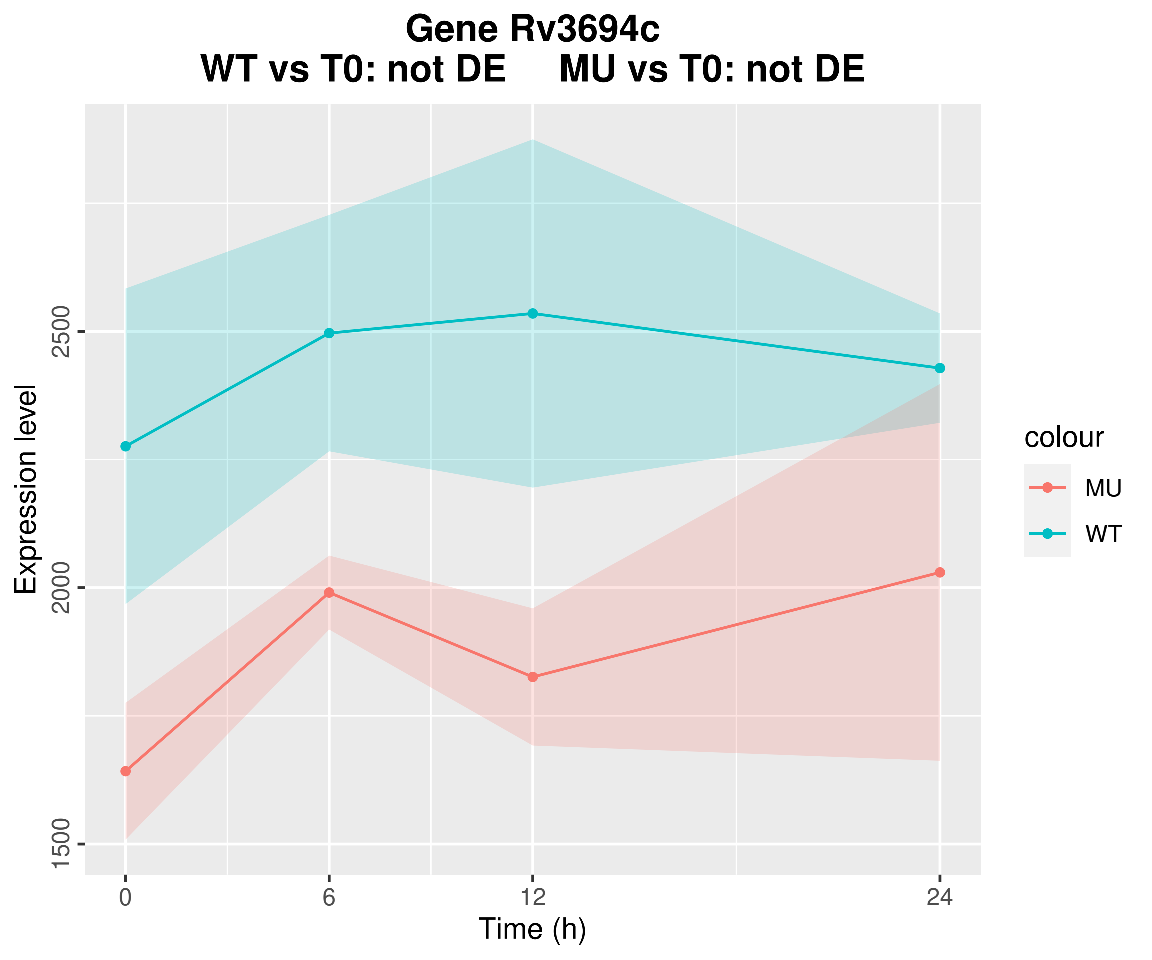

Gene Rv3694c
WT vs T0: not DE   MU vs T0: not DE
Expression level
2500
2000
1500
0
6
12
24
Time (h)
colour
MU
WT

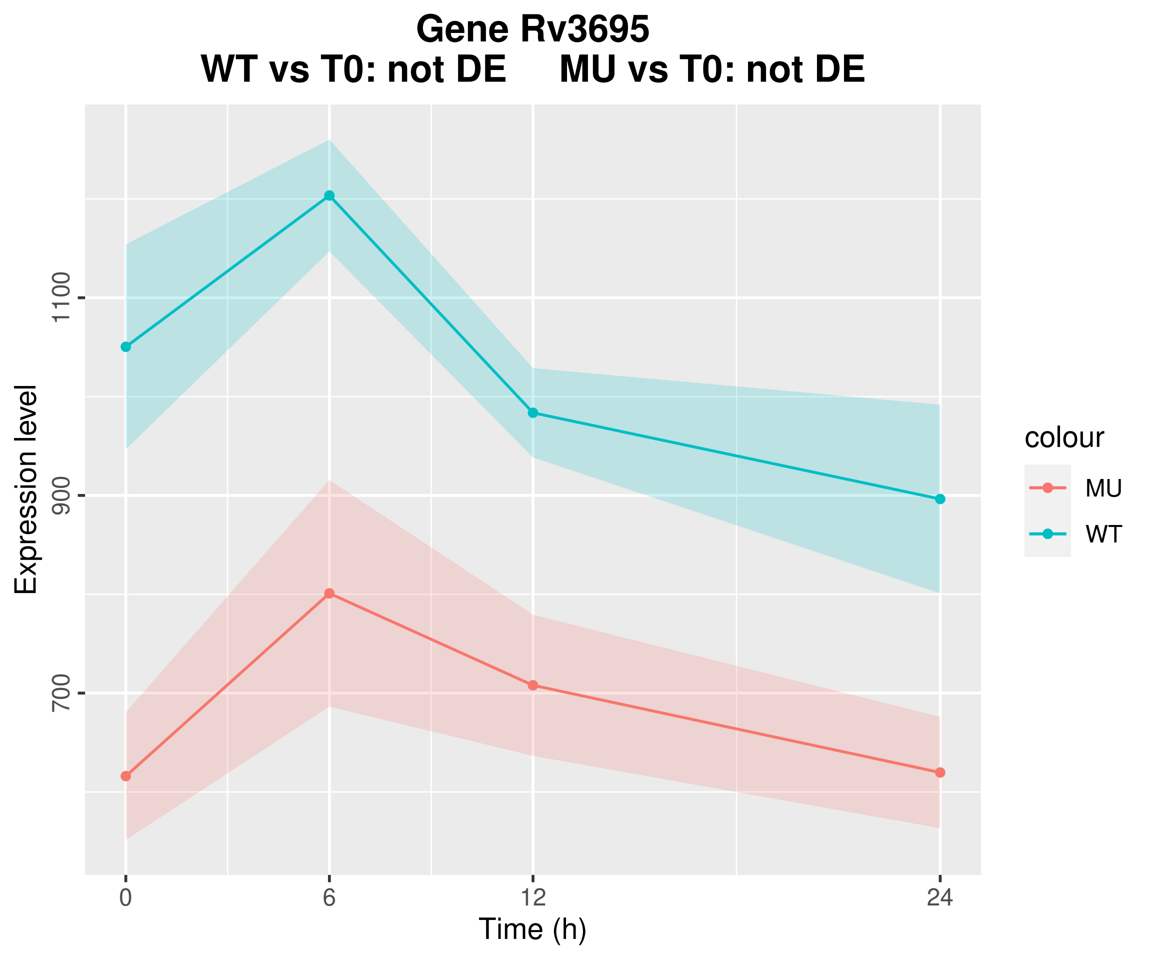

Gene Rv3695
WT vs T0: not DE MU vs T0: not DE
Expression level
1100
900
700
0
6
12
24
Time (h)
colour
MU
WT

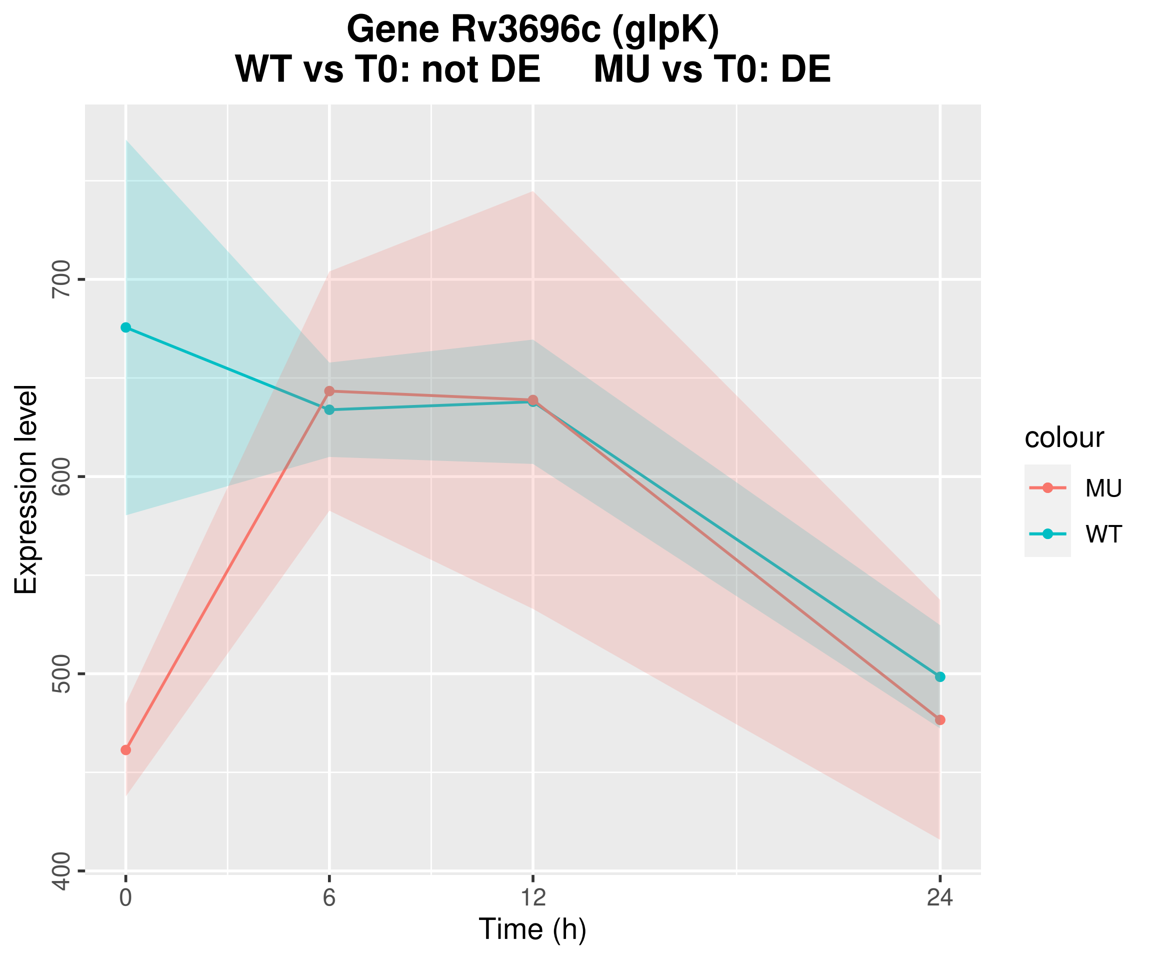
Gene Rv3696c (glpK)
WT vs T0: not DE   MU vs T0: DE
Expression level
700
600
500
400
0
6
12
24
Time (h)
colour
MU
WT

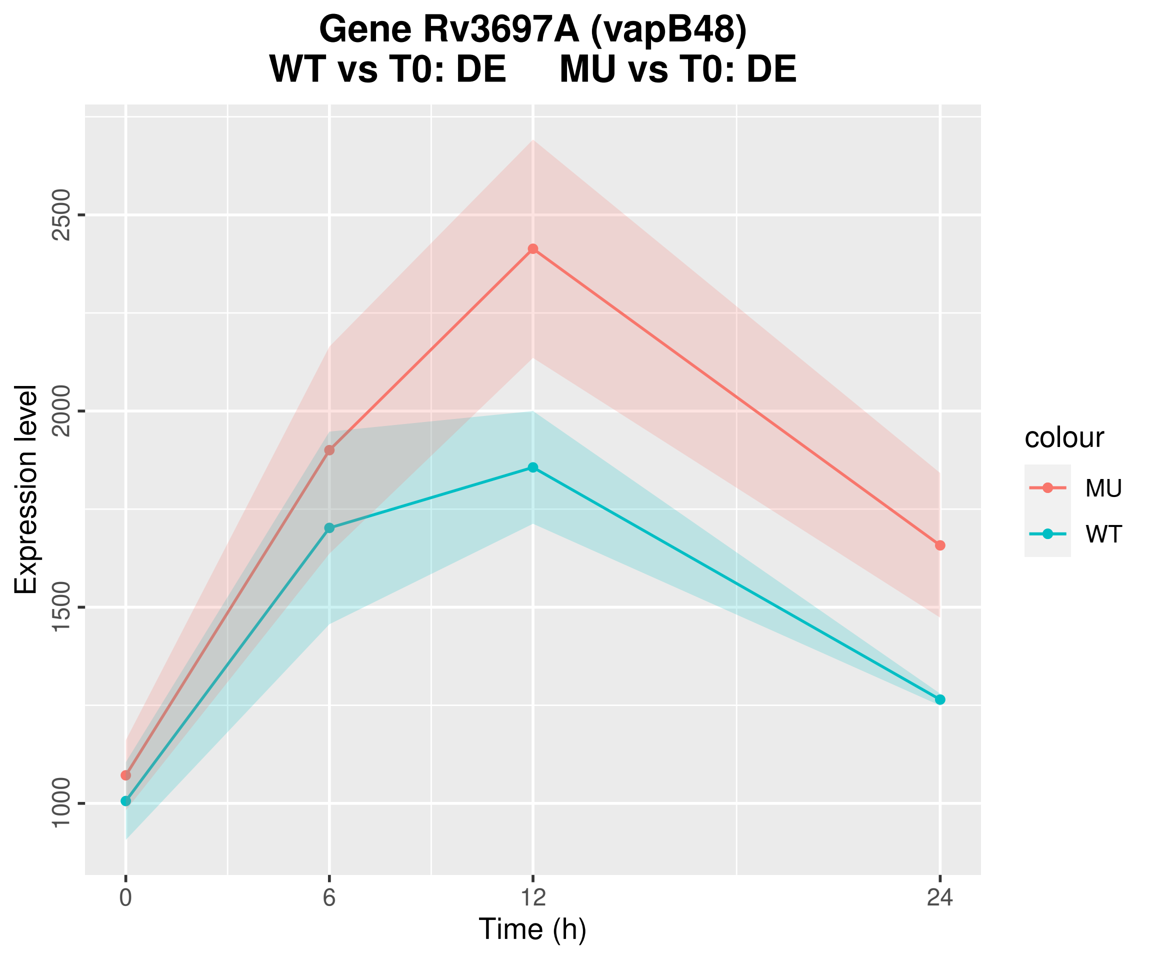
Gene Rv3697A (vapB48)
WT vs T0: DE   MU vs T0: DE
Expression level
2500
2000
1500
1000
0
6
12
24
Time (h)
colour
MU
WT

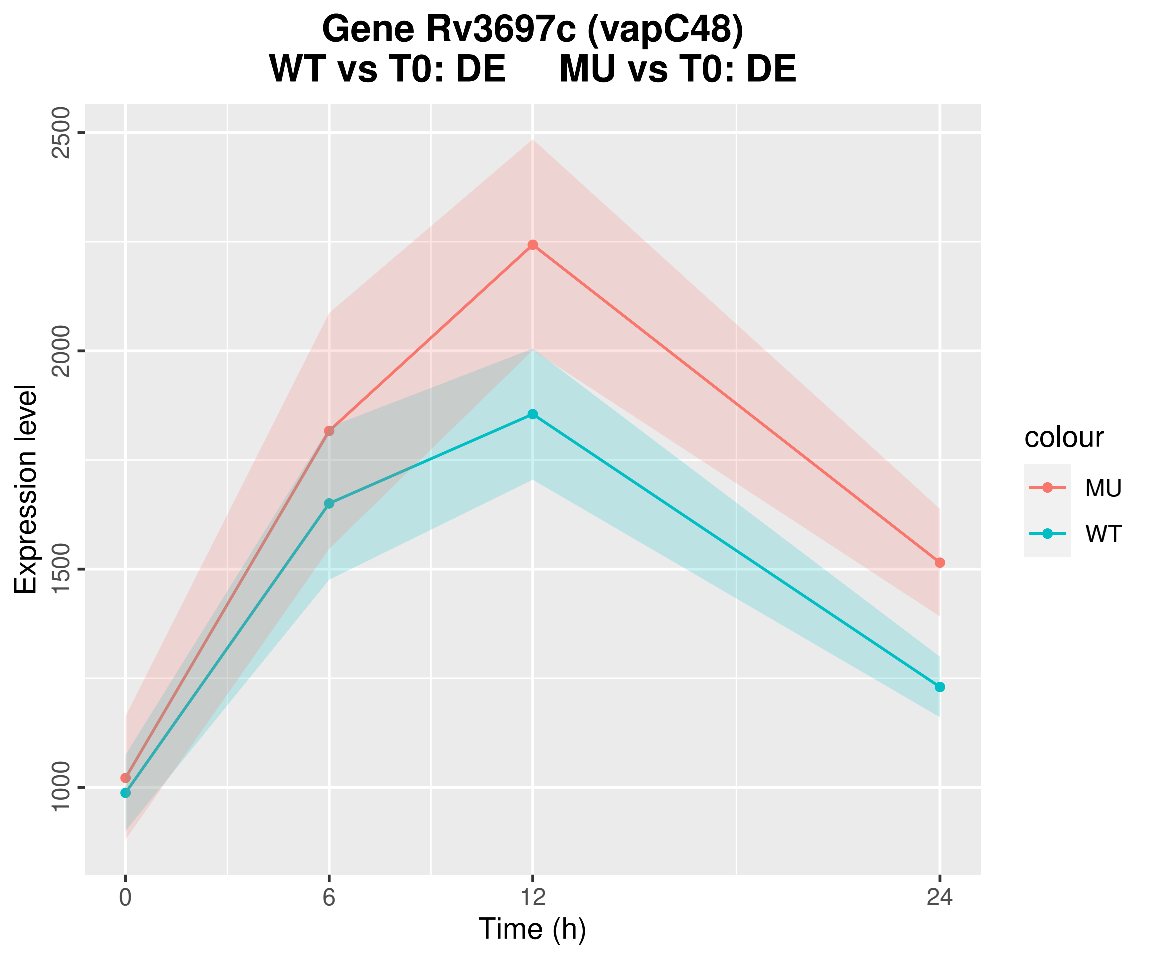
Gene Rv3697c (vapC48)
WT vs T0: DE   MU vs T0: DE
Expression level
2500
2000
1500
1000
0
6
12
24
Time (h)
colour
MU
WT

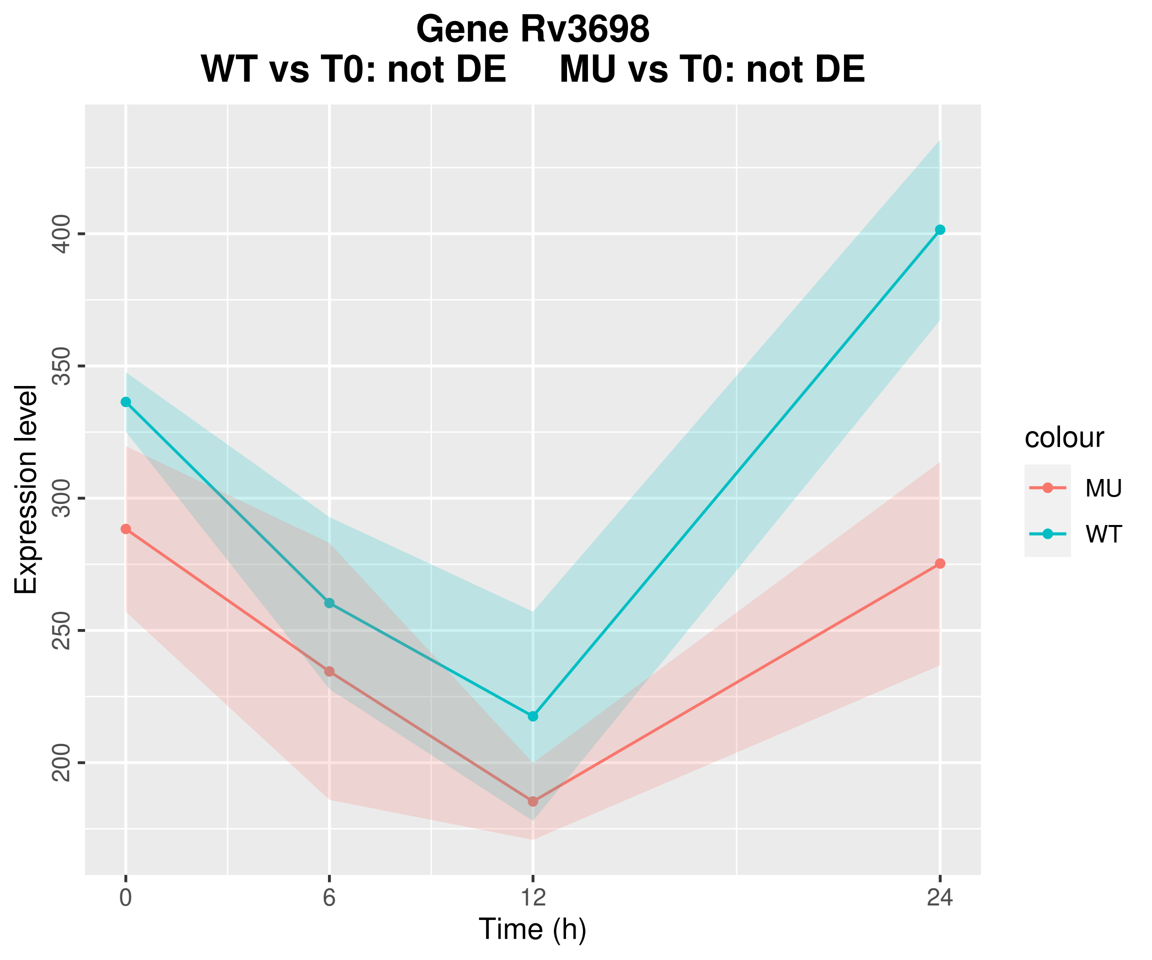
Gene Rv3698
WT vs T0: not DE MU vs T0: not DE
Expression level
400
350
300
250
200
0
6
12
24
Time (h)
colour
MU
WT

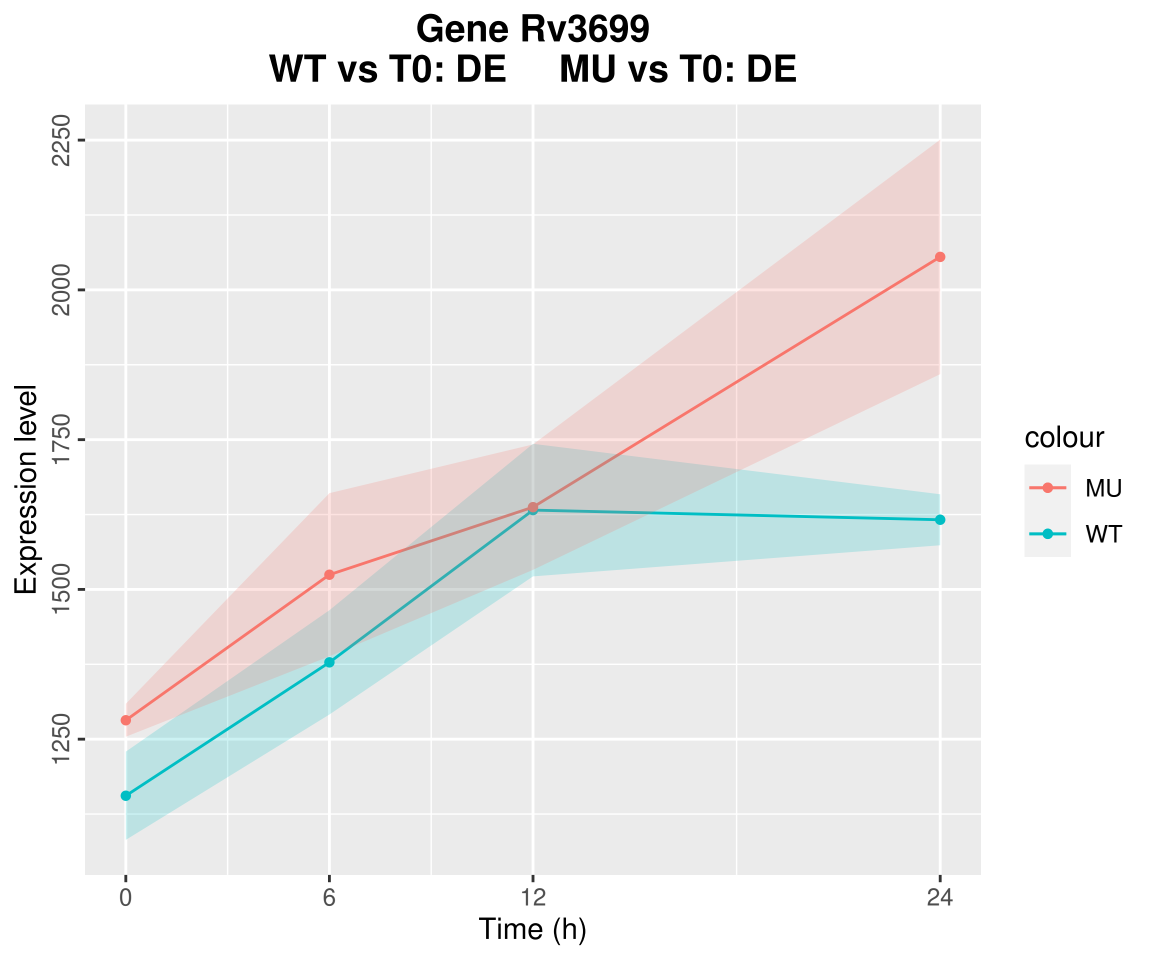

Gene Rv3699
WT vs T0: DE   MU vs T0: DE
Expression level
2250
2000
1750
1500
1250
0
6
12
24
Time (h)
colour
MU
WT

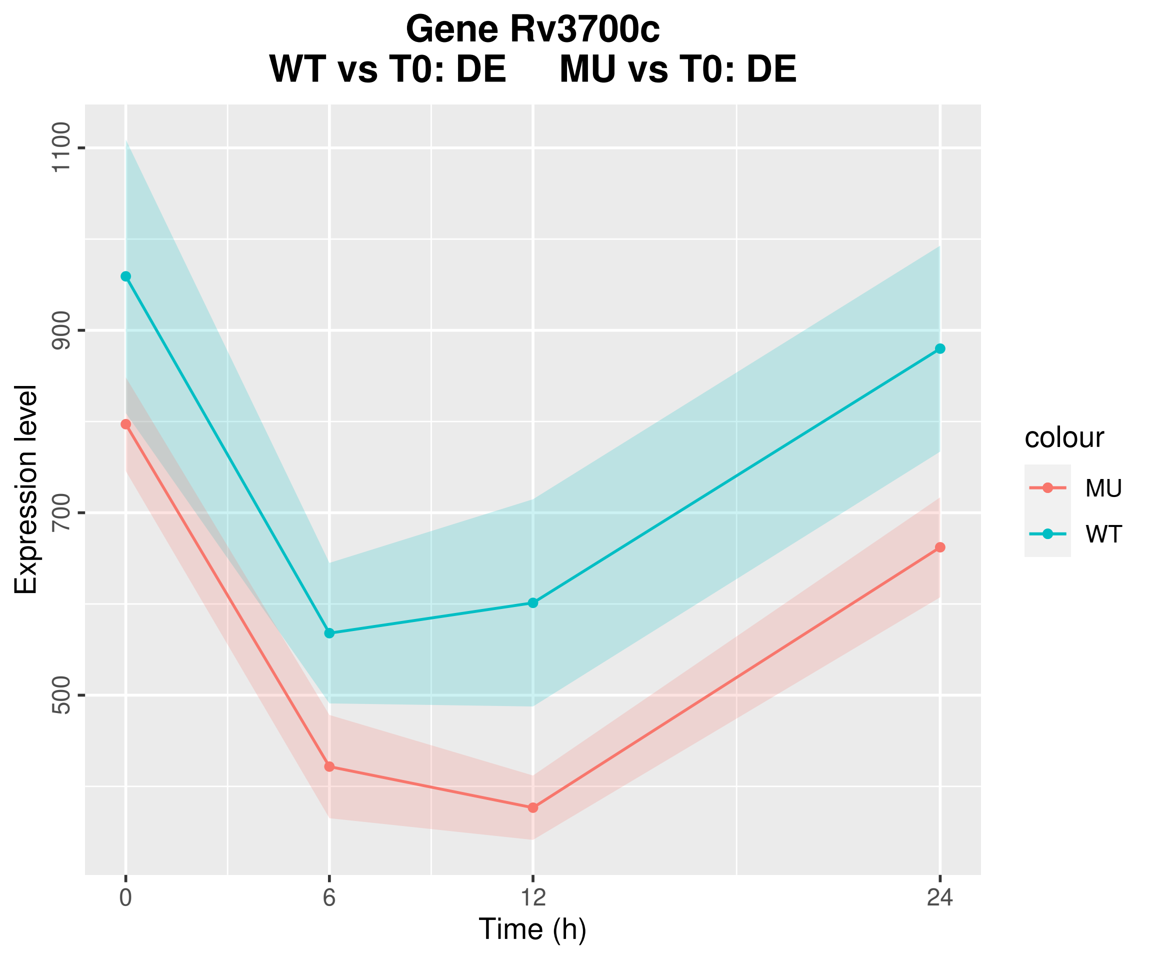

Gene Rv3700c
WT vs T0: DE   MU vs T0: DE
Expression level
1100
900
700
500
0
6
12
24
Time (h)
colour
MU
WT

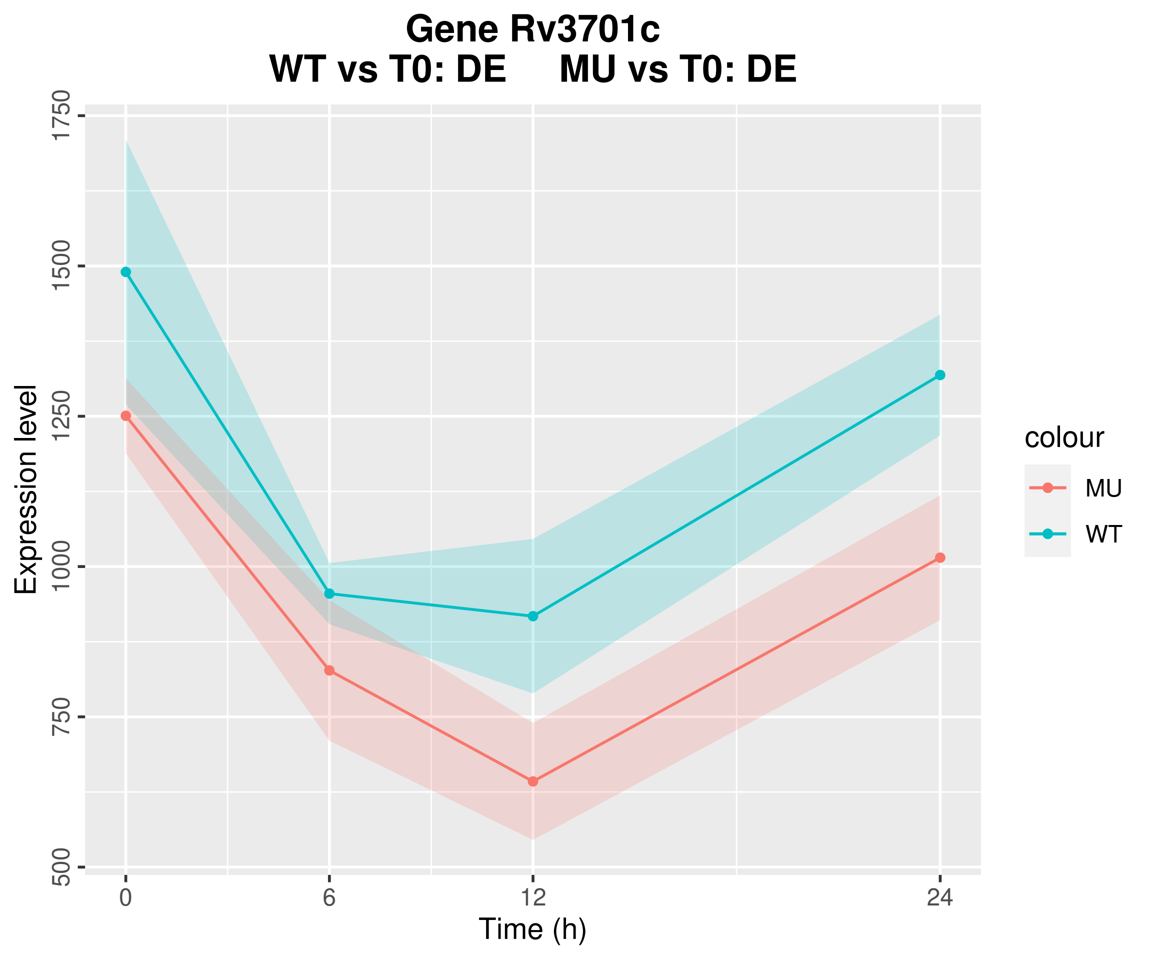

Gene Rv3701c
WT vs T0: DE   MU vs T0: DE
Expression level
500
750
1000
1250
1500
1750
0
6
12
24
Time (h)
colour
MU
WT

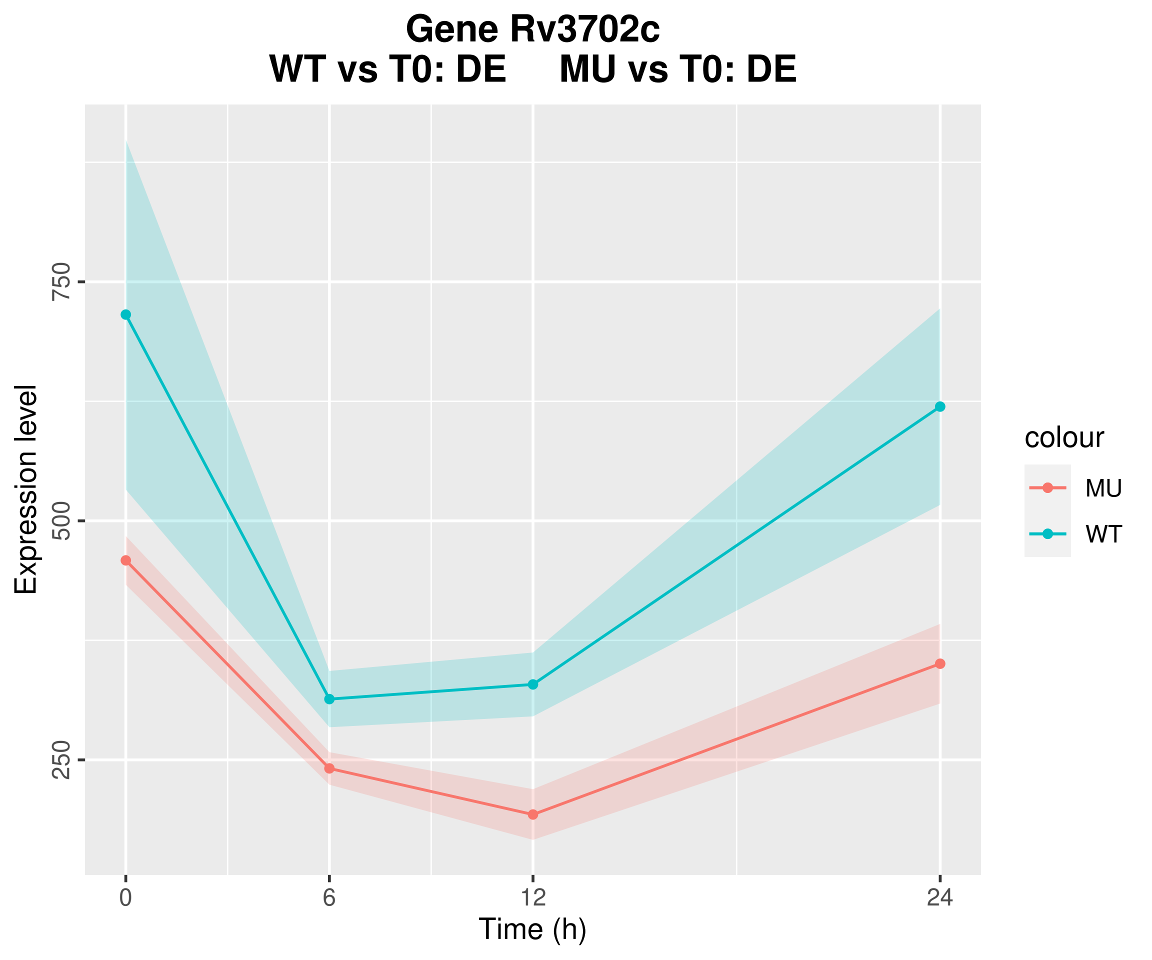

Gene Rv3702c
WT vs T0: DE MU vs T0: DE
Expression level
250
500
750
0
6
12
24
Time (h)
colour
MU
WT

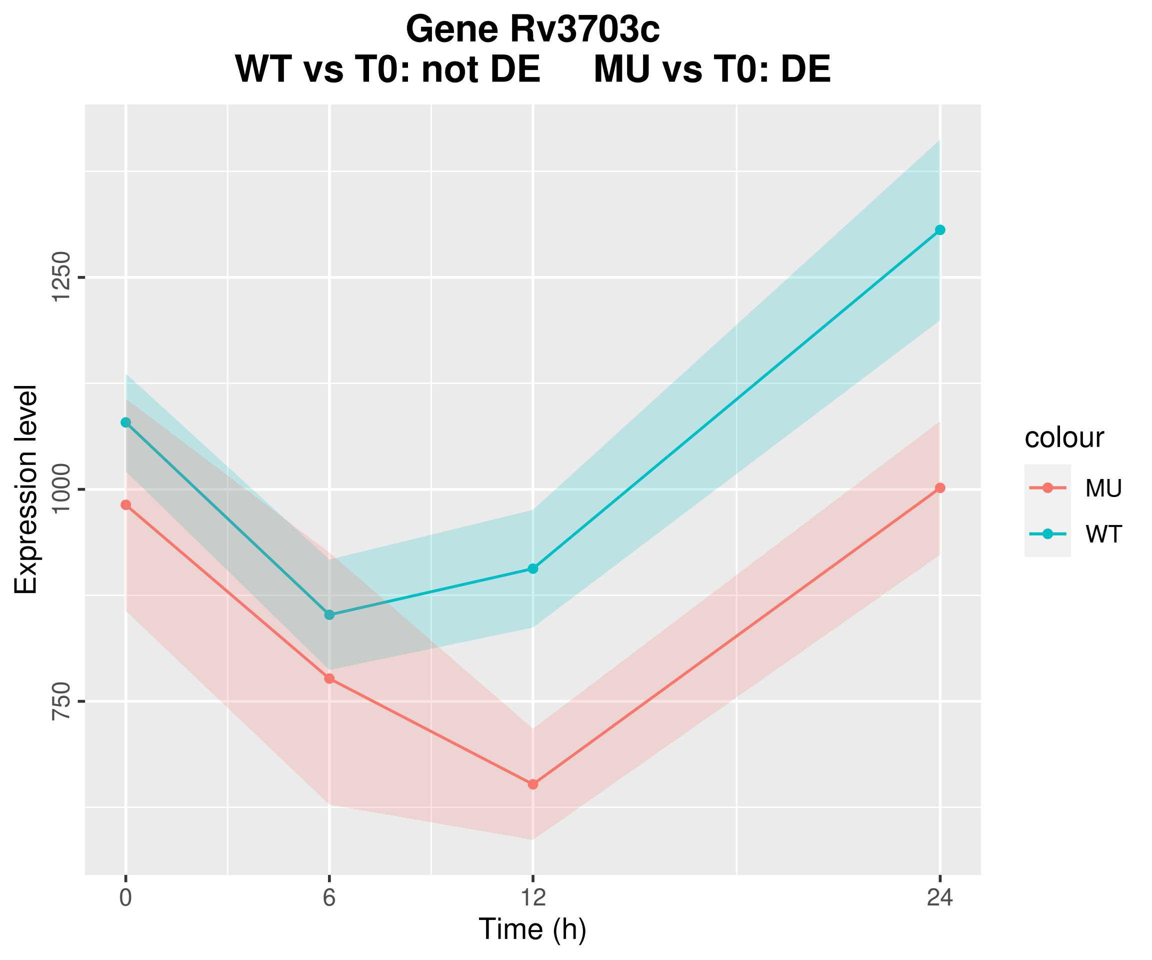
Gene Rv3703c
WT vs T0: not DE   MU vs T0: DE
Expression level
1250
1000
750
0
6
12
24
Time (h)
colour
MU
WT

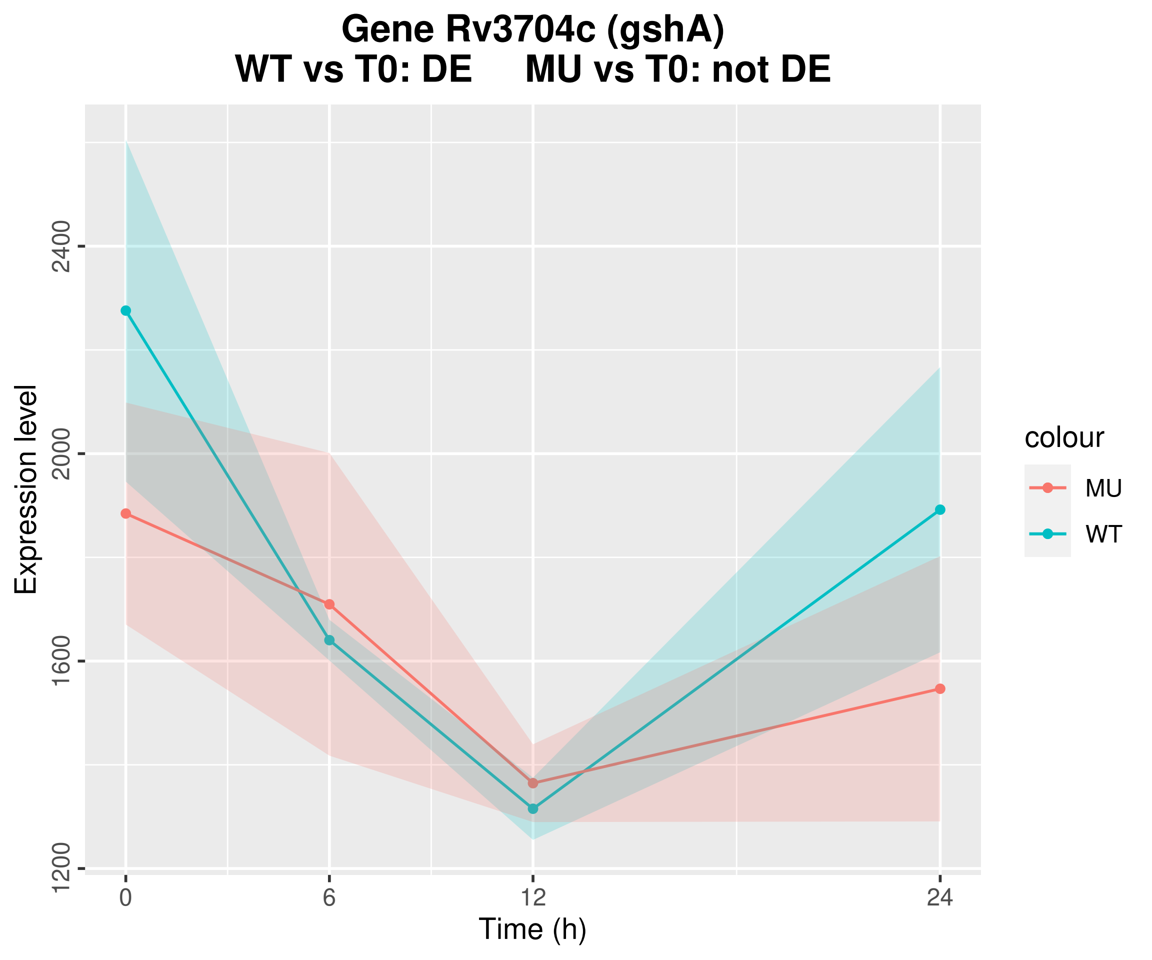

Gene Rv3704c (gshA)
WT vs T0: DE MU vs T0: not DE
Expression level
2400
2000
1600
1200
0
6
12
24
Time (h)
colour
MU
WT

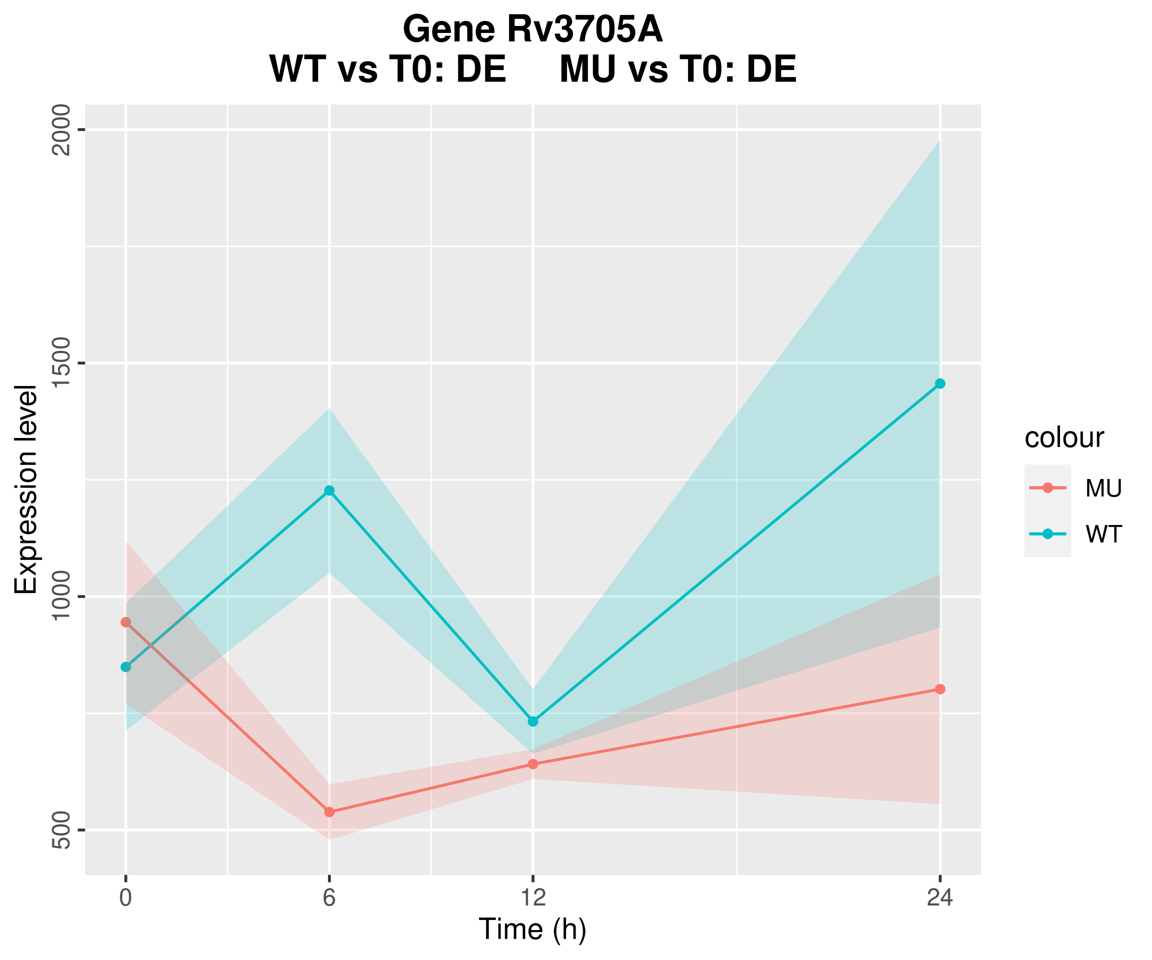
Gene Rv3705A
WT vs T0: DE   MU vs T0: DE
2000
1500
1000
500
Expression level
0
6
12
24
Time (h)
colour
MU
WT

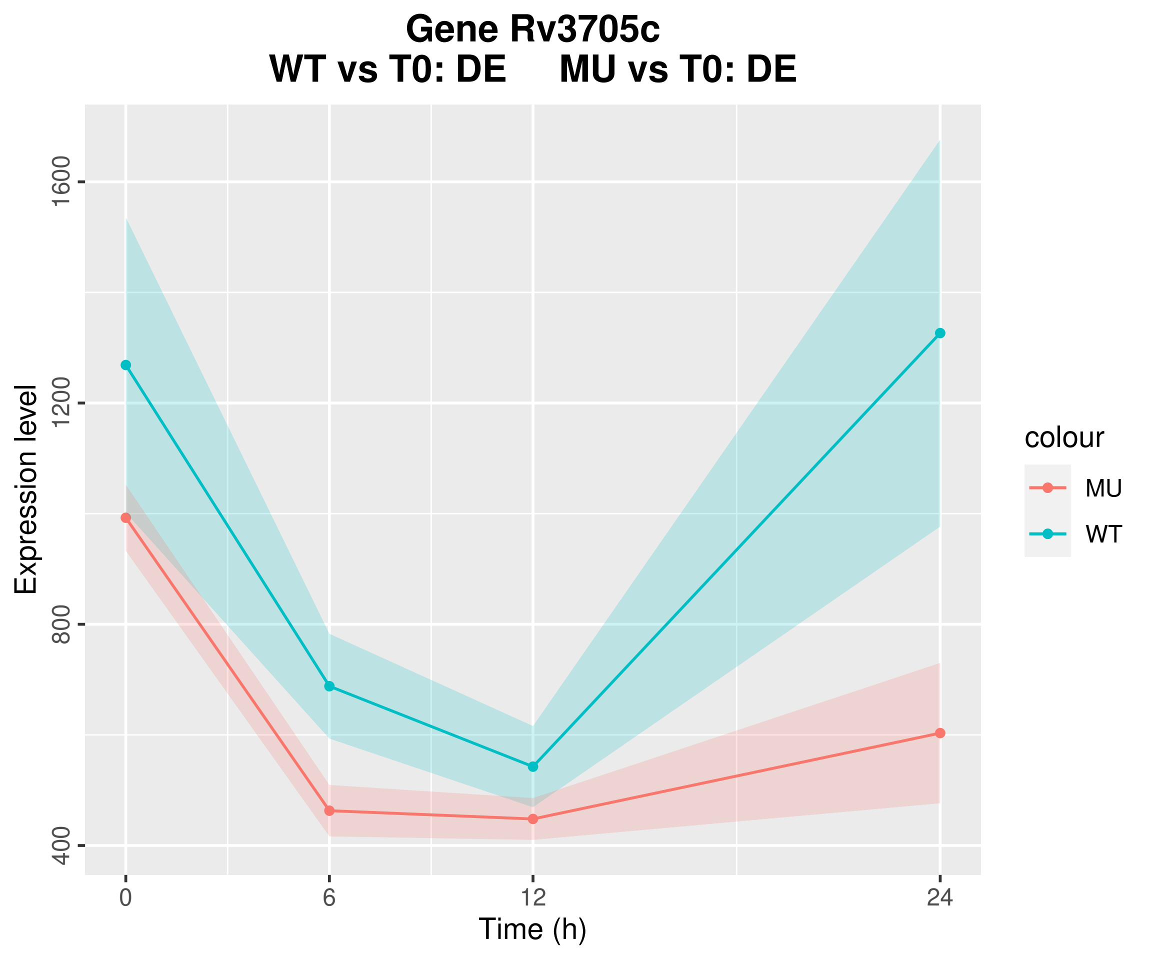
Gene Rv3705c
WT vs T0: DE MU vs T0: DE
Expression level
1600
1200
800
400
0
6
12
24
Time (h)
colour
MU
WT

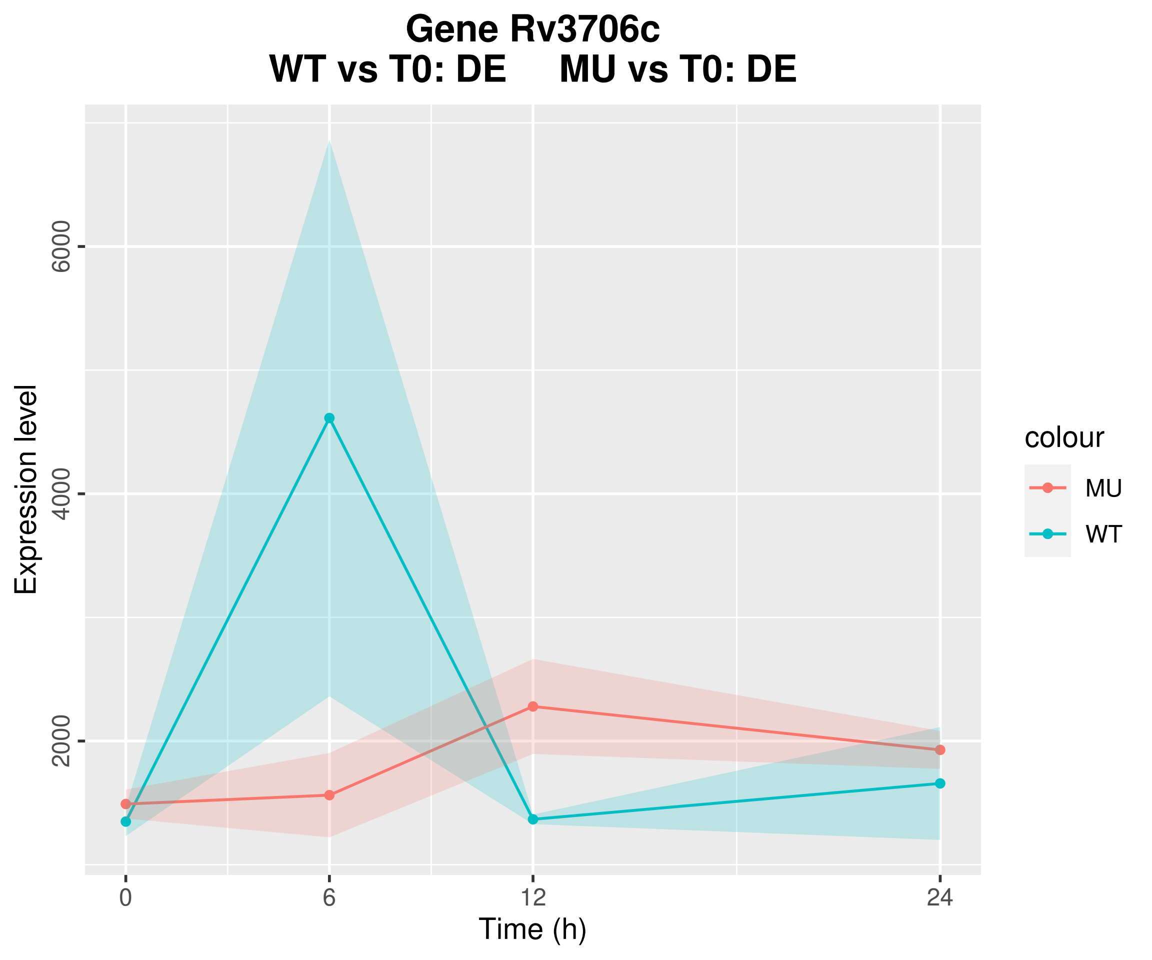

Gene Rv3706c
WT vs T0: DE   MU vs T0: DE
Expression level
6000
4000
2000
0
6
12
24
Time (h)
colour
MU
WT

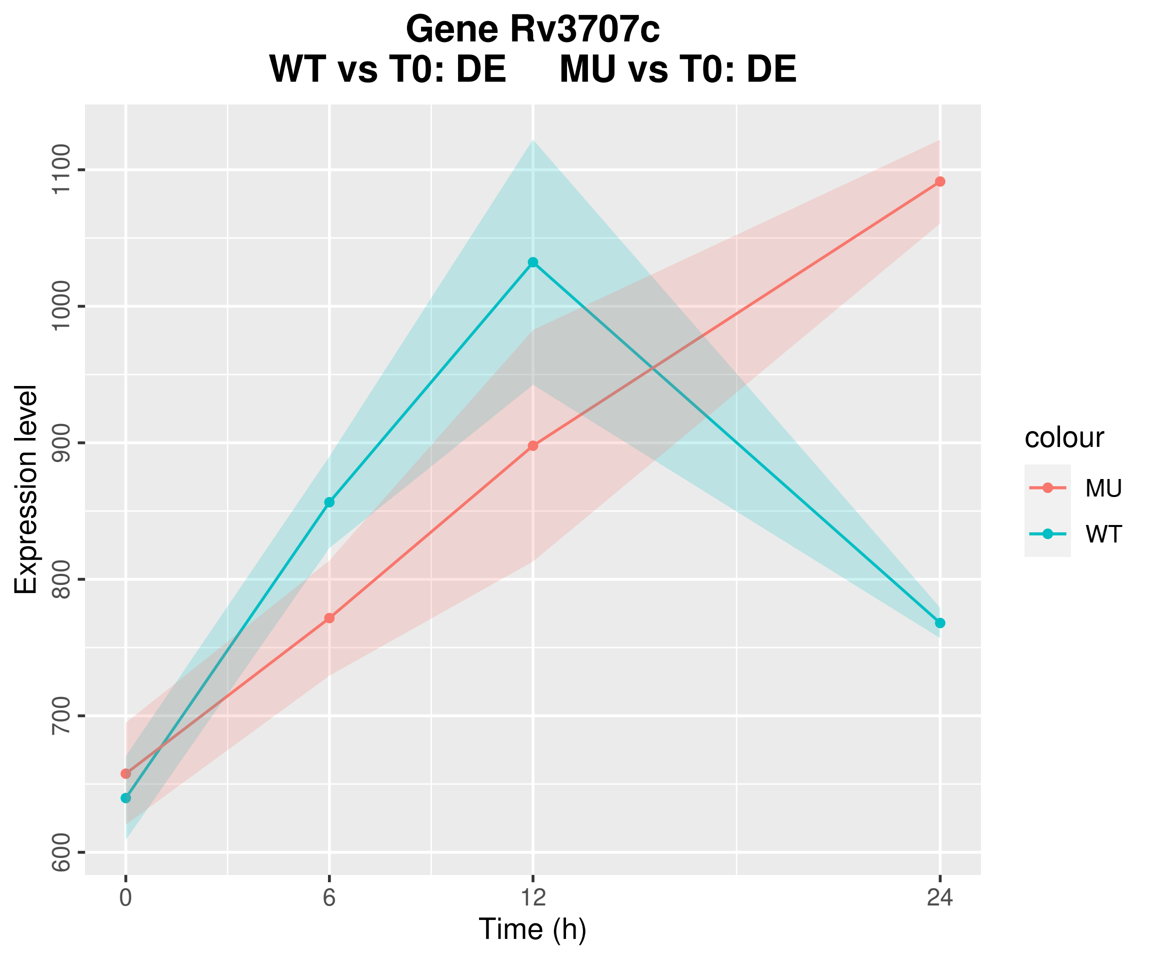
Gene Rv3707c
WT vs T0: DE   MU vs T0: DE
Expression level
Time (h)
colour
MU
WT
600
700
800
900
1000
1100
0
6
12
24

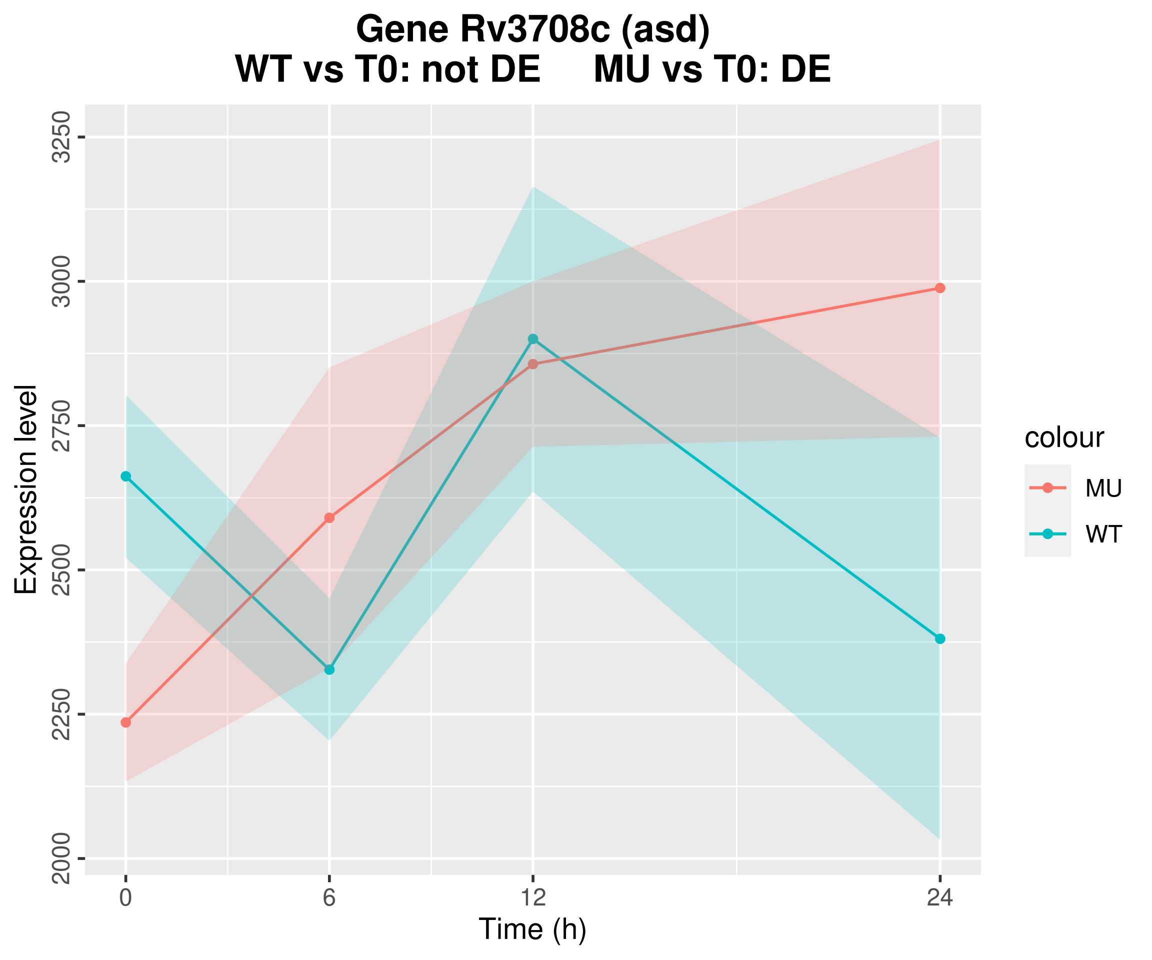
Gene Rv3708c (asd)
WT vs T0: not DE   MU vs T0: DE
Expression level
3250
3000
2750
2500
2250
2000
0
6
12
24
Time (h)
colour
MU
WT

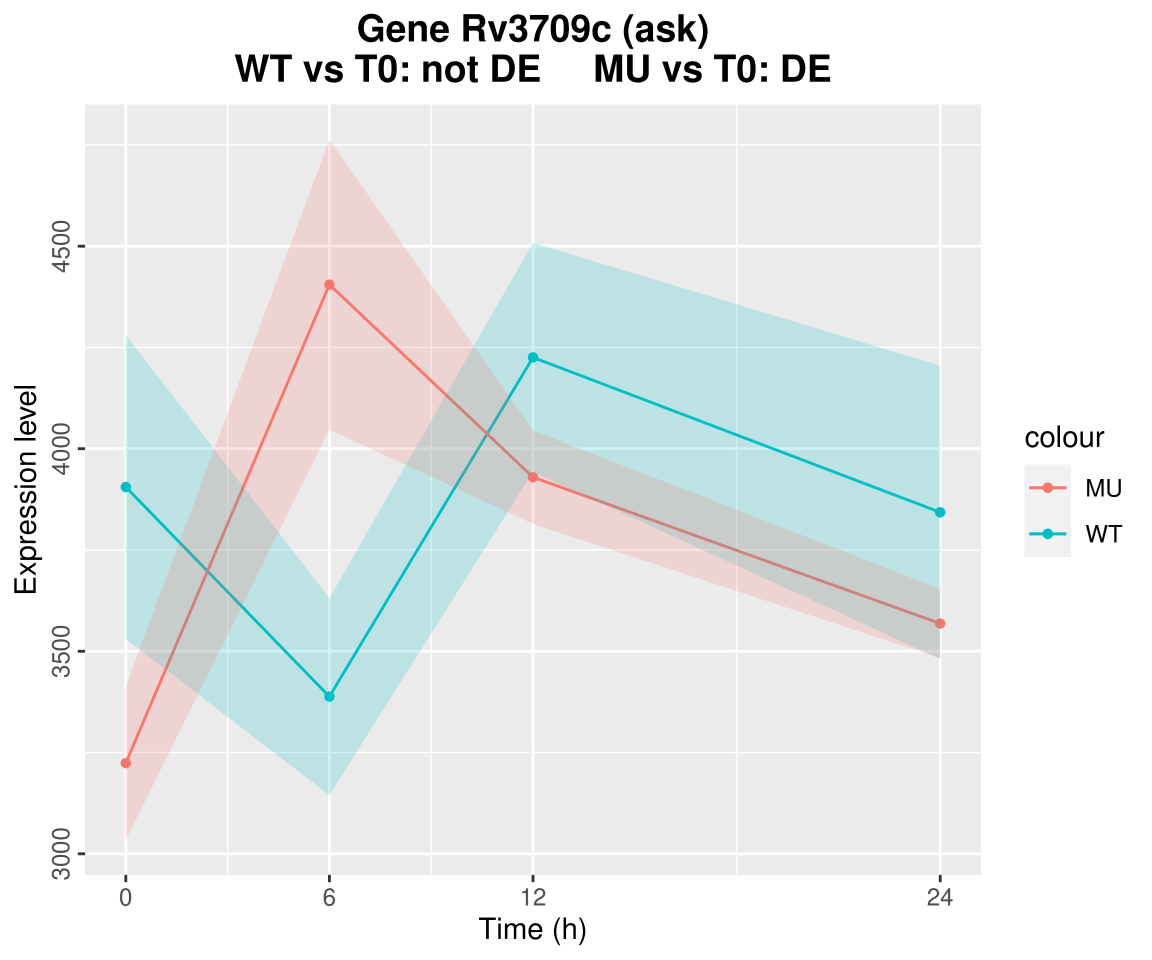
Gene Rv3709c (ask)
WT vs T0: not DE   MU vs T0: DE
Expression level
4500
4000
3500
3000
0
6
12
24
Time (h)
colour
MU
WT

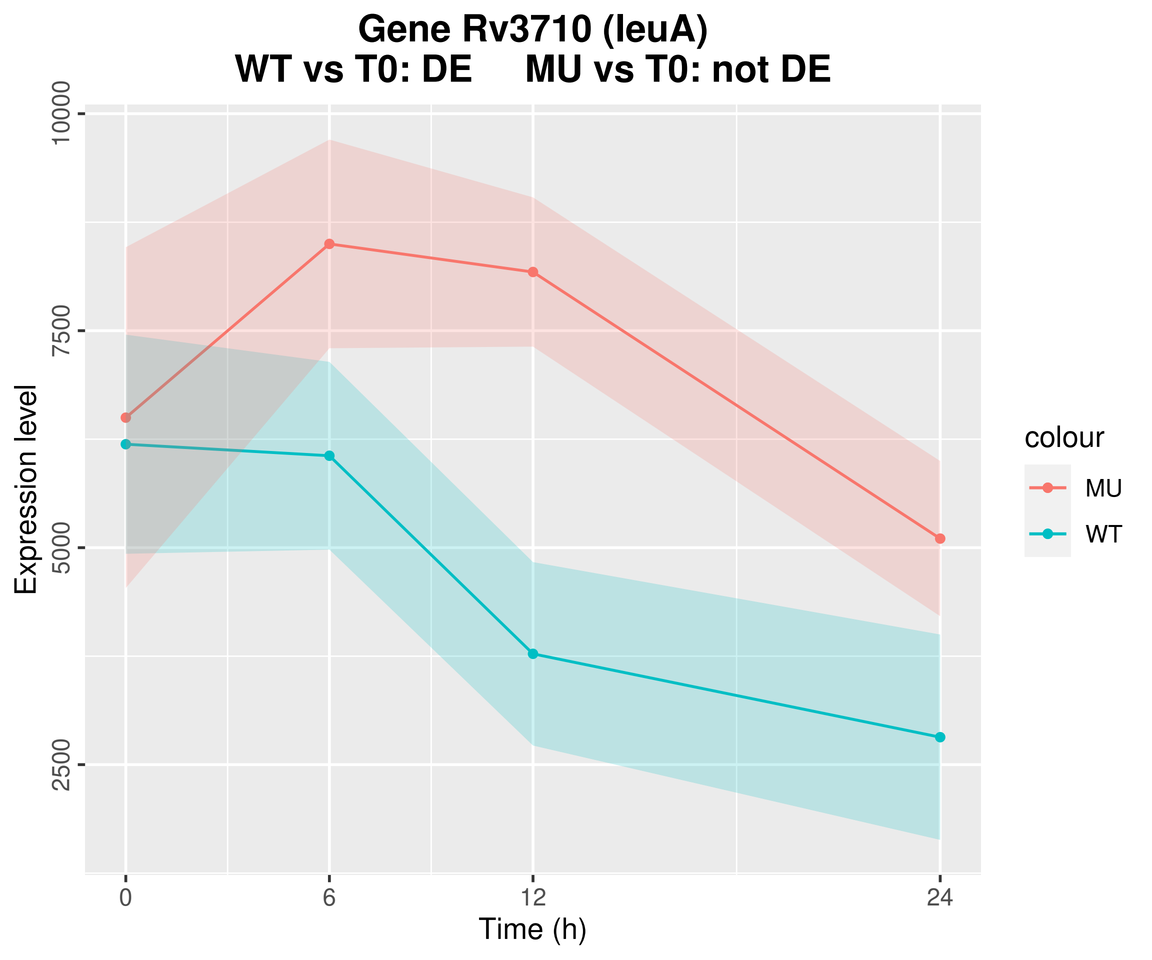

Gene Rv3710 (leuA)
WT vs T0: DE   MU vs T0: not DE
10000
7500
5000
2500
Expression level
0
6
12
24
Time (h)
colour
MU
WT

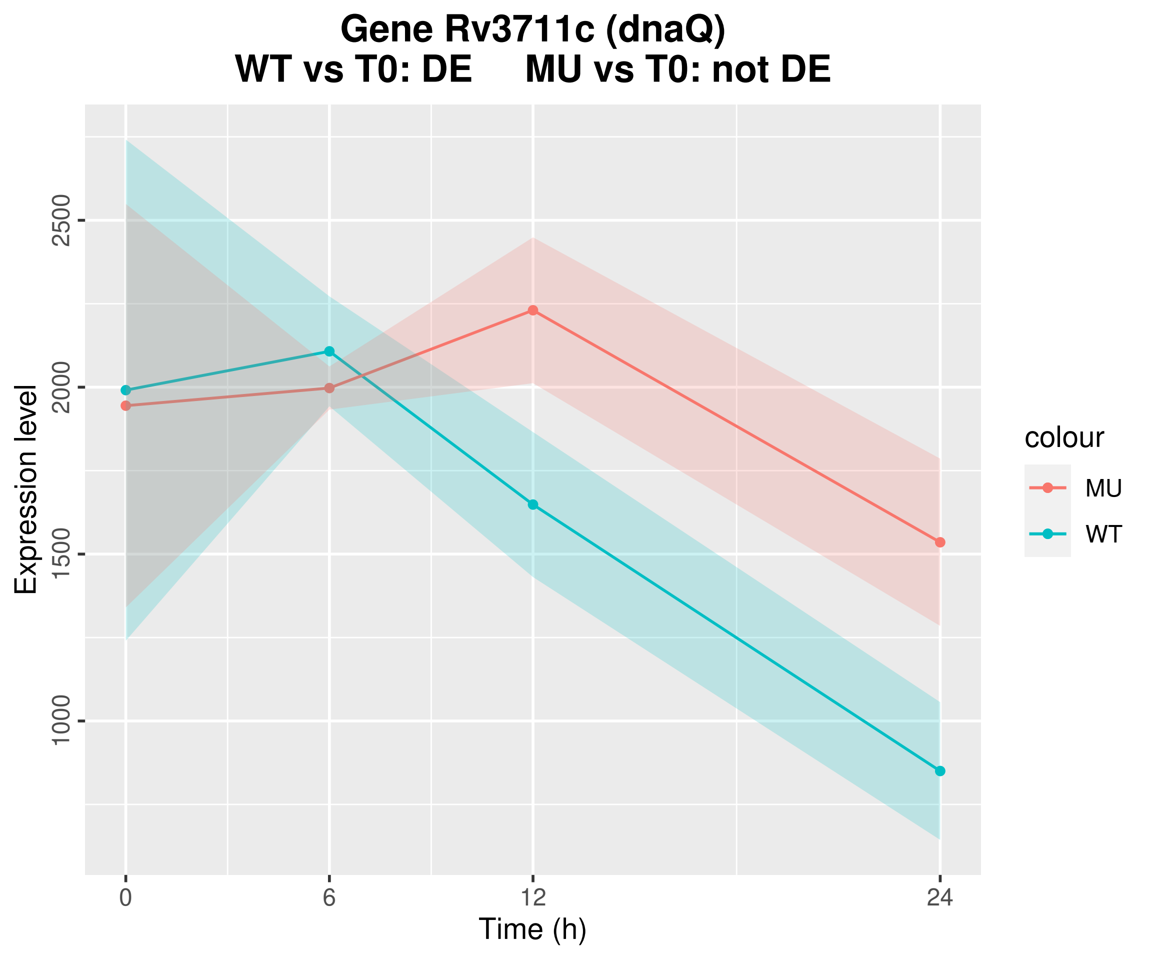
Gene Rv3711c (dnaQ)
WT vs T0: DE   MU vs T0: not DE
Expression level
2500
2000
1500
1000
0
6
12
24
Time (h)
colour
MU
WT

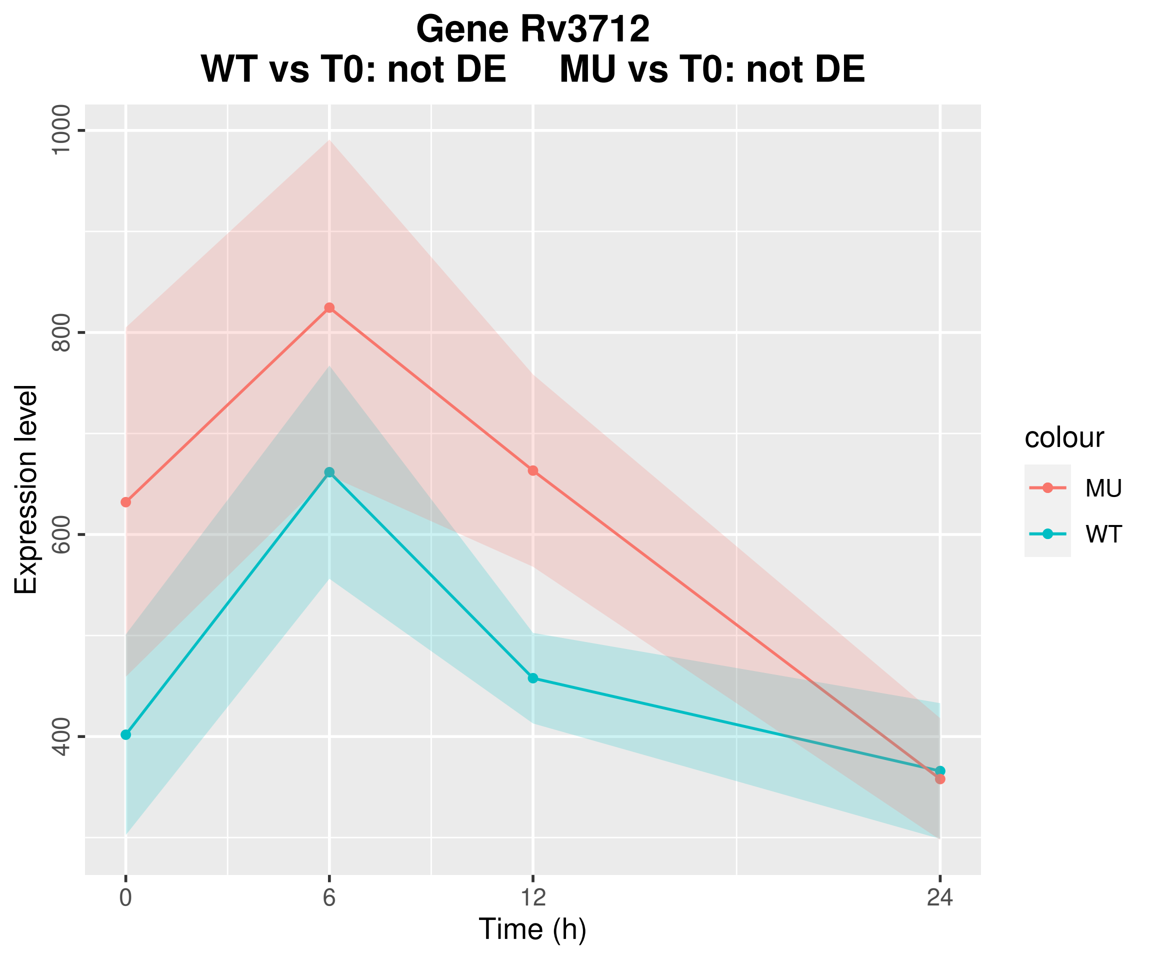
Gene Rv3712
WT vs T0: not DE   MU vs T0: not DE
Expression level
1000
800
600
400
0
6
12
24
Time (h)
colour
MU
WT

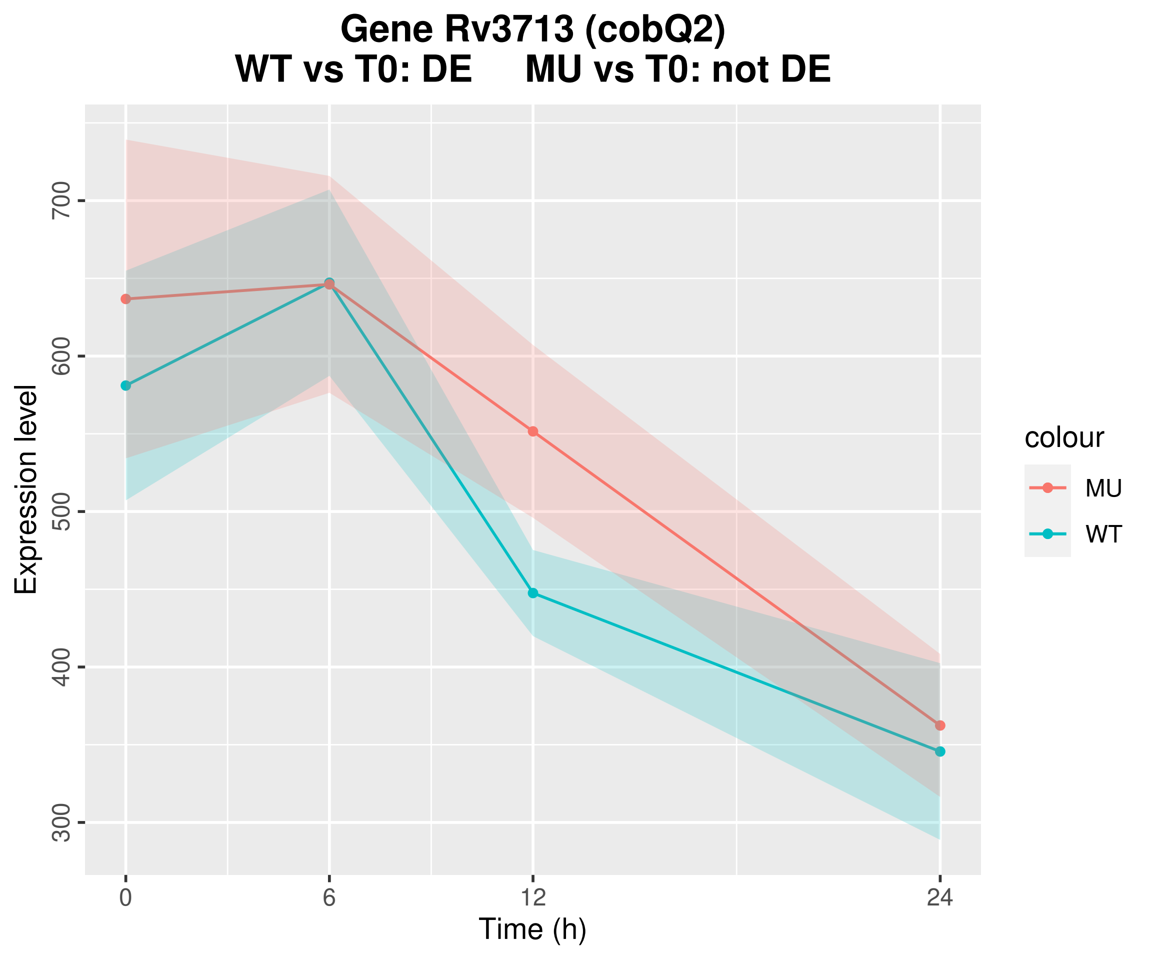
Gene Rv3713 (cobQ2)
WT vs T0: DE   MU vs T0: not DE
Expression level
700
600
500
400
300
Time (h)
0
6
12
24
colour
MU
WT

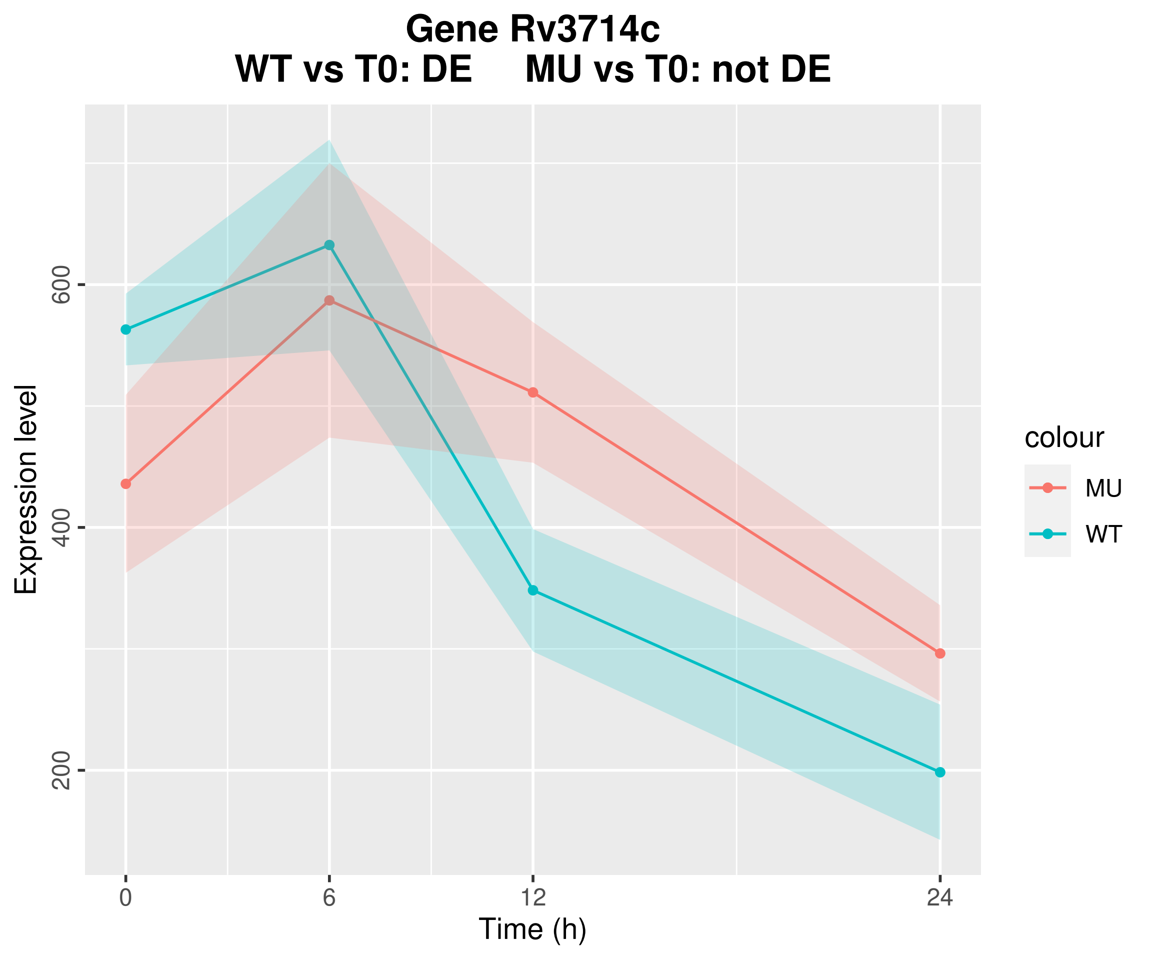
Gene Rv3714c
WT vs T0: DE MU vs T0: not DE
Expression level
600
400
200
0
6
12
24
Time (h)
colour
MU
WT

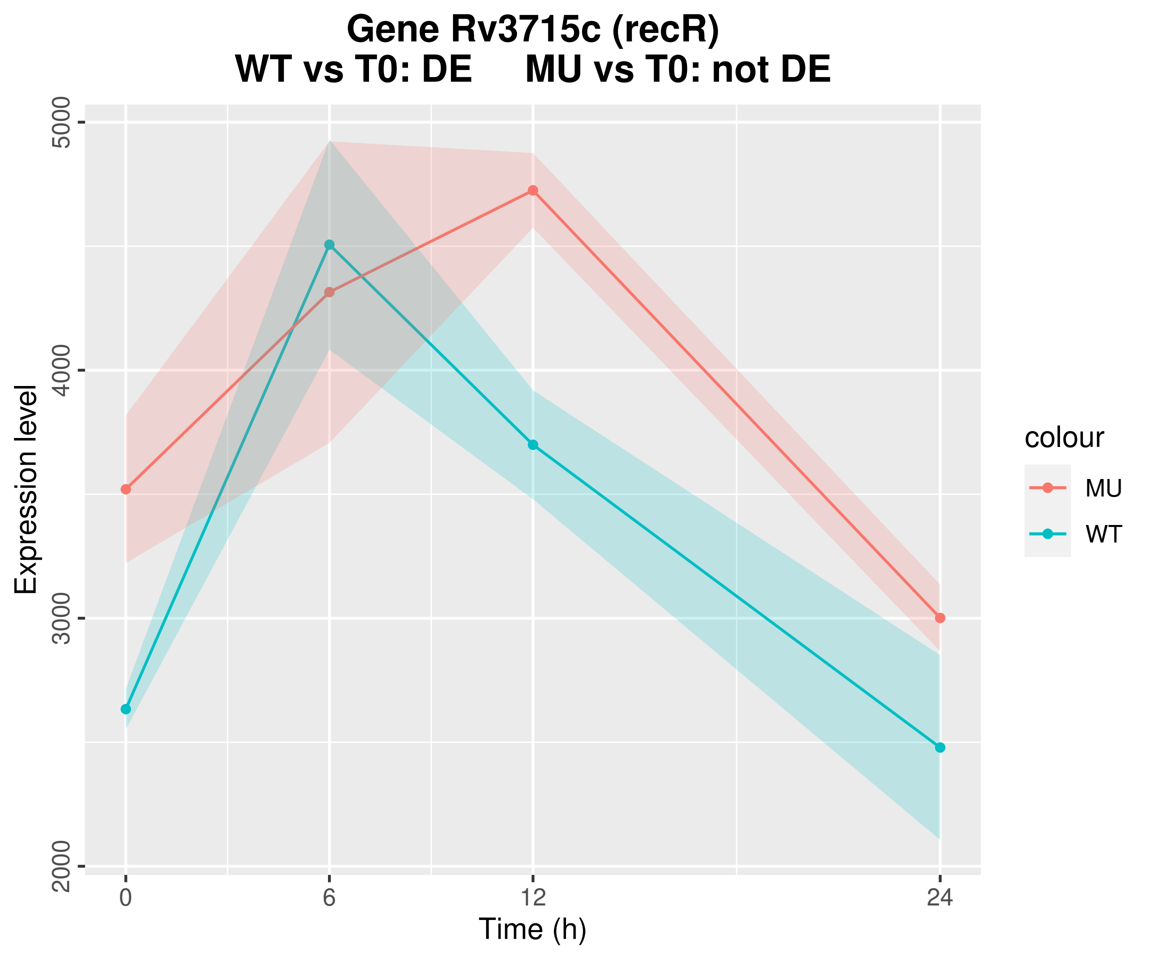
Gene Rv3715c (recR)
WT vs T0: DE   MU vs T0: not DE
Expression level
5000
4000
3000
2000
0
6
12
24
Time (h)
colour
MU
WT

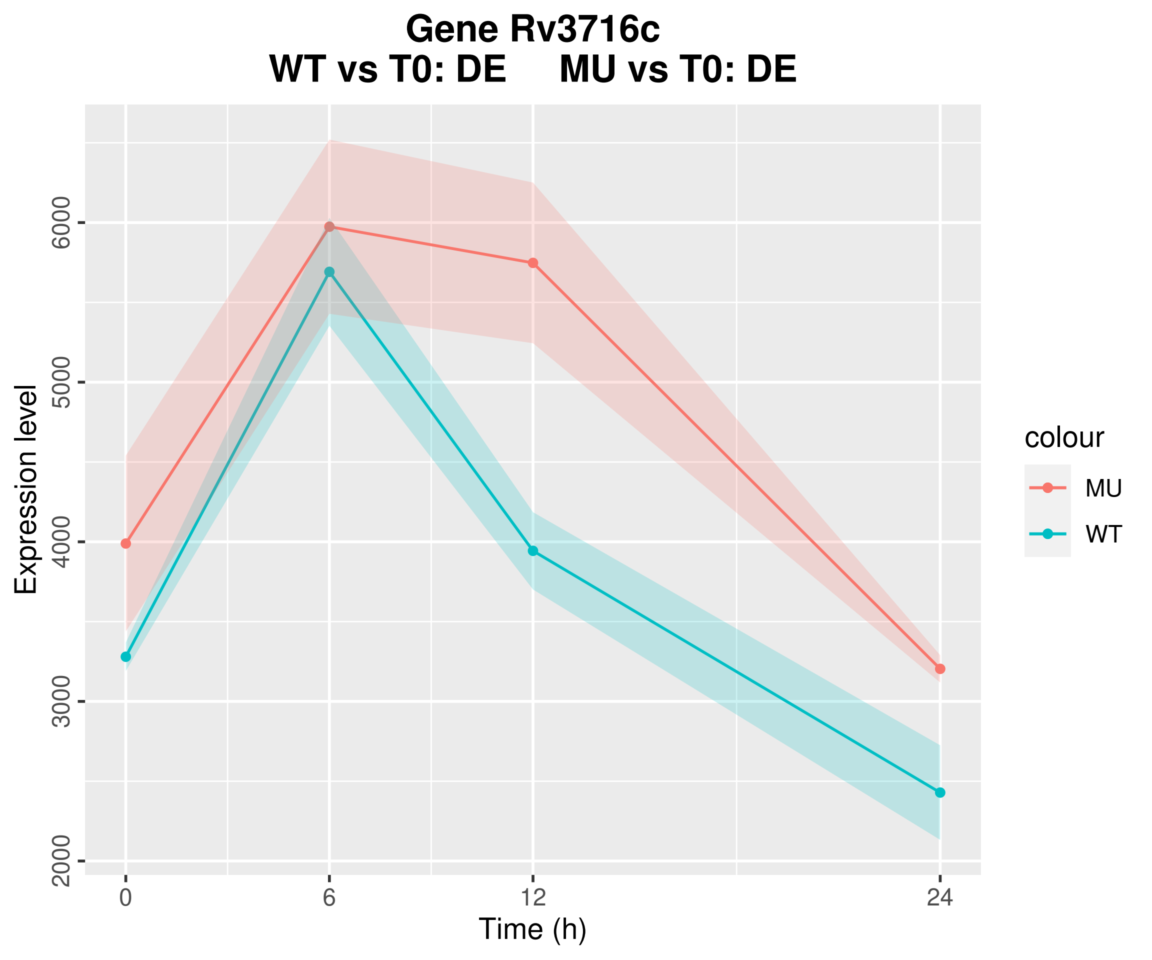

Gene Rv3716c
WT vs T0: DE   MU vs T0: DE
Expression level
6000
5000
4000
3000
2000
0
6
12
24
Time (h)
colour
MU
WT

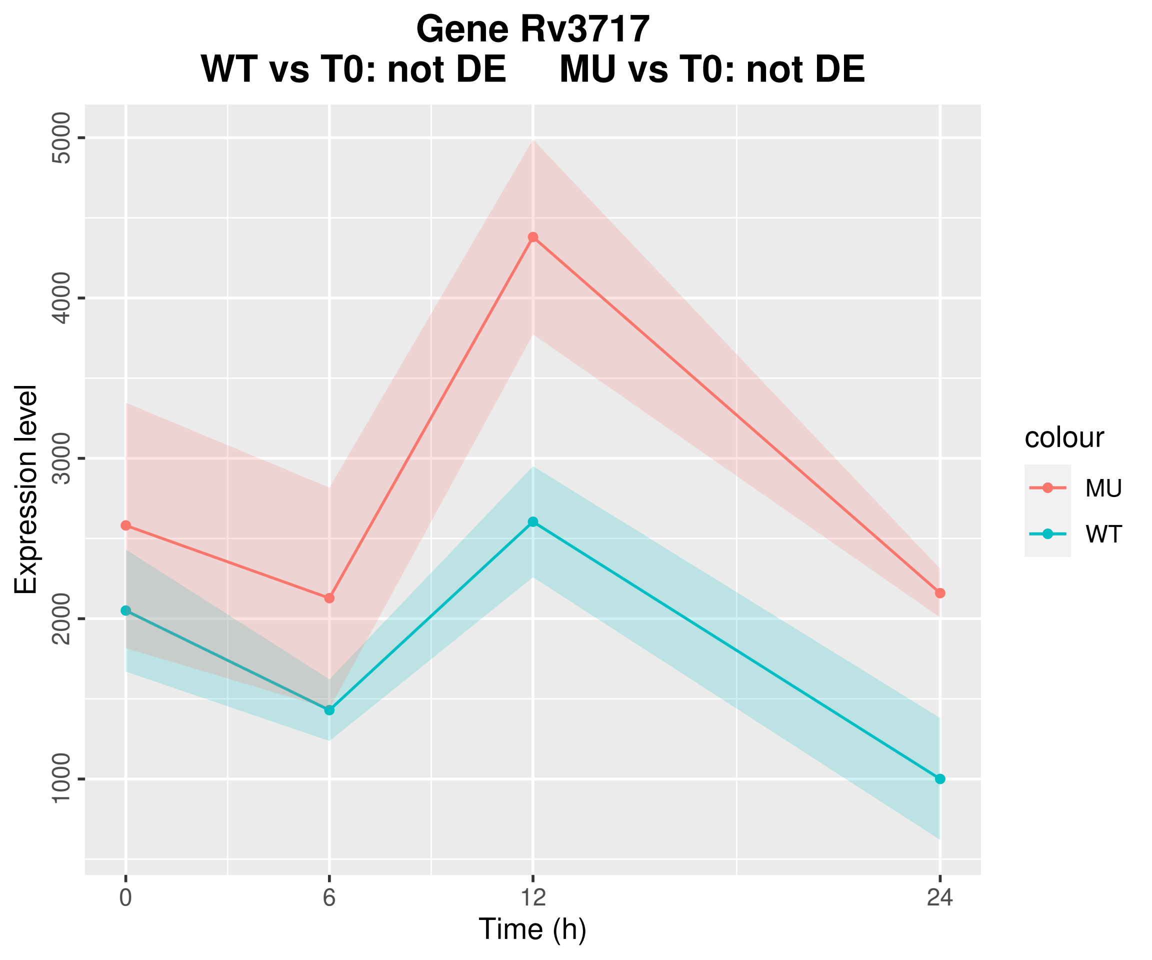

Gene Rv3717
WT vs T0: not DE MU vs T0: not DE
Expression level
1000
2000
3000
4000
5000
0
6
12
24
Time (h)
colour
MU
WT

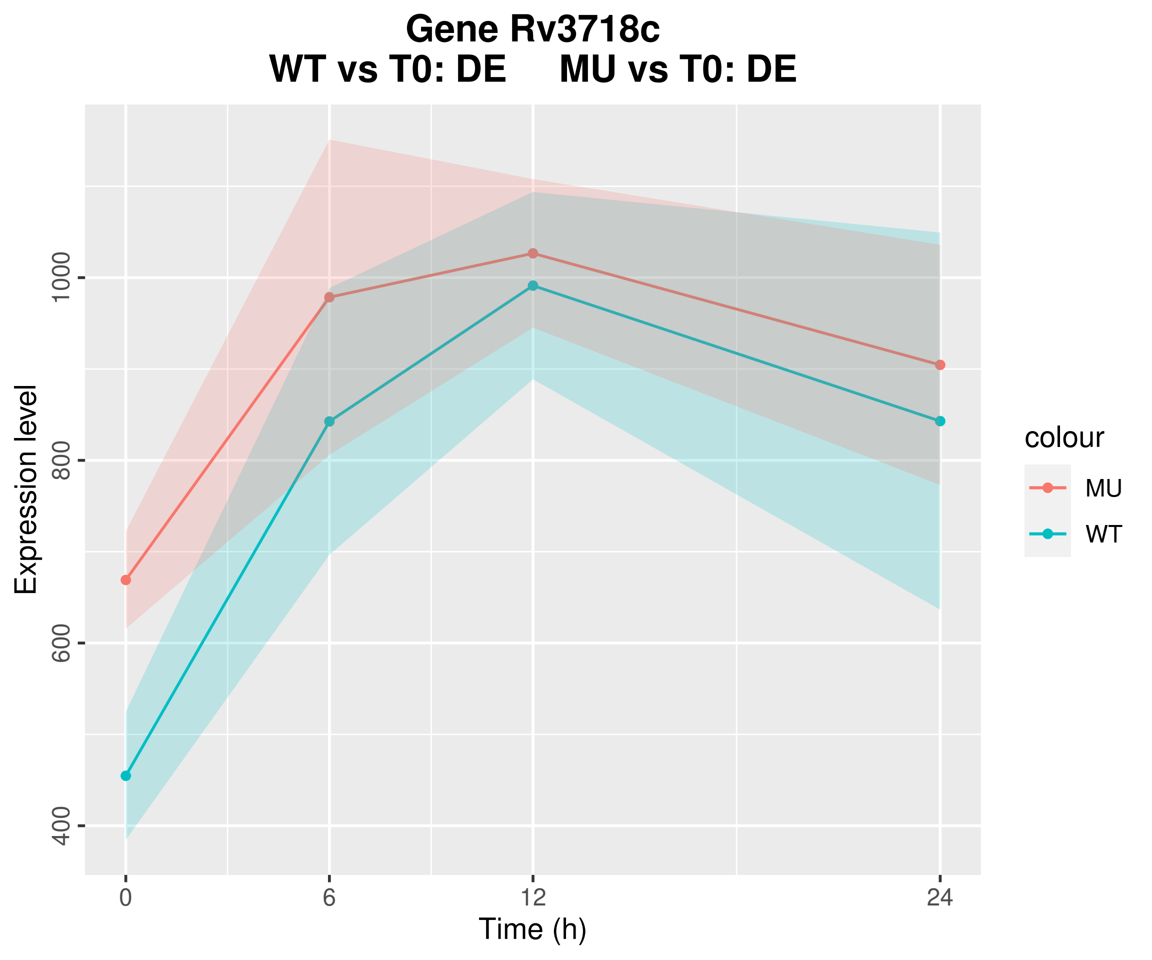
Gene Rv3718c
WT vs T0: DE MU vs T0: DE
Expression level
400
600
800
1000
0
6
12
24
Time (h)
colour
MU
WT

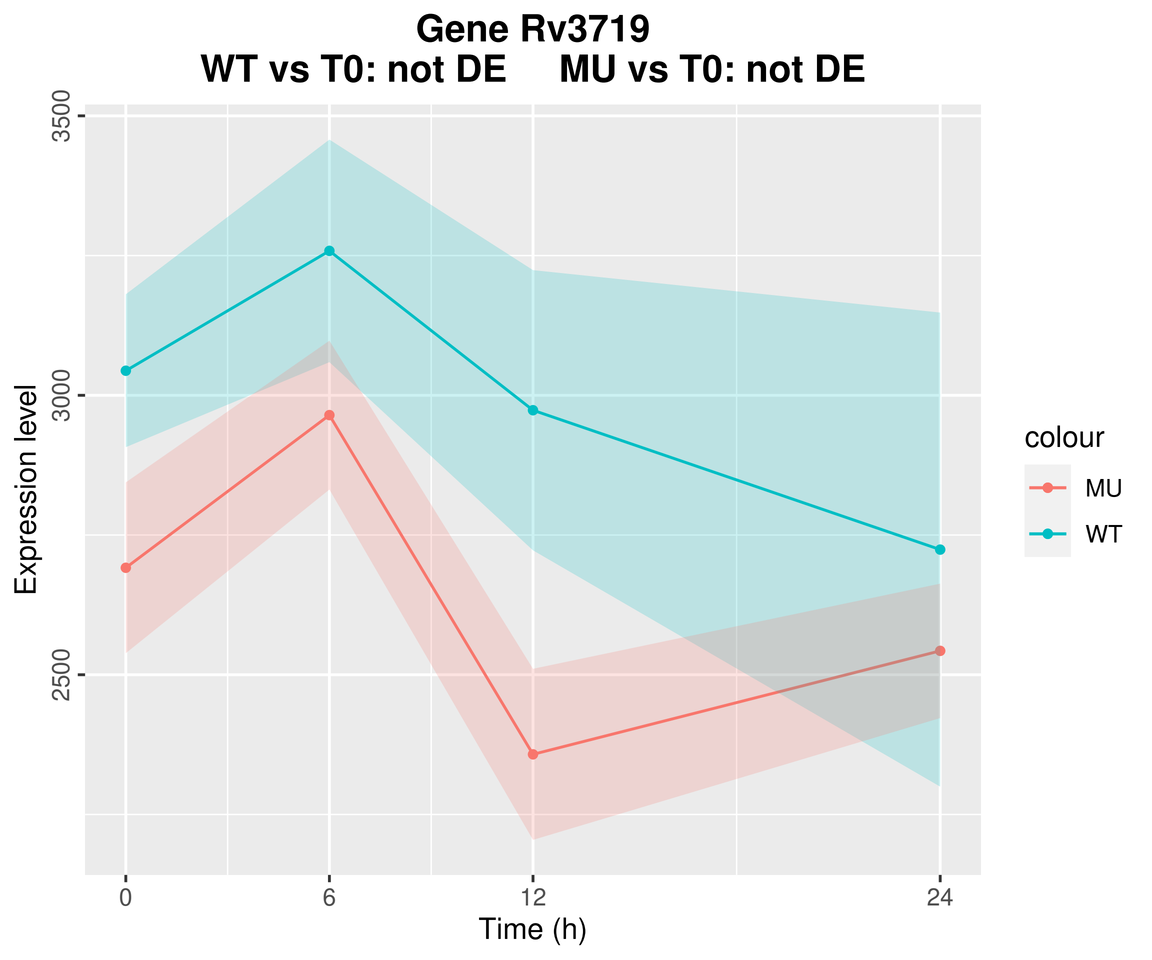

Gene Rv3719
WT vs T0: not DE MU vs T0: not DE
3500
3000
2500
Expression level
0
6
12
24
Time (h)
colour
MU
WT

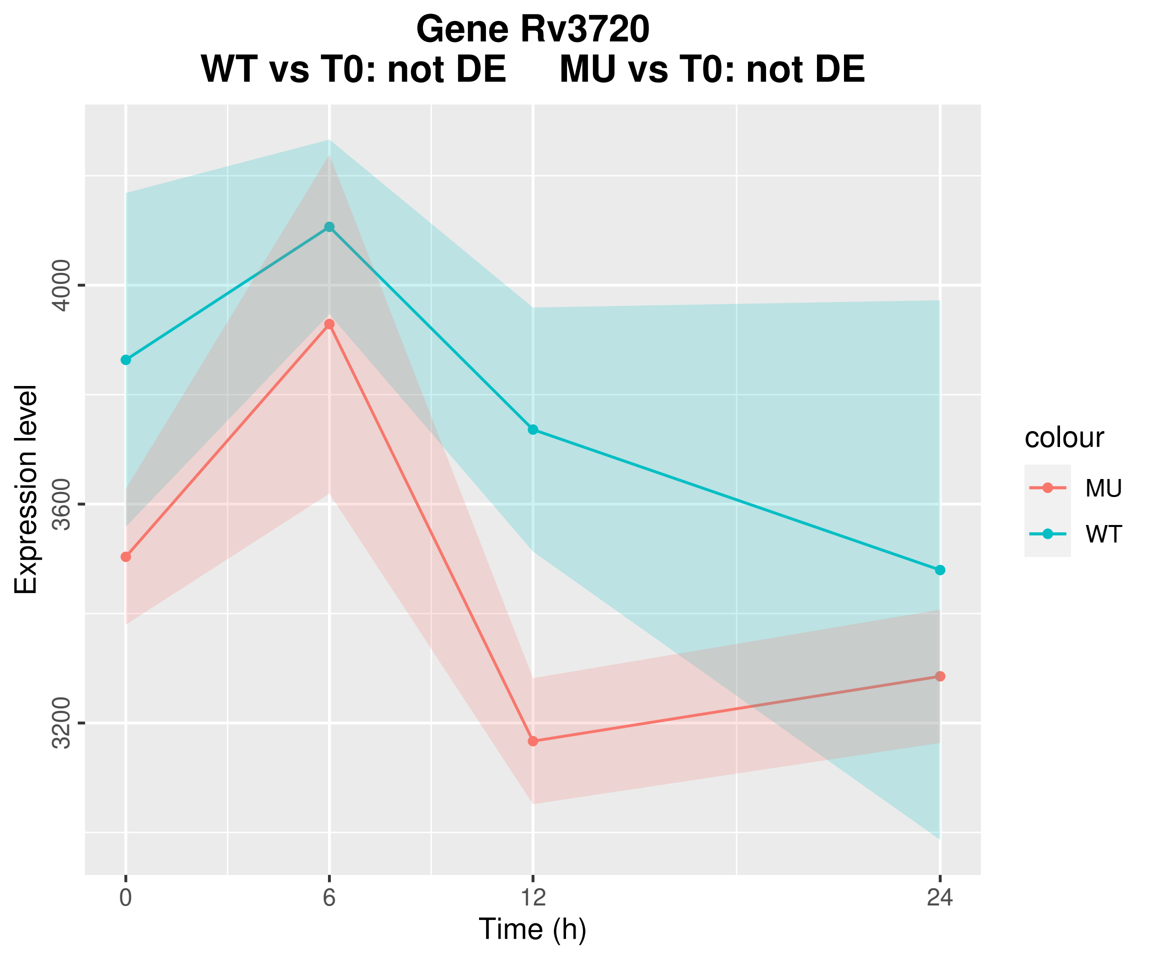
Gene Rv3720
WT vs T0: not DE MU vs T0: not DE
Expression level
4000
3600
3200
0
6
12
24
Time (h)
colour
MU
WT

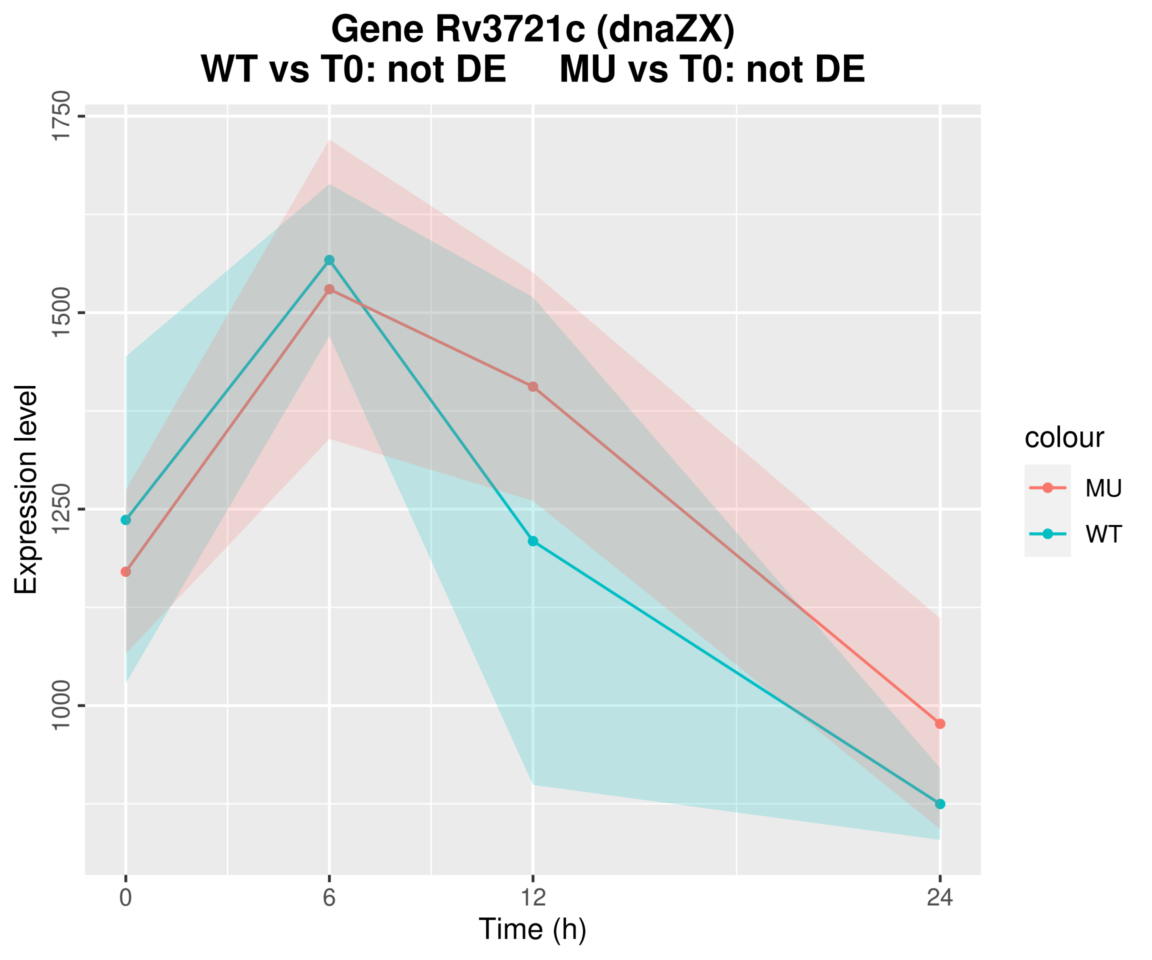
Gene Rv3721c (dnaZX)
WT vs T0: not DE MU vs T0: not DE
Expression level
1750
1500
1250
1000
0
6
12
24
Time (h)
colour
MU
WT

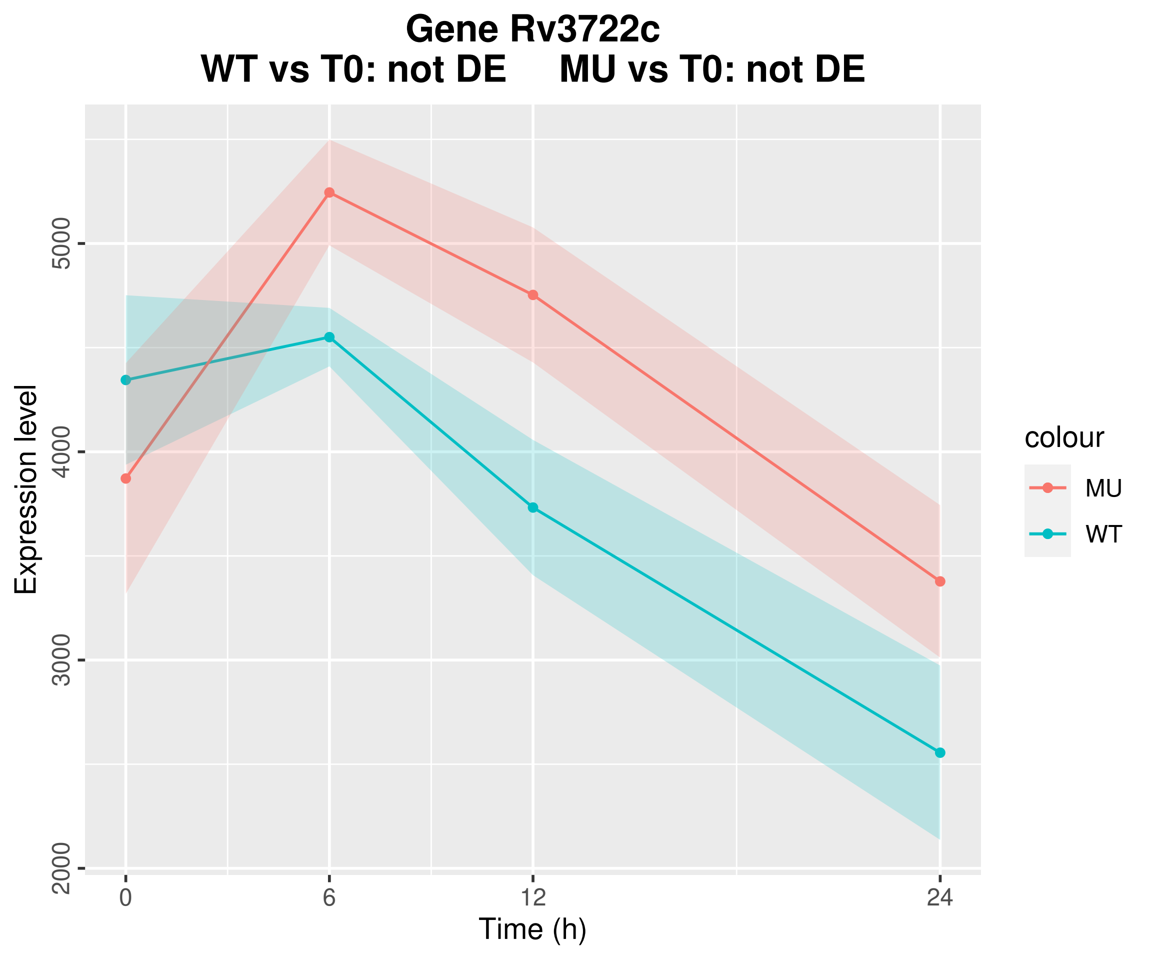

Gene Rv3722c
WT vs T0: not DE   MU vs T0: not DE
Expression level
5000
4000
3000
2000
0
6
12
24
Time (h)
colour
MU
WT

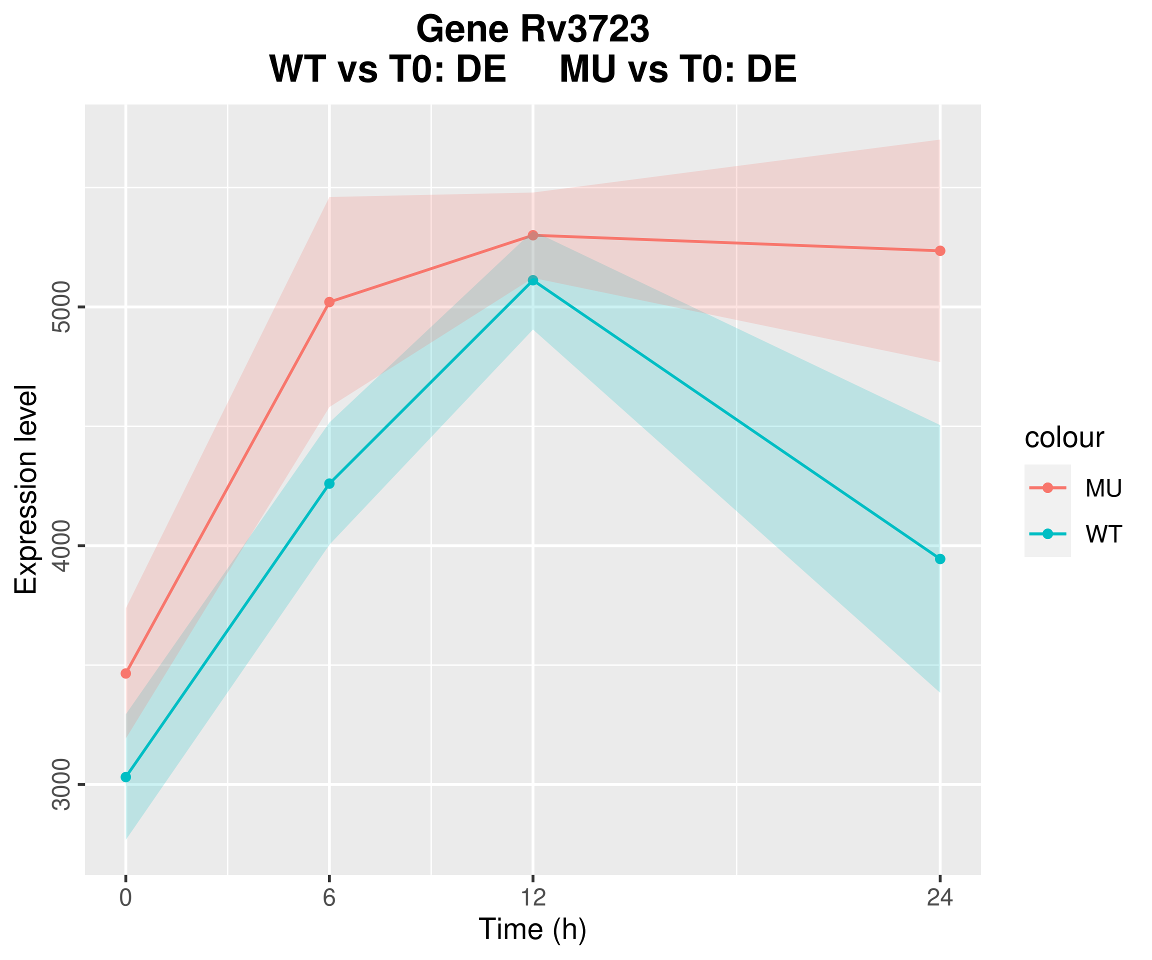

Gene Rv3723
WT vs T0: DE MU vs T0: DE
Expression level
3000
4000
5000
0
6
12
24
Time (h)
colour
MU
WT

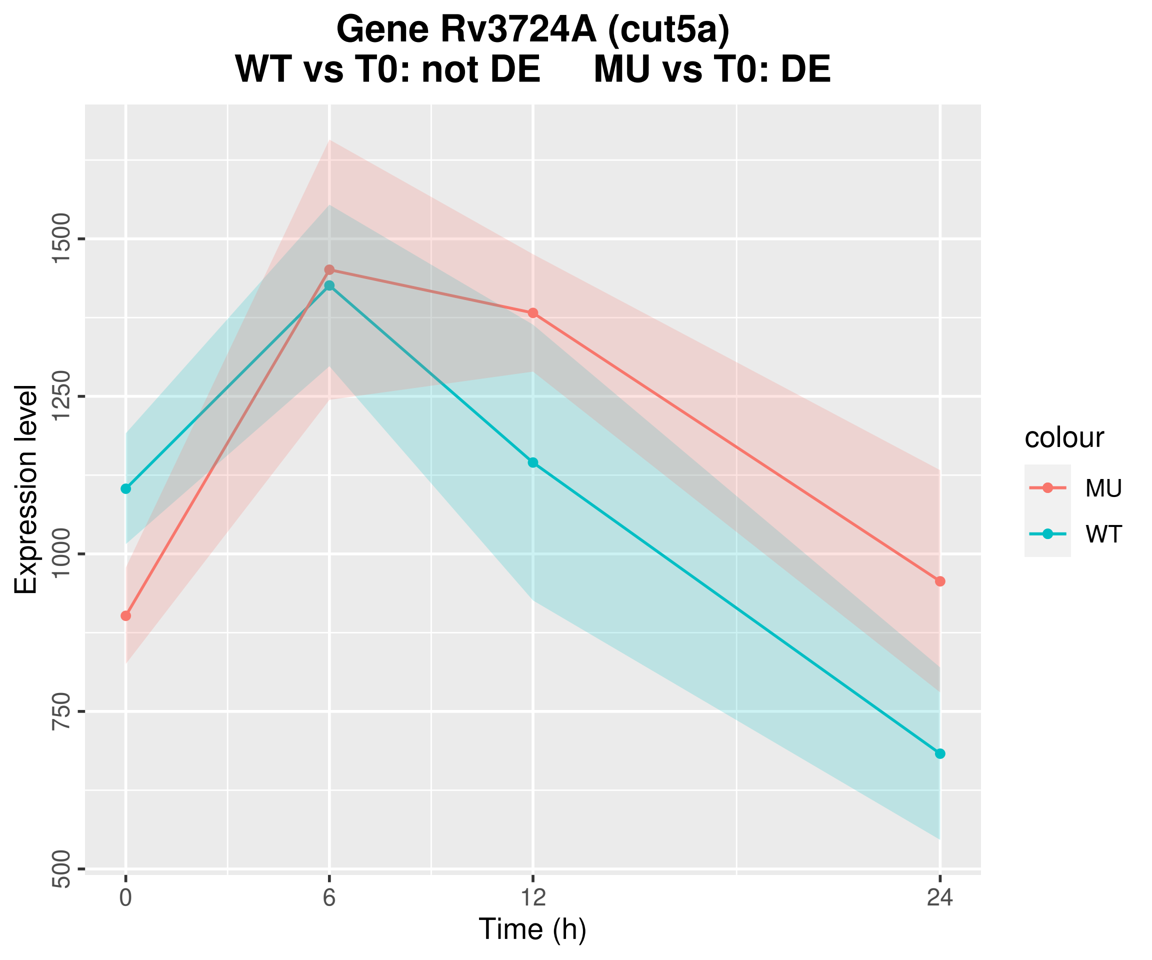

Gene Rv3724A (cut5a)
WT vs T0: not DE   MU vs T0: DE
Expression level
500
750
1000
1250
1500
0
6
12
24
Time (h)
colour
MU
WT

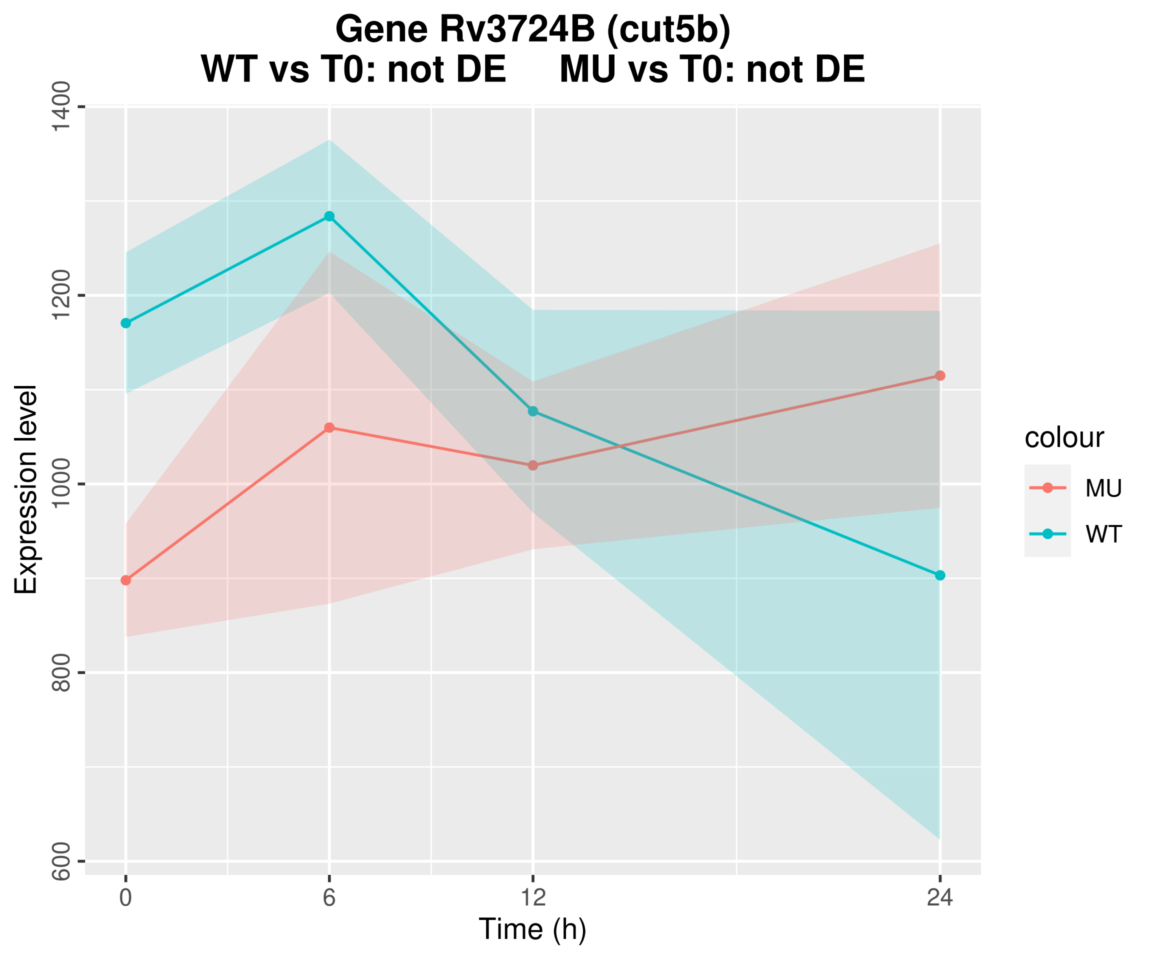
Gene Rv3724B (cut5b)
WT vs T0: not DE   MU vs T0: not DE
1400
1200
1000
800
600
Expression level
0
6
12
24
Time (h)
colour
MU
WT

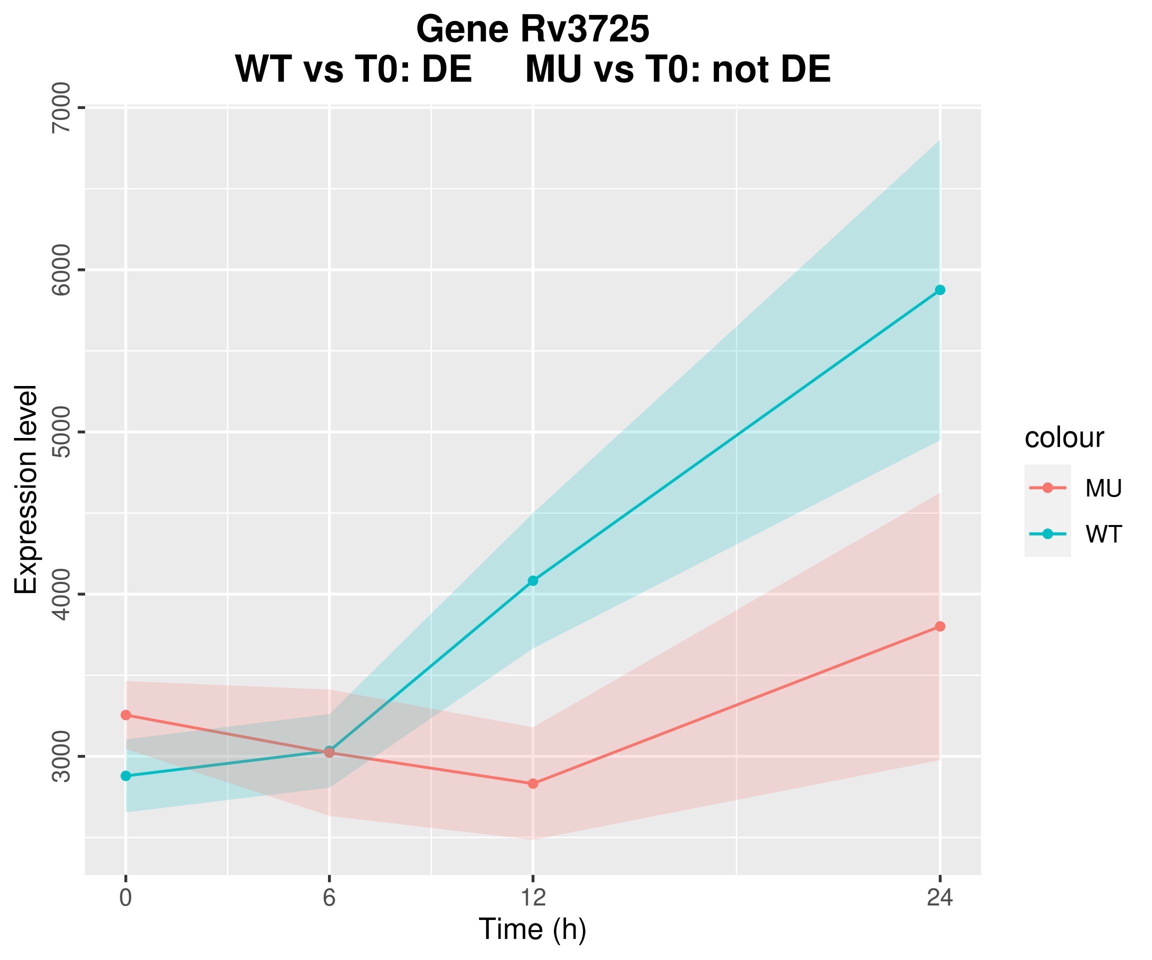
Gene Rv3725
WT vs T0: DE MU vs T0: not DE
7000
6000
5000
4000
3000
Expression level
0
6
12
24
Time (h)
colour
MU
WT

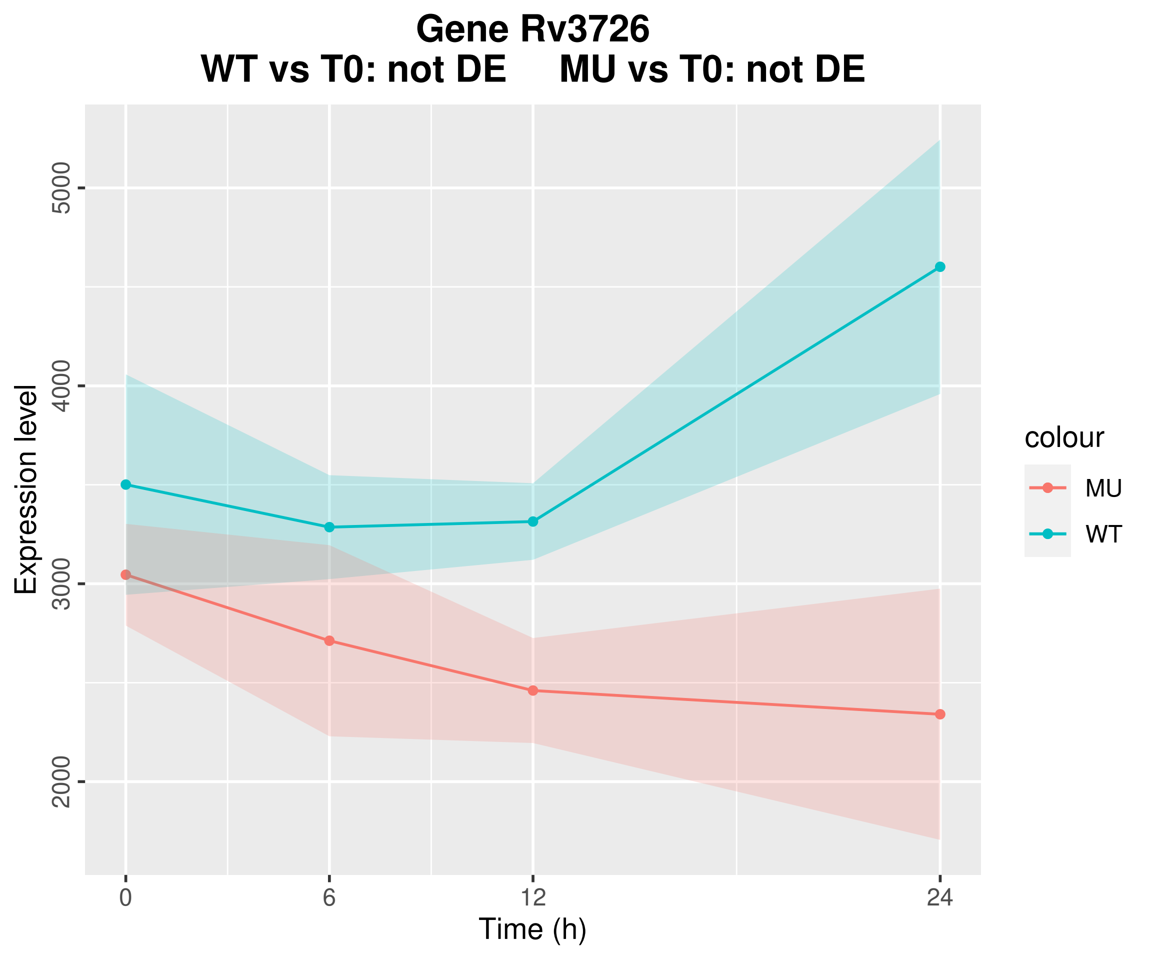
Gene Rv3726
WT vs T0: not DE   MU vs T0: not DE
Expression level
5000
4000
3000
2000
0
6
12
24
Time (h)
colour
MU
WT

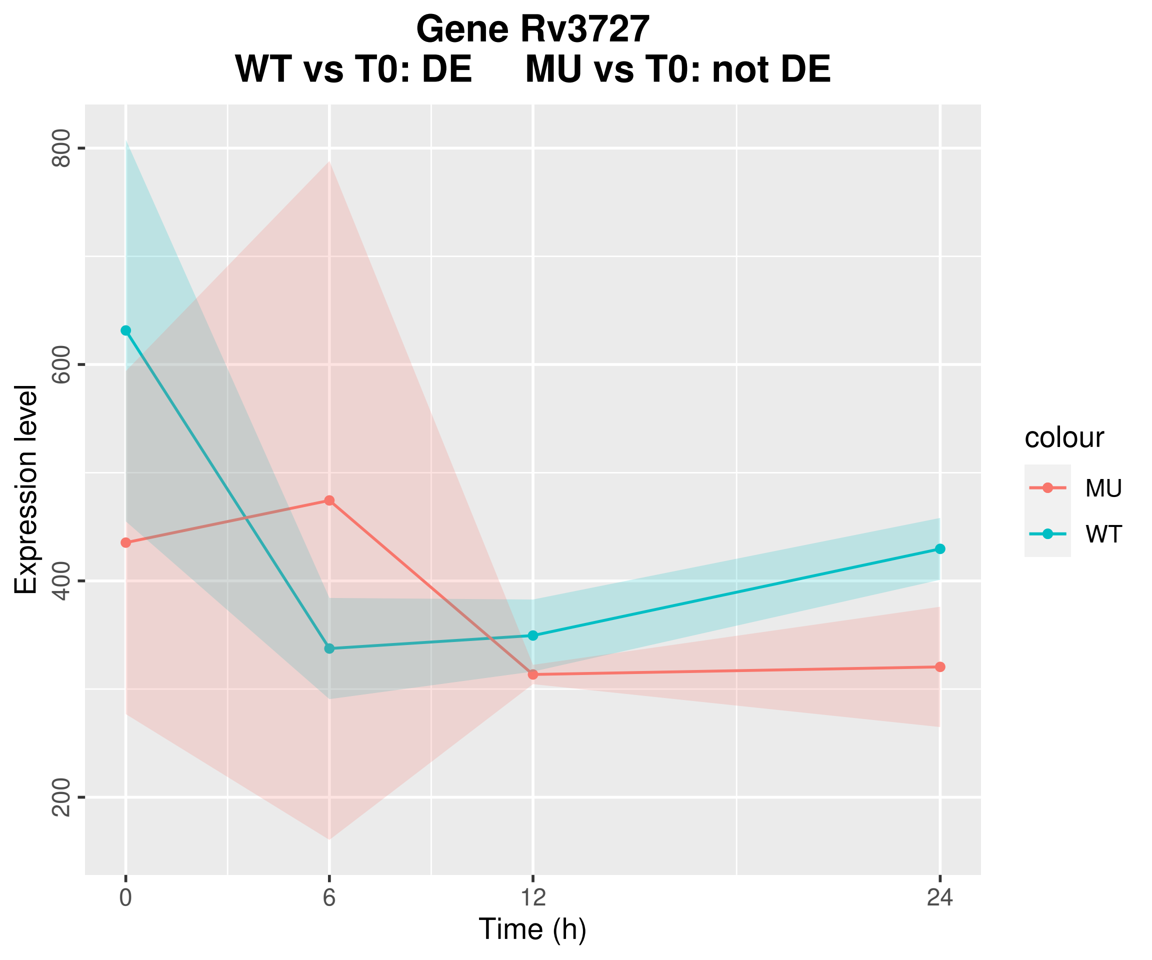
Gene Rv3727
WT vs T0: DE   MU vs T0: not DE
Expression level
800
600
400
200
0
6
12
24
Time (h)
colour
MU
WT

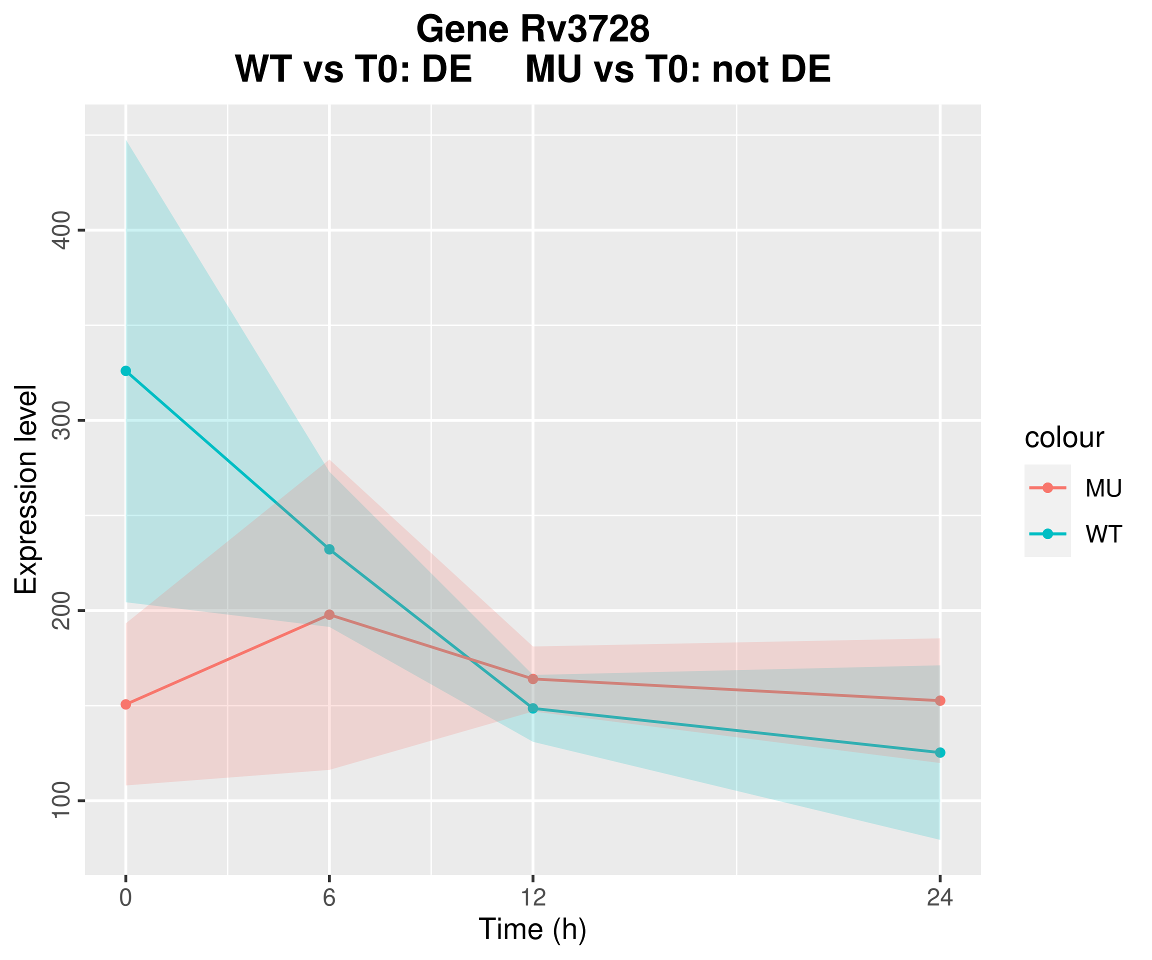
Gene Rv3728
WT vs T0: DE   MU vs T0: not DE
Expression level
400
300
200
100
0
6
12
24
Time (h)
colour
MU
WT

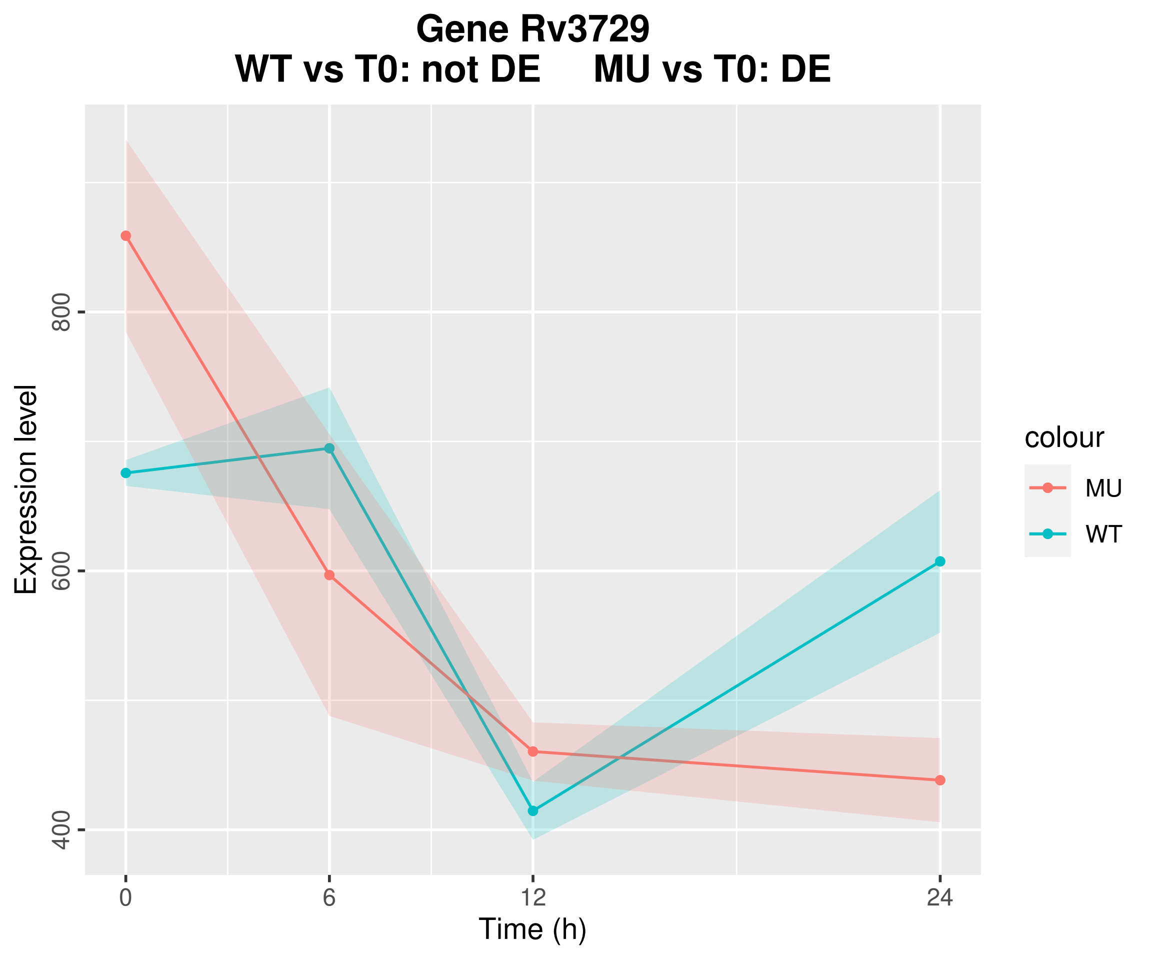

Gene Rv3729
WT vs T0: not DE MU vs T0: DE
Expression level
800
600
400
0
6
12
24
Time (h)
colour
MU
WT

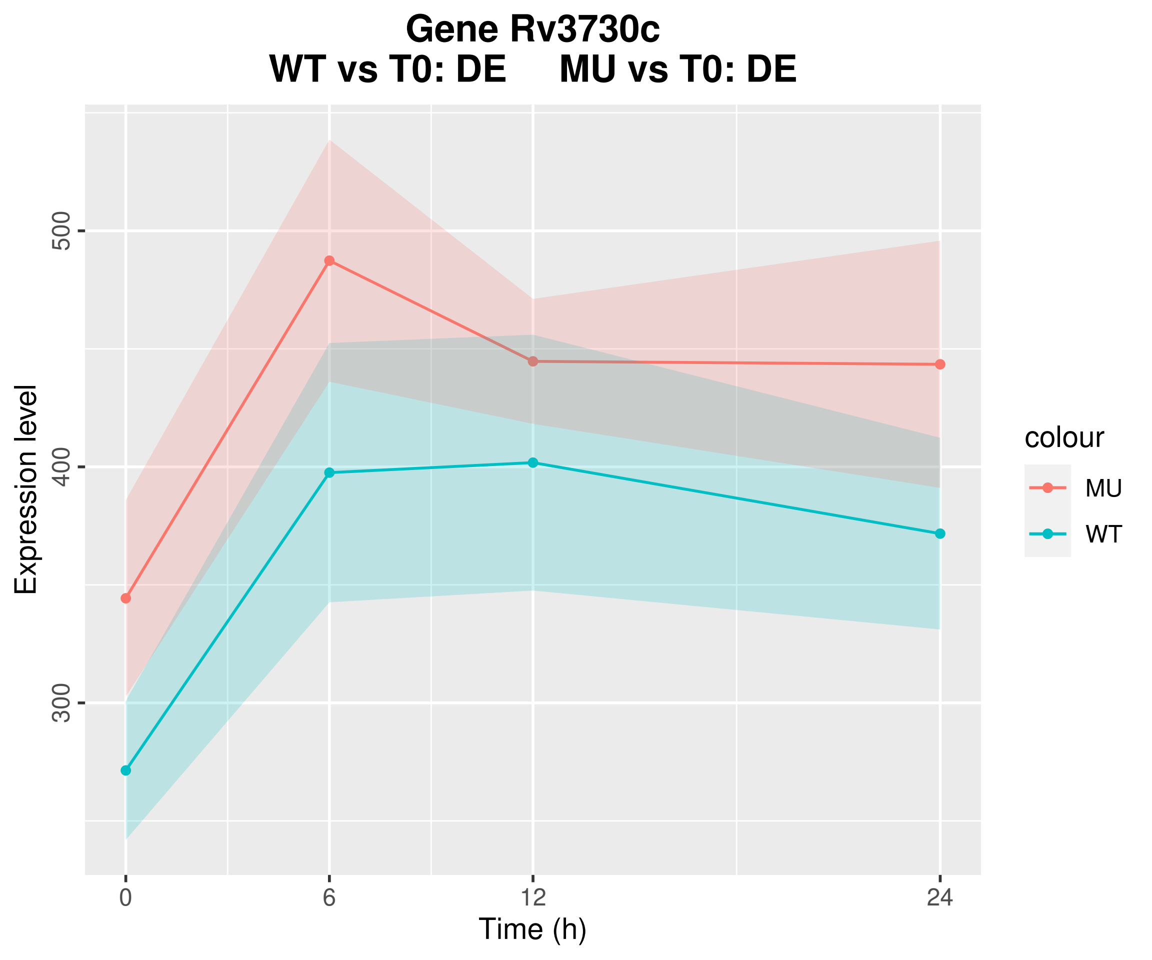

Gene Rv3730c
WT vs T0: DE MU vs T0: DE
500
400
300
Expression level
0
6
12
24
Time (h)
colour
MU
WT

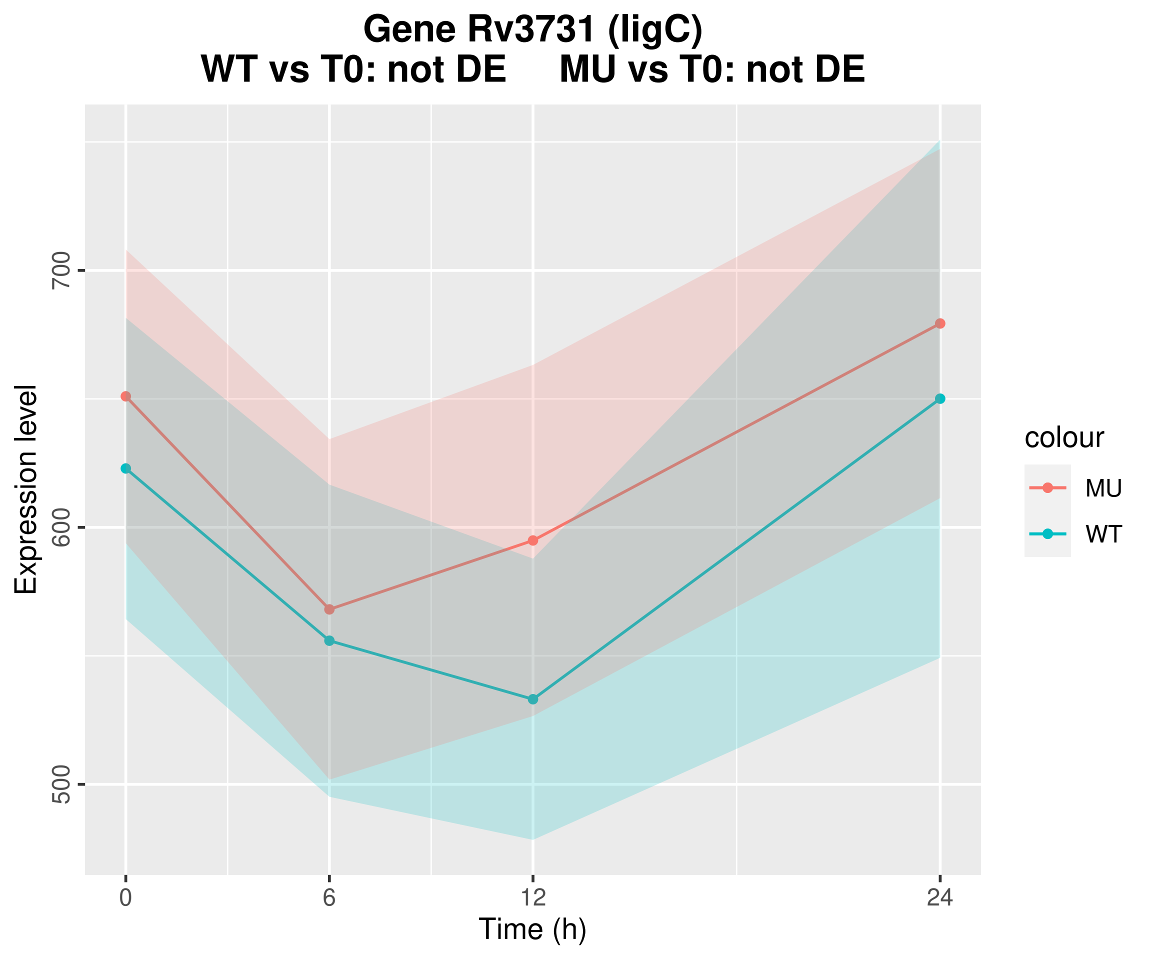
Gene Rv3731 (ligC)
WT vs T0: not DE MU vs T0: not DE
Expression level
700
600
500
0
6
12
24
Time (h)
colour
MU
WT

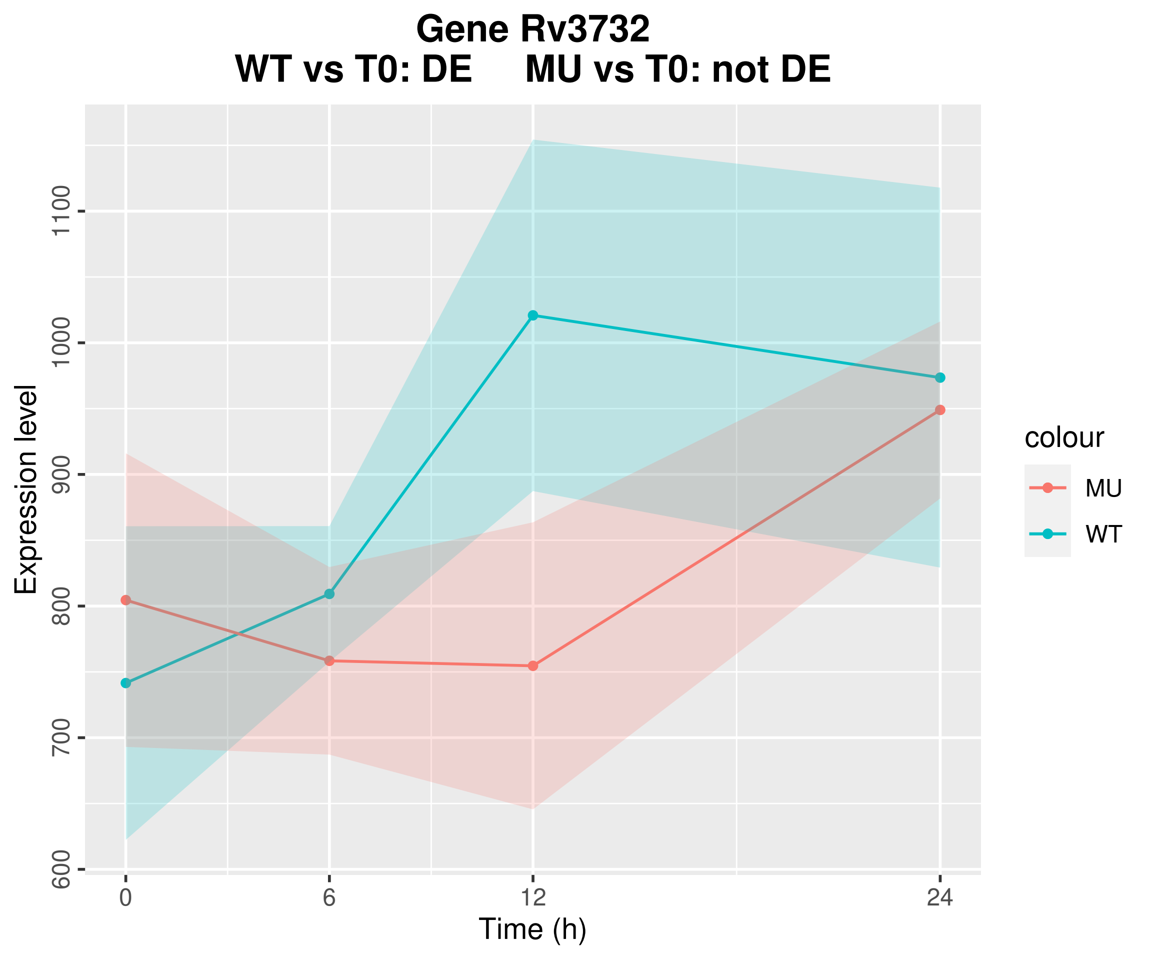

Gene Rv3732
WT vs T0: DE MU vs T0: not DE
Expression level
Time (h)
600
700
800
900
1000
1100
0
6
12
24
colour
MU
WT

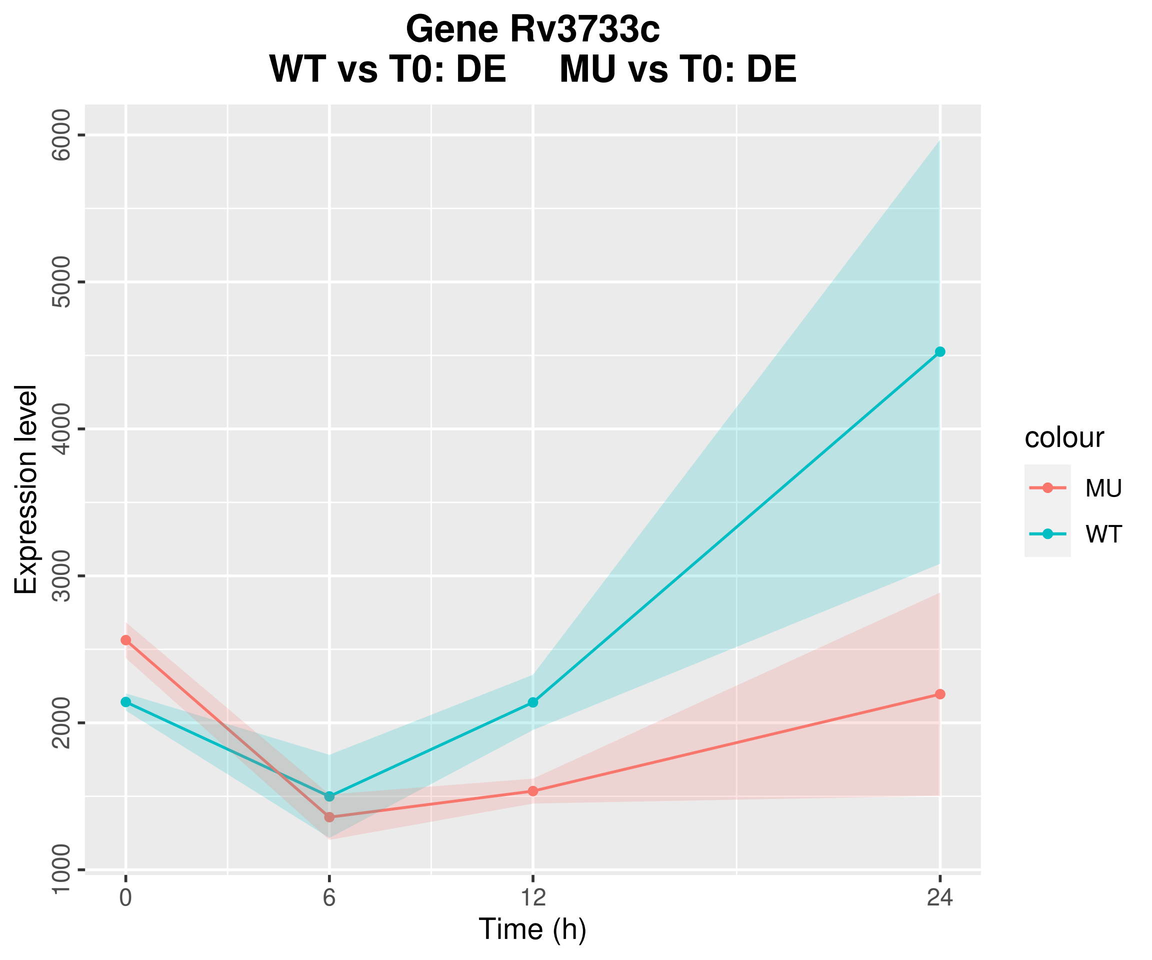

Gene Rv3733c
WT vs T0: DE MU vs T0: DE
Expression level
6000
5000
4000
3000
2000
1000
0
6
12
24
Time (h)
colour
MU
WT

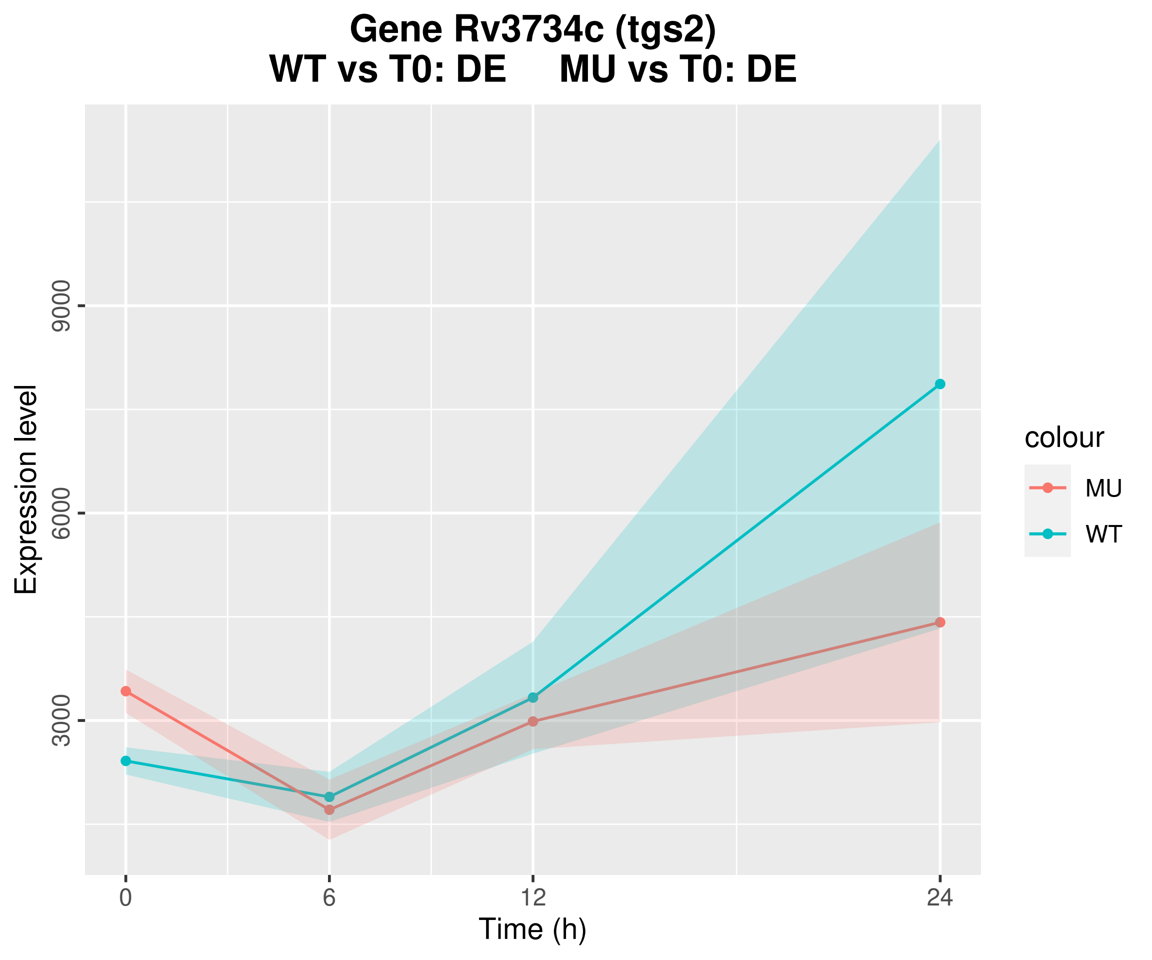
Gene Rv3734c (tgs2)
WT vs T0: DE   MU vs T0: DE
Expression level
9000
6000
3000
0
6
12
24
Time (h)
colour
MU
WT

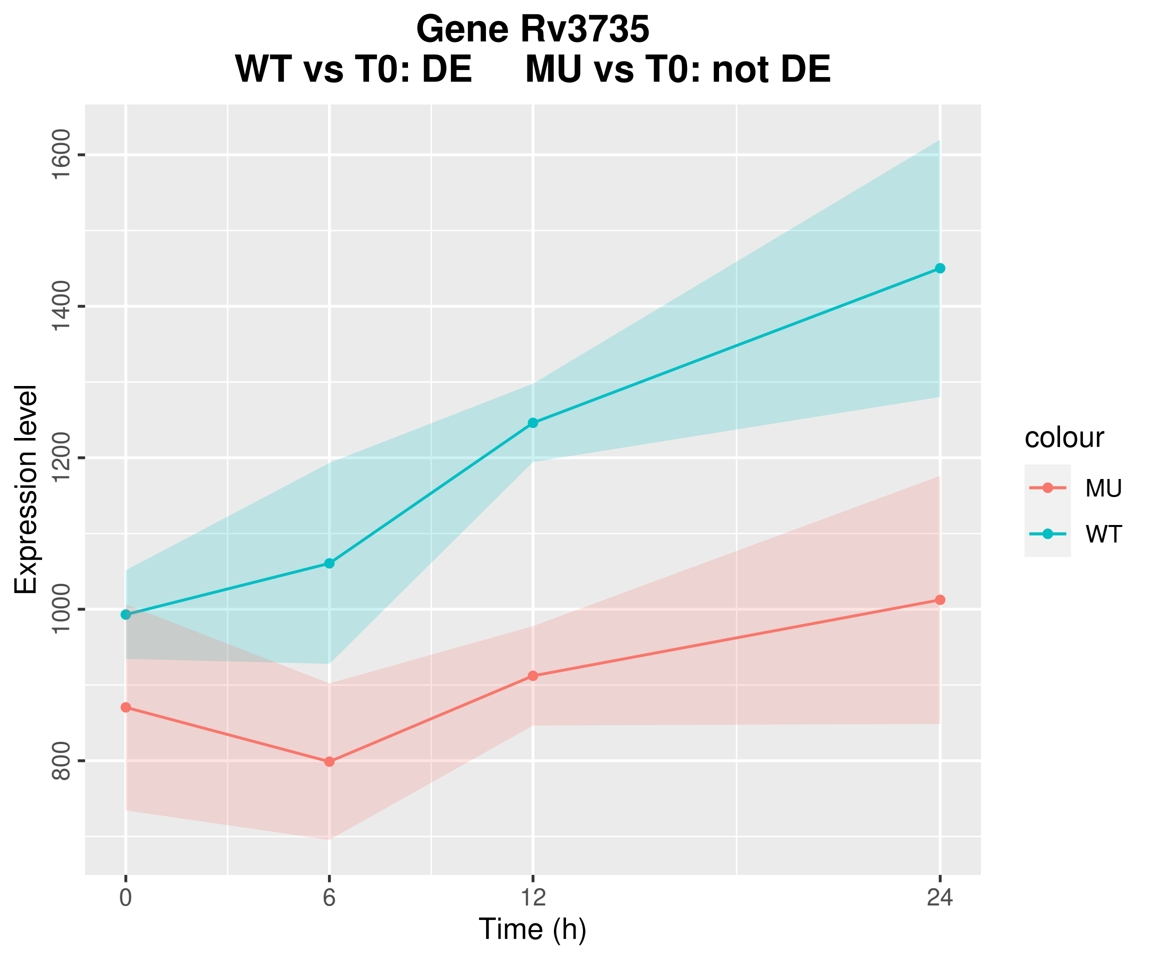

Gene Rv3735
WT vs T0: DE   MU vs T0: not DE
Expression level
1600
1400
1200
1000
800
0
6
12
24
Time (h)
colour
MU
WT

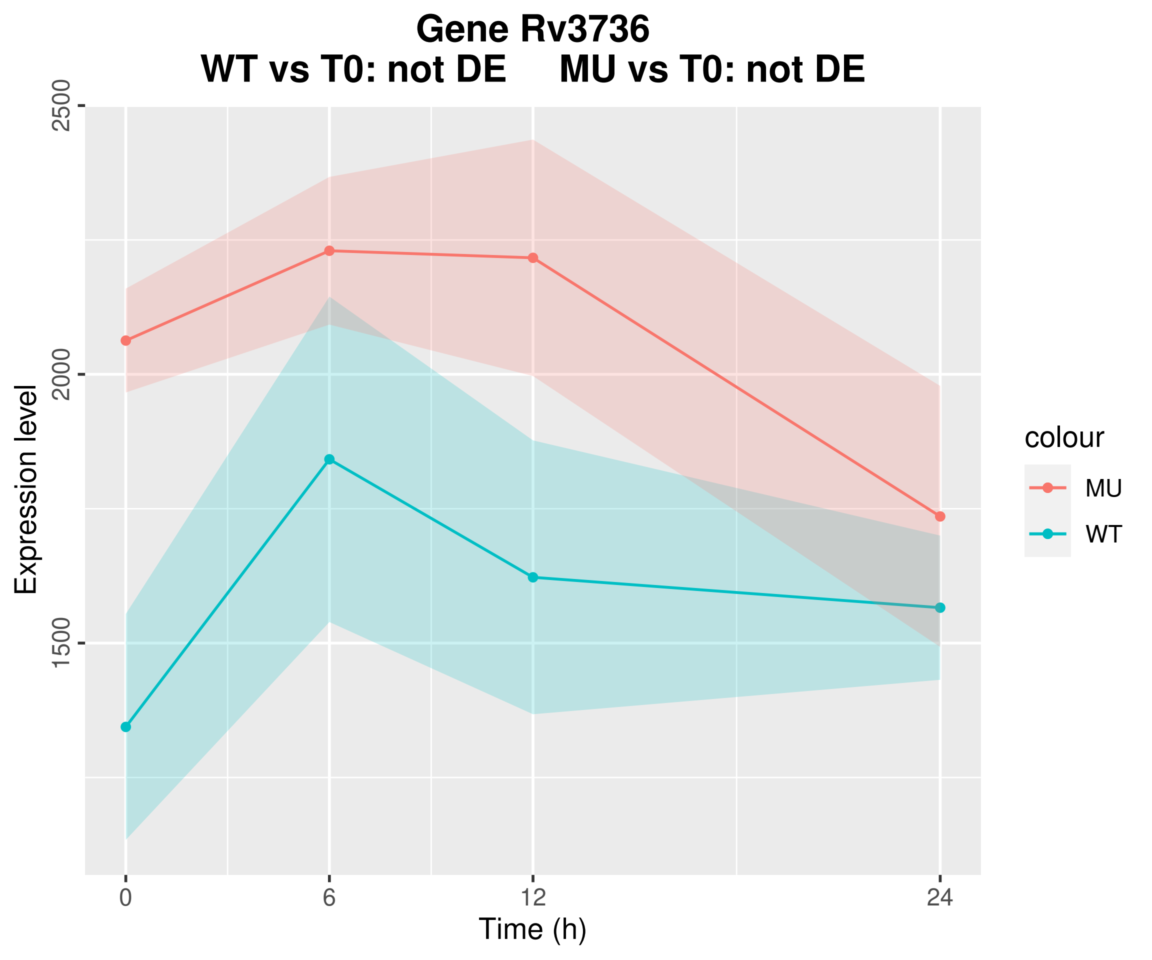

Gene Rv3736
WT vs T0: not DE   MU vs T0: not DE
Expression level
2500
2000
1500
0
6
12
24
Time (h)
colour
MU
WT

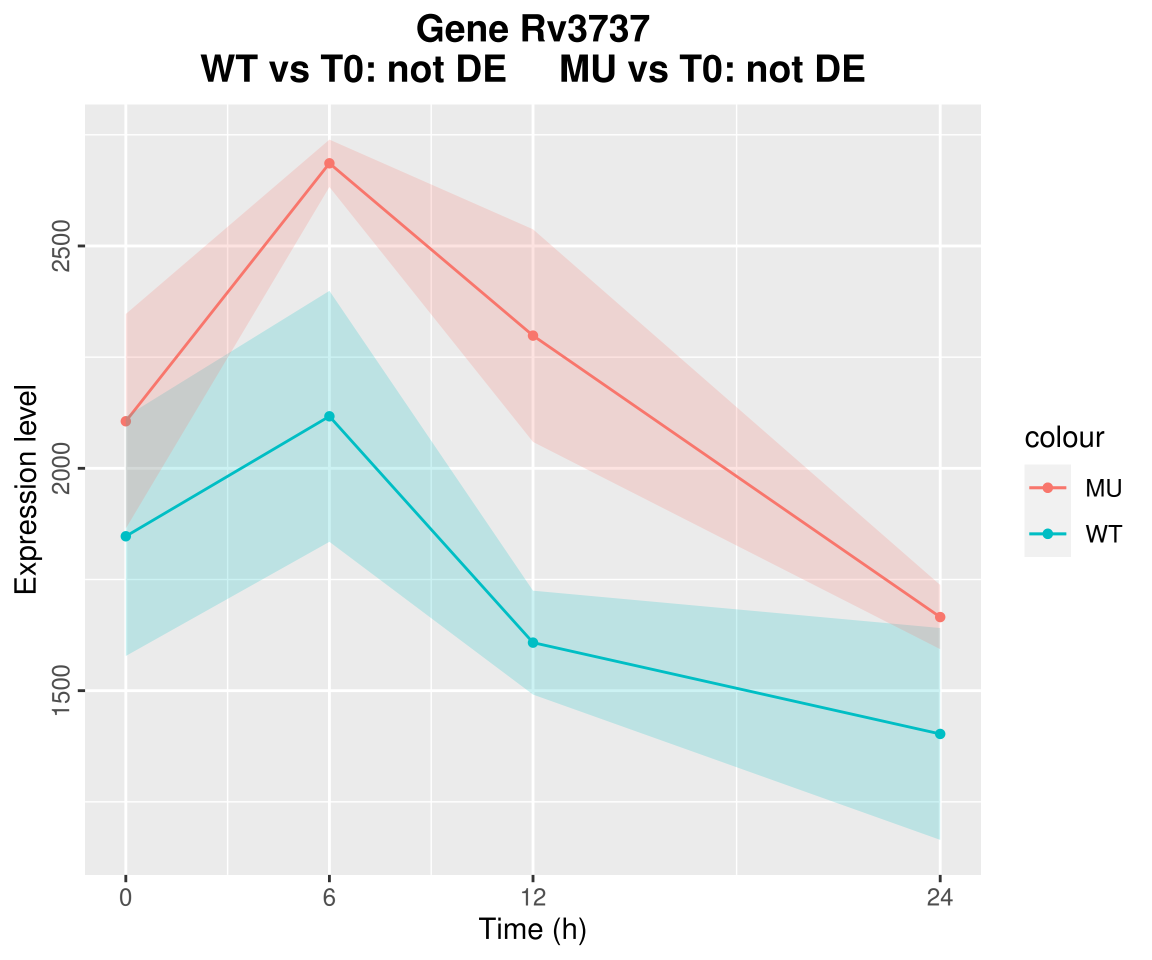

Gene Rv3737
WT vs T0: not DE MU vs T0: not DE
Expression level
2500
2000
1500
0
6
12
24
Time (h)
colour
MU
WT

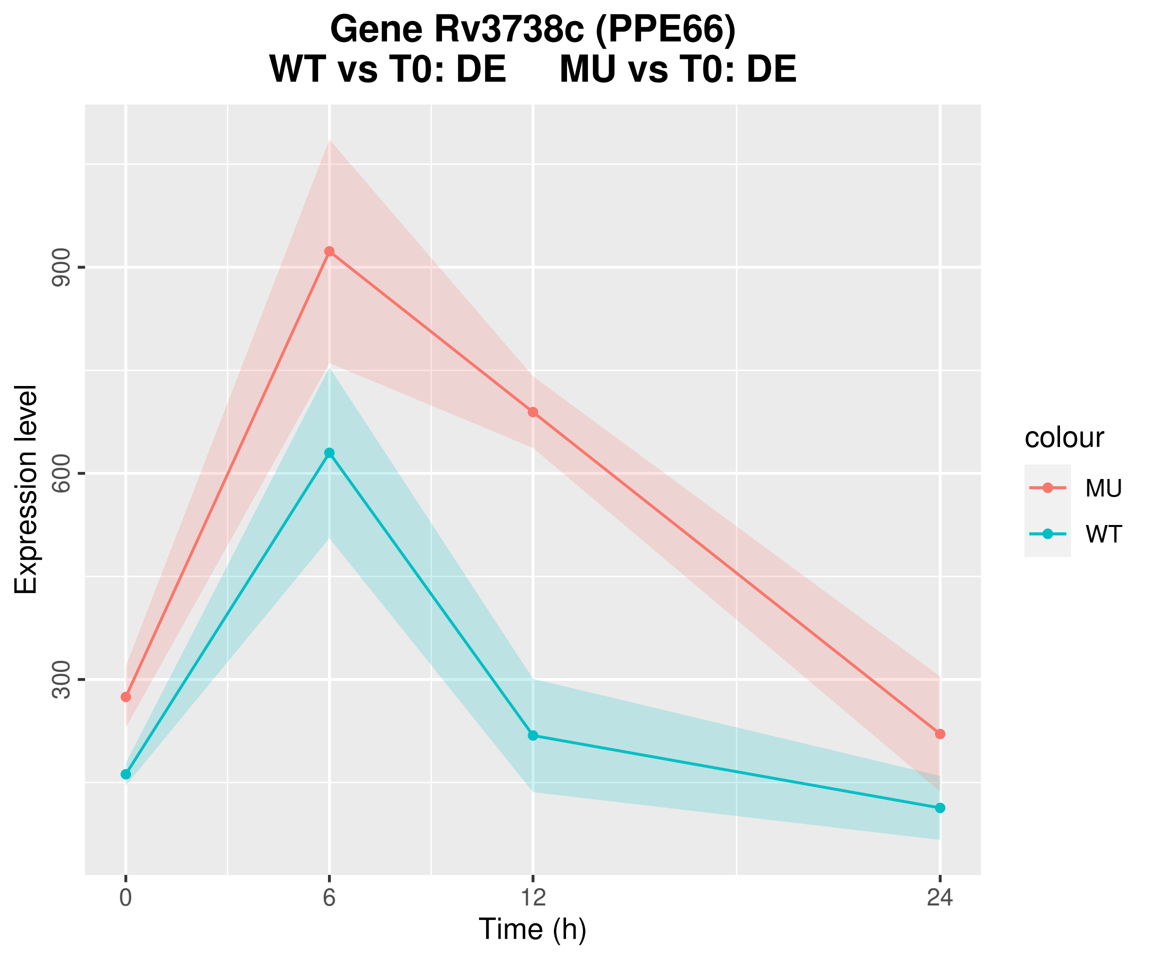

Gene Rv3738c (PPE66)
WT vs T0: DE MU vs T0: DE
Expression level
900
600
300
0
6
12
24
Time (h)
colour
MU
WT

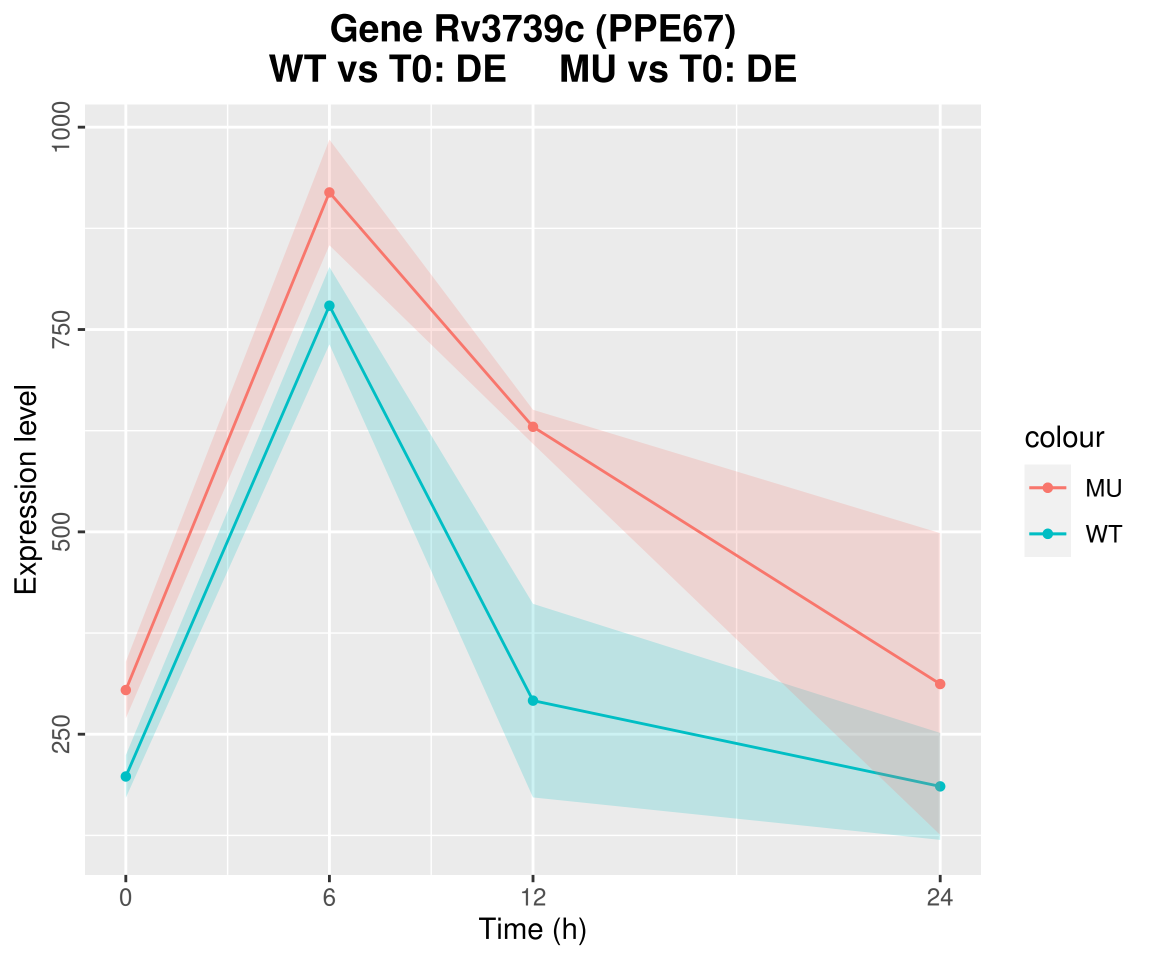

Gene Rv3739c (PPE67)
WT vs T0: DE MU vs T0: DE
Expression level
1000
750
500
250
0
6
12
24
Time (h)
colour
MU
WT

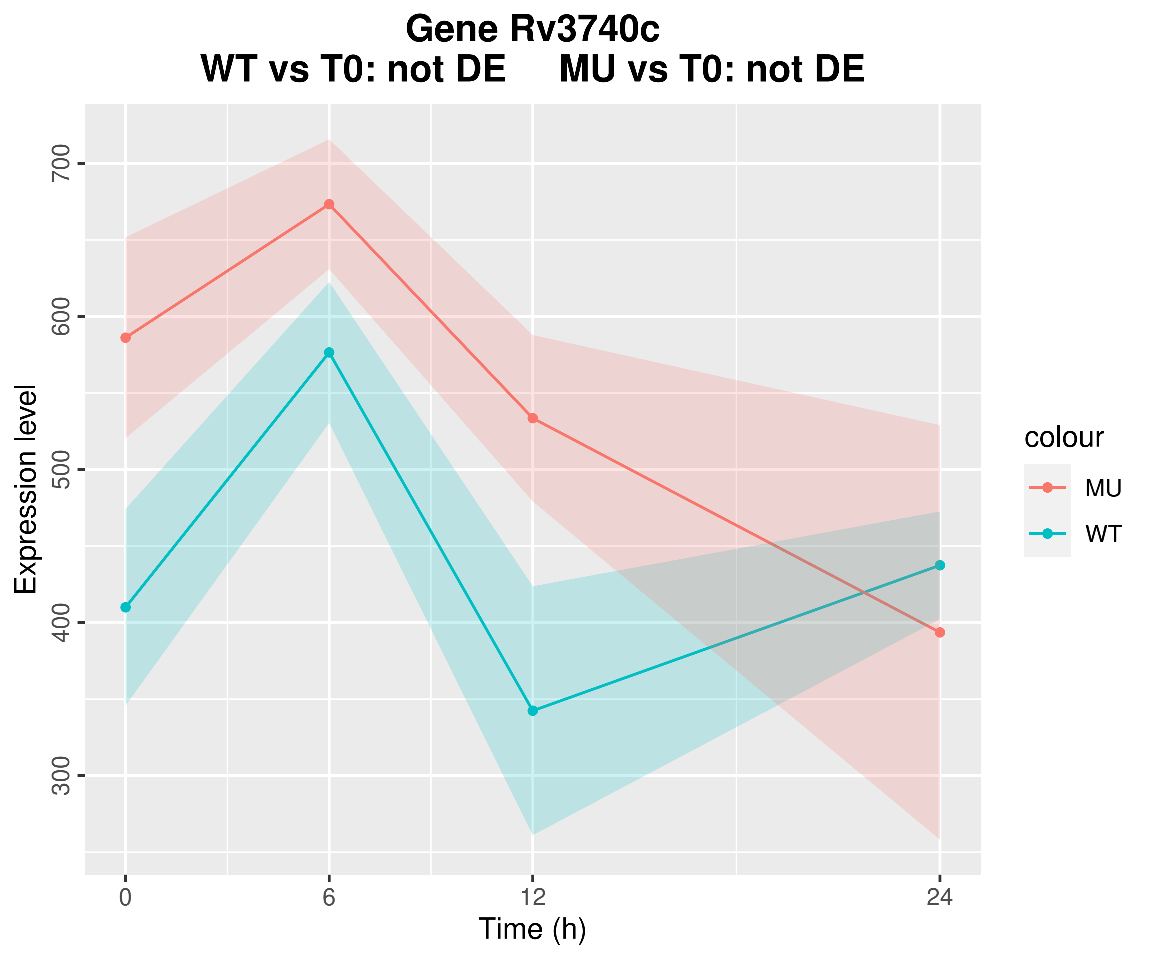
Gene Rv3740c
WT vs T0: not DE MU vs T0: not DE
Expression level
700
600
500
400
300
0
6
12
24
Time (h)
colour
MU
WT

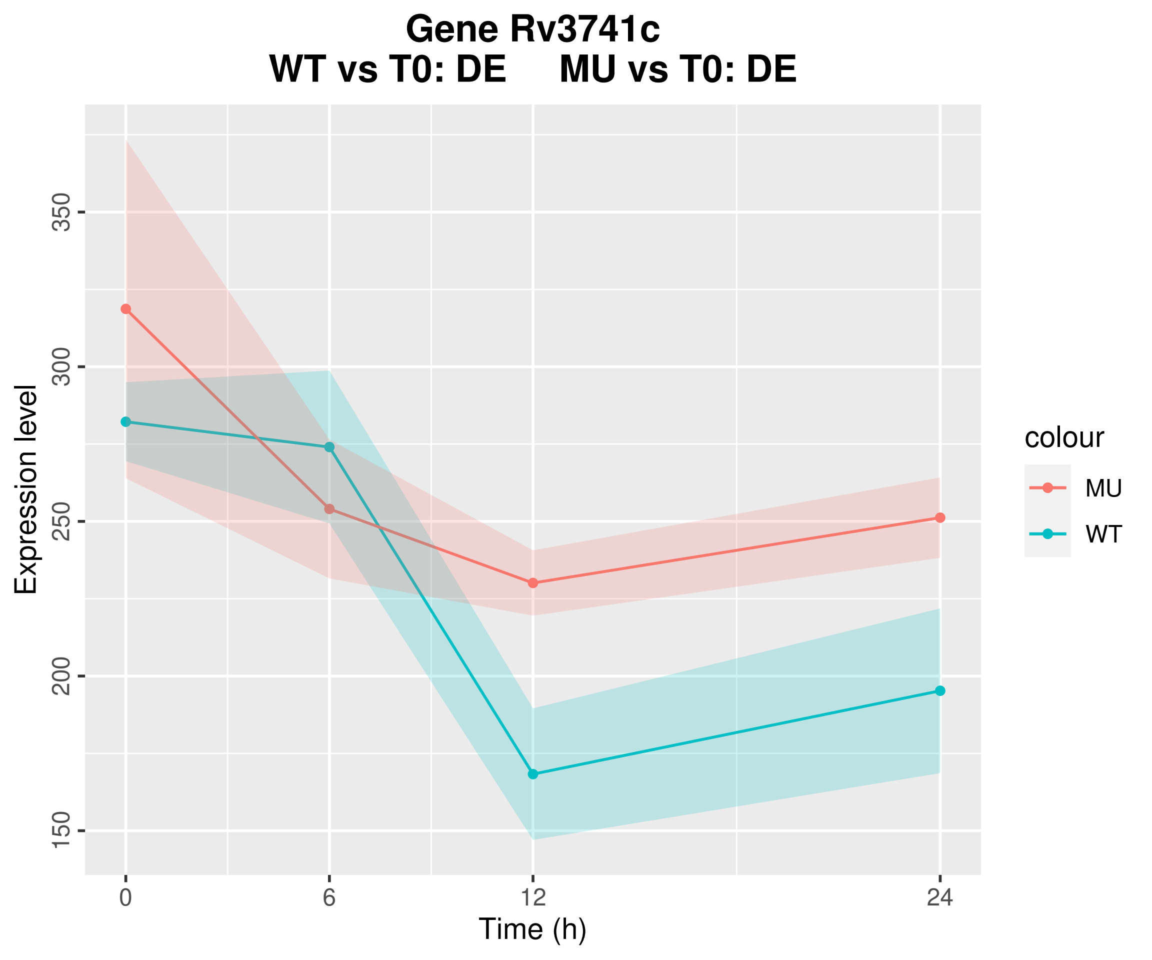

Gene Rv3741c
WT vs T0: DE MU vs T0: DE
Expression level
350
300
250
200
150
0
6
12
24
Time (h)
colour
MU
WT

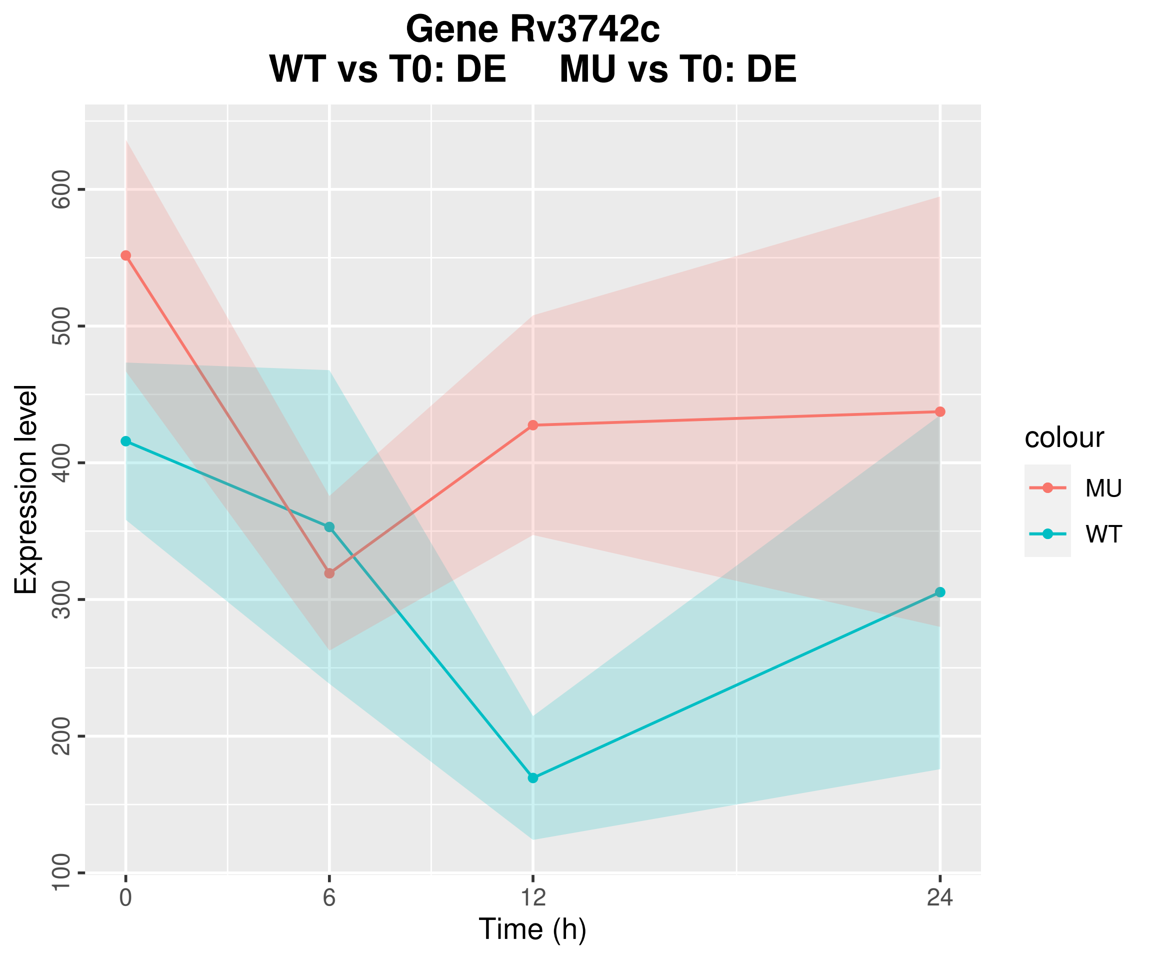
Gene Rv3742c
WT vs T0: DE   MU vs T0: DE
Expression level
Time (h)
100
200
300
400
500
600
0
6
12
24
colour
MU
WT

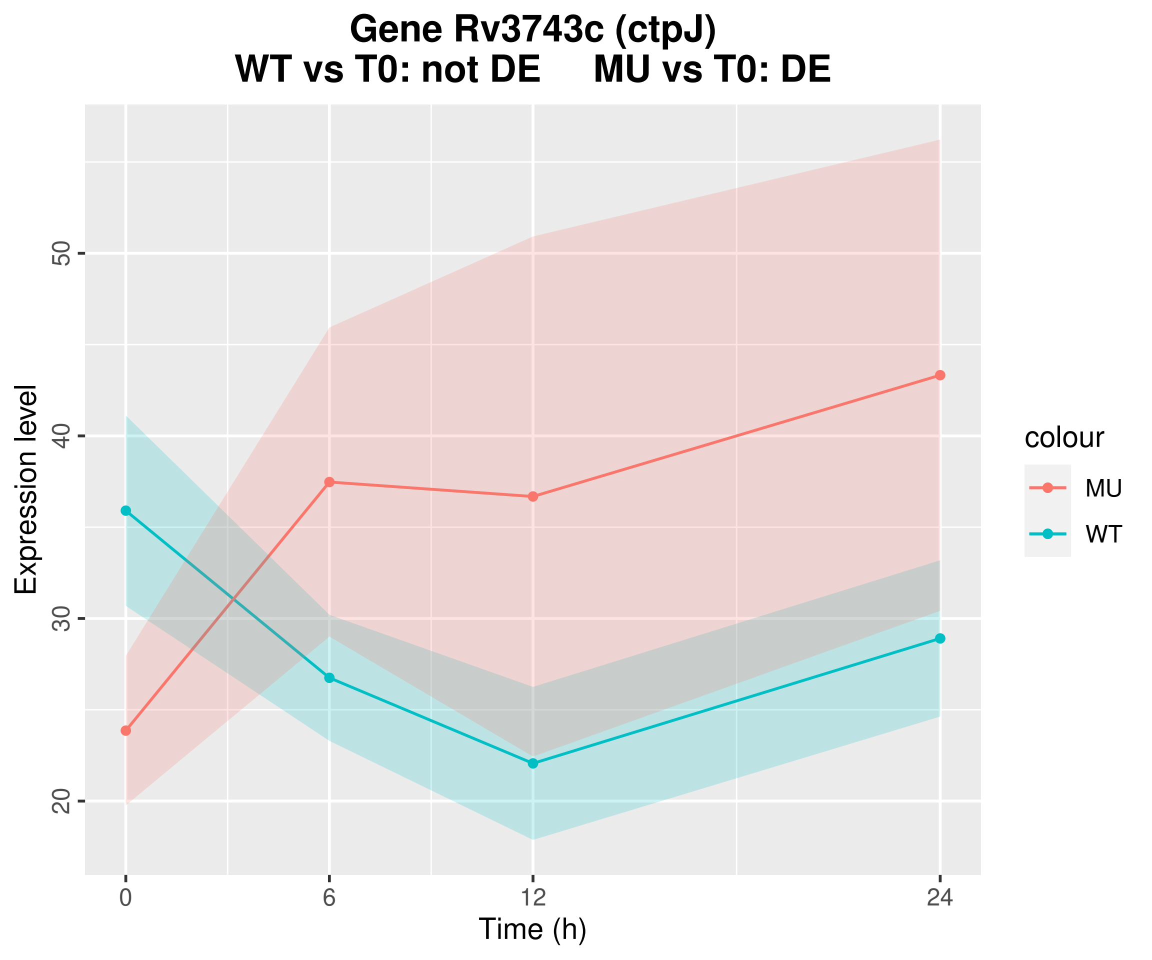

Gene Rv3743c (ctpJ)
WT vs T0: not DE   MU vs T0: DE
Expression level
50
40
30
20
0
6
12
24
Time (h)
colour
MU
WT

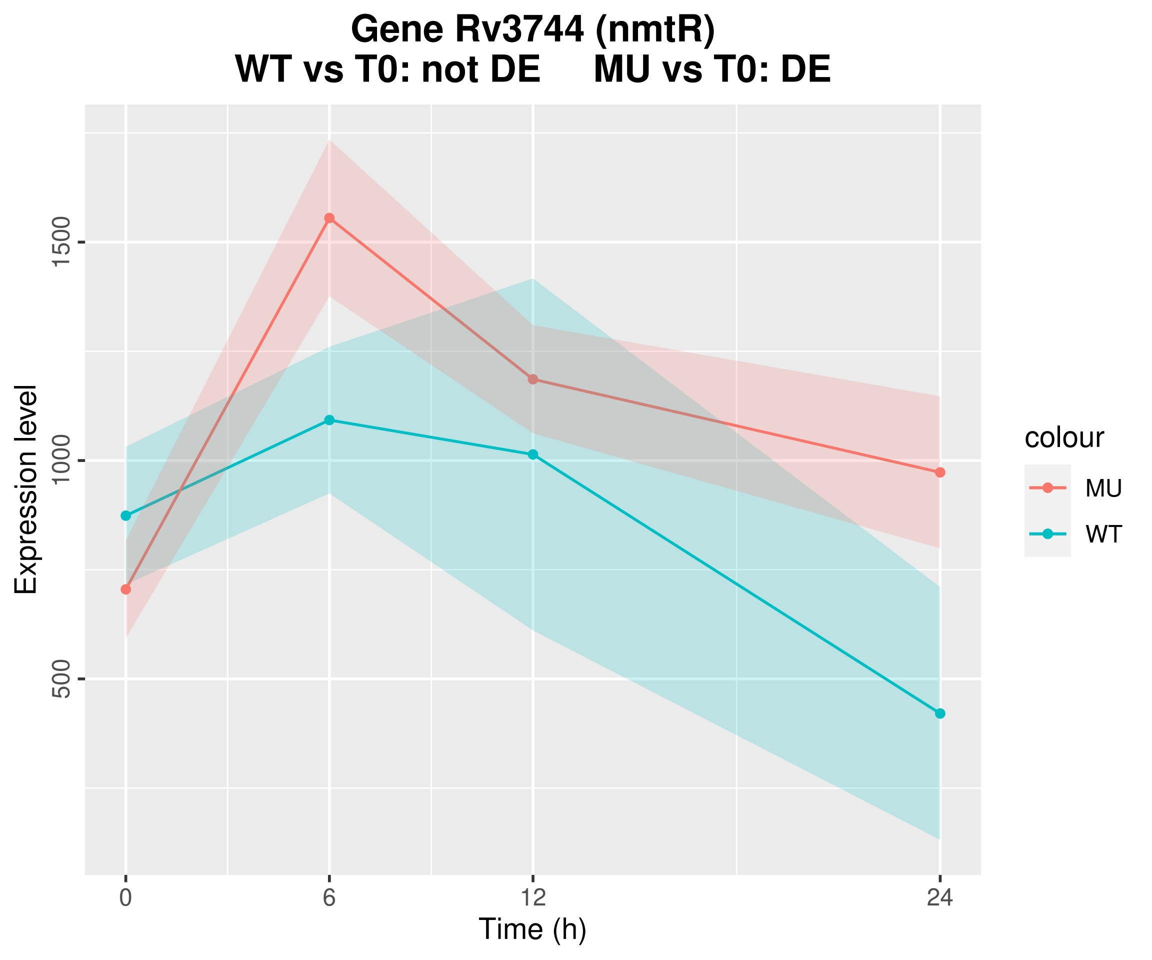
Gene Rv3744 (nmtR)
WT vs T0: not DE   MU vs T0: DE
Expression level
1500
1000
500
0
6
12
24
Time (h)
colour
MU
WT

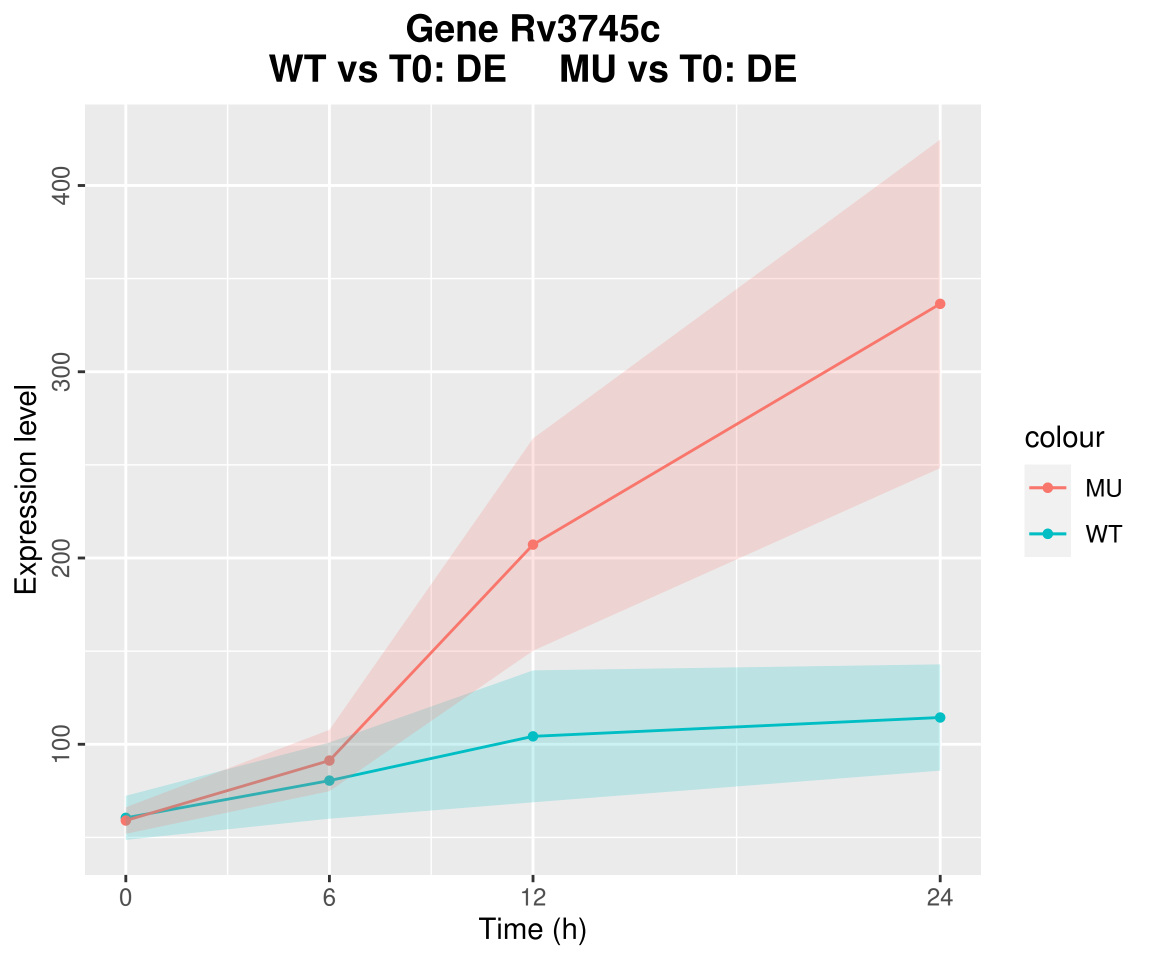

Gene Rv3745c
WT vs T0: DE MU vs T0: DE
Expression level
400
300
200
100
0
6
12
24
Time (h)
colour
MU
WT

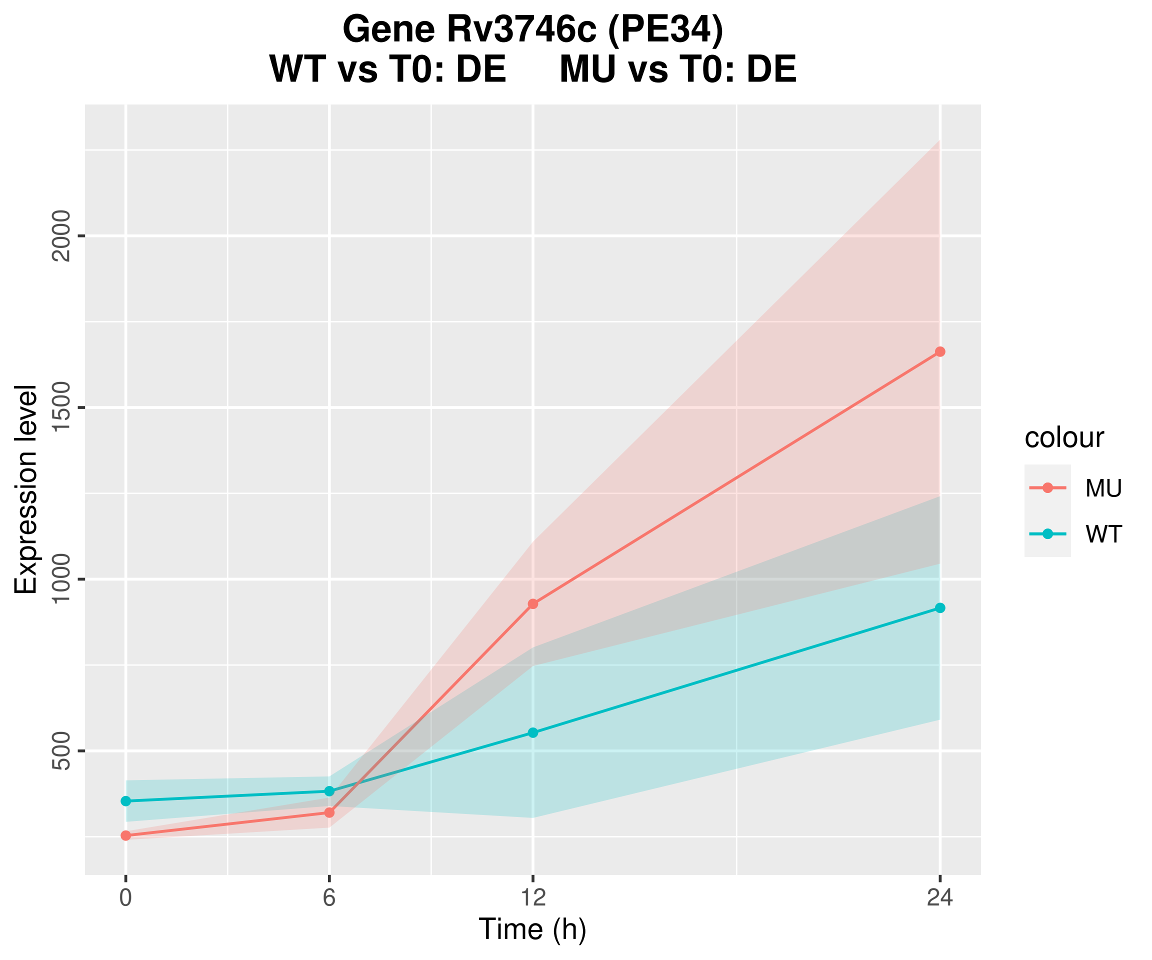

Gene Rv3746c (PE34)
WT vs T0: DE MU vs T0: DE
Expression level
2000
1500
1000
500
0
6
12
24
Time (h)
colour
MU
WT

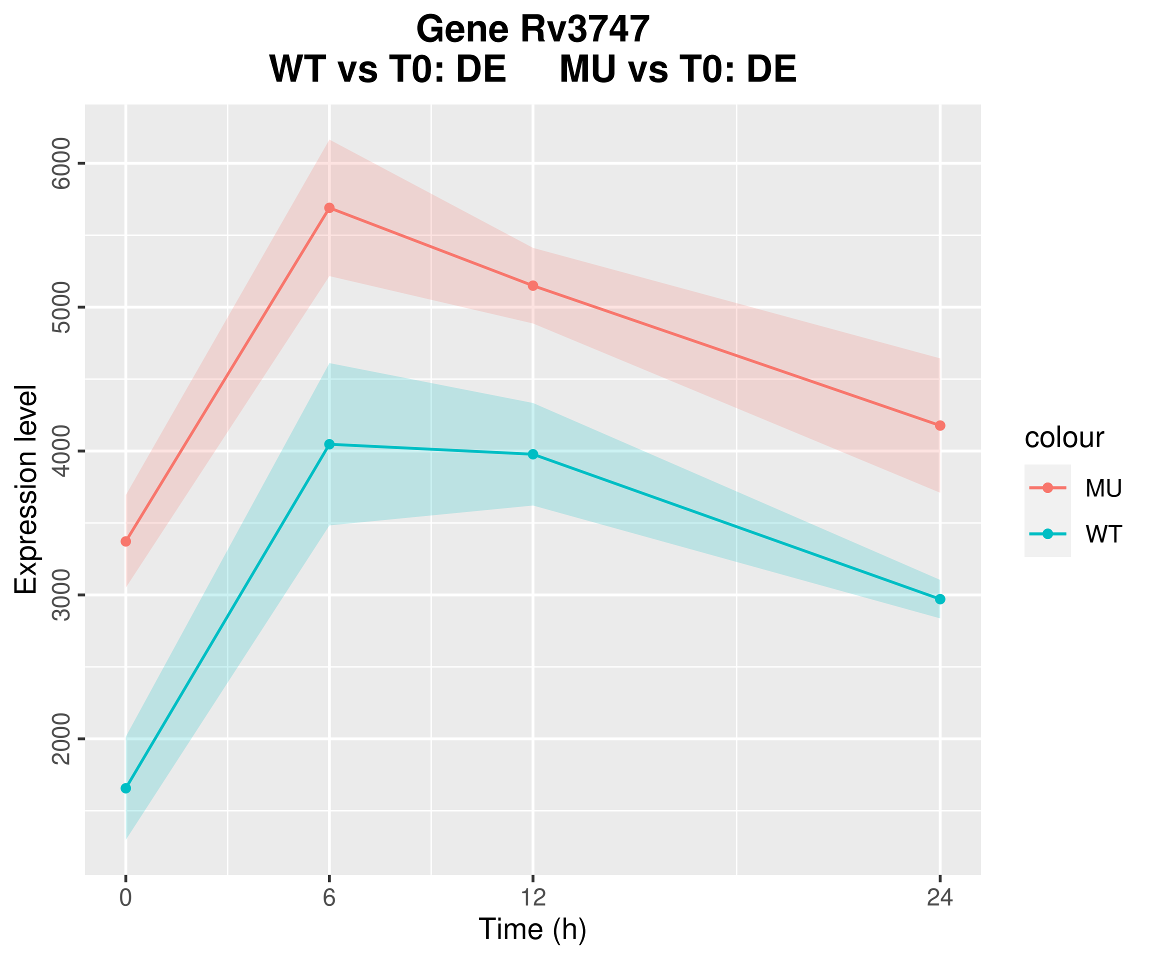
Gene Rv3747
WT vs T0: DE MU vs T0: DE
Expression level
6000
5000
4000
3000
2000
0
6
12
24
Time (h)
colour
MU
WT

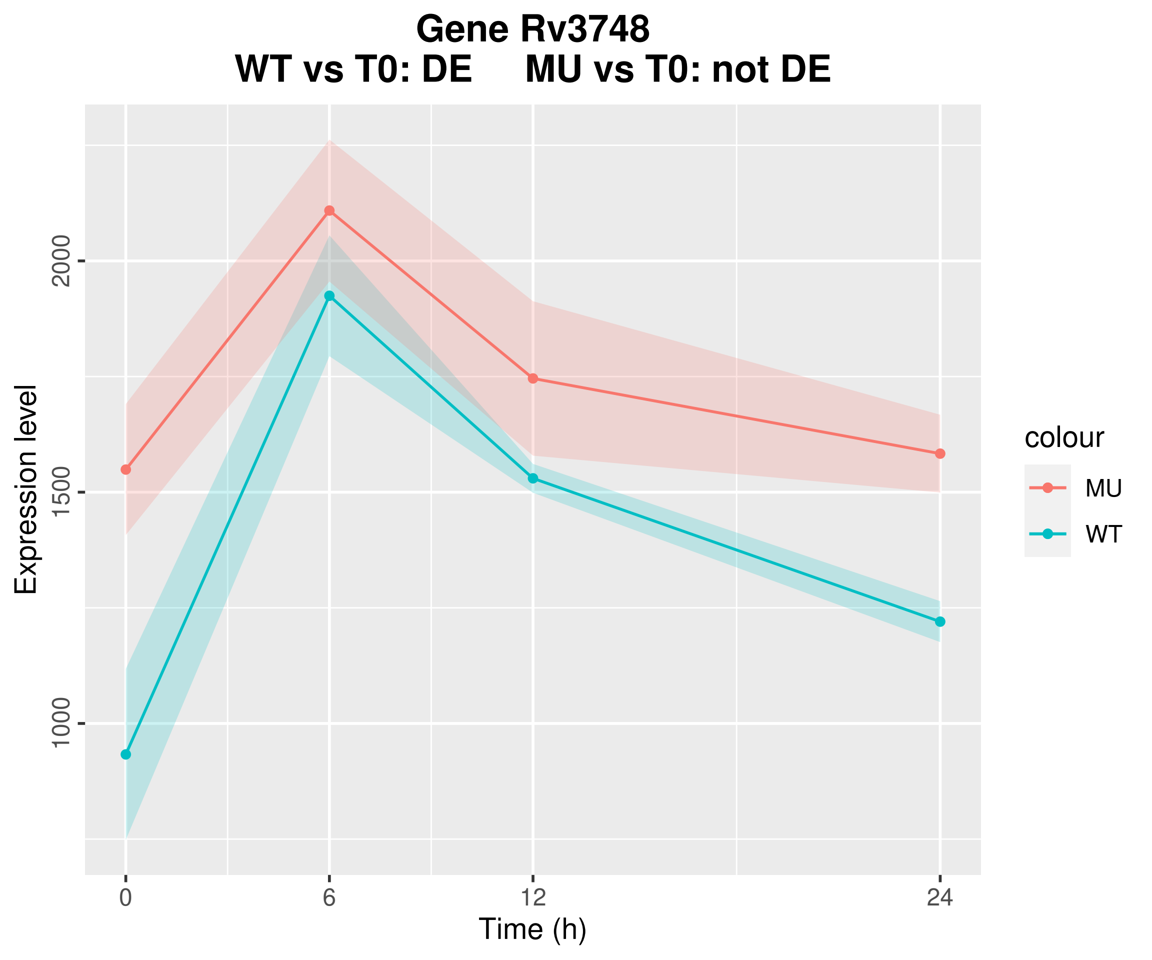
Gene Rv3748
WT vs T0: DE   MU vs T0: not DE
Expression level
2000
1500
1000
0
6
12
24
Time (h)
colour
MU
WT

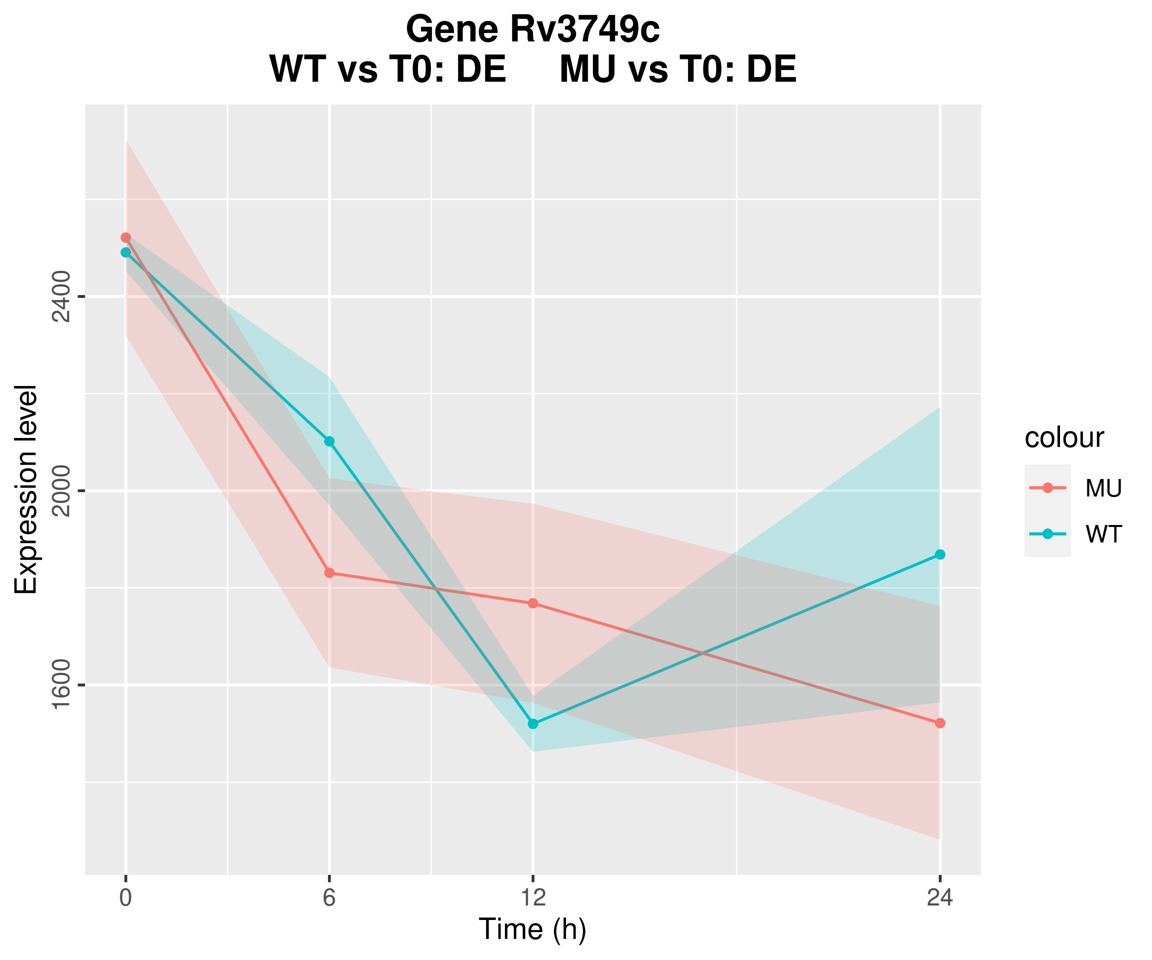
Gene Rv3749c
WT vs T0: DE MU vs T0: DE
Expression level
2400
2000
1600
0
6
12
24
Time (h)
colour
MU
WT

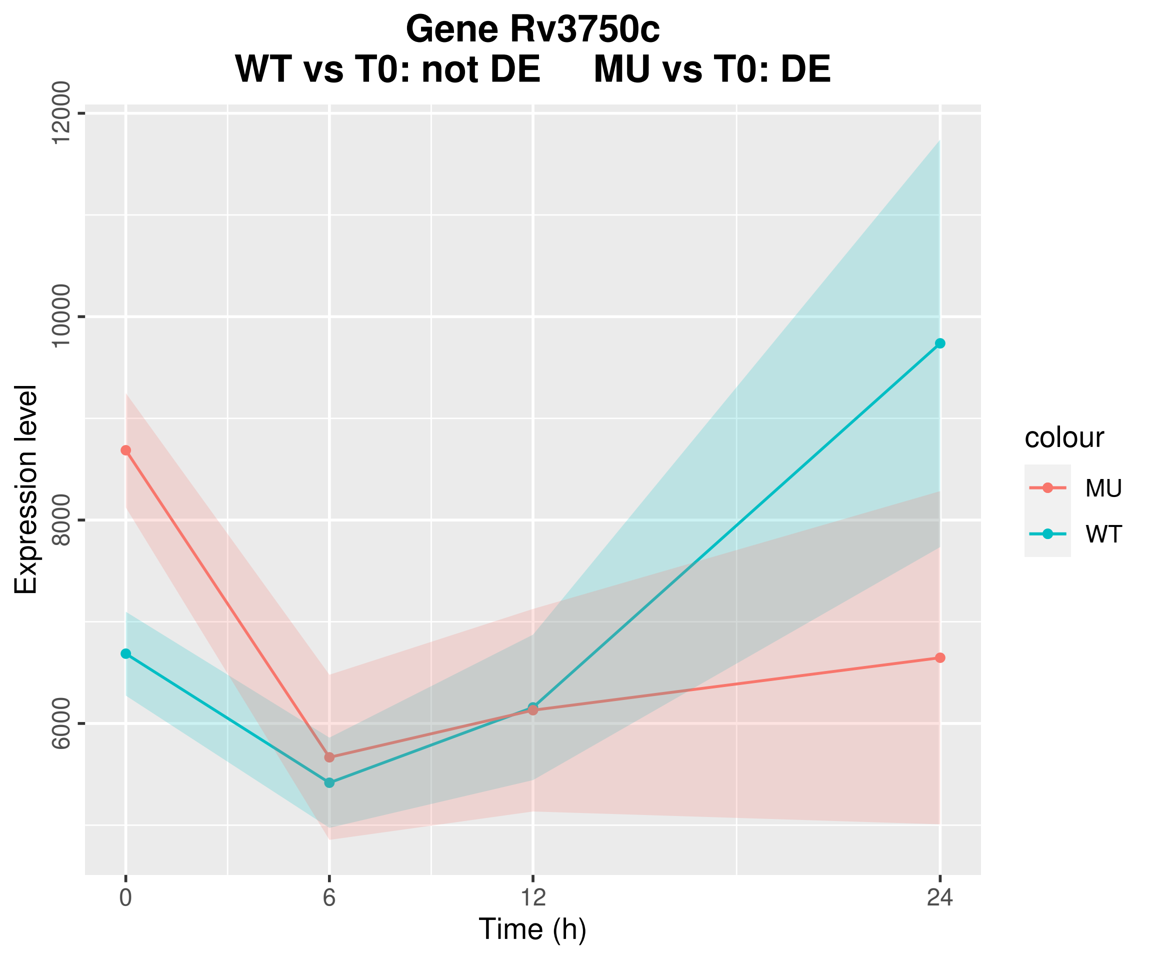
Gene Rv3750c
WT vs T0: not DE   MU vs T0: DE
12000
10000
8000
6000
Expression level
0
6
12
24
Time (h)
colour
MU
WT

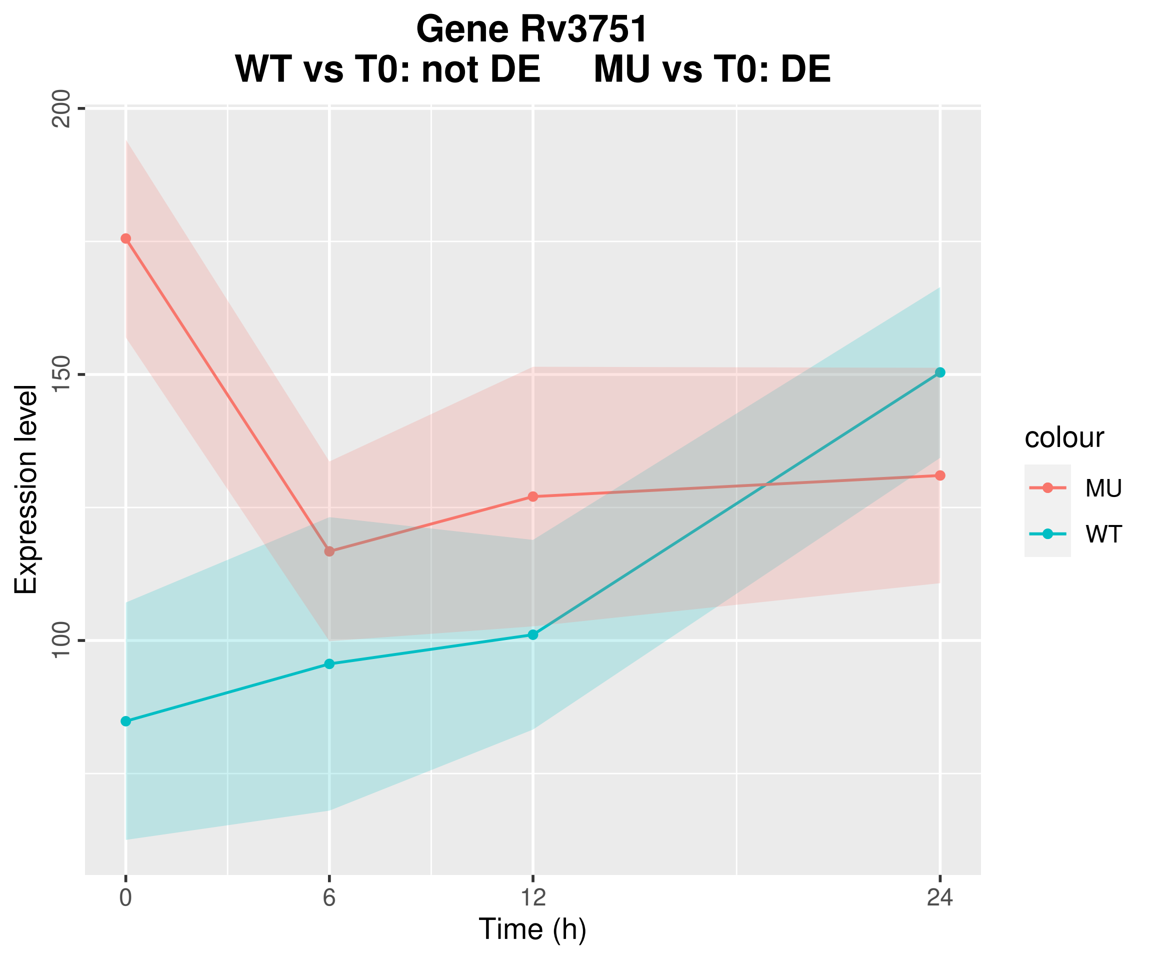

Gene Rv3751
WT vs T0: not DE MU vs T0: DE
200
150
100
Expression level
0
6
12
24
Time (h)
colour
MU
WT

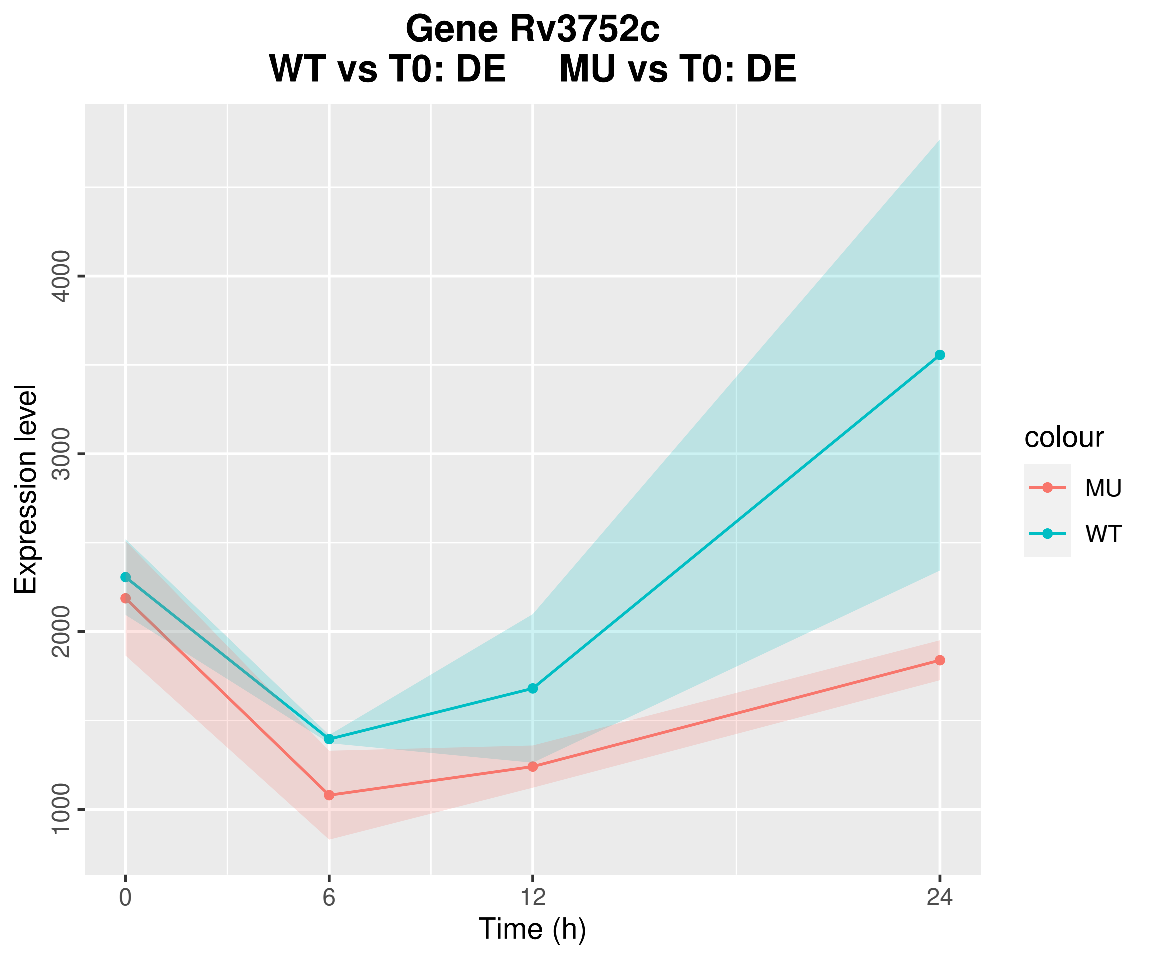

Gene Rv3752c
WT vs T0: DE MU vs T0: DE
Expression level
4000
3000
2000
1000
0
6
12
24
Time (h)
colour
MU
WT

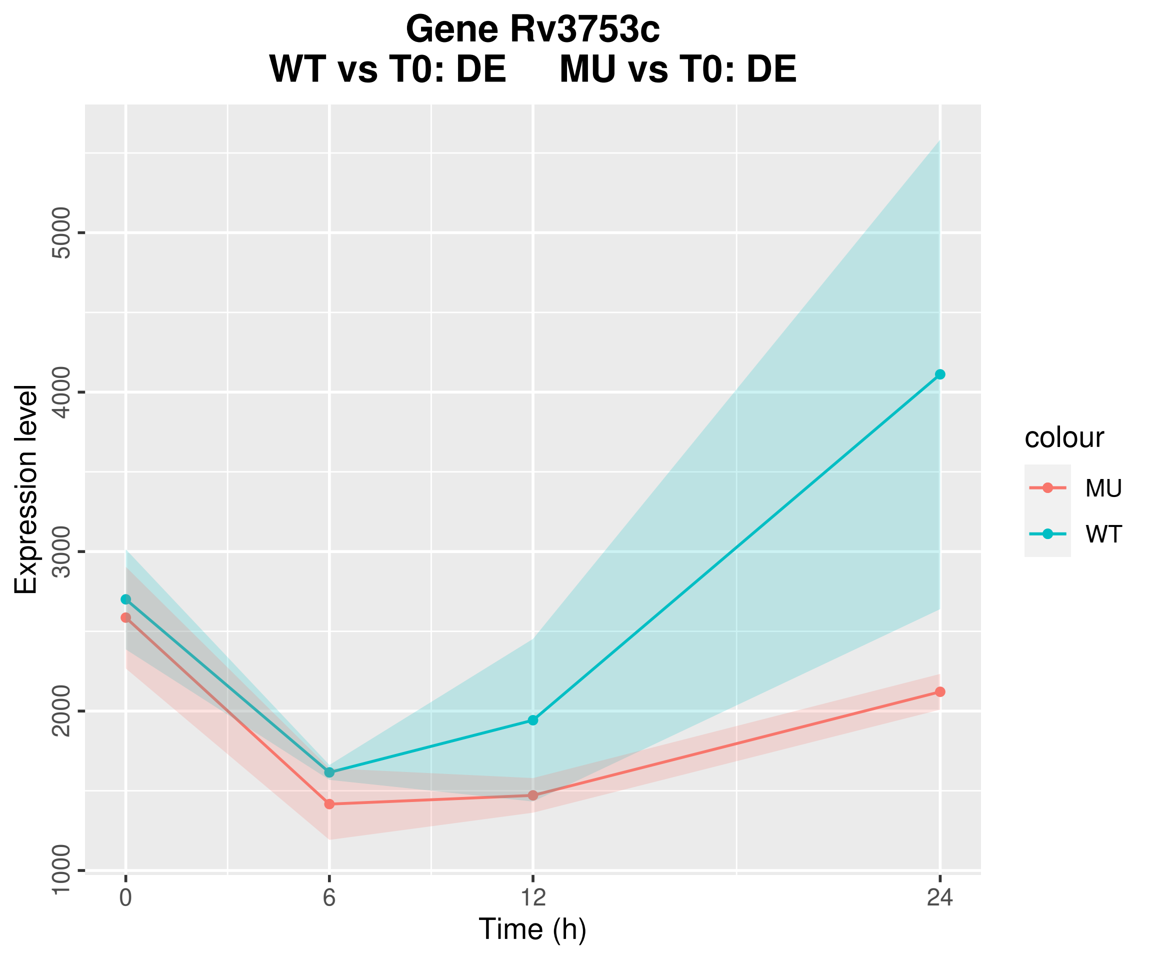
Gene Rv3753c
WT vs T0: DE MU vs T0: DE
Expression level
Time (h)
1000
2000
3000
4000
5000
0
6
12
24
colour
MU
WT

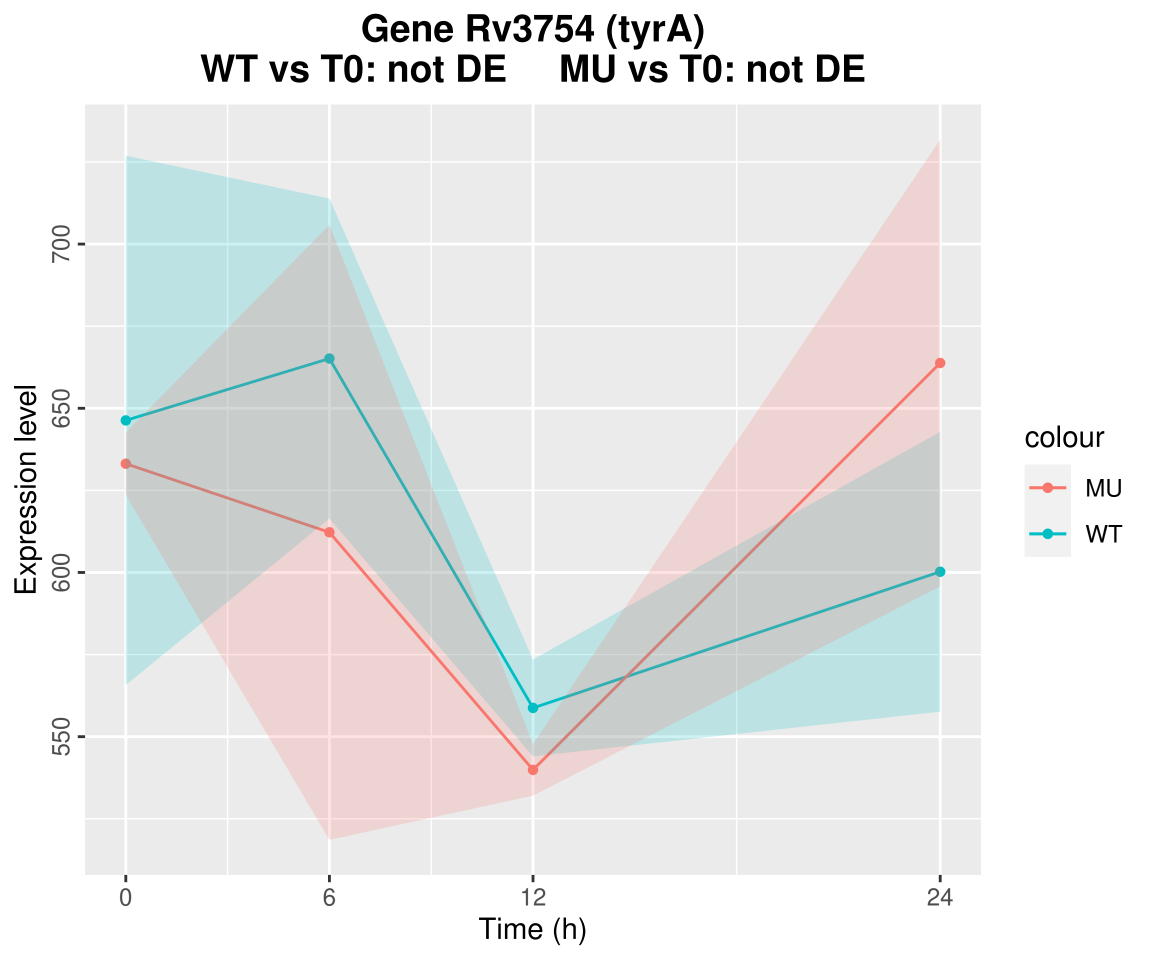
Gene Rv3754 (tyrA)
WT vs T0: not DE MU vs T0: not DE
Expression level
700
650
600
550
0
6
12
24
Time (h)
colour
MU
WT

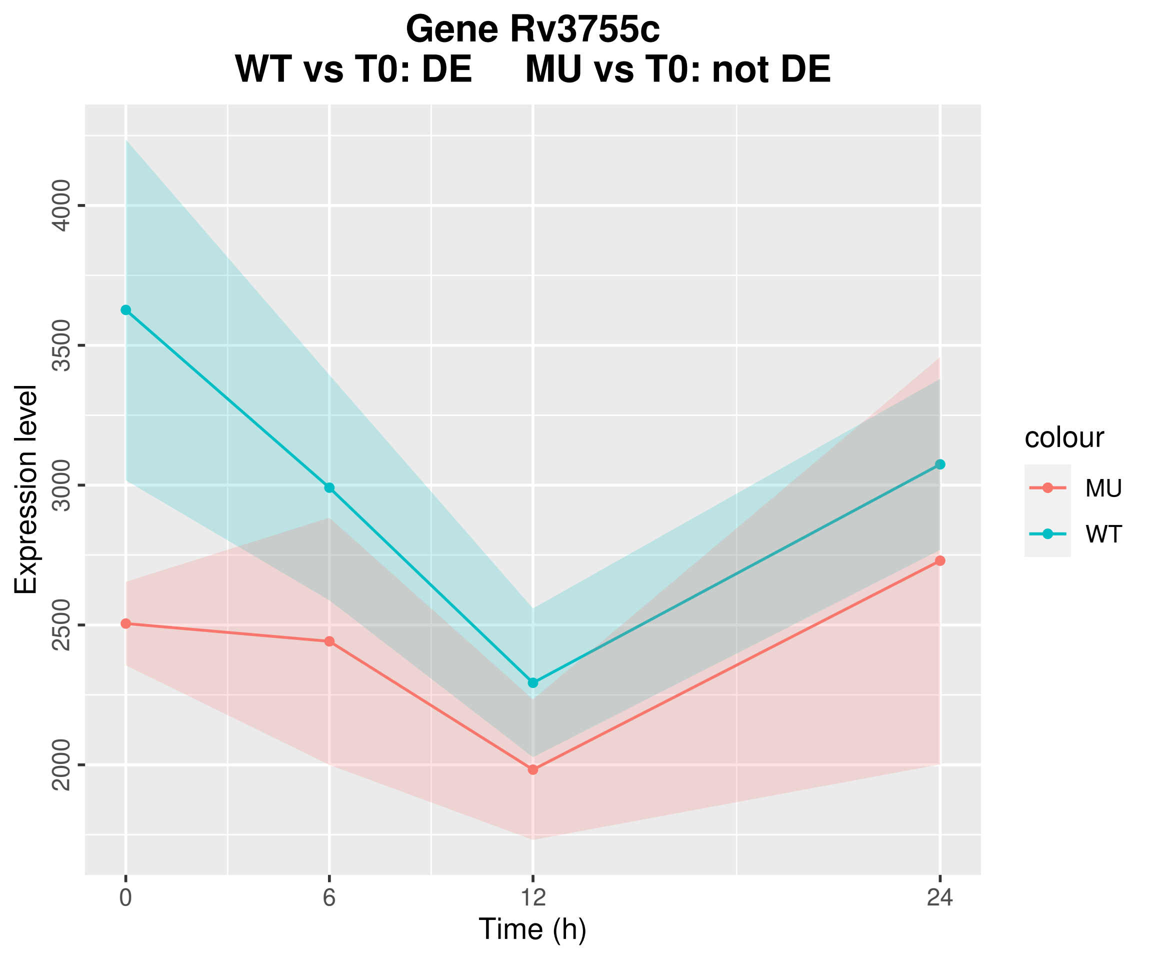
Gene Rv3755c
WT vs T0: DE MU vs T0: not DE
4000
3500
3000
2500
2000
0
6
12
24
Time (h)
Expression level
colour
MU
WT

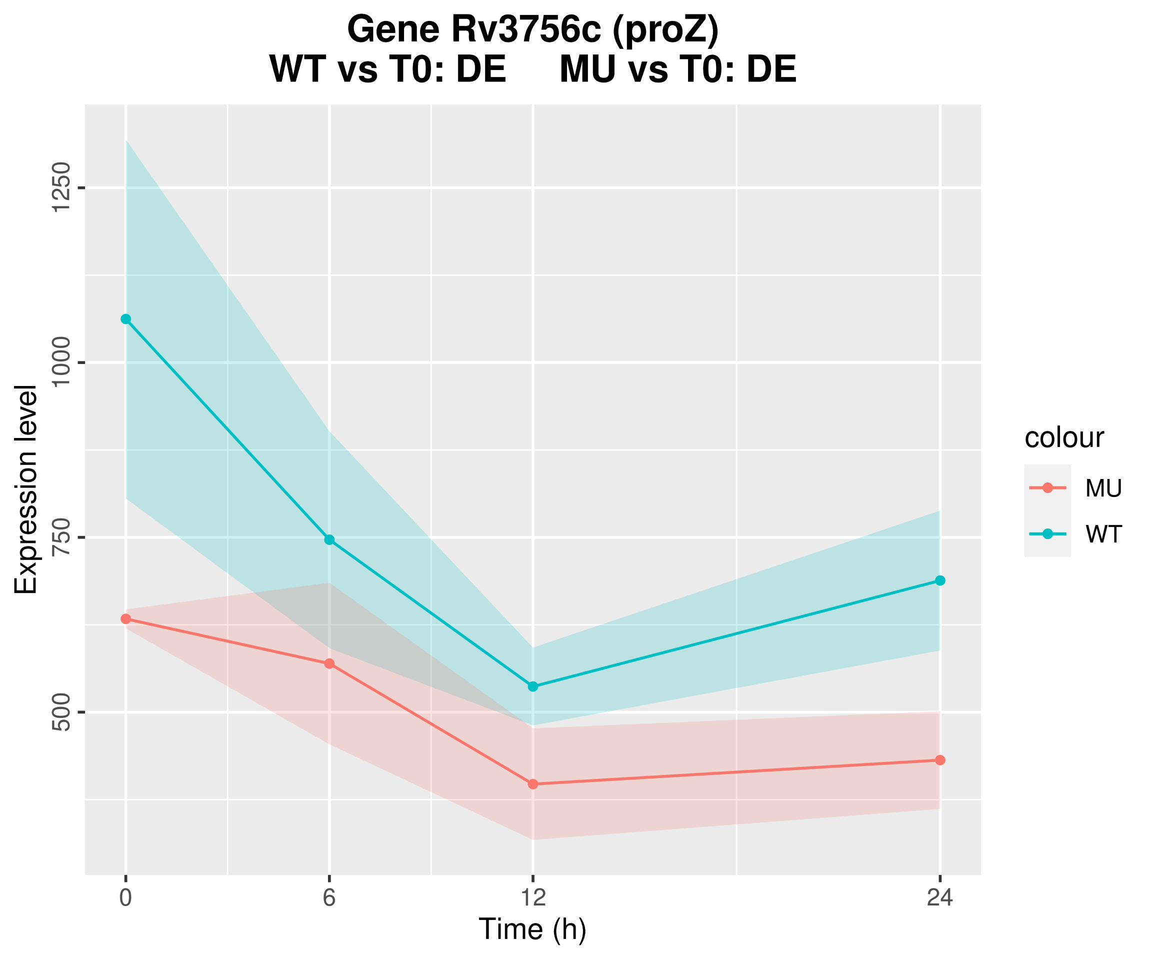

Gene Rv3756c (proZ)
WT vs T0: DE   MU vs T0: DE
Expression level
1250
1000
750
500
0
6
12
24
Time (h)
colour
MU
WT

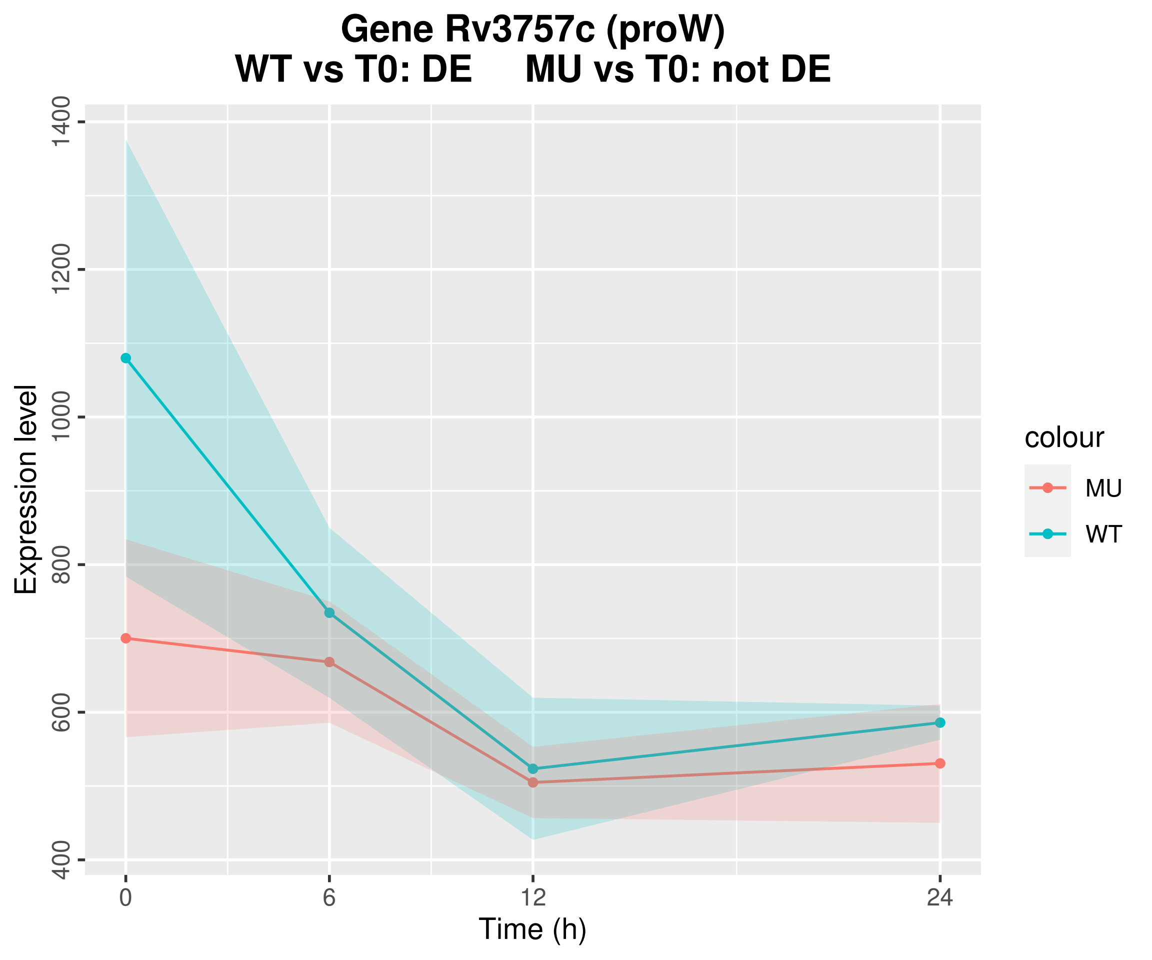

Gene Rv3757c (proW)
WT vs T0: DE   MU vs T0: not DE
Expression level
1400
1200
1000
800
600
400
0
6
12
24
Time (h)
colour
MU
WT

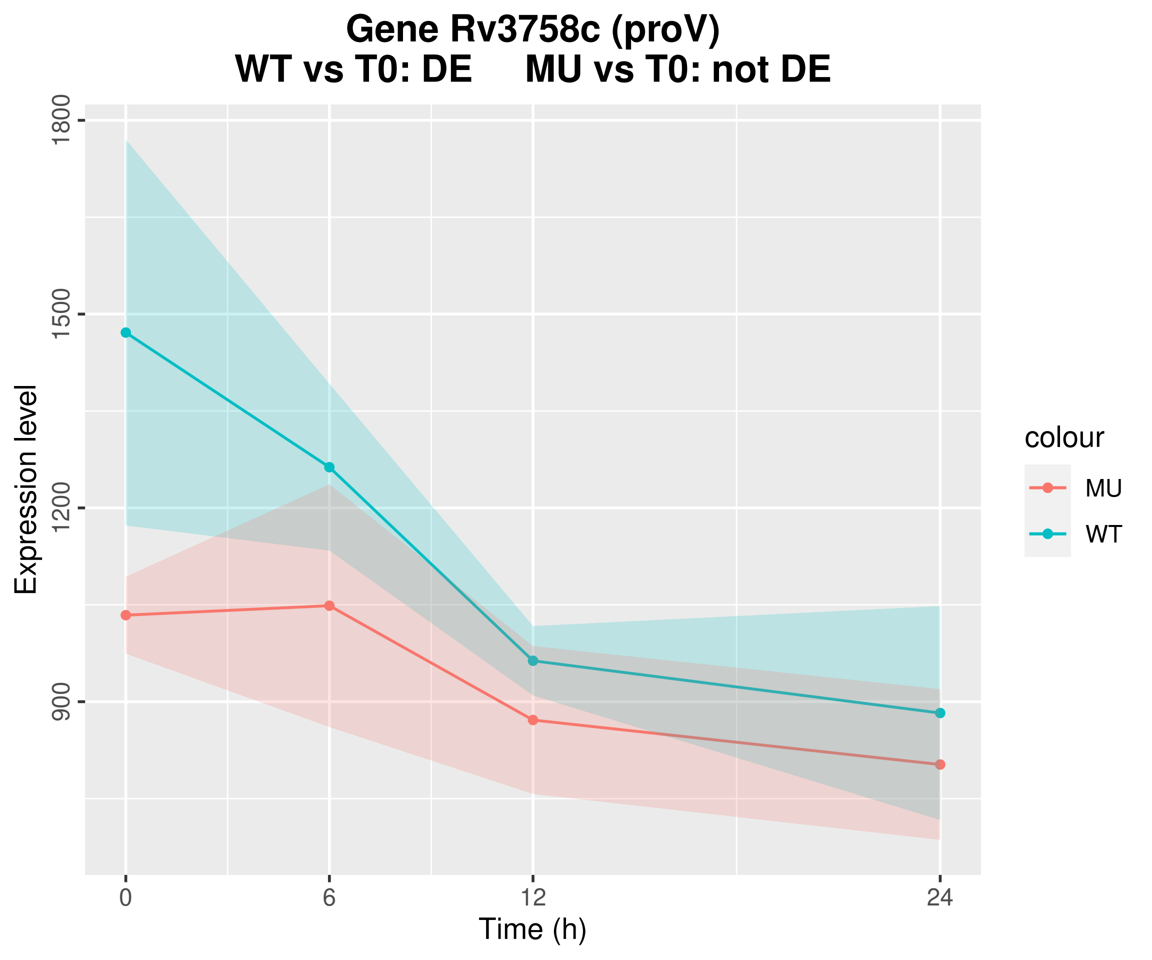
Gene Rv3758c (proV)
WT vs T0: DE   MU vs T0: not DE
Expression level
1800
1500
1200
900
0
6
12
24
Time (h)
colour
MU
WT

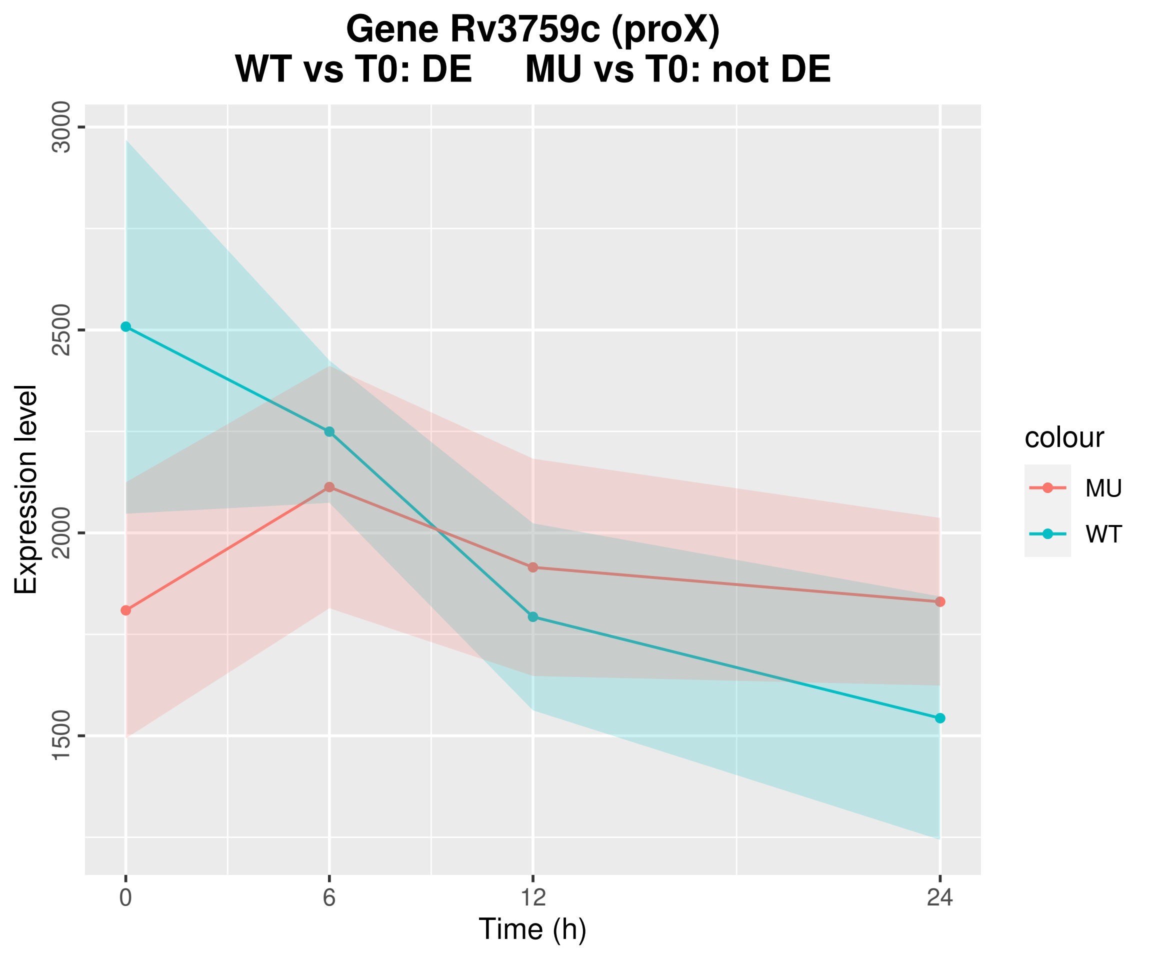
Gene Rv3759c (proX)
WT vs T0: DE   MU vs T0: not DE
Expression level
3000
2500
2000
1500
0
6
12
24
Time (h)
colour
MU
WT

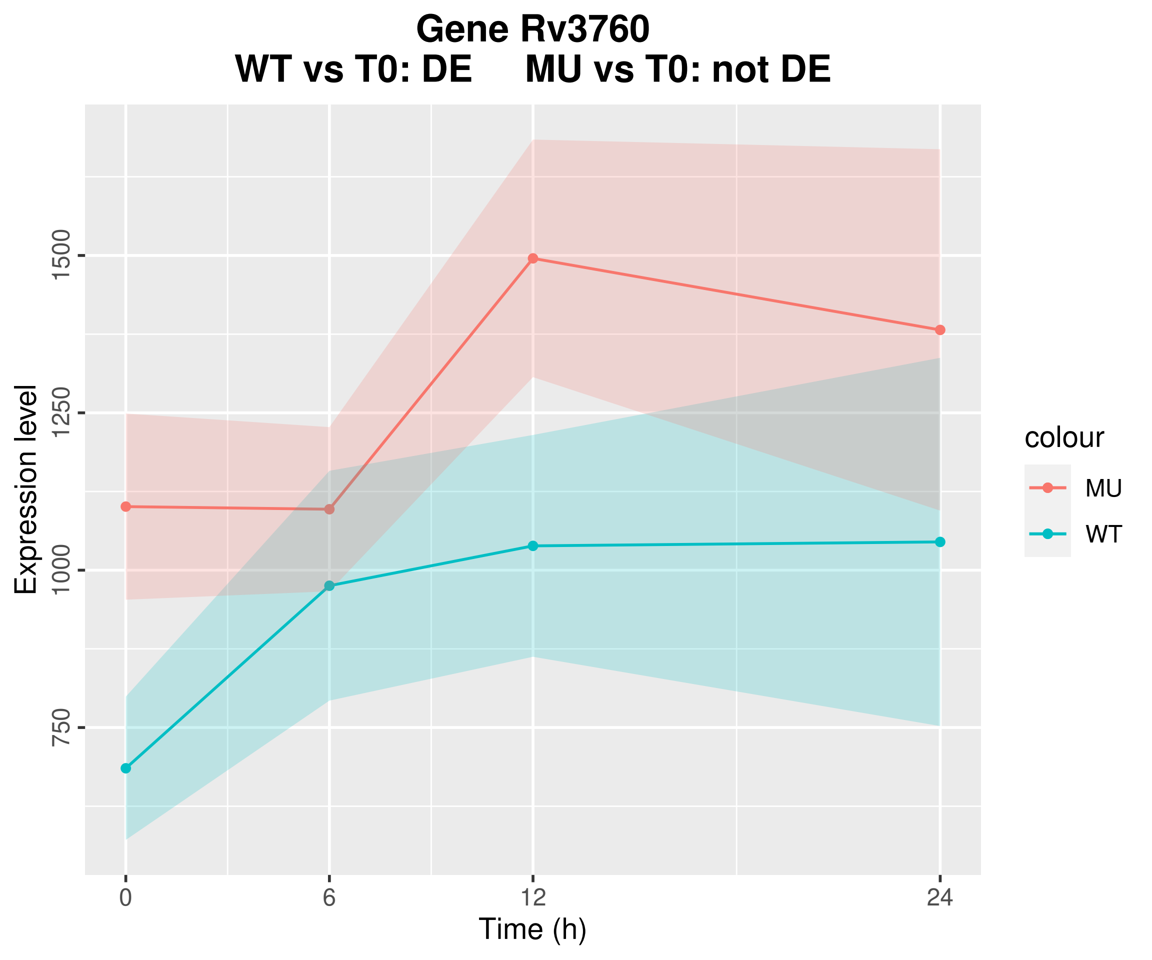

Gene Rv3760
WT vs T0: DE   MU vs T0: not DE
Expression level
1500
1250
1000
750
0
6
12
24
Time (h)
colour
MU
WT

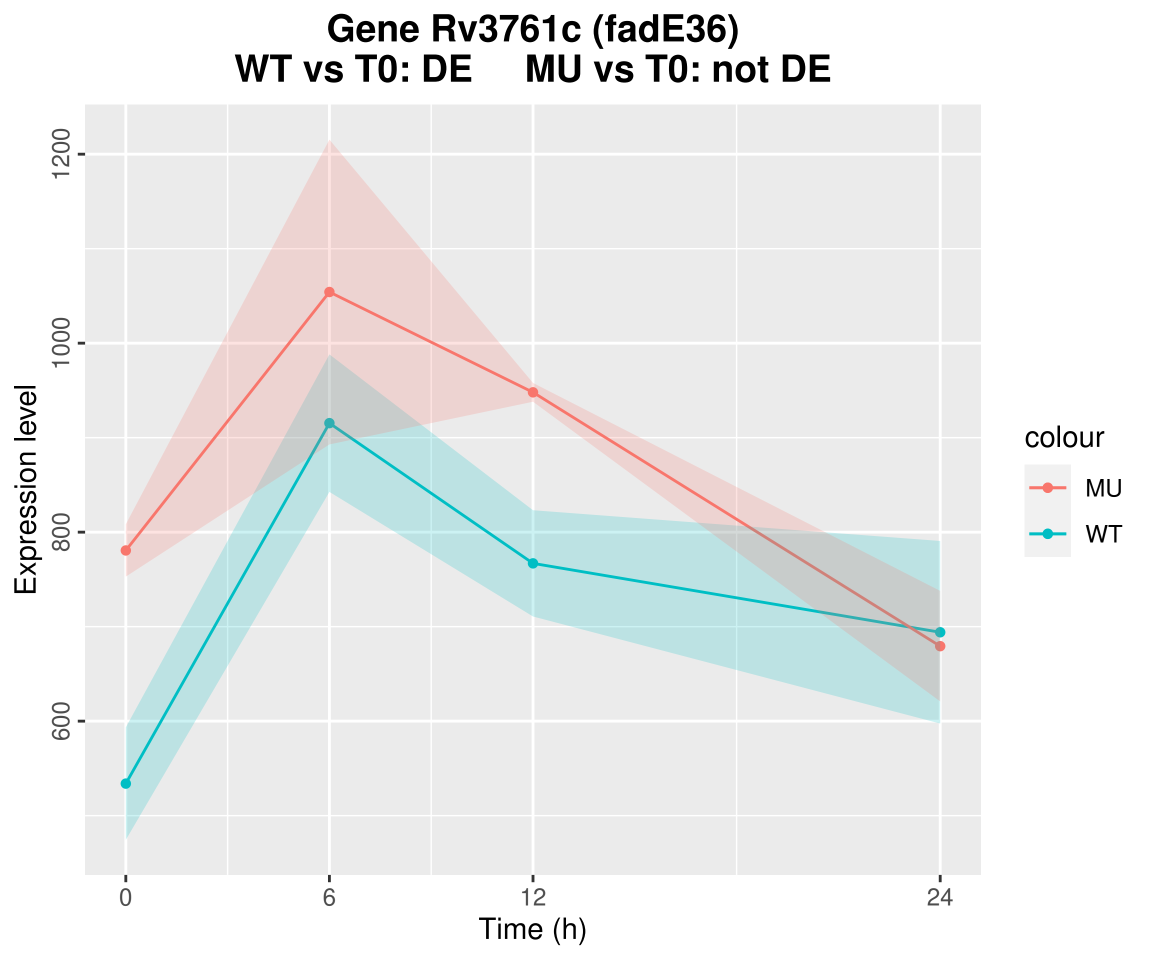
Gene Rv3761c (fadE36)
WT vs T0: DE   MU vs T0: not DE
Expression level
1200
1000
800
600
0
6
12
24
Time (h)
colour
MU
WT

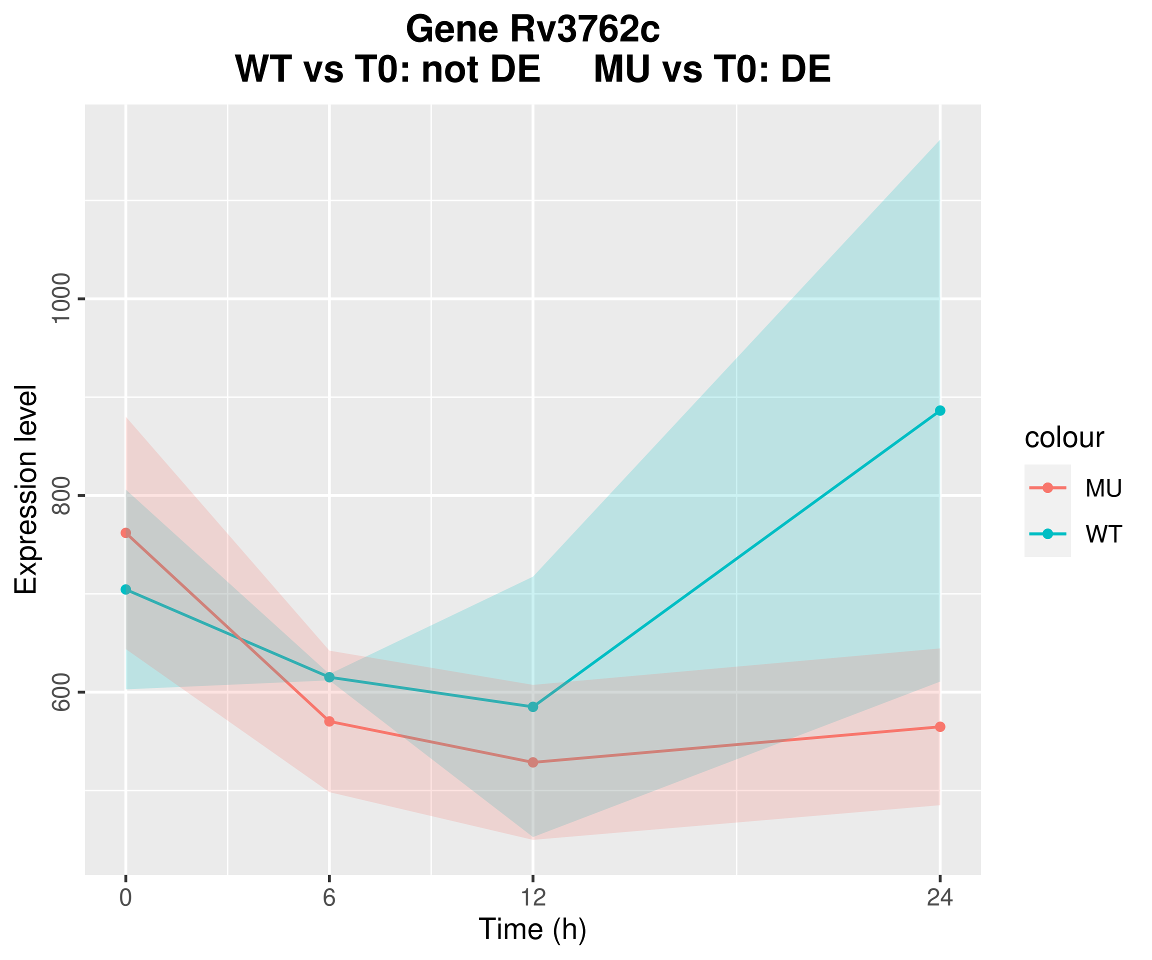
Gene Rv3762c
WT vs T0: not DE   MU vs T0: DE
Expression level
1000
800
600
0
6
12
24
Time (h)
colour
MU
WT

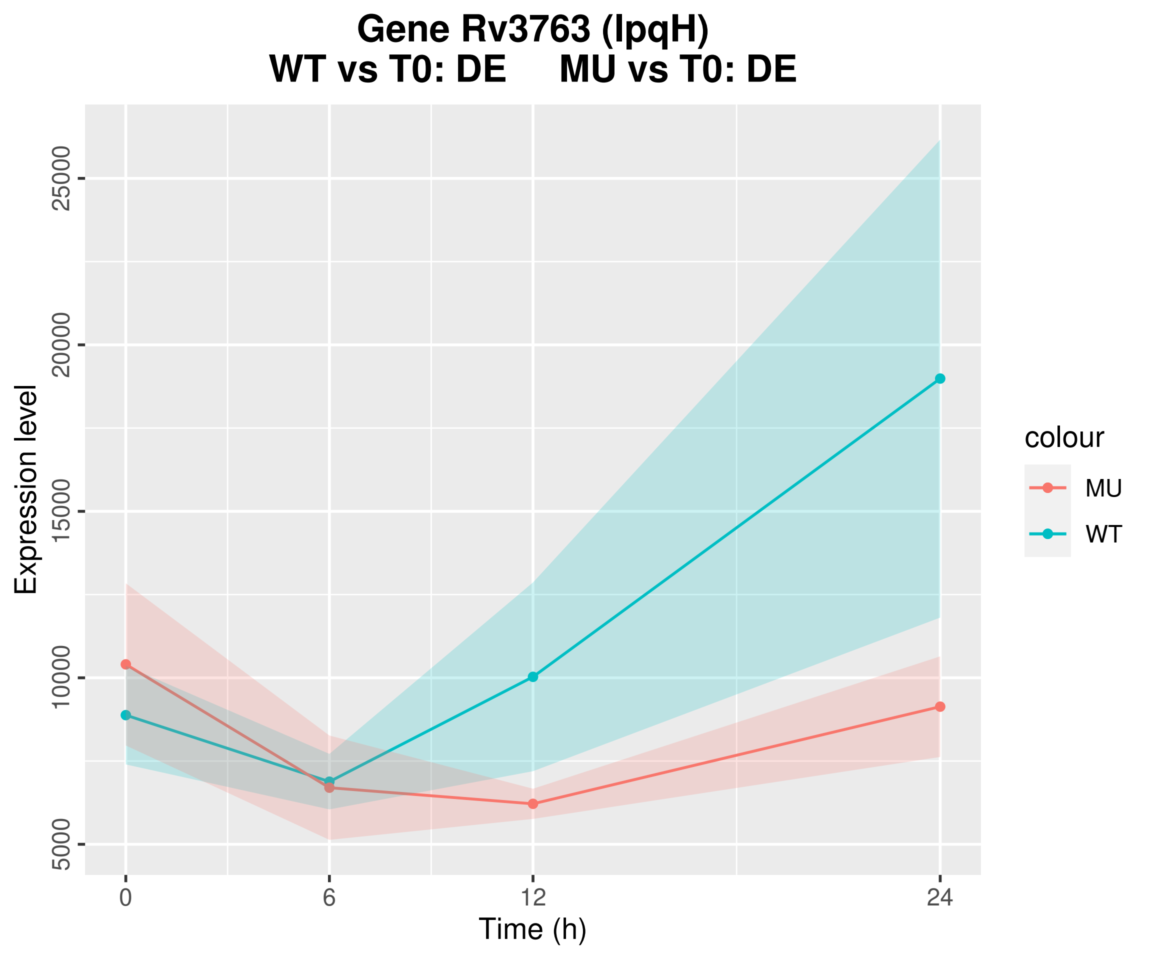
Gene Rv3763 (lpqH)
WT vs T0: DE MU vs T0: DE
Expression level
25000
20000
15000
10000
5000
0
6
12
24
Time (h)
colour
MU
WT

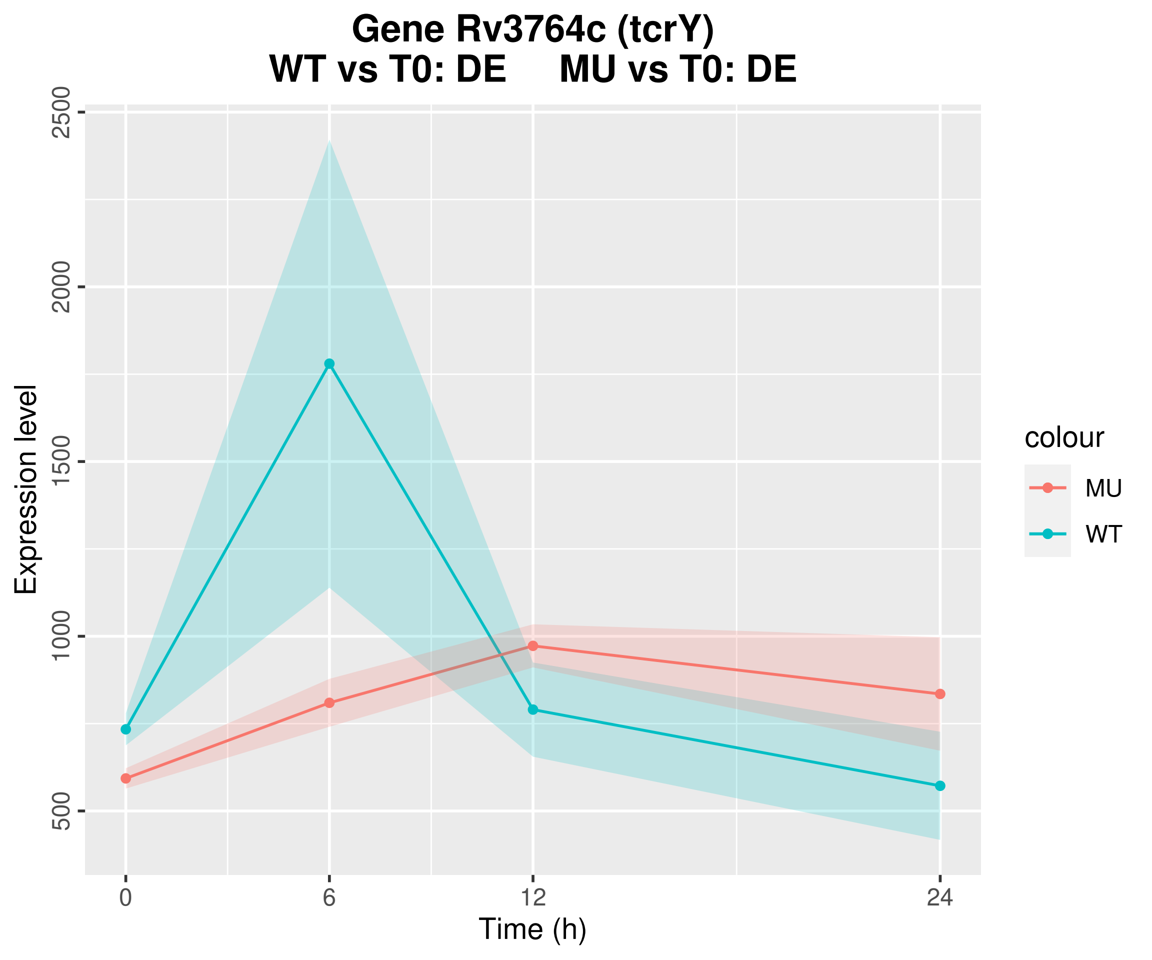

Gene Rv3764c (tcrY)
WT vs T0: DE MU vs T0: DE
Expression level
2500
2000
1500
1000
500
0
6
12
24
Time (h)
colour
MU
WT

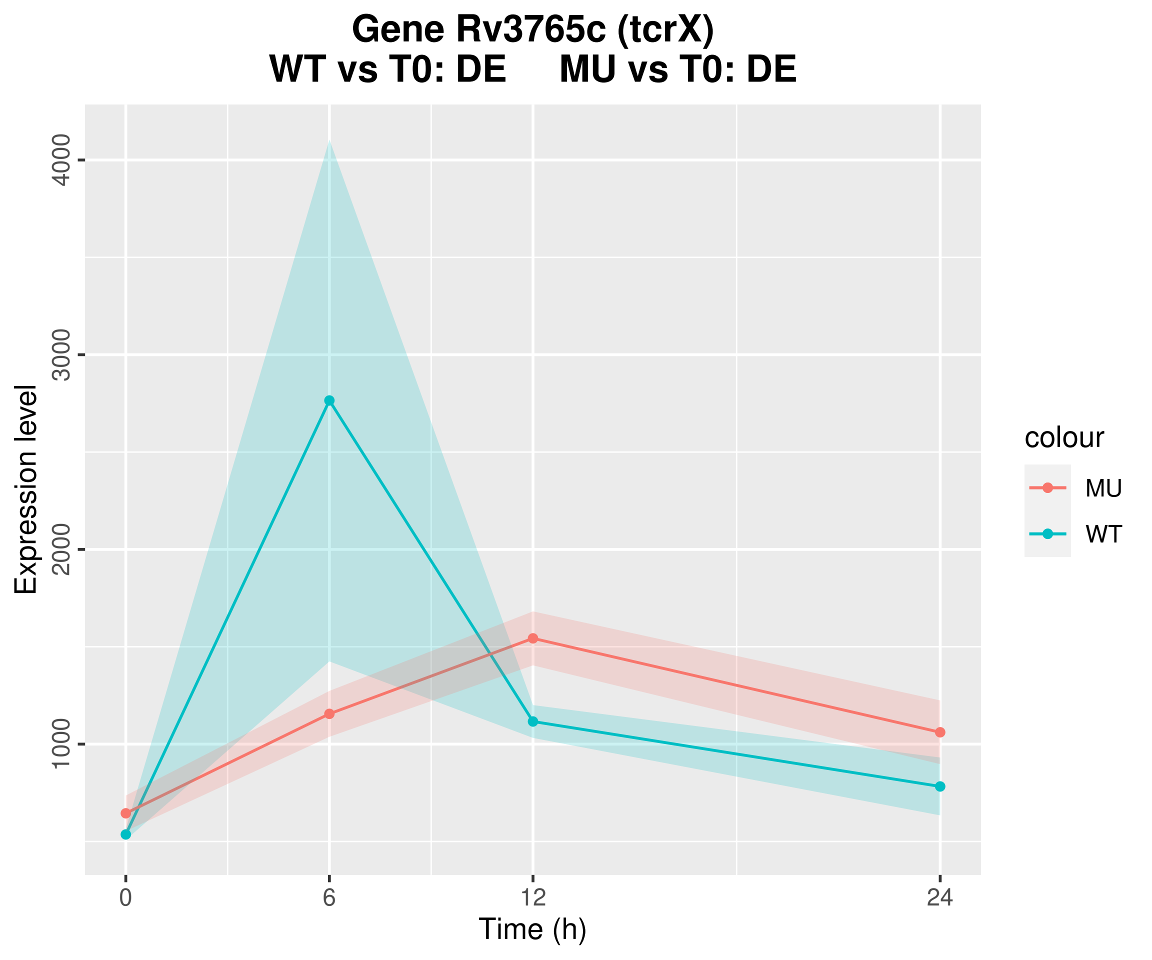

Gene Rv3765c (tcrX)
WT vs T0: DE MU vs T0: DE
Expression level
4000
3000
2000
1000
0
6
12
24
Time (h)
colour
MU
WT

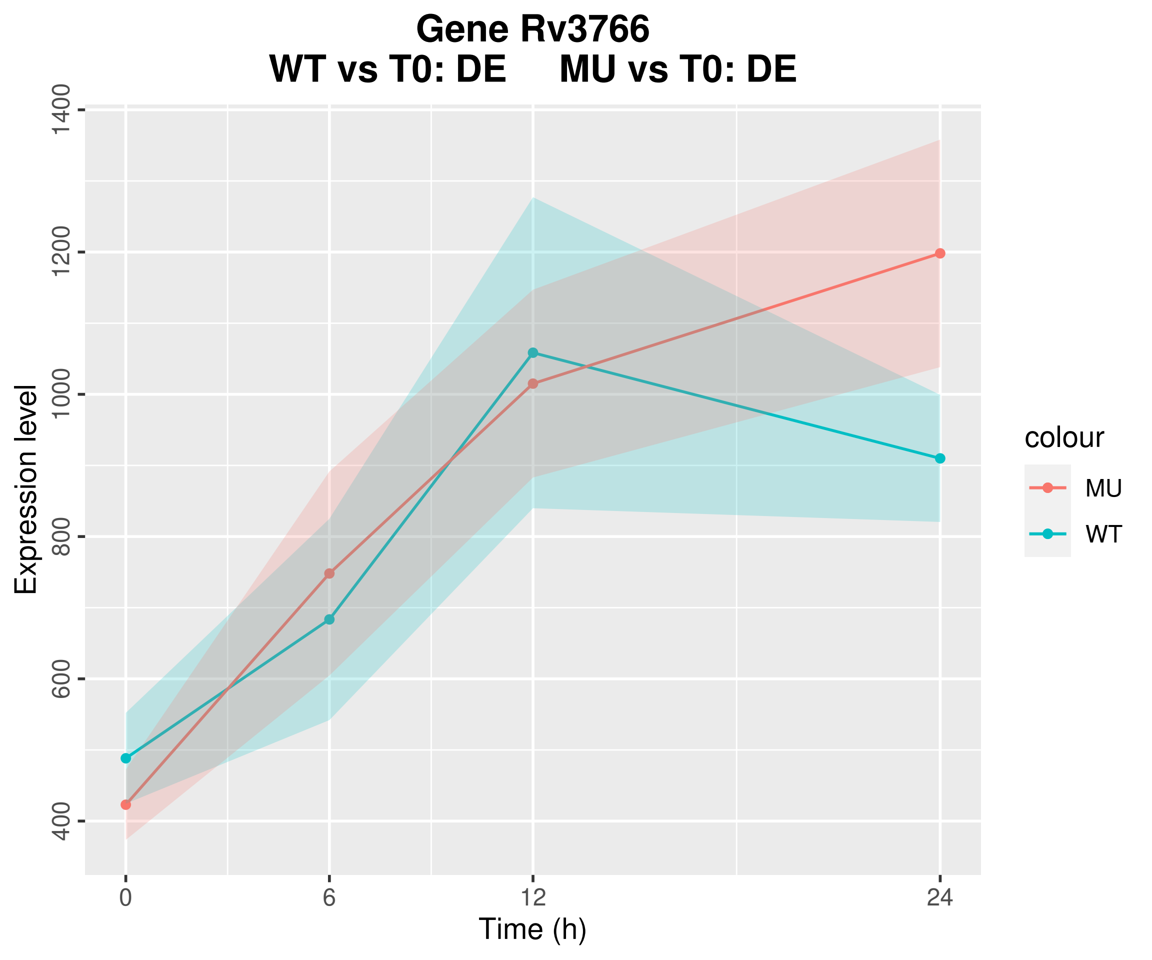
Gene Rv3766
WT vs T0: DE   MU vs T0: DE
Expression level
1400
1200
1000
800
600
400
0
6
12
24
Time (h)
colour
MU
WT

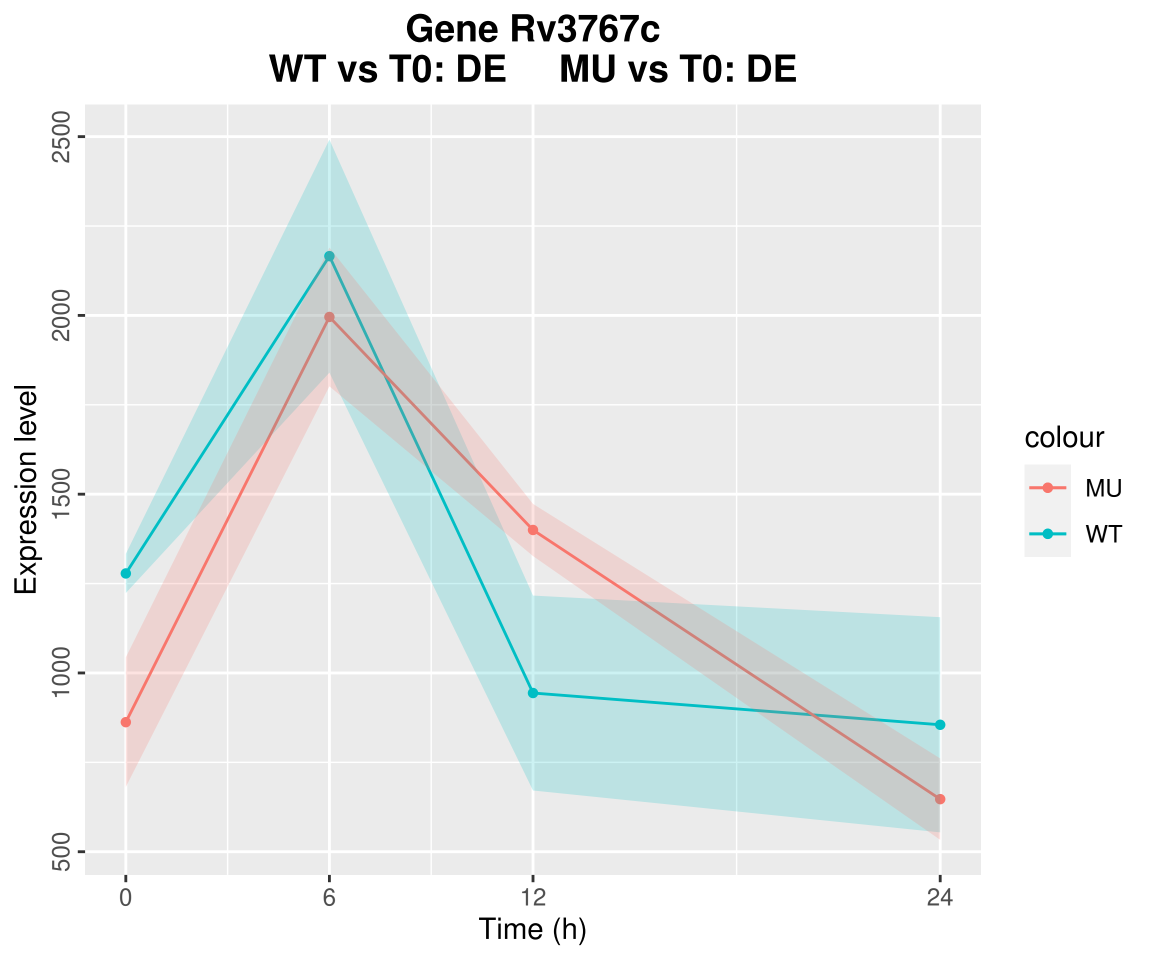
Gene Rv3767c
WT vs T0: DE   MU vs T0: DE
Expression level
2500
2000
1500
1000
500
0
6
12
24
Time (h)
colour
MU
WT

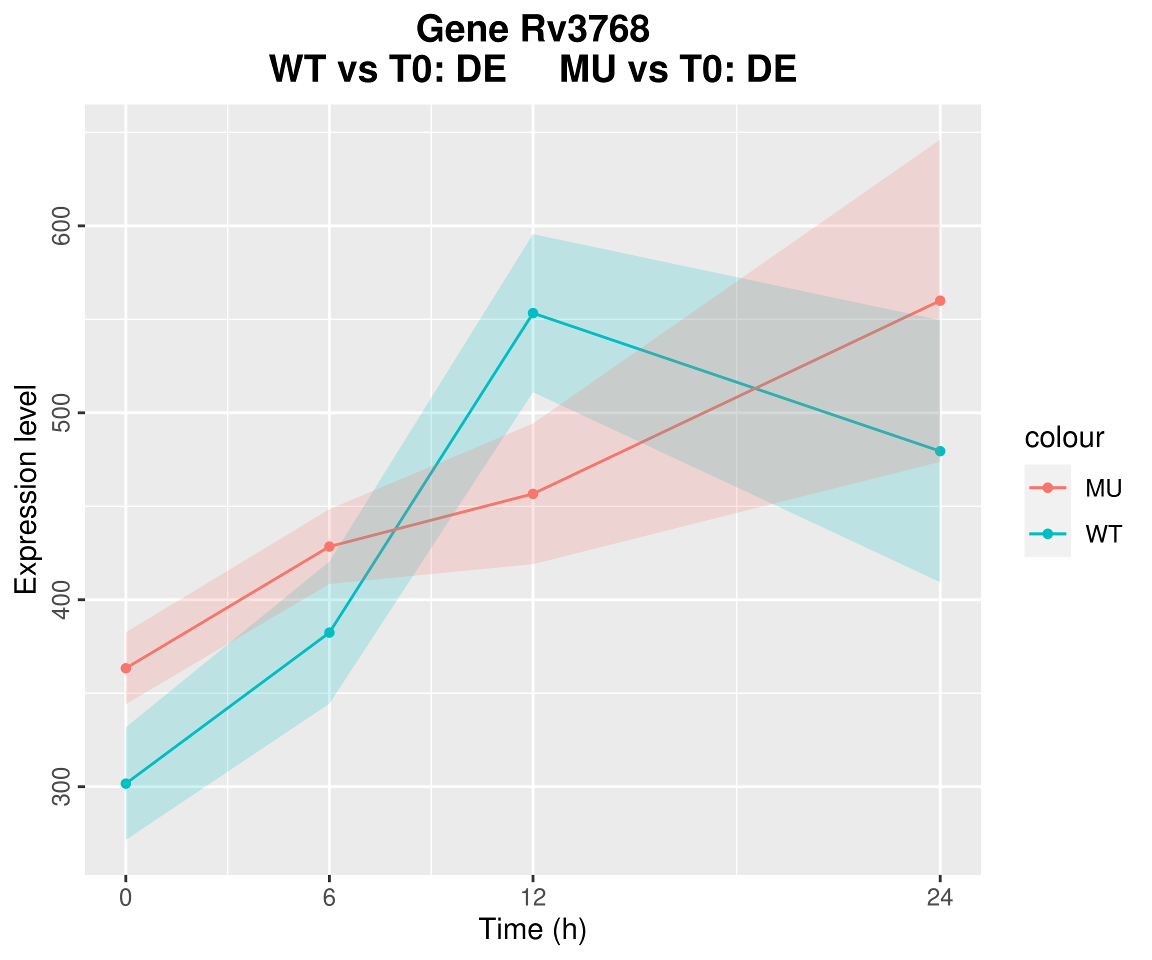
Gene Rv3768
WT vs T0: DE   MU vs T0: DE
Expression level
600
500
400
300
0
6
12
24
Time (h)
colour
MU
WT

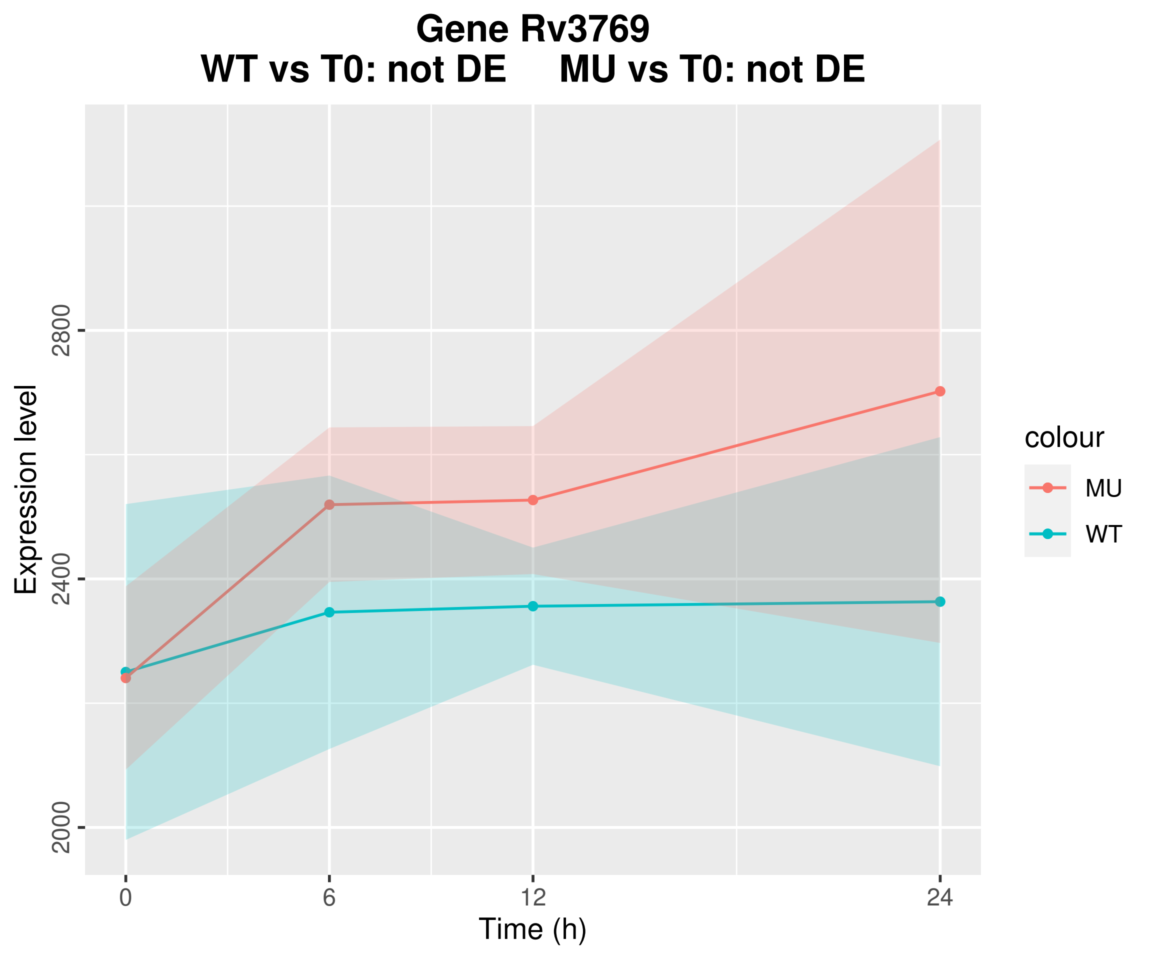
Gene Rv3769
WT vs T0: not DE MU vs T0: not DE
Expression level
2000
2400
2800
0
6
12
24
Time (h)
colour
MU
WT

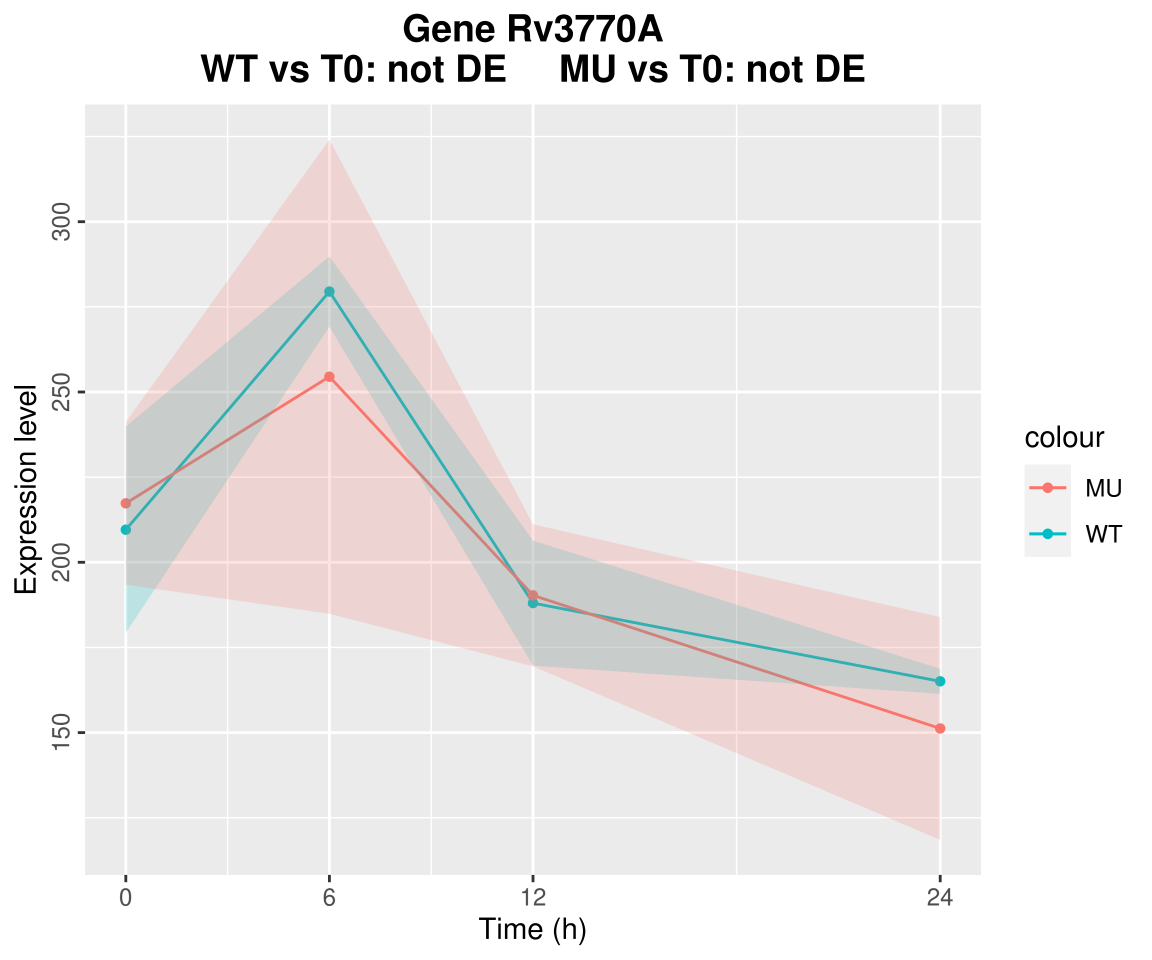
Gene Rv3770A
WT vs T0: not DE   MU vs T0: not DE
Expression level
300
250
200
150
0
6
12
24
Time (h)
colour
MU
WT

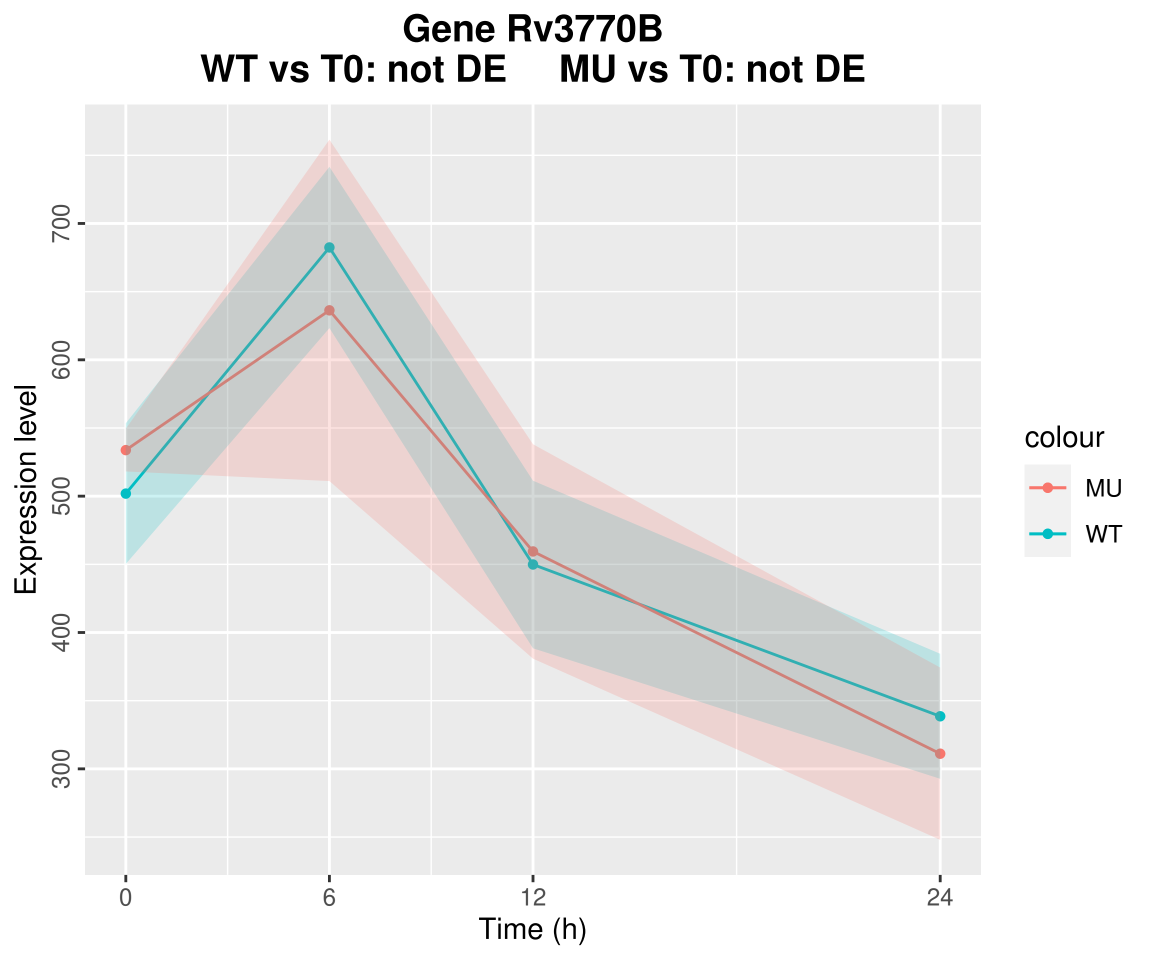
Gene Rv3770B
WT vs T0: not DE   MU vs T0: not DE
Expression level
700
600
500
400
300
0
6
12
24
Time (h)
colour
MU
WT

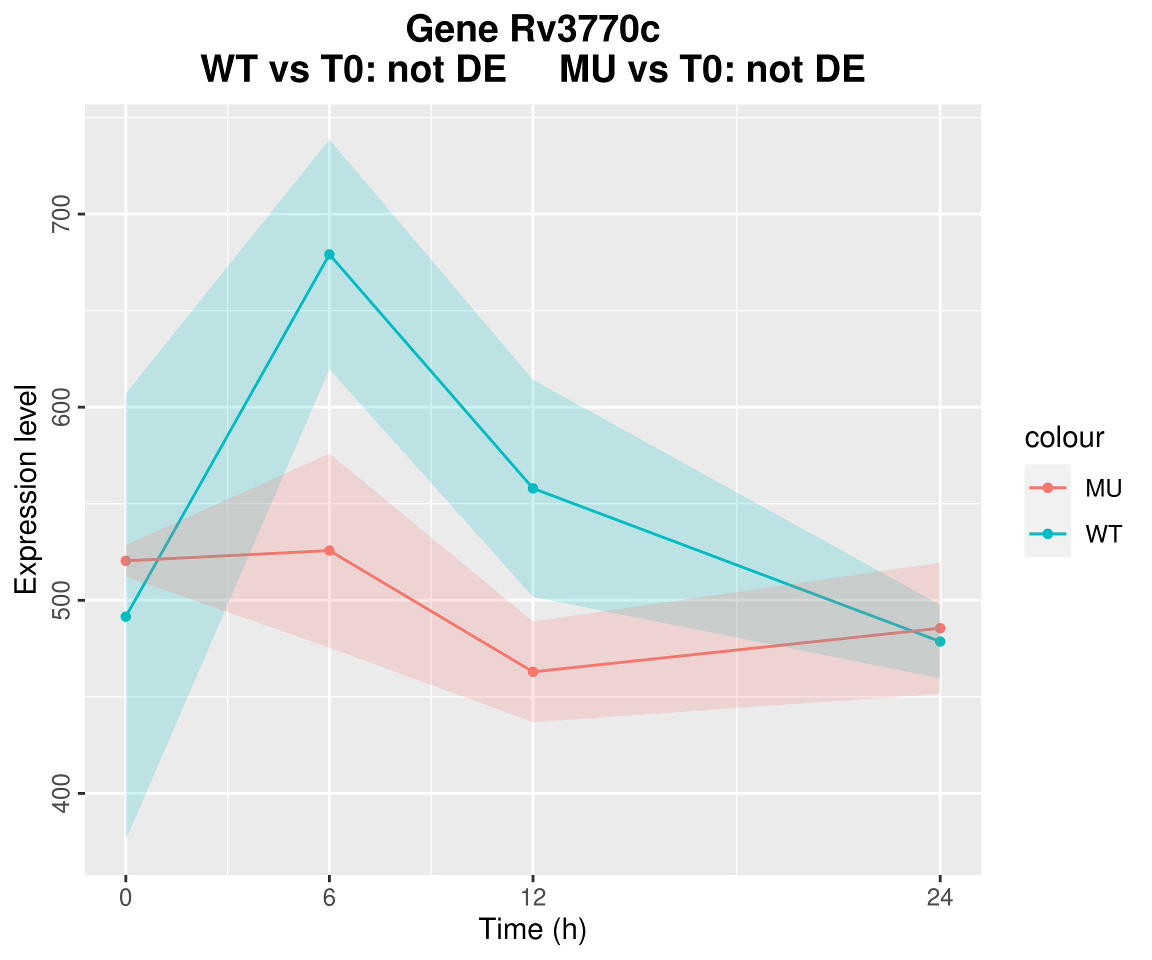
Gene Rv3770c
WT vs T0: not DE MU vs T0: not DE
Expression level
400
500
600
700
0
6
12
24
Time (h)
colour
MU
WT

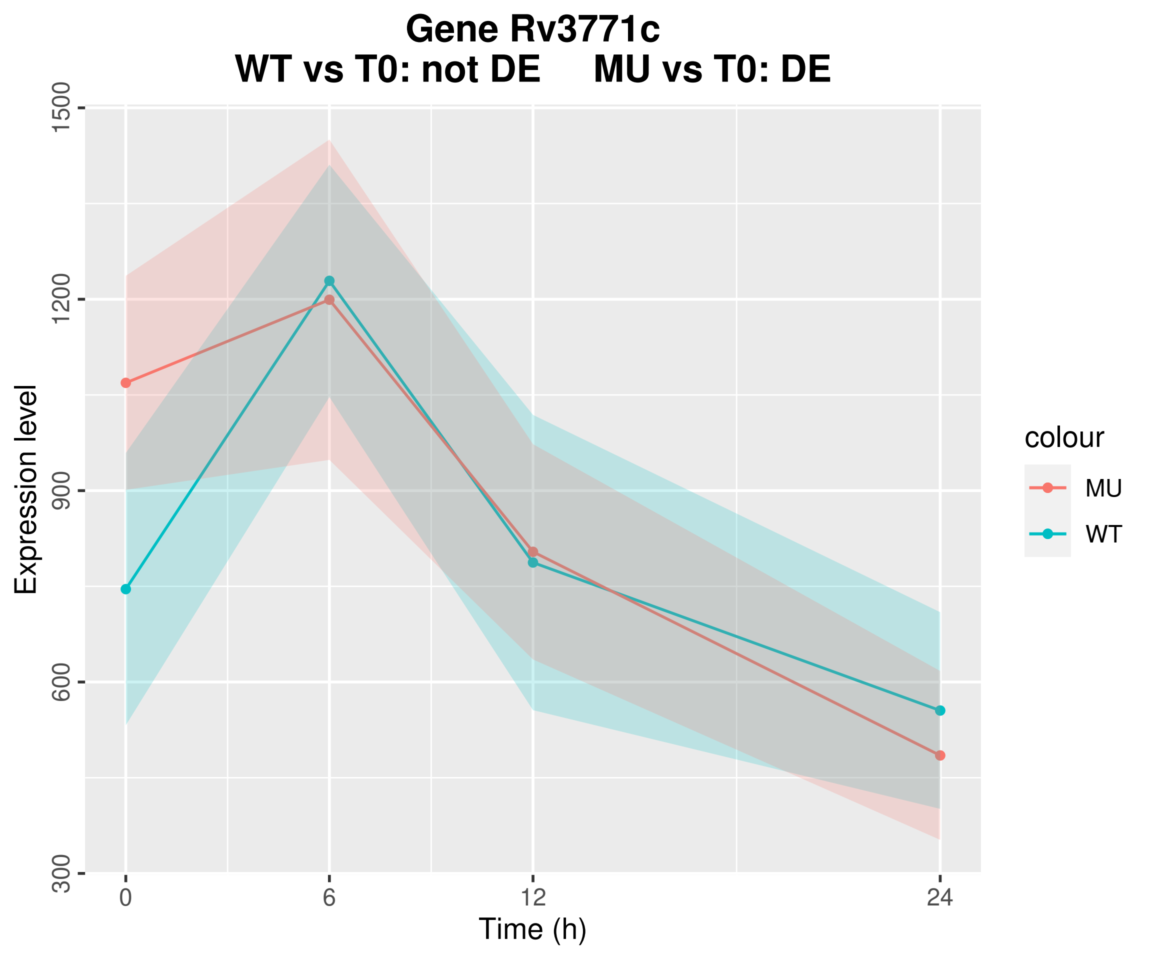
Gene Rv3771c
WT vs T0: not DE   MU vs T0: DE
1500
1200
900
600
300
Expression level
0
6
12
24
Time (h)
colour
MU
WT

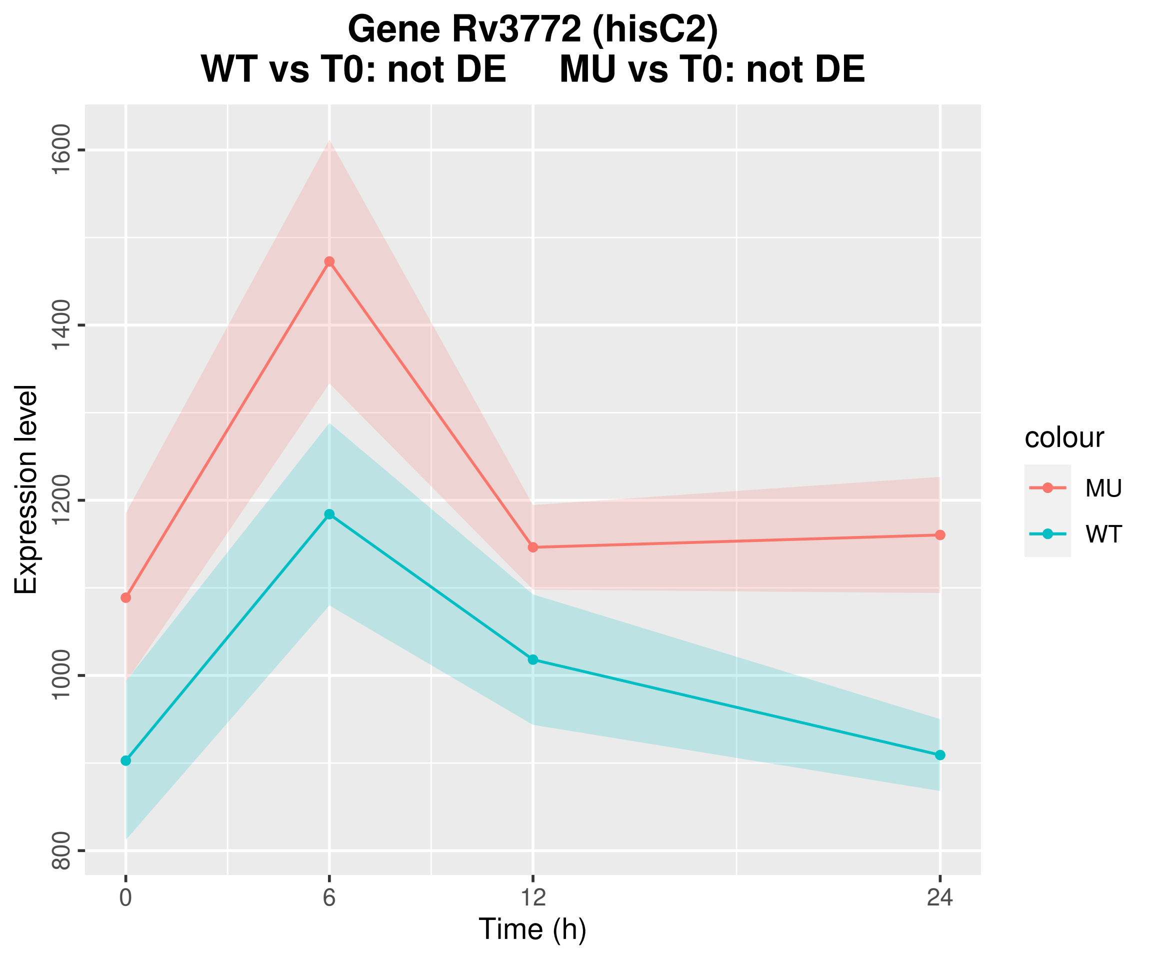
Gene Rv3772 (hisC2)
WT vs T0: not DE   MU vs T0: not DE
Expression level
1600
1400
1200
1000
800
0
6
12
24
Time (h)
colour
MU
WT

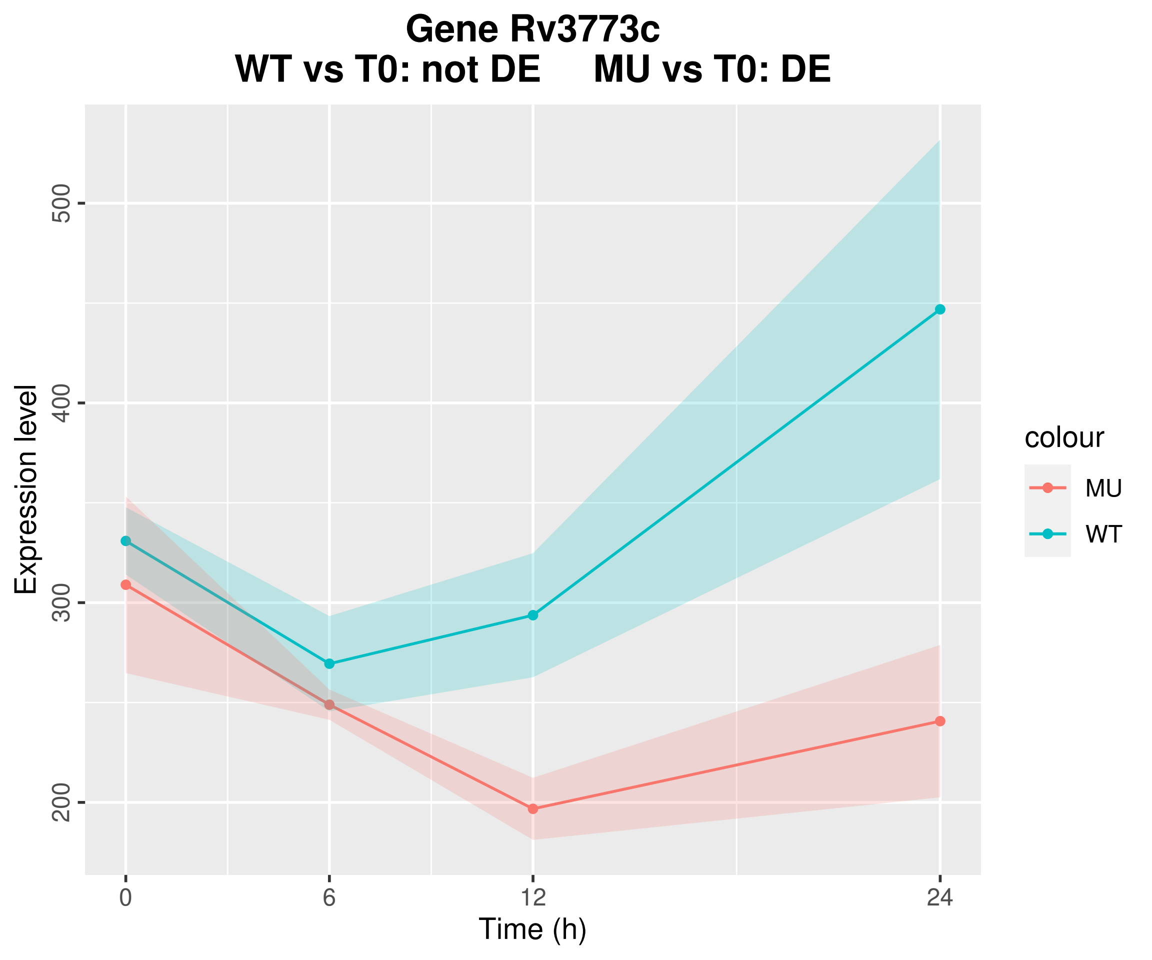
Gene Rv3773c
WT vs T0: not DE   MU vs T0: DE
Expression level
500
400
300
200
0
6
12
24
Time (h)
colour
MU
WT

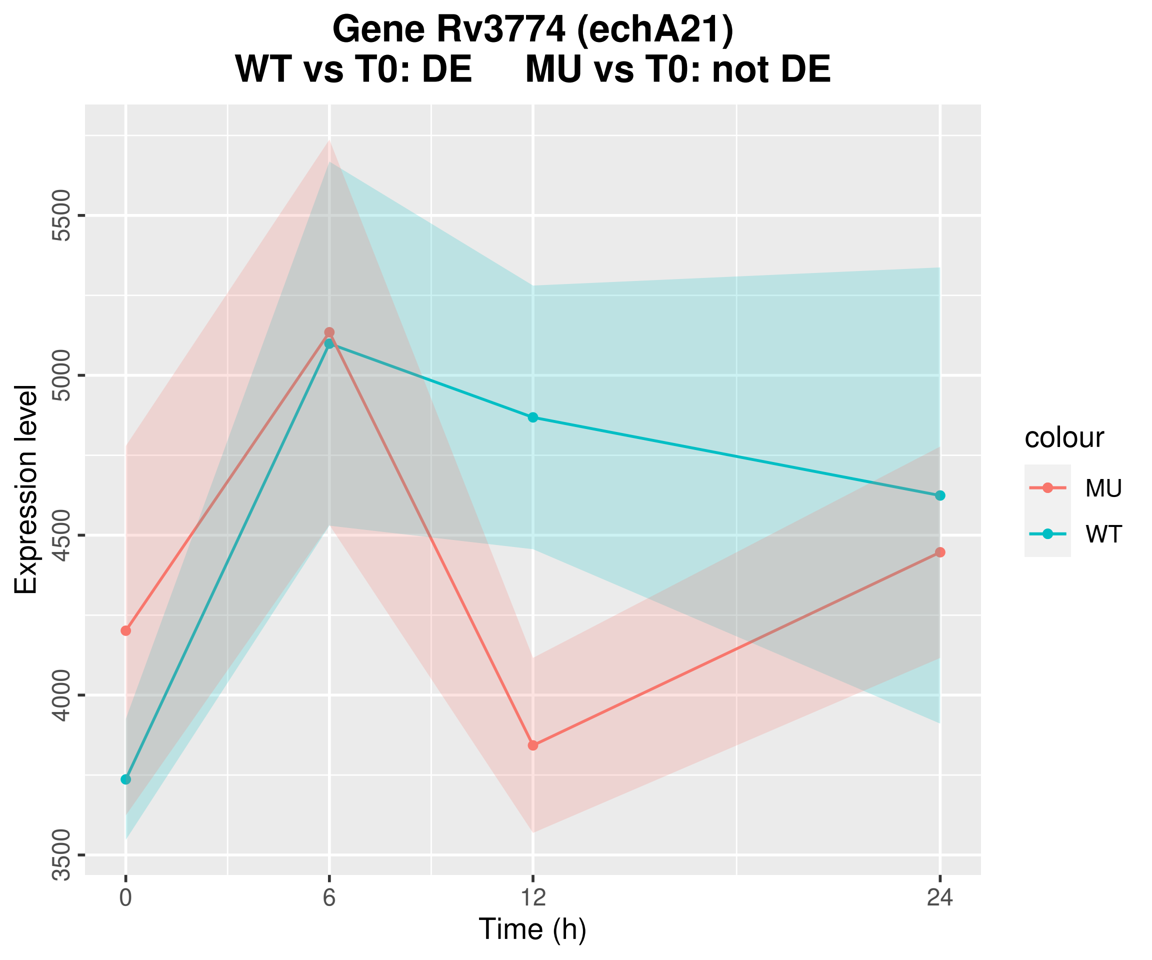
Gene Rv3774 (echA21)
WT vs T0: DE   MU vs T0: not DE
Expression level
5500
5000
4500
4000
3500
Time (h)
0
6
12
24
colour
MU
WT

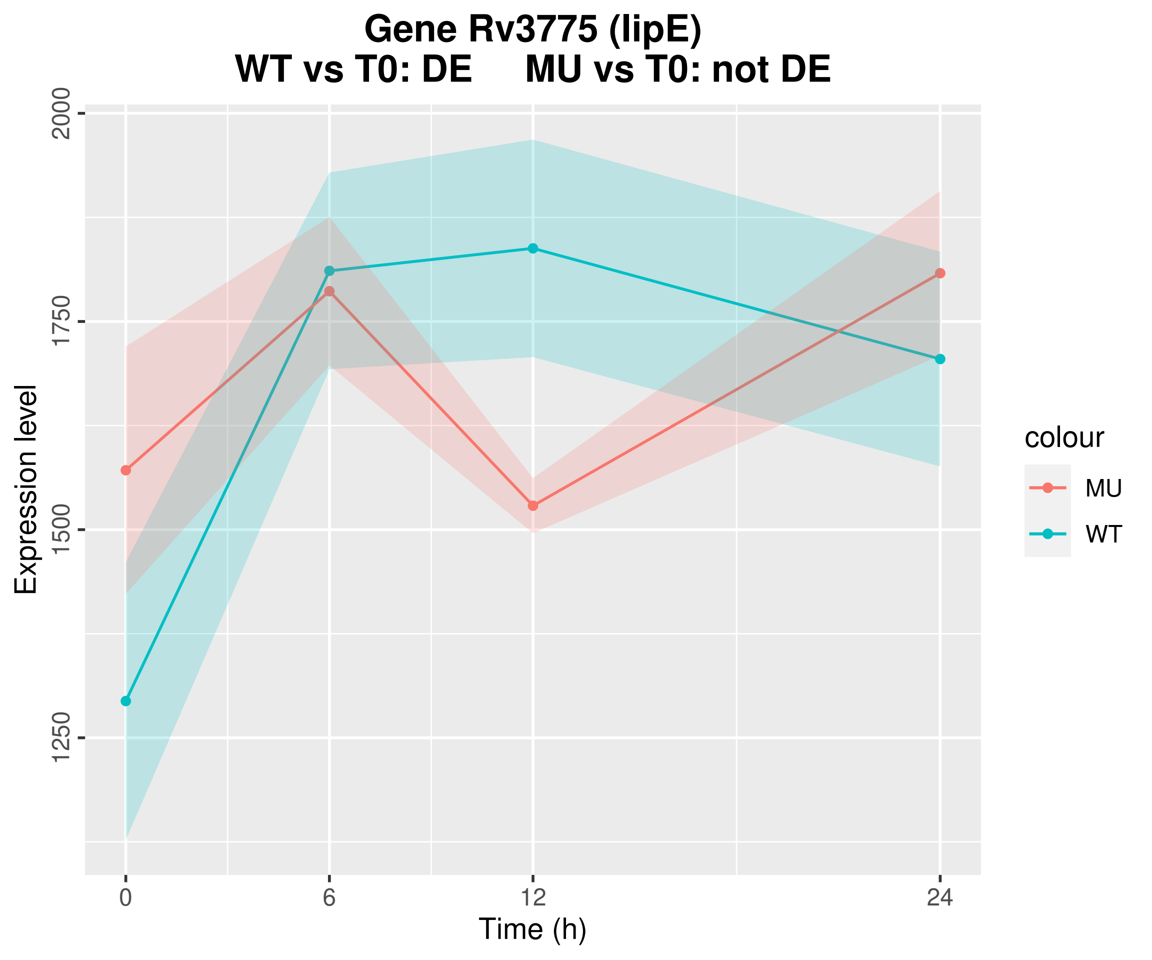
Gene Rv3775 (lipE)
WT vs T0: DE MU vs T0: not DE
Expression level
2000
1750
1500
1250
0
6
12
24
Time (h)
colour
MU
WT

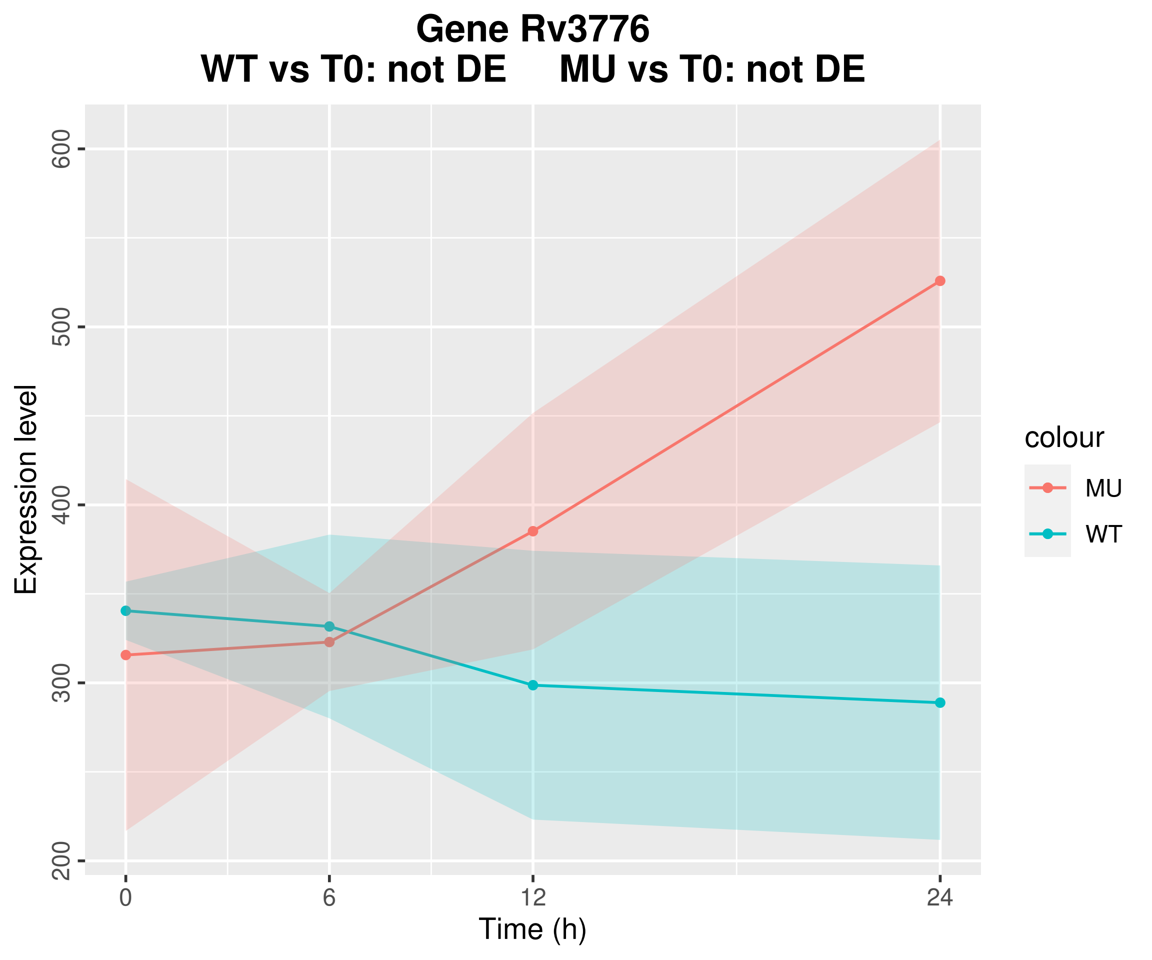
Gene Rv3776
WT vs T0: not DE   MU vs T0: not DE
Expression level
200
300
400
500
600
0
6
12
24
Time (h)
colour
MU
WT

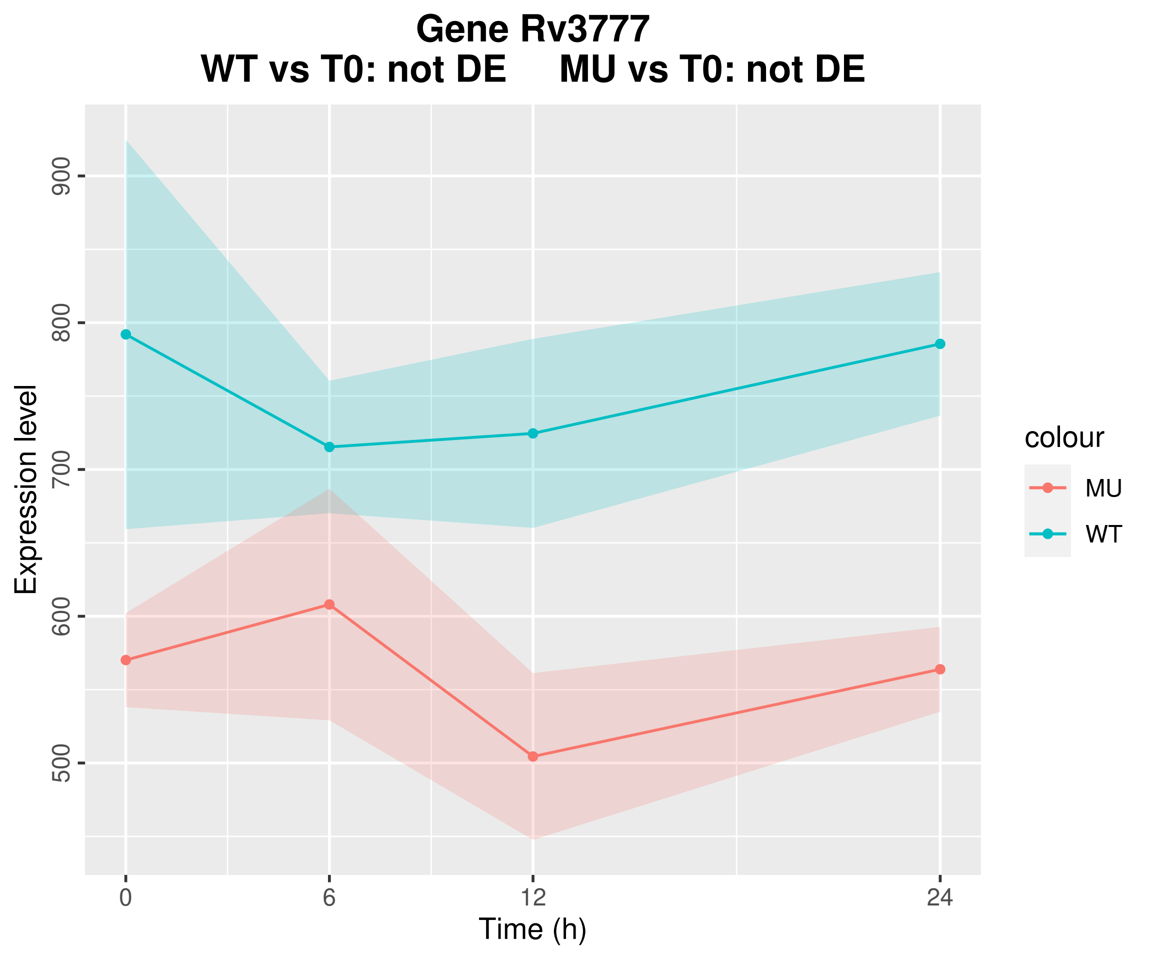
Gene Rv3777
WT vs T0: not DE   MU vs T0: not DE
Expression level
900
800
700
600
500
0
6
12
24
Time (h)
colour
MU
WT

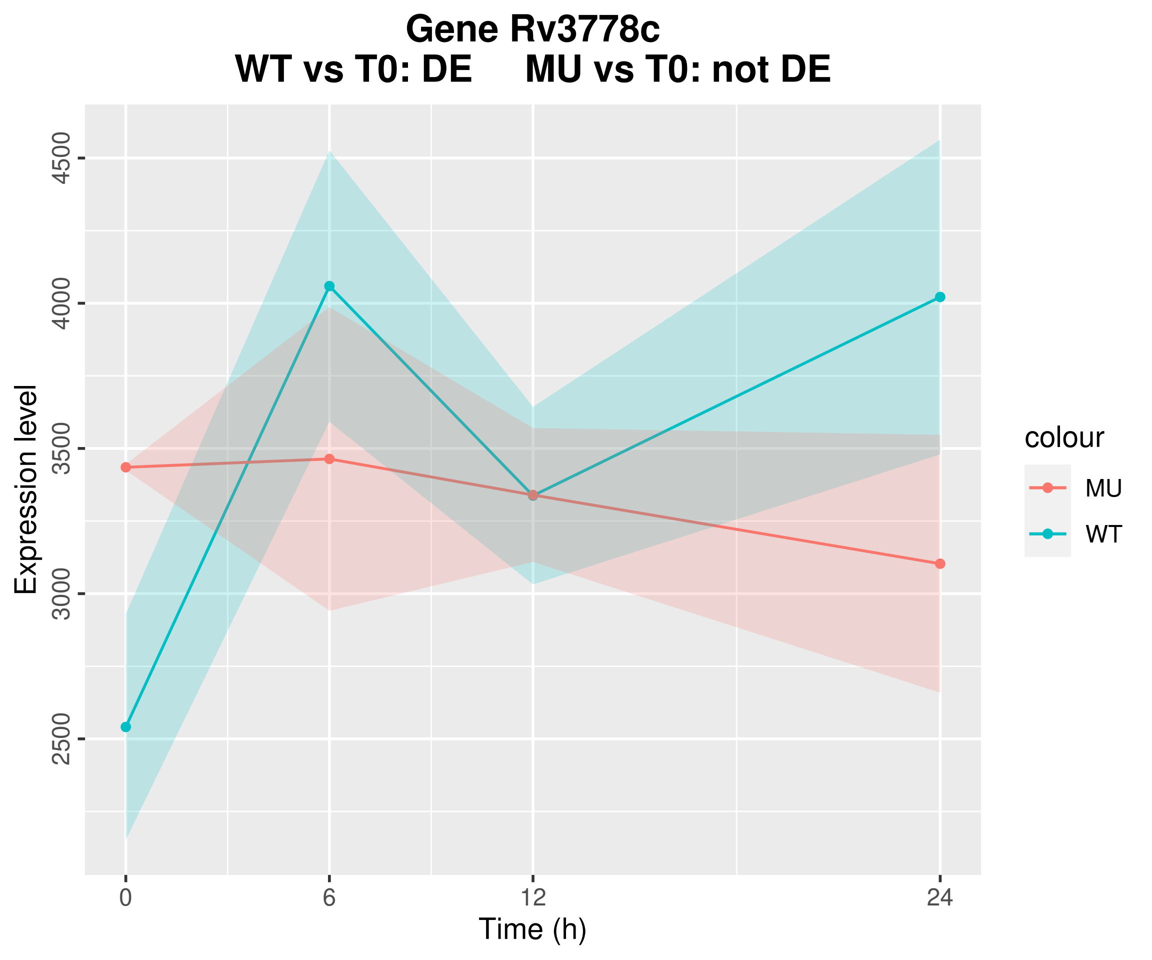

Gene Rv3778c
WT vs T0: DE   MU vs T0: not DE
4500
4000
3500
3000
2500
Expression level
0
6
12
24
Time (h)
colour
MU
WT

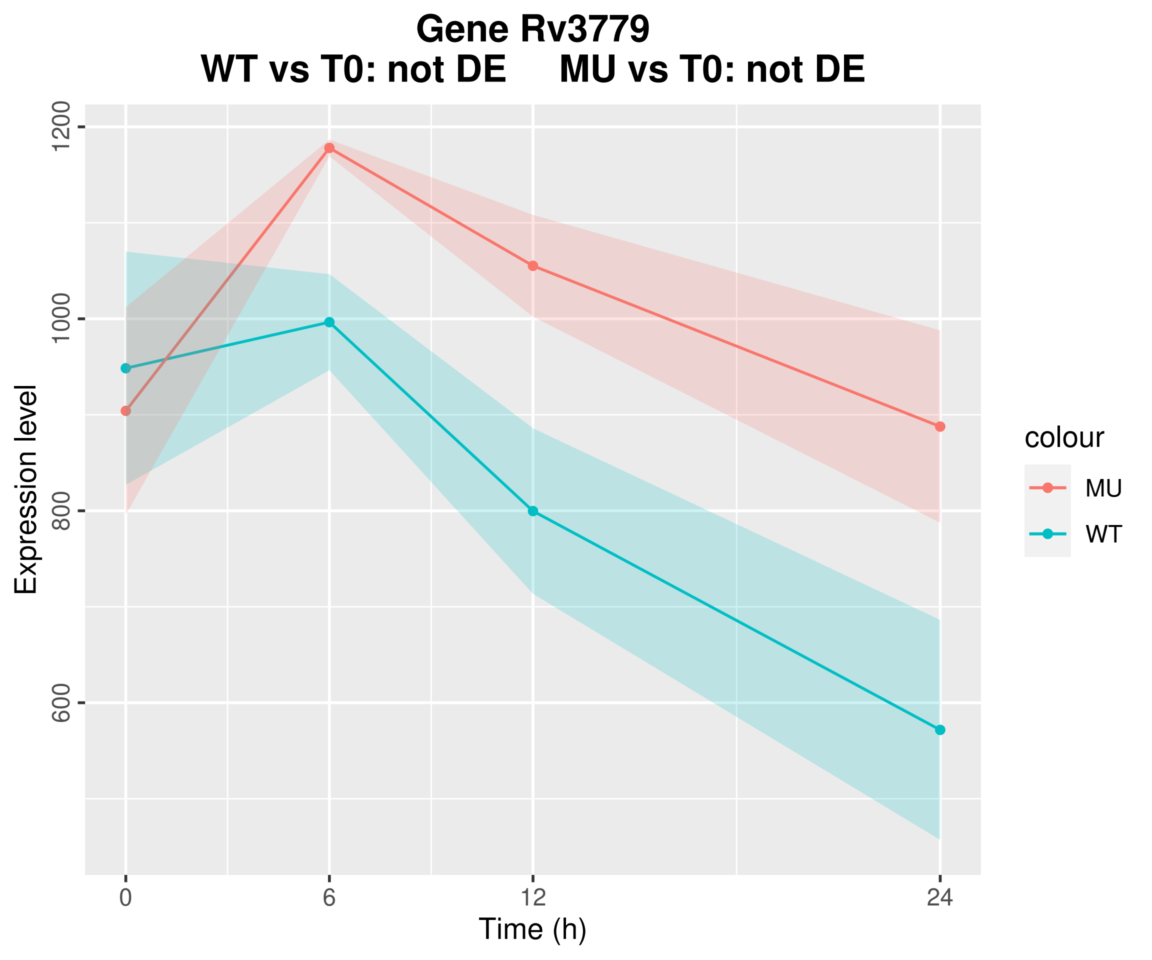

Gene Rv3779
WT vs T0: not DE MU vs T0: not DE
1200
1000
800
600
Expression level
0
6
12
24
Time (h)
colour
MU
WT

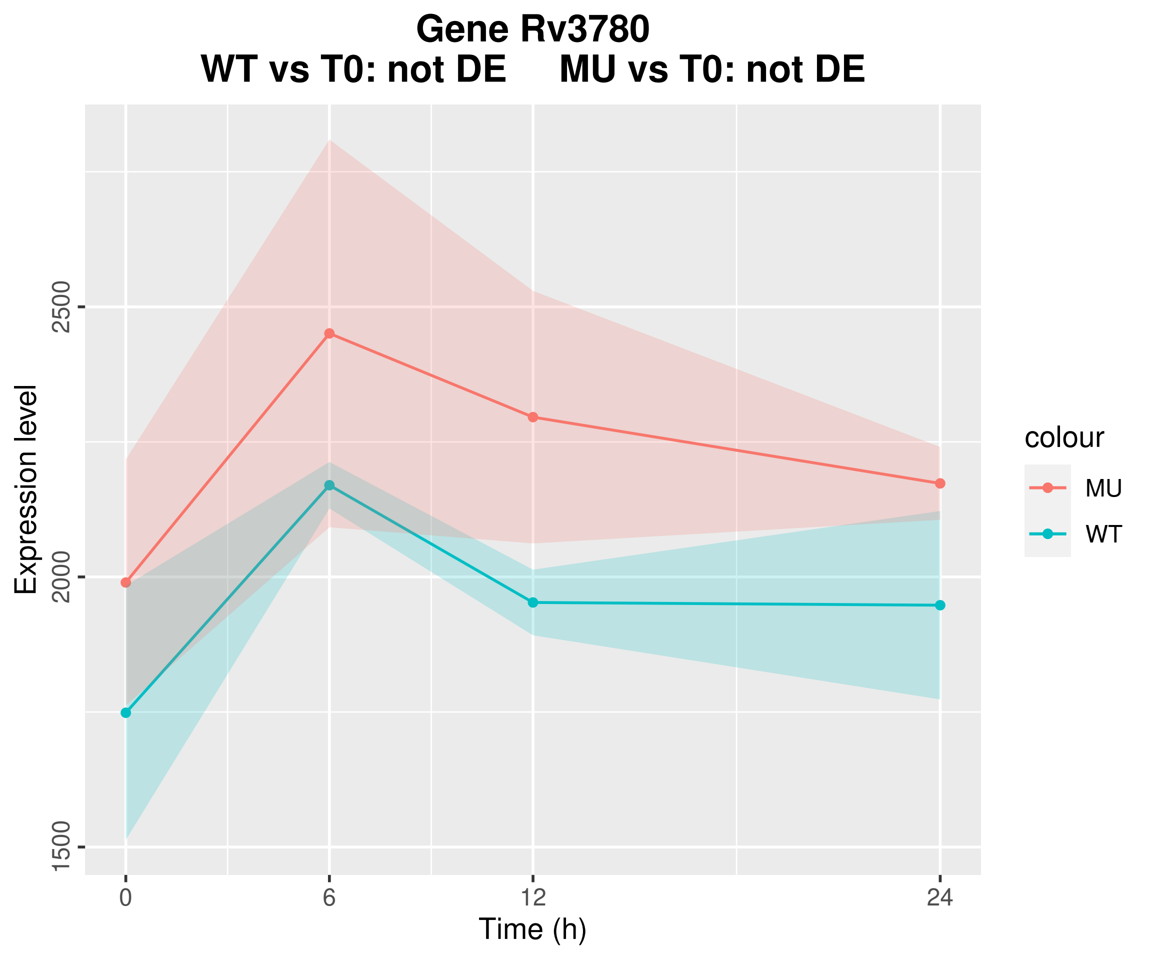

Gene Rv3780
WT vs T0: not DE   MU vs T0: not DE
Expression level
2500
2000
1500
0
6
12
24
Time (h)
colour
MU
WT

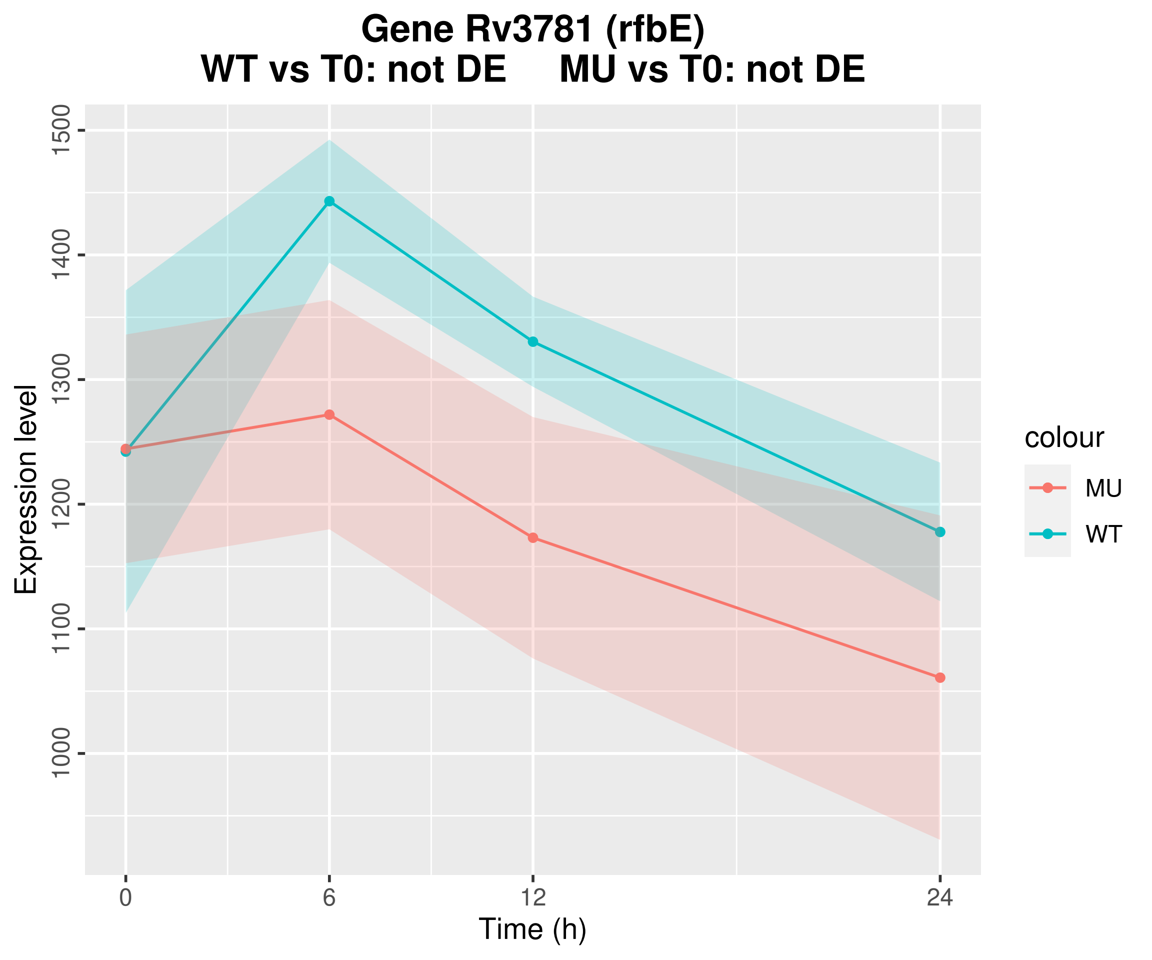

Gene Rv3781 (rfbE)
WT vs T0: not DE MU vs T0: not DE
Expression level
1000
1100
1200
1300
1400
1500
0
6
12
24
Time (h)
colour
MU
WT

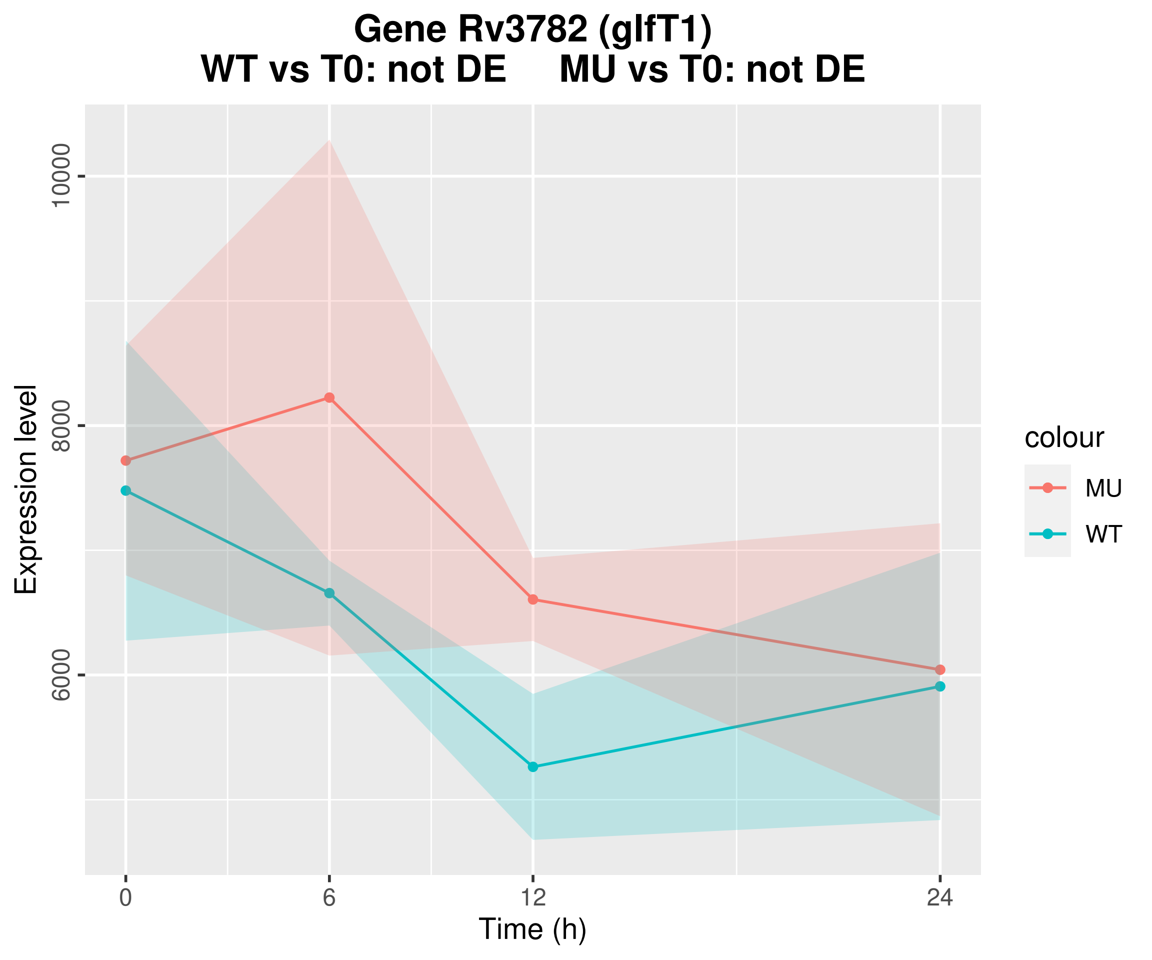
Gene Rv3782 (glfT1)
WT vs T0: not DE   MU vs T0: not DE
Expression level
10000
8000
6000
0
6
12
24
Time (h)
colour
MU
WT

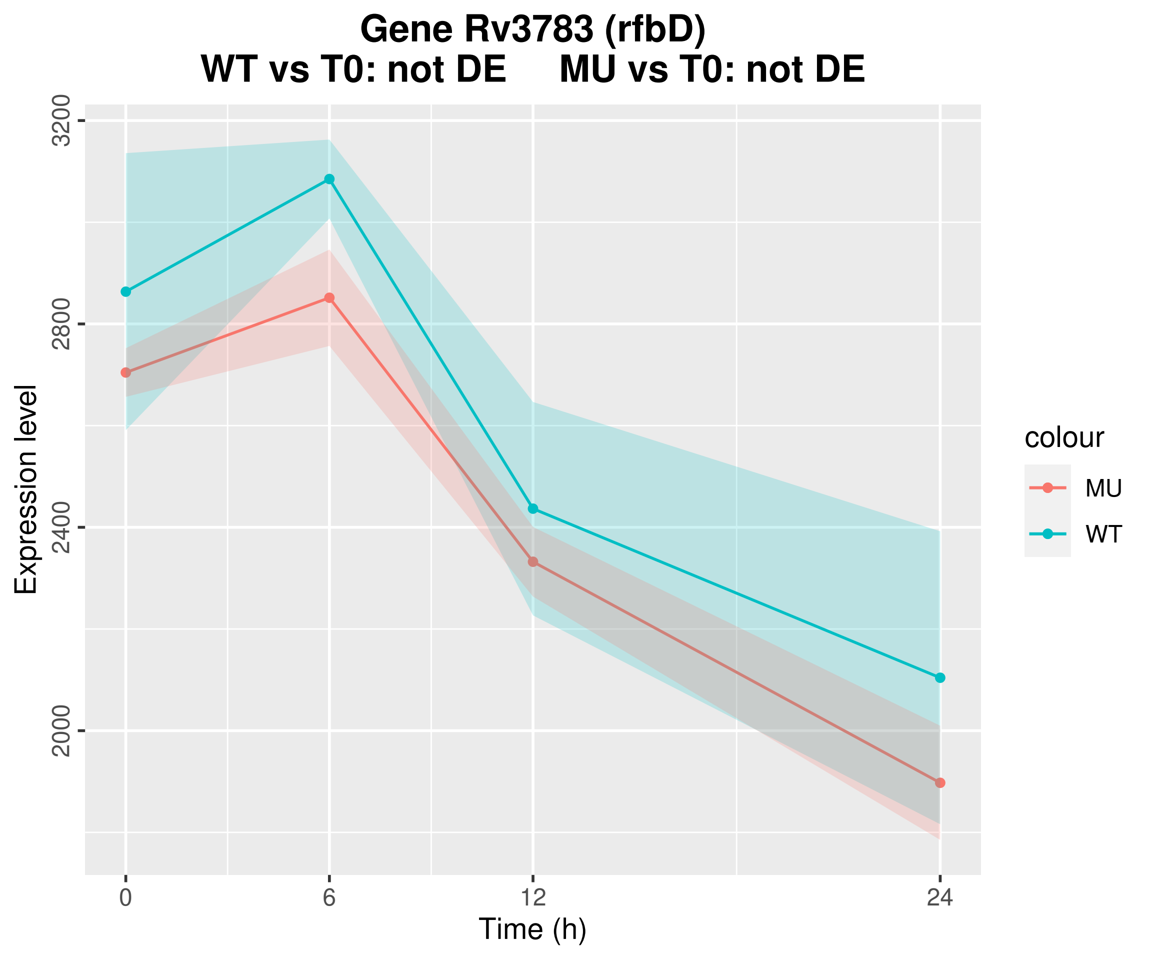
Gene Rv3783 (rfbD)
WT vs T0: not DE   MU vs T0: not DE
3200
2800
2400
2000
Expression level
0
6
12
24
Time (h)
colour
MU
WT

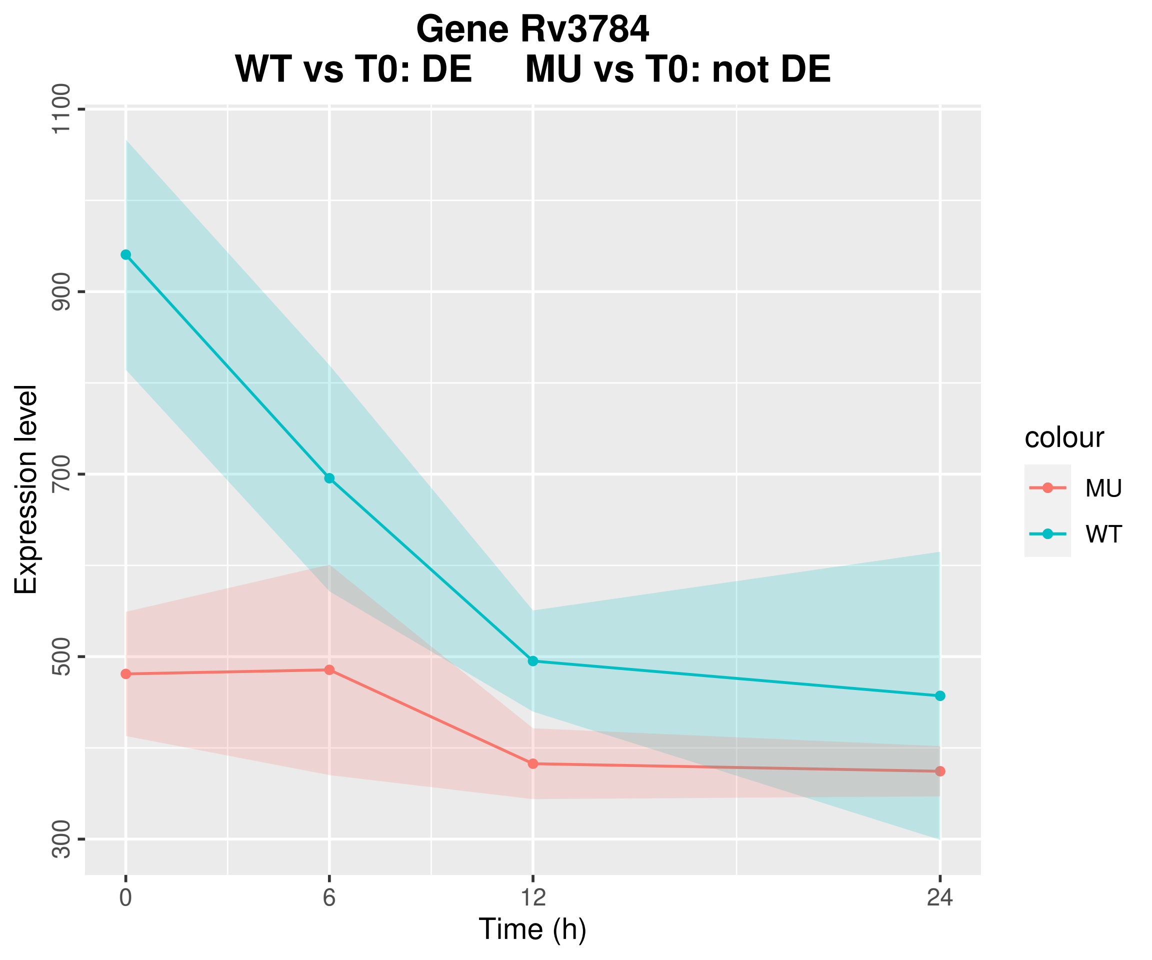

Gene Rv3784
WT vs T0: DE MU vs T0: not DE
Expression level
300
500
700
900
1100
0
6
12
24
Time (h)
colour
MU
WT

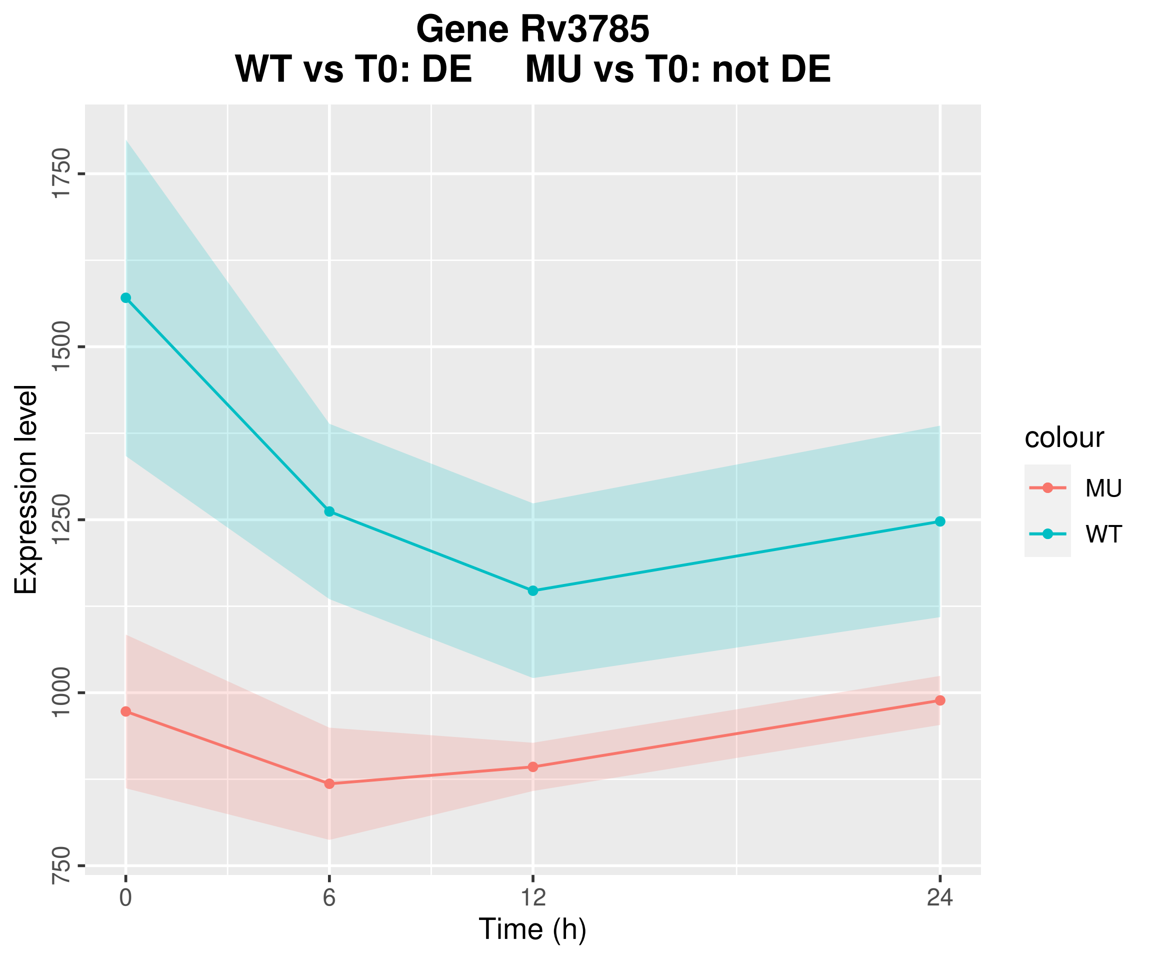
Gene Rv3785
WT vs T0: DE   MU vs T0: not DE
Expression level
1750
1500
1250
1000
750
0
6
12
24
Time (h)
colour
MU
WT

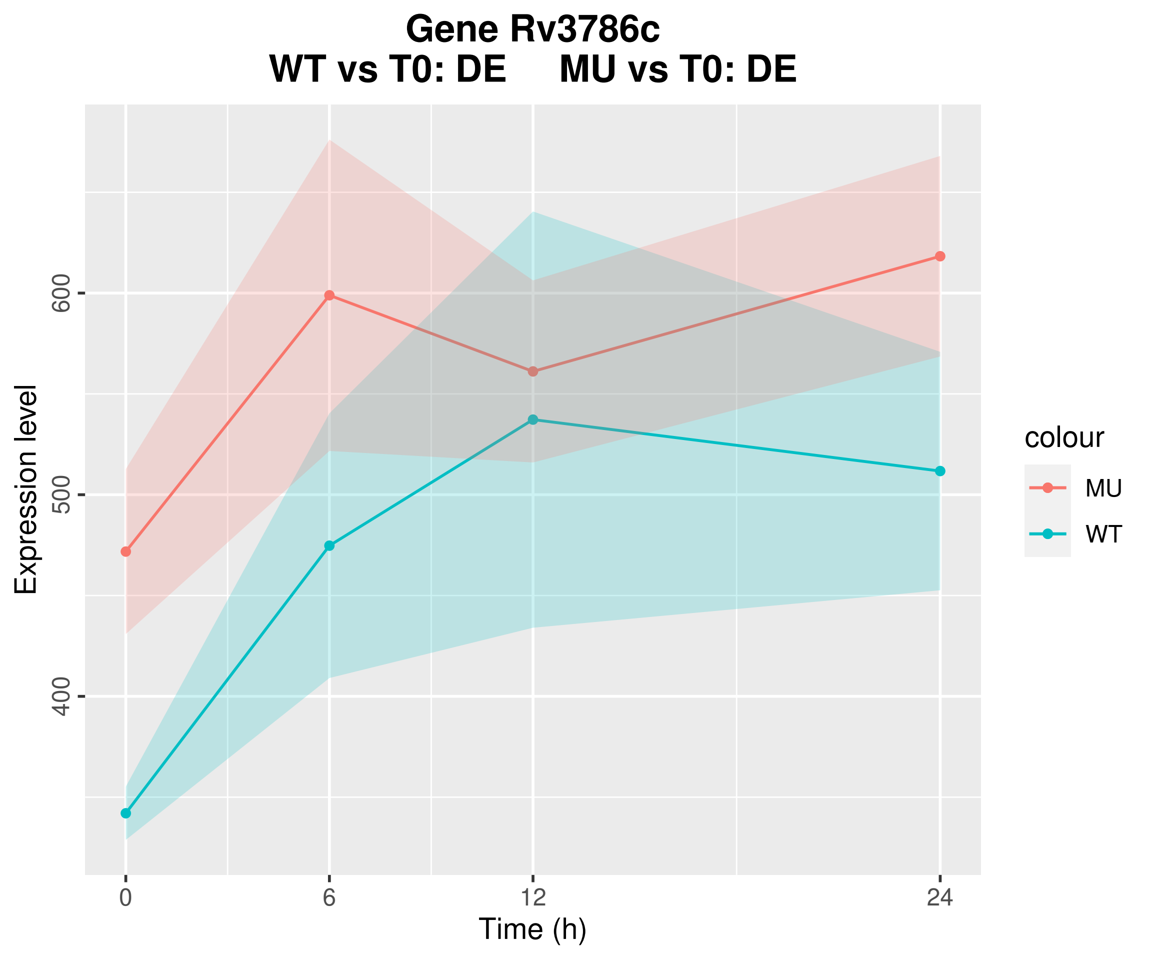

Gene Rv3786c
WT vs T0: DE MU vs T0: DE
Expression level
600
500
400
0
6
12
24
Time (h)
colour
MU
WT

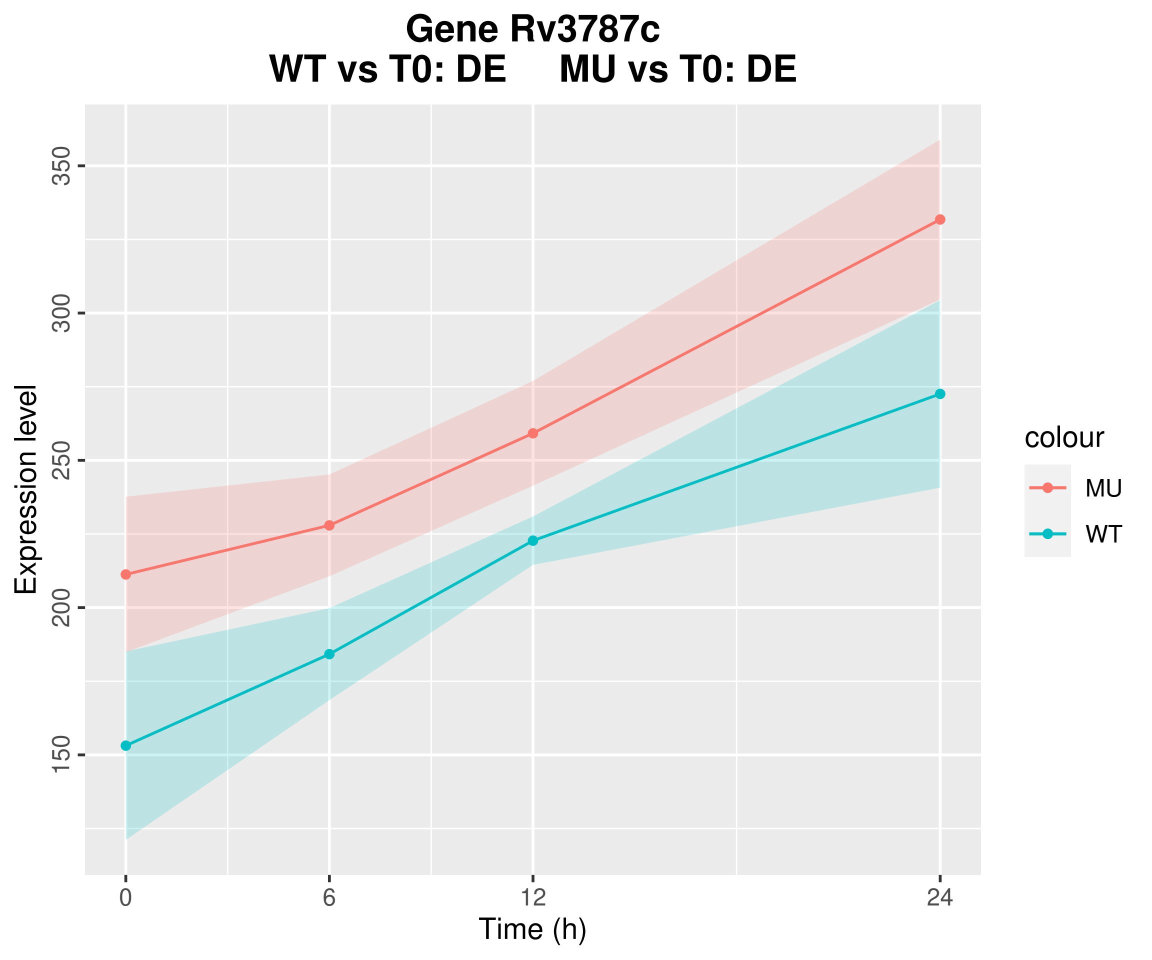
Gene Rv3787c
WT vs T0: DE MU vs T0: DE
Expression level
350
300
250
200
150
0
6
12
24
Time (h)
colour
MU
WT

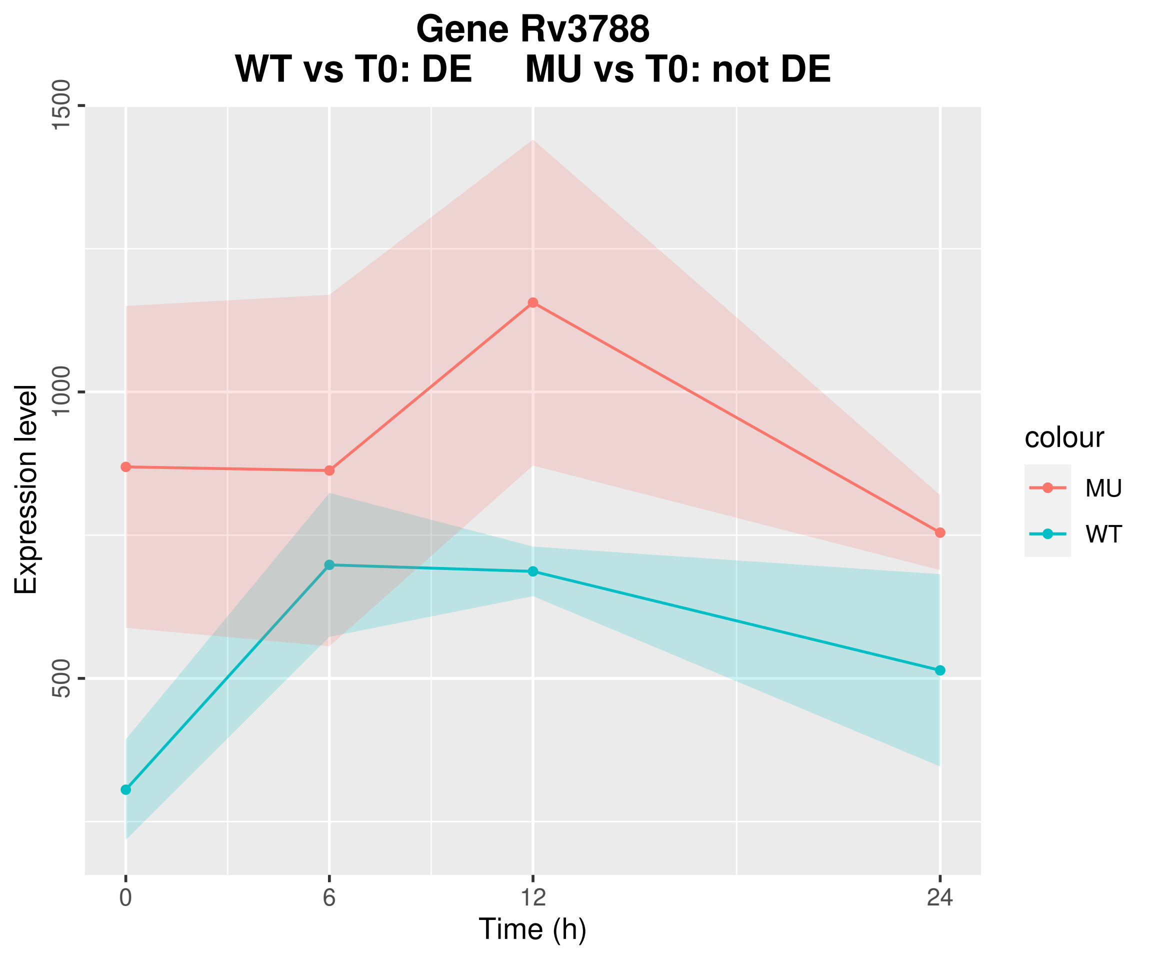

Gene Rv3788
WT vs T0: DE   MU vs T0: not DE
Expression level
1500
1000
500
0
6
12
24
Time (h)
colour
MU
WT

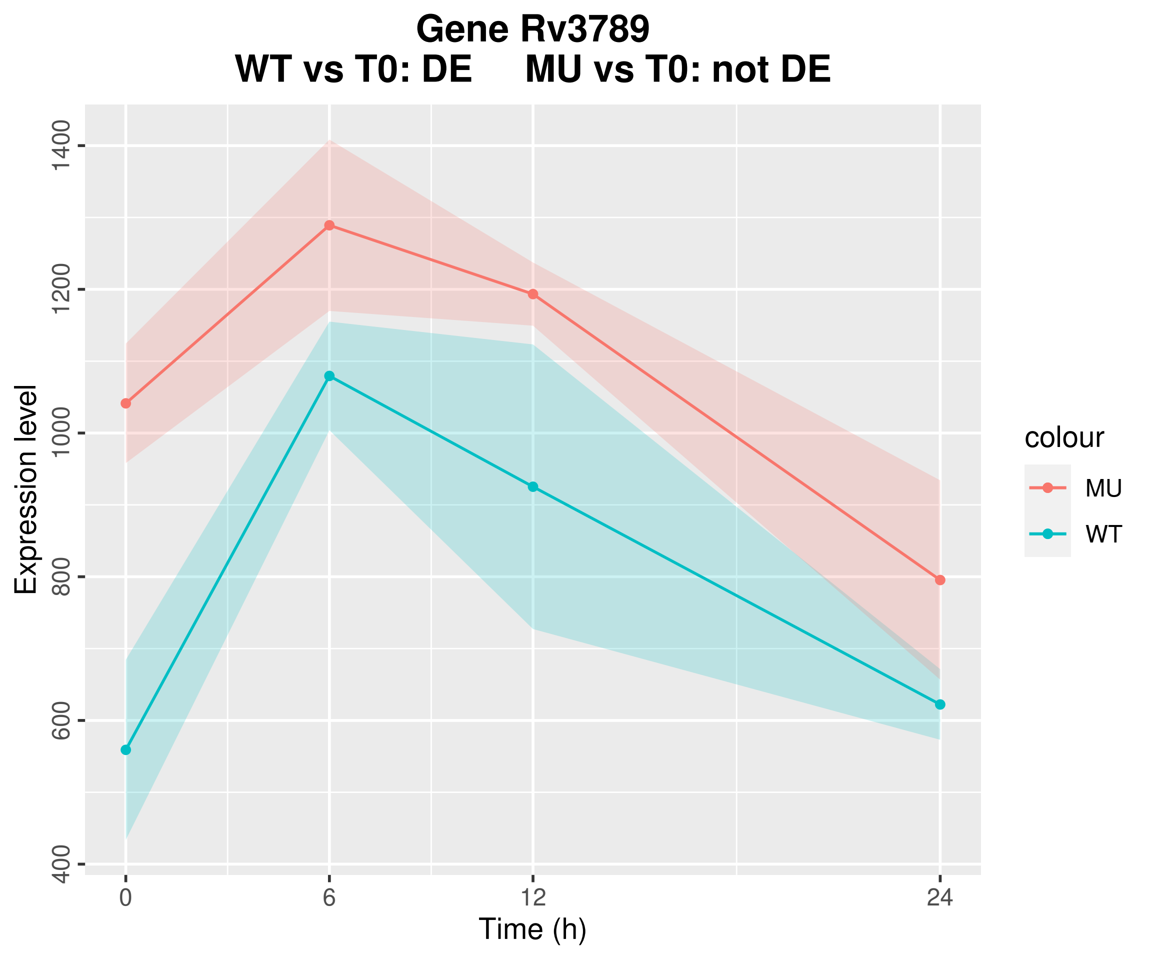
Gene Rv3789
WT vs T0: DE   MU vs T0: not DE
Expression level
1400
1200
1000
800
600
400
0
6
12
24
Time (h)
colour
MU
WT

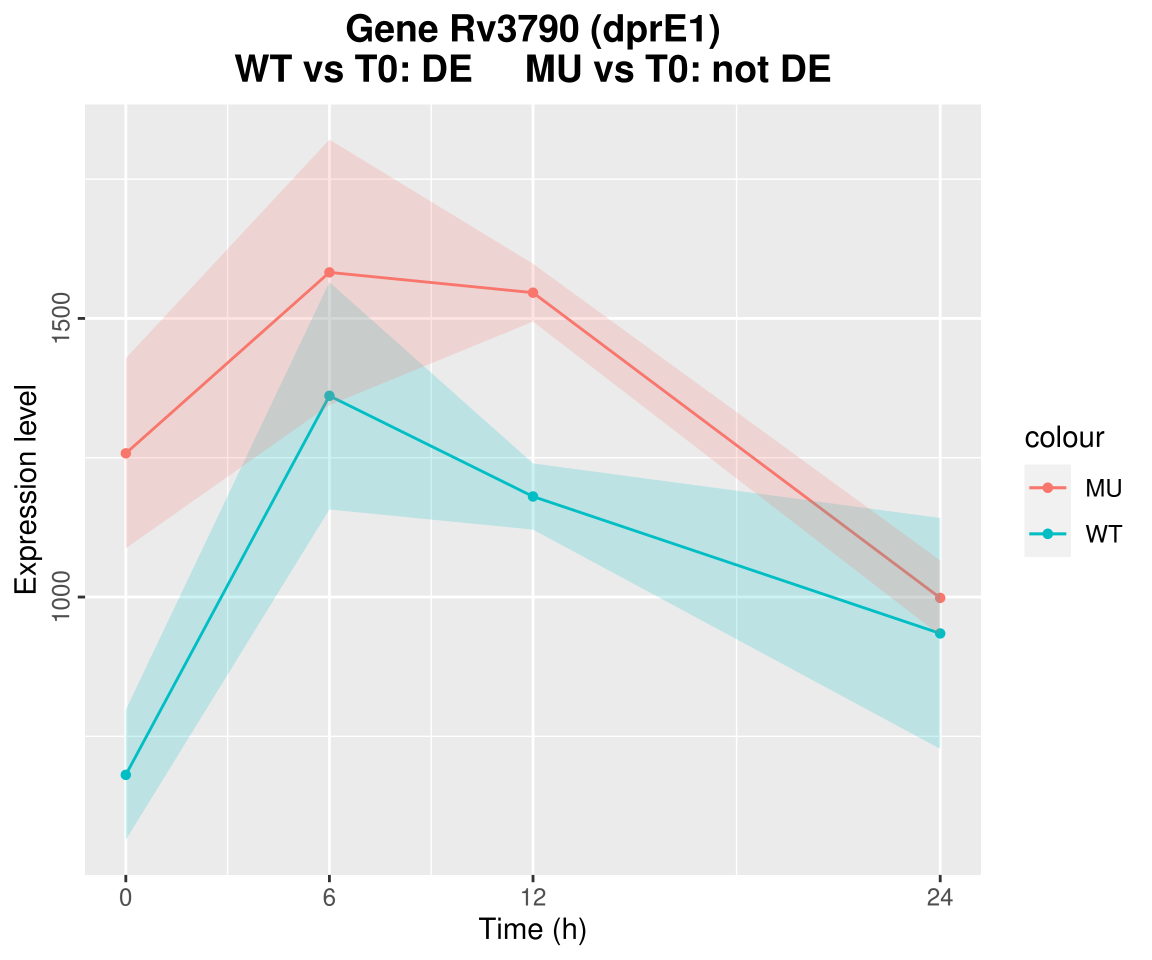

Gene Rv3790 (dprE1)
WT vs T0: DE   MU vs T0: not DE
Expression level
1500
1000
0
6
12
24
Time (h)
colour
MU
WT

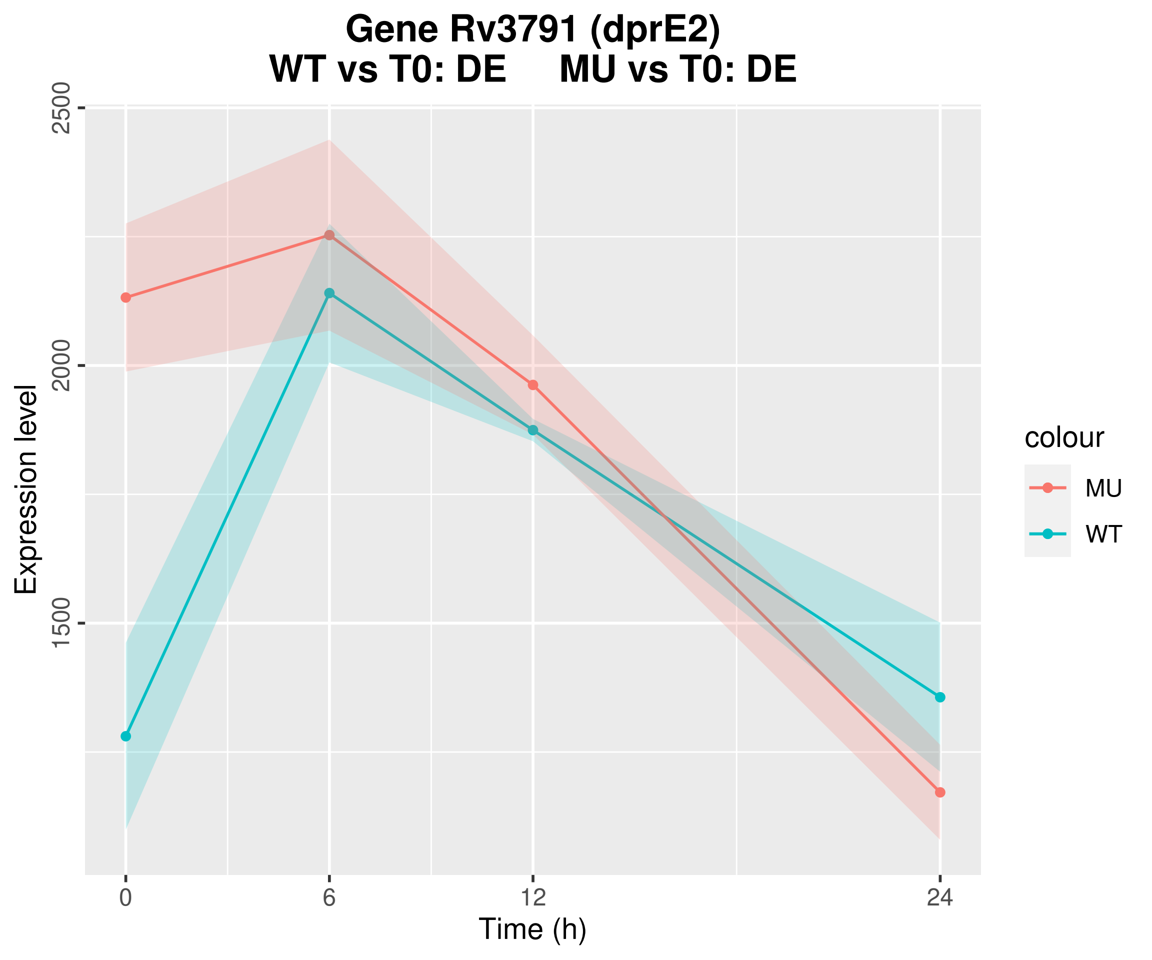
Gene Rv3791 (dprE2)
WT vs T0: DE   MU vs T0: DE
2500
2000
1500
Expression level
0
6
12
24
Time (h)
colour
MU
WT

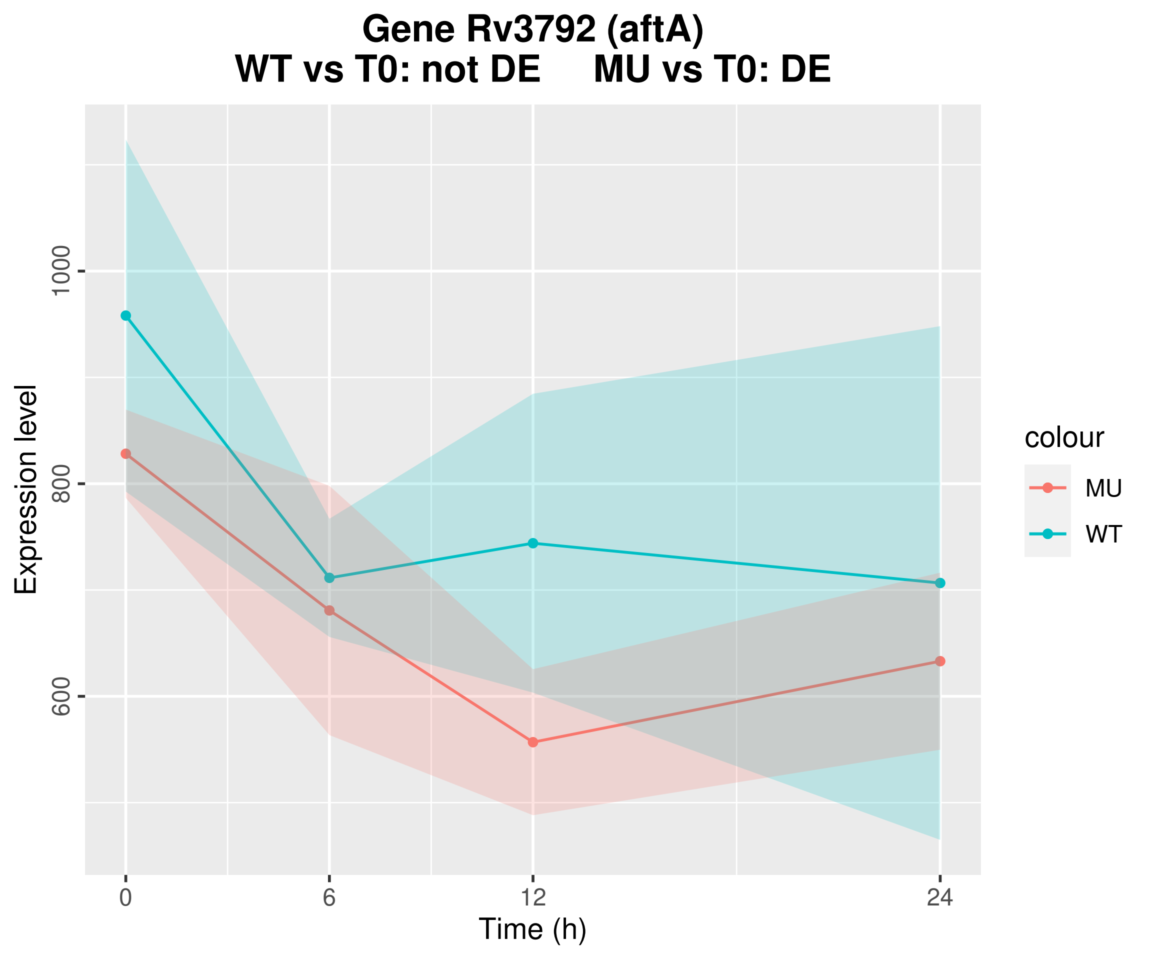

Gene Rv3792 (aftA)
WT vs T0: not DE MU vs T0: DE
Expression level
1000
800
600
0
6
12
24
Time (h)
colour
MU
WT

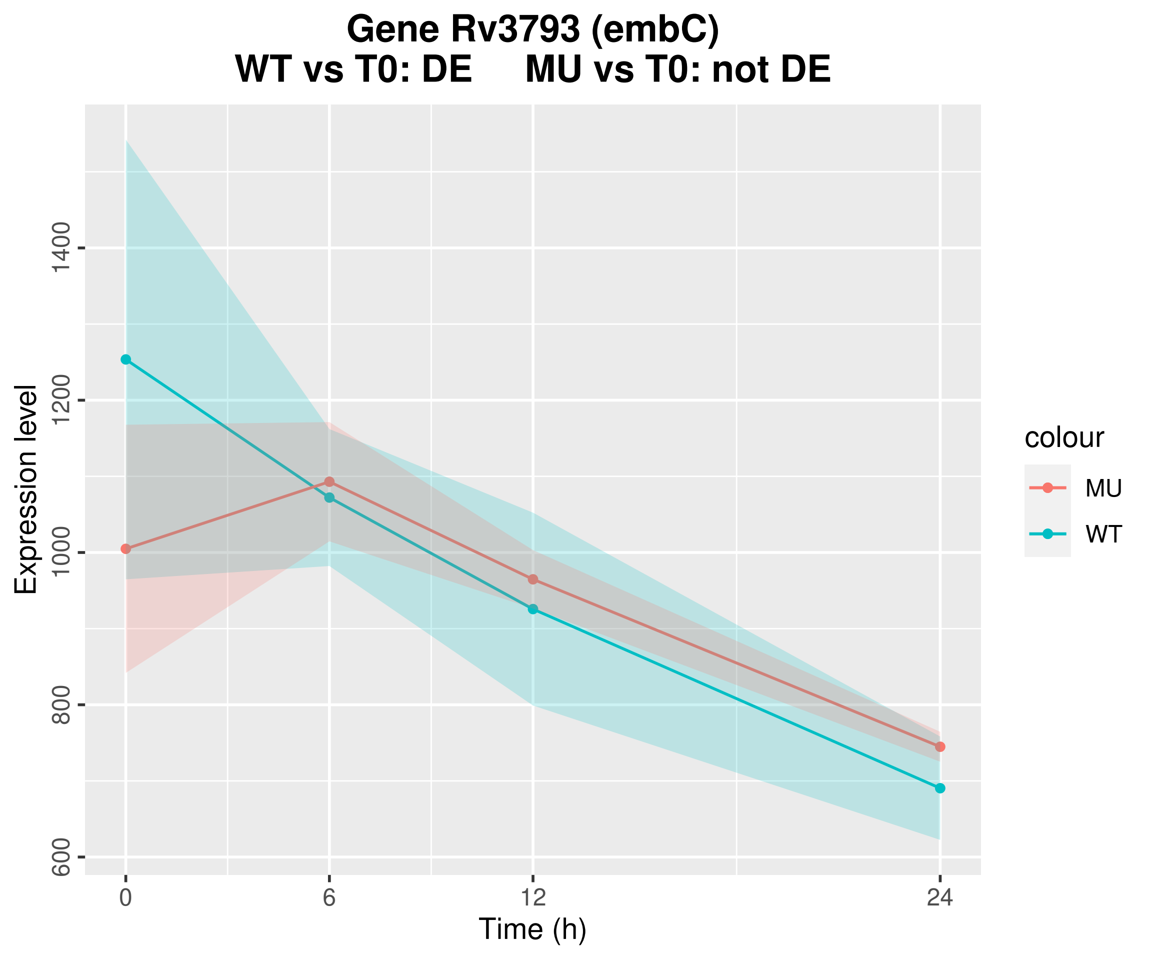
Gene Rv3793 (embC)
WT vs T0: DE   MU vs T0: not DE
Expression level
1400
1200
1000
800
600
0
6
12
24
Time (h)
colour
MU
WT

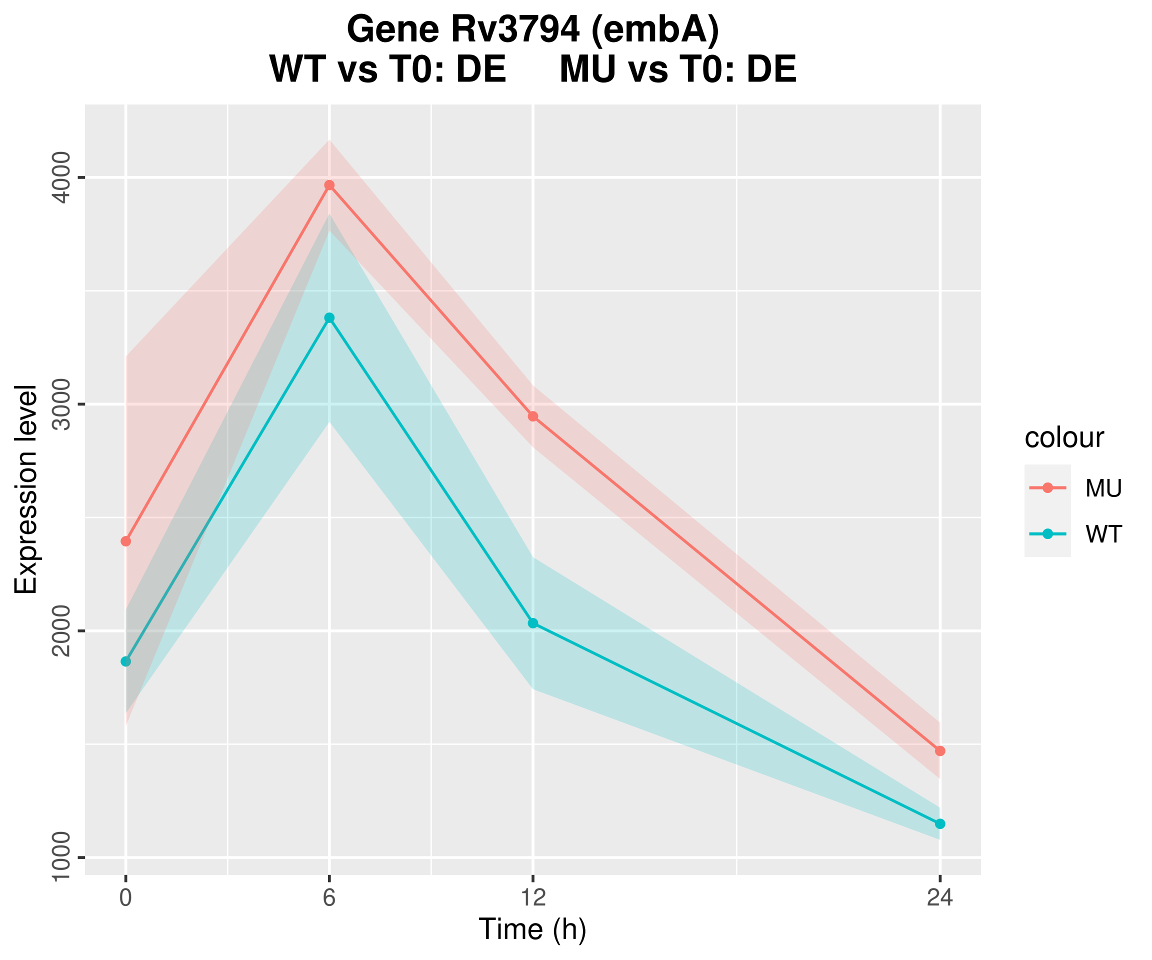

Gene Rv3794 (embA)
WT vs T0: DE   MU vs T0: DE
Expression level
4000
3000
2000
1000
0
6
12
24
Time (h)
colour
MU
WT

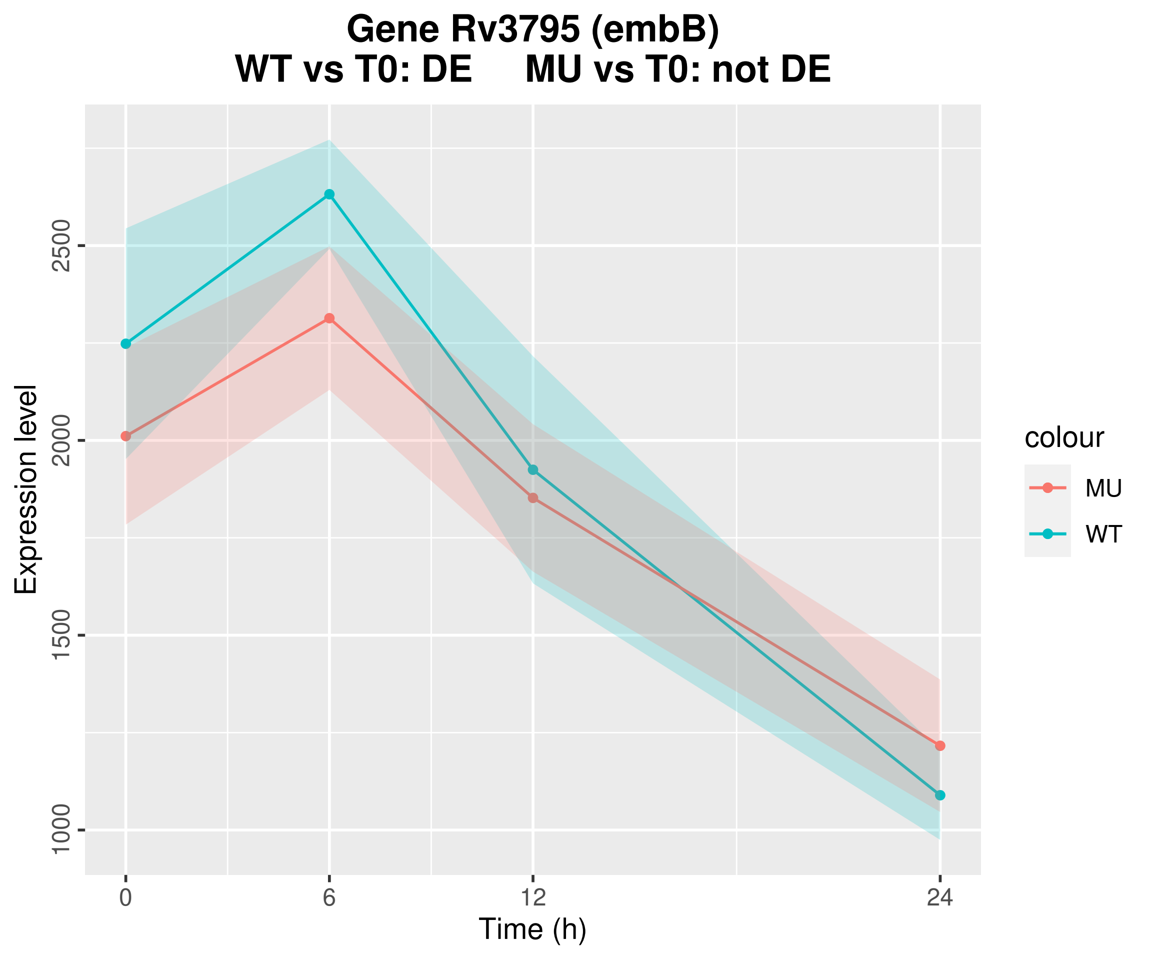
Gene Rv3795 (embB)
WT vs T0: DE MU vs T0: not DE
Expression level
2500
2000
1500
1000
0
6
12
24
Time (h)
colour
MU
WT

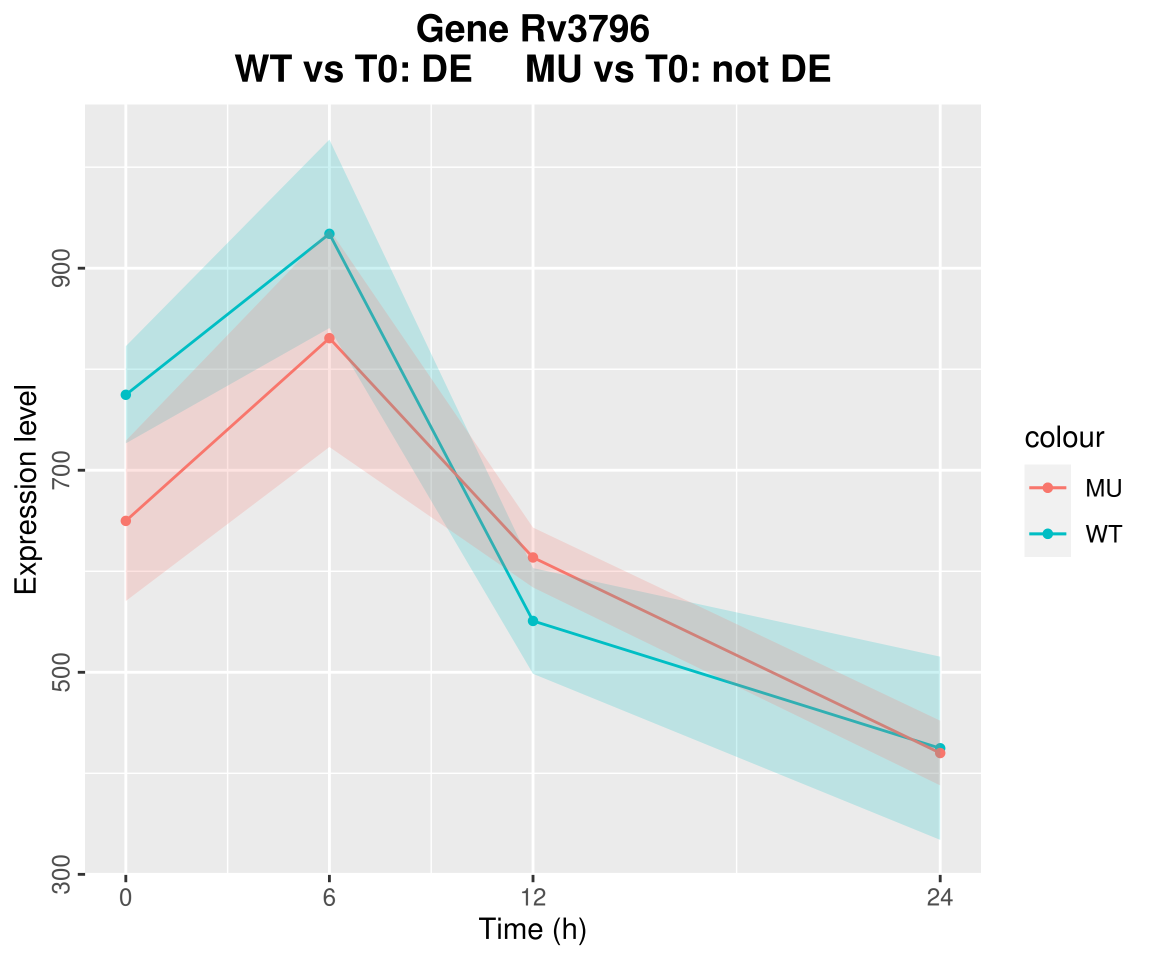
Gene Rv3796
WT vs T0: DE MU vs T0: not DE
Expression level
900
700
500
300
0
6
12
24
Time (h)
colour
MU
WT

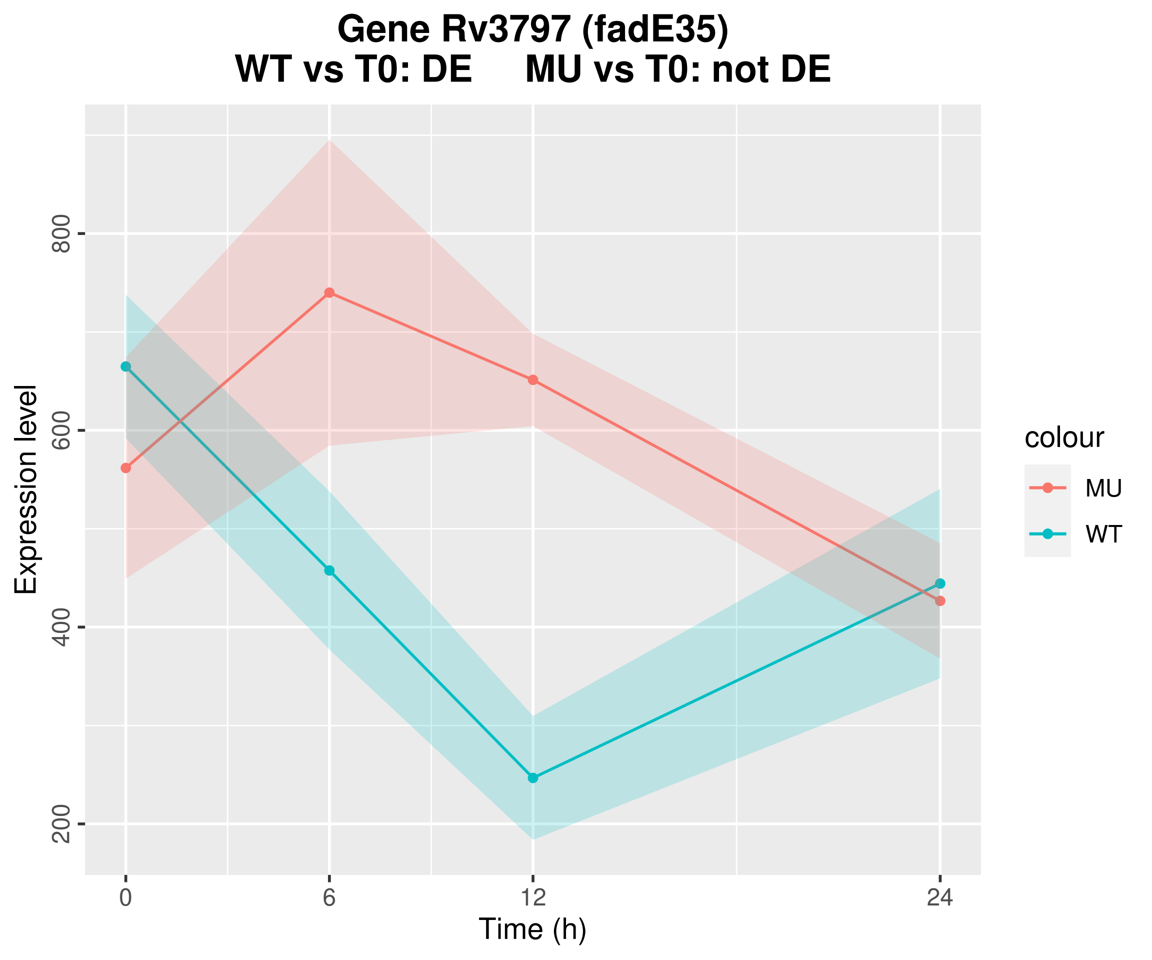

Gene Rv3797 (fadE35)
WT vs T0: DE   MU vs T0: not DE
Expression level
800
600
400
200
0
6
12
24
Time (h)
colour
MU
WT

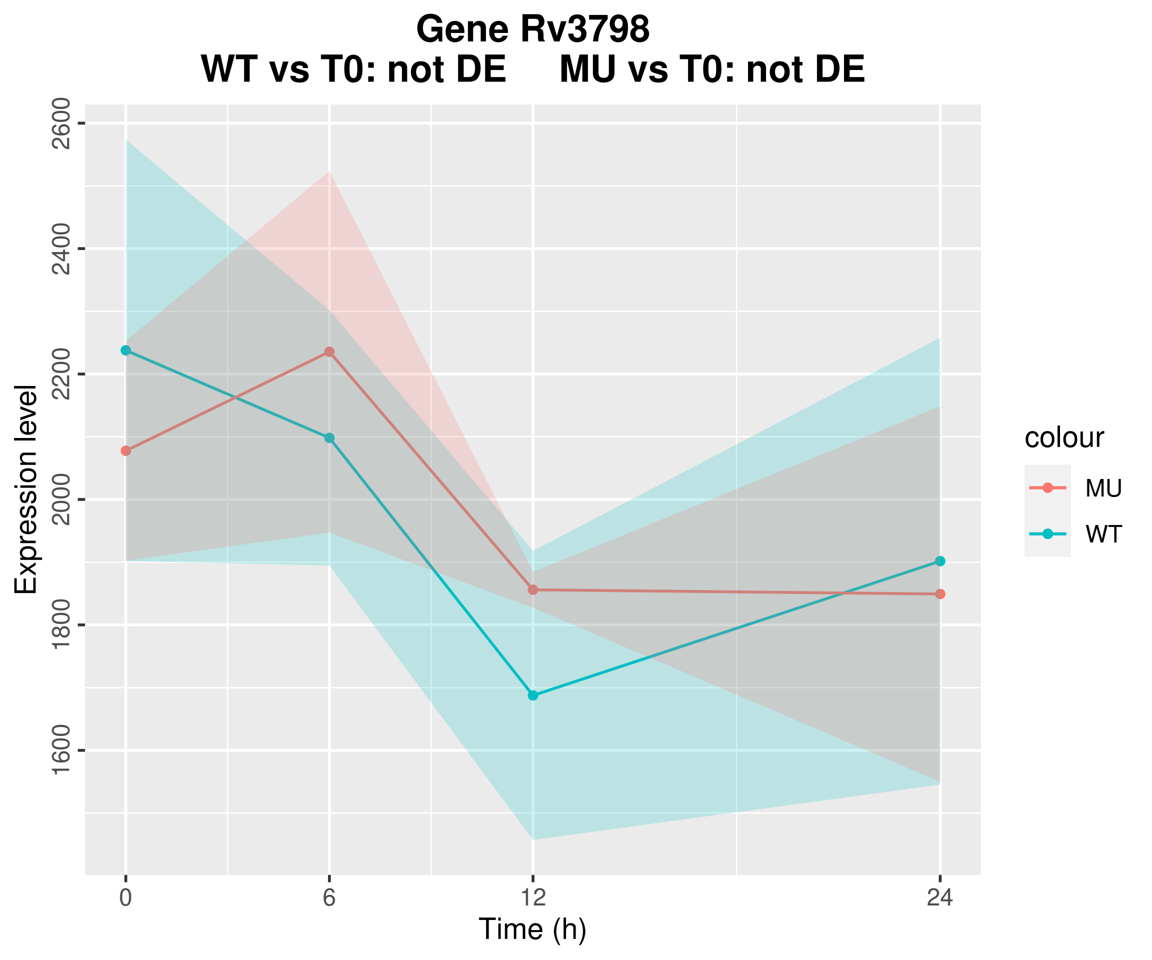

Gene Rv3798
WT vs T0: not DE MU vs T0: not DE
Expression level
2600
2400
2200
2000
1800
1600
0
6
12
24
Time (h)
colour
MU
WT

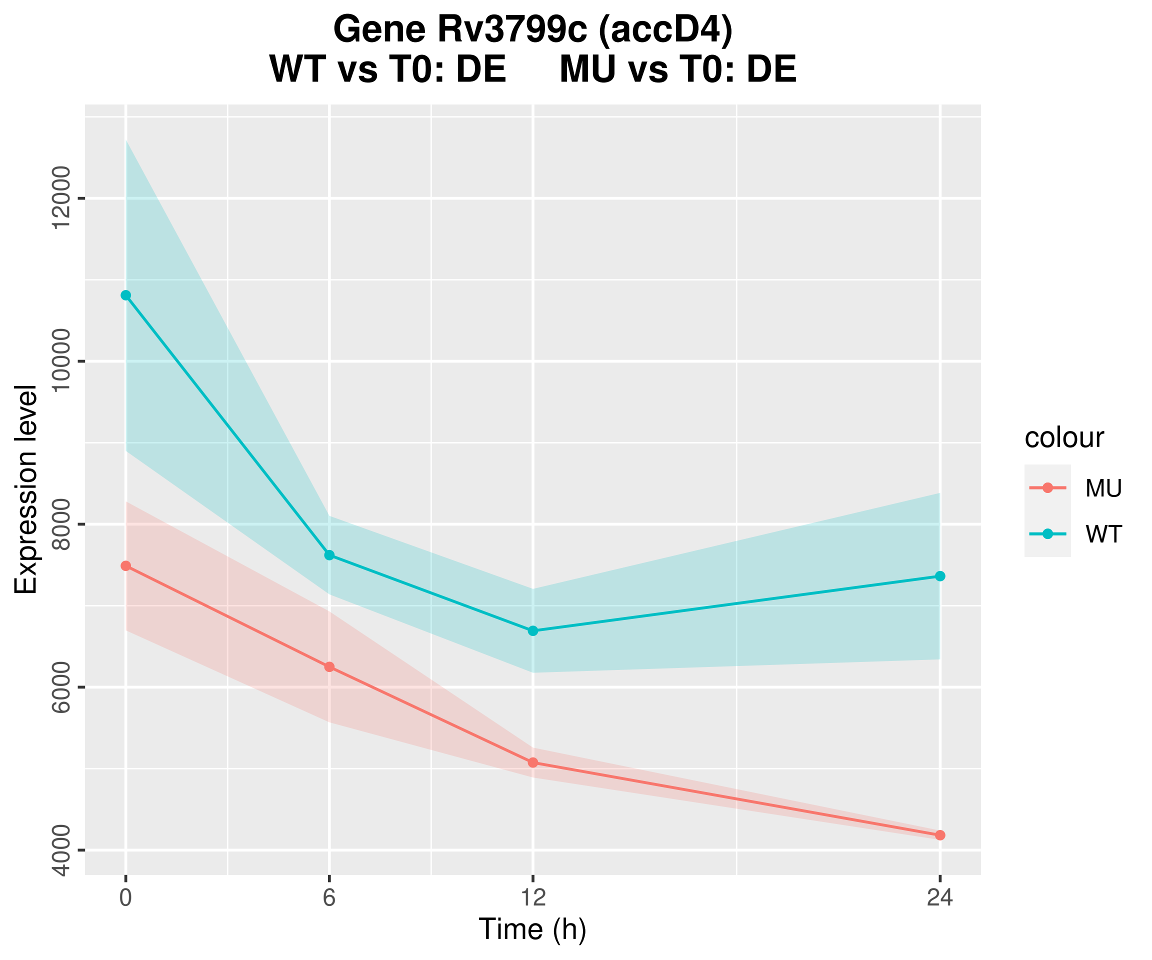

Gene Rv3799c (accD4)
WT vs T0: DE   MU vs T0: DE
Expression level
12000
10000
8000
6000
4000
0
6
12
24
Time (h)
colour
MU
WT

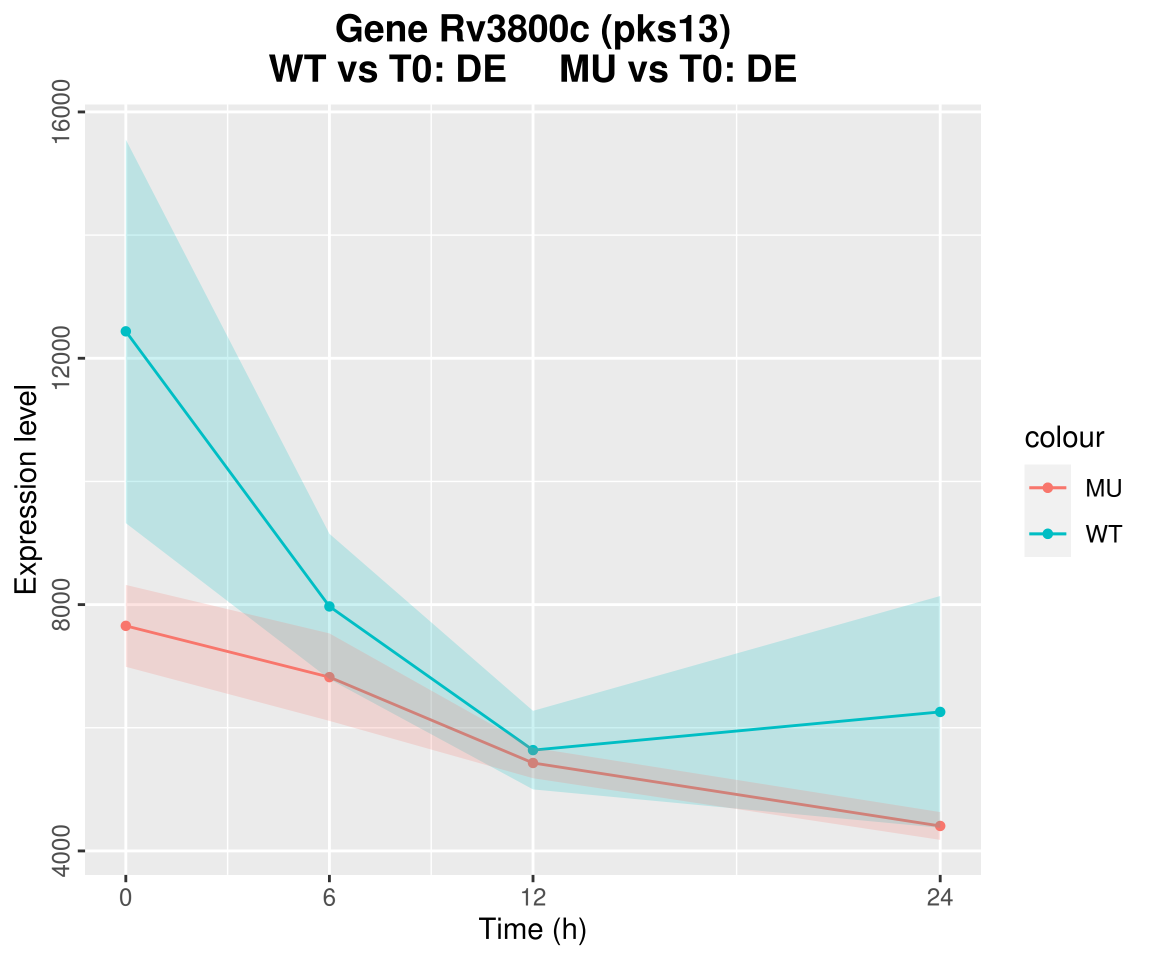
Gene Rv3800c (pks13)
WT vs T0: DE   MU vs T0: DE
Expression level
16000
12000
8000
4000
0
6
12
24
Time (h)
colour
MU
WT

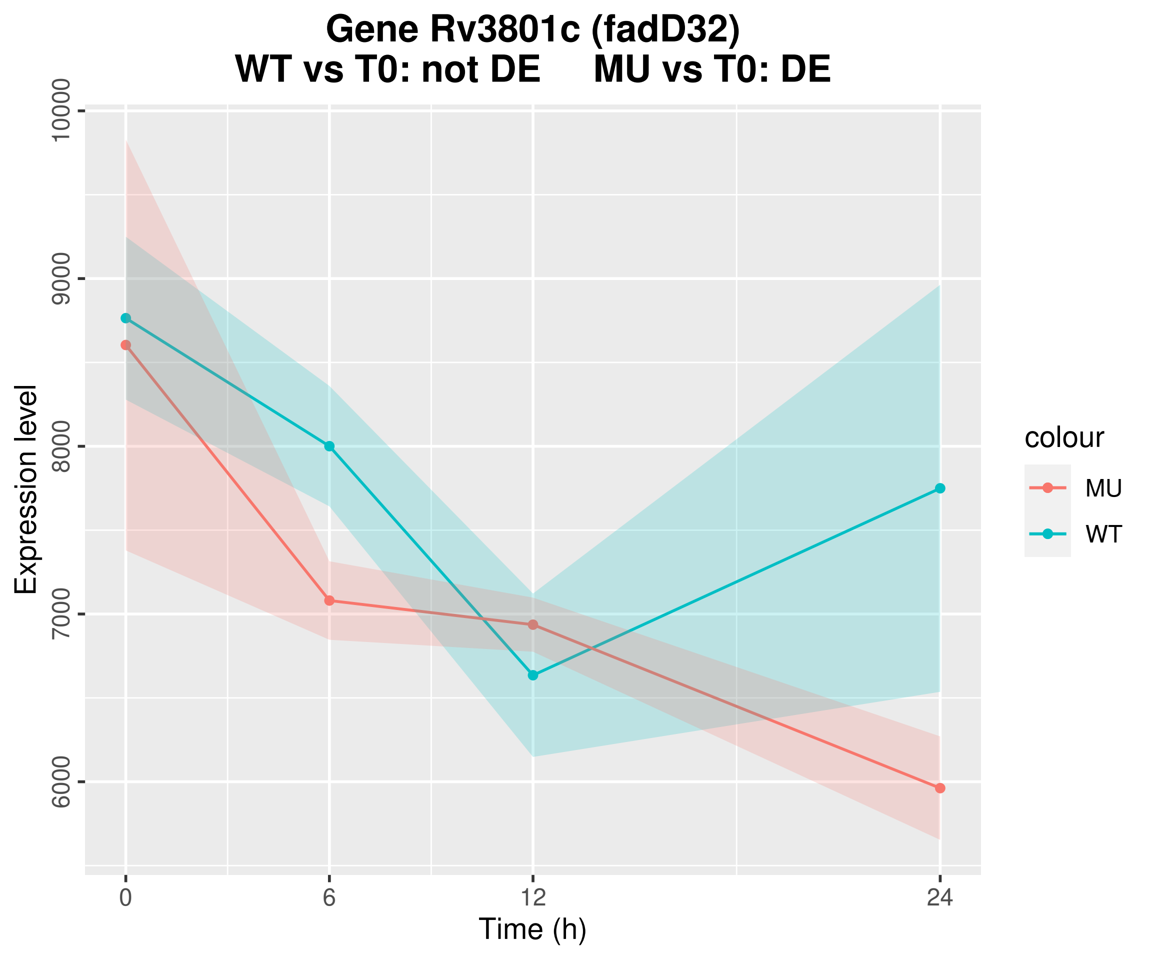
Gene Rv3801c (fadD32)
WT vs T0: not DE MU vs T0: DE
Expression level
10000
9000
8000
7000
6000
0
6
12
24
Time (h)
colour
MU
WT

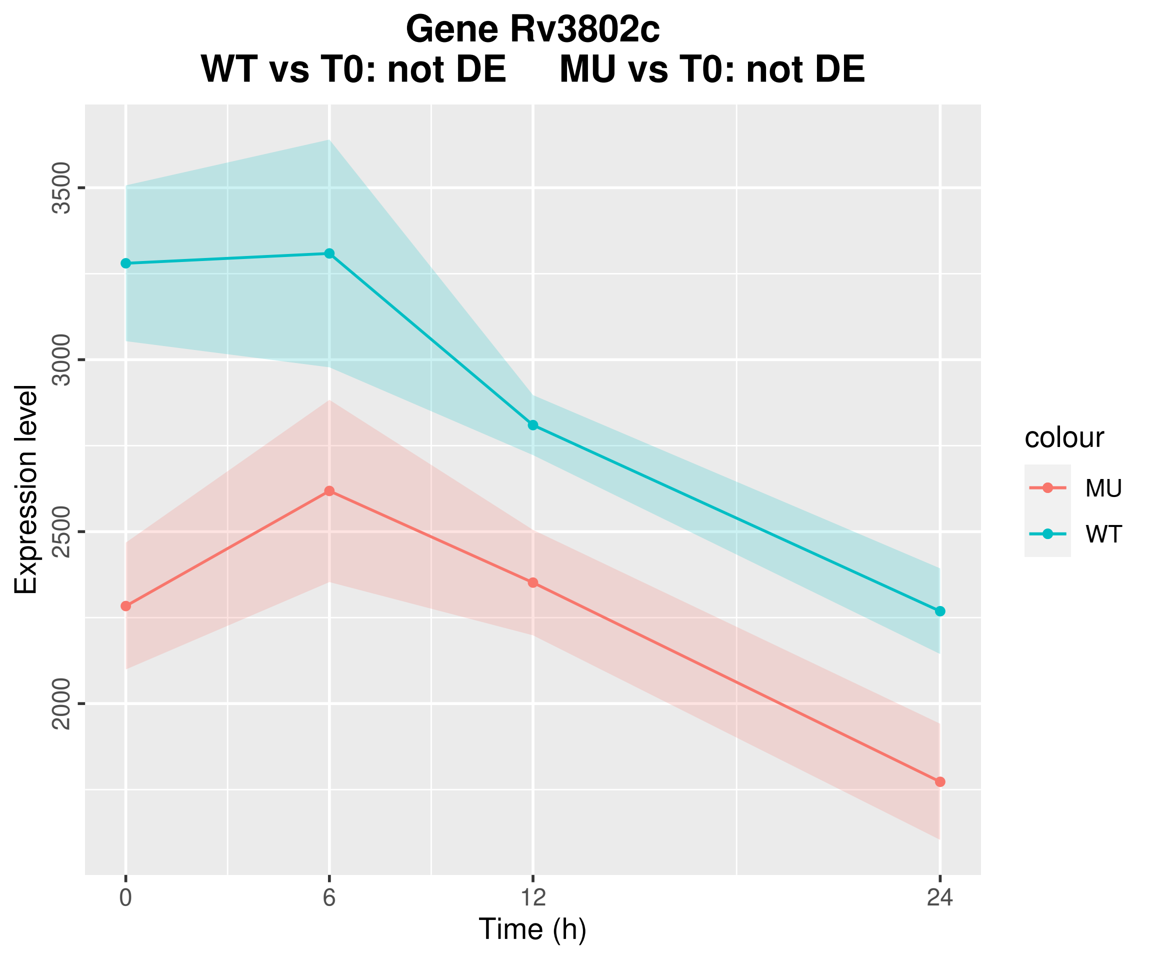

Gene Rv3802c
WT vs T0: not DE   MU vs T0: not DE
Expression level
3500
3000
2500
2000
0
6
12
24
Time (h)
colour
MU
WT

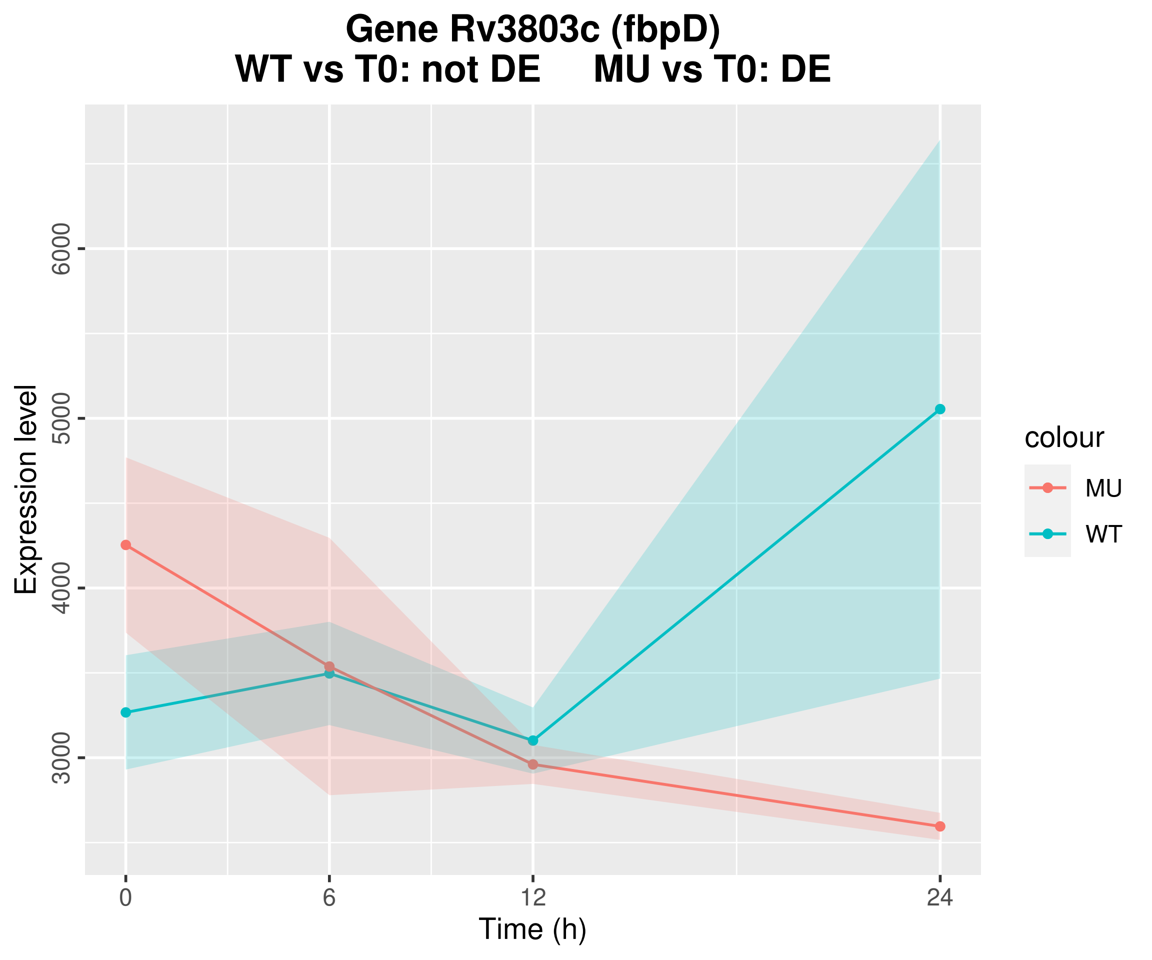
Gene Rv3803c (fbpD)
WT vs T0: not DE   MU vs T0: DE
Expression level
6000
5000
4000
3000
0
6
12
24
Time (h)
colour
MU
WT

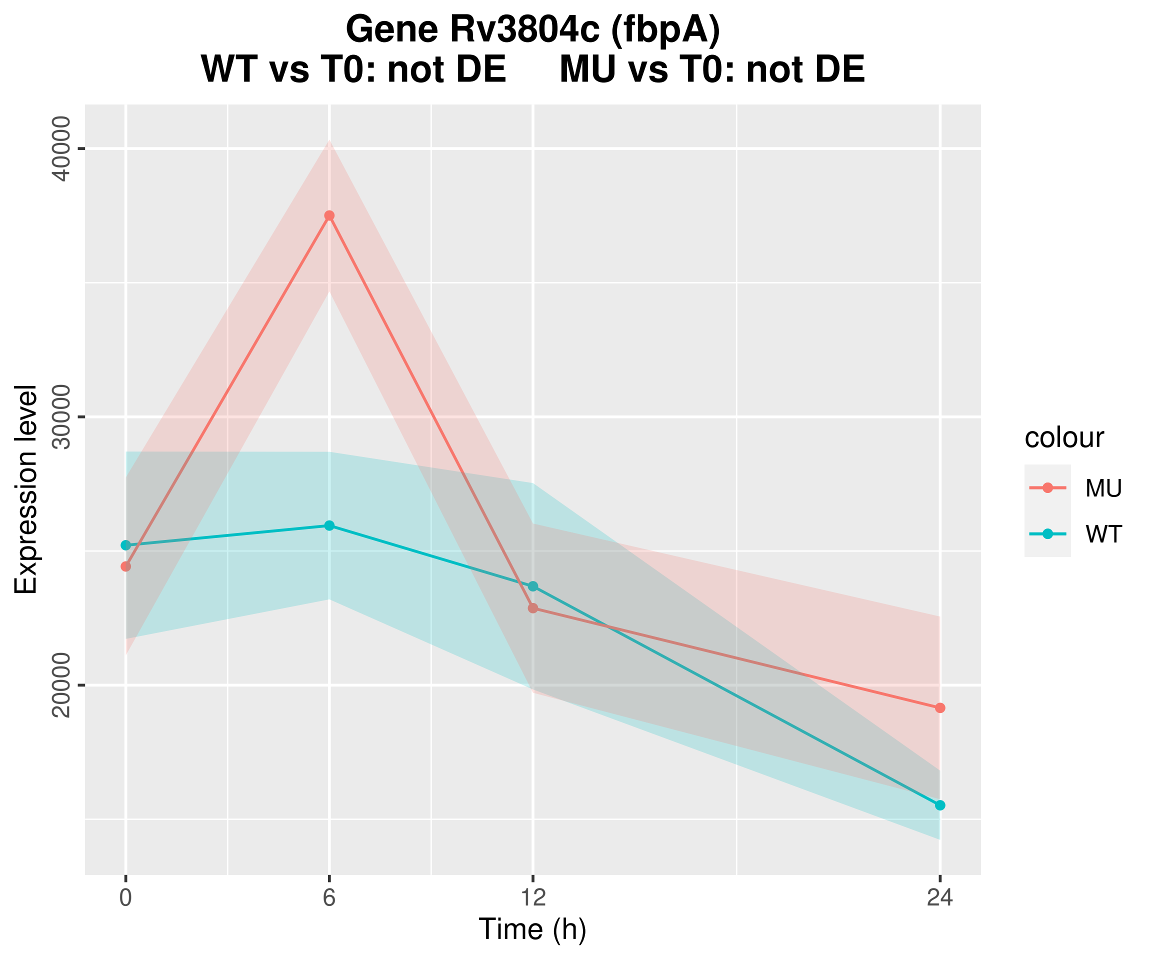

Gene Rv3804c (fbpA)
WT vs T0: not DE MU vs T0: not DE
Expression level
40000
30000
20000
0
6
12
24
Time (h)
colour
MU
WT

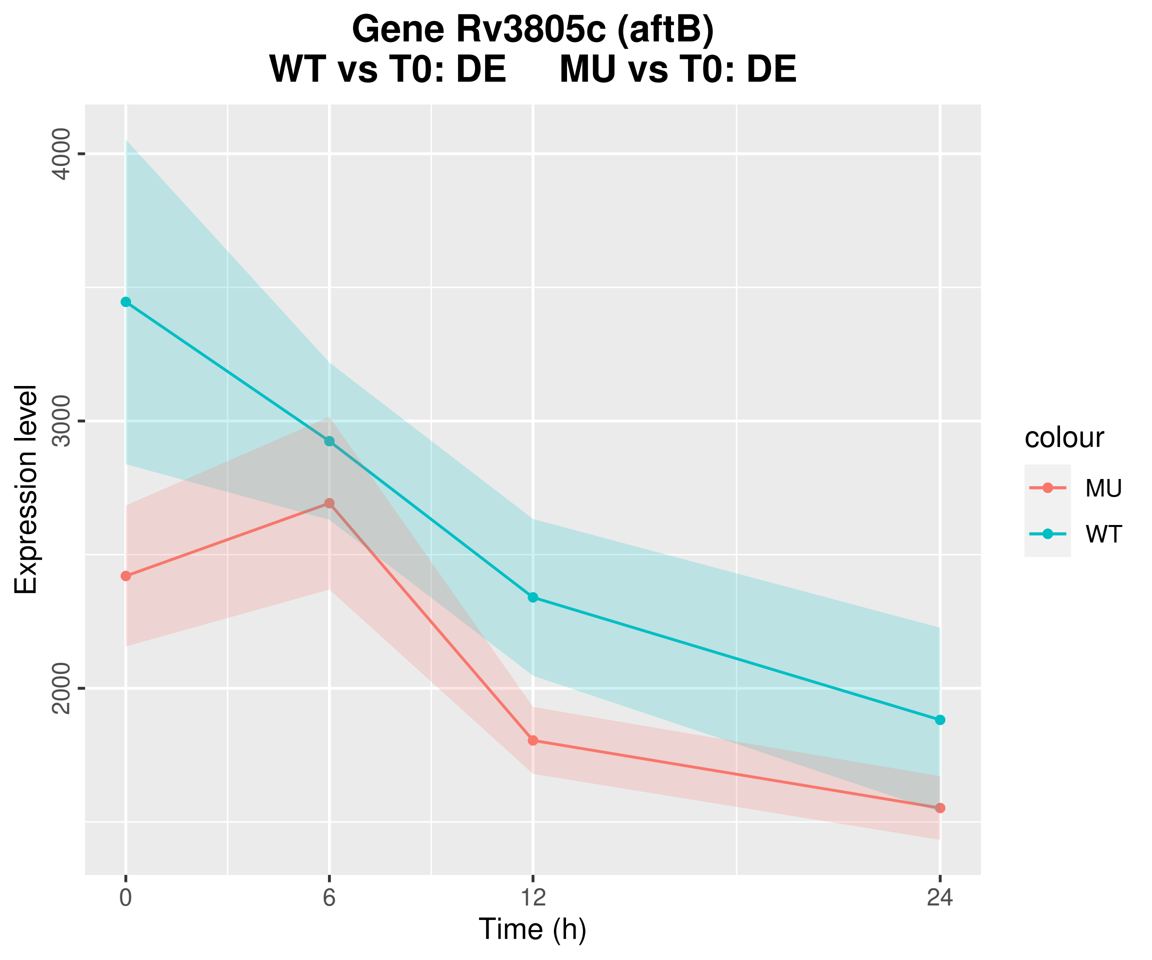
Gene Rv3805c (aftB)
WT vs T0: DE MU vs T0: DE
Expression level
4000
3000
2000
0
6
12
24
Time (h)
colour
MU
WT

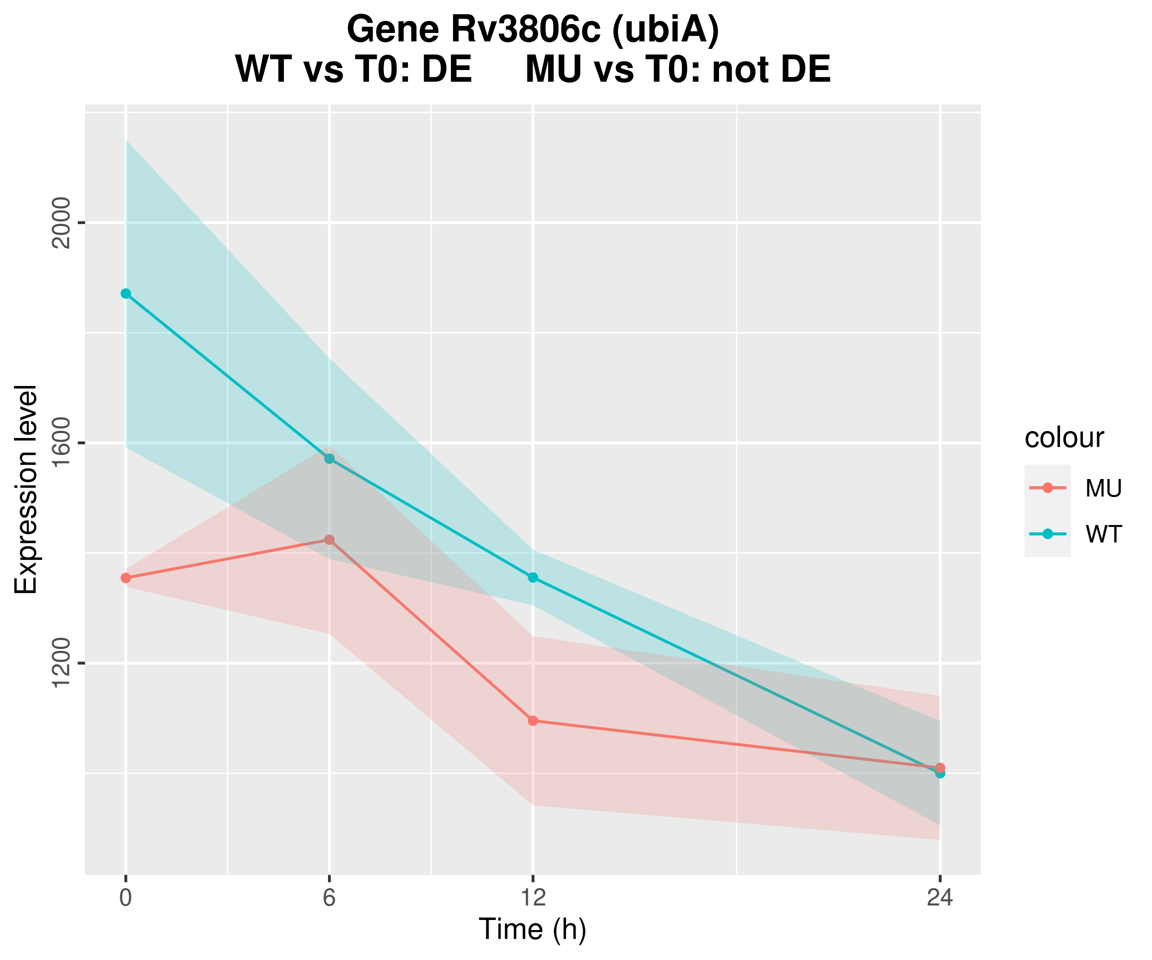
Gene Rv3806c (ubiA)
WT vs T0: DE   MU vs T0: not DE
Expression level
2000
1600
1200
0
6
12
24
Time (h)
colour
MU
WT

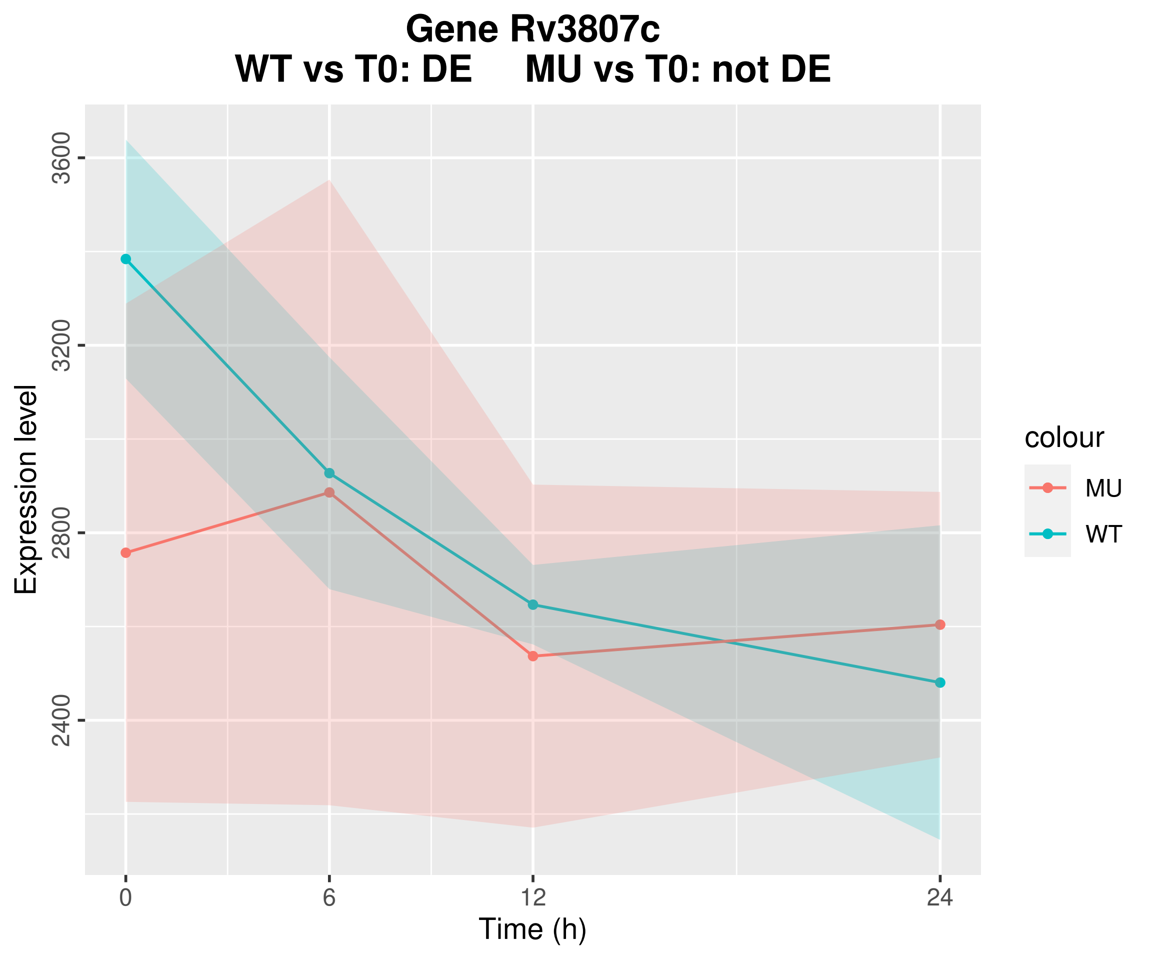

Gene Rv3807c
WT vs T0: DE   MU vs T0: not DE
Expression level
3600
3200
2800
2400
0
6
12
24
Time (h)
colour
MU
WT

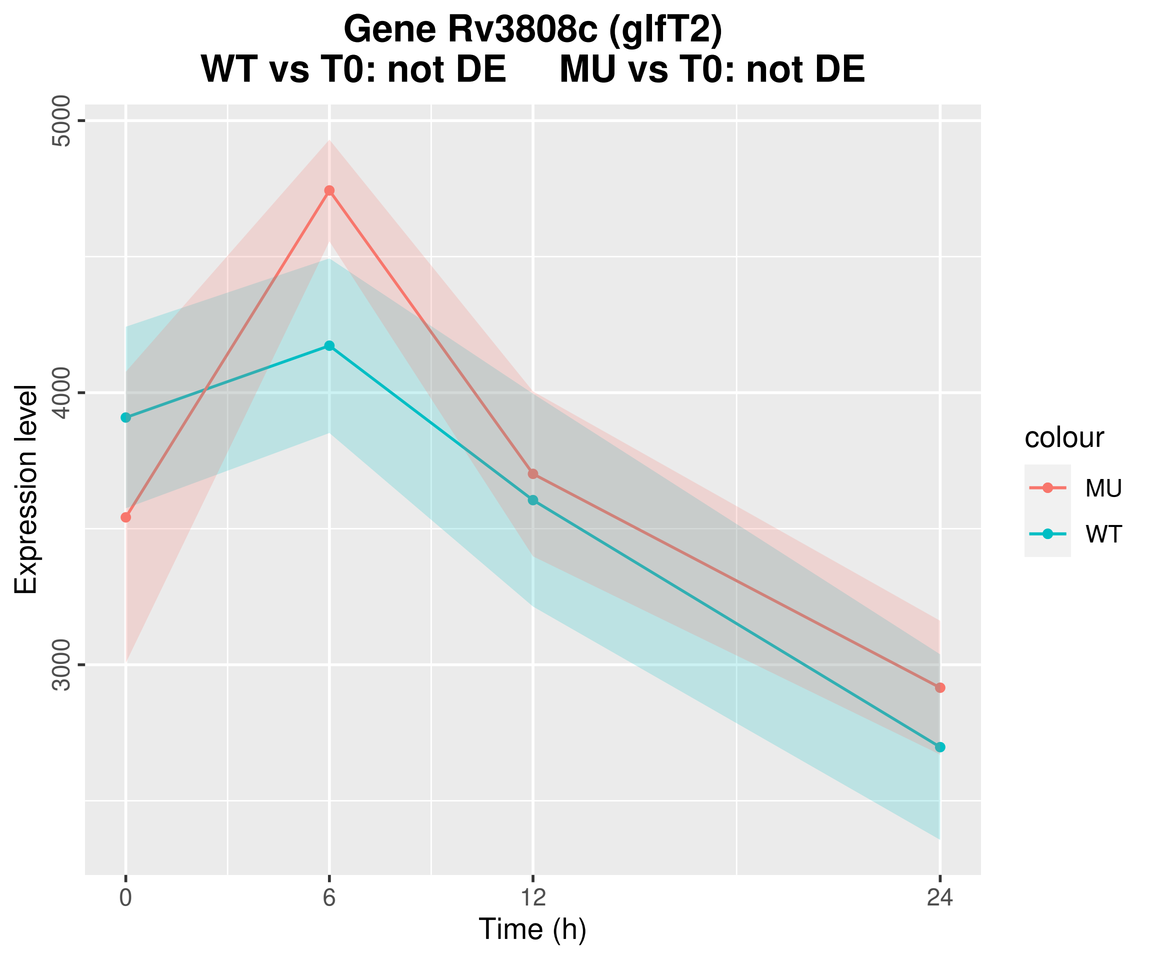
Gene Rv3808c (glfT2)
WT vs T0: not DE
MU vs T0: not DE
Expression level
5000
4000
3000
Time (h)
0
6
12
24
colour
MU
WT

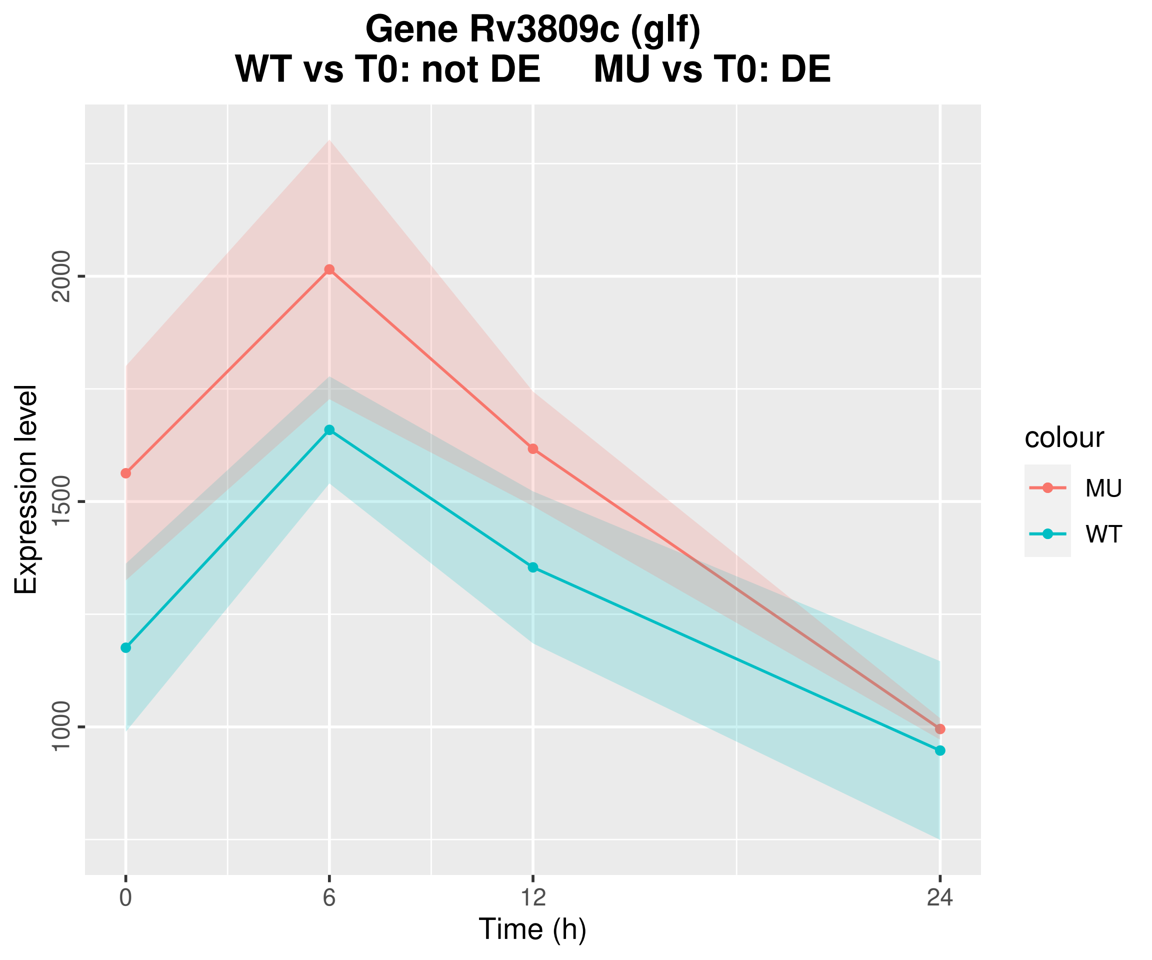

Gene Rv3809c (glf)
WT vs T0: not DE MU vs T0: DE
Expression level
2000
1500
1000
0
6
12
24
Time (h)
colour
MU
WT

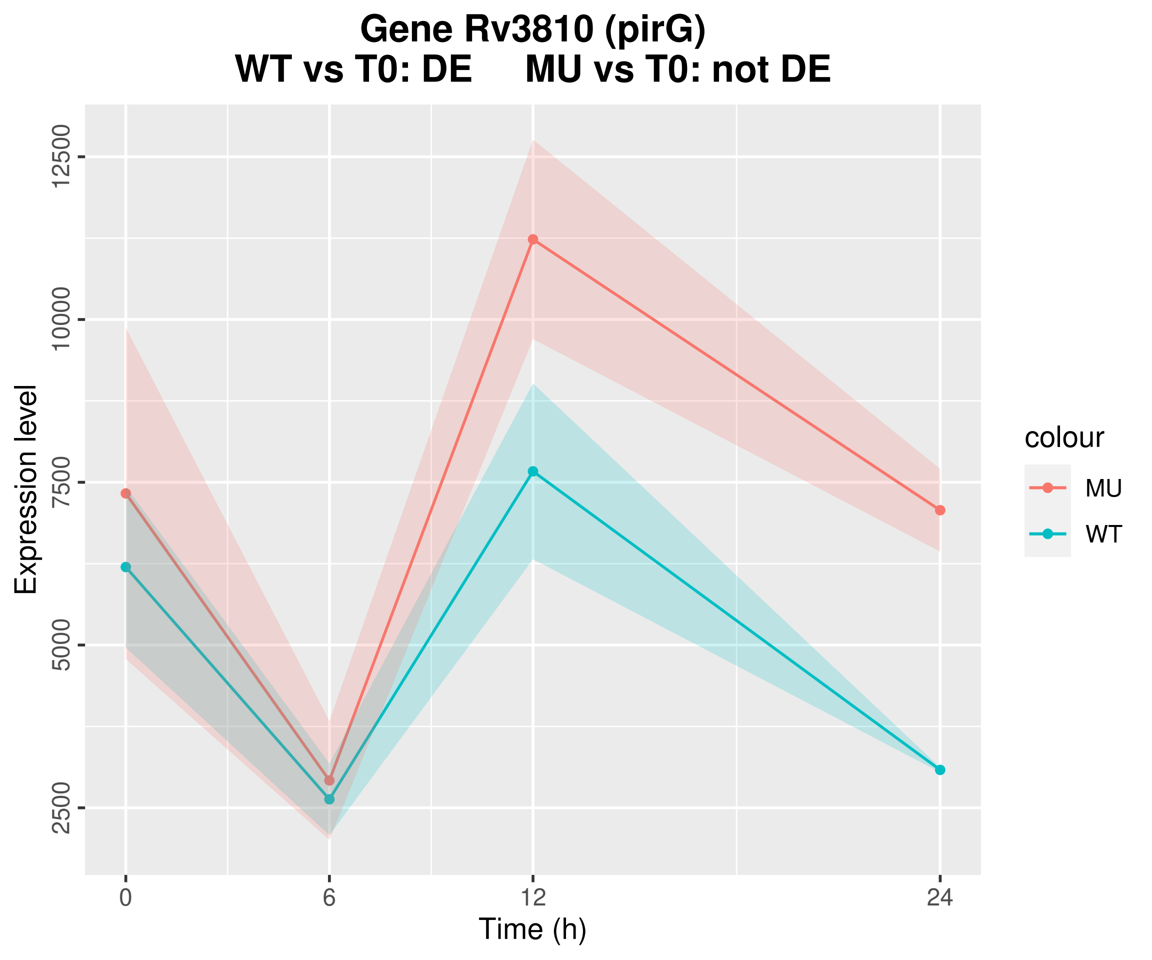

Gene Rv3810 (pirG)
WT vs T0: DE   MU vs T0: not DE
Expression level
12500
10000
7500
5000
2500
0
6
12
24
Time (h)
colour
MU
WT

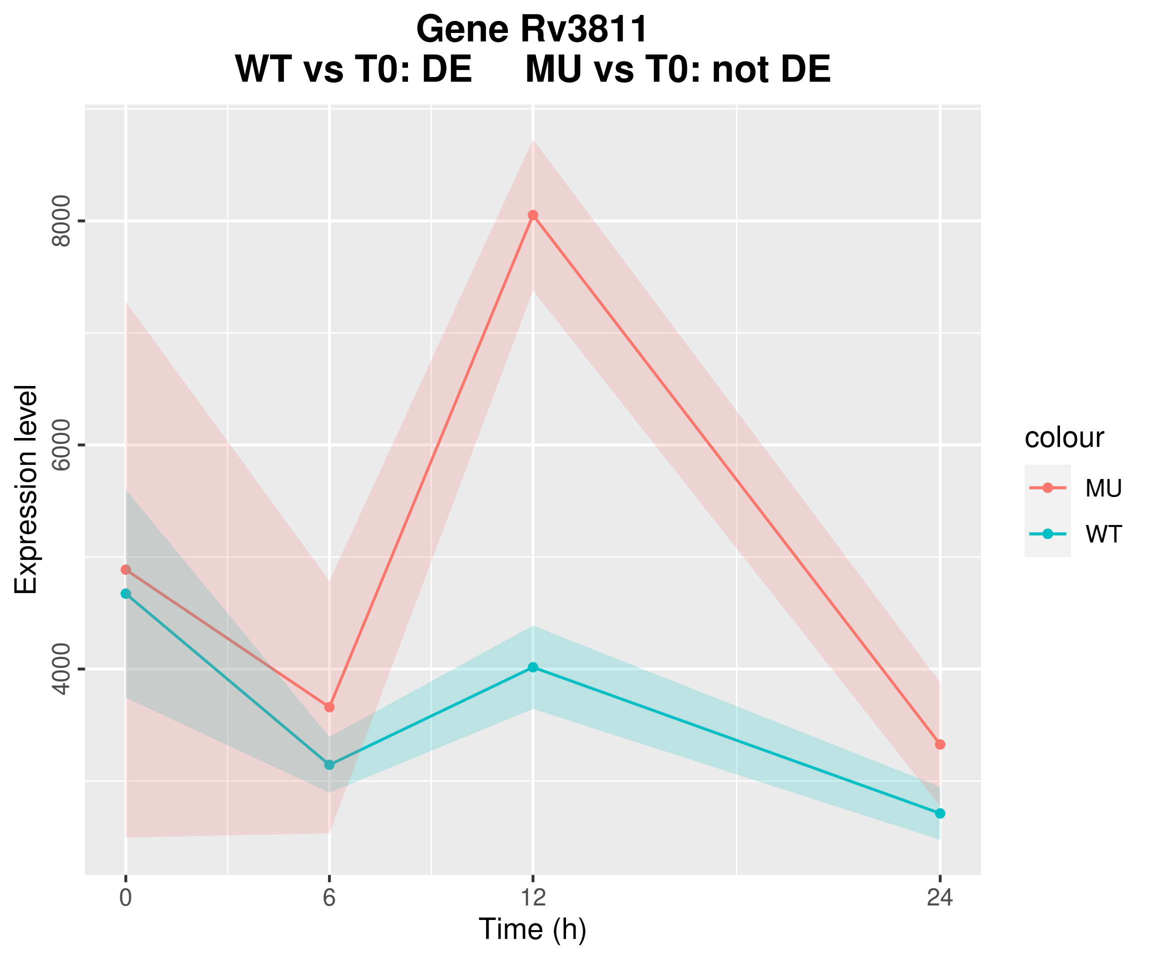
Gene Rv3811
WT vs T0: DE MU vs T0: not DE
Expression level
8000
6000
4000
0
6
12
24
Time (h)
colour
MU
WT

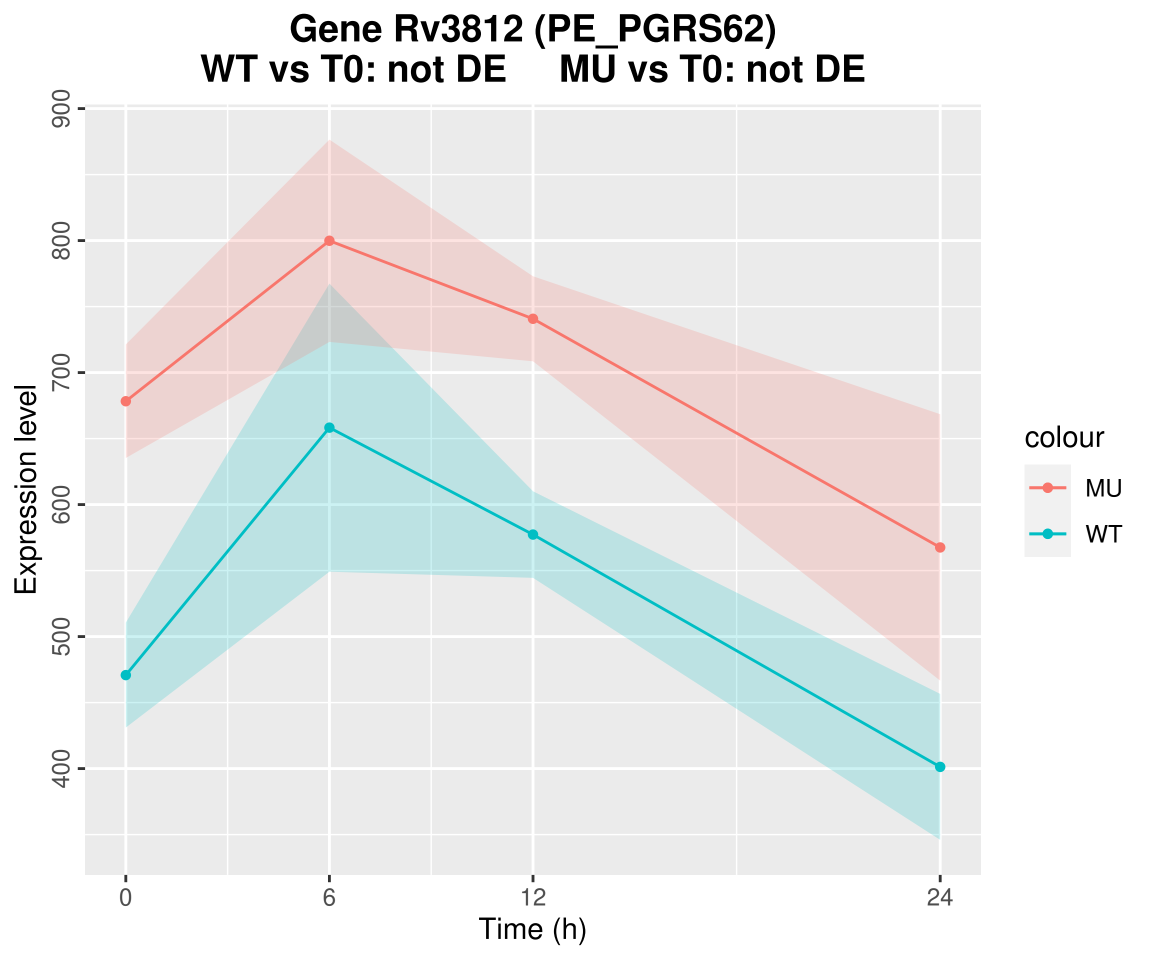

Gene Rv3812 (PE_PGRS62)
WT vs T0: not DE   MU vs T0: not DE
900
800
700
600
500
400
Expression level
0
6
12
24
Time (h)
colour
MU
WT

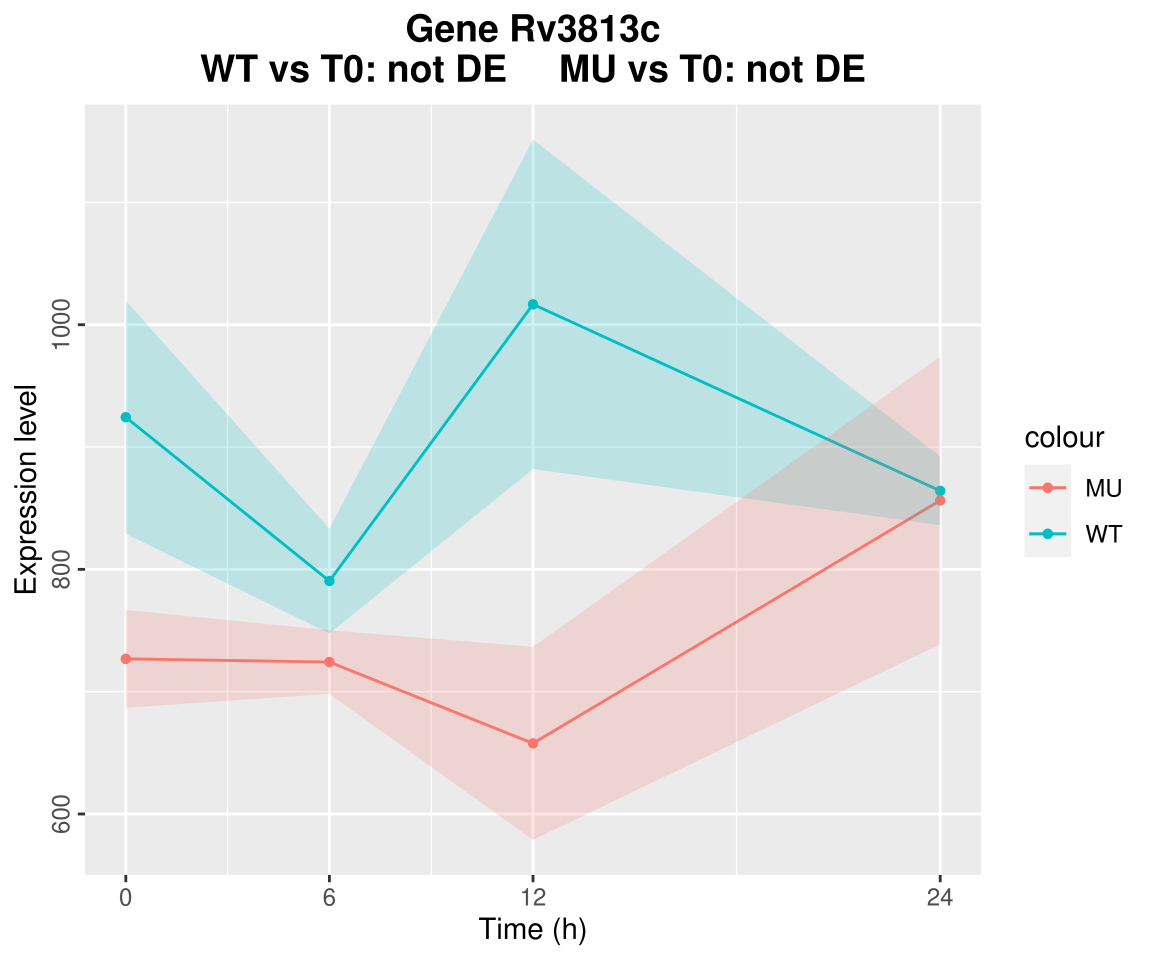
Gene Rv3813c
WT vs T0: not DE MU vs T0: not DE
Expression level
1000
800
600
0
6
12
24
Time (h)
colour
MU
WT

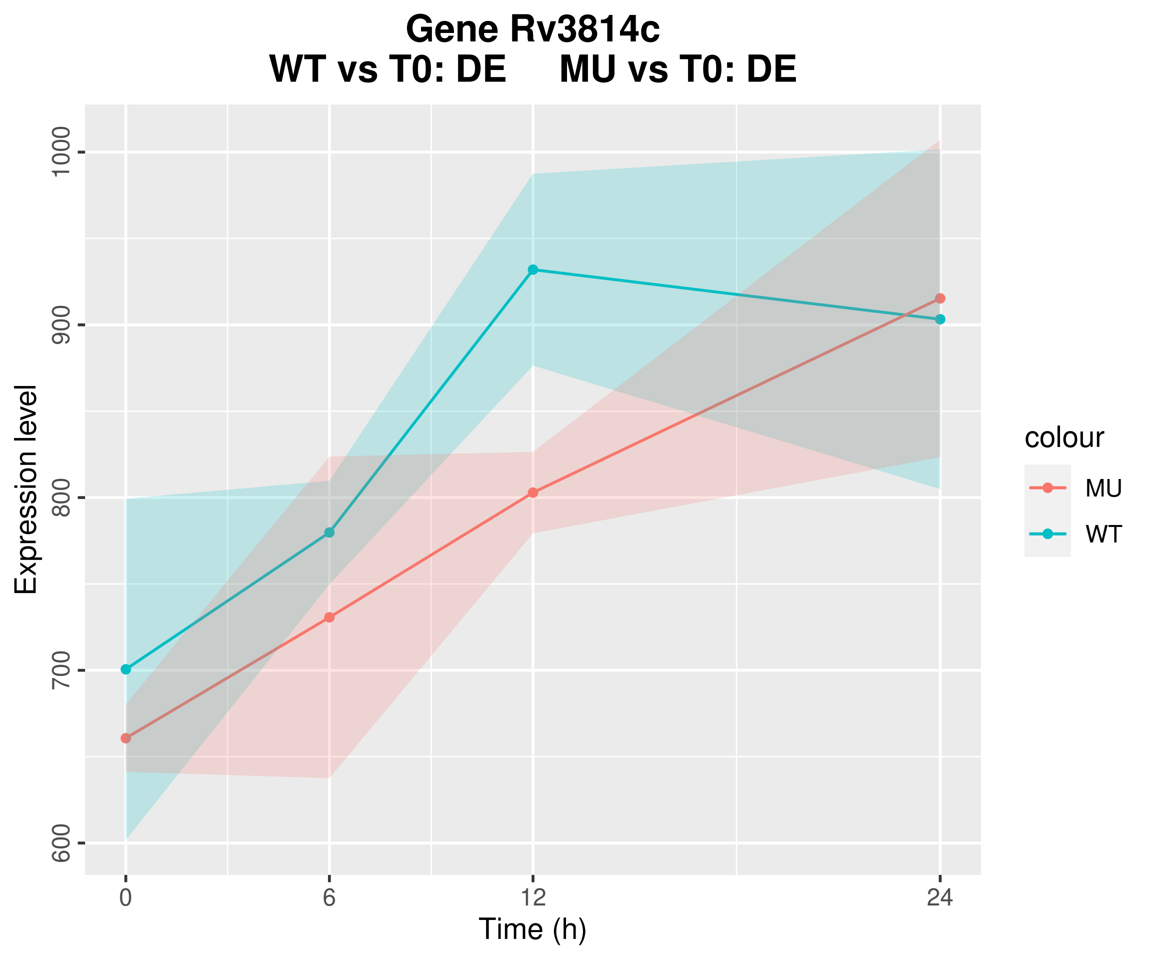
Gene Rv3814c
WT vs T0: DE MU vs T0: DE
Expression level
1000
900
800
700
600
0
6
12
24
Time (h)
colour
MU
WT

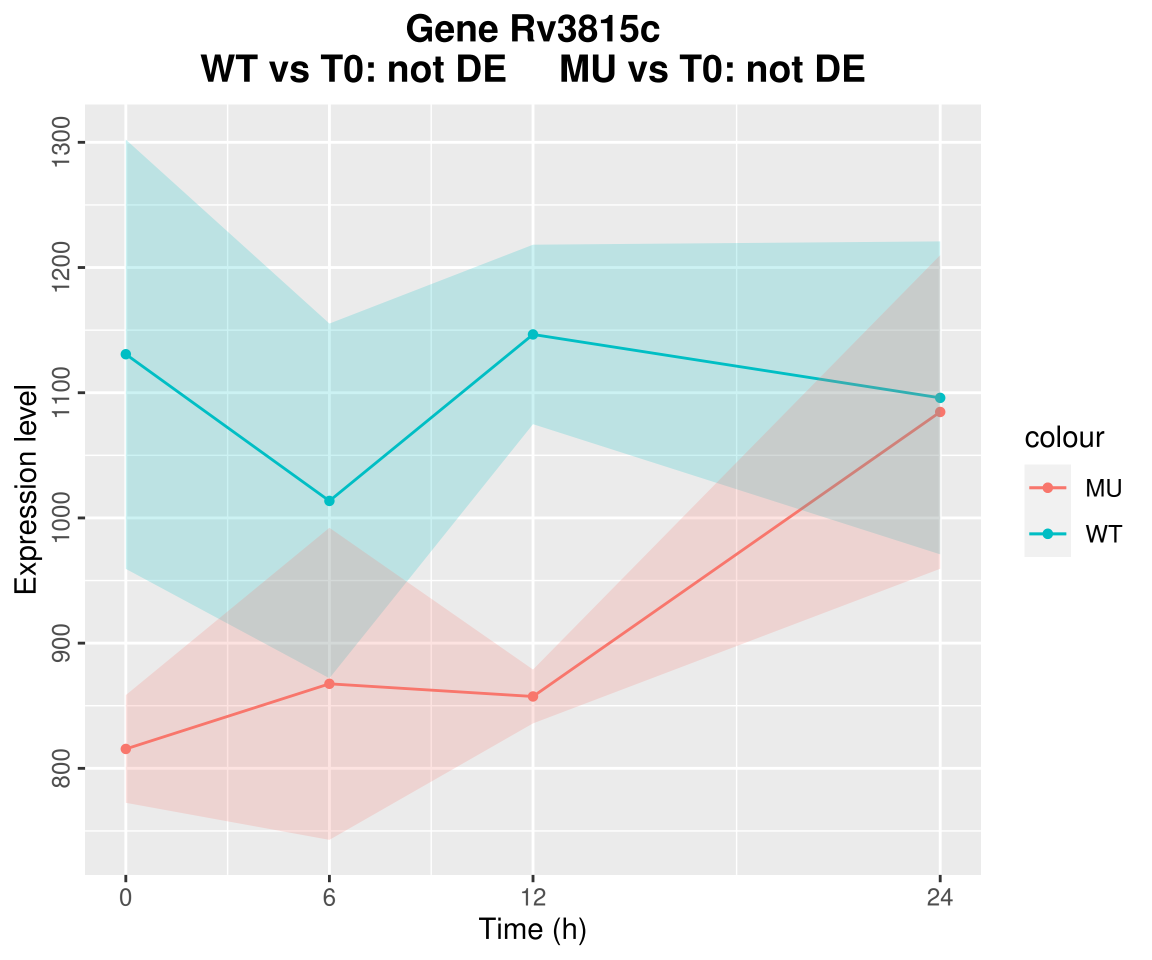
Gene Rv3815c
WT vs T0: not DE MU vs T0: not DE
Expression level
1300
1200
1100
1000
900
800
0
6
12
24
Time (h)
colour
MU
WT

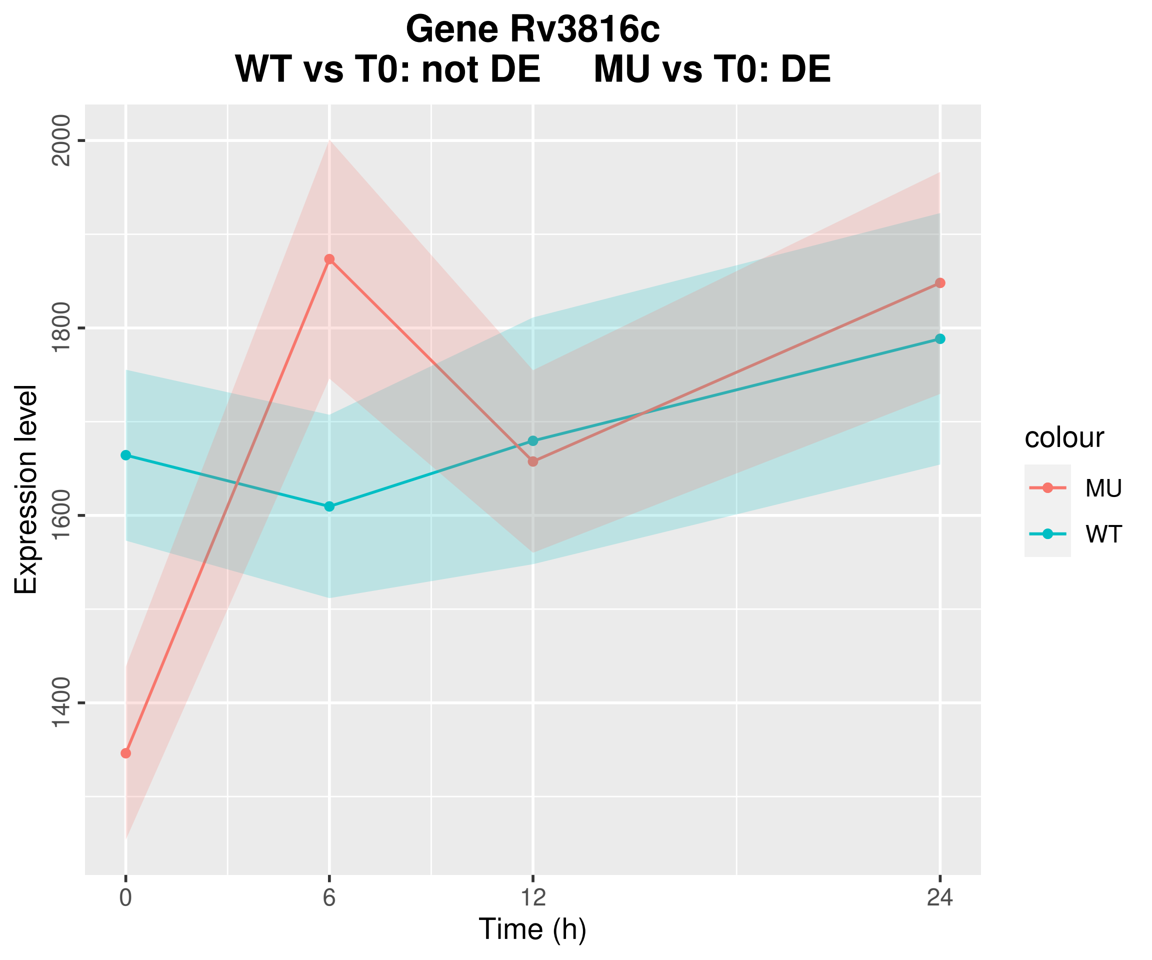
Gene Rv3816c
WT vs T0: not DE MU vs T0: DE
Expression level
2000
1800
1600
1400
0
6
12
24
Time (h)
colour
MU
WT

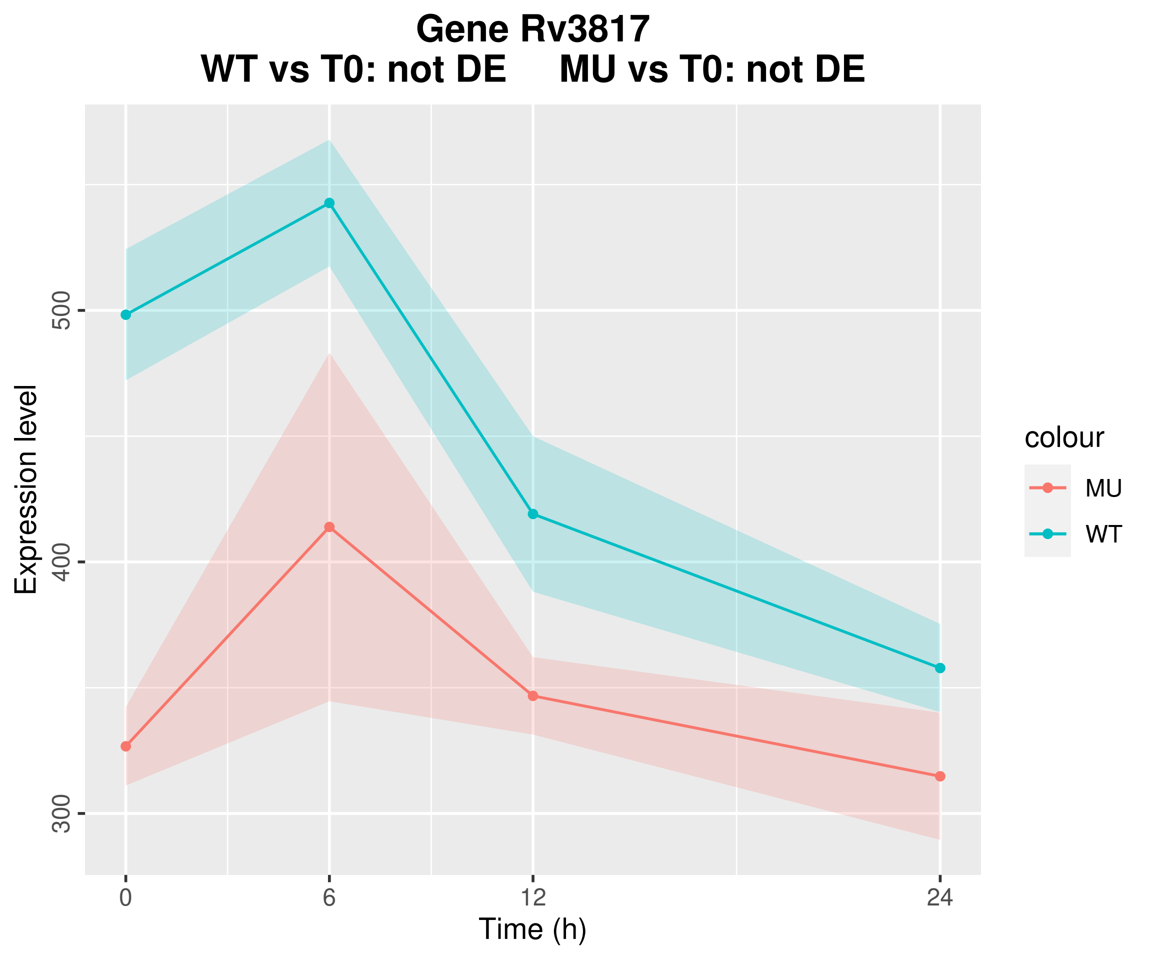

Gene Rv3817
WT vs T0: not DE MU vs T0: not DE
Expression level
500
400
300
0
6
12
24
Time (h)
colour
MU
WT

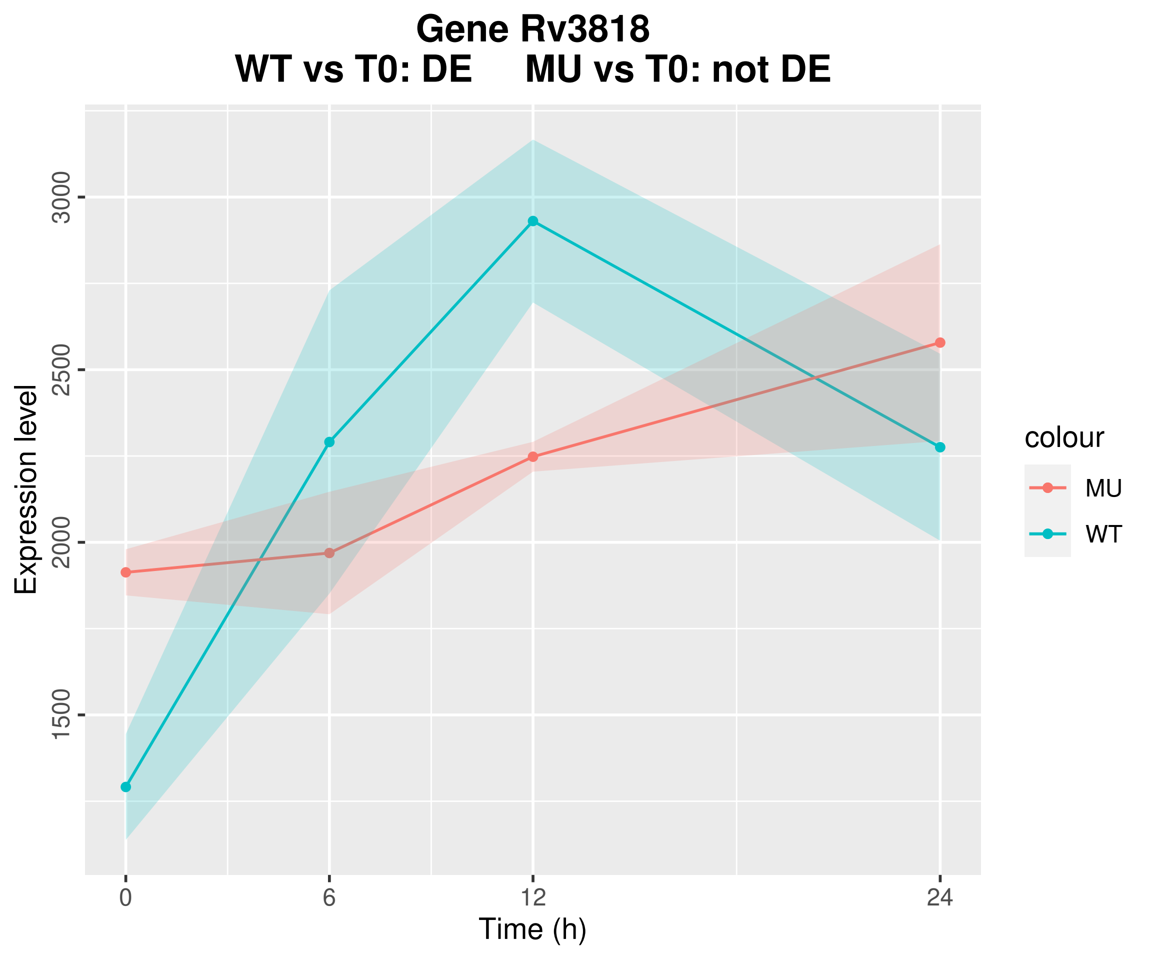

Gene Rv3818
WT vs T0: DE MU vs T0: not DE
Expression level
3000
2500
2000
1500
0
6
12
24
Time (h)
colour
MU
WT

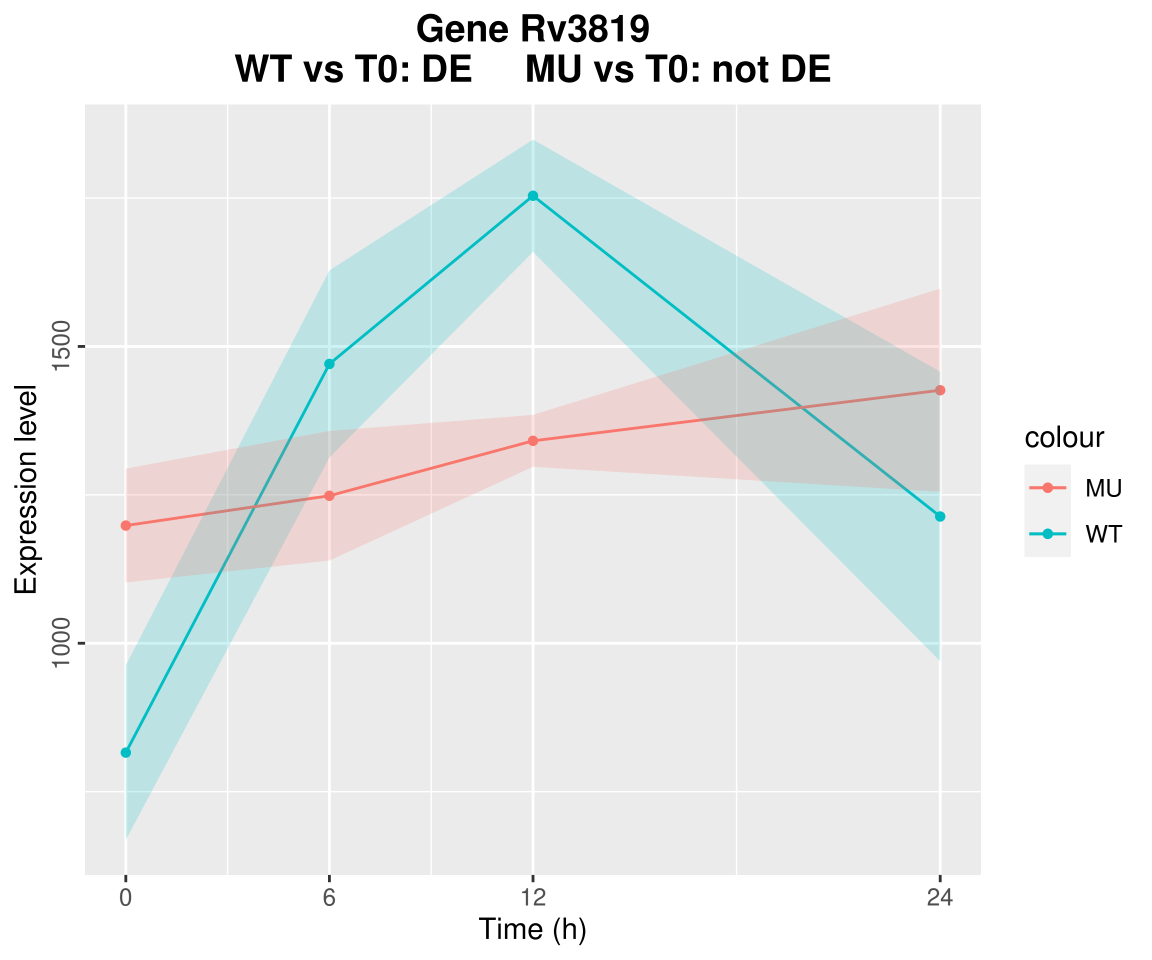

Gene Rv3819
WT vs T0: DE   MU vs T0: not DE
Expression level
1500
1000
0
6
12
24
Time (h)
colour
MU
WT

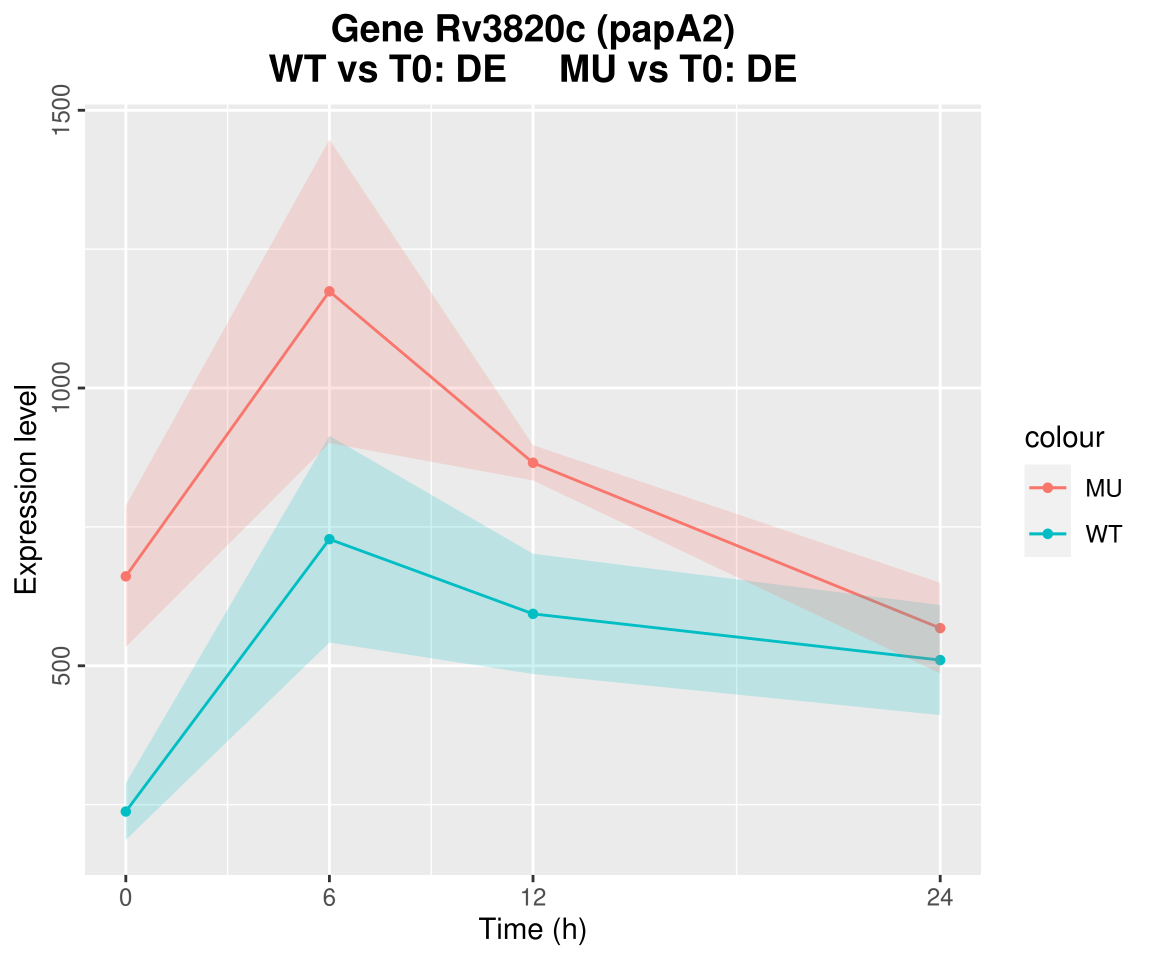

Gene Rv3820c (papA2)
WT vs T0: DE MU vs T0: DE
1500
1000
500
Expression level
0
6
12
24
Time (h)
colour
MU
WT

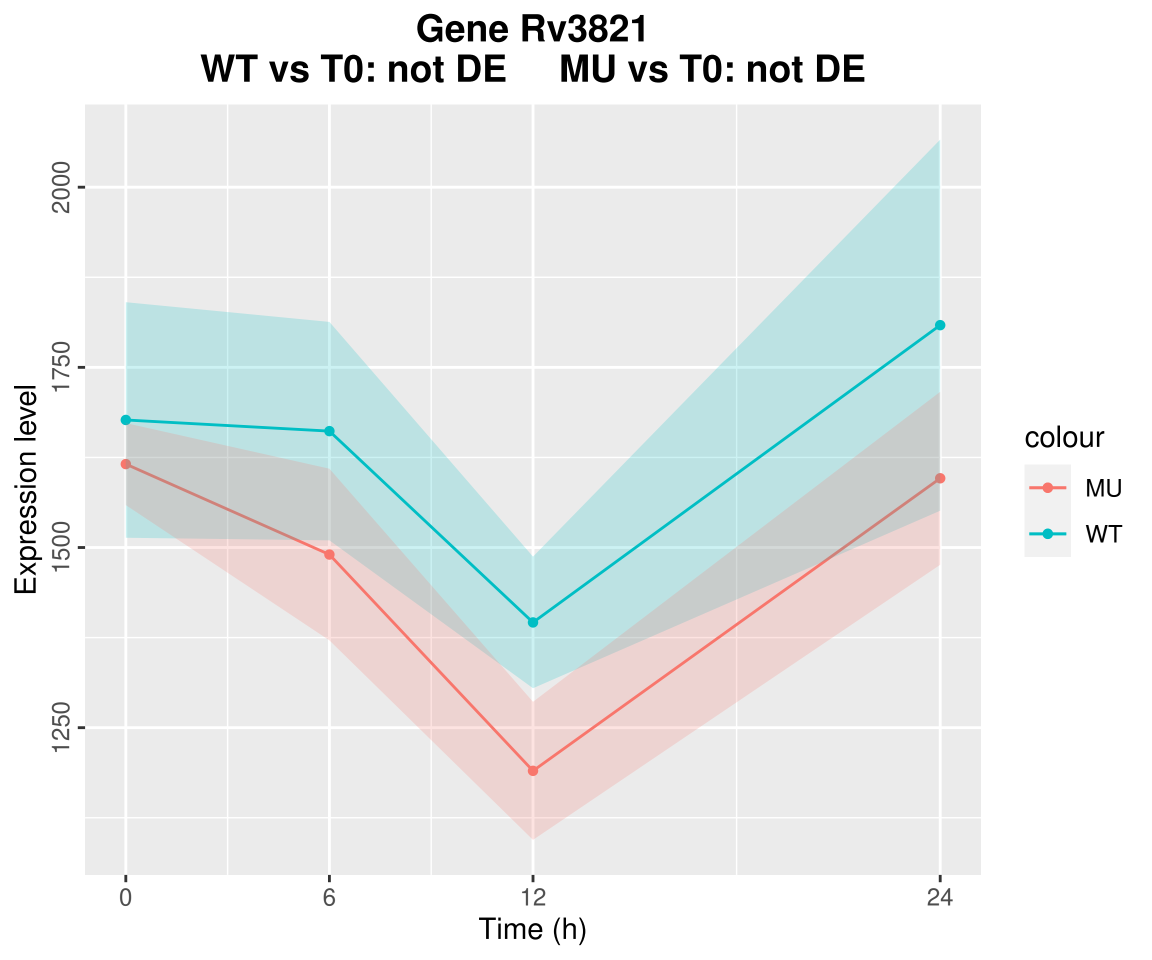
Gene Rv3821
WT vs T0: not DE MU vs T0: not DE
Expression level
2000
1750
1500
1250
0
6
12
24
Time (h)
colour
MU
WT

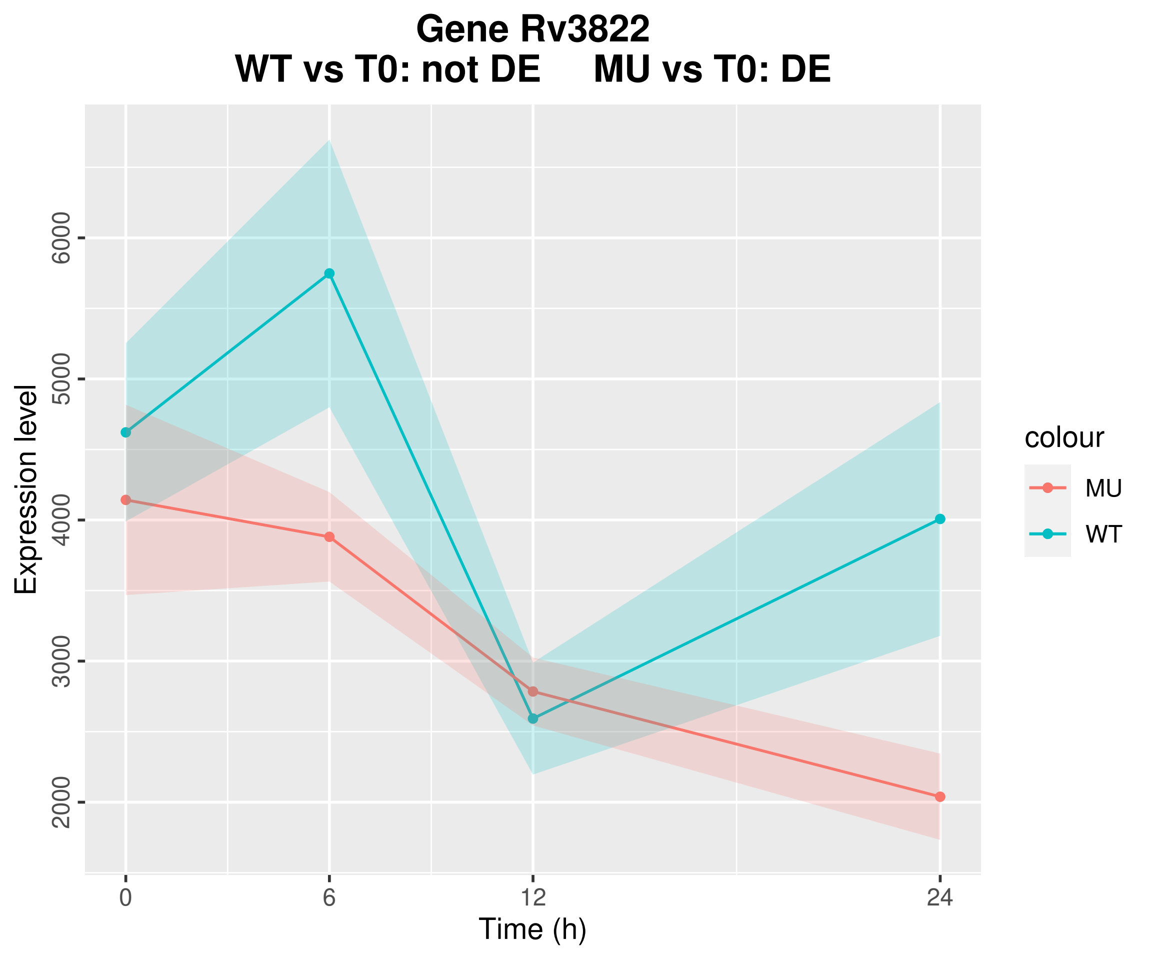
Gene Rv3822
WT vs T0: not DE   MU vs T0: DE
Expression level
6000
5000
4000
3000
2000
0
6
12
24
Time (h)
colour
MU
WT

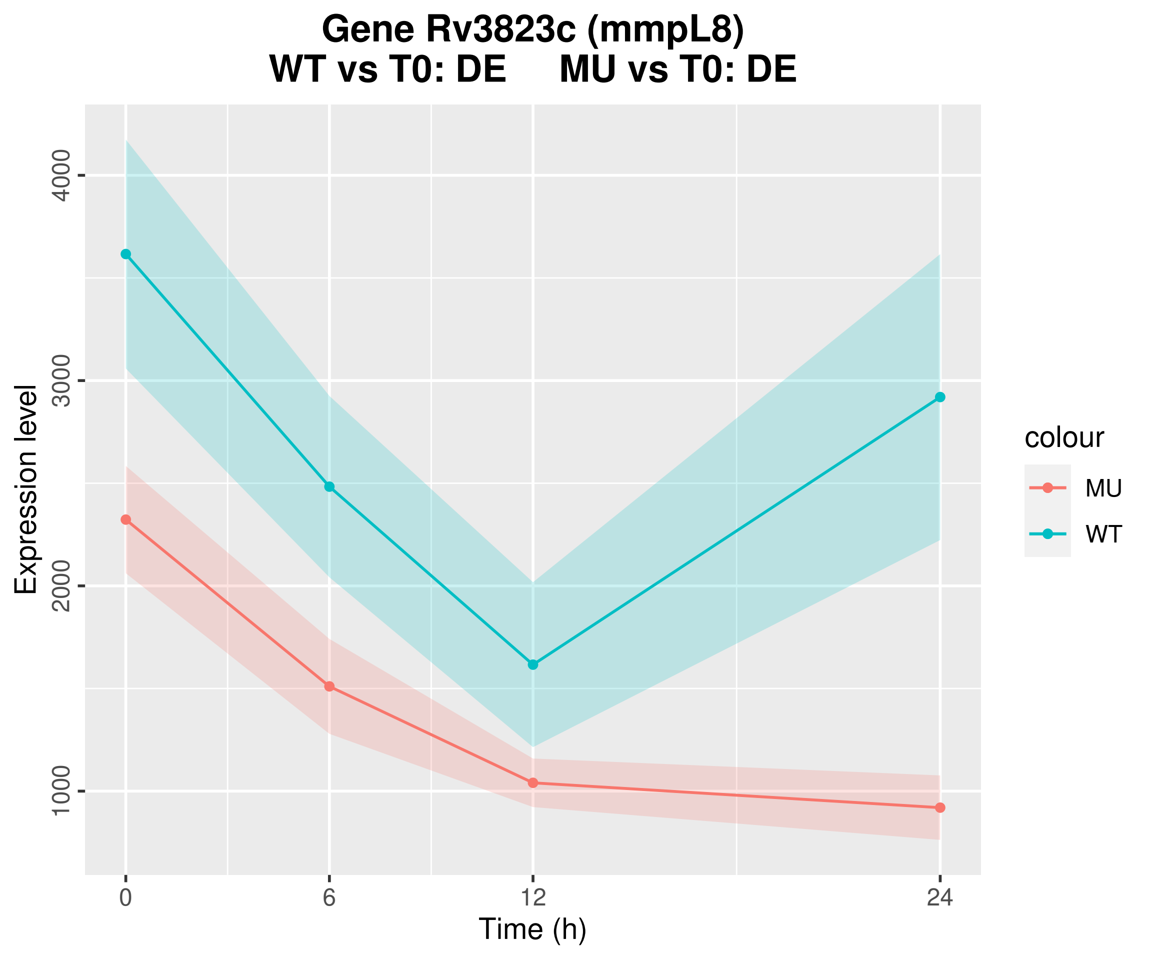
Gene Rv3823c (mmpL8)
WT vs T0: DE   MU vs T0: DE
Expression level
4000
3000
2000
1000
0
6
12
24
Time (h)
colour
MU
WT

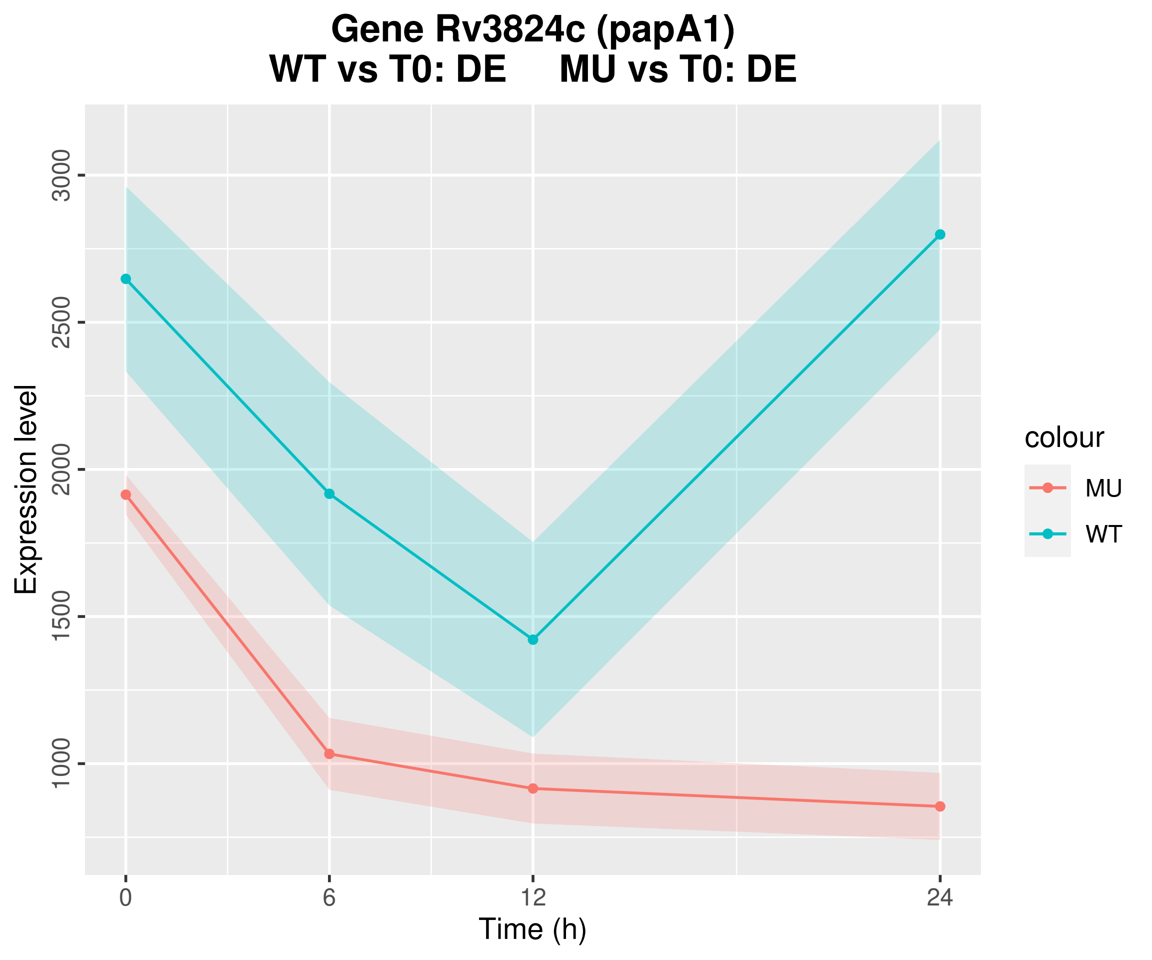

Gene Rv3824c (papA1)
WT vs T0: DE MU vs T0: DE
Expression level
3000
2500
2000
1500
1000
0
6
12
24
Time (h)
colour
MU
WT

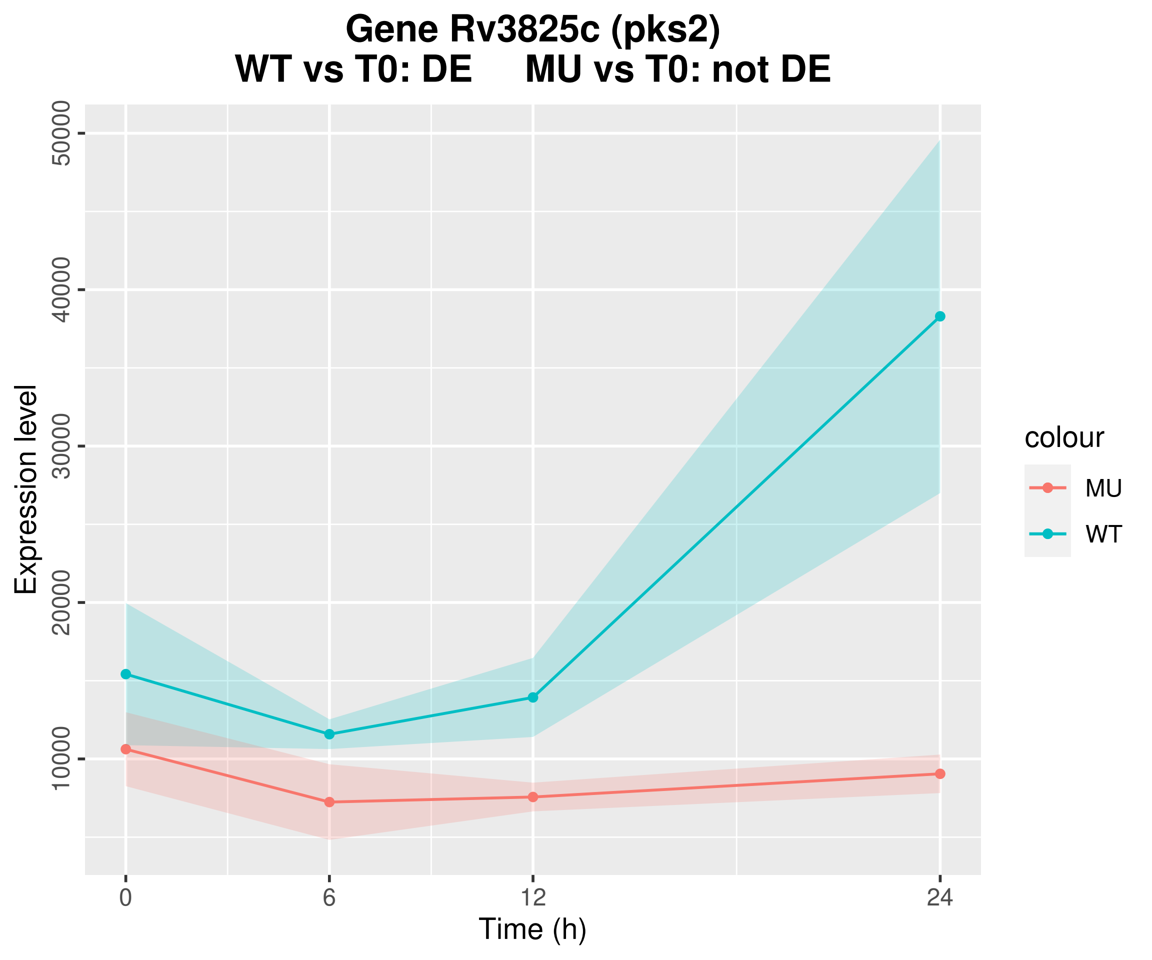

Gene Rv3825c (pks2)
WT vs T0: DE MU vs T0: not DE
Expression level
50000
40000
30000
20000
10000
0
6
12
24
Time (h)
colour
MU
WT

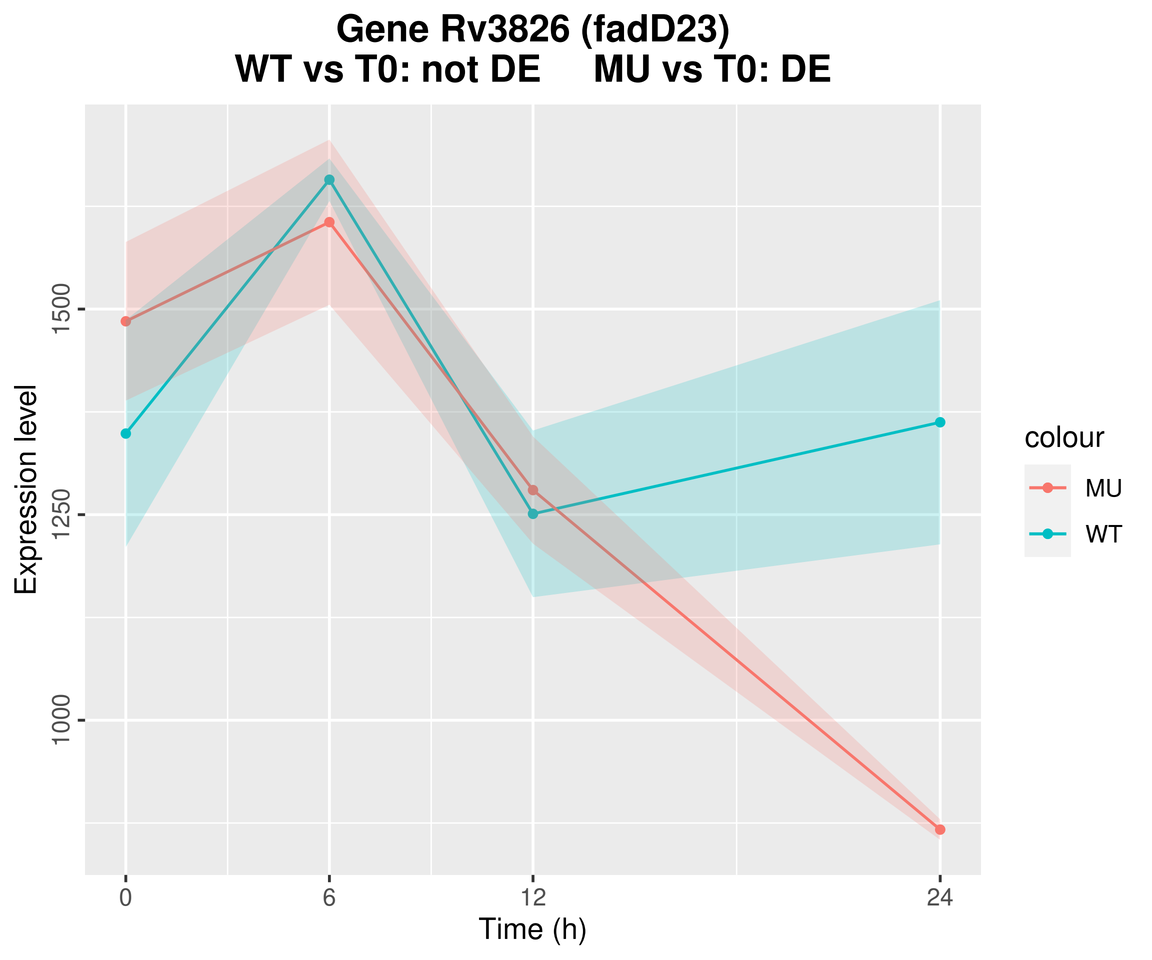
Gene Rv3826 (fadD23)
WT vs T0: not DE   MU vs T0: DE
Expression level
1500
1250
1000
0
6
12
24
Time (h)
colour
MU
WT

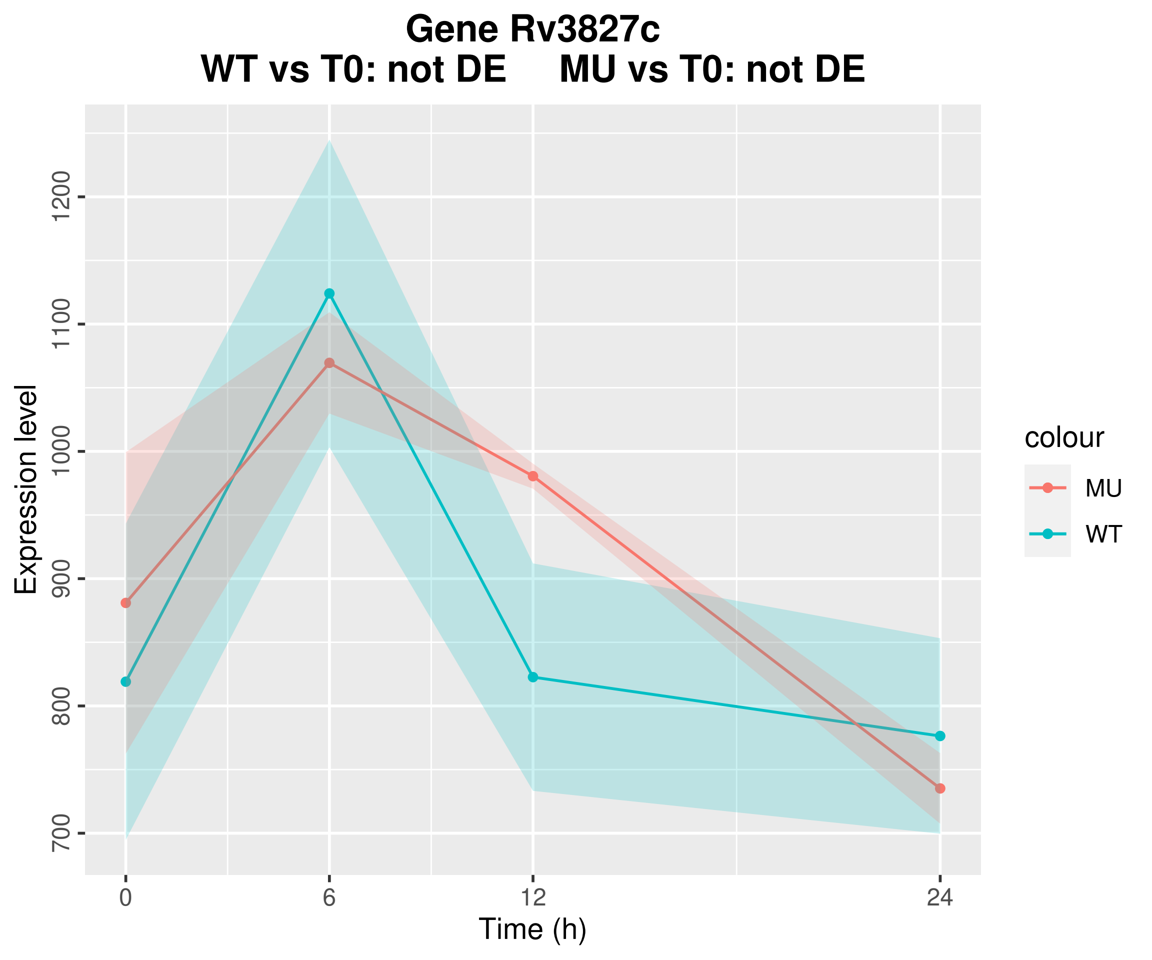
Gene Rv3827c
WT vs T0: not DE MU vs T0: not DE
Expression level
1200
1100
1000
900
800
700
0
6
12
24
Time (h)
colour
MU
WT

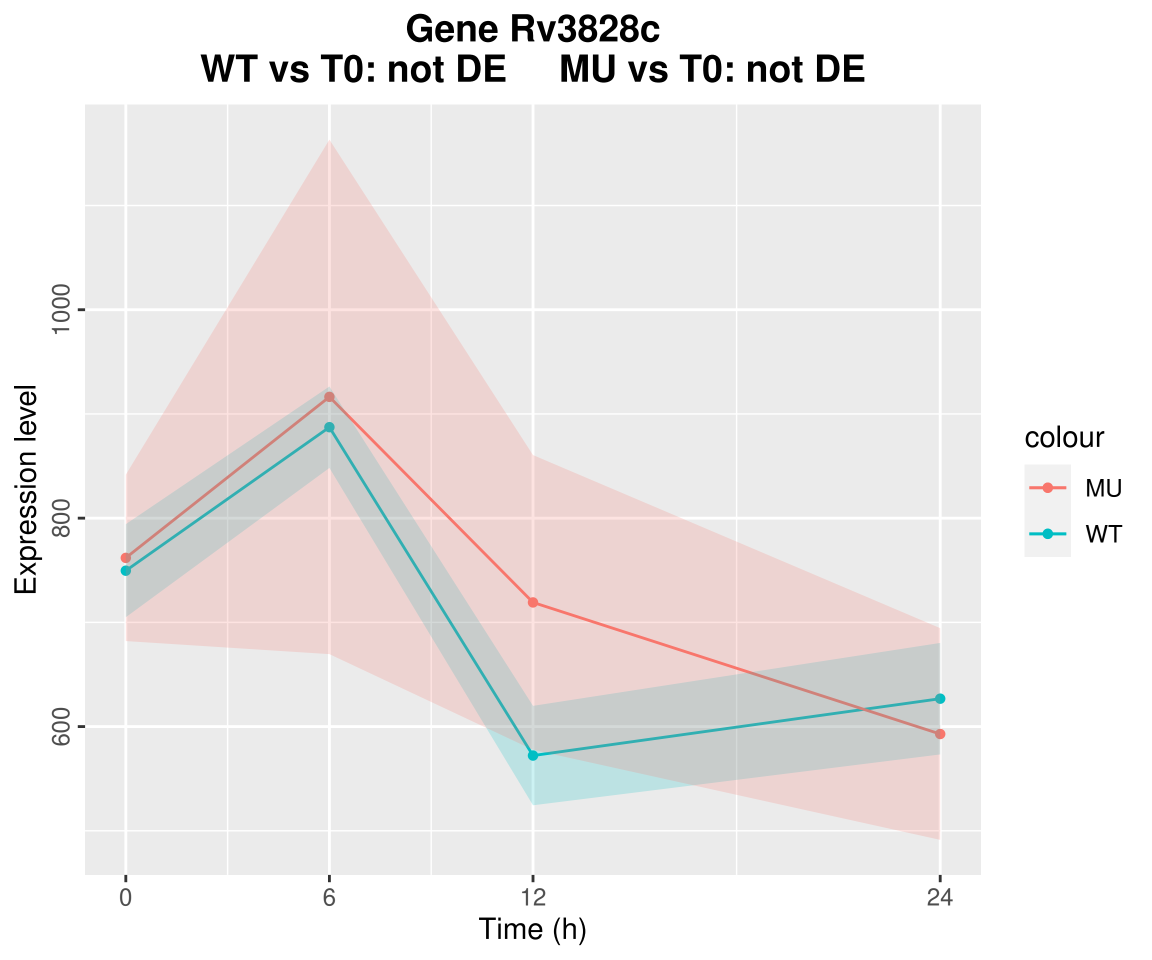
Gene Rv3828c
WT vs T0: not DE   MU vs T0: not DE
Expression level
1000
800
600
0
6
12
24
Time (h)
colour
MU
WT

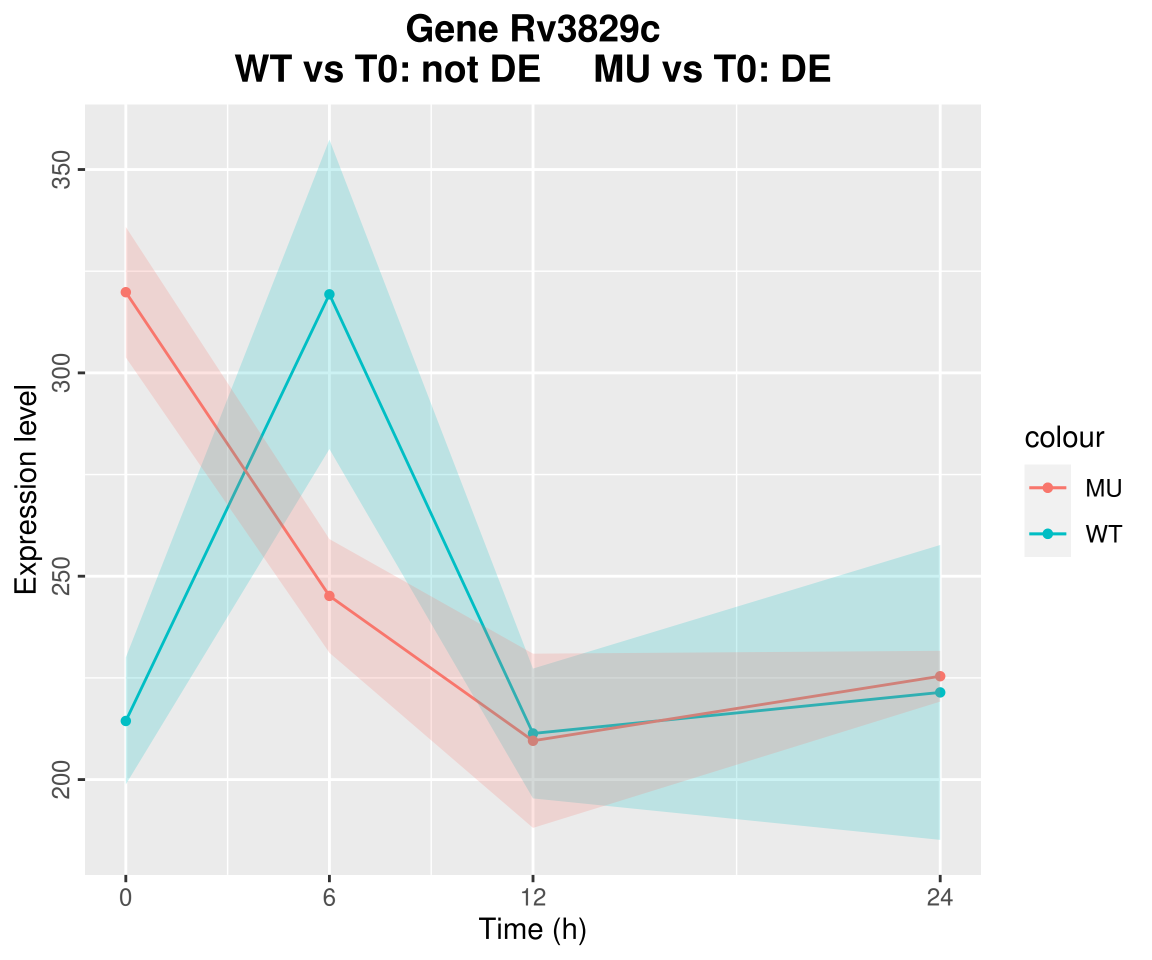

Gene Rv3829c
WT vs T0: not DE   MU vs T0: DE
Expression level
350
300
250
200
0
6
12
24
Time (h)
colour
MU
WT

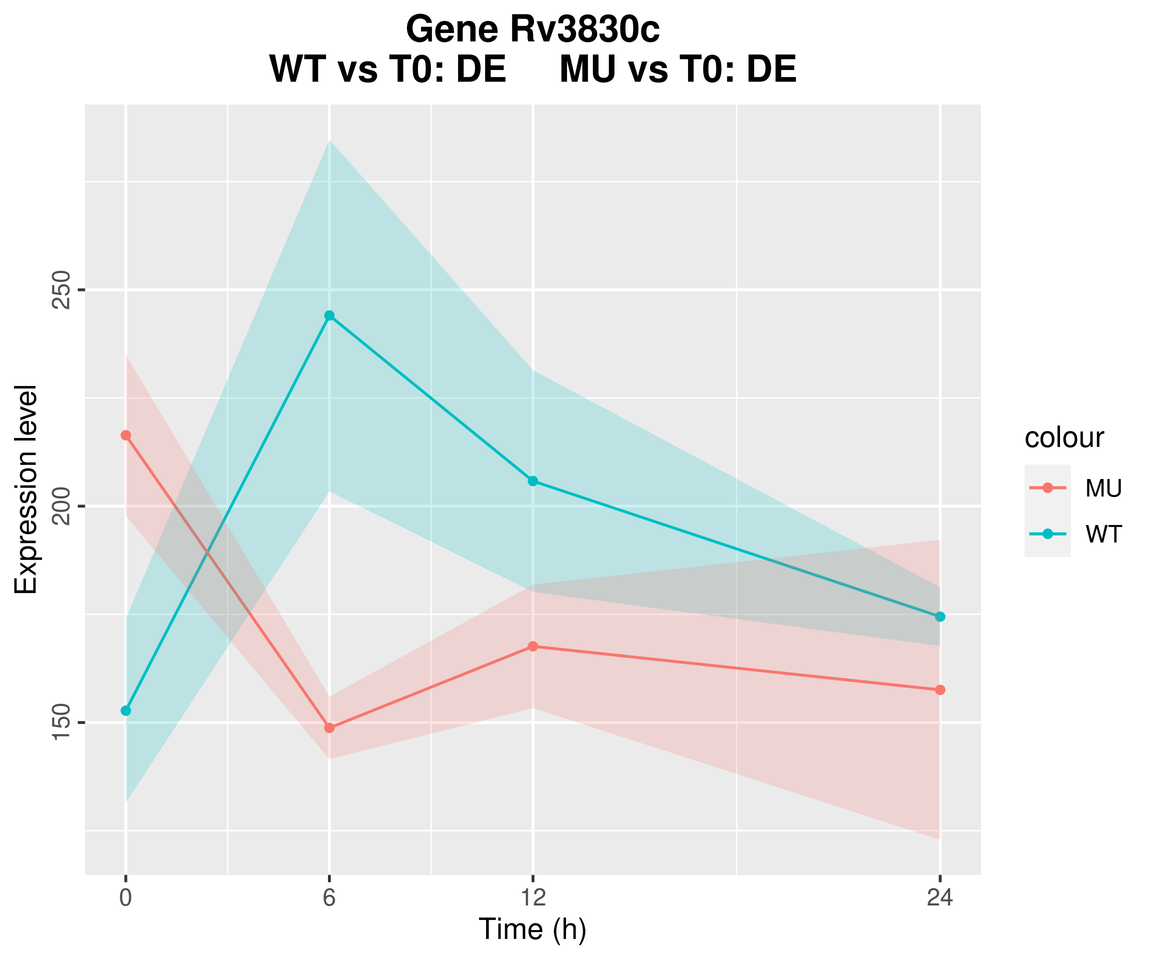
Gene Rv3830c
WT vs T0: DE MU vs T0: DE
Expression level
250
200
150
0
6
12
24
Time (h)
colour
MU
WT

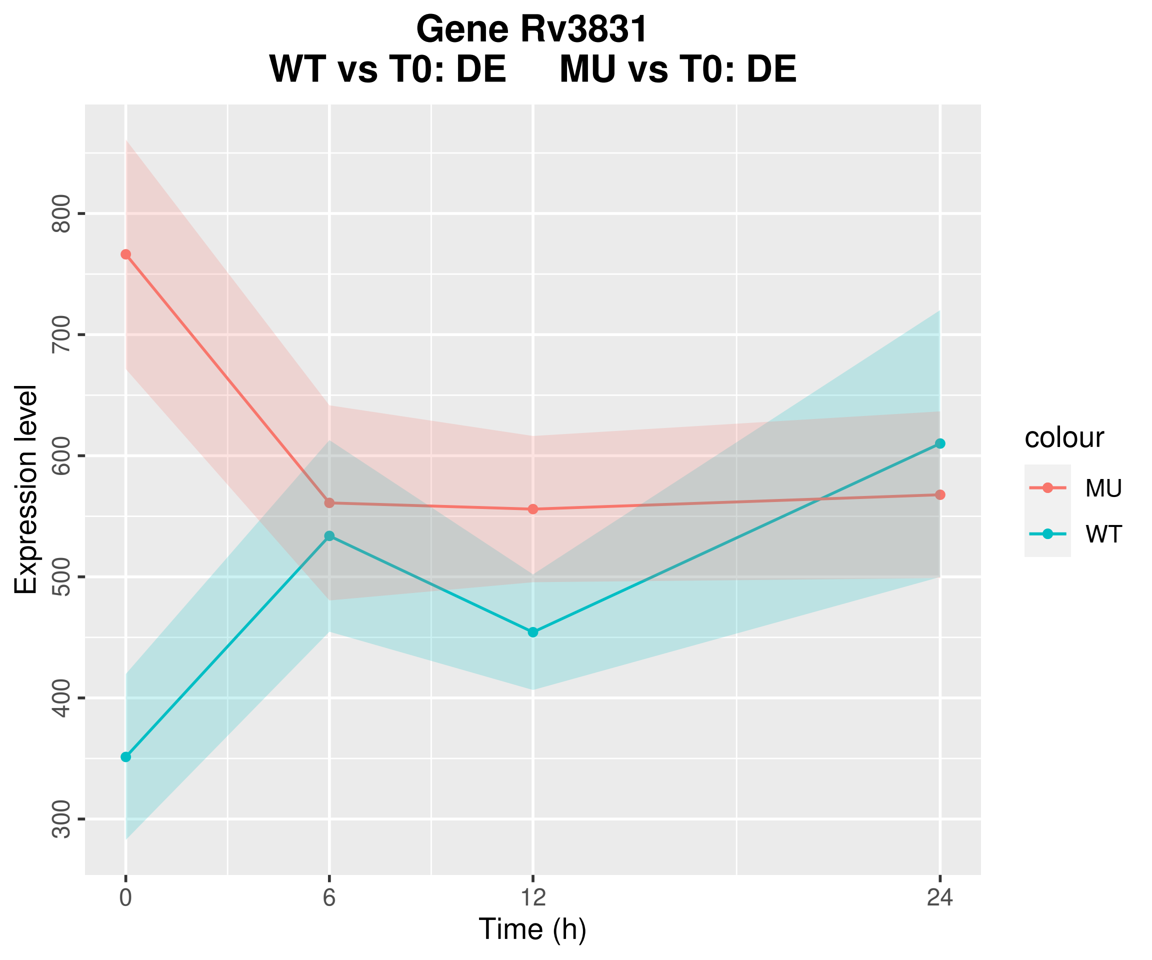
Gene Rv3831
WT vs T0: DE MU vs T0: DE
Expression level
800
700
600
500
400
300
0
6
12
24
Time (h)
colour
MU
WT

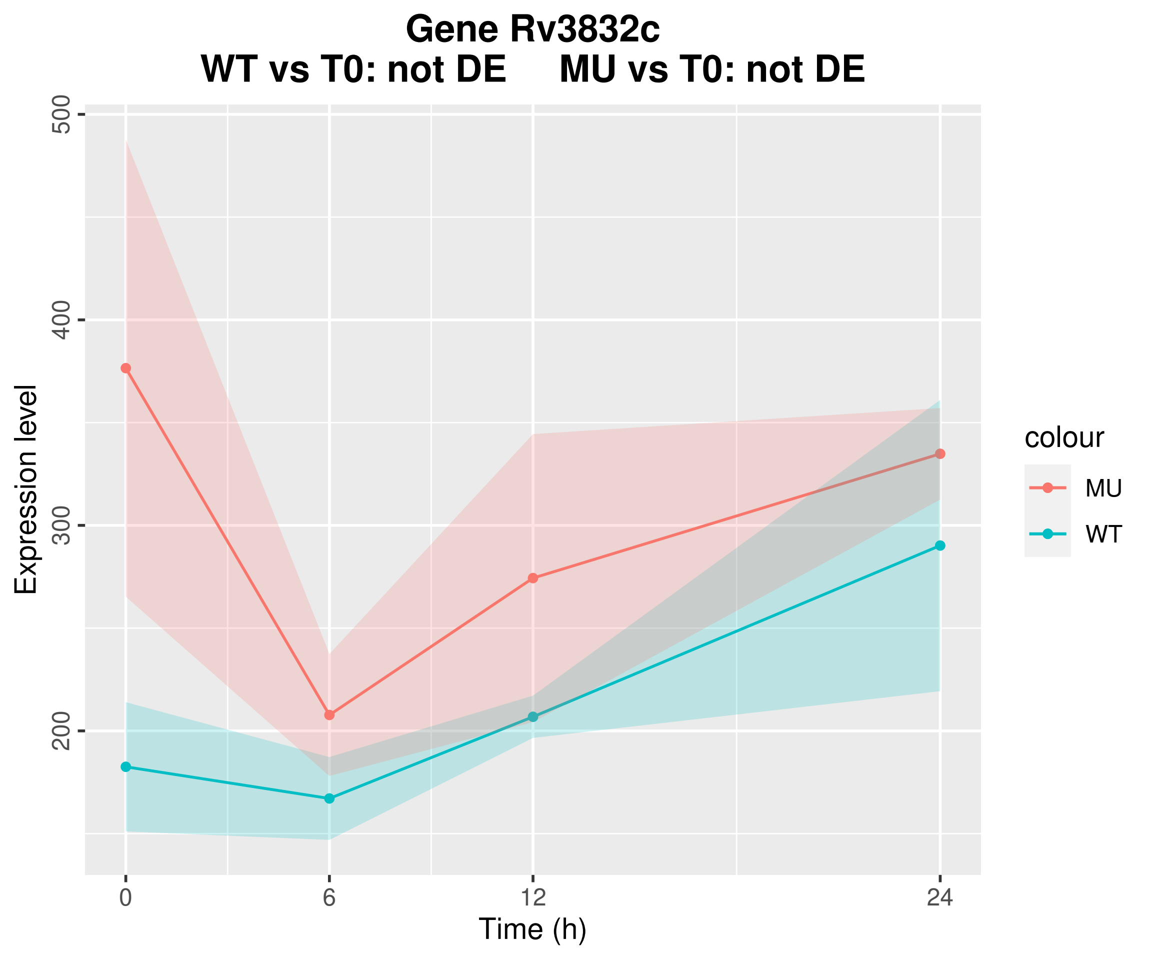

Gene Rv3832c
WT vs T0: not DE   MU vs T0: not DE
500
400
300
200
Expression level
0
6
12
24
Time (h)
colour
MU
WT

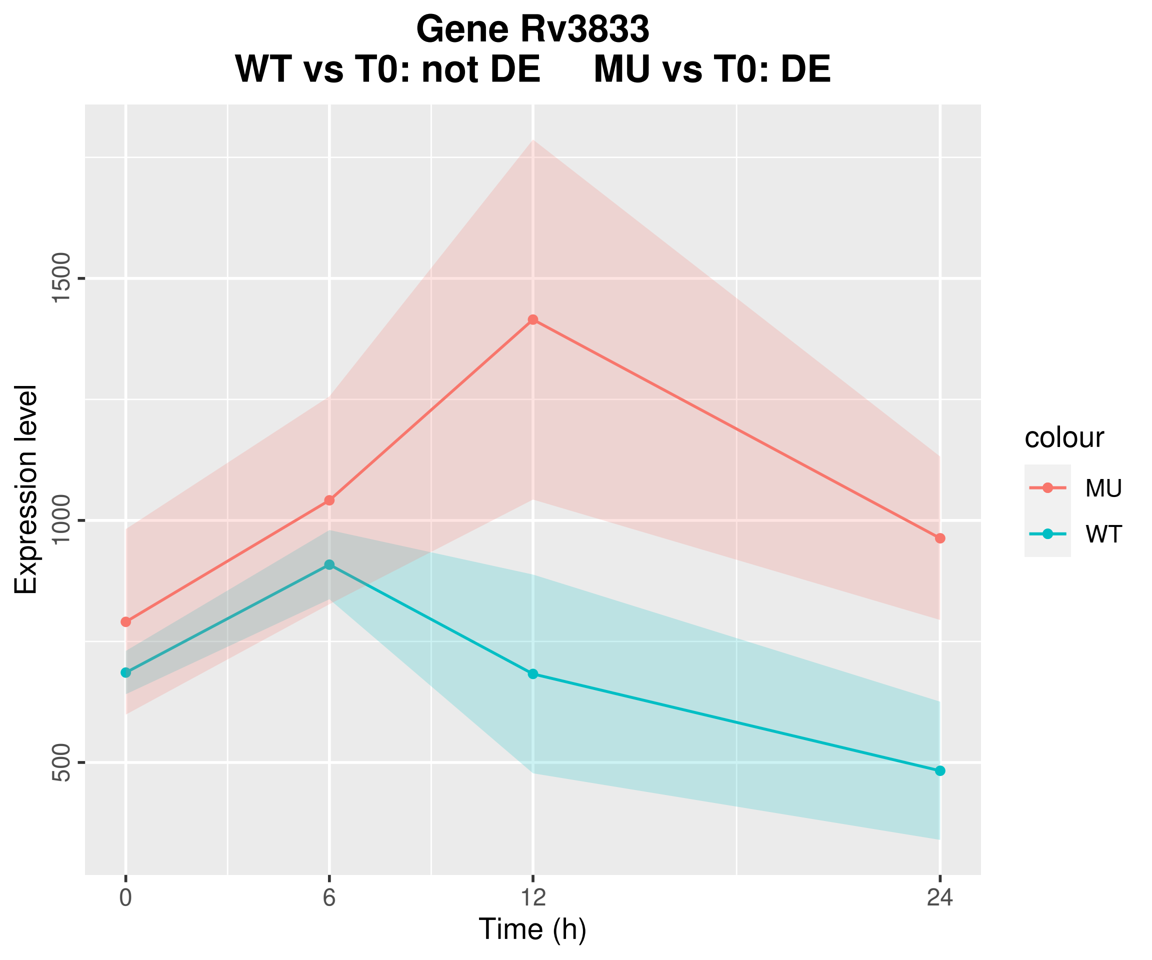

Gene Rv3833
WT vs T0: not DE   MU vs T0: DE
Expression level
1500
1000
500
0
6
12
24
Time (h)
colour
MU
WT

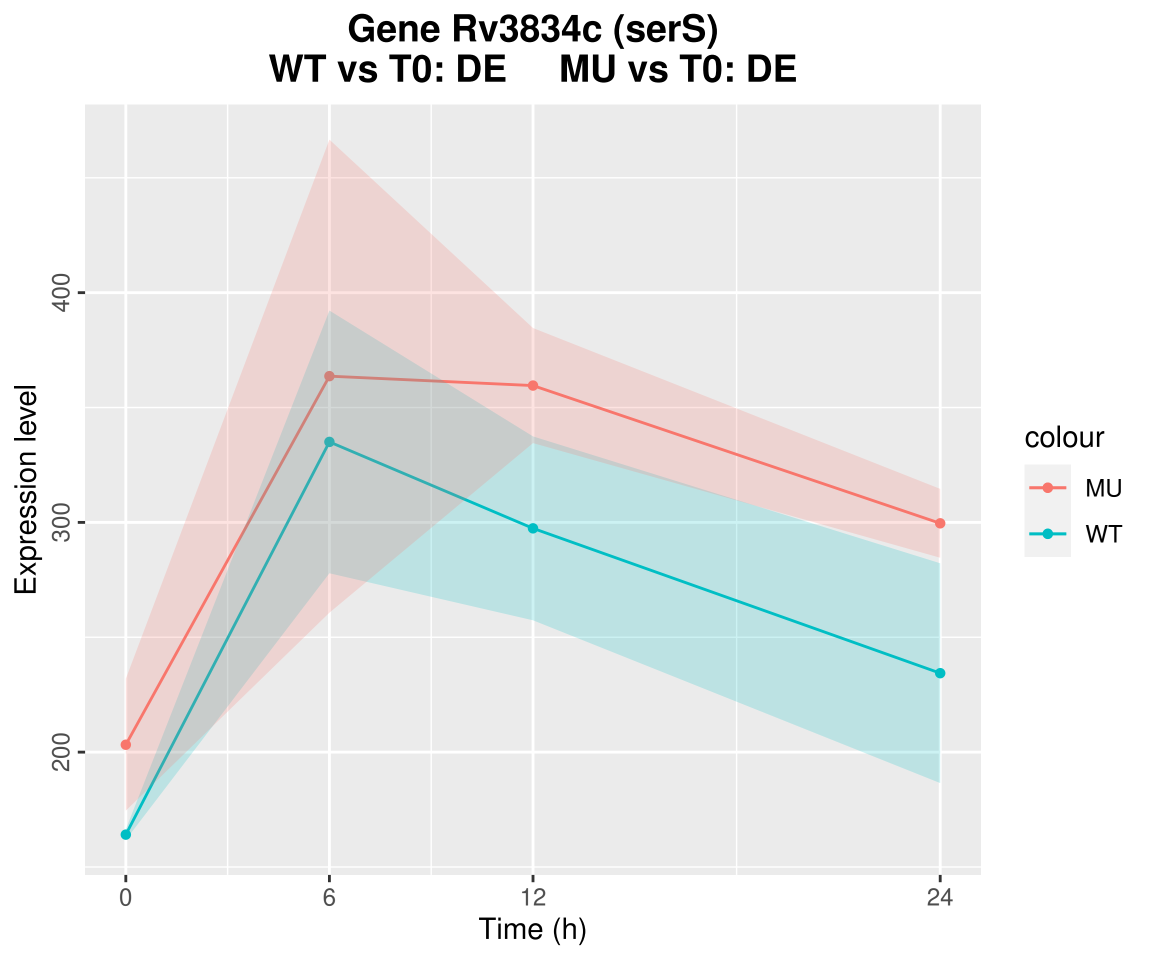

Gene Rv3834c (serS)
WT vs T0: DE   MU vs T0: DE
Expression level
400
300
200
0
6
12
24
Time (h)
colour
MU
WT

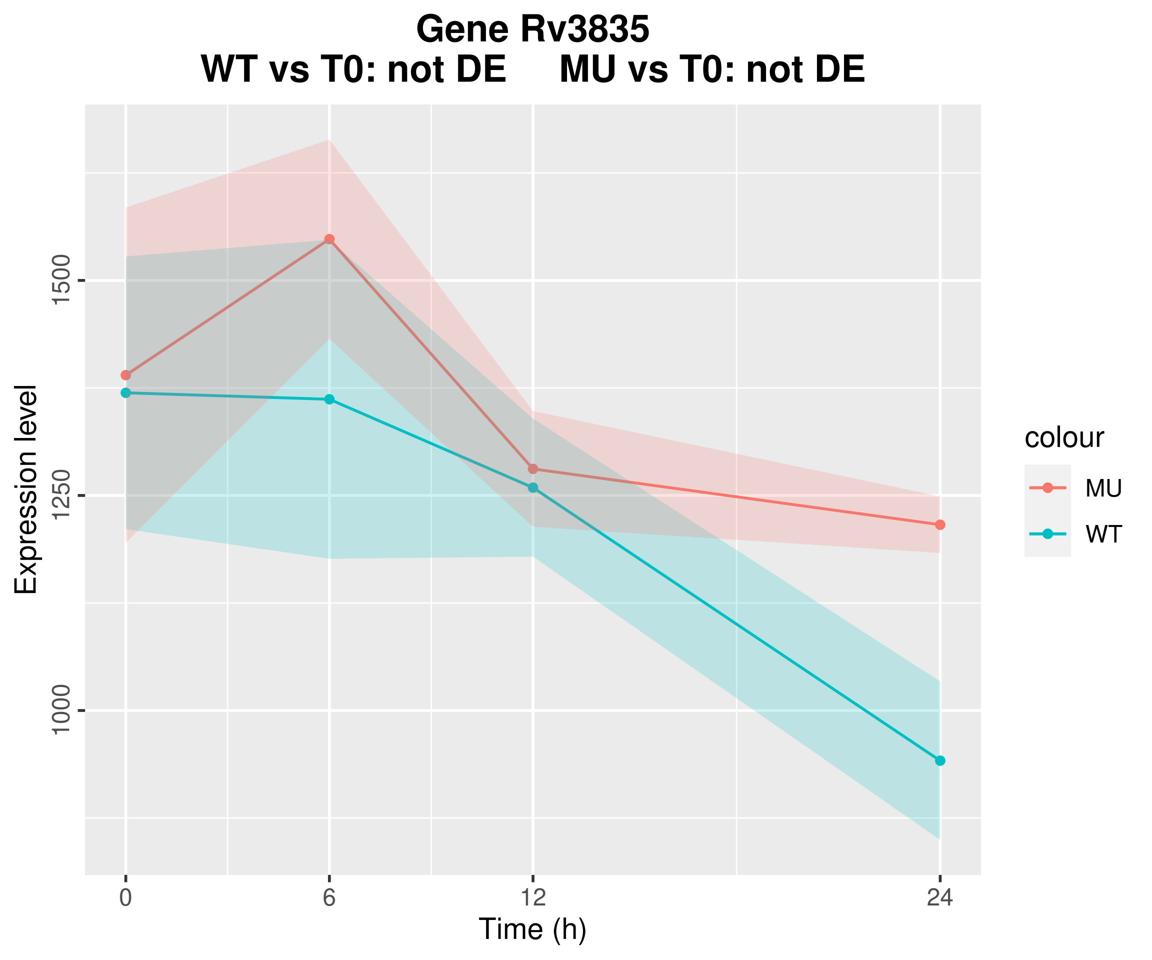
Gene Rv3835
WT vs T0: not DE   MU vs T0: not DE
Expression level
1000
1250
1500
0
6
12
24
Time (h)
colour
MU
WT

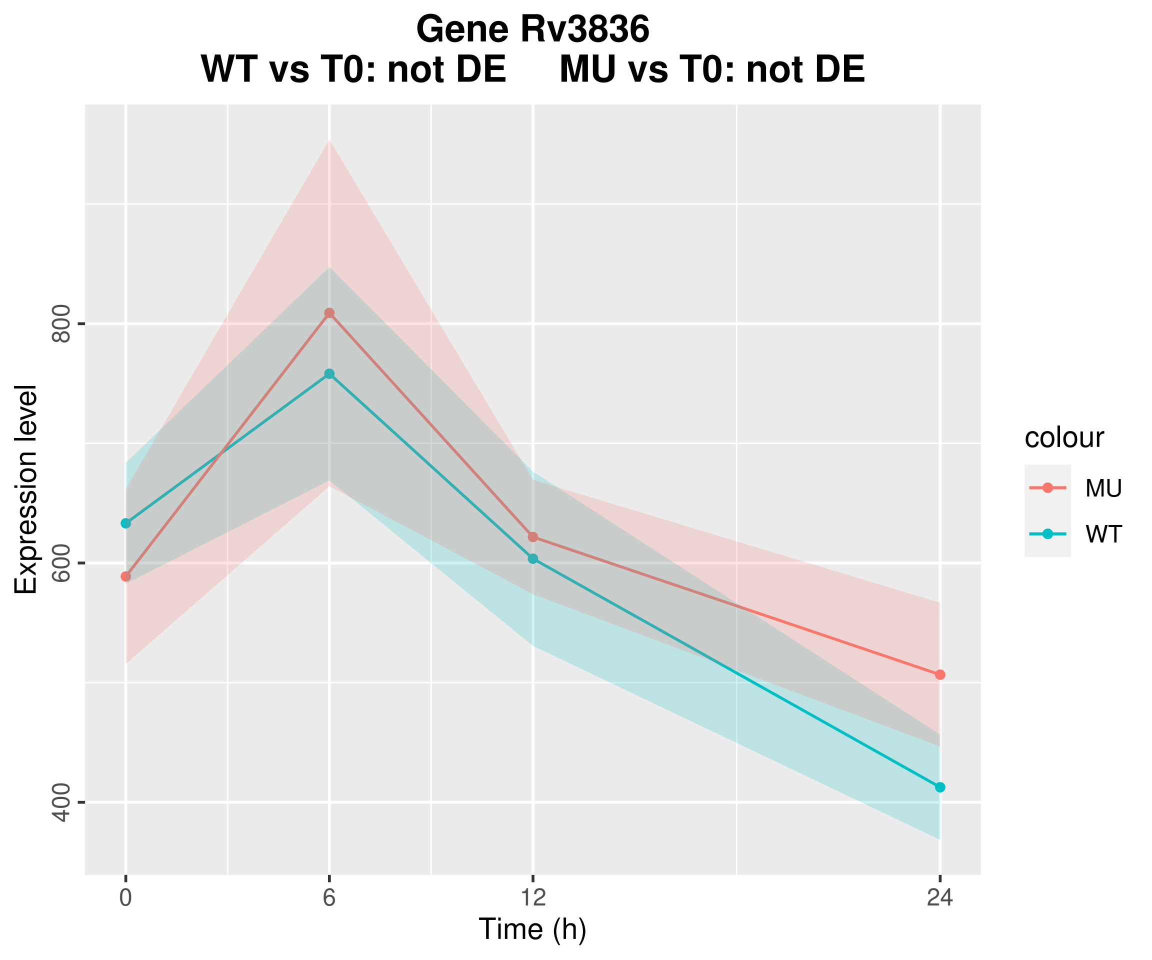
Gene Rv3836
WT vs T0: not DE MU vs T0: not DE
Expression level
800
600
400
0
6
12
24
Time (h)
colour
MU
WT

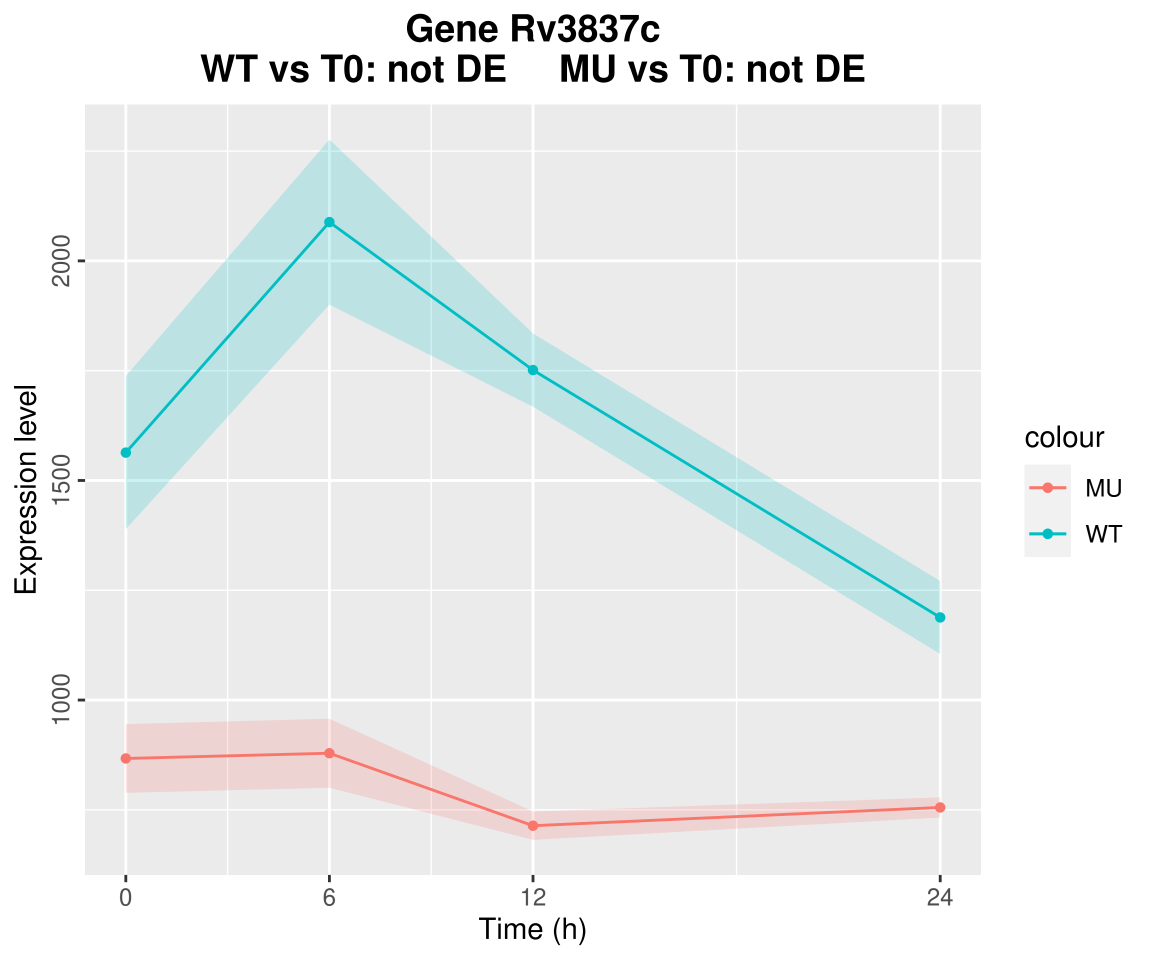
Gene Rv3837c
WT vs T0: not DE   MU vs T0: not DE
Expression level
2000
1500
1000
0
6
12
24
Time (h)
colour
MU
WT

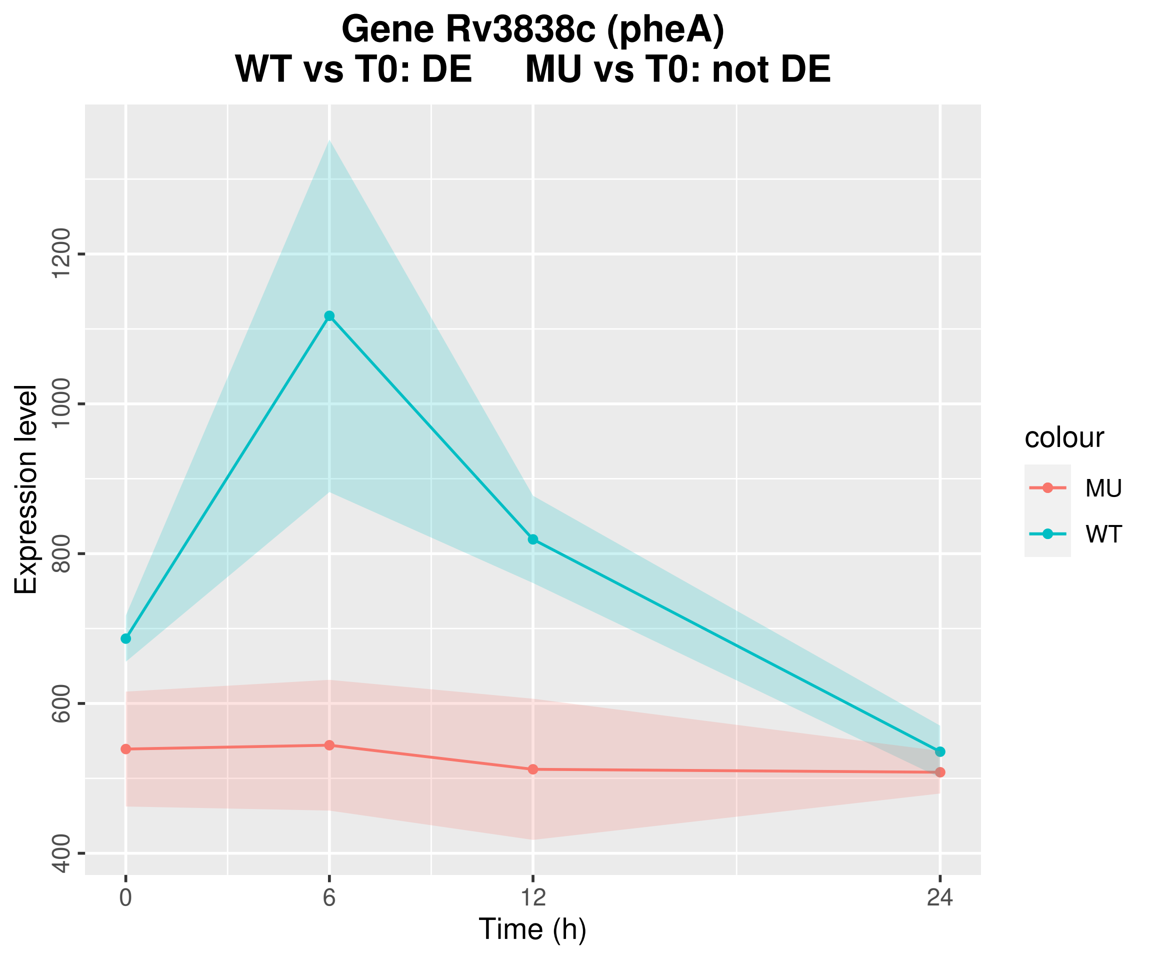
Gene Rv3838c (pheA)
WT vs T0: DE MU vs T0: not DE
Expression level
1200
1000
800
600
400
0
6
12
24
Time (h)
colour
MU
WT

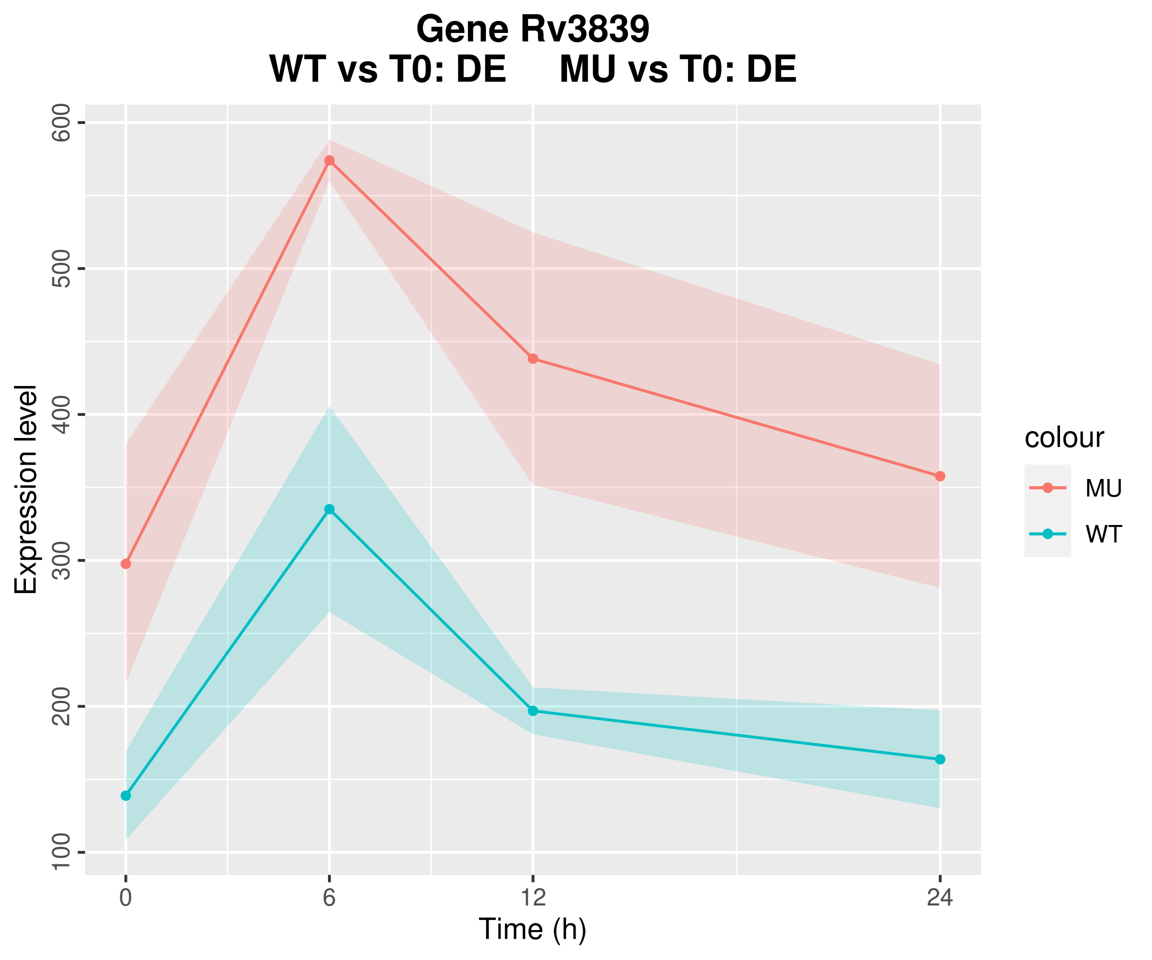

Gene Rv3839
WT vs T0: DE MU vs T0: DE
Expression level
600
500
400
300
200
100
0
6
12
24
Time (h)
colour
MU
WT

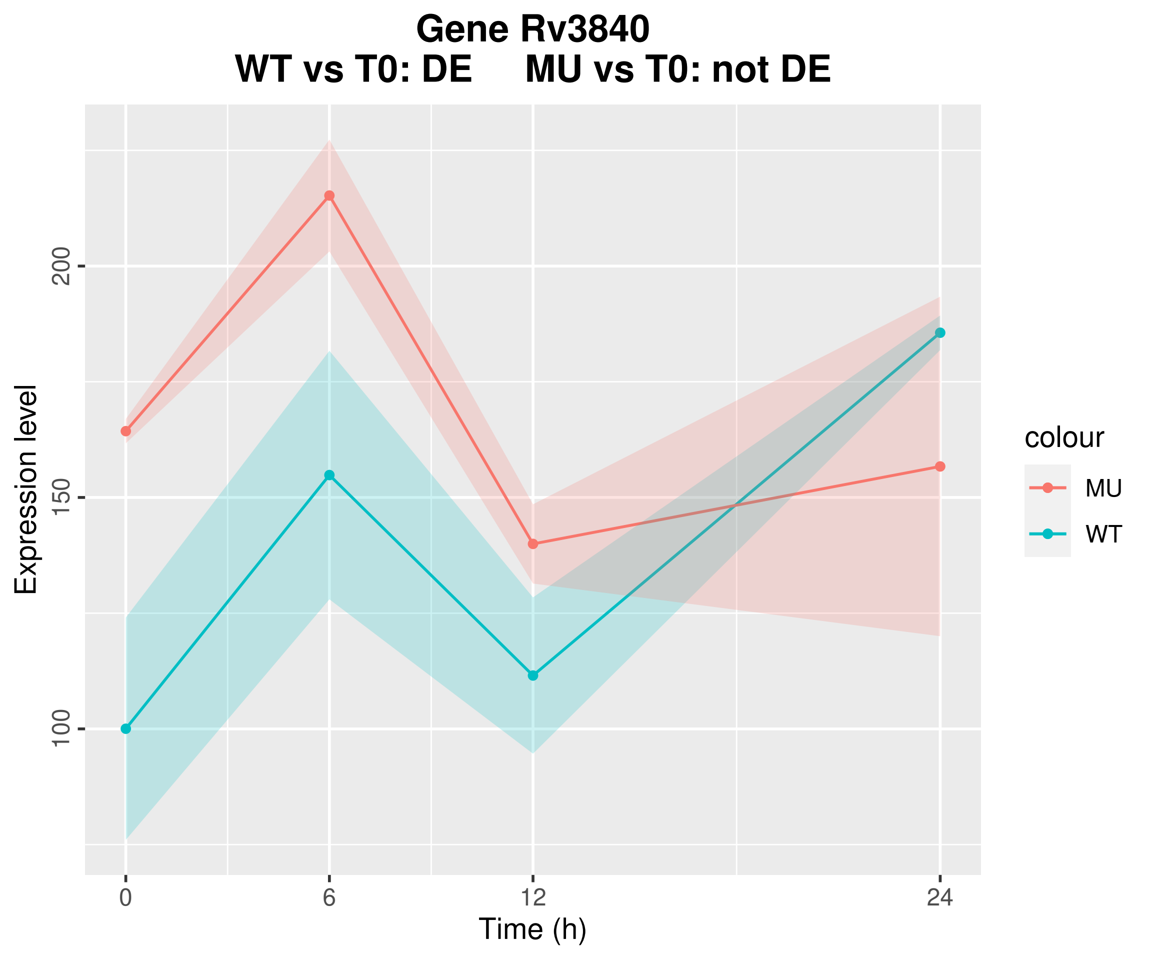
Gene Rv3840
WT vs T0: DE   MU vs T0: not DE
Expression level
200
150
100
0
6
12
24
Time (h)
colour
MU
WT

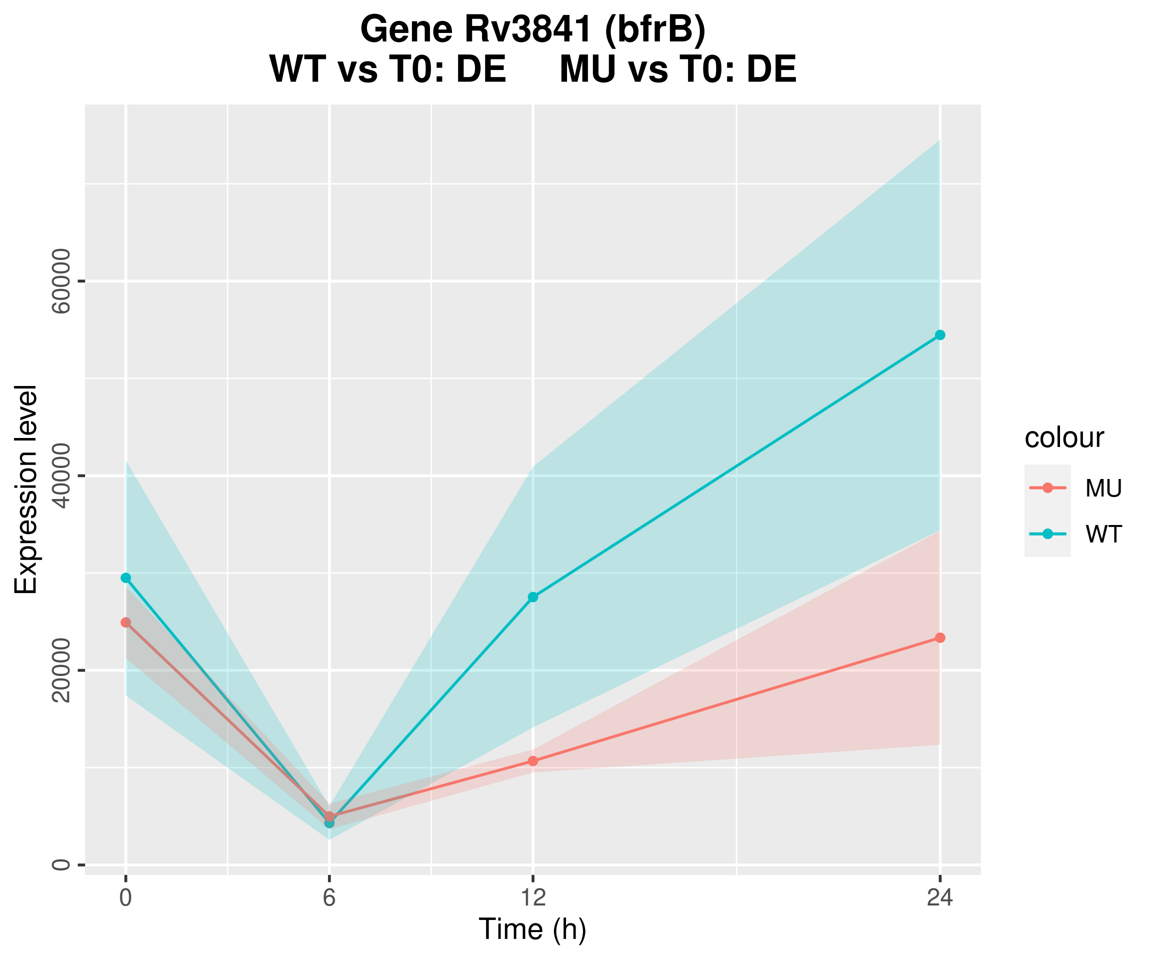

Gene Rv3841 (bfrB)
WT vs T0: DE MU vs T0: DE
Expression level
60000
40000
20000
0
0
6
12
24
Time (h)
colour
MU
WT

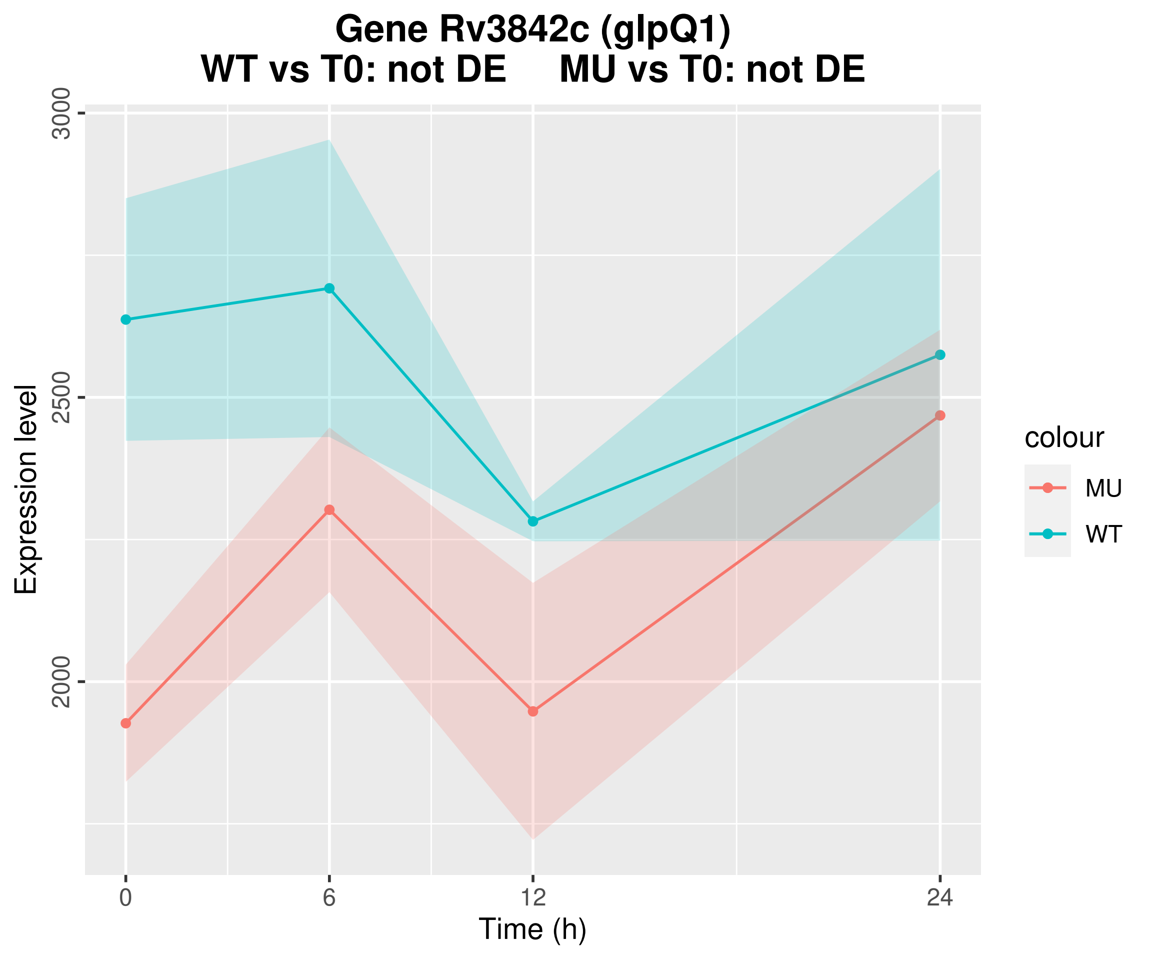
Gene Rv3842c (glpQ1)
WT vs T0: not DE   MU vs T0: not DE
3000
2500
2000
Expression level
0
6
12
24
Time (h)
colour
MU
WT

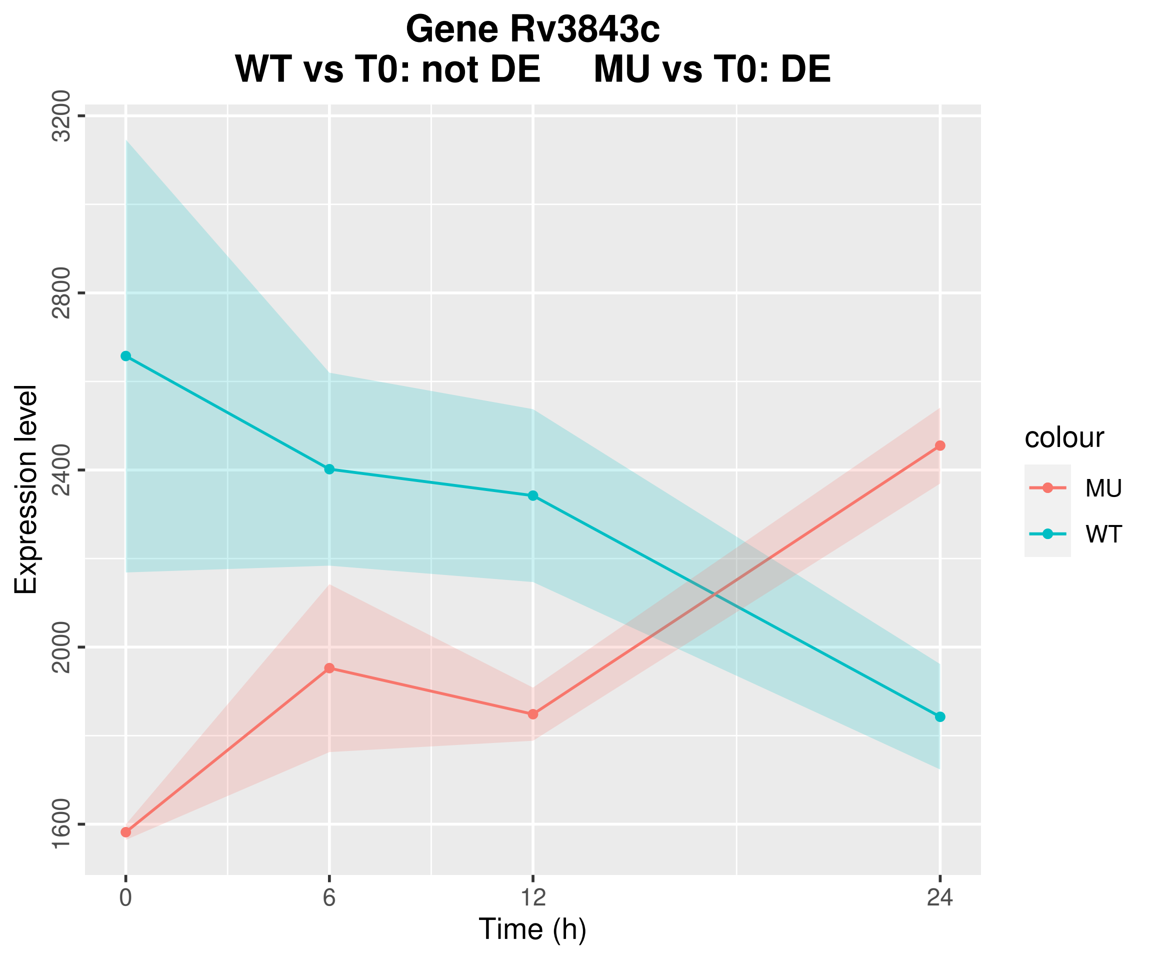

Gene Rv3843c
WT vs T0: not DE MU vs T0: DE
Expression level
3200
2800
2400
2000
1600
0
6
12
24
Time (h)
colour
MU
WT

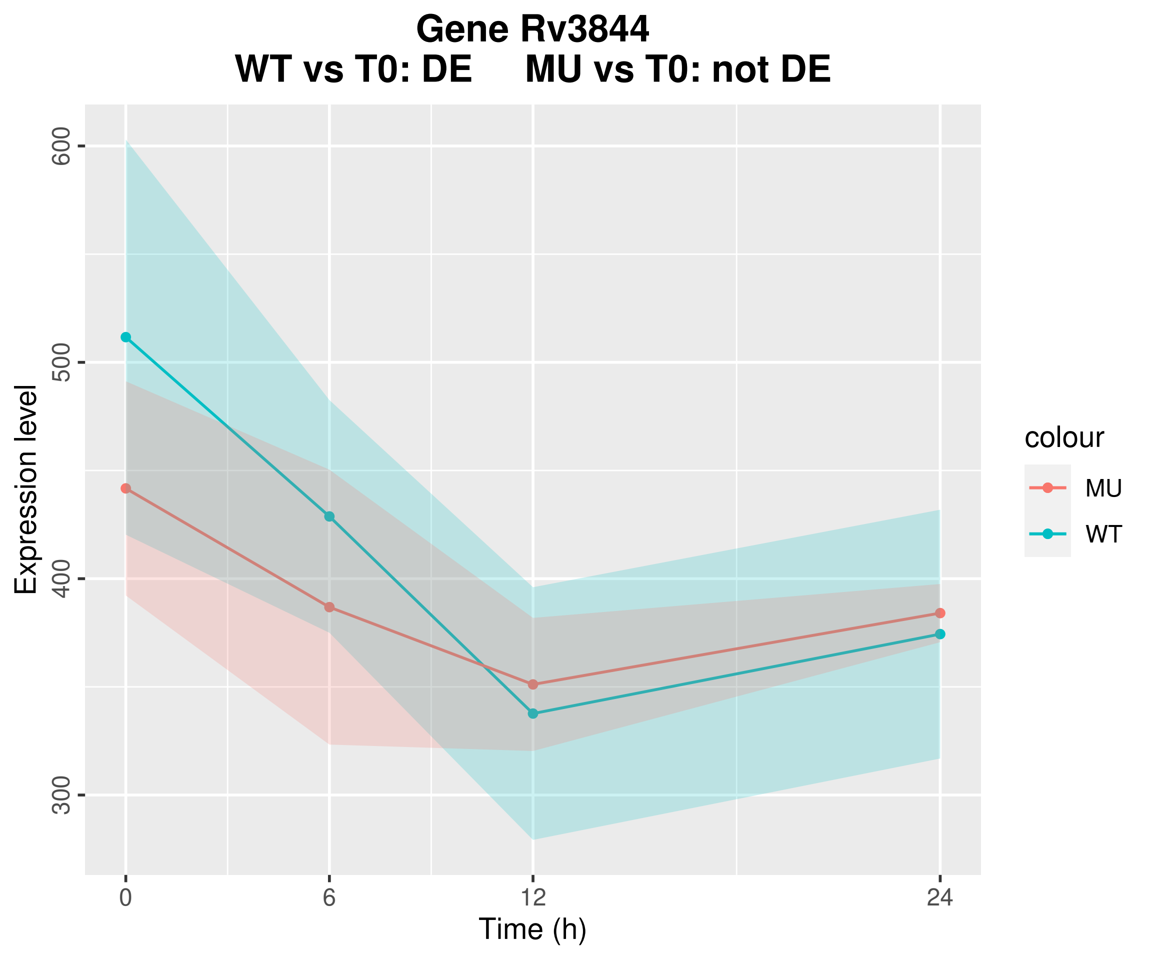
Gene Rv3844
WT vs T0: DE MU vs T0: not DE
Expression level
600
500
400
300
0
6
12
24
Time (h)
colour
MU
WT

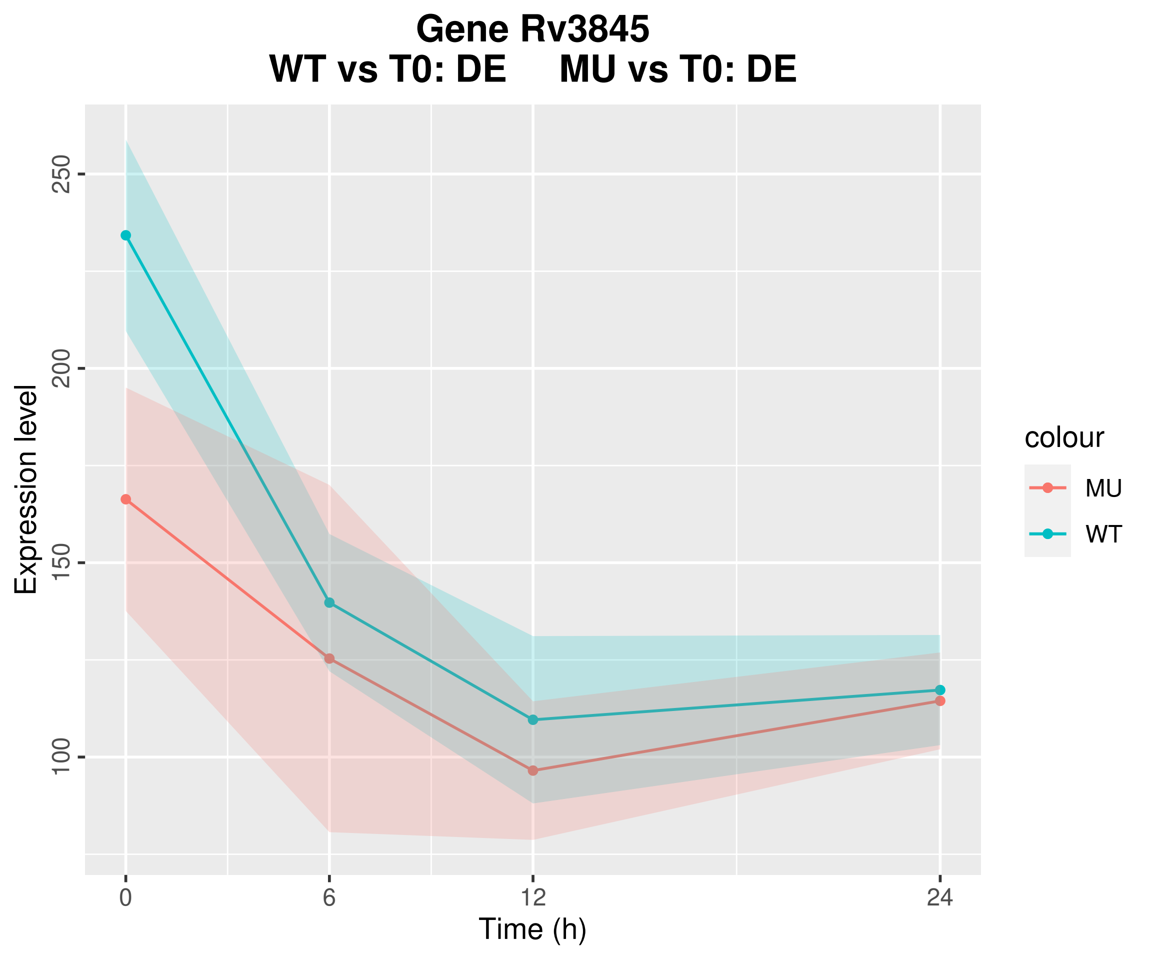

Gene Rv3845
WT vs T0: DE MU vs T0: DE
Expression level
250
200
150
100
0
6
12
24
Time (h)
colour
MU
WT

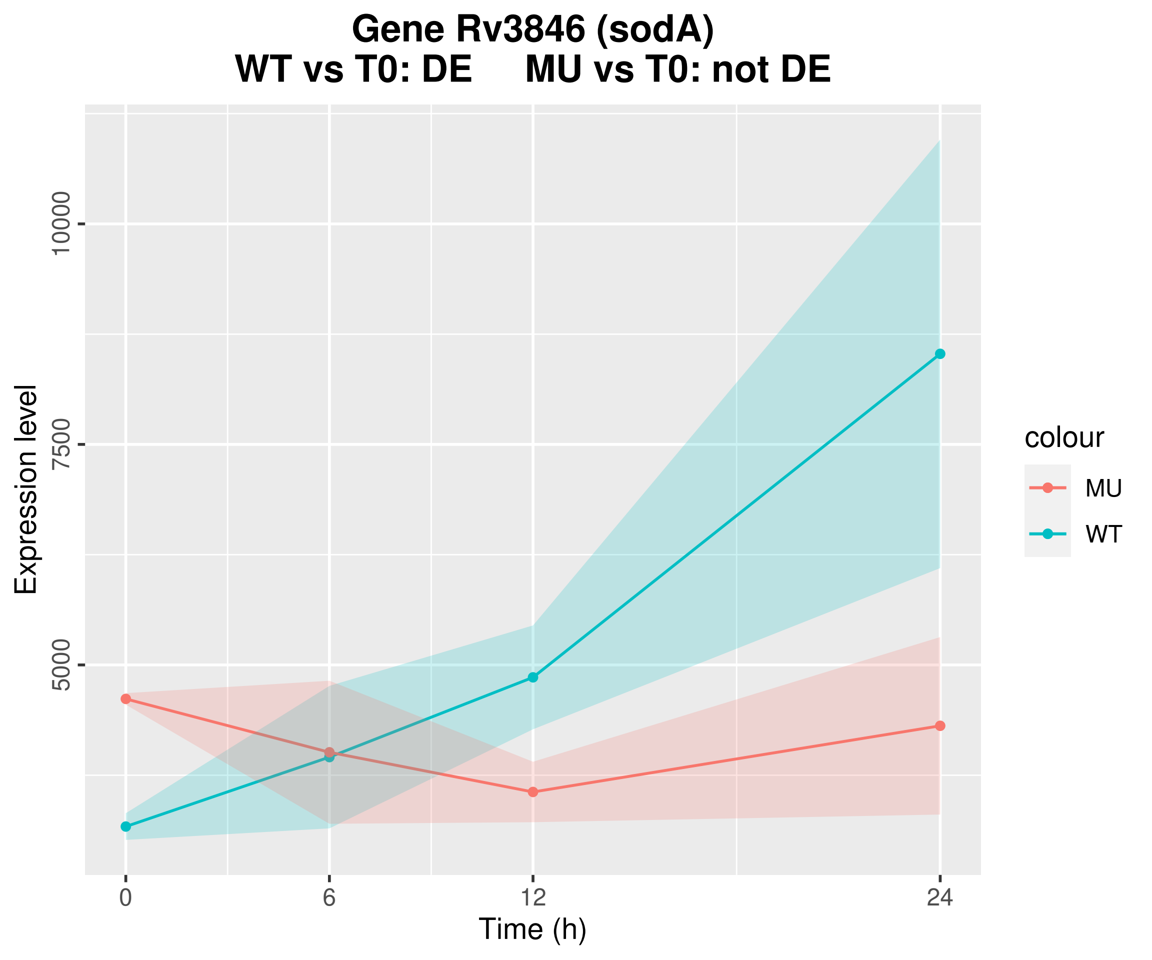
Gene Rv3846 (sodA)
WT vs T0: DE   MU vs T0: not DE
Expression level
10000
7500
5000
0
6
12
24
Time (h)
colour
MU
WT

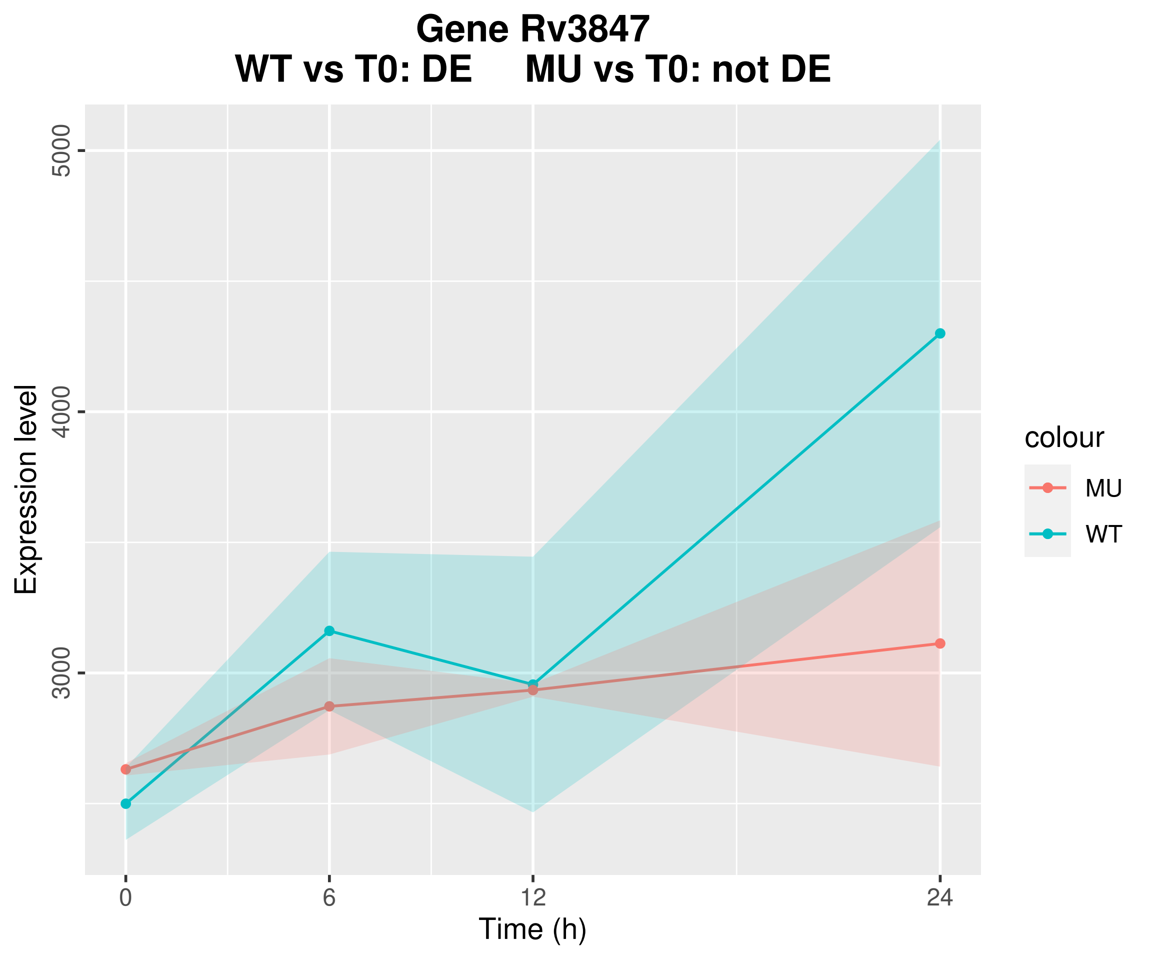
Gene Rv3847
WT vs T0: DE MU vs T0: not DE
Expression level
5000
4000
3000
0
6
12
24
Time (h)
colour
MU
WT

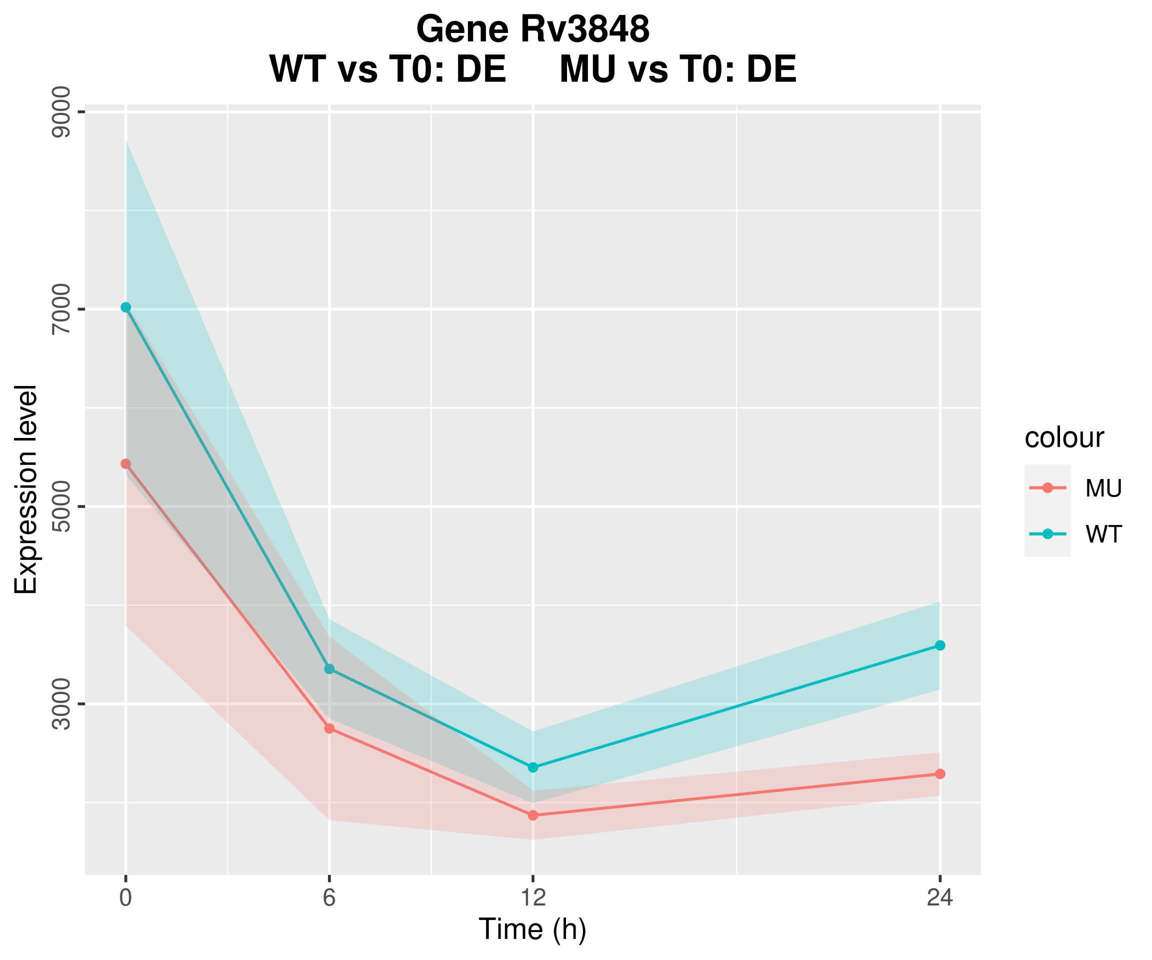

Gene Rv3848
WT vs T0: DE   MU vs T0: DE
9000
7000
5000
3000
Expression level
0
6
12
24
Time (h)
colour
MU
WT

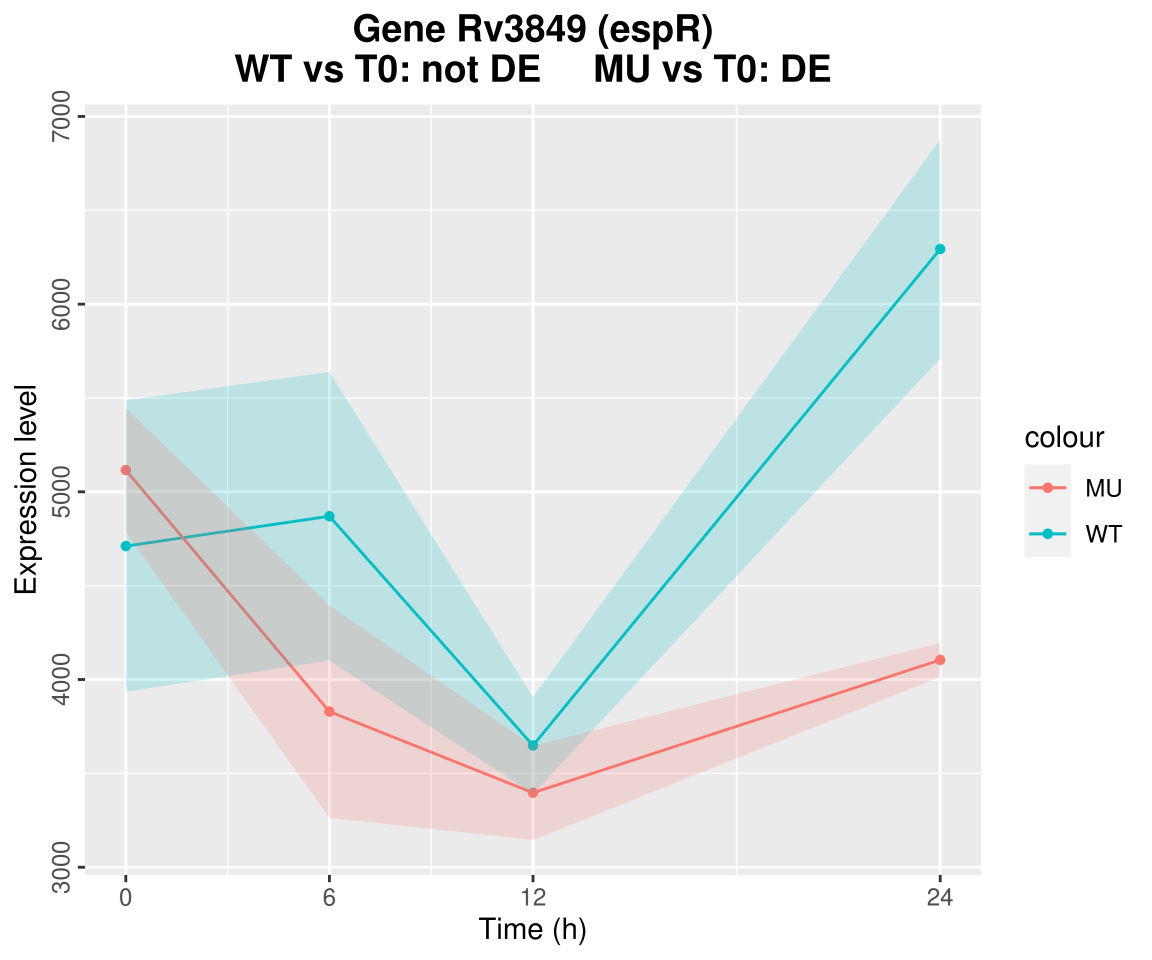
Gene Rv3849 (espR)
WT vs T0: not DE MU vs T0: DE
7000
6000
5000
4000
3000
Expression level
0
6
12
24
Time (h)
colour
MU
WT

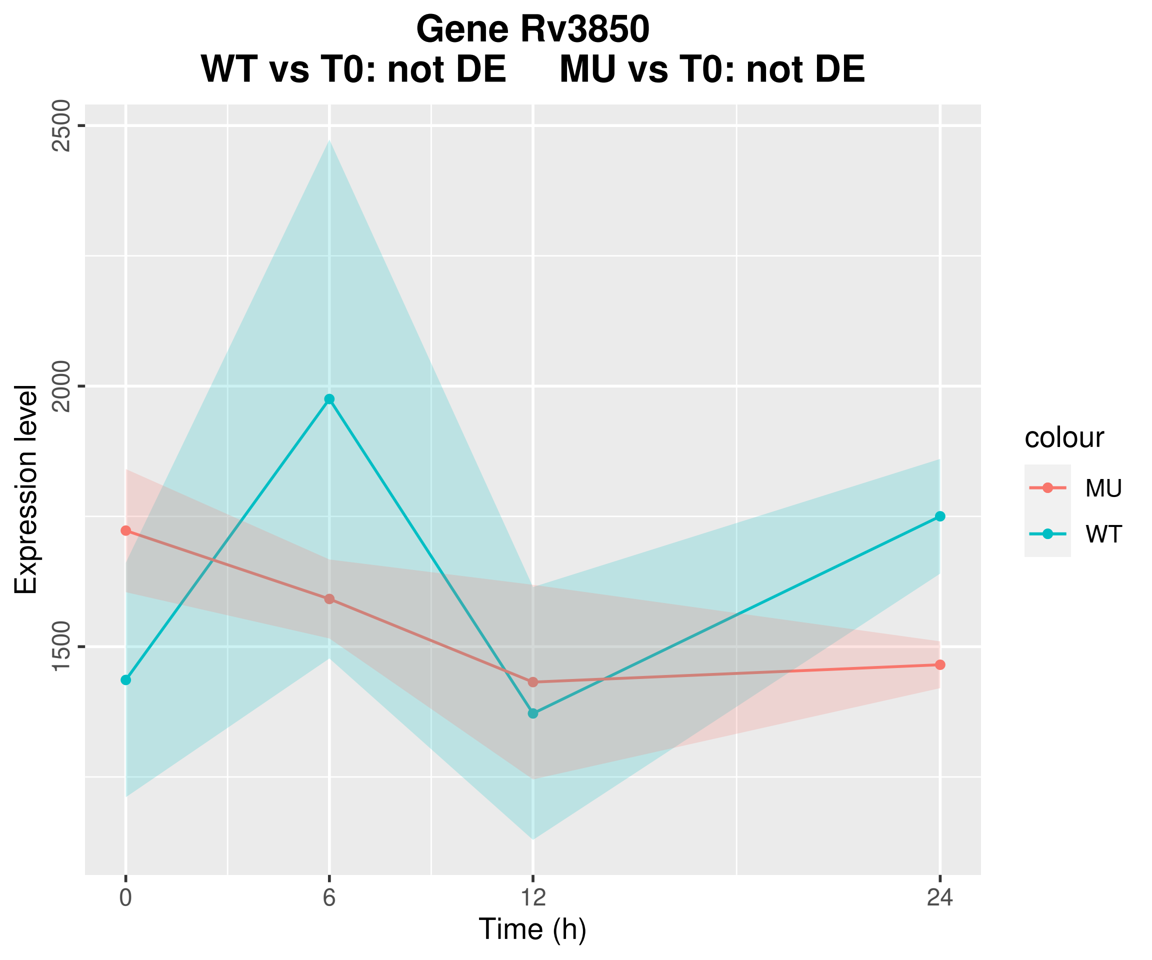

Gene Rv3850
WT vs T0: not DE MU vs T0: not DE
2500
2000
1500
Expression level
0
6
12
24
Time (h)
colour
MU
WT

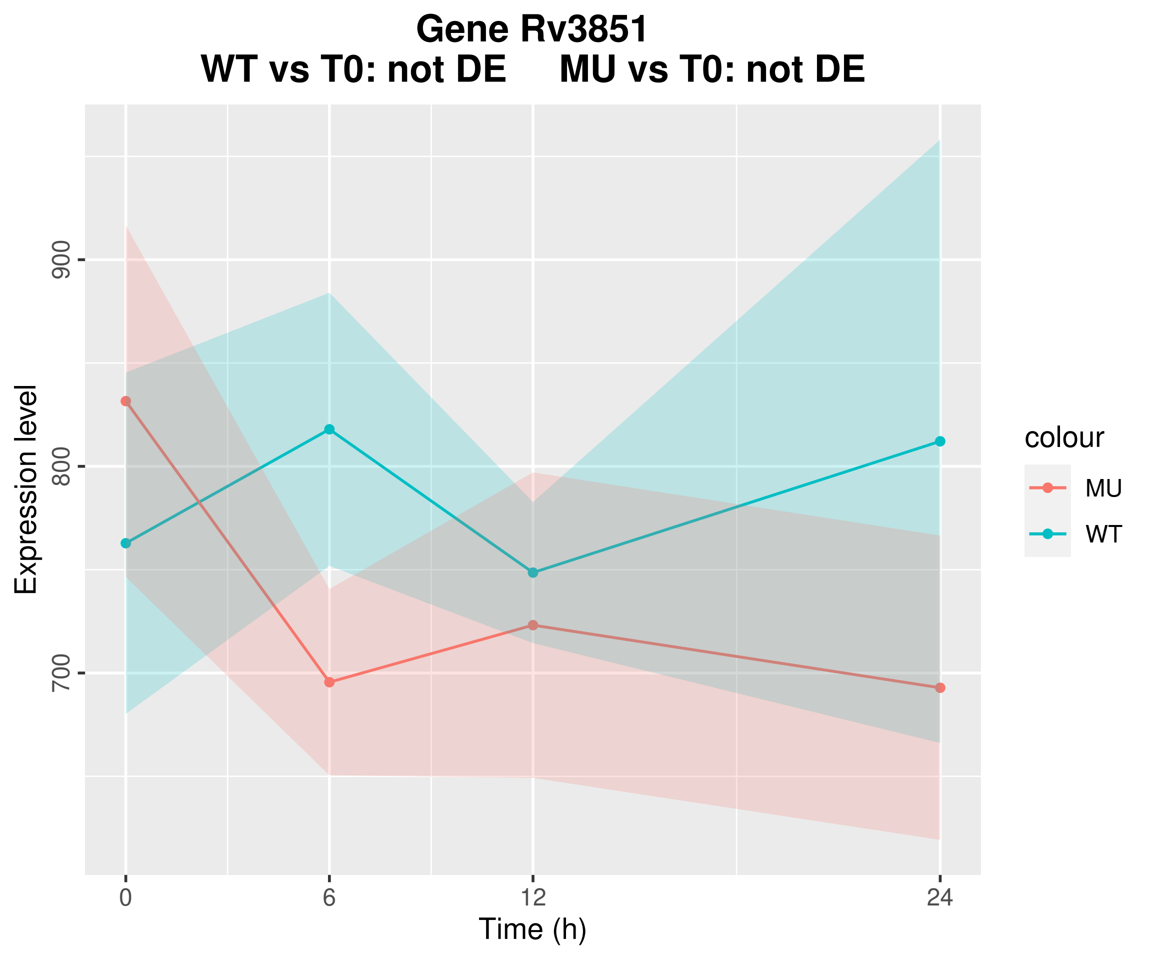
Gene Rv3851
WT vs T0: not DE MU vs T0: not DE
Expression level
900
800
700
0
6
12
24
Time (h)
colour
MU
WT

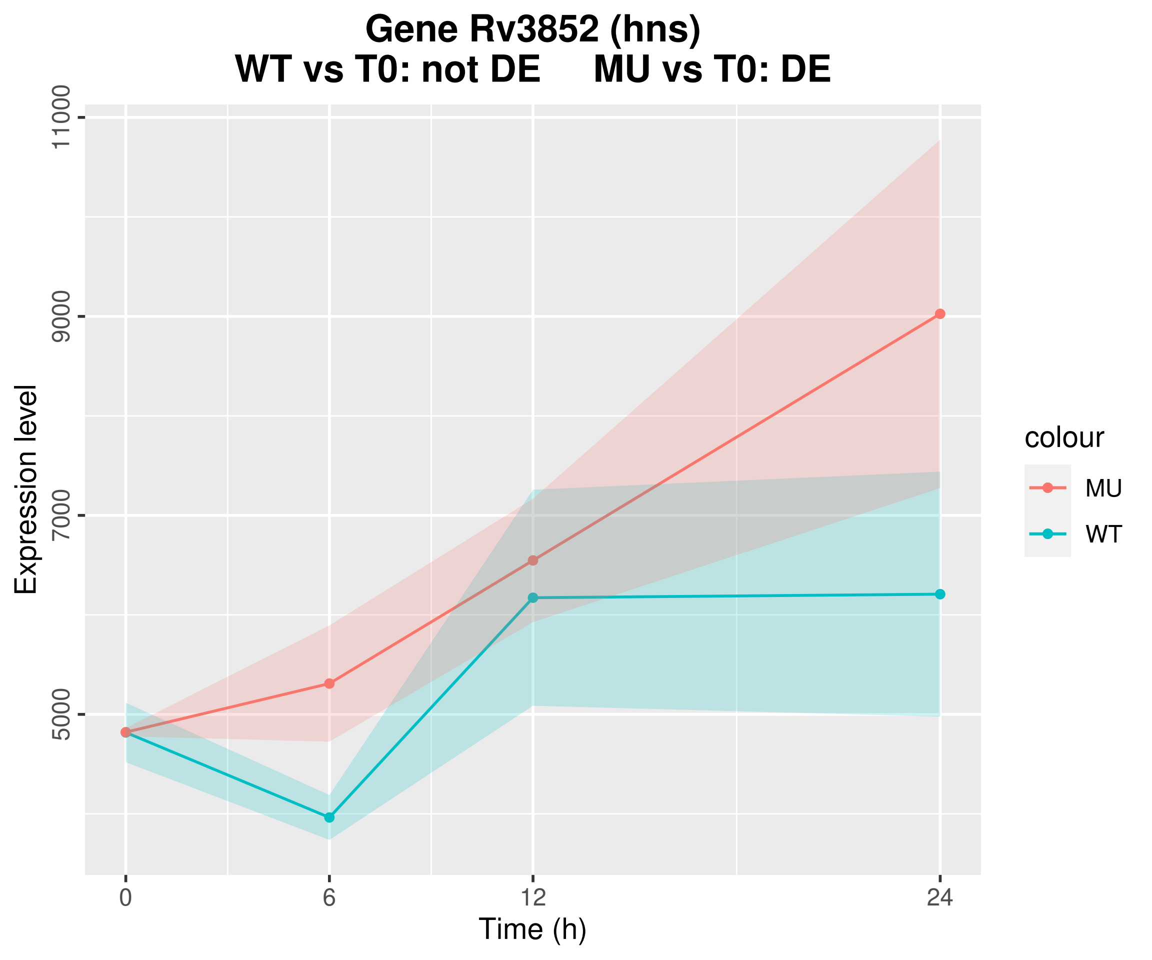

Gene Rv3852 (hns)
WT vs T0: not DE
MU vs T0: DE
11000
9000
7000
5000
Expression level
0
6
12
24
Time (h)
colour
MU
WT

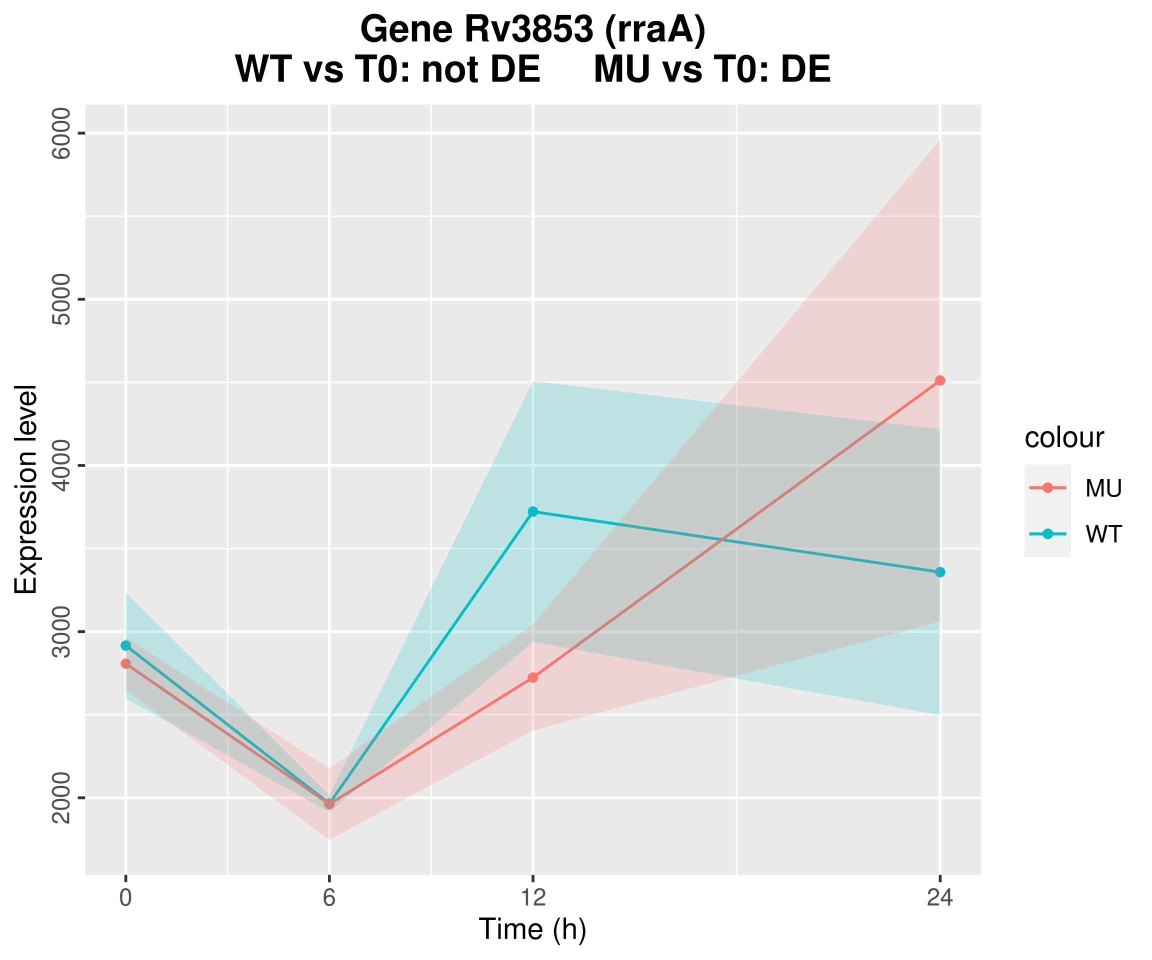
Gene Rv3853 (rraA)
WT vs T0: not DE   MU vs T0: DE
Expression level
6000
5000
4000
3000
2000
0
6
12
24
Time (h)
colour
MU
WT

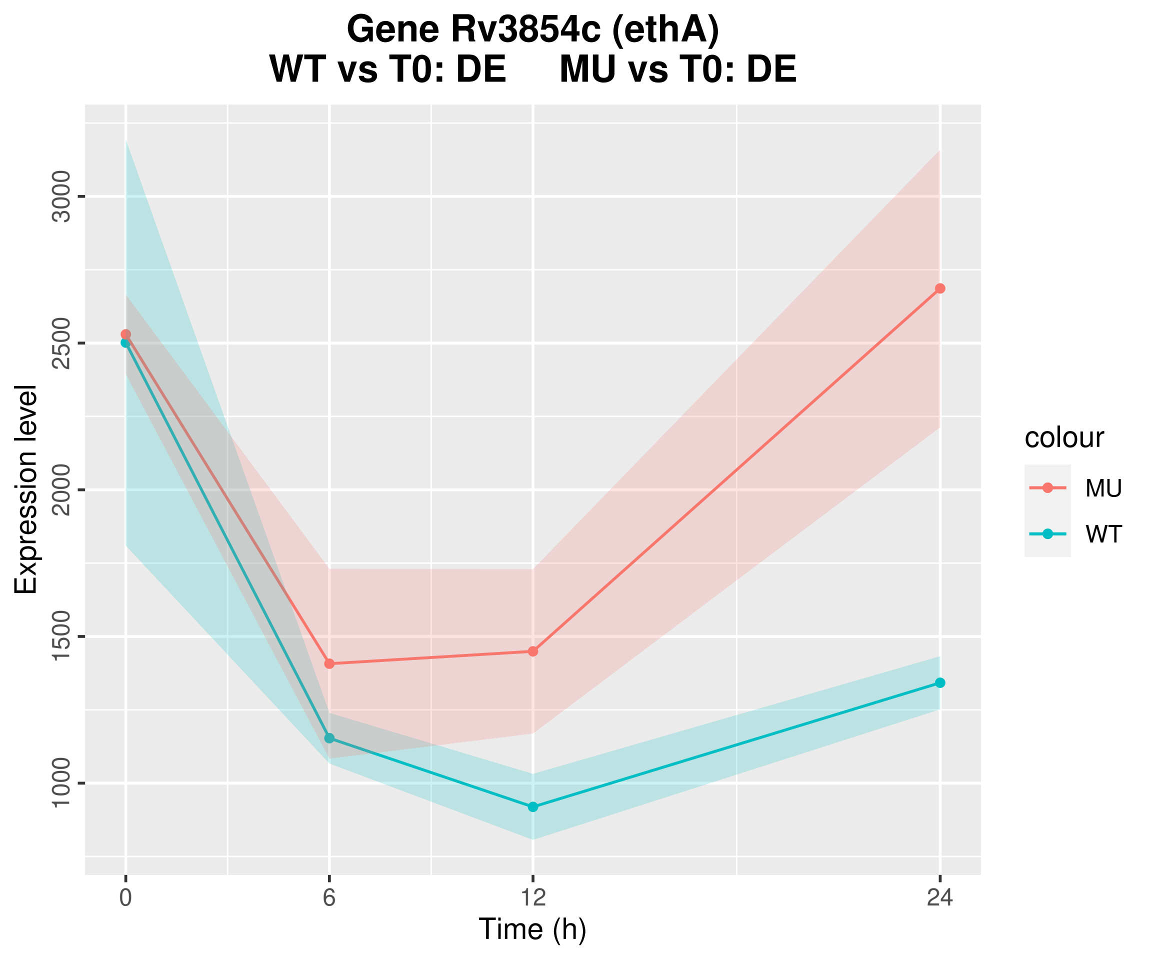
Gene Rv3854c (ethA)
WT vs T0: DE   MU vs T0: DE
Expression level
3000
2500
2000
1500
1000
0
6
12
24
Time (h)
colour
MU
WT

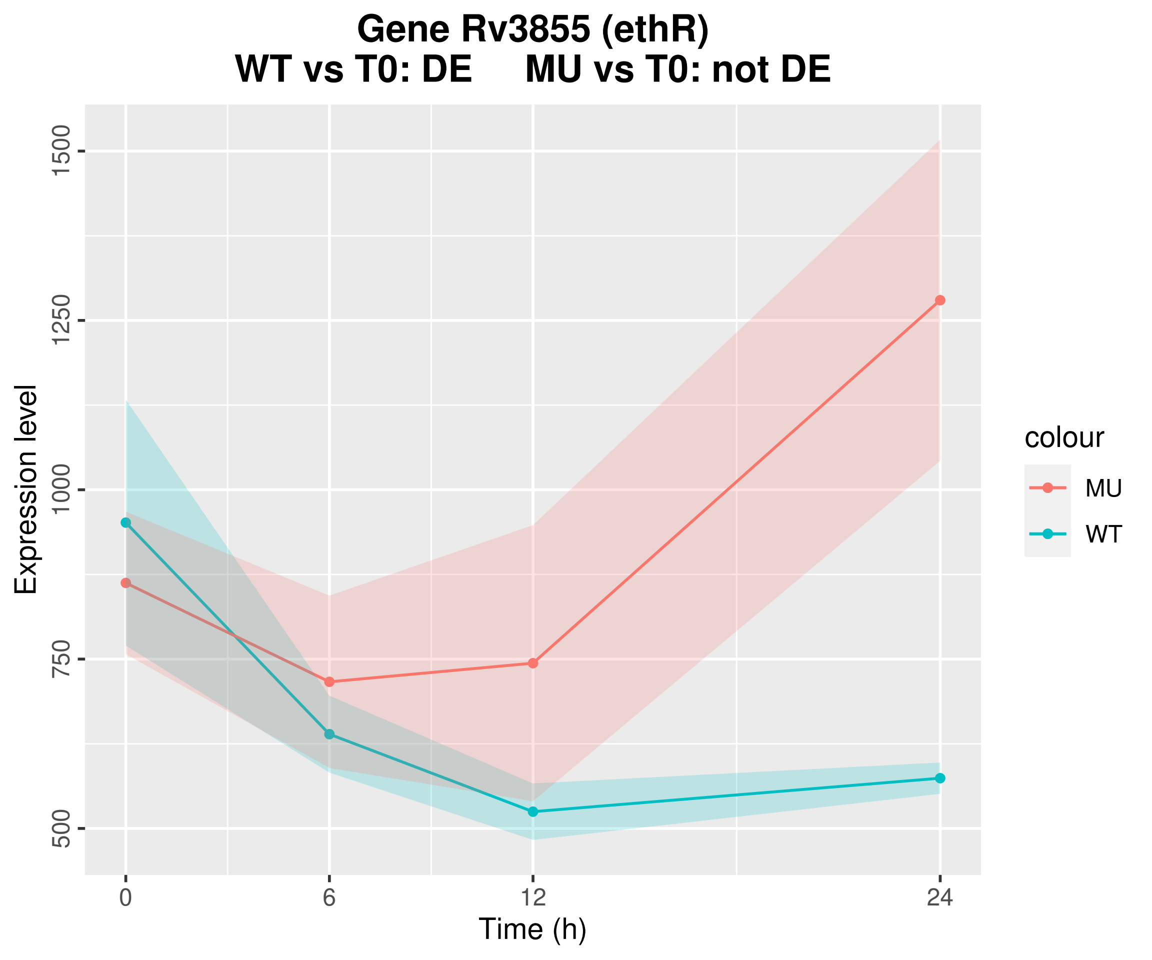
Gene Rv3855 (ethR)
WT vs T0: DE   MU vs T0: not DE
Expression level
1500
1250
1000
750
500
0
6
12
24
Time (h)
colour
MU
WT

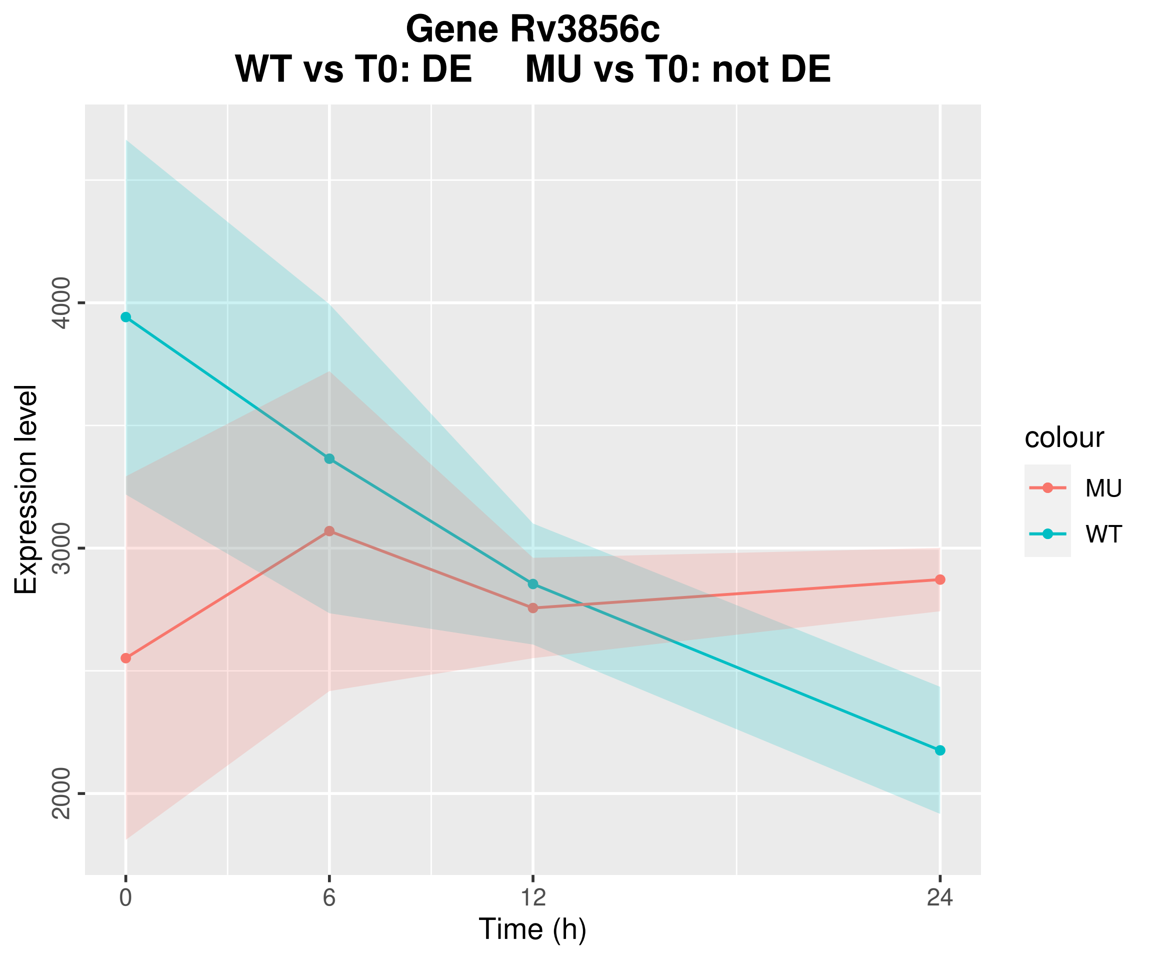

Gene Rv3856c
WT vs T0: DE MU vs T0: not DE
Expression level
4000
3000
2000
0
6
12
24
Time (h)
colour
MU
WT

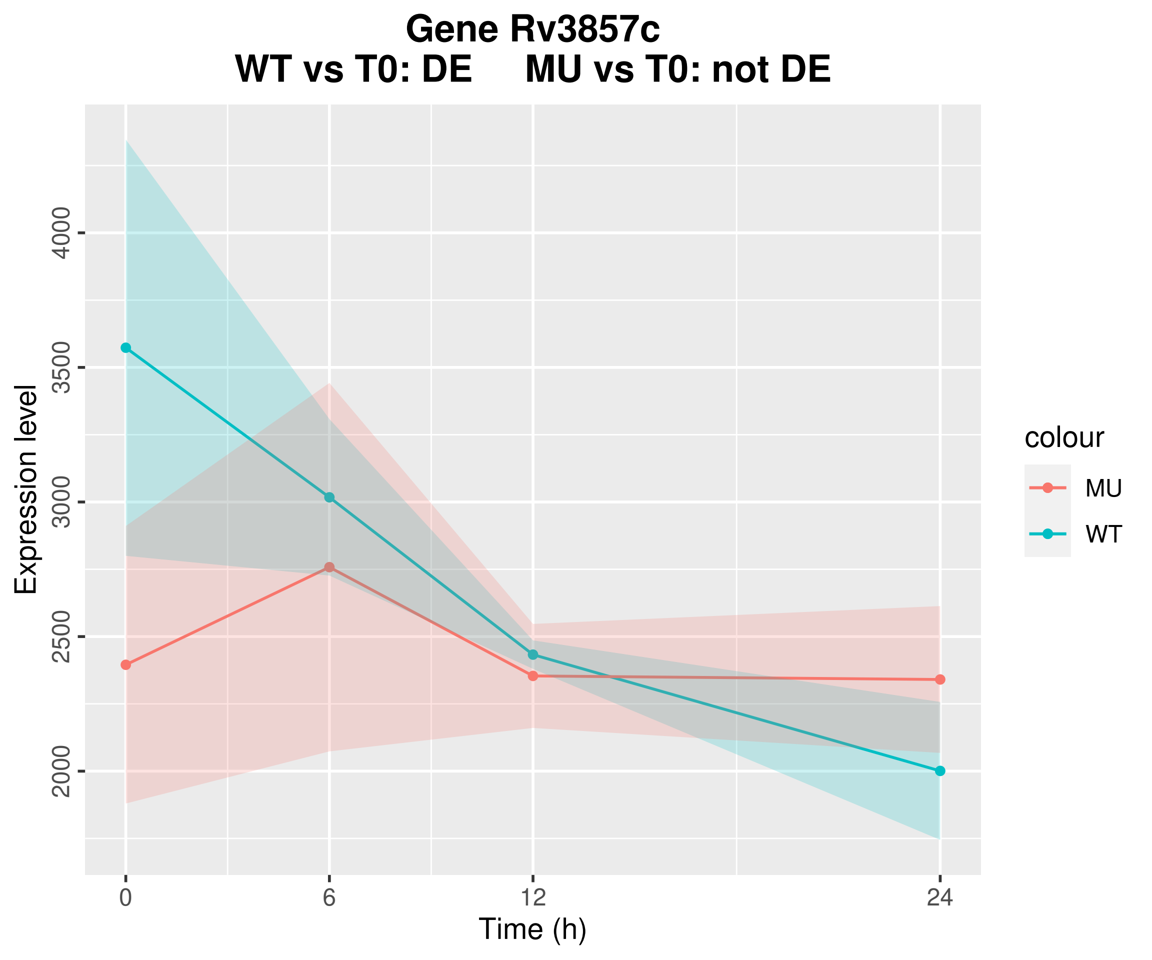

Gene Rv3857c
WT vs T0: DE   MU vs T0: not DE
Expression level
4000
3500
3000
2500
2000
0
6
12
24
Time (h)
colour
MU
WT

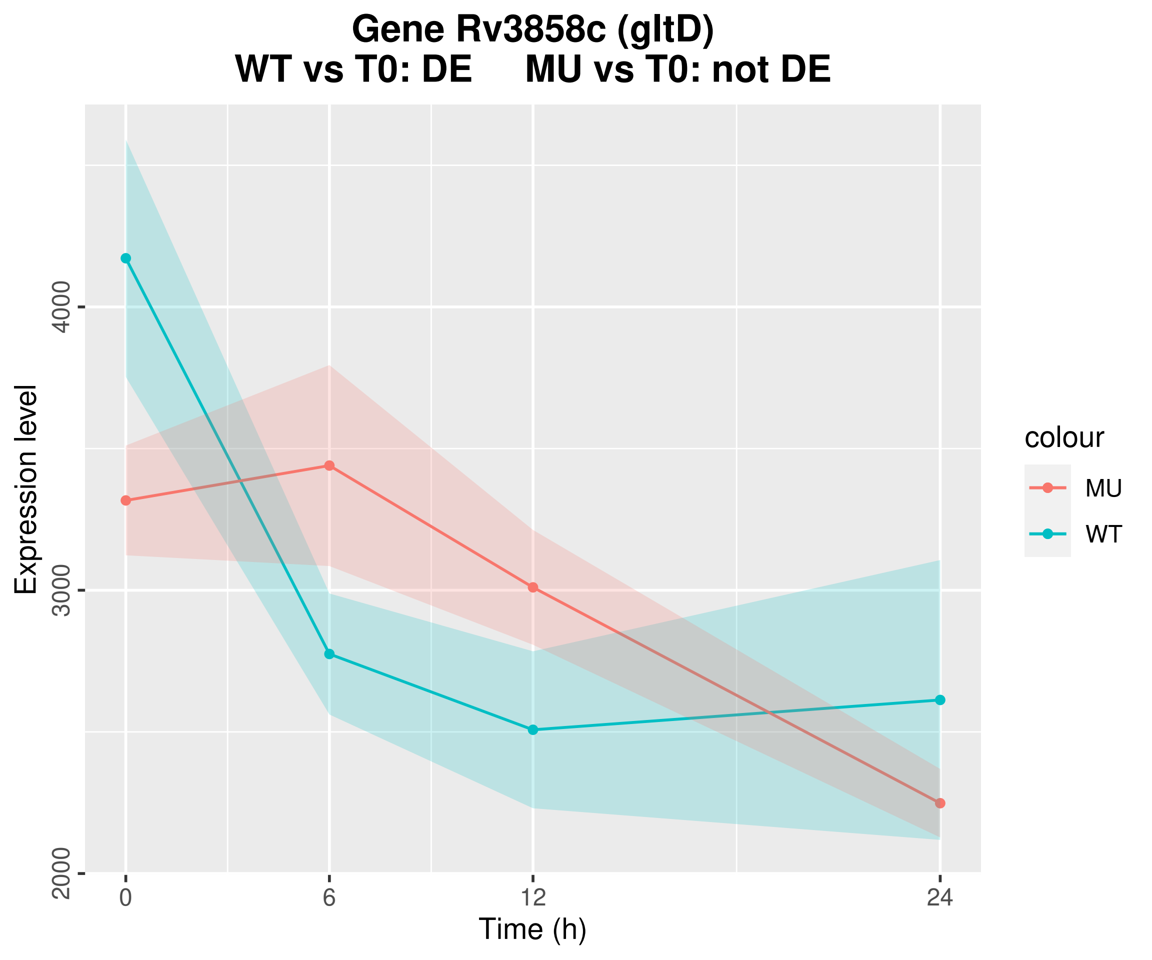

Gene Rv3858c (gltD)
WT vs T0: DE   MU vs T0: not DE
Expression level
4000
3000
2000
0
6
12
24
Time (h)
colour
MU
WT

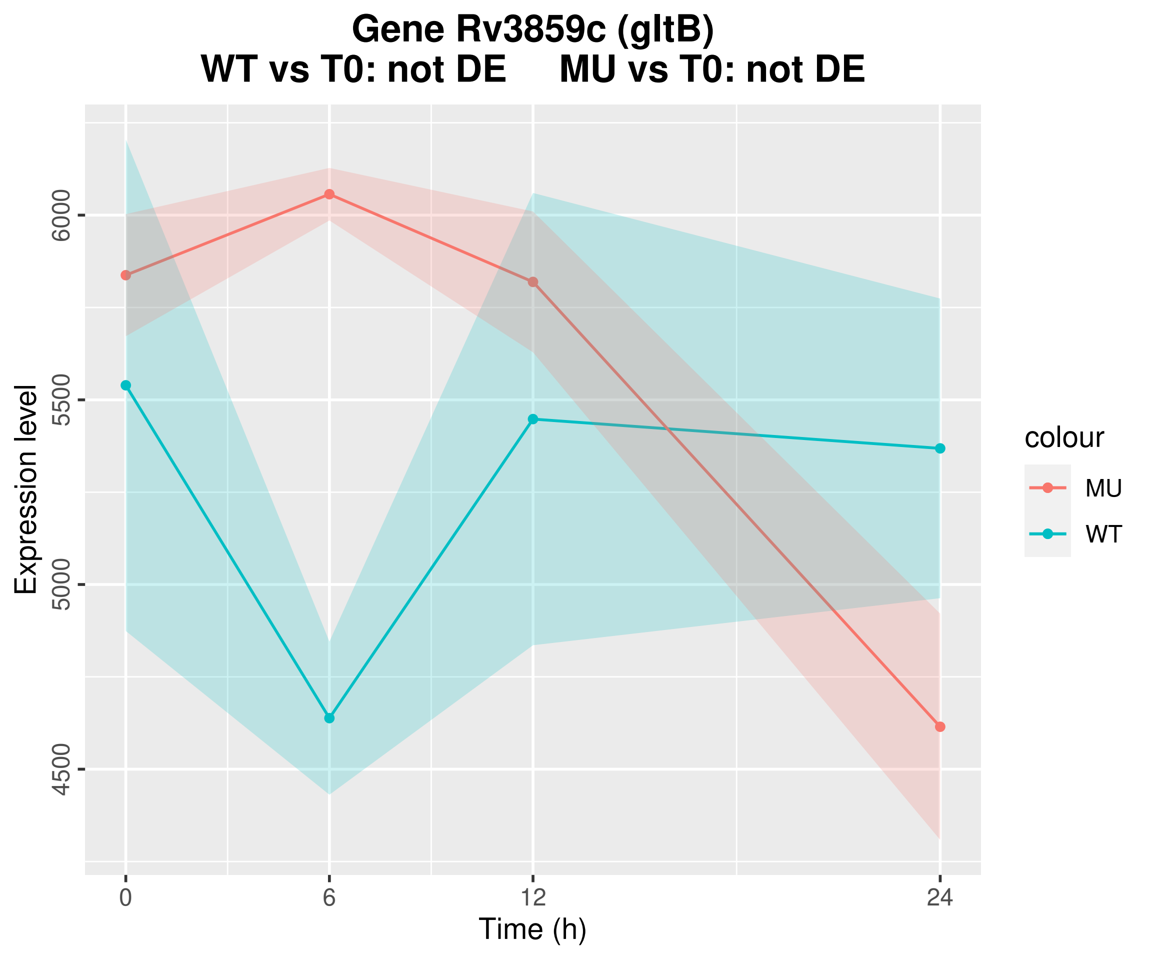

Gene Rv3859c (gltB)
WT vs T0: not DE MU vs T0: not DE
Expression level
6000
5500
5000
4500
0
6
12
24
Time (h)
colour
MU
WT

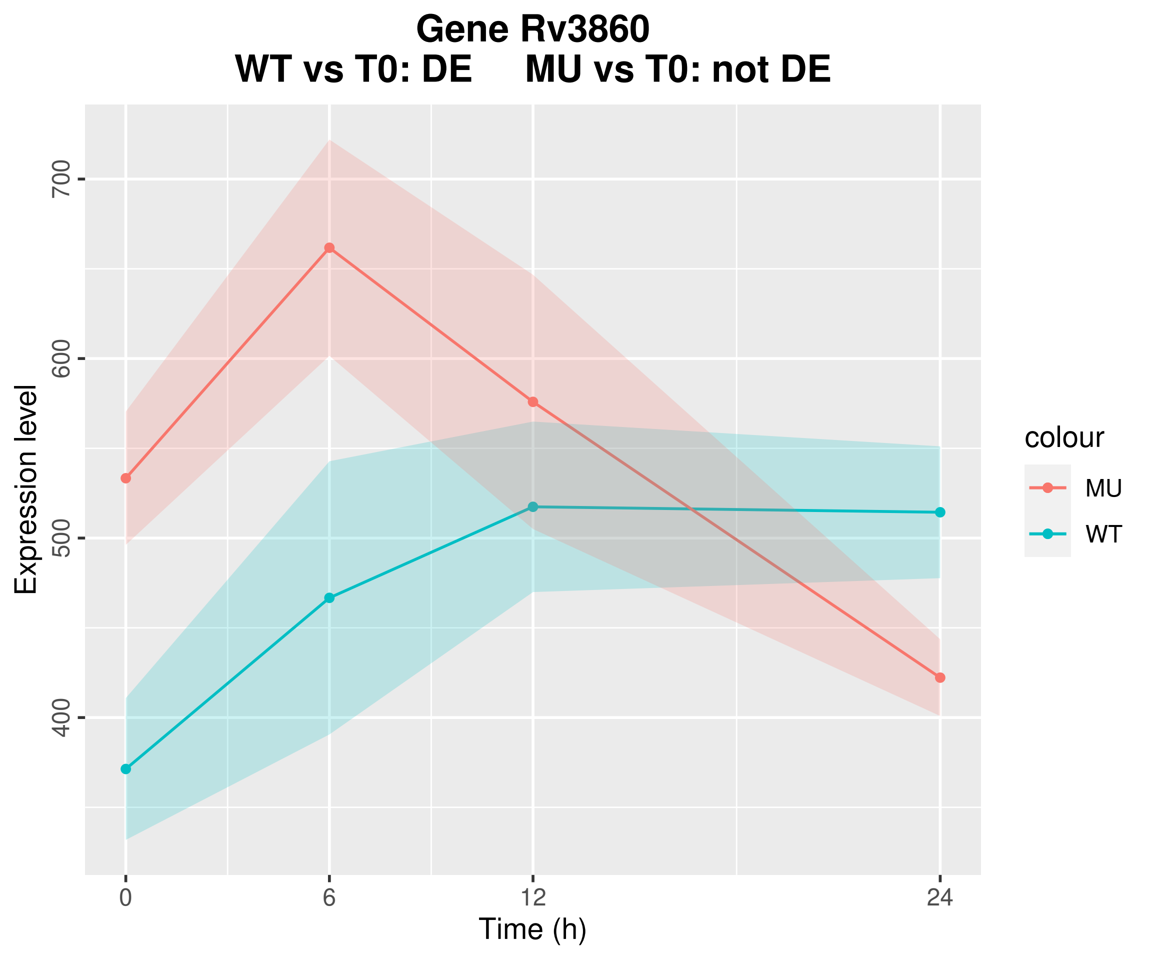

Gene Rv3860
WT vs T0: DE MU vs T0: not DE
700
600
500
400
Expression level
0
6
12
24
Time (h)
colour
MU
WT

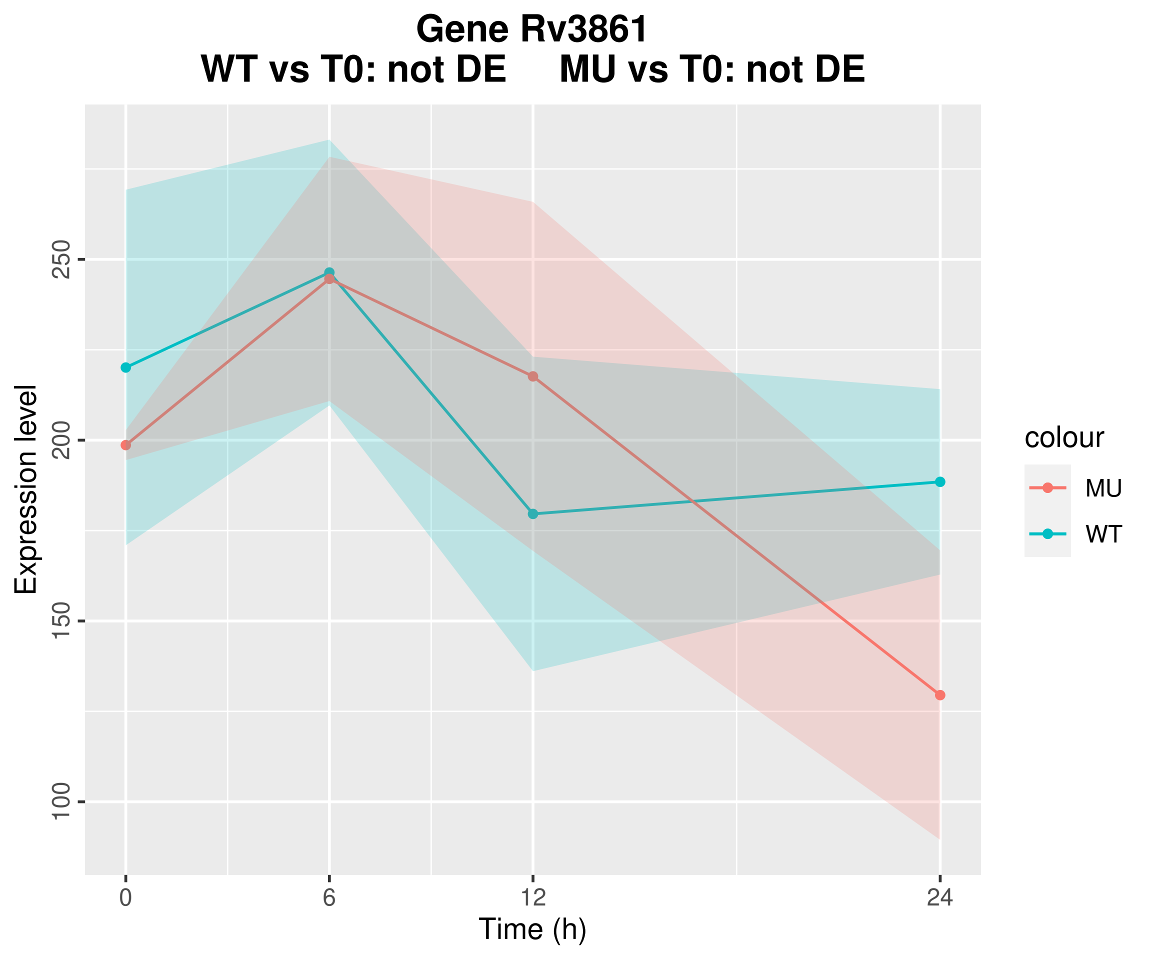

Gene Rv3861
WT vs T0: not DE MU vs T0: not DE
Expression level
250
200
150
100
0
6
12
24
Time (h)
colour
MU
WT

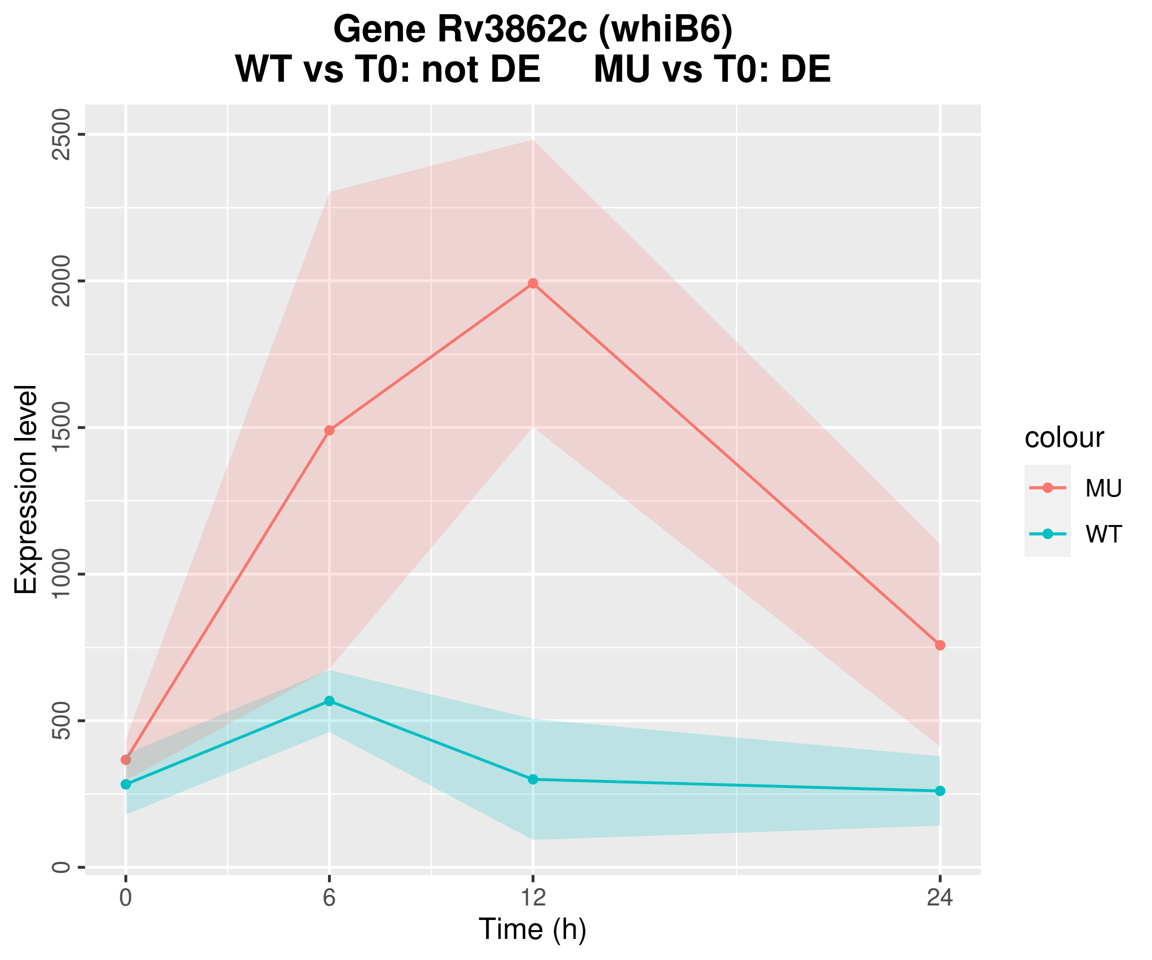
Gene Rv3862c (whiB6)
WT vs T0: not DE MU vs T0: DE
Expression level
0
500
1000
1500
2000
2500
0
6
12
24
Time (h)
colour
MU
WT

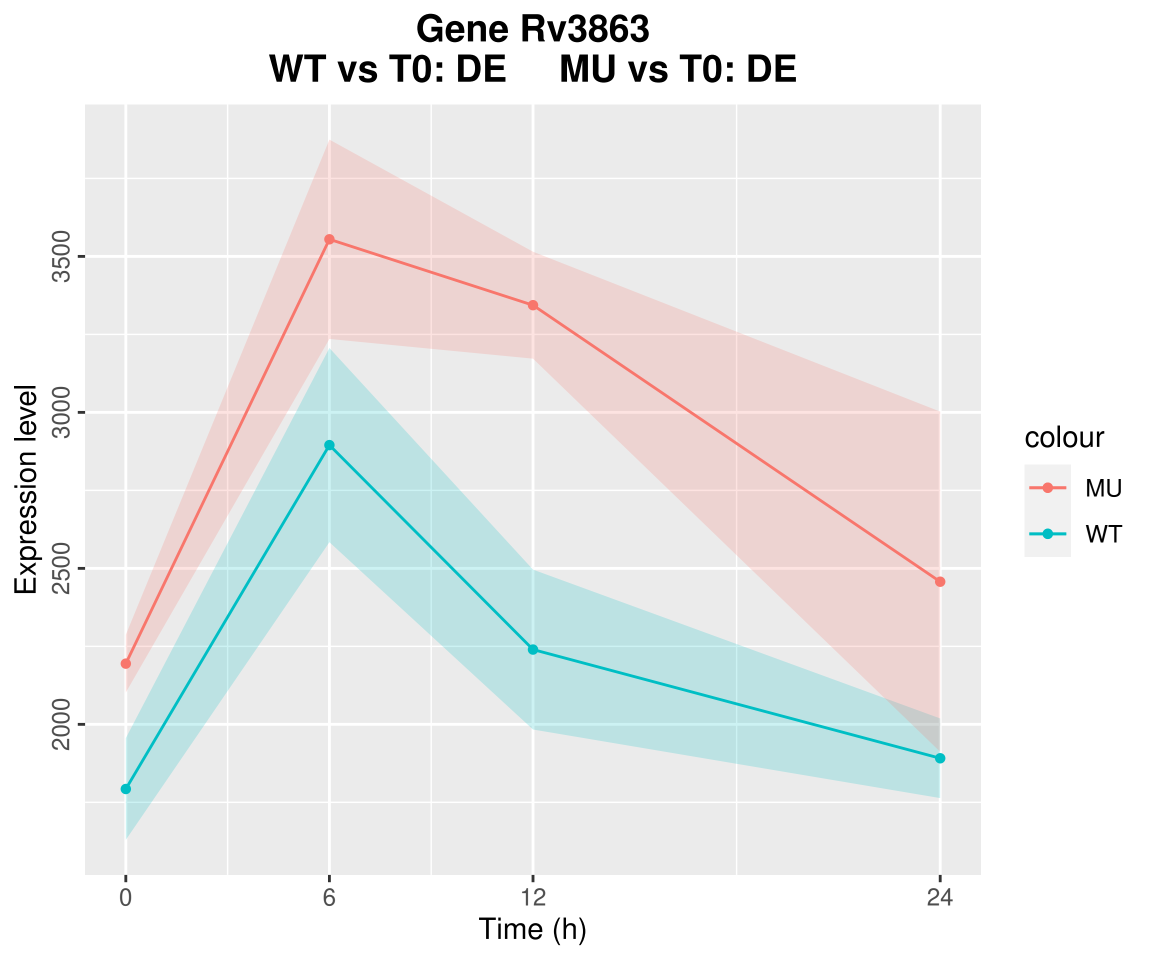
Gene Rv3863
WT vs T0: DE   MU vs T0: DE
Expression level
3500
3000
2500
2000
0
6
12
24
Time (h)
colour
MU
WT

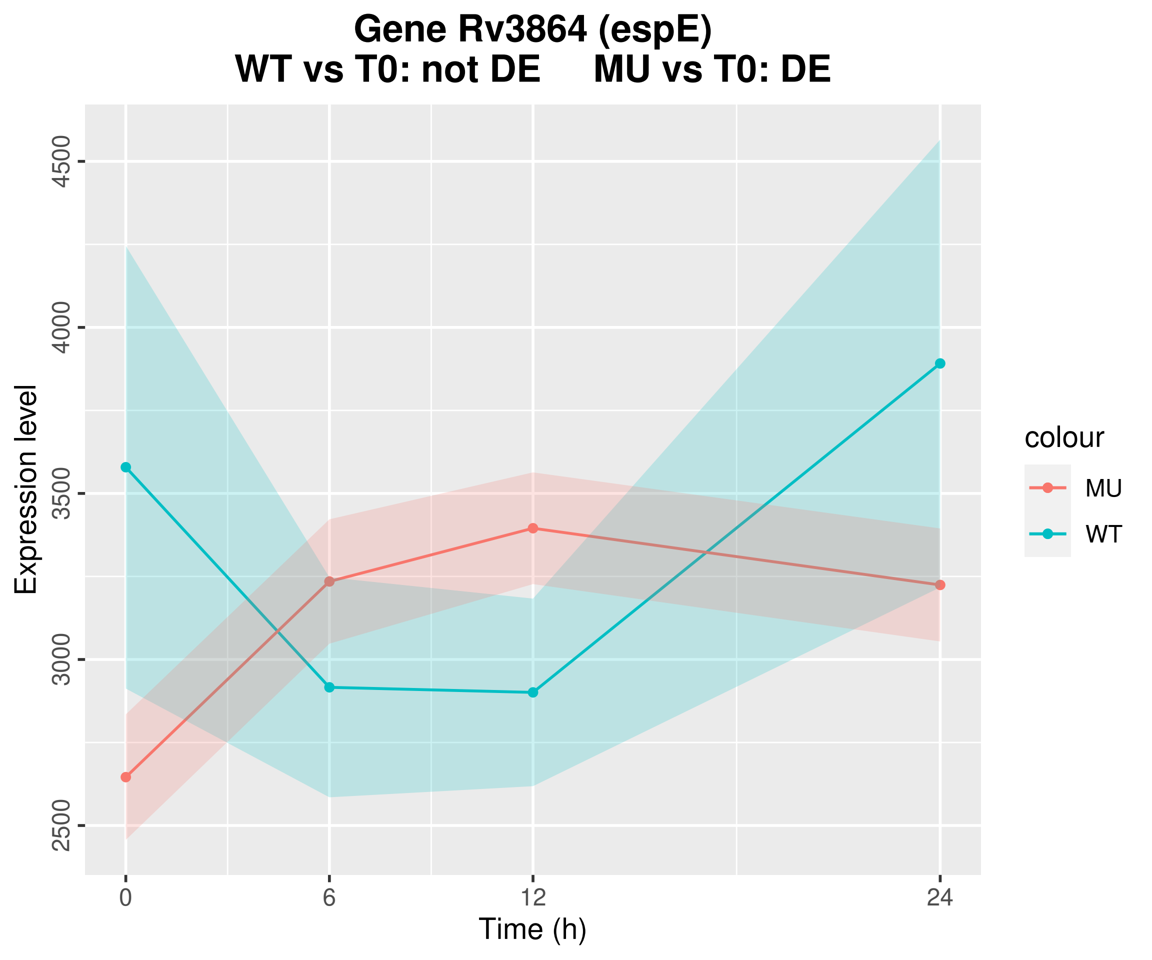
Gene Rv3864 (espE)
WT vs T0: not DE MU vs T0: DE
Expression level
4500
4000
3500
3000
2500
0
6
12
24
Time (h)
colour
MU
WT

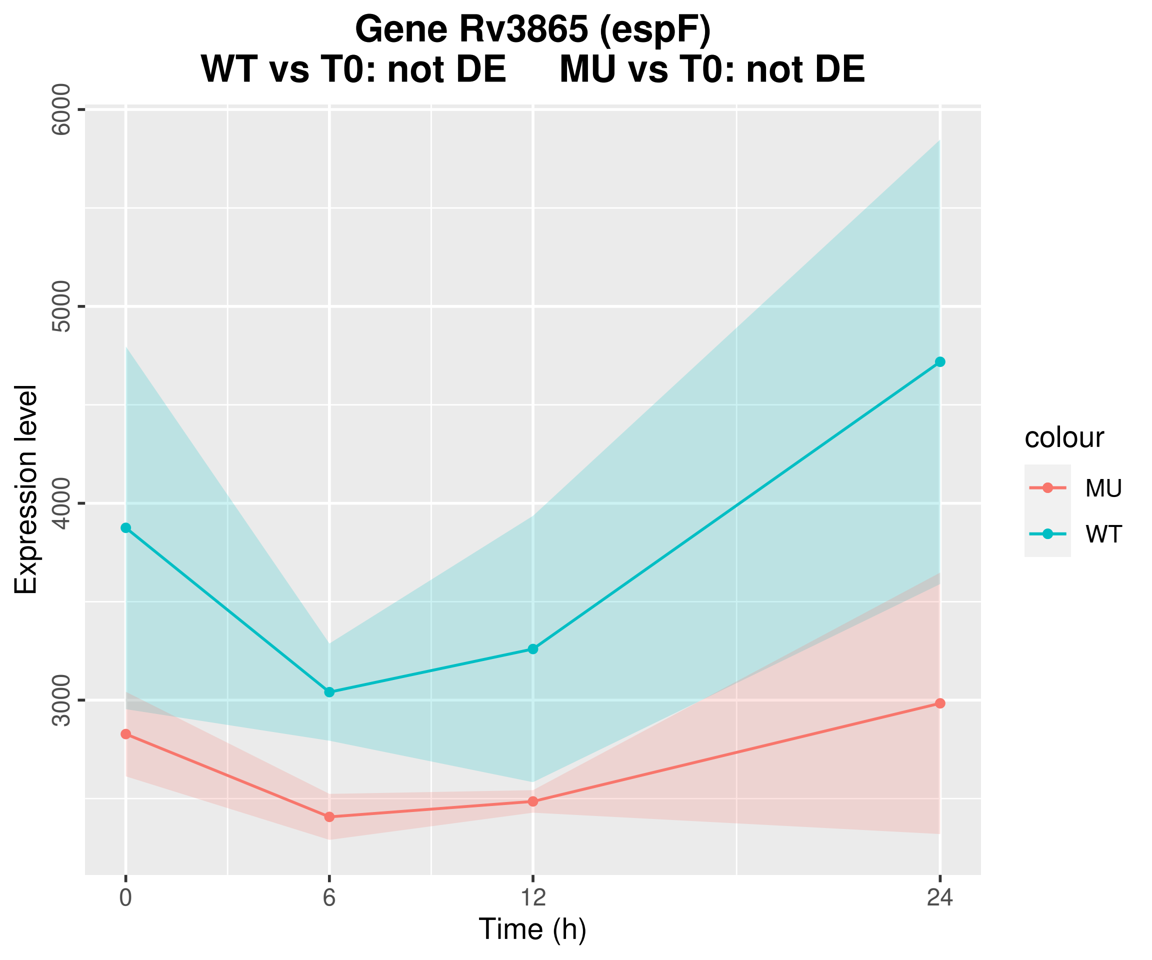

Gene Rv3865 (espF)
WT vs T0: not DE MU vs T0: not DE
6000
5000
4000
3000
Expression level
0
6
12
24
Time (h)
colour
MU
WT

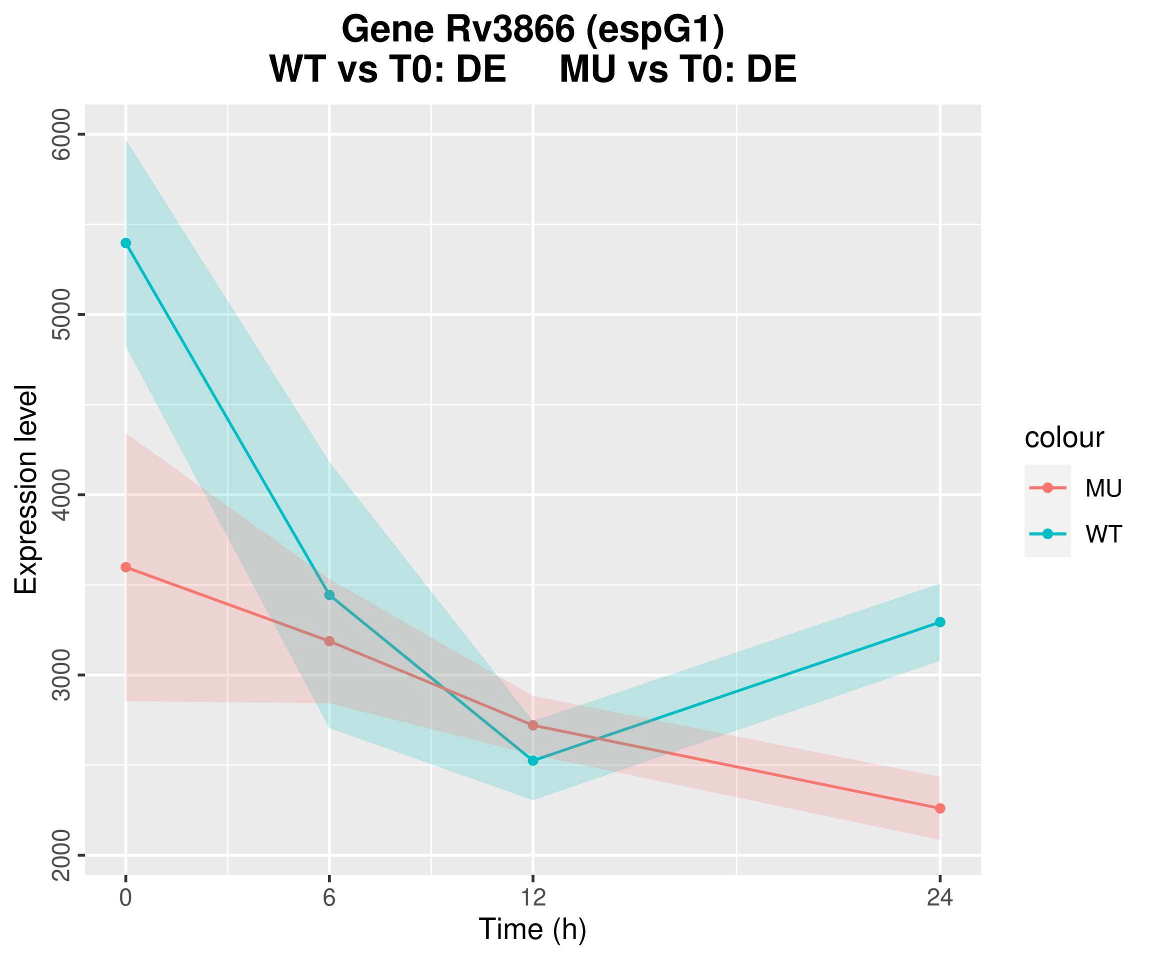
Gene Rv3866 (espG1)
WT vs T0: DE   MU vs T0: DE
Expression level
6000
5000
4000
3000
2000
0
6
12
24
Time (h)
colour
MU
WT

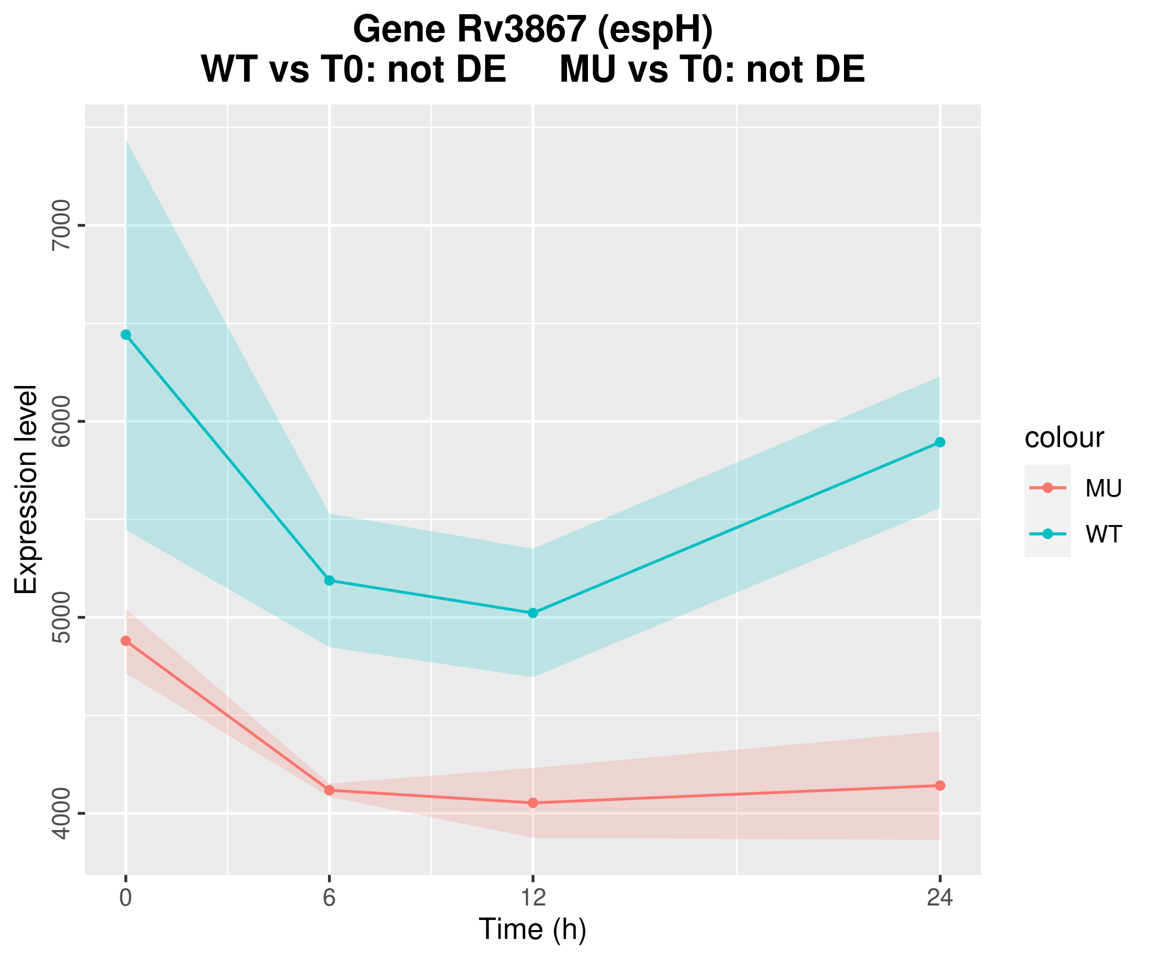

Gene Rv3867 (espH)
WT vs T0: not DE MU vs T0: not DE
Expression level
7000
6000
5000
4000
0
6
12
24
Time (h)
colour
MU
WT

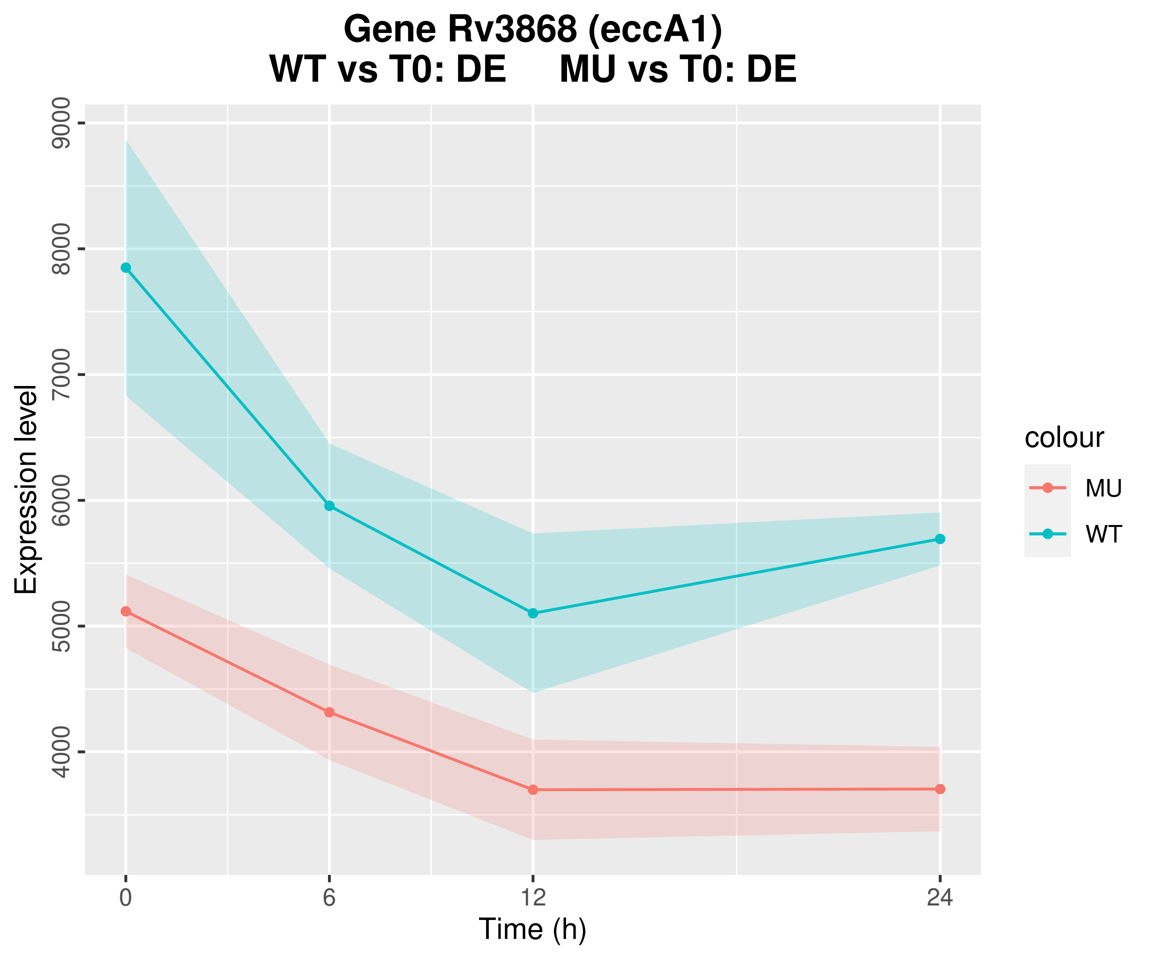
Gene Rv3868 (eccA1)
WT vs T0: DE   MU vs T0: DE
Expression level
9000
8000
7000
6000
5000
4000
0
6
12
24
Time (h)
colour
MU
WT

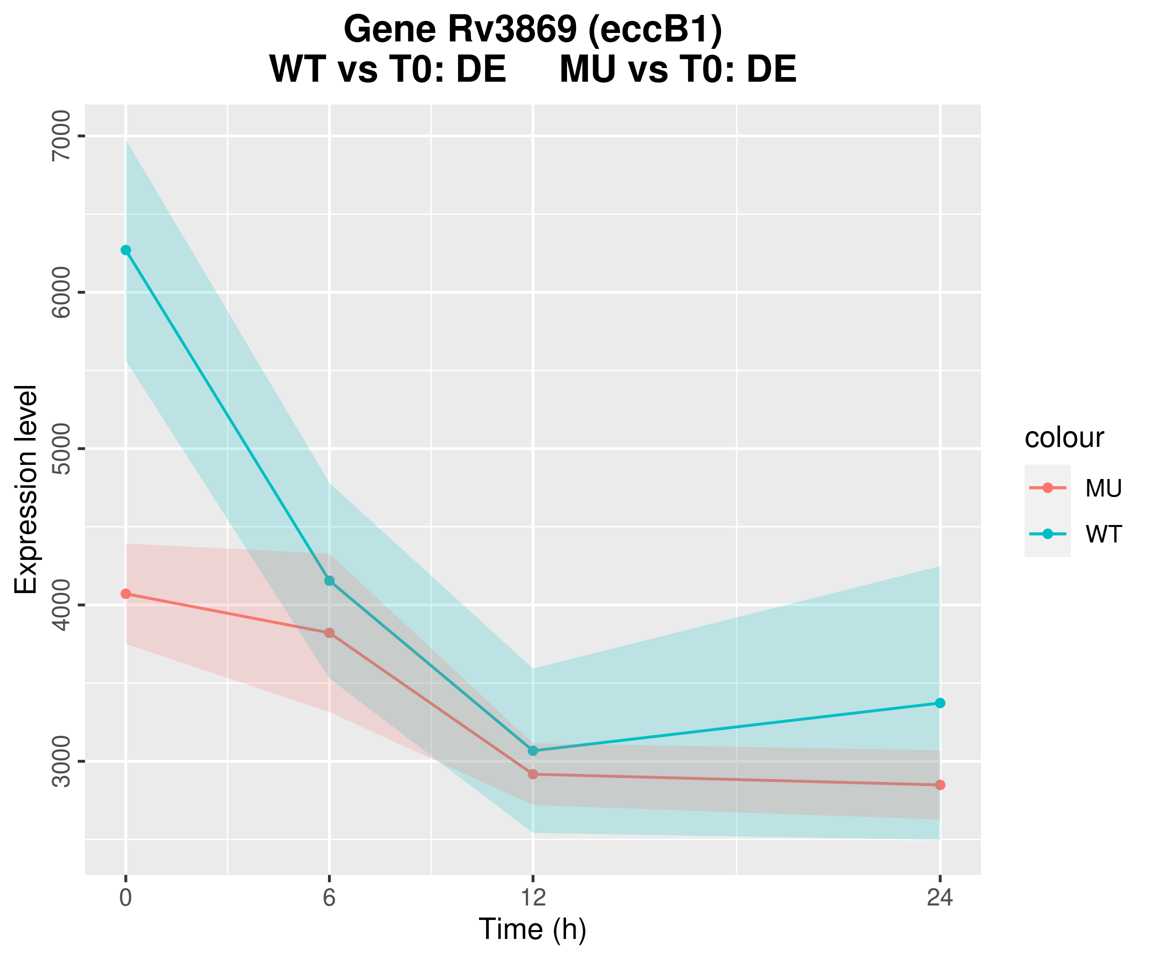
Gene Rv3869 (eccB1)
WT vs T0: DE   MU vs T0: DE
Expression level
7000
6000
5000
4000
3000
0
6
12
24
Time (h)
colour
MU
WT

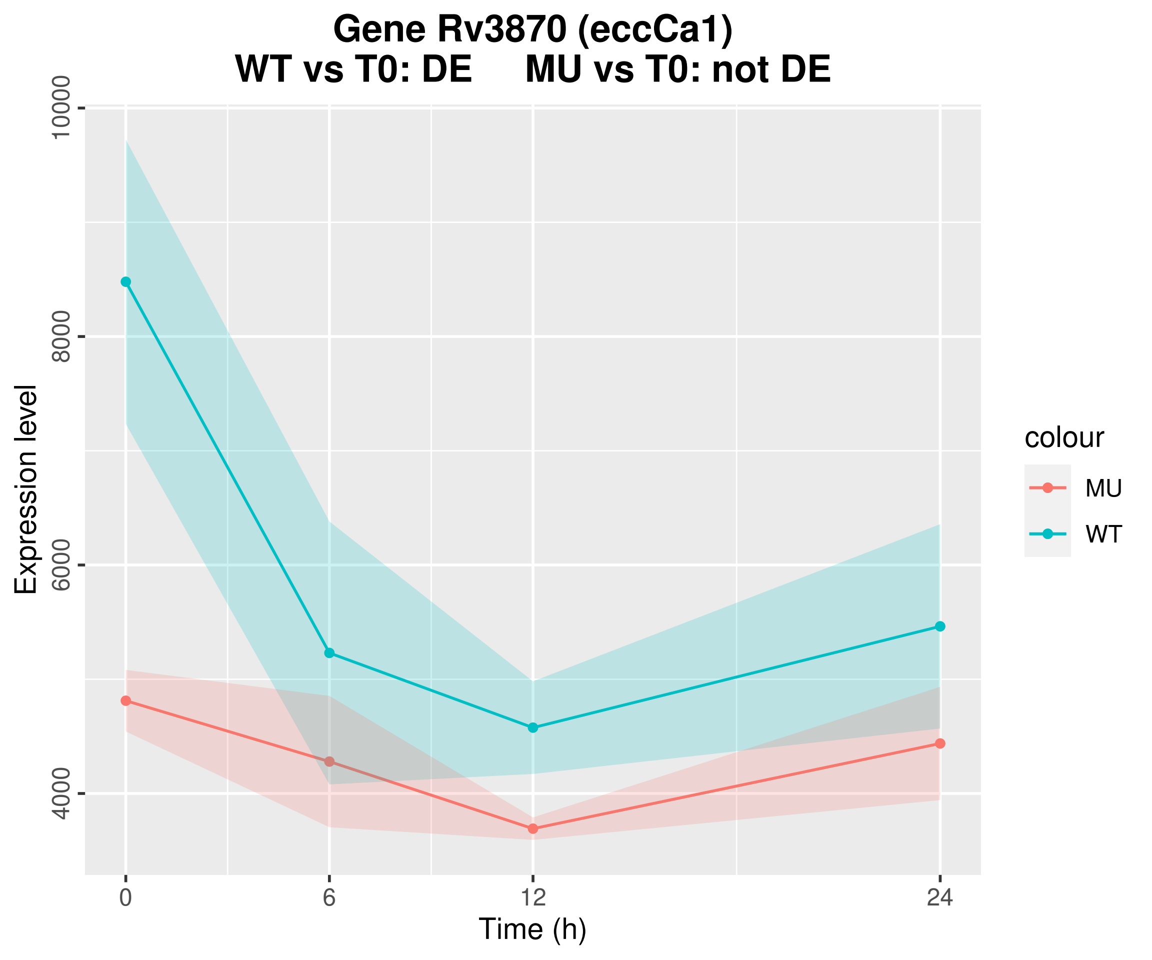
Gene Rv3870 (eccCa1)
WT vs T0: DE   MU vs T0: not DE
Expression level
10000
8000
6000
4000
0
6
12
24
Time (h)
colour
MU
WT

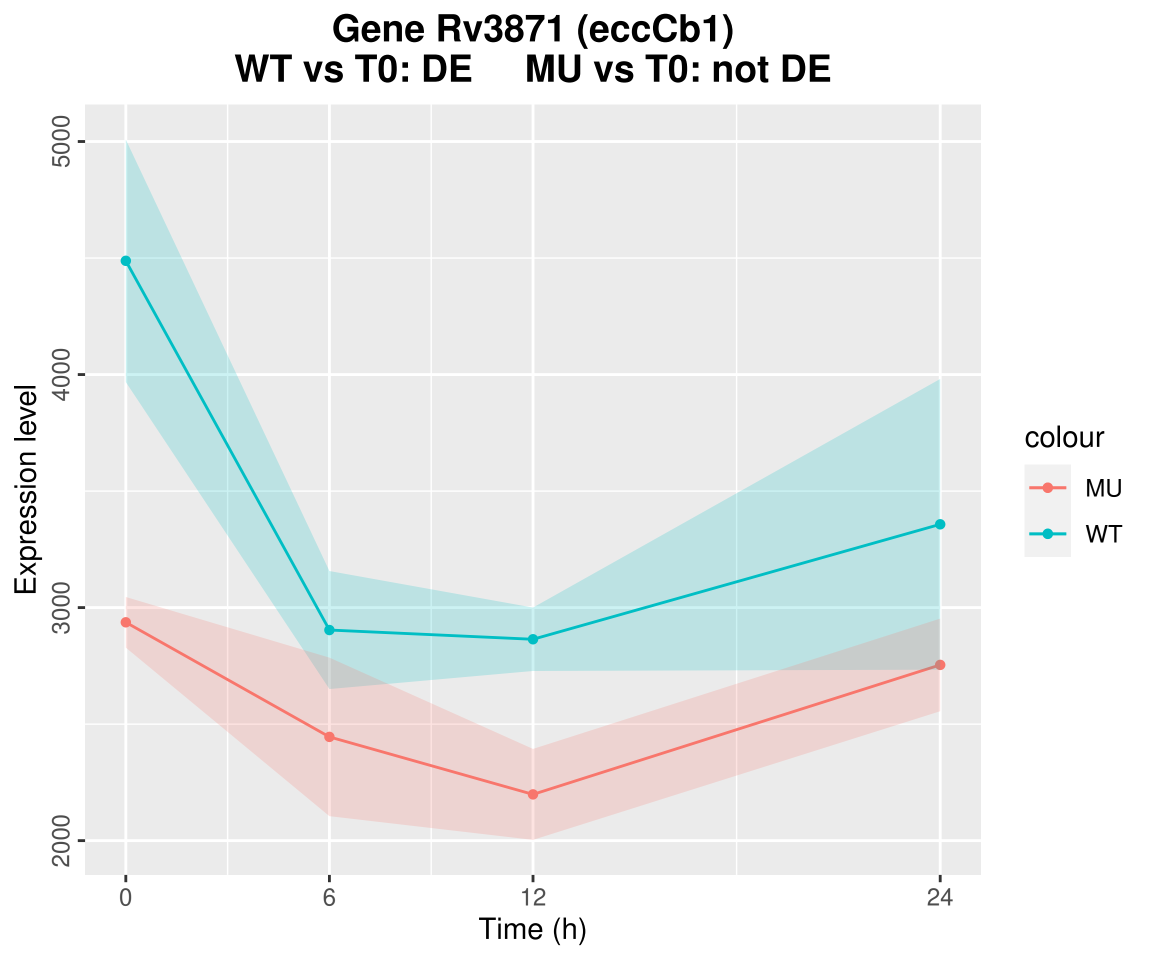

Gene Rv3871 (eccCb1)
WT vs T0: DE   MU vs T0: not DE
Expression level
5000
4000
3000
2000
0
6
12
24
Time (h)
colour
MU
WT

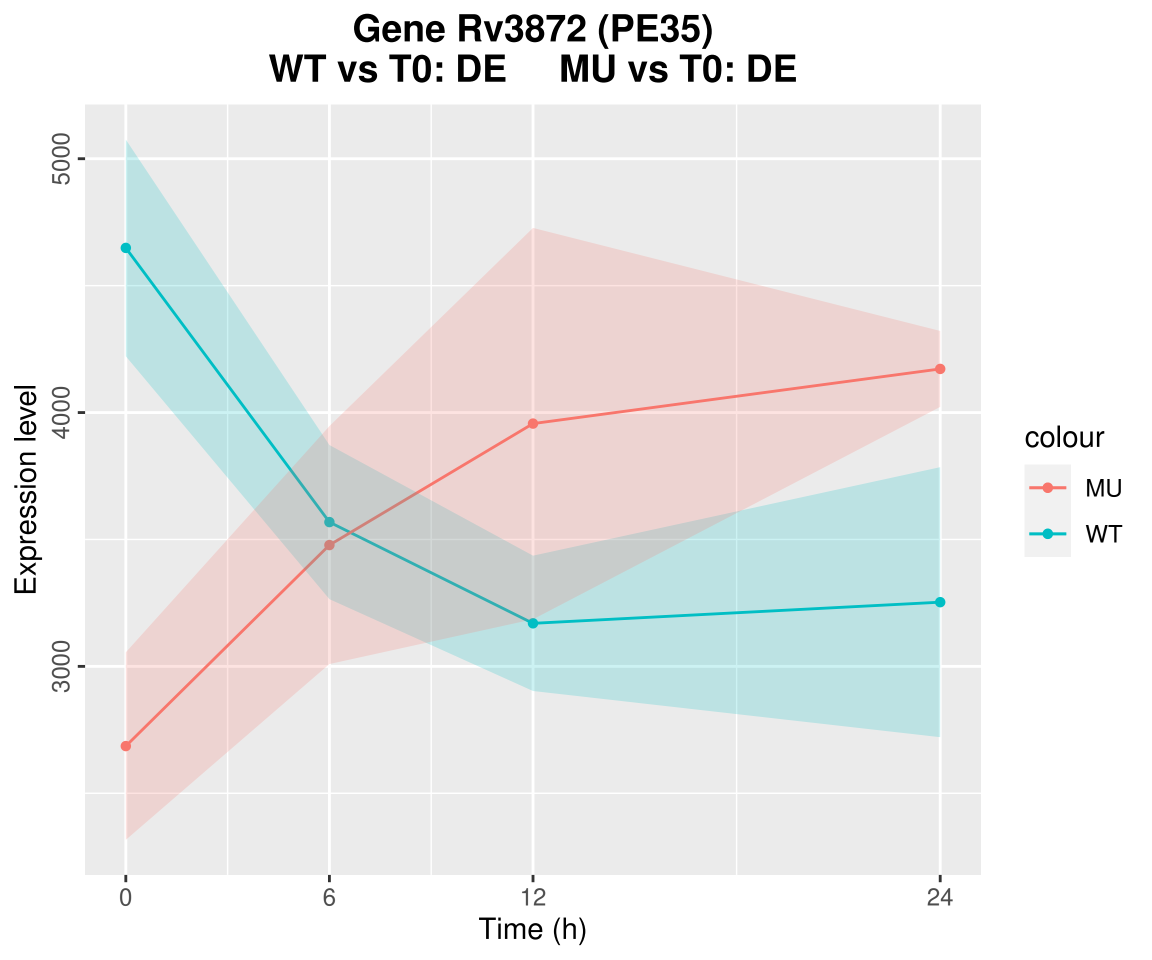
Gene Rv3872 (PE35)
WT vs T0: DE   MU vs T0: DE
Expression level
5000
4000
3000
0
6
12
24
Time (h)
colour
MU
WT

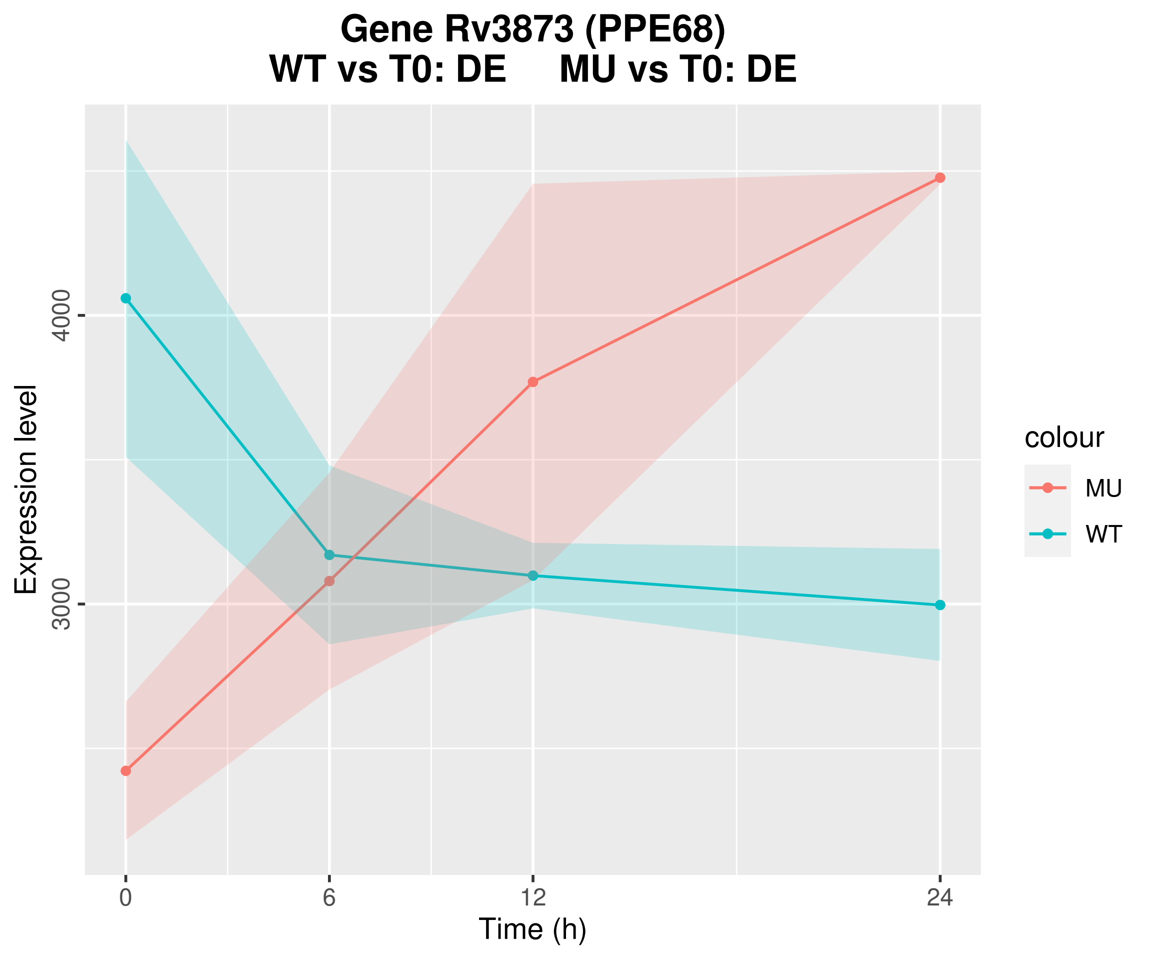

Gene Rv3873 (PPE68)
WT vs T0: DE MU vs T0: DE
Expression level
4000
3000
Time (h)
0
6
12
24
colour
MU
WT

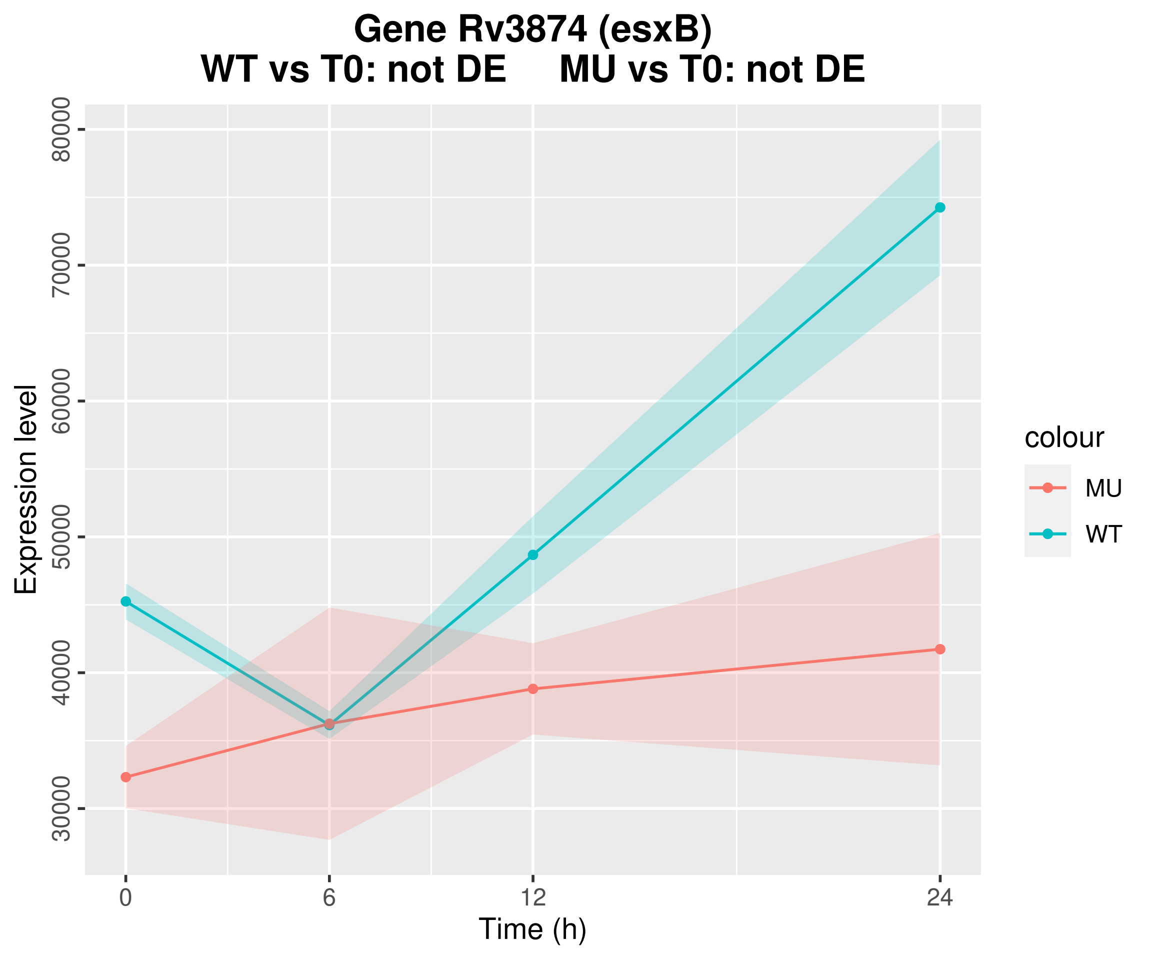
Gene Rv3874 (esxB)
WT vs T0: not DE MU vs T0: not DE
Expression level
80000
70000
60000
50000
40000
30000
0
6
12
24
Time (h)
colour
MU
WT

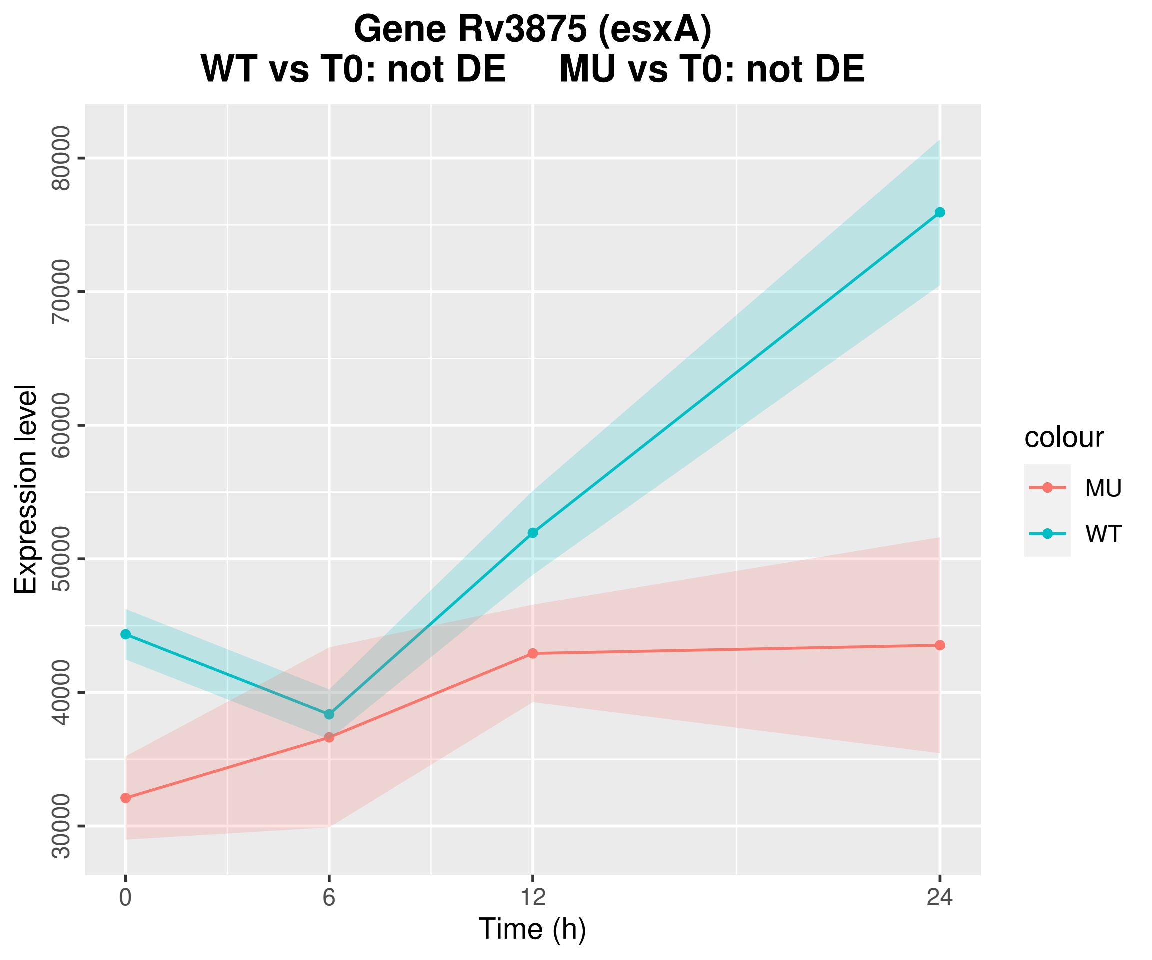
Gene Rv3875 (esxA)
WT vs T0: not DE MU vs T0: not DE
Expression level
80000
70000
60000
50000
40000
30000
0
6
12
24
Time (h)
colour
MU
WT

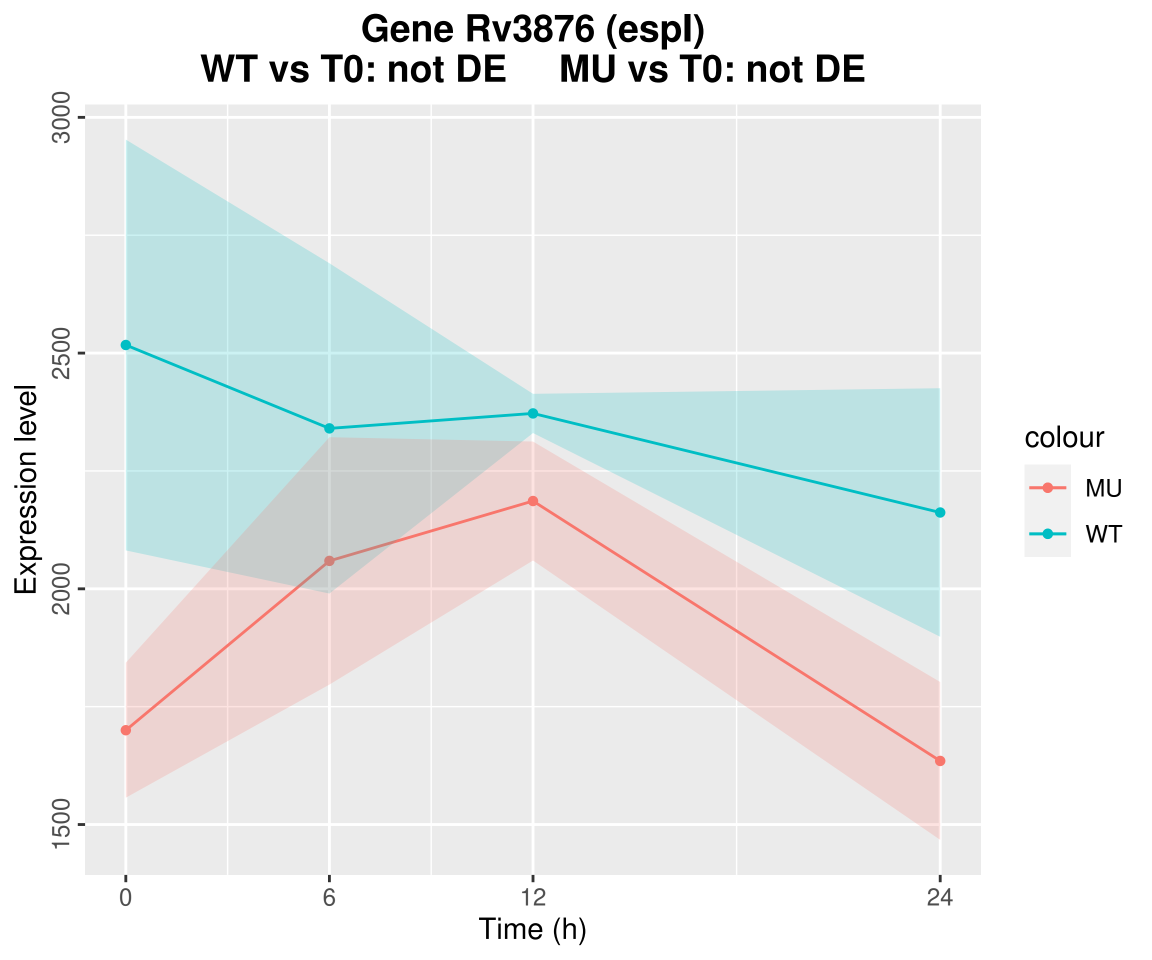

Gene Rv3876 (espI)
WT vs T0: not DE MU vs T0: not DE
Expression level
3000
2500
2000
1500
0
6
12
24
Time (h)
colour
MU
WT

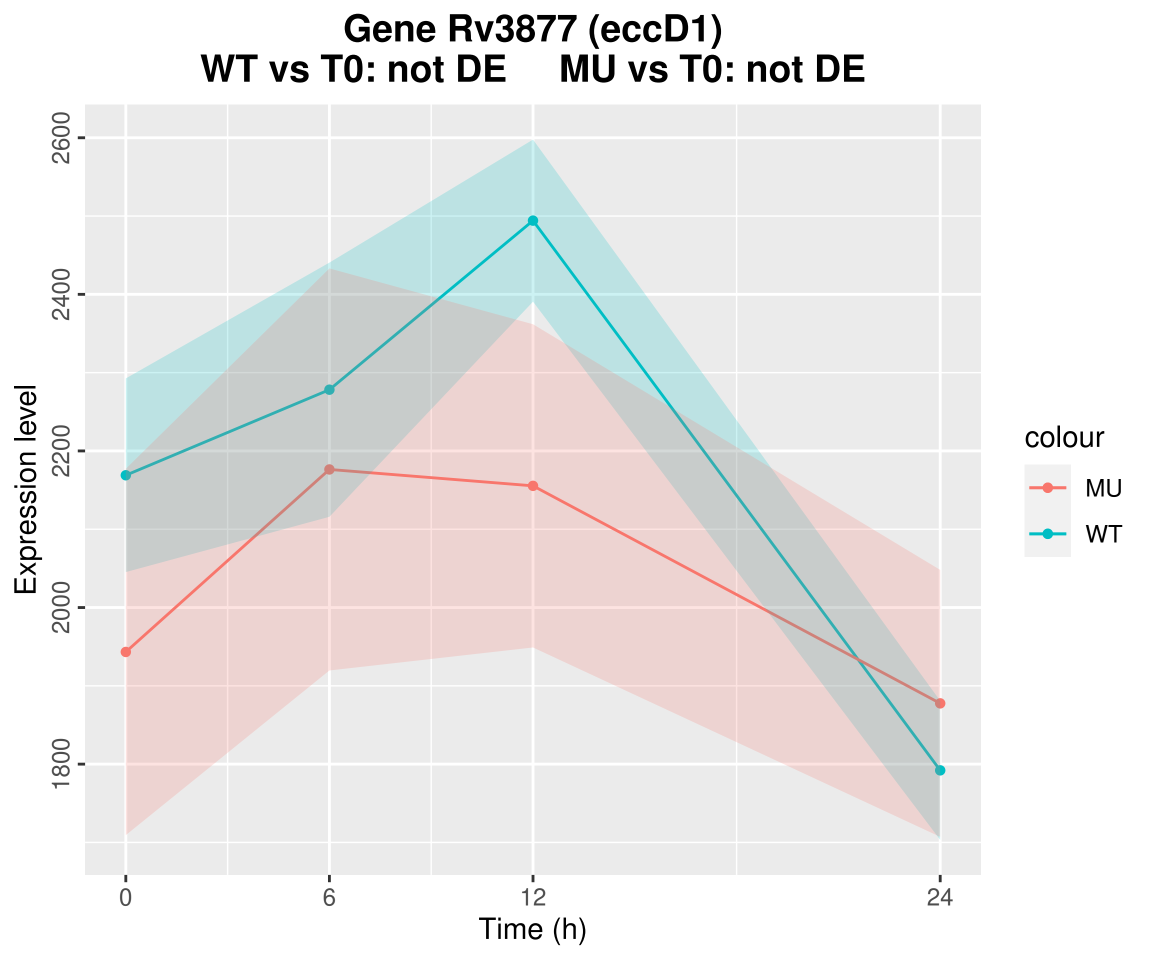

Gene Rv3877 (eccD1)
WT vs T0: not DE MU vs T0: not DE
Expression level
2600
2400
2200
2000
1800
0
6
12
24
Time (h)
colour
MU
WT

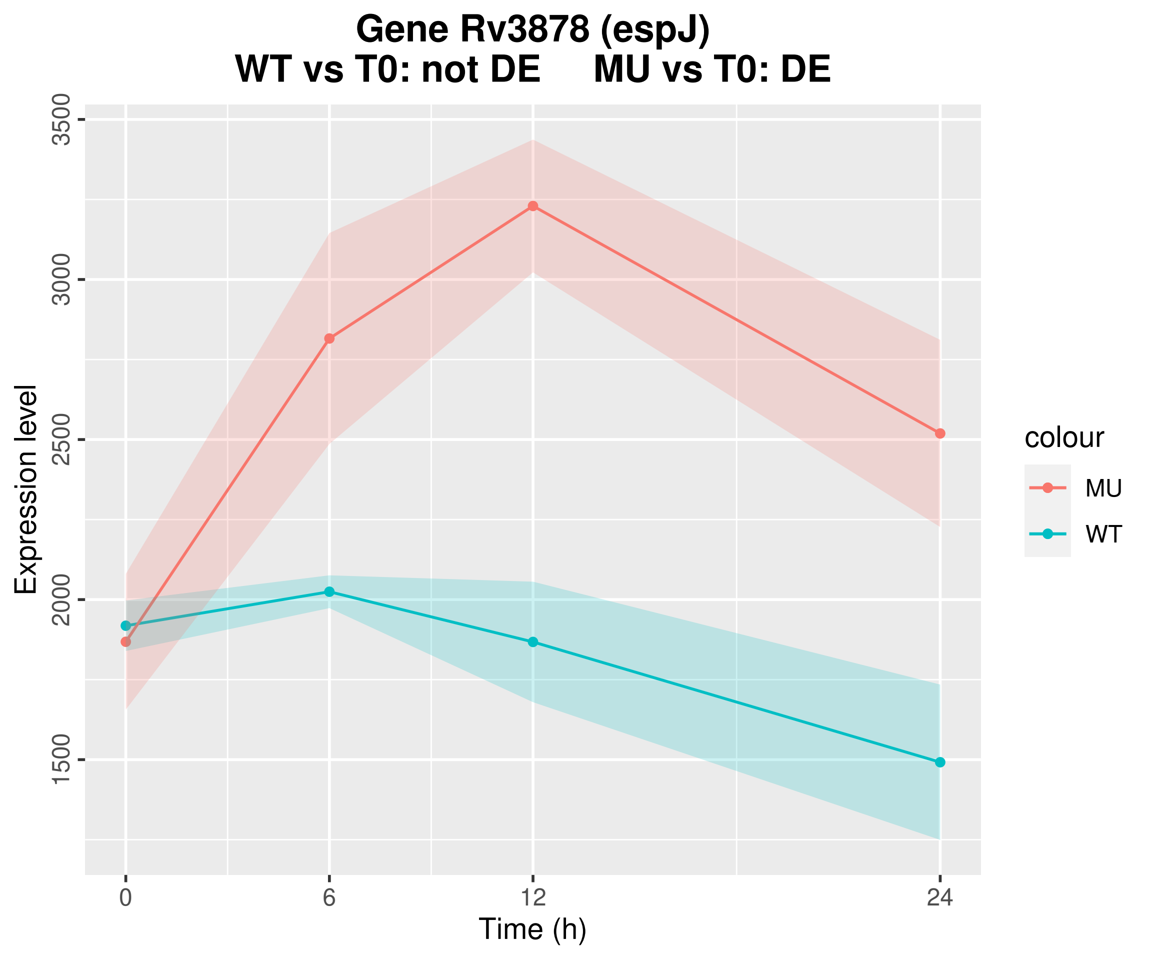
Gene Rv3878 (espJ)
WT vs T0: not DE MU vs T0: DE
Expression level
3500
3000
2500
2000
1500
0
6
12
24
Time (h)
colour
MU
WT

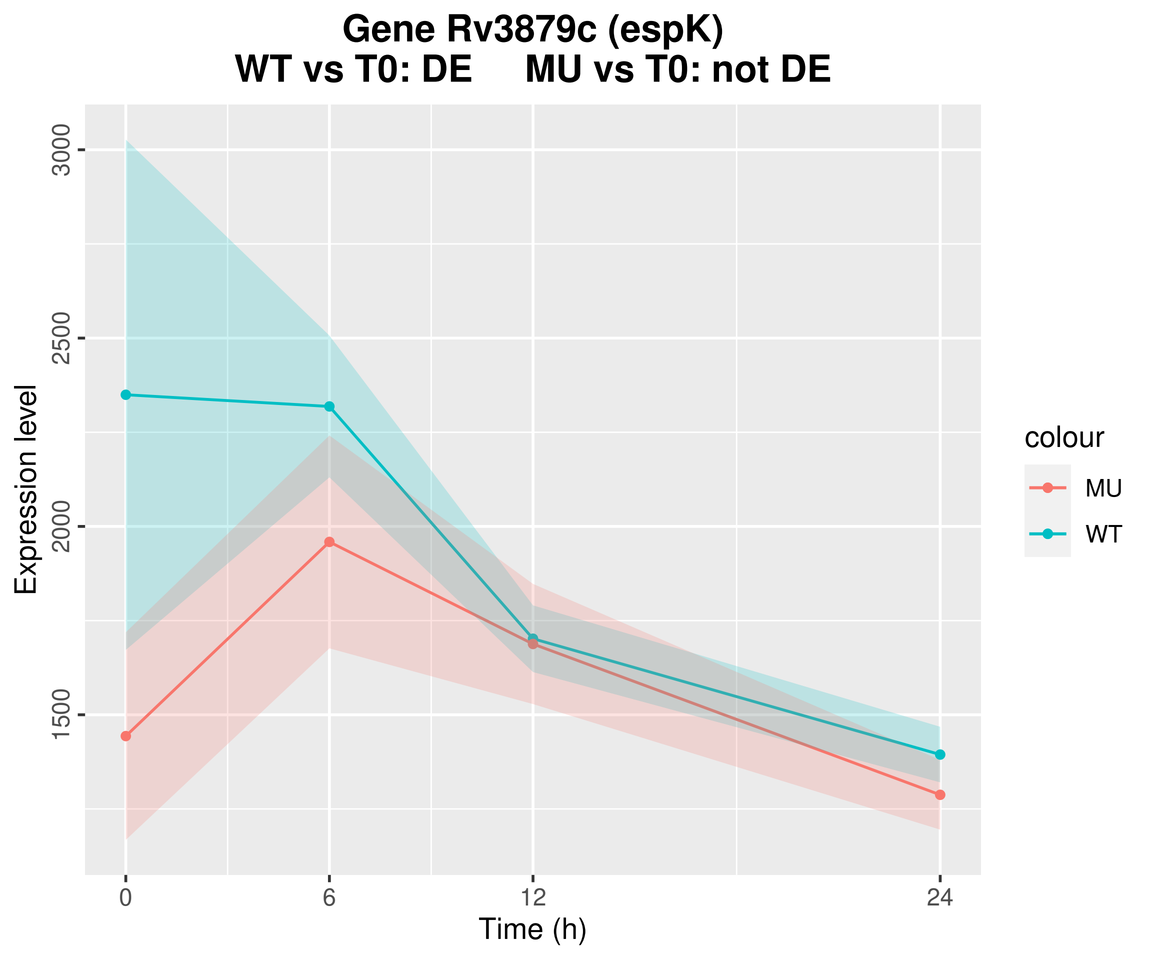

Gene Rv3879c (espK)
WT vs T0: DE   MU vs T0: not DE
Expression level
3000
2500
2000
1500
0
6
12
24
Time (h)
colour
MU
WT

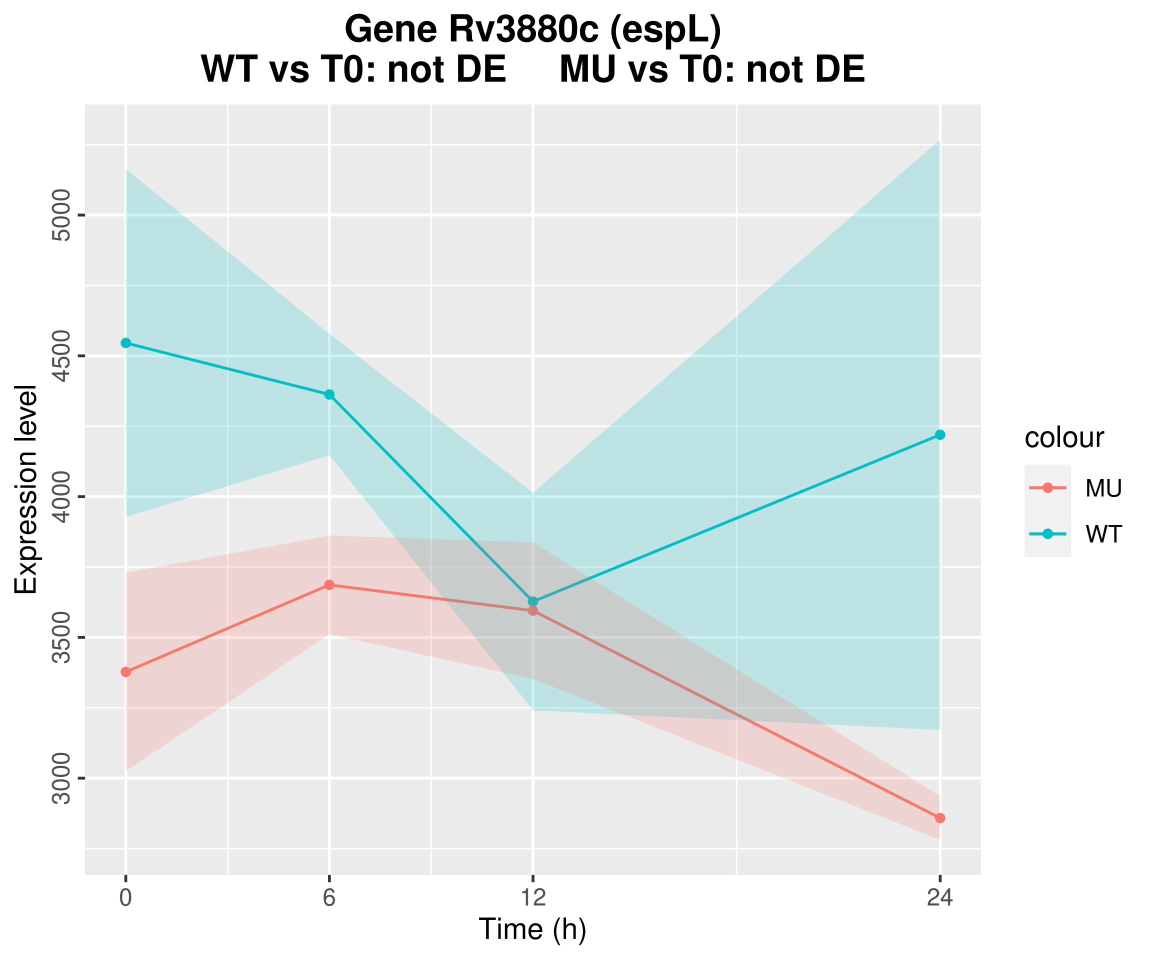

Gene Rv3880c (espL)
WT vs T0: not DE   MU vs T0: not DE
Expression level
5000
4500
4000
3500
3000
0
6
12
24
Time (h)
colour
MU
WT

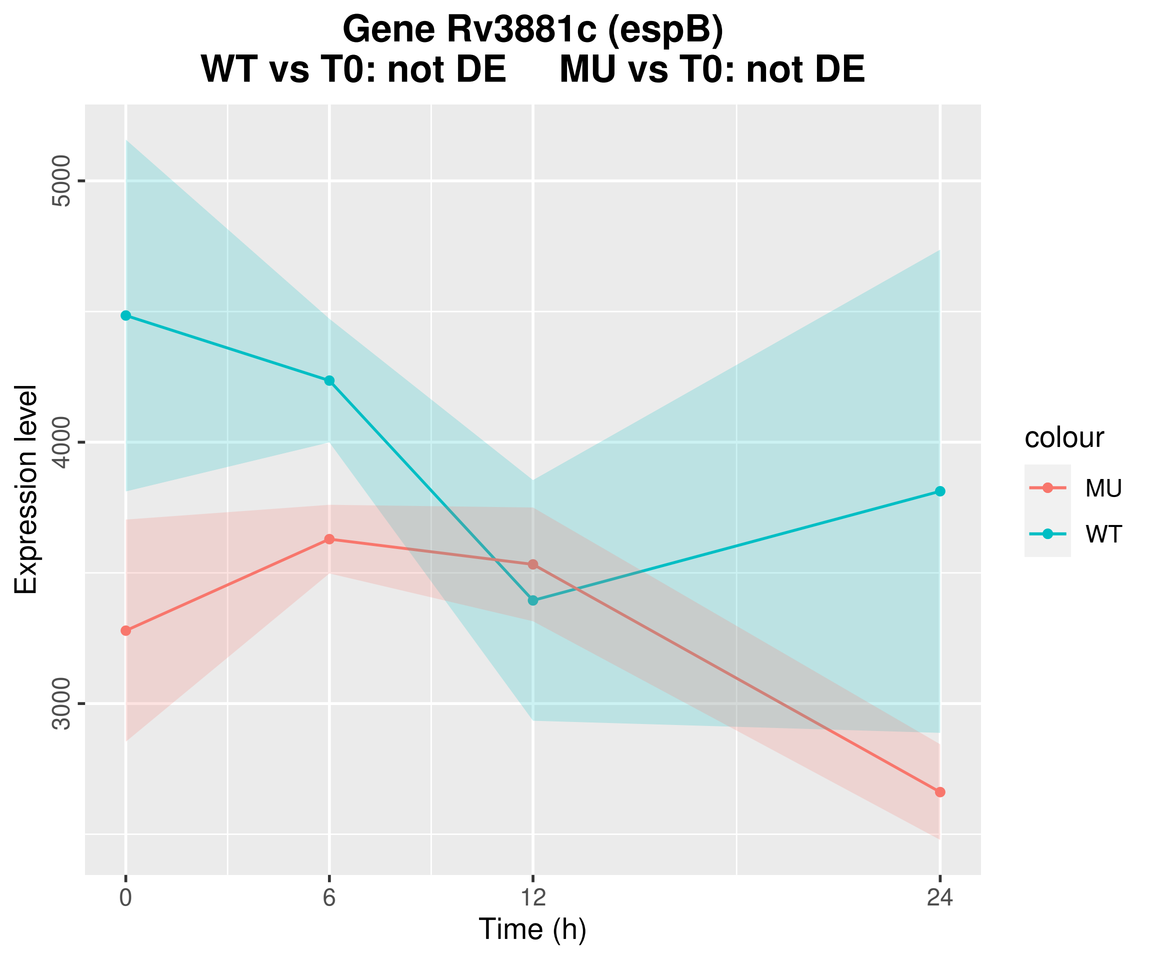
Gene Rv3881c (espB)
WT vs T0: not DE MU vs T0: not DE
Expression level
5000
4000
3000
0
6
12
24
Time (h)
colour
MU
WT

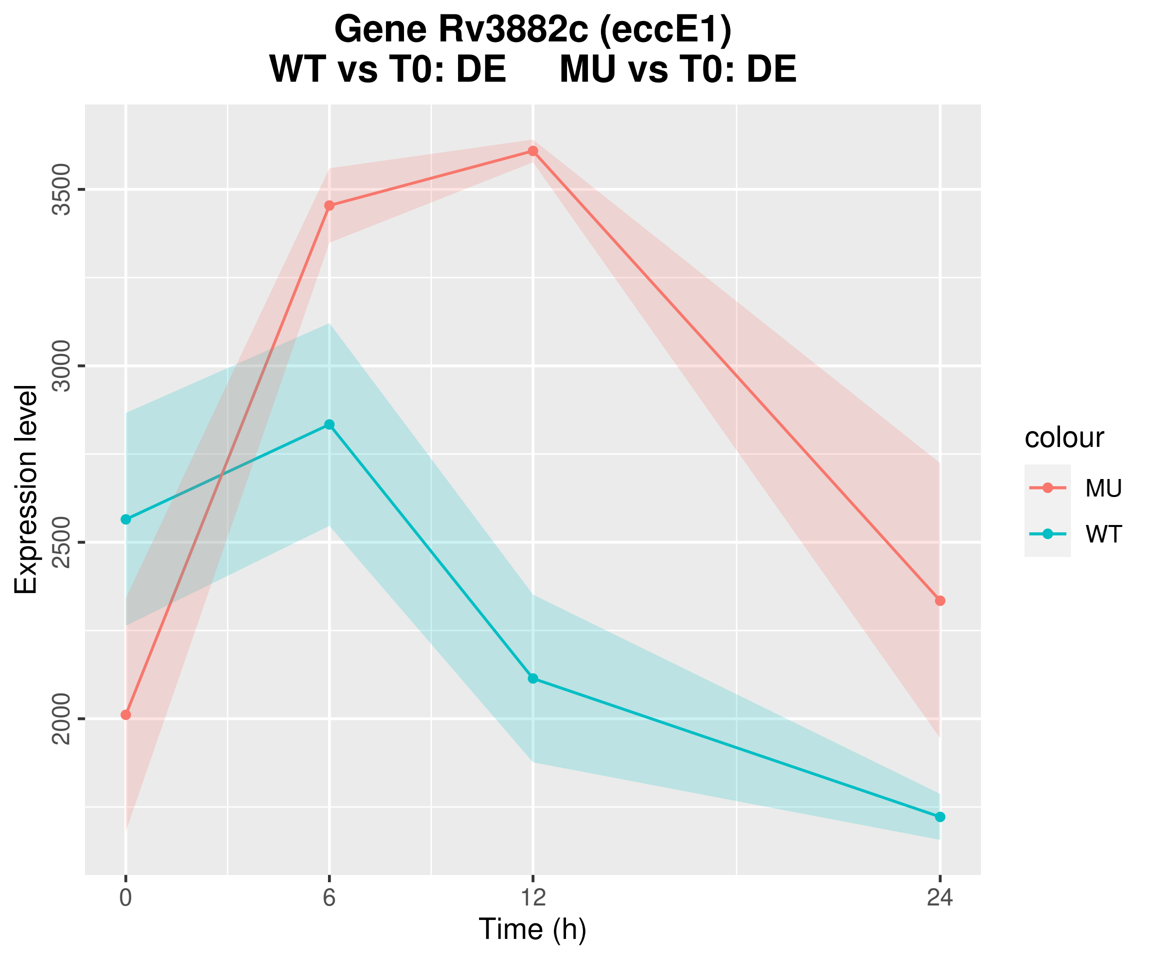
Gene Rv3882c (eccE1)
WT vs T0: DE   MU vs T0: DE
Expression level
3500
3000
2500
2000
0
6
12
24
Time (h)
colour
MU
WT

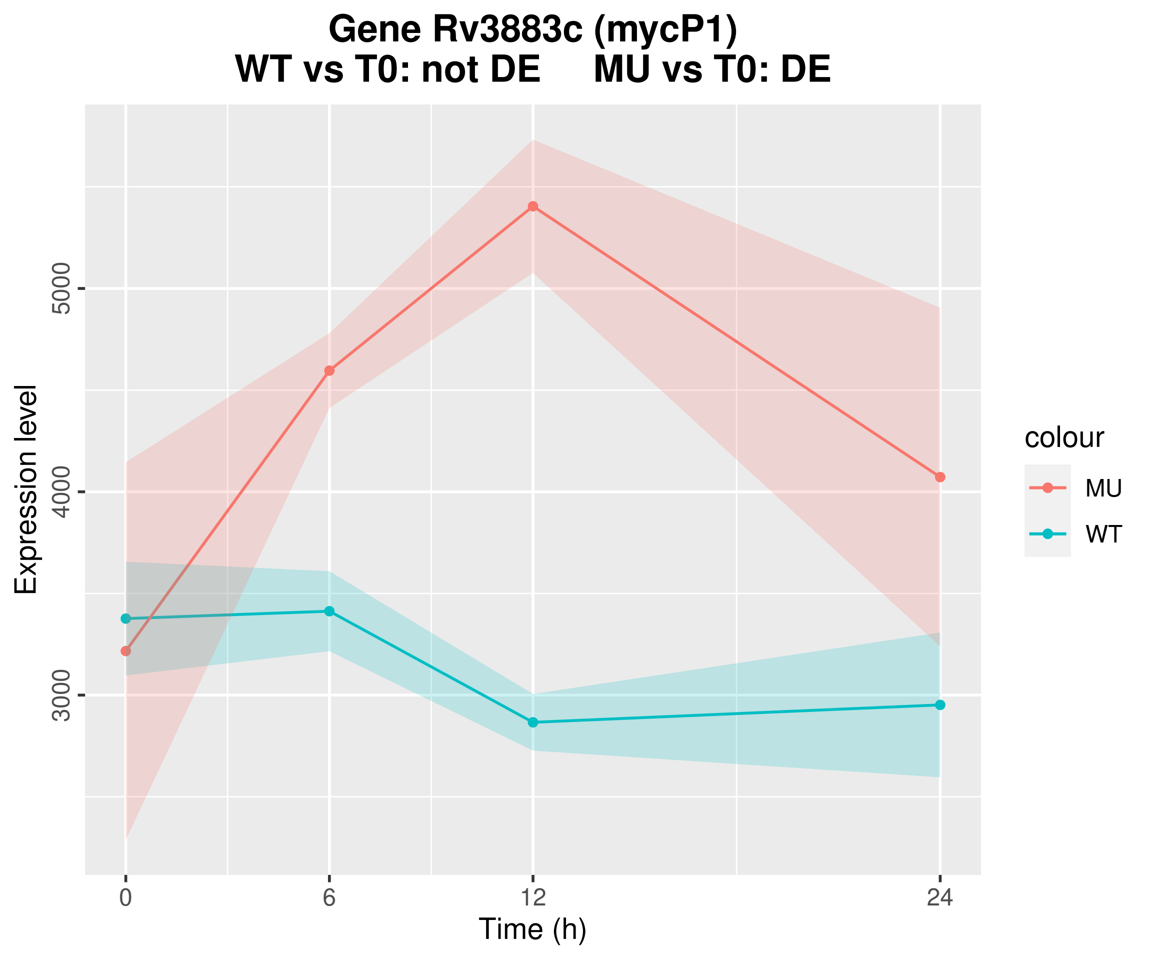
Gene Rv3883c (mycP1)
WT vs T0: not DE MU vs T0: DE
Expression level
5000
4000
3000
0
6
12
24
Time (h)
colour
MU
WT

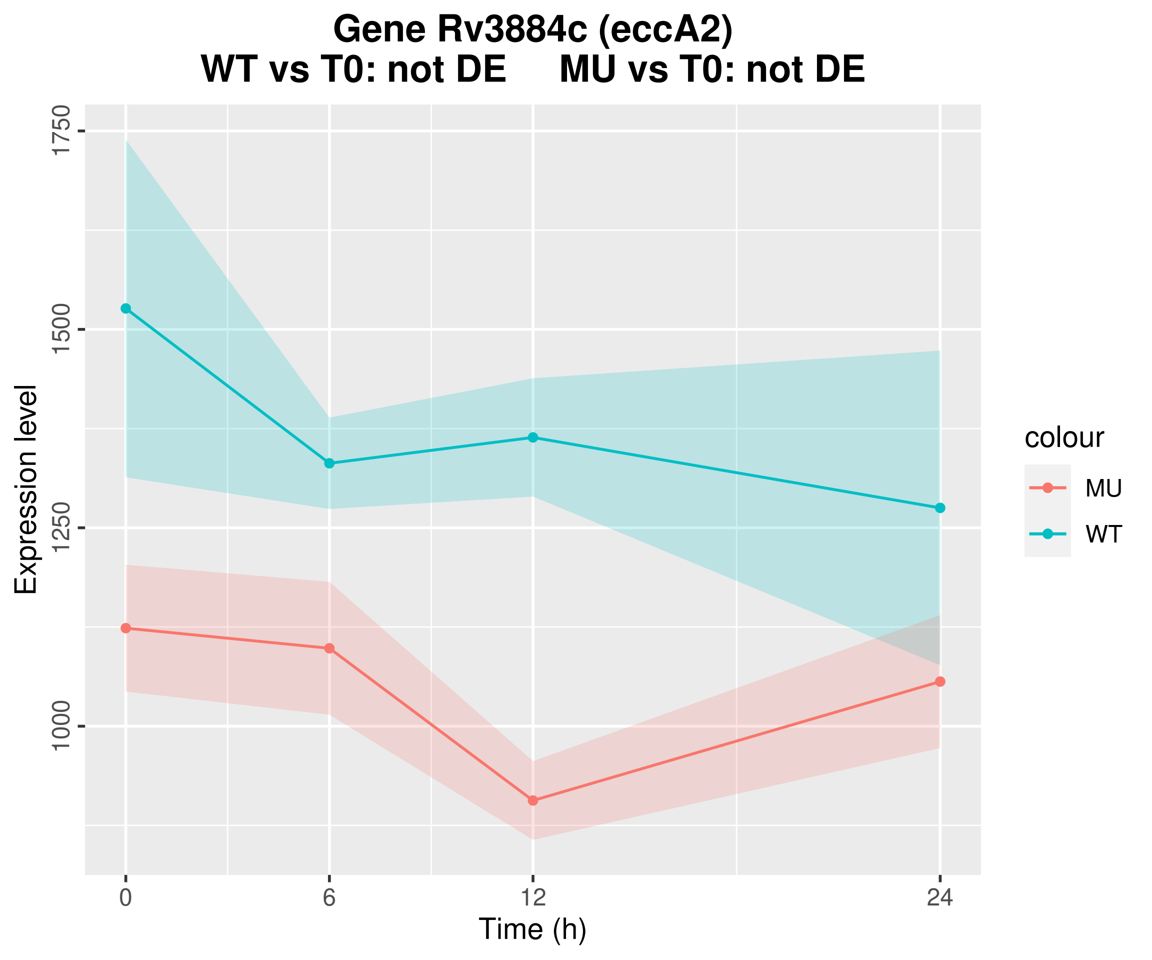

Gene Rv3884c (eccA2)
WT vs T0: not DE   MU vs T0: not DE
1750
1500
1250
1000
Expression level
0
6
12
24
Time (h)
colour
MU
WT

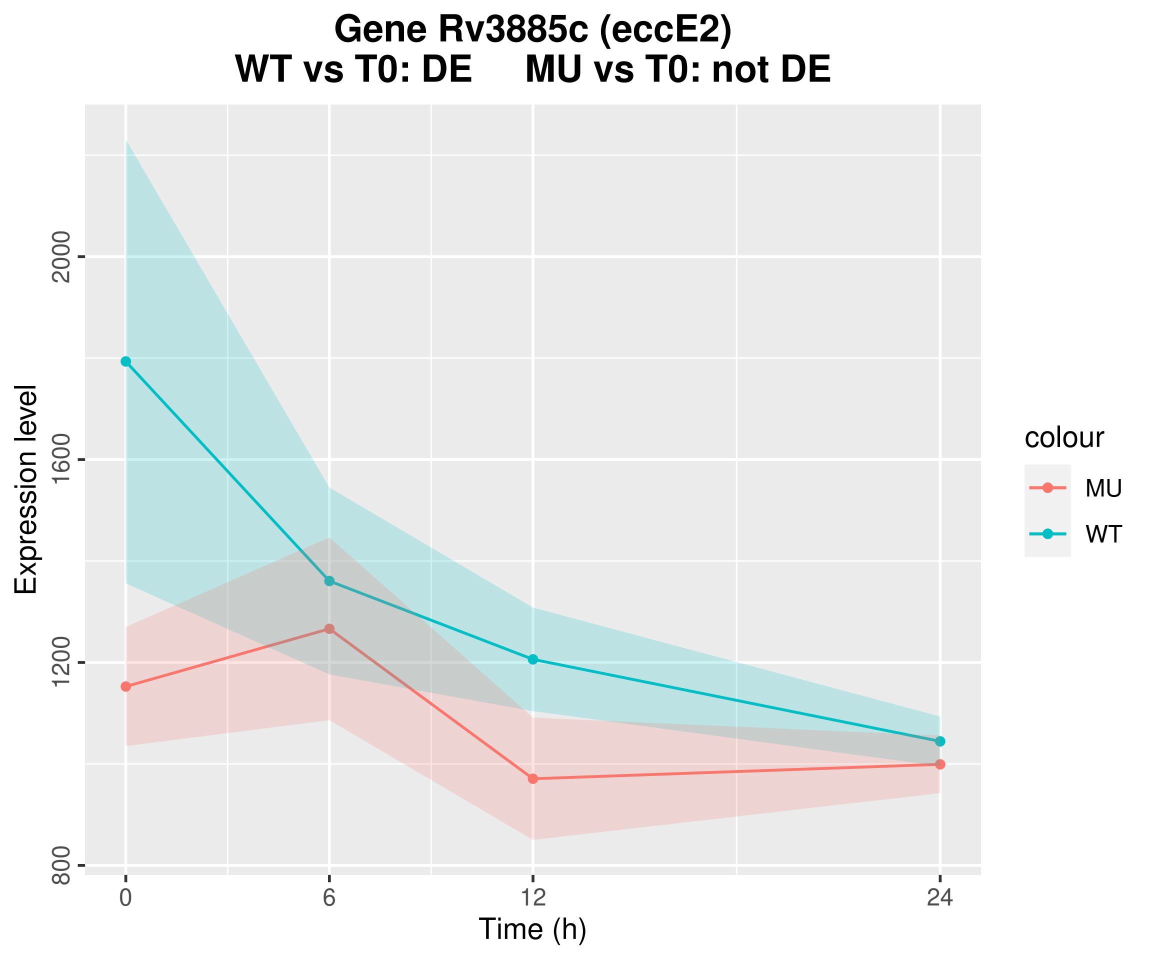
Gene Rv3885c (eccE2)
WT vs T0: DE MU vs T0: not DE
Expression level
2000
1600
1200
800
0
6
12
24
Time (h)
colour
MU
WT

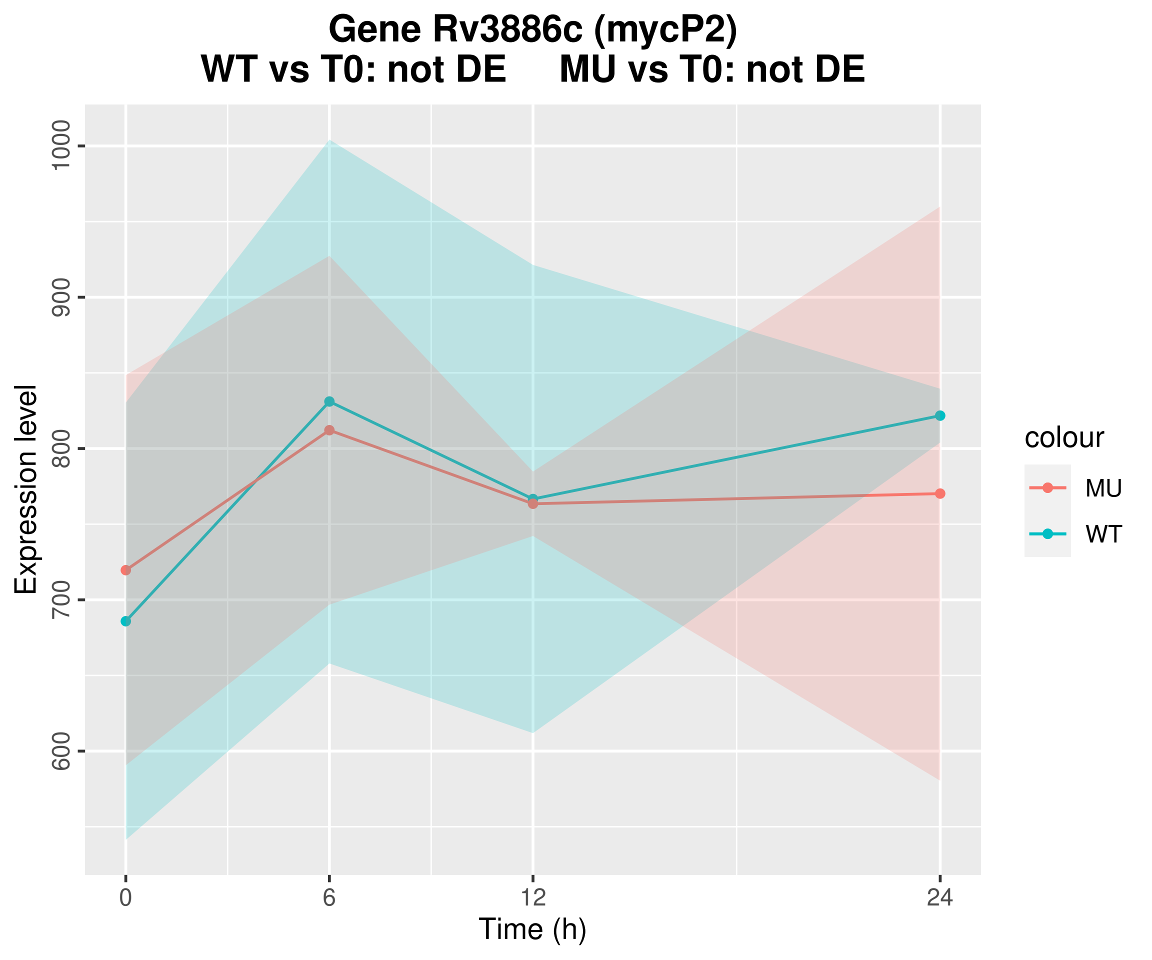
Gene Rv3886c (mycP2)
WT vs T0: not DE   MU vs T0: not DE
Expression level
1000
900
800
700
600
0
6
12
24
Time (h)
colour
MU
WT

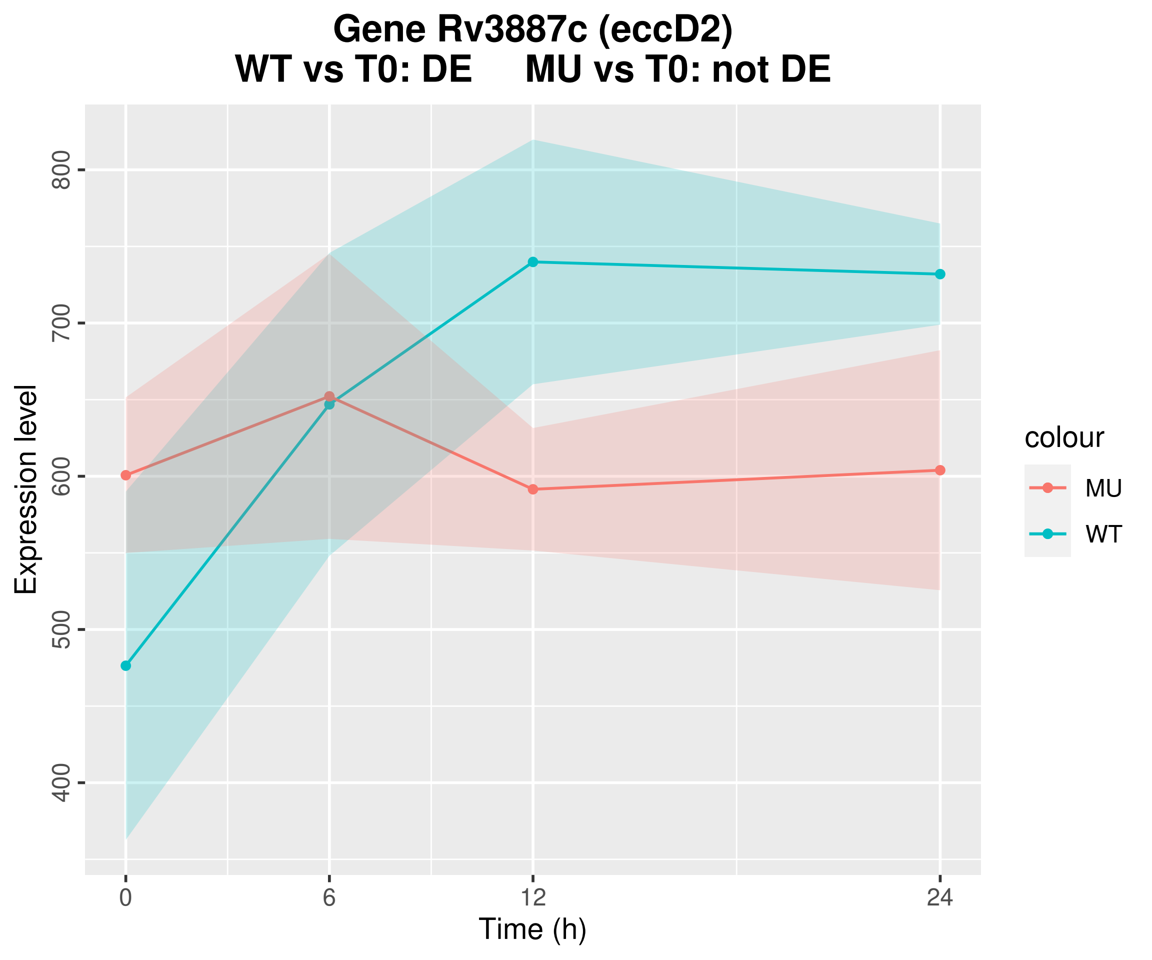
Gene Rv3887c (eccD2)
WT vs T0: DE   MU vs T0: not DE
Expression level
800
700
600
500
400
0
6
12
24
Time (h)
colour
MU
WT

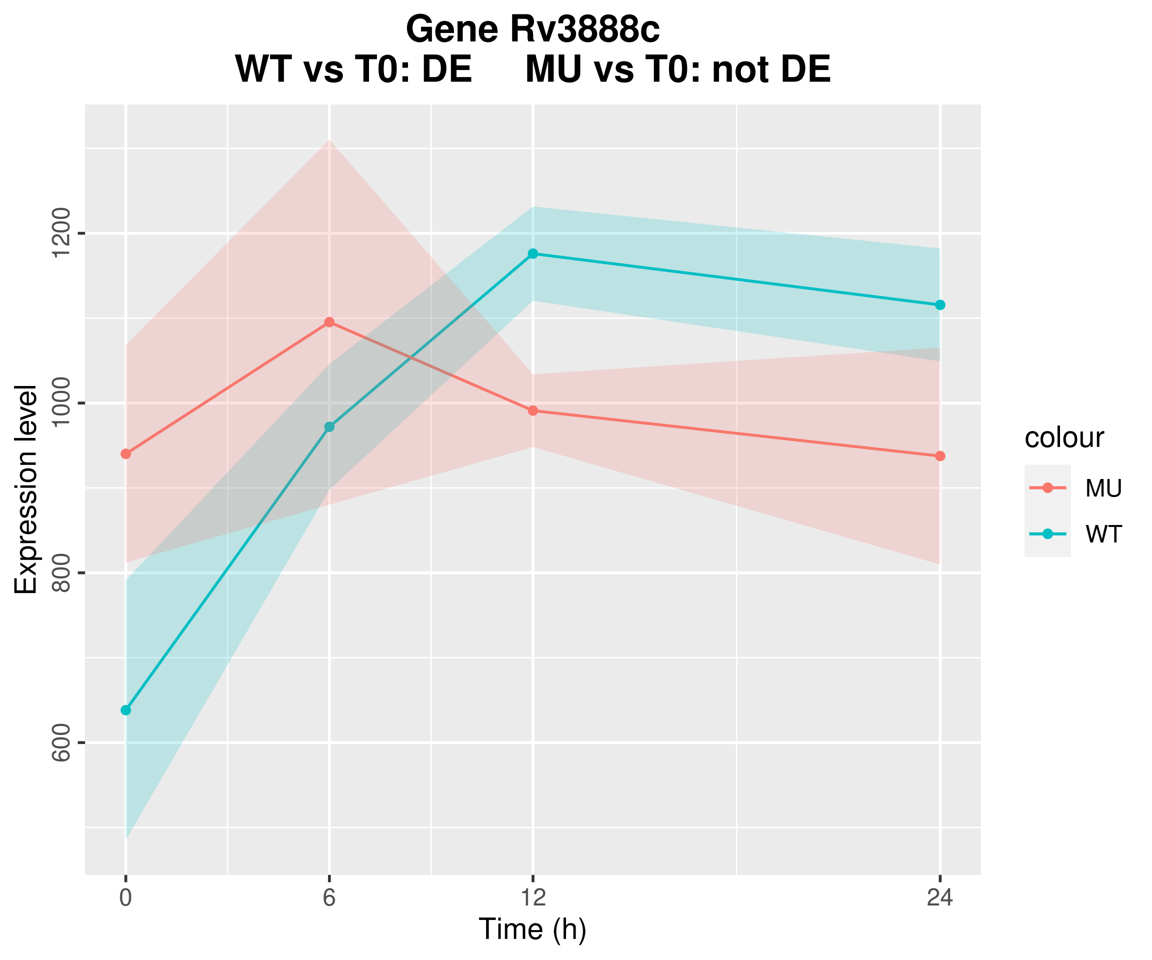
Gene Rv3888c
WT vs T0: DE   MU vs T0: not DE
Expression level
1200
1000
800
600
0
6
12
24
Time (h)
colour
MU
WT

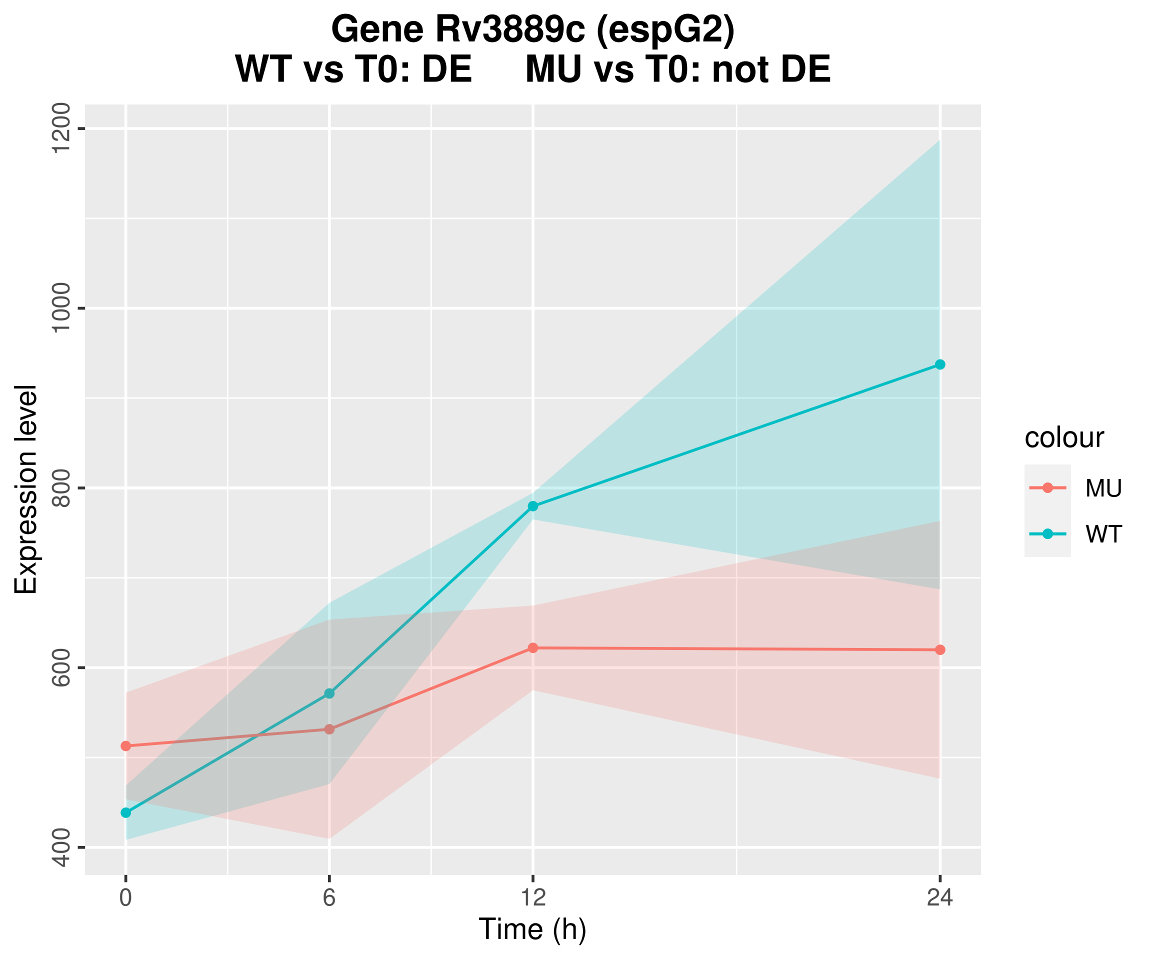
Gene Rv3889c (espG2)
WT vs T0: DE   MU vs T0: not DE
Expression level
1200
1000
800
600
400
0
6
12
24
Time (h)
colour
MU
WT

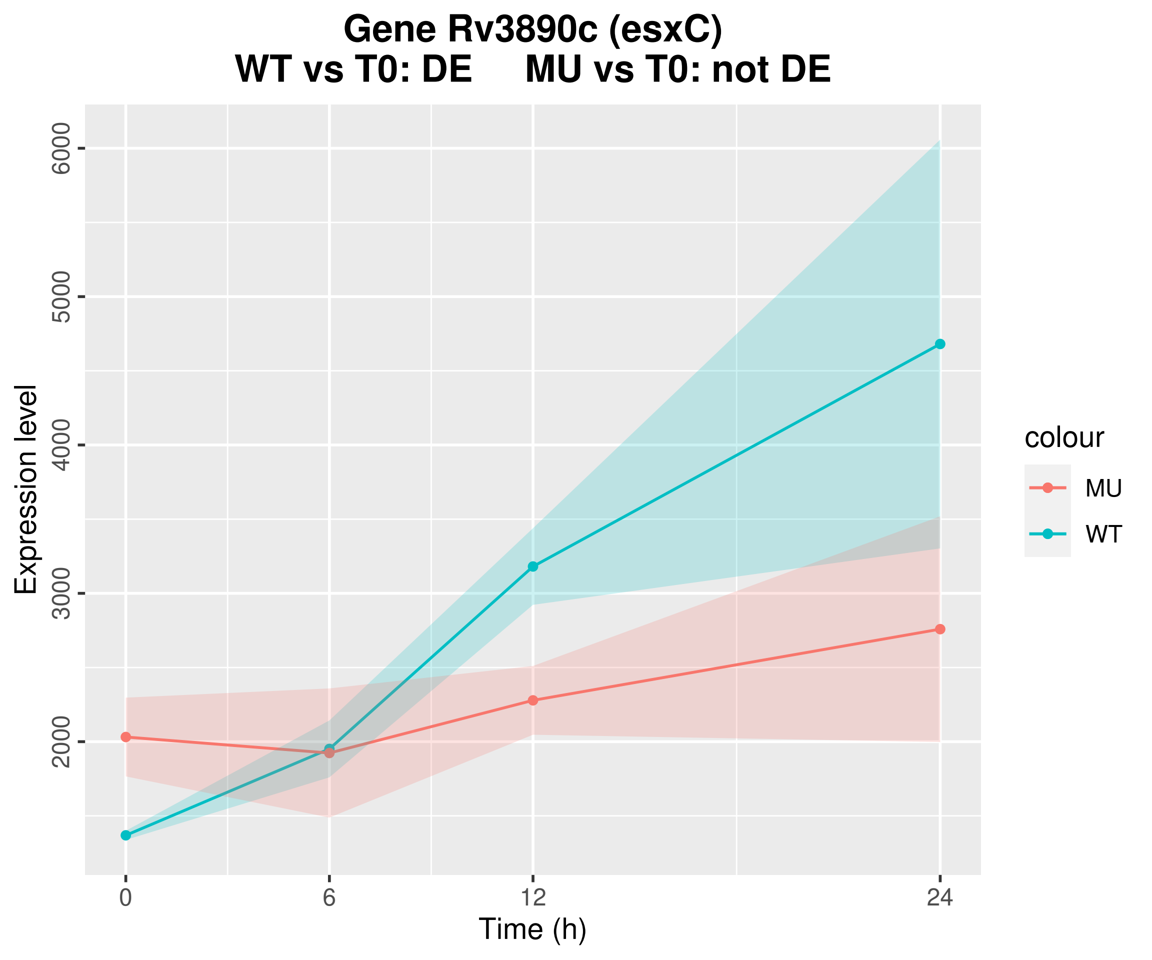
Gene Rv3890c (esxC)
WT vs T0: DE   MU vs T0: not DE
Expression level
6000
5000
4000
3000
2000
0
6
12
24
Time (h)
colour
MU
WT

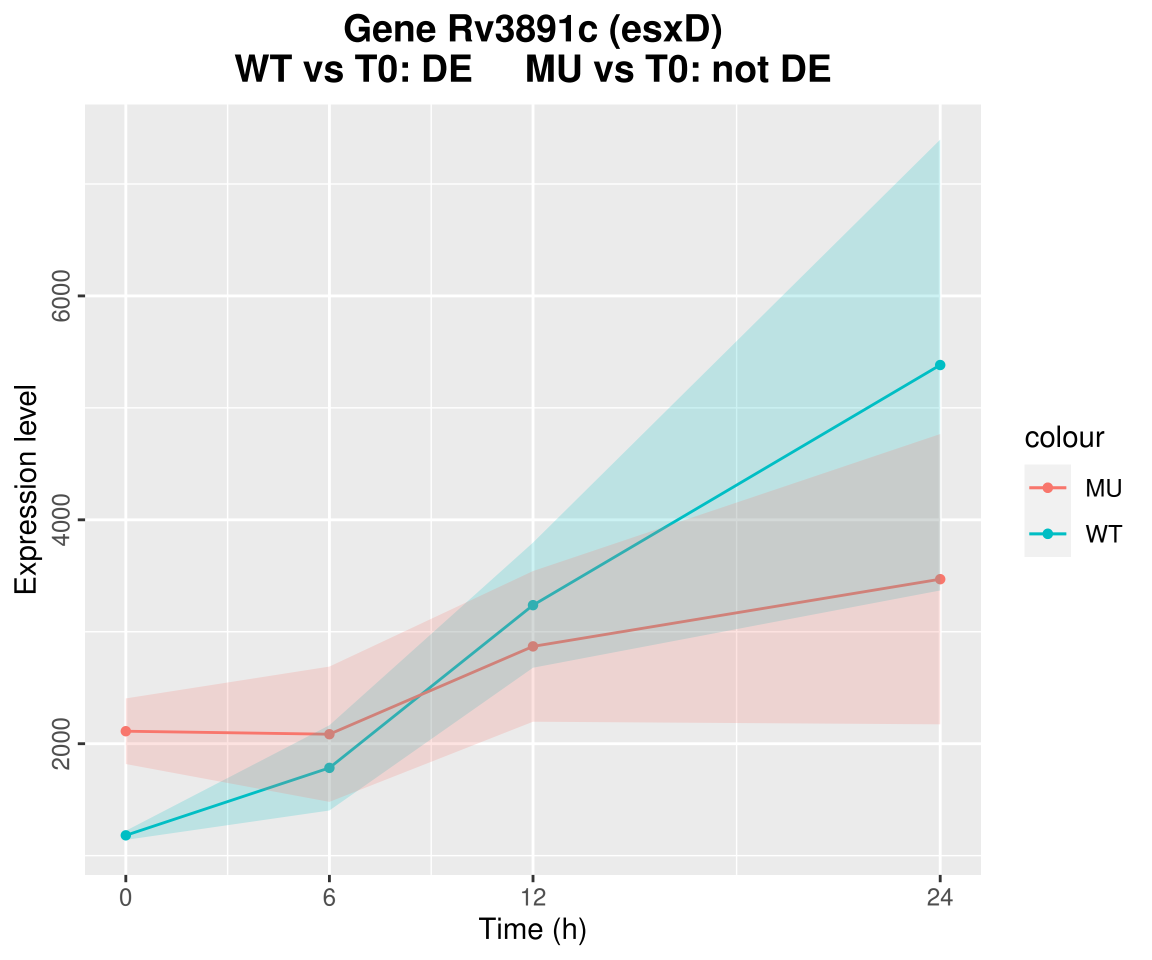
Gene Rv3891c (esxD)
WT vs T0: DE   MU vs T0: not DE
Expression level
2000
4000
6000
0
6
12
24
Time (h)
colour
MU
WT

Gene Rv3892c (PPE69)
WT vs T0: not DE   MU vs T0: not DE

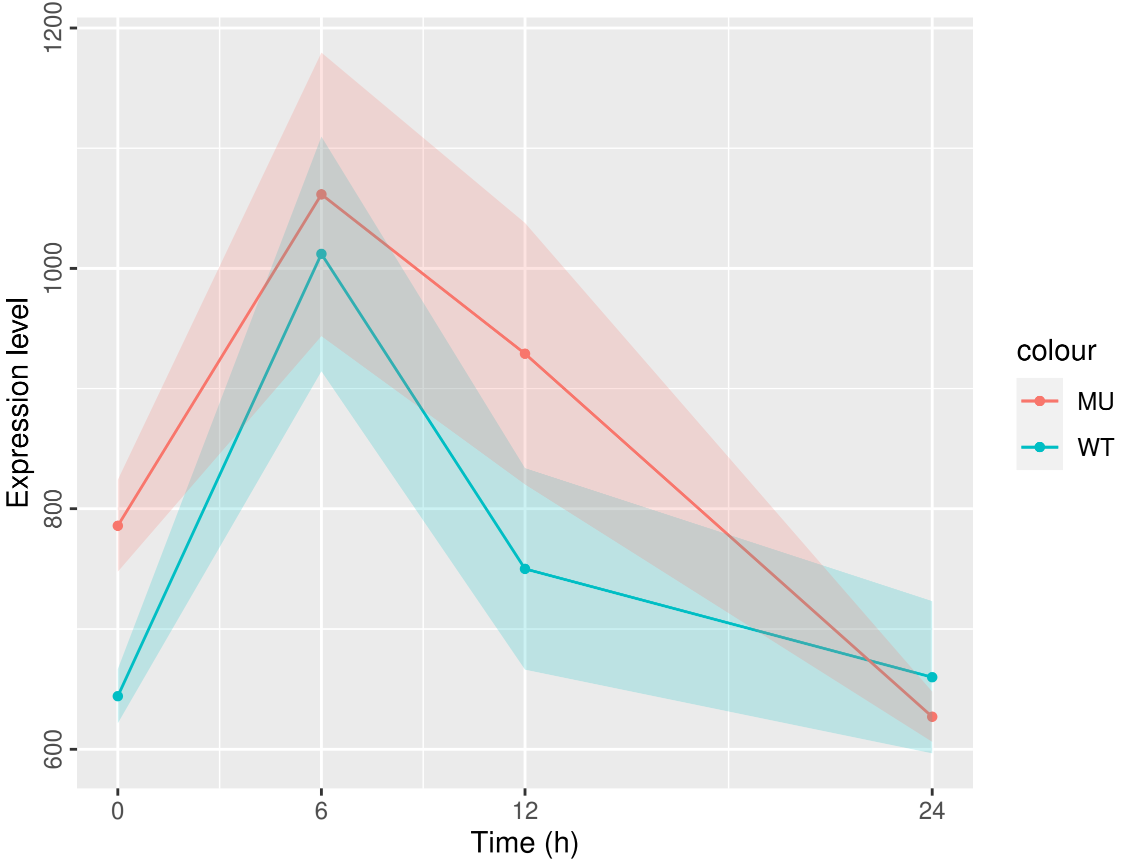

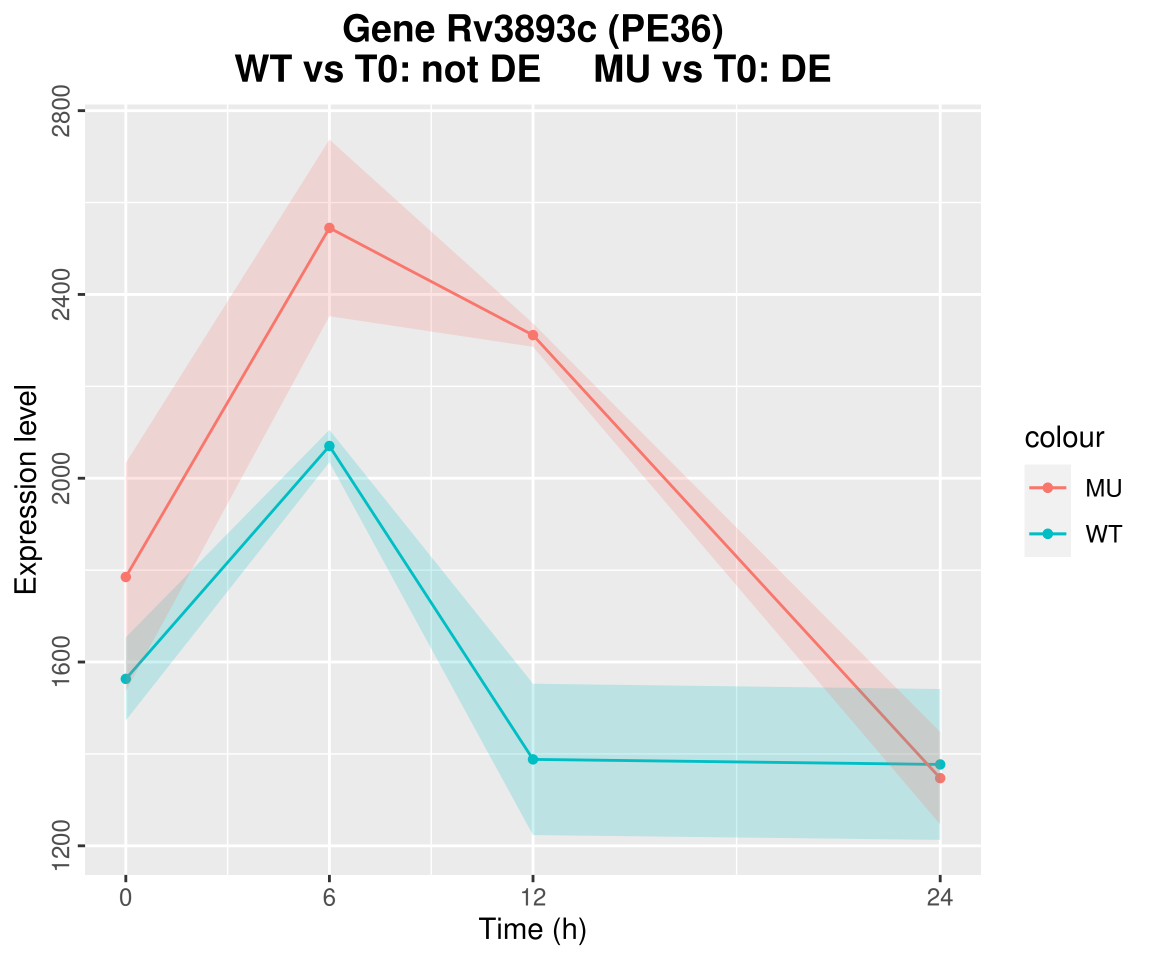
Gene Rv3893c (PE36)
WT vs T0: not DE MU vs T0: DE
Expression level
2800
2400
2000
1600
1200
0
6
12
24
Time (h)
colour
MU
WT

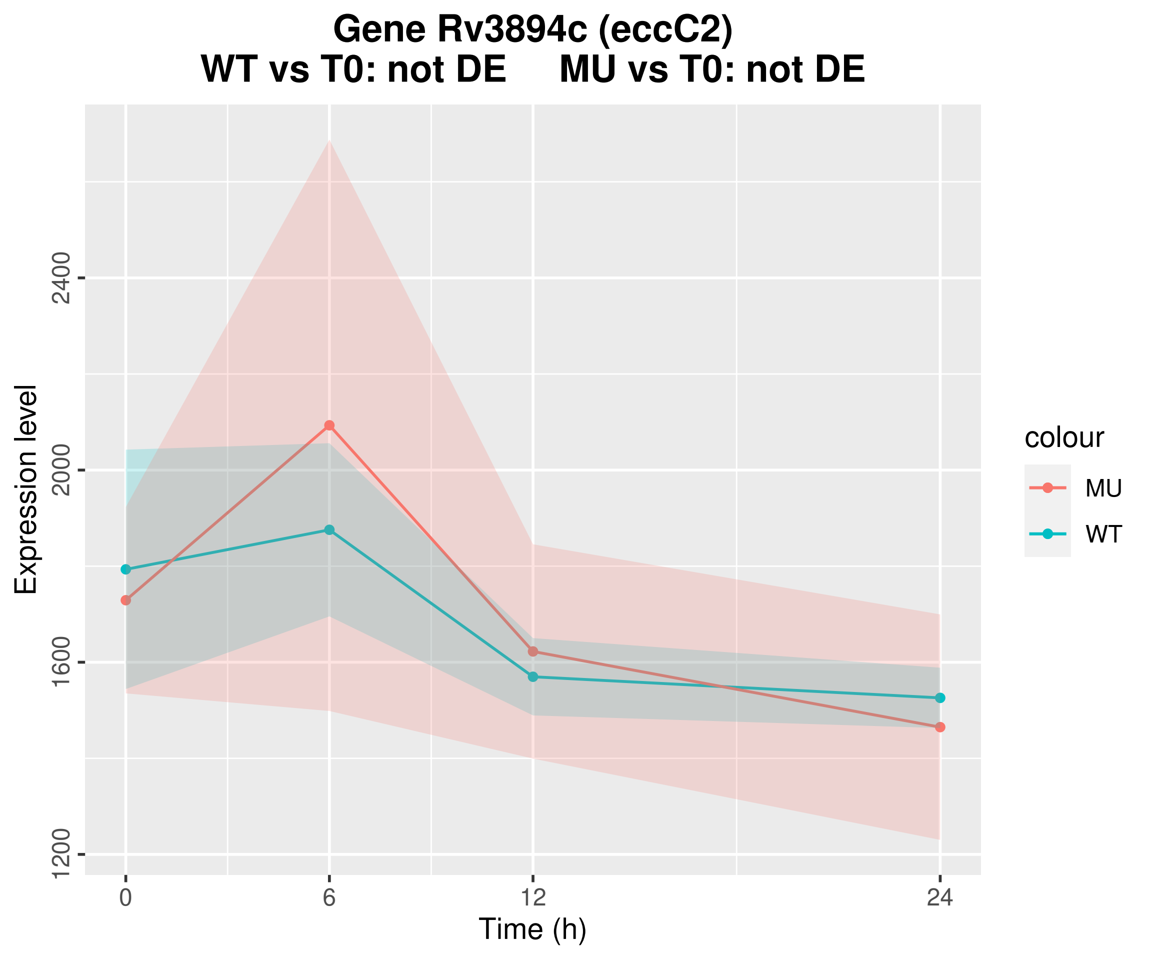
Gene Rv3894c (eccC2)
WT vs T0: not DE   MU vs T0: not DE
Expression level
2400
2000
1600
1200
0
6
12
24
Time (h)
colour
MU
WT

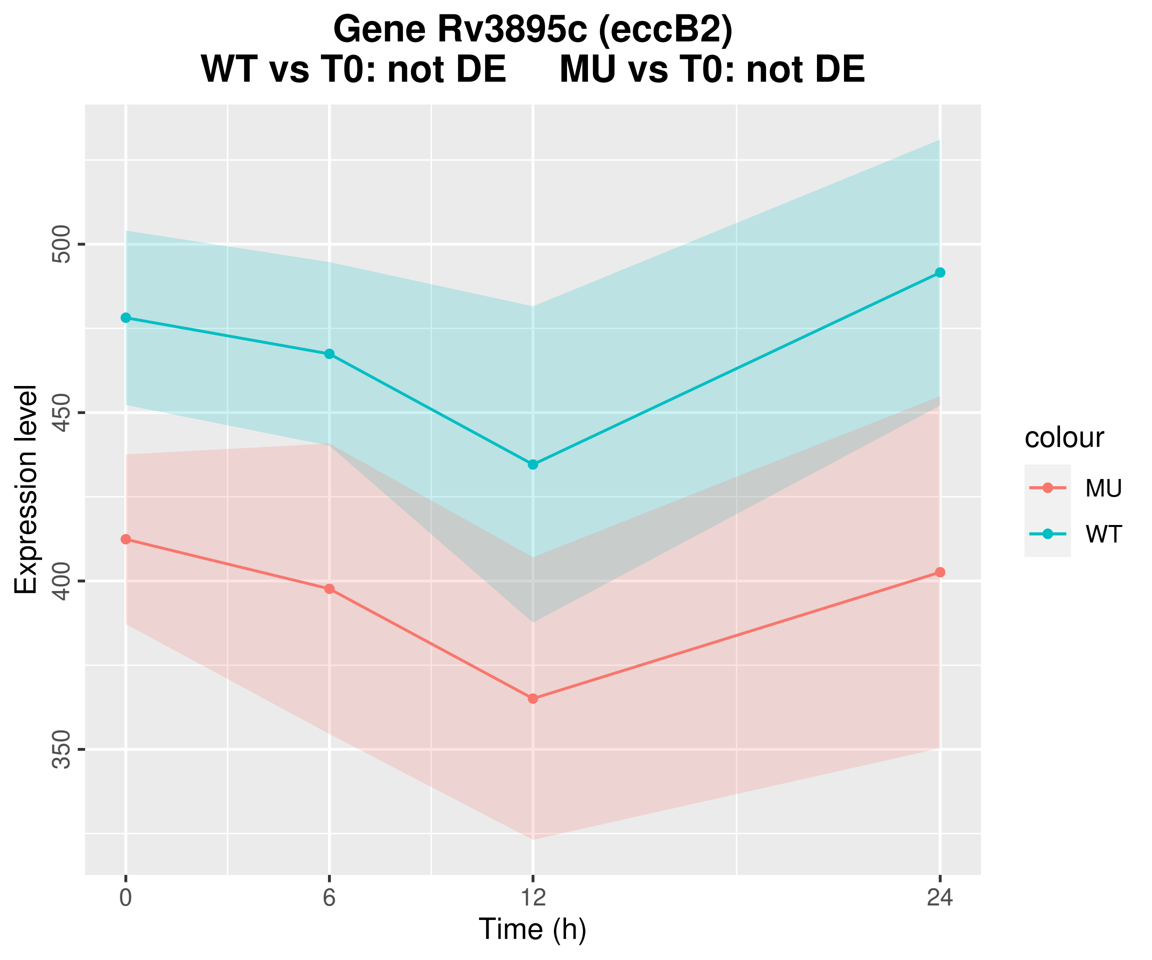

Gene Rv3895c (eccB2)
WT vs T0: not DE   MU vs T0: not DE
Expression level
500
450
400
350
0
6
12
24
Time (h)
colour
MU
WT

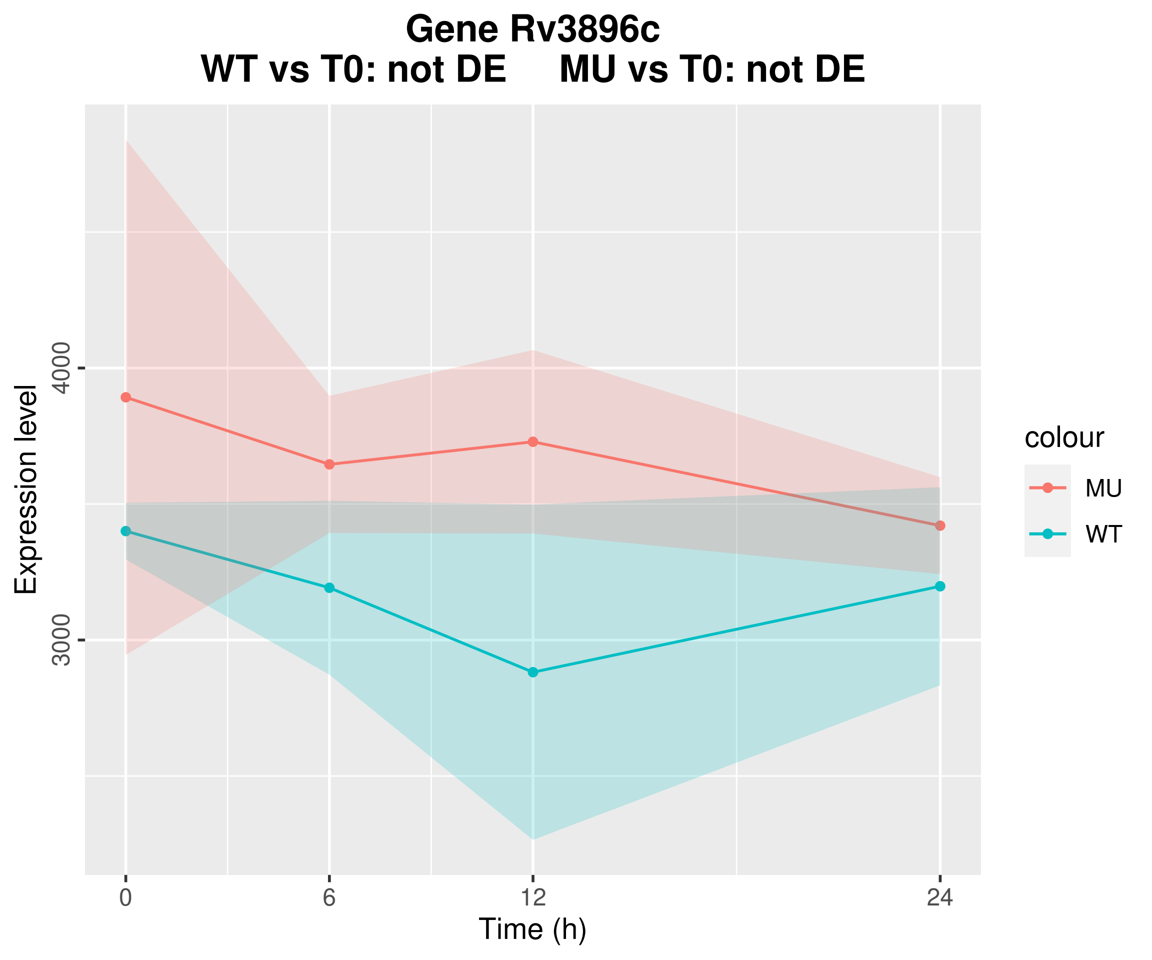

Gene Rv3896c
WT vs T0: not DE MU vs T0: not DE
Expression level
4000
3000
0
6
12
24
Time (h)
colour
MU
WT

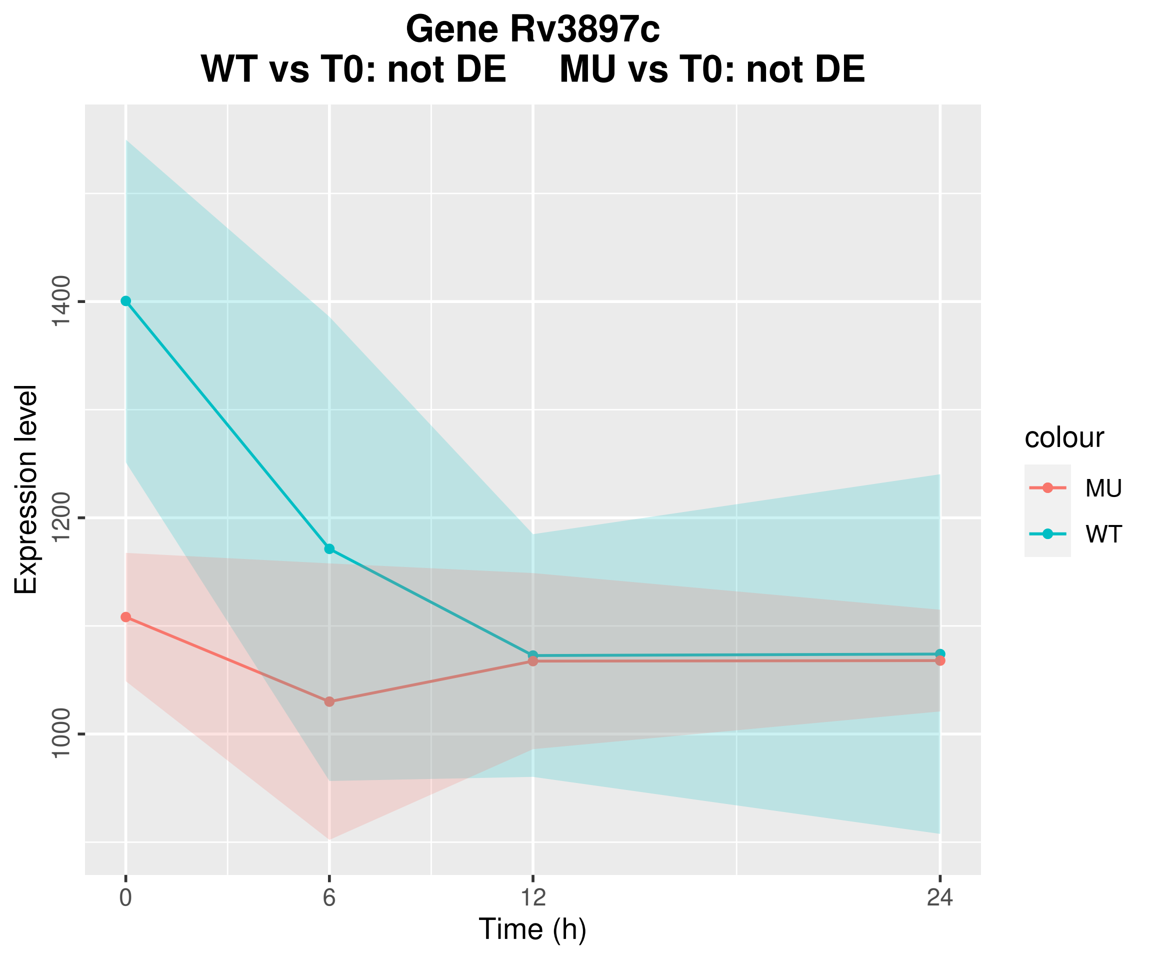

Gene Rv3897c
WT vs T0: not DE   MU vs T0: not DE
Expression level
1400
1200
1000
0
6
12
24
Time (h)
colour
MU
WT

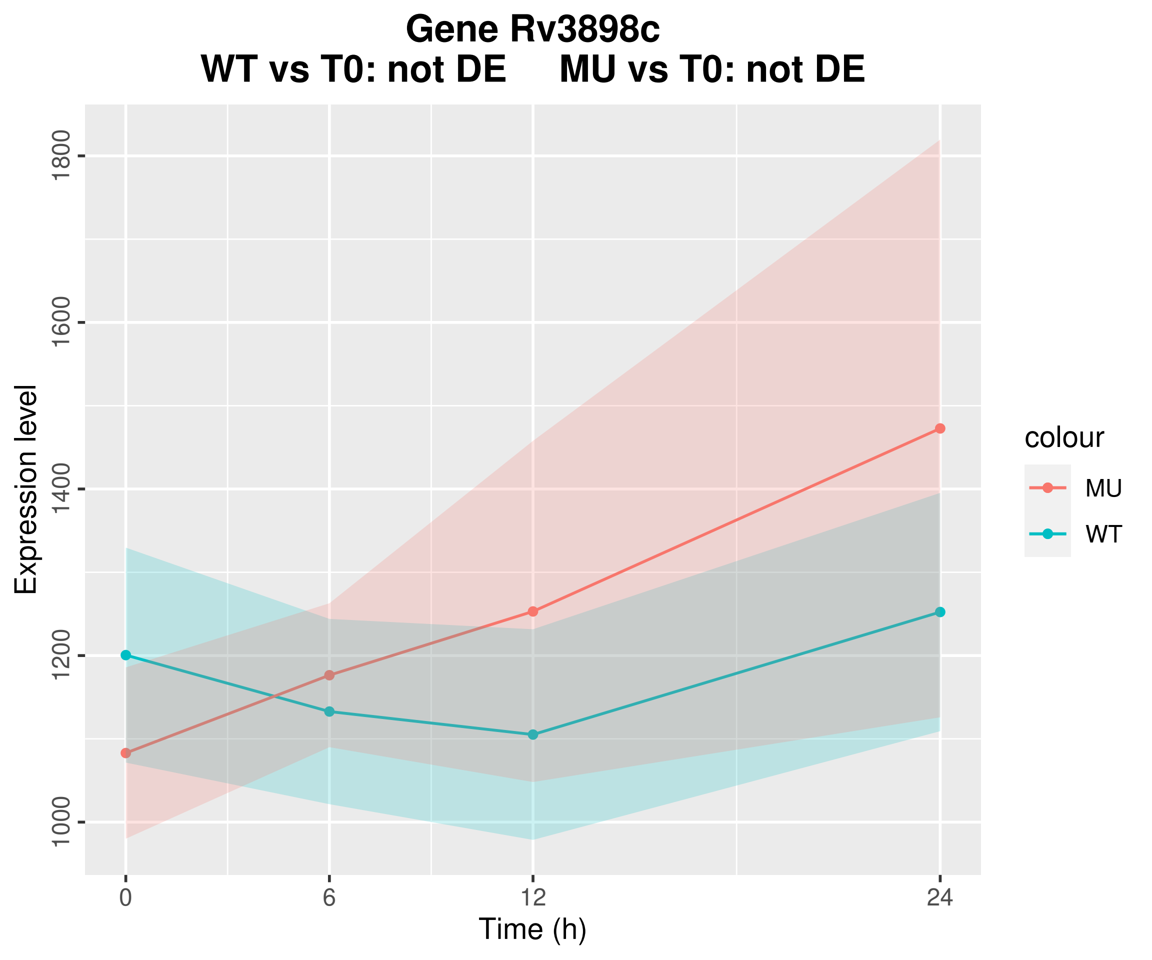
Gene Rv3898c
WT vs T0: not DE MU vs T0: not DE
1800
1600
1400
1200
1000
Expression level
0
6
12
24
Time (h)
colour
MU
WT

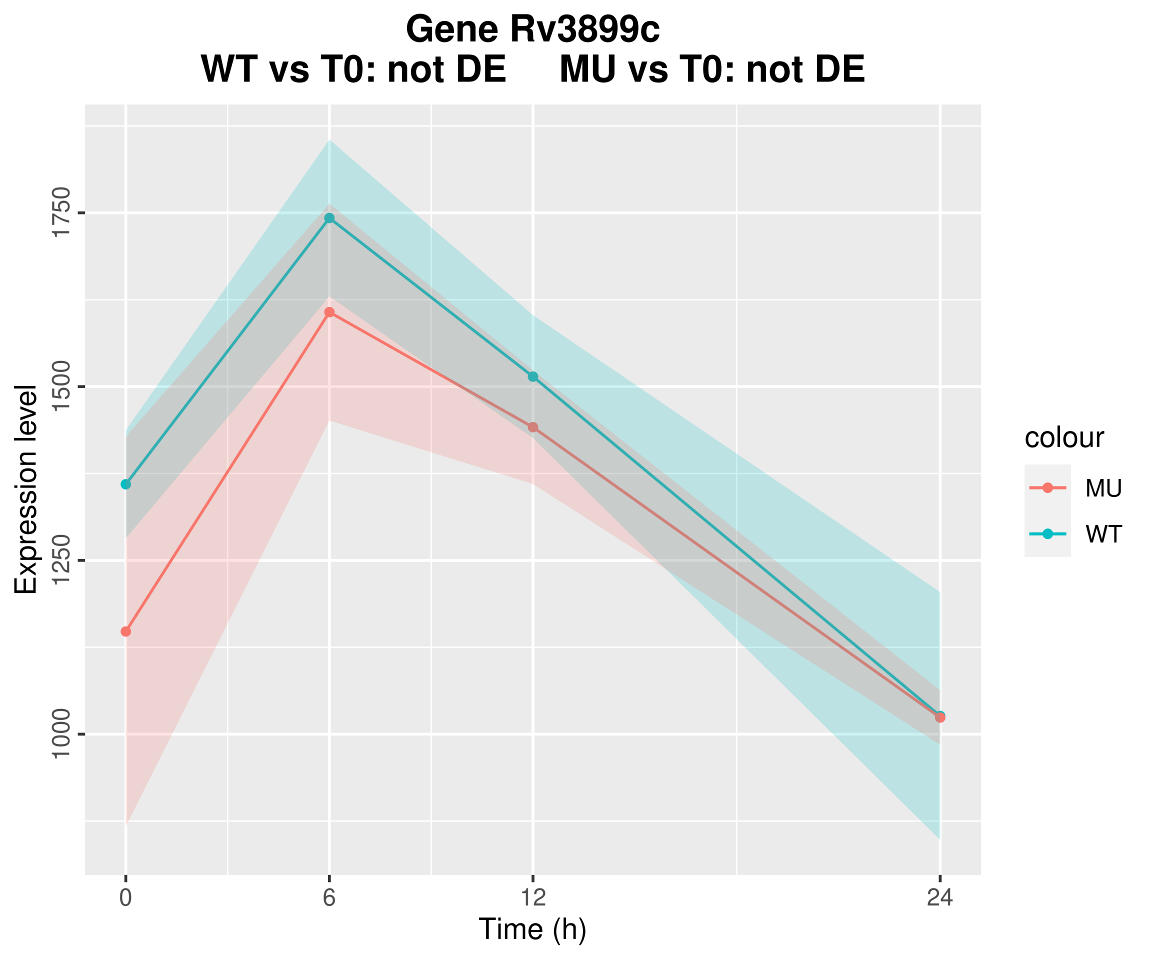
Gene Rv3899c
WT vs T0: not DE MU vs T0: not DE
Expression level
1000
1250
1500
1750
0
6
12
24
Time (h)
colour
MU
WT

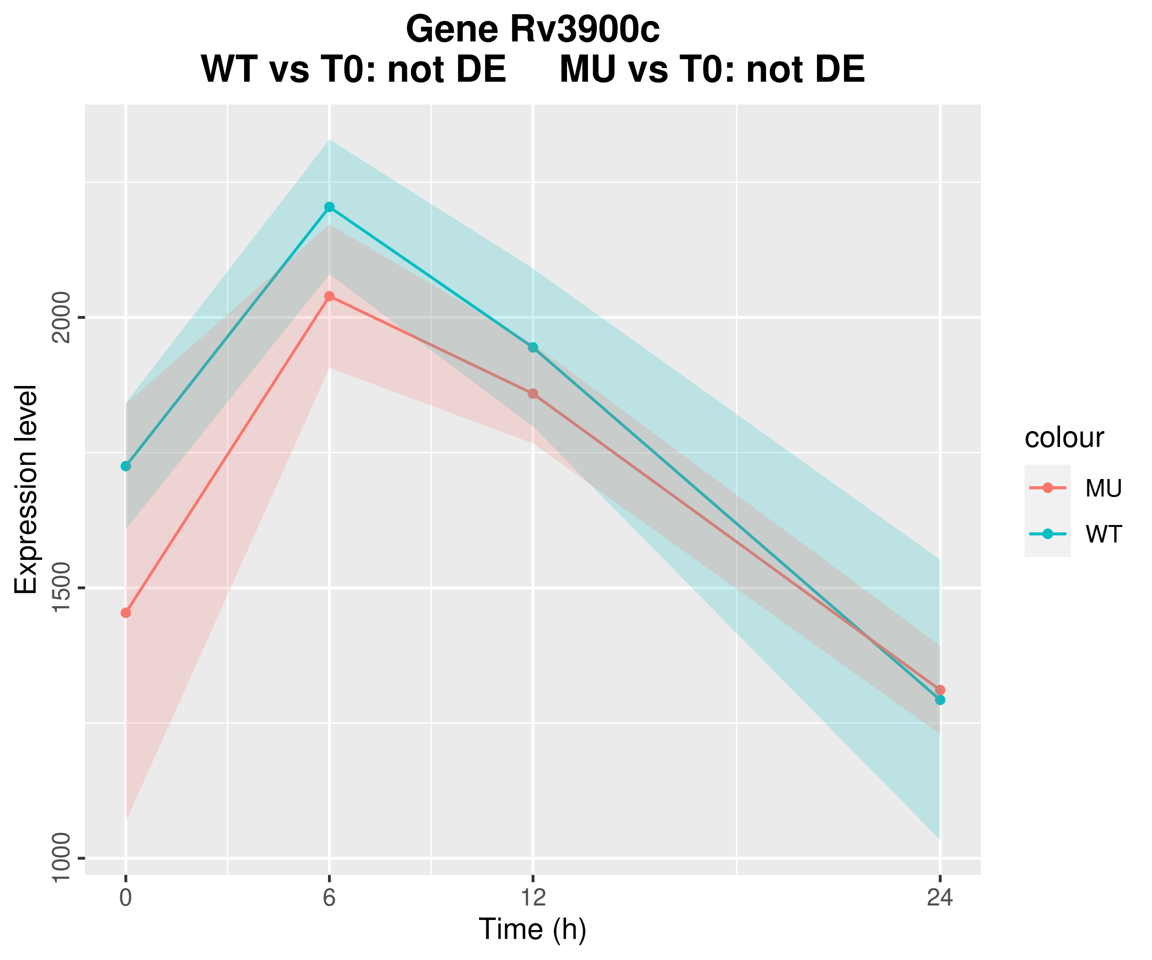
Gene Rv3900c
WT vs T0: not DE MU vs T0: not DE
Expression level
2000
1500
1000
0
6
12
24
Time (h)
colour
MU
WT

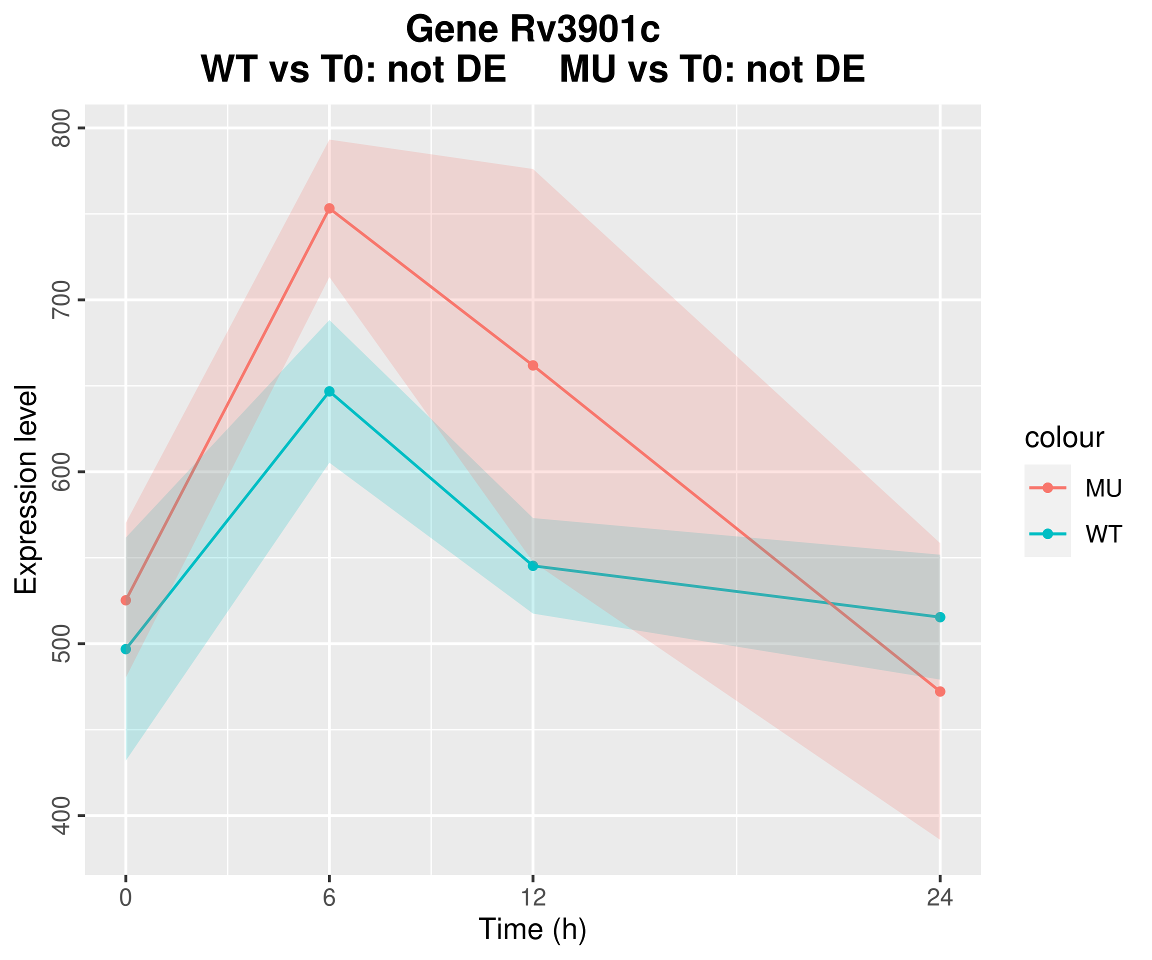

Gene Rv3901c
WT vs T0: not DE   MU vs T0: not DE
800
700
600
500
400
Expression level
0
6
12
24
Time (h)
colour
MU
WT

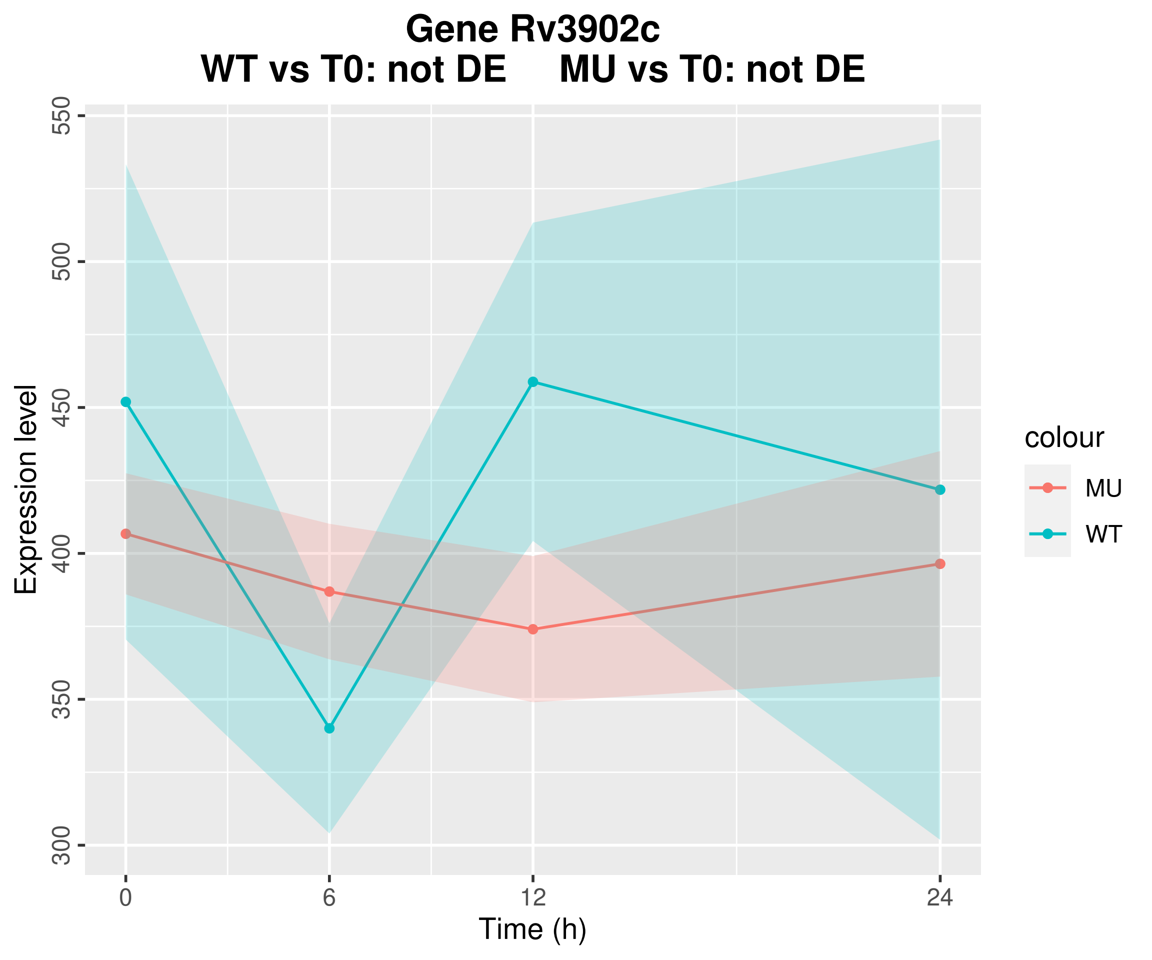

Gene Rv3902c
WT vs T0: not DE MU vs T0: not DE
Expression level
550
500
450
400
350
300
0
6
12
24
Time (h)
colour
MU
WT

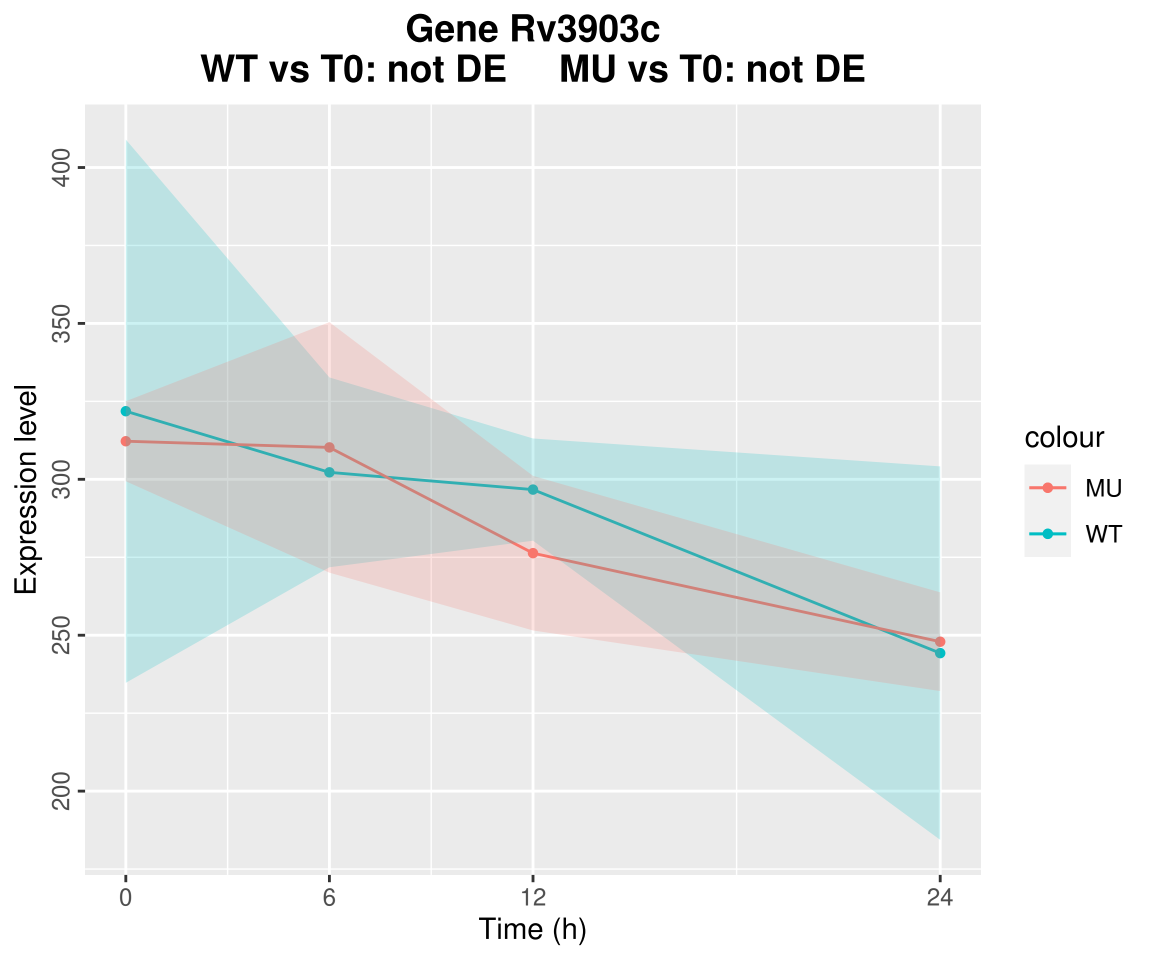

Gene Rv3903c
WT vs T0: not DE   MU vs T0: not DE
Expression level
400
350
300
250
200
0
6
12
24
Time (h)
colour
MU
WT

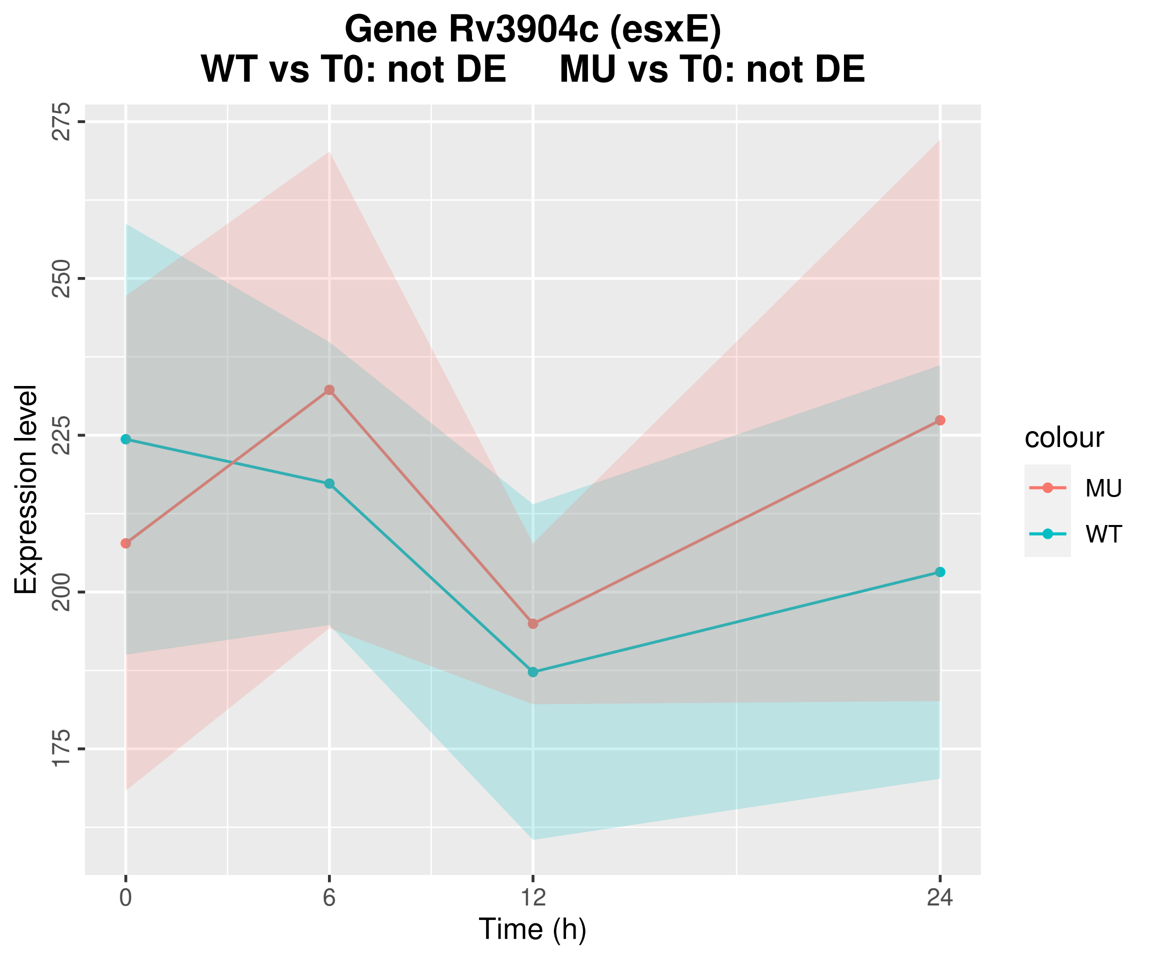

Gene Rv3904c (esxE)
WT vs T0: not DE   MU vs T0: not DE
Expression level
275
250
225
200
175
0
6
12
24
Time (h)
colour
MU
WT

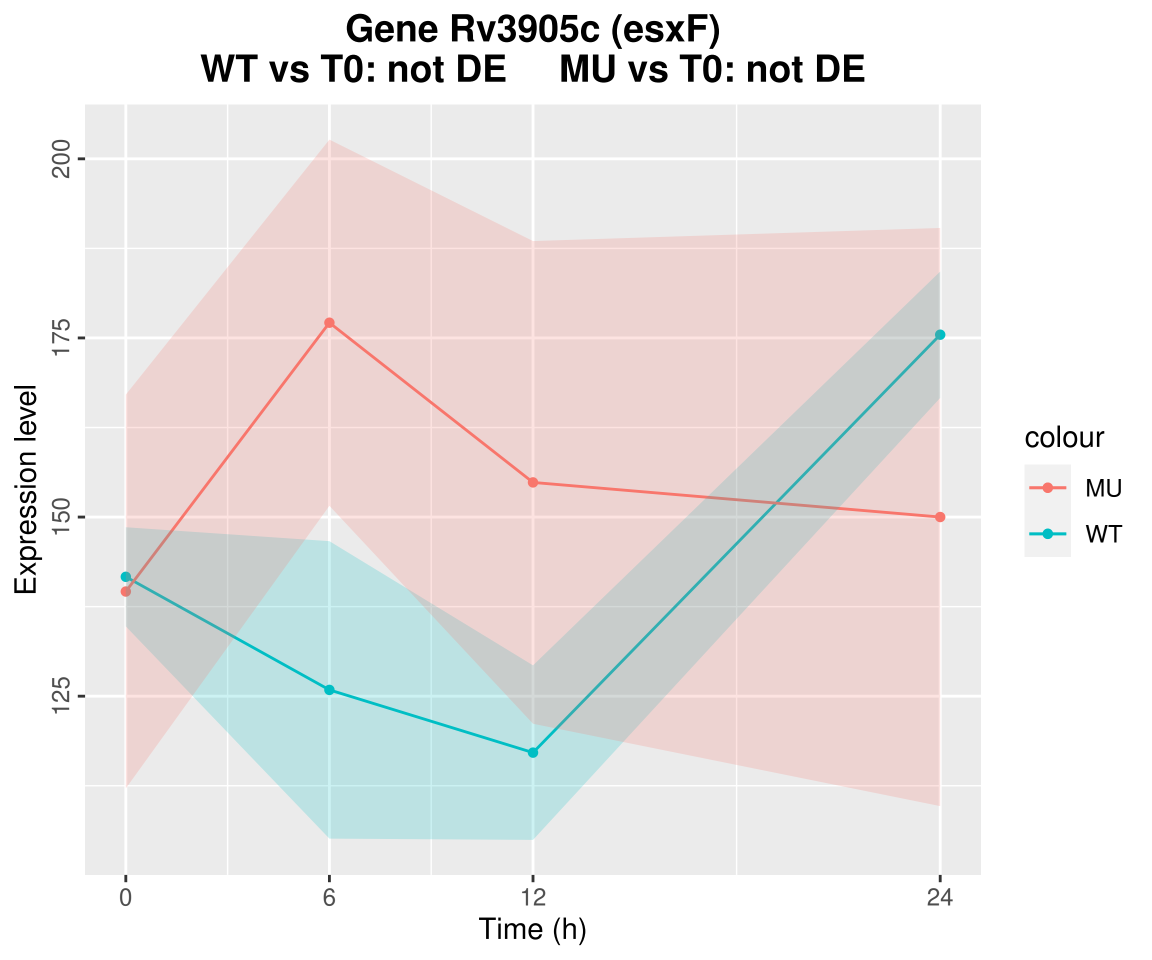
Gene Rv3905c (esxF)
WT vs T0: not DE   MU vs T0: not DE
Expression level
200
175
150
125
0
6
12
24
Time (h)
colour
MU
WT

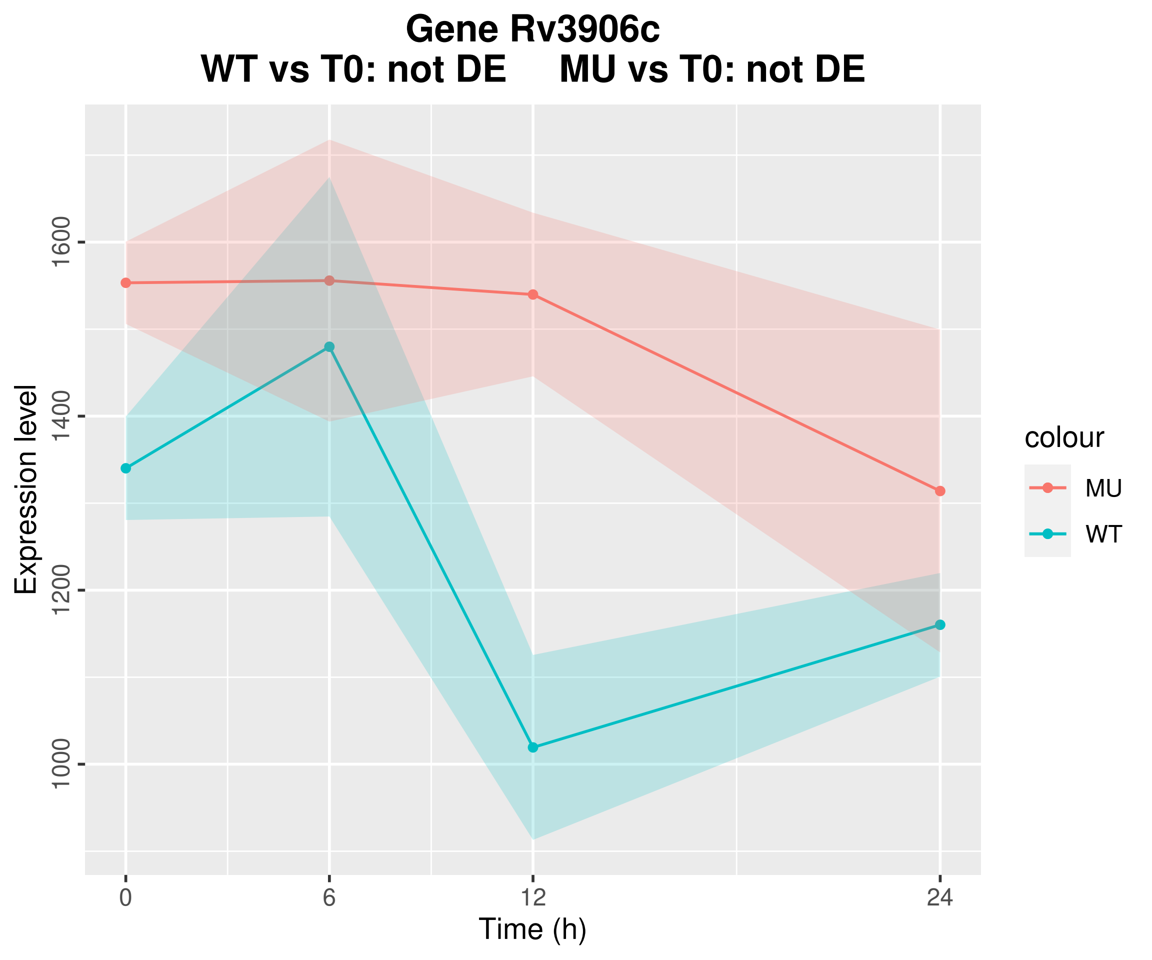
Gene Rv3906c
WT vs T0: not DE   MU vs T0: not DE
Expression level
1000
1200
1400
1600
0
6
12
24
Time (h)
colour
MU
WT

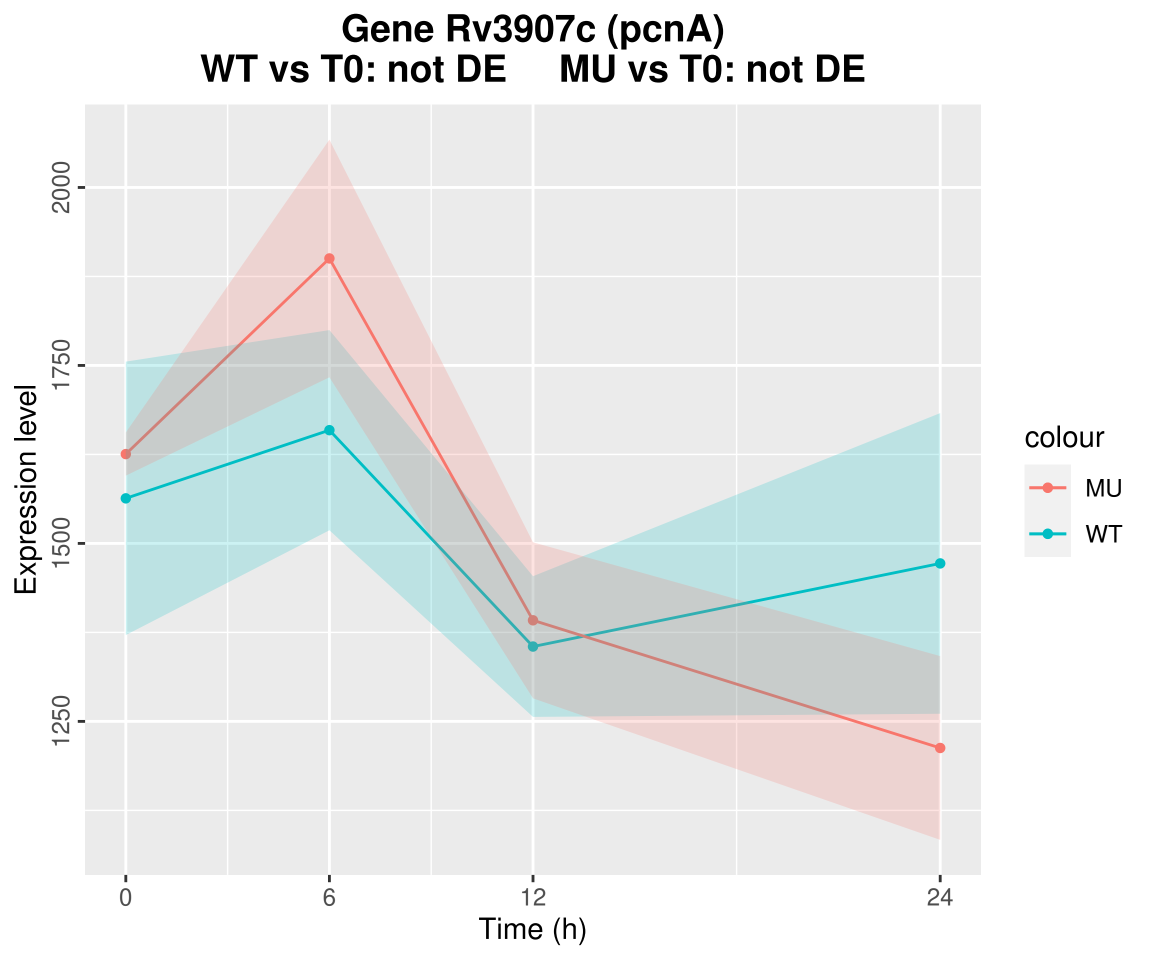

Gene Rv3907c (pcnA)
WT vs T0: not DE   MU vs T0: not DE
Expression level
2000
1750
1500
1250
0
6
12
24
Time (h)
colour
MU
WT

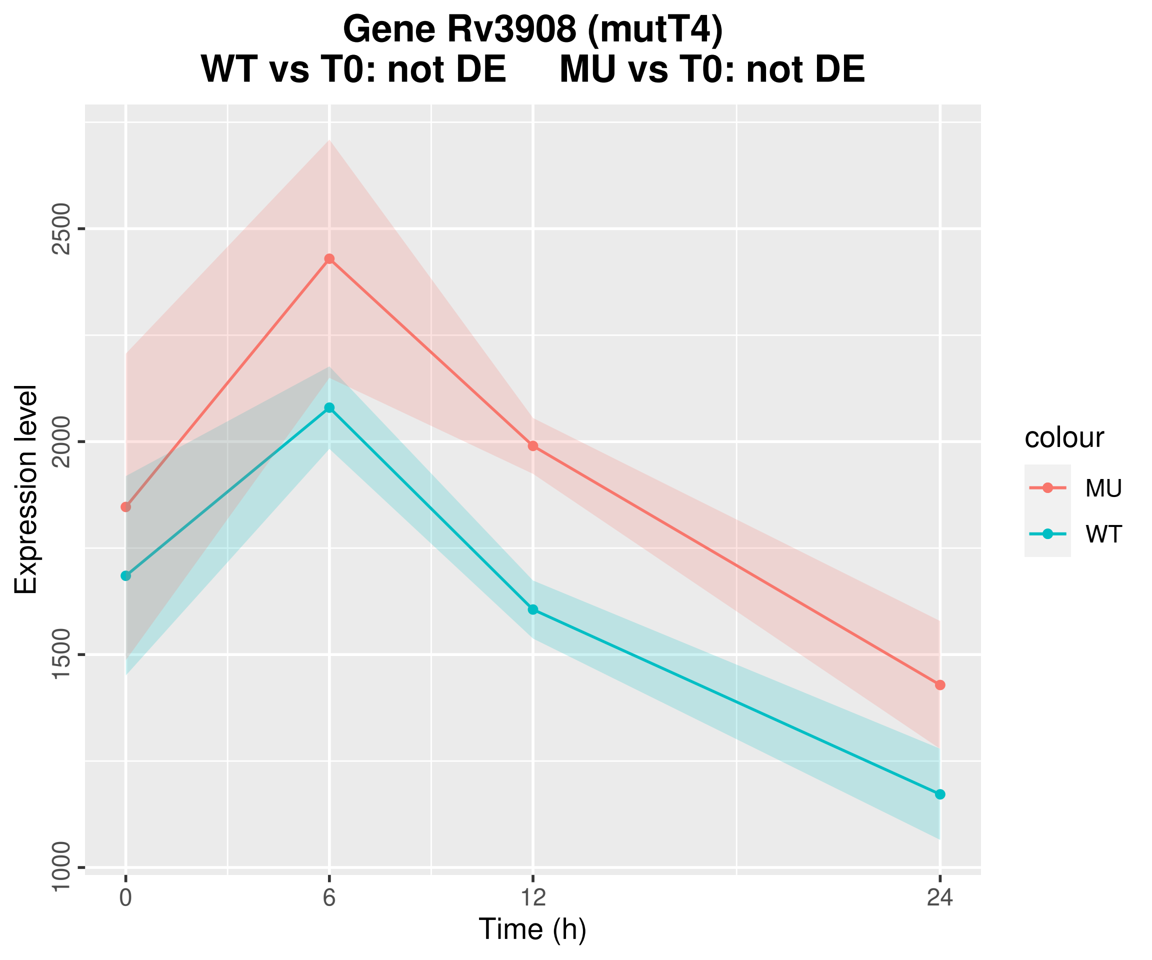
Gene Rv3908 (mutT4)
WT vs T0: not DE   MU vs T0: not DE
Expression level
2500
2000
1500
1000
0
6
12
24
Time (h)
colour
MU
WT

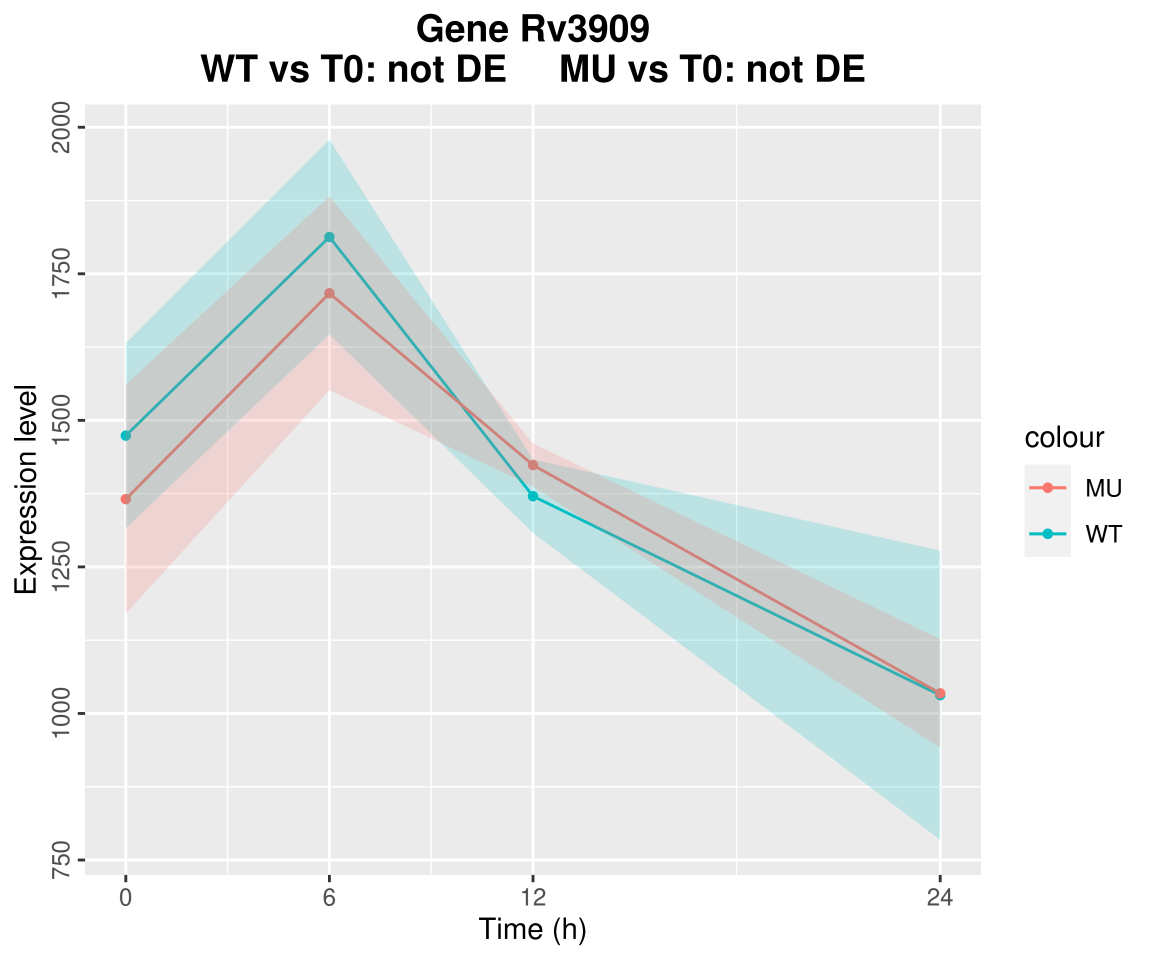

Gene Rv3909
WT vs T0: not DE   MU vs T0: not DE
2000
1750
1500
1250
1000
750
Expression level
0
6
12
24
Time (h)
colour
MU
WT

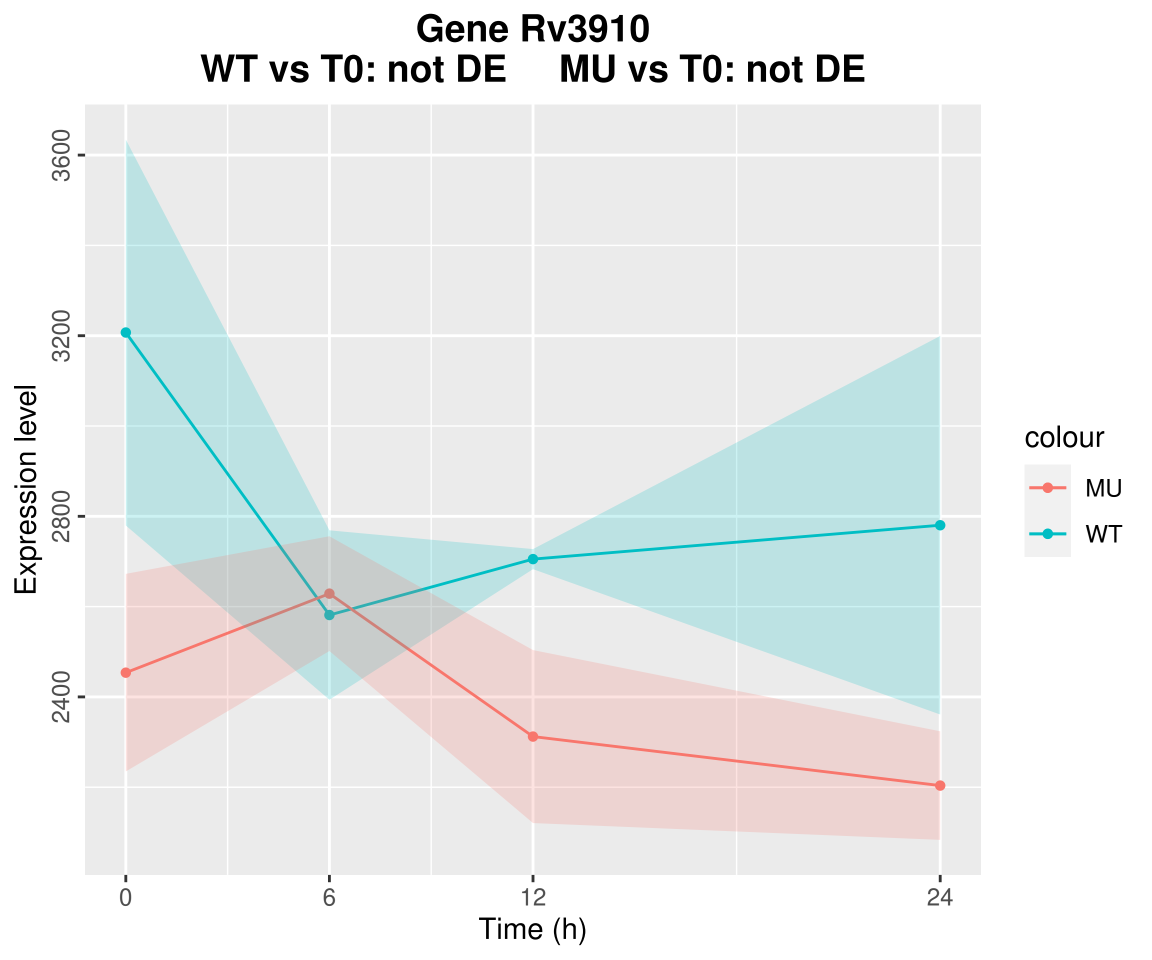
Gene Rv3910
WT vs T0: not DE MU vs T0: not DE
3600
3200
2800
2400
Expression level
0
6
12
24
Time (h)
colour
MU
WT

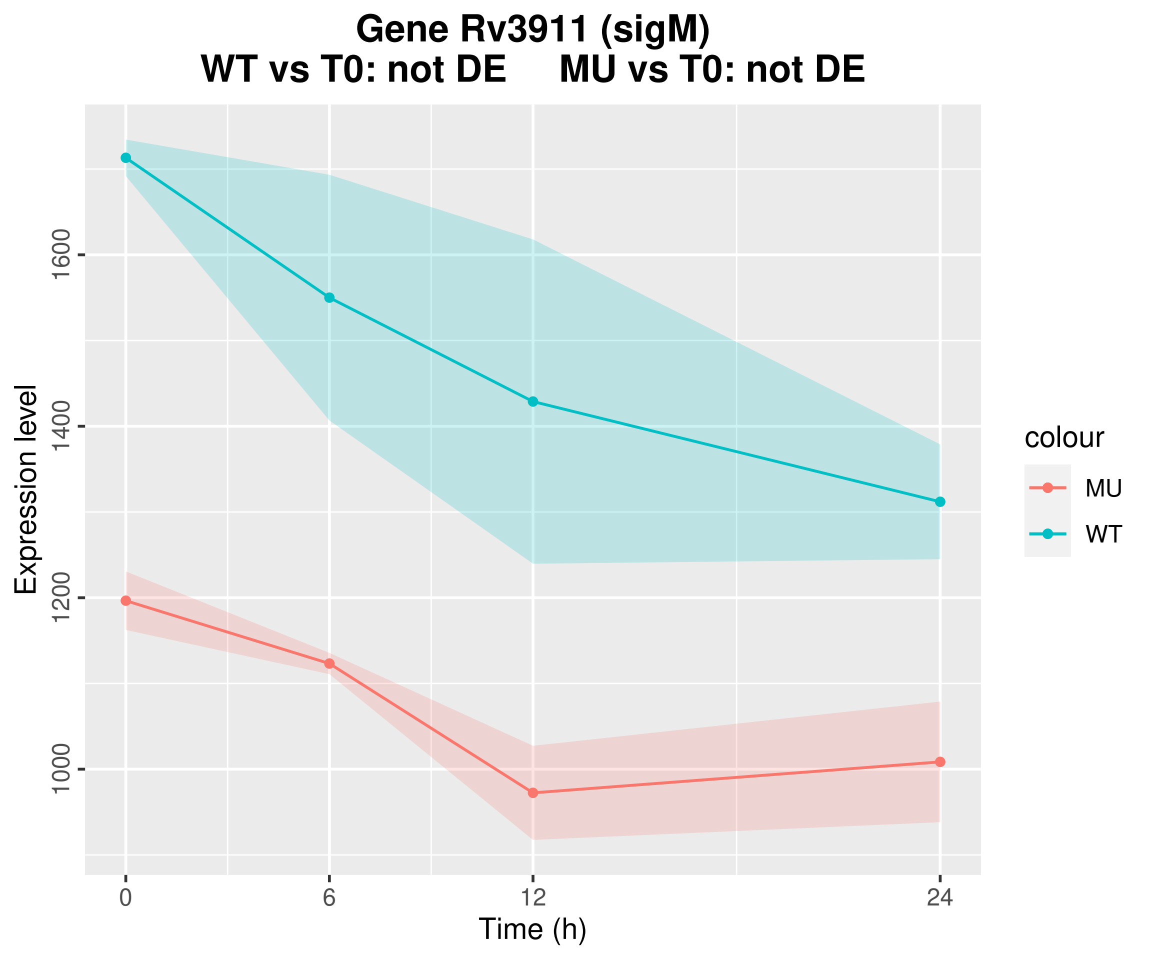
Gene Rv3911 (sigM)
WT vs T0: not DE   MU vs T0: not DE
Expression level
1000
1200
1400
1600
0
6
12
24
Time (h)
colour
MU
WT

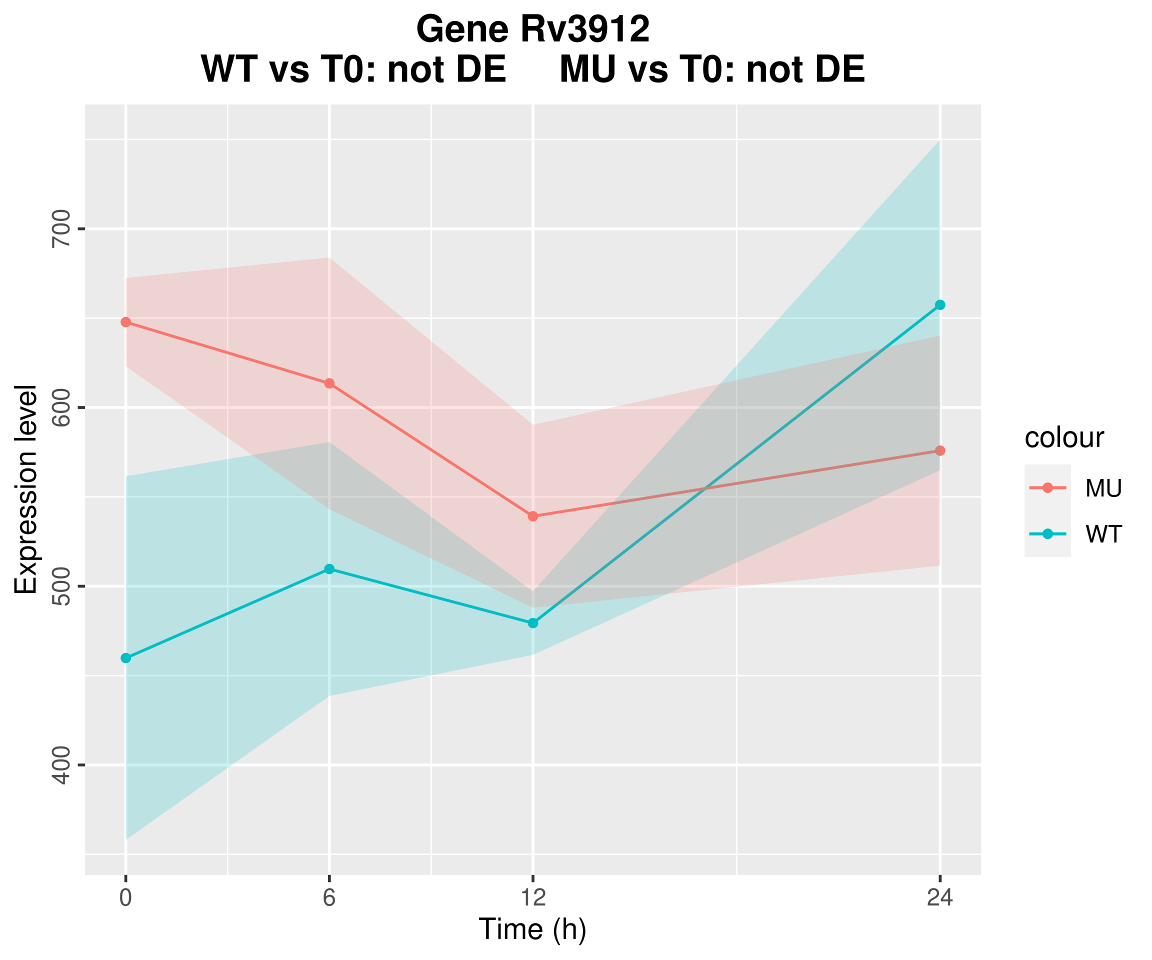
Gene Rv3912
WT vs T0: not DE   MU vs T0: not DE
Expression level
700
600
500
400
0
6
12
24
Time (h)
colour
MU
WT

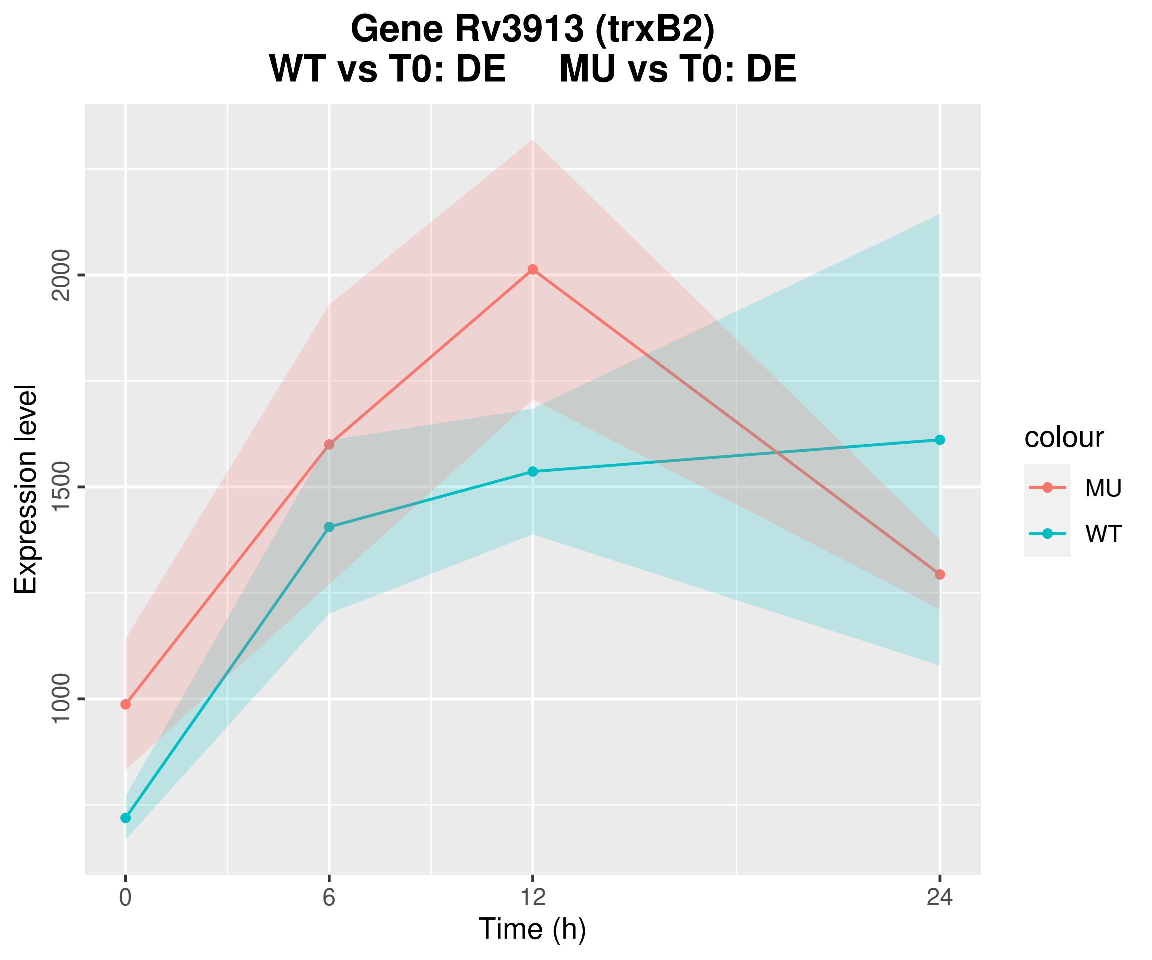

Gene Rv3913 (trxB2)
WT vs T0: DE   MU vs T0: DE
Expression level
1000
1500
2000
0
6
12
24
Time (h)
colour
MU
WT

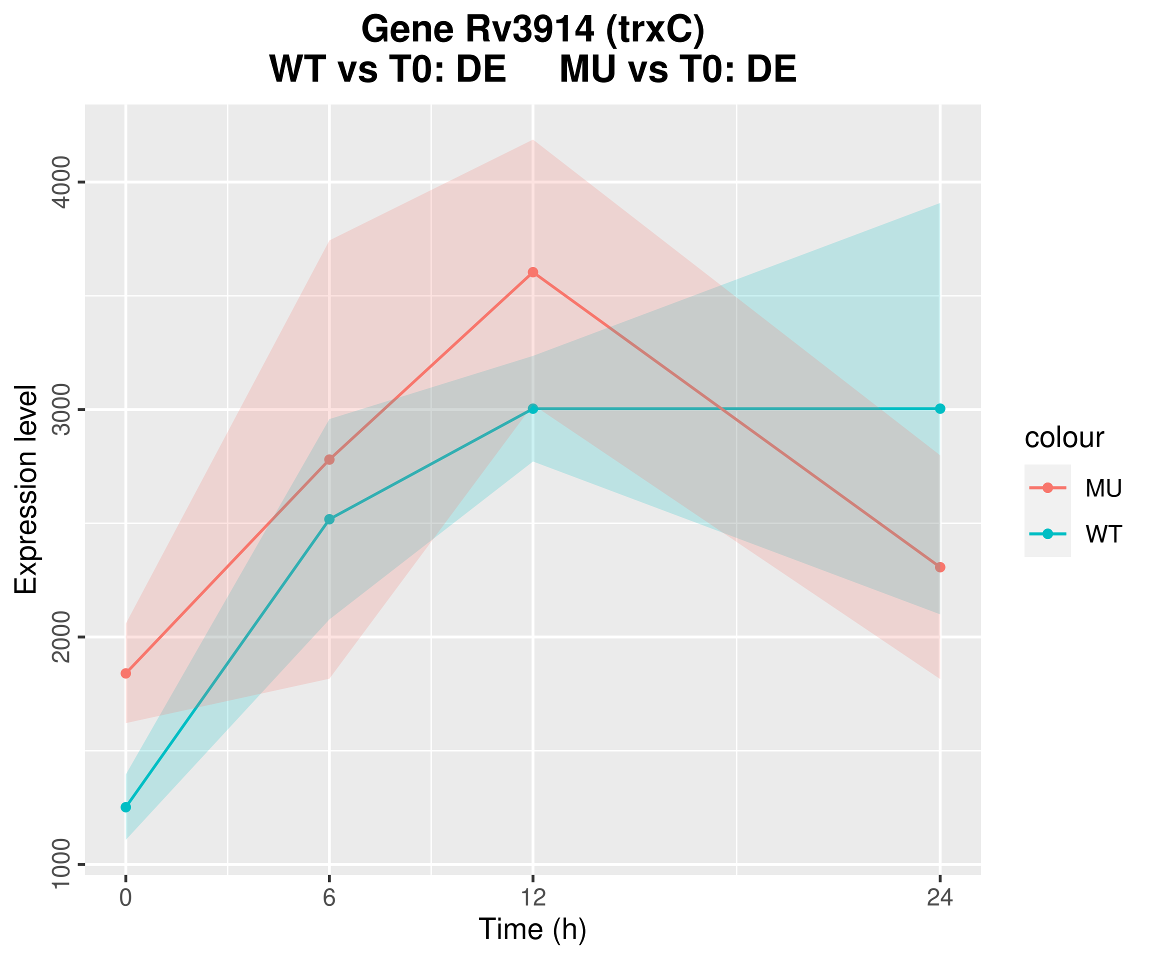

Gene Rv3914 (trxC)
WT vs T0: DE MU vs T0: DE
Expression level
4000
3000
2000
1000
0
6
12
24
Time (h)
colour
MU
WT

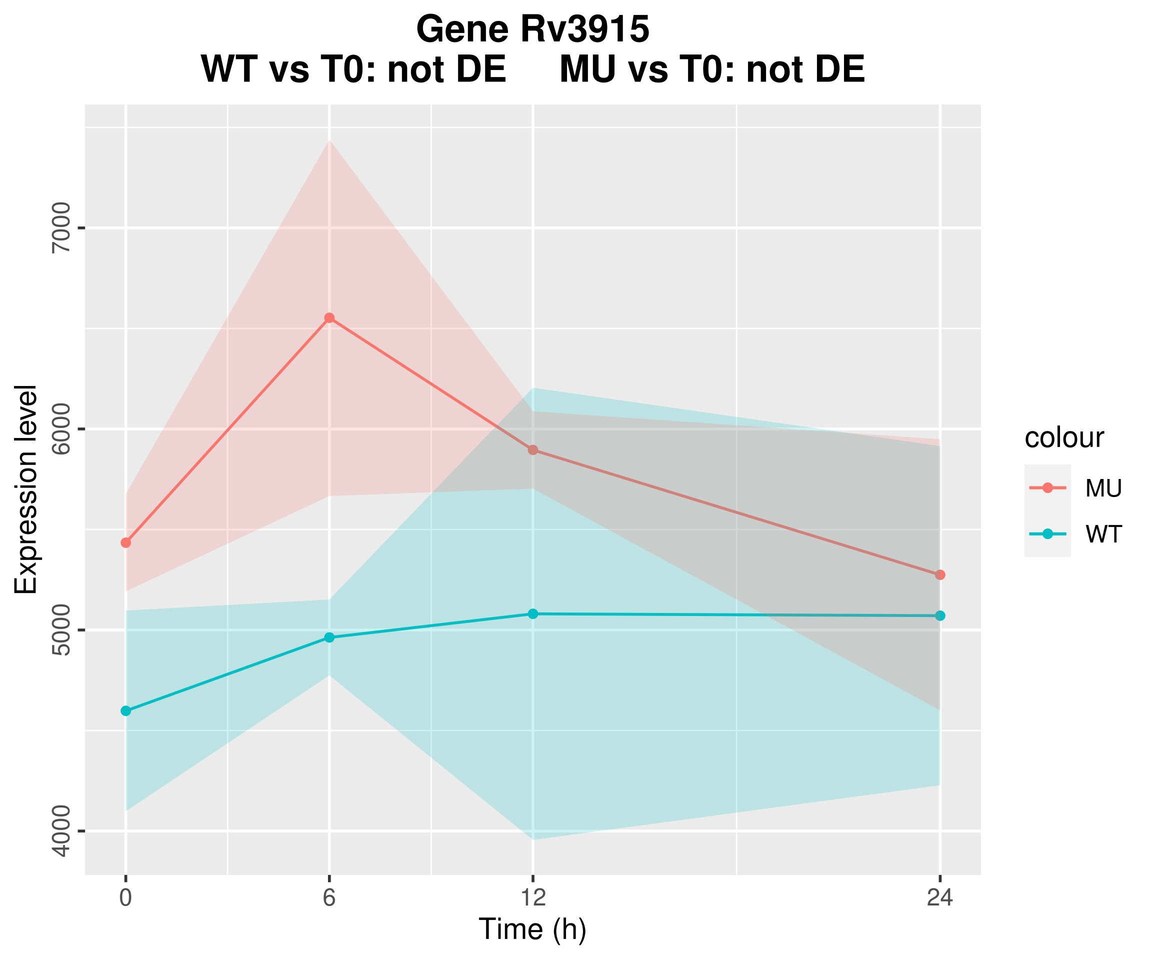
Gene Rv3915
WT vs T0: not DE   MU vs T0: not DE
Expression level
7000
6000
5000
4000
0
6
12
24
Time (h)
colour
MU
WT

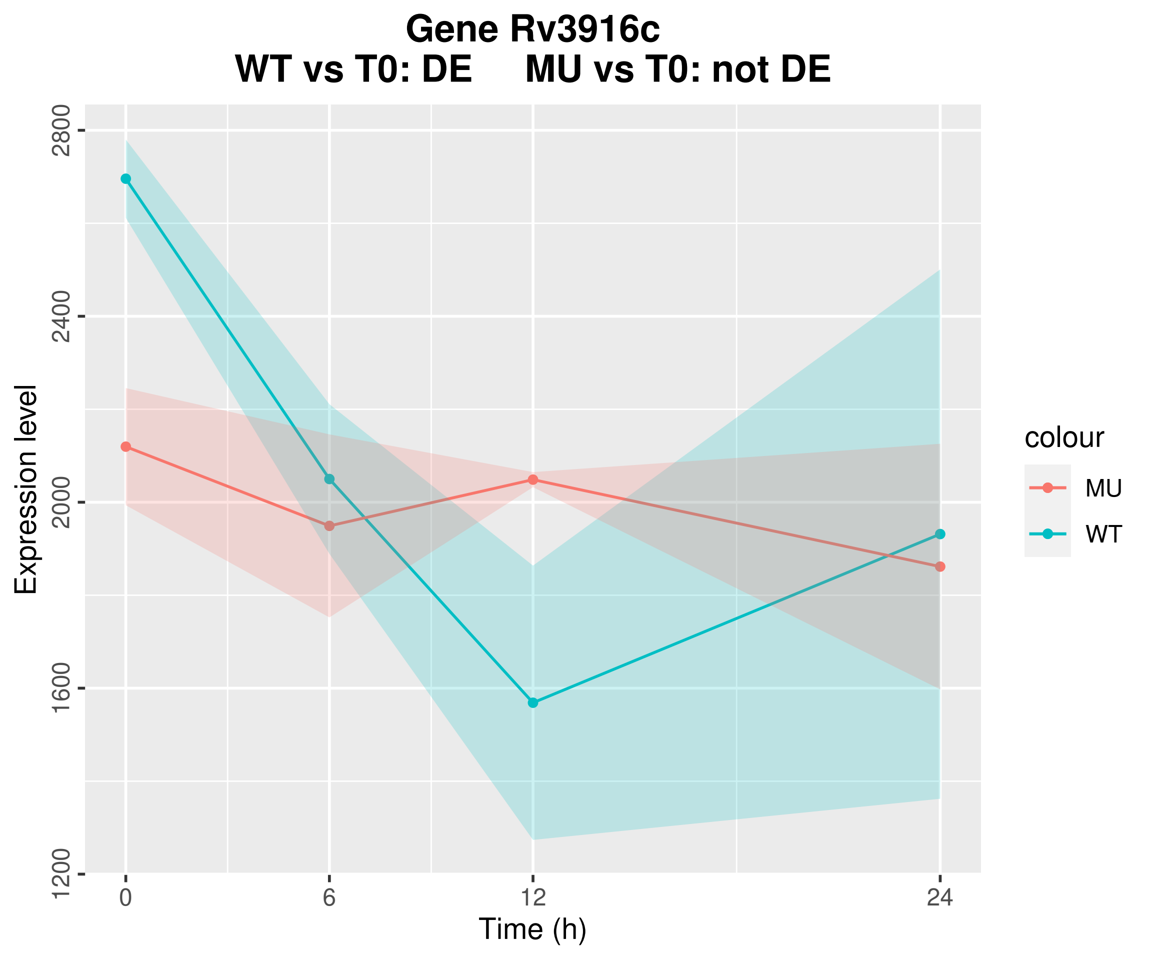
Gene Rv3916c
WT vs T0: DE MU vs T0: not DE
Expression level
2800
2400
2000
1600
1200
0
6
12
24
Time (h)
colour
MU
WT

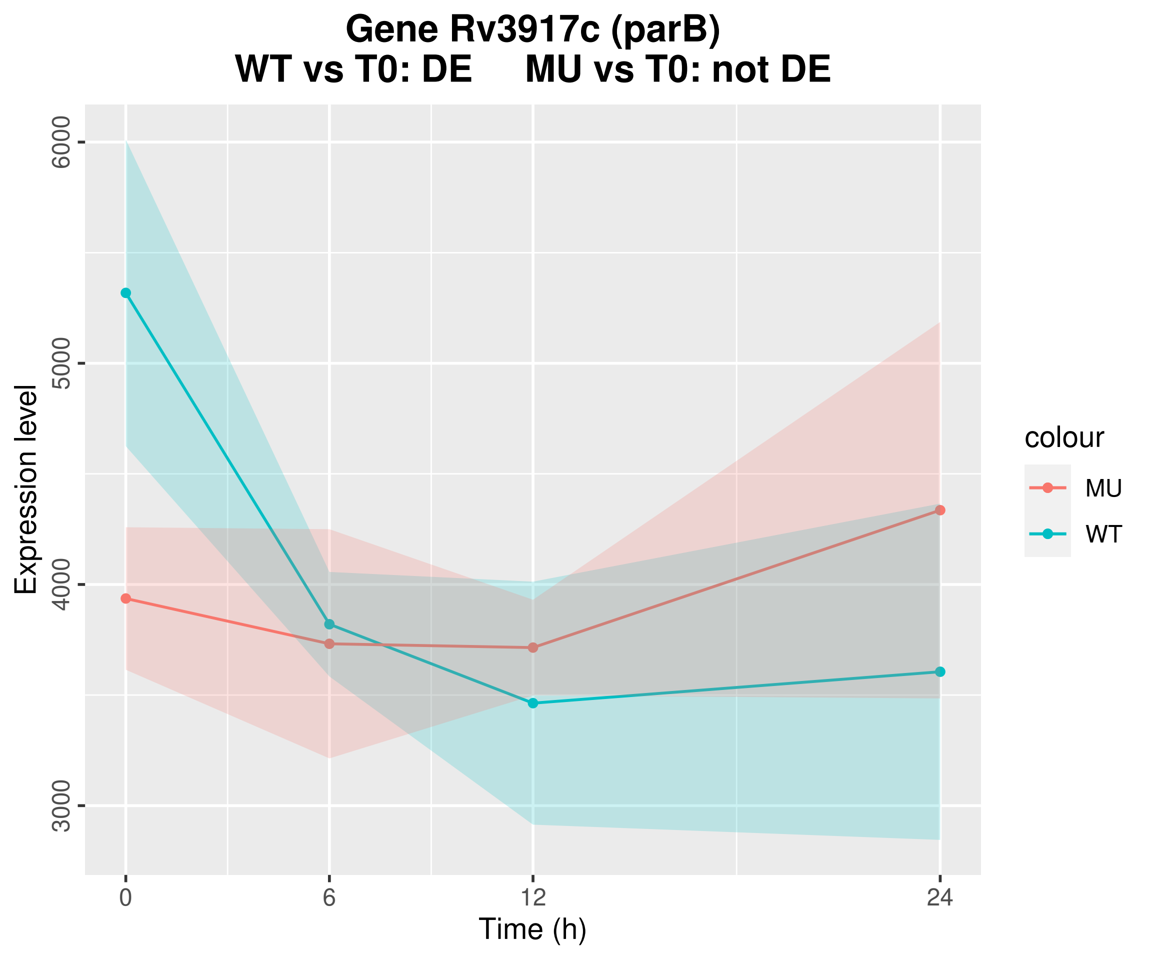
Gene Rv3917c (parB)
WT vs T0: DE   MU vs T0: not DE
Expression level
6000
5000
4000
3000
0
6
12
24
Time (h)
colour
MU
WT

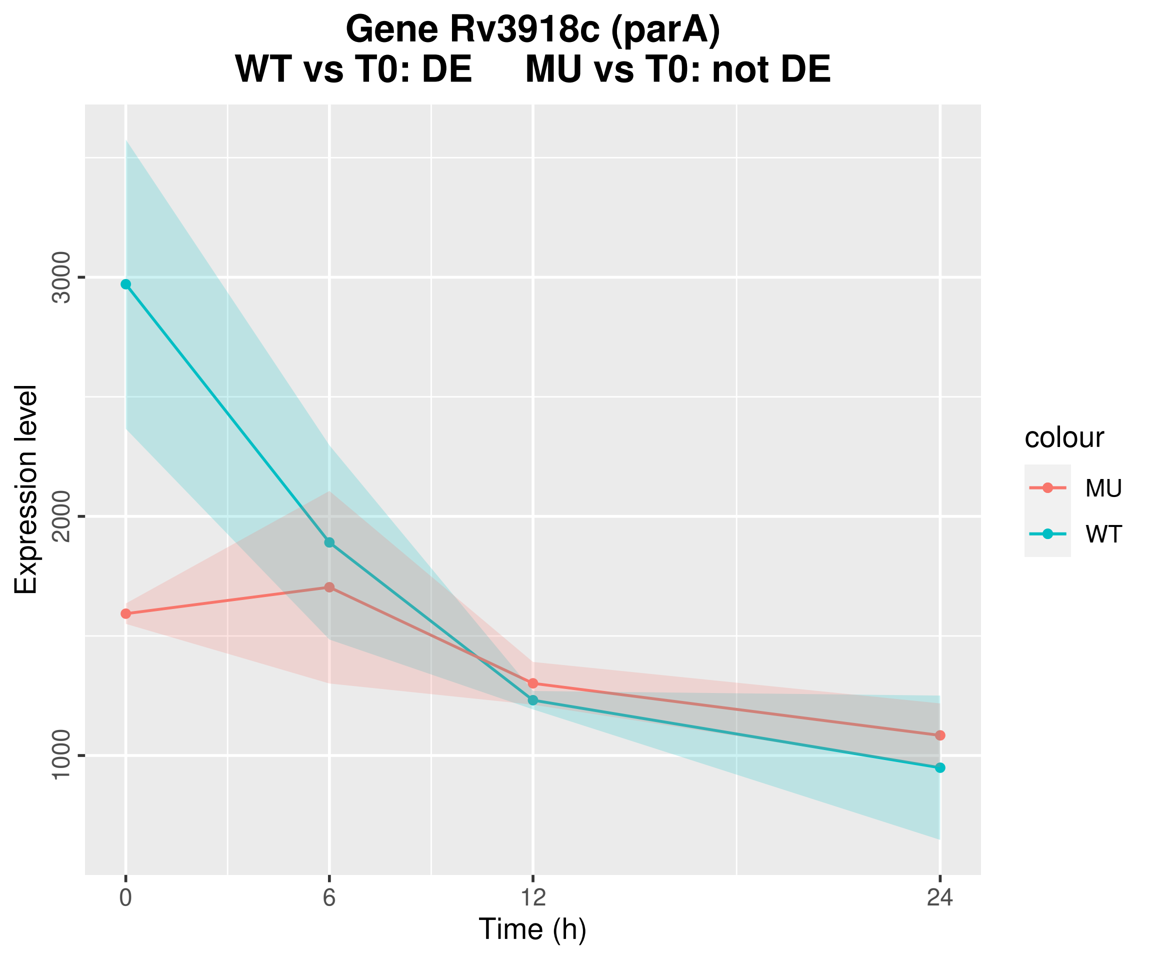
Gene Rv3918c (parA)
WT vs T0: DE   MU vs T0: not DE
Expression level
3000
2000
1000
0
6
12
24
Time (h)
colour
MU
WT

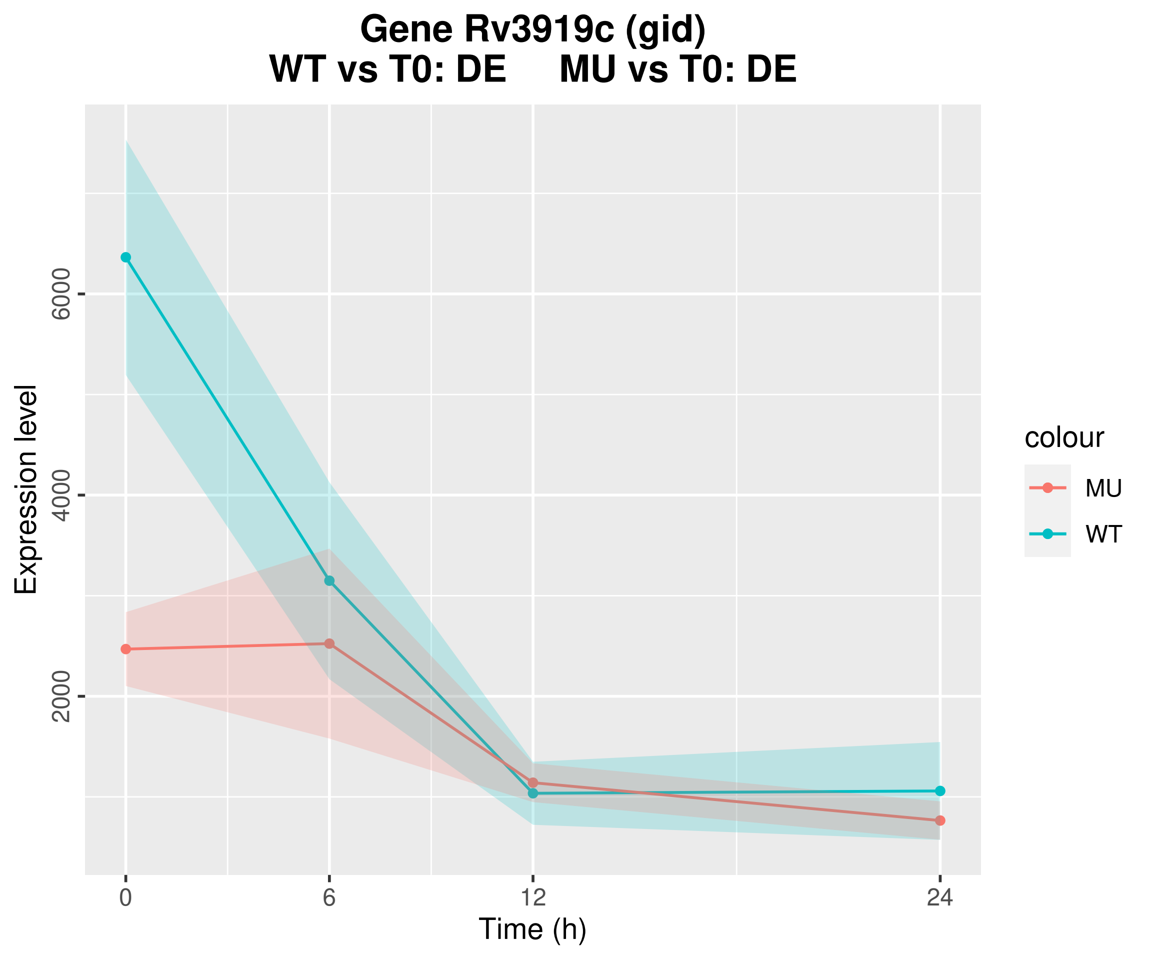

Gene Rv3919c (gid)
WT vs T0: DE MU vs T0: DE
Expression level
6000
4000
2000
0
6
12
24
Time (h)
colour
MU
WT

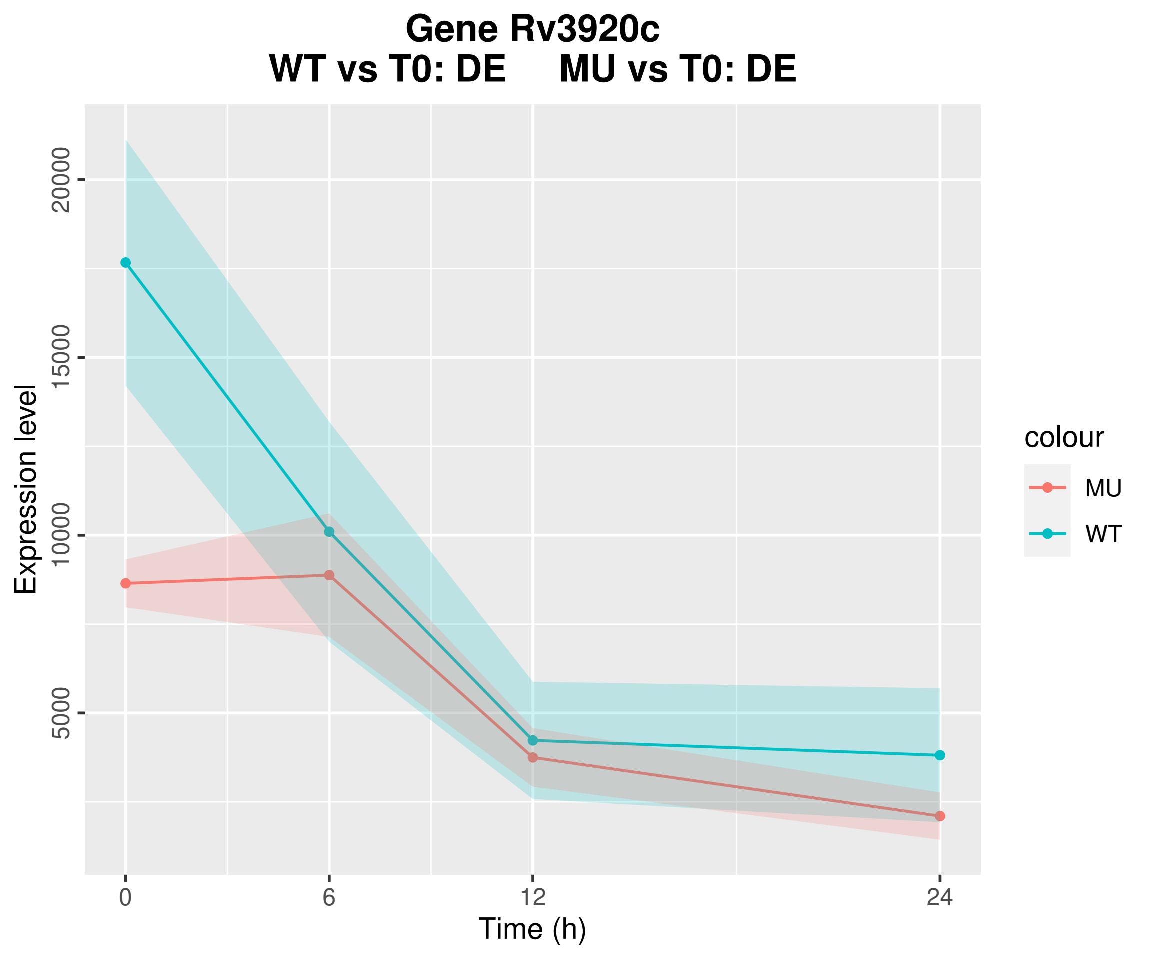
Gene Rv3920c
WT vs T0: DE MU vs T0: DE
Expression level
20000
15000
10000
5000
0
6
12
24
Time (h)
colour
MU
WT

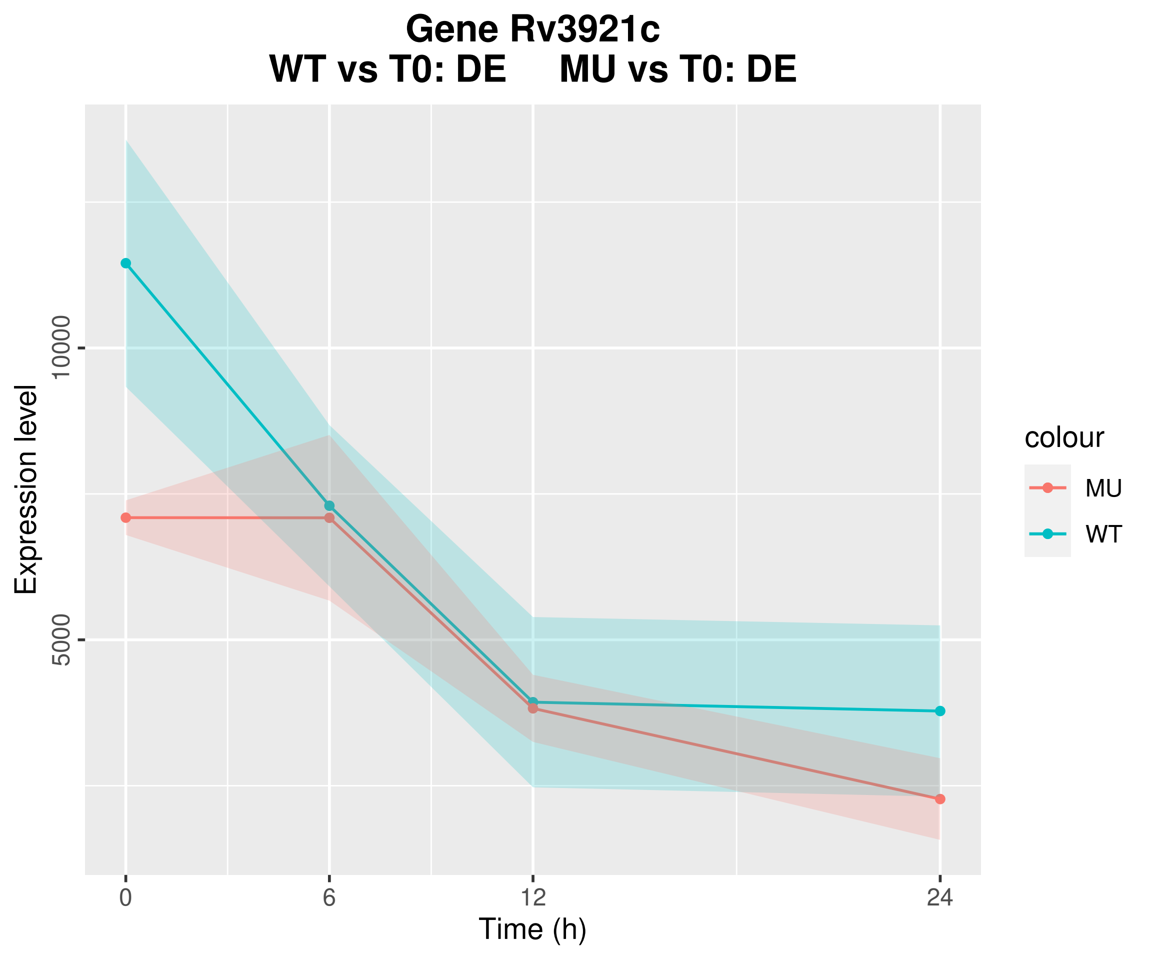
Gene Rv3921c
WT vs T0: DE MU vs T0: DE
Expression level
10000
5000
0
6
12
24
Time (h)
colour
MU
WT

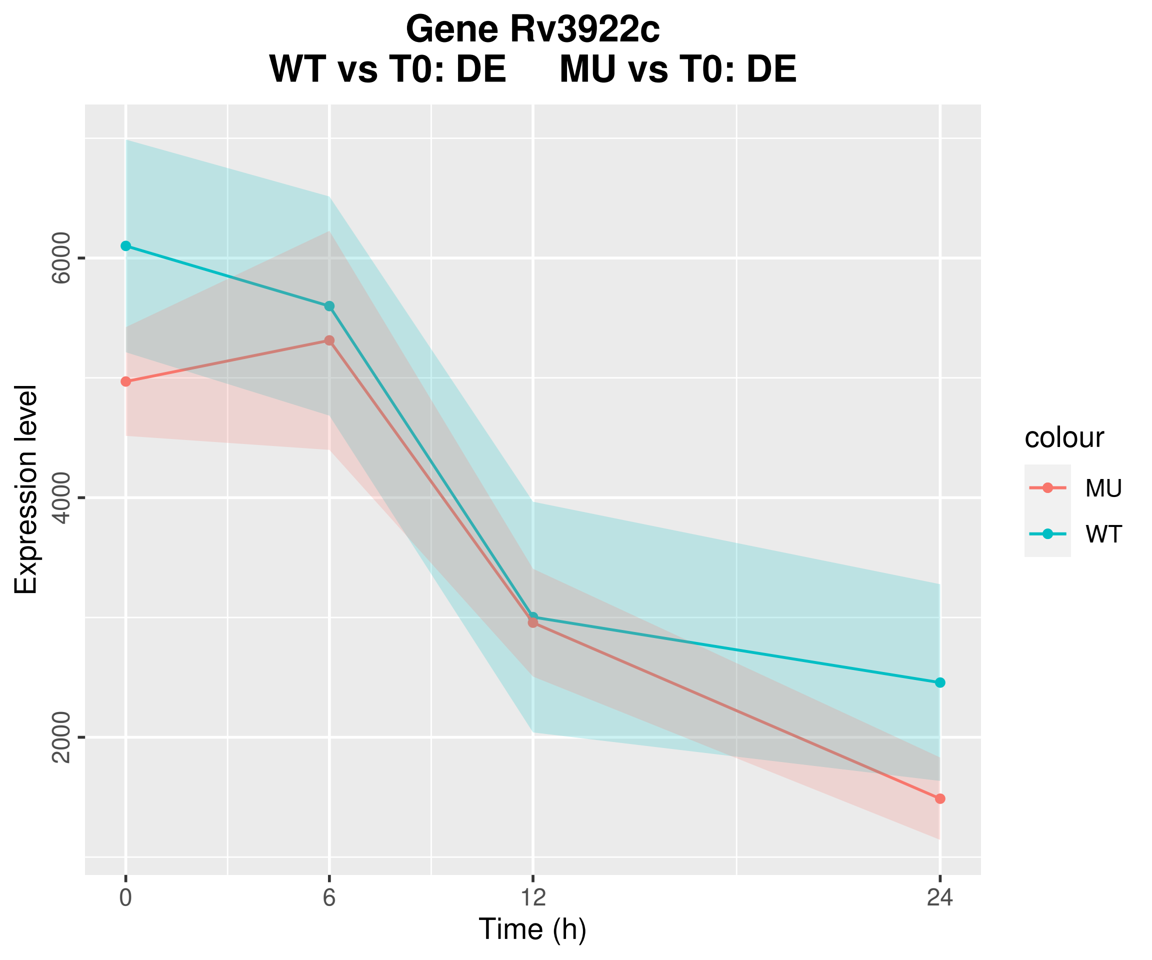
Gene Rv3922c
WT vs T0: DE MU vs T0: DE
Expression level
6000
4000
2000
0
6
12
24
Time (h)
colour
MU
WT

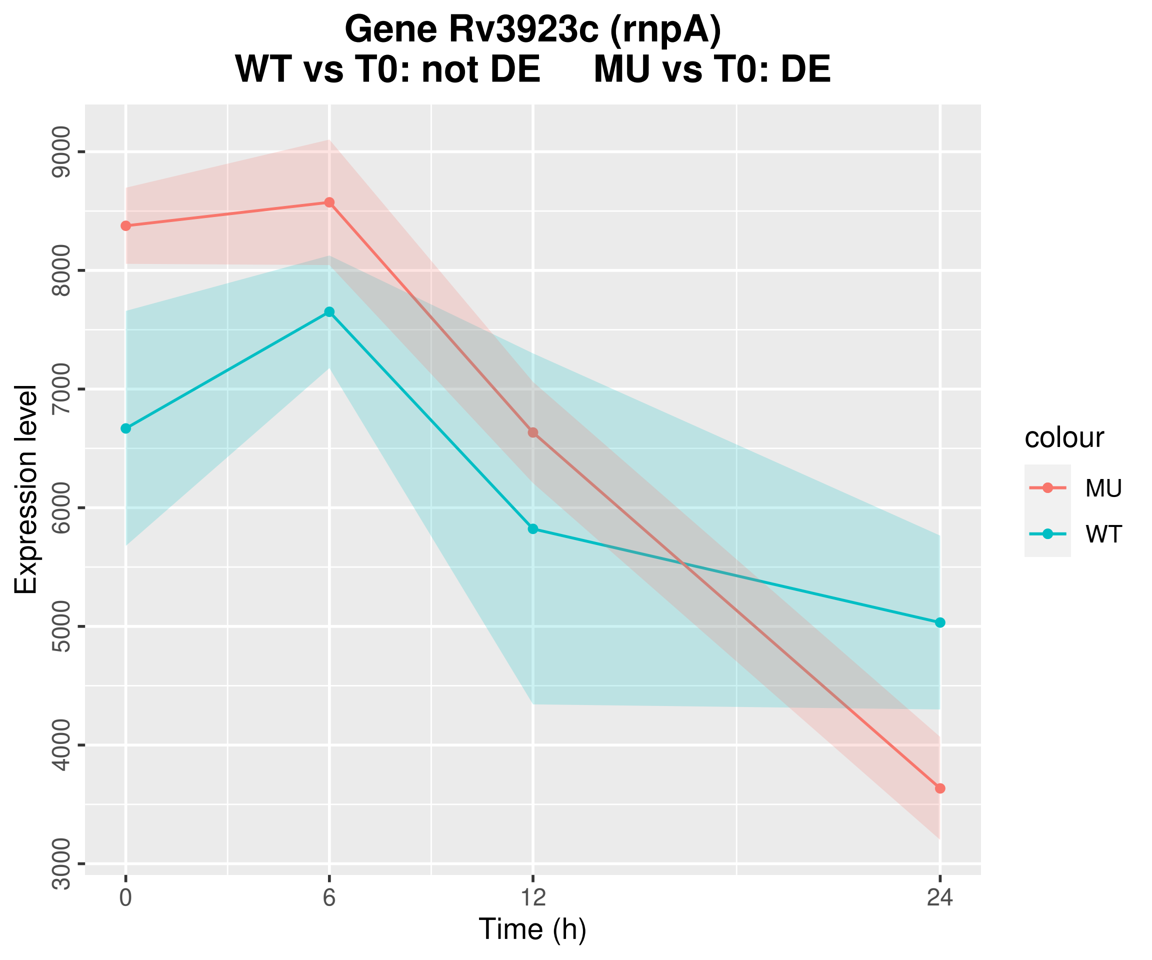
Gene Rv3923c (rnpA)
WT vs T0: not DE MU vs T0: DE
Expression level
9000
8000
7000
6000
5000
4000
3000
0
6
12
24
Time (h)
colour
MU
WT

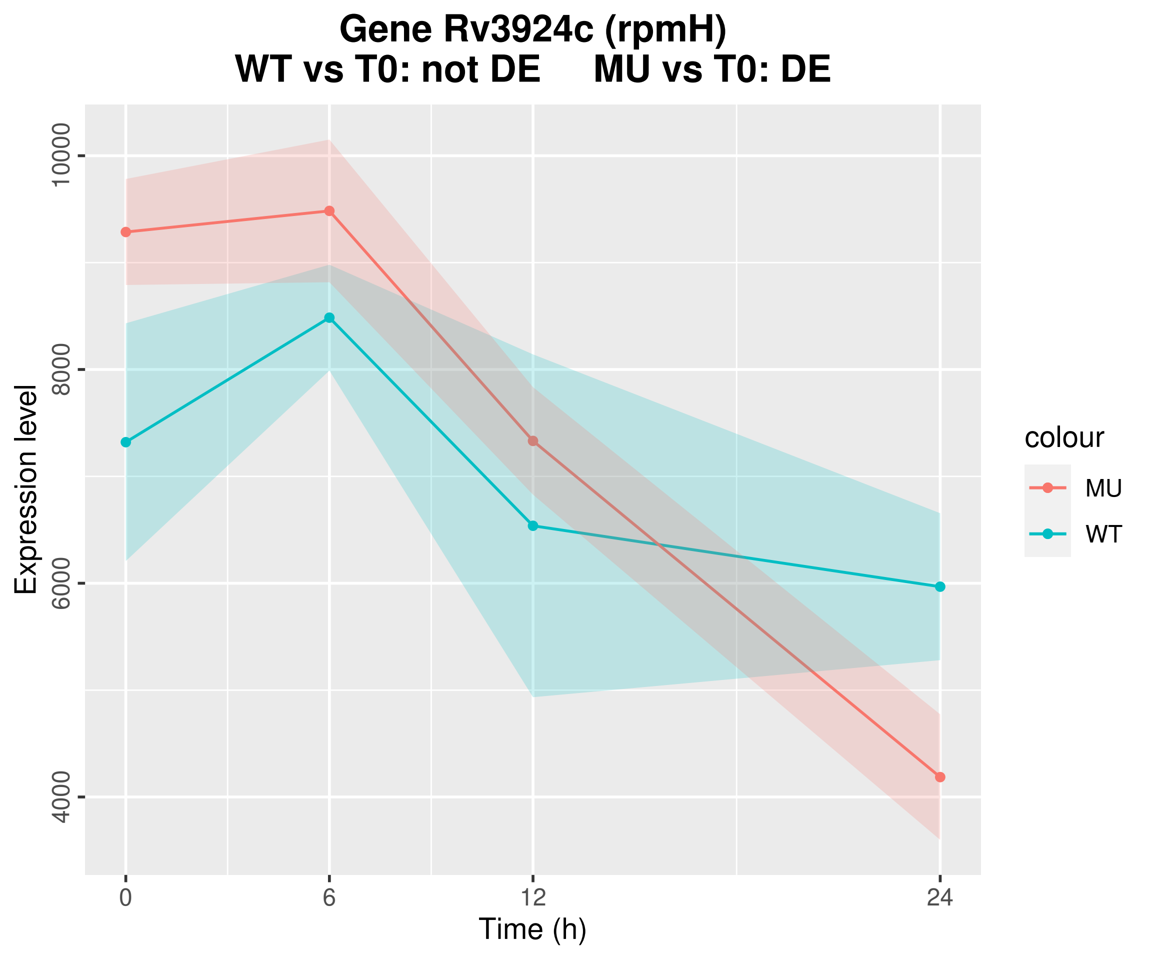
Gene Rv3924c (rpmH)
WT vs T0: not DE MU vs T0: DE
Expression level
10000
8000
6000
4000
0
6
12
24
Time (h)
colour
MU
WT
